# Supplementary material for: Immune-Related Long Non-coding RNA Signature and Clinical Nomogram to Evaluate Survival of Patients Suffering Esophageal Squamous Cell Carcinoma
Source: Front Cell Dev Biol. 2021 Mar 4;9:641960. doi: 10.3389/fcell.2021.641960 (PMC7969885; doi:10.3389/fcell.2021.641960)
Supplement: Supplementary Table 1 — Clinical information of samples from GSE53624. [file Table_1.docx]

Accession Age Sex Tobacco use Alcohol use Tumor grade T stage N stage Tnm stage Death at fu Characteristics

GSM1297240 57 male yes yes poorly T2 N0 II no 72.63333333

GSM1297310 45 male yes yes well T3 N1 III no 63.53333333

GSM1297220 71 female no no well T2 N0 I yes 0.633333333

GSM1297312 41 male yes yes well T3 N0 I no 62.86666667

GSM1297140 55 female no no poorly T3 N1 III yes 21.23333333

GSM1297242 50 male yes no poorly T4 N1 III yes 35.03333333

GSM1297222 62 male yes no poorly T3 N3 III no 63.4

GSM1297224 59 male yes yes moderately T4 N0 III yes 10.56666667

GSM1297142 50 female no no well T4 N0 III yes 45.83333333

GSM1297244 74 male no no moderately T2 N0 II yes 24.66666667

GSM1297144 50 male yes yes moderately T1 N3 III yes 17.5

GSM1297146 59 male yes yes poorly T2 N1 II yes 6.2

GSM1297226 68 male no no well T3 N0 II yes 5.066666667

GSM1297246 57 male yes yes poorly T1 N0 I yes 59.13333333

GSM1297148 54 male yes yes poorly T2 N0 II no 61.93333333

GSM1297150 51 male yes no moderately T4 N0 III yes 24.4

GSM1297152 73 female no no moderately T3 N0 II no 60.73333333

GSM1297154 51 male yes yes poorly T3 N2 III yes 15.86666667

GSM1297160 65 female no no moderately T3 N1 III yes 25.73333333

GSM1297156 45 male yes no moderately T3 N1 III yes 34.16666667

GSM1297162 54 male yes yes poorly T2 N1 II yes 22.13333333

GSM1297158 61 male yes no well T3 N0 II yes 9.766666667

GSM1297228 62 female no no moderately T3 N2 III yes 21.53333333

GSM1297164 57 male yes yes moderately T3 N0 II no 61.36666667

GSM1297166 45 male yes yes well T3 N0 II no 61.36666667

GSM1297168 39 male yes yes poorly T4 N1 III yes 15.6

GSM1297170 40 male yes yes well T2 N2 III yes 6.333333333

GSM1297172 82 male no yes well T4 N1 III yes 1.433333333

GSM1297174 53 male yes yes poorly T2 N3 III yes 8.266666667

GSM1297176 60 male yes yes well T3 N1 III yes 22.4

GSM1297178 59 male yes no poorly T4 N1 III yes 22.7

GSM1297180 58 male no no moderately T2 N2 III no 60.9

GSM1297182 62 male yes yes well T4 N3 III yes 6.633333333

GSM1297230 63 male yes yes moderately T3 N1 III yes 21.93333333

GSM1297184 49 male yes yes poorly T4 N0 III yes 6.666666667

GSM1297186 57 female no no well T2 N1 II yes 56.23333333

GSM1297188 56 male yes yes moderately T2 N0 II no 60.2

GSM1297190 72 male no no well T3 N0 II yes 47.16666667

GSM1297192 52 male yes yes moderately T3 N0 II yes 8.533333333

GSM1297194 73 male yes yes well T4 N3 III yes 13

GSM1297076 51 male yes yes moderately T4 N2 III no 60.3

GSM1297078 59 male yes yes moderately T3 N0 II yes 27.56666667

GSM1297080 59 female no no moderately T3 N0 II yes 34.66666667

GSM1297082 54 male yes no moderately T3 N0 II no 60.96666667

GSM1297212 57 male yes no moderately T4 N2 III no 60.63333333

GSM1297214 61 male no yes moderately T4 N1 III yes 19.2

GSM1297248 69 female no no moderately T2 N0 II no 63.26666667

GSM1297216 55 male yes yes moderately T3 N0 II no 61.6

GSM1297218 59 male yes no well T4 N3 III no 64

GSM1297196 75 male yes no poorly T4 N0 III yes 4.9

GSM1297198 71 female no no moderately T2 N2 III yes 32.2

GSM1297200 62 female no no moderately T2 N1 II no 62.93333333

GSM1297202 53 male yes no moderately T2 N0 II no 62.96666667

GSM1297204 62 male yes yes moderately T3 N2 III yes 37.16666667

GSM1297206 60 male yes yes moderately T3 N1 III no 62.46666667

GSM1297208 68 male yes yes moderately T3 N1 III yes 24.8

GSM1297210 57 male yes yes poorly T3 N0 II no 62.46666667

GSM1297250 59 female no no poorly T4 N0 III yes 8.366666667

GSM1297084 61 male no yes moderately T3 N1 III yes 15.43333333

GSM1297086 60 female no no poorly T3 N0 II no 61.33333333

GSM1297088 62 male yes yes moderately T4 N1 III yes 38.53333333

GSM1297090 57 female no no poorly T3 N0 II yes 23.26666667

GSM1297092 67 female no no moderately T3 N1 III yes 11

GSM1297252 67 male yes yes poorly T3 N3 III yes 10.46666667

GSM1297094 67 female no no well T3 N1 III yes 23.13333333

GSM1297096 56 male yes yes moderately T4 N2 III yes 10.03333333

GSM1297098 68 female no no moderately T3 N0 II yes 7.8

GSM1297100 66 male no no poorly T4 N1 III yes 21.43333333

GSM1297102 66 male yes yes moderately T3 N0 II no 61.9

GSM1297104 68 male yes yes moderately T3 N0 II yes 9.033333333

GSM1297232 42 male yes yes moderately T1 N1 II no 61.83333333

GSM1297106 60 male yes yes poorly T3 N1 III yes 42.03333333

GSM1297254 69 male yes yes well T2 N2 III yes 63.06666667

GSM1297108 53 male yes yes moderately T3 N0 II no 60.96666667

GSM1297110 60 male yes yes moderately T1 N0 I no 61

GSM1297112 68 male yes yes moderately T1 N1 II yes 31.5

GSM1297114 65 female yes no poorly T3 N1 III no 60.63333333

GSM1297116 51 male yes yes moderately T3 N0 II no 61.3

GSM1297256 66 male yes yes moderately T4 N0 III no 70.06666667

GSM1297118 54 male yes no moderately T4 N0 III no 61.2

GSM1297120 71 male no yes moderately T3 N1 III yes 12.56666667

GSM1297122 53 male yes yes moderately T1 N0 I yes 24.43333333

GSM1297124 62 female no no moderately T3 N0 II no 60.56666667

GSM1297126 64 male yes yes moderately T2 N0 II yes 21.26666667

GSM1297128 63 male yes yes moderately T3 N1 III yes 16.83333333

GSM1297130 57 male no yes well T3 N1 III no 69.96666667

GSM1297234 53 male yes yes well T4 N0 III no 60.76666667

GSM1297236 56 male no no well T2 N0 II no 60.5

GSM1297132 49 male yes yes poorly T3 N1 III yes 32.46666667

GSM1297258 68 male no yes moderately T3 N0 II no 72.13333333

GSM1297260 60 male yes yes moderately T4 N3 III yes 12

GSM1297262 69 male yes yes poorly T3 N1 III no 69.63333333

GSM1297300 57 male yes yes moderately T3 N0 II yes 48.76666667

GSM1297264 76 male yes yes poorly T1 N1 II yes 13

GSM1297266 68 male yes no moderately T3 N0 II no 68.7

GSM1297268 76 male yes yes moderately T3 N0 II no 68.2

GSM1297270 54 male yes yes moderately T4 N1 III no 68.6

GSM1297272 60 male yes yes moderately T3 N0 II no 68

GSM1297274 48 male yes yes well T3 N0 I no 68

GSM1297302 63 male yes yes poorly T3 N2 III yes 9.766666667

GSM1297276 69 male yes yes poorly T2 N1 II yes 0.1

GSM1297278 60 female no no moderately T3 N3 III yes 19.26666667

GSM1297134 41 male yes yes poorly T3 N1 III yes 12.23333333

GSM1297304 70 male no no moderately T4 N0 III yes 5.833333333

GSM1297280 46 male yes yes moderately T2 N1 II yes 13.46666667

GSM1297282 64 male yes yes poorly T3 N2 III no 65.86666667

GSM1297284 52 male no yes moderately T4 N1 III yes 11.5

GSM1297286 44 male yes yes moderately T4 N1 III yes 17.43333333

GSM1297288 68 male yes yes moderately T3 N1 III yes 14.36666667

GSM1297290 50 male no no poorly T3 N0 II no 65.16666667

GSM1297136 59 male yes no well T4 N0 III yes 9

GSM1297138 71 male yes yes poorly T3 N0 II yes 3.833333333

GSM1297292 53 male no yes well T3 N1 III no 65.5

GSM1297238 65 male yes yes moderately T4 N1 III no 72.53333333

GSM1297294 62 male no yes moderately T3 N0 II no 64.13333333

GSM1297296 47 male no no poorly T1 N1 II yes 26.5

GSM1297298 36 male no no moderately T3 N0 II no 65.26666667

GSM1297306 52 male yes yes moderately T3 N3 III yes 4.2

GSM1297308 53 female no no moderately T3 N2 III yes 25.16666667
