# Supplementary material for: Immune-Related Long Non-coding RNA Signature and Clinical Nomogram to Evaluate Survival of Patients Suffering Esophageal Squamous Cell Carcinoma
Source: Front Cell Dev Biol. 2021 Mar 4;9:641960. doi: 10.3389/fcell.2021.641960 (PMC7969885; doi:10.3389/fcell.2021.641960)
Supplement: Supplementary Table 4 — Clinical information of samples from GSE53622. [file Table_4.docx]

Accession Age Sex Tobacco use Alcohol use Tumor loation Tumor grade T stage N stage Tnm stage Death at fu Characteristics

GSM1296956 66.46027397 female no no middle moderately T3 N2 III yes 11.63333333

GSM1296958 64.01369863 male yes yes middle moderately T3 N0 II no 58.2

GSM1296960 50.91232877 male yes yes middle moderately T3 N0 II yes 39.16666667

GSM1296962 46.32876712 male yes yes lower moderately T3 N0 II no 57.73333333

GSM1296972 54.47945205 male yes yes middle moderately T3 N1 III yes 10.96666667

GSM1296974 56.23287671 male no no lower moderately T1 N1 II no 57.6

GSM1296976 57.09863014 male yes yes lower moderately T3 N3 III no 57.53333333

GSM1296978 44.6630137 male yes yes middle poorly T3 N1 III no 57.36666667

GSM1296980 43.76986301 male yes yes lower moderately T3 N1 III no 57.26666667

GSM1296982 67.2739726 female yes no upper poorly T3 N0 II no 57.23333333

GSM1296984 68.29041096 male yes yes lower moderately T3 N0 II yes 13

GSM1296986 60.50684932 female yes no lower well T3 N1 III yes 5.7

GSM1296988 48.40273973 male yes yes middle moderately T3 N0 II no 56.63333333

GSM1296990 54.29315068 male yes yes lower moderately T3 N0 II no 56.63333333

GSM1296992 51.9890411 female no no lower well T3 N0 I no 56.13333333

GSM1296994 58.32054795 female no no middle well T3 N0 II no 55.66666667

GSM1296964 53.99726027 male yes yes middle moderately T1 N1 II yes 11.06666667

GSM1296966 67.84383562 female no no middle poorly T3 N0 II no 55.26666667

GSM1296968 64.87945205 male yes yes lower moderately T3 N1 III no 55.26666667

GSM1296970 45.22191781 male yes yes middle moderately T2 N1 II yes 29.7

GSM1296996 66.27123288 male yes no upper poorly T3 N0 II yes 6.266666667

GSM1296998 72.24109589 male yes yes lower poorly T3 N0 II yes 25.46666667

GSM1297000 64.75068493 male no no lower well T3 N3 III yes 10.2

GSM1297002 54.57534247 male yes yes middle poorly T3 N1 III yes 13.16666667

GSM1297004 62.43835616 male yes yes lower moderately T3 N0 II no 54.73333333

GSM1297006 66.14794521 male no no lower poorly T3 N1 III yes 18.06666667

GSM1297008 53.74246575 male yes yes lower well T3 N1 III yes 36.3

GSM1297010 56.96438356 male yes yes lower moderately T3 N0 II no 54.1

GSM1297012 71.91506849 male no yes middle poorly T3 N2 III yes 16.03333333

GSM1297014 53.56438356 female no no upper moderately T1 N0 I no 52.93333333

GSM1297016 61.2739726 male no no middle well T3 N0 II no 52.7

GSM1297018 66.46027397 male yes yes middle poorly T3 N0 II yes 8.466666667

GSM1297020 62.12054795 female no no middle poorly T3 N2 III yes 4.366666667

GSM1297022 59.65205479 male yes yes middle poorly T1 N0 I no 52.03333333

GSM1297024 65.44931507 female no no middle poorly T3 N0 II yes 8.766666667

GSM1297026 51.7369863 male no no middle moderately T3 N0 II yes 1.666666667

GSM1297028 58.63561644 male no no middle well T3 N1 III no 51.3

GSM1297030 75.50958904 male no no lower poorly T3 N2 III no 51.2

GSM1297032 71.18356164 male yes yes middle well T2 N0 II no 51.1

GSM1297034 55.9890411 male no no middle poorly T3 N1 III yes 11.4

GSM1297044 57.39452055 male no no middle moderately T3 N0 II no 50.6

GSM1297046 50.94246575 male yes yes lower well T2 N0 I no 50.16666667

GSM1297048 80.95068493 male no no middle moderately T3 N0 II yes 24.46666667

GSM1297050 63.71780822 male yes yes lower moderately T3 N2 III no 49.43333333

GSM1297052 62.89863014 male yes yes lower moderately T3 N2 III yes 32.06666667

GSM1297054 62.84383562 female no no middle moderately T3 N0 II yes 25.16666667

GSM1297056 68.51780822 female no no upper moderately T3 N1 III yes 6.633333333

GSM1297058 39.53972603 male yes yes lower moderately T3 N1 III no 48.43333333

GSM1297060 68.08493151 female no no lower poorly T3 N1 III yes 11.4

GSM1297062 66.91780822 male yes yes lower moderately T2 N1 II no 48.03333333

GSM1297064 47.55068493 male yes yes lower moderately T3 N0 II no 47.93333333

GSM1297066 46.03287671 male no yes lower moderately T3 N0 II yes 6

GSM1297068 59.97260274 male yes no middle moderately T2 N1 II yes 39.16666667

GSM1297070 63.66027397 male no no middle poorly T2 N0 II yes 29.86666667

GSM1297072 78.88767123 male no yes lower poorly T3 N1 III yes 39.83333333

GSM1297074 61.33150685 male no no middle moderately T3 N0 II yes 44.93333333

GSM1297036 56.08493151 male yes no middle moderately T4 N2 III yes 39.5

GSM1297038 56 male yes yes upper moderately T2 N2 III yes 19.16666667

GSM1297040 81 male no no lower moderately T3 N2 III yes 8.5

GSM1297042 51 male yes yes upper moderately T3 N1 III yes 17.9
