# Supplementary material for: Immune-Related Long Non-coding RNA Signature and Clinical Nomogram to Evaluate Survival of Patients Suffering Esophageal Squamous Cell Carcinoma
Source: Front Cell Dev Biol. 2021 Mar 4;9:641960. doi: 10.3389/fcell.2021.641960 (PMC7969885; doi:10.3389/fcell.2021.641960)
Supplement: Supplementary Table 5 — LncRNAs obtained by GSE53622 annotation. [file Table_5.docx]

id GSM1297043 GSM1297041 GSM1297039 GSM1297037 GSM1297075 GSM1297073 GSM1297071 GSM1297069 GSM1297067 GSM1297065 GSM1297063 GSM1297061 GSM1297059 GSM1297057 GSM1297055 GSM1297053 GSM1297051 GSM1297049 GSM1297047 GSM1297045 GSM1297035 GSM1297033 GSM1297031 GSM1297029 GSM1297027 GSM1297025 GSM1297023 GSM1297021 GSM1297019 GSM1297017 GSM1297015 GSM1297013 GSM1297011 GSM1297009 GSM1297007 GSM1297005 GSM1297003 GSM1297001 GSM1296999 GSM1296997 GSM1296971 GSM1296969 GSM1296967 GSM1296965 GSM1296995 GSM1296993 GSM1296991 GSM1296989 GSM1296987 GSM1296985 GSM1296983 GSM1296981 GSM1296979 GSM1296977 GSM1296975 GSM1296973 GSM1296963 GSM1296961 GSM1296959 GSM1296957 GSM1297042 GSM1297040 GSM1297038 GSM1297036 GSM1297074 GSM1297072 GSM1297070 GSM1297068 GSM1297066 GSM1297064 GSM1297062 GSM1297060 GSM1297058 GSM1297056 GSM1297054 GSM1297052 GSM1297050 GSM1297048 GSM1297046 GSM1297044 GSM1297034 GSM1297032 GSM1297030 GSM1297028 GSM1297026 GSM1297024 GSM1297022 GSM1297020 GSM1297018 GSM1297016 GSM1297014 GSM1297012 GSM1297010 GSM1297008 GSM1297006 GSM1297004 GSM1297002 GSM1297000 GSM1296998 GSM1296996 GSM1296970 GSM1296968 GSM1296966 GSM1296964 GSM1296994 GSM1296992 GSM1296990 GSM1296988 GSM1296986 GSM1296984 GSM1296982 GSM1296980 GSM1296978 GSM1296976 GSM1296974 GSM1296972 GSM1296962 GSM1296960 GSM1296958 GSM1296956

AC093519.2 10.0896386166667 9.07875895833333 9.46190524166667 9.58157864166667 8.98227436666667 9.036003975 9.43132836666667 9.201411475 8.900308075 9.61446786666667 9.049498825 10.0460242583333 9.94714953333333 9.32990726666667 9.6076044 9.54094585833333 9.661839025 9.24370573333333 8.94592274166667 8.94836124166667 9.334967175 8.33092125 9.4470582 9.51536090833333 9.51801905 9.75280021666667 9.21916606666667 9.18598278333333 8.39364840833333 9.105193125 8.93346260833333 9.27651353333333 9.09762666666667 9.37335539166667 9.53164766666667 9.69020153333333 8.89079293333333 9.16631998333333 9.011109275 9.25094153333333 8.92760421666667 9.6529159 8.86194551666667 8.80747489166667 9.90727683333333 9.14824449166667 9.37885371666667 9.323428375 8.3215665 9.661839025 9.15604288333333 9.30083194166667 9.24658409166667 9.47543304166667 10.0478232083333 10.420672925 9.37623116666667 9.220525675 8.97615546666667 9.5521857 9.37335539166667 9.29547155833333 9.56623849166667 9.23642199166667 9.35536706666667 8.893151 8.73370975833333 9.39042674166667 8.230454425 9.66989880833333 8.84022498333333 8.77366934166667 9.54520550833333 9.24949425833333 9.10908699166667 9.24131265 8.71236105833333 9.3945379 8.77244310833333 8.426213375 8.53136013333333 9.867307775 8.8037272 8.75543470833333 8.84985913333333 8.471128425 9.24131265 9.6646531 8.59712365833333 9.1323295 8.62436223333333 9.042386175 8.32474756666667 8.4458465 8.40717060833333 8.71699468333333 8.97885180833333 8.900308075 8.7855717 9.18598278333333 8.787979925 9.06840325833333 8.45231269166667 8.72160276666667 7.53776969166667 8.83012083333333 8.53935796666667 8.45362333333333 8.33859384166667 8.78337874166667 9.15077928333333 8.72541470833333 8.62322813333333 8.20093078333333 9.57056143333333 9.2176707 9.111708925 8.54724403333333 7.86665836666667 9.44441939166667

AL138830.2 4.33106415833333 3.94318915 3.86581385 4.11509285833333 3.817257075 4.09521015 3.96873128333333 3.69672979166667 3.9705387 4.09270279166667 3.81498958333333 4.42340415833333 3.9327276 3.89974741666667 3.864937625 3.884727375 4.2871363 3.88965465833333 4.44750469166667 3.780406475 3.68631180833333 4.15484655833333 4.111010475 4.4165323 4.54275920833333 3.90232935833333 4.19519426666667 4.3750276 4.13770233333333 4.09098995 4.52785514166667 4.41717060833333 3.63373555833333 4.29154165833333 3.98423539166667 4.21399664166667 4.190498425 4.48259481666667 4.3831831 4.69199204166667 4.66132636666667 4.2871363 4.696789075 5.49272935833333 3.96958159166667 4.88903463333333 4.64653290833333 4.826730425 4.32111429166667 4.6318291 4.6241475 4.435029675 3.94837956666667 5.039107525 4.08867361666667 4.67170091666667 3.92232456666667 4.66817654166667 4.456836225 4.3831831 4.023105425 4.22423876666667 4.29792231666667 4.46857235833333 3.84629465 3.66381434166667 3.79116074166667 4.12213588333333 3.94073975 4.42684748333333 4.011089225 4.31461836666667 4.2893372 3.861448425 3.898913075 3.60891241666667 3.84547438333333 3.70428345833333 4.252842375 3.80026373333333 4.13221068333333 3.89657501666667 4.035574075 4.31304105 3.64726053333333 3.91111126666667 3.92150503333333 4.111010475 3.52420999166667 3.88880333333333 3.65665524166667 4.347124625 3.8855125 3.94763221666667 3.79800631666667 3.93419443333333 3.88965465833333 4.21564994166667 4.33331919166667 4.93534804166667 4.096636775 4.101417825 4.64031274166667 4.89586528333333 4.13691099166667 4.02802744166667 4.28643399166667 5.81853184166667 4.10685365 5.65810201666667 4.55111153333333 5.05599298333333 4.525625675 4.66661991666667 3.88389206666667 4.344009325 3.92781315833333 4.40096828333333 4.15639725 4.35821460833333

HOXB-AS3 8.5497607 5.82105976666667 6.89303899166667 7.17846976666667 6.87998445 8.618487425 7.24425825833333 7.45820561666667 6.97981448333333 7.10174751666667 7.365658075 9.9348273 6.291657675 6.05432435833333 6.21577044166667 6.50018229166667 9.856575175 6.03493583333333 7.46975303333333 6.779595125 6.18052179166667 7.188718125 6.6227255 7.75393954166667 7.53776969166667 10.4365874083333 6.6848112 6.838229925 6.42497558333333 7.10083153333333 6.74572325 6.74344500833333 6.74043675833333 9.23402224166667 11.9199419 7.03066510833333 6.85293710833333 7.48147895 6.7441857 6.68563873333333 8.245206125 7.033226925 8.02061905 7.76018400833333 9.9755856 8.20827265833333 7.62178835 7.499347125 7.94975508333333 8.00049861666667 7.46890780833333 6.67400460833333 7.27771845 6.585221025 7.16739100833333 7.56162029166667 7.35849015 7.54978125 7.625103125 8.26500145 7.92482581666667 6.690101625 6.37198143333333 7.437000375 7.48069958333333 7.05282128333333 8.61109633333333 7.581616625 7.59330443333333 7.8232377 7.595952025 7.48147895 7.86100648333333 8.37884899166667 7.41157274166667 7.18309601666667 8.26088785833333 6.73733449166667 7.49513213333333 6.37369095 5.611704975 5.6893285 7.64410754166667 8.37465215 7.14716405 7.3593209 6.595923 6.7213753 6.76567473333333 7.63212294166667 8.0070051 7.29643938333333 7.07277904166667 8.14316695 7.48714180833333 6.87998445 6.70095648333333 6.70927374166667 7.3062498 8.20827265833333 8.74159676666667 7.02662889166667 6.27620438333333 7.78611961666667 8.00143094166667 6.9982378 8.993454275 6.82479446666667 7.63799916666667 7.40711176666667 7.36490918333333 6.72671865 8.60047110833333 8.992253025 7.66352786666667 6.6189581 9.14450013333333 6.87915231666667 7.050523425 7.70049388333333

BVES-AS1 8.16110536666667 4.74100033333333 4.61689800833333 4.23511105833333 3.90713534166667 4.79683228333333 4.075722525 5.13457766666667 4.29010148333333 4.853953375 4.10854148333333 4.76129344166667 4.544983975 4.53840028333333 4.79377419166667 5.02553525 7.05999225 4.21730013333333 5.33879713333333 5.180684475 4.49520464166667 5.094703075 5.20589149166667 6.87454878333333 5.74762156666667 7.90677205 6.32934243333333 5.87078084166667 4.98278695833333 5.60489321666667 6.31742500833333 5.40623839166667 5.35864575 4.68278718333333 8.13699931666667 8.063178225 5.37246943333333 6.19334070833333 5.650153475 5.4760094 5.85430964166667 5.499379 5.18325646666667 6.66683761666667 6.9614343 7.36804055833333 6.291657675 5.22298730833333 4.93026841666667 5.08815185 5.58178744166667 5.38175511666667 4.55571135833333 6.15706108333333 5.185805625 6.78424304166667 4.227329175 5.95586469166667 5.46176480833333 5.99116495833333 4.03934740833333 5.00864485833333 5.15614551666667 5.152649175 3.91034174166667 3.7361615 3.88806908333333 3.93981401666667 3.876232825 3.9087838 3.75997265 4.08791583333333 3.95531615 4.09366275833333 4.14092098333333 4.020609225 4.26717905833333 4.436542725 5.07579073333333 4.01751818333333 4.1770019 4.12905548333333 4.880021025 7.22480836666667 3.76338045 5.01436615833333 4.09588705833333 4.45389868333333 3.8566224 5.23682500833333 3.71429205833333 4.41295244166667 5.506564725 5.04240375 4.955238375 3.877204375 4.24443253333333 5.41616288333333 4.25214924166667 5.16271053333333 5.4338253 3.75472904166667 4.95061065 5.07245089166667 6.24024199166667 5.98543355 4.61608426666667 6.29466304166667 4.41868564166667 6.74266950833333 5.24520668333333 5.47950365833333 5.00720284166667 5.24850139166667 4.50987645 4.481057225 7.60982945833333 6.86315831666667 4.70340193333333 4.403336375

AC063919.1 6.97501571666667 5.06194454166667 7.51334780833333 6.05329835833333 5.15933271666667 6.50900563333333 5.55703588333333 5.347740225 5.30937105833333 5.506564725 5.620020925 5.64469614166667 5.511999925 5.228162675 5.786330625 5.34487673333333 5.77795108333333 6.22740323333333 6.74876795 5.114099175 5.05081140833333 6.19761265833333 4.88164143333333 6.73817556666667 5.54304493333333 6.15870698333333 6.32849529166667 5.80287793333333 5.778779775 5.899280325 6.0189352 6.26883340833333 5.44621728333333 5.88213181666667 7.012070875 7.36105020833333 5.71592235833333 6.45056610833333 6.30028739166667 5.70335329166667 5.90988236666667 6.54453155833333 4.65351119166667 6.02811239166667 6.10696181666667 5.715067925 5.99706000833333 6.07758535833333 5.47188130833333 5.64108119166667 6.324552 5.29742040833333 5.78887575833333 7.1088106 5.38263980833333 6.03972224166667 5.21587950833333 5.450343875 6.28300039166667 6.37025050833333 6.6615047 5.31623470833333 7.743945075 6.79889878333333 4.59278468333333 6.62200111666667 6.17468929166667 5.72631065 6.89382319166667 5.80704883333333 4.91436364166667 6.38156883333333 5.17730950833333 5.424208775 5.89124781666667 6.01338551666667 5.80287793333333 5.36382531666667 5.20143405 5.36822230833333 6.01241375 6.64146615833333 5.72631065 6.37921816666667 5.682553675 5.87236445 5.96262355 5.09060064166667 5.42068620833333 5.08979221666667 5.74504694166667 6.812864825 5.58504371666667 5.84838258333333 6.29000819166667 6.1514684 6.05752005 6.45206855833333 5.6927165 5.48853470833333 5.986172625 5.12618295 6.41370083333333 5.345943725 5.30076113333333 4.98534708333333 6.92234795833333 5.81184100833333 5.76338288333333 6.396357 6.133595725 5.33475269166667 6.09411293333333 6.81620986666667 5.27133298333333 5.00789455 6.10696181666667 4.92694636666667 5.75521793333333 5.14319801666667

AC068587.4 7.36412529166667 6.37198143333333 6.7441857 8.308792025 7.6432255 7.27529025833333 7.2852293 7.09746866666667 7.58072930833333 6.95374109166667 7.15207910833333 6.905014675 8.59246085 7.66773091666667 6.81212784166667 7.06402211666667 6.909615425 8.26500145 7.37223949166667 6.88935775 7.724325925 7.64163629166667 6.93829808333333 7.64163629166667 7.26148395833333 6.81769201666667 7.00742828333333 7.66352786666667 7.031491525 6.94725178333333 7.86100648333333 7.1157008 7.08121445833333 6.66745250833333 6.65457601666667 5.73081749166667 7.35757096666667 7.16321075833333 7.23347658333333 7.02899554166667 6.93755019166667 7.37382095 6.79117925 6.75605898333333 7.49757348333333 6.16925395833333 6.71711986666667 7.25439505 7.02737631666667 7.15132348333333 7.14226920833333 7.009018975 7.31031474166667 7.75849498333333 6.78346969166667 6.27123641666667 7.15037268333333 6.36384354166667 7.24756645 7.12127630833333 8.27546815 7.184582075 7.66087161666667 8.54377494166667 7.744788925 7.31633914166667 7.37881741666667 8.19285094166667 8.44688928333333 7.91377156666667 7.223930825 7.36490918333333 7.80424109166667 7.47322088333333 7.87803565833333 7.24674725 7.64410754166667 7.66087161666667 5.83439000833333 7.05611735833333 7.77821760833333 7.978411525 6.96989185833333 5.903354775 6.598919125 8.320569675 8.08371099166667 7.47076813333333 8.28896994166667 6.81924664166667 7.827876425 7.41246615833333 6.825603975 5.69812770833333 7.16947939166667 7.77743810833333 9.00480995 8.11629255 7.01349440833333 7.6360768 6.05752005 8.230454425 6.857401975 5.69727409166667 6.73733449166667 7.09583709166667 6.58438846666667 6.683342625 6.37119856666667 6.96543805833333 4.843025575 5.77706405 7.04208126666667 6.280696575 5.85603955 6.4602804 6.999816375 5.62274641666667 6.182879225 6.07508890833333

BAALC-AS1 5.99926955 5.392747 5.4551887 6.39250838333333 4.74184695 6.436652525 5.077701625 5.32654480833333 5.31363400833333 6.11113416666667 5.70871841666667 5.347740225 5.2527348 5.66590344166667 6.17880248333333 5.582540175 6.968472025 5.311054825 5.42944249166667 6.21003021666667 4.78802293333333 6.71181541666667 5.926954025 5.37246943333333 5.51902839166667 5.151017225 5.31702426666667 5.75992465 5.45429715833333 6.06669943333333 4.35151680833333 5.683497175 4.75429261666667 6.272113525 6.49186828333333 6.48636519166667 6.27455109166667 5.93775123333333 6.21246914166667 4.75656106666667 6.57380151666667 5.19562818333333 5.70703833333333 6.29693088333333 6.082719625 6.80453016666667 6.19675590833333 4.301581775 5.11849680833333 5.74853151666667 6.24370750833333 5.44548114166667 6.64445649166667 6.37843725833333 5.228162675 5.96876623333333 5.47283311666667 7.576473975 5.76857745833333 5.35232301666667 7.60801480833333 6.041301775 6.47356659166667 7.61407576666667 6.238638975 6.00748140833333 6.26236639166667 5.65972369166667 7.238301775 6.430564175 5.36997850833333 6.60281753333333 5.33964168333333 6.238638975 7.64410754166667 7.90273456666667 6.21087870833333 7.18077985833333 5.299873475 7.84339445 5.71323735 6.69077578333333 7.75118009166667 6.7586539 6.127183975 5.585997275 5.80207481666667 6.20542285 4.79866623333333 7.74760565833333 6.247501925 6.45639771666667 5.70703833333333 8.24724386666667 6.91268935833333 6.15066176666667 5.28772790833333 5.91688453333333 6.102165675 6.86553285 6.27774009166667 5.01436615833333 6.43283198333333 6.66815600833333 5.16420516666667 8.17305670833333 6.66003873333333 4.854747975 5.61076979166667 6.061018125 5.41616288333333 5.893600675 8.22233045 7.107436475 5.81263556666667 5.59208724166667 6.38460903333333 8.10837065 6.62653135833333 4.85957668333333

MIR3659HG 4.89413600833333 4.10217723333333 4.107640625 4.29455464166667 4.44597125 4.68661393333333 3.92150503333333 4.19596420833333 3.85167864166667 4.252842375 3.98740066666667 4.00394323333333 3.581753225 4.181393575 5.4527323 5.32578840833333 5.457689125 4.247434625 5.41046095 4.05236036666667 3.90066126666667 4.18975846666667 4.79532921666667 5.17819575 5.98312854166667 4.111905175 4.66568316666667 4.854747975 4.94495284166667 4.80260285 5.55545125833333 5.81024634166667 4.40993569166667 5.21754135 4.934512075 4.484204225 4.67862661666667 5.13539786666667 5.23750470833333 6.14832854166667 5.26205978333333 4.70744949166667 5.33192491666667 5.85179635833333 5.31867865833333 5.33475269166667 5.706023225 5.247693175 5.56030313333333 5.25844070833333 5.858605475 5.92279354166667 5.16586305833333 6.133595725 5.59994884166667 5.54038896666667 4.33031856666667 6.1530748 5.850901925 5.83677235833333 4.10366320833333 4.39933081666667 5.08815185 4.76862936666667 3.77450598333333 4.75499598333333 3.96873128333333 4.12905548333333 4.533885625 4.48490886666667 3.81162940833333 4.61689800833333 4.007428725 3.80765118333333 4.37878591666667 5.21348641666667 4.37819051666667 4.90444880833333 4.24262859166667 4.53154068333333 3.53924516666667 4.69586463333333 4.3038327 5.165035025 5.07245089166667 4.41144705 5.11490881666667 4.56640000833333 3.870040525 4.46943080833333 5.17494715833333 5.157017525 5.20060335833333 4.6472699 5.683497175 4.37582008333333 4.073408825 4.115880825 4.038581625 5.320395475 4.75429261666667 3.8631307 4.948926475 5.333787 4.49283225833333 4.46398463333333 4.61847421666667 6.50724065 4.42260004166667 6.87679691666667 4.98442578333333 5.71839368333333 6.37447095 5.40722773333333 5.60075419166667 5.51729848333333 4.66742204166667 4.851367175 5.49108314166667 3.98974825

EXOC3-AS1 9.64169280833333 8.888205175 9.20264188333333 9.19118669166667 8.55097769166667 9.52206101666667 8.86401731666667 8.389216575 8.92128914166667 9.36648925 8.91416151666667 5.32912600833333 8.40831291666667 9.06840325833333 9.536758 8.82622243333333 9.57056143333333 8.35414590833333 9.047025875 8.77601695 8.67320644166667 8.73763425 9.41471049166667 8.9886678 9.04472984166667 9.51227434166667 9.2860314 8.8037272 8.941771525 9.16746680833333 9.46190524166667 9.04373345 8.44281486666667 9.10908699166667 9.52458273333333 9.72122880833333 8.95205770833333 8.760231975 8.95834738333333 8.99711739166667 9.20949 8.98227436666667 8.88949339166667 9.137660375 8.97615546666667 9.68325286666667 9.17652090833333 9.20949 9.02591655 9.08987286666667 9.08472375833333 9.014555675 8.62322813333333 9.0697895 9.19737939166667 9.30482685833333 8.53593740833333 9.50133671666667 9.04108516666667 9.124545075 9.558228875 8.97484515833333 10.4604604416667 9.43270350833333 9.118265275 9.52589869166667 9.48539969166667 8.64530154166667 9.8223954 8.91416151666667 8.99004123333333 7.92482581666667 8.66457645 8.981140225 10.5017410333333 9.423639975 8.596030525 9.412078125 9.97701388333333 9.81785751666667 9.37623116666667 8.94592274166667 9.03092490833333 10.7483297666667 9.40138860833333 8.6830437 8.3275657 8.675578375 8.857267525 9.82384475833333 9.791053375 8.780874675 9.16363804166667 8.99822250833333 10.0709631916667 9.24510093333333 9.24804320833333 9.97701388333333 9.83620561666667 10.6477305833333 9.19599840833333 9.17904495833333 9.62572484166667 9.820599225 9.24658409166667 10.1983029333333 9.68178754166667 9.28160753333333 9.88498314166667 8.69945335 10.401625675 10.0526391083333 10.31080925 9.61446786666667 9.93966605 9.62279060833333 9.5438982 9.32861675 10.0677716916667 8.91905655

LINC02741 9.327220125 9.73388581666667 9.936416725 9.68577309166667 10.5633610083333 9.2829911 10.5202480833333 9.7664743 9.64169280833333 9.597018275 9.50277188333333 9.49278793333333 10.1410949416667 9.3690743 9.72706908333333 9.59407011666667 9.29393176666667 9.88790173333333 8.950621275 9.52903631666667 9.3411091 9.5521857 9.09508848333333 9.37200478333333 9.35812314166667 8.577282825 9.71125059166667 9.75280021666667 9.95481326666667 9.69865891666667 9.04472984166667 9.705649125 9.5041563 9.865874625 9.071309125 9.38498160833333 10.3878914333333 9.73689675 9.27895915833333 9.50689216666667 9.56068761666667 9.42111243333333 9.61303635833333 8.94723143333333 9.09508848333333 9.75922218333333 8.68427383333333 8.94836124166667 8.98227436666667 9.29124689166667 9.27651353333333 9.03716685 9.10654574166667 9.45660805 9.32070566666667 9.33724613333333 9.045864375 9.7069395 9.072341675 9.338606375 9.691701075 9.213538675 8.901625425 9.569175825 9.69288753333333 9.36501691666667 8.87730315833333 9.57749218333333 9.11292266666667 9.09241896666667 9.16242148333333 9.59565308333333 9.49278793333333 8.93824426666667 9.29261925833333 9.5438982 9.36648925 9.43270350833333 9.338606375 9.27528178333333 9.57185823333333 8.79393961666667 9.853830825 9.42111243333333 8.926479 9.56332510833333 8.96984509166667 9.2066946 10.0220394666667 8.438679025 9.46065865 9.483981775 9.11422543333333 9.00075389166667 9.180393975 9.991570475 9.10021326666667 10.3513467583333 9.91018748333333 8.992253025 8.73487054166667 9.06439938333333 8.75089571666667 9.17152996666667 9.4302854 8.511369775 9.40775390833333 9.42234536666667 9.60008808333333 8.6830437 8.99822250833333 9.56623849166667 9.277757525 9.24370573333333 9.45660805 9.01740153333333 9.20003395833333 9.46190524166667 9.49278793333333 8.69945335

AL390198.1 11.5912221 10.0036769666667 10.0647337083333 9.791053375 9.37752135833333 9.94262806666667 8.53829400833333 10.3720129583333 9.120779125 9.58157864166667 9.54242928333333 10.4848492166667 9.616056625 10.4298254083333 10.9859995166667 10.0818728583333 10.7688333416667 9.08472375833333 10.3845893166667 10.2387529583333 10.01598095 11.1211007166667 10.011252925 10.080321975 10.1344061583333 11.2202459666667 10.2276958666667 10.401625675 9.58572294166667 10.2536886083333 10.2459336666667 9.9755856 9.588534775 9.643199125 10.6691077583333 10.8910141166667 10.3267587666667 10.332087525 10.1930602583333 10.0896386166667 10.098956 10.3390799083333 10.16579105 10.0911134416667 10.278425125 10.2093892 10.7483297666667 10.3498888833333 10.335375425 10.5880020833333 10.4979674666667 10.0425633416667 10.3702408666667 10.5410668333333 10.3123807083333 10.7872050916667 10.132787925 9.919589575 10.4511583083333 10.1138557666667 10.30417975 9.33724613333333 10.1811538916667 10.471680725 10.4681694166667 9.93026274166667 9.45099463333333 9.33115149166667 8.83768020833333 9.101427925 8.06412709166667 9.158641175 8.900308075 9.997228975 8.83768020833333 9.73114759166667 9.87742366666667 9.92732885833333 8.63451644166667 8.50568134166667 11.7230190333333 10.3684429583333 8.835077425 9.072341675 10.5805910083333 8.694797775 10.7263759583333 9.705649125 9.44156545833333 10.2045943833333 10.287159 11.5110476583333 9.74707850833333 9.74120920833333 10.5860666166667 8.301792275 9.981884775 8.49724105833333 9.91775825 9.41471049166667 10.8230366916667 7.91185755833333 9.84081016666667 10.9101974666667 8.694797775 9.9056525 9.87584709166667 8.51484585833333 10.12322705 9.9929911 9.89542063333333 8.14437105 11.0262795583333 10.7382525333333 8.77000449166667 9.99887365833333 10.5706949916667 9.51227434166667 9.60613141666667 10.2970698166667

PKN2-AS1 5.64838001666667 7.14799545 6.920697175 6.79191158333333 6.77153066666667 8.14639896666667 6.90356878333333 6.76567473333333 7.404027575 7.14716405 6.44343473333333 6.920697175 7.21511196666667 6.47872195 6.9982378 6.79655045 8.19185328333333 6.824129025 6.94396484166667 6.78272669166667 6.36790351666667 6.66605864166667 7.33905511666667 7.26970906666667 6.770698125 6.56023105833333 6.88087174166667 5.99859565833333 7.31101670833333 6.397067625 6.72205318333333 6.8314097 6.60596866666667 7.37881741666667 6.05752005 6.13124135 6.39082849166667 6.95147530833333 6.83745388333333 6.608946875 7.37881741666667 7.00071761666667 6.26957381666667 6.80665846666667 5.850901925 7.23578164166667 6.56890816666667 7.03066510833333 6.57608009166667 6.88476720833333 7.012815075 7.09835705 6.33013755 7.188718125 6.87602855 6.587379025 5.98312854166667 7.51141074166667 6.26556310833333 6.43447593333333 7.22745135833333 7.15998195 7.3846807 7.200884475 6.752753925 6.7749131 7.063179525 6.81212784166667 6.74344500833333 7.25354996666667 6.46744718333333 6.78668719166667 7.491486 7.01432623333333 6.823352775 7.928697775 8.71933655 7.505719825 7.34965898333333 6.46260194166667 7.065634 5.44621728333333 6.99413795833333 7.71998305 7.037414875 6.02811239166667 6.26313358333333 6.49099546666667 6.60281753333333 7.25701023333333 6.85293710833333 6.69403440833333 6.60596866666667 6.331672825 6.62200111666667 6.46331624166667 6.897050375 6.45953320833333 6.95691923333333 6.838229925 7.20762451666667 5.74674768333333 7.0043443 6.71557606666667 6.92880595 6.91345085833333 6.06024568333333 6.39481431666667 6.993393575 6.47650794166667 7.28603906666667 6.94396484166667 7.03837779166667 7.8519496 6.92711743333333 6.651497425 6.13868721666667 7.04581783333333 7.318718375 6.83283191666667

AC137810.1 12.6912332583333 11.4295243333333 13.1561940833333 14.003709575 8.08263128333333 15.8640624583333 11.1477633666667 11.91499415 13.89773695 12.8524193083333 15.2764462833333 14.583522125 11.1791450916667 11.4272823833333 10.0252423 13.143978525 14.6044826666667 9.73689675 10.132787925 10.9379330333333 11.8155407666667 12.4566311333333 12.6493952416667 15.1457216333333 15.0566496333333 14.7055885666667 12.2046199416667 12.52982025 10.955147375 11.900898825 11.6159368333333 11.5739352416667 15.9521644666667 14.1061019833333 13.7009408083333 13.8620215333333 10.5898562833333 13.472886275 13.9883783916667 11.93863675 13.972715675 11.6556065666667 13.3666129833333 14.0104955916667 14.633352475 12.1666511583333 11.6740143583333 11.6510632333333 11.54529205 15.663084375 14.4622164333333 13.2995032583333 11.8389506833333 10.8543733166667 11.8881455083333 13.079484475 11.6063160416667 12.315517225 12.6239992833333 13.35783415 12.3010353583333 13.4248566333333 11.6943956666667 12.552751 13.0874111666667 11.6694244 13.68323365 14.0181244166667 12.7206590083333 13.7856886083333 12.7243176833333 13.1392820916667 12.578037425 13.9495562333333 12.5234224083333 14.4412673083333 12.79731905 12.215507725 12.1362874333333 12.6943613083333 12.1711872833333 14.1766127916667 13.448561725 14.9900502166667 14.7968734083333 13.599357275 10.9901360833333 14.1398844416667 12.3062951833333 14.36243255 13.9130054833333 11.9858949166667 12.4629274416667 14.5627782916667 13.0727760333333 12.6525380333333 12.3920642833333 12.5457182583333 14.1061019833333 12.9606437 13.7723702416667 13.0457394833333 12.5689938083333 10.955147375 11.9252347666667 13.041627725 13.2203812666667 11.9739944 13.527636 14.6839186583333 12.43015245 11.113406175 12.3010353583333 13.2203812666667 14.0904534166667 12.0072618083333 12.8659456416667 13.069349375 10.36490795 13.7378356166667

MIR3976HG 4.86787458333333 4.08791583333333 4.29927855833333 4.22877676666667 3.78450821666667 4.61117119166667 4.31461836666667 4.336941625 3.93820065 4.10439639166667 4.25818273333333 4.784017825 3.77964155833333 4.46178631666667 4.204983325 4.02390940833333 4.853953375 3.81422973333333 4.8148076 3.94531450833333 3.92700760833333 4.75272380833333 5.14837419166667 5.80051365 4.89323034166667 4.08488764166667 4.54945460833333 4.68278718333333 4.73036435 4.42684748333333 4.98886558333333 5.57955060833333 3.84310781666667 4.26717905833333 4.53985004166667 4.90444880833333 4.70894576666667 5.64279376666667 5.362066125 4.95383771666667 5.646510225 4.71818525833333 5.161910175 5.91168246666667 4.36308695833333 5.38353026666667 5.493516675 5.21185403333333 4.816492275 5.47366834166667 5.4400876625 5.49191885833333 3.79392545 5.99859565833333 4.72810314166667 5.043189975 3.87234255833333 5.11849680833333 4.57012604166667 4.61689800833333 3.98267365 4.84158960833333 4.91868544166667 5.74066663333333 3.88129784166667 4.01031786666667 4.05400306666667 4.53764565 4.39514796666667 4.072707875 3.99459898333333 4.53307618333333 3.97392573333333 3.832104375 4.5308601 3.99069761666667 3.96617559166667 3.838205275 3.86669901666667 3.8566224 3.89263233333333 3.99142755 5.17494715833333 4.77715155833333 3.88965465833333 4.09183225 4.25984291666667 4.389988275 3.92150503333333 4.06889863333333 4.32535105833333 4.45823205833333 4.20416551666667 3.9517269 3.96958159166667 4.00078191666667 4.247434625 4.755783125 4.218766 5.74316985833333 4.4967147 4.0531425 5.013582075 5.35953131666667 4.57012604166667 3.9889481 4.79866623333333 6.56401094166667 4.40501044166667 6.94156313333333 4.858673825 5.61242543333333 4.758019275 5.81184100833333 4.0594979 4.564244925 4.22349475833333 4.73514765833333 4.7733425 3.96318805

AC008591.1 6.90573395833333 3.810798375 4.03934740833333 3.898913075 3.99860775833333 4.00317675833333 4.6542059 4.27153690833333 3.75646214166667 4.02639559166667 4.90848588333333 4.384745925 5.464202575 3.94531450833333 3.84463835833333 3.740256725 4.8196 4.09521015 4.45389868333333 4.164731775 4.36552305 4.29792231666667 4.08488764166667 4.675507125 4.44879201666667 4.75121364166667 4.21022225 4.076467325 4.10612433333333 4.036233975 4.3268489 4.264237 3.56110099166667 3.81422973333333 3.9834399 6.04672630833333 4.34636268333333 5.11933154166667 4.27303441666667 6.4711885 4.65659079166667 4.12429728333333 4.54800100833333 5.18232021666667 4.478284575 4.66042911666667 6.02663899166667 4.43434340833333 4.338662175 4.61186549166667 4.64492955 4.37878591666667 4.33943185 5.9442928 4.09588705833333 4.403336375 4.46466106666667 5.34230098333333 4.087125375 4.035574075 3.87234255833333 4.44947654166667 4.96673286666667 5.11849680833333 4.14619431666667 3.97718864166667 3.68467165833333 4.92935575833333 4.4967147 4.47754666666667 4.51062659166667 4.37210666666667 5.44953800833333 3.95243559166667 4.27894339166667 3.82707174166667 3.65665524166667 4.02877286666667 4.23434725833333 4.03093171666667 4.37140105 6.445841425 3.97641614166667 5.02726646666667 3.90323076666667 4.347785025 4.44679063333333 5.04820341666667 3.87165855 4.38619328333333 4.3268489 4.16702060833333 3.84629465 3.66610879166667 4.02390940833333 3.69246125833333 4.07056364166667 4.41144705 4.32611543333333 6.50428851666667 4.34636268333333 4.40096828333333 4.407147125 4.58674640833333 4.928619175 4.13615014166667 5.33879713333333 5.07245089166667 4.55798421666667 5.663186525 4.54052569166667 5.5903436 4.190498425 5.392747 5.09577138333333 4.15484655833333 4.398049275 4.23975296666667 4.15088759166667 4.46626585833333

PCA3 7.5341355 4.12508055 5.22390943333333 4.23121726666667 4.91868544166667 4.72237503333333 4.40096828333333 5.47682133333333 4.47754666666667 4.5167211 3.9705387 6.54302155 6.430564175 4.347124625 5.28331578333333 4.370597625 4.89079064166667 6.968472025 5.70066640833333 4.70831345 5.59815450833333 5.8328231 4.78566294166667 6.0323348 4.91520124166667 7.68875390833333 6.42753165 5.32408766666667 4.8116637 4.67170091666667 5.59994884166667 4.663962275 4.66231220833333 5.10595089166667 7.92754394166667 7.5606838 4.765242025 6.18946745833333 5.31273550833333 4.93534804166667 5.43787004166667 4.72477478333333 5.18501018333333 5.91328005 4.74184695 5.36997850833333 5.494444575 5.180684475 4.854747975 5.13063719166667 5.37522741666667 5.56466664166667 4.038581625 5.55464808333333 4.76218691666667 5.0768141 4.13464519166667 5.1157001 5.825172325 5.149358575 3.73698600833333 4.517575125 4.91353486666667 5.10925536666667 3.66852646666667 3.870040525 3.89729075833333 4.62007759166667 3.762448075 3.85973294166667 4.06020879166667 3.92012839166667 4.6432372 3.9889481 3.63971921666667 4.49520464166667 5.16731153333333 5.102471875 3.870040525 4.031708625 4.38845033333333 4.51592391666667 4.22056816666667 4.344009325 4.09270279166667 4.46857235833333 3.79720163333333 4.435029675 4.71676371666667 5.38263980833333 4.14479000833333 4.48781 4.38169465833333 4.67637581666667 3.97949414166667 3.94608880833333 4.111010475 4.466985075 4.1525745 5.18744376666667 4.52640311666667 4.196708 5.02165799166667 5.32654480833333 4.66231220833333 3.988166225 4.78885110833333 6.47435308333333 4.436542725 6.88611070833333 4.84158960833333 5.55790016666667 4.651163825 5.10518236666667 4.04345499166667 4.57085965 4.24046655 4.729607625 5.772683 4.73200895

LINC02197 7.493374075 8.22349021666667 8.08263128333333 7.94602126666667 8.59015729166667 7.77821760833333 8.80995673333333 8.31719618333333 8.034891475 8.27106878333333 7.93342214166667 9.06068016666667 9.19737939166667 8.16698743333333 7.75769473333333 7.67103138333333 8.5579005 8.017646775 7.12190308333333 7.60442806666667 8.53829400833333 7.77554924166667 7.71468463333333 7.51240625833333 7.23015586666667 8.15136631666667 7.73200875833333 7.08601268333333 7.093375425 7.84066634166667 7.49682960833333 7.16399434166667 7.86191561666667 7.89810335 7.4747897 7.23578164166667 7.95418985833333 7.84714276666667 7.5199474 6.76702298333333 7.42277753333333 7.64073124166667 7.833369075 7.206737225 7.23347658333333 7.76474425 7.96426395833333 7.397338075 6.89078670833333 7.3553377 7.33186256666667 7.37716343333333 7.47901903333333 7.43784024166667 7.54277829166667 6.857401975 7.96148 7.6026871 7.24089864166667 7.75849498333333 7.62092879166667 8.35637496666667 7.41157274166667 7.65472984166667 8.26714300833333 8.54062638333333 8.11629255 8.78337874166667 7.75584805833333 8.20524448333333 8.09055905833333 8.12889401666667 8.6685911 7.57468748333333 8.13273210833333 7.46714951666667 8.41697565 7.47901903333333 7.54277829166667 7.16321075833333 8.62989988333333 7.845369325 7.916501675 7.86665836666667 8.19285094166667 8.36965201666667 7.971686625 8.05651714166667 7.21336981666667 7.85737128333333 7.934282375 7.32203796666667 7.57271753333333 7.73305023333333 7.21991299166667 7.327316125 7.68954538333333 7.603532025 7.919306 7.6001855 7.68503678333333 7.23088359166667 7.8186674 7.26542881666667 7.033226925 7.69031583333333 7.68597430833333 7.02102248333333 7.348136225 7.318718375 7.28355914166667 7.33817588333333 7.002237925 7.88934271666667 7.48069958333333 7.09178105 7.66087161666667 7.92035708333333 7.44983849166667 8.58685536666667

AC117462.1 5.76427079166667 6.30112746666667 6.46260194166667 5.53239810833333 5.932662575 6.55297315833333 6.84446040833333 6.71477854166667 6.397067625 6.4048599 6.28384561666667 6.10788125833333 5.915004725 6.39399721666667 6.74876795 6.324552 7.029863175 6.03059249166667 6.713268275 6.162124375 5.597380175 6.43516865833333 7.17123928333333 6.44112955833333 6.67222361666667 6.0189352 5.90014908333333 6.87036725833333 6.28918155833333 6.47575550833333 6.35692343333333 6.9521716 6.9176246 6.58655845833333 5.941144225 6.24589635 6.76771060833333 7.19023773333333 6.37119856666667 6.96989185833333 6.99264965 6.83441448333333 6.27931271666667 6.8314097 5.80127338333333 6.2303963 6.42583693333333 6.27123641666667 6.18461388333333 6.538257625 6.50428851666667 6.64775085 6.64847380833333 7.138237575 7.48222535 6.83441448333333 6.30504875 6.967754075 6.86096935833333 6.6615047 6.31500904166667 6.4794447 6.84740460833333 5.94277945833333 6.53414359166667 6.895291875 6.529771875 6.83075820833333 6.635993725 6.84134261666667 6.67400460833333 7.30708359166667 4.98886558333333 6.3807528 6.51742765 6.40115713333333 6.59742333333333 6.25519815 6.37119856666667 7.33109959166667 5.529852675 6.3612461 6.65069284166667 7.23263500833333 6.35865758333333 6.44429526666667 6.74344500833333 6.587379025 6.41530895 5.87732999166667 6.43366564166667 6.81368843333333 6.87117053333333 6.20360416666667 6.6081855 7.35042211666667 6.86553285 7.32899926666667 6.43447593333333 7.376269825 7.02259469166667 6.77333313333333 6.58062053333333 7.76312330833333 5.28955413333333 6.61204298333333 5.364779525 6.98625575 5.93775123333333 7.24674725 6.64686755833333 6.44981341666667 6.27857845833333 6.96384525 7.005136025 6.77333313333333 7.36967581666667 6.40029824166667 7.22572939166667 6.21898574166667

LINC02778 4.57239494166667 7.29318128333333 7.24011880833333 6.47356659166667 7.70935458333333 5.695409275 7.919306 7.74841675 7.34500641666667 6.77333313333333 6.50659699166667 7.91466469166667 7.63212294166667 7.292382975 7.04281696666667 6.83895268333333 5.80207481666667 6.98302305 5.38864603333333 7.256167725 7.72932055833333 6.70536285833333 6.3612461 6.082719625 4.25516519166667 6.29000819166667 5.99620995 3.68216738333333 4.95061065 6.1367648 4.262661725 4.77485674166667 5.81446665833333 6.662197225 4.185156975 4.154054225 4.55489758333333 5.32912600833333 5.13698225 4.45750181666667 4.88416340833333 3.98423539166667 4.517575125 5.04401789166667 5.54129996666667 6.505088325 4.79605449166667 5.99448725833333 4.38845033333333 5.12699374166667 5.31195595 5.27316720833333 7.317206725 6.16676128333333 5.885364375 4.39434791666667 7.742129825 4.52640311666667 4.73757844166667 4.292405 4.63500360833333 7.17445575 5.004526525 6.23352403333333 6.88849675 5.89761375 6.25595283333333 7.77912510833333 4.75968018333333 7.327316125 7.96330909166667 5.85775243333333 7.72932055833333 6.02983153333333 6.64686755833333 7.17525139166667 6.01647188333333 5.32501348333333 6.38321685833333 5.86256211666667 8.17812686666667 6.41853091666667 5.50030605 6.102165675 6.07424019166667 7.06728804166667 6.16514798333333 7.46553646666667 6.55465186666667 5.72460895833333 5.8569379 4.04261585 4.319028675 5.64920624166667 4.4982908 5.64026789166667 6.16130541666667 4.905221075 6.80665846666667 4.59590423333333 5.27721040833333 6.272113525 4.389247575 4.52785514166667 5.62694763333333 7.12190308333333 6.3807528 5.67108565833333 6.3380145 5.86338895833333 4.43434340833333 4.99643059166667 4.00950880833333 5.28072425833333 6.65293891666667 4.85306506666667 5.2527348 4.54800100833333 4.207301975 8.03218916666667

FLG-AS1 5.10342061666667 6.76567473333333 6.64607705 6.69869794166667 6.883226375 6.061018125 6.37517364166667 7.31362185 6.32528485 6.25988588333333 5.50303829166667 5.59208724166667 5.7042258 7.805183975 6.850638675 7.29559511666667 6.102165675 6.1367648 7.903661075 6.63347905833333 7.39815565833333 7.08121445833333 7.25530323333333 6.65457601666667 7.107436475 6.05912289166667 6.29392624166667 7.445697975 6.80360636666667 6.97908095833333 7.27613468333333 6.77153066666667 5.92097311666667 4.52640311666667 6.44981341666667 6.15217918333333 6.823352775 7.08121445833333 6.39250838333333 6.77811300833333 7.344174925 7.39406969166667 7.02582578333333 8.00421275833333 6.512400125 7.0397833 8.4103435 7.68954538333333 8.290978475 6.44506816666667 7.70654949166667 6.73897381666667 5.62614020833333 7.45333459166667 7.16947939166667 7.66524353333333 6.68930803333333 7.39567078333333 6.56179321666667 7.02434959166667 6.581362225 6.23943730833333 5.54229348333333 5.23750470833333 5.42593315833333 5.020919775 5.41980085833333 5.4560179 4.82196883333333 5.18501018333333 6.1871742 5.826857975 5.93351605 5.39028695833333 5.25844070833333 6.21087870833333 4.42606766666667 4.57085965 5.71323735 5.170905675 5.2527348 6.17075145833333 5.59908800833333 5.46824720833333 5.65895484166667 5.23171054166667 4.99144798333333 5.02726646666667 5.63014134166667 5.32578840833333 5.10074249166667 5.448697 4.97972691666667 5.05599298333333 4.74274593333333 5.915004725 4.3802091 4.993870075 5.63754605 4.9625611 5.09577138333333 4.81093719166667 4.45316954166667 5.582540175 5.46824720833333 5.157017525 5.13143250833333 6.42670259166667 4.86450405 6.44192199166667 4.47471310833333 6.23442566666667 5.11752969166667 5.79314095833333 5.38175511666667 5.13457766666667 3.68978298333333 4.58895470833333 6.26704225833333 5.67701044166667

CASC2 5.867409875 5.16909541666667 4.31751935 4.185156975 4.934512075 6.539864925 3.91034174166667 4.60466838333333 4.25818273333333 4.831688925 4.95685870833333 3.967027875 3.75301153333333 4.27303441666667 5.99859565833333 4.94091306666667 6.20006668333333 4.771183175 5.74316985833333 4.879086275 4.72071955833333 5.76940198333333 5.84085751666667 5.39466070833333 6.343453175 5.82778305833333 5.11005560833333 6.35692343333333 4.34016336666667 5.26111468333333 5.8658332 5.06515519166667 4.3268489 5.86830491666667 5.76240348333333 5.95408916666667 6.19578775 5.837626175 5.24934811666667 5.34143435833333 5.35594408333333 6.32221861666667 4.71282696666667 6.45570826666667 5.26290054166667 6.104675225 5.02981615 4.771183175 5.512808975 6.22881789166667 4.771183175 5.25192735833333 4.45823205833333 6.75523526666667 4.241859075 6.11513095833333 4.72314724166667 5.46922286666667 6.033075425 5.39028695833333 4.17774023333333 3.62188045833333 6.47511975833333 4.12213588333333 5.22390943333333 6.12427160833333 4.36903601666667 4.414398275 5.679628825 4.476177125 4.247434625 4.60316775833333 3.9722338 3.70428345833333 3.81801135833333 4.38097535 6.2141109 5.03387344166667 4.32459856666667 3.9327276 4.83095916666667 5.778779775 5.35323611666667 5.29742040833333 3.636165975 5.53605506666667 6.44192199166667 4.24594024166667 3.87547831666667 4.663962275 4.15006823333333 5.74853151666667 5.13698225 4.67778629166667 4.44679063333333 3.67690364166667 6.0898961 6.86400539166667 4.79377419166667 5.926954025 6.86553285 4.32535105833333 6.22164848333333 6.64686755833333 5.486081725 3.88052193333333 4.49140346666667 5.73572338333333 5.89445190833333 6.60203268333333 4.550275675 6.01487855833333 5.65335349166667 7.24756645 4.84470445 5.86256211666667 6.95147530833333 6.20613598333333 5.76502415833333 4.0664225

LINC01115 8.44281486666667 8.28057508333333 8.66457645 8.468834925 8.33192433333333 8.32865643333333 8.21837833333333 8.57288055 8.46469871666667 8.37265579166667 8.58803331666667 8.09224021666667 8.03118996666667 8.120198675 8.18511508333333 7.501137825 8.05364296666667 8.38818113333333 7.90761645833333 7.95964986666667 8.15047776666667 8.04165159166667 8.28484028333333 8.20524448333333 8.0765578 8.20193756666667 8.13385164166667 8.09646454166667 7.94287994166667 8.00331975833333 8.3215665 8.22438401666667 8.06225145833333 8.54846981666667 8.53357196666667 8.34393171666667 8.525776075 8.43525693333333 8.41382609166667 8.389216575 8.19085505 7.99201798333333 8.2588068 8.69018435 7.8846688 7.78810495 8.27939851666667 7.9937887 7.95063335833333 8.31841858333333 8.35939504166667 8.197822625 8.58685536666667 8.43525693333333 8.33626970833333 8.23155004166667 8.18511508333333 8.11324664166667 8.070038325 8.18309575 9.214906375 8.27232419166667 8.4458465 8.527957175 9.00741491666667 8.72277565833333 8.70557491666667 8.43778974166667 8.78437034166667 8.34967031666667 8.56657345 8.31623061666667 8.14943054166667 7.9966524 8.503369575 7.882717525 8.17623105833333 7.90677205 8.20524448333333 8.51932215 8.13385164166667 8.371635775 8.33526710833333 8.32865643333333 8.36432383333333 8.15639794166667 8.54483809166667 8.37884899166667 8.13699931666667 7.952403425 9.323428375 8.53136013333333 8.43640146666667 8.70315873333333 8.81697240833333 8.599352775 8.283603725 8.52045571666667 8.33743355 8.438679025 8.504529375 8.22233045 8.45597868333333 8.36133213333333 8.03402151666667 7.45898616666667 7.91032310833333 7.78532935 7.98146166666667 7.5065832 8.236562525 8.43778974166667 8.56088768333333 8.522428575 8.33526710833333 8.57958530833333 8.503369575 8.69018435 8.27017713333333 7.863951475

AC006207.1 8.8194358 8.577282825 8.75436599166667 8.87851369166667 8.61212589166667 9.28980095 8.85242150833333 8.43217050833333 9.01843699166667 8.82622243333333 8.6830437 9.06329124166667 8.584372375 8.36432383333333 8.22733033333333 8.32566448333333 9.19875904166667 8.74401624166667 8.217333875 8.29753093333333 8.650096975 8.589042325 8.45130473333333 9.25886408333333 8.50671724166667 8.36133213333333 8.24724386666667 8.480972975 8.602930325 8.070038325 8.367490075 8.67920558333333 8.8438399 9.09508848333333 8.81089155833333 8.83136731666667 8.70557491666667 8.893151 8.83253646666667 8.56203936666667 8.45706139166667 8.47657969166667 8.13089671666667 8.473255125 8.6890691 8.74269300833333 8.576098 8.584372375 8.55097769166667 8.698399275 8.63572139166667 8.93700485 8.63814489166667 8.6890691 8.56866955 9.00075389166667 8.51593621666667 8.75543470833333 8.39699265 8.66457645 8.79763931666667 8.81206715 9.02843820833333 8.857267525 8.74523575833333 8.78209530833333 8.257703625 8.81562710833333 9.054351375 9.02317930833333 8.77244310833333 8.845210775 8.854849925 8.60414634166667 8.94464996666667 8.29753093333333 8.72889535 8.91190651666667 8.48644526666667 8.290978475 8.26088785833333 8.483114775 8.470046425 8.72541470833333 8.87730315833333 8.62078918333333 8.83253646666667 8.87974589166667 8.59015729166667 8.36542865 8.65114993333333 8.602930325 8.85242150833333 8.67196278333333 8.82622243333333 8.76267615 9.00216525 9.1468994 9.02591655 8.90815406666667 8.5427737 8.80258250833333 8.61109633333333 8.27939851666667 8.522428575 8.58305941666667 8.602930325 8.48415245 9.16483426666667 8.49932775 8.584372375 8.9288621 8.82891991666667 8.78337874166667 8.95327330833333 8.87730315833333 8.303785925 8.73252250833333 8.52145965 8.75905724166667

LINC00378 4.21647415833333 4.49076011666667 3.962497425 3.81498958333333 4.22202384166667 4.651965175 3.72622218333333 4.03017670833333 4.00317675833333 5.55949836666667 3.7651031 3.47050078333333 3.49994134166667 4.56640000833333 3.953133875 3.79634603333333 5.02478369166667 4.54354728333333 4.78488421666667 4.06485985 3.543895025 3.664550775 4.80498945 4.4967147 4.94816246666667 4.12351481666667 5.04921501666667 5.33192491666667 5.1467782 3.931009175 5.165035025 4.153363325 4.0778828 5.27222866666667 4.82759028333333 5.11849680833333 4.910233575 5.57868124166667 4.36308695833333 5.983920925 4.65727891666667 4.33777051666667 4.62007759166667 5.239299675 4.05470664166667 4.675507125 4.68347908333333 4.45532334166667 4.23746243333333 4.556397325 6.17790808333333 5.10427118333333 4.01830505833333 5.42859845833333 4.011089225 5.12618295 3.86235944166667 4.94091306666667 5.25355041666667 5.55202255833333 4.07190203333333 4.218766 5.06686440833333 4.29310653333333 3.92950448333333 4.25048369166667 3.71522014166667 3.83300729166667 3.581753225 4.49004835 3.72384201666667 4.00148819166667 3.46545674166667 3.97139808333333 3.61963650833333 3.81249149166667 3.71259308333333 4.00474495 4.31232735833333 4.04587083333333 3.37890360833333 3.414409325 4.44518198333333 5.19649501666667 3.97303310833333 3.81006580833333 4.1516504 3.86052620833333 4.087125375 4.12988213333333 4.3062682 4.05010078333333 4.24443253333333 4.21399664166667 4.35959019166667 4.31153791666667 4.283368325 4.66042911666667 4.262661725 5.54852681666667 4.072707875 3.65493706666667 4.41295244166667 4.72810314166667 4.204983325 3.73867649166667 4.29852961666667 5.22032525 3.97569303333333 5.645614725 5.66155191666667 4.73938754166667 4.16618690833333 5.64469614166667 4.17164835833333 4.61847421666667 4.40189316666667 5.17330196666667 4.79605449166667 4.06172258333333

AL161629.1 6.17554210833333 6.121685 6.50987625833333 6.29613120833333 6.08008695833333 5.57003804166667 6.324552 6.35080858333333 6.883226375 5.6868575 6.42753165 6.13600023333333 6.25070120833333 5.70335329166667 6.30112746666667 6.32373769166667 5.701530925 6.13535341666667 6.09172501666667 6.703671025 5.21672184166667 5.4370393 6.48469505 6.84134261666667 5.969534625 5.95235704166667 5.706023225 6.396357 5.51827296666667 6.30351415833333 6.39399721666667 6.75784235833333 6.31908594166667 5.8493167 6.80752626666667 6.9176246 5.867409875 6.68930803333333 6.62653135833333 6.36954999166667 5.597380175 5.448697 6.13763498333333 6.09080005833333 5.91168246666667 6.17790808333333 6.49099546666667 6.54596676666667 5.362066125 6.15706108333333 6.22349896666667 6.453551925 6.56472320833333 6.34919350833333 6.41530895 6.70438826666667 6.36384354166667 6.9918604 6.26313358333333 6.30843955833333 5.64026789166667 6.56728411666667 6.47279708333333 6.29539721666667 5.85430964166667 5.60990619166667 6.30112746666667 6.50361413333333 6.37602205 6.20696713333333 6.26791573333333 6.54071093333333 6.43516865833333 5.71592235833333 6.04838810833333 6.17075145833333 5.63936416666667 5.826857975 6.2575518 6.104675225 5.3549373 4.94570356666667 5.99926955 6.70700871666667 6.010079625 5.99779750833333 5.614181625 5.885364375 6.51330503333333 5.8328231 6.58655845833333 6.29539721666667 6.194972475 6.36633304166667 6.179749975 6.390112675 6.010079625 6.38929764166667 6.182148775 6.477957 6.581362225 6.28684615833333 6.07508890833333 5.75521793333333 6.58365504166667 5.88060724166667 6.1871742 6.31992400833333 5.24598960833333 6.6227255 5.6235969 6.04051248333333 6.42987050833333 6.88682719166667 6.50900563333333 6.585221025 6.01338551666667 6.87602855 6.64989985833333 6.352456125

AC007182.1 4.81242730833333 4.211017675 4.99219315833333 5.6868575 5.969534625 6.09974989166667 4.96503665833333 7.72082631666667 5.457689125 7.214196475 6.55935685833333 5.12983071666667 4.89918824166667 5.48522716666667 7.00361548333333 5.114099175 5.77625636666667 5.27404638333333 7.19349589166667 7.427790275 6.86715935833333 6.11361575 8.3623863 6.32221861666667 5.736606025 4.70485328333333 6.64072528333333 7.31286559166667 6.52835620833333 6.01647188333333 7.0482011 5.97375568333333 5.16731153333333 5.33093755 6.04442248333333 5.534144725 5.9607591 7.841555975 6.08667845 7.02737631666667 7.32644539166667 5.48272485833333 6.22740323333333 7.525750625 6.17880248333333 8.391481525 5.41122809166667 7.85285366666667 6.29775806666667 6.38535240833333 7.8519496 6.59515689166667 5.0923089 7.1982511 6.86786498333333 6.57456723333333 6.67136516666667 8.01279515833333 6.62118421666667 7.07043868333333 4.3566395 5.65408345833333 5.26709018333333 7.20338010833333 4.73938754166667 7.40534635 6.72671865 8.35939504166667 5.75111171666667 5.20060335833333 4.80498945 5.71234138333333 5.893600675 7.18077985833333 4.99144798333333 6.31344151666667 4.853953375 4.955238375 8.83385925833333 8.0785585 3.92700760833333 4.370597625 9.58572294166667 5.64469614166667 9.06192556666667 4.5034938 3.90409960833333 4.86787458333333 4.31533605833333 5.28955413333333 5.61847035 5.08336286666667 5.39466070833333 7.96236178333333 6.488745325 6.14913983333333 8.54724403333333 5.392747 5.858605475 5.67018873333333 4.53154068333333 4.58536020833333 4.75878485833333 6.723589425 10.888969925 5.02478369166667 6.530441325 9.288628775 6.055815425 6.86715935833333 5.46519105 7.02184869166667 8.68656096666667 7.75201335 6.64536209166667 8.11425899166667 4.57012604166667 5.771891275 5.57868124166667 5.18905345

LINC00922 6.36256870416667 5.4418135375 6.0838852875 6.00795365833333 6.54179475833333 6.27167497083333 6.30548050416667 5.82560274583333 6.58402175416667 6.027041925 5.61635895833333 5.5277243625 5.3174549 5.84884964166667 6.3063192875 5.91455771666667 5.96476334583333 6.52644000833333 7.45027837916667 6.29124821666667 5.31582855416667 5.77666020833333 6.49755714166667 7.01391032083333 6.61012195416667 5.90528075416667 4.92226553333333 5.77919802916667 5.67488022083333 6.7515430875 6.63717442916667 6.67444676666667 6.10093624583333 6.340111775 6.28266741666667 6.81327662916667 6.67016093333333 6.4061165 6.93868453333333 6.92520418333333 6.597031475 6.37806140833333 7.31142601666667 6.61165618333333 6.574184375 6.96027491666667 7.1691663125 7.37755263333333 6.519367725 6.4363180375 6.84633645 6.533790675 5.75711974166667 7.36842989166667 5.58643689583333 6.48124449166667 6.01289963333333 6.03700682083333 6.78385636666667 6.41818334583333 6.84859395833333 6.25717058333333 6.92520418333333 6.7729072875 6.46929630833333 6.46369887916667 6.59628130833333 6.51285257916667 6.23489189166667 5.92490076666667 5.82827587916667 6.2642711125 5.8032978625 6.93405621666667 6.26837457083333 6.26747899583333 5.8670189 6.59553994583333 6.51104014583333 6.6130280375 5.85972439583333 5.65526510833333 7.17076259166667 7.62629884166667 7.19786739166667 6.79848675416667 6.620806825 5.754848975 5.947081725 7.71850165416667 6.86060679166667 7.09543904583333 6.67756760833333 7.48677878333333 6.215363825 6.58248376666667 6.087059475 6.29987528333333 6.33530185416667 7.1404072875 7.75444848333333 6.47080707083333 7.40840490833333 7.2446705625 6.53786397083333 7.72466435 7.6763303375 7.15564519583333 7.14603639583333 7.2195610625 6.68211167916667 6.73295947916667 7.49556497083333 6.6339622625 6.52802937083333 6.828754125 6.46703689166667 7.01697919583333 7.33464801666667 6.2990553

AC009055.1 6.36256870416667 5.4418135375 6.0838852875 6.00795365833333 6.54179475833333 6.27167497083333 6.30548050416667 5.82560274583333 6.58402175416667 6.027041925 5.61635895833333 5.5277243625 5.3174549 5.84884964166667 6.3063192875 5.91455771666667 5.96476334583333 6.52644000833333 7.45027837916667 6.29124821666667 5.31582855416667 5.77666020833333 6.49755714166667 7.01391032083333 6.61012195416667 5.90528075416667 4.92226553333333 5.77919802916667 5.67488022083333 6.7515430875 6.63717442916667 6.67444676666667 6.10093624583333 6.340111775 6.28266741666667 6.81327662916667 6.67016093333333 6.4061165 6.93868453333333 6.92520418333333 6.597031475 6.37806140833333 7.31142601666667 6.61165618333333 6.574184375 6.96027491666667 7.1691663125 7.37755263333333 6.519367725 6.4363180375 6.84633645 6.533790675 5.75711974166667 7.36842989166667 5.58643689583333 6.48124449166667 6.01289963333333 6.03700682083333 6.78385636666667 6.41818334583333 6.84859395833333 6.25717058333333 6.92520418333333 6.7729072875 6.46929630833333 6.46369887916667 6.59628130833333 6.51285257916667 6.23489189166667 5.92490076666667 5.82827587916667 6.2642711125 5.8032978625 6.93405621666667 6.26837457083333 6.26747899583333 5.8670189 6.59553994583333 6.51104014583333 6.6130280375 5.85972439583333 5.65526510833333 7.17076259166667 7.62629884166667 7.19786739166667 6.79848675416667 6.620806825 5.754848975 5.947081725 7.71850165416667 6.86060679166667 7.09543904583333 6.67756760833333 7.48677878333333 6.215363825 6.58248376666667 6.087059475 6.29987528333333 6.33530185416667 7.1404072875 7.75444848333333 6.47080707083333 7.40840490833333 7.2446705625 6.53786397083333 7.72466435 7.6763303375 7.15564519583333 7.14603639583333 7.2195610625 6.68211167916667 6.73295947916667 7.49556497083333 6.6339622625 6.52802937083333 6.828754125 6.46703689166667 7.01697919583333 7.33464801666667 6.2990553

AP001496.4 4.83518115833333 4.98118906666667 4.84470445 5.077701625 5.513669625 5.333787 6.84275021666667 6.32934243333333 5.75363753333333 5.782225425 6.27857845833333 6.70618345833333 6.354063975 6.30591225833333 5.49191885833333 6.192534425 6.53414359166667 5.87325478333333 5.92363850833333 5.97375568333333 5.13539786666667 4.347124625 4.70744949166667 6.31177985 4.826730425 6.07927768333333 5.5439086 5.12016660833333 4.57164009166667 5.80051365 5.1157001 5.84743648333333 5.805323425 6.730005225 5.97788763333333 6.076673725 4.6241475 6.37119856666667 6.07927768333333 5.04240375 5.18149681666667 5.69101595 4.84386065833333 6.009318125 5.05353733333333 5.98543355 5.6893285 6.40568764166667 5.41046095 5.247693175 5.29908005 5.93939261666667 5.6623785 6.70852368333333 6.57760283333333 6.35865758333333 7.162304025 6.57049163333333 5.66590344166667 5.57164055 5.09385859166667 5.798958825 5.81606168333333 5.93351605 4.879086275 5.0239763 6.121685 5.85351849166667 6.20782178333333 5.629363625 6.34194690833333 6.64146615833333 6.310036575 5.782225425 6.730005225 6.22418345833333 5.63172995833333 5.55866609166667 6.30271423333333 5.16107029166667 5.29824615 4.7385841 5.27721040833333 6.20696713333333 5.524744 6.02483926666667 5.80704883333333 5.94745686666667 4.77563934166667 5.699860275 6.00842590833333 5.50565405833333 5.74674768333333 5.74853151666667 5.506564725 5.13217864166667 5.17730950833333 5.95408916666667 6.13124135 5.46922286666667 4.97724585 5.87078084166667 4.67637581666667 5.74066663333333 5.699860275 6.03387288333333 5.93939261666667 6.31908594166667 5.798958825 6.42337751666667 5.397270075 6.12427160833333 6.41530895 7.51062641666667 6.10384355833333 6.05245123333333 5.81606168333333 6.272113525 6.46821505833333 6.45206855833333

AC079340.1 6.65857890833333 8.01279515833333 7.78914439166667 8.00143094166667 8.01182280833333 7.00675840833333 8.348415775 8.322516975 8.371635775 7.39651405 7.65656303333333 8.00143094166667 8.18309575 7.82045105 7.34965898333333 7.50024405833333 7.02899554166667 8.0943338 7.23015586666667 8.09646454166667 8.11425899166667 7.48714180833333 7.48965994166667 7.65656303333333 6.81152795833333 6.85433584166667 7.3062498 6.97501571666667 6.93368999166667 7.25265295833333 7.27284798333333 7.34579063333333 7.44728600833333 7.008284125 6.442668925 6.6271591 7.529838675 8.67320644166667 7.908397225 7.41659339166667 8.09958200833333 6.94156313333333 7.012815075 7.018972075 6.753505175 6.686388325 6.8314097 8.11740648333333 7.11106645833333 7.02899554166667 6.857401975 7.376269825 8.33417815 7.51141074166667 7.51240625833333 6.94332055 8.035850625 7.91271055833333 6.71961385 7.19278388333333 7.08525161666667 7.91032310833333 6.96306144166667 7.646624025 7.528191475 7.596908525 7.76474425 7.67682356666667 7.34163923333333 7.23425756666667 7.44656456666667 7.57468748333333 7.977352125 7.1942732 7.39891643333333 7.61407576666667 7.36490918333333 7.16885323333333 7.55492436666667 7.45333459166667 7.60098480833333 7.385658775 7.89268988333333 6.98625575 7.509871575 7.29318128333333 7.69455133333333 7.53942513333333 7.05999225 6.63999905833333 7.03910078333333 6.53343775833333 6.891484925 6.883226375 6.85232785833333 7.11888511666667 7.21991299166667 7.17617635833333 7.57271753333333 7.21011905 7.08525161666667 7.30451515833333 6.797326975 6.41696196666667 7.31545595833333 6.95147530833333 6.97908095833333 7.28983956666667 7.05205246666667 6.73169263333333 6.67877244166667 7.200884475 7.06248425833333 6.51972946666667 7.51739635 7.005136025 6.35770789166667 7.33746579166667 6.87204776666667 7.6423852

AC099654.1 11.335701925 12.0287220416667 11.9277646416667 11.9068035083333 12.68791855 11.720720125 12.8804849833333 12.1550844416667 12.675133075 12.3344343166667 12.280903925 12.8948902 12.78428845 12.231089275 11.97656585 11.6813023833333 11.9966241166667 12.6390523916667 11.8177846 11.6510632333333 12.1839632166667 12.1582347333333 11.7878206 11.4154777166667 11.696738575 11.636069775 12.0640980916667 11.4345196083333 12.03386695 11.9409533916667 11.5524602083333 11.5692060833333 12.3829579166667 11.9409533916667 11.43162275 11.4345196083333 12.2760022333333 12.0315805583333 11.7482500666667 11.8086577 11.52850865 11.8522237916667 11.4272823833333 11.2665102166667 11.4295243333333 11.4466154166667 11.410632125 11.3517561583333 11.860233025 11.5204619166667 11.7878206 11.732454925 11.823121625 12.03386695 11.5912221 11.2944683583333 11.8086577 12.7941771583333 11.6862351 11.4942244666667 12.773932725 12.2950304166667 11.2388046916667 12.315517225 12.2978352666667 12.309527875 12.0099462666667 12.7644027583333 12.2649867083333 12.20732285 12.362762125 13.0217509333333 13.1811591833333 11.9252347666667 12.37994665 11.9534676 12.656069625 12.1958518666667 11.54529205 12.9135039416667 12.14966815 12.3010353583333 12.7446559166667 11.5692060833333 12.578037425 12.5079814083333 11.895484775 12.1582347333333 12.233818175 11.6335953333333 12.0533945916667 11.9175777083333 11.720720125 13.5330030583333 12.315517225 11.9510879333333 12.248342825 12.2399882333333 12.7114207833333 11.5811732666667 11.771780075 12.6273287833333 11.3036970916667 11.29925645 11.4659061 11.6694244 11.7849803666667 11.1886169916667 11.696738575 11.5912221 12.6460873916667 11.4868268416667 11.8648058416667 10.81892915 11.7092046583333 10.761090275 11.8009085 11.3258265916667 11.4868268416667 11.9175777083333

CU633967.1 10.979276575 9.491289725 10.1201558583333 9.50689216666667 8.86194551666667 9.19599840833333 9.28160753333333 9.46065865 9.32861675 9.569175825 7.8846688 9.220525675 9.10908699166667 9.78232589166667 8.901625425 9.49887978333333 11.542870275 8.33192433333333 10.3248470416667 9.15077928333333 8.46778476666667 8.61454506666667 8.09847590833333 9.53024233333333 8.94319444166667 10.5560371666667 9.50689216666667 9.412078125 8.901625425 8.99595798333333 9.54668399166667 8.26808959166667 8.618487425 9.28980095 10.632572825 9.70290516666667 9.08613931666667 8.99595798333333 9.24131265 10.6020413 9.31542745833333 9.52206101666667 9.62279060833333 10.3282105 9.72553698333333 9.92572226666667 9.2829911 9.70165576666667 10.4175013916667 9.85252195833333 8.94464996666667 9.29547155833333 8.83888513333333 9.770853875 8.730048975 9.07590878333333 9.93026274166667 9.67137481666667 9.40927853333333 9.45373374166667 10.0590324416667 9.32585686666667 10.0192491333333 8.61733391666667 11.2665102166667 8.0070051 11.0008274583333 8.63923054166667 8.14639896666667 8.81697240833333 8.473255125 9.14824449166667 8.95205770833333 10.335375425 7.55986316666667 9.96941311666667 9.55070993333333 8.28057508333333 10.1692826333333 9.41871168333333 9.95043048333333 8.37690871666667 6.59515689166667 10.2339183916667 7.52747528333333 9.18189768333333 9.31654956666667 9.41737299166667 10.5338062833333 8.503369575 9.16363804166667 8.71476503333333 9.56775843333333 8.262883775 7.953283475 7.89455625833333 7.98997320833333 10.1825522583333 8.72277565833333 10.1035939 9.82539988333333 8.33307035833333 11.019401025 10.865335225 9.02711425833333 8.95834738333333 9.58157864166667 10.0832833333333 10.30560935 9.95043048333333 8.555702075 10.424538125 8.02342083333333 10.3583768166667 8.94319444166667 8.33743355 11.2106026166667 11.5692060833333 10.1859038333333 9.983440075

SLX1B-SULT1A4 12.1191103833333 12.2273971416667 12.1348590875 12.3226481625 12.0301513 13.1244315333333 11.7547370166667 11.8842479083333 11.2817314875 12.6767967666667 13.0358386416667 12.8641698958333 12.0521124625 11.7287917875 11.9598907208333 12.5312882041667 13.3322088 11.8713966333333 11.7338009708333 12.2029893375 12.5188229791667 11.9136921208333 12.140188125 12.1272230166667 12.1246674458333 12.3529074916667 11.9798951416667 11.8995515291667 11.710465 12.1272230166667 11.8613836125 12.3226481625 12.0521124625 12.9915733208333 11.6498379166667 11.8816977791667 11.8121756375 11.3150472833333 11.6705744708333 11.9522777666667 12.0449527708333 11.9574655625 12.1087199875 12.0784995875 12.9398896333333 12.2029893375 12.0163526291667 11.8918753958333 12.4134209958333 12.4226583833333 12.0521124625 11.87663535 12.0139153875 12.083866575 11.67286945 11.9397950708333 11.8350328875 11.8073208916667 12.1802668 12.2059713958333 12.8752749875 12.2852076333333 12.4674068041667 12.0352049291667 12.6077398291667 12.5474786916667 11.6223105458333 12.732499 12.0030163666667 13.1920663041667 12.5128835708333 12.2909470791667 11.6955671208333 12.4163686833333 12.1999557708333 11.79151875 12.7531495708333 13.0710627041667 12.2525186666667 12.6288577541667 12.5378186541667 12.3584982416667 11.9979932166667 11.8274163333333 11.9726603083333 12.82376245 12.5251461666667 12.083866575 12.5509950625 12.6256640333333 12.2142624083333 13.5418565458333 13.1416303083333 11.9622608083333 12.0733533041667 11.9187598041667 11.9446598791667 11.9136921208333 11.4211996583333 12.6288577541667 12.3904551958333 12.6609051666667 14.062173875 12.4644566 12.932681075 12.4318196916667 12.5251461666667 12.6577267166667 13.1244315333333 12.0190830208333 12.8387190625 11.9522777666667 11.6683208125 11.9548048166667 11.8968445041667 12.9472122875 12.2273971416667 12.1777806583333 12.9803976708333 12.85395155

SLX1A-SULT1A3 12.1191103833333 12.2273971416667 12.1348590875 12.3226481625 12.0301513 13.1244315333333 11.7547370166667 11.8842479083333 11.2817314875 12.6767967666667 13.0358386416667 12.8641698958333 12.0521124625 11.7287917875 11.9598907208333 12.5312882041667 13.3322088 11.8713966333333 11.7338009708333 12.2029893375 12.5188229791667 11.9136921208333 12.140188125 12.1272230166667 12.1246674458333 12.3529074916667 11.9798951416667 11.8995515291667 11.710465 12.1272230166667 11.8613836125 12.3226481625 12.0521124625 12.9915733208333 11.6498379166667 11.8816977791667 11.8121756375 11.3150472833333 11.6705744708333 11.9522777666667 12.0449527708333 11.9574655625 12.1087199875 12.0784995875 12.9398896333333 12.2029893375 12.0163526291667 11.8918753958333 12.4134209958333 12.4226583833333 12.0521124625 11.87663535 12.0139153875 12.083866575 11.67286945 11.9397950708333 11.8350328875 11.8073208916667 12.1802668 12.2059713958333 12.8752749875 12.2852076333333 12.4674068041667 12.0352049291667 12.6077398291667 12.5474786916667 11.6223105458333 12.732499 12.0030163666667 13.1920663041667 12.5128835708333 12.2909470791667 11.6955671208333 12.4163686833333 12.1999557708333 11.79151875 12.7531495708333 13.0710627041667 12.2525186666667 12.6288577541667 12.5378186541667 12.3584982416667 11.9979932166667 11.8274163333333 11.9726603083333 12.82376245 12.5251461666667 12.083866575 12.5509950625 12.6256640333333 12.2142624083333 13.5418565458333 13.1416303083333 11.9622608083333 12.0733533041667 11.9187598041667 11.9446598791667 11.9136921208333 11.4211996583333 12.6288577541667 12.3904551958333 12.6609051666667 14.062173875 12.4644566 12.932681075 12.4318196916667 12.5251461666667 12.6577267166667 13.1244315333333 12.0190830208333 12.8387190625 11.9522777666667 11.6683208125 11.9548048166667 11.8968445041667 12.9472122875 12.2273971416667 12.1777806583333 12.9803976708333 12.85395155

AL157387.1 5.715067925 4.96911071666667 4.5167211 4.79605449166667 4.93534804166667 4.719880325 4.53840028333333 5.04647511666667 4.56712020833333 4.4863532 4.79770940833333 4.64653290833333 4.57549981666667 4.11283335 5.54304493333333 4.09751095 4.771183175 5.497087425 5.76584776666667 4.84158960833333 4.01031786666667 4.99965264166667 4.442953 4.94419624166667 4.80498945 4.02948560833333 5.35594408333333 5.534144725 5.18905345 5.07992535 7.20854513333333 4.78802293333333 4.83095916666667 4.89159086666667 4.67170091666667 4.57085965 5.08085140833333 5.77366633333333 5.36652833333333 5.17649486666667 5.31702426666667 4.55798421666667 5.02478369166667 5.67611049166667 4.34927778333333 5.20589149166667 5.10666243333333 7.12706315833333 4.76937243333333 5.23682500833333 4.96587955833333 6.22806264166667 5.27721040833333 5.38353026666667 4.83518115833333 5.55866609166667 5.05706839166667 6.75448656666667 5.23750470833333 4.70894576666667 7.1982511 4.43073356666667 4.95061065 4.36833938333333 4.06172258333333 4.52785514166667 4.43217184166667 4.67234389166667 4.372945875 4.31830761666667 4.51364695 5.15772628333333 4.0531425 3.80433845 4.12988213333333 4.28485988333333 4.16776561666667 5.0768141 5.1467782 3.76417531666667 4.04418303333333 4.8882791 4.25362953333333 4.826730425 4.7733425 4.10854148333333 4.94013415833333 4.35959019166667 3.84059245833333 5.498632225 4.25516519166667 4.4165323 4.55873965 4.64653290833333 4.43073356666667 4.42260004166667 4.470944925 5.34320095 4.20578401666667 5.06353930833333 4.44750469166667 3.90789943333333 5.24520668333333 5.03561549166667 4.568704925 5.01436615833333 4.53154068333333 7.50829535833333 4.70189450833333 6.6081855 4.63875195833333 5.54936804166667 5.49108314166667 4.90681635833333 4.476177125 4.59031499166667 4.10931059166667 5.51729848333333 5.490209475 5.56118513333333

AC024958.1 8.007490275 7.71850165416667 8.15590835416667 7.4659373 8.019107975 8.1865772875 7.7426097625 7.5320118125 7.66808292083333 7.88125896666667 7.46189963333333 7.58117296666667 7.41618856666667 6.90466549166667 7.29278212916667 7.64028839583333 8.2467799625 7.91983154166667 8.02391114583333 7.80212958333333 6.55650606666667 6.99376576666667 7.77953995833333 7.47783147916667 7.65425732083333 7.57855187916667 7.59006763333333 7.79768549166667 8.400597175 7.695807775 8.06271484166667 8.08129417083333 7.60567442916667 8.8056021125 8.1587090625 8.3115123625 8.057764775 8.500781275 7.81914734166667 8.46415910833333 7.86800565 8.1655284375 7.51379851666667 8.28632147083333 7.58389940833333 8.03352374166667 8.10329190416667 7.72546150416667 7.76430890833333 7.7670242 8.6081336 8.37834546666667 8.37737532916667 8.422712275 8.2938256625 8.25078265 7.78954514583333 8.3711661125 8.22585069166667 7.8897623 7.73347154583333 7.82003916666667 7.63655714166667 7.48108926666667 7.86055932916667 7.4240441 7.09043965 7.3969260625 6.94292338333333 7.6102303625 7.64899285 7.58482334583333 7.01999995416667 7.20563239166667 7.10848425416667 7.00026699583333 7.55702767083333 7.14433986666667 7.50783437083333 7.12325093333333 7.04857342916667 6.75064945 6.64490929166667 7.57855187916667 7.33702937916667 6.58099137916667 7.8551103375 7.42655255 8.183617 7.52040055416667 7.42230115 8.0056222625 7.65709280416667 8.19344144583333 7.72466435 7.50697829166667 7.53367724583333 8.02482207083333 7.35649805416667 7.877531275 7.592817075 6.96883389166667 7.25216777916667 7.43236467916667 7.6747230375 7.447697525 7.73252949583333 8.01042133333333 7.72633736666667 8.0491517 8.0890475125 8.63868771666667 8.11375281666667 8.20250818333333 8.220936525 8.00282366666667 7.96008695833333 8.00845409583333 8.1776989875 7.8083683125

AC120193.1 8.007490275 7.71850165416667 8.15590835416667 7.4659373 8.019107975 8.1865772875 7.7426097625 7.5320118125 7.66808292083333 7.88125896666667 7.46189963333333 7.58117296666667 7.41618856666667 6.90466549166667 7.29278212916667 7.64028839583333 8.2467799625 7.91983154166667 8.02391114583333 7.80212958333333 6.55650606666667 6.99376576666667 7.77953995833333 7.47783147916667 7.65425732083333 7.57855187916667 7.59006763333333 7.79768549166667 8.400597175 7.695807775 8.06271484166667 8.08129417083333 7.60567442916667 8.8056021125 8.1587090625 8.3115123625 8.057764775 8.500781275 7.81914734166667 8.46415910833333 7.86800565 8.1655284375 7.51379851666667 8.28632147083333 7.58389940833333 8.03352374166667 8.10329190416667 7.72546150416667 7.76430890833333 7.7670242 8.6081336 8.37834546666667 8.37737532916667 8.422712275 8.2938256625 8.25078265 7.78954514583333 8.3711661125 8.22585069166667 7.8897623 7.73347154583333 7.82003916666667 7.63655714166667 7.48108926666667 7.86055932916667 7.4240441 7.09043965 7.3969260625 6.94292338333333 7.6102303625 7.64899285 7.58482334583333 7.01999995416667 7.20563239166667 7.10848425416667 7.00026699583333 7.55702767083333 7.14433986666667 7.50783437083333 7.12325093333333 7.04857342916667 6.75064945 6.64490929166667 7.57855187916667 7.33702937916667 6.58099137916667 7.8551103375 7.42655255 8.183617 7.52040055416667 7.42230115 8.0056222625 7.65709280416667 8.19344144583333 7.72466435 7.50697829166667 7.53367724583333 8.02482207083333 7.35649805416667 7.877531275 7.592817075 6.96883389166667 7.25216777916667 7.43236467916667 7.6747230375 7.447697525 7.73252949583333 8.01042133333333 7.72633736666667 8.0491517 8.0890475125 8.63868771666667 8.11375281666667 8.20250818333333 8.220936525 8.00282366666667 7.96008695833333 8.00845409583333 8.1776989875 7.8083683125

AC005019.2 4.63651601666667 5.95073534166667 6.38929764166667 8.04962919166667 5.94836456666667 6.52116074166667 6.848966625 7.02582578333333 7.05534445 5.96691225 9.40775390833333 7.445697975 7.04741685833333 7.237579325 5.556241925 6.89860500833333 5.31451775 6.280696575 5.87078084166667 5.64920624166667 6.779595125 7.664424575 6.02744485833333 8.60150903333333 4.25214924166667 5.38864603333333 4.20805206666667 5.228162675 8.61968489166667 5.65650474166667 4.807510425 4.344009325 5.48030866666667 5.20324565 4.53764565 4.17325634166667 4.45973163333333 5.13457766666667 7.57184745833333 5.23837954166667 4.729607625 3.8188 5.18905345 7.91271055833333 6.53747031666667 4.76289125 6.00842590833333 4.383935575 4.32034705 10.618843275 4.58674640833333 7.21093403333333 7.26059781666667 4.78802293333333 6.06346144166667 4.35959019166667 6.170078 4.476177125 4.84075886666667 6.00243133333333 5.303993825 6.71798690833333 4.887437075 7.1702859 5.8061966 6.780345325 6.51972946666667 7.4079756 5.92614939166667 6.078446575 6.6840655 5.646510225 8.18511508333333 5.82778305833333 6.54378744166667 7.1066557 5.47188130833333 4.65659079166667 5.89207013333333 5.4567799 7.06801993333333 6.33336359166667 4.729607625 5.26378820833333 5.51981635 6.37025050833333 4.86629001666667 6.00589870833333 6.114356 5.81853184166667 5.72542938333333 4.88079474166667 4.64405129166667 5.5707947 6.587379025 4.83845381666667 5.40280533333333 4.95685870833333 6.80360636666667 4.63950710833333 5.61677996666667 6.6036039 4.40259521666667 4.28196915833333 5.54936804166667 5.84743648333333 4.81864974166667 4.83912508333333 5.332832325 6.86315831666667 4.45465283333333 5.4527323 5.39028695833333 5.67267240833333 4.92183824166667 4.86285301666667 5.34058364166667 5.03721211666667 3.93500141666667 7.093375425

STAG3L5P-PVRIG2P-PILRB 10.8407027083333 11.2613206166667 12.1666511583333 11.9277646416667 11.4058331 10.5616175083333 11.4659061 11.2665102166667 11.369137425 10.8230366916667 11.2805802833333 10.3828084833333 10.8250530166667 10.5504866333333 11.1432169416667 11.33050215 10.3720129583333 11.1409951666667 10.3632412666667 11.125139725 10.240385675 10.6634010583333 10.8271408 11.1387031666667 10.9983830416667 10.3565996833333 12.0611612416667 11.9858949166667 11.390936775 11.3471656166667 11.740874075 12.048574475 11.3761321333333 10.3928565833333 10.307287725 10.1983029333333 11.4491783416667 11.6307827666667 11.2754195166667 11.7015427416667 11.4295243333333 11.5524602083333 11.390936775 11.1551416916667 11.0122860666667 10.9101974666667 10.9901360833333 11.0146255 11.2637060666667 11.3163894833333 11.2731520583333 10.4921560416667 11.489093725 11.1951963916667 11.5333541416667 11.019401025 10.908225625 11.1211007166667 11.3110612083333 10.6535999166667 11.8854413833333 10.5447162333333 11.3638652666667 10.6653293916667 11.0122860666667 10.578428575 10.1155244166667 10.5145241083333 10.0882671583333 10.5185258166667 10.4681694166667 10.5464850833333 9.90876665 10.1120281166667 10.3845893166667 10.4033397666667 10.8589366583333 10.91632235 10.092690275 10.1967116083333 11.2944683583333 11.1886169916667 10.0647337083333 10.164300675 9.89676418333333 10.4158197333333 11.9892138666667 10.0709631916667 10.746282975 10.1489361666667 10.0349403166667 12.1232960666667 10.756939325 10.1776993666667 10.1394934333333 10.5202480833333 10.3964220916667 11.4540675166667 10.1983029333333 10.7711372583333 10.91632235 10.872244825 11.1182445333333 10.5671895333333 10.71936685 10.260350775 10.0005481 10.475071575 9.87425795 9.78377983333333 10.4941187833333 10.5410668333333 10.5050930083333 11.5260845666667 10.4902295666667 11.183487175 10.5464850833333 11.6648204916667 11.4727106916667 10.8609045666667

AL137230.2 9.826776875 10.2536886083333 10.2489068 10.5185258166667 9.70023275833333 9.9755856 9.84686086666667 10.3828084833333 10.3409099333333 10.2308420666667 10.6040700666667 9.38498160833333 9.35011415 10.3666040083333 10.56904215 10.0128890333333 10.1083804166667 10.0082598583333 10.287159 10.3666040083333 10.34257475 10.5464850833333 10.4347538666667 10.3479196583333 10.1825522583333 10.3532081333333 10.279838075 10.3459838833333 9.589758275 10.2339183916667 10.1776993666667 10.2387529583333 9.74992998333333 9.980296075 10.6594825833333 10.6383268333333 9.79859535 10.174610325 9.94385811666667 10.257033225 10.3666040083333 10.1065692416667 10.0097474333333 10.5426773833333 10.12322705 10.3549727833333 10.5145241083333 10.1542300916667 10.5338062833333 10.30417975 9.81785751666667 10.0381389583333 9.63324336666667 10.1083804166667 10.3928565833333 10.2736553416667 9.94119803333333 10.1811538916667 10.251873975 9.967859125 9.96321 9.55551340833333 10.829344675 10.8494230333333 10.6757072916667 10.36490795 10.1859038333333 10.3232071333333 11.5739352416667 10.3248470416667 9.97701388333333 9.277757525 10.8030115583333 9.346276075 10.8250530166667 10.229166925 10.3757960166667 10.279838075 10.887311375 10.6653293916667 10.2225823916667 9.48990181666667 10.7591979083333 10.420672925 9.19243765 10.5860666166667 9.548133475 9.89075863333333 9.46997115 10.36490795 10.7173483583333 10.682010725 9.72272495833333 10.3232071333333 10.0005481 9.819189925 10.4088283666667 9.58572294166667 9.70165576666667 9.24022388333333 10.398066475 9.589758275 10.3895259333333 10.4732783833333 10.7648028916667 10.0220394666667 10.2371982916667 11.496576925 10.14430765 10.6059731083333 9.423639975 10.3459838833333 10.6248927916667 11.1809665916667 10.2459336666667 10.4158197333333 9.63464729166667 11.5739352416667 10.8271408 10.8853541583333

AL158847.1 12.421258225 12.3486515916667 11.97656585 12.9135039416667 11.4703555083333 11.2685648583333 11.160332025 11.6717245416667 11.8133445166667 11.826140675 13.3438936916667 11.7773535166667 11.696738575 11.5204619166667 12.1177468833333 11.54529205 12.8659456416667 12.0799709583333 11.9808613 11.8419743833333 11.4154777166667 12.6061961916667 13.2741790333333 12.0611612416667 11.8312788416667 12.421258225 10.9836119583333 12.0904755416667 10.7015995416667 12.3402604166667 12.215507725 12.6031552333333 11.4703555083333 12.6356084 12.215507725 11.8522237916667 11.3713762333333 12.1415417666667 11.75862515 12.3920642833333 12.1550844416667 11.8854413833333 11.8753288833333 11.57888155 12.001763825 12.14966815 11.870125125 12.1815738083333 11.1650844416667 12.3185215083333 11.8625342 11.6159368333333 11.93863675 11.91499415 12.3861826333333 11.1344954166667 10.6712601416667 11.3016516333333 11.0651359666667 12.0824665333333 12.684736675 15.0566496333333 12.8524193083333 11.84692875 11.8389506833333 10.3862458416667 13.7723702416667 12.43015245 12.3371985 10.6096692083333 14.2641819916667 14.16572505 11.4032074083333 14.202140575 12.0879052583333 13.2700538583333 9.799983825 13.6342148583333 13.5553000916667 14.2461660666667 10.6878421583333 12.03386695 12.26263345 14.3428338416667 10.4316753833333 11.0536141166667 9.87742366666667 9.70973839166667 14.2461660666667 13.726268275 11.3840616416667 12.4334869333333 13.1525887583333 12.1151589416667 10.2813617166667 13.8366873666667 12.9679019583333 10.16579105 11.7252727333333 10.8271408 9.980296075 14.16572505 9.20949 12.001763825 9.91775825 12.7206590083333 11.7611345666667 11.8335796916667 9.769181275 10.8317076916667 12.233818175 12.8554837916667 9.68325286666667 11.792717175 12.012563225 15.4447775583333 9.49528248333333 10.508878025 12.43663495 10.229166925

LINC00476 6.93977528333333 7.78914439166667 7.52178743333333 7.55321126666667 7.74923976666667 7.43034844166667 8.158279725 8.01671704166667 8.02254479166667 6.794268175 7.08765801666667 7.89455625833333 8.391481525 7.31447869166667 7.845369325 7.74841675 6.957621225 7.263915325 6.49019045 7.23187408333333 7.83627273333333 7.76312330833333 6.87454878333333 7.29478473333333 7.11493935833333 6.71477854166667 7.53244018333333 6.51900598333333 7.456370675 6.780345325 6.40188925833333 7.16947939166667 7.41415456666667 6.82831530833333 6.48941536666667 7.40534635 6.88682719166667 6.77657375833333 6.72671865 6.30591225833333 5.98312854166667 7.0397833 6.47356659166667 7.13580859166667 6.98145384166667 7.10002753333333 6.442668925 6.22569964166667 7.21681046666667 6.30419145 5.99926955 6.70927374166667 7.94501785 6.87998445 6.79655045 6.643638875 6.80987979166667 5.71323735 6.44192199166667 6.95295379166667 6.9248352 7.94870053333333 6.66977713333333 6.82831530833333 6.36881363333333 6.192534425 7.13912226666667 7.7674106 6.21730323333333 6.88087174166667 6.99580511666667 7.397338075 7.859170675 6.90573395833333 7.61920535 7.5199474 6.88611070833333 7.02662889166667 6.40654535833333 7.12623191666667 7.546385025 7.25974294166667 6.41853091666667 6.68096106666667 6.6848112 6.849738 6.27380003333333 7.14716405 7.978411525 6.31742500833333 6.7441857 6.3018823 7.1552468 6.57919131666667 6.68783996666667 6.84134261666667 6.50018229166667 6.63281199166667 6.824129025 5.98543355 7.07855881666667 7.30288484166667 5.97460996666667 6.24671985833333 6.26236639166667 6.40727721666667 6.21815258333333 6.72921199166667 6.52116074166667 6.83075820833333 6.0017496 6.34768783333333 6.80987979166667 5.70703833333333 6.78970486666667 6.76239408333333 6.24370750833333 5.6623785 6.35692343333333 7.02434959166667

AC007881.2 10.5724502916667 12.1711872833333 12.16080175 12.283625975 12.8592781166667 11.2434934166667 13.5131960416667 12.6718910166667 13.2243581083333 11.880341125 12.176601525 13.6281622333333 13.5182485583333 12.5593382333333 12.0437977833333 11.9587890916667 10.9681252083333 12.6460873916667 10.9771250333333 12.3185215083333 12.4776177416667 12.0770282166667 11.7689310166667 11.2152281416667 11.0874478833333 11.6534936583333 11.183487175 11.3110612083333 11.113406175 11.6283795166667 10.2702136416667 11.43162275 11.9993623166667 11.1886169916667 10.3089135 10.4033397666667 11.6412048416667 11.125139725 10.041049 10.9656709666667 10.7830390666667 11.5719596666667 10.8746426833333 10.9656709666667 11.25019095 11.129710525 11.2613206166667 11.5310238 10.6040700666667 10.7173483583333 10.3702408666667 10.74025755 12.5969000166667 11.6740143583333 11.7435451333333 11.2021462083333 12.3010353583333 10.9920373333333 11.0122860666667 10.7946431083333 11.9993623166667 12.486478675 10.682010725 12.5969000166667 12.583994125 12.0072618083333 12.4148038083333 13.5493478666667 12.0695594916667 12.5560529333333 12.9528047916667 12.75143825 13.38044235 12.552751 12.9713989666667 12.3318273166667 12.2867892916667 11.4727106916667 11.3282592416667 11.9685451916667 13.2243581083333 13.4779890416667 12.2676890666667 10.9530714833333 11.8389506833333 12.9097414333333 12.7773959166667 12.69765135 13.339223625 11.1746153 12.2923180416667 10.763080925 10.3214407083333 11.1498746333333 11.4439581833333 12.2596429833333 11.0580407916667 11.9277646416667 12.26263345 11.6534936583333 10.4070010416667 12.9820572083333 10.307287725 10.420672925 10.6857749333333 11.8982042333333 10.7670533583333 10.5805910083333 10.61685815 10.503461525 10.7217191666667 10.1248896166667 9.983440075 10.8250530166667 10.9681252083333 11.33050215 11.823121625 9.483981775 9.87005905833333 12.5689938083333

AC024598.1 10.6096692083333 13.11410875 13.6897778 13.4629723583333 14.05850055 9.38637963333333 13.7324527083333 13.083804375 12.2649867083333 13.4340660666667 12.5327561583333 9.87299354166667 13.68323365 12.737637325 14.1559919583333 13.3856220583333 9.00741491666667 13.2450601083333 13.2741790333333 13.4340660666667 12.001763825 12.6425501583333 11.7252727333333 11.4565596333333 13.6712789833333 9.68577309166667 14.202140575 14.3129405416667 13.9495562333333 13.0531395083333 12.675133075 12.6061961916667 12.3971468083333 8.77244310833333 9.39042674166667 10.4158197333333 14.8320167 14.3328580333333 13.1943968666667 13.7078228166667 13.5182485583333 14.16572505 13.3035558583333 11.1498746333333 11.69147735 14.1559919583333 13.18555275 13.2412141666667 11.129710525 12.0799709583333 13.0217509333333 13.2243581083333 13.4152475666667 13.5553000916667 13.2784613916667 13.3711706833333 12.8804849833333 14.202140575 13.7723702416667 12.58100075 9.39312421666667 9.306047575 9.83934628333333 11.062796625 9.00480995 8.7855717 9.73689675 10.578428575 7.89997913333333 11.0740682333333 9.75922218333333 10.3337502666667 11.02834235 10.4409834125 10.176104 11.1809665916667 8.74401624166667 9.41871168333333 10.8853541583333 9.0697895 8.15235975 8.11218248333333 11.374107025 11.2613206166667 11.024274375 8.893151 8.857267525 10.3565996833333 8.83385925833333 10.1542300916667 10.2504355916667 9.78825455 9.21209545833333 10.344247175 8.26500145 9.123301475 10.1811538916667 9.72706908333333 9.627070875 8.85112088333333 9.46065865 9.5233206 9.11950295 10.0316996666667 10.4941187833333 10.0097474333333 11.5548729916667 10.260350775 10.3390799083333 10.394412525 9.991570475 11.13656485 11.5985981416667 10.0252423 9.93174519166667 12.0770282166667 10.0175973583333 10.8383688833333 10.420672925 9.52903631666667

NNT-AS1 12.309527875 10.6691077583333 10.7591979083333 10.229166925 10.3928565833333 11.28743775 9.89075863333333 10.3532081333333 10.29196065 10.8564046583333 10.9771250333333 11.2566977833333 10.752680775 10.6594825833333 10.87665705 11.1344954166667 12.3128225916667 10.6594825833333 11.361268975 11.0671957166667 10.9633547666667 10.7322398666667 11.2481652416667 10.5671895333333 10.9488262583333 12.0799709583333 10.9123222583333 10.7946431083333 10.7648028916667 10.9702919666667 10.7052053666667 10.888969925 10.61685815 11.2523367916667 11.704120775 12.3402604166667 10.632572825 10.5126549333333 10.3232071333333 10.7729963333333 10.9143603333333 10.7421405583333 11.0949364416667 11.33050215 11.696738575 11.4942244666667 11.4520079916667 11.0831276083333 11.2130362833333 11.2321633416667 10.9101974666667 10.2339183916667 11.3137050833333 11.0559555833333 11.1182445333333 11.6888272 10.7483297666667 10.9726612083333 11.1211007166667 11.2434934166667 12.6239992833333 10.3532081333333 11.3282592416667 10.7872050916667 11.9561420333333 11.50386205 10.4070010416667 11.1274628333333 11.1791450916667 10.8166201833333 11.28965795 10.9059938166667 10.3775083083333 11.1717372166667 11.6510632333333 10.7848974333333 12.0207283333333 11.55966165 10.6517451416667 11.7611345666667 11.893046575 12.1312062333333 10.927189875 11.0058729583333 11.3232895666667 11.113406175 11.6034592083333 11.3232895666667 11.3517561583333 11.7015427416667 10.9399979583333 10.618843275 11.69147735 10.6777440416667 12.1151589416667 10.9726612083333 11.183487175 11.5087194333333 10.5578671833333 12.6686352083333 11.97656585 11.3450705666667 12.737637325 11.22950995 11.394164525 12.0665318666667 11.8522237916667 11.930308275 11.6063160416667 11.1211007166667 13.6959951583333 11.0786204833333 10.9228885833333 11.3332652166667 11.5935101166667 11.6943956666667 12.5361294916667 11.2458122833333 10.6757072916667 11.5958220916667

DPH6-DT 6.45493224166667 5.18325646666667 5.387813925 3.91178589166667 4.3958178 5.6868575 4.361626725 4.93371973333333 4.95297583333333 4.779451525 4.11973251666667 5.25444665 5.20502061666667 4.38704568333333 4.22349475833333 5.11082383333333 5.24934811666667 3.898913075 4.86198561666667 4.44597125 4.40189316666667 4.23975296666667 4.25745651666667 5.53699586666667 4.50704113333333 5.69727409166667 4.24443253333333 4.252842375 4.29455464166667 5.11490881666667 4.35517875833333 4.21564994166667 4.20943228333333 4.18895399166667 5.969534625 5.9679195 4.86787458333333 4.43350984166667 5.37603039166667 6.4838525 4.83685254166667 4.69752440833333 4.51364695 5.070082275 5.208439775 5.56298029166667 4.54800100833333 4.58156329166667 4.81551458333333 4.70894576666667 4.71818525833333 4.29385256666667 4.64804604166667 4.79532921666667 4.36903601666667 4.99799986666667 4.41068774166667 4.55571135833333 4.77273383333333 4.92694636666667 4.78973365833333 4.27153690833333 4.541226475 4.353056575 4.95383771666667 5.102471875 4.20338155833333 4.7809755 4.43217184166667 4.26785038333333 4.70189450833333 4.45897225 4.71438873333333 3.980366375 4.31606005833333 4.13060835 3.924596975 3.69076226666667 3.98974825 3.92612165 4.67942881666667 3.9955461 3.94073975 4.8882791 4.04345499166667 4.86629001666667 4.6542059 3.93349674166667 3.92531665833333 5.51729848333333 3.66292545833333 4.06172258333333 4.82042135 3.89423003333333 4.11807701666667 3.62603805 3.91339629166667 4.790562675 4.179094925 5.14837419166667 4.18671215 3.75646214166667 4.43284685833333 4.43284685833333 4.292405 4.82431689166667 4.184461775 5.26290054166667 4.65963046666667 5.79569219166667 4.98534708333333 5.805323425 4.72314724166667 4.87670536666667 4.51592391666667 4.13060835 4.76049746666667 4.31751935 4.16321256666667 5.3299725

NR2F1-AS1 5.299873475 4.61847421666667 4.6700077 4.72237503333333 4.45823205833333 5.736606025 4.12988213333333 5.03074584166667 4.96673286666667 4.73514765833333 4.71758559166667 4.08559233333333 4.06020879166667 4.47242323333333 5.21098853333333 4.663962275 5.16663731666667 4.90363349166667 5.59424653333333 5.04562356666667 4.09751095 4.675507125 5.12190426666667 5.27487943333333 5.06029869166667 4.55255341666667 5.4223537 5.64469614166667 5.36997850833333 5.537859625 6.13046838333333 5.18905345 5.430247225 5.26290054166667 5.52235776666667 5.62083628333333 5.94836456666667 6.20542285 5.97867255833333 5.93939261666667 6.02483926666667 5.5439086 5.766786475 6.11996326666667 5.44471205 5.75992465 6.41370083333333 4.73514765833333 5.373446775 5.642025525 5.88961983333333 5.603294875 4.83685254166667 6.22806264166667 5.62182049166667 5.14231660833333 4.719880325 6.32528485 5.51027169166667 5.69812770833333 4.91353486666667 4.41717060833333 5.332832325 4.82348884166667 4.87816691666667 4.70744949166667 5.23259858333333 4.55798421666667 5.72460895833333 4.83845381666667 3.97718864166667 4.389247575 4.27303441666667 4.61117119166667 4.16937971666667 4.14684333333333 4.813152125 4.98971816666667 4.72810314166667 4.87364085 4.08108728333333 4.24515410833333 4.61847421666667 4.45973163333333 4.54651299166667 4.76289125 5.01921390833333 4.282727025 4.40993569166667 4.651163825 5.02478369166667 5.07839706666667 5.50303829166667 5.02165799166667 4.71676371666667 4.45823205833333 5.60738530833333 6.03972224166667 5.327405775 5.631011625 5.241814025 4.577162675 5.9244919 5.70335329166667 5.27819885 4.74735175 4.99799986666667 5.568276075 5.10162569166667 6.25595283333333 5.32912600833333 5.98312854166667 6.06024568333333 5.79054971666667 4.88341414166667 5.11238625833333 5.12016660833333 6.316535975 5.90253445833333 4.58463855

USP27X-AS1 7.2852293 5.706023225 6.15958521666667 5.87484386666667 4.47754666666667 6.05245123333333 3.82707174166667 4.31830761666667 4.48179340833333 4.88571185 5.10074249166667 5.20674935833333 4.20055220833333 4.64031274166667 5.95073534166667 6.09080005833333 8.01861823333333 4.207301975 6.88611070833333 6.02062880833333 4.78164949166667 5.09305810833333 6.80453016666667 5.52726914166667 6.14095060833333 7.70840925 6.72844050833333 6.75932524166667 4.91113061666667 6.61562239166667 5.93351605 6.16130541666667 5.33553373333333 5.93685915833333 7.387267475 7.86100648333333 5.88060724166667 6.48159381666667 5.99273749166667 6.857401975 7.01349440833333 6.587379025 6.95909085 7.52419651666667 7.072020975 7.32393866666667 7.181557425 6.156306425 6.6840655 6.70174799166667 6.43598355 6.222544625 5.37603039166667 6.43123795 6.99909050833333 7.30708359166667 4.65351119166667 6.550654 6.6279805 7.01657786666667 5.58424089166667 4.64105058333333 7.237579325 4.389988275 7.16143308333333 4.00394323333333 4.57164009166667 4.48927336666667 5.62857495 4.98886558333333 4.16550909166667 6.403442625 3.78937435833333 4.56782300833333 3.95459895833333 4.65266215 5.58504371666667 4.77273383333333 4.83614788333333 5.494444575 4.97375653333333 5.10342061666667 4.60466838333333 6.61643335 4.42914940833333 4.96911071666667 5.61505438333333 5.80704883333333 4.98534708333333 6.84740460833333 4.41784864166667 7.02662889166667 4.74898438333333 5.547570375 4.74898438333333 5.71930858333333 4.98620748333333 6.515853425 4.78164949166667 6.20451650833333 6.0420986 5.004526525 7.43866366666667 6.22349896666667 5.81184100833333 5.17730950833333 5.296452375 6.430564175 6.24024199166667 6.8961707 6.88170193333333 6.66372589166667 5.50030605 6.65373279166667 5.367325475 6.57919131666667 6.1401869 5.16271053333333 5.75111171666667 4.80100048333333

LINC02623 4.50496470833333 4.096636775 4.3268489 4.771183175 4.56033968333333 3.99379071666667 4.33403705833333 4.9202471 4.31153791666667 4.24046655 4.78488421666667 4.874362975 4.424034 4.65893614166667 4.00148819166667 3.97392573333333 4.47678545833333 4.367582 4.442953 4.05802635833333 4.39729575 3.85018766666667 4.44087261666667 4.74100033333333 4.39068023333333 4.91113061666667 4.23746243333333 4.20943228333333 4.33777051666667 4.0347939 4.90848588333333 4.23195600833333 4.74667178333333 3.89263233333333 4.45111078333333 4.09183225 4.65963046666667 5.37944853333333 4.35959019166667 4.518365025 4.858673825 5.76240348333333 4.556397325 5.1467782 5.20230749166667 4.68510404166667 4.71818525833333 4.62957980833333 5.02981615 4.70340193333333 4.62341145 4.32395948333333 3.76920943333333 5.1551752 4.30994171666667 4.43284685833333 3.7651031 4.60106575833333 4.14925433333333 4.113563875 4.33943185 4.33545854166667 5.424208775 5.20404056666667 4.68742364166667 4.79946088333333 4.19976231666667 5.043189975 5.27971544166667 4.47313854166667 4.67308416666667 4.88651261666667 5.537859625 5.03475209166667 4.25214924166667 4.49747863333333 4.96587955833333 4.41362111666667 3.77099958333333 4.574648225 3.66610879166667 4.332637675 5.34411366666667 4.15790645833333 4.60802493333333 5.00864485833333 4.72156198333333 4.6152735 4.535365525 4.338662175 5.23837954166667 4.06404305833333 4.521992575 4.034029975 4.783293425 4.42340415833333 5.06353930833333 4.83518115833333 4.51995868333333 4.65809740833333 4.758019275 5.53510280833333 4.46626585833333 4.564244925 4.56566956666667 3.84950255 4.301581775 5.39559 4.083380025 5.8328231 4.41144705 4.71050723333333 4.6472699 4.463312775 3.83468344166667 4.16702060833333 3.92612165 4.3499844 4.27817745833333 3.88309336666667

AC092902.4 9.03224780833333 9.26398391666667 9.88347994166667 9.15345658333333 10.098956 9.46481536666667 9.311047725 9.705649125 9.39994525833333 9.31935865 8.75543470833333 9.32203853333333 10.3720129583333 9.16363804166667 9.45373374166667 9.10776604166667 8.53829400833333 9.84980565833333 9.2230337 9.042386175 8.82317351666667 9.34239438333333 9.39312421666667 9.056928375 9.03716685 8.68047514166667 9.4483016 9.29792191666667 10.2260622166667 8.866571625 9.26878520833333 9.334967175 8.698399275 8.780874675 8.12305399166667 8.43311395 10.23249925 10.2339183916667 9.08861136666667 9.35812314166667 9.14824449166667 9.43592440833333 8.94592274166667 9.53278261666667 8.65575711666667 9.18189768333333 8.92027965 8.98366746666667 9.047025875 8.9709554 9.69730725833333 8.82522835 9.06439938333333 9.23402224166667 8.87974589166667 8.84739189166667 9.54939620833333 9.35946826666667 9.17402556666667 9.23135698333333 11.2434934166667 8.69140489166667 9.27253420833333 9.4483016 9.63324336666667 9.01969005 8.658204375 9.88498314166667 9.61182498333333 9.694321975 9.03833616666667 8.88575565 9.34755915833333 9.071309125 9.68178754166667 8.82317351666667 9.16746680833333 8.75314075 8.21116674166667 8.345254425 9.51389508333333 10.1360433666667 8.52340118333333 8.391481525 8.84022498333333 10.7365289583333 9.89676418333333 9.1468994 10.0509998083333 8.48948380833333 9.22419906666667 8.90815406666667 8.45706139166667 7.85554625 8.82199014166667 9.38364405 10.5107808 9.93966605 8.501312975 8.925211725 8.05052155833333 9.45947939166667 8.525776075 8.483114775 7.88367008333333 8.5982425 8.599352775 7.57377728333333 8.86517486666667 7.42182476666667 8.14539853333333 9.538153975 7.39130495 8.065151125 8.40105418333333 8.45597868333333 9.306047575 7.75769473333333 8.089532425 9.08029321666667

LINC00937 9.03224780833333 9.26398391666667 9.88347994166667 9.15345658333333 10.098956 9.46481536666667 9.311047725 9.705649125 9.39994525833333 9.31935865 8.75543470833333 9.32203853333333 10.3720129583333 9.16363804166667 9.45373374166667 9.10776604166667 8.53829400833333 9.84980565833333 9.2230337 9.042386175 8.82317351666667 9.34239438333333 9.39312421666667 9.056928375 9.03716685 8.68047514166667 9.4483016 9.29792191666667 10.2260622166667 8.866571625 9.26878520833333 9.334967175 8.698399275 8.780874675 8.12305399166667 8.43311395 10.23249925 10.2339183916667 9.08861136666667 9.35812314166667 9.14824449166667 9.43592440833333 8.94592274166667 9.53278261666667 8.65575711666667 9.18189768333333 8.92027965 8.98366746666667 9.047025875 8.9709554 9.69730725833333 8.82522835 9.06439938333333 9.23402224166667 8.87974589166667 8.84739189166667 9.54939620833333 9.35946826666667 9.17402556666667 9.23135698333333 11.2434934166667 8.69140489166667 9.27253420833333 9.4483016 9.63324336666667 9.01969005 8.658204375 9.88498314166667 9.61182498333333 9.694321975 9.03833616666667 8.88575565 9.34755915833333 9.071309125 9.68178754166667 8.82317351666667 9.16746680833333 8.75314075 8.21116674166667 8.345254425 9.51389508333333 10.1360433666667 8.52340118333333 8.391481525 8.84022498333333 10.7365289583333 9.89676418333333 9.1468994 10.0509998083333 8.48948380833333 9.22419906666667 8.90815406666667 8.45706139166667 7.85554625 8.82199014166667 9.38364405 10.5107808 9.93966605 8.501312975 8.925211725 8.05052155833333 9.45947939166667 8.525776075 8.483114775 7.88367008333333 8.5982425 8.599352775 7.57377728333333 8.86517486666667 7.42182476666667 8.14539853333333 9.538153975 7.39130495 8.065151125 8.40105418333333 8.45597868333333 9.306047575 7.75769473333333 8.089532425 9.08029321666667

AC092902.2 9.03224780833333 9.26398391666667 9.88347994166667 9.15345658333333 10.098956 9.46481536666667 9.311047725 9.705649125 9.39994525833333 9.31935865 8.75543470833333 9.32203853333333 10.3720129583333 9.16363804166667 9.45373374166667 9.10776604166667 8.53829400833333 9.84980565833333 9.2230337 9.042386175 8.82317351666667 9.34239438333333 9.39312421666667 9.056928375 9.03716685 8.68047514166667 9.4483016 9.29792191666667 10.2260622166667 8.866571625 9.26878520833333 9.334967175 8.698399275 8.780874675 8.12305399166667 8.43311395 10.23249925 10.2339183916667 9.08861136666667 9.35812314166667 9.14824449166667 9.43592440833333 8.94592274166667 9.53278261666667 8.65575711666667 9.18189768333333 8.92027965 8.98366746666667 9.047025875 8.9709554 9.69730725833333 8.82522835 9.06439938333333 9.23402224166667 8.87974589166667 8.84739189166667 9.54939620833333 9.35946826666667 9.17402556666667 9.23135698333333 11.2434934166667 8.69140489166667 9.27253420833333 9.4483016 9.63324336666667 9.01969005 8.658204375 9.88498314166667 9.61182498333333 9.694321975 9.03833616666667 8.88575565 9.34755915833333 9.071309125 9.68178754166667 8.82317351666667 9.16746680833333 8.75314075 8.21116674166667 8.345254425 9.51389508333333 10.1360433666667 8.52340118333333 8.391481525 8.84022498333333 10.7365289583333 9.89676418333333 9.1468994 10.0509998083333 8.48948380833333 9.22419906666667 8.90815406666667 8.45706139166667 7.85554625 8.82199014166667 9.38364405 10.5107808 9.93966605 8.501312975 8.925211725 8.05052155833333 9.45947939166667 8.525776075 8.483114775 7.88367008333333 8.5982425 8.599352775 7.57377728333333 8.86517486666667 7.42182476666667 8.14539853333333 9.538153975 7.39130495 8.065151125 8.40105418333333 8.45597868333333 9.306047575 7.75769473333333 8.089532425 9.08029321666667

LINC00605 7.24089864166667 6.45953320833333 6.86786498333333 6.33413388333333 5.7744326 7.18309601666667 5.63422645833333 6.11113416666667 5.850901925 6.44752274166667 6.12262255833333 5.53956186666667 5.3549373 5.70066640833333 5.96171228333333 6.51972946666667 6.15390005 6.42753165 7.3062498 6.781139 5.53699586666667 6.80752626666667 6.73249878333333 7.34892254166667 6.92405915 7.15998195 7.23088359166667 7.102382825 7.35121041666667 6.58583718333333 7.28710860833333 6.37602205 6.505088325 6.53900623333333 7.005136025 7.22572939166667 7.02662889166667 7.33580734166667 6.95992653333333 6.9464238 7.54201473333333 7.42277753333333 7.28710860833333 7.45333459166667 6.539864925 7.47235645 7.13580859166667 6.94476054166667 6.85141578333333 7.27458531666667 6.80596155833333 7.47322088333333 6.51669615 7.63892676666667 6.91920954166667 7.41941744166667 5.92530963333333 7.49427225833333 7.311835325 7.080264325 6.83745388333333 5.60913583333333 7.21758313333333 6.30112746666667 6.3612461 6.49099546666667 6.2141109 5.87646783333333 6.129619075 6.09646545833333 6.16925395833333 6.19578775 5.35953131666667 6.83210498333333 5.53510280833333 6.222544625 6.56973204166667 6.674888925 6.15468555833333 7.9017682 6.033075425 7.01657786666667 6.51151065833333 6.541436775 6.49504519166667 6.43748905 7.4747897 6.38854489166667 6.04051248333333 6.81152795833333 6.46963415833333 6.59117523333333 7.39050169166667 7.029863175 6.56098033333333 6.718754375 6.54302155 7.02184869166667 5.59277610833333 6.74266950833333 7.002237925 5.42340053333333 6.4666266 7.95522123333333 6.56261174166667 9.29393176666667 6.89382319166667 6.850638675 7.0397833 7.00978536666667 6.53500695833333 6.81368843333333 7.84438765 6.75196500833333 6.20006668333333 6.6227255 6.83895268333333 7.0570133 6.73078578333333 6.55371424166667

MEG8 5.33475269166667 4.33031856666667 4.10685365 4.25214924166667 4.49140346666667 5.22566315 5.23171054166667 4.33331919166667 3.794740275 4.69847759166667 4.403336375 5.13217864166667 4.02471265 4.35959019166667 4.233637175 4.59510340833333 4.9202471 4.484204225 5.38945345833333 4.55873965 3.88052193333333 4.20055220833333 4.44518198333333 6.20090414166667 5.06279435 4.41068774166667 5.69628823333333 4.70568649166667 4.7809755 4.49903674166667 5.18662866666667 4.727288675 4.5391327 4.27153690833333 4.650362925 4.65809740833333 4.77273383333333 5.17236361666667 5.285924575 5.09060064166667 5.81606168333333 5.20502061666667 5.24255170833333 6.338904975 4.41868564166667 5.448697 5.56298029166667 5.24020400833333 4.87504760833333 5.523243625 5.18149681666667 4.98361768333333 3.82617195833333 6.0315094 4.8571846 5.0923089 3.84547438333333 5.16029566666667 4.67942881666667 4.729607625 4.48490886666667 3.90323076666667 4.790562675 4.93689135833333 5.68514503333333 6.10052355833333 3.92950448333333 4.42260004166667 4.52857456666667 4.188201025 4.19283364166667 4.42340415833333 4.4967147 4.53840028333333 4.305532525 4.08941781666667 3.8557954 3.82884239166667 3.9955461 4.01257660833333 3.803316 3.563704825 4.564244925 4.51592391666667 4.35959019166667 4.43727345833333 3.817257075 4.42535546666667 3.88965465833333 4.88651261666667 4.58156329166667 4.96760800833333 4.226560075 4.50496470833333 4.55255341666667 4.0347939 4.27894339166667 4.63352474166667 4.12429728333333 5.24850139166667 4.541226475 3.636165975 5.08085140833333 5.43284749166667 4.66482881666667 4.02471265 5.42593315833333 6.61332625 5.537859625 6.99580511666667 4.90444880833333 5.58339051666667 5.07336385 5.867409875 4.07415490833333 4.59205544166667 4.26203231666667 4.76691938333333 4.83257791666667 3.59319884166667

AL163932.1 5.75521793333333 6.36384354166667 6.82479446666667 5.7871496 6.171399325 5.31451775 6.55849888333333 5.82190143333333 6.859502875 6.29083875833333 6.32849529166667 6.1530748 6.390112675 5.85938266666667 6.84822129166667 5.48937928333333 5.3906940625 6.37447095 6.22491186666667 6.581362225 5.17649486666667 5.734081625 5.4527323 5.83200043333333 6.29392624166667 5.47001155833333 5.736606025 5.10162569166667 5.529852675 6.61725120833333 6.11361575 6.98940225 6.75017773333333 7.31286559166667 6.1327813 5.78381885833333 6.33013755 6.94156313333333 5.64279376666667 5.625272925 5.57003804166667 6.692367775 6.39399721666667 5.37872604166667 5.32316651666667 6.25138878333333 6.72514949166667 6.55465186666667 5.87407425 6.25988588333333 6.249249475 6.91117330833333 6.75932524166667 7.34657900833333 6.62653135833333 6.343453175 7.703962625 6.839750125 7.59418351666667 6.238638975 5.43881695 6.02391205 5.597380175 6.22073784166667 6.48793390833333 5.95005389166667 6.50807960833333 6.16765155 6.45128649166667 6.74266950833333 6.69077578333333 5.860066125 6.77333313333333 5.695409275 7.297972275 5.59564198333333 5.85603955 6.01487855833333 6.35611011666667 5.95235704166667 5.77706405 4.94978725 5.70965025833333 5.611704975 6.1458196 6.16925395833333 5.843361025 6.49720533333333 5.92922820833333 5.74674768333333 6.16130541666667 5.86420291666667 5.73484704166667 6.66295756666667 5.92279354166667 6.00748140833333 6.26630801666667 6.27380003333333 7.34892254166667 6.18461388333333 5.31363400833333 6.04838810833333 5.25020996666667 5.2527348 6.20782178333333 6.41853091666667 6.36034525833333 7.20338010833333 6.331672825 7.381252125 5.75012458333333 6.88538973333333 6.321419225 6.868676375 6.957621225 6.36954999166667 6.72093702916667 6.46331624166667 6.61725120833333 7.56417009166667

CYTOR 12.1467373 11.7015427416667 12.321479575 11.3137050833333 12.2978352666667 12.4713877916667 11.2458122833333 11.43162275 12.0072618083333 12.3402604166667 11.9433354166667 10.9656709666667 12.3544155583333 11.4843161333333 13.321706325 12.0042689083333 10.6425719166667 10.929250575 12.0042689083333 11.9433354166667 12.451622875 12.520205475 12.511227025 12.3062951833333 11.9561420333333 10.6936456083333 12.5361294916667 11.8753288833333 11.6813023833333 12.14966815 12.7773959166667 12.309527875 11.3814972416667 12.6390523916667 12.614531575 10.7034530583333 11.8086577 11.7849803666667 12.37994665 11.870125125 12.223369875 11.7015427416667 11.9892138666667 12.1151589416667 12.3829579166667 12.60024735 11.9459843416667 13.041627725 12.656069625 11.496576925 12.6092834666667 12.9135039416667 12.0042689083333 12.0437977833333 11.9199419 12.1443252916667 12.14966815 12.7309384666667 12.5423167666667 12.326234125 13.38044235 13.7856886083333 12.9713989666667 13.7425781166667 11.9038701666667 12.8295815416667 13.1681379333333 12.79731905 12.9713989666667 13.5182485583333 13.2164074166667 12.873197975 13.4629723583333 13.8829970083333 12.189826275 14.03455475 12.8948902 13.2119630416667 13.6281622333333 13.010652225 12.4240585416667 11.9510879333333 13.5553000916667 14.081980575 12.877352 12.6658577166667 12.283625975 13.7135423333333 13.726268275 13.2450601083333 13.5881364666667 13.2203812666667 12.3861826333333 12.8466066333333 12.9454412416667 13.4152475666667 12.549239125 12.9345571083333 13.1648749166667 12.1524570666667 12.8659456416667 12.7873340416667 12.7446559166667 12.4566311333333 13.07608845 12.9135039416667 13.6712789833333 12.8103459166667 13.2366307916667 13.560618 13.2366307916667 12.91648245 13.1222215416667 12.898949225 13.472886275 13.2741790333333 12.4659857583333 13.6053456833333 13.2325214 13.3080989916667

LINC02785 10.7688333416667 10.6757072916667 10.979276575 11.6510632333333 11.1909946916667 10.7343611166667 11.4637255666667 10.887311375 11.2248872 10.955147375 11.2851728 11.3258265916667 11.2481652416667 10.615238775 10.5389249166667 10.5633610083333 10.4088283666667 10.8122935583333 10.4531891333333 10.5410668333333 10.471680725 10.23249925 10.4331234333333 11.0058729583333 10.6266397833333 10.61156515 10.6079206166667 10.711383025 10.7382525333333 10.632572825 10.6040700666667 10.6231101333333 11.0996982166667 10.9656709666667 10.6552609 10.6671034666667 10.8853541583333 10.746282975 10.7810321916667 10.7243055 10.5841065666667 10.4902295666667 10.4921560416667 10.5841065666667 10.4829935916667 10.1675292083333 10.011252925 10.4829935916667 10.0335859083333 10.833996275 10.8010096583333 10.935752775 10.8122935583333 10.613486775 10.756939325 10.36490795 10.5841065666667 10.5464850833333 10.5185258166667 10.4088283666667 10.9038566 11.0447636416667 10.7591979083333 11.123158875 10.6671034666667 10.682010725 11.03050155 11.1498746333333 11.0740682333333 11.0924777416667 11.1274628333333 10.9747287666667 11.1627602916667 10.7670533583333 11.0447636416667 10.7365289583333 10.927189875 10.591906075 10.7092656083333 10.7153714 10.4866972 10.4400962333333 10.81892915 10.4884789583333 10.955147375 10.881139625 11.2613206166667 11.1909946916667 11.0376671166667 10.4316753833333 11.019401025 10.8363480833333 10.8271408 10.7895709 11.3282592416667 10.8910141166667 10.9379330333333 11.0262795583333 11.1809665916667 10.595444475 10.4959997583333 11.0651359666667 10.7591979083333 10.508878025 11.0103111083333 10.61685815 10.0973630583333 10.7283400416667 10.229166925 10.5504866333333 10.6613003916667 10.756939325 10.69605555 10.5410668333333 10.9656709666667 10.6286306666667 10.5560371666667 10.869829 10.6020413 10.8010096583333

SNHG14 11.55000185 8.56203936666667 8.36872518333333 7.42945980833333 7.67772895833333 8.53473379166667 7.09583709166667 8.42421578333333 7.77912510833333 7.94397781666667 7.52905674166667 10.219194075 9.35376294166667 8.13598734166667 8.43640146666667 8.5644013 9.30083194166667 7.48641575833333 9.51389508333333 9.16111019166667 8.42525675833333 7.84339445 8.43311395 8.60414634166667 9.0818604 11.1409951666667 8.98615453333333 8.64052551666667 8.37465215 8.68193833333333 9.37471118333333 8.52045571666667 8.434168925 8.32474756666667 11.4272823833333 11.3208587916667 8.04867420833333 8.49932775 7.738549275 8.53473379166667 8.82622243333333 8.85363625833333 8.922675075 9.26153165 9.54668399166667 9.06192556666667 8.93346260833333 7.77912510833333 8.37884899166667 8.66713059166667 8.226410775 8.12103859166667 7.324744075 7.918433075 7.65036194166667 8.700744525 8.16912173333333 8.42224619166667 8.83649291666667 8.95205770833333 7.96052405 7.162304025 7.62092879166667 5.39466070833333 10.0349403166667 6.05506278333333 7.684224025 6.80987979166667 4.44750469166667 6.19099196666667 5.75676621666667 6.39786465833333 4.82853423333333 6.78424304166667 6.17554210833333 4.93610370833333 6.98061263333333 7.05282128333333 5.31623470833333 6.3554652 5.82603316666667 8.698399275 5.59815450833333 6.817034675 5.8690931 7.13734699166667 9.26648069166667 6.13600023333333 8.01671704166667 8.18103791666667 5.68514503333333 5.23342923333333 7.63212294166667 6.26791573333333 5.88131005 6.09816255 8.76400390833333 7.83074816666667 4.533885625 5.81263556666667 6.31344151666667 5.629363625 6.71961385 6.76567473333333 6.39250838333333 5.69187064166667 7.03226014166667 6.6227255 7.0250185 7.25894705 6.79191158333333 6.29775806666667 4.76862936666667 7.16885323333333 4.7677709 5.39946159166667 9.346276075 4.98704916666667 8.14748886666667 6.17790808333333

GORAB-AS1 7.4747897 7.65118818333333 7.8730469 7.58968316666667 7.743945075 7.60982945833333 7.80699265 6.999816375 7.6124014 7.919306 7.60801480833333 7.84066634166667 6.90726410833333 7.23501408333333 7.06801993333333 7.033226925 6.96632546666667 7.46633813333333 7.48965994166667 7.24756645 6.82657379166667 6.686388325 7.31362185 7.58719245833333 7.42277753333333 7.065634 7.18077985833333 7.17208088333333 7.3553377 7.46158223333333 7.60982945833333 7.70840925 7.69539535833333 7.982277475 7.526619475 7.65036194166667 7.27051901666667 7.95063335833333 7.80960054166667 7.64163629166667 7.73683083333333 7.30787071666667 7.60185643333333 7.74760565833333 7.50284468333333 7.432730975 7.43866366666667 7.4984921 7.75393954166667 7.41496745 7.72346068333333 8.00421275833333 7.52499111666667 7.774503075 7.439501125 7.41840136666667 7.75201335 7.967964725 7.51511816666667 7.2687109 7.63703748333333 8.0070051 7.51062641666667 7.97263941666667 7.52334426666667 7.743945075 7.92396325833333 7.57468748333333 8.04062745 8.28258308333333 7.96148 8.03118996666667 7.50911594166667 7.52905674166667 7.42277753333333 8.31305474166667 7.20338010833333 7.35121041666667 7.666066675 7.56764245 6.643638875 6.85141578333333 7.7430897 8.957126925 7.68954538333333 7.22915068333333 7.404027575 7.58606695833333 6.65069284166667 7.596908525 7.82133015 8.15639794166667 7.456370675 7.95883029166667 7.81329221666667 7.81050794166667 7.46975303333333 7.724325925 7.953283475 7.71373668333333 8.10558914166667 7.934282375 8.01279515833333 7.79633564166667 7.84339445 7.657622575 8.438679025 7.57001779166667 7.947814825 7.65472984166667 7.67103138333333 7.69622019166667 7.8759545 7.93342214166667 8.45362333333333 7.846293475 7.82427053333333 8.63691878333333 7.80597976666667 7.546385025

AP002358.1 4.77715155833333 5.90489263333333 5.65650474166667 6.48159381666667 5.782225425 5.40462085 5.825172325 5.362066125 5.95899999166667 5.490209475 6.16284539166667 6.02811239166667 5.85516045 5.89039591666667 5.24850139166667 6.14095060833333 5.22298730833333 6.53188953333333 5.02553525 6.03493583333333 6.53588259166667 6.08184469166667 5.29209124166667 6.58062053333333 6.42337751666667 5.54304493333333 5.41537346666667 6.338904975 5.4338253 5.56030313333333 5.38092105 6.3807528 5.56118513333333 5.21672184166667 5.29908005 4.99799986666667 5.798958825 5.858605475 5.55545125833333 4.42100515 4.9375826 6.1530748 5.451907275 5.13063719166667 6.29775806666667 6.12347004166667 5.00204984166667 4.60880553333333 5.903354775 6.949750225 6.7213753 6.01562348333333 5.50030605 4.89323034166667 4.66568316666667 4.45973163333333 4.93610370833333 4.71903925 5.41291893333333 6.50900563333333 4.79605449166667 6.20696713333333 6.57126265833333 7.50737338333333 4.990530025 6.4089881 6.40823279166667 6.55465186666667 7.27360786666667 5.95981851666667 6.48552058333333 5.96262355 6.957621225 6.25988588333333 5.13457766666667 5.4674584 6.55371424166667 6.26236639166667 5.58504371666667 6.37517364166667 5.27561860833333 5.54669463333333 6.231190225 5.941144225 5.30776938333333 6.089108925 6.41696196666667 6.06424056666667 6.061018125 5.74762156666667 6.02062880833333 6.72921199166667 5.00125610833333 5.96431428333333 5.92530963333333 5.48272485833333 7.04208126666667 5.170905675 6.22569964166667 4.48038379166667 4.75272380833333 6.14425859166667 4.49348375833333 4.70268088333333 6.779595125 5.67701044166667 5.73081749166667 5.51827296666667 4.08791583333333 6.65373279166667 6.727504025 5.06353930833333 6.02236253333333 5.26880633333333 5.28772790833333 5.18970676666667 5.362066125 5.82021451666667 5.17730950833333 5.486081725

STEAP2-AS1 8.86909838333333 6.662197225 6.58894740833333 6.92711743333333 7.26970906666667 7.50024405833333 6.43748905 6.97660320833333 6.873624625 7.08679770833333 6.80051309166667 8.480972975 7.48387143333333 7.63138205 7.27688146666667 7.36967581666667 7.63703748333333 6.72283928333333 7.67952253333333 7.40534635 6.37921816666667 7.21256346666667 7.19199751666667 7.85638375833333 7.509871575 8.6708431 7.76828564166667 7.381252125 7.21256346666667 7.82133015 7.62747078333333 7.63412013333333 6.87454878333333 6.05093405833333 8.602930325 8.710105575 7.3846807 7.69622019166667 6.850638675 7.56683574166667 6.80909233333333 7.35849015 7.32899926666667 7.32306280833333 8.18906878333333 7.080264325 7.65656303333333 6.797326975 6.730005225 7.28806855833333 6.62575970833333 6.98625575 6.86400539166667 7.63984555 7.05461925833333 7.95793864166667 7.08371145 6.930359225 8.01671704166667 7.46158223333333 9.05168943333333 8.30672025 8.16501965833333 6.50987625833333 10.14430765 8.19889111666667 5.53956186666667 6.3840179 5.97300641666667 6.3125157 6.44981341666667 7.342513875 6.436652525 7.19580808333333 6.53500695833333 5.38263980833333 7.23667846666667 7.53158344166667 7.62671015833333 6.11361575 9.94865183333333 10.1998690333333 8.100636025 7.70315380833333 6.80051309166667 9.81335845 9.44441939166667 5.3549373 7.98897931666667 8.51932215 7.64493035 6.73733449166667 7.39488855 6.781139 6.33336359166667 8.69251304166667 7.23347658333333 8.77853785833333 6.1871742 6.75017773333333 8.57406105 7.51511816666667 7.88851164166667 7.29318128333333 6.87763275 6.23943730833333 7.05461925833333 6.29539721666667 7.02899554166667 6.73498085 7.15690719166667 8.13598734166667 6.71477854166667 7.21336981666667 6.94156313333333 6.28684615833333 11.8881455083333 6.078446575 7.00742828333333 8.05052155833333

AC009522.1 4.825056575 3.98974825 4.01435405833333 4.37819051666667 4.73200895 5.25020996666667 4.60880553333333 4.88341414166667 4.37739820833333 4.61276948333333 4.68742364166667 5.07579073333333 4.58235359166667 4.61046104166667 4.7289146 4.420146625 5.677845275 4.06485985 5.430247225 4.56122548333333 4.13943409166667 5.36382531666667 4.62559538333333 6.44981341666667 5.78802209166667 4.022339075 5.52066971666667 5.42340053333333 4.67234389166667 4.36632163333333 4.73285785 4.94495284166667 4.55798421666667 5.185805625 4.90681635833333 4.568704925 4.70568649166667 5.57787028333333 4.69199204166667 4.872797375 5.22110318333333 4.6432372 5.01436615833333 5.78381885833333 6.06424056666667 5.296452375 5.38175511666667 5.86997613333333 4.70017524166667 5.03387344166667 5.02165799166667 4.58001249166667 4.5391327 6.18798263333333 4.80348209166667 6.08504715 5.84838258333333 5.44548114166667 5.04401789166667 4.8899011 4.38619328333333 4.77029946666667 4.92694636666667 4.35517875833333 3.99860775833333 4.53985004166667 4.01510810833333 4.71282696666667 4.59742191666667 4.54651299166667 4.37661541666667 5.48196351666667 4.80927436666667 4.48259481666667 4.42684748333333 3.97569303333333 4.15088759166667 4.35888175 4.09588705833333 4.24594024166667 4.52066123333333 4.66132636666667 4.18671215 4.83614788333333 4.27746985833333 4.83518115833333 5.56118513333333 4.72071955833333 4.62712253333333 4.29154165833333 4.93202185 4.398049275 4.535365525 4.45532334166667 4.05470664166667 4.43284685833333 4.0672716 5.22733176666667 4.72389136666667 5.152649175 4.89586528333333 5.43201638333333 4.86534405 5.54304493333333 4.47313854166667 4.959236325 4.70568649166667 6.46895845833333 4.32263094166667 6.753505175 4.77485674166667 5.3154224 4.49140346666667 5.728098775 4.857936675 4.58815271666667 4.42606766666667 4.6372952 4.67862661666667 4.784017825

LINC01918 8.50568134166667 8.35414590833333 8.39480550833333 8.01467125 8.09138268333333 8.59015729166667 8.51021659166667 7.92035708333333 8.42317835833333 8.20827265833333 8.48644526666667 7.89192915 8.16501965833333 8.80018586666667 8.12977375 8.13598734166667 8.273401925 8.10558914166667 8.51239310833333 8.02524268333333 7.82602878333333 7.89268988333333 8.485317225 7.94397781666667 8.02524268333333 8.237670825 8.981140225 8.27232419166667 8.323692025 8.431034975 8.53241509166667 8.33417815 8.19889111666667 8.46035811666667 8.33626970833333 8.19889111666667 8.29000288333333 8.48644526666667 8.20718200833333 8.371635775 8.25133669166667 8.42754885 8.61454506666667 8.1801409 8.206103275 8.67320644166667 9.04108516666667 8.70210218333333 8.08263128333333 7.98053493333333 8.485317225 8.41158728333333 8.01182280833333 8.790433125 8.30985033333333 8.27653735833333 8.017646775 8.7363942 8.22833255833333 8.371635775 8.65575711666667 8.63814489166667 8.43778974166667 8.08470013333333 9.2176707 8.44184676666667 8.66200190833333 8.47657969166667 8.81697240833333 8.62537700833333 8.52145965 8.80865615 8.29333656666667 8.55690941666667 8.62322813333333 8.45706139166667 8.44688928333333 8.47224078333333 8.029339375 8.25674673333333 8.97362839166667 8.70444308333333 8.36432383333333 9.08861136666667 8.33973274166667 8.50235230833333 8.33859384166667 8.31719618333333 8.1268421 8.36335646666667 8.62537700833333 8.68542795 9.042386175 8.77122099166667 8.81089155833333 8.41601444166667 8.05732331666667 9.01843699166667 8.72160276666667 8.83012083333333 8.993454275 8.45362333333333 9.24949425833333 8.66334255 8.58080123333333 8.53935796666667 8.82891991666667 8.87851369166667 8.69607195 8.98615453333333 8.33417815 9.96147485833333 8.66964434166667 8.698399275 8.36872518333333 8.43311395 8.98366746666667 8.96720605 8.69018435 7.99764143333333

AL024497.1 6.79964733333333 6.93662556666667 5.6927165 5.64366626666667 6.338904975 5.06597111666667 6.77811300833333 5.75992465 6.16676128333333 6.64536209166667 6.11513095833333 6.90356878333333 6.44903994166667 6.32765196666667 5.92772035833333 5.7939183 6.16848116666667 6.46187091666667 6.035840925 6.32934243333333 5.826857975 5.986172625 7.52253811666667 7.44810904166667 7.237579325 7.08285123333333 5.80207481666667 5.52235776666667 6.14268099166667 5.67018873333333 6.50361413333333 5.58782286666667 5.53149350833333 6.182879225 5.61505438333333 6.02663899166667 4.52712630833333 6.49790895 6.50428851666667 7.88541965 6.17288374166667 6.22881789166667 6.44981341666667 5.99187126666667 5.761496775 6.34768783333333 6.36215745833333 7.00675840833333 4.48927336666667 5.693616325 5.47950365833333 6.65857890833333 6.65697400833333 7.43109864166667 5.85351849166667 5.3154224 6.29539721666667 6.316535975 6.29466304166667 6.97415298333333 5.91774480833333 6.19938790833333 5.13539786666667 5.75278305833333 5.228162675 6.345042725 4.264237 5.52066971666667 4.77715155833333 4.81864974166667 5.79569219166667 5.42769671666667 6.93279296666667 5.603294875 5.67018873333333 4.01991520833333 4.7450973 4.19890045 6.54993728333333 4.06889863333333 5.82778305833333 5.96607250833333 6.09733190833333 5.54304493333333 5.43604663333333 5.91597829166667 4.551878575 4.58235359166667 4.93534804166667 5.01846583333333 5.941997275 5.43787004166667 6.95909085 5.35232301666667 4.435737425 3.71522014166667 4.70017524166667 4.83426329166667 5.74227275833333 5.9244919 6.35158149166667 5.48441135833333 4.61767011666667 5.01921390833333 5.68767705833333 4.44597125 5.84743648333333 6.38460903333333 5.15933271666667 6.61643335 4.58741798333333 5.38175511666667 4.8899011 5.99273749166667 5.29375345833333 4.6241475 3.81801135833333 4.44750469166667 4.5762417 5.92279354166667

AP002026.1 13.5773436583333 12.6460873916667 12.228644125 12.215507725 10.2308420666667 12.5560529333333 12.7810968666667 12.578037425 12.4540279833333 13.7009408083333 11.4565596333333 13.472886275 12.9025226333333 12.906342175 11.6063160416667 13.53902465 14.7261095416667 10.5860666166667 11.7482500666667 13.0493833666667 13.2450601083333 10.176104 13.1030363083333 13.1309825583333 13.5493478666667 13.5553000916667 12.9970547416667 12.8466066333333 11.2685648583333 12.9856740416667 12.5079814083333 12.630386725 13.2243581083333 14.0270179 13.3711706833333 13.3856220583333 12.2399882333333 13.4927346333333 12.0287220416667 12.869508175 12.5050308083333 13.041627725 13.2905536833333 12.9644897666667 13.4629723583333 13.110422675 13.083804375 11.9409533916667 13.3482488416667 12.9970547416667 12.3920642833333 10.65000145 12.0824665333333 11.125139725 12.43965125 12.8332414833333 12.83676285 12.8041650666667 13.4927346333333 13.8056241916667 8.89185349166667 7.16143308333333 11.6135579916667 7.55321126666667 12.0957082333333 6.34047511666667 9.03716685 8.88320966666667 5.61593795 8.8207743 9.6633032 5.941997275 5.31195595 5.958371875 6.696380025 6.11513095833333 10.422532775 11.1047284583333 5.761496775 8.33307035833333 6.05174113333333 6.7441857 6.27774009166667 6.09491578333333 9.01353759166667 9.85108371666667 7.0570133 8.698399275 9.03092490833333 10.0206561666667 5.63585205833333 5.87646783333333 7.57549880833333 7.931709675 6.781139 7.84438765 6.4089881 7.41333261666667 5.62083628333333 10.0395367833333 7.96236178333333 6.6279805 6.94156313333333 10.9530714833333 6.674888925 8.53357196666667 8.25567084166667 7.91466469166667 7.21758313333333 9.12586665833333 10.8054366083333 8.75197200833333 5.24598960833333 6.02159813333333 8.63814489166667 11.857596825 7.86295999166667 6.53500695833333 8.61968489166667 10.9836119583333

AL589740.1 9.31654956666667 6.9853877 6.98701886666667 6.088336625 7.23347658333333 7.84066634166667 6.696380025 7.10578959166667 7.18794728333333 7.92575334166667 6.91684939166667 8.12491806666667 7.99469491666667 6.9918604 7.41333261666667 6.16130541666667 6.85293710833333 6.74949993333333 7.52334426666667 6.512400125 6.47279708333333 6.20090414166667 6.88611070833333 6.84740460833333 7.47322088333333 9.40775390833333 7.61757204166667 6.298647425 6.88682719166667 6.6189581 7.20936795 6.63203986666667 7.13266018333333 6.44040244166667 9.94714953333333 9.81636959166667 7.181557425 7.6625944 7.60801480833333 7.91104394166667 8.01371644166667 7.397338075 7.20762451666667 8.01861823333333 7.9017682 7.2687109 7.456370675 6.9521716 6.92952805833333 7.04433776666667 7.63038558333333 7.59330443333333 6.62575970833333 6.55221608333333 6.88538973333333 7.64579728333333 7.05534445 7.352034975 7.072020975 7.09835705 6.314219375 6.723589425 6.24109811666667 6.8359128 7.51141074166667 7.1228027 7.75020911666667 6.47042564166667 7.76222746666667 7.45898616666667 6.78424304166667 5.83677235833333 6.864759375 6.75196500833333 6.40727721666667 6.09080005833333 7.1066557 6.91433144166667 7.12784525833333 5.72631065 6.12262255833333 5.99448725833333 6.94799274166667 7.24174676666667 6.78272669166667 6.49344723333333 6.64404768333333 7.00978536666667 5.04647511666667 7.4079756 6.643638875 6.95519420833333 6.48793390833333 7.34734821666667 6.96632546666667 5.82603316666667 6.36790351666667 7.30198646666667 7.134124875 6.883226375 7.45238604166667 5.958371875 6.817034675 7.291524925 6.65069284166667 6.17554210833333 7.36412529166667 6.68855885 7.5199474 8.262883775 7.37382095 7.138237575 7.72255213333333 7.363485725 6.51900598333333 5.83937101666667 6.75605898333333 8.06902854166667 6.78888389166667 7.24674725

AC027088.2 7.47026058333333 8.45975162083333 8.76710452916667 8.71874420833333 9.17712503333333 8.1127145625 9.819894575 9.41534600833333 8.72595576666667 8.541223825 9.36150873333333 10.1534230041667 7.91796035833333 8.6081336 8.0151561125 8.019107975 8.28632147083333 8.1178737875 8.17954419166667 8.37933141666667 7.81752533333333 7.46189963333333 8.21888479166667 7.66483405416667 8.22783144583333 7.378379625 8.43823438333333 7.92710977083333 7.87165699166667 7.8995164375 8.23602964166667 8.35687932916667 8.5129838375 8.7754211875 6.84633645 7.22436959583333 8.26136517083333 8.52095768333333 8.84685069166667 8.72595576666667 8.5153910375 8.1587090625 8.4889676875 8.292755375 7.70888191666667 8.27286305833333 8.00187925833333 8.05692022916667 7.78487938333333 7.54680782916667 8.46830984583333 8.02684494583333 8.71874420833333 8.55278529166667 8.4672345625 8.9305219 8.2894864125 8.66524424583333 7.77405561666667 7.95566207083333 7.57228249583333 8.124501525 7.45527234166667 8.09097087083333 8.82680041666667 7.67383879166667 8.07600020416667 8.629392475 7.87649069583333 8.55391102916667 9.55881407916667 9.0795260875 8.4793026875 7.87165699166667 7.50069094166667 7.57509314583333 7.72713566666667 7.18751969166667 7.76173494166667 7.86889685416667 7.4248928 8.73826393333333 7.389323825 7.251231075 7.3843125 8.00187925833333 8.04324084166667 6.4309010625 8.503949475 7.36062235416667 7.61535945416667 7.278958075 7.1435187125 8.027734925 7.5620857625 8.15092204166667 7.5423965125 7.52704737916667 8.0229828125 8.57126145416667 7.77056238333333 8.09181145 7.695807775 7.36694697083333 7.36216055 7.53735972083333 7.60484185833333 7.18666509166667 7.361397575 6.975415925 8.14792829166667 8.0151561125 7.10914326666667 8.470587425 7.9894762625 7.6696297125 9.57120983333333 7.46933042083333 7.64899285 8.4453242125

EWSAT1 7.47026058333333 8.45975162083333 8.76710452916667 8.71874420833333 9.17712503333333 8.1127145625 9.819894575 9.41534600833333 8.72595576666667 8.541223825 9.36150873333333 10.1534230041667 7.91796035833333 8.6081336 8.0151561125 8.019107975 8.28632147083333 8.1178737875 8.17954419166667 8.37933141666667 7.81752533333333 7.46189963333333 8.21888479166667 7.66483405416667 8.22783144583333 7.378379625 8.43823438333333 7.92710977083333 7.87165699166667 7.8995164375 8.23602964166667 8.35687932916667 8.5129838375 8.7754211875 6.84633645 7.22436959583333 8.26136517083333 8.52095768333333 8.84685069166667 8.72595576666667 8.5153910375 8.1587090625 8.4889676875 8.292755375 7.70888191666667 8.27286305833333 8.00187925833333 8.05692022916667 7.78487938333333 7.54680782916667 8.46830984583333 8.02684494583333 8.71874420833333 8.55278529166667 8.4672345625 8.9305219 8.2894864125 8.66524424583333 7.77405561666667 7.95566207083333 7.57228249583333 8.124501525 7.45527234166667 8.09097087083333 8.82680041666667 7.67383879166667 8.07600020416667 8.629392475 7.87649069583333 8.55391102916667 9.55881407916667 9.0795260875 8.4793026875 7.87165699166667 7.50069094166667 7.57509314583333 7.72713566666667 7.18751969166667 7.76173494166667 7.86889685416667 7.4248928 8.73826393333333 7.389323825 7.251231075 7.3843125 8.00187925833333 8.04324084166667 6.4309010625 8.503949475 7.36062235416667 7.61535945416667 7.278958075 7.1435187125 8.027734925 7.5620857625 8.15092204166667 7.5423965125 7.52704737916667 8.0229828125 8.57126145416667 7.77056238333333 8.09181145 7.695807775 7.36694697083333 7.36216055 7.53735972083333 7.60484185833333 7.18666509166667 7.361397575 6.975415925 8.14792829166667 8.0151561125 7.10914326666667 8.470587425 7.9894762625 7.6696297125 9.57120983333333 7.46933042083333 7.64899285 8.4453242125

TESC-AS1 6.90274688333333 6.79889878333333 6.587379025 6.40973950833333 5.87732999166667 6.3159244 5.46922286666667 6.36215745833333 6.14268099166667 6.46408151666667 5.66848611666667 5.556241925 5.09997055 6.686388325 6.51742765 6.6383855 8.16603721666667 5.05778576666667 7.60609320833333 6.05245123333333 6.25070120833333 6.91047501666667 7.62178835 7.49427225833333 6.9521716 6.92234795833333 7.43541391666667 7.0250185 6.54453155833333 6.91684939166667 7.35359963333333 6.84822129166667 6.25216388333333 6.85433584166667 7.33109959166667 6.96698138333333 6.26630801666667 6.99014773333333 6.54215274166667 6.89860500833333 6.98940225 7.35121041666667 6.93279296666667 7.60801480833333 6.74572325 7.39651405 7.54201473333333 6.4711885 7.02662889166667 6.45429151666667 6.069998 6.85232785833333 5.327405775 6.26464641666667 6.80223084166667 7.36658465833333 6.38929764166667 7.02813663333333 6.614798975 7.017380525 5.87911255833333 5.805323425 6.05752005 5.34143435833333 7.17775916666667 6.03818995833333 6.16676128333333 4.95061065 6.60666248333333 5.38446963333333 5.49108314166667 6.70772591666667 4.307075925 6.05506278333333 5.85603955 5.00789455 6.04599496666667 5.40722773333333 6.71961385 5.86830491666667 5.1467782 6.06586204166667 7.35849015 7.00591673333333 4.78638856666667 5.47447824166667 5.57701794166667 4.525625675 6.45714978333333 6.56179321666667 6.80453016666667 5.78556079166667 5.84838258333333 6.397067625 6.0000734 5.97703591666667 5.92530963333333 6.22569964166667 5.729929025 6.75784235833333 5.553828975 6.11597780833333 6.492675575 7.159138825 6.57456723333333 5.547570375 6.49720533333333 6.231190225 6.90111271666667 6.57608009166667 6.22806264166667 6.37447095 5.7939183 6.77811300833333 4.37819051666667 6.1401869 6.03894880833333 5.09997055 7.011414375 5.06686440833333

RFPL3S 9.05935188333333 9.05935188333333 9.26648069166667 9.14824449166667 9.31802425 9.323428375 9.75113850833333 9.26648069166667 9.51090269166667 9.00075389166667 9.44548651666667 9.59565308333333 9.24510093333333 8.74401624166667 8.66457645 8.545802675 9.14303199166667 9.07590878333333 8.95972230833333 8.87463969166667 8.8207743 8.72775770833333 8.82317351666667 8.81826778333333 9.14450013333333 9.11422543333333 8.74523575833333 9.136244075 8.75767668333333 8.91416151666667 8.95454636666667 8.70788464166667 9.05803846666667 8.957126925 9.34901544166667 9.124545075 9.22567063333333 9.101427925 8.90815406666667 9.05059795833333 8.75197200833333 8.45487785 8.900308075 8.80995673333333 8.53593740833333 8.24820143333333 8.5427737 8.439601225 8.918056 8.81562710833333 8.85363625833333 9.02591655 8.386019225 8.49279463333333 8.73370975833333 8.45130473333333 8.75767668333333 8.81452744166667 8.63572139166667 8.72413925 9.36501691666667 9.30212978333333 9.30352410833333 9.345108925 9.10274634166667 9.120779125 9.22186990833333 9.415981525 9.30482685833333 9.29669200833333 9.91018748333333 9.55070993333333 9.48684088333333 9.03716685 9.04108516666667 9.03833616666667 9.20264188333333 8.70444308333333 9.16242148333333 8.88575565 8.52145965 8.89185349166667 8.922675075 9.10274634166667 9.02711425833333 8.85112088333333 9.02711425833333 9.0697895 9.56775843333333 8.81452744166667 9.13898106666667 9.17152996666667 9.40927853333333 8.8870824 9.27391955833333 9.538153975 9.03966899166667 9.05935188333333 9.412078125 8.9320964 9.54668399166667 8.95579018333333 8.96984509166667 8.87730315833333 9.011109275 8.787979925 8.70210218333333 8.63572139166667 8.9709554 8.658204375 8.94047908333333 8.96238550833333 8.787979925 8.82622243333333 8.70899691666667 8.63323710833333 8.69140489166667 9.158641175 9.38637963333333 9.06555709166667

AC012184.3 12.405501075 11.8389506833333 11.9662069916667 12.0072618083333 12.37994665 12.20732285 11.5406276416667 11.9038701666667 12.1312062333333 12.215507725 12.43015245 12.0508303333333 12.0587078333333 12.1870383833333 12.248342825 12.0933481 12.9135039416667 12.0587078333333 12.2895761166667 12.1467373 12.210187275 12.2649867083333 12.2596429833333 12.1870383833333 12.368506525 12.6718910166667 12.2676890666667 12.0587078333333 12.40033825 12.309527875 12.1524570666667 12.2923180416667 11.933125475 12.2785617166667 12.5423167666667 12.684736675 12.2046199416667 12.0957082333333 12.0824665333333 12.2895761166667 12.1415417666667 12.16080175 12.0365429083333 12.215507725 12.5079814083333 12.3569700083333 12.3861826333333 12.176601525 12.210187275 12.1467373 12.038996075 12.0559423166667 12.6425501583333 12.5361294916667 12.5234224083333 12.3659453666667 12.223369875 12.2399882333333 12.309527875 12.233818175 13.1897357416667 11.6648204916667 12.351399425 11.6740143583333 12.280903925 12.69765135 11.9966241166667 12.14966815 12.2730614916667 12.1443252916667 12.2867892916667 12.37994665 12.174234975 12.26263345 12.9454412416667 12.0365429083333 12.1100895416667 12.43663495 12.4811828916667 12.46882785 11.78261185 12.2568953 12.2426995166667 11.97656585 12.5560529333333 12.2456214083333 11.2566977833333 12.5903679416667 12.60024735 12.40307625 11.8779418166667 11.8177846 12.3829579166667 12.7412194083333 12.451622875 11.7948078416667 11.9199419 12.01526755 12.283625975 11.8676643916667 12.8841629166667 11.69147735 12.8432917666667 12.048574475 12.4776177416667 13.083804375 12.7038641666667 12.3544155583333 12.877352 12.248342825 12.0174377083333 11.9940832 12.4240585416667 12.2046199416667 12.511227025 12.20732285 12.26263345 12.001763825 12.0904755416667 12.10426585

NAMA 5.52146069166667 6.60970920833333 6.26791573333333 4.78488421666667 6.40115713333333 5.10162569166667 6.859502875 6.79655045 6.768468075 4.88416340833333 6.1458196 6.63999905833333 6.78815289166667 5.38864603333333 5.47086664166667 6.608946875 5.6623785 6.44040244166667 5.09815425833333 6.63281199166667 6.63521010833333 6.22658108333333 5.51827296666667 4.76862936666667 5.13217864166667 5.38687351666667 5.33192491666667 4.88164143333333 4.72071955833333 5.70965025833333 4.93202185 4.98704916666667 5.83518768333333 5.82105976666667 6.21087870833333 5.23750470833333 4.65893614166667 5.553828975 5.23342923333333 5.13457766666667 5.10427118333333 4.35959019166667 5.364779525 5.715067925 4.56640000833333 5.28167781666667 5.10925536666667 5.16271053333333 4.7385841 5.903354775 5.513669625 4.987974525 6.70095648333333 5.39874103333333 5.95331914166667 5.26963456666667 5.25605979166667 4.97881760833333 5.332832325 4.92610205 6.86168069166667 6.67714320833333 5.34230098333333 4.7809755 5.94836456666667 4.62559538333333 6.74949993333333 6.9183608 4.84720510833333 5.42340053333333 5.866627925 6.47356659166667 7.02662889166667 5.28680903333333 6.74043675833333 6.58438846666667 4.92102411666667 4.77715155833333 5.34487673333333 6.17367879166667 5.98789045 5.88709410833333 4.519178925 4.843025575 5.580301925 5.7939183 5.22208751666667 6.438267225 6.514123925 5.04647511666667 5.16663731666667 5.15614551666667 4.222802625 5.36908168333333 5.39640219166667 5.41046095 5.42515045833333 5.03157779166667 5.151017225 5.499379 4.62007759166667 6.15066176666667 4.88258713333333 5.239299675 4.70268088333333 5.00632808333333 4.86450405 6.47279708333333 4.89159086666667 6.88170193333333 5.3495839 5.5707947 5.51827296666667 5.421489975 5.59815450833333 4.46178631666667 5.4370393 5.36908168333333 4.43284685833333 5.905668875

AC242426.2 8.206103275 9.30212978333333 8.82891991666667 9.23894454166667 8.05268059166667 11.4345196083333 8.52340118333333 9.25886408333333 9.26528025 9.01843699166667 9.65131303333333 9.50277188333333 8.31305474166667 9.3945379 8.675578375 9.6015193 14.4338157333333 8.85112088333333 8.35191391666667 9.183229075 9.43421313333333 9.74557269166667 9.87584709166667 10.260350775 9.68448639166667 8.9239143 8.91303145833333 9.07471798333333 8.17894748333333 9.06068016666667 8.36965201666667 8.71236105833333 9.24370573333333 13.1309825583333 8.54724403333333 8.27232419166667 8.75197200833333 8.77000449166667 8.70315873333333 8.43640146666667 8.85242150833333 9.346276075 9.37623116666667 9.64456304166667 9.43270350833333 9.41871168333333 9.71504586666667 8.56330485833333 9.24949425833333 9.88202425 8.5427737 8.84985913333333 9.07344920833333 9.574510875 8.93700485 9.74120920833333 8.87974589166667 8.05570605833333 9.32070566666667 9.44156545833333 8.76267615 8.25022860833333 9.610331375 8.05364296666667 8.95454636666667 8.07214884166667 8.47432581666667 8.42754885 7.14716405 8.17727110833333 8.47867590833333 7.848979625 8.78437034166667 8.700744525 8.62537700833333 8.45231269166667 8.53241509166667 8.18204448333333 8.12889401666667 8.79141695833333 8.74269300833333 9.00847981666667 10.0867346833333 7.75118009166667 9.4483016 7.89905374166667 7.239291275 8.55097769166667 7.318718375 8.79141695833333 7.57271753333333 6.723589425 7.91104394166667 9.17541009166667 7.952403425 6.3018823 8.05268059166667 6.80832605833333 8.01182280833333 8.444801925 8.78337874166667 8.20927199166667 7.39815565833333 9.936416725 6.5783546 8.62537700833333 8.40207273333333 8.02524268333333 8.28578650833333 8.62888506666667 8.86517486666667 6.9248352 7.1870921 8.24631605833333 7.84714276666667 8.39699265 7.49052953333333 5.93096713333333 8.18413825 8.70444308333333

LINC02839 4.98751184583333 4.4190647 4.2616389375 4.33297843333333 4.04968592916667 4.57049284583333 5.39512535416667 4.04634032083333 3.68856206666667 4.80220647083333 4.2083840125 4.876317675 4.18406696666667 4.179475575 5.19767359583333 4.03975270416667 4.85601532916667 4.146518825 4.8500567875 3.97096839166667 3.90994566666667 4.8732191125 4.44638094166667 4.78061298333333 6.95644487916667 5.18190851666667 4.82896159583333 5.3625774 4.74933793333333 4.5016396625 4.970233425 4.809706375 4.05435485416667 4.6123174875 4.46738564166667 4.47580400833333 4.72196850833333 4.99098900416667 4.68240405416667 4.96043188333333 6.22302179583333 4.572761225 5.107950425 5.88172093333333 4.25944933333333 5.37206293333333 5.2812010375 5.14886638333333 4.81204550416667 5.08298402916667 5.09428083333333 4.6618192875 4.2989040875 6.24067005416667 4.84511777916667 5.02128888333333 4.374684975 5.06806700833333 4.69010082916667 4.57507402083333 4.7500575625 5.47325072916667 5.60873559583333 5.98096047916667 4.93889044166667 5.43654296666667 5.054877525 5.46049224166667 5.12742854166667 5.0503892 5.3444952 6.49755714166667 4.96797017916667 4.34274152083333 4.92652420833333 4.485975925 3.79840604583333 4.99841429166667 4.98488643333333 4.14205799166667 5.26840000416667 4.86160037916667 5.35995105416667 5.74027289583333 4.15445039166667 4.76089545416667 5.23127163333333 5.9750407125 4.1350414625 5.03348041666667 4.33979760833333 5.54440451666667 5.107950425 5.1382688375 4.572761225 4.5962208875 4.86325480833333 4.6172840625 4.48384862083333 5.9288537 6.00457513333333 4.34963109166667 5.89971470416667 6.14626879166667 5.74544756666667 5.42727825 5.73446433333333 7.110687 5.61963400416667 7.30747715416667 5.6289692875 6.58248376666667 5.79012345833333 6.64811232916667 5.03768853333333 5.56958737916667 5.30031730416667 5.71193325416667 5.673988225 4.60071927083333

AC060765.2 4.98751184583333 4.4190647 4.2616389375 4.33297843333333 4.04968592916667 4.57049284583333 5.39512535416667 4.04634032083333 3.68856206666667 4.80220647083333 4.2083840125 4.876317675 4.18406696666667 4.179475575 5.19767359583333 4.03975270416667 4.85601532916667 4.146518825 4.8500567875 3.97096839166667 3.90994566666667 4.8732191125 4.44638094166667 4.78061298333333 6.95644487916667 5.18190851666667 4.82896159583333 5.3625774 4.74933793333333 4.5016396625 4.970233425 4.809706375 4.05435485416667 4.6123174875 4.46738564166667 4.47580400833333 4.72196850833333 4.99098900416667 4.68240405416667 4.96043188333333 6.22302179583333 4.572761225 5.107950425 5.88172093333333 4.25944933333333 5.37206293333333 5.2812010375 5.14886638333333 4.81204550416667 5.08298402916667 5.09428083333333 4.6618192875 4.2989040875 6.24067005416667 4.84511777916667 5.02128888333333 4.374684975 5.06806700833333 4.69010082916667 4.57507402083333 4.7500575625 5.47325072916667 5.60873559583333 5.98096047916667 4.93889044166667 5.43654296666667 5.054877525 5.46049224166667 5.12742854166667 5.0503892 5.3444952 6.49755714166667 4.96797017916667 4.34274152083333 4.92652420833333 4.485975925 3.79840604583333 4.99841429166667 4.98488643333333 4.14205799166667 5.26840000416667 4.86160037916667 5.35995105416667 5.74027289583333 4.15445039166667 4.76089545416667 5.23127163333333 5.9750407125 4.1350414625 5.03348041666667 4.33979760833333 5.54440451666667 5.107950425 5.1382688375 4.572761225 4.5962208875 4.86325480833333 4.6172840625 4.48384862083333 5.9288537 6.00457513333333 4.34963109166667 5.89971470416667 6.14626879166667 5.74544756666667 5.42727825 5.73446433333333 7.110687 5.61963400416667 7.30747715416667 5.6289692875 6.58248376666667 5.79012345833333 6.64811232916667 5.03768853333333 5.56958737916667 5.30031730416667 5.71193325416667 5.673988225 4.60071927083333

LINC01366 8.02733255833333 6.983731325 7.40534635 7.45486255 7.45333459166667 7.49513213333333 7.23578164166667 7.32203796666667 7.3839443 7.4639428 7.33429259166667 7.02737631666667 7.03837779166667 6.595923 7.61489635 7.03066510833333 7.6423852 7.52253811666667 7.51739635 7.13092705 6.78272669166667 7.02662889166667 7.21256346666667 7.26148395833333 7.703962625 7.4773374 7.529838675 7.60098480833333 7.40249240833333 7.92482581666667 7.74124910833333 7.76312330833333 7.72592023333333 7.822381675 8.25567084166667 7.725002775 7.74571144166667 7.89455625833333 7.89192915 8.00421275833333 7.57728179166667 7.80699265 7.860112175 7.58246949166667 7.72592023333333 7.934282375 7.8016903 7.92396325833333 7.525750625 8.04674969166667 8.065151125 8.16414461666667 7.73767701666667 7.845369325 8.034891475 7.88851164166667 7.746538075 8.14195365 7.696954725 7.63984555 7.54978125 6.87998445 7.54530115 7.25439505 7.24923051666667 7.746538075 7.71468463333333 6.982194575 7.84714276666667 7.48480789166667 7.23578164166667 7.44656456666667 6.83745388333333 7.04433776666667 7.48714180833333 7.1702859 7.21256346666667 7.53776969166667 7.648594075 7.12536183333333 6.78424304166667 6.63114244166667 7.42945980833333 8.3623863 7.4764098 7.14080546666667 7.28603906666667 6.96226191666667 7.02259469166667 7.46975303333333 7.76124241666667 8.26500145 7.60525565 7.80424109166667 7.931709675 7.91377156666667 7.46071238333333 7.87702689166667 7.79271326666667 7.928697775 7.55584700833333 7.558954 8.02061905 7.66918755833333 7.83433648333333 7.50206744166667 8.0765578 7.35278270833333 7.30198646666667 7.17775916666667 7.65558205833333 7.64163629166667 7.967964725 7.75769473333333 7.88646319166667 7.88934271666667 7.58246949166667 7.928697775 7.89018188333333 7.60801480833333

LINC01569 5.96876623333333 4.36632163333333 5.46824720833333 5.48441135833333 5.52817958333333 6.05827610833333 3.85340619166667 5.20324565 4.29927855833333 5.55202255833333 5.12345405833333 5.09577138333333 5.07088755 4.71903925 6.07927768333333 5.39466070833333 6.02897043333333 5.95496726666667 6.70536285833333 6.78346969166667 6.15706108333333 6.17880248333333 6.55765729166667 6.12262255833333 5.96431428333333 6.4089881 6.50724065 6.36881363333333 5.450343875 6.285311725 5.58111951666667 6.54993728333333 5.38175511666667 5.59994884166667 7.47399836666667 6.99264965 6.182879225 6.9982378 6.49433464166667 5.30328563333333 6.90726410833333 6.53343775833333 6.96989185833333 6.550654 5.93513793333333 6.6383855 4.613537925 4.18975846666667 6.68014729166667 6.54596676666667 5.34487673333333 5.63342111666667 5.62776474166667 5.71751655 5.27819885 6.07079043333333 5.44052959166667 6.141851675 6.42089168333333 6.4838525 7.02899554166667 5.296452375 8.23549675833333 5.87236445 8.273401925 7.20936795 5.72366834166667 6.0842744 7.28806855833333 6.14350423333333 5.00047423333333 7.138237575 6.01732366666667 5.35594408333333 5.87646783333333 5.77539118333333 7.48222535 7.37223949166667 5.885364375 8.09958200833333 7.36658465833333 7.96520641666667 7.70481934166667 6.57919131666667 5.17406135 6.77657375833333 7.06728804166667 5.26709018333333 6.55221608333333 6.26630801666667 6.170078 6.9982378 6.436652525 5.20060335833333 7.07767666666667 7.44478088333333 7.24833841666667 8.58559054166667 6.28846280833333 6.91520719166667 7.30708359166667 6.27123641666667 8.15235975 8.694797775 6.28684615833333 5.850901925 6.033075425 7.01432623333333 7.83885828333333 7.116464725 7.22830545833333 6.68251509166667 8.115340475 7.39488855 5.61924708333333 6.530441325 7.99469491666667 6.89860500833333 6.987780475 4.82938895833333

AL590822.1 10.219194075 9.9348273 9.93026274166667 9.68859575 10.3810533166667 9.81016945 10.2688247083333 9.4302854 10.2970698166667 10.4051703 10.17093795 10.126531775 10.2439545166667 9.54668399166667 9.67463734166667 9.04472984166667 9.43421313333333 9.89233125833333 9.42111243333333 9.32585686666667 9.60613141666667 9.65779833333333 9.26528025 9.54668399166667 9.862818575 10.041049 9.92891185833333 9.6015193 9.69865891666667 9.87299354166667 9.5341054 9.87885509166667 10.2813617166667 10.0692552166667 10.12322705 10.0053221583333 10.4810665833333 10.2030274 10.07558265 10.0723139833333 9.85252195833333 9.83458021666667 9.74259324166667 9.63464729166667 9.76226639166667 9.40271259166667 9.47971744166667 9.694321975 9.86115450833333 9.94865183333333 10.07558265 10.0738928916667 10.1998690333333 10.1378527416667 9.86849545 9.53278261666667 10.1216004333333 9.96321 9.96321 9.65427285 10.4190726416667 10.360229225 9.757653125 10.0559299333333 10.0206561666667 10.2753557916667 10.0252423 9.96147485833333 10.424538125 10.5633610083333 10.3775083083333 9.97701388333333 10.3371834416667 9.538153975 9.85953499166667 9.41328055833333 10.0559299333333 9.80276991666667 9.64606555833333 9.64872759166667 9.853830825 9.71257970833333 9.62279060833333 9.50133671666667 10.0738928916667 10.2354161583333 9.87885509166667 9.65131303333333 9.92405621666667 9.56068761666667 9.705649125 9.64606555833333 10.2736553416667 10.398066475 10.746282975 10.0943656833333 10.4331234333333 10.7092656083333 10.3479196583333 9.862818575 10.1278941 10.1138557666667 9.94714953333333 9.57749218333333 9.95043048333333 9.70973839166667 9.46065865 9.49887978333333 9.93793718333333 9.980296075 9.83620561666667 9.94385811666667 10.1020643 9.90140289166667 9.637761575 9.604456975 9.85108371666667 10.2536886083333 9.88643061666667 9.93314271666667

LINC00511 6.85141578333333 6.00748140833333 6.31344151666667 6.43516865833333 8.76400390833333 9.85953499166667 4.40993569166667 6.71093058333333 5.47283311666667 6.829893775 7.79543485 4.77418788333333 6.28768299166667 6.848966625 6.6050576 6.3840179 6.97331920833333 8.84259734166667 6.67944645 7.4754804 7.95418985833333 8.70788464166667 8.11218248333333 7.70935458333333 7.37550575 4.41362111666667 7.32393866666667 7.71898053333333 8.43217050833333 6.86637626666667 7.0339869 7.60442806666667 6.21003021666667 6.89790128333333 5.13539786666667 4.56782300833333 7.908397225 7.26312536666667 7.82133015 6.99658926666667 7.25894705 5.80207481666667 7.19508815833333 7.986151875 6.859502875 6.588167675 5.96521240833333 6.56633335 8.15235975 7.15037268333333 7.794547275 7.029863175 7.95522123333333 8.471128425 6.518184425 6.27541806666667 5.663186525 6.396357 7.69963058333333 6.662197225 11.390936775 7.080264325 9.980296075 9.84388436666667 6.683342625 10.2156287416667 7.953283475 7.84066634166667 7.00978536666667 7.45400806666667 8.41601444166667 7.24589293333333 7.32393866666667 6.7586539 8.857267525 7.363485725 8.84630949166667 9.59565308333333 8.99956494166667 8.08667318333333 6.48469505 9.74405148333333 9.10908699166667 6.95064391666667 8.27939851666667 7.1982511 5.23259858333333 7.81792510833333 10.1508267916667 8.31951934166667 7.107436475 8.80747489166667 9.35667674166667 7.51062641666667 9.70290516666667 8.20827265833333 9.63156273333333 7.94397781666667 9.508238925 9.45237763333333 8.02254479166667 5.94745686666667 7.046602975 7.87803565833333 8.68427383333333 9.10908699166667 8.97484515833333 8.28258308333333 7.188718125 9.02843820833333 8.65348820833333 8.40014016666667 10.1825522583333 7.79543485 8.103825425 7.7666378 7.565923075 9.31802425 7.908397225 6.683342625

RMST 4.56938536666667 4.26785038333333 4.03244335833333 3.90146899166667 4.436542725 6.53900623333333 4.4982908 4.765242025 4.79377419166667 5.4961009 4.179856225 5.48441135833333 4.84808040833333 4.46398463333333 4.49140346666667 5.80207481666667 7.77360815833333 4.96587955833333 5.797364775 5.09148784166667 4.13374673333333 5.33093755 4.525625675 6.43516865833333 6.09816255 4.91520124166667 4.26936089166667 5.70066640833333 5.20230749166667 4.50496470833333 4.36983928333333 4.24347425833333 4.70894576666667 5.54038896666667 5.40803009166667 6.848966625 4.21564994166667 5.47086664166667 6.453551925 4.50046304166667 5.05706839166667 4.72389136666667 4.74969148333333 5.06029869166667 5.61762540833333 6.1530748 4.548736875 4.329661925 4.3062682 6.69322991666667 4.70485328333333 4.92359158333333 4.60316775833333 6.21087870833333 3.99787199166667 5.10925536666667 5.02478369166667 4.42340415833333 6.53113324166667 5.39559 4.66817654166667 4.58741798333333 4.99470109166667 4.101417825 4.07877819166667 4.38704568333333 4.68025626666667 4.32821321666667 4.519178925 4.77563934166667 3.8566224 4.357435675 4.53840028333333 3.94394236666667 4.55329276666667 4.23287865 4.887437075 4.22805825833333 4.23746243333333 4.72810314166667 4.568704925 5.63342111666667 4.77563934166667 4.249713475 4.67467815 3.84868538333333 4.226560075 4.90276439166667 4.04680980833333 5.50122666666667 4.09521015 4.59510340833333 4.82938895833333 4.58536020833333 4.533885625 4.17164835833333 3.8855125 5.00720284166667 5.51462095 4.61432114166667 4.78566294166667 4.16618690833333 4.31074669166667 4.389988275 6.21495720833333 5.12190426666667 5.71751655 4.74020919166667 4.48927336666667 6.47511975833333 7.33659296666667 4.851367175 5.26111468333333 6.10052355833333 4.95601813333333 4.08184460833333 4.58674640833333 5.32501348333333 5.31195595 5.45934836666667

LINC00237 5.88961983333333 5.958371875 5.77706405 6.06931103333333 5.86830491666667 5.22496259166667 6.4602804 6.238638975 6.46821505833333 6.21730323333333 6.102165675 6.76771060833333 6.36555325833333 6.11282138333333 5.30937105833333 5.547570375 5.96876623333333 5.89039591666667 4.76218691666667 5.013582075 5.86830491666667 5.70965025833333 4.63651601666667 5.4786027 4.831688925 7.11263425 4.92610205 6.035840925 4.63272604166667 5.77366633333333 5.11752969166667 4.6192723 5.64279376666667 5.48780954166667 5.93685915833333 5.87236445 4.70109015833333 5.76067670833333 6.40973950833333 4.94978725 5.56030313333333 6.16848116666667 4.67637581666667 5.22032525 5.90988236666667 5.1451327 4.79377419166667 5.95331914166667 4.58741798333333 5.21185403333333 5.01921390833333 5.26880633333333 6.061018125 5.13698225 5.345943725 5.11649993333333 5.84743648333333 4.85639045833333 5.30168844166667 5.77706405 6.06269363333333 7.27458531666667 5.99273749166667 7.15755769166667 6.06177335833333 5.52726914166667 6.39956296666667 6.81539655 6.78424304166667 6.27774009166667 6.529771875 6.87602855 6.897050375 6.512400125 5.59208724166667 5.67445774166667 6.33013755 5.05265161666667 5.711525125 5.48780954166667 7.51240625833333 5.69101595 5.7871496 5.80207481666667 6.13535341666667 8.618487425 9.47971744166667 5.96431428333333 6.65293891666667 5.74925146666667 6.44343473333333 5.51633325833333 5.401166725 5.443745225 5.86997613333333 5.43881695 5.32823685 5.93351605 6.66745250833333 5.6525194 5.54580931666667 6.660735075 5.32316651666667 4.691160125 6.50269315 5.493516675 5.64920624166667 5.84406485 5.30076113333333 6.27541806666667 4.98620748333333 5.373446775 5.72460895833333 6.25294181666667 5.52817958333333 5.6883975 5.3299725 5.6868575 5.30498764166667 6.36384354166667

LINC01684 14.3528361416667 14.374704625 14.23643525 14.0270179 13.472886275 13.4053421 13.68323365 14.36243255 13.5773436583333 14.2547860083333 13.7542306333333 13.7324527083333 13.7425781166667 14.374704625 14.2285583083333 14.03455475 14.09777575 13.818712325 14.4802458 14.1766127916667 14.1559919583333 14.0518864916667 13.8760479333333 13.7927226416667 13.981121475 13.9061611 14.081980575 14.2746526833333 13.8829970083333 13.981121475 13.9195785166667 14.3328580333333 13.7723702416667 14.0518864916667 14.2285583083333 14.09777575 13.9883783916667 14.2461660666667 13.9130054833333 14.3844241333333 14.2461660666667 14.4234547 14.081980575 14.5627782916667 14.2194989166667 14.7055885666667 14.4802458 14.2116827666667 14.3328580333333 13.7856886083333 14.1307464583333 13.9195785166667 13.8425220416667 14.1307464583333 14.2116827666667 14.3932085583333 14.003709575 14.5536296416667 14.2961453916667 14.3932085583333 12.737637325 13.0493833666667 13.3856220583333 13.2278879583333 13.448561725 13.0531395083333 13.2622000833333 13.0340459666667 13.448561725 12.9644897666667 12.8218769 12.9787381333333 13.1068881 13.40047325 13.339223625 13.7599520083333 13.2243581083333 13.1561940833333 13.4779890416667 13.560618 13.2700538583333 13.0874111666667 13.89773695 13.5773436583333 13.07608845 13.1943968666667 13.18555275 13.1561940833333 12.3971468083333 13.4830368083333 14.0181244166667 12.86239415 12.7481848833333 13.1648749166667 12.7309384666667 13.2412141666667 13.20700295 13.0874111666667 13.0217509333333 13.2325214 13.2570588083333 13.4677427166667 12.7810968666667 13.83157865 13.0874111666667 13.3666129833333 13.9650040916667 13.9061611 13.3666129833333 13.61657615 13.3624533083333 13.4830368083333 13.1985289416667 13.6342148583333 13.5773436583333 13.76618685 13.4677427166667 13.507496675 13.7425781166667 13.339223625

AL590226.2 7.73104835833333 7.54723063333333 7.53942513333333 7.49052953333333 7.88851164166667 7.11493935833333 7.43034844166667 7.36105020833333 7.41578374166667 7.72082631666667 7.257981025 7.29643938333333 7.018972075 6.96462073333333 6.87454878333333 6.95519420833333 6.843581 7.95418985833333 7.44124528333333 6.57380151666667 7.01591568333333 7.33260558333333 7.11888511666667 7.18077985833333 7.50024405833333 7.565923075 7.16947939166667 7.12930466666667 7.93993941666667 8.16110536666667 7.403217625 7.20164341666667 7.33260558333333 6.82167135833333 7.80256886666667 7.37794183333333 7.808866225 7.58436099166667 7.50388626666667 7.42447915 7.27208299166667 7.48797304166667 7.32960803333333 7.3846807 7.31362185 7.072020975 6.91047501666667 7.080264325 7.040488 7.45898616666667 7.3865141 7.63212294166667 6.83283191666667 7.35849015 7.11263425 6.812864825 7.2000166 7.12784525833333 7.41333261666667 7.06648555833333 7.09583709166667 7.83074816666667 6.95519420833333 7.51584311666667 7.387267475 7.68597430833333 7.28108741666667 7.20164341666667 7.55228321666667 7.29559511666667 7.17364838333333 7.11408553333333 6.86553285 6.92711743333333 7.15998195 6.87837529166667 7.841555975 6.87837529166667 6.63347905833333 7.11334874166667 7.23501408333333 7.37223949166667 7.47076813333333 7.31101670833333 7.30947729166667 7.12048450833333 7.37881741666667 6.97744145 7.75673711666667 7.46553646666667 7.26970906666667 7.30947729166667 7.317206725 7.20762451666667 7.44205825833333 7.41941744166667 8.0797047 7.92035708333333 7.79988654166667 7.33905511666667 7.356869825 7.42945980833333 7.40711176666667 6.88682719166667 7.0043443 7.10174751666667 7.30531678333333 6.59209475833333 7.30531678333333 6.69792505833333 7.321175675 7.36174494166667 7.15755769166667 6.696380025 6.60281753333333 7.29390568333333 7.37794183333333 7.55228321666667 7.34579063333333 6.635993725

LINC02615 5.043189975 5.45934836666667 4.75968018333333 5.53870645 6.05245123333333 6.076673725 4.76862936666667 6.26957381666667 5.26290054166667 5.42593315833333 5.53699586666667 4.72071955833333 5.02726646666667 4.88258713333333 6.48089516666667 5.8690931 6.25138878333333 5.76767515833333 4.81397320833333 6.22164848333333 6.2607797 7.44381273333333 5.4760094 5.30937105833333 5.44224698333333 5.58339051666667 6.30504875 6.27541806666667 5.941997275 6.1514684 5.03475209166667 6.31500904166667 6.13124135 5.79975874166667 5.69727409166667 6.194084125 6.37921816666667 5.76857745833333 5.715067925 6.68014729166667 6.01732366666667 5.397270075 5.20404056666667 5.93685915833333 5.23171054166667 5.43604663333333 5.511999925 5.19160068333333 6.43516865833333 5.59208724166667 5.70785189166667 5.62614020833333 6.15066176666667 7.037414875 6.2141109 6.24284558333333 6.32221861666667 5.61762540833333 5.26378820833333 5.73572338333333 5.69727409166667 5.47086664166667 6.29775806666667 4.96587955833333 5.72275545833333 4.87743169166667 4.92522058333333 4.98202489166667 4.97551569166667 5.15351136666667 4.58075485833333 5.36997850833333 4.87364085 4.67234389166667 5.51827296666667 5.36652833333333 4.846343575 6.541436775 6.21577044166667 4.94734101666667 4.50782843333333 6.81924664166667 6.446663625 5.53323568333333 5.44224698333333 5.72366834166667 6.37369095 5.54038896666667 5.21348641666667 4.922692825 4.948926475 5.12190426666667 5.35685141666667 5.84489904166667 6.10384355833333 4.92781361666667 5.89609469166667 5.537859625 5.43534984166667 5.47283311666667 4.51141813333333 5.362066125 5.695409275 5.37429246666667 4.874362975 5.39874103333333 4.88079474166667 6.52835620833333 4.98118906666667 6.96698138333333 4.96503665833333 5.87484386666667 5.43469475 5.84166365 6.6279805 6.041301775 5.55202255833333 5.10925536666667 4.81791764166667 5.38523813333333

BOLA3-AS1 8.06126420833333 6.13124135 6.57216478333333 5.39559 5.10162569166667 5.85516045 5.1467782 5.37087810833333 4.816492275 6.076673725 5.47283311666667 6.85585071666667 5.494444575 5.945959 6.16444198333333 7.55664531666667 6.98701886666667 4.71438873333333 6.978243625 6.34919350833333 6.770698125 6.07424019166667 7.43866366666667 7.14716405 6.88170193333333 9.15077928333333 6.41530895 7.52905674166667 5.24598960833333 7.00071761666667 7.1066557 7.40976625 6.50183225833333 6.44981341666667 8.37690871666667 8.47992946666667 6.67136516666667 7.18234429166667 7.42447915 6.90573395833333 7.24174676666667 6.8207692 7.59418351666667 7.97263941666667 7.595952025 7.06402211666667 7.73104835833333 7.51141074166667 7.342513875 6.64146615833333 7.53854279166667 6.76405308333333 5.302465325 6.1458196 7.509871575 8.5982425 6.09572545 6.57292695833333 6.41125588333333 7.23347658333333 5.5093714 5.95235704166667 6.082719625 6.530441325 8.05820623333333 4.63950710833333 7.51240625833333 5.76427079166667 6.56023105833333 5.23587815 5.16731153333333 7.20338010833333 4.31751935 5.04085009166667 4.347124625 6.674888925 6.023112125 5.95235704166667 3.762448075 5.06686440833333 7.012070875 6.72671865 6.24589635 6.779595125 5.345943725 7.44656456666667 4.78638856666667 6.59515689166667 5.44224698333333 6.90111271666667 5.34058364166667 8.28793319166667 5.59277610833333 6.08667845 5.70785189166667 4.79866623333333 6.57993243333333 7.45158055 5.95496726666667 7.02582578333333 7.3433226 4.44230648333333 7.17277909166667 5.83122305 7.3704996 5.864991425 5.80371779166667 6.40568764166667 7.43443834166667 6.643638875 5.8658332 6.28918155833333 5.05599298333333 8.029339375 6.16444198333333 6.03387288333333 6.829893775 5.48698374166667 4.86047979166667 4.71676371666667

AL391807.1 7.55145098333333 5.12345405833333 4.33106415833333 4.663962275 4.93202185 7.717196 5.85430964166667 5.13301409166667 4.817166525 5.79658570833333 5.26625810833333 6.779595125 6.05008806666667 4.72071955833333 5.20230749166667 6.68096106666667 6.42169948333333 6.09902735833333 6.38535240833333 5.782225425 5.02647816666667 5.20589149166667 4.25048369166667 6.47575550833333 5.92195151666667 7.19111055833333 5.38175511666667 4.6700077 6.26883340833333 6.38535240833333 5.798958825 6.28233444166667 5.87325478333333 6.13939594166667 6.72671865 7.39567078333333 6.66372589166667 6.32061801666667 6.56728411666667 5.96691225 5.76427079166667 5.44782694166667 6.23943730833333 5.48698374166667 6.49099546666667 6.37999003333333 5.4567799 4.90363349166667 6.31088081666667 5.82021451666667 6.438946775 6.15390005 4.233637175 6.50269315 5.362066125 4.79770940833333 6.12793311666667 5.84406485 7.008284125 5.59815450833333 5.74066663333333 4.68278718333333 4.77273383333333 5.08490451666667 5.28868503333333 7.206737225 4.44153641666667 5.08901280833333 5.466718425 5.26013644166667 4.90681635833333 5.00377313333333 4.63431651666667 4.551878575 4.00950880833333 4.20055220833333 5.29908005 5.63342111666667 4.880021025 4.53764565 5.73081749166667 7.15207910833333 4.67091591666667 5.67018873333333 4.91520124166667 5.3212614 4.37434235 4.22877676666667 4.235972525 5.30937105833333 4.79377419166667 5.17564051666667 5.92847919166667 5.7939183 4.74735175 5.37522741666667 5.30595961666667 5.76857745833333 4.5391327 5.09577138333333 5.06194454166667 4.10612433333333 5.36997850833333 4.887437075 5.44953800833333 5.40623839166667 4.81551458333333 5.89124781666667 5.12104158333333 6.60281753333333 5.885364375 6.29000819166667 5.00377313333333 5.04921501666667 4.72810314166667 5.41980085833333 5.38353026666667 5.80704883333333 5.36997850833333 4.86708244166667

AL050403.2 5.020919775 4.10931059166667 4.663127775 4.31979769166667 4.907631425 4.525625675 5.10595089166667 5.31195595 5.53870645 5.20933644166667 5.42944249166667 5.46256163333333 5.022489825 4.26345199166667 4.887437075 4.37434235 4.90933671666667 4.95061065 4.871032675 4.73123783333333 4.63110146666667 4.75656106666667 4.58961148333333 5.09738386666667 4.939292675 4.437951325 5.74227275833333 4.82759028333333 4.79683228333333 4.48349301666667 5.42685978333333 4.80433483333333 4.45316954166667 4.96587955833333 4.45465283333333 4.48856954166667 4.83342666666667 4.81093719166667 4.74433684166667 5.21672184166667 5.32912600833333 4.58075485833333 5.1386748 5.899280325 4.329661925 5.37872604166667 5.34143435833333 5.270491775 4.83614788333333 5.12887654166667 5.11933154166667 4.71363983333333 4.50562893333333 5.826857975 4.3958178 5.03308739166667 4.49283225833333 5.10595089166667 5.070082275 4.957717925 5.22903249166667 5.21185403333333 4.57935210833333 5.86089796666667 4.248165325 4.09751095 5.60409175833333 5.62083628333333 3.81006580833333 5.00204984166667 7.99469491666667 6.34605710833333 6.30271423333333 4.249713475 4.13615014166667 4.9625611 4.6432372 4.93610370833333 4.10439639166667 3.99999136666667 5.4096421 4.389988275 4.32111429166667 4.1052673 3.78860940833333 4.60106575833333 5.17819575 4.993870075 4.5762417 4.31074669166667 4.3958178 4.6425346 4.11807701666667 3.97139808333333 4.06807705 4.12508055 5.05599298333333 4.3823314 5.13143250833333 5.30855066666667 4.466985075 5.64026789166667 5.00789455 5.36559945 4.517575125 4.29852961666667 4.67862661666667 6.58292990833333 4.63352474166667 6.91600763333333 4.853953375 5.64920624166667 4.63950710833333 5.01921390833333 4.06404305833333 4.55571135833333 4.89679370833333 4.71676371666667 4.60532013333333 5.36559945

AL135937.1 5.020919775 4.10931059166667 4.663127775 4.31979769166667 4.907631425 4.525625675 5.10595089166667 5.31195595 5.53870645 5.20933644166667 5.42944249166667 5.46256163333333 5.022489825 4.26345199166667 4.887437075 4.37434235 4.90933671666667 4.95061065 4.871032675 4.73123783333333 4.63110146666667 4.75656106666667 4.58961148333333 5.09738386666667 4.939292675 4.437951325 5.74227275833333 4.82759028333333 4.79683228333333 4.48349301666667 5.42685978333333 4.80433483333333 4.45316954166667 4.96587955833333 4.45465283333333 4.48856954166667 4.83342666666667 4.81093719166667 4.74433684166667 5.21672184166667 5.32912600833333 4.58075485833333 5.1386748 5.899280325 4.329661925 5.37872604166667 5.34143435833333 5.270491775 4.83614788333333 5.12887654166667 5.11933154166667 4.71363983333333 4.50562893333333 5.826857975 4.3958178 5.03308739166667 4.49283225833333 5.10595089166667 5.070082275 4.957717925 5.22903249166667 5.21185403333333 4.57935210833333 5.86089796666667 4.248165325 4.09751095 5.60409175833333 5.62083628333333 3.81006580833333 5.00204984166667 7.99469491666667 6.34605710833333 6.30271423333333 4.249713475 4.13615014166667 4.9625611 4.6432372 4.93610370833333 4.10439639166667 3.99999136666667 5.4096421 4.389988275 4.32111429166667 4.1052673 3.78860940833333 4.60106575833333 5.17819575 4.993870075 4.5762417 4.31074669166667 4.3958178 4.6425346 4.11807701666667 3.97139808333333 4.06807705 4.12508055 5.05599298333333 4.3823314 5.13143250833333 5.30855066666667 4.466985075 5.64026789166667 5.00789455 5.36559945 4.517575125 4.29852961666667 4.67862661666667 6.58292990833333 4.63352474166667 6.91600763333333 4.853953375 5.64920624166667 4.63950710833333 5.01921390833333 4.06404305833333 4.55571135833333 4.89679370833333 4.71676371666667 4.60532013333333 5.36559945

LINC01752 5.020919775 4.10931059166667 4.663127775 4.31979769166667 4.907631425 4.525625675 5.10595089166667 5.31195595 5.53870645 5.20933644166667 5.42944249166667 5.46256163333333 5.022489825 4.26345199166667 4.887437075 4.37434235 4.90933671666667 4.95061065 4.871032675 4.73123783333333 4.63110146666667 4.75656106666667 4.58961148333333 5.09738386666667 4.939292675 4.437951325 5.74227275833333 4.82759028333333 4.79683228333333 4.48349301666667 5.42685978333333 4.80433483333333 4.45316954166667 4.96587955833333 4.45465283333333 4.48856954166667 4.83342666666667 4.81093719166667 4.74433684166667 5.21672184166667 5.32912600833333 4.58075485833333 5.1386748 5.899280325 4.329661925 5.37872604166667 5.34143435833333 5.270491775 4.83614788333333 5.12887654166667 5.11933154166667 4.71363983333333 4.50562893333333 5.826857975 4.3958178 5.03308739166667 4.49283225833333 5.10595089166667 5.070082275 4.957717925 5.22903249166667 5.21185403333333 4.57935210833333 5.86089796666667 4.248165325 4.09751095 5.60409175833333 5.62083628333333 3.81006580833333 5.00204984166667 7.99469491666667 6.34605710833333 6.30271423333333 4.249713475 4.13615014166667 4.9625611 4.6432372 4.93610370833333 4.10439639166667 3.99999136666667 5.4096421 4.389988275 4.32111429166667 4.1052673 3.78860940833333 4.60106575833333 5.17819575 4.993870075 4.5762417 4.31074669166667 4.3958178 4.6425346 4.11807701666667 3.97139808333333 4.06807705 4.12508055 5.05599298333333 4.3823314 5.13143250833333 5.30855066666667 4.466985075 5.64026789166667 5.00789455 5.36559945 4.517575125 4.29852961666667 4.67862661666667 6.58292990833333 4.63352474166667 6.91600763333333 4.853953375 5.64920624166667 4.63950710833333 5.01921390833333 4.06404305833333 4.55571135833333 4.89679370833333 4.71676371666667 4.60532013333333 5.36559945

SLC7A14-AS1 10.5069002 5.45348568333333 5.57787028333333 5.30168844166667 5.192375575 6.660735075 5.867409875 5.99187126666667 5.57630850833333 5.05599298333333 5.38263980833333 7.009018975 8.005090175 5.92530963333333 5.05599298333333 5.36997850833333 6.85585071666667 5.56298029166667 4.36903601666667 5.141550175 5.90176635833333 5.76240348333333 6.3380145 6.06346144166667 5.27643624166667 10.3214407083333 4.95383771666667 5.65726629166667 6.56179321666667 5.05450271666667 6.15548040833333 4.35888175 4.7809755 4.44153641666667 9.11292266666667 9.705649125 4.484204225 5.506564725 5.15857075833333 4.525625675 4.74591905 5.24850139166667 6.3018823 5.12983071666667 8.577282825 5.979679875 4.48781 4.42996005 4.38169465833333 5.12104158333333 4.6472699 4.9375826 6.06177335833333 6.4089881 5.3154224 5.42685978333333 6.1514684 5.03387344166667 4.76862936666667 5.75012458333333 4.80840334166667 6.70095648333333 5.247693175 6.01732366666667 6.56890816666667 5.70503610833333 5.392747 7.00361548333333 5.36997850833333 5.270491775 6.26389580833333 6.19099196666667 6.02744485833333 5.43469475 5.43881695 5.76338288333333 5.80704883333333 4.71282696666667 5.01280093333333 5.502188825 7.41073699166667 6.492675575 4.85225948333333 5.58956383333333 5.706023225 6.73078578333333 6.13868721666667 5.39559 6.77153066666667 6.69792505833333 6.14750089166667 5.35771110833333 4.76289125 5.48441135833333 5.63754605 5.78299045 5.161910175 5.885364375 5.46176480833333 4.68510404166667 5.15933271666667 7.28275553333333 4.67467815 4.57012604166667 4.96833235 5.552968675 4.901776825 5.05525233333333 4.57786145833333 5.797364775 4.73938754166667 4.97881760833333 5.13698225 5.73824569166667 5.114099175 4.77418788333333 6.67645423333333 5.21846816666667 5.401166725 6.110329

AC015987.1 13.891343325 15.41176645 15.2938409083333 15.0566496333333 15.2087807666667 14.6145279333333 14.8320167 15.7720479333333 14.6537247416667 14.9072598916667 14.633352475 13.3711706833333 14.7261095416667 15.4265201333333 15.4265201333333 14.8320167 13.981121475 15.0891841833333 14.8946224583333 15.4447775583333 15.4607870666667 15.0891841833333 15.41176645 14.7363719916667 14.9750443166667 13.7135423333333 15.1183764916667 15.164023575 15.397048375 15.2938409083333 14.9353634666667 14.9482015833333 14.521344925 13.726268275 13.981121475 13.6897778 15.397048375 15.6405203083333 15.2087807666667 14.7468272333333 15.7422836666667 15.365320575 15.41176645 15.5936489333333 15.003829525 15.4607870666667 15.4265201333333 15.3799427166667 15.2428479916667 14.8821777416667 14.8567343583333 15.0434822083333 14.9624125 15.1942513666667 15.2428479916667 14.9624125 15.41176645 15.9041114166667 15.3471952583333 15.179006875 13.972715675 14.1477872916667 14.1766127916667 14.2194989166667 14.003709575 14.0658472 14.23643525 13.8056241916667 14.1398844416667 14.0270179 14.3428338416667 14.4234547 14.1477872916667 13.9566866 14.3328580333333 14.3844241333333 13.4830368083333 14.081980575 14.3428338416667 13.8366873666667 13.7200334416667 13.7927226416667 14.23643525 14.1847714083333 13.9963421833333 14.0904534166667 13.9495562333333 13.5773436583333 13.855751675 14.1559919583333 14.3043404916667 14.374704625 14.4338157333333 14.2850127166667 13.9130054833333 14.4234547 13.7542306333333 13.339223625 13.5021556833333 12.7941771583333 13.855751675 14.3129405416667 14.1307464583333 13.9883783916667 14.2461660666667 13.7723702416667 14.1847714083333 14.412649525 14.081980575 13.9963421833333 14.6145279333333 14.2461660666667 13.7542306333333 14.1847714083333 13.941376525 14.6145279333333 13.972715675 13.9351442666667 14.2285583083333 13.5663260666667

AL139260.1 8.59991194166667 9.70760454583333 9.32400185416667 9.69661204166667 9.14883249166667 8.94112530416667 7.96663209166667 9.49951345 9.55611895 9.21563495416667 9.03158635833333 7.38766229583333 8.53771269166667 9.56267947916667 9.7759557625 9.4020506 9.819894575 9.40066693333333 9.3213721 9.40599290833333 8.89494094166667 9.19419675416667 9.3726800875 9.8093947625 9.3456925 9.53881325833333 9.56846712916667 9.589146525 9.0028562 9.73185965833333 9.40066693333333 8.9477963375 8.8613999875 8.77794807083333 9.586437 8.86242363333333 9.15923749166667 9.7036014125 9.72772482916667 8.92942130833333 9.09961265 9.65973909583333 9.40599290833333 9.8518028375 9.1893780625 10.0044995625 9.73322877083333 10.3783373375 9.66524654166667 8.52514445833333 9.51462799583333 9.49587064583333 8.96659990833333 8.90096675 8.90746844166667 9.49587064583333 9.74051644583333 9.51462799583333 9.73622975416667 9.6167051625 8.11974702916667 8.11479973333333 8.78619112916667 8.39854170833333 8.81629975833333 8.429394375 7.52944770833333 7.97683266666667 8.96786112916667 8.14792829166667 9.046445125 7.67809828333333 7.79768549166667 8.38869885416667 8.9088324375 8.9305219 8.21888479166667 9.40851622083333 8.49666752916667 8.32616905416667 8.3146388 8.0113613375 9.08808551666667 9.49587064583333 8.42819092916667 8.71416658333333 8.12061863333333 8.28422200416667 7.90989700833333 9.21009585 8.7294721625 8.21676735416667 8.63868771666667 8.61791067083333 7.94036310416667 7.90132309583333 8.5164502375 8.39854170833333 8.8370865625 8.190340025 8.48794788333333 8.09902895833333 9.02144154583333 9.21701711666667 9.09306967916667 9.14511959166667 8.96408830833333 10.1684059208333 8.37017423333333 7.45367132916667 9.23706149583333 9.22119779166667 8.53299352916667 8.48148138333333 7.60654144583333 9.207425175 8.60099007083333 9.0905338375 9.8518028375 8.83828267083333

AL139260.2 8.59991194166667 9.70760454583333 9.32400185416667 9.69661204166667 9.14883249166667 8.94112530416667 7.96663209166667 9.49951345 9.55611895 9.21563495416667 9.03158635833333 7.38766229583333 8.53771269166667 9.56267947916667 9.7759557625 9.4020506 9.819894575 9.40066693333333 9.3213721 9.40599290833333 8.89494094166667 9.19419675416667 9.3726800875 9.8093947625 9.3456925 9.53881325833333 9.56846712916667 9.589146525 9.0028562 9.73185965833333 9.40066693333333 8.9477963375 8.8613999875 8.77794807083333 9.586437 8.86242363333333 9.15923749166667 9.7036014125 9.72772482916667 8.92942130833333 9.09961265 9.65973909583333 9.40599290833333 9.8518028375 9.1893780625 10.0044995625 9.73322877083333 10.3783373375 9.66524654166667 8.52514445833333 9.51462799583333 9.49587064583333 8.96659990833333 8.90096675 8.90746844166667 9.49587064583333 9.74051644583333 9.51462799583333 9.73622975416667 9.6167051625 8.11974702916667 8.11479973333333 8.78619112916667 8.39854170833333 8.81629975833333 8.429394375 7.52944770833333 7.97683266666667 8.96786112916667 8.14792829166667 9.046445125 7.67809828333333 7.79768549166667 8.38869885416667 8.9088324375 8.9305219 8.21888479166667 9.40851622083333 8.49666752916667 8.32616905416667 8.3146388 8.0113613375 9.08808551666667 9.49587064583333 8.42819092916667 8.71416658333333 8.12061863333333 8.28422200416667 7.90989700833333 9.21009585 8.7294721625 8.21676735416667 8.63868771666667 8.61791067083333 7.94036310416667 7.90132309583333 8.5164502375 8.39854170833333 8.8370865625 8.190340025 8.48794788333333 8.09902895833333 9.02144154583333 9.21701711666667 9.09306967916667 9.14511959166667 8.96408830833333 10.1684059208333 8.37017423333333 7.45367132916667 9.23706149583333 9.22119779166667 8.53299352916667 8.48148138333333 7.60654144583333 9.207425175 8.60099007083333 9.0905338375 9.8518028375 8.83828267083333

LINC01140 8.15341585833333 7.702242475 7.937225325 7.99573270833333 7.79075659166667 8.24724386666667 7.85737128333333 7.43621663333333 8.27017713333333 7.98146166666667 8.312024375 8.0885626 8.0743079 7.928697775 7.595060625 7.863951475 8.035850625 7.88754723333333 7.81524705 7.76222746666667 7.77743810833333 7.48222535 7.40883421666667 8.08371099166667 7.62001530833333 7.71468463333333 7.848979625 7.75934284166667 7.88754723333333 8.0943338 7.69130114166667 7.96612625833333 8.55449135833333 8.16698743333333 8.21019310833333 7.96052405 7.724325925 8.089532425 8.04674969166667 7.73389285833333 7.71898053333333 7.66087161666667 7.61757204166667 7.98424845833333 8.04570188333333 7.348136225 7.7267545 8.03026959166667 7.48222535 8.09847590833333 8.35414590833333 7.8730469 7.595060625 7.93906090833333 7.99937763333333 7.595952025 7.53590934166667 8.02524268333333 8.03218916666667 7.769180675 8.00143094166667 7.867558375 8.05570605833333 8.0099428 7.99469491666667 7.81612064166667 8.41501278333333 7.65036194166667 8.16801816666667 8.5644013 8.23251158333333 7.98700995833333 7.96426395833333 7.94198319166667 7.95883029166667 7.54723063333333 8.12206505833333 7.8730469 7.63517916666667 8.06805541666667 7.03655466666667 7.86934078333333 7.97631320833333 8.04570188333333 8.206103275 8.09847590833333 7.967964725 8.00049861666667 7.87702689166667 7.94602126666667 8.08263128333333 8.1801409 8.27017713333333 8.18615149166667 8.29000288333333 7.71373668333333 8.37265579166667 8.29333656666667 7.99764143333333 7.92129841666667 8.073175025 8.16013696666667 7.90467121666667 7.848979625 7.77912510833333 7.918433075 8.03118996666667 7.85737128333333 7.9382336 7.769180675 8.11629255 7.73578660833333 8.47432581666667 7.74760565833333 8.1092679 7.793674875 8.17213816666667 8.37884899166667 7.67103138333333 7.931709675

AC092296.2 8.46203074583333 8.12061863333333 7.759763425 7.75540274166667 8.393112975 8.12061863333333 7.77782785833333 8.19738985 7.94922780833333 8.0462257875 7.66397622083333 8.04523153333333 7.62135857083333 7.44429680833333 8.18058940833333 8.08025769583333 7.85049630833333 7.5389839625 8.29798353333333 8.01813250416667 7.7607132125 7.82375411666667 7.6772762625 8.6872986875 8.05521121666667 8.4358292 8.07159424583333 8.0705389875 8.02391114583333 8.37416044583333 8.2938256625 8.05521121666667 8.1645821375 7.485165925 9.03775150833333 9.0365854125 8.51988893333333 8.18058940833333 8.12539973333333 8.19738985 8.1625857125 7.7626753875 8.35468118333333 8.29049067916667 8.09484178333333 7.97124339583333 8.32616905416667 7.794111075 8.50183264166667 7.67809828333333 8.17954419166667 8.440177575 7.993343225 8.0373297125 7.29833710416667 7.90800684166667 7.93864725416667 8.20664264166667 8.3291413 8.06953343333333 8.41870355416667 8.5071897125 8.3802660375 7.9217374625 9.08808551666667 8.14488479166667 8.5219441125 8.0938206625 8.24576109166667 7.76430890833333 7.94036310416667 8.48588124583333 6.98899876666667 8.04422940416667 8.0056222625 7.9132410625 8.46830984583333 7.86345573333333 8.0491517 8.26236312916667 8.31253955833333 8.52738720833333 7.69838043333333 8.210679925 7.3645172375 7.99804435416667 6.63159115416667 7.24795243333333 9.23706149583333 8.3478448 8.07266193333333 7.76873315833333 9.24731365 8.23602964166667 9.01042526666667 7.67148665416667 8.93509225 8.70844077916667 7.7288436625 8.3231045 8.857816 7.64201074583333 8.689626725 9.15923749166667 9.29861491666667 7.24878446666667 8.097976325 6.79927305833333 8.33027370833333 6.8501883375 8.01718190833333 8.14792829166667 8.37316226666667 8.28422200416667 7.18906547083333 7.63262139166667 8.94112530416667 10.0152599375 8.40564610416667 7.3429182375

AC016590.1 8.46203074583333 8.12061863333333 7.759763425 7.75540274166667 8.393112975 8.12061863333333 7.77782785833333 8.19738985 7.94922780833333 8.0462257875 7.66397622083333 8.04523153333333 7.62135857083333 7.44429680833333 8.18058940833333 8.08025769583333 7.85049630833333 7.5389839625 8.29798353333333 8.01813250416667 7.7607132125 7.82375411666667 7.6772762625 8.6872986875 8.05521121666667 8.4358292 8.07159424583333 8.0705389875 8.02391114583333 8.37416044583333 8.2938256625 8.05521121666667 8.1645821375 7.485165925 9.03775150833333 9.0365854125 8.51988893333333 8.18058940833333 8.12539973333333 8.19738985 8.1625857125 7.7626753875 8.35468118333333 8.29049067916667 8.09484178333333 7.97124339583333 8.32616905416667 7.794111075 8.50183264166667 7.67809828333333 8.17954419166667 8.440177575 7.993343225 8.0373297125 7.29833710416667 7.90800684166667 7.93864725416667 8.20664264166667 8.3291413 8.06953343333333 8.41870355416667 8.5071897125 8.3802660375 7.9217374625 9.08808551666667 8.14488479166667 8.5219441125 8.0938206625 8.24576109166667 7.76430890833333 7.94036310416667 8.48588124583333 6.98899876666667 8.04422940416667 8.0056222625 7.9132410625 8.46830984583333 7.86345573333333 8.0491517 8.26236312916667 8.31253955833333 8.52738720833333 7.69838043333333 8.210679925 7.3645172375 7.99804435416667 6.63159115416667 7.24795243333333 9.23706149583333 8.3478448 8.07266193333333 7.76873315833333 9.24731365 8.23602964166667 9.01042526666667 7.67148665416667 8.93509225 8.70844077916667 7.7288436625 8.3231045 8.857816 7.64201074583333 8.689626725 9.15923749166667 9.29861491666667 7.24878446666667 8.097976325 6.79927305833333 8.33027370833333 6.8501883375 8.01718190833333 8.14792829166667 8.37316226666667 8.28422200416667 7.18906547083333 7.63262139166667 8.94112530416667 10.0152599375 8.40564610416667 7.3429182375

LINC00652 7.116464725 6.713268275 6.41370083333333 6.41696196666667 7.25439505 5.983920925 7.22153060833333 6.69077578333333 6.67222361666667 6.770698125 7.00742828333333 7.07951833333333 6.699451375 6.6840655 7.625103125 6.64445649166667 6.93442244166667 6.695532175 6.314219375 6.26556310833333 6.77248144166667 6.59279463333333 6.849738 6.10696181666667 6.858067425 7.200884475 6.56098033333333 6.45800148333333 5.78131454166667 6.56261174166667 6.619742275 6.38929764166667 6.37921816666667 6.32934243333333 7.1228027 6.5783546 6.417835775 6.48712963333333 6.5783546 6.31992400833333 6.50269315 6.26957381666667 6.78424304166667 6.21495720833333 6.28684615833333 6.38460903333333 6.50018229166667 6.05912289166667 6.54215274166667 5.8061966 6.38460903333333 5.56030313333333 6.67400460833333 6.85293710833333 6.51669615 7.181557425 5.81522059166667 6.89227535 6.36633304166667 6.31908594166667 6.81620986666667 7.00071761666667 6.64920430833333 7.4747897 6.92952805833333 6.53343775833333 8.04867420833333 7.116464725 7.27771845 6.68563873333333 7.07277904166667 7.07608708333333 7.33028253333333 6.868676375 7.44897004166667 6.45128649166667 6.93585935 6.12078816666667 6.49720533333333 6.39399721666667 8.5427737 6.19675590833333 6.86715935833333 7.10947593333333 6.72671865 6.85232785833333 8.4103435 7.95964986666667 6.993393575 5.93007595833333 6.76161903333333 6.66888841666667 6.18629009166667 6.37198143333333 6.74572325 6.57608009166667 7.8537938 6.372835675 7.029863175 7.56683574166667 6.31908594166667 7.02737631666667 5.65726629166667 6.43447593333333 6.54993728333333 5.85603955 6.07508890833333 8.243225475 8.69358161666667 8.46571488333333 6.06346144166667 7.1768951 5.56030313333333 7.5904521 6.396357 6.01241375 6.64686755833333 6.21495720833333 6.59810739166667 6.11996326666667

AC008667.1 7.95964986666667 8.14836771666667 8.30278224166667 8.17812686666667 8.24724386666667 8.3275657 8.24724386666667 8.02160229166667 8.18309575 8.16603721666667 8.25235531666667 7.93623871666667 8.397991975 8.05570605833333 8.07544260833333 8.41697565 8.25332638333333 8.660712675 8.15737470833333 7.88541965 7.88851164166667 8.16501965833333 8.38818113333333 8.18906878333333 8.303785925 7.744788925 8.35299256666667 8.17894748333333 8.31100035 8.15541876666667 8.326673625 8.697241175 8.27653735833333 8.47432581666667 8.71699468333333 7.79904711666667 8.51484585833333 8.2995344 8.395996775 8.21620083333333 8.22529060833333 8.53935796666667 8.02813729166667 7.89192915 7.8730469 7.77100474166667 7.62426479166667 8.21192885 7.94870053333333 7.947814825 7.98997320833333 8.01089986666667 8.11929538333333 8.13916790833333 8.115340475 7.77743810833333 7.4754804 8.39480550833333 7.8186674 7.82133015 8.15047776666667 7.92575334166667 8.20193756666667 8.27653735833333 7.518197225 8.84138925 7.7674106 7.70481934166667 7.98897931666667 8.23996114166667 7.85737128333333 8.13385164166667 8.04674969166667 7.96987383333333 8.16013696666667 9.20264188333333 8.386019225 8.31719618333333 8.47548560833333 8.278515525 7.66688975833333 8.18700308333333 8.81089155833333 8.1801409 8.20524448333333 8.08177765 8.15136631666667 8.05570605833333 8.0743079 7.75584805833333 8.10837065 9.16875895833333 7.73578660833333 7.80335965833333 9.1323295 8.05364296666667 8.19496856666667 8.39364840833333 8.33626970833333 8.17727110833333 7.774503075 8.485317225 8.10654075833333 7.80335965833333 8.05160935 7.91271055833333 8.15541876666667 8.02254479166667 8.11929538333333 7.54723063333333 7.56339936666667 7.59418351666667 8.81206715 7.54371946666667 8.19085505 7.76387356666667 7.77651561666667 8.522428575 7.67007186666667 7.717196

MIR124-2HG 6.36954999166667 5.523243625 6.336430425 6.05432435833333 5.81351565833333 6.41530895 5.663186525 5.94277945833333 5.87407425 6.4290253 6.26704225833333 5.64366626666667 5.08336286666667 5.523243625 6.36297995 5.50565405833333 6.49019045 5.89681745 6.54837455833333 6.129619075 6.00243133333333 6.1327813 6.19853316666667 6.07079043333333 6.63036405 6.32934243333333 6.71254476666667 6.92634120833333 6.446663625 6.49578869166667 7.05928261666667 6.58965010833333 6.09411293333333 6.19027888333333 6.88087174166667 6.93368999166667 6.64146615833333 7.31031474166667 6.91994371666667 6.92880595 6.59515689166667 6.54916510833333 6.61126938333333 6.91047501666667 6.20696713333333 6.15958521666667 6.550654 6.17880248333333 6.404149025 6.848966625 6.85651115833333 6.849738 5.66590344166667 7.1157008 6.54378744166667 7.033226925 6.09646545833333 7.12190308333333 6.86168069166667 6.71181541666667 8.017646775 6.54071093333333 7.45898616666667 6.92634120833333 6.90820158333333 7.00591673333333 6.43516865833333 6.54453155833333 6.91047501666667 6.53414359166667 6.59515689166667 6.63914835 5.89280416666667 6.39956296666667 6.93977528333333 6.10867980833333 6.14505980833333 6.77411759166667 6.70174799166667 6.6227255 6.29083875833333 6.50724065 6.30419145 6.93198700833333 7.702242475 6.31742500833333 7.005136025 6.01647188333333 6.57993243333333 6.70618345833333 7.25265295833333 7.27688146666667 7.89997913333333 7.1040313 7.854674425 7.28603906666667 7.41757468333333 7.35359963333333 6.96226191666667 8.08759711666667 7.4984921 6.397067625 6.83665193333333 7.13347761666667 6.72921199166667 5.82420564166667 6.37447095 6.89078670833333 6.29613120833333 6.78272669166667 7.526619475 6.997465825 7.92575334166667 7.22572939166667 7.14000910833333 6.6105347 7.00675840833333 7.19349589166667 7.11030754166667 6.10788125833333

U91319.1 4.44679063333333 3.95459895833333 3.957521375 4.08410715833333 3.533139575 4.13851669166667 3.67534585 3.53146109166667 3.815793275 3.801885725 3.65938206666667 3.537540825 3.73208851666667 4.164004225 4.12351481666667 3.96074574166667 4.46857235833333 3.71873703333333 4.61432114166667 4.28196915833333 3.97718864166667 3.99459898333333 4.32395948333333 4.68830681666667 4.7733425 4.53840028333333 4.40573335 4.59031499166667 4.64653290833333 4.35590056666667 4.74184695 4.61117119166667 3.81801135833333 4.096636775 4.67234389166667 5.16663731666667 4.55717808333333 5.32823685 5.208439775 6.16586921666667 5.24850139166667 4.71363983333333 5.11649993333333 5.85351849166667 4.227329175 5.247693175 5.43284749166667 5.179843975 4.58815271666667 4.934512075 4.97210654166667 4.481057225 3.782846375 5.84406485 4.344009325 4.910233575 4.32395948333333 5.015993925 4.577162675 4.675507125 3.78678109166667 3.97392573333333 5.430247225 3.94837956666667 3.47915838333333 3.6669074 4.185156975 3.543895025 3.78525670833333 3.5067492 3.801885725 3.73698600833333 3.47818535 3.61794161666667 3.64308306666667 3.67786573333333 3.40034693333333 3.7581121 3.97949414166667 3.64726053333333 3.4607158 3.98974825 4.20871595833333 4.21022225 3.72622218333333 3.67456143333333 3.66381434166667 4.3566395 4.24347425833333 4.06172258333333 3.72224865833333 4.51592391666667 4.18590468333333 3.79634603333333 3.95531615 3.988166225 4.00317675833333 4.65585600833333 3.95836893333333 5.99926955 4.45389868333333 3.6142597 5.00290588333333 5.4096421 4.4283936 3.95095814166667 4.80100048333333 6.587379025 4.29455464166667 6.52366150833333 4.78488421666667 5.32654480833333 4.62007759166667 5.04731695 4.02948560833333 4.50562893333333 4.20943228333333 4.67091591666667 4.79215016666667 3.76338045

LINC01102 4.55489758333333 3.97718864166667 3.96958159166667 4.176255425 4.09826585 4.185156975 3.76172071666667 3.94837956666667 3.924596975 3.83300729166667 3.62038586666667 3.75472904166667 3.79213375833333 4.20269309166667 4.16321256666667 3.97641614166667 4.66568316666667 4.00394323333333 4.6425346 3.981243 4.0990947 4.007428725 5.38613150833333 4.97881760833333 4.78638856666667 4.01751818333333 4.43727345833333 4.641792225 4.62182526666667 5.02818118333333 4.73675758333333 4.609492775 4.111905175 4.15790645833333 4.56782300833333 4.51995868333333 4.59432431666667 4.78973365833333 4.791365525 5.20230749166667 5.35685141666667 4.725545825 5.180684475 5.94033254166667 4.27232815 5.32501348333333 5.50030605 5.219476575 4.60316775833333 4.94171295833333 4.98118906666667 4.484204225 3.80841116666667 5.53605506666667 4.39870788333333 5.02726646666667 4.4165323 5.020076575 4.57935210833333 4.837677975 3.80110566666667 4.00394323333333 4.44597125 4.241859075 3.79116074166667 3.68398305833333 3.876232825 3.66852646666667 3.82112268333333 3.74912634166667 4.01591823333333 3.91339629166667 3.67456143333333 3.71429205833333 3.57128608333333 3.7417011 4.174726575 3.91252795833333 3.782000175 3.651463 4.29385256666667 4.20055220833333 4.71758559166667 4.275246875 3.73544420833333 3.49013763333333 3.74762774166667 4.39144765833333 3.76338045 4.056402275 3.823545625 4.5241163 4.21242450833333 3.816517125 3.992207825 4.05010078333333 4.06020879166667 4.188201025 3.794740275 5.17236361666667 4.518365025 4.17774023333333 5.05525233333333 5.49108314166667 4.49076011666667 3.99069761666667 4.82113685833333 6.65373279166667 4.29310653333333 6.56330075833333 4.783293425 5.34058364166667 4.64958116666667 5.14598393333333 4.08488764166667 4.56640000833333 4.22568374166667 4.691160125 4.78718000833333 4.14246295

MEF2C-AS1 9.536758 8.92998051666667 9.201411475 8.80747489166667 9.20949 9.12964521666667 9.21636353333333 8.71236105833333 8.901625425 8.92760421666667 8.87059211666667 9.39312421666667 8.97362839166667 8.71583128333333 9.15077928333333 8.95579018333333 8.65238185 9.24949425833333 8.993454275 8.86290175 9.06192556666667 8.941771525 8.92128914166667 8.97885180833333 9.42509784166667 9.40927853333333 9.60875009166667 9.43421313333333 9.54939620833333 9.29261925833333 9.02465829166667 9.37335539166667 9.43871485833333 9.6076044 9.44960989166667 9.65131303333333 9.67743319166667 9.43132836666667 9.42234536666667 9.3690743 8.73889361666667 9.2535473 9.137660375 9.105193125 8.74758403333333 8.95454636666667 8.84138925 9.05059795833333 9.03966899166667 9.2860314 9.26005576666667 9.26005576666667 9.455130225 9.28449495 9.38364405 9.00974125833333 8.5644013 9.14573905 9.491289725 8.854849925 9.29669200833333 9.12206050833333 9.47971744166667 8.64415260833333 8.35738369166667 9.11422543333333 8.91416151666667 9.04472984166667 8.69251304166667 9.180393975 9.03362063333333 9.01969005 8.66334255 9.1323295 9.118265275 8.85599695 9.14303199166667 9.37471118333333 8.99711739166667 8.90678281666667 8.47657969166667 8.675578375 8.84985913333333 9.18755855 9.46065865 8.84022498333333 8.93106328333333 9.04108516666667 9.3411091 9.24251089166667 8.787979925 9.50689216666667 9.3945379 9.14824449166667 9.6633032 9.53947254166667 9.32457533333333 9.62008553333333 9.47701395833333 8.93106328333333 8.94047908333333 9.16363804166667 9.40775390833333 9.15227524166667 8.80621709166667 9.170003675 9.13369941666667 8.71476503333333 8.98736196666667 9.137660375 9.26528025 8.98366746666667 8.98615453333333 8.77853785833333 9.49398269166667 9.128527525 8.70788464166667 9.51389508333333 9.46725561666667 8.835077425

LINC00639 9.45099463333333 8.33859384166667 8.16110536666667 8.57076716666667 8.47657969166667 7.91550765833333 8.17005769166667 8.32474756666667 8.37069645 8.46035811666667 8.39909144166667 8.57076716666667 8.70557491666667 8.37069645 8.22833255833333 8.213081625 8.03782043333333 9.124545075 8.55690941666667 8.197822625 8.00331975833333 8.62989988333333 8.30985033333333 8.51803561666667 7.928697775 8.029339375 8.58803331666667 8.40939095833333 6.52910926666667 8.950621275 8.93944605 8.262883775 8.323692025 7.90087799166667 8.18204448333333 8.05268059166667 8.93824426666667 8.774825425 9.02591655 8.75436599166667 8.44688928333333 8.96352476666667 8.5427737 8.17812686666667 8.602930325 8.0785585 8.16208859166667 9.111708925 8.11740648333333 7.98700995833333 7.95418985833333 8.5644013 8.03302596666667 8.05922750833333 8.30672025 7.94975508333333 8.23439215833333 8.697241175 8.05160935 8.347273825 8.322516975 8.30054675833333 8.17727110833333 8.29333656666667 8.002327575 8.87214213333333 7.89997913333333 8.13273210833333 8.9239143 8.12305399166667 7.96236178333333 8.73252250833333 8.37784194166667 8.41158728333333 8.09747674166667 8.29000288333333 8.49279463333333 8.31951934166667 8.57406105 8.511369775 7.44728600833333 7.35849015 8.82891991666667 8.43640146666667 8.37690871666667 7.86100648333333 8.24820143333333 8.49932775 5.13539786666667 7.8759545 8.527957175 8.39257754166667 8.46571488333333 8.348415775 8.2995344 8.322516975 8.65444884166667 8.25022860833333 8.76894409166667 8.19889111666667 8.02813729166667 8.274413675 8.24101278333333 8.38384043333333 8.57288055 8.25133669166667 8.54377494166667 8.9709554 8.312024375 8.42995574166667 7.83974054166667 8.26808959166667 8.11218248333333 7.68151318333333 8.01671704166667 8.00797545 7.73767701666667 8.41276785 8.19085505 7.89268988333333

AC091180.5 11.8444568666667 12.714470025 12.8466066333333 12.578037425 12.1815738083333 12.362762125 12.189826275 13.0606794083333 11.792717175 12.4744729083333 12.22065105 11.388504 12.112519875 12.421258225 12.3371985 12.2456214083333 12.112519875 12.511227025 12.708001075 12.7275736083333 12.448639725 12.2399882333333 12.7340595333333 12.8913513416667 12.4925981 11.8625342 12.43663495 12.57544625 12.6943613083333 12.7581232166667 12.8103459166667 12.4713877916667 11.9484228666667 11.8110067583333 11.9252347666667 11.7878206 12.4566311333333 12.8070736416667 12.7206590083333 12.4120381833333 12.8592781166667 12.877352 12.662426525 12.9239130666667 12.3920642833333 12.8880746083333 12.800813375 13.2741790333333 12.8554837916667 12.3971468083333 12.800813375 12.877352 12.1284072083333 12.3708273333333 12.3544155583333 12.5145401166667 12.869508175 13.11410875 12.4985912416667 12.4179335583333 10.240385675 11.9510879333333 12.4566311333333 11.9357587416667 12.0365429083333 11.4272823833333 12.3185215083333 12.1870383833333 12.16080175 11.6063160416667 11.7140151166667 11.790320325 11.9252347666667 11.5935101166667 11.6010023166667 12.4148038083333 11.13656485 11.895484775 12.617619125 12.0719435333333 11.7015427416667 10.5898562833333 12.9606437 12.0437977833333 11.9662069916667 11.9038701666667 12.3659453666667 11.745925575 11.129710525 12.03386695 12.3371985 11.9662069916667 12.2867892916667 11.9993623166667 11.8110067583333 11.797889175 11.704120775 11.75862515 12.0508303333333 12.4148038083333 12.4120381833333 12.3454957166667 12.2649867083333 12.2426995166667 12.3010353583333 12.6593838083333 12.4120381833333 12.7175457 12.3454957166667 12.37994665 12.377098625 12.6460873916667 12.1870383833333 12.2867892916667 12.0852666166667 12.75143825 12.1958518666667 12.6943613083333 12.228644125 12.0824665333333

MSC-AS1 6.07758535833333 5.945959 4.822737175 4.08184460833333 3.59138205833333 6.0420986 4.16107371666667 4.30994171666667 3.721383175 5.5754911 5.11752969166667 5.529852675 3.83468344166667 4.65809740833333 4.54430115833333 5.04562356666667 6.53188953333333 4.07877819166667 5.86256211666667 5.25117366666667 4.37739820833333 4.88571185 5.15772628333333 5.59357184166667 6.39329076666667 5.98048791666667 5.76502415833333 6.10696181666667 5.28508550833333 5.67701044166667 6.61808465 5.96262355 4.44814198333333 5.61593795 7.15037268333333 6.1401869 5.0364605 6.46187091666667 6.49720533333333 5.76240348333333 6.33336359166667 5.72016528333333 6.9176246 6.42987050833333 6.44343473333333 7.0616309 6.41125588333333 5.64469614166667 6.512400125 6.248310775 6.3554652 5.91774480833333 3.97718864166667 5.5754911 5.88439644166667 6.80832605833333 4.78885110833333 5.89207013333333 6.488745325 7.59330443333333 5.81691269166667 6.70618345833333 5.07992535 4.86875400833333 5.37693024166667 6.43962590833333 5.4223537 4.61046104166667 4.59653754166667 4.31673289166667 6.7749131 7.34657900833333 3.92864416666667 6.88682719166667 5.1551752 6.29693088333333 4.48711344166667 5.7871496 9.124545075 5.144208425 5.51827296666667 4.24347425833333 6.86946320833333 7.76312330833333 6.95374109166667 4.858673825 5.64026789166667 4.60802493333333 5.95153 6.79889878333333 5.528965775 5.70335329166667 6.39481431666667 6.56023105833333 6.77735403333333 6.6294991 4.81551458333333 6.76771060833333 5.56551444166667 5.74227275833333 7.52085370833333 4.27303441666667 5.797364775 5.77117681666667 6.42337751666667 4.88258713333333 7.28710860833333 5.430247225 6.901936475 7.52253811666667 6.35158149166667 5.7042258 6.07927768333333 7.22480836666667 7.35757096666667 6.07424019166667 7.102382825 6.43516865833333 7.658531525 4.1052673

AC109466.1 4.74471707083333 4.79727084583333 4.60757309583333 4.50529682083333 4.42215889583333 4.18857750833333 4.98078275833333 4.30745805833333 3.86190393333333 4.62137782916667 3.95794515416667 5.05131178333333 4.38434075 4.94694052916667 4.02026221666667 3.9282286625 4.63912953333333 3.9219148 4.92821639583333 4.076094925 3.79840604583333 4.70377167083333 4.34813653333333 4.62137782916667 4.8168294 4.0709043125 4.93803540416667 4.7227611375 4.6000203625 5.12060409583333 4.8830006375 4.6939016 3.74311106666667 4.08222873333333 4.66779929166667 5.0000634375 4.5796823 4.70377167083333 4.59850584166667 4.82390286666667 5.0704849125 4.54018786666667 5.02356719166667 5.74190917916667 4.161399175 5.131805575 5.068848575 5.13579774166667 4.60914915416667 4.93243676666667 4.935725875 4.60143481666667 4.48746172083333 5.33426984583333 4.52374517916667 5.94073838333333 3.74561552083333 4.94373219166667 4.42955472916667 4.54836894166667 4.564599725 4.70862960833333 4.34674365416667 4.20153883333333 4.57049284583333 3.95208124583333 4.21280651666667 4.28234809166667 3.7130492 4.32073067083333 4.42571156666667 4.49556071666667 4.4471476625 3.934597925 3.52458704166667 3.87759993333333 4.12700439166667 3.94800589166667 4.84038245416667 3.929074325 4.40889241666667 4.4549880875 4.25090944583333 4.6618192875 3.93535581666667 3.630786125 4.12700439166667 4.475072 3.66985007083333 4.44638094166667 3.989348175 4.59777915416667 4.25553867083333 3.87200055416667 4.39323452083333 3.96744667083333 4.01551317083333 4.6862413875 4.118479425 5.0894025125 4.35414409166667 3.94501249583333 4.93971341666667 5.25399853333333 4.33657136666667 3.89850427083333 4.59388868333333 6.41656400833333 4.32648216666667 6.40936380416667 4.75237628333333 5.48155119166667 5.172025925 4.93803540416667 4.1325714875 4.4205758875 4.2668479375 4.59777915416667 4.54689509166667 4.63227757083333

LINC02143 4.74471707083333 4.79727084583333 4.60757309583333 4.50529682083333 4.42215889583333 4.18857750833333 4.98078275833333 4.30745805833333 3.86190393333333 4.62137782916667 3.95794515416667 5.05131178333333 4.38434075 4.94694052916667 4.02026221666667 3.9282286625 4.63912953333333 3.9219148 4.92821639583333 4.076094925 3.79840604583333 4.70377167083333 4.34813653333333 4.62137782916667 4.8168294 4.0709043125 4.93803540416667 4.7227611375 4.6000203625 5.12060409583333 4.8830006375 4.6939016 3.74311106666667 4.08222873333333 4.66779929166667 5.0000634375 4.5796823 4.70377167083333 4.59850584166667 4.82390286666667 5.0704849125 4.54018786666667 5.02356719166667 5.74190917916667 4.161399175 5.131805575 5.068848575 5.13579774166667 4.60914915416667 4.93243676666667 4.935725875 4.60143481666667 4.48746172083333 5.33426984583333 4.52374517916667 5.94073838333333 3.74561552083333 4.94373219166667 4.42955472916667 4.54836894166667 4.564599725 4.70862960833333 4.34674365416667 4.20153883333333 4.57049284583333 3.95208124583333 4.21280651666667 4.28234809166667 3.7130492 4.32073067083333 4.42571156666667 4.49556071666667 4.4471476625 3.934597925 3.52458704166667 3.87759993333333 4.12700439166667 3.94800589166667 4.84038245416667 3.929074325 4.40889241666667 4.4549880875 4.25090944583333 4.6618192875 3.93535581666667 3.630786125 4.12700439166667 4.475072 3.66985007083333 4.44638094166667 3.989348175 4.59777915416667 4.25553867083333 3.87200055416667 4.39323452083333 3.96744667083333 4.01551317083333 4.6862413875 4.118479425 5.0894025125 4.35414409166667 3.94501249583333 4.93971341666667 5.25399853333333 4.33657136666667 3.89850427083333 4.59388868333333 6.41656400833333 4.32648216666667 6.40936380416667 4.75237628333333 5.48155119166667 5.172025925 4.93803540416667 4.1325714875 4.4205758875 4.2668479375 4.59777915416667 4.54689509166667 4.63227757083333

LINC01170 4.62559538333333 4.389247575 4.15790645833333 4.10685365 4.227329175 4.57549981666667 4.04841256666667 4.62182526666667 4.30308001666667 4.56268375 4.50423884166667 4.06020879166667 3.89974741666667 3.83300729166667 4.84000604166667 3.80841116666667 4.81093719166667 4.46778620833333 4.63272604166667 4.54575506666667 4.02639559166667 5.28331578333333 4.19890045 4.41868564166667 4.955238375 3.98974825 4.98361768333333 5.265418675 4.53463513333333 6.97164584166667 5.529852675 4.76289125 4.22349475833333 4.45532334166667 5.97703591666667 4.901776825 4.65659079166667 5.90723234166667 5.8658332 5.38175511666667 4.75356399166667 4.31232735833333 5.78469366666667 5.37246943333333 4.29792231666667 4.813152125 4.48349301666667 4.72389136666667 5.5439086 6.24510723333333 5.59277610833333 5.25605979166667 4.729607625 5.8401316 5.23837954166667 4.74274593333333 4.435737425 5.4144891 5.30498764166667 5.06114335833333 4.78802293333333 4.39068023333333 5.4223537 4.49348375833333 3.94925109166667 5.13143250833333 4.393584875 4.39651160833333 4.627926725 4.35821460833333 4.193641925 4.60880553333333 3.93739250833333 3.84132645833333 3.93654373333333 3.93981401666667 4.00317675833333 4.442953 4.36397045 4.427577425 3.886414625 4.13543773333333 4.56640000833333 4.97551569166667 4.28856184166667 4.39219723333333 4.20269309166667 4.32331949166667 4.11283335 6.412967525 4.18065260833333 5.19314300833333 4.80927436666667 4.46178631666667 5.151017225 4.41944375833333 5.52235776666667 5.84085751666667 4.82113685833333 4.89500721666667 4.916041975 3.83557885833333 5.808584625 5.451907275 4.91353486666667 3.79213375833333 4.22202384166667 5.55016594166667 4.347124625 6.51669615 5.367325475 5.32316651666667 4.95454220833333 6.12633869166667 4.62261409166667 4.262661725 4.70894576666667 5.192375575 5.729929025 4.25214924166667

AC068535.1 5.03994734166667 7.18077985833333 7.41333261666667 7.10815790833333 7.52178743333333 5.43201638333333 7.903661075 7.33186256666667 7.037414875 6.39082849166667 6.65069284166667 7.41578374166667 8.09138268333333 7.30787071666667 6.6294991 6.30919138333333 6.07079043333333 6.67714320833333 6.00671166666667 6.58292990833333 7.03066510833333 6.13454856666667 6.76915333333333 6.19027888333333 4.338662175 6.19761265833333 5.7981413 3.86919178333333 5.82262374166667 5.27404638333333 4.50496470833333 4.77715155833333 5.48698374166667 6.56472320833333 4.61046104166667 4.21242450833333 4.40189316666667 4.307075925 4.26345199166667 4.40920343333333 4.93610370833333 4.67308416666667 4.583812375 5.09738386666667 4.96337545 4.924395575 5.31363400833333 6.28768299166667 4.99292960833333 4.727288675 4.80260285 4.83095916666667 7.33429259166667 4.82042135 6.28684615833333 4.44597125 6.49433464166667 5.15351136666667 4.0594979 3.816517125 4.58961148333333 6.90431630833333 4.04512974166667 6.42828630833333 6.21730323333333 5.494444575 6.11751004166667 7.206737225 4.72637159166667 6.39399721666667 6.7213753 5.798958825 7.668434925 6.29392624166667 6.48228618333333 6.651497425 5.284267925 4.44087261666667 5.568276075 5.14837419166667 8.19185328333333 7.83433648333333 5.69013408333333 5.84406485 6.25988588333333 6.26791573333333 5.82345661666667 7.35359963333333 6.35692343333333 5.14837419166667 5.81024634166667 4.12057799166667 3.745183375 4.564954525 4.55111153333333 5.552968675 4.39068023333333 4.24046655 6.1287993 5.56466664166667 5.33475269166667 6.52835620833333 4.519178925 4.48559865 4.67637581666667 6.39956296666667 5.54852681666667 5.97703591666667 5.55866609166667 6.70927374166667 4.35888175 5.69727409166667 3.99459898333333 4.46466106666667 6.07424019166667 4.696789075 5.875661225 4.33620110833333 4.09826585 7.21336981666667

SHANK2-AS3 11.206704325 12.0799709583333 11.6740143583333 11.6740143583333 12.048574475 12.10426585 11.6159368333333 12.1073504333333 11.7299301083333 11.84692875 11.8009085 11.6307827666667 11.7611345666667 12.1312062333333 12.2540382416667 11.9277646416667 11.4520079916667 12.5457182583333 12.2261501583333 12.1415417666667 11.893046575 12.1689052416667 11.9123900916667 11.62343985 11.745925575 11.1432169416667 11.394164525 11.895484775 12.0207283333333 11.7092046583333 11.9858949166667 12.0072618083333 12.03386695 11.3590324 11.1886169916667 11.0850726666667 12.3428180916667 11.9561420333333 11.5620897083333 11.9966241166667 11.4912889416667 11.7299301083333 11.704120775 11.7092046583333 11.1321094 11.33050215 11.4345196083333 11.2272441333333 12.1550844416667 11.8202758833333 11.6813023833333 11.8155407666667 10.9462764833333 11.1477633666667 10.7895709 10.979276575 11.8133445166667 11.38647825 12.0287220416667 11.7435451333333 12.2426995166667 11.774517575 11.4822633416667 11.895484775 11.7092046583333 12.2540382416667 11.5645849916667 12.1016161833333 12.7873340416667 12.0904755416667 11.857596825 12.048574475 12.0587078333333 11.8881455083333 11.75862515 11.9858949166667 12.1985528083333 12.4629274416667 12.2867892916667 11.9789289833333 11.720720125 11.860233025 12.0879052583333 11.8133445166667 11.9561420333333 11.9409533916667 12.0640980916667 11.6486126 12.1870383833333 11.7165186083333 12.112519875 12.2181076833333 12.3010353583333 11.9662069916667 11.9587890916667 11.7661447083333 12.1958518666667 12.2130170916667 12.377098625 11.909558975 11.6763962416667 11.9409533916667 11.6486126 11.50386205 11.49913775 11.8335796916667 11.3232895666667 11.54529205 11.933125475 12.1073504333333 11.84953385 12.1443252916667 10.9747287666667 11.0008274583333 11.1026531 10.8746426833333 11.9068035083333 11.8335796916667 11.8676643916667 11.8364860833333

AC079193.2 7.21835374166667 4.75429261666667 4.80025811666667 5.8401316 6.56023105833333 5.7415456 6.29392624166667 6.4666266 6.89790128333333 6.80596155833333 5.97375568333333 5.80207481666667 6.34047511666667 5.70703833333333 5.88789478333333 5.93007595833333 6.22740323333333 6.40823279166667 6.56890816666667 6.3309624 6.41054875833333 6.13763498333333 5.9442928 6.285311725 7.00742828333333 7.25439505 7.21256346666667 5.70335329166667 5.547570375 6.79276650833333 6.46821505833333 7.32899926666667 6.768468075 7.07530188333333 7.033226925 8.19403195 6.62879606666667 6.79655045 6.39329076666667 7.27051901666667 6.30419145 5.90176635833333 6.4200423 6.33413388333333 6.66372589166667 6.7749131 6.98145384166667 6.62879606666667 6.76009966666667 5.80452428333333 6.14994006666667 6.372835675 7.58436099166667 6.88476720833333 7.13734699166667 7.13504676666667 6.98940225 6.74572325 7.36804055833333 7.21681046666667 6.59363060833333 5.93007595833333 6.07758535833333 5.71751655 7.12536183333333 6.39399721666667 5.797364775 6.16284539166667 6.25910869166667 6.52517743333333 5.4223537 4.5241163 5.30498764166667 6.00243133333333 6.41530895 5.5264404 6.09902735833333 5.86420291666667 6.00243133333333 6.00748140833333 7.42182476666667 6.67054473333333 5.75278305833333 6.331672825 6.114356 6.2575518 6.222544625 5.5912611 6.730005225 6.81075070833333 6.57216478333333 5.77706405 6.62419403333333 6.58965010833333 6.18629009166667 6.97078570833333 6.45714978333333 6.22164848333333 6.09411293333333 6.49099546666667 5.44782694166667 5.48522716666667 5.320395475 6.11282138333333 6.33974843333333 6.49186828333333 5.986172625 7.09178105 6.46408151666667 6.11916470833333 6.39560875 6.05827610833333 6.32609701666667 7.42616356666667 7.15207910833333 6.29693088333333 6.60442409166667 7.68875390833333 7.15037268333333 6.770698125

AC011477.1 9.64736453333333 10.0192491333333 9.15733181666667 9.29393176666667 9.48990181666667 9.37200478333333 9.08322639166667 9.08322639166667 9.52206101666667 8.99106136666667 8.66200190833333 9.46725561666667 8.7855717 9.62008553333333 9.980296075 9.93174519166667 10.3913684416667 8.83012083333333 9.65427285 9.71817874166667 9.6633032 10.0128890333333 9.31248745 10.1310824583333 9.49645880833333 10.1948545083333 9.78232589166667 9.951930125 9.28980095 9.26648069166667 9.52903631666667 10.2354161583333 9.78232589166667 9.85529955833333 9.89854821666667 10.16579105 9.72122880833333 9.732571725 9.64606555833333 9.65555468333333 9.92732885833333 9.8223954 10.1526159166667 10.1394934333333 9.77800786666667 9.80276991666667 10.2473472 9.639269375 9.81016945 9.67300651666667 9.466024975 9.68859575 9.75280021666667 10.2887994416667 9.9929911 10.3583768166667 9.36501691666667 9.288628775 9.828409225 9.80147330833333 9.06192556666667 9.24370573333333 8.386019225 8.06126420833333 10.2030274 9.64872759166667 8.53829400833333 8.87214213333333 8.58803331666667 8.45706139166667 8.196957075 8.74977199166667 9.137660375 9.412078125 8.79763931666667 9.09241896666667 9.97103178333333 9.124545075 8.94836124166667 8.69140489166667 8.308792025 9.604456975 8.24427536666667 9.87425795 8.957126925 9.11950295 9.981884775 9.59407011666667 9.120779125 9.865874625 8.796287025 9.40138860833333 9.919589575 10.0128890333333 9.78377983333333 8.4874442 9.29124689166667 8.34291140833333 8.39037005833333 9.574510875 10.2014392333333 9.32861675 9.630028125 10.30216785 9.767901775 9.40927853333333 10.1344061583333 7.62850093333333 9.39312421666667 9.42234536666667 9.558228875 9.83620561666667 9.83620561666667 10.31080925 9.98826495833333 9.53164766666667 9.08987286666667 8.93346260833333 9.48833739166667 9.769181275

AC211476.4 12.57544625 12.189826275 11.893046575 11.9561420333333 12.2367935833333 12.315517225 11.854762075 11.8907042166667 12.001763825 12.012563225 12.4659857583333 12.662426525 12.3062951833333 12.3318273166667 12.5938101166667 12.2130170916667 11.857596825 12.4811828916667 12.1985528083333 12.486478675 12.37994665 12.91648245 12.1582347333333 12.0695594916667 12.6784604583333 12.4897992666667 12.0437977833333 12.4629274416667 12.43663495 12.1985528083333 12.0587078333333 12.3920642833333 11.9175777083333 12.1467373 12.2760022333333 12.549239125 12.6273287833333 11.9409533916667 11.8753288833333 12.2978352666667 12.1151589416667 12.2367935833333 12.2509990916667 12.0933481 12.4925981 12.2130170916667 12.3888461083333 12.0508303333333 12.233818175 12.4270402083333 11.55966165 12.1151589416667 12.6814379666667 12.5327561583333 12.1637325833333 12.4811828916667 12.112519875 12.0933481 12.57544625 12.5689938083333 13.6959951583333 12.708001075 12.714470025 12.9417995 12.520205475 12.877352 12.526869925 12.7810968666667 12.377098625 12.4179335583333 12.9379797666667 13.2784613916667 12.233818175 12.280903925 13.07608845 12.9345571083333 13.0990734916667 13.472886275 12.5938101166667 12.9820572083333 13.1483004416667 13.4340660666667 12.1958518666667 12.360026475 12.656069625 13.2570588083333 13.3124174 12.0719435333333 13.61657615 12.5622655666667 12.7114207833333 13.6585047583333 13.2366307916667 12.6239992833333 13.0457394833333 13.41976115 13.33466465 13.6465647916667 12.583994125 13.11410875 12.6943613083333 13.0217509333333 13.1681379333333 13.1392820916667 12.700899 12.737637325 12.7548608916667 12.6390523916667 12.7243176833333 12.4713877916667 12.8804849833333 12.2649867083333 12.362762125 13.1483004416667 12.4811828916667 12.483417 13.507496675 13.0493833666667 12.3062951833333 12.79731905

LINC02655 5.50122666666667 7.52334426666667 6.97744145 8.002327575 8.43311395 6.43283198333333 8.05820623333333 8.72889535 8.10467081666667 7.22572939166667 7.81050794166667 8.34291140833333 8.70788464166667 8.350786675 7.51919369166667 7.54277829166667 6.345042725 8.135000075 6.27286545 8.86194551666667 8.49171125 8.19085505 7.32306280833333 6.57049163333333 6.45800148333333 7.11106645833333 7.63703748333333 6.59973925833333 8.00893274166667 6.798074725 5.797364775 7.22310433333333 7.95418985833333 7.00288033333333 6.3018823 5.98464866666667 7.018972075 6.55371424166667 6.47435308333333 6.37602205 6.02062880833333 6.73498085 5.74853151666667 6.22349896666667 6.25216388333333 6.59742333333333 6.061018125 7.242651775 6.39481431666667 6.16586921666667 6.635993725 6.38696694166667 8.65953305 7.53158344166667 6.89382319166667 5.875661225 8.21116674166667 6.56805765833333 6.73817556666667 6.36881363333333 6.59663961666667 7.376269825 6.63347905833333 7.75201335 7.403217625 7.22830545833333 7.702242475 8.217333875 7.12190308333333 8.08470013333333 7.36804055833333 7.74124910833333 8.84630949166667 7.04894575833333 8.20827265833333 7.63892676666667 7.25001376666667 7.54530115 7.02184869166667 7.42616356666667 7.725002775 8.00331975833333 7.43199838333333 5.57313729166667 7.30708359166667 8.51484585833333 7.5852857 8.63323710833333 7.96148 6.2575518 7.22830545833333 6.90726410833333 6.50269315 6.28300039166667 6.98061263333333 6.955970525 7.55228321666667 6.42987050833333 7.46714951666667 6.79889878333333 7.15207910833333 7.89363596666667 6.30672631666667 6.32221861666667 6.75448656666667 8.274413675 6.78346969166667 6.82831530833333 6.52770253333333 6.34272405 7.31101670833333 6.33974843333333 6.87915231666667 7.257981025 7.805183975 6.983731325 7.397338075 7.1838915 6.69077578333333 8.35414590833333

AL035461.3 11.5739352416667 11.0602668166667 11.4727106916667 11.29925645 10.81892915 11.2851728 10.8122935583333 11.4295243333333 10.9747287666667 11.3379048666667 11.4680404333333 10.65000145 10.55406855 11.176910275 11.0786204833333 11.2828826916667 13.2868729333333 10.7382525333333 11.6211812416667 11.4007975166667 11.3566719333333 11.0559555833333 11.6461752833333 11.5406276416667 11.206704325 11.6010023166667 11.167449 11.2637060666667 10.9656709666667 11.4032074083333 11.369137425 11.3086910583333 10.8543733166667 12.4334869333333 11.4389777833333 11.6603541833333 10.893006875 11.38647825 11.4032074083333 11.2613206166667 11.696738575 11.50386205 11.5524602083333 11.9484228666667 11.608832125 11.870125125 11.6763962416667 11.55725695 11.4637255666667 11.704120775 11.3110612083333 11.5811732666667 11.0901474083333 11.3137050833333 11.5935101166667 11.6510632333333 11.36658345 11.5935101166667 11.2248872 11.5835080416667 12.495555 11.7351470166667 11.9966241166667 12.1815738083333 11.49913775 10.7217191666667 12.4985912416667 11.58581685 11.9459843416667 11.4727106916667 11.75582275 12.233818175 12.2456214083333 11.2754195166667 11.1863927333333 11.9357587416667 11.4466154166667 11.2130362833333 11.8110067583333 11.354050525 11.8009085 11.1522751416667 11.4659061 12.048574475 10.9038566 11.2458122833333 12.1312062333333 11.8444568666667 10.9836119583333 11.8830544333333 12.0695594916667 12.5657299333333 11.3814972416667 11.2731520583333 12.4120381833333 11.4154777166667 11.5887081666667 11.2248872 11.43651565 12.0533945916667 12.1362874333333 12.46882785 11.8389506833333 11.6307827666667 12.448639725 11.1809665916667 11.854762075 12.486478675 12.0799709583333 11.6740143583333 11.6387066333333 11.2545716916667 11.8110067583333 12.7706567583333 11.6740143583333 11.900898825 12.2399882333333 12.0412908333333 11.5333541416667 11.5739352416667

LINC00334 5.37872604166667 4.18740718333333 4.58235359166667 4.36833938333333 4.39870788333333 4.73123783333333 4.362287525 4.924395575 4.5391327 4.846343575 4.56268375 4.84470445 4.90933671666667 4.23821421666667 4.072707875 4.39219723333333 4.79215016666667 6.32609701666667 5.81263556666667 4.84470445 4.25438255833333 4.12823880833333 4.43434340833333 4.651163825 5.826857975 4.270761425 5.15933271666667 4.80498945 5.032337825 4.99292960833333 4.924395575 4.73757844166667 4.76359273333333 4.81242730833333 5.83200043333333 5.614181625 4.69046943333333 5.47366834166667 4.58075485833333 5.10427118333333 5.17406135 4.51062659166667 5.00951006666667 5.69450579166667 4.76218691666667 5.74584819166667 6.05912289166667 5.21254813333333 5.49518486666667 5.02818118333333 5.5638092 4.90363349166667 4.64653290833333 5.72631065 5.20674935833333 5.32408766666667 5.26709018333333 6.05432435833333 5.29113221666667 5.601533025 4.72637159166667 4.436542725 5.02165799166667 4.65266215 4.62559538333333 4.96760800833333 4.42606766666667 4.72389136666667 5.21098853333333 5.53956186666667 4.76218691666667 5.37522741666667 4.91113061666667 4.67637581666667 4.185156975 4.663127775 5.44224698333333 6.71397423333333 5.59564198333333 5.524744 4.178444 4.15863169166667 4.96991525833333 4.51514020833333 4.71676371666667 4.33777051666667 4.95061065 4.73200895 5.1157001 4.70967379166667 5.552968675 5.41803970833333 5.40803009166667 5.40375725 5.44471205 5.443745225 4.60880553333333 5.241814025 4.711189975 5.40375725 4.928619175 4.51446953333333 5.69187064166667 5.185805625 5.26465755833333 4.56712020833333 6.40188925833333 6.30843955833333 5.22208751666667 6.68930803333333 5.38092105 5.695409275 5.90253445833333 6.55544135 6.0725726 5.706023225 5.74674768333333 6.208533725 6.06177335833333 4.96587955833333

TSPOAP1-AS1 6.80987979166667 11.774517575 11.6010023166667 11.696738575 10.7747006333333 10.8427097583333 10.4621870916667 11.2754195166667 11.39849905 10.6040700666667 10.9123222583333 5.69727409166667 10.0381389583333 11.9068035083333 11.5620897083333 10.9747287666667 11.394164525 10.7243055 11.354050525 11.4345196083333 11.0763715 11.7092046583333 11.9277646416667 10.0867346833333 11.0602668166667 7.44810904166667 11.234145525 11.57888155 10.9379330333333 11.5204619166667 10.80968025 11.3840616416667 11.0716730083333 11.167449 7.07277904166667 7.29870193333333 11.5359438166667 11.3036970916667 10.829344675 11.1454721416667 12.1789597916667 11.8177846 11.5912221 11.7276534666667 10.8974073083333 12.098586625 11.8036007166667 11.5237721 11.6063160416667 11.6648204916667 11.3713762333333 11.3110612083333 11.9917942583333 11.8059840833333 12.0957082333333 12.1870383833333 11.2944683583333 11.7188251083333 11.8036007166667 11.4466154166667 10.092690275 9.60317581666667 11.2130362833333 11.823121625 13.083804375 10.6040700666667 10.908225625 10.9101974666667 11.2594029083333 10.0395367833333 10.5371885333333 10.0692552166667 11.62343985 10.05421995 11.636069775 11.0447636416667 10.5165221083333 9.98678198333333 11.5333541416667 10.8446689833333 13.010652225 12.4334869333333 11.6159368333333 10.6208047916667 7.86100648333333 10.8075623083333 12.26263345 9.56775843333333 9.95651038333333 10.8853541583333 10.278425125 11.4727106916667 12.0587078333333 10.4810665833333 10.6712601416667 11.3713762333333 11.1977409666667 12.360026475 10.595444475 13.1811591833333 10.3791663666667 11.335701925 11.160332025 11.9561420333333 11.4520079916667 11.5548729916667 11.3761321333333 12.3708273333333 11.0353149833333 11.5406276416667 12.1582347333333 11.5985981416667 11.2178842166667 12.4659857583333 11.4439581833333 11.55966165 13.0874111666667 12.2456214083333 11.8779418166667 11.6135579916667

LINC00958 6.9521716 11.2710644083333 10.8363480833333 10.7382525333333 11.84953385 10.1171906166667 10.5578671833333 10.65000145 10.8746426833333 10.711383025 10.5202480833333 8.3827371 10.865335225 11.0353149833333 11.7252727333333 10.3180296083333 7.528191475 10.7830390666667 11.25019095 10.927189875 10.763080925 10.3301495666667 11.1717372166667 11.2481652416667 10.69605555 6.838229925 11.0716730083333 10.6691077583333 11.40831085 11.2637060666667 10.8383688833333 9.9348273 10.5653432666667 7.3593209 6.7586539 6.42254766666667 11.43651565 10.85192505 10.57648925 10.4604604416667 10.84728795 11.1522751416667 10.8030115583333 11.0850726666667 10.5126549333333 11.2665102166667 11.3590324 11.7435451333333 10.6343957583333 10.6613003916667 10.92060855 11.1182445333333 11.25019095 11.4154777166667 11.2944683583333 11.0716730083333 11.3517561583333 11.2665102166667 11.3060877416667 10.7243055 11.3713762333333 8.473255125 11.9917942583333 11.6534936583333 8.68193833333333 11.8110067583333 11.900898825 11.900898825 12.1467373 11.4868268416667 11.4345196083333 12.0233491166667 12.321479575 11.1387031666667 11.854762075 12.01526755 10.81892915 11.1454721416667 11.5379794583333 10.0192491333333 10.752680775 11.4032074083333 12.40033825 11.6159368333333 11.7066087916667 9.204023275 11.2458122833333 10.9419169166667 12.1073504333333 10.84728795 11.6335953333333 11.4589874916667 11.9123900916667 11.54529205 12.001763825 10.865335225 11.8389506833333 10.3164307833333 11.57888155 10.6897099583333 11.8779418166667 11.1274628333333 11.4389777833333 12.1524570666667 11.8086577 11.84953385 11.8389506833333 12.421258225 12.22065105 11.2458122833333 10.8446689833333 11.0447636416667 13.0016357 12.5870727333333 11.720720125 11.6034592083333 8.17405961666667 12.6784604583333 11.3517561583333 10.5596196916667

BX890604.2 7.20609148333333 7.52419651666667 5.60240131666667 8.05364296666667 9.05803846666667 7.78160973333333 5.879831175 6.88087174166667 5.33093755 7.354569825 7.20762451666667 6.62118421666667 8.45231269166667 7.79168211666667 6.87036725833333 7.71468463333333 7.57728179166667 8.726496825 7.52499111666667 8.67196278333333 7.44728600833333 8.59246085 7.65118818333333 7.27688146666667 8.0743079 6.760855075 9.5167557 7.51739635 7.86191561666667 7.21920913333333 8.82317351666667 8.18413825 8.64530154166667 6.87204776666667 8.33526710833333 7.25001376666667 7.34734821666667 7.793674875 7.67007186666667 7.9017682 8.015640975 7.5199474 8.99106136666667 8.29431475833333 7.73008085 8.60766696666667 8.16414461666667 8.59246085 8.75436599166667 7.931709675 8.91905655 8.77122099166667 7.01349440833333 7.86191561666667 5.59644218333333 7.30368923333333 7.62092879166667 7.20609148333333 8.618487425 8.07103965 8.51357456666667 7.01821471666667 6.231190225 7.89268988333333 8.97885180833333 7.96236178333333 7.4754804 6.382488125 6.103062825 6.49790895 7.67583710833333 7.648594075 8.72277565833333 7.57814523333333 4.91868544166667 7.32203796666667 7.46158223333333 8.60047110833333 6.727504025 8.55899596666667 6.81924664166667 7.583437825 7.25439505 7.10174751666667 7.99100168333333 6.50659699166667 10.888969925 5.21754135 7.89455625833333 7.25001376666667 8.981140225 8.01671704166667 9.14044470833333 7.55492436666667 11.02834235 7.73489795833333 7.04124730833333 8.15235975 7.44983849166667 8.23155004166667 8.18700308333333 7.38805711666667 9.00847981666667 8.115340475 7.34163923333333 8.01671704166667 7.71635294166667 7.38805711666667 8.11834109166667 6.88935775 8.94319444166667 8.33307035833333 7.2687109 7.297972275 4.2966536 7.25439505 7.48714180833333 6.73583865833333 8.66591204166667 8.5579005

AC009139.1 5.54038896666667 5.35594408333333 7.71121305833333 4.613537925 9.11687908333333 10.6796574916667 4.53228734166667 6.73733449166667 5.07245089166667 7.738549275 7.28603906666667 4.456836225 7.48480789166667 5.60738530833333 4.9956547 8.40616386666667 6.0725726 6.70438826666667 7.16885323333333 7.83530684166667 9.92405621666667 7.953283475 6.967754075 5.35594408333333 7.52905674166667 8.96098959166667 7.72751683333333 5.71234138333333 7.59936721666667 5.65972369166667 4.60532013333333 6.95064391666667 6.18946745833333 5.013582075 5.39028695833333 5.9244919 6.99658926666667 9.85252195833333 8.78437034166667 9.49645880833333 8.926479 6.727504025 5.18662866666667 5.534144725 4.98037645 9.44548651666667 7.28275553333333 4.74969148333333 7.3593209 7.80960054166667 6.27692523333333 7.17617635833333 6.27620438333333 7.90947090833333 4.62957980833333 7.848979625 9.05803846666667 7.48147895 6.36034525833333 6.23694766666667 7.311835325 6.59434034166667 6.57919131666667 4.93610370833333 13.9351442666667 7.47235645 7.57549880833333 6.321419225 6.26957381666667 7.29390568333333 7.59853308333333 7.28603906666667 5.27819885 6.67222361666667 5.78131454166667 12.1666511583333 7.28019619166667 7.6234272 6.78514255 7.6423852 13.6529585666667 10.4088283666667 5.8569379 5.31788553333333 8.62645034166667 13.9195785166667 12.6061961916667 7.62178835 8.72413925 9.23537203333333 6.86946320833333 6.88935775 7.71291500833333 6.850638675 4.71521985 7.61582255833333 5.87646783333333 14.3227822416667 5.6883975 7.14716405 7.528191475 6.55687646666667 6.75112116666667 6.6279805 5.44052959166667 7.354569825 8.22438401666667 5.57244380833333 7.87889741666667 8.22944233333333 8.40105418333333 5.51981635 7.23088359166667 7.223930825 7.6234272 7.284330325 11.704120775 5.03308739166667 6.45206855833333 7.04433776666667

LINC01927 4.91239837083333 5.12574221666667 4.91651100833333 4.90564114166667 5.14370322083333 5.03683630833333 4.70228769583333 4.580383675 5.02515947083333 5.46049224166667 4.7227611375 5.324550575 4.42571156666667 4.20457442083333 4.64364424583333 4.09945990833333 5.3491414125 4.14128700833333 5.50798936666667 4.53656142083333 4.3059003625 4.7100905125 4.76253908333333 5.1514602 4.75923252083333 4.989291875 6.3063192875 5.60534394166667 5.35639775 5.59604208333333 5.01397411666667 4.99924067916667 4.64364424583333 4.573468625 6.0327051125 7.58567632916667 5.06236944583333 5.91455771666667 4.73633866666667 4.99604264583333 5.35908853333333 4.8500567875 6.177514275 5.71366117916667 4.35264999166667 5.17606769166667 5.35544069166667 5.10035652083333 4.7347751125 5.001652975 5.64879312916667 5.2899472 4.5916078 7.485165925 4.70788147083333 5.17368165833333 5.529409225 5.054877525 5.78007172083333 5.28550504166667 4.71480429166667 5.28467671666667 4.41826714166667 4.316396475 5.1120396375 4.4771660625 4.93649753333333 4.2001572625 4.91984388333333 4.44557661666667 4.13982862083333 4.58641075416667 4.60757309583333 4.07455424166667 3.88922899583333 4.63912953333333 4.884556825 4.17809211666667 4.30047578333333 4.316396475 5.33677216666667 4.26971390416667 4.7164078125 4.4433001 4.5947138625 4.14437926666667 6.25104499583333 5.71011037083333 4.83726525833333 4.73553370416667 4.58344129166667 5.20364310833333 5.1514602 4.7124346125 5.180264225 6.60401399583333 4.42215889583333 5.31318475833333 4.77524804166667 6.1195639875 4.67594147083333 5.255702425 4.84677434166667 5.11450399583333 4.5796823 3.97680239166667 4.69237900416667 6.3162301875 4.87862659583333 6.56060569583333 5.60534394166667 5.237942125 5.02437999583333 7.5119085 5.08524204166667 5.46133727916667 7.13380124583333 4.63843010833333 4.98005168333333 4.854350675

LINC00683 4.91239837083333 5.12574221666667 4.91651100833333 4.90564114166667 5.14370322083333 5.03683630833333 4.70228769583333 4.580383675 5.02515947083333 5.46049224166667 4.7227611375 5.324550575 4.42571156666667 4.20457442083333 4.64364424583333 4.09945990833333 5.3491414125 4.14128700833333 5.50798936666667 4.53656142083333 4.3059003625 4.7100905125 4.76253908333333 5.1514602 4.75923252083333 4.989291875 6.3063192875 5.60534394166667 5.35639775 5.59604208333333 5.01397411666667 4.99924067916667 4.64364424583333 4.573468625 6.0327051125 7.58567632916667 5.06236944583333 5.91455771666667 4.73633866666667 4.99604264583333 5.35908853333333 4.8500567875 6.177514275 5.71366117916667 4.35264999166667 5.17606769166667 5.35544069166667 5.10035652083333 4.7347751125 5.001652975 5.64879312916667 5.2899472 4.5916078 7.485165925 4.70788147083333 5.17368165833333 5.529409225 5.054877525 5.78007172083333 5.28550504166667 4.71480429166667 5.28467671666667 4.41826714166667 4.316396475 5.1120396375 4.4771660625 4.93649753333333 4.2001572625 4.91984388333333 4.44557661666667 4.13982862083333 4.58641075416667 4.60757309583333 4.07455424166667 3.88922899583333 4.63912953333333 4.884556825 4.17809211666667 4.30047578333333 4.316396475 5.33677216666667 4.26971390416667 4.7164078125 4.4433001 4.5947138625 4.14437926666667 6.25104499583333 5.71011037083333 4.83726525833333 4.73553370416667 4.58344129166667 5.20364310833333 5.1514602 4.7124346125 5.180264225 6.60401399583333 4.42215889583333 5.31318475833333 4.77524804166667 6.1195639875 4.67594147083333 5.255702425 4.84677434166667 5.11450399583333 4.5796823 3.97680239166667 4.69237900416667 6.3162301875 4.87862659583333 6.56060569583333 5.60534394166667 5.237942125 5.02437999583333 7.5119085 5.08524204166667 5.46133727916667 7.13380124583333 4.63843010833333 4.98005168333333 4.854350675

AL606534.3 7.09746866666667 10.0607396166667 9.97701388333333 10.1794807166667 10.881139625 8.47772004166667 11.4154777166667 10.4884789583333 11.0874478833333 8.92998051666667 10.0316996666667 11.3110612083333 11.0786204833333 10.2387529583333 9.11422543333333 9.81494466666667 8.70899691666667 10.2619661583333 7.61667270833333 10.1930602583333 10.65000145 9.967859125 9.78825455 8.24214254166667 7.55402225833333 9.48833739166667 9.120779125 7.83627273333333 8.09138268333333 8.925211725 7.21511196666667 8.57406105 9.58024334166667 8.650096975 7.31633914166667 6.71961385 7.33817588333333 7.37456893333333 7.54978125 6.25446955 6.36633304166667 8.0743079 7.24350165 7.11334874166667 8.04674969166667 7.15132348333333 6.85232785833333 8.61348725 7.28893561666667 6.09333521666667 5.837626175 7.33817588333333 10.132787925 6.802823925 8.99106136666667 7.12868305 10.265208825 6.78970486666667 7.080264325 7.54530115 8.01279515833333 10.5145241083333 6.86096935833333 9.7484308 9.865874625 9.49645880833333 10.2276958666667 10.752680775 8.941771525 9.30482685833333 10.591906075 10.0053221583333 10.7322398666667 9.558228875 10.3089135 9.81494466666667 9.74405148333333 8.34967031666667 9.22419906666667 9.44441939166667 10.6671034666667 10.9882447083333 9.50014711666667 7.91550765833333 9.81335845 10.1930602583333 9.90727683333333 10.87665705 10.1776993666667 8.75905724166667 9.128527525 7.78810495 6.48159381666667 8.20093078333333 8.42317835833333 8.48415245 8.527957175 7.03655466666667 9.92732885833333 6.64220785 8.23251158333333 10.3282105 6.52835620833333 6.69708403333333 7.928697775 9.25094153333333 8.73487054166667 8.367490075 8.60535770833333 7.85554625 5.995352275 6.80051309166667 6.41925481666667 7.04507230833333 9.4302854 7.671941925 8.08564014166667 6.41125588333333 5.875661225 10.6671034666667

TRAF3IP2-AS1 12.6239992833333 12.9787381333333 12.9025226333333 12.9856740416667 12.451622875 13.0144948166667 12.2456214083333 12.8182497583333 12.714470025 12.7481848833333 12.8041650666667 11.49913775 12.6273287833333 13.025698125 12.9713989666667 12.4629274416667 13.3080989916667 12.7446559166667 12.8041650666667 12.8295815416667 12.78428845 13.126641525 13.069349375 13.1561940833333 12.617619125 11.93863675 12.7206590083333 12.7412194083333 12.6814379666667 12.7412194083333 13.126641525 12.9202404083333 12.630386725 13.3953731416667 12.2130170916667 11.8364860833333 12.91648245 12.8804849833333 13.0606794083333 12.7612379833333 12.7810968666667 12.8070736416667 13.0493833666667 13.1030363083333 12.5969000166667 12.9345571083333 13.0340459666667 12.800813375 13.0952688916667 12.7873340416667 13.0531395083333 13.621999975 12.9025226333333 13.20700295 13.2032715083333 13.1068881 13.0058047166667 12.7873340416667 13.126641525 12.877352 12.9202404083333 12.368506525 12.0957082333333 12.4240585416667 13.3438936916667 12.448639725 13.1722951416667 12.0904755416667 13.11410875 12.9239130666667 12.3943394583333 12.9820572083333 13.4535638583333 12.8804849833333 12.9787381333333 13.665267475 12.408692 12.7114207833333 13.0340459666667 12.7412194083333 13.4340660666667 12.8070736416667 13.621999975 13.2412141666667 13.0457394833333 12.7309384666667 13.1943968666667 12.78428845 12.14966815 12.840675275 13.2784613916667 13.33466465 13.0217509333333 13.0874111666667 13.40047325 12.6912332583333 13.0183924583333 12.9097414333333 13.7856886083333 12.6425501583333 13.74834795 12.3544155583333 12.6356084 12.873197975 12.6686352083333 12.5016594166667 12.9970547416667 13.3902474333333 13.1525887583333 13.726268275 13.0376313166667 13.3035558583333 14.2116827666667 13.0727760333333 13.2529881833333 13.025698125 12.7810968666667 13.1350912416667 12.7412194083333 12.6943613083333

AL445989.2 8.387188975 10.332087525 9.73388581666667 10.132787925 10.1120281166667 9.12724555 10.1930602583333 10.0771266166667 10.0943656833333 9.831337375 9.73556275833333 10.64050025 10.5000230916667 10.40007745 10.3371834416667 9.757653125 9.50014711666667 10.613486775 10.0381389583333 10.475071575 10.4773720583333 10.752680775 10.6208047916667 10.2175479333333 10.0784644666667 8.90815406666667 9.77392694166667 10.17093795 9.96321 9.8892648 10.0867346833333 9.94865183333333 9.90270463333333 9.36501691666667 8.01279515833333 8.51932215 9.71966379166667 9.867307775 9.757653125 9.80574389166667 10.260350775 10.0509998083333 10.0818728583333 10.096001875 9.58715105833333 10.2902993333333 9.94262806666667 9.9348273 10.1120281166667 9.84081016666667 9.72971833333333 9.97891528333333 9.83458021666667 9.85953499166667 9.64456304166667 9.7069395 10.287159 10.126531775 10.0425633416667 9.84388436666667 9.6633032 9.256048375 9.53278261666667 9.55070993333333 11.0716730083333 9.41328055833333 9.56332510833333 10.1458214083333 9.39042674166667 9.71504586666667 9.578771175 10.2854987416667 10.279838075 9.71257970833333 9.88347994166667 9.75593879166667 9.58572294166667 9.72272495833333 9.37200478333333 9.558228875 10.1489361666667 9.96941311666667 9.180393975 8.5427737 9.204023275 9.904065125 10.6343957583333 9.50689216666667 10.105187625 8.93700485 9.5341054 10.0882671583333 8.43525693333333 8.500249575 8.63097468333333 8.96465185 9.4302854 9.83774009166667 9.19737939166667 9.15983380833333 8.74977199166667 10.0607396166667 9.24804320833333 9.767901775 8.93453735833333 9.4263481 9.16242148333333 8.88103920833333 8.9106089 8.503369575 9.508238925 9.254766825 8.40939095833333 10.23249925 8.278515525 9.52903631666667 11.0397399833333 9.54939620833333 9.70023275833333 9.991570475

ADD3-AS1 5.42515045833333 6.22073784166667 6.049179025 5.47366834166667 6.0315094 5.40375725 6.27455109166667 6.40115713333333 6.64536209166667 6.24109811666667 5.87161330833333 5.257582 5.88291568333333 6.49720533333333 6.083496175 5.08336286666667 5.89039591666667 5.84406485 6.076673725 5.84166365 5.90253445833333 6.42169948333333 5.92010973333333 6.40029824166667 5.95765828333333 6.432044225 6.54596676666667 5.54490043333333 6.70927374166667 6.19675590833333 6.32934243333333 6.22740323333333 6.3840179 5.59357184166667 5.4096421 5.645614725 6.04295785833333 6.2419738 6.74731924166667 6.91920954166667 6.67877244166667 6.690101625 6.45953320833333 6.94252621666667 6.182148775 6.61725120833333 6.9853877 7.31447869166667 5.6525194 6.211679225 7.44478088333333 6.321419225 6.97501571666667 6.42987050833333 7.27935889166667 6.795014675 6.857401975 7.649391625 6.47872195 6.65618500833333 4.41295244166667 4.69350425 5.71592235833333 4.85225948333333 4.90933671666667 5.38613150833333 5.04085009166667 4.59345305 5.07492725 5.59424653333333 4.44597125 4.98704916666667 5.322240725 4.37878591666667 5.070082275 4.66661991666667 4.684258075 4.55489758333333 4.97055159166667 5.011044325 4.55571135833333 4.91203205 5.19649501666667 5.07336385 4.871032675 5.24020400833333 5.47001155833333 4.70189450833333 4.94495284166667 5.20404056666667 5.192375575 4.783293425 4.8148076 5.01028051666667 5.16909541666667 4.94816246666667 5.29824615 5.4760094 4.96673286666667 5.12104158333333 5.483555325 5.13063719166667 4.84553110833333 5.73740016666667 5.46922286666667 4.80260285 4.872797375 6.26883340833333 5.17649486666667 6.40188925833333 5.06841553333333 5.0768141 5.013582075 4.74810536666667 5.37165643333333 5.15428810833333 5.28167781666667 6.4026933 4.81864974166667 6.39329076666667

LINC00943 5.0090774625 5.07457305416667 5.57510055 5.6289692875 5.89082186666667 5.45310899166667 5.4563989 5.79531919583333 5.8130756125 4.88040788333333 5.21800475833333 5.48653273333333 5.788448925 5.4704391 4.82716035416667 7.16609550833333 5.71278936666667 5.576663225 4.81204550416667 5.08682546666667 5.64515543333333 4.373328775 5.78928647916667 6.4859428875 6.20965022083333 4.7775658625 6.08622699583333 5.53652546666667 4.86668622916667 5.2414248375 5.59080235 6.01289963333333 5.62570656666667 6.02019989166667 5.04439849583333 4.5110223625 5.64067454166667 5.10204878333333 5.20277657083333 4.95808569166667 5.77404946666667 5.12306243333333 5.87606452916667 5.95370415416667 5.41848557083333 6.05133759583333 5.55908222916667 5.19519462916667 6.4370707875 5.57992626666667 5.15566035833333 5.71278936666667 5.99230437916667 6.11073158333333 4.74772855833333 5.75787718333333 4.86910565 7.3834971875 5.104726775 6.0013075 5.54804859583333 5.3217510625 5.4506947125 4.58928309583333 4.61967494583333 5.60114360833333 4.58928309583333 5.26503811666667 5.9102806875 6.4370707875 4.60283762916667 4.69237900416667 4.8812180875 5.1120396375 5.09345835 4.4279855125 6.3264674625 5.89802375416667 4.361957125 5.053094475 4.56676010833333 4.834722225 6.1446592 5.08858232916667 5.0664177625 5.1769021875 7.9647351875 5.35817842916667 5.53462376666667 4.9541899625 5.09428083333333 4.92480807916667 5.5089590875 4.54241319583333 6.24790635 5.19767359583333 4.76324199166667 5.20277657083333 4.66861742083333 7.3709376875 6.10909092916667 5.4581056875 6.5533437 5.66196520833333 6.3313176125 4.76324199166667 4.85181332916667 6.574184375 6.2013688375 7.0447050375 5.61635895833333 6.22530575416667 5.754058775 6.18013588333333 5.35365422083333 5.11367970833333 4.57823024166667 7.0987614375 4.88946786666667 5.87947186666667

LINC00944 5.0090774625 5.07457305416667 5.57510055 5.6289692875 5.89082186666667 5.45310899166667 5.4563989 5.79531919583333 5.8130756125 4.88040788333333 5.21800475833333 5.48653273333333 5.788448925 5.4704391 4.82716035416667 7.16609550833333 5.71278936666667 5.576663225 4.81204550416667 5.08682546666667 5.64515543333333 4.373328775 5.78928647916667 6.4859428875 6.20965022083333 4.7775658625 6.08622699583333 5.53652546666667 4.86668622916667 5.2414248375 5.59080235 6.01289963333333 5.62570656666667 6.02019989166667 5.04439849583333 4.5110223625 5.64067454166667 5.10204878333333 5.20277657083333 4.95808569166667 5.77404946666667 5.12306243333333 5.87606452916667 5.95370415416667 5.41848557083333 6.05133759583333 5.55908222916667 5.19519462916667 6.4370707875 5.57992626666667 5.15566035833333 5.71278936666667 5.99230437916667 6.11073158333333 4.74772855833333 5.75787718333333 4.86910565 7.3834971875 5.104726775 6.0013075 5.54804859583333 5.3217510625 5.4506947125 4.58928309583333 4.61967494583333 5.60114360833333 4.58928309583333 5.26503811666667 5.9102806875 6.4370707875 4.60283762916667 4.69237900416667 4.8812180875 5.1120396375 5.09345835 4.4279855125 6.3264674625 5.89802375416667 4.361957125 5.053094475 4.56676010833333 4.834722225 6.1446592 5.08858232916667 5.0664177625 5.1769021875 7.9647351875 5.35817842916667 5.53462376666667 4.9541899625 5.09428083333333 4.92480807916667 5.5089590875 4.54241319583333 6.24790635 5.19767359583333 4.76324199166667 5.20277657083333 4.66861742083333 7.3709376875 6.10909092916667 5.4581056875 6.5533437 5.66196520833333 6.3313176125 4.76324199166667 4.85181332916667 6.574184375 6.2013688375 7.0447050375 5.61635895833333 6.22530575416667 5.754058775 6.18013588333333 5.35365422083333 5.11367970833333 4.57823024166667 7.0987614375 4.88946786666667 5.87947186666667

AL031686.1 10.240385675 9.89854821666667 9.94385811666667 10.0771266166667 10.3845893166667 10.3282105 10.0943656833333 10.1378527416667 10.332087525 9.99887365833333 10.2354161583333 9.78232589166667 9.64456304166667 9.79859535 10.1841002166667 10.0492420416667 11.2106026166667 9.9664556 10.6517451416667 10.29196065 10.0628765 10.0882671583333 10.1895448333333 9.974114125 10.1473463666667 10.1841002166667 10.0911134416667 9.93314271666667 10.1278941 10.1575664416667 9.93793718333333 10.0300876416667 9.65673601666667 10.1895448333333 10.3498888833333 10.4434835166667 9.610331375 9.82384475833333 10.0175973583333 9.98989775 9.96941311666667 9.78069864166667 9.90140289166667 9.95043048333333 9.80574389166667 9.97103178333333 10.0021960666667 9.53024233333333 10.092690275 10.0771266166667 9.92732885833333 10.01598095 9.33115149166667 9.56478920833333 9.84980565833333 9.77392694166667 9.62143423333333 10.0267235 9.87885509166667 9.88202425 9.83458021666667 9.9929911 10.5165221083333 9.62143423333333 10.335375425 9.87742366666667 10.4698496583333 10.1841002166667 10.0559299333333 10.2244563666667 10.5671895333333 10.6897099583333 9.96000436666667 9.65779833333333 10.0818728583333 9.83620561666667 10.006716175 10.265208825 10.3684429583333 10.92060855 9.362149025 10.2439545166667 10.1811538916667 9.991570475 9.72553698333333 9.81494466666667 9.88035836666667 9.54094585833333 10.398066475 10.0628765 10.3214407083333 10.2753557916667 10.3232071333333 9.93174519166667 9.32203853333333 10.613486775 9.75280021666667 8.85242150833333 9.32861675 9.66874293333333 9.775249875 9.9348273 10.251873975 10.132787925 10.1099622416667 9.52589869166667 9.93026274166667 10.4884789583333 9.97263284166667 9.99887365833333 10.4531891333333 10.4158197333333 9.213538675 10.1473463666667 9.74707850833333 10.30417975 9.78377983333333 10.0175973583333 7.75584805833333 9.52206101666667

AL590440.1 7.525750625 9.74259324166667 9.14942049166667 9.61303635833333 8.787979925 8.63451644166667 8.485317225 9.91313129166667 8.9239143 8.69607195 9.04373345 8.22944233333333 8.525776075 9.967859125 10.36490795 9.43421313333333 10.1508267916667 8.65694638333333 9.6529159 9.71504586666667 9.70023275833333 9.53024233333333 9.96321 9.92405621666667 10.01598095 8.796287025 9.18999519166667 8.92760421666667 8.69018435 9.34355995 9.57185823333333 8.83649291666667 9.39581173333333 8.15341585833333 9.77666165 7.356869825 8.82199014166667 9.362149025 9.44156545833333 9.81785751666667 10.7217191666667 9.205281175 9.819189925 10.17093795 9.39994525833333 9.90140289166667 10.92060855 9.90876665 9.11552891666667 10.40007745 9.32585686666667 9.28980095 9.35667674166667 9.51536090833333 10.1310824583333 10.3123807083333 9.18598278333333 9.75423146666667 9.455130225 10.044127975 7.50284468333333 7.01432623333333 8.66200190833333 7.54201473333333 7.018972075 8.12889401666667 7.15381125833333 6.608946875 8.0797047 8.55223988333333 8.86401731666667 8.426213375 7.56846155833333 6.96919575833333 8.15639794166667 6.55687646666667 8.525776075 7.67103138333333 8.22944233333333 6.97581613333333 6.09080005833333 6.89078670833333 8.94940024166667 9.1323295 7.97080016666667 7.43443834166667 7.14080546666667 7.39223886666667 7.299523825 7.84714276666667 7.36105020833333 7.71373668333333 6.92634120833333 8.005090175 7.82045105 7.603532025 7.07767666666667 7.23187408333333 7.04741685833333 9.14044470833333 7.829781175 7.92482581666667 7.49513213333333 8.02635733333333 8.28578650833333 6.42828630833333 7.24174676666667 8.48198979166667 7.33500344166667 7.62747078333333 8.57076716666667 6.91433144166667 7.671941925 7.30288484166667 7.292382975 7.92297084166667 7.15690719166667 7.581616625 7.13662455 7.00675840833333

AC006115.2 14.7109633 14.3976364916667 14.8754401291667 14.2324967791667 14.2069116708333 13.6079968583333 14.3002429416667 12.6830873208333 14.0071025833333 15.2510196333333 13.4906030791667 14.9135949708333 14.81483635 12.6672464625 13.402907675 13.1049622041667 14.9825472666667 13.6079968583333 13.4703144958333 13.4416114333333 14.0389727125 12.1827685125 13.5157223 14.3685685875 13.4653575375 15.5029783583333 15.2343050041667 14.6285630208333 14.259484 14.0389727125 13.5852119833333 13.9382603958333 15.0500659208333 13.9310886833333 16.1712486958333 15.604745325 13.976918575 14.5275544125 14.3378459375 13.6374500375 13.8396047041667 13.9531214166667 13.8945401375 14.1891447166667 14.4569724458333 12.6223498041667 12.3276188083333 13.5157223 13.3601437291667 14.5163628458333 15.0500659208333 14.9687284083333 11.5560649708333 10.7517113333333 11.2982942833333 11.6849357791667 14.0476385833333 13.6682732291667 14.5390126458333 15.2172713916667 15.4191432916667 14.7312407666667 15.3036724791667 15.2172713916667 14.6783790916667 15.3562579166667 15.3217585958333 15.4527823125 15.4356488458333 15.0371793 15.4191432916667 15.2510196333333 15.1866291208333 15.5433815958333 15.4191432916667 15.8152656333333 15.0371793 15.5828645041667 15.5227168458333 15.4527823125 14.3795643791667 14.4758763708333 14.8506082041667 14.9276467583333 15.171515225 14.4375415208333 13.6436250041667 14.6582034208333 14.062173875 15.0964940625 15.3386042 15.1247206125 14.3478349916667 15.0110329458333 13.3503720625 14.7415996125 15.0640660875 15.0245563791667 14.8627184375 15.3884955458333 14.3378459375 14.4758763708333 14.9553070416667 14.2504760375 13.7167878875 14.259484 14.7774288 13.8726803083333 15.5433815958333 15.1866291208333 14.941782525 15.0640660875 12.1348590875 12.9116226875 13.1086553875 12.2298667 15.0500659208333 15.1866291208333 14.6783790916667 13.9608453458333

ZIM2-AS1 14.7109633 14.3976364916667 14.8754401291667 14.2324967791667 14.2069116708333 13.6079968583333 14.3002429416667 12.6830873208333 14.0071025833333 15.2510196333333 13.4906030791667 14.9135949708333 14.81483635 12.6672464625 13.402907675 13.1049622041667 14.9825472666667 13.6079968583333 13.4703144958333 13.4416114333333 14.0389727125 12.1827685125 13.5157223 14.3685685875 13.4653575375 15.5029783583333 15.2343050041667 14.6285630208333 14.259484 14.0389727125 13.5852119833333 13.9382603958333 15.0500659208333 13.9310886833333 16.1712486958333 15.604745325 13.976918575 14.5275544125 14.3378459375 13.6374500375 13.8396047041667 13.9531214166667 13.8945401375 14.1891447166667 14.4569724458333 12.6223498041667 12.3276188083333 13.5157223 13.3601437291667 14.5163628458333 15.0500659208333 14.9687284083333 11.5560649708333 10.7517113333333 11.2982942833333 11.6849357791667 14.0476385833333 13.6682732291667 14.5390126458333 15.2172713916667 15.4191432916667 14.7312407666667 15.3036724791667 15.2172713916667 14.6783790916667 15.3562579166667 15.3217585958333 15.4527823125 15.4356488458333 15.0371793 15.4191432916667 15.2510196333333 15.1866291208333 15.5433815958333 15.4191432916667 15.8152656333333 15.0371793 15.5828645041667 15.5227168458333 15.4527823125 14.3795643791667 14.4758763708333 14.8506082041667 14.9276467583333 15.171515225 14.4375415208333 13.6436250041667 14.6582034208333 14.062173875 15.0964940625 15.3386042 15.1247206125 14.3478349916667 15.0110329458333 13.3503720625 14.7415996125 15.0640660875 15.0245563791667 14.8627184375 15.3884955458333 14.3378459375 14.4758763708333 14.9553070416667 14.2504760375 13.7167878875 14.259484 14.7774288 13.8726803083333 15.5433815958333 15.1866291208333 14.941782525 15.0640660875 12.1348590875 12.9116226875 13.1086553875 12.2298667 15.0500659208333 15.1866291208333 14.6783790916667 13.9608453458333

AC119674.1 8.21888479166667 8.4749057125 8.4066672375 7.69419850416667 8.29798353333333 8.56485450416667 8.43049535833333 8.01230898333333 8.21564716666667 8.653968525 8.28309340416667 8.0113613375 8.1856332875 8.26447238333333 8.48794788333333 8.4652068 8.7683520875 8.84922245 9.19059094166667 8.253910825 8.53299352916667 8.05106545416667 8.7294721625 8.237116675 8.2749409125 8.08807985833333 7.99891245416667 8.0229828125 8.2749409125 8.3691886 8.5766904125 8.09697064166667 8.13033523333333 8.5153910375 8.30932117916667 8.5219441125 8.10979551666667 8.92704160833333 8.6151378 8.478197975 8.6954348625 8.744626 8.717573275 8.7221892125 8.05521121666667 8.5153910375 8.85177119583333 8.49225294166667 8.4155136125 8.19450025833333 8.33801369583333 8.25508305416667 8.32004450833333 8.94248298333333 8.62134730833333 8.64350882083333 8.82010505 8.8739673125 8.18747397083333 8.40365409166667 7.4469252875 8.19547069583333 8.01718190833333 7.8147429125 8.24375042083333 8.33685162916667 10.5550528583333 7.757215925 8.39540114166667 8.1549084 8.20772733333333 8.02391114583333 8.33362425416667 8.2582552125 8.21154779583333 8.16856995 8.292755375 8.345719475 8.70725121666667 8.042217825 8.99165719583333 7.33540539166667 8.83955505833333 8.26447238333333 8.56385307916667 7.9738690875 7.9188695375 7.90319782083333 7.25934499583333 8.34459307083333 8.24268400833333 8.3669622125 8.61791067083333 8.7514338625 7.9952138125 8.13230399166667 9.05004839166667 8.1856332875 8.59768307916667 8.8766209125 8.400597175 8.08129417083333 8.17675108333333 8.5285699625 8.02207354166667 8.28422200416667 8.47379047083333 8.76085201666667 7.895039 8.81629975833333 8.7408531625 8.28097427916667 8.58019327083333 9.1278865375 8.30004057916667 8.2467799625 8.72712726666667 8.71064849166667 8.37316226666667 8.29604469583333

LINC01767 8.21888479166667 8.4749057125 8.4066672375 7.69419850416667 8.29798353333333 8.56485450416667 8.43049535833333 8.01230898333333 8.21564716666667 8.653968525 8.28309340416667 8.0113613375 8.1856332875 8.26447238333333 8.48794788333333 8.4652068 8.7683520875 8.84922245 9.19059094166667 8.253910825 8.53299352916667 8.05106545416667 8.7294721625 8.237116675 8.2749409125 8.08807985833333 7.99891245416667 8.0229828125 8.2749409125 8.3691886 8.5766904125 8.09697064166667 8.13033523333333 8.5153910375 8.30932117916667 8.5219441125 8.10979551666667 8.92704160833333 8.6151378 8.478197975 8.6954348625 8.744626 8.717573275 8.7221892125 8.05521121666667 8.5153910375 8.85177119583333 8.49225294166667 8.4155136125 8.19450025833333 8.33801369583333 8.25508305416667 8.32004450833333 8.94248298333333 8.62134730833333 8.64350882083333 8.82010505 8.8739673125 8.18747397083333 8.40365409166667 7.4469252875 8.19547069583333 8.01718190833333 7.8147429125 8.24375042083333 8.33685162916667 10.5550528583333 7.757215925 8.39540114166667 8.1549084 8.20772733333333 8.02391114583333 8.33362425416667 8.2582552125 8.21154779583333 8.16856995 8.292755375 8.345719475 8.70725121666667 8.042217825 8.99165719583333 7.33540539166667 8.83955505833333 8.26447238333333 8.56385307916667 7.9738690875 7.9188695375 7.90319782083333 7.25934499583333 8.34459307083333 8.24268400833333 8.3669622125 8.61791067083333 8.7514338625 7.9952138125 8.13230399166667 9.05004839166667 8.1856332875 8.59768307916667 8.8766209125 8.400597175 8.08129417083333 8.17675108333333 8.5285699625 8.02207354166667 8.28422200416667 8.47379047083333 8.76085201666667 7.895039 8.81629975833333 8.7408531625 8.28097427916667 8.58019327083333 9.1278865375 8.30004057916667 8.2467799625 8.72712726666667 8.71064849166667 8.37316226666667 8.29604469583333

AL157371.2 5.33192491666667 4.18281758333333 4.04261585 4.01435405833333 4.36552305 4.5308601 3.870040525 4.22202384166667 4.98620748333333 4.41509394166667 4.01991520833333 4.04261585 3.988166225 4.16321256666667 4.236686625 4.00885690833333 4.85225948333333 4.19596420833333 4.62007759166667 4.023105425 4.23511105833333 4.62957980833333 4.568704925 4.66817654166667 4.373711675 4.12284004166667 4.25905575 4.77798016666667 4.46178631666667 4.2650829 5.23259858333333 4.54354728333333 4.16321256666667 4.69276596666667 4.407838125 4.96503665833333 4.837677975 5.02726646666667 4.98361768333333 5.30776938333333 5.36117665833333 4.948926475 5.15772628333333 5.34487673333333 4.71818525833333 4.645792325 5.16107029166667 4.90088761666667 4.79683228333333 5.00864485833333 4.3958178 5.11752969166667 4.03709423333333 5.20143405 4.42606766666667 4.89079064166667 3.8949318 5.26963456666667 5.022489825 4.684258075 4.85639045833333 4.16321256666667 4.14317694166667 3.9517269 4.39514796666667 4.305532525 3.9722338 4.072707875 4.6432372 4.09183225 3.919283675 4.13293229166667 3.76417531666667 4.06333713333333 4.442953 3.94394236666667 4.31979769166667 4.13691099166667 3.95278473333333 4.02639559166667 4.03790625 3.97718864166667 4.42171775 4.218766 3.98423539166667 3.75141583333333 3.94925109166667 4.17090055 4.04841256666667 4.153363325 4.43284685833333 4.3268489 4.30308001666667 4.13221068333333 4.16854543333333 4.22423876666667 4.49747863333333 4.55489758333333 4.14479000833333 4.72156198333333 4.37434235 4.14022315 4.97551569166667 4.959236325 4.80260285 3.771898175 4.47543089166667 5.59424653333333 4.11509285833333 5.10427118333333 4.384745925 5.07088755 4.473850775 5.015993925 4.21730013333333 4.519178925 3.99459898333333 4.89756791666667 4.73440256666667 3.79116074166667

AC021037.1 4.675507125 6.16366735 5.54490043333333 6.25678936666667 5.85351849166667 6.691504225 5.451907275 5.42068620833333 5.01921390833333 6.36384354166667 6.35611011666667 5.8690931 4.987974525 6.33974843333333 5.28868503333333 5.58424089166667 6.71254476666667 5.17730950833333 6.09251105 5.85351849166667 5.21846816666667 6.22740323333333 5.97300641666667 5.73323451666667 6.29613120833333 5.08901280833333 6.23352403333333 5.864991425 5.84489904166667 6.21087870833333 6.12262255833333 6.21983668333333 6.07508890833333 6.1327813 5.52726914166667 5.85267908333333 5.83439000833333 6.78888389166667 5.99039410833333 6.13763498333333 6.54993728333333 5.72192319166667 6.58583718333333 6.49099546666667 5.15014486666667 6.65069284166667 6.93442244166667 6.70095648333333 6.24671985833333 6.26162493333333 6.412967525 6.05912289166667 5.71408500833333 7.11408553333333 6.74266950833333 6.78668719166667 6.01817511666667 6.80522635833333 6.74043675833333 6.52770253333333 5.351477975 5.36997850833333 7.01821471666667 5.13063719166667 5.58111951666667 5.18325646666667 4.15639725 5.39793145 5.49272935833333 5.34143435833333 5.513669625 5.86420291666667 6.6577391 5.327405775 4.78885110833333 4.44153641666667 4.80433483333333 5.19160068333333 6.09902735833333 5.63422645833333 4.97055159166667 4.16321256666667 5.354072325 5.91688453333333 4.97551569166667 5.58111951666667 5.219476575 4.826730425 5.65156180833333 5.345943725 5.311054825 4.96833235 6.33250435 5.66412521666667 5.5264404 5.3154224 6.382488125 5.93598984166667 4.826730425 4.80181009166667 5.83439000833333 5.524744 6.04838810833333 6.76319795 6.05912289166667 6.272113525 6.078446575 6.049179025 6.45493224166667 6.56401094166667 5.620020925 5.87161330833333 6.22349896666667 7.46553646666667 6.30112746666667 6.47872195 6.29539721666667 5.72631065 7.292382975 5.92097311666667

AC105383.1 4.174726575 4.45316954166667 4.21242450833333 4.32189866666667 3.84784544166667 5.38092105 4.58674640833333 4.29601435833333 4.22423876666667 4.64031274166667 4.13293229166667 5.41537346666667 4.56122548333333 4.887437075 4.49004835 3.778738375 4.31533605833333 4.10217723333333 4.329661925 4.92102411666667 4.49747863333333 3.962497425 3.94394236666667 3.93419443333333 4.437951325 5.16029566666667 3.86406553333333 3.96958159166667 4.1052673 4.283368325 5.46007473333333 4.86534405 4.20805206666667 3.94837956666667 4.94419624166667 3.92385633333333 4.26055264166667 4.46778620833333 5.1243374 4.27449745 4.900016625 4.45973163333333 4.78973365833333 5.15933271666667 4.52640311666667 4.84884604166667 4.78566294166667 4.31074669166667 5.22032525 4.7289146 4.6523136625 4.0664225 4.12823880833333 4.97210654166667 4.651163825 4.31153791666667 4.25048369166667 5.23587815 4.32111429166667 5.12983071666667 3.653206325 4.347785025 4.23195600833333 4.34016336666667 6.00589870833333 3.94763221666667 3.67534585 4.57085965 3.91034174166667 3.91753055833333 3.97788789166667 3.73368700833333 4.08108728333333 4.038581625 3.65665524166667 3.79213375833333 3.87386619166667 3.57984935 4.12508055 3.76920943333333 5.01921390833333 3.89345544166667 3.8557954 3.55213481666667 3.66852646666667 3.94394236666667 4.45973163333333 3.78365575833333 3.813370125 3.72384201666667 3.876232825 3.97303310833333 4.3062682 3.98974825 3.85167864166667 3.85254715833333 3.89185758333333 4.27449745 4.42171775 4.23511105833333 4.22877676666667 3.9087838 4.55717808333333 4.78718000833333 4.3958178 4.2871363 4.3750276 5.10595089166667 4.77636825 6.05329835833333 4.47016100833333 4.462614125 4.319028675 4.61117119166667 4.501212 4.117337075 4.0347939 4.9202471 4.44518198333333 4.73757844166667

AC096996.2 6.82167135833333 7.28603906666667 7.08453025833333 6.72921199166667 7.68875390833333 6.98940225 6.53747031666667 7.05769945 7.664424575 8.04867420833333 6.77333313333333 5.73484704166667 6.55765729166667 7.06484504166667 7.675156525 6.55221608333333 7.21093403333333 6.894577475 7.93246234166667 6.77657375833333 7.1066557 7.53942513333333 7.509871575 7.23015586666667 8.13699931666667 6.16676128333333 7.66161635833333 7.29318128333333 7.79722829166667 7.67846760833333 7.82705071666667 7.23187408333333 7.50481256666667 7.25701023333333 6.88407465833333 6.78585395 8.58559054166667 8.50671724166667 8.01671704166667 7.8016903 8.567606325 8.31623061666667 7.96612625833333 7.541139975 7.13182746666667 8.13273210833333 7.97631320833333 7.618466325 7.998447275 7.54201473333333 7.828794225 7.90947090833333 7.57001779166667 8.17405961666667 7.73389285833333 8.19085505 7.79168211666667 8.57175574166667 7.6880047 7.65558205833333 6.39560875 6.247501925 7.557410025 6.14994006666667 6.70174799166667 6.864759375 6.538257625 6.12633869166667 7.13580859166667 6.32373769166667 6.17637834166667 6.50900563333333 5.636717225 6.30351415833333 5.79658570833333 5.73484704166667 6.53667773333333 6.70852368333333 6.55465186666667 6.40654535833333 6.247501925 5.86177639166667 7.33186256666667 7.0250185 6.382488125 6.16130541666667 5.701530925 5.86338895833333 5.547570375 6.29000819166667 6.477957 6.53900623333333 6.982194575 7.04581783333333 6.91600763333333 6.65857890833333 7.11810239166667 7.25974294166667 6.860244225 6.94476054166667 7.11408553333333 6.59209475833333 6.71557606666667 6.75605898333333 7.07608708333333 6.52910926666667 6.643638875 6.88170193333333 7.22830545833333 7.499347125 6.686388325 7.32811256666667 6.883226375 6.61725120833333 5.69903179166667 6.798074725 6.337213925 6.843581 7.02102248333333 4.90276439166667

PWRN1 7.08765801666667 6.23694766666667 7.01657786666667 7.356869825 7.28983956666667 5.807844625 7.55492436666667 7.27529025833333 7.01821471666667 7.47399836666667 6.46744718333333 7.43443834166667 7.88367008333333 8.002327575 6.59209475833333 6.52208159166667 6.41925481666667 6.78668719166667 6.48089516666667 6.66526773333333 6.67054473333333 6.78970486666667 7.68334979166667 7.05127804166667 6.14750089166667 7.98700995833333 6.82010810833333 5.14051295833333 6.182148775 6.36215745833333 6.06024568333333 7.48387143333333 6.6279805 7.37456893333333 7.82045105 6.32934243333333 6.59279463333333 5.40208686666667 5.144208425 5.18905345 6.55935685833333 6.02744485833333 6.23694766666667 7.66003905 6.92952805833333 4.99882871666667 6.45206855833333 6.42254766666667 5.53605506666667 6.74795924166667 6.1367648 5.631011625 7.7430897 5.0364605 8.13699931666667 6.38460903333333 7.35278270833333 4.91698004166667 5.81853184166667 5.493516675 5.57955060833333 6.67877244166667 4.41576304166667 6.3840179 6.529771875 6.61238640416667 6.46260194166667 7.07855881666667 4.85639045833333 6.24996494166667 6.857401975 5.9244919 8.17623105833333 6.674888925 5.99187126666667 6.40823279166667 6.61562239166667 5.66155191666667 6.538257625 4.98278695833333 7.1157008 5.66761570833333 5.55545125833333 5.11933154166667 6.93279296666667 6.076673725 6.4048599 6.60596866666667 5.29908005 5.06841553333333 5.38864603333333 4.09521015 4.32331949166667 5.198900775 4.96991525833333 5.44052959166667 4.66132636666667 4.6152735 5.34487673333333 5.2527348 4.72237503333333 6.70927374166667 4.59116015833333 4.55873965 4.99292960833333 5.96521240833333 4.8148076 5.83048494166667 6.05506278333333 7.02434959166667 5.06771848333333 4.87504760833333 4.09588705833333 4.66231220833333 5.979679875 4.80927436666667 5.19649501666667 4.59966794166667 4.59510340833333 7.541139975

HOXA-AS2 8.47224078333333 8.36432383333333 8.18700308333333 8.10654075833333 7.863951475 8.2030788 7.63212294166667 8.30672025 7.43109864166667 8.18309575 8.06715831666667 7.69882669166667 7.17277909166667 7.96885535833333 8.53241509166667 8.17005769166667 7.79271326666667 7.86934078333333 8.98476900833333 8.5533307 8.51803561666667 9.072341675 8.23549675833333 8.005090175 8.77244310833333 8.95327330833333 8.64415260833333 8.28793319166667 8.46668435833333 8.90815406666667 7.94870053333333 8.74401624166667 8.38071823333333 8.237670825 8.76894409166667 9.49887978333333 8.213081625 8.20193756666667 8.0885626 8.60414634166667 8.83888513333333 8.82199014166667 8.45130473333333 8.74010955833333 8.84022498333333 9.12206050833333 8.91303145833333 8.04867420833333 9.19875904166667 8.73889361666667 8.4458465 8.49375825 8.69140489166667 8.55899596666667 8.7363942 9.04826785833333 8.438679025 8.60647770833333 8.94319444166667 8.83385925833333 9.90727683333333 6.45056610833333 7.26467686666667 6.280696575 7.88754723333333 7.45333459166667 7.72751683333333 7.96052405 6.95909085 8.07103965 6.96543805833333 7.00675840833333 6.2377944 6.38854489166667 7.457281875 7.29390568333333 8.440753925 7.79722829166667 8.21411945 8.37465215 7.15604359166667 8.05364296666667 6.99264965 7.153021525 7.21835374166667 8.2030788 6.64847380833333 5.57471 7.75934284166667 8.19285094166667 6.42430866666667 7.24350165 7.437000375 8.83136731666667 7.56501633333333 7.30029635833333 7.15456116666667 7.14151948333333 6.61204298333333 8.347273825 7.95063335833333 7.43034844166667 6.59117523333333 7.977352125 7.978411525 8.197822625 8.015640975 6.848966625 7.68954538333333 7.07530188333333 8.57508074166667 7.578958525 7.55228321666667 8.14539853333333 6.91268935833333 8.68047514166667 7.63311984166667 6.52910926666667 8.04867420833333 7.25701023333333

NAPA-AS1 13.2700538583333 14.2285583083333 14.0181244166667 13.855751675 13.9566866 13.4390764916667 13.9061611 14.521344925 14.09777575 13.8693126833333 13.726268275 12.8295815416667 13.9130054833333 14.03455475 14.2194989166667 13.7599520083333 13.560618 13.9566866 14.1131984833333 14.1307464583333 13.76618685 14.2194989166667 14.043390675 13.7009408083333 14.1131984833333 13.4390764916667 14.4412673083333 14.3528361416667 14.5536296416667 14.2116827666667 14.1477872916667 14.3227822416667 13.8489298416667 13.4340660666667 13.3166625666667 13.4053421 14.3844241333333 14.5113807666667 13.89773695 14.2641819916667 14.03455475 14.09777575 13.7927226416667 14.09777575 13.560618 14.0181244166667 14.193518025 14.12193495 13.5773436583333 13.5446884416667 13.7135423333333 13.7425781166667 13.9061611 13.9650040916667 14.3528361416667 14.0904534166667 13.9061611 14.3428338416667 14.003709575 13.855751675 13.4629723583333 13.3124174 13.6585047583333 13.07608845 12.7706567583333 12.83676285 12.8070736416667 13.2366307916667 12.800813375 13.2164074166667 13.1185208166667 13.126641525 12.840675275 13.32975295 13.32975295 13.1648749166667 13.2905536833333 13.2164074166667 13.2366307916667 13.527636 12.9606437 13.1601453416667 13.2529881833333 13.0874111666667 13.5663260666667 12.98970205 12.8804849833333 13.5182485583333 13.4152475666667 13.0340459666667 13.4629723583333 13.249060775 13.3258459833333 13.38044235 13.0952688916667 13.5021556833333 13.1897357416667 13.2366307916667 13.6342148583333 13.7425781166667 13.1601453416667 12.7175457 13.126641525 12.9749196416667 13.249060775 13.4248566333333 13.1483004416667 13.3035558583333 13.472886275 13.3624533083333 13.1943968666667 13.855751675 13.7723702416667 12.7446559166667 13.9270331 13.7599520083333 12.7340595333333 12.8841629166667 13.079484475 13.6053456833333

AL034405.1 11.8133445166667 11.3566719333333 11.4703555083333 11.0103111083333 11.8676643916667 12.189826275 10.7711372583333 11.636069775 11.4942244666667 11.8364860833333 11.5887081666667 11.1746153 11.2523367916667 11.5379794583333 11.4753252 11.4822633416667 11.9534676 11.6534936583333 11.8335796916667 11.7066087916667 11.5719596666667 11.7351470166667 11.6579532083333 12.377098625 11.8202758833333 11.6788925166667 11.4491783416667 11.5406276416667 11.7773535166667 11.7252727333333 11.6534936583333 11.7165186083333 11.3163894833333 11.7637000083333 11.7536512833333 11.9635292666667 11.792717175 11.771780075 11.4727106916667 11.6717245416667 11.5333541416667 11.6108947416667 11.7482500666667 11.9252347666667 12.0412908333333 11.854762075 11.860233025 11.9534676 11.909558975 11.792717175 11.7252727333333 12.048574475 11.9484228666667 11.9175777083333 11.9834460416667 11.880341125 11.9175777083333 11.8881455083333 11.6412048416667 12.012563225 12.4148038083333 11.857596825 11.9685451916667 11.388504 12.012563225 12.283625975 12.0365429083333 12.2978352666667 12.5938101166667 12.0099462666667 11.6862351 12.0072618083333 12.223369875 11.9534676 11.9459843416667 12.231089275 11.9917942583333 12.5234224083333 12.800813375 12.7676332 12.0559423166667 11.6510632333333 12.552751 12.01526755 11.8444568666667 11.7351470166667 12.1362874333333 12.1362874333333 12.3544155583333 12.0719435333333 12.4811828916667 12.0770282166667 11.9993623166667 12.0412908333333 12.46882785 12.1958518666667 12.01526755 11.9175777083333 12.714470025 12.0042689083333 12.368506525 11.4439581833333 12.43965125 12.7905792 12.4456003583333 12.309527875 11.9068035083333 13.1068881 12.5593382333333 12.405501075 11.7188251083333 12.3659453666667 12.2399882333333 13.0376313166667 12.1151589416667 12.1312062333333 12.0207283333333 12.7412194083333 12.617619125 12.459832675

Z68871.1 8.27802565 7.48922787083333 7.2724654875 7.40435422916667 7.40754368333333 7.93673202083333 6.62008585416667 7.99891245416667 6.76951683333333 7.54487162916667 7.35319117083333 7.880383675 8.02391114583333 8.56485450416667 7.90893406666667 6.81031525 8.09590715416667 7.73056460416667 7.72214695416667 8.050075375 7.90225138333333 8.22888744583333 8.10215072083333 7.99150983333333 8.1158165125 8.3115123625 8.1117687625 7.37341099583333 7.85146230833333 7.5389839625 7.8029642625 7.5620857625 7.04780897916667 7.01859339583333 7.70179950833333 7.80471253333333 7.50069094166667 7.90225138333333 7.85332373333333 7.86980867916667 7.240508725 7.69497334583333 8.26341354583333 7.7279418 7.5629753 7.079038575 8.31361662083333 8.14050475416667 7.8976858875 7.551036875 8.05692022916667 7.30911832083333 8.27062295833333 8.25722517916667 7.491941525 8.00187925833333 8.3802660375 7.3851697375 8.1695897125 8.12836323333333 9.8063974125 7.62219540416667 9.54168757083333 8.10979551666667 7.9738690875 8.57025190833333 8.50510535833333 8.17675108333333 8.57231814583333 8.26040965416667 8.05970320416667 8.7470021875 8.50619929166667 8.23602964166667 9.05491322916667 8.84685069166667 7.57687788333333 8.55391102916667 8.23494445833333 8.478197975 7.50949375833333 9.07289544166667 7.18751969166667 9.16423615416667 7.69419850416667 8.06271484166667 9.63079542916667 8.0791316 9.01906352083333 8.14694391666667 8.3167134 9.1397128875 7.7634984375 7.45774374583333 8.4847348375 8.46311180833333 7.22436959583333 8.5670898875 8.06175783333333 8.38869885416667 8.1625857125 7.66210537916667 8.89856694583333 8.07600020416667 8.21154779583333 8.21888479166667 8.10010901666667 8.4144194375 8.39540114166667 7.56378472916667 9.34297716666667 8.61401615833333 7.99424180833333 9.33052937916667 7.68285503333333 8.1655284375 8.68599445833333 9.12520586666667 8.78497102083333 8.70142335416667

LINC00630 8.27802565 7.48922787083333 7.2724654875 7.40435422916667 7.40754368333333 7.93673202083333 6.62008585416667 7.99891245416667 6.76951683333333 7.54487162916667 7.35319117083333 7.880383675 8.02391114583333 8.56485450416667 7.90893406666667 6.81031525 8.09590715416667 7.73056460416667 7.72214695416667 8.050075375 7.90225138333333 8.22888744583333 8.10215072083333 7.99150983333333 8.1158165125 8.3115123625 8.1117687625 7.37341099583333 7.85146230833333 7.5389839625 7.8029642625 7.5620857625 7.04780897916667 7.01859339583333 7.70179950833333 7.80471253333333 7.50069094166667 7.90225138333333 7.85332373333333 7.86980867916667 7.240508725 7.69497334583333 8.26341354583333 7.7279418 7.5629753 7.079038575 8.31361662083333 8.14050475416667 7.8976858875 7.551036875 8.05692022916667 7.30911832083333 8.27062295833333 8.25722517916667 7.491941525 8.00187925833333 8.3802660375 7.3851697375 8.1695897125 8.12836323333333 9.8063974125 7.62219540416667 9.54168757083333 8.10979551666667 7.9738690875 8.57025190833333 8.50510535833333 8.17675108333333 8.57231814583333 8.26040965416667 8.05970320416667 8.7470021875 8.50619929166667 8.23602964166667 9.05491322916667 8.84685069166667 7.57687788333333 8.55391102916667 8.23494445833333 8.478197975 7.50949375833333 9.07289544166667 7.18751969166667 9.16423615416667 7.69419850416667 8.06271484166667 9.63079542916667 8.0791316 9.01906352083333 8.14694391666667 8.3167134 9.1397128875 7.7634984375 7.45774374583333 8.4847348375 8.46311180833333 7.22436959583333 8.5670898875 8.06175783333333 8.38869885416667 8.1625857125 7.66210537916667 8.89856694583333 8.07600020416667 8.21154779583333 8.21888479166667 8.10010901666667 8.4144194375 8.39540114166667 7.56378472916667 9.34297716666667 8.61401615833333 7.99424180833333 9.33052937916667 7.68285503333333 8.1655284375 8.68599445833333 9.12520586666667 8.78497102083333 8.70142335416667

LPP-AS2 10.1794807166667 9.61446786666667 9.80705093333333 10.1216004333333 10.2551372666667 9.44960989166667 10.881139625 9.74405148333333 10.12322705 9.637761575 10.1155244166667 11.0447636416667 10.3371834416667 9.67300651666667 9.415981525 9.78995428333333 9.64736453333333 9.87005905833333 8.83888513333333 9.39994525833333 10.1344061583333 9.54520550833333 9.87742366666667 9.3411091 9.16483426666667 10.0206561666667 9.40927853333333 9.536758 9.14942049166667 9.4483016 9.17541009166667 9.51090269166667 9.85108371666667 9.48833739166667 9.96321 10.17093795 9.20264188333333 9.12964521666667 9.00354715 9.2107017 9.17402556666667 9.10274634166667 8.95205770833333 8.70899691666667 9.02205163333333 8.87059211666667 9.16242148333333 9.04108516666667 9.02465829166667 9.21916606666667 8.89912965833333 8.89438333333333 9.30352410833333 8.70661779166667 9.22419906666667 9.28160753333333 9.38498160833333 8.7204491 9.213538675 9.201411475 10.20778565 10.1575664416667 10.3479196583333 10.7946431083333 10.7767798666667 9.8892648 10.5653432666667 10.4051703 9.70023275833333 9.81335845 10.360229225 10.4829935916667 10.4434835166667 9.67137481666667 10.2635439166667 10.258686375 9.919589575 9.57749218333333 9.4263481 9.89542063333333 10.833996275 10.4791199833333 9.819189925 9.58292749166667 9.643199125 10.401625675 10.4921560416667 10.4158197333333 10.3739525666667 9.48260326666667 10.0395367833333 9.38498160833333 10.1930602583333 9.52206101666667 10.0316996666667 10.0784644666667 9.72422056666667 10.30560935 9.856575175 9.4198334 9.23135698333333 10.5107808 9.280311725 10.2970698166667 8.74523575833333 9.695916825 9.118265275 8.646559875 9.24804320833333 8.904098475 9.4470582 9.19118669166667 9.38637963333333 9.6633032 9.27391955833333 9.5984398 10.4698496583333 9.2829911 9.20003395833333 9.77666165

AP002884.3 7.33186256666667 5.69013408333333 6.09974989166667 6.17637834166667 6.46408151666667 7.04581783333333 5.7316124 5.90988236666667 5.82420564166667 6.258234125 6.15468555833333 6.72443184166667 6.96919575833333 5.83369038333333 7.072020975 6.83745388333333 6.514123925 6.97258804166667 6.50659699166667 6.60970920833333 5.50390441666667 7.23187408333333 5.15351136666667 6.48089516666667 7.18623808333333 6.38854489166667 6.87295885 7.14646856666667 7.74571144166667 6.85141578333333 6.802823925 7.368819225 6.36297995 5.8061966 6.81924664166667 5.42068620833333 7.15755769166667 6.238638975 6.285311725 7.149541075 6.57608009166667 6.18798263333333 5.71592235833333 6.987780475 6.24370750833333 6.29613120833333 6.102165675 6.982194575 6.36465791666667 6.74572325 6.26313358333333 6.03818995833333 6.55765729166667 7.77651561666667 6.20542285 6.61204298333333 6.30919138333333 6.98625575 7.682360275 7.08285123333333 7.20164341666667 4.72156198333333 6.18364681666667 5.87646783333333 5.077701625 6.97164584166667 5.367325475 6.42670259166667 6.09902735833333 6.66526773333333 5.83439000833333 7.17364838333333 6.54071093333333 6.52910926666667 6.324552 6.02811239166667 6.29613120833333 6.3807528 6.2377944 5.99116495833333 5.27316720833333 6.09733190833333 5.29034026666667 6.35770789166667 6.15217918333333 5.08815185 5.55545125833333 6.54378744166667 7.145604225 6.64775085 5.35594408333333 6.20090414166667 6.36034525833333 5.78381885833333 5.397270075 5.02553525 6.56098033333333 5.07992535 4.67467815 6.64072528333333 6.06424056666667 5.59994884166667 4.54354728333333 6.23535811666667 5.92772035833333 6.49650888333333 6.09733190833333 6.211679225 6.24370750833333 6.68251509166667 6.291657675 5.44052959166667 6.03818995833333 5.55949836666667 6.20542285 5.46922286666667 6.56973204166667 6.21815258333333 6.81769201666667 4.80661418333333

AL445588.1 6.29315451666667 5.51981635 5.20230749166667 5.50743195833333 3.89345544166667 4.22122616666667 4.275246875 4.16251111666667 3.66114276666667 3.90409960833333 3.73045055 3.73131006666667 3.707364925 4.11973251666667 4.12057799166667 3.914168525 4.75121364166667 3.87085374166667 4.75656106666667 3.90066126666667 3.91111126666667 4.13374673333333 4.45316954166667 4.67705794166667 4.96673286666667 4.14246295 4.65727891666667 4.84553110833333 4.84884604166667 4.53154068333333 4.95685870833333 4.7450973 3.78450821666667 4.17402234166667 4.86047979166667 4.34160773333333 4.70744949166667 4.62957980833333 4.63272604166667 4.80100048333333 5.20324565 4.568704925 5.0364605 5.75363753333333 4.23195600833333 5.16731153333333 5.20230749166667 5.905668875 4.80100048333333 5.04820341666667 5.08490451666667 4.67778629166667 3.753884775 5.46519105 4.34086375833333 4.92610205 4.24515410833333 4.948926475 4.463312775 4.54575506666667 5.28868503333333 4.76691938333333 5.47366834166667 5.13698225 3.90713534166667 4.11509285833333 4.35821460833333 4.711189975 4.111905175 4.22972051666667 4.02471265 4.023105425 3.94531450833333 3.93981401666667 3.66610879166667 4.0594979 3.79634603333333 4.17325634166667 4.19283364166667 3.816517125 4.40259521666667 3.85899803333333 4.4085814 4.33331919166667 3.829731875 4.16550909166667 3.861448425 4.60316775833333 5.70703833333333 4.16550909166667 4.08184460833333 4.88416340833333 4.403336375 4.05400306666667 3.9722338 3.99142755 4.44814198333333 4.246659225 3.92700760833333 5.06279435 4.72637159166667 4.06086576666667 4.96587955833333 5.33093755 5.03074584166667 4.30010035 4.95845345833333 6.488745325 4.451845525 6.74731924166667 4.87816691666667 5.62694763333333 5.12530148333333 5.61076979166667 4.04512974166667 4.51995868333333 4.42996005 4.76218691666667 4.67234389166667 3.99459898333333

C22orf34 4.97638124166667 6.57456723333333 4.06020879166667 5.681614475 6.11832703333333 6.54378744166667 6.56554306666667 5.95586469166667 5.772683 6.194084125 6.01241375 4.651163825 6.033075425 5.81184100833333 5.59357184166667 8.41929965 7.30451515833333 5.49518486666667 5.11933154166667 7.85638375833333 6.89382319166667 4.83685254166667 4.88495024166667 7.53942513333333 6.95519420833333 6.66413700833333 7.44478088333333 6.78514255 5.91867438333333 7.06728804166667 7.005136025 7.859170675 7.80256886666667 8.035850625 6.81539655 6.88682719166667 6.82479446666667 5.771891275 5.98464866666667 8.54846981666667 7.12048450833333 6.42828630833333 7.89110653333333 7.91550765833333 7.774503075 7.99100168333333 7.31788454166667 5.87646783333333 7.55664531666667 8.17213816666667 5.03994734166667 5.51902839166667 7.96987383333333 7.45238604166667 7.20609148333333 8.97484515833333 6.80665846666667 7.92482581666667 7.13004025 8.26184248333333 5.15351136666667 5.728098775 5.48441135833333 7.971686625 6.3380145 5.48272485833333 6.26630801666667 7.45898616666667 5.82961340833333 6.538257625 6.10384355833333 4.79683228333333 5.85775243333333 5.31020889166667 6.20183353333333 7.04894575833333 6.078446575 7.814238775 6.18629009166667 7.76222746666667 5.31273550833333 5.26625810833333 5.04085009166667 5.8094462 6.64847380833333 5.13063719166667 6.05667555833333 6.83210498333333 6.03387288333333 8.40414963333333 6.66295756666667 5.9124887 9.89075863333333 5.945959 5.15857075833333 6.73733449166667 6.21983668333333 5.430247225 5.36997850833333 11.9808613 6.24671985833333 6.291657675 7.80335965833333 9.03496023333333 6.78346969166667 7.770120025 6.862429025 6.42583693333333 7.98700995833333 7.6234272 5.38864603333333 6.29693088333333 7.10483954166667 7.05205246666667 7.51240625833333 7.83263275 5.761496775 7.08525161666667 7.03487794166667 8.03976036666667

LIVAR 6.66780425833333 7.00633757083333 6.4716137625 5.58826065 6.08541134583333 5.8222625875 6.9379241375 6.42540625833333 6.50546301666667 6.72093702916667 6.44865775833333 7.60654144583333 7.456826275 7.13137725833333 6.83246845 6.28882218333333 7.06360082083333 6.45913069583333 6.775319675 6.7081248 6.37559784583333 7.1691663125 7.12899385833333 6.67910944583333 6.0070965375 6.7969387125 6.73617774583333 5.70196280416667 6.8079261625 6.47473642083333 7.25039665833333 7.2446705625 6.81662227083333 7.2179684375 6.24404577916667 7.04857342916667 6.9211289375 6.52007160416667 6.74080085833333 6.2185691625 5.75151209166667 6.9954213125 6.94292338333333 6.66780425833333 6.48833961666667 6.67264684166667 6.86438238333333 6.62458650833333 6.30385280416667 6.26837457083333 6.088722775 6.94292338333333 6.16631525 6.4509263 6.72093702916667 6.7143763875 6.87956838333333 5.69057501666667 6.35911157083333 6.85102722916667 5.94073838333333 6.95803353333333 6.3707245375 6.28643380416667 5.78594570833333 5.73446433333333 5.38734372083333 6.57571095833333 6.62684522916667 7.17811446666667 6.17178580416667 6.30229826666667 6.90466549166667 6.03700682083333 6.12532919583333 6.61012195416667 6.49468991666667 6.31698049166667 5.67656046666667 6.41889286666667 6.54028792916667 5.84976845833333 6.28266741666667 6.3162301875 5.57510055 6.41964855833333 6.52007160416667 6.82122027916667 6.38970515833333 6.18757841666667 7.18116864166667 6.6432789125 5.76289318333333 6.05469357083333 6.48124449166667 6.31133033333333 6.20655155833333 6.59628130833333 6.56295625 5.65688551666667 5.98269731666667 7.39853604583333 6.5315113875 5.88749444583333 5.77836542916667 6.5240577 6.4316410875 5.70828515416667 6.56365585 6.2594972875 5.82063714166667 6.16806635833333 6.62386384166667 5.33597885 6.039335525 5.95868593333333 6.30803415833333 5.920541425 6.027041925 6.7159486875

LINC01909 6.66780425833333 7.00633757083333 6.4716137625 5.58826065 6.08541134583333 5.8222625875 6.9379241375 6.42540625833333 6.50546301666667 6.72093702916667 6.44865775833333 7.60654144583333 7.456826275 7.13137725833333 6.83246845 6.28882218333333 7.06360082083333 6.45913069583333 6.775319675 6.7081248 6.37559784583333 7.1691663125 7.12899385833333 6.67910944583333 6.0070965375 6.7969387125 6.73617774583333 5.70196280416667 6.8079261625 6.47473642083333 7.25039665833333 7.2446705625 6.81662227083333 7.2179684375 6.24404577916667 7.04857342916667 6.9211289375 6.52007160416667 6.74080085833333 6.2185691625 5.75151209166667 6.9954213125 6.94292338333333 6.66780425833333 6.48833961666667 6.67264684166667 6.86438238333333 6.62458650833333 6.30385280416667 6.26837457083333 6.088722775 6.94292338333333 6.16631525 6.4509263 6.72093702916667 6.7143763875 6.87956838333333 5.69057501666667 6.35911157083333 6.85102722916667 5.94073838333333 6.95803353333333 6.3707245375 6.28643380416667 5.78594570833333 5.73446433333333 5.38734372083333 6.57571095833333 6.62684522916667 7.17811446666667 6.17178580416667 6.30229826666667 6.90466549166667 6.03700682083333 6.12532919583333 6.61012195416667 6.49468991666667 6.31698049166667 5.67656046666667 6.41889286666667 6.54028792916667 5.84976845833333 6.28266741666667 6.3162301875 5.57510055 6.41964855833333 6.52007160416667 6.82122027916667 6.38970515833333 6.18757841666667 7.18116864166667 6.6432789125 5.76289318333333 6.05469357083333 6.48124449166667 6.31133033333333 6.20655155833333 6.59628130833333 6.56295625 5.65688551666667 5.98269731666667 7.39853604583333 6.5315113875 5.88749444583333 5.77836542916667 6.5240577 6.4316410875 5.70828515416667 6.56365585 6.2594972875 5.82063714166667 6.16806635833333 6.62386384166667 5.33597885 6.039335525 5.95868593333333 6.30803415833333 5.920541425 6.027041925 6.7159486875

AC020905.1 7.46714951666667 7.29870193333333 7.22229294166667 7.1942732 7.808866225 7.596908525 8.12408498333333 7.60442806666667 8.04962919166667 7.92396325833333 7.67007186666667 7.87702689166667 7.46553646666667 7.61582255833333 7.54028876666667 7.80424109166667 7.67007186666667 8.2214524 7.41578374166667 7.134124875 7.78355541666667 7.39651405 7.61148288333333 7.3704996 6.97744145 7.69622019166667 6.94396484166667 7.437000375 6.84523090833333 7.953283475 7.8078704 7.4773374 7.6537848 7.67007186666667 7.59936721666667 7.56919636666667 8.089532425 7.83885828333333 7.967964725 7.808866225 7.54723063333333 7.82133015 7.603532025 7.21920913333333 7.6026871 7.24923051666667 7.74841675 7.64764396666667 7.603532025 7.509871575 7.518197225 7.3704996 6.97581613333333 7.42106846666667 7.39891643333333 6.97744145 7.34734821666667 7.6423852 7.26706925 7.05282128333333 7.92575334166667 7.9266756 7.648594075 7.70654949166667 8.28484028333333 8.42421578333333 8.39037005833333 8.438679025 8.87463969166667 8.34393171666667 7.59779885833333 8.28896994166667 8.0070051 8.30985033333333 8.41501278333333 8.29555180833333 8.51932215 7.78707899166667 7.64579728333333 7.98053493333333 7.81792510833333 8.14748886666667 8.32474756666667 8.217333875 8.11629255 8.0743079 7.39223886666667 8.03118996666667 7.88080235 7.68503678333333 8.3827371 8.0743079 7.6537848 7.808866225 8.69251304166667 8.26184248333333 8.922675075 8.0885626 8.525776075 8.01861823333333 7.76018400833333 8.5533307 7.97530758333333 7.92129841666667 7.89905374166667 8.070038325 7.92754394166667 7.68054443333333 8.13598734166667 7.78258863333333 7.865741375 7.87120165 7.40149613333333 7.87702689166667 7.73389285833333 7.48387143333333 7.87211233333333 8.33859384166667 7.83627273333333 7.664424575

AF064858.1 5.42593315833333 6.28918155833333 6.85585071666667 6.95691923333333 6.76009966666667 6.13535341666667 7.60698968333333 7.17277909166667 7.28108741666667 6.4994646 6.73583865833333 7.149541075 7.50284468333333 5.81928184166667 5.92847919166667 7.26467686666667 6.35472360833333 6.45639771666667 6.33413388333333 6.50987625833333 7.34892254166667 6.48712963333333 6.768468075 6.849738 5.09997055 5.99187126666667 6.64989985833333 6.16130541666667 5.55202255833333 6.03742351666667 6.45056610833333 6.840567275 6.14913983333333 6.16765155 5.95073534166667 5.00377313333333 5.33796750833333 6.07508890833333 7.427790275 6.34194690833333 6.1327813 6.12427160833333 5.57003804166667 5.63497841666667 6.06177335833333 6.12567941666667 6.35692343333333 6.15783995 6.50361413333333 6.2141109 6.280696575 6.74731924166667 6.33494444166667 6.31742500833333 6.45056610833333 5.95681510833333 7.11888511666667 6.18052179166667 6.0725726 6.73249878333333 6.70927374166667 5.54229348333333 6.247501925 6.31742500833333 6.28846280833333 6.59363060833333 6.46484100833333 6.94332055 6.16925395833333 6.162124375 6.09974989166667 6.25294181666667 6.79889878333333 6.40188925833333 6.6271591 6.71632130833333 6.53113324166667 6.14671798333333 7.138237575 6.337213925 5.97547145833333 6.53113324166667 6.80522635833333 7.91271055833333 7.02184869166667 5.54129996666667 5.51902839166667 6.110329 6.683342625 7.43349755 6.64775085 7.2051733 6.94725178333333 7.00071761666667 6.11832703333333 6.01562348333333 5.25020996666667 5.12786334166667 6.42430866666667 5.57630850833333 7.11965454166667 6.36555325833333 5.663186525 5.13301409166667 7.04507230833333 6.42254766666667 5.99039410833333 7.18536765 6.417835775 6.59663961666667 6.987780475 5.582540175 5.92363850833333 6.17790808333333 6.97581613333333 6.699451375 7.08208855 7.79988654166667 5.465917475 6.23535811666667

CACNA1G-AS1 5.25117366666667 4.97724585 4.72477478333333 5.23497289166667 4.83614788333333 4.601803875 4.7450973 4.61046104166667 4.19596420833333 4.40501044166667 5.06279435 4.29455464166667 4.28485988333333 4.07709943333333 4.45316954166667 4.47678545833333 4.43350984166667 4.473850775 4.72237503333333 3.8204472 5.915004725 5.35864575 5.15614551666667 5.21846816666667 4.37819051666667 4.93026841666667 4.948926475 6.0898961 5.04562356666667 5.24520668333333 5.149358575 5.69101595 4.62491895 4.452546625 5.04562356666667 4.79532921666667 4.29010148333333 5.19388658333333 5.90801959166667 5.70503610833333 6.12262255833333 6.121685 5.71234138333333 6.21003021666667 4.79946088333333 5.99706000833333 5.82105976666667 5.22651561666667 4.31304105 5.33475269166667 4.65809740833333 4.719880325 4.60532013333333 4.73200895 4.97375653333333 5.48698374166667 4.30459614166667 4.64958116666667 5.611704975 5.48698374166667 4.61608426666667 4.77563934166667 4.76937243333333 4.924395575 4.44597125 4.262661725 4.39514796666667 4.61767011666667 5.33796750833333 4.393584875 4.6152735 4.393584875 4.96587955833333 4.69199204166667 4.41362111666667 4.727288675 4.71903925 4.544983975 3.898913075 4.75968018333333 3.93739250833333 4.40645795 4.79532921666667 5.20589149166667 4.29310653333333 4.62876291666667 4.34636268333333 4.15639725 4.8899011 4.72810314166667 5.16731153333333 3.9955461 3.63280979166667 3.94318915 4.85225948333333 3.69672979166667 4.14092098333333 4.31751935 4.53228734166667 4.83426329166667 4.83257791666667 5.58869843333333 5.26709018333333 5.53075733333333 4.6192723 4.087125375 4.74591905 5.08642645833333 4.049271075 5.94836456666667 4.46943080833333 4.77485674166667 5.03721211666667 5.07579073333333 4.63651601666667 4.81551458333333 3.71873703333333 4.22202384166667 4.204983325 3.56739355833333

AC004080.6 7.181557425 7.1870921 8.86194551666667 8.21411945 7.87702689166667 9.26005576666667 7.57814523333333 7.94679925 7.94501785 7.51334780833333 7.746538075 6.690101625 7.509871575 7.81329221666667 9.64736453333333 7.79904711666667 8.320569675 7.1982511 7.827876425 7.66688975833333 7.98520193333333 10.4051703 9.47141113333333 7.21336981666667 7.35849015 7.82133015 7.31286559166667 8.567606325 6.63114244166667 8.51484585833333 8.658204375 7.59779885833333 7.0497186 7.76222746666667 7.93342214166667 7.20936795 8.89800423333333 7.90947090833333 7.15381125833333 8.79393961666667 7.40249240833333 7.89018188333333 8.30575404166667 7.81524705 7.18623808333333 7.79271326666667 7.89363596666667 7.403217625 8.36965201666667 8.33973274166667 7.8078704 7.49239705 7.040488 7.54978125 8.537131375 8.55449135833333 7.67682356666667 7.77743810833333 7.92035708333333 8.038750675 8.41501278333333 6.93829808333333 9.71504586666667 7.1942732 9.33619586666667 7.363485725 7.59779885833333 10.242175025 7.769180675 8.84138925 7.71635294166667 8.49932775 8.278515525 7.72836676666667 7.78707899166667 7.96987383333333 8.796287025 8.18413825 7.75020911666667 10.4659868166667 6.96632546666667 9.101427925 8.02061905 7.437000375 7.15755769166667 9.288628775 6.66295756666667 7.381252125 8.56330485833333 7.8186674 7.23578164166667 7.53942513333333 8.42317835833333 8.33743355 7.43109864166667 8.00331975833333 8.42126873333333 7.668434925 6.65618500833333 9.71370898333333 6.86168069166667 9.11292266666667 7.491486 9.76078066666667 8.857267525 8.57175574166667 6.54596676666667 8.07103965 6.70536285833333 7.805183975 6.8993439 10.1825522583333 7.19914976666667 7.59330443333333 7.30787071666667 9.769181275 8.9886678 6.88087174166667 8.11324664166667 5.958371875

HOXA-AS3 7.181557425 7.1870921 8.86194551666667 8.21411945 7.87702689166667 9.26005576666667 7.57814523333333 7.94679925 7.94501785 7.51334780833333 7.746538075 6.690101625 7.509871575 7.81329221666667 9.64736453333333 7.79904711666667 8.320569675 7.1982511 7.827876425 7.66688975833333 7.98520193333333 10.4051703 9.47141113333333 7.21336981666667 7.35849015 7.82133015 7.31286559166667 8.567606325 6.63114244166667 8.51484585833333 8.658204375 7.59779885833333 7.0497186 7.76222746666667 7.93342214166667 7.20936795 8.89800423333333 7.90947090833333 7.15381125833333 8.79393961666667 7.40249240833333 7.89018188333333 8.30575404166667 7.81524705 7.18623808333333 7.79271326666667 7.89363596666667 7.403217625 8.36965201666667 8.33973274166667 7.8078704 7.49239705 7.040488 7.54978125 8.537131375 8.55449135833333 7.67682356666667 7.77743810833333 7.92035708333333 8.038750675 8.41501278333333 6.93829808333333 9.71504586666667 7.1942732 9.33619586666667 7.363485725 7.59779885833333 10.242175025 7.769180675 8.84138925 7.71635294166667 8.49932775 8.278515525 7.72836676666667 7.78707899166667 7.96987383333333 8.796287025 8.18413825 7.75020911666667 10.4659868166667 6.96632546666667 9.101427925 8.02061905 7.437000375 7.15755769166667 9.288628775 6.66295756666667 7.381252125 8.56330485833333 7.8186674 7.23578164166667 7.53942513333333 8.42317835833333 8.33743355 7.43109864166667 8.00331975833333 8.42126873333333 7.668434925 6.65618500833333 9.71370898333333 6.86168069166667 9.11292266666667 7.491486 9.76078066666667 8.857267525 8.57175574166667 6.54596676666667 8.07103965 6.70536285833333 7.805183975 6.8993439 10.1825522583333 7.19914976666667 7.59330443333333 7.30787071666667 9.769181275 8.9886678 6.88087174166667 8.11324664166667 5.958371875

AC004080.5 7.181557425 7.1870921 8.86194551666667 8.21411945 7.87702689166667 9.26005576666667 7.57814523333333 7.94679925 7.94501785 7.51334780833333 7.746538075 6.690101625 7.509871575 7.81329221666667 9.64736453333333 7.79904711666667 8.320569675 7.1982511 7.827876425 7.66688975833333 7.98520193333333 10.4051703 9.47141113333333 7.21336981666667 7.35849015 7.82133015 7.31286559166667 8.567606325 6.63114244166667 8.51484585833333 8.658204375 7.59779885833333 7.0497186 7.76222746666667 7.93342214166667 7.20936795 8.89800423333333 7.90947090833333 7.15381125833333 8.79393961666667 7.40249240833333 7.89018188333333 8.30575404166667 7.81524705 7.18623808333333 7.79271326666667 7.89363596666667 7.403217625 8.36965201666667 8.33973274166667 7.8078704 7.49239705 7.040488 7.54978125 8.537131375 8.55449135833333 7.67682356666667 7.77743810833333 7.92035708333333 8.038750675 8.41501278333333 6.93829808333333 9.71504586666667 7.1942732 9.33619586666667 7.363485725 7.59779885833333 10.242175025 7.769180675 8.84138925 7.71635294166667 8.49932775 8.278515525 7.72836676666667 7.78707899166667 7.96987383333333 8.796287025 8.18413825 7.75020911666667 10.4659868166667 6.96632546666667 9.101427925 8.02061905 7.437000375 7.15755769166667 9.288628775 6.66295756666667 7.381252125 8.56330485833333 7.8186674 7.23578164166667 7.53942513333333 8.42317835833333 8.33743355 7.43109864166667 8.00331975833333 8.42126873333333 7.668434925 6.65618500833333 9.71370898333333 6.86168069166667 9.11292266666667 7.491486 9.76078066666667 8.857267525 8.57175574166667 6.54596676666667 8.07103965 6.70536285833333 7.805183975 6.8993439 10.1825522583333 7.19914976666667 7.59330443333333 7.30787071666667 9.769181275 8.9886678 6.88087174166667 8.11324664166667 5.958371875

ZMIZ1-AS1 6.11996326666667 4.60712125833333 5.7981413 5.22903249166667 3.91252795833333 3.96617559166667 3.66771833333333 5.73824569166667 3.92385633333333 4.10217723333333 4.23901575 3.64308306666667 3.58244408333333 4.01672794166667 5.905668875 4.96503665833333 6.66526773333333 4.84470445 6.21577044166667 4.51514020833333 4.48927336666667 4.80260285 5.67873523333333 6.25678936666667 5.699860275 4.990530025 4.97296273333333 5.60738530833333 3.99714025 5.22298730833333 6.57456723333333 6.26630801666667 4.910233575 4.31751935 5.91411070833333 6.03894880833333 5.37872604166667 5.71057048333333 5.04164975 5.74925146666667 6.78514255 5.53239810833333 6.84446040833333 6.15958521666667 5.59644218333333 7.11493935833333 6.30271423333333 5.32654480833333 5.80207481666667 5.95586469166667 6.43962590833333 6.162124375 4.60802493333333 6.06669943333333 5.01280093333333 6.753505175 5.151017225 6.10950205 6.21657471666667 5.69101595 4.56782300833333 3.88965465833333 5.69450579166667 5.00720284166667 4.79532921666667 4.47471310833333 3.85096890833333 4.49004835 5.44782694166667 4.01991520833333 4.12823880833333 4.308553375 3.630358625 4.9424459 3.80841116666667 5.30595961666667 4.235972525 5.858605475 5.625272925 4.29310653333333 3.7417011 3.93981401666667 4.43350984166667 6.94886980833333 5.98226609166667 4.14925433333333 3.77295658333333 4.00650299166667 3.816517125 6.162124375 4.48856954166667 6.3554652 5.74066663333333 4.08941781666667 3.99860775833333 4.74591905 4.1100906 4.88258713333333 3.87386619166667 5.90253445833333 5.11238625833333 4.45973163333333 5.971264425 5.95496726666667 6.37198143333333 5.408871075 5.838559525 5.24687600833333 5.302465325 6.10539690833333 6.47356659166667 5.19160068333333 5.58504371666667 5.858605475 5.582540175 6.53588259166667 5.6753027 5.7871496 5.84085751666667 4.00474495

AC147651.1 6.54596676666667 3.83631071666667 3.877204375 5.08085140833333 4.83029645833333 5.49191885833333 4.29728523333333 4.308553375 3.72460085 5.77117681666667 4.68025626666667 4.07056364166667 3.85899803333333 4.40573335 4.69586463333333 4.53985004166667 6.91268935833333 4.64405129166667 4.9375826 4.87504760833333 4.81864974166667 3.89729075833333 4.13293229166667 5.941144225 5.34411366666667 6.18461388333333 5.27892913333333 5.528965775 6.46408151666667 5.5707947 6.12567941666667 4.9375826 4.442953 5.29742040833333 5.77706405 5.26963456666667 4.711189975 5.89280416666667 4.97296273333333 5.194761075 5.547570375 5.523243625 5.56030313333333 5.9704005 5.60075419166667 5.56118513333333 5.92279354166667 5.63585205833333 4.98278695833333 5.663186525 5.20324565 5.32578840833333 4.40096828333333 6.18052179166667 5.65408345833333 5.76067670833333 4.61046104166667 5.04240375 6.21087870833333 5.25679695833333 6.98302305 4.3318673 5.53075733333333 5.29375345833333 5.09654478333333 5.26378820833333 4.20269309166667 4.53840028333333 5.71057048333333 4.70657895833333 4.49076011666667 4.39219723333333 4.82348884166667 4.36397045 4.35821460833333 4.34848804166667 5.44052959166667 5.61315593333333 3.94073975 6.73817556666667 5.4786027 4.90276439166667 4.857936675 6.26162493333333 5.02726646666667 7.206737225 6.54071093333333 4.08941781666667 6.75932524166667 4.50562893333333 5.512808975 6.088336625 5.82778305833333 4.38169465833333 5.24687600833333 5.24850139166667 4.42469801666667 6.727504025 4.34564913333333 4.78718000833333 5.09654478333333 5.4760094 6.16586921666667 5.04562356666667 5.915004725 4.10366320833333 5.06029869166667 5.354072325 4.9202471 6.15958521666667 6.438946775 4.974631425 5.25444665 4.69847759166667 5.1243374 6.0420986 7.13734699166667 5.59208724166667 6.076673725 4.5762417

AC145207.2 14.0104955916667 14.4412673083333 14.9072598916667 14.5016827333333 14.8206556583333 14.7711244916667 13.665267475 14.8687025166667 14.1477872916667 14.7261095416667 14.3428338416667 13.7723702416667 14.193518025 14.91993005 14.7261095416667 14.202140575 14.4517284583333 14.2961453916667 14.84448205 14.91993005 15.1457216333333 14.91993005 14.7163380333333 14.6933680416667 14.7468272333333 14.6237735666667 15.1310647333333 14.84448205 14.7968734083333 14.4903909583333 15.0308763916667 15.164023575 14.2746526833333 14.3328580333333 13.6106480333333 14.0270179 14.7837331083333 15.2087807666667 14.6537247416667 14.583522125 14.8320167 14.8090170416667 14.7261095416667 14.9900502166667 14.3227822416667 14.9353634666667 14.8206556583333 15.1942513666667 14.9900502166667 14.2547860083333 15.0714825416667 14.7363719916667 14.8206556583333 15.0714825416667 14.8206556583333 15.31350405 15.1183764916667 14.9624125 15.0434822083333 15.0182363666667 14.8206556583333 14.8567343583333 14.4715069416667 14.8206556583333 13.726268275 14.8946224583333 14.8090170416667 14.521344925 14.6537247416667 14.84448205 14.7587625916667 14.8946224583333 15.0308763916667 14.672839525 14.7163380333333 15.003829525 14.5536296416667 14.9353634666667 14.7968734083333 14.7468272333333 14.0518864916667 14.402064425 15.179006875 15.2087807666667 14.8946224583333 14.3428338416667 13.981121475 14.7261095416667 14.672839525 14.8946224583333 14.7261095416667 14.3043404916667 14.7968734083333 14.8687025166667 15.2764462833333 14.84448205 14.9072598916667 14.7261095416667 15.2428479916667 14.84448205 14.8946224583333 14.4234547 15.0566496333333 15.179006875 15.003829525 14.8946224583333 15.3471952583333 15.1942513666667 14.8206556583333 15.2938409083333 15.003829525 15.2087807666667 15.4265201333333 15.259191275 15.1457216333333 15.0434822083333 14.5442613916667 14.9353634666667 14.7055885666667 14.9482015833333

KIAA0087 6.04756038333333 5.529852675 5.32408766666667 4.26124555833333 5.02912155833333 5.50390441666667 5.66848611666667 5.43534984166667 5.408871075 4.3318673 4.88079474166667 5.875661225 6.231190225 4.75968018333333 5.53699586666667 5.86420291666667 5.464202575 5.31451775 5.88131005 6.11113416666667 5.78556079166667 5.15428810833333 5.5912611 5.348698925 5.771891275 6.25369225 6.18052179166667 6.529771875 5.50390441666667 5.29908005 5.21587950833333 6.52517743333333 5.48522716666667 5.37693024166667 6.61562239166667 6.46484100833333 4.92102411666667 5.95408916666667 5.4096421 6.46895845833333 5.41201970833333 4.61432114166667 5.92195151666667 5.76767515833333 5.13619761666667 5.66590344166667 5.38864603333333 5.163476125 5.13143250833333 5.06928161666667 5.12699374166667 5.12104158333333 4.44750469166667 5.78469366666667 5.01921390833333 6.37921816666667 5.866627925 5.52066971666667 5.95005389166667 5.1157001 5.01280093333333 5.43787004166667 5.56732144166667 5.20324565 4.843025575 5.92772035833333 6.208533725 5.729929025 5.14051295833333 5.013582075 5.0923089 5.40027715 6.31829665 5.25020996666667 5.620020925 6.75112116666667 4.94419624166667 5.41710834166667 5.26290054166667 5.13380841666667 5.56466664166667 5.611704975 4.55489758333333 4.916041975 5.14760676666667 5.44953800833333 5.00548516666667 6.1514684 4.89586528333333 5.77625636666667 4.826730425 6.272113525 4.55255341666667 5.30168844166667 5.21015984166667 5.40803009166667 5.11238625833333 5.18232021666667 4.98620748333333 6.04516751666667 4.77485674166667 5.12104158333333 5.95681510833333 5.33796750833333 4.8196 5.49794591666667 5.408871075 6.4838525 5.84659308333333 6.77657375833333 5.247693175 5.61076979166667 4.99643059166667 5.6235969 5.01921390833333 5.98048791666667 5.17564051666667 5.0768141 5.12887654166667 6.17712046666667

ATP1A1-AS1 8.545802675 6.47435308333333 7.21758313333333 6.4290253 5.70239468333333 7.769180675 4.98971816666667 6.03972224166667 6.22806264166667 5.65972369166667 6.28233444166667 6.47575550833333 5.95235704166667 6.46895845833333 6.96632546666667 7.55986316666667 9.536758 5.55703588333333 7.35042211666667 7.311835325 7.04361085833333 7.18309601666667 7.45238604166667 7.46071238333333 7.76124241666667 8.13598734166667 7.87120165 7.34965898333333 6.619742275 7.4887958 6.9176246 7.52905674166667 7.15381125833333 8.04062745 8.3150991 8.46668435833333 7.06728804166667 6.82167135833333 7.06801993333333 6.80665846666667 7.43541391666667 7.50737338333333 7.75673711666667 7.8519496 8.043697625 8.20093078333333 8.34967031666667 6.843581 7.91550765833333 7.93342214166667 7.44205825833333 6.55371424166667 6.57380151666667 7.33580734166667 7.675156525 7.59232971666667 5.864991425 7.07530188333333 7.94078679166667 7.557410025 6.91345085833333 6.24024199166667 8.03782043333333 6.37447095 8.589042325 6.32934243333333 5.41122809166667 5.96352934166667 6.72283928333333 6.36881363333333 5.51729848333333 5.864991425 5.94745686666667 6.53188953333333 5.91328005 7.19663824166667 6.94476054166667 7.29390568333333 6.65618500833333 7.340722575 7.91748764166667 6.80522635833333 5.88789478333333 6.87530805833333 5.95981851666667 7.56764245 7.746538075 6.23352403333333 6.05506278333333 7.88934271666667 6.488745325 6.79117925 6.47042564166667 5.82778305833333 6.80223084166667 5.53870645 6.52208159166667 7.59232971666667 5.37522741666667 7.0357017 6.1401869 5.971264425 6.95992653333333 7.928697775 7.40883421666667 6.857401975 6.76319795 6.70772591666667 7.39329146666667 7.02259469166667 7.365658075 7.79988654166667 6.97415298333333 7.91104394166667 7.14799545 7.21093403333333 8.89438333333333 6.38929764166667 7.19199751666667 6.06758881666667

LINC02389 9.3517636 9.3213721 9.4804264125 9.44895574583333 10.6334842916667 9.53221514166667 11.05478485 10.236307225 10.6787007666667 9.9138875 10.0670721208333 10.6451122083333 10.6178507125 9.77458840833333 8.9965376875 9.4789353375 9.1966889 10.2711148208333 8.8876437875 9.32926200833333 9.92812035833333 9.7036014125 9.4020506 8.94392220416667 8.653968525 9.4933853125 8.8445253375 8.64593070833333 8.93884515833333 9.42047291666667 8.439140125 8.63868771666667 9.89609240833333 9.7036014125 9.50346409166667 9.11757217916667 9.74775465416667 9.2932755125 9.30543721666667 8.8876437875 8.67612954166667 9.13026380833333 8.5919199625 8.45755785833333 9.50756554583333 8.51079318333333 8.46619962083333 9.59329031666667 8.88387385416667 9.2567872 8.9147762 9.37940935416667 10.3954173083333 9.26342520416667 9.45443198333333 7.99150983333333 10.3625186625 9.0638453125 8.98170729583333 8.50286094166667 9.7347242875 9.9907341125 9.04529710833333 9.48329252083333 9.833823575 10.0966824666667 9.92488924166667 10.3557862333333 9.89154494583333 10.7354450375 10.5250172458333 9.89301134583333 10.4409834125 9.8063974125 10.1803173041667 9.3456925 9.9748498625 9.62778093333333 9.4530556875 9.67219066666667 10.5928407166667 10.4762218166667 9.4933853125 8.974236775 10.1146900916667 10.3625186625 10.0105001791667 10.4482290583333 10.0793932208333 8.974236775 9.44495295416667 8.4453242125 10.0655531291667 9.77864194166667 9.54316374166667 9.72904945416667 9.69661204166667 10.0763546333333 10.3172301958333 9.60529419583333 9.5020543 10.2466404333333 9.11357405 8.93509225 9.70497339166667 10.3362794333333 9.62778093333333 9.6167051625 9.66397815 9.05114369583333 9.22933481666667 8.96035595 8.96408830833333 8.73826393333333 9.77998732916667 9.0944044375 9.27963544166667 9.71743607916667 9.48469073333333 10.2496711958333

LINC02231 9.3517636 9.3213721 9.4804264125 9.44895574583333 10.6334842916667 9.53221514166667 11.05478485 10.236307225 10.6787007666667 9.9138875 10.0670721208333 10.6451122083333 10.6178507125 9.77458840833333 8.9965376875 9.4789353375 9.1966889 10.2711148208333 8.8876437875 9.32926200833333 9.92812035833333 9.7036014125 9.4020506 8.94392220416667 8.653968525 9.4933853125 8.8445253375 8.64593070833333 8.93884515833333 9.42047291666667 8.439140125 8.63868771666667 9.89609240833333 9.7036014125 9.50346409166667 9.11757217916667 9.74775465416667 9.2932755125 9.30543721666667 8.8876437875 8.67612954166667 9.13026380833333 8.5919199625 8.45755785833333 9.50756554583333 8.51079318333333 8.46619962083333 9.59329031666667 8.88387385416667 9.2567872 8.9147762 9.37940935416667 10.3954173083333 9.26342520416667 9.45443198333333 7.99150983333333 10.3625186625 9.0638453125 8.98170729583333 8.50286094166667 9.7347242875 9.9907341125 9.04529710833333 9.48329252083333 9.833823575 10.0966824666667 9.92488924166667 10.3557862333333 9.89154494583333 10.7354450375 10.5250172458333 9.89301134583333 10.4409834125 9.8063974125 10.1803173041667 9.3456925 9.9748498625 9.62778093333333 9.4530556875 9.67219066666667 10.5928407166667 10.4762218166667 9.4933853125 8.974236775 10.1146900916667 10.3625186625 10.0105001791667 10.4482290583333 10.0793932208333 8.974236775 9.44495295416667 8.4453242125 10.0655531291667 9.77864194166667 9.54316374166667 9.72904945416667 9.69661204166667 10.0763546333333 10.3172301958333 9.60529419583333 9.5020543 10.2466404333333 9.11357405 8.93509225 9.70497339166667 10.3362794333333 9.62778093333333 9.6167051625 9.66397815 9.05114369583333 9.22933481666667 8.96035595 8.96408830833333 8.73826393333333 9.77998732916667 9.0944044375 9.27963544166667 9.71743607916667 9.48469073333333 10.2496711958333

MIR3945HG 6.822540775 7.18005326666667 7.33580734166667 7.24756645 7.61667270833333 7.88171558333333 8.34393171666667 7.54530115 7.5852857 7.75201335 7.36412529166667 7.583437825 7.84801261666667 7.4639428 6.95691923333333 7.73008085 6.838229925 7.9966524 6.96543805833333 6.99413795833333 7.983242125 6.94396484166667 8.12977375 8.06412709166667 6.85503901666667 7.30198646666667 6.93755019166667 6.662197225 6.30112746666667 6.66888841666667 7.30368923333333 7.62747078333333 7.257981025 7.28603906666667 8.27939851666667 6.760855075 6.545223625 6.9606233 7.63412013333333 6.28918155833333 6.52116074166667 6.812864825 7.09746866666667 6.69708403333333 6.6294991 5.80704883333333 6.92711743333333 7.08921823333333 6.89860500833333 6.57993243333333 7.95152345 7.770120025 7.64410754166667 6.81924664166667 6.88476720833333 6.45429151666667 7.71468463333333 6.35956555833333 6.83745388333333 6.97908095833333 6.27380003333333 7.51511816666667 7.493374075 7.3704996 7.08679770833333 8.06225145833333 8.21116674166667 7.379563775 9.96941311666667 7.51141074166667 7.36105020833333 7.0250185 8.11032313333333 7.78442941666667 7.54028876666667 8.29217418333333 7.82427053333333 7.841555975 8.5644013 7.49427225833333 6.83665193333333 6.99413795833333 8.64167483333333 11.03050155 7.928697775 7.437000375 6.9248352 7.93906090833333 8.26088785833333 7.36490918333333 7.68151318333333 10.8494230333333 8.26609046666667 8.53473379166667 6.84204533333333 9.75280021666667 6.99014773333333 7.37881741666667 7.42616356666667 6.30504875 7.11810239166667 7.529838675 8.33307035833333 7.46633813333333 8.073175025 7.78707899166667 8.15639794166667 7.04281696666667 7.46305853333333 7.64410754166667 7.8500176 7.318718375 9.5341054 7.81712555833333 7.94287994166667 7.04281696666667 7.96987383333333 8.22349021666667 7.67262559166667 7.8500176

AL359313.1 6.432044225 7.0250185 7.13662455 7.27935889166667 7.012070875 6.89303899166667 7.48797304166667 6.49186828333333 6.817034675 6.52835620833333 6.343453175 6.83075820833333 7.14875900833333 7.00742828333333 6.95909085 7.20436910833333 6.541436775 7.06891925833333 7.02582578333333 7.39130495 7.21256346666667 7.56764245 7.102382825 7.3803365 7.02184869166667 7.55584700833333 7.525750625 6.93124799166667 6.823352775 7.1768951 7.297972275 7.01349440833333 6.949750225 7.73305023333333 6.8961707 6.44192199166667 6.96462073333333 7.16143308333333 7.08849513333333 6.89303899166667 6.79964733333333 7.02434959166667 6.85585071666667 8.04771451666667 6.95909085 6.73733449166667 7.14000910833333 6.70852368333333 6.74196855833333 6.79350115833333 7.153021525 6.87036725833333 7.19111055833333 7.45400806666667 7.02184869166667 6.53343775833333 6.78272669166667 6.36954999166667 6.9183608 6.57049163333333 6.47279708333333 7.11493935833333 6.32765196666667 7.07043868333333 6.43447593333333 6.80134524166667 5.92530963333333 5.95153 6.00243133333333 6.75932524166667 6.70700871666667 6.21815258333333 7.46714951666667 6.46331624166667 6.88849675 6.71798690833333 6.909615425 6.76405308333333 6.47435308333333 6.0898961 7.51062641666667 6.8961707 6.835151175 7.05205246666667 6.539864925 6.55935685833333 6.514960775 6.71397423333333 6.20090414166667 6.868676375 6.57760283333333 6.86786498333333 6.30419145 6.4711885 7.21093403333333 6.346797725 6.3018823 6.78514255 6.88759146666667 5.95765828333333 6.47872195 7.23015586666667 7.095041 6.83441448333333 6.472039025 6.77411759166667 6.6271591 6.44506816666667 6.96462073333333 6.76915333333333 6.19099196666667 5.97867255833333 6.588167675 5.620020925 6.64989985833333 6.35472360833333 5.91168246666667 5.99116495833333 6.02571480833333 6.88087174166667

AC009950.1 4.54430115833333 6.11751004166667 5.54580931666667 6.1458196 4.154054225 6.75932524166667 4.86875400833333 4.854747975 4.544983975 4.97296273333333 4.36015529166667 5.26625810833333 4.38845033333333 5.647428275 6.27455109166667 6.4711885 4.556397325 5.152649175 5.9343511 6.05912289166667 5.00204984166667 6.06177335833333 5.07992535 6.16130541666667 6.36954999166667 5.09815425833333 5.84252611666667 5.76427079166667 6.42254766666667 6.41054875833333 6.075790425 6.56472320833333 5.13698225 5.23171054166667 4.38619328333333 4.21174504166667 6.01562348333333 5.31788553333333 5.47001155833333 5.78381885833333 5.51552526666667 5.89681745 6.22164848333333 6.41125588333333 5.92279354166667 5.70871841666667 5.88789478333333 5.20759355 6.09733190833333 5.88291568333333 5.85938266666667 4.96833235 4.59813639166667 6.45206855833333 6.87915231666667 7.13662455 5.95981851666667 5.4560179 6.89382319166667 7.08121445833333 4.82938895833333 4.45605366666667 4.95141223333333 5.06427393333333 4.82348884166667 6.15390005 4.62261409166667 4.9956547 4.6152735 4.70268088333333 4.4085814 4.67308416666667 4.30459614166667 4.86945729166667 4.18740718333333 4.61276948333333 4.49348375833333 5.59564198333333 4.69199204166667 4.58961148333333 5.114099175 4.40009215833333 4.11888183333333 4.61767011666667 4.29852961666667 5.4527323 5.76067670833333 4.59278468333333 4.93848820833333 6.27455109166667 5.017674975 3.838205275 4.794510625 5.00548516666667 5.114099175 4.03093171666667 5.81780209166667 4.568704925 4.39219723333333 5.99187126666667 4.794510625 4.27006691666667 6.291657675 5.397270075 5.32912600833333 5.57003804166667 5.32408766666667 7.57549880833333 4.843025575 6.25369225 5.71751655 4.7385841 4.09013416666667 5.23750470833333 5.39028695833333 6.36633304166667 4.34564913333333 4.2650829 5.44224698333333 5.07421885833333

AL162457.1 10.2156287416667 9.6633032 10.1859038333333 10.3702408666667 10.1248896166667 11.28965795 10.4884789583333 9.77248585 9.80147330833333 9.64986950833333 10.014538925 10.3684429583333 10.2387529583333 9.45373374166667 9.50277188333333 9.5438982 9.96941311666667 9.995800175 9.415981525 9.35376294166667 9.56068761666667 9.62432968333333 9.4483016 9.45947939166667 9.37335539166667 9.68724264166667 10.0478232083333 9.84388436666667 9.65779833333333 9.97701388333333 9.79381708333333 10.0784644666667 9.919589575 11.5110476583333 9.936416725 10.0192491333333 10.014538925 9.5041563 9.60613141666667 9.78232589166667 9.183229075 9.65779833333333 9.604456975 9.180393975 9.661839025 9.120779125 9.16631998333333 9.538153975 9.04108516666667 9.26648069166667 9.38637963333333 9.61303635833333 10.0060191666667 9.6015193 9.80418235833333 9.4470582 9.46065865 9.39858201666667 9.54939620833333 9.19737939166667 9.71817874166667 10.15582795 10.265208825 10.5356204916667 9.538153975 10.5578671833333 9.87584709166667 10.2736553416667 10.0911134416667 10.2209463166667 10.30417975 10.0284963833333 10.1776993666667 9.58292749166667 9.93793718333333 9.55383968333333 9.95043048333333 9.50689216666667 9.27011996666667 9.9056525 9.35011415 9.6633032 9.69020153333333 9.548133475 10.0882671583333 10.3666040083333 10.5805910083333 10.3232071333333 9.93793718333333 9.48684088333333 9.98989775 10.3145529166667 9.94543320833333 9.68577309166667 10.1489361666667 10.1099622416667 10.0559299333333 10.2156287416667 10.1526159166667 9.79859535 9.91018748333333 10.1473463666667 10.1248896166667 9.508238925 9.584447575 9.584447575 9.76078066666667 9.40408759166667 9.43421313333333 9.6633032 10.2093892 9.39994525833333 9.67598495 9.791053375 9.92102340833333 9.853830825 9.51227434166667 9.828409225 9.37066030833333 9.9056525

SLC7A11-AS1 7.56919636666667 9.643199125 10.0425633416667 8.86290175 11.1321094 10.9101974666667 10.0192491333333 10.950912925 9.53164766666667 11.113406175 13.5230143833333 8.345254425 9.548133475 8.866571625 9.306047575 9.288628775 12.60024735 9.31542745833333 10.5860666166667 10.4829935916667 9.054351375 9.17652090833333 11.8444568666667 11.4822633416667 10.1692826333333 6.09411293333333 10.2551372666667 10.4698496583333 10.2536886083333 9.5521857 10.01598095 9.49528248333333 8.51803561666667 11.42447965 7.12706315833333 8.12206505833333 9.53278261666667 10.420672925 9.05296820833333 9.8892648 10.595444475 9.054351375 9.30964859166667 10.287159 8.25567084166667 10.591906075 10.9059938166667 11.0420795083333 9.38364405 9.74557269166667 8.80621709166667 11.22950995 8.59712365833333 8.83012083333333 8.92128914166667 8.95834738333333 10.1378527416667 11.7230190333333 10.1776993666667 10.0647337083333 12.83676285 14.5016827333333 10.4473004666667 12.1711872833333 9.27528178333333 10.9747287666667 14.4412673083333 10.56904215 10.7322398666667 9.45660805 14.374704625 14.1131984833333 10.3409099333333 14.402064425 10.761090275 14.0658472 11.5310238 12.44226215 13.89773695 12.2895761166667 8.61733391666667 8.21411945 11.3186163 13.1722951416667 11.0850726666667 8.44184676666667 9.44548651666667 8.72413925 13.7723702416667 12.6686352083333 9.22871645 13.1525887583333 12.8432917666667 11.0447636416667 10.2030274 13.68323365 10.1605282 9.54520550833333 10.711383025 9.74707850833333 11.125139725 13.5553000916667 9.974114125 12.9345571083333 11.0901474083333 11.7188251083333 11.0949364416667 12.5457182583333 10.4365874083333 12.5903679416667 10.7830390666667 13.3080989916667 10.4347538666667 11.8881455083333 10.5706949916667 13.8425220416667 10.5338062833333 14.2961453916667 14.4622164333333 9.252230075

AL645568.1 7.65213465833333 6.73969015 7.65036194166667 7.44728600833333 6.75932524166667 7.83074816666667 7.8232377 6.72205318333333 7.403217625 7.42360905 7.603532025 7.21920913333333 6.812864825 7.012070875 7.55321126666667 7.291524925 8.13273210833333 7.05611735833333 7.36257615833333 7.81712555833333 6.96632546666667 7.4747897 7.21336981666667 7.317206725 7.78707899166667 7.81612064166667 8.53357196666667 8.03026959166667 7.47399836666667 7.437000375 7.51511816666667 8.00797545 7.96236178333333 8.17894748333333 8.26088785833333 7.89632803333333 7.769180675 7.68054443333333 7.5341355 7.32811256666667 7.67262559166667 7.158396675 7.72346068333333 7.6001855 7.54028876666667 7.74760565833333 7.77279406666667 8.28896994166667 7.67952253333333 7.70049388333333 8.00049861666667 7.7530692 8.28057508333333 8.35521645833333 8.226410775 7.94679925 7.83167704166667 7.70935458333333 7.90574131666667 8.18413825 7.60982945833333 7.06648555833333 8.131875875 6.850638675 7.159138825 7.256167725 8.46469871666667 7.05928261666667 8.03026959166667 7.952403425 7.72836676666667 7.385658775 7.14475656666667 7.45238604166667 7.53321899166667 7.30531678333333 7.57095955 7.371375775 7.73578660833333 7.99289775 6.97981448333333 8.75905724166667 6.97331920833333 7.83433648333333 7.92575334166667 7.30029635833333 8.0070051 8.01089986666667 7.91748764166667 7.916501675 7.7530692 7.79543485 7.91032310833333 8.56866955 7.4984921 7.81235193333333 7.903661075 7.71898053333333 7.68954538333333 7.67682356666667 7.724325925 7.08121445833333 7.63703748333333 7.67682356666667 7.31362185 7.85737128333333 7.769180675 8.46778476666667 7.75934284166667 8.35637496666667 7.64410754166667 8.11929538333333 8.42126873333333 8.53473379166667 8.56866955 7.84438765 8.55097769166667 8.05052155833333 7.66003905 8.58199240833333

AC007663.2 7.60890624166667 5.62083628333333 4.27153690833333 4.898378175 5.11005560833333 6.06669943333333 4.682020925 5.424208775 4.71818525833333 5.71751655 5.215034775 5.23171054166667 5.06597111666667 4.2951756 5.27721040833333 6.005005 6.35006203333333 5.57471 5.52817958333333 5.51552526666667 6.59279463333333 4.41509394166667 4.76862936666667 5.759099675 6.42430866666667 6.96306144166667 5.56466664166667 5.99620995 5.00125610833333 6.598919125 5.72192319166667 5.866627925 6.18629009166667 6.04516751666667 7.725002775 7.45158055 6.11513095833333 6.87117053333333 6.15390005 6.77879458333333 5.63266520833333 5.27404638333333 6.93519288333333 6.14095060833333 7.07530188333333 6.840567275 5.18149681666667 5.01678931666667 6.57993243333333 6.104675225 6.897050375 6.24284558333333 4.70340193333333 6.02571480833333 5.13143250833333 7.04741685833333 5.42593315833333 6.42828630833333 5.90014908333333 5.905668875 5.54936804166667 5.19388658333333 5.00290588333333 5.65088844166667 5.18662866666667 5.52066971666667 5.1551752 5.47188130833333 5.48853470833333 5.464202575 5.20230749166667 5.44953800833333 4.86450405 4.97138748333333 4.85225948333333 4.47471310833333 6.192534425 5.320395475 4.63810825833333 5.09060064166667 4.81551458333333 4.68742364166667 4.393584875 5.72366834166667 4.90088761666667 5.09738386666667 4.4085814 4.39434791666667 4.60037278333333 6.32061801666667 4.857936675 5.83677235833333 5.87407425 6.79350115833333 6.25446955 5.95899999166667 5.82021451666667 5.9244919 5.33796750833333 6.36633304166667 5.490209475 5.57003804166667 5.70066640833333 6.01338551666667 6.39481431666667 4.84808040833333 4.57380974166667 6.22349896666667 5.770291875 6.619742275 6.110329 5.88131005 6.13600023333333 6.47575550833333 4.91944066666667 5.78052715833333 5.904030425 6.03387288333333 6.17712046666667 4.70189450833333

MIR4458HG 4.6690583 4.33943185 3.9087838 3.72460085 3.53146109166667 4.01351464166667 3.4309565 4.35821460833333 3.54482885833333 3.66114276666667 3.51501689166667 3.34323016666667 3.550366875 3.992207825 4.02948560833333 3.74912634166667 5.32408766666667 3.89185758333333 4.36397045 3.66023421666667 3.664550775 3.66292545833333 3.99142755 5.1386748 4.44153641666667 4.57935210833333 3.81006580833333 4.111010475 6.49578869166667 3.90232935833333 4.1770019 5.55790016666667 3.89345544166667 4.60396200833333 4.29385256666667 3.9834399 4.30308001666667 4.77029946666667 4.49140346666667 4.424034 4.92102411666667 4.437951325 4.64804604166667 5.13301409166667 4.13615014166667 4.75878485833333 4.86708244166667 4.6472699 4.43137241666667 4.695056725 4.6425346 4.21802754166667 3.653206325 4.88079474166667 4.14619431666667 4.39514796666667 3.65576101666667 4.65893614166667 4.27600955 4.35590056666667 5.243413975 4.02802744166667 5.57384081666667 3.54208803333333 3.88309336666667 3.57984935 3.753884775 3.41871389166667 3.66292545833333 3.43373805 3.45464189166667 3.54123825 3.359953025 3.56466880833333 4.34086375833333 3.55566653333333 3.64645784166667 3.60704151666667 3.65493706666667 3.51583840833333 3.464474 3.58590108333333 3.85421665833333 4.47471310833333 3.563704825 3.54296001666667 3.42291026666667 3.80110566666667 4.04418303333333 3.829731875 3.48748343333333 5.60579466666667 5.67108565833333 3.56646223333333 4.45389868333333 3.86406553333333 3.89729075833333 4.39651160833333 4.22423876666667 4.51592391666667 4.249713475 3.51501689166667 4.51062659166667 4.696789075 4.31673289166667 3.78450821666667 4.41717060833333 5.57787028333333 4.08559233333333 5.90253445833333 6.27774009166667 4.54575506666667 4.711189975 4.53840028333333 3.838205275 4.15559815 4.04261585 4.40645795 4.435737425 3.46848923333333

GMDS-DT 9.90140289166667 7.029863175 7.27458531666667 6.82831530833333 6.86637626666667 7.53854279166667 5.07088755 6.32181892083333 6.99503750833333 6.822540775 6.70772591666667 7.12536183333333 7.44205825833333 7.41941744166667 7.13734699166667 8.243225475 9.71966379166667 6.60666248333333 8.45231269166667 8.029339375 7.12536183333333 8.14007678333333 7.52419651666667 8.3150991 8.23549675833333 9.98826495833333 8.25133669166667 7.94870053333333 8.21019310833333 7.8016903 7.865741375 7.6537848 7.44897004166667 7.83074816666667 9.99423949166667 10.2387529583333 7.3553377 7.40534635 8.00331975833333 8.39037005833333 8.760231975 7.88171558333333 7.88851164166667 8.25993145 8.86194551666667 8.39037005833333 8.8207743 7.162304025 8.03026959166667 8.18309575 7.92297084166667 7.52178743333333 8.005090175 8.217333875 8.017646775 9.20003395833333 7.63038558333333 7.83627273333333 8.073175025 8.17213816666667 7.41246615833333 5.512808975 6.9853877 5.87484386666667 8.426213375 6.14994006666667 5.197291 4.83912508333333 6.703671025 6.50724065 4.79215016666667 5.43964573333333 4.63950710833333 7.8232377 6.69708403333333 6.50428851666667 7.58719245833333 7.88934271666667 6.22881789166667 6.83441448333333 8.02524268333333 7.03910078333333 6.57292695833333 7.44381273333333 6.79889878333333 8.131875875 7.06248425833333 6.63521010833333 5.511999925 8.08470013333333 6.22806264166667 6.529771875 7.16070076666667 6.00748140833333 6.61332625 6.231190225 4.83845381666667 7.20164341666667 5.82190143333333 8.0743079 8.2214524 5.81691269166667 7.92972118333333 7.846293475 5.74674768333333 7.27284798333333 7.848979625 6.93829808333333 8.28057508333333 7.107436475 8.59364526666667 7.188718125 7.81612064166667 7.24174676666667 7.04507230833333 8.257703625 8.06225145833333 7.84801261666667 7.34163923333333 6.67645423333333

RCAN3AS 11.9252347666667 12.7581232166667 12.5969000166667 12.737637325 12.4713877916667 12.0412908333333 12.1443252916667 12.43965125 12.7412194083333 12.210187275 12.098586625 10.7052053666667 11.8059840833333 12.7481848833333 12.9489833333333 12.6122431666667 12.459832675 12.5174404833333 13.010652225 12.8218769 12.620700325 12.8295815416667 12.6239992833333 12.3010353583333 12.8659456416667 11.7092046583333 12.8148064833333 12.5560529333333 13.2032715083333 12.9202404083333 12.3062951833333 13.1985289416667 12.3920642833333 12.6330816833333 11.206704325 12.0287220416667 12.5689938083333 12.8148064833333 12.2456214083333 12.486478675 12.873197975 12.8804849833333 12.9345571083333 12.6593838083333 12.552751 13.1185208166667 13.2741790333333 12.840675275 12.5457182583333 12.6330816833333 12.486478675 12.800813375 12.92711715 13.1185208166667 13.0457394833333 13.1601453416667 12.675133075 12.9345571083333 12.8913513416667 12.9856740416667 10.9726612083333 12.074763075 13.069349375 12.617619125 12.6425501583333 11.6888272 12.5689938083333 11.5719596666667 11.7140151166667 12.283625975 11.1182445333333 12.0824665333333 12.2013587333333 11.361268975 12.526869925 12.8432917666667 11.3379048666667 12.3708273333333 12.0770282166667 12.5361294916667 12.57544625 11.413218375 12.3128225916667 12.708001075 12.0072618083333 11.720720125 10.5860666166667 12.3708273333333 11.7092046583333 11.5985981416667 11.880341125 12.3128225916667 12.9679019583333 12.0665318666667 12.675133075 12.0904755416667 13.2243581083333 11.2362164916667 12.0258788416667 12.9025226333333 12.3318273166667 11.29925645 12.1388344833333 12.7275736083333 12.1312062333333 12.1100895416667 12.126038825 11.745925575 12.3062951833333 12.1151589416667 12.7706567583333 12.1177468833333 12.773932725 13.2164074166667 12.20732285 12.1284072083333 13.07608845 11.933125475 12.6658577166667 12.012563225

MYT1L-AS1 9.105193125 9.09508848333333 9.32203853333333 9.28738489166667 9.37471118333333 9.14573905 9.2535473 9.07875895833333 9.41871168333333 9.31406849166667 9.345108925 9.34239438333333 9.2676985 8.97216231666667 9.25886408333333 9.045864375 9.68577309166667 9.307161125 9.201411475 9.17274169166667 8.866571625 8.84630949166667 9.045864375 8.85242150833333 8.85599695 9.03362063333333 8.93346260833333 8.98736196666667 9.07738231666667 9.31802425 9.2829911 9.0818604 9.30482685833333 9.786734675 9.50014711666667 9.382426125 9.29393176666667 9.44548651666667 9.45660805 9.27253420833333 9.30839305833333 9.24370573333333 9.16483426666667 9.26005576666667 9.08613931666667 9.2066946 9.23642199166667 9.40661546666667 9.08613931666667 9.16746680833333 9.41328055833333 9.37066030833333 9.34239438333333 9.39704085833333 9.574510875 9.46481536666667 9.24658409166667 9.62572484166667 9.14303199166667 9.15227524166667 9.22419906666667 9.31802425 9.23253931666667 9.39042674166667 9.17541009166667 9.214906375 9.62572484166667 9.20264188333333 9.637761575 9.49278793333333 9.42893785833333 9.74259324166667 9.44156545833333 9.18189768333333 9.41871168333333 9.37996499166667 9.25886408333333 9.29930791666667 9.2535473 9.19118669166667 8.857267525 8.74758403333333 9.15495383333333 9.1349915 9.04826785833333 9.10776604166667 9.05059795833333 9.18490179166667 9.101427925 9.01740153333333 9.28449495 9.49398269166667 9.35011415 9.4483016 9.65427285 9.15733181666667 9.37335539166667 9.70973839166667 9.62008553333333 9.334967175 9.47543304166667 9.334967175 9.54668399166667 9.252230075 9.415981525 9.15227524166667 9.30083194166667 9.40661546666667 9.26648069166667 9.10908699166667 9.26005576666667 9.5052656 9.597018275 9.66037100833333 9.71257970833333 9.40775390833333 9.38903546666667 9.8223954 9.43132836666667 9.40927853333333

AL033384.1 5.01186464166667 6.390112675 4.97551569166667 6.3840179 8.274413675 7.724325925 7.21256346666667 6.170078 6.52295673333333 7.1702859 6.78970486666667 6.90726410833333 6.6050576 7.158396675 8.34967031666667 6.58292990833333 5.72542938333333 7.04361085833333 7.47158285 7.0497186 6.42497558333333 8.45231269166667 6.978243625 6.825603975 6.86786498333333 5.12016660833333 7.703962625 7.40624533333333 7.97343739166667 6.88407465833333 5.76940198333333 7.23578164166667 6.397067625 5.80452428333333 5.19160068333333 5.95005389166667 8.45487785 7.56339936666667 6.430564175 7.33260558333333 6.36555325833333 6.752753925 6.83665193333333 7.029863175 6.81075070833333 6.93124799166667 6.39250838333333 7.06248425833333 7.805183975 6.71254476666667 6.694825125 7.30708359166667 5.44548114166667 6.97078570833333 6.14832854166667 5.54852681666667 7.18077985833333 5.91067900833333 7.5852857 7.21920913333333 7.45400806666667 5.70703833333333 5.83369038333333 5.69812770833333 6.182148775 6.93977528333333 6.0725726 5.582540175 4.7677709 5.736606025 6.57292695833333 6.30351415833333 5.99448725833333 7.21758313333333 8.444801925 6.36555325833333 5.37791728333333 7.63311984166667 6.822540775 9.06840325833333 5.35323611666667 6.30762875833333 7.06248425833333 6.299463175 6.87837529166667 5.3549373 5.16420516666667 5.77961628333333 7.08525161666667 5.333787 5.582540175 6.514960775 4.898378175 6.35770789166667 5.4961009 7.39891643333333 7.43034844166667 4.436542725 5.37429246666667 5.44782694166667 4.905221075 4.37434235 5.16663731666667 7.239291275 7.45333459166667 6.28233444166667 6.13600023333333 6.588167675 5.39874103333333 6.87837529166667 5.62441374166667 6.81620986666667 6.3125157 5.74925146666667 5.55016594166667 6.0008654 5.18325646666667 5.70335329166667 8.900308075 6.660735075

AC007389.1 6.9164285125 7.58926208333333 7.20477120416667 7.47026058333333 7.5794247375 6.79233904583333 7.62219540416667 7.14995687916667 7.46189963333333 7.23623005416667 7.8880294375 7.87545125833333 7.78759197083333 7.43908239583333 7.7607132125 6.68970482916667 8.71416658333333 7.8448784875 7.69838043333333 6.7953821 7.44613127083333 7.20387460833333 7.1691663125 6.34962777083333 6.9810332375 6.69043870416667 7.2353978625 7.4469252875 7.29354348333333 7.33620015416667 7.59895015 7.39609241666667 6.9833771875 6.84095494583333 6.678382225 6.69907465833333 7.63938615833333 7.4240441 7.19067414583333 7.04086765416667 7.175713875 7.46757265 7.1160827625 6.88042809583333 6.72401063333333 6.9179927 6.9283565 7.161066925 6.84402070416667 7.05963743333333 7.37008770833333 7.4469252875 7.68747142916667 7.72713566666667 7.19544812083333 7.45859589166667 6.94364269583333 7.67383879166667 7.63088381666667 7.25397250833333 7.30666669583333 7.47867229583333 6.88646895 7.74525018333333 6.6735373375 7.3007115125 6.77773352083333 7.16275739166667 7.15170129583333 6.99866415416667 7.86345573333333 7.41201945 8.35135029583333 7.0732130375 7.35240884166667 7.10206517083333 7.752541275 6.78851839166667 7.466743825 6.9108241625 7.3386155 6.96811305 6.8577347 6.2211931625 7.00324790833333 7.15170129583333 7.30410219583333 6.95019707083333 7.46189963333333 6.9810332375 7.18751969166667 7.28938759166667 7.28152766666667 7.3207805125 7.68645623333333 7.11772604166667 6.9179927 7.54934982083333 7.3378208375 7.50526619583333 6.86991523333333 7.73630872083333 6.734577025 7.62709047083333 7.18666509166667 7.2749377875 6.96184810833333 7.29833710416667 7.00111547916667 7.19388454583333 7.1541862125 7.1804165625 7.06523952083333 7.7562925875 6.9548547 7.2454879 7.01779762083333 6.9164285125 7.53985695 6.8493523125

AC007389.3 6.9164285125 7.58926208333333 7.20477120416667 7.47026058333333 7.5794247375 6.79233904583333 7.62219540416667 7.14995687916667 7.46189963333333 7.23623005416667 7.8880294375 7.87545125833333 7.78759197083333 7.43908239583333 7.7607132125 6.68970482916667 8.71416658333333 7.8448784875 7.69838043333333 6.7953821 7.44613127083333 7.20387460833333 7.1691663125 6.34962777083333 6.9810332375 6.69043870416667 7.2353978625 7.4469252875 7.29354348333333 7.33620015416667 7.59895015 7.39609241666667 6.9833771875 6.84095494583333 6.678382225 6.69907465833333 7.63938615833333 7.4240441 7.19067414583333 7.04086765416667 7.175713875 7.46757265 7.1160827625 6.88042809583333 6.72401063333333 6.9179927 6.9283565 7.161066925 6.84402070416667 7.05963743333333 7.37008770833333 7.4469252875 7.68747142916667 7.72713566666667 7.19544812083333 7.45859589166667 6.94364269583333 7.67383879166667 7.63088381666667 7.25397250833333 7.30666669583333 7.47867229583333 6.88646895 7.74525018333333 6.6735373375 7.3007115125 6.77773352083333 7.16275739166667 7.15170129583333 6.99866415416667 7.86345573333333 7.41201945 8.35135029583333 7.0732130375 7.35240884166667 7.10206517083333 7.752541275 6.78851839166667 7.466743825 6.9108241625 7.3386155 6.96811305 6.8577347 6.2211931625 7.00324790833333 7.15170129583333 7.30410219583333 6.95019707083333 7.46189963333333 6.9810332375 7.18751969166667 7.28938759166667 7.28152766666667 7.3207805125 7.68645623333333 7.11772604166667 6.9179927 7.54934982083333 7.3378208375 7.50526619583333 6.86991523333333 7.73630872083333 6.734577025 7.62709047083333 7.18666509166667 7.2749377875 6.96184810833333 7.29833710416667 7.00111547916667 7.19388454583333 7.1541862125 7.1804165625 7.06523952083333 7.7562925875 6.9548547 7.2454879 7.01779762083333 6.9164285125 7.53985695 6.8493523125

LINC00570 8.596030525 7.63311984166667 7.76387356666667 7.37716343333333 8.42525675833333 7.61148288333333 7.89552174166667 7.14799545 8.038750675 8.03302596666667 7.68151318333333 7.79633564166667 7.73951976666667 7.68954538333333 7.62671015833333 7.4773374 8.58199240833333 7.58246949166667 7.58436099166667 7.356869825 7.43784024166667 7.297972275 7.324744075 7.41578374166667 7.7430897 7.41157274166667 8.00331975833333 7.59418351666667 8.09958200833333 7.42694153333333 8.06126420833333 7.49052953333333 7.7674106 7.947814825 7.863951475 7.9266756 7.854674425 7.97430078333333 7.794547275 7.92035708333333 7.952403425 7.702242475 7.65656303333333 7.76387356666667 7.76018400833333 7.65558205833333 7.93246234166667 7.78810495 7.557410025 7.52253811666667 7.91185755833333 7.79543485 7.59232971666667 7.64073124166667 7.83530684166667 7.87494801666667 7.53244018333333 8.02342083333333 7.84066634166667 7.74760565833333 7.94078679166667 7.558954 7.79543485 8.029339375 8.41697565 7.98053493333333 7.664424575 6.945507325 8.1591384 7.50829535833333 7.67338803333333 8.06805541666667 7.81235193333333 7.7674106 7.55584700833333 7.69455133333333 7.79543485 7.44897004166667 7.558954 7.52419651666667 7.0616309 7.02346383333333 7.4754804 7.57728179166667 8.09224021666667 7.1870921 7.33028253333333 7.33109959166667 7.39050169166667 7.31955169166667 7.78707899166667 7.625103125 7.971686625 7.72751683333333 7.78442941666667 8.59364526666667 8.15541876666667 7.919306 7.87702689166667 7.73200875833333 8.03118996666667 7.67772895833333 7.77360815833333 7.59853308333333 7.92972118333333 7.40249240833333 7.55986316666667 7.16651384166667 7.81712555833333 6.72592418333333 7.41415456666667 7.89192915 7.89997913333333 7.82602878333333 7.53776969166667 7.45238604166667 7.75020911666667 8.15737470833333 7.89997913333333 7.43199838333333

LINC00174 7.75584805833333 6.86168069166667 6.78744436666667 6.57685955 6.0017496 6.88476720833333 5.63342111666667 6.28918155833333 6.01415498333333 6.10052355833333 6.310036575 6.662197225 5.91168246666667 6.192534425 7.404027575 6.91684939166667 6.86168069166667 6.70700871666667 7.43443834166667 6.76490431666667 7.12930466666667 7.17277909166667 6.694825125 7.24833841666667 7.02434959166667 6.50583770833333 6.88257336666667 7.56255123333333 7.14716405 6.51900598333333 7.34163923333333 6.89860500833333 6.73169263333333 6.686388325 7.55145098333333 7.008284125 7.03487794166667 7.08765801666667 6.18364681666667 6.90274688333333 7.60698968333333 7.75769473333333 7.59143541666667 7.930773275 7.065634 7.6026871 7.4887958 7.18623808333333 7.5341355 7.48222535 7.14080546666667 6.64847380833333 6.28460824166667 6.97981448333333 6.48552058333333 7.70315380833333 5.86177639166667 7.165677175 7.60525565 7.23578164166667 7.11408553333333 7.58719245833333 7.20762451666667 6.895291875 6.74572325 7.69963058333333 8.57288055 6.82919294166667 6.53259255833333 5.64838001666667 6.07758535833333 7.0616309 5.53870645 5.76067670833333 6.81539655 6.05432435833333 6.683342625 6.94476054166667 6.96698138333333 6.78668719166667 7.165677175 7.14080546666667 6.63444546666667 9.45660805 6.2377944 6.58965010833333 7.42360905 5.1551752 6.162124375 6.52604935 6.061018125 6.05008806666667 7.721741775 8.22733033333333 8.33092125 9.277757525 7.134124875 8.468834925 6.14505980833333 6.56472320833333 6.89860500833333 6.78970486666667 7.24350165 10.1621651166667 8.2690851 7.76474425 8.37069645 6.90573395833333 7.02102248333333 7.493374075 8.03218916666667 8.12783245 7.08679770833333 7.44656456666667 7.68954538333333 8.31623061666667 8.038750675 8.21411945 6.58894740833333 5.51633325833333

TH2LCRR 6.18629009166667 3.83468344166667 4.24443253333333 3.84547438333333 3.56015943333333 4.101417825 3.43373805 6.15390005 3.59998825 4.826730425 3.914168525 3.861448425 3.60085248333333 4.06243171666667 4.12589258333333 4.384745925 4.41944375833333 3.653206325 4.47167149166667 3.707364925 3.905548425 3.58244408333333 3.99860775833333 8.322516975 4.56566956666667 5.761496775 4.31153791666667 5.486081725 4.3038327 4.01031786666667 6.62118421666667 4.188201025 3.815793275 4.00317675833333 7.09583709166667 5.85179635833333 4.53014425833333 4.89756791666667 4.6425346 4.39144765833333 5.161910175 4.59966794166667 5.06686440833333 5.38613150833333 4.28123426666667 4.97296273333333 5.12530148333333 7.69882669166667 6.74116495833333 4.857936675 6.28384561666667 4.17251015833333 3.73131006666667 5.1451327 4.20338155833333 4.68830681666667 4.55255341666667 4.8148076 4.435737425 4.23434725833333 3.81249149166667 3.55663424166667 4.70189450833333 3.77295658333333 3.61525770833333 3.81249149166667 4.29455464166667 4.25984291666667 4.04512974166667 3.5975309 3.77643021666667 4.073408825 3.33473813333333 3.59489521666667 3.47050078333333 3.57663446666667 3.68978298333333 4.09098995 4.075722525 3.707364925 3.41541940833333 3.3657844 3.85254715833333 4.47964904166667 3.59910355 3.737786475 4.06020879166667 3.90409960833333 3.705852925 3.77555940833333 4.22056816666667 4.85225948333333 4.21564994166667 3.71701545 3.62702168333333 4.29927855833333 4.74667178333333 4.779451525 4.087125375 4.384745925 4.366986625 3.54758195833333 5.5093714 4.90276439166667 4.98118906666667 3.85421665833333 4.56033968333333 6.53259255833333 5.29742040833333 6.27047074166667 4.427577425 4.49209699166667 4.51592391666667 4.76359273333333 4.68661393333333 5.82345661666667 4.76218691666667 5.20589149166667 4.91782031666667 4.249713475

AC003035.2 8.675578375 8.93346260833333 8.96984509166667 8.7363942 10.0692552166667 8.95834738333333 9.78523885833333 9.00480995 9.62572484166667 9.2066946 9.08861136666667 9.74405148333333 10.1020643 9.23537203333333 8.82416160833333 8.395996775 8.54062638333333 10.0478232083333 8.71236105833333 8.67668070833333 8.7855717 8.85957338333333 8.35521645833333 9.37752135833333 9.21636353333333 9.06439938333333 8.96238550833333 9.012345475 8.496094 8.79885179166667 8.67920558333333 9.118265275 9.37335539166667 8.77366934166667 8.80139076666667 8.99956494166667 9.24370573333333 8.59712365833333 8.79393961666667 8.84985913333333 8.206103275 8.28057508333333 8.44806575833333 8.0943338 8.49060618333333 7.66087161666667 8.16110536666667 8.50671724166667 9.00974125833333 9.03224780833333 9.00614454166667 9.23642199166667 9.31248745 8.80139076666667 8.33417815 7.60801480833333 9.30482685833333 8.13598734166667 8.93824426666667 8.76267615 9.57749218333333 9.33249780833333 8.89079293333333 8.75314075 8.96465185 9.42893785833333 9.0697895 9.62849099166667 9.37066030833333 9.57749218333333 9.5052656 9.10021326666667 9.826776875 9.11422543333333 8.8207743 8.71933655 9.52458273333333 9.49278793333333 8.58803331666667 8.40512834166667 8.79269821666667 8.94723143333333 8.40939095833333 8.08371099166667 9.338606375 9.03833616666667 9.72422056666667 9.08613931666667 9.04826785833333 9.05296820833333 9.03496023333333 9.50133671666667 9.45793138333333 8.94723143333333 9.88790173333333 8.97484515833333 9.40271259166667 9.36648925 9.30352410833333 8.87214213333333 8.96098959166667 9.00075389166667 9.042386175 8.05268059166667 8.81562710833333 8.63323710833333 8.87214213333333 8.60047110833333 9.10021326666667 9.00075389166667 9.08755966666667 8.60647770833333 8.99595798333333 8.02061905 8.348415775 8.56973665 9.75280021666667 9.48113538333333 9.03224780833333 10.0574939833333

LEMD1-DT 4.38845033333333 6.05506278333333 4.959236325 4.82759028333333 6.683342625 5.61315593333333 6.03894880833333 5.4551887 5.08260519166667 4.77715155833333 5.03074584166667 5.25605979166667 5.39640219166667 5.01028051666667 5.29742040833333 5.40280533333333 4.85957668333333 5.40803009166667 4.684258075 4.63352474166667 5.60075419166667 5.43110274166667 5.62857495 5.332832325 5.49272935833333 4.94091306666667 5.43964573333333 4.98704916666667 5.228162675 4.783293425 4.64653290833333 4.42996005 5.10162569166667 4.98534708333333 4.06404305833333 4.19743935833333 4.90606120833333 4.451845525 5.043189975 4.91113061666667 4.55717808333333 5.322240725 4.61276948333333 5.18970676666667 4.184461775 5.32408766666667 4.16854543333333 4.54275920833333 4.3038327 4.59031499166667 4.61432114166667 5.05353733333333 5.022489825 4.80661418333333 4.7289146 4.3750276 3.9722338 4.49432511666667 3.876232825 4.81864974166667 4.32890503333333 5.60579466666667 5.152649175 4.52276595833333 6.13454856666667 4.89413600833333 5.81853184166667 5.71323735 4.24046655 4.31153791666667 6.883226375 5.48937928333333 4.82196883333333 4.21730013333333 5.13143250833333 5.17819575 5.48937928333333 4.80100048333333 3.981243 4.70967379166667 5.284267925 6.27857845833333 5.15014486666667 4.85957668333333 4.64653290833333 4.8116637 5.22651561666667 4.241859075 6.692367775 4.97881760833333 4.27672135833333 4.27232815 4.49747863333333 4.11973251666667 4.49903674166667 4.49747863333333 4.22805825833333 4.61608426666667 4.55798421666667 5.215034775 4.473850775 4.82196883333333 4.463312775 4.6372952 4.164731775 4.41068774166667 3.93820065 5.12699374166667 4.84158960833333 5.60990619166667 5.75111171666667 5.00290588333333 4.59813639166667 4.389247575 4.26651681666667 4.12057799166667 4.55489758333333 4.22490279166667 5.23682500833333 4.58075485833333

U73166.1 10.105187625 12.3062951833333 12.0559423166667 12.2046199416667 12.1524570666667 10.615238775 11.9966241166667 11.9892138666667 12.1073504333333 11.6943956666667 11.9175777083333 10.4158197333333 11.1863927333333 11.3761321333333 11.7948078416667 10.95910865 10.0832833333333 12.1312062333333 11.7117253416667 11.8726681416667 10.8994144416667 11.3379048666667 11.167449 10.92060855 11.9433354166667 10.2141677 12.1711872833333 11.893046575 12.2785617166667 12.231089275 12.0611612416667 11.9892138666667 12.368506525 10.3913684416667 10.0097474333333 9.92102340833333 12.8041650666667 12.7612379833333 12.0207283333333 11.96099235 11.774517575 11.7689310166667 11.54529205 11.394164525 10.9859995166667 11.2130362833333 11.489093725 11.33050215 11.2566977833333 11.4942244666667 11.96099235 11.854762075 11.40831085 11.413218375 11.5765773333333 11.1951963916667 11.5062743666667 12.5016594166667 11.9993623166667 11.1211007166667 10.4681694166667 10.4570032916667 10.2536886083333 10.3004507666667 10.2736553416667 10.691603225 10.0220394666667 10.4681694166667 10.30417975 10.682010725 10.7872050916667 10.6459121416667 10.4175013916667 10.023583275 10.0492420416667 9.826776875 10.5319942416667 10.471680725 9.90270463333333 10.1825522583333 10.219194075 9.99887365833333 9.98826495833333 9.97891528333333 10.4454279083333 10.31080925 10.394412525 10.17093795 10.2551372666667 10.257033225 10.36490795 10.1171906166667 10.4570032916667 10.142733725 10.5145241083333 10.5860666166667 10.5560371666667 10.644312275 10.6757072916667 10.272016 10.1410949416667 10.5596196916667 10.11885595 9.96147485833333 9.9929911 9.87885509166667 10.0349403166667 9.75423146666667 10.2473472 10.0723139833333 10.20778565 10.2902993333333 9.97701388333333 9.826776875 9.97103178333333 9.997228975 10.1394934333333 10.2354161583333 10.0252423 9.93966605

FAM225A 11.2248872 8.90539649166667 9.19243765 9.96147485833333 10.1344061583333 9.79381708333333 9.437388275 9.73556275833333 9.61446786666667 10.260350775 9.96000436666667 10.761090275 10.5560371666667 9.50971113333333 9.48990181666667 10.272016 10.9101974666667 10.7136014583333 9.95348596666667 9.93174519166667 9.0670828 10.1526159166667 9.64606555833333 9.88347994166667 8.85242150833333 9.55939928333333 10.1248896166667 9.90140289166667 10.17093795 10.424538125 9.81785751666667 9.06329124166667 9.15733181666667 9.17904495833333 9.40138860833333 9.120779125 10.2489068 9.15733181666667 9.597018275 9.62279060833333 8.42995574166667 9.98678198333333 9.29930791666667 8.900308075 9.75922218333333 9.26648069166667 8.88320966666667 9.48539969166667 8.55899596666667 8.44688928333333 8.25993145 8.52340118333333 9.34755915833333 8.93700485 8.90539649166667 8.03026959166667 9.17541009166667 9.136244075 9.26648069166667 8.96984509166667 10.0300876416667 9.61855603333333 9.71966379166667 10.6079206166667 11.0651359666667 9.82384475833333 9.89075863333333 9.8223954 10.1811538916667 10.3371834416667 9.93793718333333 10.3549727833333 9.327220125 10.212532475 9.589758275 10.006716175 11.29925645 10.7507418916667 10.5000230916667 10.5000230916667 10.3180296083333 10.8609045666667 9.63609045833333 10.1473463666667 9.62143423333333 10.3089135 10.9747287666667 9.98826495833333 10.2260622166667 9.639269375 10.0896386166667 9.64986950833333 9.904065125 8.96098959166667 9.02591655 9.63324336666667 9.91464370833333 9.87885509166667 9.83774009166667 9.46481536666667 9.213538675 8.86517486666667 9.96941311666667 9.43132836666667 9.68577309166667 9.306047575 9.19875904166667 9.50133671666667 8.6708431 8.537131375 8.89682220833333 8.51803561666667 9.24804320833333 8.60047110833333 9.47701395833333 9.02978039166667 10.2030274 9.62279060833333 9.16631998333333 8.96984509166667

AC090772.3 8.0743079 7.21511196666667 7.725002775 7.81962728333333 7.040488 8.73370975833333 6.21003021666667 7.70654949166667 6.908912025 7.239291275 11.0786204833333 6.94252621666667 6.74043675833333 6.81924664166667 7.697934175 7.66003905 7.73104835833333 6.36034525833333 7.42862740833333 7.05999225 7.75118009166667 7.10174751666667 7.96426395833333 7.95705075833333 7.46714951666667 8.3150991 7.5341355 7.73200875833333 7.420213075 7.17123928333333 7.51334780833333 7.34500641666667 6.94476054166667 6.92317931666667 8.95972230833333 8.458054325 7.62946086666667 7.54201473333333 7.724325925 7.98997320833333 8.24820143333333 7.57184745833333 7.79543485 7.897268425 7.867558375 8.49171125 8.60535770833333 6.66815600833333 8.05364296666667 8.08759711666667 7.3846807 7.080264325 7.52334426666667 6.70700871666667 7.54808809166667 8.323692025 7.33260558333333 7.99573270833333 8.13385164166667 7.56501633333333 11.22950995 12.2509990916667 9.280311725 9.35119336666667 11.1157375666667 7.7899459 10.814470725 7.68334979166667 8.62436223333333 8.53357196666667 12.8182497583333 11.9808613 7.33342934166667 9.98826495833333 7.57989095 8.72541470833333 7.90761645833333 7.8186674 8.52145965 8.75089571666667 10.7785431666667 9.01353759166667 9.5052656 10.893006875 8.158279725 9.01843699166667 12.012563225 8.341852175 12.4240585416667 9.63464729166667 7.75020911666667 10.1542300916667 10.4810665833333 8.730048975 8.44384576666667 11.354050525 8.35845888333333 10.3913684416667 7.595060625 9.55672449166667 9.35233383333333 8.42126873333333 9.09372039166667 9.92405621666667 9.31802425 9.491289725 9.536758 8.038750675 9.59117006666667 7.73305023333333 9.81785751666667 8.994694825 8.02160229166667 9.89542063333333 9.51536090833333 11.8881455083333 10.8054366083333 8.61968489166667 11.8364860833333 7.291524925

LINC01533 4.94285702083333 6.340111775 5.94392865 4.7124346125 6.88720932916667 5.57510055 6.7271113375 6.5248156625 6.581699925 5.86048204583333 5.89885704166667 5.71104780416667 6.16479498333333 4.884556825 5.67918202916667 4.54836894166667 5.56958737916667 5.8530987875 4.60997690833333 5.37206293333333 5.89402629166667 5.50699834166667 5.592431675 5.2606255625 4.74772855833333 6.27085357916667 5.29506012916667 4.46662546666667 4.8830006375 6.076232075 5.06806700833333 5.5089590875 6.4262697625 6.22699215833333 5.14799047916667 5.20717145416667 4.94612180416667 5.04360393333333 4.883788775 4.67202240416667 5.18938010833333 5.3906940625 5.22071421666667 5.3166294875 4.25009858333333 4.89878320833333 4.75840206666667 4.8014052875 4.7164078125 5.19847848333333 4.8039084625 4.6576881625 4.56747160833333 5.2225374125 5.03603799583333 4.707014225 5.87445905833333 4.78061298333333 5.03768853333333 4.60914915416667 4.9695129875 6.2261403625 4.8411742375 4.81516109166667 6.31045869583333 5.56686944166667 6.88646895 6.17178580416667 4.10101375416667 5.65768415416667 5.3906940625 6.11073158333333 4.63843010833333 4.80619006666667 4.74142364166667 4.8395655625 5.55063607916667 4.4720473625 4.5110223625 5.20019406666667 5.23458158333333 6.04963354583333 4.55678770416667 4.30958775833333 4.45716902083333 4.8411742375 4.18180010416667 4.74772855833333 5.688863 4.658516775 4.8674785125 5.16309332916667 4.22769371666667 4.9671704375 4.492464625 5.30287547916667 4.31941318333333 4.6260152625 4.90483494166667 4.94854447083333 4.385859075 4.77524804166667 4.5924200625 4.70930977916667 4.46510397083333 4.24224383333333 4.35188010833333 5.73782292916667 4.65462964166667 6.2789455875 4.61315370416667 5.1769021875 4.46072887916667 5.31912419583333 4.32999024583333 4.36268724166667 4.5660347875 4.40889241666667 4.2816017125 5.42727825

LINC01837 4.94285702083333 6.340111775 5.94392865 4.7124346125 6.88720932916667 5.57510055 6.7271113375 6.5248156625 6.581699925 5.86048204583333 5.89885704166667 5.71104780416667 6.16479498333333 4.884556825 5.67918202916667 4.54836894166667 5.56958737916667 5.8530987875 4.60997690833333 5.37206293333333 5.89402629166667 5.50699834166667 5.592431675 5.2606255625 4.74772855833333 6.27085357916667 5.29506012916667 4.46662546666667 4.8830006375 6.076232075 5.06806700833333 5.5089590875 6.4262697625 6.22699215833333 5.14799047916667 5.20717145416667 4.94612180416667 5.04360393333333 4.883788775 4.67202240416667 5.18938010833333 5.3906940625 5.22071421666667 5.3166294875 4.25009858333333 4.89878320833333 4.75840206666667 4.8014052875 4.7164078125 5.19847848333333 4.8039084625 4.6576881625 4.56747160833333 5.2225374125 5.03603799583333 4.707014225 5.87445905833333 4.78061298333333 5.03768853333333 4.60914915416667 4.9695129875 6.2261403625 4.8411742375 4.81516109166667 6.31045869583333 5.56686944166667 6.88646895 6.17178580416667 4.10101375416667 5.65768415416667 5.3906940625 6.11073158333333 4.63843010833333 4.80619006666667 4.74142364166667 4.8395655625 5.55063607916667 4.4720473625 4.5110223625 5.20019406666667 5.23458158333333 6.04963354583333 4.55678770416667 4.30958775833333 4.45716902083333 4.8411742375 4.18180010416667 4.74772855833333 5.688863 4.658516775 4.8674785125 5.16309332916667 4.22769371666667 4.9671704375 4.492464625 5.30287547916667 4.31941318333333 4.6260152625 4.90483494166667 4.94854447083333 4.385859075 4.77524804166667 4.5924200625 4.70930977916667 4.46510397083333 4.24224383333333 4.35188010833333 5.73782292916667 4.65462964166667 6.2789455875 4.61315370416667 5.1769021875 4.46072887916667 5.31912419583333 4.32999024583333 4.36268724166667 4.5660347875 4.40889241666667 4.2816017125 5.42727825

GDNF-AS1 4.59278468333333 4.00232853333333 3.8855125 4.13221068333333 3.65225226666667 5.19649501666667 3.73544420833333 3.69829589166667 3.981243 3.93183256666667 4.0990947 3.55300464166667 3.74912634166667 3.99142755 4.37819051666667 3.81498958333333 4.63572826666667 3.79213375833333 4.64653290833333 3.81801135833333 3.83557885833333 4.05010078333333 4.30784019166667 4.50562893333333 4.82759028333333 4.05400306666667 4.52785514166667 4.65893614166667 5.21348641666667 4.38552486666667 4.74898438333333 4.55111153333333 3.67690364166667 4.02948560833333 4.09521015 4.101417825 4.51446953333333 6.05667555833333 4.45465283333333 4.6318291 4.898378175 4.45897225 4.77563934166667 5.37087810833333 4.13060835 4.90088761666667 4.81013838333333 4.75042364166667 4.67862661666667 4.91436364166667 4.89918824166667 4.56342764166667 3.946873725 5.194761075 4.21242450833333 4.66568316666667 3.803316 4.73591975 4.270761425 4.50920185 3.8188 3.51310729166667 4.34086375833333 4.42171775 3.471332425 4.484204225 3.82803336666667 4.39219723333333 3.65576101666667 3.56829485 3.70151568333333 3.61794161666667 3.755690675 3.81006580833333 3.919283675 3.575663075 4.0255905 3.77643021666667 3.78593040833333 3.595618825 4.27600955 4.21399664166667 4.21022225 3.94318915 3.757295725 3.66852646666667 4.207301975 4.46466106666667 4.23975296666667 4.06973544166667 3.72879851666667 4.49076011666667 4.01591823333333 3.77555940833333 3.86581385 3.89345544166667 3.99459898333333 5.07088755 3.68304045833333 4.8923349 4.257819625 3.64645784166667 4.74433684166667 4.82938895833333 4.31979769166667 3.80841116666667 4.42684748333333 5.72631065 4.34564913333333 6.37198143333333 4.73036435 5.35323611666667 4.40009215833333 4.783293425 3.957521375 4.36632163333333 4.00078191666667 4.45532334166667 4.39870788333333 3.66114276666667

LY6E-DT 9.24251089166667 8.485317225 7.94287994166667 8.13815014166667 6.64146615833333 7.376269825 7.05611735833333 8.525776075 6.52116074166667 8.93346260833333 7.44983849166667 7.09088685 7.43034844166667 9.19483533333333 8.20927199166667 8.82317351666667 7.13662455 8.10154305833333 8.86194551666667 9.06555709166667 8.80258250833333 9.5984398 9.37996499166667 7.10578959166667 8.74874869166667 9.16363804166667 8.24631605833333 8.84858576666667 8.04867420833333 8.94047908333333 9.4470582 8.75767668333333 8.42525675833333 6.67645423333333 9.22186990833333 8.40512834166667 7.01511199166667 9.5341054 7.33659296666667 8.38818113333333 8.40414963333333 7.62178835 9.39704085833333 8.49828676666667 8.83768020833333 9.87885509166667 9.85529955833333 8.05268059166667 8.97750675833333 9.08029321666667 9.213538675 8.08081069166667 7.80424109166667 8.54377494166667 8.96465185 9.21209545833333 8.89549855 9.53164766666667 10.1155244166667 9.82539988333333 7.68597430833333 6.49504519166667 10.1065692416667 6.57216478333333 10.5145241083333 7.06484504166667 8.15541876666667 7.73951976666667 10.6978544083333 9.57597325 8.44905829166667 7.68875390833333 4.8556402 6.77735403333333 6.48304445 8.99595798333333 9.60613141666667 9.02978039166667 9.56332510833333 6.945507325 8.88949339166667 7.66003905 8.80498713333333 9.158641175 6.88407465833333 6.46484100833333 8.51484585833333 7.67682356666667 10.344247175 8.95205770833333 7.71121305833333 9.16111019166667 6.453551925 7.39651405 6.79655045 9.77927601666667 8.03218916666667 9.03496023333333 6.336430425 10.8746426833333 8.17305670833333 4.86629001666667 9.64606555833333 6.70536285833333 9.33619586666667 9.36356514166667 5.603294875 10.2459336666667 7.28275553333333 8.480972975 8.95834738333333 8.301792275 7.99573270833333 9.78825455 7.25974294166667 7.82427053333333 8.96098959166667 11.1746153 8.39364840833333 7.827876425

AL583808.1 5.01563855416667 4.06369009583333 4.058413075 4.22612190833333 3.58629872083333 4.60570537083333 3.71043913333333 3.8168871 3.55975103333333 3.74799978333333 3.63161408333333 3.73657375416667 3.59874052083333 4.168962575 4.44557661666667 3.9691564375 5.3991013125 3.803827225 4.78602575416667 3.93535581666667 3.86450157916667 4.158269075 4.39548288333333 4.70377167083333 4.83384497916667 4.08146594583333 4.63690560833333 4.74933793333333 4.6421634125 4.7347751125 4.83806589583333 4.66271999166667 3.782423275 4.12781786666667 4.77905429166667 4.39837857916667 5.16862806666667 4.7775658625 4.6000203625 4.8830006375 5.2515505125 4.5842254625 4.95489029166667 5.73700309583333 4.18180010416667 5.2244360125 5.19519462916667 5.08298402916667 4.72516030416667 5.04604934166667 5.01397411666667 4.650763375 3.93142087083333 5.3444952 4.85350922083333 4.91394925416667 3.78635575 4.93070639583333 4.28678514583333 4.55678770416667 6.45913069583333 3.78002401666667 6.38735820416667 4.43250935 3.511708625 5.82560274583333 3.9154568875 3.82575241666667 3.77337481666667 3.998239875 3.6468591875 4.658516775 3.6920558375 3.89458091666667 3.55256972916667 3.67327606666667 3.48502324583333 3.88922899583333 3.85861335416667 4.33297843333333 3.78817005833333 6.11474347916667 4.6267788375 4.076094925 4.25944933333333 4.76820013333333 5.2820858625 4.48001641666667 3.59525702083333 4.050451075 3.84746379583333 4.5110223625 6.13979142083333 3.78159964166667 5.5939091875 4.96460901666667 3.999707925 6.109915525 3.71043913333333 5.60284809583333 7.52537087083333 3.42725754166667 4.83726525833333 5.4687350375 4.34813653333333 4.31268420416667 4.57201751666667 6.20965022083333 4.3672843125 6.74456825416667 4.99012409583333 5.4848192625 5.01482467083333 4.86982477916667 3.9760545875 4.485975925 7.36380550833333 4.60356488333333 4.30958775833333 3.74874908333333

LINC02796 5.01563855416667 4.06369009583333 4.058413075 4.22612190833333 3.58629872083333 4.60570537083333 3.71043913333333 3.8168871 3.55975103333333 3.74799978333333 3.63161408333333 3.73657375416667 3.59874052083333 4.168962575 4.44557661666667 3.9691564375 5.3991013125 3.803827225 4.78602575416667 3.93535581666667 3.86450157916667 4.158269075 4.39548288333333 4.70377167083333 4.83384497916667 4.08146594583333 4.63690560833333 4.74933793333333 4.6421634125 4.7347751125 4.83806589583333 4.66271999166667 3.782423275 4.12781786666667 4.77905429166667 4.39837857916667 5.16862806666667 4.7775658625 4.6000203625 4.8830006375 5.2515505125 4.5842254625 4.95489029166667 5.73700309583333 4.18180010416667 5.2244360125 5.19519462916667 5.08298402916667 4.72516030416667 5.04604934166667 5.01397411666667 4.650763375 3.93142087083333 5.3444952 4.85350922083333 4.91394925416667 3.78635575 4.93070639583333 4.28678514583333 4.55678770416667 6.45913069583333 3.78002401666667 6.38735820416667 4.43250935 3.511708625 5.82560274583333 3.9154568875 3.82575241666667 3.77337481666667 3.998239875 3.6468591875 4.658516775 3.6920558375 3.89458091666667 3.55256972916667 3.67327606666667 3.48502324583333 3.88922899583333 3.85861335416667 4.33297843333333 3.78817005833333 6.11474347916667 4.6267788375 4.076094925 4.25944933333333 4.76820013333333 5.2820858625 4.48001641666667 3.59525702083333 4.050451075 3.84746379583333 4.5110223625 6.13979142083333 3.78159964166667 5.5939091875 4.96460901666667 3.999707925 6.109915525 3.71043913333333 5.60284809583333 7.52537087083333 3.42725754166667 4.83726525833333 5.4687350375 4.34813653333333 4.31268420416667 4.57201751666667 6.20965022083333 4.3672843125 6.74456825416667 4.99012409583333 5.4848192625 5.01482467083333 4.86982477916667 3.9760545875 4.485975925 7.36380550833333 4.60356488333333 4.30958775833333 3.74874908333333

LINC01356 9.79240870833333 9.334967175 9.569175825 9.39994525833333 9.77927601666667 9.84834350833333 9.84258693333333 9.38364405 10.0738928916667 9.77800786666667 9.58572294166667 9.85529955833333 9.56478920833333 9.20003395833333 9.17541009166667 9.23642199166667 9.26286649166667 9.97701388333333 9.548133475 9.33115149166667 9.38903546666667 9.24658409166667 9.101427925 9.56068761666667 9.37996499166667 9.71125059166667 9.32203853333333 9.338606375 9.5052656 9.277757525 9.323428375 9.31248745 9.45373374166667 9.70165576666667 10.0818728583333 10.1473463666667 9.84081016666667 9.5521857 9.820599225 9.50133671666667 9.256048375 9.38498160833333 9.04373345 9.08029321666667 8.95454636666667 8.84259734166667 8.857267525 8.90815406666667 9.47971744166667 9.49645880833333 9.4483016 9.43132836666667 8.77244310833333 9.26878520833333 8.674394275 8.6685911 8.99711739166667 9.43132836666667 9.254766825 9.35667674166667 10.1841002166667 9.831337375 9.11552891666667 9.85797725 9.79513514166667 10.6248927916667 10.3409099333333 9.72272495833333 10.6208047916667 10.0771266166667 9.82384475833333 9.96321 9.84081016666667 9.78069864166667 9.80574389166667 9.98678198333333 10.12322705 9.936416725 9.57749218333333 9.56775843333333 9.84388436666667 9.81636959166667 9.61182498333333 9.799983825 9.80574389166667 9.66037100833333 10.401625675 9.71125059166667 9.57185823333333 9.519387125 9.53947254166667 10.0192491333333 9.88202425 10.0300876416667 10.272016 10.1811538916667 10.2702136416667 10.272016 10.0647337083333 9.9755856 9.80574389166667 9.67300651666667 9.89676418333333 9.49528248333333 9.67598495 9.16111019166667 9.6173537 9.64986950833333 10.1216004333333 9.97701388333333 9.981884775 9.71370898333333 9.23253931666667 9.59117006666667 9.07590878333333 9.03966899166667 9.63324336666667 10.1542300916667 9.51801905 9.39042674166667

AC023421.2 6.46071088333333 5.27856399166667 5.10035652083333 5.30449073333333 5.32540094583333 4.417509625 5.330455025 5.599518425 4.84264881666667 5.308160025 5.04124992083333 6.093724075 6.04404814166667 4.989291875 5.41493128333333 5.79270620833333 4.87148595833333 6.58619782083333 5.83241176666667 5.72942844166667 5.81890684166667 5.96028880833333 4.90483494166667 5.86216925416667 4.45935194166667 6.638766925 6.43482229583333 4.41474610833333 6.2741755625 5.41936614583333 5.19847848333333 4.74629541666667 4.80619006666667 5.23975184166667 6.8140698875 6.52330912083333 5.75711974166667 6.2307932625 5.77750756666667 5.8670189 4.65545469583333 4.6515645 4.55454004166667 5.271780825 4.91158133333333 4.9413130125 5.59862125833333 5.16462009583333 4.4190647 4.8053777 4.7500575625 5.18621714583333 5.30363972916667 5.169596325 4.733228875 4.6260152625 5.4219218375 4.5245276375 5.02515947083333 5.05742707916667 5.35817842916667 5.6627825125 4.28234809166667 6.02019989166667 4.90643878333333 5.25970307916667 6.027778625 5.14006110833333 4.3925407 5.4356982375 5.02865137083333 5.67742785833333 5.68553395416667 5.11367970833333 5.18862198333333 5.3089608625 5.27091237916667 4.93243676666667 5.00086517083333 5.24643280833333 5.64879312916667 6.28643380416667 5.001652975 6.03856938333333 5.19689300833333 5.94392865 5.69857975 4.98577728333333 5.5243597375 5.66017808333333 5.42024353333333 5.1538997375 4.56531204583333 5.53742774583333 5.4823441875 4.78286589166667 5.84448194583333 6.59006025 5.30639460416667 5.02437999583333 4.12625222083333 5.25718947916667 4.572761225 4.71084860416667 4.81439040416667 5.75562614583333 5.1382688375 5.40328129166667 5.35817842916667 6.1381611 4.56162244166667 5.15473165416667 6.18834422916667 4.50529682083333 5.4723572125 4.48746172083333 4.85091785 4.32928347916667 4.118479425 5.28726847083333

AC023421.1 6.46071088333333 5.27856399166667 5.10035652083333 5.30449073333333 5.32540094583333 4.417509625 5.330455025 5.599518425 4.84264881666667 5.308160025 5.04124992083333 6.093724075 6.04404814166667 4.989291875 5.41493128333333 5.79270620833333 4.87148595833333 6.58619782083333 5.83241176666667 5.72942844166667 5.81890684166667 5.96028880833333 4.90483494166667 5.86216925416667 4.45935194166667 6.638766925 6.43482229583333 4.41474610833333 6.2741755625 5.41936614583333 5.19847848333333 4.74629541666667 4.80619006666667 5.23975184166667 6.8140698875 6.52330912083333 5.75711974166667 6.2307932625 5.77750756666667 5.8670189 4.65545469583333 4.6515645 4.55454004166667 5.271780825 4.91158133333333 4.9413130125 5.59862125833333 5.16462009583333 4.4190647 4.8053777 4.7500575625 5.18621714583333 5.30363972916667 5.169596325 4.733228875 4.6260152625 5.4219218375 4.5245276375 5.02515947083333 5.05742707916667 5.35817842916667 5.6627825125 4.28234809166667 6.02019989166667 4.90643878333333 5.25970307916667 6.027778625 5.14006110833333 4.3925407 5.4356982375 5.02865137083333 5.67742785833333 5.68553395416667 5.11367970833333 5.18862198333333 5.3089608625 5.27091237916667 4.93243676666667 5.00086517083333 5.24643280833333 5.64879312916667 6.28643380416667 5.001652975 6.03856938333333 5.19689300833333 5.94392865 5.69857975 4.98577728333333 5.5243597375 5.66017808333333 5.42024353333333 5.1538997375 4.56531204583333 5.53742774583333 5.4823441875 4.78286589166667 5.84448194583333 6.59006025 5.30639460416667 5.02437999583333 4.12625222083333 5.25718947916667 4.572761225 4.71084860416667 4.81439040416667 5.75562614583333 5.1382688375 5.40328129166667 5.35817842916667 6.1381611 4.56162244166667 5.15473165416667 6.18834422916667 4.50529682083333 5.4723572125 4.48746172083333 4.85091785 4.32928347916667 4.118479425 5.28726847083333

AC079781.5 10.2643763708333 10.9668980875 10.9951368375 10.8799482708333 12.0546684541667 10.5495181458333 12.5312882041667 11.4764632583333 12.1113047083333 10.7333004916667 11.2095984041667 12.8573809541667 12.56399775 11.2190650916667 11.0409097458333 11.1875048625 10.5436968083333 11.9598907208333 9.91696874583333 11.0093021625 11.5440811625 11.37744305 10.8664585083333 10.8199753375 10.7062054458333 10.7738484833333 10.7538357416667 10.5795097916667 11.0409097458333 10.5752846666667 9.8021216125 10.6488660166667 11.068216525 10.4762218166667 9.55467654583333 10.3593030208333 10.8064994583333 10.098956 9.90071585833333 10.2068985958333 9.36846859166667 10.1753571625 10.0534295291667 9.66524654166667 10.0777955416667 9.56846712916667 9.86034475 10.237975625 10.1803173041667 10.1803173041667 9.8476021875 10.4182870166667 11.2469887625 10.4800932833333 10.5587434375 9.59329031666667 10.9217485666667 9.283743025 10.4631657541667 10.2068985958333 12.083866575 11.4740179458333 10.5059966041667 11.1780276833333 11.4377467166667 11.1285866791667 11.8377183833333 12.1677782 10.6069468625 11.3437706375 11.6522784458333 11.7837961083333 12.2663378875 10.9430338458333 12.0246139791667 11.3702568291667 11.4879602833333 11.4901913333333 10.4148400375 11.1615461583333 12.0190830208333 12.534442825 10.8395357958333 10.0716385875 10.9092115458333 11.6545501125 12.3957431333333 11.4426573 12.3415392541667 10.3345628458333 10.6468213625 11.3390265208333 10.1753571625 10.3274846333333 10.4707651916667 11.0273109541667 11.2056051791667 11.3270429166667 10.6787007666667 10.2022333166667 10.4521737208333 11.9212078625 10.92396515 10.0701092041667 10.1939573833333 10.8483554916667 10.42353545 10.3675234833333 10.4097271791667 9.9446456625 10.4374817291667 9.30282694583333 9.88119130833333 10.3291800333333 10.0433456583333 10.3854175791667 12.0707515125 10.4521737208333 10.4482290583333 11.5536666

AC004967.2 10.2643763708333 10.9668980875 10.9951368375 10.8799482708333 12.0546684541667 10.5495181458333 12.5312882041667 11.4764632583333 12.1113047083333 10.7333004916667 11.2095984041667 12.8573809541667 12.56399775 11.2190650916667 11.0409097458333 11.1875048625 10.5436968083333 11.9598907208333 9.91696874583333 11.0093021625 11.5440811625 11.37744305 10.8664585083333 10.8199753375 10.7062054458333 10.7738484833333 10.7538357416667 10.5795097916667 11.0409097458333 10.5752846666667 9.8021216125 10.6488660166667 11.068216525 10.4762218166667 9.55467654583333 10.3593030208333 10.8064994583333 10.098956 9.90071585833333 10.2068985958333 9.36846859166667 10.1753571625 10.0534295291667 9.66524654166667 10.0777955416667 9.56846712916667 9.86034475 10.237975625 10.1803173041667 10.1803173041667 9.8476021875 10.4182870166667 11.2469887625 10.4800932833333 10.5587434375 9.59329031666667 10.9217485666667 9.283743025 10.4631657541667 10.2068985958333 12.083866575 11.4740179458333 10.5059966041667 11.1780276833333 11.4377467166667 11.1285866791667 11.8377183833333 12.1677782 10.6069468625 11.3437706375 11.6522784458333 11.7837961083333 12.2663378875 10.9430338458333 12.0246139791667 11.3702568291667 11.4879602833333 11.4901913333333 10.4148400375 11.1615461583333 12.0190830208333 12.534442825 10.8395357958333 10.0716385875 10.9092115458333 11.6545501125 12.3957431333333 11.4426573 12.3415392541667 10.3345628458333 10.6468213625 11.3390265208333 10.1753571625 10.3274846333333 10.4707651916667 11.0273109541667 11.2056051791667 11.3270429166667 10.6787007666667 10.2022333166667 10.4521737208333 11.9212078625 10.92396515 10.0701092041667 10.1939573833333 10.8483554916667 10.42353545 10.3675234833333 10.4097271791667 9.9446456625 10.4374817291667 9.30282694583333 9.88119130833333 10.3291800333333 10.0433456583333 10.3854175791667 12.0707515125 10.4521737208333 10.4482290583333 11.5536666

AC010542.2 9.904065125 10.344247175 10.2339183916667 10.30417975 9.056928375 10.0818728583333 9.26005576666667 10.0005481 9.54094585833333 9.819189925 9.62279060833333 10.2970698166667 8.97615546666667 10.30560935 10.0478232083333 10.2902993333333 10.829344675 8.28578650833333 10.1344061583333 9.91617924166667 10.6517451416667 9.46997115 10.36490795 10.1913709083333 10.12322705 10.3390799083333 10.2175479333333 9.97701388333333 9.26005576666667 9.85252195833333 9.90270463333333 10.0973630583333 9.85529955833333 10.257033225 10.29196065 10.1590254833333 9.31248745 9.90727683333333 10.132787925 9.70165576666667 10.8675817916667 10.552513275 10.5356204916667 11.0786204833333 10.6998408166667 10.95910865 10.8446689833333 10.5000230916667 10.9399979583333 10.7343611166667 10.2702136416667 10.2902993333333 9.62849099166667 10.1998690333333 10.6059731083333 10.80968025 10.2354161583333 10.5145241083333 10.4829935916667 10.6572470916667 9.22995318333333 9.15495383333333 9.323428375 8.83012083333333 9.85529955833333 9.71504586666667 9.52206101666667 9.5233206 8.92760421666667 9.68325286666667 8.91674554166667 9.89075863333333 8.74401624166667 10.1859038333333 9.93966605 9.26878520833333 9.60875009166667 9.03833616666667 9.3411091 9.61446786666667 9.22186990833333 8.71356813333333 9.769181275 10.0677716916667 9.82384475833333 9.81636959166667 8.76400390833333 9.85252195833333 8.47867590833333 10.3301495666667 9.213538675 9.96147485833333 8.74010955833333 9.66874293333333 9.012345475 8.71476503333333 8.74523575833333 8.96465185 8.62190543333333 8.91190651666667 10.3583768166667 9.64169280833333 9.97263284166667 10.4316753833333 9.62572484166667 9.62572484166667 10.8609045666667 10.1201558583333 10.5050930083333 10.12322705 10.2938403083333 9.88035836666667 9.604456975 9.63324336666667 10.1728086416667 10.2060115416667 9.55070993333333 9.72422056666667 9.71669341666667 10.0097474333333

AL589645.1 4.65659079166667 3.99069761666667 3.96535873333333 4.07709943333333 3.58337008333333 4.82853423333333 3.51310729166667 4.07056364166667 3.54860440833333 4.56640000833333 3.6142597 3.60344979166667 3.54009390833333 4.18065260833333 4.14925433333333 3.93500141666667 4.70340193333333 3.76338045 4.72389136666667 4.13770233333333 3.81249149166667 4.01591823333333 4.332637675 4.56640000833333 4.771183175 4.04345499166667 4.53014425833333 4.61847421666667 4.6025075 4.33403705833333 4.696789075 4.568704925 4.00885690833333 4.076467325 4.307075925 4.235972525 4.54206718333333 4.58536020833333 4.58741798333333 4.84884604166667 5.05953466666667 5.265418675 4.86534405 5.64838001666667 4.13691099166667 7.53694975 5.228162675 4.99144798333333 4.62559538333333 4.98118906666667 4.96337545 4.574648225 3.67866685833333 5.25444665 4.29092495 4.86121514166667 3.94837956666667 4.88416340833333 4.347785025 4.53154068333333 3.80026373333333 3.83557885833333 4.43217184166667 3.67957571666667 3.48748343333333 4.1920818 3.861448425 3.653206325 3.693253925 3.96389414166667 3.43960401666667 3.4617905 3.56739355833333 3.61343018333333 3.38972336666667 3.66023421666667 3.64140023333333 3.78678109166667 3.80511266666667 3.71429205833333 3.47249425833333 3.52779533333333 4.176255425 3.95379238333333 3.69751853333333 3.67786573333333 3.75215821666667 4.35224340833333 3.89809546666667 3.99142755 3.7279162 4.384745925 3.9722338 3.74682054166667 3.85973294166667 3.86834563333333 4.00815674166667 4.03934740833333 3.705852925 5.14051295833333 4.332637675 4.13543773333333 4.75499598333333 5.01846583333333 4.30784019166667 6.59047039166667 4.61046104166667 6.073362025 4.28044521666667 6.58438846666667 4.71676371666667 5.27487943333333 4.43434340833333 4.80348209166667 3.97718864166667 4.45750181666667 4.01751818333333 4.56938536666667 4.476177125 3.51671225

APTR 12.174234975 11.3960283166667 11.169431775 11.6717245416667 11.5359438166667 11.0901474083333 11.1498746333333 11.0376671166667 11.608832125 11.0975251666667 11.2710644083333 10.3532081333333 11.33050215 11.0536141166667 11.6813023833333 11.28965795 12.6814379666667 11.1477633666667 11.43162275 11.2828826916667 11.49913775 11.3450705666667 11.3960283166667 11.4439581833333 11.6211812416667 11.6510632333333 11.0996982166667 11.3471656166667 11.234145525 11.7015427416667 11.6010023166667 11.4727106916667 11.0975251666667 11.826140675 11.9993623166667 11.2481652416667 11.0996982166667 11.0329550833333 11.109115 10.6691077583333 11.5719596666667 11.3761321333333 11.489093725 11.4843161333333 11.4413564166667 11.1717372166667 11.4466154166667 11.542870275 11.1977409666667 11.3186163 11.7773535166667 11.6034592083333 11.4912889416667 11.4389777833333 11.6211812416667 11.413218375 10.6096692083333 11.3186163 11.5719596666667 10.955147375 11.6412048416667 11.6813023833333 11.5015678666667 10.8910141166667 11.4868268416667 9.97701388333333 11.4058331 10.8407027083333 10.2473472 9.90140289166667 11.4868268416667 10.881139625 10.4732783833333 10.40007745 11.4703555083333 11.5333541416667 11.109115 11.7066087916667 10.5145241083333 10.9882447083333 11.91499415 11.0485703583333 10.7848974333333 11.5260845666667 10.4473004666667 11.496576925 11.7536512833333 10.1020643 11.860233025 11.2828826916667 10.9530714833333 10.869829 11.6307827666667 10.4051703 9.90876665 11.7092046583333 11.3232895666667 11.6763962416667 11.2272441333333 11.43651565 12.03386695 11.0146255 11.29925645 12.1204738833333 11.4179012 10.6383268333333 10.64050025 10.55406855 10.9633547666667 10.420672925 11.922473825 11.3110612083333 10.6691077583333 12.16080175 10.6572470916667 11.0467795583333 11.6888272 11.5406276416667 11.4154777166667 11.160332025

LINC02172 4.94570356666667 5.64366626666667 5.84252611666667 5.07579073333333 5.843361025 5.08815185 6.372835675 6.16925395833333 5.46922286666667 4.879086275 5.69903179166667 6.412967525 6.34768783333333 5.12699374166667 4.75878485833333 5.57003804166667 5.59424653333333 5.38263980833333 5.65485036666667 4.2737844 5.69450579166667 5.137862875 4.45030515833333 5.60738530833333 4.90088761666667 5.57471 5.19562818333333 4.83029645833333 4.78025046666667 4.52640311666667 4.95454220833333 4.79215016666667 4.86198561666667 5.97547145833333 4.94654004166667 5.152649175 4.73036435 5.06597111666667 5.6893285 5.03475209166667 5.33553373333333 4.874362975 5.40462085 5.89609469166667 4.55717808333333 5.36908168333333 6.04051248333333 5.26880633333333 5.09148784166667 5.12345405833333 5.10925536666667 5.26013644166667 4.84470445 5.528965775 4.39933081666667 5.02647816666667 4.77273383333333 5.25355041666667 5.620020925 4.53764565 4.25668654166667 4.68347908333333 4.47471310833333 4.83518115833333 5.87325478333333 4.28856184166667 3.97303310833333 6.16046391666667 4.6425346 4.98620748333333 5.98789045 4.41144705 5.568276075 5.00632808333333 4.39933081666667 4.372945875 5.07245089166667 4.7289146 3.992207825 4.2966536 5.16420516666667 4.63272604166667 4.31232735833333 4.08559233333333 4.59205544166667 5.71839368333333 5.39028695833333 4.63110146666667 4.24262859166667 5.875661225 4.09826585 4.6318291 4.11430655833333 4.41717060833333 4.94495284166667 4.59432431666667 4.29385256666667 4.63431651666667 3.90146899166667 5.29113221666667 4.47016100833333 4.37661541666667 5.00548516666667 5.37522741666667 4.52857456666667 3.97868786666667 5.04164975 6.59434034166667 4.502067325 6.91345085833333 4.84884604166667 5.67351870833333 4.52785514166667 5.37165643333333 4.06553535 4.55255341666667 4.435737425 4.72389136666667 4.68347908333333 5.786330625

PSMB8-AS1 8.54062638333333 8.98006033333333 9.45237763333333 8.78681055833333 7.9017682 10.06637255 7.82133015 9.65555468333333 8.3275657 9.41328055833333 8.62537700833333 8.85242150833333 8.077478625 9.68577309166667 8.26088785833333 11.0146255 10.5724502916667 8.19993185 9.02083145833333 9.856575175 10.041049 9.64456304166667 10.5504866333333 9.995800175 9.91018748333333 9.256048375 9.738385875 9.74259324166667 8.92128914166667 9.62008553333333 9.08322639166667 10.2854987416667 9.20815575 11.4154777166667 9.62572484166667 8.84858576666667 8.72277565833333 10.1065692416667 9.51227434166667 9.56332510833333 9.865874625 9.24022388333333 10.3684429583333 10.2339183916667 10.2045943833333 10.829344675 10.0128890333333 9.63324336666667 10.5185258166667 10.3004507666667 9.74120920833333 9.34901544166667 9.4198334 9.44548651666667 9.66989880833333 10.0021960666667 9.90140289166667 10.3371834416667 10.0365436333333 10.7034530583333 9.0697895 8.98476900833333 10.6757072916667 7.88080235 9.82539988333333 8.82622243333333 9.03092490833333 9.41737299166667 10.0395367833333 10.5485496583333 9.14044470833333 7.23263500833333 8.77366934166667 10.4434835166667 9.26005576666667 9.75423146666667 10.5240667333333 10.6552609 10.307287725 10.3123807083333 8.68542795 9.012345475 9.767901775 9.767901775 10.3123807083333 10.0349403166667 8.53357196666667 10.9184750583333 10.4158197333333 10.7173483583333 10.5410668333333 10.2209463166667 8.81562710833333 10.4921560416667 9.48990181666667 9.59251051666667 9.10908699166667 9.71370898333333 9.37623116666667 9.36086844166667 10.8830645916667 9.34755915833333 11.3761321333333 10.682010725 10.979276575 11.1909946916667 9.66989880833333 9.8223954 10.401625675 10.4698496583333 10.0509998083333 9.5233206 10.6425719166667 9.53947254166667 10.752680775 10.3164307833333 9.70023275833333 10.8746426833333 10.18760355 11.790320325

AC020916.1 10.9059938166667 9.828409225 9.45947939166667 9.73982368333333 10.2110825333333 10.632572825 9.76498495 9.72553698333333 9.45373374166667 9.661839025 9.50689216666667 9.85108371666667 9.72122880833333 9.214906375 9.33249780833333 9.20003395833333 9.27528178333333 11.3638652666667 9.77800786666667 9.639269375 9.31248745 9.37623116666667 9.14303199166667 10.23249925 9.5167557 9.87299354166667 9.97701388333333 9.92572226666667 10.6594825833333 9.78069864166667 9.96475848333333 9.52743238333333 10.0021960666667 9.604456975 9.7664743 8.56203936666667 10.7785431666667 9.97891528333333 10.2702136416667 10.1410949416667 9.87425795 9.86444535 9.74405148333333 9.49757535 9.89075863333333 9.20949 9.30352410833333 9.96147485833333 9.4198334 9.95811245 9.48539969166667 9.76360029166667 10.1473463666667 10.3666040083333 10.1794807166667 9.20264188333333 9.45947939166667 9.728380575 9.170003675 9.30352410833333 10.4262186 9.72122880833333 9.65779833333333 10.0395367833333 9.047025875 11.4703555083333 10.4531891333333 9.91775825 9.75593879166667 9.62572484166667 9.63156273333333 9.78995428333333 9.79240870833333 9.40138860833333 9.89075863333333 9.6789387 9.382426125 9.867307775 9.637761575 9.072341675 8.43640146666667 8.78337874166667 9.610331375 9.85108371666667 10.3684429583333 9.37885371666667 9.64872759166667 10.74025755 9.767901775 9.627070875 9.38364405 10.2156287416667 10.2902993333333 9.70826959166667 10.0425633416667 9.37752135833333 9.83458021666667 9.627070875 10.9814054833333 10.1967116083333 9.68448639166667 9.39704085833333 9.69865891666667 9.98826495833333 9.991570475 9.67137481666667 10.5880020833333 11.0447636416667 9.56478920833333 11.0122860666667 9.37200478333333 10.0723139833333 10.2209463166667 9.6646531 9.99887365833333 9.58715105833333 9.36086844166667 10.3532081333333 9.39704085833333 9.44548651666667

AC087636.1 6.25216388333333 6.36720153333333 6.17790808333333 6.60442409166667 6.50659699166667 6.25678936666667 6.472039025 5.9435645 6.13046838333333 6.52208159166667 6.48013065833333 6.46187091666667 6.67944645 6.43366564166667 6.21246914166667 6.23442566666667 5.63422645833333 6.69403440833333 5.50122666666667 6.213202375 6.372835675 5.7582811 6.23442566666667 5.91597829166667 6.15217918333333 5.96262355 6.2141109 6.02897043333333 6.06346144166667 6.83745388333333 5.89039591666667 5.73824569166667 6.141851675 5.80371779166667 6.09974989166667 6.1367648 6.71961385 6.515853425 6.891484925 6.20782178333333 5.92614939166667 6.48013065833333 5.8493167 5.63172995833333 6.78815289166667 6.06586204166667 5.54852681666667 6.78815289166667 5.57164055 5.51827296666667 6.4711885 6.52910926666667 6.25216388333333 5.74925146666667 5.864991425 6.02983153333333 5.88881625 6.17367879166667 6.664548125 6.03972224166667 6.6279805 7.05534445 6.04442248333333 6.25070120833333 6.36555325833333 6.795014675 6.48552058333333 6.58583718333333 7.07951833333333 6.65373279166667 6.72844050833333 6.39956296666667 7.596908525 7.153021525 6.06177335833333 5.82262374166667 6.46408151666667 6.32609701666667 6.24438405 6.28918155833333 6.13939594166667 6.23181650833333 6.41616605 6.43962590833333 6.68096106666667 6.69792505833333 6.94799274166667 6.4026933 6.29083875833333 6.22073784166667 6.79350115833333 6.45800148333333 6.62575970833333 6.21657471666667 7.04741685833333 6.61204298333333 6.23352403333333 6.74196855833333 7.13734699166667 6.76009966666667 6.614798975 7.56764245 6.64536209166667 6.10539690833333 6.52604935 6.83360518333333 6.47042564166667 6.17217228333333 6.24671985833333 5.941997275 6.32934243333333 6.20542285 5.94836456666667 5.62857495 6.58894740833333 6.52445389166667 5.90988236666667 6.50987625833333 6.59973925833333 6.65457601666667

AC098679.1 10.7883879958333 9.10842651666667 9.48469073333333 9.2458425125 9.21139857916667 9.8504446875 8.6725846125 8.98801488333333 8.8901431625 9.16423615416667 9.4503022625 10.3593030208333 9.75351584166667 8.38328876666667 9.1854422875 10.2527812916667 9.87960672916667 8.2036478875 9.01531490833333 9.87086047083333 9.68512974166667 8.99407455 9.60080369166667 9.41144194583333 9.81260468333333 10.6069468625 9.87813937916667 9.45726971666667 9.86927725416667 8.84685069166667 9.25158580416667 9.42830559166667 9.44627235833334 9.39642629583333 9.679715825 10.5850865916667 8.6601228625 9.24876873333333 9.22642191666667 9.44495295416667 9.0944044375 9.25158580416667 9.92329091666667 9.24876873333333 10.4166605625 10.1803173041667 10.1091713291667 8.99766995 9.204652225 10.0903760291667 9.33052937916667 8.4077417625 10.0342631125 9.3768762625 9.26824185416667 9.8214973125 9.52269080833333 9.36429102916667 9.9186739125 10.2527812916667 10.3836989 9.03429043333333 9.283743025 8.92198210833333 10.1701102916667 9.18815974166667 10.0701092041667 9.23706149583333 9.3517636 10.1885741916667 8.29493328333333 8.10215072083333 9.15923749166667 9.6167051625 9.52144787083333 10.1369480541667 10.4950592708333 9.3781875375 9.13301445833334 9.69229430416667 8.8019866375 10.4839214041667 9.6625711125 9.08107680833333 10.2911299916667 9.30417548333333 8.84199329583333 9.9048588125 8.30116951666667 10.5098294125 9.32653849583333 8.48694473333333 8.66135729166667 9.47349197083333 8.80684599166667 8.1158165125 7.85687752083333 9.7463256 8.871367125 8.29798353333333 8.93762455833333 8.63262070833333 9.9265255625 8.84576013333333 8.58622295416667 10.1272129375 10.7720667958333 10.4724795541667 9.804963125 9.90646466666667 10.1272129375 7.84671812083333 9.981090425 8.25184600416667 10.0825780958333 9.1759655 8.4565200375 9.3992636375 7.91421812916667 9.81565712916667

FAM198B-AS1 10.7883879958333 9.10842651666667 9.48469073333333 9.2458425125 9.21139857916667 9.8504446875 8.6725846125 8.98801488333333 8.8901431625 9.16423615416667 9.4503022625 10.3593030208333 9.75351584166667 8.38328876666667 9.1854422875 10.2527812916667 9.87960672916667 8.2036478875 9.01531490833333 9.87086047083333 9.68512974166667 8.99407455 9.60080369166667 9.41144194583333 9.81260468333333 10.6069468625 9.87813937916667 9.45726971666667 9.86927725416667 8.84685069166667 9.25158580416667 9.42830559166667 9.44627235833334 9.39642629583333 9.679715825 10.5850865916667 8.6601228625 9.24876873333333 9.22642191666667 9.44495295416667 9.0944044375 9.25158580416667 9.92329091666667 9.24876873333333 10.4166605625 10.1803173041667 10.1091713291667 8.99766995 9.204652225 10.0903760291667 9.33052937916667 8.4077417625 10.0342631125 9.3768762625 9.26824185416667 9.8214973125 9.52269080833333 9.36429102916667 9.9186739125 10.2527812916667 10.3836989 9.03429043333333 9.283743025 8.92198210833333 10.1701102916667 9.18815974166667 10.0701092041667 9.23706149583333 9.3517636 10.1885741916667 8.29493328333333 8.10215072083333 9.15923749166667 9.6167051625 9.52144787083333 10.1369480541667 10.4950592708333 9.3781875375 9.13301445833334 9.69229430416667 8.8019866375 10.4839214041667 9.6625711125 9.08107680833333 10.2911299916667 9.30417548333333 8.84199329583333 9.9048588125 8.30116951666667 10.5098294125 9.32653849583333 8.48694473333333 8.66135729166667 9.47349197083333 8.80684599166667 8.1158165125 7.85687752083333 9.7463256 8.871367125 8.29798353333333 8.93762455833333 8.63262070833333 9.9265255625 8.84576013333333 8.58622295416667 10.1272129375 10.7720667958333 10.4724795541667 9.804963125 9.90646466666667 10.1272129375 7.84671812083333 9.981090425 8.25184600416667 10.0825780958333 9.1759655 8.4565200375 9.3992636375 7.91421812916667 9.81565712916667

LINC00589 8.09847590833333 8.431034975 8.11834109166667 8.77735828333333 8.20093078333333 9.3690743 8.36432383333333 8.20718200833333 8.73763425 8.0765578 8.68542795 8.2150935 8.03683899166667 8.236562525 8.16414461666667 7.848979625 9.254766825 8.10750275 8.04962919166667 7.96885535833333 7.6360768 8.17213816666667 8.26714300833333 8.4103435 8.36542865 8.14437105 7.928697775 8.054716375 7.87702689166667 7.91550765833333 8.005090175 8.28685643333333 8.237670825 9.0697895 8.11218248333333 8.13089671666667 8.22944233333333 8.35299256666667 8.17005769166667 8.243225475 8.18511508333333 7.74760565833333 7.91185755833333 8.0601789 7.93246234166667 7.595060625 8.22529060833333 7.86191561666667 8.01371644166667 8.47867590833333 7.99469491666667 8.093307525 7.79722829166667 7.95964986666667 7.47322088333333 7.98806083333333 8.05732331666667 8.0943338 7.96612625833333 8.029339375 9.40775390833333 8.6830437 9.46847865 8.94047908333333 8.40014016666667 8.52145965 8.57958530833333 8.522428575 8.483114775 8.57958530833333 8.87463969166667 8.68047514166667 8.8194358 8.69358161666667 8.57508074166667 8.97362839166667 8.774825425 9.00741491666667 8.6685911 8.39909144166667 7.92217650833333 8.74401624166667 8.95327330833333 8.68656096666667 9.1349915 8.918056 9.6529159 8.49171125 9.10908699166667 8.53357196666667 9.1308824 8.85242150833333 8.99956494166667 8.77601695 8.888205175 9.1308824 8.78437034166667 9.06439938333333 8.95454636666667 9.00614454166667 8.8438399 8.3623863 8.22438401666667 8.62537700833333 8.348415775 8.83768020833333 7.73104835833333 8.33307035833333 8.33743355 8.322516975 8.59501004166667 8.6890691 9.2676985 8.29555180833333 8.05364296666667 8.61109633333333 8.397991975 9.17772915833333 8.86517486666667 8.71236105833333

AC005833.2 11.109115 9.983440075 10.0590324416667 10.6796574916667 10.6306232166667 9.07738231666667 10.86306385 10.4681694166667 10.3895259333333 9.95481326666667 10.2141677 10.5504866333333 10.8675817916667 10.3459838833333 9.77927601666667 10.398066475 8.91416151666667 11.0008274583333 9.983440075 10.64050025 10.3702408666667 9.33115149166667 9.80705093333333 10.6248927916667 9.20264188333333 10.6837079083333 9.98826495833333 10.4365874083333 9.79859535 10.552513275 10.5126549333333 10.2175479333333 9.17772915833333 9.02711425833333 9.55672449166667 10.7483297666667 10.4791199833333 10.42807655 10.6634010583333 10.4454279083333 10.8853541583333 10.3301495666667 9.94865183333333 9.66037100833333 10.260350775 9.83620561666667 9.412078125 10.6653293916667 9.483981775 9.53024233333333 9.52458273333333 10.3248470416667 9.97263284166667 10.12322705 10.2014392333333 9.94119803333333 10.1841002166667 10.5860666166667 8.79885179166667 9.87299354166667 9.569175825 10.2276958666667 8.922675075 10.3632412666667 9.91775825 11.0262795583333 10.2244563666667 10.4454279083333 9.73982368333333 9.64456304166667 9.80705093333333 10.3371834416667 10.7785431666667 9.661839025 9.951930125 10.3617960583333 10.0867346833333 9.54242928333333 10.3145529166667 10.05421995 9.74405148333333 14.2961453916667 10.8446689833333 10.1020643 9.991570475 10.9939867583333 9.57185823333333 9.95043048333333 9.72272495833333 9.53278261666667 9.85797725 8.96352476666667 8.85363625833333 9.610331375 9.81494466666667 9.79859535 9.18755855 9.338606375 10.2339183916667 8.35738369166667 9.183229075 10.40007745 8.700744525 9.05935188333333 9.78995428333333 9.06555709166667 10.0848290833333 10.2902993333333 10.258686375 9.57056143333333 8.40207273333333 9.980296075 9.70165576666667 8.589042325 10.1983029333333 9.5233206 10.3513467583333 8.68542795 9.124545075 9.47415811666667

LINC01471 5.983920925 6.60596866666667 6.64146615833333 6.94725178333333 7.01349440833333 5.534144725 6.98940225 6.64920430833333 7.206737225 7.02899554166667 6.74116495833333 7.1702859 6.920697175 7.242651775 6.37517364166667 6.96632546666667 6.72844050833333 7.14151948333333 6.780345325 6.83075820833333 7.43541391666667 6.74572325 6.93198700833333 5.95496726666667 6.37517364166667 6.514123925 6.4602804 6.87117053333333 6.530441325 7.005136025 7.32203796666667 7.14646856666667 7.09676788333333 7.51141074166667 6.91180415 7.18234429166667 6.67560305833333 7.17617635833333 6.57608009166667 6.7441857 6.324552 6.79655045 6.28147625 7.89268988333333 6.40188925833333 6.45800148333333 7.22310433333333 6.83075820833333 6.27692523333333 6.50900563333333 6.88087174166667 7.099165825 6.50987625833333 7.19748368333333 7.07855881666667 7.53854279166667 6.76702298333333 7.29870193333333 7.13912226666667 7.22153060833333 6.17790808333333 7.42360905 6.70700871666667 7.01349440833333 6.6848112 6.51151065833333 6.86315831666667 7.214196475 7.427790275 7.45568213333333 7.20338010833333 7.44897004166667 7.27935889166667 7.188718125 7.558954 6.90820158333333 7.28355914166667 6.65293891666667 6.51972946666667 7.214196475 7.11334874166667 7.1552468 6.80752626666667 6.694825125 6.85503901666667 7.52419651666667 7.42616356666667 6.96632546666667 7.46633813333333 6.660735075 7.41578374166667 7.23187408333333 6.56179321666667 7.14151948333333 7.20936795 7.063179525 6.585221025 6.95064391666667 7.12369916666667 6.98302305 6.63114244166667 7.69963058333333 6.91684939166667 7.47235645 6.95374109166667 6.52517743333333 7.07951833333333 6.63114244166667 6.338904975 6.883226375 6.71711986666667 7.238301775 7.37382095 7.603532025 7.47399836666667 7.63517916666667 6.9248352 7.291524925 7.47235645 7.21835374166667

MAP3K20-AS1 6.690101625 5.78131454166667 5.65485036666667 5.22208751666667 5.82603316666667 6.802823925 5.06515519166667 6.0000734 5.23497289166667 4.98442578333333 4.88258713333333 6.19578775 6.15066176666667 5.27222866666667 6.21815258333333 5.40027715 5.38175511666667 5.92922820833333 5.81606168333333 5.79975874166667 5.424208775 5.10754223333333 4.817166525 6.79964733333333 5.198900775 6.442668925 5.42068620833333 5.44471205 5.7939183 5.464202575 5.58424089166667 5.81928184166667 5.4551887 6.19175286666667 6.68783996666667 6.11996326666667 5.3212614 7.666066675 6.1287993 5.601533025 5.629363625 5.19805619166667 7.91377156666667 6.30351415833333 6.03894880833333 5.73323451666667 6.15783995 5.32316651666667 5.43881695 5.52235776666667 5.16663731666667 5.729929025 5.919367675 6.62353365 5.92772035833333 5.25444665 6.11751004166667 5.73572338333333 5.94033254166667 5.17819575 6.50583770833333 5.00632808333333 5.05953466666667 5.9343511 5.15351136666667 8.42317835833333 6.2303963 5.43881695 5.63585205833333 5.44953800833333 5.86089796666667 5.67108565833333 6.35611011666667 5.68514503333333 5.32654480833333 6.061018125 5.45348568333333 6.74949993333333 8.27017713333333 5.00548516666667 6.77879458333333 5.92614939166667 5.81263556666667 5.57868124166667 6.24370750833333 5.65972369166667 5.06029869166667 5.34487673333333 5.48196351666667 5.72892785833333 6.116700125 6.7441857 6.23442566666667 6.71961385 5.10162569166667 5.38175511666667 7.22830545833333 7.387267475 6.25294181666667 5.22110318333333 6.905014675 4.23511105833333 7.77912510833333 5.772683 7.38220016666667 7.02346383333333 7.3803365 6.76490431666667 5.69101595 6.83665193333333 4.88341414166667 5.80371779166667 7.356869825 6.80909233333333 7.26542881666667 5.18819051666667 5.50030605 6.87295885 5.71930858333333 5.96431428333333

POU6F2-AS2 5.62274641666667 5.81691269166667 7.22572939166667 6.60123596666667 6.192534425 8.5533307 6.983731325 5.932662575 5.88131005 6.404149025 7.61148288333333 6.39329076666667 5.92772035833333 5.8094462 5.95235704166667 6.36881363333333 4.462614125 5.91328005 6.41446524166667 7.525750625 6.49433464166667 6.54837455833333 5.8328231 5.28680903333333 5.35953131666667 6.17554210833333 5.47001155833333 6.0017496 6.94725178333333 6.04756038333333 7.12930466666667 5.04731695 6.19938790833333 5.85179635833333 4.816492275 5.499379 5.40623839166667 6.29539721666667 5.78052715833333 5.42769671666667 5.44782694166667 5.13539786666667 6.66605864166667 5.55202255833333 4.65659079166667 5.14319801666667 5.08815185 5.8493167 5.9343511 6.22491186666667 6.49862728333333 6.660735075 4.92610205 4.93534804166667 5.48196351666667 5.243413975 6.68855885 4.70485328333333 5.90176635833333 6.127183975 9.72272495833333 7.89268988333333 10.0647337083333 9.72706908333333 9.01353759166667 8.89682220833333 7.25265295833333 7.7430897 6.247501925 5.601533025 8.37690871666667 7.982277475 7.45400806666667 7.38805711666667 7.85097501666667 9.02465829166667 6.10052355833333 6.36633304166667 8.511369775 11.0536141166667 9.900028825 9.29792191666667 8.941771525 8.243225475 8.74159676666667 9.120779125 7.20762451666667 7.07855881666667 10.4941187833333 8.42995574166667 10.3739525666667 8.197822625 8.60860023333333 7.75769473333333 6.0420986 9.19875904166667 8.3275657 8.51932215 9.18999519166667 8.766448975 9.63609045833333 7.84438765 7.79633564166667 7.2852293 9.20264188333333 8.73370975833333 6.45714978333333 9.33989754166667 9.4198334 8.85112088333333 8.926479 8.59712365833333 7.75673711666667 5.3212614 7.659337475 5.56204226666667 8.87593866666667 6.46576575 9.820599225 6.49504519166667

AL451164.1 4.25090944583333 3.78899188333333 3.89458091666667 3.95346312916667 4.02599304583333 4.0709043125 3.74722414166667 3.53800529166667 3.9036651875 3.67570854583333 3.70115060416667 3.73823148333333 4.02983115833333 4.05506897083333 4.065978925 3.88843620833333 4.35987274166667 3.9075173875 4.43538355 3.851323775 3.63161408333333 3.70698502916667 4.18406696666667 4.247799975 4.41181262083333 3.80555362916667 3.88764135 4.24080124166667 4.0305542125 3.96498026666667 4.22314869166667 4.23082299583333 3.68856206666667 3.9612735625 4.247799975 4.14281994583333 4.0438190125 4.46662546666667 4.52167519166667 4.61479732083333 4.77905429166667 4.4447885625 4.7431708375 5.40328129166667 4.098680275 6.027041925 5.0821850875 4.65693485416667 4.380592225 4.75081864166667 4.68941043333333 4.20614402083333 3.6056146625 5.05562265833333 4.08752060416667 4.63227757083333 3.6500768 4.6267788375 4.28377235833333 4.35414409166667 3.67495364166667 3.69285759166667 4.3686877 3.6459919375 3.45929790833333 3.6111384375 4.27709560833333 3.6083625 3.78080279166667 3.59183619166667 3.69542245833333 3.7226335375 3.63925310416667 3.58795662916667 3.52737428333333 3.63241216666667 3.819202225 3.63494716666667 3.89224495833333 3.68856206666667 3.58795662916667 3.67648744166667 4.41979519166667 3.73580285416667 3.82755255416667 3.59091285416667 3.457346025 3.97680239166667 3.82078494166667 3.77337481666667 3.97525073333333 4.11770704583333 4.013045625 3.690272625 3.8570216 3.87273842916667 4.24080124166667 4.0171230625 3.6791212875 4.72595870833333 4.3770068125 3.96214940416667 4.48001641666667 5.02128888333333 4.2608991 4.33146572916667 4.518771975 5.62042860416667 4.094015125 6.13004372916667 4.48384862083333 4.6164911375 4.26751472083333 4.64449042083333 4.15211245 4.39690367916667 4.02104424166667 4.3770068125 4.36123601666667 3.8359447875

LINC02668 4.25090944583333 3.78899188333333 3.89458091666667 3.95346312916667 4.02599304583333 4.0709043125 3.74722414166667 3.53800529166667 3.9036651875 3.67570854583333 3.70115060416667 3.73823148333333 4.02983115833333 4.05506897083333 4.065978925 3.88843620833333 4.35987274166667 3.9075173875 4.43538355 3.851323775 3.63161408333333 3.70698502916667 4.18406696666667 4.247799975 4.41181262083333 3.80555362916667 3.88764135 4.24080124166667 4.0305542125 3.96498026666667 4.22314869166667 4.23082299583333 3.68856206666667 3.9612735625 4.247799975 4.14281994583333 4.0438190125 4.46662546666667 4.52167519166667 4.61479732083333 4.77905429166667 4.4447885625 4.7431708375 5.40328129166667 4.098680275 6.027041925 5.0821850875 4.65693485416667 4.380592225 4.75081864166667 4.68941043333333 4.20614402083333 3.6056146625 5.05562265833333 4.08752060416667 4.63227757083333 3.6500768 4.6267788375 4.28377235833333 4.35414409166667 3.67495364166667 3.69285759166667 4.3686877 3.6459919375 3.45929790833333 3.6111384375 4.27709560833333 3.6083625 3.78080279166667 3.59183619166667 3.69542245833333 3.7226335375 3.63925310416667 3.58795662916667 3.52737428333333 3.63241216666667 3.819202225 3.63494716666667 3.89224495833333 3.68856206666667 3.58795662916667 3.67648744166667 4.41979519166667 3.73580285416667 3.82755255416667 3.59091285416667 3.457346025 3.97680239166667 3.82078494166667 3.77337481666667 3.97525073333333 4.11770704583333 4.013045625 3.690272625 3.8570216 3.87273842916667 4.24080124166667 4.0171230625 3.6791212875 4.72595870833333 4.3770068125 3.96214940416667 4.48001641666667 5.02128888333333 4.2608991 4.33146572916667 4.518771975 5.62042860416667 4.094015125 6.13004372916667 4.48384862083333 4.6164911375 4.26751472083333 4.64449042083333 4.15211245 4.39690367916667 4.02104424166667 4.3770068125 4.36123601666667 3.8359447875

MIR9-3HG 9.87425795 9.29792191666667 8.96599376666667 9.62572484166667 8.89438333333333 8.27232419166667 9.53278261666667 8.35939504166667 8.290978475 8.94723143333333 9.31654956666667 9.27651353333333 9.757653125 10.6040700666667 8.62645034166667 8.91303145833333 8.86780791666667 8.77984139166667 8.4458465 8.32962616666667 9.01969005 9.40138860833333 8.94836124166667 8.64530154166667 7.98897931666667 8.82416160833333 11.8625342 9.27124661666667 9.071309125 9.46481536666667 9.58157864166667 8.46571488333333 8.69358161666667 9.24251089166667 8.65694638333333 8.13916790833333 9.00354715 8.48845156666667 8.60150903333333 8.33973274166667 8.56973665 9.61303635833333 9.71257970833333 8.16501965833333 9.74259324166667 8.44905829166667 10.71936685 9.87885509166667 8.40512834166667 7.420213075 9.54242928333333 9.37752135833333 9.04373345 8.92998051666667 9.520834725 8.14836771666667 8.59015729166667 8.957126925 8.700744525 9.311047725 8.89912965833333 9.89369143333333 9.79513514166667 10.420672925 9.32457533333333 11.1321094 9.01607414166667 9.91313129166667 10.1473463666667 9.06068016666667 9.68859575 9.508238925 10.4158197333333 10.0220394666667 9.56332510833333 9.415981525 9.14824449166667 9.00614454166667 9.58292749166667 9.60613141666667 9.14450013333333 9.00741491666667 9.18490179166667 10.595444475 9.0697895 9.74259324166667 10.3775083083333 9.62008553333333 9.63156273333333 8.72541470833333 9.70290516666667 9.26005576666667 9.05296820833333 9.43592440833333 8.950621275 9.90727683333333 8.45013500833333 8.61573053333333 9.07590878333333 10.3390799083333 9.02843820833333 9.637761575 10.3632412666667 9.867307775 9.83306693333333 11.2021462083333 10.2551372666667 9.74405148333333 9.58157864166667 8.27546815 8.77366934166667 12.459832675 8.60535770833333 9.35119336666667 9.08613931666667 10.2902993333333 9.82980243333333 9.88790173333333 9.67743319166667 9.68859575

LINC00924 7.42694153333333 7.10947593333333 8.32962616666667 8.29431475833333 7.61063126666667 8.303785925 7.743945075 8.2690851 7.88171558333333 8.41501278333333 7.693845675 8.90678281666667 7.85737128333333 7.61667270833333 7.793674875 9.24804320833333 7.44810904166667 8.461457375 8.63572139166667 9.28980095 8.87851369166667 7.26059781666667 9.26286649166667 8.28484028333333 8.66334255 7.953283475 7.94501785 7.94397781666667 8.577282825 7.919306 7.99573270833333 7.80597976666667 8.48415245 7.64410754166667 7.86934078333333 7.774503075 8.545802675 8.54062638333333 9.056928375 8.88103920833333 8.774825425 8.93106328333333 9.08861136666667 8.6830437 9.24510093333333 9.24251089166667 8.8037272 8.8870824 8.14316695 8.993454275 8.54483809166667 7.68597430833333 8.66334255 8.58305941666667 8.993454275 8.24631605833333 8.40315855 8.8438399 8.81697240833333 9.69865891666667 8.52918275 7.36174494166667 7.47901903333333 7.493374075 7.43541391666667 8.96720605 6.87679691666667 7.2051733 6.90274688333333 7.84438765 7.658531525 7.51240625833333 7.91466469166667 7.18794728333333 7.018972075 7.93993941666667 7.66918755833333 8.577282825 8.35939504166667 7.82045105 8.37784194166667 6.8359128 8.440753925 8.0601789 7.85824869166667 7.17917829166667 7.3593209 7.20436910833333 7.78914439166667 7.967137925 7.284330325 6.76239408333333 7.55402225833333 7.72082631666667 7.18794728333333 7.21835374166667 7.62092879166667 7.30368923333333 7.70481934166667 7.348136225 8.15235975 7.61407576666667 8.017646775 8.03782043333333 8.19496856666667 8.22438401666667 7.81612064166667 7.60185643333333 6.92317931666667 7.99937763333333 7.59232971666667 7.3839443 7.45071826666667 7.724325925 8.03782043333333 7.063179525 7.55492436666667 7.94975508333333 8.4874442 9.2535473

C8orf87 9.6633032 10.7263759583333 11.6063160416667 11.3471656166667 10.950912925 9.77666165 11.3232895666667 11.3060877416667 11.1409951666667 10.8363480833333 10.9014784333333 11.1026531 10.6671034666667 10.4033397666667 10.7591979083333 10.5410668333333 9.58715105833333 10.7549907083333 10.57648925 10.8010096583333 10.2504355916667 10.2688247083333 10.2536886083333 10.2110825333333 10.4773720583333 9.70023275833333 11.2202459666667 10.9983830416667 11.0376671166667 11.176910275 11.02834235 11.2613206166667 10.6517451416667 9.88790173333333 9.81785751666667 9.56332510833333 11.1477633666667 11.2973321166667 11.0536141166667 11.7117253416667 10.6517451416667 10.8787569166667 10.7421405583333 10.5706949916667 9.84980565833333 10.3197928333333 10.1811538916667 10.4434835166667 10.3810533166667 10.1998690333333 10.9419169166667 10.7830390666667 10.6594825833333 10.5107808 11.3060877416667 10.4902295666667 10.9613440166667 10.9228885833333 10.7173483583333 10.0559299333333 10.1859038333333 10.3964220916667 10.3702408666667 10.87665705 9.59251051666667 10.7922587916667 10.6231101333333 10.9059938166667 10.34257475 10.4866972 10.5860666166667 10.6425719166667 10.6248927916667 10.096001875 10.4331234333333 10.2354161583333 10.4138603416667 10.1526159166667 9.95811245 10.4829935916667 9.74259324166667 10.17093795 9.9929911 9.637761575 10.2753557916667 10.1216004333333 10.3913684416667 10.5485496583333 10.16579105 9.59117006666667 10.2902993333333 10.3757960166667 9.98678198333333 9.691701075 10.618843275 10.3409099333333 10.1248896166667 10.0220394666667 10.5671895333333 9.91464370833333 9.71257970833333 10.4531891333333 9.73114759166667 9.705649125 9.55551340833333 9.83306693333333 9.74259324166667 9.65131303333333 9.52206101666667 9.47971744166667 9.73689675 10.2110825333333 10.0492420416667 9.56623849166667 9.89233125833333 9.91018748333333 9.5052656 9.92405621666667 9.81335845 9.98989775

LINC00466 4.18324487083333 4.18630841666667 4.3405135625 3.84019450416667 4.20767702083333 4.061294175 4.3088935875 4.4535341125 4.17288325 3.9808046875 3.8417967625 4.34527307916667 4.34123574583333 4.4471476625 3.82928713333333 4.0382439375 4.22457077916667 4.28678514583333 4.36516523333333 3.80555362916667 4.58928309583333 3.97006014583333 4.3149772125 4.25553867083333 4.41826714166667 3.81206045 3.98778344583333 4.23007462083333 3.97263345416667 3.92495681666667 4.374684975 4.247799975 3.92741038333333 4.36268724166667 3.74133635416667 3.94356575833333 4.23861498333333 4.18251210833333 4.1357939375 4.38884895416667 4.52100952083333 4.05680977083333 4.47349465833333 4.88697484583333 3.9862313375 4.45935194166667 4.05985334583333 4.29822596666667 4.35414409166667 4.581159075 4.52100952083333 4.29559497916667 4.058413075 4.6123174875 3.94648126666667 4.19933138333333 3.48697800833333 4.37175385833333 3.85459740416667 3.93935945 3.725013725 4.02983115833333 4.1381095125 3.58380573333333 3.636626175 3.87424883333333 3.73004005416667 4.08673305416667 3.48969441666667 3.79840604583333 4.146518825 3.70622902916667 4.54241319583333 3.60652764583333 3.95278473333333 4.14281994583333 3.81539142916667 3.59525702083333 3.69628022083333 3.53800529166667 3.93228008333333 3.79677383333333 3.9282286625 4.07157350833333 3.8851199375 3.9846629625 3.53185289166667 3.93935945 3.47091660416667 3.71656358333333 3.752584875 4.14057206666667 3.76377788333333 3.61023832916667 3.6633699 3.77057298333333 3.90106512916667 3.84268744166667 4.02190916666667 4.46432285 3.99507254166667 3.8653757375 4.3548400625 4.38201302916667 4.14057206666667 3.67997409583333 3.87044713333333 4.7292611125 4.07749111666667 5.579115925 4.38133500416667 4.7371680125 3.93696812083333 4.2756282125 4.1480024125 3.998239875 3.74474362083333 4.1333395125 3.84019450416667 4.4771660625
[truncated: 16,060,986 more chars]
