# Supplementary material for: Immune-Related Long Non-coding RNA Signature and Clinical Nomogram to Evaluate Survival of Patients Suffering Esophageal Squamous Cell Carcinoma
Source: Front Cell Dev Biol. 2021 Mar 4;9:641960. doi: 10.3389/fcell.2021.641960 (PMC7969885; doi:10.3389/fcell.2021.641960)
Supplement: Supplementary Table 7 — LncRNAs associated with ESCC immunity downloaded from ImmLnc website. [file Table_7.docx]

XXbac-B461K10.4

IGF2-AS

PINK1-AS

KIAA0087

AC105760.3

DKFZP434K028

C22orf24

LINC01260

RP11-84C10.2

FAM27C

C1orf147

LINC01116

LINC01600

C10orf25

RP11-93B14.6

CMAHP

CCDC13-AS1

RP11-89N17.1

RP11-23P13.6

TMEM51-AS1

C11orf44

C15orf54

RP11-544L8__B.4

RP11-404O13.5

AC110781.3

RP5-856G1.2

C20orf197

C15orf56

MIR7-3HG

AP000679.2

MRVI1-AS1

RP11-16K12.1

AC108142.1

C5orf64

ADORA2A-AS1

AC073072.5

CTD-2144E22.5

PIK3CD-AS1

C10orf91

LINC01559

LINC01555

FER1L6-AS1

CELF2-AS1

TP53TG1

C17orf77

FAM87A

COL18A1-AS1

NCF4-AS1

LINC00315

LINC00482

LINC00313

LINC00158

LINC00846

CTD-2600O9.1

RP11-161I2.1

TCL6

AC097382.5

C22orf34

AC010969.1

LINC00319

LINC00994

LINC00943

C9orf163

AC079612.1

SCOC-AS1

MIRLET7BHG

LINC00336

RP1-253P7.4

RP11-231C14.7

AFDN-AS1

SSBP3-AS1

MUC2

RP11-560A15.3

RP11-375H19.2

LINC01317

CTD-2026K11.6

RP11-291L22.4

RP5-1096D14.6

AC144450.2

RP11-351J23.1

C1orf132

LINC00862

LINC00272

ADAMTSL4-AS1

RP11-188C12.3

ARRDC1-AS1

RP11-126H7.4

TDRG1

RP1-153P14.8

RP4-640H8.2

LINC00619

RP11-713P17.3

PSMB8-AS1

RP11-380J14.1

C1orf195

PSORS1C3

STARD7-AS1

MAMDC2-AS1

LINC01556

LINC01291

ST8SIA6-AS1

AC074391.1

FAM83A-AS1

RP11-830F9.6

RP11-863P13.4

RP11-693J15.5

RP11-352D3.2

RP11-401P9.6

RP11-812E19.3

CHKB-AS1

LINC01597

AF064858.6

LINC01531

DPP9-AS1

LINC00487

C1RL-AS1

RP11-689P11.2

HCP5

RP11-563J2.3

EWSAT1

RP1-29C18.10

RP1-206D15.6

RP1-29C18.9

RP11-474D1.3

UCA1

LINC00887

LINC02026

KANSL1-AS1

MEG3

RP11-323J4.1

RP11-1036E20.9

LINC01588

HLA-F-AS1

RP11-266K4.9

RP11-563J2.2

LINC00598

LINC00189

RP11-800A3.2

RP6-74O6.2

LY86-AS1

LINC01644

MIF-AS1

AC011298.2

RP1-32I10.10

GAS8-AS1

C1orf229

EXOC3-AS1

AC064874.1

AC112721.1

LINC01124

CYTOR

AC079305.10

RP5-1039K5.16

LINC02068

CLDN10-AS1

AP006748.1

MEG9

LINC00473

LMO7DN-IT1

AC004471.9

RP11-285G1.9

RP11-545E17.3

HLA-DQB1-AS1

RP5-919F19.5

RP11-24F11.2

AC003090.1

AC012314.20

SAMSN1-AS1

RP11-86H7.6

LINC00571

AC007879.5

RP11-54O7.3

AC006145.4

RP11-307B6.3

LINP1

IL10RB-AS1

LINC00114

DIAPH3-AS2

AC004870.4

RP1-56J10.8

XXbac-BPG181M17.4

RP11-135J2.3

AP001469.5

AC009480.3

RP11-33A14.1

AC008074.3

AC016738.4

ROR1-AS1

LINC01767

RP11-136K14.2

RP11-445P17.8

EGFR-AS1

RP4-622L5.7

ELMO1-AS1

INHBA-AS1

LINC01857

AMMECR1-IT1

RP3-369A17.4

RP3-522D1.1

RP4-798A10.4

LINC00570

MIR3681HG

C5orf66

HAGLR

AC008278.3

RP11-371A19.2

RP11-359D14.3

AC104699.1

RP1-15D23.2

AC090044.2

RP11-499O7.7

AP000697.6

RP11-336K24.5

RP11-344B5.2

LINC01527

CHL1-AS2

AC011239.1

SMIM25

AP001476.2

RP11-169K16.4

RP11-13P5.1

RP11-495P10.3

AC010884.1

AC109309.4

RP11-370B11.3

LINC01087

LPP-AS1

LINC01886

COL18A1-AS2

TGFA-IT1

HSD52

TNK2-AS1

RP11-276H7.3

AC106900.6

RP11-631F7.1

SH3BP5-AS1

RP1-50J22.4

RASAL2-AS1

GS1-174L6.4

AC007182.6

RP1-86D1.5

AC016735.1

TEX26-AS1

TMEM72-AS1

RP11-190J1.3

COL4A2-AS2

THRB-IT1

RORB-AS1

RP11-123K19.1

CDKN2A-AS1

LINC01398

AC083949.1

AC034187.2

RP11-3B12.5

RP11-448G4.4

LINC00184

RP11-82L18.2

RP5-1086K13.1

LINC01266

PGM5-AS1

AC017002.2

LINC01752

RP11-403H13.1

RP11-295G24.5

RP11-330M19.1

AC096574.4

CATIP-AS1

WWC3-AS1

AL450226.2

RP11-405O10.2

RP11-429E11.2

AC083900.1

RP11-295P9.6

CTD-2228K2.7

AC073957.15

AC012354.6

XXbac-BPG308K3.5

OSTM1-AS1

RP11-390P2.4

AC073283.7

AC093818.1

AC007362.3

INTS6L-AS1

RP1-127D3.4

LINC01770

RP11-57H14.3

RP11-1086F11.1

RP1-91J24.3

RP11-415J8.3

RP11-293P20.2

LHFPL3-AS2

AF064860.5

LINC01678

AC078942.1

TMEM246-AS1

SFTA1P

NHEG1

CTD-2384B11.2

AC104134.2

LINC01671

RP3-508I15.14

ATP13A4-AS1

LINC01107

AC002064.5

AC005237.4

AC002480.5

ZNF385D-AS1

MTOR-AS1

AC005281.2

LINC02158

RP5-997D24.3

AP001046.6

AC064875.2

KCNMA1-AS3

CADM3-AS1

AP001627.1

LINC02036

AL773572.7

SNHG23

DBH-AS1

LINC01389

MIAT

LINC00626

TRMT2B-AS1

RP11-46A10.2

LINC02072

RP5-1177I5.3

RP11-288L9.4

AC007879.4

AC000036.4

RP3-395M20.7

RP11-501J20.5

PCA3

RP4-575N6.4

RP11-69E9.1

AC009950.2

NRIR

HAR1A

RP11-10J5.1

AP001434.2

PRICKLE2-AS3

ZNF503-AS1

LINC00623

RP1-300G12.2

AC099342.1

LINC02097

AC124944.3

AP4B1-AS1

RP11-307P5.2

RP1-18D14.7

ISM1-AS1

ARHGAP26-AS1

AC093382.1

RP1-163G9.2

AC091729.8

RP4-813D12.3

CFLAR-AS1

RP11-739N20.2

AC084809.2

AC058791.1

RP11-420K8.1

RP11-506B6.6

XXyac-YX65C7_A.2

RP11-379B8.1

RP3-512E2.2

LINC01918

KIRREL-IT1

AC004744.3

RP11-445F6.2

RP11-136K14.3

PAPPA-AS2

LINC00974

RP11-137H2.4

LINC01108

RP11-589B3.6

FGF12-AS3

CTA-384D8.31

SMCR5

AC007966.1

AF127936.5

DISC1-IT1

FAM30A

RP11-706D8.3

AC109826.1

AC011893.3

LINC00840

AC005082.12

RP11-356N1.2

AC093901.1

AC135178.7

RP11-36N20.1

RP4-782L23.2

LA16c-395F10.1

LINC00161

RP1-120G22.11

AC010890.1

LINC00658

RP5-956O18.2

AC007036.6

AC105461.1

LINC00511

ITGB2-AS1

RP11-395B7.4

LINC00629

RP3-340N1.2

RP11-4C20.4

L29074.3

RP11-565P22.2

AC096574.5

LINC01630

HORMAD2-AS1

IL21-AS1

WDR11-AS1

MGAT3-AS1

AC092535.3

RP1-45I4.3

AC009518.8

RP11-445L13__B.3

RP11-203J24.8

AC017101.10

RP11-136C24.2

FAM155A-IT1

LINC01364

LINC00866

AC017074.2

AP001471.1

RP11-18B16.2

XX-C283C717.1

LINC01624

RP11-413M3.4

RP11-202G18.1

SLC2A1-AS1

AC018647.3

RP1-35C21.2

RP1-28O10.1

LINC01132

RP11-445N18.5

LINC00111

RP11-418J17.3

RP11-536C5.7

RP11-239H6.2

FAM212B-AS1

RP11-141M1.1

RP11-130C19.3

SRGAP3-AS3

TARID

RP11-276H7.2

RAPGEF4-AS1

LYPLAL1-AS1

AC133106.2

AC092839.3

AC003991.3

FALEC

LINC01649

LINC01402

RP3-467K16.4

RP11-84A14.4

AC007952.5

AL022341.3

C1orf143

EFCAB14-AS1

AC093495.4

LINC01320

LINC02048

RP11-367B6.2

PCAT6

AP001610.5

RP11-1148L6.5

AC015971.2

AC106873.4

TXNDC12-AS1

AC007040.6

HSPC324

RP11-563N6.6

AC005013.5

RP5-1091N2.9

RP11-400N13.2

RP5-994D16.9

AP006222.2

LINC01713

RP11-106M7.1

GRK5-IT1

RP13-225O21.2

LINC01013

AC006460.2

RP11-281A20.2

CCDC183-AS1

RP11-108M9.3

RP5-872K7.7

AC144450.1

AC079779.4

AC096558.1

TTC3-AS1

RP4-676J13.2

AP001065.15

RP11-145H9.3

SRGAP3-AS2

AC008781.7

RP5-884M6.1

RP13-39P12.3

LINC01672

AC003101.1

HCG22

RP11-354K1.2

MIR4500HG

RP4-784A16.2

RP11-91I20.3

RP11-404F10.2

UBAC2-AS1

RP11-544M22.8

AC097381.1

AP000355.2

AKT3-IT1

RP11-336A10.4

LINC02041

HCG23

RP4-677H15.4

HNF4A-AS1

LINC01277

RP11-88I18.3

RP11-266K22.2

RP11-80I15.4

LINC01165

MEOX2-AS1

RP11-439K3.1

VIM-AS1

CCDC26

LINC02038

RP11-84D1.1

RP11-73M7.1

RP11-168O16.1

AC009495.4

LINC00242

LINC00582

LINC00710

RP11-552D8.1

AC004691.5

XXbac-BPG13B8.10

RP4-580N22.2

AC006947.1

GK-IT1

LRRC3-AS1

SZT2-AS1

AC147651.5

LINC01715

RP11-3L21.2

RP11-12M5.3

KCNQ1-AS1

RP5-888M10.2

RP11-342D14.1

AC105053.3

RP11-52J3.2

RP11-481G8.2

RP11-479J7.2

RP5-981O7.2

RP11-390F4.10

RP1-60O19.2

RP5-1142J19.1

RP11-536K7.5

RP11-184A2.3

AC007327.5

ISPD-AS1

LINC00484

RP1-140J1.1

LINC01054

AC013460.1

RP11-142M10.2

RP4-644L1.2

UBE2Q1-AS1

AC118754.4

XIST

EMX2OS

RP11-505P4.7

OGFR-AS1

LINC01315

RP11-456H18.2

AC016999.2

DOCK9-AS1

RP11-240M16.1

TFAP2A-AS1

RP11-135A1.3

MIR181A1HG

AC093585.6

NHS-AS1

RP5-857K21.4

RP4-613B23.1

RP11-431N15.2

CTA-126B4.7

AC091177.1

TPRG1-AS2

FGF12-AS2

RP11-282I1.1

AC016738.3

RP3-508I15.19

LINC00443

RP5-997D16.2

LINC01433

SMYD3-IT1

XXYLT1-AS2

RP11-334J6.6

FEZF1-AS1

RP3-323N1.2

FAM41C

RP5-1139I1.1

AC007163.6

LINC00856

AC004160.4

SERPINB9P1

RP11-488P3.1

PROX1-AS1

AC108463.1

THAP7-AS1

AC092580.3

RP11-95M15.1

RP11-379C10.4

AOAH-IT1

LINC00102

CTB-89H12.4

AC093609.1

HM13-AS1

RGPD4-AS1

LINC01001

SC22CB-1D7.1

AC021188.4

ARHGAP26-IT1

AC012456.4

FOXD3-AS1

AC005532.5

LINC01293

LINC01614

RP5-968J1.1

RP11-486B10.3

RP11-9L18.3

AC008063.2

LINC00309

AP000356.2

HHATL-AS1

RP6-109B7.2

AC013275.2

AC105402.4

RP11-298J23.5

RP3-475N16.1

RP5-1073O3.2

LINC01468

RP1-207H1.3

AC002451.3

LINC01098

LINC00852

RP11-38L15.3

FARP1-AS1

AF127577.11

ZBTB46-AS1

LINC01510

TRIM31-AS1

AP001604.3

RP5-965F6.2

ITPR1-AS1

APCDD1L-AS1

TBL1XR1-AS1

AP000696.2

AC107072.2

CTC-490G23.2

LINC01516

RP1-45C12.1

AC011747.3

LINC01750

RP11-472M19.2

RP11-542K23.9

AC141930.2

RP4-535B20.1

LINC01353

RP11-574F11.3

RP11-374M1.2

RP1-232L24.3

RP11-344F13.1

RP5-943J3.1

AC013264.2

RP3-355L5.4

RPS6KA2-AS1

LINC01704

LINC01891

RP11-203F10.5

RP4-669H2.1

RP11-432J24.2

RP11-573D15.3

AC092652.1

LINC01611

LINC01388

LINC01819

RP11-310E22.5

RP11-527F13.1

AC067945.4

IPO9-AS1

RP5-1120P11.3

DARS-AS1

AC073316.2

RP11-281A20.1

AC079354.5

PGM5P4-AS1

PHKA1-AS1

HS1BP3-IT1

RP11-67C2.2

RP11-452K12.7

RP5-963E22.4

AC011899.10

RP11-57H12.2

EP300-AS1

AC092168.2

RP4-530I15.9

LINC01105

LARGE-IT1

RPS6KA2-IT1

DCST1-AS1

CTD-2619J13.14

RP11-149I23.3

BACH1-AS1

AP001057.1

NCOA7-AS1

LINC02043

RP11-4C20.3

XXyac-YX155B6.5

GNG12-AS1

SLC6A1-AS1

AC012485.2

RP11-557H15.4

RP1-249I4.2

AC093627.7

VIPR1-AS1

RP1-102D24.5

AC009495.3

CTB-51J22.1

RP4-794H19.1

TGFB2-AS1

RASA3-IT1

OSBPL10-AS1

RP5-1011O1.2

ST3GAL5-AS1

AC074366.3

RP11-14N7.2

AC093673.5

RP11-74C1.4

DPYD-IT1

KB-318B8.7

AC104088.1

RP5-1031D4.2

AC007386.4

AP000266.7

AC008073.7

LINC01705

AC002511.3

RP4-669P10.16

RP11-403I13.5

U52111.14

RP5-1185I7.1

AC002480.3

AC078883.3

AP001610.9

COL4A2-AS1

AC003986.6

LINC01700

CTA-212D2.2

DPYD-AS1

RP11-136K14.1

RP11-324O2.3

AP001062.9

RP11-179A7.2

VPS13A-AS1

AC034220.3

NALCN-AS1

RP11-490E15.2

AC011899.9

ERVH48-1

AC016700.2

AC005009.2

LINC01271

LINC00892

AC006042.7

RP11-301L8.2

LINC01762

AC006372.6

AC002511.2

LINC00472

RP11-220I1.2

AC007743.1

RP11-380F14.2

OSTN-AS1

RP11-482E14.1

FAM53B-AS1

TLR8-AS1

AC018878.3

RP11-235G24.3

RP1-69D17.3

CHRM3-AS2

RP4-655C5.4

RP5-881L22.6

RP11-100E13.1

AC104809.4

AC008063.3

RP1-212P9.3

STXBP5-AS1

RP1-187B23.1

SYNJ2-IT1

LINC01518

AC005162.5

FAM224A

HCG21

RP11-867G2.5

AC011294.3

DNM3-IT1

RP11-547D24.1

RP4-694A7.2

RP11-153K11.3

ERI3-IT1

LINC01137

RP11-523O18.5

AC007365.1

SLC26A4-AS1

LINC00284

LINC01765

AC098617.1

LINC01251

AP001442.2

RP13-314C10.5

RP11-168K11.3

KCNQ5-IT1

RP3-348I23.2

AC103563.8

LATS2-AS1

EZR-AS1

RP1-122P22.2

Z83851.4

RP11-305F18.1

RP11-250B2.3

RP11-354E11.2

RP11-288L9.1

RP11-379J5.5

DDX39B-AS1

RP11-310E22.4

LL0XNC01-237H1.3

RP11-364P22.1

RP4-550H1.4

LINC01675

RP3-460G2.2

AC016683.5

RP11-182N22.8

LINC01781

RP11-157D23.2

LINC01191

RP1-67A8.3

KCNH1-IT1

BOK-AS1

RP11-213H15.1

BACH1-IT3

JAZF1-AS1

RP11-52J3.3

LINC01914

AC007278.3

RP11-211N11.5

RP11-561O23.5

RP11-528G1.2

RP3-413H6.2

MAGI2-AS3

AC004231.2

ACBD3-AS1

RP1-55C23.7

RP11-285G1.2

LINC01506

RP11-37L2.1

RP5-998N21.4

CHRM3-AS1

MAPKAPK5-AS1

AL450992.2

SNRK-AS1

RP1-149A16.12

LL22NC03-2H8.4

MED14OS

PCCA-AS1

LINC01934

LINC00444

RP4-530I15.6

AC007161.5

LINC01287

C1orf140

SLC25A25-AS1

LINC00412

RP11-62F24.2

RP11-483H20.4

RP3-322G13.7

MIR155HG

SOX9-AS1

AC092839.4

RP11-124N14.3

RP11-195M16.1

SEMA3F-AS1

RP11-109I13.2

RP11-61I13.3

RP1-150O5.3

AC009303.1

ZRANB2-AS1

ID2-AS1

RP11-83A16.1

LINC00330

RP1-29C18.8

RP11-9M16.2

RP11-374P20.4

RP1-60O19.1

AC114730.2

LINC01366

LINC00601

RP5-1056L3.1

AC009495.2

RP11-145M4.2

RP1-151B14.6

DANT2

RP1-5O6.5

KDM5C-IT1

LINC01422

AF127577.8

THRA1/BTR

LINC01281

AC007556.3

AC016292.1

LINC02154

EPN2-AS1

CYMP-AS1

AC010149.4

RP11-282O18.3

RP11-342M3.5

GYG2-AS1

AC073046.25

RP4-756G23.5

RP11-63P12.7

LINC00402

LINC01871

LINC01102

AF127936.9

LINC00494

AC090952.5

AC016644.1

RP11-242G20.1

RP11-374M1.5

LINC00298

AC004593.3

LINC00607

DPYD-AS2

AL109767.1

RP11-73M7.6

HCFC1-AS1

SRGAP3-AS4

BHLHE40-AS1

RP1-60N8.1

AC073333.8

GSN-AS1

RP11-59O6.3

AC114776.3

LINC01828

RP11-626E13.1

RBMS3-AS3

RHOA-IT1

MACROD2-AS1

LINC01553

AC007405.8

RP11-123B3.2

EGOT

AC061961.2

AC018816.3

RP11-97N19.2

LINC01936

PRRX2-AS1

AC017060.1

RP6-191P20.4

LINC01447

RP11-733O18.1

RP11-89F3.2

RP11-343J3.2

RP3-523C21.2

AC104135.2

AC006369.2

AC091132.1

DIAPH2-AS1

SHANK2-AS2

AP001189.4

TLX1NB

KCCAT333

RP3-403L10.3

TRHDE-AS1

AC005220.3

LINC00479

RP11-25B7.1

RP11-320G24.1

RP11-229P13.15

LINC01134

RP11-107M16.2

RP11-296A18.6

ATG10-IT1

AC003092.1

AC130689.5

AC002401.1

LINC02195

RP11-89N17.4

AC007278.2

RP4-742J24.2

RNF217-AS1

RP11-243J16.7

RP11-162J8.3

PITPNA-AS1

CTA-221G9.7

RP11-83J21.3

RP11-69I8.2

MYB-AS1

AC073316.1

DAPK1-IT1

LINC01844

LINC01186

RP5-1184F4.5

LINC00299

LINC01563

AC024560.2

AC090617.1

RP11-944L7.4

LINC00106

LINC01554

RP4-651E10.4

RP3-329E20.2

ANKRD44-IT1

RP11-433J20.1

EDRF1-AS1

GLIS3-AS1

LINC01812

AC005550.3

RP11-6J21.2

RP5-836J3.1

AC073254.1

CNTFR-AS1

LINC01792

RP11-415D17.3

AC147651.4

TMEM26-AS1

RP1-142L7.5

AC022173.2

CATIP-AS2

AC004906.3

LINC01343

LINC01732

RP11-439H8.4

RP11-230L22.4

LINC01502

LINC01358

RP11-475I24.3

RP13-152O15.5

UNQ6494

PITRM1-AS1

MGC27382

RP11-885N19.6

LINC01522

RP11-67L3.2

RP11-554F20.1

RP4-803J11.2

LINC01684

RP11-356I2.4

PKN2-AS1

RP11-325F22.2

KCND3-AS1

RP3-359N14.2

RP13-143G15.4

AP001056.1

RP11-343H5.6

AF064858.10

FRY-AS1

AC007386.2

RP11-462G2.1

P4HA2-AS1

AF064858.11

RP11-298E2.2

RP11-54A4.2

GFOD1-AS1

AC010894.5

LINC00211

AC002454.1

AC133680.1

RP1-63G5.7

AC073130.1

DGUOK-AS1

NALT1

KLF7-IT1

SIRPG-AS1

RP3-393E18.2

PRKCQ-AS1

LINC00649

RP11-338C15.3

RP11-443B7.1

AC093110.3

AC002480.2

RP11-815M8.1

ZEB2-AS1

LINC00426

MAP3K20-AS1

AC116366.5

RP3-395M20.8

RP11-431J24.2

RP11-342D11.2

LINC00707

AC012360.6

BX470102.3

RP5-1121E10.2

AC093484.4

LINC01119

LLPH-AS1

RP4-584D14.6

SIDT1-AS1

HOXB-AS2

KMT2E-AS1

RP11-451B8.1

RUVBL1-AS1

RP4-728D4.2

PSMD6-AS2

ZBTB20-AS3

LNCSRLR

AC096579.15

RP4-753P9.3

MAGI1-AS1

ARHGEF3-AS1

RP11-430C7.5

B4GALT4-AS1

AC017002.1

AC007879.3

LINC00973

RP11-185E8.1

CTD-2313F11.1

THOC7-AS1

MME-AS1

LINC01168

RP11-430C7.4

PLCXD2-AS1

AC093627.10

LINC00886

RP11-167H9.5

RP4-714D9.2

PRICKLE2-AS2

PRICKLE2-AS1

ARHGAP31-AS1

ADAMTS9-AS1

LINC00877

IQCJ-SCHIP1-AS1

RP11-221J22.2

SUCLG2-AS1

LINC00635

PTPRG-AS1

RP4-781K5.5

RP11-641D5.2

RP11-553L6.2

AC108004.3

LINC02029

ZBTB20-AS1

TRBV11-2

RP3-455J7.4

ADAMTS9-AS2

AC002467.7

RP11-555M1.3

AC092620.2

CECR3

RP11-755B10.3

WWTR1-IT1

RP13-452N2.1

RP1-90G24.10

RP11-734K21.2

HOXB-AS4

PVRL3-AS1

LINC00996

ZBTB20-AS5

RP11-67L3.5

CTD-2377D24.4

RP11-469J4.3

RP11-274B21.14

RP5-921G16.1

AC093627.8

AC009228.1

LINC02005

ZBTB20-AS4

RP11-180K7.1

RP11-3K16.2

RP11-190P13.2

GK-AS1

RP11-231E6.1

AC110769.3

CTA-360L10.1

RP11-445N18.7

RP11-475O23.2

RP11-148K1.10

LINC01214

LINC00880

RP5-1096J16.1

LINC02000

ELFN2

RP11-167H9.4

RP11-552M11.4

RP5-839B4.8

RP11-99J16__A.2

FLNB-AS1

RP4-545C24.1

ITGB5-AS1

GATA2-AS1

RP11-734K21.3

RP11-654C22.2

FAM3D-AS1

RP11-206M11.7

HDAC11-AS1

RP11-315I20.3

AL122127.25

LINC02086

AL109761.5

RP11-613D13.8

CTD-3064M3.4

RP11-875O11.1

RP11-303E16.7

RP11-320N21.1

LINC01024

LINC00861

USP2-AS1

RP11-286E11.1

ARNTL2-AS1

RP11-15B17.1

KB-1471A8.1

LINC01585

LINC00461

NEAT1

DACT3-AS1

LINC02211

NADK2-AS1

RP11-367J11.2

LINC02202

RP11-158I9.5

RP11-18H21.1

CTD-2506J14.1

RP11-632K5.3

CASC8

RP11-325L7.1

RP11-77I22.2

RP11-13A1.1

RP11-10L7.1

RP11-461O7.1

CTD-2024I7.13

LINC00968

RP11-894P9.1

AF131216.6

RP11-1149O23.3

CACNA1C-AS1

RP11-1094H24.4

PLA2G4E-AS1

RGMB-AS1

RP11-730K11.1

RP11-700H6.1

MIR210HG

LINC01252

RP11-431M7.3

RP11-296I10.3

RP11-510M2.2

RP11-266N13.2

CTD-2235C13.2

CARS-AS1

RP11-392P7.6

CTB-127C13.1

PCED1B-AS1

NR2F2-AS1

CCAT1

RP3-510L9.1

FAM13A-AS1

RP11-446J8.1

RP11-44F21.2

RP11-148L24.1

RP11-455B3.1

TRPC7-AS1

CTB-174D11.1

RP11-74M11.2

MEF2C-AS1

LUCAT1

CTC-327F10.5

RP11-313E19.2

RP11-1191J2.4

RP11-597D13.9

LINC01197

SEMA6A-AS1

RP4-598P13.1

FGF10-AS1

RP11-479O16.1

RP11-10A14.5

RP11-247C2.2

CTB-47B11.3

RP11-768B22.2

RP11-834C11.8

RP11-158I23.1

RP11-331K21.1

HAS2-AS1

LINC02100

RP11-18D7.2

LINC01085

RP11-1079K10.3

LINC01948

CTD-2260A17.1

RP11-87E22.2

RP11-328K4.1

CTD-2215E18.2

RP11-666A20.4

C8orf34-AS1

RP11-626H12.3

LINC02065

CTD-2015A6.2

CTC-537E7.3

CTD-2135J3.3

CTD-2288O8.1

RP11-36B15.1

RP11-94H18.1

CTB-174D11.2

RP11-1334A24.6

RP5-951N9.1

CTB-113P19.1

MAST4-IT1

C5orf66-AS1

CTD-2631K10.1

RP11-290F5.1

RP11-43D2.2

PSD2-AS1

CTC-484P3.3

CTD-3080P12.3

RP11-227F19.5

RP11-452J21.2

AC195454.1

RP11-468H14.2

RP11-364P22.2

LINC02057

CTC-575I10.1

RP11-26P13.2

AC010468.2

RP11-26J3.1

RP11-317M11.1

LINC01016

LINC01060

IL20RB-AS1

CTC-563A5.2

RP11-438D8.2

RP11-502M1.2

RP13-497K6.1

LINC01091

CTD-2587M2.1

LINC01179

CTB-35F21.1

AC226118.1

CTC-537E7.1

RP11-610J23.1

RP11-281P23.2

HOXC13-AS

C5orf66-AS2

RP11-310P5.1

RP11-279O9.4

SRD5A3-AS1

RP11-89B16.1

CTD-2236F14.1

RP11-44F21.3

AC008697.1

RP11-20D14.6

RP11-223C24.1

VCAN-AS1

RP11-1101H11.1

AC145676.2

CTD-2227C6.3

PVT1

RP11-434D9.2

CTD-2175A23.1

BFSP2-AS1

RP11-814P5.1

CTC-339F2.2

RP11-541P9.3

CTC-529P8.1

RP11-677M14.3

RP11-584P21.2

RP13-884E18.2

RP11-359B12.2

RP11-380P13.1

CTD-2353F22.1

RP11-381K20.2

LINC02199

CTD-2024P10.1

RP11-267A15.3

LINC01612

RP11-122A21.2

CTB-114C7.4

RP11-305O6.3

RP5-875H18.4

RP11-94C24.8

LINC01021

CTD-2213F21.2

RP11-23P13.4

RP11-310P5.2

RP13-539F13.3

RP11-448G15.1

LINC02105

RP11-640N11.2

RP11-757G1.6

CTC-573N18.1

RP11-380D23.1

AC004066.3

RP11-47I22.2

SMAD1-AS2

RP11-440I14.2

RP11-517I3.1

CTC-228N24.2

RP11-933H2.4

LINC01182

CXXC5-AS1

RP11-138B4.1

RP11-864I4.3

RP11-392B6.1

AP000892.4

RP11-404E16.1

RP11-710F7.2

RP11-613C6.2

RP11-11N5.1

CTC-806A22.1

CTC-459M5.1

LINC01336

RP11-588L15.2

RP11-253E3.3

SMAD1-AS1

AC097467.2

RP11-297P16.4

RP11-357D18.1

AC141928.1

AC005355.1

AE000661.37

ZFPM2-AS1

RP11-582J16.4

RP11-100N20.1

HTT-AS

RP11-526F3.1

RP11-115D19.1

RP11-280G9.1

WWC2-AS1

CTD-2035E11.3

RP11-701P16.2

RP11-53O19.1

CTD-2532K18.2

RP11-478C1.7

RP11-281P23.1

F11-AS1

RP11-327O17.2

RP11-689P11.3

CTC-459M5.2

LINC00589

RP1-68D18.2

LINC00923

RP11-469N6.1

MIR3945HG

WDFY3-AS1

SMIM15-AS1

ERVH-1

RP11-81H14.2

CTC-327F10.4

LVCAT1

CTD-2516F10.2

SEMA5A-AS1

CTB-109A12.1

RP11-586D19.2

RP11-843P14.1

RP11-436H11.5

RP11-1258F18.1

RP11-109E24.1

RP11-724M22.1

RP11-381N20.1

CTD-2245E15.3

RP11-745L13.2

RP11-118M9.3

RP11-297P16.3

RP11-440I14.3

RP11-673E1.1

LINC01385

RP11-610P16.1

LINC02172

RP11-20I20.2

CTC-458I2.2

RP11-181K12.2

KB-1448A5.1

RP11-648L3.2

RP11-527N22.1

RP11-326L2.1

CTB-118P15.2

RP11-100L22.1

HOXA10-AS

RP11-706C16.7

RP11-582J16.5

RP11-1149M10.2

KB-1991G8.1

RP11-539E17.5

PCAT2

RP11-1105O14.1

RP11-10J21.4

RP1-170O19.17

RP13-923O23.6

RP11-420B22.1

RP11-731F5.2

RP11-1081M5.1

CTC-756D1.2

AC012613.2

RP11-231D20.2

LINC02159

RP11-642D21.2

CTD-2363C16.2

RP11-395I14.2

LINC02099

MIR3142HG

RP11-152P17.3

RP11-624C23.1

RP11-402L5.1

CA3-AS1

RP11-586K2.1

RP11-1080G15.1

RP11-351C8.1

RP11-723D22.2

RP11-157E21.1

RP11-875O11.3

KB-1980E6.3

LINCR-0001

CTD-2270F17.1

LINC01592

ZFHX4-AS1

KB-1615E4.2

KB-1732A1.1

RP11-981G7.3

CTC-348L5.1

KBTBD11-OT1

AL928768.3

RP11-473O4.3

RP11-893F2.13

RP11-779O18.3

AC025442.3

RP11-439C15.4

CTD-3023L14.1

CTD-2363C16.1

KB-173C10.2

RP11-177H13.2

RP11-44K6.2

CTB-140J7.2

GS1-57L11.1

FER1L6-AS2

RP11-203E8.1

FAM85B

CTB-113P19.3

RP11-10C8.2

RP11-156K13.1

CTB-33O18.3

RP11-333A23.4

MAL2

RP11-467K18.2

CTB-4E7.1

RP11-489O18.1

RP11-245A18.1

RP11-10J21.3

RP11-363E6.3

RP11-326E22.1

RP11-281O15.4

RP11-419C23.1

CTC-558O2.1

RP11-156K13.3

AC078852.1

AC005740.5

CTD-2647L4.1

RP11-642D21.1

RP11-470M17.2

RP11-661A12.4

RP11-48B3.3

CASC19

RP11-1149M10.1

CTC-558O2.2

RP11-10J21.5

RP11-43A14.1

LINC01485

LINC01933

RP11-582J16.3

RP11-115J16.1

KB-1639H6.2

CTB-120L21.1

RP11-17A4.2

RP11-473O4.4

RP11-278I4.2

RP11-44K6.4

RP11-150O12.3

RP11-134O21.1

RP11-318K15.2

MAFA-AS1

RP11-267L5.1

RP11-14I17.3

KB-1615E4.3

CTC-529G1.1

ANO1-AS2

RP11-261P9.4

RP11-560G2.1

NAV2-AS2

RP11-867G23.13

RP13-631K18.2

AP003068.9

RP11-728F11.4

NAV2-AS3

RP11-84A19.3

RP11-429J17.5

AF131215.4

RP11-673F18.1

RP11-626H12.2

RP11-22P4.2

RP11-395G23.3

RP11-356J5.4

CTD-2589M5.5

RP11-702F3.1

CTD-2560E9.3

STT3A-AS1

RP11-7I15.4

RP11-660L16.2

RP11-162D9.3

RP11-47J17.3

RP11-58K22.5

RP11-659G9.3

SENCR

CTD-2216M2.1

CTD-2005H7.1

RP13-46H24.1

RP11-334E6.3

RP13-726E6.1

CTD-2616J11.3

AC022182.1

RP11-672A2.4

CTD-2530H12.2

AP001372.2

RP11-51B23.3

CTD-2523D13.2

RP11-867G23.1

RP11-159H22.2

CTD-2516F10.4

RP11-748C4.1

RP11-23J9.5

RP11-802F5.1

RP11-326C3.7

RP11-144G6.12

LINC00678

AF131215.3

AP001257.1

RP11-1081L13.4

RP11-167J8.3

RP11-350N15.3

CTD-2547H18.1

RP13-631K18.3

CTD-2509G16.2

CTD-2589M5.4

AP000442.4

RP11-802E16.3

RP11-661A12.9

FAM66D

RP11-843A23.1

RP11-495O11.1

RP11-58K22.4

RP11-21L23.3

RP11-110I1.6

OVOL1-AS1

RP11-396J6.1

RP11-318C2.1

RP11-428C19.5

RP11-514F3.5

RP11-626H12.1

RP11-750H9.5

RP11-350N15.4

RP4-541C22.5

RP11-727A23.10

CTD-2523D13.1

MIR100HG

CTD-2005H7.2

CTD-3224I3.3

RRM1-AS1

RP11-655C2.3

RP5-901A4.1

RP11-428C19.4

RP11-96B2.1

CTD-2530H12.4

RP11-326C3.12

RP11-708L7.6

RP11-148O21.2

AP000640.2

RP11-1134I14.8

RP11-770J1.4

CTD-2562J17.2

RP11-266A24.1

AP002954.4

CTD-2531D15.4

CTD-2517M22.9

RP11-144G7.2

RP11-736K20.5

RP11-1H15.2

RP11-148O21.4

RP4-607I7.1

RP11-770G2.2

RP11-377D9.3

RP11-502N13.2

RP11-867G2.6

RP11-766N7.3

RP11-529H2.2

IFNG-AS1

RP11-757G1.5

RP11-283G6.5

RP11-428G5.5

LINC01479

AP000439.3

RP3-454B23.1

LINC00346

RP11-196H14.2

CCND2-AS2

RP11-662I13.2

RP11-286N22.10

RP11-417L19.4

RP11-392P7.7

CACNA1C-AS4

RP11-707G14.1

RP11-291B21.2

RP11-134N1.2

RP11-90D4.3

LINC01152

LINC00944

RP11-968O1.5

RP11-612B6.2

RP11-64D24.4

TSPAN9-IT1

RP11-439H13.2

RP11-771K4.1

RP11-324E6.6

USP30-AS1

RP11-664H17.1

RP13-977J11.8

RP11-338E21.3

RP11-118B22.4

RP11-794G24.1

RP11-667M19.10

RP11-363J17.1

RP11-21A7A.4

MRGPRF-AS1

RP11-598F7.6

RP13-895J2.3

RP11-274J7.2

RP11-283I3.2

RP11-75L1.1

FLJ37505

RP11-64B16.4

RP11-169D4.2

RERG-IT1

A2ML1-AS1

RP11-598F7.5

RP11-785H5.2

RP11-881M11.8

RP13-941N14.1

PLBD1-AS1

RP11-221N13.4

RP11-817J15.2

RP11-783K16.5

RP13-895J2.2

RP11-598F7.3

RP11-273B20.1

RP11-320N7.2

RP11-359J14.2

CTD-2555A7.2

RP11-729I10.2

RP11-268P4.4

RP11-486F17.1

RP11-864I4.4

RP11-266O8.1

RP11-783K16.13

RP11-996F15.2

RP11-567C2.1

RP11-54A9.1

RP11-689B22.2

CTD-2252P21.1

RP11-272B17.2

LINC01619

RP3-405J10.4

MIR3179-1

RP11-317N8.3

RP11-434H14.1

RP11-1060G2.1

RP11-190J23.1

RP3-405J10.3

RP11-290L1.5

RP11-780K2.1

RP11-793H13.8

RP1-197B17.3

RP1-71H24.1

RP11-290L1.3

RP11-359M6.1

RP5-1057I20.2

RP3-521E19.2

RP11-585P4.5

RP11-498M15.1

RP11-834C11.10

RP11-793H13.3

RP11-44N21.1

LINC01475

OVCH1-AS1

RP11-256L6.2

RP11-370I10.6

RP11-650K20.2

RP11-186F10.2

CTB-193M12.1

RP11-70F11.8

RP11-1136G11.8

RP11-845M18.6

RP11-256L6.3

RP11-769N19.2

RP11-114F10.2

RP11-493L12.5

RP11-493L12.3

RP11-46I1.1

RP11-620J15.1

RP11-219B4.6

RP1-288H2.2

CTD-2021H9.3

RP11-114H23.1

RP11-443B7.3

RP11-753H16.5

RP11-114H23.2

RP4-816N1.7

RP11-474P2.2

RP11-753H16.3

RP11-588H23.3

RP11-1105G2.4

RP11-412H8.2

RP11-394J1.2

RP11-493L12.4

RP11-161H23.5

RP11-219B4.5

RP11-570L15.1

RP11-536G4.2

MIR3180-1

RP11-1105G2.3

RP11-33N16.2

RP11-187E13.2

RP11-1029J19.5

RP11-404P21.5

RP11-665C16.6

CTD-2207P18.1

RP11-713N11.4

RP11-665C16.5

RP11-187E13.1

LINC02207

DIO3OS

RP11-862G15.2

LINC00239

RP11-638I2.9

RHOXF1-AS1

RP11-99E15.2

RP11-1070N10.3

LINC01500

RP5-1021I20.2

LINC01629

AL161668.5

LINC-ROR

RP11-369C8.1

RP11-362L22.1

RP11-725G5.2

LINC00930

RP11-509A17.3

RP11-964E11.2

RP11-386M24.3

LINC01269

RP11-218E20.3

ITPK1-AS1

CTD-2341M24.1

RP11-982M15.7

RP11-356K23.1

RP11-110A12.2

DIO2-AS1

HIF1A-AS1

RP11-944C7.1

RP11-404P21.3

RP11-255G12.3

RP11-219E7.1

RP11-7F17.3

RP11-300J18.1

RP11-76E17.4

CTD-2566J3.1

RP11-247L20.3

RP11-982M15.5

LINC01146

RP3-514A23.2

CTD-2547L24.3

RP11-270M14.5

CTD-2128A3.2

LINC01956

RP11-260M19.2

RP11-1029J19.4

RP11-47I22.1

RP11-463J10.2

RP11-696D21.2

SALRNA1

RP11-507K2.2

RP11-131H24.4

RPPH1

RP11-8L8.2

RP3-449M8.6

LINC00524

RP11-404P21.1

RP11-409I10.2

AE000662.93

ACTN1-AS1

LINC00639

FOXN3-AS2

RP11-973N13.3

RP11-77A13.1

RP11-406H23.2

RP11-973N13.4

RP11-840I19.3

RP11-1127D7.1

RP11-187O7.3

LINC00924

RP6-65G23.3

RP11-299G20.2

RP11-154B12.3

RP11-424I19.2

CTD-2647E9.3

RP11-108K3.4

RP11-56B16.5

RP11-342M21.2

CTD-3076O17.2

RP11-1008C21.1

RP11-361D15.2

RP11-815J21.3

LINC02206

RP11-617F23.1

LIPC-AS1

CTA-339C12.1

RP11-293M10.6

RP11-762H8.1

RP11-102L12.2

RP11-323I15.5

RP11-20G13.1

RP11-519G16.5

RP11-111E14.1

RP11-30K9.5

RP11-327J17.2

RP11-307C19.1

CTD-2054N24.2

RP11-64K12.9

CTD-2647L4.4

RP11-815J21.4

RP11-1069G10.1

CTD-2240J17.1

RP11-505E24.3

CTD-2308G16.1

RP11-30K9.4

RP11-158M2.3

RP11-7M10.2

RP11-158M2.5

RP11-593F23.1

RP11-265N7.2

CTC-378H22.2

CTD-2650P22.1

RP11-736N17.8

RP11-276M12.1

RP11-815J21.1

RP11-128A17.1

RP11-930O11.2

RP11-365N19.2

LINC00052

RP11-138H8.2

RP11-522B15.4

RP11-158M2.2

CERNA1

RP11-66B24.4

RP11-317G6.1

RP11-275I4.1

RP11-562A8.5

RP11-654A16.1

RP11-467H10.2

RP11-680F8.4

RP11-143J24.1

RP11-485O10.2

IQCH-AS1

RP11-343B18.2

RP11-707P17.1

LINC00677

RP11-758N13.1

CTD-3032H12.1

RP11-59H7.3

RP11-356M20.3

RP11-275I4.2

RP11-158M2.4

LINC02157

PCSK6-AS1

RP11-429D19.1

RP11-45P15.4

RP11-304L19.12

LINC02109

RP11-114H24.6

RP11-382A20.5

RP5-1142A6.5

RP5-1024G6.7

RP11-284N8.3

RP11-525K10.3

RP11-1012E15.2

CTD-3037G24.4

RP11-1100L3.8

CTD-3037G24.3

RP6-91H8.2

CTD-2651B20.7

RP11-304L19.1

IL21R-AS1

THSD4-AS1

LINC00261

AL121578.2

RP11-732A21.2

LINC01992

CTD-2524L6.3

RP11-254F7.2

RP11-42I10.1

RP11-92G12.3

RP5-1142A6.9

RP11-326A19.4

LA16c-312E8.2

RP11-258F22.2

RP11-322D14.2

RP11-394B2.6

RP11-521O16.2

RP11-863P13.6

RP11-2I17.4

RP4-536B24.3

RP11-616M22.5

RP11-432I5.6

RP11-401P9.5

RP4-676L2.1

RP11-77H9.2

RP11-109G23.3

RP11-600F24.7

RP11-973H7.1

RP11-203B7.2

RP11-27M24.2

RP11-416I2.1

RP11-382A20.6

CTD-2288F12.1

RP11-297M9.1

RP11-521I2.3

RP11-646E18.4

CTD-2583P5.1

RP11-429P3.5

RP11-96D1.7

RP11-367F23.2

NBAT1

RP11-541N10.3

RP11-405F3.4

RP11-834C11.11

RP11-85A1.3

RP11-556H2.3

RP11-1081M5.2

RP11-166B2.7

RP11-161M6.3

CTD-3193O13.1

RP11-356C4.3

RP11-271M24.2

CTD-2199O4.3

RP11-426C22.5

RP11-558A11.2

RP5-1085F17.4

RP13-379O24.2

RP11-49I11.1

RP11-615I2.2

CTD-2541J13.1

AP000223.42

RP11-592N21.2

AC012531.25

CTC-457E21.1

RP11-552C15.1

GS1-21A4.1

RP1-140K8.5

RP5-1142A6.7

RP11-23N2.4

RP11-21M24.3

RP11-403P17.2

RP11-315D16.4

RP11-368L12.1

RP11-152L20.3

RP4-529N6.2

RP11-643C9.2

AC026150.8

RP11-747H7.3

AC009133.17

RP11-255C15.3

RP11-21M24.2

Z84812.4

LINC01963

CTD-2547L24.4

CTD-2373J6.1

RP11-211G23.2

RP11-320H14.1

RP11-554A11.7

RP11-2C24.4

RP11-196G11.2

RP11-363E7.4

RP11-57H14.4

RP11-483P21.6

RP4-575N6.5

RP11-476D10.1

RP11-552M11.8

RP11-426C22.6

RP11-690D19.3

RP11-504A18.1

RP11-315A16.1

LA16c-395F10.2

RP13-122B23.8

RP4-647J21.1

RP11-546B15.1

RP1-140C12.2

RP11-84D1.2

CTD-3247F14.2

CTC-471C19.1

RP11-417E7.2

WFDC21P

RP11-252E2.2

RP11-303E16.2

RP11-7K24.3

RP11-701H24.4

RP11-179B15.6

RP11-93I21.3

LINC02178

RP11-690I21.2

RP11-734K21.5

RP11-333O1.1

RP11-496D24.2

RP11-304L19.3

RP11-20I23.6

LINC01989

RP11-356C4.5

LINC02188

RP11-863P13.5

CTD-2380F24.1

RP11-989E6.10

RP11-960L18.1

CTD-2006K23.1

RP11-830F9.7

AC140912.1

LINC02133

RP11-510J16.5

LINC02128

AC137932.6

RP11-44I10.3

RP11-389C8.2

RP11-325K4.3

RP11-554A11.5

RP11-517C16.2

RP11-276H1.2

RP11-297D21.2

RP11-863P13.3

RP11-297C4.1

RP11-378A13.1

RP11-297C4.2

PYCARD-AS1

RP11-455F5.4

VPS9D1-AS1

RP11-395N3.1

CTD-2012K14.2

RP11-378I6.1

RP11-326A19.3

RP6-24A23.7

RP11-455F5.5

LA16c-390E6.3

RP13-126P21.2

RP11-399O19.9

RP11-217B1.2

RP11-109D9.4

CTD-2576D5.4

RP11-981G7.1

RP11-731K22.1

RP11-1024P17.1

RP11-61F12.1

RP11-77K12.3

AC135048.13

RP11-757F18.5

DLGAP1-AS5

RP13-487P22.1

RP11-244O19.1

RP11-16E23.3

RP11-553K8.5

RP11-178L8.3

RP11-414J4.2

RP11-20I23.13

RP11-79H23.3

RP11-405F3.5

RP11-327F22.1

RP11-489G11.3

RP11-96K19.4

CTD-3064M3.3

BEAN1-AS1

TTC39A-AS1

RP1-168P16.1

LINC00165

SSTR5-AS1

GS1-204I12.4

RP11-669C19.1

CTC-523E23.1

AC005592.3

CTD-2354A18.1

RP4-555D20.2

RP11-44F14.2

RP11-502H18.2

LINC01996

AC144831.1

MMP25-AS1

RP11-676J12.7

RP11-700H6.4

RP11-177N22.3

RP11-1260E13.4

DKFZP434A062

LINC02185

RP11-483C6.1

RP13-638C3.3

RP11-876N24.2

LINC01978

RP11-160E2.6

AC144836.1

RP11-876N24.4

RP11-1260E13.2

LA16c-390H2.4

CTC-457L16.2

RP11-1197K16.2

RP11-353N14.3

RP5-1050D4.3

RP11-676J12.8

LA16c-349E10.1

RP11-1260E13.3

RP11-497H17.1

AJ003147.9

RP11-44F14.8

RP11-214O1.1

RP13-580F15.2

CTD-3060P21.1

RP11-1260E13.1

RP11-141J13.3

CTD-3088G3.6

RP11-876N24.5

RP5-1029F21.3

RP11-485G7.5

RP11-166P13.4

RP11-567O16.1

RP11-1055B8.2

RP11-333E1.2

RP11-149I9.2

LA16c-325D7.1

TMEM220-AS1

RP11-599B13.3

RP11-118E18.4

RP11-963H4.3

HID1-AS1

RP11-45M22.3

PPP4R1-AS1

RP11-57A1.1

RP11-321A17.4

RP11-466A19.6

RP11-285E9.6

DLGAP1-AS3

RP11-161I6.2

RP11-649A18.4

RP11-456D7.1

LINC00908

RP11-573D15.9

RP11-143J12.3

RP11-110H1.9

RP11-789C17.5

RP11-504I13.3

RP11-68I3.10

RP11-627G18.2

CTC-297N7.9

LINC02003

RP11-192H23.7

RP11-848P1.5

RP11-283C24.1

RP11-401F2.4

RP3-388N13.5

RP11-556O9.2

RP11-720L2.4

PRKCA-AS1

RP11-381P6.1

RP11-524F11.2

RP11-92B11.3

RP11-680C21.1

RP11-138I1.2

RP11-186B7.4

RP11-474I11.8

DSG2-AS1

CTD-2104P17.2

AC100830.5

RP11-888D10.3

C1QTNF1-AS1

RP11-285E9.5

RP11-227G15.2

TSPOAP1-AS1

RP11-672L10.2

RP11-70L8.4

AC010761.13

RP5-1171I10.5

RP11-466A19.8

CTD-2350C19.2

AC104984.4

CTD-2349P21.11

RP11-466A19.5

RP11-17M24.3

PCAT18

RP11-789C17.1

LINC01915

RP11-465I4.2

RP11-13N13.6

RP11-156L14.1

RP11-883A18.3

RP11-354P11.3

RP11-403A21.1

RP11-17M16.2

RP11-387H17.6

RP11-720N19.2

RP11-737O24.2

LINC00668

GACAT2

RP11-118E18.2

RP11-647F2.2

RP11-789C17.3

RP11-769O8.1

RP11-433M22.1

RP11-627G18.1

RP11-159D12.10

RP11-214O1.3

CTB-41I6.1

RP11-737O24.1

SNHG25

RP11-401F2.3

RP11-449D8.5

RP11-838N2.5

RP1-71H19.2

RP11-466A19.3

RP11-6N17.3

RP11-466A19.1

DSG1-AS1

RP1-41C23.1

RP11-173A16.2

RP11-567M16.5

CTC-499B15.6

RP11-332H18.3

CTC-215O4.4

RP11-701H16.4

RP11-820I16.3

RP11-49K24.4

AC005625.1

RP11-806H10.4

AC079466.1

CTD-2369P2.5

RP11-552F3.4

LLNLR-284B4.2

RP11-127I20.7

RP11-936I5.1

CTC-448F2.4

RP11-120M18.2

RP11-75C10.9

RP11-92C4.3

AC005757.6

CTB-30L5.1

LINC01905

RP11-1094M14.5

CTD-2369P2.4

PCAT19

RP11-839G9.1

CTD-2020K17.1

AC004490.1

LINC02081

RP11-53B2.3

CTB-31O20.8

AC006116.12

RP11-116O18.3

RP11-813F20.4

RP11-798G7.6

CTC-265F19.3

RP11-126O1.5

CTB-184G21.3

AC139100.3

RP11-1151B14.4

CTD-2008P7.9

CTC-232P5.3

RP11-75C10.7

CTD-2562J15.6

TBX2-AS1

CTB-129P6.4

RP5-951N9.2

RP11-567M16.1

CTD-2623N2.11

CTB-66B24.1

AC004637.1

CTB-25B13.12

RP11-640A1.3

RP11-1094M14.12

RP11-47L3.1

CTD-2231E14.5

CTB-186G2.4

CTD-2240E14.4

RP11-691H4.3

CTB-175E5.7

RP11-873E20.1

CTC-296K1.4

CTD-2319I12.2

AC005498.3

AC006116.15

LINC02073

RP11-78A19.2

RP11-120M18.5

RP11-13K12.5

RP11-126O1.4

CTD-2373H9.5

RP11-108P20.2

RP11-53B2.4

CTC-325H20.4

CTD-3252C9.4

RP11-53B2.1

AC005264.2

CTD-2085J24.3

RP11-383M4.6

RP11-173A16.1

CTC-503J8.4

AC104532.4

RP11-322E11.5

LINC00907

CTD-2369P2.8

AC007787.2

RP11-400F19.18

RP11-619I22.1

CTD-2587H24.10

CTD-2553C6.1

RP11-95O2.1

RP11-813F20.2

CTD-3220F14.2

RP11-691H4.4

RP11-53B2.2

CTD-2013N17.4

AC024592.9

RP11-686D22.5

RP11-49K24.8

RP11-147L13.8

RP11-718I15.1

RP11-392O1.4

CTC-265F19.1

RUNDC3A-AS1

RP11-358B23.5

RP11-426J5.2

CTD-3193K9.3

RP11-2N1.2

AC006116.24

AC004221.2

AF038458.3

CTB-133G6.2

RP4-569M23.5

CTD-2291D10.4

CTD-2126E3.4

CTC-429P9.5

CTD-2620I22.3

CARD8-AS1

CTD-2525I3.3

AC006129.2

CTC-244M17.1

CTC-429P9.2

ZNF649-AS1

RP11-678G14.4

AC003005.2

AC006129.1

SMC5-AS1

CTC-492K19.4

FENDRR

CTD-2626G11.2

CTC-471J1.9

AC006262.6

RP11-356J5.13

CTD-2587H19.2

RP11-805I24.3

RP11-572B2.1

CTD-2616J11.10

AC005523.3

RP11-678G14.3

AC003956.1

AC115522.3

RP11-316O14.1

CTD-2622I13.3

CTD-2553L13.4

CTB-61M7.2

CTD-2538G9.5

LINC01081

AC104534.2

LINC02132

CITF22-62D4.1

AC011513.4

LA16c-OS12.2

RP4-591C20.9

RP11-256I23.1

RP11-180I4.4

LINC01711

AD000684.2

MAN1B1-AS1

CTD-3187F8.14

AC007193.10

RP11-727F15.12

L34079.3

LINC01082

LINC00528

CTD-2231E14.8

CTB-92J24.3

CTD-2527I21.7

AC007292.3

CTD-3099C6.5

CTB-92J24.2

PLA2G4C-AS1

CTC-429P9.1

CTD-3032J10.3

Z69720.2

CTB-174O21.2

CTB-147C22.8

RP11-571I18.5

CTB-83J4.1

U62631.5

AC016629.3

CTD-2331H12.7

GLTSCR2-AS1

CTD-3128G10.6

AC007228.11

CTB-180A7.3

AC007292.4

CTD-3099C6.9

CTC-490E21.10

RP11-3P17.5

AP000654.4

AP006621.9

RP1-134E15.3

RP6-91H8.3

RP11-2B6.2

RP11-932O9.9

RP5-1139B12.3

RP11-20I23.8

RP11-770J1.7

RP11-2B6.3

MKNK1-AS1

SPACA6P-AS

RP11-498E2.9

RP3-426I6.5

RP11-282O18.7

RP11-497H16.9

AF003625.3

RP3-449M8.9

RP11-218C14.8

RP11-93H12.4

RP11-326C3.13

RP11-685G9.4

RP11-416A14.1

RP4-769N13.7

SCARNA2

CTC-487M23.5

MIR222HG

KB-1958F4.1

RP11-214K3.18

RP11-742D12.2

RP11-327F22.6

RP11-118F19.1

CTC-137K3.1

RP11-568J23.6

RP11-264E20.2

LINC01480

RP11-803D5.4

RP11-373D23.3

RP11-461L13.4

SMC2-AS1

POC1B-AS1

RP11-109M17.2

RP11-92C4.6

CAHM

RP11-796E10.1

RP11-342K6.3

RP11-131L12.2

RP11-230C9.3

RP11-420L9.5

RP11-355F16.1

RP11-359E10.1

RP1-142L7.9

RP11-372K14.2

RP11-701H24.5

RP4-802A10.1

FBXW7-AS1

RP11-103J8.2

RP11-103J8.1

RP11-379K22.3

RP5-856G1.1

RP11-326C3.15

RP11-171I2.5

RP11-10C24.1

NAMA

CTD-2012J19.3

RP11-171I2.4

RP11-138A9.1

RP1-142L7.8

LINC01050

RP3-523E19.2

LINC02104

RP11-701H24.7

RP1-161P9.5

RP11-710C12.1

RP11-89C3.4

RP11-10C24.3

CTD-2256P15.5

RP11-761I4.4

CTD-2541M15.3

RP11-386M24.9

CTC-428G20.6

CTD-2235C13.3

CTC-332L22.1

LINC01215

AC005740.6

CTD-2024P10.2

RP11-532F6.5

RP11-506O24.2

RP11-338I21.1

RP11-188P20.3

RP11-222K16.1

CTD-3064M3.7

RP11-884K10.7

RP11-79O8.1

RP11-557L19.1

CTC-350I8.1

RP11-44N11.3

RP11-471M2.3

RP11-367G6.3

RP11-332J15.4

CTA-363E19.2

RP4-734G22.3

CTD-2376I4.2

RP5-1126H10.2

RP11-379F4.7

CTD-2366F13.2

RP11-195B17.1

RP11-350N15.6

RP11-481J13.1

CTD-2532N20.1

RP11-347P5.1

RP11-11N5.3

CTB-181H17.1

XXbac-BPG170G13.32

RP3-406P24.4

KB-1958F4.2

RP11-531A24.7

RP11-489E7.4

LINC01749

RP11-92K15.3

LINC00551

RP11-791G15.2

RP4-594A5.1

RP11-43F13.4

RP11-359I18.5

RP11-441F2.5

RP11-192P3.4

CTC-490G23.4

RP11-44B19.1

RP3-395M20.12

CTD-2342N23.3

RP11-532F6.3

RP1-191J18.66

RP11-182L21.5

RP5-1024N4.4

RP11-415F23.3

RP11-981G7.6

RP4-535B20.4

RP11-96C23.14

RP11-54O7.17

RP11-415F23.4

RP11-255P5.2

RP11-351J23.2

RP11-480C16.1

RP11-461M2.2

AFAP1-AS1

RP11-737O24.5

RP11-359E3.4

RP11-190C22.8

RP11-190A12.8

RP11-302M6.5

RP4-539M6.21

RP11-78A19.4

RP11-259N19.1

RP4-753F5.1

CTB-161C1.1

RP11-142A22.4

RP11-53B2.6

RP11-155D18.12

RP11-357H14.17

RP4-673M15.1

RP13-1016M1.2

RP11-335L23.5

RP11-674N23.4

RP11-286H15.1

CTA-384D8.36

XXbac-B135H6.18

RP5-1042K10.13

RP3-402G11.27

RP3-428L16.2

RP5-1102E8.3

LL22NC03-N14H11.1

RP11-121A8.1

RP11-74E22.6

RP11-134L10.1

RP11-528I4.2

KB-1125A3.11

RP11-647K16.1

RP11-46J23.1

RP11-118K6.3

GS1-279B7.2

RP11-170N16.3

DGCR9

RP11-479G22.8

RP11-77E14.2

RP5-1186P10.2

RP11-130C6.1

RP11-78I14.1

RP3-508I15.20

RP11-165A20.3

RP11-25K21.6

RP11-367H1.1

AC144652.1

RP11-9N20.3

PACERR

RP11-350J20.12

GS1-293C5.1

RP11-525A16.4

RP11-449P15.2

RP11-406H21.2

DGCR10

LINC02091

RP11-11N7.4

RP3-510H16.3

RP11-20I20.4

RP11-339B21.10

RP3-402G11.25

CTD-3148I10.15

GS1-304P7.3

AP000692.10

AC006946.16

RP11-1246C19.1

RP11-713D19.1

RP11-434P11.2

LL09NC01-251B2.3

RP11-394I13.3

RP11-338K13.1

CTA-384D8.34

CTA-268H5.14

RP6-109B7.5

RP11-445N20.3

AP000350.5

RP11-314B1.2

RP5-1136G13.2

RP11-141M3.6

RP11-61L19.2

RP5-899E9.1

CTA-268H5.12

LINC02019

RP11-466F5.10

CTA-989H11.1

RP11-376P6.3

RP11-700J17.1

SFTPD-AS1

RP11-757A13.1

RP11-702B10.2

AC006946.17

RP11-54O7.18

RP11-1399P15.1

RP11-76P2.4

RP11-686O6.2

RP11-313P22.1

RP11-126K1.9

AP000230.1

RP11-80H18.4

RP11-87C7.3

RP11-426C22.7

RP11-14C10.5

RP11-59C5.3

RP11-66N24.7

RP11-405M12.4

CH17-360D5.3

RP11-133K1.11

RP11-1046B16.3

WI2-87327B8.2

RP11-81H14.1

RP11-133K1.12

RP11-603J24.21

CTD-2536I1.3

RP11-227D13.5

RP11-378E13.4

CTD-2306A12.1

RP11-340F14.6

RP11-66H6.4

CTD-3203P2.3

RP11-14C10.3

AL133243.3

RP11-272L14.2

KB-68A7.1

RP11-115D19.3

AJ011932.1

CTC-1337H24.3

CH17-189H20.1

RP4-545L17.12

RP11-326K13.5

RP11-326N17.2

RP11-93G5.1

LA16c-380H5.6

AC144831.3

RP13-714J12.1

RP11-294N21.3

RP5-965G21.4

RP5-965G21.5

RP6-159A1.4

CTD-3035K23.7

RP11-423G4.10

RP11-12G12.7

RP11-49G2.3

RP11-104N10.2

CTD-3247H4.2

RP11-986G18.2

RP11-505K9.5

RP5-1057I20.6

RP11-687F6.5

RP11-407N8.5

CTD-2129N1.1

RP1-292L20.3

RP11-49I11.4

RP11-268P4.6

RP11-478J18.2

RP5-1009E24.9

UG0898H09

RP11-8P13.5

RP11-321F6.2

CTB-1144G6.6

LA16c-329F2.2

RP11-711K1.8

RP5-999L4.2

AC008984.2

RP11-54H7.4

RP11-946L16.2

RP11-262A16.1

RP11-266K4.14

RP11-568J23.8

RP11-126O1.6

LA16c-360A4.1

RP11-759A24.3

CTD-2026K11.4

RP11-569G13.3

RP11-322E11.2

RP11-455O6.9

RP11-353N4.6

RP11-291I6.2

RP6-65G23.5

CTB-58E17.9

RP11-257O5.4

CTB-147N14.6

C18orf65

RP11-403A3.3

PICSAR

RP11-90L1.8

RP3-453C12.14

RP11-567P19.1

RP11-397O8.7

CTD-3234P18.6

CH507-254M2.2

AC006538.8

CTD-2033D15.2

CTD-2008A1.3

RP11-481J2.3

CTD-2600O9.2

AC124789.1

CTB-91J4.1

CTB-32O4.3

RP5-881P19.7

RP11-1060J15.9

RP11-121C6.5

RP11-93B14.10

LINC00540

RP11-442J21.2

RP11-139H15.5

RP11-115D19.2

CTD-2587H24.14

RP11-425M5.7

AJ011931.1

AC007950.2

RP5-1057I20.5

RP11-3D4.4

RP1-102E24.10

RP11-123K3.9

RP11-381O6.1

CTD-2515H24.2

CTD-2105E13.16

RP11-713N11.6

RP5-875H18.9

CTD-2047H16.5

CTD-2358C21.5

RP5-965G21.6

CTD-3128G10.7

RP11-616M22.12

RP13-895J2.6

AC069363.1

RP11-327J17.9

CTD-2024F21.1

RP13-554M15.8

RP11-14C10.6

RP11-785D18.3

RP11-445F12.1

RP5-1184F4.7

RP11-843B15.4

RP11-318A15.8

CH17-408M7.1

RP11-732A21.3

CTD-3035K23.6

RP11-93B14.9

RP11-278A23.4

CTD-2095E4.5

RP11-378A12.1

RP11-9E17.1

ZNF630-AS1

RP11-73M11.3

CTC-268N12.3

AC005363.11

CTC-276P9.4

RP11-115D19.4

CTD-2588E21.1

RP11-269C23.5

RP11-756G20.1

RP11-114F3.4

RP5-851M4.1

RP11-333J10.2

RP5-965G21.3

RP11-677M24.1

RP11-900F13.2

RP11-35O15.2

CTD-2240J17.2

RP11-234K24.6

RP11-70D24.2

RP11-466M21.1

RP11-736N17.10

RP11-283G6.6

RP11-632L2.2

RP11-152F13.8

LINC02139

RP5-906C1.1

RP11-172H24.4

RP11-139E19.3

RP11-42O4.2

RP11-1123I8.1

RP11-298E9.7

RP11-709D24.8

CTD-2240J17.3

RP11-74D7.3

CTD-2382E5.6

AC068831.16

RP11-108K14.12

RP11-455P21.3

RP11-227G15.11

AC000403.4

RP11-325L12.7

RP5-890E16.5

RP11-12J10.4

RP11-567L7.3

RP11-45M22.2

CITF22-49E9.3

RP3-477J10.1

RP11-108A14.1

RP11-1250I15.2

RP11-523J2.1

RP11-401P9.7

BANCR

KB-1517D11.3

CTD-2267D19.1

EPB41L4A-AS2

AC005753.1

RP11-264M12.4

CTD-2006M22.2

RP11-173D3.4

RP11-313L6.2

RP11-39H3.2

RP11-83B20.9

RP11-756P10.6

RP5-1031D4.3

RP11-797A18.5

RP3-424M6.4

Z69720.3

RP11-1430O6.1

CTA-243E7.2

C18orf15

RP11-391L3.4

RP11-250B2.4

RP11-1193F23.1

RP11-144I2.1

RP11-212I21.3

CTD-2373N4.3

CTB-22K14.1

AC007787.3

RP11-287D1.2

RP11-83B20.3

LINC01727

RP11-667F14.1

RP11-426C22.1

CTC-490E21.11

CTD-2562J17.6

RP11-517I3.2

RP11-83B20.5

RP3-442L6.4

RP11-489P6.1

RP11-894J14.2

RP11-547D13.1

RP11-374F3.5

CTD-3126B10.2

RP4-737E23.5

RP11-107E5.3

RP11-43D4.3

RP11-711M9.2

RP11-455O6.5

RP11-803D5.1

PWAR5

CTD-2231E14.2

CTA-113A6.1

RP11-175K6.2

RP11-632P5.1

RP5-991G20.6

RP13-16H11.8

RP11-2K6.1

RP11-120K19.3

RP11-212E4.1

RP11-536C12.1

LA16c-380A1.2

RP11-756J15.2

RP11-861A13.3

CH17-264L24.1

RP11-248B24.1

RP11-736E3.3

RP11-274A11.3

PRAL

RP11-16P20.4

RP11-573M3.6

RP11-170K4.2

RP3-331H24.7

RP11-329N15.3

RP4-738P15.6

RP11-83B20.6

RP11-667K14.9

RP11-566E18.1

RP11-864N7.4

RP11-776B3.1

AGPAT4-IT1

RP11-36D19.9

RP11-360N9.3

RP1-247F3.1

RP11-177C12.5

AF127577.13

RP11-95G6.1

AC130469.1

CTD-2353F22.2

CTD-2542C24.5

RP11-133K1.9

RP11-1102P22.3

RP11-394B2.5

RP11-1102P22.1

RP11-335K5.3

RP11-671J11.5

RP11-4B16.1

RP11-85K15.3

RP11-715H19.2

RP3-425C14.4

RP11-826F13.1

RP11-215P8.2

KLHL30-AS1

CTD-2576D5.1

RP11-21K12.2

RP11-575M22.1

AC083843.4

RP11-20I23.2

AC011239.2

RP11-398J13.1

RP11-445N20.2

AC006486.10

CTC-444N24.7

CTA-113A6.2

RP11-20I23.5

CTD-2357A8.2

RP11-56D16.8

RP11-76I7.1

AC005786.6

RP11-854K16.4

RP11-65L3.3

AC005514.2

RP11-637C24.5

RP11-348J12.5

RP11-182N22.9

RP1-102H19.7

AC005796.2

CTD-2527I21.5

RP11-1102P22.2

RP11-254F19.4

RP11-426J5.1

RP11-196B3.1

CTD-2081C10.5

RP11-759A24.1

RP11-389G6.4

RP11-849N15.3

CTC-304I17.6

RP11-1086I4.2

RP11-454H19.2

CTB-147C22.6

RP11-274A11.5

MAPT-IT1

RP11-485M7.2

RP11-71L14.3

CTD-2028E8.2

AP004782.1

AC006128.2

RP11-288A5.2

LINC02033

RP11-319G9.4

RP11-685B24.1

RP11-44F14.9

CTD-3222D19.9

RP11-309J13.1

RP11-642A1.2

RP11-278H7.5

RP11-60A14.1

RP11-466A19.7

CTA-212A2.1

RP3-495K2.4

CTD-2545G14.4

RP11-394B2.7

CTC-457L16.1

RP4-742C19.13

RP11-775C24.4

RP11-274A11.4

RP11-618M23.5

RP11-474C8.7

RP11-1072C15.6

RP11-513O13.1

CTD-2315M5.2

RP11-483E17.1

RP11-624A21.1

AP001962.3

RP11-360N9.2

LA16c-312E8.4

RP4-671O14.5

RP1-153P14.7

CH17-76K2.6

RP11-720L3.1

RP11-69J7.1

RP11-299G20.5

CTD-2553L13.5

RP11-196B3.2

CTC-436P18.4

RP11-76C10.3

RP11-330H6.6

AP001350.4

AC006129.3

RP11-118E18.1

RP11-832A4.7

RP11-10J21.2

RP11-540O11.8

DNM1P41

RP11-296E3.2

RP11-71H17.8

RP1-197B17.7

CTD-2001J20.1

RP11-91H12.1

RP11-907D1.3

CTD-2525P14.5

RP11-326A13.2

RP11-361H10.5

TBC1D22A-AS1

CMB9-52H4.1

RP11-83B20.7

PLAC4

RP11-133M8.3

AC093642.1

RP11-87G24.2

RP1-47M23.3

RP11-1228E12.2

RP11-463O12.5

RP11-597D13.2

RP11-331F4.5

RP11-876N24.3

RP11-314N13.9

RP11-354E23.3

RP11-867G2.4

RP11-1090M7.3

RP11-447D11.3

RP11-84A12.1

CH507-42P11.6

AD000864.6

RP11-504I13.2

RP11-49K24.3

UCKL1-AS1

RP11-638F5.2

RP11-752L20.3

RP11-174G17.3

RP11-67L14.1

RP11-470C13.4

RP11-321N4.4

RP11-574K11.26

RP5-1037N22.2

RP11-466A19.4

RP11-81A22.4

RP5-1007F24.1

RP11-560F18.1

AF131216.7

RP11-1228E12.1

RP13-554M15.7

RP11-759A24.2

RP11-358B23.6

CTD-2536I1.2

RP11-60L3.3

RP11-1110F20.1

AC074183.3

RP11-667K14.14

RP11-15F12.6

RP11-736K20.4

AC009166.7

AC000123.2

RP11-552M6.1

RP11-83B20.4

RP11-119F19.5

CTD-2256P15.3

RP11-573M3.2

RP4-695O20.1

RP5-1153D9.5

CTC-503J8.8

RP11-104F15.9

RP11-20I23.10

RP13-516M14.8

RP11-572N21.1

RP11-178L8.6

RP11-446E24.3

RP4-555D20.1

RP4-568B10.1

RP11-83B20.8

RP11-216B9.8

SALRNA2

LINC01348

SCAANT1

CH17-172B3.1

SH3PXD2A-AS1

LINC01943

LINC01232

RP13-465B17.5

CH507-513H4.6

ST3GAL4-AS1

FOXCUT

LINC00628

CTBP1-AS

CCAT2

RP13-349O20.2

LINC01395

RP11-640L9.2

TRG-AS1

ZNF638-IT1

LINC00950

RP11-900F13.3

ABALON

RP11-237N19.3

DGCR12

DBET

RP11-817J15.3

CH17-118O6.6

C2-AS1

LINC01394

RP13-465B17.4

LINC00891

SPRY4-IT1

CTD-2510F5.4
