# Supplementary material for: Immune-Related Long Non-coding RNA Signature and Clinical Nomogram to Evaluate Survival of Patients Suffering Esophageal Squamous Cell Carcinoma
Source: Front Cell Dev Biol. 2021 Mar 4;9:641960. doi: 10.3389/fcell.2021.641960 (PMC7969885; doi:10.3389/fcell.2021.641960)
Supplement: Supplementary Table 2 — LncRNAs obtained by GSE53624 annotation. [file Table_2.docx]

id	GSM1297309	GSM1297307	GSM1297299	GSM1297297	GSM1297295	GSM1297239	GSM1297293	GSM1297139	GSM1297137	GSM1297291	GSM1297289	GSM1297287	GSM1297285	GSM1297283	GSM1297281	GSM1297305	GSM1297135	GSM1297279	GSM1297277	GSM1297303	GSM1297275	GSM1297273	GSM1297271	GSM1297269	GSM1297267	GSM1297265	GSM1297301	GSM1297263	GSM1297261	GSM1297259	GSM1297133	GSM1297237	GSM1297235	GSM1297131	GSM1297129	GSM1297127	GSM1297125	GSM1297123	GSM1297121	GSM1297119	GSM1297257	GSM1297117	GSM1297115	GSM1297113	GSM1297111	GSM1297109	GSM1297255	GSM1297107	GSM1297233	GSM1297105	GSM1297103	GSM1297101	GSM1297099	GSM1297097	GSM1297095	GSM1297253	GSM1297093	GSM1297091	GSM1297089	GSM1297087	GSM1297085	GSM1297251	GSM1297211	GSM1297209	GSM1297207	GSM1297205	GSM1297203	GSM1297201	GSM1297199	GSM1297197	GSM1297219	GSM1297217	GSM1297249	GSM1297215	GSM1297213	GSM1297083	GSM1297081	GSM1297079	GSM1297077	GSM1297195	GSM1297193	GSM1297191	GSM1297189	GSM1297187	GSM1297185	GSM1297231	GSM1297183	GSM1297181	GSM1297179	GSM1297177	GSM1297175	GSM1297173	GSM1297171	GSM1297169	GSM1297167	GSM1297165	GSM1297229	GSM1297159	GSM1297163	GSM1297157	GSM1297161	GSM1297155	GSM1297153	GSM1297151	GSM1297149	GSM1297247	GSM1297227	GSM1297147	GSM1297145	GSM1297245	GSM1297143	GSM1297225	GSM1297223	GSM1297243	GSM1297141	GSM1297313	GSM1297221	GSM1297311	GSM1297241	GSM1297308	GSM1297306	GSM1297298	GSM1297296	GSM1297294	GSM1297238	GSM1297292	GSM1297138	GSM1297136	GSM1297290	GSM1297288	GSM1297286	GSM1297284	GSM1297282	GSM1297280	GSM1297304	GSM1297134	GSM1297278	GSM1297276	GSM1297302	GSM1297274	GSM1297272	GSM1297270	GSM1297268	GSM1297266	GSM1297264	GSM1297300	GSM1297262	GSM1297260	GSM1297258	GSM1297132	GSM1297236	GSM1297234	GSM1297130	GSM1297128	GSM1297126	GSM1297124	GSM1297122	GSM1297120	GSM1297118	GSM1297256	GSM1297116	GSM1297114	GSM1297112	GSM1297110	GSM1297108	GSM1297254	GSM1297106	GSM1297232	GSM1297104	GSM1297102	GSM1297100	GSM1297098	GSM1297096	GSM1297094	GSM1297252	GSM1297092	GSM1297090	GSM1297088	GSM1297086	GSM1297084	GSM1297250	GSM1297210	GSM1297208	GSM1297206	GSM1297204	GSM1297202	GSM1297200	GSM1297198	GSM1297196	GSM1297218	GSM1297216	GSM1297248	GSM1297214	GSM1297212	GSM1297082	GSM1297080	GSM1297078	GSM1297076	GSM1297194	GSM1297192	GSM1297190	GSM1297188	GSM1297186	GSM1297184	GSM1297230	GSM1297182	GSM1297180	GSM1297178	GSM1297176	GSM1297174	GSM1297172	GSM1297170	GSM1297168	GSM1297166	GSM1297164	GSM1297228	GSM1297158	GSM1297162	GSM1297156	GSM1297160	GSM1297154	GSM1297152	GSM1297150	GSM1297148	GSM1297246	GSM1297226	GSM1297146	GSM1297144	GSM1297244	GSM1297142	GSM1297224	GSM1297222	GSM1297242	GSM1297140	GSM1297312	GSM1297220	GSM1297310	GSM1297240

AC093519.2	9.004857	8.583297	8.672031	8.606200	9.087336	8.714536	8.950273	8.592859	9.812289	8.825863	8.437250	8.935159	7.626880	8.216204	8.338549	8.436002	9.372889	8.857898	8.658511	8.762566	8.665154	8.184935	9.415515	8.857724	8.969110	8.424675	8.997674	8.794756	9.183297	8.687887	8.157192	8.684477	8.934257	8.784314	8.695889	8.737644	8.575356	8.011709	8.071679	8.933305	8.936392	8.704737	8.956821	9.450783	8.944434	8.677896	8.536970	8.621747	8.962525	8.667580	8.652644	8.995493	9.677053	9.037384	8.649201	8.620648	8.849730	9.204130	8.418559	8.508125	8.548924	8.918330	9.462264	8.345381	8.695584	8.785633	7.972576	7.995191	8.047520	8.982606	8.565719	8.618776	9.007782	8.627776	8.616784	9.031417	8.470262	8.590668	8.327291	7.985306	8.990766	9.192582	8.429598	8.628819	8.672619	8.755780	8.014777	8.561770	9.179536	8.693399	8.547074	8.831633	8.682202	8.908475	8.908163	9.354532	9.813229	8.819941	8.346622	8.744959	8.729147	8.580269	8.249596	9.522089	8.297298	9.098736	8.426765	8.294115	9.272265	9.070041	9.163075	9.235036	8.102799	8.452314	8.824787	10.303096	8.639114	8.632197	8.874344	8.817289	8.746513	8.450156	8.911908	9.645034	8.296244	8.126279	8.721472	9.453237	8.660064	8.660551	8.760599	8.104486	8.564352	8.234506	8.567308	9.450135	8.196392	8.888686	8.962835	8.291199	8.994252	9.501800	8.542730	9.100998	8.717816	8.970198	8.941567	8.976368	8.706443	8.743011	8.696520	8.721334	8.151277	8.532727	8.530929	8.680808	9.021885	8.757154	9.210536	8.519151	9.032649	8.534396	9.011761	8.286088	7.971157	8.272399	8.431794	9.108933	8.706305	8.801886	8.681103	8.920759	8.641739	8.420110	8.633073	9.709524	8.561316	8.145020	7.900849	8.157804	9.071428	8.411859	8.150964	8.728390	8.372984	8.223289	8.017553	7.925527	8.801264	8.480461	8.421352	9.574876	8.363776	8.888062	8.707044	8.376752	8.527834	8.545228	8.108784	8.373727	8.111607	8.500912	8.520379	8.471529	8.871211	8.310979	8.565560	8.945222	8.530152	8.713136	8.811877	8.736112	8.880498	8.964497	9.019430	10.288341	9.087772	8.371594	9.300335	8.712812	9.487522	8.688190	9.493614	8.728680	9.022179	7.609920	8.840427	9.710638	8.887600	9.052657	9.251933	9.333386	8.522603	8.868386	8.534075	8.247398	8.428823	8.839351

AL138830.2	3.380170	3.224468	3.064388	3.283804	2.921191	3.790786	3.270447	3.169028	3.801192	3.377742	3.534249	3.691579	3.233362	3.775752	3.206777	3.275122	3.479830	3.107369	6.008419	3.522236	4.350022	3.068050	6.736595	3.573251	3.035084	2.901241	3.202543	3.431869	3.148992	3.054254	3.350047	3.861275	4.353548	3.742381	3.520242	4.025170	3.653400	3.318310	3.230192	3.002602	3.722702	3.472931	3.739547	3.281645	4.442195	5.276568	4.355051	3.758992	4.280614	4.044548	3.977212	4.663487	4.423102	3.873314	3.787788	3.080392	3.878287	2.973996	3.550424	3.102215	3.246386	3.050905	3.536093	3.061802	3.218550	3.120117	4.070467	3.176610	2.939052	3.175876	2.993540	3.099736	3.310230	3.225297	3.069362	3.268403	3.140813	2.973074	3.315690	3.409194	3.221793	3.891777	3.153544	3.466290	3.272701	4.644274	3.086732	3.180533	2.923669	3.275282	3.080594	3.193243	2.966116	3.101390	2.941574	3.028179	3.805149	3.107248	2.946960	2.960684	3.092072	3.222403	3.022786	2.917889	3.425082	3.455153	3.251420	3.289685	3.393483	3.485680	3.304598	3.539784	3.037510	3.278205	3.174211	3.186882	3.841099	3.064806	3.159296	3.099402	3.327268	3.073107	3.336436	2.953835	4.185785	2.958816	3.142180	3.406231	3.164807	3.373901	3.176851	2.998519	3.182826	4.682027	3.097255	3.477651	2.971440	4.787444	4.070266	3.749198	3.292351	5.753206	2.936817	2.914380	3.480010	3.212131	3.040379	2.968734	3.487711	3.132978	3.310854	3.108739	4.274425	3.424199	3.739136	3.726339	3.402821	3.177091	2.918581	3.416712	3.203743	3.527379	3.317763	4.212293	4.871491	4.253660	4.940144	4.855836	4.147890	5.322671	4.582230	4.051954	3.901680	3.330982	3.018930	3.475076	2.929431	3.341666	4.554107	3.866820	3.202402	3.531510	3.292351	3.276820	3.638875	4.406703	3.859612	2.957162	3.211164	2.997603	3.164434	3.405245	3.023096	3.361439	3.260153	3.211448	3.101606	3.239599	3.368755	3.461637	4.000622	3.161604	3.440673	3.417203	3.737677	3.320580	3.329443	2.877511	3.188761	3.122165	2.880130	3.442401	3.151102	3.120218	3.112510	3.048905	3.016359	3.088896	3.183307	3.139813	3.039322	2.995591	3.008249	3.758336	3.229455	3.276674	3.280717	3.404378	3.165069	3.157660	3.358975	3.012499	3.217589	3.000457	3.196648	4.517005	3.073107	3.262043

HOXB-AS3	10.102793	7.807549	7.706238	9.276233	6.690947	7.631919	8.068471	7.631140	6.944562	7.274232	6.887904	7.226540	7.065228	6.002186	8.890904	7.610841	6.513566	7.786365	7.105179	8.401432	6.534041	7.086749	12.987390	6.926667	7.483367	7.385010	8.247677	8.604216	7.217178	7.200555	7.370309	10.542394	8.045755	6.774295	7.679960	7.684733	8.654920	8.002620	8.012294	7.546612	8.171288	9.228524	7.919331	9.556934	9.101326	9.552380	7.410333	9.230442	7.463106	8.633972	10.899971	8.848337	9.569642	8.607415	9.104428	6.744976	9.421747	8.105890	8.294418	8.926440	8.668025	7.917375	12.144699	7.480750	8.588673	8.497631	8.868700	7.043666	7.500547	8.612010	8.733797	7.810660	7.534392	8.753789	9.261051	7.533908	7.545344	8.775967	8.960071	9.127188	9.314441	8.425112	9.593851	8.839043	7.922409	8.833199	8.726505	8.311618	8.785471	9.334439	9.011895	8.505652	9.097041	7.613722	9.667624	8.319126	7.715071	6.345747	6.430725	6.368693	6.657433	8.266186	6.826564	6.905419	7.526204	8.235415	9.485482	6.849484	7.715979	7.104243	8.701371	7.421823	6.598280	7.691812	6.096514	7.224175	10.576472	7.415219	6.813024	7.371725	8.282999	9.973251	8.024983	7.862315	7.734091	7.629233	13.090908	9.669719	8.068615	8.557910	7.414306	9.651648	7.252455	6.930656	7.890305	9.586230	8.144547	7.181722	6.668503	7.119635	8.388057	9.289725	9.700644	9.268311	8.606356	8.240685	5.923488	7.811267	8.978474	9.851210	7.853910	10.244721	7.917823	7.291926	8.473114	9.589818	7.359642	6.457215	9.920116	6.987484	6.876248	7.291287	8.068315	10.590376	7.806972	7.283052	7.892947	10.074846	9.379453	8.833467	8.898403	8.732389	9.274416	10.012286	8.593623	8.941854	8.584250	6.724487	9.808233	8.499478	9.411560	8.920591	9.241363	8.142995	8.866879	8.034627	7.761133	7.577148	7.573412	8.522603	8.056204	7.735797	9.055670	8.966033	8.090942	9.372113	10.865897	9.515307	10.670427	9.036048	9.300335	9.752062	10.095036	9.472625	8.034780	10.081326	8.362863	9.014249	10.558709	7.180642	9.087175	10.559179	8.798699	10.106724	11.035706	9.183929	12.593626	9.356220	7.455252	8.208762	7.696073	8.621124	8.031179	9.519632	7.153231	9.279824	6.062618	6.862821	7.232277	9.750053	8.694514	6.887904	6.870984	9.004108	7.270004	8.286241	8.094336	6.805835

BVES-AS1	6.669775	3.674319	5.118031	3.825093	3.080594	5.187008	6.639977	3.591899	3.498362	5.070805	4.465860	4.475809	4.023916	4.954166	4.226825	5.450480	4.717717	5.323834	6.851706	4.948949	5.328715	3.030591	3.184101	3.270231	5.268266	4.806374	5.546374	3.957915	5.745927	4.894172	3.725135	6.142913	5.198116	5.163477	5.989903	6.554786	6.449178	3.935805	4.756117	4.200518	5.812964	5.113550	4.714555	4.843175	6.742879	5.521370	5.817089	5.749191	5.796567	6.143754	5.255748	7.090251	4.975882	6.622253	6.498895	5.172120	7.290335	4.564688	4.461559	5.760681	5.390271	4.758070	6.258264	4.110948	4.177253	4.869995	3.389835	4.692658	3.913757	3.043133	6.038597	6.248293	4.729367	6.184157	5.448989	5.556755	6.393213	5.036741	4.167541	4.464593	4.084550	6.317064	6.847580	6.312967	7.105179	6.276841	5.780149	5.695665	4.521491	3.846089	4.964988	4.122955	4.360482	6.546478	4.295457	5.839873	6.000597	5.951091	4.986843	5.151558	4.297673	5.486905	4.949181	4.665614	4.800293	5.262870	3.456247	3.621076	3.326468	5.730493	3.806591	3.521478	3.274958	4.217947	3.494089	3.458761	3.838235	6.070817	6.419483	3.976140	3.961168	4.034530	3.801618	3.067275	6.704456	3.749198	4.240573	4.883487	3.971363	4.051282	3.449181	3.605963	6.106391	3.790356	4.791432	4.701728	3.899988	4.127649	7.234377	4.615553	3.349197	5.287363	3.074560	5.125133	6.090105	3.470157	3.066192	5.195106	4.832709	5.765200	4.622687	4.684589	5.802945	6.638539	5.174952	4.187529	3.911207	3.571510	3.328333	5.079007	4.152983	4.455648	5.414924	4.378458	5.161854	4.177655	6.081607	4.169878	6.951067	4.465424	4.821475	4.357398	8.400492	5.172120	4.551105	5.206236	4.485731	4.412646	4.313602	4.023916	6.016047	4.849503	4.144522	4.061501	3.856793	3.407494	3.815250	3.853571	5.228700	4.044097	4.871252	4.039083	7.300797	4.045411	3.392300	5.044535	3.721463	3.779151	4.346108	3.866193	4.868318	7.616037	3.755476	6.904609	4.990613	5.442939	3.936458	4.168613	3.726139	3.540932	5.049796	4.170075	5.463844	4.052367	4.781151	5.609543	3.063094	4.307910	3.906634	4.074510	3.704057	3.186761	3.025146	5.187816	4.073634	4.299617	3.658061	3.725577	5.348134	3.554282	4.251497	3.010401	3.799232	3.315404	5.390865	3.639290	5.861943	6.845709

AC063919.1	5.322096	3.789732	3.979150	4.250416	4.839385	5.874946	4.039083	4.399395	5.156018	5.166080	5.206028	5.602065	4.333986	4.815165	4.826757	4.130430	4.627574	5.217371	6.208993	4.621637	4.673384	5.793128	6.306442	4.716660	5.103393	4.777193	5.034193	5.757389	4.882858	4.703230	4.593852	4.272646	6.079900	4.463060	4.189728	4.651356	4.525013	4.268466	4.823549	4.561205	4.848888	4.609716	4.775009	4.609293	4.213858	4.879846	5.462754	5.593090	5.699132	5.393349	5.176541	4.413541	5.529728	4.666013	5.509932	5.185584	4.409815	4.996722	4.535677	4.914337	4.593634	4.797674	4.873219	4.264426	4.547921	4.722391	4.789935	3.989853	4.114367	4.513567	4.369566	4.335916	5.404435	3.804516	4.331236	3.761533	3.559545	4.230677	3.859999	4.539977	4.472756	4.599394	3.967902	5.258688	4.584786	5.353445	3.605218	4.029509	3.603661	4.562301	3.920614	3.920614	3.764496	4.495760	4.519767	4.093091	4.336340	5.173917	6.001442	4.244526	5.596256	3.611605	4.553015	4.640721	5.633578	5.184611	3.818855	4.679009	5.695665	4.935777	4.413326	5.127555	3.923504	4.841476	4.905251	6.463746	4.104098	4.838497	4.384959	6.482074	3.768830	4.187529	4.648115	5.228917	6.319613	5.782005	4.608664	4.565960	5.467532	5.227712	5.549767	5.767194	4.533520	4.599394	4.749126	4.767009	5.349462	6.189239	4.094115	4.864751	5.539006	7.018269	5.657200	5.383921	4.668615	5.808505	5.850774	5.832460	4.706614	4.404426	4.995885	5.335551	5.191894	4.289621	4.220367	4.986230	4.021312	5.320912	4.636303	5.542788	2.882424	5.277165	4.106504	3.878499	4.075160	5.852553	5.625771	4.850577	4.495551	4.450509	3.660815	3.797986	4.160569	4.190383	4.459856	5.226492	3.900421	3.360651	3.311947	2.980425	4.737391	3.312876	3.589398	4.148977	4.166893	3.047512	3.995497	4.144295	4.366579	3.614384	4.740639	6.306611	4.133608	4.403954	3.521143	4.388910	3.882986	3.828858	3.479650	4.805579	4.277970	3.934099	4.633069	4.772813	6.013683	3.730244	4.116788	3.385511	3.599132	4.169878	4.687185	4.461559	5.457696	4.785984	5.465392	5.642899	6.559099	5.641390	3.860875	6.035454	4.042312	5.284565	3.746085	4.649826	5.403504	5.281755	4.477408	4.973155	5.924523	4.691774	5.185394	3.626618	5.622975	5.352068	5.820451	5.043056	5.263095	4.570913

AC068587.4	6.031825	4.375003	5.947925	6.283289	6.869820	7.403964	6.356611	5.674760	6.165458	6.283964	6.334681	5.633010	5.606848	5.164675	6.482234	6.289442	5.289796	6.308756	7.681937	6.298576	5.222725	7.078944	5.595302	5.927411	5.889018	5.456085	6.834227	4.781346	6.695712	6.007549	6.405727	5.559465	4.436050	6.437280	5.404062	5.029321	5.214240	4.144522	5.028096	4.115070	6.290462	4.445874	5.945135	6.022897	5.804407	5.661421	6.849324	3.167789	6.469852	5.477861	5.589862	4.221462	5.059824	4.822917	6.367040	5.724374	5.457267	4.674939	6.599750	5.474461	4.287894	6.426308	5.252550	5.646519	3.922669	5.860683	4.136501	6.051126	4.446094	4.447109	5.316256	5.141933	6.625003	4.808066	5.066538	5.115798	4.492677	5.047697	4.849095	4.894383	4.236363	5.621097	5.184812	5.183414	5.321096	6.511437	4.891263	3.510886	3.722702	3.868591	4.411319	3.916834	5.066954	5.316861	6.323720	6.439708	5.662352	7.157778	7.074654	5.618176	6.549386	5.560405	5.392589	5.761020	6.107658	6.104775	6.664294	6.598280	6.777177	6.615109	6.188416	6.453304	6.474078	5.985191	7.351763	7.929878	6.374667	6.499570	5.907032	6.794137	4.146429	5.086779	4.131875	7.234690	8.927250	5.106448	7.461823	5.550148	5.715491	5.362636	6.547569	6.915418	6.370564	5.129196	5.391260	3.782082	6.343881	7.664983	6.786131	5.440433	7.131491	5.889394	10.290659	6.009832	4.161376	6.021366	4.979752	7.953560	4.622273	5.506607	5.871338	5.437645	5.566361	7.131491	6.576510	5.247625	3.501981	3.138221	6.786131	6.117144	4.028832	5.159264	3.551628	4.803917	5.030756	7.009806	3.170245	5.367584	4.651356	4.328317	4.104982	4.731438	4.456699	6.724304	5.482575	4.053199	4.926168	5.203474	4.394772	4.226387	6.248637	3.604453	4.379297	3.847173	3.684027	2.900097	4.194119	3.804710	2.887929	4.265499	4.522996	8.107120	4.061734	4.463741	4.568525	3.804926	3.246230	4.087235	3.918319	4.412855	4.845532	3.664851	5.772748	3.562491	5.847149	3.285623	4.792105	3.436438	4.016060	6.809699	4.092057	3.998094	3.918899	5.377881	4.940527	5.993385	6.023770	4.532248	6.457389	6.007165	6.051126	5.595889	5.368578	5.509568	6.544343	3.205070	5.625588	4.557891	4.841900	5.963234	4.961235	5.928833	6.239749	7.155289	7.475689	5.007858	5.013223	3.831373

BAALC-AS1	5.959156	4.060412	4.910284	4.871491	5.024248	5.976379	5.333243	4.972119	3.573059	5.644627	5.217371	4.879417	5.433353	4.726601	4.522344	5.053435	4.206388	5.372833	5.949351	4.665404	5.488756	4.632197	5.129592	4.725997	4.814772	4.712842	5.096222	4.445656	5.142569	5.195106	4.830820	4.909265	5.513706	6.010871	6.472094	4.507907	4.663683	4.910284	4.280176	4.461136	4.589492	3.182446	4.665614	5.501007	6.564495	4.576446	5.282363	5.160090	6.562847	5.659046	5.601521	6.535976	5.579830	5.589095	6.848712	4.986634	4.311473	5.688415	5.687731	5.053637	4.120971	4.564275	4.746389	4.247330	4.610996	3.951827	4.775737	4.534548	3.967228	3.452598	5.109773	4.887311	4.604402	6.155690	5.668327	5.853616	4.925961	5.384282	4.907752	4.758520	5.207233	6.781998	5.665514	6.586695	5.052996	6.139271	5.400880	6.363037	3.416052	4.747288	6.075192	3.698588	6.012483	5.046074	6.210495	5.005994	5.963956	6.008585	5.101965	5.096409	4.444552	4.366344	4.609293	5.102368	4.150618	4.403324	4.190163	4.830608	4.424630	4.858971	3.590513	4.106065	4.326137	4.628254	4.178097	4.620829	5.025838	5.503556	5.555072	8.340714	4.334841	5.651640	6.374169	6.285141	5.946886	6.348452	7.451502	5.679386	6.325254	6.707842	5.700628	5.117409	4.197310	3.919566	6.159810	6.639174	5.788863	4.882433	4.535677	5.449166	5.880506	5.841340	6.193727	8.019965	6.084054	4.387604	4.220124	5.875675	4.236160	4.403954	5.292166	6.943596	6.784344	7.821500	6.785480	5.426024	5.341041	4.576446	6.016224	5.765045	3.065217	5.761020	7.723620	8.235415	6.484623	5.796922	5.201486	8.524014	6.130528	6.501046	6.745598	7.313043	6.996619	9.369995	5.840048	5.673843	7.721304	5.765566	5.813341	4.217085	5.884422	4.607995	4.565960	5.525162	5.164280	6.327236	4.771384	4.479330	4.263344	5.654037	6.214589	5.996901	7.356999	8.520379	6.031996	5.962534	6.272706	5.962707	4.899606	7.402070	8.529222	5.798581	7.202701	6.938279	7.910028	6.685825	7.679371	6.114042	4.925093	6.712113	4.258087	7.519870	6.086241	6.676729	4.765269	6.742265	6.855573	5.752653	6.973051	5.234826	5.600779	5.701366	6.646729	6.076422	5.089194	4.749126	6.408860	5.484805	6.919882	4.210561	5.377881	5.401078	5.250184	4.808479	4.456478	5.200277	8.859114	6.738926

MIR3659HG	5.283942	4.413541	4.657491	4.959144	5.739006	5.556382	5.200690	5.243412	5.517407	5.744092	5.429568	4.913698	3.846944	5.098644	4.979544	5.377137	5.194061	4.678808	6.199294	5.617409	5.723267	5.406712	6.173696	4.941966	5.558298	5.194696	4.373932	3.998940	5.129592	4.414432	5.403869	4.582230	5.260658	4.815819	4.271174	4.188438	4.596826	4.242269	5.169098	4.485251	6.720804	4.888354	3.623653	3.916834	3.868138	6.986852	5.181177	5.249386	4.827193	4.754919	4.891867	5.166466	4.421274	5.164280	3.998329	5.436481	5.453218	3.456601	4.193224	5.318061	5.303048	5.007256	3.704471	5.733560	4.673384	5.424652	4.741069	5.256331	4.953957	5.373984	5.093948	4.299398	6.141889	5.330083	5.189746	4.785361	5.190523	5.298453	5.474055	4.740850	5.157902	5.320912	4.905873	5.313196	4.630030	4.895390	4.690299	5.339128	5.536657	6.338043	5.512606	5.650491	4.198394	4.584149	4.396359	5.280737	5.040093	4.575001	5.375957	4.786821	3.345748	3.916184	4.423335	4.493308	5.107256	5.775641	6.183618	5.635443	4.568951	5.771240	5.537228	5.392783	5.613483	5.865214	5.115151	3.114384	4.710518	5.453813	5.543524	6.844547	4.597046	4.257871	5.322870	3.830142	5.583220	4.431158	4.936406	5.312841	5.054726	4.247095	4.513162	3.420660	3.467841	3.830779	4.198394	4.981328	3.408550	4.159936	5.002715	4.815587	4.987675	5.626721	4.300892	3.650804	3.992680	3.398506	3.594018	3.998522	4.963547	4.538643	5.599491	3.698380	3.489197	3.933476	3.870092	4.634998	4.116561	5.063441	3.399682	6.402270	3.575730	3.673475	4.842115	4.230480	6.732226	5.661798	4.147052	4.710318	4.516358	4.843390	5.324027	4.123822	4.497929	3.525165	4.762713	3.797572	3.255912	4.015184	4.903797	5.293129	5.083448	5.092048	5.902912	4.924478	5.166273	6.021895	4.380693	5.086779	4.719803	4.158412	3.971168	5.561471	4.938486	3.528850	4.950633	4.858755	5.042632	5.378461	3.598578	5.282574	5.022592	5.756267	3.731296	4.375003	4.470128	4.220367	4.229251	4.858553	5.034634	5.507986	5.580774	4.303316	3.949435	4.346971	4.720656	3.577550	3.042415	5.244823	3.537245	2.995127	4.466525	4.067954	2.970823	4.770546	5.152556	5.610093	5.123104	4.373025	5.451062	4.175536	4.029752	3.828858	5.200081	3.915108	3.853359	5.406483	6.818791	5.584774

EXOC3-AS1	9.836099	10.255734	9.564664	10.023218	9.746158	9.852612	9.962417	10.271935	9.546827	9.883634	9.478926	10.022295	9.751741	9.811513	10.095931	9.533374	9.610124	9.839846	10.241662	10.101418	9.766931	10.564425	10.604741	9.836416	9.874961	10.112153	10.421948	10.121857	10.005677	9.744106	9.467350	10.119905	10.110195	10.571684	10.122304	10.096988	10.016962	10.412994	10.433959	10.192202	9.921845	10.366828	10.061641	9.726547	10.452666	10.532370	10.067808	10.347504	10.057867	10.361744	9.966801	9.926202	10.016654	10.409829	10.232060	10.273782	10.344336	10.925632	10.652361	10.509917	10.714756	9.736704	10.253926	10.337200	10.673148	10.289908	10.698977	10.131533	10.396283	10.190552	10.099349	10.429431	9.855565	10.261469	10.192512	10.356294	10.191151	10.130935	10.009895	9.449040	10.171391	9.834281	10.124584	10.161530	9.867411	9.865926	10.012765	10.019714	10.137446	10.869610	10.478878	10.622189	9.897941	9.850250	10.087772	10.132992	9.979195	9.181882	9.473227	9.591343	9.344530	9.797503	9.767395	10.210307	10.626847	9.817990	9.782680	10.332208	9.996607	9.975574	10.719585	10.119905	9.762791	10.172605	9.572138	11.258115	10.281537	9.630443	9.536731	10.323526	10.388365	9.901638	10.116055	10.340199	10.151576	10.269126	10.265002	10.765971	11.149137	10.041611	10.961076	10.067207	10.340663	10.578752	9.930320	10.485950	10.591883	9.698789	9.997359	10.562355	10.767880	11.995822	10.563692	10.861749	11.480091	10.981739	11.453855	12.099218	10.325054	9.942122	10.255889	10.667256	9.998570	11.039598	10.347657	10.280486	10.565611	11.225321	10.748747	10.717011	11.341173	9.438350	10.442759	10.669264	11.452083	10.296641	11.203158	10.489519	10.327480	10.012445	10.914011	9.908194	10.729132	10.152488	10.316937	10.403192	10.939911	11.082183	10.369246	11.921741	10.513902	11.521612	10.964890	12.447863	10.674348	11.001214	11.285485	11.333467	10.775064	10.451803	10.324612	10.516102	10.490556	9.898981	10.532043	11.855782	11.129584	10.064516	9.197498	10.749026	11.045017	11.209109	9.704886	10.172908	11.200158	10.669403	11.006688	10.397186	10.764929	10.497960	11.118854	11.070164	11.068537	10.325194	10.579659	10.612584	9.785196	9.732123	10.752942	10.289000	10.732945	11.870697	10.308784	13.040804	10.350071	9.829982	10.319814	10.824286	10.113907	10.718063	10.312288	10.589210	10.478435	10.771811	11.211573	10.985097	10.972659	11.614961

LINC02741	10.184062	9.765569	9.020771	10.040378	10.076981	11.474103	10.273200	10.724471	10.837385	10.544617	11.034048	10.684205	9.362145	9.310956	9.303094	11.361505	10.745016	9.531817	10.940064	10.587598	10.444108	10.653826	10.550859	10.043594	9.392669	10.222882	12.601240	10.783006	11.232423	11.069879	10.504236	8.995637	9.008394	10.005361	9.294904	9.446373	9.186268	9.373508	9.293154	10.141980	11.708199	9.260302	9.221273	9.855121	8.992086	9.748169	11.267954	9.380201	9.852455	9.495345	9.704576	9.349896	8.449215	8.996563	9.663280	11.091901	8.774448	8.875559	9.147530	9.029036	9.432816	10.813141	8.695584	9.689916	9.574724	10.353264	9.927545	9.421461	9.423296	10.166750	10.837978	11.439735	11.171659	10.559632	10.517707	10.224521	10.099200	10.011667	10.466097	8.774448	9.085355	9.103656	9.117278	9.349094	9.275465	9.781629	9.419282	9.404833	9.361114	9.610806	9.229997	9.925892	9.584523	9.950116	9.968341	9.869171	9.332303	9.518998	9.954431	9.596795	10.588660	9.522255	9.598476	9.762791	9.659667	10.920907	9.803650	9.910192	10.297542	11.308542	10.884619	10.224681	10.449732	11.740264	10.264852	13.145522	9.352758	9.965758	11.158191	9.745680	9.094182	8.730247	10.194166	10.482463	10.751302	10.740809	10.188159	10.345517	10.433811	10.915990	10.936702	10.340353	9.179371	10.228793	10.711830	10.264251	8.928934	9.546391	9.963485	9.483782	9.551619	10.396893	10.168737	9.994150	9.141819	11.020654	9.921845	12.264825	10.747707	10.399429	10.014151	9.412891	9.558482	9.049014	9.253595	9.642396	9.202358	9.291751	9.781478	11.511259	9.520226	8.551354	10.038097	9.191651	9.694508	11.837247	9.510060	8.988657	9.005980	9.466743	9.261194	8.447016	8.683390	9.514276	10.741574	8.763780	8.249148	8.982916	8.646046	9.284015	10.690375	9.607021	9.105197	9.023720	9.485028	9.551928	9.552669	9.533072	9.508697	10.561928	10.298160	11.955287	10.269288	9.800263	9.777523	10.069768	10.090376	10.487794	8.298809	8.710395	8.683233	9.214537	9.490785	8.637867	9.717339	9.357717	8.951168	8.871377	8.698049	9.391124	9.591971	9.276071	9.836099	10.124282	10.533504	10.122161	10.173198	9.713721	8.698049	10.022449	9.698934	9.869487	8.771577	9.347248	10.715670	10.193392	9.164187	9.790524	11.646496	9.566353	9.361114	11.589538	11.999293	10.450036	10.800584	8.926611	9.702347	11.185257

AL390198.1	6.561359	6.127825	6.919359	6.769936	7.194566	6.668503	7.489279	6.922566	7.911257	7.368285	7.192137	6.810647	7.051808	6.844206	7.635404	6.485942	6.556465	6.956132	8.888374	6.781665	7.317019	7.840159	8.135563	6.375840	6.213909	7.111770	5.336960	5.791150	6.390194	5.358153	7.174480	6.802846	7.088794	6.465215	7.638162	7.635884	7.529471	7.224502	7.689945	8.108325	6.702584	8.353369	7.081619	7.585490	7.962682	7.225755	6.635321	7.298148	7.269702	7.755300	7.153563	7.839529	7.489591	8.243691	7.156686	6.701426	7.813567	7.759598	7.597303	7.267429	7.624881	6.783864	8.228114	8.600481	7.982069	7.006419	7.420235	6.924197	6.329228	6.594520	6.398294	6.727870	6.529490	6.419799	6.416507	5.929013	6.596178	6.804613	6.664928	6.865815	7.049113	7.389651	6.665251	7.514494	7.140467	7.282114	6.931449	7.427752	7.993790	6.977696	7.155127	7.371092	7.245012	6.649358	6.595678	7.086906	7.296211	7.916296	7.238138	7.051488	7.129243	6.868069	7.057440	6.755395	7.753478	7.102997	6.886607	8.057849	7.266176	6.730623	7.508617	7.410049	7.679069	6.901111	7.216228	3.450610	7.231968	6.666388	6.425798	5.961628	6.679815	4.669502	5.735558	3.524206	5.747384	4.869173	7.269702	5.690239	6.120458	5.924711	5.203864	5.562403	5.756654	6.213909	7.477201	6.988415	4.892077	9.070041	5.556569	5.248785	6.979716	6.562355	6.533884	4.952506	6.008419	5.553982	5.457488	6.480417	4.202274	7.080486	6.198260	6.273564	6.671556	7.543733	6.324741	6.819436	6.490004	6.509741	6.524972	6.983979	5.387590	6.551642	7.105493	6.739884	5.614444	6.300676	8.760109	6.761889	6.324214	6.290806	8.988657	6.432086	6.744976	7.521779	7.191534	8.258942	7.211820	7.013620	6.468191	7.055070	6.594222	6.672354	5.889206	8.395764	6.505130	6.479304	5.305404	4.608431	6.473421	5.275329	4.948324	6.133084	6.253339	5.825312	5.762324	6.800600	6.867257	5.883706	7.363878	6.404233	6.134299	7.202701	5.594202	6.836439	6.747226	5.781271	7.766091	6.916950	6.832287	7.599840	7.758398	6.888860	5.017503	6.690804	5.917953	6.793171	6.613481	6.606716	6.984610	5.452057	7.665138	7.197441	5.561471	8.754861	5.445240	6.584714	8.831019	7.152628	4.576227	6.116308	5.836243	5.410515	5.287944	3.624828	5.592538	5.996543	7.009152	5.894592

PKN2-AS1	7.197441	5.378088	5.701880	4.218384	4.756117	5.403080	5.800809	4.614203	5.374929	5.774550	5.112361	5.756654	5.050186	4.790155	4.451198	4.949392	4.096860	5.406292	5.873510	5.303048	5.292576	5.871520	6.089390	5.078395	4.736102	4.906273	4.697431	5.500842	5.393986	5.093331	4.655999	6.547740	6.356984	5.686052	5.219974	5.045343	7.132134	5.545431	5.831751	5.761577	5.515242	5.305796	4.997172	4.878603	4.026235	5.379258	7.039382	5.220191	6.573358	4.788062	5.584774	5.539404	6.183233	5.853987	6.057813	5.179379	5.474833	5.727541	5.637139	5.597613	6.152938	5.046863	6.133618	5.347540	5.742456	6.218387	6.033281	5.527680	5.776174	6.037725	5.893709	6.395964	4.822497	6.525640	5.837861	5.445822	5.599318	6.112642	6.323204	5.209227	5.944776	5.933150	5.112145	6.006989	5.285388	6.433444	6.031288	5.731574	6.350954	6.172016	6.365202	7.224803	5.671992	6.589655	6.039639	6.619694	6.328231	5.337135	6.102054	5.462754	6.208291	5.607046	5.731574	5.733015	6.288269	5.529351	5.472537	6.474750	6.319966	5.602065	7.346294	5.198528	5.640843	5.456657	6.575670	6.136867	5.454755	5.936696	5.095809	5.892560	6.888237	6.243620	4.942813	4.098850	5.814075	5.852379	5.701880	6.152067	5.168681	5.180533	4.636718	4.567032	5.488941	5.112773	5.178157	5.627077	4.753849	5.694139	6.198588	4.590157	6.537941	7.266030	6.182544	6.308238	6.045357	5.959677	4.875077	5.805626	5.899181	5.630815	7.389954	7.184232	6.432425	7.036232	6.999064	8.209329	8.734257	8.793845	7.946776	4.536530	8.293357	5.615175	5.979978	4.935976	5.994484	7.596848	5.009311	8.177531	6.345907	5.633578	5.758326	6.058191	7.016654	6.450000	6.186736	5.925962	6.573887	6.310954	6.042616	6.826070	5.150526	6.363905	7.083448	7.181722	7.173996	7.225900	7.334115	7.010422	7.377860	7.355513	6.984911	5.126754	6.478808	6.050444	6.838192	6.467703	6.408537	6.427147	6.053723	6.380781	6.380948	6.724149	7.186189	7.737895	7.806171	6.759131	6.331981	7.372533	7.201627	6.234471	7.982864	8.512472	8.863543	8.244922	8.546584	7.174793	7.078783	7.152494	6.626630	7.604353	5.737361	5.876738	6.630470	7.519393	5.568836	7.128141	6.789423	7.152007	6.165285	7.744866	7.050856	6.808398	4.856480	4.402892	5.660863	5.975338	6.063309	5.470625

AC137810.1	12.239149	10.951475	9.469344	9.113294	6.691111	7.580396	9.673663	6.307251	6.306611	8.648892	7.111138	10.316237	8.308845	6.351290	9.087175	9.175570	8.675912	7.980180	9.914676	8.691230	8.273739	6.826891	9.868052	8.799286	9.421461	8.946451	10.511711	11.138009	12.564954	10.160896	8.101283	10.741574	9.967576	10.703580	10.596797	11.221910	11.167966	10.883700	9.458948	9.663882	8.172688	12.284727	8.757916	9.866241	9.169847	9.000419	8.655072	10.328234	11.943795	8.312374	12.062906	9.506564	9.999670	9.629681	10.305051	7.702215	10.249709	9.815273	11.716891	9.506420	11.464783	10.610931	11.437102	10.707486	9.242110	11.164048	12.050582	8.663457	8.707044	10.875313	12.087108	10.515170	8.267121	11.537024	10.283668	8.955338	9.700957	10.745154	13.688585	9.083635	9.143201	11.110723	11.205234	11.095577	10.224681	12.634097	9.492866	9.444057	10.112589	10.827827	12.471814	9.928924	9.768599	9.137471	12.934196	9.032501	12.266375	9.284296	8.568651	9.274979	8.909661	11.688994	9.641321	8.869138	8.981956	9.026892	7.688106	7.985002	9.278313	8.365932	9.245186	8.503105	9.569039	8.486586	7.069377	5.225884	11.982123	9.100099	9.087621	9.560659	9.939645	11.275733	8.450156	8.431370	8.073673	8.645888	9.363636	10.567405	7.821807	9.394200	7.347027	7.569307	6.772178	6.615435	9.113587	11.443078	7.923024	7.833540	8.259851	8.251460	9.032354	7.902121	6.575670	9.521604	10.415101	10.011809	7.092449	8.576254	9.341743	10.143394	10.847998	11.698725	10.292031	9.828267	10.435219	13.354589	12.040600	9.079626	12.209741	8.341166	9.890283	8.878315	9.660290	9.055523	7.478585	7.722083	9.065378	9.268924	10.034381	10.842407	9.258494	10.101871	10.377715	11.581405	8.893308	9.878591	9.948590	8.453070	9.748803	11.095577	9.704158	8.827010	9.218991	10.589937	11.261682	9.903042	10.532370	10.155465	10.114655	11.056896	11.532292	7.020771	12.854778	11.859791	9.957986	11.933805	12.083836	12.116834	9.785347	10.485950	8.420732	10.569842	12.016602	12.479650	10.695129	11.412382	10.446887	10.452087	11.097765	8.910839	9.825394	11.065422	11.466449	11.244791	11.522341	11.923566	11.974354	10.216690	9.304406	10.462499	7.957140	9.336024	12.117118	9.203559	7.070379	8.664994	7.372371	7.358246	7.781521	9.519152	10.934280	6.267597	6.623712	7.737577	9.688696	10.794534	10.295232	7.485352

MIR3976HG	3.259511	3.440139	3.412024	3.155801	3.406592	3.295582	3.344003	3.594204	3.886962	3.429526	3.619725	3.709474	3.375281	4.039083	3.736901	3.181376	3.642272	3.145941	5.777263	3.225976	5.427255	4.070841	6.589166	2.993254	2.965857	2.853243	3.360502	2.858041	3.424550	3.082546	3.750843	4.262444	4.009963	3.808070	4.100192	4.157373	3.732517	3.292494	3.439974	3.236658	3.489559	3.146302	3.646626	3.373274	3.816300	5.797651	4.034967	3.062355	5.186596	3.975291	3.889150	3.740764	4.471008	4.355484	4.220612	3.181086	4.285926	3.176503	3.583962	3.954808	2.856198	3.162886	4.006960	4.277739	3.582100	3.895449	2.902480	3.707684	3.501061	2.814389	3.027576	3.576521	3.550612	3.528119	2.904877	3.399199	3.445636	3.209656	3.566891	3.893080	3.485185	4.160159	3.571687	4.404200	3.520622	4.712212	3.738084	3.326154	3.766086	3.772413	3.368911	3.160172	3.936230	3.522057	3.441704	3.558731	3.896502	3.193913	3.325488	3.058828	3.087292	3.285959	3.241621	3.312092	4.723654	3.843557	3.062027	4.071257	3.428299	3.295141	2.789659	3.356572	2.963489	3.342665	3.525695	3.447280	3.066929	3.460556	3.251979	3.245524	3.606405	3.052798	3.106431	3.986286	3.767567	4.430031	4.095169	3.696522	3.289044	4.453114	3.788448	3.881295	4.461136	3.327563	3.445453	3.541317	2.945757	3.601290	3.411297	5.758146	3.759866	5.422817	4.647688	2.839365	3.781036	3.292351	4.844670	3.926528	3.586323	3.480978	4.038661	4.540679	4.128277	4.138852	3.839304	3.773031	5.190727	4.909040	4.555359	4.016060	2.953835	3.718939	3.450425	4.351327	5.632279	3.509419	2.928851	6.298082	3.904201	3.821651	3.481349	4.293542	4.559441	3.623459	3.406231	3.893535	3.127307	3.100092	3.844372	4.007400	3.300530	4.113765	3.987976	4.405609	4.535899	2.887027	3.740764	3.452065	2.774163	4.340481	3.939217	3.800534	3.363039	3.303802	3.369959	3.799662	3.473431	4.188655	3.298546	3.887402	4.621637	3.651167	3.768624	3.615151	6.204353	3.457527	3.501239	3.358308	3.617825	3.529787	3.001787	5.668880	4.752112	3.649022	4.268691	5.825139	3.654396	4.790580	5.392783	3.234074	4.090253	3.884898	2.972725	5.124915	4.022591	6.661190	3.677408	4.241625	4.341985	3.609188	3.567496	3.660212	3.765302	3.972456	3.312709	2.915154	4.840654	3.401932

AC008591.1	3.520622	3.112510	4.036699	4.013112	3.087170	3.905507	3.146645	4.487836	3.715654	3.192046	5.182198	3.204801	3.553350	3.483698	3.016992	3.915721	4.879022	2.951204	6.452268	3.037621	3.835458	3.426570	6.347604	3.585137	3.188619	2.941644	5.145926	3.130036	3.219103	3.270828	3.492579	3.224468	3.871796	3.009843	3.145036	4.055403	4.457149	4.101263	3.755678	4.329886	3.294523	3.293775	3.288984	3.118161	3.410970	4.603961	4.853955	6.079043	4.486790	3.685428	6.044651	3.456601	3.800298	3.908140	3.635673	3.015866	3.856357	3.272988	3.512937	3.591339	3.510009	2.961673	5.313012	3.726533	3.234227	4.294797	3.080487	3.260848	3.321552	3.752961	3.623459	3.447665	2.991917	3.375800	3.021680	3.859401	3.507067	3.300958	3.258402	3.480386	3.634857	3.712023	3.130736	5.291773	4.140390	4.621044	4.274648	3.803284	3.752527	4.287203	3.758791	3.045250	3.200374	3.042193	3.527379	3.757923	3.446160	3.687063	3.093613	3.144788	3.627218	3.155467	3.087756	3.063410	4.353982	3.698588	4.056076	3.744089	3.148882	3.131700	3.371537	3.862586	4.359584	3.137360	3.653027	4.169878	3.735838	2.935992	3.034354	3.772186	3.380330	3.433246	3.301457	3.262043	4.136944	3.410757	5.865953	4.072539	3.414002	4.507693	3.265578	3.239051	3.084967	2.936995	4.194775	4.874867	2.883371	3.301128	4.129602	3.405807	2.875389	5.532003	3.313017	3.786732	3.707684	3.907267	2.924098	3.510558	3.623459	4.487427	3.303468	3.094426	2.955347	3.580694	3.984336	4.124421	3.523830	3.228505	3.544884	3.117381	3.617825	3.941720	3.169995	3.615151	4.158634	3.438239	6.404233	4.444117	3.630871	6.270865	3.289377	3.537427	3.961808	4.311896	3.148035	4.840449	3.281352	3.301288	3.459121	3.243536	3.826779	5.336960	3.576726	3.976560	4.229679	3.098204	3.496154	3.939637	3.128807	3.475806	3.559545	2.963216	3.430641	3.534991	4.161376	3.564969	3.391037	3.564761	3.789934	3.437547	3.773257	3.110843	4.539078	4.216443	4.099547	3.969874	4.213858	4.096439	4.187745	4.437866	4.342860	4.081358	3.484252	3.073342	3.311024	3.165689	2.997517	3.380840	3.148514	3.641891	3.431688	3.165437	3.091429	4.760994	3.400386	4.517223	3.534455	4.345246	2.981253	4.029058	3.791856	5.802051	3.096025	3.271787	3.328333	4.113765	3.222815	3.082447

PCA3	3.195700	3.543056	3.112756	3.824156	3.265284	3.138701	3.488475	3.533103	3.472214	3.894213	3.636278	3.636664	3.477470	3.985840	4.116356	3.505145	3.591699	3.563639	5.159455	3.598390	5.529351	3.619893	6.645475	3.202823	3.617097	3.078385	4.706826	3.423315	3.685044	3.709872	3.765519	4.598327	4.439596	3.172650	3.947248	4.325941	3.720576	3.832048	3.875056	3.867730	3.497086	3.918102	3.676220	3.418603	4.597879	5.761966	5.526489	3.828431	4.693081	4.054293	4.527792	3.638690	4.577141	4.623548	5.485556	4.146648	5.016682	4.591620	4.786621	4.440482	3.969226	3.712339	6.804468	4.202047	4.145567	3.618554	4.767260	3.739547	3.316203	3.062131	3.300811	3.435083	3.696522	3.474511	3.077588	3.415541	3.483154	3.297290	3.040276	4.686349	3.497086	4.491777	3.533669	5.338103	4.070841	6.768300	3.523830	3.835677	4.763580	3.872015	3.883596	3.182446	4.787643	4.336340	3.304432	4.801966	3.889613	4.001248	3.579772	5.050593	3.720576	4.137797	3.255631	3.753824	4.857125	4.922988	3.409715	4.200744	4.248186	3.550612	3.390523	2.978043	3.148164	3.355689	3.744640	3.328676	3.012127	4.307013	3.503982	3.084269	3.725335	3.049429	3.137360	2.892149	3.751073	3.044660	3.292658	3.467841	3.186882	3.444367	3.111657	3.309363	3.274197	3.680251	3.149886	3.513915	3.130356	3.522436	3.439792	4.635852	2.887027	5.571799	3.015552	3.098204	2.997517	3.106094	2.845610	2.900365	3.608603	3.534620	3.546976	4.119882	3.157896	3.580504	3.820601	3.770710	3.755267	3.605770	3.329682	3.238603	3.843957	3.729450	3.505520	4.368077	5.307713	3.832514	3.385183	4.211867	4.143246	3.951608	3.400891	4.383150	4.600410	4.283596	3.604265	3.895449	4.034530	4.133401	4.244932	2.915706	3.314483	4.340694	4.159936	4.130430	3.908140	3.285471	3.817172	3.509038	2.813126	3.112510	3.046710	3.278841	3.027576	2.917323	3.395752	3.072994	3.161484	3.093869	3.342475	3.907688	4.657931	3.588820	3.791654	3.625812	4.848293	4.727844	3.491351	4.035158	3.734015	3.582455	3.026669	3.739768	3.463925	3.195044	3.018930	3.017403	2.958353	4.309062	3.368271	3.104774	3.053021	3.024570	2.986951	5.238653	3.279275	3.465394	3.749998	3.460369	3.243404	3.703843	3.249142	2.960127	3.253442	3.082860	2.877138	3.116393	3.205608	3.411654

LINC02197	7.570213	6.518383	7.073703	7.151233	7.208630	7.607291	6.937962	7.526374	7.915342	7.453707	7.296044	7.393824	7.451050	7.275617	6.680626	7.986694	7.415648	6.778458	5.992878	6.951703	6.875472	6.936985	8.624652	6.275725	6.726248	7.705322	8.031633	7.583336	7.520175	7.255095	8.161790	6.204353	7.157612	6.664294	7.059187	6.382496	7.014840	6.566884	6.491854	6.612157	7.721779	7.165662	6.284973	6.975929	6.534041	6.153949	8.335407	6.542210	7.507394	7.779491	6.681110	6.801251	5.617610	6.912442	6.384336	7.205967	7.538121	6.700466	6.332647	6.550996	6.681267	7.572932	7.391949	6.632896	11.260928	7.029236	7.061088	7.115180	6.544184	7.032329	7.830932	7.459959	7.860182	7.339579	7.157148	6.610796	7.488362	6.511783	6.941991	5.909357	7.313043	7.080201	6.363201	6.594520	6.102226	7.809098	8.011995	6.939832	5.402050	5.624475	6.903040	6.174379	6.568873	7.439764	7.467854	6.475242	6.380296	6.875957	7.171812	7.236582	8.363460	6.727870	7.232924	6.739227	6.864858	6.750989	7.710024	7.210174	7.885206	7.340021	7.245764	6.773823	7.173245	8.009151	7.480282	9.596485	7.333524	7.327400	7.800830	7.325321	7.358404	7.196791	8.414146	8.037595	8.118549	7.900057	7.349764	7.281300	7.743798	7.886728	7.761745	8.263083	7.513268	8.256035	8.227519	7.394755	7.972401	7.786526	6.900296	8.100252	7.125453	8.186825	7.266940	7.683826	7.082213	8.438572	7.937457	8.657423	7.647866	8.343986	6.865815	5.839182	7.496300	6.397635	6.462173	7.038434	7.234989	6.497516	6.712952	8.306963	6.248637	6.404890	7.199296	6.861228	6.232581	8.767146	7.940095	7.218267	7.780760	7.535343	7.853434	6.415636	7.066825	7.035147	7.307573	7.251999	6.102541	6.661658	6.683734	6.360207	7.281951	5.726824	6.596333	7.173526	7.235801	6.933492	7.152628	7.625464	7.321508	7.411972	8.067428	8.709005	7.457457	7.400671	6.707704	7.681002	7.208630	7.701453	6.211853	5.860307	6.330944	6.458695	6.820544	6.858491	7.954949	7.270940	6.306611	6.012629	5.855636	10.646346	6.605446	6.735632	7.186521	7.342696	7.202847	7.854375	7.825209	7.288615	6.615749	8.137100	7.521929	7.826623	7.164871	6.582457	7.889355	7.003824	6.499883	6.845709	8.073029	7.227337	7.788507	8.449532	9.889071	8.544468	7.249623	6.490623	7.618175	7.797527

AC117462.1	5.196495	5.773122	6.361202	5.704669	5.863284	5.360800	5.347361	5.453034	6.400141	5.643691	5.644822	5.283757	4.328317	5.567854	5.146754	5.762139	5.731757	5.824962	6.639342	6.073647	6.823702	6.751932	7.103141	5.491679	6.119063	5.474270	4.372359	6.797331	4.956653	4.904841	6.508471	5.881927	6.136700	4.371755	5.545624	5.820260	5.652204	6.084413	6.819280	6.721446	5.258478	6.323797	5.184411	5.222149	4.973787	5.336536	5.233225	6.140137	6.272390	5.635443	5.838803	5.755895	5.365022	5.919567	6.121129	5.008061	6.123548	6.345056	6.027838	5.810203	6.720151	5.006189	5.620566	6.451474	5.900633	6.514380	6.016723	6.088675	6.204195	7.080977	6.087995	6.717180	4.971090	6.130691	6.457563	6.149498	6.264178	6.194249	6.388847	5.879995	5.962157	5.940259	5.751013	5.729728	7.085683	6.699964	6.047635	7.007190	6.670564	6.348760	6.446533	7.188088	6.141553	6.364856	5.329117	5.719750	6.255725	4.756961	5.789578	5.404268	4.970294	5.893525	5.494698	6.519844	6.776202	5.536657	5.857773	6.747226	6.074144	5.100522	6.890454	5.750837	5.624475	5.477091	5.779447	4.497929	4.964786	5.421300	5.191797	5.654829	6.040557	5.362720	5.504676	4.496423	5.578508	5.749370	5.048938	5.286988	5.890991	5.350897	5.644055	5.047064	3.986728	4.268246	4.651943	5.695665	4.040380	5.934404	6.674294	5.519876	6.547740	8.032995	5.333817	6.319268	5.870804	5.149087	6.170824	4.987279	6.037203	6.225022	5.401255	6.098553	5.611588	6.248137	6.582944	5.905776	7.468910	6.753221	6.292186	5.518976	6.673815	5.501991	6.908917	6.340567	5.985562	6.015023	6.792176	7.842151	6.749103	6.127120	5.914751	5.396153	5.769010	5.065069	5.479634	5.641968	5.903276	5.349856	5.650665	6.227804	4.713921	6.009291	6.601343	6.093905	6.224688	6.286581	6.038597	5.503913	6.349412	6.086932	6.290462	4.940144	5.585535	5.643485	5.850948	5.515242	5.989180	6.211030	4.753849	5.913674	6.208126	6.014010	4.944128	7.417279	6.928135	6.200835	5.899364	6.288089	5.466753	5.903452	6.872750	7.222084	7.064918	6.492192	5.565029	6.708488	4.289411	6.187919	6.906051	6.133265	5.711938	5.011405	5.392395	6.663789	5.438019	6.214073	7.064759	7.570069	6.118891	7.239898	5.820451	5.101364	5.324966	5.768488	4.394058	5.389322	5.269249	5.859961

LINC02778	2.983669	3.082447	3.248272	3.523465	3.063410	4.900698	3.157189	3.181516	3.471047	3.167023	3.301128	3.118405	3.593829	3.477651	3.304598	3.396622	3.334861	2.945609	4.702801	3.150869	5.509568	3.007653	6.352424	3.198666	3.152825	3.348689	2.855168	2.949339	2.900135	3.196794	3.464122	3.260848	3.483154	2.969017	3.044137	3.901371	5.144750	3.106431	3.146072	3.074560	3.316769	3.037621	3.736479	3.090114	3.479271	4.501376	3.525165	4.641552	4.940938	3.613588	3.412222	3.257185	3.486788	4.360915	3.428492	2.981438	5.206824	3.840068	3.490484	3.671371	3.129327	3.398675	3.580146	3.124150	3.260153	3.239326	3.272250	3.172384	3.138594	2.924485	2.927073	3.948370	4.158412	3.289211	2.942527	3.240460	3.482229	4.004778	3.469431	3.449712	3.373442	3.625390	3.135127	3.843368	3.805556	6.640640	6.879726	3.206777	4.010582	3.461975	3.393483	3.055916	3.310854	3.331266	3.022471	3.428492	3.305388	2.895171	3.069602	4.082613	4.758930	3.534620	3.309363	3.407300	4.322455	4.646404	3.047106	3.115327	4.126990	3.450800	4.866199	3.826149	4.354587	3.941310	3.483534	6.749904	3.617462	2.957760	3.057495	2.968089	3.824627	2.922785	2.993919	3.312397	4.134718	3.789934	3.072259	3.133555	3.242251	3.079639	3.198532	3.243536	3.475980	3.568873	3.979371	3.257039	2.876544	3.327729	3.189576	5.281154	3.265735	5.668327	3.315404	2.996841	2.932410	4.263974	3.161721	4.064154	3.328676	3.209791	4.232240	3.371190	3.621076	3.017610	3.617257	4.702563	3.117732	3.177764	3.485680	3.343162	2.962105	3.450995	3.128807	3.712709	3.981983	3.583046	3.433974	4.409145	3.458213	3.306493	3.134492	3.413645	4.557261	3.237433	3.175549	5.025838	3.780612	3.245524	3.536689	2.994658	3.100195	3.629437	3.450425	3.433974	3.365983	3.325977	3.387908	3.215682	2.928074	2.923401	3.539784	3.056331	3.409363	3.238890	3.167789	3.891965	3.386361	3.037234	3.688091	3.470874	3.725577	3.876562	3.779991	3.600306	5.785945	5.187401	3.327122	3.784156	3.390681	3.194860	3.412911	3.909259	3.091974	3.008358	3.250421	3.630263	4.302412	3.403027	3.384151	3.736479	3.991571	3.254178	4.400471	4.688438	4.181013	2.866883	3.063094	3.264540	3.488475	4.783257	5.401834	6.517896	3.581900	3.042193	3.279752	2.942182	3.176503	3.090979

FLG-AS1	3.859999	6.787922	8.291499	6.910985	7.338002	7.901171	7.944176	9.222361	8.031937	7.445550	8.367762	7.686546	6.696500	7.556447	7.030503	8.218245	7.055070	6.641126	7.515075	8.269821	6.699651	7.265558	7.437251	7.170697	7.239898	6.748333	5.386330	6.691435	6.031651	6.967877	7.567799	7.665138	8.056526	5.035276	7.215455	8.436480	8.185409	7.189847	6.098195	6.945183	7.894662	7.049753	6.730953	7.897891	6.992917	8.056978	6.600383	6.570194	7.963752	8.383274	6.649508	7.574330	6.150169	9.217596	8.484300	7.801746	8.927706	6.535312	6.371255	6.261678	6.655645	6.547889	5.088990	7.536868	5.665514	6.182027	5.975866	7.344722	7.264952	6.528481	8.329193	7.359946	8.263240	8.244304	7.544378	7.125300	8.357843	8.757154	7.760354	6.874477	7.709848	7.168420	7.400060	7.982701	8.249148	8.508908	9.134541	7.887508	6.610125	7.831559	6.846929	6.856249	7.080486	8.020889	7.502427	7.391638	7.828691	7.481057	7.509710	4.704932	7.621190	6.579309	6.394833	6.784847	6.206431	7.058384	6.922419	6.243437	4.598327	7.999482	6.036696	6.546146	6.062774	7.481663	8.971157	6.386127	4.417754	7.285348	7.111910	3.921454	4.330333	4.634782	3.852056	4.063721	5.328715	4.094946	4.008468	4.862492	5.357589	5.527261	5.138833	4.793938	4.344597	4.644478	5.561304	5.000281	5.036116	4.014061	5.869021	5.225696	4.647688	5.925083	3.488837	4.747859	3.872419	5.751387	6.028032	4.386530	3.853571	5.065280	2.883547	5.002315	6.213909	5.408524	5.736795	5.299282	3.314962	5.091025	5.045712	5.938629	5.681029	3.900638	4.679671	5.613483	6.642091	4.026235	3.698588	6.570194	6.099247	5.521563	5.524971	5.544106	4.593406	5.410703	5.581804	5.197707	3.217434	3.428672	4.948949	4.657491	3.583962	4.944987	5.051193	4.658123	5.174521	4.464593	4.608201	4.600200	5.109134	6.717180	6.119063	5.233840	5.517407	5.421654	4.568093	7.111910	6.276841	6.150019	4.355705	7.061882	5.611787	4.159518	5.187401	5.781628	5.735745	5.520641	4.241625	3.867507	4.930806	4.335703	5.251176	3.629641	4.248826	4.760783	4.545772	5.025068	5.502762	4.129602	5.247007	3.999802	3.406943	3.331266	4.190613	4.894172	3.606610	5.658144	4.538427	5.277562	4.087434	3.679837	4.472347	5.735919	6.840955	6.607242	4.567032	6.547889	4.565960	3.962030

CASC2	4.444552	4.423542	3.912495	4.850577	3.228505	5.150526	6.750073	3.487711	3.273704	5.477682	5.620017	4.644478	4.163347	4.940938	5.104607	4.749521	3.344003	3.314962	5.116800	5.377881	4.275288	4.385646	6.173864	3.400227	3.865565	3.186612	3.558141	3.744089	4.255230	4.549075	3.516224	6.446864	5.038331	4.458852	5.290002	5.306575	5.059000	4.938279	4.641552	3.456418	4.465424	5.924919	4.079820	3.601673	4.050210	4.817923	3.976560	4.733571	5.083842	5.777806	5.124915	5.368387	5.220191	6.088324	4.455228	4.368486	4.742543	2.989807	3.478950	5.655195	5.282363	4.533520	3.554100	4.724520	4.837238	4.489766	5.153174	4.227030	5.067971	3.863232	4.875077	4.579470	4.002147	4.456478	5.046672	4.360482	4.065006	4.658241	5.057568	4.733129	4.709267	3.052050	4.530110	4.710518	5.335758	5.998849	4.682027	4.544678	3.631091	5.270048	4.696394	3.962694	4.568743	4.518062	4.955407	5.193853	4.301308	5.479076	3.995086	3.491016	3.657453	5.514823	4.195624	4.841900	5.165881	4.735340	4.259179	5.423208	3.251299	5.105042	3.449885	5.061210	3.545283	4.129377	3.910981	2.961952	3.685044	4.355913	4.183437	5.626357	4.707226	3.924576	3.088322	2.849361	5.677134	3.056542	5.297845	3.154801	4.175765	4.713705	3.378256	3.611782	5.313797	3.841530	3.525510	4.356533	3.046304	6.388174	6.628233	3.761341	4.275527	5.763590	2.929503	2.953226	5.281557	2.997895	4.135859	3.125639	5.242044	3.198153	4.295680	3.550026	5.414157	4.649179	3.903359	4.461781	3.132332	5.307327	3.728592	5.031937	3.264817	4.697431	3.277564	4.021786	4.545332	4.371963	3.022786	5.648788	5.691365	3.527209	4.970485	6.893180	5.762867	4.360255	3.977405	7.049753	6.205756	4.143455	6.519682	4.053879	4.173173	5.188407	4.111378	3.619893	3.635265	5.989356	3.613360	4.020669	4.657931	4.951665	4.158193	3.924795	5.412627	3.941517	4.408498	3.882111	4.547077	4.279959	5.805807	3.923719	6.612650	3.542277	3.616508	5.110970	3.889150	3.198410	7.219504	3.384489	3.366686	5.690981	5.752475	5.465983	3.911625	3.106541	3.945977	3.793394	2.960973	4.957935	5.896338	3.216375	5.199295	3.136008	4.533520	6.545014	3.599132	3.964222	5.977433	3.936016	3.859195	3.406943	3.630489	3.134280	3.188883	4.518473	3.708456	3.742589	5.364244	4.463741

LINC01115	10.098273	8.618151	8.705672	9.002579	8.577014	10.503929	9.365175	11.838131	12.485666	10.148412	10.605180	11.226653	8.881137	8.893308	8.674288	10.898956	10.601053	8.284696	9.177867	9.592603	8.624827	8.421803	9.833499	8.396396	8.188953	8.534236	10.543883	9.074340	11.410542	9.845979	11.364444	8.420558	8.590205	9.477832	8.831633	8.880054	8.840278	8.016172	8.188314	8.257368	10.499861	7.909740	7.694590	8.504041	7.841561	8.045919	11.507297	8.463529	9.196271	8.804706	7.847351	8.057438	7.548829	7.690710	8.030578	11.501326	7.757729	8.106950	8.944434	7.736809	8.249596	9.671780	8.206480	7.870902	8.475733	8.234802	8.419610	8.315429	8.250047	8.606664	9.489187	10.324901	10.392669	8.978474	9.529917	9.608685	9.119435	9.151539	9.502535	7.889051	7.894980	8.848779	7.806501	8.634890	8.257083	9.349719	8.174422	8.935314	8.606818	8.269361	8.130096	8.553905	8.308699	9.053766	8.830706	10.177339	9.330587	9.455713	9.757807	8.776128	10.035617	8.348402	9.356962	8.290249	8.462342	10.578905	8.922699	8.407117	8.843418	10.417364	8.788881	8.301962	9.411123	11.544838	11.711892	11.592359	7.658461	11.248503	10.863944	10.941388	8.970362	8.626257	8.876155	9.243504	10.436727	9.805321	9.221577	9.843353	9.899900	10.201399	10.231761	10.370335	8.393633	8.834942	11.539976	9.261966	8.469947	8.714198	9.292527	8.720391	9.019117	9.904261	9.504396	8.898560	8.630840	10.045720	9.252231	11.640863	9.536888	11.515004	9.122460	9.358336	9.328764	8.597896	8.832406	8.298809	8.829501	9.365340	8.503262	11.309400	8.540210	8.071533	9.139186	8.015395	8.564511	11.409515	8.786399	9.105974	8.539908	8.485224	8.245084	7.798822	8.385574	7.795703	10.084052	8.167811	8.407880	8.244777	8.133329	9.124574	9.244700	8.859721	8.579521	8.599881	8.252821	8.783210	8.658051	8.643945	8.709313	9.236897	9.903960	11.333902	9.212359	8.803036	9.304272	8.865812	9.405869	10.145963	7.870902	8.258942	10.825756	8.153614	8.575025	8.602363	9.674605	8.721903	8.690660	8.592374	8.163384	9.067393	9.080577	9.012546	9.757967	9.823607	10.071920	9.481968	9.767842	10.601209	8.900522	9.447171	8.292759	9.721686	7.981127	8.475262	11.022434	9.354243	8.634890	8.988368	11.377401	9.290321	8.468540	9.165096	10.884311	10.106999	9.755013	8.185093	10.024815	10.040378

AC006207.1	10.345357	8.638314	8.632906	8.827612	9.194946	9.315341	8.561316	9.500568	9.483782	8.639891	8.602058	8.482719	8.027164	8.223289	7.830932	9.231455	9.026158	8.024223	9.384818	8.825236	8.740108	9.387777	9.107805	8.736877	9.141010	8.672782	9.781037	9.309567	9.385728	9.383918	9.169538	8.295296	8.675156	8.608629	8.369070	8.797307	8.159927	8.216662	8.507514	8.611061	9.026460	8.913424	8.476502	8.314845	8.118397	8.073351	9.430938	9.063831	8.605743	8.276739	9.207491	8.037132	7.879149	7.996760	8.012452	9.156761	7.658000	7.916120	7.923957	7.688422	8.227204	8.812028	8.787346	8.531668	8.075788	8.026061	9.099970	8.573979	8.572024	8.617543	9.065219	8.914940	9.145331	8.783516	8.672489	9.024766	8.639275	8.757464	9.854297	8.307442	8.068901	8.121626	8.506105	8.576106	7.993159	8.790577	8.264324	8.209622	8.375408	8.839797	8.960537	8.914784	8.335407	8.624351	9.492065	8.215620	8.609545	8.367126	8.670782	8.726505	9.294739	8.364085	8.575025	8.313905	8.447805	8.938682	9.546241	8.697583	8.973624	9.401696	8.902785	9.042373	8.676199	9.698334	8.928410	11.866983	8.963871	8.908005	9.268476	8.753634	8.795216	9.069131	9.330587	10.234170	9.177565	9.185181	9.095706	9.095402	8.957149	8.795216	8.681103	8.638171	8.470408	8.436796	9.416569	8.779006	8.114390	9.363051	8.762120	9.031888	9.460158	9.312799	9.267219	9.310358	9.021427	9.485807	9.503932	10.026067	9.688230	9.214386	9.166648	8.668344	8.517941	8.534543	8.422852	8.524310	8.889464	8.958885	9.177867	9.602855	8.851528	8.376183	8.659760	8.278265	8.196235	9.664852	8.529071	8.485224	8.414300	8.339965	8.209938	7.838143	8.229190	8.034047	9.376056	8.109811	8.473114	8.076799	8.004026	8.247106	9.503615	8.775967	8.641436	8.341747	8.324142	8.705513	8.925189	8.655522	9.348808	9.206285	9.136846	9.629238	8.843862	9.006998	9.178776	8.885766	8.982606	8.733342	8.506270	8.145183	8.353996	8.237618	8.553489	8.734707	8.795667	8.322623	9.310505	8.635686	8.813641	8.353066	9.080879	8.753956	8.942784	8.858496	8.739022	8.724336	9.164484	9.283708	8.792916	8.765121	8.922996	8.729568	8.880054	8.497774	9.477368	9.579308	9.020620	8.933933	9.916997	9.132598	9.047586	9.888161	10.132853	9.241513	9.915905	8.509059	8.872943	9.461453

LINC00378	6.772683	4.731657	5.065508	5.418942	5.138025	8.307282	8.077583	6.132754	6.851706	7.973216	9.342047	9.434019	6.923207	7.475990	6.633699	5.510295	6.498041	6.074831	8.181446	5.866135	6.665395	6.733827	8.330735	5.985749	5.816007	5.769200	6.214225	5.834736	8.396474	6.882930	5.495683	6.030666	5.127355	6.428172	4.698939	5.303625	4.752349	4.551331	6.052691	5.347947	7.899568	5.442550	5.374545	5.271639	5.204869	5.048938	8.806276	3.510009	5.910088	3.333864	4.603540	5.507796	5.929174	5.438019	5.904725	8.245543	5.517603	5.442939	5.499091	5.080622	5.558862	7.754711	5.500462	6.427512	5.326544	5.224673	4.508113	5.123715	4.327452	5.360800	4.981929	5.278130	8.304531	5.015261	5.547864	4.629822	4.354817	4.944128	5.543334	5.000281	4.217728	4.635852	5.216032	4.351327	5.000471	6.402270	4.836596	5.724374	5.688595	5.295630	5.393986	5.026050	6.795092	4.069644	5.059192	5.674028	5.407741	6.721446	5.569403	5.496067	4.791226	5.476530	6.939515	6.395803	6.258431	8.095669	6.390861	5.513706	6.083882	8.553021	5.296657	6.074144	4.680289	8.705991	5.964122	5.379258	5.500043	7.780760	9.194647	6.165780	4.527792	5.199473	4.722166	4.331236	7.568694	8.137859	6.094448	6.440058	7.154973	8.512159	8.304531	5.257705	5.459239	6.403118	5.439483	6.069272	5.344374	5.430096	4.739537	5.585535	5.794218	7.438208	5.626721	5.304993	4.764426	5.797284	5.473875	8.551804	6.821014	4.819801	5.635443	3.251818	6.684561	4.767870	5.428855	4.996504	4.860231	5.927070	4.772189	7.192941	5.645429	4.710518	5.456287	5.037916	5.410127	8.344459	4.791432	5.168494	4.705302	5.009093	5.494698	5.920125	4.247541	5.339320	7.079256	4.412213	3.157189	4.775737	5.836808	4.796377	6.906715	4.935579	5.819037	5.932972	4.892508	3.491861	4.684589	4.749752	4.849289	4.369342	4.502413	8.346766	4.946094	4.195419	4.475603	4.577141	4.432882	5.163258	4.547921	4.934289	4.133401	4.084323	3.871134	4.225750	5.675129	5.327132	4.693307	5.893148	4.317138	4.722818	4.796034	6.193889	4.875077	5.790613	5.543524	5.049984	6.810976	5.077771	4.884944	4.765699	5.153174	6.589655	4.663683	5.441005	8.237449	6.167736	4.808893	5.965017	8.587697	5.448014	5.512606	3.562491	8.442096	5.878692	5.611985	5.366204	6.814288	8.111174

AL161629.1	6.312295	6.134299	6.201557	6.238948	6.796677	6.367718	5.644266	6.710559	7.163467	5.529351	5.997433	5.903090	4.792691	5.281557	6.473753	5.778004	7.105179	6.088848	7.206289	6.218215	6.507182	6.315909	9.450937	5.478043	6.232252	6.596483	6.092166	6.879572	5.709005	5.556382	6.253339	5.977611	5.966637	5.204262	5.680106	5.768830	5.842228	6.272706	6.039284	6.657265	5.563726	6.242601	5.627454	6.179318	5.593652	5.452057	6.448523	6.469350	6.830244	5.795280	6.120458	6.042616	5.659389	6.239096	6.404740	5.541466	5.609902	6.109896	6.245620	6.082836	6.720973	5.141933	7.057287	6.896873	6.440719	7.196961	6.516754	6.368368	6.568387	7.500997	6.516905	6.912945	5.646889	6.569199	6.493772	6.812869	6.693598	6.986363	6.889336	5.901849	6.123548	6.499069	6.329736	6.404576	6.466021	6.617262	6.788763	6.723847	6.810006	6.580287	6.712645	7.448561	6.358002	6.714240	6.425977	5.995352	6.939982	6.253184	5.753035	6.098195	5.646889	5.452837	5.818516	6.462676	7.116435	5.790780	6.500534	6.654016	7.300476	5.587921	7.818875	6.801251	6.877972	6.167031	6.180988	7.249321	5.886902	6.770246	6.005942	6.370383	6.240938	5.971415	5.893525	7.171315	6.320848	6.490154	6.643374	6.993859	5.868862	6.089027	5.856901	5.883154	5.651640	6.378810	6.303916	6.630936	5.986470	7.231027	6.585901	7.019207	6.655496	8.569561	6.634691	6.832787	6.952016	7.122892	7.249623	6.661840	5.947212	6.754571	6.262524	5.968975	6.365038	6.468844	6.508308	6.712466	7.311045	7.663729	7.499773	5.958590	7.127988	6.176433	7.429837	5.917953	6.595518	6.512753	6.547423	7.722684	6.188416	6.768300	6.080567	5.744656	6.209157	6.020286	5.888505	5.499091	6.267078	6.422233	6.449494	6.916950	5.286009	6.993072	7.299240	7.235142	7.031875	6.921142	6.564982	6.812404	7.298304	7.382687	7.085386	6.081461	6.719642	6.454936	6.813975	7.208783	7.166559	7.373429	6.185384	6.026064	6.781340	6.383321	6.575512	6.780543	7.940095	6.842863	6.855573	7.148992	6.618235	6.917722	7.769881	6.616246	7.297257	6.756526	6.540510	7.319961	6.022738	6.070817	6.520193	6.369034	5.982795	6.594222	6.054615	6.969671	6.180491	7.331016	6.813501	7.764971	6.372955	7.905401	6.842224	6.704931	6.736780	7.074654	6.565136	6.284297	6.218892	6.575670

AC007182.1	4.699627	6.186223	7.153391	6.851096	5.255945	6.335522	5.898847	7.693362	6.396299	6.014010	6.891582	6.338411	7.563337	7.062837	6.427147	5.341233	7.192941	7.164101	7.538276	7.708085	5.095000	8.044021	7.005493	5.844914	5.192676	4.899185	5.328100	7.491173	6.114220	5.618176	6.824823	8.437404	6.830874	4.764207	7.731029	6.428351	4.924079	7.692283	9.353052	5.807418	6.049248	7.834327	6.039123	6.913700	4.617539	5.622037	5.655012	6.416507	5.999204	6.394182	5.854171	6.064688	7.772768	8.440901	6.766987	8.321720	7.102997	5.999906	6.883240	6.820058	6.826397	6.421917	5.028289	8.551058	6.099988	5.083258	6.869668	6.696813	6.344739	6.639018	6.587033	5.070383	6.179318	7.088639	6.948428	6.283637	6.215952	8.703695	7.779666	7.699262	7.339745	7.091491	7.597166	5.257140	6.909692	7.998255	9.163879	6.661502	6.638386	6.674930	6.050444	6.295776	6.220905	7.696994	5.865783	5.737361	5.996901	5.729350	7.072747	4.206158	7.229059	5.922745	5.485941	5.106448	9.799359	6.298921	7.107883	8.876944	6.383146	6.389179	7.142409	7.377685	7.455423	4.270081	5.196495	6.753057	5.688249	7.348653	6.021366	4.922338	6.797171	6.298921	3.658626	7.143465	6.365363	10.345226	4.748070	7.375633	3.612955	9.922427	8.717346	3.440832	3.752961	3.456247	5.013002	5.186019	7.707285	3.887402	4.179668	5.089809	3.916408	5.587921	4.975882	4.253660	3.412735	5.244823	8.545954	4.773047	6.024293	5.100098	7.857395	4.990394	7.659822	8.466285	5.614049	8.195596	6.347250	6.121304	5.682541	3.501239	7.670842	4.154991	5.623704	8.433526	6.717651	11.701767	2.971964	6.391390	6.313328	5.508384	3.548191	6.002904	6.558456	9.154238	4.374352	3.827629	9.183769	6.833926	8.920300	5.344374	4.238326	5.710849	9.839396	5.072013	7.477534	4.826274	10.865608	5.333431	4.660504	8.316693	7.781356	8.774288	8.191336	6.060096	6.691766	7.082859	5.647262	6.734308	6.656310	8.140306	7.656558	7.524523	8.310979	5.172334	7.211007	11.779417	7.330879	5.610320	7.695913	5.014462	6.112317	6.821189	3.985840	8.674683	6.919186	4.593852	3.179124	5.278342	4.554945	4.161178	3.626417	3.416555	6.630313	5.068410	4.783483	6.564009	5.299860	7.833074	9.181558	6.474078	6.220415	4.014061	4.435400	5.241438	9.639954	5.697494	5.969312	6.057290

LINC00922	8.008692	7.892026	7.028135	8.025265	7.478448	8.676360	7.742874	7.636038	9.058147	8.320348	8.684334	8.314984	7.251854	7.591953	6.993072	8.466122	6.797171	7.636336	7.629887	8.176328	8.114888	7.242763	6.711199	7.507090	7.381102	8.930947	9.636219	9.102735	8.048929	8.301367	7.598679	5.815094	5.979978	6.882311	6.231727	6.087653	5.972154	4.954568	6.296916	5.452057	8.582113	5.851664	7.362762	7.366862	7.140632	7.912825	9.425721	7.805090	7.732264	7.909103	7.819040	7.526374	5.354706	5.696189	5.547320	8.685699	4.122740	2.939310	5.452248	4.742543	5.173736	8.663941	4.346523	4.983403	6.149155	5.727541	5.687890	5.821306	5.722901	5.900633	6.934730	7.097035	8.951337	6.290302	6.640481	6.158049	6.107970	5.707018	5.908115	5.740431	5.876019	6.626777	6.182027	5.879995	6.750694	7.016162	6.379289	5.663868	3.340297	5.471992	4.965208	6.071880	6.393047	7.107577	6.979716	6.727870	6.665888	6.356984	6.437732	6.326106	6.863132	5.773628	6.223823	5.846222	5.560034	7.854212	8.800210	6.382809	8.209622	9.252826	6.094089	5.560572	5.216385	9.999518	7.676806	7.598679	5.823680	7.983020	8.507231	8.596881	8.653579	8.533770	9.214963	9.211908	9.430494	9.083932	7.737895	7.315893	9.366931	9.396207	9.082741	8.888219	8.862760	8.869921	8.921659	7.996760	8.951490	8.272099	7.986694	8.954265	7.782708	8.482338	8.594703	8.277981	8.204471	9.313309	13.133580	9.480888	8.571728	8.743289	7.570519	7.094649	7.010274	6.795092	6.752329	7.016496	6.848712	7.671653	7.104070	9.701109	6.924019	6.737116	8.469344	7.763140	8.138337	10.051559	8.511664	7.761592	8.400492	8.231164	8.430558	6.781665	6.759935	6.934730	8.451544	7.986058	5.495472	6.711199	5.444298	5.328913	8.885455	4.320571	5.224298	7.465107	6.983656	6.283128	6.391390	7.281784	6.380453	7.220329	7.714645	10.563528	7.621190	7.505885	6.192830	8.693560	7.310410	6.980675	8.133472	6.982716	7.442358	7.152778	7.344870	7.421974	8.329322	7.183603	6.297720	7.050375	6.869212	6.322380	6.394833	7.255470	7.831082	7.609277	7.724989	8.165664	8.402666	6.979414	6.376310	7.510460	6.795092	7.502262	7.261055	6.684561	9.872877	8.414146	6.960860	6.383999	10.567246	7.010274	7.260440	4.717717	10.457869	9.520834	7.222406	6.605754	8.755477	9.139490

AC009055.1	8.008692	7.892026	7.028135	8.025265	7.478448	8.676360	7.742874	7.636038	9.058147	8.320348	8.684334	8.314984	7.251854	7.591953	6.993072	8.466122	6.797171	7.636336	7.629887	8.176328	8.114888	7.242763	6.711199	7.507090	7.381102	8.930947	9.636219	9.102735	8.048929	8.301367	7.598679	5.815094	5.979978	6.882311	6.231727	6.087653	5.972154	4.954568	6.296916	5.452057	8.582113	5.851664	7.362762	7.366862	7.140632	7.912825	9.425721	7.805090	7.732264	7.909103	7.819040	7.526374	5.354706	5.696189	5.547320	8.685699	4.122740	2.939310	5.452248	4.742543	5.173736	8.663941	4.346523	4.983403	6.149155	5.727541	5.687890	5.821306	5.722901	5.900633	6.934730	7.097035	8.951337	6.290302	6.640481	6.158049	6.107970	5.707018	5.908115	5.740431	5.876019	6.626777	6.182027	5.879995	6.750694	7.016162	6.379289	5.663868	3.340297	5.471992	4.965208	6.071880	6.393047	7.107577	6.979716	6.727870	6.665888	6.356984	6.437732	6.326106	6.863132	5.773628	6.223823	5.846222	5.560034	7.854212	8.800210	6.382809	8.209622	9.252826	6.094089	5.560572	5.216385	9.999518	7.676806	7.598679	5.823680	7.983020	8.507231	8.596881	8.653579	8.533770	9.214963	9.211908	9.430494	9.083932	7.737895	7.315893	9.366931	9.396207	9.082741	8.888219	8.862760	8.869921	8.921659	7.996760	8.951490	8.272099	7.986694	8.954265	7.782708	8.482338	8.594703	8.277981	8.204471	9.313309	13.133580	9.480888	8.571728	8.743289	7.570519	7.094649	7.010274	6.795092	6.752329	7.016496	6.848712	7.671653	7.104070	9.701109	6.924019	6.737116	8.469344	7.763140	8.138337	10.051559	8.511664	7.761592	8.400492	8.231164	8.430558	6.781665	6.759935	6.934730	8.451544	7.986058	5.495472	6.711199	5.444298	5.328913	8.885455	4.320571	5.224298	7.465107	6.983656	6.283128	6.391390	7.281784	6.380453	7.220329	7.714645	10.563528	7.621190	7.505885	6.192830	8.693560	7.310410	6.980675	8.133472	6.982716	7.442358	7.152778	7.344870	7.421974	8.329322	7.183603	6.297720	7.050375	6.869212	6.322380	6.394833	7.255470	7.831082	7.609277	7.724989	8.165664	8.402666	6.979414	6.376310	7.510460	6.795092	7.502262	7.261055	6.684561	9.872877	8.414146	6.960860	6.383999	10.567246	7.010274	7.260440	4.717717	10.457869	9.520834	7.222406	6.605754	8.755477	9.139490

AP001496.4	4.533715	4.765269	4.352225	4.376322	4.510286	5.455701	4.631969	6.156523	5.604749	4.583101	4.424419	3.385855	3.736479	4.505883	5.015261	4.566596	5.060442	2.908227	4.879622	4.235520	4.067119	4.748070	3.560111	4.251056	4.571161	5.677521	4.571619	4.927218	4.297904	4.527587	5.323047	4.586707	4.154991	3.912710	4.018026	5.583614	4.678389	4.910706	5.169505	4.444333	4.839385	3.155801	4.060412	3.981983	4.570485	5.430487	5.276568	3.506053	5.934947	4.653519	4.376736	4.926387	3.934099	4.840237	4.689716	4.682249	4.051084	5.179561	3.645219	4.949181	4.944783	4.363515	5.140454	5.931759	4.734819	4.566806	4.821263	5.146157	4.910706	5.288957	5.199677	5.698593	4.410703	5.404983	5.269057	4.774707	5.910785	5.404785	5.003178	5.463646	4.602523	4.881804	4.231140	4.833968	5.058787	4.626760	6.150695	5.775818	5.573143	5.436282	5.397296	5.996543	4.930625	5.245814	4.685015	4.871701	5.266469	4.388470	4.019768	4.688438	5.822404	3.779991	4.205947	4.324006	4.559007	5.166080	5.322285	5.307713	5.761388	4.821899	5.478477	5.736114	6.047635	5.266469	4.984172	7.272360	5.149493	4.884308	4.749312	5.147334	4.668615	4.713921	4.357202	5.264877	5.462582	5.326352	4.451198	5.692261	4.361722	4.088287	4.092275	4.787444	4.118088	4.373702	5.097811	5.668880	3.603661	5.016682	4.778245	5.181563	5.818516	6.844707	4.679456	5.082049	5.754786	6.456884	5.339727	5.681029	4.784334	5.012216	5.135234	4.417292	4.772601	4.679862	5.593845	5.082253	4.915972	5.373011	5.155213	5.313408	5.413577	4.656839	4.428669	4.929514	4.760385	5.166666	4.843390	5.913482	5.641575	4.737795	5.125769	4.372594	5.146157	5.559054	4.876104	4.995208	5.657036	5.216974	4.653042	4.831651	4.573081	5.202043	5.862518	5.445439	4.903797	4.935151	5.819906	5.148135	5.165102	5.703375	5.774012	4.993721	5.522694	5.885856	4.931393	6.069272	5.624269	5.524971	5.101129	4.797463	5.462754	5.222725	4.908622	5.664974	5.467155	5.664421	5.272481	6.287083	5.848261	4.862293	5.740630	4.784935	5.379656	4.766370	5.217771	4.981929	4.975059	4.698120	4.279071	5.174952	3.997047	4.025170	4.586047	5.429568	4.799428	5.973787	5.374165	5.411685	6.588530	5.789212	6.637534	8.478824	5.700263	4.467794	4.800062	4.587788	5.083066	5.080622

AC079340.1	6.420618	8.314845	7.581181	8.286241	7.385917	8.465536	8.360934	10.874275	8.037285	8.763320	8.332786	8.287349	7.384386	7.864325	7.390711	8.100093	7.638327	7.364043	9.153531	8.435693	8.541028	7.621803	6.141377	7.399762	8.051562	8.080886	7.247604	7.564569	7.777768	7.647273	8.315127	7.956035	7.473225	7.137501	7.537955	8.880343	8.572726	8.340263	8.989722	8.711476	7.824155	8.413538	8.351704	8.849578	8.443933	9.001965	7.402873	8.380819	7.335661	8.945820	7.283831	8.262290	8.196996	8.496139	8.950491	8.653117	8.301810	7.477997	7.949528	7.695045	7.935134	7.612344	6.649192	8.017844	8.703369	8.548297	8.017407	8.525740	8.033901	8.597607	7.989562	7.687949	7.790379	8.282999	7.668497	7.771269	7.737275	7.502904	7.708229	8.473429	8.040597	7.741802	7.759914	8.256785	8.505214	8.397030	8.004026	7.343668	7.525735	8.589300	8.547241	8.064734	7.727632	8.605587	6.952820	6.906870	7.911257	6.925012	7.383010	7.734420	8.597021	8.368201	7.177144	8.307119	9.949211	7.530678	8.626257	8.730470	8.384200	8.363315	7.922093	8.755780	8.144078	8.151760	7.951354	9.334599	6.833280	8.287349	7.556615	6.821815	8.810440	7.460560	7.420547	7.781671	8.454290	8.052166	7.091491	7.273344	7.924598	8.247677	7.508140	7.416867	8.528611	6.967094	7.194566	7.369060	6.375503	7.323901	7.436620	6.930656	7.107267	6.174551	7.212319	7.791742	7.962682	7.818413	7.823189	8.125103	8.069216	7.060933	7.801900	7.071647	8.390832	7.521929	8.536401	9.029816	7.837496	8.119920	7.629887	7.872609	8.408506	6.985720	8.069369	8.796419	9.637621	8.169725	6.799796	7.007341	8.345680	7.614639	7.183306	7.830635	8.185252	7.367488	7.644012	6.707995	6.906051	7.396310	7.136879	7.845183	7.847655	7.385998	7.726063	6.998180	7.038434	7.717665	9.131674	8.187900	7.633766	8.050463	7.799610	10.028734	7.806171	7.299385	8.066401	7.644929	7.628448	8.227971	6.727719	7.695315	7.465107	8.790260	6.791521	7.427752	7.868192	7.956364	6.682255	6.380296	7.044183	6.490154	7.247903	7.913572	7.924598	8.188953	7.238287	7.420070	7.200555	7.405370	7.255858	7.622388	6.948756	6.925172	7.416867	7.591191	7.824617	7.626559	7.489439	7.513425	8.754279	7.979416	8.158091	6.641471	8.454138	7.754559	8.128713	7.880499	7.493678	8.192758

AC099654.1	11.576018	11.904615	12.028997	12.114644	12.557332	13.221376	12.638870	13.012991	13.375655	13.082921	12.801793	12.808590	12.325086	12.296644	11.326901	13.079117	12.937441	11.624718	12.071131	12.627650	12.323739	12.137094	11.346810	12.264495	12.521236	12.283174	12.481140	13.022526	13.084373	12.682817	13.034495	11.632548	11.260928	11.399095	11.940136	11.940136	12.033678	11.729608	11.948032	12.056040	13.268822	11.478266	12.153901	11.839322	11.482399	11.301289	13.231650	11.708045	12.195864	11.601057	11.305602	11.329537	11.144558	11.311611	11.213047	13.137749	10.905411	11.258731	11.289041	10.623839	11.036408	13.091440	10.895803	11.551948	11.063922	11.382463	11.441607	11.295692	11.218631	11.413079	12.130685	12.472784	13.487273	12.188852	12.128873	12.084512	12.361158	11.802821	11.996542	10.957964	11.396845	11.060277	11.257082	11.535695	11.492172	11.680386	11.064379	11.183029	11.546045	11.112884	11.016201	11.380212	11.210981	11.984395	11.440762	11.792259	11.799525	12.431763	12.214094	12.694586	13.027671	12.104980	12.260507	12.386932	11.587454	13.288850	12.804049	12.262939	12.109814	13.583919	12.001025	12.854570	11.984395	13.536185	13.029353	14.720909	11.595942	12.273457	13.664472	11.787922	11.981214	11.648416	11.623310	13.319680	12.087405	12.796802	12.515826	12.796078	12.212389	12.597492	12.777583	12.195413	12.060702	11.512602	12.882226	12.239628	11.369611	11.321148	11.513777	11.608622	11.517806	11.068828	11.789270	11.854611	11.489042	12.534282	12.010496	13.330272	12.055582	12.423579	12.464689	11.657898	12.168351	11.553156	11.468535	11.774172	11.558177	12.171102	11.837404	12.943897	11.599594	11.250318	12.091525	11.543325	11.501057	12.868973	11.160805	12.402445	11.515464	11.670323	11.232005	10.772249	11.245791	11.375766	12.138659	10.961636	11.685883	11.149440	11.039023	11.224167	12.379891	11.258266	11.178065	11.112884	10.972801	11.177479	11.259428	11.054661	10.936702	12.257254	11.954230	13.073229	12.077723	11.534520	11.775216	12.200477	11.687667	11.820206	11.626596	11.485173	10.814754	11.915776	11.379904	10.651913	12.149438	11.249706	11.253526	11.081746	11.127532	11.062749	11.370366	11.902176	11.533627	11.884206	12.038655	12.202184	12.543722	12.329087	11.413847	12.005642	11.759762	11.857954	11.438226	12.600544	12.566429	13.614714	11.389096	11.551818	13.259193	11.771074	12.729457	13.470544	13.549579	12.083688	12.789055	11.646207	12.366380	12.385289

CU633967.1	9.904108	8.957785	9.646463	7.931418	8.381455	7.823644	7.710024	6.538584	7.334897	8.887749	8.051562	9.240739	8.494468	8.910236	8.795507	7.886288	8.535593	8.583923	8.702764	8.624195	8.282391	7.613414	10.120815	8.012608	10.160447	10.046469	7.250881	9.314274	8.853642	7.946027	9.126126	11.088277	10.122478	9.049275	10.061953	9.483039	8.695746	8.828725	8.867036	9.008088	8.961134	9.472477	9.080099	9.559541	10.132992	8.261106	8.876783	7.599706	7.750575	10.163187	10.430011	10.230861	10.323206	10.125235	9.937905	9.231606	10.320901	10.423614	9.877034	8.505072	10.134839	9.563450	10.190269	8.447167	8.789796	10.104477	10.326579	9.883634	10.058476	8.294418	9.936190	8.016793	9.188603	8.722201	7.532374	9.942272	9.594640	9.980743	8.234802	9.283544	8.591764	10.957536	9.165894	10.320444	10.194616	9.168150	10.280033	9.707187	9.028879	9.158502	9.821011	10.161998	9.275769	9.547611	10.249401	9.922578	9.962553	8.511237	8.626562	7.748264	9.316571	8.474166	7.536099	9.533987	8.900824	8.207388	7.026498	9.298596	8.500281	7.627042	7.992228	9.579165	7.584575	7.790379	7.219180	8.594404	10.089453	9.323989	7.774932	11.922965	8.693250	9.647368	7.248072	7.048649	8.135109	6.221062	7.673040	8.692954	9.101902	7.374716	9.453555	8.119459	5.826936	5.996901	8.232098	9.126433	9.569343	9.681259	9.010681	6.761061	8.380819	9.620568	6.850135	10.655638	9.515436	6.848549	8.973481	8.674520	8.527971	8.335866	10.317106	8.731515	9.994317	9.396514	9.321845	7.658152	7.964169	8.141326	9.121228	9.571818	10.527773	9.780558	10.488419	11.137872	9.183450	7.608529	9.330440	6.800254	10.877490	10.415564	10.416006	10.563692	11.622042	9.930158	9.688392	10.530093	10.705507	9.175420	7.967408	10.087772	8.635194	10.628379	8.395927	9.686241	10.669700	11.469730	9.795351	10.338376	8.760747	10.391503	8.514754	8.439798	8.731353	5.887979	9.945429	9.550393	10.338538	8.538157	9.464406	8.215311	10.954841	9.341743	9.939807	10.159975	8.552881	10.474815	11.388349	9.957986	9.840773	8.808130	10.474815	10.299127	11.744952	9.543795	9.813541	9.994464	8.532566	7.982864	9.070354	8.679300	8.225552	7.343018	9.619017	11.546511	7.631624	7.668974	9.789600	7.885512	5.675825	8.285774	11.660007	9.868678	6.997719	10.322128	6.625668	8.397030	8.879581	7.942940

SLX1B-SULT1A4	14.046714	13.509011	12.670521	13.725347	12.780366	12.833844	12.975099	12.252315	13.160712	12.857801	12.720078	13.164705	13.049159	12.959744	13.247129	12.732190	13.042766	12.834200	13.335001	13.151060	12.794117	13.102943	13.640364	13.272175	13.873927	13.574884	14.106916	13.008295	13.288240	13.396009	13.198919	13.941795	13.169781	14.588535	14.021820	13.484384	13.249121	13.025833	13.203792	13.228647	12.938700	13.424093	13.964894	13.275857	14.095820	13.433260	13.376076	13.063034	12.896421	13.197929	13.599502	13.407160	14.040527	13.227058	13.036697	13.011074	13.771343	13.128058	13.285244	12.761874	13.400373	13.457219	13.978080	13.375655	13.142467	13.038398	13.649396	13.040624	13.591444	13.087748	13.540385	13.540597	13.142638	13.377562	13.535967	13.427811	13.690456	13.355173	13.759917	13.574462	13.624991	14.162372	14.569736	13.037820	13.285244	13.263764	13.931123	13.297973	13.755126	12.707057	12.869225	13.389920	13.612555	13.189155	14.034970	13.410097	12.843171	13.607572	12.915887	13.161859	13.611109	12.677012	13.424093	12.353725	12.657996	12.866032	13.366676	13.003067	13.145329	12.726676	13.300869	13.259393	13.394330	13.264544	12.496680	14.108206	14.573111	13.143585	13.377337	14.298742	12.888793	13.347432	14.239437	13.849877	12.694078	14.365930	14.325742	13.608981	14.280056	13.477052	13.822664	14.384530	15.157558	13.763964	13.678771	13.578538	13.072450	13.487724	13.069482	12.996930	14.622502	13.671966	13.862105	13.405875	13.847489	13.473153	14.397321	14.117369	13.025652	13.348898	14.077463	14.060320	13.363265	13.379894	13.461577	12.878536	13.084018	13.364346	13.762967	14.012930	13.219365	13.264151	13.135653	12.910540	12.681285	13.160892	12.505911	13.274804	12.848771	13.359500	13.481256	13.225883	12.085444	14.224386	14.484873	13.791211	14.298393	13.253211	14.535929	13.893142	14.008743	13.286686	13.284435	13.717719	13.324170	13.286871	12.941926	13.628249	14.121601	13.731415	13.707724	13.939322	13.646113	13.932970	13.012290	13.440530	13.770321	13.327806	13.469871	14.073949	13.690456	13.518662	14.518957	12.916924	13.771628	13.039846	13.616612	13.810630	13.040624	14.408016	13.011946	14.108206	13.133954	13.483894	13.479517	13.953224	14.276902	13.524608	13.441594	13.948216	13.841137	14.513547	13.153806	13.239397	14.411699	14.235557	13.981851	13.938513	12.781052	13.531473	13.736836	14.232858	13.395578	14.167438	13.463591	13.986959	13.619020	13.385597

SLX1A-SULT1A3	14.046714	13.509011	12.670521	13.725347	12.780366	12.833844	12.975099	12.252315	13.160712	12.857801	12.720078	13.164705	13.049159	12.959744	13.247129	12.732190	13.042766	12.834200	13.335001	13.151060	12.794117	13.102943	13.640364	13.272175	13.873927	13.574884	14.106916	13.008295	13.288240	13.396009	13.198919	13.941795	13.169781	14.588535	14.021820	13.484384	13.249121	13.025833	13.203792	13.228647	12.938700	13.424093	13.964894	13.275857	14.095820	13.433260	13.376076	13.063034	12.896421	13.197929	13.599502	13.407160	14.040527	13.227058	13.036697	13.011074	13.771343	13.128058	13.285244	12.761874	13.400373	13.457219	13.978080	13.375655	13.142467	13.038398	13.649396	13.040624	13.591444	13.087748	13.540385	13.540597	13.142638	13.377562	13.535967	13.427811	13.690456	13.355173	13.759917	13.574462	13.624991	14.162372	14.569736	13.037820	13.285244	13.263764	13.931123	13.297973	13.755126	12.707057	12.869225	13.389920	13.612555	13.189155	14.034970	13.410097	12.843171	13.607572	12.915887	13.161859	13.611109	12.677012	13.424093	12.353725	12.657996	12.866032	13.366676	13.003067	13.145329	12.726676	13.300869	13.259393	13.394330	13.264544	12.496680	14.108206	14.573111	13.143585	13.377337	14.298742	12.888793	13.347432	14.239437	13.849877	12.694078	14.365930	14.325742	13.608981	14.280056	13.477052	13.822664	14.384530	15.157558	13.763964	13.678771	13.578538	13.072450	13.487724	13.069482	12.996930	14.622502	13.671966	13.862105	13.405875	13.847489	13.473153	14.397321	14.117369	13.025652	13.348898	14.077463	14.060320	13.363265	13.379894	13.461577	12.878536	13.084018	13.364346	13.762967	14.012930	13.219365	13.264151	13.135653	12.910540	12.681285	13.160892	12.505911	13.274804	12.848771	13.359500	13.481256	13.225883	12.085444	14.224386	14.484873	13.791211	14.298393	13.253211	14.535929	13.893142	14.008743	13.286686	13.284435	13.717719	13.324170	13.286871	12.941926	13.628249	14.121601	13.731415	13.707724	13.939322	13.646113	13.932970	13.012290	13.440530	13.770321	13.327806	13.469871	14.073949	13.690456	13.518662	14.518957	12.916924	13.771628	13.039846	13.616612	13.810630	13.040624	14.408016	13.011946	14.108206	13.133954	13.483894	13.479517	13.953224	14.276902	13.524608	13.441594	13.948216	13.841137	14.513547	13.153806	13.239397	14.411699	14.235557	13.981851	13.938513	12.781052	13.531473	13.736836	14.232858	13.395578	14.167438	13.463591	13.986959	13.619020	13.385597

AL157387.1	4.250623	4.993108	4.626343	5.054726	4.786204	4.487427	4.255890	5.748675	6.334005	4.613576	5.449750	3.895246	3.918102	4.633723	4.327649	5.234225	5.768488	4.687813	5.411287	4.901741	5.175953	5.903669	3.412420	5.028096	4.223185	4.751699	5.741912	4.817310	3.769711	3.834416	5.746855	4.408268	4.373702	4.207295	4.418927	4.109814	4.018242	3.611177	4.407819	4.797060	3.824361	4.012459	5.552203	3.587283	5.321288	5.218532	4.612954	5.296034	5.468755	5.228917	4.788883	5.943205	4.483077	4.570729	4.515053	4.336552	4.152983	4.788062	4.650252	4.083044	4.908825	4.032550	4.049784	4.195839	4.016060	4.997369	4.885388	5.254943	4.710948	5.517407	4.715607	5.154789	4.182342	5.080015	5.255133	4.921033	5.106656	4.346322	5.190131	4.279490	4.020460	4.593852	4.215554	4.485251	4.527996	5.740064	4.562514	5.224298	4.540902	4.755521	4.313602	4.459665	4.561205	5.131805	4.040380	4.530956	4.857954	4.350224	3.596508	4.006327	4.189080	4.633497	4.813900	5.413965	5.386136	4.488050	6.281614	4.622478	4.467164	4.625263	3.749198	5.325747	5.378859	4.672289	6.072060	3.760914	4.691364	4.662468	4.523673	4.796275	4.700068	4.054728	4.924879	3.368431	4.825211	3.582650	4.459665	5.483503	4.209917	5.008684	4.452895	3.426395	4.018026	4.033222	4.334415	4.282734	4.241406	5.619645	4.082834	4.853109	5.808130	5.557935	4.311250	6.833773	5.611223	4.949812	4.513363	3.687063	5.860307	5.387168	3.697777	4.107628	4.394058	3.648809	4.209917	4.389751	4.052798	4.812075	4.742349	3.741588	3.961381	4.633927	4.740850	5.354518	5.030942	4.521048	4.664093	4.369790	5.496995	4.644274	5.649908	4.242269	5.442939	4.293954	4.129806	3.715469	4.637372	4.852673	4.252568	4.606924	3.695073	4.553885	4.023461	4.743378	5.063633	4.266767	5.022393	5.364627	5.356278	4.509641	4.738648	5.359285	5.020517	7.589045	4.546452	4.939145	4.575001	4.874048	3.667908	3.786951	4.346322	4.400675	3.892646	4.901535	4.756537	4.040380	4.255230	4.389553	4.549992	3.897142	5.124493	3.555075	8.662706	3.934506	4.384713	3.478413	3.731933	5.342170	4.321207	3.605404	4.651571	5.038331	3.612579	5.122893	5.286988	6.030923	4.782850	4.613988	4.817515	3.857006	4.280176	10.708553	5.193268	4.418689	7.642308	4.126791	4.818563	4.060633

AC024958.1	8.418410	7.327719	7.105493	7.745146	7.645874	7.769577	6.826234	8.949362	8.899620	7.503695	8.069834	7.582103	6.608840	7.197600	6.599911	7.531584	8.525979	7.190783	8.436002	7.570069	8.147603	7.688871	10.113777	7.369686	7.816357	7.931268	7.316850	8.440424	7.295535	6.731923	8.526736	7.100790	6.633215	6.008585	6.644679	6.992762	6.961659	7.415512	7.806171	7.966965	7.084263	7.528744	7.267588	7.163145	7.431568	7.320128	8.183733	7.354382	7.297804	7.386163	7.675274	7.717058	6.351290	7.074821	6.816661	7.983020	6.551642	7.335219	7.343519	6.949978	7.501316	6.676256	7.761592	8.106204	7.541274	7.933559	7.133895	7.493042	7.195198	8.180684	7.509249	7.270165	7.488204	7.284600	7.470794	7.162350	7.520818	7.468472	8.589431	6.913999	7.030942	6.984753	7.245475	7.157778	7.864481	7.435698	7.648147	7.743330	7.709719	7.944176	7.654830	8.474768	6.699964	7.067141	6.845868	6.865185	7.073401	6.402607	6.596013	6.832787	6.783375	6.665571	7.098623	6.749904	8.099931	7.091977	9.118833	7.930030	9.033855	7.946185	8.753789	8.417966	8.036665	7.866510	7.809741	7.582731	7.971783	7.506496	7.066825	7.484898	7.411794	6.950312	7.392107	7.772768	8.477734	7.483209	8.786705	8.162655	8.370333	8.224788	7.989106	8.507964	7.830357	8.929545	7.695913	8.066110	7.498515	8.041935	7.457145	8.577211	8.578321	9.782831	7.978000	8.304977	9.022179	8.531228	8.258005	8.507374	7.359788	8.725114	7.068891	6.984753	6.465048	6.733689	7.362910	6.702081	7.832609	8.115470	7.910633	7.694740	7.746079	6.692759	7.700541	7.479190	7.687949	8.519296	7.784088	7.701163	7.743798	7.646968	7.599022	6.257069	6.672024	6.558456	7.619865	6.805681	6.666704	7.070868	6.771256	7.712007	6.836278	7.865564	8.032995	7.594740	7.589345	7.207692	7.296044	8.282080	8.036191	7.383010	7.329932	8.430243	7.204710	7.209083	7.253866	7.842303	7.617095	7.937457	6.802029	6.561198	7.189234	7.254021	6.956277	7.671008	7.845512	7.256941	7.681782	7.760354	7.780442	7.711235	8.469173	7.936063	8.083490	7.450892	7.230254	7.762352	6.613816	7.259651	6.858980	7.891070	6.777825	7.149287	6.285328	7.467854	8.900970	8.692458	7.964169	8.545378	9.085192	8.382834	8.081052	7.927574	8.730609	8.276122	6.826070	7.779314	7.621512	8.505802

AC120193.1	8.418410	7.327719	7.105493	7.745146	7.645874	7.769577	6.826234	8.949362	8.899620	7.503695	8.069834	7.582103	6.608840	7.197600	6.599911	7.531584	8.525979	7.190783	8.436002	7.570069	8.147603	7.688871	10.113777	7.369686	7.816357	7.931268	7.316850	8.440424	7.295535	6.731923	8.526736	7.100790	6.633215	6.008585	6.644679	6.992762	6.961659	7.415512	7.806171	7.966965	7.084263	7.528744	7.267588	7.163145	7.431568	7.320128	8.183733	7.354382	7.297804	7.386163	7.675274	7.717058	6.351290	7.074821	6.816661	7.983020	6.551642	7.335219	7.343519	6.949978	7.501316	6.676256	7.761592	8.106204	7.541274	7.933559	7.133895	7.493042	7.195198	8.180684	7.509249	7.270165	7.488204	7.284600	7.470794	7.162350	7.520818	7.468472	8.589431	6.913999	7.030942	6.984753	7.245475	7.157778	7.864481	7.435698	7.648147	7.743330	7.709719	7.944176	7.654830	8.474768	6.699964	7.067141	6.845868	6.865185	7.073401	6.402607	6.596013	6.832787	6.783375	6.665571	7.098623	6.749904	8.099931	7.091977	9.118833	7.930030	9.033855	7.946185	8.753789	8.417966	8.036665	7.866510	7.809741	7.582731	7.971783	7.506496	7.066825	7.484898	7.411794	6.950312	7.392107	7.772768	8.477734	7.483209	8.786705	8.162655	8.370333	8.224788	7.989106	8.507964	7.830357	8.929545	7.695913	8.066110	7.498515	8.041935	7.457145	8.577211	8.578321	9.782831	7.978000	8.304977	9.022179	8.531228	8.258005	8.507374	7.359788	8.725114	7.068891	6.984753	6.465048	6.733689	7.362910	6.702081	7.832609	8.115470	7.910633	7.694740	7.746079	6.692759	7.700541	7.479190	7.687949	8.519296	7.784088	7.701163	7.743798	7.646968	7.599022	6.257069	6.672024	6.558456	7.619865	6.805681	6.666704	7.070868	6.771256	7.712007	6.836278	7.865564	8.032995	7.594740	7.589345	7.207692	7.296044	8.282080	8.036191	7.383010	7.329932	8.430243	7.204710	7.209083	7.253866	7.842303	7.617095	7.937457	6.802029	6.561198	7.189234	7.254021	6.956277	7.671008	7.845512	7.256941	7.681782	7.760354	7.780442	7.711235	8.469173	7.936063	8.083490	7.450892	7.230254	7.762352	6.613816	7.259651	6.858980	7.891070	6.777825	7.149287	6.285328	7.467854	8.900970	8.692458	7.964169	8.545378	9.085192	8.382834	8.081052	7.927574	8.730609	8.276122	6.826070	7.779314	7.621512	8.505802

AC005019.2	3.283804	5.358153	3.808458	4.847648	3.967464	5.451062	3.989003	3.803066	4.057364	5.199295	4.472145	9.811828	3.908793	4.125452	4.321641	4.218793	3.440673	4.237634	7.595483	5.770690	6.377801	2.904784	5.655012	5.497164	4.221265	5.829507	7.251697	4.414658	5.374165	5.189361	3.630055	5.098046	3.065322	6.825424	4.103884	7.195198	4.337446	4.700680	4.119663	3.125395	3.771146	6.940887	7.175741	7.329623	3.399012	4.290244	4.277739	8.994252	4.615319	4.663683	10.071016	5.177742	5.587518	4.954364	8.010496	4.353548	3.770137	3.457527	5.908486	5.090008	5.602065	3.697556	3.381727	4.556230	3.091207	8.162350	9.316873	3.782305	3.831827	6.306280	4.899838	4.599178	4.443479	4.678180	4.369566	3.610550	4.924291	8.546267	8.631287	4.621436	4.537161	3.439595	10.130777	10.707761	4.708848	5.448989	5.530310	3.993280	5.356278	8.668982	6.608356	7.257558	5.906156	4.458021	10.012605	3.943276	8.303777	4.251929	4.669934	3.678004	4.827398	7.914014	4.527587	3.369785	5.014462	4.830820	3.713585	4.152749	4.208004	4.730610	5.330690	5.529351	6.640806	3.858553	3.433628	6.029076	4.134500	5.925083	3.589954	3.917040	4.779447	3.380664	4.251497	3.908140	5.634357	4.297429	3.716284	4.094749	4.733792	4.757829	5.524212	3.622868	3.851185	3.925247	4.847648	4.429795	4.316709	4.058241	4.275527	4.749752	3.290950	6.144448	4.035605	4.610588	3.910122	5.142355	4.978266	4.973155	5.742641	3.840908	3.614957	3.027977	4.595108	4.832509	4.615553	3.963586	4.013112	4.351327	4.781987	4.713069	5.406712	4.837031	3.755267	3.723511	5.492994	5.136622	4.553015	3.992018	4.199654	6.391892	4.666242	5.040502	4.950017	6.028736	4.587333	4.541317	3.337310	4.664293	5.259268	4.126095	4.499005	3.379490	4.609078	4.795182	5.251581	5.367011	4.681788	4.607765	4.557070	4.733129	4.525441	4.773477	4.851173	5.159264	3.776565	4.505883	6.022738	5.649730	3.810138	4.273323	5.392206	5.808857	5.628592	4.951447	5.432006	4.424840	5.027033	4.408268	5.547508	4.379938	5.086568	3.796720	4.793100	6.365038	4.316709	4.920418	4.573522	3.734015	3.937514	3.969670	3.513752	3.381903	3.450800	4.714337	5.060247	4.251929	4.526954	4.115928	5.576839	4.352445	5.904025	4.059980	5.028289	4.220612	5.519352	4.769928	4.122955	5.017705

STAG3L5P-PVRIG2P-PILRB	12.309669	12.806311	12.482306	12.917840	13.378453	13.120800	12.383384	14.024760	13.928111	12.939734	13.576005	13.229053	12.015060	12.311428	12.583137	14.278587	14.277581	12.450587	13.293540	14.473115	13.163201	13.043520	13.776673	12.640385	13.235191	13.500208	13.354792	14.074236	13.659121	12.505236	13.566158	11.525463	13.175984	11.817333	13.504804	13.966043	13.535732	12.676204	13.118063	13.765216	14.017264	12.927379	13.341990	13.689045	14.230892	14.181178	13.183086	13.841638	13.297375	15.399178	14.280056	13.237448	12.000568	12.748989	12.552169	13.544167	12.536742	11.963379	12.239925	12.140193	12.550549	13.478600	12.185867	13.043339	12.718533	13.323155	12.224005	12.944614	12.527930	13.065502	15.170169	14.841605	13.923216	14.466988	14.640496	13.019185	13.384576	13.226062	13.806755	12.003241	12.173094	12.896716	11.699314	12.832557	12.566772	13.697560	12.817679	13.178579	12.635767	12.579011	12.541440	13.231650	12.513855	13.414830	12.914641	13.576005	13.039676	13.614835	12.852132	13.000997	12.966367	12.774471	13.699672	12.490571	12.698356	13.678503	13.258409	12.889837	13.642281	13.867543	13.254784	13.282824	13.102201	13.789730	13.830488	13.340559	12.781728	14.032887	13.754158	12.849310	11.726308	10.955728	12.257562	12.670521	12.229725	12.060393	14.363390	13.217768	11.801628	11.658803	12.337752	12.378817	11.165378	12.352896	13.602656	12.251209	12.830155	13.868320	11.651970	12.532019	12.728615	13.226454	13.013913	11.787766	11.725723	12.618797	12.670169	13.609930	11.486012	13.517555	12.518587	12.243629	11.218189	11.872045	11.686921	11.698867	11.674592	12.196619	12.445640	13.546876	12.103454	11.107323	11.748620	11.599731	13.277050	12.303273	11.263631	11.811570	11.519647	12.203746	11.578488	11.245651	10.999997	10.888785	12.034862	11.399245	11.790004	12.083231	11.478703	12.218928	12.206044	12.569545	12.122102	12.610699	11.715417	12.016430	11.562313	10.866526	12.543245	12.173415	11.977145	13.337700	12.109967	11.916369	11.832854	11.585010	12.152993	12.770845	11.223118	11.226796	11.882808	11.184811	12.172634	11.594141	13.001778	10.942871	11.899712	12.094888	11.824133	11.837100	13.094857	12.201396	11.698568	12.207449	12.507056	12.424576	13.097319	12.074208	11.308373	11.553592	12.280960	12.647433	10.830328	12.447724	12.575245	12.549091	13.608723	13.239009	12.183786	12.880782	12.461907	12.139582	13.718689	12.129027	11.824581	12.130685	12.611352	12.060100

AL137230.2	10.617450	9.717013	10.925062	10.092624	10.483377	11.337604	11.324533	11.696817	11.194304	11.383763	11.451631	11.429574	11.045017	11.120430	10.047711	10.879299	11.403089	10.266879	10.609505	12.095947	11.259009	11.025247	9.876239	10.670884	10.189657	10.771942	9.334161	9.555229	10.074539	10.675830	10.750537	11.556411	11.142360	12.102545	11.258424	11.430579	11.463192	10.395361	10.460118	10.389435	11.222672	10.661622	11.305887	11.520985	11.283416	11.453428	10.127898	10.394618	11.470789	11.379762	11.063780	11.550016	11.050033	11.422252	11.166077	11.116371	11.368582	10.778852	11.103274	10.504682	11.015142	10.552353	10.070780	10.391640	10.886126	10.553085	10.992097	11.336278	11.096922	10.612584	11.345947	11.400910	10.632306	11.076058	11.548861	11.340546	11.793463	11.173601	11.398826	11.222057	11.282256	11.422252	11.369004	11.639628	11.549582	11.237278	11.278862	11.770488	10.824736	10.569692	10.222096	10.827110	11.146045	11.501471	11.718550	11.232575	11.107323	10.707633	11.212133	10.036868	11.252949	9.983054	10.670427	9.840309	10.516102	11.261380	10.974503	10.713869	10.448550	10.829909	11.397622	11.621293	10.218469	10.507667	11.303223	10.679432	9.102598	11.253964	10.508413	13.011590	10.218320	11.001368	11.790428	9.560659	11.025992	11.748029	9.333063	11.314528	11.723934	11.356089	12.188558	11.003790	11.824728	10.075427	10.903200	12.503725	10.100927	9.235309	11.080854	12.105743	11.223409	10.745281	11.051912	12.008706	11.908965	11.083497	9.173114	9.170296	11.495782	11.310881	11.651679	12.010940	10.963706	11.669383	11.786428	11.213657	10.424236	11.439735	10.826070	11.053493	10.891326	11.493601	11.902027	11.293819	12.218342	11.147376	9.978579	11.189473	11.907107	11.165084	11.832392	12.317479	12.606391	12.566593	11.599875	11.337458	11.691236	10.929785	10.209520	10.880620	12.161407	11.068070	11.617285	11.309113	10.596659	11.675894	11.081157	11.141784	11.537919	12.514335	12.212389	10.673716	12.196619	11.788973	11.803092	11.934585	12.380673	11.661642	12.095947	11.522184	12.301244	12.179402	11.392925	11.190758	11.403818	12.555263	12.190143	11.368020	10.694498	9.811338	11.076776	11.021682	12.424576	10.793950	11.245942	11.434496	10.655933	12.032298	10.630112	11.952893	10.404404	10.521111	10.537076	12.192594	10.354602	11.309113	10.906005	11.034454	11.864391	10.892382	11.984692	9.761826	11.063360	10.413149	11.753936	10.783006	11.518703	12.885289

AL158847.1	14.800886	14.488728	13.561728	14.435670	13.502998	14.386005	13.416705	13.299434	12.773104	11.813442	13.093311	12.607553	14.317178	13.915290	14.026270	12.695570	13.232837	11.834043	13.494882	14.686942	11.793767	10.862762	13.244529	13.033736	13.072832	13.024565	11.286261	12.037292	12.180008	13.556369	12.719368	13.553239	12.221249	13.704613	14.247926	12.024501	13.741329	14.637945	14.071794	13.754901	14.249525	12.347129	12.654999	13.859930	12.421202	12.757381	11.999458	13.900462	12.062616	14.057422	12.786304	14.313436	10.643266	15.183376	13.611591	13.447775	14.205304	12.754129	14.122743	13.629812	13.872874	13.393094	13.959054	14.288296	13.750799	14.541751	14.680064	14.164273	13.068499	14.975358	11.953478	13.028398	11.615675	12.054398	14.497531	13.233234	12.779001	13.576961	12.334777	15.164050	13.930990	13.472274	14.705283	15.161962	14.367353	13.487933	14.997957	13.839011	14.490783	13.969061	13.938772	14.073025	13.682111	13.659354	13.766279	14.159585	13.162239	13.293957	14.302591	10.879160	13.781596	14.000102	13.225108	13.404827	15.005964	12.666171	13.387105	14.937805	12.729813	13.777164	13.694470	13.762967	13.591444	11.625759	11.743625	12.637159	14.375159	13.554828	12.533129	11.860241	12.185427	12.789055	14.193358	15.356364	13.403139	14.158638	11.302336	11.882981	15.565819	13.715358	15.383684	14.736202	12.163433	14.375530	12.435794	15.056697	15.400849	11.332182	12.028551	16.377052	16.559494	16.143053	14.680965	13.942960	14.621136	12.137555	15.521068	14.756024	14.880110	12.180333	13.967702	12.641230	13.239009	12.583952	15.918070	12.752609	13.888605	13.623383	15.648843	11.294668	15.927561	12.879072	11.442790	15.564042	15.909426	11.253526	12.913586	16.175634	15.122829	15.504733	15.060407	15.603850	11.650941	12.821251	14.591979	13.475325	13.697330	14.528702	12.639389	11.740862	13.676222	14.423235	14.556844	14.423617	12.703486	13.105804	14.478214	14.706637	13.295547	15.888240	15.628044	12.778119	15.399973	12.243771	13.570563	15.429349	12.703807	14.930806	12.779334	14.655931	14.696136	12.285526	12.849653	12.919720	14.976992	13.020460	12.833284	13.167086	12.767450	11.991699	11.612110	14.347809	13.214202	13.109649	12.963794	15.184064	14.097636	13.315612	15.618883	13.018269	12.536742	11.322149	14.907590	13.407160	15.244645	14.524343	13.425768	16.033556	13.558417	15.507347	12.449765	12.729457	11.128092	15.059149	12.913034	15.347177	14.243708	11.707448

LINC00476	6.045183	5.504495	5.674591	6.589823	6.815250	6.292186	5.123510	6.584231	7.297185	5.721773	5.956634	5.677876	5.801186	5.924351	4.942813	6.285685	5.431625	6.351623	6.779578	6.462837	5.492415	7.736364	7.239061	5.834830	6.632728	6.331112	7.730087	6.047635	6.152751	5.411091	6.613637	5.046279	6.793323	6.087034	5.882835	5.291392	6.157700	5.668327	5.996198	6.231428	5.669795	6.146720	6.405727	6.357836	5.631732	6.176270	6.500881	5.320912	5.748126	6.094796	5.588122	5.990072	5.568462	6.203825	5.188817	5.141740	5.808321	6.059390	5.927599	6.160836	5.798779	5.855990	5.020329	6.322380	5.946038	6.308393	5.733384	5.946038	6.018659	5.857438	5.839528	6.284297	6.278646	5.829701	6.144094	5.920495	6.151391	5.963956	6.234306	5.408723	5.734096	5.170694	5.924523	5.630815	5.892560	6.514561	6.496512	5.581896	5.976039	5.784311	6.295776	6.145662	5.730111	5.921041	6.292836	6.593887	5.347540	7.219837	6.097354	6.373651	6.842711	5.721773	5.990781	6.200316	6.311621	5.365588	6.557965	5.993944	6.998024	6.059390	7.384238	7.095149	6.988569	5.836646	5.811306	8.646267	6.378473	6.001442	6.050288	5.755174	5.415450	6.049931	6.274392	6.912288	5.762505	6.238948	5.839182	6.566408	5.649548	5.670843	6.276203	6.232927	5.724374	5.471801	6.768967	5.354909	6.810338	6.137188	5.765200	7.036542	7.738513	7.151233	6.537774	7.260136	5.741912	6.786625	6.546804	6.458695	5.040958	5.914751	4.900045	6.747882	5.448014	5.626721	5.299069	4.873648	4.839153	5.515028	5.991827	5.380410	5.262459	6.000081	5.717116	5.392206	6.084753	6.321010	4.727036	5.331441	5.065069	5.076919	5.424652	5.592741	5.658691	6.020997	4.781151	4.835195	4.211867	5.754253	4.953147	5.336327	5.485751	5.317845	5.890639	5.480063	5.724728	4.886886	5.356651	6.019364	6.500881	5.611588	6.105503	5.888143	5.920495	5.807043	5.373609	6.152405	6.207422	5.871887	6.129664	5.451858	5.286398	4.841900	5.359485	5.826559	6.881833	5.357782	5.368759	6.106563	4.549764	5.797651	6.115120	5.281557	5.565981	4.999641	5.824047	5.506022	7.153726	5.720867	5.336536	6.095497	5.938973	6.284798	6.126955	5.428231	5.756267	5.690604	4.967357	6.045528	6.218710	7.429167	7.660627	6.777676	6.248293	6.306442	6.254020	5.621849	5.773628	5.983137

AC007881.2	9.461669	11.444835	11.050179	12.187947	12.099519	11.795267	10.964890	11.611531	11.631199	11.874475	11.535555	11.397000	11.785972	11.374333	10.681237	11.503353	11.672977	11.789270	12.859886	12.188558	12.342944	12.720580	10.474815	11.579070	11.586330	11.380384	10.086020	11.215086	10.787493	10.797174	11.713634	10.661313	11.316464	9.753292	10.977437	11.209812	11.100894	11.480649	11.186293	11.941654	11.526214	11.884784	10.922691	11.501766	11.133235	11.352031	10.848448	11.503051	11.716302	11.464337	10.795416	11.622359	11.188301	12.110108	10.940377	11.611963	11.091753	12.688875	12.057480	12.011905	11.041054	10.714317	9.582379	12.071578	11.900872	11.957549	11.901889	11.694244	10.994776	12.069547	11.613450	11.525597	11.580850	11.102976	11.853844	11.353455	10.908996	10.494828	10.906618	11.497713	11.416046	11.971606	11.647691	11.704602	11.991560	11.131351	12.283820	11.941654	12.416103	13.126344	12.238997	12.017200	11.323781	10.997597	10.454796	10.606972	11.281507	11.632692	11.164195	11.547983	12.411884	11.359717	11.672206	11.720318	13.470762	11.514555	10.947735	11.679053	12.893290	11.159203	12.749170	12.934551	12.316696	11.720318	11.665663	14.643584	10.726118	11.443990	11.711746	10.798200	10.471930	10.566197	11.588623	13.268023	9.776123	11.305447	11.937486	10.447031	10.746942	11.663576	10.768791	10.801504	10.076815	9.910027	10.934280	10.551138	11.598426	9.783775	9.688230	9.922734	12.139877	10.470285	11.250483	11.601959	8.652809	11.511703	11.644748	11.828974	9.106280	10.428516	10.490251	12.035499	9.718135	10.883860	10.460851	10.622929	8.998715	10.036097	10.758230	10.401108	10.967694	10.451084	9.450439	10.553981	10.150520	10.341556	10.295232	10.661012	8.815433	8.991935	8.913127	9.895500	9.781478	10.450036	9.643333	12.004151	11.430160	10.911904	10.208318	12.163282	10.128547	11.522791	10.821608	9.417469	9.336777	11.691236	10.093823	9.925324	9.013309	10.171845	10.214570	11.533937	10.415417	11.538067	10.026576	9.887683	9.443592	9.957845	9.658892	11.770344	11.396394	10.953350	11.107037	9.842744	11.518252	11.864391	12.109351	10.717299	10.637737	11.696453	11.351141	10.302329	9.067083	11.814926	11.250783	11.382032	11.713634	10.672708	10.489033	10.654131	10.544311	11.012108	9.748169	9.493310	10.571069	9.696776	11.084541	11.616692	9.650553	11.707306	12.348370	13.149340	12.127514	11.550311	11.693642	10.642074	10.302633	9.017607

AC024598.1	8.773990	13.486625	12.848215	13.881969	12.466727	13.575781	13.463591	14.914162	13.983511	13.880924	14.275219	13.773715	13.429511	13.024204	11.137430	14.545966	14.114654	13.242782	13.898972	14.318839	13.526138	13.493263	12.516941	13.641034	14.194332	13.241017	8.523862	14.369059	12.786674	11.571786	14.769473	12.272956	13.457219	8.540210	12.239628	13.098632	13.701651	12.990154	12.009153	12.704515	13.512856	13.223528	12.287847	13.083849	11.871132	13.257927	12.555591	12.117118	13.854876	14.084957	12.812473	14.176188	12.061208	13.446068	12.836529	12.848393	12.516941	13.448203	13.866495	12.617952	11.687212	12.231738	9.739940	12.905187	11.814644	12.364491	12.890172	13.254006	12.611838	12.400465	14.005012	13.713207	13.889925	13.295547	14.656363	12.748989	11.652428	12.546681	13.607340	12.750695	11.794389	13.094669	11.675300	13.548218	14.386005	13.297173	14.333069	14.096413	11.471518	13.314147	12.504062	12.460105	12.510296	13.905302	10.095036	12.449765	13.183285	14.440521	12.832557	13.951282	14.068626	12.765859	13.695182	13.583683	12.880949	13.195928	14.165841	14.025995	11.396394	13.902033	13.744601	12.992187	13.517777	14.030880	13.129172	11.880130	8.590817	11.887052	14.290687	8.862460	10.897614	10.656820	9.320905	9.644704	11.601212	9.659984	9.634169	11.033135	8.791209	11.124898	8.595641	11.419414	9.177266	8.922259	11.172697	10.171080	8.418259	8.941854	9.958930	9.301255	10.606321	9.245186	8.747097	11.417260	9.592273	11.276449	9.647368	9.004108	11.065101	9.537676	8.628667	10.570618	8.292759	9.185181	9.148011	10.834345	9.408019	10.115300	9.543317	9.197337	8.857582	8.215620	9.226978	9.733039	12.859356	10.885995	8.848183	9.995566	10.610648	10.023218	9.568749	9.961059	13.220765	11.158915	9.507776	10.082394	9.694205	9.894559	10.066763	9.114050	8.685237	8.685699	11.172387	9.259376	9.434621	10.849717	10.348574	9.633365	9.808072	9.802581	9.561136	11.635559	10.614503	10.948169	10.593040	11.062749	10.011528	12.747949	8.987649	10.859980	10.801645	9.596485	9.854790	9.890146	9.384818	10.487949	10.752512	10.424393	9.981975	8.585432	9.700346	9.399396	10.628861	8.859559	8.684477	9.621976	10.175472	8.871708	10.252055	10.985236	8.959155	9.996607	9.451553	9.767677	9.262432	9.991411	10.986598	10.709963	11.170020	9.952136	10.751156	8.530777	13.048973	8.680362	11.443526	8.953796	9.517921	10.525656

NNT-AS1	11.021387	9.736555	9.788049	9.913595	9.512448	9.346035	10.061198	9.063980	9.317184	9.483198	9.657788	9.537204	9.599406	9.301568	9.844563	9.774925	9.854153	9.620259	9.993987	10.070538	9.846139	10.261948	11.851271	9.598341	10.058151	10.046320	10.433959	9.722173	9.521928	9.843353	9.640574	10.265145	10.127098	10.631433	10.453574	10.382887	10.324283	9.822386	10.064516	10.193091	9.670545	10.704907	9.622457	10.457252	10.824286	10.479190	9.734386	9.936190	9.896278	10.371089	10.433224	10.496309	10.525793	10.484421	10.118987	9.784596	10.587164	10.786914	10.523853	10.761899	10.790142	9.725155	11.237709	10.460708	10.802701	10.300966	10.208000	10.039433	10.009427	10.411451	10.125679	10.156965	9.384364	10.208469	10.362213	10.171080	10.446580	10.144003	10.147640	10.090076	10.150370	10.482463	10.696036	10.487949	10.864236	10.237139	10.566949	10.668143	10.820570	10.383504	10.719260	10.733421	10.449732	10.148232	10.305371	10.082394	9.844727	9.996310	9.886402	10.193244	9.765569	9.748635	10.029801	9.766931	10.081641	9.402013	9.679564	9.740723	10.191897	9.572296	10.413447	9.899145	9.713258	9.380804	9.241228	8.590205	11.200874	9.650396	9.650895	9.279051	9.411694	10.646057	10.696919	9.056876	10.168122	9.344383	11.343861	9.152937	10.913094	9.965297	10.643415	9.860520	10.887915	10.269429	10.656389	10.638038	10.603714	10.195238	11.445862	10.304483	10.278521	11.426951	11.920823	10.758822	9.978579	10.263600	10.231313	10.683258	10.601053	10.521409	10.003213	10.678806	10.291731	11.368306	10.971013	10.534840	10.951630	10.091582	10.106142	9.692997	10.915674	9.143508	10.753411	10.060156	10.320132	8.831633	10.909294	10.840635	10.121103	10.241972	11.008580	10.902031	10.365777	11.015894	10.880179	11.305742	11.555370	10.606813	11.482953	11.062917	10.704907	11.585010	10.359626	11.580850	10.644156	10.650447	9.962091	10.352513	11.248223	10.178593	10.727937	9.816798	10.067514	10.655933	10.530093	10.781758	10.733574	10.824286	10.501539	10.704762	11.583331	10.539877	10.843773	11.858431	11.152759	10.628696	11.259294	11.525172	10.947309	12.190447	11.331478	11.013251	10.507667	10.507361	9.829982	10.754711	10.505710	10.162874	11.181871	11.170020	11.134182	11.863940	9.982906	12.365253	8.959927	10.585809	10.376060	10.398683	8.896560	10.931284	10.149179	9.698334	9.561303	11.050179	10.164877	10.345226	9.533298	11.030014

DPH6-DT	5.552989	4.139542	5.381923	4.104536	3.230636	3.781857	5.148519	3.104899	3.324325	3.833571	3.645616	3.365508	3.869240	4.211867	4.783483	4.197310	3.309058	4.638630	4.769299	4.130225	3.945762	5.078192	6.002723	3.991355	4.489549	3.718521	4.592310	5.131199	3.896502	5.079007	3.461637	6.088675	5.408923	4.450747	4.644041	5.489978	4.627811	5.117824	5.283568	4.912630	4.257422	5.457267	4.491573	4.842115	6.048741	7.263127	4.973579	5.669795	5.744656	5.932445	5.603982	6.128501	6.761235	5.426867	6.187579	4.970294	6.499069	5.780338	6.042781	5.191894	6.347421	4.067751	7.396612	4.979117	5.741365	5.465597	5.562403	5.339727	5.383335	5.747211	5.171712	4.229251	4.908622	5.435125	4.861046	4.882858	5.007457	5.205438	5.072791	5.927599	4.897460	7.413210	5.560215	6.524506	6.265543	6.080745	5.268084	5.122714	6.637206	5.555822	5.633793	5.979804	5.274097	5.266469	5.038906	4.538643	5.770778	4.444999	3.507067	3.426395	3.861945	4.136714	4.134295	4.544230	4.125253	4.484384	4.253908	4.415573	3.991355	4.032121	5.131993	4.463741	3.958117	3.497998	3.371691	4.298741	4.974843	4.325747	3.353040	5.989536	4.615968	4.796678	5.210432	3.179124	4.662858	3.822055	3.520622	3.221513	4.230916	4.300892	3.284905	3.399349	3.497287	3.135127	4.966042	3.244280	3.040061	4.327452	6.293873	3.557381	4.273784	6.927637	3.202692	4.384260	5.141933	3.545283	3.740951	3.370825	4.902978	3.814603	2.889911	4.453761	4.134295	5.749031	3.551420	3.968978	4.417533	4.806374	4.418927	5.265445	3.010865	4.536749	4.516358	4.144088	7.091816	3.982184	5.384847	4.694948	6.555305	5.011176	3.839470	4.879417	4.238752	5.670662	4.491777	6.530302	3.895640	4.811050	4.159936	4.659199	4.494905	6.465705	4.745112	6.163228	5.498902	6.386807	4.472978	3.886152	6.781998	3.922872	4.023916	3.765519	4.518062	5.381342	5.481833	5.565029	5.396732	4.752754	4.503720	4.665178	6.262353	3.865362	4.860445	5.949005	3.993280	4.847004	4.955831	7.130360	5.089194	3.251299	5.709192	3.868591	5.295832	4.052563	3.741358	3.810138	3.408177	3.424199	3.604836	3.201711	3.412024	3.406067	3.632126	6.333836	3.978243	3.988180	5.284147	4.174872	3.119498	3.763184	3.782082	5.126176	3.125273	3.173183	3.457344	4.653519	4.200088	5.141316

NR2F1-AS1	5.064681	4.713705	4.757162	5.010145	4.301736	4.952727	4.472978	5.194061	4.868117	4.559658	4.839385	4.283381	4.259390	4.643607	3.804516	5.746099	5.028096	4.201411	5.274097	5.366629	4.677740	5.983840	6.927637	4.857330	5.251176	4.569162	5.636758	5.671238	5.191503	4.556426	4.825402	4.070266	5.151735	4.660922	4.150849	4.153870	4.201411	4.686970	4.890032	4.657491	4.805960	5.050781	4.636718	5.059621	4.372594	5.378285	5.074237	5.385023	5.442356	5.013631	5.116218	3.782722	4.157572	4.645768	4.636935	5.254943	5.200488	3.447475	4.502413	3.810663	4.028609	4.908825	4.506771	5.028289	3.816090	4.998160	4.090469	4.659849	4.375003	4.560116	4.982161	4.654176	5.050593	4.590783	4.865991	5.161854	4.742152	4.434340	4.882226	4.109148	4.651131	4.933036	4.539755	4.166676	4.355265	5.052996	3.800090	4.388072	4.398284	5.006848	5.111523	4.915556	4.161178	4.346322	4.216647	4.345899	5.103586	5.560926	4.970884	4.372359	3.990448	4.497707	4.568743	4.543780	4.758520	5.358725	4.964786	4.966274	4.174872	5.630433	4.006760	4.658123	3.415052	5.162655	5.258110	3.421532	4.859838	4.699164	5.094789	4.581198	5.487095	4.490913	5.352869	4.290685	6.084054	4.552801	4.802168	5.440816	5.240664	5.140077	4.938486	4.488455	4.020460	4.292225	5.270048	5.330083	4.967997	4.702563	5.587921	5.088183	5.518396	6.078326	5.248033	5.513512	5.261653	4.919580	5.061401	5.607979	5.087179	4.892077	3.953974	4.760385	4.530110	4.935777	4.732932	4.457149	5.078192	5.547706	4.901135	5.367011	5.616848	5.056350	5.615740	4.303076	4.975473	5.115354	4.526742	5.162860	5.591192	4.773477	5.155411	3.637482	5.002927	4.208643	5.172969	4.594487	3.587872	4.929067	4.463060	4.597236	4.939739	5.219764	5.694721	4.692213	4.984377	4.844015	5.041986	4.507693	5.189553	5.133206	5.432006	5.687351	5.010742	4.417533	5.282775	4.844870	4.713491	5.170283	3.521682	4.562063	4.343705	4.627372	3.806805	4.454382	4.882020	4.541102	4.796034	4.348753	4.302876	4.242508	4.319275	4.721070	5.175953	4.716448	4.541102	5.086979	4.617539	4.666915	5.211443	4.430721	4.039309	4.482874	4.210345	3.613588	5.250184	5.231593	5.305404	5.622231	5.441793	4.247541	4.762713	3.368755	6.218387	4.860036	4.331849	4.523906	4.348753	4.816230

USP27X-AS1	7.140794	5.923308	6.457215	6.490783	6.099449	6.594222	6.604049	4.102158	4.081762	5.981511	5.965720	5.980670	6.707704	6.142569	6.272225	5.340859	5.709572	6.590146	5.059416	6.398128	6.790236	5.442356	5.701183	5.364822	6.305257	5.676561	4.792316	5.142148	5.498902	6.131542	5.439483	7.263127	7.588434	6.325780	6.958150	7.183306	7.173677	6.270170	6.342020	6.452765	5.627815	6.609828	6.327567	6.681747	6.896720	6.893364	5.306383	5.805451	7.043666	7.506496	7.013009	7.695045	7.668822	7.679817	6.400824	5.964827	7.810508	7.426317	6.812869	7.455731	7.880939	5.804026	6.716702	5.823480	6.453116	6.036353	6.490462	6.141731	6.202438	5.269057	6.736946	6.754571	5.142569	6.514720	6.901907	6.338721	6.535659	6.384819	6.463206	6.085087	7.251051	7.362606	7.507225	7.841835	7.787922	7.320128	7.232924	7.624722	6.990140	6.563515	6.707215	5.819906	6.806159	7.036077	7.072278	6.950590	6.962614	5.966279	7.431423	6.026440	5.819906	6.378641	5.686415	6.612852	4.240135	6.068056	5.094394	6.122355	4.030399	5.049157	5.211825	5.202236	3.421532	5.172533	5.518195	8.352774	6.649358	5.558298	4.270707	6.972117	5.914578	6.660691	5.121915	5.228115	6.237472	6.413357	4.701109	3.780612	5.886031	5.460645	6.171172	5.693794	5.336327	5.188216	4.930410	6.917878	4.877385	4.341124	6.622911	4.717095	4.023218	7.060280	3.682236	6.324566	5.990248	6.095497	4.036066	3.996830	6.560853	4.495125	4.853765	5.536065	5.210021	7.244210	7.194046	6.896217	5.800432	4.571814	6.567049	4.271805	4.911356	6.191997	5.375539	6.982008	5.535095	4.756961	6.183618	7.176647	6.909692	5.692261	7.210823	7.664047	5.883488	7.428515	7.337844	8.683845	7.782573	7.076870	7.427588	6.823863	6.520020	7.381252	5.623887	6.105329	7.752712	5.345734	4.954568	5.173168	5.567281	5.776908	5.483503	3.851412	6.211030	6.467861	6.190615	7.113147	5.874946	5.828417	6.563191	7.309930	7.029874	7.472494	7.274232	7.561801	6.245620	6.226757	8.039807	7.209237	5.709373	6.194433	6.044651	5.941296	5.290002	6.470882	6.175751	5.748126	5.969312	6.313672	8.326529	6.470538	6.426143	5.867417	6.387151	7.488664	4.915143	5.509932	8.129662	6.072243	4.476926	4.996956	4.485024	4.321207	5.211825	7.170405	7.965252	5.609902	6.529154	5.411091

LINC02623	3.035580	3.105977	3.470346	4.112673	5.040280	3.318310	3.182826	3.796939	3.709665	3.186474	3.906869	3.215162	3.357413	3.472563	3.828431	4.087647	3.419157	3.176379	4.723440	3.487325	3.926936	3.885739	3.092072	3.013264	4.807206	3.824627	3.828858	2.870639	3.339291	3.767567	3.451718	2.922168	3.761949	3.005824	3.083840	3.861486	3.403382	3.518201	3.123684	3.025936	3.525894	2.955347	3.397284	3.140408	3.517346	4.412213	4.208850	3.396791	4.400675	3.490659	3.350218	3.170910	3.608791	4.017194	3.535542	3.679225	4.656406	3.540150	3.633884	3.683253	2.990809	3.620269	4.021312	3.246540	3.250421	3.270368	3.142565	3.210577	3.770322	4.440018	3.232555	3.261430	3.788233	3.211727	3.050142	3.295748	3.274345	3.540515	3.477093	3.538259	3.202402	3.690137	3.204801	4.009963	3.282551	4.720456	2.995127	3.238005	3.891777	3.450610	3.154801	3.217985	3.560522	3.067628	3.728166	3.111066	3.793159	3.392134	4.630030	3.301613	3.413645	3.648387	3.470703	2.834014	4.383150	4.029277	3.844161	4.514021	4.514410	4.566368	3.806389	3.963800	4.528611	3.413453	3.418265	4.505009	3.573829	3.345748	4.094946	3.096137	3.588616	3.233219	3.646429	5.421462	3.293922	3.112133	3.495403	3.672321	3.239599	3.541895	3.303158	3.093250	3.336283	3.172804	3.207657	5.131015	2.995701	4.081964	3.230901	3.538456	4.045616	4.765079	4.167983	4.331654	3.243536	3.505862	4.235090	3.749198	3.610373	3.103006	3.114731	3.596508	3.069020	3.110252	3.584748	3.350218	3.108304	3.142315	3.751911	3.893080	2.897158	3.460186	3.545984	3.781660	4.132996	4.238087	3.281645	4.055403	3.291907	3.239326	3.072349	3.525165	4.030176	3.298388	3.473087	3.606829	3.437547	3.397284	3.595556	2.929359	3.463048	3.641091	3.313790	3.410052	3.398506	3.163655	3.442050	3.592258	2.954945	3.224468	3.137235	4.541985	3.520038	2.885220	3.199145	3.051842	3.513915	3.616696	3.134888	3.475076	3.782722	3.232555	3.531729	3.402304	4.242950	3.406400	3.368271	4.845079	3.365817	3.225846	3.211879	3.332425	3.097128	3.051738	3.542469	4.258087	3.228037	3.760284	3.136966	4.067751	3.529405	5.714759	2.991399	4.785361	4.335916	2.885561	4.009340	3.832048	4.104536	4.460059	3.642657	3.973745	3.527760	5.211443	5.711383	3.727139	3.772186	4.578828

AC092902.4	9.485177	8.230865	8.006998	9.123078	9.183929	8.753188	8.046717	9.966500	10.219870	8.783670	8.676059	8.848945	7.985306	7.839230	7.987496	9.687929	8.826002	7.952940	9.194184	9.146092	8.355382	8.841178	8.793388	8.517317	9.030985	8.701516	9.709048	8.721025	9.371660	7.806501	10.417196	8.519151	7.637692	8.586075	8.345080	8.719946	8.311134	8.961900	8.356589	8.252521	8.907026	7.792182	8.521130	8.515614	8.436480	8.493834	9.102735	7.967258	8.814990	8.170810	9.146557	8.203318	7.817798	7.868330	8.524156	8.693399	8.830099	7.528117	7.988952	7.739616	7.770647	8.417243	9.037384	8.972624	7.640322	8.608484	7.860630	9.200481	7.755586	8.620966	8.638018	8.395764	9.333063	7.927272	8.145504	8.268542	8.930315	8.147919	8.545228	8.003253	8.173323	8.632743	8.379692	8.573812	8.573979	8.804775	9.411694	8.939581	7.775738	8.317902	8.634726	8.775819	7.725434	7.982383	8.953012	8.711767	8.689429	10.001641	8.687165	8.157804	8.896105	7.841996	8.871708	7.808333	8.162073	8.513995	9.888161	8.442536	8.880210	8.527523	8.565393	8.479812	8.452630	8.375113	9.396827	9.809677	8.652809	9.087621	9.241513	9.329227	7.238746	8.602363	7.472799	9.055670	11.536302	8.127189	10.713446	8.750837	7.645570	7.858326	9.068319	8.111029	8.186986	7.343668	8.823713	7.762049	8.203908	9.325363	9.929695	8.188314	8.912971	8.798544	11.001938	8.964783	8.777381	8.713880	8.126601	10.682874	7.338770	9.519632	8.618293	7.988666	7.764057	8.782749	9.239512	7.619552	9.863013	7.606840	8.586950	8.315925	7.515856	8.519918	7.080486	8.112072	8.206040	8.866575	7.970201	9.295377	7.445068	7.693444	8.564796	7.903842	7.550519	8.677427	8.167960	7.874571	8.598963	7.585638	8.047520	7.674955	7.979579	7.564418	7.669595	7.218415	7.724989	7.133718	7.784243	7.964808	7.616653	7.834777	8.110285	10.474364	7.524066	7.961776	7.796324	7.757135	7.749980	7.782421	8.003880	8.249893	8.672180	7.951832	8.934423	8.228709	8.272834	8.620804	10.181790	8.082722	8.227971	9.858186	8.317461	7.641386	7.696224	8.584250	8.449532	8.856984	8.980147	7.206919	9.016533	7.740092	8.361105	8.080063	7.628930	7.942799	8.678667	7.608060	8.450472	8.317610	7.390405	8.979236	8.076073	9.600102	8.636158	8.942629	8.323832	7.897593	8.236218	7.367653

LINC00937	9.485177	8.230865	8.006998	9.123078	9.183929	8.753188	8.046717	9.966500	10.219870	8.783670	8.676059	8.848945	7.985306	7.839230	7.987496	9.687929	8.826002	7.952940	9.194184	9.146092	8.355382	8.841178	8.793388	8.517317	9.030985	8.701516	9.709048	8.721025	9.371660	7.806501	10.417196	8.519151	7.637692	8.586075	8.345080	8.719946	8.311134	8.961900	8.356589	8.252521	8.907026	7.792182	8.521130	8.515614	8.436480	8.493834	9.102735	7.967258	8.814990	8.170810	9.146557	8.203318	7.817798	7.868330	8.524156	8.693399	8.830099	7.528117	7.988952	7.739616	7.770647	8.417243	9.037384	8.972624	7.640322	8.608484	7.860630	9.200481	7.755586	8.620966	8.638018	8.395764	9.333063	7.927272	8.145504	8.268542	8.930315	8.147919	8.545228	8.003253	8.173323	8.632743	8.379692	8.573812	8.573979	8.804775	9.411694	8.939581	7.775738	8.317902	8.634726	8.775819	7.725434	7.982383	8.953012	8.711767	8.689429	10.001641	8.687165	8.157804	8.896105	7.841996	8.871708	7.808333	8.162073	8.513995	9.888161	8.442536	8.880210	8.527523	8.565393	8.479812	8.452630	8.375113	9.396827	9.809677	8.652809	9.087621	9.241513	9.329227	7.238746	8.602363	7.472799	9.055670	11.536302	8.127189	10.713446	8.750837	7.645570	7.858326	9.068319	8.111029	8.186986	7.343668	8.823713	7.762049	8.203908	9.325363	9.929695	8.188314	8.912971	8.798544	11.001938	8.964783	8.777381	8.713880	8.126601	10.682874	7.338770	9.519632	8.618293	7.988666	7.764057	8.782749	9.239512	7.619552	9.863013	7.606840	8.586950	8.315925	7.515856	8.519918	7.080486	8.112072	8.206040	8.866575	7.970201	9.295377	7.445068	7.693444	8.564796	7.903842	7.550519	8.677427	8.167960	7.874571	8.598963	7.585638	8.047520	7.674955	7.979579	7.564418	7.669595	7.218415	7.724989	7.133718	7.784243	7.964808	7.616653	7.834777	8.110285	10.474364	7.524066	7.961776	7.796324	7.757135	7.749980	7.782421	8.003880	8.249893	8.672180	7.951832	8.934423	8.228709	8.272834	8.620804	10.181790	8.082722	8.227971	9.858186	8.317461	7.641386	7.696224	8.584250	8.449532	8.856984	8.980147	7.206919	9.016533	7.740092	8.361105	8.080063	7.628930	7.942799	8.678667	7.608060	8.450472	8.317610	7.390405	8.979236	8.076073	9.600102	8.636158	8.942629	8.323832	7.897593	8.236218	7.367653

AC092902.2	9.485177	8.230865	8.006998	9.123078	9.183929	8.753188	8.046717	9.966500	10.219870	8.783670	8.676059	8.848945	7.985306	7.839230	7.987496	9.687929	8.826002	7.952940	9.194184	9.146092	8.355382	8.841178	8.793388	8.517317	9.030985	8.701516	9.709048	8.721025	9.371660	7.806501	10.417196	8.519151	7.637692	8.586075	8.345080	8.719946	8.311134	8.961900	8.356589	8.252521	8.907026	7.792182	8.521130	8.515614	8.436480	8.493834	9.102735	7.967258	8.814990	8.170810	9.146557	8.203318	7.817798	7.868330	8.524156	8.693399	8.830099	7.528117	7.988952	7.739616	7.770647	8.417243	9.037384	8.972624	7.640322	8.608484	7.860630	9.200481	7.755586	8.620966	8.638018	8.395764	9.333063	7.927272	8.145504	8.268542	8.930315	8.147919	8.545228	8.003253	8.173323	8.632743	8.379692	8.573812	8.573979	8.804775	9.411694	8.939581	7.775738	8.317902	8.634726	8.775819	7.725434	7.982383	8.953012	8.711767	8.689429	10.001641	8.687165	8.157804	8.896105	7.841996	8.871708	7.808333	8.162073	8.513995	9.888161	8.442536	8.880210	8.527523	8.565393	8.479812	8.452630	8.375113	9.396827	9.809677	8.652809	9.087621	9.241513	9.329227	7.238746	8.602363	7.472799	9.055670	11.536302	8.127189	10.713446	8.750837	7.645570	7.858326	9.068319	8.111029	8.186986	7.343668	8.823713	7.762049	8.203908	9.325363	9.929695	8.188314	8.912971	8.798544	11.001938	8.964783	8.777381	8.713880	8.126601	10.682874	7.338770	9.519632	8.618293	7.988666	7.764057	8.782749	9.239512	7.619552	9.863013	7.606840	8.586950	8.315925	7.515856	8.519918	7.080486	8.112072	8.206040	8.866575	7.970201	9.295377	7.445068	7.693444	8.564796	7.903842	7.550519	8.677427	8.167960	7.874571	8.598963	7.585638	8.047520	7.674955	7.979579	7.564418	7.669595	7.218415	7.724989	7.133718	7.784243	7.964808	7.616653	7.834777	8.110285	10.474364	7.524066	7.961776	7.796324	7.757135	7.749980	7.782421	8.003880	8.249893	8.672180	7.951832	8.934423	8.228709	8.272834	8.620804	10.181790	8.082722	8.227971	9.858186	8.317461	7.641386	7.696224	8.584250	8.449532	8.856984	8.980147	7.206919	9.016533	7.740092	8.361105	8.080063	7.628930	7.942799	8.678667	7.608060	8.450472	8.317610	7.390405	8.979236	8.076073	9.600102	8.636158	8.942629	8.323832	7.897593	8.236218	7.367653

LINC00605	6.373468	7.157299	7.265109	6.837524	6.462351	6.982716	7.162520	6.871129	7.001632	6.597460	6.372955	7.864481	6.113154	6.423501	7.298925	7.234989	7.142879	6.231093	7.636181	7.179077	7.644929	7.046566	8.478824	6.306280	6.734661	6.736274	5.888143	7.389033	6.743037	6.792357	7.074654	7.342373	7.374539	7.042248	6.596178	7.009963	6.974149	6.805088	6.517550	6.990140	6.343180	7.243750	6.702584	7.283831	7.506934	7.263561	6.966591	7.364513	7.736225	6.634185	6.493934	6.917243	7.166708	7.148188	8.045127	6.807572	7.662956	7.836439	7.369224	7.469869	8.026217	7.169475	7.902414	7.586459	8.573379	7.462528	8.208437	7.695178	6.988738	8.077747	7.008674	7.230254	6.684412	6.724487	6.992762	6.849972	6.745143	7.186349	7.103286	7.487562	7.211984	7.598391	7.323122	7.617692	7.286768	7.443459	6.659868	7.543116	7.861216	8.097820	7.921791	7.993319	6.903372	7.243420	7.640029	6.634515	7.277044	6.595180	6.532074	7.497706	6.493156	6.496218	5.981839	7.909264	7.795248	6.788410	7.928511	8.361560	7.526038	6.891582	7.753171	7.598858	7.309782	6.969671	6.452101	7.304121	6.672024	7.071171	6.469350	6.845047	6.302364	7.292867	7.013182	6.225900	6.717180	9.172346	6.644025	6.418153	6.524017	7.383462	11.496066	6.599911	5.419147	6.476918	6.940443	8.797467	5.189553	7.232119	6.190968	6.353603	6.824504	7.313210	6.024815	6.063309	6.012970	6.015713	6.484435	7.042884	6.556792	6.686616	6.419648	6.415159	5.905442	7.073540	6.138412	6.969980	6.772992	6.363037	6.649358	6.604877	6.784522	6.141731	6.208832	6.411997	6.647547	6.955826	6.403439	6.723381	6.580629	6.486275	6.439064	5.947925	6.616765	7.677784	7.154025	9.666547	7.547904	8.673896	6.554958	8.766990	7.313210	7.627346	6.516754	8.915861	7.553604	9.781923	6.881513	6.084585	7.239061	6.696046	6.411190	6.591021	6.662158	6.552341	6.654673	6.838038	6.994459	6.920646	7.696523	6.504481	8.429432	7.219352	6.928747	6.884082	6.937644	6.669931	6.696500	8.620804	7.471877	6.497681	7.551436	6.980014	7.473694	6.966278	6.362193	6.778938	6.298576	6.688232	6.703390	6.256420	7.548388	5.879616	10.786172	9.545320	6.755884	6.966591	10.431883	7.081775	6.914480	6.935856	6.635475	5.623364	7.072893	6.492034	6.752087	7.135790	8.805524	5.972886

MEG8	4.672936	3.548911	3.533103	3.085408	3.086827	4.165107	3.382741	3.577550	3.499242	3.428989	3.640480	4.340694	3.462345	4.124633	3.770137	3.521873	3.678626	3.081901	5.086146	4.322686	5.404435	2.885728	3.522634	3.052050	3.082860	2.858041	4.293735	4.019348	4.512448	3.577338	3.808655	4.616425	4.025591	3.868138	3.638690	4.195419	3.761124	3.522057	3.591339	3.441000	3.520441	3.634676	4.042090	3.413828	3.934099	5.870621	3.570765	5.158109	4.888964	4.053879	3.926712	3.820148	4.554340	4.447792	4.577141	4.266974	4.330993	3.502150	3.764289	4.075361	2.915804	5.775641	4.630254	3.392134	3.685860	3.644045	2.951744	3.411297	3.337310	4.652799	3.628214	4.147052	3.648387	3.446902	2.919506	3.428299	3.548744	3.395752	3.585700	3.956373	3.526862	4.759341	3.880860	4.434754	3.877385	4.540679	3.171791	3.334212	4.061298	3.864105	3.407494	3.125273	4.056495	3.314806	4.064578	3.732517	4.154543	4.588857	3.627611	2.983669	4.385646	3.644431	3.428828	4.229455	4.834368	4.700680	3.337310	3.832514	4.526742	3.733620	3.132068	3.266295	3.000143	3.644627	3.597536	4.387819	3.023191	4.757162	3.266904	3.221793	3.737677	3.051231	3.155201	3.055278	3.827201	3.357588	3.298546	3.816300	3.170383	4.018944	3.067404	3.097619	3.343649	3.339114	3.686239	3.572272	2.907021	3.493546	3.539190	4.669702	3.168276	6.117854	2.911614	2.875475	6.242444	4.184469	2.818121	3.113539	3.742196	3.590298	3.901680	4.412855	3.349361	3.508342	3.868591	4.091630	3.573829	3.568873	3.241621	3.485005	3.328072	3.771327	3.499640	4.408699	5.383335	3.770527	5.657391	4.874251	4.063276	3.882327	3.547776	4.400675	4.646207	4.114601	3.339114	3.998746	3.448389	3.347388	4.074510	2.819679	3.552388	4.258314	4.101695	4.094334	3.913138	2.926987	3.794458	3.533846	2.745328	3.116629	3.915302	3.503653	4.529657	3.189576	3.558731	4.376122	3.529232	4.107435	3.332078	4.017194	4.797882	3.699957	4.399395	4.074510	3.252661	3.498710	3.510886	3.618187	3.712125	4.054525	2.955162	3.631286	4.079378	4.918304	4.834164	3.455662	4.646000	4.295899	3.356052	5.036945	3.067842	3.577153	3.330656	5.221697	3.579575	3.188054	3.798781	3.580146	3.272865	2.987048	3.287565	3.437732	3.277869	3.136611	3.039394	2.994943	3.702258	3.421359

AL163932.1	5.531616	5.667791	5.881243	5.482575	5.758146	5.537228	4.546237	7.242424	6.237788	5.149493	5.059621	4.732932	4.028172	4.597449	4.350904	5.636210	6.941664	3.947691	5.146547	5.530479	5.957159	5.719583	7.743008	5.842228	5.434147	5.750467	5.212390	5.661986	4.391908	5.034193	5.613287	4.613576	5.615376	3.925247	5.603805	4.819395	5.119896	4.958542	5.980847	6.131372	5.221285	5.937556	4.844447	5.947212	5.082653	5.151120	5.337710	6.337381	6.260150	5.339915	5.190944	5.670314	4.034749	5.098874	5.149087	5.037736	4.010157	6.762515	5.446840	5.617970	5.747033	4.843390	5.603430	6.734817	5.776908	6.118709	6.186042	6.100310	6.069620	6.435961	5.691927	5.812237	5.453218	5.566556	5.471380	6.166683	6.626941	6.312967	6.275222	5.546772	5.962339	5.240495	5.378859	5.977794	5.935818	5.709572	6.524506	6.246949	6.862022	6.704931	6.443703	6.825914	5.611055	5.442744	5.099511	5.154379	5.559054	4.290904	4.430487	4.592522	4.459856	4.673856	4.056715	4.627811	5.989000	5.082253	6.262353	6.043642	6.485292	5.471006	6.794787	6.521055	6.824346	5.657957	5.203062	6.504398	6.120116	5.286219	4.880724	5.358153	5.878510	5.472341	4.990613	5.734828	5.389322	4.329472	5.997771	5.829913	4.599773	5.056559	4.314992	4.496847	4.170991	4.664961	5.398817	6.649670	3.949881	5.701366	5.369136	6.098553	5.302829	6.815732	6.009128	6.172854	5.675316	5.928111	5.622231	5.120292	5.288542	5.998502	4.621637	5.442160	4.185338	5.297845	5.387388	5.626357	5.838422	6.007362	5.847710	4.327873	6.650152	4.597046	6.891582	5.142956	4.603020	5.196495	6.088150	6.190268	5.824047	6.535162	5.392589	4.760599	6.174195	5.308477	4.842115	4.450965	6.556947	5.800619	5.927411	5.638273	4.538206	6.513257	6.924849	6.229537	6.366226	6.322198	6.143417	5.852202	6.784020	6.084753	5.859607	5.224876	5.892964	5.859961	6.390531	6.660520	6.345568	6.521225	5.528391	5.538833	5.648979	6.031996	5.625960	6.201713	6.026589	6.159072	6.276203	6.792176	6.466680	6.299387	7.448253	5.191120	5.310072	5.501796	5.712688	5.255554	4.928464	4.786402	4.125253	4.676249	4.697895	4.175090	5.069790	5.588508	4.738203	6.373825	5.743377	6.521545	5.328100	6.736422	7.537475	6.904609	6.034196	5.681975	5.101364	6.439408	5.416994	5.431432

CYTOR	11.846434	12.093684	13.096725	13.213993	12.334126	12.408357	12.511287	13.317644	11.675976	13.626588	13.311346	12.770158	12.653816	13.743835	11.602409	13.872874	12.245922	12.804049	13.126907	13.371459	13.136025	13.369945	12.434656	12.962718	12.564800	11.541245	12.661535	13.967420	12.183277	13.240179	13.601504	14.171283	13.321286	13.300042	13.099216	12.941758	13.080043	12.041947	13.364346	13.691937	12.333497	12.958652	13.693583	13.249887	12.917111	13.113538	12.702790	13.887810	13.753345	12.976037	13.074158	13.029905	12.926298	12.253893	13.433474	11.776985	13.637147	13.249887	12.897590	12.765130	11.794551	12.940096	14.153617	11.908190	13.256221	12.545893	12.794485	12.663235	13.247129	12.705338	12.178152	12.781558	13.704144	13.152225	13.212044	14.345644	13.505677	12.905530	12.161114	12.916749	13.748342	13.324998	13.013350	13.313179	13.930021	12.552337	12.992354	15.038723	12.723489	12.503725	12.596273	13.396009	13.203600	12.936017	13.042247	12.862205	13.231451	12.448961	12.908020	11.764040	12.186663	12.578859	11.954526	12.835806	12.614208	13.701386	12.232513	13.126528	11.323214	12.774962	12.977868	12.651235	14.135776	12.384801	13.225883	12.450257	11.213966	12.442202	12.442360	12.324636	13.719674	13.724455	12.528398	13.322545	12.853171	13.884347	12.628852	13.280648	14.125236	13.521840	13.520267	14.656836	14.859475	14.453564	12.935431	14.305623	13.852712	12.909480	12.749833	14.048831	13.479048	14.695696	12.632803	14.225717	15.113668	13.697330	12.587308	13.030425	14.272526	13.789486	14.518159	14.553109	13.507700	14.138571	13.932466	13.968540	13.724675	13.695182	13.779166	12.995256	13.568039	13.354386	13.788457	14.280707	14.756458	12.942642	13.671966	13.806501	14.739537	14.498295	14.389287	13.317644	15.066902	14.497900	13.879304	13.225296	13.889152	13.203403	13.521840	14.176803	13.562433	13.955341	13.624536	12.886172	13.772698	13.969339	13.968540	14.357698	14.089238	13.992417	13.903602	13.814296	14.471558	14.205591	14.637498	14.355885	14.622046	14.464405	14.755057	14.354516	13.749826	13.789952	14.615271	14.464001	13.802371	14.417635	14.523537	14.076027	14.120707	13.964397	14.573111	13.704846	14.681437	14.162045	14.448739	14.188386	13.467314	13.594660	14.102560	14.606651	12.944441	13.335001	14.056534	14.926494	13.648470	12.607232	13.645649	13.442646	13.370614	13.929201	13.890483	12.081830	12.670862	14.711236	13.432615	14.031471	11.989419	14.173200

LINC02785	11.447635	10.891184	10.626550	11.385568	11.233307	11.851720	10.951475	12.010330	12.035499	11.525035	10.984205	11.581405	10.787634	10.884458	10.399138	11.611094	11.699008	10.548263	12.120667	11.780607	11.505998	10.880620	12.220778	10.993754	10.799956	11.417687	12.263096	10.860414	12.523307	12.598156	11.852005	9.535499	10.734749	10.780444	11.076058	10.770741	10.912348	10.881335	11.529830	11.260782	11.948495	11.590536	10.548890	11.406899	10.442759	10.616727	12.232669	11.274308	10.945211	10.704472	11.965964	10.255889	9.881165	10.728225	10.283087	12.376134	9.970631	9.998272	10.774902	10.039141	10.875904	11.585590	10.833724	11.190179	10.859681	11.349360	11.178683	10.858370	10.599871	11.534664	12.980792	12.024345	12.261502	12.216235	11.736869	11.271312	11.232575	11.927081	12.443708	10.101418	10.021856	10.012145	10.195988	10.447803	10.070538	11.529388	10.917626	10.580067	10.446887	10.600603	10.785872	11.178065	11.041054	11.241217	11.665066	10.535450	10.766127	10.576772	10.927267	10.996398	11.193556	10.768349	11.074564	10.476015	10.956031	11.605506	11.107474	11.196379	12.006559	12.120080	11.727379	10.931888	10.846224	12.216379	11.416326	13.968272	10.685697	11.763401	11.873996	10.747238	11.310412	10.736083	11.150234	12.270085	11.582424	11.833741	11.839944	11.435100	11.405292	11.008289	11.434802	10.939339	10.832274	10.938063	11.446450	11.057630	10.424393	11.563013	10.731630	11.420870	10.892076	11.904028	11.749526	10.988542	10.923568	11.951566	10.716263	12.712441	12.142076	11.672364	10.498577	11.242082	11.564784	10.879299	10.814754	11.248808	11.338789	11.625759	11.544678	12.332077	11.348487	10.417364	11.576750	10.559328	11.064379	12.436293	10.424393	10.971621	10.888494	11.365654	10.317347	9.859459	10.545676	10.232800	11.465697	9.809058	9.994619	10.379694	10.053429	10.718958	11.490395	11.065101	11.077806	10.837802	11.015142	11.023352	11.435100	10.909711	11.178375	11.939528	11.712047	12.775824	11.946225	10.939212	11.520541	11.274308	11.735541	12.221872	9.885790	10.189200	10.140329	10.765528	10.413889	10.325054	11.702072	10.982333	10.340815	10.697935	10.588660	10.568780	11.246438	11.236568	11.511108	12.649190	11.265890	11.756782	11.617879	10.791282	10.351898	11.024955	10.921361	11.455375	10.403336	10.711413	11.910424	11.114258	10.770741	11.507007	12.529347	11.654988	11.038451	11.858578	12.804949	11.661349	11.890777	10.400807	11.016907	11.579980

SNHG14	10.294926	8.243979	8.743457	7.954949	9.128284	8.698949	10.192949	7.182354	7.049597	9.573979	9.590890	9.469018	8.968967	8.254529	8.011098	9.651500	9.740092	7.756185	7.450146	9.515907	9.787591	7.985774	10.884458	8.302103	8.280543	9.392967	10.270415	8.813057	10.349613	9.254788	10.225458	9.108455	9.722314	9.746781	9.249046	9.961205	9.657050	8.765904	8.672489	8.227971	9.856526	9.364876	9.619941	9.736555	10.645484	9.368032	10.418435	7.963592	9.899302	9.639954	9.136529	9.783615	8.659450	9.579165	9.848007	9.198284	10.254211	8.549830	8.647979	8.593456	8.752751	9.238911	9.692401	8.032238	7.752553	8.704594	7.989562	8.134218	7.395245	8.169238	9.275288	9.021121	9.862557	9.682637	9.165740	9.551928	9.355609	9.159694	9.236897	10.220494	8.145954	9.720169	9.690836	9.863484	10.727045	10.439729	9.287715	9.570131	8.095531	7.763738	8.615111	7.906928	7.201945	9.093285	9.413800	10.064990	9.848631	10.658429	9.924138	9.445907	9.719403	9.563890	8.995047	8.949983	7.900057	10.364000	8.726020	7.132916	7.822114	10.052342	8.028572	7.929278	6.498399	8.457045	8.295296	6.633699	8.823713	10.169039	9.602061	10.139112	6.112814	6.119404	6.781998	4.475158	7.094982	5.906325	10.123969	5.341422	7.545344	7.186349	8.415798	6.753389	6.815250	6.914148	9.513068	6.411029	6.316233	4.051954	8.943997	7.228420	5.785945	6.724149	6.623549	8.458622	8.234802	5.470448	6.504643	5.891332	6.396141	8.326365	7.989562	7.692283	6.050770	6.803833	10.733854	8.309320	5.259071	4.457594	6.210351	4.083044	7.629887	9.858997	7.522227	9.534134	6.384499	6.591021	8.913281	9.612400	8.434134	7.024588	7.780760	6.073115	9.834744	8.397030	9.665759	10.170756	6.499069	5.742641	7.015663	6.726070	9.381478	7.251211	5.004156	5.227496	6.091996	3.260848	6.048392	4.808479	7.397543	7.300310	7.420235	3.482425	7.537165	8.537260	7.961466	7.593195	7.410189	8.482171	9.034796	7.812921	7.951667	6.995225	8.801886	7.119964	7.918723	7.118373	9.311545	6.057995	6.376950	3.580893	4.565960	5.226103	7.547904	8.287945	8.678987	7.887351	8.912802	6.620355	7.346140	8.651781	9.045190	9.062021	6.803657	5.691561	7.920144	4.223185	3.469235	7.040810	4.971901	6.005570	5.973953	9.411987	7.342076	8.877254	5.560750	7.400671	8.894991	5.133823

GORAB-AS1	7.325954	8.019230	8.073351	8.004183	7.886288	7.833855	7.143171	8.420110	8.369861	7.549145	7.385317	7.269844	6.786300	7.407121	6.812716	8.016793	7.769286	6.790559	8.985318	7.831082	8.726020	8.865208	8.825386	7.383930	7.685180	7.851254	7.928341	7.829126	7.360429	7.500400	7.402070	7.173102	7.491020	7.273894	7.274387	7.431096	7.324214	7.769127	8.236995	7.976636	7.555981	7.944014	7.532988	7.991905	7.218571	7.279413	7.780442	7.912361	7.880939	7.724989	7.187785	7.159016	7.380825	7.890456	7.422597	8.176472	6.819593	7.784553	7.583190	7.256013	7.768978	7.610523	8.261839	8.652502	7.877792	8.016016	8.169095	8.335407	7.788999	8.464437	8.123878	8.312070	7.762841	7.823189	7.819523	8.011995	8.289790	8.056362	7.953560	7.079964	7.431096	7.260754	7.069874	7.298780	7.336755	7.174938	7.656275	7.394137	7.773375	7.890755	7.420891	8.375408	7.584267	8.008230	7.452446	7.311670	7.188396	6.903528	7.315133	7.671162	8.012742	7.539270	7.357496	7.299385	8.866122	7.270165	8.247398	8.524156	9.019117	7.871229	8.623429	7.818413	7.325321	8.201070	8.111766	8.509809	7.059634	7.597003	7.570213	7.405528	8.884058	8.711023	7.988952	8.709313	7.829686	8.060586	8.054935	8.498831	8.077902	7.817798	7.270312	7.968034	7.825370	7.741642	8.107120	8.178548	7.415648	9.332454	7.975858	8.996563	9.774107	9.844563	8.106503	8.595787	8.120535	8.525662	8.060419	8.335407	7.864481	8.238367	8.057141	8.377069	8.072573	7.380012	7.595187	8.322469	8.818873	9.222820	9.273941	8.194463	8.255295	8.107889	8.704437	7.860047	7.932329	8.163532	7.794310	8.394381	9.055012	8.533923	7.545653	8.398977	9.426185	8.454898	7.409279	7.295374	8.345381	7.894662	8.003407	8.997966	7.658000	9.751904	9.353052	8.021791	8.524310	9.690530	8.580581	9.209005	9.285069	8.581219	8.950413	8.550896	8.568189	7.977233	8.201821	9.019286	8.696372	8.936240	7.543904	7.945391	7.411972	8.251773	8.206633	8.795964	9.054717	7.841244	7.716737	8.519918	8.711023	7.973059	9.107183	8.568651	9.332000	8.371132	9.295221	8.676199	8.222383	8.231949	7.783481	8.434938	7.672732	7.472343	7.587193	8.615714	7.803104	8.801743	8.433978	9.106746	8.661319	9.649319	8.732236	8.202112	8.954553	8.112997	7.624268	8.601893	7.811119	7.734740

AP002358.1	4.781556	5.315035	4.949181	5.194696	5.611588	6.829577	5.674028	4.184469	5.484805	6.333155	5.736270	6.716702	5.716222	5.654829	5.879055	5.355495	6.117854	5.787562	5.464428	5.839364	4.395671	4.518915	6.131041	5.600779	5.660315	5.496811	6.451946	4.486156	6.527835	6.042616	5.797284	3.791429	4.750429	6.467195	5.237665	4.525882	4.863937	3.081010	4.054951	5.065069	6.102054	5.060442	4.893955	4.560331	4.499207	4.708660	7.654357	3.141935	5.972718	3.333388	5.580595	4.281018	4.720656	5.098442	6.206085	6.250316	5.517407	3.793159	5.463454	5.184812	4.838925	6.176101	4.794571	5.247007	4.754919	5.318061	4.499842	3.898693	3.357243	5.005368	4.745325	4.612057	6.414848	4.537994	4.765699	4.478450	5.032759	4.610996	5.533510	4.263078	5.138445	6.314873	4.351543	5.260658	4.414205	5.917422	4.376122	5.415278	4.165343	4.841476	5.413197	5.525734	5.324966	4.975473	6.153607	6.283964	5.966457	6.362376	5.554340	6.174889	5.728993	5.398817	5.878151	5.749370	4.543312	6.847079	5.581146	5.352272	5.134841	5.879233	4.505232	6.405898	5.752847	6.577833	7.056495	7.028923	5.200179	6.400484	6.424487	5.894247	3.803066	5.586055	6.228136	5.781452	6.721286	7.584871	6.300828	4.433495	6.731583	5.880879	7.983773	6.842050	5.665156	5.390865	5.393779	5.674591	5.714200	5.607399	4.300892	5.328913	4.983993	5.547864	7.195369	4.192784	3.135892	5.537015	4.798102	6.080567	5.137208	4.966274	5.453616	5.087383	6.098380	4.707853	3.742824	4.567032	3.114254	3.806591	5.137599	7.429837	3.063523	6.023426	3.896283	4.155180	4.330993	8.004502	5.039903	5.570130	3.050254	3.471394	4.087037	4.779250	4.346523	5.635267	6.174035	4.561205	4.084323	4.904429	3.810335	4.292692	6.075352	4.566368	5.116406	4.127424	5.091838	3.463557	4.193897	3.734799	4.892937	4.850148	6.281614	5.681611	4.677740	4.999402	4.120757	3.929129	4.538643	5.816007	3.216664	5.676185	6.837369	4.619091	4.328317	3.828051	5.468537	4.162256	5.477301	3.868138	4.644041	5.491318	4.549764	4.107829	4.664522	5.559054	5.669438	5.585157	6.256911	4.623792	5.917422	5.809630	5.718329	6.443703	4.928464	4.781987	7.662314	4.876299	5.581146	5.654640	6.236327	4.440677	5.139246	4.191032	6.621633	6.102226	6.563364	4.546237	7.091343	5.671044

STEAP2-AS1	7.594003	7.011388	7.334577	7.404412	7.562571	6.985720	7.542633	4.147890	5.612341	8.490290	8.048145	7.616037	8.414441	6.591177	6.822459	7.689945	8.417540	7.290501	5.033779	7.667444	6.452605	4.655780	6.875472	6.345747	7.614495	8.727096	8.303938	8.827314	7.596263	7.303037	7.830932	7.998563	7.778689	8.868700	8.208150	8.569098	8.509515	7.653896	8.113151	7.556139	7.439469	8.547692	7.790991	7.607143	8.991055	8.160678	8.303042	7.922721	7.440064	8.422852	8.108640	8.126124	8.104169	7.694879	7.567799	7.616815	8.643792	7.987496	8.252377	8.044166	7.438208	7.940095	7.734887	7.548686	7.730554	7.311816	7.337198	7.233534	7.278927	6.307744	6.917878	7.083005	8.113761	7.348653	7.008175	7.536246	7.239737	8.260488	7.129868	8.299261	6.480733	8.489686	7.642619	7.697916	8.987939	8.700428	7.843275	7.853280	7.027648	6.902075	7.219352	5.985916	6.951067	7.648449	7.405681	7.565037	8.174583	9.235949	7.674175	9.032961	8.968967	8.641898	8.597021	7.503840	5.752475	8.401432	7.702064	5.836808	5.775473	7.874285	3.429526	8.011995	5.952550	7.070053	6.977531	7.240071	7.628611	8.148860	8.101896	8.828225	6.727870	7.595792	6.431908	6.412890	5.364822	6.653041	9.134397	7.051012	7.382041	7.778050	7.888585	6.829577	7.498978	5.349668	8.295459	8.900672	6.322380	9.653378	9.862701	5.402259	5.677521	5.401834	4.792105	7.534064	9.253123	6.552849	9.302942	7.726229	6.594842	7.287679	7.922249	8.385284	7.828229	7.354866	9.919312	8.921205	6.909549	6.024457	7.205967	7.356390	6.935856	8.695889	7.327546	8.406518	9.052042	6.261678	10.014305	8.563284	7.496799	8.142694	9.840623	8.548297	7.068415	7.218871	8.783374	9.742277	8.909943	7.617539	9.378994	7.819973	7.232778	8.128713	6.935199	6.069104	6.387827	9.157090	6.587705	5.559054	5.020732	6.304098	6.978653	8.072444	6.533240	8.305437	7.670691	8.185737	6.312295	6.401307	8.097062	7.567498	7.348804	7.676806	8.259100	8.000883	7.353569	7.985002	10.111696	7.163769	7.367980	8.286878	4.323583	6.405052	8.039167	8.799286	8.960680	6.918220	6.804922	6.676101	7.828836	8.328131	8.104985	8.275378	6.492516	8.507964	7.114394	7.987342	7.650308	4.966720	6.872583	3.696123	9.171092	7.870902	6.957510	8.077902	6.971518	8.434296	5.998140	7.676983

AC009522.1	3.874160	3.956800	4.178097	3.885739	3.969455	4.170075	4.311896	3.329682	4.269874	5.087383	3.767157	3.603220	3.679225	3.922444	3.753370	4.538206	3.560111	3.395581	5.430487	4.133608	4.983584	5.044321	6.329228	4.786621	3.990448	3.864105	4.052563	3.956140	4.354817	5.062460	3.687880	3.826990	4.195624	4.298529	3.356906	4.086810	3.688481	3.920397	4.388910	3.671942	4.402007	2.969281	4.752973	4.000428	4.210138	5.581712	5.314008	2.986272	5.140077	4.282532	4.222293	3.633323	4.741525	4.209499	4.706397	4.731233	4.205246	2.956087	3.428299	3.779373	4.488884	4.789101	3.725135	4.027278	5.792216	3.938998	4.598551	4.325747	3.930454	3.767567	4.675355	4.505426	4.432882	4.178992	3.653225	4.662468	3.628214	3.670934	3.740951	3.809533	3.946856	4.088735	4.288978	4.389151	3.619893	4.710123	3.535542	3.486244	3.310528	3.569686	3.725335	3.185927	3.728166	4.088735	4.239223	3.573829	3.860245	3.964673	3.471226	3.759641	3.933031	4.635406	4.252167	3.934099	5.536829	4.735652	3.997882	5.197508	3.440483	4.370011	4.591620	3.875056	3.976335	4.764632	3.875478	4.418457	3.722894	3.979371	4.599178	4.027278	4.091630	4.302168	5.167486	4.734603	3.712709	3.520242	3.200504	3.478586	4.341562	4.466328	3.271619	3.461488	3.605963	4.162728	4.152088	3.480010	3.056221	5.041162	3.826779	4.288770	3.873944	5.314416	3.804710	3.772186	4.419157	4.599394	3.880460	4.334201	4.878603	3.403704	4.268466	3.560111	3.344470	3.607835	3.783345	3.712125	3.425651	4.201853	3.691158	4.451832	2.886026	4.462233	4.880948	4.265722	5.104831	5.014666	2.923401	5.118031	5.154379	4.636077	3.409363	4.156309	4.425257	4.532908	4.297429	3.815433	2.954855	3.902322	3.597342	5.943205	4.451832	4.988095	4.398938	3.843557	4.369566	4.727036	4.813900	3.888075	2.828432	4.434129	4.453114	4.908200	3.478207	3.725135	3.838658	3.781660	3.958573	3.546768	3.636664	3.819707	4.483961	3.643095	3.742020	3.594399	3.917683	3.458213	3.482975	3.082228	3.442401	3.485005	3.453144	3.764496	3.918697	3.492212	3.383664	3.235381	3.339114	3.884271	3.820807	3.541494	3.304598	5.668602	3.981076	4.974640	4.533330	3.071974	4.284509	3.712959	4.668169	4.061967	4.332259	4.453543	4.922338	4.032747	3.822877	2.956356	3.617257	4.330993

LINC01918	8.023141	7.809741	7.731800	7.968034	8.393345	9.080270	8.535315	9.484419	9.282455	9.175115	9.375451	9.047133	8.263850	8.240540	7.292562	8.904469	8.728985	8.161937	8.527214	8.742107	8.286715	8.686132	8.018603	7.757729	8.064734	8.774914	9.948751	9.123220	9.079789	8.410935	9.420683	7.741802	7.078944	7.991114	7.734740	7.834020	7.806171	7.807872	7.712175	8.295916	8.921354	7.577303	7.472343	7.459357	6.925974	7.133895	9.482261	7.959294	7.771110	7.688422	7.610677	7.693362	7.229814	7.790379	7.140322	8.986858	7.131815	7.095799	7.322163	6.897032	7.543432	8.812340	7.245923	7.649041	7.113147	7.684131	7.613414	7.460716	7.797700	7.678751	8.635047	8.454599	9.302046	8.379382	8.510434	7.971462	8.098702	7.709416	8.268231	6.636899	7.093370	7.180484	6.926972	7.467717	7.603129	7.749198	7.294130	7.613888	7.784389	7.469723	7.628611	8.134971	8.056204	7.893421	7.991262	8.023775	7.505232	8.627624	8.887283	7.919036	8.761671	7.559652	8.373576	8.212064	7.519245	8.551203	9.139637	8.266504	7.936532	8.880826	8.040597	8.310809	8.692143	9.761351	8.414729	8.744816	7.795409	8.376613	9.233160	8.712055	8.318960	8.448422	8.551804	9.685477	9.209941	9.283544	9.050083	9.254788	9.461202	9.593381	9.142279	8.850472	9.394342	8.959017	9.172346	8.950873	8.350523	8.086750	7.882174	8.427882	8.769925	8.731353	8.897010	8.854235	9.497352	9.007476	9.934782	9.375129	9.557255	10.694805	8.074127	8.307442	8.883123	8.090493	7.944322	8.062120	7.861073	8.723730	8.029643	10.778555	7.741021	7.544875	7.598391	7.976173	7.695315	10.734462	8.219328	8.479139	8.598194	7.657668	8.253736	7.675124	8.021201	7.458413	9.050770	8.669707	7.716139	7.356553	7.363410	7.960692	8.792916	7.932034	7.573879	7.733023	7.425236	8.059636	7.812017	8.307442	7.947696	8.609236	8.770226	10.862192	8.740568	8.324774	8.074418	8.835874	8.419610	8.126434	7.057287	7.272673	7.839686	8.199516	7.814763	7.812017	8.800815	7.725594	8.479305	8.079308	7.695463	8.605303	8.255434	8.578471	9.533072	9.317184	9.810261	8.145645	9.352758	9.615893	8.407426	9.803798	8.634408	8.986242	8.096125	8.897476	9.444365	8.715151	8.015092	8.386331	10.558269	7.935898	9.259376	8.814119	10.676730	9.418972	8.815582	7.958376	8.760599	9.868052

AL024497.1	5.103993	3.232699	3.002835	4.554945	3.689524	4.059769	3.967902	5.233431	3.644257	5.199677	3.894841	5.534285	3.938758	3.941517	3.719337	3.810560	3.402108	3.478586	3.603661	5.019934	4.863721	3.215682	4.110948	3.348203	3.449361	3.701392	4.504144	4.161599	5.662559	4.300892	3.727139	4.758930	3.484067	5.212965	3.424199	3.996626	4.735652	3.183074	3.748721	3.064058	4.748287	3.102819	4.125060	4.390178	5.761020	5.436108	5.143159	2.997794	5.168280	3.418075	4.590982	4.331654	4.068163	4.063079	4.205714	3.871796	4.913496	3.111657	3.140934	6.137363	2.897338	5.587518	3.491861	3.716485	3.842951	3.783976	2.861591	3.855536	3.696522	3.911625	3.598578	4.341361	4.794167	4.124861	4.190163	3.301128	4.182980	3.686432	4.139072	3.964673	4.723229	3.918319	4.281251	4.484384	4.599394	3.598945	5.712884	4.387386	3.101295	3.869456	3.542277	3.486956	3.617097	3.947935	4.099547	4.623340	4.591411	4.875077	3.944424	3.921454	5.339727	3.710057	3.779373	3.992227	3.470157	5.171481	3.006446	3.735228	3.353040	4.320790	3.098313	3.795908	3.665470	5.749744	3.717910	5.922924	4.472031	4.299617	5.231386	4.174447	3.322974	3.004165	3.282404	4.868967	3.723702	5.521763	6.207080	4.411759	5.351486	5.058371	5.780525	4.686755	3.262792	3.352380	4.466739	3.306163	4.525882	3.027576	3.499062	4.196467	3.971168	5.681213	7.229521	3.017093	4.846179	5.278130	3.163139	6.277178	3.854698	3.695073	4.366995	5.013417	5.636952	3.286231	4.413326	4.393824	3.247824	3.263098	3.008166	6.041579	3.021888	3.813804	3.464678	4.308141	5.039903	6.548199	2.902673	6.492830	4.463269	5.054079	3.411831	4.669702	4.261771	4.035847	3.973302	3.804516	3.105616	2.943473	6.367040	2.840111	4.041460	3.445079	4.447326	3.475268	3.583778	2.853354	3.553350	3.394704	2.877511	4.266350	4.334644	6.588358	4.945653	3.847370	3.473243	4.026648	4.051084	4.306150	3.633517	3.720796	4.317809	3.629032	4.230278	3.854880	3.743241	4.138852	4.059980	2.975343	3.411476	3.394523	2.966317	4.064780	4.254568	3.532537	4.802623	4.152513	5.513884	3.833118	6.206276	4.752112	3.422423	3.815038	3.763856	3.699196	7.948461	4.532248	3.317112	3.309540	4.967357	3.201711	4.504347	3.970758	6.024815	5.968975	4.246870	3.024958	4.553674	3.875056

AP002026.1	14.133856	11.186851	11.603148	11.283702	9.877183	10.086530	13.671017	9.384220	9.219434	11.504652	10.393108	11.218772	11.080729	10.589937	11.704443	11.855782	11.830475	10.511848	8.097374	11.267076	9.134694	11.188016	10.176535	10.464310	11.434496	10.085713	12.482801	9.799359	12.601601	13.071521	11.513924	13.708928	12.487307	12.356334	14.469705	13.915290	13.916047	12.352576	11.885697	12.223705	12.735569	13.541913	12.948751	13.455497	14.509550	12.969318	11.604162	11.798936	11.668318	12.614208	13.800361	13.308940	13.016071	13.658895	12.330636	10.754780	13.882222	12.629207	10.841116	12.645369	12.447571	13.595594	12.760314	10.516841	12.147509	11.763401	11.834974	12.379442	11.653340	12.465654	13.898829	13.748094	11.420288	13.384972	13.332262	13.550257	14.473933	12.099825	13.488366	12.248323	12.895646	12.807024	13.043715	13.126344	13.403666	13.157459	12.793014	11.378727	12.282721	11.869781	12.439669	12.016135	11.913381	12.702108	13.792778	13.631592	13.700156	12.783965	12.606059	11.700684	12.182961	12.682905	11.199882	10.455701	9.649319	11.722280	10.838287	9.360960	11.471808	11.959745	12.708589	9.532146	9.325661	11.557603	10.494672	9.636219	13.383479	12.153901	12.463731	9.752834	5.814250	6.744672	10.525956	3.283480	6.321320	8.334148	4.320790	5.522145	7.729343	5.357029	6.624362	7.781951	4.803917	5.083639	11.074134	4.673856	7.333384	6.015887	7.473387	8.918022	5.879233	8.033453	5.221697	4.592086	9.862391	8.416105	5.399012	12.875552	11.141637	8.530152	8.213459	7.712340	7.193586	9.650235	8.614211	9.071262	7.660314	6.350954	7.663402	5.904725	9.894119	7.348804	7.603602	9.927841	8.994888	5.375140	8.518092	12.157725	7.466956	8.838748	11.697270	8.873398	8.112848	10.361120	11.122337	8.910839	8.359117	9.273789	8.263689	6.582771	11.637074	7.075119	5.257917	6.980510	8.833342	6.332486	8.249737	6.235310	6.777825	10.161530	7.864018	5.192494	7.830357	11.978674	10.389287	8.774143	6.746121	8.764831	9.388260	7.206614	7.925527	7.580232	7.113933	7.435209	9.265515	7.144129	6.668343	9.628464	8.506737	6.257597	6.224688	8.015709	6.854303	8.187296	8.244922	7.696675	7.163940	7.036077	10.416445	5.093749	6.243786	12.460261	9.633215	5.135616	10.741254	4.818987	8.755318	9.736095	6.060246	8.700270	4.562740	4.608201	8.837982	4.775737	6.532074	7.342525	6.807739	6.824346

AL589740.1	6.193889	6.834373	6.839672	6.836129	6.816998	7.263127	7.927727	4.992675	4.707020	7.002889	6.460003	6.545320	6.156355	6.314012	5.761388	6.825914	7.364676	6.245620	6.429707	5.815647	7.046908	6.350277	8.953012	6.247463	7.542948	6.775437	8.224937	7.167633	7.913572	7.398827	6.446864	6.706060	6.694902	7.143957	7.140953	6.994160	6.958781	7.176828	6.827817	6.104268	7.472951	6.694089	6.851553	6.287083	7.389333	6.161869	8.885279	7.100178	7.611287	6.810491	6.358002	7.138283	7.073851	6.785647	6.907507	8.001204	6.555824	6.677045	6.285499	7.092126	6.553169	7.364203	9.246741	6.693087	6.780884	6.825589	6.461996	6.783375	6.182207	6.487103	7.559510	6.831026	7.502758	7.192295	6.342020	6.666704	7.052414	6.338043	5.932632	5.931261	6.206085	6.127291	5.751747	5.984867	6.467022	6.538118	5.941779	5.800619	6.249627	5.084680	5.577584	5.621097	6.185209	6.398962	5.393986	6.504316	6.506995	6.322042	6.455432	7.822399	6.331290	7.483685	6.858817	6.931748	6.298741	7.282571	6.805534	6.995829	7.180945	8.322308	6.297720	5.775473	5.178157	7.177471	6.022897	5.297474	5.018300	7.184084	7.530078	5.644627	7.342299	6.826564	6.777993	6.323204	7.817798	6.691435	6.290632	7.470962	7.283670	7.539270	5.702406	6.971038	6.036861	7.040964	7.221610	7.156379	5.085936	7.696523	7.204398	6.327724	6.306280	6.723381	6.504643	6.670564	7.478735	6.303246	5.105453	6.049248	8.162655	6.537941	6.778386	7.125777	6.650152	6.122684	6.054089	6.940736	6.105503	7.627192	6.446691	7.220994	5.525162	6.374509	7.172461	5.539772	6.612852	7.131001	6.210495	6.131041	8.111174	7.857697	6.821815	6.783375	7.780295	6.823074	8.172239	6.381820	6.861702	6.209663	6.720804	6.094972	8.989269	6.757186	7.601689	6.103589	6.267244	7.401924	6.697597	6.932872	6.689163	6.450803	7.703423	7.678105	6.942312	6.783072	6.625344	7.505885	7.673040	6.912442	6.270865	6.457727	7.157612	6.187767	5.354296	6.535659	6.195854	6.012483	5.653761	6.433281	6.592272	4.716448	5.573143	6.026766	7.292400	6.808244	5.912957	6.784522	6.358002	7.204562	6.464876	7.217482	6.647718	6.782766	6.487103	6.790236	6.807572	6.793403	5.961797	6.070155	7.309782	6.674620	6.043039	5.293315	6.197075	4.843175	5.147545	6.376157	5.920679	7.551132

AC027088.2	8.414587	9.226054	10.238166	9.499970	9.364247	10.745154	8.391617	10.071306	10.537222	8.247677	8.746045	8.964318	7.761592	8.695584	7.786221	9.240288	10.629335	8.946272	10.610931	9.369356	9.639817	9.606716	9.322724	8.595167	8.337120	8.433072	8.679595	9.380667	9.011455	8.919357	9.180474	8.263240	8.273134	8.087256	9.036048	8.667128	9.238757	8.807508	9.081035	9.070354	9.301255	8.544600	8.809523	8.394988	8.850472	8.723135	9.345751	8.205382	9.167255	8.391859	8.918624	9.352291	8.524156	9.842448	8.518570	9.047133	9.107963	8.899150	8.951490	8.567166	8.729147	8.731208	8.216812	9.601437	8.526133	10.128236	9.371801	9.684031	9.566504	11.339952	11.337754	10.552353	9.067847	11.774769	10.744255	9.249793	11.038735	9.674122	10.966093	8.111918	8.178859	8.685533	7.807549	8.727249	9.694363	9.217179	9.215763	9.361114	9.228667	9.303995	9.704725	9.157717	8.846008	8.694336	8.623429	9.173279	8.462342	9.533834	8.888996	9.851548	9.470780	9.379453	10.410577	9.596328	9.807767	9.094797	8.909516	8.835535	9.974679	9.362915	10.718371	9.295062	8.907201	9.857725	9.342515	13.333932	8.491790	8.898713	9.238440	7.959136	7.283976	10.343255	7.566103	9.851053	8.943997	9.217322	8.870392	8.652056	8.853554	8.476639	8.613295	8.034780	7.949357	7.516962	8.984120	8.337120	7.514341	10.464170	11.738357	7.671796	8.221769	9.556322	8.092006	8.300469	8.065348	10.159669	10.728981	10.940842	8.437691	8.513849	8.439638	8.429432	8.215162	8.251460	8.031326	8.755929	8.510113	8.088325	8.647850	9.551167	8.044021	10.359924	7.399130	7.878847	8.274926	9.453237	7.428515	8.794153	7.502262	8.482488	7.680701	7.114394	8.404218	7.541602	8.684334	9.128435	7.799277	7.383782	11.820064	8.036022	8.209784	9.074340	7.881227	7.192436	7.678751	7.603602	7.790829	7.958540	8.621124	8.842770	8.670032	8.778712	9.045341	10.008153	8.399723	8.931445	8.644694	9.469514	7.605625	8.485965	7.623177	7.553470	7.885206	7.810799	9.084561	8.044812	7.760209	8.363776	7.746079	7.260754	7.790829	7.633766	7.983196	8.255576	9.247803	8.800815	8.926766	8.200142	8.287044	8.181595	7.940884	8.437550	8.670197	7.368917	8.499004	8.295459	7.682089	13.151653	9.193891	9.471395	8.891442	11.004847	10.219536	9.225443	10.224681	7.370309	8.201533	8.578471

EWSAT1	8.414587	9.226054	10.238166	9.499970	9.364247	10.745154	8.391617	10.071306	10.537222	8.247677	8.746045	8.964318	7.761592	8.695584	7.786221	9.240288	10.629335	8.946272	10.610931	9.369356	9.639817	9.606716	9.322724	8.595167	8.337120	8.433072	8.679595	9.380667	9.011455	8.919357	9.180474	8.263240	8.273134	8.087256	9.036048	8.667128	9.238757	8.807508	9.081035	9.070354	9.301255	8.544600	8.809523	8.394988	8.850472	8.723135	9.345751	8.205382	9.167255	8.391859	8.918624	9.352291	8.524156	9.842448	8.518570	9.047133	9.107963	8.899150	8.951490	8.567166	8.729147	8.731208	8.216812	9.601437	8.526133	10.128236	9.371801	9.684031	9.566504	11.339952	11.337754	10.552353	9.067847	11.774769	10.744255	9.249793	11.038735	9.674122	10.966093	8.111918	8.178859	8.685533	7.807549	8.727249	9.694363	9.217179	9.215763	9.361114	9.228667	9.303995	9.704725	9.157717	8.846008	8.694336	8.623429	9.173279	8.462342	9.533834	8.888996	9.851548	9.470780	9.379453	10.410577	9.596328	9.807767	9.094797	8.909516	8.835535	9.974679	9.362915	10.718371	9.295062	8.907201	9.857725	9.342515	13.333932	8.491790	8.898713	9.238440	7.959136	7.283976	10.343255	7.566103	9.851053	8.943997	9.217322	8.870392	8.652056	8.853554	8.476639	8.613295	8.034780	7.949357	7.516962	8.984120	8.337120	7.514341	10.464170	11.738357	7.671796	8.221769	9.556322	8.092006	8.300469	8.065348	10.159669	10.728981	10.940842	8.437691	8.513849	8.439638	8.429432	8.215162	8.251460	8.031326	8.755929	8.510113	8.088325	8.647850	9.551167	8.044021	10.359924	7.399130	7.878847	8.274926	9.453237	7.428515	8.794153	7.502262	8.482488	7.680701	7.114394	8.404218	7.541602	8.684334	9.128435	7.799277	7.383782	11.820064	8.036022	8.209784	9.074340	7.881227	7.192436	7.678751	7.603602	7.790829	7.958540	8.621124	8.842770	8.670032	8.778712	9.045341	10.008153	8.399723	8.931445	8.644694	9.469514	7.605625	8.485965	7.623177	7.553470	7.885206	7.810799	9.084561	8.044812	7.760209	8.363776	7.746079	7.260754	7.790829	7.633766	7.983196	8.255576	9.247803	8.800815	8.926766	8.200142	8.287044	8.181595	7.940884	8.437550	8.670197	7.368917	8.499004	8.295459	7.682089	13.151653	9.193891	9.471395	8.891442	11.004847	10.219536	9.225443	10.224681	7.370309	8.201533	8.578471

TESC-AS1	8.141476	7.468615	7.549912	7.295865	7.358246	6.778938	7.457779	6.499228	5.722326	7.824306	7.084584	7.600626	7.139390	6.908917	6.794940	7.152494	5.474055	6.930487	7.100178	7.873058	7.897027	8.237449	8.764975	7.106131	7.672436	7.241488	7.015498	6.662158	6.868239	6.974149	6.797851	7.934327	8.277825	7.696675	7.609439	8.045289	8.011860	7.369988	7.425076	7.666698	7.651114	7.317634	7.622688	7.801599	8.268065	7.729939	7.082687	7.593360	7.283976	7.744557	7.997816	8.182181	7.995024	8.157342	7.879149	6.987484	8.295378	8.278420	7.440209	7.931121	7.764057	7.535183	7.040034	7.057617	6.925659	7.810046	7.653601	6.679298	7.517263	7.856943	7.886135	7.733473	6.816835	7.722222	7.934018	7.890146	7.598679	7.267882	7.335219	7.773688	8.059794	7.412742	8.526133	8.179917	8.312070	7.842803	8.016946	8.563599	8.039167	7.914757	8.163833	7.312895	7.413067	7.459019	7.486494	7.738981	7.154829	7.480895	6.980510	6.334497	6.775132	7.522082	7.056979	7.733324	7.663558	6.625003	7.052414	7.453866	7.649346	7.374866	7.967867	6.610967	6.683406	5.964297	6.390022	4.578613	7.351457	7.841996	7.128781	7.869842	5.966279	7.425716	7.294911	5.811028	6.883408	5.814756	5.720302	6.176773	7.726063	7.131165	7.719199	5.995014	6.173182	6.008773	6.809210	5.650297	6.983026	7.582415	8.073673	6.992259	7.671322	8.317000	6.860106	7.812017	7.637084	6.377801	6.792518	6.441540	7.336755	6.207080	7.063346	7.288615	7.220497	6.798171	8.353996	7.170236	5.758523	6.908917	6.697122	6.389356	6.478984	7.450892	6.784020	8.193845	7.400216	6.097197	7.901488	7.733781	7.604831	7.664539	8.281448	7.788217	7.424131	7.456028	6.665888	8.872170	8.140459	7.395059	8.440271	6.859613	7.305394	7.046103	6.178514	6.709750	7.632540	5.520813	6.551327	7.378634	7.087059	7.677630	7.665283	5.204262	7.292400	7.237972	7.036396	7.625771	7.253406	6.826234	7.712007	7.461032	7.303825	7.241188	6.649044	6.963071	6.470376	7.746418	8.705361	7.426143	6.483410	8.206933	5.163477	6.562847	6.714240	6.786475	6.801084	6.072576	7.381709	5.552010	6.925831	6.484623	7.017952	6.764402	6.811929	7.637692	7.652378	6.506499	7.493042	8.547074	5.679946	7.753645	6.325088	7.470014	5.137833	8.762566	6.119929	6.889336	8.239336	6.902075

RFPL3S	11.127076	9.562068	9.923196	10.264537	10.681376	11.617285	10.278824	11.181871	11.339525	10.435536	11.233464	11.110584	9.538430	9.792860	9.781775	11.035057	10.858794	8.753789	9.935441	11.398826	9.425426	9.728056	9.884535	9.442348	9.689137	10.325054	11.738943	10.606089	11.442042	10.782671	10.711692	9.218853	9.748976	10.235279	10.165028	10.321065	10.195069	9.920274	9.753921	9.913442	11.265020	9.658419	9.689437	10.143394	9.842136	10.039288	11.481782	9.351391	9.756700	9.679253	11.481500	9.611512	9.099653	9.563450	9.384665	11.099009	9.340740	8.829341	9.011278	9.027513	9.206573	10.809055	8.759794	9.222522	9.266894	9.453075	9.399848	9.505657	9.410961	9.471707	11.239944	11.204194	11.260184	11.407214	11.312487	10.568013	10.595584	10.768642	10.824430	8.701974	9.352587	9.224967	9.154950	9.400002	9.092054	10.229726	10.132992	11.153328	9.018822	9.024612	9.101177	9.110466	9.436477	10.155312	9.994941	10.203707	10.067808	9.986916	10.160896	10.003957	10.375445	10.143394	10.538660	10.506605	9.372574	11.318232	9.934782	9.519473	9.958780	11.259741	9.635782	10.084519	9.653378	11.760698	10.714458	13.329024	9.944399	10.522881	11.722608	10.632025	9.934336	9.727151	10.481536	11.737167	11.086931	10.947159	10.905252	10.487794	11.097070	10.982046	11.127958	10.218622	9.808891	9.947027	11.098087	10.507361	9.662498	10.089619	10.276834	9.406477	10.285158	9.641761	10.236834	9.980586	10.586706	11.311749	10.428979	12.393291	10.744255	10.656820	9.568410	9.966054	10.620713	10.483377	10.351898	10.290374	9.976348	10.057591	10.258179	11.569303	10.005518	9.593217	10.114982	10.074063	10.128694	11.633293	9.441595	9.915155	9.565725	10.459968	9.530543	9.211300	9.524595	9.187084	10.924627	9.187084	8.590817	9.136846	9.121085	9.105974	10.849578	9.181882	9.372889	9.429888	9.429742	9.641485	9.684838	9.575947	9.525717	10.922541	10.926688	11.772261	11.232575	10.777670	10.773731	10.328691	10.600462	11.104014	8.861351	9.328459	9.117099	9.662962	9.289425	9.307443	10.373383	10.147018	10.144761	8.911270	8.833199	9.621028	8.903060	9.386045	9.994941	10.537371	10.337340	10.400199	10.782238	10.100294	9.800263	9.917160	10.479041	10.790288	10.175472	10.129611	11.789710	10.013832	9.700494	10.556927	11.734816	9.488725	10.277608	10.265297	12.081360	10.852873	11.496207	9.501937	10.529955	11.224167

AC012184.3	12.663072	12.341197	12.570017	11.977145	12.487478	12.534127	12.677174	12.528252	11.990173	12.412694	12.471493	12.461245	12.223401	12.390459	11.946535	12.205414	12.572856	12.036088	12.585969	12.926827	12.475857	13.141902	13.101051	12.174349	12.404959	12.196766	11.997634	12.014022	12.028406	12.233426	12.354202	12.653462	12.949621	12.542605	12.937259	12.878888	12.952935	12.526938	12.443867	12.659132	12.475368	12.397063	12.814569	12.616955	12.931002	12.949109	12.402275	12.262621	12.942109	12.600026	12.573686	12.829828	13.107173	12.809121	13.097680	12.320151	13.140468	13.004475	12.296489	12.460438	12.541791	12.405277	13.266014	12.623671	12.560055	12.418490	12.655686	12.353897	12.165300	12.679231	12.395853	12.661032	12.300500	12.472784	12.798338	7.737275	13.079117	12.465329	12.697692	12.354824	12.815287	12.856932	12.645369	12.791666	12.930265	12.680777	12.705525	12.942292	12.934012	12.652437	12.659816	12.840899	12.370681	12.470791	12.460764	12.653635	12.812473	12.486329	12.607071	12.081665	12.622342	12.479650	12.117262	12.195864	13.303085	12.466153	12.514830	13.083488	12.747282	12.264187	13.127100	12.882947	12.657484	12.259420	12.775824	11.876874	12.482306	12.521236	12.261188	11.692743	12.159115	12.834529	12.249142	12.257409	11.835718	12.022508	12.115695	11.788671	11.980765	12.145773	12.435636	12.474582	12.065157	11.789556	12.183277	13.103890	11.859791	11.899712	11.883291	11.986674	12.240857	12.644314	10.242906	11.510962	11.873246	12.273308	12.103936	12.025076	12.166484	12.418655	13.046106	12.477840	13.158034	12.308880	11.870252	12.427314	12.491754	12.567105	11.446009	12.330636	12.709456	11.213197	12.798496	11.782082	12.378030	12.443196	12.649008	12.600886	12.278204	12.468921	12.161570	12.076837	13.360143	12.478661	12.306362	12.079262	11.963218	11.933500	11.570758	13.553941	12.666497	12.208223	12.564954	12.587644	12.473611	12.824043	12.389042	12.261651	12.412694	12.819221	12.229877	11.906521	12.697692	12.192905	7.688422	12.171247	12.735923	12.493046	12.072497	12.493699	12.227834	13.244951	12.348967	12.757036	12.204037	13.157459	12.169111	13.391817	12.557657	11.946077	13.019562	12.691855	13.259193	12.955360	12.161273	12.380039	11.610629	12.389516	11.643282	13.016827	12.312060	11.371738	11.760077	12.971153	12.407294	12.098451	11.809488	12.220618	12.022667	12.895995	12.178954	11.750711	12.190585	12.754938	12.604915	12.978958	12.777583	12.954050

NAMA	4.663683	4.182980	5.012216	3.939428	4.251723	5.232404	3.970288	4.233080	4.127199	3.514626	4.076598	3.863667	3.332078	4.609716	4.156948	4.475158	3.931308	4.069842	6.067528	3.750187	5.510469	4.927430	5.491318	4.145791	4.244102	3.566109	4.401806	4.575798	4.144966	4.450279	4.486379	4.564910	5.010742	4.801966	4.414893	4.415122	3.856144	3.509610	3.871553	3.098676	4.074275	4.567891	4.738874	4.884522	4.269645	6.187399	3.811858	5.222922	4.368291	4.970105	4.341985	5.037327	4.351327	4.920418	3.949881	3.782519	4.592310	3.479830	4.720656	3.933686	3.548911	3.731504	4.953562	4.267631	4.174224	4.992029	3.385511	4.006530	4.469910	3.927387	4.454188	4.097305	4.784334	4.425470	3.970970	4.245365	4.285445	4.676470	4.631969	4.267843	4.209292	5.308659	5.087580	4.243420	4.399395	5.058170	4.137156	5.061401	2.944071	4.494423	4.675819	4.916169	5.012811	4.254350	4.714961	4.013112	4.887696	4.716660	3.993280	4.126990	5.203864	4.301736	3.865767	3.738304	4.586903	4.438066	4.468417	4.102158	4.172921	3.319276	4.624008	4.426556	4.382733	3.340297	4.256083	6.861228	5.237270	3.462151	3.255150	4.476026	3.802439	4.233519	4.488660	3.925247	4.987675	4.058871	5.402456	3.672132	3.931529	5.235426	3.491016	4.320571	4.164047	5.301629	3.549833	4.978266	3.780400	4.954166	5.038113	4.785361	5.909893	5.563726	4.260212	4.720248	4.993721	4.322455	3.742824	3.728166	3.777146	4.460696	5.230355	5.052996	3.774917	5.069592	3.934506	4.391908	4.128728	4.403105	4.726811	3.400386	3.249707	4.646000	5.585535	4.215112	5.956984	3.409194	3.996401	4.330122	4.549992	4.608431	4.906492	4.022591	4.389151	3.485185	3.521682	3.716703	3.209388	4.019991	3.810560	4.218149	3.799869	3.462151	4.577141	4.210345	4.986634	2.798350	4.367861	4.027744	3.920194	4.114828	4.728702	3.231727	4.361966	4.431388	4.824132	4.495760	4.578828	4.548399	4.513363	3.846532	4.749312	4.564465	4.495760	4.843594	5.606684	3.533103	4.912395	3.152463	4.865377	4.125253	5.931759	3.996830	4.816230	4.260648	4.509201	4.712842	4.983584	3.517751	3.967228	4.787444	3.988380	4.105442	4.111801	4.291112	4.396577	4.245989	4.005441	3.504732	3.336436	4.937670	5.271047	5.804583	3.545984	4.546452	4.655780	4.888354	3.694454	3.945528

AC242426.2	11.895030	8.486125	8.037443	10.157733	7.073401	6.909858	7.640482	5.916723	4.580362	7.469405	6.377960	7.981607	7.725286	8.103712	7.460098	7.040480	5.795803	6.869059	7.679523	7.772629	6.949175	7.961466	7.217022	6.311949	6.975110	7.557866	6.412706	9.140544	7.479657	7.459810	5.752653	10.859380	8.682359	7.457145	7.712486	7.814935	8.247950	7.964325	7.645417	7.436017	6.405228	9.005015	8.168619	7.728577	8.399260	8.160080	6.437128	8.289941	8.404218	8.551354	10.963237	8.962369	8.653894	8.703369	8.450930	6.419799	8.209472	8.083646	8.390991	8.188953	8.940056	6.700968	8.373879	8.021345	7.984384	8.127189	8.224190	7.961466	7.439309	8.138337	7.689945	7.418684	6.469202	7.681937	7.336449	7.436945	8.251925	7.962682	9.535347	8.253119	9.302046	8.528920	10.789421	8.859264	9.160471	8.642515	8.787346	8.476639	8.700763	9.180314	9.022956	8.136005	8.285318	7.170697	10.555376	8.188055	8.601729	7.371577	7.128781	6.984290	6.709100	7.542183	6.899505	7.173384	6.539709	7.054266	7.807410	7.182354	7.563626	7.337348	8.438425	6.843699	6.002186	6.501873	7.125950	7.988227	12.035653	7.198684	6.754244	7.400060	5.002715	6.888860	7.971630	7.533297	5.226669	6.982081	4.889393	4.405609	7.086749	5.906509	7.214181	6.381308	6.153205	4.309458	7.821338	6.682753	6.556792	6.566408	5.947042	6.575152	7.060771	7.097357	5.696745	7.612965	7.363410	7.166091	5.840777	5.538246	7.126885	7.169643	7.007341	7.727157	6.980014	8.276122	6.055659	7.354382	5.612184	5.991295	6.422233	5.303245	7.461823	11.021387	5.324222	7.674955	7.438363	5.069790	6.622109	6.515048	7.237496	7.668822	7.304606	7.416992	7.418994	8.142400	7.309930	7.523256	7.677630	7.335828	6.844707	6.807413	6.851096	6.982716	5.870621	7.188249	7.103604	6.107118	6.054258	4.275963	7.165954	7.761745	6.149155	4.743795	7.386409	7.162193	6.949978	6.725575	5.974485	6.614638	7.811119	7.916384	6.909692	8.028572	7.527432	7.598098	7.701453	8.115024	8.486125	8.730171	7.277818	5.757968	6.221062	7.360104	6.324927	7.139220	6.617562	6.673975	6.561359	7.195034	8.834047	5.161670	5.654037	7.133232	6.844034	7.534238	5.697884	4.942399	6.484071	7.236106	3.743241	7.126261	7.554414	6.910655	5.257917	5.732672	8.431677	6.005203	8.951337	7.037948

LINC02839	3.440483	3.276202	3.066288	3.157189	2.923767	4.268049	3.434507	3.562318	3.570038	3.532537	3.708643	3.899129	3.376363	4.351108	4.038661	3.179899	3.759641	3.896720	5.191503	3.219832	5.555072	2.807995	5.468755	3.275450	3.051323	2.890164	2.923489	3.008249	3.142685	3.085290	3.797157	4.438927	3.304924	3.325316	3.738304	4.238326	3.823956	3.473978	3.268403	4.379075	3.503892	2.962105	3.610768	3.352721	3.664267	5.633992	4.199882	3.607047	4.300892	4.061055	4.115928	4.962329	4.594060	4.143246	3.978937	3.107476	4.148755	4.103439	4.064578	3.644431	3.236796	3.230063	3.527760	3.456418	3.260006	3.169527	2.764973	4.027949	3.082015	2.871110	3.295748	3.082761	3.005456	3.197292	2.909069	3.677214	3.099280	3.427805	3.138823	3.835458	3.445983	3.988618	3.215814	5.247413	3.813152	4.643607	3.377017	3.262477	2.992407	3.572687	3.207657	3.665884	3.042715	3.058514	3.017202	3.143153	3.905736	2.961473	2.944910	2.985514	4.088961	3.334371	3.039234	2.884916	4.473629	3.538047	5.598184	3.603041	3.243133	3.320580	2.784658	3.515313	3.161356	3.320743	3.364197	4.141719	3.175414	3.207173	3.153675	4.290904	5.195916	4.418223	4.582882	3.281645	6.758139	3.734186	4.252167	5.270656	4.440247	5.209621	4.598769	4.397228	4.935976	5.486729	4.483726	5.354082	5.061401	4.777623	4.905496	5.860307	3.754031	6.635160	4.057364	4.854811	4.658359	4.745112	4.343091	3.284110	5.142956	5.226103	4.772404	4.366132	5.358153	5.126176	5.524027	5.375342	5.495683	5.197099	5.019124	4.452895	5.958590	5.368387	5.384096	5.774910	6.422692	6.395482	6.228136	5.465597	6.245766	6.203457	6.057108	5.951984	5.997249	5.452837	4.177253	5.630622	6.053723	5.675995	5.357029	5.173168	4.595326	5.536264	5.611787	5.366014	5.084868	5.312841	5.280527	4.593852	5.603232	4.262655	4.574580	5.024248	4.282532	4.341562	4.468417	3.951827	4.258087	3.972872	4.147485	4.975271	5.943035	5.136227	5.448411	5.476002	4.765079	5.396153	5.141118	5.467155	4.849289	4.721287	4.713705	4.680730	4.588420	4.792105	4.453761	3.432871	4.240135	5.620566	4.671850	4.946505	4.227886	3.819056	3.832712	5.615559	5.035490	6.100152	5.199473	5.085272	4.679217	3.507618	5.228322	5.668695	4.944987	3.890045	4.069020	4.944128	4.562063	4.844447

AC060765.2	3.440483	3.276202	3.066288	3.157189	2.923767	4.268049	3.434507	3.562318	3.570038	3.532537	3.708643	3.899129	3.376363	4.351108	4.038661	3.179899	3.759641	3.896720	5.191503	3.219832	5.555072	2.807995	5.468755	3.275450	3.051323	2.890164	2.923489	3.008249	3.142685	3.085290	3.797157	4.438927	3.304924	3.325316	3.738304	4.238326	3.823956	3.473978	3.268403	4.379075	3.503892	2.962105	3.610768	3.352721	3.664267	5.633992	4.199882	3.607047	4.300892	4.061055	4.115928	4.962329	4.594060	4.143246	3.978937	3.107476	4.148755	4.103439	4.064578	3.644431	3.236796	3.230063	3.527760	3.456418	3.260006	3.169527	2.764973	4.027949	3.082015	2.871110	3.295748	3.082761	3.005456	3.197292	2.909069	3.677214	3.099280	3.427805	3.138823	3.835458	3.445983	3.988618	3.215814	5.247413	3.813152	4.643607	3.377017	3.262477	2.992407	3.572687	3.207657	3.665884	3.042715	3.058514	3.017202	3.143153	3.905736	2.961473	2.944910	2.985514	4.088961	3.334371	3.039234	2.884916	4.473629	3.538047	5.598184	3.603041	3.243133	3.320580	2.784658	3.515313	3.161356	3.320743	3.364197	4.141719	3.175414	3.207173	3.153675	4.290904	5.195916	4.418223	4.582882	3.281645	6.758139	3.734186	4.252167	5.270656	4.440247	5.209621	4.598769	4.397228	4.935976	5.486729	4.483726	5.354082	5.061401	4.777623	4.905496	5.860307	3.754031	6.635160	4.057364	4.854811	4.658359	4.745112	4.343091	3.284110	5.142956	5.226103	4.772404	4.366132	5.358153	5.126176	5.524027	5.375342	5.495683	5.197099	5.019124	4.452895	5.958590	5.368387	5.384096	5.774910	6.422692	6.395482	6.228136	5.465597	6.245766	6.203457	6.057108	5.951984	5.997249	5.452837	4.177253	5.630622	6.053723	5.675995	5.357029	5.173168	4.595326	5.536264	5.611787	5.366014	5.084868	5.312841	5.280527	4.593852	5.603232	4.262655	4.574580	5.024248	4.282532	4.341562	4.468417	3.951827	4.258087	3.972872	4.147485	4.975271	5.943035	5.136227	5.448411	5.476002	4.765079	5.396153	5.141118	5.467155	4.849289	4.721287	4.713705	4.680730	4.588420	4.792105	4.453761	3.432871	4.240135	5.620566	4.671850	4.946505	4.227886	3.819056	3.832712	5.615559	5.035490	6.100152	5.199473	5.085272	4.679217	3.507618	5.228322	5.668695	4.944987	3.890045	4.069020	4.944128	4.562063	4.844447

LINC01366	8.163962	7.970525	8.407426	8.474628	8.261839	8.106950	7.505885	9.068319	9.518073	7.893421	8.227204	8.021791	7.644012	8.214857	7.370940	8.025420	8.909943	7.616510	10.012765	8.312823	9.154798	9.534300	9.806105	8.379382	8.322308	8.528127	8.376041	8.821587	7.860474	7.315133	8.781189	7.560439	8.112229	7.858010	7.606249	7.593826	7.420736	7.998718	8.507964	8.126279	7.789294	8.158091	7.797378	7.938674	7.531429	7.445391	8.335568	7.872765	8.135425	8.074715	7.750734	7.920144	7.631455	7.944475	7.818572	8.065348	7.274847	8.026668	8.365170	7.804638	8.207850	7.378793	8.588673	8.905662	8.191485	8.471849	8.165502	8.176634	8.306046	8.545084	8.256035	8.416609	7.949038	8.085388	8.183907	7.917667	8.246033	8.133618	8.220385	7.470633	7.644771	7.730087	7.754859	7.438669	7.846394	8.560242	7.842803	8.221444	8.356438	8.340714	8.412310	8.915240	8.426454	8.001841	7.644012	7.717979	7.572330	8.065658	8.338386	8.231627	8.052786	7.804774	8.232098	8.418725	8.876783	7.635884	9.065545	9.157556	9.351391	8.066110	9.454320	8.583923	8.380819	8.482646	7.937895	7.463419	8.439951	8.315758	8.010797	7.572480	8.161158	8.158091	8.346151	8.071834	7.762841	7.913426	9.313787	8.713136	7.614639	7.983626	7.277044	7.677630	7.141928	7.646194	8.416918	8.814254	7.070868	9.041327	7.519870	8.789036	9.096285	9.218228	8.554484	8.483106	7.806972	8.215018	8.711169	8.604688	7.481512	8.550127	8.047984	8.465536	7.909425	7.801000	7.659658	7.514341	8.607415	9.165569	8.037881	7.554725	8.035245	7.939314	8.555423	7.337505	7.787161	8.141961	7.721135	8.664392	7.712340	8.291988	7.711698	7.061387	7.650473	7.715071	7.551749	7.113147	7.552843	7.992228	7.535018	8.354442	6.999824	8.457202	8.668982	8.660551	8.143925	8.085691	8.059794	8.507669	8.338060	7.789766	7.592412	7.983196	7.939622	7.725594	8.083021	8.196392	8.098995	8.354136	7.427269	7.768842	7.772173	7.870743	7.563944	8.025893	8.994888	7.983196	8.935765	8.794756	8.302736	8.125979	8.953955	8.926440	8.696984	8.975406	8.685699	9.157875	9.267981	8.582268	7.887198	8.261233	8.224339	8.459105	7.612025	8.344925	8.075009	9.262112	8.903386	8.967268	8.288870	9.144893	8.330575	7.814935	8.356438	7.870600	7.216857	8.579521	7.669290	7.633766

LINC01569	5.852014	4.685218	5.964494	5.697300	5.493183	5.924919	6.789740	5.204082	6.378810	5.205438	6.319268	5.968975	6.968033	5.260874	5.052996	6.350277	6.276999	5.221489	5.120920	7.003666	4.446299	5.887816	6.869059	5.167290	5.548432	5.079007	6.666704	3.894431	6.523509	6.422692	5.910637	5.958051	5.837134	7.603296	6.493156	6.824026	6.398294	5.739181	5.511064	5.860307	5.243609	6.555493	6.923374	6.767147	6.487103	6.052855	6.626941	6.234645	6.085920	6.596935	6.010194	6.517896	6.743835	6.847079	6.837058	5.609902	7.946493	6.412181	5.696567	5.781452	5.710849	6.714240	5.663324	5.357782	8.192596	5.708658	5.906683	4.220124	5.585889	5.743183	6.628874	6.259821	4.966918	6.193727	6.241268	5.940611	6.515374	6.053907	4.524351	6.076760	6.370564	6.640806	5.904540	6.484435	6.253184	6.969671	6.389179	6.403278	5.923113	5.703178	5.460841	5.984182	5.939145	6.494441	6.502369	6.481419	6.572093	6.257762	6.551496	5.875145	5.618176	5.947390	5.823680	5.970502	4.001044	5.018899	6.084753	5.266871	4.422881	5.750104	5.483503	5.773628	4.082186	5.977963	5.551616	6.080402	5.905080	6.941664	6.067343	6.942793	5.831051	6.675937	7.105019	6.409866	7.543733	7.375004	7.722083	8.351410	7.295699	7.534064	6.752706	6.723225	5.421654	6.017427	8.205229	7.576179	6.849652	8.505072	8.280249	6.225189	6.079900	7.006896	5.683615	7.742712	7.310802	7.391321	6.495088	7.921053	7.524523	7.225595	6.305609	6.007549	7.777466	7.307427	7.094820	7.233060	6.167909	6.379812	5.538833	6.458037	6.656772	7.942647	8.367911	6.987484	7.204398	8.229033	6.825589	7.325012	7.048173	6.561694	8.089724	6.572093	4.674518	7.724547	7.059799	10.280645	8.543841	6.596780	6.224688	6.643033	8.172688	7.739464	6.413691	7.957449	6.634691	7.489439	5.751191	5.813341	6.262702	6.970730	6.558935	5.941446	7.138754	7.164273	6.078326	7.617539	6.592272	5.335964	7.581955	7.420891	8.473263	6.724812	7.646507	7.219504	7.046248	7.942799	8.110601	5.842795	6.656942	7.459188	6.641302	5.910266	6.992762	5.528211	7.270478	7.420385	7.088794	8.057711	7.683036	7.437870	7.703286	5.893329	6.784180	7.135477	7.230254	6.878619	7.258890	7.222084	6.814127	6.638849	6.102054	5.749744	7.817936	6.326431	7.806972	6.574201	7.694121	8.149614

AL590822.1	9.916997	10.471779	10.266879	10.685116	11.030902	10.894897	10.047104	11.125919	11.621139	10.450323	10.302633	10.140452	9.806105	10.038097	9.676141	11.276168	10.840049	9.895350	11.942440	10.524734	11.182012	10.982470	11.437676	10.838706	11.055681	10.754642	10.771383	11.023215	10.941113	10.441249	10.749026	9.278749	9.583001	9.879029	9.781478	9.839071	9.922578	10.163835	10.601358	10.561470	10.671515	10.027498	10.084677	10.425603	9.789747	9.806251	10.936702	9.901485	9.968648	9.760100	9.641913	9.608835	9.462714	9.755164	9.865926	11.022755	9.262594	9.853221	9.860973	9.448879	9.890879	10.685697	10.445527	10.665039	10.052192	10.112589	10.560393	10.773291	10.417045	11.108070	11.049446	10.979225	10.989295	10.522517	10.565894	10.342962	10.551138	10.045398	10.377424	9.515518	9.605775	9.516216	9.193621	9.440482	9.495493	9.775074	9.394814	9.485177	10.064990	10.113777	10.022295	10.466973	10.046039	10.213496	9.896599	9.943047	9.712029	10.128392	10.287417	11.270879	10.556465	10.442006	10.567791	10.295850	10.849438	10.671196	11.208956	10.884458	11.640267	11.204945	11.221910	11.038010	10.584914	11.465549	10.754780	12.226312	10.200957	10.021565	10.802991	9.645810	10.708891	10.412851	10.701962	11.984395	10.690069	10.635496	11.294412	10.928906	10.645760	10.229880	10.097780	10.499007	10.110805	10.437805	10.920769	10.412851	10.076815	11.316765	9.782528	10.910730	10.814320	11.071521	11.388081	11.066743	10.165812	10.952431	10.931134	11.841136	10.544311	10.648499	10.096550	10.344770	10.381474	9.969420	10.052797	10.009427	10.543459	10.819998	10.610379	11.185403	10.428979	10.041611	11.079540	9.760895	10.059401	11.361048	10.063772	9.981518	9.945279	9.976192	9.535194	9.498268	9.792229	9.348949	10.656678	9.016697	9.627839	9.859459	9.166648	9.895662	10.742448	10.520191	10.537651	10.217724	9.987460	10.717904	10.844879	10.669700	10.720925	10.806048	10.622041	11.502190	10.445678	9.960892	10.434040	10.422993	10.246964	10.503620	9.432816	9.461985	9.679857	9.692841	9.329227	9.486434	10.519091	9.258034	8.993030	10.184839	9.861608	10.056961	10.462035	10.238620	10.555685	10.430485	10.332520	10.223615	10.395665	10.327034	10.412390	10.460997	10.716860	11.015587	10.066454	10.592969	10.920621	11.107772	10.585064	10.790561	11.522791	11.126936	10.915527	11.325845	11.693331	10.867717	10.637121	10.055298	9.971364	10.792032

LINC00511	7.267114	5.023856	5.364244	6.503012	5.753396	5.287363	4.619695	7.088639	9.247052	5.081663	7.842151	6.348760	6.607242	7.550974	5.758891	6.762196	7.958540	6.539396	7.520818	6.135173	5.921593	7.068565	4.786621	6.554321	5.648979	5.843704	7.488516	5.880149	6.717651	4.998590	8.260488	5.842057	6.022738	6.255368	5.197707	5.162468	4.981730	4.699871	5.234826	5.899553	6.096168	4.440482	5.699132	5.870430	6.375008	4.654176	6.805681	4.833968	5.992513	5.412215	5.898487	5.225509	6.064005	4.464593	6.389179	6.992106	5.916014	4.598956	6.570356	5.861573	4.329236	5.286792	3.289829	6.409018	4.388263	4.441091	4.090941	6.171172	5.174952	5.196495	4.332682	5.256947	7.311670	4.584365	5.272910	5.127943	4.782194	5.114185	5.428427	5.750104	5.097372	4.989759	5.960034	5.055944	4.771569	6.249627	4.100847	3.641891	3.797572	3.817172	3.309708	4.155406	6.541364	5.205831	5.443145	6.167552	6.423176	8.327636	7.041908	6.945345	7.532690	6.519373	7.497555	5.557350	6.666542	7.602325	8.626099	6.398385	6.611503	6.467022	5.592917	6.940736	8.686866	5.798946	7.132134	3.873734	7.690876	7.075288	7.474146	6.745941	6.388174	4.536530	5.825842	6.810491	5.765200	5.925258	8.086143	9.072827	6.867906	7.753171	6.818634	6.548685	7.316049	5.085724	8.435543	8.352148	9.140691	8.572191	6.476273	7.580396	7.351613	4.716878	9.438502	5.016263	3.961808	7.068891	6.854303	8.035245	5.096968	8.092915	7.634048	8.519459	7.630374	6.112982	6.012629	5.595302	6.136534	6.123900	5.984867	8.418107	5.149291	7.207379	7.534859	7.465716	6.127483	9.001028	4.817712	6.092703	4.721070	5.992689	6.436473	6.159451	5.766438	5.636758	4.801966	4.019348	5.991827	7.816672	4.204520	5.388534	5.706630	5.947758	7.506049	4.571619	5.078605	3.453504	6.269124	5.500266	3.764711	4.571619	5.822952	8.262928	5.411869	5.294445	6.007890	4.164047	3.789308	7.075288	6.055659	7.016822	5.684361	6.355059	7.339085	4.668385	7.041441	6.764745	3.874364	4.955407	3.884029	3.889387	3.887189	6.491222	6.136867	7.154507	8.232848	8.238523	8.693560	8.336641	7.221939	7.282895	8.326656	9.910027	4.911116	6.867257	8.225701	9.092385	6.432929	7.145660	8.995798	5.067171	7.316693	4.672398	4.958737	6.622425	6.861854	6.873370	6.586378	6.228136

RMST	5.851124	3.867730	3.241215	5.350251	3.165689	3.833785	3.197292	4.041017	5.521184	3.678427	4.506771	4.780946	4.177655	3.665068	5.548830	3.858341	3.347910	3.514432	5.122099	4.774294	4.363059	4.114172	5.306753	3.619528	5.688595	4.149177	6.104268	4.506329	5.016263	3.935574	5.624075	3.159792	3.047416	4.546452	5.107866	4.807861	3.600491	3.108874	3.951215	4.296542	3.353835	3.091863	4.016698	4.819395	4.224914	4.299823	5.386761	5.001414	4.367632	3.744452	5.183016	3.534620	3.973097	4.540468	3.395942	3.921822	3.778157	3.230901	3.734015	3.540515	4.012684	3.734015	4.701728	4.553674	3.088896	4.676909	2.911723	3.577965	3.102534	3.050905	4.193008	3.072994	3.636869	3.211309	2.912748	3.189017	3.727546	3.707485	4.845739	3.424387	3.699957	3.767990	4.660922	4.026235	5.135234	7.111770	4.388263	3.599742	3.204548	4.179668	3.131810	4.599587	3.086040	2.956909	5.188817	3.620077	3.965297	4.119882	3.628214	3.123684	3.954387	3.371366	4.020898	3.610550	4.004983	4.545772	4.115070	3.739973	3.623258	3.726139	4.209917	4.064364	3.746476	3.444014	3.444894	3.701392	4.244727	4.855836	5.297271	3.920809	3.506641	3.900421	3.183560	3.116393	4.554340	3.416390	5.473683	5.041986	3.084646	4.093924	3.023395	3.011366	3.148035	3.973522	4.214927	4.174641	3.502150	3.955259	4.539755	3.810560	3.796132	5.815816	4.021312	4.328543	3.368081	3.894213	4.002996	3.759866	3.718521	5.001085	3.281785	3.040276	3.360502	4.479106	3.672132	3.866608	3.287257	3.778359	3.849937	4.162511	3.021347	3.802243	4.384471	3.663429	4.149594	4.002780	5.308084	4.494208	4.004778	5.076507	4.512034	3.523465	3.983919	3.159921	4.081358	3.389637	3.220421	3.600306	3.484623	4.449155	4.073434	5.078605	5.254737	4.186196	3.865767	2.914747	3.559737	3.222255	3.203344	3.312876	3.889613	3.852717	3.234681	3.246230	3.944222	4.142605	3.948976	4.596165	3.132332	3.398178	3.779584	4.188438	3.804710	4.353327	4.194544	5.416994	3.326945	4.217085	3.584548	4.333986	4.962538	3.654009	3.541683	3.064058	4.177040	3.273291	3.861063	3.310854	3.171791	3.250569	3.430988	3.116871	3.059383	4.468417	4.117669	5.276946	3.231876	3.361140	3.178160	3.904662	4.456699	4.125060	3.666689	3.503653	3.014178	4.066903	3.913138	3.605963

LINC00237	5.027449	4.565327	4.488050	4.400062	4.950633	6.024991	4.103026	5.773447	5.982211	4.335462	5.482940	5.683817	3.903786	6.112494	3.880253	4.965410	5.341233	5.316256	5.359863	5.512770	4.555570	5.085936	5.761577	4.554945	4.170305	5.311070	4.345246	5.553777	4.559227	4.332454	5.761966	5.160090	4.964161	4.096860	5.330272	4.909040	4.459665	4.024123	4.940338	4.780324	5.182826	4.608201	4.592086	4.773477	4.403324	5.005368	9.195936	5.063257	4.807861	4.517005	3.989853	5.782376	3.750187	4.530956	3.613168	5.252954	4.806773	5.195106	4.867443	4.875077	4.837238	4.409371	5.404268	4.851592	5.372272	5.008885	5.603982	4.887925	5.034634	5.904025	4.808479	4.749126	5.615740	5.366809	4.831864	5.120920	5.408524	5.315869	5.263694	4.808066	4.813130	5.116800	4.786621	4.070662	4.983993	5.514046	5.574074	5.581712	5.107057	5.836443	6.159072	6.206594	4.602712	4.984377	5.206440	4.351778	5.110367	5.008061	4.921917	4.476926	4.821263	3.849064	4.388910	4.859389	4.945436	5.328489	4.774099	4.679862	6.190442	5.436894	6.346221	5.182396	5.498320	5.108708	5.378859	6.558609	4.722578	4.736346	4.853765	5.228497	4.673618	4.825015	4.936406	6.616420	6.592272	5.278530	9.277385	5.558123	5.955921	5.381923	5.759262	5.459435	8.594547	4.876104	4.950416	5.187613	5.249800	5.909516	9.445750	5.134665	6.584231	7.338002	5.442550	8.832252	7.113452	5.977257	5.826180	5.559465	5.203062	5.641968	4.050438	4.747061	4.279959	4.983403	8.134218	4.560560	5.390865	5.421654	5.427843	6.185046	5.091441	4.707447	5.307133	4.747288	4.171454	9.568254	8.590359	4.876299	4.712429	4.549992	4.046498	6.797677	5.199887	5.156247	5.711196	3.810335	5.128131	4.480635	4.404200	5.517799	5.423011	5.068188	5.289796	5.451062	5.624075	7.101874	5.228917	5.441596	5.652931	5.170899	5.396530	6.222113	5.777617	5.961292	5.145926	5.540532	5.655559	5.674760	4.592522	4.735442	5.195705	5.624670	4.980532	5.680644	5.707611	5.522331	8.477574	6.476273	5.405739	10.001021	6.400824	5.293881	5.627454	5.037736	4.933247	5.220931	4.864946	5.058371	7.492583	5.560926	5.072591	4.949604	4.837238	10.568013	5.277749	5.479076	4.803256	5.674028	5.716738	6.620183	7.612184	6.805231	6.599582	5.454402	5.725140	5.259448	5.085936	6.205920

LINC01684	14.686942	14.381001	14.806664	15.067485	14.505707	14.647558	14.829469	15.095132	14.318839	14.952794	14.905079	14.892803	14.503039	14.509168	14.025686	14.823396	14.873437	14.236540	14.268626	14.602393	14.398095	14.551021	13.510396	14.174885	14.272526	14.510745	13.944564	14.885495	14.467751	14.450216	14.864634	15.536016	15.303021	14.239134	15.116602	14.739028	14.781829	14.291337	14.536742	14.577469	14.614415	14.556424	14.833005	14.817850	15.279559	15.317503	14.577262	13.840099	15.336040	15.209040	14.633465	15.238314	15.607626	15.352487	14.826455	14.925975	15.407655	14.430074	14.269241	14.809104	14.054502	14.546394	14.236872	14.566322	14.224052	14.506817	13.817588	14.708876	14.507205	14.053306	15.293162	15.137104	14.420600	15.104224	15.005364	15.266520	15.473095	14.908102	14.637025	14.524706	14.790582	14.653700	15.113668	15.531572	15.248027	15.323382	15.688110	15.542881	13.977800	14.967376	14.122158	14.234874	14.508030	14.512377	14.904024	15.213664	15.109227	14.792525	14.977558	14.664987	14.872892	14.468143	14.634805	14.548089	14.126769	14.712563	14.327509	14.995622	13.493039	14.839682	13.844047	14.551853	14.642226	14.085269	15.168793	14.210781	13.872610	14.806664	14.554790	13.533281	13.736836	13.842996	13.966043	13.853253	14.526691	14.543000	13.392871	13.676917	13.836415	13.845880	14.103167	13.966624	13.290718	13.062840	13.929201	13.707724	13.289437	13.216405	15.026340	13.629444	13.186053	13.751062	13.070042	14.308634	14.565537	14.477449	14.235215	13.057446	13.832307	13.665236	14.139533	14.326431	14.517783	14.235557	14.260288	14.430074	14.196229	14.177726	13.809402	13.945929	13.679487	14.422502	14.299472	14.277280	14.651938	13.712440	13.148247	13.952379	14.121601	14.088628	14.213324	14.331005	14.615271	14.561327	13.780832	14.188386	14.186189	13.471830	13.826079	13.993914	14.557653	13.385185	13.617295	13.377996	13.886730	14.604561	13.750073	13.554152	13.545312	14.288296	14.316814	14.207819	14.278255	14.488728	14.341372	13.844292	14.400393	14.558412	13.376715	13.910238	14.527514	13.879568	14.180225	14.729253	14.121021	14.420258	14.540076	13.543949	13.688112	14.274191	13.522074	13.986378	14.746969	14.165527	14.054502	14.293414	13.109444	13.986668	14.032009	14.138571	13.764196	13.891542	13.739368	13.319680	13.902033	13.535306	13.823712	13.184093	13.691450	12.986468	14.271254	14.061504	14.258285	13.647535	14.324402	14.278255	14.068626	14.260288

AL590226.2	6.892688	8.053241	7.687645	7.905055	7.915342	8.728213	8.714198	9.168609	8.340415	9.442051	8.743603	8.521708	7.572932	7.876561	6.687619	9.658740	8.211887	7.444598	7.875503	8.661489	7.679523	7.525133	6.245934	8.624351	7.360270	7.995191	7.416254	8.354284	8.127189	7.969093	8.331048	6.980675	7.182354	7.308199	7.801599	7.605001	7.754859	7.561801	7.412597	8.086004	9.144597	6.631395	7.395991	7.202384	7.030503	7.282571	8.590668	7.183603	7.712007	7.491173	7.171017	7.305069	7.550519	7.808635	7.316219	8.778417	6.854156	6.943596	7.175887	6.676729	6.645317	8.352610	6.454610	7.208936	6.314181	7.294607	8.544754	8.062420	7.898519	6.925974	8.472968	9.306478	8.219027	8.398835	8.270267	7.455252	7.625771	7.677784	7.882917	6.860269	7.186349	7.301137	6.462173	6.912595	7.344870	7.326424	6.096022	6.490623	6.056734	7.012349	5.888681	6.278795	6.802210	7.516167	7.070219	7.191534	7.428055	7.718727	8.215620	7.770487	7.709848	7.762510	7.348653	6.943280	6.712645	8.193679	7.559365	7.265725	6.811147	9.427892	6.238264	6.887566	7.059799	9.029816	7.845788	8.701221	6.238775	7.618328	8.119920	7.491020	7.504157	7.495997	7.594432	7.796488	7.997230	8.224190	7.516805	7.096414	8.314211	8.139887	7.968338	7.777297	7.114246	7.504924	7.769577	7.501316	7.123062	7.375633	7.199002	7.003666	6.940135	6.953906	7.496941	7.439013	7.538121	7.820127	7.687007	8.406370	8.366232	7.528881	7.155766	6.859613	7.791742	6.933492	7.093223	6.995675	7.195520	7.534064	7.002279	8.685838	6.722898	6.540849	7.339873	7.158379	7.311816	8.643792	6.975110	6.787106	7.215612	7.187166	7.006750	6.878132	7.196301	6.646089	7.908800	6.379958	6.318078	6.331981	6.219210	6.079385	8.021345	5.992161	6.354414	6.065057	6.412522	7.226066	7.010888	7.278927	6.064179	7.510154	7.696523	8.523071	7.394443	6.792834	7.103286	7.091491	7.127214	7.444262	5.876206	6.234306	6.933776	6.288620	6.625344	6.340724	7.579940	6.169784	6.318237	5.421654	5.935640	6.275222	5.456476	6.452423	7.500701	7.136551	7.352063	7.685180	7.503062	7.903984	6.990471	7.734263	7.147212	7.228734	6.498737	5.971779	8.119024	6.986195	6.742734	6.419326	8.813954	6.140137	6.169121	6.479775	9.141334	8.121159	7.953412	6.654828	7.232439	8.247810

LINC02615	3.642272	4.757162	3.839112	5.270454	5.820796	5.481638	4.455844	5.572010	5.337511	5.293694	4.782618	3.739136	4.335916	4.764632	3.895449	5.477861	4.602105	4.430265	7.298925	5.364244	5.362147	5.367227	6.008246	4.993108	5.972340	5.302829	4.393824	5.177162	5.470625	4.858971	5.351297	4.633069	6.859440	5.185394	4.204989	4.174447	4.130430	4.639899	5.007457	4.815381	5.454755	4.960598	4.844242	4.892077	3.900187	5.809994	5.574074	3.223347	6.757487	4.012684	4.474278	3.766086	4.548146	4.406925	4.301736	5.803834	4.296754	3.583598	4.245989	4.745112	4.069644	5.285798	4.210561	5.749191	3.838658	3.598578	4.626343	4.917690	4.301102	5.164484	4.625919	4.700479	5.823129	4.133199	4.922760	4.924665	4.725774	5.004573	5.032560	4.693492	4.212963	4.560764	4.779447	4.719153	4.620143	4.963547	5.479452	4.881587	4.034313	5.218532	4.535468	4.428875	4.039083	4.971489	4.317596	4.712842	4.274018	6.410196	5.126372	4.302412	4.921470	4.979544	5.352869	4.944576	4.806164	6.109395	5.969473	5.095423	4.893336	5.675497	5.652204	5.835708	4.617086	5.754089	4.770981	5.346523	5.744826	4.976883	5.524600	4.590570	4.488884	4.044345	5.551433	5.549197	5.727541	4.772813	5.104607	5.178363	4.908825	4.939739	3.687262	4.954568	4.215554	3.969226	5.607046	4.358969	4.066279	5.231792	5.021566	5.239063	5.315232	5.855990	5.310272	5.371076	4.798317	4.658804	5.210833	5.966091	5.062843	4.185785	4.360033	5.316256	5.148135	4.280400	3.850149	3.801192	4.474750	5.326544	3.885739	5.116406	3.442756	4.653519	6.450320	4.366775	5.331246	5.878692	2.987230	5.846047	4.725569	4.914136	3.955259	4.365030	4.608201	3.641503	5.089194	4.193432	3.500876	3.761124	4.103884	3.985181	4.815819	4.734819	4.184907	4.051084	4.058241	4.851376	4.641133	4.209698	5.073004	4.511602	4.268916	5.735745	4.647912	4.634782	5.138025	5.047267	4.892731	5.067971	3.735410	4.619283	4.724734	4.113558	3.786732	4.112234	5.025271	4.356986	4.463269	3.604642	5.268480	4.880502	5.099724	4.385646	4.896431	4.406043	4.491142	4.340922	4.151259	4.890438	3.970288	5.061811	4.966918	4.232648	4.860848	5.196314	5.099097	5.708481	4.936196	4.546888	5.487463	5.374545	5.099302	5.368189	6.468519	4.909875	4.805763	4.859174	4.934074	4.521910

BOLA3-AS1	2.992993	5.651640	5.888841	5.230761	4.199007	4.602105	6.515374	3.597342	3.249984	4.635636	4.710518	5.369503	4.914945	5.006417	6.758622	4.539311	3.580893	6.109241	5.809630	4.776155	4.544458	5.362552	8.568954	4.317365	6.040904	4.710741	4.322911	3.755476	4.870826	5.242255	3.568264	6.848878	6.829577	5.634357	6.553998	6.111319	6.979258	6.536782	6.827989	6.400990	5.146342	7.151233	6.472245	6.503012	7.406485	7.003666	3.631909	6.807918	5.931759	6.847871	6.186042	7.000313	7.669290	7.307427	6.465048	4.612707	7.630676	7.841700	6.093378	7.147061	7.140162	5.547131	7.317927	6.328231	6.426645	6.356984	5.952163	5.641575	5.656663	6.493471	5.636384	5.237869	4.668169	5.864148	5.298058	5.827654	6.075028	5.651640	5.285388	6.710714	5.635631	7.039875	6.499883	7.167160	7.002106	6.775893	5.518786	6.672861	6.013309	6.128005	6.508308	6.065578	5.109345	5.877095	5.214446	5.443327	6.859613	5.652572	4.890855	5.622975	3.530322	6.223265	5.491876	6.134823	5.823680	4.374789	3.676220	5.947212	4.765079	5.237270	6.526670	3.567103	3.492031	3.211879	3.209791	3.069128	5.507004	5.031335	3.952908	3.267793	4.187994	4.525013	3.909259	3.467841	4.610790	4.403745	6.381648	3.152931	3.102636	5.115354	4.388072	3.376176	3.197292	5.658330	3.985840	3.337955	4.212293	5.933526	4.796034	3.942389	3.414352	7.374866	2.971089	6.518383	5.431041	4.291112	5.956634	3.095565	3.910773	3.304432	2.883463	4.167786	5.465983	6.419326	5.354082	5.933869	4.639678	5.579462	4.965410	5.042212	3.628614	6.345907	6.725888	5.885138	4.038230	3.274657	8.989439	3.678427	4.536749	6.222113	4.545772	5.909516	8.367601	6.770414	4.674733	8.238860	5.639354	4.903587	7.425236	5.651254	5.006417	5.520438	4.567891	4.318671	4.990613	6.202793	4.244327	4.213192	6.129010	3.154663	3.270657	4.198200	4.729367	4.406925	5.444097	4.935976	4.345679	5.773278	5.608157	4.336791	7.197441	5.932972	6.517397	7.978468	4.941773	4.531195	8.127496	3.149389	4.535677	9.272123	6.396141	3.278544	4.462868	4.953957	4.595957	4.138852	3.405895	3.777746	6.933336	5.077351	6.245620	5.499283	4.401336	7.246849	4.262655	3.705487	5.441215	4.345054	3.589021	5.588901	3.178836	4.669058	3.151361	6.084753	3.313169	6.603721	3.907267	3.316769

AL391807.1	6.092703	5.707393	4.843175	6.056734	4.367632	4.938091	5.926480	4.506771	4.821475	5.890639	5.049984	5.633193	4.525013	5.101551	4.191920	6.005570	5.926321	4.120097	5.576296	6.335850	4.572417	4.695775	7.430775	4.617937	4.548146	4.903797	7.327719	5.863827	7.139538	5.885856	6.994310	6.157532	5.557747	6.012970	6.271383	6.897209	5.920679	5.254737	5.178157	4.771972	6.316905	6.275556	5.094789	6.276382	5.604354	4.974843	6.707390	3.090979	6.451788	5.728818	6.572093	6.106746	5.965177	6.353769	6.084054	5.676760	6.654016	2.986472	4.919813	4.181660	4.726811	7.073059	6.535659	5.842965	5.136227	5.398817	4.601427	5.129196	4.393198	4.559227	6.776038	5.906838	5.278964	5.734630	6.246267	5.790965	6.633374	6.380948	6.371935	5.117203	5.286988	6.871306	6.163570	6.257931	5.776731	6.806940	5.937040	5.570508	3.404213	4.755113	4.337686	5.460233	4.638845	5.115354	6.133265	6.620820	7.337058	6.720482	5.598789	5.844038	5.194478	4.378458	5.494274	4.699871	5.282173	6.414516	4.574791	4.440882	5.774550	5.034634	4.516143	5.075261	3.922669	5.671606	4.626760	4.135625	5.435519	5.974485	6.393878	4.677314	5.458858	4.029752	5.039504	4.481472	4.442195	4.682440	4.968821	4.495125	4.195183	4.843806	5.483503	3.436352	3.205204	4.152302	5.714931	6.795248	3.545470	4.759750	4.530110	3.962484	4.737795	6.197245	3.548744	4.229863	6.312471	5.427041	4.108038	4.573962	3.959247	7.426808	4.899401	4.173784	4.482874	4.997562	4.689716	4.801966	4.124217	5.908672	4.527383	4.641957	5.242255	4.067751	6.204695	4.837448	4.632405	4.499424	3.079857	5.607046	4.250623	5.551813	4.505232	3.960729	4.494208	4.113965	5.998669	3.925015	2.971520	4.532465	3.791187	4.356986	5.423414	5.888841	6.098553	5.020329	4.983993	5.672408	4.485941	4.809298	4.511393	4.710518	4.133199	5.208635	4.890438	5.151120	5.760849	5.758891	6.119404	5.354518	4.443268	4.670600	4.881587	4.644274	4.256727	6.268961	4.776555	5.025271	4.036272	4.869173	4.715794	4.730184	5.392783	5.398443	5.339320	5.521563	5.933526	5.246412	4.624205	5.982024	4.028609	4.672505	3.644431	4.458852	3.277250	7.204710	4.817923	5.522331	4.522785	5.187401	4.389553	3.934702	4.703861	3.415206	5.646519	4.626343	4.186652	5.447016	4.907319	5.557350

AL050403.2	3.318310	3.378073	3.621281	3.013376	3.161831	4.062633	3.421705	3.468400	3.796939	4.247330	4.316709	3.815882	3.834204	4.280614	4.052563	4.362190	3.744452	3.819707	5.126176	4.022811	5.565981	3.698983	4.399610	3.765519	3.338781	2.993540	3.590704	3.010401	3.900638	3.086146	3.777942	4.085214	5.550523	3.693618	4.003453	4.208229	3.819283	3.972667	3.845666	3.185285	3.493904	3.618767	4.449838	4.160768	3.776160	6.307918	3.461300	5.161065	4.996504	4.199007	4.155406	3.994177	4.468617	4.310159	4.636718	4.118599	5.046279	3.377215	4.141946	4.180567	3.960098	3.962928	4.428669	3.751073	3.468909	3.665678	2.847233	3.859401	3.217589	2.942255	4.002780	4.168179	3.614770	3.400057	3.346247	3.562676	3.799662	3.255752	3.672321	4.126565	4.631969	4.398722	3.455487	5.090437	4.363732	5.202453	4.365248	4.209698	3.234953	3.711741	3.346402	2.968910	3.387237	4.224051	3.604453	4.979942	3.933266	4.652582	3.552011	3.518376	3.797353	3.729236	3.710908	3.267491	4.589938	5.611223	6.386127	3.805356	3.425458	4.471446	3.491185	3.470703	3.167023	3.358975	3.330656	3.947691	3.262940	4.234172	4.072539	3.121463	3.835677	3.052890	3.238755	3.062674	4.666013	3.166625	3.497086	5.259838	3.358308	3.870512	3.223899	3.463925	3.452065	3.685428	4.720456	4.268916	3.286969	3.530520	3.535907	5.863284	3.836319	5.620566	3.238311	3.054353	2.906249	3.295298	3.360006	3.619893	3.595732	3.529960	3.619327	4.337896	3.744268	3.613588	3.882986	3.878927	3.693828	3.664050	3.073679	3.345270	3.180533	3.723306	4.189728	4.276840	6.296759	3.407494	4.034530	4.983403	4.441709	4.266350	4.667960	4.194980	4.478885	4.180324	3.304280	4.164047	3.190001	3.926325	3.954387	3.056020	3.678209	4.868318	3.829744	3.740951	3.611605	2.837320	3.800090	3.381727	2.922961	3.160581	3.715250	3.177227	3.153309	3.245681	3.347031	5.402456	3.838658	4.044969	3.864506	4.276183	4.940338	4.072762	3.761949	5.032759	4.867232	3.752527	5.124300	3.056542	4.044097	3.615340	4.361722	3.869665	3.264540	3.283331	3.075075	3.230901	4.307220	3.814406	3.499964	3.958330	3.420470	4.222970	2.854722	4.984172	4.548146	6.298576	3.816516	3.568264	3.239461	3.162394	3.512007	3.897603	3.391947	3.419492	3.628019	3.881690	3.339628	3.463379

AL135937.1	3.318310	3.378073	3.621281	3.013376	3.161831	4.062633	3.421705	3.468400	3.796939	4.247330	4.316709	3.815882	3.834204	4.280614	4.052563	4.362190	3.744452	3.819707	5.126176	4.022811	5.565981	3.698983	4.399610	3.765519	3.338781	2.993540	3.590704	3.010401	3.900638	3.086146	3.777942	4.085214	5.550523	3.693618	4.003453	4.208229	3.819283	3.972667	3.845666	3.185285	3.493904	3.618767	4.449838	4.160768	3.776160	6.307918	3.461300	5.161065	4.996504	4.199007	4.155406	3.994177	4.468617	4.310159	4.636718	4.118599	5.046279	3.377215	4.141946	4.180567	3.960098	3.962928	4.428669	3.751073	3.468909	3.665678	2.847233	3.859401	3.217589	2.942255	4.002780	4.168179	3.614770	3.400057	3.346247	3.562676	3.799662	3.255752	3.672321	4.126565	4.631969	4.398722	3.455487	5.090437	4.363732	5.202453	4.365248	4.209698	3.234953	3.711741	3.346402	2.968910	3.387237	4.224051	3.604453	4.979942	3.933266	4.652582	3.552011	3.518376	3.797353	3.729236	3.710908	3.267491	4.589938	5.611223	6.386127	3.805356	3.425458	4.471446	3.491185	3.470703	3.167023	3.358975	3.330656	3.947691	3.262940	4.234172	4.072539	3.121463	3.835677	3.052890	3.238755	3.062674	4.666013	3.166625	3.497086	5.259838	3.358308	3.870512	3.223899	3.463925	3.452065	3.685428	4.720456	4.268916	3.286969	3.530520	3.535907	5.863284	3.836319	5.620566	3.238311	3.054353	2.906249	3.295298	3.360006	3.619893	3.595732	3.529960	3.619327	4.337896	3.744268	3.613588	3.882986	3.878927	3.693828	3.664050	3.073679	3.345270	3.180533	3.723306	4.189728	4.276840	6.296759	3.407494	4.034530	4.983403	4.441709	4.266350	4.667960	4.194980	4.478885	4.180324	3.304280	4.164047	3.190001	3.926325	3.954387	3.056020	3.678209	4.868318	3.829744	3.740951	3.611605	2.837320	3.800090	3.381727	2.922961	3.160581	3.715250	3.177227	3.153309	3.245681	3.347031	5.402456	3.838658	4.044969	3.864506	4.276183	4.940338	4.072762	3.761949	5.032759	4.867232	3.752527	5.124300	3.056542	4.044097	3.615340	4.361722	3.869665	3.264540	3.283331	3.075075	3.230901	4.307220	3.814406	3.499964	3.958330	3.420470	4.222970	2.854722	4.984172	4.548146	6.298576	3.816516	3.568264	3.239461	3.162394	3.512007	3.897603	3.391947	3.419492	3.628019	3.881690	3.339628	3.463379

LINC01752	3.318310	3.378073	3.621281	3.013376	3.161831	4.062633	3.421705	3.468400	3.796939	4.247330	4.316709	3.815882	3.834204	4.280614	4.052563	4.362190	3.744452	3.819707	5.126176	4.022811	5.565981	3.698983	4.399610	3.765519	3.338781	2.993540	3.590704	3.010401	3.900638	3.086146	3.777942	4.085214	5.550523	3.693618	4.003453	4.208229	3.819283	3.972667	3.845666	3.185285	3.493904	3.618767	4.449838	4.160768	3.776160	6.307918	3.461300	5.161065	4.996504	4.199007	4.155406	3.994177	4.468617	4.310159	4.636718	4.118599	5.046279	3.377215	4.141946	4.180567	3.960098	3.962928	4.428669	3.751073	3.468909	3.665678	2.847233	3.859401	3.217589	2.942255	4.002780	4.168179	3.614770	3.400057	3.346247	3.562676	3.799662	3.255752	3.672321	4.126565	4.631969	4.398722	3.455487	5.090437	4.363732	5.202453	4.365248	4.209698	3.234953	3.711741	3.346402	2.968910	3.387237	4.224051	3.604453	4.979942	3.933266	4.652582	3.552011	3.518376	3.797353	3.729236	3.710908	3.267491	4.589938	5.611223	6.386127	3.805356	3.425458	4.471446	3.491185	3.470703	3.167023	3.358975	3.330656	3.947691	3.262940	4.234172	4.072539	3.121463	3.835677	3.052890	3.238755	3.062674	4.666013	3.166625	3.497086	5.259838	3.358308	3.870512	3.223899	3.463925	3.452065	3.685428	4.720456	4.268916	3.286969	3.530520	3.535907	5.863284	3.836319	5.620566	3.238311	3.054353	2.906249	3.295298	3.360006	3.619893	3.595732	3.529960	3.619327	4.337896	3.744268	3.613588	3.882986	3.878927	3.693828	3.664050	3.073679	3.345270	3.180533	3.723306	4.189728	4.276840	6.296759	3.407494	4.034530	4.983403	4.441709	4.266350	4.667960	4.194980	4.478885	4.180324	3.304280	4.164047	3.190001	3.926325	3.954387	3.056020	3.678209	4.868318	3.829744	3.740951	3.611605	2.837320	3.800090	3.381727	2.922961	3.160581	3.715250	3.177227	3.153309	3.245681	3.347031	5.402456	3.838658	4.044969	3.864506	4.276183	4.940338	4.072762	3.761949	5.032759	4.867232	3.752527	5.124300	3.056542	4.044097	3.615340	4.361722	3.869665	3.264540	3.283331	3.075075	3.230901	4.307220	3.814406	3.499964	3.958330	3.420470	4.222970	2.854722	4.984172	4.548146	6.298576	3.816516	3.568264	3.239461	3.162394	3.512007	3.897603	3.391947	3.419492	3.628019	3.881690	3.339628	3.463379

SLC7A14-AS1	4.116995	3.126623	4.979752	3.985631	4.440882	4.173784	4.233519	4.512034	4.885177	3.242251	3.353204	3.518201	3.783976	3.509419	4.839385	3.445453	4.304213	4.044345	4.557657	3.759199	7.683512	2.945757	6.237135	3.931093	3.113210	3.889387	5.109773	3.869456	3.412735	5.267883	3.930648	4.928275	5.392969	2.898904	5.320912	3.948773	3.454582	4.597046	4.428669	4.461559	3.265735	4.304851	4.191920	3.676604	4.619695	4.553674	5.549015	4.824132	4.537161	4.973787	4.937893	4.628254	5.183414	5.101759	4.495760	3.252362	3.765519	4.669058	4.599394	5.130200	3.327729	3.673696	5.947925	4.403536	4.812075	4.152302	3.054163	3.837359	3.848851	3.799662	4.842774	4.609078	3.676812	3.719536	4.654865	3.911422	4.138230	4.073227	4.014957	4.588420	4.797463	4.251929	4.296330	4.667960	4.780742	5.089403	5.267688	3.722103	5.205618	4.745959	4.960000	3.758791	3.320254	3.750187	5.051601	4.054091	4.519978	3.611397	4.301102	3.852056	4.801357	3.422057	3.718335	3.847370	5.608157	4.255230	4.710741	4.270937	3.966608	3.831827	3.857435	4.417080	5.003583	3.898456	3.200908	5.864523	3.918102	3.278841	3.716082	4.287894	3.668095	4.665614	3.833571	6.547740	4.723654	5.181177	4.616202	4.404426	4.107221	3.643274	4.631969	3.591699	3.716933	4.107221	3.879159	4.649613	4.165107	3.532537	3.793394	4.615968	3.496333	5.244418	3.717722	3.630055	3.805149	4.290484	3.986069	4.191920	5.100918	4.846179	4.510286	4.138630	4.241625	5.278342	4.191032	3.516425	4.041233	4.227672	3.926712	4.089834	3.823524	3.932174	4.006530	4.332885	4.025170	6.366062	8.107120	4.634126	5.111719	4.872144	3.509805	4.742727	4.186652	6.112494	4.263555	5.272683	4.098433	4.645359	4.780099	4.004116	6.296097	4.740192	4.837863	5.214034	3.384000	4.194119	4.535021	3.995950	4.163583	4.367861	4.309712	3.866608	4.204520	4.606257	4.148136	4.389751	4.462632	4.056940	4.670600	5.469895	4.947941	4.542193	3.725577	4.877599	5.431227	5.031937	4.436278	5.000064	4.922128	4.194339	4.648755	4.124217	3.968315	4.410887	4.069243	4.327452	4.784935	4.350669	4.014957	4.418457	6.210188	3.679614	4.210992	4.822917	4.907542	4.408014	4.763580	4.637149	4.236984	4.551331	6.216463	6.122011	3.870092	5.495683	5.578913	4.746389	4.149177	3.393995

AC015987.1	13.349515	14.868226	15.181269	15.451210	15.456675	15.197536	15.577375	16.013271	15.225383	15.857099	15.748884	15.417001	15.391393	15.627072	14.993988	16.121338	15.647882	15.347177	16.034860	15.469767	15.784706	16.152946	14.952794	15.383684	14.801868	15.416204	13.985572	15.279559	15.123478	14.803294	15.293858	15.889470	15.739503	14.768079	15.076957	15.323382	15.258866	15.914283	15.126455	15.356364	15.345704	15.851177	15.494689	15.093138	15.069833	15.114738	15.147454	15.321092	15.572722	15.507347	14.954990	15.377722	15.511748	15.588169	14.743665	15.282381	15.564042	15.369410	15.901055	15.152524	15.405445	15.125176	14.067493	15.616895	15.292522	15.241853	15.371654	15.568457	15.347177	15.478014	15.928804	15.781709	15.352487	15.265157	15.526235	15.278819	15.084722	14.933539	14.419877	16.218720	15.584586	15.140339	14.883849	15.859538	15.781709	15.525353	15.926414	16.221987	15.553263	15.945972	15.024650	15.709527	15.768020	15.455879	14.671011	15.608551	15.398455	15.520300	15.829660	14.810097	15.533380	15.342745	15.395261	15.182677	16.172846	15.438162	15.139006	16.024403	15.547851	15.580998	15.716847	15.626034	15.812427	15.183376	15.594598	15.056697	13.632300	15.602028	15.440770	15.307946	14.468143	13.993598	13.695390	13.921593	14.668191	14.560516	13.760910	14.876538	13.966887	14.119553	14.344223	14.570991	13.404827	14.448739	14.094594	14.255787	13.885163	13.812235	14.525087	14.543837	14.607960	14.530078	13.750799	14.430444	14.046144	15.094510	14.133255	14.178659	14.560516	14.769948	13.974684	14.539639	14.443791	14.650177	14.548933	14.753586	14.183082	14.713935	14.611896	14.302275	14.461603	14.102873	15.007673	14.655466	14.724698	14.746969	13.870987	14.686942	14.385259	14.678701	14.504993	14.390000	14.810589	14.950618	15.119706	14.383815	14.400018	13.932466	14.336460	14.440880	14.573507	15.137730	14.773706	14.446360	14.395843	14.389287	14.284842	14.222202	14.658149	14.625115	14.842135	14.087732	15.039342	14.637498	14.712131	14.742751	14.778050	15.268645	13.860992	14.650177	14.698400	14.722869	14.505707	14.731754	14.674591	14.616154	14.090137	13.992417	13.870710	14.038211	14.388223	13.970475	14.656836	14.834547	14.306647	14.798373	14.255787	14.226329	14.460072	14.589784	13.703898	13.775677	13.918854	14.343879	14.276055	14.616554	14.878681	14.963955	14.410783	14.415465	13.728857	13.828188	15.015687	14.684100	14.641807	14.687871	14.491152	13.845633

AL139260.1	9.540026	9.476414	10.285158	9.659506	10.302633	9.643333	10.376965	10.494370	9.428521	10.257870	10.223316	9.769520	9.422970	10.384236	9.520226	9.733039	10.027337	10.319358	10.777970	10.062587	10.674816	10.046779	9.799644	9.839071	8.395689	8.462629	8.571866	8.950273	8.180535	9.126584	9.713091	9.950580	10.659191	9.984899	9.835522	10.020621	10.420096	9.924735	10.222567	10.657698	9.336631	9.861767	9.400283	9.973102	9.770486	10.028734	9.533834	10.006772	10.377715	9.734545	10.096988	9.881488	10.013557	10.360365	10.554613	9.919633	10.754335	10.347657	10.570126	9.966500	9.956464	8.838597	8.811217	10.334961	10.396283	9.904543	9.483198	10.501072	10.236196	10.905697	9.992041	10.245297	9.662346	10.062130	10.554299	10.042830	10.566197	10.310064	10.316078	10.347353	10.108529	10.256330	10.130357	10.221812	10.270009	10.266163	10.558269	10.596035	10.213953	10.727937	9.939807	10.829597	10.016345	10.102793	9.750518	10.530253	10.351729	9.563299	9.819984	9.616680	9.741675	10.074063	9.852983	10.424393	11.703716	9.463307	8.313127	11.041360	10.624589	9.912662	10.528668	9.803650	9.985333	9.253295	9.748309	9.112263	9.666547	9.708602	9.674912	11.057038	9.600803	8.977562	9.638262	7.350547	8.932113	8.735642	7.949190	10.052026	8.829501	9.495797	9.096736	7.375167	8.456594	8.613295	9.130300	8.858205	8.154551	8.700270	8.878011	9.779527	9.417469	10.075003	8.854389	7.463738	7.840460	9.423296	8.568954	7.430775	9.267822	9.584671	9.202497	9.023558	9.766473	9.619482	9.623390	10.102954	9.736856	9.902880	9.790662	8.172546	9.240585	8.469947	9.759027	9.723440	9.829822	9.284296	8.913424	9.698334	9.511660	9.417469	8.899923	9.328916	9.472171	10.026868	8.972624	9.557406	10.335836	9.439413	9.773259	9.807767	8.764223	9.663731	10.738688	10.466251	9.556482	9.951526	9.764810	9.373655	10.796577	9.662803	9.553903	8.017690	9.785948	9.570720	9.731377	9.583773	10.246336	9.832460	9.384818	9.788660	10.228793	10.138967	8.991496	9.301568	9.661397	9.836740	9.550393	8.791835	8.984267	8.844175	9.729654	9.294739	9.177266	9.485177	9.213676	8.801121	7.353126	10.050017	8.920759	9.149407	8.669426	9.030423	9.200174	9.435228	8.521130	8.049077	10.780147	10.266592	9.637621	9.083635	9.282941	6.522990	8.384808	7.722222	9.782304	10.858937	10.438091	9.384070

AL139260.2	9.540026	9.476414	10.285158	9.659506	10.302633	9.643333	10.376965	10.494370	9.428521	10.257870	10.223316	9.769520	9.422970	10.384236	9.520226	9.733039	10.027337	10.319358	10.777970	10.062587	10.674816	10.046779	9.799644	9.839071	8.395689	8.462629	8.571866	8.950273	8.180535	9.126584	9.713091	9.950580	10.659191	9.984899	9.835522	10.020621	10.420096	9.924735	10.222567	10.657698	9.336631	9.861767	9.400283	9.973102	9.770486	10.028734	9.533834	10.006772	10.377715	9.734545	10.096988	9.881488	10.013557	10.360365	10.554613	9.919633	10.754335	10.347657	10.570126	9.966500	9.956464	8.838597	8.811217	10.334961	10.396283	9.904543	9.483198	10.501072	10.236196	10.905697	9.992041	10.245297	9.662346	10.062130	10.554299	10.042830	10.566197	10.310064	10.316078	10.347353	10.108529	10.256330	10.130357	10.221812	10.270009	10.266163	10.558269	10.596035	10.213953	10.727937	9.939807	10.829597	10.016345	10.102793	9.750518	10.530253	10.351729	9.563299	9.819984	9.616680	9.741675	10.074063	9.852983	10.424393	11.703716	9.463307	8.313127	11.041360	10.624589	9.912662	10.528668	9.803650	9.985333	9.253295	9.748309	9.112263	9.666547	9.708602	9.674912	11.057038	9.600803	8.977562	9.638262	7.350547	8.932113	8.735642	7.949190	10.052026	8.829501	9.495797	9.096736	7.375167	8.456594	8.613295	9.130300	8.858205	8.154551	8.700270	8.878011	9.779527	9.417469	10.075003	8.854389	7.463738	7.840460	9.423296	8.568954	7.430775	9.267822	9.584671	9.202497	9.023558	9.766473	9.619482	9.623390	10.102954	9.736856	9.902880	9.790662	8.172546	9.240585	8.469947	9.759027	9.723440	9.829822	9.284296	8.913424	9.698334	9.511660	9.417469	8.899923	9.328916	9.472171	10.026868	8.972624	9.557406	10.335836	9.439413	9.773259	9.807767	8.764223	9.663731	10.738688	10.466251	9.556482	9.951526	9.764810	9.373655	10.796577	9.662803	9.553903	8.017690	9.785948	9.570720	9.731377	9.583773	10.246336	9.832460	9.384818	9.788660	10.228793	10.138967	8.991496	9.301568	9.661397	9.836740	9.550393	8.791835	8.984267	8.844175	9.729654	9.294739	9.177266	9.485177	9.213676	8.801121	7.353126	10.050017	8.920759	9.149407	8.669426	9.030423	9.200174	9.435228	8.521130	8.049077	10.780147	10.266592	9.637621	9.083635	9.282941	6.522990	8.384808	7.722222	9.782304	10.858937	10.438091	9.384070

LINC01140	7.637243	7.194888	7.160752	7.731181	8.810910	8.028111	7.040480	8.443781	8.050788	7.635884	7.247314	7.864173	7.009806	6.950590	6.554476	7.576516	8.008692	7.094649	7.907241	7.198061	7.107418	8.128115	9.023720	7.580232	7.662149	7.913282	7.932034	8.034188	8.071679	7.661829	8.280091	7.018269	7.591030	7.150580	7.016496	6.977046	7.169340	6.935051	7.445236	7.726686	7.707451	7.486779	7.049113	7.430138	7.265404	7.177969	7.855309	7.119964	7.267429	7.263271	7.391638	6.873688	6.710407	7.028306	6.652886	7.710341	7.306185	7.008175	7.374236	7.072588	7.133895	7.311816	7.378933	7.543582	7.245629	7.354382	7.222702	7.260136	7.311187	7.673040	7.467854	7.396166	7.733473	7.423685	7.189847	7.483209	7.319649	7.160752	7.747797	6.875616	6.894955	7.116904	7.031545	7.196961	7.379718	7.100637	7.112679	7.388420	7.203012	7.588584	7.502904	8.008852	7.128470	7.149622	7.535343	7.188088	6.907507	7.297185	8.011995	7.319795	7.547747	6.966591	7.568409	7.335828	8.454290	7.511709	8.150964	7.460098	8.559151	7.837337	8.209472	8.000110	7.641687	8.378612	7.626412	9.837182	8.036507	7.564875	7.879300	7.346294	7.897749	7.504456	7.960849	9.139811	8.371749	7.929719	8.596272	8.033141	7.701304	7.732580	7.897307	8.004347	7.290824	8.779155	7.563777	8.261392	7.418220	8.206480	7.249623	8.129345	8.703369	8.992256	8.567616	8.433371	7.494577	8.215914	8.478049	8.565719	7.625464	8.489514	7.617855	8.182032	7.443000	7.233060	7.155127	7.399762	7.861216	8.545378	8.110900	8.159168	7.731029	7.242596	7.820127	7.019054	7.088478	8.151277	7.620128	7.580861	7.429995	7.239557	7.074170	6.694902	7.118530	6.880073	7.463901	6.943118	6.954382	7.376276	7.029566	7.438046	7.579940	7.707451	7.899568	7.698822	7.653754	7.412915	7.592104	7.657521	8.221604	7.434700	7.661681	8.510749	7.644473	7.443459	7.507394	7.742874	7.634962	7.885997	7.290824	6.909077	7.172935	7.476608	7.282895	7.420736	8.239020	7.466791	7.225755	7.526374	7.601380	7.805727	8.561316	7.474745	8.083795	7.855600	8.095372	8.358156	8.385284	8.563599	7.354709	8.330258	7.935728	8.083021	7.552684	8.508449	8.096280	8.261557	7.778689	8.051562	8.482018	8.638314	8.147768	9.452602	8.715151	8.205382	8.142249	7.889355	7.547904	7.710641

AC092296.2	8.413235	8.518715	8.897149	8.994020	9.243185	8.827314	9.266095	10.221812	9.994941	9.130010	9.383032	8.700916	7.941974	8.513701	8.739929	9.640265	8.966956	8.611061	9.480408	9.461061	8.442096	9.635073	10.602394	9.082889	9.868678	9.939807	9.534899	9.465521	8.974524	8.612329	9.706104	8.856529	9.246593	8.643630	8.757006	8.971604	9.204578	9.121228	9.171237	8.802019	9.615752	8.132684	8.822202	9.364876	9.354532	9.501800	8.892991	9.583773	9.069888	10.109128	9.285515	9.485965	8.908005	9.036629	8.296702	9.101649	9.215611	8.576857	8.289196	8.221915	9.238262	8.701974	10.113060	8.495049	7.854837	9.135166	9.073896	8.799150	9.460288	8.804313	9.407255	9.683738	9.456162	9.098890	9.188910	9.083201	8.765121	8.731790	8.497483	9.741675	9.088689	9.171885	8.658051	9.372726	9.606560	9.563002	8.474768	9.408019	8.420558	8.062420	8.548453	8.669134	9.070041	9.386045	8.655793	9.286903	8.802612	9.627241	8.813641	9.318053	8.948886	8.943081	9.556322	9.014091	9.006123	9.209617	9.893788	9.009822	8.386809	9.671933	7.727325	8.463989	8.264002	9.529758	8.742107	8.644089	7.776338	9.315204	9.478293	9.573678	9.150795	9.098736	9.939949	9.233328	8.808720	8.436649	10.889072	9.350193	8.910236	9.684363	9.179536	7.886728	8.811877	8.828881	8.975575	9.186600	8.482967	9.968492	8.578321	8.306186	9.451258	9.997492	8.721025	10.064990	9.512448	9.637322	9.431743	8.495049	9.480095	10.476151	8.831175	8.987164	9.103956	8.591764	8.925189	9.595225	9.194647	9.254788	9.005349	9.655028	9.027048	9.185181	10.209210	9.444994	10.257553	9.049437	10.334339	9.087336	10.544926	9.520834	9.756864	8.679595	9.462413	8.608629	8.997674	8.682359	8.605743	8.269199	8.873698	9.205043	8.887442	8.987805	8.372685	8.029041	8.983807	8.862310	9.108288	10.757656	8.452630	9.980109	9.624150	9.197188	9.388562	9.160629	8.709631	9.326293	9.289113	9.200933	10.222956	9.158340	9.706401	8.530464	9.357409	9.266095	9.302345	8.049384	9.766182	8.585756	8.311618	8.756076	8.538773	9.629681	10.159386	9.101649	10.039433	8.477734	9.757339	9.406640	8.713570	8.884828	8.946123	9.827223	8.927086	7.610523	10.127592	9.479495	8.129022	7.695315	9.469843	8.827483	8.350047	8.233737	9.179081	9.910027	8.725420	8.762422	8.872790	9.769058

AC016590.1	8.413235	8.518715	8.897149	8.994020	9.243185	8.827314	9.266095	10.221812	9.994941	9.130010	9.383032	8.700916	7.941974	8.513701	8.739929	9.640265	8.966956	8.611061	9.480408	9.461061	8.442096	9.635073	10.602394	9.082889	9.868678	9.939807	9.534899	9.465521	8.974524	8.612329	9.706104	8.856529	9.246593	8.643630	8.757006	8.971604	9.204578	9.121228	9.171237	8.802019	9.615752	8.132684	8.822202	9.364876	9.354532	9.501800	8.892991	9.583773	9.069888	10.109128	9.285515	9.485965	8.908005	9.036629	8.296702	9.101649	9.215611	8.576857	8.289196	8.221915	9.238262	8.701974	10.113060	8.495049	7.854837	9.135166	9.073896	8.799150	9.460288	8.804313	9.407255	9.683738	9.456162	9.098890	9.188910	9.083201	8.765121	8.731790	8.497483	9.741675	9.088689	9.171885	8.658051	9.372726	9.606560	9.563002	8.474768	9.408019	8.420558	8.062420	8.548453	8.669134	9.070041	9.386045	8.655793	9.286903	8.802612	9.627241	8.813641	9.318053	8.948886	8.943081	9.556322	9.014091	9.006123	9.209617	9.893788	9.009822	8.386809	9.671933	7.727325	8.463989	8.264002	9.529758	8.742107	8.644089	7.776338	9.315204	9.478293	9.573678	9.150795	9.098736	9.939949	9.233328	8.808720	8.436649	10.889072	9.350193	8.910236	9.684363	9.179536	7.886728	8.811877	8.828881	8.975575	9.186600	8.482967	9.968492	8.578321	8.306186	9.451258	9.997492	8.721025	10.064990	9.512448	9.637322	9.431743	8.495049	9.480095	10.476151	8.831175	8.987164	9.103956	8.591764	8.925189	9.595225	9.194647	9.254788	9.005349	9.655028	9.027048	9.185181	10.209210	9.444994	10.257553	9.049437	10.334339	9.087336	10.544926	9.520834	9.756864	8.679595	9.462413	8.608629	8.997674	8.682359	8.605743	8.269199	8.873698	9.205043	8.887442	8.987805	8.372685	8.029041	8.983807	8.862310	9.108288	10.757656	8.452630	9.980109	9.624150	9.197188	9.388562	9.160629	8.709631	9.326293	9.289113	9.200933	10.222956	9.158340	9.706401	8.530464	9.357409	9.266095	9.302345	8.049384	9.766182	8.585756	8.311618	8.756076	8.538773	9.629681	10.159386	9.101649	10.039433	8.477734	9.757339	9.406640	8.713570	8.884828	8.946123	9.827223	8.927086	7.610523	10.127592	9.479495	8.129022	7.695315	9.469843	8.827483	8.350047	8.233737	9.179081	9.910027	8.725420	8.762422	8.872790	9.769058

LINC00652	5.744988	5.379465	5.471198	5.861573	5.937556	6.016557	5.871137	6.367217	6.133265	5.541466	5.090658	5.104607	5.345157	5.790965	5.241057	5.631552	5.706824	5.548626	5.418549	5.138833	3.978937	6.133084	6.615919	5.016263	6.205198	5.252954	7.159318	5.879823	5.614444	5.699868	5.165881	5.699868	5.637139	5.829507	6.326106	6.028563	6.284798	5.923488	5.865582	6.030252	5.445055	6.099807	5.982024	5.788126	6.158888	5.116218	5.909697	5.349077	5.882650	6.186042	5.716576	6.606208	5.784855	5.801873	5.808130	6.273716	6.287413	6.160317	6.620820	6.446077	6.163391	6.067343	6.546318	5.921773	7.236268	6.403750	6.414516	6.137188	6.154988	6.024991	5.887816	5.946205	6.170998	5.967336	6.241436	6.118891	6.168430	6.208650	6.193370	6.109572	6.405375	6.414516	5.765200	5.621468	6.122868	6.201199	6.192830	6.486107	6.004284	5.929174	5.944425	6.122355	6.153453	5.975696	5.948634	5.544882	5.211625	5.776731	6.535976	5.991631	6.712279	5.720302	5.858504	5.693412	5.571259	5.934404	5.872632	5.427467	6.298401	5.969657	6.081078	6.246787	6.385776	5.939707	5.000281	8.139887	6.656465	5.653667	5.414727	5.133626	5.400334	6.036861	5.807238	7.310106	6.245437	5.952550	5.957159	5.739713	6.605973	5.861398	5.092048	5.746099	5.393986	5.950215	5.866471	5.507796	5.124710	5.871137	5.571612	5.945684	6.313672	6.274562	5.829507	6.336018	5.489572	6.634360	6.863293	6.754726	5.974996	5.631732	6.151749	6.689329	6.668017	7.390553	5.990697	6.981168	6.230922	6.143072	5.901681	6.709910	5.646889	6.266890	6.234964	6.307087	5.494903	6.748935	9.154005	6.363037	5.789578	4.512448	5.868862	3.901467	6.491380	6.042616	6.425281	8.155791	6.150695	6.229193	4.039945	5.796387	6.290632	5.485941	5.740241	6.115959	6.072060	6.328750	5.740630	6.128820	6.190787	5.739713	6.021706	6.921935	5.664974	8.038673	6.073290	6.384499	6.719133	5.702406	6.340247	5.797835	7.168868	5.441417	5.757968	4.964590	7.018597	6.311124	10.128078	5.701366	5.731757	5.826762	6.050621	7.328040	6.120635	5.846222	5.298058	5.795280	5.883597	7.016333	5.640502	5.850774	6.253184	7.442516	6.149498	5.900980	6.303916	6.000261	5.794384	5.302239	6.456215	6.366712	6.340056	7.929278	6.325419	6.305958	6.620518	7.562281	5.077351	6.726582

AC008667.1	8.030875	8.361415	8.226898	8.272099	8.445759	8.858496	7.917823	9.118187	9.388260	8.702595	9.329705	8.493381	8.057990	8.351110	7.435698	8.808130	8.733493	8.074418	8.439490	8.070916	8.355850	9.366931	8.483714	8.394381	8.060100	8.135425	8.559301	9.289571	8.659306	8.843418	9.139186	7.773375	7.636774	8.137259	7.530078	7.816672	7.526038	7.830932	7.792490	8.149782	8.747415	7.903686	7.949357	7.958992	7.407121	8.196996	9.060660	8.114390	7.723151	7.925680	7.750575	7.481057	7.202536	7.509424	7.521779	8.934423	7.289562	7.015993	7.920886	7.279886	7.828982	8.553905	6.560853	8.171446	7.346868	7.763738	8.251297	8.133472	7.918883	8.802316	8.700125	8.380662	9.697083	8.445923	9.093285	7.843764	8.092315	8.187900	8.549976	6.946617	7.300797	6.896873	7.208153	7.076870	6.977224	7.678751	7.218109	7.337058	6.706060	7.590442	7.200399	7.475528	7.607888	8.079763	7.790829	7.632388	7.592559	8.027797	8.726942	8.211601	9.022477	8.232693	8.602194	8.218076	8.224643	8.640980	9.329557	8.578625	8.203475	8.896861	8.037285	7.893570	8.065177	9.180017	8.522603	10.966093	7.956035	8.284075	9.057511	7.598098	7.925527	7.511385	8.021631	9.851210	8.439951	8.717495	9.382546	8.980580	8.218245	10.134533	8.717495	8.679746	8.444854	8.918624	8.111029	8.347074	7.868615	7.934793	7.648891	7.613722	7.838627	7.482753	8.085924	8.082554	7.454147	8.950569	8.708722	8.720391	8.148697	8.906422	8.116849	8.011098	8.836925	7.595483	7.269393	7.734740	7.573718	7.904130	7.296707	8.950413	7.375167	6.894312	7.612344	7.190158	8.647531	9.745999	7.504612	7.220170	7.442078	7.614780	7.535627	6.410859	7.322163	7.033591	7.533611	6.547889	6.596333	7.264952	6.585901	7.192137	8.117164	6.867727	7.244210	7.254931	7.072417	7.299697	8.009468	7.642619	7.597003	8.040436	8.302596	11.200874	7.728725	7.691024	7.777610	7.268785	7.695178	8.583923	6.360702	7.115967	6.926148	7.503220	7.221773	6.639977	8.041408	6.921278	6.700295	5.969157	6.407708	7.248929	6.980170	7.077360	7.747180	8.331995	7.977373	7.994571	8.972184	9.352150	7.589205	9.451406	7.954791	8.621889	7.878989	7.154664	8.647397	8.567308	7.572780	7.223889	9.973251	7.249473	7.798494	7.852191	9.536430	8.974524	9.620086	7.627346	8.820230	8.647089

MIR124-2HG	6.290462	5.826559	6.024627	5.659389	5.639555	6.118183	5.546573	6.236812	6.656942	5.520989	5.482022	5.445055	4.125890	5.239264	4.810612	5.064862	6.590464	5.353254	6.390363	5.186207	6.212521	6.250481	8.402976	5.273296	5.949516	6.185384	4.844242	6.433951	6.185209	5.202236	6.863293	5.281972	5.974825	4.901135	5.218154	5.817445	5.934404	5.750467	6.106391	6.754070	5.598184	6.438569	5.000890	5.675497	5.753035	5.422452	5.767194	6.355910	5.828040	5.564660	6.014345	5.955394	4.918089	5.407741	4.792890	5.212965	5.467155	6.137520	6.326106	5.998314	6.216124	4.792316	6.335850	6.613481	6.357152	6.457727	6.012970	6.048901	6.390194	6.729992	5.764109	5.571799	4.826543	5.985373	6.009651	5.831224	6.335680	6.337715	6.267757	5.492597	4.873439	5.650297	5.312218	5.983672	6.055823	6.090612	5.462582	6.313497	6.493615	6.893180	6.316062	6.955972	5.633793	5.709005	5.169712	5.700628	5.661421	4.728702	5.289371	5.136622	4.179876	5.101129	5.035696	5.574273	6.532414	6.039284	6.872429	6.426975	6.925659	5.350897	7.245923	6.018321	6.752888	5.295428	6.219210	7.144444	6.306280	5.851850	5.068188	6.152067	6.733993	5.939326	6.083687	6.480893	6.200479	6.145501	6.823863	7.185042	5.885856	5.890109	6.059726	5.811119	5.325555	5.834109	5.933869	6.603417	5.522511	7.409279	6.725130	6.214759	6.894638	7.894497	6.771880	6.384666	6.626000	5.829333	6.671872	7.031249	5.798581	7.436620	6.993391	6.520543	5.751568	5.868125	6.396792	6.150019	6.674620	6.974641	7.263889	6.272706	6.969337	5.596256	7.348188	6.069272	6.075192	6.587371	6.208126	6.686143	6.375321	6.611997	6.037539	5.654433	6.491065	5.275538	5.718155	5.219974	6.402768	6.098896	6.378320	7.313859	5.761388	7.251999	7.234690	6.486107	6.718122	6.697278	6.425798	6.696196	7.134041	6.254868	6.017258	5.695458	6.209157	5.962157	6.369883	6.565136	6.997719	6.757650	5.425814	6.326589	6.364548	6.228136	5.961797	6.362867	6.424487	6.672528	6.594381	6.495744	6.737778	6.329915	8.021201	6.804613	6.789904	6.652886	6.588698	7.299545	6.514561	6.582283	6.092548	4.018708	6.523859	5.705784	5.485008	7.367653	5.901504	7.739294	6.571007	7.520520	6.140652	7.620747	6.186409	6.410385	6.443539	6.176600	6.722898	6.499069	6.352940	5.676561

U91319.1	3.544508	3.531510	3.259434	3.102534	2.988814	3.178289	3.616132	3.452065	3.416390	3.380500	3.459846	3.312397	3.690955	3.806591	3.438735	3.209109	3.656217	2.977408	4.589492	3.216236	5.021566	2.839050	3.538259	2.949012	2.900266	2.888826	3.482425	2.985514	3.194728	3.075527	3.560111	3.911846	3.778554	3.098565	3.502755	4.045411	3.602466	3.291768	3.435083	3.266295	3.451547	3.682236	3.620269	3.352877	3.804309	5.834830	3.542870	3.047001	4.926387	3.844161	3.850572	4.002996	4.515287	4.249931	4.089190	3.199768	4.241406	2.915154	2.910624	4.056940	2.850348	3.197854	3.941069	3.316628	3.574404	3.515884	2.901417	3.461815	3.211573	2.974354	3.134377	2.985886	3.300958	2.979637	3.183432	3.370658	3.044544	2.955435	3.032233	3.844577	3.478413	4.156076	3.616322	4.197310	3.476161	3.816300	3.029190	3.279903	3.202239	3.657866	3.450610	3.038852	3.667489	3.344305	2.991831	3.032337	3.944838	3.277712	3.380840	3.022268	3.273130	3.233635	3.026197	2.913548	4.556009	4.012899	3.327268	3.549833	3.022890	3.301772	2.883012	2.910186	3.010401	3.354861	3.615523	2.993919	2.960877	3.185151	3.259434	3.147174	3.704272	3.142565	3.169527	2.974354	3.800298	2.937825	3.224050	3.420840	3.131467	3.318958	3.061114	3.006446	3.191065	3.184892	2.970823	3.565517	2.884435	4.501816	3.344305	4.292225	2.813763	5.295630	2.883803	2.826566	2.844900	3.086264	2.893899	2.967039	3.529787	3.413828	3.385855	3.692563	3.144215	3.414002	3.765884	3.656029	3.452065	3.383393	3.103641	3.199655	3.396622	3.654009	3.500876	4.321835	5.330483	3.530322	2.902480	5.141740	3.699776	3.721463	3.532151	4.324651	4.446490	3.610154	3.107476	3.844785	2.876946	2.811222	3.203743	2.786208	3.315887	4.054951	3.877841	3.903970	3.722298	2.863919	3.530135	3.267345	2.882057	3.063753	3.049007	3.287873	3.000357	3.050142	3.344003	3.044761	3.090739	3.115938	3.268254	3.856144	4.655105	3.669264	3.622868	3.504165	3.137848	3.320089	3.516964	3.004637	3.499640	4.986033	2.922961	3.313496	3.456795	3.307309	3.079971	3.000262	2.939310	4.004337	4.046032	3.015098	3.036937	2.928241	2.837600	4.945885	4.716023	3.388949	3.508342	3.920809	3.201820	3.145941	2.984694	3.159554	3.254505	3.913358	2.964728	3.006352	3.186612	3.388600

LINC01102	3.609700	3.600306	3.273291	3.126752	2.977322	3.204261	4.269645	3.411476	3.441340	3.358469	3.501981	3.506053	3.377896	3.934506	3.654009	3.213260	3.608603	3.068252	3.850978	3.242519	5.081245	3.569466	5.044934	3.149389	2.922660	2.956250	3.281645	3.023793	3.300530	3.264674	3.681431	4.065006	3.890274	3.364521	3.629032	4.110501	3.700152	3.460369	3.585521	3.507239	3.499802	4.010582	3.674319	3.405410	3.852056	5.894592	3.537834	3.191611	6.122355	3.989435	3.895449	3.874364	4.566165	4.410887	4.149407	3.204261	4.293954	2.964561	2.956990	3.764064	2.872264	3.215034	4.072762	3.376527	3.604265	3.565159	2.874221	3.272250	3.274494	3.058021	3.230761	2.998429	3.323165	3.090979	3.234227	3.385360	3.042603	3.013264	3.122045	3.865968	3.485520	4.200744	3.610969	4.116995	3.502531	3.875478	3.134492	3.495222	3.313984	3.676604	3.328221	2.989318	3.690345	3.373733	3.003120	3.046004	3.977212	3.325163	3.158788	3.084157	3.010582	3.281206	3.054656	3.077267	4.439596	3.362333	3.418434	3.628398	3.056331	3.329019	3.852937	3.018287	3.015270	3.386033	3.667109	3.310854	2.977243	3.201577	3.277400	3.171050	3.814603	3.152112	3.192551	3.054863	3.860460	3.321058	3.230761	3.445079	3.127814	3.316944	3.152112	3.012040	3.239897	3.313790	3.249707	3.528850	2.929130	3.164434	3.385511	4.331654	3.176138	4.884944	3.059176	2.840883	2.886374	3.130470	2.909136	3.311317	3.584353	3.678826	3.424917	3.787366	3.281503	3.528493	3.815433	3.750428	3.663429	3.504352	3.234379	3.238755	3.884687	3.686042	3.473243	4.391046	5.413792	3.459491	2.942182	6.031113	3.929574	3.837146	3.696923	4.390400	4.639063	3.649813	3.112756	3.902531	2.883463	2.838906	3.544145	2.800127	3.340445	4.197752	3.991131	3.950977	3.782519	2.845741	3.518911	3.387908	2.917967	3.082998	3.063094	3.313017	3.174752	3.076552	3.362151	3.146072	3.145815	3.144458	3.283804	3.853571	4.746858	3.687679	3.625598	3.535542	3.170136	3.334212	3.462151	3.059814	3.523264	3.493904	2.872066	3.332238	3.488475	3.350218	4.264426	3.544508	2.991100	4.113121	3.247376	3.048062	3.059715	3.102425	3.099621	4.760599	3.260848	3.483534	3.571337	3.480010	3.232441	3.382407	3.438735	3.039515	3.278841	3.102425	2.908590	3.033286	3.214200	3.422630

MEF2C-AS1	8.003253	7.654357	7.682592	8.156599	8.371908	8.186673	7.330879	9.210851	9.145331	7.991593	8.074267	7.731957	7.565945	7.919036	7.425716	8.049234	8.774914	7.754859	8.946451	7.521441	8.383127	8.847553	9.392378	8.202867	8.330575	8.402976	8.378322	8.874499	8.055259	7.363727	8.814849	7.291767	7.379556	7.685331	7.616815	7.671958	7.672732	7.674327	8.256475	8.557313	7.818572	8.406518	7.849606	8.151610	7.902121	7.830789	8.034188	7.849325	7.862606	7.596093	7.697302	7.446170	7.181248	7.375955	7.354215	7.900849	7.417566	7.872765	7.880362	7.562431	7.971157	7.513425	8.240396	8.843862	7.890146	8.211447	7.901642	8.037881	7.619865	8.573058	7.621041	7.731332	8.056835	7.492285	7.758072	7.787317	7.889501	7.459810	7.926812	7.533448	7.417279	7.411359	7.326749	7.420235	7.956970	7.502758	7.457297	7.641095	8.192758	7.973373	8.007160	8.693560	7.651592	7.477534	7.567799	7.750438	7.595187	8.129345	7.922875	8.218245	8.266671	7.610677	7.918129	7.728256	8.211137	7.856943	9.086559	8.162655	8.916955	8.365500	8.853927	9.017440	8.437092	8.436936	7.952630	8.477251	8.519296	7.930369	8.032533	7.800225	8.049077	7.596093	8.348987	8.768535	8.320792	8.178706	8.823713	8.478049	8.318216	8.119314	8.015092	8.301664	7.968034	8.213302	7.902121	8.573379	7.901019	9.223120	7.389033	8.693709	8.754570	9.365664	8.560242	8.791593	8.572726	8.276739	9.097364	8.698484	7.819973	8.576857	8.599566	7.996613	7.287828	7.509989	7.569604	7.321353	8.246522	8.712812	8.600963	8.412762	8.249440	7.671958	8.102045	7.607291	7.698370	8.436154	8.096900	8.087256	7.755152	7.618931	7.870600	7.242922	7.241488	7.303979	7.898958	7.125129	7.638944	7.864791	7.218710	8.096450	7.599534	8.521422	8.324630	8.108945	8.039489	7.910185	7.749652	8.056835	8.236995	7.777904	7.775580	8.622183	7.625612	7.594133	7.706238	7.906169	7.774785	7.984242	7.311670	7.280042	7.386548	7.290668	7.323456	7.673686	8.247810	7.384238	7.378793	8.149015	7.997816	8.191012	8.510590	7.897027	8.063486	8.124953	8.074267	8.195596	8.453070	8.697502	7.834160	8.287784	7.418994	8.117615	7.658152	7.901809	8.429432	9.336323	7.898958	8.566155	8.733026	8.910236	8.480778	8.771440	9.120947	8.140171	7.584728	8.234355	7.633766	8.249440

LINC00639	7.605306	7.783924	7.792348	8.124795	8.504471	8.463830	7.925680	10.176456	10.020041	8.807664	8.640041	8.757756	8.477734	8.314845	8.211744	8.450156	8.856984	8.177391	8.189742	7.946027	7.775738	7.636181	7.161102	7.576358	8.059483	8.891767	8.566718	9.077642	8.296702	8.047832	9.284604	7.646818	7.756016	7.807549	7.857697	8.420110	8.292455	7.948001	8.468700	8.067133	8.404997	8.014477	8.098103	8.339021	8.082255	8.279940	9.205043	8.314845	7.978305	8.318352	7.600158	8.329950	7.568853	7.963592	8.191798	8.865668	7.734581	7.045786	7.593195	7.551749	7.662653	8.122600	7.530827	8.044966	7.903197	8.144078	7.520351	7.870285	7.801746	7.912959	7.882013	8.126434	8.226603	7.984078	7.876139	7.957608	7.817143	7.905227	7.717516	7.847655	7.437870	7.663402	7.611602	7.734263	7.605082	8.345080	7.576995	7.473547	7.227337	8.165029	7.860319	7.796942	7.541891	8.280399	8.145020	7.727632	8.216204	8.415946	8.995047	7.902878	9.100248	7.756814	8.097219	8.121626	9.060510	8.357224	9.832155	9.134397	8.439638	8.631128	7.868475	9.199695	8.986084	8.819478	9.226345	9.474009	8.037443	8.643164	8.626257	7.871375	8.240987	7.787467	8.195917	9.360161	8.547074	8.442536	7.708703	8.534396	8.383585	8.737472	8.332786	8.816206	8.317157	9.126584	8.614955	8.373727	8.406518	7.450892	7.544875	7.830076	7.638001	7.416404	8.190378	8.178407	8.173935	8.567907	9.364727	8.925972	8.236995	8.908005	7.714215	7.922093	8.256329	7.752553	8.085086	8.344925	8.192439	8.307612	7.870451	9.113294	7.741197	7.832927	8.069677	8.310979	8.691979	9.493614	7.153231	7.789160	7.893724	7.866197	8.226324	7.589205	7.715374	7.671958	8.470095	7.002726	6.754906	7.280541	7.310260	7.494271	8.340131	7.508788	7.603766	7.202384	7.090101	7.505395	7.899114	8.013547	7.606999	7.944096	8.181304	9.070803	7.925836	7.857542	7.858931	7.796942	7.967552	8.013217	7.119964	7.378634	7.422597	8.078556	6.940135	7.186675	8.257688	7.400060	7.245475	6.211381	7.182510	7.125950	7.492285	7.278770	7.793702	8.206331	8.104330	7.941504	8.405459	8.434607	7.241639	8.888062	7.765616	8.028111	7.617381	7.532852	8.417837	9.328764	8.469173	7.697302	9.029335	7.501657	8.903213	8.815433	9.534899	8.632743	8.525979	8.066831	8.570053	8.564511

AC091180.5	13.155345	13.619482	13.388628	13.891833	13.390537	13.411645	13.801126	13.952110	12.801968	13.714416	13.649396	13.479279	13.420393	13.624759	13.075520	13.815559	13.449721	13.215415	13.336226	13.704144	13.440127	13.817826	11.569606	13.421709	13.525049	13.534199	12.072185	13.577378	12.721275	13.235191	13.191256	13.918056	13.960811	13.584387	13.635698	13.653913	13.600149	13.384372	12.906948	13.053836	13.650975	12.616477	13.846147	13.845352	13.381340	13.844830	12.817593	12.986294	14.129877	14.105943	13.625467	14.158638	14.028483	13.906117	14.005012	13.390157	13.896143	13.272567	12.759589	13.347873	12.800391	13.318278	12.014173	12.716248	12.717320	13.324998	12.698701	13.103175	13.061923	13.005778	13.646573	13.700412	13.496693	13.594881	13.660327	13.372722	13.837713	13.581115	13.570563	14.587290	14.090692	14.021820	13.936877	14.084808	14.129527	13.806245	13.818636	13.996150	13.042576	13.667931	13.246311	13.390537	13.825287	13.939615	13.328213	13.745828	13.943207	13.263764	13.525933	13.025290	13.607340	13.489501	13.361622	13.057276	13.299046	13.243546	12.822489	13.265367	13.032476	13.582997	12.992898	13.120423	13.017536	13.408151	14.114029	13.098245	12.142942	13.570340	12.985374	13.337481	13.805968	13.071874	13.074158	12.729636	13.780832	12.533949	11.783668	12.233426	12.734879	12.767083	12.462754	12.621674	12.600707	12.988891	12.460438	12.773448	11.889157	11.789411	13.341166	13.151837	13.023099	12.371021	11.763724	13.012474	13.330689	13.819403	13.316023	11.841289	13.362235	12.146729	12.480123	12.647613	12.831161	13.015361	12.978391	13.484618	12.711326	12.572521	12.664778	12.925613	12.447268	13.196137	13.688814	13.877971	13.904321	12.590461	11.879384	12.793186	13.840878	13.421261	13.365165	13.482140	13.623383	13.373992	13.239205	13.465295	12.548754	12.752782	13.236652	12.597317	11.949752	12.977506	12.805624	11.898226	13.054711	12.636992	12.608022	12.851777	12.266993	12.884726	13.117722	12.485499	13.277634	12.919349	13.133751	12.965824	13.432385	13.362235	12.695910	12.974908	13.855947	13.915790	13.733881	13.243931	12.596981	13.800082	13.383910	12.379442	13.153006	12.818568	13.076654	13.272175	13.466605	13.331689	12.810934	12.903047	11.290392	13.211099	12.409200	12.969318	11.909265	12.352259	12.678191	12.384330	12.032445	11.930776	13.977501	13.110780	13.175795	12.423432	12.052072	12.691189	13.166715	13.196931	13.731668	12.598323	13.312746	13.209096

MSC-AS1	7.326266	5.909697	5.681420	5.965177	3.293126	5.568836	5.960936	5.157073	4.511393	5.158207	5.252732	4.678180	5.779623	5.148333	5.289592	5.587157	5.577218	4.940761	4.920011	5.904540	6.464876	5.737715	6.604561	4.236984	5.275128	4.721961	5.207639	5.432612	5.033779	6.895583	4.227258	7.617855	7.679669	5.983328	7.255858	6.971967	6.732375	5.759262	6.045528	6.169288	5.635849	7.019054	6.748634	6.221248	7.631140	5.786478	4.947749	6.857061	6.850296	6.807095	6.242601	7.848996	7.492285	7.202097	6.820386	5.500266	7.377194	6.821189	4.395671	6.098896	6.106037	6.621465	8.408805	5.805451	6.927488	5.690430	6.170824	6.091820	5.991118	6.419648	6.874986	6.348934	4.888751	7.090413	6.227975	6.950755	6.270170	6.901754	6.172347	7.483367	6.987783	7.406172	7.157612	7.145355	7.028135	7.237820	6.436473	6.664782	6.262852	5.655559	5.423011	5.883154	6.620820	6.350277	6.381308	6.539082	6.984911	6.422546	5.116218	4.392978	4.310828	5.965720	5.332847	4.766823	5.124300	6.085087	5.423414	5.944425	5.336327	4.927430	6.427847	5.202650	3.406943	4.791656	4.871916	3.116275	5.461798	5.701366	4.955831	6.475585	6.973815	6.317926	4.717292	3.231565	5.414727	4.120296	6.409018	8.339807	7.568694	5.134841	4.294363	8.546755	5.329287	6.344566	4.579470	9.111930	6.021175	6.927637	8.747736	5.821481	8.096768	7.029717	3.178423	5.421874	8.822972	4.605637	3.197721	3.852056	7.915342	4.765269	7.662314	7.003666	7.865090	6.390022	7.467417	8.070641	5.815647	6.846929	6.442193	6.899004	5.859961	6.494441	7.956793	6.873056	7.761430	4.217947	7.646194	7.544076	9.516216	8.636910	7.657521	6.843510	9.231299	7.916120	8.009296	8.136815	5.849536	4.734603	5.867788	7.238594	5.411287	6.762196	7.718880	4.998777	6.621633	6.571789	5.670490	7.794928	5.864523	6.094796	7.957304	4.768671	8.189259	8.385284	6.489496	8.259705	7.375490	6.806316	8.263388	7.777145	6.526178	8.997674	8.174901	8.203624	5.856204	8.559772	8.484300	6.867088	6.222275	5.283372	7.016822	6.824667	8.003583	6.438660	6.809369	6.711340	5.451663	6.435274	7.217178	7.730239	5.663683	4.018026	6.893841	7.152494	6.246624	6.921935	4.052798	8.109811	5.632451	6.481906	5.691365	3.114384	4.847214	6.636437	6.214917	5.606325	8.821736	5.257333

AC109466.1	3.593240	3.722298	3.751278	4.182980	3.497463	6.933931	4.340694	4.891473	4.521717	3.483890	5.306198	4.369566	3.887402	3.998329	3.593829	3.572084	4.568093	3.327563	5.718155	3.364197	5.155411	4.429095	5.334014	3.354699	4.133820	4.236160	3.476529	4.283174	4.237440	4.471008	4.972542	3.860875	4.214696	3.142685	3.655820	4.120971	3.841707	3.485860	4.337446	4.401594	3.418075	4.442638	4.241625	3.361140	3.687679	5.574445	4.273115	4.789720	4.503938	4.147052	4.131034	5.763933	5.252954	4.121617	5.092892	4.386530	4.089190	3.097128	3.727364	3.306834	4.631969	3.636076	3.595009	4.634359	4.855023	5.384282	2.858775	4.482648	4.379733	4.528611	4.329886	4.349640	3.640693	4.769928	3.724930	3.744089	4.538427	4.456699	4.886886	3.789934	3.946628	4.171454	3.846944	3.983505	4.804933	4.804112	4.447792	5.291967	5.308286	4.732302	4.686559	5.659956	3.070362	3.916620	3.306163	3.537427	4.204289	3.322813	3.370658	3.049429	4.730815	3.266581	3.557581	3.117845	4.493786	5.357782	4.533330	3.604642	4.469910	4.510286	4.369123	5.436481	4.452047	4.472347	4.504144	7.585638	4.251723	3.962694	3.535179	4.195839	4.022811	3.463379	3.544884	3.582100	5.500842	6.681747	3.913757	4.413541	5.536264	4.221682	5.860863	4.265101	4.799201	3.834204	4.506555	4.309946	3.770527	4.095615	3.947691	4.394513	4.210561	6.555125	3.340106	4.591411	3.899988	5.658509	4.323583	4.281473	4.654410	3.685237	3.393310	3.257039	3.278696	3.702855	4.198394	4.354587	4.196670	4.453761	4.742152	5.342170	3.308743	3.808277	3.934506	4.119882	5.129993	5.778004	3.614384	4.055845	4.351327	4.219035	5.238461	4.428669	4.210345	4.484599	5.258478	4.471916	2.949867	4.229863	4.763775	5.065069	4.471008	4.732080	3.848186	4.603961	4.616876	2.832031	4.459269	4.395908	3.233108	4.888117	4.334201	4.603126	4.509201	4.484173	4.568306	4.994379	4.855214	4.961439	4.261771	4.358517	4.500504	4.351108	3.731709	4.350669	3.919774	4.545552	4.655999	4.744249	4.544230	4.188223	6.040557	3.614582	3.878499	3.784800	3.758791	3.647203	4.276612	3.496333	3.246386	3.254178	3.284276	3.661202	3.167932	4.882433	3.908140	3.962248	4.321641	4.714127	4.675590	4.490913	4.335291	4.783483	5.361740	4.692213	5.399603	4.694331	4.769494	3.751278

LINC02143	3.593240	3.722298	3.751278	4.182980	3.497463	6.933931	4.340694	4.891473	4.521717	3.483890	5.306198	4.369566	3.887402	3.998329	3.593829	3.572084	4.568093	3.327563	5.718155	3.364197	5.155411	4.429095	5.334014	3.354699	4.133820	4.236160	3.476529	4.283174	4.237440	4.471008	4.972542	3.860875	4.214696	3.142685	3.655820	4.120971	3.841707	3.485860	4.337446	4.401594	3.418075	4.442638	4.241625	3.361140	3.687679	5.574445	4.273115	4.789720	4.503938	4.147052	4.131034	5.763933	5.252954	4.121617	5.092892	4.386530	4.089190	3.097128	3.727364	3.306834	4.631969	3.636076	3.595009	4.634359	4.855023	5.384282	2.858775	4.482648	4.379733	4.528611	4.329886	4.349640	3.640693	4.769928	3.724930	3.744089	4.538427	4.456699	4.886886	3.789934	3.946628	4.171454	3.846944	3.983505	4.804933	4.804112	4.447792	5.291967	5.308286	4.732302	4.686559	5.659956	3.070362	3.916620	3.306163	3.537427	4.204289	3.322813	3.370658	3.049429	4.730815	3.266581	3.557581	3.117845	4.493786	5.357782	4.533330	3.604642	4.469910	4.510286	4.369123	5.436481	4.452047	4.472347	4.504144	7.585638	4.251723	3.962694	3.535179	4.195839	4.022811	3.463379	3.544884	3.582100	5.500842	6.681747	3.913757	4.413541	5.536264	4.221682	5.860863	4.265101	4.799201	3.834204	4.506555	4.309946	3.770527	4.095615	3.947691	4.394513	4.210561	6.555125	3.340106	4.591411	3.899988	5.658509	4.323583	4.281473	4.654410	3.685237	3.393310	3.257039	3.278696	3.702855	4.198394	4.354587	4.196670	4.453761	4.742152	5.342170	3.308743	3.808277	3.934506	4.119882	5.129993	5.778004	3.614384	4.055845	4.351327	4.219035	5.238461	4.428669	4.210345	4.484599	5.258478	4.471916	2.949867	4.229863	4.763775	5.065069	4.471008	4.732080	3.848186	4.603961	4.616876	2.832031	4.459269	4.395908	3.233108	4.888117	4.334201	4.603126	4.509201	4.484173	4.568306	4.994379	4.855214	4.961439	4.261771	4.358517	4.500504	4.351108	3.731709	4.350669	3.919774	4.545552	4.655999	4.744249	4.544230	4.188223	6.040557	3.614582	3.878499	3.784800	3.758791	3.647203	4.276612	3.496333	3.246386	3.254178	3.284276	3.661202	3.167932	4.882433	3.908140	3.962248	4.321641	4.714127	4.675590	4.490913	4.335291	4.783483	5.361740	4.692213	5.399603	4.694331	4.769494	3.751278

LINC01170	4.940144	6.580790	4.901535	5.446009	4.950838	6.079043	6.348934	5.211825	4.978070	6.192527	5.565029	5.212182	4.410020	5.449750	4.488884	5.558672	4.526742	5.276363	5.241057	5.212774	5.884779	5.472537	5.000684	4.640321	5.430487	4.746858	4.286569	5.005368	5.501007	5.581896	4.762246	4.768483	5.708658	4.978266	5.421300	5.083066	4.764426	5.306753	5.091025	5.739353	6.447691	3.320089	5.044934	5.130792	3.892199	4.857743	5.667420	5.034634	4.967357	4.805149	4.998590	5.954733	5.809809	6.751932	5.548830	5.882290	3.846532	5.186596	3.682691	4.248186	4.504570	4.083256	4.839616	5.164897	4.860848	5.635100	5.262672	4.933461	5.371479	5.414727	5.949174	6.137520	6.002723	5.920495	6.014177	5.838422	6.039824	5.774910	5.506022	4.975059	4.702801	5.113154	4.737795	4.867232	5.123919	4.970485	3.698983	5.155607	4.167541	5.287944	5.359285	5.304609	5.252144	5.617787	4.980930	5.111523	4.864751	5.146958	4.781151	4.564910	4.441091	5.392783	5.155607	5.173168	5.437457	6.017781	5.351860	5.567109	5.184611	6.859440	5.414727	5.214240	4.458439	6.571479	5.262253	4.296116	4.572849	4.791432	5.185007	4.929514	6.445240	4.820465	5.106012	4.402892	6.723695	5.128561	4.948530	4.778641	5.736795	5.600944	5.110367	4.383610	4.457789	4.623340	5.028289	4.905873	3.908793	5.621097	5.372272	6.086738	5.268667	6.085920	5.131199	5.310272	5.985562	5.138445	5.740431	6.120967	6.471578	4.289203	4.681788	5.249800	5.386549	5.048723	4.801756	4.362190	5.349273	5.541842	5.573704	6.367558	4.570033	5.536829	5.816187	3.806591	4.442195	6.317250	3.890511	5.545822	4.358758	5.013840	5.230761	5.331849	6.531764	4.374567	5.922745	3.832282	4.602311	4.619929	4.723015	4.717292	4.725774	6.027664	4.912630	5.694721	5.667049	5.495885	5.384644	5.779106	5.705254	6.426806	6.246949	6.122868	5.695092	4.974416	5.935284	5.911336	6.421107	6.136363	4.685015	4.702350	4.988095	3.800534	3.894646	5.244823	4.358088	4.416195	5.445634	4.938930	4.885836	4.485024	5.407741	5.176738	5.804210	5.733384	4.886247	4.456478	3.473243	5.167290	5.519352	4.991195	4.789935	4.622273	4.203136	5.345357	6.043642	5.350897	6.091996	5.202453	6.559425	5.136022	4.527150	5.284366	6.060983	5.462933	4.541985	4.997172	5.235030	6.219399

AC068535.1	3.101859	3.128093	2.941296	3.305084	3.827629	4.840237	3.275450	3.036164	3.408550	3.311459	5.693593	3.596331	3.205479	3.729032	3.655026	3.538259	3.358816	5.450652	4.451198	4.033635	5.774175	3.771535	3.391764	3.044660	3.052485	3.884271	2.859930	3.057595	2.926799	3.385855	3.525165	3.117616	4.481723	3.085166	4.192585	3.994643	3.543602	3.195847	3.022068	2.995701	3.418603	2.950816	3.672507	3.230901	3.401235	4.211867	3.266021	5.091641	5.672603	3.852717	3.788884	4.971704	3.825521	4.472978	3.595183	3.516425	3.811402	3.950322	3.931529	3.608409	3.036058	3.019493	3.484451	3.934099	3.189017	3.112510	3.069240	3.152335	3.060140	2.885728	3.886152	3.264238	3.105977	3.633884	4.116788	3.287257	3.902101	3.755044	3.154801	3.909010	4.773477	4.064364	3.133914	3.773677	3.222099	5.314008	5.629710	3.860653	3.817375	3.418786	3.374715	3.400227	3.611782	3.498710	3.350579	3.897785	3.366512	2.860134	2.954506	3.393310	5.230559	3.200255	2.948842	2.909854	4.337007	3.685860	3.777553	4.166251	4.862684	3.180813	5.710123	4.550664	5.269846	3.202692	3.106766	7.126590	3.449885	3.219103	3.132846	3.030200	3.186612	3.153417	2.956521	4.459459	4.720656	3.763184	3.111657	3.352380	3.696307	5.499654	3.436171	3.962030	3.577338	3.456978	4.053199	3.284905	4.827193	3.324679	3.416052	3.708077	3.738741	5.161480	3.279590	3.771736	2.921006	4.664093	3.620269	4.355265	3.508161	3.163906	3.765302	5.213818	3.129094	3.394523	3.688091	3.565704	4.301736	3.016992	4.325277	3.182173	2.862495	3.528493	3.571337	5.161480	4.092057	3.209109	4.066279	5.244823	3.778757	3.732967	3.341397	3.620269	4.039720	3.788048	2.992100	4.469699	3.718738	3.777553	3.575730	2.966669	3.104774	3.446902	3.307309	3.300244	3.981983	3.058412	3.300530	3.130149	2.911444	3.638690	3.662649	3.066396	4.048031	4.890032	3.258846	3.276507	4.460269	3.140813	3.978030	3.529405	3.810138	3.207511	3.984782	3.328072	5.418549	3.095565	3.353835	3.536283	3.332425	3.104560	3.652214	3.236383	3.106321	3.021968	3.845423	4.622478	4.568743	5.059192	3.188883	4.276612	3.415052	3.568486	3.699957	4.708459	3.609968	3.492212	3.915505	3.250276	3.333725	5.493365	6.521225	8.588177	3.148640	3.142064	3.622668	2.906249	3.271619	3.209656

SHANK2-AS3	11.932456	11.954526	11.767509	12.215771	12.425577	13.070763	12.298858	12.815796	13.526328	12.816972	13.042247	12.696624	12.112840	11.807225	11.268654	12.776712	12.746911	11.435314	12.280654	12.679736	11.329368	12.076353	11.280469	11.356381	12.487141	12.829828	13.407961	12.912866	13.035259	12.860767	12.940455	11.498901	11.466898	12.034420	11.698422	11.779259	11.944663	11.398216	11.866700	11.733781	12.966194	11.594299	11.904326	11.971923	11.641765	11.515780	13.228465	11.250930	11.704017	11.584703	11.393514	11.204194	11.345621	11.340687	11.275177	13.065305	11.147664	11.175962	11.130778	10.920323	11.038010	12.976952	10.870351	11.157291	10.963237	11.015291	11.436941	11.692880	11.400440	11.497575	12.093060	12.355628	13.098440	12.184989	12.187471	12.110262	11.997151	11.730489	11.989104	11.409205	11.349651	11.416927	10.900269	11.456523	11.500773	11.665066	11.561285	11.663282	11.016056	11.113613	10.852098	10.859380	11.557455	11.839782	11.912357	11.964779	11.425646	12.396312	12.201865	13.058112	12.539305	11.758591	12.457385	11.863798	11.757691	13.110956	12.398188	11.331478	11.731098	13.192203	11.051636	12.340875	11.935497	13.436646	12.928845	13.944041	11.455375	12.646754	13.681146	11.957851	12.320942	11.869641	12.430104	13.254784	12.913792	13.025099	13.301878	12.858461	13.190080	13.121579	12.921144	12.613710	11.803385	11.674592	13.065502	12.388886	12.011560	11.861688	12.366380	11.251658	12.057308	11.363862	12.166942	12.228465	12.197526	12.677497	13.006514	13.635217	12.741631	13.051634	11.923703	12.084324	12.347129	12.124642	12.226464	11.992461	11.599324	12.018571	12.069478	13.411878	12.156958	11.746557	12.053155	11.603568	11.622042	13.399078	11.615814	11.621743	11.340239	11.409809	11.322149	11.501545	11.161862	11.768414	12.579338	11.028357	11.290706	11.000620	11.047235	11.348194	12.998952	11.079395	11.752033	11.152318	11.067334	11.291432	11.816748	11.341907	11.422716	12.141427	12.445794	13.680191	11.988788	12.397685	12.375821	11.932304	11.956666	12.073577	11.347581	11.508193	11.359302	11.263347	11.656596	11.587606	12.294973	11.463192	11.456977	10.982470	11.099443	10.806201	10.856734	11.890041	11.930928	12.200621	12.420008	12.109069	12.802671	12.250568	11.813291	12.181736	12.386142	12.599699	11.741301	11.433898	13.143030	12.929922	11.240345	11.099868	13.448408	11.115175	12.175303	12.838440	13.585542	12.269467	12.657171	11.165378	12.805624	13.018633

AC079193.2	5.588122	5.209227	5.717116	6.001097	6.116129	5.951816	6.177822	6.132434	6.839672	6.181859	5.859782	5.870804	5.836243	5.070805	4.455015	6.605592	6.467195	6.015023	6.123195	6.505130	5.897757	6.339728	7.065404	6.518720	6.593887	5.702980	6.914949	5.431041	6.141731	5.035911	7.053976	4.104297	6.871946	6.551816	6.047817	6.000758	6.269812	6.000081	6.117144	6.696813	5.777077	6.289289	6.154306	6.398962	6.720973	4.638012	6.132591	5.750837	5.722522	5.214240	6.189910	6.245766	5.873876	6.827338	6.112982	4.484599	5.914578	6.508622	6.381466	5.801382	6.175069	6.066254	6.556152	6.050770	6.058527	5.533321	5.456476	5.482205	5.131199	6.189430	5.848814	6.105134	6.571326	6.197741	6.072243	5.892560	6.279314	6.299226	5.900633	6.903209	6.539247	6.159451	5.828960	6.175069	6.666542	6.530621	6.653693	5.887621	7.087205	6.367040	6.610796	7.011235	5.852379	5.740812	5.351297	6.786475	5.504495	7.159784	5.531616	6.054797	5.327319	5.993056	6.175587	4.676249	5.877095	5.884422	6.485460	6.730471	6.863914	6.361514	6.310424	6.678007	5.823851	6.355158	5.238461	5.583994	6.052366	5.975866	7.096262	5.360411	5.697684	5.508191	5.687183	4.726203	5.514823	4.125452	7.350988	6.851706	6.738587	4.663487	7.210504	4.442432	4.404666	3.776955	5.850948	6.367558	6.899975	5.399603	5.569758	4.853765	6.787922	6.951856	7.356854	5.661421	6.347250	6.526356	5.931085	5.624864	5.330483	7.347461	4.700068	7.400671	5.276162	5.899887	7.339253	5.963410	6.922099	6.375321	6.506005	5.267480	6.145662	5.588508	6.469674	4.655105	4.404426	4.733340	6.131898	6.297720	5.824962	6.603879	6.462173	5.266656	6.346571	5.294248	5.145729	5.803115	5.855085	6.203650	4.838497	5.700263	5.057174	6.352776	6.220905	6.054797	6.080065	5.936504	5.514453	5.303420	5.712313	5.774910	6.467861	4.884736	6.004284	5.519185	6.152236	6.543856	6.026945	5.762139	6.781209	5.780710	5.892386	6.115636	6.009128	6.629515	6.419483	6.332981	6.464703	6.961340	6.503995	6.982395	7.125623	5.423208	5.431041	5.531254	6.249627	5.318802	6.552999	3.560924	6.256249	4.925961	5.401460	5.682715	4.691987	6.256249	6.237625	6.373651	6.125675	7.215778	4.891263	6.294903	7.324535	6.002372	6.408366	5.170495	4.099547	6.546953	4.447569	6.510260

AC011477.1	9.874083	9.295221	9.265650	9.117278	9.187084	9.289571	9.452450	8.455195	9.144456	9.073438	9.169228	9.073896	9.207491	8.925972	9.675373	9.287859	8.886372	9.333538	9.778460	9.445444	9.341170	9.595374	10.445678	9.698934	9.614989	9.364092	9.510060	8.597021	8.958743	9.309408	8.672339	9.375451	9.938052	9.660142	9.981518	9.820554	9.817863	9.925756	10.186829	10.289908	8.699378	10.512586	10.293860	9.809677	10.145498	9.799359	8.432106	9.759952	9.474607	9.809845	9.743643	9.953197	9.544712	9.834281	9.531817	8.654155	9.981518	10.241044	9.712489	10.034063	10.407984	9.166801	10.677766	9.793310	10.006166	10.068275	9.759333	9.541580	9.238102	9.966054	9.474903	9.626458	8.793066	9.551777	9.423772	9.629238	9.505342	9.738061	9.565725	9.756057	9.829507	9.515307	9.759952	9.698789	9.752686	10.022905	9.707187	9.725305	10.292343	10.033608	10.027195	10.100633	10.255126	9.563148	9.925892	10.112665	9.818886	9.633215	9.078706	9.536570	9.443123	9.683738	9.137787	9.484419	10.427336	8.578625	9.564183	10.138059	10.332520	8.860469	10.383504	9.774181	9.887831	8.769925	8.622673	6.937460	10.260726	9.069888	8.892214	10.874430	9.492544	9.348642	9.242889	8.257856	8.727096	8.229190	9.296921	7.659976	8.362192	9.252538	9.157407	8.525523	8.393345	8.616164	9.371195	8.856378	9.365340	9.989367	9.274698	8.721757	8.868076	9.392967	9.116516	9.456955	8.125564	8.641137	8.710395	8.507514	8.317753	8.519771	9.696489	9.549294	9.674273	9.894559	9.844876	9.953492	9.754059	9.806528	9.886715	8.730911	9.971512	10.302784	10.296016	9.568254	8.847874	8.297469	9.265061	9.160629	9.250083	9.073136	9.967099	8.850610	10.105829	9.662174	8.800503	9.345894	8.988657	9.220517	9.919156	9.848007	9.123078	11.178995	9.457860	9.529591	10.602257	8.745107	9.541895	9.353931	10.086994	9.499181	9.394200	8.552439	9.443912	9.422051	9.057198	9.351997	9.098890	9.810719	11.086626	9.807593	9.272705	10.270875	9.873016	9.332164	9.984732	8.857274	9.986838	10.605791	9.741831	10.513902	10.554299	10.635038	9.723772	9.739786	9.773557	9.190561	9.351090	9.782680	9.241660	9.186105	9.365980	9.703107	9.349567	9.936797	8.789321	9.312647	9.129842	8.848031	8.272535	9.958130	9.249360	9.663115	8.731651	9.079331	6.793483	9.457860	8.891903	8.778562

AC211476.4	11.911322	12.108644	12.135906	12.463562	12.690337	12.400969	11.817639	11.964028	13.063805	12.353569	12.755993	12.364182	12.548754	12.486329	12.245455	12.148094	12.772931	12.171247	12.626503	12.589445	12.412540	12.518430	12.744389	12.105127	12.820415	12.735923	13.614204	12.487990	12.484713	12.286918	13.247713	11.929992	12.180812	12.676682	12.116974	11.961457	11.959745	11.876433	12.705000	12.465490	12.372913	12.499786	12.321255	12.131436	12.214434	12.277746	12.810174	11.931845	12.171102	12.134671	11.929392	12.057308	11.896631	12.247046	11.999926	12.505236	12.100426	12.344088	12.304346	12.420088	12.186842	12.486157	12.842437	12.556219	12.646567	12.086064	12.427016	12.320942	12.327331	12.640723	12.203451	12.262160	12.718533	12.029310	12.369200	12.356573	12.012961	11.841289	11.764040	11.740264	12.447724	12.395993	12.041521	12.119577	12.353897	11.997933	12.278351	12.352896	12.674301	12.480968	12.419436	12.153299	12.146584	12.156789	12.151088	12.701417	11.888571	12.800035	12.614041	12.956616	12.379731	11.968715	12.723135	12.349580	12.642737	12.497024	12.929753	12.913978	12.723135	12.763272	12.547624	13.183285	12.691020	12.819748	12.652596	12.977133	12.539475	12.254686	13.394129	12.793186	12.259261	12.452025	13.306600	13.601268	12.993251	13.119075	14.755534	13.798010	13.354386	12.783797	13.727121	13.932970	12.150335	12.595766	13.807309	13.165501	13.891542	14.036663	13.164898	12.736959	13.469871	13.555677	13.645878	12.718016	12.876249	12.448476	13.260449	13.644259	12.620642	13.838218	12.935833	13.254784	11.881927	12.584309	12.777426	12.259736	12.299354	13.344050	13.335408	13.692656	13.608723	13.090537	12.798847	12.479954	12.093684	13.254395	11.804981	12.759782	12.031682	12.327640	13.134732	12.764791	12.057775	12.696084	13.399078	12.533129	12.874855	12.903931	12.592167	12.905355	13.530568	13.028809	12.934196	12.784513	12.626503	13.257008	12.310934	12.515826	13.162623	13.178186	13.066209	13.216008	12.442202	12.817505	12.686046	12.140678	13.286686	12.415626	12.279300	12.811445	12.888611	12.662414	12.609346	12.383214	13.638949	12.099378	12.940276	12.740227	12.226923	12.451389	13.215415	13.297753	11.969922	12.322207	12.820415	12.964739	13.713891	12.318233	12.887748	12.618117	13.326420	13.249121	12.626987	12.577039	13.420393	13.580155	13.040220	12.910540	12.873402	12.914980	12.854570	12.549566	13.240382	12.533788	12.546069	13.446264	13.268822	12.949809

LINC02655	5.679852	4.630478	5.417579	5.424047	5.672945	5.565784	3.668671	8.142105	9.479239	5.028096	6.610796	6.261164	4.149177	6.344739	5.409120	5.374545	7.291614	6.100152	7.822714	5.548432	6.083332	6.740540	7.693984	5.769929	6.085920	6.081078	6.226055	7.424306	5.020938	4.758930	7.029236	3.942389	4.795599	5.571799	5.111153	5.839364	5.278766	4.968621	6.373313	5.998849	4.640721	6.303063	5.935640	6.856580	5.908843	7.114394	5.389874	5.197929	5.514453	3.699384	5.057174	4.543088	5.014044	4.720037	4.743378	6.030506	4.157572	3.432871	5.702980	4.887311	5.127943	4.438496	4.571814	7.437870	4.921690	4.646621	2.919864	5.188600	5.165102	6.528665	4.966918	5.264877	5.733384	4.933247	5.490169	4.864543	5.589862	5.108923	4.797249	4.470544	4.863307	4.650040	5.962534	6.464369	4.695972	5.991827	6.564660	4.991612	5.204869	5.138833	4.812075	5.465983	5.176137	5.178778	4.970105	5.903452	5.214240	7.691511	6.769450	6.960210	6.904609	4.509201	6.242444	5.854561	5.870061	5.451663	9.003202	7.368285	7.667017	5.931921	7.708553	7.583021	6.417972	6.677849	7.131658	7.486494	6.656772	6.545166	5.500266	4.926168	4.966918	5.131805	4.973998	6.529490	5.820610	5.207435	7.582103	7.584728	5.782376	5.309215	5.581712	5.074237	5.454083	4.998160	5.925599	6.420618	5.725344	7.085231	4.895390	6.980847	7.073703	7.502758	6.131372	4.765904	4.542193	6.595852	7.417279	6.514226	4.793518	7.053976	6.189761	5.107057	6.443210	5.118253	5.679002	5.226103	5.736795	6.930818	5.848984	5.426670	5.658691	5.212965	6.518878	4.944128	6.913398	5.021154	5.673669	6.172526	3.732140	3.971793	4.373702	5.069990	4.071884	4.601011	5.084680	4.763580	3.174610	5.519352	4.279959	4.914136	3.945977	5.350464	7.260903	5.609543	5.209429	3.184892	4.712429	5.327897	5.835708	5.192676	5.091838	6.184157	5.062258	5.013631	5.160301	4.880280	5.004156	4.713491	4.128953	3.989853	4.417292	6.678466	5.433001	4.759133	7.074317	4.632837	5.197099	5.964122	6.212680	5.556180	5.744826	5.434147	5.357975	5.209429	5.391069	5.758891	7.535780	6.641471	5.267082	6.000758	6.043120	5.522331	6.085431	5.293505	4.847004	8.268700	7.148341	6.826732	6.029259	6.836278	7.313538	5.867214	6.874986	6.193889	6.463206	6.289442	6.044989	4.416874

AL035461.3	14.197518	12.163756	12.676354	12.983527	12.955541	12.401457	12.819748	12.026746	11.910004	12.332876	12.563831	12.491562	12.733547	11.907107	11.900004	12.434966	12.221727	12.148544	11.578338	12.340685	12.172634	11.086782	11.077953	11.838207	11.157147	12.616315	12.494332	11.672678	12.454375	12.529678	11.911924	12.774141	12.701221	12.973483	12.705699	12.552494	12.629517	12.267304	11.723465	11.706874	12.514995	11.579215	10.982910	12.531515	12.433717	12.489908	12.323101	11.662670	12.539123	12.425254	12.797845	12.262315	12.723489	12.681457	12.502885	12.825230	12.203451	11.199882	12.145451	11.945155	11.661349	12.298219	11.860703	11.348194	11.976069	11.961308	11.506569	11.627501	12.054832	11.514222	12.515666	12.582441	12.215055	12.621342	12.500751	12.316075	12.157396	12.680252	12.557657	11.443675	12.446937	12.679547	13.019003	13.092197	12.739334	12.721110	12.962142	12.199062	11.880705	12.225678	11.786118	11.596548	12.626671	12.378353	12.968566	12.726341	12.557332	11.928470	12.785766	11.742737	12.255202	11.715283	12.144058	11.989871	11.454294	12.449933	11.795267	11.892007	11.645041	12.316396	11.678914	12.311900	11.990484	12.161407	11.866837	11.965245	13.224119	12.775483	11.944663	13.133580	13.056178	12.992703	13.369328	12.994008	13.042247	12.937441	13.155145	13.472510	13.549579	12.881502	13.417523	13.369945	12.395853	12.507872	12.792841	13.399963	12.634942	12.715054	13.824511	12.214094	12.219378	11.918508	13.456599	13.446470	13.299219	13.490594	12.554292	12.902705	12.674134	11.965086	12.849137	12.847005	12.793758	12.174197	13.011244	12.949458	12.324058	12.540801	11.820950	13.407961	11.703267	12.096818	13.356786	12.680949	13.087953	12.982957	11.487873	12.786841	12.744741	13.009749	13.795380	12.941572	13.422986	13.269040	13.618777	14.018727	12.307454	12.553137	12.445640	11.305887	13.462926	11.365974	11.686785	12.121934	11.892609	11.807078	12.290138	12.060393	11.928150	12.905001	12.978042	12.739892	12.633466	12.920784	13.199727	13.533281	13.455025	13.094296	12.105127	12.668989	13.229238	13.547331	13.064161	13.730157	13.411210	13.212614	14.557653	11.916369	12.503725	12.268074	12.517769	12.182961	13.376481	13.410314	13.163401	13.172295	12.842271	12.236813	12.886000	13.813000	12.626987	12.509651	12.179561	12.431923	12.907332	12.638003	12.701928	12.228152	12.155866	12.266375	12.751886	14.133559	12.181736	13.650755	13.249693	12.227205	12.942109	13.000629

LINC00334	4.325277	4.876507	4.890855	4.308348	4.221883	5.355495	3.501629	7.264197	6.062618	3.754459	4.004337	4.266557	3.437038	3.844785	3.596508	4.327221	6.538445	4.001936	5.746275	4.420677	5.547508	5.382725	4.717717	4.304213	4.182761	4.094535	4.332057	5.269846	3.727364	3.696923	5.799467	4.046238	4.136278	3.756928	4.092887	4.132996	5.885687	4.560560	4.709267	5.148725	4.891473	4.806374	3.720796	3.366686	3.991816	7.054921	3.808277	5.035062	4.107435	4.279959	4.102823	4.497707	4.378241	4.244102	4.123175	3.725775	4.078461	4.087647	4.764426	4.575798	4.548399	3.764496	4.617539	5.206028	4.844015	5.314218	4.569828	4.599178	5.247413	5.319944	4.086810	4.536530	3.673284	5.131397	4.725144	4.794982	4.726407	5.182198	4.849503	4.712429	4.332057	5.137208	3.917902	4.923433	5.162655	5.431432	4.509201	5.758523	5.521947	6.193198	6.121129	5.995662	4.404200	4.235322	3.734389	4.664293	4.568306	2.941020	4.119452	3.947935	3.389127	3.765302	3.715250	4.000224	5.272683	4.297673	6.234645	4.850771	5.449370	4.363515	5.440433	6.686786	5.437070	3.890045	4.584365	4.918304	4.899001	4.533330	4.605418	5.114561	5.233225	4.911992	4.539078	4.727241	5.806871	3.838432	6.727555	5.984697	4.580993	4.004778	3.729835	3.038227	4.735238	3.939428	4.303540	6.636899	3.610969	4.375472	4.062858	5.148725	5.777263	6.992420	3.645219	4.470334	4.563629	4.692213	4.924478	4.356325	4.267631	6.000081	3.523465	3.535381	3.902322	3.702441	4.035385	5.342778	4.456478	4.801357	4.454821	4.389553	3.444894	4.506329	3.400726	4.151044	7.060771	4.683975	5.166466	4.697895	4.477408	4.199237	4.596165	4.093293	4.298741	3.500685	3.812109	3.681816	3.931529	4.108917	4.440018	4.978266	3.755267	5.168681	5.473286	5.181177	5.512606	4.198610	4.116561	5.143789	5.398443	4.556230	4.356108	4.371120	5.086779	4.195624	5.347143	4.792890	5.552381	5.274097	4.045188	5.480462	5.221285	4.536749	4.219250	4.470128	4.278181	4.823549	5.164064	5.570508	5.926133	5.904025	6.107820	4.274873	4.206158	4.695359	4.067751	4.191700	3.673077	4.662255	3.870715	3.249584	3.625812	4.051282	3.726752	4.910501	4.444999	6.615919	5.319005	5.617970	4.018242	5.453034	6.197582	4.835195	4.307440	4.743795	4.877599	5.887073	5.512948	4.820845

TSPOAP1-AS1	12.194595	12.043801	11.773277	12.652080	12.903734	12.354202	12.385972	12.592652	12.456759	12.734044	12.795726	12.197990	12.875552	12.354202	12.011096	12.801613	12.635955	12.207137	13.045174	12.468921	12.920968	13.357004	11.284152	12.268379	12.653975	12.783624	11.294819	12.853682	12.133995	11.915341	13.113930	11.965086	11.880861	11.967332	12.497024	12.181582	12.707057	12.254206	12.896716	12.600363	12.629032	12.723310	12.083542	13.254784	11.919972	12.861151	12.076202	12.128415	12.413808	12.996930	12.650904	12.805449	12.313009	12.759968	11.718080	12.125545	11.685541	12.567752	12.587805	12.686750	12.849490	11.934741	8.215914	12.782775	12.317479	11.495210	12.684030	12.934891	12.622511	12.730151	12.252315	12.813513	12.669498	12.601063	12.694078	12.008851	12.513371	12.084810	12.376451	12.236505	12.163120	12.594923	12.295732	12.777426	12.624672	12.775154	12.826118	12.909649	12.623334	12.988128	11.913381	13.193477	12.643905	12.218342	12.302166	12.629207	12.405594	12.345161	12.568720	12.741631	12.017633	11.698277	12.340373	12.777426	13.431034	12.874685	13.100874	13.210340	12.821781	12.706040	13.614714	13.092940	13.167086	12.583137	12.165300	11.676245	12.435325	12.082273	12.435950	12.192751	12.210352	13.232466	12.215771	12.136334	11.938632	11.468535	13.645649	11.620399	11.641173	11.841598	12.464195	12.042551	12.752950	11.476974	11.912208	12.218206	12.847695	13.474856	13.680915	12.506071	13.739103	13.233234	13.422807	12.822669	7.331331	11.914759	11.865618	11.980765	12.030634	12.770845	11.508193	12.232669	11.466592	12.326361	12.971329	11.869930	11.189611	13.049903	12.491754	12.211598	12.694911	13.009202	10.085546	13.073797	12.838773	12.084512	12.820920	12.566593	12.704327	12.258961	11.795418	12.406661	12.619822	11.050179	12.936720	14.588535	13.243931	12.463227	12.773792	13.240382	11.320099	13.699890	12.641718	12.177372	10.957831	12.806823	12.456759	12.168975	10.810803	13.177598	12.185131	11.759040	11.594455	12.065618	12.065925	11.250318	12.298858	12.975277	11.953709	12.051190	12.396166	12.463883	11.973443	11.968566	12.354041	12.369546	12.442847	11.331034	11.535555	14.063237	12.296782	12.760481	12.504387	11.756948	11.975261	12.555263	11.918646	12.649190	12.599699	11.514083	14.401146	13.248301	11.186992	11.903261	11.795725	12.188415	12.033077	13.304537	11.604311	13.709428	12.508819	12.571167	12.305725	13.907695	12.059357	11.936694	11.669538	12.485326

LINC00958	8.671577	11.596704	11.680103	11.677730	12.010792	11.659265	11.731839	11.379601	11.018827	11.968868	11.560680	11.001656	11.432097	11.923703	11.231267	11.602409	10.760858	11.151555	10.931888	11.950952	10.954685	10.982910	8.363017	11.081305	11.624998	11.356537	9.245031	10.704472	10.613473	10.734015	11.676245	11.497575	11.408599	11.244028	11.603716	11.676108	11.135665	12.002055	11.459062	11.715142	11.138168	10.563844	11.328644	11.366980	10.308933	12.025727	10.111998	10.774607	11.872355	11.718698	11.341779	11.514083	11.971451	12.268515	11.730047	11.058092	11.670022	12.029925	11.660429	11.913078	11.537179	10.769710	7.903984	11.257393	12.100117	11.626908	11.734522	11.306805	11.876585	11.311166	11.422382	11.752750	11.213966	11.967950	12.024036	11.008883	11.962445	11.562171	11.734663	11.238299	12.102545	11.796745	11.661180	12.091837	12.156499	11.693032	12.217152	12.281610	11.147376	11.932006	11.731098	11.762513	11.709255	12.705163	10.863229	11.403244	11.958284	11.779120	11.514705	11.574347	11.557144	11.775216	11.175825	11.571786	12.218048	11.221771	10.791439	11.534802	11.404839	11.392925	12.346315	10.492203	12.220306	11.434353	12.338722	11.032989	8.588986	10.417364	11.076482	11.555370	13.248097	12.869775	11.955741	11.279887	11.522936	11.749227	11.198701	11.463911	12.421050	12.505236	10.180413	11.480510	11.919358	12.470874	11.472498	10.789138	10.124584	9.404986	12.162058	11.177032	10.773135	7.939461	10.946533	12.464858	12.421993	11.669088	12.706881	11.661642	12.034566	10.643878	12.277123	11.579070	12.786841	12.319355	10.939475	11.922470	12.277746	12.736077	11.982902	11.925502	12.307380	10.471030	13.030100	12.283174	10.947735	12.257562	9.981518	11.509511	12.712770	12.384483	11.764791	9.178480	12.561046	11.134465	8.656980	13.407160	12.356248	12.724498	12.304945	12.687779	11.600441	12.379442	12.063042	11.769600	12.343108	13.111562	12.011560	13.179374	9.866387	11.693488	11.582863	11.380384	12.293591	10.977161	12.954599	12.377877	12.616658	12.342613	11.035559	12.556686	12.860948	13.307926	12.690828	13.174582	11.914277	13.003067	14.036663	12.602572	12.163592	7.791129	12.946442	11.936861	12.288638	11.704750	12.377391	11.920396	11.986813	13.224930	12.549091	12.950183	11.313224	12.224476	11.551818	11.767668	10.296336	12.222955	11.358100	11.707159	12.148257	7.744116	10.559019	8.527214	10.621452	8.189568	12.535268	11.860241	12.300959	12.842271

BX890604.2	7.280541	6.846171	7.785744	6.402954	8.187744	6.704635	7.258890	6.716859	8.373268	7.940418	7.345331	8.380972	6.579129	7.933104	7.160111	6.649670	7.331485	7.650640	8.130869	7.200242	6.021895	6.027116	3.853359	6.898041	8.305140	7.188718	7.000485	5.441596	7.010741	7.426641	7.675124	7.225135	7.160111	8.014321	7.473850	7.966334	7.694590	7.001942	6.340875	7.138922	7.162692	7.082365	7.827891	7.408829	7.914144	7.887956	6.175417	7.429530	8.754431	8.052014	6.361202	7.495847	8.383274	7.696675	8.045582	7.629708	8.406370	8.156894	6.960356	7.742712	6.506005	8.241603	5.592169	6.774469	6.934730	7.469081	6.609496	7.551590	6.814751	6.657757	7.448876	7.051964	8.151117	7.415371	7.724697	7.042423	8.174583	7.171812	7.332577	7.635566	7.266176	7.639235	7.346731	7.487887	8.118721	7.899412	6.081971	7.906019	7.191904	6.461005	7.012518	6.594690	6.877345	7.234543	7.146904	7.938524	8.630690	9.231170	7.805396	6.729161	8.221769	7.275940	7.058536	7.574659	6.470882	7.917975	9.124740	6.645317	8.585291	6.629998	6.958469	7.719642	7.944014	7.413508	9.385879	6.926316	6.594381	7.417279	7.114581	7.688567	6.688707	7.951508	6.409686	8.120986	6.685988	6.939515	7.903197	7.307127	8.442687	7.125950	9.631529	6.769450	7.323456	6.379812	6.291515	7.715374	7.305069	7.383930	7.599840	5.299860	5.316256	6.403439	6.801084	8.131342	7.260304	6.603253	5.676369	6.655645	7.272830	7.126885	7.124824	6.702249	8.134813	7.237972	8.350354	7.649794	6.423741	6.497040	7.441605	6.339056	6.580967	7.811119	7.619552	8.622673	8.228550	5.709925	8.193845	8.924600	8.205533	6.011402	7.201627	8.517642	7.654215	8.079617	7.131491	8.881463	8.982606	6.847079	8.497483	6.084236	7.477534	7.240393	6.720650	7.358404	7.738827	6.585387	7.184736	7.024084	6.463884	7.482584	7.219352	8.234201	7.865090	7.346434	6.994003	8.312674	7.087205	7.910938	7.854059	7.964652	7.843121	7.680124	7.196624	8.707677	7.542332	5.598789	8.779006	7.449040	6.478155	7.542183	7.121194	6.471216	6.979716	7.447919	7.759279	8.775061	10.171699	7.433286	6.783714	7.917375	7.344115	6.768454	7.148656	7.133718	8.326035	9.715949	6.877654	7.976325	6.033822	6.448680	7.212644	7.018100	6.906870	9.368473	6.959558	7.170697	7.454307	6.721446

AC009139.1	4.705969	8.919067	6.484435	3.940865	5.320335	5.223707	4.824325	8.736729	3.264817	7.240235	5.403080	5.139018	6.135334	4.034749	4.550441	3.015552	3.413453	4.982161	5.046672	5.351486	6.262169	3.684424	6.456884	3.884501	3.660415	3.595732	5.156673	5.152764	5.792732	6.601017	4.409815	6.673975	7.881227	7.152007	5.917082	4.368705	6.021175	5.501007	6.550859	2.945757	5.590809	4.678389	6.328231	4.264426	3.905507	5.167290	6.495744	7.278927	4.893955	4.059534	6.647877	5.447613	7.724989	4.906492	6.277687	6.337219	8.862151	2.908776	6.260320	6.068594	5.323834	5.954371	9.369059	4.402892	7.052870	5.169309	2.830622	3.859999	6.313672	4.584365	5.965720	4.304427	5.821481	5.217960	4.446690	7.940884	5.319391	9.598799	6.860269	6.566408	6.834227	7.183306	6.136867	4.900919	4.947941	5.690981	7.153391	5.523836	7.551749	4.105442	6.080902	5.552010	7.451050	7.121335	5.673483	4.813536	4.514603	5.011599	4.465643	3.049958	8.602194	7.141116	4.556426	4.621238	4.016509	4.276612	6.482902	4.214482	3.230636	4.784747	3.237433	5.085724	4.395213	3.857435	4.144966	3.578380	4.448244	6.363201	4.503720	6.552682	6.185046	5.579830	5.590622	7.380012	4.740639	5.377137	14.073307	3.922224	6.203297	6.139271	8.771295	5.263694	3.884687	3.385360	6.443034	4.994379	9.822386	7.088975	10.727782	4.123822	6.111319	7.343018	7.298474	6.148479	7.465257	4.728936	3.079184	5.013631	6.417818	6.583920	8.140022	9.049275	5.401078	7.277818	6.112642	8.443325	8.021935	6.449821	6.289289	4.567032	7.600924	6.579309	5.321096	7.494871	6.247463	4.323583	8.915570	6.476576	6.939832	6.045528	7.400216	8.883738	6.442684	7.706091	8.128554	12.703807	6.607708	5.658509	6.978487	5.199677	6.673327	5.250586	6.007743	7.994267	8.513238	5.769010	5.464428	5.173357	5.752653	7.508140	7.017144	4.088735	6.162716	8.375886	8.967589	7.740395	6.431572	7.413210	7.272830	7.522082	6.957350	5.800809	7.465716	6.495585	5.965177	6.981481	7.707918	7.938385	7.571715	11.430746	4.620829	6.380781	6.323204	5.896175	5.917588	6.806475	8.073965	4.356325	6.350625	5.922559	12.784513	4.839385	7.268785	5.615376	5.272049	3.074230	4.915972	5.413577	4.602712	6.421107	5.079407	3.915505	4.538874	3.939637	4.109814	6.319104	5.411685	7.147857

LINC01927	5.279748	4.786821	5.296854	5.345157	4.704932	4.177040	3.993074	5.469134	5.065719	4.286131	4.471008	3.965491	3.549833	4.168179	4.336791	4.124421	4.751098	3.561768	5.189959	5.872991	4.865575	5.348703	7.061387	4.144750	4.434129	4.199654	4.329006	5.625588	4.339423	4.123175	5.036945	4.175304	4.856703	4.134067	4.436502	4.953347	4.065006	3.461815	4.312966	4.722166	4.696606	4.715794	4.666013	4.178097	4.194980	5.491117	4.761215	4.288978	5.114965	4.650703	5.431807	5.443514	4.582452	4.229863	4.489992	4.723874	4.407357	4.493308	5.779447	6.173182	4.725774	4.804112	4.983403	4.445874	4.277079	4.113339	4.445874	4.736757	5.051969	5.083842	4.804325	5.544882	4.239223	4.998379	5.341799	5.106448	5.752475	4.985191	5.877950	4.235322	3.880658	3.994643	4.542656	4.661386	4.631548	6.037539	3.690137	4.631548	5.195497	4.285215	4.272236	4.027084	4.264426	4.658123	4.527996	4.758520	4.624606	3.991131	4.510512	4.660271	3.652825	4.628485	4.773254	4.579040	4.735652	4.642591	5.731388	5.036534	5.190944	4.271174	4.559877	4.779250	5.514243	4.809096	3.836911	3.849721	5.260447	4.646834	4.423102	4.140390	4.368291	4.579696	4.574580	4.162932	4.391494	3.491512	5.679386	4.788062	4.028172	4.067119	3.235794	4.066903	3.501629	3.607467	3.603661	5.074842	3.155076	5.032354	7.707285	4.998379	5.475801	6.471216	3.972667	3.962928	3.906188	3.919345	3.796319	4.267396	4.936617	4.752349	3.418972	4.244526	5.033574	4.895790	3.615932	3.518728	4.508979	4.032337	4.158634	5.585889	5.069790	3.860245	4.506771	4.224260	5.099511	5.064275	3.945762	4.586707	4.875280	3.767157	3.989853	5.112773	4.389553	3.547999	3.528493	7.508617	4.439375	5.676951	5.759620	4.240349	3.300244	4.741289	5.177536	4.082186	3.879790	4.650918	4.588420	5.241057	4.867022	4.388470	4.644478	4.561427	4.427665	4.668615	4.776982	5.212584	5.286988	5.576665	3.748721	4.240135	4.612282	4.031703	3.790786	4.676014	5.295428	4.369566	7.043506	4.351778	4.601427	4.451832	4.258947	4.378241	4.589492	4.816027	4.529888	3.528493	3.224468	4.990394	3.502755	3.517751	4.787871	4.152302	3.780830	5.113751	4.382956	5.292942	4.800722	4.995208	4.901952	4.135625	4.348963	5.581146	4.367199	4.412213	3.732326	5.027033	4.338129	4.250189

LINC00683	5.279748	4.786821	5.296854	5.345157	4.704932	4.177040	3.993074	5.469134	5.065719	4.286131	4.471008	3.965491	3.549833	4.168179	4.336791	4.124421	4.751098	3.561768	5.189959	5.872991	4.865575	5.348703	7.061387	4.144750	4.434129	4.199654	4.329006	5.625588	4.339423	4.123175	5.036945	4.175304	4.856703	4.134067	4.436502	4.953347	4.065006	3.461815	4.312966	4.722166	4.696606	4.715794	4.666013	4.178097	4.194980	5.491117	4.761215	4.288978	5.114965	4.650703	5.431807	5.443514	4.582452	4.229863	4.489992	4.723874	4.407357	4.493308	5.779447	6.173182	4.725774	4.804112	4.983403	4.445874	4.277079	4.113339	4.445874	4.736757	5.051969	5.083842	4.804325	5.544882	4.239223	4.998379	5.341799	5.106448	5.752475	4.985191	5.877950	4.235322	3.880658	3.994643	4.542656	4.661386	4.631548	6.037539	3.690137	4.631548	5.195497	4.285215	4.272236	4.027084	4.264426	4.658123	4.527996	4.758520	4.624606	3.991131	4.510512	4.660271	3.652825	4.628485	4.773254	4.579040	4.735652	4.642591	5.731388	5.036534	5.190944	4.271174	4.559877	4.779250	5.514243	4.809096	3.836911	3.849721	5.260447	4.646834	4.423102	4.140390	4.368291	4.579696	4.574580	4.162932	4.391494	3.491512	5.679386	4.788062	4.028172	4.067119	3.235794	4.066903	3.501629	3.607467	3.603661	5.074842	3.155076	5.032354	7.707285	4.998379	5.475801	6.471216	3.972667	3.962928	3.906188	3.919345	3.796319	4.267396	4.936617	4.752349	3.418972	4.244526	5.033574	4.895790	3.615932	3.518728	4.508979	4.032337	4.158634	5.585889	5.069790	3.860245	4.506771	4.224260	5.099511	5.064275	3.945762	4.586707	4.875280	3.767157	3.989853	5.112773	4.389553	3.547999	3.528493	7.508617	4.439375	5.676951	5.759620	4.240349	3.300244	4.741289	5.177536	4.082186	3.879790	4.650918	4.588420	5.241057	4.867022	4.388470	4.644478	4.561427	4.427665	4.668615	4.776982	5.212584	5.286988	5.576665	3.748721	4.240135	4.612282	4.031703	3.790786	4.676014	5.295428	4.369566	7.043506	4.351778	4.601427	4.451832	4.258947	4.378241	4.589492	4.816027	4.529888	3.528493	3.224468	4.990394	3.502755	3.517751	4.787871	4.152302	3.780830	5.113751	4.382956	5.292942	4.800722	4.995208	4.901952	4.135625	4.348963	5.581146	4.367199	4.412213	3.732326	5.027033	4.338129	4.250189

AL606534.3	4.844447	4.461559	4.184247	6.759287	8.330575	8.919658	5.429745	8.288418	8.813954	4.747061	5.569403	6.773975	5.822952	5.677332	5.201083	4.436278	6.316233	5.421300	5.994834	4.783483	6.563191	5.677332	7.819040	4.749752	4.998160	6.165458	4.572211	5.601521	5.580041	6.995225	5.642536	6.131041	6.945345	6.522990	4.986843	7.030189	6.411029	5.088591	4.999641	5.837310	5.070180	3.481349	4.332454	5.841877	5.515838	5.278530	8.440271	5.921041	7.830635	4.804715	4.755113	5.835534	4.083676	6.935051	5.531616	5.863467	4.716878	3.394523	5.076102	5.781628	5.183621	5.113938	4.315840	5.972886	5.322671	5.366014	5.092686	5.316075	4.868967	4.532908	6.308756	6.920987	4.499842	5.825489	5.320912	5.431227	7.101115	6.587371	5.904380	6.660520	6.858189	5.117619	5.545245	5.805110	6.591326	6.509917	9.711248	6.892517	5.211243	5.370515	5.660863	5.665927	4.932831	6.313497	7.070379	6.665571	6.448680	6.662960	8.065034	8.373123	10.355524	5.680106	5.829913	5.858684	6.240272	7.613266	5.039903	6.258431	8.372533	5.392969	8.473729	8.194144	8.584534	6.257931	6.318421	13.489728	7.221773	6.719302	5.608330	6.187067	4.290904	6.301676	5.283182	10.262533	7.125453	7.747952	5.265665	7.281951	6.386980	5.987381	7.259651	8.482967	7.814030	6.987783	8.000720	6.986518	7.540513	4.203616	5.068410	7.692592	7.749822	6.410859	7.730715	8.260173	6.786940	9.596007	7.823335	9.460449	4.502191	6.368195	7.772016	8.236375	8.567616	6.308756	6.659868	7.313210	6.655174	5.235840	7.265404	7.370309	3.448178	5.008283	5.876401	6.225900	4.811675	7.535484	6.276203	6.905093	6.715371	4.549075	6.077446	5.094394	7.057930	7.910344	6.540336	8.086143	2.978889	6.503995	5.313196	6.536782	5.767752	3.796939	5.217771	4.843806	6.242601	5.000281	5.206824	4.722578	3.974446	6.551642	6.796677	4.008468	6.915103	7.604046	5.378859	7.050856	6.811443	6.226399	8.219328	4.873845	5.096409	7.010571	8.180074	7.139694	9.140861	7.568409	4.925505	6.124083	6.174035	4.631116	5.763225	5.232814	5.188014	6.472740	7.014074	8.210515	8.957785	7.706992	5.397646	9.110639	8.172546	7.195686	8.955481	6.761889	6.582607	5.969157	5.520641	6.338043	5.990072	8.996119	9.900438	11.159497	7.283526	8.093260	9.648868	6.360873	6.643535	5.251390

TRAF3IP2-AS1	13.191043	12.417829	12.558955	12.863311	12.674839	12.565285	12.724498	13.112985	12.922594	12.958452	12.585646	12.537757	12.718193	12.937994	13.051023	12.847364	12.807870	12.785598	13.025652	12.878010	12.486329	13.761667	11.802240	12.507380	12.545714	12.764108	11.820064	12.942292	12.161570	12.239319	12.858461	12.967107	13.001582	12.741823	12.730151	12.798338	12.710992	12.776543	13.378878	13.348898	12.472784	13.458952	13.086878	12.774646	12.504387	12.731681	12.460607	13.271981	12.845626	13.136216	13.054008	13.039489	12.909315	12.769062	13.161296	12.855512	12.847695	13.650975	13.451174	13.633991	13.479517	12.452025	12.879237	13.502602	13.230666	13.263573	13.086086	13.093911	12.708762	13.377337	12.620010	12.579838	12.505754	12.624672	12.750339	12.619661	12.832199	13.008849	12.716063	12.822839	12.563000	12.557817	12.876415	12.993251	12.664096	12.795036	12.872130	13.281038	13.023845	13.266591	13.015726	13.208477	12.958452	12.910006	12.698531	12.743377	12.710821	12.580335	12.889319	12.945707	12.794317	12.665284	12.658166	13.004678	13.255784	12.681634	13.367062	13.593974	12.467788	12.581823	12.542438	13.359500	13.390336	12.488616	12.956811	11.985558	13.488586	12.704678	12.549408	11.697997	12.363568	12.351789	11.642694	12.392060	12.100873	13.124799	12.660869	13.580404	12.214434	13.220585	12.556362	12.723310	12.980619	13.040804	12.067345	12.964557	12.231416	12.606059	13.286686	12.194595	13.303685	11.755885	12.885092	13.539286	12.812654	12.960501	12.650060	12.333192	12.419287	12.565127	13.173253	13.468126	13.763467	12.332724	12.366707	13.287464	13.014650	13.040624	13.574462	12.695090	13.101611	12.907514	13.731149	13.697560	12.519877	12.833655	12.901614	12.745055	13.138899	13.362235	12.384984	13.225108	13.832809	13.098245	11.765403	13.887810	12.976403	13.134359	13.610375	14.021288	12.380673	13.913715	14.107553	12.945001	12.608516	13.167086	13.153220	13.587916	13.528551	12.465995	12.685364	12.956811	13.153629	12.348967	12.623334	12.949621	12.919349	12.881322	12.718690	12.672419	12.682475	12.902509	12.594107	12.685861	12.702451	13.132807	12.606221	13.145143	13.048184	11.468830	13.180932	12.793936	13.241017	13.004678	12.691524	13.157459	12.033841	12.316396	12.333192	12.602739	13.654386	12.618117	12.650533	13.038580	12.018090	13.689737	12.929922	13.052245	13.177797	12.694911	13.555053	11.676671	12.469069	11.728547	13.509723	13.034495	12.400465	12.624672

AL445989.2	9.490307	9.760421	9.788977	10.130482	10.415855	11.077806	9.992195	11.065268	10.919103	10.951940	10.764037	10.379863	10.571684	10.225000	9.402461	10.578284	10.387783	9.782680	10.922234	11.136838	10.309103	11.229610	10.477695	9.820277	9.812137	10.022905	10.967556	10.196749	10.279430	9.731833	11.028666	10.266592	10.046469	10.162874	10.072356	10.170273	10.059401	9.973866	10.129611	10.206592	10.716117	9.997954	10.055756	9.869780	9.598639	9.930780	10.370786	9.711867	10.534405	10.410721	10.058151	10.417364	10.163835	10.425603	10.075275	10.707919	10.153274	10.319973	10.593179	9.866086	10.130189	10.358566	8.731651	10.609923	9.863922	9.886092	10.318172	10.050017	10.076815	10.351116	10.220494	10.612891	10.906775	10.306115	10.736390	10.168271	10.243835	10.038692	10.105829	10.089927	9.454926	10.131092	9.750979	10.690215	10.280189	9.956772	10.094118	10.104182	9.974195	10.204906	10.070853	10.200381	10.336292	9.889689	10.203849	10.379105	10.311711	10.673881	10.795970	10.488568	10.673148	9.947027	10.145201	10.005677	10.707919	10.911171	10.957378	10.590962	10.486410	10.437361	10.700309	10.898956	10.362972	11.034313	10.579044	11.526932	9.656714	10.241513	11.214512	10.405472	9.481820	8.544600	10.776525	11.094441	10.136690	9.739008	11.787474	10.682266	9.974049	10.456314	10.245152	10.308483	8.264623	10.231595	10.157277	11.004248	10.190858	11.945767	10.207204	10.090978	10.570126	9.921992	10.169963	8.401741	8.540524	10.685541	11.515926	10.835875	9.254659	10.867134	10.090524	10.084209	9.556781	9.017157	8.938096	9.278587	8.887600	9.603163	9.973693	9.982906	9.424072	10.035161	9.172647	9.565121	9.294591	10.286370	8.709631	9.780402	9.024914	9.063225	9.946708	9.300643	9.791757	9.392213	9.169074	10.488104	9.591500	10.041156	9.161680	9.312969	9.875925	10.010197	9.673171	8.863054	8.732552	10.011809	9.670545	9.150322	9.166801	9.507629	9.982756	10.705507	9.739631	9.924583	9.673010	9.306026	9.028257	9.625683	8.626257	8.952716	9.553448	8.736877	9.069131	9.139811	9.747864	9.280452	9.944110	8.797944	8.685097	11.475722	8.751131	8.777808	9.180169	9.308341	10.488419	10.500314	10.987791	10.303411	9.404197	9.941815	10.145498	11.002938	8.937504	8.446387	10.398977	10.032116	10.852403	9.414552	9.231906	9.204423	10.095615	9.928630	11.402937	9.651972	10.040543	9.346191	9.073896	9.850414

ADD3-AS1	3.124906	4.471008	5.617409	4.507461	4.773888	5.066954	4.473189	6.175920	5.948816	6.252335	4.650040	4.167541	3.897142	4.154789	3.203743	5.305404	5.720120	3.539584	5.379059	4.984987	3.994643	5.184212	7.018100	4.407357	4.990800	4.794982	5.001920	5.312428	4.896218	4.444781	4.141719	3.528493	3.520622	3.952698	5.424256	5.017318	4.611221	4.027500	4.804325	4.657286	4.585408	4.879622	5.082862	5.115354	4.961642	4.705969	5.493884	3.254330	5.586787	5.554537	5.082049	5.858872	5.104607	5.462398	4.526520	4.984172	4.302412	3.220989	4.655105	4.536948	5.519715	4.946727	3.627611	5.301434	4.512448	5.225301	5.213361	5.334554	5.277165	6.227479	4.832709	5.106852	5.233026	4.990394	4.596619	4.938279	5.593652	4.551957	4.951026	4.431388	5.834830	4.692658	4.436050	3.957709	4.772189	5.887268	4.572417	4.322056	3.721910	4.602914	4.143455	4.972542	4.815165	5.322671	4.442195	4.717502	4.195419	5.202453	4.283174	4.490913	4.430930	4.850577	4.426343	3.925247	5.268846	5.192286	5.882462	4.393599	5.059192	5.271843	5.863088	5.416410	5.048505	5.368578	5.558298	4.816230	4.951665	4.589696	4.875476	3.335003	3.306340	4.171454	3.880460	4.619695	4.951226	3.960098	4.135395	4.204289	5.765908	4.276612	3.795711	4.161376	4.087434	3.995744	4.280176	3.124150	4.541747	4.043878	4.300245	3.536093	4.488050	6.106910	5.499857	3.865968	4.278859	4.463060	4.679671	4.254130	4.354817	3.396622	4.114601	4.290685	3.800090	4.761419	3.556578	3.847173	3.980200	4.198394	4.424419	4.216015	4.416195	4.001044	5.050399	4.121617	4.465643	5.350251	3.066396	4.961870	3.875056	3.465216	4.295457	5.061015	4.336791	4.368291	4.460477	3.707302	3.206265	3.069717	4.360707	3.100427	4.822497	3.925898	3.912495	4.238326	4.186196	3.285202	3.855323	4.386748	3.364854	4.105841	4.074943	5.063052	4.156076	3.554492	4.370684	4.208643	4.468417	4.128728	3.769711	5.696938	4.199007	3.463925	3.765884	3.496914	4.367199	3.990254	3.657453	3.486039	3.867287	3.772832	3.981754	4.181446	4.092696	3.834416	4.236984	4.359819	4.648115	3.870092	3.566286	4.249931	4.430265	4.240767	3.916834	4.828464	4.535677	4.842988	4.458238	4.160365	5.326937	5.740431	5.591589	4.238752	5.478242	4.614203	4.407357	4.035158	3.756928	4.216443

LINC00943	4.774707	4.396141	3.642453	5.860683	4.256960	4.867689	4.559877	4.548399	4.276612	5.026469	3.605046	3.537245	4.912191	4.292475	3.862994	5.453987	3.618187	5.910459	5.853247	5.709925	5.327510	6.101704	7.154192	5.650101	5.412057	4.003237	6.839827	3.411654	4.751901	5.429568	4.803256	5.212390	6.751276	7.223587	5.744553	5.322671	4.681159	4.498338	4.656839	6.047817	3.759641	5.458276	6.809524	6.340875	5.026050	5.858872	5.753756	5.729540	7.293816	5.325747	5.013840	5.335131	5.947585	5.123104	5.312218	4.922338	4.337007	5.681420	6.827484	4.368486	4.677106	3.834204	4.775942	5.077771	5.278342	4.464384	4.665824	5.196906	3.792048	5.356752	4.159518	4.381597	4.835606	6.127825	4.867232	6.054258	4.683330	4.405609	5.187816	5.906683	5.505443	4.623340	5.900068	5.307898	5.144161	5.059416	6.118370	5.261653	5.150726	4.174224	4.475809	5.443899	4.314773	5.334186	6.363905	5.678637	3.979593	5.090008	4.602311	5.285197	4.732510	5.442939	4.585408	5.268266	4.890438	4.957517	5.317463	5.886377	4.543088	4.039720	5.576296	4.969692	3.605218	4.807861	4.586903	5.794762	4.949812	3.939637	4.851376	4.854590	4.483961	4.423983	6.064868	4.730610	4.318222	5.308477	6.525640	6.237788	4.613162	4.626343	3.166091	3.462669	6.406553	5.024458	7.197753	5.459635	3.248272	7.093674	6.084925	5.122893	4.540902	5.676185	4.104536	4.266350	8.172379	4.168403	3.374551	7.020142	4.805763	6.010539	5.973236	5.943205	4.506771	5.854561	4.223852	5.580041	4.883281	5.118253	4.505232	5.454178	4.635852	6.327073	5.107866	4.965626	5.386549	5.937761	2.848170	6.810338	4.374125	5.068188	6.058707	5.880149	5.467532	5.393349	4.638012	4.349824	5.759442	6.867405	4.938930	4.984377	3.685044	5.460841	5.580595	5.900980	4.867902	6.183618	4.816027	3.754459	4.916624	4.558102	4.428669	4.614899	4.790369	5.671418	5.153174	4.906273	4.556647	3.823524	5.875319	6.122011	4.762713	6.095497	6.713250	4.750654	4.628037	5.633578	5.632657	5.644822	5.438019	3.917040	5.357029	5.782921	6.930178	5.362720	5.438752	3.913138	4.582030	6.093560	4.162019	6.468027	4.354159	3.997047	5.942682	7.772173	4.350467	4.845739	4.497496	4.729562	4.259179	5.485008	5.838236	5.948086	5.176324	4.376736	4.850976	3.816963	4.204520	5.638080

LINC00944	4.774707	4.396141	3.642453	5.860683	4.256960	4.867689	4.559877	4.548399	4.276612	5.026469	3.605046	3.537245	4.912191	4.292475	3.862994	5.453987	3.618187	5.910459	5.853247	5.709925	5.327510	6.101704	7.154192	5.650101	5.412057	4.003237	6.839827	3.411654	4.751901	5.429568	4.803256	5.212390	6.751276	7.223587	5.744553	5.322671	4.681159	4.498338	4.656839	6.047817	3.759641	5.458276	6.809524	6.340875	5.026050	5.858872	5.753756	5.729540	7.293816	5.325747	5.013840	5.335131	5.947585	5.123104	5.312218	4.922338	4.337007	5.681420	6.827484	4.368486	4.677106	3.834204	4.775942	5.077771	5.278342	4.464384	4.665824	5.196906	3.792048	5.356752	4.159518	4.381597	4.835606	6.127825	4.867232	6.054258	4.683330	4.405609	5.187816	5.906683	5.505443	4.623340	5.900068	5.307898	5.144161	5.059416	6.118370	5.261653	5.150726	4.174224	4.475809	5.443899	4.314773	5.334186	6.363905	5.678637	3.979593	5.090008	4.602311	5.285197	4.732510	5.442939	4.585408	5.268266	4.890438	4.957517	5.317463	5.886377	4.543088	4.039720	5.576296	4.969692	3.605218	4.807861	4.586903	5.794762	4.949812	3.939637	4.851376	4.854590	4.483961	4.423983	6.064868	4.730610	4.318222	5.308477	6.525640	6.237788	4.613162	4.626343	3.166091	3.462669	6.406553	5.024458	7.197753	5.459635	3.248272	7.093674	6.084925	5.122893	4.540902	5.676185	4.104536	4.266350	8.172379	4.168403	3.374551	7.020142	4.805763	6.010539	5.973236	5.943205	4.506771	5.854561	4.223852	5.580041	4.883281	5.118253	4.505232	5.454178	4.635852	6.327073	5.107866	4.965626	5.386549	5.937761	2.848170	6.810338	4.374125	5.068188	6.058707	5.880149	5.467532	5.393349	4.638012	4.349824	5.759442	6.867405	4.938930	4.984377	3.685044	5.460841	5.580595	5.900980	4.867902	6.183618	4.816027	3.754459	4.916624	4.558102	4.428669	4.614899	4.790369	5.671418	5.153174	4.906273	4.556647	3.823524	5.875319	6.122011	4.762713	6.095497	6.713250	4.750654	4.628037	5.633578	5.632657	5.644822	5.438019	3.917040	5.357029	5.782921	6.930178	5.362720	5.438752	3.913138	4.582030	6.093560	4.162019	6.468027	4.354159	3.997047	5.942682	7.772173	4.350467	4.845739	4.497496	4.729562	4.259179	5.485008	5.838236	5.948086	5.176324	4.376736	4.850976	3.816963	4.204520	5.638080

AL031686.1	11.267197	9.824324	10.347954	10.072505	10.150520	11.426795	11.176414	11.675026	10.450445	11.522263	11.787766	11.425803	10.582604	10.704617	9.733183	11.197968	10.817007	10.100633	9.306478	11.253242	9.203996	9.994790	8.964783	9.924891	9.564266	10.106724	10.295699	10.766711	10.519091	11.071366	10.825019	10.814754	10.325669	11.125919	11.337014	11.456254	11.503353	10.816868	10.667558	10.750836	11.074426	10.602963	10.356625	10.395504	10.440967	10.463100	11.215233	10.008945	10.740051	10.828572	10.645044	10.747396	10.265433	10.758389	10.405917	11.093843	10.378655	10.221095	10.286654	10.247848	10.647119	10.980857	9.599094	10.138059	10.232190	9.795801	10.253361	9.721843	10.134688	9.992195	11.016496	10.979805	11.342184	10.869744	11.069879	10.976864	11.170765	10.908996	11.114554	10.078682	9.434921	9.706564	9.908340	10.377424	10.328856	10.761899	10.368062	10.621738	9.860973	9.985333	9.885790	10.074213	9.881018	10.845325	11.030306	11.074704	10.498125	10.704328	11.402792	9.945742	11.161727	10.462807	10.045094	10.438848	10.223316	11.181427	10.795970	10.231014	8.859422	11.117105	9.265364	11.111588	10.523247	11.046498	11.028512	9.837342	10.162738	10.713869	10.800888	11.232423	9.967259	10.479505	10.433811	9.863484	11.282256	11.219673	9.814640	10.753860	11.073555	10.601209	11.379904	10.971457	10.071306	9.996472	10.132299	10.351898	10.175625	8.755477	10.899971	9.911272	10.287574	9.468552	10.091582	10.604891	9.828423	11.135202	10.957536	11.788671	11.743759	10.916587	9.926476	9.070196	11.457713	11.063641	11.437102	10.958111	10.854786	10.364435	10.254638	10.197070	10.933966	10.371545	10.828144	10.737617	10.757526	11.370520	9.640574	11.519395	10.709812	11.076330	10.962078	11.022131	11.082627	10.392057	11.873552	10.554299	10.756330	10.427919	10.344466	10.335092	10.853742	10.393702	10.343395	10.159975	9.837182	9.674273	9.756219	10.417533	10.201258	11.526788	11.447213	10.327183	10.880917	10.866362	10.975823	11.045293	10.983350	11.080441	9.558784	9.438669	10.575116	10.158173	9.715343	10.209812	10.726410	10.428065	9.687291	9.635073	9.102892	9.298143	9.330903	9.284449	11.001938	10.525491	10.181458	10.921515	10.007217	11.122655	11.282105	11.123360	9.566691	9.964730	10.290520	10.030393	10.914172	10.299712	9.260456	9.568588	11.378286	9.118833	10.619400	10.360365	10.229726	11.410252	10.052342	11.472792	11.058092	10.569204

AL590440.1	10.188009	9.985333	10.443193	10.226509	9.508226	9.598341	11.082337	9.929076	8.808720	10.148232	10.779119	11.089762	10.339163	11.095709	10.574965	10.424711	11.055538	10.650874	9.090933	10.471779	9.601111	8.018603	9.908039	10.302178	11.675026	11.396845	9.491411	11.141336	11.166523	9.989516	10.209666	10.528219	10.563986	9.771440	11.133549	10.888350	11.185858	11.367280	10.943468	10.251447	9.785948	10.958864	10.697783	10.677766	9.786827	10.852247	9.781184	11.072707	10.629019	10.794089	10.697351	10.910445	10.979805	10.609210	11.250783	11.296440	11.605367	11.782962	11.880130	11.471518	12.397227	10.061953	10.699115	10.429581	9.860973	11.913683	10.684823	10.128078	10.142906	10.295699	9.937098	10.376671	10.407254	10.573635	10.019714	10.041915	10.523247	10.850917	10.560567	11.040450	9.811172	11.228875	11.183899	11.278409	11.436814	11.070472	10.869324	11.303088	10.856734	11.017339	10.882649	9.993095	10.316379	10.760858	10.601523	10.925790	11.102534	9.956906	11.223409	10.850468	10.296801	11.311317	9.968029	10.204906	9.054152	10.449177	11.247077	10.377715	9.726215	10.236667	9.218710	10.525043	10.722674	9.701109	9.528337	10.236049	10.941833	11.158629	9.802880	7.668974	8.164723	8.613447	7.868330	8.237926	7.330232	8.084751	6.521703	8.156894	7.517109	8.890238	8.167657	9.187379	10.357076	7.831235	8.429123	9.216553	7.276417	8.494761	8.166131	8.435693	7.461972	8.425591	7.031545	9.278447	7.225445	8.633518	7.425554	8.342015	10.011809	7.726838	9.718286	7.966042	10.248333	10.583331	9.372726	9.057026	8.666965	8.732710	7.060771	7.694121	8.367279	7.612498	9.429598	8.049851	9.175115	8.953479	8.995798	9.700800	7.171971	8.247810	8.587096	10.126478	9.253123	9.521928	7.939154	8.412310	11.696817	10.743665	9.067710	10.204762	9.294119	9.006263	8.877700	8.791835	10.300344	8.448899	8.312531	6.967400	10.989737	8.400184	7.446975	9.452150	8.353209	8.961134	8.575963	9.203412	8.717201	9.619319	10.992097	6.853497	10.358877	9.299232	8.573979	7.563186	8.262447	10.495573	8.431198	9.696933	9.371510	11.158629	7.672436	8.129662	7.928511	8.487060	8.930315	9.750518	7.550222	8.910988	10.402145	8.038673	8.926766	8.340263	7.645097	8.460937	8.627031	6.996619	8.584972	8.154862	8.356589	7.166559	8.995047	7.200694	8.744057	8.635856	8.998577	6.352776	9.651348	7.934793

AC006115.2	15.796320	12.874491	14.251813	13.487496	13.833047	14.096118	14.989019	13.321286	14.302959	14.788691	13.637384	14.674118	14.378131	12.731001	13.307488	14.670049	15.116030	14.277280	10.727937	14.764819	10.290216	10.992855	12.580656	13.715603	14.197180	14.940922	15.762943	15.625168	15.350928	14.501441	15.104927	14.309634	10.862314	14.464777	15.502234	14.651534	14.143339	13.134732	14.107221	13.596087	15.377722	14.528293	15.489513	15.480428	16.013271	16.044226	15.529882	13.790706	14.867085	14.318185	14.070098	14.658149	15.524473	12.502721	14.280559	14.426320	15.988198	12.506387	12.474915	12.352576	13.266411	16.245035	13.281221	11.099443	11.353312	11.885850	12.713284	11.125198	10.789859	12.194431	15.982133	13.904457	15.090679	14.573899	15.527160	13.768548	13.696837	15.095132	13.165282	12.673786	12.995633	16.092758	13.635468	12.800565	14.313722	15.157558	14.451322	15.435694	12.383858	11.506409	12.135587	11.852005	11.816438	13.067904	13.040624	14.118926	13.559500	14.543837	15.047189	15.306516	14.546394	15.230680	14.964550	14.738102	10.314618	14.670049	14.010352	10.844098	12.347575	15.029282	11.573596	11.933343	12.248492	14.958920	14.551021	14.808146	14.770475	15.265827	15.826441	14.477842	12.239925	15.165299	14.293075	15.899823	14.845201	15.135878	16.298008	15.153835	14.475985	15.697564	14.775142	15.247395	14.557264	15.384452	15.357170	15.087640	14.433465	11.419997	13.313356	11.227828	11.895030	12.327002	14.164900	13.761927	13.933244	15.003643	14.257035	15.571867	14.713469	16.253204	15.801695	15.041784	15.979533	15.239718	15.950994	16.751734	17.434795	13.779642	13.211006	15.064607	13.761156	16.174278	16.292480	16.377052	14.806121	15.213664	16.819885	14.764819	16.233280	16.202822	15.729053	15.205421	16.051180	15.676954	15.370867	15.511748	14.012342	12.871579	14.135776	14.459642	14.981620	10.189657	11.368860	13.449931	13.855680	12.896716	14.202755	13.459166	12.328915	15.013920	16.543736	14.553963	16.210808	15.414655	15.593684	15.826441	15.068646	15.919342	15.573696	15.758639	14.573111	15.416204	16.648016	16.720589	14.900158	15.502988	15.434895	12.957891	13.282220	13.984682	12.259261	15.795114	15.785756	16.052430	15.271483	16.226917	16.907711	14.701092	14.768542	16.199785	14.300251	15.491291	15.031025	11.187573	16.769750	12.556686	10.310511	10.981607	14.576434	12.202831	14.298038	13.327985	14.581385	14.715375	15.074047	14.824917	16.134222	14.164594

ZIM2-AS1	15.796320	12.874491	14.251813	13.487496	13.833047	14.096118	14.989019	13.321286	14.302959	14.788691	13.637384	14.674118	14.378131	12.731001	13.307488	14.670049	15.116030	14.277280	10.727937	14.764819	10.290216	10.992855	12.580656	13.715603	14.197180	14.940922	15.762943	15.625168	15.350928	14.501441	15.104927	14.309634	10.862314	14.464777	15.502234	14.651534	14.143339	13.134732	14.107221	13.596087	15.377722	14.528293	15.489513	15.480428	16.013271	16.044226	15.529882	13.790706	14.867085	14.318185	14.070098	14.658149	15.524473	12.502721	14.280559	14.426320	15.988198	12.506387	12.474915	12.352576	13.266411	16.245035	13.281221	11.099443	11.353312	11.885850	12.713284	11.125198	10.789859	12.194431	15.982133	13.904457	15.090679	14.573899	15.527160	13.768548	13.696837	15.095132	13.165282	12.673786	12.995633	16.092758	13.635468	12.800565	14.313722	15.157558	14.451322	15.435694	12.383858	11.506409	12.135587	11.852005	11.816438	13.067904	13.040624	14.118926	13.559500	14.543837	15.047189	15.306516	14.546394	15.230680	14.964550	14.738102	10.314618	14.670049	14.010352	10.844098	12.347575	15.029282	11.573596	11.933343	12.248492	14.958920	14.551021	14.808146	14.770475	15.265827	15.826441	14.477842	12.239925	15.165299	14.293075	15.899823	14.845201	15.135878	16.298008	15.153835	14.475985	15.697564	14.775142	15.247395	14.557264	15.384452	15.357170	15.087640	14.433465	11.419997	13.313356	11.227828	11.895030	12.327002	14.164900	13.761927	13.933244	15.003643	14.257035	15.571867	14.713469	16.253204	15.801695	15.041784	15.979533	15.239718	15.950994	16.751734	17.434795	13.779642	13.211006	15.064607	13.761156	16.174278	16.292480	16.377052	14.806121	15.213664	16.819885	14.764819	16.233280	16.202822	15.729053	15.205421	16.051180	15.676954	15.370867	15.511748	14.012342	12.871579	14.135776	14.459642	14.981620	10.189657	11.368860	13.449931	13.855680	12.896716	14.202755	13.459166	12.328915	15.013920	16.543736	14.553963	16.210808	15.414655	15.593684	15.826441	15.068646	15.919342	15.573696	15.758639	14.573111	15.416204	16.648016	16.720589	14.900158	15.502988	15.434895	12.957891	13.282220	13.984682	12.259261	15.795114	15.785756	16.052430	15.271483	16.226917	16.907711	14.701092	14.768542	16.199785	14.300251	15.491291	15.031025	11.187573	16.769750	12.556686	10.310511	10.981607	14.576434	12.202831	14.298038	13.327985	14.581385	14.715375	15.074047	14.824917	16.134222	14.164594

AC119674.1	8.397483	8.582845	8.391617	8.357368	7.636038	9.364092	9.580724	9.297703	8.341166	8.988657	8.862905	8.803515	8.757154	8.934093	8.162515	8.962369	8.590359	8.180074	9.134862	8.773214	9.027842	8.767294	8.516237	8.068471	8.548924	8.366808	7.736364	9.174045	9.050843	8.819040	8.269199	8.583923	8.166286	7.525428	7.827764	8.104330	8.038201	8.460937	8.792758	8.483714	9.562068	8.829886	7.725594	8.384656	7.605162	7.859428	8.895157	8.943236	8.717495	8.380503	8.199678	7.716427	7.995659	8.127337	8.671736	8.621889	7.627192	8.314984	8.509971	7.737275	8.419610	9.139186	9.015152	8.698199	8.231164	8.442240	8.822360	8.301065	8.233424	8.574419	8.768535	8.707834	9.349567	9.010403	8.402217	8.343367	8.484449	7.956200	8.089401	7.721779	7.190316	7.685180	7.613107	7.990179	8.423466	8.239020	7.532374	8.310809	8.206933	8.167229	7.747180	8.301521	7.926641	8.088613	7.760531	7.773840	7.608834	7.344115	7.517109	7.714645	7.713903	8.176328	8.006397	8.272834	8.659306	9.506715	9.430349	8.567027	8.629901	9.162916	8.622365	8.825708	8.429897	9.120357	8.578471	7.472022	8.008230	7.980345	8.324630	7.935444	8.442390	8.788881	7.864627	7.720828	9.606243	7.874736	7.559214	7.674175	9.396057	8.763929	8.608181	8.787683	7.390104	8.338225	7.833074	8.414300	7.514932	8.524156	7.772173	8.320945	7.823834	8.979397	7.282257	8.219103	8.486274	8.844814	8.102344	7.705019	8.910236	7.688871	7.731332	8.319278	8.019671	8.035245	7.220497	7.700687	8.797307	8.722507	8.524156	8.311294	8.361723	7.348015	8.141961	7.784088	8.064117	8.281286	8.564352	8.551647	8.324142	8.716113	9.062460	7.569307	8.242770	8.425755	8.354136	7.595624	8.120836	8.365788	7.856494	8.209048	8.357526	8.935314	8.410617	8.221444	8.485373	8.901108	8.110900	8.103403	8.346622	8.321873	8.134813	8.352148	8.754719	7.570213	8.163242	8.101434	8.482967	8.287945	7.135309	8.119607	7.858326	7.667444	7.535627	8.570349	8.477574	7.585638	7.835968	8.086143	7.632540	7.433914	8.212373	7.723319	8.577737	8.116384	7.363410	10.754173	6.116491	8.314539	7.971938	7.358404	7.589045	7.454005	7.776496	7.793259	7.742387	8.317610	8.355223	8.347074	7.741489	8.167506	8.383748	8.188467	8.798246	8.590817	7.944014	8.369704	8.805667	8.387588

LINC01767	8.397483	8.582845	8.391617	8.357368	7.636038	9.364092	9.580724	9.297703	8.341166	8.988657	8.862905	8.803515	8.757154	8.934093	8.162515	8.962369	8.590359	8.180074	9.134862	8.773214	9.027842	8.767294	8.516237	8.068471	8.548924	8.366808	7.736364	9.174045	9.050843	8.819040	8.269199	8.583923	8.166286	7.525428	7.827764	8.104330	8.038201	8.460937	8.792758	8.483714	9.562068	8.829886	7.725594	8.384656	7.605162	7.859428	8.895157	8.943236	8.717495	8.380503	8.199678	7.716427	7.995659	8.127337	8.671736	8.621889	7.627192	8.314984	8.509971	7.737275	8.419610	9.139186	9.015152	8.698199	8.231164	8.442240	8.822360	8.301065	8.233424	8.574419	8.768535	8.707834	9.349567	9.010403	8.402217	8.343367	8.484449	7.956200	8.089401	7.721779	7.190316	7.685180	7.613107	7.990179	8.423466	8.239020	7.532374	8.310809	8.206933	8.167229	7.747180	8.301521	7.926641	8.088613	7.760531	7.773840	7.608834	7.344115	7.517109	7.714645	7.713903	8.176328	8.006397	8.272834	8.659306	9.506715	9.430349	8.567027	8.629901	9.162916	8.622365	8.825708	8.429897	9.120357	8.578471	7.472022	8.008230	7.980345	8.324630	7.935444	8.442390	8.788881	7.864627	7.720828	9.606243	7.874736	7.559214	7.674175	9.396057	8.763929	8.608181	8.787683	7.390104	8.338225	7.833074	8.414300	7.514932	8.524156	7.772173	8.320945	7.823834	8.979397	7.282257	8.219103	8.486274	8.844814	8.102344	7.705019	8.910236	7.688871	7.731332	8.319278	8.019671	8.035245	7.220497	7.700687	8.797307	8.722507	8.524156	8.311294	8.361723	7.348015	8.141961	7.784088	8.064117	8.281286	8.564352	8.551647	8.324142	8.716113	9.062460	7.569307	8.242770	8.425755	8.354136	7.595624	8.120836	8.365788	7.856494	8.209048	8.357526	8.935314	8.410617	8.221444	8.485373	8.901108	8.110900	8.103403	8.346622	8.321873	8.134813	8.352148	8.754719	7.570213	8.163242	8.101434	8.482967	8.287945	7.135309	8.119607	7.858326	7.667444	7.535627	8.570349	8.477574	7.585638	7.835968	8.086143	7.632540	7.433914	8.212373	7.723319	8.577737	8.116384	7.363410	10.754173	6.116491	8.314539	7.971938	7.358404	7.589045	7.454005	7.776496	7.793259	7.742387	8.317610	8.355223	8.347074	7.741489	8.167506	8.383748	8.188467	8.798246	8.590817	7.944014	8.369704	8.805667	8.387588

AL157371.2	4.074728	4.089384	3.726533	3.507422	3.676420	3.311317	4.087647	4.318222	3.511432	3.662866	4.366579	3.767775	3.531287	3.946628	3.775313	3.814603	3.896720	3.779373	4.365030	4.049098	3.995290	4.647039	5.383142	3.976335	4.027949	3.973302	4.157168	4.770142	4.296754	3.742196	4.005218	3.668001	3.680841	4.024123	4.008667	3.964886	3.767372	3.618011	3.892646	3.647203	4.253225	4.210992	4.704697	4.298965	4.701302	5.432006	3.933902	4.789520	3.497086	5.211243	4.488884	4.155406	5.176961	4.416195	4.449608	4.515737	4.077825	4.278181	3.755476	3.749398	4.091630	3.972872	4.336791	3.912272	3.992680	4.056272	3.721214	4.137598	4.478450	4.196900	4.119452	3.765884	3.768196	3.654812	4.097968	4.340922	3.844161	3.939637	4.251723	4.093706	3.591134	3.702258	3.190933	4.438717	3.556393	3.619528	3.035276	3.550812	3.810767	3.961596	3.442941	3.711741	3.537062	4.235520	3.573059	3.443103	3.852263	3.969874	4.029752	3.451172	3.460735	3.782722	4.107221	3.764064	3.488107	3.998746	4.505426	4.292225	3.921645	4.205947	3.605770	3.638275	3.698196	3.947935	3.790356	3.114145	3.733827	3.417386	3.788884	3.545073	4.024321	3.903359	4.131450	4.179211	4.302633	3.528312	3.409888	3.433819	4.353548	3.853131	3.992680	3.840679	4.140191	3.961168	3.320420	3.356732	3.923084	4.199448	3.681040	4.467794	4.133608	4.043645	4.393824	4.318222	4.574160	3.970758	5.004573	3.442575	4.334201	3.429526	3.429348	3.547577	4.002351	3.830142	3.561360	3.636869	3.486428	4.412646	3.556033	4.237634	4.410020	3.622433	4.235959	4.036699	5.071631	3.990448	4.168403	3.653225	4.141946	3.852717	4.100847	4.909040	4.344597	3.541895	4.278181	3.668862	3.518376	3.960729	3.938536	4.110281	4.603754	4.485731	4.018482	3.940672	3.691947	3.419896	4.243420	4.136096	4.903587	3.994643	3.945317	5.165298	3.590928	3.635857	4.466525	3.891573	3.661019	4.147052	3.528850	3.915969	4.080922	3.777746	3.248272	3.658843	3.415387	3.461116	3.624461	3.627415	3.598945	3.587872	3.625812	3.542469	4.453967	3.928910	3.571687	3.435445	3.282855	3.871134	3.793589	3.819912	3.527038	4.066688	3.753370	3.549307	5.098046	4.185785	3.745044	4.051496	4.785776	3.494846	3.306834	3.352877	5.268084	4.092696	3.412024	3.980864	3.489197	5.793310

AC021037.1	5.586972	6.375008	6.605592	6.758965	5.964297	6.241596	6.657598	6.672695	5.454402	6.321693	6.095321	6.360544	6.333320	6.931129	6.534525	5.619454	6.275058	6.468342	6.785647	7.306028	6.985556	7.759759	5.883320	6.576008	5.660863	5.903276	5.543158	6.483079	5.566754	5.506607	6.390194	7.513578	7.701163	5.688249	6.651763	6.689974	7.197441	6.981935	7.758398	6.898358	5.744826	7.336755	7.252299	6.366062	6.543690	6.952016	6.279147	7.227793	6.326261	7.141280	7.183775	6.875957	6.872429	7.729788	6.793969	5.817964	7.340836	8.277230	7.757279	7.929278	7.809896	5.611787	6.478640	7.446500	7.552843	6.838662	7.749980	6.879422	7.287679	7.163940	6.933021	6.795092	5.752847	7.026166	6.902230	7.011554	6.824667	6.717341	6.570672	6.777825	6.718962	6.160979	7.066045	7.463901	7.328659	6.925659	7.357154	7.017782	8.100567	8.027797	7.607143	6.984610	6.687119	6.539082	6.610626	6.298921	7.011554	5.757763	6.709750	6.080065	5.507796	6.673497	6.016047	6.816500	8.402370	6.872750	6.403592	6.333836	6.138754	5.887621	7.783317	6.887075	6.328905	5.669438	6.422858	5.863653	6.920499	6.224688	5.874564	5.230986	5.842965	5.332458	5.265665	4.626760	6.256249	5.385582	5.877095	5.873345	6.504161	4.995885	5.643284	5.339523	7.083787	4.896655	6.241436	6.104430	4.748940	6.321505	5.812805	5.573890	7.062373	7.885047	4.988308	5.254349	6.644836	5.517407	6.019182	5.721773	5.915113	5.382322	4.769928	5.032136	5.659219	6.570821	4.861435	6.497681	7.547276	7.044183	6.414516	5.862518	7.813567	7.482906	6.118709	6.839180	6.249790	4.996722	6.842050	6.962293	6.267913	6.438911	6.674449	5.911153	7.562869	5.848261	6.802029	7.342525	7.757729	6.986195	7.608374	8.445923	4.899001	8.962369	7.462608	7.499603	6.792991	7.641095	6.566884	6.719642	6.779254	6.403904	5.926670	5.051783	6.926316	6.246267	6.284297	5.570335	6.424977	5.534897	6.267078	6.412347	7.702677	6.327414	7.107099	7.004886	5.630622	6.341865	7.675274	6.852835	6.506319	7.670222	8.761230	8.106503	6.229710	5.504676	6.185545	5.373609	5.121125	4.221462	6.879422	5.555427	6.180152	7.736081	5.708658	8.191967	6.341388	6.910985	6.809524	8.210515	5.146342	7.276265	5.693593	6.589655	5.441005	5.348318	6.030746	6.525818	6.049583	7.168563

AC105383.1	3.103194	3.241792	3.541120	3.103641	2.971777	4.282318	3.138823	3.224749	3.632530	3.283615	4.087647	3.119753	3.267793	3.648809	3.329348	3.108516	3.330169	2.838906	4.081358	3.449181	3.751463	3.755044	3.067954	3.429179	2.949261	3.630688	3.342475	3.869665	3.462866	3.239599	3.682236	4.088092	3.819283	3.252820	3.333551	3.986510	3.399682	3.826564	4.802623	3.846532	3.434900	3.331917	3.532537	4.238991	4.959144	4.969692	3.409032	3.019669	3.668862	3.521478	3.231876	4.218793	3.588064	4.175536	4.310828	3.804926	4.081133	4.058241	6.438911	3.774502	2.954241	3.267491	3.702040	3.993074	3.432717	3.426570	3.370996	3.954387	3.915108	2.899911	3.018363	3.849489	3.608025	2.816925	2.901241	3.325633	3.819283	4.034749	3.486244	3.550026	3.527209	3.684424	3.948370	4.819179	3.803679	3.492579	4.413763	3.990914	4.208643	5.048303	3.802439	4.442859	3.729450	3.637482	3.393833	3.191453	3.447665	4.228110	4.286998	3.053432	3.428828	3.679024	3.239185	3.248143	4.539078	4.887089	3.976560	4.205490	3.247085	3.050905	3.682691	3.353204	3.713585	3.180407	3.461975	4.376322	3.387383	3.512383	3.173699	3.254178	3.319115	2.976862	3.034896	3.432363	4.075361	3.643475	3.155331	7.426143	2.955162	3.099736	3.332583	3.104899	3.464825	2.934112	3.009843	3.198410	2.779964	3.220137	3.236086	3.357764	3.452949	3.072069	3.604265	2.872967	4.400248	3.498362	3.702441	3.948976	3.397284	3.485860	3.420840	3.716703	3.063869	3.181767	4.317138	3.396622	3.155688	4.708270	3.170910	4.067317	3.197423	3.564191	4.194544	4.216869	4.442432	4.140191	2.875432	3.869875	3.252820	3.101390	3.083374	3.797760	4.319054	3.891777	3.675783	3.763395	3.972667	6.188082	3.682453	3.745894	3.234227	3.776955	3.799232	3.712339	3.619528	3.394158	3.258539	3.671130	2.865258	2.966219	3.478413	3.822675	3.726946	3.943719	3.999159	3.550612	3.701577	3.527576	3.303975	3.612759	3.998094	3.522634	3.750428	3.890274	3.052684	2.999944	4.064154	3.888075	5.330860	3.871796	3.885110	4.481034	3.514088	3.123193	3.340650	3.029190	3.395031	4.579245	3.193243	3.134024	3.288923	3.216664	3.342302	4.861435	3.675783	3.950977	3.938536	3.601477	3.058711	3.510009	3.491512	4.370219	3.415875	3.314311	3.379837	3.621667	3.286535	3.211448

AC096996.2	6.023595	8.430243	8.289196	8.734257	8.569098	9.034634	8.167506	9.088528	8.271201	8.846470	8.203624	7.893118	7.897027	8.743457	7.497414	8.692458	7.995502	8.047349	9.874507	7.914604	10.202804	9.880256	8.612010	8.773214	9.033411	7.968034	6.938279	9.098890	8.303620	8.093562	8.126922	7.749198	7.575743	6.545727	7.434079	7.420235	7.829763	8.216056	8.697583	8.521422	9.089149	7.922093	8.092170	8.225251	7.550519	7.586137	9.189333	7.881547	7.486340	8.293964	7.588120	7.741197	8.136815	8.161613	7.942495	8.596272	7.579513	8.588507	7.904729	7.422117	7.623954	8.747246	6.508622	9.157249	7.563777	7.685481	8.313277	8.259547	8.267307	8.397030	7.848701	7.952777	9.110639	8.092604	7.970847	7.743500	7.786526	7.535018	7.669595	6.953765	7.127053	6.872429	6.908452	6.849813	7.316219	6.846929	5.727185	6.856433	6.922265	7.687491	6.239587	7.087205	7.400364	7.663101	6.622109	6.839672	6.873531	7.243579	7.154192	8.279032	7.303979	8.374648	8.623749	8.730911	9.117278	8.725564	8.893154	9.650396	8.795062	9.635213	9.221723	8.028874	7.987496	9.373655	8.796126	6.307251	6.555824	7.550222	8.790577	6.142569	7.086906	7.113452	7.917375	7.333066	8.622365	7.159964	6.155690	7.206289	7.023127	7.038903	6.589823	6.870481	6.988569	6.939212	7.272673	6.817639	6.505827	6.465872	6.942312	7.789934	7.659388	7.408829	7.020611	7.175574	6.885672	7.052870	7.197600	7.105801	7.911574	7.014228	6.473421	6.500369	7.190930	6.941502	6.631246	6.733689	6.774295	7.362762	6.707538	7.872919	7.042423	7.087997	7.285199	7.186039	7.463106	8.637555	6.989049	6.494280	7.445861	6.834845	6.769775	6.972602	7.610523	6.888237	7.475221	6.065668	6.719816	6.593887	6.147950	6.159072	7.571434	7.204235	7.373429	6.577162	6.738926	7.035762	7.669595	7.393824	7.222846	6.881833	7.012038	8.554202	7.063971	6.356073	7.186189	7.025864	7.195520	7.170547	6.151554	6.620518	6.467861	6.729672	5.560926	5.850948	6.098026	5.749191	5.258293	5.241641	6.382153	5.067171	6.468519	6.518553	6.798331	6.635961	6.715517	5.430849	6.334005	6.885519	7.034521	6.761558	6.607057	7.115667	6.749904	5.904197	8.832570	7.278613	7.865882	7.491814	8.872790	6.534701	6.733213	5.313196	9.209310	7.249782	6.839012	6.639977	6.858980	7.909583

PWRN1	4.627372	3.834416	6.382809	4.850372	4.862492	6.498895	4.688438	6.758456	6.698233	4.769928	5.083066	5.404608	6.787415	5.023623	3.592078	4.990800	4.886247	5.692666	4.283596	5.293315	3.972227	6.331112	3.136114	6.009651	5.260447	3.234953	6.319795	3.663429	6.392190	4.523234	5.161670	5.872814	4.395213	5.494080	5.358907	6.655000	4.722391	4.132328	4.249061	4.002351	3.853814	4.511823	5.971051	5.212390	5.898668	5.030546	6.552341	3.208736	5.881243	4.210138	6.196567	3.247981	4.662049	4.390632	5.617024	5.452837	7.004100	4.156309	5.351089	6.132434	5.444097	5.503367	5.376928	6.084753	3.677408	4.720656	5.214446	6.620976	4.657710	5.427467	3.382935	4.817096	5.474055	5.739533	4.537161	3.345748	5.455513	5.405352	4.599587	4.175304	5.360611	3.730665	5.146547	4.519350	6.013848	6.059561	7.178144	4.262444	4.298115	4.465643	4.937295	4.138401	7.302722	5.216196	4.899606	6.127825	6.687772	7.942495	6.601343	4.838712	6.682255	5.298852	6.665571	4.820845	6.919027	9.247803	8.732389	6.551327	6.432929	4.116995	6.099247	6.722579	8.290722	4.100192	7.245330	8.741801	6.202616	5.773447	7.253406	4.272646	3.730048	3.497998	3.559932	6.609828	6.545484	4.918512	3.662244	4.559441	4.524558	5.845087	3.830779	4.462440	4.245569	4.190613	5.099511	4.455228	4.238537	3.526294	3.539371	3.972227	4.172695	2.955993	3.343473	3.984979	3.633128	5.924351	4.533715	6.173864	3.508512	4.299823	5.291392	4.594913	4.953957	3.291431	3.742589	6.237316	4.027278	4.143246	3.705674	3.797572	3.603835	4.643182	3.440673	4.295899	4.573522	6.865185	6.973051	5.831751	3.397833	3.847370	3.091207	4.168613	4.392582	4.649422	3.839470	5.855821	4.086136	4.947342	4.048661	3.777553	4.484173	3.960968	4.033222	3.807012	3.881295	3.607467	4.532908	3.915108	2.993158	5.066111	4.426343	6.687456	4.784935	5.348318	3.319115	4.875077	4.381821	3.710484	4.432246	3.672690	4.061501	7.944780	6.768300	4.279959	5.668880	4.094115	3.383293	4.578414	4.382536	3.997680	2.899082	4.399847	4.220124	4.066688	4.373025	5.379465	6.421746	9.090065	3.226592	5.773278	5.106012	5.416606	5.815647	5.065719	5.313196	3.866394	4.253430	5.274756	3.871134	6.436133	7.430474	8.918759	4.062409	5.530117	6.306926	3.180672	3.544145	3.572500

HOXA-AS2	6.814438	7.775107	8.214240	7.396166	8.482792	7.959136	7.831401	7.363410	8.974964	8.126601	7.934974	7.606840	7.728109	7.901171	7.620128	8.317000	7.714780	7.157299	8.062120	8.066401	8.429897	8.085541	8.895958	7.964491	8.567450	7.825691	7.651747	7.364043	7.882013	7.645417	8.616473	7.937457	8.298658	7.820271	8.443484	8.286878	8.595787	7.532374	7.776496	7.763447	8.264458	8.056204	8.041861	7.908800	8.256188	8.601729	7.990331	7.918129	7.541740	8.572331	8.413384	8.249737	8.877113	8.438572	8.029503	7.762049	8.644401	8.582268	8.101896	8.145954	8.826140	8.203010	8.949195	8.360170	7.981300	7.646673	8.252222	8.150217	7.584112	8.373879	7.927727	7.995341	8.073351	7.748264	8.185252	7.738513	8.328445	7.460397	7.731332	8.182981	7.993319	8.132684	7.843275	8.294269	8.410780	8.123251	8.023141	7.795703	9.177420	8.032533	8.177391	8.292455	8.223437	8.340131	7.326110	8.437550	7.712966	8.208900	7.489903	7.779491	7.229369	7.703286	7.347389	7.832020	7.923817	7.441605	8.202701	8.216356	8.212373	8.026668	8.893784	8.372378	7.406331	8.256329	7.207080	7.446500	6.128501	7.693526	8.030875	7.455423	5.959677	7.287679	8.511528	5.609716	6.657433	7.432657	7.449204	8.714844	7.421349	7.133232	5.986093	6.431908	6.527507	5.692261	9.011607	7.214668	5.915847	6.439892	7.005961	6.824965	7.156379	8.030875	6.230244	7.591653	5.519715	7.127053	5.077569	7.164273	7.693823	8.853927	5.520641	6.450968	7.768377	6.822918	6.683070	7.470633	6.583596	6.576349	7.326424	6.711340	6.977224	7.142720	6.621138	6.812101	6.592444	6.825914	6.658538	6.360207	6.471216	7.582415	6.870984	7.080815	6.584561	8.423166	7.678459	7.428055	5.804747	7.827291	5.963776	7.224175	8.254529	7.436471	7.312895	8.482646	6.830405	6.687772	6.693760	6.213566	7.856647	6.832940	6.683902	6.716023	6.772178	7.285048	7.022815	7.459810	7.926479	7.263271	8.459576	6.840811	6.951549	6.750073	7.232778	6.917410	7.334577	7.112679	6.605022	8.175380	7.631624	7.075602	7.743966	8.190228	6.887252	6.730784	6.403278	6.749904	7.461972	6.018512	6.780543	6.789577	6.509590	6.837369	6.756046	7.485047	6.806000	7.778213	9.875126	6.746121	7.004729	6.766987	6.008419	5.276946	8.501568	6.832615	6.682427	6.163228	6.639497	6.674131

NAPA-AS1	14.045286	14.539639	14.240086	14.464777	14.356594	14.316814	14.386005	14.484052	14.550161	14.429371	14.547186	14.447515	14.198488	14.404753	14.156390	14.565911	14.679130	14.447515	14.543000	15.004214	14.332395	14.833576	14.205591	14.157364	14.326082	14.405128	13.430372	14.354516	14.254105	14.095205	14.371589	14.066603	14.203084	13.774477	14.739993	14.649747	14.822343	14.649747	14.984580	15.088208	14.563436	14.947411	14.729253	14.407352	14.517397	14.632175	14.269241	14.439683	14.435274	14.724698	14.495949	14.779929	14.726520	15.000238	14.517783	14.491542	14.661345	14.960579	14.887119	14.565911	14.893861	14.445611	14.285845	14.946400	14.642226	15.224770	14.792018	14.822949	14.655466	15.270155	15.848852	15.579100	14.501857	15.103033	14.942582	14.331365	14.877613	14.693813	14.853259	14.867085	14.657723	14.484873	13.954619	14.539165	14.484873	14.396205	14.735782	15.107990	14.881255	14.830506	14.821872	15.064607	14.658618	15.204490	13.828722	14.697497	14.045844	14.311014	14.186537	14.553109	14.303277	14.528293	14.423617	14.480594	14.583170	14.403999	14.575515	14.487923	14.517028	14.355522	14.477842	14.876067	14.795023	14.347441	14.880110	14.225335	13.976899	14.976992	14.319544	13.399078	13.688348	13.625907	13.871812	13.673865	13.932204	13.803853	13.068719	13.425542	13.706318	13.777435	13.700412	12.968955	13.800361	13.384972	13.460920	13.393094	12.996223	13.316216	12.773448	13.143030	13.994473	13.773464	12.991245	12.939392	12.939042	13.896143	13.423212	13.622196	13.669109	13.610850	12.979696	12.957359	13.468573	13.931643	13.405475	13.833047	13.597268	13.713891	13.834567	13.929742	13.993914	12.983527	13.681376	13.713891	13.857811	14.025686	13.213993	13.319284	13.300682	13.753859	13.576247	13.156520	12.892075	13.364346	13.913967	13.613971	13.710918	13.680663	13.665978	14.171915	13.704846	13.776435	14.311709	13.922935	13.801661	13.530801	14.121601	13.078931	14.270558	13.542577	13.641034	13.940443	13.785834	13.574462	13.854876	13.314762	13.751062	14.000643	13.530801	13.293957	13.907439	14.412818	13.351805	14.194648	13.231246	13.780832	13.310353	14.253766	13.977800	13.627288	14.316430	13.567112	13.498487	14.216003	13.559834	13.153006	12.855773	13.302463	13.595345	13.313356	12.909129	12.795726	13.106410	13.579444	13.941234	13.040426	13.384372	14.227650	13.950442	13.028031	13.646573	13.029905	14.308984	13.375462	13.028031	13.721501	13.450944	13.620154

AL034405.1	12.455483	12.184698	12.137865	11.704750	11.930334	11.837992	12.294671	11.904757	11.507746	12.243307	11.939528	11.975261	11.900292	11.995512	12.220778	11.896778	12.036237	12.428003	12.364975	12.252154	12.476025	12.546681	12.850884	12.127196	12.012819	12.128733	12.012048	11.527903	12.084672	12.051649	11.752925	12.482141	12.192450	12.086652	12.479302	12.403076	12.352099	12.758584	12.395544	12.571330	12.262939	12.438036	12.299016	12.459117	12.441586	12.321432	11.671357	12.184541	12.136334	12.106371	12.199524	12.363731	12.082273	12.536272	12.522051	11.857830	12.345161	12.288317	12.221427	12.442360	12.567588	12.129935	12.854406	12.306362	12.086204	12.120220	11.982123	12.227667	11.995974	12.525777	11.940136	11.971773	11.749834	11.959299	12.019013	11.951988	12.247505	11.881010	12.085897	11.815237	12.297752	12.029310	12.142076	12.406051	12.495360	12.198594	11.958709	11.818235	12.722115	12.208079	12.023112	12.326361	11.901464	12.268220	11.851556	12.224317	12.335876	11.874773	12.067502	11.773425	12.075117	12.298055	12.014764	11.857195	13.537270	11.934117	12.372433	12.710821	13.095248	12.044248	12.956616	12.249599	12.265583	12.006089	11.986356	10.924627	12.236952	12.132348	11.697417	12.585328	13.047238	12.561379	12.464531	11.693803	12.932954	12.013103	12.475857	13.064722	12.609687	12.706190	12.534911	12.666963	12.199062	12.243629	11.995202	12.957359	12.752429	13.432385	13.240382	12.647084	12.685200	13.068904	11.852632	12.115080	12.730491	11.572225	12.383214	11.778816	12.678360	12.265122	13.051023	12.765130	12.887037	12.696084	12.445002	12.465329	12.812829	13.625684	12.799200	13.102010	13.090158	12.791666	13.176594	12.690509	12.915887	12.393629	12.922049	12.364491	13.216575	12.975455	12.958825	12.668162	12.626987	12.547774	12.131138	12.822839	12.277908	13.017369	12.407615	12.322207	12.911260	12.726851	13.764461	12.600707	12.019164	12.984090	12.284891	12.272493	12.737801	12.286918	13.064722	12.346629	12.382100	12.497991	12.414483	12.905001	13.524838	12.537583	12.028718	12.369370	13.305359	11.825192	12.203451	13.144955	12.833284	13.232837	12.734379	12.756348	12.604249	12.611187	12.906255	12.244853	13.295754	12.836529	12.052224	12.432090	11.913228	12.980105	12.471030	13.129561	12.360678	11.822914	12.228631	13.418183	11.869930	13.391215	12.920784	12.710992	11.733178	13.292760	12.762553	13.186829	11.663877	13.181319	11.776688	13.023845	12.275923	12.373685

Z68871.1	7.034226	6.513895	6.981935	6.040731	5.955921	6.364856	6.729161	6.002723	7.265404	6.510098	6.755554	6.160979	6.545484	6.308928	6.980170	6.646563	6.422546	8.598050	6.507989	7.175090	5.580219	7.005812	7.307573	5.962157	6.013134	5.947758	7.207379	5.415835	6.561359	6.335031	7.288615	7.285348	7.776826	6.927973	7.901337	7.741642	6.829419	6.493315	6.313497	6.980170	6.749580	7.045467	7.899893	7.308044	7.343668	6.764402	7.105493	7.519393	8.039807	7.212966	6.654673	7.532374	7.490073	7.186675	8.169560	6.962768	7.412291	7.227002	7.024588	6.898683	6.130691	6.633547	7.192436	6.376639	7.091977	6.752554	6.308073	7.024588	6.826732	6.608681	6.543039	6.610626	6.906393	6.886288	6.977046	6.394679	6.883579	6.881513	6.280180	6.890454	7.367653	7.543904	7.085535	7.445861	7.249175	7.891377	6.185209	7.560752	7.288615	6.765059	6.422858	6.111965	6.919882	7.146759	6.770091	6.819116	7.699767	7.424306	7.409893	7.073236	6.900296	6.699964	6.750364	7.093674	6.567898	8.559151	6.512094	6.736946	6.746276	6.324405	5.897574	7.585809	5.851124	6.382325	7.563186	4.907542	6.775273	7.143631	7.164573	7.676347	7.535627	7.248072	7.054595	5.116218	7.928678	6.212846	8.354587	8.760915	7.819359	7.791586	6.817639	7.159784	7.408053	7.654830	7.557866	8.613599	9.914982	6.874477	8.709164	7.245629	7.534543	8.049527	2.958915	6.256093	6.570356	7.249782	6.977046	7.010112	7.828691	8.109379	7.811416	9.488302	8.004347	8.555103	8.687597	7.336449	6.806159	7.537619	7.123846	7.113000	7.374392	8.691230	8.164571	8.332937	8.702764	7.635884	8.931119	8.391617	7.596408	7.843764	9.018370	8.691536	8.042486	7.608684	7.565352	9.086267	8.809674	8.356756	8.638018	7.585336	7.361977	9.137632	7.020942	7.196791	7.370624	8.308242	7.677630	7.199608	8.231014	8.016016	7.933104	8.111174	7.780760	7.312433	6.975438	7.299697	8.423009	6.893978	7.221773	8.293964	9.824482	6.623405	7.849468	8.529384	8.744816	7.564260	8.112848	8.274926	7.063002	8.734549	7.171017	7.176647	8.384354	7.144281	6.707538	8.351110	8.513380	8.062273	8.744959	8.706598	7.927415	7.170697	7.576358	8.210228	7.556615	9.321385	8.857440	8.093869	6.509917	7.110840	8.199516	6.709910	7.101269	7.553935	7.480582	7.492043	7.843927	6.126045

LINC00630	7.034226	6.513895	6.981935	6.040731	5.955921	6.364856	6.729161	6.002723	7.265404	6.510098	6.755554	6.160979	6.545484	6.308928	6.980170	6.646563	6.422546	8.598050	6.507989	7.175090	5.580219	7.005812	7.307573	5.962157	6.013134	5.947758	7.207379	5.415835	6.561359	6.335031	7.288615	7.285348	7.776826	6.927973	7.901337	7.741642	6.829419	6.493315	6.313497	6.980170	6.749580	7.045467	7.899893	7.308044	7.343668	6.764402	7.105493	7.519393	8.039807	7.212966	6.654673	7.532374	7.490073	7.186675	8.169560	6.962768	7.412291	7.227002	7.024588	6.898683	6.130691	6.633547	7.192436	6.376639	7.091977	6.752554	6.308073	7.024588	6.826732	6.608681	6.543039	6.610626	6.906393	6.886288	6.977046	6.394679	6.883579	6.881513	6.280180	6.890454	7.367653	7.543904	7.085535	7.445861	7.249175	7.891377	6.185209	7.560752	7.288615	6.765059	6.422858	6.111965	6.919882	7.146759	6.770091	6.819116	7.699767	7.424306	7.409893	7.073236	6.900296	6.699964	6.750364	7.093674	6.567898	8.559151	6.512094	6.736946	6.746276	6.324405	5.897574	7.585809	5.851124	6.382325	7.563186	4.907542	6.775273	7.143631	7.164573	7.676347	7.535627	7.248072	7.054595	5.116218	7.928678	6.212846	8.354587	8.760915	7.819359	7.791586	6.817639	7.159784	7.408053	7.654830	7.557866	8.613599	9.914982	6.874477	8.709164	7.245629	7.534543	8.049527	2.958915	6.256093	6.570356	7.249782	6.977046	7.010112	7.828691	8.109379	7.811416	9.488302	8.004347	8.555103	8.687597	7.336449	6.806159	7.537619	7.123846	7.113000	7.374392	8.691230	8.164571	8.332937	8.702764	7.635884	8.931119	8.391617	7.596408	7.843764	9.018370	8.691536	8.042486	7.608684	7.565352	9.086267	8.809674	8.356756	8.638018	7.585336	7.361977	9.137632	7.020942	7.196791	7.370624	8.308242	7.677630	7.199608	8.231014	8.016016	7.933104	8.111174	7.780760	7.312433	6.975438	7.299697	8.423009	6.893978	7.221773	8.293964	9.824482	6.623405	7.849468	8.529384	8.744816	7.564260	8.112848	8.274926	7.063002	8.734549	7.171017	7.176647	8.384354	7.144281	6.707538	8.351110	8.513380	8.062273	8.744959	8.706598	7.927415	7.170697	7.576358	8.210228	7.556615	9.321385	8.857440	8.093869	6.509917	7.110840	8.199516	6.709910	7.101269	7.553935	7.480582	7.492043	7.843927	6.126045

LPP-AS2	10.272760	9.270915	9.027842	9.591051	10.000549	10.969399	9.860599	10.659191	11.206577	10.135138	10.548745	10.713006	9.813834	9.513664	9.193324	10.043446	10.610648	9.141665	9.994619	9.857725	9.191817	9.863922	10.399284	9.440330	9.334765	10.030084	11.060112	10.090524	10.577542	10.306414	10.494077	9.310802	9.549005	10.094402	9.324612	9.342047	9.243185	8.867333	9.491898	9.516679	10.129307	9.727909	9.098132	9.165569	9.436629	9.151110	11.110584	9.685622	9.350940	9.177420	9.199210	9.141158	9.053617	9.522409	8.862612	10.238002	9.350193	9.587642	9.226210	9.507924	9.822224	10.101581	10.472696	9.579000	9.640875	9.577451	9.696489	8.902638	9.444839	10.046469	9.579624	9.642396	10.585228	9.513967	9.567958	9.596795	9.637166	9.409559	9.566504	9.608373	9.516216	9.315503	9.215920	9.485177	9.222522	9.619017	9.867411	9.291914	10.449732	9.909568	10.089619	10.128831	9.603604	9.523175	10.373661	10.054496	9.273625	10.104326	10.537651	10.057120	11.261983	9.340437	9.137949	8.928332	9.220659	10.434589	9.768766	9.703934	10.435394	10.427471	10.099670	9.646619	9.528337	11.065268	10.334644	14.516636	9.756381	10.078848	10.935492	11.125488	9.678286	9.947176	9.498268	11.925351	10.098728	11.517507	11.689898	11.018683	10.964156	10.511086	11.195021	11.626169	10.366828	10.453422	10.907937	11.360602	10.520951	10.412690	9.560491	10.448243	11.160805	10.635345	9.939340	10.062899	10.641916	11.184811	10.173056	11.928304	10.574965	11.077366	9.816047	10.732503	10.300503	9.632285	10.043161	9.511205	9.610292	10.503929	10.295699	11.492028	10.414333	9.326595	9.720786	9.688079	9.294436	11.502051	9.402928	9.617475	9.652594	9.373655	10.551898	9.511023	9.374473	9.432358	10.942871	10.541389	10.155626	9.769520	9.247960	10.700162	10.287417	10.806918	9.598476	10.111696	9.678939	9.837964	9.464406	9.549938	10.623218	9.602218	10.182552	11.008883	10.200086	10.666359	9.637462	9.865926	9.846608	9.646463	9.519781	9.510392	10.356921	9.673171	9.678789	9.719718	10.817152	10.186664	8.786090	10.853742	9.985333	9.701109	10.626550	10.455864	10.078848	9.990112	11.319133	11.307706	11.398515	10.680952	10.370017	10.730315	11.317780	9.986408	9.968648	10.597077	10.478807	9.839071	10.224681	10.578433	11.836797	10.581306	10.887345	11.748493	11.788373	11.347581	11.759996	9.592749	10.078682	10.673881

AP002884.3	5.877950	5.539594	5.650297	6.341705	6.833280	6.773490	6.149155	5.226859	6.826070	6.452765	6.673975	6.867906	6.401780	5.823680	5.293315	6.389356	5.758146	6.976098	5.858142	7.123215	5.896338	5.271047	5.826762	6.835648	7.046406	6.634185	7.169943	6.361699	6.649358	5.867417	7.256173	5.319005	6.548360	6.491699	6.461507	5.513154	6.126045	5.730853	5.851850	6.059027	6.988104	5.689182	6.670097	6.376000	6.792357	5.172120	6.941340	5.913302	5.985034	5.701366	6.027838	5.432417	6.615919	6.276695	6.497681	6.456040	6.330441	4.041233	6.752706	5.929695	4.900484	6.727241	5.037736	6.203650	5.363851	5.543334	4.672936	5.459048	5.232814	4.885618	5.900980	5.895320	6.727719	6.012132	6.420618	6.174728	5.574617	6.265036	5.918522	6.294553	6.064005	6.311949	5.671418	6.089390	6.541196	6.818290	6.572737	6.282611	3.670528	5.559465	5.425023	4.893955	5.994297	5.553777	6.079385	6.376795	5.416410	7.884753	6.485132	7.057617	7.015827	6.510415	6.924368	6.304265	5.447832	6.701426	5.575395	6.371084	5.487997	6.849972	3.947248	7.507990	6.549863	7.040172	6.583596	5.965347	6.482556	7.061882	7.783009	4.193008	5.489978	4.817515	5.931422	5.417778	5.315439	5.900633	6.948756	6.470712	4.283596	5.416994	6.888567	5.008172	4.698520	2.974731	6.331112	5.740431	6.217326	4.283381	6.026440	5.597810	4.279959	5.634528	7.031875	5.044934	4.874651	6.400316	5.561304	5.053206	4.958737	6.786940	5.936334	6.235988	5.249579	6.355577	5.486905	6.049248	3.674319	4.795599	5.235235	4.892077	3.006794	5.516844	5.306198	5.575947	4.043878	5.140676	3.278841	5.825842	4.536530	4.232847	6.379471	5.193075	4.690748	6.054977	4.720037	3.530322	3.927841	5.522331	4.907752	4.651131	5.604559	4.530110	4.850148	4.622478	5.817445	3.084646	5.129592	4.454595	2.933891	5.141118	5.857270	5.003178	4.815587	5.620566	5.730301	5.594019	4.582452	5.491318	5.640130	5.635849	6.405559	5.807238	6.218038	5.761196	6.832287	5.067778	6.579465	3.454582	4.434540	4.427447	4.084103	5.116611	4.581620	5.800432	5.292942	4.956861	7.715526	5.824779	6.030587	5.115354	6.024120	6.814127	5.324768	4.591620	5.818516	4.151044	4.979117	4.452439	5.985373	3.615523	5.910637	5.482022	5.614444	5.233633	4.837655	5.599318	6.272225	6.014517

AL445588.1	3.332425	3.443103	3.299460	3.107143	3.173311	3.875870	3.428672	4.581198	3.554680	3.520806	3.678004	3.807616	3.330982	3.882327	3.680636	3.292205	3.854056	3.462669	5.025654	3.197292	5.231197	2.831876	3.839470	2.907395	3.096273	2.983345	3.208343	3.107025	3.037993	3.058119	3.757311	4.394513	3.659062	3.103641	4.920208	4.155600	3.739136	3.320254	3.074874	2.951744	3.411476	3.147783	3.586121	3.346564	3.690345	5.684727	3.486039	3.176138	4.202274	4.220367	4.085430	3.993947	4.394772	4.149594	3.977405	3.122165	4.080922	4.171867	3.503315	3.641503	2.888542	3.102889	3.472563	3.282239	3.514264	3.382065	2.941574	3.212842	3.188883	2.870431	3.143041	2.971703	3.207803	3.187273	3.150753	3.358619	3.055089	2.973723	3.014748	3.803477	3.477651	4.058241	3.420470	4.272029	3.428299	5.146157	2.914278	3.246100	3.771953	3.629032	3.272103	2.939586	3.191065	3.267491	2.997034	3.029377	3.814003	3.000046	3.491351	3.358816	3.132332	3.271149	3.022268	3.075743	4.509419	3.816963	3.498184	3.566109	3.163387	3.587283	3.368271	3.671130	3.244978	3.294696	3.489376	3.071974	3.186045	2.983861	3.140672	3.801192	4.811050	3.858770	3.536093	4.166469	5.408923	3.446532	3.664050	3.761124	3.963386	4.252787	3.788048	3.734589	3.844372	4.494423	3.454582	4.336791	4.558305	3.688884	4.804112	4.466966	3.432871	5.463106	3.656029	4.069644	3.970970	3.313340	3.725577	3.526077	4.598956	3.585700	4.414893	3.219233	4.578189	4.839829	4.456699	4.532041	5.024657	4.256282	4.017593	4.042312	2.967290	4.402680	4.375223	4.152749	5.061015	4.021539	3.360502	5.184411	4.511929	5.088183	5.018503	5.231593	5.735202	4.855214	3.785850	4.840654	4.205947	4.882020	4.659661	4.883876	3.927163	4.594487	4.510736	4.553885	4.482874	2.892069	4.745112	4.307013	3.147523	3.522634	3.652414	3.732755	3.604642	3.674117	3.832514	3.551811	3.617462	3.727937	3.984336	4.803039	5.614049	4.784543	4.165577	4.735652	5.655947	4.812075	4.829534	4.096648	4.736346	4.483519	4.228324	4.263344	4.315607	4.259390	3.800298	3.744089	3.240460	4.768483	3.990448	3.761533	3.593435	3.245376	3.489018	4.905684	4.477155	4.232435	3.545073	3.817794	4.017194	3.910981	4.628705	3.637869	3.789308	3.588064	3.373274	3.926936	3.990448	3.708262

C22orf34	5.098442	5.470823	5.195106	5.198528	4.544458	4.916624	4.344818	5.066344	3.997680	4.777623	3.632126	5.278342	4.626760	4.866199	4.060851	4.671850	3.982623	5.411685	5.044535	4.564910	5.198116	3.850978	3.322172	5.229116	4.216015	4.275963	4.774907	2.879381	4.079820	6.266717	3.728814	4.557261	5.693030	6.283455	5.706824	5.405925	4.157992	3.969670	4.434340	5.612899	5.193268	5.702804	6.144094	5.943556	6.525307	5.555640	4.147276	6.181161	5.811119	5.749744	5.393177	5.786123	7.573718	4.214696	4.012263	4.781766	6.080065	4.422440	4.131875	6.723049	7.311966	4.624831	4.989358	4.986634	5.009920	4.877984	4.869580	4.336124	6.108518	5.280737	5.168280	5.667420	3.487527	6.481749	5.631017	7.645097	3.983064	6.363201	4.556840	5.601135	5.161272	3.882327	5.623887	5.886031	6.570524	6.271204	5.649730	6.542050	4.103884	4.376122	6.389179	5.499857	4.605637	5.728084	6.516401	4.932450	4.871916	3.501981	3.408870	6.606890	3.509610	3.263968	4.380378	4.695775	4.694948	5.010954	4.576227	4.034967	4.982563	3.303802	3.872419	4.727455	3.593015	4.321835	3.686239	5.983672	5.445439	5.285388	4.497281	8.324932	5.572010	5.508384	6.283455	5.048105	5.304215	4.843594	3.957915	5.418743	7.814319	4.739299	4.522996	4.705969	7.859735	5.565213	4.817515	5.033779	6.099449	3.871796	5.372833	5.997433	4.752112	6.075352	4.265499	4.701728	9.783615	4.604611	4.190163	3.402108	6.213207	5.351089	4.734819	6.570038	5.869386	6.209329	5.069790	5.562403	5.623704	5.732672	5.839182	5.947390	5.992335	5.948086	5.761020	5.020938	5.487827	4.652372	5.441417	6.002186	5.478242	6.093031	5.761196	6.905732	5.607979	4.998969	5.959510	5.788289	5.459435	5.421874	7.950164	7.098623	6.022066	5.214626	6.192351	6.566103	6.625344	5.712313	5.733384	6.933021	6.626777	7.305872	5.890991	4.710123	5.933698	6.733368	8.230097	4.782194	7.099247	5.163477	6.083169	5.233026	5.759977	6.344898	6.232414	6.563364	6.247783	6.383408	6.104087	5.269846	5.720120	5.081029	5.773278	4.889393	5.499484	5.811679	5.063633	4.358758	3.242102	5.457696	9.801645	4.622687	3.604028	6.160979	6.054977	6.172693	5.752653	6.849813	4.899185	5.275538	4.334644	5.250790	6.880073	4.016698	4.448486	4.921470	5.770307	5.686221	6.623882	8.060100

LIVAR	6.542552	5.347540	6.060246	5.743007	5.956452	6.464703	5.399789	6.586378	6.720973	6.560853	5.432797	6.005203	5.940259	5.734096	4.227886	6.081807	6.193889	6.160836	6.069104	6.175920	5.580041	7.366400	6.556465	6.293873	6.282450	6.288089	6.158574	6.469674	6.210351	5.433546	6.728222	3.618767	5.642738	6.163391	5.787200	6.226232	5.881571	5.976204	6.325946	7.139694	7.065404	6.184684	5.738273	5.442550	5.861774	2.890514	6.046399	5.424256	5.689353	5.807418	5.409725	4.843390	5.083842	6.170659	5.662741	5.950553	5.657391	5.372272	6.673497	4.930410	5.480262	6.623080	4.731657	6.403592	4.957084	5.684727	6.039284	5.343190	5.932632	6.661190	5.902366	6.348278	5.757389	6.011402	6.073804	5.521947	5.938282	5.063633	5.501596	5.276568	5.106233	5.176541	6.218215	5.073843	6.443376	5.769929	5.544106	5.710123	5.504676	5.639932	6.171172	6.059390	5.389322	5.669981	5.640329	5.919034	4.980930	7.482442	6.810819	5.581341	5.941446	6.365895	6.314683	6.063646	7.053011	5.441987	6.199832	6.735790	6.762515	6.495088	7.091343	5.384460	5.736968	6.007743	6.174195	6.506661	6.537292	6.167552	6.440386	5.510469	5.657957	5.454938	5.623172	6.594842	5.795443	5.366809	5.997605	6.495088	5.343190	5.581535	5.644627	5.847710	5.416806	5.226103	5.770487	5.751191	6.439064	5.816732	5.455882	6.122355	6.451641	6.409176	5.571612	5.755704	5.500842	5.903831	6.107820	6.827174	5.767564	6.492653	4.212512	5.559465	5.943900	5.662741	5.979585	5.088990	6.132918	5.714561	5.433353	6.276695	5.465597	4.622899	6.018659	4.983217	2.956087	6.057290	4.216443	6.108169	5.668159	4.057364	5.369136	3.568264	5.232211	4.785361	5.124300	5.415640	5.421112	5.432219	4.411106	4.313806	5.668695	4.035847	5.594934	5.620195	5.419147	5.471380	6.101704	5.583614	5.994834	5.434567	5.869213	6.354724	5.173736	5.313408	5.215041	6.027664	5.573143	5.629885	4.571619	4.654618	5.186596	6.298921	4.412646	5.236029	5.940438	5.461616	4.622076	5.734276	4.973998	5.816919	5.746099	5.274932	5.453034	5.712313	6.343517	5.250989	6.463206	5.598592	5.152347	6.145055	5.491876	5.792377	4.859838	6.012391	6.224688	5.810576	5.528973	5.995187	6.953765	5.895493	4.870422	6.282271	7.206919	6.727241	5.792732	6.089564	5.541466	5.309024

LINC01909	6.542552	5.347540	6.060246	5.743007	5.956452	6.464703	5.399789	6.586378	6.720973	6.560853	5.432797	6.005203	5.940259	5.734096	4.227886	6.081807	6.193889	6.160836	6.069104	6.175920	5.580041	7.366400	6.556465	6.293873	6.282450	6.288089	6.158574	6.469674	6.210351	5.433546	6.728222	3.618767	5.642738	6.163391	5.787200	6.226232	5.881571	5.976204	6.325946	7.139694	7.065404	6.184684	5.738273	5.442550	5.861774	2.890514	6.046399	5.424256	5.689353	5.807418	5.409725	4.843390	5.083842	6.170659	5.662741	5.950553	5.657391	5.372272	6.673497	4.930410	5.480262	6.623080	4.731657	6.403592	4.957084	5.684727	6.039284	5.343190	5.932632	6.661190	5.902366	6.348278	5.757389	6.011402	6.073804	5.521947	5.938282	5.063633	5.501596	5.276568	5.106233	5.176541	6.218215	5.073843	6.443376	5.769929	5.544106	5.710123	5.504676	5.639932	6.171172	6.059390	5.389322	5.669981	5.640329	5.919034	4.980930	7.482442	6.810819	5.581341	5.941446	6.365895	6.314683	6.063646	7.053011	5.441987	6.199832	6.735790	6.762515	6.495088	7.091343	5.384460	5.736968	6.007743	6.174195	6.506661	6.537292	6.167552	6.440386	5.510469	5.657957	5.454938	5.623172	6.594842	5.795443	5.366809	5.997605	6.495088	5.343190	5.581535	5.644627	5.847710	5.416806	5.226103	5.770487	5.751191	6.439064	5.816732	5.455882	6.122355	6.451641	6.409176	5.571612	5.755704	5.500842	5.903831	6.107820	6.827174	5.767564	6.492653	4.212512	5.559465	5.943900	5.662741	5.979585	5.088990	6.132918	5.714561	5.433353	6.276695	5.465597	4.622899	6.018659	4.983217	2.956087	6.057290	4.216443	6.108169	5.668159	4.057364	5.369136	3.568264	5.232211	4.785361	5.124300	5.415640	5.421112	5.432219	4.411106	4.313806	5.668695	4.035847	5.594934	5.620195	5.419147	5.471380	6.101704	5.583614	5.994834	5.434567	5.869213	6.354724	5.173736	5.313408	5.215041	6.027664	5.573143	5.629885	4.571619	4.654618	5.186596	6.298921	4.412646	5.236029	5.940438	5.461616	4.622076	5.734276	4.973998	5.816919	5.746099	5.274932	5.453034	5.712313	6.343517	5.250989	6.463206	5.598592	5.152347	6.145055	5.491876	5.792377	4.859838	6.012391	6.224688	5.810576	5.528973	5.995187	6.953765	5.895493	4.870422	6.282271	7.206919	6.727241	5.792732	6.089564	5.541466	5.309024

AC020905.1	7.473547	7.457779	7.252455	7.633165	8.076799	8.053241	7.784088	8.959927	8.639417	7.909103	8.090173	7.785009	7.837640	7.795703	7.185698	8.433371	8.250993	7.263889	7.857244	8.006998	7.917667	8.639417	7.377540	7.671486	7.566427	8.538466	8.014946	8.008692	7.955709	8.083951	7.546301	7.103764	7.309276	7.106294	6.962124	7.272497	7.368750	7.292079	7.615270	7.416992	8.213124	7.252922	7.330395	7.400671	7.210504	8.013547	8.454436	7.963752	7.872609	8.382997	7.148992	7.217178	7.153231	7.260304	7.244060	8.582555	6.689649	6.987484	6.895743	6.738587	6.842402	7.832020	7.067588	7.473850	7.257257	6.828774	7.692283	7.285498	7.531737	7.751504	7.929278	7.811416	8.184186	7.635566	7.841244	7.746868	7.664681	7.254305	7.523591	7.075460	6.943755	7.086906	6.752888	7.022488	6.704142	7.508140	6.720973	6.919027	7.093995	7.114731	6.675937	7.350547	7.273273	7.708703	7.674467	7.312275	7.274847	7.325643	8.462629	7.534703	7.977373	7.248226	7.536401	7.674175	7.199002	7.827449	7.601067	8.459410	8.134382	8.441329	7.436945	7.500092	7.801072	8.613599	7.750887	8.555262	7.480117	7.433592	8.151906	7.539896	7.943715	7.890928	8.062120	9.303557	8.767930	8.210366	7.624722	8.052786	8.537828	8.433072	7.859428	8.401741	7.830502	8.376467	8.125256	7.906479	7.807713	8.937007	7.606101	8.150217	8.213302	8.053687	8.565872	8.251925	8.245874	8.736423	8.617997	9.145633	8.447016	7.664983	7.572480	7.463738	7.684279	6.939982	7.326749	7.262670	7.885358	7.877178	7.526204	8.586509	7.460716	7.261207	8.076511	7.370150	8.136005	9.182433	8.260003	7.934793	8.363165	7.514786	8.147026	7.131001	7.467980	7.065558	8.176472	6.661502	7.063661	6.930002	6.565283	7.466365	8.097820	7.281623	7.312596	7.365458	7.267272	8.122441	7.871660	7.731640	7.764971	8.021201	7.769428	9.221577	7.685039	7.749037	7.282895	7.904884	7.939959	7.620888	7.024249	6.967877	7.437391	7.340507	7.090578	6.384819	7.777904	6.763286	6.276999	7.236268	7.010112	6.939673	7.283976	7.636629	7.719036	7.668974	7.455587	8.642660	7.928678	8.073176	7.446170	8.387119	7.532530	8.117460	7.563026	7.561665	8.773068	7.800516	7.774614	7.600778	9.052510	8.120836	7.354709	8.686736	9.163879	9.084689	7.875198	7.730554	7.999482	8.457339

AF064858.1	5.504866	5.993944	6.231589	5.093331	5.070805	5.460645	5.560572	4.407357	4.667368	5.256134	4.205947	4.355265	4.559658	4.221883	5.121915	4.309946	5.477861	4.556230	3.800090	5.179750	4.771384	4.876299	5.649908	4.418689	4.339614	4.915772	6.415636	5.026050	4.694111	5.890818	5.640130	5.575213	7.426808	6.850296	6.841294	7.246377	4.803466	5.595302	5.168280	5.380613	5.735022	5.497735	5.997605	5.633010	5.276751	6.793787	3.749398	5.071631	6.994310	6.242601	5.945313	5.803834	7.875964	5.809809	7.562431	4.366775	6.966936	5.481638	6.734147	5.955394	6.276695	5.727904	7.071967	5.196121	5.962339	5.353254	6.405228	5.245221	5.803115	4.787871	5.490389	5.217569	5.714005	5.941126	6.207603	5.603232	5.945861	6.667041	6.180653	6.509278	5.735022	7.292562	6.086582	6.248637	6.351290	6.588854	6.669775	7.431882	5.645803	5.896175	5.070383	6.822140	5.904197	7.245012	6.141020	6.206085	7.186521	5.534087	5.016682	4.337446	5.601329	5.142148	5.363470	5.381734	6.414695	5.679760	4.428477	5.715491	5.744826	4.388677	5.317463	4.765079	4.163583	4.523673	5.698775	6.787592	5.097591	5.533140	3.864726	5.384096	6.407869	5.888841	6.184495	5.125344	5.427041	5.305796	5.012415	5.503158	5.475801	7.329294	5.451468	4.547511	4.417533	5.686221	6.337546	6.926316	4.615319	3.779584	3.983713	4.362606	4.904203	6.402607	5.535685	4.152088	7.369686	5.504312	4.742349	4.022811	5.536657	5.929695	7.409279	6.990471	7.709559	5.789407	6.625003	6.764402	5.335758	5.619275	6.810819	5.707018	5.764682	4.855836	5.977090	6.521225	6.562022	5.798581	4.731438	5.633010	6.584884	8.329650	7.080341	7.475689	7.520175	6.881346	4.280400	5.368578	6.338556	7.048173	6.535471	7.106294	6.463746	7.137983	6.062618	5.223543	7.264952	6.885035	5.984369	6.191997	5.837488	5.084868	6.717502	7.860474	5.605914	6.563011	6.304937	6.473753	6.725575	6.154306	6.303063	5.565981	6.216463	7.878847	7.350396	8.268542	5.288139	7.557083	6.409866	5.530683	6.478315	6.253010	7.449365	4.290484	7.205802	6.471216	7.499773	6.253184	4.580993	6.410196	4.176172	4.836172	4.447792	4.691187	6.524815	5.672408	5.714005	6.075903	5.295016	5.612522	5.973236	4.941966	4.884944	3.849284	4.138230	4.737795	6.941182	6.394997	7.349921	6.011945

CACNA1G-AS1	4.900277	4.787036	5.103183	4.236363	5.665330	5.653667	6.578654	4.363059	4.340238	5.977963	6.561513	5.462754	5.052996	5.064071	3.932805	4.666013	4.143859	5.283942	4.639269	4.813130	5.397471	5.951091	5.568071	4.481034	5.074842	5.164280	4.598956	5.298453	5.477682	5.224480	4.293954	5.660509	5.956452	5.278530	5.625588	4.663287	5.400334	5.897574	5.548626	5.559465	5.812038	5.372272	5.361740	5.398631	4.564070	4.535677	6.739227	6.130691	6.177466	3.548744	5.118253	5.024657	5.350251	5.780710	5.272267	5.723646	5.812591	6.404074	4.620375	5.986470	6.023065	5.691927	5.857096	5.145729	6.033450	5.597613	6.209157	5.100098	4.934698	5.746275	5.194696	5.334740	6.255368	5.621097	5.664600	4.694111	5.220735	4.857743	4.951888	6.058527	5.284147	5.330483	4.837448	5.357782	5.064478	5.958775	4.108917	4.861224	6.047988	5.910637	5.760681	5.361935	4.514021	5.503158	4.701513	4.371755	5.245997	4.713491	5.408923	4.840449	4.666471	4.775323	4.661386	5.582087	4.823549	5.841158	4.267631	4.157992	5.583614	6.307584	4.795822	4.816461	4.489327	5.488546	5.204082	6.508946	4.515053	5.846949	5.479266	4.716241	4.813536	4.198200	4.621436	5.826180	5.212584	5.842057	3.872015	3.952908	6.337219	6.362867	5.914377	4.407357	5.171907	3.884687	4.773047	4.191700	5.287944	4.411994	4.784747	3.788048	4.681462	5.813148	4.982780	5.749031	6.217501	4.578613	4.870422	5.412215	5.647068	3.730665	4.175536	3.053347	5.072591	4.925093	4.500729	5.683992	3.691158	4.756329	4.326548	5.718496	3.319425	4.616662	4.151892	4.466739	4.297904	6.625668	8.264324	3.804926	3.562676	4.747288	3.372431	3.632741	4.297673	4.131450	5.378859	4.549553	3.214073	3.500497	4.471699	3.991571	5.722901	3.613780	4.539078	4.321207	4.357870	3.103265	4.497929	4.341124	5.945861	5.064071	4.071686	6.028736	5.141531	3.972456	4.076394	5.039504	4.615319	4.576920	4.057171	4.537365	5.318802	4.941365	7.412915	6.588698	5.045524	5.313603	4.698120	3.178836	3.504959	3.701819	3.413099	3.795499	5.186207	5.192676	4.404898	4.412855	3.966797	3.800534	4.463269	4.542417	3.989228	4.165343	4.073227	4.527150	5.650665	3.072674	3.875478	4.162932	5.909697	5.031741	3.751689	4.824132	5.921216	5.741546	6.128005	3.512576	5.479452	5.211443

AC004080.6	5.709757	7.373908	7.582572	8.743603	9.478846	8.052496	7.995977	8.950873	7.351763	9.107963	7.503220	9.304406	8.815301	9.564819	7.364834	9.085673	7.312433	8.455668	9.062320	9.254350	8.696372	10.092323	9.533695	8.675156	7.671958	7.644929	6.866463	8.020889	7.274556	7.712340	8.836925	7.246521	8.447639	8.021631	7.880784	9.192277	8.507070	8.764223	9.316873	8.363776	8.089246	8.649359	7.686546	6.724651	7.087352	7.733781	9.098294	9.277081	7.948606	9.008088	8.479473	8.880498	7.709559	9.957211	7.372973	8.671577	7.408527	8.550896	9.613345	8.154391	7.941504	7.534703	7.844360	9.079479	8.849250	8.472170	8.143298	8.021631	8.888062	9.478293	8.969436	8.267918	8.038029	7.288120	8.997519	9.181715	7.862606	8.022679	8.043711	9.226836	7.569307	8.095831	7.202536	8.800815	8.913879	9.044099	8.524461	9.464857	9.070503	9.869000	8.896105	8.427087	7.819523	9.484723	6.749816	8.849097	7.501480	7.156379	8.263850	8.621435	7.474745	7.914604	7.013910	8.699691	8.387119	8.145954	7.571574	9.950580	7.144444	7.660314	9.847208	10.469499	8.619393	7.050856	7.535484	10.075427	7.545653	7.614780	7.242119	7.443459	6.574654	7.007040	8.256628	8.177095	5.805451	9.237192	6.755073	5.869386	8.063024	8.289502	7.048505	6.434113	7.520666	7.352207	8.454138	7.826623	7.160590	6.456884	6.729309	7.083939	9.795202	9.459026	10.311130	8.333092	6.507989	8.815760	9.068809	7.305240	7.647559	6.239749	5.639734	6.587534	6.705266	7.572330	8.032238	6.789740	8.523071	7.856494	7.422117	6.864078	6.973051	7.872919	5.766438	7.981758	8.564961	6.263018	8.406370	8.641137	6.931748	6.541524	8.092915	9.499476	8.760273	10.052486	7.246377	6.380125	9.982213	9.003636	7.268627	7.824155	9.469843	7.693673	8.289941	8.981810	5.545431	7.983486	7.779974	7.656423	7.525576	8.216975	7.630676	7.465866	7.087059	6.417479	7.383930	8.424232	8.034627	7.563186	8.274332	8.743900	8.643479	8.973923	7.720980	6.527992	8.642817	7.201019	8.715458	7.948606	7.914144	10.441420	7.357496	8.436316	7.050375	6.217668	6.406070	7.029717	6.079385	7.396166	8.701516	8.935159	8.319729	8.234802	8.149308	10.881335	7.019371	7.531429	10.250277	5.977794	7.036695	8.608484	7.538769	10.256478	8.025893	10.052635	9.037684	6.159451	9.256070	6.438240

HOXA-AS3	5.709757	7.373908	7.582572	8.743603	9.478846	8.052496	7.995977	8.950873	7.351763	9.107963	7.503220	9.304406	8.815301	9.564819	7.364834	9.085673	7.312433	8.455668	9.062320	9.254350	8.696372	10.092323	9.533695	8.675156	7.671958	7.644929	6.866463	8.020889	7.274556	7.712340	8.836925	7.246521	8.447639	8.021631	7.880784	9.192277	8.507070	8.764223	9.316873	8.363776	8.089246	8.649359	7.686546	6.724651	7.087352	7.733781	9.098294	9.277081	7.948606	9.008088	8.479473	8.880498	7.709559	9.957211	7.372973	8.671577	7.408527	8.550896	9.613345	8.154391	7.941504	7.534703	7.844360	9.079479	8.849250	8.472170	8.143298	8.021631	8.888062	9.478293	8.969436	8.267918	8.038029	7.288120	8.997519	9.181715	7.862606	8.022679	8.043711	9.226836	7.569307	8.095831	7.202536	8.800815	8.913879	9.044099	8.524461	9.464857	9.070503	9.869000	8.896105	8.427087	7.819523	9.484723	6.749816	8.849097	7.501480	7.156379	8.263850	8.621435	7.474745	7.914604	7.013910	8.699691	8.387119	8.145954	7.571574	9.950580	7.144444	7.660314	9.847208	10.469499	8.619393	7.050856	7.535484	10.075427	7.545653	7.614780	7.242119	7.443459	6.574654	7.007040	8.256628	8.177095	5.805451	9.237192	6.755073	5.869386	8.063024	8.289502	7.048505	6.434113	7.520666	7.352207	8.454138	7.826623	7.160590	6.456884	6.729309	7.083939	9.795202	9.459026	10.311130	8.333092	6.507989	8.815760	9.068809	7.305240	7.647559	6.239749	5.639734	6.587534	6.705266	7.572330	8.032238	6.789740	8.523071	7.856494	7.422117	6.864078	6.973051	7.872919	5.766438	7.981758	8.564961	6.263018	8.406370	8.641137	6.931748	6.541524	8.092915	9.499476	8.760273	10.052486	7.246377	6.380125	9.982213	9.003636	7.268627	7.824155	9.469843	7.693673	8.289941	8.981810	5.545431	7.983486	7.779974	7.656423	7.525576	8.216975	7.630676	7.465866	7.087059	6.417479	7.383930	8.424232	8.034627	7.563186	8.274332	8.743900	8.643479	8.973923	7.720980	6.527992	8.642817	7.201019	8.715458	7.948606	7.914144	10.441420	7.357496	8.436316	7.050375	6.217668	6.406070	7.029717	6.079385	7.396166	8.701516	8.935159	8.319729	8.234802	8.149308	10.881335	7.019371	7.531429	10.250277	5.977794	7.036695	8.608484	7.538769	10.256478	8.025893	10.052635	9.037684	6.159451	9.256070	6.438240

AC004080.5	5.709757	7.373908	7.582572	8.743603	9.478846	8.052496	7.995977	8.950873	7.351763	9.107963	7.503220	9.304406	8.815301	9.564819	7.364834	9.085673	7.312433	8.455668	9.062320	9.254350	8.696372	10.092323	9.533695	8.675156	7.671958	7.644929	6.866463	8.020889	7.274556	7.712340	8.836925	7.246521	8.447639	8.021631	7.880784	9.192277	8.507070	8.764223	9.316873	8.363776	8.089246	8.649359	7.686546	6.724651	7.087352	7.733781	9.098294	9.277081	7.948606	9.008088	8.479473	8.880498	7.709559	9.957211	7.372973	8.671577	7.408527	8.550896	9.613345	8.154391	7.941504	7.534703	7.844360	9.079479	8.849250	8.472170	8.143298	8.021631	8.888062	9.478293	8.969436	8.267918	8.038029	7.288120	8.997519	9.181715	7.862606	8.022679	8.043711	9.226836	7.569307	8.095831	7.202536	8.800815	8.913879	9.044099	8.524461	9.464857	9.070503	9.869000	8.896105	8.427087	7.819523	9.484723	6.749816	8.849097	7.501480	7.156379	8.263850	8.621435	7.474745	7.914604	7.013910	8.699691	8.387119	8.145954	7.571574	9.950580	7.144444	7.660314	9.847208	10.469499	8.619393	7.050856	7.535484	10.075427	7.545653	7.614780	7.242119	7.443459	6.574654	7.007040	8.256628	8.177095	5.805451	9.237192	6.755073	5.869386	8.063024	8.289502	7.048505	6.434113	7.520666	7.352207	8.454138	7.826623	7.160590	6.456884	6.729309	7.083939	9.795202	9.459026	10.311130	8.333092	6.507989	8.815760	9.068809	7.305240	7.647559	6.239749	5.639734	6.587534	6.705266	7.572330	8.032238	6.789740	8.523071	7.856494	7.422117	6.864078	6.973051	7.872919	5.766438	7.981758	8.564961	6.263018	8.406370	8.641137	6.931748	6.541524	8.092915	9.499476	8.760273	10.052486	7.246377	6.380125	9.982213	9.003636	7.268627	7.824155	9.469843	7.693673	8.289941	8.981810	5.545431	7.983486	7.779974	7.656423	7.525576	8.216975	7.630676	7.465866	7.087059	6.417479	7.383930	8.424232	8.034627	7.563186	8.274332	8.743900	8.643479	8.973923	7.720980	6.527992	8.642817	7.201019	8.715458	7.948606	7.914144	10.441420	7.357496	8.436316	7.050375	6.217668	6.406070	7.029717	6.079385	7.396166	8.701516	8.935159	8.319729	8.234802	8.149308	10.881335	7.019371	7.531429	10.250277	5.977794	7.036695	8.608484	7.538769	10.256478	8.025893	10.052635	9.037684	6.159451	9.256070	6.438240

ZMIZ1-AS1	4.828889	4.650703	4.951026	5.754786	6.602610	5.939707	5.951816	4.533330	5.486535	6.372787	5.671044	5.640502	5.356463	5.187816	4.652372	6.162053	4.344134	4.860036	4.492238	5.954888	5.863467	5.088183	6.316736	5.124300	5.707611	5.572745	4.047376	5.223543	5.332046	5.115354	5.455882	5.684541	4.576446	4.024737	3.852488	5.250002	5.841877	5.078813	4.403324	3.137111	5.613677	4.235959	5.188216	5.961292	5.441215	5.988291	4.441091	5.439680	6.277988	5.637335	4.539543	5.307530	4.952506	3.499422	6.231918	5.331849	5.716576	3.064168	5.185394	4.628485	4.675590	5.239632	4.743173	4.104747	4.441709	4.573081	4.771972	5.114561	5.295428	3.027882	5.802237	4.426343	5.613082	4.936851	5.237066	4.367632	5.131015	4.441288	5.210230	5.321288	5.245026	7.025223	5.523280	6.551816	5.566556	6.104959	4.087871	6.081265	5.228700	5.893148	5.410898	4.175536	5.337335	5.255133	5.051783	6.153607	6.092548	5.812964	5.701880	5.263904	3.647551	4.986033	5.105234	5.321484	4.042090	5.391837	4.170551	3.903127	3.502755	5.090210	3.194728	5.209826	4.324870	4.462440	4.730184	2.983861	5.225509	6.088523	6.245120	4.811450	5.165881	4.085922	5.842057	4.348963	5.192874	4.164473	4.951026	4.298529	5.910785	5.406923	4.680510	4.511823	3.672507	4.496423	5.627815	4.073227	3.544693	5.020133	7.308199	5.192874	4.914529	6.191130	4.728469	7.193101	9.331538	3.497840	4.321207	5.175953	5.474270	5.240495	4.940527	6.261499	5.708310	5.187401	4.669934	5.694139	4.511602	4.990394	4.554945	5.715666	4.799428	5.010542	6.912113	4.566596	5.881412	5.007036	4.931589	6.319613	5.496273	5.413006	5.683817	5.362905	5.632079	5.774175	4.230073	5.247625	3.100817	4.655780	5.389488	4.786621	4.169878	4.271805	4.846179	5.332255	5.274932	4.752973	5.350251	4.906166	4.888751	4.970294	4.746858	5.070383	5.429745	5.002715	4.418927	4.794770	4.831864	5.051969	4.971489	5.609716	6.831645	4.608431	5.445634	5.171481	6.302900	6.560069	6.564660	3.509205	5.102775	5.012415	5.322096	4.795393	4.711800	4.574160	5.109541	4.494905	4.991830	3.942152	4.747673	4.539977	4.441288	4.012263	4.782417	4.497281	4.669702	3.591899	4.610371	4.728272	5.587921	4.135395	3.366868	3.611605	3.908575	4.053659	3.839112	6.048392	5.947212	3.904426

AC147651.1	7.408672	4.750654	4.613576	5.506211	5.875675	6.804922	5.384644	5.581712	6.266717	5.747211	5.436894	5.532739	5.331647	4.398722	3.400386	5.215220	5.861943	6.660691	7.403034	5.109345	5.760849	5.500043	7.052109	4.453761	5.830666	5.948634	5.817802	6.129664	6.176949	4.779065	6.332318	5.760681	4.098630	5.989717	5.490755	5.092471	4.351778	6.198428	5.123314	4.748729	4.788469	5.990613	5.277165	5.846771	5.874946	4.614899	5.783971	5.893525	5.311070	5.269656	6.219047	5.131993	6.371774	4.376947	5.880687	4.672733	5.902203	5.761773	6.090105	6.030746	5.547131	5.130587	5.127555	5.836646	5.873704	5.603982	5.145926	5.077351	5.342558	5.297077	4.456699	5.345357	5.078813	5.186207	5.304609	5.343992	5.386330	5.585157	5.440614	4.532908	5.464226	5.765730	7.309440	5.279964	5.208018	5.998669	3.721214	5.863467	5.343606	5.524600	5.788863	5.367584	5.271428	4.967792	6.149697	5.654829	5.631190	4.747061	5.438752	5.729176	3.516224	5.191332	5.325747	5.176738	6.107820	5.076314	6.053381	5.434751	5.884422	4.960407	5.631017	5.706630	5.841877	5.101129	5.350035	6.315570	7.968486	6.625166	4.776982	6.074144	5.753589	5.137013	5.230986	7.148188	7.246079	5.688415	7.194717	7.308348	7.154192	6.046399	6.525135	6.119556	3.840275	5.530117	6.905093	7.014228	6.934574	6.172188	5.457267	4.170305	7.587332	7.653754	5.880506	7.526669	5.783423	3.685044	5.600431	5.095809	5.902746	6.117492	5.275754	4.491993	6.264178	6.470376	6.630470	4.112673	6.853335	5.932286	6.851706	5.194061	5.445055	4.567244	7.288452	6.420124	4.367199	5.257140	6.899975	5.137404	5.993385	6.314873	5.554340	7.195198	4.144522	5.254737	5.176324	5.749929	5.798581	5.879616	6.307251	5.973624	5.057972	5.540532	5.871338	5.780710	5.771071	5.983672	4.921258	5.050399	6.010871	5.807043	4.686755	5.034412	4.380795	5.671606	5.640329	5.000890	6.880073	5.901849	3.919566	7.129553	5.705784	7.524222	5.046279	6.822459	6.907201	5.018899	6.487592	5.368189	6.015887	6.318752	6.460347	5.859607	5.784669	6.175587	5.575947	5.775818	6.765217	5.305796	8.038673	6.269643	7.432820	5.633992	4.192784	8.214100	4.896655	6.822304	6.283128	6.080402	4.677740	5.315035	4.861046	5.383539	5.403080	7.933245	6.617746	6.608840	7.782573	5.455701

AC145207.2	14.300589	14.466587	14.495138	14.563436	14.509938	14.299113	14.273526	14.051810	14.231500	14.463188	14.371917	14.236872	14.303599	14.368027	14.159585	14.246286	14.608380	14.508773	14.972581	14.154529	14.512377	14.320552	14.743181	14.189577	14.301922	14.347441	14.653700	14.483264	14.100097	14.059470	14.485225	14.885495	14.184313	14.333706	14.177090	13.763467	14.324726	14.508030	14.611896	14.596200	14.239437	14.316111	14.594880	14.353021	14.650647	14.186537	14.207485	14.166159	14.189577	14.147617	14.091572	14.356236	13.995849	13.664721	14.302959	14.289961	14.918365	14.687432	14.625993	14.859475	14.131743	14.493157	13.880400	14.202755	14.316814	14.704362	14.164594	14.629113	14.173886	14.269566	13.829453	13.971862	14.229504	14.274191	14.160557	14.115590	14.255462	14.507205	13.521387	14.285224	14.590670	14.513178	14.239437	14.267351	14.295373	14.064122	14.062109	14.628138	14.274531	14.286202	13.897223	14.224386	14.093399	13.876318	13.794844	14.455390	14.188094	14.510745	14.209806	14.581809	14.148848	14.617785	14.531879	14.664523	14.858502	14.533492	13.933765	14.855412	14.742275	14.304308	14.636601	14.405515	15.041784	14.104742	14.756024	13.105616	14.262592	14.791560	14.455390	14.070910	14.960579	14.180553	14.436781	14.297379	14.434912	14.610103	14.476349	15.190719	14.461603	14.848968	15.001364	15.133964	14.956666	14.971474	14.569736	14.981620	14.961694	14.825429	14.766663	14.985105	14.828938	15.427861	15.021020	15.469004	14.668633	14.533864	15.072836	14.362657	14.659090	14.316430	14.498295	15.244645	14.508030	13.976074	14.711236	14.458184	14.086526	15.505597	15.056697	14.668191	14.140212	15.185427	14.934577	14.699677	14.725604	14.199474	14.097959	14.158638	14.989606	14.422502	14.543837	15.036921	15.241129	14.926494	14.661796	14.087427	14.553562	14.822343	14.229183	14.616554	14.807649	15.260285	15.344956	13.810158	14.316111	14.430074	14.328965	14.459642	14.233539	14.170304	14.406946	14.819862	14.344919	14.193049	14.582286	14.419519	13.870453	14.713935	14.680064	15.246670	14.743665	15.092451	14.716286	14.933539	14.759633	14.367353	14.608827	14.642226	14.616554	14.731220	14.899137	14.406566	14.786789	14.640110	14.463188	14.278587	14.813710	14.722869	14.612714	15.321822	13.818086	14.887119	14.511123	14.915189	14.409900	14.812187	14.985629	15.036300	14.449082	14.993988	14.752647	13.744349	14.167781	13.741572	14.355885	15.062228	14.387857	14.707989

KIAA0087	5.141531	4.584561	4.929751	4.985191	4.386959	4.866428	4.736757	3.821651	6.132434	5.076102	5.714759	4.890226	3.965297	3.815433	3.691374	3.992227	5.005368	4.067751	5.745522	4.682853	5.237270	5.956634	7.252922	4.796034	4.736757	5.766438	4.603961	5.047697	3.998329	4.467164	4.836806	4.709907	5.082049	4.661156	4.585625	5.552778	4.200088	5.199110	6.235988	6.256911	4.462233	7.542477	5.214446	5.022393	4.854383	5.795999	4.909682	5.358725	5.457054	4.812916	4.762246	4.257422	4.789520	4.348297	4.006960	4.656195	4.221265	4.818987	4.533330	4.807440	4.879022	3.972227	5.361147	5.384644	4.482874	4.542417	4.504144	5.093331	4.424419	4.941572	4.275288	4.259179	4.938279	4.679456	4.508763	4.540205	3.885110	5.475614	4.281251	4.834783	4.851376	4.509419	4.289621	5.084041	5.113938	5.475801	5.468537	5.208635	5.289165	4.884088	5.669061	5.397863	4.691364	4.764873	4.580993	4.947342	4.429095	5.705436	4.547511	4.139961	4.348753	4.852460	3.732517	4.776361	5.319391	5.754786	5.127355	4.021786	4.334415	4.992229	5.362349	4.872577	5.384096	4.776555	3.894646	5.502197	4.814353	4.348075	4.432687	4.944987	4.163837	4.098433	4.522996	3.289829	5.447016	4.766370	4.967125	5.633382	5.033779	4.855023	5.201863	4.562063	3.957709	3.368081	4.113121	4.601427	3.684027	5.445822	4.425470	5.141118	4.878379	6.638708	4.528201	4.607348	5.902563	4.513162	5.262870	4.915972	4.926168	4.660271	4.703636	3.449554	4.077444	4.853765	5.175557	4.621044	5.305197	6.597774	5.895831	4.865170	7.653147	4.973155	4.754468	4.566368	5.266469	5.268846	7.563478	4.802376	4.974416	4.311250	4.431388	4.321207	4.487427	5.184212	4.778032	4.018242	5.528031	4.101695	5.614049	5.296854	4.412414	4.957295	5.563155	5.160478	4.890438	4.094334	4.440677	5.230559	5.693030	4.576446	4.921258	4.658804	5.014852	4.787444	4.333078	4.463060	5.756839	4.654176	4.062858	5.010145	4.617724	4.893127	4.968821	5.594934	5.416020	5.247413	5.389689	4.865170	4.590982	8.362543	5.612522	4.638630	5.377319	5.045882	4.700882	4.395671	4.749752	4.328317	3.956576	4.636077	3.567669	3.771535	4.658359	5.146958	5.780710	4.449155	4.597046	5.118456	4.775942	5.536460	4.049784	4.402892	5.320716	4.600410	3.968092	4.554548	4.007400	4.393198

ATP1A1-AS1	9.351391	8.649523	8.406806	7.985912	7.783924	7.456680	8.261839	6.491380	6.369034	7.689945	7.536401	6.924197	7.191824	7.994418	8.121159	7.473087	6.960860	7.747653	6.564337	7.944176	6.643709	7.016496	7.739775	7.919491	7.854689	7.216709	7.264789	7.441756	7.828229	8.115905	6.577340	9.000419	8.839797	8.196996	8.610302	8.589143	8.681429	8.396241	8.343224	8.006259	7.686546	8.583613	7.903028	8.429598	8.787017	8.357526	6.466356	7.612965	8.036665	8.170358	8.765747	8.471683	8.899150	8.793982	7.912361	7.485213	9.226836	9.154319	8.324297	8.907201	9.301890	7.945557	7.946342	7.559365	9.124574	7.893570	9.052042	7.921791	9.035594	8.156255	8.735320	8.471849	7.004242	7.760697	8.285627	8.043864	8.890530	7.758541	8.836326	7.962366	7.929278	8.145338	8.701221	9.110179	8.903213	8.575963	8.691536	8.385123	8.049687	8.556238	8.481554	7.696523	8.482967	7.940255	9.319298	8.759794	8.400640	7.090251	7.224502	7.753171	5.703574	8.417966	7.816672	7.994730	6.403439	7.012195	6.112642	6.609496	6.428172	7.406634	8.633684	6.334340	6.934887	7.597303	7.715979	4.162019	9.123673	7.060771	6.718122	8.310673	7.938385	8.195435	6.995990	5.948433	6.126600	6.068771	6.477812	6.169288	6.968714	7.360429	8.340131	5.928111	6.580287	7.366862	7.140632	7.683036	7.335968	6.647222	7.714346	6.994310	7.004886	7.184970	7.184084	7.250091	7.944322	6.816191	7.234047	6.640806	8.067133	6.354414	7.234209	8.926272	7.997674	7.895767	9.598034	7.579177	8.469804	8.318651	7.935898	6.620820	8.067133	7.645719	7.566103	7.693526	7.608684	6.470376	7.755300	8.107736	8.096450	8.305299	8.878154	8.928332	11.123080	8.572331	7.218710	9.319298	8.778417	8.493243	8.743748	7.401308	7.966042	7.906308	7.329623	8.397333	8.569732	7.789614	7.628611	7.916296	8.860469	8.182820	8.033303	6.534701	7.681937	8.347200	7.883365	9.286431	8.146426	7.772318	7.696675	7.194888	7.130189	7.380361	7.870902	9.340437	7.596408	7.914144	9.362614	7.804933	7.813075	7.478884	8.129950	9.489997	7.626255	7.339873	7.837808	8.232255	5.672408	6.724651	8.608784	7.015993	9.350323	7.144750	8.886372	8.611857	6.323377	5.582847	7.558763	5.829913	6.762196	6.748935	6.151907	9.174484	6.580113	7.627673	5.921773	8.015395	7.143465	8.318216

LINC02389	9.630443	9.774645	9.733340	10.114501	10.407984	10.981739	8.757756	11.164944	11.980027	9.680950	9.686383	9.491096	8.969436	8.925347	8.402059	10.458339	10.925062	8.721903	9.057511	9.814640	8.757916	9.511023	9.221577	9.285962	9.303709	9.999817	11.531684	10.209666	10.531004	10.366528	10.661160	8.374041	8.516237	9.763401	8.987805	9.172960	9.206866	9.846295	9.672393	9.252081	10.188009	9.513068	8.457490	9.170296	8.729876	9.030848	11.605218	8.522011	9.540496	8.622822	8.651036	8.163242	7.912658	8.521708	8.039326	11.101213	7.719509	8.191175	8.343510	8.131342	8.126763	10.969857	9.688696	9.602061	8.107430	8.078855	8.963724	9.470011	8.628077	9.189192	9.656270	9.736421	10.873539	9.089930	9.241954	9.871223	9.720011	9.268924	9.464406	8.393192	8.498529	8.341455	7.752712	7.873506	8.398835	8.633518	9.231170	8.472968	8.302435	8.410935	8.112700	8.726942	9.238440	9.436629	10.056961	8.843116	8.534236	9.369204	10.134839	10.866824	10.533808	9.243504	9.518843	9.565422	9.898075	10.131533	11.228716	9.975738	10.581000	11.162156	9.838908	10.341269	10.096988	11.733178	10.302178	12.947738	10.177481	9.898224	11.313224	11.018364	10.678072	10.339746	10.411625	11.993060	10.772399	10.446887	11.852935	10.596938	10.249543	9.985649	10.182107	9.954281	9.539708	9.942272	10.982768	10.301733	10.023543	9.279219	9.296135	9.805482	10.372281	9.101829	10.123807	9.676141	8.935314	11.024358	10.468732	12.092757	10.668858	11.415128	10.331914	9.598341	9.884840	9.214386	9.498564	9.576673	11.379601	10.133901	9.667979	11.533627	9.898694	8.224479	9.498731	9.181558	8.999951	12.035336	9.353931	9.386834	8.997966	9.370599	9.085192	8.513995	9.209310	8.646197	10.029332	8.702764	8.566718	8.984549	8.296548	8.955627	10.046931	9.036629	9.329557	9.156011	8.739179	9.403550	9.721234	9.863318	9.015924	9.948435	10.157573	12.203886	9.867261	9.874226	9.884227	9.950116	9.869780	9.729350	8.987489	8.636601	8.492586	8.409690	9.102283	8.984879	10.021399	8.677427	8.394381	9.122611	9.032354	8.866575	9.010403	9.553589	9.743043	9.716241	9.909707	9.955081	10.466388	10.349760	9.346191	10.652206	10.659191	10.809503	9.796404	9.419441	11.017200	10.751302	9.835366	9.521779	11.938348	10.328083	10.985538	11.518100	13.128058	11.158191	10.383188	9.935596	10.171845	10.642239

LINC02231	9.630443	9.774645	9.733340	10.114501	10.407984	10.981739	8.757756	11.164944	11.980027	9.680950	9.686383	9.491096	8.969436	8.925347	8.402059	10.458339	10.925062	8.721903	9.057511	9.814640	8.757916	9.511023	9.221577	9.285962	9.303709	9.999817	11.531684	10.209666	10.531004	10.366528	10.661160	8.374041	8.516237	9.763401	8.987805	9.172960	9.206866	9.846295	9.672393	9.252081	10.188009	9.513068	8.457490	9.170296	8.729876	9.030848	11.605218	8.522011	9.540496	8.622822	8.651036	8.163242	7.912658	8.521708	8.039326	11.101213	7.719509	8.191175	8.343510	8.131342	8.126763	10.969857	9.688696	9.602061	8.107430	8.078855	8.963724	9.470011	8.628077	9.189192	9.656270	9.736421	10.873539	9.089930	9.241954	9.871223	9.720011	9.268924	9.464406	8.393192	8.498529	8.341455	7.752712	7.873506	8.398835	8.633518	9.231170	8.472968	8.302435	8.410935	8.112700	8.726942	9.238440	9.436629	10.056961	8.843116	8.534236	9.369204	10.134839	10.866824	10.533808	9.243504	9.518843	9.565422	9.898075	10.131533	11.228716	9.975738	10.581000	11.162156	9.838908	10.341269	10.096988	11.733178	10.302178	12.947738	10.177481	9.898224	11.313224	11.018364	10.678072	10.339746	10.411625	11.993060	10.772399	10.446887	11.852935	10.596938	10.249543	9.985649	10.182107	9.954281	9.539708	9.942272	10.982768	10.301733	10.023543	9.279219	9.296135	9.805482	10.372281	9.101829	10.123807	9.676141	8.935314	11.024358	10.468732	12.092757	10.668858	11.415128	10.331914	9.598341	9.884840	9.214386	9.498564	9.576673	11.379601	10.133901	9.667979	11.533627	9.898694	8.224479	9.498731	9.181558	8.999951	12.035336	9.353931	9.386834	8.997966	9.370599	9.085192	8.513995	9.209310	8.646197	10.029332	8.702764	8.566718	8.984549	8.296548	8.955627	10.046931	9.036629	9.329557	9.156011	8.739179	9.403550	9.721234	9.863318	9.015924	9.948435	10.157573	12.203886	9.867261	9.874226	9.884227	9.950116	9.869780	9.729350	8.987489	8.636601	8.492586	8.409690	9.102283	8.984879	10.021399	8.677427	8.394381	9.122611	9.032354	8.866575	9.010403	9.553589	9.743043	9.716241	9.909707	9.955081	10.466388	10.349760	9.346191	10.652206	10.659191	10.809503	9.796404	9.419441	11.017200	10.751302	9.835366	9.521779	11.938348	10.328083	10.985538	11.518100	13.128058	11.158191	10.383188	9.935596	10.171845	10.642239

MIR3945HG	6.273716	5.907765	6.479464	5.489780	6.387666	6.370223	5.269057	6.178831	6.133452	4.652372	6.249790	5.557350	5.873704	5.642156	5.148725	5.389141	5.614444	5.631190	4.745325	5.102586	4.616876	6.072743	6.162362	5.182603	5.374165	5.491117	6.642422	6.052366	5.356463	5.565388	4.707020	7.066825	7.636629	5.862318	5.113751	5.633578	5.855990	4.554733	4.816230	5.220376	5.344374	4.856270	5.769200	6.221944	4.987279	4.926830	5.682715	5.091237	6.940287	5.797459	6.634185	5.228497	6.301676	5.005368	6.895266	6.093732	6.962768	3.098676	6.413207	6.081807	5.663324	6.122172	6.619878	5.672198	5.379836	6.378320	4.868318	5.884247	5.683252	5.609543	4.971305	5.571459	5.626160	5.815457	4.962098	6.453478	6.203297	8.292913	6.407375	5.841877	6.048575	6.088848	7.059481	5.546160	5.276363	7.488204	6.749103	6.645317	3.414533	5.160478	4.665178	5.992335	6.610626	6.320848	6.190442	5.478477	6.357670	5.827305	6.595518	5.595686	6.906051	5.274932	5.408524	5.653503	6.831645	5.991295	7.032025	5.812237	6.899672	5.084041	6.290129	5.926480	6.198772	5.700984	6.838662	7.978147	6.300828	6.380453	5.567473	6.099247	6.931129	6.565283	5.075898	7.169340	6.935695	7.780760	6.163918	5.719936	6.115788	7.788679	6.809699	6.839672	5.974825	5.863653	6.614638	5.775099	6.826732	5.313797	5.810403	5.608956	6.468519	6.910160	6.741451	5.888143	6.967570	7.971306	6.524506	6.058707	5.637335	6.076577	8.461720	7.306185	6.951549	6.082328	6.237788	7.367488	6.722079	5.750837	6.873531	6.511783	6.798652	6.057995	6.863457	7.224502	6.082166	6.524017	4.827398	6.614141	6.440230	8.508601	6.625668	6.810491	8.010662	6.417161	5.100098	5.879616	5.330083	6.299881	6.226232	7.171477	6.798331	5.728468	6.260984	5.749031	6.276382	6.055495	7.751504	7.979734	6.134299	5.914194	7.198379	7.585336	6.539396	5.493723	7.600158	6.965797	6.404074	7.296211	7.630212	7.208153	6.063123	9.875925	8.416918	6.062264	7.309276	7.721779	6.640640	5.119275	6.471389	6.860916	8.287044	6.859944	6.444900	6.874986	6.918844	7.210174	7.272982	8.811877	5.796183	6.912288	6.258431	5.635932	6.430895	6.607406	5.860123	6.895426	6.552176	6.293010	6.253184	7.654830	6.684412	5.906156	6.268961	6.498041	7.077825	6.323058	5.858327	7.171017

AL359313.1	4.233943	4.973998	4.983403	5.218747	4.997562	5.539006	4.950017	5.148725	5.970151	5.298266	5.354518	5.140676	4.636935	4.824132	3.601673	5.156441	4.767260	5.493530	9.057845	4.704262	4.932210	6.400824	7.324693	5.331441	6.300025	5.472912	5.652761	5.733384	5.468334	4.780536	5.340663	5.631375	5.885856	5.487463	5.397471	5.144534	5.333817	4.827398	5.460233	5.653303	4.843806	5.195317	5.444863	5.413965	5.650491	6.521703	5.564296	6.665571	5.673669	4.850372	4.674733	4.514410	5.008061	5.556954	4.904841	5.102166	5.420915	3.331586	4.728053	5.324222	5.264099	5.039717	4.666242	5.707790	4.209698	5.279336	4.938708	4.542193	4.481255	5.199677	4.691987	5.021985	5.130200	4.613377	4.545332	5.051783	4.971489	4.370684	4.460696	5.568071	4.968204	4.485024	4.416652	5.132818	5.448784	5.592356	5.335333	4.608201	4.311473	4.535232	4.461347	5.373984	5.283568	4.769494	4.693307	5.037736	5.315439	6.319966	5.909357	5.301434	6.467861	5.217771	4.761419	4.988511	5.750286	5.675664	5.763409	5.865783	6.052855	5.297845	5.950728	5.801382	5.887979	5.890639	5.981184	6.663954	5.630622	5.528031	4.979752	4.586047	4.741718	4.694948	5.391069	4.872577	5.410127	5.201083	5.359285	4.616662	4.948949	4.963948	5.436282	5.722901	4.666684	5.241641	4.982359	4.392978	5.031741	6.550701	4.238087	5.594019	5.696389	6.270170	5.193652	6.224175	4.585201	4.794770	5.413965	5.588697	4.474278	4.988511	5.845087	5.553381	5.597613	4.348753	5.029742	4.739990	4.587990	5.171907	5.230761	4.826274	4.885618	5.052568	5.166666	4.941365	6.268096	5.663513	4.228110	5.166466	3.979980	4.411994	3.902945	3.723903	5.627262	4.500504	5.095423	4.952935	3.437226	4.736955	3.936663	5.018899	5.087383	4.126990	3.649022	4.572417	4.121850	2.967290	3.817375	3.999159	4.709048	4.521910	4.314992	5.499857	4.379297	4.409371	4.657286	4.598769	4.255015	4.201853	4.767009	3.623459	4.308576	4.222766	4.816230	4.255667	5.396337	4.250189	4.430265	5.276363	4.330557	4.612707	5.126963	4.450509	4.580787	4.744005	4.968821	5.817802	6.572737	5.638981	4.276183	5.675497	4.689059	4.911116	5.428638	5.016059	5.168494	5.267688	5.761196	4.895587	5.658330	6.474906	5.885332	8.380355	5.616498	5.442550	5.352869	5.145152	5.046279	4.762246

AC009950.1	3.021888	4.131034	5.291008	4.518473	4.773477	4.145567	5.069189	3.289829	3.705075	4.284509	4.964161	4.532041	4.596385	3.841530	5.435317	4.714127	3.440314	4.888117	5.872251	4.178992	4.170991	2.923401	4.408498	4.098180	5.765200	4.179211	3.898221	4.347207	3.550026	4.100192	3.585521	4.996504	6.011945	4.366995	6.107290	5.183016	5.074237	4.135625	4.071686	4.683097	4.374567	3.159038	4.936617	5.451663	5.034193	4.679862	5.355901	3.445983	5.117409	5.375342	5.695092	5.079600	6.237135	5.872632	6.009468	3.783146	6.202255	5.195705	3.408374	4.811255	4.399157	4.502005	3.565350	3.566109	4.554548	4.363938	4.759133	4.162728	5.151558	3.773875	4.711587	5.146547	4.709478	5.438573	5.322870	5.694530	5.049796	4.808479	4.615553	5.436894	4.240349	5.877095	4.644693	4.400062	4.973155	5.582847	4.193897	4.538874	3.502150	4.714337	4.217298	3.501239	4.479330	5.196121	4.746389	6.015023	5.396922	5.454938	3.656622	5.109134	3.548744	5.348703	4.873219	5.299668	4.214696	5.356081	3.615725	3.571891	3.084859	4.166893	2.928153	3.789732	3.428828	4.283814	5.118860	3.510558	3.494846	4.146429	4.278859	3.173949	3.249707	3.746283	3.572687	3.351071	3.365338	3.332078	3.938758	3.047744	3.902722	3.073894	3.239185	4.687586	3.304924	3.969670	3.484252	3.190658	3.947935	3.155076	3.722103	3.679614	3.944424	5.848448	3.551628	3.412024	4.478041	3.704057	3.829065	3.450800	3.438428	3.355340	4.004337	3.918319	3.267793	4.622687	3.995497	3.478586	3.039623	3.246827	3.050905	3.134142	3.131125	4.565758	4.701513	4.571394	4.443268	4.510286	3.307599	5.230761	3.906188	4.933652	3.163387	4.918304	4.359819	4.770981	4.127852	3.695478	5.298453	3.162054	5.017906	3.067954	3.211727	3.601673	3.375281	4.832709	4.389151	3.100934	4.173575	3.522236	3.046894	3.228776	3.236086	3.144331	5.033991	4.420222	6.195488	3.830333	4.745325	3.573251	5.984369	3.463925	4.659199	5.589666	4.273323	5.657036	3.790356	4.027744	3.885300	3.311535	3.994643	3.375064	3.525510	4.026036	3.214885	4.261080	4.336340	4.063276	3.608025	3.634285	3.914226	2.924830	3.595938	5.380410	3.906869	4.639678	4.439375	3.234227	3.235083	3.268690	3.071207	2.881962	3.041680	3.913993	3.162054	3.388778	4.888117	3.585898	3.260848	3.319776

AL162457.1	14.274870	9.338896	9.500267	11.734962	10.743513	11.400278	10.147476	11.451020	12.079851	11.738062	12.244853	11.500204	10.679279	10.242296	9.600027	10.493458	11.346391	9.731236	10.780444	9.985187	10.041915	10.471332	11.828835	10.015244	10.230861	11.174150	12.028718	11.060999	11.446009	10.770306	11.828693	11.248361	9.109712	10.469201	9.235309	9.487673	9.415037	9.330587	9.884840	10.055298	11.206863	9.892101	9.670699	9.970631	9.498106	9.818272	12.507547	9.276071	9.900076	8.969436	11.480786	9.057993	8.288418	8.798544	8.782909	11.643705	8.534684	9.111762	9.121540	9.068474	9.352074	11.443990	10.080574	10.069492	9.361550	9.675852	9.494561	9.336944	9.242578	10.213349	9.703107	9.864543	11.549154	9.341898	9.548845	9.918845	9.771131	9.541747	12.017489	8.870565	10.048802	8.827314	10.476151	9.018966	9.004704	9.377318	9.323513	9.205823	9.771440	9.738831	9.384220	10.364578	9.585307	9.760100	11.932141	9.783615	9.366774	10.352828	11.178065	10.701056	11.082044	9.468725	10.216834	9.848968	10.183009	11.102824	10.930245	10.216098	11.752925	11.616246	11.292805	10.637586	10.398387	12.431277	11.074065	13.334349	11.565671	10.748441	12.193531	11.210101	10.125679	9.991411	10.440967	11.988461	11.417391	11.715283	11.747192	10.958719	11.315577	11.582863	11.897073	11.272361	10.796728	11.042718	11.327340	10.641061	10.512425	11.277646	10.211657	10.871143	10.627292	11.398667	11.069585	10.635345	10.121103	11.321288	11.424939	12.949293	10.953768	12.342480	10.348265	10.148077	10.202804	9.468105	9.770001	9.685777	10.342153	10.614651	10.503462	12.253114	10.215186	9.538600	10.342477	9.478608	9.878741	12.400465	9.631239	9.754711	9.282455	10.054496	9.479088	8.687013	9.095560	9.130166	10.553234	8.849578	9.151110	9.529145	9.744747	9.736555	10.488104	9.920741	9.718135	9.957845	9.835686	10.043446	9.520834	10.030841	10.132147	10.070208	10.150520	13.101611	9.761660	10.140152	9.689137	9.925599	10.132853	10.362545	9.546391	8.947669	9.092981	9.441911	9.711552	9.080577	10.828444	9.078242	9.093582	9.937579	9.679086	9.711552	10.566491	10.163031	10.367141	10.680054	11.057484	11.037724	11.207013	10.697935	9.558934	11.372021	10.467548	11.510826	9.902118	10.985236	11.830475	11.182859	10.008647	10.735064	11.712469	11.474238	10.759120	11.816015	13.576005	11.550609	11.013094	9.962244	10.550696	11.474531

SLC7A11-AS1	13.318885	9.810408	9.356658	10.707919	8.947836	10.079690	10.626098	11.672364	10.269126	9.851210	10.155626	9.955081	10.843616	11.251075	9.057352	10.134839	10.635198	8.975115	10.963392	11.074426	9.039784	9.650087	8.667128	8.889622	8.537828	10.016803	8.157342	9.545767	10.418738	11.765719	10.308628	11.927081	10.764174	11.865618	9.591500	10.387951	9.659667	10.650301	11.029707	10.594114	9.578851	8.810595	9.041919	10.021399	9.142130	10.588224	9.280909	10.295091	10.219095	10.193562	10.491887	10.356463	9.542983	10.641644	9.740576	10.899668	9.930473	9.057511	8.837234	9.628003	8.640670	12.030784	8.501222	9.761826	9.850577	9.674122	9.599253	9.543140	11.022434	9.430494	10.016190	11.899287	8.506105	10.819087	10.199765	10.134060	10.239706	11.442042	10.547653	10.893147	11.076776	11.564336	12.302631	11.072990	10.616277	10.671967	12.160336	11.544514	10.234797	10.043446	9.115721	9.740576	9.919633	10.517413	10.776828	10.122625	9.836740	10.478589	11.115020	8.354442	11.613605	11.320390	9.768766	8.834349	12.438515	10.604444	7.413356	11.893367	10.420414	10.557083	9.036324	8.289633	11.036408	10.016345	9.202358	9.801046	10.849009	11.031948	10.383040	11.488900	11.856087	9.758574	9.168451	13.461357	13.847197	13.758124	9.002437	12.990709	14.773263	12.623496	13.198527	13.650755	9.182666	13.686442	9.161231	14.957224	13.358864	7.048016	9.816653	14.321264	14.539639	14.472293	9.899900	10.352666	15.172005	11.291291	14.169692	13.189888	14.342816	12.467952	12.910727	14.007596	11.651970	10.569539	14.034970	11.993963	11.722134	12.007616	12.360524	11.145141	13.408151	13.360596	10.175472	13.978627	14.528293	8.925660	8.421655	14.413643	14.099791	14.462773	14.464405	13.088702	11.511259	10.508722	13.981326	10.516242	11.424053	10.986127	11.031349	10.893753	8.882342	12.497991	10.953197	10.255734	8.832900	11.451147	12.591670	14.144293	10.980998	14.570106	14.019284	11.585439	14.269900	12.954599	12.148544	14.229857	12.060702	13.429298	8.851059	12.624833	11.859940	13.216575	12.676354	11.680103	12.320466	12.208987	9.636374	9.157875	12.026746	10.862604	11.275464	10.505710	13.158777	12.766737	11.768847	14.621136	13.432615	13.931123	14.414362	10.363418	8.562976	7.842803	12.565935	9.633519	12.366882	11.145736	13.260641	14.176188	9.355751	11.479023	9.802880	8.670956	7.988227	13.893951	12.926482	13.248301	14.029080	12.378188

AL645568.1	7.781203	7.274066	7.456825	7.595187	7.537785	7.603445	7.200242	8.178706	8.241455	7.078474	7.521618	7.042248	7.050084	6.910830	6.990315	7.185369	7.752712	7.212144	8.199029	7.352977	7.367178	7.529175	8.612986	7.640962	7.436017	7.788217	8.227358	8.335866	7.099873	7.052261	7.819822	7.152778	8.229033	7.046406	7.226223	7.720231	7.555345	7.495847	7.514055	7.990498	7.440209	7.476161	7.701304	8.092315	7.540820	6.903209	8.970829	7.769428	7.727157	7.572630	7.767463	7.738827	7.683512	7.453866	7.493195	6.667856	7.544378	7.986694	7.446170	7.820856	7.700541	6.904766	7.874891	7.592717	7.479029	7.726686	7.368431	7.536569	9.814319	7.667305	7.318527	7.067750	7.252922	7.418833	7.520020	7.690235	8.216204	7.498515	7.824617	7.828836	7.304762	7.626701	7.498978	7.356854	7.836137	7.956657	7.861985	7.939154	8.047520	8.030875	8.206933	8.366808	7.552226	7.348503	7.595792	7.467854	7.652974	6.738763	6.577833	7.120267	6.119404	6.782927	7.057146	7.167633	7.643849	7.365150	7.549287	7.188899	8.103097	7.249473	7.937592	7.521618	7.526374	7.205220	7.278770	7.243579	8.421514	7.078318	7.636483	7.557866	7.696675	7.820271	7.459019	7.675124	7.889199	7.220994	7.809896	8.123421	7.608977	7.131001	7.049913	7.388100	6.913240	7.073993	7.369846	8.323519	7.249008	8.129817	8.148697	7.794622	7.850645	8.571418	7.857100	7.457944	8.180988	7.899257	7.844523	7.535941	7.375004	8.459410	7.532225	8.095531	7.361058	7.735340	8.029333	7.604509	8.131953	7.722684	8.709631	7.654519	8.592526	8.478374	8.886537	7.542797	7.355023	8.333829	7.876419	8.190857	8.203475	8.065802	8.147919	7.938524	7.692283	7.872133	6.833118	7.990179	8.169095	7.808635	8.091424	8.290087	7.329294	8.114241	7.983196	7.940095	8.056978	7.927272	8.198081	12.432257	8.314211	7.853434	7.821963	6.818962	7.856943	8.001992	7.929428	8.562835	8.044166	8.311931	7.937457	7.450282	8.170037	7.840310	7.663886	8.230865	7.919983	8.285007	8.193995	8.182981	8.147026	8.526581	8.893154	8.433526	8.042008	7.762671	7.792640	8.021631	7.500997	7.181093	7.509989	6.597946	7.385763	6.942471	7.788842	7.812165	7.288615	8.164264	7.552684	8.779155	7.202847	8.576857	7.979579	9.114833	7.168099	7.919331	6.744168	8.263388	7.064300	7.552843

AC007663.2	5.452642	6.094796	5.808321	5.715301	5.720120	5.721949	5.842614	5.783252	7.015335	5.542439	5.745163	4.901535	4.742945	5.651060	5.816007	6.007890	5.604154	5.840959	6.197075	5.846583	5.691168	5.234225	6.977046	5.160478	5.968070	6.006989	6.809699	6.479620	6.509101	5.925083	6.156523	6.448680	6.748786	5.330483	6.754571	5.462754	6.706060	6.382153	6.053723	6.017083	6.546953	6.550531	5.532003	6.235310	5.740241	7.208936	7.488204	5.622037	6.039123	5.853436	5.818145	6.088523	6.359697	6.885835	5.379059	6.249479	5.785415	6.296916	5.485941	5.850596	6.223644	6.624661	6.910655	5.974996	6.011759	5.552587	6.608356	6.219210	6.097863	5.959854	6.683902	6.754404	6.299534	6.006099	6.185718	6.281779	6.498576	6.116491	5.949174	5.360226	5.096594	6.056734	5.839528	6.240584	6.630313	6.033281	5.689540	5.535863	5.241252	5.498722	6.636123	5.467351	5.437070	5.574617	5.105833	5.539969	4.419593	5.499484	5.289592	5.917236	4.788690	6.143417	6.260150	5.928111	5.236428	6.157198	5.837310	5.805451	4.917062	6.565605	6.306781	5.253360	4.742727	6.537292	6.006989	5.298852	5.332458	5.349077	6.483079	5.270454	5.638080	5.813892	6.038087	5.934753	5.974298	4.279959	5.854561	6.713426	5.095809	5.002508	4.750429	3.960098	5.042632	5.173168	5.675316	5.593652	4.515925	5.118642	5.588122	4.683764	4.972948	6.005022	5.266871	5.483684	5.302043	5.156018	5.570508	5.118860	6.304614	5.989903	5.195705	4.984580	5.218532	5.797459	5.265055	5.435125	5.043462	5.846949	5.358725	5.973067	5.353060	6.080902	5.382322	4.404426	6.627577	7.270643	5.913674	5.398631	5.886031	4.821065	5.260230	4.609293	6.104959	5.634357	6.353092	3.781660	5.497353	3.872654	4.160365	5.106012	6.473593	5.561114	5.636952	5.739353	4.815587	5.621670	5.705050	6.258431	5.877609	5.910637	6.077446	6.259130	5.709757	5.910459	5.760681	6.093378	5.968264	5.923827	4.864340	4.801541	5.315660	4.933461	5.132404	5.387789	4.571619	5.226103	7.517578	5.215041	4.965827	5.411091	5.026259	4.408936	5.392206	5.236029	5.056350	4.433919	4.399847	4.412855	5.490389	4.354362	5.855636	5.439863	5.556004	5.359108	6.271705	5.953089	5.140892	5.243216	6.280180	5.018300	4.997172	3.557169	6.054615	5.777437	5.862898	5.855636	5.260447	6.330096

MIR4458HG	3.084269	3.175876	2.941729	2.972058	2.883547	3.059943	3.141816	3.039947	3.292959	3.096025	3.254330	3.006239	3.200632	3.453144	3.318473	3.060878	3.232699	2.997133	4.251298	3.316203	5.505652	3.064517	2.985231	2.944347	3.025453	2.940245	3.010212	2.848679	3.164694	3.012499	3.462151	3.066396	4.408699	3.182313	3.085489	3.800534	3.294849	3.107814	3.188761	3.186761	3.381552	3.008683	3.481536	3.156053	3.613360	4.720248	4.963547	3.235381	4.675173	3.344944	3.140544	3.047416	3.463048	3.973522	3.632948	3.047744	4.047589	3.873072	3.457869	3.762166	2.882424	3.080099	3.739768	3.158273	3.370267	3.382935	3.366868	3.147914	3.184892	2.857923	2.971345	2.909563	3.157896	2.877304	2.906663	3.291100	2.991507	2.989405	2.955609	3.542578	3.229042	3.605404	3.337787	3.868591	3.272865	5.156870	2.879294	3.243404	4.216443	3.545073	3.175876	3.127099	3.806168	3.139936	2.902673	2.918499	3.392300	3.978461	3.779768	3.109814	2.880718	3.284110	2.925616	3.172928	4.416874	3.943276	2.875812	3.168060	2.957334	3.096025	3.912934	2.822736	2.877138	3.172384	3.169414	2.843076	3.124029	3.051231	3.165069	3.022175	3.245109	2.915706	3.004535	2.839050	3.456073	2.896339	3.046004	3.169283	3.256748	3.041967	2.916007	2.975703	3.074765	3.092801	3.026124	3.106189	2.894688	3.260848	3.861063	5.232814	2.925111	4.994800	3.035971	2.919416	2.881700	2.863017	3.020720	2.872562	3.347910	3.114254	3.627023	3.834608	3.006794	3.092544	3.527038	3.213805	3.161219	3.219233	3.115938	3.154426	2.941487	3.533311	3.193392	3.971571	4.339197	4.587990	3.016572	5.015261	3.062027	3.008488	2.963837	4.631548	4.118354	3.383293	3.023898	3.713156	3.737108	3.173430	3.687880	2.938338	3.186045	3.806389	3.446902	3.595183	3.554680	3.436171	3.338435	3.283173	2.854479	3.464825	2.948263	3.139069	2.871232	2.878010	3.243970	2.969894	2.959467	3.008577	3.126752	3.521478	3.803679	3.808655	3.480010	3.361296	3.664267	3.406764	3.363712	3.907267	3.448389	3.236383	3.129702	3.464122	3.179124	3.026197	2.924028	3.039515	2.929130	3.775550	3.134377	3.014839	2.971185	3.090979	2.767014	4.769928	3.726752	2.857028	3.145266	3.264389	3.087647	2.961762	2.757952	2.850826	3.110353	2.988170	2.893352	2.839637	3.081010	3.206265

GMDS-DT	7.885668	6.571166	6.310954	5.602452	5.297077	5.868488	6.895583	3.948773	4.547719	6.265208	6.059900	6.353267	6.248802	6.090612	6.534701	6.307918	5.956634	6.157532	6.738272	6.297246	6.578323	6.055991	9.131538	6.146884	6.930487	6.002372	7.417717	6.638032	6.577086	6.324741	6.553169	6.634850	7.027169	6.625846	6.675937	6.647062	7.008016	6.688383	6.917569	6.659868	6.011945	7.436323	7.193101	6.365038	7.115031	5.215840	6.680133	7.546964	7.283387	6.886757	6.923052	7.344419	7.644624	7.197441	7.334897	5.755895	7.244060	7.794750	7.806972	7.721135	7.966495	5.997961	9.108455	7.694590	7.445236	7.344722	7.121496	6.612650	6.129664	6.873205	6.558609	6.098380	5.831929	6.520361	5.467155	6.191997	5.711196	6.079720	6.373651	7.068063	6.709100	7.667752	7.325012	6.877654	7.136114	7.044666	5.832637	6.323720	7.268489	6.891257	7.529936	6.749103	6.070980	6.085087	6.320684	6.560692	6.764745	6.673975	5.569032	6.567049	5.946702	6.681110	6.110433	6.544343	6.756046	6.181324	5.603982	6.849972	6.194961	5.806871	6.640640	6.839990	6.048575	4.897681	5.013840	3.423646	8.799907	6.153949	6.104959	5.562034	5.483503	6.098380	5.266656	3.297766	4.734819	5.625408	6.600383	4.211207	5.043056	4.740639	6.123031	5.250790	6.258961	3.095811	5.987742	5.046472	6.406726	6.962923	4.807639	5.780710	6.968370	7.310410	6.179989	4.465424	5.378088	4.863512	5.401834	3.848646	5.814756	6.698709	5.469490	5.001920	3.846089	6.154648	4.620143	6.062086	4.679217	5.580774	5.734276	4.218585	5.955221	6.380948	3.457694	4.291335	4.358295	4.767656	6.930178	5.522694	6.104959	5.296657	6.402954	6.388356	4.528201	6.755554	5.768648	6.706394	7.300159	6.965964	6.890454	6.175250	5.893525	6.669475	6.892994	7.277975	7.082859	5.075463	5.757587	4.055179	7.514341	5.631190	6.289971	4.474750	5.089194	5.565213	5.604946	5.455513	5.409120	5.821481	7.272190	5.529168	6.679132	5.907765	5.254163	5.087383	4.626343	5.210833	4.073869	6.800931	6.310111	6.273564	6.567744	5.217569	4.679217	4.418689	5.374545	5.049157	6.895426	4.019991	6.467022	3.916620	6.194961	6.276695	5.914942	5.816919	4.914529	4.828047	6.708319	4.743795	4.955831	5.886902	5.575751	5.358537	4.275963	5.541080	4.263344	6.780704	5.533321	4.458238

RCAN3AS	11.084541	11.013401	11.053092	10.873702	11.916831	11.887052	11.664015	12.653093	12.120820	12.294973	11.881590	11.865618	11.617561	11.665819	11.189884	11.901166	12.068901	11.491172	11.787922	11.961308	11.037869	11.945613	10.616727	11.540128	11.320543	11.663120	10.491133	12.147944	11.223559	11.296869	11.839013	11.567979	11.596548	10.810258	11.325255	11.795586	11.830768	11.405292	11.587764	11.744491	11.711892	11.027041	12.265271	11.967801	11.763724	11.964164	11.561422	10.841568	11.861406	12.103782	11.853542	11.826085	11.865134	11.820512	11.717338	11.319280	11.807225	11.225775	11.822149	11.439584	11.709538	11.615530	9.466126	11.384967	10.890139	10.701056	11.188301	11.056421	11.109254	11.005699	11.282690	11.680103	11.824728	11.428860	11.517507	11.217736	11.487873	11.219233	11.560243	11.667871	10.966241	11.464200	11.583181	11.736404	11.909419	11.472366	11.869049	12.185585	11.072280	11.094441	10.868295	11.050179	11.131914	11.405292	11.101364	11.422789	11.672977	11.845386	11.449574	11.486387	12.117551	11.465624	11.280614	12.046769	11.948669	11.714683	12.147040	11.656178	10.754335	11.945301	11.397159	12.189898	11.899712	11.877610	11.759614	9.871373	10.496640	11.684367	11.930467	11.213047	10.850333	10.518248	10.780444	10.890438	11.743340	11.449574	10.831970	11.789270	10.885995	11.607591	10.802397	10.033478	10.062899	10.750836	11.085280	10.385937	10.721789	10.119603	11.675026	10.536771	10.296641	10.120507	11.799679	11.145141	9.947962	10.943027	11.471231	11.498149	10.979949	11.025555	11.694395	10.788558	11.869930	11.240073	11.127958	11.811705	9.797194	11.459486	10.589350	10.601053	10.453118	11.118406	12.018249	11.727140	11.557144	12.006245	9.694363	11.529079	11.691697	11.266027	10.943897	11.172234	12.516941	10.934442	10.112915	11.190903	10.070853	11.700373	11.516227	12.084672	11.142502	11.365508	10.412994	10.463260	9.962834	10.887634	10.406956	10.782381	9.717177	10.797454	10.623514	11.478703	11.308100	11.279663	11.272662	10.912644	10.555535	11.021387	10.828572	10.864387	12.189815	11.894727	10.432020	11.951260	11.420572	11.570621	10.890583	10.883860	9.701277	10.079182	10.981739	9.966953	11.166077	9.922286	10.434445	11.027915	11.613311	11.693032	10.839445	10.182714	10.195069	10.926544	10.810258	10.653521	11.246365	11.455650	10.377424	10.240163	12.325410	10.659325	10.888785	8.057141	11.449574	10.686008	10.978923	10.715070	10.859380	11.241643

MYT1L-AS1	10.461885	9.505499	9.566857	9.607774	9.830772	10.224836	9.285962	10.513768	10.665327	9.770486	9.866689	9.626458	9.165262	9.616997	9.331216	9.819482	10.427634	9.619017	11.201594	9.714221	10.584051	9.992041	11.420135	9.801200	10.002558	10.229726	9.943490	10.655009	9.768599	9.667023	10.502419	8.642660	9.094633	8.754861	9.136223	9.259079	9.357553	9.589501	10.085861	10.289000	9.863484	10.132441	9.312325	10.002270	9.411694	9.710638	9.912200	9.742594	9.483782	9.344218	9.663577	9.403399	9.010977	9.399241	9.421602	9.867109	8.956821	9.753292	9.766182	9.504879	9.959230	9.550248	10.801787	10.551284	10.190104	10.233845	10.273491	9.971364	9.798602	10.799798	9.607163	9.739328	9.756057	9.447632	9.604383	9.747254	9.876085	9.789600	9.952574	9.342047	9.415969	9.493455	9.451098	9.418832	9.346950	9.304702	9.396978	9.553448	10.308483	10.112915	10.013258	10.688872	9.470011	9.545005	9.497806	9.456162	9.375129	8.587856	9.035284	9.314738	9.191651	9.109712	9.262911	9.574283	10.528361	9.667023	10.577542	10.277457	11.047235	9.974821	11.029872	10.653668	10.486856	10.399900	9.857107	10.640900	10.768187	9.530244	9.753292	9.635491	10.057277	9.726699	10.246964	10.789138	10.187258	9.525226	9.944847	10.506461	9.940550	10.122918	9.628464	10.077125	9.521160	10.188617	9.687929	10.340663	9.391590	11.097335	9.293796	10.553981	10.232331	11.338197	10.040543	10.323048	10.081162	9.944110	10.674952	10.541837	9.954431	10.488419	9.132751	9.508068	9.435860	9.358941	9.431886	9.633365	10.064832	10.632606	10.496787	10.524141	10.746326	9.540974	10.650157	9.574724	10.011226	10.693482	9.639042	9.589330	9.580110	9.896130	9.436929	9.220054	9.666547	9.335710	9.778460	9.021121	9.677525	9.906961	9.473843	10.225000	9.694205	10.704472	10.697494	10.590679	10.388515	10.533808	10.286654	10.163352	11.073280	9.815427	9.854464	10.587598	9.765726	9.484491	9.959383	10.027644	10.093965	10.091891	9.235176	9.382862	9.589501	9.687778	9.318981	9.554494	9.912045	9.674754	9.369517	10.438091	10.129611	10.082987	10.945054	9.778003	10.212263	10.014305	9.905132	10.284884	9.369517	9.491096	9.283708	10.147018	9.184093	9.820866	9.600803	10.431267	10.099038	10.524581	10.269714	10.798060	11.069134	10.990768	10.567716	10.823244	10.907937	9.689297	9.584671	10.008153	9.667023	10.162874

AL033384.1	5.429406	4.921258	3.701159	4.839829	6.159451	7.521618	4.759750	6.201016	6.792834	6.746568	7.252607	5.570880	5.609716	6.441219	5.420358	7.553313	6.185718	5.971051	5.823680	7.314813	4.564910	6.843037	6.088324	5.276162	5.746855	5.859241	6.218892	5.643883	6.263018	4.409815	7.840933	4.748507	4.495551	5.868488	4.803039	4.739775	5.448014	3.482229	4.124217	4.319720	7.299385	4.467997	5.076919	4.842335	5.199887	5.679760	7.348503	3.661837	5.904380	4.616662	3.663429	4.092057	5.517407	5.420915	5.265665	6.297553	6.327896	4.759341	4.714555	4.101263	4.903414	6.717021	3.949658	5.954371	4.447326	4.255015	4.266557	5.373011	4.421986	4.138630	5.055139	5.914578	6.904609	5.660863	5.912590	4.618177	5.331849	5.411287	5.416994	5.085506	6.166148	4.457789	5.095809	5.946702	6.137696	5.501991	6.366865	5.702228	5.229330	4.384260	4.156749	4.169012	4.763361	5.000281	5.779803	6.839012	3.690742	7.734420	6.653518	6.199473	5.467155	4.917062	6.035254	5.426425	4.594913	7.128302	6.248137	5.835886	4.798741	7.127841	4.756537	5.427467	3.797353	7.015163	6.086091	7.728256	3.310230	6.679815	7.044822	8.425886	4.877177	3.930246	6.615435	4.524351	5.280527	6.128661	3.582276	7.097035	6.250316	8.037595	5.306198	3.318958	3.943958	3.822254	10.912200	5.336143	6.060246	3.317974	3.613168	3.871796	6.253184	5.418549	4.459856	3.331917	3.893761	6.581645	5.052375	6.180491	3.722894	5.698420	6.182027	5.285388	4.049098	5.056167	4.136501	4.973376	4.584786	3.578788	4.214482	5.074237	3.793159	3.684027	4.957517	6.340247	5.083639	6.278158	3.066288	5.912590	4.648966	4.656406	4.066903	4.272029	4.561847	5.498523	4.125660	3.839304	4.680948	3.763184	4.051282	5.622407	3.735838	4.915556	5.841340	4.502191	4.196239	3.836533	4.605023	4.193432	2.843630	6.219753	5.670148	6.069439	5.410127	6.176270	5.127160	4.888117	5.995662	5.382725	4.444999	6.442361	4.478041	4.502191	7.033252	4.534790	4.521910	5.475033	4.034313	4.256960	4.397228	3.687679	4.610790	4.907970	3.436697	6.421425	5.702980	4.939947	5.441215	5.328489	5.005573	3.316628	3.915721	4.642787	3.227317	4.963547	6.702249	5.749031	4.934698	5.057972	7.147061	4.353327	3.926325	3.837575	6.549699	4.579914	6.932551	4.117895	7.545980	5.831751

AC007389.1	13.425768	7.977519	7.727632	7.398027	7.825209	8.305594	7.659388	8.758075	8.650279	7.416551	8.092315	7.491333	6.831962	7.466203	6.956582	8.077902	8.364386	6.927315	8.751808	8.043558	8.252377	8.718419	8.455053	7.808635	7.434700	7.928816	7.288615	8.955174	7.970042	8.041861	7.653896	6.854303	7.170864	6.502522	7.405370	7.465866	7.663263	7.892473	8.184797	7.817798	8.234802	7.518806	7.103604	7.220994	6.861228	7.519107	7.847351	7.073059	8.305299	6.911787	7.061243	7.490073	6.989840	7.401461	7.187316	8.440271	7.027807	7.555801	7.355811	7.097967	7.552380	7.703146	7.337505	7.839369	7.664213	8.019965	7.835074	7.989271	8.144078	8.577147	8.983343	9.069452	8.222845	8.684928	9.246593	8.011860	7.965874	7.889666	8.474935	7.037948	6.738430	7.041594	7.020611	7.526963	7.687645	7.386409	7.656124	8.391462	7.165954	8.123421	7.872765	8.214857	7.876139	8.118397	7.335828	7.292718	8.102949	7.292246	7.165039	7.512022	8.395613	7.525133	7.552843	7.870600	8.255721	7.989106	7.785295	8.236995	8.190071	8.444544	9.151539	7.842803	7.487562	8.581670	8.230395	11.180206	7.698229	7.535343	8.289036	8.161308	7.833855	7.364513	7.052716	7.792112	8.216505	8.026217	7.186835	7.301614	7.999946	7.882760	7.723774	7.178144	7.078154	6.976239	7.861216	7.483367	6.002904	8.019824	7.352520	7.875662	7.636483	8.658361	7.485352	7.593519	7.391165	8.206040	7.884152	8.462777	8.312674	6.909393	7.212319	7.034830	7.669761	7.468757	7.729343	7.364043	7.898660	8.302279	7.808938	8.445618	8.171446	7.552537	7.702215	6.978170	7.993159	8.031021	7.038119	7.751360	6.867727	6.840632	6.576181	6.619535	7.020142	6.448351	7.498353	6.523154	7.348503	6.842863	6.934087	7.299240	7.162049	7.750575	7.729788	7.465563	7.527432	7.691511	8.001992	8.068615	7.923664	8.278869	7.827291	8.735642	8.110120	7.992068	7.386840	7.578233	7.980345	8.292759	6.472402	6.577833	7.169643	6.916362	6.331457	6.653213	7.377540	8.001204	7.049597	6.487433	7.131658	7.539104	7.694121	7.658000	7.894811	7.563944	7.603602	7.598247	7.430932	7.409893	7.422282	7.808494	7.100637	7.201315	6.946451	7.697302	7.385763	7.388571	8.118721	8.378160	8.411859	8.853927	7.169340	7.915673	9.123377	8.267453	8.532116	7.118530	7.736364	8.081951

AC007389.3	13.425768	7.977519	7.727632	7.398027	7.825209	8.305594	7.659388	8.758075	8.650279	7.416551	8.092315	7.491333	6.831962	7.466203	6.956582	8.077902	8.364386	6.927315	8.751808	8.043558	8.252377	8.718419	8.455053	7.808635	7.434700	7.928816	7.288615	8.955174	7.970042	8.041861	7.653896	6.854303	7.170864	6.502522	7.405370	7.465866	7.663263	7.892473	8.184797	7.817798	8.234802	7.518806	7.103604	7.220994	6.861228	7.519107	7.847351	7.073059	8.305299	6.911787	7.061243	7.490073	6.989840	7.401461	7.187316	8.440271	7.027807	7.555801	7.355811	7.097967	7.552380	7.703146	7.337505	7.839369	7.664213	8.019965	7.835074	7.989271	8.144078	8.577147	8.983343	9.069452	8.222845	8.684928	9.246593	8.011860	7.965874	7.889666	8.474935	7.037948	6.738430	7.041594	7.020611	7.526963	7.687645	7.386409	7.656124	8.391462	7.165954	8.123421	7.872765	8.214857	7.876139	8.118397	7.335828	7.292718	8.102949	7.292246	7.165039	7.512022	8.395613	7.525133	7.552843	7.870600	8.255721	7.989106	7.785295	8.236995	8.190071	8.444544	9.151539	7.842803	7.487562	8.581670	8.230395	11.180206	7.698229	7.535343	8.289036	8.161308	7.833855	7.364513	7.052716	7.792112	8.216505	8.026217	7.186835	7.301614	7.999946	7.882760	7.723774	7.178144	7.078154	6.976239	7.861216	7.483367	6.002904	8.019824	7.352520	7.875662	7.636483	8.658361	7.485352	7.593519	7.391165	8.206040	7.884152	8.462777	8.312674	6.909393	7.212319	7.034830	7.669761	7.468757	7.729343	7.364043	7.898660	8.302279	7.808938	8.445618	8.171446	7.552537	7.702215	6.978170	7.993159	8.031021	7.038119	7.751360	6.867727	6.840632	6.576181	6.619535	7.020142	6.448351	7.498353	6.523154	7.348503	6.842863	6.934087	7.299240	7.162049	7.750575	7.729788	7.465563	7.527432	7.691511	8.001992	8.068615	7.923664	8.278869	7.827291	8.735642	8.110120	7.992068	7.386840	7.578233	7.980345	8.292759	6.472402	6.577833	7.169643	6.916362	6.331457	6.653213	7.377540	8.001204	7.049597	6.487433	7.131658	7.539104	7.694121	7.658000	7.894811	7.563944	7.603602	7.598247	7.430932	7.409893	7.422282	7.808494	7.100637	7.201315	6.946451	7.697302	7.385763	7.388571	8.118721	8.378160	8.411859	8.853927	7.169340	7.915673	9.123377	8.267453	8.532116	7.118530	7.736364	8.081951

LINC00570	8.249737	8.017257	7.869083	8.086750	8.350957	8.809523	8.042654	9.120196	9.007159	8.314692	8.893945	8.164115	7.833692	7.913572	7.501152	8.579234	8.562682	7.463265	8.693097	7.839084	8.708137	8.830706	9.160153	7.981918	8.041861	8.653423	8.550430	8.913879	8.446695	8.479665	8.649523	7.574967	8.146426	7.556927	8.001687	7.916925	8.114390	8.132097	8.453220	8.377983	8.866122	8.008303	7.912825	8.472816	8.117928	8.038029	8.899150	8.378467	7.924902	8.212373	8.357368	8.132391	7.654357	7.889992	7.669450	8.840879	7.894980	8.057573	7.565801	7.565945	8.069216	8.452168	8.392095	8.310361	7.927574	9.029501	8.052166	8.036665	8.105442	8.425886	8.338549	8.025737	8.642211	8.152367	8.153907	8.190071	8.181140	7.921630	8.238367	7.535183	7.745146	7.658461	7.348804	7.444435	7.694590	7.621347	7.798655	7.831717	8.112848	8.058121	7.961155	8.507231	7.908184	8.076073	7.725594	7.908644	7.783481	7.816519	7.942495	7.778213	8.237305	7.561200	7.875198	7.924902	8.507811	8.468110	8.938239	8.286878	8.850318	8.895756	8.878315	7.993159	8.195596	8.993336	8.395144	10.392965	8.613906	7.935728	8.785633	7.752553	8.567166	8.307442	8.601432	9.963969	9.244397	9.026613	8.148080	10.341850	8.986709	9.126663	8.628667	8.481866	8.311463	8.780550	8.310809	8.392258	8.219027	8.870721	7.650640	8.743011	8.921804	9.226836	9.119287	8.824170	9.133674	8.915861	9.202190	9.206285	9.307595	8.608629	8.892827	8.560695	8.535943	8.558709	7.927727	8.137100	8.426298	8.833199	8.473863	10.100454	8.782271	7.782573	9.407561	8.325415	8.573650	12.523307	8.467956	7.817308	8.382372	8.755318	8.106503	7.553153	8.146572	7.627192	8.566024	7.755464	7.847801	7.550222	7.466031	8.199678	8.743149	8.155151	8.234201	8.162515	9.219741	8.360634	8.422572	8.719354	8.573236	8.355532	8.263240	10.398231	8.484598	8.069216	8.164571	8.308845	8.572024	8.436480	7.533225	7.591499	7.806171	7.696675	7.517109	7.759138	8.511835	8.055590	7.377364	8.094921	7.860910	8.056835	8.488015	8.301664	8.673896	8.519151	8.893638	8.206187	8.624652	9.664186	7.908951	8.448582	7.708703	8.212669	8.170198	8.239783	9.317338	9.350764	8.213942	8.808590	10.391503	8.770072	8.201668	8.998577	9.682033	9.875764	9.594640	8.478824	8.310512	9.267822

LINC00174	8.494761	7.779314	7.622541	8.722201	8.930315	8.570650	8.164882	8.581371	9.086111	8.311463	9.014249	8.412020	8.378322	8.676661	8.124646	8.729427	8.804918	8.024837	9.990754	8.874966	9.317184	9.743792	8.549357	8.073965	8.222227	9.164789	9.008722	9.016068	8.280091	8.161158	10.169039	8.347074	8.862905	9.009822	7.854983	8.063817	8.343986	7.428367	8.568189	8.120379	8.063661	8.587856	8.278120	8.046403	8.000110	8.126763	8.662549	7.615127	8.290249	8.516062	8.146260	8.343986	8.285627	8.973923	8.092915	8.547074	8.317753	8.196850	8.444854	8.275673	8.685393	7.889199	7.891690	8.606818	8.533318	8.327809	7.696675	7.745467	7.553011	7.565945	7.794157	8.037595	8.431198	8.209472	8.390063	8.107430	7.766387	7.935134	8.322623	7.935134	8.306636	8.022249	8.181886	8.703075	8.329322	8.512760	8.508449	8.937952	9.157090	8.545954	8.121159	8.996563	8.526736	8.431198	8.699378	9.055670	8.242770	9.069592	8.593456	8.693560	8.574264	7.769734	8.618776	8.471373	8.407253	9.115266	9.464238	8.746205	8.552881	8.558851	9.326153	8.899620	8.797944	8.215914	8.736262	6.373651	9.335710	8.736112	9.371801	9.101487	8.741172	6.955501	9.110179	8.079921	8.073176	10.202314	10.911465	11.243746	8.272834	11.433749	9.063687	11.193953	8.374334	10.334168	10.989151	9.395272	8.918624	8.996270	9.034153	9.492065	10.873089	8.057990	7.595483	8.085541	9.209780	8.639584	10.356139	10.663706	11.068675	10.131092	8.630044	8.470408	8.158525	7.862754	7.915502	7.631455	7.665421	10.913576	9.803956	8.337894	9.293482	8.498349	9.028257	9.876562	8.517642	8.800662	7.184381	7.511709	11.047235	10.950287	8.764379	7.921935	12.075438	7.941974	9.417014	9.350620	7.960216	9.122611	7.903984	8.755176	9.623069	11.715283	8.057282	9.306026	9.168451	10.628223	8.044341	7.608529	8.063661	7.422439	8.296548	8.763780	9.985950	8.246193	7.788507	9.334765	8.559612	10.122304	7.921791	8.369861	8.841804	8.728213	8.595951	8.305140	8.937335	8.145338	9.233328	9.497504	9.013164	8.921205	10.202155	9.816047	9.642061	11.007562	10.775641	8.394685	9.607163	9.302664	7.883682	8.314984	9.884840	9.004108	7.423039	10.248163	8.328604	11.022131	10.624726	7.544875	8.143298	8.039489	7.107267	6.051477	8.462629	6.969499	8.289633	10.825019	10.679584	10.065569

TH2LCRR	7.120590	7.802054	9.016942	3.006886	3.485185	4.326994	3.204548	3.190259	3.818633	3.417386	4.271805	3.350579	3.718738	3.671565	5.746099	3.465394	3.246827	2.984243	5.018300	4.385189	4.262444	4.234619	3.575730	3.837575	3.088004	2.994863	4.955010	2.960243	3.554492	5.794218	3.678826	5.470448	4.813130	5.599318	8.396552	3.973745	3.347388	3.268982	3.366167	3.502755	3.468909	2.965270	4.764207	3.194444	5.373801	5.190727	4.369123	8.572024	5.626914	5.610093	2.860671	4.386959	5.286594	4.148755	8.119314	3.124783	8.305897	5.214626	3.832712	3.975516	3.325163	5.089194	12.429308	3.619528	6.030085	5.356651	3.434507	3.494265	3.614384	2.876167	3.082761	3.191743	3.223626	4.230073	3.648190	6.209329	3.905736	4.448013	3.653615	7.223728	3.325488	6.219210	3.447839	5.113938	5.748312	3.610373	3.069240	5.655195	5.669251	4.104747	4.571992	5.076314	4.482874	5.861216	6.747722	5.945684	9.035125	3.581900	3.838432	3.067954	3.011939	4.109814	3.524981	3.536283	4.632083	4.689517	3.625812	3.282710	3.427251	3.138106	5.048505	3.473087	2.937314	3.234074	4.692443	3.094426	3.993280	3.984979	3.389637	6.386455	5.462582	5.484060	4.088092	3.256478	6.173023	3.239740	3.152335	3.949658	3.605770	4.061298	4.231567	4.437866	4.019991	3.231727	3.378073	3.444894	2.878713	7.223425	5.022964	4.464978	5.996198	6.885835	3.755267	2.942671	4.200518	4.249061	3.358469	3.098076	5.472912	3.913757	6.883579	4.126337	3.848851	5.059824	4.484173	4.593634	3.418786	3.382584	5.816919	3.673284	6.520703	5.251944	4.358295	4.374352	4.801148	4.743795	7.217959	4.146648	5.388935	6.337033	5.531254	5.899028	5.254943	5.664974	3.440832	4.325528	8.144245	3.652825	4.023692	5.338729	4.278634	4.845532	4.732302	5.929339	4.381821	5.599151	5.877095	4.603540	4.950017	3.242519	5.419794	4.036699	3.660010	4.593634	5.821680	5.118456	3.600877	3.467279	4.130630	4.846376	4.990613	3.840468	5.809052	6.660184	3.303625	5.092275	6.502026	5.798946	4.983584	4.248826	4.839616	3.656838	5.032949	5.591589	4.203616	4.627160	3.925461	4.141946	3.888075	3.984336	3.189156	4.749126	3.969874	5.231197	5.749370	4.630030	5.916363	5.470077	3.690559	5.435714	3.126509	2.886566	3.516064	3.723107	4.478041	4.602914	3.853814	3.803066

AC003035.2	8.122600	7.789614	8.306486	8.636601	9.351550	9.226978	7.924902	9.013634	10.231014	8.850032	8.575504	8.504777	8.306963	8.272099	8.097966	9.231033	9.117099	8.094770	8.651781	8.898245	8.196996	8.245543	8.126763	8.566293	8.694983	9.302803	10.119753	8.736571	9.438948	8.356756	9.854951	7.422754	7.733625	8.620648	8.422111	8.170037	8.248548	8.311931	8.455195	8.556238	9.038186	8.409551	8.592374	8.817738	8.626562	8.513995	9.517845	7.749198	8.197780	8.206780	8.043107	8.000418	7.438844	7.954492	7.858931	9.114050	7.882174	7.632388	7.809098	7.808333	7.466203	9.394814	7.319795	8.040277	7.447919	8.006846	7.837979	8.054480	7.621512	8.006846	8.131646	8.137701	9.525550	8.116545	8.892991	8.044166	8.129345	7.751051	7.922875	7.423979	7.879916	7.703286	7.130837	7.303584	7.615728	8.100858	8.210651	7.822714	7.344269	7.040964	7.361219	7.044183	7.516020	7.829434	7.832020	8.443933	8.041233	9.210014	8.564961	9.291432	9.397313	8.499968	9.278749	8.644846	8.463224	9.341743	9.944399	9.139637	9.516518	9.687451	7.794750	9.297703	8.995967	9.694205	9.420386	10.267776	8.533168	8.811379	10.204147	8.574878	8.348548	8.239020	9.031263	9.848160	8.679458	8.197159	9.894559	9.394660	8.924459	7.910938	8.821432	8.516237	8.213942	7.701899	9.508068	8.897303	9.378827	9.710793	8.767446	8.384808	8.148697	6.910016	9.145926	7.962059	7.949357	9.130010	8.776916	10.328691	7.816357	9.713565	9.084407	9.029197	8.559451	8.638802	8.239336	8.109965	8.568189	8.800062	8.638314	9.778769	8.505967	8.495213	8.467638	8.386977	8.039807	9.662174	7.856494	8.311773	7.878416	7.822714	8.262447	7.755152	7.951508	8.437092	8.571418	7.851729	7.990643	8.012155	7.632856	7.693526	8.451235	7.631000	7.668055	8.027463	7.837808	8.440743	7.912658	8.108640	7.350077	8.259547	8.230097	9.165262	8.328299	8.573979	8.089246	8.003583	7.928816	8.158705	7.715071	8.215162	7.603602	7.495361	7.820271	7.889051	8.707227	7.684432	10.909151	8.111607	7.690085	8.585603	7.212144	7.706387	8.261975	7.721135	8.632336	8.418725	9.083352	8.785931	8.092448	8.944434	9.142743	9.524895	8.486274	8.083254	9.522089	10.099038	9.151539	8.191012	9.677830	7.647714	9.483198	9.207170	9.380201	9.228821	8.203475	8.286403	8.353838	8.715629

LEMD1-DT	3.267491	3.162054	3.003333	3.579772	4.665178	5.016059	3.225437	3.739374	3.388600	3.284757	5.355303	3.617825	3.593637	3.903127	3.250849	3.112376	3.437547	3.131365	3.775550	4.051954	4.442432	3.834608	5.994659	3.129814	3.346247	3.027882	3.065438	3.509805	3.122513	3.148035	3.632310	4.052167	6.386807	3.342824	5.692082	5.073004	3.695280	3.258846	3.260848	3.195162	3.432543	3.782722	5.217771	3.099736	5.439680	6.902551	3.611973	6.062355	4.837655	4.017194	4.028832	5.412417	4.909265	3.851853	3.527379	3.006446	3.864105	3.353204	3.880019	5.555822	3.851185	3.269604	3.439974	3.069827	3.155331	3.110727	2.929281	4.745760	3.014748	3.092690	3.405895	3.304129	3.554282	3.402924	3.030894	3.607835	2.946892	3.818855	3.154050	3.405245	3.134142	3.711134	3.068474	4.739990	3.036842	5.356278	3.638065	3.039515	4.850976	3.251299	3.824156	2.938071	3.165304	3.122871	3.074437	3.261151	3.568486	3.653027	3.996830	3.304924	5.082452	4.519767	3.058828	3.858138	3.937075	4.275090	4.860036	3.245817	3.143041	3.221513	3.056447	3.890731	3.825093	3.864949	3.899349	6.087306	3.085822	3.011939	3.504959	3.077373	3.235083	3.281352	2.971853	4.605847	5.579830	3.234681	5.409725	3.381903	3.102750	5.158305	3.146768	3.761533	3.239051	3.047512	3.074008	3.400227	3.126623	3.165192	5.724545	3.831179	3.095565	6.516754	4.157992	3.189710	2.953586	3.480978	3.337623	2.959195	3.425082	3.432044	3.708643	4.702563	3.244844	5.644822	4.048661	3.745473	3.266021	3.178699	3.062576	3.198410	3.674530	7.125950	5.375140	6.050117	6.934730	3.328333	5.300214	5.537646	4.123402	4.089608	5.199887	5.391837	3.805556	3.195416	4.428056	3.436697	4.131875	3.786494	5.928643	3.634676	3.075974	3.422057	3.321225	3.242102	3.111995	2.920528	5.915478	3.114979	4.420222	3.171902	2.934808	3.075184	3.004535	2.949165	3.223215	2.944259	4.176392	3.028570	3.760508	3.309363	3.711134	3.102215	5.482767	3.283804	4.474527	3.156400	3.705864	3.018085	3.237709	4.801541	2.939236	2.940658	3.039394	3.038319	3.843155	3.475442	3.576932	4.092696	3.776565	4.313602	6.213566	2.980514	3.335812	4.299617	5.849182	5.000471	3.268690	3.458955	3.081779	3.039234	3.837146	4.620143	3.189850	4.245775	5.132613	3.054044	3.006162	3.224468

U73166.1	11.091311	15.369410	14.409900	14.896544	15.059149	15.348633	15.212349	16.391445	15.490401	15.476405	15.423337	15.234869	14.422502	14.691055	13.883273	15.880276	15.839623	14.485225	13.644958	15.792922	13.903602	14.291671	13.064543	15.161962	15.149334	15.417001	12.203886	15.848852	15.419437	14.873437	14.978704	13.679951	14.706135	12.266676	15.140997	15.646933	15.515982	14.750726	15.568457	15.619793	15.640499	14.644889	15.628044	14.763822	16.180048	15.905774	15.251620	16.393332	15.249434	16.990930	15.805918	16.357477	15.132734	16.091387	14.916232	15.818695	13.852202	15.008818	14.326082	14.484873	14.241749	15.406237	11.745676	14.537935	13.875509	14.827462	15.017444	13.995291	14.562573	14.458915	16.416212	16.711084	15.442379	16.119818	16.079155	14.938834	15.321092	15.285115	14.861942	15.292522	14.184313	14.485961	13.647795	15.037486	15.737267	14.893861	15.534337	16.001371	12.870661	13.589099	13.618283	13.613971	14.496348	15.354130	13.631382	14.218945	14.719949	15.126455	14.331727	15.739503	14.922259	15.320436	15.433373	15.083595	13.140875	15.297462	14.563014	13.123808	13.265809	16.264810	13.223145	14.800886	14.561753	15.455879	15.819800	15.277400	10.981739	15.550613	15.584586	10.516102	11.634790	11.030306	11.639016	12.819396	13.674088	11.880566	12.707731	11.603290	11.692880	11.724086	11.526057	11.438510	11.228419	11.045577	13.702414	11.604311	11.027621	11.100603	11.487432	11.132065	11.440178	11.080287	12.228465	11.403536	11.327922	13.831246	12.423432	12.784157	11.850817	11.802080	10.730021	11.056587	11.075319	11.075896	11.233616	11.838566	11.272361	12.583792	11.496673	12.578683	11.136838	10.695437	11.197241	11.406614	14.896544	12.423279	11.968413	11.629239	12.071423	11.954390	11.853235	10.439434	10.764768	10.729876	12.028997	10.351116	10.649536	10.535450	10.813727	10.664741	12.134970	10.661925	11.027763	10.838131	10.822512	11.147241	11.247766	10.912940	11.324220	12.028252	11.555799	14.023893	11.446152	11.312768	11.898092	11.097635	11.158476	13.014469	10.420249	10.174868	11.795267	10.160896	10.601825	10.570283	11.171363	10.760108	11.017339	10.408136	10.631271	10.728676	10.660719	10.934731	11.314073	10.954993	11.399407	11.165803	11.707755	11.080123	11.333902	11.339682	11.775928	12.099673	11.230061	10.837527	12.824043	11.722134	11.045739	12.017344	12.976403	11.114868	11.327191	11.918344	14.990649	11.610786	11.468075	10.241972	11.234915	12.018432

FAM225A	10.577682	9.713408	10.505574	10.685541	11.098860	12.666334	10.938211	13.353095	13.514012	11.869488	12.614041	12.332077	12.474915	9.779969	10.009742	11.945155	13.005039	9.420386	7.695178	11.720464	8.192439	8.860321	7.794750	9.081509	9.549005	13.017023	13.136608	12.407294	11.863940	12.180333	13.983800	10.464739	10.953197	11.998560	11.171227	11.561576	11.266314	8.375113	8.051562	7.860319	12.157396	8.249596	9.892101	10.899381	9.390506	10.974369	13.592841	8.109965	11.142648	9.761509	9.683409	9.744106	8.515175	9.251636	8.986242	12.895995	9.894275	7.351296	7.733324	7.946493	7.393824	11.969628	8.739929	8.571281	7.694121	7.714505	7.472188	7.329294	8.462927	7.418684	10.973802	11.081604	12.488312	10.136400	11.084833	11.901889	10.642536	10.490413	11.150814	8.489064	10.468439	9.302046	9.287241	9.007159	10.048648	11.039883	11.007562	11.003228	7.603602	7.695178	8.114888	8.605139	9.960448	11.483981	13.095970	11.756172	11.050779	12.798338	13.006339	11.550159	12.702957	10.557792	11.070018	9.342816	8.196072	12.015985	9.809845	8.540372	9.226054	12.000733	8.696372	11.251510	9.088211	13.363072	12.495360	13.768030	9.156597	12.551723	13.142552	11.413991	11.076625	10.923714	10.767508	11.996843	13.090537	12.556686	15.240441	12.477354	12.182810	12.443708	12.570198	12.571856	10.251030	11.537619	12.316696	11.829732	10.457715	9.087336	12.106988	9.523327	9.635073	9.288951	10.541101	10.517413	11.649898	12.148397	12.980792	13.123620	12.724991	13.449081	11.626309	11.506409	11.818995	10.815965	12.545714	11.464934	9.274416	8.590817	9.360471	13.322740	8.869598	10.099818	10.989429	10.254975	10.666208	13.435155	9.367092	10.754642	10.014741	10.891048	10.521258	9.240450	10.153421	9.158035	11.559519	11.559648	9.089320	8.380173	9.836099	8.926766	10.890583	9.163229	9.248124	8.568356	8.333675	9.672249	8.342909	10.215794	10.106438	10.757376	11.222202	13.945662	10.648499	11.396102	11.608893	10.731478	10.901875	11.245651	9.683246	10.322892	9.806105	9.286274	10.755737	11.466748	11.917748	10.632025	10.996548	9.405418	8.871377	9.121979	10.048802	9.702347	11.714242	12.425904	12.556996	12.328136	13.952379	12.171247	10.381341	13.462463	12.748800	11.710430	9.794237	10.433661	13.332466	10.598790	8.570494	9.351997	12.426059	9.540184	11.960063	9.284449	14.832004	13.119075	11.981974	8.890904	12.008090	12.845812

AC090772.3	6.978944	8.560535	7.615580	8.702129	8.025737	8.147919	8.528767	6.638849	7.046103	8.453841	8.955627	8.532566	8.381590	7.859735	8.555749	8.395927	8.264623	8.211744	8.956821	8.479665	7.808938	7.290668	9.550248	8.202269	9.269835	8.984549	8.543841	9.114516	8.909799	8.836181	7.871375	8.816206	8.978474	9.775229	8.950129	8.321580	8.159168	8.295916	9.099806	8.791374	7.889992	8.897010	8.690507	8.517004	8.933305	10.056502	8.490573	9.236434	8.814706	8.883900	9.145041	8.997042	8.537994	8.806574	9.326153	8.669568	8.588986	8.172842	9.380364	9.321044	8.509971	6.571326	10.224521	8.609851	10.805904	8.982281	9.959667	8.050619	10.443501	7.794465	8.729568	7.400060	7.949190	8.153614	8.418725	8.695277	7.525289	9.117571	8.666526	8.797307	8.167075	8.477408	9.413800	8.657423	8.698199	8.924735	9.766473	8.996270	8.891290	9.227888	9.700346	7.728725	9.205492	8.086300	9.061269	8.156255	8.090338	7.311187	7.274387	8.373422	7.628930	8.527834	8.494612	8.443484	7.279413	7.764659	7.599995	7.942495	7.717220	7.619865	6.945652	8.361560	7.911734	7.943394	8.596115	5.708822	8.773506	8.357685	8.047832	9.320484	9.421008	9.510217	10.565474	9.843038	8.833908	9.034002	9.912507	8.345080	14.182770	10.228793	12.324636	13.578284	10.314435	11.969628	9.125034	12.297906	12.034131	11.938203	10.308933	10.934589	12.594761	12.857801	10.103273	12.584309	12.717320	9.768599	11.041054	9.860824	11.612874	9.772646	9.405273	11.012393	9.986683	8.885915	11.572813	10.201859	9.386205	10.738523	12.073275	9.855121	13.360143	11.896778	10.127429	11.409053	14.280410	7.847503	10.845927	14.208831	10.839289	10.891628	11.771398	13.096345	10.222414	9.752370	13.433260	11.850666	11.104718	12.131737	10.641355	10.284731	9.194647	10.420715	11.393969	9.466743	11.514845	12.225366	9.541747	10.433508	10.657248	11.866219	10.384381	8.706443	10.686008	10.004749	9.151110	9.931101	9.979042	12.442059	9.704430	10.225742	10.184839	10.131679	9.761509	10.298003	12.273123	9.540665	11.212599	9.938377	10.791738	11.585010	8.851230	10.196916	9.228524	9.066139	9.436336	12.454528	10.403638	9.696322	13.818903	10.900123	12.007014	9.098599	11.633293	11.111735	12.915341	9.429598	10.329500	13.740121	8.488318	10.042222	11.188446	10.973947	7.154341	14.378131	11.284886	11.664624	10.820134	10.190703

LINC01533	4.437866	4.932831	4.742152	4.559227	5.461998	6.050621	5.681213	4.545552	6.416842	6.002723	5.093749	5.538246	3.642453	5.367778	4.211207	5.589095	5.211243	5.239451	5.819564	5.645034	5.734630	6.033281	3.860245	6.640307	6.460347	5.301032	5.689540	5.666294	6.644836	5.824601	3.643475	4.479106	3.726533	4.452678	5.178571	4.440018	4.389553	4.406043	5.577775	4.446299	6.551816	4.542193	5.632831	5.437831	5.475412	5.199677	5.687890	4.905496	5.561471	4.446490	4.779910	3.796526	4.256083	5.568253	4.378241	5.941954	3.780612	4.850372	4.470334	3.983281	3.487527	5.869386	5.931261	4.937295	4.624415	3.330169	4.274018	5.301032	4.291994	4.834971	4.987279	5.324027	5.366629	4.511393	4.270707	4.553238	5.140454	4.231140	5.449553	4.841044	3.707485	4.878825	3.146427	4.416874	4.204758	5.560215	4.945220	3.775101	4.489766	4.668615	3.881910	3.081673	4.376523	4.847004	4.544904	5.164897	4.374567	5.822222	4.681366	6.416995	6.498399	5.501391	5.356854	5.866471	4.353652	5.499091	5.588901	5.691927	5.971960	6.660184	5.871685	5.894247	3.603661	6.712466	4.935777	8.912217	4.486980	5.350464	6.192527	5.954888	4.220124	4.253225	3.336283	5.854171	4.014957	6.592947	4.348297	4.314044	4.263344	3.540150	4.223393	5.997605	4.212293	4.405609	4.749521	3.470703	3.821253	4.698520	4.575394	4.965626	5.473107	6.264502	4.329236	4.566165	3.212552	6.350954	3.659429	5.736449	3.583046	3.327915	5.126176	3.514432	4.752973	5.876401	3.703635	3.850353	3.978030	4.209499	4.248379	4.494905	3.460735	5.820088	4.247095	4.134067	4.558102	3.649216	4.657931	4.804933	4.369566	4.051954	4.116561	4.747673	5.013631	3.745044	4.166469	5.643485	4.818759	4.200088	4.039083	3.176379	4.469265	4.087037	4.572639	3.876342	3.551213	3.843761	3.719536	3.390681	3.041777	3.740582	4.238537	3.095811	3.516786	4.674939	3.516425	4.236160	3.711546	4.415978	4.292692	3.744452	4.347401	3.134992	4.408699	3.854698	5.608157	4.318023	3.543602	4.226387	3.637482	5.612184	2.915428	4.895790	3.909259	3.110843	4.138014	4.827721	5.556382	4.099547	3.973522	5.602258	4.817310	4.035847	4.848706	4.779910	5.103586	4.005218	5.733728	4.120517	3.556033	5.608157	4.831864	6.296264	5.252550	5.846222	5.214626	4.019348	3.647373	3.384652

LINC01837	4.437866	4.932831	4.742152	4.559227	5.461998	6.050621	5.681213	4.545552	6.416842	6.002723	5.093749	5.538246	3.642453	5.367778	4.211207	5.589095	5.211243	5.239451	5.819564	5.645034	5.734630	6.033281	3.860245	6.640307	6.460347	5.301032	5.689540	5.666294	6.644836	5.824601	3.643475	4.479106	3.726533	4.452678	5.178571	4.440018	4.389553	4.406043	5.577775	4.446299	6.551816	4.542193	5.632831	5.437831	5.475412	5.199677	5.687890	4.905496	5.561471	4.446490	4.779910	3.796526	4.256083	5.568253	4.378241	5.941954	3.780612	4.850372	4.470334	3.983281	3.487527	5.869386	5.931261	4.937295	4.624415	3.330169	4.274018	5.301032	4.291994	4.834971	4.987279	5.324027	5.366629	4.511393	4.270707	4.553238	5.140454	4.231140	5.449553	4.841044	3.707485	4.878825	3.146427	4.416874	4.204758	5.560215	4.945220	3.775101	4.489766	4.668615	3.881910	3.081673	4.376523	4.847004	4.544904	5.164897	4.374567	5.822222	4.681366	6.416995	6.498399	5.501391	5.356854	5.866471	4.353652	5.499091	5.588901	5.691927	5.971960	6.660184	5.871685	5.894247	3.603661	6.712466	4.935777	8.912217	4.486980	5.350464	6.192527	5.954888	4.220124	4.253225	3.336283	5.854171	4.014957	6.592947	4.348297	4.314044	4.263344	3.540150	4.223393	5.997605	4.212293	4.405609	4.749521	3.470703	3.821253	4.698520	4.575394	4.965626	5.473107	6.264502	4.329236	4.566165	3.212552	6.350954	3.659429	5.736449	3.583046	3.327915	5.126176	3.514432	4.752973	5.876401	3.703635	3.850353	3.978030	4.209499	4.248379	4.494905	3.460735	5.820088	4.247095	4.134067	4.558102	3.649216	4.657931	4.804933	4.369566	4.051954	4.116561	4.747673	5.013631	3.745044	4.166469	5.643485	4.818759	4.200088	4.039083	3.176379	4.469265	4.087037	4.572639	3.876342	3.551213	3.843761	3.719536	3.390681	3.041777	3.740582	4.238537	3.095811	3.516786	4.674939	3.516425	4.236160	3.711546	4.415978	4.292692	3.744452	4.347401	3.134992	4.408699	3.854698	5.608157	4.318023	3.543602	4.226387	3.637482	5.612184	2.915428	4.895790	3.909259	3.110843	4.138014	4.827721	5.556382	4.099547	3.973522	5.602258	4.817310	4.035847	4.848706	4.779910	5.103586	4.005218	5.733728	4.120517	3.556033	5.608157	4.831864	6.296264	5.252550	5.846222	5.214626	4.019348	3.647373	3.384652

GDNF-AS1	3.037030	3.248412	3.155801	3.014178	2.939990	3.797572	3.397833	3.434507	3.443277	3.465394	3.632741	3.696923	3.291431	3.708077	3.273291	3.185419	3.738525	4.411106	4.690963	3.218550	4.503720	2.878713	3.399349	2.848033	2.990442	2.997603	3.041967	3.022890	3.091524	3.308743	3.598158	3.830142	3.703843	3.127205	4.446299	4.059096	3.644627	3.275122	3.043913	2.865368	3.313984	2.967113	3.445983	3.279422	3.535542	5.258891	3.166776	3.031851	4.423758	4.442432	4.170991	3.839304	4.151892	4.104297	3.801618	3.047969	5.694332	3.975291	3.579178	3.461116	2.957162	3.234227	3.301457	3.163139	3.357933	3.216664	2.982236	3.129327	3.064517	2.862378	3.083140	2.941808	3.073211	3.244432	3.103750	3.310230	3.038319	2.965952	2.997218	3.628214	3.389835	4.004562	3.371691	4.069842	3.408374	4.204758	2.943473	3.232441	3.606610	3.453834	3.176610	2.864434	3.081441	3.180813	2.952203	2.999766	3.694034	3.117943	3.049429	2.962274	3.021553	3.239185	3.094079	3.178028	4.251723	3.725335	3.124671	3.224190	3.023990	3.206112	3.204019	2.983258	3.090739	3.232699	3.240314	3.042930	3.032050	3.011063	3.081779	3.002140	3.321848	3.075306	3.054353	2.959101	4.424419	2.988075	3.283480	3.488475	3.217434	3.492212	3.196929	3.021347	3.159038	3.066619	2.968632	3.684214	3.924386	3.385183	3.352721	3.814003	2.846808	5.341623	2.822736	2.861935	2.934195	2.886653	2.920166	3.029377	3.464678	3.446722	3.477266	3.077933	3.186339	3.438067	3.760284	3.729835	3.327915	3.084157	2.856077	3.103006	2.854242	3.506641	3.273291	3.888699	4.460059	3.115700	3.027789	3.908140	4.385878	4.096439	3.736280	3.905294	4.162728	3.396948	3.010303	5.271639	3.828629	3.147640	3.151361	2.890356	3.127099	3.274958	3.582100	3.560309	3.288923	2.943386	3.224618	3.053347	2.844634	3.062481	3.013864	3.049338	3.029190	3.010039	3.276674	3.046804	3.140408	3.089121	3.200908	3.753824	4.275750	3.416052	3.582650	3.429348	3.229324	3.475076	3.396129	3.370825	3.320089	3.238005	2.840570	2.988326	3.220989	3.146903	3.043798	2.928153	2.962645	3.456247	3.247981	2.977498	3.110727	3.031471	2.884071	4.610790	3.452427	3.092072	3.154426	3.231876	3.107938	3.449554	2.964653	2.944727	3.154426	2.987431	2.982814	3.170136	3.018540	3.162277

LY6E-DT	6.254554	6.819280	6.884549	7.653601	7.853763	7.133718	7.794622	3.662035	4.428056	6.911631	7.099426	6.154472	6.977046	5.401834	6.689974	7.568409	6.426143	4.896846	4.301736	7.286286	5.633578	3.738741	4.942999	6.170149	4.248826	7.057617	5.392206	4.773047	6.834227	7.830932	5.159865	8.785931	8.248095	7.904884	7.969250	7.800683	9.174484	5.456853	5.399789	4.049784	7.782708	4.519978	5.367584	8.242186	8.894109	8.403614	4.523673	6.231257	7.132134	8.708137	8.303777	9.032354	8.718419	8.659760	7.754268	6.874986	9.148637	6.821498	5.429406	7.939314	5.793655	6.663285	3.515313	3.617257	5.841706	6.804922	5.926871	7.514055	6.933336	3.826779	8.393485	7.857100	6.803329	8.015237	7.936228	8.287349	7.101718	6.909234	7.508788	6.493772	7.973975	8.422572	8.710249	8.891442	9.352291	7.922249	7.393024	8.127830	5.176137	5.617787	6.295437	5.668327	7.221939	9.116189	7.364986	6.652567	8.288257	7.502904	6.713426	5.834287	4.927430	6.911468	6.647718	5.506211	3.607261	5.183414	3.814815	4.956023	3.520441	6.893364	3.065854	4.283814	3.925898	5.712884	4.967357	3.475442	3.387237	6.913700	6.637380	9.635352	6.826564	7.424306	8.747415	5.963061	7.796942	8.087401	7.552537	5.932802	8.965547	7.513109	4.588420	7.123215	7.598858	6.251495	6.314683	8.499478	3.748346	5.786653	9.144893	4.299398	3.857006	5.558298	9.660763	5.344963	7.130525	9.983819	7.568265	7.895952	8.687013	6.924197	7.123215	6.354724	6.632032	9.860677	6.660184	8.193053	6.242766	4.802376	5.867788	7.637383	4.966488	5.607219	8.606037	9.304702	8.436796	3.650223	6.199832	8.233292	8.738564	9.652899	6.498235	11.293238	8.400034	7.650137	8.221915	10.582172	6.864398	7.903509	7.942176	4.694331	8.427236	3.533103	7.710796	4.841476	6.041931	7.507990	6.754571	5.292378	6.321505	7.701163	9.263696	5.750104	7.597003	6.772337	8.078230	6.303063	6.302189	10.080285	6.053214	8.520379	10.427471	9.484565	8.788120	11.219673	7.402245	7.178144	8.439018	5.603033	5.918859	4.961439	6.339386	5.491679	7.788999	7.140023	8.414587	8.047693	6.802686	7.392419	7.509710	6.741635	4.489766	6.476742	5.033779	4.063389	6.801411	3.243811	6.856249	5.145729	6.269812	2.861260	4.215112	3.067524	7.465716	5.985916	4.820657	3.460369	9.404833	9.342664

AL583808.1	3.745674	3.604453	3.032138	2.976312	3.140052	3.912710	3.392489	3.952908	3.495604	4.053015	3.666887	3.834808	3.299913	4.111616	3.983505	3.194728	3.645020	3.950106	4.694525	3.493734	5.276363	2.871646	4.443074	3.353521	2.940422	3.155938	4.661156	3.114254	2.905881	3.078974	4.232008	4.382303	3.273704	3.123311	4.119034	4.144522	3.764064	3.594018	3.742381	2.899003	3.410422	2.959101	3.517551	3.198002	3.666461	5.439305	4.066688	3.458025	3.939217	4.082186	4.039945	3.890045	4.414893	4.158864	3.916834	3.308406	4.096439	3.092443	3.915505	3.594018	2.947900	3.008249	3.318122	3.574995	4.461136	3.758992	4.517223	3.731296	4.036272	2.876858	2.946892	2.918243	3.330982	3.202087	2.916473	3.516224	3.431688	3.605586	3.469235	3.769062	3.396129	4.108703	4.358295	4.359375	3.455153	4.783894	3.193531	3.619143	2.984597	4.413763	3.993522	4.343091	3.166354	3.136258	3.073894	3.129814	3.840468	3.622868	2.929615	2.968089	2.962645	3.290793	3.320580	3.623258	3.789308	4.099547	4.263344	3.995744	3.716933	3.312397	3.347031	3.916408	3.038635	3.319276	3.271149	3.193913	3.293775	3.148764	3.684027	7.538769	4.921690	3.407300	5.458276	5.520055	5.997605	4.021312	6.222113	6.594690	5.804407	4.297220	3.724528	4.502413	3.413645	3.536689	3.001475	3.620461	5.761773	3.422249	3.442756	4.556009	6.332486	5.940259	5.149493	4.627811	4.983217	3.985840	3.545812	6.602156	3.582650	4.029058	5.747033	6.070506	3.184762	3.496731	4.432687	4.778032	5.222149	3.934932	4.379938	7.168563	4.532465	4.050645	5.468334	4.092275	5.040958	4.282532	3.568681	5.236428	4.290904	4.905496	4.687391	4.085430	4.326137	3.475806	5.452642	3.681221	4.187745	4.300044	4.653624	5.696022	4.895184	6.256249	4.648115	4.855214	4.572849	4.597879	4.211867	4.942614	2.916007	3.873314	2.957685	5.971051	3.239599	3.341666	3.775313	3.510558	3.377564	3.437397	3.266440	5.145547	4.454821	6.034026	3.741358	3.569850	5.042632	3.528850	4.736346	2.907021	4.557261	3.420058	5.637531	3.112860	3.115819	3.593015	3.011063	4.437643	3.777553	5.586787	5.529921	2.976768	3.198153	6.061355	2.953408	6.946451	5.963776	5.533876	6.566103	5.703574	3.301457	4.803039	6.634185	4.313602	3.455318	4.329472	4.921917	5.751191	3.234824	6.819436

LINC02796	3.745674	3.604453	3.032138	2.976312	3.140052	3.912710	3.392489	3.952908	3.495604	4.053015	3.666887	3.834808	3.299913	4.111616	3.983505	3.194728	3.645020	3.950106	4.694525	3.493734	5.276363	2.871646	4.443074	3.353521	2.940422	3.155938	4.661156	3.114254	2.905881	3.078974	4.232008	4.382303	3.273704	3.123311	4.119034	4.144522	3.764064	3.594018	3.742381	2.899003	3.410422	2.959101	3.517551	3.198002	3.666461	5.439305	4.066688	3.458025	3.939217	4.082186	4.039945	3.890045	4.414893	4.158864	3.916834	3.308406	4.096439	3.092443	3.915505	3.594018	2.947900	3.008249	3.318122	3.574995	4.461136	3.758992	4.517223	3.731296	4.036272	2.876858	2.946892	2.918243	3.330982	3.202087	2.916473	3.516224	3.431688	3.605586	3.469235	3.769062	3.396129	4.108703	4.358295	4.359375	3.455153	4.783894	3.193531	3.619143	2.984597	4.413763	3.993522	4.343091	3.166354	3.136258	3.073894	3.129814	3.840468	3.622868	2.929615	2.968089	2.962645	3.290793	3.320580	3.623258	3.789308	4.099547	4.263344	3.995744	3.716933	3.312397	3.347031	3.916408	3.038635	3.319276	3.271149	3.193913	3.293775	3.148764	3.684027	7.538769	4.921690	3.407300	5.458276	5.520055	5.997605	4.021312	6.222113	6.594690	5.804407	4.297220	3.724528	4.502413	3.413645	3.536689	3.001475	3.620461	5.761773	3.422249	3.442756	4.556009	6.332486	5.940259	5.149493	4.627811	4.983217	3.985840	3.545812	6.602156	3.582650	4.029058	5.747033	6.070506	3.184762	3.496731	4.432687	4.778032	5.222149	3.934932	4.379938	7.168563	4.532465	4.050645	5.468334	4.092275	5.040958	4.282532	3.568681	5.236428	4.290904	4.905496	4.687391	4.085430	4.326137	3.475806	5.452642	3.681221	4.187745	4.300044	4.653624	5.696022	4.895184	6.256249	4.648115	4.855214	4.572849	4.597879	4.211867	4.942614	2.916007	3.873314	2.957685	5.971051	3.239599	3.341666	3.775313	3.510558	3.377564	3.437397	3.266440	5.145547	4.454821	6.034026	3.741358	3.569850	5.042632	3.528850	4.736346	2.907021	4.557261	3.420058	5.637531	3.112860	3.115819	3.593015	3.011063	4.437643	3.777553	5.586787	5.529921	2.976768	3.198153	6.061355	2.953408	6.946451	5.963776	5.533876	6.566103	5.703574	3.301457	4.803039	6.634185	4.313602	3.455318	4.329472	4.921917	5.751191	3.234824	6.819436

LINC01356	10.748141	10.815810	10.896417	10.704200	10.990476	11.437819	11.248361	11.116825	11.442182	11.491895	11.407065	11.371284	10.590962	10.584201	10.436727	11.131351	10.891777	10.214264	10.023218	10.847257	10.070853	9.541895	10.268511	10.149179	10.476151	10.890583	11.507297	11.164195	12.013558	11.732422	11.142360	10.027195	9.812607	11.367880	10.684506	10.565474	10.609789	10.837802	10.126943	10.120961	11.652576	10.129759	10.491566	10.276978	10.389118	10.291882	11.796289	10.285761	10.265002	10.428516	10.126326	10.190552	10.042830	10.191151	10.118840	11.765088	10.146111	9.801046	9.412285	9.804548	9.735158	11.938632	11.833741	9.743493	9.823738	9.773869	9.890283	10.203404	10.212581	10.132585	10.996710	11.093692	11.907433	10.959032	10.897486	11.088431	10.921942	11.094004	10.664741	9.583001	9.497955	9.994464	9.424977	9.753620	9.764347	9.903186	9.603754	9.658892	9.604383	9.298930	9.128284	9.628464	10.150370	10.556615	10.326429	10.253644	9.936498	10.899829	10.592750	11.274151	10.924780	10.664468	10.950728	10.946219	9.826316	11.637826	10.130357	10.344466	10.358409	11.826245	9.870429	10.009742	10.443501	12.023884	11.013672	12.297109	10.016962	11.083077	11.963054	10.933529	11.120015	11.379762	11.372309	11.608459	11.341014	11.692447	11.554325	11.581538	11.804392	11.690470	11.423597	11.398068	10.992855	11.565671	11.475722	11.453428	10.696641	10.424236	10.900123	10.231761	9.896439	10.412232	10.921206	10.992556	11.194032	11.295264	11.179610	12.779681	11.866983	11.334043	10.922234	10.716420	11.533180	10.716263	10.762808	10.846377	11.308373	10.902471	10.734462	12.130980	10.620579	10.826216	10.936553	10.510937	10.675082	12.104382	10.420414	10.189059	11.129277	10.793359	10.740494	10.374399	10.638914	10.489958	11.820950	11.198103	10.173953	9.913442	9.941976	10.400645	12.052072	10.906300	10.144911	10.098577	10.319057	10.507219	10.702123	10.639693	10.447803	11.014855	11.589820	12.082923	11.330144	11.248503	11.253098	11.343391	11.680103	11.096779	10.554929	9.826316	10.371089	9.937579	10.412549	10.496309	10.864387	9.962991	9.815723	10.281995	10.216407	9.889840	10.338376	10.650157	11.567573	11.221025	11.077806	10.758086	12.195580	11.160065	11.089903	11.481367	10.851641	11.430579	11.130935	10.371240	11.817639	10.699421	10.689930	9.970361	12.162501	10.480656	10.159076	10.588378	11.969338	10.842134	10.971168	10.389594	10.962371	11.909566

AC023421.2	6.811147	5.284565	4.993108	6.380296	5.601135	7.865730	6.606374	6.231727	6.141731	7.744995	8.491478	7.898223	6.139091	5.363851	4.453967	7.330158	7.448253	4.530956	5.405925	7.666067	4.784124	5.243809	5.835886	4.355913	5.257917	5.339320	7.417421	6.535312	8.328604	6.664613	6.362867	4.917266	4.829094	7.289104	6.791693	6.085920	6.321505	4.872775	4.957295	3.837782	7.262822	2.952581	5.214034	4.814353	5.472171	5.450854	9.154798	5.859241	5.825139	4.716023	3.745044	4.235959	4.139301	4.277307	4.449838	7.871982	5.133404	3.027480	3.586905	3.484067	3.093041	8.228867	4.708660	3.869015	4.183437	4.654410	4.235322	4.616425	4.370890	4.372594	5.930416	6.166868	8.120696	6.387306	5.828960	5.834109	6.049762	5.342558	5.337890	4.842988	4.180567	3.884898	4.318023	4.319720	4.009963	5.374929	3.950551	3.463210	2.977056	3.156400	3.490484	3.392134	4.084981	5.490389	5.787748	6.417972	5.114185	8.006998	7.491660	6.606716	7.822263	6.187067	6.097512	5.358537	5.251581	7.831559	4.411549	5.454755	4.538427	7.964808	4.669934	4.143033	4.844870	8.115024	7.080977	8.568189	5.131397	8.086300	7.960849	6.670249	5.372063	4.639269	5.490755	5.792044	6.190787	7.689797	8.620345	6.232414	6.702081	7.656711	7.837337	7.292246	5.219369	4.869784	7.616951	5.737892	4.999402	4.981142	5.614814	4.133820	4.854590	5.777617	4.950416	5.635631	5.391069	7.196961	5.817089	7.724844	5.650854	6.962124	6.132434	5.635100	7.339745	5.601705	4.862492	6.625166	4.519116	4.850976	4.372155	7.053976	2.910533	4.493107	3.757114	4.137363	5.053865	7.547120	5.125133	5.465211	4.011026	3.780400	3.512208	3.744836	4.023017	4.333078	7.117230	4.725774	3.060046	3.302053	3.371691	2.968734	7.120267	3.289532	3.127814	3.215305	4.170551	2.849361	4.021539	3.599742	5.562034	5.196697	5.881412	6.266387	5.559256	6.128501	5.069189	6.134823	4.914337	6.150503	4.252568	3.614582	4.113339	3.364197	3.967464	3.540150	6.261843	3.388949	5.110367	2.923134	3.973097	3.328510	3.158396	4.689517	6.188929	6.855733	7.191688	6.764101	8.633518	7.680701	5.251176	6.703693	7.298148	7.017952	4.960214	3.745674	7.089306	3.108051	4.131246	3.748721	7.228420	4.809737	4.701302	6.032733	6.674449	7.148828	7.695913	5.821481	7.583967	6.189910

AC023421.1	6.811147	5.284565	4.993108	6.380296	5.601135	7.865730	6.606374	6.231727	6.141731	7.744995	8.491478	7.898223	6.139091	5.363851	4.453967	7.330158	7.448253	4.530956	5.405925	7.666067	4.784124	5.243809	5.835886	4.355913	5.257917	5.339320	7.417421	6.535312	8.328604	6.664613	6.362867	4.917266	4.829094	7.289104	6.791693	6.085920	6.321505	4.872775	4.957295	3.837782	7.262822	2.952581	5.214034	4.814353	5.472171	5.450854	9.154798	5.859241	5.825139	4.716023	3.745044	4.235959	4.139301	4.277307	4.449838	7.871982	5.133404	3.027480	3.586905	3.484067	3.093041	8.228867	4.708660	3.869015	4.183437	4.654410	4.235322	4.616425	4.370890	4.372594	5.930416	6.166868	8.120696	6.387306	5.828960	5.834109	6.049762	5.342558	5.337890	4.842988	4.180567	3.884898	4.318023	4.319720	4.009963	5.374929	3.950551	3.463210	2.977056	3.156400	3.490484	3.392134	4.084981	5.490389	5.787748	6.417972	5.114185	8.006998	7.491660	6.606716	7.822263	6.187067	6.097512	5.358537	5.251581	7.831559	4.411549	5.454755	4.538427	7.964808	4.669934	4.143033	4.844870	8.115024	7.080977	8.568189	5.131397	8.086300	7.960849	6.670249	5.372063	4.639269	5.490755	5.792044	6.190787	7.689797	8.620345	6.232414	6.702081	7.656711	7.837337	7.292246	5.219369	4.869784	7.616951	5.737892	4.999402	4.981142	5.614814	4.133820	4.854590	5.777617	4.950416	5.635631	5.391069	7.196961	5.817089	7.724844	5.650854	6.962124	6.132434	5.635100	7.339745	5.601705	4.862492	6.625166	4.519116	4.850976	4.372155	7.053976	2.910533	4.493107	3.757114	4.137363	5.053865	7.547120	5.125133	5.465211	4.011026	3.780400	3.512208	3.744836	4.023017	4.333078	7.117230	4.725774	3.060046	3.302053	3.371691	2.968734	7.120267	3.289532	3.127814	3.215305	4.170551	2.849361	4.021539	3.599742	5.562034	5.196697	5.881412	6.266387	5.559256	6.128501	5.069189	6.134823	4.914337	6.150503	4.252568	3.614582	4.113339	3.364197	3.967464	3.540150	6.261843	3.388949	5.110367	2.923134	3.973097	3.328510	3.158396	4.689517	6.188929	6.855733	7.191688	6.764101	8.633518	7.680701	5.251176	6.703693	7.298148	7.017952	4.960214	3.745674	7.089306	3.108051	4.131246	3.748721	7.228420	4.809737	4.701302	6.032733	6.674449	7.148828	7.695913	5.821481	7.583967	6.189910

AC079781.5	10.046174	8.666374	8.909370	9.773869	10.744560	10.391055	9.149117	10.630554	11.518846	9.972631	9.986107	10.063327	10.021399	10.182552	9.423138	10.455701	10.108388	9.872723	11.495210	10.061353	9.811988	11.478703	11.808575	10.072673	10.365154	10.444582	10.978775	9.578383	10.673307	9.517921	10.715970	8.924600	9.001028	10.221812	9.552669	9.390643	9.255736	9.679709	9.377152	9.930320	10.133737	9.824177	9.768915	9.823152	9.586696	9.317901	10.405770	9.044566	9.244397	9.213447	9.484419	9.351550	9.128931	9.153228	9.422970	10.206899	9.651040	9.433441	9.542515	9.142743	9.225274	10.373383	9.387777	10.360811	9.113294	9.134862	9.194034	9.056578	8.576857	10.080850	9.025547	9.597725	10.846377	9.093137	9.669565	9.262594	9.602546	9.093884	8.986399	9.356816	10.104790	9.426808	9.224522	9.576673	9.662962	9.410338	10.300812	9.384500	9.967099	8.594879	9.313309	9.643918	9.190703	9.184855	9.835366	10.422850	9.237643	11.170020	10.460269	10.616870	11.439430	9.643474	9.918091	9.171092	10.389118	10.230524	11.048986	11.013401	12.118645	10.151110	11.963379	11.181727	11.017781	11.202068	10.071016	13.973310	10.869032	10.177785	11.199022	10.739904	9.667624	9.111112	10.981887	12.370359	9.574876	10.753411	12.636117	11.510387	10.483985	10.719585	10.554448	11.050179	9.917940	10.941388	11.480371	11.395495	11.129440	12.364654	9.625524	10.657396	11.709681	10.897879	10.941388	9.832155	8.842443	11.121141	11.248954	11.759614	8.603763	11.206137	11.150531	10.350071	10.168737	9.826170	9.267219	9.383185	9.616048	9.764510	10.174725	11.267512	9.522409	9.775681	9.493782	9.647662	8.785931	10.691016	8.956370	9.474302	8.874648	9.676894	9.975738	9.114833	8.789796	9.441595	9.505499	10.097471	9.674273	9.736555	9.001665	9.752686	10.081162	9.710316	9.511361	9.123526	9.354532	10.287254	8.876783	9.280151	9.096576	9.482261	9.847374	10.524141	10.047711	9.932019	9.334599	9.389155	9.753620	9.127357	9.595225	10.178271	9.803650	9.443739	10.243523	9.647063	11.051636	9.883787	10.164130	10.049407	9.454926	9.821458	10.318172	9.172195	8.819040	9.686241	11.140371	11.253380	12.052666	10.819847	9.920741	10.976265	10.725657	11.085876	9.246741	9.784891	10.822371	10.860851	11.664771	9.950421	9.745228	12.014918	11.560840	12.269152	11.431952	10.419803	11.211287	10.105679	10.064236	10.249835

AC004967.2	10.046174	8.666374	8.909370	9.773869	10.744560	10.391055	9.149117	10.630554	11.518846	9.972631	9.986107	10.063327	10.021399	10.182552	9.423138	10.455701	10.108388	9.872723	11.495210	10.061353	9.811988	11.478703	11.808575	10.072673	10.365154	10.444582	10.978775	9.578383	10.673307	9.517921	10.715970	8.924600	9.001028	10.221812	9.552669	9.390643	9.255736	9.679709	9.377152	9.930320	10.133737	9.824177	9.768915	9.823152	9.586696	9.317901	10.405770	9.044566	9.244397	9.213447	9.484419	9.351550	9.128931	9.153228	9.422970	10.206899	9.651040	9.433441	9.542515	9.142743	9.225274	10.373383	9.387777	10.360811	9.113294	9.134862	9.194034	9.056578	8.576857	10.080850	9.025547	9.597725	10.846377	9.093137	9.669565	9.262594	9.602546	9.093884	8.986399	9.356816	10.104790	9.426808	9.224522	9.576673	9.662962	9.410338	10.300812	9.384500	9.967099	8.594879	9.313309	9.643918	9.190703	9.184855	9.835366	10.422850	9.237643	11.170020	10.460269	10.616870	11.439430	9.643474	9.918091	9.171092	10.389118	10.230524	11.048986	11.013401	12.118645	10.151110	11.963379	11.181727	11.017781	11.202068	10.071016	13.973310	10.869032	10.177785	11.199022	10.739904	9.667624	9.111112	10.981887	12.370359	9.574876	10.753411	12.636117	11.510387	10.483985	10.719585	10.554448	11.050179	9.917940	10.941388	11.480371	11.395495	11.129440	12.364654	9.625524	10.657396	11.709681	10.897879	10.941388	9.832155	8.842443	11.121141	11.248954	11.759614	8.603763	11.206137	11.150531	10.350071	10.168737	9.826170	9.267219	9.383185	9.616048	9.764510	10.174725	11.267512	9.522409	9.775681	9.493782	9.647662	8.785931	10.691016	8.956370	9.474302	8.874648	9.676894	9.975738	9.114833	8.789796	9.441595	9.505499	10.097471	9.674273	9.736555	9.001665	9.752686	10.081162	9.710316	9.511361	9.123526	9.354532	10.287254	8.876783	9.280151	9.096576	9.482261	9.847374	10.524141	10.047711	9.932019	9.334599	9.389155	9.753620	9.127357	9.595225	10.178271	9.803650	9.443739	10.243523	9.647063	11.051636	9.883787	10.164130	10.049407	9.454926	9.821458	10.318172	9.172195	8.819040	9.686241	11.140371	11.253380	12.052666	10.819847	9.920741	10.976265	10.725657	11.085876	9.246741	9.784891	10.822371	10.860851	11.664771	9.950421	9.745228	12.014918	11.560840	12.269152	11.431952	10.419803	11.211287	10.105679	10.064236	10.249835

AC010542.2	11.658189	11.387625	11.744798	11.713327	11.630454	10.924005	11.845235	10.989151	10.635496	11.547236	11.168397	11.207783	10.987180	11.243314	11.383634	11.491594	10.929712	11.130031	12.456759	11.618765	11.694395	11.711018	9.200336	11.683496	11.783598	11.768847	11.417099	11.175522	11.241643	11.853395	11.405143	11.920823	11.768560	11.041648	11.925051	11.647837	11.810068	11.749984	12.218494	12.378188	11.562313	12.676852	11.391292	11.434496	11.741869	11.165803	11.217158	12.065618	11.058784	11.454008	11.649619	11.800745	11.902027	11.581405	11.127076	11.248361	11.353017	12.351197	11.864994	11.982413	12.197990	11.705942	13.209700	12.583137	12.341379	11.706405	12.240559	11.493891	11.734381	12.264028	11.662821	11.284747	10.888202	11.339525	11.545607	11.860555	11.774016	11.704316	11.615241	11.939846	11.737635	11.872355	11.683496	11.549648	11.777620	11.966878	11.812156	12.089816	12.505403	11.993963	12.248989	12.578521	11.613311	11.442790	11.642537	11.462914	11.531239	11.659107	11.539677	10.994167	11.038163	11.380992	11.226506	11.414563	11.745095	11.079831	11.870252	11.200580	12.548924	11.605506	9.521307	12.066571	11.690040	10.771383	11.080441	9.948751	12.384330	11.440637	10.994463	10.410869	10.591557	11.606677	11.729465	9.650553	10.029478	10.785727	10.765383	11.670899	10.780734	10.402284	9.882574	10.680952	11.246940	10.818491	11.538956	11.859653	10.013258	11.302336	11.037267	10.890897	11.459486	8.280832	9.700030	11.579980	12.327958	10.607687	9.273471	9.796331	11.395640	11.543627	11.564784	11.230487	10.346580	10.950587	10.733421	11.612110	11.929527	12.087259	12.280813	10.336136	11.964481	10.653995	10.550253	10.706272	10.203707	8.075788	10.915384	10.684506	10.918229	10.905697	11.575856	11.141784	11.228558	11.782660	11.608622	10.606089	11.724539	11.048256	10.837075	12.036393	11.768414	11.696386	12.088732	12.052072	12.226768	11.678161	11.001214	10.967834	12.441914	11.285485	10.954078	9.743493	10.932934	11.586749	11.454008	11.750117	11.931067	11.093843	11.990637	11.633293	10.399429	11.054506	11.433898	11.590688	10.999696	11.077953	11.653482	12.480642	11.875072	11.195021	12.135587	11.393682	11.122494	11.051785	11.420572	11.302634	11.257082	11.311460	10.886269	11.475562	10.517868	10.143844	11.345947	12.326688	10.632306	10.554929	10.855244	11.315434	9.660763	8.986399	11.786428	10.134688	10.691815	10.207843	10.826359	11.596981	11.540550	11.817489

AL589645.1	3.000357	3.195295	3.017496	2.986165	2.874316	4.526322	3.331266	3.257454	3.341822	3.417894	3.580504	3.605586	3.286231	3.789104	3.831827	3.146302	3.527576	3.700152	4.786204	3.104112	4.903414	2.825096	4.119452	3.032852	3.038434	2.846004	3.013073	2.865639	3.151643	3.058412	4.174012	3.065747	4.627811	3.049223	3.263345	4.268916	3.666689	3.209388	3.273883	2.888014	3.366686	2.926174	3.665884	3.257185	3.626812	5.318242	3.257771	3.172139	5.035276	3.758566	3.779151	3.612955	4.281683	4.082186	3.838658	3.115462	4.256083	2.935480	3.725775	3.708456	2.972902	3.087647	3.632310	3.082860	3.319115	3.353669	2.855957	3.355179	3.173051	2.845610	2.998688	2.895906	3.066288	2.902002	2.901598	3.308061	3.037030	2.952746	2.961386	3.767990	3.364693	4.049324	3.366868	4.394991	3.468909	4.756537	3.152112	3.297612	3.202543	3.473431	3.277138	3.262477	3.491185	3.097736	2.922883	2.944910	3.786278	3.214341	3.558530	2.950339	3.083140	3.249707	3.003522	3.387383	4.377580	3.862777	3.081556	3.424717	3.055377	3.283480	2.771095	2.897975	2.924280	3.284757	3.040188	3.080690	2.932571	2.988814	3.184101	3.017983	3.310528	2.980607	3.038042	3.080487	5.460043	2.965857	3.173699	3.278379	3.177618	3.385511	3.139324	3.015950	3.188883	3.451547	2.960516	3.489710	3.227892	3.431525	3.269604	4.224051	2.818845	4.978711	2.931594	2.892787	2.803798	2.954506	3.017878	2.928747	3.544326	3.283173	3.329990	3.344944	3.009272	3.211879	4.560560	3.694878	3.168786	3.259588	2.888289	3.142918	2.863298	3.804926	3.294849	4.032992	4.872775	3.188468	3.178289	4.967125	3.795069	3.760706	3.435445	4.021539	4.290484	3.460924	3.051115	3.707077	2.946804	3.339114	3.556578	2.891799	3.186761	3.640480	3.330982	3.496731	3.538645	2.855606	3.698588	3.272404	2.851496	3.020128	2.978709	3.060046	2.949867	2.918048	3.266440	3.038635	3.094765	3.016683	3.235659	3.748721	4.354587	3.421018	3.736479	3.580694	3.556033	3.496154	3.465935	3.052890	3.360160	3.437038	3.224050	3.322974	3.133555	3.038520	2.950188	2.936912	2.920907	3.697989	3.233362	3.219514	3.038755	2.920907	3.265284	4.845952	3.662649	3.104112	3.397284	3.261430	3.164186	2.901334	2.789659	2.870546	3.215034	2.963327	3.060878	2.938239	3.008075	3.313017

APTR	12.988700	12.418166	12.568405	12.770516	12.853341	12.153445	12.015060	12.331121	13.477273	12.599183	12.767450	12.334279	12.744026	12.382432	11.917597	12.443867	12.987390	12.123320	12.705163	12.774962	12.583137	12.526786	12.891207	12.231096	12.479954	12.674301	12.128873	12.574386	12.266375	12.059510	13.087211	12.809121	12.778119	12.452816	12.292654	12.527089	12.402445	12.517447	12.413496	12.088271	12.491075	11.929527	12.045149	12.554786	12.317939	12.731336	12.243145	11.766175	13.290071	12.503202	12.822669	12.716248	12.600707	12.446416	12.455483	12.499786	12.922772	12.245314	12.329562	12.362103	12.554786	12.249289	12.611506	12.351035	12.578521	12.130980	12.171733	12.656343	12.236059	12.502227	12.237727	12.849653	12.466981	12.521399	12.732697	12.351330	12.619661	12.263096	12.641550	12.184698	12.345812	12.726510	12.697518	13.007579	12.991998	12.768875	13.002707	13.107335	12.683500	12.772604	12.772931	12.673439	12.523149	12.535932	12.614041	13.124180	12.920968	12.529854	13.032306	12.721811	12.435325	12.085752	12.339191	12.223705	12.592493	13.133580	13.189888	12.917665	12.890856	12.324058	13.147891	12.335103	12.991998	12.645205	12.726341	11.794698	13.070763	12.372273	12.252783	12.796448	12.432257	12.270901	12.487141	11.968715	12.389189	11.944822	14.111384	12.773964	12.958452	12.181736	13.459608	13.039136	11.750711	11.920531	13.090908	12.461067	12.460764	13.605255	12.383858	12.180008	12.258508	12.757557	12.802136	11.995045	13.132807	12.839105	13.164705	12.646251	12.247673	12.768334	11.537619	13.221167	11.742441	12.328285	13.083488	12.015827	12.743020	12.284575	11.848060	12.591334	12.330024	12.599341	12.801093	13.156161	12.228964	12.630012	12.284891	13.084780	12.547000	11.730335	13.333079	12.230187	11.930143	12.699525	12.367990	12.544739	11.789113	12.466153	12.638694	12.063646	12.124049	11.812441	12.312544	12.839995	12.491562	12.565623	11.877464	12.604915	12.293129	12.920968	12.517769	11.132779	12.039995	12.656189	11.906665	12.184846	13.206127	12.429784	11.506569	11.587454	13.039136	11.917282	12.511596	12.379442	13.484139	12.263875	13.673646	11.820064	12.226617	12.253893	13.271222	12.529519	12.437077	11.555223	12.667828	12.879072	13.197929	12.069172	12.753795	12.909823	13.261202	12.101924	11.718826	12.670010	12.404343	13.474856	12.981496	12.991998	11.795854	13.053130	11.352182	13.222566	11.903881	13.283833	13.136985	13.046278	13.119075	12.197841

LINC02172	4.840237	3.954387	5.763765	3.626008	3.209518	3.616696	4.777193	4.084981	3.786278	3.501061	3.866394	3.803066	3.669497	4.253009	4.022370	3.901467	3.730048	3.737498	5.144161	3.261735	5.482205	2.947624	6.138053	3.760284	3.772186	4.524788	3.577965	3.593240	3.126509	4.269404	3.999159	5.195106	4.886667	4.044097	5.424454	4.200312	5.625588	4.912630	4.926387	4.240135	3.684214	4.986033	3.909010	3.412024	5.092471	5.690604	4.001248	5.325555	4.787036	4.386303	4.266147	4.650482	5.878333	4.309263	5.202855	3.213397	5.578136	5.095208	4.076598	4.651356	4.381162	4.053199	6.116663	5.022202	6.236812	4.410264	2.857145	3.800966	4.198200	4.139961	4.893955	4.465207	3.144788	4.917266	4.938279	4.711800	7.487725	5.432006	4.334201	5.807949	5.720302	4.641957	4.146217	4.590157	6.242113	4.868967	5.968975	5.237472	4.836376	5.039101	4.792691	5.166666	4.835195	4.444552	3.758992	4.471008	4.263078	3.084859	3.121586	3.520242	4.957084	3.319776	3.547999	3.337147	4.725774	4.010802	4.501816	3.650983	4.405609	3.336976	5.017503	4.034749	4.228532	3.345113	3.304280	5.255554	4.174872	3.872223	3.234227	3.394158	3.571687	4.367632	3.776160	3.690345	4.095823	3.947248	3.789934	3.763395	3.225038	3.519105	3.318632	3.072563	3.433070	3.548394	4.494905	3.787581	3.232304	3.841530	3.456795	4.749126	3.641891	6.169121	3.352380	3.037944	6.986041	3.970080	3.560309	3.317112	4.488660	3.912072	4.104297	3.426395	3.430804	4.784543	3.876779	4.914730	4.578828	3.988380	4.408699	3.487910	3.687880	3.712339	3.769308	4.376523	5.294827	3.280419	5.597008	4.123626	4.560560	4.231803	3.832923	4.675590	4.486585	5.149493	3.174332	5.717993	3.163139	3.827419	3.982623	3.010212	4.179668	4.380590	5.131015	4.639678	4.156749	2.846290	3.790356	4.433495	2.938338	3.992680	4.283381	3.161074	4.793710	4.157572	3.786732	7.402709	4.553238	4.079591	4.099296	4.944128	4.683975	3.658626	4.176606	5.391260	3.738741	5.338509	4.169878	5.375140	4.130031	4.095392	4.130225	3.413828	3.294372	3.490659	3.669057	3.661403	4.857743	3.743889	3.321225	3.432543	3.253718	2.981253	3.035276	4.997369	3.753824	4.459856	3.691947	3.561567	3.224345	4.837448	3.560111	4.918935	3.796526	3.140672	3.270828	4.582230	3.855740	3.387578

PSMB8-AS1	10.099038	10.344614	10.832125	9.569803	9.514568	9.913122	10.087473	8.431048	8.086750	8.812917	9.443739	9.354397	10.011528	8.446086	9.476585	9.965758	8.688665	9.787132	7.695762	10.415564	8.048473	10.275916	8.626721	10.558269	9.072667	8.437404	10.578592	7.810194	9.319298	11.682169	7.404585	11.206863	12.454055	12.151881	11.575095	11.252070	10.422850	9.686684	9.275993	9.932019	9.107183	10.183009	11.057782	10.778555	10.825316	11.227693	8.633227	10.541677	10.614231	10.738842	11.243131	11.034179	11.300099	11.053352	10.882223	8.815901	11.460712	10.495855	9.539381	10.050349	10.355208	9.431265	11.096172	9.555843	10.291882	9.861608	9.979195	10.171549	10.588804	9.924291	10.659469	10.336891	8.812483	10.829597	10.816113	11.860414	10.552052	11.405727	10.767431	11.108217	11.312627	11.346092	11.072137	10.638914	10.743513	10.633501	11.254400	10.949961	10.761158	10.005677	10.967106	10.821454	10.263600	11.430440	11.767362	11.221910	11.043725	9.476893	9.324307	9.277385	9.024467	10.097624	9.375129	9.025237	9.469668	9.065545	8.327291	9.959832	8.336641	8.203908	9.963485	8.095973	8.029041	8.614047	10.253644	8.162350	9.378542	9.604056	7.985454	10.572908	10.516533	11.764203	10.166750	8.565719	8.680653	10.615426	7.762198	9.156011	10.588378	10.015244	8.951656	8.673896	10.997288	8.275513	11.147807	11.111735	8.844175	8.119024	10.955728	8.421040	10.493743	9.835818	7.700373	6.566263	10.730762	9.779969	7.778353	8.244058	10.674193	10.287723	11.221910	11.135362	9.892413	11.941196	9.387333	10.993145	9.574876	9.266894	10.274257	8.194144	10.053753	10.584613	11.010198	10.540309	11.275177	8.511392	10.300015	11.171363	10.887345	11.701916	11.133688	11.622846	11.379443	12.024345	10.130482	10.875011	10.746036	10.057867	10.371998	10.721933	10.270720	10.250420	11.030306	11.109254	10.619249	10.114821	10.690069	9.880715	11.439584	10.819554	10.897742	7.778353	10.416284	12.090445	11.969922	11.070018	10.717586	9.891957	12.256489	11.288336	11.866384	11.424641	12.048425	12.067039	10.336136	11.442637	10.652063	11.218031	10.917191	9.873790	11.286577	10.517868	11.513170	11.324962	11.161265	10.969318	9.981518	11.116077	9.758430	10.903200	9.893477	8.303777	11.359717	11.306661	9.520997	9.138103	10.069768	9.642697	8.707044	10.188159	9.645966	5.796753	9.798110	8.898560	11.589947	10.223028	11.233924	10.772988

AC020916.1	9.667023	10.445039	10.027803	10.561160	10.074846	10.900269	10.428516	13.003596	11.118110	11.068392	10.670884	10.849875	10.249099	10.550997	11.206291	10.737016	11.493459	10.661313	11.947446	10.880179	11.125774	11.062443	10.863522	10.230680	10.542688	11.162013	10.692263	9.788049	10.700919	9.545320	10.894314	9.414244	9.467673	9.297703	9.738061	11.527185	11.058092	10.688592	11.294105	11.005550	10.336744	11.105301	10.923714	11.444404	10.941524	11.039164	10.789717	11.568999	9.767533	11.410401	9.700644	10.609075	10.043161	10.345672	11.135510	11.176130	10.801504	10.221670	10.591114	9.969125	10.663292	9.440966	9.922427	10.973802	10.838984	11.404110	9.996163	10.963237	10.120667	10.954841	9.963319	9.904409	10.199612	10.203849	9.914854	9.704430	10.071163	9.485177	10.004584	10.580695	9.508697	10.659191	10.230378	10.577237	10.345357	10.484745	10.228505	9.723772	10.308933	11.397315	10.797295	10.949054	10.448715	11.001368	9.848007	9.703426	10.378655	10.796121	10.544926	11.003664	11.407356	11.132205	10.468311	11.059966	12.066571	10.157733	11.873097	11.150531	11.723465	10.611388	10.898182	11.926124	10.801045	10.477389	10.807688	11.266465	11.409964	11.167966	10.439875	9.880715	10.905411	9.183929	10.170273	11.632692	10.496309	11.136100	10.442903	10.456314	10.113060	10.420871	9.839233	11.508929	9.933222	11.025555	11.581666	10.095346	10.561618	10.547812	8.799601	10.980712	11.263631	10.911465	11.633888	9.821619	9.888772	10.557516	10.732241	10.834345	9.465805	10.228938	10.514215	10.429581	10.378185	9.032049	10.608143	11.391155	10.062437	10.690696	10.367597	10.881335	10.822371	8.864687	10.103273	11.106728	11.330144	11.116223	9.552380	9.354397	10.082245	9.687291	9.337273	9.359225	9.367092	9.149548	10.105230	8.521708	9.315341	9.934635	9.044254	10.924005	9.301723	10.658429	10.310211	9.330903	9.426037	9.549005	11.188446	10.391769	9.627241	9.988415	9.930637	12.206196	9.656882	8.737644	10.152953	9.579952	9.327512	10.382558	8.582845	9.432202	9.485342	11.051479	9.751277	8.931445	9.874810	10.059546	8.776457	9.077935	10.080716	9.171885	10.305812	10.341712	10.239706	11.059091	10.617597	10.327183	11.234493	11.294959	9.527395	10.435833	9.465672	10.891628	9.176364	9.540026	10.195682	11.266775	9.763401	10.565474	10.997755	10.204147	11.654225	10.843773	10.747087	9.660909	10.233257	10.816421	9.816348	10.435833

AC087636.1	4.799860	5.173357	5.313196	5.356463	5.801873	4.778245	4.755730	6.010016	6.167031	4.523906	4.717717	3.868380	4.535021	5.134841	4.523456	5.193268	4.714127	4.712006	5.915478	5.407929	6.878463	5.554340	6.336018	5.139678	5.549397	5.160676	5.794762	5.512770	5.388347	5.146342	5.110367	4.931393	4.552590	4.911553	5.094583	4.930625	4.974416	5.252550	5.081029	5.580774	4.961870	5.076102	4.559877	4.504347	4.802168	4.991195	6.588206	4.633723	5.955069	4.653285	5.059824	4.772404	3.887853	5.192874	4.241625	5.504676	4.840860	4.713921	4.632624	4.336791	5.903669	5.533140	5.801186	5.834830	4.765079	4.801756	5.533321	5.398817	5.453987	5.530117	5.212390	5.323834	5.058787	5.139865	5.112145	5.017906	5.191120	5.272267	5.234034	5.036116	4.957517	4.875476	4.478041	4.731438	4.915143	4.879622	4.819179	4.661156	5.243412	4.846579	4.775112	5.646170	5.479634	4.849289	5.151558	4.873845	4.947539	5.535863	5.851310	6.114404	5.919744	4.652164	4.875692	5.057772	5.550148	5.538051	5.668508	5.472537	6.130858	5.277938	6.379471	6.642863	5.862703	5.423414	5.346143	6.869212	5.388158	4.867232	5.154379	4.631548	5.577584	5.760681	5.484613	7.921359	4.355484	4.586903	5.442160	5.537433	4.935151	4.288341	4.394513	5.080404	5.444863	5.206824	5.568253	5.216600	4.481977	5.819037	5.488546	6.152405	6.521055	6.099807	5.454755	5.492994	5.343795	5.149703	5.847321	5.943373	5.046074	5.403080	5.487292	5.073004	5.258293	5.353445	4.886886	5.111933	5.515242	5.934404	5.852202	4.987279	5.261427	4.686126	4.786402	4.057800	4.547719	6.889496	4.320370	4.863103	5.079803	4.656839	4.736102	3.732967	5.199110	5.273690	5.755531	4.379297	3.658626	4.122955	4.313402	5.623522	5.647262	5.341041	5.479452	4.736955	5.521763	5.731220	5.602258	5.510295	5.270048	5.608956	5.237270	5.917953	5.703574	5.418355	5.192286	5.299282	5.422062	4.958361	5.450854	5.022393	4.274225	4.739299	5.429221	4.909040	5.304807	4.515508	4.274873	6.058707	5.503367	4.701940	5.582288	5.614247	5.123104	4.998160	4.955209	4.849690	5.944425	4.877385	5.536829	5.387388	5.395965	5.171481	5.508191	5.402259	5.576665	6.605904	5.581896	4.964786	5.750104	6.370064	6.812716	7.533448	6.137696	6.405559	6.760278	5.266258	4.740192	5.513154

AC098679.1	8.827010	9.103509	8.217923	9.643632	8.144245	8.523545	9.192889	7.858621	8.227663	8.734872	8.814706	9.188009	9.049437	8.549674	9.335123	7.985161	7.734091	8.695418	9.540818	8.009296	8.700270	8.647850	11.772691	8.760747	8.812183	8.976665	9.827055	9.982379	10.060156	8.255129	9.104114	9.318351	9.418245	9.182515	9.460752	9.254029	8.718743	8.404521	9.848007	8.900227	8.617997	10.422252	9.405140	9.204578	9.183929	8.807358	9.578851	10.309258	8.563284	9.475967	9.870094	9.379881	9.233946	8.805667	8.369555	8.265397	9.538600	9.331538	9.181095	9.517138	9.529758	9.401376	10.665611	9.676005	9.681259	9.448879	9.652120	8.288418	8.828225	8.916172	9.279525	8.726647	9.003490	8.349595	8.860035	8.888062	8.448422	9.058310	9.551464	9.467673	9.136846	9.401214	9.270612	9.309714	8.680202	9.374798	9.516518	9.018370	10.001164	10.069917	9.922734	9.239986	8.998715	8.700428	9.658106	8.151117	8.505802	9.454459	8.768231	8.746649	8.797797	8.541900	8.148523	8.391772	8.039326	8.366526	8.378322	7.710165	8.533168	8.247398	8.648119	8.774603	8.778562	9.293796	7.473087	7.180484	9.356076	8.403614	9.866241	9.197498	8.776916	9.060051	9.122924	8.659902	8.535756	9.464698	10.983770	10.601209	7.996613	9.381126	8.094631	8.858952	9.658575	9.232384	8.222067	8.935159	8.154862	8.746649	7.446661	8.160080	9.628290	9.015314	7.876419	10.186206	11.036851	8.220554	8.000418	9.243968	8.571281	10.382558	9.406320	10.337919	8.918330	8.951656	8.280399	8.822822	8.457202	10.067357	9.373989	9.587157	9.377622	8.941716	8.699834	8.111766	8.118397	8.326972	9.145795	8.056204	10.116055	9.706104	8.758681	8.894686	8.286088	9.371967	9.114196	9.098736	8.758828	8.417243	8.979397	10.096842	9.148011	9.263538	9.011126	9.753777	10.013689	10.463567	8.732389	9.084076	9.367092	8.388820	9.013164	9.442801	8.765121	9.815723	9.595374	9.404032	9.136379	8.827917	9.873647	9.919156	8.127977	9.061578	10.371089	9.732580	9.339624	9.508842	10.541677	10.240599	10.876200	8.707677	10.109280	9.505030	9.550096	9.623524	9.592749	9.777842	10.207843	9.483938	8.333537	9.280909	8.339021	8.294728	9.776589	8.614955	7.835530	9.794095	7.766856	7.138446	8.034340	9.225443	10.211984	8.790410	9.360799	7.772925	8.383906	10.056502	7.895860	8.864142

FAM198B-AS1	8.827010	9.103509	8.217923	9.643632	8.144245	8.523545	9.192889	7.858621	8.227663	8.734872	8.814706	9.188009	9.049437	8.549674	9.335123	7.985161	7.734091	8.695418	9.540818	8.009296	8.700270	8.647850	11.772691	8.760747	8.812183	8.976665	9.827055	9.982379	10.060156	8.255129	9.104114	9.318351	9.418245	9.182515	9.460752	9.254029	8.718743	8.404521	9.848007	8.900227	8.617997	10.422252	9.405140	9.204578	9.183929	8.807358	9.578851	10.309258	8.563284	9.475967	9.870094	9.379881	9.233946	8.805667	8.369555	8.265397	9.538600	9.331538	9.181095	9.517138	9.529758	9.401376	10.665611	9.676005	9.681259	9.448879	9.652120	8.288418	8.828225	8.916172	9.279525	8.726647	9.003490	8.349595	8.860035	8.888062	8.448422	9.058310	9.551464	9.467673	9.136846	9.401214	9.270612	9.309714	8.680202	9.374798	9.516518	9.018370	10.001164	10.069917	9.922734	9.239986	8.998715	8.700428	9.658106	8.151117	8.505802	9.454459	8.768231	8.746649	8.797797	8.541900	8.148523	8.391772	8.039326	8.366526	8.378322	7.710165	8.533168	8.247398	8.648119	8.774603	8.778562	9.293796	7.473087	7.180484	9.356076	8.403614	9.866241	9.197498	8.776916	9.060051	9.122924	8.659902	8.535756	9.464698	10.983770	10.601209	7.996613	9.381126	8.094631	8.858952	9.658575	9.232384	8.222067	8.935159	8.154862	8.746649	7.446661	8.160080	9.628290	9.015314	7.876419	10.186206	11.036851	8.220554	8.000418	9.243968	8.571281	10.382558	9.406320	10.337919	8.918330	8.951656	8.280399	8.822822	8.457202	10.067357	9.373989	9.587157	9.377622	8.941716	8.699834	8.111766	8.118397	8.326972	9.145795	8.056204	10.116055	9.706104	8.758681	8.894686	8.286088	9.371967	9.114196	9.098736	8.758828	8.417243	8.979397	10.096842	9.148011	9.263538	9.011126	9.753777	10.013689	10.463567	8.732389	9.084076	9.367092	8.388820	9.013164	9.442801	8.765121	9.815723	9.595374	9.404032	9.136379	8.827917	9.873647	9.919156	8.127977	9.061578	10.371089	9.732580	9.339624	9.508842	10.541677	10.240599	10.876200	8.707677	10.109280	9.505030	9.550096	9.623524	9.592749	9.777842	10.207843	9.483938	8.333537	9.280909	8.339021	8.294728	9.776589	8.614955	7.835530	9.794095	7.766856	7.138446	8.034340	9.225443	10.211984	8.790410	9.360799	7.772925	8.383906	10.056502	7.895860	8.864142

LINC00589	8.366808	8.440743	8.581670	8.490290	8.468110	8.671094	8.692789	8.891903	8.249737	8.662390	8.417837	8.466587	7.994418	8.078556	7.599534	8.092170	8.379067	7.762671	7.882013	8.319415	8.008852	8.468400	8.296090	8.138502	7.693526	8.306800	8.447340	8.693881	8.523071	9.060051	7.852039	8.210084	8.147162	8.933782	8.434607	8.723284	8.473429	8.388365	8.414877	8.424530	8.643164	8.133176	8.567450	8.397774	8.616628	8.903845	8.816517	8.722657	8.221156	9.058786	8.616015	8.437404	8.353996	8.580730	8.204777	8.985166	8.058865	8.380503	8.041562	8.247950	8.345535	8.496279	8.640980	7.908479	8.614358	8.038673	8.644694	8.489686	8.759488	8.563905	8.771856	8.663941	8.415646	8.543687	8.553764	8.571281	8.630690	8.818569	8.581670	7.748566	7.927272	8.047832	8.296996	8.072872	8.229033	8.117009	7.832609	8.307920	8.072303	8.229359	8.017102	8.029643	8.244457	8.770850	8.506585	8.143161	7.986694	7.748877	8.540872	8.337585	8.677280	8.457045	8.306800	8.751471	8.443154	8.471218	8.092315	8.005774	8.382213	8.884356	8.142995	8.336175	8.075475	8.725272	8.370333	9.834586	8.527674	8.203158	8.596418	9.221723	9.411410	9.125500	9.099806	9.748803	9.751741	9.470633	8.693097	9.195936	9.594314	9.479952	9.262594	9.245688	8.908317	9.224202	8.652353	9.955696	8.483274	8.785314	8.926766	8.785782	9.317901	9.411410	9.423296	9.391424	9.832913	8.913583	9.216696	10.075727	10.105679	8.989583	8.445159	8.967105	9.469018	8.857898	9.625053	8.758540	9.079184	9.310654	9.091411	10.010785	9.261665	8.883123	9.583159	9.685927	9.997209	10.001795	9.079626	9.151539	10.620438	10.071781	9.495797	9.465521	9.585599	8.721025	9.630159	8.848183	9.641913	8.840733	9.369204	9.408176	9.079184	9.932177	9.255128	8.916955	8.513701	10.025121	9.083201	9.473843	8.825708	9.219741	9.488302	9.546241	9.606097	8.889464	9.293796	9.671337	10.820704	9.033552	8.497774	8.927706	8.942784	8.893945	8.362863	9.585599	8.736423	9.129389	8.603917	8.931776	8.915416	9.402928	9.016533	9.170296	10.870351	9.019286	9.060510	9.044421	8.963120	9.743792	9.381956	9.399241	8.639417	8.517795	9.525717	9.161365	9.461985	9.469192	8.890530	9.216398	10.490101	8.885455	9.341453	9.100248	9.371801	9.598341	9.169683	8.557608	9.198437	10.212263

AC005833.2	9.347857	10.853180	9.858997	11.008580	10.376671	12.555422	11.939377	11.498901	10.943322	13.000090	12.307924	12.626178	10.720056	11.145577	10.980120	10.845782	10.640470	10.459258	12.157396	11.359439	11.214237	11.016496	10.659620	10.406224	11.186151	11.390861	9.719099	10.436727	11.493313	11.527331	11.332913	10.294312	10.739457	9.279672	9.844727	10.867581	10.783168	10.767880	11.178222	11.144404	12.202018	10.767880	9.616997	9.993540	9.424212	9.429122	11.359717	9.933063	10.561928	9.599698	9.376837	9.814480	10.395050	11.141047	11.494500	12.804949	10.825756	10.132720	10.712259	10.354903	10.941833	11.167246	9.225751	10.990603	11.390418	11.583022	10.430163	11.079687	10.491272	11.525740	11.079831	10.785414	11.624718	11.196818	10.507987	10.224206	10.313708	10.248629	10.847688	11.100303	9.963157	10.719260	10.390607	11.413991	11.853844	11.733178	11.001508	9.997954	10.734915	11.678602	11.774619	11.668837	11.181128	11.872954	10.046469	9.657205	10.950121	10.037337	10.692551	10.547653	11.410252	10.850174	9.915301	11.002085	12.209741	11.532717	11.539250	11.394876	11.455650	12.005783	11.319960	11.197531	10.516533	12.287067	10.861007	13.177797	8.879418	11.442933	11.200580	9.336631	11.518252	9.130629	9.533530	11.550016	11.076058	11.261231	11.267354	10.149023	10.081486	11.716302	10.058151	10.001321	9.708142	10.179032	9.703426	11.858578	8.986560	8.780406	8.448422	9.363205	9.577775	9.288170	9.595856	9.017015	8.921205	11.258115	10.155158	10.707032	10.119753	10.067514	10.425603	10.995221	11.384067	9.263984	10.660719	11.860241	10.122012	10.589516	10.108989	10.620123	10.896881	8.233897	9.143958	9.613799	10.029332	11.472498	9.661702	10.361430	9.275465	9.502704	8.529985	9.610570	9.989959	9.454180	9.358480	9.362011	8.002306	9.261349	9.255589	10.662229	10.070706	9.673342	10.294615	9.285515	8.807202	9.141971	11.956506	10.637273	9.873790	11.264092	10.741115	11.853395	10.595136	9.091411	11.089443	10.476626	9.449677	11.339952	8.710706	9.167845	9.337859	11.621139	9.413036	8.965870	10.885415	10.861593	8.937007	8.838597	9.970187	11.842962	10.126326	11.592678	9.906511	12.825230	10.441113	10.310359	10.385788	10.577237	9.440640	10.205213	9.975902	9.811172	9.415515	9.685477	10.372919	10.299864	9.671636	10.520042	11.705224	10.756176	9.830156	13.083113	12.051190	10.604302	11.187873	10.680952	9.555843	11.305008

LINC01471	6.915897	6.203457	5.833722	6.430725	6.613319	6.679465	6.358329	7.650787	7.198532	6.694411	6.990874	6.227645	5.361536	5.475412	5.749031	5.991118	7.418994	6.456040	7.257558	6.353769	6.935199	6.744337	8.680202	6.053569	6.320848	6.783864	5.632279	7.285807	5.988641	6.333155	6.684249	6.071333	6.242113	5.119073	5.462754	5.912590	6.032910	6.247297	6.635160	6.811147	6.537292	6.612852	5.605540	5.838997	5.474637	7.260754	7.287828	6.428351	6.218387	6.441052	7.137983	6.261843	6.292672	5.755704	6.121636	6.933021	5.180956	6.434442	6.232414	6.224856	6.878780	6.101332	6.609006	6.751613	6.661190	6.646089	6.365363	6.404740	6.676256	7.278613	6.312967	6.166510	6.666875	6.439561	6.798807	6.445076	6.522018	6.912288	6.923052	6.032553	6.101888	6.469202	6.335177	6.695877	6.436133	6.662321	6.260479	6.881987	7.036077	7.582415	7.219663	7.729485	5.950046	6.919713	5.896175	5.861943	6.336540	5.296854	6.196731	5.935459	5.523280	5.373424	5.909011	6.020997	6.738272	6.430895	7.637847	6.300519	6.698709	7.688261	7.409893	7.413508	6.841755	6.685006	6.388685	7.075119	7.421509	6.446077	5.957324	6.626475	6.851233	6.437280	6.627098	7.482123	7.303037	6.605303	7.062837	7.094174	6.693087	6.887252	6.669144	6.537292	6.405052	6.356259	5.878692	7.463265	6.237788	7.302565	6.549699	7.503062	7.008016	8.434938	6.778153	6.795092	7.631140	6.932252	7.105179	6.205198	7.163769	6.935051	5.472912	5.682715	6.004284	6.135334	6.113504	6.277503	6.297720	7.250720	6.943118	6.929221	6.997868	5.904025	6.571007	6.145662	7.370309	6.991290	6.327073	6.477635	6.862341	7.302884	6.497206	6.385953	6.092548	5.953430	7.282413	5.513333	6.578323	6.532579	6.351789	7.011065	6.495893	7.090101	7.271730	7.027965	6.878304	6.751438	6.939515	7.018436	7.688703	6.749580	6.592806	6.911310	6.721128	6.447691	6.723538	6.959401	7.212483	7.299385	6.259821	6.256420	6.934574	6.925341	6.352776	6.745598	6.439561	7.118530	6.896720	7.273416	7.125129	7.247008	8.112848	6.190442	7.133386	6.536782	6.353092	6.437583	5.599318	5.444298	5.524212	5.862318	5.745927	6.415472	6.006620	6.392020	6.720973	7.816813	6.875616	7.097357	7.218571	7.521137	7.083939	7.697302	6.824667	6.977391	6.845047	7.359016	6.552682	7.312433

MAP3K20-AS1	5.052568	5.872251	5.725140	5.456085	5.440816	4.105649	5.141316	6.236149	5.641209	5.188994	5.549584	4.897894	5.684924	4.763361	5.052375	6.074503	6.201713	5.674028	6.189096	5.538448	5.541648	5.734630	7.085989	4.974843	4.560560	5.645429	5.742456	5.425023	5.583801	4.618177	5.649548	6.607708	6.293693	6.737455	5.626914	5.445240	4.633497	4.706826	4.466525	5.818676	6.016557	5.166894	6.257931	5.706450	6.314181	5.927937	6.295776	5.825674	5.832637	5.263694	8.179776	5.965177	6.707995	5.789893	6.403278	6.246624	5.694139	5.184611	6.304098	5.205618	6.123900	4.862073	7.158238	4.911789	5.877786	5.621097	5.018503	5.809052	5.850080	5.395166	5.317069	4.842558	5.514243	5.712313	5.655195	6.210188	5.451468	6.071153	6.521385	6.265036	6.413357	6.492368	5.612899	6.483577	6.167203	6.653041	5.802411	5.816007	5.193075	5.526878	5.765566	5.549967	4.982161	5.174138	7.282571	6.288089	5.870430	6.854303	5.597810	6.882160	6.366712	6.037539	6.454774	5.566361	6.505475	6.424825	7.326424	6.252335	5.766251	4.439375	4.657286	6.666704	4.900277	5.384847	7.097815	4.503720	4.332259	6.912595	4.439818	6.208291	6.592110	4.204989	3.943719	4.232648	4.694948	6.092872	7.036542	6.549386	4.718124	5.217960	4.733792	5.455513	3.950770	3.835886	5.487657	7.220329	4.569828	7.807111	5.441793	4.831039	5.672771	6.137188	5.342778	4.549075	7.505232	5.261427	4.178524	5.668508	4.868967	5.260874	7.834020	6.608356	6.535312	5.732104	4.334841	5.642899	3.714240	5.975338	6.221944	6.853155	5.497353	5.563547	5.700818	6.238438	5.431807	7.014515	4.810612	5.654640	5.815183	6.057447	5.981511	6.311621	7.211007	6.486761	5.676760	4.927218	5.832460	6.100488	5.625771	9.021575	4.270937	7.046248	7.232924	5.393177	5.425023	6.122684	6.133974	6.080567	4.726407	6.059900	6.095497	5.806134	7.033749	5.092471	6.119929	4.948324	5.852202	5.851399	6.393878	6.232581	6.051295	6.882311	6.673024	6.050621	5.271843	7.018752	5.564851	5.306575	5.778374	4.159936	7.107731	5.635631	6.762817	7.448724	7.069707	5.908291	5.640661	7.196961	6.062264	7.150091	7.106131	4.187745	5.373011	5.158305	4.869580	8.316384	5.370703	5.486353	6.707390	4.596619	5.674946	5.202453	5.063876	4.392359	7.325321	5.821680	8.023925	6.034380

POU6F2-AS2	5.734276	4.263344	4.229863	4.634571	3.630688	3.627611	2.987328	5.147334	5.817964	4.533520	3.891153	4.799646	3.095098	4.005674	4.182342	4.954166	5.287167	4.640524	5.405537	3.360160	5.200892	5.688971	8.493080	4.003453	4.167115	3.358816	5.266656	4.510736	4.221883	4.112439	3.496154	5.657957	5.153779	6.883408	5.410318	5.035276	4.824325	3.988380	4.173385	3.989435	4.784543	3.339777	4.135166	4.561847	3.729640	5.660509	5.873185	3.062769	5.265665	4.757595	2.834204	5.972524	3.919566	5.622592	4.414205	3.919774	5.533140	2.988244	2.871110	3.364044	4.366579	3.132332	4.833111	3.770910	6.764402	3.967902	3.943502	3.755267	5.176541	2.947036	4.962329	4.154789	4.340039	3.595732	5.636210	3.628835	4.591411	4.945328	4.031073	3.373901	5.116406	3.661202	4.727660	5.073004	4.640321	5.651254	6.504481	5.721394	4.815819	4.059980	3.795711	3.481157	3.545812	4.255667	4.756961	4.581826	5.631375	5.634913	5.507386	6.429367	5.943205	3.877841	5.624670	4.727241	6.091481	4.342640	4.931393	3.732140	3.547179	3.359328	4.226166	5.356278	3.647203	4.099749	5.469490	6.950916	4.526520	5.432797	5.333817	8.965706	4.897234	6.434113	7.783924	6.056932	4.877385	5.272683	8.109379	9.255278	8.171288	4.799646	7.626255	6.043958	5.596256	4.189270	8.199817	8.347659	7.945391	6.226570	5.543701	5.869547	5.536264	7.293658	7.326903	6.125147	6.690947	5.462754	6.401461	5.905949	6.469350	3.308242	8.484010	6.925494	6.221600	7.702677	7.594133	7.308348	5.631190	6.334855	5.116800	7.401771	5.361351	7.880939	8.231793	6.783864	8.614955	4.662049	6.461996	6.908604	6.753911	7.735196	8.686419	6.543218	5.112958	7.229521	6.466680	8.323100	5.982211	6.922419	5.102586	8.167960	3.220706	6.153453	6.339056	8.016641	6.833118	8.332937	4.271174	4.829983	5.202650	5.980467	7.008826	5.134841	5.557143	7.915342	6.281614	6.360376	7.951667	5.853616	3.956026	7.787467	3.883794	6.951067	7.352207	6.940736	8.510590	9.295850	8.323832	6.876846	4.842335	4.893762	3.924166	4.720037	4.980326	7.416404	6.478155	8.222383	7.113933	8.507964	8.887600	5.050976	6.826732	8.807358	6.422692	9.291751	6.596780	6.808244	4.927430	4.191252	5.594019	3.948773	6.365038	5.502762	4.366344	8.641436	6.858980	7.078639	5.301839	8.265248

AL451164.1	5.076102	3.267949	2.975343	3.205479	3.022360	3.922669	3.261577	4.628909	3.282551	3.193913	4.070266	3.388600	3.227892	3.835458	3.349361	3.178289	3.849489	3.138823	6.409505	3.262792	4.563408	3.704674	4.848706	3.321552	3.077719	3.431869	3.320901	3.546768	3.156291	3.247824	4.243420	5.478043	3.438920	3.234953	3.401235	4.212746	3.595938	3.844372	4.449608	3.655630	3.444894	3.778757	3.409363	3.529594	3.630871	5.544684	3.781438	3.622237	4.682027	3.444367	3.389306	3.619143	4.036272	4.014711	3.750187	3.098204	4.086810	3.488107	4.314773	3.747497	2.982124	3.112510	3.671754	3.175025	3.373442	3.698588	2.958724	3.509610	3.256478	3.870715	3.436524	2.933097	3.222539	3.037828	3.997446	4.093706	3.060657	3.165304	3.316355	4.147485	3.215034	3.701819	3.395581	4.058646	3.190794	3.506434	3.142565	3.529043	3.345400	3.337623	3.477093	3.885739	3.541895	3.121823	3.122628	2.921753	3.681816	3.187677	3.269945	3.074560	2.896516	3.106648	3.244432	3.421705	3.036164	3.490858	3.009424	3.391037	3.236957	3.296369	3.352877	3.726533	2.864434	4.083256	3.386886	3.229919	3.369248	3.135782	3.196264	4.779250	3.354861	3.469619	3.051529	3.214885	4.634571	3.094313	3.672869	3.228654	3.481909	4.114367	3.007968	3.294372	3.377017	3.111404	2.990083	3.825737	2.959013	4.461781	3.267949	3.958796	3.236796	5.382123	3.285471	2.951119	3.778157	3.437904	4.914337	3.043601	3.911422	3.504959	4.329886	3.487138	3.044226	3.232235	3.866608	3.567285	3.260708	4.570485	3.859821	3.805149	3.895449	3.514088	3.212415	4.041871	5.132194	3.347388	3.222099	4.819801	3.270993	3.286535	3.328510	3.878927	4.131875	3.406067	4.288556	3.752330	3.573430	2.997419	3.744089	4.406247	3.232021	4.561637	3.437397	4.298741	3.877385	2.962457	3.923084	3.809533	2.998519	2.982627	2.975923	3.187142	3.360006	3.609803	4.122955	3.565159	3.582100	3.656838	3.767157	3.542469	4.076094	3.448007	3.573430	3.966797	3.037724	3.289211	3.903586	3.360967	3.314806	3.750428	4.080249	3.278993	3.188054	4.147052	3.410052	3.042603	3.183197	3.732967	3.413099	3.138823	2.971440	3.207657	3.233108	6.043642	3.736070	3.063410	4.013585	3.431688	3.441518	3.437397	3.177892	2.978709	3.955259	3.241215	6.165285	3.501061	3.725577	3.931308

LINC02668	5.076102	3.267949	2.975343	3.205479	3.022360	3.922669	3.261577	4.628909	3.282551	3.193913	4.070266	3.388600	3.227892	3.835458	3.349361	3.178289	3.849489	3.138823	6.409505	3.262792	4.563408	3.704674	4.848706	3.321552	3.077719	3.431869	3.320901	3.546768	3.156291	3.247824	4.243420	5.478043	3.438920	3.234953	3.401235	4.212746	3.595938	3.844372	4.449608	3.655630	3.444894	3.778757	3.409363	3.529594	3.630871	5.544684	3.781438	3.622237	4.682027	3.444367	3.389306	3.619143	4.036272	4.014711	3.750187	3.098204	4.086810	3.488107	4.314773	3.747497	2.982124	3.112510	3.671754	3.175025	3.373442	3.698588	2.958724	3.509610	3.256478	3.870715	3.436524	2.933097	3.222539	3.037828	3.997446	4.093706	3.060657	3.165304	3.316355	4.147485	3.215034	3.701819	3.395581	4.058646	3.190794	3.506434	3.142565	3.529043	3.345400	3.337623	3.477093	3.885739	3.541895	3.121823	3.122628	2.921753	3.681816	3.187677	3.269945	3.074560	2.896516	3.106648	3.244432	3.421705	3.036164	3.490858	3.009424	3.391037	3.236957	3.296369	3.352877	3.726533	2.864434	4.083256	3.386886	3.229919	3.369248	3.135782	3.196264	4.779250	3.354861	3.469619	3.051529	3.214885	4.634571	3.094313	3.672869	3.228654	3.481909	4.114367	3.007968	3.294372	3.377017	3.111404	2.990083	3.825737	2.959013	4.461781	3.267949	3.958796	3.236796	5.382123	3.285471	2.951119	3.778157	3.437904	4.914337	3.043601	3.911422	3.504959	4.329886	3.487138	3.044226	3.232235	3.866608	3.567285	3.260708	4.570485	3.859821	3.805149	3.895449	3.514088	3.212415	4.041871	5.132194	3.347388	3.222099	4.819801	3.270993	3.286535	3.328510	3.878927	4.131875	3.406067	4.288556	3.752330	3.573430	2.997419	3.744089	4.406247	3.232021	4.561637	3.437397	4.298741	3.877385	2.962457	3.923084	3.809533	2.998519	2.982627	2.975923	3.187142	3.360006	3.609803	4.122955	3.565159	3.582100	3.656838	3.767157	3.542469	4.076094	3.448007	3.573430	3.966797	3.037724	3.289211	3.903586	3.360967	3.314806	3.750428	4.080249	3.278993	3.188054	4.147052	3.410052	3.042603	3.183197	3.732967	3.413099	3.138823	2.971440	3.207657	3.233108	6.043642	3.736070	3.063410	4.013585	3.431688	3.441518	3.437397	3.177892	2.978709	3.955259	3.241215	6.165285	3.501061	3.725577	3.931308

MIR9-3HG	7.880939	7.751051	8.068901	7.993011	8.578625	9.139186	8.229359	8.895756	9.082741	8.364386	8.588024	8.467956	8.837687	9.508697	7.949190	8.292137	8.707834	8.536813	7.994877	7.900849	7.675274	8.427398	8.396077	7.325321	7.792182	8.702764	7.765302	8.039022	7.784243	9.611185	8.854235	8.431530	8.636313	9.318197	7.884890	7.997674	8.434770	7.720677	7.747490	9.181391	8.421514	7.801000	8.476374	7.467717	7.752553	7.824001	9.686684	9.527862	7.897170	7.742243	7.253245	8.073673	7.999175	7.877495	7.126739	8.545084	8.238691	7.966965	7.763140	8.310512	8.400034	9.866534	7.985161	7.802216	8.688665	8.112072	8.042316	7.684432	9.690530	8.117316	8.628373	8.591468	10.307712	9.217026	8.059955	7.762998	8.437404	8.052786	8.003253	7.592865	8.105290	7.394443	8.392872	7.738981	8.985166	7.710499	7.710341	9.318981	10.167376	9.227425	10.095931	10.824286	9.885790	8.817145	8.735952	9.357553	7.982069	9.365816	9.150489	7.696523	8.680053	7.748566	7.867093	7.541602	8.374648	8.145020	8.040770	8.398520	8.736729	8.049851	9.647967	9.374473	9.060792	9.104114	9.644234	9.931382	8.329193	7.697463	8.595641	11.730804	8.201070	9.441595	9.501613	10.127268	9.842888	10.282619	8.513380	8.065177	10.543304	9.865593	9.064741	9.074979	9.264743	9.000251	8.035070	8.736262	10.625496	8.586817	8.708855	8.985166	10.586401	8.920300	11.114868	9.612099	8.038363	8.858496	11.266616	9.874961	10.327332	8.467060	9.030848	9.151857	8.414729	9.595531	8.244137	8.264324	8.925815	8.258791	8.727096	9.550559	9.440966	8.516378	7.631624	9.319127	8.588177	10.037024	9.817412	9.592125	9.147183	8.370991	8.934093	9.879639	9.215117	9.108775	7.917531	10.159823	11.895908	10.235279	11.086626	10.793498	12.922424	9.725155	9.010252	9.418692	8.367433	8.765904	9.249793	8.783374	10.336136	8.688503	8.928003	10.477996	8.697270	8.539248	8.416754	8.773990	8.250212	7.722369	8.315586	9.250707	9.909868	9.629238	8.733640	10.865293	10.320280	8.545084	10.700162	10.365777	9.774780	11.636925	12.111443	9.781326	10.063175	10.101264	11.366830	9.002906	9.743643	9.074813	8.245874	9.450295	9.939017	11.618169	9.045642	9.148972	9.733039	8.817145	11.379300	8.863852	8.962369	10.187407	9.200481	11.489199	10.450766	9.367092	9.487987	10.834345	10.637121	9.174793

LINC00924	6.980847	7.840605	7.637084	7.682889	5.716401	6.500881	8.242770	6.603721	6.626161	6.950139	6.610287	6.190615	7.581955	5.450480	6.289971	6.363201	6.529490	6.692278	6.522817	6.158733	5.918859	5.874564	7.637084	5.645625	7.200863	7.710341	6.759935	6.296097	7.613414	8.729291	7.013182	8.172997	8.153757	7.403034	8.488015	8.816052	8.282853	7.534238	6.411190	6.591954	7.308493	6.785812	8.761671	8.035393	8.774914	8.142995	7.797527	7.510154	7.143020	8.468252	8.681905	8.312218	8.844653	7.816968	8.409240	7.041908	8.820838	6.989197	6.812101	8.419942	7.568853	8.180074	8.454138	6.688080	7.263127	7.109818	6.257597	7.005042	6.502522	6.328750	7.980661	7.421823	7.800683	7.701163	8.309320	9.202010	7.996300	8.703980	7.631140	8.625594	7.670691	8.961134	7.030651	7.752712	8.647237	8.050463	9.295850	7.700850	8.146104	7.285661	7.029236	7.340836	9.162764	8.131825	7.345155	7.886885	7.711542	7.896712	6.601179	6.909858	7.817936	6.790559	6.273200	5.786478	6.080745	6.860269	6.873205	6.571622	5.869888	6.453958	6.292186	6.894312	6.851397	5.892386	6.115788	7.759138	6.397635	6.672861	6.468342	6.709100	7.349921	6.696046	7.667305	5.548626	6.365895	6.779754	6.310111	6.498737	5.897408	6.169784	5.599491	6.936341	4.523673	5.581896	6.390687	6.501233	5.257917	5.366408	5.915478	5.735919	6.039639	7.927574	5.258891	6.442889	7.345011	6.636437	5.866135	6.646901	7.522227	7.153563	8.191798	7.057440	7.315739	7.505885	7.636629	7.594133	7.008175	6.068237	6.596935	6.317575	6.212338	7.272830	7.157778	7.776203	7.439764	6.374667	6.475585	6.584231	8.253439	8.199981	7.968338	7.835680	7.316534	8.277093	7.086906	8.185892	5.654640	6.292024	7.583967	6.818634	7.747337	7.420891	6.129996	6.772178	6.781503	6.275556	6.706060	6.173696	6.188416	7.476459	7.008175	6.924368	7.029566	8.140306	8.743011	7.025077	7.929428	7.311966	7.779827	6.937304	8.529985	6.636123	7.747653	7.935444	7.183603	8.276415	6.816998	7.397868	7.143171	6.569361	7.322331	7.898797	8.551647	7.531429	7.717058	6.752888	7.176042	6.015887	5.725140	7.208153	6.453805	5.795803	4.759341	5.434949	5.951633	6.483741	5.917236	5.893329	5.339320	5.783606	6.679815	7.374716	5.255133	6.626475	6.159451	6.737942	5.670314	6.089564

C8orf87	11.049446	11.678914	11.509940	11.739081	12.140193	12.338554	11.784334	12.484861	13.003067	12.302166	12.655512	12.093684	11.264699	11.763874	10.860274	12.764791	12.379891	10.845031	12.350849	12.268991	11.750569	12.280226	12.832557	11.417994	11.488461	12.410313	12.342178	12.813004	12.796632	12.143742	12.314304	10.229100	10.812838	11.106155	11.742152	11.364289	10.833302	10.606255	11.341779	11.812156	13.125202	11.553024	12.271395	12.934551	12.414153	12.768697	13.126907	12.482801	11.589394	13.707975	12.122018	11.804689	10.121103	10.553536	10.740494	12.657171	9.982457	10.343395	10.940980	10.625804	11.093405	12.446758	10.660719	11.426361	11.164654	11.628369	11.226941	10.723422	10.984949	11.766473	11.854155	12.895646	12.796802	11.533180	11.806329	11.161862	11.396845	11.489935	11.396102	10.117917	10.631433	10.614968	9.999961	10.546727	10.683185	11.431806	10.627927	10.702740	10.941247	10.343685	10.988997	11.330459	10.873822	11.458920	11.317780	11.627636	11.174973	11.487280	11.632987	11.938632	11.794910	11.222510	11.910424	11.149750	11.423001	12.813876	11.966551	11.284296	12.384984	12.630012	12.488771	11.499775	11.915924	13.035077	12.832376	14.205886	10.524431	13.075325	12.981137	11.036699	10.600462	10.442148	11.105301	12.508519	11.486239	11.744952	11.623726	11.361505	11.551514	11.915487	11.742737	11.320543	10.720925	10.947309	12.028097	11.154511	10.250277	10.583475	10.104632	11.117676	10.581438	11.291005	11.208645	10.496003	10.436435	12.515153	11.781211	12.602397	11.012515	11.755741	10.698366	10.928598	11.352461	10.199926	10.208624	10.550253	11.031948	11.011686	10.740809	12.334126	10.662229	9.676450	10.991775	11.271480	11.937773	12.659466	9.916692	10.345226	10.518615	10.703381	10.145661	9.250874	9.902261	9.639347	10.976116	9.105657	9.227425	9.879639	10.072505	10.346580	11.583602	10.307568	10.203098	10.213496	10.157125	10.240313	10.369106	10.374844	10.525043	10.812696	11.036553	12.814750	10.654734	10.500010	10.772399	10.376501	10.771060	11.054506	9.331832	9.722628	9.887683	9.946536	10.252055	9.768131	11.285784	10.021565	9.728219	10.139853	10.010612	9.968820	10.810803	10.892382	11.332042	11.677143	11.812728	11.740862	11.647837	11.276012	9.965460	11.133235	10.759397	11.568841	10.244721	10.126326	13.231451	11.438654	10.778689	11.730047	12.755481	11.186851	10.911171	12.237727	13.006158	11.471231	12.041947	10.462340	11.102976	11.602546

LINC00466	3.062027	3.068364	2.953662	2.940730	3.001092	2.948513	3.242655	3.099517	3.336602	3.250709	3.466667	3.475806	3.212271	3.740380	3.168786	3.076108	3.407115	3.063198	4.402233	3.184228	4.006760	2.919506	4.547077	3.019585	3.208736	2.983669	3.178289	3.008683	3.007450	3.015774	3.606829	3.189289	4.778245	3.074560	3.396948	4.030852	3.598945	3.127678	3.063293	3.041326	3.392629	3.157430	3.375281	3.922669	3.391393	3.928725	4.374125	3.774707	4.789720	3.892199	3.862994	4.802838	3.629852	3.908360	3.377215	2.932052	4.178524	3.693618	3.770322	3.492758	3.627218	2.960516	3.302377	3.042415	3.103006	3.041777	3.014468	3.219103	3.309872	3.071095	2.910186	2.779964	2.998784	2.920440	2.977243	3.219103	2.946960	2.939310	2.954687	3.385855	3.145941	3.653615	3.741588	3.416207	3.049338	5.850428	3.283950	3.098454	3.481157	3.928725	3.008959	2.934370	3.005456	2.984327	2.892599	2.961952	3.322496	3.019211	2.996841	2.935217	3.166625	3.186474	2.966746	3.501981	4.284974	3.588270	2.946625	3.118529	3.008249	3.118273	4.465860	3.208467	3.013264	3.407494	3.009947	3.868380	3.088208	2.943219	3.042065	3.010125	3.114521	2.927608	3.119999	3.033387	3.108608	3.066192	3.114731	3.294372	3.086586	3.235234	3.103396	2.986651	3.178028	3.002910	2.936732	3.355506	2.954133	3.327268	3.215305	3.541317	2.866588	4.737607	2.939052	3.044311	2.930079	3.062769	2.923214	3.127559	3.350383	3.381552	3.279275	3.083374	3.087865	3.313790	3.726946	3.648387	3.101045	3.034792	2.958614	3.168786	3.012214	3.728166	3.301909	3.536283	3.829180	4.110743	3.828051	4.333078	3.928910	3.875478	5.012216	3.346871	3.905958	3.156927	2.916572	4.514214	3.544508	3.646626	3.441340	3.385685	3.012127	3.286969	3.250993	3.182313	3.060561	3.010303	3.316489	3.109934	3.192946	2.925707	2.876759	2.967819	2.933007	2.920361	3.181376	2.950816	3.041027	3.006045	3.136736	3.339114	3.649813	3.120613	3.367240	3.233362	4.720037	3.245240	3.215305	3.307309	3.231044	3.056331	2.945170	2.989807	3.010401	2.968089	2.989222	3.032967	3.060451	3.076658	3.151248	2.922701	3.017302	2.970911	2.948079	4.640524	3.952908	2.904234	3.118879	3.330807	3.016806	3.567669	3.787788	3.553152	3.500336	2.928005	2.899544	2.960243	2.943756	3.094079

AC096543.1	3.062027	3.068364	2.953662	2.940730	3.001092	2.948513	3.242655	3.099517	3.336602	3.250709	3.466667	3.475806	3.212271	3.740380	3.168786	3.076108	3.407115	3.063198	4.402233	3.184228	4.006760	2.919506	4.547077	3.019585	3.208736	2.983669	3.178289	3.008683	3.007450	3.015774	3.606829	3.189289	4.778245	3.074560	3.396948	4.030852	3.598945	3.127678	3.063293	3.041326	3.392629	3.157430	3.375281	3.922669	3.391393	3.928725	4.374125	3.774707	4.789720	3.892199	3.862994	4.802838	3.629852	3.908360	3.377215	2.932052	4.178524	3.693618	3.770322	3.492758	3.627218	2.960516	3.302377	3.042415	3.103006	3.041777	3.014468	3.219103	3.309872	3.071095	2.910186	2.779964	2.998784	2.920440	2.977243	3.219103	2.946960	2.939310	2.954687	3.385855	3.145941	3.653615	3.741588	3.416207	3.049338	5.850428	3.283950	3.098454	3.481157	3.928725	3.008959	2.934370	3.005456	2.984327	2.892599	2.961952	3.322496	3.019211	2.996841	2.935217	3.166625	3.186474	2.966746	3.501981	4.284974	3.588270	2.946625	3.118529	3.008249	3.118273	4.465860	3.208467	3.013264	3.407494	3.009947	3.868380	3.088208	2.943219	3.042065	3.010125	3.114521	2.927608	3.119999	3.033387	3.108608	3.066192	3.114731	3.294372	3.086586	3.235234	3.103396	2.986651	3.178028	3.002910	2.936732	3.355506	2.954133	3.327268	3.215305	3.541317	2.866588	4.737607	2.939052	3.044311	2.930079	3.062769	2.923214	3.127559	3.350383	3.381552	3.279275	3.083374	3.087865	3.313790	3.726946	3.648387	3.101045	3.034792	2.958614	3.168786	3.012214	3.728166	3.301909	3.536283	3.829180	4.110743	3.828051	4.333078	3.928910	3.875478	5.012216	3.346871	3.905958	3.156927	2.916572	4.514214	3.544508	3.646626	3.441340	3.385685	3.012127	3.286969	3.250993	3.182313	3.060561	3.010303	3.316489	3.109934	3.192946	2.925707	2.876759	2.967819	2.933007	2.920361	3.181376	2.950816	3.041027	3.006045	3.136736	3.339114	3.649813	3.120613	3.367240	3.233362	4.720037	3.245240	3.215305	3.307309	3.231044	3.056331	2.945170	2.989807	3.010401	2.968089	2.989222	3.032967	3.060451	3.076658	3.151248	2.922701	3.017302	2.970911	2.948079	4.640524	3.952908	2.904234	3.118879	3.330807	3.016806	3.567669	3.787788	3.553152	3.500336	2.928005	2.899544	2.960243	2.943756	3.094079

ARHGEF7-AS2	4.744878	5.186207	5.338103	5.410703	4.782194	4.340238	4.856270	4.677961	4.591187	4.323808	4.754703	5.446840	4.547921	4.391694	4.520611	4.677535	5.689713	5.203864	4.567683	4.450965	4.007805	4.193224	5.214446	3.908360	5.008283	4.640929	3.880460	4.888751	4.342640	4.023461	5.568253	5.286594	4.546670	4.571814	5.266871	5.256728	4.954364	5.271224	5.412818	5.555072	4.888964	5.910088	5.483145	4.067751	5.356651	4.179668	3.737677	5.807043	5.247211	6.156355	4.172286	5.822404	6.248637	5.016059	5.466753	3.665068	6.008962	5.450296	4.643812	5.644448	4.987279	5.320535	6.180152	5.571799	5.336960	5.938799	5.142569	5.827305	4.719803	4.783690	5.352484	4.735238	4.793710	5.122315	5.243216	4.901741	4.948949	5.086568	5.302426	5.438382	5.212013	6.292024	4.180769	5.364435	5.786290	6.080567	3.407660	6.086738	6.193370	5.869213	5.358339	6.245120	4.664522	4.512913	3.854271	4.527150	5.383142	5.360800	3.962030	4.379938	3.137469	4.752754	4.972948	4.434129	4.276377	3.911846	5.029939	5.375539	5.229116	4.749977	4.965626	5.078395	5.230986	4.374352	4.454382	3.551811	5.412215	4.296330	4.287426	4.382536	4.856053	4.169878	4.008227	3.565704	5.184212	3.296065	4.448486	4.871491	3.895246	4.879225	4.593406	3.489018	3.815038	3.499062	3.552211	5.359285	3.839112	4.111173	4.230916	3.516620	5.656461	5.807783	3.989853	3.727364	4.098630	3.664638	3.642881	3.250849	4.796873	4.764426	3.545812	3.380664	4.267843	4.373702	4.653729	4.223606	4.627574	5.022393	4.567683	3.778554	4.812712	4.184907	3.342139	3.932381	3.937294	3.836533	6.017610	4.196022	5.093749	3.951215	5.110765	5.838997	4.052798	4.513162	4.628909	3.456795	5.574989	4.384260	3.595938	3.534455	4.454821	5.280957	5.445822	4.823117	5.290806	4.381390	5.317845	5.135234	5.038113	4.722391	4.449838	3.759435	4.500504	4.547293	5.112773	4.522996	4.869381	5.211443	4.213645	4.519978	5.095809	5.133206	3.815667	5.468945	4.756329	5.155607	5.282173	5.852553	5.257140	5.495098	6.477288	4.329006	4.791016	4.592701	4.229003	3.585340	3.081779	3.364854	3.822254	3.152573	3.533669	3.386208	3.633323	4.619476	3.953135	5.556180	4.617539	5.034634	4.243194	4.335916	4.207761	3.824627	4.109593	4.147890	3.828431	5.586055	4.329006	4.134295

INHBA-AS1	6.114492	6.395482	6.519531	6.563856	7.659658	8.074715	6.793969	6.884549	6.859119	6.312295	5.979585	6.411686	6.492988	6.962614	5.645429	6.414848	6.559759	7.478448	6.992762	6.179318	6.527835	7.394443	7.830789	6.797677	6.415816	6.570356	6.848046	6.588530	6.545166	7.366400	6.566263	6.561694	7.573412	6.707215	6.685825	6.858345	6.479464	6.573050	6.446215	6.610287	6.417644	6.464543	6.917410	6.609186	6.591797	7.970525	6.683231	7.234543	6.726421	6.715079	6.049931	6.598774	7.031402	6.760608	6.029573	6.907980	6.554476	6.102725	6.152579	6.087838	6.247611	6.596935	7.338317	6.993391	6.725739	6.430553	6.858980	7.103764	6.482556	6.644356	6.940135	6.876248	6.653361	6.595007	6.428706	6.564009	6.729992	6.650608	6.514869	6.261991	6.272537	5.718155	6.409866	6.543856	8.132838	7.079432	6.830549	6.055134	6.631873	6.316062	6.240938	7.826301	6.766039	6.654176	6.667531	6.226570	5.572561	6.636899	7.795089	7.148490	8.173460	6.811769	6.457215	6.627417	6.814751	7.044973	6.635006	6.918220	8.046799	6.744337	7.764815	7.949190	7.097357	6.824965	6.692925	10.313708	6.863627	6.025164	6.329228	6.156030	6.840313	7.075460	6.621298	9.145795	7.491020	7.464507	6.390022	6.957191	6.496218	6.806000	6.516230	7.359312	7.044666	7.200242	6.979258	6.577491	7.741021	6.406399	6.404576	7.342224	7.706992	7.739616	7.103604	7.560752	6.374509	8.022249	7.372828	7.158379	7.251051	6.785321	7.727632	8.136662	7.651452	6.942312	6.838519	6.845217	7.303037	6.660032	7.119015	6.702584	6.812869	6.949175	6.213566	6.431244	5.977611	7.404412	7.587332	6.499069	6.863914	6.675761	6.821014	6.694411	6.769936	6.741127	6.787106	7.252455	6.295600	6.563856	6.489335	6.778458	7.078474	6.773662	6.868396	7.075288	6.809210	6.520879	7.440361	6.950755	6.859613	7.326749	7.283831	7.098469	6.864858	6.985072	6.705103	6.853335	6.964669	6.668017	6.598774	5.639734	6.493615	6.863457	6.892994	6.844206	7.738827	6.616937	5.640130	7.362150	6.809524	7.207379	6.992106	7.008016	6.756707	6.747722	7.125777	7.128470	7.648004	7.111620	7.084741	7.683680	6.830707	6.746725	7.377685	7.269078	7.046566	6.268440	6.876407	6.597115	7.453093	8.132838	8.222537	9.781923	7.829434	8.006397	8.986858	6.682255	6.521545	6.037378

AL353633.1	6.838192	6.134154	6.011218	6.258790	5.646519	6.272390	5.319005	7.431258	6.709910	5.968975	5.926133	5.719583	4.962098	5.554340	5.930243	5.748864	7.135309	6.661015	6.895743	5.809630	6.927973	6.035818	8.369704	6.119757	6.408366	6.269124	5.624864	6.714240	4.885836	5.101129	7.175887	4.159287	6.256740	4.524788	6.174889	6.160979	6.308238	6.712799	6.477812	6.670249	5.195497	6.786300	5.272049	6.494926	5.776731	6.354414	5.695665	6.532898	6.291862	5.844412	6.368863	6.922885	6.063823	5.904897	5.832995	5.126559	5.595686	6.277988	6.163391	6.200316	6.999064	5.211443	7.772699	7.520987	7.316850	7.170405	6.790559	6.580629	6.761558	7.439309	6.417161	6.608183	5.551433	6.674294	6.639497	6.508946	6.835808	6.702900	7.157612	6.476742	6.187221	6.600216	6.433951	6.329383	6.449821	6.704797	6.396973	7.094649	7.533611	7.367028	7.504008	7.940740	6.164933	6.529490	6.159451	6.068056	6.428172	3.651989	3.553536	4.575611	3.198271	5.066111	5.386761	5.899181	6.703546	5.863994	7.077646	6.696500	6.830874	5.593090	7.400216	6.783224	7.076523	5.896541	5.957324	5.025654	7.003210	5.970502	5.214830	6.694902	6.386980	5.652931	6.176949	5.458449	6.583759	5.317249	6.599069	6.804613	5.493365	6.422390	5.675497	4.507461	4.113965	6.878304	5.482575	7.212319	6.264325	6.953300	6.440058	7.303979	5.938973	8.550581	6.075028	6.490623	6.668179	6.074831	6.587534	4.913908	5.976039	7.311495	4.939739	5.426024	5.026469	6.301328	6.554958	6.360207	6.807255	6.966936	6.985556	5.505443	7.234838	6.396610	7.548532	5.921968	6.802353	6.341865	6.765874	6.202616	6.664928	7.111770	6.911143	6.236971	6.137868	5.613847	5.722522	5.838053	6.867906	6.542552	6.741451	7.365307	5.518786	7.961924	7.901171	7.665583	7.304277	7.049753	6.767312	7.209395	7.806324	6.485460	6.539555	6.619878	6.862503	7.467259	6.925831	7.028768	7.283387	7.526823	6.440386	6.445076	7.059962	7.029236	5.892386	6.986363	6.749727	6.824346	7.095312	7.665583	7.314660	7.493510	8.746649	6.238775	6.768967	6.600383	6.135515	6.368043	4.100847	3.193913	4.975882	4.291112	4.915556	9.053125	5.734096	6.752252	6.046736	7.730402	7.331642	7.463901	5.448014	7.547747	6.649044	6.761727	5.885495	5.709192	4.982563	7.509850	6.315909	5.885138

AP001767.3	7.141750	7.223425	7.411359	7.369060	6.722079	6.626161	7.415512	5.545624	7.088794	7.950478	7.188552	7.117886	7.084741	7.069874	6.782450	6.911631	6.676879	7.380825	6.693274	6.983494	6.190268	7.753027	8.581825	6.876549	6.630766	7.355349	7.331016	6.878780	6.678007	6.979258	6.985390	7.295535	7.647109	7.699767	7.972255	7.693526	7.309276	7.240235	7.337505	7.494871	6.822304	7.874571	7.493195	7.734887	8.129662	7.602658	7.230114	7.503220	7.442212	7.741642	7.806014	7.446500	7.557379	7.671162	6.546318	6.876248	8.202784	8.220237	7.661380	7.526038	7.917823	7.459019	8.932840	7.518351	7.574816	7.107099	7.009806	7.245475	7.381252	7.665283	7.440515	7.106590	6.522990	7.288783	7.444765	7.315270	7.481815	7.045786	7.268785	6.479137	7.853434	7.664681	7.553537	7.480444	7.607725	7.753321	7.451641	7.772318	7.224035	6.828319	7.289259	7.132916	7.266319	7.679669	7.277363	7.439916	7.416254	7.370474	7.353422	7.834479	6.986852	7.433286	7.203798	7.312743	7.630374	6.522655	6.727241	7.437546	7.974303	7.176504	7.477997	6.888567	6.796203	6.976898	7.390858	6.553514	7.753478	7.159604	7.166993	7.657521	7.069044	7.200694	7.753171	7.043338	6.903040	7.987793	7.387462	6.645932	7.869232	8.596881	8.186508	6.639497	6.640143	7.808778	7.580698	8.094336	8.599881	7.056010	7.464963	7.838778	8.398520	8.143925	6.914949	7.087666	7.428515	7.067588	8.280692	6.403904	6.580790	6.975438	7.801445	8.510905	8.144245	8.100858	8.586667	7.588434	7.573412	7.259489	8.205229	7.380825	7.643358	7.676347	8.253887	8.435543	8.246193	6.737116	8.267621	7.940418	7.597003	8.768535	8.679458	8.122926	7.584419	7.207847	6.329228	8.143925	8.587391	8.560387	8.205382	8.230249	6.757042	8.831175	8.010496	8.180988	8.582268	6.656131	7.732264	8.682812	7.938222	8.581371	8.388218	7.125777	7.847503	7.845347	8.201668	8.201533	7.355349	7.750125	6.861077	8.446244	8.533620	7.905551	8.058996	8.811548	8.738729	7.394285	8.894837	7.154664	7.600485	8.856221	7.320594	8.795964	7.993319	7.537013	7.617539	8.139275	7.808778	7.112839	8.445759	7.635404	8.738410	8.512924	7.391479	7.908951	7.268335	7.209572	7.997958	7.310875	5.974825	7.754711	6.021175	7.247314	8.308999	7.860047	8.626721	8.315429	8.213459	7.654357

RAB30-DT	7.141750	7.223425	7.411359	7.369060	6.722079	6.626161	7.415512	5.545624	7.088794	7.950478	7.188552	7.117886	7.084741	7.069874	6.782450	6.911631	6.676879	7.380825	6.693274	6.983494	6.190268	7.753027	8.581825	6.876549	6.630766	7.355349	7.331016	6.878780	6.678007	6.979258	6.985390	7.295535	7.647109	7.699767	7.972255	7.693526	7.309276	7.240235	7.337505	7.494871	6.822304	7.874571	7.493195	7.734887	8.129662	7.602658	7.230114	7.503220	7.442212	7.741642	7.806014	7.446500	7.557379	7.671162	6.546318	6.876248	8.202784	8.220237	7.661380	7.526038	7.917823	7.459019	8.932840	7.518351	7.574816	7.107099	7.009806	7.245475	7.381252	7.665283	7.440515	7.106590	6.522990	7.288783	7.444765	7.315270	7.481815	7.045786	7.268785	6.479137	7.853434	7.664681	7.553537	7.480444	7.607725	7.753321	7.451641	7.772318	7.224035	6.828319	7.289259	7.132916	7.266319	7.679669	7.277363	7.439916	7.416254	7.370474	7.353422	7.834479	6.986852	7.433286	7.203798	7.312743	7.630374	6.522655	6.727241	7.437546	7.974303	7.176504	7.477997	6.888567	6.796203	6.976898	7.390858	6.553514	7.753478	7.159604	7.166993	7.657521	7.069044	7.200694	7.753171	7.043338	6.903040	7.987793	7.387462	6.645932	7.869232	8.596881	8.186508	6.639497	6.640143	7.808778	7.580698	8.094336	8.599881	7.056010	7.464963	7.838778	8.398520	8.143925	6.914949	7.087666	7.428515	7.067588	8.280692	6.403904	6.580790	6.975438	7.801445	8.510905	8.144245	8.100858	8.586667	7.588434	7.573412	7.259489	8.205229	7.380825	7.643358	7.676347	8.253887	8.435543	8.246193	6.737116	8.267621	7.940418	7.597003	8.768535	8.679458	8.122926	7.584419	7.207847	6.329228	8.143925	8.587391	8.560387	8.205382	8.230249	6.757042	8.831175	8.010496	8.180988	8.582268	6.656131	7.732264	8.682812	7.938222	8.581371	8.388218	7.125777	7.847503	7.845347	8.201668	8.201533	7.355349	7.750125	6.861077	8.446244	8.533620	7.905551	8.058996	8.811548	8.738729	7.394285	8.894837	7.154664	7.600485	8.856221	7.320594	8.795964	7.993319	7.537013	7.617539	8.139275	7.808778	7.112839	8.445759	7.635404	8.738410	8.512924	7.391479	7.908951	7.268335	7.209572	7.997958	7.310875	5.974825	7.754711	6.021175	7.247314	8.308999	7.860047	8.626721	8.315429	8.213459	7.654357

CA3-AS1	6.749427	5.294069	5.101965	5.844745	4.923626	4.713705	4.796678	5.567109	5.412818	4.638196	4.288106	4.413541	3.872015	4.174641	3.940672	5.004156	4.968821	5.335551	6.032910	5.066749	5.180129	5.010542	4.775532	4.903414	4.432882	5.031741	4.011026	5.394196	4.549553	4.455844	5.963956	4.953347	5.481833	3.955463	6.265874	5.602850	4.648318	5.032949	5.628214	5.204262	4.598956	5.847907	4.366995	6.200659	4.258314	5.065280	4.091186	5.737539	4.824782	5.536065	6.234806	5.389488	5.287566	6.379636	5.041986	4.647688	5.142569	4.013825	5.588697	4.883876	5.769576	4.362400	5.473107	6.127660	5.188014	5.906325	4.628485	5.164280	5.029113	4.438286	5.459635	4.721070	4.368291	5.332046	5.480262	5.536065	5.465983	6.009291	5.796922	5.586258	5.393349	5.474055	5.703743	6.223644	5.568462	5.967890	5.757214	6.143417	5.920125	7.031101	5.976903	5.634192	4.730610	5.318242	5.478477	5.507386	5.162860	4.691364	4.212080	3.756112	2.922883	4.322056	4.725358	4.481723	5.425814	3.812109	5.438382	4.969692	4.911553	4.312513	5.350672	5.811491	5.082452	4.094115	4.399395	3.214073	5.362552	4.905873	4.120971	4.475158	5.369136	4.259179	4.438717	3.653027	4.469699	3.258539	4.643607	6.060096	3.203454	4.125890	3.269432	3.353521	3.508878	3.034680	3.784156	4.817923	4.304851	5.720507	5.415640	4.642591	3.973522	6.908123	4.443479	4.268916	4.655316	5.375342	4.022591	3.330982	5.321484	5.204669	3.366868	4.125660	4.122295	5.846583	5.017503	6.472574	4.293735	6.064343	4.629125	3.568681	5.435917	6.596178	6.352110	3.899548	4.346322	4.704057	4.671850	4.506555	5.967336	6.061355	4.938091	6.207268	6.395163	5.636210	4.736547	3.500685	5.289796	5.104607	4.703430	6.704456	4.611221	6.882311	6.325088	6.114764	5.765566	5.718684	5.533321	5.210833	5.530310	4.554340	3.842101	3.524784	5.709192	4.265308	6.105329	6.529826	6.341388	6.254020	4.517005	6.808084	5.958405	7.639389	4.283596	5.851489	4.274873	6.948756	6.182710	6.095497	7.107099	6.141195	6.247965	4.260648	5.434147	5.202650	3.929129	3.779151	2.964653	3.633323	3.202692	3.095195	3.035084	3.280580	3.514792	5.839528	3.418265	6.284973	5.353675	5.716576	4.698722	5.052996	5.092471	4.408936	3.936016	3.539970	4.848293	4.945653	6.442684	4.514021

AC005394.2	3.697132	3.592078	4.495125	4.257871	4.115265	5.112145	3.139813	5.662741	5.932972	3.212842	3.380330	3.497463	3.329019	3.586905	3.232167	4.235090	5.297474	3.828431	4.867232	4.136278	4.948142	4.320147	4.989154	3.781660	4.317138	4.777623	3.775101	4.437866	3.336283	3.809905	3.998522	3.053347	3.156795	3.315690	4.950633	4.491142	3.529232	3.575186	4.391908	3.929129	3.591699	3.072259	3.847173	4.428669	3.364370	4.052167	4.430721	3.851299	4.161815	3.701159	3.659062	4.892077	3.724101	3.869456	3.346564	3.712709	3.784585	3.399199	3.707302	4.639269	4.410703	3.664267	4.698520	4.991195	4.426343	4.932450	4.150003	4.221883	4.647039	4.749126	3.972667	3.675168	3.315547	3.805556	3.635440	4.204989	4.508979	4.832062	4.930410	3.471394	3.696307	4.299199	3.836113	3.841099	4.248611	4.393198	4.636718	4.230073	5.255358	5.870975	5.444863	4.466328	3.138823	4.466094	4.151468	3.315118	3.274494	2.954241	3.211573	3.201577	3.795499	3.357764	4.140843	3.692766	4.610588	4.177655	5.837861	4.149177	4.657491	4.573962	5.139018	5.671606	4.824782	4.368291	4.238991	5.760681	4.609716	4.505883	3.613168	4.141298	3.647203	4.541524	4.007805	4.518915	4.519767	4.455844	5.017104	5.270048	3.435445	3.143738	3.771327	3.483534	3.478770	3.041777	3.357764	4.765699	4.063954	5.311819	4.336552	4.569828	3.875266	5.893148	4.177253	4.442432	4.707447	4.224260	4.306150	3.843557	3.432543	3.788048	3.242816	3.115819	3.078754	4.451832	3.659062	3.553536	3.044544	3.841099	4.178312	3.636076	4.040595	3.370267	4.388910	3.489635	3.889387	4.556230	3.914887	3.182446	3.701577	3.650021	4.078269	3.393483	3.902322	3.146903	3.809533	3.401420	3.354861	3.578380	3.604836	4.222970	3.069020	4.508113	4.113339	4.407819	4.538206	3.040595	3.868380	5.436682	3.414177	4.114172	3.526294	3.820148	3.491669	3.479650	3.998940	4.158412	4.063502	4.588651	3.675783	3.354520	3.815038	3.078974	3.476529	4.149805	4.546888	3.811621	4.261771	5.104831	4.848293	5.246614	4.877177	3.065538	3.967025	3.527209	3.276359	4.192784	3.275282	3.201711	3.146302	3.283480	3.371691	3.583598	3.477093	4.694948	3.807841	5.526878	3.995086	4.194119	3.895047	6.535017	5.654240	5.168072	4.001479	4.283381	3.938016	5.088396	3.754254	3.666689

AC005394.1	3.697132	3.592078	4.495125	4.257871	4.115265	5.112145	3.139813	5.662741	5.932972	3.212842	3.380330	3.497463	3.329019	3.586905	3.232167	4.235090	5.297474	3.828431	4.867232	4.136278	4.948142	4.320147	4.989154	3.781660	4.317138	4.777623	3.775101	4.437866	3.336283	3.809905	3.998522	3.053347	3.156795	3.315690	4.950633	4.491142	3.529232	3.575186	4.391908	3.929129	3.591699	3.072259	3.847173	4.428669	3.364370	4.052167	4.430721	3.851299	4.161815	3.701159	3.659062	4.892077	3.724101	3.869456	3.346564	3.712709	3.784585	3.399199	3.707302	4.639269	4.410703	3.664267	4.698520	4.991195	4.426343	4.932450	4.150003	4.221883	4.647039	4.749126	3.972667	3.675168	3.315547	3.805556	3.635440	4.204989	4.508979	4.832062	4.930410	3.471394	3.696307	4.299199	3.836113	3.841099	4.248611	4.393198	4.636718	4.230073	5.255358	5.870975	5.444863	4.466328	3.138823	4.466094	4.151468	3.315118	3.274494	2.954241	3.211573	3.201577	3.795499	3.357764	4.140843	3.692766	4.610588	4.177655	5.837861	4.149177	4.657491	4.573962	5.139018	5.671606	4.824782	4.368291	4.238991	5.760681	4.609716	4.505883	3.613168	4.141298	3.647203	4.541524	4.007805	4.518915	4.519767	4.455844	5.017104	5.270048	3.435445	3.143738	3.771327	3.483534	3.478770	3.041777	3.357764	4.765699	4.063954	5.311819	4.336552	4.569828	3.875266	5.893148	4.177253	4.442432	4.707447	4.224260	4.306150	3.843557	3.432543	3.788048	3.242816	3.115819	3.078754	4.451832	3.659062	3.553536	3.044544	3.841099	4.178312	3.636076	4.040595	3.370267	4.388910	3.489635	3.889387	4.556230	3.914887	3.182446	3.701577	3.650021	4.078269	3.393483	3.902322	3.146903	3.809533	3.401420	3.354861	3.578380	3.604836	4.222970	3.069020	4.508113	4.113339	4.407819	4.538206	3.040595	3.868380	5.436682	3.414177	4.114172	3.526294	3.820148	3.491669	3.479650	3.998940	4.158412	4.063502	4.588651	3.675783	3.354520	3.815038	3.078974	3.476529	4.149805	4.546888	3.811621	4.261771	5.104831	4.848293	5.246614	4.877177	3.065538	3.967025	3.527209	3.276359	4.192784	3.275282	3.201711	3.146302	3.283480	3.371691	3.583598	3.477093	4.694948	3.807841	5.526878	3.995086	4.194119	3.895047	6.535017	5.654240	5.168072	4.001479	4.283381	3.938016	5.088396	3.754254	3.666689

AC005381.1	3.697132	3.592078	4.495125	4.257871	4.115265	5.112145	3.139813	5.662741	5.932972	3.212842	3.380330	3.497463	3.329019	3.586905	3.232167	4.235090	5.297474	3.828431	4.867232	4.136278	4.948142	4.320147	4.989154	3.781660	4.317138	4.777623	3.775101	4.437866	3.336283	3.809905	3.998522	3.053347	3.156795	3.315690	4.950633	4.491142	3.529232	3.575186	4.391908	3.929129	3.591699	3.072259	3.847173	4.428669	3.364370	4.052167	4.430721	3.851299	4.161815	3.701159	3.659062	4.892077	3.724101	3.869456	3.346564	3.712709	3.784585	3.399199	3.707302	4.639269	4.410703	3.664267	4.698520	4.991195	4.426343	4.932450	4.150003	4.221883	4.647039	4.749126	3.972667	3.675168	3.315547	3.805556	3.635440	4.204989	4.508979	4.832062	4.930410	3.471394	3.696307	4.299199	3.836113	3.841099	4.248611	4.393198	4.636718	4.230073	5.255358	5.870975	5.444863	4.466328	3.138823	4.466094	4.151468	3.315118	3.274494	2.954241	3.211573	3.201577	3.795499	3.357764	4.140843	3.692766	4.610588	4.177655	5.837861	4.149177	4.657491	4.573962	5.139018	5.671606	4.824782	4.368291	4.238991	5.760681	4.609716	4.505883	3.613168	4.141298	3.647203	4.541524	4.007805	4.518915	4.519767	4.455844	5.017104	5.270048	3.435445	3.143738	3.771327	3.483534	3.478770	3.041777	3.357764	4.765699	4.063954	5.311819	4.336552	4.569828	3.875266	5.893148	4.177253	4.442432	4.707447	4.224260	4.306150	3.843557	3.432543	3.788048	3.242816	3.115819	3.078754	4.451832	3.659062	3.553536	3.044544	3.841099	4.178312	3.636076	4.040595	3.370267	4.388910	3.489635	3.889387	4.556230	3.914887	3.182446	3.701577	3.650021	4.078269	3.393483	3.902322	3.146903	3.809533	3.401420	3.354861	3.578380	3.604836	4.222970	3.069020	4.508113	4.113339	4.407819	4.538206	3.040595	3.868380	5.436682	3.414177	4.114172	3.526294	3.820148	3.491669	3.479650	3.998940	4.158412	4.063502	4.588651	3.675783	3.354520	3.815038	3.078974	3.476529	4.149805	4.546888	3.811621	4.261771	5.104831	4.848293	5.246614	4.877177	3.065538	3.967025	3.527209	3.276359	4.192784	3.275282	3.201711	3.146302	3.283480	3.371691	3.583598	3.477093	4.694948	3.807841	5.526878	3.995086	4.194119	3.895047	6.535017	5.654240	5.168072	4.001479	4.283381	3.938016	5.088396	3.754254	3.666689

MAP4K3-DT	8.147603	8.830404	8.334148	8.785633	8.706305	9.433441	8.866731	9.404833	8.115470	9.236267	8.974670	8.856845	7.794310	8.369704	6.968869	9.773259	8.664235	7.526669	7.540820	8.843862	7.462771	8.775967	9.325221	7.934018	8.065034	8.631600	7.911881	8.831175	8.890530	8.818068	8.472666	8.471849	7.976016	8.173625	7.817936	8.368477	7.978789	8.238226	7.968783	8.019529	9.863624	7.655466	7.128302	8.040436	7.994104	8.212526	8.960537	8.189259	8.652972	7.770487	8.295020	8.185093	7.471425	7.738048	8.072719	9.131387	7.605001	7.511240	8.139429	7.593195	7.443164	8.953170	8.257221	7.481352	7.372828	7.762998	7.835236	7.955250	7.950164	8.163833	9.380048	9.497352	9.557862	8.987164	9.220517	8.669282	8.947521	8.462038	8.849892	7.566103	7.571434	7.880784	7.348334	7.981450	7.700231	7.964491	6.922566	7.914905	7.520987	8.217923	7.957304	7.775417	7.802510	8.836612	8.262136	8.167229	7.804165	7.750575	7.914905	8.539575	7.907089	8.235581	8.366526	7.750125	7.972915	9.219906	8.444705	7.760354	8.127063	10.328083	8.176634	7.784389	7.518041	9.995272	8.826560	6.589655	7.942332	8.582697	9.181882	8.986399	9.661702	9.281837	8.847714	8.780870	9.421896	8.939900	7.892325	8.586667	9.346648	9.275288	9.952895	9.301410	7.078474	6.911143	8.421352	9.282762	7.946027	7.985002	9.614826	7.863243	8.384047	8.888996	8.207229	7.516478	9.746469	9.828423	9.003636	9.304141	10.281231	7.308044	7.629708	8.527674	9.445907	8.215758	8.991795	7.503062	8.345535	8.488318	8.064421	11.032397	8.031937	7.474299	9.299232	7.869232	8.815133	10.389118	7.376415	8.744503	8.724169	8.806427	8.247106	8.899923	8.228709	7.492437	9.874226	8.180232	7.574019	7.266472	7.374069	7.281157	9.580724	7.694740	7.564569	7.693207	7.645570	7.649346	8.797014	8.324774	8.516685	9.228039	10.370786	10.110042	9.686684	9.047133	9.125313	9.607774	9.981835	9.824324	6.954068	7.415948	8.114241	7.287083	6.843859	7.743500	8.576701	7.437104	7.946637	7.070868	6.958150	7.844862	7.482906	7.634048	9.728386	8.911416	8.303462	8.407880	6.885991	7.974303	8.719785	8.103712	7.716737	8.008532	7.121043	7.005493	9.208546	8.394089	7.879149	7.842958	10.614085	7.786365	6.674131	7.320294	10.098577	9.245828	9.249650	7.225755	8.633832	10.220952

THUMPD3-AS1	10.273046	9.073438	10.068414	9.846464	9.851053	9.226054	9.756700	9.603163	11.267736	9.141010	10.037488	10.040698	9.836740	9.130300	9.368622	10.016654	9.525226	9.149407	10.272099	10.279731	8.120696	9.520363	9.987460	9.904543	9.052979	10.003957	9.850742	8.982916	10.050816	9.608227	10.567549	9.607021	9.584068	10.364725	10.277608	10.328691	10.122918	9.587009	9.243810	9.387625	9.688849	10.198715	9.547157	10.720767	10.571069	10.310064	9.897193	8.974524	10.236988	9.644393	10.179183	9.567637	9.603459	9.888772	10.146111	9.468552	10.052192	9.434327	9.006707	9.381628	9.161680	9.915761	10.127429	9.618393	9.163554	9.393908	9.119735	9.394059	9.083038	9.254187	10.142906	9.851053	9.476585	9.754543	9.992951	10.152342	9.924891	9.756381	9.730932	9.600803	10.202012	10.023382	10.048025	9.786976	9.989806	10.422993	10.468588	10.146709	9.603459	9.395127	9.757004	9.147352	9.253123	9.928321	10.170585	10.795127	10.196594	10.922691	10.184062	10.293540	10.475118	9.873170	10.246494	9.873486	10.010461	9.817412	8.836326	9.444994	10.263444	9.833499	9.619017	9.195450	9.355898	9.787132	10.039917	7.677137	10.247418	10.082692	10.138502	9.403225	8.887749	10.150520	9.329390	7.947234	9.952278	9.629990	10.472219	10.465797	8.821128	9.109255	9.353931	9.721996	8.636601	7.892783	9.954924	9.981055	9.569977	10.583044	11.077806	8.458106	10.356463	9.876719	8.819941	7.992858	10.033023	8.682676	9.136379	9.017440	8.759174	10.298823	9.971512	9.726547	10.527773	10.035941	10.226509	10.089002	10.034852	9.243504	9.462714	9.574584	9.525056	9.787425	10.430163	10.106724	10.091114	9.442662	8.623592	9.958459	8.940204	9.591343	10.031960	9.917481	9.725448	9.661397	9.119892	10.316237	10.321983	8.742540	9.383185	9.556781	9.178776	9.764810	9.090513	9.348949	9.470474	10.076651	9.363205	9.496124	9.291914	9.903042	10.136988	8.747578	9.870596	10.332208	9.652422	9.517769	10.052954	9.541437	9.521160	10.565026	10.625962	9.827965	10.486117	10.288200	10.060308	10.145201	10.581438	9.570720	9.027677	11.340546	8.970055	9.572296	10.296166	10.086358	10.577989	9.973693	10.724767	9.895199	10.198715	9.238600	10.638766	10.115901	10.067357	10.217418	10.747707	8.636601	9.535347	9.515156	8.082554	9.164187	8.080215	9.211605	8.899452	10.037784	9.527862	9.523327	9.919961	9.385584

PVT1	7.398827	8.094026	8.248095	8.203318	9.214227	8.506270	7.881547	8.225701	8.752281	8.201379	8.827175	8.553172	8.813362	8.334459	8.702920	8.135425	9.159549	8.301367	9.783445	8.149614	7.887659	9.059425	7.930369	8.126434	8.414729	8.897627	8.782984	7.901974	8.702764	8.293964	9.228524	7.382041	8.181886	8.697420	7.968783	7.883850	7.749652	8.079921	8.150452	7.775891	8.041723	7.640791	8.266356	8.258487	8.316068	8.348118	7.914014	8.243979	8.653270	7.961155	7.810660	7.842645	8.257221	8.098702	8.974230	9.096148	8.179917	7.952777	8.281921	8.067577	7.221773	8.606200	6.911932	8.326972	8.272099	8.777533	7.343018	7.829434	7.959604	7.367347	7.800516	7.634048	8.641277	7.522817	7.873058	8.429740	7.664378	8.043261	7.566262	8.367911	7.590442	8.744354	7.704547	7.646194	7.552226	8.155151	8.215758	8.229359	7.385010	7.521779	7.143631	7.413067	8.516378	8.147026	8.162350	8.832406	8.638802	8.946763	8.995337	9.000419	9.442348	8.027463	8.910839	8.337740	8.359865	9.234409	8.635856	8.867627	10.073625	8.368909	8.580435	8.925032	9.638103	8.838141	9.879153	10.027958	7.450589	8.723135	8.511664	9.427590	8.665298	10.100454	9.711867	10.014741	8.910839	8.426021	8.212526	9.019712	9.689001	8.803984	8.773664	9.502845	9.028879	9.611185	8.263240	8.880826	10.032414	9.902118	7.817635	9.464087	9.491096	8.152212	8.866575	9.010103	9.615452	8.258326	10.199612	10.071163	8.254367	9.594166	9.110310	8.566155	9.049437	9.128284	8.232389	7.633766	10.074685	9.335503	8.518854	8.761974	8.040770	9.630443	8.166131	9.148812	8.902003	9.375753	6.886757	8.742697	8.020708	8.579079	8.586387	9.978744	8.354284	9.245982	8.991352	7.821963	9.408800	9.523644	8.113464	8.719507	8.563599	8.627031	8.228114	7.925376	8.674211	8.360324	8.504625	9.251933	7.737895	8.888996	7.895121	9.786976	8.384047	8.562976	9.849598	8.067722	9.172647	8.328445	7.889831	7.858176	9.897656	7.952156	8.781511	8.952566	9.240911	8.968822	9.728526	7.077031	8.414001	8.917875	8.418870	8.363626	8.833467	8.648567	8.860924	9.074979	9.692242	8.950129	9.431886	8.897149	8.819333	9.421602	7.919491	9.426037	9.039625	12.207603	9.815584	8.950129	8.865961	9.187229	8.760747	11.101499	8.997674	9.770486	9.334373	9.642061	8.921354	9.333538

BGLT3	2.986272	3.099977	2.947532	2.971853	3.030074	4.181237	3.123562	3.122165	3.172503	3.158914	3.270368	3.193913	3.184490	3.496731	3.169872	3.056447	3.290281	4.044741	4.146648	3.085714	4.056076	3.653615	4.743795	3.096025	3.302676	3.373442	3.072994	2.966669	3.120117	2.999567	3.547776	3.246540	3.099853	2.989987	3.181767	3.892448	3.498889	3.066192	3.224050	3.103123	4.298741	3.249984	3.248272	3.090417	3.390175	4.462868	5.577407	3.938111	3.035084	4.685922	3.505344	3.418265	4.749126	3.840275	3.374715	2.990342	3.886748	3.182569	3.591339	3.637869	3.562491	3.000262	3.512749	4.243194	3.192163	3.236086	3.006045	3.274958	3.145152	3.064168	2.900822	2.866092	3.009751	2.908590	3.379996	3.726339	3.151870	2.908776	2.927701	3.445636	3.138823	3.666461	3.065121	4.030399	3.187406	5.055944	3.139187	3.197423	3.438735	3.275122	3.137848	3.257039	3.217115	3.527760	2.952475	2.968089	3.365983	3.145941	3.501239	2.955040	2.948932	3.137608	2.950272	2.811222	4.132328	2.926633	2.948161	3.216951	3.133804	3.145372	2.954042	2.899623	2.953498	3.150262	2.955435	3.198410	2.923669	2.922168	3.063094	2.975807	3.166091	2.917889	3.002733	2.893450	3.363531	2.898436	3.050060	3.062355	3.005456	3.060353	2.979074	2.969823	3.088322	3.020128	2.921570	3.240032	2.969017	3.258230	3.132978	3.593829	2.859452	5.254943	2.984694	3.152463	2.900172	3.034213	2.900459	2.857923	3.299460	3.345113	3.802439	3.102534	2.937997	3.088102	3.626008	3.493366	3.025052	3.188332	3.043913	3.570038	3.219233	3.340297	3.140813	3.548394	4.217947	5.265445	3.951398	3.136008	3.784800	3.477093	3.237709	3.751689	3.902101	3.172139	2.933248	3.470703	3.220137	3.441704	3.571687	3.316203	3.039135	3.549478	4.795599	3.322974	3.370455	3.032967	3.577444	3.265284	3.194444	2.915804	2.926455	2.964653	2.900722	3.165946	3.794458	2.974907	2.979449	2.968264	3.108304	3.357076	3.716082	3.049958	3.519668	3.406943	3.641293	3.283015	3.307599	3.289973	3.264389	3.265442	3.322974	3.107586	3.052485	2.995495	2.935992	2.890735	2.894612	3.450064	3.138348	2.945514	2.989987	2.900004	2.886294	4.585625	2.935072	2.942527	3.195162	3.333551	2.994217	2.914017	2.939990	3.256478	3.095013	2.932169	2.926540	2.944169	2.923134	3.097963

DHRS4-AS1	14.450917	15.048391	14.702498	14.634337	14.452088	14.454290	14.947964	14.248223	14.234569	14.637945	14.324726	14.610103	14.584001	14.676953	14.144611	14.471558	14.406227	14.293761	14.530263	14.759178	14.690192	14.563014	14.529498	14.479473	14.760083	14.887600	14.717187	14.396941	14.751210	14.649747	14.487150	14.461989	14.413643	14.580207	14.697958	14.664070	14.708876	14.832004	15.009362	14.870791	14.716286	14.945774	14.478648	14.504600	14.686942	14.395124	14.808644	14.714881	14.544632	15.093809	14.185875	14.582286	15.071599	14.992275	14.744131	14.911437	14.659533	15.143286	15.063409	15.017982	15.047833	14.867085	15.045993	14.992829	15.037486	15.234869	15.098818	15.160798	14.735321	15.038723	14.999084	14.800437	14.537158	14.418012	14.589784	14.917362	15.230680	14.487923	14.602856	15.072836	14.426320	14.453564	14.493555	14.729702	14.480594	14.477088	14.014363	13.818636	14.428245	14.907125	14.564295	14.779929	14.361581	14.946885	14.222846	14.484499	14.789144	14.055969	14.327509	15.040623	14.305307	15.053711	14.532252	14.556844	14.450917	14.286202	14.756942	14.828938	15.171380	14.649251	14.607960	14.416904	14.530652	14.743665	14.463605	14.551021	14.407352	14.839161	14.451322	14.335070	14.340690	14.289302	14.270558	14.494741	14.484052	14.821349	14.229183	14.167438	14.630361	14.264573	14.566322	14.707094	14.477088	14.223156	14.334703	14.256071	14.184052	14.451725	13.854600	14.378512	15.587282	14.338926	14.465165	14.560516	14.267351	14.275877	14.475985	14.731220	14.514288	14.385259	14.013491	14.105943	14.257993	13.844544	13.995849	13.800876	14.518159	14.471172	14.470097	14.600408	14.584760	13.630442	14.087427	14.494365	14.545966	14.637498	13.902528	14.498295	14.076889	14.064771	14.188678	15.028728	14.378131	14.475183	14.733504	13.742612	14.484873	14.471966	13.949622	14.369411	14.784831	14.508368	14.456976	14.411336	14.500265	14.673241	14.415465	14.680520	14.124606	14.454664	14.346713	14.532252	14.321264	13.765490	14.110411	14.384530	14.365930	14.225335	13.680915	13.652033	14.452476	13.672469	13.576005	13.607817	14.324060	13.944829	13.588617	14.519413	14.104742	13.888334	14.658149	14.390000	14.271885	13.916329	13.944308	14.491542	14.033170	14.283789	14.799897	14.421778	14.396941	14.440081	13.861539	15.584586	14.533864	14.497164	15.701693	14.636601	14.839682	14.260943	14.162992	14.035571	14.669062	14.863545	14.104048	14.758705	14.343514	14.684603

AL359232.1	8.217593	8.944736	8.773664	8.388365	8.953955	9.316571	8.791835	8.371594	8.294728	8.907026	8.602820	8.750677	8.327636	8.149158	8.044812	9.401696	8.235581	8.077583	6.894802	8.442390	7.574330	7.567185	7.950317	8.092315	8.549225	8.527214	9.598034	8.980298	9.822688	9.731525	8.948886	7.975543	8.219624	8.714844	8.719946	8.214394	8.879252	8.526736	8.883274	8.445759	9.466126	9.261349	7.821662	8.232693	8.367911	7.778689	9.582219	7.811119	8.099298	7.639084	7.414920	7.932481	8.508291	8.039807	7.824779	9.360799	8.295378	8.503865	8.098253	7.957304	8.093260	9.770821	9.728699	8.478049	8.676059	8.054480	8.060250	8.286715	8.034917	7.919331	8.389600	8.250993	9.456470	8.422421	8.312979	8.254820	8.571418	8.483880	7.941974	8.021631	7.814466	8.112700	7.655283	7.704063	7.887198	8.189742	8.160080	7.964808	8.064117	7.663729	7.596551	7.947845	8.245084	8.357224	8.424530	7.945865	7.902121	9.050240	9.191345	8.650119	9.365175	8.482967	8.669134	8.537536	7.013910	9.123377	8.108325	7.873982	7.934176	9.386992	6.836129	7.929428	8.188055	8.966033	8.860469	11.193097	8.037881	8.792288	9.511205	8.954400	9.123220	8.637074	8.607266	10.023543	9.516988	9.485177	9.384220	9.463167	9.702032	9.207491	9.789292	9.356076	9.040712	9.620259	9.418832	8.426929	8.287349	7.340993	8.932113	7.405064	8.291041	7.118055	9.026010	9.174793	9.974365	9.501800	9.468105	10.004584	9.550393	9.556170	9.174484	9.294739	9.077786	8.596881	8.777381	8.505072	8.343224	9.742277	8.768392	9.726052	8.277390	8.056042	8.826560	8.602194	8.156894	10.006772	8.153471	8.529825	8.203010	8.407722	8.029333	8.969905	9.552825	8.743603	9.475200	9.791422	8.432106	7.959912	8.184337	8.387588	9.774645	9.298930	8.565249	8.418036	8.481708	8.633073	8.237618	8.411404	7.749198	9.111602	9.102439	10.275761	8.831940	8.555749	8.563436	8.563436	8.712976	8.779006	8.215620	8.031937	8.308548	8.195255	8.173625	8.406806	8.925815	8.202701	7.906019	8.405606	8.607266	7.583649	7.894196	8.243979	9.038327	8.862000	8.777381	8.924735	9.036629	9.725155	8.747882	9.313468	8.652972	9.171571	8.439951	7.958214	9.576986	8.326972	7.863555	7.660947	9.234252	7.500400	8.260003	9.075596	9.558934	9.401214	10.122625	9.345280	9.355284	9.664852

AL354714.1	6.770915	7.331953	7.402070	7.057930	6.373651	7.362303	7.347692	7.642308	7.791273	7.436471	6.274562	7.064759	6.563191	7.341765	6.420945	6.971038	7.464963	7.069219	7.051964	7.405370	6.264006	7.335828	9.134397	6.720650	6.472574	7.081450	5.996901	7.155127	6.384666	6.832615	7.713118	7.325797	7.309112	7.530378	7.054425	6.556152	6.759769	7.049262	7.502112	7.543264	7.022026	7.421041	6.293351	6.445919	6.464543	6.314526	6.794137	7.322955	7.072747	6.911631	7.033252	7.213260	6.945183	7.662002	6.799796	6.899169	7.227941	7.357964	7.490559	7.576995	7.543582	6.650152	6.351290	7.188718	7.114883	7.439160	7.638327	7.240719	7.197133	7.091816	8.082112	7.703146	6.990632	7.774614	7.979874	7.361219	7.349431	7.321353	7.648612	7.594432	7.389651	7.229059	7.479657	7.178765	7.156379	7.354709	7.371092	7.641842	7.478884	8.129817	7.542183	7.586605	6.830244	7.197133	6.892054	6.691766	7.097035	6.208126	6.493471	6.659030	5.733384	7.293485	7.115667	7.291140	6.907201	6.310424	7.041120	6.395639	7.333659	6.926505	7.642619	7.449836	7.121945	6.706548	7.368917	6.518383	6.720650	7.188552	6.440719	8.044489	7.231492	8.219328	6.531604	7.247752	7.218109	6.555824	7.037167	8.034780	7.721625	7.062047	7.308803	5.902563	5.852907	5.438382	5.814929	7.231821	7.651278	8.009151	7.104405	6.570672	6.917878	8.171919	7.046908	6.727870	6.986699	6.660184	8.706305	6.524506	7.293816	7.811119	6.760754	7.303511	7.712340	7.695913	7.541740	6.703844	7.139538	6.858817	7.428515	7.732409	7.668348	6.825914	7.276265	7.825691	6.494756	7.568409	7.026811	7.079746	6.512277	7.014074	6.011759	7.344115	6.068056	5.451468	6.891740	7.128302	7.363230	7.673529	10.167524	8.217923	6.602455	6.915261	7.315429	6.767961	7.043839	7.499287	7.729193	7.446500	7.370150	8.418870	7.823644	7.407269	7.997230	7.609920	7.548238	7.178923	8.024983	7.491173	5.826559	7.972915	7.186349	7.741489	7.341605	6.614323	6.745598	7.771269	8.583923	7.364043	6.920025	8.620648	7.796780	6.381466	6.855890	7.691350	7.541740	7.461507	6.409176	7.151068	7.541891	5.553381	10.460420	7.371092	6.662005	6.258112	6.787592	7.935298	6.295933	7.730863	6.918372	7.161880	8.013058	7.468910	6.914786	7.556139	6.156030	6.631873	6.855086	6.734990

AC109927.1	3.285202	4.093091	3.556975	3.935574	3.437732	4.822715	4.578189	4.783045	3.833985	4.541102	3.804710	4.074510	3.787581	4.564688	4.325277	3.642272	3.339114	4.277739	4.124030	4.788883	3.791856	3.369092	5.804210	3.509038	3.154050	3.852263	4.467997	3.002910	4.406483	4.008667	3.417742	3.655630	5.690604	4.223852	3.137360	3.861275	4.200518	3.471876	3.262940	3.923719	4.470128	3.413828	3.256050	3.714654	3.441340	4.767656	4.433285	5.351860	4.755113	3.545470	3.502367	3.400545	3.848646	3.839470	3.476336	4.033858	4.931393	3.091325	3.337310	3.561768	3.225182	4.052798	3.602660	3.128450	3.278205	3.288352	3.067061	3.801826	3.597536	3.049099	4.024941	3.787161	4.017194	3.808458	4.215317	4.274425	3.912934	3.836319	3.952698	3.497840	3.926712	3.679225	4.207761	3.869875	3.182173	4.649613	5.118253	3.386208	3.470503	3.362672	3.554886	3.285202	3.770527	3.587651	4.255015	5.492218	4.115928	3.586323	4.458439	4.172494	4.830197	3.637684	3.686638	3.833320	4.270510	4.120296	4.027949	4.205947	3.223347	4.421729	3.074675	3.499422	3.792920	4.394058	3.589021	6.004097	4.697895	4.550441	3.757114	4.263078	3.971363	3.838432	4.473629	4.284260	5.698420	4.812075	3.269945	3.095195	4.588420	4.847214	5.162291	4.238537	4.408936	3.987976	4.333762	3.277564	3.523264	3.244137	4.441288	3.382935	4.372359	5.751387	4.070266	3.804108	4.002147	4.499642	4.601214	4.879022	5.287566	3.247085	4.134500	4.705113	4.396789	3.903970	3.772413	3.959460	3.063972	4.370011	3.538985	5.511434	3.361618	4.372814	3.769910	3.677808	4.348753	4.862073	3.515884	4.844870	3.417295	3.386886	3.212697	3.617462	3.913564	3.601857	4.532465	4.941572	3.114254	3.137740	3.427805	4.852460	4.789520	3.681816	3.495025	3.463557	3.453668	3.086264	3.809104	4.149594	3.090316	4.908396	4.124861	5.228497	4.811255	3.844372	4.462017	4.351108	4.347825	3.874606	3.844372	3.420255	3.766740	5.069189	3.470874	3.893535	4.830197	3.735838	3.329682	3.302377	3.810138	3.746675	3.166213	3.970288	4.269156	4.144966	5.429221	4.050878	4.478450	3.785421	3.950770	4.462868	4.129377	4.004337	4.499642	4.682249	4.397453	3.693618	3.968740	3.299913	5.396530	3.291272	3.939843	4.477408	4.250835	4.101480	4.329006	5.297845	5.145352	5.563155

AC063948.1	4.254130	5.140892	5.153391	4.818345	5.144750	5.093331	5.457488	4.103884	5.266067	4.392767	4.801541	4.821065	4.951026	5.881754	5.215041	5.092048	4.485503	5.637695	5.532003	6.025164	4.648966	5.240856	6.054797	5.172533	6.472916	5.656663	5.382123	4.546011	4.436995	5.018110	3.706050	5.292766	5.688415	5.128768	5.901681	6.246624	5.594579	5.567659	4.677106	4.796480	3.656029	5.332046	7.328193	5.286398	6.332318	6.242601	3.690955	7.382041	6.662500	5.402259	5.520055	6.710559	5.976562	6.037203	6.574042	5.473286	7.068415	6.945031	5.804026	6.420945	6.242601	5.202453	6.020286	5.491876	6.149325	5.999541	5.818327	4.889600	5.621291	5.213169	5.027227	5.507191	4.524788	5.101759	5.982412	5.698946	5.385023	4.800293	4.548399	6.086091	5.031741	5.906683	6.477105	5.869021	6.069439	6.417479	5.686221	6.856737	4.320571	5.355688	5.859961	4.391694	6.061895	5.632079	6.034728	5.878692	5.618933	5.907938	5.599867	6.992762	5.130587	5.877786	5.829507	5.321907	7.852796	5.248033	5.481638	5.170102	4.637372	4.324870	4.641133	5.425814	5.250383	4.508113	4.948142	4.579040	5.065719	4.743378	4.409145	5.267268	5.736616	6.065925	5.890109	5.657200	6.225022	5.409725	5.441215	4.922338	5.517224	6.040904	5.979585	5.031335	5.894762	6.184157	5.437645	5.632279	6.119757	6.606716	6.450803	5.956812	6.262524	7.103438	5.331849	7.412455	6.151749	4.970294	5.081245	4.980126	5.811679	4.363938	6.157700	5.837134	6.270338	5.911868	6.672197	6.023065	5.835886	5.855990	6.148479	5.309215	5.877950	7.960374	6.397453	6.723381	7.171971	5.067971	7.709122	7.260304	6.707538	6.332154	7.002889	6.318078	5.882650	6.928293	6.221062	7.242922	7.457944	6.163054	6.951549	6.694563	5.703923	6.280526	6.397635	7.207847	6.802029	6.706217	5.964297	6.580451	6.150019	5.826180	5.957159	5.827305	5.928111	6.316736	6.619366	6.009291	5.977433	5.286594	6.438240	6.249133	7.214347	6.890454	6.257931	7.184084	6.572438	6.353439	8.010496	6.139446	6.450636	6.726738	5.950379	6.306442	6.042288	6.713426	6.131372	6.117854	6.045183	5.862318	7.365150	6.420124	6.473421	6.650608	6.065235	8.791046	6.524348	5.801715	6.276999	6.077620	4.803466	5.606848	5.709757	5.009920	5.514643	6.877175	5.093331	5.247625	5.679946	6.095667

AC016766.1	3.009751	3.110964	2.997517	2.972991	2.901165	3.475806	3.174211	3.167789	3.210436	3.183560	5.113154	3.111066	3.176379	3.457694	4.631116	3.084967	3.423478	2.893176	4.365913	3.134280	3.554680	3.010483	3.213675	2.869702	2.994217	2.961386	3.014925	2.932255	3.678427	2.998262	3.425827	3.182446	3.968740	3.013959	3.064058	3.869665	3.368594	3.061915	3.167932	3.014748	3.274958	3.053432	3.358619	3.315887	3.480978	4.694331	4.092887	3.078385	3.433819	3.588616	3.428139	3.323489	3.465394	3.949658	3.502150	3.019853	3.899349	3.607835	3.175025	3.542469	3.028960	3.026197	3.483534	3.142565	3.282239	3.248829	3.207511	3.108304	3.090545	2.907490	2.969982	2.907199	3.029060	2.905795	2.995701	3.240032	3.001092	2.906489	2.961557	3.460015	3.238168	3.675584	3.292959	6.087475	3.229611	4.652164	2.878010	3.207657	3.838884	3.417203	3.134769	2.953408	3.320089	3.105977	2.915154	2.946804	3.437547	3.175288	3.065854	2.919416	3.069947	3.152931	2.938155	2.863100	4.295235	3.410052	2.952746	3.074437	2.955694	3.056542	2.874047	2.905321	2.933165	3.122871	3.082648	2.850697	2.928427	3.002557	3.059383	2.984795	3.159437	2.965763	3.001873	2.848304	4.486379	3.524404	3.122628	3.195044	3.026845	4.824555	2.981173	2.964370	3.075974	3.017496	2.929202	3.341666	2.848837	3.305084	3.167274	3.239461	2.908133	4.985607	2.846808	2.895276	2.911614	2.864740	2.880718	2.983258	3.257771	3.236383	3.305388	3.280580	2.961862	3.003333	3.610373	3.404378	3.076339	3.203454	2.984795	3.084157	2.965377	3.430641	3.142064	3.764916	4.044741	3.960729	2.995910	3.577750	3.441518	3.292351	3.188761	3.483698	4.043645	3.289532	2.980688	3.578183	3.549094	2.988326	3.323817	2.931258	3.110054	3.520622	3.496333	3.463379	3.384322	3.214729	3.265735	3.122398	2.892414	2.953586	2.953130	2.996090	2.898622	2.938155	3.178423	2.994943	2.982627	3.020044	3.096907	3.512937	3.837575	3.313790	5.069387	3.336436	3.396948	3.387578	3.344639	3.615151	3.325007	3.177227	2.932169	3.112229	3.123311	3.037128	2.962457	2.909854	2.914185	3.446902	3.120835	3.434709	2.989807	2.883971	2.809993	4.619929	3.184361	2.916383	3.029060	3.169283	2.988814	2.845063	2.825610	3.141052	3.078861	2.954774	2.848033	2.923214	3.010212	3.100195

SLFNL1-AS1	10.959584	10.256188	9.689752	10.053263	9.559089	9.771440	10.320901	8.949507	9.580581	9.640875	10.115619	10.113777	9.917640	9.822386	10.863064	10.096264	10.181309	10.211080	8.236065	10.121857	8.868076	9.545767	10.843943	9.841226	10.703444	10.595136	10.867134	10.099670	9.941340	9.852455	9.942734	10.678516	10.658275	10.899381	10.386880	10.246336	10.227300	10.254638	10.899103	10.890897	9.725448	11.083077	10.516399	10.582460	11.049872	10.021565	10.528361	11.104875	10.095615	10.488713	10.822224	10.510787	10.504838	10.625496	10.568780	9.742743	10.687519	10.065419	11.107474	11.445862	11.382315	9.777842	11.059536	10.820861	11.450945	11.150980	10.591407	10.485631	10.569055	10.709812	9.798436	9.784276	9.356515	10.071631	10.005200	10.085231	10.078202	10.307130	10.337472	10.849717	10.578592	10.517560	10.816113	10.668712	10.684971	10.577828	10.642678	10.625804	9.966500	11.113773	11.311749	9.930780	10.529955	10.228061	10.536920	10.465042	10.308026	10.242906	9.961344	10.704762	9.881488	10.377566	10.248333	10.271317	9.176798	9.962834	10.137918	10.244867	9.492217	9.554494	8.292455	10.371390	10.156509	8.944584	9.579308	9.915301	11.602845	10.292188	9.983355	11.991869	10.085861	9.830616	9.915155	9.290484	9.619319	9.760421	10.796271	9.800887	9.533530	9.916997	10.265433	9.960892	11.622683	11.098696	10.844240	10.521739	9.527862	8.004823	10.973802	9.275288	9.826316	8.775819	9.534899	10.861911	11.037558	9.804101	9.049938	9.604702	9.359225	11.047517	10.934280	10.711339	8.860183	10.421303	10.179183	10.812246	10.182714	11.338491	10.761158	9.757967	11.704017	10.119905	10.912644	10.198291	10.135620	10.004584	11.337903	9.979491	9.923526	10.376347	10.900123	10.111382	10.569382	10.863522	9.962553	11.225487	10.404404	10.997755	11.412033	11.682616	10.728524	11.110584	11.134615	11.014277	10.889218	12.533788	10.244867	10.569692	11.746252	9.568899	10.319973	9.736555	10.150237	10.515780	9.979195	10.749604	10.075883	10.380154	10.897742	10.710821	10.980857	10.823846	10.443810	11.062597	10.342797	9.995566	10.436890	10.148575	11.023522	11.166369	10.249259	10.408443	9.995098	9.535972	10.231014	10.412994	10.173953	9.991577	10.947735	9.867261	11.030608	10.757656	10.418587	8.851683	9.471554	9.046071	10.521568	9.202653	9.092226	8.296702	11.190758	10.568476	9.428670	9.688392	9.660604	12.193677	10.868443	9.308341

AC007780.1	11.434944	9.993095	10.672285	10.584201	10.989882	10.790007	10.887915	11.243882	12.091066	11.021537	10.925491	11.120500	11.418124	10.760711	9.552071	10.683473	10.410869	10.540467	11.026609	11.236266	10.682874	11.689738	10.788558	11.161418	11.233464	11.981504	11.385992	11.500573	10.223453	10.738842	10.669403	10.001021	10.921361	10.735629	11.185858	11.503353	11.470479	10.762958	10.924932	10.726580	10.796577	11.244028	10.747396	10.852403	11.770623	11.213821	10.189346	10.845325	11.059966	11.140921	11.237000	10.962648	10.404404	11.132065	10.978030	10.057740	11.359302	11.071222	10.876767	10.935769	11.199385	11.279433	11.147104	11.000920	10.220026	10.487949	11.053797	9.466593	10.271167	10.975823	10.926836	11.202837	10.663706	11.403089	11.317631	10.784668	10.873395	11.078407	11.009906	10.363720	10.295699	10.297542	11.239356	10.857343	11.105870	10.537651	11.687667	11.129723	11.047954	10.146257	10.960321	10.693316	10.945509	10.743352	11.407356	11.188894	10.541837	11.575095	10.793806	11.436541	11.016629	11.020512	10.976116	11.070628	11.371135	9.756549	11.685720	10.692399	11.572071	10.511571	11.128877	10.799059	10.734462	10.703174	9.919633	8.691384	11.352031	10.713717	10.717904	11.705650	9.336777	11.072990	10.893606	11.677143	10.163518	10.385019	10.801045	11.085876	10.552645	10.317425	11.365974	11.785972	9.622288	9.334014	10.352051	9.896747	11.173855	9.806528	10.397475	9.679709	11.811570	10.225137	11.174150	10.863674	10.850333	10.360509	11.870089	10.888932	9.873486	10.380306	10.873250	11.763241	10.612891	10.605498	11.539250	11.759188	10.148077	10.384099	10.729414	10.768036	10.857505	10.363280	10.983770	12.371190	10.788244	9.996027	10.493458	11.585304	11.336873	11.434639	11.077953	9.977682	11.355206	11.222510	9.274416	10.895475	11.567272	10.148575	11.550461	10.752648	11.020795	9.873938	10.056961	10.002558	10.223453	10.471490	10.977161	10.334961	10.660403	11.165803	11.711327	9.765411	11.618317	11.882808	10.630554	11.346523	11.212902	11.184811	10.725060	10.851497	10.273634	11.541101	11.888108	10.788704	10.691964	11.617427	10.948319	11.676671	10.328234	9.855744	9.869000	10.928007	11.029412	11.623999	11.722134	11.706718	12.024501	9.737007	10.766265	10.536920	11.149440	11.331187	11.132510	10.636673	10.200957	10.100633	9.567473	11.117105	10.422706	11.934741	10.630981	8.539743	10.547178	10.252193	8.137701	11.021084	10.680952	9.915602

Z97192.1	9.498874	11.206137	11.070628	10.852403	11.159921	11.259576	10.542131	12.198897	11.566982	11.414710	11.082761	10.378811	10.181623	10.803584	9.621679	12.070082	11.238772	10.986432	11.253964	11.466592	11.162605	11.416927	10.507824	11.318521	11.312627	10.635496	11.122951	11.462482	11.289771	10.883094	10.423614	9.485652	9.471395	10.277741	10.134533	10.130935	9.936051	10.801045	10.670580	11.065422	11.916981	10.665912	10.155626	10.391357	9.711397	10.319057	11.977304	10.234334	10.238782	10.776077	9.522255	10.159823	9.418110	9.817412	9.373989	11.188301	9.663731	9.687929	10.335694	9.416708	9.626458	11.217158	9.744273	10.718205	9.548845	9.585450	11.033609	11.322294	10.278971	10.864090	11.802954	11.637826	11.905178	10.861160	11.178065	10.860851	11.857195	10.934280	11.232861	9.207170	9.778003	9.331538	8.872006	9.594008	9.612878	9.843788	9.903641	9.510701	10.217279	10.034852	9.674605	10.559179	10.694662	11.174150	9.598476	10.086189	10.062277	9.824324	9.960750	10.592327	10.352051	10.917489	11.131082	11.048552	10.106859	11.686785	10.918816	11.174295	10.783006	12.027347	11.062006	10.591268	11.333064	12.228964	10.770741	11.756331	10.950866	10.448243	11.819746	9.827819	10.318904	9.774484	10.553536	11.527756	10.708724	9.975902	12.887380	10.965488	12.066401	10.798335	10.135620	10.807365	9.914854	11.033295	10.775210	10.275187	10.947592	11.219233	11.305008	10.638329	10.475871	10.418738	10.628696	11.004248	9.958930	11.849732	10.937294	11.960844	10.933681	10.341850	9.556934	9.923681	10.448243	10.011086	10.398977	9.374798	10.629019	10.337340	11.085135	11.615402	10.790693	11.582720	10.096550	9.782986	9.979491	11.507607	11.238007	10.723285	11.482100	9.448726	10.821164	9.218710	10.192202	8.859884	10.119603	9.400733	9.105050	10.507361	9.141819	9.462714	11.205984	10.105095	10.081486	9.665614	9.114355	10.327627	10.920907	9.690836	10.384236	11.260318	10.612294	11.744952	9.974513	10.159386	10.722360	10.820704	10.613036	11.210101	8.833056	9.619626	9.852612	9.071115	9.549938	9.313619	10.516981	9.964114	9.178174	9.876562	9.326903	9.410193	10.896568	10.027644	10.227300	9.737303	9.761509	10.964294	10.058151	9.851968	9.731680	10.032414	12.570358	10.263444	10.219377	9.944691	11.143394	10.749317	10.806779	10.291284	11.492440	10.926240	10.125390	11.946535	12.255523	10.392827	10.383819	11.382463	10.685116	11.026742

PARD6G-AS1	6.792176	8.105589	8.656538	8.309468	8.514437	8.794153	8.255721	8.209622	8.152212	8.183583	7.645097	7.830357	7.906479	7.783157	7.428055	8.404680	7.386840	7.861363	8.337740	8.624652	8.066831	8.419689	7.964950	7.893570	7.873058	7.596263	7.199772	7.648612	8.724336	8.635194	8.193679	6.896050	7.182036	7.406010	7.130033	7.177471	7.341605	7.026662	7.838627	7.160590	8.645720	7.345981	7.422754	7.289877	7.643684	6.951703	8.160532	7.488806	7.062687	7.933559	7.095312	7.263271	8.006102	8.213942	7.462608	9.159233	7.414000	8.511237	7.500092	7.622248	7.734887	8.163962	7.533908	7.895952	7.397708	7.882612	8.548297	8.184337	8.204629	7.873982	8.344769	8.439347	8.402370	8.202269	7.878989	7.632540	8.023465	7.728256	7.788507	7.648283	7.736955	7.644929	7.735196	8.149015	7.792804	7.554088	7.821662	7.776496	7.796780	7.708553	7.587332	7.235310	7.873205	7.936532	7.270940	7.574494	6.968204	7.299081	7.639389	7.922567	7.429167	7.574330	7.943235	8.247398	7.758398	8.026527	7.545191	8.175070	8.632606	8.157042	7.543733	7.691024	7.690392	8.079308	8.019077	9.170158	7.341765	7.722369	8.097820	6.672354	8.427236	8.070314	7.977373	8.942629	9.007324	8.684477	7.503538	7.410915	8.479979	7.917227	7.480582	7.940255	7.932184	7.242261	7.592260	7.003210	8.027463	8.522603	8.477734	8.299261	9.145633	7.728876	8.348987	7.974303	7.643210	7.456502	8.604528	8.519918	8.942008	7.026811	6.560227	7.190316	8.947669	7.164101	7.224035	7.025864	8.202413	7.958836	6.953594	9.018670	7.110988	6.557284	7.014228	7.944941	6.805534	8.917548	9.576986	7.694590	7.771110	7.240719	6.492653	8.157641	7.888733	7.023420	9.006123	8.546912	8.930788	7.089929	7.664047	7.497555	7.873506	8.067722	7.487887	7.255549	7.464195	8.841178	8.492264	8.006998	7.068891	8.418259	8.684928	8.954868	8.284855	7.099087	7.827616	8.052786	8.031021	8.671249	7.340356	8.181140	7.690876	8.157342	7.175395	7.705159	7.455252	8.548453	9.579000	7.314004	7.002445	7.954949	7.250091	7.849468	6.820232	7.808333	7.636629	7.787467	6.999064	7.473850	7.655962	6.978010	8.353066	7.216228	7.918883	6.549699	7.617231	7.167160	7.827449	8.259246	8.239474	7.119181	7.126261	8.887600	8.585918	8.337120	8.224937	7.982540	8.246938	8.052014

LINC01299	7.550059	5.795999	5.661986	6.050288	7.506195	7.507394	7.671486	7.406634	7.715526	5.900251	6.510415	7.181248	6.144448	5.844038	6.064179	6.612157	6.570194	6.359697	4.638413	6.145930	5.676561	5.667601	7.703743	5.310882	5.934753	5.930243	7.047064	6.211683	6.367558	6.623405	7.607888	6.381648	6.160658	7.217816	6.188764	6.105843	6.396792	5.544106	5.733207	5.225089	6.423337	5.565213	5.352484	5.033991	5.678263	6.321167	7.090101	5.451257	6.851706	5.824601	5.541648	6.139091	5.390063	6.145662	5.826559	6.990471	6.076077	4.110047	5.249386	5.450296	5.561665	6.492192	4.863721	5.216385	5.496608	6.255895	5.473683	5.525554	5.395166	6.014010	5.747033	6.778153	6.201557	6.002372	6.463376	5.516256	6.305257	6.299881	6.417644	5.611223	5.707018	5.827845	5.714385	5.814756	5.888505	7.025223	7.002726	5.998849	5.587352	5.605125	5.293129	5.423011	5.124112	6.831169	7.711380	6.644174	6.463039	7.243901	7.637243	6.774130	8.168133	5.650854	5.933150	6.848878	5.659751	6.449333	6.188929	6.663285	6.250803	6.412181	5.268667	6.543039	7.185042	7.483685	6.284973	10.348720	6.177655	6.659365	8.078697	6.925494	5.716929	5.682541	6.034196	8.701068	6.946970	8.803984	8.362692	9.398779	6.566103	6.350795	6.533725	7.048173	5.842614	5.071631	8.680959	6.670097	6.316408	4.619091	5.495472	6.177655	5.057568	6.418325	5.860683	5.820260	5.896885	7.134361	6.841126	7.249175	6.408201	8.131342	6.905576	6.595180	6.987008	6.546804	6.272050	6.383651	5.731757	5.404268	4.764632	7.965718	5.660509	5.653503	4.639899	5.041365	6.374349	7.492437	4.702139	6.250316	6.226399	3.594204	5.987381	3.661019	5.483684	5.958962	6.922419	6.504643	4.027084	4.540679	4.886247	5.216385	7.351613	4.918512	4.641333	5.549967	5.502762	4.236770	4.891068	5.399206	5.050186	6.076760	6.541364	5.982957	6.040358	7.036868	5.561665	6.416338	6.607057	6.316559	6.236490	5.356278	5.352869	5.719936	5.803115	6.059216	7.557235	6.332819	5.874564	4.592901	4.847648	4.247745	5.608956	5.696656	5.888681	6.851233	7.097357	6.424977	8.026826	6.419483	6.254868	7.542477	6.419326	6.797851	7.132761	5.186207	6.700808	6.998767	6.404740	5.780525	6.310954	5.118253	6.686616	7.409279	8.295020	7.391321	9.003955	6.107820	7.585179	7.663558

RHOXF1-AS1	4.671443	5.097372	5.689869	5.436282	4.806374	4.603341	5.779623	5.704486	4.765468	5.763933	5.630433	5.843332	5.792044	4.115928	4.654865	6.257421	5.578727	4.941966	4.794167	5.190131	4.115070	5.657575	6.610455	4.595957	5.965720	4.944987	5.644055	5.504117	5.764306	5.460645	5.178363	5.512009	6.271536	5.237665	5.922559	5.575579	5.716576	5.705254	5.019124	5.432797	5.902563	5.355688	5.355303	6.312121	4.633497	6.042449	4.790807	4.778032	5.901849	6.171677	6.038250	5.913863	6.319104	4.927846	5.111719	5.426219	4.924079	4.790807	5.497164	5.442939	6.415011	5.092275	5.216032	5.441987	5.701366	5.807238	5.982957	5.963234	5.620566	5.727185	6.198588	5.829701	6.090105	5.806670	5.778189	5.718329	5.861398	5.757033	5.062667	5.469895	4.938930	5.721042	5.826376	6.205198	6.107290	5.622231	5.346523	4.573081	4.481034	4.926387	4.527996	4.759341	5.233026	5.605914	5.995187	6.100310	5.781067	6.675596	5.454938	4.753194	4.366579	5.476909	6.154825	5.114355	5.848448	5.231197	6.220415	5.646889	5.250002	5.693412	5.402645	5.742830	6.111791	5.949673	5.095617	3.557381	5.841158	4.444999	4.900277	5.811871	5.772380	5.471006	6.007165	4.489115	6.014686	4.166251	5.916177	5.381734	4.499005	5.304038	5.025466	4.295235	4.210992	4.405372	5.985562	6.064179	5.017503	5.588697	8.184054	5.369314	6.072060	6.179672	4.821475	5.149703	5.187816	5.017503	5.396530	5.207639	5.679386	5.021154	5.193853	6.474078	5.747563	6.032173	4.978070	5.421462	5.003178	5.160478	5.301032	5.707393	5.700066	5.717290	6.839336	5.195497	5.969657	5.471992	6.265389	5.595130	6.218892	6.172188	5.736114	6.001794	5.377702	5.103791	4.980930	6.635321	6.093560	5.756267	6.220581	5.819906	5.354909	6.119227	6.449656	6.247611	5.969473	6.064688	6.100310	5.988091	6.206431	5.779986	6.047108	5.971415	5.821481	5.855085	5.749191	5.608542	6.043307	5.247007	5.297648	4.410703	5.703743	5.956256	5.316667	5.798779	4.262444	5.654829	4.735884	4.577767	4.978070	5.313012	5.348318	5.517999	6.058347	5.571063	5.424256	5.211625	5.089598	5.097372	5.058586	4.429095	5.204082	4.940938	4.708848	5.340097	5.153573	6.416166	5.431041	6.333488	5.161065	4.921033	4.970105	5.214830	5.838236	5.103183	3.523264	6.038961	5.472912	5.771240

AC098868.1	3.334035	3.725577	3.045780	2.979449	2.870220	3.055476	3.094537	3.125011	3.244280	3.111293	3.213675	3.072887	3.162507	3.539784	3.519870	3.289211	3.271149	3.131810	4.202047	3.008075	4.129176	2.937218	6.669632	3.006045	3.017610	3.064705	2.912944	2.847893	2.983258	3.104462	4.582882	4.986428	3.648603	4.847873	3.183074	3.835027	3.729032	3.642881	3.256050	3.181767	5.283182	3.054765	3.399012	3.175876	4.073227	6.623712	3.621076	3.014839	5.046863	4.351778	3.148035	3.187273	4.531195	3.963386	4.443074	3.078861	4.091852	3.092914	3.266904	3.695073	2.955892	3.534249	3.600306	3.118879	3.270087	3.688091	3.002331	3.551811	3.336602	2.957247	3.333219	3.527928	3.125532	3.440673	3.533103	4.036699	3.394523	3.170383	3.353367	3.986286	3.402485	3.742824	3.904201	5.257333	3.277250	4.775112	4.326994	3.821651	3.656217	3.683833	3.472743	3.253277	3.788048	3.284599	4.007615	3.902722	3.514792	3.152463	3.260291	3.158396	3.290950	3.134769	2.956161	2.805927	4.415349	3.394875	2.909480	3.254178	3.056861	3.118062	3.321058	3.218685	2.843334	4.495328	3.149759	4.188883	2.864111	2.960421	3.044421	5.201083	3.273291	2.939501	3.021347	3.086146	3.460735	3.275282	3.054547	3.124783	2.968632	5.208841	2.936439	3.196929	3.100934	3.231565	6.426975	3.150130	2.967652	3.273291	3.063293	3.655630	2.879470	7.005199	2.928074	2.920694	2.835017	3.154050	2.820479	3.278696	3.349703	4.213405	3.167149	3.606405	3.342475	3.109333	3.583962	3.748119	3.007067	3.242391	3.074008	4.428669	3.005736	3.474511	3.213948	4.031479	6.657433	3.332238	2.928747	4.641333	3.140052	3.049223	3.035174	3.664050	4.161599	3.398178	3.334035	3.725775	3.059176	3.017878	4.092586	2.959101	3.659231	3.637482	3.330807	4.576446	3.598578	4.602914	3.504165	3.309363	2.897338	3.330982	2.968632	3.343813	3.333065	3.114979	4.253225	3.510886	3.753157	3.021968	3.375633	4.560560	3.982847	3.482596	3.529405	3.456073	3.405895	5.239264	3.409194	3.399199	3.297612	3.334035	3.247085	4.578828	3.150262	3.093488	3.200147	2.921832	3.162886	3.603220	3.372595	2.965952	2.976586	2.930628	3.014268	4.803917	3.101390	2.906924	3.888276	3.174870	3.214200	3.350383	3.386361	2.823537	4.110948	2.999669	3.150262	2.856904	4.364149	4.855023

AC021074.3	2.988516	3.595556	2.958437	2.981735	3.094313	3.067732	3.115578	3.143041	3.214341	4.463269	3.328221	3.288352	3.177227	3.670112	3.410970	3.116393	3.261151	3.184361	3.243262	3.069827	4.369123	3.682453	8.321274	3.012317	2.984327	2.849998	3.045148	2.867173	3.246386	3.029761	3.652626	5.536065	3.294849	2.992100	3.243262	3.835677	3.517346	3.071765	3.254178	4.072539	3.393310	3.115327	3.312397	3.112983	3.566286	5.107057	4.554340	3.197164	4.173385	3.289685	3.283173	3.384000	3.873072	3.898920	3.608025	3.059943	3.998940	3.074874	3.040188	3.584949	2.986372	3.085408	3.468209	3.070452	3.250569	3.370455	2.913243	3.225437	3.199768	2.933891	2.944727	3.338122	3.155467	3.304129	2.915428	3.232167	2.975518	3.263834	3.006984	3.448612	3.201300	3.727139	3.320901	4.135859	3.226440	4.674273	3.221235	3.252236	3.196929	3.209388	3.188193	3.181086	3.499802	3.068050	2.908676	2.916383	3.577965	3.175677	3.311611	2.929202	3.131810	3.115819	2.953300	2.815060	3.469619	3.444014	4.609495	3.303802	3.161356	3.148640	3.213260	2.793161	2.808409	3.234953	3.176261	3.024084	2.891462	3.046710	3.194035	2.991703	3.244699	2.921905	3.017496	3.016806	3.462494	3.045148	3.071974	3.125011	2.990536	3.096798	2.994298	3.135127	3.145815	3.154285	2.949012	3.173311	2.986843	3.012591	3.139568	3.824361	3.124552	6.952175	2.935072	2.902673	2.815765	2.991208	2.838503	3.049852	3.382935	3.441000	3.890951	3.364994	2.952386	3.119247	3.588616	3.465570	3.019944	3.195567	3.017930	3.148164	3.084646	3.420255	3.129913	3.891965	4.749312	4.394772	3.043520	4.079378	3.157958	3.213397	3.186882	3.727139	4.056715	3.343813	3.653615	3.670739	3.105858	2.936817	3.456418	2.900991	3.189408	3.456418	3.188332	3.350047	3.500497	2.912468	3.501981	3.350741	2.934535	3.137740	3.112756	3.118879	3.152463	2.892692	3.201577	3.031257	3.105287	2.973807	3.103641	3.503802	4.007174	3.363362	3.580146	3.464678	3.542686	3.309058	3.377896	3.054163	3.177227	3.317617	3.156053	3.283331	3.113899	3.028768	2.911012	2.920528	2.972326	3.512749	3.142315	2.951849	2.972991	2.886842	3.088637	3.793394	3.115109	2.974981	3.237122	3.295582	3.087059	3.266751	4.273323	2.782823	3.136611	3.004431	2.869807	2.891462	3.053243	3.256478

AC005746.2	12.030634	12.127514	12.085752	12.186508	12.045726	11.655606	12.177029	12.007161	11.394579	11.948032	12.262785	12.101029	12.396312	12.651910	12.632134	11.851418	12.142518	12.196161	12.928312	12.228152	12.870929	13.139689	13.151653	12.190447	12.368451	12.059052	11.899125	12.240224	11.681278	11.725122	12.035653	12.461907	12.581982	12.406200	12.327798	11.920823	12.278662	12.301868	13.036157	12.621510	11.841449	13.292351	12.248989	12.438343	12.406816	12.389516	11.909709	12.682475	12.224619	12.537757	12.489908	12.554292	12.671548	12.817345	12.475368	11.860241	12.210507	13.108876	13.375462	12.974569	13.531718	11.488318	13.172095	13.348067	13.111379	13.069852	12.926298	13.044091	12.772756	13.160892	12.472957	12.193053	11.881320	12.340875	12.510794	12.768875	12.393464	12.633304	12.516134	12.607315	12.250724	12.439820	12.246874	12.490240	12.252154	12.428658	12.760647	12.731336	13.413997	13.113355	13.024023	13.434297	12.886514	12.137705	12.451069	12.025251	12.125254	12.126428	12.615979	12.150924	12.147660	12.183450	11.836176	12.561046	12.732358	11.941813	12.243629	13.120033	12.682302	11.682920	13.305150	12.769602	12.360207	11.288746	12.234041	10.729132	12.746742	11.976069	11.673716	12.318871	10.948982	12.063646	12.224317	11.562614	12.104382	11.455967	11.778520	12.240396	12.494832	11.210844	13.015726	12.674493	12.708589	11.733316	11.939528	12.669662	13.196931	13.162064	11.922034	13.557270	13.493480	13.421932	12.462229	13.137935	12.736450	11.348194	12.244688	12.040452	12.089045	12.233736	11.960220	12.843348	11.947588	12.286447	12.883111	12.221092	11.543325	12.785412	13.105424	12.139103	13.213195	13.464002	12.196906	12.531515	12.369546	10.772100	13.295984	12.156344	11.973443	11.824884	12.548071	11.703111	11.797346	11.507746	12.798496	13.307696	12.933477	12.756522	12.680421	12.077128	12.745728	13.446068	12.895455	13.242782	12.737801	12.534620	12.709993	12.333192	13.053301	12.340685	12.504387	10.964294	12.373685	12.706881	12.233142	11.928470	11.882981	12.707907	12.553295	11.925662	11.794841	12.117118	12.078970	11.854889	12.554619	12.253114	12.539646	12.971153	12.275032	12.781558	13.045554	13.109074	11.955287	11.761931	14.097093	12.468601	11.952744	11.981504	12.917665	11.802080	12.494508	12.768610	12.141761	13.325998	11.294819	11.547236	12.393629	13.417523	11.127669	13.287052	12.687955	12.043199	11.528321	11.807531	11.620849	13.228263	12.151563	12.017489

OIP5-AS1	13.580621	13.571674	14.049746	14.106283	14.152378	13.893395	13.951850	13.701861	13.772698	14.005012	14.099512	13.821463	14.185875	13.953485	13.311915	13.711946	13.869352	13.744601	13.048590	13.932970	13.459378	13.050819	12.851230	13.869642	13.410994	14.238833	13.940443	13.406964	13.677133	13.634745	12.905897	13.933523	13.693359	14.048218	14.384169	13.869088	14.384169	13.995849	13.767473	13.862844	13.866741	13.727608	13.851451	14.260288	14.477449	14.168090	13.650975	12.738673	13.653439	14.271565	13.819912	14.407674	14.544632	13.995849	13.477273	14.010352	14.411336	14.172567	14.464001	13.834843	13.653197	13.692412	12.809121	13.270823	13.185843	13.794844	13.389060	13.544622	14.063237	12.885092	14.118279	14.330345	13.869642	14.328965	14.174543	14.146475	14.371589	14.193699	13.937151	14.226329	13.229868	14.189262	14.292726	14.091572	14.543837	14.372657	14.536366	14.620267	13.199903	13.876596	13.580155	13.354977	13.330272	14.102234	13.852424	14.145530	13.852712	14.212093	14.346364	14.101917	13.832307	13.709428	14.009020	14.243388	12.495001	14.103780	13.686701	13.314147	12.969511	13.782568	12.674576	14.192430	13.761409	13.693813	13.411645	14.086854	13.329024	14.142398	13.766016	13.402704	13.229238	12.826659	12.840899	13.314543	13.371030	13.150661	14.058874	14.241406	13.125419	13.602656	13.563376	13.721765	12.675008	12.198594	14.330677	13.556609	13.548009	12.173896	13.742334	12.051938	12.705875	12.408357	13.202193	12.634450	13.134908	13.060802	13.703898	13.674334	13.408793	13.055794	13.093911	12.579166	13.104248	13.273969	14.405515	13.779166	13.541684	12.901113	13.462926	13.289639	12.941369	14.233539	14.192430	13.713659	13.459822	13.183505	12.437743	14.042006	13.987492	13.661499	13.384142	13.239764	14.060320	13.616838	13.469650	13.927324	13.144764	12.607714	14.192726	12.907332	13.113147	12.838923	12.629849	13.174008	13.307696	13.197314	12.282721	12.390623	12.237565	13.677795	13.439894	13.783328	13.940978	13.862844	13.535095	13.720180	13.596345	14.095514	13.941517	12.903734	12.850884	12.946630	13.827679	13.132988	13.156161	13.667672	13.487724	12.579663	12.742681	13.033913	12.396485	13.055429	12.930094	14.043175	13.315612	13.862600	14.039072	13.134908	13.648695	13.478168	14.389287	13.640816	13.360372	11.978826	13.285244	13.355364	13.450541	13.081212	14.006448	12.620818	13.570340	13.823712	13.553691	14.120707	13.237061	13.610599	13.386677	12.116671

AP005233.1	6.437583	5.139678	4.729562	5.687731	6.730784	6.605904	6.166510	5.552381	6.277988	6.093732	6.045357	7.076052	5.788486	5.723813	5.941446	6.041080	5.996352	5.744988	6.358161	6.008419	5.479634	6.781998	6.280716	5.912957	6.046222	5.869547	6.993072	6.651927	6.880224	7.562721	5.954174	5.802411	6.859119	7.271258	5.846583	5.570130	5.033574	4.798980	5.240060	6.057995	7.295699	5.625960	5.995500	5.232404	4.885177	5.847321	7.450282	5.040958	6.730471	4.221682	4.577568	3.480575	4.819179	4.878825	5.183414	6.453304	4.751098	4.950416	4.951226	5.231998	4.598956	6.307087	4.486980	5.958962	5.693030	4.421506	4.640113	4.935976	4.928648	4.741069	5.692082	5.246802	6.292836	5.394196	5.581896	6.210017	4.736547	5.085066	5.233840	5.358153	4.459459	4.163148	4.824132	4.792691	4.330775	5.620917	4.512247	4.664293	4.924879	4.249492	5.167290	5.214626	5.184212	5.290806	6.195488	5.931595	5.700818	6.667856	6.897209	5.911679	6.936495	5.609543	5.485210	5.009920	5.878510	6.546804	6.331457	6.401610	6.464703	6.249133	5.963956	6.487895	6.557781	6.982716	6.438911	9.202653	5.954546	6.117316	6.307403	6.774295	5.322477	5.377508	6.104959	9.330748	6.846929	6.964223	6.228480	5.946038	6.746121	6.604700	7.355649	6.314368	7.226540	6.764101	6.196190	6.018512	6.063123	6.366226	6.188243	6.242269	6.760754	6.524017	5.947042	5.953623	5.510681	6.302728	6.853000	7.789934	7.360750	5.853816	6.677686	7.161578	6.855890	5.479850	5.784491	5.394196	6.048206	5.932445	5.622037	7.980968	5.786653	5.333050	3.591699	4.698939	5.388935	7.644012	6.174379	6.848381	3.851185	3.667710	5.501991	4.421274	5.275956	5.961628	6.413534	5.936696	4.931772	5.545078	4.271585	4.355913	6.511783	4.382733	5.531818	5.049157	4.971704	4.415978	4.719803	5.027874	5.973624	6.029076	5.285388	6.407708	5.727541	6.086241	5.989536	5.161272	4.989560	5.107256	6.712952	5.066749	4.686970	5.015854	5.659956	4.546452	6.775273	5.107256	4.081133	5.853071	4.393198	4.974640	5.477301	5.732857	5.143372	6.071333	6.349083	7.636336	6.791693	5.880328	5.463454	6.777993	6.341049	5.813892	6.248977	6.004467	7.405064	7.992551	6.531910	5.929013	7.004570	6.305094	9.044421	10.202804	7.805872	6.686786	9.578531	5.654037	5.600604	5.965532

AC114296.1	5.409725	6.288089	6.131199	6.798807	7.605952	7.709416	7.717665	5.349077	7.230410	7.411494	8.317902	7.666067	7.865882	7.355953	8.044341	7.372685	6.553821	7.564735	7.481663	7.574967	6.167552	6.324566	5.562964	6.610796	7.466956	7.485213	5.860480	6.620661	8.257083	7.274066	7.959450	6.080235	6.299387	6.705732	7.168260	6.487753	6.276841	6.433787	7.099873	6.025527	7.816055	6.325254	6.213743	6.228660	6.165625	5.169895	7.170697	6.588358	7.127683	7.029566	5.389689	6.208471	7.576179	7.241803	6.382496	7.747014	6.610125	6.492034	7.688703	6.240938	6.522172	7.833239	4.683975	6.567229	7.268030	7.301775	6.137868	5.981355	5.807043	5.513333	6.853796	6.619366	8.318352	6.632032	6.846482	6.416995	5.852014	6.051295	5.984867	6.752087	5.757389	7.518492	6.529826	6.315909	6.164262	6.743344	6.672024	5.621097	7.372060	7.423039	6.003613	5.900808	5.657391	6.267597	5.697116	6.786777	6.925341	7.850049	7.362910	7.071494	7.644170	7.010274	6.951067	6.473421	5.620195	7.756185	6.738763	6.768300	6.970435	7.847801	5.691927	7.089448	5.697116	7.687007	7.735493	7.503220	5.066749	7.298474	7.844523	5.862898	5.869213	6.097691	6.380125	7.289259	8.724336	7.499939	6.634691	6.550026	7.298304	7.595034	7.620747	7.662002	6.469202	6.085431	6.339386	6.814751	6.608020	6.242601	6.762969	6.959726	5.744281	6.801722	6.945974	9.439867	5.903831	6.692759	8.352610	7.731957	6.282271	6.097197	5.727373	7.726063	6.283128	6.785647	6.010016	7.170697	5.456085	6.373651	6.314012	7.543116	6.321882	6.716203	5.726450	6.942471	4.820024	7.632540	5.817445	7.528881	6.334497	6.489666	6.123379	6.324053	6.053381	5.869021	6.503995	4.054293	6.972602	6.383495	6.113851	4.085922	7.471118	5.081663	5.364627	5.912042	4.997962	6.859440	6.405727	5.390655	5.052790	6.876549	6.284639	8.411708	6.867559	5.274315	6.395639	7.071013	5.306198	6.974953	7.964169	6.030587	7.709559	8.117928	6.488220	5.591589	6.576349	6.662005	5.946702	6.400484	5.390655	8.569732	5.375957	6.101332	6.007165	6.542552	6.476422	7.509115	7.981127	7.052414	6.892352	6.699341	6.881513	7.478735	5.870621	4.601657	7.603908	6.392873	5.884247	7.804477	7.891543	8.448582	5.844038	7.441933	7.866197	7.711380	6.977855	9.027513	8.005145	9.169378

AC090015.1	5.212182	5.116800	4.892077	5.900068	5.865382	6.332154	6.215597	5.759794	6.295088	6.916190	6.079207	6.366543	6.604049	6.619878	5.641209	6.361202	5.512948	6.142569	5.801382	5.766650	4.542873	6.647877	6.310111	6.133795	5.423414	6.016557	6.101011	5.675497	6.479464	6.176949	6.998024	5.400711	4.827193	6.294903	5.586787	5.258688	5.661228	4.757595	5.957866	5.871338	6.818111	4.846579	6.122868	4.913698	6.128820	5.538833	7.134838	4.871701	6.605446	6.004648	5.027227	5.940958	4.350022	6.333836	6.006620	6.336018	5.943900	4.590783	5.593460	5.043914	5.175557	6.783375	5.652022	6.003768	4.647255	5.039101	3.904662	4.691774	5.374929	5.069087	5.110560	5.438019	6.685661	4.949812	5.363098	5.019510	4.928464	4.883487	5.114185	5.344374	5.250184	4.811255	4.799860	6.189910	6.106203	5.556382	6.299534	7.062687	4.452895	4.345054	3.982401	6.164933	5.221908	6.031113	6.308579	6.062086	4.555570	7.024421	7.049113	5.879425	7.015163	5.639555	5.538246	5.313196	6.124083	5.823304	6.241930	6.573722	6.148479	6.696813	5.569758	6.813657	6.889664	6.739082	5.471801	6.821344	5.989180	6.135515	6.696813	6.278646	4.323583	4.916860	6.208291	6.892517	6.460672	6.468342	5.684924	6.128005	6.637869	5.533321	7.007495	6.067163	6.314012	4.690116	5.815457	5.472341	6.401461	6.560227	4.353117	5.961292	6.866291	6.676570	5.156441	4.517644	6.487433	6.135834	5.635267	6.892837	5.567473	6.236490	6.361202	5.528031	6.397786	5.196495	5.361935	4.886886	3.717910	4.098630	4.871701	5.810937	3.522057	5.606848	3.556975	5.580595	4.888552	7.166708	5.489345	6.107118	4.501617	4.519767	6.163918	5.449926	4.912191	8.066401	5.404062	5.384460	4.514410	6.595180	4.703861	3.829515	6.528989	4.479959	4.842115	4.944576	5.024042	3.678427	4.598327	4.414205	4.727660	4.864751	5.458276	7.234377	4.588197	4.562063	4.756537	4.488884	4.732932	4.944783	4.665178	4.306150	4.516566	4.876299	5.406483	5.140077	5.582847	4.741289	4.659428	4.850976	4.463269	6.886446	4.680730	4.683330	4.510054	5.244418	5.644448	6.278646	7.237496	5.929876	4.924665	6.208993	5.267268	7.010274	5.453987	4.976495	6.492192	3.999802	6.614789	5.204460	6.774295	5.717116	6.358855	5.985749	6.535017	7.311340	6.603417	5.551244	6.039284	6.024120

ATP6V0E2-AS1	7.179851	6.603417	6.846321	7.077031	7.214504	7.563944	7.338924	7.424453	7.875198	7.700231	7.556767	7.357154	7.127683	7.428825	6.255895	8.084927	7.172144	6.596333	6.648533	7.555653	6.853335	7.595034	6.699341	7.256941	7.525891	7.466791	7.993460	7.326424	7.958691	8.261975	8.012742	6.190787	6.296264	7.199937	7.130525	6.483410	6.438911	6.397966	7.145355	7.481057	7.570962	7.099873	6.382153	6.611684	6.244951	5.860307	8.099606	6.194799	7.192436	6.474078	5.899713	5.658509	5.969983	5.712490	5.391260	8.216812	5.389874	5.667420	6.166312	5.698593	6.321505	8.196996	6.441696	6.664928	5.975338	5.681788	6.284973	6.152579	6.261843	6.686616	7.044339	7.022815	8.387874	6.938118	6.676256	7.412132	7.188396	6.860578	6.681577	5.462213	6.123195	5.951091	6.067163	5.980467	6.115466	6.059216	5.525913	6.188764	6.350795	6.213566	6.169288	6.381984	6.559257	7.039538	6.881346	7.391321	6.048392	7.320440	7.400837	6.915418	6.513895	6.439561	6.851706	6.480733	6.975438	7.665421	7.856797	7.117559	7.136114	8.448740	6.167031	7.268335	6.995829	8.478049	7.105801	7.661530	7.485700	7.469241	8.504326	7.330879	6.965640	7.022655	7.763738	8.050019	8.029783	7.652204	8.098548	7.672577	8.246658	7.622838	7.963443	7.649509	7.225135	6.927973	8.283450	7.507556	7.067906	8.156082	7.410915	7.102355	7.751051	6.807413	7.827891	7.761283	7.092916	7.500852	7.524066	8.836181	8.321274	8.103403	6.876549	6.535017	6.822304	7.247314	6.760608	7.027807	6.840955	7.528278	7.361058	8.108499	6.766516	6.587705	6.871784	6.436133	6.177655	8.760109	6.955501	7.111460	6.897032	6.615109	6.674294	6.090612	5.863994	5.927772	7.927415	5.714759	6.576684	6.156680	5.761196	6.017427	8.256475	6.509428	6.535162	6.214225	6.370223	7.033904	6.707704	6.803991	6.730471	7.181722	7.270312	8.545522	7.363410	6.746568	7.260754	7.707128	7.270794	7.033904	6.069808	6.261678	6.284143	6.044295	6.329072	5.945313	6.694724	5.901504	6.235988	6.724651	5.993944	7.606249	6.414193	6.561036	7.245475	7.089781	7.351457	6.465215	7.398827	6.627260	6.887904	7.120888	6.961817	7.174638	6.212680	6.526670	8.414146	8.037595	6.545484	7.278613	8.772484	5.934593	7.199296	6.754070	9.050550	8.197159	7.325643	7.086749	7.825370	8.156419

AC015853.3	6.254711	5.953430	6.249294	6.204695	7.342853	7.522372	6.296097	6.735475	8.324774	6.388514	7.063971	6.572093	6.514380	6.339386	7.030340	6.951703	7.047385	7.087505	7.094820	6.854459	6.841294	7.484272	7.082071	6.364215	6.909077	6.997424	7.224803	6.976396	6.585224	6.150337	7.528744	5.860307	6.291686	6.238775	6.016224	5.775473	5.630622	5.935986	6.514869	6.255368	6.604561	6.536469	6.590464	5.362905	7.073059	6.595678	8.051400	4.540468	6.009128	6.271869	6.542050	6.290806	6.573209	4.817096	6.961011	6.616593	6.315909	6.677849	5.888319	6.293693	6.775740	6.732713	6.547258	6.679465	5.941446	6.094274	5.891684	6.930002	5.534285	6.265874	5.613082	6.240584	7.187785	6.001274	5.727904	6.306280	5.616498	6.054797	5.647837	6.386127	6.673180	6.899330	6.001621	6.093031	5.886377	6.207422	6.312471	6.220905	6.164933	5.896007	5.780525	6.337877	6.199995	5.973067	6.485942	6.954382	5.767564	7.872292	6.701426	7.590734	7.564418	6.354258	7.539270	6.919713	6.948600	7.990014	7.466791	6.814751	7.277975	6.904150	7.711088	7.524066	7.540966	6.559257	7.989271	5.899553	6.855890	6.999669	7.189847	6.533725	7.044339	6.067343	6.214759	7.414000	7.322163	6.015023	7.173677	6.955026	6.039824	6.697122	6.427678	6.686295	6.055991	6.950916	6.580113	7.050375	7.050084	6.581133	6.414516	6.709596	7.306346	6.643191	7.345155	5.857438	7.435851	6.951067	7.168420	6.441696	6.211030	7.130688	7.707451	7.421041	6.887409	6.594222	6.306114	6.322380	5.958405	6.608840	6.497516	7.827891	5.934593	6.346891	7.080815	6.994765	6.599750	8.752751	5.561471	5.681611	6.317575	6.945502	6.402270	6.680133	6.259130	6.026440	6.383146	5.695665	6.989049	6.168779	5.729176	6.674449	6.415636	6.671556	6.539864	6.415636	6.178996	6.153116	5.924919	5.662352	6.241930	5.608760	6.231727	6.905252	6.448008	5.704486	6.597629	5.833722	5.946541	6.356807	6.566103	6.942312	6.272853	7.377860	6.952820	7.208630	6.604224	6.808714	7.076870	6.295088	6.717651	6.158049	6.640640	6.667699	6.455878	6.893364	6.832787	6.778314	6.874639	6.912595	7.156070	7.321353	7.558311	6.615435	5.914377	6.389695	7.030045	8.274471	6.877972	7.006419	7.083939	7.311816	6.454774	5.245413	6.340567	5.733911	6.440869	6.376474	7.869975	6.576349

LINC01551	4.594487	4.934289	4.873439	4.572211	5.535095	6.123031	5.044934	4.686349	4.904429	5.598592	5.614981	5.418942	5.446840	4.937479	4.383610	4.634359	4.888117	4.144750	4.004337	4.962733	7.383462	4.641133	4.520391	4.677961	4.305947	5.212965	5.560034	3.313017	6.180324	6.208832	4.228532	5.024248	4.569356	4.993534	5.654829	5.514823	5.510880	4.597449	4.678808	5.286219	4.634571	4.910284	5.191503	3.882548	5.520438	5.715491	5.937556	3.216664	4.895790	4.301736	4.325747	3.732140	4.402233	5.397646	4.377797	6.315570	4.128728	3.637058	3.514967	4.406247	3.169872	5.976730	6.955501	4.185988	3.954599	4.004337	4.768671	4.812278	4.792691	4.844670	5.242044	5.189746	4.360255	4.628485	4.751297	4.486379	4.918735	4.864141	4.438286	4.028365	4.099547	5.192286	3.283331	4.530110	3.128450	3.644045	3.899129	4.196467	5.410127	5.138445	4.533920	4.594913	4.888552	5.578314	5.129196	4.962733	4.011238	5.213361	6.499409	3.530715	6.600383	5.324222	6.028906	4.610996	5.008684	5.201664	3.269270	4.397017	5.053637	5.743183	4.389969	5.674028	5.388347	5.864148	5.643883	8.134218	5.420552	5.233225	5.236428	6.479304	4.972119	5.046672	4.976292	6.221429	4.733992	5.965177	5.422062	4.510512	5.551616	5.937210	5.302654	5.060038	6.712466	5.316667	5.163477	5.715491	4.268246	4.137598	4.659428	8.519771	4.287894	4.525222	4.439375	5.433546	4.237440	4.922562	4.966918	6.385776	5.389689	5.299488	4.279071	4.585625	5.364822	5.994659	4.726203	5.219974	5.419369	4.966720	3.869240	4.882858	4.525882	4.521491	4.065226	4.310828	5.264099	5.620917	8.289941	6.016224	3.696123	4.374352	3.559157	4.539977	4.937893	4.612491	6.137696	5.903090	3.681611	3.624039	5.412627	2.973074	6.698709	5.016903	3.965491	3.898221	3.792920	3.145372	6.140293	4.325941	4.014957	5.013417	4.256727	4.350467	5.119499	4.810612	3.828629	5.415835	4.769077	4.304213	6.727389	3.599926	4.583926	4.889393	3.768431	6.146017	4.797249	4.451198	6.545648	6.142058	6.021540	6.869059	7.414000	7.473387	6.377288	5.294445	5.557350	5.352272	6.207603	5.353254	4.451413	5.484426	4.061501	5.329694	5.683055	7.291614	5.783252	3.869665	4.379075	4.090696	5.964494	6.387306	6.324566	7.274232	6.387827	6.702249	7.156070	4.330993	4.947124	5.513512

AL080284.1	7.955250	6.680307	6.659526	6.857373	8.746205	8.489686	7.834777	7.720538	7.723928	8.228419	7.340680	8.236218	6.370383	5.724374	6.022738	7.822114	8.179637	6.237625	6.173023	6.737629	6.614789	5.739713	7.660947	5.484266	6.242766	6.062774	8.715005	8.216505	9.546085	6.950312	7.333384	5.960569	5.907399	6.369883	6.397786	6.947794	6.662321	6.067343	5.827479	6.198084	7.982069	5.887816	4.616876	4.903797	4.270937	7.277515	8.697270	6.571622	6.326915	6.571326	5.639354	7.290824	5.643883	4.972338	5.849536	7.717365	4.930625	5.604254	5.661605	5.459239	6.147950	7.907702	6.196023	6.311292	6.796039	6.837708	5.985034	6.109241	6.012483	6.351789	6.484623	6.785321	8.389429	7.334897	7.099087	6.591640	6.947942	6.990803	7.021281	6.100310	6.137188	6.096514	6.267913	5.573704	6.429874	6.757330	6.048575	6.834845	6.787592	6.845392	6.505475	7.440662	6.059561	7.254764	6.260824	6.457563	5.915113	7.892168	6.993221	6.809854	7.998563	6.416667	6.704456	7.011554	6.113325	9.145493	6.764745	7.025384	6.021366	7.843121	7.171656	6.830405	6.542716	8.133329	8.029333	11.138902	6.756526	8.083490	8.310071	8.188791	6.003941	5.962157	6.875616	8.802612	6.916640	7.976016	6.712952	6.807413	6.893180	6.494926	7.976173	6.728047	5.626559	6.962124	8.051400	6.840313	6.155690	7.768377	6.672861	6.183960	5.885856	7.886728	5.185806	6.449494	5.973236	7.872457	6.651927	8.249596	6.102725	6.626317	6.392873	6.035818	6.650937	5.594019	6.248293	6.315398	6.154472	5.827654	6.326261	7.648449	5.769929	5.540357	5.125344	4.513567	7.579650	7.250720	6.240427	5.517603	7.473225	5.971232	7.155289	5.705254	5.145926	5.527261	7.223889	5.230162	5.538641	5.897057	5.387789	5.987193	8.171607	6.678634	6.322563	6.640806	6.697122	6.012132	5.923827	5.971594	6.288952	5.786290	6.425798	7.050375	6.560853	6.669775	6.256093	6.380125	6.804768	6.513727	5.910785	5.514643	5.959677	6.210017	5.562964	6.156355	6.861393	6.177466	6.135834	6.779254	6.445324	6.338411	7.568100	5.897057	6.230244	6.124277	5.991459	6.417644	7.297031	6.905576	5.476002	6.426975	6.492988	5.515655	6.042288	6.269643	7.586605	6.521864	6.090960	6.159981	7.201468	6.730953	6.298082	6.725888	8.141802	7.475689	8.008692	6.592623	6.664928	6.671706

CSPG4P1Y	12.069615	12.740770	13.256418	13.573342	13.473575	14.447111	13.092393	14.231871	14.539165	13.862356	13.349515	13.077040	13.623869	14.483652	12.285826	13.920769	13.767728	14.942038	13.743347	13.557035	13.429924	13.858073	13.268023	13.613971	14.892803	14.339249	12.968209	14.288989	12.765660	12.890515	13.381148	12.595412	14.073025	11.858897	13.192884	12.570358	14.535541	13.311346	13.150661	13.826360	13.607340	12.224164	15.005964	13.829710	12.969891	12.799450	13.169407	12.972212	13.375462	14.821872	13.164330	14.069818	13.473575	13.659861	14.180845	13.993598	14.250487	14.611416	13.097867	14.418012	14.914667	14.314071	11.568715	14.302275	12.664946	13.075888	13.177797	14.531467	13.780832	13.698756	14.330345	14.007021	15.081140	13.493953	12.268074	12.745897	13.629180	12.227834	12.242226	12.821082	15.023469	13.162064	12.604579	14.419877	12.986294	12.769987	12.658981	14.113158	13.146420	13.332882	13.643752	13.776185	13.210441	13.413378	12.171565	13.692881	14.401522	14.491542	13.244951	14.581385	13.820166	13.356586	13.948216	13.915790	13.427811	13.112389	14.043788	13.261408	13.345293	12.781401	13.410097	14.963955	13.628490	14.005898	15.889470	13.845086	12.426059	13.684527	14.354835	11.502051	11.714008	12.110262	12.402762	13.387976	13.029529	12.565623	13.350346	12.861867	12.868696	12.304791	12.699705	13.078004	12.990324	12.332724	12.698021	12.235457	13.616405	12.492085	11.327922	12.571167	12.474271	12.242226	13.270211	13.304119	12.253272	11.668471	12.800035	12.617952	11.808281	12.697518	11.600441	12.551868	11.352601	12.201396	11.434639	12.216095	11.954979	11.817411	11.905620	12.889156	11.704169	12.296644	11.826393	12.494020	12.037461	12.604249	12.809830	11.861688	12.135906	11.916369	12.504387	11.348941	11.606112	12.470469	12.793936	12.218342	12.574744	11.974500	12.396914	12.514165	12.532655	12.156499	12.396914	12.869060	11.748921	11.564497	12.137398	12.107410	11.668018	11.717191	11.450569	14.265286	11.833741	10.999226	11.608315	11.730195	11.203302	11.736262	11.555531	12.611021	11.954526	11.380384	12.456759	11.385706	12.321093	11.871756	12.410794	12.299522	12.005642	12.075117	12.706541	12.035185	11.658189	12.396312	12.122371	12.830155	13.208700	11.728087	12.339350	12.364339	12.763759	13.091804	11.862156	11.765403	12.936194	12.983333	12.821603	12.551723	12.911640	12.057775	13.087953	13.616405	13.740367	13.141663	12.507212	11.652130	12.804429	12.397227

CSPG4P2Y	12.069615	12.740770	13.256418	13.573342	13.473575	14.447111	13.092393	14.231871	14.539165	13.862356	13.349515	13.077040	13.623869	14.483652	12.285826	13.920769	13.767728	14.942038	13.743347	13.557035	13.429924	13.858073	13.268023	13.613971	14.892803	14.339249	12.968209	14.288989	12.765660	12.890515	13.381148	12.595412	14.073025	11.858897	13.192884	12.570358	14.535541	13.311346	13.150661	13.826360	13.607340	12.224164	15.005964	13.829710	12.969891	12.799450	13.169407	12.972212	13.375462	14.821872	13.164330	14.069818	13.473575	13.659861	14.180845	13.993598	14.250487	14.611416	13.097867	14.418012	14.914667	14.314071	11.568715	14.302275	12.664946	13.075888	13.177797	14.531467	13.780832	13.698756	14.330345	14.007021	15.081140	13.493953	12.268074	12.745897	13.629180	12.227834	12.242226	12.821082	15.023469	13.162064	12.604579	14.419877	12.986294	12.769987	12.658981	14.113158	13.146420	13.332882	13.643752	13.776185	13.210441	13.413378	12.171565	13.692881	14.401522	14.491542	13.244951	14.581385	13.820166	13.356586	13.948216	13.915790	13.427811	13.112389	14.043788	13.261408	13.345293	12.781401	13.410097	14.963955	13.628490	14.005898	15.889470	13.845086	12.426059	13.684527	14.354835	11.502051	11.714008	12.110262	12.402762	13.387976	13.029529	12.565623	13.350346	12.861867	12.868696	12.304791	12.699705	13.078004	12.990324	12.332724	12.698021	12.235457	13.616405	12.492085	11.327922	12.571167	12.474271	12.242226	13.270211	13.304119	12.253272	11.668471	12.800035	12.617952	11.808281	12.697518	11.600441	12.551868	11.352601	12.201396	11.434639	12.216095	11.954979	11.817411	11.905620	12.889156	11.704169	12.296644	11.826393	12.494020	12.037461	12.604249	12.809830	11.861688	12.135906	11.916369	12.504387	11.348941	11.606112	12.470469	12.793936	12.218342	12.574744	11.974500	12.396914	12.514165	12.532655	12.156499	12.396914	12.869060	11.748921	11.564497	12.137398	12.107410	11.668018	11.717191	11.450569	14.265286	11.833741	10.999226	11.608315	11.730195	11.203302	11.736262	11.555531	12.611021	11.954526	11.380384	12.456759	11.385706	12.321093	11.871756	12.410794	12.299522	12.005642	12.075117	12.706541	12.035185	11.658189	12.396312	12.122371	12.830155	13.208700	11.728087	12.339350	12.364339	12.763759	13.091804	11.862156	11.765403	12.936194	12.983333	12.821603	12.551723	12.911640	12.057775	13.087953	13.616405	13.740367	13.141663	12.507212	11.652130	12.804429	12.397227

CR786580.1	5.495472	6.521864	7.017144	7.737895	7.694590	8.500912	7.938844	7.995191	8.453070	8.569261	8.707227	7.661380	8.575963	7.504456	7.976016	8.175833	7.525576	7.568100	6.755554	8.300034	5.362905	7.118842	5.830666	7.721466	8.124795	8.889622	8.255576	6.952016	8.475899	7.635106	9.091579	7.179077	8.506105	8.918624	8.230097	7.878566	7.189556	6.221429	6.793483	7.575291	7.675274	7.050084	8.295756	8.403138	7.855139	7.911114	7.758241	6.412522	6.843510	7.532530	7.215455	7.732726	7.619993	7.848996	6.825914	7.527604	8.041562	7.003520	6.745774	7.602148	6.118370	7.246521	5.267480	6.950916	7.366238	6.278158	6.696046	5.599318	6.827338	5.434949	7.049262	6.986195	8.380819	7.021413	7.689026	7.585977	7.797234	7.743966	7.340176	7.588584	8.134662	6.854771	7.785913	7.401924	7.956511	8.244777	9.216398	7.755300	5.791497	6.218038	6.279570	6.626161	7.713603	7.511538	7.523415	8.880343	7.485352	9.307273	8.224051	8.011860	9.392669	7.648749	8.174901	6.999064	5.317249	7.993159	7.708409	6.178514	6.796372	7.354382	6.396973	8.620804	8.167811	8.082398	6.790858	11.740562	7.046566	8.101146	7.712658	6.899330	7.177144	7.274556	7.593028	8.020288	5.750104	9.946210	8.451859	8.369555	9.153228	9.293154	8.419453	8.905380	8.673441	6.776533	8.426149	8.469344	9.742434	6.217326	7.310728	6.502846	8.910988	6.034026	6.349235	9.517921	10.094258	8.444090	10.183317	9.675373	7.347027	11.030608	8.021631	9.335435	7.165196	7.220008	10.397613	7.182971	6.614141	6.889496	8.257856	8.787683	9.380364	8.039167	6.808714	8.426298	7.386079	7.945709	5.582672	8.963428	6.645317	6.276525	8.840879	8.477574	7.091636	8.224788	8.359575	8.266671	7.137662	7.520520	8.335713	7.294607	8.115905	5.328913	6.746725	6.223463	7.744413	7.092603	4.592701	6.649358	4.877984	8.388218	7.366550	10.396283	7.898958	8.623284	7.347027	8.111029	7.383297	7.712007	8.899452	8.095831	8.034493	8.032084	8.744057	9.074813	9.276833	7.457620	8.913879	6.842863	7.192616	7.519107	7.160265	8.940056	6.438240	7.747490	8.829820	8.420110	10.349459	8.280249	8.849097	9.628948	6.644356	11.286424	8.038832	5.779623	8.898099	9.832315	5.834109	4.210774	7.044666	6.528989	9.728056	10.938762	7.968189	10.129148	8.832732	8.303042	7.512640	7.952630

AC002470.2	9.080099	8.911416	8.772007	8.918918	9.263696	9.165740	8.621124	9.428521	9.309714	8.767146	9.364092	9.264136	8.537536	9.075755	8.925189	9.442200	9.746158	8.740710	9.345116	8.860035	9.334161	9.086111	10.840472	8.716906	8.800503	9.046071	8.856378	9.557255	8.675156	8.371132	9.657640	8.800815	8.768833	9.054399	8.606664	8.808882	8.881902	9.195114	9.056426	9.493614	8.928787	9.101487	8.224643	8.833199	8.429598	8.875702	8.949039	8.540060	8.618626	8.562976	8.739179	8.767146	8.410935	8.803036	8.753188	8.736423	8.886993	8.756854	9.003048	8.953955	9.557714	8.529384	8.973069	9.113163	9.521779	9.932177	9.317465	8.871852	9.336472	9.691309	9.036933	8.975115	8.876783	9.042516	9.227589	8.857724	9.163554	9.293796	9.560158	8.517477	8.248407	8.835236	9.918392	8.896265	8.929239	8.946123	8.875260	9.059743	9.026158	9.073286	9.210693	9.713408	9.259079	8.868386	9.142130	8.812917	9.072055	8.150655	8.963573	8.456594	8.073351	8.485965	8.657576	8.628513	9.200336	8.852566	9.048228	9.073756	9.581615	8.840124	10.254815	9.206130	9.080577	8.864761	8.685237	9.197498	8.862310	9.050240	8.963428	9.240288	9.053125	8.710706	10.070853	9.533221	9.278749	9.211300	9.303246	9.844876	9.376209	9.892101	10.102954	9.412451	8.740267	10.524883	9.965013	9.552229	9.749575	9.060510	8.981956	9.546675	9.787767	10.912789	9.640265	9.706401	9.250083	9.567165	9.989210	10.082245	9.393593	10.061791	8.950728	9.272858	9.298453	9.455567	9.791578	9.294436	9.078706	10.093823	10.113777	9.892413	9.540332	8.440424	9.802880	8.973923	9.121839	9.830156	8.907026	9.615291	9.005162	9.675214	8.985474	9.295525	9.096009	9.368622	9.696933	9.328916	9.798436	10.195547	9.323513	10.441420	10.084367	10.027337	9.393908	9.676294	10.695882	9.593696	9.337427	10.791282	9.620259	9.313619	9.823468	9.990754	9.452733	8.888996	9.084561	10.036268	9.308487	10.050200	8.191336	8.756532	10.749026	11.027185	8.511664	8.516857	9.688696	9.356658	10.849009	9.059573	9.063831	9.358630	9.736704	10.544926	9.597096	10.035391	9.246132	9.747254	9.024612	9.151401	10.212729	8.895157	8.985166	8.875113	8.827483	9.224038	9.829671	10.143394	10.133901	9.938377	9.330587	10.137918	8.830255	8.635361	9.459261	9.922889	9.492544	9.378827	9.638262	9.346335

AC010132.1	4.652164	5.076507	5.021782	5.849897	5.913302	6.409176	5.386136	5.741744	5.454178	5.211243	5.741546	5.552778	5.413577	4.890855	4.817923	5.580595	5.328100	5.167076	4.914529	5.101965	4.877599	6.474588	4.968821	4.838925	5.471006	5.850596	6.277838	6.187399	5.511627	6.159627	4.932210	5.937388	5.789735	5.132818	6.566553	4.734422	5.869547	5.425023	5.233225	4.880724	5.436108	4.806164	4.921258	5.532203	5.026650	5.705599	6.246787	5.040093	5.720302	5.424454	4.529888	5.766650	5.437272	4.932634	5.821680	6.617562	4.619283	4.877599	4.231140	3.746283	4.824325	5.848984	6.043958	5.287167	4.586266	4.713491	5.383921	5.572941	5.671992	5.328489	5.871887	5.531417	5.669795	5.368578	6.217146	5.903090	5.689869	5.200081	5.408923	4.136944	4.609716	4.801541	4.509851	5.892225	4.539543	5.835534	5.268266	4.921033	4.407357	4.755912	4.642979	4.873845	5.190727	5.521763	5.658691	5.282775	5.411091	5.577038	6.335031	6.011036	6.422233	5.577775	5.600431	5.515838	5.408524	6.344049	5.484613	5.537228	6.007362	5.849182	4.975473	6.051653	5.836962	5.785945	6.380781	7.852191	5.716039	5.191332	5.994484	5.270048	5.568644	5.645243	5.547508	6.633547	6.099988	6.202793	5.762324	5.432219	5.851664	5.679002	5.865214	5.992513	5.532003	7.064300	5.782736	5.072202	4.916860	5.576296	4.869995	5.174952	6.379289	6.041579	5.434949	6.096346	5.375342	6.587207	6.064868	6.646258	6.450968	5.030756	6.386455	5.472341	6.040731	6.164262	5.431041	6.138578	6.024991	5.519715	5.866471	6.107970	5.791497	5.212390	5.972886	5.090658	5.220376	6.524506	5.849706	5.458649	4.500267	4.556426	5.809809	5.425386	4.516358	5.427041	6.227645	5.394196	5.013223	4.584149	4.372594	5.165298	5.628020	5.279537	5.013631	4.797882	4.513800	5.662352	5.809994	5.645429	5.534285	5.873185	5.651254	7.032489	5.430849	5.924351	5.473286	5.967171	5.891332	5.541080	5.153573	5.185200	4.667960	5.345157	5.010327	4.132996	6.143417	5.082049	4.514824	4.116788	4.545772	4.633723	4.947749	5.129592	5.172969	5.520438	5.150526	6.181859	5.711196	6.361202	5.578314	5.977611	5.540532	5.655012	5.624075	4.347616	6.360702	4.829983	5.121515	4.654618	6.795557	5.287944	6.213909	8.232995	7.000485	6.678326	6.748017	5.126559	5.383335	5.507191

LINC01450	3.376704	3.636869	3.238467	3.250993	3.665678	4.042312	3.235659	3.888504	4.481472	3.347210	3.454178	3.694034	3.208073	3.710057	3.360160	3.674948	4.319940	3.546557	3.213675	4.312513	5.386136	3.223498	6.161322	3.740582	3.377742	3.480575	3.273704	3.229042	3.389127	3.110154	3.703843	4.121850	4.969038	3.322496	4.890855	3.980615	4.137156	4.124633	3.837146	3.386728	3.358469	4.131671	3.410757	4.145187	4.831039	5.622975	4.732080	4.482874	3.701159	4.067751	4.013585	4.876299	4.398722	4.032121	3.809721	3.753370	4.022173	5.961292	3.815250	4.386303	3.634676	3.468909	4.129377	3.964673	3.489710	3.357933	2.924830	4.817515	3.540150	3.199655	3.053021	2.889992	3.596936	3.126045	3.132715	3.226157	3.349361	3.526862	3.650223	3.420362	3.149520	3.858770	3.072783	3.852937	3.416555	5.868862	4.746389	3.972872	3.281942	3.789308	4.468831	4.035605	3.050364	3.109568	3.129094	3.318958	3.801618	3.020720	3.069947	3.671130	3.325791	3.758566	3.130865	3.168920	3.007755	3.922224	3.631286	3.583224	3.783976	3.276977	4.521910	4.666915	3.795908	3.340650	3.819912	4.481255	3.604265	4.890438	3.260006	3.616879	3.981754	3.749587	3.664851	3.486428	3.637261	4.265101	5.308659	7.429685	3.759641	5.664777	3.161831	3.890045	3.567669	4.310159	3.953338	6.418153	3.664851	3.492758	4.310159	3.761124	3.465394	6.109395	3.379837	3.485185	3.172014	3.711134	4.328317	4.328766	3.493640	4.555359	3.681221	4.384260	4.410264	4.106504	3.705864	3.749777	3.701819	3.503315	3.598158	5.860480	4.264190	3.789732	4.170075	4.882660	5.184611	4.814772	4.616662	3.543244	4.210345	4.056495	3.792920	3.994177	4.098180	3.606610	3.259296	4.289822	5.641575	3.764289	3.837575	3.972030	3.158665	4.008227	3.826149	3.564009	3.503653	2.942671	5.168882	3.298088	3.368911	3.038755	3.126509	3.951827	3.227610	3.126876	3.193531	4.018944	4.051722	3.851853	4.648318	5.378285	3.984565	3.819912	3.543602	3.515144	6.192527	3.905294	3.800966	3.193103	3.859401	3.839470	4.619476	3.013959	3.147914	3.140052	3.730244	4.404426	5.151351	3.463210	3.290793	4.573962	3.476529	3.941921	3.085714	3.038139	3.604642	4.008227	3.678626	3.473610	4.372594	4.167983	4.228324	3.497463	3.325488	3.953338	4.086354	4.066061	5.561471	5.078192

AC093627.4	9.135312	7.176334	8.566155	8.606037	8.599414	9.027197	8.479139	9.215448	9.790211	8.278869	9.086422	8.934423	8.468110	8.274612	8.217766	8.156082	9.172960	8.549674	8.305738	7.946776	7.973521	8.081792	7.918723	7.790379	8.308999	8.920132	8.210824	8.693709	8.686866	8.625761	9.012226	8.826140	8.910839	8.542882	8.192439	8.669707	8.909370	8.454138	8.591139	8.076646	8.567907	7.841244	7.424921	8.204055	7.775738	7.872765	9.455567	8.484766	8.477896	8.743900	8.127496	8.052166	8.607729	9.068164	7.933723	8.877113	8.929545	8.921964	8.296244	6.190615	8.370333	8.481092	7.610220	8.380007	9.135773	8.763320	8.350957	8.275513	9.025547	8.642817	7.992704	8.335568	8.202269	8.064267	8.975271	8.959613	8.042154	8.763615	9.020771	8.464437	7.555205	7.819359	9.007935	7.989271	8.195107	8.487060	8.176794	7.952777	7.786851	8.423166	8.282551	8.151906	8.077123	9.005162	9.538600	8.979083	8.364848	8.320945	9.336944	8.320792	8.886993	7.277044	8.169880	8.525523	8.488914	8.390832	8.596272	8.792595	8.695746	8.476374	8.500737	8.484010	8.304977	9.509295	8.925815	9.825553	9.056119	8.622520	8.706158	9.686819	7.893878	9.964423	10.140152	9.096736	8.603763	8.987939	7.930519	8.058121	8.412310	9.819984	9.406320	8.235581	8.373123	8.234355	8.214394	7.866510	9.022327	8.243241	8.176247	7.990967	8.384200	8.158860	8.496726	9.204729	8.082255	8.807664	8.759794	9.696173	8.564200	8.692458	8.266965	8.303042	8.632906	7.819973	8.691979	8.357224	8.163242	8.498687	8.308074	8.934567	8.055892	6.422692	7.778984	7.647414	7.775107	9.433276	7.860182	8.870392	8.193208	7.856355	7.293973	10.428816	8.136662	8.759647	9.144456	11.313353	9.801346	10.374263	5.866135	8.113464	7.717365	7.058232	8.224788	8.937648	9.298143	8.492264	8.467060	8.220385	9.761826	7.690876	8.632743	9.746308	8.106503	9.513366	8.990911	7.760209	8.263850	8.647531	8.166936	7.160752	9.482103	8.009628	7.739294	7.517884	8.456594	8.396396	9.179081	8.444854	8.377069	8.678199	8.358620	7.703584	8.302435	8.899923	9.096576	8.410935	8.819941	8.633227	8.491946	8.855773	7.213260	7.639235	9.096576	8.317157	8.494468	7.399457	9.381628	8.036507	8.962975	8.209938	7.904130	8.855472	10.872478	9.096285	8.648726	9.116670	8.372228	7.924598

LINC01780	3.016359	4.606700	3.171902	2.956087	3.174332	4.444781	3.196648	3.118062	3.263834	3.234542	3.415541	4.002351	3.161074	3.544508	4.189270	3.031075	4.815381	3.045889	4.590982	3.165816	3.717910	4.037145	3.440139	2.954506	3.135394	3.489559	3.550612	2.946625	2.999469	3.384997	4.438286	5.944073	3.469235	3.783575	3.125273	3.925898	3.432871	3.821032	3.008075	2.960790	3.330982	2.955435	3.948773	4.020669	3.403382	6.059726	4.605235	3.730665	4.273784	4.718328	4.034080	3.294060	3.573430	4.321019	4.006114	3.187793	3.708077	3.957014	3.682035	3.569850	3.516786	3.482229	3.422630	3.595732	3.139568	3.054547	4.030852	3.589211	4.362190	2.921570	2.904689	2.844207	3.000357	3.308406	2.939052	3.903586	3.503653	3.104345	3.168541	3.374235	3.775550	3.605046	3.050364	3.847556	4.393599	3.100328	3.320089	3.161604	3.873944	3.870938	3.893761	2.940141	4.002351	3.991131	3.443636	3.251132	3.237122	3.484623	3.752126	3.916408	3.012965	4.007174	3.436697	2.932649	5.148333	3.825093	2.903558	3.071207	3.846944	3.346730	3.923290	3.158396	2.991298	4.537161	3.034402	3.788048	2.987859	3.260006	3.901276	3.442220	4.319054	2.965543	3.695698	3.228185	4.711587	4.382956	3.492401	3.188883	3.438239	3.167149	3.582650	3.432363	3.818633	5.426219	3.210577	4.553238	3.115819	3.845207	3.207657	3.376176	3.938998	4.743570	3.417203	2.980688	3.941517	3.300811	4.513162	3.143488	3.570961	4.483726	5.071631	3.318473	4.273558	3.736479	3.633323	3.470157	4.001479	3.878499	3.694253	3.413645	2.889992	4.750654	4.071053	3.558934	5.872991	4.988308	4.833968	3.327563	5.210637	4.290484	3.223746	3.381371	4.490665	4.392359	3.205732	3.401604	3.863667	3.523465	3.446722	4.312729	3.344639	3.416052	4.120971	4.138852	4.373702	3.216802	3.925693	3.217861	2.945757	3.142180	3.264817	3.125639	3.202823	3.134492	3.893761	3.631286	3.049223	3.217861	3.355030	4.311668	3.636869	3.079410	4.551331	4.803678	3.577750	3.943502	3.842951	3.655026	4.013337	9.158783	4.092696	4.296542	4.126337	3.803896	4.034967	3.872223	3.241215	3.420058	4.016290	3.160581	4.193897	5.776559	3.408711	5.293694	3.599742	3.903786	3.952050	3.517163	3.670112	3.186882	3.484623	4.916169	4.565960	3.334678	3.567669	3.434340	3.339114	4.395213

AC243964.3	9.798264	8.829025	9.099653	9.130300	8.799286	8.367911	8.905230	8.489514	8.380662	8.769584	8.794756	8.765596	8.463529	8.358315	8.837687	8.139115	9.219434	8.681429	7.721779	8.818873	8.292455	8.579234	8.249737	8.126124	7.557702	8.646914	8.454436	8.737163	8.635528	8.692954	9.239370	9.310505	9.256834	9.166498	8.699095	8.500281	8.698199	8.453530	8.196392	8.255129	8.858058	8.046717	8.036507	8.587856	8.966199	8.404521	7.972255	8.180232	9.743348	8.797307	8.933148	8.745438	9.242889	8.960834	9.505657	9.075755	9.051002	7.817936	8.247106	8.492438	7.936999	9.237192	9.403399	8.057141	8.411096	8.918918	7.635884	8.111766	8.258639	7.657521	8.223754	8.524461	8.468844	8.573650	8.369224	7.834020	8.425886	8.952418	8.618776	8.653894	9.212359	9.502229	9.376353	9.168150	9.150322	9.018822	8.836027	9.202653	7.693051	7.701453	7.633902	7.776998	8.495364	9.297552	8.266021	8.650408	9.558482	8.887600	8.785314	8.607116	9.226978	8.602527	8.517004	8.932263	9.339197	8.866879	8.098401	8.942331	8.370991	8.544600	6.671872	7.471425	8.492438	8.014946	8.906422	6.517896	8.899923	9.337708	8.036983	8.869289	10.500161	9.740576	9.396514	9.980904	9.930936	10.334799	8.310979	9.657347	9.781478	9.501025	9.973251	9.583467	10.273930	10.734015	8.085844	10.350375	8.907201	7.939314	10.648931	9.829507	9.712029	8.950728	9.562068	8.988078	10.167666	9.933222	9.811172	7.867093	10.093823	10.802991	11.300099	9.690083	10.371545	9.788490	10.073446	9.897941	8.975271	10.080574	9.477056	10.085713	8.923712	9.365664	10.102338	10.018797	9.630595	9.540974	8.199678	9.635632	10.370938	10.258842	10.332208	9.912045	10.383819	9.724538	9.411269	9.363051	9.253455	9.399241	9.790059	10.297862	9.772502	9.500882	10.380306	8.625761	8.424675	10.306560	8.964176	9.906665	8.816052	8.845859	9.769058	9.620259	9.465964	8.484010	9.623678	10.231920	9.929695	9.649935	9.084407	9.593060	10.047711	10.977437	9.782227	10.919567	9.690233	10.222722	9.696322	8.712212	8.766232	9.709211	9.926476	9.664041	11.103722	10.575116	9.766026	9.353052	8.207086	10.220796	9.834281	11.857035	9.032049	9.122460	9.318351	10.309258	9.535836	9.348480	9.197337	9.125034	9.346035	7.544378	9.367576	7.147061	7.427903	9.440177	10.154056	9.712939	9.542983	10.704038

LINC02472	2.919675	3.117732	2.949076	2.970632	2.926088	3.033913	3.235794	3.106541	3.256343	3.326945	4.193897	3.922002	3.207511	4.270303	3.461815	3.162886	3.347539	3.663842	3.107369	3.177618	4.664747	2.865464	5.390063	3.127424	3.235794	2.922619	3.025936	2.987972	3.172928	3.042930	3.607655	3.688884	2.944727	3.032657	3.303975	3.899349	3.655026	3.281206	3.296210	3.005642	3.364044	3.341822	3.211164	3.094537	3.401932	5.212584	3.238755	4.222090	5.066749	3.728166	3.790356	3.769513	4.106979	3.754254	3.504732	2.993726	3.772413	3.316355	3.552782	3.552978	3.392134	3.015461	3.398506	3.011754	3.142064	3.231727	2.865464	3.296210	3.178160	3.042930	2.917720	2.861148	3.059176	2.925247	2.967466	3.156400	2.953498	2.922348	2.926362	3.320580	3.162886	3.727364	3.079971	4.173385	3.220989	3.185927	2.894780	3.230901	3.023296	3.184613	3.166091	3.185015	3.001787	2.978982	2.904037	2.937662	3.592616	3.138106	3.178836	2.942927	2.865464	3.161074	2.977791	2.893630	3.010672	3.022360	3.043724	3.313496	3.008683	3.226440	2.902569	2.911283	2.945514	3.231044	2.982540	2.933598	2.978148	2.967652	3.146189	2.940060	3.169527	2.913548	3.001976	2.861366	3.335311	2.945448	3.101606	3.202543	3.125395	3.879570	3.134377	2.982540	3.195416	3.189289	2.961862	3.341148	3.222099	2.960600	3.295881	4.044097	2.844337	5.739896	3.004535	3.064168	2.871339	2.876946	2.904877	2.869702	3.442575	3.404213	3.301772	2.983458	3.013456	3.209518	3.638275	3.675388	3.231727	3.205070	2.990174	3.128332	3.294995	3.335963	3.115819	3.608409	4.838091	3.158535	4.291559	4.742727	3.819707	3.825314	3.585137	3.600679	3.765704	3.194161	2.943386	3.428492	3.430280	3.440139	3.530135	3.137360	3.062481	3.366512	3.124425	3.181516	3.270526	2.872870	3.614770	3.349518	3.136856	2.937913	2.931868	3.007450	2.943972	2.913024	3.117138	2.956087	3.024851	2.965857	3.080690	3.451366	3.905507	3.109691	3.595938	3.462494	2.926987	3.484451	3.346247	2.978230	3.191194	3.342302	3.181767	3.021451	3.001390	2.976862	2.944437	2.907863	2.885929	3.193531	3.167650	2.944628	3.000357	2.906315	2.832031	3.070681	3.004804	3.099517	3.247824	3.403206	3.109208	2.869702	2.819158	3.077719	3.149886	2.933248	2.830622	3.054547	2.943824	3.205479

AC025423.4	12.226148	12.804949	12.893478	12.613560	12.968566	12.566268	12.516941	12.909129	12.415626	12.323253	12.771214	12.449603	12.612348	12.797127	12.405117	12.763103	12.527775	12.419436	12.258675	12.816305	12.422459	12.160822	11.243314	12.506071	11.972222	12.357184	12.217765	11.987250	12.156499	12.611352	12.304791	12.432421	12.481977	12.611352	12.734538	12.672605	12.691855	12.365907	12.298540	12.171733	12.511128	12.386589	12.813004	12.937089	12.769062	12.344233	12.601063	12.435325	12.459772	13.131924	12.663909	12.675008	12.619138	12.906787	12.603413	12.708953	12.671375	12.299183	12.169579	12.300012	12.045877	12.344853	12.260195	12.419759	12.394267	12.462928	12.285680	12.595412	12.415458	12.435481	12.673286	12.729124	12.473114	12.911640	12.833456	13.155545	13.030100	12.418490	12.588951	13.077603	12.587968	12.650060	12.916404	12.892609	12.596273	12.624672	12.685514	12.919527	12.546681	12.506387	12.371489	12.735398	12.613710	13.132988	12.823360	12.940096	12.671375	12.354541	12.425577	12.373685	12.389829	12.735048	12.707214	12.834869	12.502069	12.539305	12.854034	12.397858	12.243771	12.583137	11.938785	11.995822	12.625160	12.313325	12.972757	11.857513	11.917886	13.006339	12.378969	14.223156	12.247353	12.933125	13.383693	11.949587	12.681971	11.816305	13.922935	11.949752	13.044646	11.988788	12.630986	11.782817	12.270578	11.616843	12.241433	11.848352	12.161407	12.281444	12.838923	12.281277	12.473611	12.262315	12.630185	11.578205	12.244367	12.948910	12.437743	12.530838	12.491562	12.369546	12.467321	11.898369	13.352991	12.867268	12.954789	12.483749	11.970367	11.983055	11.840994	12.269467	12.744026	13.130760	12.558326	12.621674	12.386932	12.096818	12.697321	12.833106	12.855334	12.645369	12.481303	13.675974	12.060534	12.374349	12.613037	13.202580	12.796960	12.396914	13.353095	13.881710	12.098604	12.440954	12.211771	12.395067	12.837405	12.563972	12.559720	12.269936	12.595587	12.699016	12.964368	12.111742	12.304038	12.806146	12.918224	12.263397	12.824882	12.854978	12.232212	12.445298	13.043715	12.090726	12.943738	12.378643	11.641173	12.473611	12.882781	12.089348	11.892168	12.601913	12.087562	12.508187	12.203451	12.512588	12.380187	12.294510	12.061997	11.819362	12.847695	12.016883	12.655512	12.240857	12.854219	11.913837	12.615346	12.901976	12.712524	12.810731	12.576234	11.987098	12.020355	12.346785	12.477509	13.174367	13.068499	11.996401	12.447416	11.988461

AL450322.1	8.181595	8.320503	8.246193	8.393485	8.645888	9.023129	8.314211	9.557714	9.752370	9.287859	8.448422	8.543369	8.374648	8.981042	7.873818	8.951337	8.630690	8.345680	8.308242	8.572191	8.860924	8.483106	8.096900	8.605587	9.158502	8.027318	8.835394	8.466587	9.132598	8.932840	8.614047	7.899114	7.976790	8.162959	8.243401	8.378612	8.478522	8.336025	8.555423	8.622365	8.707526	8.164882	8.427087	8.149462	8.029186	8.421205	8.956668	8.386026	8.429598	8.555749	7.785295	8.006846	8.024983	8.110120	8.120986	8.879890	7.983196	8.113311	8.302103	7.733023	8.321580	8.752751	8.327972	8.408218	8.305140	8.345999	8.695418	8.437550	8.196546	8.494761	8.076073	8.323100	8.901108	8.079308	8.392410	8.428965	8.216056	7.909425	8.222067	7.710952	8.137859	8.070916	7.745934	7.623618	7.810799	7.833074	7.544875	7.966042	7.751504	7.768842	7.621803	7.763738	8.024983	8.352921	8.044341	8.294728	8.079159	8.551958	8.491478	8.652056	8.932113	8.391462	8.441781	8.562835	8.814706	8.908396	9.399848	8.993942	8.940945	9.008394	8.697583	9.139031	8.868076	9.642543	9.239678	10.183454	8.376902	8.273739	8.774603	8.532428	8.708279	8.460937	8.553905	9.684197	8.345680	9.495630	9.547923	8.815133	9.312969	8.562241	8.299261	8.856984	8.871542	8.499325	8.833467	8.583923	8.022090	8.642055	8.220099	8.967900	8.317610	8.306800	8.731515	9.453727	7.777768	8.606664	8.957459	9.508697	8.568356	8.852566	8.591616	8.371908	8.686132	8.364848	8.024072	8.101896	8.620966	9.010820	8.190710	9.184546	8.486274	7.787764	8.385428	8.132097	8.188055	9.587157	8.342612	7.855139	8.646337	8.150523	8.009151	7.848701	8.351557	7.967116	8.698049	7.837979	8.078065	8.271494	7.290501	8.596272	8.963282	8.978636	8.363935	9.547923	9.080099	8.972768	8.399403	8.549830	8.244457	8.211137	8.223899	9.875764	8.093407	7.916471	8.323832	8.225251	8.477734	8.384497	7.876282	7.991114	8.316232	8.180232	7.746418	8.089724	8.255721	7.709719	7.805246	8.033303	7.822263	7.545035	9.156761	7.981300	8.406370	8.372685	8.430079	8.360634	9.169996	9.328299	8.409398	8.739630	8.694983	8.773664	8.219933	9.852765	8.905538	10.019581	8.811070	8.227519	9.659354	8.461565	9.812449	9.263235	9.869407	8.833199	9.286274	8.511835	9.042516	8.913879

FOXD3-AS1	9.653534	9.509295	9.296135	9.779075	10.160447	10.173348	9.789292	10.108388	10.437507	9.646619	9.693450	10.235279	9.854297	9.217443	9.142279	9.582379	10.119603	9.214386	9.882077	9.535836	9.711706	10.090224	10.576472	9.582540	9.871973	10.333610	10.762352	10.633212	10.805904	10.204611	10.063772	9.195778	8.933305	10.159669	9.636374	9.521779	9.792860	9.827819	9.620259	9.695415	10.215186	9.617475	9.521604	9.603010	9.797967	9.186600	10.629019	9.561136	9.579000	9.243810	9.042653	9.033552	9.220963	9.608994	9.325070	10.398977	9.127188	9.296921	9.371195	9.091411	9.294436	10.145351	10.074685	9.509923	9.150322	9.189785	9.532764	9.166351	9.226054	9.636997	9.914396	9.735774	10.267489	9.644704	9.574876	9.317184	9.649472	9.642543	9.737303	8.400970	9.020172	9.003490	8.734401	8.588177	8.895467	9.421160	9.068319	8.825536	9.162611	8.690809	8.844490	8.843862	9.081657	9.359075	9.754388	9.360161	9.224202	9.598956	10.529664	10.061049	10.995221	9.830915	9.726848	9.417638	9.551318	9.807593	10.490710	9.772809	10.416284	10.220177	9.899900	10.349613	10.372281	10.610379	9.605154	13.339537	10.172908	9.755013	10.347055	9.276833	10.080285	10.089453	10.009096	11.717191	10.249835	10.964018	10.713869	10.717299	10.772249	10.495855	11.039733	11.085733	11.970814	10.838841	10.612584	10.652524	10.354160	10.394618	10.304338	10.639852	11.380538	10.466534	10.893881	11.241360	11.314073	10.716709	11.053643	11.673108	10.379694	10.787634	10.136690	9.640875	10.122789	10.128997	10.757526	10.026576	10.258997	10.099349	10.216690	10.865149	9.881326	9.841511	9.782831	10.283957	9.777068	10.867997	10.825019	10.700036	9.920741	9.794394	9.958780	10.285304	10.742017	10.300503	10.708068	9.661074	10.218028	9.361114	9.611805	9.769677	10.913094	10.018949	10.307712	9.310045	9.341602	10.425295	9.456011	9.564183	9.929695	9.952895	10.046625	11.534802	9.731680	10.258842	9.593851	10.020621	10.045559	9.865768	9.449194	9.689001	9.949060	9.235620	10.297240	10.373979	10.051261	9.764963	9.970187	9.833794	9.419441	11.427665	9.863624	9.482261	10.113777	10.326281	10.555843	10.175472	11.182712	10.525956	10.365006	11.410967	10.750219	10.607118	10.058151	10.715670	11.165939	10.420556	9.269228	9.965900	11.032989	10.464310	11.185995	12.467649	10.945949	11.289617	10.668285	10.475566	9.368032	10.715826

AC073864.1	6.923538	5.886203	6.462837	6.090105	6.238438	6.368195	6.641126	7.515375	7.010741	6.291686	6.698079	6.939673	6.561198	6.722418	5.832637	6.780059	6.102386	6.658225	6.079720	7.612641	5.896885	7.733625	6.689803	6.571007	6.470882	6.730471	6.328905	6.959726	6.385607	6.801865	6.453805	6.752407	6.080235	6.418823	6.248637	6.411029	6.373313	6.598280	6.707842	6.664782	6.649192	6.646404	6.557284	6.559099	6.776038	4.905496	6.452765	7.385010	6.239749	6.848224	7.277363	6.625166	6.407375	6.784020	6.092703	6.426975	6.351290	6.610626	6.553821	6.437128	6.570672	6.589006	5.221107	6.968033	5.828214	6.742265	6.727389	5.778924	6.537774	6.009291	6.446533	6.555125	6.354181	6.956719	6.676729	6.112145	6.475585	6.611844	6.970890	6.941837	6.681747	5.826008	6.885035	7.191688	6.820058	7.328360	6.671061	7.173526	6.349577	6.136700	7.095641	6.608356	6.391555	6.300351	6.973051	5.993753	6.027116	6.066254	6.501553	6.493471	6.956874	6.506499	6.457035	6.741794	6.849972	6.015023	6.322198	6.716392	7.480750	6.830549	7.753027	6.469852	7.027491	6.450320	6.551327	8.721757	6.815409	6.283964	6.631099	7.727475	6.039824	7.580396	5.686778	7.100946	6.479620	6.832128	7.931730	7.487256	6.777825	6.792834	7.142561	7.030189	6.390531	6.513727	5.998502	6.012483	6.635961	9.300643	10.136546	7.502112	7.690876	7.523591	6.737942	8.136495	6.824177	6.815567	6.762817	7.018894	6.543690	5.712490	6.620355	6.142404	6.447349	6.493156	6.855573	7.015004	6.008773	5.907218	7.019538	6.965004	6.524664	8.732236	5.995839	6.997868	4.567891	7.420547	8.665949	6.216805	6.250956	6.615919	8.241293	6.283964	4.774099	5.800232	6.749727	7.163769	8.106793	6.707704	5.775290	5.873185	7.939314	4.733992	6.109732	6.236650	5.609902	5.854373	6.444900	6.007890	5.778189	6.676256	7.236735	7.137662	6.536628	7.262516	6.165625	7.459959	7.526204	7.376878	6.145662	6.519214	6.238775	5.280957	6.395482	6.911310	7.065558	6.050288	9.122924	5.360034	5.519352	7.504008	6.395316	6.197419	6.359531	6.662662	6.049071	6.800600	7.703743	6.193018	6.990315	6.745598	6.842050	6.366226	6.635796	7.158379	7.008826	6.766184	5.643691	8.102799	7.322799	8.060419	6.817477	9.926926	6.956277	7.120590	6.960210	5.990613	6.724980	6.553998

LINC01951	7.688703	7.429837	7.649509	7.888271	9.372405	8.764687	8.049077	8.602058	9.729026	7.639530	8.126124	8.165664	8.297298	7.943235	7.811416	8.593306	8.686278	7.812790	8.267121	8.159780	8.003880	8.020116	9.021575	8.341166	8.371282	8.560859	8.479812	7.749652	8.378761	8.087867	8.465978	7.881713	7.724392	8.032389	7.585336	7.722516	7.565945	7.434079	7.739464	7.527936	8.346473	7.853910	7.522227	7.951220	7.986868	7.339745	8.475093	7.114246	8.051705	7.421509	6.699964	7.685481	7.401461	7.866197	7.768688	8.476914	8.131953	8.081473	7.849748	7.564875	7.372533	8.351263	8.379542	7.245012	7.382348	7.500852	7.644012	7.578077	6.767805	7.976484	8.050019	8.315586	8.931119	7.624268	7.893724	7.663886	8.290249	7.979874	7.506641	7.835968	7.996613	7.685481	7.592717	7.944630	8.056835	8.022388	8.862905	7.819040	7.550369	7.263271	7.518967	7.210664	8.457490	7.356249	7.879615	8.637555	7.499287	9.177093	8.090022	8.997209	10.027037	7.891690	8.010496	7.395847	7.433592	8.558556	7.569764	7.560439	9.245501	7.991746	8.717976	8.796419	8.526736	8.684928	8.162515	13.257195	7.763284	8.382834	8.800956	8.561770	7.503062	7.647866	7.768842	10.511993	7.009488	8.891442	8.498063	8.890098	8.574419	8.087091	8.308999	8.808882	8.459410	7.872457	9.220351	8.622365	8.225403	8.879747	7.761592	7.621347	8.959155	9.070964	8.176949	8.537828	7.808494	9.822224	8.387874	9.866387	7.145053	7.986058	8.029186	7.563337	7.882013	7.731957	7.635247	7.583190	7.756185	7.282895	7.957608	8.663004	7.040326	6.890923	7.658152	7.297331	6.648704	8.437250	7.858621	7.581340	7.142250	6.838038	7.353902	6.872271	7.993159	7.967712	8.324932	8.290249	7.520020	7.192436	7.997547	7.157148	8.676820	8.015395	6.985241	7.614214	7.468910	7.705945	7.007495	6.948428	7.167473	7.855444	7.434866	8.147162	7.828836	7.966042	7.542948	8.055178	8.176167	7.848550	8.019965	7.981918	7.202252	7.003824	8.311294	8.154862	8.854235	7.484585	7.604509	7.716737	7.349431	7.190458	6.897684	8.215758	6.931597	7.654519	7.793259	8.635361	9.838268	7.742874	7.943093	9.288025	8.892827	8.078556	7.945709	7.471570	8.197934	7.912515	7.952777	8.436649	7.622388	9.044099	8.903998	10.256330	8.435693	9.287550	9.804849	7.425999	9.000858	7.199002

AL662791.1	3.894841	3.858553	3.182955	3.376176	3.155201	3.493155	3.753157	4.273323	3.428828	3.436845	3.517751	3.704272	3.418265	3.662866	3.221098	3.313340	3.706050	4.274018	4.945220	3.585898	4.594060	3.673900	4.771384	2.866196	3.428492	3.336283	3.406067	3.078033	3.063523	3.560111	3.599742	4.448914	4.472556	5.092686	3.620663	4.078904	3.615523	3.472373	3.410422	3.730244	3.298088	3.488296	4.323129	3.399349	3.622237	5.610683	3.666279	5.789735	4.850372	4.240972	3.552782	3.646235	4.217517	4.280176	4.361519	3.765098	5.443899	4.963356	4.038230	3.932604	3.838007	3.458409	3.823524	3.543818	4.233725	3.474890	3.547999	3.514432	4.407127	2.880050	3.730447	2.971853	3.163529	3.075743	3.369248	3.318632	3.188761	3.587872	3.229611	4.343705	3.427086	3.750428	4.066061	4.327873	3.907475	4.883281	2.910921	3.360651	4.380980	3.735228	3.511083	3.425827	3.378791	3.212966	3.464122	4.163837	4.119452	3.554492	4.703636	3.061915	2.916007	3.800747	3.689738	4.036500	4.493308	3.326945	3.031164	3.857219	3.266440	3.199019	3.384652	4.029966	3.225182	3.178028	3.420255	2.884071	3.776955	3.023691	3.257771	6.084054	3.934301	4.010802	4.961642	3.299620	4.668169	4.126791	5.210637	3.760082	5.009920	6.543039	5.120098	3.219514	4.071257	5.766251	3.230063	3.938536	4.716660	3.499964	3.410579	3.889613	4.286794	5.029113	4.568093	3.781857	3.500163	3.083946	3.176503	3.248272	4.106504	3.458409	3.983281	6.409342	5.725538	7.605625	5.600431	4.875692	4.167325	4.781766	3.909692	3.216951	3.319596	6.151211	4.707020	4.756117	5.628403	3.579772	6.250151	5.623172	4.918935	5.975531	6.266039	5.517407	4.352665	4.473629	3.622868	7.599995	4.483961	4.593634	4.553674	5.671044	4.701728	4.962733	4.002249	4.617086	3.735018	3.590513	3.616696	5.667049	2.847233	3.164325	3.600306	3.952908	3.103544	5.430849	4.703636	4.449155	5.686778	3.275589	4.367399	3.884501	5.598387	5.145152	4.308141	5.761020	5.329500	4.785776	6.981609	4.104098	3.601673	4.783483	5.029321	4.885618	4.829317	4.385646	4.980732	3.894841	3.105727	5.762690	4.491993	4.488254	4.790369	4.408699	3.311024	4.900277	4.504789	4.840654	3.934301	4.443890	3.976140	3.192314	3.632741	3.964886	4.208004	3.689939	4.758070	3.350218	4.096648	5.551244

KRT73-AS1	9.820554	9.841075	9.830772	10.319671	10.711967	11.086626	10.143394	11.482684	11.800445	10.619535	11.076625	10.558709	10.307568	10.281995	9.891644	10.986598	10.952734	9.949359	10.944920	10.532684	9.878300	10.622485	9.879639	10.283247	10.304338	10.820288	11.082761	11.022755	11.374203	10.465346	11.577639	9.270309	9.323358	9.895662	9.462714	9.558026	9.779527	9.483497	10.218950	9.873016	11.077209	9.783941	9.599698	9.966216	9.722467	9.493002	11.484580	9.733183	9.501613	9.687451	9.187848	9.599698	9.058600	9.554189	9.319592	11.333467	9.243650	9.654867	9.609304	9.432358	9.298596	10.701664	9.340284	10.183317	9.467510	9.436174	9.540818	9.790211	9.566202	9.717969	9.829507	9.905450	11.260034	9.645034	9.682352	9.659217	9.674912	9.592125	9.640574	9.506420	9.445750	9.140691	9.137318	9.418245	9.498564	9.616838	9.641321	9.756549	9.314274	9.596951	9.292212	9.561904	9.820128	9.706564	9.791261	9.941496	9.389608	10.607990	10.677043	11.065719	11.045883	9.805157	10.454947	10.191313	9.782227	11.341014	10.737331	10.074846	10.800096	11.211843	9.695569	10.947159	11.103121	11.522791	10.935332	13.021558	10.259615	10.543163	12.042411	9.864242	10.293236	9.669428	10.536625	11.769600	10.357076	10.805904	10.410131	10.905852	10.462807	10.904808	10.280486	10.804313	10.120195	10.281686	10.895631	10.633662	10.542688	9.965013	9.583324	10.356772	10.806201	9.332454	10.939212	10.022143	9.734702	10.640759	10.853314	11.809331	10.143844	11.057630	10.279430	10.219240	10.391055	9.448419	9.578383	9.721087	9.587330	10.549491	9.879639	11.588326	9.618556	8.922566	9.785797	9.811011	9.603163	12.222032	8.798246	9.387777	9.518231	9.645490	9.474447	9.026892	9.579457	9.663577	10.811801	8.700428	9.378682	9.588735	8.975737	9.812767	10.589073	9.749266	9.776425	9.316571	9.523949	9.497029	9.845979	9.723277	9.636066	9.782831	9.819984	12.224164	10.096699	9.246431	9.871673	9.713091	9.665912	9.838114	9.662648	9.272858	9.222961	9.316873	9.215448	9.260302	10.265725	9.584367	8.937648	8.994735	9.206130	9.049134	9.591343	9.864242	9.772353	10.024815	10.158948	10.107303	11.055538	10.850333	10.016962	10.137446	10.098434	10.776378	9.974679	8.727249	11.086626	10.945347	9.761351	9.769225	11.822299	9.624616	10.612438	12.410614	11.770058	10.816421	11.475722	9.883041	10.634740	10.648046

AC062021.1	2.939501	3.130994	2.961281	3.224618	3.776777	3.644833	3.179770	3.134769	3.222471	3.288352	3.433819	3.478207	3.143382	3.758791	3.561567	3.100195	3.327729	3.544326	3.278205	3.089357	4.613377	2.873923	5.944243	3.035479	2.960337	2.833615	2.921753	3.555652	2.945514	3.071765	3.618554	3.520242	3.104004	3.007147	3.225038	4.606485	3.589211	3.932381	3.268690	2.987590	3.354699	3.074342	3.686432	3.113539	3.520806	5.183414	3.771953	3.397123	4.621436	3.425458	3.487711	3.547999	3.959039	3.748346	3.579949	3.048798	3.910981	3.055377	3.249584	3.554680	3.010483	3.069128	4.466328	3.025843	3.195162	3.304280	4.298299	3.272701	3.183560	2.927843	3.270526	3.270526	3.083703	3.747084	2.872870	3.195295	3.328676	3.273434	3.017983	4.080493	4.199237	5.711017	3.229455	4.284744	3.201820	4.854590	5.705599	3.296210	5.240664	3.196264	3.186045	3.144331	4.781987	3.028867	3.383664	3.357076	3.620461	3.971168	5.031937	3.402304	5.425587	3.145506	3.085822	2.851000	3.191888	3.101606	3.009356	3.456247	3.624828	3.191453	5.324966	3.971363	5.921041	3.240594	3.212131	5.924182	3.113539	3.010039	3.183972	3.004804	3.192046	3.169872	3.102750	4.074275	4.146648	3.302053	3.094210	3.154426	3.577338	3.196648	3.082015	3.721910	3.688674	3.260708	3.260291	3.274958	3.152335	3.030200	3.200037	4.007174	3.258079	5.475033	3.136389	4.221049	3.686432	3.269604	3.060771	3.873734	3.622433	3.399682	3.772413	3.197854	2.968089	3.133205	3.692972	3.575355	4.000428	3.207511	4.316287	3.276674	3.033581	3.394158	3.124906	3.820601	4.806773	3.597934	3.286969	4.587788	3.381903	3.449181	3.347539	3.690137	4.784543	4.000845	3.292351	4.336552	3.101167	3.039234	3.452771	3.505688	3.156795	4.832709	3.117261	3.252098	3.917040	4.205490	3.791429	3.525894	2.939990	3.721214	4.325277	3.415541	3.837359	3.909466	3.157660	3.643095	3.729032	3.398852	4.346523	3.568873	6.236650	4.155600	4.399157	4.218149	4.554733	5.122893	3.484623	4.079378	3.798389	4.121187	4.225572	4.446690	3.176379	3.507789	3.903586	4.120097	4.598769	3.782722	3.159554	4.608664	3.624461	3.644627	4.046238	3.325163	3.085822	3.032050	3.670112	3.924576	3.107702	4.508763	5.019934	5.616310	3.154548	4.343705	4.912630	2.958164	3.004261	3.259434

AC020663.1	7.043839	6.336540	6.247129	6.059216	6.257597	5.915291	6.552682	7.349764	6.447841	6.868396	6.742430	6.613319	6.360873	5.217371	4.845952	6.696500	6.392707	5.721042	6.453304	6.678786	5.948433	6.768807	7.724547	5.889945	5.749191	6.492653	6.599750	6.035454	6.110433	6.349577	6.769612	7.338621	7.899733	6.406726	6.606716	6.910016	6.632208	6.462351	6.626777	5.872814	6.164766	6.493156	7.197133	6.305436	6.608518	6.266222	5.917767	7.295374	7.313210	6.846929	6.771880	7.013769	6.483249	6.764910	6.532242	6.170998	7.032959	6.582607	6.052855	6.245286	6.101173	6.149498	6.864398	6.513413	6.303063	6.382653	6.542210	5.993219	5.860480	5.879823	6.938731	6.156847	6.264678	6.701586	6.782927	6.527507	6.399293	6.213566	6.644989	7.032798	6.109395	6.908452	7.338002	7.101874	7.507090	7.314004	6.940443	7.158379	6.458853	6.203297	6.293873	6.269984	6.471578	6.924368	6.837708	6.901263	6.518553	6.535813	7.437391	6.231093	5.677876	6.294553	6.937644	6.247129	6.489031	6.300025	6.368195	6.567229	6.057447	6.436771	5.490755	5.859782	5.695283	6.472916	5.702406	3.475806	6.475242	6.409866	6.710559	6.443376	6.138928	6.197911	5.994297	5.580219	5.882107	5.782921	6.654828	6.598280	6.125147	6.187221	6.201016	5.237270	4.308576	4.646207	6.394679	6.747557	5.634192	6.653361	7.006750	5.153573	6.512094	7.429995	5.649730	5.335758	7.677137	5.963234	5.745522	5.777806	6.098721	7.129090	6.076577	6.946970	6.489335	6.507989	6.794137	6.608356	5.514243	6.233766	6.071333	5.715301	6.190787	7.046566	5.894762	6.514869	6.333488	5.651640	6.040557	7.183448	6.706548	6.101173	6.759287	7.813386	6.581133	6.526516	6.161869	7.711380	7.156070	5.975696	6.445240	5.961797	6.086417	7.537785	6.101534	5.501007	5.930416	5.635100	6.206911	5.802237	6.241268	6.805391	6.251495	6.289971	6.755554	6.564827	6.408030	6.759287	6.281963	6.249479	6.678786	6.513257	6.175069	7.596093	6.577011	7.638162	6.488381	6.354258	7.265880	6.477635	5.910637	6.421746	6.164413	6.118621	6.296097	6.515723	6.702745	5.931261	5.841877	6.752554	6.342844	5.895133	6.230595	5.779623	5.367401	7.247008	6.369883	6.482390	6.381820	7.378634	5.825842	5.131199	4.856053	4.625504	6.549177	5.224480	5.121711	6.035454	6.176433	6.110611

LINC01391	3.647373	3.689322	3.359154	3.177091	3.296988	3.398506	3.332891	4.103651	3.747497	3.449975	3.654812	3.613360	3.285959	4.066061	3.876342	3.234953	3.736280	7.236888	4.690520	3.269270	5.547320	2.797548	6.852010	3.237709	2.946251	2.949946	3.098076	3.134643	3.225297	3.096526	3.761949	3.443636	3.841707	3.578380	3.662430	4.175536	3.762370	3.521143	3.571687	3.501239	3.557169	3.989435	3.707077	3.422057	3.907084	5.945313	3.779768	3.731504	5.066111	4.069644	3.980864	3.914887	4.606057	4.477837	4.188223	3.210436	5.462933	2.930955	2.930800	3.873734	2.871866	3.238168	4.115709	3.421880	3.646235	3.595556	2.853836	3.360160	3.346247	3.180813	3.134992	3.007450	3.310696	3.022068	3.288775	3.399529	3.054547	3.072563	3.025936	3.964886	3.563815	4.196467	3.643475	4.322686	3.491185	4.880948	2.904877	3.303975	3.316944	3.781438	3.377215	3.184762	3.521873	3.404877	3.016992	3.081673	3.997047	3.569850	3.415052	3.223498	3.371537	3.313340	3.119866	3.194860	4.607348	3.376866	4.033222	3.791429	3.080195	3.343649	3.012965	3.666279	4.102823	4.525222	4.286569	4.963752	3.446902	3.160172	3.289532	3.180407	3.933686	3.208207	3.269121	4.456478	3.878499	2.959741	3.435630	3.443636	4.930410	3.886551	3.798389	3.174870	3.706865	3.455662	3.370658	4.331456	7.413845	3.403532	3.448800	4.829983	3.629641	6.201380	3.905077	4.578613	3.174610	2.973255	3.279133	3.099736	3.592821	3.582100	3.394158	3.736901	3.374079	3.579178	4.981142	3.803066	3.737108	3.483534	3.229119	3.304432	3.745674	3.716284	3.502531	4.448705	5.459844	3.540515	3.150130	5.530879	4.113965	4.000845	5.748485	5.171289	4.710741	3.680841	3.117616	5.199473	6.513088	2.793797	3.590298	2.777877	3.351386	4.252167	4.067751	4.007174	3.818223	2.816359	3.678427	3.499802	2.954687	3.073566	3.074342	5.237472	3.059490	3.088772	3.367694	3.053021	3.176019	3.161949	3.320089	3.944838	5.966091	3.723511	3.668479	4.093706	3.677408	3.469619	4.242727	3.055916	3.623070	3.565063	2.975163	3.241621	3.517346	3.390862	3.150634	3.126165	3.124783	4.202274	4.259179	3.930017	3.326468	2.950634	2.842344	6.297082	4.195839	3.398506	3.754254	3.601857	3.281352	2.920076	4.812075	4.358969	4.393599	5.068793	4.584561	3.842751	3.209109	3.699776

LINC01473	6.320141	5.101551	5.411498	5.815816	5.971051	5.788863	4.977057	7.044502	6.530971	5.281154	5.938282	5.744449	4.479552	4.510948	4.053015	5.938629	6.527025	4.530746	6.901263	5.809240	7.357319	6.454454	7.364043	5.983507	6.119227	6.139091	6.091996	6.552999	5.603033	5.109134	6.936495	4.457789	4.637149	4.850976	4.736547	4.781151	5.061593	5.866650	6.219047	5.951633	5.674417	5.875145	5.212965	5.027033	5.234034	5.175953	5.489143	4.893555	5.906156	5.302829	5.397646	5.268480	4.868737	4.343480	4.908825	5.010327	4.447109	5.915113	5.439680	5.114771	5.724545	4.865575	6.094614	6.212161	5.536065	5.273498	5.393558	5.634913	5.810937	5.972886	5.506022	5.540532	5.770104	5.552778	5.814250	6.052691	5.869712	6.033636	6.118183	5.074842	5.019934	4.575001	5.412417	6.110778	5.870430	5.874564	5.545624	6.184870	5.895493	6.188082	6.036011	6.043307	5.096968	5.612184	5.493884	4.982359	5.305994	5.261064	4.305328	5.872991	5.534481	4.802376	5.140892	5.276363	5.882835	5.766251	7.776338	5.894247	6.317250	5.314416	6.477985	6.571931	6.122011	5.647837	6.257069	5.189553	6.205198	6.035254	5.739006	6.129182	5.355794	5.322285	5.357975	5.680842	5.998849	4.837863	7.154025	6.357836	5.004573	5.342348	5.067171	4.393198	4.289203	4.809510	5.887621	6.349945	5.161480	7.084910	5.717648	6.751134	6.107970	7.797700	6.344049	5.399987	6.040904	5.638458	6.023770	5.984867	5.239063	6.099247	5.202453	5.833332	4.235322	5.055139	4.944783	5.293505	5.860863	6.200479	6.344739	5.588508	6.292024	5.549015	4.664961	4.826274	4.813536	5.329117	4.881804	5.867788	5.580219	5.194061	5.863653	5.017906	4.931205	4.994155	5.002508	5.327510	5.870804	5.817622	5.794569	6.453116	4.663683	6.118709	6.261164	5.855821	5.464226	5.774012	5.928833	5.778374	6.666875	5.760509	5.489572	5.338729	5.604749	5.659219	6.086091	5.914751	5.958590	6.076077	5.234034	5.307713	5.135616	5.664421	5.834643	5.693962	6.162545	5.580595	5.863827	6.164105	5.472912	6.016224	6.829577	5.879425	6.184870	5.701880	5.359285	6.250630	6.146884	4.908825	4.923203	5.651254	5.523096	5.072402	5.255748	5.563905	5.585356	7.757438	5.753589	6.655979	5.551244	6.547889	6.036181	6.405052	5.269057	5.458649	4.486980	5.926480	5.413006	5.308286

KIF25-AS1	3.993727	3.481349	3.696307	3.097510	2.969281	3.143866	3.302221	3.508161	3.422423	3.378073	3.499964	3.317974	4.096860	3.749198	3.393483	3.199539	3.701392	2.957078	4.917690	4.758717	4.902978	4.342640	3.195971	2.892599	2.901334	2.925438	3.091098	2.974825	3.198532	4.175536	3.609384	4.113765	4.819801	3.390005	3.493155	4.028832	3.566383	3.194035	3.375800	3.213948	5.391260	3.608791	4.617086	3.343649	3.756112	5.741365	3.480575	2.914278	4.901535	3.836745	3.802243	3.707302	4.378001	4.205714	4.034967	3.188761	6.189239	3.139813	2.969108	3.626008	2.868595	3.457163	7.678909	3.317445	3.563042	3.498710	2.981438	3.219976	3.403382	2.907863	3.153195	3.311317	3.271303	3.138106	3.146768	4.401806	3.050591	3.024181	4.082384	3.826779	3.462494	4.110743	3.607835	4.181446	3.415875	4.097537	2.892414	4.659849	3.503137	3.646235	3.303625	2.991399	3.723903	3.327268	3.669497	3.016470	3.886748	3.266581	3.128684	2.952851	2.890272	3.729640	3.007147	2.920528	4.613988	3.292205	5.150726	3.471047	3.005943	3.265127	2.845492	2.965658	3.023990	3.318632	3.523658	2.894327	5.258293	3.121463	3.230761	3.107586	3.618554	8.103712	3.162886	2.878713	3.724305	2.947624	3.259725	3.454010	3.139324	3.377564	3.071333	3.080777	3.170383	3.157765	2.967039	3.607261	2.875475	5.214626	9.470011	4.165795	4.194980	5.027874	2.855056	2.820203	2.879209	2.906828	2.891974	2.883284	3.528493	3.471876	3.442575	3.634676	3.221235	3.408711	3.744452	3.625008	3.304432	3.363192	3.090979	4.958542	3.316769	10.286805	3.396129	4.254130	5.199473	3.186612	2.853467	4.994379	3.730876	3.658843	3.505145	4.227030	4.355913	3.578183	3.097843	10.928906	3.034597	2.840727	3.298691	2.808844	4.138014	4.104747	3.910122	3.908793	3.720796	2.930172	3.421969	4.436502	4.789935	3.078861	3.520622	3.262344	3.235083	3.030691	3.344796	3.052890	3.091325	3.661837	3.260708	4.313602	4.570485	3.661635	3.624648	4.498125	3.192699	3.403382	4.534351	3.209656	3.494647	3.458409	2.904877	3.335311	3.552388	3.302676	3.059814	2.952123	2.938338	3.941720	3.228037	3.030788	9.012996	2.917967	2.841712	4.974204	3.216664	5.202855	3.428989	3.360160	3.166625	2.816668	2.858268	2.919761	3.217115	3.072446	2.881013	3.213260	3.164694	3.339444

LINC00271	5.999019	4.240972	3.966177	5.521370	5.350251	4.776772	5.032354	3.897372	5.399987	5.341041	5.985373	5.546959	6.129339	4.929067	4.827617	5.183621	3.833785	5.621097	4.560116	5.771240	4.692443	4.304851	3.529405	5.366809	4.752349	5.921968	5.577407	4.983002	5.327707	5.610320	6.920646	5.725344	4.931984	6.074503	5.670843	4.642591	5.287944	4.434754	4.030852	4.800062	4.234408	4.680510	5.666294	5.007036	5.724545	5.667246	5.161480	3.960729	6.203650	5.767949	6.014517	6.191829	6.067709	6.141195	4.797463	4.603540	5.994297	4.920418	5.848984	5.501596	4.625504	5.146342	3.803679	3.502531	4.360033	4.923847	4.832287	4.545772	4.129602	4.164473	5.151735	5.275754	5.390063	5.196906	5.342558	4.595765	4.874251	4.634126	5.027663	4.770748	6.196567	4.546888	5.294248	5.758891	5.726450	5.846949	5.621468	5.748312	4.163347	5.156018	5.038906	3.354007	4.773888	4.806580	5.955069	5.745747	5.116611	6.920987	5.740064	5.314218	5.124493	5.464037	5.448989	5.408723	4.591859	4.301736	4.755113	3.595367	3.256748	4.794571	3.482229	3.692171	4.651571	5.520055	4.263766	5.518786	6.040557	4.968621	6.338219	5.116018	4.216647	3.828858	4.484799	3.777351	4.161178	4.792316	7.752132	5.940777	5.393177	4.996504	4.839829	4.955407	4.294363	4.710123	6.340056	5.629710	6.503174	4.293954	5.648403	4.355705	4.385646	5.695665	5.917422	5.218947	5.692666	3.945528	6.267418	5.381153	6.357670	7.127371	4.228779	5.440816	5.564486	5.651640	5.824779	5.543158	4.337446	4.162019	4.023218	4.889600	3.556033	6.429182	5.333915	5.362349	5.133206	5.151943	4.244526	6.595852	5.807949	4.067541	6.372450	5.354296	5.712111	6.371417	4.470544	6.624196	6.778458	5.731757	5.980847	5.679386	5.291392	4.029058	4.532465	4.583926	4.965208	4.513800	4.827617	4.428875	5.248396	6.127483	5.298852	4.274425	5.979208	4.732732	4.412646	4.928862	6.149498	4.667150	5.453218	5.680644	6.270170	6.533240	4.232008	5.817964	6.076577	6.032910	7.022026	4.247330	4.381162	5.820796	4.068580	4.963547	4.351993	4.605847	5.456476	6.197741	6.854771	4.206826	5.565581	5.709373	7.602148	5.152964	3.870938	4.957731	4.541317	3.777146	3.792266	3.700948	3.835245	3.347910	4.096230	5.286792	4.706826	5.529351	4.886886	4.976883	5.922745	5.907032

AC233280.1	4.241181	4.143646	4.841693	3.972227	5.538051	5.645429	4.786402	5.419596	5.835000	6.555493	5.234225	5.183414	5.865783	5.638800	5.371258	5.524027	5.354296	5.772933	5.642738	4.669702	6.250481	6.979414	2.961168	5.871338	5.183621	5.259448	4.655551	5.483684	5.081245	5.433353	6.963876	4.183437	4.085214	4.396141	5.665514	5.146342	4.515287	4.851173	4.898779	5.796753	5.226492	4.830197	4.817515	4.290904	5.104831	5.407741	4.610790	3.169155	5.909357	4.864543	5.348890	4.461136	4.000622	6.148819	4.658804	4.982161	4.778843	4.321835	4.800293	5.350672	5.567473	6.545648	3.901874	5.618556	4.000224	4.814569	5.779447	4.584149	4.614433	4.398722	4.694111	5.184411	4.831039	4.970105	5.497164	4.222970	4.830608	4.286998	4.584786	4.979544	4.926168	3.979044	5.660863	5.233026	4.920011	6.117316	5.441987	5.802945	3.811201	3.952698	5.746099	4.504144	4.226582	5.043266	4.928275	5.647662	4.916860	6.285141	6.185885	5.824047	6.070817	5.196697	4.763986	4.644478	5.747936	5.063441	6.051126	5.701183	5.796922	6.052855	5.310505	5.653503	6.197582	5.500462	6.624196	6.425455	5.083842	5.095423	5.667961	4.388910	3.849937	4.394513	4.034967	6.730623	5.247211	4.249700	4.464171	3.952050	5.877609	4.106504	4.442638	4.774707	5.630261	5.238461	4.872775	4.371560	5.368189	4.475158	4.211644	4.614095	6.741451	5.424841	5.497735	4.531413	4.101263	4.260010	5.444097	5.291576	5.222725	6.104959	4.666684	3.992473	4.811050	5.288957	4.422881	4.430487	3.653615	4.405609	4.667368	5.279336	3.016157	3.667303	4.105442	4.454382	5.021566	5.580219	4.004983	5.744092	3.389637	4.426112	5.696189	5.004365	5.439113	4.273115	4.551752	4.909040	3.314311	2.971520	4.683764	5.182396	5.291194	5.607219	5.284147	4.697895	4.494208	4.098630	3.994432	4.179668	2.818845	4.234838	4.275963	4.870222	4.030399	4.411759	3.928725	4.190383	3.886152	4.621851	4.106504	4.729138	4.364615	6.265874	4.755320	3.981983	5.683425	4.194775	4.980732	3.412911	3.430988	7.086749	4.003905	4.080493	4.118816	4.425470	4.223185	4.970105	5.947758	4.927430	5.510469	5.939707	5.007256	5.586055	3.906188	7.190009	5.299668	5.396337	5.433001	6.146017	4.787643	5.580219	5.526678	7.567340	4.487836	7.977839	6.764246	4.817712	4.995664	4.612954

AL136988.2	11.605660	11.731533	11.595221	11.879544	11.649159	11.352321	11.536751	12.082750	11.430746	11.897073	11.942282	11.868313	11.655906	12.613710	11.172547	11.381887	11.181128	11.616116	11.717044	11.309686	11.641173	12.771018	12.173726	11.807983	11.764203	11.432696	11.316005	12.018090	11.248076	11.994731	11.164048	11.794698	11.739222	12.243771	11.733781	11.464934	11.412105	11.637961	12.241742	12.145311	11.588472	11.928915	11.829413	11.780148	11.932456	11.562614	11.455816	11.799525	10.992250	11.608893	11.769304	11.741168	12.091683	11.277350	11.892007	11.689592	11.691697	11.701916	11.816892	12.046313	11.588775	11.528777	12.342480	12.156958	12.476535	12.011409	11.735962	11.285928	12.181281	11.938632	11.721223	11.233924	11.533775	11.383209	11.446601	11.616246	11.783598	12.234198	11.802240	11.214660	11.600906	11.363862	12.117551	11.412105	11.951428	11.410682	11.635707	11.833001	11.438510	11.764948	11.664327	11.639395	11.325112	11.713495	11.565388	11.333751	10.791885	10.783168	11.727818	11.365804	11.557455	11.296731	11.660141	11.568715	11.788973	10.643415	12.041232	12.424072	11.703035	11.169002	11.939528	11.447943	11.758591	11.392179	10.951475	10.837243	11.668608	11.785826	10.914915	11.842489	12.191674	12.494508	12.493861	11.851418	11.503051	12.607398	12.413808	12.457538	11.396538	12.531515	12.224619	13.182111	13.148784	12.733879	11.488900	12.952366	12.010940	11.878463	12.884027	12.382744	12.755658	12.015376	12.723985	13.266997	13.578768	11.957393	10.821767	11.042557	12.852304	11.721674	12.152396	12.801093	11.926124	12.300500	11.826829	12.486970	12.381309	13.541913	12.563485	12.463072	12.522828	11.818076	12.098917	12.182961	12.144699	11.695468	11.967950	11.879689	13.297173	12.833106	11.920531	11.929674	13.937428	12.310609	12.771543	13.137749	11.979444	12.485843	12.311740	13.333932	12.523476	11.989871	12.771214	11.668018	12.406200	12.965441	11.931707	12.558955	12.300335	11.215233	12.085596	11.266930	12.948751	12.251358	12.715418	12.471342	12.432907	12.391894	12.503389	12.562827	12.636458	12.828929	12.433717	12.565935	12.284575	13.297375	12.471970	12.399011	12.814055	13.709428	12.068749	12.701417	13.576247	12.134671	13.170169	12.933669	11.238299	12.561718	11.981434	13.531252	11.162452	12.325086	12.910176	13.124995	11.227229	12.375982	12.070678	12.051330	11.538542	11.923414	13.941795	12.673286	11.330738	11.290392	11.405580	13.206525	11.401523	12.604080

TMEM72-AS1	3.270231	4.168179	3.622668	4.303765	3.931529	3.173430	3.329843	4.584996	4.226825	3.448991	3.601477	3.738525	3.559545	4.144750	4.064578	3.591339	3.527379	3.618554	5.654433	3.692766	5.255945	4.656195	5.724200	4.051282	3.133914	3.334529	4.384959	4.909483	3.871346	3.163906	3.833571	4.489327	3.657270	3.472931	4.444117	4.088500	3.741358	3.833118	4.389751	3.921822	3.571510	5.064275	3.636664	3.342824	3.878719	5.634913	4.105841	3.037621	3.817590	3.787366	3.796939	3.780400	4.262444	4.232240	4.384959	3.266295	4.199237	3.427251	3.678427	3.858138	2.969823	3.803896	5.162291	4.531822	4.551957	3.800747	2.885728	4.260868	3.253277	3.902722	3.980864	3.420255	3.812920	4.281018	3.980200	3.667489	4.168179	4.081358	4.315607	3.785850	4.113121	4.033413	4.243194	4.306150	4.517223	3.737871	3.518201	4.008008	5.268480	5.810576	4.747474	4.569586	3.750428	3.417570	3.174065	3.380840	3.863442	3.993522	3.325007	4.592086	3.676604	3.637058	3.858553	3.149886	3.945317	4.013112	4.992892	4.260648	4.142379	3.739136	4.174872	4.217298	4.396141	3.886152	3.661635	3.706254	3.877385	3.838235	3.284757	3.808898	4.657066	3.372078	4.006960	3.894646	3.803679	3.162994	4.201411	3.831827	3.431688	3.420840	3.481157	3.258992	3.368911	3.799232	3.590298	3.435897	3.160050	5.126559	3.415052	4.778245	3.571157	4.297673	3.886152	2.864651	4.228324	3.092544	2.829957	3.886962	4.211406	3.933266	3.497840	3.694253	3.266150	3.533669	3.780830	3.774917	3.430988	4.214927	3.981290	3.836911	3.755267	3.637684	3.793394	4.261993	5.252144	4.084550	2.904133	4.090253	3.756112	3.747923	3.589398	4.161815	4.436278	3.567669	3.332078	3.833985	3.315887	3.332891	3.879790	2.855056	3.385360	3.802855	4.464384	4.403105	3.706050	2.865464	3.645616	3.558934	2.871971	3.130736	3.168395	3.542076	3.538985	3.636471	3.638690	3.939428	4.021995	4.135395	3.265442	3.811621	4.494208	3.705075	3.722513	3.573829	3.625205	3.740951	4.021106	3.056976	4.695359	4.474088	4.612707	3.450236	3.271787	3.121240	3.677001	3.210181	3.405245	3.922669	3.281352	3.068147	3.132218	3.245817	3.270657	4.137797	4.259390	4.428056	3.742020	3.784800	3.520038	4.930410	4.087434	4.154991	3.358816	3.501239	3.611605	4.255446	3.780830	4.964988

AC106038.1	3.162277	4.172921	3.689939	3.258402	3.227169	3.711546	3.520038	3.638275	3.356732	3.234379	4.091852	3.641091	3.862377	3.702855	3.082761	3.555846	3.555255	2.870014	4.383833	3.388778	4.652164	3.894213	2.963837	3.190259	2.858432	3.260560	3.333065	2.922883	4.169878	3.044226	3.668288	4.402680	3.711920	3.494456	4.049098	4.148555	3.468909	3.113210	3.288506	3.244844	3.412735	3.360160	3.509205	3.575527	3.642881	5.316861	3.836745	2.856424	3.812109	3.723702	3.523465	3.434709	3.843761	4.200088	4.795822	3.133674	4.067954	3.828629	3.063523	3.658433	2.901917	3.495782	3.613588	3.275122	3.478950	3.438067	3.193392	3.294203	3.493734	2.866692	3.563639	3.684214	3.204421	3.891153	4.225364	3.776955	3.718335	3.187273	3.385360	3.657270	4.231353	3.890511	3.526678	3.813595	4.273784	4.050645	4.390632	3.838007	3.951398	3.542076	3.204137	2.841922	3.718521	3.254330	3.579178	3.593435	3.677408	4.438066	3.865565	3.467841	3.246954	3.651989	3.450064	3.124425	4.539977	4.393824	3.121586	3.207511	3.652825	3.114865	3.908360	3.163794	2.986272	3.295582	4.726203	4.966918	3.176610	3.613588	3.038227	3.232304	3.438920	3.047106	3.676604	4.198394	3.591899	3.320580	4.647912	3.911207	3.606173	3.552978	5.662165	3.150988	3.752726	2.968562	3.631286	3.448991	4.679217	3.824627	3.661635	3.497086	3.423315	2.893899	3.828431	2.938528	3.427639	3.975291	3.388778	4.089608	3.389637	3.513374	3.493155	4.655551	3.203085	4.702350	4.956023	3.535542	3.163139	3.303468	3.113210	3.770137	3.155467	4.500504	3.316489	4.443268	4.688212	3.902531	2.805152	4.072220	3.583778	3.358816	4.361722	5.381551	4.344818	3.978030	3.529232	4.737997	3.706665	2.911822	3.461975	2.846128	3.269432	3.671565	3.802663	3.789513	3.637684	3.128578	3.125395	3.777746	2.828839	4.286998	4.031890	3.201160	3.255752	3.706468	3.619327	3.251979	3.065747	3.620077	3.259296	3.746283	4.256083	3.587089	3.547999	4.931984	5.427041	4.063502	6.797851	3.628398	3.368594	3.257943	2.808409	3.303300	3.343324	3.192046	3.019853	3.694034	3.628019	4.058646	4.025591	3.634285	4.055845	3.455153	3.014925	5.278766	3.404378	3.214600	3.843155	3.218416	3.096025	3.541683	3.565350	4.807206	3.291431	3.808655	3.917257	3.416555	3.463557	4.991612

LINC00534	3.162277	4.172921	3.689939	3.258402	3.227169	3.711546	3.520038	3.638275	3.356732	3.234379	4.091852	3.641091	3.862377	3.702855	3.082761	3.555846	3.555255	2.870014	4.383833	3.388778	4.652164	3.894213	2.963837	3.190259	2.858432	3.260560	3.333065	2.922883	4.169878	3.044226	3.668288	4.402680	3.711920	3.494456	4.049098	4.148555	3.468909	3.113210	3.288506	3.244844	3.412735	3.360160	3.509205	3.575527	3.642881	5.316861	3.836745	2.856424	3.812109	3.723702	3.523465	3.434709	3.843761	4.200088	4.795822	3.133674	4.067954	3.828629	3.063523	3.658433	2.901917	3.495782	3.613588	3.275122	3.478950	3.438067	3.193392	3.294203	3.493734	2.866692	3.563639	3.684214	3.204421	3.891153	4.225364	3.776955	3.718335	3.187273	3.385360	3.657270	4.231353	3.890511	3.526678	3.813595	4.273784	4.050645	4.390632	3.838007	3.951398	3.542076	3.204137	2.841922	3.718521	3.254330	3.579178	3.593435	3.677408	4.438066	3.865565	3.467841	3.246954	3.651989	3.450064	3.124425	4.539977	4.393824	3.121586	3.207511	3.652825	3.114865	3.908360	3.163794	2.986272	3.295582	4.726203	4.966918	3.176610	3.613588	3.038227	3.232304	3.438920	3.047106	3.676604	4.198394	3.591899	3.320580	4.647912	3.911207	3.606173	3.552978	5.662165	3.150988	3.752726	2.968562	3.631286	3.448991	4.679217	3.824627	3.661635	3.497086	3.423315	2.893899	3.828431	2.938528	3.427639	3.975291	3.388778	4.089608	3.389637	3.513374	3.493155	4.655551	3.203085	4.702350	4.956023	3.535542	3.163139	3.303468	3.113210	3.770137	3.155467	4.500504	3.316489	4.443268	4.688212	3.902531	2.805152	4.072220	3.583778	3.358816	4.361722	5.381551	4.344818	3.978030	3.529232	4.737997	3.706665	2.911822	3.461975	2.846128	3.269432	3.671565	3.802663	3.789513	3.637684	3.128578	3.125395	3.777746	2.828839	4.286998	4.031890	3.201160	3.255752	3.706468	3.619327	3.251979	3.065747	3.620077	3.259296	3.746283	4.256083	3.587089	3.547999	4.931984	5.427041	4.063502	6.797851	3.628398	3.368594	3.257943	2.808409	3.303300	3.343324	3.192046	3.019853	3.694034	3.628019	4.058646	4.025591	3.634285	4.055845	3.455153	3.014925	5.278766	3.404378	3.214600	3.843155	3.218416	3.096025	3.541683	3.565350	4.807206	3.291431	3.808655	3.917257	3.416555	3.463557	4.991612

LINC01878	3.683833	3.855323	4.871491	4.873439	5.529728	4.996956	3.727139	4.773888	5.260039	4.640721	4.761419	3.915505	4.514214	5.666110	4.724322	5.369136	3.718521	4.248826	5.149291	4.365717	5.641019	7.303037	3.733411	5.314846	5.505847	5.427255	4.136278	5.544106	4.678590	3.689738	4.787242	4.429095	5.242255	4.941966	3.945977	4.210138	3.797157	3.645219	3.796319	4.308348	5.019323	5.110765	4.436502	3.931917	3.925247	5.966637	4.631333	3.910981	3.953135	4.173173	4.004337	4.026873	4.644478	4.611414	4.543312	4.394513	4.491346	3.522236	4.290484	4.415349	3.031257	4.274425	4.238752	4.837031	3.757728	3.785223	2.914833	3.738525	3.614174	3.289685	3.318310	4.144295	4.951226	3.445260	3.263968	3.464678	4.309062	3.870296	3.876562	4.009111	5.606848	4.304213	5.928111	4.313602	4.214482	3.925693	5.547864	3.793794	3.941189	4.088500	3.612379	3.860653	4.525652	4.153649	4.214253	4.528407	4.007400	5.966457	5.722705	4.321019	5.350464	3.640480	5.321701	4.831465	5.818327	4.249492	5.838997	4.501376	5.361740	3.839470	5.245413	5.604354	5.407524	5.216196	3.812109	6.209840	4.335291	5.074630	5.490582	3.890511	4.047589	3.574031	8.031490	5.697884	4.052798	3.264817	3.480575	3.855323	3.199655	3.651376	4.644041	3.263968	4.476026	3.672869	6.128820	4.182761	4.616876	3.409194	3.551811	4.879846	4.420003	3.089684	4.955831	3.808898	3.426395	5.044730	7.136879	7.124664	4.235090	3.977012	3.538645	4.015628	3.503982	3.661837	3.894646	3.857006	3.915108	3.647986	3.676420	3.462866	5.084868	4.499005	3.993727	4.486790	5.495098	4.496204	3.199019	4.276377	4.351993	4.071257	3.856144	4.463060	4.852018	5.286594	3.937075	4.503720	3.113321	5.160676	4.153415	4.383833	4.529888	4.388677	4.328092	4.150204	3.937922	2.863812	3.718738	3.593637	3.008858	3.560309	3.814191	4.451610	4.068580	3.923504	3.797572	4.000224	3.422423	4.127199	3.765302	4.677961	4.918089	5.660863	3.858971	4.638845	3.300392	3.822877	3.485520	3.559545	3.701577	3.614770	3.622038	3.378791	3.564009	3.427251	3.917902	4.289203	5.865953	4.922760	3.650021	3.510009	4.302412	4.284044	4.627372	5.123919	4.333986	6.233086	5.293881	4.171213	6.237625	4.994800	5.670662	6.028563	5.265055	3.356572	3.902722	4.444117	4.300892	3.494456

AC093865.1	3.683833	3.855323	4.871491	4.873439	5.529728	4.996956	3.727139	4.773888	5.260039	4.640721	4.761419	3.915505	4.514214	5.666110	4.724322	5.369136	3.718521	4.248826	5.149291	4.365717	5.641019	7.303037	3.733411	5.314846	5.505847	5.427255	4.136278	5.544106	4.678590	3.689738	4.787242	4.429095	5.242255	4.941966	3.945977	4.210138	3.797157	3.645219	3.796319	4.308348	5.019323	5.110765	4.436502	3.931917	3.925247	5.966637	4.631333	3.910981	3.953135	4.173173	4.004337	4.026873	4.644478	4.611414	4.543312	4.394513	4.491346	3.522236	4.290484	4.415349	3.031257	4.274425	4.238752	4.837031	3.757728	3.785223	2.914833	3.738525	3.614174	3.289685	3.318310	4.144295	4.951226	3.445260	3.263968	3.464678	4.309062	3.870296	3.876562	4.009111	5.606848	4.304213	5.928111	4.313602	4.214482	3.925693	5.547864	3.793794	3.941189	4.088500	3.612379	3.860653	4.525652	4.153649	4.214253	4.528407	4.007400	5.966457	5.722705	4.321019	5.350464	3.640480	5.321701	4.831465	5.818327	4.249492	5.838997	4.501376	5.361740	3.839470	5.245413	5.604354	5.407524	5.216196	3.812109	6.209840	4.335291	5.074630	5.490582	3.890511	4.047589	3.574031	8.031490	5.697884	4.052798	3.264817	3.480575	3.855323	3.199655	3.651376	4.644041	3.263968	4.476026	3.672869	6.128820	4.182761	4.616876	3.409194	3.551811	4.879846	4.420003	3.089684	4.955831	3.808898	3.426395	5.044730	7.136879	7.124664	4.235090	3.977012	3.538645	4.015628	3.503982	3.661837	3.894646	3.857006	3.915108	3.647986	3.676420	3.462866	5.084868	4.499005	3.993727	4.486790	5.495098	4.496204	3.199019	4.276377	4.351993	4.071257	3.856144	4.463060	4.852018	5.286594	3.937075	4.503720	3.113321	5.160676	4.153415	4.383833	4.529888	4.388677	4.328092	4.150204	3.937922	2.863812	3.718738	3.593637	3.008858	3.560309	3.814191	4.451610	4.068580	3.923504	3.797572	4.000224	3.422423	4.127199	3.765302	4.677961	4.918089	5.660863	3.858971	4.638845	3.300392	3.822877	3.485520	3.559545	3.701577	3.614770	3.622038	3.378791	3.564009	3.427251	3.917902	4.289203	5.865953	4.922760	3.650021	3.510009	4.302412	4.284044	4.627372	5.123919	4.333986	6.233086	5.293881	4.171213	6.237625	4.994800	5.670662	6.028563	5.265055	3.356572	3.902722	4.444117	4.300892	3.494456

LINC02145	5.569403	4.960817	5.381734	5.302426	5.669061	6.419483	5.639734	6.011759	5.981021	6.537292	6.470882	6.184870	5.693030	5.723646	5.987550	5.960749	4.987082	5.627815	4.782618	5.469704	5.878510	5.217569	4.256493	5.790268	5.199677	5.592741	6.647222	5.678435	6.808244	6.273370	5.912957	5.309441	5.424652	5.833722	5.499091	5.485387	4.505232	5.186019	5.281755	4.315607	6.471216	5.216600	6.002186	5.192286	5.669438	5.288335	6.448351	5.055944	6.093378	6.204353	5.625588	5.626914	4.866199	5.125769	5.369930	6.828463	5.298266	5.414539	5.612184	5.066344	4.291112	7.478135	5.703574	4.568743	5.190944	5.293505	5.206028	4.911992	5.306383	5.204869	5.497164	5.555072	5.825312	5.455882	5.628798	5.365800	4.995442	4.915556	5.308084	4.692874	5.231386	5.114355	4.833968	3.969226	4.222533	5.038906	3.620663	4.094115	5.339915	4.975473	4.211406	4.046498	4.998590	5.311259	5.444298	5.605914	5.383539	6.010016	6.081078	3.913993	5.770487	5.535095	5.574359	5.698228	5.207639	6.622253	5.203864	6.121304	4.989759	6.720151	4.549553	5.690430	5.609543	6.552682	5.890639	5.656461	4.119034	6.168083	7.064609	5.427843	5.461399	5.019719	6.286218	6.303246	6.651927	6.749103	6.437425	5.513706	7.461663	6.812101	7.617692	7.020298	5.612522	5.927411	5.624864	6.101534	7.394285	6.361860	6.272853	5.437645	5.385402	5.158488	6.973051	5.460043	5.755895	6.511783	6.925012	8.499004	6.397130	6.144448	5.533510	6.353937	5.814250	6.028736	6.458853	5.037135	5.006189	6.510260	5.304038	6.736595	4.988699	5.569403	5.426219	5.394569	4.868737	6.876248	5.217371	6.330441	5.348703	5.779986	5.700263	4.144522	6.197075	4.832287	6.720650	4.074510	4.310828	5.514823	4.412213	4.078461	6.933492	6.316736	5.334186	7.134208	4.803917	3.340445	4.529657	5.141933	3.370267	5.991459	5.625058	6.662321	5.392969	5.138251	6.045528	5.014044	5.265256	6.264325	4.162728	5.411869	7.747180	3.861705	4.623548	3.742020	5.788677	6.091996	4.827193	3.587651	4.472556	5.416020	3.173569	4.759964	5.705599	5.278530	5.697116	5.571459	6.695389	5.534087	4.472347	6.244951	5.491318	6.678326	5.227496	5.007858	7.448876	6.309443	5.509173	5.502582	7.049913	5.051969	6.316736	5.455882	6.780224	6.723049	5.789578	6.892688	7.160752	6.443703

AL450998.3	11.228274	11.421337	11.654988	12.315273	12.292362	12.199524	12.261347	12.550390	12.256799	12.110554	12.344708	12.256489	12.408357	12.837237	11.989871	12.067188	12.145157	12.281444	13.102010	12.473611	12.597651	12.794485	12.862774	12.486157	12.324636	12.140840	11.713327	12.116357	11.566391	11.654225	12.525969	11.838717	11.976391	12.221872	11.659417	11.899287	12.089667	12.583792	12.712606	12.737126	11.984838	12.679231	12.111299	12.042272	12.197056	12.102695	11.579668	12.118645	11.759353	11.852632	11.429272	11.939377	11.788973	12.121584	11.977595	12.038942	12.037911	12.219214	12.175134	12.362443	12.321897	11.937174	12.380518	12.708077	12.346950	12.017200	11.933193	11.735962	11.971773	12.016290	11.421630	11.413991	11.923566	11.653340	11.944085	11.543468	11.694395	12.028865	11.602692	11.941813	11.632245	11.623005	11.467784	11.869781	11.807364	11.900148	12.236059	12.369887	12.012208	11.739666	11.624718	12.093373	11.621293	11.661642	11.126061	11.777140	11.672829	12.413496	12.183450	12.021503	12.366548	12.247673	12.079262	12.510794	13.126712	12.077128	12.670010	13.182307	12.933307	11.929992	12.840179	12.812113	13.286480	11.686355	12.120977	10.671652	12.011560	12.068295	11.889157	12.024345	11.820206	11.798936	12.593292	11.966406	11.271942	12.586624	12.093826	13.267817	12.518430	12.589286	11.401220	11.788222	12.407615	12.549244	11.513041	12.140678	11.854007	12.699345	11.751007	12.840179	12.580172	12.551868	12.135587	12.399323	12.762553	11.688416	12.326856	11.301743	12.004721	12.519526	12.197990	12.598661	12.780199	11.837992	11.213047	12.547774	11.990950	12.707057	12.068126	12.046914	11.706718	12.007474	12.547310	12.369200	12.085120	12.711163	11.943350	11.956053	12.719524	12.160178	12.086064	11.749227	12.170949	12.155089	11.205541	11.828835	11.894727	12.141279	11.990173	12.927977	12.236505	12.586980	12.255020	11.936861	12.354824	12.576234	12.378030	12.027045	12.166779	11.367880	11.488318	11.664624	12.177989	11.936777	11.576476	11.857954	11.988461	11.862748	11.640267	12.265271	12.593292	12.723489	11.994409	12.222032	12.287067	12.056828	11.518252	13.266215	12.109661	11.159782	11.912061	11.126207	11.876433	12.001025	11.885697	11.856880	12.281925	12.594107	12.247353	11.675164	12.110108	12.108644	12.274074	12.789412	11.666726	12.767982	12.647433	12.472784	12.190289	12.687272	12.306362	11.398826	11.297513	10.739457	11.739966	12.688955	11.389966	12.120667

AL590867.1	3.648603	3.608603	3.313790	3.190259	3.021235	4.031273	3.387739	3.386535	3.518728	3.492579	3.636869	3.821651	3.298546	4.167541	4.034967	3.233940	3.737108	3.710276	4.727455	3.283015	5.488194	3.778554	5.224480	3.274802	3.222539	3.038578	3.077719	3.327729	3.206525	3.095013	3.725775	3.864506	3.543244	3.108051	3.981754	4.186897	3.799443	3.607655	3.523658	3.409363	3.510727	4.079591	3.666066	3.449181	3.828431	5.843704	3.718738	5.563726	3.841099	4.119254	4.167541	5.597613	4.604611	4.413089	4.152302	3.179770	4.259773	3.096798	3.795908	3.674735	3.317763	3.198410	3.986510	3.312397	3.485520	3.405574	2.803798	3.321225	3.209388	3.375064	3.118405	2.944169	3.278544	3.316489	3.290793	3.374402	3.357413	3.471876	3.103870	3.886347	3.490280	4.239453	3.516425	3.982401	3.443636	4.416652	3.624648	3.252973	3.002248	3.632741	3.431159	2.911537	3.006708	3.308242	3.003425	3.114384	3.973973	3.268548	3.103870	2.971623	3.089903	3.339444	3.078974	3.162507	4.417754	3.888075	3.500685	3.631499	3.061222	3.344944	3.393310	3.564191	3.223091	3.396455	3.618187	3.677808	3.037944	3.142565	3.267648	3.179770	3.797157	3.183972	3.204941	3.145941	4.781346	3.297937	3.268254	3.535381	3.529232	3.487325	3.777553	3.237267	3.420255	3.593637	3.129913	3.697556	3.319425	3.907475	3.464489	4.732302	2.771095	5.405739	3.068364	2.993827	3.213397	3.587089	3.111404	3.287565	3.596331	3.537427	3.896720	3.424199	4.771198	3.770527	3.878061	3.869015	3.854056	3.397123	3.153309	3.247223	3.823735	4.905873	3.530715	4.344368	5.403869	3.872419	4.169649	4.128728	5.763590	4.288556	5.392206	4.360482	4.619476	3.608025	3.098676	3.825521	2.894239	3.388059	4.248826	2.963327	3.311947	4.058446	3.874160	3.736070	3.487138	2.776734	3.712709	3.609803	3.352560	3.084037	3.027183	3.617825	3.279422	3.090114	3.348845	3.835458	3.213524	3.167396	3.316047	3.847370	4.762051	3.616696	3.607467	3.452771	3.467670	3.370996	3.416207	2.909854	3.498889	3.445636	3.494265	3.320420	3.423646	3.332726	3.371691	4.084323	3.227738	3.773031	3.270368	3.025936	3.130994	3.209242	2.947724	4.749521	4.080720	3.533311	3.659231	3.576726	3.767372	3.287419	3.942389	3.218112	3.292658	3.346067	3.645020	3.213524	3.245240	3.417203

AL358134.1	3.648603	3.608603	3.313790	3.190259	3.021235	4.031273	3.387739	3.386535	3.518728	3.492579	3.636869	3.821651	3.298546	4.167541	4.034967	3.233940	3.737108	3.710276	4.727455	3.283015	5.488194	3.778554	5.224480	3.274802	3.222539	3.038578	3.077719	3.327729	3.206525	3.095013	3.725775	3.864506	3.543244	3.108051	3.981754	4.186897	3.799443	3.607655	3.523658	3.409363	3.510727	4.079591	3.666066	3.449181	3.828431	5.843704	3.718738	5.563726	3.841099	4.119254	4.167541	5.597613	4.604611	4.413089	4.152302	3.179770	4.259773	3.096798	3.795908	3.674735	3.317763	3.198410	3.986510	3.312397	3.485520	3.405574	2.803798	3.321225	3.209388	3.375064	3.118405	2.944169	3.278544	3.316489	3.290793	3.374402	3.357413	3.471876	3.103870	3.886347	3.490280	4.239453	3.516425	3.982401	3.443636	4.416652	3.624648	3.252973	3.002248	3.632741	3.431159	2.911537	3.006708	3.308242	3.003425	3.114384	3.973973	3.268548	3.103870	2.971623	3.089903	3.339444	3.078974	3.162507	4.417754	3.888075	3.500685	3.631499	3.061222	3.344944	3.393310	3.564191	3.223091	3.396455	3.618187	3.677808	3.037944	3.142565	3.267648	3.179770	3.797157	3.183972	3.204941	3.145941	4.781346	3.297937	3.268254	3.535381	3.529232	3.487325	3.777553	3.237267	3.420255	3.593637	3.129913	3.697556	3.319425	3.907475	3.464489	4.732302	2.771095	5.405739	3.068364	2.993827	3.213397	3.587089	3.111404	3.287565	3.596331	3.537427	3.896720	3.424199	4.771198	3.770527	3.878061	3.869015	3.854056	3.397123	3.153309	3.247223	3.823735	4.905873	3.530715	4.344368	5.403869	3.872419	4.169649	4.128728	5.763590	4.288556	5.392206	4.360482	4.619476	3.608025	3.098676	3.825521	2.894239	3.388059	4.248826	2.963327	3.311947	4.058446	3.874160	3.736070	3.487138	2.776734	3.712709	3.609803	3.352560	3.084037	3.027183	3.617825	3.279422	3.090114	3.348845	3.835458	3.213524	3.167396	3.316047	3.847370	4.762051	3.616696	3.607467	3.452771	3.467670	3.370996	3.416207	2.909854	3.498889	3.445636	3.494265	3.320420	3.423646	3.332726	3.371691	4.084323	3.227738	3.773031	3.270368	3.025936	3.130994	3.209242	2.947724	4.749521	4.080720	3.533311	3.659231	3.576726	3.767372	3.287419	3.942389	3.218112	3.292658	3.346067	3.645020	3.213524	3.245240	3.417203

AC008555.4	3.229611	3.056976	3.083503	4.487427	4.359819	5.159865	4.508979	3.189408	3.377394	3.890274	3.437038	4.420461	3.756928	3.830333	3.288923	3.056655	3.405718	3.235083	4.478450	3.060561	5.363470	3.791856	5.391260	3.676420	3.196504	3.137979	3.839112	3.664851	3.489197	6.594381	3.664851	3.745148	6.368368	3.583046	4.647475	4.633497	4.140191	3.087292	3.107702	3.920194	3.827843	3.352062	4.553466	3.619528	4.047376	6.611684	3.454372	5.312635	4.261080	5.158488	3.824361	4.926168	4.877783	3.879790	4.042742	3.675983	4.479959	3.585700	3.830333	3.529232	3.657644	2.888451	3.265866	3.001687	3.308406	3.015552	2.962105	3.161721	3.399850	3.115819	3.187544	3.331129	3.503137	3.351217	4.231567	3.537062	3.290448	3.730048	3.267345	3.421184	3.313496	3.396948	4.831651	3.699776	2.930729	5.410318	4.780099	3.964673	3.375281	3.231044	3.667489	2.940918	3.828244	3.817172	3.876112	3.906869	4.225364	4.389553	3.898000	3.994432	5.507986	3.779584	3.853359	3.614582	4.306815	3.946856	2.944992	4.146648	3.106189	3.091524	3.129580	4.479106	3.586490	3.097843	3.902722	6.644513	3.599539	3.858341	2.984795	3.739973	4.427875	4.785984	5.390655	5.440238	5.495472	5.580595	3.597342	4.334841	4.955209	4.571619	5.449750	5.525913	4.383721	4.203381	4.330333	4.254130	4.046032	3.618945	4.444781	5.556382	3.790786	5.867214	4.371755	4.643391	4.064578	4.212512	3.796526	4.787871	6.156355	4.338539	4.544678	5.250989	5.275329	4.809737	4.888117	4.640321	4.902554	4.517005	4.344134	4.204289	3.311774	4.402007	4.259584	4.549312	6.626161	4.679009	4.606257	5.175953	4.288106	4.682853	4.959791	5.240275	5.506401	5.478043	4.698722	4.935579	4.964389	4.828889	4.320571	4.811675	4.407819	4.890226	4.926387	4.961018	4.526110	4.667368	4.325069	3.954387	4.671850	4.089190	3.818223	4.191032	4.087871	4.172921	4.191032	3.868783	4.177463	4.216869	4.646834	4.712429	5.400880	4.809737	5.401834	4.891867	5.226492	6.073804	4.644041	5.105634	4.513363	4.288341	4.564465	4.532908	4.404898	4.569828	4.066061	4.554733	4.897234	5.166894	4.098630	5.859070	4.248186	4.269404	4.513363	4.666915	4.263555	4.530516	5.069790	4.081762	3.965731	4.307220	3.683162	4.656631	4.053879	5.474055	6.423652	4.395908	4.601911	4.005674

AC008555.2	3.229611	3.056976	3.083503	4.487427	4.359819	5.159865	4.508979	3.189408	3.377394	3.890274	3.437038	4.420461	3.756928	3.830333	3.288923	3.056655	3.405718	3.235083	4.478450	3.060561	5.363470	3.791856	5.391260	3.676420	3.196504	3.137979	3.839112	3.664851	3.489197	6.594381	3.664851	3.745148	6.368368	3.583046	4.647475	4.633497	4.140191	3.087292	3.107702	3.920194	3.827843	3.352062	4.553466	3.619528	4.047376	6.611684	3.454372	5.312635	4.261080	5.158488	3.824361	4.926168	4.877783	3.879790	4.042742	3.675983	4.479959	3.585700	3.830333	3.529232	3.657644	2.888451	3.265866	3.001687	3.308406	3.015552	2.962105	3.161721	3.399850	3.115819	3.187544	3.331129	3.503137	3.351217	4.231567	3.537062	3.290448	3.730048	3.267345	3.421184	3.313496	3.396948	4.831651	3.699776	2.930729	5.410318	4.780099	3.964673	3.375281	3.231044	3.667489	2.940918	3.828244	3.817172	3.876112	3.906869	4.225364	4.389553	3.898000	3.994432	5.507986	3.779584	3.853359	3.614582	4.306815	3.946856	2.944992	4.146648	3.106189	3.091524	3.129580	4.479106	3.586490	3.097843	3.902722	6.644513	3.599539	3.858341	2.984795	3.739973	4.427875	4.785984	5.390655	5.440238	5.495472	5.580595	3.597342	4.334841	4.955209	4.571619	5.449750	5.525913	4.383721	4.203381	4.330333	4.254130	4.046032	3.618945	4.444781	5.556382	3.790786	5.867214	4.371755	4.643391	4.064578	4.212512	3.796526	4.787871	6.156355	4.338539	4.544678	5.250989	5.275329	4.809737	4.888117	4.640321	4.902554	4.517005	4.344134	4.204289	3.311774	4.402007	4.259584	4.549312	6.626161	4.679009	4.606257	5.175953	4.288106	4.682853	4.959791	5.240275	5.506401	5.478043	4.698722	4.935579	4.964389	4.828889	4.320571	4.811675	4.407819	4.890226	4.926387	4.961018	4.526110	4.667368	4.325069	3.954387	4.671850	4.089190	3.818223	4.191032	4.087871	4.172921	4.191032	3.868783	4.177463	4.216869	4.646834	4.712429	5.400880	4.809737	5.401834	4.891867	5.226492	6.073804	4.644041	5.105634	4.513363	4.288341	4.564465	4.532908	4.404898	4.569828	4.066061	4.554733	4.897234	5.166894	4.098630	5.859070	4.248186	4.269404	4.513363	4.666915	4.263555	4.530516	5.069790	4.081762	3.965731	4.307220	3.683162	4.656631	4.053879	5.474055	6.423652	4.395908	4.601911	4.005674

TAT-AS1	5.448989	6.495893	5.919402	6.562847	6.857697	6.568544	5.487657	7.685039	7.093370	5.913482	7.589045	6.374003	6.955026	6.389179	5.700263	6.224337	7.186835	5.929695	6.457563	6.107290	6.533725	7.138446	6.892054	5.759064	6.400824	6.601510	5.999204	6.124801	6.534041	6.716859	6.914480	4.965410	6.590464	6.279486	5.782921	5.792931	5.161670	5.764109	5.740812	5.924711	5.194263	7.199452	6.498235	5.586055	5.036945	6.169451	7.093223	6.882462	7.106294	5.577038	6.811443	5.914578	5.244612	5.571799	5.622975	7.145969	4.783690	5.852202	7.151564	5.406712	6.340567	6.233766	6.581803	6.639819	5.868125	6.620976	6.539396	5.815094	5.452837	6.205920	5.834830	5.766251	5.612184	5.506401	5.542603	5.967336	6.103589	5.900433	6.467861	5.779106	5.534897	5.827126	5.846583	5.831408	4.979752	6.689163	6.877175	5.552203	7.368591	7.101269	7.310728	6.707842	5.892040	5.792377	7.792348	5.109134	5.602065	5.311259	7.181567	6.788922	6.646901	5.112361	5.535863	5.808677	6.854156	5.591378	6.718634	7.016162	6.947121	5.977433	7.295535	7.666363	7.129553	6.870984	5.765566	7.890599	6.526356	6.050444	6.019002	5.942682	7.733172	6.577340	6.038961	8.264324	7.288615	7.267114	6.568544	6.453644	6.931597	9.317616	7.389491	6.821344	7.776338	7.321353	6.059726	6.780884	7.165801	7.071647	5.551433	7.010888	7.415795	8.039489	7.234838	7.099873	7.092603	6.527992	7.144913	7.061088	6.558111	7.410771	6.721286	5.569403	6.558766	6.532738	6.266890	6.490623	6.127483	7.017144	6.244135	6.842402	6.703546	6.496048	5.955069	5.337890	6.534372	7.556139	7.478298	6.521385	6.259130	6.308238	6.445240	5.174138	5.602645	5.274756	6.323541	5.423414	5.947925	6.234806	5.255554	6.095667	6.196567	7.401924	6.497681	6.097354	5.691739	7.159318	6.086091	7.265558	7.152928	5.756654	5.965347	10.302329	6.025877	6.581645	5.448784	6.454610	5.833177	7.265725	6.445408	6.213566	6.429015	6.940887	6.797498	5.796183	6.263844	5.566934	7.326749	7.187785	6.321505	6.203825	6.924197	5.959510	5.892040	6.057447	6.034026	6.405375	6.050288	8.001992	6.976741	7.013620	6.715863	8.144245	6.377960	8.856984	6.564495	7.052261	7.074965	7.507849	8.498198	7.447439	7.797093	8.011709	8.123563	7.344722	7.271730	6.156355	6.182888	7.911734

LINC01189	13.093911	13.670308	12.569208	11.882808	10.674034	10.983494	12.325086	10.277608	9.731680	11.383909	10.684665	10.676118	11.603568	10.980565	11.943188	11.268947	11.218911	11.489358	9.418385	11.741168	10.482931	9.356816	10.837243	10.743957	10.200957	11.435520	12.104520	10.448109	11.708808	13.022716	10.861593	12.390459	13.066984	12.600707	12.914980	12.592167	12.429938	12.116671	11.449574	11.999597	11.217885	12.421501	12.253731	11.909858	13.337083	12.275923	10.664020	11.880705	13.489501	12.170325	13.010893	13.128611	13.630696	12.872324	12.787348	11.724236	13.380959	12.107264	11.533482	12.583952	12.037148	13.271981	14.485225	10.519506	12.679909	11.926285	12.035812	12.069326	12.337443	11.727061	12.934551	12.629675	11.111015	12.589445	12.205891	12.694078	13.302074	14.768992	12.704678	11.827113	12.922049	14.387470	12.621510	13.152801	12.541440	12.533294	13.340354	12.921703	11.798508	11.943350	12.107410	12.159431	13.649857	12.899883	13.431034	12.650709	13.107525	11.675491	11.033441	11.708199	11.556411	12.224005	11.572225	11.402351	11.023071	10.771811	11.287321	11.639316	10.281837	10.528059	10.305675	10.221376	10.332208	10.801205	10.207514	11.602845	12.612348	11.883726	9.937249	12.755816	11.194590	12.742860	11.937329	8.126124	10.204906	11.321723	10.536920	10.547653	9.983976	9.761826	9.113163	11.134615	9.935109	7.664047	11.663576	11.354634	10.358232	8.356909	11.475428	8.937156	9.283708	10.101418	10.194330	9.149853	11.701443	11.205836	9.430938	9.955843	11.185403	10.880917	12.197526	10.112589	10.834982	12.283965	10.198884	12.913034	10.926688	11.675647	12.231261	8.672031	12.219537	11.655757	9.890432	11.876151	11.900732	9.562541	11.518565	11.565671	11.868456	11.462019	12.547774	12.511128	12.372577	14.228587	10.088700	12.942843	11.796151	11.397000	11.765403	11.556994	12.016602	10.402900	11.003077	11.878180	13.296766	11.981504	12.203133	12.038794	12.674301	12.226923	12.800233	8.248407	13.337481	13.548900	13.189888	13.746570	13.174957	12.383533	13.047049	11.473822	12.127196	10.988997	12.411264	12.305101	11.522936	12.657324	11.933805	11.918508	13.944308	11.225924	11.670323	13.102010	11.969628	10.977739	13.198527	11.606238	11.592678	10.613754	11.070018	10.503158	10.809503	12.304038	12.730151	10.886712	10.245297	11.255159	9.828875	9.263062	9.990112	10.115619	10.701804	11.641765	10.025602	11.359874	9.764510	12.034708	10.494224	8.853185

AC022168.1	14.415078	13.159931	13.660563	14.237480	14.538753	14.437151	13.252643	15.203769	15.360997	14.829469	15.101143	14.291025	14.689299	14.134512	14.062109	13.971275	15.319671	14.547650	15.837350	14.744972	15.050118	15.887107	16.115572	14.619411	15.066902	15.030442	15.546202	14.972581	14.739993	13.900462	15.044189	13.357611	13.629444	14.669583	13.917771	14.109120	13.836415	13.849656	14.761042	14.494741	14.350910	15.098171	14.148848	14.666375	14.132654	14.236249	14.993988	14.053306	14.514288	14.217951	14.224715	13.983511	13.945662	13.811422	13.448408	15.336040	13.687645	14.178659	14.222202	14.318839	13.966624	14.519413	14.465165	15.153835	13.842481	14.321264	13.791211	13.928944	13.590518	14.043788	14.138571	13.742612	14.407352	13.590983	13.812737	14.108830	13.501531	13.817826	13.311915	12.785225	13.435563	13.601504	13.376715	12.962142	13.656014	13.444169	13.158422	13.280056	13.951282	13.415034	13.666468	13.839834	13.608509	12.715895	13.260249	13.325421	13.576247	14.627696	14.604960	15.340435	14.750281	13.618534	14.317493	14.244036	15.010559	14.281068	15.339763	15.521068	15.830739	14.378131	14.975923	14.751698	15.411549	14.782312	15.139712	14.678701	15.612324	14.584760	15.250206	14.411336	14.626808	13.771102	14.814210	15.131490	14.896010	15.260285	15.089479	15.478793	14.746490	15.086549	15.048970	15.028728	15.372489	15.410054	15.230037	15.009362	15.283070	15.187361	13.278020	15.482965	15.537762	15.456675	15.235562	14.316111	14.294067	14.794023	14.762371	15.199621	14.545097	15.735297	14.596200	14.612308	13.779882	13.788457	13.639188	13.878453	14.409528	14.453564	14.594501	15.363968	13.815048	13.731415	13.997851	14.013234	14.171613	15.245371	14.262921	13.817588	13.772698	14.178034	14.585219	13.770321	14.340345	14.646701	14.257035	14.035241	13.745828	14.424884	13.509479	14.349252	14.740482	14.882312	14.691055	14.817850	14.456152	14.132353	14.253596	14.258285	14.564707	14.044362	14.196229	15.751009	14.240420	14.065689	14.021288	14.456152	13.945117	14.008169	13.519100	13.564364	13.467719	13.768030	14.369059	13.759659	14.995049	13.764981	13.552300	14.260943	14.166778	13.753345	14.288622	13.868567	14.079189	14.394753	14.813710	14.682292	15.585396	15.007673	13.961884	15.248690	14.932997	15.467330	14.281401	14.249525	15.103652	15.521068	14.383074	14.561753	15.666334	14.883369	15.272298	14.764819	15.788853	14.228587	14.587290	14.052673	14.425417	14.636141

FAM182B	6.642091	8.242186	8.901108	8.166444	9.357409	8.810751	8.578178	8.770683	8.493981	8.093562	8.083021	8.112229	7.154664	8.444090	8.392568	8.243618	8.303777	8.336175	8.767294	8.452928	8.429270	8.539092	7.573089	7.977839	7.692731	7.957608	5.843146	5.999712	6.976564	8.315925	7.764057	8.686736	8.488165	9.293634	8.778417	8.852413	9.351391	8.689429	8.910390	8.864910	8.159472	8.109664	8.643945	9.009822	9.332620	8.916489	7.509249	8.655072	8.551354	9.183143	8.053099	9.042079	8.937952	8.693560	8.674520	7.655796	9.042970	8.694037	9.435704	8.860183	9.773557	8.130580	7.008826	9.545005	11.982268	8.463989	9.076729	8.938096	9.330903	8.628373	8.435543	8.767370	7.915057	8.745107	8.747578	8.798699	8.947836	8.837390	8.382213	9.293321	9.245688	8.858351	7.834927	9.042970	8.394988	8.650408	9.243185	9.093811	10.609789	9.192889	8.696520	9.168008	9.585768	8.457490	8.572894	9.041919	8.251152	8.386977	8.827612	8.664085	8.686736	8.076211	7.735340	8.886682	8.137554	8.333242	7.766091	8.225701	9.689916	7.360104	10.695882	8.999654	8.793388	7.848400	8.008852	12.233290	8.824170	8.301208	7.633323	8.140757	7.135790	8.365788	11.282980	9.897496	9.868052	7.606840	8.800956	7.214976	8.092006	6.956132	6.870481	8.094921	7.867408	8.570204	8.494911	9.076223	8.789321	6.025877	7.443608	7.836896	9.881630	7.174638	9.386045	9.876877	6.570821	10.868738	9.957686	8.010942	8.185409	8.453765	8.338549	7.071171	8.629423	9.226210	9.309714	9.645339	7.826149	7.654215	8.275964	6.922566	6.759611	6.581301	10.804313	9.441108	8.700916	6.675596	8.927562	8.407117	8.215479	8.007915	9.863318	7.571715	9.011761	8.569261	7.125300	9.406851	7.814319	10.492047	7.452446	9.474607	10.719738	8.406081	8.981042	7.672128	7.815906	7.765933	8.003097	6.111465	6.358684	7.315270	7.533448	5.445055	8.951656	7.595792	9.626163	8.458444	7.344115	8.535756	8.277390	8.760109	7.857395	8.138502	8.555749	8.047031	8.743748	9.281202	5.565388	8.738265	7.705631	10.176874	7.990498	8.840579	7.511709	8.710550	9.246741	7.990643	8.353690	8.070476	7.232626	7.940884	7.990179	8.973345	7.926319	9.152311	7.640322	7.094174	7.919819	7.240393	7.186986	9.723772	9.048698	11.457713	7.897307	8.044489	9.233946	10.595736	11.462167	6.298242

AC004949.1	3.089007	3.149648	2.980968	3.100817	3.043913	3.772413	3.294849	3.116275	3.384489	3.342475	3.553719	3.531098	3.238890	3.666887	3.867935	3.073894	3.403382	4.408268	5.156018	3.271463	4.443074	2.870546	3.036937	2.996841	2.918874	3.251132	2.927229	2.835200	3.297612	3.045058	6.130858	2.984795	3.819476	3.056976	4.973579	3.959460	4.975882	3.292205	3.082998	2.883185	3.386535	2.875573	3.469235	4.672505	3.586121	4.714337	3.267648	5.112773	4.215112	3.657270	3.648387	4.778641	3.998746	3.949881	3.753157	3.074125	4.763986	5.448593	3.719337	3.563815	2.884539	3.067954	3.499422	3.276977	3.241215	3.253582	4.284044	3.216236	3.080285	2.829344	3.476705	3.125639	3.122628	3.251299	2.881304	3.299913	3.174211	3.261306	3.364044	3.646035	3.290051	3.886347	3.313984	4.167786	3.394330	3.508342	4.282734	3.267061	3.474326	3.909259	4.116995	3.535542	3.670528	3.093869	3.107025	2.940141	3.646035	3.815433	3.051422	3.136966	3.351555	3.273570	3.204421	3.179263	4.397453	3.146768	3.161831	3.286382	3.145036	3.230636	3.190933	3.645431	2.963995	3.243404	3.093613	4.059769	2.867589	3.019119	3.177227	3.041326	3.231305	2.950339	2.989318	3.131810	3.516964	2.958268	3.123193	3.324499	3.144562	3.342302	3.114145	3.121101	3.145662	3.471558	3.047106	3.323489	3.131700	3.322974	3.214600	3.876965	2.845063	5.029742	2.949520	2.845194	2.847758	3.225038	2.807495	3.333065	3.499242	5.888841	3.195567	3.680445	3.053243	3.996401	3.679614	4.431596	3.141697	3.078974	3.026669	3.284447	2.799671	3.535381	4.838497	3.939843	4.434966	3.206112	3.021553	3.834608	3.524206	3.551213	4.393824	3.848186	4.125660	3.784156	3.043601	4.849690	3.094660	3.413453	3.438239	2.862495	3.178028	3.470503	3.195295	3.370267	3.338945	2.936912	3.421184	3.129580	2.840270	3.027789	3.225437	3.108516	3.259588	3.100817	3.273130	3.333551	3.384000	3.353521	3.199539	3.616322	4.059769	3.387908	3.644257	3.504538	3.050060	3.329538	3.430280	3.208073	3.286102	3.276820	3.081122	3.429179	3.134992	3.030280	2.954431	3.138221	3.385511	3.304280	3.190933	3.135394	3.104345	7.382687	3.138823	4.811050	3.432543	2.984053	3.292205	3.266581	3.160172	3.335169	3.386208	5.556755	3.182173	2.972426	2.976134	2.855389	3.045148	3.285471

IL12A-AS1	5.872814	9.271368	10.359626	9.781326	9.152311	9.778612	10.360811	10.533962	9.996607	11.332767	9.954745	10.617300	9.930158	10.584342	8.634408	11.037396	9.886092	10.251030	11.489935	10.531275	10.848721	10.832125	9.556004	11.666120	11.276012	10.465495	4.869381	10.755886	8.966199	8.524461	11.295264	8.941567	9.725608	5.440238	9.385584	9.438812	9.990578	9.825874	10.752225	10.739617	11.014118	10.501539	8.101596	9.059909	9.373508	9.065994	9.947027	9.819984	11.020219	10.721496	9.585768	10.333876	9.003351	11.181275	8.725272	8.393485	9.649168	10.696499	10.265874	8.651036	10.340353	9.286592	7.993159	9.769225	8.830873	10.026399	10.276224	9.652899	8.485965	10.697935	10.271167	9.830318	9.834281	9.535194	11.002649	9.358788	8.853185	8.721757	10.141648	9.363352	9.808891	9.744273	9.720935	10.247700	11.050340	9.320194	9.725752	10.821608	9.655028	10.756330	10.621593	9.329705	9.463454	10.177785	6.535162	9.168451	10.084677	9.688696	8.814119	10.747561	8.665298	10.266747	9.863484	11.832712	10.529215	9.509159	10.053598	10.353584	8.641277	9.924735	10.156509	11.197663	10.426073	10.419658	9.815273	6.605592	6.243786	9.062763	11.491752	5.952163	6.816351	6.419156	5.561304	5.580041	6.730148	5.424256	5.594934	5.589300	5.540532	5.399789	4.998777	5.564851	5.871338	4.351327	6.986699	6.028393	5.726450	6.792518	7.505232	5.814929	7.131658	7.954492	5.138445	6.378320	7.167777	5.966637	6.519038	4.866428	7.631767	6.566408	5.697884	6.096861	5.774550	6.844034	6.973051	6.992420	7.254305	7.983918	6.966936	5.769010	7.458716	6.082664	7.522372	6.473242	7.153563	5.066749	7.161880	7.862455	6.856433	6.739227	6.115636	6.175751	6.716392	5.493530	5.282363	6.256740	6.636899	5.636016	6.308238	6.731923	5.347361	6.556947	7.139854	7.101570	6.886288	7.116435	6.177317	5.810403	7.077190	6.412347	6.213566	6.869995	6.339728	6.556302	6.283128	6.050444	6.502026	7.051177	5.775290	6.502369	7.556615	6.510098	5.653667	7.204398	6.813024	6.491222	8.053534	6.985720	6.659365	6.845709	6.768454	7.005493	6.690459	6.271204	5.916177	6.993546	5.403080	6.089923	6.721907	5.819384	5.985749	6.428865	6.158733	7.005199	5.780899	6.271383	7.346294	7.902271	5.879425	7.203950	6.601671	6.645149	8.227358	5.369713	4.167541	6.005570	5.807418	5.888505

LINC01501	3.140189	5.470077	2.908396	2.944169	2.852017	4.112927	3.195416	3.049958	3.248706	3.192699	3.373077	3.268818	3.159156	3.517163	3.209109	3.032050	3.307599	3.097370	4.423102	3.008166	3.820148	5.810403	3.885110	5.016059	3.165304	2.944727	3.046403	2.924280	2.985598	3.885110	3.508161	3.286102	5.972154	4.833328	4.304213	4.380590	3.458025	3.074230	3.045250	2.960877	3.332726	2.887027	3.886551	3.106895	4.106979	6.595518	3.383146	3.669702	3.932174	3.647986	3.553719	3.311317	3.511432	3.863232	3.351217	2.946126	3.765704	3.609803	3.648387	3.560522	4.607348	3.282404	3.390523	3.844577	3.068701	3.114922	3.076229	3.133914	3.084859	2.955040	3.597156	2.840570	3.002449	3.123088	2.827512	3.205608	3.092072	3.044544	3.201021	3.400227	4.585625	3.902722	3.044948	3.766740	3.147783	3.089357	3.507422	3.177764	3.698380	3.740951	5.097811	3.095680	3.211309	2.997218	3.262192	3.918899	5.145352	2.925007	3.020820	3.290588	3.104462	4.084748	2.948932	2.915340	4.080720	3.859612	2.910624	3.116871	3.262627	3.087865	4.066903	3.173430	2.926455	3.642453	3.857219	4.670370	3.209927	2.914747	3.044226	4.104536	3.190933	4.144966	3.366167	3.067524	4.379938	2.920528	3.064517	3.148640	3.047204	3.134280	3.024654	3.145662	3.099280	3.027882	3.032657	3.251669	2.967466	3.327915	4.209499	3.444014	3.106541	5.088396	3.499640	3.017698	2.891716	3.396948	2.877915	3.135394	3.427639	3.247981	4.667764	4.489549	3.371190	3.924386	3.618011	3.467486	3.046004	3.042415	2.954431	3.132978	5.529728	3.387908	3.146539	3.648387	6.194249	3.116070	3.666689	3.234681	3.574031	3.505862	3.218815	4.753418	3.907900	3.167932	2.929615	3.417386	3.527209	3.495222	3.471394	3.205732	3.036658	3.391037	4.199237	5.103393	3.170619	3.092801	4.738648	3.163906	3.015461	4.785984	2.903112	2.954855	2.927229	5.391433	3.147914	3.102750	3.028768	3.002835	3.100685	3.399199	3.663078	3.068701	3.441340	3.313340	3.237433	3.218112	3.290793	3.506641	3.257943	3.199296	3.131365	3.102750	3.021035	2.998077	2.986651	3.107143	3.389835	3.166354	3.128450	2.924747	2.999285	2.885392	3.650804	6.313838	3.594809	2.881962	3.152702	3.714654	3.334371	4.173385	3.338781	2.877138	3.099736	3.457694	3.373442	2.908396	2.922619	3.085489

TRAM2-AS1	10.022905	8.881137	8.266671	7.659517	7.908033	7.545344	8.978636	6.246624	6.243249	7.854212	8.042486	7.951997	7.936848	7.618765	8.077902	8.167506	7.741021	8.076799	7.155448	8.478522	6.631099	7.659822	8.010187	7.806501	8.050957	8.129817	8.048777	7.729031	7.917531	8.480778	6.521545	9.952136	9.346191	9.492722	9.333538	9.618236	9.483938	8.737163	8.518570	8.021791	8.450767	8.550127	8.970668	9.449820	9.411196	9.205341	7.490867	8.593456	9.217443	9.173578	9.761195	9.390059	9.669719	9.722467	9.478766	7.738048	9.648561	9.472477	9.323814	9.538430	9.368318	8.236836	9.384220	7.503840	8.415017	8.924017	9.348480	8.367762	8.775501	7.938222	9.417014	8.944285	7.288120	9.205189	8.802888	8.981810	9.507486	9.260456	9.215448	9.328138	9.413036	9.286747	9.751437	9.685152	9.711552	9.820866	9.484876	9.437883	9.517921	8.412762	8.801264	8.200588	9.468552	9.033112	9.594939	9.525226	9.293634	8.294728	8.594791	8.553172	7.139854	8.884508	8.365170	8.722507	6.979561	7.406485	5.328291	6.843699	6.557284	7.748405	7.521929	6.701120	7.127053	7.026996	7.363727	7.266636	9.229278	8.278584	7.062210	9.140105	8.298062	8.600335	7.478884	5.316861	6.835648	8.282999	8.982281	6.999376	8.610302	9.348949	8.782589	8.595469	9.038669	8.045755	11.446601	9.388406	8.499478	8.383906	8.949666	6.649508	8.918022	9.224522	7.104714	8.444854	9.562249	7.923181	7.269546	6.803329	8.879418	7.621041	8.221604	9.021730	7.701001	9.205341	10.299419	9.444674	9.257116	9.109255	8.634259	7.054770	8.710550	10.519506	8.955775	8.961900	9.261665	4.819801	9.655676	9.523949	9.110466	9.317338	8.756854	9.344082	9.034796	9.793010	8.749551	9.941169	10.217652	8.799440	10.194030	9.291592	7.907241	9.550248	8.211273	8.715458	8.771152	8.945066	8.583923	8.406370	8.281598	8.995047	9.205823	6.296759	9.406320	9.676294	8.654016	10.213643	9.789901	9.310802	9.944548	9.247657	8.756688	9.346191	9.405140	9.515156	9.315204	8.856845	9.545166	9.895500	8.517165	9.333838	8.354756	11.651384	9.117278	9.113163	8.826140	9.116189	8.574878	8.683845	9.874356	8.551958	9.385126	8.351263	8.739325	8.457783	7.049753	6.324741	7.422597	9.186923	4.760157	7.873058	6.865105	9.245186	6.250956	9.400155	8.551804	8.659306	8.990029	7.414306

LINC02829	8.725420	7.812165	8.576701	8.594703	8.924735	8.853642	8.775819	9.106746	9.793762	8.375725	8.514907	8.452011	7.341605	8.097820	8.069216	7.888585	8.783210	7.758072	8.339643	7.820127	8.438572	7.914014	9.570876	7.930670	8.224643	8.168776	8.246522	8.191798	8.413076	9.265944	8.833199	8.714536	8.572191	8.228550	8.871708	8.396241	8.798095	8.502947	8.239177	8.778562	8.323980	8.938979	7.907702	7.700541	8.233142	8.679595	8.706598	8.200672	8.633972	8.041562	9.006556	8.402976	8.407426	8.157042	8.602527	8.308548	8.947063	8.940056	7.877349	8.669568	9.197337	7.749652	9.928779	8.541337	9.485965	8.496279	7.984384	8.374334	8.657895	9.095706	8.893154	8.320030	8.326365	8.568651	8.379382	7.965252	8.744656	8.666306	9.008722	8.629744	8.549357	9.298286	8.761068	8.498831	8.728985	8.415321	8.740267	8.948426	8.474459	8.727572	8.798858	9.922286	8.284696	8.243979	9.227589	8.775212	9.109422	7.161729	8.944895	7.372371	9.570429	7.888733	7.935898	7.595034	8.491331	8.360473	8.536545	8.083021	8.712812	8.582980	9.532764	8.644551	8.679746	9.688696	8.158230	12.233892	9.291287	7.977839	8.397483	8.109516	7.725750	8.494612	8.097062	9.481968	8.285318	8.943551	8.338225	8.628077	8.395764	8.021935	8.790890	8.363460	8.358315	8.950569	8.408506	8.518092	7.753792	7.893118	7.519245	8.296244	8.222992	9.527862	8.041723	8.334459	8.067133	8.861063	8.379846	9.188603	8.551203	8.583467	8.728842	8.032238	8.123878	7.777768	7.540217	8.107889	8.455668	8.487552	8.362379	8.746346	8.549225	7.379253	8.456424	7.429837	8.491331	9.102283	7.937139	7.958836	7.712007	8.432106	7.965553	7.414765	7.473992	8.153907	8.314692	7.782573	8.272535	7.568100	8.289196	8.119024	7.750125	8.063486	8.443933	8.664235	8.493981	7.908033	8.433371	8.211273	8.463370	8.002152	8.032238	10.278521	7.916620	8.914940	8.111326	8.172842	8.407880	8.549523	9.052979	7.579023	8.123096	7.899257	8.776601	7.944475	9.887037	8.210824	7.827891	8.493691	8.773664	7.938222	8.685097	8.558383	8.093407	8.604373	8.497483	8.728390	9.317025	8.841804	7.261207	8.816206	7.327546	7.843275	8.062577	7.654357	8.182032	8.235894	7.885821	8.296244	8.914640	9.336631	8.573520	9.544271	9.780722	10.247562	9.467041	8.126434	8.011860	8.407722

AC021573.1	3.782519	5.004978	4.542656	4.662858	4.810612	5.897757	4.610790	5.204869	5.267883	5.286219	5.984549	5.131601	4.908396	4.764873	3.992473	4.346108	4.020669	4.058446	4.133401	4.951226	4.065417	5.519876	4.642979	4.526322	4.614662	4.403536	5.248396	4.878171	5.422452	5.351089	4.944576	3.907900	4.093483	5.334554	4.332885	3.894646	3.499802	3.388249	5.156247	2.977056	5.568644	4.543088	4.850577	4.330993	5.487997	5.203474	5.870621	3.561948	4.832287	3.825737	3.891965	3.626417	4.353327	3.743475	3.676420	5.673307	3.824361	2.902203	3.340980	3.196929	3.158535	5.722326	3.799869	5.185007	4.261329	3.940444	2.874799	4.572211	4.186196	3.790356	4.377152	4.791432	5.828600	4.809737	4.251298	4.134295	4.012684	3.889387	4.014061	3.423840	3.215682	3.846309	3.149648	3.839112	3.458025	3.372751	3.430072	3.544145	2.986165	3.352205	3.866608	3.544508	4.690520	4.382536	4.111378	4.117207	3.698196	4.797463	5.472912	4.992029	4.949604	4.493308	4.886461	4.068386	3.654009	5.691168	3.202239	4.111173	5.508191	6.022066	5.027449	4.663487	3.910773	6.122011	5.752102	6.049248	3.672321	4.718543	5.830469	4.220612	5.996725	4.189497	4.134951	5.468537	5.470625	5.740241	5.131015	4.676014	5.525162	5.941126	5.318615	5.529728	4.384713	4.233519	4.789935	3.409888	4.652164	4.987473	4.074728	3.552211	4.591620	5.290806	4.882226	4.355484	6.414193	4.624831	5.490755	5.236029	4.809096	4.017403	4.291112	4.263555	4.824325	4.224260	4.085922	3.580694	4.399847	5.030342	4.485024	5.722134	3.186045	3.912072	4.575213	5.387990	5.409021	6.377801	3.637869	3.634857	3.829065	3.862994	3.981754	3.979779	3.803066	3.691777	5.209227	3.439246	2.911822	3.117381	3.014925	3.034513	5.486905	4.417754	3.741358	4.383833	3.274197	2.868491	3.854486	3.988826	3.128450	4.350467	4.126337	6.307251	4.090033	3.676220	3.987353	3.861275	3.757311	3.982401	3.529043	3.471558	3.936856	3.733620	3.497840	3.564394	4.541102	3.607261	3.675584	2.922430	3.395581	3.993947	3.980864	4.448013	5.130404	4.488050	4.134718	4.864751	5.103993	5.895133	3.791429	5.384644	4.343924	4.662049	3.390175	4.446690	5.853071	4.092887	4.604402	3.952263	5.806670	4.497929	3.775101	3.592419	7.115335	5.153174	5.836962	3.031742	5.053637	5.076919

AC016876.3	12.535101	11.787766	12.091986	11.699156	10.978775	10.650579	12.286161	8.161790	8.743748	11.202677	10.771942	11.186151	11.470648	10.784221	11.340687	11.311032	10.963865	11.498757	9.845032	10.879160	10.983634	10.343685	11.342331	11.275035	12.499786	11.667717	13.093498	11.785542	11.987566	12.223567	11.478857	12.142227	12.138949	11.653049	12.170325	12.063952	11.678446	11.916526	11.576476	11.526648	11.821698	11.998395	11.474238	11.229016	12.545533	11.663731	12.385620	10.949499	11.635875	11.410105	11.530622	12.093684	12.261028	11.881462	11.733626	11.344451	12.460105	11.986052	11.270443	12.271395	11.940596	12.034862	13.175795	11.495210	12.028865	11.890777	11.566982	11.361662	11.355342	11.283569	12.001437	11.821627	11.538688	12.192131	11.635559	11.864242	11.780607	11.630904	11.704169	11.708507	12.152682	12.387757	12.314450	12.246555	12.574922	11.844802	12.189499	11.870697	11.915924	11.338197	12.381475	11.913984	11.599023	11.739362	12.063646	12.059200	11.946386	11.494352	11.156239	11.952744	10.999088	11.558020	11.342184	10.372148	10.171845	11.581538	10.113060	10.759397	11.407214	11.614795	10.152488	9.727021	9.369204	10.622336	10.335836	9.423296	11.865300	11.958426	12.037614	11.090592	10.960176	12.472635	11.432256	9.673342	9.771758	11.656735	10.721933	10.988232	10.128392	10.422850	10.577828	11.488021	11.109107	9.927545	12.309519	10.669542	10.626696	11.482247	11.442637	10.425753	11.201594	9.740241	8.704594	10.897349	12.336360	10.311282	8.894109	10.818491	11.901752	12.162658	11.493313	11.316613	10.096699	11.573114	10.531733	11.589394	11.046049	9.333838	11.721525	10.168271	10.747853	10.911687	10.512657	10.765826	10.129148	9.794550	9.947176	10.892843	11.000920	11.300978	11.262723	11.515004	10.800888	12.264668	12.144058	12.009009	11.140921	10.366240	11.397159	11.035981	12.901456	10.057591	10.683473	11.941196	11.585590	10.641213	10.900409	10.888061	11.342940	10.968756	11.336433	8.618626	11.472951	12.192594	11.681278	11.897804	11.436404	10.886421	12.453577	11.834209	11.030608	11.221474	12.553621	12.365559	11.049601	11.694395	11.423450	12.599341	12.307454	10.949194	11.460244	11.204945	11.184036	11.341779	11.676971	11.009768	12.010041	10.379694	11.779120	10.535606	10.288200	11.014430	11.301743	11.664472	11.004703	8.175070	10.903502	10.601358	9.455567	10.979225	10.762958	7.403812	9.778003	10.717586	10.042521	11.341633	11.293672	10.616727

AL662884.3	9.090065	8.944434	9.178174	9.301890	9.187848	9.315818	8.965087	9.949803	8.187593	8.723730	9.191963	8.720391	8.800353	8.973768	9.162455	9.006858	8.744205	8.847874	8.867627	9.555526	8.876155	9.133976	9.507186	8.384354	8.680506	9.447790	7.811267	8.713286	8.228550	8.568786	8.739630	9.059743	8.227971	8.963871	8.902324	8.997042	9.224668	8.879747	9.259835	8.462629	8.957459	8.949039	8.474628	8.811548	8.753188	9.000858	8.590817	8.680808	8.395764	9.191181	8.721903	9.075896	9.053453	9.388107	8.837982	9.112719	9.120492	9.354834	9.314604	9.353778	9.394660	8.628955	8.866575	9.207939	9.225274	8.898245	9.521459	9.325501	9.278587	8.700763	9.129684	8.901410	8.829188	9.119435	9.308181	8.724655	9.002906	9.027348	9.264136	9.151252	9.048313	9.003955	8.949507	9.183143	9.002733	8.553322	9.045924	9.595708	8.800353	9.523811	9.109255	9.135312	8.613447	8.729568	8.925660	8.815582	8.727096	8.781189	8.648119	8.981494	8.277093	8.829341	8.352774	8.569415	8.760191	9.142130	8.116545	9.144893	8.427882	8.804313	9.195614	8.961744	8.639891	8.870081	8.234051	6.508308	9.705663	8.826430	8.309911	9.028564	8.630690	8.010187	8.720086	8.379542	8.297298	8.304531	9.516679	7.872133	8.402513	9.583919	9.573194	8.060923	8.581371	8.776128	8.769138	8.158230	8.470262	8.344300	8.067949	9.414865	9.316100	9.741359	8.142539	8.090022	10.358566	8.458269	9.494403	8.338549	8.267453	7.721135	7.692126	8.425886	8.596115	8.734085	8.624991	8.028572	7.908184	8.932414	8.551354	9.549146	7.738981	8.924735	8.408648	7.973975	8.958885	8.622822	8.218549	8.837234	7.937295	7.379401	8.808720	8.457045	9.375295	9.385584	7.675274	9.533072	8.560242	9.426344	9.136529	7.946342	8.739022	10.505574	9.714868	8.879747	10.001641	8.638314	8.818569	8.660551	9.452017	8.004026	7.905856	9.137318	8.401291	8.254205	8.323250	9.753457	8.903845	9.212359	8.519607	8.446548	9.156286	8.919658	8.562976	8.069057	8.945066	8.761068	9.320034	7.802652	8.286878	9.781629	8.954400	8.416754	8.885279	9.458185	8.127189	7.591191	8.060586	9.413487	8.155006	8.432106	8.905804	9.665161	8.029643	8.984120	8.802888	7.700850	10.009427	7.782109	8.515903	8.207086	7.405370	8.448422	8.958268	8.599566	8.818873	7.767009	8.834648	8.339965

AC006148.1	4.190383	3.309363	4.257644	3.792048	3.572883	4.387386	3.569466	3.566286	4.259390	3.294696	3.383848	3.757923	3.341315	3.684609	3.397123	3.777553	3.901467	4.430487	3.370101	3.791856	4.819605	3.825314	5.286792	3.785421	3.524010	3.743241	3.661202	3.658061	3.646035	3.313984	4.036699	4.164047	4.463946	3.303158	4.298965	3.936458	4.289621	3.877169	4.154543	4.190822	3.883794	4.320571	3.393833	3.251979	3.465757	5.715666	3.849937	5.449370	4.096007	4.028609	5.079007	3.960968	4.315607	4.051496	3.791856	3.270368	4.121617	3.716284	3.923950	3.936458	3.836745	3.340818	4.321835	4.134295	4.119882	3.466290	3.073797	3.776955	3.743889	4.007400	4.568525	3.838658	3.457163	4.349409	3.618945	3.437547	4.010802	4.410264	4.439142	3.502367	3.158396	3.807012	3.847173	4.096439	4.076598	3.997882	3.810767	4.173784	3.691579	5.099724	4.759750	4.711373	4.263974	4.007615	3.452427	3.828051	3.984565	3.515884	4.268691	3.155938	3.318473	3.682236	3.872223	3.805962	2.972426	3.687483	4.131450	4.038661	4.235090	3.870092	5.543701	4.143455	4.530956	3.695478	4.782194	3.692766	5.076507	3.298088	3.097255	3.326783	3.456601	3.959460	3.401932	3.261007	4.734422	4.009758	3.107369	3.751073	3.740182	3.823300	5.047909	4.030399	3.725775	3.169028	3.298691	4.123822	3.215955	3.758791	3.468400	3.992878	3.797760	6.013483	3.078385	3.854698	4.142173	4.115483	3.237433	4.278181	3.484067	3.727139	3.365338	4.136278	3.087522	4.063954	3.668862	4.216015	3.797353	4.158864	3.572883	4.099749	4.602311	3.493546	3.334529	3.855094	5.280527	3.936663	4.788883	3.575527	4.146850	5.383142	3.744452	3.904426	4.111378	3.307599	3.608025	3.664851	3.863442	3.917683	4.082186	3.590298	3.475634	4.166676	4.205246	3.894841	3.703843	3.157539	3.746872	3.620269	3.624828	4.357202	3.778157	3.784800	3.957460	3.967025	3.414533	4.312729	4.467794	4.434540	3.134643	3.405057	3.959039	3.588064	3.594809	3.850353	4.263344	3.719536	3.508701	3.609188	4.481034	5.337135	5.743007	3.945083	4.353327	4.309458	5.027663	3.882548	3.480575	3.982847	4.835798	2.990442	3.096025	3.279275	3.056767	2.986843	3.544145	3.506641	3.787788	4.227481	3.960968	4.283596	3.422630	4.283814	3.904846	3.389306	3.583046	4.578414	3.140672	3.631909

AC011997.1	3.679024	3.859401	5.186207	3.320901	3.892863	5.728635	4.781987	3.606405	3.796319	5.067971	3.179124	5.525913	4.995885	4.756961	3.013864	3.933476	3.291431	4.066279	5.398239	4.502632	3.984132	2.899623	5.755174	3.823956	3.033186	2.855056	3.817590	4.504144	3.780400	5.187613	3.692171	6.184870	6.428009	6.660520	5.091740	5.092275	4.064780	4.026422	3.136611	3.104774	3.672507	3.030788	5.828417	4.623340	5.062667	6.365725	3.082998	5.690430	4.951888	3.326945	3.149648	5.542439	5.187613	6.549020	3.354007	3.754031	4.774707	4.641771	2.914185	3.081216	4.196900	3.271463	5.550338	4.246430	7.143793	4.937295	3.224190	3.743889	6.653693	4.808685	5.368759	4.647255	4.514021	4.620829	4.758717	4.896655	4.913908	5.990425	4.450965	4.493107	5.517799	4.601657	4.305530	4.158412	6.239250	4.728053	5.127355	5.855451	3.780400	4.773688	4.990182	4.848706	4.788469	6.660691	4.882226	4.662255	4.967577	4.492238	5.773799	3.071765	5.149087	4.622273	3.116871	3.326289	3.138701	3.975516	4.105200	3.655248	4.575001	3.385855	3.146903	4.031073	4.480424	3.650223	3.210577	8.300613	4.552801	5.397296	3.798781	7.215612	7.532690	7.005651	6.932089	5.999906	7.658152	6.906870	7.182036	8.225251	8.386497	7.804774	5.973953	6.597303	5.926670	6.821014	9.807438	8.506737	6.968555	7.843927	8.625448	5.346143	7.079746	7.028638	4.775323	4.844447	4.660717	6.911310	7.237972	8.535756	6.687619	8.681103	7.464647	6.214073	6.889820	7.822714	7.119340	7.485863	8.016331	7.958043	6.230434	8.288718	5.901504	7.820856	7.050231	8.259547	7.971462	6.534701	6.267418	8.284375	6.438911	6.243437	8.590668	7.507225	6.698233	6.235819	7.247454	6.707215	9.163879	7.371577	6.395163	8.147296	6.817639	8.789321	7.904584	6.704299	5.355303	7.926948	5.523836	6.222275	7.187472	8.037595	7.277818	6.821498	6.762051	6.676101	6.805088	6.438750	8.698784	4.657066	6.386626	7.701001	6.706060	7.761133	7.323742	8.928934	5.892225	6.102725	7.762352	6.995064	7.061705	5.901152	9.103050	7.662149	7.343361	5.889945	6.270170	7.485863	6.997719	8.412605	7.612641	6.143590	6.093905	6.202073	6.668017	6.611312	6.400824	9.740576	9.190561	7.959136	6.668017	4.322056	7.057930	7.684279	6.815893	7.620601	7.981450	8.347960	8.137408	8.370663

AC009126.1	12.365730	12.003998	12.922772	11.892609	11.944822	12.514165	9.016068	10.912348	8.905538	11.896910	12.631817	9.152161	12.277596	11.531829	11.840994	9.605775	8.981810	11.955891	12.056973	11.767362	9.355751	9.690083	12.999705	12.064410	9.671636	11.914124	9.634626	10.852730	9.172804	10.427471	11.217158	10.000862	13.902821	13.495100	10.289759	9.723120	12.106206	13.064722	11.939225	9.716865	11.743625	12.495694	13.050076	12.532185	9.867886	12.547466	11.203593	11.612110	12.084672	9.547000	12.401619	12.520900	13.077040	12.673960	9.253123	11.822002	9.924735	12.712606	11.930467	12.799536	12.474915	11.781211	13.221572	12.149284	12.754938	12.268825	9.009334	9.769520	12.464048	12.301868	13.121579	12.172787	11.362843	9.807917	12.168975	10.648195	13.096155	13.163768	12.461067	10.047564	12.772251	12.766912	12.684911	11.998716	12.221569	12.242226	13.073430	12.509321	13.452455	12.017927	9.936498	10.509173	9.679997	13.574462	13.439251	13.456369	12.670692	9.774925	11.840994	12.118961	8.716431	12.186990	11.844337	11.449124	12.492245	11.743625	10.430794	11.568275	11.578788	10.985679	10.649241	11.627501	11.147104	11.744798	10.018340	11.496673	12.135755	11.867445	10.813574	12.585498	14.079189	13.756638	11.663282	11.019158	12.904642	9.735326	11.506274	8.909057	12.759968	12.300335	8.545228	10.860728	13.247129	12.390459	10.275187	10.539729	11.494786	12.705338	11.658340	8.610302	9.783615	13.180561	9.471077	6.564660	13.492817	9.352587	9.406773	9.359995	9.965154	13.532157	10.674348	12.894731	11.438654	10.930669	8.440271	13.093911	13.411431	12.742336	9.817117	11.629710	12.277746	11.942282	12.037148	9.161081	12.003241	11.452540	11.187721	12.084810	9.411694	12.637320	11.841289	13.927588	10.849009	10.315629	14.221891	9.556635	13.144955	12.696267	13.079851	12.493861	12.040600	11.652576	13.346566	12.310302	12.007014	9.509444	9.452450	11.761613	12.620010	12.583792	12.230037	11.992622	9.117878	13.193698	10.127592	13.139865	13.174008	12.455008	10.930535	13.220585	12.513208	12.550714	13.647299	13.285446	10.750069	13.655038	12.371021	13.839834	13.072064	8.261557	11.299529	9.564183	13.627523	13.296189	13.382177	11.298834	9.515750	13.705084	11.898226	10.473776	10.287723	11.498007	13.225108	13.807854	11.415437	12.992354	13.851943	14.088628	12.718016	10.502146	13.734845	11.887512	12.810174	7.141928	13.435563	12.204356	10.449177	12.905702

LINC00624	9.031263	8.522159	8.463529	8.719047	8.475262	8.628955	8.829820	8.182981	9.091411	8.785471	8.994888	8.909661	8.781959	9.032809	8.901561	8.740411	8.906121	9.516845	9.641913	8.697270	8.213942	8.627031	8.701516	8.996563	9.154648	8.500281	8.771856	8.580435	9.343762	8.211447	8.841178	8.904614	8.538466	8.837390	8.615560	8.749391	8.862310	9.180782	9.107963	8.500737	8.711476	8.591139	8.643630	8.614358	8.865812	8.640510	8.384808	8.423166	8.564352	8.418107	8.505802	8.590515	8.798544	9.107805	9.139490	9.012701	9.063687	9.131674	9.298286	9.362011	8.750190	8.525385	8.040770	8.430715	8.304834	8.786090	8.477896	8.634726	8.368619	7.640791	7.888271	8.221444	8.812183	8.208005	7.969404	8.198553	8.395764	8.520064	8.222690	8.051259	8.450767	8.740108	8.737644	8.852719	8.926272	8.578104	8.445923	8.728075	7.949691	8.406229	8.400640	6.923712	8.286088	8.457624	8.234802	8.573979	8.734872	8.940204	8.686736	8.589745	8.890098	8.805667	9.190243	8.537113	7.905856	9.051585	8.200588	9.044566	7.872919	9.147027	6.992259	8.734707	9.314274	8.739179	8.964176	7.647273	9.873938	8.985474	9.258494	8.924876	9.005162	8.149782	8.858952	9.424518	9.720345	9.137949	10.527605	9.598172	9.953948	9.547475	9.675373	9.486874	9.655812	9.874810	8.355063	10.045253	11.042851	8.593456	10.508560	8.700763	9.761660	8.958885	9.637621	9.502999	10.480506	8.663627	9.669110	9.073438	9.657496	10.242440	9.302803	9.877347	9.538277	9.365025	9.950260	8.998099	9.538600	9.774181	9.824636	10.227130	9.354532	12.132348	9.002437	9.034153	9.729026	9.333214	9.072507	9.489336	9.407868	9.603010	8.965870	10.570618	9.704576	9.516374	9.052657	9.837811	10.369561	9.866387	9.244109	8.901561	8.662072	7.921053	9.305574	9.013781	8.959765	9.113896	8.031021	8.789796	8.149924	8.176634	8.614358	9.633073	8.536545	8.043558	8.405459	8.928003	8.684630	8.588347	8.082255	9.069131	9.406930	8.839797	8.954704	9.454030	8.909057	9.166952	10.065121	7.942647	8.594242	9.470328	7.431568	8.525064	8.633369	8.618939	9.178480	9.904409	9.568588	9.846464	9.872421	8.872478	9.148637	9.117730	8.696676	7.781203	9.702796	10.821920	9.251483	7.973373	9.317754	7.072747	9.671482	10.857343	9.246132	9.278749	9.416264	10.257553	8.739022	9.967099

H19	10.624904	12.439334	12.333497	11.126207	9.847521	10.884619	11.343861	13.298825	11.105158	11.284886	11.885261	11.480371	10.684665	10.281686	10.634437	10.960936	14.241749	11.275177	10.863674	10.809966	9.879639	11.128382	13.278020	11.787619	14.037255	11.578488	14.000355	12.264028	12.200477	10.238002	11.487721	12.019164	10.370639	12.649718	11.909858	11.187721	13.471598	12.656681	12.650709	13.451381	11.849423	11.304114	11.669383	10.757072	13.416705	13.132093	13.084018	11.878180	14.100390	11.769146	11.836941	12.781558	11.762513	11.752315	11.942202	11.488318	11.905051	12.882781	11.380538	13.781330	12.279446	13.755904	15.704556	12.797127	14.186189	10.845782	12.742146	12.099062	14.213010	12.034708	12.040600	14.660429	11.502750	12.023550	11.736262	11.924435	12.085444	12.590794	12.272044	10.754780	14.003878	12.578859	12.970784	12.164047	12.297429	11.501620	11.731098	12.005180	11.371423	11.029412	11.472498	11.366540	11.295264	16.357477	10.910730	11.239356	14.769473	12.697321	11.955437	12.167578	12.853171	12.166192	11.360602	12.017927	11.072137	12.000326	12.264495	13.581602	12.462754	11.192513	12.095640	12.176872	10.930976	11.689281	11.742152	12.073734	14.661796	12.128415	12.045007	11.827966	14.833005	12.775319	14.954990	13.496693	11.444260	13.491711	13.211496	14.543837	13.350346	16.036135	13.844292	13.437511	11.844950	12.685514	15.708514	13.232837	12.274241	11.630904	11.808281	10.159237	12.614041	13.514888	16.305150	12.258961	12.542605	12.505754	14.636601	15.378429	11.601212	13.724455	14.823396	12.725852	15.369410	14.228587	15.084166	13.116011	12.735923	15.323382	14.693813	12.237727	14.035241	14.292381	12.777426	13.773464	13.300495	14.919494	13.560400	13.550705	12.877302	14.707094	14.225335	12.773104	13.047627	14.124606	15.560443	15.874614	13.662003	15.578257	14.042604	11.695777	14.654622	11.965086	11.152612	13.327985	16.127121	12.045877	13.274601	13.248925	11.294265	14.023284	13.311346	14.477842	13.900992	12.716418	13.868567	12.885476	14.053607	12.712283	14.476349	13.306600	11.905178	14.310666	13.294543	15.628044	16.294338	14.154830	12.837405	12.697859	15.680087	12.950547	12.041372	15.236212	12.421050	13.349098	14.871778	11.701443	13.740121	15.632923	12.246079	11.832095	9.953948	16.342113	14.128019	11.258854	15.616895	15.897567	11.424357	10.654131	14.430444	14.698400	12.011409	11.323494	10.940687	10.755577	15.883639	14.685534	15.069269	15.630002

AC069287.1	9.163879	11.700081	11.772400	11.262129	11.797931	11.701300	10.995680	12.964739	11.360307	11.133393	11.127236	10.804597	11.153927	10.944920	10.108076	12.298219	11.770782	10.123969	10.741115	13.141902	10.520951	11.259889	9.691309	10.894314	10.931284	10.789568	10.623514	11.307971	11.320998	11.835276	11.129584	10.589937	11.246940	10.273491	12.613710	11.686489	12.349434	11.624998	12.262160	11.318521	11.653482	11.399554	10.949348	11.619805	10.908098	10.832583	10.863380	11.916672	11.654988	12.593292	11.340546	11.533482	10.111224	10.534840	9.898981	11.926602	9.881018	10.793216	10.592028	10.443347	10.255271	11.433898	8.841504	11.028205	11.058248	10.587449	11.420288	11.000770	10.587598	10.745574	12.435794	12.752073	11.353164	12.818205	12.067039	11.388150	11.944227	12.199224	11.811133	10.266295	11.050779	10.459392	10.760108	11.227537	11.849090	11.693331	12.873035	12.871579	10.510212	11.417687	10.377106	10.245014	10.713869	11.546356	10.517131	11.036408	10.887033	10.572597	11.209812	11.687959	11.039307	10.768187	10.236515	10.980262	10.027195	11.279592	10.739904	10.711554	10.975234	11.724977	10.777248	11.060112	11.164797	11.416462	11.257970	12.575393	10.210139	11.586749	11.290706	10.099038	12.098761	10.868738	10.879893	12.364654	11.007280	10.843616	10.659325	10.198572	10.554613	10.511711	10.348265	10.742594	10.149811	10.345071	11.241571	10.667694	10.670884	10.311282	10.936997	10.221516	10.930245	10.026399	10.983634	10.636526	9.267376	11.094152	10.692703	11.320998	11.328057	10.970588	10.414939	10.535767	11.146797	11.357096	10.943897	12.131586	10.756929	11.742737	10.211984	11.189187	10.559632	9.096285	10.759828	10.374549	10.480213	11.101364	9.229567	10.817007	11.332182	11.332619	9.682033	9.549938	9.852765	9.102598	10.666208	9.172960	9.576225	10.075727	10.086358	9.182973	10.877350	10.061488	10.697351	10.743957	10.385470	10.853896	11.348648	10.230524	9.584367	11.018523	11.437253	11.584703	12.112986	10.266879	11.169583	11.531086	11.650785	12.247673	9.450937	10.696036	10.084209	11.060858	9.924891	10.386712	10.966969	13.101809	11.217158	9.512302	10.632025	8.893784	9.954924	10.189508	10.827984	11.393373	10.521111	10.324760	10.609075	10.590228	10.180413	10.245440	9.674273	9.934938	9.608994	8.998577	11.084401	10.094705	10.129759	10.235436	11.548425	10.299864	10.823396	11.543325	11.967332	10.585064	11.324079	9.805636	11.137136	10.658003

LINC02661	6.960528	7.815434	7.210325	7.338002	7.423202	7.491020	7.564260	8.293964	7.330084	7.580698	7.205220	7.371577	6.685825	7.258388	6.503995	7.433448	7.007190	6.916190	7.824466	7.255390	7.047698	7.749198	7.291287	7.506792	6.932722	7.587193	7.432040	7.887040	7.336449	7.518967	6.930002	7.301137	6.920646	7.389802	7.191094	7.159168	7.175574	7.097191	7.721625	7.418065	7.166993	7.021564	7.289104	7.487887	7.153076	6.892994	7.795703	7.538276	7.038434	7.642002	7.245629	6.906222	6.737942	7.206919	7.561801	7.584575	6.328231	7.011554	6.455588	6.482902	6.275556	7.636038	6.778938	6.713250	7.073993	6.836278	8.035707	7.249008	7.690710	7.511385	8.092604	7.921494	7.395245	7.688703	7.877641	7.588434	7.491489	7.183306	7.705631	6.240763	6.337877	6.435274	6.338886	6.764576	6.232252	7.230254	6.497368	6.688548	6.699040	6.652734	6.401461	6.479926	7.157464	7.303037	7.157612	6.774963	7.049430	6.628063	7.178605	7.353126	7.499773	7.580071	7.408672	7.319485	6.638849	7.193418	7.642151	7.363878	7.460716	7.826149	7.221610	7.179231	7.153076	7.533611	7.151398	9.036629	6.985720	7.084741	7.362606	7.076359	7.917667	7.681937	8.043407	8.174583	8.142845	7.819822	7.488664	7.095149	8.271789	7.756974	8.012155	8.003583	7.490242	7.437870	7.116435	7.664047	7.842803	8.230545	7.147390	7.656558	8.148080	7.886885	8.234355	8.347074	8.200142	7.881084	8.390063	8.324142	8.010350	7.719036	7.249782	6.949028	7.692126	7.284600	7.583190	7.032025	6.932401	8.117615	7.638623	8.313592	7.665583	7.365622	8.423320	7.425855	7.676648	8.652204	8.463989	7.052109	7.675274	7.480750	6.950916	6.760278	8.322469	7.302415	7.986694	6.933492	6.886446	6.848549	6.704797	7.057617	7.597626	7.407903	6.852172	6.833773	7.345981	7.922249	7.530827	7.753947	7.867736	8.031633	8.102045	8.155706	7.647273	7.563777	7.310410	7.636928	8.020116	7.756185	6.209329	6.587033	7.041120	6.804613	6.320848	6.502522	7.697916	6.551986	6.357670	7.015663	6.559586	6.780543	6.961969	7.348953	7.438208	7.455423	7.242763	7.757438	7.246849	7.089781	8.031179	7.724222	7.748119	8.061955	7.522082	6.226757	8.526581	7.184381	7.272024	7.583498	8.744057	7.347870	7.708553	8.235581	8.079159	8.582268	7.594277	7.155766	7.526963	8.256188

AL035696.3	4.305530	3.951608	4.653285	4.124030	4.703430	4.192585	4.581198	3.612180	5.641575	4.323583	4.338757	4.166676	3.686638	4.340238	5.184611	5.883706	4.685922	5.096781	5.153391	3.821840	5.586787	5.151943	7.304606	4.162728	5.087783	3.607467	4.114601	4.016290	4.084103	3.584146	4.325277	6.244951	4.482201	4.133199	3.771327	4.758717	4.509851	4.425910	4.908622	4.229251	4.071053	4.197752	4.470544	4.433285	5.081663	5.739896	3.798389	5.274932	4.863307	4.134951	4.093091	5.140892	4.314773	4.206826	5.448214	3.764289	4.301102	5.063441	4.559658	4.312308	4.430930	3.710276	5.324027	5.852907	4.716878	4.812712	2.973996	5.031937	3.988826	5.281557	4.312513	4.512913	3.810335	4.368291	4.455015	4.279071	3.675983	4.105649	4.081964	3.940865	3.742589	5.040737	4.568951	5.045882	3.979980	5.018503	3.456978	5.579658	3.536493	3.938758	4.250623	4.217085	4.314274	4.525441	4.050645	4.329886	4.343924	5.725915	5.032949	4.056715	4.487427	4.425695	4.432464	5.274756	5.204869	4.285445	3.921822	5.784311	4.694331	3.551024	5.274756	3.894646	3.430804	4.299823	4.993534	3.590125	4.661814	4.667368	4.010157	3.677597	4.171213	4.207537	4.260010	4.276840	3.867507	3.621479	3.623869	3.693202	3.232555	3.489559	3.463557	3.131266	3.496914	3.734799	5.084041	4.993721	4.073434	3.893990	3.630871	4.857538	4.433078	5.905278	4.222293	3.365983	3.466108	3.831827	4.361519	3.319425	3.594809	4.374352	6.035818	5.787378	3.946628	3.698788	4.340694	3.876779	6.152751	5.315232	4.808066	3.407850	4.238326	4.765468	4.514021	5.654640	5.384847	3.906869	3.817794	6.181161	4.745112	4.502413	3.881295	4.164884	5.791150	3.818855	3.424550	3.881073	3.318473	4.031073	4.190613	4.167541	3.818633	5.457488	4.979752	4.902149	4.650918	2.946892	4.085214	4.118088	3.217728	4.831465	4.200963	3.580319	3.473087	3.742196	4.068580	3.409531	4.419593	4.252367	3.334861	3.845888	4.771765	4.001688	3.739136	3.645020	5.308286	3.584548	4.392978	3.710057	3.747923	3.589211	4.187529	4.539543	4.389151	4.110281	3.424717	4.184247	4.237440	6.144967	4.236984	3.808655	3.838007	4.424840	4.066061	4.967125	4.512247	4.940144	4.641333	5.764109	4.056076	4.462017	3.921454	5.680207	3.597725	4.212746	4.242508	4.742727	3.821032	3.833785

AL021368.4	13.017706	11.116371	12.288317	12.999705	13.527682	12.994903	12.157572	11.723179	13.709682	12.749319	13.051243	12.960120	13.909712	13.455726	12.778642	12.977674	12.665284	12.786494	12.717483	13.125775	11.631959	13.539933	13.992685	12.859527	13.612309	13.588874	14.109785	13.076654	13.324386	12.332224	13.543058	11.642235	12.475857	13.254006	12.767450	12.594269	12.395231	12.038203	12.671375	12.896716	12.788897	13.118063	13.498035	12.362753	12.831161	12.413496	13.136798	11.897222	12.027347	12.587474	12.568243	12.218048	12.346629	11.858736	11.559235	12.860559	12.466646	12.633795	13.602862	13.313966	12.745055	13.216776	12.428658	13.034304	13.370400	12.508187	12.600544	12.398339	11.687522	11.722441	12.489908	12.384483	13.091997	12.194744	12.709456	12.482141	12.188936	11.916216	12.422968	12.066401	12.473959	12.364654	11.927388	12.385972	11.830901	12.637828	12.618964	12.452025	11.853844	11.849270	12.115229	11.719874	11.881927	11.975737	12.709108	12.908749	11.541804	13.205336	12.668810	13.854600	13.416087	12.868341	13.258023	13.021558	12.218342	12.446101	12.964557	12.680421	13.344471	12.528710	12.560565	13.560621	13.046106	13.199119	12.398695	12.864781	14.114029	12.945176	13.750073	13.359283	10.910132	12.407139	13.603336	13.559941	12.470709	13.111982	14.969696	13.755369	12.882947	12.646916	13.579900	14.041399	13.576715	12.590626	14.344919	12.645882	13.551881	13.781822	12.910540	12.669837	13.616144	12.947892	14.270244	13.023480	12.538617	12.342178	13.543737	14.329679	11.921879	14.683638	13.325714	13.704144	12.444671	13.860992	12.348689	12.455171	12.766030	12.925613	12.667488	14.383815	12.397379	12.586457	12.549244	11.749984	11.493459	12.857098	13.266215	12.493861	11.915487	11.862600	12.792507	12.202831	11.648556	12.229099	12.989438	13.142104	12.949809	13.085901	13.131724	12.832035	13.427333	11.799242	12.044706	12.983333	12.903231	12.308401	11.799846	11.358396	11.754080	12.378188	12.217605	13.453090	12.327958	13.045554	12.029621	12.725496	12.459627	12.062451	12.184541	12.583792	12.150176	11.980630	13.231246	12.480968	13.790935	12.482634	13.333079	12.373381	12.254351	13.720180	12.260677	12.543078	12.070398	12.475535	13.090343	12.082273	13.206525	13.172095	12.776199	13.022344	13.886983	14.605414	12.708077	12.820600	12.759247	13.501308	12.382262	12.357368	12.014173	12.515324	13.064161	13.978346	13.181119	12.901113	12.324942	13.572169	12.852645	12.919527

AC007922.4	6.712279	6.080745	5.790446	6.750212	5.915291	6.214073	5.098644	7.410476	6.697766	6.066083	6.271204	5.578913	4.489327	5.229535	4.303540	6.394353	6.723049	5.418942	6.380781	5.683252	5.388727	6.806609	6.898842	5.758146	6.536628	6.279147	6.115959	6.728999	6.273900	4.988308	5.548432	5.108299	5.460645	5.208018	5.119896	5.435317	5.087783	5.620378	5.944425	6.925831	6.229889	6.034196	4.926168	5.617787	4.978266	5.651060	5.883706	6.314873	5.853247	5.761388	6.886446	5.669251	4.676014	5.314629	4.769077	5.969815	5.244018	5.388158	5.955741	6.015023	6.212521	5.624573	5.865953	5.839699	5.171289	5.430849	6.163570	6.129831	6.317412	6.807918	5.953976	5.912230	6.055326	5.518195	5.529921	6.904150	6.489335	6.109896	6.500881	5.540894	5.146342	5.375758	5.622231	5.460459	5.136803	6.278303	5.225301	5.429745	6.077122	6.437425	6.042288	6.335850	5.517407	5.750104	5.627454	5.292378	5.663683	4.622478	4.611640	6.078492	5.159668	4.476479	4.960000	5.456287	6.448680	6.092366	7.199153	5.694139	7.014664	5.900251	5.958405	7.052870	6.363905	6.891084	6.548360	5.467740	6.941837	5.912785	6.041931	5.880149	6.167909	5.748312	6.439408	6.085087	6.430211	5.440816	6.288620	6.024627	5.679946	5.439113	5.902912	5.460043	5.153573	5.953089	5.950898	6.565927	5.527848	6.611684	5.158668	6.635160	6.506661	7.563777	6.440386	5.999906	6.203457	6.340247	6.677849	6.371084	5.588697	6.274070	5.254163	5.010145	4.813130	4.910706	5.207836	5.247007	5.814929	6.497368	6.247297	6.710559	6.153607	4.720656	5.973440	4.563629	5.179183	6.687119	5.843870	5.636952	5.998314	6.141731	5.577038	4.642400	5.374545	4.539977	5.329117	4.864543	2.965002	5.795803	5.704669	6.325946	5.344963	5.974298	6.393213	5.324222	5.529921	5.955221	6.011588	6.404074	6.731754	5.719936	5.624864	6.629846	5.422452	5.119499	6.753737	6.180324	6.216306	6.562527	4.970105	5.242044	4.919813	5.471006	5.280157	5.282173	6.118370	5.725727	5.387789	5.946205	5.875319	5.691927	6.714755	5.532941	5.971779	5.559669	5.094394	5.432797	4.736102	6.146192	5.176137	5.226103	4.628254	5.415835	4.624415	6.271204	6.208471	6.921443	5.441987	6.538118	6.861393	6.155871	6.347762	6.540510	6.632390	6.304098	5.264485	6.560538	5.778004	6.145315

AC024224.2	7.337058	10.444263	9.672692	10.926976	9.092540	10.636826	10.509324	13.140875	10.011809	11.283846	10.478137	10.872782	9.345116	10.266747	9.946536	10.006010	9.857253	9.804238	11.516358	10.711967	10.713575	9.907741	7.996465	9.240288	10.244125	9.868372	7.891070	9.184546	8.532874	8.751471	10.593479	9.396668	9.906054	8.486586	9.418110	11.156096	10.575699	10.371390	11.107920	10.798335	9.629836	10.762044	10.369246	11.059685	10.155465	10.860126	8.302435	11.420720	9.452298	11.682169	9.758720	10.757808	10.497091	10.692703	11.383209	10.703174	10.836326	9.755615	10.415417	10.004105	10.555685	8.396396	8.138502	10.726741	11.082627	10.950728	9.884994	10.690375	9.918999	10.766421	9.764810	9.728699	8.757154	10.285451	9.451258	9.301410	9.201691	9.028112	9.696173	11.262567	9.757807	10.219240	10.383504	10.657248	11.009768	11.092058	10.127429	9.452602	9.957369	11.358554	11.462325	10.749317	9.716701	10.667694	8.218549	8.005145	10.473309	9.271660	9.354991	9.625854	11.340239	10.536625	8.778111	10.613186	12.302809	8.557010	11.791206	11.179915	10.328691	9.904700	10.032710	11.226653	10.700036	9.077935	9.922286	9.893168	8.109085	10.602394	8.300756	9.140257	10.952015	8.537536	9.214667	9.587330	9.612257	10.145201	8.084126	9.034328	8.848183	9.868052	8.023315	9.025237	8.261557	8.207388	8.410935	8.851981	7.384537	7.882461	8.138337	8.675783	8.429897	8.220237	7.960064	8.368619	8.572585	9.128602	8.524156	8.754431	8.685533	8.262784	9.513820	10.428979	10.062587	7.935134	10.170585	11.048256	8.739022	9.682776	9.461985	8.498687	10.105679	8.367126	9.443123	10.505842	11.256930	8.615851	8.264787	8.300756	10.060006	9.381628	7.917069	9.614087	9.212057	9.179371	8.174901	7.412291	7.676983	9.268311	8.570349	10.599871	8.273893	8.553322	9.721686	9.187084	8.194802	8.861204	11.229765	9.994790	9.229567	9.648718	9.316571	9.424825	9.729505	8.304234	9.821458	9.498268	8.670782	9.936797	8.306800	8.985776	8.721185	11.370366	8.659160	8.055096	8.826140	10.171699	8.014014	7.937457	9.801811	8.077276	9.204729	9.569977	8.483274	10.217581	8.103552	8.124953	7.778846	8.874814	8.888374	7.649648	7.938524	7.887508	8.525523	7.542183	8.246033	9.863624	8.868843	9.483198	10.122304	8.859422	9.696633	10.567716	9.340740	7.812644	9.965154	10.442006	9.094042	9.411829

ZFAS1	12.050270	11.834521	11.810536	11.816748	12.166049	13.173445	13.262398	12.567262	11.981504	13.036888	12.973655	12.615027	12.553295	12.454709	12.449269	12.967107	12.619661	11.619805	11.842962	13.239764	12.522828	13.101611	11.965386	11.788373	11.096245	11.731098	12.026457	11.419699	12.626503	13.146881	12.541440	13.195709	12.350213	12.855167	12.700529	12.918772	12.612527	11.605953	11.986513	12.013873	12.992703	12.212235	11.918799	12.248657	11.771536	12.556507	12.356099	11.210844	13.146323	12.533129	13.067170	12.667306	12.788738	12.950361	12.740227	12.536900	11.835574	12.313176	11.898839	12.220929	12.465329	12.810174	12.271395	12.302476	12.499125	11.989104	12.141594	12.353087	12.052817	12.739334	12.463883	12.951673	12.630012	13.178186	13.255365	13.465295	12.569043	12.774962	12.807870	12.801968	12.250390	13.026917	12.510296	13.333719	12.845970	13.019185	12.892258	12.085120	12.632463	12.106702	12.182658	12.501102	12.719193	12.631637	13.408793	13.546876	13.180750	12.681285	12.802136	11.469730	12.397379	11.989871	11.620681	12.024797	12.906948	13.054008	12.570017	12.713284	12.325410	12.740585	12.989438	12.007938	11.960368	12.649882	13.059695	12.308229	11.486239	12.694415	11.792548	13.554388	13.873413	12.830155	11.704169	12.222032	13.176786	12.909649	13.355563	12.618964	13.085544	13.395578	13.914229	12.532185	13.407361	13.148421	12.792190	13.593974	11.509511	13.445237	13.036697	13.499351	13.206525	12.789960	10.487353	11.752033	13.467905	13.693813	13.018816	13.501741	13.033913	13.394726	12.936910	12.538957	13.610375	13.146515	13.322965	13.087036	12.399627	12.467488	12.201865	13.321286	12.242991	13.457642	13.838475	12.304191	12.597317	13.053642	13.660563	13.353964	12.824203	12.865869	13.479279	13.525049	12.714355	13.366847	13.941517	14.323669	12.238835	12.919886	12.652080	12.423741	13.767473	12.771018	12.751371	13.070763	13.423212	13.056546	12.202988	13.104454	12.888611	13.980804	13.642031	13.136216	13.379474	13.194312	13.152404	13.955482	13.693813	13.635940	12.638694	11.832712	12.293129	13.198141	13.052613	13.514460	13.473987	12.600707	13.944564	12.538079	12.645722	11.669857	13.059695	12.814928	13.907177	13.039136	12.769250	12.920968	12.780366	13.470544	13.286871	13.986378	13.279851	12.098027	12.695910	12.594269	12.532185	13.950442	12.734213	12.832911	13.028986	13.594434	12.748645	13.504804	12.572856	13.718425	13.363072	12.811936	13.494652	13.759150

LINC01018	7.889199	5.884608	6.389527	5.394962	4.405149	4.976292	7.275147	7.033749	3.211011	6.384666	5.539594	6.019364	6.050444	6.075714	5.222525	5.783785	5.681788	5.274097	4.848706	6.657757	7.228566	5.219369	6.792991	4.380153	5.562964	6.352940	6.637049	5.716929	5.630622	5.840959	4.737156	7.037782	5.050399	7.059020	7.431258	8.320503	7.295217	7.084584	5.875319	5.559669	6.402607	5.448214	6.712466	5.626914	8.210824	6.689803	5.895133	6.344224	7.663729	6.338886	6.786940	7.595792	7.737418	6.565136	7.467854	5.946038	8.252957	7.135615	5.757214	7.353733	5.950215	6.255895	6.808244	4.218384	5.709192	5.781818	5.771842	5.731934	5.375957	4.893955	6.213374	6.399293	5.759620	7.262516	6.688383	5.782376	6.751780	5.769200	5.741744	7.125777	6.071153	6.840459	7.591348	7.434866	7.891377	7.042884	6.079562	6.674294	6.040169	6.073115	5.957686	4.644041	5.844038	7.076359	6.442193	6.282954	7.517109	5.842228	6.304937	7.255240	4.901535	7.431882	6.400141	6.806770	5.265872	5.682541	4.894172	5.414350	3.387739	6.121473	3.599132	3.514792	7.409741	5.343606	6.042781	4.462440	6.936015	6.753737	5.009920	9.687451	4.698722	7.472188	4.825211	3.255912	5.484613	6.676570	9.245031	3.118642	4.750198	4.675355	6.238082	4.795599	6.535976	3.217861	5.181974	3.672690	5.607979	6.541025	8.634560	6.620976	5.361147	6.158402	4.120296	4.801541	4.308348	7.104070	4.982563	5.106012	5.342989	4.863103	4.692874	3.385511	6.345747	7.428825	10.031627	6.423013	7.172616	4.061734	4.209499	5.156673	3.611605	9.065840	3.605046	6.839827	7.091028	4.548635	8.908475	9.162916	5.108098	4.971090	7.438669	9.233946	5.029113	7.004242	5.973787	9.997062	9.871373	4.394772	8.428351	6.128336	5.899553	4.467794	3.395942	5.709925	4.882858	6.049762	5.405925	4.598956	3.689939	5.940259	4.778641	5.071019	6.305436	5.451257	4.721287	6.068594	5.888505	6.251818	6.926316	5.626559	6.173182	6.925974	8.294728	5.515655	4.556426	5.676561	8.576254	5.573143	5.758891	7.069044	4.164884	5.082049	5.577218	5.993577	6.413207	5.346523	4.026648	4.713285	8.043711	3.956800	7.449527	7.086292	5.602850	7.511538	5.537433	4.511602	6.777676	5.105042	5.081245	3.815433	3.523465	10.597246	3.717333	8.264002	4.670819	5.298453	6.500534	4.638196

LINC02636	3.472041	3.802855	4.316287	3.823735	4.880280	4.178992	3.586490	4.653285	4.353756	4.723440	5.154192	4.112439	4.357202	3.862777	4.053199	4.215778	3.734799	3.258539	5.796753	3.916184	4.721287	2.950734	2.920166	4.641771	3.839304	3.977212	4.625263	4.747673	4.499642	4.871035	4.407819	4.091186	4.117207	4.694111	3.775101	4.821263	3.559545	3.809721	3.389306	3.217271	4.116561	2.994217	3.592258	3.560720	3.984132	5.685687	4.222293	2.831611	4.798541	3.473610	3.351555	3.285326	4.054525	4.247330	4.040380	5.241252	4.262240	3.015270	3.091098	3.841327	2.745328	4.115070	3.908140	3.264389	3.554492	3.550424	2.957247	3.215552	4.013112	2.781925	5.083066	4.392767	3.566286	4.052563	4.639479	3.832048	3.767372	4.204520	3.588270	3.823109	3.422057	3.989228	3.613588	4.302412	3.480978	4.771765	3.319596	3.315547	3.810335	3.663078	3.324325	3.107025	4.191032	3.687880	3.957709	3.608224	3.846532	3.955463	3.342824	5.756839	4.316287	3.854880	3.264389	4.754919	4.657491	5.073638	5.245596	3.517751	3.330982	3.576521	2.796100	3.430804	5.306753	3.725775	5.556569	5.427255	3.197854	4.510512	5.078395	4.943402	4.916860	5.819727	4.519569	6.124083	5.577038	4.862492	3.490280	5.165102	6.120967	6.179672	5.779280	4.709478	3.955259	4.267396	5.036116	5.800993	6.218215	3.242251	4.680068	4.072762	3.810767	5.452057	6.246949	5.702980	4.465860	4.734603	5.332847	5.867214	5.344783	6.649670	4.235322	4.023461	5.975168	4.478658	6.524815	4.645359	4.928862	3.826564	3.708838	5.408923	4.212080	4.925093	3.418972	6.284639	6.158213	5.824047	2.761677	5.493723	3.109451	3.134024	4.198200	6.005942	4.476926	3.619528	5.873876	3.895246	6.359889	2.873153	3.687063	6.648363	4.757390	3.988618	3.691579	3.884898	3.975729	3.577338	4.919813	3.647986	2.764973	5.792044	5.384644	4.072539	5.306198	3.859821	4.129377	4.330557	5.942139	3.986510	4.156749	3.814603	5.052996	3.699384	3.710057	3.579178	5.527261	3.748966	3.480782	3.377017	3.499802	3.460735	2.957857	4.925961	4.148977	6.018659	5.022592	4.908825	4.422222	5.319749	5.304038	5.400520	5.197099	3.264112	6.066254	5.033372	6.271869	5.264099	3.491185	4.162511	5.032354	3.137848	4.939947	3.722103	4.663074	8.145338	4.766370	3.111885	5.065508	7.194222

AC007092.1	3.133321	3.280580	2.962645	3.211011	3.786494	3.754647	3.156400	4.006327	3.346247	3.097128	3.320580	3.170512	3.515884	3.528663	3.515313	3.093983	3.705259	3.033286	4.229679	3.128951	3.996401	3.533669	3.311317	2.973520	5.430849	3.500163	3.309872	4.139542	2.997133	3.674530	3.514088	3.498184	4.302168	3.019944	3.268254	3.825093	3.299180	3.219976	3.652214	3.338286	3.428989	4.736547	3.564394	3.226157	3.675983	5.048505	3.961381	3.317278	6.398294	3.424817	3.138594	3.007968	3.812109	4.082834	3.805753	3.104345	4.114828	4.220612	3.752330	3.897142	2.858268	3.138106	3.896934	3.226736	3.484252	3.465935	3.521682	3.162748	3.221947	2.835017	3.040061	3.525695	3.226592	3.077475	2.883547	3.338619	3.457163	3.490484	3.206648	3.649813	3.295444	3.664050	3.438239	3.981290	3.364994	4.544904	4.658123	3.254505	4.452047	3.647551	3.216236	3.390523	4.038661	3.204548	3.135892	3.094079	3.472743	3.092977	4.044548	3.521682	4.310159	3.131949	2.898333	2.900266	4.529888	3.487325	5.381153	3.238005	3.616508	3.151870	4.186427	3.311459	3.636278	3.207039	3.423478	5.505053	3.616879	3.454010	3.164807	3.076658	3.373733	3.288506	3.028480	3.994857	3.647002	3.854486	3.245376	3.257943	2.994298	3.087292	3.672132	3.359328	3.225182	3.214073	3.039322	3.085938	2.910020	3.241347	3.165816	3.598945	3.121463	6.921278	3.271463	4.526322	2.936539	3.892863	3.526294	3.948370	3.510205	3.181516	3.276202	4.084748	3.017610	3.241947	3.535725	3.223498	3.363362	3.325791	3.210048	3.415875	3.149126	3.596729	3.269432	4.099063	4.656195	3.306975	2.995701	6.164766	3.100561	2.996285	2.939586	3.947691	4.237224	3.472931	3.063626	3.771953	4.094115	3.534991	3.899349	2.831066	3.239740	3.984336	3.635857	3.801618	3.682236	3.610550	3.366336	3.356906	2.826793	3.536493	2.985598	3.189710	2.904234	3.182446	3.307309	3.337787	3.430641	3.150130	3.185015	3.647766	3.934702	3.483154	3.565704	3.396129	3.765519	3.068050	3.376866	4.100847	3.534047	3.293775	3.001185	3.628019	3.274494	3.080690	2.929615	3.143269	3.497840	4.045411	3.701392	3.353204	3.409194	4.244932	3.168541	4.883487	3.166490	5.720507	3.690559	3.189576	3.161219	4.439596	3.925461	5.623704	3.353204	3.532733	3.633687	2.882613	3.426919	3.967702

LINC01793	3.133321	3.280580	2.962645	3.211011	3.786494	3.754647	3.156400	4.006327	3.346247	3.097128	3.320580	3.170512	3.515884	3.528663	3.515313	3.093983	3.705259	3.033286	4.229679	3.128951	3.996401	3.533669	3.311317	2.973520	5.430849	3.500163	3.309872	4.139542	2.997133	3.674530	3.514088	3.498184	4.302168	3.019944	3.268254	3.825093	3.299180	3.219976	3.652214	3.338286	3.428989	4.736547	3.564394	3.226157	3.675983	5.048505	3.961381	3.317278	6.398294	3.424817	3.138594	3.007968	3.812109	4.082834	3.805753	3.104345	4.114828	4.220612	3.752330	3.897142	2.858268	3.138106	3.896934	3.226736	3.484252	3.465935	3.521682	3.162748	3.221947	2.835017	3.040061	3.525695	3.226592	3.077475	2.883547	3.338619	3.457163	3.490484	3.206648	3.649813	3.295444	3.664050	3.438239	3.981290	3.364994	4.544904	4.658123	3.254505	4.452047	3.647551	3.216236	3.390523	4.038661	3.204548	3.135892	3.094079	3.472743	3.092977	4.044548	3.521682	4.310159	3.131949	2.898333	2.900266	4.529888	3.487325	5.381153	3.238005	3.616508	3.151870	4.186427	3.311459	3.636278	3.207039	3.423478	5.505053	3.616879	3.454010	3.164807	3.076658	3.373733	3.288506	3.028480	3.994857	3.647002	3.854486	3.245376	3.257943	2.994298	3.087292	3.672132	3.359328	3.225182	3.214073	3.039322	3.085938	2.910020	3.241347	3.165816	3.598945	3.121463	6.921278	3.271463	4.526322	2.936539	3.892863	3.526294	3.948370	3.510205	3.181516	3.276202	4.084748	3.017610	3.241947	3.535725	3.223498	3.363362	3.325791	3.210048	3.415875	3.149126	3.596729	3.269432	4.099063	4.656195	3.306975	2.995701	6.164766	3.100561	2.996285	2.939586	3.947691	4.237224	3.472931	3.063626	3.771953	4.094115	3.534991	3.899349	2.831066	3.239740	3.984336	3.635857	3.801618	3.682236	3.610550	3.366336	3.356906	2.826793	3.536493	2.985598	3.189710	2.904234	3.182446	3.307309	3.337787	3.430641	3.150130	3.185015	3.647766	3.934702	3.483154	3.565704	3.396129	3.765519	3.068050	3.376866	4.100847	3.534047	3.293775	3.001185	3.628019	3.274494	3.080690	2.929615	3.143269	3.497840	4.045411	3.701392	3.353204	3.409194	4.244932	3.168541	4.883487	3.166490	5.720507	3.690559	3.189576	3.161219	4.439596	3.925461	5.623704	3.353204	3.532733	3.633687	2.882613	3.426919	3.967702

AC007100.1	3.133321	3.280580	2.962645	3.211011	3.786494	3.754647	3.156400	4.006327	3.346247	3.097128	3.320580	3.170512	3.515884	3.528663	3.515313	3.093983	3.705259	3.033286	4.229679	3.128951	3.996401	3.533669	3.311317	2.973520	5.430849	3.500163	3.309872	4.139542	2.997133	3.674530	3.514088	3.498184	4.302168	3.019944	3.268254	3.825093	3.299180	3.219976	3.652214	3.338286	3.428989	4.736547	3.564394	3.226157	3.675983	5.048505	3.961381	3.317278	6.398294	3.424817	3.138594	3.007968	3.812109	4.082834	3.805753	3.104345	4.114828	4.220612	3.752330	3.897142	2.858268	3.138106	3.896934	3.226736	3.484252	3.465935	3.521682	3.162748	3.221947	2.835017	3.040061	3.525695	3.226592	3.077475	2.883547	3.338619	3.457163	3.490484	3.206648	3.649813	3.295444	3.664050	3.438239	3.981290	3.364994	4.544904	4.658123	3.254505	4.452047	3.647551	3.216236	3.390523	4.038661	3.204548	3.135892	3.094079	3.472743	3.092977	4.044548	3.521682	4.310159	3.131949	2.898333	2.900266	4.529888	3.487325	5.381153	3.238005	3.616508	3.151870	4.186427	3.311459	3.636278	3.207039	3.423478	5.505053	3.616879	3.454010	3.164807	3.076658	3.373733	3.288506	3.028480	3.994857	3.647002	3.854486	3.245376	3.257943	2.994298	3.087292	3.672132	3.359328	3.225182	3.214073	3.039322	3.085938	2.910020	3.241347	3.165816	3.598945	3.121463	6.921278	3.271463	4.526322	2.936539	3.892863	3.526294	3.948370	3.510205	3.181516	3.276202	4.084748	3.017610	3.241947	3.535725	3.223498	3.363362	3.325791	3.210048	3.415875	3.149126	3.596729	3.269432	4.099063	4.656195	3.306975	2.995701	6.164766	3.100561	2.996285	2.939586	3.947691	4.237224	3.472931	3.063626	3.771953	4.094115	3.534991	3.899349	2.831066	3.239740	3.984336	3.635857	3.801618	3.682236	3.610550	3.366336	3.356906	2.826793	3.536493	2.985598	3.189710	2.904234	3.182446	3.307309	3.337787	3.430641	3.150130	3.185015	3.647766	3.934702	3.483154	3.565704	3.396129	3.765519	3.068050	3.376866	4.100847	3.534047	3.293775	3.001185	3.628019	3.274494	3.080690	2.929615	3.143269	3.497840	4.045411	3.701392	3.353204	3.409194	4.244932	3.168541	4.883487	3.166490	5.720507	3.690559	3.189576	3.161219	4.439596	3.925461	5.623704	3.353204	3.532733	3.633687	2.882613	3.426919	3.967702

AC007179.2	3.133321	3.280580	2.962645	3.211011	3.786494	3.754647	3.156400	4.006327	3.346247	3.097128	3.320580	3.170512	3.515884	3.528663	3.515313	3.093983	3.705259	3.033286	4.229679	3.128951	3.996401	3.533669	3.311317	2.973520	5.430849	3.500163	3.309872	4.139542	2.997133	3.674530	3.514088	3.498184	4.302168	3.019944	3.268254	3.825093	3.299180	3.219976	3.652214	3.338286	3.428989	4.736547	3.564394	3.226157	3.675983	5.048505	3.961381	3.317278	6.398294	3.424817	3.138594	3.007968	3.812109	4.082834	3.805753	3.104345	4.114828	4.220612	3.752330	3.897142	2.858268	3.138106	3.896934	3.226736	3.484252	3.465935	3.521682	3.162748	3.221947	2.835017	3.040061	3.525695	3.226592	3.077475	2.883547	3.338619	3.457163	3.490484	3.206648	3.649813	3.295444	3.664050	3.438239	3.981290	3.364994	4.544904	4.658123	3.254505	4.452047	3.647551	3.216236	3.390523	4.038661	3.204548	3.135892	3.094079	3.472743	3.092977	4.044548	3.521682	4.310159	3.131949	2.898333	2.900266	4.529888	3.487325	5.381153	3.238005	3.616508	3.151870	4.186427	3.311459	3.636278	3.207039	3.423478	5.505053	3.616879	3.454010	3.164807	3.076658	3.373733	3.288506	3.028480	3.994857	3.647002	3.854486	3.245376	3.257943	2.994298	3.087292	3.672132	3.359328	3.225182	3.214073	3.039322	3.085938	2.910020	3.241347	3.165816	3.598945	3.121463	6.921278	3.271463	4.526322	2.936539	3.892863	3.526294	3.948370	3.510205	3.181516	3.276202	4.084748	3.017610	3.241947	3.535725	3.223498	3.363362	3.325791	3.210048	3.415875	3.149126	3.596729	3.269432	4.099063	4.656195	3.306975	2.995701	6.164766	3.100561	2.996285	2.939586	3.947691	4.237224	3.472931	3.063626	3.771953	4.094115	3.534991	3.899349	2.831066	3.239740	3.984336	3.635857	3.801618	3.682236	3.610550	3.366336	3.356906	2.826793	3.536493	2.985598	3.189710	2.904234	3.182446	3.307309	3.337787	3.430641	3.150130	3.185015	3.647766	3.934702	3.483154	3.565704	3.396129	3.765519	3.068050	3.376866	4.100847	3.534047	3.293775	3.001185	3.628019	3.274494	3.080690	2.929615	3.143269	3.497840	4.045411	3.701392	3.353204	3.409194	4.244932	3.168541	4.883487	3.166490	5.720507	3.690559	3.189576	3.161219	4.439596	3.925461	5.623704	3.353204	3.532733	3.633687	2.882613	3.426919	3.967702

AC226119.1	10.951170	10.845031	10.856734	11.068675	11.307832	11.221771	11.123986	11.093692	11.293950	11.427522	11.248503	11.118254	10.913714	10.960321	11.373342	11.298381	11.331187	10.905852	11.713779	11.659107	11.380538	11.510090	11.663120	10.453877	10.606661	10.959158	11.334490	10.341269	10.808891	10.907937	11.616996	11.286424	10.723422	11.120843	11.009483	11.032989	11.057630	10.752648	11.142502	11.240625	11.109567	11.169165	11.147949	11.119870	11.130492	11.056273	10.620851	11.063360	11.131914	11.190322	11.126783	11.509649	11.154939	11.163334	10.951332	11.074564	11.024358	11.290706	10.860851	11.295264	11.170765	11.225024	11.261231	11.190903	11.292037	11.051785	11.042247	11.155520	11.069292	11.289463	11.167837	11.135945	10.783006	11.273089	11.378139	11.152165	11.269690	11.219974	11.006117	10.872038	10.734749	11.124285	11.099443	11.484725	11.468386	11.068971	11.064664	11.393514	11.587163	11.546807	11.342060	11.507445	10.876634	11.403962	10.941247	11.179915	11.054370	11.284004	11.464934	10.934589	11.080123	10.859529	11.089609	11.281661	11.897946	11.098248	10.630255	11.169880	11.689126	11.201144	11.979749	11.582863	11.016496	10.757138	11.167837	11.456392	11.241079	11.031212	10.979372	11.609621	11.467492	10.985538	11.320543	11.567413	10.963865	11.608020	12.203133	11.931223	11.754375	12.207292	11.584703	12.096669	10.432922	11.509511	11.796151	12.215941	11.568841	12.158494	11.212599	11.160666	11.886889	11.588775	10.619105	11.369916	11.924965	10.835128	11.400599	10.494828	11.031349	11.178535	11.957549	11.809935	11.811860	11.265456	11.642056	11.055964	10.452666	11.891078	11.559784	11.549582	11.643568	12.687444	10.756043	11.212744	11.832244	11.658803	12.145773	11.350393	11.771822	11.362688	12.365418	11.513405	11.431058	10.957536	11.104579	12.527089	11.797058	11.252221	10.689190	11.527480	11.699314	11.968715	11.273397	11.190615	11.039883	11.579668	11.128797	11.303523	11.342560	11.928150	11.139199	11.446601	12.259261	12.035812	11.673261	11.469421	11.802080	10.889985	11.513924	10.914603	11.861260	10.963108	11.202229	11.404558	11.310412	11.499211	12.307765	11.515310	11.495353	11.850039	12.441436	11.492313	10.925062	11.160065	12.804429	11.672529	11.177918	11.556100	11.854889	11.223249	12.563000	10.587449	11.642235	11.899423	10.899381	11.888725	11.618016	11.136541	10.453118	11.562464	10.993001	11.600290	11.010491	11.102534	11.487573	11.212133	11.546206	12.215055

AL031674.1	5.971232	5.027449	4.672733	4.684809	4.310159	4.015408	3.944654	6.180816	5.121125	4.330557	3.298088	4.356986	3.219103	4.371341	3.323979	4.883086	5.346143	3.051231	5.421462	4.479749	4.351327	5.174521	6.907352	4.300892	4.535899	4.338539	4.341124	5.116218	4.226166	4.950218	5.849706	4.142379	3.499964	3.810767	4.335703	4.117669	3.503482	4.552590	5.062460	4.514410	4.934501	5.312218	3.813595	3.890731	4.128508	7.174480	4.108703	4.569162	4.913496	5.134262	4.403745	3.278544	4.858971	3.948568	3.662244	3.653400	4.049324	4.512913	4.885618	4.937670	4.980126	4.232648	5.286594	5.330690	5.356854	5.082452	5.747211	5.435125	5.470448	6.012970	4.423542	4.920011	4.124861	4.748070	4.819605	4.649179	5.139865	5.102775	5.284366	4.797463	4.479959	3.705674	3.192314	4.893762	4.613988	3.418434	4.170305	4.505883	7.796632	5.714005	5.382725	5.808321	4.402680	4.248186	3.803679	4.194119	4.449382	4.156749	3.112133	4.224672	3.022268	4.422440	4.171454	4.667368	4.909040	3.734389	5.986628	4.680510	5.166666	4.802376	5.392783	5.382508	4.725144	4.502867	3.893535	3.079184	5.250002	4.292692	4.106979	5.128131	5.087179	4.501816	4.790369	4.009111	4.932831	4.144966	5.307713	4.309712	4.291335	3.665267	4.760783	3.407660	3.150988	3.494647	3.801826	5.447412	3.765098	4.971901	4.639063	3.798781	5.039101	6.802846	4.313806	4.218149	4.598327	4.033635	4.430721	3.996193	5.185200	5.162655	3.389637	3.517163	3.904662	4.081552	4.030852	4.094535	4.780946	5.140257	4.677535	4.376947	5.536657	4.110281	4.199448	4.047376	7.099247	4.330333	3.010672	4.854590	5.026650	5.130587	5.762505	4.289203	4.947749	3.933266	5.068986	3.725135	3.304129	3.669702	4.326994	5.347361	4.352445	5.427255	5.697494	5.105234	6.410035	5.378285	5.168681	5.170899	5.662912	4.376523	5.019719	4.028172	5.381551	4.442638	4.742349	5.524971	5.054726	5.419794	3.961596	5.568462	4.015628	5.058371	3.797157	4.470334	3.784800	4.914945	4.536102	6.299226	4.806984	5.201863	5.877440	4.240135	4.552801	3.907267	4.054525	3.709474	3.762780	3.648190	5.815094	4.021539	4.745760	4.540902	4.446889	4.166676	4.481034	5.785779	4.374352	5.362905	4.470334	5.327132	5.056350	3.894841	4.435851	4.117437	3.317112	5.113938	4.248611	5.200081

LINC01119	6.069980	7.147212	6.160836	5.758717	5.628214	5.209030	6.552849	5.176961	4.526520	5.732104	5.514243	5.586972	7.324535	5.536065	6.416667	5.445439	5.239849	5.971415	6.396299	6.656772	5.973440	6.557111	9.038018	6.458853	6.482234	6.302189	6.266222	6.451330	6.052025	6.056734	5.557747	6.907043	7.485700	6.233583	6.474906	6.748173	6.609828	6.336540	6.962293	6.746442	5.689353	7.052414	6.869358	5.991827	7.425236	5.698420	8.216812	7.549912	7.241025	6.065393	6.557781	6.902716	7.505395	6.806316	7.516962	5.520813	6.933336	7.422439	7.345011	7.965718	7.577914	6.940135	10.339897	6.675415	7.591953	6.761400	6.898200	6.941837	5.929339	7.262342	6.809524	5.779106	6.007890	6.737629	6.750212	6.764745	6.125852	7.099566	5.816187	7.403964	6.728667	7.611602	7.031402	7.390553	7.165039	6.283637	7.344870	7.328976	7.654215	7.621942	7.545191	7.713269	7.146288	6.597774	6.167736	6.965964	7.096562	6.805231	6.310763	6.377801	5.496995	6.430725	5.907569	6.161144	6.724651	5.868862	5.552010	6.483410	6.039123	5.595130	9.449820	5.866135	6.031996	4.934923	5.018696	5.018110	5.961797	6.121820	6.024457	5.904197	7.901488	6.590464	6.653361	4.971305	5.216196	5.758891	6.733993	6.913084	4.805149	7.049262	5.769576	6.743344	7.688261	6.725278	5.706824	7.334280	6.054258	5.631886	7.535183	6.271204	7.260440	7.081450	4.094749	6.760102	8.711913	5.563155	3.691374	5.161854	6.244796	5.868488	6.620518	7.904280	6.112814	6.380296	6.257931	7.409130	6.962124	7.360270	8.378322	6.091996	7.013910	6.823372	5.849182	6.586878	5.278766	6.890278	8.857724	6.383651	7.419145	7.206445	6.248802	6.744976	9.422207	7.964950	5.213818	7.956657	6.934258	6.940736	7.555981	8.596115	8.362192	7.901974	6.984911	7.159454	7.991262	7.832150	7.695045	7.200399	8.427398	6.715696	6.688383	5.005774	7.759279	7.743330	6.320684	7.136551	8.084588	6.600383	8.233737	7.473225	8.219476	8.094770	7.553313	8.177678	5.829913	7.951508	8.039167	8.551958	9.006858	7.116743	8.997966	6.877654	8.584839	5.752847	6.958150	7.106770	7.401622	5.725140	7.041908	7.896560	6.704931	5.416410	6.231918	7.796171	5.569032	6.036353	7.508474	6.595180	6.195129	10.002425	6.539555	5.289592	3.742824	5.255945	5.787378	6.746725	7.322331	5.687890

DNAJC27-AS1	3.137360	3.248982	3.467279	2.991507	3.812713	3.099736	3.803284	3.200908	3.326154	3.049767	3.302999	4.030852	3.233782	3.611177	3.510886	3.610154	3.230761	2.975243	4.175304	3.064705	4.019768	3.133459	3.818633	2.978709	2.927701	3.297612	2.939822	3.215955	3.430280	3.165437	3.666279	3.708456	4.094946	3.279275	3.890511	3.891777	3.377742	3.053432	3.258230	3.301128	3.447665	3.141417	3.538985	3.231305	3.672321	4.817515	5.308659	2.945373	4.429795	3.264238	4.291559	3.473610	3.614174	4.543995	3.769711	3.094765	5.080814	3.697989	3.322496	3.801408	2.881617	3.121586	3.734799	3.198002	3.720146	3.455153	3.256478	3.105616	3.211727	2.858540	3.051004	3.096410	3.232304	3.300671	2.884539	3.327729	3.241947	3.206777	3.517751	3.630489	3.478586	3.706665	3.458858	3.860653	3.279133	4.144088	3.748346	3.252098	4.128953	3.485375	3.190532	3.047625	4.029509	3.179263	3.895246	3.213805	3.471726	3.141935	3.141547	3.164562	3.760508	3.840275	2.954328	3.016157	4.577985	3.841707	2.914278	3.739768	3.075635	3.102314	4.740639	3.193392	2.856198	3.228037	3.497998	5.558298	3.384830	3.104899	3.209656	3.053740	3.345270	3.485005	3.033707	3.595938	3.589954	3.028867	3.436352	3.232304	2.947380	3.069240	3.281785	3.342302	3.591134	3.209109	3.149253	3.078974	2.877038	3.537062	3.082546	3.582844	3.417570	4.935976	3.026034	2.865368	3.572084	3.065854	2.803334	3.174752	3.421184	3.410052	3.785637	3.953545	3.063410	3.248412	3.598775	3.280243	3.066728	3.251979	3.171175	3.195416	3.046403	3.568873	3.275887	4.097968	4.569162	5.216196	2.851496	3.999384	3.003425	3.030788	2.972426	3.849489	4.328092	3.473243	3.039394	3.988380	3.537632	3.074125	3.771327	2.846687	3.253117	3.794458	3.518911	3.703237	3.656838	3.255912	3.273883	3.339777	2.844900	2.987048	2.979165	3.209242	2.937738	3.130616	3.300671	2.991100	3.003614	3.050905	3.180141	3.580146	4.003453	3.526294	3.538985	3.402652	3.485005	3.138823	3.373733	3.775550	3.384489	3.261430	2.989102	3.614384	3.258846	3.125532	2.941020	3.905507	3.417742	3.792048	3.252236	3.592616	3.287257	2.948767	2.932827	4.925093	3.201021	2.900822	3.240032	3.180933	3.117381	4.213645	3.098911	3.092914	3.127678	3.998746	3.725775	2.860021	3.142685	3.278993

AC110772.2	3.954808	3.283615	3.881295	3.612955	4.208004	4.856703	3.657866	3.263968	3.323165	5.082049	5.629161	3.059943	3.667710	3.650983	5.114965	4.582030	3.268108	4.942999	4.150618	4.686755	5.680106	4.093706	6.228660	3.655026	3.040703	5.077351	5.825312	4.150303	6.390861	3.818420	4.661386	4.770981	4.105442	5.851124	5.953976	5.166466	4.436502	7.306970	3.313340	4.525441	4.066061	4.476238	4.457789	4.762486	5.559256	4.979752	3.829065	3.196264	3.697132	4.493546	4.650918	5.632831	4.687586	4.746389	5.388158	4.398484	4.415122	4.341788	3.567496	3.900638	4.014061	6.034881	4.738648	4.324223	4.032747	3.567103	3.509038	3.961808	3.235939	2.886374	4.853955	3.550231	4.597236	5.446617	5.064478	3.347210	3.296519	3.847741	4.754919	4.524143	6.269459	5.699132	4.005441	4.942399	5.270257	4.985191	3.965102	4.172089	4.431596	4.065846	4.007805	4.089608	3.909010	4.570033	3.827419	6.167379	5.267082	6.706217	3.160705	3.808458	5.319572	3.867287	4.676249	4.089384	4.602712	4.474750	3.733620	4.185785	4.950416	3.625598	3.795908	3.662430	2.902002	5.351582	5.473470	3.529405	3.456073	5.439483	5.196314	3.236796	3.809104	2.985514	3.680841	3.253442	3.613999	4.805579	3.149886	3.726339	3.308242	3.613588	2.912842	3.257316	3.144918	3.877385	3.802855	3.132218	4.343091	3.232829	3.447839	4.735442	3.040276	2.863017	3.168670	2.920528	3.984565	4.346762	3.221664	3.215429	3.500497	4.434540	3.530520	4.500042	3.781438	4.781346	3.604265	3.400726	7.219043	3.320743	3.234542	3.207928	3.409888	3.619893	3.408374	4.277970	4.544904	3.474700	2.925438	3.909914	3.430072	3.214885	3.062131	3.931093	4.407357	4.564465	3.691579	3.786494	4.255890	3.360967	3.899129	2.890430	3.865767	3.952050	3.882111	3.833785	3.686870	3.570038	3.323165	3.385360	2.857366	3.498184	3.079971	3.261151	4.203381	4.774907	3.320743	3.083140	3.946420	3.086586	3.513915	10.060156	4.660049	3.520242	8.891442	3.475634	3.070579	3.058412	3.379837	4.088961	3.484795	3.284110	3.724930	5.510880	4.623548	4.699164	5.275329	3.023296	3.210048	3.961168	3.223215	3.877169	3.093613	3.134377	3.024958	4.932634	3.132068	8.041861	4.886886	3.553719	3.123779	3.028960	3.366512	2.869515	3.190259	3.113441	3.980405	2.926892	7.029396	4.376122

LINC01136	3.361439	3.594809	3.590513	3.409194	4.435637	3.841899	4.340039	4.786821	3.660607	3.308061	3.609384	3.559157	3.551628	4.080249	4.957935	4.042312	3.564394	3.006528	4.731233	3.749998	5.233026	4.285926	2.870737	4.011437	4.311047	3.685623	4.766605	2.823310	3.232021	4.846376	3.814603	5.550523	4.344818	3.820377	4.778843	4.149594	3.706865	3.355506	3.588432	3.547776	3.528119	3.710057	4.927218	3.846532	3.865565	5.824962	3.337147	2.872443	3.938111	3.823300	3.578380	5.762505	4.837863	4.863721	4.246657	3.695478	4.552801	3.669264	3.634474	4.066688	2.833451	3.477651	4.095169	3.403382	3.849937	3.668862	4.190163	3.421705	3.502367	4.006327	3.629234	3.148383	3.468724	3.421359	3.795499	4.534548	3.942615	3.644431	3.628614	3.937709	3.499964	4.149177	3.645219	4.376322	3.527576	3.852056	3.041132	3.327268	4.254568	3.806805	3.385511	2.965168	4.345246	3.897372	3.642272	3.529960	3.903586	4.100192	4.052563	3.222539	4.115070	3.486244	3.243404	3.725135	4.856480	4.031890	3.798589	4.181013	3.288642	4.376736	4.563629	4.863937	5.992161	3.733411	4.606485	6.207080	3.882986	3.165192	3.422249	3.384322	3.751278	3.630055	3.145815	5.191120	4.207063	3.317974	3.590928	3.479469	3.286102	3.391037	3.335465	3.394158	3.312568	4.380980	3.214463	3.392796	2.868195	3.382065	3.434709	4.500729	3.723702	2.805927	3.565159	3.770527	3.189289	4.254797	3.204261	3.497998	3.819056	3.604453	4.286343	4.982563	5.118860	4.855836	3.828051	3.723903	3.466290	3.563639	3.304758	3.764496	3.485185	4.187106	3.519290	4.420222	5.360226	3.311459	2.772872	4.276183	3.548569	3.376527	5.142569	4.393599	4.667368	4.465860	3.116629	4.188883	3.522634	3.182446	4.094115	2.757952	3.358117	4.217947	4.073227	4.125452	3.941517	3.032564	3.648809	4.037989	2.769141	3.130616	3.160939	3.688293	3.443447	3.034402	4.786621	3.350383	3.630688	3.992473	3.327407	5.674028	4.676470	3.735228	3.775945	3.608025	3.871134	3.856567	3.872862	3.748721	3.648603	3.542870	2.862176	3.875266	3.499062	3.360502	3.401604	5.789212	4.417292	5.142569	3.821840	5.132995	4.084748	3.239051	3.923611	5.214034	3.503802	3.235381	3.725135	3.444367	3.299460	4.841476	4.159074	7.377364	3.627218	5.646519	5.658691	3.729236	3.300530	3.437547

LINC02682	10.862762	10.627143	10.877350	11.354029	11.836322	11.545312	9.944548	12.289352	12.625698	10.830328	11.441892	10.701355	10.885551	10.834982	9.969266	11.316909	12.152682	10.223767	11.159921	11.039023	10.856320	11.507746	11.336433	9.932911	10.844403	11.245791	12.567105	11.214951	11.471379	11.792414	12.381631	9.711867	9.755469	10.410577	10.222882	9.979650	10.053263	10.257400	10.666811	10.481855	11.132510	10.573790	10.617007	11.823375	10.476950	11.352876	12.331442	10.091891	11.240485	10.181167	10.075134	9.724389	9.408958	9.736242	9.380969	12.511911	8.994576	9.967099	9.620086	9.666225	9.713721	11.524427	11.003228	10.974369	10.027958	10.144761	10.323048	10.513768	9.834894	10.738376	10.914318	10.807528	11.686029	10.104477	10.286511	10.872641	10.915990	10.243674	10.226354	9.592273	10.043446	9.813386	9.559249	9.646315	9.698334	9.915301	10.662229	9.688079	10.400338	10.084519	9.920116	10.721226	10.586401	10.695129	11.819904	10.251906	9.852765	10.569984	12.416578	12.062305	11.737635	9.885790	10.257870	10.370179	10.901113	10.933681	11.705064	11.467920	12.706715	11.672604	11.756636	11.875511	11.636032	12.215332	11.583602	14.065689	11.594299	11.156708	11.902176	10.870038	11.500503	11.785542	11.635098	13.076484	11.662361	11.588926	12.301709	12.747949	11.556839	11.203735	11.051205	12.220306	11.446896	11.495782	12.213629	11.627766	11.358695	11.536144	10.652947	11.431952	12.108177	11.437102	11.741168	11.020059	10.349176	11.902027	11.554168	12.997893	11.377693	12.488616	11.416603	10.943746	10.714015	10.215948	10.661925	10.328083	11.037558	11.949439	11.162307	12.335403	10.941247	10.339746	12.044097	10.426707	11.266180	12.525283	10.995680	10.568922	10.510068	11.106454	10.204075	10.049254	9.952136	9.720646	10.883860	9.573371	9.605775	10.789717	9.629531	10.280033	11.712614	10.733261	10.607548	10.413149	10.518462	10.931134	10.289908	11.057038	10.364867	11.048835	11.029265	13.184665	10.722975	10.901875	10.746642	10.963392	10.760252	10.705664	10.448849	9.828718	9.934336	10.144003	10.509773	10.125390	12.014319	9.535499	10.647894	10.984035	10.836162	10.611997	11.060705	11.437819	11.897652	10.925632	11.975098	11.668018	11.804981	11.524099	10.432310	11.980316	11.403536	11.668763	11.006688	11.197663	11.932902	12.396485	11.011245	11.013967	13.086475	12.041676	12.312392	13.165894	13.440958	12.770845	12.524622	10.746036	11.442790	11.566391

AL359762.3	6.404576	5.555072	6.174889	6.298082	6.938425	6.933336	6.433281	5.067971	5.458089	7.069377	6.814915	6.518878	7.242596	6.985720	6.737942	6.668503	5.050399	6.671706	5.646889	6.854940	6.352776	4.221883	3.587089	6.041416	6.167736	6.239917	6.646901	5.742641	6.807572	5.715666	6.511935	6.636745	7.010741	7.522372	6.944237	6.956719	6.812716	6.142404	5.461042	6.582944	6.344898	5.574989	7.061243	6.456542	7.118530	6.143417	6.103756	6.003768	5.902912	6.318925	6.386980	6.534841	7.290501	6.767312	6.847723	6.097354	7.139220	6.197582	6.005381	6.887409	5.304215	7.149139	5.045343	5.300411	4.901741	5.295630	5.105234	5.566754	5.494903	5.044730	5.779106	6.123900	6.897032	6.516230	5.987193	6.162362	5.986628	6.282611	5.941779	7.085989	6.523509	6.195305	7.126590	6.251818	7.292400	6.540336	6.631395	7.016162	5.184411	4.950218	5.707790	4.811884	6.421917	6.274070	5.747563	6.750842	5.976379	8.101896	6.595180	6.743835	5.994659	7.418516	7.374392	6.566408	4.666915	6.615749	4.503080	5.376740	4.360255	6.998925	3.630688	3.858770	6.017258	6.579946	6.771409	3.458409	5.631552	6.285141	6.799796	5.655749	4.864946	5.386549	6.119757	3.224190	4.638845	5.518396	5.869547	6.617262	3.890045	5.513333	4.215778	4.352445	5.844914	4.470784	7.742537	5.634192	6.258961	5.106233	7.076205	4.465860	3.912495	3.175677	5.467944	3.561768	5.347947	4.262886	3.346402	4.993314	4.086810	7.393824	7.047064	6.310605	7.142561	6.522817	5.226669	6.106037	4.619929	4.419800	4.088735	5.328100	4.142802	6.908604	6.298082	5.218747	4.692443	4.953147	5.146157	5.781452	5.047064	5.489978	6.256911	5.880687	5.737892	6.434267	5.590423	6.617107	5.261653	5.477492	5.027663	4.484173	7.176828	4.038891	4.088961	5.362349	4.675819	5.422817	4.769713	4.171454	4.696606	5.029742	5.078192	3.374402	5.365392	5.767383	5.401255	5.191894	5.514243	5.397471	7.220008	7.127512	6.312967	7.020142	6.948600	6.313838	5.093331	5.787010	5.434367	4.891666	4.860848	4.328092	3.775101	4.887505	3.984782	5.256331	6.170311	5.329117	6.900296	6.099633	6.198966	5.194696	6.606374	6.224501	5.534087	3.951215	5.193853	5.072202	5.086364	4.332259	5.106656	3.141052	3.739973	3.251669	4.834368	3.432203	3.956140	5.583994	5.990959	5.067563

AC005906.2	5.239451	5.573528	5.614247	5.724545	6.131717	5.696189	5.354518	5.426024	5.413577	5.244217	5.421112	4.992029	5.416410	4.939947	5.184812	5.176324	5.290002	5.715301	4.695359	5.256533	6.290462	6.511783	5.133626	5.342989	5.070805	5.225696	5.832808	6.223992	5.795999	5.603982	5.451468	5.525913	5.909182	5.541080	5.394781	5.271047	5.183216	5.252144	5.857438	5.694721	5.418161	5.455127	5.743007	5.717483	5.645803	4.388470	5.955069	5.814075	5.868676	5.630622	4.990394	5.534481	5.534897	4.589267	4.939337	5.655749	4.906492	3.057545	5.207639	3.415696	5.151351	5.504676	5.100522	5.169098	5.425587	5.115570	5.651451	5.609716	5.598001	6.046564	5.602065	5.406292	5.221489	5.408149	5.179925	5.107646	5.493530	5.253951	5.459635	4.926830	4.484799	4.940144	4.776982	5.150726	4.259584	5.563905	4.498550	3.746085	6.197075	4.855214	5.354706	5.178778	4.893127	5.726824	5.219369	5.308084	5.183216	5.696022	6.594690	5.719936	6.139091	5.478682	5.648788	5.743007	5.693412	5.623172	5.880506	5.991459	6.652567	5.468537	6.972442	6.694243	6.428172	5.449926	5.640502	7.418220	5.681611	5.117203	5.287944	5.280957	6.006820	6.050946	6.224501	7.712340	6.569550	6.434799	5.594202	5.766090	6.145662	5.900980	5.836054	6.111878	6.060983	6.624362	5.227278	5.883154	6.654016	5.711383	5.400520	6.234471	6.973051	5.971051	6.381820	6.490307	6.033636	6.074670	6.615279	5.910785	6.249627	5.701700	6.420256	5.802237	6.247129	5.697684	5.571259	5.393779	5.720686	6.209157	6.044131	6.632728	5.495098	5.235426	6.305257	5.461042	4.296754	6.878304	5.503367	5.491117	5.743733	5.534678	6.049248	5.985034	5.525162	5.449553	5.953274	7.234047	5.797651	5.606131	3.354520	5.864878	6.257762	5.249579	5.513154	6.172526	5.575751	6.125302	5.790613	6.059726	7.318527	5.966803	6.138754	6.433086	5.541274	5.496995	5.642899	5.737892	5.554340	5.786123	4.999837	5.353882	5.769744	5.297474	6.731754	5.161065	6.569708	5.121125	4.598769	5.993753	5.664777	5.555257	5.834830	5.654037	6.149697	5.688775	5.927411	5.999712	5.922745	6.373141	5.779623	6.510098	6.079900	6.054615	5.664777	4.871252	6.273370	6.155690	5.624670	6.295776	6.701120	6.941664	7.096414	11.955437	6.752407	6.772519	7.001632	5.984369	5.748312	5.842795

AC080129.1	3.355179	5.634192	4.264426	5.201083	4.500504	5.640130	4.423758	5.607399	6.031288	4.042742	4.753418	4.579245	3.914226	4.899185	4.184058	4.927846	5.724200	4.725997	6.011402	5.355303	5.529168	6.494756	6.031288	3.618554	4.040146	4.195419	3.977797	4.597046	4.547293	4.602712	5.754982	5.386970	5.908291	4.491993	6.368863	5.302426	4.486379	4.628705	5.434751	6.659030	4.011026	5.783606	5.710849	5.648576	5.253360	5.975338	4.470544	5.674028	5.665725	5.800809	5.007858	6.219926	4.079591	7.323297	5.489780	3.902945	5.482022	3.055709	6.015713	4.899838	5.857616	3.886152	3.188761	6.416995	6.003241	6.682079	6.384336	5.937210	6.228838	6.461996	5.649360	6.234806	4.979752	5.098442	5.408923	5.180533	5.031543	6.110971	5.483318	5.840428	6.334340	5.766251	4.615553	5.923113	6.504965	5.631017	5.550523	5.614981	5.702406	6.774469	6.742107	6.630632	5.435519	5.033372	4.302876	4.554548	5.795443	4.729746	4.209072	4.004778	5.823129	5.076314	4.524143	5.073004	5.951435	4.911553	5.775473	5.716039	5.685292	4.183639	6.762515	6.149498	6.133452	3.650804	5.719750	8.086750	4.082834	5.471198	3.926712	4.384260	4.240767	5.296440	5.783785	6.054258	5.427843	5.351297	3.991131	5.014252	4.371963	5.951633	5.587921	5.950898	6.096022	5.917236	6.097354	4.810162	5.468755	5.247821	4.730184	5.182396	5.361740	6.699791	5.285388	4.947124	5.363287	6.123900	4.423983	5.257917	5.006189	5.045882	5.387388	5.343190	6.349412	6.024627	5.172769	5.216797	4.859174	5.964657	5.589666	5.346739	5.429745	5.540166	4.979942	5.924351	6.119404	5.888319	5.296854	4.848502	5.641019	5.734630	5.428231	5.350897	4.619283	8.901410	4.551538	5.071831	6.040169	7.140632	5.404268	7.809741	5.632831	5.577218	5.353445	5.603033	5.941296	6.487103	6.357498	5.034412	6.131542	4.885618	5.181372	5.867615	5.492799	5.206028	5.286792	5.007256	4.729562	5.463106	5.248396	8.392872	6.361699	5.683055	5.774720	5.187613	6.593414	6.692115	5.112773	6.445578	8.068759	5.588122	6.159273	5.605327	6.393047	5.292576	5.795803	5.567473	6.351457	5.204082	3.920614	5.717483	5.656663	4.532248	5.305994	5.721949	4.836596	5.935459	8.708855	5.757763	5.408333	6.218710	7.098469	7.323901	4.960817	5.178157	5.848984	4.869381	5.305197	4.565960

AL358215.1	4.206388	3.806389	3.274657	2.969108	3.512383	4.128728	3.372905	3.286678	3.640900	3.388422	3.524206	3.558339	3.379304	3.722298	3.299620	3.161484	3.468400	3.495782	4.980930	3.436845	4.600612	3.158149	3.512749	3.082345	3.624039	3.361140	3.207343	3.236957	3.267949	3.425458	3.473243	4.134718	5.113352	3.508701	4.425257	4.019348	4.571992	3.617097	4.384713	3.455153	3.936458	3.760082	3.735018	3.209109	3.543981	5.212774	3.141697	5.101129	3.296210	4.142605	3.954187	5.671238	4.343091	4.150003	3.678209	3.154930	5.366204	4.993314	4.418223	4.042528	3.677597	3.495403	6.799452	3.295582	3.497641	3.435989	3.763184	4.270510	3.801618	2.965075	3.274802	3.033479	2.994477	2.956161	2.994026	3.638474	3.242519	4.060633	3.029478	3.714449	3.358117	3.799007	3.116070	4.268916	3.479650	5.165298	4.464384	3.424029	4.592522	3.776565	3.547577	3.832048	5.881927	3.497287	3.115819	2.956909	3.404877	3.192946	4.038230	2.970823	2.952475	3.224618	2.971440	2.916877	4.421071	3.480575	3.089786	4.208643	3.297455	3.268982	2.856302	3.502944	3.259725	3.462866	3.830333	3.285805	3.694253	2.981829	2.988814	3.425827	3.412024	3.512576	3.167274	3.660010	4.996722	3.256210	3.562130	3.335626	3.187010	3.319425	3.528312	3.132332	3.189289	3.081673	3.267793	3.467279	3.630489	3.461300	3.417894	3.977405	2.965377	5.508966	4.515737	3.505520	3.258697	3.156291	3.792920	3.245966	3.561139	3.274657	3.442220	3.608791	3.282239	4.230278	3.720995	4.474088	3.947691	3.514626	3.396300	3.458761	3.721214	4.114601	3.283950	3.803477	4.686559	4.281683	4.806984	3.447839	4.378669	3.995744	7.902414	4.663894	4.212080	3.295748	3.242102	4.657710	4.963356	4.353117	4.159074	3.350218	3.941921	4.906899	3.896934	3.867507	3.596508	4.257186	4.551538	3.990914	2.976862	3.351900	3.072161	3.764064	3.088322	2.918334	3.950770	3.125395	3.355689	3.576521	4.181013	3.751463	3.949881	3.101859	3.662866	4.087434	3.809313	3.247223	3.398675	4.324870	3.688884	3.865968	3.520622	3.045474	3.158535	3.050591	2.969894	2.968362	2.988244	4.078669	3.304598	3.015866	3.094313	3.013169	3.128332	4.837863	3.575730	3.004535	4.029966	4.038891	3.171902	3.210048	3.392134	3.064605	3.587089	3.600306	3.454372	3.416712	3.261885	3.925461

AC025272.1	7.755009	8.510434	6.177466	6.855890	6.484435	6.169288	6.350111	6.323377	5.392206	5.828214	5.400880	7.794310	6.917878	4.947342	4.986843	6.614141	6.481577	5.821866	7.362910	6.350954	5.847149	6.970119	6.663632	6.596935	5.240060	6.409018	8.136323	9.024467	8.387588	8.331361	6.454454	7.700231	7.406634	7.250720	6.149155	6.102226	6.623246	6.811299	6.295933	6.188588	5.855990	7.506342	6.752407	5.777263	5.386761	6.263525	6.488708	7.718880	6.501718	5.734630	7.957608	6.616420	6.804151	5.957686	6.793323	5.640843	5.412818	6.008419	7.505088	6.111965	7.100946	5.165881	7.069377	7.249782	5.966637	6.822459	8.106204	6.112317	6.834688	7.192295	6.639342	5.966457	5.816007	7.022026	6.087137	6.734308	6.779417	7.280692	7.672279	6.109076	6.468191	6.252502	7.094491	7.302884	6.065393	6.569550	5.633010	4.957731	6.958315	7.655962	7.433753	6.903040	7.035000	6.903692	7.638327	6.122355	6.869358	4.975271	5.733560	5.180318	6.145843	6.952325	6.306114	5.083842	6.805681	5.413382	7.320886	5.652761	6.376639	6.257597	6.043120	6.302900	6.454936	5.647262	5.989536	6.897530	7.143171	5.941446	4.866626	6.415159	6.754571	6.689163	6.687772	6.296590	5.692666	6.177466	6.983026	5.717822	5.667961	5.414157	5.536264	5.684541	5.800031	5.002123	6.009832	6.266553	5.000064	7.204235	8.813498	5.999712	7.007847	7.025548	5.112361	5.492799	5.709757	6.460003	5.563726	6.146192	8.034340	6.403278	6.374667	5.649908	5.599318	6.459196	4.551752	6.200316	6.290129	6.520193	7.150257	5.548054	6.339882	6.377960	6.090277	6.267597	5.741185	5.473683	6.541524	5.826180	6.162362	6.202438	7.177313	6.077284	5.932445	6.703085	7.387317	7.550369	6.110611	6.165458	6.039824	6.512277	7.334280	5.789407	6.861854	6.109395	7.182510	6.531278	6.953906	6.174195	6.760754	6.334164	6.568705	5.854925	6.446215	7.258388	7.351296	6.444367	6.786940	6.097512	6.629691	5.621097	5.786822	5.967524	6.627907	6.558290	5.459435	6.382325	5.211443	7.218710	7.542035	6.048206	6.488381	6.111618	6.529653	5.846393	6.996308	5.597810	5.524027	6.165970	5.250586	5.641968	6.063480	5.483684	6.928135	6.270865	6.142058	6.411836	6.688080	6.290129	6.626000	7.245629	6.586060	7.920740	7.601231	5.373984	4.752349	6.516230	5.056559	6.364380

AL162458.1	5.202453	5.924182	5.877095	6.282611	5.524212	6.303405	6.159539	3.801618	4.337231	6.428535	5.675825	5.560926	6.523859	5.863088	5.384460	5.852724	5.730853	6.782450	6.269286	5.833523	5.594934	6.294060	6.622744	6.682909	6.387827	6.014686	6.426806	6.644356	4.547511	6.211030	6.335850	6.641921	6.972892	6.133618	6.491222	5.999204	6.761558	6.545978	6.868899	6.242941	5.821306	5.977611	5.835193	6.019002	6.267078	4.652582	6.531278	5.514823	6.429707	6.618065	6.245437	6.605173	6.505475	7.130033	6.330096	5.598001	6.731107	7.017782	6.827338	7.001459	6.421917	5.935986	6.169288	6.211381	5.754786	6.295088	5.823304	5.416211	6.151554	5.637695	6.298741	6.361860	5.332046	6.472094	5.561471	6.008053	6.256911	5.800232	5.883488	6.892352	6.828774	6.540849	7.137983	7.026011	6.952325	6.091316	6.584884	6.762515	6.176101	6.593567	6.506661	4.927430	5.879233	6.503655	6.450636	6.491065	5.886723	6.653833	6.869995	5.769576	4.850577	6.613170	6.349761	6.790708	6.353769	5.639932	4.476479	6.565283	5.708658	6.120803	6.044821	4.759750	3.998329	5.822053	6.292672	2.975807	5.659580	5.706129	5.934947	5.883488	6.760102	6.331636	6.340567	5.903669	5.990072	6.165285	5.245705	6.353769	5.504117	5.797651	6.386127	4.596165	6.071153	5.689540	6.557617	6.517234	5.647837	5.938973	5.806670	6.215952	7.103286	7.410049	4.659010	5.760333	6.671872	6.554321	6.966278	4.613787	6.710064	5.096409	6.358329	7.153076	6.193564	6.936164	5.734458	6.499728	7.153726	5.971594	6.515893	6.116129	6.148819	5.597008	7.612641	7.305553	4.206826	6.579465	6.707842	6.494116	5.467351	6.880073	8.897010	6.500696	7.207529	6.790236	6.098380	7.474601	7.701001	7.088639	6.502026	6.696046	6.186736	7.233701	6.415159	5.787010	8.489362	5.001729	6.322896	7.159318	7.779491	6.329915	5.961628	5.317069	6.148301	5.931921	6.035254	6.732875	6.477451	4.812916	6.412347	6.342339	7.626255	8.721757	6.180988	7.169340	7.703896	7.439610	8.415946	5.254349	6.526670	6.642551	6.185718	6.961340	6.536628	5.932632	5.914377	6.265874	6.183233	6.215781	7.050084	7.094174	6.690628	8.504625	6.104268	8.703539	5.753934	4.916860	5.844038	5.514243	4.909265	5.146342	3.349361	4.626343	5.898668	5.352662	7.764057	6.995990	6.875472	7.996145

LINC02249	2.909650	3.063972	3.517979	2.949423	3.774707	4.348075	3.200255	3.460015	3.330982	3.296369	3.469431	3.551811	3.172928	3.672507	5.272267	3.034513	3.317445	3.536879	3.639493	3.090979	4.466966	2.856077	5.130200	3.121240	3.159921	3.343813	2.865258	3.161604	2.953130	3.216520	3.602837	3.175876	4.104536	3.026393	3.274197	3.910352	3.589398	3.116070	3.098911	2.894087	3.340297	2.995591	3.190933	3.362333	3.357588	4.628705	4.074275	3.828629	4.105200	3.637482	3.695698	4.580573	4.040380	3.763184	3.389455	2.972619	3.537062	4.604801	3.616132	3.998940	3.372263	3.356732	3.964450	3.718939	3.078505	3.130994	3.491351	3.469235	3.416712	2.993726	2.945514	3.065854	3.205204	2.886294	2.899003	3.167149	3.669919	3.156795	3.178536	3.382935	3.159437	3.747923	3.026669	4.173173	3.205070	3.120117	3.566891	3.215552	3.047744	3.109451	3.102113	3.187677	3.085290	2.957247	3.508701	2.928509	3.448007	2.890735	3.903586	3.779151	4.366132	3.156147	3.054254	2.827972	3.440139	2.958067	2.980425	3.350579	4.554945	3.194590	5.389322	4.500729	4.346108	3.195162	3.068364	5.659219	3.822675	2.911101	3.104004	2.942671	3.116629	3.550424	2.979074	5.217569	4.359584	3.414002	5.159865	3.439246	3.110054	3.219672	3.107938	3.280419	3.650983	4.748507	3.389127	3.306975	3.166490	3.144215	3.178423	3.888504	4.024737	5.633010	3.188619	3.014660	2.844337	3.970521	3.408711	3.495604	3.443277	3.391947	3.270526	3.924166	2.993629	3.186045	3.646035	3.607467	3.039843	3.390353	2.887371	3.126623	2.961473	3.290281	3.710484	3.484451	5.611588	3.847370	3.933476	3.133914	3.722702	3.737108	3.447097	3.570961	3.830779	3.931529	3.107143	6.022244	3.145941	3.451269	3.392300	3.173311	3.062027	3.227169	3.515313	3.115462	3.157539	2.874597	3.561567	3.245109	3.092072	2.923946	2.932321	2.983166	3.184613	3.271619	3.134888	3.469965	3.276507	2.969540	3.349518	3.417042	3.838235	3.043601	3.897603	3.448178	3.246100	3.327407	3.341822	2.981829	3.795499	3.211727	3.228037	3.066834	2.987590	3.016850	2.937218	3.448007	3.810335	3.357243	3.166213	3.425082	3.915108	3.550026	4.176172	3.878287	2.964561	3.006162	3.214600	3.321848	3.059060	4.389354	4.311896	6.498399	3.122628	3.966797	4.166006	3.029277	2.905083	3.167650

TMEM161B-AS1	7.126410	7.036396	7.332112	7.421675	7.814612	7.072747	6.744672	7.335363	6.920814	7.038903	7.107418	7.214504	7.044183	7.124824	7.112839	7.162520	6.454936	7.748119	6.248466	7.237496	6.635006	7.431742	7.102823	7.444912	7.612641	7.470477	6.207781	7.084102	6.855086	7.084910	7.268335	7.244857	8.534684	7.781356	7.755300	8.258791	7.637383	7.410476	7.259331	7.437704	6.480089	7.032798	8.199981	8.086591	8.083646	8.251925	7.506496	7.572780	7.595944	7.401308	7.683826	7.726996	7.367653	7.300159	7.881227	7.196624	7.621512	8.248863	6.897684	8.395764	7.320128	7.197600	7.072588	6.889336	6.363544	7.599840	7.849748	6.743037	7.190316	7.047856	6.883408	6.858189	6.838347	6.818962	7.085839	7.947076	7.203331	7.098777	6.915261	7.246216	7.965718	7.266472	7.772318	7.192616	7.853910	8.137408	7.894811	7.921630	7.452925	7.359642	7.266775	6.159273	7.325954	7.044973	7.525576	8.193517	7.638623	8.072162	7.760697	7.412597	7.808938	7.246079	7.052870	6.908123	7.906643	6.477635	7.777466	7.321353	7.484743	7.249008	7.290668	7.849748	8.083490	6.985556	6.843037	5.624269	8.040277	7.076870	6.845392	6.931924	6.749580	7.394137	7.473694	5.696389	7.487256	7.168868	6.086582	5.538833	5.683252	6.015887	7.849325	6.799953	6.729672	5.228322	7.283976	7.182354	8.079921	7.038273	7.865730	7.556139	6.944078	7.581658	5.615740	6.252157	7.583498	5.133823	7.144590	6.045714	6.931129	6.322896	7.075119	7.126885	8.516857	8.014014	7.699912	7.236268	6.912442	6.951549	6.787255	8.299713	6.179820	7.723319	8.089724	7.906786	7.985774	7.078474	8.248700	8.656980	7.457457	7.146440	8.338225	8.076073	5.901152	7.355649	7.322955	7.654980	8.305897	7.373586	8.764520	6.039284	6.433951	6.267757	6.090277	6.702584	7.694879	6.839336	6.474750	7.003068	6.494116	7.341765	6.465705	7.401308	6.375503	6.537941	6.965325	6.090277	7.389491	6.151211	6.532074	7.709416	7.661681	7.081923	7.324214	8.009151	8.044812	8.061071	8.839199	6.000758	7.049113	7.630032	6.316408	7.357319	6.186409	6.308238	6.567229	7.011725	6.487592	7.837050	7.961301	5.505443	6.898041	6.610125	6.630936	6.552518	6.239749	6.448351	7.158238	7.347389	5.799127	6.942312	6.497516	8.327291	6.176101	7.568557	5.002508	6.781665	7.100327	6.343349

DLEU1	9.912359	11.004546	11.253964	11.124898	11.323494	10.351729	11.398515	10.233543	7.413067	10.638329	10.322128	10.783617	10.519506	11.054075	11.231863	9.880559	10.246336	10.858937	9.964114	11.205541	10.925940	11.147664	10.716860	10.199050	9.367251	9.279219	8.723582	8.777808	8.892214	10.235733	9.346191	11.794980	11.598591	10.830911	11.164797	11.698867	11.766326	11.738805	11.929674	11.388081	10.037937	11.895619	11.256056	11.664926	11.455214	11.958709	8.553764	11.304710	11.558020	11.773141	11.648993	12.129478	11.826536	12.004151	11.370978	9.756549	11.924002	12.499288	11.550159	12.155089	11.918948	10.274257	11.230338	11.715417	11.219382	11.187132	11.674871	11.820802	11.631808	11.799373	11.279433	11.974803	9.910027	11.614054	11.929833	11.636639	11.843867	11.501471	11.313224	11.282105	11.968868	11.683357	11.574347	12.204037	11.941654	11.682779	11.873552	11.924435	11.869342	11.941976	11.512020	12.160822	11.670323	11.792548	11.180206	11.882525	11.509511	9.979042	10.935174	10.070060	10.086677	11.283569	10.616435	11.293950	12.133274	9.828718	9.731236	11.933193	10.780147	9.344981	12.517931	10.288521	10.794845	9.528337	10.512279	8.294115	10.218179	10.124758	8.487552	11.518703	10.520340	11.222510	10.105230	9.094797	9.745680	9.255278	8.397483	9.357717	11.036408	10.047865	9.539708	11.540550	9.168150	9.334161	9.349896	10.649376	10.582028	10.525491	10.095773	10.059104	10.078848	11.051205	8.541337	10.898787	10.743957	9.796404	9.070041	6.726582	9.326448	7.526374	10.257400	10.676730	10.983634	11.980316	10.726118	10.574229	11.205836	11.359582	10.354160	9.667179	11.143986	10.752648	10.193562	10.624119	11.612573	8.026527	11.222202	11.168548	12.805624	10.905549	10.950587	11.557603	11.530297	11.840382	9.440482	11.687372	11.720774	11.064802	12.138794	11.795120	9.239370	11.536600	11.106019	11.523392	11.206577	11.865134	10.378509	11.816438	11.053227	11.304855	10.270574	7.366550	11.013825	11.430746	11.641016	10.868599	12.123755	12.137865	9.853064	11.002234	11.059091	11.180206	10.061488	10.713151	10.763571	11.878918	11.578639	11.357672	10.553379	12.443867	11.736567	11.299384	11.708045	9.616360	9.768444	10.670015	8.451090	10.737937	10.895968	10.328083	11.070913	8.202701	11.257836	10.598160	7.563944	10.479190	11.827669	10.630981	6.991611	11.306046	9.594314	10.121388	8.741333	11.645346	10.527913	10.447955	10.912644	8.858812

DLEU2	9.912359	11.004546	11.253964	11.124898	11.323494	10.351729	11.398515	10.233543	7.413067	10.638329	10.322128	10.783617	10.519506	11.054075	11.231863	9.880559	10.246336	10.858937	9.964114	11.205541	10.925940	11.147664	10.716860	10.199050	9.367251	9.279219	8.723582	8.777808	8.892214	10.235733	9.346191	11.794980	11.598591	10.830911	11.164797	11.698867	11.766326	11.738805	11.929674	11.388081	10.037937	11.895619	11.256056	11.664926	11.455214	11.958709	8.553764	11.304710	11.558020	11.773141	11.648993	12.129478	11.826536	12.004151	11.370978	9.756549	11.924002	12.499288	11.550159	12.155089	11.918948	10.274257	11.230338	11.715417	11.219382	11.187132	11.674871	11.820802	11.631808	11.799373	11.279433	11.974803	9.910027	11.614054	11.929833	11.636639	11.843867	11.501471	11.313224	11.282105	11.968868	11.683357	11.574347	12.204037	11.941654	11.682779	11.873552	11.924435	11.869342	11.941976	11.512020	12.160822	11.670323	11.792548	11.180206	11.882525	11.509511	9.979042	10.935174	10.070060	10.086677	11.283569	10.616435	11.293950	12.133274	9.828718	9.731236	11.933193	10.780147	9.344981	12.517931	10.288521	10.794845	9.528337	10.512279	8.294115	10.218179	10.124758	8.487552	11.518703	10.520340	11.222510	10.105230	9.094797	9.745680	9.255278	8.397483	9.357717	11.036408	10.047865	9.539708	11.540550	9.168150	9.334161	9.349896	10.649376	10.582028	10.525491	10.095773	10.059104	10.078848	11.051205	8.541337	10.898787	10.743957	9.796404	9.070041	6.726582	9.326448	7.526374	10.257400	10.676730	10.983634	11.980316	10.726118	10.574229	11.205836	11.359582	10.354160	9.667179	11.143986	10.752648	10.193562	10.624119	11.612573	8.026527	11.222202	11.168548	12.805624	10.905549	10.950587	11.557603	11.530297	11.840382	9.440482	11.687372	11.720774	11.064802	12.138794	11.795120	9.239370	11.536600	11.106019	11.523392	11.206577	11.865134	10.378509	11.816438	11.053227	11.304855	10.270574	7.366550	11.013825	11.430746	11.641016	10.868599	12.123755	12.137865	9.853064	11.002234	11.059091	11.180206	10.061488	10.713151	10.763571	11.878918	11.578639	11.357672	10.553379	12.443867	11.736567	11.299384	11.708045	9.616360	9.768444	10.670015	8.451090	10.737937	10.895968	10.328083	11.070913	8.202701	11.257836	10.598160	7.563944	10.479190	11.827669	10.630981	6.991611	11.306046	9.594314	10.121388	8.741333	11.645346	10.527913	10.447955	10.912644	8.858812

MCM3AP-AS1	9.529428	9.734237	9.819657	9.513223	10.283247	9.957369	9.434019	9.401532	10.679279	9.645490	9.854464	9.583001	9.362011	9.573371	9.883787	10.043161	9.717177	10.511086	10.430630	9.718454	9.445907	10.096550	9.472171	9.924291	10.146709	9.826170	9.625683	9.615893	9.455087	9.554494	10.183759	9.508545	9.667023	9.596485	9.999817	9.740092	10.012922	9.738519	10.065121	10.615566	9.992041	9.996607	9.784423	10.211224	10.136837	9.637322	9.171571	9.479637	9.837660	9.855744	9.415969	9.690611	9.908965	9.983976	10.112741	9.757807	10.023382	10.213496	10.047564	10.155465	10.012605	9.577451	9.667979	9.873486	10.213038	9.791103	9.603315	9.686521	9.961059	9.989210	9.183450	9.575634	9.632445	9.755615	9.497029	9.448101	9.482720	9.273625	9.145493	9.382710	9.910666	9.822688	9.667979	9.997492	9.864543	9.709999	10.075134	9.941976	10.139993	10.368062	9.808891	9.997062	9.922734	9.705031	9.297246	9.744936	10.060453	9.983819	10.186968	9.951078	10.054805	9.576986	10.214264	9.974195	10.314129	9.919156	9.767080	10.607990	10.492998	9.947962	10.159823	10.083270	10.096550	9.940701	10.172297	11.086028	9.754543	9.795351	10.177038	10.700757	10.040077	9.393908	10.335529	9.854621	11.365359	10.120339	12.210507	11.348487	11.166077	10.348429	10.532527	11.068392	10.066011	10.596659	11.089762	10.336453	11.670022	11.250318	10.179942	10.524431	10.110805	10.761000	10.207514	10.482002	11.098860	9.328138	10.809338	10.738842	10.012145	11.839473	10.380008	10.491272	10.017871	11.536302	10.993001	10.333876	10.981465	10.832876	10.904667	10.584613	10.720056	11.297951	11.053092	11.276728	11.047378	11.440893	10.752793	11.080441	9.766473	10.489183	10.621738	10.751617	10.170130	10.028430	10.848448	10.790142	10.872038	10.863810	10.018340	10.542688	9.835366	11.215233	10.448550	9.569642	10.521739	9.767842	9.891491	10.586129	10.644156	10.079519	9.089471	10.346123	10.134533	10.104632	9.871071	10.332062	9.823607	10.016803	9.205189	9.993251	9.786390	9.743949	9.862854	10.246494	11.331759	10.698977	11.393088	10.315490	10.299419	11.655757	11.098411	10.724009	9.485807	10.360069	10.516841	11.112724	10.773291	10.747853	10.849009	9.460158	11.128092	10.641778	9.886562	10.957536	10.374695	11.409661	10.801504	11.015753	10.494224	10.803737	9.525858	11.809935	9.949211	11.440039	10.676888	11.608893	9.914676	10.825609

AP001469.3	9.529428	9.734237	9.819657	9.513223	10.283247	9.957369	9.434019	9.401532	10.679279	9.645490	9.854464	9.583001	9.362011	9.573371	9.883787	10.043161	9.717177	10.511086	10.430630	9.718454	9.445907	10.096550	9.472171	9.924291	10.146709	9.826170	9.625683	9.615893	9.455087	9.554494	10.183759	9.508545	9.667023	9.596485	9.999817	9.740092	10.012922	9.738519	10.065121	10.615566	9.992041	9.996607	9.784423	10.211224	10.136837	9.637322	9.171571	9.479637	9.837660	9.855744	9.415969	9.690611	9.908965	9.983976	10.112741	9.757807	10.023382	10.213496	10.047564	10.155465	10.012605	9.577451	9.667979	9.873486	10.213038	9.791103	9.603315	9.686521	9.961059	9.989210	9.183450	9.575634	9.632445	9.755615	9.497029	9.448101	9.482720	9.273625	9.145493	9.382710	9.910666	9.822688	9.667979	9.997492	9.864543	9.709999	10.075134	9.941976	10.139993	10.368062	9.808891	9.997062	9.922734	9.705031	9.297246	9.744936	10.060453	9.983819	10.186968	9.951078	10.054805	9.576986	10.214264	9.974195	10.314129	9.919156	9.767080	10.607990	10.492998	9.947962	10.159823	10.083270	10.096550	9.940701	10.172297	11.086028	9.754543	9.795351	10.177038	10.700757	10.040077	9.393908	10.335529	9.854621	11.365359	10.120339	12.210507	11.348487	11.166077	10.348429	10.532527	11.068392	10.066011	10.596659	11.089762	10.336453	11.670022	11.250318	10.179942	10.524431	10.110805	10.761000	10.207514	10.482002	11.098860	9.328138	10.809338	10.738842	10.012145	11.839473	10.380008	10.491272	10.017871	11.536302	10.993001	10.333876	10.981465	10.832876	10.904667	10.584613	10.720056	11.297951	11.053092	11.276728	11.047378	11.440893	10.752793	11.080441	9.766473	10.489183	10.621738	10.751617	10.170130	10.028430	10.848448	10.790142	10.872038	10.863810	10.018340	10.542688	9.835366	11.215233	10.448550	9.569642	10.521739	9.767842	9.891491	10.586129	10.644156	10.079519	9.089471	10.346123	10.134533	10.104632	9.871071	10.332062	9.823607	10.016803	9.205189	9.993251	9.786390	9.743949	9.862854	10.246494	11.331759	10.698977	11.393088	10.315490	10.299419	11.655757	11.098411	10.724009	9.485807	10.360069	10.516841	11.112724	10.773291	10.747853	10.849009	9.460158	11.128092	10.641778	9.886562	10.957536	10.374695	11.409661	10.801504	11.015753	10.494224	10.803737	9.525858	11.809935	9.949211	11.440039	10.676888	11.608893	9.914676	10.825609

AL445070.1	3.162394	3.260848	3.033581	3.167274	3.384830	3.514967	3.282710	3.250126	3.383146	3.365508	3.533669	3.549657	4.150401	3.748541	3.803066	3.171671	3.512576	3.385855	4.819179	3.100092	4.746162	3.352721	3.796521	3.301288	2.942671	3.154050	3.469619	4.580993	3.864949	3.347910	3.570961	3.404038	5.144750	3.269604	3.232829	4.564275	3.617653	3.176379	3.344944	2.989405	3.400545	2.954241	4.444781	3.606405	3.721910	5.452453	3.448800	5.324406	3.710276	3.620663	3.581694	3.436524	4.177655	4.133401	3.935574	3.449885	4.740639	2.947532	3.519668	3.784800	2.861935	3.122628	3.812307	3.169645	3.448178	3.460369	2.918581	3.330807	3.203743	4.653285	3.061684	2.919585	3.151102	2.883284	2.876946	4.466966	3.044868	3.066084	2.972326	3.804926	3.384997	4.041460	3.514264	4.366344	3.482975	3.589579	3.472214	3.307167	5.620566	3.610969	3.310373	3.565159	3.870296	3.188332	3.221793	2.939745	3.825314	3.267491	4.568525	3.588820	3.789732	3.239051	3.007845	2.934273	4.907542	3.681431	3.025453	3.448389	3.641701	3.263273	3.907084	4.061967	4.084550	3.296669	3.226736	5.811306	3.478013	3.063293	3.214600	3.163906	3.394158	2.990809	3.053850	4.048661	5.803658	3.456247	3.159038	3.308552	3.127678	3.316355	3.707302	3.924166	3.173183	3.424387	3.096657	3.407994	3.059277	3.429708	3.249455	4.096648	3.267491	4.883487	3.125920	2.850107	2.801210	3.176851	4.612707	3.469807	3.549307	3.202239	4.530516	3.671565	3.957225	3.193796	4.802168	3.601673	3.177342	3.346402	2.983166	3.160705	2.877304	4.408699	3.377896	4.200312	5.009920	3.218278	5.358725	4.009963	3.421532	3.426202	3.230192	4.076394	4.358295	3.537834	3.417042	5.381153	2.937088	3.054656	3.629234	2.821982	3.248412	3.881073	3.528312	3.706468	3.685623	2.911444	3.654812	3.303079	6.695231	3.039135	2.990624	3.145036	2.929028	3.404038	5.044730	3.042314	3.081673	3.040703	3.282097	3.765704	4.380153	3.579772	3.723511	3.583046	3.528119	3.439102	3.475076	3.270657	3.471394	3.464489	3.220706	3.858770	3.228359	3.110964	2.969281	3.283950	3.686239	4.701109	3.230063	3.085166	3.579359	3.026761	3.821651	4.992229	3.591134	3.016572	3.440832	3.249707	3.423478	4.597655	5.057174	4.158864	3.225437	3.294849	4.458439	2.982725	3.105015	3.343649

AC097500.1	4.710741	4.873845	5.448014	5.103393	5.211825	5.366204	5.297077	6.214073	6.481071	5.066344	4.547293	5.638981	4.785776	4.951888	5.178571	5.848095	5.516256	6.256093	5.020517	5.914578	7.948606	5.520438	7.038903	5.481833	5.104212	6.028032	6.207422	5.092471	5.967706	4.066061	5.665514	4.951026	6.101173	5.648788	4.895003	5.088183	4.406043	5.167486	4.700882	5.811119	5.188600	5.418942	6.200170	5.491509	5.112773	5.628020	4.635852	4.434966	8.209188	5.178157	5.473683	4.560764	4.728936	4.730409	5.047697	4.759964	4.927632	6.046564	5.588221	5.188014	5.057568	4.898117	5.226669	4.546670	4.906899	4.507693	5.185394	5.428231	3.994643	4.198610	4.592310	4.261329	5.904025	4.290026	4.740192	4.683553	4.324651	4.245775	4.727660	5.055345	4.210345	5.570335	4.781556	5.504495	4.563408	5.528031	3.981290	4.780324	3.400057	4.771972	5.091237	4.444333	5.627077	4.818759	4.717095	5.756267	5.898668	6.365531	5.563726	6.386455	5.286594	5.767383	5.800809	5.638630	4.423542	6.704142	6.254190	5.862898	6.233928	5.697884	4.950017	5.620566	5.685865	4.859838	4.698722	3.869665	5.281557	5.643691	6.621955	6.271705	3.408374	5.307713	5.925258	4.648115	5.184812	5.750837	7.193734	6.092703	5.240060	3.910981	6.307087	3.731709	4.360915	3.337455	5.380410	3.344161	6.779905	6.392190	4.971704	7.217959	5.610320	6.056359	4.077226	3.839648	5.289796	3.580319	3.385511	5.019934	3.513752	5.390063	4.047183	6.978010	4.222090	4.276612	4.875280	4.325941	5.127160	5.413006	5.908291	5.047064	5.465211	5.973440	4.584786	4.558533	5.242838	3.998940	4.420461	8.149462	4.147052	4.001044	5.944776	3.660212	4.112234	3.740951	4.575798	5.698228	6.325088	4.885618	4.176392	5.666110	4.695972	5.119073	4.659199	5.097372	4.907970	3.190142	4.525013	3.520441	3.312092	4.813729	4.655551	5.333050	5.135818	3.980864	4.081133	3.594809	5.241641	4.661814	5.132995	4.811450	4.663287	5.659580	5.268266	3.546357	5.436108	3.277564	5.789735	3.320743	5.063876	4.863512	5.028887	5.827654	4.361109	4.332057	4.601911	5.303625	6.559586	5.077136	5.571063	3.961381	6.685988	5.593090	4.479330	3.010961	6.729818	8.144863	5.567659	4.958542	4.512683	4.510948	4.859389	6.212521	3.416712	4.405609	3.943048	5.516256	4.072332	5.425587

AC048352.1	10.556299	9.955381	9.700800	10.277457	10.389743	10.939769	10.150159	10.399138	10.938063	10.862048	10.737773	10.578433	10.192680	9.833499	9.581160	10.824430	10.601972	10.032568	10.174575	10.105985	10.268977	9.498564	9.813689	10.278197	10.231313	10.318904	11.792112	10.795256	11.599875	10.527605	10.930096	9.273303	9.248899	10.369246	9.509006	9.454320	9.392967	9.613345	9.837507	9.543959	10.895043	9.222670	9.483497	9.469018	9.458028	8.997209	10.962078	9.573116	9.566057	9.409404	9.182813	9.128931	9.005510	9.228368	9.046851	10.948319	8.716747	9.269228	9.325363	9.113027	8.981494	10.966697	10.095773	9.651648	9.214963	9.070041	9.511815	9.416111	9.327210	9.367576	9.916212	9.872723	11.588623	9.732752	9.768291	9.879153	9.733780	9.719876	9.659354	8.979999	9.376209	9.291751	8.861847	9.125182	8.965870	9.382546	8.860924	8.734257	9.037545	8.896560	8.788276	8.851683	9.379881	9.592125	9.562068	9.562674	9.252538	10.006772	10.068558	10.492047	10.699884	9.832460	10.177956	10.052635	9.691463	10.952894	10.528938	10.247991	10.092323	11.263943	8.912356	9.790792	10.008647	11.868313	10.649097	12.080298	10.347802	10.523247	11.431661	10.422850	10.210775	10.016345	10.105829	11.252514	10.426540	10.858370	10.688294	10.970868	10.849149	10.644015	10.658726	10.726580	9.991246	10.239105	10.936398	10.165185	9.954115	10.599565	10.002270	10.037024	9.198284	9.322286	10.952814	10.698520	9.837182	10.552052	10.470285	11.704443	10.319671	10.844240	10.139112	10.155626	10.173663	9.585599	9.470923	9.557789	9.969713	10.511234	9.760255	11.366691	9.831681	9.296135	10.088070	9.505192	9.370750	11.412947	10.018488	9.560968	9.757639	9.477984	9.459710	8.972908	9.829180	9.349896	10.437361	8.994888	9.598799	9.488153	9.082132	9.239068	10.727937	10.052486	9.742743	9.133527	9.490307	9.854621	9.821926	9.664186	9.598257	9.991411	10.130189	11.592521	10.142602	9.759184	10.037488	9.993380	9.908116	9.967099	9.095706	9.087482	9.411694	9.094484	9.123220	9.249501	9.846464	8.961589	8.947219	9.311708	9.194339	9.293321	9.225274	9.336944	10.115901	9.902411	10.408604	9.962091	10.676585	10.654872	9.829180	10.640900	10.221670	10.776975	9.735326	9.653882	10.882081	10.419503	10.223316	9.507341	11.652576	9.358480	9.739183	10.352051	11.578639	10.373383	10.532370	9.835818	10.586252	10.614968

AC092957.1	6.599069	5.080622	5.250790	5.038906	4.462440	4.289411	4.138852	6.235310	6.495744	4.710123	5.009311	4.431806	4.095823	4.227886	4.704484	5.299860	6.041579	4.575798	6.699341	5.184212	4.932831	5.708481	8.728213	5.117824	5.111332	5.584774	5.903090	5.755704	4.518262	3.384489	6.449017	5.039315	3.262627	3.930017	4.993938	5.252360	4.688212	5.529921	5.955069	6.267078	4.416195	5.825139	5.282574	4.908825	5.823129	5.115354	4.787444	5.649152	4.517865	5.050976	5.267480	4.903414	4.805579	4.688212	4.882433	4.619091	5.354909	5.563547	5.033991	5.253951	5.966091	4.303540	7.923498	5.731220	4.832509	5.804210	5.626559	5.210432	5.075463	6.066429	5.186403	4.911992	4.519767	5.039903	5.167486	5.007256	5.500266	5.489143	5.662165	5.053206	5.241057	5.413197	4.818345	5.344783	5.227918	5.135038	6.104268	5.618375	5.766438	6.069439	5.257140	6.118183	5.366408	5.249169	4.833968	5.505443	4.871035	4.996722	4.315607	5.395166	4.785361	4.955831	5.042632	5.116998	6.092166	4.137598	6.702081	5.351297	6.679637	4.735652	5.905278	5.070584	5.306575	4.824555	5.601886	5.111933	5.586787	5.509757	4.656631	6.004648	5.046863	4.935362	5.207435	4.543555	5.099511	4.683975	8.665616	6.240427	4.418457	4.837863	4.909040	5.450296	4.363059	4.797674	6.142756	6.126226	4.324430	7.817635	6.190095	5.234225	5.550863	7.205658	6.422546	5.509173	5.605914	5.194915	5.231197	5.649152	4.371341	7.493360	4.521717	4.046498	3.738525	4.863937	5.850774	5.443145	5.944073	6.217501	6.445749	4.619283	6.221248	5.457054	5.592917	5.656663	4.327221	4.693712	5.765566	5.018696	5.631017	5.687890	4.811675	5.237270	5.403080	5.705946	4.146648	5.948272	5.788863	5.626914	5.516058	6.096346	4.496204	5.769200	6.053723	5.706450	6.236149	6.023595	5.236233	5.638458	6.031113	5.354706	5.546573	4.234408	5.525913	5.688249	5.048938	5.804026	6.137019	5.876566	5.116018	4.950633	5.495885	5.215041	5.300632	6.194249	5.267082	5.851124	6.305257	6.450151	6.368195	5.807596	6.519531	5.477301	5.335758	5.726105	5.707018	5.600431	6.046399	4.891666	5.221285	5.272049	7.404585	6.027309	4.692874	5.533140	4.783894	6.829419	5.524600	6.537292	4.481034	6.590824	4.562514	5.934404	3.906634	5.270656	3.573534	5.968070	5.978335	4.507693

AC013643.2	12.508357	11.946386	12.099218	12.222322	12.340029	11.812156	11.903881	11.145141	10.712868	11.905051	11.880130	11.807837	11.902491	12.122996	11.723321	11.798508	11.745962	11.998560	11.483981	12.458016	11.613910	11.004703	10.947309	12.255523	12.537583	12.313810	11.869200	11.835426	11.998998	11.732727	11.791025	12.196161	12.197990	11.530788	11.893367	11.824884	11.809762	12.064108	12.277746	11.888108	12.248989	11.953033	11.564033	11.635559	11.873552	11.954825	11.911179	11.982123	11.908646	11.862748	11.611675	11.929077	11.718244	12.052817	11.627214	12.206351	11.864688	12.090589	12.414625	12.027939	11.887650	12.036088	11.688416	11.841912	11.912793	12.028865	12.194595	11.731995	11.944381	11.268654	11.823688	11.639167	11.909419	11.722280	11.741588	11.560528	11.795854	11.703111	11.775784	12.502885	12.342480	12.350849	12.194595	12.467321	12.535770	12.204194	12.457084	12.486643	11.695305	12.672605	12.577702	11.745525	11.903572	11.642694	11.701443	11.983360	11.945301	11.359439	11.990637	11.305602	11.430746	11.337082	11.112018	11.387167	10.731203	12.011905	11.749984	11.118560	11.802240	11.848200	10.128831	11.911633	11.924151	11.816748	11.842489	11.514555	12.389189	12.092137	11.967950	11.859051	12.423579	12.140038	12.249758	10.377715	11.854889	10.776378	11.761159	10.918816	11.284886	11.684367	12.004567	11.881462	10.612741	11.464636	11.526932	11.544982	10.912789	11.850968	11.324079	12.080159	10.595005	10.705664	12.107264	11.348487	12.268675	11.772691	11.170020	12.262939	11.680518	11.294959	11.099157	11.847904	12.015985	11.317854	11.927863	11.937016	11.721674	12.238050	12.289963	12.572856	12.304346	11.778520	12.137398	11.962757	11.438954	12.149134	12.313810	12.156958	11.944525	11.190322	12.092757	11.198701	11.366830	12.526615	11.446732	11.012108	11.689126	11.448683	12.012048	10.909711	12.675868	11.847129	11.679053	12.480800	11.710591	11.731685	11.576321	11.217736	10.410277	11.863196	12.279737	12.099673	11.719874	11.330144	11.224167	12.087259	11.939225	11.966716	12.146883	11.913837	11.201442	12.380353	12.297752	11.855782	12.091683	12.087710	11.664926	11.453855	12.277596	12.986119	11.838566	11.295979	10.711413	11.346092	11.209250	11.736718	10.621887	11.396845	10.816719	11.255473	11.309839	10.230378	11.049734	10.627787	11.071521	12.388554	11.375164	11.999153	10.871747	10.631731	11.030014	11.881010	11.479943	11.678294	12.715418	12.162362	11.923414	11.953180

FAM245A	3.190933	3.067628	3.760914	3.893990	5.625058	5.559864	4.991405	5.320335	5.640130	3.084157	3.842751	4.072108	4.284974	4.589492	4.523673	3.985401	3.254330	3.753370	4.635636	3.379675	3.951398	3.618554	6.685342	3.260560	2.983458	3.364994	4.482420	3.703237	4.059534	4.515508	3.589398	3.995744	4.616662	4.319720	4.340694	3.914658	4.787871	2.940060	3.228359	4.150618	3.756928	2.994658	3.244137	4.030176	3.908575	4.644478	4.751493	3.249455	5.897408	2.946892	2.901241	3.172650	3.409888	4.726407	3.340106	4.475603	3.897372	3.170245	3.087392	5.601135	2.985121	3.680841	3.455318	4.765904	3.861063	3.218815	3.004073	3.180009	3.950322	2.905236	4.550879	4.654865	3.809905	4.680510	4.257422	3.197854	4.603961	3.973745	3.809721	4.976495	4.474945	3.075635	3.581694	5.270257	4.645768	4.832927	6.161697	4.183837	3.644431	3.314652	3.528493	3.526294	3.511629	3.638474	4.825841	4.637149	4.512913	4.804933	4.549312	5.289796	7.088332	4.051282	3.557756	4.753418	4.198610	4.117895	3.410970	5.021566	5.283568	4.177253	4.638196	4.148136	5.384282	4.402464	4.114601	9.899302	3.840068	3.777351	4.042312	3.936663	5.177346	4.469469	4.454382	7.492895	5.567281	6.284798	4.801357	5.273109	4.900919	5.182396	5.142569	6.420437	6.065925	5.679002	5.366809	4.926387	4.975271	4.923626	4.874048	6.085754	6.354105	6.956277	4.059534	5.776731	4.627811	6.894638	5.700066	5.657575	5.250790	4.910501	5.944605	5.261864	6.139789	4.777623	5.290806	5.633010	5.615913	4.945436	5.559256	5.580595	5.839699	4.793100	5.087993	5.344189	5.791497	5.748312	5.804210	5.976204	5.672603	6.017610	5.432612	5.592538	5.775818	5.617787	5.232404	6.231727	5.583801	5.649152	6.135673	6.162870	4.676014	5.795108	5.476348	5.787200	5.581341	5.715116	5.051783	4.894601	5.837310	5.246412	5.335333	5.373011	5.665725	5.741912	4.581198	4.937082	4.915143	4.806374	5.941954	5.276162	6.065235	5.500266	6.110778	5.670314	6.167379	5.793851	5.534285	6.121129	5.621468	5.301233	5.848984	5.221908	4.817515	5.577775	5.776559	5.804026	6.182888	5.826008	4.441972	7.099707	5.409120	5.184611	6.500369	6.162545	4.922988	5.014852	5.380786	4.856480	4.676681	6.333320	6.100152	7.373908	6.000261	6.318421	7.814763	4.702139	4.788270	4.500267

LINC01138	8.638648	7.690085	8.282999	8.955775	9.640719	9.239678	8.472487	7.985002	10.236353	8.777808	9.333838	8.940945	9.477671	8.499816	8.516685	9.348320	9.281986	8.397169	9.083932	8.874177	7.938222	9.106902	9.595856	9.415969	8.322775	9.851548	10.510212	9.192120	9.770486	8.998577	9.690384	8.181446	8.914022	9.956160	9.052817	9.187084	8.474459	7.657826	7.452761	8.838141	9.179536	8.411404	9.461373	8.992171	8.560695	8.506105	9.641171	6.852835	8.692458	8.409551	8.387433	8.286545	8.223754	8.625139	8.459410	8.722345	8.598050	8.145338	7.978633	7.943093	7.483828	9.122611	8.062420	8.496139	7.415371	7.015335	7.079256	7.826149	6.720650	7.446975	7.752553	8.132536	9.707995	8.315586	8.268065	8.466587	8.212064	8.115622	8.339505	8.696520	8.468700	8.149782	8.768535	8.283766	8.834349	9.046701	8.425261	8.226603	7.436323	7.185369	7.277658	7.592104	7.749351	8.296388	9.225591	9.737168	8.734401	9.895199	9.074340	9.681108	9.450937	8.927404	9.283248	8.784884	8.158381	9.597413	8.435252	8.772914	9.071262	9.285069	7.480750	9.585147	9.358630	9.236267	9.788049	9.725752	8.329486	9.099653	9.495201	7.834777	8.288418	8.342758	9.117571	8.976221	6.826891	9.319893	9.908965	9.575947	8.694983	8.969751	9.352445	8.942487	9.696933	8.501395	10.374263	10.018641	8.275241	8.247245	8.876622	7.264630	9.513820	7.753792	9.140544	7.409130	9.706882	8.675471	6.706701	10.512279	8.375886	11.536144	9.594314	10.459678	9.046541	9.173745	9.081509	8.478522	7.637383	8.042654	7.820703	10.735480	7.525891	10.546423	8.502947	7.677784	8.213942	9.046071	7.878847	7.960692	7.811708	9.198113	8.236218	9.027348	9.449354	8.965547	9.666724	8.695418	7.567799	8.275964	7.250233	8.402513	8.326365	7.553604	7.921494	8.613766	7.570519	7.675427	7.040480	7.206289	7.562431	7.277975	7.485863	8.781959	8.351263	8.878011	8.495694	8.160678	7.506049	8.073029	9.358336	8.465383	7.391023	8.994416	9.423469	9.325829	9.467194	8.448582	9.334307	8.115622	8.527214	7.567799	8.421962	8.595167	8.469008	8.715629	9.675550	10.284425	10.123198	10.539572	9.533987	10.240017	10.548890	8.138337	9.076571	9.373508	7.816813	9.869327	8.876306	8.322153	8.168936	7.365150	9.939949	10.796271	9.383185	7.029396	8.849250	8.652809	8.646482	9.972329

LINC02694	11.705354	11.782817	11.261682	11.642537	11.354335	12.275329	11.814486	11.908646	12.168351	12.230786	12.207137	12.201238	11.861688	11.852935	11.136974	11.869049	12.131274	10.915527	11.312487	12.309519	11.359302	10.659045	12.289963	10.916445	10.829909	11.426062	12.495360	11.379601	12.117551	12.033231	12.216524	11.090874	10.673307	11.589672	11.299384	11.411961	11.353895	10.764309	10.866981	10.557223	12.236647	10.457105	10.408443	10.862460	10.491272	10.708724	12.580656	9.998570	11.032245	10.238936	10.487949	10.080574	9.934938	10.202951	10.144761	12.456129	9.691000	9.858336	9.777366	9.836257	10.389287	12.183786	11.400440	10.614358	10.375288	10.462179	10.509917	10.817887	10.344466	10.893304	12.196008	12.118493	12.358156	11.948032	11.934585	11.770207	11.822149	11.831962	11.970213	10.090671	10.659325	10.595136	10.014305	10.512879	10.397912	10.956934	10.437038	10.432156	10.098434	10.077279	10.002425	10.633822	11.006400	11.863940	11.525172	11.762074	10.884165	11.300543	11.814345	11.939377	11.851720	11.688574	12.006245	10.997755	10.417533	12.063353	10.723643	11.157291	11.591601	12.351330	11.187132	10.396893	10.206741	12.381803	11.764948	13.394547	9.861608	12.237727	12.409843	11.734962	12.216235	11.623726	12.143103	12.615027	12.695570	11.982583	11.563602	12.201396	12.449269	12.773792	12.404804	12.363880	11.820950	11.672364	12.040293	11.672076	11.070628	11.183767	11.536302	11.233464	11.221910	11.651679	11.497855	11.038451	10.771657	12.190585	11.568999	12.938173	12.168674	12.042106	11.435100	11.541375	12.234834	11.342479	11.373342	11.651082	11.460855	11.398352	11.053797	12.722641	10.951940	10.318172	11.414134	10.812401	11.322294	12.707731	9.957526	11.612110	10.897614	11.044735	10.370639	9.960750	10.490710	10.145661	12.210352	9.454180	9.632445	9.910666	9.651195	10.432020	11.841745	10.575556	10.618351	10.569842	10.419038	10.999088	11.280614	10.888642	10.911904	12.137255	11.942873	12.939392	11.947128	11.709829	11.952291	11.748620	12.099378	12.240075	9.916529	10.783006	10.819554	10.729414	10.557516	10.556772	11.982123	10.764606	10.326281	10.548263	10.393702	10.088070	10.908849	11.541804	12.274074	12.651748	12.463389	11.589071	12.044097	12.026601	11.362843	12.458956	11.710741	12.534911	10.922541	10.252493	12.713119	11.524887	10.668712	10.789421	12.832911	11.513777	10.802095	11.316765	12.612867	12.256640	12.052362	10.256478	11.923268	13.001778

ITGA9-AS1	15.246007	14.168734	13.900711	14.201164	13.709428	14.082208	14.448739	12.802136	14.157364	14.616154	13.647062	14.412450	14.051518	13.224119	13.488148	14.511568	14.678334	13.964639	12.327002	14.465534	12.962142	12.101494	13.556139	13.693359	13.949893	14.517397	15.736354	15.171380	15.085910	14.414735	14.696136	13.581357	11.806190	14.082507	15.025188	14.374427	13.815559	12.927582	14.135120	14.098263	15.161343	14.388615	14.907125	14.428245	15.503877	15.344243	15.331635	13.473788	14.263241	13.873120	13.522074	14.149795	14.466061	12.831697	13.352500	14.350910	14.847322	13.094857	12.495176	12.937441	12.930619	16.033556	15.250206	12.868242	12.351330	12.107582	12.787522	12.739334	11.740562	13.234805	15.224770	13.511061	14.917864	14.045286	14.861942	13.393700	13.598139	14.678334	13.209908	12.635767	13.182875	15.407655	13.404601	12.494420	13.479279	14.595757	13.757880	14.392936	12.420436	11.923414	12.976230	12.512753	11.873996	13.108685	13.099406	13.781086	13.399301	14.027387	14.505707	15.190029	14.017548	14.630796	14.732223	14.575995	11.629391	14.448911	14.692440	13.739103	14.196861	14.917362	12.710502	12.697518	12.586302	14.968533	14.059748	14.878115	14.641807	14.996793	15.672165	14.183722	13.585102	14.892277	14.884408	15.726974	14.672821	14.938328	15.922928	14.894400	14.348197	15.438162	14.578917	15.091892	14.913079	15.552451	15.248027	14.763822	14.188986	13.045174	13.036330	13.639891	12.856932	13.013710	14.242737	13.515355	13.473369	14.803294	14.058288	15.502988	14.573507	15.926414	15.386744	15.008260	15.527160	14.784831	15.607626	16.169798	16.897220	13.841923	13.704846	14.965129	13.692881	15.601071	15.543645	15.805918	13.925119	15.080534	16.572650	14.324402	15.763886	15.681084	15.234130	14.128314	15.683269	14.640937	15.116602	14.431229	13.510177	12.853832	14.826455	13.934369	14.850021	11.846434	12.970971	14.422880	14.064122	12.788738	14.515058	14.657723	13.072631	14.489554	15.987023	14.627696	15.745908	14.890760	15.291679	15.454339	14.586497	15.787848	15.281689	15.553263	13.597687	14.730705	16.143053	15.763886	14.521823	14.689299	14.479473	12.997893	13.791984	15.048970	12.909129	16.026974	15.609450	15.779491	14.969696	15.975682	16.538902	14.572654	14.614415	15.705567	14.111066	15.367177	14.854894	12.256151	16.474625	14.113158	12.814750	12.491754	14.659974	13.121375	14.957224	13.693359	14.742751	14.382065	14.583563	14.601973	15.632923	14.129209

AL031658.1	16.456985	15.379850	15.706649	15.815434	15.882641	16.234936	16.048355	15.883639	15.760765	16.076368	15.915538	16.163437	16.001371	15.781709	15.450314	15.873355	15.924163	15.547001	15.476405	15.779491	15.340435	14.736679	15.160128	15.404616	15.499684	15.616895	15.911833	16.036135	16.443550	16.111440	15.954463	15.871113	15.059742	15.536844	15.715793	15.558628	15.988198	15.887107	15.729053	15.219191	16.412035	15.731087	15.813386	15.663417	15.890581	15.934855	16.182924	15.729053	15.978326	15.781709	15.726974	15.637542	15.682114	15.249434	15.531572	15.726000	15.717865	15.308698	15.256065	15.451967	15.131490	16.152946	15.598287	15.224051	15.368664	15.507347	15.368664	15.589896	15.315239	15.195519	16.127121	16.115572	16.259687	15.998443	15.994548	15.997211	15.837350	15.482081	15.604793	15.390024	14.993419	15.419437	14.887600	15.655519	15.517756	15.790919	15.430200	15.589896	15.044721	15.191395	15.128463	14.913617	15.333793	15.186033	15.278819	15.682114	15.630940	15.756462	15.827482	15.728021	15.854635	16.020683	16.057790	15.730084	15.082962	16.430830	15.420214	15.979533	15.310201	16.355654	14.926494	15.481254	15.756462	16.369009	15.964494	15.613151	15.810180	16.472279	16.156164	16.175634	15.903390	15.478014	15.847680	15.916773	16.261246	16.086016	16.010487	16.184460	16.131397	16.259687	16.171220	15.966981	16.011919	15.570171	15.983265	15.817557	15.462495	15.385183	16.064222	15.205705	15.124619	14.888617	15.836242	15.784706	15.897567	15.724946	16.010487	16.207472	16.308700	15.809175	15.380594	15.745908	16.213991	16.115572	16.370743	15.985841	15.985841	15.984477	15.942235	16.161910	15.656594	15.948483	15.874614	15.970695	15.932498	16.249977	15.448092	15.934855	15.953297	15.455879	15.418602	15.526235	15.738421	15.458388	16.562284	15.484605	15.412346	15.043521	15.160798	15.304417	16.483130	15.113035	15.173276	15.078176	15.189377	15.455879	15.379121	15.523618	14.817306	16.263142	16.228514	16.178602	15.998443	15.613151	16.005250	15.958303	15.989529	15.979533	15.227331	15.371654	15.505597	15.343482	15.268645	15.487850	15.889470	15.437228	15.363968	15.059742	15.153250	15.222515	14.938834	15.607626	15.854635	15.852280	15.521068	15.875766	15.789877	15.903390	16.433023	15.819800	15.852280	15.924163	15.204490	15.168793	16.066984	15.626034	15.966981	15.186033	16.358433	14.856471	15.411549	15.292522	16.125498	15.978326	16.160600	15.732189	16.196709	16.228514

AL160408.6	5.722522	6.402115	6.255038	6.092366	6.515723	7.051012	6.770742	6.489666	6.343517	6.801570	7.136406	7.042589	6.848878	6.549863	6.566408	6.778767	6.282611	7.159454	5.188014	6.454137	5.396922	6.753389	6.351290	6.353267	5.139865	6.201557	6.273200	6.363722	6.374349	6.759131	5.984369	6.369034	6.414041	6.502846	6.439561	6.290979	6.566103	6.006989	6.422858	6.319104	6.869995	5.693030	6.077122	5.729728	5.887816	5.021782	6.784847	6.003073	7.039698	6.137188	6.023946	6.898683	6.415472	6.788763	6.894955	6.588854	6.476918	6.614638	5.980285	6.767312	6.693274	6.669144	4.563408	6.141377	6.347604	6.268440	5.904897	5.674417	6.051653	5.419147	6.592272	6.328572	6.591797	7.105493	6.639819	6.586228	6.517234	6.452605	6.207946	6.429015	7.226387	6.946970	6.482715	6.645317	7.318389	7.171164	6.451788	7.145053	3.481349	6.837058	6.316905	5.567659	6.930656	6.907043	6.828154	6.398128	6.556627	6.965487	6.963071	6.432086	7.203012	6.533240	6.421746	7.025864	6.066803	6.438569	7.114246	5.739006	4.830410	6.383321	5.466753	5.609146	6.611503	6.242941	7.149940	9.465964	5.921216	6.549547	6.811607	5.708658	5.080404	6.526001	6.284143	7.550222	7.091187	6.214073	6.940592	5.029542	7.642151	8.050788	7.548686	6.139789	6.293351	6.904927	5.858684	6.072576	6.804922	4.365913	5.760681	5.069387	5.996725	5.920679	6.464703	5.180533	5.518195	6.898683	6.529490	6.354414	6.469512	4.606057	5.821306	5.867214	6.869212	7.116743	6.184870	6.267597	5.398443	6.983822	5.836054	7.085386	5.176541	5.524971	6.310264	5.870621	5.454582	7.033428	3.174479	6.240087	6.383651	5.359672	8.184337	5.885856	4.765699	6.534372	5.726450	8.335259	7.422439	5.396530	7.262980	6.636582	7.379718	7.466791	5.003376	5.286398	5.869888	6.006436	5.503367	5.374347	4.465424	6.123195	6.113696	6.646563	6.196731	5.813148	6.137868	6.245437	5.950898	6.158049	5.326168	8.018297	7.443943	6.506319	6.037029	6.052025	7.219837	5.435519	6.096861	3.252362	5.355114	6.274896	5.111523	6.082836	6.038781	7.194566	6.734479	8.657128	7.277975	6.668503	6.288774	6.701586	6.625166	7.233231	6.438750	6.581952	6.825424	7.061882	5.814250	4.505426	6.024815	5.658878	5.601329	6.636280	7.320128	7.416992	8.213459	6.539709	6.390363	6.746880

AC104024.1	4.774907	7.031715	6.737455	3.866608	4.145382	4.717717	5.435317	3.667908	3.952908	3.524404	3.595009	4.808685	4.762246	4.279291	5.476909	5.915113	5.368387	4.580573	4.562063	3.828051	5.451858	5.689713	4.018026	6.237472	4.623792	4.012459	5.998669	4.553466	4.691774	7.612789	3.790786	6.192675	6.351290	7.272673	5.147132	5.636210	4.974843	5.119275	3.790551	5.264677	4.567453	6.069620	4.676249	5.309863	6.346571	6.012132	5.015261	6.755245	6.666388	4.924665	5.667420	5.281372	6.054447	5.418161	6.834227	4.049551	4.269874	7.093370	3.876965	6.632208	6.045183	4.648755	4.398284	4.402007	7.513747	6.588206	4.793938	5.445055	5.750104	6.960356	5.833523	5.104831	4.313806	5.723098	5.565029	7.324386	4.304213	5.318615	6.218387	7.737107	5.387168	6.725130	6.612852	5.486905	5.947212	7.531584	4.316078	3.590704	7.318693	6.657757	7.405226	6.070506	5.138445	5.271224	6.601343	5.876918	7.136114	3.915108	4.251723	3.680036	3.975942	6.377960	4.000622	3.572687	5.297077	3.837359	5.076102	4.477408	3.988826	4.337231	4.941773	4.274018	3.503482	3.492031	4.589696	5.275754	4.795182	4.529657	4.842335	5.004978	4.221682	3.858138	5.598387	5.170283	4.687391	4.701109	3.419157	3.731092	4.027278	4.521491	4.482648	4.456478	4.528825	5.499654	3.918319	5.374742	4.046498	3.833320	4.104982	4.716878	5.043266	5.806871	4.178992	4.220612	4.540679	4.247095	4.432246	4.281018	7.418369	3.644045	6.901432	3.671754	4.465424	5.942491	4.813130	5.335131	4.787871	4.261993	4.334841	4.557891	6.939054	5.390271	5.077771	6.051653	5.355303	4.803256	4.955624	5.532567	4.237860	5.380192	4.851173	5.057174	4.566368	3.954599	3.999992	3.875659	6.275222	5.873704	6.556627	4.871252	4.447326	6.487895	4.542873	5.153997	6.124461	3.823956	5.429045	4.438717	6.813657	4.840654	4.470334	4.889825	4.358295	5.837861	6.545320	4.036699	3.654197	4.352445	6.273716	4.445100	5.001521	4.126337	5.395166	4.617539	4.094115	5.379656	4.130430	4.228324	4.103651	4.323376	4.875692	6.438911	4.716241	5.326352	3.911625	4.423102	4.275963	4.749977	3.547999	3.492401	4.146217	3.760914	5.424454	4.538427	5.621097	4.991007	4.138630	4.758930	4.567453	5.216797	3.586706	4.577985	5.082862	4.352225	4.132328	3.279903	3.590704	5.748312

C21orf62-AS1	8.095215	7.063002	7.239737	6.809210	7.195840	6.981330	7.358559	6.568387	6.520703	6.314683	6.813820	6.620034	6.512753	6.656942	5.646170	6.937799	6.541025	6.481906	7.959604	7.146904	6.613022	7.718121	7.451958	6.520543	6.715079	7.227642	7.513578	6.819436	7.021281	6.945652	6.812869	7.467717	7.383627	7.264337	7.695463	7.464806	7.404264	7.349096	6.988415	6.904304	6.579309	7.516020	7.570962	7.782421	7.805559	7.630529	6.691940	7.001307	7.774011	7.726838	7.632243	7.825839	7.988792	7.941663	7.692436	6.328572	8.126124	7.499939	6.245120	7.887812	8.040436	6.583114	7.814179	7.038434	7.356249	7.619230	6.864858	7.160265	7.356249	7.533758	7.533154	7.497876	6.930656	7.572932	7.306028	7.710499	7.362446	7.443314	7.702525	7.543582	7.309782	7.497706	7.783481	8.001370	7.494731	7.503695	7.715979	6.816500	6.547423	6.789904	7.492437	6.458352	7.181888	7.335968	8.022679	7.951997	7.707598	7.114246	6.828774	7.137353	7.303511	7.666363	7.000313	6.554636	7.439309	6.537120	6.988248	6.889972	6.769936	6.517234	7.538121	7.517425	6.965325	6.608356	6.280002	7.645097	7.824617	7.026011	6.523509	6.857697	6.065057	7.795409	6.794294	6.276052	6.094274	6.181161	6.591640	7.037948	6.309443	6.226232	5.899713	6.851233	7.010274	5.814075	7.319152	6.516230	6.755073	6.912751	6.593887	6.898513	6.740986	6.693444	7.089781	6.384336	7.849468	6.901907	6.624509	6.533884	6.260652	7.365622	6.302543	6.543690	6.604700	7.871982	6.061708	7.217482	6.622109	6.684249	5.984697	6.620183	6.595347	7.341300	7.569307	6.689492	5.676185	6.948257	7.268335	7.499450	7.813567	6.947279	7.019207	7.041278	6.751780	7.577914	7.414619	8.604836	6.577833	5.632451	7.125950	6.693760	6.484786	6.754906	6.623712	7.032329	7.435533	6.411368	6.999995	6.408860	7.009640	6.639977	6.137019	6.001097	6.834227	7.841835	7.168868	6.592444	7.026498	6.816835	7.912959	6.823214	8.393192	6.487592	7.266775	7.224338	7.094332	7.272673	7.121630	6.731583	6.506499	6.775740	6.533404	6.935051	6.409686	6.629998	7.284304	6.678634	6.691269	6.411368	6.622253	6.019182	7.084263	7.366862	6.427331	6.394030	6.319966	6.165458	6.406893	6.527507	6.414695	6.732083	7.420070	7.540513	6.706217	6.256420	5.443145	7.137804	6.114580	6.126791

AP000223.1	6.360207	5.303420	6.425612	5.708310	6.017610	6.066803	6.706060	4.856703	4.765269	6.679465	5.994659	5.994484	5.905949	5.598950	5.973067	6.065758	4.157373	6.612937	5.206440	6.548685	4.758717	5.652572	6.890278	6.009291	6.032361	6.414193	6.592444	5.415640	6.411526	5.907569	5.806483	7.271866	7.205220	6.910985	6.553169	6.620518	6.504813	5.887073	5.626160	5.796183	5.706129	5.934225	6.256420	5.904197	7.398357	5.535274	7.568409	4.540902	6.844875	6.779417	6.339882	7.030795	6.717965	6.621633	6.798171	5.412627	7.076870	6.516401	6.119404	6.626000	6.219926	6.944407	6.551816	5.771660	5.685501	5.760681	5.397471	5.847149	5.718329	5.571063	6.084413	6.332318	5.682349	6.535813	6.412706	6.284297	6.364380	5.832808	5.867047	6.370223	7.013250	6.621955	7.115967	7.241025	6.971359	6.888237	6.753389	6.904609	5.826008	5.356854	6.287255	5.466381	4.766147	6.398633	5.921041	7.008506	6.984122	6.816835	6.145315	5.838629	5.903276	6.840811	6.215255	7.023584	5.539594	6.317575	3.871796	5.788126	5.139865	6.242269	6.324741	3.920397	4.243194	5.692843	5.934593	5.902746	5.807949	6.393549	6.068771	4.678389	6.959726	4.849503	5.044120	4.174641	5.534481	4.253660	5.779623	5.733015	4.034530	5.614619	3.564761	5.153573	5.544882	4.557261	5.927772	4.726811	4.991007	5.103183	7.495527	4.095392	4.897894	5.074044	4.885618	4.299199	7.285661	5.499857	4.791016	6.263018	4.986230	5.599867	5.715301	5.369314	6.553169	6.724980	5.024657	6.345907	4.708660	3.435630	3.421359	5.638458	3.385360	6.406553	5.956091	4.458635	5.115354	6.423830	7.507849	6.221429	5.055944	4.802376	6.287255	4.279490	5.509173	5.716576	6.842050	7.983341	5.348890	4.570485	7.254305	2.826095	6.804468	4.089190	3.933686	4.478885	6.775273	5.047909	3.832048	3.626618	2.939822	4.307689	3.897603	3.902101	5.071425	6.084925	4.798102	5.226103	5.045524	4.672936	6.652734	5.957324	6.159451	6.117854	6.562355	6.257247	5.784124	4.793518	5.334186	5.407122	3.693618	6.864398	4.853955	4.008886	6.070155	5.656282	6.059027	5.945684	4.996090	5.488546	4.821263	4.915143	5.772006	5.439305	6.612650	5.077771	6.327073	3.720796	5.805266	5.209621	5.119896	6.197911	4.522996	3.967702	5.150938	4.525652	7.388420	4.802376	6.448187	5.451257

AC138819.1	8.830873	6.400824	6.389695	7.290175	6.468342	7.612965	6.297553	5.717116	8.250515	5.889394	6.766039	6.039824	6.178831	5.197099	6.713105	7.163317	6.320511	5.624475	7.381393	7.739929	6.053723	7.171164	10.893304	6.993072	6.480417	7.353422	7.839084	5.041771	5.995187	6.869510	4.391694	6.748017	7.646194	6.716023	7.425397	7.001784	7.946637	6.657757	6.501473	7.534703	6.261678	9.011126	8.046561	7.852796	8.694827	7.127988	6.927637	7.483685	7.236106	7.360598	7.940095	8.275673	6.791521	6.576349	7.188088	5.374929	6.969980	6.005203	6.835477	6.559257	6.917090	5.394569	7.467564	7.030651	8.657743	7.044666	6.844206	5.859070	5.437457	6.685504	6.598102	6.019537	5.375957	6.241268	7.205967	7.024748	6.619878	6.576684	6.020626	7.731029	7.502589	6.858648	7.516962	6.688080	6.852479	7.985454	6.492192	6.137520	7.230410	7.700687	6.801251	7.049913	7.384386	6.327724	7.552684	7.678603	7.623778	7.795703	7.392569	6.687456	7.384238	7.172303	7.015663	6.192830	7.341144	7.039059	6.593717	7.578853	7.557702	6.775740	6.809854	7.344870	6.814915	6.538445	6.623080	8.152212	8.959613	6.714591	6.937962	7.000648	6.470538	5.756839	4.808479	6.132261	8.834047	4.541102	4.352445	6.398476	4.663894	5.516844	5.140676	6.286581	4.836172	3.721463	7.067906	4.083676	4.974843	4.873219	9.487831	8.136815	10.332682	9.819818	4.927218	4.926830	3.304280	5.875675	3.801826	5.420746	4.783045	7.576358	8.625298	6.629515	5.491679	8.105747	9.730932	5.470251	4.837448	4.364149	6.202971	5.299668	8.759006	5.497164	5.204869	5.862703	6.751438	4.765079	6.523680	8.943551	4.730184	4.307910	4.699401	9.320034	4.712429	5.093109	5.839364	6.504481	5.393986	5.169098	7.898958	4.975882	5.269057	7.590292	4.770332	6.237472	6.171506	4.096860	4.742727	3.497287	2.923401	7.309440	4.675355	4.343091	5.286398	6.566720	5.920679	4.704484	5.198116	5.059621	5.362147	5.496273	4.947749	6.051477	6.023426	3.594204	5.354706	4.088287	4.083676	5.112562	5.604154	4.801966	5.604354	4.791873	4.633497	4.165577	4.635852	5.203062	9.462714	5.168280	7.996613	5.426425	5.629533	4.492888	5.407524	6.430895	6.876407	10.012765	5.345937	11.061124	5.305593	10.171845	7.308348	7.553771	5.337135	4.748507	7.157856	5.725344	6.302364	4.488660

LINC01584	3.330807	3.655026	3.294060	3.529405	3.779373	4.589046	4.768875	3.793159	4.575611	4.772404	4.191471	3.804710	3.876965	4.126990	3.984565	3.970758	3.592258	3.097510	4.375472	3.526862	6.177991	4.860231	2.840406	4.423102	3.929129	3.973097	3.891965	4.164884	3.884271	4.567453	3.746085	4.651356	5.075261	4.205246	4.753194	4.048215	3.542686	3.776777	3.823300	4.237224	4.061298	3.163529	3.840275	3.713156	3.792471	5.544106	4.852882	2.846290	3.874364	3.523055	3.105727	4.012459	4.269874	4.291335	4.453114	3.881910	4.252167	3.615151	5.181372	3.889613	2.792523	4.204289	4.610996	4.570485	5.176961	4.014251	3.911207	3.939428	3.604265	3.275887	4.068386	4.474088	4.366995	3.709474	3.558731	4.130031	3.922002	3.852717	3.686239	4.464384	3.664638	4.015408	3.576726	4.519116	3.687483	3.798195	4.690963	3.330982	5.192099	4.481723	3.689524	4.301736	4.611640	4.310159	4.248826	3.596508	4.198200	3.418434	3.671754	3.401046	4.400675	4.196900	3.282710	5.199473	4.719153	4.116788	3.789104	4.075568	3.576726	4.014957	3.619725	4.304639	4.459269	3.878287	4.079820	5.046472	3.826149	3.148640	3.808070	3.547366	4.384959	3.814815	3.879570	4.608853	4.570252	3.795069	4.335082	4.029752	4.382536	4.438717	4.158412	4.160569	4.143859	3.561139	4.037774	3.214200	3.691947	3.289685	5.119499	4.268049	4.654410	2.801819	3.853131	3.990254	5.920125	3.857435	3.983505	4.058871	5.206236	3.981290	5.183414	4.191471	4.336552	5.062027	4.123626	4.085214	4.258087	4.123175	4.535468	3.936856	5.111332	4.294152	4.281683	4.416195	5.145547	5.022393	4.987884	5.448989	3.178160	4.971305	4.807639	4.807639	4.796275	4.503080	3.978243	3.944424	5.447513	5.140077	5.320716	4.691187	3.697556	4.505426	4.655780	5.092686	4.771972	4.809948	4.424630	4.328317	5.808857	3.759435	4.722818	3.708456	3.518376	4.089384	4.217298	3.693618	3.889387	3.617097	3.528312	4.318671	4.441288	4.529888	4.512683	4.072967	4.291994	3.989853	3.831827	5.133206	4.387604	4.518062	3.961808	3.864506	5.147334	4.618177	4.332454	4.937893	4.164473	4.200744	4.290685	4.344818	4.581421	3.478207	4.752754	5.887979	5.245026	3.312397	3.861705	5.281154	4.074067	4.701940	4.976883	6.801411	3.539584	4.457149	4.147276	4.086810	3.352721	3.658246

AC016526.2	6.462508	6.403439	6.330096	5.940611	6.014848	5.978520	5.827654	6.962442	6.819436	5.724374	6.159981	6.004467	5.297271	5.624670	5.857096	5.847321	7.315893	5.929013	7.454763	5.910266	6.918673	6.368520	8.041063	6.642700	6.794137	6.771077	6.320684	7.113308	5.242044	5.483503	7.258388	5.523096	5.719583	5.647464	5.924711	6.071709	6.126226	6.536952	6.685988	6.998767	5.814250	6.872750	6.127291	6.697597	5.987005	5.328913	5.964827	6.126791	5.814929	5.808677	6.444202	6.067883	5.819218	6.307584	6.235310	5.486905	6.484071	6.619535	6.778458	6.706394	7.105493	5.963410	7.619552	7.121945	7.163145	7.086134	6.879880	6.584561	6.618387	7.165801	6.320848	5.892386	6.099988	6.110246	6.189096	6.475089	6.761727	6.829747	7.016162	6.502676	6.327073	6.521864	6.438065	6.806940	6.713749	6.074831	6.276203	6.957676	7.477847	7.429837	7.297185	7.633475	6.333488	6.423337	5.941126	6.266890	6.246267	5.418355	5.680106	5.021154	4.517005	5.614619	5.686221	5.754089	6.773823	5.542603	6.982874	6.314526	6.619878	5.823680	7.321205	7.168563	7.426641	5.450652	6.225380	3.626008	6.667531	6.316233	5.741365	6.522347	6.707042	6.023247	6.155509	5.824416	6.433086	4.630694	6.204695	6.854459	5.367584	6.665078	6.942144	4.991830	5.577407	6.910016	5.986282	7.675124	6.300676	6.947794	6.140137	7.088794	6.682255	7.911114	6.435459	7.042248	6.868239	5.900068	6.667203	4.605235	5.979585	7.011065	5.655195	5.818676	5.831751	6.319268	6.542716	6.161697	5.970326	7.125129	7.177645	5.004573	7.138922	6.337715	7.863083	5.780525	5.801382	6.414516	6.077284	5.508567	6.435793	7.050084	5.972154	6.173519	6.800931	6.226232	5.395571	5.952550	6.742265	7.023919	6.624661	7.208301	5.868125	7.258046	7.552843	7.278613	7.101416	7.183603	6.790559	7.089448	7.602658	6.239917	6.480576	6.473421	6.491380	6.136867	6.710407	6.839508	7.070554	7.191984	6.457035	6.918673	6.955662	6.839827	6.606041	7.021564	5.642738	7.369224	6.755724	7.586137	7.124348	7.007040	8.709776	6.189910	6.835477	6.838834	6.351290	5.750286	6.062086	5.570335	4.993314	5.794384	5.256134	5.958590	5.859782	6.850296	5.801873	7.160446	7.196002	7.166846	5.991459	7.003383	6.891257	4.797674	5.037327	6.001794	5.625960	7.369846	6.327567	6.609496

LINC02649	8.685533	7.970993	8.251297	8.022679	8.393192	8.583773	7.769734	9.426344	9.444994	8.193517	8.473114	8.581371	7.649962	7.905856	7.477201	7.884753	9.086788	7.721779	9.012701	7.697916	9.154157	8.186673	9.988894	8.464289	8.340131	8.646623	7.873818	9.224813	8.411545	8.117460	9.426185	6.981330	7.634048	7.165039	7.365307	7.885997	7.683968	7.866510	8.375113	8.513238	8.363017	8.402513	7.719347	7.761133	7.611123	7.917823	8.891290	7.862157	7.975543	7.778353	7.859101	7.953099	7.243579	7.739616	7.872133	7.904729	7.505885	7.648749	7.762049	7.916120	8.364085	8.183127	8.355223	8.557469	8.298809	8.496873	8.108026	8.048473	7.886288	8.674050	7.963443	7.926147	8.291346	7.782857	7.871229	6.262524	8.092006	8.073965	8.282853	7.659517	7.764503	7.833855	7.753792	8.155472	7.885668	7.843587	7.959912	8.209048	8.738729	8.833056	8.395299	9.297703	7.836439	7.928816	7.503695	7.608060	7.917531	7.179381	7.901488	6.672024	7.762841	7.560582	7.366100	7.550519	8.676059	8.257528	9.412451	8.682529	9.080879	8.443632	9.107805	9.162133	9.487831	8.943236	8.524770	8.937007	8.402822	8.043261	8.066532	8.294877	8.143761	8.165811	8.075160	8.757756	8.585918	8.240987	8.900522	8.582268	7.723151	8.046561	8.285774	8.182181	8.397333	9.400733	7.768086	9.132911	7.480282	9.667179	7.546146	8.812340	7.845026	9.661074	8.430558	8.827314	8.368619	8.336025	8.574419	8.423320	7.912825	8.889773	7.560139	7.713903	8.131953	7.251697	7.693984	7.501316	7.849748	8.442687	8.323674	8.633073	8.505652	7.801746	7.966199	7.812644	8.106503	8.764087	7.825991	8.139115	7.820127	7.917375	7.944475	7.369060	8.158525	7.384857	7.893878	7.290024	7.493678	7.805246	7.817478	8.446086	7.940884	8.455053	8.389906	8.543369	8.465219	8.126279	7.981758	8.085388	8.482338	7.857244	7.804165	8.591916	7.731029	7.601841	6.608020	8.037132	8.197780	8.426149	7.434700	7.491173	8.005774	7.881392	7.901019	7.925059	7.824306	8.280692	7.945709	8.674683	8.384047	8.373123	9.145192	8.022090	8.058996	7.839369	7.997958	8.181595	7.646673	7.564103	7.274847	8.382675	8.056362	7.473087	7.443164	8.349281	8.250515	9.146716	8.720713	8.972624	8.638171	9.250083	8.680362	9.542810	9.021885	8.531075	8.082554	8.181140	8.009151	7.910344

LINC02656	8.685533	7.970993	8.251297	8.022679	8.393192	8.583773	7.769734	9.426344	9.444994	8.193517	8.473114	8.581371	7.649962	7.905856	7.477201	7.884753	9.086788	7.721779	9.012701	7.697916	9.154157	8.186673	9.988894	8.464289	8.340131	8.646623	7.873818	9.224813	8.411545	8.117460	9.426185	6.981330	7.634048	7.165039	7.365307	7.885997	7.683968	7.866510	8.375113	8.513238	8.363017	8.402513	7.719347	7.761133	7.611123	7.917823	8.891290	7.862157	7.975543	7.778353	7.859101	7.953099	7.243579	7.739616	7.872133	7.904729	7.505885	7.648749	7.762049	7.916120	8.364085	8.183127	8.355223	8.557469	8.298809	8.496873	8.108026	8.048473	7.886288	8.674050	7.963443	7.926147	8.291346	7.782857	7.871229	6.262524	8.092006	8.073965	8.282853	7.659517	7.764503	7.833855	7.753792	8.155472	7.885668	7.843587	7.959912	8.209048	8.738729	8.833056	8.395299	9.297703	7.836439	7.928816	7.503695	7.608060	7.917531	7.179381	7.901488	6.672024	7.762841	7.560582	7.366100	7.550519	8.676059	8.257528	9.412451	8.682529	9.080879	8.443632	9.107805	9.162133	9.487831	8.943236	8.524770	8.937007	8.402822	8.043261	8.066532	8.294877	8.143761	8.165811	8.075160	8.757756	8.585918	8.240987	8.900522	8.582268	7.723151	8.046561	8.285774	8.182181	8.397333	9.400733	7.768086	9.132911	7.480282	9.667179	7.546146	8.812340	7.845026	9.661074	8.430558	8.827314	8.368619	8.336025	8.574419	8.423320	7.912825	8.889773	7.560139	7.713903	8.131953	7.251697	7.693984	7.501316	7.849748	8.442687	8.323674	8.633073	8.505652	7.801746	7.966199	7.812644	8.106503	8.764087	7.825991	8.139115	7.820127	7.917375	7.944475	7.369060	8.158525	7.384857	7.893878	7.290024	7.493678	7.805246	7.817478	8.446086	7.940884	8.455053	8.389906	8.543369	8.465219	8.126279	7.981758	8.085388	8.482338	7.857244	7.804165	8.591916	7.731029	7.601841	6.608020	8.037132	8.197780	8.426149	7.434700	7.491173	8.005774	7.881392	7.901019	7.925059	7.824306	8.280692	7.945709	8.674683	8.384047	8.373123	9.145192	8.022090	8.058996	7.839369	7.997958	8.181595	7.646673	7.564103	7.274847	8.382675	8.056362	7.473087	7.443164	8.349281	8.250515	9.146716	8.720713	8.972624	8.638171	9.250083	8.680362	9.542810	9.021885	8.531075	8.082554	8.181140	8.009151	7.910344

MCPH1-AS1	14.371917	14.898599	14.898046	14.963955	14.524343	15.040623	15.102359	16.016830	14.778050	15.718870	15.160798	15.498917	15.133964	15.237652	14.619813	15.644970	15.472304	14.975358	14.952229	15.465655	15.219191	15.258165	13.898577	15.112372	15.113668	15.513476	13.192104	15.274457	14.736202	14.891226	15.373253	14.739993	14.534260	12.869416	14.601973	15.062814	14.575515	15.007673	14.557264	15.510860	15.480428	14.439683	14.726960	14.294396	13.791726	14.460851	14.830506	15.148056	15.438975	14.952794	14.249198	14.769473	15.617837	15.384452	15.493839	15.482965	14.773706	15.083595	14.625551	14.681870	14.708876	15.304417	13.192705	15.148056	14.521080	15.033365	14.914667	14.682292	14.721418	15.168107	15.527160	15.490401	15.279559	15.242555	15.399178	14.541332	15.244645	15.071599	14.871315	15.674067	15.009958	14.729253	13.349308	15.301621	15.300250	14.838593	13.986959	14.811620	14.110109	14.844672	14.601973	14.704362	14.788691	15.674965	14.061195	14.730211	15.775426	15.123478	15.056095	15.638552	15.335238	15.452790	14.731220	15.260285	15.849957	15.155102	15.465655	16.029650	14.800437	14.953863	14.746490	15.250968	15.068646	15.443248	15.740567	14.965129	12.833456	15.247395	15.253922	13.209291	14.874974	13.595124	13.287261	14.966246	14.313436	14.590224	12.962142	13.403370	12.696084	13.577611	13.003243	12.573019	12.578521	12.423279	13.595345	12.260677	12.419600	12.531002	12.258508	13.363482	13.027092	12.632463	12.887748	12.434656	12.715418	14.746969	13.270023	13.833309	13.930296	13.832307	13.954342	13.270211	15.251620	14.801392	12.928492	14.731220	13.186246	12.996042	13.138899	13.984981	13.556834	11.933974	13.052613	13.426653	14.359381	14.826972	12.420594	13.048001	12.945351	13.253022	11.632395	12.618964	12.464996	12.502391	13.335613	10.781910	11.637826	12.378643	12.371190	13.366847	13.740602	12.570358	13.418509	12.510140	13.694716	12.864398	14.051810	13.363274	13.495100	14.123349	13.828942	14.869310	14.043788	13.574051	14.576819	12.950547	13.356786	14.729253	12.047379	13.055989	13.864108	14.902407	13.886983	11.924594	13.896685	14.905582	11.874918	12.713284	12.926643	11.256216	12.476380	13.502404	12.447268	13.953224	13.341361	12.868885	12.608516	14.364077	12.539305	12.434341	13.273776	13.148784	12.726021	12.225366	14.042604	12.733547	12.868696	13.854876	15.273761	14.480925	12.558955	13.168641	14.878115	13.185635	14.557653	12.875731	14.319186	13.728857

LINC02382	4.399610	3.158396	2.992302	3.977012	3.504538	3.630871	3.249584	3.479469	3.450800	3.342302	3.507067	3.803896	3.222403	3.939428	5.671238	3.018838	3.437226	3.678626	4.592522	3.170383	4.589492	2.967375	4.077019	3.116871	2.935557	2.927156	2.861591	2.904966	3.242251	3.041967	3.638275	3.105616	4.369123	3.106431	4.302876	4.054951	3.976335	3.228654	3.182446	3.796132	3.888699	3.021136	3.542469	3.326154	3.170512	6.397786	3.287873	3.378974	3.925461	3.399682	6.592947	4.791656	3.618767	3.943048	3.685428	3.223091	3.998746	3.588820	4.016935	6.871306	3.271303	3.028768	3.444894	3.063410	3.177342	3.159296	3.001475	3.180672	3.053740	2.955254	3.054163	3.369613	3.064806	3.510205	2.876246	3.270087	3.292494	3.886748	4.015628	3.622038	4.832509	6.003768	3.281206	4.585625	3.115214	3.458955	5.526878	3.488296	3.525510	3.397284	3.027678	2.961557	4.285926	3.039843	4.306356	3.363890	3.435989	3.257771	4.018026	3.636471	4.788062	3.214885	2.995591	2.850107	4.173784	3.101983	2.966843	3.427979	4.816890	3.170512	4.646621	4.226387	5.114771	4.000622	3.357588	6.765392	3.320901	3.034305	3.745674	4.129377	3.239897	3.798781	3.360327	5.030342	5.076102	4.956231	3.105510	3.435989	5.494505	6.098896	7.468145	3.468909	4.095169	5.394962	3.937922	3.341480	7.029077	3.374079	3.216520	3.982623	6.952973	5.809052	6.148664	6.445240	3.870512	4.515508	3.751689	4.833748	3.473243	3.317763	3.217271	4.307440	3.189017	4.551218	9.865136	3.696923	3.261577	3.151248	3.026761	3.795304	5.418161	3.553917	3.399850	7.430932	6.458196	3.569850	3.336436	8.124035	5.862318	5.562589	4.037357	5.006627	4.047589	3.348550	3.018363	4.018708	5.600431	3.854698	10.023382	3.072783	3.139187	3.397123	3.230761	3.297612	3.180009	2.980607	3.355030	3.122269	3.014178	3.270828	4.548870	3.680841	3.366167	4.145382	3.257771	6.167552	3.718335	3.372263	3.225182	5.095617	6.521864	3.411297	4.187745	3.658433	3.655026	3.123779	3.292054	3.286969	3.805556	5.715850	3.516964	4.031479	3.072349	3.064806	3.720995	6.083510	6.221155	3.402485	6.930332	3.688674	3.470874	3.205070	4.227030	7.522516	4.938708	4.238537	3.346564	4.103884	3.742381	8.110601	5.215622	6.499570	3.854698	6.531764	6.805391	2.952475	3.059176	3.781660

AP002793.1	7.212810	7.983918	7.193101	7.294297	7.776826	8.077423	7.800516	9.645648	10.076981	7.587193	8.245699	8.056042	7.039223	7.116743	7.065404	7.686842	8.591289	7.646968	7.071494	7.523920	7.457779	7.880784	7.470962	7.413067	7.795703	8.868386	9.039625	8.684172	7.794622	7.855750	8.018297	6.917410	6.901599	8.154078	7.421349	6.917569	6.760102	6.474906	6.597629	6.786625	7.537331	7.135949	7.256013	6.677201	6.738926	6.293351	8.219027	7.268627	7.120747	6.613816	6.721610	6.601343	6.599911	6.498737	6.024627	8.146874	5.771424	6.406726	6.688845	6.453116	6.534841	7.662478	6.820848	7.415948	6.994459	6.665725	6.781340	6.724487	6.673180	6.942312	7.409279	7.014664	8.207388	7.405370	7.263127	7.065074	6.954704	6.979258	7.365458	5.898487	6.332154	6.244453	6.006820	6.041748	6.448187	6.906552	6.289787	6.725278	6.647547	6.922419	6.680626	7.039538	6.614789	6.697597	7.626255	7.295217	7.097357	8.002152	8.333675	8.020889	8.509059	7.631455	7.555345	7.170080	6.561036	8.432903	7.561200	7.328193	7.449989	8.842936	7.127214	7.964652	6.926148	9.527862	8.712362	7.879068	7.086585	8.446548	7.461663	7.561058	8.240540	7.185698	7.671162	8.009910	8.370828	8.558383	9.007935	8.721025	7.830215	7.858176	7.853595	8.233292	7.139071	8.347200	7.958214	7.971462	7.464195	7.023420	7.108862	7.744995	7.102187	8.348688	7.294755	7.774932	7.735196	8.203624	8.632197	8.360170	7.959136	8.538157	8.146426	6.856249	8.308548	7.446661	6.568544	6.652567	6.799271	7.301462	6.875616	8.136969	6.683231	7.009152	6.910490	6.503485	6.390687	8.449532	7.184232	7.167777	6.601982	6.703085	6.774963	6.456375	6.744337	5.832460	7.256786	5.920125	6.333320	6.618065	5.802582	6.301676	7.681937	6.734990	7.312433	7.317492	7.030340	7.196624	6.980337	7.170547	7.191384	7.263271	6.691609	8.907661	7.363230	6.884549	6.951402	7.013910	7.118842	7.517578	6.185046	6.046918	6.513895	6.355752	5.929174	6.310954	7.580861	6.129505	6.491525	6.747557	6.331981	6.451151	6.852010	6.779905	7.144281	7.725750	7.366400	7.703743	8.186038	8.648726	7.377860	8.164115	8.345680	8.074267	7.161102	5.973953	8.965236	7.589490	7.449040	7.401008	10.199050	7.253698	7.742243	5.870975	10.308933	7.837808	7.442078	7.193734	9.894559	6.958927

PTPRG-AS1	3.945083	3.954808	3.408711	4.834572	3.741358	4.116356	4.448486	3.707077	4.232847	4.244727	3.896283	3.898000	5.442744	4.224051	4.894383	4.260868	5.305404	5.905278	6.168600	4.220124	4.483519	5.356278	8.028718	4.084103	4.395671	5.341041	5.184019	3.636664	5.701366	3.923950	5.316075	4.624415	4.882433	5.109966	5.682349	4.097537	4.900919	5.125769	5.075463	5.363098	4.170551	6.157700	5.157266	4.481723	5.629710	4.967792	4.177463	5.464226	4.758717	4.187529	4.861840	5.100098	5.072402	4.707853	5.152556	4.807639	5.031937	4.434754	4.576446	5.165688	4.701302	4.717292	6.816500	5.585889	5.185394	4.347207	3.121463	4.861224	3.645431	4.656406	3.040931	3.732326	5.341137	3.639699	3.914443	4.032747	3.793589	4.159518	4.000224	5.822053	5.608542	4.509851	4.731233	4.478885	3.703446	5.699682	5.702406	4.421986	3.850777	4.397453	4.084550	5.165688	4.441709	3.774917	3.496154	5.093331	4.750429	6.133084	4.184469	5.434949	5.301629	5.303625	4.919813	4.464171	5.847521	5.722326	3.607047	5.459635	6.390022	4.442195	6.383651	5.921968	4.630905	3.673077	4.923203	5.323834	5.197929	4.548399	5.549967	3.488475	4.191032	3.364370	4.047589	3.479830	4.547511	3.893535	6.495088	5.753206	4.131875	4.286569	4.312966	4.081133	4.098630	3.566286	5.532394	4.175765	5.637139	5.695092	3.938998	3.806591	6.871479	5.827845	4.465207	4.083256	5.661986	3.741804	3.586490	3.896720	3.927841	5.300214	6.078831	6.691940	4.089190	4.259773	3.983064	5.148725	3.671942	4.855023	6.090277	4.378241	3.456073	6.303742	3.683070	5.287944	4.399395	4.254568	4.607348	3.915721	4.412855	4.221049	5.159865	4.302633	4.218384	3.823300	4.700680	3.782082	4.181870	4.028172	3.661837	5.621097	3.899788	6.138053	6.297403	5.557563	5.811119	3.057796	4.105649	3.849284	4.504789	3.360006	4.063276	4.447326	4.585408	4.198815	4.051722	4.343480	4.097105	4.527587	5.872074	5.721590	4.281683	4.896017	5.371076	5.064071	4.282318	4.156749	4.511393	5.283568	6.143926	6.203650	4.427011	4.367199	5.346337	5.599491	5.386330	5.198116	6.774963	5.671044	3.944222	5.635267	5.470251	5.206824	5.286988	7.201165	4.464384	6.799632	3.432543	5.577218	3.173828	5.342989	5.969657	5.088591	3.177091	2.943661	4.178992	5.171084	4.581198	5.198932

AC005740.5	12.670692	13.061734	13.474422	12.907514	12.915514	12.847527	12.888444	12.988336	12.051938	12.438849	13.007394	12.595412	12.641908	12.751371	12.279737	12.712606	12.493535	12.046914	10.618196	12.971491	12.131586	12.541791	10.378348	12.277448	11.860703	12.485499	12.088432	12.140678	12.495856	12.766912	12.570678	12.778288	12.520217	12.576557	12.909649	12.753644	12.845970	12.564301	12.594107	12.141427	12.813176	12.028997	12.299695	12.861696	12.044248	12.701928	12.829828	12.211933	11.957549	12.625698	12.448174	12.231903	12.202348	12.928845	12.405594	12.803190	12.168975	12.211771	12.301868	12.544554	12.246079	12.890688	11.934585	12.002645	12.515979	12.338722	12.441436	12.094888	12.139723	12.067188	12.749657	12.733221	12.675868	12.556834	12.972030	12.724498	12.588458	12.648312	12.654817	12.492404	12.445794	11.869781	12.340685	12.878352	12.786494	12.767266	12.588621	11.960063	12.165300	12.193677	11.962182	11.566261	12.598484	12.519877	12.325086	12.633940	12.434806	12.361945	13.110603	12.032445	12.480968	12.490737	12.405442	12.183786	11.683357	12.794485	12.217152	11.238920	11.353755	12.693398	10.438251	11.505243	12.397858	12.599503	11.698277	12.158785	11.728873	12.714173	12.613211	12.876066	13.402926	12.381631	13.512208	13.393700	13.291948	12.901798	13.607817	13.651246	13.874711	12.461067	13.738842	13.960526	13.076484	12.889667	12.733709	13.379894	12.934369	11.931223	12.503389	12.323571	12.051034	11.878043	11.716302	12.964171	13.706318	13.185635	13.745592	12.650709	12.945707	12.662746	12.899561	13.258409	13.347432	12.735569	12.532804	12.602572	12.451713	12.534620	12.434490	14.155728	13.146687	12.560216	13.714879	14.253099	13.110603	13.855137	13.069852	13.142299	13.010893	12.352259	13.383479	12.683500	12.728950	12.182512	13.224516	12.059948	12.048596	12.357829	12.051649	12.078970	13.131537	12.100117	12.097254	12.410173	12.029158	12.790316	13.670308	13.257008	12.621510	12.873750	13.287648	13.422807	13.624759	12.380187	12.720405	13.120597	13.445036	12.813513	12.516623	11.956737	12.737126	12.467649	12.615500	11.984692	12.611680	13.345736	13.638076	12.046618	12.202348	12.077435	12.308401	12.706881	13.025290	13.362663	12.891887	13.041546	12.743196	12.562209	12.804429	13.233234	13.309538	12.130069	12.177372	11.898092	13.645649	13.432842	12.304637	12.174829	13.613276	10.445813	12.066401	13.071521	12.250070	13.777971	13.866741	12.654306	13.671017	14.332395

LINC01435	3.169283	3.118273	3.555420	3.422630	3.409715	5.254349	3.801192	4.115265	3.356906	3.303975	3.495604	3.686638	3.187406	3.885300	3.306834	3.552588	4.204289	3.330807	4.808260	3.870092	4.494208	4.364615	5.901312	3.208983	3.211309	2.924280	3.465394	3.057495	3.778757	6.229710	3.624828	3.934702	2.977243	3.446341	4.458021	4.010356	3.684609	3.439595	3.205070	3.058330	3.424029	3.517551	4.151892	3.227317	4.016509	7.523112	3.731504	4.252568	3.973745	3.941921	4.768288	4.120517	4.822317	3.729032	4.867443	3.919566	3.704674	4.400471	6.908762	4.091630	3.663842	3.428139	4.410703	3.537632	3.078754	3.651573	3.424199	3.381552	3.327122	3.223626	3.828629	3.616132	2.985231	3.306659	2.970911	3.785421	3.210577	3.433435	2.969198	4.433919	3.548394	3.597342	4.312308	4.867443	3.058514	4.512913	3.627611	3.398178	2.975243	3.859401	3.607655	3.474700	3.525695	3.302053	3.822254	3.263098	3.548569	4.379297	3.875266	3.614957	3.248829	3.448612	3.002248	2.845492	3.118529	3.801192	3.560522	3.381371	4.046498	3.516964	4.449155	3.771953	3.366868	3.155801	3.348550	4.068163	4.081552	3.001092	3.127678	3.340297	3.288352	3.018363	3.275887	3.550424	4.963118	3.128217	3.616132	3.332238	3.264112	3.271787	3.161356	3.462151	3.539190	3.981076	3.449361	3.752961	3.068701	3.641701	3.420255	4.288341	3.796526	5.942325	3.748721	3.846089	3.570961	3.227169	3.447475	3.299022	6.010016	3.416712	3.318473	2.974532	3.580319	4.061055	3.714240	3.724305	3.445079	3.091737	3.309058	3.658626	3.402108	3.439792	3.820148	3.661019	6.936015	4.228779	4.337896	3.818633	4.041871	4.129806	4.031703	3.612379	3.653400	4.610790	3.820148	3.407300	3.321552	6.319439	3.409194	3.418603	3.476161	4.645156	3.162507	4.208456	3.090007	2.912105	3.460735	3.330497	3.506244	3.293126	3.275122	4.262444	3.670314	3.471558	3.562491	3.099736	3.806591	3.124671	3.564969	3.299620	4.598327	3.169155	4.218585	3.339936	4.129806	4.325277	3.236658	2.926799	3.562864	3.503315	3.000745	3.897785	3.008959	3.067628	3.634857	3.807402	4.001044	3.197591	3.178028	3.799232	3.370996	3.348689	3.263968	3.215682	3.900187	3.073342	3.813350	3.465935	3.826564	4.737391	4.025364	5.701880	4.283174	3.589021	4.249492	3.267648	2.978793	3.187142

C1QTNF1-AS1	4.483961	4.624606	5.033574	3.482425	4.304213	6.242444	3.402821	4.251929	5.214830	3.512576	3.687880	3.874160	3.958796	4.166469	4.579914	3.479830	4.341124	5.507986	4.796480	3.450800	5.322285	3.919345	5.953623	3.099853	3.897372	3.184101	2.886206	2.939745	3.347210	6.201713	3.824871	5.033779	5.927070	4.665404	6.150337	5.431625	4.653042	4.019768	4.476026	4.202047	3.470157	4.461347	5.595686	3.658626	4.141719	5.323047	3.655026	6.406726	4.742543	4.999641	4.315607	5.057568	6.199473	4.880724	5.775290	3.082345	5.516471	5.902563	4.009111	3.342139	4.053015	3.886748	5.443327	4.821263	4.972948	5.050593	2.771095	5.460043	4.742727	4.309946	5.018110	4.127852	3.050060	4.724937	3.933686	4.951665	4.784747	4.771198	4.224260	4.607348	4.771198	5.942868	3.514967	5.479634	3.816516	5.064862	5.254541	5.562403	3.292054	5.167871	4.910706	5.315869	5.002715	4.630905	3.973097	3.677597	5.170283	4.270303	4.500267	4.257186	5.669251	4.267631	3.525165	4.504347	4.678590	4.224260	4.577328	4.050878	4.494905	3.272103	4.392128	7.141116	4.803917	3.457344	4.192341	8.694827	4.234619	4.235728	2.959101	3.906634	5.735202	5.530117	3.487527	5.219764	6.358161	4.460921	4.305947	5.131805	3.410237	4.546670	4.498338	4.532248	7.277201	5.427843	4.118599	5.163477	5.136022	3.438428	4.429329	4.760157	4.202480	6.185046	4.385646	5.568836	4.849690	5.151120	5.237066	4.275090	5.451468	4.407819	4.164884	5.346523	5.052375	5.480621	5.157073	4.706826	3.977012	5.069189	4.869580	3.610154	2.860021	6.583759	6.671392	4.328317	4.988511	3.957014	6.329228	4.613787	6.093732	4.993534	5.024458	6.416842	5.395571	6.291862	3.575355	6.306442	6.103246	3.819476	6.059726	6.628233	3.883367	7.250720	5.467155	4.816890	6.385607	2.997691	5.529921	5.008684	4.570729	5.135234	5.155213	3.717333	5.424256	4.733340	4.946727	5.744988	5.321484	5.897757	5.895320	4.889393	5.814756	5.045882	6.022066	5.927937	5.055751	6.766833	4.626343	4.981730	5.329287	4.940144	5.368387	4.719803	5.053435	4.389354	5.671606	5.282775	5.230761	5.176541	3.689524	5.301434	6.195854	3.637869	5.615175	4.762713	3.457344	5.629161	4.385189	3.445983	5.785779	5.152964	7.438512	7.300310	3.255631	5.170283	5.918522	5.507599	4.697431	6.932089

AC097359.2	7.749198	7.887198	7.583805	7.704063	6.909393	6.884549	7.873818	7.669450	5.045524	7.795703	7.792043	7.586459	7.288297	7.381884	7.454455	7.036232	6.328070	7.030189	5.557935	8.115905	6.395803	6.527507	7.564735	6.704797	5.501195	6.489172	5.605914	5.570880	6.379958	7.229521	5.440614	8.451235	8.427882	7.333524	8.078556	8.042654	8.114718	7.498194	7.155448	7.102506	7.087666	7.338002	7.829277	7.351140	7.500701	7.494271	5.994834	7.851877	8.062577	8.117928	8.363017	8.307920	8.477574	9.203704	8.101146	7.228566	8.575025	7.954492	7.160752	8.526432	7.724697	7.061088	7.467717	6.833118	6.959166	7.530523	7.259980	7.000814	7.281784	6.936341	8.132536	7.645874	6.104775	7.860047	8.027797	7.778689	7.532374	8.052634	7.562125	8.451400	8.386497	8.520064	7.975388	8.468844	8.553905	8.612493	8.919508	8.672941	6.481577	7.600778	7.791273	6.289123	7.805727	7.633165	8.260954	8.003880	7.907550	6.786475	7.484133	7.134838	6.294553	7.551590	6.720650	6.383999	7.475990	6.456712	5.795108	7.134838	4.146429	6.572438	4.320147	6.557781	5.794569	5.942325	6.546642	6.773743	7.340680	7.471713	5.855990	6.826397	7.325492	7.294297	6.571326	5.469311	7.651887	6.277838	5.407524	5.297845	7.396612	7.075119	6.392523	7.240393	6.942312	7.201019	5.959677	6.595347	5.723267	3.639699	6.847580	5.545245	6.525135	8.217923	5.142956	5.984182	7.810194	6.437896	4.482420	4.063721	7.382198	5.648576	7.549912	7.520351	8.851831	8.557010	7.663729	8.429123	8.142105	6.300828	6.280526	6.278646	6.270170	8.518715	7.620447	7.547429	7.457145	6.374509	7.426143	7.666860	7.941504	8.371594	8.088613	8.104810	8.483714	8.857274	7.007659	8.892522	7.785744	6.983494	7.881392	8.365652	7.950929	7.284141	7.130189	6.747055	8.435693	6.252335	7.251537	7.830932	7.969404	7.724222	7.637692	5.014252	8.087711	7.712486	7.812644	7.978147	8.141040	7.250720	8.646623	8.164723	8.990170	8.225862	7.883850	9.526785	7.664681	8.667886	9.102439	6.939212	6.909692	7.509989	7.316219	8.293216	8.053853	7.996465	7.515075	7.287083	5.529728	6.364856	8.518570	7.271564	7.251211	7.433753	6.627417	6.794463	6.231257	4.521910	5.384096	5.858872	5.088591	4.226387	6.225189	3.803679	5.884057	6.358161	7.503695	6.916792	8.004991	7.648004

KIF9-AS1	7.471877	7.943715	7.716737	7.877938	7.164573	6.629051	7.897027	7.542035	7.330723	8.313592	7.270004	7.633766	7.300797	7.708085	7.782421	6.827028	7.662478	7.843587	7.578853	7.383159	9.170919	7.392865	8.799005	7.337058	7.817798	7.522372	7.555055	7.302565	7.332577	7.214976	7.313210	8.860035	7.956035	6.846171	8.248548	7.780597	7.710024	7.586750	8.081951	8.494761	7.706238	6.617262	8.320792	7.301614	7.840159	7.486025	7.229814	7.860474	7.681302	8.044021	8.236218	8.163384	8.125256	7.830789	8.549674	7.359312	8.249893	8.708432	7.910028	8.541182	7.539738	7.678105	8.781348	7.785158	7.241025	8.181815	7.511861	6.936806	7.862916	6.824177	7.676648	7.377194	6.611684	7.944322	7.469723	7.726532	7.561521	7.881864	7.705490	8.266504	8.593456	8.559301	8.291041	8.374816	8.295756	8.206480	7.736081	8.889162	7.634821	7.457779	7.575291	7.220329	7.645719	7.631000	7.468910	7.507708	7.573412	7.447140	7.561521	7.142250	7.258388	7.854375	7.545191	7.441130	8.495049	7.084102	6.818111	7.581501	7.313210	7.313376	6.520543	6.858648	6.751276	5.919402	6.643191	3.828629	6.989049	8.221444	8.591764	6.307251	6.925172	7.121335	6.310264	5.435917	6.180988	5.140892	6.113851	5.902366	8.039022	6.636437	6.517896	6.001621	6.316559	7.421349	6.303742	6.066254	6.949978	7.578077	7.374392	10.428662	6.311781	8.555103	5.846047	8.333242	7.985161	5.723098	6.466853	4.832709	6.592272	6.221429	6.902551	7.600158	8.558992	7.039875	7.020447	7.054770	6.848878	6.988104	7.248226	6.668179	6.605446	8.984412	6.644989	6.880224	7.553935	7.781810	5.947390	7.223266	7.085535	7.372973	7.347217	8.349747	8.096606	7.173245	6.257931	8.390063	7.082071	7.281001	7.755863	6.203650	8.847553	7.085535	6.450484	6.713926	7.189234	6.472094	6.869995	6.966591	6.811443	7.792348	6.870310	7.572932	6.385143	6.793483	7.487395	7.206919	6.965487	7.289259	7.638327	7.249927	7.397106	6.944724	7.424306	8.379382	7.284456	7.139390	8.026668	6.895743	6.626475	7.545653	7.549287	6.496218	6.586695	6.637049	6.484786	6.457727	5.331647	6.862821	6.819593	6.102054	7.642151	5.337710	5.520438	6.222113	7.640177	6.113325	5.898668	7.162193	6.508146	6.720151	5.914751	6.606890	4.986634	5.848448	6.012299	6.905732	6.487592	9.309261

AC073359.1	3.122513	3.217115	3.499242	4.764873	2.961386	3.867935	3.402652	4.309712	3.381015	3.337147	6.625506	3.893314	3.216236	4.759341	3.470346	3.173051	3.891573	4.861224	4.386094	3.322010	6.919027	4.130225	5.897408	4.094946	3.469063	3.026966	2.913340	3.225710	3.911422	3.295141	4.846376	4.407819	5.453218	3.050905	4.209292	3.997237	3.727738	4.023916	4.157992	3.365338	3.547999	4.155180	3.700564	3.217728	3.427979	5.503158	5.273498	4.626121	4.581421	4.031890	4.011621	4.662858	4.729746	5.327897	4.151676	3.019759	3.931741	3.833118	3.886748	3.764711	3.744640	3.003820	3.728363	3.141935	4.213858	3.560720	2.936188	3.322010	3.795711	3.294849	3.872015	3.585340	3.439102	3.058021	5.564109	3.167650	3.810767	4.705113	3.827419	4.044969	3.485860	2.812834	3.071644	5.389689	3.355340	5.150938	6.524815	3.519870	4.044097	4.051954	3.918899	3.362333	3.414002	3.362672	3.711134	4.627372	4.398066	3.045578	3.514432	3.255474	3.745044	3.182826	3.414533	3.194161	7.279568	3.727364	3.235939	3.926325	3.290793	3.276032	3.801192	3.532333	3.170910	3.254990	3.057068	5.071831	3.253277	3.015270	4.030642	3.016261	3.308061	3.406231	3.744089	3.299460	3.923290	3.353521	3.684817	3.370267	3.147032	4.470334	3.173828	3.802439	5.362147	3.190658	3.501239	3.421532	3.970080	3.394704	3.413099	5.901504	3.559737	6.268605	3.680636	3.325977	5.049361	3.852488	3.253718	3.949193	3.488475	4.732732	3.945083	3.509805	7.145355	4.432246	3.703237	3.774707	4.729562	3.675983	3.597934	3.428139	4.343705	3.465570	3.281645	3.714449	5.155009	3.166213	4.730610	3.411297	4.184058	4.072108	3.796526	3.674319	4.107829	4.126095	2.966487	3.865565	3.923290	3.880658	3.903786	4.729562	3.059277	3.872654	3.497998	4.151044	3.430804	2.956161	3.769910	3.567285	3.615151	3.884898	3.509419	4.212963	4.268466	4.742152	3.136114	4.560966	4.339822	3.222255	3.451718	3.365338	7.880939	3.114023	3.474326	5.352869	4.497496	3.747084	8.101146	3.150811	4.956023	4.021539	3.576932	2.991620	4.797674	4.361722	3.541683	4.367399	3.949881	3.411118	3.205479	3.083277	4.342189	3.518201	3.382584	5.786290	3.017983	3.286102	6.110971	3.533311	3.162054	3.445983	4.833968	4.612707	3.252661	3.050060	5.934593	3.267949	3.367694	3.712339

AL627309.1	9.246975	11.121888	11.676518	11.547537	11.905620	11.743340	10.961364	12.848393	11.282980	11.008736	11.079831	10.815666	11.057038	11.013094	10.425458	12.149438	11.706258	9.964730	10.809503	12.937259	10.238166	11.467630	9.998272	10.719260	10.711830	10.385173	10.557083	11.218772	11.350258	11.764203	11.029110	10.099818	10.971168	10.186029	12.314450	11.384067	11.809037	11.540839	11.963547	11.385706	11.615530	11.234215	10.953350	11.565808	10.832721	10.937758	11.060705	11.453567	11.468830	12.385139	11.338491	11.344607	9.758720	10.357076	10.194030	11.922034	10.276070	11.082486	10.510068	10.026067	10.195988	11.347723	8.747415	10.763121	10.992855	10.539254	11.833609	11.095424	10.583768	10.388515	12.404343	12.569374	11.373199	12.776712	12.246555	11.236126	11.831355	12.236647	11.857513	10.852730	10.726580	10.881487	10.576472	10.519232	11.647240	11.635559	12.553472	12.593626	10.566051	10.974225	10.402004	10.143067	10.662229	11.574024	10.522806	10.725946	11.013535	10.634437	11.201757	11.214379	11.088131	10.928007	10.469815	11.083792	10.496144	11.220589	10.523247	10.562777	11.007714	11.733316	10.590962	11.206863	11.164654	11.343546	11.259294	12.565784	10.179942	11.850968	11.206137	10.091114	11.630009	10.812975	11.043892	12.440954	11.219233	10.785414	10.571069	10.061641	10.509173	10.580246	10.266163	10.700036	10.202804	10.490251	11.150814	10.589073	10.597545	10.318616	10.785872	10.034536	11.161862	10.270720	10.886421	10.520492	9.225119	11.102224	10.564425	11.330738	11.282541	10.832274	10.127898	10.399900	11.006688	11.463192	10.629643	12.297906	10.794980	11.329687	10.313234	11.152895	10.595288	9.157407	10.739904	10.367907	10.608759	11.288477	8.906569	10.665460	11.218485	11.349211	9.615149	9.330289	9.654195	9.241660	10.652809	9.297852	9.720786	10.108529	9.735774	9.227589	10.825459	9.897193	10.343685	10.704762	10.241816	11.327777	11.277927	10.210307	9.455567	11.077806	11.270443	11.555669	12.106044	10.455381	10.863944	11.455214	11.730195	12.264668	9.999366	10.382254	10.414630	10.763276	9.452450	10.285603	10.946533	12.739508	11.066165	9.564348	10.151883	9.065378	9.754870	10.165338	10.786766	11.249397	10.294008	10.443960	10.703888	10.540467	9.909395	10.161066	9.704158	10.084367	9.734090	9.629531	11.037724	9.802412	9.936797	10.245297	11.502051	10.231158	11.074858	11.507007	11.909566	10.548890	11.214808	9.662648	11.214032	10.559481

CTBP1-AS	8.073029	7.745934	8.393936	9.066762	9.537676	8.231627	7.854837	9.312969	10.351574	8.176949	8.286715	9.017440	8.746957	9.448726	8.655072	8.622520	8.592063	9.188603	9.118515	8.458106	8.290249	10.128831	8.586667	9.945591	9.783445	9.112102	9.822073	8.398835	8.214701	7.803407	8.474628	7.676806	8.062420	9.300797	8.624991	8.162350	8.466427	8.444544	9.165894	9.161517	8.326818	9.173745	9.603163	9.207170	9.236267	9.087175	7.883226	8.907201	8.193517	8.449681	8.400184	8.347801	8.181595	8.055590	8.644254	7.843275	8.527523	8.908163	9.413934	8.482018	8.930159	8.557608	8.906269	9.606560	8.803350	8.992714	8.660218	8.524014	8.003583	8.934423	7.884152	8.514597	8.883433	8.286403	8.577014	8.325256	8.279347	7.807263	8.020288	8.464743	8.601276	8.038673	8.478374	8.189101	8.009151	8.456424	8.464437	8.557010	8.414001	8.284855	9.216398	8.105290	8.955627	8.207850	8.540706	9.395428	8.308242	11.028799	8.663767	9.169847	9.361262	8.920300	9.288485	8.940056	9.146557	8.077583	10.555984	9.644234	9.607484	8.352921	8.820544	9.697083	9.694834	8.550896	8.787190	10.465042	9.717806	8.789635	8.210366	8.144863	7.749492	7.946637	9.333386	9.747720	7.887508	7.691511	9.081509	8.804313	8.555423	8.034493	8.868700	8.711169	8.661319	8.969110	8.360934	8.554809	8.527674	8.773366	8.190228	8.264002	9.933882	8.570650	10.938762	8.673745	7.768842	8.680959	8.427087	8.759794	7.571121	8.388820	8.900522	8.584534	9.101487	8.295459	7.744413	8.491179	8.823411	9.444517	9.098890	7.755724	8.899150	8.864465	9.207632	8.754861	8.564352	7.478448	8.743900	7.839230	8.263544	8.374973	8.090493	8.345080	7.810508	8.718581	7.001632	8.358315	8.268065	9.356365	8.153471	8.551058	8.221444	9.159093	9.068474	9.685622	8.675628	8.507811	8.741801	8.607876	8.567308	7.569912	8.101742	8.492586	8.293964	8.233424	8.028874	7.966655	7.870600	8.352309	8.600815	8.654468	8.303042	9.086706	8.881463	7.946185	8.882036	8.076365	8.776297	8.703369	8.855773	9.876877	8.336479	8.846939	8.549674	8.856984	9.226526	8.924459	11.177770	8.257528	8.062120	9.215448	8.775819	9.459408	8.885766	8.570802	8.403921	9.904851	9.323193	8.522454	7.726996	8.573979	9.067393	9.229431	8.657423	8.695584	9.101177	9.057511	8.622365	8.331532

BDNF-AS	4.720456	4.643812	3.903586	3.804516	3.737290	4.488884	4.821688	3.411831	3.279133	3.900187	4.042742	3.963170	4.305090	3.657644	4.539543	4.271174	3.389637	5.462933	5.511064	3.982623	5.924182	2.885929	7.760826	4.461136	4.842558	4.389751	4.155826	4.077825	3.463379	4.133608	3.444894	4.854383	5.480262	4.892731	5.334554	5.038906	4.921033	4.937082	4.464593	4.659199	4.109365	4.855023	5.138833	4.479552	5.743559	4.561427	3.294372	5.710503	5.342778	4.711373	4.721070	6.735475	4.574160	4.882020	4.244102	3.791856	5.005774	5.462213	4.680068	5.070805	5.191503	4.596826	5.898129	3.800090	5.831929	4.853765	4.251056	4.155826	4.055845	4.037570	4.630694	3.618767	4.178750	4.135859	4.217517	3.872862	3.873530	4.412414	4.103651	5.099896	3.908140	5.382123	5.287944	5.215840	5.739713	5.938973	4.047803	4.584561	4.892281	4.705113	5.251176	4.738874	4.727036	3.852263	5.206643	4.555570	4.974416	5.505053	4.266557	3.580146	4.538874	4.805763	4.398938	4.475377	3.806389	3.659637	3.083140	3.186612	4.404666	3.877608	3.633687	4.225973	3.907475	3.210577	3.217115	5.382725	5.142772	4.144522	3.525332	3.425651	3.520441	3.387237	3.304432	4.051722	4.635636	3.312092	4.493107	3.804108	3.201711	3.257316	5.547131	3.222815	5.183016	2.982627	4.871035	3.378073	7.350691	4.637149	5.085272	5.128561	5.227278	6.493615	5.616848	3.182826	5.715666	3.908575	3.399682	5.951984	3.474511	3.264238	3.279133	4.144295	3.412420	4.803678	6.240584	4.129806	3.233782	4.166006	6.300025	3.153417	6.681267	3.688674	4.563852	6.573050	4.240573	3.166776	4.421986	6.854771	4.756117	4.046733	4.831039	4.792890	3.885739	4.569162	3.724930	6.013134	6.723847	4.000622	7.296707	3.495025	3.720995	4.977248	3.303300	4.174872	4.347207	2.913243	4.452047	3.143613	3.406067	6.356984	6.078831	3.095417	3.087392	3.407300	3.435630	4.210992	3.136389	3.383293	4.614662	5.046672	4.429565	3.700948	6.915103	4.942999	4.083468	3.622038	3.344470	4.893762	4.643812	6.438400	3.088322	3.431688	3.255912	3.431159	3.133459	3.769062	5.074630	3.392955	5.077569	5.516654	5.294827	3.699196	3.952698	4.238991	4.848293	3.087170	3.167529	5.684361	3.537632	3.062769	4.415573	6.937962	3.462669	5.552010	3.090545	4.020223	3.464292	3.198895

AC103796.1	4.720456	4.643812	3.903586	3.804516	3.737290	4.488884	4.821688	3.411831	3.279133	3.900187	4.042742	3.963170	4.305090	3.657644	4.539543	4.271174	3.389637	5.462933	5.511064	3.982623	5.924182	2.885929	7.760826	4.461136	4.842558	4.389751	4.155826	4.077825	3.463379	4.133608	3.444894	4.854383	5.480262	4.892731	5.334554	5.038906	4.921033	4.937082	4.464593	4.659199	4.109365	4.855023	5.138833	4.479552	5.743559	4.561427	3.294372	5.710503	5.342778	4.711373	4.721070	6.735475	4.574160	4.882020	4.244102	3.791856	5.005774	5.462213	4.680068	5.070805	5.191503	4.596826	5.898129	3.800090	5.831929	4.853765	4.251056	4.155826	4.055845	4.037570	4.630694	3.618767	4.178750	4.135859	4.217517	3.872862	3.873530	4.412414	4.103651	5.099896	3.908140	5.382123	5.287944	5.215840	5.739713	5.938973	4.047803	4.584561	4.892281	4.705113	5.251176	4.738874	4.727036	3.852263	5.206643	4.555570	4.974416	5.505053	4.266557	3.580146	4.538874	4.805763	4.398938	4.475377	3.806389	3.659637	3.083140	3.186612	4.404666	3.877608	3.633687	4.225973	3.907475	3.210577	3.217115	5.382725	5.142772	4.144522	3.525332	3.425651	3.520441	3.387237	3.304432	4.051722	4.635636	3.312092	4.493107	3.804108	3.201711	3.257316	5.547131	3.222815	5.183016	2.982627	4.871035	3.378073	7.350691	4.637149	5.085272	5.128561	5.227278	6.493615	5.616848	3.182826	5.715666	3.908575	3.399682	5.951984	3.474511	3.264238	3.279133	4.144295	3.412420	4.803678	6.240584	4.129806	3.233782	4.166006	6.300025	3.153417	6.681267	3.688674	4.563852	6.573050	4.240573	3.166776	4.421986	6.854771	4.756117	4.046733	4.831039	4.792890	3.885739	4.569162	3.724930	6.013134	6.723847	4.000622	7.296707	3.495025	3.720995	4.977248	3.303300	4.174872	4.347207	2.913243	4.452047	3.143613	3.406067	6.356984	6.078831	3.095417	3.087392	3.407300	3.435630	4.210992	3.136389	3.383293	4.614662	5.046672	4.429565	3.700948	6.915103	4.942999	4.083468	3.622038	3.344470	4.893762	4.643812	6.438400	3.088322	3.431688	3.255912	3.431159	3.133459	3.769062	5.074630	3.392955	5.077569	5.516654	5.294827	3.699196	3.952698	4.238991	4.848293	3.087170	3.167529	5.684361	3.537632	3.062769	4.415573	6.937962	3.462669	5.552010	3.090545	4.020223	3.464292	3.198895

AC091059.1	12.207603	11.406614	11.370366	11.339812	11.261682	11.538822	11.699769	12.380831	11.813001	12.080733	12.089348	11.329537	11.670323	12.064848	10.863810	12.035336	11.264849	11.127669	12.543245	11.689592	11.236266	12.052362	12.860559	11.328200	11.521468	11.336873	11.580987	11.749526	11.076482	11.578918	12.192450	11.659107	12.281444	11.404390	11.724086	12.026020	11.914277	11.435520	12.051330	11.695625	11.306355	12.537925	11.798796	11.745962	11.896055	11.785397	12.105607	11.335682	12.375982	11.975594	11.620849	11.745676	11.510541	12.060393	11.756331	11.271942	12.202348	12.317768	11.818403	12.040781	12.506387	11.613450	13.045554	12.473286	11.616246	11.503209	12.051649	11.417687	11.260034	12.015680	11.925502	12.111299	12.294356	11.919514	11.928150	12.015212	11.995430	12.576063	12.800565	11.465833	11.781799	11.812013	11.351456	11.736262	11.972520	11.962317	11.825487	12.666497	12.288476	11.658633	11.997004	12.692342	11.818854	11.962614	12.191422	11.943939	12.511911	11.888571	11.643705	12.233736	11.644012	11.463470	11.173138	11.695305	11.878463	12.138794	12.623671	12.822312	11.326605	11.351965	12.883285	11.968244	11.468075	11.847279	11.276012	10.311562	11.826972	11.566103	11.177032	11.199598	11.084541	10.687045	10.808261	9.363785	9.856025	9.720646	10.201110	10.520787	9.562398	10.387620	10.216548	9.698632	10.246336	8.609684	11.437522	10.145498	9.849598	10.186206	10.367294	10.664468	10.928007	10.775210	7.959450	10.066011	10.345226	10.962224	9.133821	8.730609	10.888642	11.279290	11.440486	10.477996	12.113904	11.322294	11.242082	11.400910	10.900409	10.600915	10.950866	9.381799	11.643128	10.280645	10.170425	11.368020	10.737331	11.379601	10.779258	11.572225	11.159497	10.737016	11.028205	10.490251	10.581882	11.335396	10.745016	11.131914	11.092378	10.551284	10.791282	11.233464	10.644015	11.470648	11.305447	11.729158	12.132968	10.162595	11.394279	10.272270	11.586469	11.278862	11.517367	10.220637	11.356381	11.528035	11.696238	11.232005	10.603714	11.801189	11.300099	10.708399	11.123509	11.084984	11.418543	10.704617	10.218179	11.521285	10.928598	11.919091	10.931591	10.598160	11.309839	11.574797	11.565952	11.601212	11.184501	10.613617	10.909711	9.876562	11.908965	10.853896	10.913253	10.925790	10.518938	11.360449	9.972329	11.407838	11.772550	12.007161	11.608893	11.793165	10.311130	9.017607	10.589073	10.368806	11.550739	11.394657	10.782833	9.914535

AC011287.1	3.623070	3.103870	3.023492	2.958164	5.595490	2.973621	3.249291	3.710276	3.242251	3.143153	3.180533	3.419492	3.448991	3.248272	3.165304	3.350218	3.371537	2.982627	4.855630	3.129702	3.424717	3.300811	4.109365	4.469910	2.823537	5.963234	4.385646	3.916408	3.164562	5.709757	3.180933	4.653042	3.682035	5.994834	3.111995	3.587651	3.042065	2.959467	3.186612	3.407994	3.224898	3.107586	3.263680	3.136008	3.314962	4.548399	4.191032	3.301457	3.340818	3.517551	3.451547	4.517644	3.667489	3.697132	3.487325	3.204019	3.559346	3.807616	3.058228	3.056861	3.006617	3.136114	3.584548	3.080195	5.951263	3.046004	3.147523	3.049429	2.927396	2.954431	2.815060	3.169527	2.988516	3.168920	2.952942	3.199539	3.103123	3.043316	2.955040	3.331432	3.531098	3.552782	5.906325	3.589021	3.481349	3.156291	3.543056	3.248143	3.715868	3.541895	3.041223	3.089684	3.438239	5.128768	2.950734	3.271787	3.336976	2.920694	3.031471	3.419704	2.961473	3.161356	2.883463	3.601290	3.666461	3.276359	3.207803	3.078754	3.147411	2.858775	3.226736	2.903112	3.270368	5.103791	3.109691	3.666887	2.895725	3.543244	3.039947	6.089564	3.235794	3.761533	8.026826	2.948513	3.941921	8.678502	10.328381	5.445822	5.141933	5.032949	4.562514	3.360160	3.007067	3.036058	4.848706	10.811523	2.960421	10.397613	3.542686	3.179520	6.796372	3.170136	2.877304	8.023141	3.087392	2.961952	2.891716	5.592169	3.619725	3.056331	3.406231	3.696741	3.751463	3.091524	3.459671	7.050084	6.524815	3.192046	3.018540	3.116514	3.032233	3.987558	3.139813	3.458025	4.111173	3.105510	3.171791	3.466886	3.333725	4.065006	9.463760	4.378001	3.739870	3.245966	4.005674	3.406231	3.658061	8.026061	6.675596	3.643475	3.252513	3.785223	3.761730	6.424487	6.537120	8.257368	3.346871	5.876918	2.996541	7.755152	3.185151	4.019991	3.140934	3.625205	3.182446	3.204801	8.554956	7.695315	3.268818	8.664838	3.633128	7.980027	3.349518	3.696307	3.167650	3.246100	8.549225	7.936848	3.563815	3.045250	3.472214	3.210733	3.459671	3.058954	3.351555	3.043601	3.788233	3.264389	5.751387	2.962645	5.550863	3.783575	5.488546	7.914757	3.238311	4.069644	3.279590	3.019025	8.388680	2.932410	3.267648	5.350897	3.402485	3.081556	10.180089	4.063954	9.878449	3.066084

LINC00642	5.265256	4.211406	4.399847	4.031479	3.678004	4.206388	4.309062	5.941954	6.124641	4.192341	4.125253	5.239063	3.174065	4.503080	3.148764	4.267631	5.295428	3.026305	5.099511	4.095615	3.800966	4.352225	3.979371	4.478885	3.192946	4.415122	3.987558	8.076211	3.618361	3.762166	6.282271	4.994999	5.788486	3.580146	5.676951	4.604801	4.169012	6.283964	5.593845	5.093331	4.027949	5.168681	4.385646	3.691374	3.399349	6.514720	5.177162	5.946886	4.438286	4.915556	4.828672	5.320125	3.702653	5.416211	4.839153	3.680636	4.275288	3.899988	4.639479	4.682027	5.089598	3.900869	6.835965	5.358153	5.059824	5.102586	4.415978	4.547293	5.121125	5.153573	4.730610	4.950218	4.047589	5.091641	4.731438	4.767870	5.186207	5.384096	4.623340	4.740192	3.857435	5.116218	3.607655	5.104433	4.694111	4.483726	4.587333	5.601521	5.304993	6.096514	6.182888	5.677134	4.537365	4.309062	4.080493	4.130031	3.413099	3.664638	3.644833	3.309872	3.173430	3.742824	3.900869	4.099063	4.767465	4.469265	6.207268	4.555359	5.115354	4.131034	5.640329	5.358339	5.233225	3.965297	5.292378	4.232435	4.170075	4.850148	3.936016	5.421112	4.613377	4.312111	5.100721	3.735018	4.912630	3.864105	4.598089	6.276052	4.141508	4.491142	10.020187	4.020460	3.504732	3.868591	3.223215	5.402645	3.749777	3.762780	3.433070	4.023218	4.157992	6.269812	4.668169	4.206826	3.915505	3.531729	5.503721	4.364402	3.455662	5.804210	4.273558	5.106852	3.799869	5.099097	4.613988	4.494905	4.688859	5.092471	4.517865	3.515884	5.417778	4.526954	3.786037	3.613588	6.532414	3.978461	5.289796	4.787871	4.956653	5.338929	4.891263	4.914730	5.606684	5.460645	4.074067	3.390175	6.222275	4.296542	4.530746	6.722418	4.465424	6.371595	5.919034	4.511602	7.691511	6.073976	5.030546	4.760599	4.887696	4.727241	5.483318	4.342640	4.947342	3.957225	4.844870	5.346739	4.882433	5.069189	4.590570	5.576839	5.297271	4.857743	4.203849	4.999837	3.679614	5.263283	5.173168	4.863307	5.632657	5.856716	5.951263	4.698120	4.609716	4.654865	3.373733	4.121617	4.773688	3.697556	4.259584	3.605046	3.156686	8.238523	3.748966	4.441288	3.608025	6.568873	4.734422	5.780899	4.187529	5.392206	4.061055	4.096230	3.379490	4.604402	4.507461	8.266356	6.185046	4.079143

NUTM2A-AS1	9.728386	10.410869	10.581147	10.285603	11.240625	9.922889	10.721933	10.487353	12.058559	9.834894	11.180978	10.825164	10.750069	10.981887	10.704472	10.466673	10.425753	10.734749	10.575261	10.639693	10.567549	10.389287	10.193244	9.777068	10.888350	11.190467	10.985236	10.263285	10.062755	10.820438	10.285908	10.407254	10.609356	10.347130	10.691964	10.023218	10.401108	10.710534	10.218469	10.860851	10.021399	10.032710	10.059259	10.029183	8.892055	10.651628	10.389435	10.047403	10.338699	10.374399	9.936952	9.770001	10.260093	10.419946	10.669403	10.197653	10.492682	10.018949	10.136400	10.411931	9.940247	10.334339	9.949956	9.733626	10.400645	11.046498	10.197218	9.885942	10.284425	9.771440	10.149948	10.029019	10.185134	10.292031	10.004426	10.015553	10.507508	10.609789	9.883634	11.164944	11.146797	9.927545	11.124819	10.550397	9.836416	10.108989	10.992556	11.195935	10.272099	10.725946	10.813141	10.454346	10.489800	10.400807	10.402586	10.737168	9.330000	10.857655	10.340815	10.018641	10.729132	10.487949	9.901638	9.959383	10.396893	9.805002	10.127592	10.705209	11.658340	10.111696	10.260726	9.380526	11.191787	10.824882	10.126162	12.095797	10.708891	10.718371	10.160284	9.813689	11.074288	9.970502	9.921845	10.302329	9.914232	10.540943	11.716739	11.776985	9.212976	11.373199	10.174725	10.854645	10.891628	10.730762	11.126936	10.479041	10.683914	11.244350	10.769710	11.227537	10.454651	10.265579	7.006419	10.205978	11.205836	10.203404	10.754933	10.319814	10.278197	10.710393	10.742017	11.176950	10.660556	10.276541	9.598476	10.624589	10.650732	10.743513	10.505710	11.041648	10.083424	9.471554	10.957229	9.405273	10.266020	11.079831	9.679406	10.448550	11.141493	9.649786	9.981055	10.181458	10.888061	10.524883	9.175420	10.077279	9.679086	9.576825	11.005994	9.739328	9.762791	10.160896	9.373989	10.691348	11.119567	11.486562	10.215648	11.150531	9.664527	10.606255	10.233109	10.367456	10.848296	10.152036	10.311421	10.719908	11.939377	10.990030	11.283569	10.995960	9.844727	11.537024	10.973509	9.167845	9.951386	11.213047	12.078045	10.843161	11.293950	10.725060	11.114554	10.314618	11.024661	11.219673	11.189611	9.380969	11.910424	10.689640	9.576517	9.594166	11.099157	10.382558	10.031468	10.699884	10.548890	10.660096	11.497421	10.784818	10.781605	11.080729	8.999495	11.214951	10.526109	10.369707	11.851556	10.780871	10.475718	10.548595

LINC01901	3.043133	3.093488	3.028480	2.969823	2.886935	4.825015	3.267491	3.222255	3.240314	3.324679	3.471726	3.405057	3.202948	3.520622	3.043405	3.066396	3.514432	2.993354	4.454188	3.343649	3.841327	2.908051	3.364370	2.890616	3.000457	2.950634	2.978230	3.004535	3.060657	3.009843	3.397123	3.245524	3.312092	3.191888	4.520391	3.945528	3.471558	3.067524	3.093613	3.672321	3.236796	3.034991	3.790147	3.100561	3.309218	6.563856	4.002147	5.051393	3.304758	3.809313	3.781857	4.577234	3.807201	3.876112	3.497463	3.781036	3.723702	3.350741	3.439974	3.353367	3.203454	2.998784	3.303975	3.076788	3.382935	3.101859	2.998611	3.131467	2.992302	4.317138	2.968455	3.042415	2.972326	2.933007	3.105154	3.221098	3.152232	2.923588	2.962457	3.460735	3.247533	3.789513	3.177618	3.832923	3.262344	3.241621	3.130994	3.179899	5.560034	3.298691	3.088896	2.921191	3.094765	3.848646	2.920694	2.965377	3.513752	3.372263	3.660815	2.959101	3.005564	3.181767	3.066619	2.915428	4.234172	2.919864	3.335311	3.091974	3.124150	3.128093	3.461488	2.977056	3.037030	3.137111	3.022360	2.988612	2.967375	2.948767	3.013565	3.269121	3.134492	2.988326	2.997603	2.963678	5.761966	2.943972	3.168786	3.275739	3.933031	3.303802	3.159671	2.978612	3.098454	2.955694	3.073566	3.491016	2.910921	3.336436	3.243262	3.382741	2.868089	4.053433	3.226736	2.893983	2.907490	2.988422	2.919953	2.962180	3.308901	3.247223	3.336784	5.341233	3.043405	4.260212	4.280814	3.566891	3.921050	3.108516	2.873384	3.078033	2.940324	3.602357	3.902322	4.574791	6.413534	3.625390	4.974843	3.432871	3.857435	3.737498	3.987558	3.562864	3.910773	3.235234	3.817172	3.399682	3.292809	3.160321	3.126980	3.046101	3.048905	3.314806	3.378434	3.316489	3.175414	2.985886	3.283173	2.990174	3.011642	2.976679	2.954855	2.963489	2.935217	3.597934	3.527928	3.013675	3.440314	3.019759	3.129094	3.519870	3.878499	3.180533	3.487325	3.364521	2.961058	3.298231	3.335963	4.791873	3.220706	3.646429	2.910921	3.005736	3.128450	3.036263	2.989896	2.907677	3.032233	5.216600	3.147783	2.945681	3.012499	2.900991	2.849469	4.589046	2.927229	3.068701	3.057375	3.186612	3.002557	3.132715	3.096657	5.218532	3.095293	2.936439	2.837918	3.008577	2.951744	3.089684

LINC01902	3.043133	3.093488	3.028480	2.969823	2.886935	4.825015	3.267491	3.222255	3.240314	3.324679	3.471726	3.405057	3.202948	3.520622	3.043405	3.066396	3.514432	2.993354	4.454188	3.343649	3.841327	2.908051	3.364370	2.890616	3.000457	2.950634	2.978230	3.004535	3.060657	3.009843	3.397123	3.245524	3.312092	3.191888	4.520391	3.945528	3.471558	3.067524	3.093613	3.672321	3.236796	3.034991	3.790147	3.100561	3.309218	6.563856	4.002147	5.051393	3.304758	3.809313	3.781857	4.577234	3.807201	3.876112	3.497463	3.781036	3.723702	3.350741	3.439974	3.353367	3.203454	2.998784	3.303975	3.076788	3.382935	3.101859	2.998611	3.131467	2.992302	4.317138	2.968455	3.042415	2.972326	2.933007	3.105154	3.221098	3.152232	2.923588	2.962457	3.460735	3.247533	3.789513	3.177618	3.832923	3.262344	3.241621	3.130994	3.179899	5.560034	3.298691	3.088896	2.921191	3.094765	3.848646	2.920694	2.965377	3.513752	3.372263	3.660815	2.959101	3.005564	3.181767	3.066619	2.915428	4.234172	2.919864	3.335311	3.091974	3.124150	3.128093	3.461488	2.977056	3.037030	3.137111	3.022360	2.988612	2.967375	2.948767	3.013565	3.269121	3.134492	2.988326	2.997603	2.963678	5.761966	2.943972	3.168786	3.275739	3.933031	3.303802	3.159671	2.978612	3.098454	2.955694	3.073566	3.491016	2.910921	3.336436	3.243262	3.382741	2.868089	4.053433	3.226736	2.893983	2.907490	2.988422	2.919953	2.962180	3.308901	3.247223	3.336784	5.341233	3.043405	4.260212	4.280814	3.566891	3.921050	3.108516	2.873384	3.078033	2.940324	3.602357	3.902322	4.574791	6.413534	3.625390	4.974843	3.432871	3.857435	3.737498	3.987558	3.562864	3.910773	3.235234	3.817172	3.399682	3.292809	3.160321	3.126980	3.046101	3.048905	3.314806	3.378434	3.316489	3.175414	2.985886	3.283173	2.990174	3.011642	2.976679	2.954855	2.963489	2.935217	3.597934	3.527928	3.013675	3.440314	3.019759	3.129094	3.519870	3.878499	3.180533	3.487325	3.364521	2.961058	3.298231	3.335963	4.791873	3.220706	3.646429	2.910921	3.005736	3.128450	3.036263	2.989896	2.907677	3.032233	5.216600	3.147783	2.945681	3.012499	2.900991	2.849469	4.589046	2.927229	3.068701	3.057375	3.186612	3.002557	3.132715	3.096657	5.218532	3.095293	2.936439	2.837918	3.008577	2.951744	3.089684

AL592464.3	5.367227	6.542863	6.600383	6.580287	7.071494	6.512602	6.840632	7.795863	7.115503	6.911310	6.354574	6.139789	6.145843	6.204195	6.390022	6.875324	6.732713	6.458853	6.206757	6.489496	8.343667	7.089781	7.862455	6.336018	7.043506	7.092294	6.595180	7.243253	6.457889	6.216805	6.801865	5.774550	6.480249	5.827126	6.074831	6.487433	6.675238	6.621138	7.351140	6.946138	6.575670	6.924849	6.823537	6.927488	7.278770	6.803657	6.596013	7.107418	7.062373	7.210504	6.464369	6.948756	6.553514	6.831311	6.488864	6.758139	6.666875	7.263889	6.508768	6.752252	6.714097	6.619192	5.872074	6.792991	6.786131	7.013469	6.951703	6.977391	6.680133	7.169031	6.915897	6.933776	6.645475	7.053011	6.626630	6.457035	6.423830	5.900808	6.311124	6.083510	5.555640	5.813148	5.868488	5.671992	6.619694	6.119404	4.885618	6.458853	6.822609	6.629051	6.590303	6.894135	6.286834	6.778153	6.510942	6.109896	6.615749	6.447183	7.197600	6.683564	7.183928	6.800770	6.416995	6.609956	6.416842	6.056734	7.618931	7.161260	7.462608	7.026498	7.193889	6.621955	6.329736	6.950755	6.453805	7.115503	6.154648	6.342505	6.341865	5.537015	6.341388	6.287752	6.263844	8.552114	6.332647	6.305436	7.092126	5.539969	5.930416	5.920679	5.930924	6.801251	7.007847	7.043839	5.841877	5.622037	6.279147	7.772768	6.740220	6.949175	6.499570	7.518806	6.538740	6.797498	6.102054	6.075352	7.089448	6.296590	6.013683	5.702804	5.471198	5.971960	5.344963	5.452453	5.170102	5.350464	5.977611	6.490783	5.584968	6.845868	5.933348	5.971232	6.243953	5.127555	5.883320	6.996945	5.985916	5.762867	5.860863	5.323238	6.241930	4.495974	5.842965	5.525162	5.969815	6.513895	5.885138	5.549015	6.457389	5.081456	6.439253	5.735022	5.780149	6.039824	5.725538	5.898297	6.131372	6.063309	6.584073	5.875675	5.614444	7.133232	5.585157	5.471598	5.812805	5.123715	5.584968	5.590242	5.570508	5.471006	5.412627	5.071831	5.233026	5.113751	6.504161	5.040502	5.607597	6.184870	5.943205	6.780059	6.576181	5.929174	6.439253	6.283289	6.203650	6.854620	6.607406	6.650301	5.626914	6.902396	6.428351	6.081461	5.389322	6.089564	6.561513	6.442193	6.569550	6.098195	7.045786	7.207245	6.385280	7.643358	7.380184	7.171477	6.892517	6.278795	6.175920	5.714200

AL592464.1	5.367227	6.542863	6.600383	6.580287	7.071494	6.512602	6.840632	7.795863	7.115503	6.911310	6.354574	6.139789	6.145843	6.204195	6.390022	6.875324	6.732713	6.458853	6.206757	6.489496	8.343667	7.089781	7.862455	6.336018	7.043506	7.092294	6.595180	7.243253	6.457889	6.216805	6.801865	5.774550	6.480249	5.827126	6.074831	6.487433	6.675238	6.621138	7.351140	6.946138	6.575670	6.924849	6.823537	6.927488	7.278770	6.803657	6.596013	7.107418	7.062373	7.210504	6.464369	6.948756	6.553514	6.831311	6.488864	6.758139	6.666875	7.263889	6.508768	6.752252	6.714097	6.619192	5.872074	6.792991	6.786131	7.013469	6.951703	6.977391	6.680133	7.169031	6.915897	6.933776	6.645475	7.053011	6.626630	6.457035	6.423830	5.900808	6.311124	6.083510	5.555640	5.813148	5.868488	5.671992	6.619694	6.119404	4.885618	6.458853	6.822609	6.629051	6.590303	6.894135	6.286834	6.778153	6.510942	6.109896	6.615749	6.447183	7.197600	6.683564	7.183928	6.800770	6.416995	6.609956	6.416842	6.056734	7.618931	7.161260	7.462608	7.026498	7.193889	6.621955	6.329736	6.950755	6.453805	7.115503	6.154648	6.342505	6.341865	5.537015	6.341388	6.287752	6.263844	8.552114	6.332647	6.305436	7.092126	5.539969	5.930416	5.920679	5.930924	6.801251	7.007847	7.043839	5.841877	5.622037	6.279147	7.772768	6.740220	6.949175	6.499570	7.518806	6.538740	6.797498	6.102054	6.075352	7.089448	6.296590	6.013683	5.702804	5.471198	5.971960	5.344963	5.452453	5.170102	5.350464	5.977611	6.490783	5.584968	6.845868	5.933348	5.971232	6.243953	5.127555	5.883320	6.996945	5.985916	5.762867	5.860863	5.323238	6.241930	4.495974	5.842965	5.525162	5.969815	6.513895	5.885138	5.549015	6.457389	5.081456	6.439253	5.735022	5.780149	6.039824	5.725538	5.898297	6.131372	6.063309	6.584073	5.875675	5.614444	7.133232	5.585157	5.471598	5.812805	5.123715	5.584968	5.590242	5.570508	5.471006	5.412627	5.071831	5.233026	5.113751	6.504161	5.040502	5.607597	6.184870	5.943205	6.780059	6.576181	5.929174	6.439253	6.283289	6.203650	6.854620	6.607406	6.650301	5.626914	6.902396	6.428351	6.081461	5.389322	6.089564	6.561513	6.442193	6.569550	6.098195	7.045786	7.207245	6.385280	7.643358	7.380184	7.171477	6.892517	6.278795	6.175920	5.714200

AL009179.1	7.064918	8.196850	7.692731	8.290571	7.707918	7.721625	7.862455	7.276724	7.455423	7.444435	8.137259	7.590884	8.208290	8.504041	8.629570	7.479732	8.228419	8.344925	8.685393	8.208005	7.151233	7.303825	6.168779	7.722222	7.516962	8.371282	8.009296	6.451641	7.086906	7.951220	8.577147	8.751471	9.095237	9.401532	8.918759	8.393039	8.191012	8.542271	8.243097	8.118549	7.712007	8.212820	8.272998	8.443484	7.901171	8.801264	6.711048	7.240558	8.545084	8.868537	8.254971	8.903998	8.585603	8.783516	7.569464	7.064759	8.703980	9.073896	8.829820	9.234881	8.529222	6.810006	5.703375	8.194308	9.126584	8.246792	8.249009	8.548593	8.878485	7.684733	8.621889	8.340857	7.112374	8.275241	8.587697	8.461880	7.938997	8.260645	8.451544	8.992872	8.948154	8.521866	9.060362	9.168755	8.725877	8.483559	9.102283	8.917711	9.241805	9.332454	8.845102	8.319729	8.656538	7.977373	8.274612	8.594404	8.399107	8.012608	8.214394	8.008376	7.377685	7.985774	7.976484	7.945391	7.559214	7.866036	7.037782	8.364702	7.558623	6.916640	7.995191	8.342169	7.819822	6.960528	8.161613	7.416404	6.956277	8.140171	7.474745	10.558269	9.441268	8.410303	9.810864	8.170358	9.430349	9.289571	9.786976	8.663004	9.914081	8.247950	9.803340	8.417243	9.450295	8.998577	8.280832	5.032560	9.301723	10.215186	7.774290	7.068728	8.567761	9.666547	9.639347	9.632128	8.447968	7.619091	9.286747	7.382041	8.640041	10.048505	9.304559	9.630301	9.548072	10.483072	9.662648	8.177391	8.776297	7.620289	9.004108	10.042994	9.288791	6.677849	9.111274	8.576106	10.117312	8.810595	10.113777	9.562846	9.441911	10.184516	9.876085	9.227124	9.866830	8.388365	8.718897	11.126207	10.313050	9.831828	10.789859	9.144285	8.961900	8.773068	9.348642	10.103567	8.507514	10.217418	7.643516	8.430406	7.289104	7.778846	8.637555	8.821898	10.062437	10.027037	9.327985	8.600963	9.550393	9.396514	9.843946	9.777366	10.524883	9.548541	9.231455	10.346123	9.208692	9.528007	10.107612	9.428831	8.998099	11.264551	9.725448	9.955843	9.890146	9.910192	9.116026	8.971304	8.971304	7.893421	10.252933	9.333538	8.743289	8.853796	7.842468	9.418832	8.405306	9.400283	9.694363	8.835394	7.173245	8.682951	10.324283	10.305051	7.936848	10.219240	10.165649	9.460752	10.744413	8.757622

AC074091.2	10.282778	8.767294	8.184797	9.015016	9.114050	8.710550	9.647662	7.718569	6.903040	9.103050	9.168451	8.736729	9.525550	8.834192	8.853327	9.158942	8.827010	9.024612	7.797700	9.179234	8.127063	7.633902	8.573379	8.218403	9.068809	9.074192	9.462868	8.674991	8.619728	8.733953	8.759006	9.841361	9.820277	9.404032	9.781184	9.797503	10.037644	8.533168	8.088467	8.111918	8.957952	8.737472	8.122765	9.670699	9.856355	9.511815	8.437691	7.797769	9.149548	9.805636	9.626936	9.872877	9.669890	10.205513	8.216356	7.855750	10.212131	9.832155	8.959765	9.601437	9.150168	8.719507	8.137100	7.564875	8.220099	7.884890	9.037384	8.802888	9.076223	8.051848	9.674605	9.849917	8.626389	9.726848	9.792860	9.284296	10.133459	9.055523	9.876877	9.621360	10.135620	9.295221	10.186500	10.215794	10.211375	9.741831	9.835366	9.967417	8.981350	8.696372	9.042970	8.643164	8.828074	9.813229	10.225283	9.810261	9.041181	9.143350	9.380364	9.178480	7.307127	9.300486	9.076571	8.257083	7.121194	8.937800	6.242269	7.030795	6.787592	8.675628	8.720555	6.580790	7.229212	8.387288	8.010187	6.024627	9.113447	8.777808	8.318960	9.253455	8.426610	8.239783	8.268065	7.426963	8.351704	8.752132	9.389757	9.821926	9.146716	8.338861	9.539381	9.106595	8.421514	7.174321	9.305718	9.162133	8.242495	8.203158	10.868295	7.180642	7.202536	7.304277	9.005980	9.059743	9.057845	8.399107	8.168776	7.486779	8.404071	7.920144	8.330882	9.354532	8.160220	8.898856	9.870914	8.975910	7.736081	7.890146	8.039807	8.701221	8.302895	7.994267	8.534543	8.591289	8.930003	7.296370	9.712939	9.631674	9.458185	8.966798	9.756057	9.543959	9.620259	9.382862	9.046541	10.696919	9.280598	9.234252	9.055523	9.377775	8.570802	7.695178	6.815893	7.935134	8.248548	8.426298	8.171919	9.157875	7.959136	9.426037	9.241070	6.709432	9.056876	10.124419	9.458628	9.010103	9.511023	9.237341	9.785048	9.532917	9.690691	8.859264	9.212818	9.246132	8.990766	9.221273	10.331914	8.800815	7.481815	9.209310	8.432586	8.885279	8.328907	8.243837	8.743748	9.977842	8.455746	8.428035	10.189508	8.883900	10.498864	9.318981	8.008376	7.741802	8.020288	6.695877	8.385428	7.070379	7.294130	7.383782	6.355752	10.173663	7.562869	9.882077	8.086143	8.882957	8.764223	8.189568

LINC01532	3.887402	6.023247	4.031703	3.829515	3.876112	4.935579	4.535899	4.805358	3.741157	4.087235	4.644930	3.857219	3.887853	4.426792	3.382935	3.416712	4.905873	3.321225	5.754089	2.990174	4.103884	3.555255	7.981918	4.112439	3.025532	2.981901	4.643391	3.639699	5.047267	3.608224	4.429565	5.630815	3.089121	3.410422	4.242950	4.740850	3.459121	4.034749	4.426112	3.430988	4.621851	3.417570	4.348963	4.865170	3.293775	5.226103	4.866199	5.044321	4.505883	3.175158	4.341124	4.124421	4.022370	3.708262	4.237440	3.726946	3.890045	4.194119	5.850080	3.541494	3.104899	3.483534	5.143159	6.331290	4.647039	4.158864	4.624205	3.877841	3.422249	4.498788	4.295457	3.647986	4.840860	3.952908	2.918405	3.748541	4.191032	3.741358	3.688293	3.272404	3.282239	3.591899	4.517429	4.093706	3.940037	3.156927	3.077475	3.659429	3.159671	3.239897	4.530309	4.151044	3.230351	2.965377	3.203085	3.790356	3.526294	5.369713	4.481723	3.297133	3.935366	3.101606	3.232829	4.039309	6.259290	4.490216	4.530746	3.538259	4.548146	4.066688	3.401046	4.254568	3.888075	4.390178	3.372595	4.621637	4.225364	3.798589	3.341480	5.074237	7.151398	4.201183	5.064071	5.721042	6.493934	6.395803	5.229330	6.403118	5.847710	7.906479	5.061210	6.266387	4.557891	4.792890	4.980732	5.698775	5.276568	4.984580	4.522120	4.047803	5.063257	6.638032	4.925093	5.251581	5.550338	5.951263	6.293351	6.205020	5.413006	6.514053	5.146342	3.124906	4.296754	3.957709	4.344597	5.014044	5.355901	5.263904	5.516844	7.472951	4.739775	4.734206	6.284798	5.256728	4.809510	6.888567	4.019991	4.147890	5.343386	6.123900	5.224094	3.935805	3.729236	5.309663	6.028393	3.532733	3.236658	5.196697	3.453144	4.763580	4.349640	5.057568	6.834522	4.523906	4.845079	4.349824	4.179668	3.722298	4.038661	4.476479	3.957014	5.238060	5.605717	3.961596	4.614899	5.571063	4.752528	4.983993	4.360707	4.011437	3.731504	4.050878	4.770142	4.890855	4.418689	4.707447	4.115483	4.847414	4.975882	4.467583	5.563905	4.328766	5.441793	5.214830	5.531417	4.804325	6.943280	6.089748	4.720248	5.789212	3.423840	5.025271	4.597879	6.513257	5.320335	5.054726	4.567032	7.084910	7.181888	3.731092	4.418927	4.956231	4.276183	5.458276	5.577218	4.763775	5.612341	5.187613

AC067942.4	2.956250	3.164945	2.981538	2.982978	2.924485	4.084748	3.212131	3.980200	3.246386	3.295444	3.426395	3.531287	3.200908	3.836533	3.610550	3.171791	3.359844	3.711334	3.228037	3.143382	4.764632	2.897683	6.005022	3.083503	3.192699	2.916572	3.016895	2.932490	3.142443	3.044010	3.624039	3.874606	4.693492	3.012317	3.285045	3.846309	3.646235	3.264817	3.424029	3.156556	3.357076	3.466667	3.939843	3.129702	3.520966	6.525818	3.243536	3.844577	3.313638	3.523830	3.583415	3.699957	3.943276	3.822877	3.629032	3.055187	3.980200	3.233782	3.449361	3.684424	3.160939	3.068900	3.513915	3.070579	3.238168	3.362672	2.898242	3.308901	3.225710	3.324325	2.970632	2.892414	3.091974	2.898732	2.959741	3.882760	2.977873	2.910533	2.994563	3.475634	3.218815	3.771535	3.314652	4.260424	3.266440	3.315269	2.896701	3.251299	3.099736	3.288775	3.236658	3.230351	3.274802	3.031355	2.913548	2.930079	3.667710	3.223498	3.858770	3.201160	2.880411	3.152573	2.976228	2.917227	3.046101	3.346067	3.042193	3.330300	3.055916	3.208983	2.885639	2.920788	2.908676	3.250421	3.058828	2.883971	2.974907	3.025146	3.187677	2.976679	3.229611	2.942101	3.027977	3.078263	4.530746	2.933891	3.036658	3.200037	3.092690	3.207928	3.103544	2.975807	3.214463	3.296669	2.962366	3.312092	3.238168	3.460015	3.262627	4.122295	2.861366	5.137013	2.972811	3.029190	2.870127	2.930079	2.875668	2.955254	3.439974	3.416207	3.925898	3.151974	2.984243	3.183197	3.607467	3.637058	3.240460	3.356052	3.084646	3.117943	3.466476	3.520441	3.154930	3.854271	6.073976	3.156556	3.745894	3.436697	3.514967	3.538985	3.459846	3.649917	3.947460	3.310033	3.000046	3.616132	3.318122	3.218685	3.687679	2.971964	3.150130	3.502755	3.221664	3.328860	3.496731	2.903731	3.643851	3.398011	3.010672	2.967039	2.945091	3.055187	2.926362	2.910264	3.601083	2.972619	2.997034	2.980607	3.111532	3.544508	4.072967	3.379490	3.634676	3.490280	2.983166	3.487910	3.377017	3.030378	3.269121	3.431343	3.187793	3.160827	3.049338	3.003614	2.927608	2.925007	2.912576	3.613780	3.165554	2.983345	2.988516	2.907112	2.844337	3.096025	3.091207	3.106094	3.265442	3.349197	3.183560	2.855829	2.907763	2.869807	3.163139	2.962891	2.923059	3.047204	3.015655	3.261151

LINC00575	2.956250	3.164945	2.981538	2.982978	2.924485	4.084748	3.212131	3.980200	3.246386	3.295444	3.426395	3.531287	3.200908	3.836533	3.610550	3.171791	3.359844	3.711334	3.228037	3.143382	4.764632	2.897683	6.005022	3.083503	3.192699	2.916572	3.016895	2.932490	3.142443	3.044010	3.624039	3.874606	4.693492	3.012317	3.285045	3.846309	3.646235	3.264817	3.424029	3.156556	3.357076	3.466667	3.939843	3.129702	3.520966	6.525818	3.243536	3.844577	3.313638	3.523830	3.583415	3.699957	3.943276	3.822877	3.629032	3.055187	3.980200	3.233782	3.449361	3.684424	3.160939	3.068900	3.513915	3.070579	3.238168	3.362672	2.898242	3.308901	3.225710	3.324325	2.970632	2.892414	3.091974	2.898732	2.959741	3.882760	2.977873	2.910533	2.994563	3.475634	3.218815	3.771535	3.314652	4.260424	3.266440	3.315269	2.896701	3.251299	3.099736	3.288775	3.236658	3.230351	3.274802	3.031355	2.913548	2.930079	3.667710	3.223498	3.858770	3.201160	2.880411	3.152573	2.976228	2.917227	3.046101	3.346067	3.042193	3.330300	3.055916	3.208983	2.885639	2.920788	2.908676	3.250421	3.058828	2.883971	2.974907	3.025146	3.187677	2.976679	3.229611	2.942101	3.027977	3.078263	4.530746	2.933891	3.036658	3.200037	3.092690	3.207928	3.103544	2.975807	3.214463	3.296669	2.962366	3.312092	3.238168	3.460015	3.262627	4.122295	2.861366	5.137013	2.972811	3.029190	2.870127	2.930079	2.875668	2.955254	3.439974	3.416207	3.925898	3.151974	2.984243	3.183197	3.607467	3.637058	3.240460	3.356052	3.084646	3.117943	3.466476	3.520441	3.154930	3.854271	6.073976	3.156556	3.745894	3.436697	3.514967	3.538985	3.459846	3.649917	3.947460	3.310033	3.000046	3.616132	3.318122	3.218685	3.687679	2.971964	3.150130	3.502755	3.221664	3.328860	3.496731	2.903731	3.643851	3.398011	3.010672	2.967039	2.945091	3.055187	2.926362	2.910264	3.601083	2.972619	2.997034	2.980607	3.111532	3.544508	4.072967	3.379490	3.634676	3.490280	2.983166	3.487910	3.377017	3.030378	3.269121	3.431343	3.187793	3.160827	3.049338	3.003614	2.927608	2.925007	2.912576	3.613780	3.165554	2.983345	2.988516	2.907112	2.844337	3.096025	3.091207	3.106094	3.265442	3.349197	3.183560	2.855829	2.907763	2.869807	3.163139	2.962891	2.923059	3.047204	3.015655	3.261151

ZBED3-AS1	6.128661	3.326783	3.048272	4.011026	3.779768	5.268480	3.250993	3.778757	3.824156	3.376866	3.546976	3.962484	4.273115	4.463518	3.805556	3.705259	3.556216	3.300671	5.443514	3.655248	4.883876	2.880520	7.767780	2.954431	2.882920	3.774315	3.255150	4.227886	3.876112	3.521143	3.651791	3.869665	5.506022	3.388249	3.276359	4.852235	3.652414	3.214463	3.416712	3.087392	3.426736	3.003120	4.255015	3.595732	3.773875	5.639734	3.857006	5.223352	3.792920	3.677001	3.614957	3.505520	4.254350	4.667563	3.973522	3.332238	5.171712	2.949776	4.574373	3.859612	2.843897	3.885739	3.910773	3.658843	3.520242	3.518376	3.527928	3.348056	3.242102	2.809656	3.546557	3.796132	3.190001	3.236086	2.831611	3.360006	4.444999	4.001248	3.983064	3.833118	5.364822	4.084748	4.672733	4.621238	4.588197	3.632948	6.521545	3.298691	3.623070	3.686042	3.503315	4.571394	3.943719	3.238311	4.750429	4.884308	4.252167	4.409815	3.990914	4.543088	6.369725	3.998522	3.829935	3.537427	4.626946	3.841099	3.476529	3.550612	5.869386	3.630489	6.185209	3.183839	4.837863	4.116148	3.613360	8.967105	5.555164	3.567669	3.233635	3.458581	3.479650	3.195847	4.124030	3.738084	4.448486	5.322870	4.350904	3.648809	3.121823	3.321058	5.631190	5.145152	6.014517	3.367694	5.515838	3.440139	4.894383	6.608681	3.395752	4.716023	5.514453	5.568071	4.242508	5.232404	3.265442	6.023770	3.933031	5.997089	3.568264	4.449608	4.829094	4.773477	3.607655	4.156948	5.129592	3.901062	3.229919	3.420470	3.526862	4.755730	2.903488	4.780099	3.407300	4.280614	5.162655	3.851412	5.733384	4.099972	3.435083	3.435630	3.930246	4.159287	4.471446	4.977248	4.603341	6.302189	2.940730	4.419800	3.696522	2.934625	3.872419	3.999384	3.747084	3.829515	3.761124	2.921460	3.689738	3.372078	2.787050	4.053659	4.005218	3.186339	3.598578	4.712212	3.570590	3.995950	3.141186	3.808898	5.686968	3.833571	4.482648	3.822877	6.139271	4.604611	4.529240	4.472756	5.874764	3.525165	3.537834	4.806580	3.270657	3.707684	3.289532	3.386728	4.519116	4.502632	5.276751	4.664961	3.477266	3.843957	3.682453	4.689517	5.436481	5.030546	3.714240	3.241947	3.627415	3.339291	3.296822	6.296916	4.738874	6.662960	3.256748	4.138014	4.304427	3.231727	3.640119	3.369613

AL133467.3	8.733172	7.691511	7.513898	7.515856	7.688106	7.512640	6.478984	9.220659	9.100841	7.036396	7.249623	7.162049	6.093732	6.360376	6.847079	7.790829	8.910839	7.358858	8.131825	7.643210	8.613766	9.145926	8.774288	7.437704	7.006116	7.068891	7.744413	8.243979	7.104070	7.005199	8.289335	6.993391	7.731640	7.216228	7.245629	7.164423	7.180790	7.978305	7.789160	8.295756	6.756872	7.946185	7.866655	7.798982	7.158850	7.535018	7.134995	7.921935	8.144245	7.716737	7.815116	7.833540	6.764576	7.604509	7.265880	7.212644	6.935051	8.034493	7.852497	7.664681	7.749037	6.987484	7.217482	8.217118	7.185042	7.916768	8.041233	8.064885	7.882321	8.773506	7.690392	7.789465	7.338770	7.482286	7.669290	7.871660	7.908340	7.738209	8.112848	7.483988	7.452118	7.558311	7.404412	7.727325	7.357154	7.777904	7.492126	7.858176	7.961466	7.937139	7.911257	8.507964	7.733625	7.626701	7.131815	7.437870	7.674467	6.891419	7.329134	7.462448	7.053478	7.419924	7.443314	7.417872	8.932263	7.084584	8.945521	8.125849	8.937335	7.318079	9.512761	8.409690	8.330105	7.635736	8.107279	7.638327	8.253272	7.659388	7.667897	7.669595	7.452446	7.131658	7.055864	7.755863	7.145355	7.135790	8.253887	8.432903	6.956132	6.825424	6.591640	6.888237	6.574822	7.071340	7.358111	8.176634	6.733368	8.144389	6.949641	7.611123	8.181304	8.616310	7.036868	6.989522	6.841755	7.560294	7.706537	8.010187	6.659694	8.163962	7.575743	7.448092	6.761235	6.988909	7.086134	6.945345	7.749351	7.961301	7.958992	6.486107	7.881713	7.969565	7.704712	6.769612	7.465257	7.375562	7.594003	7.879300	7.579513	7.524222	7.026340	6.457563	6.653213	6.415816	7.377364	7.033252	7.290024	7.541891	7.198061	7.930805	7.328976	7.431096	7.689026	7.643684	7.373908	7.754421	7.664047	7.480444	8.229507	7.433753	7.501152	7.385917	7.233867	7.739134	7.652041	7.724547	7.639084	7.864627	7.045129	7.230711	7.415648	7.230864	7.454307	7.010888	7.992858	7.259818	7.308199	8.121469	7.487887	7.778530	8.444402	7.732109	7.889992	7.571280	7.966199	8.047349	6.960860	6.971663	7.562281	7.046566	6.917090	6.708319	7.162692	7.998109	6.963224	8.576401	7.832609	8.536401	7.435209	8.934723	8.328762	7.673841	7.730239	7.311187	6.884396	7.873506	7.148023	7.418684

TCL6	8.733172	7.691511	7.513898	7.515856	7.688106	7.512640	6.478984	9.220659	9.100841	7.036396	7.249623	7.162049	6.093732	6.360376	6.847079	7.790829	8.910839	7.358858	8.131825	7.643210	8.613766	9.145926	8.774288	7.437704	7.006116	7.068891	7.744413	8.243979	7.104070	7.005199	8.289335	6.993391	7.731640	7.216228	7.245629	7.164423	7.180790	7.978305	7.789160	8.295756	6.756872	7.946185	7.866655	7.798982	7.158850	7.535018	7.134995	7.921935	8.144245	7.716737	7.815116	7.833540	6.764576	7.604509	7.265880	7.212644	6.935051	8.034493	7.852497	7.664681	7.749037	6.987484	7.217482	8.217118	7.185042	7.916768	8.041233	8.064885	7.882321	8.773506	7.690392	7.789465	7.338770	7.482286	7.669290	7.871660	7.908340	7.738209	8.112848	7.483988	7.452118	7.558311	7.404412	7.727325	7.357154	7.777904	7.492126	7.858176	7.961466	7.937139	7.911257	8.507964	7.733625	7.626701	7.131815	7.437870	7.674467	6.891419	7.329134	7.462448	7.053478	7.419924	7.443314	7.417872	8.932263	7.084584	8.945521	8.125849	8.937335	7.318079	9.512761	8.409690	8.330105	7.635736	8.107279	7.638327	8.253272	7.659388	7.667897	7.669595	7.452446	7.131658	7.055864	7.755863	7.145355	7.135790	8.253887	8.432903	6.956132	6.825424	6.591640	6.888237	6.574822	7.071340	7.358111	8.176634	6.733368	8.144389	6.949641	7.611123	8.181304	8.616310	7.036868	6.989522	6.841755	7.560294	7.706537	8.010187	6.659694	8.163962	7.575743	7.448092	6.761235	6.988909	7.086134	6.945345	7.749351	7.961301	7.958992	6.486107	7.881713	7.969565	7.704712	6.769612	7.465257	7.375562	7.594003	7.879300	7.579513	7.524222	7.026340	6.457563	6.653213	6.415816	7.377364	7.033252	7.290024	7.541891	7.198061	7.930805	7.328976	7.431096	7.689026	7.643684	7.373908	7.754421	7.664047	7.480444	8.229507	7.433753	7.501152	7.385917	7.233867	7.739134	7.652041	7.724547	7.639084	7.864627	7.045129	7.230711	7.415648	7.230864	7.454307	7.010888	7.992858	7.259818	7.308199	8.121469	7.487887	7.778530	8.444402	7.732109	7.889992	7.571280	7.966199	8.047349	6.960860	6.971663	7.562281	7.046566	6.917090	6.708319	7.162692	7.998109	6.963224	8.576401	7.832609	8.536401	7.435209	8.934723	8.328762	7.673841	7.730239	7.311187	6.884396	7.873506	7.148023	7.418684

AC087521.1	15.423337	15.746888	15.129069	13.376280	12.649538	11.755427	13.624759	11.954825	11.882216	13.021558	12.047981	13.382177	12.642737	12.715232	14.884408	12.517113	14.697497	13.707975	12.344564	11.414134	12.684200	12.788897	15.076369	12.256640	13.853818	13.359821	14.558412	13.462463	14.224877	15.020432	12.626671	14.138248	13.295547	13.749075	13.749826	13.530118	13.360596	13.702665	13.652718	13.262596	13.534637	14.574718	14.690655	13.588617	16.128614	14.295679	13.301249	15.973135	16.114305	14.580994	14.231187	14.853259	14.794550	13.707975	15.204490	14.553109	14.902407	14.111066	13.163671	15.014487	13.320668	15.025759	16.387390	13.576482	14.668633	14.593195	12.993460	13.475325	12.627335	12.650060	14.193049	12.730828	13.624065	13.982948	12.416578	13.792498	12.832557	15.011645	13.386257	15.487850	13.784079	16.452541	13.593974	13.883013	13.281421	13.398018	12.795726	14.004466	13.844544	12.998772	13.015899	13.470952	14.422133	12.860392	14.302275	13.080043	15.472304	13.473987	13.121958	14.776105	13.791211	13.817328	14.524706	14.882312	13.453926	14.114654	14.590670	13.873120	14.413643	12.980619	13.310550	13.019739	12.486494	12.018432	13.238812	13.773212	15.158834	14.592389	13.756377	13.540385	13.641300	13.399764	13.004306	13.672956	11.855112	12.558148	13.809161	12.226768	12.090589	12.234346	12.659650	14.342484	13.068499	13.532157	12.969695	13.140875	12.718193	12.388737	10.822512	12.232212	13.234805	13.810879	12.242693	12.591334	14.840176	13.125419	11.850817	13.113930	13.365784	13.171908	14.681870	13.213003	12.928492	11.774910	12.157572	13.557270	13.113147	12.714534	14.312732	12.680252	14.454664	12.939558	13.143772	14.749262	13.296386	11.989104	15.242555	12.624160	14.187463	13.161096	13.594881	13.330907	14.350910	15.102359	13.171328	13.210738	14.350242	13.296386	13.106614	14.198488	14.956144	13.308350	12.110693	12.724823	14.390000	13.065843	13.198725	12.868696	13.164133	13.066209	13.992417	12.152235	13.054711	13.850714	13.820478	13.029714	13.709428	13.445662	13.512526	12.706541	13.216008	12.088879	14.185552	13.069482	12.500257	12.166337	13.464641	12.598484	14.836045	13.328825	12.789412	12.647261	14.622906	13.005406	13.839552	14.036135	13.132270	12.134507	12.902873	13.724675	13.678771	13.851451	14.726053	12.226923	13.824511	13.135455	11.609343	12.371963	12.829461	13.538127	14.392936	12.150502	11.092206	12.927582	11.960063	14.037568	11.911024	12.516134

AC087521.3	15.423337	15.746888	15.129069	13.376280	12.649538	11.755427	13.624759	11.954825	11.882216	13.021558	12.047981	13.382177	12.642737	12.715232	14.884408	12.517113	14.697497	13.707975	12.344564	11.414134	12.684200	12.788897	15.076369	12.256640	13.853818	13.359821	14.558412	13.462463	14.224877	15.020432	12.626671	14.138248	13.295547	13.749075	13.749826	13.530118	13.360596	13.702665	13.652718	13.262596	13.534637	14.574718	14.690655	13.588617	16.128614	14.295679	13.301249	15.973135	16.114305	14.580994	14.231187	14.853259	14.794550	13.707975	15.204490	14.553109	14.902407	14.111066	13.163671	15.014487	13.320668	15.025759	16.387390	13.576482	14.668633	14.593195	12.993460	13.475325	12.627335	12.650060	14.193049	12.730828	13.624065	13.982948	12.416578	13.792498	12.832557	15.011645	13.386257	15.487850	13.784079	16.452541	13.593974	13.883013	13.281421	13.398018	12.795726	14.004466	13.844544	12.998772	13.015899	13.470952	14.422133	12.860392	14.302275	13.080043	15.472304	13.473987	13.121958	14.776105	13.791211	13.817328	14.524706	14.882312	13.453926	14.114654	14.590670	13.873120	14.413643	12.980619	13.310550	13.019739	12.486494	12.018432	13.238812	13.773212	15.158834	14.592389	13.756377	13.540385	13.641300	13.399764	13.004306	13.672956	11.855112	12.558148	13.809161	12.226768	12.090589	12.234346	12.659650	14.342484	13.068499	13.532157	12.969695	13.140875	12.718193	12.388737	10.822512	12.232212	13.234805	13.810879	12.242693	12.591334	14.840176	13.125419	11.850817	13.113930	13.365784	13.171908	14.681870	13.213003	12.928492	11.774910	12.157572	13.557270	13.113147	12.714534	14.312732	12.680252	14.454664	12.939558	13.143772	14.749262	13.296386	11.989104	15.242555	12.624160	14.187463	13.161096	13.594881	13.330907	14.350910	15.102359	13.171328	13.210738	14.350242	13.296386	13.106614	14.198488	14.956144	13.308350	12.110693	12.724823	14.390000	13.065843	13.198725	12.868696	13.164133	13.066209	13.992417	12.152235	13.054711	13.850714	13.820478	13.029714	13.709428	13.445662	13.512526	12.706541	13.216008	12.088879	14.185552	13.069482	12.500257	12.166337	13.464641	12.598484	14.836045	13.328825	12.789412	12.647261	14.622906	13.005406	13.839552	14.036135	13.132270	12.134507	12.902873	13.724675	13.678771	13.851451	14.726053	12.226923	13.824511	13.135455	11.609343	12.371963	12.829461	13.538127	14.392936	12.150502	11.092206	12.927582	11.960063	14.037568	11.911024	12.516134

LINC01749	7.499287	10.790561	10.681966	11.137430	10.366983	11.119708	10.456786	11.539103	11.132065	11.295540	10.632913	11.181727	10.120507	10.805904	10.260405	11.155348	10.675082	10.342317	12.348814	11.081604	11.434496	11.485014	9.259376	10.526714	11.016907	10.778399	8.178407	10.521739	10.480656	9.934023	11.297951	10.207052	10.137918	7.996465	9.675698	9.904700	9.595374	10.408604	10.681966	11.070164	10.933966	10.134374	9.492394	10.117917	8.581064	8.787346	9.785504	10.232190	10.383188	9.597413	9.366459	9.807593	10.114655	10.609923	10.584613	11.543627	10.016345	10.256634	10.641916	9.945429	10.339585	9.954586	8.298501	10.971168	10.897486	11.261074	10.705664	10.497236	10.207843	11.271173	10.546267	10.571684	10.359776	10.326880	10.567866	9.704886	10.238936	9.947336	9.918999	10.644592	9.816348	10.038692	9.367092	10.461579	10.640470	10.850174	9.303995	9.415203	10.758822	11.032834	10.955883	11.105870	10.171699	10.824882	8.245699	8.925815	10.612584	10.070060	10.024815	10.539877	10.969399	10.927713	10.099818	10.596797	12.076508	10.163835	11.940454	11.417994	11.322294	11.001791	11.193393	11.553156	10.894745	11.091311	11.004248	9.903802	7.679960	10.906618	10.409375	7.659658	10.955140	8.180074	9.030556	9.565885	9.703426	9.154950	7.315588	8.968517	8.223133	9.497955	7.551132	7.443608	7.613107	7.926948	8.597472	7.527128	7.470633	7.946637	7.615728	9.252826	9.042224	8.286088	8.678667	7.748264	7.508617	10.582900	9.092678	8.624652	9.001514	8.191967	9.252538	9.397811	10.190703	7.961301	9.258917	10.060910	8.833342	9.412138	8.916489	8.656980	9.833202	7.736660	8.821277	8.762269	9.323027	9.868842	7.534703	9.125313	8.465536	8.904760	7.334280	8.847386	9.041468	7.871660	8.006998	6.352940	7.316850	8.547241	8.385428	9.722628	8.538466	9.891332	9.795507	8.371749	7.944475	8.536970	10.963865	9.833356	9.479371	10.209367	9.770967	10.448409	8.996270	7.733926	10.174432	9.481968	8.058720	10.004264	7.730554	8.457202	8.543687	10.200528	7.694438	7.248533	8.591289	9.030278	7.171477	7.567050	8.755780	6.872583	9.087336	9.994317	8.165664	10.717299	8.218871	7.844214	7.052716	9.195614	8.420558	7.488051	7.823644	8.368201	7.711235	7.113452	9.067242	9.324766	8.912971	10.101264	10.965351	9.562068	9.150322	7.843425	11.102534	8.168459	9.115266	10.152177	8.031633	9.579792

LINC01950	3.218550	3.427251	3.072563	3.049429	2.916669	4.016060	3.371366	3.449554	3.471226	3.441180	3.624828	3.621479	3.384322	4.036922	3.665678	3.180141	4.363298	3.144788	5.013631	3.228505	5.195497	2.839800	4.770546	2.975163	2.987972	2.870014	3.023793	2.856669	3.215034	3.082345	3.747923	3.906634	5.125344	3.093613	4.450747	4.809510	3.742381	3.348380	3.471394	3.170910	3.486956	3.122628	4.444999	3.364197	3.819056	5.983137	3.740380	4.633069	4.589492	3.981517	3.913564	3.788448	4.466525	4.352893	4.072539	3.177467	4.644693	3.085822	3.505862	3.947248	2.879294	3.160321	4.001936	3.298831	3.586706	3.559545	2.882716	3.393310	3.282710	2.823310	3.076229	3.013777	3.227610	2.913447	2.937825	3.395942	3.079741	3.046804	3.070262	3.894646	3.487711	4.168824	4.128508	4.469469	3.519492	4.563408	3.289044	3.329348	3.663078	3.775550	3.378073	3.207511	3.829744	3.273130	3.035084	2.977679	4.445656	3.220637	3.830142	3.036658	3.034402	3.295748	3.067275	2.811599	4.702563	3.220989	3.073342	3.703446	3.053850	3.348550	2.776734	3.472041	2.950272	3.358469	3.392489	3.275122	2.939501	3.111180	3.250569	3.094079	3.595183	3.016034	3.185285	2.982627	4.340694	2.967819	3.249455	3.430072	3.172804	3.410970	3.109934	3.191743	3.288191	3.279422	3.600306	3.561139	2.945840	3.476336	3.422057	4.486379	2.819158	5.249800	2.888899	2.843897	2.804663	3.051640	2.872654	2.896976	3.585898	3.476911	3.467670	3.812109	3.089121	4.104982	3.838432	3.783575	3.955463	3.458213	3.082860	3.298231	2.942255	4.157781	3.442575	4.352225	5.544882	3.917257	2.968264	4.696830	3.934932	3.858341	3.527038	4.282318	4.558305	4.081133	3.097255	3.896283	3.060451	3.026966	3.807841	2.809993	3.296369	4.111173	3.890951	3.939428	3.809721	2.872176	3.758791	3.450064	2.782823	3.059943	3.193243	3.225182	2.963579	2.938438	3.363712	3.121586	3.124671	3.098911	3.295881	3.890731	4.635216	3.917683	3.770137	3.619725	3.335465	3.492579	3.506641	3.285202	3.621281	3.541683	3.046004	3.487910	3.355506	3.163794	2.987590	2.955162	3.319276	4.146429	3.308743	3.032564	3.062992	2.989896	2.895725	5.110165	3.394158	3.045148	3.674530	3.506879	3.218815	2.767014	3.258697	3.350916	3.263834	3.054163	2.989987	2.911101	3.155331	3.398360

PCOTH	7.873982	9.591661	9.609659	9.577618	9.207939	9.444365	9.776906	8.945521	8.285627	9.058147	9.183769	9.614388	9.433582	9.828423	10.337340	8.352610	9.072827	9.860350	9.987767	9.942734	9.458339	9.222961	8.986709	9.147855	8.817738	8.954265	7.606691	7.828982	8.464122	8.645720	9.598956	10.234648	9.587330	7.966965	9.658248	9.780804	9.667801	9.861128	10.148077	9.776425	9.254187	10.313234	9.633839	9.385126	9.683095	10.037937	7.895121	10.171241	9.330440	9.522255	9.833794	10.354444	9.859301	10.559328	10.229572	8.706598	9.885624	10.815666	10.247418	10.400338	10.334644	9.175262	12.253579	10.141980	10.009427	11.025992	10.805904	10.052342	9.921681	10.314129	9.916055	9.958459	8.592526	9.535499	10.195828	9.089471	9.633073	9.341898	9.922889	10.401855	10.300168	10.488419	9.954115	10.628696	10.325523	10.081641	10.245152	10.793359	10.513463	10.624904	10.331758	10.156062	10.126478	9.464698	9.963485	9.956160	9.710793	9.826170	9.185481	9.085969	8.979537	9.165894	9.432358	9.617636	11.075613	9.111274	9.338896	10.285761	10.165812	8.268700	10.629335	9.385879	9.762143	7.630838	8.181446	9.024177	8.948886	9.101829	8.723284	7.989848	8.944584	7.437704	8.036665	8.272606	8.481866	6.876695	8.531075	7.387462	8.159927	8.017690	8.297915	8.454898	7.149940	8.588347	7.576848	7.624575	7.662002	10.039433	8.985620	8.103403	8.061229	9.797342	6.826070	8.469173	8.551058	7.926319	7.810345	8.133176	8.108184	6.666053	8.517165	8.404071	8.851230	8.616628	8.613148	8.177531	9.166801	8.469173	9.380969	7.735196	8.908475	9.551464	7.273416	9.048542	9.419282	7.339085	8.584087	8.804636	8.890833	8.060764	9.085823	9.481353	8.539092	9.310505	8.095084	9.037384	9.381478	8.262447	10.427336	9.639817	9.103656	9.786097	8.423166	9.060362	8.716580	8.990911	8.659450	8.716277	8.789485	8.339021	8.053391	7.264197	8.440099	8.681750	9.172804	9.037850	8.421040	9.520226	8.908475	8.969270	9.261966	9.024321	7.938524	9.028257	8.409992	8.885915	9.964264	9.395428	9.259682	8.386026	9.218401	8.471036	8.457783	9.013468	7.574967	7.729193	7.696834	7.980493	8.562100	7.486025	10.292644	7.880784	8.758828	8.769584	6.611684	9.164484	8.571129	9.112263	6.428351	9.707187	7.540067	9.512448	7.242424	8.048777	7.356705	8.330575	8.460319	9.241954

C1QTNF9-AS1	7.873982	9.591661	9.609659	9.577618	9.207939	9.444365	9.776906	8.945521	8.285627	9.058147	9.183769	9.614388	9.433582	9.828423	10.337340	8.352610	9.072827	9.860350	9.987767	9.942734	9.458339	9.222961	8.986709	9.147855	8.817738	8.954265	7.606691	7.828982	8.464122	8.645720	9.598956	10.234648	9.587330	7.966965	9.658248	9.780804	9.667801	9.861128	10.148077	9.776425	9.254187	10.313234	9.633839	9.385126	9.683095	10.037937	7.895121	10.171241	9.330440	9.522255	9.833794	10.354444	9.859301	10.559328	10.229572	8.706598	9.885624	10.815666	10.247418	10.400338	10.334644	9.175262	12.253579	10.141980	10.009427	11.025992	10.805904	10.052342	9.921681	10.314129	9.916055	9.958459	8.592526	9.535499	10.195828	9.089471	9.633073	9.341898	9.922889	10.401855	10.300168	10.488419	9.954115	10.628696	10.325523	10.081641	10.245152	10.793359	10.513463	10.624904	10.331758	10.156062	10.126478	9.464698	9.963485	9.956160	9.710793	9.826170	9.185481	9.085969	8.979537	9.165894	9.432358	9.617636	11.075613	9.111274	9.338896	10.285761	10.165812	8.268700	10.629335	9.385879	9.762143	7.630838	8.181446	9.024177	8.948886	9.101829	8.723284	7.989848	8.944584	7.437704	8.036665	8.272606	8.481866	6.876695	8.531075	7.387462	8.159927	8.017690	8.297915	8.454898	7.149940	8.588347	7.576848	7.624575	7.662002	10.039433	8.985620	8.103403	8.061229	9.797342	6.826070	8.469173	8.551058	7.926319	7.810345	8.133176	8.108184	6.666053	8.517165	8.404071	8.851230	8.616628	8.613148	8.177531	9.166801	8.469173	9.380969	7.735196	8.908475	9.551464	7.273416	9.048542	9.419282	7.339085	8.584087	8.804636	8.890833	8.060764	9.085823	9.481353	8.539092	9.310505	8.095084	9.037384	9.381478	8.262447	10.427336	9.639817	9.103656	9.786097	8.423166	9.060362	8.716580	8.990911	8.659450	8.716277	8.789485	8.339021	8.053391	7.264197	8.440099	8.681750	9.172804	9.037850	8.421040	9.520226	8.908475	8.969270	9.261966	9.024321	7.938524	9.028257	8.409992	8.885915	9.964264	9.395428	9.259682	8.386026	9.218401	8.471036	8.457783	9.013468	7.574967	7.729193	7.696834	7.980493	8.562100	7.486025	10.292644	7.880784	8.758828	8.769584	6.611684	9.164484	8.571129	9.112263	6.428351	9.707187	7.540067	9.512448	7.242424	8.048777	7.356705	8.330575	8.460319	9.241954

AC006055.1	7.753171	8.292913	8.326529	7.683036	7.745467	7.800683	7.142250	7.640791	7.196301	6.997121	6.871633	6.644025	6.188588	6.800512	6.921772	7.839529	7.783157	6.320684	7.661380	7.681302	8.272399	7.970201	8.064117	7.057617	6.850776	7.225135	7.400060	7.100946	7.156988	8.104486	7.318389	6.664453	6.705266	6.396141	6.918047	7.052414	6.888402	7.470633	7.701304	7.245475	7.782573	7.341605	7.570213	7.881084	7.579023	7.584728	7.156070	8.280543	7.109184	8.073812	7.353902	7.565037	7.102823	6.812241	6.824667	7.824001	6.653833	7.719509	7.281951	7.191384	7.516962	7.619699	8.140611	7.741944	8.435693	7.858176	8.853327	8.427236	8.499478	8.480944	8.248700	8.279636	7.353902	7.958214	7.957304	8.126279	8.336979	7.977373	7.966965	7.296370	7.240071	7.256941	7.204710	7.509424	7.400060	7.533448	6.694724	7.504008	8.164423	8.012742	7.740543	8.325256	7.927895	7.728725	7.210664	7.041908	6.875163	5.402259	5.974655	6.818111	5.976039	7.292562	7.178765	7.340176	7.923498	7.421823	9.394342	7.584267	8.161466	7.728413	8.033453	7.344419	7.554088	7.658461	7.151855	6.715371	7.755724	7.139854	7.282571	7.229212	8.969436	8.292137	7.639084	7.370624	8.815301	6.629353	7.323297	7.687797	7.032657	6.937304	6.705442	6.834227	6.686143	7.305553	7.883075	8.166604	6.289442	7.903197	8.485081	8.394089	7.937295	8.558383	7.539578	7.799143	7.551590	8.210228	7.627673	7.877023	8.899150	7.518351	6.188243	5.994659	7.241803	7.080486	7.047698	6.802210	7.704236	7.846086	7.267588	8.350047	7.704236	8.068023	8.739630	7.921935	7.862916	8.117780	9.032204	7.191688	8.826560	7.985002	7.912039	7.456182	7.849905	6.828463	8.337740	7.196961	7.827291	7.823491	7.510936	7.887508	7.972088	8.592063	8.598516	8.519009	7.734581	9.625053	9.156149	9.139637	9.336472	8.539403	8.675628	7.586750	8.501719	8.051116	8.365337	8.287349	8.642660	8.360473	7.365938	7.289724	8.217923	7.313699	6.767805	7.963592	7.317019	7.532056	7.800367	8.014946	7.894811	7.943560	9.003490	7.856045	7.939314	7.419618	7.132428	6.643709	6.507989	5.814075	7.385465	6.467195	7.712966	6.534841	7.696523	8.052166	7.758989	9.342205	8.864310	8.430406	8.415166	8.070916	7.380361	7.309782	8.022388	7.570962	7.106453	7.766232	7.214668	8.502624

AL020995.1	14.206207	14.150431	13.968272	13.979980	14.388615	13.961354	13.934369	14.188986	14.036962	13.991278	14.204014	14.003265	14.326082	14.397321	14.613963	14.052067	14.149487	14.153617	14.443429	13.680430	14.103780	14.455390	14.208831	13.959663	13.909973	14.298038	13.282417	13.722979	13.953781	13.750540	14.216655	14.640937	13.739872	14.074554	13.910783	14.233886	14.228587	14.412818	13.962461	14.492360	14.036962	14.472714	13.981851	14.198163	14.182169	14.229183	13.662957	14.447515	14.567967	14.375530	14.175520	14.189262	14.425606	14.254782	14.182477	14.329679	14.574718	13.891833	14.117673	14.466224	13.847489	13.971862	13.949893	14.199812	14.293075	14.794023	13.760910	14.672364	14.250802	13.995849	14.096118	13.811679	14.043461	13.868836	14.324402	13.873413	13.995561	14.066903	13.976074	14.378512	14.648021	13.297973	14.328233	14.628138	14.241084	14.011503	14.409528	14.453564	14.571778	14.499877	14.666375	14.346007	14.816832	14.257321	14.062947	14.353744	14.283789	14.149487	14.429732	13.996707	14.344223	13.695872	14.341026	14.258622	15.024650	14.188986	14.699677	14.749262	14.625551	14.172267	14.765753	14.526310	14.686498	14.020995	14.405128	13.388411	14.144912	13.442646	13.829453	14.515458	14.297708	14.562573	14.899624	14.720909	14.368341	14.716740	13.757393	14.636601	14.608380	14.371917	14.440880	14.324726	14.644025	14.691512	14.364816	14.528293	14.631248	14.776105	14.965129	15.091892	15.039969	15.042927	15.203034	14.725162	14.609256	14.817306	14.793559	13.897223	14.290687	14.691512	14.174885	14.729702	14.455390	14.570991	14.080973	14.262592	14.357698	15.132054	14.657265	14.692440	14.417282	14.563436	14.633465	14.258622	14.756942	14.599518	13.779166	13.931123	14.138248	14.427017	14.214354	14.300589	14.275557	14.713029	13.906657	14.519853	15.057918	14.818366	14.541332	14.843666	14.498295	14.677419	15.022274	14.637945	14.188986	15.125176	14.541751	14.389287	14.647121	14.079493	14.742275	14.236540	14.801392	14.386354	14.583170	14.564295	14.541751	14.588954	14.167438	14.039346	15.084722	14.305307	14.218603	15.056095	14.747454	14.924899	14.938834	14.716286	14.938834	14.468143	15.038723	14.523537	14.427855	14.379907	14.034063	14.658149	14.374090	14.284537	14.419519	14.090418	14.469284	13.971570	13.983245	14.777592	14.432746	14.972581	15.265827	14.691512	14.218945	14.683169	14.554790	14.490783	14.394408	14.387085	14.613963	15.085293	15.431836	14.466988

LINC01106	10.618976	10.524000	10.369561	11.042091	11.322916	11.326682	10.729132	12.342322	11.755885	11.177335	11.087512	10.984344	10.364292	10.713151	10.300812	10.980857	11.001508	10.514059	11.598591	10.845627	11.066449	11.384967	11.341014	10.509017	10.901270	11.281969	11.707448	10.804169	11.126352	10.703444	11.589239	9.881488	9.720646	10.271020	10.067664	11.006117	10.752793	10.596344	11.005256	10.882649	11.160511	10.713151	10.865897	10.835128	11.091311	10.879160	11.976069	10.743052	10.162874	10.850468	10.300503	10.476626	10.131679	10.313861	10.792626	11.267660	10.437507	10.356139	10.275187	10.393847	10.461142	10.801353	10.574079	10.954214	10.852403	10.882791	10.328381	10.720621	10.320132	10.976695	10.668143	10.749156	11.355934	10.782833	10.534558	10.141187	10.269288	9.915761	10.155777	10.307130	9.946377	9.927841	9.950580	10.093506	10.396441	10.313708	10.257261	10.003818	10.009271	10.794089	10.590376	10.394473	10.160284	10.732656	10.429717	9.888466	10.227300	11.170321	10.993001	11.386601	11.591314	10.779848	10.898647	10.830197	11.485939	11.114406	11.367432	10.990768	11.481643	11.418410	11.031068	11.471087	10.978627	11.553024	10.875592	13.031373	11.315434	11.062917	11.574797	10.363720	11.287476	10.549196	10.929640	12.533294	11.635258	11.290061	11.018990	11.101499	11.645189	11.263041	11.290061	10.770448	10.948753	11.137722	11.836470	11.123670	12.009735	10.979516	9.466126	10.393847	11.299108	10.855398	11.225775	10.898036	10.731351	11.393088	11.637961	12.146584	11.123986	12.381803	10.209520	10.384715	10.709207	9.891332	10.853314	10.853742	10.484898	10.581306	10.078051	11.766326	10.478878	10.869610	10.125998	10.715522	11.224607	12.299183	9.738519	10.378034	10.144304	10.001946	10.844714	10.261469	9.693891	9.726848	11.329984	9.137632	9.704576	10.298493	9.436629	10.282924	11.697997	10.474973	10.673442	10.621312	9.534899	10.115300	10.860728	10.683631	10.321819	11.247355	10.555376	12.538957	10.777392	10.334012	10.495118	10.666359	10.833152	10.484421	9.730779	9.210231	9.475674	10.480956	9.364412	10.293860	10.782833	9.935758	10.448849	9.721843	9.658740	9.500727	10.197958	9.879782	10.503158	10.786013	10.076365	10.508413	11.851271	10.433295	10.692859	11.529530	10.501072	11.817030	10.262095	9.888161	11.972983	11.039023	10.136690	10.265874	12.276073	10.986046	11.269855	12.050270	12.060100	11.462019	11.363568	10.380759	10.321391	11.769304

LINC01123	10.618976	10.524000	10.369561	11.042091	11.322916	11.326682	10.729132	12.342322	11.755885	11.177335	11.087512	10.984344	10.364292	10.713151	10.300812	10.980857	11.001508	10.514059	11.598591	10.845627	11.066449	11.384967	11.341014	10.509017	10.901270	11.281969	11.707448	10.804169	11.126352	10.703444	11.589239	9.881488	9.720646	10.271020	10.067664	11.006117	10.752793	10.596344	11.005256	10.882649	11.160511	10.713151	10.865897	10.835128	11.091311	10.879160	11.976069	10.743052	10.162874	10.850468	10.300503	10.476626	10.131679	10.313861	10.792626	11.267660	10.437507	10.356139	10.275187	10.393847	10.461142	10.801353	10.574079	10.954214	10.852403	10.882791	10.328381	10.720621	10.320132	10.976695	10.668143	10.749156	11.355934	10.782833	10.534558	10.141187	10.269288	9.915761	10.155777	10.307130	9.946377	9.927841	9.950580	10.093506	10.396441	10.313708	10.257261	10.003818	10.009271	10.794089	10.590376	10.394473	10.160284	10.732656	10.429717	9.888466	10.227300	11.170321	10.993001	11.386601	11.591314	10.779848	10.898647	10.830197	11.485939	11.114406	11.367432	10.990768	11.481643	11.418410	11.031068	11.471087	10.978627	11.553024	10.875592	13.031373	11.315434	11.062917	11.574797	10.363720	11.287476	10.549196	10.929640	12.533294	11.635258	11.290061	11.018990	11.101499	11.645189	11.263041	11.290061	10.770448	10.948753	11.137722	11.836470	11.123670	12.009735	10.979516	9.466126	10.393847	11.299108	10.855398	11.225775	10.898036	10.731351	11.393088	11.637961	12.146584	11.123986	12.381803	10.209520	10.384715	10.709207	9.891332	10.853314	10.853742	10.484898	10.581306	10.078051	11.766326	10.478878	10.869610	10.125998	10.715522	11.224607	12.299183	9.738519	10.378034	10.144304	10.001946	10.844714	10.261469	9.693891	9.726848	11.329984	9.137632	9.704576	10.298493	9.436629	10.282924	11.697997	10.474973	10.673442	10.621312	9.534899	10.115300	10.860728	10.683631	10.321819	11.247355	10.555376	12.538957	10.777392	10.334012	10.495118	10.666359	10.833152	10.484421	9.730779	9.210231	9.475674	10.480956	9.364412	10.293860	10.782833	9.935758	10.448849	9.721843	9.658740	9.500727	10.197958	9.879782	10.503158	10.786013	10.076365	10.508413	11.851271	10.433295	10.692859	11.529530	10.501072	11.817030	10.262095	9.888161	11.972983	11.039023	10.136690	10.265874	12.276073	10.986046	11.269855	12.050270	12.060100	11.462019	11.363568	10.380759	10.321391	11.769304

BX664727.3	9.173578	10.836911	11.054223	11.130181	12.535440	11.896350	11.561422	13.033913	13.955201	12.996396	12.368898	12.160962	11.753524	11.567694	10.945347	12.971662	12.585159	10.923426	11.717496	12.279737	10.998620	10.852403	10.157573	12.138659	10.759553	12.432090	10.813574	12.266835	12.490737	10.303411	12.681634	10.819395	10.954078	10.124282	11.551046	11.883726	11.620096	9.176047	10.171241	11.290218	12.434341	10.209812	11.250318	11.905620	11.695921	11.308691	11.613018	11.003940	12.182961	11.525035	11.455375	11.817939	11.128662	11.904904	11.731240	12.196619	11.410252	10.544617	9.557406	10.468887	10.564716	11.125340	8.853185	11.105005	9.250394	10.471930	10.197958	10.993595	10.094258	10.157733	11.531829	12.022296	12.491075	11.101922	11.509057	9.877034	11.044169	10.416749	11.235506	9.975129	12.373862	11.289319	10.815666	11.376800	11.439584	12.225678	12.604080	12.209897	9.594314	11.155939	10.291426	10.147175	10.419351	11.542870	10.327627	12.789228	12.417049	13.092572	12.526615	12.051938	12.553621	11.619947	11.278240	11.167246	10.413149	12.833655	11.686785	11.529216	11.000137	12.215771	10.316937	12.330024	12.922237	12.844209	12.933307	12.869060	8.190710	12.110108	13.420820	9.093884	8.120535	8.445159	8.243691	9.551619	9.810561	9.204423	9.711248	8.856221	9.677525	8.594547	9.854297	9.087029	8.797467	7.448405	11.076058	7.778353	9.503777	7.943875	10.067207	9.895500	8.550751	8.682812	9.695858	8.239020	8.208290	10.186500	11.040311	12.002645	8.221296	10.582745	9.282148	9.917791	8.902489	10.089305	11.293530	8.827765	6.764246	8.192439	8.060586	9.474159	7.976250	9.795202	9.022711	9.162455	9.554332	8.918918	9.122293	10.606661	7.765302	8.448899	9.931531	9.285198	8.354912	9.700957	9.560158	9.288951	9.080413	7.691187	9.262911	11.033754	9.077494	9.994150	8.066831	7.271105	7.828542	6.280526	8.105747	7.894196	7.120438	9.801645	9.428521	10.880771	8.424386	10.100294	8.666821	8.861204	8.175218	9.728056	8.249737	8.608629	10.025761	8.914940	9.213755	9.715628	9.994317	9.073438	9.432961	8.659306	8.365652	9.725679	8.513849	7.668657	8.461405	8.344925	9.155870	9.711248	10.725657	13.813249	8.839951	9.491898	8.279940	9.796717	8.703980	11.094860	9.400283	7.747337	9.092054	9.467673	8.324297	8.671249	10.955290	11.115623	12.050270	11.796425	8.718419	7.584728	8.899620	9.730779

NPSR1-AS1	3.556216	3.274494	3.503802	4.014711	3.700152	5.872814	3.982847	3.873072	4.819801	3.627611	3.808458	4.313187	4.628254	4.393599	5.086979	3.587283	3.847741	4.077019	6.085431	3.801192	5.576123	4.288341	5.359863	3.700948	4.487427	3.865767	4.295680	3.719948	4.160365	6.554476	3.792266	4.502005	5.294248	5.813523	4.989560	4.700068	4.963752	3.476529	3.447475	3.955721	3.492401	3.227610	4.293542	3.407300	3.419492	5.479452	5.229742	5.210833	3.529405	3.871796	4.113121	4.642400	4.298529	4.679217	3.937514	5.137208	4.155826	3.324164	5.776731	6.038597	4.018026	4.541985	4.078077	3.985401	6.257931	4.135395	3.512749	4.019768	4.179448	3.948370	4.152302	4.405609	3.855740	4.065635	5.336960	3.520441	4.530956	4.792890	3.890045	3.825965	4.155180	4.520187	3.338945	4.706826	3.939428	4.663074	5.228497	4.262655	4.646834	3.508342	3.531510	4.407819	3.368431	4.388470	4.059534	3.998522	4.032121	3.705674	5.100098	3.847973	4.447569	3.518201	3.618011	3.193913	4.263974	3.955463	3.212131	4.005674	4.663487	3.967228	4.457358	4.892077	3.714025	3.936663	3.959671	6.123379	4.457149	4.063276	3.880019	5.795443	4.789935	6.895893	6.889972	5.859422	5.812038	5.817622	5.831051	9.188910	6.687309	6.542716	8.196922	6.293010	5.245026	5.777617	7.151398	6.009651	7.930519	7.296877	5.993753	5.285608	9.180624	6.245120	7.225295	6.456542	3.775945	5.822138	6.088150	6.523680	5.556755	4.214696	4.631763	6.403904	5.234034	4.671238	7.106932	5.543524	5.785779	5.268667	5.788863	5.401255	5.822222	4.636718	4.537806	7.814612	5.597418	5.728468	5.551433	6.260150	6.623246	5.958405	5.391260	6.571622	9.225274	6.002372	5.813148	4.890642	7.111770	7.218267	10.491412	7.059799	4.622478	6.343349	5.432797	7.728413	4.248611	7.025864	4.986843	4.670370	3.069717	7.768086	8.723880	6.284143	5.970859	6.223463	4.782194	7.295374	6.993859	5.242458	4.327221	7.376122	4.711800	4.791016	5.168280	6.199995	5.991118	7.447919	5.784669	6.046222	6.417161	3.807402	4.895390	6.673180	5.161670	6.107820	5.079007	5.714561	6.265036	5.846583	5.973787	6.750842	6.426308	6.042781	4.895003	5.182603	4.866199	7.365785	5.450652	7.291452	5.490755	8.694675	6.567413	6.034026	5.208422	6.589006	5.477301	6.668343	7.263889	5.631552

LERFS	9.750360	3.339628	3.094537	8.243837	4.077444	4.536303	8.852855	3.343813	6.074503	8.511392	7.463593	3.626008	3.413645	8.028251	8.427236	9.117571	3.615151	8.398367	9.002906	8.568651	10.053117	3.986286	8.682047	4.151044	7.649794	7.993626	6.852662	6.099247	6.862022	3.474700	7.341449	8.659902	8.643164	3.380330	8.134382	4.074067	3.692869	8.712812	3.649813	8.353066	3.368081	8.330735	7.719199	4.012899	9.365510	5.324768	4.322275	6.688548	4.353548	9.016697	8.879890	8.700428	9.536303	9.614532	4.053879	3.958117	8.781813	9.285515	8.287349	4.603540	9.113742	3.907084	9.067242	9.361876	9.749575	9.645339	9.190703	9.666076	3.764496	4.744249	3.398011	3.798589	7.976790	8.145020	8.235265	7.931883	8.063661	8.690660	7.781047	9.895040	9.093285	4.443695	3.207511	8.859114	5.443704	8.579659	6.615435	4.932450	9.782528	4.525882	4.963118	4.876960	5.386761	8.191798	8.231014	7.690085	4.364402	8.255295	4.643182	7.195840	5.682879	3.329348	3.346247	4.781987	9.463760	3.414881	7.851095	9.698167	8.650567	4.077226	5.712111	4.479749	8.481265	7.128302	3.349518	8.813057	3.823300	8.083021	3.214341	9.094797	3.442575	3.425827	10.185134	5.077771	3.437038	9.322286	3.256210	8.509059	10.702740	4.780099	3.182955	5.013631	5.570880	3.678826	7.308960	3.588064	9.062181	5.188216	9.230147	9.867109	6.061172	6.837217	4.137797	9.503469	8.444854	8.372533	5.605914	8.830706	3.562676	8.087867	6.090612	9.003636	3.722103	8.774754	3.770710	3.777942	8.670629	3.719536	6.390022	3.162277	9.901638	5.522886	4.837655	10.558107	4.790155	4.073634	5.063876	4.877783	9.052200	8.270421	6.392190	9.859596	9.865593	5.466948	3.554282	6.641748	10.181943	7.010571	4.136501	6.469350	3.982184	9.169228	11.230646	9.055814	9.117878	10.680518	10.823846	3.355877	3.105510	4.014461	4.035158	3.493366	9.684838	8.671094	6.898683	6.126226	9.873647	8.822507	10.310359	6.425281	4.255446	4.140390	8.329650	4.865575	5.622781	3.595009	4.072108	11.206436	3.649639	3.670112	5.388727	3.814191	6.251304	5.426219	5.611055	3.584146	8.207548	4.132328	3.946856	4.510054	3.197164	4.484599	3.512937	6.352110	3.032456	9.754059	10.098728	7.175090	3.805753	3.696307	5.915666	5.035911	7.064300	4.146648	8.582268	3.383293	4.429329	3.178423

LINC00092	6.898200	6.647718	6.588358	6.592806	6.056548	6.190615	6.826397	6.949641	6.057724	6.348100	6.348278	5.751933	5.807043	5.934947	6.590464	6.416842	6.520020	6.356611	5.369136	5.970859	5.894247	6.283455	4.705737	6.181161	6.272537	6.332486	6.186409	7.005812	6.453805	6.408030	6.253010	6.537941	6.533076	6.020084	6.925659	6.705103	6.659189	6.744515	6.742107	6.936985	6.479464	7.157778	6.895893	6.851397	6.806316	6.074503	6.343705	6.708631	5.706630	6.559586	6.912442	6.699040	7.222846	6.796677	5.960936	6.655496	6.492830	6.929221	6.012299	7.039382	7.130688	6.491222	6.807739	5.815816	6.612852	6.733213	6.664782	6.356259	6.390022	7.020611	6.565927	6.159810	6.805681	6.865024	6.477635	6.290632	6.545320	6.322896	6.534213	6.179165	6.155339	6.115466	6.490919	6.642700	6.190615	6.379122	5.007457	7.024922	6.786940	5.951633	6.185718	6.084585	6.267597	6.444714	6.534841	6.157372	6.270865	5.624670	6.399974	6.175587	5.231998	6.539864	6.689163	6.714396	5.523672	6.362694	6.228480	5.566934	6.369198	6.876846	6.163918	6.322380	6.079720	6.489829	6.017258	4.471226	6.482234	6.414516	6.028219	6.127483	6.507182	6.125492	6.447349	6.044651	6.969819	6.395163	6.466680	6.178147	6.841126	6.482234	6.655000	5.997249	5.687539	6.787106	5.645429	6.777676	6.695877	6.317412	6.994160	5.757033	6.318925	7.214504	6.271705	6.315037	7.136255	5.896338	7.405852	6.561036	6.886288	6.360044	4.955831	5.972154	6.468191	5.946702	7.211509	5.708658	6.337546	6.624833	6.338721	7.018100	7.770177	5.319944	6.479137	6.476576	6.307087	7.154973	6.255368	6.565283	6.055495	5.815094	6.182378	6.899823	5.256728	4.897460	6.458853	5.415123	5.750467	5.091237	5.686415	5.505652	6.565762	5.290178	6.076077	6.429547	6.058347	6.607556	5.819727	6.453116	6.593255	6.319795	5.940611	7.404121	6.459041	5.847710	5.996352	6.333155	6.410859	6.380453	4.717095	4.843594	4.976495	5.089598	4.645564	4.526954	6.469024	4.665404	6.340724	5.554151	5.320535	5.607789	5.764306	5.893148	6.609828	6.070155	5.946702	5.657752	5.307133	6.315570	7.298780	6.099807	6.190268	6.539709	5.761020	5.642899	7.055864	7.054921	5.790077	6.306611	7.158083	6.334005	5.856542	5.601886	6.576865	7.410189	5.510295	6.261164	6.212161	7.030651

AL136171.2	3.384830	3.386361	3.231044	3.279903	3.968092	5.613287	4.545552	5.199473	4.262444	4.308141	4.205714	4.061734	4.057171	3.996401	3.526294	3.707302	3.749998	3.374715	4.724520	3.232021	5.089598	4.486379	3.546768	3.592419	3.888276	3.290281	4.176392	3.831179	4.063276	4.808685	3.544508	3.746872	5.355303	3.807841	4.383833	4.084323	3.686870	3.307167	3.190794	2.918581	4.014461	3.367536	3.723306	3.344470	3.642453	5.550523	4.978711	5.007457	4.553674	4.095615	4.087871	4.561637	4.461781	4.090941	3.953753	4.526742	4.047183	3.124425	3.592419	3.369613	3.085714	4.125253	3.537834	3.180672	3.380664	3.251818	2.862495	3.333065	3.062481	2.998077	3.437732	4.186427	4.420876	3.514792	3.561567	3.499422	4.044345	3.214341	3.412024	3.779584	3.438239	4.127424	3.448178	4.145791	3.425827	3.777553	3.843155	3.215814	3.051842	3.496154	3.219377	2.947036	3.098676	3.801192	4.006530	3.540716	4.395671	3.521682	4.962098	4.401806	5.238860	3.750428	3.573829	3.236383	4.450065	4.219685	3.406067	3.614957	3.337955	5.399012	4.048881	3.427979	3.198532	4.573522	4.401806	8.251602	3.204421	4.067954	4.263555	3.207928	3.497287	3.227317	3.301128	5.016059	5.018503	5.555822	3.122871	3.473978	4.338539	3.491669	5.191503	5.373424	4.256960	4.776772	4.954364	3.704057	4.394058	3.486428	3.386886	4.336791	3.501795	3.224898	4.744878	3.828244	3.949881	5.255945	4.668831	5.809630	3.833118	3.415696	3.662430	4.572849	5.265055	4.427225	3.786037	3.772413	3.409194	3.500163	2.883284	4.795599	3.038434	3.573059	3.394704	4.045188	5.022964	5.407929	3.414881	4.011621	4.294797	4.166251	3.799232	4.144088	4.167115	3.476161	4.515287	3.600306	3.000927	3.138221	3.082124	2.930244	4.850771	3.553350	3.637058	3.585521	3.316355	2.837759	3.468909	3.056118	2.960877	3.291272	3.520038	4.506555	3.349518	3.668862	3.304280	3.384830	3.700762	3.122269	3.261430	3.805753	4.489115	3.470874	3.617257	3.441518	5.133823	3.258697	3.381903	2.957425	4.971489	3.345907	2.900365	3.017403	3.527760	3.254990	3.269121	4.277307	5.705436	3.865968	3.236242	4.564275	4.057171	3.749198	4.137797	4.844870	4.141298	3.458213	3.412420	3.677408	5.820260	3.978030	3.897372	4.546452	5.449926	5.490389	6.871129	3.200632	4.145985	4.438286

AC008574.1	10.542996	10.181790	9.735002	9.966351	10.184062	10.046174	8.753789	10.828144	11.130492	9.518073	9.392525	8.978787	9.354834	9.261505	8.962224	10.190858	10.209812	9.787767	10.982768	9.839233	10.387334	11.195313	10.736862	9.823468	9.724077	9.832913	11.082627	9.941976	9.815890	10.689640	10.436289	8.901108	9.242420	9.740576	9.307738	9.050083	9.122460	9.833053	9.998570	9.850414	9.813834	9.919633	10.015553	10.223615	9.418537	10.033181	10.085546	10.851641	9.333538	9.533987	9.698474	9.137949	8.602194	9.104114	8.875559	10.077903	8.325568	9.040712	9.332303	8.872320	9.194647	9.498949	10.260570	10.488263	10.029650	10.396283	10.062277	9.994464	10.025761	10.368958	10.167524	9.967725	9.677215	9.596328	9.969420	10.471332	9.804849	9.977682	9.858496	8.940204	9.330289	9.337427	9.151110	8.829658	8.833772	9.108128	8.822202	9.102439	9.997209	9.519313	9.418692	10.121247	10.023858	9.682776	9.252538	9.235458	9.021885	9.262432	9.706564	10.121388	10.028112	9.470780	9.038835	9.510547	10.653668	9.204268	10.818334	10.533504	11.203593	9.958620	10.604741	10.128997	10.129533	10.421638	9.570876	11.270728	9.915905	9.276233	9.785196	9.682352	10.307427	10.670015	11.009167	11.582266	10.716117	9.550699	10.334339	10.739298	9.873790	9.885942	9.573835	10.676888	9.925456	10.087156	10.783312	10.385324	10.232649	11.070769	9.681259	10.863810	11.423308	11.257836	10.433508	10.183168	9.573835	10.518778	10.312584	11.116961	11.481940	11.186151	10.780734	10.010929	9.354397	10.298660	9.909395	10.204454	10.252493	10.967556	10.538071	10.226672	11.781504	10.834204	10.782076	10.409829	11.156846	10.534263	12.140193	10.046320	10.213953	10.731932	11.111876	9.052352	9.705031	8.993942	9.832913	8.621889	9.464857	9.965977	9.686383	9.825091	9.655511	10.900269	10.713006	10.650447	10.246644	11.036851	11.116371	10.370938	11.353895	10.784818	10.486856	10.658893	10.194758	11.083497	11.091172	10.653379	10.005200	10.261790	9.784276	9.485652	9.614684	9.721549	9.250237	9.344383	10.209520	9.249208	8.925514	10.221376	10.143238	10.090224	10.554140	10.511993	10.478589	10.221951	10.327627	10.039288	10.325356	9.987620	9.961955	10.260405	10.227300	9.835818	10.574965	10.572294	11.000306	11.201014	10.221095	11.005405	11.252514	11.148096	10.988852	10.665460	11.335983	11.155348	9.618393	10.116680	9.623840	10.271935

AC005726.4	10.589350	11.782082	11.757091	11.356381	11.826672	11.990038	11.648129	12.617631	11.939377	11.389835	12.579663	11.867283	12.428494	12.295263	11.706258	11.965674	12.697518	12.517931	12.874685	12.708077	12.092757	13.077813	12.372433	12.002488	12.350849	11.790568	9.669719	12.147801	10.393702	11.474379	11.464200	12.253114	12.114935	10.900542	11.581815	12.033231	12.379442	12.179561	12.270412	12.275467	11.623310	12.452958	11.539526	12.292501	12.177847	12.201709	11.767815	12.679736	12.937994	12.182211	11.735820	12.490240	11.953784	12.316396	12.650373	11.708808	12.389993	13.218179	12.214256	12.580335	12.713119	11.078978	12.142942	12.684724	12.415458	12.571685	12.629517	12.070991	11.924304	12.364975	11.883578	11.704169	12.262009	11.539976	11.742441	11.555942	11.696238	11.201301	11.445502	12.454709	11.890208	11.962317	11.603009	12.482960	12.420264	11.822772	12.490065	12.418490	12.454528	12.833456	12.384801	13.009941	11.665969	11.499627	12.092757	11.321148	12.430939	12.297109	11.998857	12.080298	12.107738	11.628487	11.887800	12.285680	12.132968	11.878918	12.951673	12.617792	11.826672	12.149134	13.037619	12.764987	12.950935	12.064994	12.476380	10.867436	11.381146	11.470178	10.774471	10.914011	12.319676	12.683833	10.133309	10.723571	11.054223	11.796904	9.106595	12.759418	12.240396	11.112153	10.692703	12.153299	9.619319	11.665355	10.315490	12.002055	10.520492	11.345621	12.629517	10.948465	12.297752	12.338888	12.794859	11.830627	12.912699	11.687068	12.371021	10.495424	11.781504	9.404364	12.155557	12.894031	12.428658	12.665988	12.687096	11.791025	11.183899	12.526938	12.724991	12.226768	12.680777	10.227300	12.475688	12.711789	12.523632	12.379442	11.963218	12.313176	12.728294	12.422163	12.557506	11.990637	13.031373	11.646207	11.672977	12.560728	12.088271	13.019562	12.021503	13.238812	11.062917	12.336677	12.556219	12.279139	12.462402	11.843396	11.797346	12.884726	12.428983	12.415936	11.416927	12.058410	11.848060	11.292947	11.585156	12.386932	12.288785	11.647837	11.745962	11.996132	11.838717	12.548924	10.989151	10.315931	12.035653	12.397858	11.990798	10.896417	11.939089	13.705084	13.569877	12.002055	11.556248	10.356294	12.046024	12.854406	10.635922	10.325194	11.435658	10.999538	12.149284	11.130181	12.196619	9.451098	9.950260	13.325811	11.470648	12.567417	11.701300	12.791154	10.656244	9.614756	11.352321	11.313927	12.633633	13.073797	12.034566	11.241786

GHRLOS	9.397984	5.822404	6.090277	7.273894	6.485292	6.355577	6.381820	6.238082	6.234964	6.110433	6.899004	6.724009	6.435961	6.795407	6.277338	6.650152	5.598001	7.083939	6.672354	6.389527	6.405228	6.848224	6.864858	6.909393	7.130688	6.536782	7.637243	6.827174	7.316049	6.056359	7.180716	6.048392	6.276382	6.536130	6.221429	5.903669	5.886902	6.063646	6.246949	5.979394	7.060771	6.142913	6.215090	6.081078	6.064005	6.096022	6.616082	3.888075	5.490933	5.754089	6.353769	6.229889	6.808398	6.561513	5.900068	6.624509	5.943556	5.241641	6.480893	6.212338	6.194433	6.608020	6.012629	7.032329	5.982024	5.564109	5.783606	6.018321	5.980847	5.683252	5.842413	5.727009	7.031249	6.607883	5.875848	6.336194	5.858872	5.555822	6.254379	6.122524	8.019965	6.016047	7.136406	5.907032	6.302728	6.069808	6.243786	5.677521	5.565029	6.290302	6.228136	6.353937	5.696389	5.957324	7.079890	6.759131	5.714005	6.972755	6.673024	6.102725	5.777077	5.971594	6.382496	6.235139	6.464205	6.889972	5.621097	6.412890	6.548531	6.831799	6.150883	6.064179	6.292519	7.469723	5.893525	3.896934	9.788660	6.596333	7.058867	6.462676	5.579462	5.435917	6.770091	5.393558	6.401610	5.931759	7.379401	6.351948	6.349945	6.363905	6.159451	5.350251	6.049762	5.188014	6.255368	5.544106	6.905419	7.701741	6.256249	5.556954	7.617692	7.040326	6.895117	5.834287	5.871520	5.741365	6.551153	6.316233	5.526086	6.999064	6.945652	7.494731	6.150337	5.821960	5.713648	5.838236	6.371595	6.314526	6.643374	6.360207	6.677201	5.884422	6.749727	5.071631	5.840777	6.413860	3.975082	6.839336	5.611389	6.089748	6.126045	6.510765	6.119227	6.071333	6.017610	7.178449	5.395965	6.117854	5.599491	5.740064	6.334855	6.458037	6.490004	6.395163	5.493183	6.182027	6.059900	5.938973	6.164593	5.683252	6.092872	7.258729	6.745292	5.873704	6.170311	5.473470	5.318061	5.984182	5.821680	6.362867	6.622911	5.912413	5.739896	6.123548	6.353603	5.572745	5.764109	6.302543	6.280526	7.250881	6.328750	6.035624	6.942471	6.297553	6.583759	6.919027	6.400990	5.949005	5.938282	5.778374	5.834461	5.773122	6.209157	6.197075	7.770020	6.609956	6.491222	7.031545	6.088150	5.897057	5.254737	4.361109	6.776373	5.498722	4.960214	6.305257	5.723646	6.321882

LINC02243	4.309062	6.048901	6.762969	5.906325	6.218553	6.397288	5.388534	7.001784	7.566262	6.436296	6.184495	5.429928	5.723098	5.483684	6.160498	6.054089	7.114731	6.245934	7.511087	6.359358	6.169288	6.684412	3.875870	6.472916	6.528989	6.237788	4.576446	6.318752	4.836596	4.214482	6.698079	4.032337	6.074503	3.251420	6.324566	5.894419	6.484623	6.723538	6.651763	6.514869	6.148979	6.240938	5.887268	6.172693	6.260150	5.346143	5.031133	6.537617	6.528481	6.672528	5.379656	6.743511	6.373651	6.147950	6.900141	4.779447	6.340056	6.530462	6.075352	6.813820	6.386807	6.105329	5.069592	6.681424	6.099807	6.936660	6.480249	6.711340	6.336858	6.538740	6.151044	6.522018	6.376474	6.349761	6.184157	5.868297	6.520879	5.963776	6.448680	5.672945	7.258388	6.878619	5.843332	6.391724	6.798652	5.991295	6.234645	7.314813	6.391555	7.166846	6.785647	6.473421	6.023065	6.184684	4.780536	6.690947	6.457727	6.635639	5.576123	6.017966	4.526322	6.043642	6.042781	6.710064	6.080235	6.111319	7.080815	6.121473	6.013848	6.208993	6.490783	7.383782	6.455739	6.611844	6.282611	3.096798	5.885856	6.720151	6.296759	5.347143	5.968636	5.549584	5.267883	4.545772	5.770865	4.031890	5.807949	6.587705	4.988511	4.527996	4.451413	3.398852	3.362862	5.109773	4.801966	6.493772	4.361722	5.116611	5.294069	5.450109	4.175304	3.423141	4.880948	4.168403	4.616876	4.399847	4.526520	4.155600	5.303048	6.283637	4.649826	4.625919	4.653285	5.638458	5.723468	5.421112	5.861398	5.749031	6.031825	3.397663	6.198588	4.826069	4.833536	5.972524	5.882835	5.065915	5.851124	5.223904	5.736968	5.844745	5.331054	5.540710	4.364402	4.599394	3.879377	4.510512	5.637531	5.064478	5.867417	5.879233	5.047486	5.924711	6.413054	6.789577	5.792044	6.088675	6.120116	5.712490	6.439408	5.854373	6.132918	5.101551	5.211443	5.012811	5.853816	5.484613	5.844914	6.230595	4.523234	6.021706	6.448008	5.735202	4.602105	5.474270	4.180324	5.756267	6.190442	6.502026	6.370564	5.714561	6.776202	4.845739	5.195317	5.453409	4.804508	4.738203	5.140454	3.792920	4.319054	4.309458	4.101929	4.573305	3.782305	3.341985	4.863937	6.527668	5.150938	5.625960	5.547320	5.278130	5.467740	4.128277	4.787036	4.220843	3.443447	6.584391	5.191120	4.826975

LINC01278	10.817456	10.480071	10.489348	10.408918	10.396893	9.728699	10.881780	9.721549	10.566794	10.165490	10.300812	10.715522	10.534405	10.726741	10.866362	10.163693	11.096025	10.905411	10.177481	10.319358	10.071781	9.158634	11.963218	10.501981	11.452975	10.983350	10.777392	10.538660	10.514372	10.111224	10.621312	10.791003	10.642391	10.797454	10.908849	10.980402	10.778555	10.769094	11.493752	11.226360	9.974195	11.664926	11.260318	11.077064	11.757848	10.965807	10.635922	11.116825	10.659929	11.075744	10.975234	11.265315	11.087821	10.960028	11.124128	10.549645	11.157753	11.161418	11.186293	11.553090	11.457713	10.167210	12.237422	11.144712	11.499480	11.660429	10.674816	10.998487	10.612294	10.850333	10.258347	10.218780	10.177785	10.459101	10.253644	10.405472	10.252635	10.573479	10.209812	11.001214	9.736555	10.943897	10.754173	10.896417	11.046663	10.714317	10.597077	11.028057	10.804745	11.457713	11.500069	10.667558	10.800421	10.481176	10.415709	10.821164	10.878106	11.031948	10.390190	11.410967	10.624589	10.790693	10.580695	10.804745	10.109280	10.408136	11.201301	10.600754	10.432310	10.177785	10.284105	11.327340	10.603402	9.578695	9.922132	9.485652	11.779993	10.871301	10.298660	10.317106	10.555685	9.536888	10.627617	9.406930	10.073916	9.537676	11.116514	9.290819	10.683631	10.620713	8.024072	10.820861	8.953170	10.569984	11.780465	10.668998	10.580549	10.229100	11.016496	9.333694	9.735326	10.404707	7.854212	10.893606	11.164122	9.549146	10.301432	9.038186	10.191151	11.567979	10.454651	11.052063	10.890897	10.559019	11.058092	10.663570	10.674034	11.651828	10.586252	11.396102	11.017653	10.929328	11.263347	9.567790	9.622911	8.630532	11.227093	10.864843	10.678664	10.964294	11.331890	9.776425	9.516374	10.675990	11.004977	11.491172	10.985390	11.433163	11.357821	11.388220	11.664771	11.266027	10.670427	11.879226	11.650203	8.951490	9.889523	10.201399	11.960524	9.670057	10.818042	9.708142	9.871223	10.262977	9.974980	10.095773	9.252371	10.070060	10.995069	10.395201	11.700848	10.544790	10.056794	11.525035	10.550537	10.843022	11.344308	10.795127	11.584232	11.376076	10.628861	10.782533	10.369707	10.887784	10.633979	9.994941	10.838131	11.221623	11.473964	10.801939	10.750993	10.992855	10.315931	11.544838	10.266295	9.857414	8.495213	10.182411	8.074558	9.684672	11.080441	11.352461	9.892557	9.976047	9.496425	11.420720	10.519634	10.106438

LINC02245	4.483726	5.597008	3.997680	4.538643	4.853109	3.911422	4.574373	4.349640	3.438239	4.382536	4.091630	4.479330	5.337511	3.875870	4.237224	5.038717	3.783976	3.531729	4.867902	3.646035	5.158305	5.466171	3.937709	5.790613	4.647039	4.332057	5.830666	3.537632	5.163875	5.249800	3.951398	5.545822	5.594741	6.049248	4.957731	4.119034	3.690559	4.007805	3.704471	4.355705	4.196239	5.822053	6.979258	4.830197	4.292225	5.841877	5.171712	5.377137	4.703636	3.975082	4.156309	4.454382	4.571814	4.340039	4.375003	4.667563	4.217298	2.877038	3.044660	4.133199	4.186897	5.342348	3.928045	3.254014	4.358517	4.536303	4.954364	3.512937	4.839385	3.686432	5.802945	4.279704	4.452895	4.724322	5.068986	5.438573	4.066688	4.360482	4.464171	4.540205	5.381153	4.148755	4.789315	4.323583	3.433246	4.616876	4.676909	3.361618	3.051640	3.603420	6.446215	3.602466	3.844785	3.801408	5.696745	5.056559	5.257705	5.354706	5.673307	5.155607	4.524788	5.021566	4.276612	4.416417	4.290904	5.265872	4.281251	3.538985	4.746858	4.479330	4.225750	3.946420	5.076507	3.757509	5.059416	5.966279	4.679862	4.251929	3.917683	5.224673	5.442744	4.961870	5.385942	6.633699	5.005994	5.131805	5.175953	4.711163	5.687731	5.450296	5.178157	6.264678	4.380980	5.934048	5.387168	4.756117	5.300034	5.399411	4.630254	4.940338	5.095208	4.866199	6.211381	5.237665	4.023218	5.380613	5.594741	5.285608	4.916860	4.710123	6.527507	6.117492	5.857773	5.238461	5.181563	5.139865	6.795557	5.077670	5.552381	5.496608	6.029427	7.358858	5.448214	4.585851	5.354909	5.704288	5.135234	5.453218	5.122315	5.419794	5.690061	5.132194	4.580573	5.272267	5.583220	4.937295	5.803658	5.905278	5.533876	5.221489	4.922760	5.452453	4.969692	5.405352	5.150726	6.069272	5.621849	5.153573	6.399129	6.229015	5.280349	6.005381	5.151735	5.584380	5.371883	4.636503	4.965626	4.954166	4.388072	5.272267	5.493530	5.013840	5.534481	5.072591	6.239250	5.574617	4.968621	4.954780	4.347616	5.116800	4.605847	5.280157	5.829507	6.143590	5.974996	6.453116	6.258431	5.724934	5.607219	5.518786	5.976903	5.119499	5.651254	4.693307	5.431227	5.074630	5.398042	5.308659	4.912630	5.425023	5.359108	5.712111	4.485251	6.754571	5.882650	4.249268	5.953274	3.966797

LINC00608	9.252081	10.246494	10.523247	9.622602	10.956204	10.484898	9.858186	10.814320	10.354297	9.924291	9.809526	9.616513	9.781478	9.322724	8.991352	9.581160	9.805798	8.847553	8.986399	9.547000	9.257270	9.083496	8.843711	9.575176	9.484419	9.951526	10.022295	9.557862	9.972959	10.670884	9.992041	9.089780	9.304406	9.755312	10.004887	9.805157	9.820421	10.890718	9.936352	9.757807	10.010929	8.995183	9.771907	9.947336	9.266732	9.237796	10.631587	9.739183	10.301273	9.636846	9.428985	9.143652	9.924583	10.040378	9.800112	10.682725	9.076866	9.535194	9.223414	9.169228	9.314738	10.242602	9.655361	9.456470	9.954745	9.984426	10.289000	10.424856	9.979650	9.571204	10.159076	10.346735	9.918554	10.177187	9.972017	10.188159	10.224681	10.152036	9.791422	8.796854	8.917391	8.676820	8.501395	8.799150	9.412451	8.741333	9.081198	8.991632	8.761378	8.856845	8.346622	10.241662	10.546889	9.715949	9.891644	8.914189	9.239370	9.087482	10.993300	9.943787	11.156096	10.196135	9.593696	9.460752	8.992256	9.627839	9.307880	9.632756	10.271242	10.461142	9.465521	10.657698	10.016027	10.740809	10.801939	14.495949	9.925044	9.664693	9.850250	9.404833	10.938338	10.902031	9.855744	13.113355	10.115901	10.690535	9.827521	10.188159	9.981684	10.211375	10.314277	10.375288	9.917330	10.408443	9.548217	9.535124	10.014459	9.258639	9.434169	9.482562	9.407561	9.504879	10.026399	10.191897	9.393123	11.147376	10.516678	11.185699	10.670732	9.984109	9.952432	10.963237	10.862460	10.155465	9.841670	10.019869	11.280333	10.324445	9.854012	10.786621	9.771605	9.440330	11.156400	9.755913	9.724995	11.333064	10.190552	9.764347	10.170585	10.018168	9.446549	9.968648	10.778128	9.717806	9.827819	9.451258	10.185134	10.262244	9.423296	10.070706	10.409829	9.932019	9.906813	10.241972	10.552052	11.576321	10.767583	10.500010	9.757177	10.522062	11.028357	11.500204	10.381054	10.060910	10.388365	10.635345	10.547653	10.265297	9.199838	9.018370	9.146408	9.870914	9.148313	9.858186	9.686521	9.265792	8.511835	8.854885	8.683233	12.413338	10.715970	9.485652	9.921529	9.589501	10.272441	10.822512	10.228061	10.195238	10.218469	10.585512	10.009271	9.926202	9.901485	8.993030	10.402432	9.641321	9.580581	9.295979	11.639316	9.913924	10.755886	12.761697	11.427665	11.989574	12.610869	9.464406	10.092469	9.578078

AL355490.2	5.956984	6.121636	6.730953	6.280339	7.621942	7.860780	6.144272	7.755586	7.482753	6.894638	6.697597	7.614214	7.467717	6.765217	6.524190	6.996619	7.165350	6.602455	6.414516	7.017480	6.273200	6.168083	3.126876	6.262852	6.400316	7.534859	6.453116	6.296759	7.181888	7.772095	7.104714	7.230565	5.936504	6.169121	6.892994	6.607556	6.899975	6.896720	6.409686	7.120438	6.388847	6.638386	7.094174	6.878132	7.760531	7.526521	6.869358	7.058706	6.948428	7.851095	6.297403	6.993072	6.764101	6.563101	6.892688	6.872750	7.021281	7.124170	6.198260	6.808558	6.750364	6.212680	6.285057	6.676879	6.259647	6.678326	7.224338	7.496941	6.721610	6.694243	7.012195	7.375490	6.291343	7.282257	7.355811	6.729672	6.823074	6.543376	7.057146	7.101874	6.034196	6.882766	6.065057	6.367718	6.605592	6.140137	6.645475	7.572480	5.822952	6.461153	6.829419	6.422546	6.322896	6.874639	7.165525	6.723847	7.164573	6.671556	6.620976	7.240235	6.974320	7.555801	6.899975	7.197292	6.349577	8.542119	5.812591	7.414920	5.545431	7.013318	6.029915	5.959347	6.383146	6.621465	9.894275	8.965385	6.202971	7.967408	6.612494	6.128661	6.401307	5.924919	5.990248	8.743457	7.177645	6.038781	6.515542	6.519038	7.770487	6.042106	9.301255	9.475967	5.840959	5.995500	6.332486	7.180790	6.159451	6.256420	6.755724	5.617223	5.923210	6.095667	5.929339	5.913302	7.405681	5.949174	7.613569	9.026892	8.405306	6.278983	7.374392	5.357029	6.811443	6.730471	6.743835	6.335850	6.323720	6.294903	7.204873	6.512439	6.641126	6.521225	6.719475	7.626091	7.847351	7.261055	6.862666	7.950786	7.610371	6.744168	6.985072	7.283387	7.388100	6.151907	6.925012	7.248533	6.538274	5.896175	6.788096	6.614965	6.320141	6.680626	6.439408	6.440547	6.616593	7.350988	7.094820	6.194249	5.579285	7.854059	8.242037	6.448187	7.452925	7.004100	6.438569	6.707995	6.807918	7.764815	7.154192	6.748786	7.604670	6.330610	5.956812	6.681903	5.960936	6.663285	7.956657	5.579658	6.196190	6.758287	5.944425	6.122172	6.706217	6.623549	6.249294	8.288103	8.068759	5.574445	8.358960	6.356073	7.784243	6.377801	6.213207	5.358153	10.048962	6.442531	7.944014	6.522655	6.319613	5.661228	4.729367	4.516358	6.318570	9.406640	8.228550	6.501046	8.963282	5.609146

AL035443.1	3.169028	3.738084	3.230192	3.245966	2.956161	3.479650	3.158396	3.102636	3.705674	3.241215	3.309540	3.563462	3.392629	3.567496	4.611855	3.666279	4.051084	3.345113	4.568634	3.193913	4.193432	3.527928	4.314526	3.506879	3.989435	3.719536	3.138106	3.531510	3.313340	3.640310	3.325163	3.934932	3.202948	3.006794	4.408699	3.818223	3.593637	3.394704	3.973973	3.366686	5.117203	4.069243	3.257316	4.858360	3.452598	5.582288	4.357634	6.218215	3.307309	4.099972	3.782941	3.984782	4.202709	3.847173	3.689524	3.080487	4.024941	3.343324	3.559157	3.737108	3.492579	3.034402	3.876965	3.398178	3.377017	3.592419	4.315399	3.572687	3.622668	3.148992	3.323165	3.156795	3.048487	2.961862	3.069717	3.566891	3.541895	3.268982	3.531287	3.354520	3.119618	3.690955	3.145266	3.981983	3.302999	3.324325	3.012317	3.273291	3.376866	3.778359	3.254990	3.877841	4.513162	3.220285	2.932490	2.962733	3.739374	3.230494	3.588270	3.035361	3.129702	3.392300	3.003234	2.894780	2.988170	3.073894	3.231305	3.348380	3.072259	3.256611	3.738741	5.686415	3.580146	3.471876	3.091524	3.063972	4.704697	3.035580	3.093041	3.024958	3.931917	3.411476	3.314143	3.207511	4.082613	3.104112	3.153544	3.679024	3.957915	3.579359	3.542076	2.979742	3.110727	3.065028	3.171671	4.555796	3.144562	3.616508	3.654396	3.669919	3.857219	3.715868	3.294696	3.852488	3.041680	3.103265	2.962817	3.125273	4.760157	3.107702	3.302535	3.187925	2.976134	4.334201	3.578788	3.618187	3.505145	4.177253	3.211727	4.809096	4.259584	4.420876	5.173553	3.781236	5.137013	4.278403	6.181510	3.417570	3.833571	3.706665	3.583224	4.873219	4.369790	3.273704	3.100195	3.601290	3.482786	3.528850	3.793159	3.873944	3.260848	3.983919	3.824627	3.567883	3.604028	4.944576	4.003453	3.604265	3.266581	3.373077	3.165069	3.714240	2.959653	3.122513	3.852056	3.411654	3.416390	3.763005	3.335963	4.386303	3.817375	3.121586	3.527038	4.295680	2.989506	3.592078	3.369613	3.274958	3.596131	3.528663	4.000428	3.960968	3.600877	3.081779	2.976312	3.009424	3.023296	3.655248	3.612579	2.969646	3.212966	3.055187	3.011754	3.020401	3.052798	3.391393	3.305865	3.865362	3.423840	4.102375	5.540894	3.142180	4.097759	3.234681	3.248143	3.796132	3.281503	3.767157

AC138123.1	3.111066	3.993074	3.782941	4.249061	4.388072	4.694111	4.321207	4.074943	4.366775	3.718738	3.866820	3.230063	3.151974	3.441518	3.288191	4.395431	4.356533	3.167149	4.887696	3.930876	3.893990	4.535021	2.993919	4.021312	3.176019	4.197524	3.912710	4.236770	4.376736	4.433495	3.919774	3.636076	4.421986	3.870092	4.728702	4.409608	3.275739	3.205859	3.862377	4.745959	3.927615	4.397228	4.233519	3.930454	3.479469	4.636077	4.338539	5.264485	3.915969	4.275527	3.284905	4.157168	3.671942	3.818420	3.490858	4.255890	4.694331	4.190613	4.136944	3.789934	4.776555	4.549553	3.753613	5.145352	3.930648	3.835677	4.013337	4.129377	4.207761	3.704887	4.059329	3.858341	4.504789	4.009546	3.503137	3.843155	5.405739	3.786732	3.816516	3.541895	3.858971	4.117207	3.114521	4.513162	4.300454	3.836113	4.864946	3.608224	4.962733	4.129377	4.503080	4.383385	4.597236	4.098630	3.910352	4.487189	3.825965	4.113339	4.060633	4.687813	5.694332	3.897603	4.044097	4.234838	4.731233	4.760599	4.651753	4.292918	5.785597	4.806773	5.787200	4.686970	5.259071	4.114172	3.172928	7.181406	4.107221	3.365508	3.873530	3.789104	4.341562	4.654618	4.807861	4.964161	4.661814	4.872577	3.833571	4.449155	4.702350	3.745473	3.640900	5.124915	4.244727	3.785421	4.207761	4.425910	4.101929	4.803466	4.390178	5.034193	5.060442	4.701940	4.575611	4.391494	4.462017	4.831651	5.030127	5.030546	4.822102	3.826564	5.072402	4.275963	4.579470	4.738421	4.850577	4.811255	4.391494	5.257333	5.524212	4.752528	3.298088	4.198815	4.228110	4.559441	4.184692	4.892281	5.584588	4.825015	4.944576	4.238537	4.648115	4.933854	5.090437	4.505426	4.964988	5.434367	5.391260	4.986634	4.611221	5.430663	4.547511	4.489549	5.022202	4.592522	5.199887	4.527996	4.114601	4.746858	4.950218	4.875077	4.234172	5.104433	4.647255	4.883876	3.945083	5.419369	4.345246	4.319275	3.838432	3.560522	5.122714	4.365248	4.168824	4.566368	4.663287	4.272646	4.212080	4.112927	5.223707	3.879159	4.326548	4.601214	4.354362	4.354817	4.628037	4.451413	5.142569	4.860445	4.912630	4.810162	4.299617	4.543780	4.944987	5.690604	4.658582	4.629822	5.101129	4.826069	4.874651	5.450296	6.246351	6.685825	4.266350	4.010356	4.520611	3.928272	4.536530	5.016903

AC138123.2	3.111066	3.993074	3.782941	4.249061	4.388072	4.694111	4.321207	4.074943	4.366775	3.718738	3.866820	3.230063	3.151974	3.441518	3.288191	4.395431	4.356533	3.167149	4.887696	3.930876	3.893990	4.535021	2.993919	4.021312	3.176019	4.197524	3.912710	4.236770	4.376736	4.433495	3.919774	3.636076	4.421986	3.870092	4.728702	4.409608	3.275739	3.205859	3.862377	4.745959	3.927615	4.397228	4.233519	3.930454	3.479469	4.636077	4.338539	5.264485	3.915969	4.275527	3.284905	4.157168	3.671942	3.818420	3.490858	4.255890	4.694331	4.190613	4.136944	3.789934	4.776555	4.549553	3.753613	5.145352	3.930648	3.835677	4.013337	4.129377	4.207761	3.704887	4.059329	3.858341	4.504789	4.009546	3.503137	3.843155	5.405739	3.786732	3.816516	3.541895	3.858971	4.117207	3.114521	4.513162	4.300454	3.836113	4.864946	3.608224	4.962733	4.129377	4.503080	4.383385	4.597236	4.098630	3.910352	4.487189	3.825965	4.113339	4.060633	4.687813	5.694332	3.897603	4.044097	4.234838	4.731233	4.760599	4.651753	4.292918	5.785597	4.806773	5.787200	4.686970	5.259071	4.114172	3.172928	7.181406	4.107221	3.365508	3.873530	3.789104	4.341562	4.654618	4.807861	4.964161	4.661814	4.872577	3.833571	4.449155	4.702350	3.745473	3.640900	5.124915	4.244727	3.785421	4.207761	4.425910	4.101929	4.803466	4.390178	5.034193	5.060442	4.701940	4.575611	4.391494	4.462017	4.831651	5.030127	5.030546	4.822102	3.826564	5.072402	4.275963	4.579470	4.738421	4.850577	4.811255	4.391494	5.257333	5.524212	4.752528	3.298088	4.198815	4.228110	4.559441	4.184692	4.892281	5.584588	4.825015	4.944576	4.238537	4.648115	4.933854	5.090437	4.505426	4.964988	5.434367	5.391260	4.986634	4.611221	5.430663	4.547511	4.489549	5.022202	4.592522	5.199887	4.527996	4.114601	4.746858	4.950218	4.875077	4.234172	5.104433	4.647255	4.883876	3.945083	5.419369	4.345246	4.319275	3.838432	3.560522	5.122714	4.365248	4.168824	4.566368	4.663287	4.272646	4.212080	4.112927	5.223707	3.879159	4.326548	4.601214	4.354362	4.354817	4.628037	4.451413	5.142569	4.860445	4.912630	4.810162	4.299617	4.543780	4.944987	5.690604	4.658582	4.629822	5.101129	4.826069	4.874651	5.450296	6.246351	6.685825	4.266350	4.010356	4.520611	3.928272	4.536530	5.016903

AC009630.1	13.134908	13.151653	13.112597	13.065668	13.253211	12.922049	13.049903	12.936452	12.413004	13.054365	12.976769	12.829461	13.018269	13.050446	13.172863	12.782224	12.787348	13.030251	12.301868	13.055989	12.046618	12.642396	12.403886	12.811111	13.079117	12.790143	12.535616	12.597148	12.401457	12.799704	12.916212	13.367062	13.572169	12.709826	13.372935	13.144371	13.092940	13.346348	13.603829	13.063231	12.919156	12.728615	13.198527	13.088340	13.203986	13.252042	12.779167	13.302870	13.021352	13.482140	13.025652	13.321898	13.338927	13.609219	13.039489	13.174957	13.437511	12.670692	13.124799	13.383249	13.147466	12.831516	12.773792	12.716063	13.095248	13.328825	12.899883	13.170352	13.206710	12.683500	13.457857	13.212236	12.641390	13.018087	13.053642	13.309969	13.257830	13.152020	13.148968	12.311900	12.285826	13.307048	12.986119	13.475103	13.393903	13.341776	13.328825	13.409232	12.610699	12.879072	13.004862	12.538079	12.878188	13.391817	12.977133	13.173050	13.227239	12.682648	13.144955	12.677669	12.981325	12.959932	12.769062	12.771018	13.029714	12.949621	13.216008	13.271981	12.155089	12.759782	12.933669	12.699525	13.020807	12.576063	13.099767	12.499454	12.782063	12.572521	12.487141	12.903047	13.348475	13.119650	12.475368	11.933343	12.542124	13.615908	13.292951	12.860065	12.951286	12.841587	13.486198	13.035435	12.463389	12.396166	12.854034	12.607398	12.690509	12.877659	12.942843	12.764791	12.693903	12.723310	13.203792	12.147357	12.727241	13.154762	12.950005	13.376280	13.048778	12.331584	12.562670	12.940455	13.597930	13.229650	13.198725	13.287648	13.016643	12.788040	13.054365	12.650709	12.441914	12.895811	14.094287	13.483216	13.163939	13.128244	13.854373	13.590299	13.304119	13.548671	13.345088	13.405029	13.155970	13.659121	12.516941	13.028986	12.586817	12.844915	13.403569	13.022716	12.866895	12.869775	12.733221	13.074351	13.434924	12.616955	13.386677	13.052443	12.389829	12.829643	13.335001	12.615820	13.647795	13.303472	12.882226	13.558652	13.245910	13.097680	12.254351	12.220472	12.831697	13.245152	13.305570	13.151461	12.847527	13.233400	13.159327	12.409358	13.086292	13.003067	12.490065	12.815455	12.887037	13.309752	13.718167	12.733221	12.537248	13.388838	13.601738	12.598323	13.005956	13.017536	13.413148	11.848060	12.639889	13.531931	12.950935	12.764425	13.340998	12.739334	12.180333	13.517315	12.401128	13.426653	13.698011	14.006716	14.784831	12.214739

LINC01357	13.804379	14.655931	14.231187	14.181178	13.939054	13.881710	14.408756	14.059166	14.045286	14.304961	14.151049	14.090418	13.884891	13.924569	13.542327	14.741814	14.037892	13.725347	14.566731	14.203084	14.571403	14.299871	14.243708	14.348910	14.240086	14.202148	14.359381	14.243388	14.654144	14.628630	14.063831	13.376280	13.603098	14.005012	13.947375	13.793026	13.892647	14.079189	14.214704	14.152064	14.616972	14.220230	13.751062	13.841137	13.763702	13.556834	14.423235	13.893142	14.000936	14.024155	13.396455	12.879950	13.491043	13.586012	13.265163	14.675040	12.968393	13.728107	13.668871	13.366218	13.614956	14.621589	14.137016	13.934635	13.790935	13.980518	14.162706	14.406227	14.034368	14.392936	14.800437	14.601201	14.213670	14.069818	14.343152	14.495949	14.662329	14.077200	14.260288	13.423212	13.224119	13.199903	12.702957	13.380743	13.241797	13.681631	12.787348	12.880604	13.481700	13.615204	13.259393	13.773961	13.367886	14.030880	13.547331	13.731149	13.586976	13.337899	13.462029	14.746969	13.666705	14.644443	14.229504	14.224052	14.040253	13.860992	14.217951	14.695210	14.241749	14.702065	14.471966	13.736592	13.540173	14.616554	14.236249	14.278587	13.453292	13.954619	14.408375	13.592174	14.266988	14.113444	14.103476	14.234212	13.723478	14.464405	14.310316	13.939913	14.482860	14.262283	14.380635	14.275219	14.084957	14.210781	14.290297	13.679373	13.553239	14.087427	13.530801	13.912944	13.808112	13.907998	14.404753	14.205886	13.786118	13.904184	14.095820	14.769948	14.447111	13.966887	13.556139	14.022116	14.011814	13.684034	13.895060	13.627746	14.114980	14.253099	14.010653	14.449082	14.132032	13.401636	13.831022	13.389060	13.604077	14.447934	14.407674	14.033777	13.503677	13.347208	12.726510	12.882947	13.364746	12.903734	14.564295	12.634275	13.348067	13.383910	13.168263	13.330066	14.513547	13.730157	13.657952	13.589804	13.626588	13.954061	14.064122	13.970725	13.642281	14.379577	14.295055	14.329679	14.025686	13.872874	13.995291	14.247926	14.298038	14.176495	12.853682	13.039136	13.154391	13.017536	12.975277	13.052774	13.856758	12.620500	12.515324	13.505465	13.272175	13.635217	13.625907	13.734845	14.355885	13.739872	13.741572	13.953485	13.517989	13.885693	14.651534	13.767258	14.419877	14.265936	13.591444	13.741572	14.087427	13.947085	13.815559	13.817826	14.699677	13.777435	13.485749	13.893681	14.580207	14.173200	13.815786	13.669109	13.786878	14.466587

AC132217.1	13.694245	13.283442	13.311346	13.363673	12.056205	11.317122	13.223925	11.109863	10.756043	12.689295	11.608020	12.190143	13.111562	11.579215	13.146023	12.617792	12.542124	12.310451	11.392045	12.068901	11.109863	9.898374	13.047442	11.354634	12.999330	12.278662	14.418742	13.699672	13.638302	12.947549	13.375239	13.506799	12.452642	12.869060	13.895620	13.098814	13.279851	12.224905	13.709911	13.549809	13.671278	13.939615	13.301878	13.412931	14.645337	13.448203	13.647535	12.154051	13.189521	12.616477	12.433552	13.251240	13.723956	12.640533	12.931521	12.523982	14.648824	13.554152	13.188209	13.513328	12.984802	14.817306	16.079155	13.053130	13.065502	11.933343	13.358268	12.591506	11.384067	12.874301	13.955482	12.843685	12.792190	13.491043	13.683082	12.386450	13.474637	14.305938	12.570678	12.540801	13.277050	14.982214	13.357219	12.566593	13.015726	13.539933	12.986468	13.872088	12.609852	11.959449	13.087748	11.953784	10.950433	12.430104	12.275467	13.114296	13.347873	13.772698	13.008664	13.768298	12.806661	13.783587	13.934369	12.642396	11.381596	13.768548	12.854978	12.303440	13.498714	12.614041	12.339350	10.968475	9.875282	12.267304	11.931845	12.114644	14.541332	13.372722	13.964397	12.495520	12.555905	13.643032	14.065689	14.840176	13.336016	13.698756	14.795023	13.310755	12.814409	14.448739	13.568953	14.175228	14.032280	15.099414	14.096118	13.147891	13.286686	12.995839	11.347880	13.097867	12.632803	13.229868	13.030425	12.789777	12.780538	13.568039	12.401781	13.884891	13.363072	14.782312	14.771836	15.098818	14.496768	13.783587	14.522678	15.303728	16.128614	13.120996	13.257008	13.520479	12.473785	14.879649	14.396563	14.511941	11.873849	13.233610	14.949869	12.933307	14.378131	14.756458	14.113728	13.935487	15.470625	14.237480	14.184313	14.168428	14.100390	13.176981	14.813167	14.465534	14.018427	12.179561	13.723726	14.726960	14.344582	13.455497	14.344582	14.399625	13.311346	13.571462	15.284471	11.466217	14.732623	14.016992	14.509550	14.506070	13.177184	15.184749	14.855412	15.347177	12.927379	13.996150	15.871113	15.333793	13.218380	14.003265	14.197518	12.533636	13.514460	14.686004	12.319852	15.476405	14.487547	14.615271	13.556609	15.125176	16.080456	12.855334	13.580864	14.609701	12.620010	14.334703	13.573121	12.693740	15.649863	12.063188	10.981301	12.221872	12.503725	12.819943	14.732623	12.606892	10.251178	11.863798	13.308756	14.361168	14.282078	12.100117

NIFK-AS1	12.243471	11.642983	11.550891	11.788373	11.735252	11.132065	12.166337	10.658429	11.704602	12.048913	11.896631	11.560243	11.663576	11.821859	11.796904	11.713188	11.747596	12.045007	12.219839	11.876151	11.982268	12.432750	13.224320	12.208851	12.618964	12.229877	13.465295	12.093060	11.928776	11.567979	12.109661	11.785972	11.565808	12.261028	12.127025	12.102241	11.877315	11.859202	12.186842	12.301550	11.702812	12.327640	12.169426	12.049673	12.596461	12.196471	12.229570	11.603148	11.244638	12.043646	11.977304	12.105607	11.880566	12.160962	11.279136	11.370215	11.915487	12.712948	12.374817	12.646251	12.685861	11.631664	13.019903	12.293733	12.018734	11.876288	12.325731	11.809037	12.016290	12.027196	11.774910	12.019013	11.563737	11.539250	11.833448	11.724391	12.045007	11.665214	11.996703	12.061519	12.410794	12.001304	12.107881	12.259890	12.464048	11.478113	12.345482	11.973905	12.581481	12.313325	12.921519	12.436448	11.670177	11.869342	12.082436	12.185131	11.515630	11.866080	11.673868	12.111007	11.255761	11.585010	11.474379	11.451479	11.006400	11.390710	11.482100	11.899859	12.049973	11.520092	12.220306	11.841449	11.569749	11.930776	10.902781	11.311460	12.512428	11.662223	12.061039	11.933343	11.512602	11.988461	11.940295	12.864781	11.559918	12.018090	13.713207	12.211265	12.812829	11.427239	13.460684	12.618196	12.360369	11.941124	12.914157	12.677669	13.899912	13.158034	11.187721	12.817345	12.828755	12.507212	12.644551	13.326420	12.996223	11.486239	12.664778	12.809830	11.623447	13.167690	11.365508	13.165501	11.498149	11.711892	12.148979	11.487573	11.876433	12.045443	12.528710	12.615500	12.345482	12.651748	11.628369	11.299108	11.937016	11.906521	13.264960	11.704316	12.009448	12.063353	12.902330	11.598732	11.486562	12.564459	12.478001	13.405475	12.749999	12.607553	12.286301	12.664433	12.030069	12.330170	11.903405	12.813876	12.722462	11.875371	11.864391	12.108933	11.944525	11.782370	11.938053	11.968715	12.250568	12.504062	11.551514	12.095489	12.348206	11.624998	12.550893	11.744056	12.063353	11.880269	12.357184	12.020215	13.780832	12.198897	12.881502	13.394129	12.863485	12.982760	12.413177	13.239591	11.911633	11.365203	11.775216	12.433076	12.632621	12.366220	12.387604	11.958855	13.210542	12.672938	11.837689	13.509723	12.366380	12.672605	12.336677	11.824133	11.639167	12.378030	11.703111	13.465527	12.211598	11.997933	11.330317	12.919156	12.995633	12.282721

SNHG27	6.227804	6.731107	7.202384	7.943093	8.937800	8.356909	8.134971	7.864018	7.500701	8.732878	7.784243	7.877495	8.350679	8.215914	7.708703	8.081336	7.684131	7.939154	6.224688	8.996119	6.093732	7.205507	2.997691	7.993460	7.316378	8.227971	6.657757	8.773832	8.338225	7.799143	8.147768	7.860780	7.491960	7.823189	8.799907	8.746346	8.617837	7.375318	7.418684	7.598247	9.055012	7.043666	7.865564	7.964169	7.938222	8.509809	8.005930	7.256786	8.502791	8.781189	8.620345	9.506420	8.983483	8.744057	8.468844	7.875346	8.298501	7.266940	7.884296	7.438512	8.379382	7.994877	5.700263	8.236218	7.197292	7.412291	7.260136	7.543582	7.640791	7.367488	8.557775	8.433222	8.299127	8.504625	7.803104	7.838479	8.627316	7.443459	7.345830	8.296090	8.170358	8.008532	8.106950	8.703980	8.565872	8.139429	8.802612	9.066762	6.659030	6.940887	7.085839	7.207692	7.726369	8.886372	8.844331	8.828725	7.528117	9.170296	9.326293	8.618939	9.066762	7.386409	6.963561	8.055425	6.806940	7.745934	8.161308	6.581645	6.817639	7.511538	6.242269	8.069994	8.133767	9.447171	9.605311	10.122304	7.335968	8.028417	8.127337	6.484435	6.080402	7.723928	7.399610	9.448726	8.753634	8.690022	9.473694	7.844360	7.548829	7.350077	7.839993	8.191967	7.432187	7.680418	7.045129	7.454005	7.805559	6.352110	9.376353	8.047693	7.538121	6.089923	7.369846	7.059799	7.910344	7.479190	9.799359	7.889199	7.470321	7.682746	9.332770	7.705322	7.720677	8.444252	8.566718	8.033453	7.074170	6.940887	7.455423	9.810261	6.985072	9.632923	8.220099	8.881137	9.301096	8.059636	6.420945	8.482488	7.922721	8.137259	9.279219	9.453075	8.106349	8.291988	7.071814	7.970369	7.094332	8.476201	6.455739	7.241803	8.311463	7.408991	7.331787	6.169288	7.241488	6.914636	6.340247	7.737739	7.660627	9.305574	7.667444	8.121469	8.222383	8.221296	7.651114	8.342612	7.322799	7.220840	8.033303	7.228105	8.513380	8.216662	7.315739	8.775967	8.333537	8.610302	8.838304	7.344269	6.387306	7.264041	7.284141	7.032959	8.214556	9.023558	9.118515	7.678284	7.912825	8.092604	8.561316	9.325070	7.065558	8.038522	7.473992	6.331112	8.058274	8.007316	5.196495	6.273716	7.827449	6.780704	7.538942	8.447968	8.690354	9.655511	9.712641	7.493966	7.600485	7.886135

LINC02028	5.582087	3.981754	4.848888	4.680948	4.533920	3.956373	4.850148	4.288978	5.382508	5.118860	4.492238	4.514214	5.064071	3.658246	4.825015	4.799428	4.501617	4.932210	4.091404	4.841900	3.610768	4.829761	3.037828	5.132613	4.809948	5.117824	3.848412	3.670528	4.624831	3.991355	4.518062	5.286792	4.705737	4.063276	4.246657	4.766147	3.302832	4.579914	3.700373	5.250989	4.890438	3.779584	3.770527	3.444014	5.352484	4.431596	5.392969	4.881587	4.304213	4.279291	5.678637	4.991007	4.711800	4.536102	5.659580	5.164484	4.137797	5.408723	4.753636	5.032136	5.250989	5.192676	3.789732	4.417754	3.277564	5.169505	3.478013	4.749752	5.142569	3.221793	4.720037	5.442550	5.489143	4.491142	4.856270	4.187994	4.730610	3.742589	4.747673	4.524351	5.545822	4.265722	5.522511	5.711575	3.792266	5.544482	4.669702	4.889825	3.974322	5.283568	4.093293	3.029573	4.363732	4.032121	4.315840	4.070266	5.179750	5.607789	5.177346	4.551957	3.946628	5.173357	5.214830	5.820965	4.427225	4.976081	4.452678	5.233225	3.604453	5.270454	3.445636	4.837238	4.635636	4.957517	4.332885	5.701880	5.590622	4.350467	5.731220	3.949193	3.230761	3.815433	4.410264	4.368077	3.443822	3.667710	4.405149	5.631886	6.484961	4.526520	3.211727	3.975942	3.516224	4.951888	4.385646	3.095013	5.298453	3.233514	5.788126	3.558141	4.725358	2.961473	4.958929	4.115265	5.848814	4.653958	4.008008	4.175090	3.366167	5.129196	3.962248	4.227030	5.233633	6.784020	6.116663	4.124633	3.897603	3.696923	4.605418	4.631969	3.045673	6.355425	3.683253	6.051126	4.160365	5.119275	2.952291	5.012617	3.089591	3.488475	6.452936	5.574445	4.154789	5.483145	4.371560	5.238461	6.837217	4.674273	4.488884	7.147212	3.349197	4.424630	3.602249	4.288106	3.881910	6.636899	4.157373	5.102586	4.589492	4.013585	6.033087	4.826975	4.049098	4.392128	4.307689	6.114764	6.154306	3.961168	5.471380	5.680842	7.182971	5.359863	3.267061	3.348845	4.568093	5.704106	6.254190	3.704057	6.260320	3.937922	4.388677	3.359328	5.589095	3.560522	4.697895	4.256493	6.299534	5.314629	5.665725	5.192099	5.823304	3.892199	4.478244	4.766370	3.816718	4.967792	7.053316	3.213397	4.744005	3.604453	5.723813	5.430290	3.920194	5.253360	5.666110	2.935901	4.984779	3.816090

SNHG11	11.056896	11.278715	11.139121	11.373199	11.545901	11.019905	11.146945	11.081604	11.080123	11.000770	11.384387	11.214660	11.569895	11.529981	11.881590	10.877350	11.284004	11.498297	12.681285	11.275872	12.032911	12.359894	12.633466	11.545312	11.711161	11.655452	10.980857	11.051479	10.838984	11.023522	11.757091	11.708338	11.341907	11.607297	11.494352	11.536600	11.246365	11.225321	12.018432	11.635707	11.122494	12.115080	11.677874	11.584392	11.554781	11.950357	10.917928	11.471518	11.378421	11.351898	11.676671	11.534947	11.309548	11.652730	10.876331	11.402055	11.379601	12.046769	12.015376	11.927709	12.315928	11.112442	12.602220	12.226464	12.361792	11.398515	11.795267	11.982268	11.490395	11.806047	11.291576	11.309839	10.944027	11.047517	11.474696	11.393969	11.093554	11.357096	11.317473	11.498901	11.859341	11.509057	11.460104	11.931556	11.413847	11.595353	11.969051	11.772691	12.846651	12.341197	12.179561	12.772441	11.883430	11.525892	11.389687	11.999597	11.188596	11.680972	11.806639	11.361944	11.069731	10.977591	10.954523	11.149440	12.922594	11.077953	11.637393	12.001171	12.510296	10.966241	12.807530	12.290442	11.672364	11.177770	10.764768	9.904108	12.249758	11.119708	11.203158	11.487721	12.205131	11.576476	12.230643	11.909419	11.121300	11.225775	11.676671	11.498757	11.059231	11.611243	12.038655	11.351302	12.023550	12.395544	11.376521	12.240995	11.345947	12.795036	11.080590	12.643422	13.057827	13.139689	9.624778	12.000733	12.202018	11.695305	12.021084	11.752925	11.364157	11.849578	12.041521	12.303112	11.596548	11.776541	12.092910	11.709538	12.281762	12.653975	12.286760	12.395993	12.263695	12.272044	12.833844	12.090445	11.820064	11.300543	13.343265	11.693954	11.519246	11.938053	13.543266	11.607591	11.223118	12.348206	12.562365	12.897423	12.808400	12.761183	12.167097	12.454210	12.664248	13.147685	12.134970	12.641718	13.369728	12.009735	11.677583	11.714834	12.908228	12.122532	11.335546	10.988689	11.592521	11.886732	11.419262	12.316396	11.963054	11.321595	12.270578	11.477417	11.550311	12.300335	11.908965	11.520985	12.663909	12.020926	13.073797	12.686920	12.582141	12.493699	13.147264	12.630185	11.563303	11.672977	11.257536	11.992764	11.504805	11.758737	12.053155	12.047525	12.095335	11.310266	11.922622	14.840681	11.129277	12.260507	13.152801	12.936720	10.577989	13.135275	11.430015	12.678191	11.139199	12.041372	11.726896	11.813591	12.462754	11.781940

LINC02369	7.449204	7.658320	7.710024	7.599022	7.623954	7.954325	7.837337	8.245084	8.604996	7.898958	8.638018	8.115024	7.277658	7.152628	8.298205	7.566103	8.334307	7.534859	8.812483	7.554895	8.886223	8.384965	9.458185	7.418516	7.457297	8.507374	7.832466	8.903553	8.048777	7.665421	8.114888	6.945974	7.196961	7.270940	7.382853	8.204055	7.475221	7.718569	7.823032	7.963752	7.804315	8.102493	7.112221	7.652204	7.285348	7.406634	8.279032	7.262146	7.480117	7.358246	7.500701	7.557235	6.885195	7.138922	7.160938	8.263850	6.736780	7.558171	7.625771	7.381252	7.756185	7.490559	7.817143	8.022991	7.974303	8.087091	7.787922	7.639084	7.839529	8.413538	7.741802	7.824306	7.561362	7.646042	7.987342	7.742537	7.926147	8.152526	8.342466	7.397259	7.351296	7.549434	7.652674	7.569604	7.493195	7.784389	7.817308	7.820409	8.422572	8.265082	8.181595	8.842121	7.590734	7.486025	7.470014	7.607888	7.709416	6.807739	7.274704	7.283052	7.621347	7.180945	7.522817	7.605777	8.304531	8.015395	9.797342	8.313433	8.869598	7.977519	8.546267	7.822571	7.917227	8.356589	7.824466	7.840933	7.374866	7.957140	8.439178	7.790686	7.949190	7.860780	7.677944	8.236218	8.376752	8.243097	8.376902	8.216975	8.052166	8.183907	8.489064	7.811708	7.854059	8.512760	8.114560	8.267918	7.458243	9.163879	7.544235	8.876944	8.628077	9.676294	8.550581	8.226013	8.342466	8.616940	9.030689	8.743289	7.780597	8.368767	7.474898	7.590584	7.756656	7.803407	8.272248	7.858931	8.185409	8.821587	8.613599	8.479979	8.406229	7.673196	8.686570	7.711542	7.900057	8.701068	7.721304	7.820409	7.746562	7.847219	7.369686	7.042723	7.593028	7.381393	8.021345	7.327894	7.840310	7.594432	7.632690	8.267453	7.335363	8.503262	8.385870	8.304234	8.187593	8.364235	8.128270	8.655793	8.663180	8.091866	7.906479	8.674520	8.036022	8.055590	8.057990	8.200421	8.338386	8.681429	7.195686	7.333384	7.800068	8.063817	7.370940	7.897027	8.310979	8.174583	7.814763	8.544468	8.118240	8.294728	9.084076	8.320348	8.541182	8.462777	8.674211	8.355382	7.676648	7.858176	7.905856	8.073029	7.619091	8.182181	7.627512	7.993159	8.250672	9.735468	8.278584	9.003955	8.615418	8.905075	7.651887	8.345227	8.775967	7.844862	7.565801	7.944780	7.972401	8.192439

AL359636.2	3.791429	3.593435	3.796720	3.520966	3.347910	5.173168	5.294623	3.608603	4.051084	5.310700	4.687813	4.392978	3.938111	4.463946	3.638065	5.166466	3.773677	3.025453	4.784935	4.430031	5.298266	3.117616	3.461975	3.742381	3.495025	3.730665	4.279490	4.196239	4.775532	5.362147	3.949193	4.330333	3.876562	4.785984	4.625504	4.120296	3.827843	3.302053	3.433974	3.970758	5.703178	3.745252	3.644045	4.282532	3.807841	5.850774	4.877783	3.001582	5.031133	3.966608	3.902531	3.804108	4.497496	6.906870	4.110948	5.704288	4.259390	3.390523	3.036937	3.796526	2.854479	5.574074	4.345899	3.386208	3.635857	3.567496	2.988075	3.562130	4.245989	4.311473	5.141316	5.301434	5.144534	5.317069	5.182826	4.900698	4.788690	4.692658	4.191252	3.907900	3.519290	4.185785	3.636869	4.287203	3.821651	3.912710	4.466525	3.501061	3.689322	3.748966	3.525332	3.057375	3.821651	4.032747	3.330982	3.477651	4.191700	3.611782	4.011437	4.156749	3.508701	4.129602	4.243640	3.555075	4.708660	4.704057	3.632530	3.758566	3.404378	5.753756	3.665267	3.709872	3.114521	5.395965	4.345054	3.924795	3.357933	4.398722	4.380153	4.272457	3.755678	3.931308	3.321552	3.814603	6.211030	4.113339	3.295881	4.715607	4.961439	4.649179	4.418223	3.429708	3.663429	3.308242	5.177954	3.670314	2.908227	3.687483	5.304807	4.548870	4.974416	5.478043	4.476926	3.800534	4.711800	4.782850	4.147276	4.456478	6.274724	3.945528	3.483890	3.779584	5.347947	3.993522	4.613377	3.895246	3.454010	3.757923	3.162277	4.211644	5.036312	3.671754	5.348318	4.336552	5.341041	5.032354	2.879860	5.540166	3.943958	3.825521	4.563408	4.316930	7.373908	3.760706	5.743559	3.866193	3.179000	2.849469	3.531098	3.821840	5.118642	4.633723	4.048215	4.023218	3.808277	2.921280	3.786278	3.432203	2.877915	6.147780	5.524779	4.395908	5.936334	4.604402	4.787036	5.023196	4.819395	4.709267	3.299460	3.910981	4.712006	3.702258	3.670314	3.519870	3.784371	3.567103	3.443447	3.326289	3.591134	4.789520	2.932919	4.104747	3.657866	3.598578	3.364994	3.422793	3.250276	4.206158	4.779065	4.238087	3.788669	3.359328	4.093091	5.071019	3.729236	3.453834	3.976140	3.924166	4.655999	4.138014	4.114601	3.260006	4.136096	4.261329	4.055179	3.202823	4.351327	5.038113

AL023913.1	6.869510	6.345056	6.602610	6.490462	6.585224	6.631246	5.749744	7.812644	7.805090	6.223096	6.792176	6.281779	6.005942	6.174728	5.539006	6.098721	7.696224	6.139446	8.287505	6.512277	7.439916	7.760697	9.058476	6.957510	7.234047	7.255549	6.952973	7.590139	6.147780	6.016224	7.482584	6.171845	6.299709	5.930243	6.676101	6.082328	6.159072	6.594072	7.210664	7.142720	6.311124	6.795092	6.673180	6.530971	6.457215	7.277201	6.847422	6.613637	7.066988	6.400990	6.769775	6.686143	5.364822	6.385448	6.290462	6.495585	5.419147	6.655645	7.008016	7.020771	7.078318	5.977794	7.558171	7.294607	6.731923	6.789085	6.878937	6.687119	6.740220	7.112068	6.085754	6.546953	6.518205	6.149155	6.331290	6.474078	7.064918	6.790236	7.230254	6.179989	6.314873	6.364856	6.494605	6.451641	6.711340	6.587881	7.054266	6.825119	7.290501	7.054770	6.940443	7.606389	6.539709	6.843699	6.342844	6.535976	6.337715	6.349412	6.749103	6.259290	6.710714	5.755704	6.269286	6.151907	7.386079	6.252335	7.571434	6.483249	7.877349	6.589328	7.916925	7.826070	6.884715	6.645932	6.835309	6.807739	7.491814	6.820848	7.297031	6.738926	6.888947	6.595678	6.532414	7.431096	6.854303	6.940443	9.435543	8.888996	7.006896	7.257719	6.764576	7.561665	6.201890	6.979561	7.206128	7.693823	6.691609	8.915570	6.662158	7.931730	7.662653	8.635194	8.933782	7.247752	7.303657	7.696994	8.440099	8.215620	6.914636	8.323597	8.176472	7.824779	5.960569	7.854534	6.594520	6.881674	8.280832	8.925660	8.247677	7.771722	8.085086	6.738109	7.464349	6.577833	6.955662	7.077190	7.169475	7.600485	6.666216	7.165954	7.094332	5.961446	6.082990	6.629691	6.586378	6.021175	7.274387	7.429167	6.732226	7.395245	6.191492	7.629075	7.633165	7.537619	6.950916	7.578551	7.142409	7.455878	7.411794	6.421107	7.086585	6.620034	6.631560	6.881033	7.020298	7.114394	7.033428	7.495361	6.112317	6.534841	6.733047	7.448405	6.372450	7.051012	7.818250	7.148656	6.826397	7.821963	7.581658	7.641842	7.810345	7.694438	7.745783	7.079746	7.489761	8.230097	7.946185	7.796942	6.744515	7.669290	7.041441	7.639530	6.079385	7.173838	7.061387	9.044254	6.994003	8.170810	7.087830	8.344769	8.579823	8.450472	7.246377	6.681747	6.325088	7.732798	6.794787	7.670842

SNAP25-AS1	6.869510	6.345056	6.602610	6.490462	6.585224	6.631246	5.749744	7.812644	7.805090	6.223096	6.792176	6.281779	6.005942	6.174728	5.539006	6.098721	7.696224	6.139446	8.287505	6.512277	7.439916	7.760697	9.058476	6.957510	7.234047	7.255549	6.952973	7.590139	6.147780	6.016224	7.482584	6.171845	6.299709	5.930243	6.676101	6.082328	6.159072	6.594072	7.210664	7.142720	6.311124	6.795092	6.673180	6.530971	6.457215	7.277201	6.847422	6.613637	7.066988	6.400990	6.769775	6.686143	5.364822	6.385448	6.290462	6.495585	5.419147	6.655645	7.008016	7.020771	7.078318	5.977794	7.558171	7.294607	6.731923	6.789085	6.878937	6.687119	6.740220	7.112068	6.085754	6.546953	6.518205	6.149155	6.331290	6.474078	7.064918	6.790236	7.230254	6.179989	6.314873	6.364856	6.494605	6.451641	6.711340	6.587881	7.054266	6.825119	7.290501	7.054770	6.940443	7.606389	6.539709	6.843699	6.342844	6.535976	6.337715	6.349412	6.749103	6.259290	6.710714	5.755704	6.269286	6.151907	7.386079	6.252335	7.571434	6.483249	7.877349	6.589328	7.916925	7.826070	6.884715	6.645932	6.835309	6.807739	7.491814	6.820848	7.297031	6.738926	6.888947	6.595678	6.532414	7.431096	6.854303	6.940443	9.435543	8.888996	7.006896	7.257719	6.764576	7.561665	6.201890	6.979561	7.206128	7.693823	6.691609	8.915570	6.662158	7.931730	7.662653	8.635194	8.933782	7.247752	7.303657	7.696994	8.440099	8.215620	6.914636	8.323597	8.176472	7.824779	5.960569	7.854534	6.594520	6.881674	8.280832	8.925660	8.247677	7.771722	8.085086	6.738109	7.464349	6.577833	6.955662	7.077190	7.169475	7.600485	6.666216	7.165954	7.094332	5.961446	6.082990	6.629691	6.586378	6.021175	7.274387	7.429167	6.732226	7.395245	6.191492	7.629075	7.633165	7.537619	6.950916	7.578551	7.142409	7.455878	7.411794	6.421107	7.086585	6.620034	6.631560	6.881033	7.020298	7.114394	7.033428	7.495361	6.112317	6.534841	6.733047	7.448405	6.372450	7.051012	7.818250	7.148656	6.826397	7.821963	7.581658	7.641842	7.810345	7.694438	7.745783	7.079746	7.489761	8.230097	7.946185	7.796942	6.744515	7.669290	7.041441	7.639530	6.079385	7.173838	7.061387	9.044254	6.994003	8.170810	7.087830	8.344769	8.579823	8.450472	7.246377	6.681747	6.325088	7.732798	6.794787	7.670842

LINC00623	7.921630	7.476161	7.391165	7.821963	8.099606	8.675912	8.182032	7.945865	8.299554	8.616940	8.383127	8.279188	7.891853	7.923498	7.895271	8.303620	7.648283	8.045919	6.773975	7.886728	7.567185	7.186039	7.485047	7.180642	7.171315	7.959136	8.654316	8.195435	8.835394	8.640510	8.215479	7.556139	7.796780	8.188180	7.873357	7.688567	7.440807	7.421823	7.087352	7.598098	8.680959	7.411494	8.018445	7.627346	7.743966	7.516320	9.079479	8.119173	7.704063	7.915502	7.490242	7.477847	7.357319	7.621803	7.440515	8.498349	7.177969	7.185369	6.396299	7.249782	7.043039	8.855177	7.228105	6.613976	7.067750	6.958469	6.715222	7.023584	7.444262	6.570524	7.477201	7.553771	8.618939	7.385010	7.460716	7.574019	7.212319	7.270478	7.398357	7.333524	7.206767	7.097191	7.390104	7.532225	7.317634	7.482286	6.928747	7.209733	6.382153	6.951067	6.523859	6.549386	7.359173	7.923957	8.135700	7.696379	7.406955	7.847503	8.451544	7.630374	8.099931	7.884753	7.916768	7.697463	6.629051	8.866122	7.347533	7.521277	6.688383	9.097512	6.242601	7.163769	7.322163	9.052510	8.123096	9.744747	7.227500	7.863083	8.902489	7.668055	8.505214	8.052321	8.412161	8.913424	8.474768	9.088985	8.140022	8.231627	9.474231	8.849892	9.338762	8.076646	9.451098	8.972481	8.828551	8.517642	8.596418	8.013862	8.321274	8.275673	8.184186	7.247168	8.004347	8.473263	9.323513	8.178250	8.320945	10.119753	9.211908	9.832460	8.238691	8.836612	9.167255	8.221008	8.608181	7.725142	8.344925	8.669872	7.980180	9.752532	8.177966	8.982132	8.155934	7.903984	8.370333	9.499476	9.128435	8.472816	8.913424	8.547692	8.306963	8.357224	9.747100	8.009468	9.707662	7.494577	7.989848	7.557702	7.985774	8.613599	8.973923	7.614945	8.237152	7.911114	7.824155	8.258942	7.804933	8.390991	7.712007	7.683680	7.847801	9.312647	8.237152	7.984536	8.138022	8.119920	7.707451	7.832466	8.322308	7.979874	7.763284	8.060764	8.992714	9.050240	8.585128	7.867093	8.932414	7.661235	8.851831	7.818250	8.313592	8.383440	8.672031	8.672180	8.707227	8.637723	8.620493	8.824787	8.884828	8.840733	8.868537	8.027241	8.349433	8.359575	9.455425	8.269361	8.289633	7.666067	9.376056	6.443539	8.379692	8.069369	9.575947	9.135773	8.920452	8.442390	8.309176	9.977682

AC242842.1	7.921630	7.476161	7.391165	7.821963	8.099606	8.675912	8.182032	7.945865	8.299554	8.616940	8.383127	8.279188	7.891853	7.923498	7.895271	8.303620	7.648283	8.045919	6.773975	7.886728	7.567185	7.186039	7.485047	7.180642	7.171315	7.959136	8.654316	8.195435	8.835394	8.640510	8.215479	7.556139	7.796780	8.188180	7.873357	7.688567	7.440807	7.421823	7.087352	7.598098	8.680959	7.411494	8.018445	7.627346	7.743966	7.516320	9.079479	8.119173	7.704063	7.915502	7.490242	7.477847	7.357319	7.621803	7.440515	8.498349	7.177969	7.185369	6.396299	7.249782	7.043039	8.855177	7.228105	6.613976	7.067750	6.958469	6.715222	7.023584	7.444262	6.570524	7.477201	7.553771	8.618939	7.385010	7.460716	7.574019	7.212319	7.270478	7.398357	7.333524	7.206767	7.097191	7.390104	7.532225	7.317634	7.482286	6.928747	7.209733	6.382153	6.951067	6.523859	6.549386	7.359173	7.923957	8.135700	7.696379	7.406955	7.847503	8.451544	7.630374	8.099931	7.884753	7.916768	7.697463	6.629051	8.866122	7.347533	7.521277	6.688383	9.097512	6.242601	7.163769	7.322163	9.052510	8.123096	9.744747	7.227500	7.863083	8.902489	7.668055	8.505214	8.052321	8.412161	8.913424	8.474768	9.088985	8.140022	8.231627	9.474231	8.849892	9.338762	8.076646	9.451098	8.972481	8.828551	8.517642	8.596418	8.013862	8.321274	8.275673	8.184186	7.247168	8.004347	8.473263	9.323513	8.178250	8.320945	10.119753	9.211908	9.832460	8.238691	8.836612	9.167255	8.221008	8.608181	7.725142	8.344925	8.669872	7.980180	9.752532	8.177966	8.982132	8.155934	7.903984	8.370333	9.499476	9.128435	8.472816	8.913424	8.547692	8.306963	8.357224	9.747100	8.009468	9.707662	7.494577	7.989848	7.557702	7.985774	8.613599	8.973923	7.614945	8.237152	7.911114	7.824155	8.258942	7.804933	8.390991	7.712007	7.683680	7.847801	9.312647	8.237152	7.984536	8.138022	8.119920	7.707451	7.832466	8.322308	7.979874	7.763284	8.060764	8.992714	9.050240	8.585128	7.867093	8.932414	7.661235	8.851831	7.818250	8.313592	8.383440	8.672031	8.672180	8.707227	8.637723	8.620493	8.824787	8.884828	8.840733	8.868537	8.027241	8.349433	8.359575	9.455425	8.269361	8.289633	7.666067	9.376056	6.443539	8.379692	8.069369	9.575947	9.135773	8.920452	8.442390	8.309176	9.977682

LINC01934	4.403954	4.059329	3.699776	4.618879	3.387739	4.583101	5.256331	3.659429	3.524981	3.425277	6.210351	3.730447	4.092275	3.872223	4.288556	3.204680	4.574791	4.448244	4.882020	4.662653	4.980732	4.625705	6.189761	3.882986	3.113321	3.083503	4.765904	2.978612	3.854271	6.018512	3.791429	6.182378	6.763922	5.635849	7.044502	5.347143	3.684424	4.678389	3.372905	5.177346	3.463379	3.652626	6.319613	4.700068	3.971793	5.759977	3.283480	4.685218	4.830820	4.284744	4.984377	5.632279	5.775641	4.897894	4.628705	3.883794	5.608157	4.577568	3.620854	4.874437	2.858041	3.952483	3.840068	3.932381	3.673900	3.584949	3.341666	3.857435	4.052367	3.987760	4.393824	4.261993	3.723306	4.743173	4.622478	5.263904	4.655780	4.227258	4.934289	5.238860	5.778374	5.180737	5.688775	5.000281	5.164484	5.096222	5.483503	3.961808	4.400248	4.403745	3.535381	3.927841	5.068986	5.224480	5.452642	5.520232	5.491509	3.679225	3.181900	3.299620	4.675819	5.250586	3.631909	4.417080	5.061811	3.337623	3.121240	3.917040	3.365338	3.241074	3.864506	3.473243	3.172255	3.325163	3.823735	5.548243	3.889613	3.510886	3.225437	4.318222	4.436995	3.864506	4.098433	3.223626	3.715868	4.201623	3.342302	3.559737	3.354007	5.740241	3.336283	4.044097	4.388677	3.243536	3.822483	4.342860	2.884071	4.193432	5.601521	4.202709	4.448244	5.493530	3.261885	3.286969	3.359328	4.495328	3.736479	5.043056	4.945436	3.631909	4.929972	3.821032	3.523055	7.016162	4.263555	4.367199	5.940063	4.543780	4.442195	3.563815	3.353204	5.128561	3.948976	4.316930	5.184019	3.385183	2.837600	6.450484	4.327649	4.073634	4.342419	5.131397	4.552175	3.611177	4.291335	4.573305	4.438286	4.183437	3.948773	2.875070	4.267631	3.910557	4.126095	4.082834	3.830958	3.675983	4.196467	3.442575	2.807495	4.976883	4.239223	3.285471	3.963386	5.213361	4.953764	3.574592	3.755476	3.285326	4.384959	3.898000	4.785776	5.068602	4.907319	5.415123	4.943402	4.055845	3.555255	4.344368	3.576726	3.488662	3.342824	5.162860	4.432687	4.775323	4.389553	4.904203	3.911846	4.182548	4.424419	4.447569	3.731296	3.230636	5.774175	5.042632	3.956373	3.414177	3.425458	4.154991	3.285623	4.806984	4.526742	3.526294	3.199539	3.558934	3.862377	3.356572	3.200632	3.312709

AC022509.2	10.388103	11.339812	11.252221	11.346092	12.048596	11.261231	11.101072	10.857955	10.352828	11.117676	10.591883	11.059685	10.632913	11.460998	10.951332	11.394433	10.238329	11.121750	12.002347	11.638275	11.844337	11.330317	10.347954	11.370520	10.769869	10.728085	9.607926	10.487647	10.744869	10.542996	10.348265	10.922099	11.494936	10.035004	10.894314	11.058656	11.284596	11.145002	11.642694	11.714683	11.285631	11.717792	11.015291	11.106019	10.798488	11.183899	10.156965	11.137290	11.055964	11.039164	11.003940	11.004546	11.316464	11.484271	10.703735	10.684506	11.058092	8.299412	11.594141	10.832422	11.709994	10.993754	11.094860	11.880421	11.308833	11.547384	11.638582	11.588775	11.187418	11.631664	11.055681	11.193873	11.094860	11.330888	11.386452	11.106454	11.423308	11.013251	11.404839	11.058248	11.477264	11.076192	11.044022	11.245386	11.330738	11.309982	11.010198	11.558489	12.002950	11.684797	11.503932	12.253731	11.216126	11.182712	10.817752	10.670159	11.263347	10.889830	10.826070	10.590962	10.692703	11.022755	11.038163	11.409205	11.863352	11.039023	11.202757	11.884500	12.154051	10.969544	12.264345	11.707017	11.216559	10.922388	10.983080	10.206432	10.084209	10.873539	10.270720	8.928003	10.731932	9.851548	9.188155	7.325492	9.425426	8.928486	9.381126	9.605466	9.658575	8.807508	7.486641	8.191175	8.509809	8.926611	10.775064	8.689568	8.488318	9.876562	11.331613	8.385714	9.840929	8.989722	6.450320	8.453383	11.194728	9.219146	6.666388	7.002587	9.432202	7.904729	9.905132	10.289278	9.668144	9.385584	9.404833	10.111224	9.807001	10.390190	10.417667	9.070503	9.926621	9.469344	8.447497	9.106280	9.577290	7.980814	10.194166	8.997042	10.781158	9.714868	9.157090	9.450439	9.828115	9.952136	9.756381	10.855566	7.350547	8.825708	9.506715	9.903332	9.549440	9.601437	11.051785	10.871585	10.733261	10.420249	9.712784	9.267666	10.158811	10.361430	10.069917	8.548140	9.787289	10.563986	10.576019	10.012922	10.415101	10.163835	9.968179	10.142065	10.132853	10.132147	10.204611	10.489348	8.799286	9.599562	9.855744	11.375453	10.651028	9.168755	11.303668	9.145633	9.836571	10.160131	9.041626	8.734872	8.702764	9.868222	7.442078	9.099191	10.194030	7.535018	10.034063	9.796567	7.937457	9.546241	10.927410	10.026868	8.844331	10.366684	9.691158	8.177531	10.202012	7.177969	9.695127	8.804154	9.570720	7.976484

AC024145.1	10.388103	11.339812	11.252221	11.346092	12.048596	11.261231	11.101072	10.857955	10.352828	11.117676	10.591883	11.059685	10.632913	11.460998	10.951332	11.394433	10.238329	11.121750	12.002347	11.638275	11.844337	11.330317	10.347954	11.370520	10.769869	10.728085	9.607926	10.487647	10.744869	10.542996	10.348265	10.922099	11.494936	10.035004	10.894314	11.058656	11.284596	11.145002	11.642694	11.714683	11.285631	11.717792	11.015291	11.106019	10.798488	11.183899	10.156965	11.137290	11.055964	11.039164	11.003940	11.004546	11.316464	11.484271	10.703735	10.684506	11.058092	8.299412	11.594141	10.832422	11.709994	10.993754	11.094860	11.880421	11.308833	11.547384	11.638582	11.588775	11.187418	11.631664	11.055681	11.193873	11.094860	11.330888	11.386452	11.106454	11.423308	11.013251	11.404839	11.058248	11.477264	11.076192	11.044022	11.245386	11.330738	11.309982	11.010198	11.558489	12.002950	11.684797	11.503932	12.253731	11.216126	11.182712	10.817752	10.670159	11.263347	10.889830	10.826070	10.590962	10.692703	11.022755	11.038163	11.409205	11.863352	11.039023	11.202757	11.884500	12.154051	10.969544	12.264345	11.707017	11.216559	10.922388	10.983080	10.206432	10.084209	10.873539	10.270720	8.928003	10.731932	9.851548	9.188155	7.325492	9.425426	8.928486	9.381126	9.605466	9.658575	8.807508	7.486641	8.191175	8.509809	8.926611	10.775064	8.689568	8.488318	9.876562	11.331613	8.385714	9.840929	8.989722	6.450320	8.453383	11.194728	9.219146	6.666388	7.002587	9.432202	7.904729	9.905132	10.289278	9.668144	9.385584	9.404833	10.111224	9.807001	10.390190	10.417667	9.070503	9.926621	9.469344	8.447497	9.106280	9.577290	7.980814	10.194166	8.997042	10.781158	9.714868	9.157090	9.450439	9.828115	9.952136	9.756381	10.855566	7.350547	8.825708	9.506715	9.903332	9.549440	9.601437	11.051785	10.871585	10.733261	10.420249	9.712784	9.267666	10.158811	10.361430	10.069917	8.548140	9.787289	10.563986	10.576019	10.012922	10.415101	10.163835	9.968179	10.142065	10.132853	10.132147	10.204611	10.489348	8.799286	9.599562	9.855744	11.375453	10.651028	9.168755	11.303668	9.145633	9.836571	10.160131	9.041626	8.734872	8.702764	9.868222	7.442078	9.099191	10.194030	7.535018	10.034063	9.796567	7.937457	9.546241	10.927410	10.026868	8.844331	10.366684	9.691158	8.177531	10.202012	7.177969	9.695127	8.804154	9.570720	7.976484

AF127577.4	7.455587	7.084263	7.698822	7.495847	7.457145	7.486938	6.883240	8.386331	8.936240	7.594432	7.708847	7.565801	7.440987	7.616653	7.266775	7.410333	7.896560	7.671958	8.453220	7.567642	7.595034	8.129022	6.420124	7.362910	7.690085	7.945245	8.450472	8.153159	7.855600	7.179381	7.902121	6.937142	7.370309	7.692283	6.806159	6.807095	6.426645	6.388174	7.258729	7.371092	7.408527	7.243901	7.210823	7.493825	7.014840	7.051012	7.571280	7.119340	7.066362	6.960210	7.176989	6.798955	6.542379	6.833595	8.040436	7.727475	6.825270	6.055134	6.014010	6.793171	6.872271	7.330395	6.676101	7.538443	6.522347	7.322799	7.036542	6.849324	6.712645	7.140953	6.837524	7.061088	7.850213	7.166091	7.039538	6.472916	6.846482	6.938890	7.140322	6.728831	7.169340	6.803009	7.031101	7.340356	7.114883	6.990944	7.793702	7.254455	6.931748	6.807739	6.421107	7.098777	6.894955	7.060427	7.180642	7.321205	7.664213	7.686252	7.548388	6.986852	8.403614	7.081849	7.680124	7.264197	8.017844	7.933559	8.796854	8.485660	8.320710	7.616815	7.456182	7.864481	8.563905	8.224339	9.582846	7.523749	7.660137	8.029503	7.666067	7.715374	7.430932	7.858699	7.809422	8.219476	7.666215	7.893570	7.526823	8.461405	8.020116	7.897170	7.459810	8.047984	7.898797	7.173102	7.872919	8.228114	8.175683	8.116691	7.360429	7.994571	8.275378	7.387779	8.222067	7.726838	7.049430	7.998868	7.893878	7.786851	7.270165	8.854719	7.879615	8.439347	8.634112	7.620128	6.905419	7.187472	6.946970	7.515375	7.297031	8.269978	7.033591	6.867405	8.614503	7.303187	7.444262	8.478522	6.665725	6.943923	6.717502	7.414000	6.857547	7.359312	7.801145	7.324535	7.313043	7.053478	6.378641	7.267882	5.976039	7.698069	7.345331	7.216857	7.359788	6.977531	6.843699	7.199772	6.998483	6.890278	7.846711	6.689329	8.160678	8.313127	7.532771	7.131815	6.846321	6.713749	7.245629	7.898958	6.985072	7.804165	6.977696	7.482753	7.489439	7.439469	7.863083	8.247106	6.924538	7.204235	7.371424	6.816191	6.998925	7.198837	7.285199	8.525817	8.337894	8.336333	8.485826	8.149015	7.210325	8.444705	7.201165	7.857697	7.506792	7.914298	7.864173	9.394342	8.332937	8.149924	8.500912	7.504760	8.407880	7.606691	8.439018	7.831082	7.621512	7.505088	8.279940	7.941504

AF127577.2	7.455587	7.084263	7.698822	7.495847	7.457145	7.486938	6.883240	8.386331	8.936240	7.594432	7.708847	7.565801	7.440987	7.616653	7.266775	7.410333	7.896560	7.671958	8.453220	7.567642	7.595034	8.129022	6.420124	7.362910	7.690085	7.945245	8.450472	8.153159	7.855600	7.179381	7.902121	6.937142	7.370309	7.692283	6.806159	6.807095	6.426645	6.388174	7.258729	7.371092	7.408527	7.243901	7.210823	7.493825	7.014840	7.051012	7.571280	7.119340	7.066362	6.960210	7.176989	6.798955	6.542379	6.833595	8.040436	7.727475	6.825270	6.055134	6.014010	6.793171	6.872271	7.330395	6.676101	7.538443	6.522347	7.322799	7.036542	6.849324	6.712645	7.140953	6.837524	7.061088	7.850213	7.166091	7.039538	6.472916	6.846482	6.938890	7.140322	6.728831	7.169340	6.803009	7.031101	7.340356	7.114883	6.990944	7.793702	7.254455	6.931748	6.807739	6.421107	7.098777	6.894955	7.060427	7.180642	7.321205	7.664213	7.686252	7.548388	6.986852	8.403614	7.081849	7.680124	7.264197	8.017844	7.933559	8.796854	8.485660	8.320710	7.616815	7.456182	7.864481	8.563905	8.224339	9.582846	7.523749	7.660137	8.029503	7.666067	7.715374	7.430932	7.858699	7.809422	8.219476	7.666215	7.893570	7.526823	8.461405	8.020116	7.897170	7.459810	8.047984	7.898797	7.173102	7.872919	8.228114	8.175683	8.116691	7.360429	7.994571	8.275378	7.387779	8.222067	7.726838	7.049430	7.998868	7.893878	7.786851	7.270165	8.854719	7.879615	8.439347	8.634112	7.620128	6.905419	7.187472	6.946970	7.515375	7.297031	8.269978	7.033591	6.867405	8.614503	7.303187	7.444262	8.478522	6.665725	6.943923	6.717502	7.414000	6.857547	7.359312	7.801145	7.324535	7.313043	7.053478	6.378641	7.267882	5.976039	7.698069	7.345331	7.216857	7.359788	6.977531	6.843699	7.199772	6.998483	6.890278	7.846711	6.689329	8.160678	8.313127	7.532771	7.131815	6.846321	6.713749	7.245629	7.898958	6.985072	7.804165	6.977696	7.482753	7.489439	7.439469	7.863083	8.247106	6.924538	7.204235	7.371424	6.816191	6.998925	7.198837	7.285199	8.525817	8.337894	8.336333	8.485826	8.149015	7.210325	8.444705	7.201165	7.857697	7.506792	7.914298	7.864173	9.394342	8.332937	8.149924	8.500912	7.504760	8.407880	7.606691	8.439018	7.831082	7.621512	7.505088	8.279940	7.941504

DLX6-AS1	4.614899	5.455701	6.098026	5.691739	5.676760	7.181888	6.190968	6.719816	8.405606	6.961340	6.231257	6.215090	6.843699	6.269459	5.121307	5.449926	5.998669	6.169965	6.771561	6.009651	5.546573	6.648197	6.946451	5.477861	5.919034	5.572010	5.770690	6.618065	6.228480	6.129505	5.647464	6.277687	7.320440	5.329694	6.042449	5.781818	5.304609	5.113751	5.772748	5.839873	5.559864	5.484805	6.813820	5.697684	5.544296	6.339386	6.444202	5.301629	5.680842	5.036945	4.895587	4.957084	6.318421	6.024627	4.871701	6.914558	5.741912	5.610492	5.671418	5.592538	6.270689	5.359108	5.034847	6.743511	6.182710	5.970151	4.740420	5.814075	6.944407	5.805975	5.549397	6.063309	5.961118	5.417579	6.586695	5.665156	5.305593	6.344898	5.861573	4.853550	4.798741	5.352869	4.842988	5.588508	5.980847	6.609956	6.967400	6.453644	6.179165	5.681420	5.890818	5.646696	6.418153	5.711017	5.006848	5.855821	5.924711	5.544296	6.393377	5.357204	5.947925	5.390063	5.989536	5.607219	7.073703	5.840048	6.795726	6.269643	5.795625	5.866471	6.863771	6.128820	6.229015	6.273038	7.347533	7.427119	5.083639	6.064179	6.021706	5.092048	6.404890	6.820707	5.727722	8.953955	9.310802	9.259991	8.419013	8.959613	10.348851	6.947625	9.711248	8.167811	5.749191	6.559759	6.798331	8.381126	10.190269	9.057845	6.538915	6.764402	9.517296	8.534236	6.803329	8.731353	6.986195	6.255529	8.418259	8.046561	7.703743	7.678603	9.081198	8.551058	5.994297	7.803260	9.473557	6.781665	7.111288	6.511935	8.550896	8.191175	9.755615	10.678806	6.186223	9.575333	8.788120	8.655658	8.355063	9.486874	7.599534	7.589045	6.678173	9.003802	6.074144	8.325088	7.415512	5.741546	8.752281	7.704547	8.698049	9.365816	5.860480	7.247008	8.433978	7.075119	9.194647	5.812038	8.312374	7.761133	6.037029	9.298596	8.557910	6.859786	7.334421	7.830502	7.472022	7.178765	8.962224	6.707042	5.901312	7.095799	5.506211	7.656423	6.269459	6.457389	9.069131	8.406370	5.029542	5.011793	6.026589	8.664235	5.966988	7.740253	6.262524	7.050084	5.719393	8.926272	8.440271	5.980140	11.020219	6.219210	8.323832	5.410703	6.993859	10.199328	7.920886	8.154232	6.816998	9.273471	6.804922	8.149308	6.812404	7.581501	6.120458	10.840049	9.081657	8.623429	8.978474	6.349412

AC244453.2	7.476608	9.151857	8.563746	9.387940	9.347711	8.832732	9.513068	10.001021	10.325821	10.177481	10.280033	9.372405	9.710473	10.506461	8.861351	9.662962	9.926775	9.766182	9.332303	10.190552	7.702064	9.148637	7.135309	9.879782	8.864142	9.256667	6.136033	9.591051	8.544600	7.445391	10.581000	9.177343	9.178024	6.754906	9.352897	8.525817	9.495957	9.571519	9.145633	9.104114	9.149548	8.236375	8.926942	9.343442	8.495049	8.890530	8.245391	9.173279	9.450937	10.044497	8.938979	9.611660	8.791046	9.559852	8.861351	8.262447	9.325363	9.416873	8.033303	8.820838	8.707677	9.291287	7.226387	8.307762	9.297087	8.051259	8.615278	8.959155	8.774914	8.089878	8.497774	9.275769	10.169200	9.508697	9.982592	8.524918	8.258326	8.527214	8.914940	9.410961	9.561452	9.081657	8.794067	9.585922	10.245866	9.505806	10.190703	10.251612	8.042316	9.255589	8.728680	8.518412	8.478049	9.568749	8.001370	9.222218	9.478926	10.868882	8.850032	9.929233	10.416591	9.487673	9.841825	9.571980	8.963573	9.338178	9.281202	8.560695	9.302664	9.661702	8.758681	10.540943	10.292953	10.149344	8.346304	9.264575	7.487256	8.331048	9.982125	8.499181	8.880498	7.562431	11.787766	7.674779	6.854940	6.152236	8.574739	8.144863	7.235475	7.371577	7.592260	6.452423	8.084435	5.226669	8.586239	7.271105	7.981758	9.499181	6.469202	5.655361	6.665078	7.186039	6.566720	6.306280	8.439490	7.298148	7.249927	7.524985	8.092448	10.436000	7.192778	8.106204	7.114093	8.482792	8.100093	7.648283	9.215294	6.110778	6.970435	6.713926	8.290249	11.322294	7.816055	8.430715	8.644694	7.128470	11.271637	7.042248	7.479964	9.180782	7.758843	8.612810	6.996945	10.202804	10.424711	8.219775	8.092448	8.640510	7.996465	8.061672	6.670398	6.377471	7.127214	8.788445	8.759174	7.154664	7.038273	7.351296	7.005199	7.452274	7.076686	9.806105	8.015395	8.387729	7.579023	7.205220	6.838519	8.251152	8.633518	8.231627	8.291499	7.941504	9.285069	8.749699	8.073510	7.826953	12.194595	7.518657	8.546755	6.721446	7.606389	8.161001	7.321205	8.482967	8.100252	8.898403	8.452479	9.094042	7.872609	7.137983	9.120791	6.197741	8.104330	8.887600	6.241762	7.838309	9.820277	7.264952	5.652761	5.797284	8.143459	9.899599	8.449850	7.203178	8.306186	7.572330	7.812489	7.540217

AC244453.3	7.476608	9.151857	8.563746	9.387940	9.347711	8.832732	9.513068	10.001021	10.325821	10.177481	10.280033	9.372405	9.710473	10.506461	8.861351	9.662962	9.926775	9.766182	9.332303	10.190552	7.702064	9.148637	7.135309	9.879782	8.864142	9.256667	6.136033	9.591051	8.544600	7.445391	10.581000	9.177343	9.178024	6.754906	9.352897	8.525817	9.495957	9.571519	9.145633	9.104114	9.149548	8.236375	8.926942	9.343442	8.495049	8.890530	8.245391	9.173279	9.450937	10.044497	8.938979	9.611660	8.791046	9.559852	8.861351	8.262447	9.325363	9.416873	8.033303	8.820838	8.707677	9.291287	7.226387	8.307762	9.297087	8.051259	8.615278	8.959155	8.774914	8.089878	8.497774	9.275769	10.169200	9.508697	9.982592	8.524918	8.258326	8.527214	8.914940	9.410961	9.561452	9.081657	8.794067	9.585922	10.245866	9.505806	10.190703	10.251612	8.042316	9.255589	8.728680	8.518412	8.478049	9.568749	8.001370	9.222218	9.478926	10.868882	8.850032	9.929233	10.416591	9.487673	9.841825	9.571980	8.963573	9.338178	9.281202	8.560695	9.302664	9.661702	8.758681	10.540943	10.292953	10.149344	8.346304	9.264575	7.487256	8.331048	9.982125	8.499181	8.880498	7.562431	11.787766	7.674779	6.854940	6.152236	8.574739	8.144863	7.235475	7.371577	7.592260	6.452423	8.084435	5.226669	8.586239	7.271105	7.981758	9.499181	6.469202	5.655361	6.665078	7.186039	6.566720	6.306280	8.439490	7.298148	7.249927	7.524985	8.092448	10.436000	7.192778	8.106204	7.114093	8.482792	8.100093	7.648283	9.215294	6.110778	6.970435	6.713926	8.290249	11.322294	7.816055	8.430715	8.644694	7.128470	11.271637	7.042248	7.479964	9.180782	7.758843	8.612810	6.996945	10.202804	10.424711	8.219775	8.092448	8.640510	7.996465	8.061672	6.670398	6.377471	7.127214	8.788445	8.759174	7.154664	7.038273	7.351296	7.005199	7.452274	7.076686	9.806105	8.015395	8.387729	7.579023	7.205220	6.838519	8.251152	8.633518	8.231627	8.291499	7.941504	9.285069	8.749699	8.073510	7.826953	12.194595	7.518657	8.546755	6.721446	7.606389	8.161001	7.321205	8.482967	8.100252	8.898403	8.452479	9.094042	7.872609	7.137983	9.120791	6.197741	8.104330	8.887600	6.241762	7.838309	9.820277	7.264952	5.652761	5.797284	8.143459	9.899599	8.449850	7.203178	8.306186	7.572330	7.812489	7.540217

LINC01931	5.268480	5.302043	5.008684	5.475614	4.903587	5.053637	4.011825	5.325747	5.257917	5.629358	6.034559	4.826975	5.113938	5.506814	4.179448	5.005573	5.960403	4.669702	6.096861	5.213361	5.096594	5.530683	5.767564	5.131397	4.728702	5.208231	5.303625	5.152347	4.756537	4.092696	5.735022	4.946505	5.040737	4.909040	5.177742	4.735884	5.446009	5.706129	5.919744	5.385942	5.319749	5.628403	5.850256	5.154192	4.780742	4.908200	6.629846	5.251176	4.683097	4.699871	4.894172	4.668169	5.412627	4.680948	5.954888	6.277687	4.866626	3.489559	4.843175	5.118642	4.810612	4.511823	6.157198	5.879823	6.308579	5.706630	6.054615	5.806670	5.838997	5.791150	5.574273	5.045149	6.253339	5.447016	6.023595	5.859422	5.242838	5.400149	5.287363	5.108299	5.470625	4.991195	4.595108	4.793308	5.181372	5.404983	4.685218	6.550701	5.668880	5.477091	5.732104	5.765379	5.158863	5.151558	5.998669	4.895003	5.112562	4.821688	4.799646	5.115798	4.117895	4.789720	5.510681	5.744826	6.149325	5.279964	6.268096	5.673483	5.787010	7.619091	5.692666	6.174035	5.437831	5.944776	4.910284	4.122955	5.809240	5.422633	4.649179	4.856480	5.693794	4.721510	4.724734	4.214253	5.392969	3.926528	5.538246	5.821481	5.001521	5.561665	4.088092	4.838712	5.366408	4.186652	5.747033	6.053214	4.011026	5.852202	4.656631	4.575798	4.463060	5.352068	4.645359	4.530746	4.676681	4.367399	4.991612	4.458635	4.099063	5.597418	4.605418	4.847648	4.367632	4.498788	5.443327	5.030342	5.953274	6.419962	5.386330	4.971704	6.170311	6.200170	5.302426	5.327510	4.791016	5.410318	4.879417	5.212013	4.680730	5.436894	4.897681	4.273558	5.715666	5.550863	5.833332	4.709693	4.794571	5.171289	4.609944	5.582288	4.383150	6.294060	6.306611	5.462213	5.183216	6.390022	5.791150	6.022066	5.617409	6.069272	4.776361	5.974298	5.754786	5.504312	5.697884	5.218154	6.002372	4.957084	4.764426	5.362720	4.958542	4.686970	4.870615	5.302043	4.576446	4.869580	6.456542	5.757763	5.439483	5.390865	6.024815	5.241641	5.169505	5.999906	4.998590	5.429568	4.603961	5.307530	5.178999	3.955259	4.367399	4.881587	4.621436	5.835368	4.927632	6.961011	5.154192	5.781628	6.372278	5.201863	5.002123	6.217146	5.131805	4.100634	3.519668	5.355303	4.929303	4.572417

AL133166.1	3.167149	3.090114	2.964728	3.049338	2.986372	3.392300	3.241347	3.075527	3.379675	3.241487	3.467279	3.506641	3.209242	3.839885	3.414533	3.054765	3.396948	3.216236	7.902571	3.182313	4.233943	3.799662	4.316491	3.099621	3.737498	3.003001	3.050591	3.413645	3.007845	3.019025	4.301736	3.179124	4.571161	3.087392	4.563169	4.042997	4.286131	3.161356	3.923084	3.110252	3.416052	3.329990	3.438067	3.462494	3.432203	3.825737	3.256210	3.847173	3.182313	3.902322	3.884271	3.586323	3.531510	3.905736	3.412222	3.226157	3.819707	3.742824	3.913757	3.586323	3.707485	3.232021	3.329843	3.048798	3.104345	3.628019	3.035479	3.458213	3.048062	3.121240	3.492031	4.303540	3.242972	3.711134	2.958816	3.212552	3.746283	3.206407	3.535725	3.444894	3.485375	3.643475	3.078385	3.351071	3.009067	4.244526	3.903127	3.353669	3.494647	3.270231	3.013565	3.393144	3.088102	3.635857	3.054765	3.343473	3.333388	3.013376	3.441000	3.004535	3.903586	3.188468	2.973908	2.826793	4.328317	2.938239	2.939668	3.192699	3.099517	3.126509	3.247223	3.612579	3.176379	4.154312	3.035580	3.617257	3.107369	2.968562	3.392134	3.197854	3.145662	2.934535	2.953835	3.014178	3.266021	3.034402	3.104667	3.334678	3.082860	3.245376	3.118273	3.060561	3.219233	3.136736	3.134024	3.331129	3.023990	4.177655	3.202402	3.710276	2.869606	3.582650	3.061340	3.145036	2.949012	3.115938	2.917130	3.166213	3.365817	3.406943	3.261306	3.207803	3.131810	4.028832	3.734589	3.690345	3.143382	3.101726	3.029656	3.187273	3.127307	3.449361	3.314652	3.608603	3.984336	3.204548	3.878927	3.304280	3.921822	3.899988	3.512749	3.314483	3.925693	3.179398	2.932255	3.884501	3.527576	3.792920	3.618187	3.456073	3.044010	3.302535	3.241621	3.192314	3.104899	3.029060	3.467086	3.137848	3.314483	3.231565	3.128450	3.004637	3.358816	3.171311	3.185682	3.485680	3.093250	3.043798	3.351386	3.313984	3.655248	3.154663	3.363890	3.401604	3.174332	3.167023	3.183560	3.319276	3.251299	3.080285	2.972725	3.039322	3.033076	2.976768	3.394875	3.081216	3.213397	3.160172	3.252661	3.112376	3.049767	3.045673	3.251540	4.656839	2.953586	2.897780	3.172139	3.335963	3.044761	3.122974	3.559932	3.664851	3.437397	2.938071	2.888289	2.974354	2.970441	3.168171

AL135878.1	3.167149	3.090114	2.964728	3.049338	2.986372	3.392300	3.241347	3.075527	3.379675	3.241487	3.467279	3.506641	3.209242	3.839885	3.414533	3.054765	3.396948	3.216236	7.902571	3.182313	4.233943	3.799662	4.316491	3.099621	3.737498	3.003001	3.050591	3.413645	3.007845	3.019025	4.301736	3.179124	4.571161	3.087392	4.563169	4.042997	4.286131	3.161356	3.923084	3.110252	3.416052	3.329990	3.438067	3.462494	3.432203	3.825737	3.256210	3.847173	3.182313	3.902322	3.884271	3.586323	3.531510	3.905736	3.412222	3.226157	3.819707	3.742824	3.913757	3.586323	3.707485	3.232021	3.329843	3.048798	3.104345	3.628019	3.035479	3.458213	3.048062	3.121240	3.492031	4.303540	3.242972	3.711134	2.958816	3.212552	3.746283	3.206407	3.535725	3.444894	3.485375	3.643475	3.078385	3.351071	3.009067	4.244526	3.903127	3.353669	3.494647	3.270231	3.013565	3.393144	3.088102	3.635857	3.054765	3.343473	3.333388	3.013376	3.441000	3.004535	3.903586	3.188468	2.973908	2.826793	4.328317	2.938239	2.939668	3.192699	3.099517	3.126509	3.247223	3.612579	3.176379	4.154312	3.035580	3.617257	3.107369	2.968562	3.392134	3.197854	3.145662	2.934535	2.953835	3.014178	3.266021	3.034402	3.104667	3.334678	3.082860	3.245376	3.118273	3.060561	3.219233	3.136736	3.134024	3.331129	3.023990	4.177655	3.202402	3.710276	2.869606	3.582650	3.061340	3.145036	2.949012	3.115938	2.917130	3.166213	3.365817	3.406943	3.261306	3.207803	3.131810	4.028832	3.734589	3.690345	3.143382	3.101726	3.029656	3.187273	3.127307	3.449361	3.314652	3.608603	3.984336	3.204548	3.878927	3.304280	3.921822	3.899988	3.512749	3.314483	3.925693	3.179398	2.932255	3.884501	3.527576	3.792920	3.618187	3.456073	3.044010	3.302535	3.241621	3.192314	3.104899	3.029060	3.467086	3.137848	3.314483	3.231565	3.128450	3.004637	3.358816	3.171311	3.185682	3.485680	3.093250	3.043798	3.351386	3.313984	3.655248	3.154663	3.363890	3.401604	3.174332	3.167023	3.183560	3.319276	3.251299	3.080285	2.972725	3.039322	3.033076	2.976768	3.394875	3.081216	3.213397	3.160172	3.252661	3.112376	3.049767	3.045673	3.251540	4.656839	2.953586	2.897780	3.172139	3.335963	3.044761	3.122974	3.559932	3.664851	3.437397	2.938071	2.888289	2.974354	2.970441	3.168171

AL080276.2	10.755577	11.130181	11.142070	10.429717	10.090376	10.249401	10.897742	10.198291	8.290249	10.470880	10.161839	10.220177	10.174868	10.700757	10.709373	10.470880	9.586696	10.620285	10.437655	10.940377	10.501981	11.187132	9.523327	10.509626	10.086825	10.028734	9.251636	9.255589	9.406320	10.505273	8.969110	10.531581	10.584342	10.899829	10.897417	10.844714	10.745574	10.851359	10.219536	10.337046	10.177339	10.390190	10.541990	10.530395	10.787927	11.007562	9.419282	10.711692	10.651028	11.102976	10.464013	10.768642	11.348941	11.410542	11.456392	10.247271	11.263631	11.338351	10.843477	11.146191	11.289617	10.238166	11.807364	10.783768	10.706734	11.064379	10.954214	10.716420	10.764458	11.049601	10.952091	10.963108	9.466897	10.809503	11.041512	10.957536	11.045577	10.785111	10.812975	11.315712	11.140228	11.146644	10.922691	11.402644	11.300255	10.478807	11.190467	11.386601	11.447359	10.836911	10.854038	11.622534	11.267660	11.403690	10.746642	10.955728	10.683914	9.306308	10.100136	10.115901	10.204906	10.952734	9.992352	10.523247	11.619033	10.070373	9.693309	11.164048	10.850618	9.870596	10.636091	9.586539	10.081326	9.479088	10.232495	8.793229	10.086189	10.647283	8.703539	9.872579	10.265433	10.809966	9.441108	8.596272	9.605154	9.657205	9.062460	9.400155	9.622752	9.724077	9.794550	9.554938	9.738216	9.848797	9.831828	10.103883	9.569490	10.464013	10.740649	10.288521	10.523560	9.194946	9.783941	10.712868	10.534405	10.096406	9.319592	8.445759	10.172011	8.478824	9.939497	10.086677	11.099157	10.227905	10.553085	10.449732	10.500161	9.397811	10.017560	9.720935	10.147330	10.307130	11.169437	11.295839	11.253098	8.727249	10.560245	10.324445	12.056828	10.815965	10.529524	11.239944	12.016745	10.870835	9.291136	10.834204	11.479490	10.499007	11.416326	10.840472	9.765411	11.343391	10.967834	10.498125	10.410131	10.724311	10.817152	11.056896	11.068828	11.352031	10.930738	9.028112	10.877054	10.396893	10.686158	10.980998	11.261074	10.575398	11.031212	10.912200	11.092206	10.841416	10.734015	11.286882	9.658892	11.213347	11.079978	11.235980	10.782833	11.023655	11.753661	11.097215	11.265170	10.711413	10.331914	10.182257	8.632197	9.327661	10.373227	10.538968	10.246494	9.518998	10.395960	11.113033	8.224643	9.058147	10.918390	11.774172	9.778317	11.291891	9.778915	9.948751	8.659450	10.124118	10.331612	10.494077	10.626257	9.584671

DUXAP8	7.561200	7.514634	7.783157	7.769286	8.490573	7.840159	7.143957	8.654016	8.813641	7.404585	7.591191	7.545653	7.584419	7.207080	7.280692	7.822865	8.566420	7.412742	8.399882	7.652378	8.369555	8.071679	9.758866	7.684886	7.821500	8.454290	7.915196	8.436316	7.187241	7.653754	8.968822	6.629202	7.393516	7.609920	7.119015	7.101718	7.234690	7.267272	7.651592	7.937742	7.487725	7.673196	7.235310	8.151906	7.141280	7.185698	8.369861	7.331485	7.627673	7.257882	7.406485	7.320594	6.792518	7.351914	7.082213	7.802216	6.703844	7.247454	7.649041	7.512950	7.599022	7.748566	8.033614	8.352011	8.192133	8.081792	7.850360	7.533908	7.598098	8.041408	7.520351	7.416992	7.603602	7.452446	7.608834	7.820563	8.015866	7.906928	7.894029	7.443772	7.471877	7.416551	7.350547	7.471570	7.781521	7.397708	7.608208	8.178859	8.188055	8.502160	8.275804	8.909516	7.492285	7.740704	7.818572	7.711235	7.360909	7.227941	8.179776	7.654980	7.877178	6.878619	7.203950	7.132134	8.015092	7.310568	8.895467	8.283307	8.797467	7.511861	8.817585	8.825386	8.578625	7.989708	8.072719	9.360471	7.962059	7.416404	7.685039	8.802470	8.598194	8.198239	8.833056	10.223028	8.524461	9.222522	9.245349	8.338549	10.046320	8.897303	9.125500	7.989562	8.888219	8.799766	8.554809	9.408477	9.595374	9.721843	7.845788	8.827765	10.144304	9.187229	8.661319	9.106902	9.083932	8.319872	8.998256	9.463307	8.663180	8.690022	8.256035	9.315503	7.845347	7.078154	8.337276	7.636038	7.763140	8.520528	8.552114	10.232331	8.240685	8.095215	9.550248	7.518197	7.814030	9.011761	7.233867	7.718880	7.771722	8.546755	8.445308	8.389906	7.445068	8.182665	7.694438	7.543432	7.986529	8.726020	7.771722	8.333242	8.013377	8.394089	9.160471	9.005980	9.571980	7.797378	7.774290	8.743289	8.756688	8.271789	7.860474	9.141971	8.614211	7.751360	8.131646	8.665298	8.404218	8.280995	7.887812	7.373908	7.449836	8.302736	7.649648	8.012452	9.883634	8.766397	9.306026	8.527971	8.669282	8.246658	9.632756	8.834349	8.924459	8.006706	8.914940	8.663767	8.895157	9.229700	8.275513	8.576545	8.703539	9.563299	7.786526	8.903060	8.108945	9.140105	8.037595	8.357685	7.996145	9.353627	9.824019	9.591971	9.182973	8.622183	8.689858	8.172997	9.194034	9.201089

LEF1-AS1	5.781452	6.989994	6.756046	7.191534	6.194626	5.744092	7.350831	6.487592	6.341049	6.218387	6.838662	6.774295	7.048345	7.042589	7.335511	6.028736	6.363544	7.821807	8.371594	7.499603	6.195674	8.259547	6.198966	6.803009	6.294400	6.731428	7.043506	5.885687	6.192830	7.286449	7.378490	7.893570	8.314211	7.828691	7.641095	7.124170	6.465215	7.567340	8.224051	7.270478	6.831311	7.317336	7.480282	7.126885	6.350277	7.135790	5.048303	7.825209	7.385317	7.461194	7.034373	7.706091	7.587048	7.907411	7.456028	6.817477	7.659080	7.455423	7.895952	7.800683	7.625464	6.429707	7.608060	7.123519	8.038832	7.282114	7.537013	6.751932	7.977839	7.249623	7.610220	6.802515	6.230595	6.501873	7.579796	7.345653	6.728379	7.976484	6.815893	7.674955	7.216069	7.576179	7.744247	7.250881	7.516962	7.400060	8.158860	7.659822	8.593782	8.384200	7.474898	8.604373	7.617692	6.416338	7.338770	7.252299	6.730953	6.942632	6.783072	6.049248	5.755895	6.622744	6.399654	6.681903	8.185252	6.367558	5.148725	7.684432	7.944176	5.753589	8.469008	7.505088	7.013318	5.278342	6.016900	6.243437	6.293693	5.847149	6.638849	6.853335	8.852935	9.176514	7.516637	7.099426	6.032173	8.060419	11.704895	8.723582	8.109516	8.255129	8.898713	7.901337	9.095402	7.234690	7.111770	9.024914	9.772051	11.229016	10.549196	8.715629	9.759184	10.007656	6.484247	9.317025	10.369246	8.343060	7.298925	7.849606	8.443006	7.873058	7.182971	9.523644	7.421675	7.531429	8.227358	8.603917	7.970993	7.744557	9.964264	7.154973	9.057845	9.023129	7.422597	8.912971	8.629123	6.599666	9.434327	8.844962	9.109868	8.453220	9.090357	8.779928	8.993336	9.356962	7.818413	11.956358	9.268476	8.087562	9.186268	9.402928	6.560378	9.276381	7.860474	8.704893	8.893638	8.774288	8.956217	8.355382	9.025712	8.204934	8.938682	7.443943	9.025392	8.976956	7.927415	9.262267	9.123809	8.716580	9.341602	9.022623	8.379382	8.980884	9.339487	9.512448	8.858656	8.953170	9.668932	10.637121	9.634765	9.863778	9.639654	8.915861	9.188009	8.495845	9.186105	7.905856	8.842601	8.157342	8.902489	8.939900	11.387475	8.096606	8.964030	11.027337	7.246079	7.433129	8.166604	9.422277	6.371417	10.720767	8.869289	8.141183	6.109395	8.446869	7.336598	8.227519	6.481749	7.560439

GAS6-AS1	13.838218	8.993336	9.751120	11.203446	10.083424	8.971604	8.557775	8.436316	10.466388	8.443006	8.971009	8.767777	9.319744	8.453530	8.396241	9.666076	10.409686	8.462342	9.271815	9.973102	8.679147	10.600462	10.985966	10.611543	9.269982	9.667331	10.678967	9.073598	9.173899	8.638495	10.825918	9.982592	10.400960	10.907218	9.495345	10.018168	10.013832	8.768080	9.668780	9.962417	8.894991	9.392213	10.016803	9.990425	9.792563	10.147330	9.870914	10.007217	9.996472	9.571668	11.785826	10.275333	9.018670	9.390797	9.458028	8.429598	9.603904	10.002721	9.333063	10.122478	9.898527	7.780295	9.288325	10.110954	9.775681	10.274257	9.690691	9.286431	9.824019	11.239635	10.637586	11.035559	8.997519	11.166964	10.783906	9.584068	9.752217	9.393908	12.080159	8.478374	10.270875	9.372256	10.576019	9.374473	9.822841	9.836257	9.787911	9.816798	9.598639	10.212581	10.114066	10.289278	9.118977	10.454796	11.801044	10.562644	9.373508	10.209520	9.205043	10.126943	8.508449	9.465805	9.511205	9.132751	8.972481	9.043309	9.303419	8.900522	9.856355	8.244137	10.154986	10.018641	9.529591	10.504682	9.293955	8.414877	10.390905	9.184707	9.405273	9.716241	6.667203	10.044168	11.329537	8.424967	10.550253	8.615111	8.178859	8.749551	10.061953	11.261529	7.450737	9.442348	7.718421	8.193517	10.065419	8.509216	11.134615	9.701109	8.569561	6.506499	9.024612	7.870132	8.450472	10.009427	9.342816	7.921494	7.509564	7.926641	8.875559	9.496877	10.739617	11.178683	8.067428	8.921804	10.425603	8.567616	8.041233	10.826808	10.234480	7.358858	8.015709	8.780550	7.769881	9.128284	8.528920	6.920175	8.052945	9.551318	7.396775	9.437744	8.908475	8.402976	7.331642	8.924459	11.176868	8.796854	10.294926	10.014741	8.312674	9.334161	8.539743	9.304141	8.890976	11.289188	10.373383	7.008983	8.041861	9.331538	8.707526	9.313309	10.174103	10.518938	11.570177	10.311282	8.414441	11.290061	8.012294	11.927388	8.679147	9.667475	7.927895	11.044169	9.628799	8.279940	11.635707	10.671515	10.026067	9.074052	10.439729	6.767312	11.616246	8.120535	10.024497	9.547157	8.993798	13.184665	10.362833	11.575527	11.795725	8.305738	7.909425	8.685533	8.103852	7.884152	7.614495	10.441113	11.068537	8.928643	8.272998	8.897794	6.338219	7.529779	9.430938	9.986251	9.025867	9.465672	10.050969	10.113319

AC091133.3	14.406946	13.214418	13.147891	13.828722	13.932694	14.830972	14.460851	14.333069	14.744545	14.704765	14.874974	14.597809	14.345644	13.788993	13.242980	14.659974	14.551462	13.472510	13.530118	14.216321	13.370191	13.234611	13.337083	13.640816	13.952110	14.285845	14.940393	14.418742	14.908102	14.557653	14.028211	12.976769	13.275436	14.340690	14.160557	13.770853	14.101624	13.201795	13.505465	13.344888	14.868226	13.410767	13.825287	13.128244	13.374219	13.341166	14.960048	13.231451	13.407261	13.459166	13.423212	13.289244	12.478336	12.455171	12.894925	14.967376	12.672242	12.817679	12.685364	12.713455	12.722641	14.716740	12.660319	12.787861	12.563000	12.760481	12.926643	13.060084	13.048001	12.947738	14.364475	14.405128	15.017444	14.294729	14.314071	13.873644	13.844292	13.724916	13.483216	12.662065	12.940635	12.772931	12.399784	12.927977	13.124180	13.330066	13.078931	13.045554	12.417049	12.344853	12.362273	12.388237	13.249512	13.559082	13.685040	13.738340	13.249693	13.788457	14.110411	14.726053	14.083435	13.651504	14.061795	13.744349	12.392356	14.642705	13.057446	12.785412	13.393903	15.022274	12.827524	13.361393	13.504360	15.078176	14.676953	15.693501	13.595345	14.546813	15.219810	14.103780	13.480608	13.390765	13.994733	14.646701	14.864634	14.957224	14.824917	14.710793	14.824917	14.857987	14.920608	14.907590	13.927849	13.968540	14.779929	14.332725	13.703646	13.330495	13.899661	13.526786	13.560400	13.325208	14.315778	13.785091	13.883550	14.401854	14.607572	15.497254	14.562573	14.194648	13.577611	13.860196	14.356594	14.231871	14.302591	13.962183	13.327985	13.742612	13.717944	15.201679	13.450752	13.662957	13.331903	13.216575	13.524838	14.987284	13.418399	13.413378	13.460034	13.362873	13.231246	12.705525	12.554619	12.943191	14.824917	12.624487	12.649718	12.673786	12.538254	12.821082	14.764819	12.691020	12.663072	12.838440	12.811770	13.055072	13.006847	13.229238	12.873035	14.277280	14.349898	15.198897	14.624663	14.128019	13.867818	13.943473	14.092821	14.011814	12.723223	12.860065	12.794117	12.623671	13.189155	13.034304	14.027670	12.957538	12.871943	12.560381	12.566268	12.682992	12.453577	13.628249	13.992685	13.849077	14.162706	14.041399	14.062109	13.948216	14.653700	14.074236	14.015006	14.480594	13.418183	12.478971	14.853812	13.499351	12.637159	12.862948	15.260285	12.872852	13.219767	14.037568	15.362494	14.535115	14.695210	13.140268	14.469705	14.855908

ZSCAN16-AS1	9.347399	8.788596	8.665460	8.651036	8.701516	8.253582	8.847714	7.671796	7.369686	8.370333	8.476914	8.436002	8.673596	9.662803	9.540332	8.697420	8.529384	9.488153	10.365940	9.089471	10.217279	9.643474	9.520679	8.753340	8.779006	8.546912	6.991290	8.770683	7.732409	8.493243	8.410456	9.695858	10.058151	8.320792	9.238262	9.158340	9.252981	9.507486	9.829982	9.736242	8.482018	10.095931	8.604836	8.946909	8.509809	8.814119	8.360324	9.674605	9.729192	9.270915	9.455263	9.903802	9.698167	9.545005	9.542983	8.603452	9.160000	10.023543	10.202479	10.064236	10.482463	8.177095	10.308784	10.173198	10.344088	10.004105	9.925324	9.354397	9.550096	10.083270	9.564026	9.237796	8.525979	8.949507	9.180474	9.605311	9.153080	9.437883	9.230297	9.737303	9.370301	9.348320	9.612099	9.981361	9.653378	9.682637	10.192680	9.810719	10.737937	10.693482	10.082987	10.679894	8.991935	9.207939	9.543140	9.284164	9.240739	8.409690	9.544433	8.527971	8.196072	8.535005	8.089401	9.650396	10.132147	8.571583	9.818140	10.297862	8.840427	8.477896	10.915527	8.887442	9.239831	7.042723	8.923143	6.737629	9.986994	8.295459	8.005597	8.252750	8.887920	8.070785	9.089780	6.912751	8.895309	7.126885	5.544882	6.114580	8.206187	6.665725	8.497483	6.587705	8.971604	8.340263	7.640791	7.875043	8.779318	9.067544	7.204873	10.348851	9.046235	10.151269	7.752866	8.574878	8.644551	8.456103	9.180782	5.172120	8.261704	8.318796	8.829820	8.994735	8.908772	9.858261	8.482646	8.816989	8.196072	9.524275	9.093737	8.362863	9.236267	8.951958	8.331848	7.734740	9.000251	8.152212	9.584068	9.274259	8.561770	9.071115	9.921369	8.585756	9.158783	9.354243	7.514055	9.008878	9.545615	9.841825	8.966033	9.742277	8.471218	10.845168	9.980743	10.030841	10.153109	8.911908	9.583919	8.923868	10.225898	8.775212	7.765779	8.240987	8.827765	8.680362	9.252371	9.024005	8.782589	9.006556	9.509295	8.881902	10.014459	9.145795	8.492102	9.179234	9.484092	9.392820	10.278824	9.998417	9.629098	9.180782	10.186361	8.786239	8.791046	9.669428	9.082741	8.738106	7.162520	8.973069	9.379453	7.589345	9.165262	8.792140	9.068809	10.918947	7.284600	8.052786	10.374549	8.632336	7.800225	9.224967	7.805396	5.531254	7.208783	8.999207	9.898224	9.048398	8.188180	10.002111

NBR2	7.053815	7.705019	9.343930	7.896881	8.346927	7.477675	8.094184	7.889051	9.176364	8.248548	7.485863	8.076073	7.037948	7.852952	6.342505	8.274926	9.478608	6.736595	7.509424	7.390858	6.084413	9.187694	6.957997	6.768967	6.979858	7.293485	7.284141	7.304121	7.814030	7.186349	7.224035	7.589490	7.676648	6.692115	7.957449	8.038832	7.303187	7.832314	7.738668	8.231793	7.096876	7.509564	8.262784	7.898223	9.010252	8.765277	7.117886	7.802360	8.333380	9.365025	8.446695	8.983047	7.670062	8.920911	7.842151	8.212972	7.995341	8.466728	8.484010	8.259398	8.163103	7.763900	7.311966	8.376752	7.689797	9.433117	8.142105	8.042486	8.024837	10.244125	7.783481	8.514294	7.512950	8.601569	8.047176	7.363410	9.139637	8.429270	8.825090	7.902721	7.877178	7.032025	6.775437	7.453707	8.398367	8.508291	6.907201	7.620888	8.352459	8.305140	8.574581	8.342169	8.348548	7.830932	6.850776	8.127977	8.152526	8.088915	8.024223	8.306186	7.799277	7.834020	8.178108	7.712966	9.141158	7.635106	8.565393	7.613722	8.805216	7.356854	9.353778	7.885206	8.192295	7.627512	7.980661	5.300823	6.104268	7.973828	7.123370	6.560069	6.459852	6.680785	6.511109	6.262169	7.290824	7.346731	6.152579	5.915666	7.717365	6.970435	7.497414	6.191829	7.166091	5.768136	7.128933	7.658461	6.638849	5.139018	5.983507	5.580405	6.912595	6.404233	6.274237	5.726824	6.022574	6.521545	6.469202	6.632728	6.591177	5.630433	5.777263	6.482234	6.646729	6.675596	8.218076	5.898487	7.241967	6.945345	6.804305	5.574617	6.593887	6.860578	6.946451	7.045467	7.964325	6.189430	6.430725	7.802510	6.472402	6.676570	6.606540	6.862666	7.045129	7.267882	7.090251	6.935371	8.057849	6.227479	6.872429	6.456040	7.095799	7.044973	6.477451	6.736780	6.770414	6.695712	6.053907	6.440058	7.188718	7.233867	7.361839	6.290462	8.016793	6.449494	6.275884	6.074831	7.265109	7.763738	6.917090	7.943093	6.719302	6.154648	5.439483	5.356081	6.086241	6.003417	6.147950	8.007627	7.769428	8.593306	6.134823	7.744247	5.738433	6.120635	6.717810	7.296211	6.534213	6.483249	8.556858	6.918844	7.012688	7.703743	6.068771	7.433286	7.542183	7.307876	5.976379	8.244457	6.250956	5.679386	5.446617	5.061015	7.203798	6.612650	4.928060	6.796372	6.251652	6.303916

AP000550.1	12.764425	13.056363	13.099216	13.041374	12.921327	13.257008	13.012991	13.352500	13.482590	13.630916	13.361000	13.357413	13.406084	13.452250	12.868696	13.483665	13.448871	13.337899	13.811422	13.597474	13.616838	13.658201	13.194717	13.337700	13.463136	13.199119	12.623163	13.700893	12.846490	12.985549	13.529678	13.039307	13.029169	13.078931	13.400373	12.943011	13.440736	13.105991	13.662003	13.795129	13.581357	13.588617	13.394129	13.408374	13.154016	13.388838	13.135275	13.544379	13.087393	13.426006	13.147891	13.500645	12.932588	13.095248	13.442236	13.551398	13.118872	13.490363	13.646573	13.567112	13.433879	13.279655	12.636300	13.716792	13.162423	13.350971	13.198919	12.848047	12.849996	13.466605	13.871812	14.020402	13.240382	13.655507	13.631803	12.919527	13.300869	13.495327	13.163768	12.975643	12.971153	12.787522	13.245910	13.514224	13.442036	13.073608	12.916212	13.285050	13.406084	13.141081	12.827524	13.410314	13.021558	13.210542	12.488771	12.906948	12.998439	13.257609	13.483665	13.384972	13.521840	13.333300	13.085733	13.413573	14.117033	12.927582	13.363874	13.917477	13.797198	13.570764	13.721200	13.376917	13.543949	13.551187	13.334349	13.119840	12.805797	13.411431	13.378652	12.647433	12.733709	12.011905	12.699870	12.901113	12.527434	13.020460	12.903395	13.139114	12.810174	13.127299	12.443027	13.457435	12.754466	13.017706	12.904469	12.411574	12.947179	12.738848	12.425904	12.769602	12.973815	13.349098	12.981137	12.818398	11.858897	13.352695	13.205236	12.868513	12.918970	12.824203	12.939212	13.822206	13.242189	12.514492	12.927379	13.020993	12.784685	13.969061	13.171908	12.732697	12.991245	13.150496	13.260641	13.402063	13.164133	13.124576	12.758764	13.111562	13.196137	13.085544	12.884558	12.969511	13.873120	13.066597	12.028097	12.648668	12.752782	12.912180	12.331584	13.267817	12.900773	13.322325	13.413378	12.773104	12.389189	12.630661	12.875210	13.145720	13.342844	12.787861	12.714355	13.047238	12.743196	12.358076	12.671216	12.581640	12.833456	13.249319	11.922787	13.018816	13.325421	12.699705	12.302809	12.837755	13.054365	13.022716	13.899912	13.026553	12.336986	12.379891	13.410097	12.818889	13.174367	13.270630	12.220150	13.052774	12.596096	13.135275	13.107335	13.282824	12.893290	12.875043	12.674660	12.828755	12.844764	12.502227	13.642784	13.120597	12.881322	13.502805	12.450102	12.416578	13.372935	12.401781	12.964739	13.397092	12.109351	12.993071

AC108860.2	9.900685	8.731790	8.741484	8.953719	8.487060	9.326293	9.509769	9.668298	9.151110	9.353052	9.229431	9.424518	8.817434	9.346191	8.275804	9.262112	8.789036	8.715941	8.225701	9.642850	8.863543	8.233897	5.927070	9.424518	8.135109	8.631443	8.337276	10.127268	9.230592	8.652644	9.040866	9.313619	9.439867	9.312170	9.060792	9.306959	9.363051	8.836326	8.617394	8.152684	9.648405	8.561461	8.581533	9.137787	8.414877	9.565885	9.190703	9.438350	9.373508	9.692692	9.849294	9.269982	9.406773	9.613042	9.484248	9.739328	9.481198	8.716580	9.320484	8.699239	9.287550	9.070354	6.010351	8.327636	8.885766	8.535756	9.137166	8.634112	9.248755	8.644846	9.615291	9.645339	9.441595	9.133075	9.522089	9.284919	9.598476	9.279993	9.751741	9.107052	8.718581	9.205189	9.422970	10.042994	9.446213	9.729654	9.688230	9.424977	7.895121	8.788596	8.803665	8.457202	8.610765	9.761826	9.266732	9.930780	9.207170	8.865514	9.421461	8.181446	8.779928	8.863054	8.452314	8.413693	8.515175	9.024467	8.527834	7.830076	7.841085	9.594486	8.543687	7.945100	8.065348	9.474760	8.918918	8.776297	8.922106	9.430029	9.247506	9.981975	9.074507	8.670782	8.487552	8.226898	9.616513	9.036468	9.884227	9.980904	10.657396	9.304702	12.091837	9.715472	9.322003	7.581340	9.242578	8.996270	9.852455	9.793310	8.560535	8.978474	9.413335	10.241972	8.352610	10.288341	7.101570	9.435860	9.914232	9.968820	9.552979	9.630443	8.511664	10.095931	10.011809	9.547475	11.006400	8.745271	9.323027	9.151110	8.658051	10.225000	10.361744	9.963319	10.302178	10.442903	10.756176	10.532207	10.429581	10.678215	10.282310	9.745841	10.743802	10.216834	9.229700	10.345357	10.486716	9.353339	10.551898	9.736856	10.212581	9.707187	9.681259	9.779222	9.391124	10.551284	9.188455	9.596328	9.903491	9.150168	9.147855	9.929996	10.325054	10.055298	9.610570	10.522954	9.063980	9.252538	10.112450	9.848968	8.606664	9.345116	10.452087	9.811988	9.543317	9.642224	10.692399	10.742017	11.451020	9.243504	8.268392	11.106592	9.641761	8.983807	9.073598	9.118833	9.964578	10.186829	10.801504	9.148313	10.142294	8.339807	9.102283	9.851053	8.547074	10.338376	10.457715	8.135040	9.174945	9.668780	10.094875	8.460319	8.208762	10.632913	9.617944	11.085434	10.361430	7.380825	11.087667	10.976418

LINC01634	8.031326	8.174272	8.514154	8.215162	8.535756	8.376902	7.668657	9.412745	9.788199	8.037443	7.949357	7.639694	7.723319	8.289335	7.745299	8.805380	9.188455	8.329650	10.065121	8.587391	9.234409	8.186342	10.266747	8.921964	8.854885	8.588507	8.016946	9.216856	7.972576	7.434700	9.118025	6.522172	7.463419	6.579129	7.719642	7.857244	7.911114	8.588177	8.892214	9.074656	7.997674	9.023860	7.892473	8.619883	7.991905	8.291499	8.012452	8.825708	7.753027	8.400640	8.692143	8.675783	7.940418	8.301367	7.980968	8.235894	7.980968	8.712976	8.752132	8.698630	9.057993	8.034627	9.681699	9.716701	9.189333	9.061578	8.627316	8.760109	8.499181	9.382710	7.835387	8.280399	7.944014	7.915342	7.956970	8.305594	8.585603	8.517477	8.384965	8.249893	8.208599	8.305897	8.352011	8.312218	8.441625	7.726838	8.412020	8.535166	9.068977	9.146408	8.965547	9.478293	8.588830	8.422852	8.218403	8.056835	7.359016	6.736422	6.471216	7.981127	5.835708	7.369846	7.560439	7.975858	8.813498	8.335866	9.304141	8.682047	10.143394	8.479139	9.612716	9.194184	8.927250	8.582555	8.132391	7.588584	8.961589	8.225403	8.594547	8.132838	8.610009	8.522315	8.447968	9.034328	8.615278	7.394137	8.658361	9.033262	7.935728	8.169560	7.398027	7.847503	7.771722	8.085086	8.644996	9.321630	8.155006	9.637784	8.312674	9.446549	8.220099	10.222096	8.680053	8.739179	8.575504	8.567027	8.973923	8.200757	7.938844	9.140403	7.040172	7.736809	7.242763	7.989562	8.136815	8.193370	8.918624	9.072203	9.314604	8.000110	9.345280	8.135563	9.079479	8.366666	8.548453	8.759647	8.766838	7.705019	9.168906	9.136379	8.669872	8.210651	8.761825	8.306963	7.915822	8.126922	8.749551	8.949039	8.763472	9.440330	7.906169	9.760895	9.740417	9.506865	9.148972	9.169378	8.990614	8.878485	9.583001	8.034627	8.219624	8.130096	8.557010	7.890928	8.552881	8.837234	8.801575	8.640980	8.192912	8.491179	8.602983	8.535756	8.281286	8.868700	7.596093	8.911133	8.435100	9.154481	9.239678	8.918330	9.895820	8.396552	8.529692	8.470875	8.299713	7.718421	6.629051	6.890454	7.270794	6.366543	7.371725	7.719642	7.869975	8.831175	8.067428	9.280749	8.808720	9.515307	8.704437	9.614243	9.336777	8.724655	8.557608	7.587048	7.650473	9.099806	8.510905	8.725272

AL353651.1	8.491946	8.704893	8.271654	8.789485	8.727734	9.079789	7.908479	9.476741	9.281062	8.536680	8.429598	8.304834	8.130096	8.067272	7.409429	9.142441	8.370991	7.870743	8.064885	9.043152	7.868192	7.601067	6.227645	8.809052	8.992404	8.568651	9.015459	9.429598	8.819333	9.379298	8.231309	7.766700	7.968189	8.054626	8.115759	8.221008	8.300189	8.130869	8.003253	7.898958	8.728390	7.757279	8.139887	8.390673	7.850793	7.817308	8.869457	8.264324	8.177531	8.529692	7.701163	7.598858	7.169797	7.344722	7.203012	9.105505	6.787592	7.161260	7.286929	6.944237	7.244382	8.590817	7.438844	7.671725	7.053815	7.139220	7.884296	8.102949	7.994877	7.855600	8.350208	8.398665	8.918759	7.916120	8.235894	8.342015	8.343224	8.231164	8.126922	7.831872	8.018297	7.393665	7.449836	7.243579	7.045294	7.569464	7.837050	7.382348	7.690876	7.618023	7.431568	7.720677	8.068315	8.126763	7.817798	8.046880	7.574330	7.730863	8.133019	8.418725	8.808882	7.899733	8.270121	7.833239	6.754070	8.561461	8.080215	8.275513	8.317610	8.929856	7.922409	8.437844	8.451859	9.456011	8.317000	11.251075	8.329805	8.202867	8.636910	8.701068	9.298766	8.504625	9.128119	10.103717	9.399241	8.805073	8.520379	8.388680	8.599266	9.071601	8.585432	9.331061	8.480944	8.529222	9.305431	8.458776	8.990334	8.741634	8.678987	8.532116	8.018603	7.765933	9.220819	9.339034	8.929856	9.446063	10.051862	10.101581	9.890580	8.728985	8.633518	7.841085	8.431048	8.318960	8.255576	8.296388	8.770989	8.749391	8.559612	9.438502	8.196072	8.047520	8.703835	8.002306	8.131646	9.495957	8.422713	8.172239	8.602058	7.814466	7.847351	7.164719	7.610677	7.388571	9.180169	7.078783	7.439160	7.530985	6.939358	7.196624	9.143652	7.609133	8.146710	7.453707	7.280216	8.211137	8.086750	8.097219	8.035393	8.235265	8.350208	9.864397	8.186986	8.528442	8.211447	8.516857	8.252222	8.237305	8.500912	7.859735	7.687159	8.452314	7.777297	8.462629	8.619393	7.399296	6.879880	8.034627	7.962531	7.589490	7.683351	8.078697	8.369861	8.283150	8.782589	8.394089	8.812917	8.573979	7.935728	8.650870	8.210366	8.753475	8.618151	6.864533	9.804101	8.389600	7.979109	7.999789	10.329779	8.413076	9.188910	9.831828	10.267029	9.341898	9.107805	7.901337	8.426454	9.145192

AL353651.2	8.491946	8.704893	8.271654	8.789485	8.727734	9.079789	7.908479	9.476741	9.281062	8.536680	8.429598	8.304834	8.130096	8.067272	7.409429	9.142441	8.370991	7.870743	8.064885	9.043152	7.868192	7.601067	6.227645	8.809052	8.992404	8.568651	9.015459	9.429598	8.819333	9.379298	8.231309	7.766700	7.968189	8.054626	8.115759	8.221008	8.300189	8.130869	8.003253	7.898958	8.728390	7.757279	8.139887	8.390673	7.850793	7.817308	8.869457	8.264324	8.177531	8.529692	7.701163	7.598858	7.169797	7.344722	7.203012	9.105505	6.787592	7.161260	7.286929	6.944237	7.244382	8.590817	7.438844	7.671725	7.053815	7.139220	7.884296	8.102949	7.994877	7.855600	8.350208	8.398665	8.918759	7.916120	8.235894	8.342015	8.343224	8.231164	8.126922	7.831872	8.018297	7.393665	7.449836	7.243579	7.045294	7.569464	7.837050	7.382348	7.690876	7.618023	7.431568	7.720677	8.068315	8.126763	7.817798	8.046880	7.574330	7.730863	8.133019	8.418725	8.808882	7.899733	8.270121	7.833239	6.754070	8.561461	8.080215	8.275513	8.317610	8.929856	7.922409	8.437844	8.451859	9.456011	8.317000	11.251075	8.329805	8.202867	8.636910	8.701068	9.298766	8.504625	9.128119	10.103717	9.399241	8.805073	8.520379	8.388680	8.599266	9.071601	8.585432	9.331061	8.480944	8.529222	9.305431	8.458776	8.990334	8.741634	8.678987	8.532116	8.018603	7.765933	9.220819	9.339034	8.929856	9.446063	10.051862	10.101581	9.890580	8.728985	8.633518	7.841085	8.431048	8.318960	8.255576	8.296388	8.770989	8.749391	8.559612	9.438502	8.196072	8.047520	8.703835	8.002306	8.131646	9.495957	8.422713	8.172239	8.602058	7.814466	7.847351	7.164719	7.610677	7.388571	9.180169	7.078783	7.439160	7.530985	6.939358	7.196624	9.143652	7.609133	8.146710	7.453707	7.280216	8.211137	8.086750	8.097219	8.035393	8.235265	8.350208	9.864397	8.186986	8.528442	8.211447	8.516857	8.252222	8.237305	8.500912	7.859735	7.687159	8.452314	7.777297	8.462629	8.619393	7.399296	6.879880	8.034627	7.962531	7.589490	7.683351	8.078697	8.369861	8.283150	8.782589	8.394089	8.812917	8.573979	7.935728	8.650870	8.210366	8.753475	8.618151	6.864533	9.804101	8.389600	7.979109	7.999789	10.329779	8.413076	9.188910	9.831828	10.267029	9.341898	9.107805	7.901337	8.426454	9.145192

AC090618.1	5.325189	5.404785	5.859782	5.522145	5.273109	5.902022	5.469704	5.519352	4.590157	5.791335	6.020461	5.802751	4.617539	5.658878	4.856921	5.730111	5.561471	5.731037	4.950416	5.533321	6.196023	6.727870	6.000081	5.101364	5.573704	5.459844	5.509757	5.581341	5.957866	5.504866	4.938486	6.086417	5.529534	5.139018	5.893525	6.182027	5.636384	5.523280	5.457267	5.182826	6.084753	5.260874	6.322380	5.403273	6.268440	5.142355	5.678435	6.066609	5.302426	6.501718	5.870804	6.893364	5.835000	5.523672	5.845265	6.737629	6.245286	5.631552	5.303048	5.862318	6.826891	5.466753	5.794762	5.601329	5.405739	5.923113	5.194478	5.399012	5.349462	5.376333	5.758891	6.202971	5.670148	5.693412	5.442939	5.486138	4.914337	5.198932	5.811871	5.969157	5.681611	5.639162	5.430290	4.719590	5.663513	6.026064	5.002927	5.250002	5.787378	5.706450	5.899713	5.066538	5.286009	5.562034	5.382508	5.812591	4.258714	5.508191	5.753756	5.153779	4.813130	5.224298	5.581535	6.049418	5.964827	5.846583	5.031335	5.485210	5.302829	5.719750	4.701940	5.224298	3.577153	6.029738	5.027227	5.123314	5.068602	5.631886	5.217771	4.819605	5.039315	5.413382	5.117824	4.979752	5.864357	5.252732	4.099749	4.392767	5.457891	5.526489	5.677134	4.721287	5.673483	4.059769	5.167076	5.383142	5.222725	4.428265	4.890855	5.090437	5.314218	5.642899	4.892731	5.283942	5.452642	5.385402	5.673483	5.523672	6.101332	4.527587	4.361722	3.931093	5.360977	5.410703	5.769744	4.792105	5.398817	5.598387	5.519533	5.764682	5.449553	5.877950	5.159865	6.254554	4.762913	5.526489	6.265874	5.029542	5.398631	5.431432	5.896007	4.549992	5.025466	5.136022	6.143926	5.682349	2.953662	4.366775	5.427467	5.485941	5.268667	3.845888	5.283757	4.686126	5.506211	3.032744	4.258947	5.204669	5.492994	5.284990	5.971779	5.917588	4.759526	4.798541	5.562403	4.863937	5.385023	5.377319	5.023196	5.052375	5.143372	4.878171	3.793159	4.781766	4.146217	4.981328	4.749126	5.295216	5.313196	4.758930	5.159668	5.281972	4.928060	4.905496	5.312635	3.868380	5.216032	5.272683	4.847214	5.167687	4.368077	5.332656	5.725727	4.986033	8.192295	3.963170	4.263078	5.386549	6.085087	3.971793	5.209621	5.273690	6.121304	4.617086	4.311473	5.296440	5.224094	5.468537

NOP14-AS1	9.112102	7.619552	8.094770	8.050181	7.377364	7.379718	8.313277	7.672732	8.133925	7.758989	7.495029	7.844679	8.295916	7.575743	8.068315	7.998718	7.719779	8.022679	7.984536	8.052166	7.399296	9.678939	10.279115	7.883682	8.494468	7.774290	8.621747	8.118397	7.861985	8.039167	7.976016	8.563284	8.377526	9.236097	8.100858	8.477080	8.172842	8.342758	8.169238	8.568651	7.986373	8.681103	8.471683	8.333380	8.998715	8.079617	7.533611	8.386977	7.934648	8.509809	8.461880	8.658361	8.309606	7.945557	8.245874	7.392419	8.274038	8.780406	8.328299	8.854885	8.701068	8.219624	8.564664	8.598194	8.276915	8.865068	8.619728	7.856943	8.153471	8.236836	8.346622	7.860630	7.173102	8.078065	8.111454	7.992398	8.229943	8.015092	8.277531	8.575356	8.336479	8.821432	8.717201	8.824316	8.552276	8.498831	8.251297	8.302435	8.833772	8.269030	8.099775	8.504625	8.047176	8.367126	8.245874	8.202701	8.285931	8.151610	8.343986	8.235415	7.573718	8.832252	8.020441	7.659080	8.755477	7.158238	8.139719	8.028572	8.516237	7.270312	8.761974	7.720538	8.075160	7.559652	7.908644	6.981481	9.131804	8.596418	7.695045	8.687887	9.036324	7.883226	7.659388	6.816998	6.903372	9.147530	8.149614	8.344141	8.768833	9.068809	7.579940	8.016483	7.757576	8.558229	8.816369	9.323193	7.414619	7.482123	7.555503	8.441329	10.082093	8.893482	7.658000	8.194802	7.334897	7.625612	8.461565	7.103919	8.387874	8.763929	8.714362	10.033321	10.414186	8.787683	8.299713	9.059279	8.473114	9.569490	8.494150	8.670340	8.400184	8.502465	10.058771	7.938047	8.732236	9.187379	8.791835	7.661530	7.498194	10.104477	8.902638	8.128410	8.946909	8.365652	7.360750	9.326448	8.855318	9.209617	8.159617	9.186753	8.142249	11.544838	9.573194	7.920144	10.497236	8.361105	9.555843	9.130781	8.622673	7.616951	8.238086	8.613906	8.562976	10.426856	8.528611	8.274332	8.731208	8.423009	8.459410	9.554938	9.932497	10.263600	8.574878	9.386205	9.406477	9.702032	8.210515	8.705991	8.310213	8.529985	9.504085	8.426298	9.250394	8.231793	8.060419	8.583613	8.532116	9.262112	8.271789	10.313397	8.610158	8.145645	8.191175	8.254205	7.378634	9.833499	7.367178	8.527214	7.722369	8.576545	7.538443	6.200479	7.222702	6.550996	11.886584	8.445618	8.549073	10.688592

AQP4-AS1	4.261993	4.151468	3.630263	3.687483	4.421986	4.274225	3.340980	3.275282	3.987558	3.099621	3.699957	3.084157	3.072887	4.162019	3.041027	4.160768	3.259725	2.911101	4.414658	4.095392	7.300476	4.899606	2.945275	3.561948	2.916065	3.837359	3.730665	3.806389	3.739136	3.708262	3.457163	6.352776	4.904018	3.653615	4.701728	3.792471	6.152405	4.824132	3.150513	3.168786	3.321367	3.019211	4.291559	3.031966	3.668288	4.518915	4.330557	5.336327	5.287363	4.057579	3.948773	3.086586	3.300530	5.954546	3.432871	3.781236	5.591378	3.885300	3.229324	3.662649	4.799201	4.070467	4.242727	3.797572	3.299325	3.450064	4.631116	3.999159	4.714337	4.844242	4.401806	3.806389	3.050697	3.876112	4.296330	4.306815	4.149594	4.044345	4.115070	4.207537	4.084748	3.534249	3.242102	3.685428	3.577965	3.234379	5.308084	3.983919	4.118816	4.662255	4.403954	3.911625	4.723440	4.785032	4.395671	4.114828	3.296369	3.682453	3.480010	4.555796	5.783971	4.296330	3.394523	3.746085	4.312111	3.009660	3.542686	4.012899	4.700479	3.931741	3.950106	4.691987	4.985191	4.161178	3.679421	7.032025	3.837359	3.915302	3.351386	3.844577	4.069842	4.199007	4.211406	5.079803	4.008227	3.706468	3.043233	3.918319	4.278859	3.820601	3.556033	5.188014	4.229455	4.043878	3.313638	3.565350	3.784800	3.967702	3.693202	6.889820	5.267883	2.917227	4.706397	4.681788	4.551957	4.084103	4.705113	3.922669	4.064780	3.185419	5.593845	3.572272	4.827825	4.730409	3.530322	5.670843	5.146547	3.436845	4.821688	4.667150	2.955040	4.498125	4.163148	3.847370	4.033635	4.876104	5.001307	5.377319	4.194980	3.673077	4.053199	3.434147	6.392355	4.605235	3.696522	5.440238	3.783146	3.035580	3.522853	5.415123	4.267396	3.834808	4.024515	3.802439	3.994643	3.820601	4.238326	4.327452	4.384713	4.065006	3.964011	3.783771	4.008227	3.679837	4.159074	4.184907	4.301102	4.168403	4.202047	3.479830	3.702040	4.124421	3.797353	4.264426	3.700373	4.642176	3.771736	3.857006	3.930246	4.159074	4.528611	4.526742	4.435180	3.867935	3.803679	3.681611	4.708061	3.608791	3.848186	4.977057	4.723654	3.546768	5.213607	4.625504	3.770322	3.722513	4.136501	3.795908	3.923084	4.424630	5.848261	7.578551	4.540468	4.515925	4.403324	3.721214	3.947054	4.069020

LNCTAM34A	9.860030	10.076651	10.081787	10.540943	10.636237	10.841262	10.412390	11.144256	11.385844	10.720621	10.986127	11.021682	9.706401	10.479347	9.898527	10.605498	10.793806	9.844400	10.809816	10.447656	10.300168	10.644015	10.533504	10.522363	10.480213	10.713151	11.561877	11.207935	11.052063	10.513159	11.156994	9.403709	9.688392	10.420871	9.939645	10.049864	10.084209	10.145201	10.393108	10.286060	11.014582	10.067980	10.522062	10.147175	10.099983	10.159386	11.130935	10.214880	9.880256	10.329171	9.897496	9.940101	9.670057	9.807001	9.789600	11.058656	9.472923	10.111998	10.046779	9.771286	10.024497	10.491272	10.506316	10.499570	9.980743	10.002425	10.456157	10.029650	10.215042	10.543604	10.368662	10.446580	11.103121	10.263119	10.376965	10.156965	10.162442	10.000412	10.139112	9.452298	9.595374	9.452150	9.385437	9.468870	9.366931	9.644234	9.336631	9.515000	9.759652	9.700268	9.320329	9.728056	10.056061	10.056660	9.897339	9.909249	9.483782	10.571850	10.315346	10.528219	10.551435	10.280645	10.554613	10.497395	10.629643	10.727782	10.645484	10.650447	10.644015	11.048552	10.150520	10.680054	10.743352	11.193556	10.472068	12.010496	10.058619	10.236667	11.123080	10.385788	10.096842	10.197218	10.882649	11.455214	10.646637	11.007861	11.043290	11.125198	10.891048	10.798060	10.826808	10.805904	10.655479	11.058784	10.482608	10.533656	10.270415	11.111448	10.050656	10.520340	11.214660	10.537222	11.236428	10.715970	10.370938	10.827682	11.115929	11.418410	10.689337	11.000620	10.072068	10.474064	10.465495	9.983675	9.836416	9.927545	10.261790	10.617007	10.254503	11.359874	10.257704	10.054345	10.398534	10.108827	10.380759	11.500204	9.989516	9.933063	10.247562	10.088700	9.901638	9.484876	9.884098	9.648129	10.609923	9.325221	9.737911	10.209812	9.492722	10.434742	10.692859	10.358232	10.553689	10.288341	10.116364	10.418435	9.996917	10.319510	10.548595	10.068414	10.226065	11.846978	10.064074	9.865299	9.931856	10.018340	10.242602	10.169652	9.327512	9.434327	9.634765	9.564348	9.569490	9.269228	10.246802	9.392669	9.234252	9.701427	9.607021	9.793010	9.780722	9.843788	10.349459	10.062130	10.694969	10.778852	11.234215	10.522517	10.105679	10.405317	10.379105	11.110444	10.304626	10.259775	11.266465	10.942565	10.281096	10.163693	11.585740	10.161998	10.562644	11.013825	11.575238	10.609657	10.719738	10.402432	10.289759	11.086179

MIR34AHG	9.860030	10.076651	10.081787	10.540943	10.636237	10.841262	10.412390	11.144256	11.385844	10.720621	10.986127	11.021682	9.706401	10.479347	9.898527	10.605498	10.793806	9.844400	10.809816	10.447656	10.300168	10.644015	10.533504	10.522363	10.480213	10.713151	11.561877	11.207935	11.052063	10.513159	11.156994	9.403709	9.688392	10.420871	9.939645	10.049864	10.084209	10.145201	10.393108	10.286060	11.014582	10.067980	10.522062	10.147175	10.099983	10.159386	11.130935	10.214880	9.880256	10.329171	9.897496	9.940101	9.670057	9.807001	9.789600	11.058656	9.472923	10.111998	10.046779	9.771286	10.024497	10.491272	10.506316	10.499570	9.980743	10.002425	10.456157	10.029650	10.215042	10.543604	10.368662	10.446580	11.103121	10.263119	10.376965	10.156965	10.162442	10.000412	10.139112	9.452298	9.595374	9.452150	9.385437	9.468870	9.366931	9.644234	9.336631	9.515000	9.759652	9.700268	9.320329	9.728056	10.056061	10.056660	9.897339	9.909249	9.483782	10.571850	10.315346	10.528219	10.551435	10.280645	10.554613	10.497395	10.629643	10.727782	10.645484	10.650447	10.644015	11.048552	10.150520	10.680054	10.743352	11.193556	10.472068	12.010496	10.058619	10.236667	11.123080	10.385788	10.096842	10.197218	10.882649	11.455214	10.646637	11.007861	11.043290	11.125198	10.891048	10.798060	10.826808	10.805904	10.655479	11.058784	10.482608	10.533656	10.270415	11.111448	10.050656	10.520340	11.214660	10.537222	11.236428	10.715970	10.370938	10.827682	11.115929	11.418410	10.689337	11.000620	10.072068	10.474064	10.465495	9.983675	9.836416	9.927545	10.261790	10.617007	10.254503	11.359874	10.257704	10.054345	10.398534	10.108827	10.380759	11.500204	9.989516	9.933063	10.247562	10.088700	9.901638	9.484876	9.884098	9.648129	10.609923	9.325221	9.737911	10.209812	9.492722	10.434742	10.692859	10.358232	10.553689	10.288341	10.116364	10.418435	9.996917	10.319510	10.548595	10.068414	10.226065	11.846978	10.064074	9.865299	9.931856	10.018340	10.242602	10.169652	9.327512	9.434327	9.634765	9.564348	9.569490	9.269228	10.246802	9.392669	9.234252	9.701427	9.607021	9.793010	9.780722	9.843788	10.349459	10.062130	10.694969	10.778852	11.234215	10.522517	10.105679	10.405317	10.379105	11.110444	10.304626	10.259775	11.266465	10.942565	10.281096	10.163693	11.585740	10.161998	10.562644	11.013825	11.575238	10.609657	10.719738	10.402432	10.289759	11.086179

AP000802.1	5.444097	5.550691	6.310424	5.383539	6.710407	5.780525	5.366408	6.765059	7.282257	5.125769	5.337710	5.260230	5.157704	5.690061	4.795393	5.309663	6.101173	5.026259	5.817964	5.015854	4.753194	5.981355	6.638849	5.471801	5.821680	5.911515	5.935818	6.127120	5.967524	5.139678	5.909357	4.782194	5.300411	4.721510	5.162468	5.672408	5.058170	5.705436	5.345937	5.842795	7.042589	5.823129	4.739537	4.366579	5.683425	4.594714	5.907399	5.921216	5.360411	4.397017	4.309458	5.971051	3.530520	4.213645	5.232618	5.561840	5.244612	3.105154	4.565222	5.454178	5.495284	6.103414	7.803715	5.421654	4.518915	5.549584	4.976292	5.292378	4.459665	4.597449	4.791873	5.149945	5.856204	5.434367	5.508191	4.756759	5.270257	5.532567	4.990182	5.704486	5.643691	5.512009	5.313797	5.299282	5.772933	6.028563	5.744826	5.765908	5.942682	4.227030	5.412057	5.917236	4.944576	5.259071	5.525554	5.486138	5.002123	6.335177	6.008053	6.479926	6.211381	4.784543	5.124112	5.499857	5.531254	5.423847	6.333488	5.856542	6.072576	5.948086	5.856380	6.141195	6.301328	6.523680	5.982957	4.604611	5.879616	5.623364	6.253339	5.658509	5.653667	5.795108	4.984987	6.793634	4.583101	4.918935	5.940063	6.802846	5.371076	5.461998	4.554548	5.212584	4.147276	4.017194	5.417778	6.268096	4.422659	5.293694	4.348753	4.625705	4.664093	6.428865	6.240087	5.238461	6.465872	5.095423	6.326261	5.613287	5.253951	5.907399	4.271174	4.925961	4.938708	4.569586	5.068986	4.308348	5.625408	6.004648	5.041578	6.260984	5.696189	3.605770	3.328072	4.204989	4.412855	5.864357	5.605540	4.400888	3.135255	3.107586	4.226825	3.595556	6.190095	5.651451	5.351297	4.445656	5.179183	4.216443	4.461347	4.597046	5.696389	5.655559	5.390271	4.544458	4.868526	4.983993	4.189080	4.966918	4.759750	4.667960	5.209621	5.097372	5.486138	4.954780	5.096594	5.222525	5.314218	5.529168	5.676760	4.761835	4.577328	4.910706	4.829761	5.526286	5.996901	5.052996	5.055546	6.393714	5.501796	5.295016	6.159627	4.920208	5.653503	5.332255	4.685684	5.407524	5.853436	5.603430	6.267244	6.437425	4.390400	5.181974	5.120098	6.077446	4.746622	6.880697	5.625771	5.412627	5.347143	5.855636	5.440238	5.503367	5.730674	4.478885	4.844447	5.927411	5.152145	5.105833

AL354696.2	9.181558	7.873506	7.542477	8.133618	9.332620	9.301568	9.005349	8.222227	10.076508	9.999670	9.878156	10.153732	9.477984	9.175420	8.081052	9.586696	8.443933	8.821898	7.607143	9.036629	7.340176	8.870889	7.914604	9.744936	9.169996	10.595736	10.553843	9.555370	9.751590	8.826297	9.777523	7.686094	7.113000	9.328138	8.129662	7.851729	8.155934	7.897749	7.591653	7.704877	9.125182	7.617855	8.173166	7.516478	7.494871	7.753792	10.014151	7.008016	7.814935	7.192137	7.613722	7.067302	7.067141	7.130837	6.664134	9.721843	7.654830	7.128141	7.497876	6.839180	7.360270	9.748169	6.497857	7.197441	6.570672	6.807255	7.398827	7.367028	6.697122	7.135615	8.445159	8.535005	10.522806	8.248548	8.617088	8.092915	8.550581	7.578551	8.088325	7.494577	7.941178	7.158850	7.781356	7.612498	7.171164	7.896881	8.481708	7.416551	7.286140	6.313142	7.092603	7.078944	8.113605	8.324142	8.632606	8.752885	7.742243	9.725305	9.152161	9.690384	10.177339	8.674683	8.920591	8.597472	7.624107	9.182666	8.485373	7.788365	9.062021	9.865593	7.785295	10.481389	9.696776	11.521903	9.358026	11.137575	9.047586	9.444674	11.011245	9.739008	8.303042	8.775212	9.197036	11.080854	9.228210	10.620438	10.458804	10.054975	11.077518	10.350675	10.441855	10.955883	9.616211	9.573835	10.205361	10.238329	9.316571	9.007324	8.147603	8.117164	8.782589	7.627042	9.909868	9.635917	9.262112	8.858812	10.687354	11.465549	9.254499	10.203481	10.005677	8.972481	10.168427	8.491478	8.527214	8.469008	9.422496	8.887521	8.535943	12.024501	8.445469	8.441329	8.173166	7.695315	7.419775	11.674147	7.815587	7.981450	7.870600	8.152839	7.955563	7.834927	7.827120	7.988366	8.783670	8.277825	6.843037	8.146104	7.200863	7.947541	9.526925	7.464647	7.400060	7.642308	7.649346	7.752132	7.102355	8.433681	7.421349	8.953796	8.656754	11.290218	8.881902	8.714536	8.266810	8.888686	9.678939	8.986858	8.013689	8.075788	7.920886	8.871852	8.355532	7.981450	9.533695	8.292608	8.457783	8.337585	7.621653	8.273592	7.987940	9.771758	9.552071	9.377775	9.939949	9.515907	11.037001	10.536345	9.144597	10.838287	9.920741	9.850742	8.681905	9.190561	10.558269	10.296166	8.910543	7.338470	10.833896	8.388218	9.930780	9.938377	10.908849	9.285661	9.644393	8.347493	10.267640	9.991411

LINC02401	4.668831	3.146645	2.997034	2.979346	3.046304	3.011262	3.120613	4.034313	3.211164	3.083840	6.773490	3.020309	3.172650	3.479271	3.000927	3.070040	3.344639	2.854942	4.056495	3.102113	4.086570	3.720796	3.037128	2.875187	2.927608	3.275739	3.012591	2.901516	3.048798	4.581421	4.716878	3.427251	3.508512	3.195847	3.133804	3.845207	3.325316	3.009751	3.205978	3.143269	3.341480	3.158665	3.407494	3.131266	3.538645	4.683975	3.175677	2.952291	3.538645	3.403206	3.249984	3.229755	3.404710	3.970521	3.556393	3.045250	3.982184	3.472373	2.994863	3.546167	2.987230	3.060561	3.515144	3.309708	3.317445	3.323165	3.216802	3.049767	3.109333	3.678626	3.383664	3.022360	3.108051	2.887198	2.981253	3.264540	3.030378	3.240744	3.049958	3.457694	3.215955	3.623258	3.378791	5.830863	3.186339	4.860445	3.333219	3.203743	3.831572	3.384322	3.581487	3.520806	3.531287	3.133674	3.206407	2.939668	3.451718	3.179263	3.086040	2.914017	2.956521	3.115578	2.923767	2.825356	4.390400	3.048487	2.949681	3.097035	3.322172	3.032744	3.273704	5.561114	2.908133	3.143488	3.220137	3.262627	3.500685	3.056221	3.113778	3.812920	3.199296	2.963327	3.009751	2.911723	3.334371	2.888014	3.425996	3.189850	2.959563	6.398294	2.930628	3.031650	3.081122	2.927305	2.924028	3.229324	2.811599	3.224749	3.107369	3.204548	2.922168	2.964183	2.851754	2.865464	2.900459	2.941808	2.862605	3.029190	5.454938	4.209917	3.322172	3.471558	2.995127	3.037128	3.582650	3.323817	3.016683	3.220421	3.057265	3.129094	3.053523	3.468209	3.159671	3.871346	4.166251	3.107248	2.879575	3.724930	3.165946	3.106321	3.092690	3.547577	4.094749	3.340818	2.992993	3.672321	3.395399	2.908499	3.354007	2.904133	3.172255	3.563042	3.528119	3.518376	3.475634	3.216664	3.460015	3.152112	2.907677	2.968264	3.108410	3.079530	2.884269	2.928929	3.219514	3.050591	3.002910	3.024084	3.103006	3.478207	3.831827	3.409531	4.443074	3.319276	3.353521	3.106541	3.325488	3.577750	3.293126	3.182826	2.931766	3.223215	3.171902	3.080901	2.952851	2.943569	3.009272	3.534620	3.109333	3.003897	3.022268	3.023492	2.980688	4.704484	3.047204	2.915428	3.053145	3.184361	2.996175	3.288191	5.801715	3.661403	3.078141	3.003522	2.853243	2.920907	3.078263	3.144788

TBC1D8-AS1	7.452118	6.572895	7.238287	7.192778	7.054595	7.342696	7.083939	7.329932	7.608208	6.962923	6.956874	7.344722	7.140322	6.516230	5.867047	7.025077	8.039167	6.943118	7.251697	7.623778	6.792518	6.516569	6.724980	6.032553	6.207080	7.150750	6.856249	6.162716	6.709910	7.332577	6.782610	7.431568	6.979858	7.744247	7.803562	7.892783	7.606389	7.224338	7.287366	7.255858	6.776373	7.150909	7.386079	6.996945	6.705103	7.169797	6.698079	6.800425	7.640322	7.643210	7.734740	7.566739	7.465716	7.948606	6.730314	7.309610	7.959757	7.496300	6.683406	7.165801	7.256473	6.818962	6.866135	6.818634	7.344870	6.918372	7.215129	6.675761	7.500092	6.846482	7.836281	7.667600	6.469852	7.777466	8.124336	7.132283	8.080543	7.641842	7.809896	6.633374	7.764659	7.150415	7.208002	7.569764	7.745146	7.888585	8.244457	7.758843	7.493195	7.317172	7.034067	7.359173	7.268627	7.315429	7.574494	7.403034	7.132591	6.708631	7.478448	7.382525	7.144750	7.139220	6.769450	7.471256	7.390405	6.804468	6.931748	5.648979	7.621803	6.001794	7.168714	7.558907	7.383782	6.714591	7.239898	8.427398	7.529626	6.786300	6.595180	8.699971	6.822754	7.790829	7.406331	8.077747	7.581181	6.858491	7.840159	7.887040	8.221915	7.588434	8.549976	7.653754	6.817820	6.643860	7.835530	9.568410	8.371749	6.922265	8.133925	7.393203	6.671061	6.730953	7.122273	7.532988	8.247677	8.290571	7.033749	8.109965	8.003880	7.652521	7.986529	7.829126	7.695913	7.908479	8.668344	7.687159	8.396077	7.836741	8.034627	7.198684	7.776998	8.159472	7.148656	7.243420	7.797527	7.810799	7.960216	8.065348	8.398224	8.179171	7.778353	7.792954	8.669134	7.806014	8.235581	9.302803	8.368477	7.785439	7.628126	7.394137	7.544702	8.126922	7.077988	7.695178	7.310728	7.890755	6.895743	7.971783	6.994459	8.183407	7.895271	6.600064	8.453383	9.396207	7.524523	8.537994	8.457938	8.305738	7.509249	8.416609	8.520218	7.998718	8.117316	7.730239	8.997674	8.524156	8.120379	8.214240	7.623618	7.780907	7.779666	8.106793	7.456332	7.458170	7.679233	8.798699	7.698069	7.827616	8.266671	7.192697	8.934093	8.094476	8.402513	8.458106	6.784180	7.496477	6.910328	7.561521	6.255368	7.359489	8.162279	7.420891	6.427847	8.072005	7.761907	7.673686	7.407740	7.452274

AC011525.1	3.555075	5.577038	4.855630	4.403954	4.196467	5.275956	5.369136	3.157189	3.262792	4.542417	3.731933	3.171050	3.144215	3.964886	3.072161	3.744268	3.854486	2.876858	3.999802	3.109333	4.052167	4.756117	3.190409	3.542076	2.876081	2.912748	3.428139	2.867977	5.134262	5.740630	3.543818	3.359648	3.930246	4.073634	3.149189	3.833320	5.550863	3.071207	3.179770	3.240594	5.061593	3.068578	3.485680	3.128217	3.607467	4.695359	4.172695	3.104462	3.595183	3.375064	3.125138	3.064806	3.288506	4.035385	3.587872	5.416211	4.012459	4.986634	3.220421	3.722513	2.897158	5.420746	3.668479	3.886551	4.257186	3.372905	4.794361	5.442939	5.207435	3.974872	4.316709	5.273895	4.231140	4.480635	4.119663	3.556578	5.268084	4.002996	4.029752	3.463379	4.031479	3.549657	3.881295	3.600877	3.183307	5.326168	3.859401	3.221513	4.252367	3.494647	3.130253	2.942255	4.587788	4.106740	3.170136	3.541120	3.337310	3.150988	3.117381	4.373025	3.799007	5.138648	4.657491	4.512913	4.394274	4.175090	6.667699	3.762565	4.159287	5.512009	3.312568	3.824361	3.934506	4.500042	4.424419	5.667791	3.310373	3.814406	4.696606	3.948147	5.678811	4.966488	4.392978	4.056940	6.287577	5.036741	3.077828	3.183693	4.900698	3.872654	3.959460	4.366132	3.825521	3.935165	3.384322	4.163347	2.831066	3.198410	3.563232	5.217569	4.809737	5.577775	3.992680	4.837238	4.483519	3.727139	4.053433	4.573522	6.415159	3.290448	3.278696	3.766514	4.082384	3.086264	3.547366	5.298266	3.975942	3.209927	3.693392	4.736547	2.987511	3.527928	3.158273	3.965491	4.212746	4.078904	2.920622	3.771535	3.089007	3.000745	2.981438	5.533510	4.169230	3.694034	5.763225	3.714654	5.317463	3.030991	3.613780	2.858268	5.367227	3.725976	5.965017	3.660212	3.531937	4.803466	5.093109	6.030746	3.157896	5.489780	5.352662	3.379675	4.867689	4.034530	3.478950	5.591982	4.671443	3.874839	3.460369	3.537245	3.790668	3.465570	3.386361	3.529405	4.458852	3.056861	3.325316	3.935366	3.400227	3.158665	2.912656	4.449382	4.191920	3.063753	3.577338	3.399529	3.046403	3.649216	5.590622	3.207803	4.009340	3.560111	4.107221	4.702563	4.809096	6.646089	5.145729	3.845207	6.322896	3.432203	4.079820	5.802582	4.085214	5.109966	3.559545	2.865368	4.289822	5.256533

AC011525.2	3.555075	5.577038	4.855630	4.403954	4.196467	5.275956	5.369136	3.157189	3.262792	4.542417	3.731933	3.171050	3.144215	3.964886	3.072161	3.744268	3.854486	2.876858	3.999802	3.109333	4.052167	4.756117	3.190409	3.542076	2.876081	2.912748	3.428139	2.867977	5.134262	5.740630	3.543818	3.359648	3.930246	4.073634	3.149189	3.833320	5.550863	3.071207	3.179770	3.240594	5.061593	3.068578	3.485680	3.128217	3.607467	4.695359	4.172695	3.104462	3.595183	3.375064	3.125138	3.064806	3.288506	4.035385	3.587872	5.416211	4.012459	4.986634	3.220421	3.722513	2.897158	5.420746	3.668479	3.886551	4.257186	3.372905	4.794361	5.442939	5.207435	3.974872	4.316709	5.273895	4.231140	4.480635	4.119663	3.556578	5.268084	4.002996	4.029752	3.463379	4.031479	3.549657	3.881295	3.600877	3.183307	5.326168	3.859401	3.221513	4.252367	3.494647	3.130253	2.942255	4.587788	4.106740	3.170136	3.541120	3.337310	3.150988	3.117381	4.373025	3.799007	5.138648	4.657491	4.512913	4.394274	4.175090	6.667699	3.762565	4.159287	5.512009	3.312568	3.824361	3.934506	4.500042	4.424419	5.667791	3.310373	3.814406	4.696606	3.948147	5.678811	4.966488	4.392978	4.056940	6.287577	5.036741	3.077828	3.183693	4.900698	3.872654	3.959460	4.366132	3.825521	3.935165	3.384322	4.163347	2.831066	3.198410	3.563232	5.217569	4.809737	5.577775	3.992680	4.837238	4.483519	3.727139	4.053433	4.573522	6.415159	3.290448	3.278696	3.766514	4.082384	3.086264	3.547366	5.298266	3.975942	3.209927	3.693392	4.736547	2.987511	3.527928	3.158273	3.965491	4.212746	4.078904	2.920622	3.771535	3.089007	3.000745	2.981438	5.533510	4.169230	3.694034	5.763225	3.714654	5.317463	3.030991	3.613780	2.858268	5.367227	3.725976	5.965017	3.660212	3.531937	4.803466	5.093109	6.030746	3.157896	5.489780	5.352662	3.379675	4.867689	4.034530	3.478950	5.591982	4.671443	3.874839	3.460369	3.537245	3.790668	3.465570	3.386361	3.529405	4.458852	3.056861	3.325316	3.935366	3.400227	3.158665	2.912656	4.449382	4.191920	3.063753	3.577338	3.399529	3.046403	3.649216	5.590622	3.207803	4.009340	3.560111	4.107221	4.702563	4.809096	6.646089	5.145729	3.845207	6.322896	3.432203	4.079820	5.802582	4.085214	5.109966	3.559545	2.865368	4.289822	5.256533

GATA6-AS1	13.663476	5.299069	5.608956	9.436629	3.800747	3.585898	5.402456	4.058018	4.092696	4.434754	4.158193	4.748940	3.668479	3.658626	4.585201	3.921257	4.495125	2.892787	5.159068	4.134500	4.277507	3.813350	7.628769	3.677808	3.375800	3.904426	5.839528	5.137833	6.237788	5.030942	4.111801	9.863624	5.640329	5.042847	5.809240	4.398484	3.980200	5.318061	4.707447	4.722818	4.256083	5.493884	5.425587	4.715794	6.798004	5.596446	4.928648	5.108299	4.927632	5.276946	9.235309	6.274724	6.424158	4.109814	6.606041	3.867507	6.491065	3.174332	4.400248	5.522511	4.517005	6.564009	7.733625	4.879417	5.703923	5.174138	4.883876	4.472145	4.203381	4.537597	5.378642	3.813595	4.871491	4.785130	5.064071	4.989759	3.801192	5.470077	7.700231	5.569032	9.816798	6.845868	10.972346	5.545431	4.910501	4.423542	4.324430	5.595889	4.839616	5.902746	5.259448	5.342558	4.583727	4.580573	10.751755	4.371120	4.651131	5.963956	4.817923	3.132846	5.134051	4.426112	5.258110	4.753194	4.071461	4.307910	4.742945	5.362720	4.097968	3.378974	4.204054	4.451198	3.385685	6.083687	4.676014	2.946960	13.557270	6.044476	3.738084	8.176328	5.486905	6.193018	5.395965	3.960518	3.724732	4.280814	4.969038	4.941966	3.214463	4.713921	6.057290	4.377580	4.474750	5.811679	5.132404	4.663487	3.216951	5.267268	5.945135	5.532941	4.183437	5.488756	3.129327	2.851000	6.432761	3.527576	4.451610	3.661202	4.573305	5.311259	5.673307	5.997249	5.309863	5.402456	4.312729	4.643812	4.701109	5.010542	5.696567	4.306150	4.222090	5.027449	3.443447	6.233086	5.126176	3.862377	5.072591	4.527996	5.211243	6.780384	6.503485	4.825610	4.272029	6.935695	5.322477	6.248802	3.757311	4.606485	5.236641	5.079407	5.468755	5.142772	4.795822	4.984172	5.642156	5.367401	4.074728	3.972667	4.493992	4.302876	3.884687	3.309218	5.324966	5.422633	4.581620	4.993721	6.185545	4.660049	6.218038	5.607597	5.534481	7.163317	4.477618	4.571992	5.134841	5.836443	4.973787	5.147729	6.250151	6.084753	5.888681	5.546573	5.311617	6.874005	4.787036	4.194980	4.678180	5.276162	3.869015	6.775437	3.187544	4.974204	4.699871	4.378669	3.427805	4.617539	6.434267	4.650482	3.179124	4.782417	4.544904	4.278403	3.261151	5.020329	3.543056	6.324741	3.648387	3.624648

ZKSCAN7-AS1	8.113151	7.601067	7.536569	7.850793	7.833855	8.246792	8.533021	6.835808	7.470321	8.248863	8.520218	8.218720	8.207229	8.044166	7.701453	7.497876	7.424608	7.677294	7.677630	7.710499	7.161260	7.294462	9.418832	7.750438	7.627673	8.296548	7.934793	7.757729	8.176634	8.219775	7.524066	7.946493	8.385714	7.803562	8.035557	8.129345	8.339505	7.715526	7.510294	7.520351	8.246033	7.958540	7.180178	7.639694	7.529779	7.355811	8.347349	7.041120	7.260903	7.172144	7.720828	7.160446	7.705322	8.123421	7.222979	8.064267	8.060100	7.657040	7.204235	7.902271	8.023141	8.140611	7.949845	7.527358	7.735797	7.327400	7.555055	7.530078	7.231189	7.438669	7.705798	7.933884	7.908479	7.613722	7.909883	7.755586	7.696675	7.676806	7.968189	7.406955	8.087091	7.541274	7.517109	7.637084	7.999017	7.968783	7.929878	7.079100	7.350077	7.449836	7.679960	7.252455	7.214347	7.643516	7.900541	8.033901	7.308493	7.640482	7.811267	7.377540	7.980968	7.714346	7.619699	7.326266	7.057617	7.773840	6.965162	7.383930	7.825526	8.239177	7.861985	7.897891	7.268335	8.398977	6.903372	10.046931	8.308074	7.825370	8.076799	8.104010	7.662478	7.994571	8.302279	7.717665	8.390063	8.375886	8.764087	7.545191	8.699971	9.093137	8.944146	8.403771	8.408506	7.984536	7.303979	8.374816	8.618151	7.893570	7.327400	7.024748	7.562869	8.553625	6.679298	8.532273	8.423320	8.090805	8.326972	8.417392	8.694827	8.197638	7.533908	8.345381	8.085844	8.237926	8.836027	8.656689	8.518092	7.574174	8.095531	8.377526	7.848400	6.852172	8.151117	7.206767	6.378130	8.605447	8.080719	7.871824	7.087205	7.772173	7.020611	7.829277	8.105140	8.121626	8.778562	8.962062	7.334577	6.920814	7.607725	8.030420	8.309176	6.476273	7.002889	7.550974	7.861073	7.138120	7.023420	7.120267	7.241639	7.794310	8.010042	8.101146	8.083490	8.342909	7.803260	8.461880	7.995024	8.098401	7.841996	8.307442	8.050313	8.124646	8.072444	8.819941	8.236218	7.627834	7.006116	7.802510	7.868917	7.961616	7.690876	7.854059	8.290249	8.219933	8.390368	8.033141	8.227204	7.944176	7.442516	8.401432	8.152062	8.490573	7.323122	8.135851	7.897749	6.701902	7.476312	6.798331	8.471529	7.603129	8.236218	9.287241	8.047693	8.090649	8.405771	8.732389	8.240832	8.912971

AC144548.1	12.240857	13.605486	12.962718	13.369728	13.641034	13.567588	13.569652	13.684034	14.255152	13.796160	13.733881	13.633991	13.489501	14.053607	13.209096	14.081566	14.002993	14.248223	14.473115	14.193358	14.578102	14.743665	13.417129	14.480594	14.559252	13.819912	13.160146	14.248871	13.027092	12.919527	14.415078	13.350346	12.876931	13.269247	12.821962	12.920057	13.383029	13.797744	13.942960	13.967990	13.887810	13.392871	13.775933	13.387343	12.937441	13.880137	13.335001	13.336430	13.446068	13.654386	13.068499	13.476835	13.439680	13.072064	13.633730	13.330066	13.242002	13.632300	14.008743	13.291131	13.889152	13.759150	11.882660	13.728607	13.302074	13.842755	13.850447	13.282626	13.454152	13.920234	12.979696	13.431034	13.903602	13.422596	13.686701	12.875210	12.678900	12.543722	12.899561	13.574051	13.231839	12.741823	12.998772	13.525511	13.685514	13.108685	12.978577	13.886983	13.166504	13.347657	13.226454	13.767473	13.109444	13.529678	12.055422	13.196712	13.492381	13.766279	13.154569	14.022985	13.662723	13.457219	13.686919	13.959054	14.529101	13.347208	14.613963	14.723372	14.438602	13.895060	14.220551	14.001811	14.277280	13.974138	13.556609	12.465995	12.142800	13.476158	14.257035	13.154762	12.547000	13.263369	14.214354	13.974945	13.938513	14.073025	14.247611	13.648013	14.588093	14.419877	14.479473	14.359381	15.223282	15.193465	13.325811	12.920598	14.818887	14.440081	11.847451	14.581385	14.801392	15.323382	13.240605	13.383693	14.117990	12.998439	14.629113	13.335001	13.779642	13.030625	13.670785	13.445036	14.012650	12.849310	12.473452	12.170476	13.749588	13.812737	13.553456	14.352710	13.488148	15.069269	14.215623	13.227239	14.483652	14.371917	13.338927	13.505905	13.954934	13.161477	13.994473	13.412114	14.357698	13.933765	12.911088	12.346468	13.762421	13.941795	14.103780	13.373784	13.246311	13.685973	14.422502	13.512420	13.290718	13.340998	13.601738	13.927324	13.310353	13.631592	13.733641	14.203084	13.607817	12.401299	12.748645	13.398659	13.935214	13.434297	12.117864	13.190645	13.812477	11.955591	12.863843	11.628650	13.731903	12.922424	13.504804	12.401457	13.216008	12.820105	13.131924	13.345934	13.372065	14.299113	13.868320	12.747792	13.848547	13.306393	13.589099	13.701136	13.277634	14.116167	13.071141	13.192705	14.660894	14.116761	14.022116	13.546876	14.049457	13.704613	13.382177	13.812477	13.818636	14.346007	13.920769	14.073025	13.270630	13.417523

LINC02465	5.178157	3.294203	3.618767	3.353521	3.014748	3.929346	3.628398	3.956576	3.414881	3.992018	3.782519	3.775752	3.636869	4.015856	3.529405	4.327873	3.643475	3.782941	4.935362	3.751073	6.993710	2.970992	5.441215	4.784747	3.831373	3.547776	3.948976	3.818855	3.227169	3.747923	4.746858	4.451198	4.369790	3.345907	4.317365	4.041233	4.106979	4.591187	3.794028	3.765884	3.743241	4.132093	3.992878	3.613780	3.386886	5.824234	5.179925	5.602645	3.230761	3.983505	3.930017	3.958796	5.285608	3.600491	4.001688	3.217985	5.022393	3.813595	5.766090	3.666279	3.783771	3.360327	4.533330	4.319502	3.747084	3.772413	4.638630	3.786732	3.633323	3.286969	3.582276	3.400891	3.027084	3.082228	3.627023	4.333307	3.295298	3.264389	3.224618	5.678637	3.479271	3.431607	2.998429	3.843155	3.994857	4.581826	2.987672	3.647551	3.570038	4.227258	3.381552	3.044948	4.596165	3.753824	3.690742	3.940037	4.275750	3.364994	3.337623	3.231430	3.003234	3.366512	3.880253	2.901334	2.946892	3.105287	4.834368	3.723511	3.297133	4.055620	3.301128	3.841707	3.131266	3.236796	4.343091	2.963914	4.762913	2.967208	3.112510	3.767775	4.462233	3.940672	3.728363	2.973723	4.661156	3.374235	3.707077	4.125060	3.776160	4.381821	3.589398	3.409194	3.845423	4.592086	3.681816	4.747673	3.928045	4.470784	4.311896	6.256740	3.564009	4.215112	4.303986	3.331586	3.834808	3.644431	3.475980	3.045578	5.311259	4.508542	4.027744	4.531615	4.469265	5.123715	4.638845	4.933461	5.103393	4.279490	4.290904	3.530135	4.742945	4.446690	4.653729	4.694734	6.068237	4.938708	4.718747	4.229679	4.839385	5.313012	4.387173	5.799127	5.508966	4.759133	3.596508	4.832927	5.288335	5.757214	4.918089	4.545122	3.764916	4.811884	4.961439	5.067563	4.492238	5.092471	4.703861	4.494423	4.744005	3.796720	3.629852	3.828244	4.057171	3.753613	4.646404	3.626213	4.002553	3.757509	5.314416	4.916406	5.346739	4.846791	4.180769	4.621436	3.401932	4.677740	4.093924	3.984565	4.726407	3.729640	3.765704	4.500042	4.397228	4.258714	4.354817	3.504538	3.142794	5.483145	4.121401	3.690742	3.366512	3.262192	3.385183	2.960337	3.725976	5.122315	4.504570	4.520391	4.061055	3.189156	3.859195	3.264982	3.660815	3.646626	3.190259	4.022173	4.318671	3.833118

ZNF582-AS1	7.855139	7.610979	7.612184	7.373586	7.565037	9.952895	9.361262	8.809052	9.541580	9.757967	10.129759	9.084689	8.618151	8.472017	8.607266	8.975910	9.667979	8.140757	8.676509	8.256329	7.789934	7.939622	9.400155	8.228867	8.287349	8.728535	9.107052	9.130445	9.668449	8.594547	8.395613	7.838941	7.668348	8.022827	7.700074	7.801900	8.284855	7.776055	7.513747	8.023465	9.343609	8.189101	7.240235	7.565037	8.165963	8.012452	10.381341	7.906019	7.866357	7.748723	7.227941	8.381284	7.821963	7.740543	7.893724	10.515780	8.166936	8.087867	6.943118	8.050788	8.451544	9.300797	8.620186	7.566262	7.439309	8.096125	7.402552	7.916120	6.812241	7.211984	7.941974	8.506737	10.193710	8.357999	8.334459	7.956200	8.303328	7.311187	7.778689	8.177531	7.728876	7.971462	7.908340	8.047349	8.135851	8.251602	7.275617	7.545824	7.583498	7.788679	7.758989	7.789160	7.763140	8.028718	8.192133	9.005828	8.121305	8.871708	8.583923	8.898099	9.419361	8.060764	8.214556	7.825991	7.358858	9.455087	8.535462	7.628611	8.439347	10.078682	8.702595	8.277666	6.409866	10.308167	9.908340	10.431097	6.969499	9.074813	10.111838	9.876416	5.366204	7.065715	6.924197	7.756499	8.781348	9.044869	7.819822	7.272673	7.926948	8.260812	8.414001	10.049101	7.955709	7.964808	8.939581	6.447183	7.178923	5.574074	6.681903	6.235988	6.683406	7.181722	7.380184	6.995675	7.008506	8.377215	7.627346	9.066139	7.084741	8.023925	6.963561	7.582883	7.504456	6.711499	6.716392	8.175070	7.519176	7.856355	7.092916	8.180074	6.714591	6.111791	5.101759	5.814929	5.999712	8.386497	6.749255	6.922969	6.145315	6.147242	6.953906	6.409342	6.205920	7.797234	7.903197	8.152839	5.513154	5.385023	6.283128	6.390531	8.360934	4.836376	4.661156	6.229383	7.080648	5.449166	6.164766	6.255895	5.661421	7.023420	7.332899	9.814948	7.267429	7.666067	7.085989	7.675427	6.759769	6.878780	7.648147	6.257421	5.966988	8.055425	7.282571	6.914148	8.183407	5.669251	5.969983	7.516020	7.149622	7.551900	5.958775	7.561058	7.264495	7.218267	8.038522	7.866036	8.201220	8.552881	6.445240	7.819523	7.021413	8.453383	7.180790	6.275725	8.382372	6.195674	6.303405	6.291686	8.449384	6.852662	7.836896	8.433072	10.567405	8.200278	8.124646	6.858267	7.573568	7.667897

AL355922.3	7.337844	9.088375	8.626389	7.504760	7.548238	7.277515	7.188249	7.618627	7.592104	7.166260	6.883579	7.124824	7.084102	7.007341	6.996945	7.183448	6.790070	6.823372	8.049234	6.864398	5.997433	8.209784	7.403178	6.838038	7.169643	6.853155	8.474628	7.733473	7.553771	8.819791	6.866773	9.068809	10.176535	8.097062	9.486110	8.142539	7.930949	7.733324	7.966655	7.476921	7.185369	8.184186	7.712966	6.613022	6.903827	8.032995	8.338549	7.795703	8.759334	7.878566	9.213906	8.535462	10.144304	7.060427	8.236065	7.605162	8.698484	8.764520	7.283831	8.205229	9.357110	8.029783	11.586469	7.115031	9.097199	9.743643	9.509006	7.329461	10.524581	7.407903	7.073059	7.411359	7.162049	7.484743	8.283307	10.296336	7.972255	11.059091	8.304234	9.348949	8.761230	9.941340	8.162655	8.604528	9.659667	6.686616	10.320751	8.282080	9.794550	9.813541	8.326656	10.702889	9.840456	9.786097	9.003955	7.536868	8.200588	6.541196	6.994003	7.226066	7.917227	6.566263	6.978010	6.758287	7.524675	7.146440	7.188088	6.912751	7.558311	7.278159	8.437404	7.915673	7.835236	7.464507	6.332981	9.989367	7.458716	6.910160	7.306970	8.128554	8.775501	10.542394	7.510460	8.667742	7.761430	8.246938	7.346579	8.101146	7.395991	7.023277	7.382041	7.349268	8.164571	7.108192	8.364848	6.906870	6.788763	7.793394	8.374334	7.614214	9.370896	9.197498	8.097374	7.623954	9.745544	8.673745	7.230254	7.411794	7.992398	8.053853	10.575116	9.167845	8.797944	8.830404	7.852796	8.893945	9.192737	7.638786	10.839888	7.382687	9.425577	9.265944	8.480861	9.849917	9.205975	7.352207	9.004108	8.675471	10.554299	10.356625	10.030230	10.234648	9.558329	11.681866	8.522763	9.178174	10.382403	9.034153	9.604210	10.835284	9.001818	9.680465	8.065802	10.916445	12.085291	9.911891	10.221236	8.721185	11.329984	8.490573	8.332147	6.907507	9.857725	10.339450	11.217158	9.986994	11.770940	8.397632	11.270579	9.476893	9.823738	9.795943	11.316765	9.635491	8.240685	10.107468	10.073289	11.135818	13.434924	9.989516	10.455381	10.720204	9.558642	9.661551	9.947176	8.324297	7.118214	8.251602	8.561461	8.449215	7.411794	7.229814	10.300966	9.819657	7.240393	7.267429	8.788881	7.574019	7.231968	9.276233	9.134109	10.425753	7.498818	7.799910	8.085924	9.615291	6.998925	6.791208

AL355922.4	7.337844	9.088375	8.626389	7.504760	7.548238	7.277515	7.188249	7.618627	7.592104	7.166260	6.883579	7.124824	7.084102	7.007341	6.996945	7.183448	6.790070	6.823372	8.049234	6.864398	5.997433	8.209784	7.403178	6.838038	7.169643	6.853155	8.474628	7.733473	7.553771	8.819791	6.866773	9.068809	10.176535	8.097062	9.486110	8.142539	7.930949	7.733324	7.966655	7.476921	7.185369	8.184186	7.712966	6.613022	6.903827	8.032995	8.338549	7.795703	8.759334	7.878566	9.213906	8.535462	10.144304	7.060427	8.236065	7.605162	8.698484	8.764520	7.283831	8.205229	9.357110	8.029783	11.586469	7.115031	9.097199	9.743643	9.509006	7.329461	10.524581	7.407903	7.073059	7.411359	7.162049	7.484743	8.283307	10.296336	7.972255	11.059091	8.304234	9.348949	8.761230	9.941340	8.162655	8.604528	9.659667	6.686616	10.320751	8.282080	9.794550	9.813541	8.326656	10.702889	9.840456	9.786097	9.003955	7.536868	8.200588	6.541196	6.994003	7.226066	7.917227	6.566263	6.978010	6.758287	7.524675	7.146440	7.188088	6.912751	7.558311	7.278159	8.437404	7.915673	7.835236	7.464507	6.332981	9.989367	7.458716	6.910160	7.306970	8.128554	8.775501	10.542394	7.510460	8.667742	7.761430	8.246938	7.346579	8.101146	7.395991	7.023277	7.382041	7.349268	8.164571	7.108192	8.364848	6.906870	6.788763	7.793394	8.374334	7.614214	9.370896	9.197498	8.097374	7.623954	9.745544	8.673745	7.230254	7.411794	7.992398	8.053853	10.575116	9.167845	8.797944	8.830404	7.852796	8.893945	9.192737	7.638786	10.839888	7.382687	9.425577	9.265944	8.480861	9.849917	9.205975	7.352207	9.004108	8.675471	10.554299	10.356625	10.030230	10.234648	9.558329	11.681866	8.522763	9.178174	10.382403	9.034153	9.604210	10.835284	9.001818	9.680465	8.065802	10.916445	12.085291	9.911891	10.221236	8.721185	11.329984	8.490573	8.332147	6.907507	9.857725	10.339450	11.217158	9.986994	11.770940	8.397632	11.270579	9.476893	9.823738	9.795943	11.316765	9.635491	8.240685	10.107468	10.073289	11.135818	13.434924	9.989516	10.455381	10.720204	9.558642	9.661551	9.947176	8.324297	7.118214	8.251602	8.561461	8.449215	7.411794	7.229814	10.300966	9.819657	7.240393	7.267429	8.788881	7.574019	7.231968	9.276233	9.134109	10.425753	7.498818	7.799910	8.085924	9.615291	6.998925	6.791208

AL355922.1	7.337844	9.088375	8.626389	7.504760	7.548238	7.277515	7.188249	7.618627	7.592104	7.166260	6.883579	7.124824	7.084102	7.007341	6.996945	7.183448	6.790070	6.823372	8.049234	6.864398	5.997433	8.209784	7.403178	6.838038	7.169643	6.853155	8.474628	7.733473	7.553771	8.819791	6.866773	9.068809	10.176535	8.097062	9.486110	8.142539	7.930949	7.733324	7.966655	7.476921	7.185369	8.184186	7.712966	6.613022	6.903827	8.032995	8.338549	7.795703	8.759334	7.878566	9.213906	8.535462	10.144304	7.060427	8.236065	7.605162	8.698484	8.764520	7.283831	8.205229	9.357110	8.029783	11.586469	7.115031	9.097199	9.743643	9.509006	7.329461	10.524581	7.407903	7.073059	7.411359	7.162049	7.484743	8.283307	10.296336	7.972255	11.059091	8.304234	9.348949	8.761230	9.941340	8.162655	8.604528	9.659667	6.686616	10.320751	8.282080	9.794550	9.813541	8.326656	10.702889	9.840456	9.786097	9.003955	7.536868	8.200588	6.541196	6.994003	7.226066	7.917227	6.566263	6.978010	6.758287	7.524675	7.146440	7.188088	6.912751	7.558311	7.278159	8.437404	7.915673	7.835236	7.464507	6.332981	9.989367	7.458716	6.910160	7.306970	8.128554	8.775501	10.542394	7.510460	8.667742	7.761430	8.246938	7.346579	8.101146	7.395991	7.023277	7.382041	7.349268	8.164571	7.108192	8.364848	6.906870	6.788763	7.793394	8.374334	7.614214	9.370896	9.197498	8.097374	7.623954	9.745544	8.673745	7.230254	7.411794	7.992398	8.053853	10.575116	9.167845	8.797944	8.830404	7.852796	8.893945	9.192737	7.638786	10.839888	7.382687	9.425577	9.265944	8.480861	9.849917	9.205975	7.352207	9.004108	8.675471	10.554299	10.356625	10.030230	10.234648	9.558329	11.681866	8.522763	9.178174	10.382403	9.034153	9.604210	10.835284	9.001818	9.680465	8.065802	10.916445	12.085291	9.911891	10.221236	8.721185	11.329984	8.490573	8.332147	6.907507	9.857725	10.339450	11.217158	9.986994	11.770940	8.397632	11.270579	9.476893	9.823738	9.795943	11.316765	9.635491	8.240685	10.107468	10.073289	11.135818	13.434924	9.989516	10.455381	10.720204	9.558642	9.661551	9.947176	8.324297	7.118214	8.251602	8.561461	8.449215	7.411794	7.229814	10.300966	9.819657	7.240393	7.267429	8.788881	7.574019	7.231968	9.276233	9.134109	10.425753	7.498818	7.799910	8.085924	9.615291	6.998925	6.791208

AC005323.2	3.638275	3.229755	2.986165	2.969724	2.971255	3.510009	3.337623	3.142685	3.439246	3.372751	4.107829	3.679614	3.319596	3.774315	4.003905	3.067954	3.393833	3.786494	4.780742	3.063626	4.743570	3.011939	2.943972	3.158914	3.229042	3.006239	3.078385	2.900822	3.121586	3.049541	3.535381	3.989435	4.437234	2.968562	3.539784	4.024123	3.550424	3.366868	3.224190	3.265866	3.430072	3.058330	3.717722	3.234227	3.542469	4.830608	3.273570	4.100634	3.503982	3.823524	3.659429	3.266021	4.279071	3.963586	3.785850	3.091098	3.935165	4.305738	4.234619	3.853359	2.998688	3.035687	3.763620	3.097370	3.332425	3.266904	3.177618	3.250849	3.380840	2.832736	3.034025	3.022471	3.104667	3.098911	3.038227	3.337623	3.088102	3.027358	2.960516	3.716082	4.480424	3.551420	3.097035	4.622899	3.322172	3.475806	3.462669	3.200147	4.169012	3.612955	3.150262	2.944169	3.729236	3.074125	2.946541	3.120467	3.527038	2.960684	3.451883	3.151248	4.137156	3.213260	2.963052	2.865853	4.516566	3.115214	3.143382	3.305531	3.026482	3.260708	2.814697	3.352721	3.155201	3.220568	3.307309	5.115798	3.376176	2.998262	3.136258	3.367066	4.815819	3.885300	3.870938	3.930246	4.671443	3.425651	3.179520	3.777746	3.904846	4.262655	3.545984	4.277079	3.946628	5.095208	3.581694	4.550664	4.364149	3.640693	4.498788	4.567891	3.985631	2.939668	3.546768	4.179876	4.326548	4.060178	3.652626	3.164945	4.586903	3.768830	4.230480	4.751297	4.690748	4.685684	4.688032	4.773888	5.184019	4.879022	4.613377	3.850978	5.275956	4.596826	4.787643	4.656406	4.628705	3.761124	4.826274	4.976700	5.155213	5.260447	4.834971	5.061210	5.198733	4.828464	3.640310	5.021154	5.056350	5.290178	4.688212	4.577985	4.017403	5.205070	4.910082	5.158488	4.660271	4.876104	4.548146	4.156076	4.822102	3.828431	3.698588	3.905077	3.706865	3.567669	3.937514	3.590513	3.826779	3.629234	4.165107	5.445055	5.566556	4.822497	4.467583	4.828464	3.963800	4.682632	4.472756	5.191894	4.617724	4.436278	4.523673	4.653042	4.522120	4.175765	3.934702	3.805556	4.122526	5.079600	4.078461	4.020669	3.640693	4.129602	4.427011	5.021154	3.308242	4.381821	4.529888	4.060633	3.920194	3.854698	3.397663	3.680445	3.725976	3.533846	3.305459	3.901874	3.923290	3.788448

MYHAS	3.638275	3.229755	2.986165	2.969724	2.971255	3.510009	3.337623	3.142685	3.439246	3.372751	4.107829	3.679614	3.319596	3.774315	4.003905	3.067954	3.393833	3.786494	4.780742	3.063626	4.743570	3.011939	2.943972	3.158914	3.229042	3.006239	3.078385	2.900822	3.121586	3.049541	3.535381	3.989435	4.437234	2.968562	3.539784	4.024123	3.550424	3.366868	3.224190	3.265866	3.430072	3.058330	3.717722	3.234227	3.542469	4.830608	3.273570	4.100634	3.503982	3.823524	3.659429	3.266021	4.279071	3.963586	3.785850	3.091098	3.935165	4.305738	4.234619	3.853359	2.998688	3.035687	3.763620	3.097370	3.332425	3.266904	3.177618	3.250849	3.380840	2.832736	3.034025	3.022471	3.104667	3.098911	3.038227	3.337623	3.088102	3.027358	2.960516	3.716082	4.480424	3.551420	3.097035	4.622899	3.322172	3.475806	3.462669	3.200147	4.169012	3.612955	3.150262	2.944169	3.729236	3.074125	2.946541	3.120467	3.527038	2.960684	3.451883	3.151248	4.137156	3.213260	2.963052	2.865853	4.516566	3.115214	3.143382	3.305531	3.026482	3.260708	2.814697	3.352721	3.155201	3.220568	3.307309	5.115798	3.376176	2.998262	3.136258	3.367066	4.815819	3.885300	3.870938	3.930246	4.671443	3.425651	3.179520	3.777746	3.904846	4.262655	3.545984	4.277079	3.946628	5.095208	3.581694	4.550664	4.364149	3.640693	4.498788	4.567891	3.985631	2.939668	3.546768	4.179876	4.326548	4.060178	3.652626	3.164945	4.586903	3.768830	4.230480	4.751297	4.690748	4.685684	4.688032	4.773888	5.184019	4.879022	4.613377	3.850978	5.275956	4.596826	4.787643	4.656406	4.628705	3.761124	4.826274	4.976700	5.155213	5.260447	4.834971	5.061210	5.198733	4.828464	3.640310	5.021154	5.056350	5.290178	4.688212	4.577985	4.017403	5.205070	4.910082	5.158488	4.660271	4.876104	4.548146	4.156076	4.822102	3.828431	3.698588	3.905077	3.706865	3.567669	3.937514	3.590513	3.826779	3.629234	4.165107	5.445055	5.566556	4.822497	4.467583	4.828464	3.963800	4.682632	4.472756	5.191894	4.617724	4.436278	4.523673	4.653042	4.522120	4.175765	3.934702	3.805556	4.122526	5.079600	4.078461	4.020669	3.640693	4.129602	4.427011	5.021154	3.308242	4.381821	4.529888	4.060633	3.920194	3.854698	3.397663	3.680445	3.725976	3.533846	3.305459	3.901874	3.923290	3.788448

AL121845.1	7.139390	7.676806	7.723319	7.764204	7.495213	7.682746	7.714346	8.496424	7.773210	8.158705	7.488204	7.691976	7.515221	7.624268	7.190458	8.000277	7.735493	7.419464	7.816357	7.615888	7.293155	8.239639	6.871784	7.948319	8.115323	8.104985	7.993790	8.485531	8.076646	7.580544	7.876561	7.272360	7.854375	7.281623	8.055590	7.524675	7.816519	7.585179	7.718273	7.449679	8.269199	7.320594	8.012895	7.449365	7.204710	7.356553	7.983626	8.170037	7.340507	7.767159	7.568694	7.840933	8.047031	7.582415	7.739616	8.039326	7.798494	7.551900	7.387934	7.723928	7.341300	8.094336	7.440064	7.115180	7.346579	7.470168	8.004026	7.416867	7.745783	7.832466	7.889666	7.695178	8.004347	7.714645	7.721625	7.518657	7.760531	7.615728	7.498273	7.372533	7.535018	7.968034	7.884438	7.620601	7.666215	7.594740	7.205049	7.403034	7.186349	7.017630	7.272190	7.172935	7.360909	7.838627	7.242261	7.228105	7.133232	7.387934	7.208301	7.501815	6.928293	7.754559	7.787764	7.222846	7.639530	7.860474	8.431048	7.420385	6.868239	8.450004	6.910160	7.503695	7.470477	8.243241	7.570519	5.228322	7.143465	7.519719	8.129662	7.822865	8.017553	7.769734	7.853595	7.724844	7.976790	7.755464	7.959294	7.993945	8.040277	7.740862	7.944941	8.765904	9.495345	7.796171	7.396775	7.908800	7.043839	10.020187	8.422262	8.205706	8.268392	8.350523	7.915673	8.641739	9.165569	8.436480	7.852497	7.471118	8.042154	7.750282	7.647414	7.221773	7.507849	8.016016	7.438363	7.431882	7.419464	7.729485	7.138283	7.771423	7.075460	7.398965	8.016793	6.871129	7.952475	8.296090	8.307119	7.070379	7.967867	8.618151	8.486586	8.160678	8.392410	8.027797	8.048929	8.476201	8.024532	7.397708	7.581018	7.783625	7.925836	7.285348	7.954791	7.443772	8.128713	7.972915	7.612344	7.476161	7.703423	7.534543	7.464195	9.145041	7.693823	7.726838	7.474601	7.794928	8.187149	7.518806	7.453707	8.081052	8.293663	8.291041	7.565801	8.318502	7.309610	7.799277	7.063002	7.632540	7.188718	7.723476	7.489903	7.200082	8.210651	7.848092	7.892783	6.896050	7.079256	7.592412	7.822263	8.132536	6.673327	7.856943	7.337058	8.484922	8.416918	7.471118	7.007190	7.858470	8.656980	7.103764	7.809422	8.315429	8.617997	7.686094	6.632208	7.891219	7.955709	8.497631

AC109361.1	5.245997	5.752475	6.113696	5.649152	5.972886	5.519185	5.093749	6.202971	6.674294	6.279314	6.518720	6.009832	6.592947	6.102226	5.372272	5.792732	6.336359	6.678007	6.794787	6.056734	5.696567	5.827126	3.286969	6.203825	5.127943	5.418549	5.384282	5.073437	5.822404	4.775532	6.140137	5.698228	5.715116	7.180027	5.656116	5.790965	5.910637	4.583727	5.689182	4.857743	6.309083	5.100098	6.349412	5.959347	5.304807	4.704697	5.923308	4.981730	4.048031	6.786625	4.700479	6.433444	6.239749	5.216974	5.595130	6.009651	6.322042	4.578189	5.779986	5.300034	5.705436	5.628798	5.840959	5.941126	4.947539	5.288749	4.689279	6.007549	5.208422	3.233635	5.088183	4.705302	6.295600	5.053435	5.529728	5.466948	4.812712	4.994379	5.086568	6.187767	4.823117	5.931422	5.386970	5.681611	6.734817	6.545166	6.078492	6.240427	4.243194	5.023623	4.849906	5.074630	5.805975	4.749977	5.408333	6.434619	6.011218	7.049597	6.416338	6.098380	6.778314	5.377702	6.130349	5.692666	5.592356	6.535017	6.367040	5.769929	6.677352	5.241057	6.087995	5.941296	6.006099	5.731934	5.408723	6.208993	5.137208	6.468027	5.721394	7.084584	6.154825	5.128988	6.976317	6.286020	5.073004	4.864751	6.811299	7.143957	5.336536	5.259448	6.328401	6.108974	4.928862	4.745760	6.846929	6.595852	7.491660	5.705254	6.014177	6.436616	7.030045	4.928862	5.000684	3.293775	5.448014	4.182548	4.396141	4.877385	4.937082	7.073401	6.170149	7.009488	5.425386	5.390063	5.448784	5.824234	3.655820	4.809298	4.278403	7.507990	3.147523	6.173696	6.713105	5.303625	5.286988	4.183437	4.374789	4.911789	6.008053	3.923504	5.698593	6.489829	5.678435	5.632657	4.715794	8.162959	4.522344	6.235139	3.931917	5.846047	5.623364	5.633992	6.598614	5.548054	6.265543	5.937761	4.775112	3.704471	4.773477	4.256282	5.284990	4.723229	5.767564	5.829913	5.518396	5.281557	5.872251	5.329881	5.405155	6.496679	5.727541	7.475046	7.239393	6.093207	6.292519	5.878692	6.215090	5.075669	6.577999	5.569943	4.803678	6.027664	5.202236	5.955394	5.900633	5.683817	6.975438	6.504643	5.336143	5.779106	6.179820	6.243249	5.239063	6.036181	3.955912	6.302728	7.168563	5.244612	4.441288	6.429367	5.573143	6.064519	4.135859	4.939337	7.336125	6.184321	7.816205	5.281557

CLEC12A-AS1	4.021312	4.066483	3.682035	3.704674	4.166251	5.035490	3.333551	4.400675	4.420003	3.431159	3.628835	3.765884	3.649639	5.162860	3.874606	3.628614	3.812307	4.398484	5.252360	3.719129	5.001729	4.049784	2.961386	3.575186	4.073634	3.426395	4.276612	4.090469	3.843957	3.583046	3.702653	3.632126	4.468199	3.462494	4.263078	4.114601	3.700762	3.871796	3.275122	4.836806	3.793589	2.954945	4.033635	3.910773	3.755678	5.808321	3.300811	2.974981	3.774502	3.823956	3.966797	4.740850	4.382733	4.768875	4.149594	4.065635	4.183437	3.114731	3.645020	3.767990	2.859452	3.969874	4.075568	3.992878	3.855934	4.069243	2.868709	3.662244	4.099749	4.512448	3.760284	4.416417	3.457694	3.859612	4.301945	3.803679	4.462632	4.095169	4.037357	3.958796	3.473978	4.096007	3.499964	4.348753	3.466886	4.921033	4.710318	3.982623	3.453144	4.754255	4.433701	3.819283	3.793794	3.995744	3.365338	3.392134	3.851619	3.712339	3.704471	4.400248	4.983993	3.418603	3.319276	3.723702	4.595765	4.128277	4.405149	4.160569	4.212080	4.427875	5.081029	4.668385	5.850256	4.463269	3.880658	7.140794	4.072967	3.209388	3.850777	4.041871	3.545812	4.238752	3.864726	5.184812	4.445201	4.728272	3.990254	3.853814	3.676812	3.518376	4.221049	5.617024	4.008468	4.185118	4.340922	4.694525	4.063502	4.819179	4.122071	4.303076	4.969897	3.745894	4.353548	3.990448	3.979980	5.244217	4.006327	5.255358	3.849721	4.265722	3.430468	4.468417	3.373274	3.414352	3.801618	3.817794	3.747923	4.007805	4.478658	4.180324	2.850826	4.221883	3.872223	4.243194	6.607242	4.142605	2.916669	4.072539	4.844870	4.250623	4.506112	4.202047	4.469469	4.173784	4.235728	5.358907	3.034305	3.780212	3.605218	3.014061	4.296116	3.839648	3.650983	3.735410	3.875870	2.857248	4.463060	3.893535	3.318122	3.657453	4.745112	3.514264	4.018708	3.672132	3.521300	4.443268	4.397017	4.080493	3.943276	3.984979	4.468617	3.586490	3.903359	4.024123	4.964988	4.345054	3.639080	3.138475	3.967702	4.197752	3.805753	3.887189	4.036500	3.487138	3.643851	4.513567	4.640929	3.912072	3.822483	4.615751	4.522785	4.098180	5.054532	5.001920	3.884029	4.710518	4.067119	4.192784	4.659849	5.057355	6.121304	10.717743	5.137013	4.027744	5.216797	3.921454	3.920194	3.773031

C2CD4D-AS1	8.897960	10.479041	9.458948	10.310511	10.208162	10.822815	10.093506	10.512728	9.888617	10.362213	10.659325	10.657396	11.177770	10.633979	10.205978	9.251933	10.257261	10.466673	10.618828	11.394279	10.199926	11.683069	10.438539	9.662014	9.972182	10.002111	10.437119	10.674193	10.102629	10.377424	10.398534	9.889366	10.013258	10.056660	10.535843	9.648272	9.712489	10.486856	11.013251	10.269288	9.748635	10.472844	9.444365	10.090671	10.188617	10.223174	9.978433	10.001321	10.352360	9.644870	9.864242	10.152953	10.369851	10.687354	9.494887	11.104718	9.112719	10.895475	11.636925	10.134688	10.194616	9.466743	9.428048	10.438690	9.985333	10.518030	10.429581	10.734915	10.099349	10.500761	10.250055	10.685853	10.506461	10.287089	10.815965	9.718135	9.386992	11.082337	9.933063	10.301733	10.418738	10.192949	10.893881	10.227757	9.599094	9.730269	12.051034	10.030393	11.036123	11.260928	10.204002	10.240017	9.916835	9.903259	10.357958	9.687929	10.032116	9.761351	10.240898	10.274723	10.499425	9.502999	9.880868	10.262977	10.525491	9.836571	10.188771	11.284152	11.456254	10.211224	11.094860	10.483072	10.357811	10.570453	9.941169	11.295540	9.175262	11.417687	9.782831	8.987404	11.017912	9.729026	9.730779	12.040929	11.005839	11.337301	9.496268	9.417796	11.124285	11.334043	10.183600	10.739457	10.353264	10.673442	9.369356	9.573678	10.830197	9.978433	9.603604	11.125638	11.890634	11.069879	10.102629	10.292500	9.540974	10.191313	11.706091	10.479505	10.038692	10.258179	11.935497	11.647387	10.706106	11.419414	10.663292	9.838114	10.770016	11.553440	11.093692	10.719585	12.142518	8.545522	10.002883	11.552276	10.411012	11.285631	9.284449	11.698867	9.401376	11.812873	9.348480	11.497855	9.889840	10.583626	10.697067	8.520957	12.551723	11.844021	11.524099	11.012393	10.085231	10.465656	11.006550	10.232190	10.364292	11.039457	12.227505	10.859758	9.912962	10.499276	12.260037	12.196766	11.081157	11.207310	10.205059	9.341028	10.376060	10.250573	9.498731	11.649619	10.093965	11.281969	11.840095	9.666225	11.135945	11.349211	9.670699	11.105584	12.073426	9.369995	10.724767	10.334012	10.714015	12.381157	11.206436	10.954367	10.302178	10.357958	9.766473	9.995732	9.653882	9.927224	10.365940	10.299712	10.491272	10.420249	10.340048	11.451777	11.383500	12.213183	10.112915	10.173348	10.965940	10.689778	10.150081	10.364867	13.062840	9.947176

AC107464.2	6.589166	6.831311	6.894802	7.156686	7.595792	6.740402	5.548626	6.810647	6.926505	5.819384	6.238599	5.739181	5.212013	6.001621	5.779986	6.524664	6.373468	6.742734	7.121194	6.391212	6.600872	6.937799	6.363037	6.540510	6.322896	6.486275	6.507640	6.367718	6.252648	6.998626	6.707538	6.407533	6.998767	5.922158	5.866135	5.875145	5.784311	6.619366	6.812404	6.296097	6.601098	6.381308	6.017966	5.666470	5.550338	5.882835	6.413691	6.228838	6.648884	5.758523	5.836646	6.974641	6.027309	5.737157	5.616676	6.587881	5.892040	6.257069	5.978520	5.689713	6.133618	6.352424	6.580790	6.599582	6.077122	6.500881	5.965532	6.306114	6.357670	6.347250	6.143754	6.031651	6.380296	6.324214	6.039123	7.028306	5.562770	5.607789	5.966637	5.974485	5.496067	6.275884	5.628798	5.598001	6.198260	6.060443	5.359285	6.190968	6.265208	6.451151	6.378130	6.509741	5.845843	6.116491	5.715666	5.801555	6.581476	6.228307	6.436771	6.239426	4.737997	5.614814	5.909893	6.289442	7.138922	6.240763	6.486107	6.576865	7.437870	6.828154	6.936495	5.889758	5.629358	6.535471	6.289123	4.269156	6.113504	6.157700	6.104605	6.827174	6.969337	6.592444	7.063346	7.883365	7.148341	5.337335	6.897032	6.525640	5.859782	6.133452	5.512770	5.174727	5.120694	5.772380	5.956984	6.538584	6.657757	7.753947	6.481906	6.877000	6.598102	7.180484	7.081142	5.917588	6.220581	6.112642	6.233243	6.299534	6.827028	6.762817	6.539709	6.504643	5.894762	5.830277	6.010871	5.696745	6.730314	7.002106	6.433444	6.273564	6.761727	6.004842	6.188929	5.832460	6.215090	6.797498	6.216306	6.733993	5.960936	5.979585	6.532898	5.746680	6.003768	5.310072	5.932102	5.136622	6.043958	5.856204	5.658691	6.474428	6.016224	6.572895	6.852835	6.549699	6.313838	6.400316	6.206431	6.859119	6.623405	6.006820	6.126600	6.704456	6.364065	5.713463	7.163317	6.118533	5.925258	6.354574	5.697684	6.083687	6.345056	6.226940	5.790077	5.884422	6.298741	5.950728	6.146017	6.164766	6.427147	6.585224	6.900460	5.927599	6.467703	6.388847	6.002186	6.249294	5.880328	6.299387	6.224856	5.698054	4.857538	5.843870	5.905949	7.426143	6.401461	6.110778	6.958624	7.188249	6.900625	6.654828	5.080015	4.644693	6.199294	7.073993	5.858872	6.725435	6.305094	5.841340

LINC01060	5.227496	3.943958	4.294152	3.020820	3.808070	4.586482	5.285798	4.343705	4.150204	3.451366	4.587333	5.915666	3.475076	3.902322	5.077771	4.236363	6.155169	4.302168	7.522660	4.979544	4.935151	2.866196	3.406592	4.334644	3.489710	3.599132	3.166907	4.227481	3.229197	3.048168	3.658626	5.728276	5.614619	4.400062	5.642738	5.627262	3.653615	4.325528	4.906706	2.823310	3.939428	4.433701	4.446690	4.165107	3.235659	5.109345	3.659062	5.684176	4.270510	4.184907	6.199832	5.835000	4.946937	5.847907	3.469431	4.240349	4.191700	2.966116	3.727546	3.427639	2.978509	3.773677	5.735558	3.332078	3.105510	3.619143	4.420003	5.659956	3.302535	4.534351	4.204758	3.169645	4.071461	3.245308	5.559054	5.471380	5.866298	4.129176	3.533311	4.717717	3.575730	6.335031	6.732083	4.892937	3.021771	3.422630	5.177742	4.258087	5.310505	4.115709	3.159226	4.305090	5.026469	4.041233	3.432543	4.202480	3.663078	4.640524	5.747211	3.177091	4.236770	3.488837	4.719590	4.060633	3.570590	3.060878	3.955721	3.333864	3.444014	3.762166	3.570590	3.949658	3.520806	4.154543	4.012459	5.460645	3.643851	4.337231	3.829935	5.266656	7.116435	6.971198	5.058586	5.837134	5.621849	5.837488	7.161880	8.284855	4.597655	8.154232	5.808321	6.552341	4.007174	4.772189	3.645431	9.823152	5.667049	4.292225	8.535005	4.258714	3.869015	3.279133	3.548394	5.069387	4.043645	3.837359	3.447097	4.227030	4.875897	5.419369	6.535813	7.006116	6.636437	5.450652	6.607883	5.814075	5.432612	6.156355	5.056350	5.849182	2.844483	5.364244	6.580287	6.127291	5.776908	4.560966	3.468909	6.880869	6.803657	7.994267	6.849029	5.157902	10.575398	7.563944	5.287363	7.113000	3.488837	3.430988	3.270526	4.333544	6.615749	7.303037	3.997882	3.134888	5.304215	4.691364	6.093560	4.538643	3.389455	4.872144	5.314008	4.085680	7.206128	5.925083	5.416606	6.467195	7.183448	6.479775	6.010016	5.641575	9.715628	8.514154	6.183618	7.156225	6.594690	8.365788	5.341623	7.574330	5.016475	3.873530	5.803834	5.002315	6.290632	7.068063	8.231949	5.438573	6.236149	8.793679	6.164413	6.463206	6.295519	7.681937	7.601986	4.029277	4.341562	4.663487	3.478586	3.244978	4.517005	3.856793	5.610492	5.221285	3.419704	5.783423	8.961744	6.105503	5.723098	5.960034

LINC00698	5.320125	6.789423	6.523680	6.496512	7.249473	6.905576	6.395482	7.894662	7.405370	6.881674	6.843037	6.529826	5.998502	6.825589	5.803302	7.182830	6.635961	6.265874	8.098401	6.969180	6.841442	7.861673	3.880460	7.116904	7.250091	7.294911	6.934413	7.126885	6.416995	6.685988	6.806159	6.752706	6.499228	6.042958	6.725278	6.822609	6.753389	6.758790	6.965640	6.776864	7.011235	6.139446	7.307876	7.000485	6.731107	7.553313	7.178293	7.559510	6.629515	7.407740	6.695877	6.704456	6.841126	6.681747	6.500881	7.064451	6.842402	6.716859	6.342505	6.243437	6.132434	6.907669	6.921935	6.761889	6.634515	6.681903	7.299385	7.042589	7.232778	7.339873	7.035923	7.050231	7.136406	6.564171	6.966132	6.444537	6.687772	6.404740	6.749580	6.407869	6.335522	6.298921	6.079385	5.694907	5.970684	6.067343	6.596780	5.955561	6.510765	6.322725	6.163738	6.706701	6.645635	6.951242	6.389179	6.536130	6.084413	6.707215	7.450429	6.705732	7.508140	6.641471	6.826070	6.794940	7.563186	6.488381	8.186342	7.480444	7.178293	7.239393	7.390251	7.431096	7.355953	7.280042	6.981609	8.967765	6.371084	6.309083	7.051964	5.942491	6.890923	6.917090	6.586378	8.142400	6.571326	6.703844	6.708631	6.459532	7.061243	6.438569	6.439253	7.339407	6.580967	6.958624	7.201315	6.286581	6.894955	6.885195	6.350625	7.683512	7.055251	7.212483	7.951667	7.243901	6.576181	7.405681	7.380184	7.250881	6.866933	6.931924	6.791839	6.352940	6.932089	6.839990	6.356807	6.650152	7.579513	7.399914	6.692425	7.416084	6.573209	6.342844	6.409505	6.803329	7.299081	7.332112	6.660184	6.403750	6.923538	6.709432	6.349083	6.670097	6.742107	6.298576	6.620355	6.567898	6.075028	6.354414	5.626160	6.251982	6.892517	6.828927	6.803657	6.739082	5.823680	6.856433	6.988415	7.090251	7.015993	6.533404	6.629691	7.472188	6.207268	6.208471	6.258619	6.339551	6.178831	6.795726	6.203457	6.269124	6.512602	6.463552	5.838997	6.118709	6.636899	5.773628	5.900251	6.548199	6.133452	6.411997	6.282954	6.616765	6.796039	6.433086	6.849484	6.609329	6.891740	7.448561	6.214589	7.052716	6.328905	6.913084	6.860916	6.761061	6.931597	7.236423	6.791839	7.101115	7.534064	6.767961	7.574019	8.562976	7.785913	7.392419	7.546796	6.144094	6.690947	7.121496

AC007566.1	7.191384	7.066045	6.956582	7.654056	7.913572	8.535462	7.442212	7.768377	8.279032	7.742537	7.981918	7.456680	7.470321	7.519870	7.047214	7.779666	7.276417	7.410476	8.048929	7.555503	7.986529	7.742387	7.602969	7.298624	7.296211	7.587332	8.683701	7.485213	8.404997	7.875964	8.417684	6.904609	6.722579	7.589345	7.125129	6.823863	7.109658	7.096876	6.995064	7.143631	8.037881	6.555305	7.129243	6.695877	6.499228	6.354105	8.409992	6.774786	6.985556	6.622744	6.765874	7.840605	6.757827	6.654828	6.454454	7.974303	6.500534	5.869386	6.296590	6.151907	6.239096	7.909425	7.050231	7.139854	6.657265	6.323541	6.976898	6.969337	6.664294	7.115335	7.363878	7.558018	8.248548	7.092603	7.196002	6.985072	7.451050	6.909077	7.090496	6.204695	7.201019	6.301513	6.457889	6.065578	6.049931	6.623549	7.897027	6.817315	5.922924	5.719750	5.538448	6.101173	6.565450	6.784180	7.232119	7.462298	6.628549	7.770961	8.016793	8.215018	8.811379	7.218710	7.566262	7.143793	8.051848	7.836439	7.768537	7.429685	7.849606	8.273458	7.162520	7.535343	8.024072	8.834047	7.349268	11.508491	7.235637	7.483988	8.342466	7.263718	7.714929	7.445550	8.415321	8.983646	8.661006	8.370503	8.059636	8.693881	8.603609	8.156736	8.168619	8.664994	8.179009	7.766387	8.535943	7.745934	7.842645	8.039167	7.781047	8.561316	8.339505	7.957140	8.396721	7.995659	7.983020	8.751471	8.891594	9.308181	8.170037	8.598657	7.862455	7.442823	7.929719	7.271258	7.200242	7.207847	7.396775	7.379095	7.236268	9.135924	6.648533	7.033749	7.395991	6.612852	6.588854	8.771577	6.978816	7.525891	7.422754	6.843859	7.906019	6.983656	7.210174	7.446661	8.252679	7.349921	5.878151	6.944562	6.407210	6.859786	8.377828	7.144129	7.525289	7.381884	6.896388	7.254764	7.142561	7.276888	7.657668	7.608374	7.586300	9.121540	7.443314	7.675897	6.958927	7.588888	7.329932	7.055070	7.028135	6.834522	6.844875	6.597629	7.606249	6.275556	8.227971	6.657110	6.287255	6.613816	6.427331	6.504481	6.587534	7.260136	7.527936	7.787607	7.754421	7.873667	9.239986	8.346622	7.406798	8.324142	7.907878	8.049234	7.662002	7.592865	8.711618	8.024223	7.383627	7.160446	9.286431	7.362446	8.248095	9.308633	9.082889	8.119770	8.828074	6.853000	7.638623	8.538318

STEAP3-AS1	11.818235	10.709812	10.448409	11.354180	11.829878	11.618317	10.846224	11.153181	11.242375	11.311032	11.377987	11.652891	11.625150	11.532579	11.410105	11.006688	11.292947	11.547842	11.707017	11.432403	11.604311	10.497826	10.154668	11.475276	11.281661	11.586607	11.502476	11.246511	11.346523	10.500604	11.977451	11.145141	10.634740	10.954993	10.752225	10.777970	11.017653	11.350844	11.466898	11.164195	11.256343	10.988377	10.986598	11.077953	10.919423	11.226506	12.240559	10.861307	10.920323	10.746795	10.932934	10.568922	10.862314	10.674816	10.854330	11.551948	11.067913	11.564634	11.531987	11.639628	11.342640	11.140636	11.729303	11.946674	11.821698	11.206137	10.999386	10.853180	11.074564	11.069445	10.617597	10.727474	11.662821	10.414333	11.202548	10.559481	10.822512	10.839735	10.505113	10.857034	10.939616	10.643569	11.620681	11.126061	11.162013	10.684060	10.951782	11.232126	11.560987	11.543787	11.292037	11.143538	11.277024	10.757526	10.771504	11.239797	10.598624	11.761931	11.097765	11.938203	11.357390	10.652361	11.304260	11.843106	11.669383	11.682616	11.813591	11.193097	11.038010	11.843253	10.567246	12.218928	12.000568	11.323915	11.318368	11.901608	12.547774	11.305148	11.990637	12.841413	11.158191	10.954367	12.290765	11.620399	10.697641	13.128798	10.798200	12.796263	12.456759	12.505580	12.751886	12.737468	12.559887	12.546379	12.599949	11.922622	12.905187	13.802855	12.038357	10.947159	12.118005	11.111876	12.797496	12.702190	13.108505	11.726597	11.925351	12.020495	11.474930	11.492172	11.357672	11.972983	11.405878	11.357821	11.847451	10.410577	11.624303	12.658790	11.275035	13.082545	11.748029	14.460448	10.889381	11.721084	11.761011	12.575902	11.567272	11.962037	12.193677	11.712895	11.784487	12.124203	13.602191	11.352601	11.135665	10.395665	12.651235	12.568065	11.346391	12.606564	11.570177	11.684959	12.048425	12.648482	12.485843	12.064261	11.129584	11.830322	12.969891	11.611243	10.839289	12.307454	12.240995	11.277927	11.159639	11.327497	11.893668	11.238920	11.152465	11.627636	12.006698	10.954078	11.307971	11.567694	13.101611	11.655296	12.242693	13.374445	11.731098	11.070628	12.380187	12.575393	12.002950	10.752793	11.629083	11.939987	12.434039	11.193873	12.125096	11.624862	11.272950	12.918224	11.466898	12.136795	11.869342	12.302809	11.616692	11.784487	12.379891	11.770058	12.421202	10.938913	11.525035	10.869744	10.717011	11.978674	12.440302	12.098604

MIR3681HG	3.560522	4.484911	3.872862	4.499207	4.357398	4.590982	5.128988	4.579696	3.366512	7.002587	3.997882	5.504495	5.454582	4.962329	4.375003	5.522145	5.081029	4.018482	6.748935	5.100098	4.385646	4.823740	2.901641	4.347401	4.415978	4.929751	4.840449	5.671803	5.490755	4.072762	3.913564	3.779151	5.031133	4.630905	5.251581	4.365248	4.526954	4.397856	5.045149	4.468617	4.853765	3.002449	5.366204	5.528583	5.414157	5.216114	4.850771	4.390400	4.100634	6.443880	3.557169	4.873439	3.757728	5.281372	4.633927	5.153573	4.210992	3.663659	4.721287	4.202709	4.580787	5.150345	3.414002	4.458021	4.376736	4.360033	4.675173	4.279071	4.331849	4.765468	4.993721	4.537365	4.897681	4.957517	4.441091	4.151259	4.505009	4.144966	4.234619	4.429329	4.000224	4.270510	4.401806	3.983713	3.325488	4.311047	3.269757	3.792048	3.751463	3.468909	4.835984	3.941921	4.736955	4.509641	4.574160	4.927218	4.189963	4.324430	4.516358	3.795711	3.774707	4.885618	4.565553	4.679862	4.863103	4.490665	4.009546	4.375887	3.952263	5.583220	3.690955	4.369566	3.646429	4.012032	4.658804	4.183837	3.520441	5.092048	4.478041	3.986951	5.679946	4.384043	4.280614	4.668831	4.535677	4.688032	4.506555	4.055620	6.466524	4.998969	4.831039	4.818987	4.655316	4.395431	4.742727	5.912042	4.388263	4.225572	8.458269	4.443268	3.859999	2.885728	4.392767	3.989853	5.143789	4.513567	4.780324	3.862994	4.199882	3.525332	3.904846	3.277138	5.055546	4.670157	4.319940	4.716448	3.709665	5.633010	4.585625	4.852018	2.902856	4.266974	4.435637	4.937479	4.436390	5.053865	2.860463	3.963800	6.332318	4.724734	4.281894	3.783976	6.551642	3.905507	4.956023	4.212746	3.546976	4.233080	4.538874	4.276183	5.684541	3.423141	3.799869	4.427225	4.882226	5.025654	4.849095	4.358088	4.666471	4.042312	4.209917	5.455127	4.686349	3.679614	4.004778	4.514214	4.706614	4.998160	4.564688	4.641133	4.253009	5.157073	3.523465	4.184692	3.344470	4.280176	3.887630	3.511083	3.864506	3.994857	3.955912	4.330333	5.208841	5.069592	5.242255	3.595367	3.557169	4.737391	4.682027	4.342419	4.658804	4.307910	3.770137	4.638413	4.855023	3.043601	3.671754	3.780400	5.344963	3.067172	4.151892	3.752330	4.917062	5.089598	4.318222	3.732755	4.856480	5.171907

AC005871.2	6.187919	7.699912	8.735793	7.372217	6.666875	5.189553	8.432259	4.055179	3.432203	6.976239	5.501796	5.800031	8.009628	4.366579	8.091578	6.444714	5.726824	6.562022	8.045127	6.495744	4.835798	6.379958	8.284855	5.874405	6.988248	6.475242	4.922562	4.900277	6.888075	7.344583	7.824779	8.791046	8.812483	7.708085	8.442390	8.657895	8.490141	7.863395	8.597472	8.305140	6.078651	8.942934	8.519009	8.162811	9.074507	7.938222	4.353117	8.586817	7.243086	8.590063	7.647273	8.609545	9.193324	9.140257	7.430932	5.439305	9.378682	9.420843	8.050181	8.962835	8.709005	7.698520	10.693316	7.934176	9.443123	8.323832	8.481554	8.212064	8.436316	8.118091	8.994416	7.965409	7.013469	8.413235	8.686570	8.036665	7.959604	8.612810	7.972915	8.687165	7.867574	8.781813	8.506430	8.833772	8.891903	8.608181	9.102118	9.076729	9.615752	8.235265	8.459259	8.966496	9.136846	7.843425	6.899330	7.729031	8.669426	7.661829	6.951549	7.518967	6.757978	7.635404	7.557531	8.560387	6.889496	6.092703	3.282404	6.002186	7.348188	5.630077	8.494468	5.789735	4.069243	3.726139	5.051393	5.677521	4.026235	6.361860	6.093378	5.150162	7.102823	6.724812	5.342348	4.861638	3.811402	3.930454	8.035707	3.396455	3.617653	3.368911	4.323808	5.779106	6.247611	3.784800	4.346971	3.305865	5.943035	3.102314	3.562491	4.169428	4.642979	3.196384	2.890356	2.948079	6.564337	4.197752	3.176261	3.277712	3.964450	4.711587	6.654673	4.891068	5.589862	6.596780	6.405052	7.739929	4.907319	6.838347	9.984109	3.218685	5.951984	4.885388	3.608025	6.555824	4.882020	3.264112	10.188009	5.296034	5.812591	6.389356	7.510294	5.604354	7.461663	7.562721	5.170694	8.124176	6.781998	4.434129	7.693362	6.116663	4.877599	5.368387	4.068163	6.043307	6.309772	6.673327	6.196567	4.638630	2.881501	6.763125	5.627625	6.718122	5.885495	7.050375	6.920335	4.293146	4.583727	6.212338	9.636701	6.433951	6.203297	8.495049	6.489335	6.241930	4.537597	7.197753	4.868967	7.960849	7.798822	6.785321	6.416667	6.657265	6.119227	8.557469	7.157464	4.091630	4.936617	6.944879	4.018242	7.617692	4.835984	6.979258	5.977257	9.229123	3.225437	3.106321	4.497929	6.862173	3.242519	7.788679	6.697122	4.197310	4.525882	3.618011	4.899001	4.639063	3.428492	3.462669

EMX2OS	6.187919	7.699912	8.735793	7.372217	6.666875	5.189553	8.432259	4.055179	3.432203	6.976239	5.501796	5.800031	8.009628	4.366579	8.091578	6.444714	5.726824	6.562022	8.045127	6.495744	4.835798	6.379958	8.284855	5.874405	6.988248	6.475242	4.922562	4.900277	6.888075	7.344583	7.824779	8.791046	8.812483	7.708085	8.442390	8.657895	8.490141	7.863395	8.597472	8.305140	6.078651	8.942934	8.519009	8.162811	9.074507	7.938222	4.353117	8.586817	7.243086	8.590063	7.647273	8.609545	9.193324	9.140257	7.430932	5.439305	9.378682	9.420843	8.050181	8.962835	8.709005	7.698520	10.693316	7.934176	9.443123	8.323832	8.481554	8.212064	8.436316	8.118091	8.994416	7.965409	7.013469	8.413235	8.686570	8.036665	7.959604	8.612810	7.972915	8.687165	7.867574	8.781813	8.506430	8.833772	8.891903	8.608181	9.102118	9.076729	9.615752	8.235265	8.459259	8.966496	9.136846	7.843425	6.899330	7.729031	8.669426	7.661829	6.951549	7.518967	6.757978	7.635404	7.557531	8.560387	6.889496	6.092703	3.282404	6.002186	7.348188	5.630077	8.494468	5.789735	4.069243	3.726139	5.051393	5.677521	4.026235	6.361860	6.093378	5.150162	7.102823	6.724812	5.342348	4.861638	3.811402	3.930454	8.035707	3.396455	3.617653	3.368911	4.323808	5.779106	6.247611	3.784800	4.346971	3.305865	5.943035	3.102314	3.562491	4.169428	4.642979	3.196384	2.890356	2.948079	6.564337	4.197752	3.176261	3.277712	3.964450	4.711587	6.654673	4.891068	5.589862	6.596780	6.405052	7.739929	4.907319	6.838347	9.984109	3.218685	5.951984	4.885388	3.608025	6.555824	4.882020	3.264112	10.188009	5.296034	5.812591	6.389356	7.510294	5.604354	7.461663	7.562721	5.170694	8.124176	6.781998	4.434129	7.693362	6.116663	4.877599	5.368387	4.068163	6.043307	6.309772	6.673327	6.196567	4.638630	2.881501	6.763125	5.627625	6.718122	5.885495	7.050375	6.920335	4.293146	4.583727	6.212338	9.636701	6.433951	6.203297	8.495049	6.489335	6.241930	4.537597	7.197753	4.868967	7.960849	7.798822	6.785321	6.416667	6.657265	6.119227	8.557469	7.157464	4.091630	4.936617	6.944879	4.018242	7.617692	4.835984	6.979258	5.977257	9.229123	3.225437	3.106321	4.497929	6.862173	3.242519	7.788679	6.697122	4.197310	4.525882	3.618011	4.899001	4.639063	3.428492	3.462669

AC021078.1	12.106044	12.460764	12.952737	12.672419	12.863485	12.794859	12.540801	13.571924	12.981325	12.650709	12.916924	12.691189	13.187814	13.135842	13.024751	12.472480	13.087953	13.202774	12.774646	12.991828	12.289963	12.790316	11.411231	12.784870	12.561046	12.730828	11.150531	12.500257	11.669233	11.833001	13.269649	13.093127	13.410097	12.498635	13.265809	13.487032	13.456140	13.464002	13.768298	13.644498	12.252621	13.217568	13.397792	13.213993	13.256614	13.208899	11.981815	13.183086	13.091080	13.547331	13.146323	13.540597	13.583453	13.660789	13.607340	12.917665	13.828188	13.755369	14.243065	13.949083	13.460282	12.103128	12.444832	13.376076	13.126712	13.749340	12.987965	13.171526	13.111982	12.773272	12.553785	12.623163	12.185427	12.722115	12.574034	12.594761	12.695415	13.332882	12.725496	13.271410	12.947003	13.163201	13.160536	13.268822	13.182875	13.016071	13.443521	13.526138	12.606059	13.391817	13.291349	12.800391	13.029529	12.885092	12.232669	12.858824	13.416505	13.379894	13.208899	12.912866	13.365165	12.888269	12.915514	13.236251	13.229238	12.855334	12.586624	13.622440	12.374518	12.532804	12.248989	13.667672	13.686208	12.270578	13.208899	12.218494	12.954985	13.203986	12.564626	12.129637	13.144182	11.742737	11.688852	11.491752	11.697417	11.953478	11.099868	11.964932	11.360018	11.822613	11.615675	11.291145	11.875371	11.356381	11.815555	12.691705	11.926923	10.433811	12.430780	12.086504	11.825339	10.979225	12.386450	11.506274	11.628369	12.259577	11.435100	10.829442	11.418269	12.043340	12.473611	12.079262	12.927010	13.102201	12.865672	12.759247	12.671728	13.424902	12.700204	11.786268	12.205573	13.256221	13.248737	13.350971	13.057096	12.262009	12.351495	12.677828	13.499122	13.022526	12.927769	13.175184	13.379067	12.925613	12.193531	12.986841	13.982948	12.887748	13.403139	13.119075	10.823092	12.648482	12.894372	13.003777	12.852645	12.962912	13.223234	12.539475	11.891730	12.131138	12.019903	11.951566	12.550714	11.552276	12.654127	12.284891	12.497831	12.816305	12.733879	12.784870	12.251358	12.947549	12.436907	12.685053	12.081830	13.712440	13.021956	11.937627	12.841587	12.097725	12.707907	12.207137	12.827872	12.917111	11.903881	12.375286	11.267354	12.243471	12.256640	12.437588	12.083542	11.546206	12.235907	11.775503	11.289463	12.423579	12.863311	12.913978	12.253893	11.580132	13.282824	12.162952	11.950952	11.493752	12.562209	13.095579	12.016883	11.575387

AC068631.1	13.736123	14.050321	14.535541	14.065689	14.321619	14.069209	13.874185	14.015578	14.079189	14.205304	14.258285	13.929475	14.160839	14.244331	14.289302	14.047634	14.068933	13.961354	15.091311	13.857001	14.842680	14.620267	14.427017	14.172267	13.846674	14.116481	13.548009	14.249198	13.802610	13.532379	14.439328	14.118926	14.027079	13.282220	13.575781	13.583239	13.783082	14.113158	14.464777	14.197180	13.919105	14.314424	13.772955	13.895889	13.744863	13.980804	14.151049	13.823177	13.716537	13.761409	13.859406	13.782074	14.073025	13.956868	13.930296	13.717490	14.379181	14.401146	14.525897	14.039659	14.119258	13.585542	14.094001	15.001900	14.265614	14.375159	14.050321	14.441978	14.251440	14.015006	13.595842	13.981594	14.012079	13.959948	13.985840	13.657694	14.380267	13.746068	13.522531	14.101917	14.147348	14.158320	14.024155	14.012079	13.986378	13.920769	14.073307	14.155124	14.809575	14.872324	13.802610	14.553963	14.273182	13.918056	13.981075	14.242079	14.179251	14.230892	14.107553	13.963822	14.080659	13.790935	13.975250	14.201495	14.680965	14.023284	14.392613	15.068646	14.418363	14.204014	14.797410	14.709387	14.424221	14.060320	14.194332	13.561973	14.378131	14.039346	14.003541	13.965466	14.169985	13.697560	14.630361	14.208161	14.121867	13.817064	13.578055	13.980251	14.121322	13.942362	14.430826	13.884089	14.293761	13.768298	13.956042	13.973576	14.496348	14.847322	13.907695	14.872324	15.206355	15.305145	14.308984	14.965129	13.879304	14.037255	14.406946	13.883273	13.720180	13.491499	13.848547	13.879568	13.939054	13.879568	14.002993	13.517315	13.920769	14.304308	14.324726	14.277581	14.260288	13.891028	13.610599	13.890768	13.727608	14.038510	13.942960	13.895620	13.327590	13.048778	13.822921	13.578538	14.043788	13.916329	14.104388	13.346348	14.297708	13.659596	14.007021	14.035241	14.508368	14.838593	14.488728	14.470097	14.087732	14.402538	13.552782	13.665978	14.409900	13.972313	13.702414	13.630442	13.383249	13.747708	13.784808	13.308155	13.686919	13.725868	13.659861	13.984082	13.841137	14.128868	14.287592	14.022116	13.866239	14.002723	14.204626	14.880655	14.360038	14.987284	14.838593	13.912401	13.930021	13.673865	13.651246	13.432729	13.939615	13.741329	13.732138	13.959663	13.445237	14.336805	13.550946	15.054906	14.319890	14.527106	14.453914	14.935700	13.873120	14.958920	14.359066	14.174222	14.031471	13.988071	13.794332	14.466587	13.749826	14.285533

AC129807.1	3.136503	3.172928	2.994658	3.212697	2.885728	3.021451	3.061915	3.174332	3.192163	3.045058	4.414432	2.998262	3.187142	3.478013	3.026034	2.985413	3.504165	2.866792	3.850149	3.081335	3.660010	3.047315	3.350741	2.928074	3.024851	2.875475	2.978433	2.913842	3.070143	2.998161	3.535907	3.619143	3.480575	2.987779	3.202948	3.841327	3.845207	3.005456	3.280419	3.238168	3.362151	3.411831	3.390862	3.138946	3.535907	6.113851	3.201021	3.074008	6.432425	3.351071	3.250126	3.254178	3.607261	3.941720	3.555846	3.060771	4.192784	3.188054	3.062769	3.595367	3.126294	3.063972	3.579359	3.167932	3.330807	3.358308	3.155201	3.125771	3.131700	2.989717	2.979742	2.912944	3.125395	3.342139	2.956087	3.256478	3.339291	2.880634	3.351386	3.467841	3.176379	3.611973	3.335812	3.640119	3.175414	4.230480	4.486790	3.916834	3.674948	3.359844	3.138106	3.032564	3.562491	3.117732	3.448800	3.130616	3.484623	3.055089	3.088896	3.287257	3.603220	3.098204	2.979637	2.769141	4.390632	3.472743	2.957599	3.181767	4.516358	3.074125	4.545552	3.270368	4.462017	3.368431	3.219233	4.008008	3.299620	3.058412	3.130253	3.038227	3.242655	2.954506	3.018459	3.100328	3.346564	2.873577	3.076552	3.141816	2.929503	3.039322	2.904234	2.963416	3.079083	2.942527	3.679225	3.174065	2.827017	3.175876	3.079971	3.302221	3.316489	3.100427	2.888372	2.930955	4.450279	2.937314	2.872066	2.997133	3.240032	3.353521	3.328676	3.459278	2.941371	3.072887	3.581299	3.339114	3.006446	3.270657	3.115819	3.370996	3.358308	3.462669	3.184490	3.853131	6.169121	3.136503	2.952123	6.529154	3.127678	3.116275	3.085166	4.259584	4.058871	3.326783	2.990915	3.683253	5.320335	2.963760	3.463557	2.978793	3.176379	4.426556	3.524981	3.538047	3.534455	3.161949	3.330169	3.204137	2.974245	2.944534	2.954328	3.101045	3.169028	3.215682	3.220285	3.134377	3.187406	3.029761	3.452261	3.413277	3.796319	3.339114	3.407115	3.353204	3.372595	2.964816	3.347910	3.789934	3.291272	3.710057	3.289211	3.256343	3.168920	3.106895	3.209927	3.042065	3.655820	3.560111	3.126752	3.044311	2.969646	2.881882	3.722894	4.747288	3.068806	2.946126	3.131125	3.267949	3.002449	5.010145	5.114185	5.357589	3.096273	3.013675	2.955347	2.912748	3.078861	3.159437

AC009264.1	6.492653	8.495213	9.540496	8.672619	8.946763	8.125256	6.884396	9.279051	9.904261	7.139220	8.290722	7.591953	7.579796	7.797700	6.250316	8.129817	9.989046	7.058536	8.132684	8.152526	8.027961	9.974195	8.938979	6.792669	6.659189	7.576179	7.684131	7.925680	8.026826	7.508788	8.380503	8.046880	8.521866	7.121630	8.309760	8.066676	7.546796	8.179171	8.442840	8.866575	7.555345	8.075929	8.582555	8.575025	8.866575	8.918759	8.510113	8.921804	8.818068	9.714385	8.736112	9.481353	8.140306	9.328621	8.183260	8.697583	8.564961	8.203908	9.226696	8.716277	8.676199	7.624268	8.425591	9.122460	8.709164	9.698632	8.947836	8.685838	8.892214	10.427634	8.623429	8.742859	7.591653	8.683845	8.410456	8.060335	9.518998	8.617997	8.657423	8.178250	7.389333	7.439469	7.249623	7.727960	8.911573	8.909943	8.218403	7.780597	8.655375	8.529384	8.435252	9.228821	8.262784	8.389600	6.930178	7.829763	8.438425	7.914298	8.458444	8.328445	8.787190	8.466728	8.308845	7.745467	9.664930	7.161420	9.454926	8.721472	9.552530	7.472343	10.341269	9.073136	9.777366	7.380528	9.148812	11.188596	7.440807	8.711913	7.215778	7.057769	7.999482	7.478448	7.396775	8.853796	8.262784	8.007915	7.605306	8.124953	8.217593	9.781775	8.391312	8.779775	7.891690	8.701221	7.177144	6.842576	8.017982	7.200694	6.719133	7.115823	8.415017	7.197133	7.511861	7.468757	6.139619	8.481265	7.496300	9.385126	6.912288	7.261516	7.507090	7.139390	6.807413	6.512094	6.921278	7.189399	7.547904	7.462608	7.315429	8.170959	6.743511	6.263683	6.908762	6.905252	8.164264	8.522159	6.498576	7.474444	7.169340	7.050231	7.093370	6.220089	7.167473	6.681577	7.843927	6.851553	6.160151	6.559899	6.162362	6.851706	7.574330	6.897684	6.964752	6.409686	6.528837	6.819891	6.761061	7.918277	7.696379	6.718452	7.130837	10.909711	6.904304	6.621633	6.851096	7.680418	6.806000	8.718897	6.286020	6.484071	6.371084	7.887040	6.430211	6.202971	7.839529	5.665927	6.162545	6.138928	6.970435	5.914751	6.930985	6.902396	6.724009	6.703085	6.381466	7.778213	7.932184	8.292608	7.367980	7.785913	7.838941	8.253119	7.136551	7.673686	7.422439	7.466651	7.425554	8.344141	9.176047	7.928181	7.826953	9.977682	8.180988	8.121626	8.713286	7.734091	7.320886	8.197638

LINC01785	8.162515	8.936547	8.660394	8.506430	8.858351	9.200620	8.894256	9.899599	9.430938	9.078406	8.726175	8.379228	9.149696	9.002124	8.814706	9.314604	8.777234	8.766693	9.739498	9.355284	9.099347	9.403225	8.963428	8.881137	8.832900	8.340857	8.725420	8.639584	9.442051	9.020946	8.939127	8.820386	8.772771	8.124336	8.860765	8.773990	8.646337	9.273865	9.463027	8.884508	9.099653	8.812633	8.856682	8.817585	8.785471	8.806276	8.947364	9.486434	8.991352	9.172960	8.518570	8.888374	8.911270	8.992256	8.657283	9.680619	8.535315	8.947993	8.948426	8.461720	8.713717	9.092540	9.250874	9.125034	8.874814	8.689141	9.140691	9.083352	8.889309	8.824932	8.818569	8.401134	9.154648	8.295296	8.338861	8.836925	8.580126	8.808590	8.239020	8.987939	8.594088	8.680202	8.564796	8.557608	8.744205	8.312674	8.395299	8.635277	8.702764	8.685097	8.201668	8.612161	8.773068	8.609545	7.810799	7.986868	8.437983	8.110601	8.445618	8.604836	8.557469	8.795349	8.358156	8.430558	9.388406	9.308341	9.500267	9.421747	8.853327	9.776589	8.367279	9.106435	9.046988	9.656270	9.440805	9.525550	8.071381	8.790410	8.960223	8.752281	10.882507	9.302345	9.135166	9.365510	9.752834	9.355444	7.965252	9.688849	9.262432	9.365664	7.846711	9.686981	9.513820	9.578695	9.086559	10.087619	8.539984	9.785504	9.710638	9.196444	10.342962	8.501851	9.345894	10.390024	9.362145	9.756700	8.906727	9.239831	10.119137	9.559541	9.676753	9.116189	9.177093	9.676753	9.012062	9.565422	9.274098	10.906775	9.267981	9.450439	9.406773	9.046851	10.130482	9.533530	9.662962	10.339310	9.110466	8.820838	10.116982	10.200957	9.212976	9.384583	10.935634	8.836612	10.455568	9.455263	9.867109	9.343609	8.709631	9.781256	9.883787	10.012765	10.332815	9.155178	8.748492	9.764810	9.531152	9.746469	9.223579	8.916172	8.990170	9.812767	8.759006	8.561939	9.757967	9.919312	9.199695	8.749391	9.462868	9.965611	10.149811	10.002111	9.231299	9.904409	9.083201	9.533072	9.010820	9.011455	10.219536	7.859886	10.458502	8.370991	9.477368	9.902734	8.699543	8.404836	8.343367	8.690022	8.964318	10.019305	9.750518	7.692126	8.446086	9.703711	8.941854	10.069768	9.839846	9.513068	10.073841	8.729427	9.762460	9.120947	9.430651	8.994888	9.851210	8.951958	9.537522	10.143394

AC068234.2	9.877509	9.594314	9.139031	9.284604	8.388057	8.682047	9.337859	7.950786	7.938047	8.794316	9.202358	8.960071	9.055978	8.616784	8.851528	8.952716	10.096988	8.820386	10.154836	8.272248	9.675852	9.993540	12.691342	8.390510	11.533180	10.017413	11.499354	10.882649	9.598034	9.132447	9.024321	9.573538	9.123673	9.694834	9.468870	9.276833	9.369356	9.271368	10.306994	9.144893	9.127646	10.606255	9.768444	9.005828	10.459392	9.085969	10.121103	10.447340	9.941340	9.229567	9.186268	9.822999	9.660142	9.396668	9.776123	9.543795	10.284884	10.059845	9.725448	10.545824	10.145800	10.454196	12.859886	10.808891	10.175472	9.955381	9.541437	8.958268	9.150017	9.906344	8.918170	8.920132	9.152637	8.867920	8.300756	9.069592	9.135166	9.526630	8.894400	9.748470	9.517602	10.095931	9.572138	8.722821	9.387940	8.981042	9.501474	9.927841	10.872927	9.223414	9.857414	9.376678	9.426344	9.032354	8.644694	10.160131	9.897656	9.723440	9.068319	9.730932	8.970055	8.631759	9.440177	9.460288	10.802095	9.373508	9.191505	9.918702	10.274257	8.917391	9.759333	9.330151	9.194946	8.687013	9.114516	7.741944	11.171227	9.696322	9.790373	8.061229	10.211984	9.261194	9.042079	11.599182	8.929239	9.651117	9.067242	9.103956	9.073438	10.103883	10.619249	11.047654	10.860851	10.650012	10.184062	10.315777	9.176798	10.308628	8.732389	10.702889	11.823838	11.633590	9.285357	11.016907	12.844764	9.044254	9.793460	8.731515	8.786864	10.282924	9.296921	10.084677	9.044099	8.468252	8.724496	9.417638	9.191505	10.676585	10.357076	10.206147	9.809677	9.394059	9.660142	9.657640	8.830099	8.958107	10.182411	8.282714	10.167376	10.397331	9.921845	9.070803	11.476411	10.241513	9.691000	12.502721	9.969125	9.841075	9.744106	10.490710	10.887784	10.915826	10.592028	9.762628	10.111382	10.043738	9.299855	9.472171	9.698167	8.546267	9.590890	9.547475	9.315204	8.832570	9.408477	10.072356	9.479495	9.287393	10.595584	9.173279	9.408330	9.138891	9.303709	11.188016	9.212214	9.747791	9.451861	10.903646	10.878700	11.677730	10.561773	9.445145	10.320901	9.384818	9.710793	9.104746	9.365175	8.519459	9.009654	11.831658	8.777073	10.298333	9.082741	11.089903	9.231606	9.679253	9.485177	9.710793	9.508226	10.943606	10.895043	8.242770	8.920911	8.457490	8.717346	11.577194	8.149015	8.904922

AL591543.1	3.193531	3.190409	3.046804	2.947532	2.908972	3.103123	3.367397	3.222961	3.454178	3.445453	3.634068	4.991007	3.300075	4.109814	4.128508	3.161949	3.572883	3.983713	4.619283	3.185682	5.170495	3.601290	4.354817	3.592078	3.237557	2.938626	2.972247	2.950121	3.099621	3.064168	3.720576	3.506053	3.241487	3.126165	3.673696	4.111378	3.765884	3.338781	3.765302	2.998519	3.954387	3.289973	3.523658	3.352205	3.590704	4.817310	3.302221	5.159068	3.459846	4.040595	4.059534	3.859999	4.166469	4.031273	3.794881	3.060140	4.282532	3.199145	4.102375	7.518351	3.541120	3.047744	3.364283	3.032233	3.156927	3.163906	2.845345	3.304758	3.077588	2.989807	3.017496	2.777877	3.058711	2.917967	2.951204	3.277250	2.992905	2.971964	2.967113	3.723511	3.311774	3.995497	3.312266	4.160569	3.242816	4.079591	3.221664	3.192699	3.006617	3.314483	3.153417	2.978889	3.191194	3.013376	2.916669	3.120954	3.752726	3.197164	3.067732	2.960243	3.193103	3.262043	3.031650	2.979346	4.226166	3.108164	3.478950	4.803256	2.988244	3.277564	2.843630	3.842553	3.074874	3.293922	3.122398	4.108038	2.977135	3.025052	3.187273	3.087756	3.293126	2.999190	2.988422	2.893814	3.598158	2.988814	3.201427	3.467086	3.205859	3.474700	4.433285	3.070452	3.354520	3.701577	3.009660	3.538259	3.424029	3.400545	3.306834	4.458852	3.121719	4.919371	3.084037	3.031742	2.884632	3.100328	2.880815	3.100427	3.552782	3.481909	3.366686	3.267345	3.223347	3.583962	3.804926	3.815038	3.342824	3.230063	2.947986	3.464122	3.047315	3.558141	3.425082	3.950977	4.840860	3.254679	3.710484	3.707190	4.221462	4.150204	3.758992	3.848412	4.184058	3.396300	3.195295	3.843957	3.046203	3.927615	7.067452	3.195567	3.153056	3.284905	3.194590	3.250126	3.191322	2.835200	3.603041	3.177618	3.056221	3.033286	2.899378	3.080392	2.991399	2.933330	3.268982	3.003333	3.133459	3.033286	3.261885	3.638275	4.233943	3.450064	3.674632	3.466476	3.243970	3.480386	3.362333	2.933891	3.257185	3.291352	2.928074	3.133459	3.045250	3.001976	3.012774	2.931963	3.030691	3.174211	3.248272	2.946451	3.048487	2.940324	2.876544	4.579040	3.106094	3.171433	4.908200	3.333864	3.361296	3.116871	3.094426	3.320743	3.378618	2.974981	3.111657	3.070785	3.039135	3.322010

AC107881.1	5.259838	3.932381	4.426112	3.642881	5.092275	3.732036	3.720796	5.277938	4.936851	3.988180	4.309062	3.787161	3.261885	4.122526	3.997446	3.644045	4.399610	3.965731	6.126425	3.375457	5.110165	3.847741	4.907542	3.801192	3.639699	3.683640	3.459121	3.870512	3.419157	3.271463	4.713921	3.850978	4.376736	3.353367	3.663078	4.073634	4.087235	4.279490	4.758520	4.158864	3.442050	3.419157	3.430641	5.203669	4.641133	4.829761	4.329006	3.855323	3.302377	3.988826	4.043645	3.890511	4.062858	3.887630	3.660607	3.572084	3.844372	3.368911	4.351327	3.780212	4.498338	3.731709	4.535468	4.689905	4.778843	4.798980	3.894213	4.273558	3.841327	4.365030	3.642881	3.404710	3.062481	3.360651	3.964886	4.074943	4.307220	4.843175	4.408936	3.523465	3.705487	3.867507	3.212415	3.979593	4.890855	4.440882	4.034313	4.767260	4.727036	5.285608	4.983993	5.905278	2.962366	3.417042	3.232021	3.262940	3.984782	3.112633	3.402108	3.439974	3.279275	3.222099	3.009272	3.997446	5.057972	3.371537	5.738626	3.968740	4.924665	3.711546	5.399206	5.174521	5.427660	3.890511	4.870826	4.373262	5.687634	3.827843	3.475634	4.443479	4.130822	3.694253	3.574228	4.914529	3.622038	2.981735	4.507907	4.807861	3.190259	3.451547	3.216802	3.063410	3.363362	3.566681	3.127205	4.750198	3.413828	3.767567	3.640119	4.405372	4.270510	3.905294	3.201942	3.554886	3.614957	3.846532	4.081552	4.211406	3.543602	4.244727	3.358388	3.628214	3.291616	3.566891	3.771327	3.811621	3.650804	4.512034	4.346108	3.422057	3.206648	3.477093	5.952163	4.699164	4.835606	3.659231	3.953753	3.441180	4.173575	4.138401	3.783146	3.654009	3.962248	3.287111	3.342475	4.013585	3.211573	3.839648	3.609188	3.321367	3.542469	5.175767	4.625919	5.097811	4.729367	4.314992	4.163347	4.211207	4.692874	3.469063	3.198895	3.306659	3.241347	3.103870	4.086136	4.310387	4.632405	4.875692	3.207928	3.526481	4.083676	3.363192	3.604836	4.001248	4.057579	3.573251	4.892077	5.552778	4.935579	5.006417	6.175250	3.016572	3.528493	3.005091	3.193659	3.596936	3.121348	3.109333	3.219976	3.470346	3.307751	3.046612	3.580319	3.410970	3.093983	5.946541	4.635216	5.065280	3.527928	5.746099	5.008684	4.090253	4.246657	3.981754	4.191700	5.540532	3.790356	3.662244

TP53TG1	12.475688	13.254596	13.600599	13.226062	13.388411	13.374445	13.429298	13.003777	12.820920	13.298414	13.188586	13.273354	13.548671	13.620619	13.462029	12.765859	13.005956	13.175795	13.475544	13.619020	13.711410	13.123071	13.016439	13.129172	12.952737	13.225499	12.536567	12.974569	12.393126	12.808222	13.169964	13.317644	13.492817	13.764461	13.610599	13.436214	13.432615	13.357821	13.462242	13.326618	13.374839	13.045913	13.398659	13.262797	13.367661	13.535095	12.951673	13.221376	13.252042	13.357611	13.055429	13.531007	13.687645	13.663761	13.114121	13.348289	13.174367	13.868320	13.734624	13.703414	13.583239	12.810934	13.224119	13.154391	13.850447	13.595594	13.357821	13.349953	13.684527	13.426259	13.532839	13.426653	12.966913	13.282417	13.656748	13.515560	13.656241	13.261613	12.976769	13.309969	13.095970	13.145720	13.221672	13.729117	13.613516	13.157276	13.528984	13.520707	13.344260	13.545103	13.325616	13.151837	13.469230	13.337277	13.089053	13.543266	12.987020	12.413974	13.304119	13.018087	12.891207	13.284032	13.325616	13.063231	13.115442	13.152404	13.015361	13.231246	13.025290	13.003777	13.459166	13.741572	13.377131	12.747949	12.959197	12.445148	12.716779	12.857438	12.931713	13.789486	13.041374	13.114655	14.088933	13.006847	12.240559	12.856573	13.635698	13.270023	13.134452	12.638694	13.372935	13.412114	13.039136	13.255166	14.394046	13.517555	13.407769	13.718916	12.710169	13.193086	12.912180	12.758584	13.342844	12.852132	13.414635	13.109444	12.865869	12.749999	13.149739	12.973105	13.313356	13.466605	12.726021	12.859701	12.570017	13.359500	12.902705	13.479279	12.580656	12.943369	13.199300	13.025476	13.465968	13.223145	12.762299	12.925780	12.677497	13.452889	13.149535	13.348289	13.382177	12.851592	13.479279	13.643515	13.162836	12.791154	13.214995	13.420820	12.870321	13.237061	13.915790	13.028398	13.009202	13.628249	13.382399	14.121322	13.066403	12.742146	12.703137	13.345516	13.371858	12.742336	13.358268	13.531252	13.256221	13.316841	13.746814	13.654386	13.818086	13.647417	13.460034	13.474422	12.982400	12.990709	13.683790	13.019365	13.022885	12.825422	12.946838	12.638546	13.777688	13.433682	13.183285	12.884027	12.513519	13.578768	13.031762	12.725852	13.581115	13.312545	13.424487	13.127100	12.845812	12.553951	12.580172	12.253114	12.776199	13.377562	12.830477	12.546069	13.215415	12.210975	12.953485	12.836701	13.038781	13.767473	14.157670	13.003936

AL162414.1	8.049234	8.454290	8.306636	8.415017	8.382213	8.348688	7.904431	9.047293	8.957952	7.751504	7.985774	7.423685	7.387168	7.921630	7.700373	8.037881	8.632470	7.893878	8.958743	8.290887	8.789635	8.900824	10.013106	8.731061	8.591468	8.440424	11.460712	8.873841	8.454599	8.237152	8.896716	7.887351	8.541825	7.827291	8.140757	7.966655	8.062577	8.365016	8.893308	8.853185	8.801425	8.784314	9.265515	8.813954	9.009028	8.309468	9.119287	8.256188	7.900849	8.007316	8.143761	8.356138	8.339021	8.131825	8.105290	8.076799	8.560859	8.452479	8.830404	8.316530	8.712812	8.037443	9.804390	9.309714	8.719946	8.609851	8.360634	8.589300	8.147447	9.249208	8.165502	8.232693	7.967258	8.057711	8.425112	8.432259	8.328907	8.600963	8.571129	8.991632	8.633227	8.478374	7.961155	8.076365	8.283307	8.057438	8.543212	8.453383	8.766072	9.005980	8.856081	9.653064	8.184797	8.607876	8.343667	8.743748	8.410935	7.287754	7.870451	7.552537	8.064885	8.394237	7.967116	7.754859	8.761068	7.628126	8.949830	8.674520	9.117099	7.856943	9.684672	8.660855	8.602194	8.143607	8.063024	8.958268	8.655960	8.104810	8.174113	8.799766	8.513238	9.334944	8.891594	8.748492	8.182340	8.148080	8.446387	8.659760	7.674779	7.674779	7.450892	7.985621	9.120492	8.175531	7.999017	8.513548	7.571876	10.115138	9.096148	8.931939	9.537522	10.218622	8.173460	8.688353	8.452928	9.123809	8.504777	9.100248	9.130010	9.246741	9.341317	7.904130	7.584419	8.332304	7.877938	8.012155	9.057026	8.838304	9.807299	8.704893	9.052979	9.601111	9.340883	7.871513	8.305299	8.646623	8.426021	8.310673	7.918277	8.379542	8.324297	9.067083	8.009296	7.800830	8.400333	7.592717	8.233142	8.753475	7.912658	8.295296	8.077276	8.678199	9.775518	9.027677	9.365664	9.180938	8.909516	8.178108	9.262267	8.637723	8.513548	8.037595	7.972915	8.708722	8.619728	8.294728	8.501222	8.369861	8.675783	7.843425	8.277390	8.292137	9.024321	8.487552	8.635686	8.096900	8.932113	9.665313	9.803340	8.309911	9.461531	8.293064	8.749866	8.398367	9.459862	8.524770	7.925226	7.943560	7.309610	8.225089	7.730087	7.855600	9.088375	9.528476	8.216056	8.723582	8.477251	9.019712	8.312674	9.854951	8.718269	8.865514	8.589143	8.073965	7.977373	9.192889	7.778050	8.519607

LINC01520	4.209698	3.562864	3.702653	3.491016	3.330300	3.169872	3.425996	4.732080	4.187106	3.462866	3.691777	3.800090	3.512576	4.257871	4.234838	3.228776	3.677001	4.169230	5.126372	3.445804	5.600944	2.953408	3.193913	3.232021	3.345400	3.098454	2.960127	3.641891	3.227451	3.097736	4.334201	4.697660	5.160301	3.149253	3.794881	4.206826	4.300892	3.586490	3.610154	3.936663	3.533846	4.070060	3.717910	3.466886	3.808458	5.728084	3.342824	3.791429	5.756267	4.189497	4.094334	3.895246	4.585625	4.430721	4.133820	3.187406	4.288341	4.198610	4.345679	4.094749	3.402652	3.169414	4.071884	3.565890	3.882760	3.575527	3.018287	3.803284	3.283015	2.881445	3.171433	3.209109	3.567103	3.290129	2.950938	3.616322	3.700564	3.321225	3.822877	3.958573	3.503315	4.507907	3.541120	4.371963	4.161376	4.971901	3.254844	3.901467	4.128277	3.818855	4.308141	4.044345	3.978030	3.277712	3.054254	3.110054	3.924386	3.332425	3.690345	3.136856	3.133555	3.412583	3.100561	3.529405	4.855423	3.442575	3.442050	4.154991	3.238467	3.350047	3.105977	4.196022	4.238537	3.381180	3.631713	3.351900	3.177467	3.288923	3.258402	3.241074	3.743475	3.080392	3.117261	3.134643	3.805556	3.039843	3.272404	3.551024	3.222255	3.522634	3.203344	3.133459	3.437904	3.772832	2.985886	3.607467	3.186612	3.515480	3.460556	4.860231	2.868818	2.972138	3.035174	3.062674	3.024851	3.718335	2.957685	3.045780	3.611177	3.616508	3.574228	4.041871	3.311774	3.678209	3.876342	3.859401	3.819912	3.571891	3.313340	3.258402	3.980864	3.761533	3.558934	4.345246	5.341233	3.299460	3.379837	5.691561	4.411994	4.180105	3.750428	4.386748	4.609944	3.636471	3.106648	3.889854	4.016698	4.221462	4.224672	3.000143	3.309363	4.177040	4.085922	4.064154	3.832923	2.966116	3.759866	3.476529	2.852017	3.116393	3.020931	3.297290	3.297766	3.024851	3.407994	3.404378	3.186339	3.585700	3.360967	3.907900	4.695153	3.642453	3.788448	3.603661	3.762565	3.499242	3.469235	3.698983	3.685860	3.727738	3.535381	3.596131	3.420840	3.177227	3.029761	2.951849	2.953300	4.256493	3.384489	3.043798	3.282710	2.988326	2.952581	5.218154	3.241621	3.702258	3.818223	3.536283	3.310033	2.938701	3.157304	3.830958	3.271787	3.106094	3.070262	3.210306	3.246695	3.418265

AC131157.1	6.371595	6.913240	6.710407	7.210664	8.443632	8.341593	7.177313	7.853114	7.955563	7.129553	7.332899	6.926825	7.434079	6.426806	6.663632	6.500369	6.843205	6.150337	6.064688	6.459852	5.129196	6.407533	8.261839	6.599069	6.760913	7.201627	7.013318	6.770414	6.826397	7.872765	6.912288	6.414193	6.656772	7.660314	7.083939	6.999824	6.805835	6.364380	6.746880	6.321882	7.492895	6.381820	6.549177	5.953430	6.026945	6.781814	7.758541	6.860916	7.415371	6.301676	6.038961	6.688383	6.454774	6.654176	5.719393	7.728876	6.241596	6.390861	6.494605	6.010194	5.850080	7.416707	6.939673	6.235819	7.301137	6.338556	7.162049	7.112068	7.355811	6.828927	6.925494	7.136255	7.016822	7.068565	6.848381	7.154507	6.981609	7.123846	6.563515	6.615435	6.887409	6.939515	6.680307	5.799305	5.819384	6.565136	6.598774	6.430725	6.747557	6.417161	6.295260	6.598280	6.650779	7.213873	7.538769	6.945183	6.207268	7.160446	8.600481	7.363727	9.162292	6.642091	6.915752	6.669775	6.398128	7.454005	6.323541	6.378473	7.212319	7.077190	6.073290	7.987940	6.916950	7.289562	7.169797	11.405292	7.864018	6.802515	6.998925	8.332609	6.897862	8.298658	7.948163	10.489519	7.726838	9.167255	8.142400	7.722994	8.402744	8.163833	8.467193	8.303462	7.497876	8.074860	7.003068	8.445759	7.500092	7.234209	7.418369	6.446864	7.345830	7.517109	9.276953	7.509710	8.020574	8.176399	7.662956	9.038327	7.725286	7.023127	8.133618	7.838778	7.855444	8.143761	7.229974	7.231345	7.805872	7.148188	8.020288	8.573979	6.911143	8.176167	7.677630	6.797008	8.956987	8.881137	6.640971	7.188718	7.060597	7.490073	6.479620	7.064451	7.336125	8.893638	8.256628	7.059634	6.904227	8.320195	5.783971	7.267429	10.088368	6.517896	7.806972	8.161001	6.328905	8.364848	7.245475	7.243750	7.512022	7.401461	6.979414	8.587391	7.596551	8.351557	7.032657	7.582103	7.649041	7.290988	7.418516	8.232098	6.995225	7.846866	6.868741	6.279828	8.205080	7.531429	6.537617	7.325012	7.347389	7.186835	6.670564	6.549547	7.066199	7.149940	7.570962	8.522763	8.321274	7.196156	7.503840	8.335081	7.892168	6.373313	8.416105	7.581658	7.764336	7.083159	7.145206	6.991455	7.680124	7.799277	8.933148	10.398977	8.207229	9.912359	10.605340	6.701902	8.509971	7.822263

LINC00637	5.172533	5.817622	5.499283	5.522331	6.472094	6.494116	5.437457	6.453958	6.006099	5.926133	6.571007	6.238775	5.783971	4.903206	6.010016	5.975338	6.331290	5.145352	5.729904	5.621291	5.612706	6.254020	5.332847	5.543701	5.294248	5.956984	6.739567	6.125492	5.178363	5.866135	6.574986	4.394772	5.701183	5.721228	5.387990	5.726273	5.383723	5.172334	5.950728	5.203864	5.465020	5.453409	6.371084	5.560215	5.744656	5.828960	6.120635	6.194626	5.895133	4.952935	5.814929	5.305404	5.374545	5.654640	5.399411	6.052691	5.182603	4.280400	5.906683	4.659428	4.559877	5.780899	5.840428	5.655361	5.580041	5.315035	5.796753	5.494505	5.519876	5.252954	5.296440	5.133404	5.661986	5.492218	5.751568	5.367584	5.649908	5.082653	4.944128	4.431596	5.521370	5.595889	5.272910	4.397017	4.410264	5.442939	5.166080	5.030127	3.527038	4.698120	4.908825	4.780099	5.990072	5.693220	6.546804	5.993385	5.524600	5.242838	6.996945	6.185545	6.186901	5.452248	6.159451	5.628798	6.256249	5.808677	5.835193	5.946886	6.565605	5.778004	5.709005	6.219210	5.539772	6.556627	5.739533	7.258561	5.880506	5.333630	5.903831	5.396153	5.834287	5.516844	5.501195	7.536868	6.054258	6.292672	5.873510	5.824779	6.026945	6.146017	6.238264	5.788677	5.736968	6.210495	5.906683	6.877972	6.524972	5.372063	5.908672	5.462582	5.928289	6.265713	6.488062	5.591192	5.429928	6.557781	6.793969	5.644055	5.319944	7.922249	5.427843	5.977090	5.995500	5.447016	5.543701	5.290575	6.014848	6.223463	6.194626	6.025527	5.900808	5.849182	5.786653	6.336194	5.487657	6.469512	5.491117	5.316472	5.620195	5.674417	5.501596	4.644930	5.118031	5.300034	4.972948	4.286998	2.957162	5.736270	4.276183	5.130404	6.047817	5.940777	5.516844	5.012216	5.166894	5.479266	5.219764	5.237472	6.656772	5.564296	5.396922	6.335522	5.351089	5.692082	4.592701	4.949392	5.204262	4.896655	4.821688	4.829317	5.875675	5.279336	4.723874	5.965720	6.267418	4.287894	5.493183	4.599394	4.790155	5.977433	5.480063	5.721042	5.447832	5.248785	6.091996	5.547131	5.801186	6.787255	5.706450	6.176773	6.117492	6.748173	5.955394	7.304922	5.367986	6.142404	5.071425	5.386136	6.707042	5.623887	6.072576	6.638539	7.465563	7.121790	6.688383	5.595130	5.999019	6.194961

AL132712.1	5.172533	5.817622	5.499283	5.522331	6.472094	6.494116	5.437457	6.453958	6.006099	5.926133	6.571007	6.238775	5.783971	4.903206	6.010016	5.975338	6.331290	5.145352	5.729904	5.621291	5.612706	6.254020	5.332847	5.543701	5.294248	5.956984	6.739567	6.125492	5.178363	5.866135	6.574986	4.394772	5.701183	5.721228	5.387990	5.726273	5.383723	5.172334	5.950728	5.203864	5.465020	5.453409	6.371084	5.560215	5.744656	5.828960	6.120635	6.194626	5.895133	4.952935	5.814929	5.305404	5.374545	5.654640	5.399411	6.052691	5.182603	4.280400	5.906683	4.659428	4.559877	5.780899	5.840428	5.655361	5.580041	5.315035	5.796753	5.494505	5.519876	5.252954	5.296440	5.133404	5.661986	5.492218	5.751568	5.367584	5.649908	5.082653	4.944128	4.431596	5.521370	5.595889	5.272910	4.397017	4.410264	5.442939	5.166080	5.030127	3.527038	4.698120	4.908825	4.780099	5.990072	5.693220	6.546804	5.993385	5.524600	5.242838	6.996945	6.185545	6.186901	5.452248	6.159451	5.628798	6.256249	5.808677	5.835193	5.946886	6.565605	5.778004	5.709005	6.219210	5.539772	6.556627	5.739533	7.258561	5.880506	5.333630	5.903831	5.396153	5.834287	5.516844	5.501195	7.536868	6.054258	6.292672	5.873510	5.824779	6.026945	6.146017	6.238264	5.788677	5.736968	6.210495	5.906683	6.877972	6.524972	5.372063	5.908672	5.462582	5.928289	6.265713	6.488062	5.591192	5.429928	6.557781	6.793969	5.644055	5.319944	7.922249	5.427843	5.977090	5.995500	5.447016	5.543701	5.290575	6.014848	6.223463	6.194626	6.025527	5.900808	5.849182	5.786653	6.336194	5.487657	6.469512	5.491117	5.316472	5.620195	5.674417	5.501596	4.644930	5.118031	5.300034	4.972948	4.286998	2.957162	5.736270	4.276183	5.130404	6.047817	5.940777	5.516844	5.012216	5.166894	5.479266	5.219764	5.237472	6.656772	5.564296	5.396922	6.335522	5.351089	5.692082	4.592701	4.949392	5.204262	4.896655	4.821688	4.829317	5.875675	5.279336	4.723874	5.965720	6.267418	4.287894	5.493183	4.599394	4.790155	5.977433	5.480063	5.721042	5.447832	5.248785	6.091996	5.547131	5.801186	6.787255	5.706450	6.176773	6.117492	6.748173	5.955394	7.304922	5.367986	6.142404	5.071425	5.386136	6.707042	5.623887	6.072576	6.638539	7.465563	7.121790	6.688383	5.595130	5.999019	6.194961

CCDC18-AS1	11.223409	10.690696	10.680668	11.072137	10.951170	10.658003	10.376811	10.630402	11.728395	10.557368	11.059966	11.073706	11.080854	11.240485	11.185858	11.251075	11.426795	11.140774	12.326856	11.145736	11.527903	11.716588	12.958279	11.434353	11.891560	11.531086	12.806823	10.944749	11.591011	11.011088	11.629546	10.505574	11.238152	11.767815	11.014430	10.900700	10.336292	10.663292	11.177620	11.400278	10.730315	11.495500	11.653194	11.297194	11.337604	11.266314	11.305447	11.119415	10.542840	10.538517	10.776676	10.599941	10.647894	10.778128	10.605932	10.716117	10.806633	11.124739	11.190322	10.986908	11.353312	10.347353	12.147040	11.892457	11.162452	11.057484	10.727782	10.681966	10.688872	10.930388	10.746482	10.359015	10.774471	10.437038	10.677631	11.226796	10.384381	10.597404	10.832274	10.964018	11.109567	10.917928	11.109422	10.693482	10.745880	11.285033	11.046276	10.480506	11.203593	10.745421	10.988542	11.420427	11.020059	10.464600	11.397622	11.343546	10.772399	11.638582	10.984949	11.509649	11.117393	10.656678	11.100158	10.997597	11.323494	10.969544	11.358695	11.717496	12.338106	10.822371	11.644595	11.686630	11.445261	11.006688	11.081604	9.576986	12.316558	11.228207	11.588472	11.301597	10.998209	11.615675	11.682169	12.326688	10.141487	11.306499	12.451069	12.716779	11.049446	11.304710	10.341118	11.997634	11.627636	11.560080	12.450102	11.966878	13.000090	12.262315	10.997454	12.712770	11.908190	11.289319	12.981868	11.243470	12.506563	10.891326	12.297271	11.263206	11.038878	13.381550	11.826672	11.993647	11.617879	11.468075	10.928598	10.451232	10.384236	12.459940	12.005939	11.963054	10.847257	11.636639	12.161570	10.866824	10.782076	11.970814	11.555531	10.702258	11.240345	10.934589	10.746942	10.685116	11.598873	10.978333	10.127592	11.298091	12.231738	11.409053	11.241643	12.131436	11.295400	12.696084	11.477417	11.666576	11.194864	11.992298	11.141047	11.128382	11.213047	11.064957	10.869032	12.361945	11.228140	11.802954	11.498007	11.552116	11.758300	11.042851	11.626752	11.704895	11.769304	11.680678	11.296282	11.314073	11.923268	11.338924	10.885843	11.950074	11.345621	10.940980	11.786724	11.741588	10.768642	11.305742	11.352321	11.739525	12.437588	11.789556	11.158915	10.623370	12.282409	12.181119	11.537919	11.070164	11.202677	12.013715	11.111448	11.719572	12.254351	11.853688	11.325255	11.854155	11.189332	10.970588	11.115623	11.209956	10.824430	11.888248

LINC00886	12.629207	13.001399	13.280841	13.221376	13.337481	13.113355	13.048590	12.901614	13.454152	13.154939	13.356398	13.112784	13.348289	13.543266	13.572418	13.056909	13.232267	13.835083	13.855680	13.446715	13.904184	13.716086	13.229238	13.420393	13.027671	13.468361	13.352500	12.846314	12.936194	12.677174	13.929201	13.319284	13.038398	12.857967	12.437077	12.652254	12.798177	13.118268	13.321475	13.483894	13.199300	13.122511	11.963379	12.707057	12.293129	12.332876	12.846651	12.633304	13.140068	12.305259	12.633633	12.710332	12.965824	13.108876	13.147466	13.083488	13.296569	13.151060	13.261809	13.221771	13.257609	12.956427	12.807363	13.187612	13.411431	13.772186	13.003402	13.019562	12.864041	13.575112	12.454709	12.925434	13.029169	12.863135	13.043905	12.636300	12.832730	12.521236	12.719907	13.452889	13.434710	13.403996	13.333509	13.417523	13.433474	13.136703	13.193275	13.213390	13.885163	13.600149	13.413573	13.789231	13.198822	12.954237	12.380187	13.107335	13.385833	13.248301	12.964911	13.100310	13.348067	12.871579	13.384972	13.153006	14.119553	13.272778	13.453926	13.939054	14.168428	13.265367	13.420610	13.549809	13.771102	13.255365	13.311915	12.781052	12.787861	13.628713	13.291530	13.644498	13.600359	13.035974	13.333079	13.625234	13.568039	13.631382	13.077603	13.896685	14.057105	13.723478	13.852424	13.274601	13.766279	14.241084	13.336430	13.605004	14.206497	13.924569	12.704167	13.862105	13.813787	14.092524	13.706089	14.148532	13.440736	12.951859	14.162992	13.546876	13.374641	13.956288	13.745828	13.399078	13.472510	13.040804	13.256221	12.769602	13.702414	14.143610	13.599935	14.092188	13.873644	12.954420	13.115624	12.563000	13.108876	14.076027	13.181502	13.397318	12.788576	13.025290	13.157657	13.043715	13.318676	13.533746	13.087563	12.761354	13.640816	13.739368	13.501531	13.852424	13.261991	13.779882	14.223479	13.955201	13.391817	13.835083	13.543266	13.554388	13.315805	13.205915	12.869060	13.862356	12.813692	12.783624	12.906615	13.014108	13.426460	12.883285	13.204762	13.500882	13.372935	13.155744	13.699452	13.667422	13.419947	13.660103	13.875509	13.778222	13.764707	13.966334	14.256694	13.606633	13.570987	13.464641	13.447352	13.211293	13.650308	13.819912	12.683833	13.835875	13.135275	12.739166	12.805972	13.947085	13.866741	14.696136	14.240420	14.147348	13.833834	14.045422	13.745098	13.794078	13.424295	13.642784	13.673412	13.430372	13.258409	13.731668

UMODL1-AS1	4.450279	4.657931	4.542193	5.097170	5.022964	5.886377	5.267688	4.696606	4.522785	4.816890	5.029939	5.749370	4.926605	5.049157	5.380017	5.331647	3.725577	4.935777	4.673167	5.027874	4.994155	4.636718	4.260424	5.015854	4.654410	4.637598	4.723654	5.233026	5.168072	5.139865	4.123822	5.491509	5.186403	4.203381	4.016060	5.462933	4.165107	4.293146	4.970105	4.356767	4.729746	4.940338	4.949181	6.160498	4.416874	4.867443	4.862901	4.797882	3.963586	3.432871	4.535021	5.227060	4.474088	4.470784	4.156309	5.242646	4.037774	2.959563	5.393558	3.297290	4.502867	4.922988	5.993385	4.958542	5.251581	4.746858	4.078461	5.470823	5.253548	4.531195	5.667791	4.920418	4.960407	5.011405	4.769713	4.712842	4.500729	4.905496	4.895587	4.270937	3.285471	4.645156	3.007755	4.327452	4.559877	6.180816	3.319425	4.159074	3.876779	4.956452	4.703861	5.074842	4.538874	5.024657	3.992680	4.679009	4.397017	5.259448	6.331636	4.477618	5.093749	4.344134	5.301629	5.446009	4.413089	5.292378	5.404608	5.672771	5.542037	5.418355	5.639162	5.255554	4.648966	5.532739	4.662468	6.471216	5.416020	4.613377	6.043120	4.766370	5.290178	5.330272	4.842115	4.812075	6.372955	5.758717	4.781987	5.419147	5.576665	5.715116	5.537015	4.937082	6.705732	9.246132	5.518786	4.169428	4.886038	5.636952	5.446840	5.772748	4.090696	5.239632	6.154472	5.800619	6.032910	5.110165	6.162716	4.587555	5.953089	5.294827	5.501596	5.831582	5.774012	5.395571	5.906325	5.381342	5.416410	6.512439	5.559465	6.584714	4.990394	5.019719	6.683902	5.216974	4.548399	6.361699	3.397284	5.887073	5.702980	4.718328	5.792216	5.664974	5.104607	4.865788	6.232252	5.239063	5.962534	5.139246	4.402233	6.220905	5.503158	7.057617	6.304265	5.344963	4.855423	5.974118	5.762324	5.669438	5.837134	5.813892	5.789578	5.909182	5.480621	4.671443	5.374929	5.149703	4.837031	4.845952	4.550664	5.268480	4.400062	5.283182	4.485731	5.289592	6.372955	4.473629	4.815587	5.004156	4.631763	5.037529	5.393779	6.068929	6.449178	5.749744	5.600779	5.521947	5.136441	6.420256	5.963587	5.965720	5.547864	4.850577	4.526322	6.920499	5.019323	5.753035	6.034380	6.338721	6.351948	5.716401	5.120496	5.018110	6.275884	5.073437	5.553982	4.770142	4.716660	5.665927

LINC01506	7.547120	7.042589	7.694438	5.783971	5.506401	5.835193	5.839699	5.873510	5.828417	5.421462	5.159865	4.790369	5.020732	5.133823	5.802411	6.016557	5.101364	6.297720	7.432187	5.781818	6.254554	7.451363	7.410476	5.734630	6.472402	5.264677	6.785812	6.505645	5.763035	7.511538	5.756839	7.632094	8.511528	6.114580	8.564352	6.749103	6.714591	7.159318	6.871784	6.958469	6.111319	7.705019	6.325254	5.515655	6.807255	7.726063	6.405559	6.843859	8.324142	7.385917	7.736225	7.937295	8.246792	7.206767	8.211273	5.660140	8.797014	8.366367	6.549177	6.804922	7.687491	6.455107	10.322742	6.473593	8.452168	8.023141	7.441605	7.109503	8.203475	7.180484	6.306280	6.418492	5.632657	7.203798	6.310264	8.553625	7.168420	7.970688	7.510785	8.867772	7.343668	7.925226	7.440987	7.240071	7.744703	6.320511	7.765467	7.666536	8.421205	8.367126	7.094085	9.570876	8.161158	8.993639	7.633475	7.982383	7.714780	6.585732	5.177162	5.300034	5.962707	5.476169	5.903452	5.743377	5.881754	5.581896	5.620378	6.191665	6.760447	6.250803	7.486025	5.766090	5.816548	5.801382	5.183414	6.681424	7.426476	5.299282	6.289603	5.519352	7.159016	7.180642	6.517061	5.772933	5.598387	5.128371	4.933461	5.484613	4.876299	5.602452	5.308286	4.954364	6.881192	6.294903	5.863467	5.989000	5.443899	6.396299	5.918696	6.567744	6.801251	6.939673	5.446840	5.413792	6.798482	6.721610	5.947758	5.470077	7.312129	6.353937	8.103097	8.462927	6.425612	7.467564	5.383539	7.520666	7.320728	6.992762	8.182665	6.766184	8.851230	6.259986	6.723381	8.320030	7.449679	6.280919	7.417717	6.472574	8.375113	9.141010	8.233424	8.498831	8.196850	9.866830	5.523672	6.485786	8.668500	7.735034	8.393039	9.552979	6.635321	9.469018	7.284881	8.214240	9.018966	7.507849	9.554646	9.385285	9.904987	6.740044	7.110154	6.433281	7.314004	6.910016	9.478136	7.316378	8.228550	7.594133	8.092448	7.204235	7.797527	8.850610	9.119435	8.078697	5.734276	8.435395	7.652974	8.347074	9.774329	7.874736	8.771577	7.769577	8.565560	8.233737	9.441911	5.646519	5.432219	5.373200	5.477861	7.076359	5.368189	5.799127	8.110900	7.307573	6.104268	5.530479	6.366062	6.125147	6.097354	7.856208	6.784675	5.789735	6.094274	5.179561	5.489572	8.249148	5.103393	6.073804

TMEM252-DT	7.547120	7.042589	7.694438	5.783971	5.506401	5.835193	5.839699	5.873510	5.828417	5.421462	5.159865	4.790369	5.020732	5.133823	5.802411	6.016557	5.101364	6.297720	7.432187	5.781818	6.254554	7.451363	7.410476	5.734630	6.472402	5.264677	6.785812	6.505645	5.763035	7.511538	5.756839	7.632094	8.511528	6.114580	8.564352	6.749103	6.714591	7.159318	6.871784	6.958469	6.111319	7.705019	6.325254	5.515655	6.807255	7.726063	6.405559	6.843859	8.324142	7.385917	7.736225	7.937295	8.246792	7.206767	8.211273	5.660140	8.797014	8.366367	6.549177	6.804922	7.687491	6.455107	10.322742	6.473593	8.452168	8.023141	7.441605	7.109503	8.203475	7.180484	6.306280	6.418492	5.632657	7.203798	6.310264	8.553625	7.168420	7.970688	7.510785	8.867772	7.343668	7.925226	7.440987	7.240071	7.744703	6.320511	7.765467	7.666536	8.421205	8.367126	7.094085	9.570876	8.161158	8.993639	7.633475	7.982383	7.714780	6.585732	5.177162	5.300034	5.962707	5.476169	5.903452	5.743377	5.881754	5.581896	5.620378	6.191665	6.760447	6.250803	7.486025	5.766090	5.816548	5.801382	5.183414	6.681424	7.426476	5.299282	6.289603	5.519352	7.159016	7.180642	6.517061	5.772933	5.598387	5.128371	4.933461	5.484613	4.876299	5.602452	5.308286	4.954364	6.881192	6.294903	5.863467	5.989000	5.443899	6.396299	5.918696	6.567744	6.801251	6.939673	5.446840	5.413792	6.798482	6.721610	5.947758	5.470077	7.312129	6.353937	8.103097	8.462927	6.425612	7.467564	5.383539	7.520666	7.320728	6.992762	8.182665	6.766184	8.851230	6.259986	6.723381	8.320030	7.449679	6.280919	7.417717	6.472574	8.375113	9.141010	8.233424	8.498831	8.196850	9.866830	5.523672	6.485786	8.668500	7.735034	8.393039	9.552979	6.635321	9.469018	7.284881	8.214240	9.018966	7.507849	9.554646	9.385285	9.904987	6.740044	7.110154	6.433281	7.314004	6.910016	9.478136	7.316378	8.228550	7.594133	8.092448	7.204235	7.797527	8.850610	9.119435	8.078697	5.734276	8.435395	7.652974	8.347074	9.774329	7.874736	8.771577	7.769577	8.565560	8.233737	9.441911	5.646519	5.432219	5.373200	5.477861	7.076359	5.368189	5.799127	8.110900	7.307573	6.104268	5.530479	6.366062	6.125147	6.097354	7.856208	6.784675	5.789735	6.094274	5.179561	5.489572	8.249148	5.103393	6.073804

LINC02326	5.242646	3.642272	3.172928	3.166213	2.953929	3.229611	3.396455	3.505862	4.131450	3.470874	3.637869	3.710670	3.304924	3.709060	3.348689	3.158914	3.743889	4.361722	4.804112	3.216108	4.560331	3.819912	3.033286	2.848964	3.074874	3.028960	3.177091	3.030691	3.134280	3.247981	3.617257	3.973745	4.670370	3.132978	3.998940	4.352893	4.036066	3.328072	3.070362	2.897158	3.309058	2.981364	3.450425	3.288352	3.543244	5.275754	3.175549	3.090417	3.619725	5.952712	4.032121	4.679671	4.182548	4.114828	3.809905	3.046304	3.835027	4.152302	3.736175	3.534620	2.957599	3.390353	3.322172	3.428492	3.556033	3.809533	3.026761	3.141935	3.484451	2.861014	3.090114	3.596131	3.072674	3.345748	2.998889	3.309218	3.532733	3.893761	3.475442	3.638065	3.803284	3.999384	3.389637	4.106065	4.086354	4.796034	4.304427	4.285445	3.717910	4.290904	4.373025	4.548399	3.094079	3.187925	2.954774	3.125011	4.732302	3.456247	3.225561	3.045361	4.152749	3.238755	2.970513	3.012591	4.222090	3.075635	3.449885	3.614384	3.417042	3.184228	4.115070	4.631116	3.846309	3.236383	3.283480	4.422659	3.311459	3.022360	2.917967	4.633277	3.362333	3.087756	3.051323	2.940324	4.077825	3.630871	3.579359	6.638032	3.220421	3.499964	3.200773	3.027977	3.160939	3.115327	3.533492	3.686638	3.437732	3.375633	3.349197	3.855934	3.000046	2.981538	2.822427	2.899544	2.965763	4.270707	3.035687	2.976312	3.463379	3.468909	3.499062	3.089121	3.210865	3.481536	3.987976	3.743241	3.418972	3.120348	2.883880	3.101167	2.869606	3.508342	3.283480	3.903127	4.495760	3.121463	3.064517	3.855094	6.247611	4.100847	3.953974	3.953974	4.179876	3.402108	3.014660	3.516786	4.012263	3.334678	3.269757	2.890616	3.323817	3.285471	3.632310	3.623258	3.375064	2.977243	3.252098	3.116871	2.844207	3.179000	3.495604	3.048487	3.902945	4.784935	3.278544	4.197114	3.658061	3.429348	4.109593	3.767775	4.274425	3.429348	3.592616	3.569466	4.196670	3.923504	3.910352	3.472373	3.845423	3.982623	3.981290	2.994477	3.222539	3.154801	3.153056	3.564191	3.448389	3.565159	3.265735	3.124671	3.045889	3.842751	2.888990	4.593146	3.046710	3.637869	3.476161	3.217434	3.691947	3.033804	5.416211	3.468400	3.155331	3.125011	2.920528	3.345113	3.030280	5.014044

LINC01187	3.081556	3.872654	3.402821	4.185118	3.422423	4.037774	3.181650	5.201083	5.090210	4.075160	3.362499	3.459278	3.118879	4.484173	3.039394	3.786732	3.425827	3.001390	4.589938	4.145567	3.799007	4.809510	3.554981	3.350741	3.564969	3.393833	3.589211	3.969226	5.077771	2.999469	3.403206	3.185419	3.053619	4.167325	4.840237	3.891153	3.450425	3.748721	3.117138	4.324223	3.243811	3.102636	3.226873	4.332259	3.325163	4.345899	3.798195	3.317445	4.256493	4.408014	3.702258	4.954780	3.753613	3.809533	3.530906	4.150401	3.714449	3.273704	3.513915	3.393995	3.387739	3.677408	3.334035	4.681788	3.152335	3.926325	3.176503	5.180737	3.686432	4.473189	4.154789	3.465024	3.670528	3.484795	4.232435	3.613168	3.975291	3.580146	3.125138	3.418265	3.823300	3.832712	3.108739	3.776955	4.128728	4.353756	4.046238	3.286102	3.391578	3.259859	3.071553	4.777193	3.106541	3.029190	3.316047	3.511629	3.425458	3.618767	4.671443	3.347210	3.772186	3.155938	4.154789	3.780212	4.227030	2.919250	5.377702	4.248826	4.720037	4.137598	3.501629	3.132068	3.010303	3.666279	3.177091	3.438735	3.855536	3.921645	2.982350	3.481721	3.770527	4.370011	4.735652	3.461300	3.851412	3.928725	3.765519	3.657866	4.340922	4.666684	3.714879	4.339822	4.067751	2.952475	3.544145	3.401604	3.212966	4.016290	3.391037	4.314274	3.893535	3.525332	3.066288	4.708061	4.480635	4.614899	4.595326	5.140892	3.281037	3.242102	3.297766	3.039726	3.368081	3.108410	3.637869	3.525165	3.071864	3.117381	3.924386	3.449361	3.004306	4.199237	4.158412	3.484623	5.028694	4.560560	3.357764	3.362333	3.868138	3.647551	4.279704	3.485860	3.841707	3.184892	4.385878	3.372078	3.240744	3.276507	3.217115	3.180407	3.086361	3.351071	3.561948	3.267648	3.234379	3.015361	3.462669	6.275222	3.156927	3.221098	3.855536	7.273273	3.348689	3.516786	3.159554	4.139301	3.190001	3.246827	3.472041	3.435989	3.745044	3.101295	3.457254	3.400227	3.283804	3.106094	3.302832	3.252236	3.207803	3.676812	3.093751	3.581900	3.051004	3.649216	3.444194	3.658626	3.629032	5.500043	3.409888	4.205490	3.841327	6.469024	4.010356	4.602105	3.890045	3.136114	3.308406	6.242601	5.379059	3.405895	3.006984	4.032337	4.570033	4.203381	3.919566	3.051004	3.660815	4.309946

PPFIA2-AS1	8.768688	7.140794	6.435126	7.028923	7.012518	8.137100	6.929077	9.848797	9.018527	7.247752	8.795507	8.938834	8.769138	7.168260	7.396166	8.964936	9.078876	6.680932	6.440547	7.766091	6.537460	6.371255	7.590139	7.308348	6.708319	9.418537	10.015727	10.092047	8.269199	8.225862	9.750825	6.558766	7.786677	8.769584	6.948756	7.148656	7.838309	7.429000	6.904927	6.961011	8.295156	6.960210	7.514055	8.188620	7.434223	7.226852	8.653728	6.776202	7.422597	6.642863	6.960210	8.044166	7.090413	7.459810	6.533240	9.055161	7.701304	6.500213	5.645988	7.616186	6.163918	8.905380	6.977224	6.807918	6.177991	5.879055	6.573722	7.353422	6.234306	6.686952	7.696523	8.303178	8.169398	7.514207	7.035147	7.890456	7.153726	6.966591	7.752866	5.911679	6.804613	8.456897	6.777676	7.310568	6.619878	7.119340	8.193995	6.879726	6.113154	5.966803	6.060983	6.026945	7.274994	7.875819	8.341022	7.617692	8.363776	8.261839	8.424232	8.433526	8.517477	6.957191	7.566739	7.804017	6.155871	9.459862	8.901863	6.600383	7.609761	7.658918	6.908123	6.311444	7.972576	8.676199	8.661167	9.763723	7.190783	8.354756	8.739325	8.731061	7.847503	6.912113	7.816055	8.914331	8.577568	8.482488	10.026868	8.733797	8.060586	8.783833	9.353198	10.510355	7.563626	9.207170	9.307880	8.451693	7.188396	7.293658	6.933179	7.101115	7.919671	7.770487	7.678603	7.200863	8.530464	8.736112	10.519232	9.399241	8.163532	11.083949	7.608529	8.888851	8.815582	7.114731	7.131165	7.970042	8.038832	8.109085	7.365622	10.140329	7.135615	7.566427	8.324142	7.141433	6.979561	8.942784	6.748483	7.303979	6.809699	6.698709	8.566875	7.152154	7.950164	6.912595	7.983341	7.232778	6.708953	6.443539	6.838038	7.145969	8.815433	6.917090	6.978653	6.877345	6.581952	6.886446	7.348953	7.269237	7.290988	7.787764	8.072303	9.923845	8.235738	7.355513	7.729788	7.495029	7.582883	7.772629	6.557617	6.781665	6.966132	6.967570	8.600639	7.225135	8.377983	7.718121	7.339253	6.607242	6.697442	6.350277	6.682079	8.103712	9.139186	8.745271	8.265248	9.479088	9.468399	8.622962	7.353902	8.524310	7.553771	8.169560	8.112376	6.846000	9.776270	9.970187	6.828463	7.415795	8.879747	7.599022	6.903692	8.507964	8.971882	8.657743	8.252070	7.256173	8.757006	8.310361

AC018781.1	2.999567	4.956653	3.855094	4.222293	4.519569	3.751689	4.529462	4.700680	3.981983	3.979980	4.053659	3.671942	5.379059	5.165102	3.242519	3.830561	4.548870	3.535542	6.319104	4.720858	4.511823	5.082452	3.000046	3.591134	3.594603	4.609293	2.831449	3.188619	3.827843	4.003237	4.546237	5.326352	5.537646	4.727455	4.716023	5.040958	3.460369	4.894812	6.378810	4.127649	3.301613	5.178778	4.426792	3.661202	3.789732	5.368189	4.239453	5.924351	3.184892	3.499422	3.000046	4.423758	4.189497	6.017427	4.888354	4.853765	4.440018	4.887089	4.695568	4.083256	5.101129	2.986472	3.919015	4.817096	6.625846	5.213169	4.852882	4.781151	5.322477	5.332255	4.863721	3.838884	3.973097	3.398360	3.681221	3.951215	4.090033	4.747859	3.979980	5.054726	3.333864	3.303802	5.273690	4.836376	5.368759	4.633277	5.157704	4.179668	4.696606	4.935976	4.049551	4.468417	3.379996	4.318454	3.324679	4.610588	4.314526	4.274425	5.806670	4.023916	5.690430	3.478770	4.172494	4.263974	5.217371	3.600679	3.195416	3.504959	5.636016	4.408936	6.021540	4.665178	5.466171	3.176741	3.238168	6.959245	4.245569	4.574373	3.160450	3.393310	6.164105	3.229455	3.904662	5.757214	3.313169	4.262886	3.021451	4.913262	6.385607	6.993546	4.723874	5.184611	4.425257	5.372063	3.257771	5.115151	5.426867	3.402821	3.514967	5.042212	5.462398	4.228324	4.191471	4.955831	3.244569	5.613082	4.511602	4.116995	5.229951	3.131949	4.954166	4.715187	6.936495	6.104605	4.672398	4.636303	3.974446	5.674760	5.542603	3.136856	4.176606	3.596331	3.293467	3.847556	5.951263	4.436995	4.820255	5.567109	6.194067	7.081775	5.114561	6.569199	7.445861	4.559441	3.339291	3.987558	6.898683	5.182396	4.205947	4.080922	4.511823	8.142694	6.147780	4.057800	3.792471	6.695389	6.356984	5.869386	3.140408	4.844870	5.422633	4.293735	6.835143	3.726946	3.270657	4.380795	3.226157	5.092471	4.501816	6.921935	5.789012	5.085724	3.669919	3.522853	6.486942	5.518976	4.376523	4.435637	3.843957	3.476161	6.733538	4.595108	5.699482	7.044183	5.036945	4.779065	4.886461	9.042224	3.425082	4.981524	3.372595	4.486156	5.265256	4.933652	4.119034	4.925505	3.113651	5.552778	6.137520	6.086738	5.892964	7.304439	3.136389	4.478041	6.888075	4.561847	5.947390	4.897894

AC091730.1	4.463518	3.652214	4.398722	3.758136	3.453504	5.195106	4.729562	3.791187	4.294590	4.386094	6.112982	3.843957	3.703635	4.092057	3.217434	3.775550	4.509851	3.392955	5.406712	4.558767	7.285807	4.479959	4.788062	4.336124	3.909914	4.162511	3.547999	4.486379	4.107829	3.514432	4.123402	4.949392	4.808893	3.485860	4.593634	5.017906	3.878927	4.379733	4.422659	4.327873	4.279291	4.634998	4.433495	3.677408	4.492888	6.342505	4.548870	4.933461	3.138106	4.039309	4.119882	4.598327	4.924879	4.595957	3.534249	4.124633	5.186207	5.903831	4.735021	4.270707	3.469807	3.921050	3.664638	4.305328	3.997680	4.782417	3.890511	4.583521	3.743677	4.184469	4.383610	4.050878	4.277970	4.125253	4.025811	3.640480	4.243194	4.168403	4.160569	3.258992	3.751689	3.615932	3.911207	4.001248	4.239674	3.199768	3.636278	3.590928	3.176019	3.995086	3.827201	3.824871	6.011945	3.985840	3.639493	3.512749	5.030127	4.174641	3.889387	3.183197	3.747497	3.918319	3.572084	4.384713	6.475585	3.455318	4.498550	3.537834	4.019768	4.536303	3.489018	4.009546	3.597156	4.061734	4.148136	3.786037	3.315404	3.842331	4.048439	4.448486	3.378791	3.765098	3.701577	3.356380	4.932634	3.410579	3.523264	3.213675	3.330300	5.246802	3.747084	3.386208	3.500685	3.048363	3.301909	3.869015	3.641701	3.943048	4.412414	5.844412	3.830142	4.362190	3.889613	3.191453	3.283950	3.613780	3.997237	3.208598	3.434147	3.852488	3.242655	3.031851	3.118405	4.419800	3.509419	3.851412	3.403027	4.330333	3.114145	3.971168	3.991816	3.417894	3.233219	4.474088	6.424158	4.279291	4.475809	3.213524	3.757311	3.731504	3.551024	3.692766	3.801408	3.192551	3.838884	4.239674	3.538047	3.485680	3.661403	3.225038	4.319720	3.719536	3.882327	3.398178	3.560309	2.972619	3.657270	3.541120	3.414002	4.303316	3.446160	3.783976	3.548911	3.574404	3.772413	3.553350	3.975516	4.494208	3.043520	3.336976	3.719129	3.119124	3.514088	3.512749	3.232021	4.419157	3.357588	3.104462	3.308242	3.610373	3.462494	5.434367	3.492212	3.584548	3.552388	3.836745	3.654812	4.268466	3.148882	3.103870	3.186045	3.768830	3.478586	5.064275	3.509805	4.586707	3.256050	3.708838	4.264190	3.675168	3.258402	3.348203	4.828672	3.204421	3.241947	4.392582	3.765302	3.291272

PTPRD-AS1	5.085066	5.438573	5.930243	5.059000	3.210577	3.183560	6.008962	3.131810	3.252362	3.279133	4.595557	3.655456	5.448989	4.263078	3.771535	4.503938	3.360651	3.690742	4.579914	5.211243	4.417292	2.883880	2.965763	3.554282	3.051640	3.179000	3.515480	2.853596	4.394991	5.905606	3.488837	7.317172	6.117492	4.454188	6.122684	7.223425	6.431572	6.021706	4.587333	5.025838	3.590125	6.228660	5.029321	6.359193	6.555657	5.866471	3.212697	4.591187	6.581301	6.688383	7.446825	8.154391	7.268335	7.376584	6.575343	3.275450	6.663467	6.104087	3.659062	6.009128	6.269984	5.724374	4.988095	3.383293	5.399411	4.592701	4.842335	4.377152	4.023218	5.181372	6.638849	5.695092	4.637598	6.372955	6.605592	6.068594	5.436481	5.126963	5.916532	7.209395	5.589862	6.890763	6.178831	6.790858	6.380125	5.813703	6.020997	6.420618	3.371366	4.332454	4.796678	3.769308	3.604642	5.240664	5.640502	6.738272	7.539270	4.783894	3.270526	5.638273	3.124671	4.934698	4.914730	3.858553	4.343924	4.045411	2.984859	3.271463	2.936269	3.295444	3.739768	3.023492	2.898622	3.961381	5.060442	2.966669	2.891009	4.300454	3.559545	2.921191	3.299022	5.582672	3.261430	2.842927	3.354520	7.021100	3.085408	3.155801	7.899733	3.630688	5.460043	7.335219	3.100427	3.427251	2.956087	3.291838	6.389356	3.376176	3.466886	3.847370	6.155509	2.976959	2.945514	2.930244	6.348278	2.944169	2.827017	2.971520	6.730471	3.173828	4.817515	4.179876	3.305865	4.428477	7.511240	7.004100	3.187273	3.222403	4.696194	3.104004	3.215814	3.794679	3.474326	5.754253	5.849182	3.138106	5.840777	9.249208	4.884944	8.512612	4.661156	8.704285	4.545332	6.971518	3.649432	3.870296	2.996005	3.318632	6.059216	3.046203	3.955721	4.346108	3.233362	3.542870	4.402007	2.985985	3.635440	5.829144	6.934574	5.710664	6.729309	3.330656	6.566720	6.406726	5.408333	6.583596	5.345734	5.840959	5.936160	4.810612	4.858553	4.834971	5.695283	6.703546	3.026669	7.091491	3.542578	3.190142	4.325277	3.965972	7.447288	3.394704	3.193243	5.050399	5.233840	3.650427	4.028832	5.500462	9.774329	2.938626	3.006617	3.190001	4.956861	4.784124	3.625390	3.736280	3.270087	6.313672	3.096273	2.815060	3.173051	2.864018	3.182446	9.563002	3.060561	2.888372	4.150204	3.649639

LINC02125	12.009892	9.952895	10.250420	10.359924	10.369561	10.628531	10.246644	10.543883	11.417534	10.465495	10.914915	10.835128	10.309726	10.209666	9.819984	10.835875	10.643569	9.991577	7.693823	10.926096	7.704386	7.720377	7.524985	10.235733	10.377424	10.827984	11.759040	11.170765	10.625804	10.413733	10.916445	10.578433	9.507486	10.302633	10.371240	10.415709	10.354764	10.535767	9.887355	9.802726	10.288034	9.614989	10.115300	10.248333	10.202804	10.041611	10.775210	10.397835	9.868842	9.925599	10.262831	10.133459	9.897193	9.820554	10.031316	10.575398	9.479239	9.289882	9.271660	9.529917	9.622911	10.573479	8.105290	8.014164	8.356290	9.274259	9.358192	9.095560	9.454926	9.156924	10.117147	10.179942	10.847998	10.487949	10.447656	10.484421	10.403775	10.489348	10.797903	9.502078	9.006051	10.175307	10.131679	10.285761	10.174103	10.205821	10.205513	10.323968	8.422852	9.124887	8.891442	8.232995	9.072667	10.609210	10.714168	10.573635	10.215042	10.790846	10.334496	10.523105	10.675241	9.964578	10.521111	10.243068	7.837337	10.701207	10.549040	8.133472	8.161158	10.662371	7.862916	10.089927	10.815522	12.137705	10.849438	11.078978	10.837385	10.833010	11.189746	10.470586	10.046469	10.403638	10.496942	10.496003	10.144304	10.334339	10.866362	11.140371	10.219240	10.555535	10.513768	10.254371	9.835965	9.778915	10.977437	10.635198	9.611938	7.724844	10.719260	7.540820	7.777466	7.862455	9.516071	10.147930	11.236722	10.677631	11.020512	10.567866	10.353584	10.887784	10.323048	9.642850	10.742311	10.225458	9.961344	10.519634	10.655314	10.113319	9.920909	10.499861	9.897794	10.595005	10.859103	10.379552	9.960604	10.561470	9.876239	10.145498	10.226509	10.147476	10.662983	10.302633	10.106586	10.334168	10.421803	11.000620	9.790373	9.196602	9.703584	9.966653	10.701355	8.614880	8.144863	8.489977	9.547759	9.541895	9.478766	9.467673	10.164597	10.256793	10.449732	10.845782	10.641061	10.526885	10.598624	10.564878	10.746036	10.490556	9.612878	9.087930	10.092773	10.211984	10.654131	10.932789	10.192512	10.377106	10.589798	8.412161	9.635352	9.647662	8.571583	9.127357	10.604741	10.794534	10.743352	10.541677	11.066310	10.633212	10.494961	10.702585	10.140602	9.973403	10.307273	8.072573	10.739768	11.242535	8.101596	8.069531	10.370017	8.025420	9.564026	10.989295	11.046807	9.727909	10.376501	10.613036	10.656389	10.179183

AC007036.1	9.033552	8.753634	8.813498	9.131232	9.188603	9.426344	9.096433	9.877034	9.594640	9.261818	9.213755	9.182046	8.883590	8.562835	8.374973	9.163075	8.935611	8.568189	9.230592	8.802612	9.089149	8.739022	9.171408	9.072055	8.913049	9.323814	9.137166	9.717177	9.546675	9.323358	9.452733	8.039489	7.928181	8.733493	8.327291	8.294728	8.691670	8.574878	8.704285	8.496581	9.447310	8.300613	8.160678	8.424087	8.023465	8.087711	9.647967	8.483274	8.579234	8.509664	7.973521	8.023465	7.717220	7.980814	7.785744	9.692841	7.648004	8.054935	7.774143	7.583649	7.901171	9.326740	8.447639	8.819333	7.951832	8.206331	8.821128	8.645281	8.524608	8.865812	9.072667	9.110639	10.067980	9.077494	9.026158	8.987164	8.880667	8.728842	9.061269	7.860047	7.965252	8.125564	7.744866	8.424675	8.011566	7.977373	7.990815	8.452314	8.125979	8.350523	8.079159	8.802470	8.290249	8.405306	8.636601	8.198553	7.837979	8.301521	8.652502	8.758395	8.699971	8.642515	8.800503	8.935930	8.350047	9.221273	9.297703	8.609391	9.300940	9.883183	8.871542	8.960071	8.643945	10.209210	9.081348	10.398387	8.721615	9.136672	9.733183	9.235309	9.194034	9.072354	9.493310	9.820866	9.582699	9.403709	8.724496	8.997674	9.393593	9.711552	9.295377	9.447475	8.535756	9.365340	8.977700	8.976956	9.010103	9.222961	8.460319	9.071601	8.140757	8.803984	9.365664	9.198592	9.040712	9.090065	10.302329	9.918554	9.529758	9.146716	8.361723	8.094921	9.372256	8.525662	8.612493	8.498063	8.684477	9.356658	8.450156	9.838745	8.696520	8.358960	8.559301	8.121305	8.682359	10.307568	8.561617	8.212064	8.280249	8.264787	8.493691	7.866800	8.108325	7.685946	9.158942	7.806171	7.847351	7.872292	7.522964	8.477251	9.347097	8.399557	8.243097	8.287945	7.864481	8.876016	8.857133	8.810440	8.549976	9.229700	8.959613	10.312432	9.478766	9.009822	8.880054	8.928172	9.107052	9.123673	7.948163	7.977093	8.234051	8.142694	8.247398	8.247398	8.427882	8.198239	7.826786	8.109085	8.033453	8.066110	8.667281	8.428664	8.766990	8.684928	8.938682	8.634112	8.722345	8.897960	8.970362	8.919206	9.000709	9.388862	8.647089	8.423166	9.434019	9.086422	8.953955	8.437550	9.909395	8.490141	8.448740	9.052352	10.653251	9.157556	9.190703	8.065970	9.630595	9.959667

DTX2P1-UPK3BP1-PMS2P11	10.607408	9.667331	10.068875	11.065562	11.894583	10.961076	10.090224	11.229920	13.033027	11.401656	11.794980	11.272222	11.823222	11.510698	10.965036	11.395640	11.497713	11.702518	12.083083	11.491172	10.859681	11.908802	10.564425	11.912357	12.421829	12.153151	12.966913	11.539526	11.644748	10.723716	12.823533	9.497955	10.068558	11.570455	10.660235	10.417979	10.307712	10.334961	11.303223	11.458006	11.116077	10.862913	11.067474	10.797595	10.288842	11.235828	12.040781	10.229880	10.119452	10.493142	10.488568	10.294147	10.181623	10.294615	10.376210	11.002234	10.604891	10.977879	11.272515	10.609356	10.799798	11.522341	9.425577	10.848448	10.030084	10.135934	10.787056	10.171080	9.957211	10.612741	10.204611	10.695129	11.868900	9.953047	10.689190	10.338699	10.006772	9.974821	10.052192	10.007378	10.796728	10.452826	10.466973	10.464170	10.254975	10.392366	10.415101	10.606813	9.634169	10.369246	10.812104	9.622130	10.858937	10.151718	10.545078	11.539378	10.086020	12.404804	11.390710	12.203961	11.516068	11.094152	11.816305	10.035941	10.574965	11.369466	12.855684	10.669868	11.196672	11.897073	10.299569	12.034708	12.049673	12.192131	11.235675	13.065305	12.091683	11.260483	12.715895	11.382315	10.028734	10.695590	12.000733	12.489075	10.806048	10.999088	14.160839	12.853682	11.701608	11.246365	12.269302	12.681457	10.881198	11.243882	12.796078	11.920674	13.160712	13.369728	10.965488	10.603565	12.125403	10.222567	12.592823	11.559235	10.356772	11.019158	11.627357	13.026553	10.382728	13.214995	12.039701	12.235761	10.801353	10.895631	10.715670	10.409077	10.589210	11.580850	11.852176	12.385787	10.843330	10.758822	11.174007	10.415101	10.630981	12.454210	10.557654	10.668429	9.844727	10.595136	11.461123	10.505428	10.026226	11.053092	10.643734	10.716709	10.965807	11.856406	10.597545	10.684350	11.377843	10.424856	10.759553	11.535555	10.621738	10.981739	10.063932	10.147858	10.582172	10.713869	11.009325	11.775928	10.205513	10.837243	10.259158	10.192816	11.265386	10.774339	10.585975	11.359155	11.357390	10.784367	11.730489	9.939340	12.147944	10.055903	11.170765	10.385173	11.214379	11.751751	10.262677	12.007161	10.143702	10.940687	11.945155	12.060872	13.650539	11.196086	10.991621	11.321595	12.606727	12.378353	9.949662	10.555064	13.020460	12.705525	10.955581	10.697935	12.054237	10.343395	11.460396	12.224905	12.082750	11.515630	10.942265	11.968715	11.817939	11.663731

AC107223.1	5.659046	4.904203	5.805110	5.033991	4.872775	4.390400	5.121307	5.664600	5.776908	5.510681	4.461559	4.898566	3.296822	4.402233	3.786951	4.984779	5.775641	4.292692	4.664093	5.783971	5.305404	4.809948	6.766987	6.551327	4.879417	5.422633	4.884088	5.211443	5.707611	4.114367	5.713082	3.111404	4.550879	4.456263	4.539977	5.926133	5.193268	5.393986	5.687890	6.190095	5.137833	6.209840	5.928833	5.354706	5.096222	4.436050	5.425587	3.147523	4.730409	4.627372	5.711196	5.488194	5.377881	5.089403	3.408550	4.027744	5.457696	4.771569	5.163875	5.638458	5.568253	5.019510	6.639977	6.234806	5.447016	5.878692	5.339320	4.981328	6.117316	5.450854	4.670157	5.182603	5.781067	5.271047	5.096781	5.805626	5.238653	5.646696	5.826008	5.642536	5.213169	5.635849	5.331647	6.174551	6.255725	5.380962	5.830114	6.479464	6.468027	5.917082	5.928111	6.183047	5.349273	5.657752	5.809994	6.131717	6.018512	5.276363	4.449155	5.233225	4.205714	4.242950	4.218384	4.622478	5.141531	4.415122	6.256911	4.761630	6.240584	4.675355	5.141531	4.903206	5.920857	4.755113	4.830820	6.079720	5.440816	5.341041	5.624670	5.002123	5.115354	5.532941	4.546452	4.069444	3.240594	4.211867	5.815271	5.920317	3.660415	3.815433	3.823524	3.906869	3.286535	2.932255	5.373200	5.735376	3.591517	4.274425	6.046399	4.848293	4.335703	7.219663	6.469202	4.230278	5.172769	5.585889	4.047589	4.556009	3.377394	8.233737	3.570246	4.690116	3.575186	5.091838	5.278530	5.204869	5.317463	5.782546	5.778189	4.007174	6.336858	5.141316	6.010871	4.011621	3.844991	4.709693	4.423983	4.531413	4.890438	5.122508	4.726203	3.474700	4.157992	3.727364	3.486788	5.173917	4.828672	4.720248	5.699307	5.472537	5.003956	5.865783	5.723098	5.848261	5.703178	5.765200	4.941572	6.048741	5.734828	4.984779	5.045712	5.291008	5.083066	5.229535	5.406483	5.432417	6.620034	6.693929	5.070383	4.934923	4.541524	5.628020	4.938279	5.874232	4.413763	5.460043	5.177346	5.538448	5.733911	5.185200	6.450000	4.859838	5.043914	4.960214	5.356081	5.022770	4.833328	4.587091	4.046238	3.872419	6.169121	3.566480	4.115483	4.633277	3.938536	6.570524	4.370451	5.790965	3.811402	5.286219	4.049098	5.783423	3.863232	3.675168	3.635673	4.759341	5.127753	6.386807

AC010319.4	8.946763	9.118833	9.431743	9.164933	8.614047	8.672619	8.773664	7.393365	8.685393	8.002934	8.938239	8.333829	9.402928	9.707662	8.820838	8.689294	8.129817	9.510139	8.094770	9.810261	9.415969	10.189059	9.221410	8.738564	9.117730	8.154078	10.038839	7.966655	8.511528	9.349409	8.683701	9.442348	10.243374	10.249259	9.591971	9.015314	8.742469	9.363934	8.992714	9.668618	8.525523	10.181790	9.969420	9.039156	9.227589	9.073136	8.581371	10.030393	8.773832	9.448263	9.614243	9.532294	9.874810	9.123220	8.802316	8.071679	8.975910	10.031960	10.210307	10.077750	9.533530	8.272399	10.140602	10.044168	10.430794	8.849097	10.049101	10.612438	9.448726	9.967099	8.633369	8.541028	9.370141	9.325501	8.579659	10.028112	8.526275	8.875412	8.660855	9.625356	9.689916	9.121228	9.578531	9.306635	8.929856	8.614955	9.606097	10.007529	10.621452	9.518373	9.869780	11.342184	9.573979	9.768291	9.898075	9.460588	9.179371	8.943236	9.033855	8.631287	8.362192	8.656820	9.103349	8.725114	8.669134	8.874177	8.745107	9.755312	9.479952	8.122287	10.725800	9.684838	8.888525	7.984242	8.919658	8.675705	10.368062	8.709468	8.779634	11.131496	10.211224	10.299419	11.005550	8.696520	10.041915	10.723422	8.763929	8.070641	10.460269	10.425006	8.185578	7.926641	11.455058	9.826316	10.247562	11.075613	8.707973	9.981361	10.557368	11.842797	10.494370	10.232060	8.790729	7.548829	11.096025	10.199926	10.013689	10.334496	10.164130	10.307862	9.658248	8.683390	9.439260	10.808596	7.483988	9.556482	10.207688	11.291724	9.909395	8.069057	10.367597	11.047086	8.905804	10.075003	9.920116	9.192889	10.154836	7.971783	10.378509	10.431883	9.340740	10.856874	8.450626	10.320751	10.094705	10.305051	11.141047	11.454736	9.509606	10.636826	10.457105	9.699709	11.620252	11.448972	10.032856	10.737773	9.659835	8.909943	11.924435	8.927706	10.559792	10.064516	8.874814	10.091421	10.244125	8.356909	8.272998	7.653463	10.873972	10.618351	10.657546	10.520191	10.482160	10.971775	10.635922	9.819818	9.728699	10.821920	11.276880	9.753292	12.273457	10.082093	10.153109	9.944847	10.104477	8.310673	8.935611	11.153618	8.732710	9.085022	9.430029	10.961636	10.609923	11.286577	10.029478	10.472372	13.140676	10.707761	9.350764	10.064236	10.757808	7.742387	9.477511	7.499060	10.029478	11.836941	10.683771	10.452240

BISPR	8.946763	9.118833	9.431743	9.164933	8.614047	8.672619	8.773664	7.393365	8.685393	8.002934	8.938239	8.333829	9.402928	9.707662	8.820838	8.689294	8.129817	9.510139	8.094770	9.810261	9.415969	10.189059	9.221410	8.738564	9.117730	8.154078	10.038839	7.966655	8.511528	9.349409	8.683701	9.442348	10.243374	10.249259	9.591971	9.015314	8.742469	9.363934	8.992714	9.668618	8.525523	10.181790	9.969420	9.039156	9.227589	9.073136	8.581371	10.030393	8.773832	9.448263	9.614243	9.532294	9.874810	9.123220	8.802316	8.071679	8.975910	10.031960	10.210307	10.077750	9.533530	8.272399	10.140602	10.044168	10.430794	8.849097	10.049101	10.612438	9.448726	9.967099	8.633369	8.541028	9.370141	9.325501	8.579659	10.028112	8.526275	8.875412	8.660855	9.625356	9.689916	9.121228	9.578531	9.306635	8.929856	8.614955	9.606097	10.007529	10.621452	9.518373	9.869780	11.342184	9.573979	9.768291	9.898075	9.460588	9.179371	8.943236	9.033855	8.631287	8.362192	8.656820	9.103349	8.725114	8.669134	8.874177	8.745107	9.755312	9.479952	8.122287	10.725800	9.684838	8.888525	7.984242	8.919658	8.675705	10.368062	8.709468	8.779634	11.131496	10.211224	10.299419	11.005550	8.696520	10.041915	10.723422	8.763929	8.070641	10.460269	10.425006	8.185578	7.926641	11.455058	9.826316	10.247562	11.075613	8.707973	9.981361	10.557368	11.842797	10.494370	10.232060	8.790729	7.548829	11.096025	10.199926	10.013689	10.334496	10.164130	10.307862	9.658248	8.683390	9.439260	10.808596	7.483988	9.556482	10.207688	11.291724	9.909395	8.069057	10.367597	11.047086	8.905804	10.075003	9.920116	9.192889	10.154836	7.971783	10.378509	10.431883	9.340740	10.856874	8.450626	10.320751	10.094705	10.305051	11.141047	11.454736	9.509606	10.636826	10.457105	9.699709	11.620252	11.448972	10.032856	10.737773	9.659835	8.909943	11.924435	8.927706	10.559792	10.064516	8.874814	10.091421	10.244125	8.356909	8.272998	7.653463	10.873972	10.618351	10.657546	10.520191	10.482160	10.971775	10.635922	9.819818	9.728699	10.821920	11.276880	9.753292	12.273457	10.082093	10.153109	9.944847	10.104477	8.310673	8.935611	11.153618	8.732710	9.085022	9.430029	10.961636	10.609923	11.286577	10.029478	10.472372	13.140676	10.707761	9.350764	10.064236	10.757808	7.742387	9.477511	7.499060	10.029478	11.836941	10.683771	10.452240

LINC01127	7.205507	10.256330	9.405418	8.054162	6.952175	6.527835	7.180328	6.575845	7.528744	6.961011	8.471218	8.734401	8.220715	7.026498	9.040712	6.753737	7.720231	8.149614	7.017952	7.836281	7.236423	6.621465	4.510512	7.089152	7.548388	8.285007	7.087059	6.040904	6.045528	7.816357	6.325946	8.190071	8.795062	9.380201	8.727901	8.409240	7.333384	7.598858	8.609684	7.991262	6.760447	6.222763	7.781047	7.520818	7.451958	10.244867	6.541196	7.647109	7.808494	8.187744	8.781813	7.267734	8.254367	9.643014	9.362011	6.982716	7.978789	7.103919	7.545824	9.008800	8.076646	7.754268	6.659365	6.202793	7.338924	8.642211	7.510936	7.178605	9.564348	7.489116	7.254455	7.520987	7.800068	7.821500	7.776417	8.011098	9.006556	10.124758	8.557910	8.911133	9.074979	9.059743	7.656886	7.397106	7.500547	7.593826	8.439951	8.256329	8.803828	8.479979	8.303620	7.644473	8.621266	8.179476	8.409992	8.352459	7.932481	6.552341	7.915502	7.281157	6.423176	7.767922	7.465483	7.634200	8.421803	6.306611	6.907043	7.550222	7.648004	6.177822	6.955501	5.681029	6.032733	6.346068	8.216204	7.955121	7.147559	7.379556	7.051334	8.621747	13.738842	11.387475	6.881346	7.551132	7.839230	7.073059	6.812869	9.269690	8.704893	11.058784	11.878180	8.481414	6.643033	12.575563	6.711800	9.513967	10.333744	11.618540	9.484092	8.427711	7.390553	7.439309	7.967258	7.759598	9.572587	9.533221	7.371092	6.424310	8.363776	6.408698	8.360473	10.454196	9.002124	8.870565	9.930158	7.587332	7.719509	10.818648	9.166648	7.823834	7.688703	7.136255	7.572480	9.049134	12.907514	7.003666	6.752706	8.905538	8.055096	11.714834	7.802054	9.056728	12.072972	9.245828	7.094649	8.420732	8.454436	9.572587	9.650895	8.001042	8.836925	6.923374	8.160386	7.134361	9.723772	6.500696	8.162350	9.766182	9.429446	8.525817	9.214667	9.602218	9.957986	7.158679	9.056272	11.832854	11.067334	9.992644	11.575527	8.106349	9.421602	8.280399	7.884438	8.976221	9.072667	9.288025	7.405370	7.881713	8.707834	11.289463	10.494224	7.959294	8.621435	8.654016	10.878246	9.097041	7.397543	10.027498	8.362379	6.672197	8.457624	6.698709	8.217766	6.156355	7.940095	5.830114	7.393824	7.969720	7.794310	6.467861	5.408923	7.103286	8.185578	9.760100	8.525817	8.208290	8.983483	9.454030

AC025419.1	3.142180	3.349518	3.055709	3.029573	3.056655	3.100817	3.381903	3.433435	3.453144	3.471726	3.636471	3.685044	3.363712	3.998940	3.703843	3.192946	3.669497	3.416712	5.099724	3.223347	5.193268	2.814389	2.968562	3.016806	2.905401	2.797548	2.977966	2.880634	3.016992	3.079410	3.704272	3.441340	3.504352	3.038042	4.328543	4.152749	3.746283	3.371190	3.397480	2.998784	3.460556	3.023492	3.590928	3.310696	3.773257	5.730674	3.292959	3.384997	3.684424	4.033635	3.989667	4.994379	4.469049	4.219469	4.016509	3.681221	4.246870	3.024654	3.733827	3.900421	2.944534	3.129211	3.931529	3.448612	3.515884	3.486956	2.817488	3.415206	3.249291	2.836829	3.034123	2.906315	3.145372	2.892982	2.939668	3.377215	3.347031	3.058514	2.994943	3.872419	3.464825	4.143033	3.483534	4.422440	3.516786	3.611782	4.598089	3.318958	3.459121	3.724732	3.355689	3.246386	3.556975	3.143964	2.943120	2.973807	3.910352	3.133804	4.047376	2.968264	3.308061	3.300530	3.028570	2.824573	4.717717	3.111066	3.104560	3.643095	3.483154	3.411476	4.432687	4.159936	3.009356	3.341666	3.198532	5.033779	2.958268	3.644833	3.223215	3.068050	3.508512	3.007845	3.086264	3.426202	3.644833	3.202823	3.390523	3.654197	3.200632	3.436524	3.154159	3.201820	3.349011	3.999384	3.083503	3.605218	3.064058	3.512576	3.422966	4.486790	2.803334	2.944910	3.141417	5.941446	6.153116	3.322329	2.859012	3.879159	3.583598	3.402108	3.428139	4.008468	3.955912	3.972227	3.839648	3.795711	3.395212	4.202274	2.979549	3.312397	2.895437	3.676604	4.056272	4.271174	5.223134	3.853359	3.289532	3.989667	4.112008	4.010157	3.643274	4.225750	5.511256	3.569274	3.352877	3.831572	3.023596	3.279590	3.745044	3.316489	3.250849	4.020669	3.791187	3.819283	3.719129	5.399789	3.789308	3.401235	2.821633	3.050142	3.002248	3.136856	3.274345	2.939146	3.338619	3.150003	3.144107	3.076552	3.501795	4.483726	4.992229	3.551213	3.773481	3.617257	3.068364	3.449712	3.690955	3.178289	3.583415	3.526481	3.546976	3.334678	5.128561	3.118161	3.181516	3.318310	3.500336	4.059329	3.298546	4.599178	3.311317	2.936539	3.578183	5.066538	3.116070	3.075184	3.623359	3.491861	4.111616	4.657491	5.414727	3.994432	3.259588	3.008577	3.164325	2.952203	3.129913	3.370101

LINC02454	3.142180	3.349518	3.055709	3.029573	3.056655	3.100817	3.381903	3.433435	3.453144	3.471726	3.636471	3.685044	3.363712	3.998940	3.703843	3.192946	3.669497	3.416712	5.099724	3.223347	5.193268	2.814389	2.968562	3.016806	2.905401	2.797548	2.977966	2.880634	3.016992	3.079410	3.704272	3.441340	3.504352	3.038042	4.328543	4.152749	3.746283	3.371190	3.397480	2.998784	3.460556	3.023492	3.590928	3.310696	3.773257	5.730674	3.292959	3.384997	3.684424	4.033635	3.989667	4.994379	4.469049	4.219469	4.016509	3.681221	4.246870	3.024654	3.733827	3.900421	2.944534	3.129211	3.931529	3.448612	3.515884	3.486956	2.817488	3.415206	3.249291	2.836829	3.034123	2.906315	3.145372	2.892982	2.939668	3.377215	3.347031	3.058514	2.994943	3.872419	3.464825	4.143033	3.483534	4.422440	3.516786	3.611782	4.598089	3.318958	3.459121	3.724732	3.355689	3.246386	3.556975	3.143964	2.943120	2.973807	3.910352	3.133804	4.047376	2.968264	3.308061	3.300530	3.028570	2.824573	4.717717	3.111066	3.104560	3.643095	3.483154	3.411476	4.432687	4.159936	3.009356	3.341666	3.198532	5.033779	2.958268	3.644833	3.223215	3.068050	3.508512	3.007845	3.086264	3.426202	3.644833	3.202823	3.390523	3.654197	3.200632	3.436524	3.154159	3.201820	3.349011	3.999384	3.083503	3.605218	3.064058	3.512576	3.422966	4.486790	2.803334	2.944910	3.141417	5.941446	6.153116	3.322329	2.859012	3.879159	3.583598	3.402108	3.428139	4.008468	3.955912	3.972227	3.839648	3.795711	3.395212	4.202274	2.979549	3.312397	2.895437	3.676604	4.056272	4.271174	5.223134	3.853359	3.289532	3.989667	4.112008	4.010157	3.643274	4.225750	5.511256	3.569274	3.352877	3.831572	3.023596	3.279590	3.745044	3.316489	3.250849	4.020669	3.791187	3.819283	3.719129	5.399789	3.789308	3.401235	2.821633	3.050142	3.002248	3.136856	3.274345	2.939146	3.338619	3.150003	3.144107	3.076552	3.501795	4.483726	4.992229	3.551213	3.773481	3.617257	3.068364	3.449712	3.690955	3.178289	3.583415	3.526481	3.546976	3.334678	5.128561	3.118161	3.181516	3.318310	3.500336	4.059329	3.298546	4.599178	3.311317	2.936539	3.578183	5.066538	3.116070	3.075184	3.623359	3.491861	4.111616	4.657491	5.414727	3.994432	3.259588	3.008577	3.164325	2.952203	3.129913	3.370101

AC079248.1	9.091411	7.781521	7.922093	7.976325	7.410333	7.610220	6.886607	8.757006	8.950873	7.199153	7.462948	6.940592	6.394182	6.556302	6.767480	7.378008	8.164115	6.813657	9.128284	7.314980	8.372078	8.947669	8.808432	7.413210	7.883365	8.083179	7.548986	8.301962	6.667531	6.718290	8.578029	6.850610	7.371577	6.480893	7.233867	7.298780	7.369531	7.733781	8.069677	8.167229	7.041278	8.033745	7.112523	8.053534	7.587048	8.038832	6.734990	7.520818	7.804165	7.524066	8.998715	7.356854	6.916498	7.264337	7.115967	7.120888	6.824823	7.416992	7.551749	7.423979	8.121626	6.745143	8.168776	8.297469	8.036507	8.408039	8.081792	8.058274	7.914144	8.472170	7.783924	7.745783	6.851397	7.452274	7.409576	7.602325	8.061955	7.912515	8.222845	7.270312	7.422282	7.484585	7.534859	7.355953	7.263127	7.464349	7.663263	7.663729	8.035070	8.232848	8.039489	8.751471	7.529175	7.715526	7.511087	7.186835	7.258046	5.807043	6.490004	6.930656	6.601827	6.964223	7.361839	7.227500	8.557313	6.813975	9.080721	7.872292	9.169228	7.344870	9.300940	8.083795	7.712966	7.707285	7.956793	7.801445	9.312017	7.670842	7.118530	7.733473	7.938047	7.947387	7.629708	7.626412	8.013547	6.907043	7.902414	8.205706	7.180178	7.287232	6.922731	7.284738	6.827817	6.638708	7.773530	8.159472	6.998180	9.049757	7.405681	8.516237	8.634259	9.330289	7.909264	8.130729	7.833239	8.300756	8.235738	7.547276	6.966132	8.520957	6.942960	7.285048	7.080037	7.281001	7.759279	7.176828	8.597172	8.587540	8.841949	6.966591	9.087482	7.532852	8.361249	7.900239	8.373422	7.398827	7.605777	8.368201	8.069677	8.024380	7.646818	7.060771	7.623954	7.091491	7.006419	8.139581	7.697615	7.855750	7.689652	8.137100	6.127291	8.752751	8.540872	8.435100	8.262928	8.433222	8.627465	8.377215	8.684928	8.279347	8.375265	7.491333	7.737895	7.648966	7.865090	7.939622	8.269978	8.351704	6.960860	7.294911	7.700541	7.609439	7.120108	7.534859	7.860630	7.591030	7.536099	8.072573	7.881713	7.946493	9.008088	8.053534	8.306339	8.052166	7.801599	8.055892	6.993546	7.216228	7.512640	6.533076	7.150091	7.257719	7.278463	8.213603	6.770414	8.687887	7.919983	9.205975	7.787467	9.169074	8.088613	8.718195	7.713603	7.604670	6.547423	8.139887	7.343668	7.243086

SNHG4	4.277507	4.665178	4.998590	5.358537	5.208422	5.458449	5.419596	3.821455	5.578508	7.333384	5.658144	4.707633	5.535863	5.160880	6.213374	4.407592	6.220089	6.550531	5.139018	5.105453	4.630254	5.862133	6.166510	5.177346	6.015535	5.440433	5.482940	4.832509	4.399395	4.288978	5.309663	4.430930	4.742727	5.405352	4.538643	4.299199	4.588857	4.844242	5.209030	5.563335	5.600431	4.476675	5.228917	4.670819	4.535468	5.517025	4.018482	4.686755	4.358969	4.184469	4.688212	4.033222	4.674733	4.960000	6.383146	4.485503	5.431625	5.402864	5.649152	4.896846	5.507599	5.351860	3.520806	5.128768	5.489143	5.339320	4.697431	5.055546	5.102586	4.899606	4.483077	4.944347	5.321096	4.622076	4.606257	3.812518	4.187106	4.008468	4.184907	4.908200	5.615376	5.844745	5.663106	5.674591	4.475809	5.201290	4.216232	4.241625	3.634857	4.253430	4.793100	4.481255	5.550863	4.225973	4.052167	5.586787	5.474055	6.129664	4.773477	5.339727	5.581341	4.664522	5.151351	5.083258	5.805807	5.100721	5.499091	5.370132	5.388347	4.903797	4.792523	5.673843	6.014010	4.753194	6.294223	4.556426	4.254568	5.078395	5.338103	5.972524	5.268480	5.232211	5.640661	4.702563	6.198966	6.083510	4.886886	5.815457	7.507708	6.014848	7.028923	5.900633	5.410898	5.730111	3.951608	5.715301	7.122109	6.577340	4.856480	5.374545	5.443145	5.591378	5.098046	6.356807	6.095860	3.679024	6.783550	5.485210	4.703636	4.883086	5.899181	6.012483	6.162870	5.053637	5.631375	4.197752	5.585157	6.044821	6.282111	7.572932	5.490169	6.609828	4.841265	5.475412	4.851173	6.413534	2.868709	6.228307	4.070662	4.830820	5.549197	6.580287	4.302168	5.562589	5.102586	4.125253	5.790965	6.343881	5.978520	5.969983	4.229251	3.566109	4.909875	7.630374	5.948634	4.226166	4.724322	6.087838	4.528407	6.364695	5.245997	6.945502	4.582452	4.021539	4.692213	4.003905	5.592917	4.949604	4.573522	5.913125	5.341799	5.094168	5.628989	4.659849	5.084680	6.146192	8.284075	3.822675	5.392969	5.995187	5.487292	5.084680	5.121711	4.580787	6.094972	6.192830	6.587534	6.282271	5.447016	4.434540	5.053206	6.204195	4.755320	5.519185	5.515028	8.272248	5.657200	6.017781	5.621097	5.154379	4.664522	5.228322	5.509173	4.912395	4.008008	5.775290	5.973787	5.828040

AL590648.2	9.249650	10.632025	10.159823	10.483523	10.073625	10.426073	9.663577	11.244638	10.773135	10.499007	9.968648	9.803036	9.139490	9.461202	9.040231	10.977591	10.323206	8.993942	9.603163	9.959383	10.020947	10.060453	9.399241	10.394473	9.815273	10.483815	9.628948	10.673716	10.005518	10.783470	9.987919	9.018064	9.391124	10.192512	10.111696	10.388663	10.152953	10.259775	10.063327	10.196135	10.618196	9.483497	9.572739	9.777842	9.495345	9.413487	10.507219	10.145661	10.149344	10.002883	9.843644	9.874660	8.897960	9.530075	9.394342	11.199162	8.907201	10.135934	9.957369	9.823152	9.459408	10.401393	8.693709	9.253595	10.350819	9.809058	10.077440	9.852904	10.098434	10.271317	10.524431	11.123230	10.623514	10.847688	10.294147	10.479505	10.242440	10.805904	10.437805	8.909516	8.921804	8.658677	9.378994	8.866731	8.927250	9.011607	11.029265	9.278901	8.953796	9.118025	9.348320	9.600645	9.930936	10.104632	9.739183	9.064741	9.661238	9.076223	9.193184	10.940539	9.519473	10.204002	10.427919	11.128520	9.423772	10.252193	10.205670	9.797657	9.856025	10.946219	9.726215	9.509006	9.913768	11.021233	10.506163	10.586845	8.469008	9.386992	10.737488	10.816868	11.311460	10.471930	10.580549	10.705507	9.992352	10.045877	8.702595	9.049275	9.864543	9.745999	9.627241	9.328764	9.419599	9.578078	9.715026	9.236739	9.136672	8.771577	9.132278	9.896882	9.736095	10.634289	10.060602	9.038018	9.128435	10.587449	10.026399	10.391357	11.243601	9.168150	9.232049	9.008878	11.452234	10.109727	11.477980	10.194616	10.464013	10.421303	9.452905	10.279584	9.880256	8.797014	9.957211	10.695437	10.008811	10.795839	8.999495	10.083133	10.114501	9.944847	9.936636	9.272401	9.650235	9.061269	10.867857	8.096768	9.863013	10.445527	10.228649	9.125817	10.437954	8.980580	9.489187	10.197653	9.897040	9.815584	10.894897	9.945894	10.115451	10.501981	10.808261	10.808891	10.805632	9.688079	10.589937	10.187258	10.989737	10.916152	8.743149	8.960071	8.572331	10.287417	8.342466	8.404521	9.184246	12.105743	8.826707	9.122762	9.047757	8.596115	9.389608	9.893962	9.564664	11.123825	9.297552	9.502375	8.930947	8.863543	10.216978	8.990029	9.634319	10.171845	11.072856	8.149782	10.360437	8.861847	9.606408	10.268218	10.916152	9.635073	9.300940	10.271020	11.038878	10.286654	9.738374	9.167108	9.593060	10.451232

AC107208.1	3.157049	3.293922	3.733620	3.025748	2.993254	3.152573	3.468038	3.130356	3.285045	3.182173	3.239740	3.366336	3.191743	3.810881	3.458761	3.173569	3.336436	3.596508	6.288774	3.213260	4.680068	3.163259	4.012263	3.185151	3.492956	3.092914	2.967208	3.086481	3.084646	3.041680	3.581299	4.273115	5.516471	2.985413	3.339444	3.815882	3.662035	3.449885	3.678209	3.557581	4.099296	4.294797	3.374715	3.181900	3.592078	5.710503	3.674948	4.506555	3.493904	3.666689	3.565159	3.835886	3.926936	3.959039	4.096860	3.111762	4.136501	3.810767	6.515893	3.976560	3.609384	3.079298	3.953545	3.275887	3.472563	3.524784	3.175288	3.364854	3.314143	3.182695	3.988180	3.361618	3.123779	2.915238	2.935655	3.171902	3.116514	2.932827	2.943316	3.507957	3.684027	3.657043	3.381552	4.195183	3.729236	3.334371	3.443822	3.254844	3.711920	3.605218	3.487325	3.300671	3.401604	3.471558	3.038520	2.934989	3.737498	3.266904	3.748541	3.186045	2.985121	3.646801	3.066084	3.054044	2.953586	3.173051	3.185419	3.411654	3.033913	3.237433	3.128332	3.002733	3.091624	3.251299	3.138106	3.597536	3.049958	3.074675	3.189408	3.294523	3.555846	2.970360	3.183693	3.113096	3.726339	3.261577	3.094660	3.240032	3.413645	3.072994	3.026966	3.618945	3.503137	3.202239	3.565350	3.275887	3.176610	4.632405	3.341822	4.046238	3.216802	3.351217	3.045250	3.365508	3.272404	3.167023	2.959195	3.171558	3.373589	3.385511	3.357413	3.321552	2.960790	3.218550	3.569066	3.645826	3.595009	4.145567	3.337147	3.707485	4.605235	3.494265	3.258992	3.986951	5.299860	3.719948	4.479552	3.644529	3.568074	3.436845	4.401099	3.676420	4.014711	3.889150	3.344944	4.602105	3.998746	6.424645	4.113339	3.202087	3.167650	4.078669	3.807201	3.750187	3.770527	3.275589	3.736280	3.523264	3.243811	3.209242	3.217434	3.074675	3.300392	2.934989	3.114521	3.178160	3.541895	3.253442	3.718939	3.792701	3.989003	3.415696	3.813804	3.615523	3.009356	3.240314	3.352062	3.604836	3.561360	3.558934	3.450064	3.363531	3.151361	3.075859	3.485680	3.088427	3.234542	4.389969	3.748966	3.016359	3.427639	3.118766	3.514264	2.955993	3.229919	3.249291	3.370455	3.486788	3.147783	3.344161	3.021451	3.427251	3.171791	2.993450	3.198410	3.230351	3.256210	3.224345

LINC00963	10.950433	10.401393	10.412994	9.706727	9.938201	9.601275	10.450191	10.220026	9.652899	9.888772	10.087473	10.581306	10.321065	10.794980	10.607831	9.624616	9.575947	10.582900	10.596659	9.869327	10.028734	11.096621	10.394763	10.029332	10.582028	10.200663	9.836571	10.218028	9.525550	9.840929	9.581160	10.526564	10.649097	10.407984	10.904808	10.625051	10.820704	10.561160	10.958111	11.053227	9.818731	11.117105	10.811801	10.423456	10.970868	11.030608	9.926202	11.604162	10.427336	10.456939	10.850174	10.817302	10.902906	11.002085	11.503797	9.990936	11.100452	11.683496	11.367432	11.614652	11.443382	9.694508	11.996703	11.245651	11.734231	11.771231	11.501620	10.241816	10.797016	10.938612	10.254063	10.477695	9.437883	10.336891	10.071163	10.456786	10.392669	10.740345	10.639693	10.335381	10.388103	10.889381	10.894469	10.429581	10.561314	10.657698	10.422252	10.367747	10.802397	10.987180	11.430015	10.759553	11.132065	10.661925	10.890293	10.602394	10.760404	9.678789	10.190413	9.816047	10.023382	10.504682	9.704725	10.355208	11.254561	9.251027	11.086028	10.829909	9.774329	9.356220	10.491412	10.820570	11.147807	9.318351	9.709371	9.358336	11.388220	9.756549	8.991194	10.210629	9.837342	10.802095	9.901638	9.691158	9.849598	10.398977	9.621194	9.645648	10.097780	10.495573	9.685777	11.209109	10.217895	11.164195	10.322594	10.041915	10.505273	9.946536	9.972631	10.254503	11.099577	11.050340	10.918947	11.673410	11.344019	9.544844	10.250726	9.850577	9.896599	10.226672	9.914854	10.068275	9.554047	10.681521	11.220119	10.586560	11.077806	11.219673	11.203158	9.243504	11.383909	10.100927	9.922132	10.844403	11.073138	9.220054	10.109582	10.655314	10.723866	10.177481	10.362833	11.050779	11.308691	11.326134	9.918629	10.138215	11.546206	11.099157	11.428987	10.827682	10.231448	11.948336	11.203593	12.035336	11.553024	11.014582	10.231595	11.222510	10.992556	10.781447	10.413889	9.730779	10.405317	9.968029	10.177956	10.581306	10.673442	10.603105	10.704907	11.352182	11.254129	10.690375	10.190703	10.957088	11.071366	9.994941	10.402145	11.456392	11.075744	11.288046	11.083368	11.140512	11.093119	9.942430	10.132853	10.789859	9.959230	10.106724	11.239490	11.115929	9.532917	10.123342	10.850018	10.408443	9.820128	10.586401	10.414470	10.001164	9.793460	10.599709	11.095144	9.930320	9.428192	10.654734	8.960071	11.404241	9.243504	10.732367

AL391056.2	10.950433	10.401393	10.412994	9.706727	9.938201	9.601275	10.450191	10.220026	9.652899	9.888772	10.087473	10.581306	10.321065	10.794980	10.607831	9.624616	9.575947	10.582900	10.596659	9.869327	10.028734	11.096621	10.394763	10.029332	10.582028	10.200663	9.836571	10.218028	9.525550	9.840929	9.581160	10.526564	10.649097	10.407984	10.904808	10.625051	10.820704	10.561160	10.958111	11.053227	9.818731	11.117105	10.811801	10.423456	10.970868	11.030608	9.926202	11.604162	10.427336	10.456939	10.850174	10.817302	10.902906	11.002085	11.503797	9.990936	11.100452	11.683496	11.367432	11.614652	11.443382	9.694508	11.996703	11.245651	11.734231	11.771231	11.501620	10.241816	10.797016	10.938612	10.254063	10.477695	9.437883	10.336891	10.071163	10.456786	10.392669	10.740345	10.639693	10.335381	10.388103	10.889381	10.894469	10.429581	10.561314	10.657698	10.422252	10.367747	10.802397	10.987180	11.430015	10.759553	11.132065	10.661925	10.890293	10.602394	10.760404	9.678789	10.190413	9.816047	10.023382	10.504682	9.704725	10.355208	11.254561	9.251027	11.086028	10.829909	9.774329	9.356220	10.491412	10.820570	11.147807	9.318351	9.709371	9.358336	11.388220	9.756549	8.991194	10.210629	9.837342	10.802095	9.901638	9.691158	9.849598	10.398977	9.621194	9.645648	10.097780	10.495573	9.685777	11.209109	10.217895	11.164195	10.322594	10.041915	10.505273	9.946536	9.972631	10.254503	11.099577	11.050340	10.918947	11.673410	11.344019	9.544844	10.250726	9.850577	9.896599	10.226672	9.914854	10.068275	9.554047	10.681521	11.220119	10.586560	11.077806	11.219673	11.203158	9.243504	11.383909	10.100927	9.922132	10.844403	11.073138	9.220054	10.109582	10.655314	10.723866	10.177481	10.362833	11.050779	11.308691	11.326134	9.918629	10.138215	11.546206	11.099157	11.428987	10.827682	10.231448	11.948336	11.203593	12.035336	11.553024	11.014582	10.231595	11.222510	10.992556	10.781447	10.413889	9.730779	10.405317	9.968029	10.177956	10.581306	10.673442	10.603105	10.704907	11.352182	11.254129	10.690375	10.190703	10.957088	11.071366	9.994941	10.402145	11.456392	11.075744	11.288046	11.083368	11.140512	11.093119	9.942430	10.132853	10.789859	9.959230	10.106724	11.239490	11.115929	9.532917	10.123342	10.850018	10.408443	9.820128	10.586401	10.414470	10.001164	9.793460	10.599709	11.095144	9.930320	9.428192	10.654734	8.960071	11.404241	9.243504	10.732367

CRNDE	14.032887	13.164330	12.936361	12.350213	13.428242	12.734697	12.759968	12.016602	13.232656	13.169964	13.114466	12.923310	13.275630	12.949109	12.796448	13.101246	12.880949	12.824360	13.173050	13.170547	12.514165	13.158236	10.277128	12.458490	12.563656	13.065843	11.998089	12.900943	13.160146	12.396775	13.205135	13.070948	12.838440	13.375239	12.373219	13.048973	13.192705	12.663909	12.689676	12.975003	13.177797	12.587805	12.505580	12.927582	12.447100	13.114655	12.740932	12.658626	12.683500	12.738848	13.232267	12.982760	12.991998	12.556996	12.852304	12.987020	12.828585	12.981325	13.576482	12.611506	13.038781	12.989260	9.764038	12.667996	12.697321	13.253798	13.065113	13.127492	13.181502	13.169042	12.530524	13.068719	12.831697	12.648843	12.086955	12.932855	13.270023	12.591670	12.929922	13.556609	13.076070	13.347873	13.201614	13.484618	13.650539	13.347004	12.783624	12.906428	12.241146	12.908398	12.685861	12.461245	12.795726	12.831516	13.195507	12.880116	13.025652	12.978769	13.216213	12.963458	12.977133	12.197683	13.144182	12.967785	12.920057	13.069852	13.572645	12.763614	12.701599	13.024924	12.864781	13.846147	13.079851	12.985929	12.810001	12.724156	13.835083	13.200096	12.806661	12.523632	12.077128	12.182366	12.985929	12.965441	13.667422	13.377893	14.143610	13.422596	13.110603	12.650060	14.018427	13.389060	13.850161	11.732880	13.003777	13.087563	14.086854	13.406084	13.201413	12.383858	12.513066	12.399170	12.300959	12.855512	13.659354	12.706881	13.250094	12.929387	12.725852	11.730636	13.354386	13.228843	11.586749	12.905355	12.780890	12.373862	13.044264	12.545063	12.281273	13.852979	12.423579	12.783797	12.849137	12.598156	12.144213	13.088340	12.655347	13.158236	12.979696	11.162452	12.818568	12.380673	11.980478	13.227239	12.983527	12.897590	13.191043	12.594761	13.682594	13.046278	12.570017	11.081888	12.077573	13.304338	12.554944	13.232053	11.970814	12.180653	12.672776	12.980105	12.914314	12.109069	12.594923	12.373219	11.902027	12.456759	13.417727	12.555591	12.234198	12.934369	11.635407	12.767801	12.514664	11.559388	12.918404	12.885476	13.611868	12.021503	11.956209	12.240559	12.086504	12.465654	12.731336	12.322814	12.955360	13.314357	12.686920	12.373381	12.556219	11.866837	14.238479	12.666814	12.316848	12.738484	12.699016	13.500645	13.085733	12.065618	11.861545	12.175782	13.076484	12.818205	12.686046	13.054557	13.191466	12.149438	13.108505	12.703486

AC105389.2	10.638766	10.375600	10.926394	10.116521	10.511571	10.356772	10.443501	11.392045	10.463567	10.565611	10.754335	10.431566	10.055446	11.015142	10.043161	10.513312	10.247271	10.518615	9.776589	10.956655	10.363564	11.221771	9.002292	9.962703	9.842744	10.095615	9.037545	10.868295	9.311872	9.414865	9.209476	10.959734	10.761456	8.124953	10.589798	11.232423	11.092968	10.967244	10.532841	10.597077	10.240313	10.645622	11.409053	11.315712	11.859051	11.271173	9.911427	10.709515	10.809966	11.458006	11.406317	11.481940	11.061273	10.938612	11.123080	10.075427	11.056273	10.750993	11.447786	11.263943	11.144860	9.649021	11.089443	10.104632	9.879312	10.755737	10.614802	10.299712	10.300662	10.928442	10.499717	10.402738	10.779119	10.646637	10.713575	10.760108	10.530696	10.864236	10.994776	10.877792	10.717161	10.726410	10.695729	11.026609	10.630708	10.683914	11.039023	11.127958	10.296801	10.692703	10.370938	10.388515	10.797903	10.915674	10.479922	10.322594	10.601671	10.075427	10.800584	9.814948	10.679432	10.402004	9.647368	10.470285	9.779667	10.503158	11.792707	10.303257	9.236897	9.731236	10.152953	10.911171	11.345621	9.269228	10.582460	8.732236	9.865136	10.428377	9.181391	12.000733	8.806738	9.362915	11.943795	8.289502	8.744057	8.289941	6.693087	5.715301	9.588264	8.875559	8.242339	11.653194	9.961344	8.466587	8.912217	12.453263	9.167693	6.969499	10.312904	7.171017	7.503220	8.537536	8.028111	9.375129	7.585490	10.153421	8.371749	6.595007	7.922093	7.035923	8.536970	9.785797	7.237820	10.251030	10.534558	10.372281	8.472017	9.191505	9.999208	7.863395	9.243968	11.868456	8.437404	10.109887	10.592327	5.030756	9.374309	9.488302	9.421160	10.955581	9.541747	9.899742	9.292820	10.052797	9.157407	9.809526	9.270142	11.036408	9.340740	12.113307	9.046235	9.583773	6.803991	9.630914	9.378381	10.097156	7.990967	10.568013	10.360509	9.922734	8.072573	7.669595	10.636673	9.439867	9.794721	10.589660	8.853642	10.180089	9.864397	10.704907	8.894400	11.985558	8.934877	8.593456	9.839396	9.833356	9.192737	9.513967	10.196293	7.756974	8.376324	8.278740	9.867261	11.300978	8.582697	10.368806	8.959155	8.043558	10.416006	7.985306	7.308199	8.366526	8.381906	7.336598	6.858980	7.093070	8.697583	9.045496	5.614247	9.022477	8.510827	7.670842	8.706158	7.798982	6.831474	8.744057	11.739666	6.628874

AL731702.1	6.027116	6.847871	8.192596	6.673497	8.191798	7.058867	7.461032	7.428367	8.408039	7.420547	7.451641	6.514226	7.400671	7.837979	7.966042	7.042723	6.185545	7.578551	8.729427	7.289104	7.576995	7.097666	9.637166	7.585336	7.821963	7.540366	6.440719	5.290178	7.112374	6.972302	7.459660	8.047176	7.391321	6.541025	7.237820	7.230711	7.707285	7.306185	7.120590	7.558907	7.495997	8.255721	7.330723	8.756854	8.395299	7.829763	6.274977	8.841504	7.783317	7.719642	7.306970	8.274770	7.433914	7.075119	8.949362	8.033453	7.637243	8.262615	7.467980	7.268933	7.549912	6.648884	7.323901	7.085535	8.021791	8.279636	7.380184	7.037634	7.311670	8.073673	7.004100	6.587371	6.668343	7.272190	7.325643	7.230410	7.585017	5.985373	7.030942	8.537385	8.015709	8.562395	7.671958	8.479812	7.563626	8.211887	7.231968	9.025237	8.267621	8.210651	7.605625	8.458269	8.075788	6.797851	6.941991	7.504305	8.672339	7.795409	6.822918	7.459660	8.370159	7.346579	7.579650	8.210366	7.835968	8.372078	6.682753	8.277666	7.938997	7.384691	8.151442	8.979397	8.292608	6.994765	8.645720	4.403324	7.094491	8.065802	7.319485	5.013417	5.100318	5.578314	6.622744	7.264337	4.881587	6.565450	7.527284	8.132391	9.802581	5.384847	5.953089	9.180017	3.757509	7.375318	7.342853	8.582845	8.101434	6.883579	7.299697	10.090076	9.519313	9.098599	8.043107	7.667152	4.458852	7.048016	7.824466	8.362054	7.856797	4.411759	4.132771	6.311781	5.812591	6.216306	8.052786	5.872251	7.237038	5.654240	10.288200	5.459048	9.278901	6.190442	5.925599	9.228967	9.787289	7.488204	7.632094	9.989959	6.999376	8.026668	9.137471	8.620648	8.311618	7.531429	10.314618	6.304265	7.844679	7.148828	6.075192	5.724374	6.703085	8.270740	8.534396	7.442516	4.604190	4.667764	6.383495	6.733689	8.107120	7.879615	7.630838	3.859612	6.901432	5.792732	6.258961	8.689858	7.125129	8.312531	8.191644	7.821006	7.709416	5.698775	7.008983	5.779986	7.613414	7.111138	8.174746	6.300828	6.340056	5.584179	5.478477	6.870984	5.814250	4.025811	4.195839	8.447497	9.341453	4.855630	9.348808	6.873056	6.994918	4.770748	7.363410	7.437391	8.673275	8.853327	8.003880	8.658051	5.078395	6.014517	5.206440	4.249492	6.253660	6.488381	4.185118	9.208388	7.678603	6.020997

LINC02609	4.647688	3.161074	3.475076	3.833785	3.690742	3.813595	3.640119	3.852717	3.213188	3.683640	4.673167	3.207427	3.985401	3.527928	3.018736	3.670528	3.967702	2.873295	4.693081	3.514088	3.737871	4.167541	5.754253	3.810560	3.036937	4.256493	4.642176	3.533669	3.706050	4.160768	3.596131	3.659820	3.544326	3.604453	3.198153	3.888276	4.599883	3.652214	3.555075	4.294797	3.360006	3.364370	3.936230	4.226825	4.065226	4.687813	6.016047	3.018363	3.488662	3.349361	3.223215	3.205204	3.477093	3.975729	3.547776	3.763395	4.025591	3.325316	3.054254	3.606829	3.081441	3.799232	4.430031	4.139961	3.579575	4.072762	3.956800	3.487910	3.661837	3.972456	3.729450	3.239326	3.613360	3.443636	4.157992	3.265578	3.460015	3.432363	4.418002	3.474511	3.188883	3.606610	4.124861	3.625390	2.981173	5.021154	4.398722	3.675168	3.778757	3.367892	4.521287	4.434340	3.600877	3.286535	3.737871	3.633323	4.072332	4.178097	3.656029	4.475158	3.961168	3.445453	3.956576	3.955259	4.438927	3.410237	7.476921	4.439375	3.833571	4.338339	3.682453	4.111173	3.839112	3.052172	3.285959	4.482201	3.735228	3.707077	3.846944	3.859999	3.781036	4.040813	3.838007	4.443074	4.044741	4.529462	5.289165	3.755678	4.762486	4.438496	4.142802	3.386535	4.954780	4.049784	3.754254	3.739768	4.360482	5.924182	4.970691	3.278696	4.808479	5.572010	4.340238	4.002780	4.874867	4.477618	3.676420	4.256493	3.374079	5.398443	3.534455	3.547179	3.664440	4.249061	4.497063	4.535899	4.805149	3.684214	3.569686	4.404426	3.287419	4.568306	3.303158	4.329886	4.301736	5.942682	2.912576	3.684214	3.117845	3.091429	4.372359	5.059000	4.101263	3.926325	4.287666	3.691579	4.428477	2.954328	3.478013	2.955694	3.718118	4.282734	4.403745	3.560924	3.582455	3.229455	3.297612	4.406043	2.981538	4.000428	3.273704	4.990182	3.889854	3.665884	3.329538	4.167983	3.710484	4.120757	3.460186	3.433628	3.808277	3.452949	3.808458	5.492799	4.583521	2.970090	4.531195	3.529043	3.295582	4.832287	5.149087	3.605404	3.183560	3.681816	3.799232	4.295235	3.979150	3.762565	5.244612	3.699776	5.226305	3.878287	4.623113	4.849095	3.756526	7.717516	4.891867	3.731092	4.857330	4.465207	4.416652	4.831465	4.539078	3.236086	3.880658	3.736901	3.720796	4.971704

LINC02788	4.647688	3.161074	3.475076	3.833785	3.690742	3.813595	3.640119	3.852717	3.213188	3.683640	4.673167	3.207427	3.985401	3.527928	3.018736	3.670528	3.967702	2.873295	4.693081	3.514088	3.737871	4.167541	5.754253	3.810560	3.036937	4.256493	4.642176	3.533669	3.706050	4.160768	3.596131	3.659820	3.544326	3.604453	3.198153	3.888276	4.599883	3.652214	3.555075	4.294797	3.360006	3.364370	3.936230	4.226825	4.065226	4.687813	6.016047	3.018363	3.488662	3.349361	3.223215	3.205204	3.477093	3.975729	3.547776	3.763395	4.025591	3.325316	3.054254	3.606829	3.081441	3.799232	4.430031	4.139961	3.579575	4.072762	3.956800	3.487910	3.661837	3.972456	3.729450	3.239326	3.613360	3.443636	4.157992	3.265578	3.460015	3.432363	4.418002	3.474511	3.188883	3.606610	4.124861	3.625390	2.981173	5.021154	4.398722	3.675168	3.778757	3.367892	4.521287	4.434340	3.600877	3.286535	3.737871	3.633323	4.072332	4.178097	3.656029	4.475158	3.961168	3.445453	3.956576	3.955259	4.438927	3.410237	7.476921	4.439375	3.833571	4.338339	3.682453	4.111173	3.839112	3.052172	3.285959	4.482201	3.735228	3.707077	3.846944	3.859999	3.781036	4.040813	3.838007	4.443074	4.044741	4.529462	5.289165	3.755678	4.762486	4.438496	4.142802	3.386535	4.954780	4.049784	3.754254	3.739768	4.360482	5.924182	4.970691	3.278696	4.808479	5.572010	4.340238	4.002780	4.874867	4.477618	3.676420	4.256493	3.374079	5.398443	3.534455	3.547179	3.664440	4.249061	4.497063	4.535899	4.805149	3.684214	3.569686	4.404426	3.287419	4.568306	3.303158	4.329886	4.301736	5.942682	2.912576	3.684214	3.117845	3.091429	4.372359	5.059000	4.101263	3.926325	4.287666	3.691579	4.428477	2.954328	3.478013	2.955694	3.718118	4.282734	4.403745	3.560924	3.582455	3.229455	3.297612	4.406043	2.981538	4.000428	3.273704	4.990182	3.889854	3.665884	3.329538	4.167983	3.710484	4.120757	3.460186	3.433628	3.808277	3.452949	3.808458	5.492799	4.583521	2.970090	4.531195	3.529043	3.295582	4.832287	5.149087	3.605404	3.183560	3.681816	3.799232	4.295235	3.979150	3.762565	5.244612	3.699776	5.226305	3.878287	4.623113	4.849095	3.756526	7.717516	4.891867	3.731092	4.857330	4.465207	4.416652	4.831465	4.539078	3.236086	3.880658	3.736901	3.720796	4.971704

AC002066.1	3.280866	3.425458	3.548911	3.518201	3.178536	3.445453	4.461347	3.534455	4.566368	3.831373	3.928725	3.598945	3.269270	3.727937	3.384830	3.145152	3.751073	2.963216	4.597046	3.431688	4.751493	4.791656	7.509115	3.090316	3.936663	3.561768	3.279422	3.859821	3.564573	3.166625	3.805753	4.205490	3.642272	3.414533	4.665614	4.040813	3.578183	3.272988	3.777351	4.112439	3.400057	3.278379	5.493884	3.329682	3.725577	7.379718	3.741157	2.839800	5.336960	3.922669	3.805556	3.751073	4.172286	4.221265	3.996626	3.166776	4.163583	4.148136	2.936269	3.624461	2.837918	3.159921	4.335462	4.511172	3.836533	3.859401	3.037234	3.921050	3.790786	2.849361	3.234681	3.711134	3.700152	3.329843	3.106189	3.357588	4.234619	3.836113	3.509610	3.783146	3.997680	4.076809	3.591699	4.669058	3.915969	5.256331	3.901276	3.938998	3.811402	4.449155	4.052167	3.957225	3.724528	3.502367	3.312266	3.363039	3.829065	3.659062	3.275887	3.477266	3.436845	3.254178	3.685860	2.891627	4.557070	3.274197	6.596333	4.208850	4.050645	4.053659	3.284905	3.455487	3.753157	3.389637	3.740582	3.657866	3.638275	3.429708	3.175414	3.085290	4.297220	3.141186	3.767775	3.932604	3.661635	3.390005	3.290588	4.902978	3.807616	4.809737	3.855323	3.859612	3.363192	4.917062	4.374125	4.126337	3.211448	3.590513	3.366336	4.018482	3.246230	5.151351	3.149126	2.801819	4.367399	3.207657	3.191453	3.041879	3.413277	3.632948	3.491185	3.610373	3.254679	4.189080	3.743677	3.632948	3.232167	3.573829	3.498362	3.976335	3.017403	5.166466	3.692563	4.220124	6.622590	4.232435	2.804194	5.197929	3.888906	3.686638	3.569066	4.123402	4.343480	3.974446	3.079184	3.803679	3.494456	2.831266	3.292959	2.787050	3.273883	3.716082	4.533920	3.904846	3.972456	2.968089	3.740182	3.791654	2.804194	3.250849	3.826564	4.073227	3.848412	3.355877	3.510886	3.852937	4.448013	3.566891	3.323651	3.949658	4.511602	4.078077	3.583046	4.545772	3.940444	4.210561	3.442756	3.477470	3.846944	3.441518	5.168494	3.503137	5.924711	3.275739	3.851853	3.260006	3.927615	3.946203	3.245109	3.710057	3.206648	3.756526	3.476705	4.891666	3.532333	6.110060	3.617825	4.284509	3.808458	3.428672	3.747700	3.850978	3.297455	3.788448	3.581102	3.179398	3.808277	3.991816

MIR99AHG	8.406973	9.159549	10.791439	9.938377	10.620851	9.706727	10.236515	9.248671	10.380607	10.051559	10.231158	10.035467	10.613754	10.161066	10.379863	10.727170	9.783941	10.035161	11.376591	10.353264	10.831500	10.803584	12.161724	10.510632	10.225137	10.785727	9.533221	9.998125	11.052514	9.734545	11.454008	10.101089	9.721381	9.013309	10.006313	9.719876	10.088070	9.705954	10.009587	10.112589	10.411451	10.216690	10.030684	10.487949	10.641213	9.784423	10.103883	10.292031	10.007811	10.436435	9.911427	10.991323	10.476015	10.464170	10.192816	10.390190	10.328083	10.172605	9.683738	9.920576	10.509173	10.468588	10.813141	10.389435	10.123198	10.333610	9.973403	9.734702	9.279219	10.141818	10.332520	10.126788	10.798200	10.197218	10.321391	9.872723	9.678939	9.628799	9.965758	10.091582	9.341602	10.639071	10.101264	10.592463	10.472696	10.898647	10.803584	10.339746	10.577237	10.182107	10.151110	10.611842	9.927691	9.718772	9.519473	10.686890	10.553536	11.431505	10.118219	10.617300	10.564425	10.113627	10.583893	9.900517	9.205043	10.451538	10.847998	9.683095	11.057327	10.255734	11.248223	10.774758	9.617309	10.430953	9.779969	9.170296	7.916296	10.216098	10.981465	9.340740	5.828214	9.953787	9.190397	7.537165	8.869757	10.251178	10.727045	9.823468	8.000720	9.106280	6.094614	7.899114	9.343930	4.946094	9.551619	6.885035	7.470014	10.799956	11.650510	9.031417	9.533834	9.467958	9.508545	7.057287	10.198572	9.296135	7.668348	9.512608	8.444544	8.710094	8.303938	9.487347	7.309276	8.625594	8.147162	9.700346	9.708902	7.554088	7.479190	9.576673	8.823280	9.424825	8.431048	9.296921	7.629075	8.799286	9.033411	8.347493	8.490290	9.104906	8.518092	8.261975	7.376878	9.150640	10.852730	9.378381	7.941031	8.192133	8.550581	7.864325	8.143459	7.085071	6.599911	8.606356	8.424967	7.787922	8.478374	7.282413	10.681103	7.757898	9.226696	8.875559	8.750190	9.342205	9.409267	8.353369	7.892783	9.295062	9.765127	8.263850	7.674467	7.819359	9.590890	9.123526	8.024983	9.025237	10.668858	9.516518	9.363205	6.219753	6.481749	8.981956	8.415496	9.695275	9.170296	9.356515	10.605340	9.008878	7.592260	7.919191	8.444854	9.716397	7.746079	8.637867	7.708703	10.155158	9.134862	7.442823	6.917569	8.294571	9.179536	8.034493	8.953479	7.134995	10.300168	7.682889	10.489958	10.470736

LINC02770	5.936160	6.438569	6.548360	6.478984	6.278646	8.804154	6.949978	7.406485	5.709005	6.480089	6.101011	6.440058	5.426670	6.442361	5.514046	6.966936	6.174728	5.841158	6.375158	6.529154	6.553821	6.859944	7.746256	6.228136	6.224856	6.371935	5.952342	6.229383	6.463206	7.472951	6.202255	6.316062	5.668695	5.248396	6.242444	6.094614	6.392873	6.548037	6.406893	5.896338	7.141280	6.046054	6.073647	5.870061	5.890305	6.216805	6.403592	6.389008	6.254868	7.687797	5.778551	6.298242	5.589300	5.966988	5.872251	6.824026	5.169309	6.267418	5.695854	5.299860	6.195674	6.431572	6.213374	5.875848	6.156680	6.137696	6.601510	6.880073	6.632896	6.494116	7.786526	7.417421	6.569708	7.330879	7.921494	6.347421	7.004729	6.392873	6.861077	5.397296	6.227645	5.514643	5.619100	5.544106	5.992513	5.005368	6.338411	6.202616	5.732104	6.453644	5.946886	5.860683	6.289442	6.146720	5.720867	6.186042	5.621291	6.327073	5.834643	6.227294	6.097354	6.461996	6.016394	6.396973	6.031288	6.872894	6.846929	6.008773	6.205920	7.214976	6.734479	6.327896	5.912957	7.311495	6.793969	6.956719	5.756083	6.455432	6.315398	5.850596	5.968264	5.854561	5.923827	6.431739	9.110310	6.151211	5.720507	5.248396	6.235642	5.829507	6.391212	5.928833	6.024120	5.784311	6.363905	6.002904	5.253951	6.422080	5.815094	6.638386	6.891902	7.315893	6.819593	6.456215	5.819037	6.344898	6.151907	6.687309	6.788410	5.699868	6.166148	5.271843	5.844914	5.810937	6.190268	5.793485	6.007549	6.053214	6.317412	6.477451	5.993944	6.166148	5.559669	5.282173	7.424453	6.422390	5.377881	5.740630	7.174321	5.851850	5.443327	6.056179	4.732510	5.357204	6.142058	5.888319	5.854925	6.293351	5.107426	6.115466	6.441853	5.756083	6.520020	6.110971	5.747563	6.162362	6.253010	6.204845	6.029076	6.703546	6.446362	6.566103	6.080567	6.406553	5.913125	6.130691	5.998669	6.507640	5.413006	4.894172	5.669795	6.064868	5.776559	5.746680	4.828464	5.888143	5.541842	5.684727	5.788289	5.559669	5.724545	5.977794	5.869888	5.724200	6.046736	4.971305	6.481749	5.374347	6.103246	5.234417	5.818870	6.021366	5.686052	5.781818	6.577011	7.196156	6.168963	6.485635	6.329228	6.153294	5.761966	8.128868	7.699612	6.978170	5.779280	5.869021	6.649670	6.276695

AC009365.3	3.353521	3.965972	3.287730	3.676220	4.356767	3.791856	3.387383	4.749752	4.072108	3.466290	3.808277	3.791654	3.412024	4.213645	5.306753	3.248706	3.683451	3.311024	4.872775	3.261577	5.377508	2.864862	6.389857	3.792471	3.601290	4.043227	3.897372	2.877393	3.279275	3.494265	3.802024	4.441499	3.827419	3.137608	4.647039	4.196670	3.782082	3.825737	4.390178	3.256343	3.594399	3.291768	3.824871	3.896056	3.824156	5.761577	3.968740	3.069827	5.030342	4.103884	4.048215	3.880019	4.499424	4.376322	4.067317	3.626618	4.272884	3.386973	3.776362	3.917902	2.906081	3.162277	3.923504	3.799869	4.963356	4.028172	2.855606	3.931917	4.276840	2.844900	3.123904	3.890731	3.259859	3.191743	2.910104	3.406764	3.519870	4.259773	3.957709	3.884029	3.489376	4.172494	3.564009	4.353327	3.727364	3.766295	5.339128	3.333065	3.499062	4.064154	3.719743	3.430468	3.761533	3.261577	3.666461	3.561360	3.938111	3.218112	3.292809	3.762166	4.301945	3.305233	4.065417	2.840727	4.699871	4.887696	3.701392	4.338129	3.952698	3.470503	5.150345	4.749312	4.925093	3.376363	3.558934	6.352776	4.427447	3.140544	3.261306	3.456978	3.638875	3.839112	3.463379	4.288341	3.810767	4.209499	3.368431	3.723511	3.201820	3.490659	3.482786	3.516064	3.800298	5.369503	3.170770	4.000224	4.427011	3.439246	3.535381	4.647475	3.849284	5.763409	4.552175	5.221107	3.322641	4.648966	4.083468	4.053015	3.683070	3.570423	3.516425	3.764496	4.669702	4.444117	3.870938	3.830779	3.532925	3.409363	4.889825	3.410757	2.992210	3.738525	4.088500	4.357398	5.303245	4.120097	2.936439	5.196697	4.190383	4.091404	3.709474	4.308141	4.572417	5.888505	3.201820	3.886347	3.207928	3.383664	3.839470	4.032992	3.306340	3.985401	3.879790	3.953338	4.456047	2.840406	3.697132	3.454178	2.809656	3.076788	3.213397	3.266904	3.384000	3.484451	3.382935	3.676220	3.696307	4.077226	3.702040	3.890045	4.642176	3.666279	3.870512	3.620461	5.167871	3.689322	3.566286	3.133914	4.656631	3.850572	3.702040	3.437226	3.364044	3.194860	3.105015	3.751463	3.922444	4.020460	3.335812	3.720576	3.161219	3.626213	4.603540	5.081456	3.219514	4.000224	3.701819	3.779373	3.235659	5.536657	5.490169	5.231386	3.271463	3.311179	4.452678	4.285690	3.182313	3.418075

AC093159.1	6.954230	6.985880	7.827616	7.690085	9.020620	8.242339	8.549225	7.961924	9.944985	7.367347	7.595624	8.452168	9.426185	8.157804	8.197159	8.659902	9.138750	7.694879	9.575333	8.150523	8.467127	8.936240	7.946637	9.089471	8.668344	8.059157	6.674449	7.539270	7.859735	6.568387	8.534543	8.810910	7.645570	7.088332	8.678046	7.779666	7.516478	8.608484	8.336479	8.544002	8.996430	8.273739	6.950755	7.678603	7.501657	8.392872	7.749037	8.272248	8.831339	8.469173	6.541364	8.886993	7.003520	8.401902	8.620493	8.259246	7.638001	7.345653	9.133821	8.184642	9.112102	6.752706	5.917236	8.192295	9.012546	9.759027	8.216662	8.008230	8.004183	8.875702	8.195596	8.035393	7.253245	8.236995	9.084861	7.328812	8.231949	8.111918	8.101742	9.130781	8.259246	9.394200	7.665138	9.028257	8.300907	9.338178	8.324774	9.071428	8.679908	7.630529	8.096768	8.392729	8.282853	8.563436	6.616937	7.692886	8.290249	8.361892	9.985187	8.648119	7.913282	8.469498	8.284375	9.327512	9.729974	9.351844	8.992714	9.700030	8.636601	9.190703	8.296244	9.057352	9.020620	8.217923	7.386991	5.337135	6.967094	8.742397	8.056526	6.288447	6.289603	5.631190	6.142242	6.754906	6.791839	5.637695	6.402768	6.026589	6.879880	6.703996	6.370564	6.617903	5.545624	6.136534	9.752686	6.612852	5.287769	6.187399	6.524190	9.037545	7.474898	7.967552	7.430932	6.049248	6.165285	6.282111	6.982874	8.889464	6.100310	6.216805	5.971779	7.146440	7.072417	6.607556	6.121473	7.309276	6.730623	6.538915	7.713269	6.377625	7.713603	5.572010	6.751932	10.559179	6.602789	6.952175	5.978683	8.283918	7.016333	6.833280	6.437128	7.043338	6.382496	6.233583	5.667246	5.494080	6.187067	6.766184	6.347920	6.733538	6.096514	7.961004	6.767961	6.959726	6.338219	6.879422	6.897378	8.465055	6.841755	7.552537	7.847219	6.516905	7.405370	7.896712	6.703237	7.248226	6.538445	8.003880	6.838662	6.279147	8.297767	5.952712	5.947212	6.259647	7.315270	6.713926	6.537941	8.722507	6.186736	5.898668	7.362762	7.805727	6.608183	7.396775	8.127674	6.439408	6.140472	7.488806	6.312644	5.049595	7.381252	6.381308	6.850460	6.142569	7.316049	6.974149	6.751134	6.978653	6.414360	7.140023	6.055659	6.340875	7.083620	5.899028	6.130691	7.693673	7.207379	6.483410

AC233992.1	14.560951	13.703898	14.020108	13.867818	14.060320	13.532613	13.713659	13.646573	13.668871	13.483216	13.590518	13.639891	13.819661	13.731903	14.121021	13.806501	13.628713	13.802855	14.545966	13.866741	14.157364	14.263585	15.266520	13.996448	13.461357	13.519583	14.105360	13.453090	13.605949	13.579662	13.927324	14.169985	13.625234	13.540808	13.484384	13.508794	13.622440	14.234212	13.783082	13.935762	13.791726	14.165841	13.488803	13.671748	13.693583	13.342327	13.689737	14.245947	13.555459	13.158422	13.411431	13.768298	13.288424	13.260449	13.589804	13.772438	14.044663	13.013913	13.915290	13.791595	14.203355	13.563867	14.810589	14.193358	14.568485	14.117673	13.443956	14.095205	13.706089	14.302959	13.237824	13.443082	13.771102	13.628936	13.764461	13.967702	13.491711	13.380743	13.533746	14.037892	13.956288	13.617542	13.517555	14.292020	13.382599	13.371858	13.901790	13.901790	14.564295	13.900187	13.966624	14.245669	14.256351	13.593974	13.576715	14.146140	13.782074	13.635217	13.989848	13.866741	13.801889	13.970475	14.122158	13.876872	14.319186	13.824511	14.427447	14.726520	14.553109	13.679001	14.818366	14.125236	14.247254	13.778222	13.957131	13.464192	14.625115	13.848828	13.579215	13.308547	14.954990	14.392249	14.870791	14.625115	14.571403	14.472714	14.678701	14.737586	14.706135	14.558039	14.568889	14.667309	15.011117	14.735321	14.256694	14.552678	14.708439	15.375547	14.019845	15.418602	15.272298	15.451210	14.494365	15.305847	14.918365	14.144293	15.042927	14.611021	14.171283	14.972581	14.236249	14.318185	14.486754	14.459642	14.255462	14.167438	14.063565	15.337535	15.041266	14.416536	15.035758	14.325416	14.751210	14.396563	14.627696	14.627696	13.884891	14.338582	14.559663	14.456152	14.054182	14.195897	14.820339	14.456152	14.460448	13.955482	14.800886	14.517397	14.329971	14.335422	14.565911	14.902407	15.202396	14.556424	14.509550	14.917362	14.431588	14.571403	14.558412	14.492765	14.446360	14.640937	14.473933	14.497531	14.101917	14.481669	14.731220	14.381329	14.096118	14.025054	14.522236	14.511123	13.747078	14.950163	14.796433	14.482460	14.799897	14.868819	14.922259	15.015078	15.403893	14.350592	14.780890	14.368027	14.163659	14.435670	14.567967	14.511941	14.061795	14.419090	14.135120	14.300251	14.247926	15.585396	14.378847	14.767089	15.514297	15.317503	14.611021	15.241853	14.708439	14.763822	14.004726	14.050643	14.523924	14.264573	14.562573	14.932454

ZNF503-AS2	9.897656	9.856355	10.608316	10.158031	10.879442	10.330692	9.760564	9.945742	10.807817	10.025602	9.842448	9.711552	10.254211	11.342479	10.095036	10.297097	9.795943	10.670159	10.711413	10.586401	11.059375	10.490251	10.256330	10.464170	10.800584	10.220346	10.036640	9.746469	9.270748	9.028879	11.346810	10.396585	10.086994	9.626458	10.450191	10.186829	10.395050	10.508722	10.819554	11.016907	9.831389	10.497236	9.898827	10.228208	10.334644	10.532043	9.923526	9.893477	9.308024	10.417045	10.187258	10.307273	10.152177	10.447031	9.657205	9.571204	10.895475	9.804238	10.577397	11.166523	11.344019	9.791578	9.801645	10.949809	10.498280	10.067357	10.880317	10.841416	10.315198	10.709053	9.701744	10.082544	9.903802	10.107612	10.581438	9.438812	11.038010	9.719718	10.060910	10.341990	10.096988	10.010612	10.268977	10.947159	10.358566	10.216548	10.423303	10.790142	10.960769	10.454651	10.815522	11.229610	10.566346	10.363870	10.453118	10.700452	9.640101	10.478435	10.511993	8.107736	9.889523	9.537835	10.054975	10.021856	10.309726	10.201859	11.161265	10.734602	10.936835	10.008153	11.233616	10.973802	10.909151	10.031790	9.891032	7.679371	10.660096	9.818272	9.830772	10.096699	9.225910	10.671810	11.186429	10.991775	11.502905	10.521111	10.397331	10.960028	12.719723	9.594314	11.203302	9.239986	9.084076	8.536093	11.586188	9.192582	11.314216	12.234834	11.006688	8.978023	11.582424	11.199882	11.357241	11.328644	8.953642	9.497649	8.897960	8.486274	9.578383	10.013258	9.069592	10.167210	9.921529	9.919156	10.095036	10.338699	10.251612	9.267981	11.013672	11.203302	10.499007	9.235036	9.645339	10.785111	9.704886	9.486588	10.215042	10.130189	10.519634	10.463567	10.913253	10.252635	9.848797	10.654131	10.200381	11.572679	11.054661	10.504977	11.203446	9.880112	10.495573	9.587330	10.425458	11.833609	10.295385	11.930143	10.243674	9.428192	9.157407	10.141818	10.134987	9.177716	9.875439	10.626696	10.445210	9.653700	10.029952	10.475871	10.376965	10.415101	10.551138	11.102385	11.187132	10.149344	11.938203	10.735785	11.339682	10.847549	10.867436	11.667440	9.117730	10.533175	9.343930	10.792335	11.004092	10.292953	12.282409	9.796717	9.050083	10.423140	10.720767	10.120036	9.356515	9.347557	10.641061	11.376371	14.172567	10.908236	8.971882	11.478703	10.219377	8.822507	10.191313	10.744115	8.660855	10.849009	12.226148	8.655658

AL135908.1	3.093983	3.341315	3.221235	3.021035	2.989506	3.701577	3.120835	3.251540	3.290518	3.086361	3.337787	3.367892	3.309363	3.776955	3.295141	3.174479	4.297429	3.020128	3.014468	3.177227	4.495551	3.076437	5.036741	3.112633	3.792920	2.989605	3.309058	2.937913	3.194590	3.053243	3.725335	4.626946	3.679225	3.022268	4.147276	3.927387	3.565517	3.264389	3.499242	3.490659	3.469807	4.007805	3.470346	3.195044	3.629852	5.526878	3.296519	3.761124	3.547179	3.556975	3.417894	3.566681	4.265722	4.111616	4.164884	3.110353	4.102158	3.716933	4.996956	3.828051	3.171671	3.124286	3.829515	3.236957	3.461488	3.507422	3.035971	3.218112	3.261577	3.033581	3.041027	2.947624	3.299913	2.924387	2.969198	3.315690	3.078385	3.022471	2.946960	4.922562	3.219976	3.700564	3.344796	3.951215	3.280243	3.562864	2.948767	3.248562	3.623459	3.382935	3.227317	3.024570	3.689939	3.157304	2.932827	2.938239	3.664050	3.260006	3.147032	3.097035	2.894688	3.097619	2.973434	2.813126	3.320743	3.273291	3.103750	3.431688	3.045780	3.214463	2.994563	3.451718	2.949261	3.249846	3.481157	2.902203	2.956804	3.123904	3.222255	3.023395	3.447839	2.945275	3.061222	2.871971	3.887402	2.910264	3.141052	3.263834	2.971440	3.094765	2.979449	3.021035	3.184361	3.080594	2.960684	3.918102	2.897589	3.051004	3.228359	3.885110	2.923134	5.218337	2.984243	3.023096	2.925521	2.933510	2.892692	2.982448	3.458581	3.547179	3.472373	3.650983	3.016992	3.340106	3.631499	3.557381	3.289532	3.423315	3.269432	3.209656	4.225750	3.545073	3.240178	4.037145	5.118456	3.242519	3.277400	3.743889	3.390175	3.322010	3.289829	4.082724	4.218585	3.432543	3.010961	3.766295	3.785421	4.709478	3.868783	2.976228	3.247680	3.908575	3.643475	3.729835	3.715250	3.041132	3.504538	3.455894	2.998343	2.987511	2.996629	3.233782	2.940324	2.916473	3.299180	3.065747	2.970179	3.007561	4.214051	3.551024	4.049098	3.368594	3.576932	3.472373	3.050591	3.337787	3.346564	3.337147	3.355340	3.395581	2.977589	3.356380	3.263555	3.109451	2.938338	2.928074	3.015774	3.891153	3.232699	3.020128	2.970272	2.976228	2.753408	3.434147	3.204421	3.183432	3.343001	3.444894	3.158149	2.916788	3.007067	3.819476	3.764496	3.069128	2.851879	3.019759	3.150377	3.294203

LINC01980	3.775550	3.570038	4.820845	3.427251	3.475634	4.078461	3.297133	4.784747	4.809737	4.121850	3.757311	3.851412	3.411118	4.300245	4.209292	3.247376	4.667150	3.910981	4.992029	3.990254	5.621291	3.936016	3.585700	3.786732	3.232555	3.986069	6.934258	3.534991	3.297290	3.671942	4.207295	4.806580	3.995744	7.278613	3.773481	4.175090	3.791187	3.463925	3.683070	4.157373	3.467486	4.252167	3.604028	3.410970	3.739374	5.763225	3.322641	3.391201	3.770527	3.952483	3.444367	4.409608	4.291559	4.356986	4.026873	3.624648	4.270937	4.038661	4.106979	4.073434	3.588432	3.517979	3.976140	3.777146	5.552587	3.585898	2.919675	3.597536	4.303765	2.960877	3.655820	3.230901	3.354345	3.090632	2.837184	3.372905	3.573638	4.375223	3.120348	3.902531	3.984782	3.169527	4.169012	4.418002	3.436171	3.711334	3.440832	3.877608	3.764711	4.138630	3.730244	4.121401	4.745112	4.707447	3.825093	3.385855	3.666279	2.959828	3.884687	3.376176	3.089786	3.787161	3.283173	3.153417	4.186897	3.789934	4.895184	4.768483	4.165343	3.229611	3.544693	4.069444	3.147411	3.314483	4.524143	3.993947	3.930876	4.329472	3.565159	7.495361	3.680445	7.452118	6.808084	7.166091	8.500592	6.951242	9.812137	10.235279	8.683233	6.998328	7.467717	4.702139	5.282775	4.529240	8.156736	8.906727	8.220099	6.795557	5.496448	6.754404	6.707704	3.569686	7.423039	4.780742	6.710243	3.992227	3.574795	7.375004	6.457727	8.956668	7.508788	6.853335	8.288418	3.646801	7.490867	3.833118	5.834461	4.418689	6.039824	8.299261	4.770981	6.253840	3.814603	5.346943	5.528211	8.569415	5.819906	7.050375	4.840025	6.392190	7.390858	7.240235	4.682249	6.456040	7.882461	3.886551	4.964988	5.552778	4.509201	6.738272	7.762841	4.058018	5.724934	5.731037	3.982401	5.918146	4.107435	4.801541	2.901917	5.607219	6.627417	8.125401	4.881804	6.031651	6.313672	5.106852	6.618065	4.912822	4.697230	7.493678	7.395399	6.597774	6.880073	3.556216	6.972302	6.871946	7.984384	3.448612	5.656838	3.567103	3.754459	7.100497	5.588122	7.268179	7.244210	8.131646	8.313053	8.353690	5.723098	6.084585	8.118240	6.314873	5.307133	7.277044	9.223728	9.194792	7.647559	5.942491	6.865815	4.567244	6.472916	4.018708	5.312014	8.013217	8.027961	7.132283	8.895157	7.626255

BRWD1-AS2	13.802610	9.734852	9.516845	9.727021	10.006313	10.194914	9.609148	11.012454	10.809816	9.813689	10.305962	10.015553	9.698632	9.983520	10.286805	10.269714	10.766127	9.845032	9.836892	9.876085	9.659506	10.704617	9.566353	9.895040	9.741675	9.865926	10.321672	10.703174	9.986107	9.995732	10.247122	9.281202	9.269835	9.644557	9.474009	9.419599	9.333386	9.493154	9.976047	10.457422	9.925456	9.593217	9.492722	9.408176	9.177867	9.530388	10.489183	9.815427	11.319554	9.684363	9.689001	9.589014	9.343442	9.555229	9.870429	10.622189	9.204887	9.978283	9.502229	9.244246	9.758866	10.089778	9.698167	9.843353	9.549294	9.663577	10.187701	9.903332	9.521604	10.105381	9.830616	9.869327	10.367747	9.768599	9.826469	9.691000	9.728699	9.871223	10.187118	9.419138	9.735623	9.574876	9.169683	9.781326	9.491735	9.427590	9.332164	9.942884	10.004105	10.006922	10.012445	9.924431	9.977842	9.827055	10.141187	9.542370	10.263285	9.460223	9.843946	10.369106	10.152807	9.435860	9.465354	9.879955	9.710156	10.980120	10.323206	9.858997	10.025602	10.222096	10.137446	10.668858	10.205821	10.522954	10.778399	11.935187	10.936234	9.970631	10.465042	10.072068	10.150520	10.312731	10.133901	10.973367	10.423927	10.501830	9.584367	10.144161	10.493600	10.209520	10.258038	10.213953	9.536731	10.741115	10.202155	10.041611	10.161228	9.175728	9.605311	9.798110	10.006313	10.978333	10.307862	10.280332	10.139408	11.106894	10.567716	10.584473	9.747411	10.006772	9.517769	9.217895	10.642536	9.518073	10.238936	9.553903	10.078682	10.338699	10.117756	11.267660	9.959230	9.254350	9.901158	9.824636	10.189954	11.122655	9.243650	9.422970	9.334161	9.625998	9.414244	10.014606	9.796567	9.117278	10.143702	9.504734	10.290812	9.766182	9.102439	10.238620	10.159386	9.885790	9.828718	9.531003	10.727170	9.894872	9.960892	10.038406	10.002883	9.714536	9.902261	11.607439	10.129759	9.750672	10.285020	9.613799	10.211080	10.785583	9.414400	8.646770	9.994317	9.375451	9.115266	8.899303	9.306635	9.822386	10.691815	9.762301	9.437418	10.344336	10.013689	10.123062	10.223316	10.113907	10.120195	9.800263	9.923356	9.825874	9.869327	9.892861	10.483985	10.212412	9.834281	10.371390	10.686890	10.231761	10.257870	10.072985	11.160953	10.163693	10.726410	10.364578	10.902031	10.311562	10.525956	10.036565	10.483232	10.268218

AL161443.1	9.990112	9.879955	9.725903	9.286274	9.614826	10.011667	10.385324	10.258997	10.233407	10.357244	9.670859	9.932341	9.836099	10.274568	9.920274	9.346950	9.282148	10.009895	9.390971	9.374625	9.434327	8.647397	9.323656	9.881777	9.279993	9.421602	9.196896	9.547923	9.996310	9.678129	9.474760	9.758117	9.369517	8.894400	9.746469	9.283708	9.938377	9.953047	9.747580	9.446710	9.975902	9.615149	8.716431	8.795062	8.743748	9.095706	9.185481	9.457241	9.263384	8.699239	9.119139	8.667886	9.090065	9.041468	8.975910	9.885790	8.489226	8.674365	9.054864	8.811379	8.898403	9.241660	8.950728	8.909199	8.668982	8.612161	9.536570	9.318499	9.630159	9.538904	9.771286	9.711248	9.888002	9.472625	9.598956	9.824482	9.967417	9.696024	9.369675	9.063070	8.986560	8.649966	8.905804	9.190703	8.654316	8.890530	9.011761	8.944434	9.277081	9.475967	9.274698	9.145192	9.452298	9.871517	8.898099	9.351844	9.311096	9.168609	9.204130	9.104906	9.569490	9.438812	9.415969	9.642697	9.911740	9.752532	9.360799	8.622183	9.355898	9.712784	9.743643	9.949359	9.764038	10.204002	9.717969	9.625524	10.121548	9.041327	8.915570	9.798602	10.005518	10.086677	9.486271	9.692075	9.481049	10.100294	9.312647	9.897339	10.215648	9.312017	10.022449	9.896439	10.387334	9.468399	8.776457	9.175115	9.305858	9.839560	8.064734	9.193324	8.712976	9.915602	10.426856	8.104330	9.372889	9.402162	10.005361	9.893477	9.882240	9.950421	8.963573	8.314211	9.813541	9.650895	9.220054	9.258034	9.972796	9.864397	9.662498	10.366528	9.972475	8.353066	9.464238	9.105197	9.475061	9.912359	9.480095	9.665759	9.097512	9.463598	8.893945	8.990911	9.134397	8.560387	10.194475	8.922407	8.061379	8.789635	9.068649	9.181882	9.480244	8.846625	9.499324	8.969751	8.350957	9.395272	9.503777	9.167554	9.710473	9.591343	10.043446	10.315629	9.351550	9.121392	9.999961	9.606716	9.748470	9.381126	9.155110	8.770072	9.039784	9.328138	8.854389	8.973205	8.726942	9.305574	8.933782	9.183598	9.030423	8.981654	9.082581	9.552825	9.563744	9.701427	9.106435	8.564961	8.414300	9.415969	9.730122	9.842056	8.760915	8.712055	9.672549	9.376209	10.859835	9.417325	8.402976	9.310879	9.757807	9.652120	9.922734	9.145493	10.110348	10.021856	8.911573	10.473776	9.404197	9.472022

AC009226.1	3.341666	3.232441	3.282710	3.336976	3.739374	3.498710	3.326154	3.292054	3.540716	3.374551	3.587651	3.697989	3.281352	4.003905	4.120097	3.283015	3.478207	3.709872	4.758717	3.530520	4.810612	3.649216	3.031543	3.309363	2.996841	3.453834	3.216520	2.879676	2.963216	3.201021	3.647551	3.369785	4.012684	3.645431	3.646235	4.069644	3.697351	3.317617	3.266581	3.857006	3.459278	3.274802	3.597934	3.853814	3.653615	4.624205	3.304598	3.223746	3.713806	3.954187	3.949881	3.552011	3.913757	4.023017	3.844161	3.087059	4.093706	3.716485	4.091630	3.723702	3.084967	3.325791	3.591339	3.176261	3.290129	3.749777	3.563042	3.255474	3.427251	2.901684	3.122628	3.491016	3.138701	3.392796	3.277400	3.317112	3.582844	3.502367	3.316203	3.712709	3.348550	3.873072	3.417894	3.764064	3.241417	4.383610	4.773254	4.004116	3.616879	3.589954	3.459671	3.623459	3.655248	3.246954	3.191322	3.726946	3.671371	3.393995	3.501795	3.371366	4.592901	3.229324	3.558339	3.524010	4.513800	3.167023	3.581900	3.300392	4.447326	3.237557	4.094749	3.576521	3.760706	3.571510	3.313340	6.021175	3.211164	3.451883	3.203604	3.118062	3.411831	3.113651	3.085290	3.955721	3.713156	3.482596	3.429708	4.085922	3.872419	3.736901	3.370996	3.373077	3.713806	3.689102	4.506329	3.379304	3.746283	3.859401	3.333864	4.158634	3.176741	2.973074	3.540335	4.809298	3.695280	4.132328	3.679225	4.341985	3.541683	3.306163	3.283480	3.828858	3.369248	3.589211	3.757509	3.717722	3.419492	3.238467	3.466886	3.627611	3.014178	3.618361	4.166251	4.068163	4.740850	3.609596	3.144107	3.991816	3.902531	3.920397	3.503137	3.847973	4.164473	3.888906	3.065854	3.878499	3.338286	3.923290	3.813595	2.958915	3.216108	3.555420	3.338619	3.473243	3.362333	2.987048	3.389306	3.192314	2.919864	3.130865	3.317617	3.533846	3.574795	3.612955	3.304598	3.989003	3.215814	3.648387	3.931418	3.589021	4.057171	3.537834	3.588270	3.570038	4.370890	3.522436	3.370996	3.317974	3.748966	3.222673	3.488296	3.407850	3.674735	3.381015	3.781660	3.824627	4.144750	3.432717	3.229919	3.812109	4.016698	4.189270	4.257644	4.844447	3.168276	3.011063	3.830142	3.255631	3.488296	3.368911	4.511393	6.807572	3.243970	3.374715	3.711134	2.978793	3.596131	3.324499

AC097518.2	3.649432	3.586706	2.988612	3.035867	3.452065	3.097963	3.781438	3.647002	3.404378	3.353204	3.545812	3.885512	3.223746	4.059980	3.694034	3.363712	3.428492	3.745894	3.384151	3.319276	4.912191	3.672321	5.785597	3.329843	3.477651	3.029478	3.530906	3.430988	3.542277	3.719948	5.465597	4.049784	4.565553	4.871916	3.644257	4.047589	3.743475	3.348203	4.081133	3.171902	3.444194	3.770910	3.393310	3.613168	3.463557	4.866527	3.796526	4.272884	5.332458	3.977405	4.020460	3.888504	4.015856	4.631548	3.553350	3.571157	3.822877	3.541494	3.909259	3.549307	3.771146	3.660212	3.312709	4.062633	3.493155	3.699591	2.868491	3.256611	3.087756	3.548911	2.942012	3.087522	3.078861	2.930955	2.996285	3.173051	2.912656	3.114865	3.146302	3.928725	3.142443	3.720995	3.124150	3.694454	3.025146	4.057364	2.973434	3.097370	2.966219	3.162054	3.504959	3.854271	2.901165	2.938155	2.997517	3.198271	3.616696	3.796720	3.946420	3.097370	2.953586	3.587651	3.152232	3.370825	3.110353	3.099402	3.849387	3.299620	3.886152	3.778359	3.567883	3.102636	5.304993	4.138230	4.154789	2.924925	3.202948	3.528493	4.286998	3.708077	3.225437	3.261577	3.292494	3.089684	3.514967	3.169872	3.520242	3.401766	3.147640	3.351386	3.468038	3.316489	3.391201	3.331764	3.085603	3.399682	3.274657	3.818420	3.334371	4.468617	3.137740	5.529534	3.353669	3.185015	3.249291	3.072069	3.737498	3.128578	3.526294	5.074237	3.349874	3.885512	4.567683	3.538456	3.743889	3.785850	3.422630	4.109814	3.501061	3.193913	3.699089	3.444725	4.016060	3.694253	4.849289	3.248143	4.338539	5.371479	4.119882	4.098850	3.756526	3.541120	3.898693	3.205204	3.330656	3.447475	3.437732	3.818633	3.662649	3.474700	3.594603	3.841707	3.324679	3.869875	3.139324	2.864326	3.527760	3.994432	3.625008	3.138475	3.157660	3.057912	2.973344	3.095680	3.173430	3.154663	3.293922	3.354345	3.657866	3.366336	3.864949	3.222673	3.497998	3.317617	3.189850	3.713156	3.399349	2.926987	3.490484	3.704272	3.501981	3.775313	3.823735	2.985985	3.537834	3.005736	2.907677	4.093706	3.197591	2.951652	3.336118	3.036263	2.991208	3.167789	3.432717	4.799860	3.300958	3.466290	3.370267	3.256478	3.152112	3.110485	3.788048	3.072259	2.899721	3.133205	3.713156	4.258947

AC005753.2	9.126433	10.088700	9.760421	9.825091	10.477090	10.497826	9.933712	10.995960	11.204776	10.346735	10.579352	10.595584	9.216247	9.733183	9.233946	10.014741	10.762352	9.423613	11.263206	9.278447	10.991471	10.798335	11.660141	9.613799	9.724839	10.485474	8.662549	11.537331	11.035706	10.083571	10.359472	8.352459	9.485482	9.032354	10.776828	11.289771	9.253595	9.517138	10.137745	10.715970	11.273851	10.521409	10.373383	10.794534	10.717743	11.571323	11.032834	10.667694	9.871807	11.940454	10.635038	10.796271	8.763929	9.401070	9.202653	10.339380	8.891129	8.962224	9.186435	9.329853	9.795034	10.583044	8.884973	10.225137	9.940701	10.718958	9.879312	9.293796	9.448101	10.763875	10.133149	12.496521	10.602549	10.184516	10.613473	9.219434	9.824324	9.984270	9.826469	9.377930	8.619238	9.676599	8.841178	8.968967	8.962369	9.765726	8.807970	9.612257	9.560968	9.441911	9.696776	10.265579	9.470328	10.179183	9.579952	9.699709	9.683246	9.146259	9.063380	9.551619	9.392213	9.531300	10.136400	9.639817	10.046039	10.861911	10.308324	9.824482	11.068675	11.003511	11.143986	10.057591	10.786766	10.950728	11.294540	12.071732	7.909264	12.084965	10.794089	9.469192	8.996891	7.852039	8.479305	8.957303	10.234170	10.145498	9.372113	9.618709	10.244422	10.230378	10.571372	9.225751	8.858812	8.177835	9.625195	9.137632	8.069057	8.829341	8.421514	8.809828	8.699834	9.590740	8.274926	8.264458	8.318502	11.143830	9.047921	10.637586	9.650553	9.271518	8.540372	8.861679	9.787425	8.632470	9.453555	8.126601	8.820230	8.918471	8.522315	10.659191	8.921058	7.868192	8.858205	9.569197	10.787634	11.063641	8.243691	8.912356	8.982281	8.561010	8.609069	7.593826	9.318053	7.506049	9.782986	7.026996	7.869548	7.854837	7.807263	8.393936	9.748635	8.684630	8.730763	8.624032	8.375886	8.084927	8.460790	8.229801	8.756456	8.938834	9.750211	10.321225	8.685393	9.267666	8.524770	8.039959	9.138891	9.251188	7.329779	7.348953	9.259236	8.068615	7.508140	7.728413	8.688665	8.478824	7.878416	8.009018	8.009910	8.009628	9.084689	8.404521	9.104590	9.276523	8.864614	8.821128	8.731515	8.932990	8.183260	8.778865	8.352309	8.366808	7.681614	8.173460	10.340663	10.074213	9.097822	10.537222	10.500010	8.819184	8.367911	8.783516	10.622336	8.954265	9.586854	8.689568	9.418245	10.233257

AP002761.2	6.778767	7.210823	6.991935	7.117059	7.366705	8.184487	7.462608	8.412310	8.073510	7.634654	7.587048	7.355513	7.641532	7.899257	7.711088	7.903686	7.142409	7.153391	7.925226	7.655132	7.542633	8.029925	6.424487	7.492437	7.629887	7.606389	6.969499	7.969565	7.700373	7.587802	7.761133	6.842224	6.852662	6.794294	6.741451	7.002279	7.261675	7.485700	7.699612	7.129553	7.978000	6.968555	7.250319	7.367347	6.749103	6.802515	7.549287	7.625771	6.783224	7.909583	6.983026	7.354866	6.918220	7.115967	7.147857	8.074715	6.835808	7.403660	7.320294	7.195198	7.271866	8.144078	7.047698	7.620747	6.792669	7.283831	8.080543	7.785913	7.461345	7.663101	7.269702	7.194888	8.175989	7.198684	7.147710	6.800111	7.146904	6.574201	6.711800	6.589328	6.733213	6.308579	6.321320	6.249479	5.979208	6.664134	5.622037	6.151044	6.323377	6.307744	6.092548	6.090782	6.820707	6.855733	6.725278	6.705442	7.291614	7.161260	7.849748	7.938844	7.681782	7.506342	7.377860	7.424306	7.884438	7.804477	8.202112	7.925836	7.573718	8.462198	7.062837	8.386809	7.556447	8.250361	7.992228	7.826473	6.817820	6.853634	7.814319	6.023595	6.597115	6.426975	6.716023	7.662314	7.391949	7.445391	6.647062	6.802353	7.711698	8.531531	7.005042	7.849905	7.127683	7.805727	7.028135	6.976239	7.497876	7.255095	6.252502	8.207548	8.225089	6.733827	7.281001	7.059481	6.763766	7.034373	7.456502	7.662814	7.507556	6.967094	6.551327	6.165780	7.438363	6.226232	6.347920	5.847321	6.276841	6.780384	6.491065	7.659822	6.816998	6.269459	6.275725	6.356259	7.090101	7.559819	6.625668	6.964524	6.978653	6.869668	6.654347	7.505088	6.734147	6.440386	8.268871	6.032553	6.668816	6.635639	6.300519	6.227645	7.290175	6.898513	6.893689	6.676879	6.505827	7.254611	7.022488	7.128781	7.047698	7.060125	7.166846	8.455668	6.379812	5.640329	6.349577	6.781209	6.115636	6.629202	6.080402	5.983328	6.334855	5.740431	5.516058	5.204869	6.490154	4.481723	5.237869	5.495098	5.199295	6.766660	5.679946	6.128820	6.234130	5.720686	6.130349	6.894638	6.893180	6.663285	7.563186	7.152154	6.947452	6.808558	6.949322	6.641604	7.619402	7.221148	6.818634	7.779974	7.664539	6.749580	7.774785	7.532530	8.208005	7.912039	7.360270	6.809699	6.989522	7.124976

AC099668.1	9.552071	8.727572	8.512159	9.001028	9.346950	8.940654	8.726942	8.814849	9.910968	9.032961	9.566353	9.696322	8.737644	9.700346	9.342515	9.294591	9.643632	9.692546	10.110042	8.997966	8.430887	9.353198	8.630365	9.937753	10.350819	10.781158	9.579792	9.225591	9.045496	8.408039	9.700644	8.674991	8.406370	9.330151	8.246938	8.309606	8.945669	8.723135	9.042224	8.456268	8.969110	8.961440	8.375886	9.339959	8.320348	8.963871	8.937800	8.247398	8.954265	8.718419	8.603917	8.230703	7.979579	8.922699	8.853327	9.144597	8.604528	8.639746	9.198763	9.144597	8.463830	8.503715	7.966042	9.269228	9.744429	9.177867	8.239177	8.995047	7.800225	8.528611	7.997547	8.401902	8.863054	7.882917	8.625298	7.882461	8.230249	7.881084	8.444402	8.276257	8.098995	8.499181	8.631915	8.730470	8.637074	9.542983	8.931289	8.094336	7.391949	9.169378	8.934723	7.549747	8.445759	8.132838	8.924600	9.010403	8.691670	9.436174	9.343124	9.702032	9.486434	8.555262	9.584367	9.078242	8.147095	9.564026	10.438091	9.235458	9.226696	9.095402	8.798095	9.904543	9.351550	9.761826	9.312017	9.793310	10.655933	9.333063	9.311545	9.276679	8.297469	8.165963	8.676509	9.730779	8.152993	9.548708	11.155073	8.575504	9.474231	9.171885	9.683888	8.142539	8.881137	8.549976	9.661397	8.575191	9.775518	10.249984	9.666724	8.686419	9.417175	9.235309	10.417533	9.776270	9.697083	8.831339	10.673716	9.473089	8.143459	8.677119	8.800353	8.796555	8.413235	8.828392	7.660314	8.027164	8.265560	8.833056	8.644551	9.643474	7.552684	8.274471	8.105747	7.915822	8.329193	9.253595	7.354866	9.131387	8.184797	7.766091	7.740543	8.185409	7.597626	8.410780	8.434607	9.203256	8.348402	9.167255	9.212214	8.409551	8.824486	9.005015	8.368909	10.110195	9.160000	8.015395	8.075632	7.473547	7.925527	7.914604	7.504008	10.510494	8.119459	7.892633	7.563026	8.112700	8.487233	8.421514	8.074267	7.803562	8.203475	8.241293	8.848779	7.845347	10.034692	8.033614	9.256667	8.257221	8.344769	8.619099	7.256473	9.331832	7.810799	8.634560	8.076211	8.833908	10.638627	8.862905	9.939645	8.494911	10.967244	10.195682	8.345381	8.682676	9.460158	10.024169	9.478608	7.861073	8.967765	8.518242	8.876622	9.088985	9.524895	9.030128	8.914640	9.238102	9.838745	8.203755

LINC01697	14.241084	13.966624	14.564295	14.672821	14.599518	14.952794	13.697097	14.530652	15.467330	14.414022	14.425228	14.207145	14.478214	14.373693	13.845880	14.467367	15.028163	13.924569	14.487923	14.626390	14.014363	14.301922	14.866612	14.120170	14.550582	15.018571	15.386012	14.427017	14.854342	14.616554	14.900158	13.517777	13.104454	14.348910	14.645753	14.430074	14.425606	14.303944	14.880110	14.512793	14.722869	14.846797	15.125176	14.929736	15.196216	14.852694	15.229335	14.159893	14.259951	14.889685	14.288622	14.289302	13.754901	13.682827	13.647299	15.110454	14.034368	14.492360	13.987240	14.237798	14.012342	15.213005	14.887119	14.636601	13.468126	13.802610	14.406227	14.548933	13.468361	14.508773	14.353395	14.329971	14.815284	13.818348	14.275219	14.277280	14.353021	13.840099	13.941517	13.444604	14.178338	14.036962	13.311729	13.742612	14.160557	13.582290	14.216655	14.265614	13.848828	13.170352	13.834567	14.137944	13.825046	13.944308	14.386698	13.870453	13.316023	14.303277	14.642705	15.424949	14.671925	14.103167	14.328233	14.438602	14.282749	14.789144	14.819862	14.734398	15.336040	15.081140	14.412450	14.795023	14.145227	15.122829	14.274531	15.945972	15.161962	14.708439	15.422546	14.398878	14.547186	14.494741	14.484873	15.267864	14.568889	14.968533	15.524473	14.979310	14.506441	14.258950	14.329971	14.906627	14.477449	14.467751	15.239037	14.680520	14.541332	14.707094	14.268626	14.571778	14.257321	14.552678	14.737586	14.008461	13.502404	14.473115	14.741363	15.616895	14.529498	15.104927	14.539639	14.567967	13.789231	14.205886	14.300954	14.320921	15.016809	14.544276	14.295679	15.123478	14.079765	14.451322	14.444149	14.527899	14.358722	14.906118	14.930234	13.886983	14.354835	14.340345	14.626808	13.711167	13.672215	13.650539	14.851142	13.303085	13.869642	13.845633	13.354386	13.621305	14.524706	13.461577	13.626821	13.763702	13.530118	13.619696	13.915536	13.867259	13.281130	14.102873	14.297379	15.209040	14.174543	14.121601	14.072863	14.427447	14.080659	14.166466	14.093713	13.828450	13.158236	13.538360	14.277280	13.675754	14.535929	13.096527	13.570563	13.868836	13.887531	13.812235	13.819661	14.274531	13.970183	13.726632	14.248871	14.671925	14.944787	14.278941	14.384530	14.600827	14.940922	15.101738	14.046144	13.980518	15.003643	14.783853	14.051810	14.328600	15.092451	14.397321	14.959508	15.313805	15.651828	14.797410	14.303944	14.360038	14.463605	14.548542

AP001042.3	3.156400	3.062882	3.232304	2.932255	3.866608	5.193652	3.273434	3.614957	3.307751	3.383146	3.570246	3.690955	3.363192	3.819707	3.818855	3.054547	3.406496	3.811402	4.030852	3.192444	4.701728	2.834582	4.436502	3.526862	3.047625	2.841362	3.320254	2.902856	2.933695	3.043724	3.629437	5.987381	6.856249	3.063626	3.398675	3.975082	3.658061	3.140544	3.048062	3.583598	3.361439	2.914747	3.275739	4.214051	3.401046	4.357398	3.781660	6.949175	3.124783	3.749398	3.840468	3.608409	5.579093	5.338729	3.518728	3.674948	3.723306	3.042314	3.745894	4.456047	3.225976	3.010483	3.141697	3.995744	3.036164	3.596936	2.833801	5.236029	3.497641	5.414539	2.914659	3.026845	3.550812	3.298546	4.065006	3.409888	3.113441	3.326945	3.127559	3.495782	3.216375	3.861063	4.623113	6.078831	3.243404	3.265578	3.006399	3.219514	4.855836	3.099402	3.802024	3.035580	3.143866	3.689939	2.992513	3.072161	3.555255	3.083946	4.639269	3.988180	3.472931	3.201942	3.539190	3.196648	3.739768	2.988422	5.154607	3.250849	3.244280	3.811621	3.106895	6.409505	2.938795	3.713156	3.062674	4.863721	2.913922	2.841712	3.128807	4.659010	3.120613	3.097963	3.071553	3.553719	5.791671	3.735228	3.575730	3.267648	3.170245	3.374235	3.175158	3.487711	3.865968	3.441180	3.226873	3.394523	3.317617	3.253718	3.627841	4.062858	3.220137	5.153573	3.702441	2.925438	3.341148	3.435630	3.171671	3.896934	3.486956	3.387578	4.288978	6.164933	3.062131	3.327915	3.702855	3.686870	3.061802	2.983258	2.832360	3.144458	2.866491	3.349518	3.311611	3.578380	4.245989	3.785011	6.022897	3.250276	3.877169	3.907084	3.536689	5.427467	5.551616	3.639290	3.128684	3.380170	3.012317	4.777623	4.831864	3.072259	3.070885	3.419322	4.504570	3.199019	3.477825	2.830622	3.175025	3.283331	5.742830	3.212552	4.630478	4.044548	3.290129	3.543981	3.440314	3.264112	3.129453	3.267949	3.167529	3.503482	3.990661	4.366995	4.845532	3.461815	2.945091	3.487711	3.367694	2.921832	3.073448	3.952263	3.985840	3.109008	3.849284	2.954774	3.471726	3.239461	2.960877	4.922128	3.659062	3.625390	3.273130	3.115214	3.051323	4.189963	3.002910	5.084460	4.023017	3.636471	4.141508	3.560111	6.246949	3.193392	3.609803	3.531729	4.366132	3.424717	4.513800	3.226873

AP001042.1	3.156400	3.062882	3.232304	2.932255	3.866608	5.193652	3.273434	3.614957	3.307751	3.383146	3.570246	3.690955	3.363192	3.819707	3.818855	3.054547	3.406496	3.811402	4.030852	3.192444	4.701728	2.834582	4.436502	3.526862	3.047625	2.841362	3.320254	2.902856	2.933695	3.043724	3.629437	5.987381	6.856249	3.063626	3.398675	3.975082	3.658061	3.140544	3.048062	3.583598	3.361439	2.914747	3.275739	4.214051	3.401046	4.357398	3.781660	6.949175	3.124783	3.749398	3.840468	3.608409	5.579093	5.338729	3.518728	3.674948	3.723306	3.042314	3.745894	4.456047	3.225976	3.010483	3.141697	3.995744	3.036164	3.596936	2.833801	5.236029	3.497641	5.414539	2.914659	3.026845	3.550812	3.298546	4.065006	3.409888	3.113441	3.326945	3.127559	3.495782	3.216375	3.861063	4.623113	6.078831	3.243404	3.265578	3.006399	3.219514	4.855836	3.099402	3.802024	3.035580	3.143866	3.689939	2.992513	3.072161	3.555255	3.083946	4.639269	3.988180	3.472931	3.201942	3.539190	3.196648	3.739768	2.988422	5.154607	3.250849	3.244280	3.811621	3.106895	6.409505	2.938795	3.713156	3.062674	4.863721	2.913922	2.841712	3.128807	4.659010	3.120613	3.097963	3.071553	3.553719	5.791671	3.735228	3.575730	3.267648	3.170245	3.374235	3.175158	3.487711	3.865968	3.441180	3.226873	3.394523	3.317617	3.253718	3.627841	4.062858	3.220137	5.153573	3.702441	2.925438	3.341148	3.435630	3.171671	3.896934	3.486956	3.387578	4.288978	6.164933	3.062131	3.327915	3.702855	3.686870	3.061802	2.983258	2.832360	3.144458	2.866491	3.349518	3.311611	3.578380	4.245989	3.785011	6.022897	3.250276	3.877169	3.907084	3.536689	5.427467	5.551616	3.639290	3.128684	3.380170	3.012317	4.777623	4.831864	3.072259	3.070885	3.419322	4.504570	3.199019	3.477825	2.830622	3.175025	3.283331	5.742830	3.212552	4.630478	4.044548	3.290129	3.543981	3.440314	3.264112	3.129453	3.267949	3.167529	3.503482	3.990661	4.366995	4.845532	3.461815	2.945091	3.487711	3.367694	2.921832	3.073448	3.952263	3.985840	3.109008	3.849284	2.954774	3.471726	3.239461	2.960877	4.922128	3.659062	3.625390	3.273130	3.115214	3.051323	4.189963	3.002910	5.084460	4.023017	3.636471	4.141508	3.560111	6.246949	3.193392	3.609803	3.531729	4.366132	3.424717	4.513800	3.226873

FTX	12.611838	12.289811	12.472784	12.270578	12.180967	13.374219	13.396889	12.640215	13.045554	13.449721	13.453090	13.179374	13.346566	12.563000	12.243919	13.099956	12.690670	12.133719	11.390561	12.862587	11.588196	11.865134	10.813866	12.092606	11.762074	12.602912	13.410314	12.358636	13.772438	13.431034	11.680518	12.163592	11.912931	12.803011	12.027808	12.173255	11.516227	11.682317	11.305887	11.474531	13.423660	11.186429	11.001508	11.797058	11.641926	11.280469	13.717719	11.545765	12.148397	11.502476	11.250318	11.419850	10.937758	11.521468	11.292209	13.835331	10.933084	10.741413	11.365508	10.939212	11.124424	13.178784	11.759472	11.146352	11.119708	11.744491	11.060112	10.840635	10.629643	11.009325	12.685861	12.652934	13.300869	12.379731	12.280960	12.765306	11.852324	12.277123	11.998716	11.475562	11.427813	11.756331	11.117536	11.160666	11.558935	11.845386	11.305602	11.354771	11.315434	11.066875	11.217302	10.944920	11.846574	12.482960	12.250882	12.452958	11.515780	12.728769	12.820920	12.962524	13.169042	12.081981	12.135429	12.220618	10.957964	13.422370	12.476862	10.745154	11.590838	13.615437	9.908039	11.989871	12.129637	13.473987	12.818889	12.710992	11.334490	13.509232	13.805698	12.312392	11.750569	11.783439	11.941351	11.984838	12.777077	12.692342	12.355778	12.383384	13.020637	13.348898	13.610144	12.362103	12.013715	11.759472	12.924903	11.868313	11.630454	11.064379	13.113729	10.990603	11.659564	10.053598	12.414625	11.638582	11.622846	13.059891	11.570893	12.801254	12.833844	11.012583	12.089045	11.476828	12.160484	11.918646	11.567272	11.440893	10.966399	10.590541	11.303088	13.531252	10.779848	10.891326	11.316765	11.166822	10.735207	12.811111	10.765247	11.903720	10.727937	11.597416	10.772100	10.662816	11.162156	11.068537	12.685688	10.564137	10.667694	11.082486	10.792913	11.241932	12.582966	11.472792	11.223118	11.126352	11.535109	11.528035	10.770448	10.816113	10.715670	11.935813	11.991560	13.811679	11.943044	11.869781	12.311428	11.698422	11.579521	12.082436	11.635258	11.666271	11.077518	11.964028	11.207783	11.817862	11.448088	11.103871	11.008142	10.968909	11.054661	10.789859	10.486117	12.086504	12.360678	13.107173	13.073608	11.151852	12.868341	13.191043	11.755581	12.388554	12.244999	11.958855	11.699769	11.555058	13.453723	12.798678	11.040012	11.322916	13.078555	10.000549	11.916369	11.612729	13.301047	11.776985	12.161114	11.316464	11.989104	12.962524

AC007749.1	5.025271	4.785984	4.603540	5.014462	5.378859	4.931205	4.297673	6.245120	5.799127	3.877608	4.774099	4.239223	4.095392	4.774707	4.513800	4.871701	5.580774	5.143570	6.800931	5.009721	5.301839	4.892508	4.101061	5.042847	5.439680	5.323047	5.461399	5.523672	4.586482	4.640929	5.803658	3.405057	4.127424	3.664638	4.176172	4.139301	4.424224	5.175953	5.306575	5.039717	4.182342	5.148725	5.152556	5.337511	4.749521	5.833177	4.625919	3.955022	4.704932	5.356278	5.281755	5.228322	4.602712	4.371963	5.029113	4.741718	4.221682	2.929615	4.599587	4.121850	5.559465	4.540902	5.312635	5.390458	6.054615	5.496067	4.626946	4.778450	4.401806	5.394962	4.762246	4.510054	4.627574	4.530746	5.068602	4.253660	4.540679	4.832287	5.281755	4.396141	3.796939	4.223852	5.062258	4.216232	4.503503	5.531254	5.478477	5.008283	4.051084	5.009093	5.278530	6.570524	4.428669	4.785776	4.983217	4.257186	5.385942	4.583310	5.111332	5.344189	4.479959	4.337231	4.845952	5.176324	4.761835	4.587333	5.877950	4.875897	5.661421	4.456929	6.249627	5.963499	5.681029	4.737391	5.076919	5.404435	5.580774	5.062667	4.497707	4.356533	4.724734	4.795822	4.990182	5.989180	4.388072	4.396577	5.632079	5.366408	5.221697	4.778843	4.343285	4.202480	4.916624	4.980930	4.501816	5.425215	5.487997	5.252144	4.847648	5.056350	5.327319	3.694034	5.981021	4.991007	4.729956	6.397453	6.024120	5.284147	4.470128	5.486905	3.817172	3.355030	3.953974	4.187529	4.439596	4.883487	5.652572	5.810203	5.376740	4.958929	5.344374	4.798541	5.178363	4.359159	5.356463	5.462933	3.859999	4.094946	5.366629	4.417080	4.886247	4.353327	4.590157	4.863721	4.039083	3.796939	4.719803	4.676249	4.271401	5.355688	4.508113	5.899364	5.727541	4.856053	5.159865	2.793161	4.400248	4.859608	4.744249	4.543312	4.305090	5.344963	4.362400	4.463060	4.575611	4.919371	4.798980	5.396337	4.339822	4.106504	4.737795	4.163837	4.160768	4.458021	5.396732	4.173173	4.291112	5.002508	5.178999	5.375758	7.039059	4.045833	4.281683	4.063079	5.161854	4.974416	4.561427	4.573729	3.751463	4.909875	5.971960	8.357685	4.990394	4.662653	4.520611	5.881051	4.822317	4.794361	5.025466	6.223823	6.112814	7.133232	5.609543	5.263095	4.842988	5.381551	4.878603	4.707633

AC078878.2	4.233299	4.791226	4.703861	4.358088	5.919921	5.114355	4.788469	6.127291	5.711750	4.478244	5.245413	4.908200	4.276377	4.385646	3.747084	4.664747	6.097021	5.952163	5.804407	4.689279	5.332458	5.586055	4.242269	7.275147	5.225089	5.710664	4.803039	5.619275	4.612707	4.413993	5.348514	4.361519	4.909265	3.666066	4.680510	4.133820	4.241181	5.865057	5.057972	4.932450	4.644693	5.287944	4.162511	5.823480	3.950551	5.708822	5.306575	5.381342	4.768671	4.897460	5.627262	5.248606	4.429095	4.249061	4.256282	5.399789	4.113121	5.082862	3.419704	4.071461	4.565553	4.726407	4.979117	5.552203	5.387168	5.927411	4.723654	4.896017	5.181765	5.136622	4.963547	4.930625	5.125562	4.905496	4.886461	5.251581	5.721773	5.778728	5.417191	4.802168	5.048105	4.177463	4.468417	5.017104	5.081029	4.555796	6.094796	5.206643	5.369713	6.202616	5.841706	6.117672	4.244102	4.667150	4.700479	4.820657	4.553674	4.671850	5.281154	3.877608	4.871916	4.324430	4.225572	4.887089	4.775112	4.601657	6.453478	4.981524	6.084236	4.766605	6.244301	5.594202	6.555125	5.219149	5.498722	6.283964	4.565553	6.274237	5.119275	5.120496	5.141118	4.662653	4.158634	5.843332	4.514410	4.477618	4.954166	5.250586	4.905496	4.541317	4.987884	3.672690	3.573829	4.357870	4.191700	5.894762	4.911789	4.470128	4.427875	4.963752	5.174521	6.034728	6.825589	4.858150	5.558298	5.607979	4.655316	5.916723	4.484384	5.376154	4.795393	4.957084	4.609495	5.101364	3.982184	4.879417	5.791497	5.385402	5.261653	5.185394	5.815816	4.365486	6.976898	4.610157	5.103393	5.297845	5.567659	4.788690	4.711587	5.264297	4.682632	4.120757	4.299398	3.923084	4.492677	4.257871	4.384959	4.200518	3.365160	4.653042	4.773477	4.976495	5.580957	4.658359	5.798779	3.460735	4.669702	5.062027	5.806483	4.747474	4.957295	5.295832	4.928464	4.647688	5.416020	5.193437	5.882835	5.321484	4.655999	4.557457	4.612954	4.900277	4.606257	5.404268	5.424652	5.700984	5.303625	5.442550	5.819906	5.235636	6.419483	4.989560	4.370451	4.813130	4.801357	5.141531	4.377152	5.622975	3.903786	4.713921	4.192784	4.403105	4.244526	4.912395	4.276840	6.389695	5.281154	4.983993	6.929852	5.813341	5.907218	6.492034	5.554716	5.449166	5.787200	5.063441	4.862901	5.100098

AC087294.1	5.250383	5.444863	5.444666	5.157266	6.768454	6.087475	5.602065	5.229535	6.129010	6.112494	6.083510	6.287915	6.502522	6.303573	5.783423	5.384460	5.480462	6.908452	6.119227	5.969983	4.588857	6.375503	3.251979	6.536952	6.829419	6.629051	5.859070	6.180988	5.548054	5.868676	5.570700	5.966637	6.034380	5.708481	6.353937	5.933698	6.162362	6.511935	6.474078	6.826397	4.871701	5.983328	6.439708	5.861943	6.386455	6.290462	5.295016	5.626559	5.563547	5.730674	5.631190	6.351948	6.344049	5.884608	5.933698	5.629161	6.879880	6.001097	6.278795	6.205388	6.671230	6.131199	4.122071	6.381308	6.732375	6.093207	5.580405	5.589095	5.632079	6.098195	4.933247	5.069990	6.346571	5.595490	6.133084	4.969468	4.984987	5.476348	5.396530	5.805110	6.212521	5.846222	5.959156	6.279314	5.519715	6.063309	5.691739	7.362606	3.937922	5.614049	6.020084	5.999712	5.689182	5.821866	5.379656	6.048741	6.070506	6.868569	5.793128	6.414695	6.359531	5.959510	6.042449	6.432929	5.962707	5.334938	5.291008	6.919713	6.254190	6.215781	5.831751	7.319485	7.211007	5.220376	6.324927	6.739567	6.269286	5.300632	5.633382	6.098026	5.658144	5.173168	6.038250	7.186835	6.311949	5.126754	6.247463	5.993577	6.342674	6.015713	6.607242	5.894072	5.985562	6.256588	5.373609	5.476348	6.700141	7.161729	6.125147	5.181177	7.151068	6.453478	7.192778	6.636437	6.749103	5.322671	6.518205	5.489345	6.265713	5.472171	5.890991	5.717822	6.893180	7.299240	7.236735	5.825489	6.032173	7.096715	6.530621	5.552587	6.372450	6.033450	6.609828	7.054770	6.607057	5.603614	5.532203	6.660368	7.775417	5.616498	6.601982	6.750212	6.147780	5.880328	5.678435	7.330723	6.192351	6.083169	7.308803	6.757186	5.647662	5.370703	6.181510	7.014664	6.148301	5.964494	5.739006	5.849897	6.410196	4.986033	5.408923	6.112494	5.538833	5.636384	5.223904	5.684361	5.117203	5.752475	5.792044	6.580287	5.950215	6.742265	6.041748	6.117854	6.106746	6.695069	6.948600	4.501170	5.391260	6.938279	5.427843	5.925429	4.989560	6.139271	5.828774	6.511783	7.155448	5.580041	7.481057	6.004097	6.479304	6.092548	5.921773	5.678637	6.277178	4.891473	7.221939	6.656942	5.410898	5.725538	6.482390	5.316256	4.919371	7.352063	6.647062	6.656942	5.744988	5.989356

LINC00265	12.603735	12.424399	11.927863	12.580507	12.786304	12.837237	12.164205	12.838440	14.050019	12.401781	13.153423	12.842271	12.886514	12.098166	11.959893	13.020101	13.305786	12.181896	12.712770	13.131924	11.862452	12.626178	12.576723	12.653093	12.734213	13.176379	14.102873	12.862405	13.159327	12.567752	13.400373	11.808731	11.833903	12.901798	12.680252	12.563000	12.373531	11.875677	11.965245	12.600182	12.750174	12.497991	12.971662	12.816808	13.142467	12.559435	12.862405	11.620096	12.711326	12.588458	12.531673	12.324636	12.493360	12.437883	12.416267	12.936910	12.469565	12.096234	11.974500	12.022667	12.116506	12.997678	12.087108	12.431923	12.008851	12.258212	12.085120	11.859940	11.528777	12.049360	12.412212	12.716779	13.155345	12.202678	12.729813	12.553295	12.377714	12.159115	12.436907	11.492313	12.778288	12.504387	12.102820	12.368604	12.453577	12.426059	12.533949	12.355477	11.805598	11.432540	11.800594	11.946821	12.337312	12.295732	12.708224	13.131924	12.233576	13.551649	12.846490	13.738603	13.258800	12.434806	12.540801	12.508819	12.071423	12.973105	13.234411	12.511911	12.909315	12.613710	12.312060	13.356586	13.099019	13.241587	13.019562	13.652033	12.811936	13.147466	13.770103	13.403370	12.175782	12.351330	13.312162	13.212614	12.719368	12.979897	14.505307	13.157459	13.005589	12.820105	13.218984	13.389682	11.946966	12.230187	13.600359	13.162623	12.656833	12.676354	12.394589	11.747030	12.462229	12.268991	12.796802	12.154928	12.313639	12.747949	12.827695	13.986959	12.005783	13.687645	12.970971	13.062098	12.459458	12.688794	12.681634	12.299016	12.150924	12.160484	12.231738	12.986294	12.343108	12.319355	12.941000	12.439334	11.953784	12.854406	11.606822	12.374518	11.873996	12.264187	12.621674	12.211122	11.769915	12.457385	12.764600	12.880949	11.878180	12.110262	11.741007	11.969338	13.109859	12.326688	12.090118	12.434806	12.583952	11.878774	11.439735	11.962903	11.846709	12.491398	12.666171	13.314543	12.365108	12.786304	12.534620	12.327798	12.745055	12.491754	11.688705	12.989438	12.332224	12.926482	12.958080	12.328450	13.400180	11.945613	12.235309	12.196906	11.909419	12.025251	11.899423	12.673286	12.305259	12.735398	12.889837	13.346348	14.031176	13.167478	12.901456	12.983527	13.080831	13.278412	12.223401	12.544739	13.170749	12.874491	12.480642	12.096962	12.735217	12.307305	12.644060	12.543245	13.121772	12.143273	12.048284	12.514664	13.453519	12.796263

AL844175.1	3.271303	3.608791	4.026036	3.229611	3.274958	3.258992	3.631713	3.391393	3.727139	3.426570	3.558731	3.763184	3.329538	3.613999	3.214073	3.364521	3.996401	4.216647	5.945313	4.622076	4.484799	3.698788	3.158535	2.868936	4.202921	3.833118	2.933598	3.110727	3.310230	3.028179	3.511834	4.118354	5.562770	3.104345	5.378285	3.997446	3.491861	4.239674	3.590125	4.828889	3.251299	3.189156	3.658626	3.234379	3.189156	5.334014	3.963170	3.830142	4.933854	4.074275	6.736780	4.781987	3.518376	4.131034	5.116406	3.056118	4.871035	5.331647	4.083916	4.338757	3.100817	4.818563	3.663659	4.172695	4.012899	4.377797	3.444194	5.247821	4.046032	3.987353	4.357870	4.731657	2.869316	2.972619	3.916620	4.304639	5.024657	4.045188	4.160159	5.730674	5.511434	3.907900	6.244796	5.550523	4.006530	3.277869	5.787200	5.118031	4.142802	3.596729	5.145547	3.398675	3.180009	3.770137	3.702441	3.650021	4.599993	4.549992	3.937514	4.530956	3.749777	3.681431	4.253908	4.676681	4.317365	3.608224	3.056861	4.723229	5.002715	3.651791	6.381466	3.815433	3.208983	3.351726	4.059980	5.657036	3.575527	3.643475	3.535907	5.701880	3.388600	3.244137	4.875077	5.572941	3.235659	5.460841	3.253718	3.412024	3.492579	4.325277	6.082499	3.062769	3.248272	3.041559	2.984959	3.599539	9.764510	3.361618	3.505145	6.034380	8.320195	5.247413	2.838045	3.992878	3.687262	3.353040	5.841158	4.539977	3.461815	3.382741	3.500876	3.241792	3.143153	4.114828	3.723511	7.566901	3.727937	3.395581	8.667281	3.571891	4.518062	3.533669	3.245240	7.081923	4.608431	3.117616	5.635631	9.171571	4.813900	8.611378	4.403954	9.228667	4.236770	4.429565	3.181516	3.571687	11.432854	3.853814	6.052025	2.956704	4.113339	4.701728	5.824234	3.837146	4.412646	3.465394	3.620269	3.277400	2.885929	4.803917	8.446244	4.745325	2.993354	4.213192	3.545470	5.124112	4.068797	4.915556	8.735793	6.387151	4.160365	3.654396	6.556627	3.462669	5.599491	3.699776	3.433435	7.349268	6.873688	3.421532	3.579949	3.038319	3.220706	5.795999	4.197752	4.407127	8.212820	4.015856	9.410338	3.127424	3.166490	3.306493	3.395399	4.732302	4.779910	3.008358	3.083277	8.090805	3.143269	5.256947	3.542469	4.808893	3.744268	4.442638	4.706614	3.290793	8.241887	3.209388

CYP4F26P	10.203849	8.660855	8.561316	9.104276	9.032809	8.908916	7.947541	10.063627	10.049254	8.629123	8.544468	8.546912	7.869083	8.464289	8.143607	8.769925	9.641913	8.544600	10.250874	8.550430	9.887355	9.088985	10.272099	8.937952	9.069452	9.220517	8.977562	9.853854	9.012062	7.928954	9.916692	7.931418	8.009910	7.254455	8.230249	8.412161	8.490431	8.812340	9.034634	9.445444	8.177241	9.548217	8.324142	9.347097	8.503865	8.701221	8.424087	9.035740	8.316068	8.529071	9.878300	8.621747	7.928954	8.327463	8.246522	8.207850	8.012608	8.528127	8.924017	8.448133	9.146092	8.004823	9.136672	9.604702	9.024177	9.533530	9.293634	8.928643	8.919206	9.802113	8.425436	8.310979	8.506430	8.189742	8.419013	8.755780	8.940204	8.826707	9.496731	8.222383	8.466728	8.508601	8.622183	8.645434	8.651326	8.463370	8.767446	9.018966	9.317184	9.428192	9.357110	9.862854	8.449384	8.598050	8.893154	8.347074	8.411404	7.919491	8.031790	8.369070	8.047520	7.926641	8.495845	8.430406	9.352758	8.191175	10.096406	9.267511	10.115138	8.853327	10.192949	9.549766	9.500105	9.311096	8.772914	10.066454	9.949359	8.872170	8.712506	8.962224	8.685533	8.304977	8.828225	9.339347	8.746346	8.105290	9.162455	9.227738	8.335713	8.398835	8.214857	8.114888	8.111607	8.461246	9.191181	9.454607	8.808720	9.900517	8.287195	9.988100	8.888062	10.244562	9.167108	9.372726	8.732389	9.231906	9.780886	9.199210	7.950929	9.877830	7.966334	8.390991	7.708847	8.298658	8.590205	8.376041	9.222820	9.520066	9.430029	8.305140	9.683738	8.571281	9.736555	8.796555	9.046851	8.634408	8.905662	8.617394	8.841504	9.243968	8.797166	8.094026	8.410935	8.275089	7.845650	7.858470	8.419942	8.776457	8.267307	9.045341	7.736364	9.580426	9.539063	9.256514	9.292527	9.016697	8.862460	8.873398	9.806251	8.616784	8.569261	8.968219	8.322016	8.210515	8.775819	8.814119	9.003802	9.152019	8.243979	8.337120	8.638018	8.602666	8.332304	8.756380	8.728213	8.835707	8.786864	9.296761	9.246593	9.172491	10.149344	8.352610	8.554054	8.637555	8.588347	9.255442	8.326196	8.451090	8.057141	7.886288	7.643210	8.398071	8.234355	8.710550	8.322153	10.108228	9.288025	9.980249	8.939581	10.159669	9.108128	10.069172	9.503469	8.757756	8.420110	8.995798	8.612329	8.446869

HOTAIRM1	10.659325	13.049325	13.253617	12.719045	13.669861	13.534858	13.038207	13.138696	13.794844	13.665482	13.459822	13.728355	13.086086	13.345934	12.537425	13.344050	13.688112	13.479279	13.907998	13.147466	13.855947	13.464002	14.172567	13.954619	13.842204	13.450346	12.940635	14.085609	13.409465	13.019903	13.936877	12.282721	13.440327	12.202348	12.957891	13.125775	13.614482	13.214797	12.950183	13.406084	13.415858	13.178372	13.123418	12.625000	13.084373	12.839799	13.505677	13.158422	13.644720	12.845274	12.976403	13.217360	13.088702	13.019903	13.495791	13.211853	13.323791	14.220230	12.606727	12.833284	13.191685	13.268023	13.428458	13.260048	12.447724	13.120243	13.750320	13.145911	12.862205	13.469871	13.096912	13.282626	13.545103	13.343055	12.792190	12.983527	12.779167	12.953654	12.649718	12.829461	12.660319	13.177997	12.351946	13.677795	13.548218	13.063231	13.302266	13.928111	13.360816	13.544379	13.932466	13.477934	12.263544	12.962331	12.413004	13.312545	13.384142	13.482375	13.040990	13.744863	12.953101	12.744741	13.121772	13.274388	12.984259	13.589555	13.124995	13.023480	13.198725	13.528551	14.514695	13.615204	12.845274	13.703179	13.964108	12.301392	10.980712	12.879237	13.922935	12.245775	12.138182	11.141047	13.345934	11.505101	11.215233	13.233234	11.723608	12.242991	12.551558	12.626178	12.394426	12.476380	12.299016	12.881690	13.229868	12.823859	12.838923	11.982123	12.016290	11.223716	13.206334	13.741329	11.868033	12.766196	12.688955	13.227872	10.764458	12.200007	12.471814	12.722641	12.074208	12.119110	12.522505	11.485173	11.649619	11.612874	11.624581	12.173726	12.076508	13.240817	11.773277	11.522478	10.580067	12.617952	12.147509	11.314528	11.833153	12.162807	11.589394	11.640863	12.049973	11.306805	11.914600	12.083384	13.039846	10.890718	10.406784	11.855631	10.652206	12.604249	12.553137	12.622821	12.352259	12.687955	12.118493	11.555058	11.101364	11.743182	11.808575	11.884048	11.232005	12.280960	11.733934	11.741168	12.147660	11.460998	12.402123	12.362443	12.410614	11.116077	11.878180	12.734213	11.499627	11.296282	12.561217	12.039243	11.900584	11.830627	12.343913	11.478113	13.004862	12.174829	12.235907	12.559575	11.517660	12.439504	12.624672	13.330066	12.536567	13.455025	11.243131	12.754129	11.531829	13.389263	12.364809	13.348289	14.078621	12.434490	11.956809	11.585889	11.391155	11.210981	13.534637	12.258058	12.462402	11.397159	13.015899	12.138659

AC092112.1	7.447592	7.737577	6.460187	7.632690	8.692789	9.026158	6.806475	8.413384	8.278120	6.612650	6.650301	8.022249	7.117886	6.676101	6.929686	6.962442	5.776731	7.562721	5.132818	7.331953	6.407052	6.095149	3.100934	7.006896	6.421425	6.659694	8.262290	6.852172	7.297185	8.447968	6.583270	7.867408	8.405459	9.262594	7.919036	7.607888	7.829918	6.650457	6.433086	6.838038	5.943035	6.853977	8.149462	7.471118	6.971038	7.469869	7.557379	7.605777	8.147919	7.132134	7.511087	7.666363	7.482286	7.674015	6.832940	7.434394	6.941182	7.222084	7.247314	6.719133	6.858036	6.658391	5.686052	7.025699	9.929378	6.787758	6.909393	6.372612	7.127512	7.001632	7.749822	8.201533	6.361202	7.688871	7.065481	8.022679	8.082722	8.347960	7.790093	7.627834	8.561770	6.837369	7.226699	7.217959	7.937895	7.643684	9.855565	7.845347	7.679523	7.349096	7.800068	7.419145	7.461972	8.776128	8.551058	8.153471	7.929108	7.790829	8.562241	8.356589	10.530253	7.691660	6.719982	7.193101	6.918525	6.685342	5.967524	7.067141	8.138502	5.910088	7.554414	8.386497	9.299007	7.565352	7.741340	13.453090	7.871229	7.010888	6.584884	9.768915	5.952712	9.446549	8.028718	11.203885	6.667699	10.835430	7.867887	8.917875	8.427398	7.015335	8.278869	9.337427	8.533473	8.217766	9.490469	7.153563	8.313592	6.892994	9.407099	8.479305	6.932401	5.026830	9.050697	9.210997	7.096414	10.914011	8.259246	11.660731	7.556927	8.848488	10.256330	8.248245	8.235738	8.982281	8.729734	8.203755	8.074715	6.594072	7.375167	8.576106	7.145969	8.638802	7.070219	7.363878	7.211984	8.054935	10.434742	8.128554	8.001992	7.957608	9.692841	8.603763	8.346473	8.785471	7.490559	9.586539	12.292819	9.564348	8.412605	9.488153	7.794465	7.421509	7.108364	8.960537	7.623470	9.895350	7.164273	7.175232	8.478229	8.675912	8.735952	8.990467	8.870081	9.909707	8.236375	9.214054	8.578321	7.443164	10.672422	7.312275	8.540872	8.531668	9.155395	10.907218	10.219095	8.630690	7.564103	8.998715	8.191012	6.661840	7.694879	8.070641	10.524284	10.255569	9.697251	8.990614	9.880112	9.002124	7.769881	9.794095	10.290812	10.685266	9.614532	11.897513	6.896050	8.313277	7.816055	6.887075	6.891902	8.067272	10.175160	11.967030	10.367141	9.298766	11.112018	6.887904	9.186105	9.212057

UBA6-AS1	10.179183	15.559524	16.487541	15.005364	15.555940	15.437228	15.103033	16.105830	15.942235	15.278078	15.237652	15.407655	15.895253	16.228514	15.192041	14.769948	15.756462	15.541137	15.474785	16.115572	15.814383	16.036135	14.908102	15.840619	15.942235	15.758639	13.780832	15.247395	14.636601	14.416187	15.565819	15.789877	15.693501	14.570991	15.441531	15.847680	15.472304	15.301621	15.601071	15.990738	15.586347	15.495462	15.916773	15.576365	13.373144	15.943548	15.521877	15.639403	15.209711	15.921788	15.006579	15.480428	16.161910	15.661481	15.360997	15.973135	16.313885	16.331210	15.770228	16.028244	15.998443	14.921174	12.709826	16.107193	15.263762	15.566607	15.579100	15.941539	16.290771	16.037601	14.137944	16.047049	15.192041	15.050118	15.704556	15.325830	16.454666	15.955729	14.531049	16.377052	15.431049	15.510860	15.399973	16.134222	15.922928	15.411549	16.064222	15.873355	15.545439	15.936069	15.654597	16.206017	14.759633	15.757552	15.034008	15.366350	15.707533	15.357970	15.755343	15.883639	15.453625	15.215025	15.536844	15.256743	15.987023	15.520300	15.913059	15.568457	15.077583	15.744893	15.773226	15.767032	15.903390	15.106780	16.087400	15.185427	13.359085	16.207472	15.481254	11.074134	11.016056	12.997305	12.917482	11.126499	14.068367	13.732652	10.689027	13.628249	13.618017	13.751547	11.700541	10.666510	10.243068	9.413800	15.366350	9.378381	12.449451	8.560859	10.655314	13.201208	9.393908	12.818008	13.781086	9.128766	9.632756	14.719949	14.954990	13.716792	11.611382	11.597416	10.291426	12.563162	11.878918	11.997933	11.273543	13.970475	14.692917	13.150306	9.866689	10.599709	12.047224	10.011007	10.717743	15.392156	13.619696	12.929565	11.683496	13.760169	10.250277	9.823152	11.190758	12.732523	9.538430	13.952936	12.185716	9.824482	11.301442	14.123986	12.503046	10.856874	10.283957	12.876931	12.655175	12.081513	9.799143	9.765260	13.610599	11.144712	12.192292	14.942582	11.830475	14.080973	9.509159	10.549791	13.833551	10.362972	10.134060	14.401522	9.437744	13.879059	13.493480	9.993380	11.932902	10.045720	10.617741	11.359439	9.852765	10.141818	13.088141	9.874810	11.660885	14.975358	10.222882	10.676585	10.513768	11.195637	14.491152	11.947805	9.957211	9.972631	12.090445	12.703137	12.712770	8.519918	14.361168	13.023845	15.735297	14.563436	12.829989	12.476025	10.565894	10.140452	14.809575	10.135777	13.343460	13.581357	10.582604	11.207013

AC026333.3	10.478435	12.929565	13.287464	13.302074	13.655038	13.412720	12.962142	14.203696	12.541262	13.793821	13.858854	12.640533	13.905564	14.395502	12.920413	14.274531	13.285050	13.533066	14.427447	13.947375	13.789730	14.480189	12.334126	13.381340	13.767473	13.903911	10.171080	13.793821	12.245314	12.557817	13.731149	12.591825	13.495791	11.995512	12.737468	13.756127	13.588156	12.873750	13.924569	14.083435	13.858073	13.327590	14.058288	13.354589	12.333670	13.661260	12.569545	12.692683	14.303599	13.746570	12.362103	13.351592	14.120452	14.245669	14.127698	13.846933	13.984981	13.853253	13.749588	13.775677	12.867783	13.180160	12.137555	14.656363	13.840099	12.391255	13.551398	14.559252	13.835602	13.513556	13.239302	13.477934	13.477273	12.879237	14.253427	12.713993	14.522678	14.462773	13.975250	14.928143	14.143339	13.344050	12.482634	13.928691	13.263764	13.401636	13.701136	14.128019	14.095049	14.100390	13.202977	13.724455	14.429371	14.042877	12.661535	13.988980	13.487933	13.397318	13.296970	13.734372	13.965466	12.586624	13.021168	14.257035	14.421398	13.763209	14.393289	14.693813	12.286447	13.268822	14.506817	13.658668	14.215623	13.499351	12.967881	12.857438	9.825243	13.242002	12.916049	15.348633	13.347432	12.588785	12.384984	11.848789	13.962183	12.942109	9.263847	10.246802	11.904326	12.869960	10.359182	10.482463	11.602116	11.901752	12.054832	11.346092	10.409534	9.784596	11.187873	11.983502	11.501897	11.467784	12.110693	10.429581	9.817117	14.575107	13.302266	11.118254	11.758886	11.456523	12.000568	12.564800	14.552270	12.258675	12.939392	12.572023	11.976679	12.649362	11.846836	12.106846	13.054365	9.837507	13.736123	13.067904	12.821251	11.694244	9.663115	13.173812	12.198274	13.650539	12.211265	12.864211	12.481464	13.095783	10.855566	9.005015	13.811158	13.483216	12.608353	14.773263	12.997893	13.413148	12.931331	13.686442	14.383451	12.936361	13.449081	13.377131	12.381157	13.180160	12.716965	12.156344	12.494508	12.039701	14.309961	12.236952	13.342644	13.420393	10.626098	12.517283	13.225108	12.960870	12.901976	13.984082	11.880705	13.202774	10.240737	11.508193	11.779120	12.010041	13.842996	13.076654	12.636634	13.659121	11.900148	12.644718	9.316241	11.687522	11.569303	11.793022	12.058239	12.474271	12.547466	11.440637	12.164205	12.336360	13.644958	13.774941	12.045877	12.223705	12.875552	10.108529	13.062098	10.213194	12.427164	13.555053	13.415241	10.506765

LINC02624	7.233383	7.641687	7.845650	7.778689	7.976173	7.817143	7.383159	8.396077	8.239336	7.343967	7.486938	7.388883	6.590464	6.975929	6.992596	7.486025	7.890599	6.909077	8.705991	7.240071	7.934974	8.640510	9.152473	7.480444	8.268871	7.957140	7.739464	7.988792	7.581181	7.673040	7.866962	7.155448	6.986363	6.653213	7.127683	7.298474	6.859263	7.145969	7.513109	7.917531	7.584267	7.349921	7.313043	7.814612	7.189399	7.498978	7.716139	7.454455	7.427752	6.954230	7.166091	7.036868	6.896720	7.156225	7.568557	7.890928	7.272024	7.385465	7.686094	7.287971	7.790524	7.287366	7.762198	8.150052	7.582415	7.783009	7.879615	7.848848	7.904130	8.357224	7.768537	7.814935	7.717516	7.555801	7.499060	7.509424	8.071834	7.997387	7.865390	6.986363	7.317336	6.928432	7.289724	7.214181	7.268933	6.827989	8.243097	7.230114	7.726532	7.385155	7.387168	8.004502	7.462608	7.651747	7.416254	7.414920	7.079100	7.345331	7.849606	7.763602	8.656980	7.369686	7.572780	7.428367	8.068615	7.165525	8.192758	7.904884	8.781348	7.766700	9.092833	8.440099	8.315429	8.060764	7.724844	9.616513	7.598098	7.274387	7.962059	7.022655	7.715828	7.606101	7.615888	8.999804	7.362446	7.706860	7.541602	7.703423	7.214035	7.456680	7.469081	7.739775	7.295217	7.461032	8.034493	7.575291	7.541891	8.444252	6.744003	8.259851	8.682812	8.425112	8.079480	8.394089	8.085086	8.491478	8.050788	9.035284	7.387618	7.826623	7.548082	7.509710	7.546301	7.326266	7.458563	7.183306	8.248095	8.312674	7.980345	7.920886	8.165188	7.614945	8.699378	7.554725	7.725594	8.186986	7.167325	8.173935	7.501815	7.425554	7.087059	6.897607	7.205372	7.116277	7.599377	7.429346	7.123215	7.465107	6.902075	7.583190	7.809422	8.111607	8.116691	8.014777	7.537013	7.732870	7.662814	7.679069	7.790829	8.190541	8.098702	8.159168	7.602658	7.589649	7.581955	8.015092	7.709122	8.169238	7.312895	7.088794	7.053815	7.529471	7.226699	7.325171	8.075160	7.216709	6.442531	7.959136	7.876139	6.994310	8.163833	7.465257	7.673196	7.546964	8.107889	8.163103	8.462777	7.614214	7.698520	8.107279	7.467854	7.642151	7.524066	7.792043	7.665583	8.187149	7.971938	8.435835	7.986868	9.004561	8.799286	10.136250	8.279347	8.174113	7.733781	7.277658	7.327546	7.694438

LINC00923	3.892199	4.842774	4.416874	4.539543	4.572211	4.845079	4.519569	4.859608	4.970485	7.052716	4.681366	4.608431	4.467366	5.025654	3.105403	4.495974	4.884308	4.755113	4.626946	4.654618	4.791432	5.639555	2.911822	4.645768	4.602914	5.191894	5.529921	4.611855	4.238537	4.226582	5.143159	3.254505	3.914658	4.401806	4.953764	3.752527	3.979150	3.728166	3.957014	4.919371	4.993938	3.009521	4.700680	3.694878	5.106448	4.505883	5.532003	3.431343	4.274425	3.408711	3.169645	4.539078	3.393651	4.623340	3.493366	5.182396	4.939947	4.025364	4.980326	3.723903	3.631286	4.943891	6.212338	4.594913	3.315690	4.023916	3.577153	4.284044	4.611855	4.748729	3.536879	4.582030	5.160880	4.162728	3.903786	4.211207	4.694331	3.884687	4.349640	3.908793	3.700152	3.525510	4.942189	3.972872	4.144088	3.171175	4.164047	3.823524	4.229455	4.518683	4.502632	3.986728	4.212963	4.081358	4.327873	4.703430	3.240460	5.141740	5.080622	5.235840	4.714555	5.116611	4.708848	4.975658	4.845952	3.811621	4.848502	4.348542	5.657752	4.718963	5.247007	5.385211	5.160090	4.993534	4.460696	4.664522	4.431158	4.774907	4.679862	6.739567	5.066538	4.518915	3.858553	4.891473	3.828629	4.741525	4.603540	5.051969	6.961500	4.583101	5.574989	4.473189	6.195674	5.520813	4.852018	4.882660	4.024941	4.393599	3.690345	4.162728	6.188082	2.899721	4.581620	4.707020	4.235520	4.396577	4.657491	4.591620	4.014251	6.638849	4.310387	4.942999	4.981142	5.767564	3.494456	3.228037	5.242044	4.506555	4.500267	5.044730	2.949611	5.379656	4.391494	4.099547	4.063079	5.279964	4.456047	5.876566	3.157430	3.048581	2.994658	3.480782	4.106299	3.295582	4.128277	7.049262	3.933686	4.525652	3.640693	2.894612	4.495974	5.200690	3.638690	3.520441	5.037736	3.537632	3.660010	4.363938	2.943316	3.794238	4.275090	6.263018	4.375223	3.794458	4.139072	4.201183	4.052167	4.294152	3.818420	3.486039	3.668288	5.465211	4.993534	3.980200	4.701302	3.570246	4.126565	3.955022	3.796526	5.549397	4.271585	5.406110	4.519569	5.085066	5.731934	4.604402	5.049157	5.886031	5.674591	5.188216	6.004467	4.570729	4.321835	4.673167	5.404268	5.053435	3.682236	3.984132	5.593282	5.620917	5.384282	6.450484	5.519185	4.348753	4.452895	4.598769	3.995086	5.251776

LINC02532	8.213774	3.253582	2.997982	3.548569	3.263273	3.117845	3.213260	3.124671	3.365338	3.191065	3.401420	3.173828	3.267648	3.545283	3.844991	3.088208	3.292054	3.257454	4.371859	3.268403	4.238326	3.019404	6.670564	2.966669	2.915340	3.645219	3.187793	2.803334	3.091974	3.040595	3.458581	3.147294	4.233943	3.400726	3.872419	3.813804	3.366868	3.144788	3.267214	3.170383	3.403027	2.978148	3.546167	3.280072	3.676420	5.084041	3.272546	2.948429	3.765302	3.345748	7.373429	3.053243	3.915108	4.019991	3.872419	3.142685	4.141508	3.481536	3.656622	3.790973	2.822427	3.122747	3.813350	3.168395	3.445804	3.451883	3.182044	3.201942	3.177618	2.797125	3.100328	3.075635	3.178028	2.871536	2.874704	3.331586	3.023793	3.057161	2.962547	3.711334	5.499654	3.818855	5.209621	4.177040	3.442575	4.134718	3.644627	3.282855	4.067751	3.592616	3.244844	3.168171	4.106979	3.201427	4.216015	3.133674	3.655630	3.242102	3.243536	3.083703	3.184490	3.168920	3.037418	2.843198	4.540468	3.195971	2.988326	3.311774	3.546976	3.171175	2.817488	3.617825	3.103544	3.248982	3.286535	4.138630	8.313127	3.222099	3.212552	3.336784	3.360967	2.970992	3.026482	3.309872	3.654396	3.075859	3.106541	3.291907	3.047315	4.188438	2.978433	3.023492	3.407994	3.904846	2.986472	3.158788	3.011939	3.292959	3.155938	3.748541	3.300075	2.774163	3.328676	2.849225	2.858775	3.033286	3.226440	3.091207	3.477651	3.060451	3.222673	4.048031	5.488371	3.674319	3.553719	3.276820	3.224050	3.293775	3.107586	3.167396	2.906663	3.591339	3.314483	4.124861	4.736547	3.207928	2.871110	4.051722	3.062576	4.032550	2.973723	4.002996	4.222090	3.509805	3.087756	3.826990	3.304280	3.299913	3.746476	2.800651	3.252661	3.883170	3.534455	3.724528	3.666689	3.177091	3.415541	3.254990	2.796100	3.035580	2.986558	3.799007	2.902673	2.884539	3.305084	3.012499	3.021451	3.022567	3.209791	3.700373	4.076809	3.677001	3.645020	3.506879	3.214885	3.292054	3.436524	3.707302	3.456795	3.340650	3.068364	3.707874	3.263418	3.126876	3.202087	3.434709	2.937088	3.784800	3.192946	3.093147	3.061340	2.989318	3.163794	4.912822	3.191743	2.977679	3.304924	3.090860	3.879790	4.114828	4.479330	3.136856	3.176379	3.031075	3.491185	2.931506	3.142315	3.319425

SLC25A5-AS1	7.312433	5.680106	6.870813	6.453958	6.323720	6.218215	7.328360	5.577584	5.700818	6.347421	6.466853	6.125675	6.578487	6.702417	6.532074	6.576684	5.727009	6.460003	6.655979	6.604561	5.720686	5.980285	5.319749	6.175417	6.484247	6.195129	6.734990	5.627815	6.474428	6.134299	5.897227	7.658623	7.498661	7.012688	7.105647	7.332417	7.387618	7.005651	6.392190	6.540186	6.437128	6.970890	7.118842	6.865185	7.433914	4.580362	6.692425	7.271564	6.779417	7.278770	7.157778	7.669912	7.463738	7.734263	7.421675	5.930243	8.061229	7.106294	6.521864	7.298925	7.414765	6.491525	7.204562	6.592272	6.275884	6.877490	6.769290	6.974792	6.633056	6.675083	6.824346	6.346221	6.703390	7.186189	6.783224	6.622253	7.041120	6.743202	6.476102	7.344419	6.732375	7.229369	7.176647	7.579177	7.616951	6.688548	6.913084	7.352977	6.708790	6.853497	6.125852	6.132918	6.432086	7.214830	6.648041	7.224035	6.901599	7.162520	6.891902	7.194046	5.607046	7.075460	6.930332	7.122578	6.406893	6.437732	5.352869	5.714200	4.993108	6.644679	5.766438	6.202793	5.273296	5.605125	6.489666	3.510367	7.172144	6.620661	6.470538	6.717965	5.675995	5.746482	6.656942	4.302633	6.445240	5.603982	7.314660	7.660474	6.597629	6.098721	6.825119	6.225900	5.324597	6.025877	6.651258	6.554786	6.933776	6.269124	7.961776	5.268266	6.097197	7.402873	5.255133	5.553182	6.303246	4.853955	6.243953	5.993577	6.972442	6.405375	6.255895	8.600963	6.723049	7.341925	7.816055	6.670915	5.698228	6.199473	5.627358	6.665078	6.280919	7.404910	6.876846	7.432040	5.482205	5.905278	8.120155	7.662002	7.719642	7.247168	8.385870	7.292562	6.546478	7.369531	6.475585	8.341593	7.844214	6.783072	7.506934	6.424158	6.828774	7.845347	7.177645	6.938279	6.770915	7.096715	7.145969	6.643709	7.535941	7.041120	7.645269	5.310700	7.074170	8.209048	6.242941	6.872271	7.858621	7.008674	6.683406	7.395714	8.888374	6.771711	6.596935	6.529490	6.700466	7.076686	8.235894	6.432597	5.696745	6.170998	5.322477	6.100838	6.322563	6.473421	5.770865	6.645635	7.174321	6.079043	8.462629	5.716039	8.199363	6.707390	6.464205	7.493510	6.869059	4.697660	5.195317	6.491525	5.351297	5.325555	4.816688	5.101759	5.055139	6.273716	4.339822	7.108364	6.258961	5.679946

AC113414.1	7.122892	7.786221	6.325780	7.687159	5.640661	6.777177	7.383930	6.404740	4.434340	8.565560	6.293873	6.843699	6.881674	6.848381	7.169475	7.168563	7.212966	7.835387	8.061071	6.439561	7.560294	5.794931	2.988814	6.066973	7.432496	7.396166	5.027874	6.477812	7.886288	6.355910	7.267429	7.822114	6.346393	5.694332	5.687183	5.902563	6.187919	5.994484	7.406634	6.059216	7.367347	6.332318	5.175953	6.926148	5.873704	6.845868	4.675355	6.737116	6.033087	6.261499	5.661986	6.265543	6.797331	5.401645	5.721042	7.083787	5.650854	6.523154	6.972117	6.355256	6.582283	6.882620	6.160979	5.064681	3.924166	7.349096	6.672354	7.584267	6.853497	6.811929	6.438750	5.227278	6.640143	6.213374	6.211030	7.219837	5.406712	6.539709	4.590982	6.409342	6.001442	7.123215	6.453644	7.750887	5.727373	6.815732	7.083787	5.926871	3.175876	7.214181	6.459041	6.354574	3.674117	6.954382	6.254190	4.342640	3.963586	6.470203	6.801570	6.396299	6.604877	6.569881	7.089306	6.756207	6.422080	6.162870	6.515542	7.269078	5.782186	7.143020	5.423847	8.300907	4.175969	7.017144	6.707538	4.550879	6.228660	7.524066	7.526963	6.408860	8.232255	5.664256	6.577833	5.357589	7.386247	6.697442	5.749561	4.864946	8.646914	4.994379	5.722901	7.627042	6.042958	8.068023	7.090251	7.872292	8.160532	8.485224	5.506607	7.813224	5.629161	3.152825	6.543376	6.990944	8.864465	5.223543	7.201019	7.173245	6.937962	7.784873	6.219210	6.465872	7.953706	6.471578	6.306442	5.622781	6.920814	8.515759	6.454936	7.996465	5.946205	6.221600	7.137353	6.567049	6.911143	6.021895	5.472722	6.401307	6.594381	4.174224	6.452101	4.974204	6.038087	5.947758	7.942332	5.799849	6.743667	5.990613	7.043199	6.751134	6.391043	6.731428	6.046736	4.905684	7.799754	6.309927	7.561945	7.120747	6.882311	6.124083	5.412818	7.513268	5.678435	5.702406	7.242922	4.721510	7.487101	5.151943	6.061895	7.421974	6.989679	4.927632	6.030085	6.150503	6.946970	6.664928	6.171677	2.985037	6.877490	5.886377	7.457297	4.299398	6.467545	6.888860	4.227672	4.499207	4.869173	5.315035	7.877349	5.757968	6.514561	5.838997	6.095497	6.670915	6.638207	9.127357	7.630529	6.550996	6.454454	5.452642	7.319961	3.432363	7.585017	5.905278	5.577584	7.372685	6.970435	8.032856

AL031666.2	5.923488	6.886135	7.662478	6.923867	6.983178	6.868396	6.024457	8.090942	6.674778	6.632896	6.040557	7.104405	5.753206	5.762139	7.174793	7.034373	6.722726	6.236812	7.166559	6.493772	7.082365	7.710165	6.144617	6.173864	6.338043	5.847907	6.062933	7.329932	4.905873	5.231998	6.506831	5.690061	7.452599	4.887696	6.565762	6.145144	6.693929	6.169784	7.233867	7.091187	8.055892	7.017952	6.943280	7.226387	6.956719	7.359489	5.370515	6.913084	6.644174	7.670222	7.016983	7.166260	6.441853	7.138120	7.284456	5.839528	7.182665	7.779827	7.035286	7.079256	7.825991	6.091481	6.678634	7.774143	6.558456	7.784553	7.372533	7.299385	7.486779	8.314845	8.102949	8.513380	6.096022	7.955709	7.800830	7.069219	7.299081	7.625330	7.755300	6.715079	7.206919	6.908123	6.449821	6.818634	6.530798	6.146884	7.454307	8.430715	6.702584	7.167473	7.243420	7.861985	7.326749	7.743966	5.861573	6.047635	7.622920	5.329117	6.434619	6.821815	6.331457	6.970890	6.913856	7.253698	7.948757	5.179925	7.116576	7.540513	7.154192	6.553821	8.563599	7.224972	7.388729	5.880506	7.569161	5.809052	5.572010	7.203798	6.156355	5.583994	5.933348	6.142913	5.834109	7.291287	6.536782	4.663487	5.529168	5.434147	5.060247	5.882650	5.731757	4.867022	5.550691	6.477985	7.139220	5.346523	5.260039	5.861216	4.865575	6.514226	5.602645	3.471047	5.971594	5.499654	5.399308	6.504316	6.080745	5.230761	4.510054	6.063823	5.707393	6.374837	5.968070	6.722579	4.627574	5.451858	6.153116	6.240272	5.779623	7.666363	5.755704	5.163875	6.559586	5.923308	6.628549	5.950898	6.167736	5.437272	6.645932	6.581476	6.037203	5.694721	6.011218	6.180816	4.388910	6.106391	6.719302	7.191824	5.306753	7.050534	5.580595	7.505088	7.086292	5.404435	7.169943	5.927937	6.550859	6.109572	6.863132	6.570194	6.828154	4.530309	6.735311	5.014252	6.350954	5.513333	6.483079	7.773530	5.578314	6.036011	9.069273	5.467351	5.277938	7.414619	6.011036	7.434700	6.927488	5.452453	6.081078	5.712111	7.385610	5.747211	7.376878	5.051393	5.549197	6.409176	5.295832	6.134823	5.236233	6.932252	5.587352	7.159964	6.004842	6.605446	6.124801	7.573879	6.378473	7.164719	6.194799	6.540026	8.133767	6.242601	5.432612	4.991195	6.027309	5.892768	5.569576	5.945313

AL049870.3	10.870835	9.302664	10.111540	10.161998	10.680518	9.992041	10.614651	9.909114	10.856320	10.294147	10.335381	10.487353	10.458804	10.200381	10.198884	10.747396	9.912507	9.936952	10.657698	10.489348	10.601053	10.044788	12.294356	9.985035	10.902906	10.752512	11.767049	10.427634	11.144404	9.793920	11.571323	9.991732	9.874810	10.057277	10.262677	10.513902	10.558709	10.243523	10.494672	10.563692	10.254063	11.008736	10.327480	10.431566	10.493142	10.257553	11.255473	9.956906	9.126126	9.931696	9.985649	10.432545	10.098120	10.169200	9.790524	10.281385	10.527040	10.869477	10.477553	10.846814	11.011552	10.716860	11.585740	10.312731	10.528938	10.521409	10.219240	10.081967	9.734545	10.798780	10.055597	10.306560	10.609657	10.271935	10.079519	10.242440	10.564279	9.742594	9.988728	10.337771	10.300966	9.762628	10.332682	10.278664	10.869477	10.366828	10.475430	10.540467	11.015291	10.291426	10.895475	10.693627	10.054057	10.096699	10.446580	10.514059	9.417796	11.070312	10.286370	10.797903	9.867109	10.473309	10.149023	10.339897	10.062437	10.147330	10.181623	10.141487	10.903200	10.579502	11.531829	9.906054	9.378827	10.778399	9.256667	11.100603	10.865897	10.666208	11.261380	10.576772	8.934723	9.136065	9.789747	9.608538	8.972908	9.643918	9.753292	10.451395	9.964867	9.517296	10.301432	9.830915	9.389757	9.499324	11.249023	10.262244	9.318053	9.966801	9.516374	10.863674	9.849770	10.731781	9.933712	10.448715	9.554189	9.782986	9.519152	10.993300	9.112102	11.096621	9.335123	9.394342	8.930159	9.431265	10.023543	9.657788	10.466810	9.904409	9.343124	9.551928	9.433441	9.335569	9.843495	9.010681	9.068164	10.185582	8.712976	9.544271	8.904922	8.763170	10.103883	9.337859	8.734707	9.487347	10.001946	9.932777	8.837234	9.318053	8.918471	9.415969	10.515623	9.939949	9.503469	9.586539	9.664527	9.411123	8.777533	8.866879	10.175018	9.341453	9.537676	9.143508	9.259991	9.839071	9.417469	9.832615	9.405556	9.129229	10.766265	9.650087	9.789747	9.735326	9.610727	9.666872	9.870094	9.689001	9.570429	10.788398	9.857107	10.139689	9.318827	9.759027	9.327512	9.666076	9.898981	9.051148	11.098411	9.607325	9.565121	9.947336	9.604530	9.621360	9.164187	10.797595	10.054805	8.705672	9.287859	10.425603	9.891801	10.504406	9.219146	7.983773	10.553689	9.697544	9.128435	9.916692	9.944847	9.627691

AC022784.1	12.037148	11.317056	11.548287	11.610052	11.809762	12.652934	12.190447	12.432421	12.762381	12.631160	12.841413	12.743706	11.872205	12.003100	11.597568	12.080159	12.389676	11.628487	10.808753	12.327168	11.803385	11.816173	10.920184	11.780465	11.585156	12.064848	13.264544	12.546069	12.788040	12.292362	12.291548	11.213507	10.930976	12.131737	11.612874	11.978981	11.615814	11.879689	11.357821	11.502750	12.572521	10.819087	11.184951	11.196379	11.142502	11.155666	12.730491	11.187721	11.552587	11.214808	10.966969	10.633212	10.736862	10.463567	10.603870	12.527592	10.377566	10.934280	10.637121	10.794682	10.481689	12.664618	9.604383	10.798488	9.899599	11.174295	11.417836	11.222202	11.318087	10.565026	11.652730	11.729465	12.844209	12.135121	11.940295	11.761312	11.910288	11.709110	11.770344	10.426540	10.502569	10.750389	10.288677	10.680668	10.862604	11.325845	10.681815	10.426540	10.425458	11.723766	10.790846	10.596659	11.265456	11.760077	11.146797	11.847451	11.917748	12.368604	11.830627	12.485005	12.575563	11.633888	12.177192	12.042106	11.108144	12.539475	12.463562	11.704017	10.894745	12.813342	10.954841	11.348194	11.778816	13.306393	12.599014	12.275177	10.959032	12.347129	13.439680	12.116671	11.404390	11.284371	12.074353	12.503725	12.728769	12.725496	12.902873	12.892258	13.179765	12.608353	12.806661	12.743020	11.851856	12.011560	12.430780	12.641550	11.473665	10.628696	10.901113	11.297358	10.978775	10.455568	11.598873	11.377987	11.253526	11.790286	12.294510	13.210112	12.273762	13.049541	11.437676	11.551660	12.156499	12.058559	11.216126	11.336717	11.693331	11.440039	11.084693	12.835254	10.796271	10.697494	11.609049	10.598160	10.777827	13.129366	10.591268	11.371284	11.000306	10.796577	11.089443	9.734852	10.169500	10.512279	12.344233	9.672692	10.412086	10.249099	10.225370	10.449467	12.726341	10.505428	10.635198	10.049407	13.281038	10.845627	10.833896	11.072856	10.119137	11.093256	11.656735	13.128058	12.112064	11.762513	11.479023	12.059200	11.854455	11.240625	10.322594	10.549791	10.638482	10.693912	11.072707	10.860728	11.690932	10.988689	10.657396	10.140907	10.925491	11.487721	10.572740	11.595476	11.942720	12.307454	12.709993	12.114204	12.694239	12.333973	11.508636	12.635273	11.821698	12.109513	12.448328	10.572597	13.315805	12.364654	11.301874	10.298660	12.844050	10.431097	11.197531	11.384967	13.147891	11.574642	11.435242	11.151411	12.465329	12.752782

LINC01159	6.566408	6.282450	6.322563	6.363544	7.359946	6.467022	6.135673	7.098929	7.109818	6.318078	6.272706	6.446864	5.433001	6.543856	5.378285	6.163918	7.511385	6.077620	6.813024	6.002530	6.268096	7.332577	7.458243	7.086585	7.256648	6.715222	6.104775	7.212810	6.239587	5.915113	6.499728	4.718328	5.985749	5.904025	6.356437	5.900251	5.818676	5.838053	6.643191	6.760608	6.358329	6.015535	6.300828	6.073290	6.861077	6.730471	6.123031	5.487095	5.290178	5.961292	6.102541	6.835143	5.085506	5.409314	4.615121	5.858504	5.478682	6.056548	5.762139	5.864878	6.333836	6.253010	7.011725	6.825746	6.144967	6.503995	5.798204	6.167031	6.114764	6.902551	5.912957	6.477105	6.104605	6.055659	5.750837	5.151351	6.157372	5.806670	6.469202	6.127660	5.203062	5.882650	5.337710	5.925083	5.979804	5.588697	5.588122	6.116308	6.577162	6.652077	6.556302	6.704797	5.969473	5.883889	5.817622	5.828774	5.250790	7.943235	5.911336	6.641921	6.833118	5.465392	6.018659	6.420776	6.367558	5.946365	7.572330	5.659046	7.501815	6.609956	6.791521	7.567498	6.693929	6.626777	6.371935	5.757033	7.434558	6.483741	6.549699	7.031175	6.041080	5.591589	5.494903	7.326266	5.769200	5.290806	6.521545	5.976204	5.626559	5.694721	5.457696	5.597008	5.496067	5.502582	5.478682	6.408366	4.579470	6.662960	5.758891	5.711196	6.371774	6.868396	6.023770	6.288447	6.124801	5.342348	6.692595	5.141316	5.509568	5.879233	4.479330	4.781151	4.492888	6.305609	5.244217	5.069790	5.183812	6.272050	6.186579	5.663868	6.363905	6.533240	5.309441	5.605540	6.506499	5.693962	7.743660	4.852460	5.316667	5.291773	5.993219	5.988641	5.376333	4.373702	4.993314	6.948099	5.491509	4.994155	5.528211	6.317575	5.585717	5.753035	6.011402	5.556569	5.854373	5.686052	5.710503	6.123195	6.278795	5.545624	5.592741	6.114764	5.379656	5.179561	4.949604	6.027838	6.071530	6.202438	5.300632	4.552354	5.317845	5.061811	5.248785	5.552010	4.904316	5.284147	5.656663	6.491380	5.946205	8.076646	6.715696	5.536264	5.550863	5.352068	4.991612	5.879616	6.481237	4.587990	4.271174	5.568462	4.730610	4.947749	4.784747	5.157902	5.785597	6.831311	5.763590	6.607708	6.500881	6.495088	6.515048	6.666216	6.572438	6.913999	5.954174	7.027648	5.763933	5.858142

AC018730.1	6.566408	6.282450	6.322563	6.363544	7.359946	6.467022	6.135673	7.098929	7.109818	6.318078	6.272706	6.446864	5.433001	6.543856	5.378285	6.163918	7.511385	6.077620	6.813024	6.002530	6.268096	7.332577	7.458243	7.086585	7.256648	6.715222	6.104775	7.212810	6.239587	5.915113	6.499728	4.718328	5.985749	5.904025	6.356437	5.900251	5.818676	5.838053	6.643191	6.760608	6.358329	6.015535	6.300828	6.073290	6.861077	6.730471	6.123031	5.487095	5.290178	5.961292	6.102541	6.835143	5.085506	5.409314	4.615121	5.858504	5.478682	6.056548	5.762139	5.864878	6.333836	6.253010	7.011725	6.825746	6.144967	6.503995	5.798204	6.167031	6.114764	6.902551	5.912957	6.477105	6.104605	6.055659	5.750837	5.151351	6.157372	5.806670	6.469202	6.127660	5.203062	5.882650	5.337710	5.925083	5.979804	5.588697	5.588122	6.116308	6.577162	6.652077	6.556302	6.704797	5.969473	5.883889	5.817622	5.828774	5.250790	7.943235	5.911336	6.641921	6.833118	5.465392	6.018659	6.420776	6.367558	5.946365	7.572330	5.659046	7.501815	6.609956	6.791521	7.567498	6.693929	6.626777	6.371935	5.757033	7.434558	6.483741	6.549699	7.031175	6.041080	5.591589	5.494903	7.326266	5.769200	5.290806	6.521545	5.976204	5.626559	5.694721	5.457696	5.597008	5.496067	5.502582	5.478682	6.408366	4.579470	6.662960	5.758891	5.711196	6.371774	6.868396	6.023770	6.288447	6.124801	5.342348	6.692595	5.141316	5.509568	5.879233	4.479330	4.781151	4.492888	6.305609	5.244217	5.069790	5.183812	6.272050	6.186579	5.663868	6.363905	6.533240	5.309441	5.605540	6.506499	5.693962	7.743660	4.852460	5.316667	5.291773	5.993219	5.988641	5.376333	4.373702	4.993314	6.948099	5.491509	4.994155	5.528211	6.317575	5.585717	5.753035	6.011402	5.556569	5.854373	5.686052	5.710503	6.123195	6.278795	5.545624	5.592741	6.114764	5.379656	5.179561	4.949604	6.027838	6.071530	6.202438	5.300632	4.552354	5.317845	5.061811	5.248785	5.552010	4.904316	5.284147	5.656663	6.491380	5.946205	8.076646	6.715696	5.536264	5.550863	5.352068	4.991612	5.879616	6.481237	4.587990	4.271174	5.568462	4.730610	4.947749	4.784747	5.157902	5.785597	6.831311	5.763590	6.607708	6.500881	6.495088	6.515048	6.666216	6.572438	6.913999	5.954174	7.027648	5.763933	5.858142

AC009365.1	4.879846	4.736102	4.747859	6.035454	6.441219	6.350277	5.140257	6.779417	7.164423	6.853335	6.546642	6.794940	6.711965	5.986093	4.862492	5.009311	6.466021	5.011405	5.993577	5.069387	6.055134	4.700680	7.166404	5.178157	4.680289	6.838834	6.389179	5.738433	5.269846	6.571789	7.444912	4.638413	3.782722	5.116218	4.443074	4.778641	5.443704	5.055751	4.205714	4.411319	6.403118	4.923847	3.815667	4.801541	4.682853	5.169098	8.568786	4.477155	5.183016	4.058241	4.040146	4.281251	5.608157	3.812109	4.478244	6.846765	3.926325	3.620854	3.924795	3.607835	3.846729	6.743202	4.281683	4.311250	4.373025	4.911116	4.507242	4.607765	4.252568	3.993727	4.955010	5.299488	6.547107	4.097759	4.901952	5.400711	4.517223	4.470128	4.262886	3.335465	3.960098	3.659231	3.098204	3.323316	3.608224	4.867232	4.042312	5.875848	4.886038	3.945977	3.957915	4.920418	5.037916	5.269249	6.343180	5.000471	4.809096	5.962707	7.569764	6.772683	6.483577	5.298058	5.847321	5.065280	5.045149	5.567473	4.951026	4.993108	4.204289	6.038781	4.864543	6.404233	5.960569	7.539270	7.066662	7.256648	5.460459	6.028219	6.132434	5.229330	5.674028	4.963356	6.467022	7.360909	7.028638	6.142404	5.981511	5.804407	7.900057	6.569361	7.561801	6.910655	6.770414	6.736595	5.351677	5.807783	6.347920	5.532941	4.888354	5.144352	4.284744	5.494903	7.249321	4.945653	5.552989	6.296420	7.237677	6.649192	6.965325	8.156255	5.391260	5.266469	5.743559	4.691187	5.032136	5.073229	5.939145	4.721287	4.680068	7.747797	4.377580	3.518376	4.500504	5.052568	5.033372	8.619883	4.559441	5.708481	4.183639	4.550441	4.302876	5.641019	3.732517	4.066061	5.726644	3.507422	3.514626	3.885739	3.754459	4.132771	6.162053	4.718328	4.352665	4.418927	3.907475	3.472373	4.636303	5.113550	3.990448	5.464226	5.381551	8.016331	4.579696	4.865377	5.364244	4.498550	5.240060	5.135616	3.428672	3.296210	3.738939	3.881690	3.387383	3.328860	6.323541	3.598945	5.225089	5.144534	3.311024	3.955463	5.013840	4.717928	4.984172	5.804583	5.397296	5.749191	6.934887	7.160446	5.475801	7.554247	5.920317	6.322896	5.640843	5.197306	7.508474	5.208018	3.874839	4.775737	6.517061	5.382123	6.515893	7.210041	9.130166	7.966334	6.953906	4.933036	6.674778	6.158402

AC123595.1	8.752885	7.319152	7.469644	7.744866	8.427236	9.314604	8.529825	9.024766	8.960071	8.675783	9.206285	9.398637	8.621584	8.025580	8.078855	8.222227	8.683089	7.704712	8.464590	8.016331	8.419013	8.510749	9.517296	8.343667	8.168296	9.000566	9.682637	9.111112	9.827675	8.900227	8.722971	7.309610	7.622087	8.523545	7.489591	7.536868	7.789614	7.425999	7.731800	7.801000	8.685533	7.812644	7.182036	8.090173	7.745299	7.467564	10.003058	7.195840	8.149614	6.980170	7.342853	7.039698	7.064918	7.280383	7.861363	9.211908	7.446975	7.531277	7.527775	7.410189	7.597468	8.759950	7.961301	8.025893	7.658461	8.095531	7.625612	7.495029	7.622541	8.064574	7.896112	7.995502	9.322724	8.028874	7.964325	8.018445	8.139719	8.196546	8.244457	7.370785	7.518351	7.520175	7.409576	6.911787	7.347027	7.966334	8.052496	7.623778	7.677460	7.850793	7.751210	8.285007	8.248863	8.289633	8.648726	8.316693	7.862754	8.313277	9.072055	8.467638	9.524275	7.287232	8.125709	7.779974	8.104652	8.987805	8.768535	7.947541	8.902785	9.229700	8.791665	8.371594	8.250672	9.689605	8.996430	12.752609	7.938047	8.811703	8.939423	8.830099	8.373960	7.607291	7.840605	10.568013	9.241805	9.932911	9.895350	10.361120	9.202497	10.293707	9.776425	10.585380	9.406024	9.267059	9.194034	9.340437	8.629123	9.063831	8.140611	8.560387	8.899303	9.625854	9.826316	8.970829	9.105657	9.739786	9.884994	11.649756	8.486274	8.926121	9.217443	8.983646	9.322286	8.421962	8.368477	8.729427	8.995637	8.744959	9.123377	11.435808	8.176794	7.408527	8.748785	8.225701	7.628126	10.549196	7.921935	9.047757	7.826301	8.397483	7.522082	7.816357	8.389753	8.104652	9.045789	8.005930	7.767315	7.926147	7.205220	8.767930	8.791995	8.098995	8.055749	7.732726	8.354136	8.324630	8.146710	8.706747	8.285774	8.226166	8.543687	10.849009	8.607569	8.717495	8.303462	8.616473	8.656397	8.463370	8.371132	8.197638	7.715669	8.475419	8.263850	7.770324	9.786539	9.083352	7.758072	8.032995	8.114241	8.594703	8.852270	9.344831	9.483198	9.456638	9.871373	10.207843	9.724689	9.446852	8.335081	9.923051	9.716865	9.820554	8.264623	8.240832	9.645648	9.616513	7.788365	8.728213	10.903357	9.194946	9.585922	9.632756	10.809055	10.152807	10.556772	7.881084	9.462868	9.142279

LUCAT1	8.752885	7.319152	7.469644	7.744866	8.427236	9.314604	8.529825	9.024766	8.960071	8.675783	9.206285	9.398637	8.621584	8.025580	8.078855	8.222227	8.683089	7.704712	8.464590	8.016331	8.419013	8.510749	9.517296	8.343667	8.168296	9.000566	9.682637	9.111112	9.827675	8.900227	8.722971	7.309610	7.622087	8.523545	7.489591	7.536868	7.789614	7.425999	7.731800	7.801000	8.685533	7.812644	7.182036	8.090173	7.745299	7.467564	10.003058	7.195840	8.149614	6.980170	7.342853	7.039698	7.064918	7.280383	7.861363	9.211908	7.446975	7.531277	7.527775	7.410189	7.597468	8.759950	7.961301	8.025893	7.658461	8.095531	7.625612	7.495029	7.622541	8.064574	7.896112	7.995502	9.322724	8.028874	7.964325	8.018445	8.139719	8.196546	8.244457	7.370785	7.518351	7.520175	7.409576	6.911787	7.347027	7.966334	8.052496	7.623778	7.677460	7.850793	7.751210	8.285007	8.248863	8.289633	8.648726	8.316693	7.862754	8.313277	9.072055	8.467638	9.524275	7.287232	8.125709	7.779974	8.104652	8.987805	8.768535	7.947541	8.902785	9.229700	8.791665	8.371594	8.250672	9.689605	8.996430	12.752609	7.938047	8.811703	8.939423	8.830099	8.373960	7.607291	7.840605	10.568013	9.241805	9.932911	9.895350	10.361120	9.202497	10.293707	9.776425	10.585380	9.406024	9.267059	9.194034	9.340437	8.629123	9.063831	8.140611	8.560387	8.899303	9.625854	9.826316	8.970829	9.105657	9.739786	9.884994	11.649756	8.486274	8.926121	9.217443	8.983646	9.322286	8.421962	8.368477	8.729427	8.995637	8.744959	9.123377	11.435808	8.176794	7.408527	8.748785	8.225701	7.628126	10.549196	7.921935	9.047757	7.826301	8.397483	7.522082	7.816357	8.389753	8.104652	9.045789	8.005930	7.767315	7.926147	7.205220	8.767930	8.791995	8.098995	8.055749	7.732726	8.354136	8.324630	8.146710	8.706747	8.285774	8.226166	8.543687	10.849009	8.607569	8.717495	8.303462	8.616473	8.656397	8.463370	8.371132	8.197638	7.715669	8.475419	8.263850	7.770324	9.786539	9.083352	7.758072	8.032995	8.114241	8.594703	8.852270	9.344831	9.483198	9.456638	9.871373	10.207843	9.724689	9.446852	8.335081	9.923051	9.716865	9.820554	8.264623	8.240832	9.645648	9.616513	7.788365	8.728213	10.903357	9.194946	9.585922	9.632756	10.809055	10.152807	10.556772	7.881084	9.462868	9.142279

AC063952.4	7.671958	7.774290	7.672898	7.864481	7.796171	7.965409	7.792043	7.894980	7.879758	7.815116	7.578705	7.334577	6.988569	7.032182	6.750212	7.368431	7.560294	7.166559	7.898519	7.324386	7.760826	7.743008	8.157493	7.264789	7.076359	6.632390	6.713250	7.742874	7.934018	7.863243	7.204235	7.569604	7.145969	7.174163	6.940592	6.891084	6.989994	7.433753	7.284738	7.478448	8.186342	7.338470	6.010871	6.757042	6.199995	5.929339	7.680267	6.681110	7.367028	6.823214	6.888567	6.808885	6.813340	6.887741	6.970730	7.713435	6.455432	6.928432	7.064759	6.573050	7.572185	7.573718	7.418684	7.492285	7.364513	7.270940	7.612184	7.681614	7.682404	7.924598	7.693207	7.527284	7.747797	7.531887	7.491660	7.270165	7.652041	7.389191	7.329134	6.443880	6.704635	6.223463	6.531278	6.532074	6.598102	7.645570	7.223266	6.823537	7.845183	7.336280	7.139694	7.489591	7.029566	7.100497	6.300993	6.505130	7.319961	6.994459	7.254021	7.352823	7.567340	7.328360	7.208301	7.571876	7.823335	8.196072	7.510785	7.441933	7.747014	8.508449	8.351865	7.299545	7.720377	8.448899	7.308493	8.424087	7.705159	6.978653	7.421675	7.525428	8.099298	7.784389	8.253582	7.813224	8.348402	7.558907	7.878260	7.760354	8.050463	8.139719	7.160938	7.382198	7.199937	6.623549	7.641386	7.923339	8.524156	8.712976	7.794750	8.248548	8.433816	9.106435	8.553322	8.234656	7.590734	7.747490	7.719509	8.618293	8.442390	9.280151	7.685039	7.329461	7.848245	7.099247	7.776338	7.000814	8.336333	8.424530	7.642619	8.276257	7.815434	7.299697	7.679817	6.868899	7.356553	8.826140	7.253698	7.947845	7.509710	7.383010	6.913084	7.246377	7.559652	7.167777	8.593010	7.444912	7.920589	7.443943	7.799910	8.541658	7.942940	8.734257	8.129187	7.818875	7.811416	7.913282	7.964808	7.904431	8.414300	8.011709	8.059715	7.811708	8.295296	7.851877	7.635247	7.877938	7.906928	7.741489	7.000648	7.109503	7.688261	7.047549	6.907507	7.565801	8.128115	7.204562	7.398965	8.006846	7.651278	7.636702	7.871982	8.027961	7.689026	7.482286	7.135309	7.582415	7.624268	8.017257	8.238086	7.392716	7.783772	7.763284	7.655796	8.456103	8.148697	7.895121	8.199029	8.511528	8.454898	8.806276	8.288718	9.495201	8.417392	7.621942	7.207379	7.284738	7.876282	8.605139

LINC02591	9.120492	8.163242	8.270740	9.065840	9.623069	9.241805	8.774754	9.744273	10.325523	8.832570	9.529758	9.655511	9.229431	9.523949	9.046071	9.333838	8.884356	8.920376	9.701888	9.107332	9.000110	9.209157	10.563219	9.822841	9.503777	9.558642	10.484898	9.169228	9.296610	9.109868	9.041181	8.274332	8.636910	9.823884	8.842601	8.476914	8.656689	8.646197	8.454599	9.227589	8.988507	9.145493	9.515307	8.745107	9.103800	8.674520	9.369517	9.080413	7.796324	8.504326	8.167811	8.546419	8.763615	8.826297	8.479139	9.076866	8.783210	9.276523	9.305277	9.150322	9.405140	9.174793	9.588576	9.354094	8.688190	8.742540	8.792288	8.223133	8.266356	8.948291	8.572726	8.380355	9.696173	8.767446	8.716747	8.921205	8.331700	8.211273	8.586509	8.467473	8.433526	8.516685	8.277390	8.417684	8.600049	8.474628	7.901974	8.875113	9.755013	8.867036	9.224522	9.592749	9.073136	9.162133	9.135773	9.313619	8.193679	9.345450	9.180017	9.007632	8.712506	9.204729	8.952859	9.000858	8.756854	8.982132	9.581892	8.718121	9.390506	9.125660	9.166498	9.819337	9.583919	9.674273	8.672489	9.827675	8.971445	8.619728	9.625356	9.543140	9.001818	8.316232	9.465179	9.966500	8.794316	9.074340	10.256330	8.939581	8.728213	9.656426	9.179848	9.608075	9.552669	9.590587	9.474159	9.627839	8.618293	7.550369	8.666821	8.531668	9.559999	8.587856	9.125817	8.801575	8.755477	9.338465	9.113742	9.688696	8.576545	10.241203	8.914189	9.157249	9.513501	8.477574	7.667897	8.650870	8.761974	9.820128	8.729291	9.524275	8.938096	9.008558	8.598050	8.558851	8.577277	9.408800	8.166604	7.323595	8.407117	8.366088	8.476639	7.949038	8.859114	8.982441	8.819333	8.199029	8.346927	8.794606	7.760209	8.799005	9.111930	8.687318	8.993030	9.389757	9.136672	9.073286	8.204471	8.473581	9.000709	8.184797	8.412468	9.681392	8.958589	8.670197	9.042516	8.673745	8.157342	8.850032	9.542810	8.823280	8.233737	9.229700	8.848031	9.621360	8.993181	7.652974	8.614804	9.709848	9.592125	9.890580	10.215948	8.989863	9.283544	9.323513	9.874356	8.590668	9.485807	9.531982	8.511835	9.485965	9.646315	9.196271	8.827612	9.066608	9.592440	9.877830	8.161466	7.959912	9.367879	8.903998	9.312017	10.027195	10.086994	8.765596	9.285962	9.697396	8.356290	9.922132

AC008496.2	8.409551	9.161517	8.554484	8.577568	8.804154	7.640177	8.419453	7.517578	8.252070	8.244609	8.533021	8.685533	8.408805	8.295296	9.149853	8.718121	8.269361	8.833199	8.861063	8.646197	7.987181	7.416084	11.187132	8.958743	9.180474	8.958268	8.528611	9.414865	9.271969	8.423166	8.362692	9.380526	8.585918	9.810408	8.724169	8.987939	8.958107	8.581670	8.752751	8.849730	8.472968	9.176514	8.664085	9.163879	9.280452	8.823713	9.683246	8.935611	8.772629	9.219906	8.947993	9.062021	8.976368	8.744959	9.126967	8.573058	9.128119	8.949830	9.377622	9.122924	9.567009	8.417684	11.017510	9.220054	10.141980	9.038327	9.159233	8.750986	9.173279	8.997364	8.651625	8.643311	8.472170	8.750022	9.010403	8.818241	8.788120	9.285661	9.117099	9.374798	8.980884	9.388862	9.172960	9.573835	9.351550	9.262432	9.353052	8.892055	9.671188	9.042079	9.447790	9.117099	9.099806	8.180838	9.107492	9.218853	9.182515	8.663180	8.938979	9.028112	8.338060	8.723284	8.680202	8.780870	8.397169	7.889992	8.515034	8.405306	9.037684	8.180074	9.072827	8.747415	8.476201	8.007472	7.671486	8.400801	8.161308	8.852719	8.198081	9.689916	8.562241	9.576986	8.405459	9.343762	8.990467	10.128694	9.574283	8.446548	8.968673	8.573236	10.386256	8.842121	10.007378	7.640322	10.402738	9.095237	10.111382	9.653700	10.466673	9.564507	8.555909	9.084243	9.876877	10.022295	8.410617	9.205649	7.230864	9.999048	8.776601	9.492544	8.850472	8.439951	8.271201	9.552825	9.763085	9.207491	8.595167	9.048698	8.967435	8.314211	9.677215	8.631600	10.347055	9.138437	8.575191	8.602194	10.555843	9.262911	8.936079	9.201231	9.492217	9.095560	9.836099	9.721549	10.475718	10.988232	11.369466	10.040698	10.021565	10.855566	9.612400	9.750825	9.414865	10.046547	10.842875	10.682122	9.060051	8.405459	9.771907	8.368201	9.485965	8.190710	9.738831	10.404103	8.782909	9.744747	9.400002	8.785633	10.683331	10.373661	10.557368	9.954924	9.699083	11.495210	10.021101	10.428516	10.197958	10.776828	10.276224	9.456308	9.941815	9.741675	8.094921	8.863706	8.624508	9.447310	9.415515	9.475363	10.277301	8.423629	8.712812	10.112741	9.809845	10.196293	7.206919	8.789174	8.523382	10.526885	7.385317	9.832758	9.695713	9.246132	7.651114	9.082581	8.975406	9.279384	8.952418	8.444090

TPT1-AS1	8.384047	8.748036	8.564200	8.504041	8.710321	8.907026	9.136223	8.838748	8.980298	8.677427	8.927633	8.979999	8.950728	9.568410	8.786399	9.274259	9.113027	8.626257	9.195114	9.494730	8.129187	9.212976	10.065419	8.680653	9.412599	8.947836	9.019117	9.028112	8.614211	8.243401	9.398469	8.807046	8.886073	8.667742	9.003802	8.952859	9.381126	9.437249	8.935611	9.028879	8.647850	9.185636	9.099191	9.182515	9.419599	8.981810	8.434442	8.824932	9.277548	9.310654	9.291592	9.130445	9.399085	9.222961	9.366138	8.630840	9.447790	9.615452	9.159694	9.344981	9.201691	8.908772	9.755469	9.334014	8.774914	9.424518	9.068319	8.738729	8.311463	8.647693	8.866274	8.692143	9.388107	9.077347	9.213270	8.453689	8.714536	8.800956	8.943081	9.473089	9.179692	9.196896	9.107963	9.311872	9.537039	8.797797	9.602366	9.581892	9.008878	9.174641	8.705991	8.767611	9.122611	8.915240	8.503412	8.929856	9.185636	9.522722	8.670197	9.325661	8.874648	9.097364	9.209476	8.917711	8.947993	9.326595	9.286592	9.809978	8.697899	9.189785	8.923429	8.891290	9.648718	8.785471	8.837843	6.111319	8.897627	8.641277	9.634626	8.284855	9.131973	8.245084	7.630838	8.305738	7.558478	8.128410	9.754226	8.310809	8.522763	8.469173	8.854389	9.020018	7.903509	7.989562	8.894256	8.501568	8.011098	8.499325	8.190378	8.412161	8.396241	7.912515	8.485224	8.081192	9.212520	8.063661	8.443081	7.547593	7.634486	7.957883	8.271947	8.787017	9.593060	8.663767	7.858326	8.878315	8.478374	8.831497	8.605303	6.950448	7.724547	9.133380	9.597882	8.676360	8.451400	8.037881	8.296244	8.207548	10.134987	9.645648	8.932558	8.767777	9.945139	8.990766	7.930030	9.638720	8.990170	8.238691	9.401696	8.226324	8.546108	9.410488	8.633972	8.620028	9.137632	9.055814	8.327636	8.152993	8.776297	8.453383	8.489362	8.324932	9.000251	8.415166	8.275673	9.151401	8.740108	9.627387	9.467827	9.204423	8.709164	9.754711	8.906569	9.380048	7.858326	8.959155	9.374798	8.789796	8.787683	8.325568	9.337110	8.905804	9.065994	8.415321	8.569898	8.861679	9.341602	8.729291	8.503105	7.796324	10.191313	8.360634	8.460633	8.002769	7.514634	9.732580	8.887600	8.022090	7.985454	8.655793	7.204022	5.666656	7.494498	7.247008	8.196705	8.538773	8.474316	7.546301

Z84485.1	7.575603	7.550974	7.834160	7.580698	7.708229	8.083021	7.416254	7.533611	8.468252	8.279188	8.290087	8.052945	7.308348	7.948001	7.364365	8.370828	8.824316	7.865227	8.695889	7.678459	7.338770	8.365016	7.724844	8.366367	7.782708	8.493080	9.248587	7.376415	7.870902	7.312275	8.701668	7.380012	8.002934	8.481866	8.023925	7.625330	7.460241	7.139071	7.488806	7.585336	7.736809	7.272497	7.977678	7.990967	7.610371	8.021791	8.464289	6.484786	7.280541	7.628292	6.889174	8.134382	7.691511	8.161158	7.788217	7.895952	7.308348	6.623080	7.663558	7.433129	6.665078	6.942144	6.940592	7.126590	6.672197	6.428009	7.155921	6.880550	6.543526	7.188899	6.832787	7.028470	8.163679	6.861393	7.633622	7.306970	7.150750	7.211176	7.228420	7.674628	8.148242	7.885512	7.481815	7.456502	7.696523	7.919819	8.008692	7.990815	6.698709	7.736955	7.300310	7.323595	8.086448	7.078783	7.347870	8.266356	8.023315	8.467956	8.388820	8.095831	7.568853	7.182354	7.842958	6.992106	7.028638	7.992704	8.427563	8.317461	8.052014	7.479807	7.287366	8.394988	7.391638	8.301810	7.619230	6.152751	7.380825	8.072872	8.130096	8.865208	7.255390	8.005298	8.515332	8.755929	7.984242	7.743798	11.498609	7.514055	9.895199	7.442676	8.183127	7.452118	8.852566	5.124493	11.001938	9.354675	7.979579	7.137501	6.639174	7.824306	8.862760	10.045559	8.526901	7.148828	7.446975	8.151760	5.960212	7.391949	7.698822	9.629990	9.218055	9.262112	8.252821	9.183450	8.208599	7.659517	8.299554	8.059157	7.242261	8.817585	6.540026	8.382213	9.069273	6.967400	7.728413	8.494150	7.086134	7.919036	6.463552	5.983137	8.467638	8.685237	7.005812	8.365170	8.069369	9.644070	8.733797	7.723476	7.389191	6.626475	7.381539	8.670032	7.146133	6.818791	8.196072	6.769936	6.903692	6.797498	7.517578	6.718290	6.531278	9.877347	7.745467	8.407253	7.403964	8.980884	7.337505	7.619091	8.040436	8.539743	8.807508	8.328604	9.854621	8.233737	8.366957	9.432816	9.337708	7.508140	8.409838	8.839496	7.671958	10.657396	7.431568	8.294571	8.519607	7.890305	8.941716	10.607990	8.490724	6.578148	9.400578	9.526925	5.971594	8.637389	8.546912	8.720555	8.937648	7.456825	7.570827	7.290668	7.324386	6.579309	8.347801	7.265404	8.089401	6.424645	9.299855	7.386991

LINC02291	3.050254	3.027480	2.936817	2.935139	3.059715	2.952386	3.183074	3.709872	3.335465	3.675168	3.462866	3.693618	3.149520	3.806168	3.471558	3.399012	3.348845	3.529405	4.453325	3.762166	5.414157	2.869906	5.332656	3.148992	3.183074	2.874704	2.936632	2.983166	3.040482	3.009521	3.665267	3.833320	3.052798	2.926799	3.450236	4.235322	3.904662	4.650703	4.148341	4.639899	3.375999	2.984145	4.345679	4.182761	3.270087	4.116356	3.218416	4.903206	2.963416	3.552011	3.532537	5.364435	4.587091	4.281251	4.228110	2.957247	4.631548	3.190532	5.392969	4.718543	3.422249	3.069020	3.792266	3.514626	2.995806	4.051084	3.565517	3.441704	3.002733	4.145567	3.989853	3.533846	2.926088	3.364370	3.713156	3.151777	3.404710	3.276507	3.280866	4.390178	4.693921	4.809510	4.251929	5.120292	4.754468	3.126294	4.096860	4.171867	5.113154	4.446690	3.961596	3.882327	4.242269	3.999992	3.244432	3.482074	3.372905	3.822055	3.435445	3.087170	4.300454	4.006760	3.152112	3.132846	3.456795	3.385685	2.963678	3.202239	3.606610	3.117616	3.262627	4.299617	3.206112	3.090007	2.904234	5.236641	2.919585	3.340445	3.016157	3.329538	4.525882	3.791654	3.674735	3.031257	4.613787	4.330775	3.377896	4.570252	3.877608	4.659428	3.791856	3.968740	4.125660	4.923626	3.616696	4.658123	4.464593	4.207295	4.397017	5.394781	3.811201	5.318615	3.748346	3.930017	4.047376	3.649813	3.591517	3.066619	4.660922	4.398284	4.718124	4.579696	4.525441	4.631969	4.966274	4.808479	5.171712	4.828464	4.584996	4.024737	5.466381	4.445656	4.960000	4.775942	4.141508	4.261771	4.045188	5.231386	5.215422	5.639734	5.220931	5.818145	5.662165	4.977448	3.716284	4.931205	5.834461	5.224673	5.338509	5.276162	4.009963	5.551616	5.382725	4.825610	4.909265	5.462398	4.731011	4.571394	5.443899	3.967228	3.812518	4.147890	3.959460	3.811201	4.271401	3.722894	4.095823	3.905507	4.543312	5.145926	5.777806	4.972948	4.797674	5.151943	3.766740	4.854590	4.482648	5.641390	4.720858	4.295017	4.823944	4.139961	4.420461	4.348075	3.979593	3.731296	4.560560	5.157073	4.363298	3.893535	3.540150	3.716284	4.234838	6.200316	4.021995	4.570033	4.761835	4.738203	4.219685	4.091852	5.953089	4.519350	4.553466	4.654176	3.257598	3.608025	3.784585	4.010802

TTN-AS1	8.698199	8.574878	8.681103	8.359725	8.352610	7.719509	8.185093	7.287828	8.275089	7.657668	8.162959	8.383585	8.858812	8.240244	8.335259	8.373879	7.179077	8.478374	7.943093	8.142995	8.233292	7.702215	9.615149	8.390063	8.465833	8.484152	8.702920	8.203549	8.295916	8.072444	8.013217	8.209472	8.452011	7.456502	8.562835	8.352774	8.597896	8.342015	7.999482	8.160840	8.188791	8.480144	8.269978	8.525523	8.943847	8.382834	8.365652	8.234201	8.687887	8.422262	8.024983	8.397774	9.152937	8.997042	8.511835	7.990967	8.904614	9.082279	8.383440	8.367433	8.417243	8.482171	9.303557	8.991496	8.574739	8.436796	8.097665	8.405459	8.049077	8.069057	7.735797	7.801299	8.405932	8.365788	7.825066	8.534075	8.304234	8.401432	8.111029	8.764223	8.719507	9.139811	8.865961	8.721185	8.771295	8.432586	8.561617	8.955174	9.275616	8.589904	8.528611	8.350047	8.641591	8.231627	7.894343	8.666239	8.929856	8.719354	8.351034	8.791665	8.578764	8.376467	8.392258	8.517004	7.490716	7.866655	8.448133	7.847036	7.715374	7.981300	7.622838	8.432106	8.594879	8.083490	8.546755	5.065915	8.186038	8.425112	8.531668	9.459408	8.231014	8.966199	8.461720	8.154232	8.593010	8.783516	5.792931	9.413800	7.998109	8.568651	8.499181	8.279490	5.602850	7.935579	8.634112	5.778374	9.199695	10.918527	7.972741	5.867615	7.435698	8.603452	8.927086	8.952252	7.870451	8.231949	8.883900	8.937800	8.512159	8.163833	9.568254	8.749551	5.786478	8.219177	9.437418	8.402822	8.661167	6.268789	8.471849	9.699709	10.283087	9.147686	6.282111	8.576545	8.118549	8.718121	9.113896	8.073351	9.336323	8.807664	8.271350	9.124281	8.659760	8.772484	9.022327	9.629098	9.547923	9.155715	8.606356	9.860973	7.203644	9.165409	9.271214	8.837390	8.738877	8.639891	8.330735	8.723730	8.973345	8.014164	8.722345	4.819605	9.781923	7.457297	8.938239	8.644551	8.610765	8.506105	8.105747	9.404986	9.667331	9.245828	9.567325	9.682193	9.520601	7.637243	9.291914	9.677525	8.757916	9.259991	10.756632	9.510060	8.778562	7.689945	7.803857	6.487258	8.277981	9.279672	9.260903	9.053125	9.527862	9.375602	7.736809	8.159617	7.815434	9.944691	7.363567	8.409992	8.526581	7.843121	8.601893	6.796677	7.871824	8.230865	9.216856	9.275769	10.020041	7.645874

STXBP5-AS1	3.602249	4.977448	5.360977	6.215426	6.041239	5.349856	5.093948	5.874232	5.560034	8.178250	6.196731	5.252732	5.874764	6.880384	4.528407	6.262524	4.444117	6.007743	4.009546	7.878416	5.702980	7.260591	6.849484	6.226940	6.053723	4.151676	4.096648	6.351115	5.531069	4.581826	5.771842	5.995500	7.377685	4.111378	5.524600	6.326431	6.046222	5.958590	6.282784	5.482767	5.892964	5.193075	5.944776	5.316667	5.035911	4.626343	5.287363	5.759620	7.576848	6.260984	6.347421	6.109395	6.995990	7.025699	6.689492	4.659010	6.599750	5.163477	6.668647	5.982024	6.165970	6.463552	3.298831	6.499570	4.254568	6.038250	7.275773	7.513747	5.285608	6.196567	6.327236	7.279886	6.299075	6.412706	6.271536	6.962614	4.247095	5.350035	5.954174	5.407929	6.469512	6.333155	6.080065	5.939707	7.348804	5.709757	6.497206	6.627907	5.324768	6.590824	5.565981	5.661798	7.274387	6.022738	5.475033	5.986093	7.208002	7.839369	6.527992	5.621291	6.183411	5.316256	6.349412	6.057635	7.218109	6.352600	5.104212	7.244210	4.080249	5.540710	4.510512	5.695092	5.337335	5.458276	6.739227	3.522057	3.988380	5.077975	5.771071	3.070681	5.014252	3.003333	4.045188	3.496333	3.314311	3.736675	2.892332	3.785421	3.360006	4.573729	3.051738	3.077719	3.117616	3.122974	3.717722	3.466667	2.930628	3.228037	3.567285	3.510558	3.820807	4.713285	2.894780	3.077475	6.350446	3.841530	2.930332	3.037724	3.484795	3.157189	3.884029	5.476909	6.413534	3.661202	3.584949	5.885856	3.310033	3.149886	2.854942	3.422423	3.292351	3.942389	3.418786	3.901062	4.201623	4.962329	3.628835	3.875870	5.675497	4.251056	3.640310	3.968092	4.395431	3.313638	2.969108	3.470874	3.013565	3.632530	3.393483	5.304215	3.497998	3.295748	3.682876	3.289685	3.347388	4.520187	6.539709	3.045361	3.658061	3.079083	3.176503	5.590423	4.918304	3.053523	6.067528	3.555846	3.714879	4.274425	3.154548	5.556755	4.734206	7.221939	3.338435	3.563462	3.285326	4.894601	3.408711	2.977679	5.532394	3.500685	4.166469	3.965297	3.678427	3.137111	3.426736	3.086732	3.640310	5.415278	3.156291	5.248966	3.023296	3.407850	3.483890	4.799201	2.947900	3.223498	4.687586	3.270828	3.158914	4.044741	3.483337	3.635673	4.979544	2.863708	4.358295	3.094765	3.235083	3.755678

AL138916.1	3.602249	4.977448	5.360977	6.215426	6.041239	5.349856	5.093948	5.874232	5.560034	8.178250	6.196731	5.252732	5.874764	6.880384	4.528407	6.262524	4.444117	6.007743	4.009546	7.878416	5.702980	7.260591	6.849484	6.226940	6.053723	4.151676	4.096648	6.351115	5.531069	4.581826	5.771842	5.995500	7.377685	4.111378	5.524600	6.326431	6.046222	5.958590	6.282784	5.482767	5.892964	5.193075	5.944776	5.316667	5.035911	4.626343	5.287363	5.759620	7.576848	6.260984	6.347421	6.109395	6.995990	7.025699	6.689492	4.659010	6.599750	5.163477	6.668647	5.982024	6.165970	6.463552	3.298831	6.499570	4.254568	6.038250	7.275773	7.513747	5.285608	6.196567	6.327236	7.279886	6.299075	6.412706	6.271536	6.962614	4.247095	5.350035	5.954174	5.407929	6.469512	6.333155	6.080065	5.939707	7.348804	5.709757	6.497206	6.627907	5.324768	6.590824	5.565981	5.661798	7.274387	6.022738	5.475033	5.986093	7.208002	7.839369	6.527992	5.621291	6.183411	5.316256	6.349412	6.057635	7.218109	6.352600	5.104212	7.244210	4.080249	5.540710	4.510512	5.695092	5.337335	5.458276	6.739227	3.522057	3.988380	5.077975	5.771071	3.070681	5.014252	3.003333	4.045188	3.496333	3.314311	3.736675	2.892332	3.785421	3.360006	4.573729	3.051738	3.077719	3.117616	3.122974	3.717722	3.466667	2.930628	3.228037	3.567285	3.510558	3.820807	4.713285	2.894780	3.077475	6.350446	3.841530	2.930332	3.037724	3.484795	3.157189	3.884029	5.476909	6.413534	3.661202	3.584949	5.885856	3.310033	3.149886	2.854942	3.422423	3.292351	3.942389	3.418786	3.901062	4.201623	4.962329	3.628835	3.875870	5.675497	4.251056	3.640310	3.968092	4.395431	3.313638	2.969108	3.470874	3.013565	3.632530	3.393483	5.304215	3.497998	3.295748	3.682876	3.289685	3.347388	4.520187	6.539709	3.045361	3.658061	3.079083	3.176503	5.590423	4.918304	3.053523	6.067528	3.555846	3.714879	4.274425	3.154548	5.556755	4.734206	7.221939	3.338435	3.563462	3.285326	4.894601	3.408711	2.977679	5.532394	3.500685	4.166469	3.965297	3.678427	3.137111	3.426736	3.086732	3.640310	5.415278	3.156291	5.248966	3.023296	3.407850	3.483890	4.799201	2.947900	3.223498	4.687586	3.270828	3.158914	4.044741	3.483337	3.635673	4.979544	2.863708	4.358295	3.094765	3.235083	3.755678

CT69	5.559256	5.114355	4.981142	4.187745	3.595009	5.397296	3.814003	3.169645	6.026255	4.379938	5.128371	7.005651	5.873185	5.272267	4.077019	5.462754	4.253009	4.937893	4.157781	4.969692	4.195624	5.119896	4.771765	3.664851	3.242972	3.216664	4.577985	6.367217	6.823863	6.614789	3.690742	4.221462	7.284304	8.227663	7.043666	6.648197	6.112642	4.562740	3.069362	3.028768	3.583962	2.818522	4.240972	3.679421	5.728276	4.080021	3.147914	3.922444	5.687890	5.946541	4.308576	4.846791	6.222275	5.690239	5.696938	6.199294	6.609329	2.976586	5.735202	7.844679	5.052157	4.582030	4.288106	4.899001	7.557702	4.539977	4.957295	4.935151	6.108677	4.249700	5.640130	5.533510	2.905987	6.249967	6.029915	5.737539	5.863994	6.549699	5.433353	4.845739	6.437896	5.231593	4.479749	3.923504	4.924879	6.560853	6.905252	6.103246	3.137608	3.690345	4.012032	4.975658	5.395166	7.367807	5.282173	5.971960	5.730853	4.418689	5.673109	4.374567	3.173699	4.340039	3.446160	5.133823	5.629533	4.586707	4.981524	4.592701	3.514626	5.400711	4.368705	3.203604	3.682691	3.118062	4.438927	3.754254	4.813900	6.797331	7.154829	7.639530	6.439408	6.396792	5.854925	3.618767	6.735952	5.311819	3.486039	8.159168	7.245173	6.597115	9.588576	5.607597	5.535685	4.987279	7.953099	6.395482	5.888143	3.859821	5.605540	6.329072	6.191492	3.931093	6.239749	3.090417	5.115570	6.463552	10.179480	10.291284	8.788742	3.530322	5.976903	7.866962	7.569604	8.484598	8.512760	8.418725	6.124801	4.520391	6.952016	4.693307	7.592559	4.956452	5.913302	9.473557	3.960518	4.416195	4.030176	7.851095	8.658207	6.190615	7.211176	7.653754	7.191534	7.408204	6.058191	5.076919	10.563370	9.139490	12.157725	6.971814	5.496995	7.617855	8.237618	7.758694	5.355901	7.830932	8.579659	6.661840	4.952305	8.439178	7.993159	4.002996	8.607266	8.261704	7.095312	8.479473	7.696994	5.711196	7.353422	8.263240	6.942960	6.465705	4.965208	6.871306	7.839369	9.395906	8.399331	6.515893	5.195705	3.153056	5.549767	5.873345	7.800225	5.899028	6.227804	5.553381	4.872775	5.116218	7.725434	3.501795	5.223352	3.082345	7.592104	9.156924	5.734096	8.131646	5.638981	8.337585	8.260003	10.451084	4.647912	2.876246	3.512749	5.186794	6.143417	8.617543	9.267666	10.075003

LINC01010	5.559256	5.114355	4.981142	4.187745	3.595009	5.397296	3.814003	3.169645	6.026255	4.379938	5.128371	7.005651	5.873185	5.272267	4.077019	5.462754	4.253009	4.937893	4.157781	4.969692	4.195624	5.119896	4.771765	3.664851	3.242972	3.216664	4.577985	6.367217	6.823863	6.614789	3.690742	4.221462	7.284304	8.227663	7.043666	6.648197	6.112642	4.562740	3.069362	3.028768	3.583962	2.818522	4.240972	3.679421	5.728276	4.080021	3.147914	3.922444	5.687890	5.946541	4.308576	4.846791	6.222275	5.690239	5.696938	6.199294	6.609329	2.976586	5.735202	7.844679	5.052157	4.582030	4.288106	4.899001	7.557702	4.539977	4.957295	4.935151	6.108677	4.249700	5.640130	5.533510	2.905987	6.249967	6.029915	5.737539	5.863994	6.549699	5.433353	4.845739	6.437896	5.231593	4.479749	3.923504	4.924879	6.560853	6.905252	6.103246	3.137608	3.690345	4.012032	4.975658	5.395166	7.367807	5.282173	5.971960	5.730853	4.418689	5.673109	4.374567	3.173699	4.340039	3.446160	5.133823	5.629533	4.586707	4.981524	4.592701	3.514626	5.400711	4.368705	3.203604	3.682691	3.118062	4.438927	3.754254	4.813900	6.797331	7.154829	7.639530	6.439408	6.396792	5.854925	3.618767	6.735952	5.311819	3.486039	8.159168	7.245173	6.597115	9.588576	5.607597	5.535685	4.987279	7.953099	6.395482	5.888143	3.859821	5.605540	6.329072	6.191492	3.931093	6.239749	3.090417	5.115570	6.463552	10.179480	10.291284	8.788742	3.530322	5.976903	7.866962	7.569604	8.484598	8.512760	8.418725	6.124801	4.520391	6.952016	4.693307	7.592559	4.956452	5.913302	9.473557	3.960518	4.416195	4.030176	7.851095	8.658207	6.190615	7.211176	7.653754	7.191534	7.408204	6.058191	5.076919	10.563370	9.139490	12.157725	6.971814	5.496995	7.617855	8.237618	7.758694	5.355901	7.830932	8.579659	6.661840	4.952305	8.439178	7.993159	4.002996	8.607266	8.261704	7.095312	8.479473	7.696994	5.711196	7.353422	8.263240	6.942960	6.465705	4.965208	6.871306	7.839369	9.395906	8.399331	6.515893	5.195705	3.153056	5.549767	5.873345	7.800225	5.899028	6.227804	5.553381	4.872775	5.116218	7.725434	3.501795	5.223352	3.082345	7.592104	9.156924	5.734096	8.131646	5.638981	8.337585	8.260003	10.451084	4.647912	2.876246	3.512749	5.186794	6.143417	8.617543	9.267666	10.075003

LINC00867	3.005371	3.510886	2.994298	4.192784	3.898920	4.181660	3.720350	3.416052	3.274802	3.463048	3.905736	3.878927	4.584996	4.721961	3.048363	2.983458	3.494089	4.367199	4.341788	4.319275	3.780212	4.365717	5.600062	2.942843	2.960684	3.425651	2.883185	4.525222	2.893899	3.746675	3.463925	4.458439	4.936617	4.624205	4.397856	3.950770	4.177867	3.776362	3.743475	2.855606	3.277712	4.891867	3.232021	4.039945	3.695280	4.214253	3.927841	3.147783	3.272988	4.562514	3.835245	4.604402	5.325747	8.024983	4.069444	3.226736	5.217371	3.626417	4.798980	3.409032	4.510948	3.503137	4.655105	5.486729	4.733129	3.899988	3.591899	4.405609	3.986069	3.734589	4.462233	4.440247	2.960684	3.796939	3.174610	3.210733	3.686638	3.888699	3.351071	3.854056	4.887696	4.721510	3.731933	3.987558	4.145985	5.724545	5.487292	4.570485	3.540932	4.126990	5.106012	3.998094	3.905736	3.997047	3.661837	4.759526	3.961596	5.337710	4.602914	3.509610	4.351108	3.689524	4.260868	4.762486	4.060633	2.902569	3.014660	4.278859	4.354587	3.506641	4.767870	4.092057	3.332425	3.075184	3.147523	6.121636	3.251132	3.562130	3.534620	3.255631	4.648318	3.442050	3.080690	4.902978	3.154663	5.001085	4.422222	3.309218	3.754254	3.346730	3.297133	4.188223	6.479620	3.957014	4.260212	3.473610	4.700882	3.453834	3.642657	3.561948	3.818855	3.131467	3.260291	2.875187	3.290588	3.766514	3.978716	4.194339	3.425458	3.833785	3.314962	3.764711	4.298529	4.348542	3.675388	3.611605	3.544884	3.001475	2.895812	3.436171	2.936188	4.868967	4.194775	3.520622	3.681816	4.197968	3.169414	3.358469	4.100847	3.830333	4.265722	4.579696	4.515053	3.838432	3.508161	6.479304	3.508512	3.921645	5.188014	4.029966	3.693392	4.409145	3.978030	3.287873	4.365030	5.366408	4.327452	3.008488	2.963678	3.922669	3.601857	2.951964	5.529534	3.733411	3.530520	3.511834	4.138630	3.728814	4.149177	5.128371	4.142605	4.016509	4.729562	5.093331	4.102158	6.040358	8.173625	4.951447	4.319054	10.924627	5.261427	3.781236	4.703430	3.391201	4.111173	3.540150	3.702653	3.499802	3.681816	4.209499	4.381597	3.220706	3.663078	4.370890	2.913640	4.696394	4.179211	4.201183	4.320370	4.084103	4.306599	6.363722	3.732517	3.923950	5.236428	3.013777	4.205490	3.152112

AC108721.2	2.911012	3.050254	2.926987	2.940324	2.888372	3.332726	3.222539	3.086827	3.238755	3.309058	3.492401	3.650021	3.185015	3.735838	3.461975	3.101726	3.306659	3.571891	5.369503	3.128684	4.486980	2.843334	4.877385	3.108874	3.118529	2.870639	3.003522	2.946804	3.010748	3.030894	3.610550	3.378434	2.927396	3.918899	3.332891	3.899548	3.620663	3.152931	3.075527	2.878538	3.359328	3.018838	3.169645	4.029058	3.327407	4.586047	3.550026	3.909914	3.010865	3.689102	3.786494	3.647373	4.051084	3.669264	3.368755	2.952475	3.572687	3.145941	5.305197	3.334035	3.373589	3.309363	3.155467	2.934195	3.028960	3.075184	2.842927	3.233219	3.080195	3.026761	2.884737	3.051939	4.169878	3.248143	2.933330	3.146645	2.965763	3.034991	2.916788	3.264674	3.130865	3.689102	3.002331	4.038443	3.142064	4.750654	3.929346	3.194035	2.950339	3.049660	3.061561	3.088208	2.956704	2.938963	2.890430	3.079639	3.468038	2.915529	3.248143	2.939913	3.608409	3.153675	2.958268	2.865464	3.131949	3.462866	2.989896	3.242655	3.170512	3.194035	3.365646	2.860463	2.931158	3.203454	2.961473	3.637058	2.931868	2.929202	3.718521	2.938795	3.090545	2.899169	2.959195	2.835561	3.178028	2.961862	3.086948	3.183074	3.123193	3.254844	3.232699	2.976312	3.173311	3.187793	3.013959	3.306340	3.184892	4.196670	3.210436	3.906427	2.830622	3.998522	2.993827	2.981829	2.833801	2.908972	2.885392	3.187273	3.420255	3.406400	3.272404	2.952031	4.573962	3.234953	3.638065	3.642657	3.069240	2.984795	2.868936	3.131467	2.979074	3.266021	3.432203	3.433819	4.332259	3.166907	4.029277	3.068199	3.794458	3.843368	3.526294	3.530906	3.682691	3.939843	2.912656	3.280866	3.173949	4.989759	3.245817	3.178028	3.047315	3.102636	2.978230	3.030788	3.059943	2.846952	3.489376	3.203344	3.156147	2.915706	2.919761	3.890731	2.976768	2.902569	3.122513	2.942671	3.046501	2.939990	3.159921	3.364197	3.770710	3.029872	3.552782	3.410422	3.497998	3.272546	3.303975	2.912019	3.055089	3.159156	3.116871	3.005564	2.965270	2.943219	2.950522	3.029961	3.312876	3.057495	3.149126	2.841922	2.997603	2.989506	3.032744	3.271787	2.972991	3.028179	3.191194	3.332425	3.075743	2.870842	2.978043	3.524010	3.124425	2.924674	2.876167	2.971623	2.912211	3.261306

AC007359.1	4.765699	3.207039	2.996005	3.897372	2.981253	5.938973	3.263834	3.526862	3.309708	3.335003	3.463210	3.674319	3.243133	3.919345	3.612579	3.203743	3.410757	6.161322	3.076552	3.225846	4.909682	2.963837	4.862901	3.222815	3.499802	3.082546	3.036842	3.182313	3.187925	3.222961	3.616132	5.706450	2.994298	3.193796	3.426570	3.918899	3.717529	3.482786	3.593015	3.364044	3.364994	4.145985	3.289532	3.168395	3.412735	6.657929	3.243133	4.620375	3.206777	6.247783	3.896934	4.802168	4.037033	3.859821	3.604642	3.034025	3.949658	3.786732	3.915721	3.864949	3.805556	3.640119	3.732755	3.145266	3.288642	3.347031	2.966669	3.366167	3.260006	3.218960	2.996629	2.940060	3.036937	3.114521	3.020044	3.367536	2.962645	3.048689	2.939668	3.428492	3.161219	3.718738	3.148882	4.205246	3.268254	3.726139	2.918405	3.223347	3.260006	4.329472	3.436524	3.266904	4.448244	3.682453	3.136736	2.957960	3.674735	4.682027	3.518201	3.095680	3.273434	3.170136	3.006794	2.955254	2.926799	3.064806	3.229755	3.373733	3.030894	4.384260	2.939052	4.321835	3.100561	3.246827	2.997419	2.917227	3.061561	3.006708	4.008227	4.124421	3.286828	3.258846	3.681611	2.916877	6.261499	2.956436	3.731296	3.292959	3.136611	3.257454	3.159156	2.998784	3.251420	3.298388	2.968910	3.413828	5.882107	2.938239	3.377896	4.239453	3.031355	3.902945	3.067172	3.367066	3.055600	2.881304	2.996175	2.874986	3.473810	3.426570	4.892508	3.037234	3.068701	3.329682	3.650983	3.749587	3.591699	3.528493	3.222255	3.143041	4.380153	3.402485	3.228654	3.710484	6.182544	3.183074	4.713285	3.284905	5.876918	3.936663	3.977012	5.236860	3.829065	3.214200	2.977679	4.198610	5.712688	3.878927	4.008667	3.491185	3.452949	3.784371	3.498544	3.439792	4.353982	2.996175	3.728592	3.463048	3.450800	2.994563	2.925809	2.990263	2.974081	3.265578	3.455153	2.970992	3.061802	3.001475	3.113778	3.455894	3.942850	3.191453	3.620461	3.472743	2.938528	3.480211	3.312876	4.277079	3.450610	3.502367	3.257771	3.981983	3.469619	3.026482	2.962817	2.918966	3.003707	3.553917	3.376363	3.483698	3.304924	2.925247	3.016895	2.940245	3.019669	3.312568	3.332078	3.490858	4.109365	2.883880	3.560720	2.948609	3.169872	2.937088	2.883463	3.284447	2.980514	3.309363

TTTY6B	4.765699	3.207039	2.996005	3.897372	2.981253	5.938973	3.263834	3.526862	3.309708	3.335003	3.463210	3.674319	3.243133	3.919345	3.612579	3.203743	3.410757	6.161322	3.076552	3.225846	4.909682	2.963837	4.862901	3.222815	3.499802	3.082546	3.036842	3.182313	3.187925	3.222961	3.616132	5.706450	2.994298	3.193796	3.426570	3.918899	3.717529	3.482786	3.593015	3.364044	3.364994	4.145985	3.289532	3.168395	3.412735	6.657929	3.243133	4.620375	3.206777	6.247783	3.896934	4.802168	4.037033	3.859821	3.604642	3.034025	3.949658	3.786732	3.915721	3.864949	3.805556	3.640119	3.732755	3.145266	3.288642	3.347031	2.966669	3.366167	3.260006	3.218960	2.996629	2.940060	3.036937	3.114521	3.020044	3.367536	2.962645	3.048689	2.939668	3.428492	3.161219	3.718738	3.148882	4.205246	3.268254	3.726139	2.918405	3.223347	3.260006	4.329472	3.436524	3.266904	4.448244	3.682453	3.136736	2.957960	3.674735	4.682027	3.518201	3.095680	3.273434	3.170136	3.006794	2.955254	2.926799	3.064806	3.229755	3.373733	3.030894	4.384260	2.939052	4.321835	3.100561	3.246827	2.997419	2.917227	3.061561	3.006708	4.008227	4.124421	3.286828	3.258846	3.681611	2.916877	6.261499	2.956436	3.731296	3.292959	3.136611	3.257454	3.159156	2.998784	3.251420	3.298388	2.968910	3.413828	5.882107	2.938239	3.377896	4.239453	3.031355	3.902945	3.067172	3.367066	3.055600	2.881304	2.996175	2.874986	3.473810	3.426570	4.892508	3.037234	3.068701	3.329682	3.650983	3.749587	3.591699	3.528493	3.222255	3.143041	4.380153	3.402485	3.228654	3.710484	6.182544	3.183074	4.713285	3.284905	5.876918	3.936663	3.977012	5.236860	3.829065	3.214200	2.977679	4.198610	5.712688	3.878927	4.008667	3.491185	3.452949	3.784371	3.498544	3.439792	4.353982	2.996175	3.728592	3.463048	3.450800	2.994563	2.925809	2.990263	2.974081	3.265578	3.455153	2.970992	3.061802	3.001475	3.113778	3.455894	3.942850	3.191453	3.620461	3.472743	2.938528	3.480211	3.312876	4.277079	3.450610	3.502367	3.257771	3.981983	3.469619	3.026482	2.962817	2.918966	3.003707	3.553917	3.376363	3.483698	3.304924	2.925247	3.016895	2.940245	3.019669	3.312568	3.332078	3.490858	4.109365	2.883880	3.560720	2.948609	3.169872	2.937088	2.883463	3.284447	2.980514	3.309363

AC008175.1	4.765699	3.207039	2.996005	3.897372	2.981253	5.938973	3.263834	3.526862	3.309708	3.335003	3.463210	3.674319	3.243133	3.919345	3.612579	3.203743	3.410757	6.161322	3.076552	3.225846	4.909682	2.963837	4.862901	3.222815	3.499802	3.082546	3.036842	3.182313	3.187925	3.222961	3.616132	5.706450	2.994298	3.193796	3.426570	3.918899	3.717529	3.482786	3.593015	3.364044	3.364994	4.145985	3.289532	3.168395	3.412735	6.657929	3.243133	4.620375	3.206777	6.247783	3.896934	4.802168	4.037033	3.859821	3.604642	3.034025	3.949658	3.786732	3.915721	3.864949	3.805556	3.640119	3.732755	3.145266	3.288642	3.347031	2.966669	3.366167	3.260006	3.218960	2.996629	2.940060	3.036937	3.114521	3.020044	3.367536	2.962645	3.048689	2.939668	3.428492	3.161219	3.718738	3.148882	4.205246	3.268254	3.726139	2.918405	3.223347	3.260006	4.329472	3.436524	3.266904	4.448244	3.682453	3.136736	2.957960	3.674735	4.682027	3.518201	3.095680	3.273434	3.170136	3.006794	2.955254	2.926799	3.064806	3.229755	3.373733	3.030894	4.384260	2.939052	4.321835	3.100561	3.246827	2.997419	2.917227	3.061561	3.006708	4.008227	4.124421	3.286828	3.258846	3.681611	2.916877	6.261499	2.956436	3.731296	3.292959	3.136611	3.257454	3.159156	2.998784	3.251420	3.298388	2.968910	3.413828	5.882107	2.938239	3.377896	4.239453	3.031355	3.902945	3.067172	3.367066	3.055600	2.881304	2.996175	2.874986	3.473810	3.426570	4.892508	3.037234	3.068701	3.329682	3.650983	3.749587	3.591699	3.528493	3.222255	3.143041	4.380153	3.402485	3.228654	3.710484	6.182544	3.183074	4.713285	3.284905	5.876918	3.936663	3.977012	5.236860	3.829065	3.214200	2.977679	4.198610	5.712688	3.878927	4.008667	3.491185	3.452949	3.784371	3.498544	3.439792	4.353982	2.996175	3.728592	3.463048	3.450800	2.994563	2.925809	2.990263	2.974081	3.265578	3.455153	2.970992	3.061802	3.001475	3.113778	3.455894	3.942850	3.191453	3.620461	3.472743	2.938528	3.480211	3.312876	4.277079	3.450610	3.502367	3.257771	3.981983	3.469619	3.026482	2.962817	2.918966	3.003707	3.553917	3.376363	3.483698	3.304924	2.925247	3.016895	2.940245	3.019669	3.312568	3.332078	3.490858	4.109365	2.883880	3.560720	2.948609	3.169872	2.937088	2.883463	3.284447	2.980514	3.309363

TTTY6	4.765699	3.207039	2.996005	3.897372	2.981253	5.938973	3.263834	3.526862	3.309708	3.335003	3.463210	3.674319	3.243133	3.919345	3.612579	3.203743	3.410757	6.161322	3.076552	3.225846	4.909682	2.963837	4.862901	3.222815	3.499802	3.082546	3.036842	3.182313	3.187925	3.222961	3.616132	5.706450	2.994298	3.193796	3.426570	3.918899	3.717529	3.482786	3.593015	3.364044	3.364994	4.145985	3.289532	3.168395	3.412735	6.657929	3.243133	4.620375	3.206777	6.247783	3.896934	4.802168	4.037033	3.859821	3.604642	3.034025	3.949658	3.786732	3.915721	3.864949	3.805556	3.640119	3.732755	3.145266	3.288642	3.347031	2.966669	3.366167	3.260006	3.218960	2.996629	2.940060	3.036937	3.114521	3.020044	3.367536	2.962645	3.048689	2.939668	3.428492	3.161219	3.718738	3.148882	4.205246	3.268254	3.726139	2.918405	3.223347	3.260006	4.329472	3.436524	3.266904	4.448244	3.682453	3.136736	2.957960	3.674735	4.682027	3.518201	3.095680	3.273434	3.170136	3.006794	2.955254	2.926799	3.064806	3.229755	3.373733	3.030894	4.384260	2.939052	4.321835	3.100561	3.246827	2.997419	2.917227	3.061561	3.006708	4.008227	4.124421	3.286828	3.258846	3.681611	2.916877	6.261499	2.956436	3.731296	3.292959	3.136611	3.257454	3.159156	2.998784	3.251420	3.298388	2.968910	3.413828	5.882107	2.938239	3.377896	4.239453	3.031355	3.902945	3.067172	3.367066	3.055600	2.881304	2.996175	2.874986	3.473810	3.426570	4.892508	3.037234	3.068701	3.329682	3.650983	3.749587	3.591699	3.528493	3.222255	3.143041	4.380153	3.402485	3.228654	3.710484	6.182544	3.183074	4.713285	3.284905	5.876918	3.936663	3.977012	5.236860	3.829065	3.214200	2.977679	4.198610	5.712688	3.878927	4.008667	3.491185	3.452949	3.784371	3.498544	3.439792	4.353982	2.996175	3.728592	3.463048	3.450800	2.994563	2.925809	2.990263	2.974081	3.265578	3.455153	2.970992	3.061802	3.001475	3.113778	3.455894	3.942850	3.191453	3.620461	3.472743	2.938528	3.480211	3.312876	4.277079	3.450610	3.502367	3.257771	3.981983	3.469619	3.026482	2.962817	2.918966	3.003707	3.553917	3.376363	3.483698	3.304924	2.925247	3.016895	2.940245	3.019669	3.312568	3.332078	3.490858	4.109365	2.883880	3.560720	2.948609	3.169872	2.937088	2.883463	3.284447	2.980514	3.309363

HCG20	16.357477	15.553263	15.847680	16.289097	15.736354	16.835817	15.509129	15.228590	16.835817	15.501368	16.359390	16.026974	16.220278	15.790919	16.138567	15.939590	16.691628	15.999973	16.452541	16.160600	16.379042	15.584586	16.777348	15.859538	16.541277	16.748238	17.391548	16.192130	15.488676	16.942750	16.556694	14.165208	14.733504	16.168373	16.450253	16.452541	16.217060	16.508802	16.759174	16.580235	15.831819	16.516525	17.377026	17.572102	16.572650	17.595049	16.426704	16.375050	16.363316	16.968008	17.042023	16.922680	16.485350	16.186008	15.961903	16.627037	16.196709	15.969476	16.624262	16.580235	16.174278	16.351585	16.097256	17.021223	15.971905	16.271502	16.077730	16.308700	15.554200	16.860748	16.088816	16.109922	16.635635	15.993114	16.243477	16.439291	15.871113	15.332400	15.382940	14.822949	15.871113	16.269830	15.490401	15.578257	16.437172	15.152524	17.199116	16.124210	15.698607	15.888240	15.714733	16.032263	16.069654	16.412035	17.027876	16.135616	14.816832	16.570086	17.434795	16.973803	16.851732	15.303728	15.928804	15.858328	16.538902	15.901055	16.459030	16.990930	17.595049	16.292480	16.281614	16.416212	16.624262	16.773335	15.536016	16.807646	16.807646	16.887432	16.476725	17.170843	16.957850	16.714243	16.957850	16.990930	16.973803	17.322495	17.076555	17.199116	17.113525	17.048853	17.189676	17.434795	17.135902	17.002792	17.048853	17.189676	16.979770	17.309944	16.990930	17.144930	17.015068	17.042023	17.048853	16.962425	16.711084	17.069256	17.076555	17.161247	16.887432	17.055536	16.777348	17.391548	17.262291	17.209463	17.128044	16.917430	16.827810	17.309944	17.273415	17.069256	16.839687	17.595049	17.621635	17.229824	17.297234	16.997234	17.209463	16.874939	17.170843	17.199116	17.422071	17.391548	17.152637	17.170843	17.218552	17.273415	16.691628	17.144930	16.957850	16.641548	16.815163	16.796364	16.937351	16.803816	16.827810	16.887432	16.720589	17.170843	16.076368	17.170843	17.120651	17.229824	17.170843	16.985483	17.098194	16.860748	16.897220	16.865778	16.985483	16.759174	16.979770	16.784906	17.309944	16.952421	17.285107	16.672020	17.199116	16.685246	16.784906	16.922680	16.613490	16.855860	17.170843	16.823927	16.962425	17.297234	17.152637	17.285107	17.322495	17.309944	17.469225	17.391548	16.370743	17.170843	17.090717	17.048853	17.487844	16.811523	17.452318	17.076555	17.128044	17.262291	17.161247	17.250717	16.947470	17.008331	17.170843	17.152637

AC073115.2	9.903641	7.383930	8.131953	7.923498	9.083201	10.886421	9.395906	10.179636	11.699156	10.475566	10.994776	10.774902	10.726580	9.657050	8.613766	9.978744	11.223249	8.566024	8.019824	9.824177	7.520987	8.805216	7.122734	8.008692	8.430406	10.126943	10.799503	9.828875	11.705650	9.681259	10.863064	7.429346	7.982864	9.802581	8.334638	8.458776	8.546912	8.223437	7.741944	7.997387	10.688294	7.708847	7.451502	8.205080	7.233383	7.636928	11.260318	6.762363	8.842121	7.503695	7.843425	6.984448	6.872750	6.963224	7.607888	10.518030	7.215303	6.554786	7.141928	6.692278	7.007495	10.941833	7.304121	7.928816	7.439013	6.642091	6.603417	7.084263	7.032489	7.051636	8.357999	8.735164	11.182012	8.362692	9.052979	8.301208	8.677593	8.261106	8.792595	7.258223	7.439916	7.706091	7.449040	7.461032	7.182191	7.743500	7.968034	7.575443	6.626317	6.660691	6.828154	7.621803	8.037285	8.330882	9.669428	9.533072	8.020116	10.691964	9.987919	10.194475	10.842557	8.198709	8.160678	7.924758	7.609592	11.256626	8.404680	8.236522	8.729291	11.446732	8.258942	8.996430	8.325722	12.119577	10.865747	12.633940	8.980451	10.840049	11.942720	10.328691	8.042570	8.860035	8.157342	10.628379	10.586252	10.765826	11.625303	11.011552	11.878180	10.883417	11.431505	11.326605	9.308024	9.279219	10.477843	10.687204	9.575634	8.846470	8.554202	7.784873	8.535315	7.622688	8.738646	7.836587	9.236897	10.021101	9.543634	12.168033	9.180624	11.439584	9.292212	9.146092	10.167812	9.145041	8.745107	8.965385	8.902924	8.188055	8.360473	11.330888	7.732580	7.052716	8.131027	7.183603	7.040480	11.408599	7.365458	8.170666	7.424453	8.253272	10.226833	7.140467	7.384238	7.810046	9.800263	7.742387	7.100031	7.741197	6.436616	8.171288	10.790693	7.031715	7.384386	8.177241	7.161420	7.185369	6.857226	7.881227	7.090879	8.590063	8.852855	12.169426	8.721025	9.163554	8.796126	8.764831	8.938834	9.803956	7.204095	8.746045	7.417135	7.819973	8.885766	8.210366	9.310956	8.055096	7.642308	7.105801	7.464507	8.155151	8.212669	8.503865	8.923429	9.807001	10.381054	11.009628	11.654537	10.076651	8.573520	10.738842	8.853927	8.600963	7.786067	8.138337	11.771822	9.232773	9.604702	7.768537	11.777466	8.118549	8.918022	9.566691	12.092606	10.613340	10.651778	8.316232	11.421793	9.776745

AC092652.2	4.157572	5.437831	5.046472	4.789720	4.638845	5.119073	4.185785	4.159287	3.742589	3.414177	4.414205	3.769910	4.518683	3.836745	3.824871	5.539969	3.420255	5.827479	4.288556	4.948324	4.806984	6.071333	6.941340	6.352424	4.934074	4.066061	6.573209	4.159936	4.998969	5.682541	4.586482	3.923719	5.362147	6.363201	5.677134	4.872775	4.353548	3.968978	3.949881	5.081456	4.867689	3.878287	6.354904	5.563335	5.098644	4.809096	4.916406	3.299770	4.234838	4.589267	4.566165	5.398443	5.847907	4.718963	5.679760	4.151892	5.178999	2.971964	3.663429	5.537646	4.073434	4.236984	4.197114	4.936406	4.819801	4.537994	4.245156	4.233299	4.830410	5.176961	4.885618	4.728936	3.823109	4.961642	4.714127	5.843610	4.552175	4.852018	4.962538	5.405925	5.160676	5.103993	5.245814	4.354159	5.411287	4.966720	5.304215	4.457149	2.918966	4.321019	4.068580	4.364149	5.383142	4.535021	5.571063	5.731220	3.588616	4.210561	4.579245	4.925093	4.944128	5.677332	4.875476	4.566596	4.720248	4.695568	4.914529	4.378001	4.936196	4.600798	5.659956	4.678590	4.177040	3.766295	4.404426	4.288770	4.802376	4.549764	4.366579	4.963547	3.813595	4.229863	4.979544	3.882760	4.711163	3.707302	3.953135	4.117895	3.606610	3.654812	3.834416	3.311774	4.077627	3.451883	4.064578	3.421705	4.524143	4.773047	4.676909	4.149407	5.262064	5.810403	4.338129	3.865362	3.416712	4.241406	3.736070	4.330122	3.947054	3.841327	5.300214	4.722578	3.686870	4.179211	3.659637	4.022591	3.121463	4.532248	5.070805	4.877599	2.854479	5.010327	4.715187	4.494423	4.479749	5.581146	3.409194	3.347723	4.033413	5.391638	4.521491	5.174521	4.081358	3.944424	4.235322	3.449885	4.791226	3.393483	3.523465	3.912710	4.515287	3.174479	4.777405	5.010145	4.145382	4.744878	4.352665	3.614582	3.068578	4.487189	4.483623	4.583926	4.326994	4.728272	5.082049	3.567669	4.186897	3.887853	4.887925	4.746389	5.438573	4.714127	5.224876	4.311473	3.740951	4.194544	4.390178	2.980688	4.625705	3.669264	3.709269	4.682249	4.242727	4.248186	5.236029	5.154789	4.752528	4.915357	3.948773	4.224051	4.143455	4.309946	4.950838	3.719948	4.388910	3.720576	4.611640	4.745112	4.733792	4.652372	4.453543	6.049762	3.897785	3.869875	3.352877	3.762370	3.604265	4.036699

OSER1-DT	10.284261	9.541437	9.537204	9.990270	9.201231	8.831339	9.884535	9.082581	8.676661	9.075596	9.109712	9.134862	9.044566	9.390643	9.385126	9.349094	9.326293	9.429122	9.323989	9.523949	9.372113	10.158948	10.776525	9.267376	9.952574	9.086559	8.741634	9.670216	8.470095	9.132598	8.980298	9.724538	9.531300	9.585768	9.532449	9.800580	10.073145	9.796260	9.772051	9.940701	9.173114	10.580067	9.543959	9.823738	10.315047	10.110501	8.841949	10.369411	9.065840	9.830616	9.811665	10.250874	10.084677	10.254503	10.165968	8.772007	10.130357	10.988852	10.748279	10.404404	11.442042	8.604528	10.887634	10.237708	11.668018	10.128392	10.753252	9.996472	10.008153	10.925632	10.161675	10.010322	8.888851	9.866534	10.025761	9.623390	10.129611	9.775681	10.084209	10.135620	9.798110	10.013106	10.093356	9.904108	10.143394	9.668618	9.819657	10.213349	10.156509	10.666063	10.665039	10.704762	10.325356	9.996027	10.260870	10.054345	9.534300	8.760426	8.895603	9.505192	8.753956	9.647229	8.883900	9.702498	9.033411	8.599566	9.528969	8.861847	9.420386	8.751961	10.356625	9.699238	9.634626	8.661167	8.668823	9.555229	10.718205	9.166044	8.547842	9.515000	8.969905	9.649630	9.651040	9.764655	8.897303	8.427087	9.457396	9.494561	9.543317	8.495845	8.946451	10.421638	9.337273	8.914189	9.409559	9.271969	9.141819	10.155465	9.941976	9.917640	9.938377	10.297710	9.093137	10.221516	10.625962	9.362614	9.714055	9.172195	8.597021	9.056272	9.090859	9.268147	8.582980	9.958305	8.812777	9.968179	9.682497	9.346806	9.768766	9.779667	10.330253	9.183143	9.747580	9.761660	9.699554	8.707834	12.278204	9.911740	9.438039	9.319298	10.511711	9.264430	9.325501	10.242742	9.966953	11.191346	10.654274	10.407984	10.492682	10.939475	9.240739	10.339746	9.418245	10.977591	10.305812	10.064236	10.064832	9.696633	11.616996	10.044031	9.697251	8.959017	9.759652	10.188159	9.305574	10.219240	9.516374	9.782528	10.452240	9.022179	10.537517	9.516071	9.172804	9.564966	9.601901	9.337708	10.944165	9.999670	9.934023	11.637244	10.696336	10.202155	9.872723	9.740092	9.371967	9.710793	9.201089	8.202555	10.858655	8.470551	9.925599	8.883123	9.968029	9.990754	8.469498	8.985928	9.287715	9.593381	9.206719	9.672249	9.513820	10.315047	9.239678	9.564348	8.500281	9.940101	9.503469	8.775501

ALOX12-AS1	5.033574	12.878352	12.203133	13.768777	12.643586	13.817826	14.452088	14.401854	14.270558	13.795380	14.191449	14.407674	14.187463	13.712951	13.292351	14.152064	14.657265	14.157045	12.906787	14.218603	11.525892	14.150116	11.086626	14.222846	14.031741	13.635698	5.035490	14.274870	13.165696	11.983055	14.520255	12.409200	11.865455	8.502624	11.939528	13.004678	13.599935	14.239437	13.368506	13.323361	13.881196	13.112197	11.727061	12.100117	12.459627	12.105433	12.107881	13.290292	12.914484	13.398659	12.141427	13.197314	11.159782	14.607132	13.004126	13.782334	13.326420	12.800938	13.099956	10.922234	12.075117	13.184288	6.675596	13.094857	12.623824	13.932466	12.513371	12.579011	11.691697	11.974354	13.474198	12.620010	13.901234	12.951111	13.397092	12.165154	10.784070	12.373075	12.187471	14.365930	12.862405	14.213010	12.640533	14.164900	13.604783	13.617769	14.266988	13.902268	11.816015	13.372298	13.649396	11.823989	12.126882	12.320942	8.489838	11.653340	13.513556	13.929475	13.070763	13.067170	14.138911	13.946797	12.846490	14.091290	14.140212	13.337083	12.950935	15.342745	12.779681	13.990442	12.789777	14.216975	13.680430	14.013234	13.461144	12.786993	7.741944	13.343265	14.119258	3.798195	8.576857	8.624991	8.083646	10.631433	10.608452	10.162595	7.843587	8.526432	9.426987	9.917940	9.914232	9.519473	9.387474	7.982701	10.002111	8.752885	11.031349	4.648755	9.001665	7.556767	9.246593	7.109503	9.831389	10.209061	7.066045	7.264041	9.321845	8.393633	7.440209	8.749551	9.337273	8.997519	7.416992	6.636123	11.249550	9.285515	9.778460	8.905380	10.501693	8.529384	11.233001	6.543039	7.870743	9.891801	12.572187	7.247454	7.479497	10.264772	10.703735	10.024337	7.992858	12.151731	8.920132	8.166286	7.743660	8.017257	9.224202	10.406784	8.159020	6.727870	9.899439	6.343011	8.695746	8.733493	8.373576	7.176828	11.135665	7.755724	6.803657	9.326903	11.761159	10.643569	9.611660	8.222227	7.595187	7.868036	7.120108	10.940377	8.259246	9.794721	9.725608	8.261975	8.803828	11.048256	10.695283	9.452602	8.188314	6.343517	9.241805	7.789766	6.132754	10.437507	6.925494	7.771423	7.916768	9.018064	8.411404	7.484898	8.098858	8.310809	8.146104	7.244534	9.241070	10.355676	9.695858	11.822914	10.678072	9.391590	8.341593	10.526253	6.768967	5.149945	12.910006	8.612161	9.494887	9.112425	11.609621	8.293805

AC040977.2	5.033574	12.878352	12.203133	13.768777	12.643586	13.817826	14.452088	14.401854	14.270558	13.795380	14.191449	14.407674	14.187463	13.712951	13.292351	14.152064	14.657265	14.157045	12.906787	14.218603	11.525892	14.150116	11.086626	14.222846	14.031741	13.635698	5.035490	14.274870	13.165696	11.983055	14.520255	12.409200	11.865455	8.502624	11.939528	13.004678	13.599935	14.239437	13.368506	13.323361	13.881196	13.112197	11.727061	12.100117	12.459627	12.105433	12.107881	13.290292	12.914484	13.398659	12.141427	13.197314	11.159782	14.607132	13.004126	13.782334	13.326420	12.800938	13.099956	10.922234	12.075117	13.184288	6.675596	13.094857	12.623824	13.932466	12.513371	12.579011	11.691697	11.974354	13.474198	12.620010	13.901234	12.951111	13.397092	12.165154	10.784070	12.373075	12.187471	14.365930	12.862405	14.213010	12.640533	14.164900	13.604783	13.617769	14.266988	13.902268	11.816015	13.372298	13.649396	11.823989	12.126882	12.320942	8.489838	11.653340	13.513556	13.929475	13.070763	13.067170	14.138911	13.946797	12.846490	14.091290	14.140212	13.337083	12.950935	15.342745	12.779681	13.990442	12.789777	14.216975	13.680430	14.013234	13.461144	12.786993	7.741944	13.343265	14.119258	3.798195	8.576857	8.624991	8.083646	10.631433	10.608452	10.162595	7.843587	8.526432	9.426987	9.917940	9.914232	9.519473	9.387474	7.982701	10.002111	8.752885	11.031349	4.648755	9.001665	7.556767	9.246593	7.109503	9.831389	10.209061	7.066045	7.264041	9.321845	8.393633	7.440209	8.749551	9.337273	8.997519	7.416992	6.636123	11.249550	9.285515	9.778460	8.905380	10.501693	8.529384	11.233001	6.543039	7.870743	9.891801	12.572187	7.247454	7.479497	10.264772	10.703735	10.024337	7.992858	12.151731	8.920132	8.166286	7.743660	8.017257	9.224202	10.406784	8.159020	6.727870	9.899439	6.343011	8.695746	8.733493	8.373576	7.176828	11.135665	7.755724	6.803657	9.326903	11.761159	10.643569	9.611660	8.222227	7.595187	7.868036	7.120108	10.940377	8.259246	9.794721	9.725608	8.261975	8.803828	11.048256	10.695283	9.452602	8.188314	6.343517	9.241805	7.789766	6.132754	10.437507	6.925494	7.771423	7.916768	9.018064	8.411404	7.484898	8.098858	8.310809	8.146104	7.244534	9.241070	10.355676	9.695858	11.822914	10.678072	9.391590	8.341593	10.526253	6.768967	5.149945	12.910006	8.612161	9.494887	9.112425	11.609621	8.293805

EP300-AS1	6.955501	4.947539	5.149945	5.859607	5.869213	4.957295	6.077620	4.983217	6.574042	6.278478	6.675415	6.450636	5.904725	5.978520	6.762817	6.572269	5.355114	6.446215	6.971198	6.340724	6.230244	7.178144	3.495403	6.427147	6.564660	5.404785	5.604946	5.881927	6.669931	5.072202	5.441215	5.076919	5.366014	6.170481	4.948324	5.514453	6.116835	4.342419	5.043462	5.375342	7.027807	5.539213	5.729176	5.483876	5.911515	5.949005	5.730111	3.840468	5.915113	5.143159	5.622975	5.845645	5.850774	5.237665	6.770742	6.097197	5.937556	5.097811	5.988091	5.795108	4.084323	5.896338	4.512034	6.174551	5.731037	5.387789	5.154192	5.304038	5.554151	5.416606	5.160676	6.087838	6.038961	5.605125	5.436682	5.842228	4.980326	5.599491	4.969692	5.433001	5.890458	6.456375	5.596819	5.536460	5.725915	6.530302	5.741365	5.967706	4.328092	4.863721	5.754614	4.925961	6.439253	5.334373	5.758146	6.872429	6.590635	7.851729	7.045619	6.090277	6.722079	6.694724	7.527775	6.720482	6.424977	6.628397	6.361202	5.936863	6.335850	5.846949	6.188764	7.821963	6.853634	5.975168	6.909077	6.210495	7.460716	7.326424	7.436620	4.636303	4.674939	3.862377	5.223134	5.037327	4.813320	4.484173	3.608224	5.469704	3.947691	4.516358	5.134665	4.961235	3.627218	4.379075	5.454582	3.819912	5.650101	4.441091	5.129796	4.861046	6.155339	3.000262	6.334340	4.166676	3.868783	6.674131	6.267913	5.277375	3.550026	4.185338	5.420746	6.154472	5.559465	4.755730	4.926605	4.511393	3.711920	3.302535	4.669934	5.331441	3.636471	3.963170	4.307689	4.812486	5.311445	5.491679	3.721688	5.001085	4.690520	4.365913	5.523672	4.293345	4.542656	5.549967	4.639269	5.038331	4.539311	5.605540	4.063502	2.888542	4.663894	4.346523	4.435851	4.167325	3.876965	4.594487	4.078461	3.883367	4.485731	4.202047	5.652389	5.563335	4.666242	4.899838	5.164484	3.409194	3.790973	4.603540	5.006848	5.848814	5.249579	5.261254	5.681029	5.345157	5.059000	4.988308	4.597879	3.980615	3.680445	3.920194	3.342139	5.081663	3.998746	5.453987	5.782736	5.899553	6.954230	5.787378	3.938758	5.171289	5.750837	5.304807	5.045149	5.164675	5.190727	4.002780	6.115788	4.008886	4.605418	3.685044	4.811450	3.731092	5.732857	4.023916	4.001044	5.951263	4.914730	4.243870

AL035658.1	6.955501	4.947539	5.149945	5.859607	5.869213	4.957295	6.077620	4.983217	6.574042	6.278478	6.675415	6.450636	5.904725	5.978520	6.762817	6.572269	5.355114	6.446215	6.971198	6.340724	6.230244	7.178144	3.495403	6.427147	6.564660	5.404785	5.604946	5.881927	6.669931	5.072202	5.441215	5.076919	5.366014	6.170481	4.948324	5.514453	6.116835	4.342419	5.043462	5.375342	7.027807	5.539213	5.729176	5.483876	5.911515	5.949005	5.730111	3.840468	5.915113	5.143159	5.622975	5.845645	5.850774	5.237665	6.770742	6.097197	5.937556	5.097811	5.988091	5.795108	4.084323	5.896338	4.512034	6.174551	5.731037	5.387789	5.154192	5.304038	5.554151	5.416606	5.160676	6.087838	6.038961	5.605125	5.436682	5.842228	4.980326	5.599491	4.969692	5.433001	5.890458	6.456375	5.596819	5.536460	5.725915	6.530302	5.741365	5.967706	4.328092	4.863721	5.754614	4.925961	6.439253	5.334373	5.758146	6.872429	6.590635	7.851729	7.045619	6.090277	6.722079	6.694724	7.527775	6.720482	6.424977	6.628397	6.361202	5.936863	6.335850	5.846949	6.188764	7.821963	6.853634	5.975168	6.909077	6.210495	7.460716	7.326424	7.436620	4.636303	4.674939	3.862377	5.223134	5.037327	4.813320	4.484173	3.608224	5.469704	3.947691	4.516358	5.134665	4.961235	3.627218	4.379075	5.454582	3.819912	5.650101	4.441091	5.129796	4.861046	6.155339	3.000262	6.334340	4.166676	3.868783	6.674131	6.267913	5.277375	3.550026	4.185338	5.420746	6.154472	5.559465	4.755730	4.926605	4.511393	3.711920	3.302535	4.669934	5.331441	3.636471	3.963170	4.307689	4.812486	5.311445	5.491679	3.721688	5.001085	4.690520	4.365913	5.523672	4.293345	4.542656	5.549967	4.639269	5.038331	4.539311	5.605540	4.063502	2.888542	4.663894	4.346523	4.435851	4.167325	3.876965	4.594487	4.078461	3.883367	4.485731	4.202047	5.652389	5.563335	4.666242	4.899838	5.164484	3.409194	3.790973	4.603540	5.006848	5.848814	5.249579	5.261254	5.681029	5.345157	5.059000	4.988308	4.597879	3.980615	3.680445	3.920194	3.342139	5.081663	3.998746	5.453987	5.782736	5.899553	6.954230	5.787378	3.938758	5.171289	5.750837	5.304807	5.045149	5.164675	5.190727	4.002780	6.115788	4.008886	4.605418	3.685044	4.811450	3.731092	5.732857	4.023916	4.001044	5.951263	4.914730	4.243870

AP006216.1	8.121961	7.925376	8.555588	7.553604	7.610523	7.096262	8.292759	6.606208	6.082990	7.356705	7.031715	7.389491	7.429995	7.680560	7.614945	7.495213	6.544343	8.028874	5.898847	7.976016	6.842224	7.639389	4.252568	7.830215	7.478448	6.956277	7.337058	6.040731	7.195034	7.289562	6.870163	8.361560	8.709005	7.681937	8.179009	8.309468	8.387729	8.398520	7.609133	6.673180	7.631321	6.843205	8.208437	7.724062	8.369861	8.425591	6.805231	7.712966	7.526204	8.307119	7.999175	8.648892	8.691086	8.364702	8.785047	7.328193	8.903697	8.014477	6.771256	8.148380	7.720828	7.647714	7.758398	6.579946	6.939212	8.076211	6.876112	7.406485	7.634486	6.960691	8.503262	8.289036	6.998328	8.839043	8.415646	8.436480	8.441475	8.081629	7.805727	8.580730	8.517795	8.897627	8.644254	8.968822	8.834502	8.181446	7.849748	8.909661	7.498353	6.904609	8.301664	6.993391	8.131342	8.348402	8.268700	8.240987	8.277390	7.467106	7.616653	7.235310	7.193418	8.098253	7.684886	8.182493	7.481663	6.889035	6.615435	6.941664	6.598102	7.242261	6.772683	5.593845	5.734630	7.338317	7.014228	5.668880	6.742879	8.058996	7.311816	6.550701	7.103919	6.889035	7.025384	4.892937	7.568100	5.702228	6.570356	5.606131	7.106131	5.793485	7.358111	6.928747	5.928463	5.957495	7.296044	6.389695	6.509428	6.283128	8.617543	6.165970	6.069620	6.114042	7.209878	7.077190	6.802210	6.396792	6.014345	5.818870	6.805534	6.066609	6.029573	7.030651	7.302415	7.736364	7.946027	7.688567	7.326266	6.663632	6.404576	7.061882	5.662165	8.092315	7.573089	7.706387	8.103712	6.316736	8.711023	8.053853	8.314539	7.173526	8.348402	7.554568	7.850213	7.736364	6.505130	7.909425	8.019965	5.670662	7.900239	5.955221	7.139220	6.892352	5.562770	6.146352	7.789160	6.201199	6.826732	6.026440	6.076260	7.500400	7.636483	5.684727	7.272024	7.800830	7.359642	7.286929	7.845788	7.647866	7.964491	7.760978	8.749699	8.462038	8.264623	8.068175	7.442078	6.755073	9.203996	6.207268	5.855990	8.319558	7.016162	7.416707	7.153563	7.639389	7.087205	6.404740	6.669775	7.035147	6.797171	6.710714	7.889051	7.612641	7.475689	6.304098	7.483367	6.000418	7.169943	6.455878	5.375758	6.120967	4.169230	5.107866	6.480089	7.842645	6.735311	6.450320	8.360784	6.912113

CLRN1-AS1	4.631116	5.624670	5.636952	4.991007	5.007648	4.798317	5.002123	5.844038	5.410318	4.510736	5.069387	4.913496	4.224672	5.275329	4.617086	5.306198	5.461399	5.328489	5.686601	5.552010	4.298741	5.917953	5.810751	5.856716	5.461230	4.962538	4.760157	6.159627	4.116561	4.508542	5.267688	4.158412	4.850976	4.556426	4.508763	4.690116	6.258112	5.798946	4.997369	5.307133	4.881152	4.510948	4.942614	5.673307	4.806580	5.910976	4.535899	6.180324	5.100318	5.638630	5.165102	6.348278	6.328905	5.167076	6.040731	4.736102	6.234964	5.938799	5.255358	5.743916	6.532579	5.109966	5.462933	5.522511	5.928111	6.052527	5.774363	5.553579	5.026830	5.378088	5.207233	5.414727	4.834572	5.289592	5.060634	5.294623	5.493183	4.696394	4.898566	5.939522	5.445634	5.686415	6.531604	5.154192	5.241252	4.264885	6.511268	5.350464	5.980847	5.782186	5.478682	5.631732	5.184611	5.528211	4.639479	4.953764	4.924291	4.886461	5.726644	4.373474	4.242727	4.238326	4.340039	5.700818	4.918304	4.629822	5.937388	5.599867	5.449926	4.650482	4.956861	5.697494	5.149087	4.901535	6.131542	4.307013	5.580219	4.754057	4.311473	5.251776	6.922808	5.799127	5.728276	5.672408	5.724200	4.444333	5.236860	5.616107	5.919744	5.310072	4.306150	5.272267	5.461042	4.489766	5.312428	6.039824	6.655496	5.926133	5.188600	5.431807	5.717116	8.059157	5.722705	5.550691	5.122893	4.681159	6.681903	4.739080	5.630815	5.141740	4.182342	3.595732	5.736616	5.403273	5.350251	6.006099	5.891163	5.481638	5.083842	5.860683	5.000684	6.662321	6.449255	6.775586	6.259469	5.459239	6.390861	6.053569	7.308199	5.741185	5.618375	6.786777	4.602914	5.421462	4.129602	5.510117	6.684561	6.168430	6.769775	11.558020	4.771765	5.527848	6.151211	5.273895	4.961642	6.376000	5.962339	5.175953	5.076726	6.895893	4.602311	5.363664	5.030546	3.990057	5.537646	5.456657	6.358684	4.743173	6.168963	6.589823	6.170311	6.189761	4.445656	5.295016	3.943958	7.093818	5.404435	5.122099	5.341978	5.781271	5.802411	5.146754	5.117619	4.679009	6.276525	4.863937	4.434129	5.809052	5.244823	3.904662	4.187994	4.291335	5.952913	4.636718	4.867232	5.843146	5.672603	4.928464	6.031288	5.285798	5.251776	5.959677	5.935459	7.125950	4.339614	5.730674	4.594714	5.605540

SIAH2-AS1	4.631116	5.624670	5.636952	4.991007	5.007648	4.798317	5.002123	5.844038	5.410318	4.510736	5.069387	4.913496	4.224672	5.275329	4.617086	5.306198	5.461399	5.328489	5.686601	5.552010	4.298741	5.917953	5.810751	5.856716	5.461230	4.962538	4.760157	6.159627	4.116561	4.508542	5.267688	4.158412	4.850976	4.556426	4.508763	4.690116	6.258112	5.798946	4.997369	5.307133	4.881152	4.510948	4.942614	5.673307	4.806580	5.910976	4.535899	6.180324	5.100318	5.638630	5.165102	6.348278	6.328905	5.167076	6.040731	4.736102	6.234964	5.938799	5.255358	5.743916	6.532579	5.109966	5.462933	5.522511	5.928111	6.052527	5.774363	5.553579	5.026830	5.378088	5.207233	5.414727	4.834572	5.289592	5.060634	5.294623	5.493183	4.696394	4.898566	5.939522	5.445634	5.686415	6.531604	5.154192	5.241252	4.264885	6.511268	5.350464	5.980847	5.782186	5.478682	5.631732	5.184611	5.528211	4.639479	4.953764	4.924291	4.886461	5.726644	4.373474	4.242727	4.238326	4.340039	5.700818	4.918304	4.629822	5.937388	5.599867	5.449926	4.650482	4.956861	5.697494	5.149087	4.901535	6.131542	4.307013	5.580219	4.754057	4.311473	5.251776	6.922808	5.799127	5.728276	5.672408	5.724200	4.444333	5.236860	5.616107	5.919744	5.310072	4.306150	5.272267	5.461042	4.489766	5.312428	6.039824	6.655496	5.926133	5.188600	5.431807	5.717116	8.059157	5.722705	5.550691	5.122893	4.681159	6.681903	4.739080	5.630815	5.141740	4.182342	3.595732	5.736616	5.403273	5.350251	6.006099	5.891163	5.481638	5.083842	5.860683	5.000684	6.662321	6.449255	6.775586	6.259469	5.459239	6.390861	6.053569	7.308199	5.741185	5.618375	6.786777	4.602914	5.421462	4.129602	5.510117	6.684561	6.168430	6.769775	11.558020	4.771765	5.527848	6.151211	5.273895	4.961642	6.376000	5.962339	5.175953	5.076726	6.895893	4.602311	5.363664	5.030546	3.990057	5.537646	5.456657	6.358684	4.743173	6.168963	6.589823	6.170311	6.189761	4.445656	5.295016	3.943958	7.093818	5.404435	5.122099	5.341978	5.781271	5.802411	5.146754	5.117619	4.679009	6.276525	4.863937	4.434129	5.809052	5.244823	3.904662	4.187994	4.291335	5.952913	4.636718	4.867232	5.843146	5.672603	4.928464	6.031288	5.285798	5.251776	5.959677	5.935459	7.125950	4.339614	5.730674	4.594714	5.605540

EHMT2-AS1	6.561513	5.993056	5.940777	6.288447	6.068594	5.657391	5.158488	5.466753	5.703923	5.326544	5.404983	5.686601	5.051393	3.976140	5.231998	6.011759	5.299488	4.762913	5.021154	5.738273	5.482940	5.196314	3.613588	5.402645	5.687731	5.641575	6.922099	6.179165	6.012629	6.000081	5.501991	5.373200	5.400149	5.690061	5.762505	5.539594	5.227060	5.486729	5.434949	5.400880	5.880506	6.090441	5.998669	5.014462	4.932450	5.901312	6.523509	4.895790	4.366132	4.385418	5.598789	3.942389	5.352068	4.388263	5.052790	6.101704	4.539078	5.281755	5.028887	4.424224	5.193268	5.957686	5.126176	5.121915	4.101929	5.386970	6.019724	6.024120	5.717648	6.058191	6.155169	5.867214	5.929013	5.659219	5.388727	5.702043	5.485387	5.211625	5.977963	4.240135	5.176137	5.009311	5.371076	4.725144	4.034749	4.669934	3.762565	3.986069	3.092690	5.137833	5.085724	4.719153	5.096023	4.842335	5.952712	5.100318	5.098046	4.853955	5.953430	6.103061	4.940144	5.884957	5.778551	5.551616	5.539772	5.925962	5.461616	5.616498	5.598789	6.158049	5.420552	6.216805	6.081971	6.126045	4.897681	6.371935	7.455081	5.446207	5.972340	4.830820	5.992513	5.689353	6.030587	6.831962	5.822776	4.902368	5.317069	5.550523	5.622407	5.392206	5.449166	5.013417	4.946937	5.121915	5.781452	5.314008	4.865377	4.890855	5.380410	4.756961	5.264297	3.206407	5.785597	5.539772	5.720686	6.001621	5.853071	5.814075	5.810937	5.217960	4.964786	4.650703	5.380613	5.227918	4.710948	5.152145	5.089809	5.935818	5.222327	6.001274	4.056715	5.261864	4.957295	4.414205	5.826936	6.300351	3.774502	4.141946	5.085506	5.289371	3.795711	4.876299	4.604801	4.008667	6.014517	4.443479	2.870639	4.597046	4.006327	4.179668	6.220905	4.708270	5.474055	4.900919	5.100918	5.834830	5.995839	6.057447	5.604559	5.438752	5.431041	6.608356	5.582483	5.053637	5.358153	5.675664	5.230559	5.998502	3.962694	5.203062	4.998777	4.626946	3.916620	3.924166	4.332259	4.335703	4.325941	2.960790	4.121850	4.844870	4.344368	4.677314	5.105634	5.094394	4.660049	4.712212	4.232008	4.809737	5.246412	4.891666	5.579830	6.345056	5.465983	5.016682	6.180653	4.962938	5.035490	5.248785	6.523509	5.525734	5.563726	5.322096	6.168263	5.953780	6.365895	5.166080	5.040958	5.953274

LINC01765	9.263062	8.148080	8.345080	8.403771	8.631287	8.514907	8.307762	9.696173	10.801353	8.236522	8.417092	8.481092	8.205229	8.385574	8.072796	8.532727	9.238102	8.099606	8.706907	8.392729	8.093070	8.073029	9.109255	8.630218	9.210851	8.908772	9.151539	9.117571	8.260488	7.888733	9.355134	8.527523	8.053534	7.713903	8.130869	8.212972	7.584871	8.239020	7.951354	8.321274	8.312823	8.353838	7.846711	7.603766	8.233570	7.907089	8.538318	8.367126	8.277230	8.347074	8.455668	8.351110	8.381284	7.951067	8.455510	8.193370	8.193679	8.736571	8.318216	8.532428	8.758681	8.284696	8.992714	8.788120	8.370008	8.459707	8.394237	8.235738	8.274926	8.930630	8.301208	8.530152	8.341747	8.220715	8.476374	8.891903	8.665460	8.372533	8.386026	8.487395	8.501395	8.585918	8.599566	8.269676	8.692290	8.570802	8.716431	8.602363	9.008249	8.670629	8.849250	8.846008	8.206933	8.440901	8.418107	9.058147	8.122926	9.205823	8.331361	8.749091	8.674365	7.599995	8.547692	8.482488	8.538466	8.069057	9.247361	7.754268	9.344981	8.455966	9.063831	8.663457	9.002579	9.084561	9.544116	10.672285	9.076223	9.120056	8.999207	7.976636	8.056978	8.253736	7.853434	9.025392	8.247463	8.200142	8.174272	8.931119	7.981607	8.073351	8.474628	8.650279	7.964491	8.312531	8.697124	8.446086	7.588434	8.368767	7.603129	8.676972	7.899568	8.671736	8.686419	8.450626	8.284375	8.695889	8.425755	8.538925	7.795248	9.607926	7.851095	8.027961	8.103236	7.934327	7.577760	7.372371	8.067133	8.176949	8.141644	8.488914	8.017553	7.433753	7.920290	7.792348	7.743330	8.694675	8.065177	7.703423	8.090493	8.182493	7.896267	7.573412	8.425112	7.910938	7.934176	7.380184	8.256628	8.002152	7.846238	8.449850	8.058121	8.586509	8.154862	8.068615	8.286545	8.527214	8.306800	8.350523	8.557313	8.081629	7.748877	8.677896	8.017844	8.420110	8.466427	8.397333	8.820386	8.298205	8.488318	7.766526	8.172239	8.114241	7.997547	8.309911	8.568189	8.292913	7.918723	8.673896	8.362379	8.073812	8.968219	7.936388	8.621266	8.506105	8.738729	8.253582	8.575963	8.185409	7.324693	8.206331	7.464647	7.614639	7.985454	8.310979	8.053853	8.917093	7.766387	8.663941	9.091908	9.078557	9.188756	9.673965	9.368032	8.130096	8.371426	8.585128	8.709631	8.311294

AC010230.1	7.217816	9.965013	10.131841	9.410488	8.931622	9.618393	9.387333	11.851418	8.693709	10.065419	7.845347	10.012445	10.756471	8.542271	9.650553	10.717904	10.181623	10.538968	8.028718	8.026826	9.509444	10.593946	7.962366	9.769830	8.896105	9.199695	8.080543	8.147162	8.395927	9.598799	10.820288	8.394089	7.342224	7.657521	9.575333	9.593851	9.082279	9.850414	7.440515	9.648272	10.639071	8.509809	7.697764	7.592717	7.349096	9.903960	9.849770	9.956772	9.545320	8.979397	8.792439	8.138022	9.001818	8.019824	9.690530	9.507703	7.602325	8.717976	8.813641	8.110438	8.727572	7.924442	7.307876	8.931776	8.938386	8.495049	8.994576	7.193889	9.008558	9.017754	9.582846	8.058996	11.083949	8.538157	8.694514	9.150489	7.455423	8.393485	8.308399	9.520363	8.205382	7.796780	8.656980	10.292953	9.379735	8.897476	10.009096	8.906727	8.457783	8.060250	7.302237	7.540217	8.440271	8.392872	7.624722	8.506430	9.610435	9.350940	9.919470	9.381478	9.505969	9.568749	7.680267	9.834281	10.615566	10.031008	10.240163	10.454039	8.227815	9.944548	9.407719	10.044031	10.251030	11.416927	8.595641	8.527214	6.966591	8.896716	9.835965	7.888585	10.637121	9.203412	8.289335	10.331914	9.661551	8.973481	7.925059	8.107279	8.280399	7.765933	8.227204	8.285774	7.049753	8.003407	8.267776	7.443943	8.579521	7.938674	7.620128	8.727249	9.255442	7.928511	7.777466	7.039223	7.192941	9.594314	7.708409	8.138502	8.893308	8.860469	7.659658	6.671706	10.417829	10.251178	7.816205	9.648718	8.143761	7.244210	8.035393	8.882494	8.275241	7.494271	7.740092	7.703423	10.458181	9.546085	8.256785	8.284229	8.761378	8.944895	7.371267	7.884753	7.349682	7.857542	7.859591	6.371595	7.335044	7.863861	7.496300	8.628155	8.028417	7.224803	8.759647	7.360909	9.222961	8.190071	7.126739	8.973413	8.592859	9.059425	7.867408	11.238772	8.249596	7.791899	9.882726	7.113763	7.931572	8.893154	7.630676	7.636483	8.361415	10.419946	9.530828	7.860047	8.367126	11.534947	6.886135	7.864018	7.312433	6.713250	6.895266	7.727960	7.390553	7.331331	7.831082	7.038741	7.174480	9.395272	7.582883	7.470477	7.746079	7.134672	7.401622	7.469565	9.172195	8.436316	8.365500	8.159316	11.624581	10.971168	7.600924	8.116691	10.801504	8.049687	9.881018	7.489591	8.689858	8.625939

LINC01957	7.217816	9.965013	10.131841	9.410488	8.931622	9.618393	9.387333	11.851418	8.693709	10.065419	7.845347	10.012445	10.756471	8.542271	9.650553	10.717904	10.181623	10.538968	8.028718	8.026826	9.509444	10.593946	7.962366	9.769830	8.896105	9.199695	8.080543	8.147162	8.395927	9.598799	10.820288	8.394089	7.342224	7.657521	9.575333	9.593851	9.082279	9.850414	7.440515	9.648272	10.639071	8.509809	7.697764	7.592717	7.349096	9.903960	9.849770	9.956772	9.545320	8.979397	8.792439	8.138022	9.001818	8.019824	9.690530	9.507703	7.602325	8.717976	8.813641	8.110438	8.727572	7.924442	7.307876	8.931776	8.938386	8.495049	8.994576	7.193889	9.008558	9.017754	9.582846	8.058996	11.083949	8.538157	8.694514	9.150489	7.455423	8.393485	8.308399	9.520363	8.205382	7.796780	8.656980	10.292953	9.379735	8.897476	10.009096	8.906727	8.457783	8.060250	7.302237	7.540217	8.440271	8.392872	7.624722	8.506430	9.610435	9.350940	9.919470	9.381478	9.505969	9.568749	7.680267	9.834281	10.615566	10.031008	10.240163	10.454039	8.227815	9.944548	9.407719	10.044031	10.251030	11.416927	8.595641	8.527214	6.966591	8.896716	9.835965	7.888585	10.637121	9.203412	8.289335	10.331914	9.661551	8.973481	7.925059	8.107279	8.280399	7.765933	8.227204	8.285774	7.049753	8.003407	8.267776	7.443943	8.579521	7.938674	7.620128	8.727249	9.255442	7.928511	7.777466	7.039223	7.192941	9.594314	7.708409	8.138502	8.893308	8.860469	7.659658	6.671706	10.417829	10.251178	7.816205	9.648718	8.143761	7.244210	8.035393	8.882494	8.275241	7.494271	7.740092	7.703423	10.458181	9.546085	8.256785	8.284229	8.761378	8.944895	7.371267	7.884753	7.349682	7.857542	7.859591	6.371595	7.335044	7.863861	7.496300	8.628155	8.028417	7.224803	8.759647	7.360909	9.222961	8.190071	7.126739	8.973413	8.592859	9.059425	7.867408	11.238772	8.249596	7.791899	9.882726	7.113763	7.931572	8.893154	7.630676	7.636483	8.361415	10.419946	9.530828	7.860047	8.367126	11.534947	6.886135	7.864018	7.312433	6.713250	6.895266	7.727960	7.390553	7.331331	7.831082	7.038741	7.174480	9.395272	7.582883	7.470477	7.746079	7.134672	7.401622	7.469565	9.172195	8.436316	8.365500	8.159316	11.624581	10.971168	7.600924	8.116691	10.801504	8.049687	9.881018	7.489591	8.689858	8.625939

LINC00240	6.033450	7.655630	7.616815	7.222237	7.514207	6.842402	8.075009	6.375503	5.643691	7.147212	7.341300	6.592272	7.731332	6.874005	7.483988	7.341449	6.970286	7.831559	6.318752	7.127512	7.702832	7.913863	6.127825	6.860751	7.349096	6.992596	6.610967	6.673815	6.879422	6.901263	6.497681	7.829763	8.055096	9.610435	8.165811	7.147710	7.597626	7.765140	7.917531	7.487101	7.694121	7.871513	8.086591	7.530985	7.942647	7.711380	5.904811	8.888062	7.747653	8.423936	8.315284	7.906928	7.726229	8.476201	8.435693	7.059962	8.173460	8.064734	8.771577	8.553172	8.499816	7.023127	8.485826	8.287505	10.497682	8.958107	8.317753	7.715828	8.842121	8.059636	7.670222	7.218871	6.841755	7.228880	8.079016	7.941031	7.489279	8.828225	7.633026	8.282551	7.944322	8.372078	7.933723	7.906928	7.818875	7.893421	7.923339	8.499968	9.641025	8.521276	8.823280	7.979256	8.479979	8.012294	7.400060	7.810660	8.354284	6.544263	7.427752	7.049913	6.799953	7.561665	7.499773	7.415648	7.873506	7.060933	6.444900	7.938047	7.688703	7.066825	6.835808	7.144590	7.244382	6.782766	7.188552	5.646889	7.012195	7.320594	6.407533	9.403879	8.252521	9.246281	9.610570	7.437251	8.713717	8.468540	9.010252	7.524675	9.483938	8.551958	8.490141	7.357319	8.264002	9.266572	10.405164	7.958836	9.838589	8.477896	8.191012	9.126126	8.537113	8.342315	6.642551	8.670197	8.468110	8.874344	8.394381	8.703217	8.870081	8.547241	7.723620	8.729734	9.872579	10.271020	7.644624	9.326153	8.581825	10.264104	8.049851	9.446373	10.328083	10.479922	10.546423	9.700957	8.900076	7.713603	10.318616	9.697251	9.544271	9.626311	10.909986	9.109564	8.959927	10.487949	8.293216	10.170425	9.908039	9.807299	10.525956	10.673572	9.704430	11.392765	9.716241	11.327051	10.094402	9.828875	9.083038	9.041013	8.998099	8.755780	9.900685	7.570365	9.131232	9.191817	9.392378	10.941987	9.361876	8.252957	10.020041	9.639042	10.053117	9.568749	8.139581	10.094118	9.810408	9.049437	9.778612	9.942574	10.121857	10.646958	10.758681	10.900409	10.289000	10.604741	9.296135	8.510590	7.083448	9.941976	8.807046	8.409240	9.026613	9.645648	9.679564	9.570720	8.631443	10.451937	10.010033	8.350047	7.820409	7.232702	9.071601	8.514437	7.677545	7.474898	11.026307	11.053493	8.166936	9.799644

LINC01942	10.410577	11.650785	11.721853	11.546955	11.369466	12.421202	11.905325	12.144846	12.216095	12.038655	11.564187	11.393088	10.921942	11.018523	10.643266	12.508357	12.284727	10.561773	11.057934	11.868765	10.985815	10.059259	11.842962	11.494786	11.406029	11.659417	12.413004	11.535848	11.924594	12.002950	11.488461	9.876877	10.577397	10.624276	10.999088	11.398515	11.228716	11.603568	11.581815	11.196672	12.410460	11.068971	10.756043	10.613036	12.588458	10.407254	12.873402	10.707486	10.894314	10.353264	10.041471	10.525793	10.050514	10.788074	10.351729	12.667996	9.922734	10.443347	10.148232	9.971065	10.382728	11.882381	11.618016	10.710534	10.525043	10.754335	11.557144	11.403690	10.887194	11.225775	12.468111	12.809121	12.160336	12.634942	11.938785	11.215233	11.747030	11.462482	11.629083	10.144003	9.962091	9.761038	9.358941	10.030393	10.922691	10.821308	10.253789	10.795127	10.214424	10.451803	10.286961	10.095615	10.471635	10.990768	9.902261	10.526885	10.505273	10.166113	10.925355	12.653635	10.726495	12.063494	11.639796	11.900292	10.566949	12.044248	11.082183	10.937605	10.935020	12.489402	10.721496	11.048103	10.816868	12.070827	10.865149	10.994322	10.048962	11.377987	12.231738	10.094118	11.347581	10.850468	10.995532	11.058092	11.608175	11.015753	10.739768	10.918671	11.042091	11.255761	11.029412	11.175052	10.455244	10.825918	11.985255	10.681376	10.245014	10.628223	10.171391	10.406371	9.726365	10.746326	10.607831	10.705811	10.949662	10.878998	10.806353	11.240345	11.403244	10.450933	10.054204	10.458502	11.094991	10.100633	10.684823	10.422096	10.359776	11.057327	10.570126	11.444404	10.799266	10.288200	10.495716	10.397036	10.476626	11.250483	10.791282	10.139559	10.414333	9.916055	9.786539	9.952724	10.483671	9.805636	11.733026	9.827223	10.220177	10.096406	9.684363	10.002491	12.005939	10.310657	10.324127	10.102474	9.923051	10.610648	11.089609	10.759553	10.475566	11.031068	10.974793	11.623726	11.519320	10.071306	10.728981	10.951940	10.736862	11.239797	9.358192	9.204268	9.508226	9.292368	8.768080	9.690691	10.476626	10.237443	9.759499	9.846759	9.657952	9.728859	10.089453	10.050200	10.918527	10.376060	10.165490	9.814069	9.387777	10.567716	10.907218	10.160131	10.960176	11.046942	10.735207	9.724077	11.335396	10.154056	10.240737	10.291882	11.652130	10.605030	10.104477	10.702740	12.348060	10.953621	10.735207	10.092469	10.416142	11.741732

AC108868.2	6.808558	6.757330	6.902551	6.770091	6.854620	7.208630	6.241436	8.100858	8.070476	6.716392	6.461507	6.351290	5.427255	6.294060	5.923113	6.431739	7.824617	5.919744	8.453841	6.526178	7.073540	7.069219	8.819333	6.759287	7.277658	7.239898	5.970859	7.994877	6.501046	5.993944	8.042154	6.097863	6.268605	5.665725	6.866621	6.468342	6.516401	7.158540	7.093370	7.602969	6.356437	6.961340	8.315925	6.678634	6.085920	6.034728	6.271383	7.088173	6.869668	6.363722	6.571007	6.637692	5.704106	6.226940	5.708822	6.092548	6.003768	6.933931	7.412915	7.009640	7.823491	5.836443	7.195198	7.356999	6.923538	7.228734	7.090725	7.131001	7.051334	7.897027	6.837708	6.723225	6.662158	6.592272	6.502846	6.825270	7.025548	7.018436	7.183775	6.664294	6.505475	6.837708	6.541196	6.961969	6.946970	6.172016	6.980014	7.464806	7.783924	7.782275	7.776635	7.959450	6.767147	6.337715	6.538274	6.503812	6.734308	4.516782	5.760167	5.538833	5.701530	6.043810	5.999906	5.932102	7.333066	6.360702	8.019077	6.635160	8.015395	6.835808	8.047832	7.515856	7.361695	7.023838	7.004100	7.861363	7.295865	6.690628	6.277503	7.293658	7.125453	7.938674	6.843510	8.091866	7.334577	6.693274	9.653378	7.717665	6.595852	6.378641	6.941033	6.854303	6.333155	6.181161	7.166260	7.845933	6.309443	14.141470	7.695178	6.917243	6.832447	8.454739	7.082529	7.697302	7.509182	7.435375	7.593670	6.731923	6.683902	8.241734	6.220581	6.173696	6.037539	6.715696	6.783375	6.825914	7.429000	7.358641	7.534859	7.221302	7.578395	13.644720	6.738763	6.271204	6.084585	6.634850	7.045786	7.052561	6.976898	7.119808	6.755554	5.894072	6.187399	6.335357	6.244453	6.241762	6.927637	7.139071	7.269393	7.723620	5.926480	7.694279	7.546146	7.123215	7.338317	7.264789	7.022169	6.995064	8.983646	6.956132	6.839180	6.213743	6.787255	7.265880	7.073851	7.445236	7.554725	7.307573	6.868569	6.549020	6.847079	6.880224	6.703844	7.306970	6.865815	7.323456	7.193263	8.816369	7.712340	11.553894	8.317000	6.956429	6.800600	7.030503	6.885035	6.762196	5.960034	5.519715	5.767949	5.860123	7.883516	7.778050	6.232581	7.323122	6.169784	8.176472	6.986363	8.183733	6.913240	8.322016	7.651278	7.858776	7.157148	7.389954	6.796372	7.480282	6.883757	6.513088

LINC01184	9.223728	7.427903	7.361977	8.435100	8.571796	8.406806	8.195107	8.680808	9.615291	8.386809	8.611061	8.996270	8.944736	8.160840	7.647109	9.129229	8.307762	7.832150	6.956874	8.561617	6.830549	7.665583	5.911336	8.243837	7.047385	8.243097	9.367419	7.448724	8.574419	7.654215	9.175262	8.294269	8.512159	9.264276	8.224190	8.102493	8.131825	7.733625	7.417421	7.482286	8.442687	7.237349	8.311773	7.393365	7.688261	7.882013	8.610624	7.199772	8.032680	7.725142	8.492438	7.715828	8.105747	7.695762	7.751682	8.091424	7.944014	7.329134	7.708229	7.864627	7.316049	8.432586	7.298624	7.021413	7.726838	6.671556	6.908917	7.442516	7.911412	7.025077	7.933245	8.135264	8.836027	8.404354	8.489064	8.377526	8.056042	8.280543	8.023465	8.269676	7.774932	7.939622	8.173625	8.209938	8.265861	7.989271	8.373576	8.487552	6.933336	7.338002	7.172935	6.936660	7.605952	8.284375	8.568356	8.950273	8.076511	9.625524	8.981195	8.403459	8.598516	8.195435	8.389906	6.933776	6.526516	7.925989	6.404740	7.809818	6.735311	8.438425	5.653667	6.944879	7.225295	8.376613	8.014625	6.535017	8.050788	8.431198	9.220198	8.799907	7.440662	7.674467	8.402513	8.277390	9.001352	8.055749	9.768599	9.461531	8.862151	8.133618	9.474009	8.547241	7.949528	6.913553	9.545767	9.323989	8.576106	6.773975	8.653728	7.225295	6.964835	5.573704	8.653728	6.681267	8.500737	8.292455	7.624881	9.063980	7.337058	9.618091	9.063225	9.340437	9.098132	8.437550	8.477251	8.321274	8.529222	8.036022	7.054921	9.281062	7.521618	9.548379	6.684067	8.077123	8.800062	9.174484	7.810046	9.157249	8.838748	8.427882	8.747246	8.057141	8.800210	9.032501	8.388820	9.315654	8.073029	7.864173	7.685796	7.164719	8.252679	7.342224	7.247604	8.329486	7.096715	7.131815	8.421205	8.643311	7.390251	8.319729	7.973216	8.173323	9.050083	9.059573	8.470262	8.085691	8.528362	8.221444	8.839638	7.948319	9.012546	8.344459	8.660064	8.356290	8.756688	9.593381	9.770314	7.220994	7.713903	7.478298	8.007316	7.993011	8.944584	8.798095	8.942331	9.029335	10.086530	9.775382	8.248700	8.868229	9.619777	7.841561	7.598098	7.444119	8.167506	7.496149	8.266671	7.038273	8.636313	5.838997	6.236149	6.660691	8.396552	8.470551	7.881084	7.242922	8.435543	9.434019

AL392086.3	7.500244	7.123370	7.164573	7.128302	8.133925	7.657040	6.746442	8.524770	8.095215	7.365938	7.391949	7.012349	6.747557	6.902716	6.915897	7.215455	8.232848	6.909692	8.410136	7.154341	7.815906	7.585017	8.324469	7.037328	7.469723	7.914905	6.397130	8.075160	6.708953	6.582944	7.748566	6.637206	6.547258	6.180988	6.325419	6.673649	6.673327	7.140322	7.366550	7.726369	7.189399	7.425397	6.528481	6.976564	6.479926	6.910490	8.091263	7.087666	7.142250	6.754571	6.900947	7.098130	6.613319	6.751438	6.898683	6.891902	6.620355	7.271258	7.152007	7.026166	7.728413	6.639819	7.299081	7.695045	7.361839	7.709719	7.268335	7.058706	7.367807	7.797234	6.918844	6.979858	7.089152	7.112839	7.230864	7.166404	7.254611	7.373586	7.363230	6.848046	6.864533	6.774786	6.601982	7.212483	7.269237	6.879880	7.413067	7.701163	7.828542	7.978789	7.856797	8.268231	6.944237	7.223266	7.012518	6.773662	7.194222	6.137868	7.423039	6.882311	7.324066	6.355577	6.792176	7.187619	7.975232	7.166993	8.158091	7.354382	7.884753	7.492126	8.311294	7.237496	8.038522	7.558311	7.712966	9.564664	7.018100	7.244382	7.185203	7.260136	7.416404	6.919542	7.171164	9.815273	7.886288	7.349764	8.215620	7.899257	7.198837	7.481815	7.518967	7.452761	7.267734	7.796780	7.082687	8.000720	6.794463	8.612986	6.696653	7.879463	7.490073	8.592063	7.911734	7.652974	7.716737	7.614214	8.216204	8.637074	6.667041	7.339873	7.032798	7.096114	7.148656	6.484961	6.757330	6.815567	7.387462	7.978000	7.799425	8.146710	7.722222	6.736422	7.495527	6.833435	7.341765	8.161001	6.978944	6.910572	7.301775	7.434558	6.584073	6.676570	6.830874	6.593887	6.619366	6.566408	6.914636	7.282571	7.004100	7.572330	6.949028	7.500701	7.646341	7.319152	7.438512	7.241025	7.308348	7.440515	8.202112	7.142250	6.993859	7.772629	7.185042	7.436323	7.298925	7.098623	7.405370	7.768377	6.896554	6.812869	7.082289	7.135167	7.114731	7.299240	7.527284	7.750125	7.393665	7.702677	7.544076	7.552380	8.595322	7.397399	8.239783	7.420547	7.416084	8.428190	7.578853	7.503695	6.800931	7.566575	7.130688	7.189063	7.319006	8.128270	7.309276	8.325568	7.512491	8.183127	7.975543	7.895121	7.258223	8.374648	8.534075	7.930212	9.202653	7.305069	7.190316	7.156379

LINC02665	7.500244	7.123370	7.164573	7.128302	8.133925	7.657040	6.746442	8.524770	8.095215	7.365938	7.391949	7.012349	6.747557	6.902716	6.915897	7.215455	8.232848	6.909692	8.410136	7.154341	7.815906	7.585017	8.324469	7.037328	7.469723	7.914905	6.397130	8.075160	6.708953	6.582944	7.748566	6.637206	6.547258	6.180988	6.325419	6.673649	6.673327	7.140322	7.366550	7.726369	7.189399	7.425397	6.528481	6.976564	6.479926	6.910490	8.091263	7.087666	7.142250	6.754571	6.900947	7.098130	6.613319	6.751438	6.898683	6.891902	6.620355	7.271258	7.152007	7.026166	7.728413	6.639819	7.299081	7.695045	7.361839	7.709719	7.268335	7.058706	7.367807	7.797234	6.918844	6.979858	7.089152	7.112839	7.230864	7.166404	7.254611	7.373586	7.363230	6.848046	6.864533	6.774786	6.601982	7.212483	7.269237	6.879880	7.413067	7.701163	7.828542	7.978789	7.856797	8.268231	6.944237	7.223266	7.012518	6.773662	7.194222	6.137868	7.423039	6.882311	7.324066	6.355577	6.792176	7.187619	7.975232	7.166993	8.158091	7.354382	7.884753	7.492126	8.311294	7.237496	8.038522	7.558311	7.712966	9.564664	7.018100	7.244382	7.185203	7.260136	7.416404	6.919542	7.171164	9.815273	7.886288	7.349764	8.215620	7.899257	7.198837	7.481815	7.518967	7.452761	7.267734	7.796780	7.082687	8.000720	6.794463	8.612986	6.696653	7.879463	7.490073	8.592063	7.911734	7.652974	7.716737	7.614214	8.216204	8.637074	6.667041	7.339873	7.032798	7.096114	7.148656	6.484961	6.757330	6.815567	7.387462	7.978000	7.799425	8.146710	7.722222	6.736422	7.495527	6.833435	7.341765	8.161001	6.978944	6.910572	7.301775	7.434558	6.584073	6.676570	6.830874	6.593887	6.619366	6.566408	6.914636	7.282571	7.004100	7.572330	6.949028	7.500701	7.646341	7.319152	7.438512	7.241025	7.308348	7.440515	8.202112	7.142250	6.993859	7.772629	7.185042	7.436323	7.298925	7.098623	7.405370	7.768377	6.896554	6.812869	7.082289	7.135167	7.114731	7.299240	7.527284	7.750125	7.393665	7.702677	7.544076	7.552380	8.595322	7.397399	8.239783	7.420547	7.416084	8.428190	7.578853	7.503695	6.800931	7.566575	7.130688	7.189063	7.319006	8.128270	7.309276	8.325568	7.512491	8.183127	7.975543	7.895121	7.258223	8.374648	8.534075	7.930212	9.202653	7.305069	7.190316	7.156379

LINC01645	5.404268	3.317617	3.072994	3.013864	2.912019	3.162165	3.396622	3.395752	3.512576	3.475634	3.648387	3.810335	3.382241	4.204758	3.971793	3.216951	3.704887	3.541895	4.968204	3.248272	5.357204	2.847488	3.134280	3.045474	3.037944	2.912656	3.134142	2.910452	3.159554	3.159156	3.767372	3.976766	5.511064	4.830820	3.664851	4.203381	3.775313	3.443447	3.461637	3.160050	3.494647	3.370267	3.787161	3.412583	3.759435	5.679203	3.879377	3.272250	3.704674	4.112927	3.897142	3.922872	4.314274	4.298965	4.031890	3.164562	4.255667	3.032564	3.902531	3.880860	2.848679	3.134024	3.864105	3.233362	5.612522	3.867287	2.831876	3.369092	3.404213	2.884827	3.026034	2.861366	3.425996	2.776734	2.951013	3.378618	3.027480	2.949012	3.001475	3.853814	3.452427	4.148977	5.673307	4.372814	3.360327	3.714879	2.945170	3.277250	3.291272	3.689322	3.281785	3.012317	3.547999	3.378073	2.924925	2.993354	3.931917	3.078974	3.599926	2.973344	2.923401	3.307309	3.018930	3.136389	4.604801	3.212010	3.657866	3.619528	3.199655	3.326154	2.825823	3.092443	3.073107	3.359512	3.441340	3.236242	5.101129	3.049660	3.180672	6.213743	5.597418	5.224298	5.339320	3.504959	3.780830	3.110615	3.261151	3.515480	3.207511	5.191894	3.946420	3.074560	3.401604	3.499422	2.979944	6.528665	3.323979	3.469965	3.435630	4.636935	3.178160	2.992302	2.925707	4.164473	3.211164	2.896516	3.668288	3.134142	3.589211	3.507239	3.453144	5.125562	3.470874	3.566480	5.741912	5.822222	3.532333	3.426202	3.057375	3.271619	3.027084	4.773888	3.489710	5.198733	5.273109	3.749998	3.139936	5.980467	4.230916	6.036861	3.757923	6.382809	4.487836	4.723229	5.406483	3.849064	8.111029	3.570590	3.826990	7.575127	3.272701	3.909010	3.727546	3.895047	4.384959	4.923203	3.712339	3.376527	2.857593	4.047589	3.517979	3.215429	6.352110	3.300671	3.351386	4.842335	5.002508	3.097619	6.009468	4.687185	4.576920	3.742196	5.123314	4.809737	4.120757	8.433978	4.799201	3.431688	3.557381	5.793851	3.027183	6.318925	3.265284	6.573050	3.024570	3.100934	4.830410	4.748070	3.303802	3.032744	3.070982	4.218793	3.530322	4.990182	3.193796	4.327221	3.645826	3.508342	3.223091	3.377394	3.201942	6.414041	3.268254	8.606356	3.014660	3.130036	4.756759	4.993534

AL136114.1	5.404268	3.317617	3.072994	3.013864	2.912019	3.162165	3.396622	3.395752	3.512576	3.475634	3.648387	3.810335	3.382241	4.204758	3.971793	3.216951	3.704887	3.541895	4.968204	3.248272	5.357204	2.847488	3.134280	3.045474	3.037944	2.912656	3.134142	2.910452	3.159554	3.159156	3.767372	3.976766	5.511064	4.830820	3.664851	4.203381	3.775313	3.443447	3.461637	3.160050	3.494647	3.370267	3.787161	3.412583	3.759435	5.679203	3.879377	3.272250	3.704674	4.112927	3.897142	3.922872	4.314274	4.298965	4.031890	3.164562	4.255667	3.032564	3.902531	3.880860	2.848679	3.134024	3.864105	3.233362	5.612522	3.867287	2.831876	3.369092	3.404213	2.884827	3.026034	2.861366	3.425996	2.776734	2.951013	3.378618	3.027480	2.949012	3.001475	3.853814	3.452427	4.148977	5.673307	4.372814	3.360327	3.714879	2.945170	3.277250	3.291272	3.689322	3.281785	3.012317	3.547999	3.378073	2.924925	2.993354	3.931917	3.078974	3.599926	2.973344	2.923401	3.307309	3.018930	3.136389	4.604801	3.212010	3.657866	3.619528	3.199655	3.326154	2.825823	3.092443	3.073107	3.359512	3.441340	3.236242	5.101129	3.049660	3.180672	6.213743	5.597418	5.224298	5.339320	3.504959	3.780830	3.110615	3.261151	3.515480	3.207511	5.191894	3.946420	3.074560	3.401604	3.499422	2.979944	6.528665	3.323979	3.469965	3.435630	4.636935	3.178160	2.992302	2.925707	4.164473	3.211164	2.896516	3.668288	3.134142	3.589211	3.507239	3.453144	5.125562	3.470874	3.566480	5.741912	5.822222	3.532333	3.426202	3.057375	3.271619	3.027084	4.773888	3.489710	5.198733	5.273109	3.749998	3.139936	5.980467	4.230916	6.036861	3.757923	6.382809	4.487836	4.723229	5.406483	3.849064	8.111029	3.570590	3.826990	7.575127	3.272701	3.909010	3.727546	3.895047	4.384959	4.923203	3.712339	3.376527	2.857593	4.047589	3.517979	3.215429	6.352110	3.300671	3.351386	4.842335	5.002508	3.097619	6.009468	4.687185	4.576920	3.742196	5.123314	4.809737	4.120757	8.433978	4.799201	3.431688	3.557381	5.793851	3.027183	6.318925	3.265284	6.573050	3.024570	3.100934	4.830410	4.748070	3.303802	3.032744	3.070982	4.218793	3.530322	4.990182	3.193796	4.327221	3.645826	3.508342	3.223091	3.377394	3.201942	6.414041	3.268254	8.606356	3.014660	3.130036	4.756759	4.993534

LINC02582	3.196264	3.388249	3.970080	3.708838	3.534455	8.037132	4.869173	4.002553	3.785011	3.951398	5.510469	6.437896	6.553514	3.487138	6.110433	3.848412	3.879570	3.191611	4.218585	2.988612	4.348075	2.993158	4.118354	3.498362	5.115798	3.391201	4.439142	7.983626	4.139542	3.541683	3.971571	3.814406	5.104212	8.591139	3.113321	7.396310	3.592419	3.979980	3.257316	5.665514	3.491185	6.502522	4.183837	3.085290	4.160965	5.900251	4.132093	5.496811	8.027164	5.375539	6.571326	3.101726	5.886203	3.059490	3.345400	3.280580	4.043227	2.938155	2.877393	7.599706	4.929751	3.335963	4.592901	6.357498	3.902531	7.580232	3.078385	3.840908	3.547366	4.244327	6.896873	9.135773	7.761907	4.399157	2.851394	6.705732	5.107646	4.450747	7.240558	5.940438	4.093091	4.278859	8.257856	4.149594	3.942850	5.110165	6.032733	5.907399	3.676220	6.971198	3.290281	4.058018	3.753157	7.909264	6.690947	5.523096	5.425215	4.433285	6.162545	3.644045	5.773447	3.104667	3.657866	3.250849	6.064343	3.134643	6.794940	4.016060	4.156535	4.212512	3.824627	5.138833	6.178147	3.966392	6.774469	9.059116	4.454188	4.876960	9.086706	3.787581	3.686239	4.984580	4.178992	3.986951	13.448203	10.173663	4.188223	4.132771	6.953458	10.435833	11.952588	12.573184	3.472214	12.608353	4.841693	5.355688	9.501173	3.599742	3.031164	6.550188	4.341361	6.626317	3.401766	9.848310	6.847580	5.740431	13.164516	4.439818	6.014686	6.082836	5.724934	5.709373	14.147923	6.263844	11.979596	5.567281	6.954230	5.621849	11.107037	4.627160	12.325329	4.628254	4.207761	9.315965	10.447194	5.154192	5.343386	12.401619	10.011976	11.991560	4.613787	10.566051	7.488664	7.363410	4.029509	3.953135	9.258034	7.812644	12.686212	6.757827	6.951067	4.374352	12.559114	3.483890	12.724823	3.089591	7.475046	7.271730	2.960790	11.915341	14.092821	13.785091	5.240856	4.477618	11.975594	8.330575	5.198528	12.582308	10.858370	3.914226	3.987976	13.541684	5.165688	5.178571	9.585450	10.615704	6.396610	3.431869	12.644874	3.279275	9.334161	8.520218	6.332981	12.218206	10.708399	9.766931	5.317845	11.538542	7.075119	3.601857	3.534249	5.131397	5.001729	12.309839	6.945031	12.467788	3.924795	4.363298	7.853763	11.579376	5.131993	7.787010	3.141052	11.455816	13.578055	4.848086	8.926611	14.143339

AC073869.1	11.021537	10.952734	11.207310	11.647837	11.902934	11.561285	11.071222	11.171659	11.711018	11.204334	11.731533	11.475722	11.957688	11.762513	11.653773	11.402351	11.708507	11.201594	12.297271	11.793022	11.843396	12.308073	12.253731	11.647091	11.241932	11.540839	12.384018	11.025247	11.082486	11.135510	12.047677	11.851856	10.966826	11.867445	11.360307	11.434496	11.395196	11.242830	11.731098	11.087512	11.389096	11.401919	11.725426	11.658633	11.652286	11.529216	11.250318	11.337754	10.838572	11.475989	11.271942	11.383763	11.555942	11.341633	11.380842	11.421024	11.242375	11.473380	11.814206	11.707017	11.353164	11.519102	11.490395	11.909709	11.616116	11.484876	11.374064	11.465833	11.353017	11.395495	10.898787	11.041944	11.192072	11.153465	11.173855	11.316909	11.406317	11.168835	10.915229	11.238152	11.392463	11.216707	11.459334	11.246224	11.398216	11.065268	11.106728	11.475428	11.871756	11.224891	11.118110	11.382171	11.273685	11.171227	11.173855	11.526932	10.967395	11.761613	11.502051	11.682920	11.636032	11.082486	11.670323	11.896631	12.218642	11.758435	11.710886	11.929527	12.062769	11.298091	11.897222	11.969051	11.906809	11.045017	11.325255	11.146945	11.211143	11.776541	11.778816	11.924002	12.036237	11.816173	12.146258	12.020053	12.613710	11.553592	13.483216	13.121579	12.946087	12.398510	12.022667	12.759247	12.728453	12.672242	12.427016	12.625160	11.424053	13.398242	12.060246	12.381950	12.985929	13.064903	12.528398	12.903231	12.418326	11.968244	12.625864	12.893290	11.515780	12.536900	11.931845	12.168818	11.912793	12.108177	12.371963	11.418543	12.333040	12.111443	12.072662	12.768334	12.005488	13.570340	12.535616	12.427016	12.781892	12.456454	12.608672	11.765719	12.537583	12.406661	12.894731	12.530368	12.727048	12.563831	12.173415	12.437418	12.662914	12.383064	12.039543	12.512753	12.229877	12.855334	12.490403	12.098166	11.979884	12.213780	11.896910	12.295263	12.238197	11.786572	11.625150	12.268825	12.680777	11.817489	11.616996	11.496506	12.389993	12.105607	12.238835	12.032445	12.240857	11.796425	12.416103	12.195413	12.494832	12.075900	12.709456	12.178468	12.114497	12.002196	12.477677	11.848789	12.168033	12.120220	12.023550	12.254045	12.441748	12.526303	12.135429	12.647084	12.642396	12.592823	12.320466	12.461548	12.556062	12.646251	12.808400	12.356573	12.009735	12.564124	12.432257	11.801784	11.511560	11.401523	11.830901	12.359421	11.975737	12.688641

LINC02068	13.793821	11.572521	11.831216	12.919156	13.297173	13.870710	12.261188	13.006688	14.314792	13.433055	13.698011	13.510627	13.121772	12.251358	11.588472	13.081962	13.379894	11.847129	11.637244	13.282417	10.794980	13.119075	11.575387	12.750518	12.818398	13.772698	14.956144	13.331903	14.098889	13.493714	13.829710	11.394433	10.624354	13.921593	12.616315	12.275467	12.278204	11.516497	11.582567	12.323571	13.545103	11.764040	12.497333	12.270412	11.896481	11.948032	14.304625	11.220409	11.912512	11.528321	11.723465	11.100452	11.182712	11.771822	11.267512	13.863866	11.229610	11.251510	11.960368	11.437676	11.299384	13.774703	10.991775	11.938053	11.171659	11.253671	11.274739	10.854645	10.903791	11.159054	12.333497	12.629358	14.104048	12.128415	12.226617	12.534911	12.611838	12.265122	12.542773	11.578488	12.126428	11.387167	11.530788	11.311611	11.883291	11.977304	13.166504	11.620849	10.580396	10.522954	10.770156	10.845782	11.145002	12.095797	12.548405	12.849137	11.640106	13.883013	13.329024	13.965466	14.086526	12.077723	12.731164	12.314144	11.357096	13.573342	13.436214	11.908190	12.262939	13.785359	11.363568	13.114655	13.109444	14.630361	13.364551	15.637542	13.274601	13.680915	14.848968	13.644259	12.394426	12.491754	13.180160	14.618611	13.350346	14.257321	15.346400	14.868819	14.148532	13.772186	14.244331	13.843254	12.864781	12.532185	15.026340	14.031176	13.167290	12.631325	12.873750	11.480931	13.202193	11.297513	13.759917	12.254351	12.535440	13.778222	13.939322	15.421067	12.676040	14.803294	13.886983	13.194618	12.922424	12.912699	12.910910	12.949809	11.895183	12.309839	12.362103	14.675040	11.603886	12.231416	12.607232	11.880705	11.442042	14.235215	11.837992	12.571685	11.493313	11.855782	12.121934	11.566391	12.107881	11.914277	13.437980	11.976530	11.965386	12.318072	11.406183	12.037911	13.555053	11.360449	12.020926	11.745676	11.538688	11.938934	11.087964	11.445125	11.014277	12.889837	13.043339	14.357312	12.895811	13.440327	12.581481	13.228263	12.853341	12.721811	12.822141	13.081778	12.326030	12.217765	13.354589	12.434490	13.889403	12.190585	12.261502	11.667291	11.820802	11.498757	11.488318	12.177847	12.492884	13.053474	13.726121	13.790457	14.749773	14.126434	12.772251	13.851451	13.567112	14.690192	12.772251	12.016745	14.503811	14.365218	11.352182	11.006838	14.417635	11.609621	13.820166	14.478214	14.418363	13.926780	13.881969	12.415936	13.983245	14.083435

LINC00421	6.504316	6.355425	7.349599	7.104070	7.661530	6.476102	5.880149	6.981609	7.488806	5.601705	6.662807	6.393213	6.813024	6.564495	6.729161	6.127660	6.920025	6.514226	6.565762	5.958775	5.782005	6.833773	3.523465	6.391724	6.565605	6.690296	6.864246	6.307251	6.355425	6.502676	6.837369	5.834287	5.614444	6.261164	6.220415	5.964297	6.000418	7.014364	6.523154	5.720507	6.863914	5.900433	5.952163	5.891684	5.976039	5.468334	7.981607	6.308238	6.918372	6.368520	5.733384	6.039985	6.672528	6.031825	6.201890	6.994459	5.898847	6.621633	6.833118	6.736422	7.256786	6.466185	7.701304	6.618235	6.368520	6.827484	7.179381	6.335680	6.219399	7.503840	6.309772	6.921443	6.517234	6.566408	6.816039	6.053907	6.619694	6.212338	6.873056	6.791354	5.745747	6.524190	6.122524	7.284600	5.780525	5.727722	6.266717	6.000261	7.011235	6.675238	5.869712	5.819727	6.433787	6.851233	6.937304	6.258264	6.838662	6.882930	7.322799	6.739227	7.668203	5.775099	6.445578	6.782296	6.588854	6.607883	5.855636	6.813820	7.355178	7.048505	6.419326	6.665888	7.117391	7.306655	6.999995	8.970055	6.553821	6.807739	6.538274	5.975338	5.985562	6.755073	6.468027	8.634726	6.246435	6.794137	6.291515	6.733538	6.218710	6.571007	6.617903	7.522817	7.669912	6.934730	6.612494	6.268961	6.995380	6.194626	5.908115	6.416166	6.769936	6.150503	7.034373	6.872429	6.370900	7.601067	7.037167	7.156686	6.596637	6.739082	5.512770	5.303420	6.669144	7.204710	6.292519	6.205756	6.704797	6.452936	6.388356	7.743660	6.075714	5.984182	5.721590	5.562223	5.548830	8.278420	7.901974	6.828610	6.549386	6.085087	5.948634	7.608977	5.689182	5.625408	6.698079	5.815647	6.867559	6.520703	6.803168	7.207379	6.579946	6.543856	6.664453	6.337381	6.128501	6.579778	6.129010	6.326431	7.679233	6.755245	6.752087	7.572780	6.400141	6.524190	5.978848	6.526516	5.912230	7.205220	6.970286	5.371258	5.724934	5.724019	7.024748	6.585060	6.293693	5.853987	6.123195	5.903090	7.171164	5.872446	5.873704	5.968975	7.198684	6.250803	6.171845	6.624362	7.207379	6.094274	6.919542	7.589979	5.898668	6.888237	6.406726	8.619393	6.904447	6.689163	5.750467	7.783625	6.833118	6.648041	7.053815	7.996465	8.032680	7.312129	7.577607	6.540336	6.685825	6.647877

AF228727.1	8.273739	9.242420	9.235949	8.206187	7.312129	7.549912	8.354136	7.873982	7.405852	7.616951	7.484272	8.361723	8.089558	7.406172	8.943081	8.129187	7.928816	9.581758	6.915103	7.779666	6.679132	8.075475	8.873552	8.815582	8.541825	8.273306	8.704594	8.392729	8.699239	9.374798	6.966758	9.324141	9.609822	9.043152	9.857253	9.773401	9.307880	8.928332	8.106048	8.809674	7.579940	9.726215	9.315503	8.845859	9.535836	9.150795	7.707285	10.245152	9.600027	9.056876	9.742128	9.300003	9.653238	8.979691	9.913519	7.610371	10.137149	8.768990	8.974230	9.803036	10.044168	7.724844	9.812137	7.968338	9.200001	9.955223	9.192889	8.511664	9.369517	9.606408	8.900672	8.577424	7.695762	9.135018	8.464904	9.442348	9.011455	9.645490	9.685152	9.376837	8.953479	10.385788	9.577910	9.437579	9.235458	9.454459	9.069131	8.898245	8.574419	9.211454	10.111224	9.012701	7.956364	9.668932	10.272900	9.389757	9.487987	7.644473	7.466031	8.474013	7.419305	8.592681	8.030333	8.262136	7.635566	7.680560	7.214668	7.618175	7.219043	7.470794	8.004502	7.609761	8.098858	7.207080	7.566262	7.511709	8.555423	8.143161	7.178144	7.763447	7.643053	9.152311	7.694740	7.451502	7.512491	7.398027	6.545320	6.483079	7.650308	7.516478	7.795863	7.401771	7.058867	7.810948	7.623325	7.010422	8.050957	6.901907	7.429837	7.271405	7.354060	8.117780	7.433914	7.299240	8.272399	7.814935	7.498039	7.597778	7.892633	7.333971	8.689429	8.104486	7.972088	8.499968	8.055259	8.917548	9.445907	7.165801	8.354756	7.033749	9.063225	7.772318	8.262290	8.867036	7.975388	7.287366	8.850907	9.138891	8.592374	9.351997	8.493834	8.436796	8.627169	9.691000	7.918723	8.223754	9.007632	8.166286	9.485807	9.660604	8.645568	7.976016	7.723151	8.498063	9.489658	8.033901	9.540665	9.536303	9.512911	8.862310	8.680653	7.769286	8.254049	8.977416	9.550559	8.306800	9.018527	8.112848	8.906727	8.427398	9.290023	8.418870	9.708602	8.847386	7.154192	8.977263	8.332304	8.061672	8.715458	9.453555	7.401461	7.480582	8.603917	8.881298	9.022798	7.373269	7.620747	6.249133	9.371195	7.554895	6.996467	6.942793	9.282762	7.328360	7.474745	6.548685	7.672898	7.814763	7.644771	7.680267	7.263561	7.967116	7.755724	7.613414	7.032657	8.526736	6.670564	7.575127

AL672310.1	6.346891	7.262980	6.803991	7.064451	7.122109	7.595624	8.227663	8.482646	7.691812	8.670782	8.100408	7.688567	8.050957	8.213302	7.339085	8.574581	7.761133	7.792348	8.931445	8.001370	8.360009	9.370447	6.164105	7.488362	7.852796	7.369686	6.266039	7.845650	7.256473	7.201165	8.633227	6.854771	8.469008	7.504456	7.788070	8.201973	8.526901	8.219328	8.713413	9.076571	7.541740	8.809052	7.316219	7.591030	7.270478	7.737418	8.031790	8.563129	8.096125	8.465536	6.969980	8.758395	7.462608	9.159846	8.385714	7.677630	7.990498	6.781503	8.232995	8.828225	9.067393	7.654678	5.961118	8.784314	7.690710	7.820856	8.731790	8.155622	8.348987	8.271350	8.394832	8.151906	8.208900	7.679371	8.204349	7.668348	7.844214	7.890599	8.070785	7.826786	7.751504	7.437704	6.762969	7.901337	8.420732	6.781047	7.576031	7.425397	7.512793	7.838941	7.613266	8.138807	7.077031	8.383585	7.676983	7.012349	7.461345	7.443943	8.228867	7.127053	8.070314	8.108325	7.340507	8.260488	8.729734	7.453233	8.869289	8.555749	7.521929	8.312218	8.304669	8.358470	8.412762	7.875819	8.103852	9.644234	7.031545	7.256173	8.226166	7.352662	6.545484	6.018321	7.001459	7.293155	6.934258	6.946970	5.705254	7.413210	6.347920	7.488806	6.267244	6.803481	7.128141	6.935856	6.532242	6.757650	7.723476	6.138578	6.225189	6.677849	6.735148	5.892040	6.649044	6.296759	6.603253	7.499142	7.027169	6.278158	6.959245	6.708790	5.418161	7.485213	9.578851	6.622590	7.009488	7.832020	6.745143	7.695913	7.748264	7.005961	6.756526	6.014010	7.187166	7.093509	7.366238	8.043107	7.717058	6.877490	7.261359	6.848046	6.889174	6.538584	8.626257	6.661015	6.783550	6.091133	5.544684	6.785005	7.277975	8.630992	7.207529	6.883083	7.915822	7.796324	7.385155	7.248533	8.323388	7.920290	7.402245	6.415636	6.746725	7.802216	6.810338	6.821014	6.943755	7.801000	6.934887	7.410333	6.761889	7.064918	6.733213	6.993546	5.765200	6.646901	6.022244	7.596408	5.601521	6.494756	5.716401	5.864709	6.579946	6.635796	6.810819	8.302279	5.913482	6.684706	6.099633	7.397708	6.220581	6.851233	6.708319	6.632208	6.382968	7.245629	6.413207	8.083951	7.250233	7.122734	7.901809	7.095641	7.007495	8.983483	7.826149	7.434079	9.240911	7.268335	8.111454	6.921772

AC139099.2	6.346891	7.262980	6.803991	7.064451	7.122109	7.595624	8.227663	8.482646	7.691812	8.670782	8.100408	7.688567	8.050957	8.213302	7.339085	8.574581	7.761133	7.792348	8.931445	8.001370	8.360009	9.370447	6.164105	7.488362	7.852796	7.369686	6.266039	7.845650	7.256473	7.201165	8.633227	6.854771	8.469008	7.504456	7.788070	8.201973	8.526901	8.219328	8.713413	9.076571	7.541740	8.809052	7.316219	7.591030	7.270478	7.737418	8.031790	8.563129	8.096125	8.465536	6.969980	8.758395	7.462608	9.159846	8.385714	7.677630	7.990498	6.781503	8.232995	8.828225	9.067393	7.654678	5.961118	8.784314	7.690710	7.820856	8.731790	8.155622	8.348987	8.271350	8.394832	8.151906	8.208900	7.679371	8.204349	7.668348	7.844214	7.890599	8.070785	7.826786	7.751504	7.437704	6.762969	7.901337	8.420732	6.781047	7.576031	7.425397	7.512793	7.838941	7.613266	8.138807	7.077031	8.383585	7.676983	7.012349	7.461345	7.443943	8.228867	7.127053	8.070314	8.108325	7.340507	8.260488	8.729734	7.453233	8.869289	8.555749	7.521929	8.312218	8.304669	8.358470	8.412762	7.875819	8.103852	9.644234	7.031545	7.256173	8.226166	7.352662	6.545484	6.018321	7.001459	7.293155	6.934258	6.946970	5.705254	7.413210	6.347920	7.488806	6.267244	6.803481	7.128141	6.935856	6.532242	6.757650	7.723476	6.138578	6.225189	6.677849	6.735148	5.892040	6.649044	6.296759	6.603253	7.499142	7.027169	6.278158	6.959245	6.708790	5.418161	7.485213	9.578851	6.622590	7.009488	7.832020	6.745143	7.695913	7.748264	7.005961	6.756526	6.014010	7.187166	7.093509	7.366238	8.043107	7.717058	6.877490	7.261359	6.848046	6.889174	6.538584	8.626257	6.661015	6.783550	6.091133	5.544684	6.785005	7.277975	8.630992	7.207529	6.883083	7.915822	7.796324	7.385155	7.248533	8.323388	7.920290	7.402245	6.415636	6.746725	7.802216	6.810338	6.821014	6.943755	7.801000	6.934887	7.410333	6.761889	7.064918	6.733213	6.993546	5.765200	6.646901	6.022244	7.596408	5.601521	6.494756	5.716401	5.864709	6.579946	6.635796	6.810819	8.302279	5.913482	6.684706	6.099633	7.397708	6.220581	6.851233	6.708319	6.632208	6.382968	7.245629	6.413207	8.083951	7.250233	7.122734	7.901809	7.095641	7.007495	8.983483	7.826149	7.434079	9.240911	7.268335	8.111454	6.921772

AL732372.1	6.346891	7.262980	6.803991	7.064451	7.122109	7.595624	8.227663	8.482646	7.691812	8.670782	8.100408	7.688567	8.050957	8.213302	7.339085	8.574581	7.761133	7.792348	8.931445	8.001370	8.360009	9.370447	6.164105	7.488362	7.852796	7.369686	6.266039	7.845650	7.256473	7.201165	8.633227	6.854771	8.469008	7.504456	7.788070	8.201973	8.526901	8.219328	8.713413	9.076571	7.541740	8.809052	7.316219	7.591030	7.270478	7.737418	8.031790	8.563129	8.096125	8.465536	6.969980	8.758395	7.462608	9.159846	8.385714	7.677630	7.990498	6.781503	8.232995	8.828225	9.067393	7.654678	5.961118	8.784314	7.690710	7.820856	8.731790	8.155622	8.348987	8.271350	8.394832	8.151906	8.208900	7.679371	8.204349	7.668348	7.844214	7.890599	8.070785	7.826786	7.751504	7.437704	6.762969	7.901337	8.420732	6.781047	7.576031	7.425397	7.512793	7.838941	7.613266	8.138807	7.077031	8.383585	7.676983	7.012349	7.461345	7.443943	8.228867	7.127053	8.070314	8.108325	7.340507	8.260488	8.729734	7.453233	8.869289	8.555749	7.521929	8.312218	8.304669	8.358470	8.412762	7.875819	8.103852	9.644234	7.031545	7.256173	8.226166	7.352662	6.545484	6.018321	7.001459	7.293155	6.934258	6.946970	5.705254	7.413210	6.347920	7.488806	6.267244	6.803481	7.128141	6.935856	6.532242	6.757650	7.723476	6.138578	6.225189	6.677849	6.735148	5.892040	6.649044	6.296759	6.603253	7.499142	7.027169	6.278158	6.959245	6.708790	5.418161	7.485213	9.578851	6.622590	7.009488	7.832020	6.745143	7.695913	7.748264	7.005961	6.756526	6.014010	7.187166	7.093509	7.366238	8.043107	7.717058	6.877490	7.261359	6.848046	6.889174	6.538584	8.626257	6.661015	6.783550	6.091133	5.544684	6.785005	7.277975	8.630992	7.207529	6.883083	7.915822	7.796324	7.385155	7.248533	8.323388	7.920290	7.402245	6.415636	6.746725	7.802216	6.810338	6.821014	6.943755	7.801000	6.934887	7.410333	6.761889	7.064918	6.733213	6.993546	5.765200	6.646901	6.022244	7.596408	5.601521	6.494756	5.716401	5.864709	6.579946	6.635796	6.810819	8.302279	5.913482	6.684706	6.099633	7.397708	6.220581	6.851233	6.708319	6.632208	6.382968	7.245629	6.413207	8.083951	7.250233	7.122734	7.901809	7.095641	7.007495	8.983483	7.826149	7.434079	9.240911	7.268335	8.111454	6.921772

AC019117.3	5.425814	4.485731	5.101551	7.146288	3.664267	4.242950	3.823109	3.722298	4.067119	3.825314	3.316489	3.909259	4.910082	4.556647	3.404556	4.033413	3.457869	4.841476	3.156795	4.549553	6.699501	5.194478	4.621238	4.076598	4.065846	3.936230	5.040958	4.065635	4.211406	4.172921	3.882986	7.323742	5.561304	6.256420	5.747936	3.971168	5.266656	4.867689	4.009758	3.774502	5.197929	3.998746	5.840428	4.667764	4.814569	5.658509	4.754255	4.012263	3.590704	4.412646	6.208993	5.184812	5.254349	5.426670	3.697351	3.971571	5.558862	3.913358	6.806609	3.909259	5.317845	4.513363	7.501316	4.164473	8.219624	5.719936	4.227672	5.006627	5.277938	4.932831	4.410264	4.126095	3.765884	3.778955	4.701513	4.077226	4.553466	5.034412	5.026050	4.424630	4.836596	4.231567	5.521563	4.928464	3.238890	4.607149	5.223543	3.722298	7.488362	4.879622	3.240744	3.989667	5.257140	5.109134	4.996504	4.917690	4.782850	5.201486	3.458025	3.376704	4.424630	4.584561	4.853109	3.934506	5.228322	3.299022	4.402892	5.350897	4.256493	3.984336	3.926325	4.356533	4.619091	3.382584	3.620663	5.239849	4.927218	3.867507	4.733340	3.990057	4.115709	5.628592	7.254764	4.587555	5.272481	5.159865	4.938930	9.844400	4.323808	5.800619	6.624196	5.690793	6.579465	3.630688	7.772768	8.203908	4.356767	4.198394	6.078831	6.261678	5.500670	3.468209	3.433628	3.785850	5.364627	6.666388	4.900045	4.169428	4.316930	4.912822	7.441444	5.022592	8.901252	6.778627	4.015408	7.006750	6.432761	5.179925	4.263344	11.094004	5.816548	3.635673	10.150822	6.253010	5.940259	6.187221	4.851592	3.792920	7.174793	5.541466	6.152751	6.211030	5.334554	7.405064	5.419596	6.765874	5.782546	8.360784	4.617326	9.975129	6.776533	6.234130	6.031825	8.213603	6.115959	8.489226	6.530302	6.699040	7.439610	4.463060	4.357202	5.588901	4.834164	6.586878	5.818327	6.544667	7.580698	4.437234	6.678326	5.213818	8.812633	6.394506	7.296127	4.449155	8.070314	6.411836	3.370267	4.365602	5.164675	4.459856	5.520055	5.684176	6.937644	4.767870	8.025265	4.883686	5.008885	5.654829	4.101061	5.814929	4.442638	10.617007	5.660315	10.647894	3.699591	8.109245	4.815819	5.453034	6.166683	3.605218	6.287577	6.751932	5.514453	5.308659	8.380819	4.718328	6.285328	10.347657

AC019117.2	5.425814	4.485731	5.101551	7.146288	3.664267	4.242950	3.823109	3.722298	4.067119	3.825314	3.316489	3.909259	4.910082	4.556647	3.404556	4.033413	3.457869	4.841476	3.156795	4.549553	6.699501	5.194478	4.621238	4.076598	4.065846	3.936230	5.040958	4.065635	4.211406	4.172921	3.882986	7.323742	5.561304	6.256420	5.747936	3.971168	5.266656	4.867689	4.009758	3.774502	5.197929	3.998746	5.840428	4.667764	4.814569	5.658509	4.754255	4.012263	3.590704	4.412646	6.208993	5.184812	5.254349	5.426670	3.697351	3.971571	5.558862	3.913358	6.806609	3.909259	5.317845	4.513363	7.501316	4.164473	8.219624	5.719936	4.227672	5.006627	5.277938	4.932831	4.410264	4.126095	3.765884	3.778955	4.701513	4.077226	4.553466	5.034412	5.026050	4.424630	4.836596	4.231567	5.521563	4.928464	3.238890	4.607149	5.223543	3.722298	7.488362	4.879622	3.240744	3.989667	5.257140	5.109134	4.996504	4.917690	4.782850	5.201486	3.458025	3.376704	4.424630	4.584561	4.853109	3.934506	5.228322	3.299022	4.402892	5.350897	4.256493	3.984336	3.926325	4.356533	4.619091	3.382584	3.620663	5.239849	4.927218	3.867507	4.733340	3.990057	4.115709	5.628592	7.254764	4.587555	5.272481	5.159865	4.938930	9.844400	4.323808	5.800619	6.624196	5.690793	6.579465	3.630688	7.772768	8.203908	4.356767	4.198394	6.078831	6.261678	5.500670	3.468209	3.433628	3.785850	5.364627	6.666388	4.900045	4.169428	4.316930	4.912822	7.441444	5.022592	8.901252	6.778627	4.015408	7.006750	6.432761	5.179925	4.263344	11.094004	5.816548	3.635673	10.150822	6.253010	5.940259	6.187221	4.851592	3.792920	7.174793	5.541466	6.152751	6.211030	5.334554	7.405064	5.419596	6.765874	5.782546	8.360784	4.617326	9.975129	6.776533	6.234130	6.031825	8.213603	6.115959	8.489226	6.530302	6.699040	7.439610	4.463060	4.357202	5.588901	4.834164	6.586878	5.818327	6.544667	7.580698	4.437234	6.678326	5.213818	8.812633	6.394506	7.296127	4.449155	8.070314	6.411836	3.370267	4.365602	5.164675	4.459856	5.520055	5.684176	6.937644	4.767870	8.025265	4.883686	5.008885	5.654829	4.101061	5.814929	4.442638	10.617007	5.660315	10.647894	3.699591	8.109245	4.815819	5.453034	6.166683	3.605218	6.287577	6.751932	5.514453	5.308659	8.380819	4.718328	6.285328	10.347657

HIPK1-AS1	10.809658	9.753620	9.675373	10.102022	10.819847	11.775503	10.819998	11.139043	11.076924	11.163045	11.540693	11.624718	10.170756	10.442439	9.285962	10.493600	10.651913	9.807593	10.734749	9.990936	10.657546	10.225283	10.663292	10.344770	9.826917	10.640470	10.982333	11.715866	11.206436	11.253814	10.870211	8.741333	9.387333	10.102954	9.542515	9.676599	9.878741	9.478608	10.067980	10.243835	11.254400	9.754711	8.941567	9.598341	9.055670	9.471707	11.993963	9.447632	9.736856	9.068319	9.293321	9.207016	8.426149	9.135618	9.151252	11.285190	8.683701	7.312433	9.296431	9.189635	9.889366	10.959032	9.509444	9.672692	9.610435	9.747720	9.643474	9.564026	9.454607	10.296801	10.427471	10.627787	11.218031	10.745421	10.318464	8.642515	10.110195	10.057120	10.228577	8.764687	8.946198	8.918170	8.951490	9.214537	9.326740	9.302345	9.799962	9.941651	9.599253	9.710556	9.749575	10.125679	9.688392	9.957526	10.120667	9.978130	9.387940	9.371510	10.929640	6.183618	11.071521	9.665161	9.926202	9.969713	10.373661	11.321595	11.137575	10.035315	10.862762	11.440762	10.661925	10.034381	10.667979	12.304503	10.986127	13.941517	9.603904	10.627617	11.824728	10.749767	10.402738	10.179032	10.659620	12.634450	11.544245	11.631046	10.465042	10.578284	11.234071	11.454008	11.483549	11.320998	10.545972	10.473622	10.967556	10.276224	9.629378	10.457252	9.773557	10.453574	10.372919	10.686008	11.544838	10.156215	10.392827	11.658059	11.836026	12.263324	11.118110	11.116223	9.749429	10.173663	10.396585	9.784102	9.881777	10.091891	10.217133	10.600754	10.538810	11.881320	10.265725	8.826560	10.163352	9.154481	10.206287	12.067983	9.583773	9.714695	9.337427	9.604210	8.980580	8.635194	9.123526	9.011607	11.045293	8.652972	7.214181	9.173578	9.174191	9.810261	11.064957	9.918091	9.703851	9.669890	9.903491	9.981684	10.180720	10.054345	10.198425	10.514215	10.439584	12.165750	10.409375	10.499570	8.717665	10.050349	10.531581	10.508124	8.962062	9.191963	9.304840	9.905601	9.644234	9.318827	10.729414	9.610435	9.214807	9.752686	9.629378	9.516845	10.479789	10.851782	10.810113	10.754495	11.364604	10.876491	11.021817	10.826359	6.456542	10.544160	10.051862	11.877032	9.928472	9.915448	11.849886	11.671644	9.902261	10.462499	12.900238	10.732656	10.456003	11.752181	12.775483	11.523082	12.031083	9.937753	10.903791	11.329368

AC006059.5	11.519785	11.259083	11.379157	12.344708	12.805120	12.313325	11.674592	12.488929	13.554610	12.306066	12.785412	12.733547	12.305259	12.084512	11.777298	12.873402	13.073967	12.625513	12.221249	12.107738	11.020654	9.601742	11.146045	12.439669	12.839995	12.933477	14.038211	12.497024	13.345088	12.349121	13.783328	10.202804	10.750219	12.594761	12.093514	11.573736	11.574181	11.643568	11.442486	11.118854	12.662414	11.161727	12.955178	12.200941	12.304346	11.927388	13.230081	10.974369	11.483981	11.187873	10.819395	10.779848	11.060112	11.039457	11.148096	12.991054	11.048835	10.824430	11.656893	11.749227	10.377270	12.630506	10.462179	10.948465	10.150520	11.142800	10.993595	11.444971	10.208318	9.961344	11.868033	12.056973	13.325208	11.537919	11.584232	11.827669	11.738357	11.232575	11.333064	10.807688	10.985679	11.114090	10.592175	10.872181	11.020219	11.470479	11.538956	10.515170	9.430794	10.180871	10.651183	8.899452	11.205682	10.927410	11.308969	12.317768	11.252070	13.848828	12.384645	14.030557	13.110956	11.953330	13.440530	12.083542	10.652663	12.879950	12.764425	10.634289	11.345196	13.279019	9.522567	12.673113	12.254045	13.415858	12.731001	14.760083	11.785542	12.899372	13.728107	11.386742	11.280187	11.233307	11.738510	13.091080	11.202548	12.127025	13.613516	12.568878	11.943486	11.495782	11.798061	12.191674	12.095039	11.383354	13.312973	11.649471	11.320390	12.715740	11.912061	10.600317	9.316873	8.058121	12.162058	10.888785	10.285908	13.730659	11.744056	13.835331	11.055235	12.726021	12.249758	11.775784	11.392925	11.261983	10.805332	11.051054	11.069879	11.294819	11.524887	12.636634	10.293094	11.656596	11.282256	10.897742	10.192057	12.637828	10.394618	10.562500	10.321522	10.404248	10.510212	10.093823	10.510494	10.476319	12.040929	10.119905	10.037185	10.810938	9.600344	10.156673	11.930928	9.702651	10.128392	10.492371	10.474513	11.086256	10.584613	10.177956	9.133976	11.442637	11.583602	12.369200	11.300978	11.263041	11.342184	11.326759	10.937143	11.082044	10.919103	10.294459	9.927990	10.238166	10.789859	9.649168	11.635258	9.861128	9.436174	9.670859	10.296948	9.885790	8.415646	10.978478	11.128092	11.791206	11.593733	11.563013	12.443867	11.866983	11.570035	12.263875	12.762924	12.997678	11.768259	10.443347	12.448328	12.006559	10.190552	9.505499	12.692342	9.477511	12.571081	13.005956	13.007031	12.026311	11.881590	11.238772	11.879850	12.017344

NSMCE1-DT	6.812404	7.479964	7.652674	6.103246	6.154122	5.887451	7.504305	4.701940	6.074144	6.247463	6.207946	6.461996	6.048741	6.400484	7.273736	6.438750	5.322477	6.356259	6.812550	6.588854	6.519531	6.028906	7.004395	5.585535	6.384167	6.481071	6.601982	6.362193	6.026945	7.817635	6.104605	7.917667	8.828392	7.269546	8.241603	7.864018	7.042589	7.583805	7.348804	7.622087	7.660790	7.477997	7.317172	6.917569	7.224338	7.572932	5.875497	8.457339	8.684010	7.459959	8.419307	8.292455	8.421352	8.058274	8.388982	5.698593	8.202413	8.418725	7.103286	7.638623	7.578077	6.365725	9.690691	6.411686	8.233570	7.830357	7.578705	7.040810	7.219837	7.425999	7.548388	7.402709	5.658144	7.719199	7.999482	8.321873	6.553821	8.082255	7.902121	8.209938	7.735797	8.851230	7.644473	8.103552	7.607580	7.497273	9.040231	8.670340	8.730171	8.496279	8.751961	8.578321	7.580698	7.731332	8.348402	8.370503	8.338549	6.565450	6.172188	6.511614	6.575343	7.073236	6.561198	6.546478	6.001621	6.164105	5.999204	6.869820	6.951402	5.618176	6.703996	6.672695	6.146720	5.141118	6.396792	8.416918	7.338317	6.124801	6.366226	7.264337	6.858189	7.104243	6.603879	6.248637	4.037357	6.296916	5.329694	7.117230	6.888402	5.892040	4.521287	5.874946	6.513566	4.764207	8.198709	6.233766	4.866428	4.805579	5.994297	6.062086	6.775132	7.544235	4.178312	5.401460	7.738827	6.510942	5.186794	5.639162	7.098929	7.335661	7.402552	7.729637	6.868396	7.913282	5.552010	6.842050	6.386127	7.791742	7.120590	6.672861	6.471216	7.526963	6.866463	6.386980	7.020611	7.178923	6.704299	6.453304	7.171812	8.201070	7.339873	7.423367	7.232119	7.911881	6.455588	7.193418	7.611728	6.825347	7.601231	7.330395	6.767961	8.901561	7.081775	7.721135	7.639389	7.680124	7.583805	5.729728	7.554725	6.044131	6.025164	4.682440	7.287679	7.355023	8.492264	6.616082	7.644624	7.343892	8.441197	7.402873	7.966495	7.275617	7.939788	7.578077	6.753389	8.219775	6.912945	8.338861	8.418410	6.481906	8.549523	6.689974	7.008016	7.675274	6.942793	5.858872	5.720507	5.495683	5.960403	6.377288	5.931085	5.579285	7.692436	6.692115	3.923719	5.146157	8.083795	5.983507	6.722726	7.053011	6.348278	7.095474	5.943556	5.193437	7.190009	6.449821	6.585732	6.887566

LINC02219	11.775928	10.730465	10.430630	11.696988	11.019313	10.878998	10.319510	11.822149	12.235309	11.126352	10.958253	10.936997	10.389876	10.928751	10.460269	11.269690	11.403690	10.680952	11.496207	11.106317	11.193393	11.466296	11.578338	11.082761	10.945211	11.216126	11.296591	11.556411	11.481075	10.556772	11.746871	11.229920	9.969420	10.157125	10.319973	10.593329	10.402145	11.122337	11.033441	11.180812	11.144787	10.848151	10.845927	10.938471	10.386256	10.419503	11.476828	11.516950	10.625962	10.734462	10.793498	10.488104	10.001795	10.043315	10.236667	11.415282	9.724995	10.601671	10.714458	10.494224	10.623514	10.820134	11.513924	11.401919	10.810938	11.004847	11.087082	10.922983	10.395050	11.445714	11.069731	10.905852	11.011245	10.354602	10.655784	10.578905	10.827682	10.123062	10.639993	10.270875	10.262977	9.731833	10.330253	10.091731	10.196594	10.641500	9.751357	10.102022	10.758970	10.518184	10.422396	10.763276	10.591883	10.397186	10.467548	10.295850	10.326118	10.398815	10.738842	11.282391	11.030164	10.699718	11.006998	10.873089	11.429874	11.031212	12.294510	10.966969	11.529671	11.381887	11.291576	11.334350	11.445581	11.134465	11.491019	11.549716	11.562464	10.712709	11.235350	11.686630	10.648931	11.016629	11.504506	11.531086	10.904376	10.882649	10.857034	10.821920	11.988628	10.763720	11.432854	11.023804	11.600013	11.984838	10.706880	10.383504	11.344171	11.615965	10.574514	11.107474	12.003376	12.704678	11.985888	10.812104	9.937098	11.324533	11.149918	11.972822	11.413703	11.440637	10.049101	9.924431	10.326735	10.237443	11.049127	9.719099	11.099868	10.725060	10.776378	11.104579	11.493018	10.883700	10.514841	10.316523	11.052514	11.444695	11.171500	11.089609	10.202155	10.057867	11.799097	10.635777	9.963157	9.546675	12.002645	9.808735	10.645484	11.509649	11.696817	10.386089	11.172982	10.905852	10.742155	10.377715	10.206432	10.558425	10.715522	10.376671	11.280895	10.899971	11.103415	11.686191	9.929530	9.782073	10.301432	10.191626	10.285451	10.641916	9.793762	9.476585	9.533530	9.477221	9.661397	10.363870	10.389118	9.556482	9.874507	10.840472	10.187701	10.819395	10.709659	11.950810	10.762808	10.702889	10.552793	10.357381	10.305962	10.485254	10.950587	11.112018	10.700452	11.083644	10.445361	11.249245	12.269936	12.161407	10.469045	12.429483	11.722749	12.226002	11.285343	11.744276	11.384527	12.432750	10.530093	10.969695	10.395504	11.093554

LIPE-AS1	9.824324	10.087299	10.252352	10.194030	10.407694	10.324901	9.984579	10.511711	10.789285	10.280332	10.196594	10.277301	10.054204	10.963392	10.118219	9.918392	10.263944	10.630112	10.888061	10.274087	11.295979	11.080287	11.613910	10.776828	10.868137	10.408753	10.225742	11.076125	9.656579	9.929076	11.226941	10.114208	10.054975	9.995098	9.656579	9.755913	9.959230	10.442286	10.569539	10.653102	10.117613	10.670015	9.955081	10.001482	9.873016	10.355676	10.274402	10.589660	10.219240	9.908194	9.823307	10.006772	10.278350	9.967259	10.728225	10.625655	10.343540	10.732656	10.347353	10.497826	11.027621	10.171080	11.215845	10.806353	10.741863	10.623981	10.586706	10.642988	10.148722	10.851206	9.818140	9.831535	10.377270	9.908340	10.215321	10.002558	10.454796	10.117756	9.981055	11.375024	10.268511	9.958130	9.949060	10.411451	10.091731	10.149179	10.261006	10.259465	10.945211	10.583044	10.645344	11.833001	10.307568	10.239105	9.970033	9.900225	10.195238	10.269429	10.635922	10.237865	10.396893	9.718945	9.961344	10.074373	11.655906	10.189954	11.009021	11.311749	11.140636	10.340199	11.217736	10.513312	11.234624	10.271093	10.980565	11.727527	9.915155	10.027498	10.122304	10.720354	9.730932	10.192949	10.926688	11.174893	9.951078	10.007217	9.781037	10.136546	10.206287	9.127188	10.932636	9.741359	9.985649	10.316937	9.300176	9.400437	10.870835	9.438350	8.404680	10.409829	11.415437	11.658962	11.327777	9.984732	9.491096	10.043446	11.023937	10.450933	9.777523	10.058771	9.554047	9.554260	9.379735	9.077210	8.721025	9.878449	10.059401	9.769058	10.448109	10.246644	10.538660	10.216978	9.244547	9.707187	10.119452	10.099038	10.271475	9.706104	9.616997	9.199535	9.281837	10.135620	9.687451	9.824950	9.470474	10.245440	10.175307	10.622632	10.336744	11.560528	10.225137	10.092773	10.809816	10.171241	9.384500	10.928442	10.059698	9.312491	9.445303	9.699238	9.755013	10.298333	8.941567	9.513967	9.623524	9.032809	9.781775	9.544433	9.504396	9.582699	9.730122	10.293860	9.556170	8.987020	9.989516	9.674912	10.261311	10.353428	10.003818	11.246365	10.196439	9.746922	9.731085	9.772051	9.957211	9.274836	10.233543	9.886715	9.931531	9.590255	9.496877	10.089927	9.579792	10.571372	10.979079	9.606243	11.857830	10.949054	9.668449	10.481102	10.498577	10.355373	10.581438	11.165668	10.004584	9.441108	10.781158	9.488153

LINC02029	3.186761	3.422423	4.983584	3.073894	3.190658	3.002049	3.383664	3.357588	3.445453	3.452427	5.104831	3.732755	3.701819	3.867935	4.388072	3.617825	3.713585	3.172384	4.486379	3.214341	4.471446	4.983584	3.263273	2.872761	2.932490	3.395212	3.951827	3.052050	3.341148	4.018026	3.631286	3.827843	5.632279	4.256282	3.745894	4.062633	3.664638	3.243676	3.674948	3.562864	3.311317	3.032564	3.867935	3.629852	3.483154	4.891473	3.714449	2.985231	3.608224	4.150204	4.038230	3.851853	5.062460	3.130994	5.497353	4.411106	3.729640	5.703923	3.644045	3.269270	2.868818	3.788233	6.022738	3.740764	3.814003	4.981524	3.283950	3.805753	4.076394	2.911012	3.643274	2.894612	3.349874	3.371691	3.217861	3.846729	3.375800	4.407127	3.450610	4.980532	3.869240	4.827617	3.321367	4.117207	3.823956	4.368705	3.502150	4.158634	3.240460	3.547577	3.409531	3.764916	4.165795	4.939145	4.175969	3.900187	4.032992	3.644833	4.727844	3.063869	3.427639	3.681221	3.938324	3.486244	4.043645	3.632310	5.169505	3.749998	3.228359	3.106895	2.809301	3.444194	3.108516	3.222539	3.368512	3.473978	3.020517	4.098433	2.837457	3.406400	6.114220	3.957225	3.016359	3.582455	3.285045	3.289973	3.270368	3.503982	3.344944	7.300310	3.212131	3.420058	4.055403	6.778767	3.223626	5.048505	3.434340	3.344003	3.539970	3.786037	3.241947	3.105154	4.044345	3.339444	3.463048	3.994432	3.430641	3.038434	4.333544	5.146958	3.441180	5.220735	6.885035	4.282734	3.762780	4.026422	3.285202	6.542050	2.837918	5.774910	2.886294	3.503137	3.681611	3.793394	4.267843	4.164473	3.003001	3.811402	4.591411	4.521910	3.774917	4.902978	7.855444	3.387578	4.371120	3.442050	5.921216	3.285471	3.493734	7.074170	3.682691	9.904851	5.534897	3.370996	4.162019	7.229521	4.520187	4.175969	2.895437	3.068474	3.262192	4.440018	4.857125	3.395942	5.334740	4.136944	3.606829	3.719948	6.490462	5.516844	6.614638	3.790147	4.382956	6.334164	3.173311	6.852172	5.028694	6.208471	4.509851	3.695929	7.014840	4.619283	7.310106	5.702980	5.648576	5.845446	3.324499	6.266039	3.790973	8.236689	3.927387	6.337381	3.204548	5.584968	3.746283	5.472341	3.530135	4.262655	4.512448	3.459278	6.438065	3.230351	3.150262	4.471699	6.359531	3.150988	4.191252	6.852835

LINC00880	3.186761	3.422423	4.983584	3.073894	3.190658	3.002049	3.383664	3.357588	3.445453	3.452427	5.104831	3.732755	3.701819	3.867935	4.388072	3.617825	3.713585	3.172384	4.486379	3.214341	4.471446	4.983584	3.263273	2.872761	2.932490	3.395212	3.951827	3.052050	3.341148	4.018026	3.631286	3.827843	5.632279	4.256282	3.745894	4.062633	3.664638	3.243676	3.674948	3.562864	3.311317	3.032564	3.867935	3.629852	3.483154	4.891473	3.714449	2.985231	3.608224	4.150204	4.038230	3.851853	5.062460	3.130994	5.497353	4.411106	3.729640	5.703923	3.644045	3.269270	2.868818	3.788233	6.022738	3.740764	3.814003	4.981524	3.283950	3.805753	4.076394	2.911012	3.643274	2.894612	3.349874	3.371691	3.217861	3.846729	3.375800	4.407127	3.450610	4.980532	3.869240	4.827617	3.321367	4.117207	3.823956	4.368705	3.502150	4.158634	3.240460	3.547577	3.409531	3.764916	4.165795	4.939145	4.175969	3.900187	4.032992	3.644833	4.727844	3.063869	3.427639	3.681221	3.938324	3.486244	4.043645	3.632310	5.169505	3.749998	3.228359	3.106895	2.809301	3.444194	3.108516	3.222539	3.368512	3.473978	3.020517	4.098433	2.837457	3.406400	6.114220	3.957225	3.016359	3.582455	3.285045	3.289973	3.270368	3.503982	3.344944	7.300310	3.212131	3.420058	4.055403	6.778767	3.223626	5.048505	3.434340	3.344003	3.539970	3.786037	3.241947	3.105154	4.044345	3.339444	3.463048	3.994432	3.430641	3.038434	4.333544	5.146958	3.441180	5.220735	6.885035	4.282734	3.762780	4.026422	3.285202	6.542050	2.837918	5.774910	2.886294	3.503137	3.681611	3.793394	4.267843	4.164473	3.003001	3.811402	4.591411	4.521910	3.774917	4.902978	7.855444	3.387578	4.371120	3.442050	5.921216	3.285471	3.493734	7.074170	3.682691	9.904851	5.534897	3.370996	4.162019	7.229521	4.520187	4.175969	2.895437	3.068474	3.262192	4.440018	4.857125	3.395942	5.334740	4.136944	3.606829	3.719948	6.490462	5.516844	6.614638	3.790147	4.382956	6.334164	3.173311	6.852172	5.028694	6.208471	4.509851	3.695929	7.014840	4.619283	7.310106	5.702980	5.648576	5.845446	3.324499	6.266039	3.790973	8.236689	3.927387	6.337381	3.204548	5.584968	3.746283	5.472341	3.530135	4.262655	4.512448	3.459278	6.438065	3.230351	3.150262	4.471699	6.359531	3.150988	4.191252	6.852835

LINC01588	7.104714	7.553935	7.674467	7.355513	7.452446	7.837640	7.355513	7.953875	8.770989	7.769127	7.961155	7.201019	7.023757	7.247008	6.787592	8.132536	7.767009	6.703390	7.845512	7.313538	7.912039	9.371510	6.777993	7.217816	7.556447	7.732870	8.928332	8.335259	7.435375	7.765302	8.149158	7.237820	7.867408	7.613569	7.008175	6.796203	7.410632	7.144913	7.562571	7.484585	7.517738	7.184551	7.557531	7.811267	6.658538	7.141116	8.025265	7.114883	7.247314	7.316850	7.156988	7.427426	7.221454	7.526374	7.263413	7.765302	6.906051	7.245629	6.990803	7.344722	7.032959	7.660627	7.079582	7.774932	7.319152	7.120747	7.376878	7.189556	7.133547	7.724307	7.115335	7.291140	7.998255	7.346434	7.435698	7.632388	7.254455	7.363230	7.214504	7.100946	7.699122	7.405226	6.821344	7.566262	7.812489	7.359016	7.502904	7.697615	7.300310	6.920499	6.711800	7.209083	7.261207	7.381709	7.569764	7.263271	7.278463	7.685652	7.599203	7.462948	6.904304	7.194134	7.314180	7.582415	7.678603	7.303347	8.283450	8.088168	7.930369	7.795559	7.475837	7.957883	8.624991	8.062875	8.157042	7.011235	6.938279	7.558907	7.724547	9.339624	7.123846	8.038832	8.002306	7.940740	7.625771	7.320728	7.255858	10.952265	7.291614	8.577014	7.323122	8.003880	6.436296	7.657214	7.398027	7.980027	6.680785	7.439610	6.215090	7.061387	7.228566	7.367980	7.319152	7.263561	7.402245	7.557702	7.798320	7.360104	7.280216	8.058274	7.310728	6.979858	7.512793	7.008344	6.534041	6.700626	8.504625	7.781047	6.809369	8.188055	6.734308	7.066825	8.467060	6.264325	6.685006	8.689294	6.261678	6.626000	7.216228	7.102355	7.336449	6.577999	7.950317	8.217766	6.974641	6.483249	7.027169	7.386840	6.613170	7.000313	9.206866	7.814466	7.685331	6.961969	8.345227	7.580396	7.192941	7.228880	7.687159	6.949641	6.734479	9.105810	7.325954	7.450737	7.159168	7.879300	7.718727	7.361534	7.366100	7.964030	8.175833	7.051808	7.182354	7.289724	7.642764	8.842290	7.646042	7.631321	6.405898	6.194626	7.439160	7.009322	7.261987	7.416707	7.876419	6.962293	6.581952	8.457339	6.411526	6.664613	7.305394	8.230097	6.747882	7.810345	7.359016	7.760531	7.585490	6.707390	9.032501	7.245764	8.033141	6.858345	8.935453	7.870902	7.858176	6.816351	9.076571	8.583613

AC092604.1	10.833010	7.204562	7.632690	8.613148	7.917069	9.357110	7.508960	8.450004	8.854719	8.022991	7.786677	8.646197	7.492583	7.187785	7.257882	7.606389	8.104985	6.637206	7.776496	7.838627	7.418369	7.306185	7.565945	7.175887	7.175887	7.448405	10.075573	7.948757	8.782122	8.374187	8.469643	7.336280	6.941340	8.599881	7.624410	6.914636	8.218549	7.104562	7.688261	7.316049	7.937139	7.277658	6.841294	7.272982	6.511437	6.198966	8.312218	6.778153	7.189713	6.792991	6.983178	6.653361	6.124461	6.605592	6.717651	8.387433	6.058347	7.023277	7.088173	6.789740	7.250720	9.233800	7.476921	7.962531	7.288783	7.472951	7.383010	7.606840	7.199772	7.700687	7.889992	8.248245	8.308699	8.043407	7.813882	7.642917	8.072573	7.963443	7.797234	6.803168	7.483531	6.821498	7.069044	6.700295	7.597944	6.520020	7.852039	8.121469	7.853595	8.183260	7.678909	8.368201	7.091977	7.439309	8.168619	7.586459	6.819280	7.273273	8.003097	8.387433	8.790890	7.503695	7.531887	7.874427	7.619865	8.778111	7.752553	7.567942	7.905709	8.221769	8.012452	8.377215	8.155006	9.221723	8.722060	11.980027	9.258639	9.290023	9.115417	8.805073	7.430775	7.810508	7.540820	9.192889	8.333380	8.339965	8.170358	8.288257	8.281921	8.105589	8.471529	8.357999	7.819523	7.757576	8.222383	7.998109	7.144750	7.456028	7.927895	7.911881	7.439469	8.206633	7.910483	7.933245	8.188791	9.184246	8.489514	9.881488	8.097062	8.712055	8.277825	7.683680	8.323388	7.656124	7.593670	8.175380	8.064117	8.065503	8.225862	8.611378	7.385610	6.791839	7.930212	6.868741	7.266775	8.559772	6.962293	7.154829	7.708979	7.760052	7.040810	7.019207	6.871129	6.877972	8.396077	7.053316	6.761727	8.065802	6.876112	7.501657	8.560859	7.766856	7.732580	7.559214	7.830076	7.092126	7.664378	7.315429	8.848945	8.112700	8.382213	8.697899	8.298809	9.141010	8.050313	8.266888	8.102493	7.891853	7.196457	6.763125	6.936806	7.368131	7.057440	7.325321	8.082255	7.368285	7.192941	7.838309	7.863243	7.148992	8.262290	7.248533	7.895415	7.785009	7.980027	8.094921	8.208150	8.111607	7.779491	8.087867	7.884753	8.390063	8.324297	6.957510	8.443325	8.289502	7.710165	7.475221	8.134662	8.090173	8.219775	10.558564	9.161517	8.581959	9.007935	7.658320	8.530929	8.216356

AC090241.2	12.754620	6.706394	7.645570	10.859980	7.942940	7.662314	7.778530	7.793544	7.421823	7.588434	8.209048	7.712658	6.952663	6.873531	7.247454	7.381709	7.432347	7.109818	7.382853	6.911787	6.664294	7.271866	7.184232	7.013182	6.929370	7.144444	7.755863	8.333986	8.079617	7.488051	7.251051	10.342962	6.671230	7.213873	6.869820	6.759287	7.121043	6.760913	7.012518	6.714928	7.818413	6.879572	6.954230	6.808084	6.929531	6.515893	8.715151	6.767147	6.335680	6.924368	13.225499	7.071494	6.116129	6.660520	6.003613	7.714645	5.965532	6.639497	6.520020	6.100310	6.547423	8.185578	6.693760	7.055251	6.518205	6.800770	6.875792	6.621633	6.925172	7.462140	7.309782	7.274994	8.013217	7.207379	7.148490	6.915595	7.047698	6.653693	9.919156	6.341705	8.754719	5.959854	8.652204	5.812591	5.958228	6.421573	6.301676	6.245620	6.069808	7.289724	6.344739	6.739567	6.835808	7.024922	10.537076	6.825746	6.177466	7.049913	7.927574	7.562431	7.832609	7.176504	7.347533	7.242119	7.050534	7.423685	7.493360	7.351140	7.047549	8.328445	7.003383	7.387080	7.140023	10.104182	7.431423	8.882792	11.624998	7.158379	8.516062	9.064290	6.429182	7.255858	8.401902	8.428035	7.551436	7.875964	7.551288	6.100488	7.748264	7.711380	7.910787	7.578233	7.206445	7.828542	6.525640	6.672024	7.166846	6.553514	6.749727	7.065715	8.028334	7.916296	7.710641	6.875472	7.095799	7.488806	7.637537	8.238860	7.391797	6.842050	7.916768	7.435533	7.412915	6.678964	6.199995	6.729818	6.832615	6.833926	6.461507	7.556139	6.643860	5.870061	6.973815	6.655174	6.236149	8.057849	6.682909	7.718121	6.918525	10.930096	6.779754	6.038597	6.187067	6.034026	7.186349	5.971051	6.095860	6.143234	5.831051	6.257597	7.868330	6.228136	5.947758	6.488062	6.225022	6.616593	6.635006	7.035147	6.629515	6.751276	7.002279	7.825066	6.755395	6.612157	6.325088	6.642863	6.658538	7.955865	5.753589	6.421425	6.223992	6.724009	5.633793	5.527477	7.221773	6.101534	5.339915	5.909516	5.821481	8.547074	6.157372	6.623549	7.073059	8.520666	6.650937	7.603766	7.940418	7.318693	7.187943	7.124976	6.666216	7.506496	6.687933	6.137019	8.549976	6.370733	5.853071	7.565494	8.122287	7.457297	7.244686	8.474768	9.076866	8.272399	7.956035	8.216356	6.803329	7.433286

AC079385.1	9.149696	9.066450	8.931289	8.737163	8.947669	9.385437	8.732878	10.238166	10.214570	9.155715	8.878315	8.766546	8.599266	9.267981	8.828392	9.565422	9.531817	9.036629	11.067036	9.527548	10.231761	10.302486	10.162738	9.159093	9.336777	9.262112	9.326448	9.419441	9.162292	9.028410	9.866387	8.075632	9.030278	7.909264	8.485531	8.720713	8.705991	9.380526	9.683246	9.720935	9.321044	9.330587	8.888525	9.249936	8.702595	8.964652	9.114516	9.890580	9.084689	9.541747	9.379881	9.189936	8.886073	8.964318	8.858812	9.490469	8.637867	9.666872	9.617155	9.214386	9.554494	9.056578	10.104326	10.187701	9.792404	9.749888	9.871373	9.569490	9.397158	10.243374	8.867333	8.756219	9.234881	8.393785	8.583138	9.003636	9.138103	8.763170	8.848337	8.543841	8.659902	8.656397	8.273739	8.689858	8.516533	8.844490	7.961301	8.239920	9.398779	9.291287	8.755019	9.631674	8.934877	8.609851	8.079159	8.241455	8.797944	7.713903	8.173935	8.864011	7.892168	8.983194	8.922566	8.937335	10.463392	9.005510	10.713575	10.147330	10.731932	9.795801	10.479789	9.971216	9.980249	9.944548	9.454030	8.909943	9.623678	8.789485	8.841949	8.976069	9.237035	8.675783	9.225443	9.092385	9.340123	8.382051	8.999951	9.526496	8.745587	8.963428	8.362192	8.685838	8.714674	9.474607	9.210997	9.703107	8.942784	10.124584	8.570053	10.083894	9.800421	10.435536	9.380969	9.271368	8.884058	9.462714	9.337273	9.490144	9.036324	9.639042	7.704877	8.958743	8.135563	8.460162	8.636158	8.436649	9.298930	9.870429	9.405722	9.157407	9.823152	8.918330	9.386364	8.938239	9.110639	9.515750	9.694042	9.174191	9.497649	9.453875	9.350323	8.888851	9.060792	8.702129	8.796854	8.177835	9.433117	9.816348	9.025867	9.461373	9.002733	10.609075	10.270279	9.799962	9.639513	9.765726	9.873170	9.547611	10.335998	8.671094	8.705361	9.875601	8.451544	8.192133	9.126126	8.927857	9.035892	8.953796	8.418107	8.489226	8.873841	8.539403	8.294877	8.543369	8.450307	8.338225	8.016016	9.241070	9.031124	8.798544	9.789901	8.422713	8.718581	8.478824	8.456424	8.588024	7.542035	8.342169	8.331995	8.203475	8.488758	8.558556	8.640041	9.511815	9.075896	9.999817	9.827965	9.754059	10.010322	10.011976	9.728613	9.794875	10.322594	9.080879	8.779775	9.247960	8.486435	9.272123

MEG3	11.450138	9.030848	9.478926	9.252684	9.307880	7.220994	8.246265	5.959854	6.617107	8.703695	7.255095	11.199162	9.917330	7.873205	10.464739	8.986560	10.475286	9.890879	10.932033	8.767930	9.317616	10.603870	13.502805	9.118187	9.575333	9.316100	12.101494	10.263444	12.342785	8.983952	10.723716	8.771856	7.801299	9.016869	8.530634	8.833056	8.538466	9.591051	9.435860	9.322724	9.856025	10.626401	9.238911	9.027513	10.255271	9.259236	10.050514	9.914535	9.018218	9.170762	9.321704	9.161231	8.941412	9.258777	9.825553	9.614087	9.199210	10.209666	11.220264	10.160284	9.228368	11.993505	12.010940	10.420715	9.261665	9.817564	9.715628	9.053453	7.710641	9.383918	9.693584	8.941854	10.214570	9.360643	8.717976	7.975388	7.954325	8.685982	10.066011	10.573345	9.525633	10.217581	9.946708	9.103656	8.873698	9.533221	9.127794	7.555055	9.292992	10.089619	9.987919	9.065038	9.461061	7.376276	10.426707	8.753188	10.557792	11.692447	10.092047	9.786258	10.892843	9.676599	10.797174	10.437038	10.055446	10.563370	10.872478	9.122762	12.277596	8.848337	11.095294	10.075883	11.275601	8.659160	8.002620	8.561173	11.454433	10.888785	9.885942	8.502624	8.675471	8.689429	8.945966	9.443437	6.683734	9.192429	9.843644	10.263119	7.794622	9.515829	8.278265	9.217742	10.369106	9.436336	7.707764	9.644870	8.895958	8.593623	6.237625	8.539908	11.153465	9.997647	8.934723	10.268218	11.984250	9.270612	7.690559	9.224967	6.754571	10.667256	10.012445	10.845325	8.913583	6.510601	7.752132	9.435228	8.902489	9.627691	9.967417	7.952777	9.352897	7.810799	8.274186	9.222067	6.301676	9.389914	11.730949	7.173102	8.451400	8.145645	7.446500	7.895271	7.897027	9.260739	8.502947	7.435209	8.721615	9.407719	7.403034	9.143652	8.902489	8.077276	8.511066	8.987489	8.644996	8.301664	8.969436	8.305897	8.356756	7.615270	9.116670	6.779905	10.167973	8.474013	8.091263	9.680465	8.957459	9.591500	9.381478	9.633073	7.429167	9.318660	10.618043	9.177093	9.607325	9.321556	8.659160	9.524895	10.479041	13.333189	8.872943	9.501025	9.188306	10.748441	10.011976	10.717161	12.827344	9.889840	7.856943	12.600363	7.914298	10.205513	9.413487	8.416105	9.490307	8.997819	7.411972	7.458098	6.625166	9.389005	11.032834	11.102534	5.075463	7.260440	8.269199	10.269856	8.238860	7.077031

AL117190.1	11.450138	9.030848	9.478926	9.252684	9.307880	7.220994	8.246265	5.959854	6.617107	8.703695	7.255095	11.199162	9.917330	7.873205	10.464739	8.986560	10.475286	9.890879	10.932033	8.767930	9.317616	10.603870	13.502805	9.118187	9.575333	9.316100	12.101494	10.263444	12.342785	8.983952	10.723716	8.771856	7.801299	9.016869	8.530634	8.833056	8.538466	9.591051	9.435860	9.322724	9.856025	10.626401	9.238911	9.027513	10.255271	9.259236	10.050514	9.914535	9.018218	9.170762	9.321704	9.161231	8.941412	9.258777	9.825553	9.614087	9.199210	10.209666	11.220264	10.160284	9.228368	11.993505	12.010940	10.420715	9.261665	9.817564	9.715628	9.053453	7.710641	9.383918	9.693584	8.941854	10.214570	9.360643	8.717976	7.975388	7.954325	8.685982	10.066011	10.573345	9.525633	10.217581	9.946708	9.103656	8.873698	9.533221	9.127794	7.555055	9.292992	10.089619	9.987919	9.065038	9.461061	7.376276	10.426707	8.753188	10.557792	11.692447	10.092047	9.786258	10.892843	9.676599	10.797174	10.437038	10.055446	10.563370	10.872478	9.122762	12.277596	8.848337	11.095294	10.075883	11.275601	8.659160	8.002620	8.561173	11.454433	10.888785	9.885942	8.502624	8.675471	8.689429	8.945966	9.443437	6.683734	9.192429	9.843644	10.263119	7.794622	9.515829	8.278265	9.217742	10.369106	9.436336	7.707764	9.644870	8.895958	8.593623	6.237625	8.539908	11.153465	9.997647	8.934723	10.268218	11.984250	9.270612	7.690559	9.224967	6.754571	10.667256	10.012445	10.845325	8.913583	6.510601	7.752132	9.435228	8.902489	9.627691	9.967417	7.952777	9.352897	7.810799	8.274186	9.222067	6.301676	9.389914	11.730949	7.173102	8.451400	8.145645	7.446500	7.895271	7.897027	9.260739	8.502947	7.435209	8.721615	9.407719	7.403034	9.143652	8.902489	8.077276	8.511066	8.987489	8.644996	8.301664	8.969436	8.305897	8.356756	7.615270	9.116670	6.779905	10.167973	8.474013	8.091263	9.680465	8.957459	9.591500	9.381478	9.633073	7.429167	9.318660	10.618043	9.177093	9.607325	9.321556	8.659160	9.524895	10.479041	13.333189	8.872943	9.501025	9.188306	10.748441	10.011976	10.717161	12.827344	9.889840	7.856943	12.600363	7.914298	10.205513	9.413487	8.416105	9.490307	8.997819	7.411972	7.458098	6.625166	9.389005	11.032834	11.102534	5.075463	7.260440	8.269199	10.269856	8.238860	7.077031

AL117190.2	11.450138	9.030848	9.478926	9.252684	9.307880	7.220994	8.246265	5.959854	6.617107	8.703695	7.255095	11.199162	9.917330	7.873205	10.464739	8.986560	10.475286	9.890879	10.932033	8.767930	9.317616	10.603870	13.502805	9.118187	9.575333	9.316100	12.101494	10.263444	12.342785	8.983952	10.723716	8.771856	7.801299	9.016869	8.530634	8.833056	8.538466	9.591051	9.435860	9.322724	9.856025	10.626401	9.238911	9.027513	10.255271	9.259236	10.050514	9.914535	9.018218	9.170762	9.321704	9.161231	8.941412	9.258777	9.825553	9.614087	9.199210	10.209666	11.220264	10.160284	9.228368	11.993505	12.010940	10.420715	9.261665	9.817564	9.715628	9.053453	7.710641	9.383918	9.693584	8.941854	10.214570	9.360643	8.717976	7.975388	7.954325	8.685982	10.066011	10.573345	9.525633	10.217581	9.946708	9.103656	8.873698	9.533221	9.127794	7.555055	9.292992	10.089619	9.987919	9.065038	9.461061	7.376276	10.426707	8.753188	10.557792	11.692447	10.092047	9.786258	10.892843	9.676599	10.797174	10.437038	10.055446	10.563370	10.872478	9.122762	12.277596	8.848337	11.095294	10.075883	11.275601	8.659160	8.002620	8.561173	11.454433	10.888785	9.885942	8.502624	8.675471	8.689429	8.945966	9.443437	6.683734	9.192429	9.843644	10.263119	7.794622	9.515829	8.278265	9.217742	10.369106	9.436336	7.707764	9.644870	8.895958	8.593623	6.237625	8.539908	11.153465	9.997647	8.934723	10.268218	11.984250	9.270612	7.690559	9.224967	6.754571	10.667256	10.012445	10.845325	8.913583	6.510601	7.752132	9.435228	8.902489	9.627691	9.967417	7.952777	9.352897	7.810799	8.274186	9.222067	6.301676	9.389914	11.730949	7.173102	8.451400	8.145645	7.446500	7.895271	7.897027	9.260739	8.502947	7.435209	8.721615	9.407719	7.403034	9.143652	8.902489	8.077276	8.511066	8.987489	8.644996	8.301664	8.969436	8.305897	8.356756	7.615270	9.116670	6.779905	10.167973	8.474013	8.091263	9.680465	8.957459	9.591500	9.381478	9.633073	7.429167	9.318660	10.618043	9.177093	9.607325	9.321556	8.659160	9.524895	10.479041	13.333189	8.872943	9.501025	9.188306	10.748441	10.011976	10.717161	12.827344	9.889840	7.856943	12.600363	7.914298	10.205513	9.413487	8.416105	9.490307	8.997819	7.411972	7.458098	6.625166	9.389005	11.032834	11.102534	5.075463	7.260440	8.269199	10.269856	8.238860	7.077031

LINC00158	5.145547	3.309540	3.529594	3.736070	3.170245	3.984336	3.386033	3.514088	4.081964	3.478207	3.964886	3.754254	3.890274	3.720995	3.580504	3.451883	3.771736	5.158863	4.951447	3.802243	4.767870	4.804325	3.344003	3.894213	3.206648	3.347031	4.011621	3.513374	4.363515	3.878927	3.601290	3.658843	3.096657	3.332726	3.511249	4.050761	4.209698	3.217115	3.040931	3.681431	3.783976	3.009843	3.381727	3.569274	4.144522	5.364040	3.769062	3.040188	3.649119	3.858341	5.908672	5.482205	4.173575	4.082384	3.853359	4.333986	3.911422	3.619893	3.382584	3.391947	2.906249	4.490426	3.790973	3.602660	3.315404	3.238467	3.643095	3.826358	3.730447	3.154426	3.232441	3.109814	4.071884	3.346247	3.101606	3.718118	3.432871	3.348056	3.503982	3.689939	3.408550	4.063079	3.417042	4.164264	3.436524	4.082186	2.902002	3.365983	3.382065	3.448178	3.197164	3.768830	3.949881	3.507422	3.721910	3.180263	3.756326	3.873944	3.498710	3.332078	3.134280	4.145567	3.287257	3.260848	4.309712	3.363712	4.811675	3.667908	3.327915	4.130031	3.143153	3.218550	3.195567	3.884501	3.952698	2.977966	4.118816	3.183839	3.395942	3.670739	3.938536	4.135859	4.058241	3.717137	5.100098	3.499640	3.520966	4.022370	3.849064	5.211443	3.857006	4.554548	3.948370	5.234826	3.388059	3.901467	4.511172	4.472556	4.700479	4.028609	4.639269	4.967577	3.664050	3.888276	4.833968	3.493904	4.687586	4.597449	5.265872	3.681611	3.451366	3.101295	3.729640	4.970691	3.756928	3.734799	3.245376	3.692766	4.237440	5.420358	2.877138	4.715607	4.102611	4.006327	4.654618	5.274315	3.000262	3.905736	5.646696	5.179561	5.413577	5.373801	4.129602	3.676220	4.808685	3.546768	4.584786	3.588432	3.077424	4.466525	4.864543	4.719590	5.010327	4.856921	3.317445	4.968421	4.631333	4.163837	2.862823	3.982847	3.725976	5.093948	3.691158	3.391947	3.827629	3.504165	3.995290	3.663264	3.649216	4.439375	4.379938	3.670934	4.007174	3.445453	3.518911	3.484252	3.404038	3.190001	3.613588	4.058871	3.481721	3.834808	4.088287	4.543780	3.717529	3.233108	3.825737	5.169505	3.816516	3.806168	3.106766	3.575355	3.990254	4.671751	5.013840	3.611605	3.618187	4.008667	4.871035	3.362862	3.390681	3.339114	4.949604	3.703843	3.561567	3.879570	3.993727	5.376928

RAPGEF4-AS1	5.064071	5.331647	4.762913	5.286792	8.029783	5.191503	5.131397	5.291194	5.438019	5.407929	5.036741	5.175155	4.977448	5.378461	4.619695	5.758326	5.030546	5.934048	5.044120	5.093551	4.768671	5.396337	4.524143	5.341623	5.529168	5.001521	5.547131	5.579093	5.429406	5.172334	5.383921	5.667246	5.728468	5.418743	4.040146	3.956800	4.575213	4.776982	5.359672	5.606325	6.988569	4.960817	5.805975	5.208841	4.927430	7.250566	5.123314	5.357204	5.174138	6.218710	4.978711	5.537646	5.579658	4.831864	4.554107	5.168882	3.703446	3.244699	5.066749	4.472978	3.950106	5.908291	5.052996	5.380192	3.382241	4.996288	5.330272	4.917062	5.051601	4.659849	5.449553	4.865377	5.194061	4.715396	5.100721	4.946094	4.778245	4.794167	5.165298	4.955624	5.838422	4.729956	4.403105	4.259390	4.051282	4.835798	4.963948	4.428265	4.260868	4.962538	5.030942	5.348703	5.167076	4.678808	4.991195	4.925093	4.482201	5.260874	4.474945	4.693492	4.919813	4.290026	5.046672	4.465643	4.109365	4.916406	5.945514	4.816461	5.847907	5.665330	5.425215	5.648979	5.472537	5.639162	4.363298	6.630154	4.946505	4.606485	5.441987	5.812805	5.920679	5.750286	5.583994	8.294115	6.492034	6.673024	6.892837	7.188899	6.129747	6.181161	5.893525	7.131326	5.643883	5.305593	4.870615	6.055495	7.415080	7.067141	5.442550	4.991830	8.119920	5.063257	7.640962	6.611844	5.951435	5.462398	5.623172	6.291862	6.781047	5.974485	6.150503	5.728276	5.537228	5.000684	5.281755	5.389689	5.123715	5.568836	6.390687	7.571715	5.617024	5.609146	5.403869	4.793938	7.083620	6.155871	5.275956	5.125344	6.193018	5.400149	5.269057	6.646089	5.218154	5.294827	6.029915	4.335703	5.417778	5.943556	5.078192	5.543910	6.377960	5.901152	6.879422	5.359285	5.224876	6.143072	5.062460	5.217960	5.039903	5.607789	5.644266	6.046564	6.042958	5.132613	5.168280	5.631732	5.341799	5.930066	5.745522	6.493934	5.154607	5.368578	5.044321	5.092275	5.459239	5.182826	4.599394	5.068793	5.478682	6.266387	6.131041	5.466753	5.323834	5.504312	5.674417	5.452057	7.012195	5.967890	5.514243	5.946038	6.240272	5.391433	5.300823	5.372272	6.046222	7.163317	5.421654	4.816230	5.895664	6.077953	6.738926	7.751682	6.092166	5.667246	5.441417	4.907752	6.504965	6.783072

AL031963.2	5.789212	6.349235	6.448187	6.662005	8.080543	8.703980	6.892517	7.634337	7.592717	7.302237	7.555653	8.137701	6.861077	7.638327	8.177966	6.598774	6.891902	7.654357	8.000277	6.680932	7.790379	7.759138	7.481352	7.147212	7.520351	7.188249	7.923181	7.901019	7.151068	7.146288	6.681903	6.649826	6.586695	7.099087	6.138928	6.374667	6.792669	6.820848	6.870984	6.031651	6.864078	6.910985	7.002445	6.844383	6.876695	6.489829	7.789160	6.794137	7.437870	5.962157	6.940736	7.127988	6.814915	6.889336	6.622590	7.269393	6.907814	6.722898	6.839508	6.871129	6.782296	5.761388	5.779106	7.272830	7.190458	7.181093	6.964378	6.819891	6.685661	6.887409	6.419962	6.780384	6.587033	6.996467	6.938569	6.519373	6.706060	6.544667	6.710064	6.569199	7.066045	7.232119	6.921935	6.859440	7.112839	6.880384	7.742537	6.660184	7.354382	7.088794	6.941340	7.552843	7.092126	7.253165	7.325954	7.116743	7.015163	7.436323	8.254672	7.262342	8.404997	6.651431	6.502208	7.104405	7.183140	7.117559	6.176773	7.300797	7.763738	7.023420	8.171446	8.127189	8.461565	7.322163	7.518197	11.390271	7.089781	6.825424	7.466203	5.950379	6.578654	6.900296	7.030045	9.369675	8.769925	7.867408	6.977696	7.402552	7.729788	7.137662	7.997081	7.671796	7.752553	8.869598	7.512950	7.361695	8.107575	8.070785	6.504813	7.456825	8.095973	9.122611	8.290887	7.613722	6.076577	7.821163	8.747246	7.929878	6.664928	7.347870	7.355349	7.362446	7.719036	7.229369	7.357496	6.556465	8.140611	8.160840	6.871129	7.312129	7.197133	6.760447	7.286449	7.109658	6.735952	8.018756	7.259185	7.659517	7.104714	6.734990	6.969819	6.984122	6.750212	6.711340	6.376310	6.915418	7.025864	7.231189	6.731583	7.159604	6.718806	7.530239	7.587048	6.920646	6.825589	6.808558	6.640971	6.784344	6.135334	6.691609	6.367881	7.984242	6.735148	6.941182	6.368043	6.222589	6.596935	6.833118	6.381130	6.554958	7.012195	7.031875	7.482753	7.185042	8.230395	7.058384	6.875616	7.261822	6.941340	7.117886	7.398506	7.241639	7.289724	7.577148	7.815906	8.442687	7.923664	7.864791	6.888402	8.021631	7.505553	7.294462	7.604831	6.746725	7.169943	7.848092	7.781203	8.173323	8.236995	8.416918	8.721615	9.366612	8.314984	8.270121	9.384364	7.213561	7.012038	7.234047

PCAT19	7.041278	7.789465	7.630529	7.109184	7.290175	7.246521	7.713749	7.239061	8.046403	7.447919	7.113763	7.089152	7.150257	7.423979	6.891740	7.640029	7.067750	7.126107	8.045755	7.184898	7.283526	8.549225	8.326196	7.556447	7.677137	7.613888	8.125256	7.725594	7.698669	7.860474	7.183140	7.211984	7.505232	7.384091	8.314984	8.133925	7.905709	7.804477	7.961776	7.777904	7.700074	8.151760	7.816672	7.628611	7.902414	7.503220	8.045127	7.941178	7.456332	7.892947	7.467717	8.041063	8.132838	7.906786	7.889666	7.855600	8.059794	7.753947	7.606840	7.801900	8.055096	8.132838	9.034153	8.067877	7.759759	7.656711	8.282080	8.046247	8.059636	8.315925	7.519393	7.703896	7.768688	7.411972	7.542332	7.682746	7.499142	7.596093	7.431742	7.355649	7.159454	7.773375	7.308348	6.783072	6.865962	6.949475	6.547569	6.961817	7.551288	6.843510	7.173677	7.532852	7.645570	7.596263	7.367488	7.245330	7.075460	7.311816	7.418684	7.973216	7.715237	7.784873	7.779142	7.630032	8.612161	7.688106	8.690809	8.627316	8.766546	7.879300	8.420395	8.002934	7.620601	7.819359	7.403660	8.143459	7.699912	6.994003	7.648004	7.339253	7.889199	7.691511	7.403812	8.033141	7.347217	7.176334	7.274704	6.947452	7.393824	7.314180	7.076359	8.147296	7.327894	7.325797	7.591953	7.229521	7.492126	8.441197	7.149139	7.712007	8.671909	8.327463	7.890456	7.776496	7.111138	7.757279	7.814935	7.989420	7.567942	7.378180	7.683351	7.036695	7.654980	7.591111	7.367807	7.689354	7.518041	7.452599	7.368917	8.152367	7.798982	7.431882	7.622838	7.550974	7.458243	8.204629	7.797998	7.150580	7.944014	7.835968	7.998718	8.000418	7.983486	8.400184	7.587974	8.130580	8.058865	7.807549	7.917227	7.721779	8.040277	8.630690	8.193370	8.022249	7.732264	8.450004	8.207229	8.144078	8.615560	7.576687	7.716894	8.315758	7.354866	7.397106	7.754859	7.400671	7.522964	7.568853	7.319649	7.232626	7.964030	7.468305	7.129387	6.906393	6.767480	6.382968	6.835477	7.586605	7.345011	7.629392	7.335968	7.330723	7.861521	7.744413	7.545035	6.591177	6.985880	7.507990	7.471118	7.975543	7.741340	7.762510	7.707918	8.079921	8.133329	8.095215	7.858326	7.598098	8.478824	8.708432	8.070168	7.989106	7.975388	7.847503	7.139390	8.058121	7.100497	7.495527

LINC01350	6.172854	5.739533	5.409519	5.993944	6.720973	7.309112	6.765217	6.502026	7.275303	7.102187	7.267588	7.365938	7.136114	6.278795	6.067163	7.003666	6.625668	6.601827	5.578314	6.425455	5.113154	6.705442	3.623459	6.651597	6.328401	7.199153	7.495997	7.064918	7.419464	6.654673	7.022026	5.270048	5.270656	6.959086	5.949673	5.656838	5.633793	6.046222	6.189239	6.060803	6.841294	5.920679	5.783606	5.336739	4.944576	5.757968	7.451363	5.431807	5.687731	5.688775	5.088807	5.789212	5.025654	5.086779	5.276751	6.891740	4.684175	4.742349	6.315909	4.858553	5.037529	7.515075	4.431596	5.728635	4.678808	5.401645	5.747936	5.905949	5.266067	5.523476	6.178831	6.546318	6.765392	5.954888	6.328231	6.153607	6.134666	5.354082	5.737539	5.641968	5.645625	4.817096	4.683553	5.619849	5.055139	5.359863	5.835534	4.946727	6.469512	5.210230	5.434949	5.570700	6.232414	6.327414	6.194961	6.196904	5.968264	7.195198	7.132761	7.532374	7.582246	6.515723	6.849180	6.728222	5.653667	7.190616	7.536401	6.234130	6.743344	7.287232	6.299387	7.120438	6.581952	7.695045	7.019995	8.450156	6.895426	6.932089	7.506792	6.743202	6.136033	6.333836	5.855085	7.776496	7.054118	7.282895	7.192436	6.496218	7.358858	7.576687	7.761592	8.112700	7.763284	7.403964	7.013469	6.677045	6.946970	6.278158	5.692082	6.186223	6.551986	4.365248	7.151855	6.632896	5.231792	6.214917	7.179851	7.671008	6.227294	7.302884	6.949814	6.755554	6.927315	5.699482	5.990072	5.300214	5.917236	6.273716	6.155690	7.919114	5.497164	5.431227	5.798204	5.465392	5.958405	7.545191	5.185584	5.885138	4.921917	4.378864	6.087137	4.848706	5.319191	4.672733	6.130610	5.635267	4.773047	5.881243	4.818759	5.199887	6.606716	4.720656	5.485751	4.984377	4.903414	5.750104	5.349273	6.211853	5.089403	6.152751	6.587033	8.028874	5.901152	5.865214	5.890109	6.069980	5.659046	5.993056	5.532739	5.570508	5.079007	5.784669	5.794218	5.056973	6.733827	5.329881	4.517223	6.209840	5.121307	6.491380	5.728276	6.605303	6.635475	6.341563	8.052786	6.967570	7.855139	8.222067	6.454774	7.526521	6.434113	7.683826	6.333238	5.907765	7.282413	7.408527	6.070155	5.765730	8.052634	6.185885	7.038119	8.412020	7.456028	7.285199	6.699341	6.494926	7.266775	7.151564

AC011481.2	11.402644	10.703034	11.244199	11.218911	12.725151	11.964164	10.624904	12.331442	13.110410	11.494786	11.405878	11.171500	10.834345	11.271780	10.956655	11.731839	11.778966	11.434802	11.657473	11.200874	11.306661	11.351456	10.792913	11.789113	11.829567	11.748493	12.716597	11.859051	11.751007	11.448683	11.777466	10.989737	10.818932	11.341475	11.029556	11.427239	11.342795	11.245512	11.681573	12.313639	11.244638	11.772400	11.977304	11.332913	11.723766	11.706559	12.064690	11.673410	10.874859	11.278998	11.076776	10.931745	10.461885	10.381341	11.075007	11.604584	10.718205	11.292947	10.948319	11.060999	11.255007	11.351898	10.805332	11.364771	11.094152	11.538956	11.610492	11.442790	11.146797	11.824884	11.241786	11.651970	11.694244	11.514705	11.623005	11.196379	11.359582	11.133855	11.178375	10.943027	10.906618	10.712868	10.718513	10.887492	10.840472	10.658275	11.787474	10.771942	11.066875	11.253098	10.993754	11.804689	11.284596	11.141336	11.028968	10.844714	10.954214	11.877032	11.615675	12.923310	12.355477	11.305148	11.986953	11.938934	11.758737	11.511703	12.186842	11.735252	12.231096	11.520394	11.616246	12.338259	12.371644	12.251510	11.847451	15.269335	11.883726	11.307400	12.398188	10.767583	10.605649	11.900732	11.558177	14.167438	11.238299	11.656893	13.117722	12.408357	11.306661	11.157753	11.629870	11.202995	11.273851	11.226653	12.688477	11.670323	11.162605	11.711161	11.505696	10.974225	11.136541	11.189332	12.436614	11.553024	10.954214	12.571510	11.919811	13.616838	11.304260	11.870853	11.482399	11.769458	11.672076	11.298970	12.442059	11.420720	11.494211	12.025076	12.394751	11.885548	12.533788	11.976836	11.803092	12.505754	12.282255	12.280366	11.742598	10.974503	11.285485	10.862913	11.873246	10.537651	10.490996	10.459968	11.754542	10.531581	11.501057	11.385252	10.804745	12.005488	11.144404	11.104280	11.582863	11.762657	11.434046	12.153299	11.650646	11.031349	11.726175	11.890634	12.230324	12.061519	12.254045	12.396485	11.525035	11.651082	11.969485	11.372593	10.812551	10.639071	10.536345	10.431883	11.193393	10.758534	12.206813	11.379443	10.304910	11.429417	11.131212	11.416773	11.165084	11.543167	10.664468	11.498757	11.397159	12.313639	12.778642	11.214098	12.258058	11.730335	12.315273	13.074351	12.569545	11.279136	12.220472	12.419928	11.938934	11.711018	11.959893	11.501057	12.660501	13.237061	12.450751	12.587157	13.477482	11.257393	11.201442	12.056681

ZNF213-AS1	6.736422	6.241762	6.609496	5.930750	6.740701	6.783375	6.386980	5.934593	6.697923	6.011759	6.095149	6.200659	6.432929	6.541874	6.485786	6.544521	5.894762	6.505645	6.329383	7.025223	6.655000	6.055823	5.720302	6.424158	6.326261	6.507640	6.513727	5.374347	5.719583	6.765217	5.723267	7.110500	7.893118	6.705266	7.036396	6.456375	7.146904	6.249133	6.038781	6.664134	6.102386	6.799271	6.882160	6.600383	6.498041	6.739567	5.588122	7.464647	6.500369	6.769290	6.729161	7.253245	7.043338	7.349921	7.009963	6.149155	7.030045	7.532530	7.091187	7.357496	6.964524	6.364065	7.210325	6.214759	7.344722	6.365363	7.384857	6.820544	6.711800	7.261516	6.686616	6.695877	5.993385	6.862341	6.803168	6.905732	6.615749	6.415306	5.784124	7.162865	6.341221	7.574659	7.036077	7.145511	7.258890	6.852172	6.828927	7.435209	7.757898	7.347692	7.447592	7.128141	6.573722	6.617107	7.274387	6.984911	7.123519	6.179318	6.752407	6.097512	5.668327	6.080567	6.241098	6.593255	7.210174	5.693593	6.087137	5.823480	5.705050	5.708913	6.728222	5.688415	5.297271	6.833435	5.973236	7.194385	6.581952	6.218710	5.605914	6.522018	6.096022	7.112839	6.647718	7.176042	6.410859	6.539555	5.858327	6.904927	6.796372	6.598280	7.115667	5.602258	6.085087	6.014848	6.634691	6.399465	6.174379	5.644627	7.045786	6.950590	6.930178	7.343817	5.549584	6.479137	7.111770	6.480089	6.953139	6.268268	7.097191	5.991996	5.705946	7.550222	6.797498	7.208301	5.753934	6.725575	6.638207	6.197075	6.814438	6.728532	7.050084	7.650787	6.878304	6.912595	7.132761	5.885332	6.909393	7.041120	6.973206	6.751134	7.928341	7.348953	6.283289	7.026166	6.652077	7.659822	7.293017	7.916296	7.672732	7.670691	7.690085	7.030942	6.878132	7.437251	5.996352	6.870656	6.509428	6.199832	7.452446	7.284304	6.828774	5.911153	6.794940	6.987484	6.868741	6.629353	7.045129	5.943721	6.989049	6.841294	8.152839	7.134995	6.817820	7.358559	6.494756	7.051177	9.277238	7.702064	7.198837	8.039167	7.235637	6.759442	6.291686	7.018100	6.574654	7.088478	5.598184	6.481071	7.291287	5.965017	6.313838	5.873510	6.842863	8.333986	5.937761	6.083169	7.234989	7.059020	5.503721	6.090441	5.722326	5.637695	7.444912	6.649826	7.621041	7.350077	7.022334	5.996025

AC084024.3	13.850447	13.323685	14.077200	13.765490	13.915536	13.600599	13.809906	13.781086	13.044264	13.117358	13.645878	13.686919	13.896959	13.803574	13.589555	13.442036	13.898284	13.579444	14.362657	13.710693	13.982141	13.881710	14.065366	13.436852	13.348475	13.248522	13.213586	13.128058	13.195218	13.368187	13.998144	14.125236	13.981594	13.964108	14.092821	13.595594	13.597268	14.078902	14.172267	13.762178	13.648470	13.937428	13.387795	13.281605	13.239397	13.021352	13.459378	13.403761	13.447352	13.301455	13.073608	13.574249	13.844544	14.082814	13.301455	13.930570	14.009307	13.762711	13.189708	13.565499	13.438613	13.535095	14.100719	14.120170	14.269241	13.741329	13.704613	13.855412	13.530341	14.111384	13.605717	13.493714	13.481700	13.541252	13.468361	13.959054	13.593519	13.460282	13.251437	13.918854	13.619020	14.055969	13.370832	13.836966	13.843499	13.659121	13.970475	13.746068	14.315778	13.694470	13.638076	14.089539	13.457857	12.933669	13.225685	12.970060	13.623148	13.711946	13.917771	13.266014	13.750799	13.520934	13.270630	13.406280	14.950618	13.702916	13.358673	14.747900	14.190840	13.716086	14.434184	14.025995	13.807854	13.503914	13.454346	12.886000	14.156390	13.822921	13.530341	13.759150	14.002151	13.748962	13.454586	13.240817	13.826855	13.109649	13.978885	15.387598	14.764339	13.844047	13.788715	13.876318	13.055072	13.470762	13.790457	13.674594	13.541252	14.328233	13.971275	14.408016	14.477088	14.385642	13.396218	13.453926	13.539286	14.032009	12.906787	14.028776	13.975517	13.627746	13.307696	13.636921	13.889152	13.803322	14.110411	14.132032	13.995003	14.174885	14.220230	13.885693	14.365930	14.070098	14.559663	14.118926	14.797410	14.024760	14.090994	14.190235	13.902033	14.590670	13.760392	14.078621	13.826079	14.229504	13.434924	13.368685	14.468896	14.687432	13.841373	14.301613	13.812477	14.872324	14.435274	14.068082	13.932970	13.825546	14.230892	14.217951	13.939913	13.960811	14.102873	13.479517	13.922935	14.335422	13.844544	13.815559	14.136402	13.858620	13.463363	13.951850	13.280648	13.944829	14.089838	14.008169	13.969621	13.812235	13.979690	13.949622	14.475183	14.302275	14.322301	13.801126	14.489914	14.116481	14.911437	13.743597	13.688585	14.662761	14.256071	13.969908	13.544622	14.322988	13.846403	14.323669	13.956584	14.219589	14.435274	14.567126	12.960870	14.399253	13.541684	13.979146	13.154665	14.235215	14.332725	13.628713	13.951559	13.652481

DGUOK-AS1	12.263250	12.458956	12.435149	12.374817	12.563831	12.236059	12.412056	12.353395	12.686212	12.111443	12.464048	12.410313	12.501897	12.373219	12.757557	12.221092	12.307305	12.626503	13.047238	12.286161	12.525969	12.873402	12.798177	12.441117	12.058559	12.287532	12.455483	11.908348	11.816892	12.473286	12.648843	12.673113	12.537757	12.844580	12.790143	12.482465	12.313176	12.467952	12.724316	12.752429	12.228465	13.041546	12.306218	12.306995	12.633304	12.779001	12.170015	12.658790	12.584475	12.488616	12.594269	12.592167	12.823360	12.578859	12.904297	12.278977	12.664096	13.259620	12.980105	12.881502	13.120423	12.301392	13.405255	12.953847	13.305359	13.009749	12.844764	12.836168	12.526786	13.172863	12.275765	12.384180	12.110693	12.434181	12.512264	12.546528	12.360062	12.193677	12.146258	12.941000	12.584810	12.667828	12.662414	12.574210	12.646567	12.340526	12.840269	13.051243	13.471830	13.000906	12.973999	13.404601	12.721811	12.591825	12.690337	12.607071	12.537925	12.313176	12.542279	12.164845	12.374817	12.146729	12.437247	12.487478	13.319680	12.376134	12.809655	13.073430	12.871377	12.320466	13.050446	12.948055	12.958080	12.066401	12.256938	11.595768	12.925940	12.209741	12.123320	13.291530	12.823533	13.061923	13.283232	13.244322	12.695910	13.056546	13.486814	13.320905	13.409232	12.600363	13.678008	13.488586	13.305786	13.575329	12.708224	14.165527	13.918854	13.728857	13.215627	13.052774	13.695624	13.794603	12.949458	13.288044	13.478390	12.814055	13.440958	12.406051	12.835254	13.351397	12.680949	13.488366	12.590117	12.445481	13.380743	12.425577	13.219174	13.772698	13.635698	13.873120	13.707232	13.736123	13.169593	12.891207	13.208700	12.847857	14.022985	12.856393	13.303472	13.038207	12.799704	13.389482	13.608852	13.314970	13.074750	13.950442	13.458723	13.361000	13.572887	13.961884	13.121579	14.329308	13.974396	12.962331	13.262596	13.771628	13.001582	13.395578	13.646327	13.235816	13.271787	9.833053	12.800745	13.368069	12.682992	12.659650	13.627059	13.009581	13.069482	13.464641	13.296569	13.164330	13.280056	13.103890	12.713455	13.242189	13.638949	13.827679	13.345736	13.689979	14.022408	13.228465	13.300682	12.955541	12.679062	13.249319	13.072631	13.282824	13.346151	13.457435	12.826659	12.841260	12.954050	13.848311	13.043339	13.717719	14.002435	14.149163	12.758764	13.444821	13.334586	13.669633	12.456916	13.402926	12.952935	13.833309	13.517315	13.326797

AL356804.1	14.486754	13.677364	14.048831	13.885427	13.922401	13.634622	13.733881	13.986102	13.223528	13.744601	13.641524	13.672469	14.071478	13.870167	13.945929	13.550257	13.471598	13.973310	13.950998	14.044056	13.803086	14.461232	13.659354	13.813525	13.272382	13.567588	12.610024	13.627059	13.288424	13.327384	13.193698	14.247926	14.144912	14.358378	13.989252	13.888605	14.233212	14.272202	14.189262	14.140212	13.782334	14.461989	14.322632	14.150431	14.077200	14.185552	13.265367	14.260288	13.767021	14.072083	14.243388	14.193358	13.820478	13.681855	13.482140	13.471189	14.214704	14.554379	13.959663	14.616154	14.264253	13.429701	13.699452	14.025054	14.282078	14.080659	13.969621	14.248223	14.129877	14.331727	13.844047	13.983800	13.465295	13.589555	14.115260	13.889152	13.761667	13.686442	13.544870	13.923778	13.942960	13.432615	14.109446	14.182770	14.212381	13.753087	13.693113	14.050321	14.221208	14.315778	14.010076	14.208161	13.856506	13.361189	13.587916	14.029975	13.938227	13.872610	14.003541	13.452455	13.541051	13.643752	13.370400	13.832809	14.233539	13.749826	14.097959	14.267647	14.149487	13.694956	14.628630	13.991573	14.240420	13.524608	13.340354	12.918404	14.690655	13.427110	13.050076	14.117033	14.544276	13.806245	14.187776	13.575112	13.879304	13.802371	13.456599	14.057105	14.247926	14.408756	14.187463	14.040253	14.284537	14.048218	13.559082	14.791097	14.018727	14.202473	13.760666	14.412071	13.783848	14.111066	14.446360	14.605414	13.970725	13.995003	13.854373	12.898463	13.998713	13.750073	14.035874	14.085609	13.874711	14.348197	14.223479	13.982398	13.992417	14.705283	14.447934	13.505253	14.255152	14.336460	14.408016	14.620267	14.767089	14.387085	14.355522	14.047634	14.433465	13.964108	14.487150	14.394408	14.934577	14.726960	14.199131	14.475583	14.317493	14.783339	13.886983	14.479847	13.883273	14.953342	14.855908	14.219589	14.277581	14.557264	14.550161	14.049457	14.531467	14.151714	14.214354	13.270211	14.658149	14.138571	14.089539	14.365566	14.366997	14.497900	14.264949	14.220880	14.746969	14.164900	14.318504	14.384877	14.213324	14.942038	14.484873	14.730211	14.199474	14.240757	14.538340	14.368027	14.584760	14.405515	14.011503	14.112576	12.784870	14.093117	13.975517	14.517783	14.370128	13.860196	14.557264	14.672364	13.624536	14.280707	14.595344	14.178659	14.069516	14.115260	14.548933	13.964108	13.644498	14.535929	14.331727	14.240420	14.509168	14.501441

AC091588.1	7.408829	3.624039	3.755044	5.477861	3.459121	3.381371	3.154050	3.164325	3.760284	4.110281	4.390178	6.365038	3.728814	4.297904	3.669497	3.690345	3.281206	3.413453	4.703636	3.647203	5.220931	3.723903	6.381130	3.390862	3.558731	3.759641	3.887402	3.503653	4.345899	4.053433	3.813804	3.048744	3.710057	3.416880	4.262886	3.761730	3.389127	4.350669	3.121348	2.952851	3.755044	2.910921	4.001479	3.659429	4.658804	4.320790	3.256050	3.060353	3.906427	3.152702	6.652077	3.977012	4.667150	3.652214	4.479749	3.348845	4.313187	3.125639	3.528312	5.608956	4.272884	5.138833	4.336340	3.866193	3.208467	3.246695	3.018540	3.455487	3.069128	3.682876	3.621076	3.802663	3.721463	3.540335	3.441518	3.930876	3.346247	3.365646	4.134067	4.389969	5.043683	4.494423	5.153997	4.161599	4.090253	3.968978	2.957960	3.240178	4.270081	4.687185	3.160705	3.292959	3.686432	3.775550	5.093749	3.681816	4.194544	3.859612	3.052684	4.124633	3.695929	4.283814	4.070060	4.432882	4.350669	4.377797	3.576932	4.265101	4.095169	4.124861	3.332425	3.181376	2.934989	5.863284	4.647912	5.498722	6.538274	4.172089	4.309712	5.565029	4.304213	4.093706	3.476336	3.256748	3.363712	3.513752	3.679421	3.869665	5.434949	3.880019	6.773312	5.208635	3.740182	3.753370	4.529040	3.909259	3.531098	4.204520	4.259584	4.488455	3.980200	5.115354	3.452598	3.948147	4.769494	3.455318	4.196239	3.898693	3.821032	3.064168	4.529888	3.600120	4.092057	3.764711	3.533846	3.887630	3.958330	3.781036	3.966608	3.525695	2.868818	3.888075	4.072762	4.004337	4.148341	4.114601	3.028570	3.599926	4.900484	6.079720	4.082186	4.819605	5.098874	4.565327	4.544678	3.810663	3.065950	4.766370	5.583801	2.974081	3.481721	4.537994	5.028491	3.453668	3.849937	3.030074	4.120097	4.446889	2.994477	3.731709	3.886551	3.714449	3.880658	3.697132	3.718738	3.529043	3.688481	4.111378	3.306340	3.505344	4.636077	4.296330	4.085430	4.318671	3.568486	3.448800	3.813804	4.973155	4.220367	3.738084	3.641503	3.809104	4.527383	3.469965	3.685428	3.491669	3.180813	3.444725	4.460696	3.726139	3.852056	3.800090	4.546237	4.756961	3.899129	3.558141	4.443890	4.058241	5.357204	3.050905	3.356732	4.886461	4.627160	5.425386	3.382584	3.797157	3.091974	3.756928

AC009041.2	9.883634	10.634289	10.555535	10.580695	10.888202	11.242830	10.292786	12.287847	12.015985	10.858370	10.824286	10.542131	10.373979	10.799059	10.401239	11.380692	11.433305	10.508413	12.286161	10.870979	11.485467	11.591914	11.835276	10.876331	11.258115	11.182859	10.884458	11.550016	10.791439	10.599413	11.440039	9.478766	9.817117	9.538754	10.193091	10.370786	10.245440	10.728676	11.213966	11.139782	11.009628	10.897194	10.846224	11.188596	10.540943	10.827110	11.051479	11.267197	10.466810	11.069445	10.667558	10.625655	10.239877	10.566197	10.653668	11.279734	10.085395	10.842134	10.845782	10.468732	10.873539	10.825019	11.434353	11.641016	11.131914	11.164797	11.384067	11.298525	10.952265	11.667567	10.585512	10.697641	11.325845	10.330253	10.556465	10.524000	10.496309	10.266020	10.437199	10.157733	10.094705	10.228938	9.972959	10.167376	9.925599	10.188920	9.790662	10.086530	10.831803	10.798335	10.450036	11.073138	10.443646	10.387032	9.782528	10.073145	10.215486	9.779375	9.719403	10.995532	10.201110	10.547030	10.591268	10.828444	11.844183	10.890293	12.381475	11.403089	11.899287	11.443990	11.762942	11.692007	11.427813	11.918019	11.030902	10.891777	10.768349	10.358719	11.031349	9.651040	10.870038	10.465346	10.549491	11.285784	10.913411	10.488419	10.964592	11.037001	10.462499	10.580549	10.331296	10.754173	10.290216	10.825019	10.683771	11.032397	10.190413	12.041676	10.184516	11.333206	11.123230	11.666875	11.135945	11.160233	10.867283	11.204776	10.986739	10.972500	10.716566	10.890897	9.444517	9.904851	10.332961	10.160730	9.961516	9.971216	10.668143	11.172387	10.776828	11.017339	10.945949	10.284582	10.840198	10.460708	10.875163	11.331187	10.658003	9.867411	10.786329	10.591557	10.315777	10.368958	10.469815	9.977992	10.931284	9.883943	10.765692	10.637586	10.310657	10.820861	10.930535	11.337151	11.424641	11.226653	10.761605	11.325255	11.156239	10.932329	11.260483	10.426073	10.447340	11.708649	10.158664	9.992805	10.525956	10.347055	10.433224	10.545676	9.767080	9.650732	10.354297	10.125998	9.677379	9.759333	10.072505	9.986994	10.128831	10.885415	10.439434	10.264251	11.199598	10.071631	10.304483	10.026726	9.895971	10.046469	9.621515	9.607774	10.154986	9.974821	10.179803	10.487497	10.422993	10.987180	10.909711	11.329368	10.687354	11.251510	11.891411	11.578918	11.090176	11.109863	11.888108	10.220952	10.241972	10.492850	10.098577	11.238457

AL390294.1	4.648318	5.722134	5.442939	4.883686	4.871491	4.051282	5.009311	6.027116	6.107658	4.729138	5.253360	4.268916	4.685218	5.229330	6.261843	5.142355	5.893709	6.366543	5.584774	5.366204	5.991827	5.767016	3.597536	5.473683	4.842115	5.190332	4.909875	5.277562	4.703636	4.454382	6.304614	4.772189	5.783785	4.712006	5.542232	4.916169	4.868967	5.380962	5.477492	5.712313	5.333630	5.462213	5.129196	5.337335	4.607765	5.728276	4.170551	6.146192	5.009920	5.698054	5.516844	5.550338	5.412417	4.749126	5.834461	4.607995	5.359672	6.609828	5.362905	5.361147	5.690604	4.349824	6.486942	6.406553	6.055823	5.838236	5.823851	5.984182	5.511434	6.299534	5.456085	5.059621	4.577141	5.393177	5.559465	5.501195	5.542788	5.476909	5.781271	5.742641	5.162091	5.852379	6.290302	5.698593	6.081078	4.463269	4.501816	5.779106	6.767639	5.974485	6.295088	6.159810	5.500842	5.141933	4.943641	5.024657	5.603033	3.844991	4.794982	3.497840	3.097843	4.670600	4.595957	5.299860	6.303405	4.009963	6.635160	6.203297	4.995664	4.828047	5.639555	5.898668	5.735022	4.560966	5.558672	2.976768	5.167290	4.972749	4.405822	5.078813	5.321907	4.800293	4.946291	4.067954	4.509201	3.201577	6.482556	5.593652	3.675783	3.894213	3.874364	3.234227	6.923867	4.787036	3.685860	5.922158	4.726407	4.769494	5.134841	4.856053	4.583727	5.020133	4.493992	5.465494	4.596619	6.166868	4.592522	3.224749	4.762913	5.865783	3.379996	5.673669	3.435272	5.110970	4.032121	4.516566	5.866650	5.434567	5.057772	3.987353	5.324597	4.867689	5.124300	5.956812	5.171481	4.001248	6.030923	4.886247	5.687890	5.711575	5.336960	5.272267	5.016682	4.059329	5.117619	3.867730	6.383321	5.303245	6.470026	7.237677	4.225572	6.527992	6.242269	6.675761	6.688383	5.648403	6.045183	5.522511	6.905252	4.845532	5.184411	4.484173	5.113352	5.645625	5.780899	5.432219	5.819384	5.655195	5.105634	5.590809	5.919921	5.217371	4.698939	5.606848	3.659231	5.867047	5.772933	6.460518	5.601705	5.674591	6.251495	4.515737	5.080195	5.861216	5.179183	3.264238	3.187677	5.624475	5.185394	4.225572	4.200088	3.525332	4.177463	6.441853	4.358295	6.591465	5.582847	5.822222	4.561637	5.053637	6.069104	5.682879	4.433078	4.383610	4.144966	5.971232	6.512277	4.284509

GATA3-AS1	4.648318	5.722134	5.442939	4.883686	4.871491	4.051282	5.009311	6.027116	6.107658	4.729138	5.253360	4.268916	4.685218	5.229330	6.261843	5.142355	5.893709	6.366543	5.584774	5.366204	5.991827	5.767016	3.597536	5.473683	4.842115	5.190332	4.909875	5.277562	4.703636	4.454382	6.304614	4.772189	5.783785	4.712006	5.542232	4.916169	4.868967	5.380962	5.477492	5.712313	5.333630	5.462213	5.129196	5.337335	4.607765	5.728276	4.170551	6.146192	5.009920	5.698054	5.516844	5.550338	5.412417	4.749126	5.834461	4.607995	5.359672	6.609828	5.362905	5.361147	5.690604	4.349824	6.486942	6.406553	6.055823	5.838236	5.823851	5.984182	5.511434	6.299534	5.456085	5.059621	4.577141	5.393177	5.559465	5.501195	5.542788	5.476909	5.781271	5.742641	5.162091	5.852379	6.290302	5.698593	6.081078	4.463269	4.501816	5.779106	6.767639	5.974485	6.295088	6.159810	5.500842	5.141933	4.943641	5.024657	5.603033	3.844991	4.794982	3.497840	3.097843	4.670600	4.595957	5.299860	6.303405	4.009963	6.635160	6.203297	4.995664	4.828047	5.639555	5.898668	5.735022	4.560966	5.558672	2.976768	5.167290	4.972749	4.405822	5.078813	5.321907	4.800293	4.946291	4.067954	4.509201	3.201577	6.482556	5.593652	3.675783	3.894213	3.874364	3.234227	6.923867	4.787036	3.685860	5.922158	4.726407	4.769494	5.134841	4.856053	4.583727	5.020133	4.493992	5.465494	4.596619	6.166868	4.592522	3.224749	4.762913	5.865783	3.379996	5.673669	3.435272	5.110970	4.032121	4.516566	5.866650	5.434567	5.057772	3.987353	5.324597	4.867689	5.124300	5.956812	5.171481	4.001248	6.030923	4.886247	5.687890	5.711575	5.336960	5.272267	5.016682	4.059329	5.117619	3.867730	6.383321	5.303245	6.470026	7.237677	4.225572	6.527992	6.242269	6.675761	6.688383	5.648403	6.045183	5.522511	6.905252	4.845532	5.184411	4.484173	5.113352	5.645625	5.780899	5.432219	5.819384	5.655195	5.105634	5.590809	5.919921	5.217371	4.698939	5.606848	3.659231	5.867047	5.772933	6.460518	5.601705	5.674591	6.251495	4.515737	5.080195	5.861216	5.179183	3.264238	3.187677	5.624475	5.185394	4.225572	4.200088	3.525332	4.177463	6.441853	4.358295	6.591465	5.582847	5.822222	4.561637	5.053637	6.069104	5.682879	4.433078	4.383610	4.144966	5.971232	6.512277	4.284509

AL050349.1	3.851853	3.338781	3.043520	3.836745	3.162748	3.133914	3.456073	3.277869	3.413453	3.320089	3.549833	3.415541	3.330656	3.763620	3.931741	3.289829	3.461488	3.314806	4.684809	3.434147	4.754057	2.918874	2.835200	2.979074	2.909854	2.869606	3.062131	2.814389	3.292809	3.063626	3.616508	3.554100	5.233431	3.370267	5.125769	4.016509	5.192676	3.212697	3.373589	3.184101	3.444725	3.027084	3.589211	3.335003	3.747301	5.482394	3.665470	2.923059	3.825521	3.611397	3.491016	3.300392	4.261329	4.189497	3.995290	3.171311	4.842335	3.199296	3.672321	3.862994	2.832957	3.146427	3.893535	3.268548	3.515144	3.506879	3.000143	3.289044	3.308242	3.092544	3.251420	3.115819	3.228654	3.159038	2.905401	3.379996	3.096526	2.990536	3.893080	4.100192	3.400891	3.938758	3.546557	4.338129	3.497641	3.718335	3.085603	3.302676	3.881295	3.657644	3.304758	3.396129	4.095615	3.243404	3.152112	2.937088	3.824361	3.279590	3.563042	3.068578	2.919585	3.228037	3.007067	2.915706	5.394569	3.238005	3.081673	3.476336	3.058619	3.277138	2.913137	3.089248	2.910020	3.306975	3.402304	3.233362	3.778955	3.110054	3.234681	3.400726	3.480010	2.994747	3.278379	3.514967	3.721214	2.954608	3.185546	3.367066	3.117616	3.335169	3.046101	3.123779	3.175414	3.521143	2.962274	3.332078	3.021888	3.378073	3.302999	4.102611	2.867782	2.825356	3.263418	2.830622	2.821065	3.007653	2.851120	3.249707	3.549478	3.269945	3.370658	4.157373	3.142794	4.950218	3.726339	4.784747	3.786951	3.379490	3.102113	3.192826	2.926892	3.649639	3.391947	4.240135	5.057355	3.246827	2.861014	4.129602	3.350047	3.306163	3.136966	4.193224	4.407592	3.575186	3.190794	4.759133	4.854590	3.239740	3.794679	2.796100	3.284447	4.139072	3.650223	3.840679	3.746675	2.981829	3.578183	3.334529	3.911846	3.376363	3.010303	3.229455	2.958437	3.295748	3.429179	3.044137	3.299180	3.088208	3.626812	3.790973	5.167871	3.620077	3.725335	3.575730	3.117616	3.679421	3.950106	3.503653	3.509610	3.543602	3.904201	3.697132	3.320743	3.129814	2.944727	3.105858	2.941197	4.017832	3.522436	3.029573	3.020931	2.918966	3.094765	5.867047	3.213260	3.087522	3.463740	3.208073	3.208073	2.848679	3.044948	2.864548	3.269270	3.211309	3.889613	2.913548	3.154930	3.365817

LYPLAL1-DT	4.988308	4.772404	4.388072	4.858553	3.871134	3.570590	7.557235	3.480386	3.415206	4.958737	4.277307	5.153391	6.298242	5.178999	4.648115	4.848706	3.992227	5.850256	4.185988	4.660717	4.896218	4.630905	3.160111	4.000845	5.905776	5.018696	5.217371	4.731873	5.670314	5.292576	4.365486	6.219210	5.833523	5.619849	5.703178	5.877609	6.145662	5.811871	4.795599	4.579696	4.153870	4.742945	7.314813	5.427041	7.094982	5.804920	5.359672	7.082365	5.538833	6.197911	5.424256	6.639018	6.464369	6.369883	6.371935	5.335551	6.659868	7.182665	5.631732	6.824504	6.153607	4.486156	6.393047	3.946856	4.741718	6.358684	5.475224	5.145547	5.275538	3.929574	5.625408	5.142772	4.964988	5.527072	4.904429	5.327319	5.474055	4.950416	3.960968	6.821344	5.454178	5.981021	6.394506	6.261164	6.715517	4.394058	5.406923	5.705599	5.816732	4.821899	5.728468	4.046961	4.817096	5.772555	5.278130	5.921041	6.004284	6.205756	5.211443	4.851592	3.692563	6.164413	5.210021	5.314629	4.295017	4.915772	3.783146	3.480978	3.331586	4.320147	3.644627	3.328676	3.048363	4.191920	3.612579	3.050142	3.364521	5.274932	4.672062	5.450480	3.862377	4.486379	4.457789	3.083840	5.568836	5.240856	3.980615	5.074237	4.218585	3.310528	3.230901	5.651060	3.371537	3.282855	5.278130	5.628989	4.829317	3.789308	5.706450	4.187293	5.358153	3.054459	3.949881	4.209917	4.465643	3.328860	6.754404	3.164807	4.828889	4.565960	2.986843	3.529043	4.486379	5.726824	3.713156	6.236149	3.661635	3.383146	3.734799	3.764064	3.450425	7.010422	4.773477	5.590809	5.309024	3.284599	5.763035	5.880687	4.766605	4.881804	4.825402	5.496811	4.623113	5.736114	5.591982	6.603417	6.598614	2.871436	5.111523	4.202047	5.616310	5.015654	3.816718	3.844372	4.680068	5.569943	4.316930	3.920397	3.762780	4.706397	4.221265	3.309708	3.760284	5.914751	4.504570	4.627574	3.180407	3.260006	6.872271	5.106233	5.511627	5.594202	3.488837	4.707853	3.132715	5.189186	4.588197	3.838658	4.685922	4.525882	3.022360	4.107435	5.508769	4.167115	3.974446	3.336283	5.100918	4.118354	6.035254	3.048363	3.728166	4.542193	4.102375	4.693492	4.495125	3.420255	3.444542	4.347401	3.272546	3.007349	3.480010	4.438496	3.274494	4.493786	3.071095	3.922002	3.207803	3.608224

TLX1NB	5.481833	4.861638	4.744674	5.047064	5.469490	6.133974	4.380153	5.718855	5.502384	4.453543	4.590365	4.480184	4.597236	5.384644	4.279490	4.844447	5.982024	4.467997	5.834461	4.857743	4.637372	6.080065	6.459694	5.020133	5.214446	5.321288	4.825402	5.631017	4.572849	4.686970	5.204082	3.492669	6.016047	5.756654	4.826543	3.988826	4.296542	4.453114	4.547511	5.069387	5.129592	4.425910	4.751699	3.760914	3.953545	6.136033	4.606700	4.894812	4.945653	4.360255	3.400227	4.447109	3.725335	4.116788	4.567891	5.273296	5.591192	3.417203	4.624606	4.424840	4.795182	4.748729	4.465643	3.862994	4.293345	3.511083	4.365717	4.723654	5.188817	4.986634	5.010327	4.716878	4.986428	5.215840	4.587555	4.635636	4.912822	5.392395	5.118031	4.751901	4.685218	4.408014	6.154122	4.375887	5.217960	3.612180	4.198394	4.411319	5.518786	5.254737	5.625408	6.032733	5.313797	5.318061	4.907099	4.931984	4.476026	4.372359	5.032759	4.674939	5.945684	4.127852	4.808260	4.970884	5.349462	6.391724	5.731037	4.917896	5.578508	4.879225	5.746680	6.580113	5.216797	5.113154	5.548830	8.279636	6.335031	4.713491	4.306815	4.865575	5.055345	4.979337	5.090840	6.286581	5.274525	5.653503	5.465020	5.396153	4.886667	5.690793	5.469222	5.866298	5.498320	5.773799	5.387590	5.561114	5.145152	6.141553	4.125060	4.296116	5.733384	5.360611	6.114580	5.612341	5.275538	5.462933	5.495284	5.398443	4.262886	4.837655	4.502005	5.001307	5.476722	4.639479	3.715067	3.981076	7.104405	4.987279	5.119499	5.778728	3.374402	4.491993	3.752527	4.242950	6.054089	5.568836	5.338325	4.143033	3.305233	3.212010	4.260212	7.476608	4.358969	8.559451	4.809948	10.803872	8.300907	4.975473	4.451413	5.576482	5.133823	4.860639	4.885388	6.404233	4.459459	4.652799	4.324006	5.234225	4.528201	5.484426	4.761419	5.484613	4.359819	4.321835	4.735442	6.673180	4.589938	5.221489	4.402233	7.258388	4.453219	6.460844	5.000281	4.803466	4.643391	3.528119	4.279291	4.353756	4.804112	6.011759	6.295933	4.882433	5.199887	6.110611	5.418161	5.127160	4.214482	5.180956	4.823328	4.913262	6.383999	4.536102	4.163148	6.014517	6.690459	5.508966	5.749191	5.575751	5.780710	5.762505	5.322477	8.028251	5.360977	6.410385	6.484786	5.869712	7.280383	5.089598

AP000781.1	13.405029	13.108685	13.409465	13.355364	13.311157	12.941000	13.504804	12.545219	11.996843	13.029353	13.116791	13.127100	13.392871	13.553691	13.725623	12.926298	13.204762	13.798279	12.864398	13.149535	13.236032	13.773212	13.053474	12.994178	12.633940	12.616658	12.667122	12.278662	12.814409	13.118268	12.649882	14.214354	13.321898	13.998449	13.504598	13.282417	13.548218	13.705820	13.784553	13.518195	12.941572	13.226454	13.494652	13.389482	13.347432	13.242607	12.568878	13.107335	13.308155	13.163401	13.221977	13.423660	13.537063	13.167690	13.563147	12.975099	13.811422	13.461577	13.375655	13.574051	13.330066	13.285661	13.128058	13.276661	13.821718	13.568953	13.593744	13.816046	13.266591	13.410314	13.124799	13.468792	12.837080	13.550061	13.712695	13.361622	13.675309	13.202193	13.344888	13.580621	13.452684	13.185249	13.650308	14.025054	13.543949	13.043715	13.424703	13.350761	13.466396	13.578768	13.094478	13.312364	13.719938	13.483445	13.500424	13.686701	13.651765	13.288424	13.370191	13.208477	13.160892	13.317644	13.724455	13.829979	14.267647	13.116791	12.293129	13.855412	13.256418	12.797679	13.788457	13.371858	13.856506	12.644233	13.022716	12.269856	13.777164	13.288424	12.219692	13.632774	14.104048	13.821718	13.646573	13.525933	13.970183	13.749340	14.009539	14.145840	14.603287	14.090418	14.396563	14.684100	14.403650	14.660429	13.083849	14.089238	13.950183	14.073949	13.736123	13.936042	14.252485	14.285845	14.038793	14.267647	14.294396	13.116011	13.919400	13.311915	13.917477	13.382599	14.176803	14.992829	14.152378	13.925407	14.315126	13.746068	13.669633	14.039346	14.100390	14.331005	14.188386	14.314071	13.749340	13.899116	14.016266	13.349953	14.578479	14.002151	14.130795	13.813787	13.912944	14.203355	14.696565	14.101624	13.609930	14.419877	14.573899	14.226630	14.390000	14.153617	13.586012	13.820727	13.935487	13.916329	14.128314	14.021820	13.841373	14.445242	13.413890	14.103780	14.082507	13.230081	13.985840	13.912401	13.937690	13.863595	14.289643	13.954934	13.547331	13.747829	14.000936	13.526786	13.100874	14.272867	14.556424	13.950998	14.789655	14.013234	13.679951	14.584760	14.412450	13.781330	14.122447	13.845880	13.827410	13.477052	13.494410	14.945774	14.343152	14.268271	14.582713	13.608281	13.989564	13.876596	13.356199	13.680191	14.048218	13.834843	13.415442	13.815786	14.413643	14.197518	12.669498	13.719437	14.159585	14.056534	13.860733	14.432746

DLGAP1-AS4	4.107628	4.083676	4.317596	3.959460	3.767567	4.502413	5.661798	5.503721	5.904897	5.159865	3.584353	4.776982	4.481723	4.492677	5.330083	5.680307	5.342170	6.437425	6.238775	6.368368	3.413453	4.677961	3.682035	4.692658	5.392017	5.036312	5.848261	5.934593	4.987082	5.028694	6.733689	3.122871	5.780710	4.160365	4.486790	3.772413	5.210432	6.245286	4.176172	4.930410	5.442550	4.353117	4.218384	4.587555	4.844242	6.848046	5.893709	3.126165	4.182119	4.752112	3.200374	3.108410	4.631763	3.931308	3.997446	3.703446	3.581299	5.657391	3.008488	3.176138	2.917130	4.950218	3.525165	3.540335	3.283331	4.613162	4.051496	4.722818	4.155406	4.144522	4.387386	4.933036	4.805763	5.064862	4.673856	4.053879	3.764916	4.268691	4.812075	5.421654	5.504495	4.934698	4.876507	5.460043	5.189361	4.587091	5.842965	5.763409	4.088287	3.824627	5.158668	3.769910	3.480386	4.406043	3.957014	5.273109	4.348963	6.378810	3.080594	5.459844	5.462933	5.691365	6.041579	6.626941	5.147545	5.627077	4.507907	4.204989	4.392582	5.912957	6.096861	5.606500	5.327132	4.262240	6.078326	5.913482	4.521717	5.409217	6.713426	4.304851	3.187273	5.463106	3.523465	5.284771	3.932174	4.191471	10.101264	4.687813	3.711741	3.704674	5.117409	4.606257	3.614582	3.278841	4.556840	5.030756	6.593567	9.207016	9.297703	3.183432	5.023196	6.615749	9.417014	4.215554	4.525882	6.042958	8.547399	5.506607	4.330775	4.255667	3.243536	6.464703	3.264112	4.018482	3.523465	3.325488	4.217728	3.202948	3.971571	3.342302	2.961168	4.144295	3.270657	4.946291	3.649022	4.972948	2.971623	7.824617	3.508342	3.073679	4.181446	8.281598	4.042742	3.298831	6.449656	7.623618	4.773688	5.814075	7.451641	3.885300	4.256960	4.164473	4.510948	4.065846	3.565159	3.461116	3.777553	4.770748	2.899911	3.916834	3.952050	4.864751	4.239223	4.756759	4.046032	3.800534	3.986728	4.809510	4.383833	5.073437	4.235090	3.535179	7.022026	4.318222	3.737498	3.768196	5.495284	3.835245	4.135166	4.217085	3.932381	3.667908	3.531098	3.078141	4.237224	3.494456	5.466171	3.476911	4.721961	3.660815	8.193208	6.661190	4.640929	4.615553	6.221062	7.349921	3.849284	8.415496	5.753206	6.315207	5.683817	6.366378	4.241852	5.833332	3.164434	6.563515	4.032747	5.622975

AL049649.1	3.886962	3.087170	4.053879	2.974981	3.182826	3.422057	3.041132	3.152573	4.421071	3.069128	3.123562	3.136008	3.101983	3.424917	3.018085	2.956615	3.299325	2.887571	4.041017	2.817805	3.774502	4.760385	3.253442	2.955694	3.703446	3.005736	3.093041	2.929281	2.998889	3.279903	3.444014	3.456601	6.228838	3.319276	4.377580	4.913039	3.241347	3.179124	3.247981	3.180141	3.283015	3.308242	4.289822	4.420461	3.406592	4.576920	4.196467	3.335465	4.654410	3.364370	3.278379	3.256748	3.528578	3.459121	3.800534	3.247085	3.928272	3.113651	2.937314	3.470503	4.816461	3.271149	4.136096	3.136258	7.526038	3.301909	2.977589	3.136611	3.866394	2.985037	3.876965	3.233362	3.254990	3.409888	2.876355	3.563639	3.673900	3.872654	3.159156	3.424550	4.051722	3.198895	4.060178	3.676220	3.915108	3.201021	4.732732	3.863232	4.610790	3.904662	3.131266	4.321019	4.917478	4.575394	3.221235	3.701819	3.417386	3.584353	5.375758	4.380153	4.628909	4.123626	3.862377	3.281503	4.344368	3.012671	2.945091	3.746283	3.295881	2.996175	3.581102	3.481721	4.521491	3.414352	3.236086	5.560926	3.229042	3.181086	3.839885	3.003425	4.554340	3.777553	3.286969	4.239674	3.299180	3.935805	3.066192	9.748635	3.028867	5.015854	3.709872	4.662858	3.527760	3.268690	3.933476	3.209242	2.871110	3.299460	6.342339	3.252098	3.677214	3.093488	3.773257	4.462233	2.893630	3.915969	3.274802	3.331586	3.795069	3.261885	3.823735	6.087137	4.532041	3.934301	4.608664	5.979208	3.004535	3.491185	3.997882	4.346762	3.232304	4.563629	9.406640	4.736346	4.192585	4.071884	3.001976	3.663842	3.166490	3.148882	3.101726	3.525510	5.915113	6.317575	3.124783	4.535021	5.905278	5.916532	4.640929	5.166273	3.603220	4.449382	3.645826	6.068409	3.467486	6.239426	3.338286	5.898487	2.986741	4.158193	4.146850	4.149177	4.159936	5.166080	4.348075	6.139789	5.028096	4.662255	5.648979	5.670843	5.704486	4.176825	3.398178	3.384151	4.583727	5.851310	6.248802	4.806164	5.484266	3.570246	3.661202	4.260212	5.607219	3.594603	6.239096	4.406247	4.316078	5.344963	4.074275	5.021566	4.077825	6.119227	4.012459	4.699871	3.016572	5.675129	3.639909	3.503137	4.510512	4.309062	5.215041	6.449656	3.272546	3.947460	4.543555	3.269604	3.282097	6.277338

AC096570.2	8.539575	8.843566	8.529825	8.931119	9.166952	8.800662	8.097528	10.132720	9.884227	9.034964	8.394237	8.280692	7.849606	8.513995	7.803562	9.691940	9.132751	8.349890	9.741022	8.489362	9.546085	9.807299	9.290321	8.437550	8.760273	8.493381	8.423320	9.697083	8.271201	8.180988	9.562398	8.060250	8.056204	7.457297	8.170666	8.339021	8.610473	8.540872	8.963871	9.166952	8.718897	8.793229	9.039625	8.028111	7.845650	8.064734	9.202802	9.049014	8.199981	8.314053	9.680298	8.579079	8.044812	7.982383	8.368909	8.659902	7.796488	8.426765	8.361723	7.975543	8.946451	8.594879	8.273458	9.386205	8.478982	9.056119	9.087175	9.324307	9.131804	9.952432	9.008878	8.819941	9.175876	9.161680	9.333214	8.633073	8.745107	8.746649	8.580435	7.801445	7.883226	7.774143	7.991114	8.156599	8.264002	8.031326	7.931730	8.347801	8.735952	8.890976	7.866962	9.488153	8.658828	8.685097	7.536868	8.298658	8.090022	7.959294	8.447968	8.734401	8.672619	8.655793	8.593623	9.008394	9.358941	8.620648	10.011809	8.935765	9.369059	9.308024	9.660909	9.304702	9.122924	9.358788	9.369204	8.565110	8.794460	8.347659	8.831940	8.151277	8.409551	7.772629	8.272248	9.166498	8.526133	7.694279	8.593623	8.280995	8.104486	7.959294	7.684733	7.768234	7.867574	8.342241	8.220554	8.285774	7.906928	9.029335	7.830215	8.742249	8.969905	8.991935	8.113934	8.301521	7.867574	8.787683	8.450004	8.424087	7.801000	8.466122	7.423685	7.483531	7.632388	7.264789	7.206614	7.296211	8.092170	8.204471	8.086591	8.399403	8.005298	7.510154	7.824617	7.486641	7.919331	8.606818	7.997547	7.879149	7.839844	8.340263	8.048777	7.245330	7.550974	7.192295	7.542183	7.050689	7.444435	7.779974	7.326903	8.008692	7.667152	8.117164	8.027626	8.113311	7.771269	8.026061	8.198709	8.419769	8.372836	8.114241	7.872133	8.953642	7.893878	7.807111	7.880066	8.032389	7.976484	8.222383	7.158679	7.149940	7.754711	7.939461	7.168714	7.650959	7.838627	7.331787	7.173526	8.372228	8.049687	8.131490	9.111274	8.187900	8.246658	7.831717	8.496424	7.834327	7.892473	7.736514	7.619699	8.217593	8.057438	8.195435	7.640029	7.970201	8.233897	8.597328	8.012294	9.135312	8.795349	8.875260	8.735484	9.584839	9.718616	8.357685	7.412291	8.174422	7.645719	8.438131

AC096570.1	8.539575	8.843566	8.529825	8.931119	9.166952	8.800662	8.097528	10.132720	9.884227	9.034964	8.394237	8.280692	7.849606	8.513995	7.803562	9.691940	9.132751	8.349890	9.741022	8.489362	9.546085	9.807299	9.290321	8.437550	8.760273	8.493381	8.423320	9.697083	8.271201	8.180988	9.562398	8.060250	8.056204	7.457297	8.170666	8.339021	8.610473	8.540872	8.963871	9.166952	8.718897	8.793229	9.039625	8.028111	7.845650	8.064734	9.202802	9.049014	8.199981	8.314053	9.680298	8.579079	8.044812	7.982383	8.368909	8.659902	7.796488	8.426765	8.361723	7.975543	8.946451	8.594879	8.273458	9.386205	8.478982	9.056119	9.087175	9.324307	9.131804	9.952432	9.008878	8.819941	9.175876	9.161680	9.333214	8.633073	8.745107	8.746649	8.580435	7.801445	7.883226	7.774143	7.991114	8.156599	8.264002	8.031326	7.931730	8.347801	8.735952	8.890976	7.866962	9.488153	8.658828	8.685097	7.536868	8.298658	8.090022	7.959294	8.447968	8.734401	8.672619	8.655793	8.593623	9.008394	9.358941	8.620648	10.011809	8.935765	9.369059	9.308024	9.660909	9.304702	9.122924	9.358788	9.369204	8.565110	8.794460	8.347659	8.831940	8.151277	8.409551	7.772629	8.272248	9.166498	8.526133	7.694279	8.593623	8.280995	8.104486	7.959294	7.684733	7.768234	7.867574	8.342241	8.220554	8.285774	7.906928	9.029335	7.830215	8.742249	8.969905	8.991935	8.113934	8.301521	7.867574	8.787683	8.450004	8.424087	7.801000	8.466122	7.423685	7.483531	7.632388	7.264789	7.206614	7.296211	8.092170	8.204471	8.086591	8.399403	8.005298	7.510154	7.824617	7.486641	7.919331	8.606818	7.997547	7.879149	7.839844	8.340263	8.048777	7.245330	7.550974	7.192295	7.542183	7.050689	7.444435	7.779974	7.326903	8.008692	7.667152	8.117164	8.027626	8.113311	7.771269	8.026061	8.198709	8.419769	8.372836	8.114241	7.872133	8.953642	7.893878	7.807111	7.880066	8.032389	7.976484	8.222383	7.158679	7.149940	7.754711	7.939461	7.168714	7.650959	7.838627	7.331787	7.173526	8.372228	8.049687	8.131490	9.111274	8.187900	8.246658	7.831717	8.496424	7.834327	7.892473	7.736514	7.619699	8.217593	8.057438	8.195435	7.640029	7.970201	8.233897	8.597328	8.012294	9.135312	8.795349	8.875260	8.735484	9.584839	9.718616	8.357685	7.412291	8.174422	7.645719	8.438131

AL162724.2	9.379453	6.643535	6.082990	5.660863	4.728272	4.342640	5.979394	3.556578	4.552354	6.943755	4.977057	5.494903	5.587352	5.915291	6.018827	5.425215	5.191894	6.045357	4.680289	4.935151	3.659062	5.498722	6.989840	5.417979	6.043810	5.391837	6.488864	5.141531	6.079900	6.097354	5.987923	6.620355	7.140794	7.014664	6.536628	5.753934	5.654433	5.767564	4.297429	5.867047	6.702745	5.686415	6.338886	5.794931	6.648704	5.174333	5.041162	7.077988	6.551496	5.258110	6.208832	7.992228	6.627577	5.645988	7.221773	5.316256	7.113308	5.679386	6.296420	6.448842	6.387497	5.380786	5.705946	5.343606	6.210495	8.624991	5.408923	5.840428	9.091411	5.355303	7.816519	4.680948	5.377319	5.732104	7.882917	6.173023	7.850645	8.214100	5.940611	8.288103	6.083169	6.346068	6.139974	6.654347	6.386807	6.690804	4.619476	5.957324	3.965731	5.505053	6.333155	6.960860	5.866298	5.438019	8.968056	5.961974	7.241488	5.296657	7.035624	6.032361	3.880658	5.829701	5.557143	6.250316	5.517407	5.263904	5.052568	5.932286	5.038331	5.472912	3.349703	4.923847	3.790786	4.654410	5.752653	2.942927	6.370064	7.446013	5.357397	7.155448	6.640307	6.626161	6.115636	4.227481	5.564109	5.844230	5.090658	5.917236	8.845102	5.846135	5.872251	6.008246	6.976098	8.033141	5.730493	6.044989	6.463884	5.257333	4.997562	5.037916	6.383321	4.454382	6.276999	6.423176	9.060510	4.023916	6.208471	4.555570	6.466680	8.904922	6.325419	5.946702	5.930416	6.336711	7.496149	6.696500	7.632540	5.652761	7.129553	5.997433	5.005774	6.075028	5.418743	6.294553	6.154988	4.675590	6.197075	6.756046	6.478984	6.299387	8.055096	7.323456	6.893530	6.979414	6.746442	5.973787	6.060246	6.591797	6.232252	6.188764	5.160478	7.030340	5.612341	5.736449	9.123809	5.431227	6.168083	10.573635	5.638273	9.325829	5.611588	6.115466	6.236327	9.002733	6.270170	10.119603	7.954175	5.781818	10.133459	6.123379	6.768454	6.125675	6.543690	7.763738	6.664134	6.140137	6.744168	4.491777	6.790559	4.758717	6.323720	6.103414	6.652567	7.399610	6.204695	6.275556	6.318421	9.043957	7.801445	6.823537	5.489345	7.092603	8.555909	7.160590	6.504316	6.269984	4.775942	5.346943	4.891473	3.447475	5.295630	3.213675	5.507191	5.942139	4.549764	6.516905	5.830666	6.383146

LINC00303	9.456011	9.582699	10.706418	9.052657	9.883943	11.908190	8.415166	11.342184	11.186429	9.659506	9.689916	9.569119	9.474009	10.675830	7.976484	9.587494	8.522454	8.370333	8.830706	9.830156	9.676599	11.228140	9.433117	9.540665	9.381310	10.122304	12.212531	12.036847	9.864397	9.266894	8.662706	9.938377	7.921494	9.421302	8.969905	10.229726	9.198592	8.839043	8.823048	9.884840	11.374203	8.548140	9.133075	9.041919	8.807046	10.182411	12.085444	9.533834	9.861767	8.034917	8.968369	8.544915	8.187744	7.457620	8.547997	9.884840	9.575176	8.776916	7.764503	9.514276	8.678987	10.436000	9.020946	8.573650	8.606960	8.740108	10.458023	10.280189	10.469201	10.516678	9.314604	9.523949	9.909868	10.590376	11.212744	9.831535	11.376659	11.228875	10.904667	7.501316	7.632094	7.322955	7.649041	8.105890	8.045582	8.934567	7.774011	7.504157	8.348688	8.170501	8.870231	9.328138	9.999670	10.980998	10.689474	10.636387	8.723135	8.134218	10.131242	8.572894	10.041770	9.316873	10.309258	10.312135	9.118187	9.519473	9.861294	8.577882	9.261966	9.851372	9.079789	8.980451	10.555376	10.810677	10.930096	11.626020	8.500592	9.328299	11.913228	9.182973	8.519296	10.394618	9.535972	10.517868	10.401542	10.812696	9.384364	8.770383	11.137872	10.733727	11.291724	9.885942	10.110042	9.204578	12.828063	8.513238	10.727474	10.369106	8.545228	9.839709	9.667979	9.772950	11.579822	8.873253	9.149407	11.383354	11.037001	10.824736	10.926836	10.553379	8.793388	8.880982	10.457422	9.001665	9.360643	9.157249	11.200158	8.999207	9.395748	9.958620	10.502281	9.253295	8.876461	11.006117	8.882195	11.449715	8.853642	10.614503	8.135563	8.206040	9.172346	7.963443	8.127977	8.081336	10.232124	9.627540	8.401741	9.123220	8.504177	8.342466	9.026613	8.871708	9.759027	10.632749	8.469643	8.826560	9.919803	8.900970	9.197337	10.666588	11.190043	12.078200	8.882644	9.167255	10.769710	9.253455	9.323027	9.751120	8.396872	8.841026	8.162350	7.665583	7.762352	7.618023	9.834586	7.871982	7.610076	8.454138	8.039167	9.136846	8.570802	10.238002	9.507924	9.442200	10.000862	9.878300	11.643868	9.085192	9.994619	9.102892	9.712029	9.465521	11.023215	9.112263	11.739222	9.910666	9.526496	9.781775	11.378855	9.823468	8.948426	10.330692	11.687068	9.323513	10.454196	9.825394	9.601901	9.508697

AC105219.1	5.342989	4.835984	5.419596	5.520055	5.545822	3.677001	5.651842	4.270081	3.732140	4.666013	6.253840	5.337710	4.781556	5.839364	5.119896	4.902368	3.415696	4.900919	5.190523	6.030587	6.459532	6.269124	5.264877	5.413006	5.691927	4.305328	5.829507	5.306198	5.287944	5.129592	5.844038	6.657929	6.153116	5.809052	5.681975	5.048105	5.895493	5.658878	6.089027	5.225884	4.162019	6.078831	5.593845	4.530746	5.169505	5.221697	5.564109	5.002123	6.702745	5.628214	5.398042	6.096514	5.722134	6.243437	4.647912	4.067317	6.250630	7.169171	6.399465	6.074670	6.088150	4.954364	6.600216	5.770487	6.696363	5.497735	5.944425	4.795599	5.844745	6.105329	5.767752	5.455701	6.011218	5.706129	5.508384	5.524404	5.060815	5.574989	6.924019	5.974298	5.746482	5.767383	6.305762	5.946541	5.906838	6.278478	5.442160	5.813703	6.932252	5.218337	6.035624	6.620976	6.684561	5.154379	5.439113	5.988641	4.693081	5.555640	5.463844	5.465983	3.424387	5.267480	6.135173	5.682156	5.746482	5.221107	5.446009	6.777993	4.967792	5.218747	6.282271	5.658509	4.397228	4.827617	6.539082	3.486244	4.788469	4.639269	5.753934	6.173182	6.999824	5.834109	8.016483	6.334855	6.025877	6.505475	5.798581	6.622425	6.396462	6.951067	6.895743	6.211683	6.124801	2.978889	4.142802	6.293544	4.838497	7.845788	6.070323	5.818516	7.535484	6.162716	5.403080	7.681453	6.459041	4.026235	7.786067	6.347604	5.622407	6.080567	6.305958	6.371417	6.161322	6.829927	6.392873	6.506995	7.443608	7.056979	6.235468	5.257917	7.522227	7.539270	6.051840	6.008585	5.981021	5.838629	2.863017	7.748877	7.067141	6.019537	6.842224	7.004570	7.509989	6.687119	5.339523	6.062264	8.665791	5.899364	8.385714	5.859607	6.256093	6.339228	7.644473	7.253406	7.237820	8.500912	4.125890	6.435274	6.381984	7.759138	6.399974	8.315127	6.186736	6.805534	5.060634	4.840237	7.030340	6.837856	6.427512	7.434079	6.508768	6.985556	5.055139	6.949175	7.391165	7.182510	5.919921	6.521055	5.008463	7.149940	6.550364	6.271204	5.915478	5.319944	5.292166	5.625771	4.519350	7.501152	5.329287	4.622273	5.728818	7.311966	5.183812	7.965252	5.875848	6.388847	7.347389	6.893978	7.565494	6.455262	7.153877	4.432246	5.742456	6.223644	6.261164	5.184812	6.206911	7.440064

AL358394.3	4.194980	4.215554	4.067317	4.099749	4.909040	3.885110	4.861224	4.682632	5.790268	4.752973	5.668327	4.260648	4.504789	4.668385	3.387908	5.047486	5.419988	2.978793	4.554107	4.536102	6.332486	4.263974	3.613168	3.367066	2.871971	3.813350	5.377881	3.470703	5.179750	4.581198	4.895587	4.494905	4.121401	4.921258	4.806580	4.744005	4.471226	3.857663	3.798781	4.691187	4.942399	3.949193	4.301308	4.691187	4.796480	4.612057	4.297429	2.984959	5.655361	4.316491	2.861148	3.004637	4.930625	3.707684	4.829983	4.263555	5.103586	4.173173	4.304213	3.888699	2.836657	7.359642	4.372359	4.264647	3.301772	4.481723	3.248143	4.026873	3.390523	3.832048	3.811402	4.698520	5.589666	4.493308	4.753194	4.122295	4.403536	4.410703	4.383610	5.007858	4.444333	5.202855	4.491142	4.706397	5.287769	4.331456	4.120517	4.087037	4.139961	3.758336	3.295748	3.757728	3.977797	4.283596	4.723229	5.797835	5.209429	6.277687	4.769928	4.940144	4.903414	4.675590	5.021154	4.518262	4.543995	5.269249	3.848412	3.813350	3.289685	5.001920	2.925616	4.984172	4.472978	4.520611	6.117144	4.815819	3.930017	5.048505	3.942850	5.641968	6.054977	5.738626	5.703178	5.361351	5.811871	5.231792	5.535095	6.272390	5.147334	5.798946	5.173357	5.044535	4.722578	4.446490	6.114928	5.503721	4.659010	4.884944	5.433181	4.438496	5.073437	6.239917	3.901062	4.840025	4.766823	5.728084	4.571992	5.050781	5.830469	6.086738	6.712279	5.884779	6.800425	5.504866	5.516471	5.411091	5.794569	5.454938	5.214446	5.631190	5.653667	5.809994	6.434113	4.921033	4.392128	6.107970	2.867885	5.824962	4.966488	5.872446	5.497735	8.019529	6.260150	5.844572	5.440614	6.964524	5.525352	5.073004	4.768089	5.995500	7.272497	5.783785	5.615913	5.692261	5.974996	4.898357	5.869547	5.392969	5.797459	4.989759	6.742879	6.198260	6.038781	5.059000	5.293129	5.169895	5.494505	5.348703	4.920418	5.559864	6.297901	5.605914	6.043642	6.079562	5.396922	6.246949	5.971415	5.813892	5.398239	5.379059	5.486138	4.480424	5.175351	5.789578	6.543526	6.785647	6.657265	6.711199	5.773447	6.395163	5.834830	6.150019	5.537848	5.250184	6.236149	6.000081	6.147780	5.138025	5.206643	3.558731	4.992892	4.738648	5.818145	4.492238	4.959576	5.435714	5.868862	4.872144

AC012588.1	8.555588	6.191829	3.021680	5.105042	3.456073	3.321705	3.096526	3.187273	3.303468	3.658843	4.073634	5.752102	5.203669	3.647766	4.103026	5.386761	3.229324	2.967652	5.164280	4.117669	4.126791	3.123193	4.525441	4.466525	3.587089	2.982978	4.985822	5.563547	5.746482	4.068580	3.578788	6.906051	3.721463	4.957935	4.430930	3.969455	3.513374	4.670370	3.521873	3.473431	4.438286	7.976173	4.775737	4.587091	3.622433	5.598001	3.381552	6.962923	6.156195	3.599742	7.236582	3.598578	4.264647	4.772601	6.610455	3.131125	4.106740	3.460735	7.351140	3.720995	6.225900	5.086779	3.880253	6.070667	3.472214	6.084585	7.200555	3.204421	3.221235	5.961797	3.693392	3.200037	3.247981	3.912272	3.023596	3.300392	3.718521	4.027500	7.221148	3.392134	3.182826	6.649358	7.767619	7.013182	5.187816	7.116277	5.061015	3.253442	4.918304	7.853434	8.630532	3.780400	4.217947	3.191743	7.614780	3.250849	6.200170	3.452427	4.434754	3.216664	3.587872	5.474461	3.075974	2.872066	5.343795	3.269757	4.431806	3.384151	3.460735	3.229755	3.249455	4.445874	5.686415	3.275450	3.440314	5.181372	9.449989	3.276032	3.234953	5.452642	3.552782	2.962817	6.740220	3.465394	3.688884	2.899814	3.115578	3.291431	2.966487	3.100328	3.321225	3.486428	3.135892	3.211448	3.658843	3.151361	2.874133	3.761341	3.427441	3.619528	3.471394	4.927632	3.602066	2.960877	2.926362	3.220568	3.185285	3.310854	3.506434	3.393995	5.069189	3.658626	4.067119	4.067317	3.577338	3.504352	3.463557	3.452065	3.257039	4.477837	5.026050	4.139301	3.392489	4.052167	5.160478	3.382584	3.584949	5.268084	3.358816	4.154082	3.325163	4.086810	4.227886	3.495604	3.036658	3.763184	3.499964	3.478950	3.735018	3.072563	4.568093	3.978716	3.948976	3.728166	4.271585	3.411654	3.424029	3.379120	3.332238	3.450425	3.000143	3.227892	3.023793	2.952123	3.283331	3.160172	3.214885	4.039945	3.197721	3.464678	6.347250	3.894431	3.300811	5.321096	3.772832	3.666689	3.362672	3.288506	3.425458	3.463210	2.996732	3.330497	3.274345	4.800722	3.059383	3.710670	3.369613	4.047376	3.513374	3.473431	4.209917	3.015024	3.689524	3.290281	3.208343	4.597655	3.904426	3.415696	3.165946	3.858770	4.522344	5.805975	3.175549	3.063972	3.691777	5.968975	3.169414	3.290950

PCAT18	8.555588	6.191829	3.021680	5.105042	3.456073	3.321705	3.096526	3.187273	3.303468	3.658843	4.073634	5.752102	5.203669	3.647766	4.103026	5.386761	3.229324	2.967652	5.164280	4.117669	4.126791	3.123193	4.525441	4.466525	3.587089	2.982978	4.985822	5.563547	5.746482	4.068580	3.578788	6.906051	3.721463	4.957935	4.430930	3.969455	3.513374	4.670370	3.521873	3.473431	4.438286	7.976173	4.775737	4.587091	3.622433	5.598001	3.381552	6.962923	6.156195	3.599742	7.236582	3.598578	4.264647	4.772601	6.610455	3.131125	4.106740	3.460735	7.351140	3.720995	6.225900	5.086779	3.880253	6.070667	3.472214	6.084585	7.200555	3.204421	3.221235	5.961797	3.693392	3.200037	3.247981	3.912272	3.023596	3.300392	3.718521	4.027500	7.221148	3.392134	3.182826	6.649358	7.767619	7.013182	5.187816	7.116277	5.061015	3.253442	4.918304	7.853434	8.630532	3.780400	4.217947	3.191743	7.614780	3.250849	6.200170	3.452427	4.434754	3.216664	3.587872	5.474461	3.075974	2.872066	5.343795	3.269757	4.431806	3.384151	3.460735	3.229755	3.249455	4.445874	5.686415	3.275450	3.440314	5.181372	9.449989	3.276032	3.234953	5.452642	3.552782	2.962817	6.740220	3.465394	3.688884	2.899814	3.115578	3.291431	2.966487	3.100328	3.321225	3.486428	3.135892	3.211448	3.658843	3.151361	2.874133	3.761341	3.427441	3.619528	3.471394	4.927632	3.602066	2.960877	2.926362	3.220568	3.185285	3.310854	3.506434	3.393995	5.069189	3.658626	4.067119	4.067317	3.577338	3.504352	3.463557	3.452065	3.257039	4.477837	5.026050	4.139301	3.392489	4.052167	5.160478	3.382584	3.584949	5.268084	3.358816	4.154082	3.325163	4.086810	4.227886	3.495604	3.036658	3.763184	3.499964	3.478950	3.735018	3.072563	4.568093	3.978716	3.948976	3.728166	4.271585	3.411654	3.424029	3.379120	3.332238	3.450425	3.000143	3.227892	3.023793	2.952123	3.283331	3.160172	3.214885	4.039945	3.197721	3.464678	6.347250	3.894431	3.300811	5.321096	3.772832	3.666689	3.362672	3.288506	3.425458	3.463210	2.996732	3.330497	3.274345	4.800722	3.059383	3.710670	3.369613	4.047376	3.513374	3.473431	4.209917	3.015024	3.689524	3.290281	3.208343	4.597655	3.904426	3.415696	3.165946	3.858770	4.522344	5.805975	3.175549	3.063972	3.691777	5.968975	3.169414	3.290950

AF254983.1	3.030074	3.425996	3.000556	3.504959	3.339291	4.958361	3.252513	3.331764	3.327915	3.172928	3.409194	3.196648	3.377017	3.700564	3.198666	3.111015	3.372751	2.968172	4.593634	3.211164	4.329672	3.450425	6.122011	2.950938	3.417386	3.186339	2.940495	2.995591	3.035174	3.776777	3.664440	4.619476	4.232008	7.425397	3.585700	4.053659	3.454010	3.491351	3.497287	3.609968	3.441874	3.910773	3.605770	3.242102	3.701159	5.443704	3.244569	4.236363	3.753370	3.903586	3.416052	3.193659	4.223393	4.289203	3.870938	4.891473	4.008468	4.966720	4.159287	4.073869	3.077267	3.135664	4.047376	3.360160	4.062197	3.536093	3.885512	3.245376	3.281645	2.884071	3.103265	3.429348	3.199019	2.926987	3.398675	3.369613	3.196648	3.010401	3.056221	3.754254	3.391393	3.770710	5.549767	4.169012	3.411831	3.467086	3.661635	3.746872	4.741289	3.781236	3.259296	2.881882	3.857911	3.254505	3.452949	3.387739	3.492758	3.456795	3.386208	3.234542	4.801148	3.325488	2.844634	2.942255	4.629125	3.238890	3.060657	3.300244	3.328072	3.192699	2.881501	3.243133	4.234172	3.792471	3.579575	8.050463	3.060353	4.096860	3.186180	3.005091	3.534249	8.041861	5.766650	4.731873	3.658433	5.113938	3.223091	3.335311	3.199019	3.180141	3.384997	3.689322	3.565517	3.836113	4.244327	3.262192	2.880411	3.337310	3.282855	3.752126	3.041027	5.874564	2.897877	3.238890	3.163387	5.496067	3.375457	4.713705	3.556578	3.487527	3.716082	4.027949	3.534991	3.400227	8.209329	3.507422	3.798781	5.193853	3.360327	3.852717	4.013585	3.637058	3.307751	4.145567	4.899185	4.022370	3.535179	3.995290	3.860653	3.284447	3.063869	4.202274	4.441972	3.497840	6.514869	7.300012	4.931984	4.035605	4.177040	2.915529	3.243676	4.166251	4.037989	4.048031	3.812713	4.070060	3.550812	3.457694	2.844483	3.039031	3.711741	3.206407	3.107369	8.325415	3.342665	3.200632	3.023990	3.149126	3.240744	3.794238	4.143646	3.661202	3.645826	3.380500	5.136441	3.175783	3.371366	4.427225	3.673077	3.409888	2.846952	3.434147	3.346730	3.549307	3.619528	3.557581	4.916169	4.227481	3.946203	3.358816	6.634691	7.179851	3.624039	4.975473	3.177342	3.009660	3.115462	3.293629	3.179770	3.027789	3.483698	4.430031	3.976335	3.651573	4.888354	3.103006	10.106142	3.249584

CASC8	3.352877	4.119452	5.084247	3.731092	4.187529	4.800062	4.212080	4.431596	3.918697	4.230073	3.926325	3.669057	4.533920	4.604801	5.841340	4.110047	3.331129	4.815819	4.686559	4.117895	6.328070	3.849937	5.729904	4.400248	4.583101	4.099296	5.135234	4.113558	3.471726	5.347143	3.752961	4.600410	3.972030	4.971090	4.482201	4.009546	3.712339	4.018026	4.206614	3.683451	4.419362	2.907863	4.830820	5.186207	3.397480	5.533140	3.647986	6.988569	4.816230	4.337686	5.047486	4.427011	4.049977	4.705969	4.820845	4.918512	4.571394	3.298691	4.399610	7.945391	4.343924	4.759341	4.499642	4.189963	5.657752	4.189270	2.903914	5.154192	4.332057	3.781857	4.227258	4.329236	3.992878	3.964673	4.180769	4.113339	4.758311	4.779250	4.092474	3.898693	3.873944	3.893080	4.894812	4.863512	5.079007	3.576726	4.072967	4.468199	3.816516	4.244932	3.586121	3.768196	4.134295	5.966457	3.897372	4.158412	4.538643	3.480386	3.872419	3.656424	3.919774	4.899401	4.109365	4.021106	4.437643	3.749587	4.389354	4.993938	3.651791	4.488455	4.934698	4.447792	4.419362	5.026469	4.881587	4.562301	4.389151	4.256493	3.782941	5.843146	5.259838	6.197419	5.983137	4.688642	8.555103	6.061533	6.859944	4.490913	6.515374	5.858684	5.529728	4.000622	5.962707	5.931595	3.685428	5.892964	6.018321	4.983789	5.324597	4.723229	4.571161	6.740540	4.667960	9.072055	4.627372	5.291967	8.395144	4.832927	6.617746	3.911625	5.217960	5.310882	5.633578	5.865382	4.970884	3.813152	4.924665	5.784669	5.416211	4.968421	8.494612	4.327873	6.005753	6.869995	7.991593	5.650297	6.240938	6.300519	6.063123	6.559759	5.280157	6.100152	5.236428	6.733827	5.263473	3.856793	6.666053	7.994267	12.800035	5.696189	5.956452	5.203062	5.988091	6.231918	5.188817	6.440386	5.508966	6.853335	3.295298	4.898117	6.873056	5.966091	5.694530	6.497857	6.037378	6.290632	5.205438	5.880328	5.074842	5.276568	7.341449	4.717717	6.030746	4.820845	4.141946	5.396732	6.664613	3.478950	5.033779	4.216443	5.273895	5.500842	6.746725	5.175767	6.468683	5.808130	4.098850	5.139865	4.322455	4.955209	8.903553	4.253660	4.517865	7.323901	5.011599	8.090173	4.658123	4.565960	6.701426	9.068649	5.000890	6.351789	5.403273	6.906222	7.184898	7.324535	6.681267	8.472487

LINC01541	3.192826	3.212842	3.056655	2.952581	2.911537	3.113441	3.398675	3.268108	3.478586	3.482786	3.667303	3.817794	3.328860	4.174224	4.146429	3.185819	3.628398	4.019151	4.666242	3.077475	5.300034	2.830429	5.711383	3.141186	3.166354	2.922961	3.115819	2.947380	3.122871	3.074675	3.737108	3.677001	5.046074	3.137235	3.688884	4.137156	3.786951	3.397480	3.286231	2.934882	3.461637	3.186761	3.551213	3.369959	3.635265	5.096409	3.883367	3.459491	3.532333	4.102823	4.105442	3.909010	4.295457	4.094749	3.872223	3.072563	4.067751	3.186821	4.099749	3.630688	3.439246	3.062131	3.377017	3.032852	3.178536	3.168786	2.811599	3.317445	3.075410	2.948161	3.024753	3.064290	3.550231	2.923669	2.955162	3.309540	3.004535	2.976862	2.975923	3.756526	3.357764	4.059980	3.342665	4.224260	3.298831	3.595009	2.951849	3.329682	2.953408	3.354171	3.173051	2.931258	3.145815	3.030074	2.923767	2.984327	3.821840	3.195567	3.030788	3.034513	2.938878	3.283331	3.028960	2.992100	4.195419	3.128807	3.178536	3.418972	2.999083	3.297290	2.803798	3.117381	3.080099	3.292351	3.156556	2.956356	2.985231	3.217728	3.201711	3.089248	3.320743	3.008166	2.992993	2.853596	3.641293	2.994747	3.234074	3.498362	3.381727	3.512383	3.228776	3.095293	3.392955	3.711546	2.974981	3.598775	3.435630	3.410052	4.841900	4.577985	2.809993	5.926133	2.992407	2.956436	2.866196	2.893176	2.875573	3.111995	3.574592	3.491861	3.397284	4.271805	3.255752	3.600120	3.828629	3.837146	3.418434	3.211448	2.896251	3.208073	2.966219	3.592616	3.439595	4.036500	4.958361	3.593637	3.597536	3.783345	4.321207	4.213405	4.475158	3.967464	4.249931	3.445260	3.042415	3.639080	3.033286	3.905507	3.650619	3.095565	3.173569	3.282855	3.202692	3.265284	3.178836	2.797548	3.619143	3.172804	2.978709	3.045578	2.917130	3.906869	3.004804	2.943824	3.294523	3.015270	3.152702	3.045361	3.276820	3.714449	4.353756	3.490098	3.704272	3.509610	3.034305	3.545283	3.404378	2.894981	3.275589	3.308901	2.882159	3.104899	3.066619	3.016850	3.018736	2.937997	3.026482	3.187010	3.272701	2.980773	3.060140	3.146903	2.969982	4.561427	3.364521	3.189017	3.450800	3.375633	3.381180	2.797548	3.240931	3.367536	4.021312	2.982350	2.883547	3.080195	3.230063	3.348380

ELOVL2-AS1	5.434751	6.242941	5.760167	5.591982	5.702610	5.921216	6.136700	5.050976	5.007256	6.013848	6.052366	6.287752	5.147729	4.952305	4.168403	5.122315	4.216232	5.539213	4.942999	5.305197	5.423616	4.613576	6.299226	5.264099	5.008684	5.909697	5.139678	5.089809	5.874564	6.465215	4.727844	4.546670	6.264325	5.441215	4.911789	5.480621	5.740064	4.761835	5.584179	4.979942	5.988291	3.755267	5.174727	5.277938	5.318802	5.217151	5.971594	5.365022	4.744467	6.038597	5.311259	4.974640	5.370132	4.825841	5.114185	6.032361	5.408923	3.405245	4.136714	5.151558	5.529728	5.561471	5.137833	4.464171	5.652389	5.164484	5.423208	5.343795	5.433353	5.824779	5.695458	5.739713	5.885332	5.637335	5.445634	5.443704	5.447412	5.312841	5.422259	5.374545	5.672198	5.633578	5.292766	4.547077	4.646834	5.040093	5.245413	4.649613	4.319502	5.430290	5.047486	5.030942	5.902022	5.665156	5.420746	5.417191	4.902978	4.924478	5.686968	5.294623	6.001794	5.338509	5.397106	5.409120	5.566556	6.043642	6.736422	5.282363	5.817964	5.950553	5.989000	5.413382	5.254163	5.692666	5.497164	7.369531	4.893955	5.586972	5.263904	6.422858	6.187399	6.359019	5.944243	5.787562	6.735790	6.442531	4.669934	3.771736	6.395163	6.127660	6.349577	6.120458	6.347920	5.151735	5.227060	4.725144	5.157266	6.008419	5.809240	5.489143	5.163875	6.991455	5.961446	7.203012	6.853796	5.547706	5.791866	6.324566	6.648704	4.769077	5.090210	5.281154	6.347250	4.946291	6.208832	5.662559	5.918696	5.330860	5.667791	6.830549	4.560966	5.223904	6.237316	4.837031	5.119073	6.795092	6.481237	4.790369	5.915666	5.375758	6.060803	5.312218	5.874052	5.359108	6.839672	5.849182	6.544021	5.103393	5.825489	5.471198	6.472740	5.407741	4.780536	5.923827	5.923827	5.834287	5.213607	5.694721	5.818327	5.614247	5.432219	6.612852	5.632831	5.168072	5.302426	5.426670	5.384096	5.413792	5.134262	5.269469	6.739227	5.367584	4.900045	5.575213	6.087390	5.211052	4.397228	5.524212	5.490933	5.703743	5.188407	5.395166	5.957159	6.174551	5.633010	6.178514	5.665330	5.575213	6.212846	5.987193	5.782005	5.165688	5.713648	5.783088	6.444714	5.779623	5.386136	4.978070	6.732375	6.068409	6.576008	6.526267	6.381648	6.201199	6.023065	4.728272	7.057146	6.170481

KLHL7-DT	11.651226	11.428576	11.484580	11.958571	12.502885	12.376134	11.224167	12.100117	13.413378	12.061997	12.319186	12.116025	12.217605	12.151398	11.748325	12.368451	12.268991	11.923414	13.530568	11.970367	12.627995	12.454210	13.895060	12.494332	12.903734	12.439014	13.310550	12.375286	12.864977	12.091683	12.557178	10.438998	10.578905	12.079262	11.163914	11.014855	10.842875	11.137136	11.627063	12.015528	12.366548	11.778217	11.617561	11.661349	11.625463	10.959032	12.873035	11.114713	11.251225	10.731478	10.830613	10.590541	10.888061	10.997454	10.867717	12.514165	10.862192	11.309113	11.711018	10.942718	11.199162	12.449269	12.682992	12.425406	11.476126	11.324220	11.362983	11.579070	10.757376	11.734522	11.069585	11.361662	12.890172	10.988997	11.000306	11.064219	11.428261	10.968609	11.098087	10.751617	11.125488	10.823396	10.170273	10.609356	10.845031	11.100894	11.554325	10.477236	11.335983	10.599248	11.157897	11.323062	11.234915	10.837385	11.196379	11.805598	11.059091	12.253272	11.703111	13.158966	13.197125	11.871756	12.338888	11.698867	12.192594	12.590794	12.610024	12.642818	14.082208	12.639201	13.318885	12.614854	12.438849	13.252250	12.487643	15.065209	12.174197	12.225203	13.392871	11.864688	11.548736	11.966878	12.179402	13.560400	12.130980	12.387604	13.480172	12.917482	12.620642	12.039995	12.446101	12.711326	12.367368	12.107410	13.349098	12.028865	12.811445	13.540385	11.239944	12.769791	12.666814	12.493360	13.189708	12.276225	11.320848	12.704167	12.400969	14.069209	11.261380	13.068294	12.640533	12.656343	11.758435	11.543627	11.506720	11.434639	11.720935	12.186663	12.247193	13.003243	11.780760	11.309262	11.487129	11.220119	10.857034	13.132807	11.719874	11.282541	10.582324	10.846071	11.480371	10.752372	10.990030	11.143107	12.046618	11.639628	11.357672	11.789856	10.816421	11.378571	11.941813	12.109967	11.937486	11.964164	11.362553	11.743482	11.167110	11.081007	11.450869	11.467339	11.455214	12.634942	11.497575	11.545901	11.093119	11.644166	11.380212	11.310712	11.369313	11.031349	11.155211	10.744707	11.580709	11.109422	12.378730	10.658726	10.821164	11.757386	11.682031	11.869049	11.862600	11.580709	10.946082	11.416773	12.088271	12.347281	13.155744	12.113600	11.731533	12.200157	13.045913	13.271026	12.061682	12.703315	13.029905	12.977674	12.401781	12.429637	12.849996	13.258986	12.908579	13.779166	12.887380	12.292362	12.279590	11.239205	12.166049	12.365108

LINC00205	6.217833	6.509428	6.759287	6.638207	7.297649	7.155448	6.676570	7.223587	7.349599	6.619535	6.521703	6.477105	6.591797	6.722079	7.098301	6.553750	7.066199	6.514561	6.244796	6.194067	6.455107	7.390553	8.652502	6.611503	6.719816	6.880697	7.734887	6.873531	6.957676	6.877809	7.090879	6.226940	6.363201	6.531278	6.269812	6.423176	6.518553	6.245120	6.695069	6.839672	6.769450	6.241762	6.813657	6.459372	6.914480	5.708117	7.451786	6.883408	6.276695	6.914321	6.208126	6.472916	6.729672	6.424825	6.499409	7.137983	6.209329	6.688548	6.984610	6.874639	6.141889	6.995990	7.274704	6.763922	7.173996	6.942144	7.062528	7.026340	6.687933	7.020142	6.453478	6.561513	7.187316	6.916033	6.657929	6.575152	6.303916	6.627907	6.416166	6.280716	6.081265	6.701120	6.388514	6.305257	6.404740	6.202971	5.075261	6.271028	6.091820	5.934404	6.184684	6.577340	6.850930	6.559425	6.817820	6.466853	6.406241	6.951067	7.438844	7.260591	6.935695	6.628397	6.867257	7.029077	6.855573	7.400837	7.609920	7.216398	7.203950	6.931597	6.921772	7.103141	7.154507	7.095312	7.267734	7.832466	7.133718	6.722579	6.896050	6.455878	6.918372	6.654673	7.271730	8.041861	7.151564	7.259980	7.610371	7.535018	7.605625	7.735656	7.081142	7.996465	8.272834	8.161613	7.780442	7.335828	7.231821	7.003961	7.168099	7.730863	7.926812	7.802652	7.985161	7.351457	9.168906	7.306812	7.798494	7.795863	7.128470	7.953412	6.877972	7.322955	7.141280	6.575343	6.617107	6.865815	6.830874	7.435698	7.139854	7.949845	6.155871	6.734990	6.560692	7.375633	6.867559	7.997230	7.666215	6.701586	7.520520	7.493678	7.670222	7.344269	7.565198	6.824504	7.873667	8.205229	7.365622	7.249623	6.623405	7.015993	7.550974	6.534701	7.231492	7.021726	7.038741	7.402070	7.044502	7.375633	6.970435	6.606716	6.724651	8.582845	7.384537	6.894802	6.519214	7.280383	6.824965	6.694724	7.349268	6.900141	6.820386	6.708319	7.185875	6.747397	7.228420	6.835648	7.035000	6.607883	6.841442	7.315429	6.233086	7.126410	6.927315	6.983328	7.130033	7.143793	7.584419	7.503840	7.376584	7.409893	7.457457	7.909425	7.430288	7.760697	7.580232	8.112700	6.933179	7.205658	8.427398	6.599421	7.666698	7.602148	7.390405	7.300476	7.475528	7.277818	6.779094	8.130096

AC131212.1	8.700428	7.503220	7.816357	8.266504	8.868990	7.885997	7.256786	8.518092	9.765885	7.670691	8.546755	8.799601	9.058147	8.436316	7.594582	8.392410	8.301962	8.166936	9.678939	8.267621	7.753947	8.925032	7.975388	7.444262	8.899923	9.049437	10.171241	8.340857	8.211887	8.182340	9.342205	7.104714	7.107418	8.242339	7.018100	6.636280	7.340993	7.343668	8.135700	8.036022	8.977860	7.648612	7.699122	7.328812	7.396943	7.409429	9.863922	7.348804	8.175531	6.965004	7.708085	6.802029	6.760913	6.892352	6.774963	9.694983	6.524815	7.070554	7.459505	6.202438	6.596483	8.582845	7.033591	8.149462	6.635160	7.651114	8.109085	7.241488	7.499603	7.597944	7.368917	8.205080	7.921218	7.540217	6.836582	7.337348	7.102506	6.503995	7.856355	5.912590	6.243437	6.678326	7.094174	5.703375	6.520543	7.361219	6.498399	5.818327	6.813024	6.682909	6.449821	6.647062	7.977519	8.129492	8.249893	7.851569	7.051636	8.360934	9.423296	7.962682	9.232220	8.013689	8.856682	8.021201	7.945391	8.017690	9.212673	7.944780	9.187084	8.657423	8.350679	9.570429	7.833385	9.305431	8.966798	10.394763	9.351090	8.464122	8.373727	7.186349	8.332609	7.996145	7.875198	10.509324	8.436480	9.004266	10.629794	8.668179	9.313309	9.359541	9.396376	10.584473	8.437404	10.062587	10.173505	7.616186	9.725155	9.717497	7.798494	9.487987	8.601276	10.864986	11.049446	9.088528	7.511709	9.809208	10.491272	11.329368	9.361876	10.845325	8.947364	8.222845	8.192133	8.845571	6.726248	7.925680	9.162916	9.547475	8.516533	10.137446	8.532727	6.517896	7.172616	7.319006	7.506934	10.154836	7.618175	8.096280	7.140794	7.321508	7.910633	7.423039	7.205220	7.125300	8.387588	6.054353	7.582415	8.173625	6.227645	6.799110	7.435851	7.750438	7.705945	8.268871	7.225445	8.154688	7.064300	8.306636	6.841294	7.347533	8.784314	9.778915	6.848381	6.429874	6.819593	7.024249	7.065404	8.005443	6.571479	6.482234	7.197133	7.335219	6.108518	7.007659	8.343367	6.570672	6.476576	7.349096	7.482286	8.388680	7.144281	7.911257	8.419769	7.701592	8.675912	8.469498	9.275465	9.808570	6.925974	9.231455	8.674365	10.679432	6.552176	7.183306	10.172011	10.078202	7.666860	8.884973	10.406784	8.227663	10.417979	10.440659	10.197070	9.555676	8.422421	7.941031	9.051002	8.303777

AC012368.1	7.268335	7.112374	4.914945	5.112145	7.520666	6.995225	5.672198	9.226054	6.061708	7.677944	5.808505	8.055425	6.839672	7.390553	6.029738	7.923024	7.842303	5.980285	7.551436	8.685699	6.948257	5.726450	4.740850	7.423830	7.981450	5.126963	6.857373	6.575152	7.467980	9.006707	5.430096	7.178293	6.361035	8.848945	7.544235	5.911515	5.480817	7.530523	4.983993	6.970286	8.224788	7.001307	7.792804	5.608760	5.802051	5.213607	8.226898	7.466203	7.282413	7.555503	7.788217	8.167657	6.754404	7.370940	8.337432	5.838422	6.031113	7.746708	7.477201	7.275464	7.783772	6.945974	6.722898	7.469869	7.625330	6.838192	5.443704	7.645719	7.956793	6.092548	6.902551	5.222525	7.539578	5.264297	5.566754	6.649044	8.141802	7.179683	5.450652	8.063024	6.241596	7.701163	5.685865	7.932633	8.418410	8.495694	7.967867	7.951508	6.921278	7.490867	7.437870	7.492744	5.953430	5.215220	7.108059	8.372378	6.797498	8.526133	6.470203	8.099439	7.269078	7.791129	7.905227	8.056526	6.536288	8.454739	7.768978	5.742641	6.685661	7.136255	5.890991	5.999019	7.504456	7.335511	8.694514	3.993522	7.082365	8.757622	7.275940	8.148242	6.184157	5.764499	5.625058	5.907032	7.895952	6.083169	11.814780	5.303814	8.444252	6.506176	10.164279	9.576225	5.735745	4.626343	6.221944	6.049931	8.991496	7.281623	8.210515	4.863307	5.299488	6.954704	8.684334	8.460790	3.752330	3.360967	8.680808	7.652521	8.524310	5.198116	8.178407	6.215426	9.224038	7.544235	8.072719	5.145352	8.362054	3.752330	7.245475	6.190095	7.905055	7.875043	5.022964	6.480417	4.580148	7.476921	8.187744	7.463593	7.137353	8.566155	7.995502	7.610841	7.172935	8.796696	5.220931	3.574404	8.125979	8.969905	8.004991	8.975271	7.214668	7.707764	8.783374	7.012349	6.935371	6.384666	7.969093	8.122926	7.355178	10.073145	6.671556	8.230249	3.406400	4.841693	6.909393	8.124176	7.698822	5.759707	8.398835	6.625668	6.836582	4.537365	9.059573	7.955865	7.718121	7.884753	8.256628	6.074144	7.381709	8.274038	7.468145	6.363388	4.244727	7.411070	9.457241	5.844572	9.714055	6.290462	8.295020	6.766660	9.856526	6.399465	9.526630	5.941954	10.856013	10.348720	6.132754	5.291194	5.638273	8.215620	3.856793	5.070584	7.034067	10.032414	4.378864	8.856984	10.675990	6.007549

SMIM10L2B-AS1	9.489658	9.705173	9.806251	9.881488	10.277896	9.775518	9.743197	9.504396	9.979650	9.798264	10.006631	9.905601	9.754711	10.108989	10.065268	10.608143	9.240911	9.930473	11.256056	10.163518	10.263119	10.517707	11.631352	10.022449	9.822073	10.436590	9.581892	9.292527	9.344981	9.082435	10.957536	9.950116	9.947489	9.041626	9.955843	9.929233	10.258512	10.144304	9.850250	10.015875	9.964730	10.537651	9.493002	10.045253	10.107149	9.696633	9.326595	10.173953	9.710638	9.870914	9.696173	10.210307	10.165649	10.601209	10.149344	10.223905	9.986994	10.508560	10.610379	10.441113	10.048346	9.372405	9.972631	10.671515	9.738831	9.874660	10.403949	9.755164	9.782073	10.642678	10.126943	9.816653	8.954265	9.723440	9.566691	9.840623	9.962553	9.508545	9.486588	10.798200	9.848474	10.007983	9.861608	10.167812	10.163693	10.136250	10.589660	10.166903	10.739904	10.530547	10.259615	11.286882	10.214880	9.641171	9.868222	9.895199	10.056350	9.472923	10.235733	10.142602	8.861679	9.755013	10.086825	10.455864	10.041323	10.216690	9.579792	9.750053	10.088070	9.441108	10.951940	10.693482	10.620851	9.081509	9.627839	6.630313	9.893017	9.537039	9.395272	10.066907	8.118549	8.030875	9.021575	8.168296	9.702796	9.561597	10.025602	7.953560	8.785782	8.643945	11.057327	7.483209	9.292061	8.941567	10.077279	8.453530	9.881018	10.034381	9.473387	8.922699	11.362843	10.526253	10.166903	9.086267	8.504041	10.587164	10.327332	7.466031	8.199201	9.331538	8.222845	8.319872	8.006259	10.075275	10.420556	9.061269	8.565393	8.782589	9.067393	7.428206	8.960071	8.021201	8.015555	9.955081	9.013164	7.909740	9.979195	9.371510	8.194463	8.378612	10.240599	9.726365	8.877113	9.012401	8.918471	10.195547	11.180978	9.578531	10.021399	8.638802	10.571531	9.872275	9.311872	8.367911	8.629256	8.825863	8.610302	8.345999	9.361550	9.833794	9.111930	8.915416	8.271350	8.270594	8.823574	9.001028	9.587642	9.183143	9.455263	9.155560	10.022591	9.391895	9.762301	9.432358	7.955250	8.820970	10.550997	9.073286	9.154950	9.000858	9.179536	8.372228	8.498349	8.441197	8.266671	8.545801	10.168890	8.757464	10.899253	8.251773	10.052192	10.344466	8.437092	10.538212	9.632923	9.067847	9.408477	10.135460	7.615580	9.749113	8.745438	7.115335	9.460905	8.352774	7.098301	8.885279	8.732710	7.731957

AP001347.1	7.973216	9.399085	9.896130	8.027797	9.916212	9.054864	9.812289	8.477251	8.930003	9.636374	9.311096	9.391747	8.691086	10.145061	7.856943	9.878449	8.991194	8.441329	8.508449	9.759333	9.206427	8.571728	9.121839	9.292212	9.019864	8.395457	8.199029	8.247106	9.319893	9.684363	9.922427	9.163408	8.991935	8.366808	9.092833	9.633683	10.440659	8.416105	8.298809	9.286903	9.493154	8.555909	9.058786	9.231754	9.141010	9.728859	9.446710	8.955627	10.652524	8.545084	9.033411	10.086189	9.455713	8.756854	9.546241	9.311408	9.617475	9.366138	9.185335	9.107647	9.055357	9.283248	9.603010	8.465978	8.098858	8.615714	9.483039	9.527248	8.938834	9.142589	9.388562	9.398469	9.976192	9.447951	9.472625	10.093356	9.477671	8.874966	9.379881	9.959089	10.602115	10.285451	8.485531	9.895500	10.504838	9.816798	9.424212	10.545972	8.886223	10.227583	8.382997	9.461373	9.970502	8.842770	8.518715	9.155870	9.567790	9.847208	8.258639	8.959322	8.043711	8.834942	8.186038	9.920424	9.359995	9.454768	8.217766	8.216662	8.907201	9.539708	9.216398	9.266572	7.971157	9.235176	9.479952	9.603904	7.249473	8.700270	9.713565	10.174575	8.509809	9.252684	8.396552	8.136815	8.346927	8.347200	8.235110	7.554088	8.042316	8.178108	7.964808	8.182665	7.373586	8.095215	8.250047	8.586239	9.678129	8.869457	9.275288	8.018297	8.382051	8.446086	8.080382	8.244304	7.999482	8.125256	7.774290	8.825863	9.583612	10.015727	7.784942	7.490073	8.016641	8.500441	8.600192	8.714362	8.851683	8.387119	8.528920	8.670471	8.601729	8.256933	8.397928	7.815116	8.119459	8.693250	9.822073	8.507964	8.524014	7.941178	8.179776	8.207086	8.397483	8.711913	8.921354	8.842443	8.616628	7.851877	8.193370	8.167657	8.110900	9.205341	8.183733	7.980345	8.162515	8.621747	8.506350	8.279787	9.345450	9.025712	8.928332	8.615714	8.320348	8.992086	9.111274	8.662072	9.468250	8.616940	9.139031	8.533318	8.588986	7.734740	8.188791	9.776425	7.742387	8.292379	7.830357	8.677280	8.479473	8.546108	8.160678	8.652972	8.440424	8.021935	8.341455	8.239336	8.458106	7.864173	8.458444	8.388218	8.014090	8.137259	9.160629	9.408800	9.063380	6.966432	7.967712	9.002906	8.780406	8.526736	8.425755	9.716865	9.303246	8.947596	6.782610	7.910938	7.605952	8.632197

LINC00629	4.031273	3.119498	2.991399	2.953226	2.863202	3.533492	3.264674	3.359512	3.301288	3.367240	3.514967	3.472743	3.173051	3.619327	3.867730	3.074675	3.417742	3.671565	4.676014	3.020309	4.521910	2.850582	2.944727	3.032657	2.957425	2.774163	3.483337	2.848033	3.125395	3.035276	3.496333	2.929202	4.201411	3.204261	3.192699	3.907900	3.549833	3.096798	3.968740	2.873704	3.309540	2.889447	3.496731	3.867061	4.107628	4.729367	3.717137	3.033804	3.394158	3.287419	3.562491	5.210637	3.981983	3.904846	3.699591	3.079083	4.010356	2.955162	3.705674	3.587283	2.943219	3.205978	4.373932	3.024181	3.211573	3.259588	2.872066	3.284110	3.173311	2.845888	2.997330	2.881501	3.053850	2.800651	2.887198	3.255631	3.106094	3.136966	3.072446	3.662035	3.282404	3.913138	3.304758	4.300044	3.409715	3.390523	2.930172	3.217434	3.274657	3.343649	3.176969	3.150988	3.584146	3.148992	2.901598	3.557581	3.648387	2.981364	2.936995	2.970992	2.898333	3.195847	3.054163	2.877138	4.344134	3.901062	3.017496	3.287111	2.942182	3.228916	3.700152	2.871646	2.905236	3.226592	2.993450	3.192046	3.232167	2.970823	3.048062	2.988326	3.204680	2.958532	5.015459	2.928005	3.428828	3.078033	4.218585	3.224618	6.138754	3.307479	3.099517	3.826564	3.369785	3.489376	2.943316	5.379465	3.226298	3.574228	3.142443	3.926528	2.837457	2.966317	3.691777	3.643274	6.159627	3.660607	2.819412	2.914747	3.475980	3.787161	3.201160	4.265934	3.026966	3.187544	3.660415	3.541895	3.062769	4.190163	2.878010	3.117004	2.840570	3.461815	4.507242	3.950106	4.452439	3.348689	3.047106	3.632948	5.130587	3.497641	4.516782	3.789934	4.120971	3.384997	3.035276	3.612759	2.941644	3.358619	3.468909	2.896885	6.114580	3.478207	3.164807	3.332238	3.393483	4.606257	3.561139	3.167396	2.865853	3.003425	3.186761	3.042514	4.117437	2.901917	3.221235	3.322329	3.810560	3.819476	5.092892	3.605404	4.101929	3.357413	3.678626	3.535907	3.019493	4.514410	6.726738	3.107369	3.442575	4.939947	4.974204	3.386361	5.146547	3.580146	3.616508	3.418603	3.723511	5.482575	3.343473	4.251056	5.364244	4.666684	3.572272	4.790807	3.904426	3.029761	3.287257	3.104560	3.126509	2.922081	2.962547	2.864434	3.173699	2.944808	3.045148	2.902569	2.989987	4.420677

SNHG1	10.681237	9.492544	10.134213	10.735940	11.715283	10.697641	9.946044	9.191963	10.421638	10.655479	11.456977	10.225601	10.299127	10.404862	10.470130	10.610513	11.315292	10.331758	10.870667	10.581752	9.749888	10.407551	10.281537	10.667256	10.934280	10.868443	10.561010	9.656270	11.446299	10.480362	11.701916	10.331003	10.608316	11.552438	9.980904	10.252933	10.039917	9.156149	9.267511	9.331691	10.888202	9.233160	9.564026	9.866387	9.351997	10.256188	10.269429	9.424072	11.222672	9.428363	9.942734	9.982457	9.584068	9.269835	10.734171	10.677766	10.123658	9.689297	9.499476	9.907428	8.757154	10.746036	9.036791	9.797967	9.371801	8.987164	9.514711	9.236897	9.384364	9.904700	9.198437	10.087299	10.980857	9.569803	9.795202	10.292953	10.068114	10.144304	9.646619	9.107647	10.047711	10.417045	10.063327	9.372256	9.878002	10.522222	10.147175	10.098434	9.481049	9.428670	9.394972	9.772809	9.256358	9.994317	10.228938	10.939616	11.059231	11.602276	11.040450	11.239944	11.295264	9.803650	11.046049	9.770821	11.241643	11.579521	9.849435	10.929785	11.316305	11.106019	10.534263	11.042718	10.861307	11.063922	11.610786	9.548217	10.048346	11.201144	11.123986	12.712770	10.942565	11.122655	12.140038	10.897879	10.947592	11.245085	12.157572	12.734213	11.775928	12.360678	11.210392	10.966557	11.033295	10.847998	11.062749	11.930467	11.852632	12.833456	11.123986	10.516533	11.018683	10.436000	10.741254	10.319671	11.129584	9.230297	11.933422	12.044855	10.354602	11.867283	11.913539	10.797903	12.520550	10.541990	11.281809	10.236515	9.310654	10.507219	10.316237	12.088879	9.791905	10.565026	11.174150	9.797029	11.101499	11.494063	10.015392	11.529216	9.568410	10.447656	11.168109	11.211287	9.741981	10.216258	10.606523	10.881641	10.788836	11.277927	10.194475	10.548349	10.880917	10.847104	10.471176	10.487647	11.198701	10.803872	9.666547	10.326735	10.000412	10.775932	10.499717	11.495782	10.890139	10.046469	10.555231	10.958864	12.002196	10.118547	8.917875	10.651028	10.824882	10.786473	10.841416	10.846071	11.211714	11.093256	11.852470	9.931696	10.330850	10.511848	10.710821	9.882399	10.882081	10.365777	11.862005	11.345196	12.331121	12.493699	11.665969	11.698422	12.484861	11.334043	10.096406	11.029556	11.417099	12.824882	11.258585	10.349010	10.105230	10.730156	10.632170	12.767266	10.768791	11.465983	12.180168	10.345517	12.375982	11.695777

CR382287.2	4.486790	3.041027	2.920166	3.595556	6.090441	3.990448	3.943048	4.695153	4.468831	4.143033	3.812518	3.395399	4.785361	3.428989	3.067732	3.524206	4.302412	4.389969	4.909682	4.168613	6.589823	3.607047	3.213805	4.026036	4.355484	3.860653	3.220831	4.687185	3.797760	3.921822	3.964886	5.261864	5.927411	4.030399	5.327319	3.853359	4.235090	3.043520	3.084646	2.989222	3.263968	3.008249	3.275450	3.800298	3.358117	6.210495	4.145985	3.470503	3.712529	5.017104	4.569162	5.000684	3.385944	5.161480	3.310373	4.621238	5.048938	3.565890	5.004761	3.502531	3.237557	3.821455	3.397663	3.465216	3.607047	3.486614	3.658843	3.412911	3.058228	2.988326	3.376527	3.227892	3.554886	3.849937	3.871346	3.177342	3.621667	3.418786	3.206265	4.380153	4.194775	3.557966	3.035867	5.331441	4.126791	4.965626	4.298965	3.923290	3.777746	4.170794	3.072161	3.548394	3.245109	3.949435	3.786037	3.546768	4.056076	3.416555	6.250803	4.703861	4.542873	3.741588	3.762565	3.608224	4.232435	3.730048	2.914475	3.706665	4.659010	3.588064	4.128728	4.620829	4.654176	4.225364	4.139301	6.131542	4.592522	4.147681	3.006528	5.121711	3.078635	3.897142	3.999384	6.696363	4.429095	4.201853	5.649730	4.607995	5.257705	3.091624	4.314773	3.728814	3.778359	4.054951	3.995950	3.230761	4.546670	5.692478	3.998522	6.226232	5.541080	3.122398	4.230278	4.699871	3.421359	3.993947	4.630254	3.854056	3.366686	3.499062	4.016060	4.779673	3.813152	5.412057	4.260212	3.391393	3.028867	3.102314	3.474326	3.775101	2.952031	3.329538	4.281894	3.507239	6.252648	3.713806	3.402485	3.251818	3.859821	3.491185	4.875692	4.573081	5.089598	3.456418	3.420660	4.627372	5.216032	3.414177	4.430265	3.088427	4.359159	3.412024	4.899401	4.124217	3.238755	3.210048	3.745674	3.871553	2.988516	3.944424	3.548911	4.569828	4.464978	3.584548	3.475634	3.326945	3.264238	3.298088	3.882327	4.332885	3.589021	4.204289	4.701513	4.226166	4.975882	3.840068	4.746622	3.588064	3.835677	3.423840	4.358295	3.643095	3.893314	3.583415	4.021312	3.828244	4.331849	4.561847	5.599318	4.445656	3.466886	5.980467	4.295899	4.592901	3.825737	3.153805	3.672690	3.477825	3.077933	4.573962	3.981983	6.006820	4.982780	6.001097	6.069980	3.455662	4.145791	3.444894

AC084816.1	3.326945	3.149253	3.042065	2.974245	2.882613	2.985598	3.109116	3.194860	4.220612	3.290448	3.296065	3.534455	3.148279	3.392489	3.070143	3.070785	3.395399	3.021680	3.918319	3.308901	3.907475	2.887669	3.560924	3.288016	3.064919	3.241792	3.251818	2.995806	3.309058	3.004352	3.203901	3.334678	3.150634	3.209242	3.922669	3.772832	4.281683	3.021771	3.246386	3.006886	3.223626	3.115938	3.288984	3.100092	3.168920	4.910183	3.126980	3.080392	3.282097	3.607047	3.667109	3.451718	4.104747	3.706468	3.571510	3.089474	3.862165	2.930628	3.342665	3.331266	3.214729	3.036549	3.517751	3.297133	3.247085	3.211164	2.961557	3.210306	3.311179	3.016470	2.831611	3.326945	3.022998	2.931044	3.024654	3.203211	3.073211	2.986272	2.919761	3.476705	3.199916	3.761533	3.224749	3.896934	3.299913	3.307903	3.470346	3.368081	3.152931	3.303975	3.115700	3.076284	3.251818	3.090316	2.984419	2.953929	3.643095	3.103544	3.004909	3.078033	3.148514	3.143964	3.848646	3.200632	4.095392	3.024459	3.150634	3.170619	3.351071	3.192946	2.885821	3.656029	3.500497	3.169763	3.034213	3.309708	3.196794	3.080099	3.075743	4.077019	3.219377	3.067061	5.753035	3.567103	3.234681	4.087235	3.098454	3.570765	4.455432	3.172384	4.835401	3.286382	3.048363	2.979346	2.924334	3.364521	4.300892	3.196648	3.171311	4.483961	2.858432	3.581299	5.455513	2.948842	2.885470	3.097736	2.917819	3.355689	3.292351	3.083277	3.273291	3.151870	3.010961	3.084037	3.573829	3.482074	4.890032	3.223899	3.130736	3.150513	5.358153	3.377742	4.005218	5.876401	4.414205	3.093371	6.649991	3.465570	3.710276	3.557169	3.314311	3.799007	3.778554	3.271619	3.254844	3.468038	2.939913	3.100195	3.132068	3.059814	3.112510	3.566480	3.410757	3.389455	3.332891	2.956615	3.442220	3.021968	3.049767	6.967094	7.420070	3.431343	2.923134	2.966030	3.175351	3.001687	3.006162	4.488254	3.125532	3.445079	3.845666	3.177467	3.492579	3.421018	3.286678	3.133804	8.250047	3.053347	3.226592	4.494685	3.060451	3.094537	3.126623	3.076909	2.977873	3.199768	3.094426	3.301128	3.126980	3.070785	3.070885	9.067544	3.449554	4.520611	3.494456	3.180009	3.146768	3.224190	3.030591	2.857822	3.705487	5.343992	3.132332	2.965075	3.059943	4.098850	3.002049	3.146903

AC087235.2	3.326945	3.149253	3.042065	2.974245	2.882613	2.985598	3.109116	3.194860	4.220612	3.290448	3.296065	3.534455	3.148279	3.392489	3.070143	3.070785	3.395399	3.021680	3.918319	3.308901	3.907475	2.887669	3.560924	3.288016	3.064919	3.241792	3.251818	2.995806	3.309058	3.004352	3.203901	3.334678	3.150634	3.209242	3.922669	3.772832	4.281683	3.021771	3.246386	3.006886	3.223626	3.115938	3.288984	3.100092	3.168920	4.910183	3.126980	3.080392	3.282097	3.607047	3.667109	3.451718	4.104747	3.706468	3.571510	3.089474	3.862165	2.930628	3.342665	3.331266	3.214729	3.036549	3.517751	3.297133	3.247085	3.211164	2.961557	3.210306	3.311179	3.016470	2.831611	3.326945	3.022998	2.931044	3.024654	3.203211	3.073211	2.986272	2.919761	3.476705	3.199916	3.761533	3.224749	3.896934	3.299913	3.307903	3.470346	3.368081	3.152931	3.303975	3.115700	3.076284	3.251818	3.090316	2.984419	2.953929	3.643095	3.103544	3.004909	3.078033	3.148514	3.143964	3.848646	3.200632	4.095392	3.024459	3.150634	3.170619	3.351071	3.192946	2.885821	3.656029	3.500497	3.169763	3.034213	3.309708	3.196794	3.080099	3.075743	4.077019	3.219377	3.067061	5.753035	3.567103	3.234681	4.087235	3.098454	3.570765	4.455432	3.172384	4.835401	3.286382	3.048363	2.979346	2.924334	3.364521	4.300892	3.196648	3.171311	4.483961	2.858432	3.581299	5.455513	2.948842	2.885470	3.097736	2.917819	3.355689	3.292351	3.083277	3.273291	3.151870	3.010961	3.084037	3.573829	3.482074	4.890032	3.223899	3.130736	3.150513	5.358153	3.377742	4.005218	5.876401	4.414205	3.093371	6.649991	3.465570	3.710276	3.557169	3.314311	3.799007	3.778554	3.271619	3.254844	3.468038	2.939913	3.100195	3.132068	3.059814	3.112510	3.566480	3.410757	3.389455	3.332891	2.956615	3.442220	3.021968	3.049767	6.967094	7.420070	3.431343	2.923134	2.966030	3.175351	3.001687	3.006162	4.488254	3.125532	3.445079	3.845666	3.177467	3.492579	3.421018	3.286678	3.133804	8.250047	3.053347	3.226592	4.494685	3.060451	3.094537	3.126623	3.076909	2.977873	3.199768	3.094426	3.301128	3.126980	3.070785	3.070885	9.067544	3.449554	4.520611	3.494456	3.180009	3.146768	3.224190	3.030591	2.857822	3.705487	5.343992	3.132332	2.965075	3.059943	4.098850	3.002049	3.146903

AC087235.1	3.326945	3.149253	3.042065	2.974245	2.882613	2.985598	3.109116	3.194860	4.220612	3.290448	3.296065	3.534455	3.148279	3.392489	3.070143	3.070785	3.395399	3.021680	3.918319	3.308901	3.907475	2.887669	3.560924	3.288016	3.064919	3.241792	3.251818	2.995806	3.309058	3.004352	3.203901	3.334678	3.150634	3.209242	3.922669	3.772832	4.281683	3.021771	3.246386	3.006886	3.223626	3.115938	3.288984	3.100092	3.168920	4.910183	3.126980	3.080392	3.282097	3.607047	3.667109	3.451718	4.104747	3.706468	3.571510	3.089474	3.862165	2.930628	3.342665	3.331266	3.214729	3.036549	3.517751	3.297133	3.247085	3.211164	2.961557	3.210306	3.311179	3.016470	2.831611	3.326945	3.022998	2.931044	3.024654	3.203211	3.073211	2.986272	2.919761	3.476705	3.199916	3.761533	3.224749	3.896934	3.299913	3.307903	3.470346	3.368081	3.152931	3.303975	3.115700	3.076284	3.251818	3.090316	2.984419	2.953929	3.643095	3.103544	3.004909	3.078033	3.148514	3.143964	3.848646	3.200632	4.095392	3.024459	3.150634	3.170619	3.351071	3.192946	2.885821	3.656029	3.500497	3.169763	3.034213	3.309708	3.196794	3.080099	3.075743	4.077019	3.219377	3.067061	5.753035	3.567103	3.234681	4.087235	3.098454	3.570765	4.455432	3.172384	4.835401	3.286382	3.048363	2.979346	2.924334	3.364521	4.300892	3.196648	3.171311	4.483961	2.858432	3.581299	5.455513	2.948842	2.885470	3.097736	2.917819	3.355689	3.292351	3.083277	3.273291	3.151870	3.010961	3.084037	3.573829	3.482074	4.890032	3.223899	3.130736	3.150513	5.358153	3.377742	4.005218	5.876401	4.414205	3.093371	6.649991	3.465570	3.710276	3.557169	3.314311	3.799007	3.778554	3.271619	3.254844	3.468038	2.939913	3.100195	3.132068	3.059814	3.112510	3.566480	3.410757	3.389455	3.332891	2.956615	3.442220	3.021968	3.049767	6.967094	7.420070	3.431343	2.923134	2.966030	3.175351	3.001687	3.006162	4.488254	3.125532	3.445079	3.845666	3.177467	3.492579	3.421018	3.286678	3.133804	8.250047	3.053347	3.226592	4.494685	3.060451	3.094537	3.126623	3.076909	2.977873	3.199768	3.094426	3.301128	3.126980	3.070785	3.070885	9.067544	3.449554	4.520611	3.494456	3.180009	3.146768	3.224190	3.030591	2.857822	3.705487	5.343992	3.132332	2.965075	3.059943	4.098850	3.002049	3.146903

PRICKLE2-DT	6.842711	5.545245	5.143570	5.489572	5.006189	5.665927	5.519185	5.356278	5.596256	6.233243	6.074144	5.387388	6.746880	5.466568	4.766147	5.653503	5.018110	5.289796	4.762913	5.160301	6.605904	4.791432	5.611223	5.163669	5.479452	5.217371	5.966988	5.357782	5.720507	6.049931	4.858150	7.333524	7.035286	5.905442	6.572438	5.479452	5.264485	5.511823	5.438382	5.371883	6.272853	5.769576	5.856380	4.887925	5.070383	4.679009	5.854373	3.276032	4.486585	5.718684	5.794931	6.207268	5.940259	6.373313	5.275329	5.770690	5.466381	5.630622	5.627815	5.723646	5.946038	5.780149	5.576839	5.168681	5.777263	5.031543	5.836243	5.448593	5.655947	4.960000	5.133206	5.425386	6.307251	5.650665	5.584588	5.874564	5.504866	5.502197	5.216797	5.345357	5.664600	5.232211	6.157532	4.891666	5.865953	4.557891	6.058527	3.530520	4.789101	5.867615	5.328913	4.650918	5.675995	5.945313	6.026064	5.399411	5.527477	5.642156	5.381734	5.564660	5.261064	5.557143	5.467155	5.319391	3.798195	6.498399	5.629533	5.067361	5.164064	6.300519	4.052798	4.458635	4.554340	6.093732	5.407741	5.482767	6.986518	5.698593	6.046222	6.926972	8.295916	8.609236	6.429015	6.344049	6.648533	8.006998	6.402607	6.841609	7.521929	7.854689	6.837369	7.915822	8.003407	7.388100	5.926480	7.813738	7.482906	7.655283	7.141750	7.200082	8.631915	7.418369	6.330944	9.007324	7.427119	6.907201	7.098623	6.352600	7.828383	7.021874	7.563478	7.856045	7.488204	7.506641	7.774143	8.781348	7.981127	7.077503	9.549005	6.997424	8.003407	8.647397	7.369988	7.522964	5.765379	7.840776	6.626941	5.005573	8.893945	8.101283	7.967712	8.799601	8.414729	10.110653	6.516905	8.362863	8.601123	7.470168	7.895271	8.737317	5.972886	7.412132	6.524815	8.707044	9.244547	9.062320	7.796942	7.912658	8.573058	7.510460	8.286403	6.497681	8.752584	7.616362	7.019054	8.801425	9.105974	7.243665	8.472487	8.791374	6.954230	8.472666	9.271071	9.623390	8.205706	8.825386	7.418684	8.647397	9.648405	9.628003	7.748119	8.133925	9.092054	7.258046	8.442840	7.889501	8.128115	7.763900	6.604877	8.580581	6.191665	7.293973	8.297612	7.418684	6.368043	6.463552	4.535468	6.123548	7.154507	6.742879	8.057849	4.947941	6.248977	6.117672	7.039382	9.131674	6.807255	6.745450

MIRLET7BHG	5.508966	6.866135	6.756707	6.480893	6.225189	6.633215	7.065889	7.019538	7.112068	6.905093	6.977224	6.562527	6.758287	6.933776	6.927315	6.457889	6.961969	6.307584	7.046755	6.821815	6.679972	7.541740	6.494756	6.175587	6.626777	7.472343	6.006620	6.576865	6.741127	6.617903	7.225595	5.892768	7.375801	6.242113	6.917878	6.378641	6.813501	6.982244	6.864533	7.122191	6.974477	6.126600	6.045883	6.474239	6.292024	5.211625	6.627907	6.763922	6.172347	6.497368	6.797498	5.732486	6.341705	6.752407	6.637206	6.608020	6.529995	6.835143	6.867257	6.974477	7.019371	6.913084	4.726203	6.952479	6.552682	6.796039	6.783224	6.640307	6.529154	7.347870	6.591465	6.725739	7.275617	6.619878	6.858980	6.923207	6.869820	6.312471	6.854940	6.800254	5.558123	6.200659	6.535659	7.196624	6.612494	6.724651	6.787255	6.862022	6.805681	7.059481	6.902396	6.938890	6.458853	6.280919	5.809052	6.535813	5.854737	7.014515	6.720151	6.052527	6.698541	6.349761	6.851096	6.930487	7.983341	6.530462	7.715828	6.835477	7.267429	7.328515	6.989840	6.795886	6.310424	7.317927	6.993391	5.006417	5.880879	6.831098	6.293010	5.771071	6.100838	7.566739	6.521545	6.203457	6.055134	5.323637	7.205967	6.344566	8.268065	7.336125	6.129339	7.086749	4.637149	6.785005	6.890454	6.266717	6.255895	7.201165	6.353937	7.367653	6.916640	6.061533	6.257931	5.722901	5.397296	6.172854	6.485786	5.259268	5.679570	6.336194	5.059824	5.835708	6.269286	6.847871	5.163258	5.968636	5.190332	6.558766	6.780384	6.143590	5.541466	7.886135	6.093378	5.270656	4.734819	5.831224	5.411685	5.111332	5.347947	5.043266	6.735311	5.028887	4.523456	5.552010	5.228700	6.137363	6.401780	5.343795	6.307584	6.019364	7.694590	5.861216	6.234130	5.580774	5.330860	5.494698	6.023595	5.726824	6.325088	5.943900	6.921278	5.857270	5.714385	4.857743	5.747776	6.413207	7.808179	7.339745	4.844015	6.393549	5.905080	5.667961	5.191894	5.564851	5.792544	5.835534	5.593652	5.589666	5.812038	6.441696	6.468191	5.886723	5.695283	6.508622	5.906838	6.626777	5.783088	5.879425	5.734458	4.717095	6.547569	6.767805	3.960518	5.036312	7.241488	6.707390	6.360544	6.714928	6.501718	5.728993	4.467997	5.433353	7.422893	6.430211	5.072591	6.150019	7.772016	5.286594

AP000866.2	8.461246	8.410780	9.028725	9.355751	8.805380	9.735468	9.999817	9.738061	10.517868	10.742902	9.985804	9.488725	9.432672	9.462413	8.222845	10.019135	9.600645	9.715792	9.360471	9.338762	9.228667	10.045094	5.765730	9.404197	9.155715	8.884828	6.423652	10.421948	8.035707	8.430079	10.532207	8.887749	9.914854	7.048016	9.166044	9.747720	10.507219	9.108933	8.826140	10.023382	9.145331	8.710094	8.910089	8.341313	8.063024	8.396396	9.594640	8.788881	10.293860	9.202959	9.125500	9.773557	8.564352	9.635352	9.167413	9.213755	9.052657	9.572296	9.085022	9.108933	9.266415	9.099030	6.055659	9.099653	7.491020	8.397928	8.813057	8.680653	8.809382	7.606999	9.602061	9.743643	10.872332	10.082987	9.206285	8.963120	9.096895	8.818720	8.633684	8.507070	8.533318	8.512159	8.681905	9.735158	10.269429	8.979397	9.803484	9.866241	7.582572	8.801425	8.673596	8.076966	8.300613	9.869632	8.076365	8.811379	9.569977	9.505192	9.568588	9.224522	9.679253	9.362458	9.217443	10.257870	9.388703	9.742594	8.937648	8.516857	6.494605	10.064686	8.022090	9.394814	9.123377	10.014459	10.561160	9.244855	7.973665	8.284523	9.440177	6.876695	6.467545	7.259980	7.684592	7.215455	8.133925	7.928181	7.017480	7.251854	8.525229	7.717365	7.652378	7.787607	6.853497	5.935986	8.554202	7.255549	7.316534	5.828417	7.568694	6.395163	6.645149	5.074842	6.673815	6.129339	6.609006	7.053011	7.340680	6.689974	7.077190	7.877938	7.712816	8.239020	7.287083	7.685039	8.749391	8.724336	7.128933	6.403592	7.153563	7.452599	6.140652	7.163467	6.342844	7.240393	8.000883	7.988792	7.177645	8.769297	7.792640	7.575127	8.162811	7.540966	8.204934	7.757898	6.981935	7.573089	7.830076	6.286020	7.327719	6.269984	7.306185	4.609293	6.125492	5.877440	6.293351	5.784124	6.872117	6.760447	6.005942	8.154232	8.179476	9.167845	8.375886	7.610371	7.446825	7.419924	7.467417	7.801746	6.922099	6.885035	8.326365	7.662653	8.075009	8.590359	7.992398	7.645417	8.099606	5.003178	5.989000	7.268489	6.288269	6.206594	8.054626	7.250881	7.452118	8.072573	8.184337	7.602495	7.048649	7.786365	7.639530	8.411859	8.549976	6.787592	8.361249	5.398817	6.346393	6.231727	8.124795	4.894812	5.495885	8.111918	9.330440	8.869138	7.055251	6.140472	7.006419	7.368750

AC013268.5	8.252070	14.371236	10.653826	13.478390	12.904642	13.922401	13.261024	13.717490	13.651246	14.223759	13.743835	13.812235	12.804235	13.242390	9.221723	14.228894	12.905702	13.217987	10.876200	14.309339	10.453574	11.606379	10.149488	14.140840	13.115442	13.709428	9.249046	12.099673	12.988517	8.101596	13.312973	10.270415	11.538224	8.900377	15.130285	12.957538	11.777298	12.715740	11.104426	12.886695	14.417635	11.560840	8.755780	9.696489	8.047176	8.631600	10.935332	9.983819	12.579997	9.707507	9.118833	9.850742	10.053909	11.213657	8.784001	13.631128	9.585307	9.967880	10.096988	10.378348	10.528515	13.336430	8.930003	10.375445	9.933063	11.970814	12.352099	10.844879	11.115175	11.312768	13.606202	14.858502	13.644958	13.217148	13.481256	11.346253	12.555759	13.582741	12.742336	11.156994	11.836026	11.345331	9.388107	12.761183	13.261809	11.842489	10.465042	10.630402	9.889071	10.915674	10.980565	9.245828	10.289132	12.666814	10.549347	10.901565	12.003376	9.726547	10.985679	12.766382	11.701767	12.533636	12.559575	12.639201	10.101089	12.443196	11.942584	9.952432	10.120507	13.959663	10.657248	12.807024	10.127268	13.876318	13.041185	12.699016	8.113605	13.867818	13.063034	7.621512	10.558425	7.656558	9.365664	9.528007	12.166779	9.459408	8.610158	8.512310	9.291287	9.367251	8.823853	8.801575	8.773068	8.249148	11.704750	8.534396	9.035428	7.709719	9.150952	8.090173	9.290649	8.747578	9.734545	8.646696	9.488302	12.634617	8.833978	9.015016	7.729193	8.541028	7.826786	8.314845	9.609659	10.193562	9.854464	9.060051	10.096699	8.938682	8.955174	10.111087	8.715790	7.305553	7.965553	7.979950	8.533168	9.937753	7.684131	9.571980	7.907411	7.678459	7.442516	10.785271	8.025893	6.927150	8.428190	7.093370	7.447439	8.067877	8.194634	7.852039	8.503550	8.014946	10.184971	7.470168	8.137859	8.492818	9.286431	8.462927	8.836481	10.749156	11.000620	12.066401	9.003351	8.336025	9.715472	8.763170	9.143060	11.602276	7.997958	8.715941	9.693891	7.928954	8.678828	8.502324	8.228265	8.092170	7.256786	7.795248	7.810427	7.803857	8.433072	8.450697	8.618293	8.592063	8.162959	8.287044	7.431568	8.136815	7.994571	8.050788	8.249009	8.621435	8.423166	8.171766	9.357553	8.113151	8.088467	10.163835	10.660235	8.974375	8.490868	8.078697	13.652481	8.358078	9.249360	9.041181	9.204578	8.445017

AC140479.4	8.252070	14.371236	10.653826	13.478390	12.904642	13.922401	13.261024	13.717490	13.651246	14.223759	13.743835	13.812235	12.804235	13.242390	9.221723	14.228894	12.905702	13.217987	10.876200	14.309339	10.453574	11.606379	10.149488	14.140840	13.115442	13.709428	9.249046	12.099673	12.988517	8.101596	13.312973	10.270415	11.538224	8.900377	15.130285	12.957538	11.777298	12.715740	11.104426	12.886695	14.417635	11.560840	8.755780	9.696489	8.047176	8.631600	10.935332	9.983819	12.579997	9.707507	9.118833	9.850742	10.053909	11.213657	8.784001	13.631128	9.585307	9.967880	10.096988	10.378348	10.528515	13.336430	8.930003	10.375445	9.933063	11.970814	12.352099	10.844879	11.115175	11.312768	13.606202	14.858502	13.644958	13.217148	13.481256	11.346253	12.555759	13.582741	12.742336	11.156994	11.836026	11.345331	9.388107	12.761183	13.261809	11.842489	10.465042	10.630402	9.889071	10.915674	10.980565	9.245828	10.289132	12.666814	10.549347	10.901565	12.003376	9.726547	10.985679	12.766382	11.701767	12.533636	12.559575	12.639201	10.101089	12.443196	11.942584	9.952432	10.120507	13.959663	10.657248	12.807024	10.127268	13.876318	13.041185	12.699016	8.113605	13.867818	13.063034	7.621512	10.558425	7.656558	9.365664	9.528007	12.166779	9.459408	8.610158	8.512310	9.291287	9.367251	8.823853	8.801575	8.773068	8.249148	11.704750	8.534396	9.035428	7.709719	9.150952	8.090173	9.290649	8.747578	9.734545	8.646696	9.488302	12.634617	8.833978	9.015016	7.729193	8.541028	7.826786	8.314845	9.609659	10.193562	9.854464	9.060051	10.096699	8.938682	8.955174	10.111087	8.715790	7.305553	7.965553	7.979950	8.533168	9.937753	7.684131	9.571980	7.907411	7.678459	7.442516	10.785271	8.025893	6.927150	8.428190	7.093370	7.447439	8.067877	8.194634	7.852039	8.503550	8.014946	10.184971	7.470168	8.137859	8.492818	9.286431	8.462927	8.836481	10.749156	11.000620	12.066401	9.003351	8.336025	9.715472	8.763170	9.143060	11.602276	7.997958	8.715941	9.693891	7.928954	8.678828	8.502324	8.228265	8.092170	7.256786	7.795248	7.810427	7.803857	8.433072	8.450697	8.618293	8.592063	8.162959	8.287044	7.431568	8.136815	7.994571	8.050788	8.249009	8.621435	8.423166	8.171766	9.357553	8.113151	8.088467	10.163835	10.660235	8.974375	8.490868	8.078697	13.652481	8.358078	9.249360	9.041181	9.204578	8.445017

AC112229.4	8.252070	14.371236	10.653826	13.478390	12.904642	13.922401	13.261024	13.717490	13.651246	14.223759	13.743835	13.812235	12.804235	13.242390	9.221723	14.228894	12.905702	13.217987	10.876200	14.309339	10.453574	11.606379	10.149488	14.140840	13.115442	13.709428	9.249046	12.099673	12.988517	8.101596	13.312973	10.270415	11.538224	8.900377	15.130285	12.957538	11.777298	12.715740	11.104426	12.886695	14.417635	11.560840	8.755780	9.696489	8.047176	8.631600	10.935332	9.983819	12.579997	9.707507	9.118833	9.850742	10.053909	11.213657	8.784001	13.631128	9.585307	9.967880	10.096988	10.378348	10.528515	13.336430	8.930003	10.375445	9.933063	11.970814	12.352099	10.844879	11.115175	11.312768	13.606202	14.858502	13.644958	13.217148	13.481256	11.346253	12.555759	13.582741	12.742336	11.156994	11.836026	11.345331	9.388107	12.761183	13.261809	11.842489	10.465042	10.630402	9.889071	10.915674	10.980565	9.245828	10.289132	12.666814	10.549347	10.901565	12.003376	9.726547	10.985679	12.766382	11.701767	12.533636	12.559575	12.639201	10.101089	12.443196	11.942584	9.952432	10.120507	13.959663	10.657248	12.807024	10.127268	13.876318	13.041185	12.699016	8.113605	13.867818	13.063034	7.621512	10.558425	7.656558	9.365664	9.528007	12.166779	9.459408	8.610158	8.512310	9.291287	9.367251	8.823853	8.801575	8.773068	8.249148	11.704750	8.534396	9.035428	7.709719	9.150952	8.090173	9.290649	8.747578	9.734545	8.646696	9.488302	12.634617	8.833978	9.015016	7.729193	8.541028	7.826786	8.314845	9.609659	10.193562	9.854464	9.060051	10.096699	8.938682	8.955174	10.111087	8.715790	7.305553	7.965553	7.979950	8.533168	9.937753	7.684131	9.571980	7.907411	7.678459	7.442516	10.785271	8.025893	6.927150	8.428190	7.093370	7.447439	8.067877	8.194634	7.852039	8.503550	8.014946	10.184971	7.470168	8.137859	8.492818	9.286431	8.462927	8.836481	10.749156	11.000620	12.066401	9.003351	8.336025	9.715472	8.763170	9.143060	11.602276	7.997958	8.715941	9.693891	7.928954	8.678828	8.502324	8.228265	8.092170	7.256786	7.795248	7.810427	7.803857	8.433072	8.450697	8.618293	8.592063	8.162959	8.287044	7.431568	8.136815	7.994571	8.050788	8.249009	8.621435	8.423166	8.171766	9.357553	8.113151	8.088467	10.163835	10.660235	8.974375	8.490868	8.078697	13.652481	8.358078	9.249360	9.041181	9.204578	8.445017

LINC01094	4.994379	6.986041	6.879422	7.658461	5.722901	7.984691	8.565393	6.276841	6.644356	7.964808	8.651326	7.858176	8.262928	8.291988	7.254455	8.464289	7.927415	7.621942	7.683351	9.221273	6.484247	8.839797	4.356108	7.459505	6.854459	7.808333	5.573354	7.906479	7.403034	7.753321	7.438844	8.192912	8.692789	6.609186	8.005298	7.544235	8.348402	7.921791	8.455053	7.115031	7.259489	7.609133	7.381102	7.618765	7.472188	7.837640	6.989679	8.014625	7.644929	9.022623	8.431048	8.748190	8.039959	9.360161	7.800225	8.031179	8.159472	7.429167	7.922249	7.906928	7.820856	6.756872	6.232252	6.709750	7.168420	9.037684	6.739884	6.849652	7.512190	6.356984	8.115176	7.352520	7.581181	7.933104	8.526133	7.823834	6.725278	8.228419	7.679523	8.452011	8.280995	8.728680	8.795216	8.652502	9.238440	8.336333	9.695569	8.387119	7.026996	8.361560	7.229212	6.958315	7.869407	7.842645	7.613722	6.991111	8.060764	7.541740	7.908033	7.078474	8.026381	7.808028	6.885835	7.721928	7.922567	8.012742	6.944562	6.446864	6.762969	8.395457	7.386991	6.908604	6.625506	7.021726	7.361695	7.680852	4.468831	7.819523	6.709258	7.851415	8.370503	8.267776	8.043864	5.483876	7.833540	8.202867	4.981730	6.407052	7.388100	8.212526	5.534087	6.845868	7.701899	5.638458	7.126261	6.612494	6.136033	7.120590	8.844814	7.596551	9.097364	6.424977	6.462676	6.422546	10.075427	7.905055	7.094491	6.769290	8.376902	5.174138	8.729876	8.824643	7.289877	8.782122	8.170666	10.238782	7.391797	8.743900	9.362915	7.269237	8.168619	8.972481	7.987181	9.107963	8.295296	6.207268	7.512950	7.936696	9.765127	9.637166	8.426149	9.212057	9.093884	10.384099	8.170501	9.089471	7.338924	7.545491	8.425112	9.525056	6.472916	7.285661	7.436154	8.020441	10.016190	8.110438	8.925815	8.492438	8.496279	8.184054	8.669426	6.504481	9.994317	9.225910	8.861847	8.896716	8.713136	8.242624	8.813362	9.926202	8.389280	10.497236	9.791422	9.951687	7.660474	9.853381	8.378322	8.344605	9.435623	6.852320	8.131646	8.055590	8.342169	9.544271	8.381455	7.628611	6.928920	8.500123	7.457457	6.758456	6.016047	8.375568	8.309911	6.716203	5.988091	6.469852	6.696046	6.761889	7.592104	6.775132	6.172188	4.663287	7.268489	6.363905	7.725286	9.268924	6.210351	6.124083

AC020978.5	11.992764	11.848789	11.828254	11.854611	12.290442	12.085291	11.831507	12.166049	11.410967	12.198437	12.009153	11.914892	11.815237	12.289811	12.157109	11.969197	11.736869	12.380596	12.181582	12.159874	12.075900	12.406816	11.687522	12.245455	11.985717	12.025076	10.648931	12.004151	11.356245	11.378727	11.583914	11.747480	11.899574	11.223559	11.491895	11.602116	11.781940	11.653194	11.824728	11.847129	12.053308	11.557144	11.798213	11.679352	11.477840	12.104683	11.344019	11.684367	11.508636	11.886584	11.727221	12.038357	11.958138	11.921595	11.571480	11.485173	12.038055	12.196619	12.005642	12.138794	11.993505	11.733178	10.903357	11.800594	11.622188	11.709681	11.756489	11.944984	11.798654	11.630009	11.228140	11.491752	11.998089	11.494646	11.563886	11.556994	11.782817	11.031948	11.437253	11.938632	11.888248	11.960368	11.822458	12.005028	12.206509	11.980478	11.921437	12.109967	12.069772	12.247833	12.001025	12.177192	11.518703	11.561877	11.408446	11.807983	11.525597	11.807225	11.435242	11.354634	11.803385	11.594601	11.646067	11.934741	11.931223	11.677583	12.461067	11.925195	12.524622	12.131274	12.409998	12.090726	12.407456	12.157109	11.771231	10.982333	11.985558	11.769600	11.491895	11.461731	11.504078	11.491172	11.990332	11.161418	11.091598	11.382618	10.019581	11.144558	10.650447	10.797016	10.824581	11.326759	11.020219	10.512879	10.470285	10.748602	11.355342	11.589071	10.589660	11.350698	11.021233	11.481782	10.345672	10.969060	11.286882	10.870667	11.731380	9.976663	10.982768	10.038406	11.553299	11.261231	11.157450	10.858234	10.869032	11.127532	11.382901	11.147376	10.854176	11.572813	11.301597	11.325112	11.524738	11.620543	11.290061	10.804025	11.627063	11.680252	11.472234	11.264402	11.534520	11.209109	11.216410	11.595221	11.012272	10.756632	11.119415	11.658633	11.147807	12.216235	11.287162	10.293094	11.603148	11.545153	11.767589	11.735962	11.256930	11.429723	11.208087	11.372450	11.294265	10.796867	11.102224	10.716860	10.933379	11.044022	11.253671	10.817007	11.305292	11.978981	11.016776	11.967186	10.967556	10.961636	10.712414	11.753214	11.869342	11.701767	11.316160	11.758886	11.781799	11.350100	11.192513	11.227693	11.143538	11.150075	9.109255	10.693627	10.931284	10.286805	11.065879	9.887355	10.880771	11.925502	11.259156	11.323781	12.295577	11.743921	11.377693	11.758300	11.245386	11.452687	11.180344	11.545765	11.276591	11.740264	12.074208	11.021682

AL008633.1	10.227757	11.412815	11.421630	10.792768	10.157573	10.802540	11.859940	9.596485	8.472017	10.932789	10.320604	10.557792	9.669565	9.780111	9.645034	11.676671	11.654225	9.146408	8.498687	9.555229	9.394200	9.281361	11.048690	9.602218	11.552884	10.954078	12.835254	10.882081	12.619138	11.993060	9.701575	10.015875	8.917093	10.700036	11.310881	11.012393	10.944749	11.723934	10.445361	10.017560	12.091066	9.962417	9.028725	8.048145	9.336944	7.963443	13.555264	8.786399	10.609210	8.638314	8.805963	9.521604	10.224367	9.726052	10.996548	12.604579	9.933222	10.359182	9.051877	10.401393	9.826780	12.384645	12.893478	10.333451	9.936636	10.859681	10.752065	10.829303	9.431265	10.033321	12.372577	11.983360	12.126285	12.346315	11.311749	10.886562	11.527049	11.169583	10.237443	9.753141	8.578920	9.400578	8.710861	9.903491	10.679735	9.938201	9.097199	9.127646	8.994100	9.021275	9.422207	8.330882	10.116828	10.636387	9.689605	10.292786	10.076034	9.095942	9.704158	12.334431	9.286114	11.943350	11.270443	11.123509	8.531668	11.471808	9.784596	9.265364	9.163229	12.345161	8.719047	8.197638	7.806501	11.439106	9.061101	8.025420	8.922407	11.429272	12.002806	9.001818	11.128729	11.599731	10.346580	9.680141	10.302329	10.689337	8.109811	8.503550	10.054805	9.893017	9.410032	10.889511	8.812917	9.133674	12.134176	10.242440	7.909883	8.463686	9.684031	8.757464	9.233645	9.523811	8.149158	11.007562	10.280806	11.659709	8.939279	11.186151	11.786428	9.834586	9.534899	8.489226	11.081157	10.146788	9.857725	10.959158	11.141336	10.214424	10.159386	11.332767	10.121103	8.513701	8.339807	8.389906	8.063817	12.303903	8.156082	9.269539	9.019430	8.873698	9.108604	9.803484	10.506905	10.949662	12.441914	9.657640	9.485652	8.642211	9.743120	9.573194	13.640603	10.521568	9.880715	9.457098	10.504236	10.397759	10.744255	9.748635	9.607325	11.690625	11.871889	11.126639	12.010184	11.256783	10.642988	11.629391	11.567413	10.649097	10.449732	8.517477	9.309567	8.575025	9.242889	10.279891	9.271368	9.470923	8.813641	9.053617	10.054496	8.921804	8.180535	10.579659	11.004092	10.248940	10.063327	8.864465	8.178407	8.749699	10.884311	9.080413	10.236834	11.336580	10.954214	7.653896	10.736390	7.981450	8.472319	8.652502	11.337301	9.069452	7.311187	7.199772	10.529215	8.258005	8.509216	9.450783	10.141048	11.447496

AP002433.1	5.297271	4.349640	4.647039	4.514021	5.421112	4.866626	4.306815	5.344585	4.469049	4.619929	4.746389	4.855423	4.483077	5.020329	3.491861	4.844870	5.297845	4.178750	5.603033	4.740420	4.118816	5.337135	3.258402	5.159068	4.661814	4.747061	5.396922	4.685452	5.337890	4.271174	3.807616	5.725140	4.855023	4.984779	5.040958	5.039903	4.392582	4.622899	4.299398	3.767157	4.472556	5.188817	4.235959	4.448914	4.117437	7.648612	4.254568	3.012411	3.460735	4.796480	2.776734	3.153195	3.650619	4.943402	5.085506	3.543602	4.935362	3.234824	3.283950	3.717722	4.154991	5.280737	4.064578	5.040502	4.701728	4.563169	3.076108	3.807012	4.712842	4.455844	4.412855	5.079803	4.688032	4.953347	5.057355	4.660922	5.155607	4.823549	4.738203	4.794770	4.175536	3.682876	5.146157	4.126095	5.333431	5.148333	5.989000	5.272910	3.704272	4.417080	4.657710	4.551105	4.149594	4.744674	4.105442	4.319940	3.936856	5.175767	3.608409	4.027744	4.273784	5.050399	4.774907	4.882433	4.410887	3.376527	5.101129	4.026873	4.844015	5.553579	4.760783	5.625960	5.558862	4.505663	4.400675	5.986915	5.012811	4.785984	4.956861	4.460269	4.434129	3.375800	4.085214	4.731657	4.870422	3.516786	4.805763	4.379733	3.671371	3.941720	3.725335	3.900869	3.460369	3.211011	4.938708	5.475801	2.996468	4.493786	5.201486	3.704272	4.152088	3.873530	4.702139	4.016060	4.199237	4.565758	4.648966	4.553238	3.671565	4.063276	4.725358	3.792701	3.362499	4.037145	4.916406	3.936663	3.799662	4.321641	4.006760	5.396922	5.198528	3.508161	4.674273	4.023218	7.410632	3.761949	2.906249	4.674939	5.434367	9.974679	3.948976	3.732755	4.283814	3.734389	3.468909	3.742824	3.180933	3.066834	3.685623	3.472563	4.233519	4.459073	4.795082	4.541985	3.668862	3.076108	3.660212	4.506771	3.438735	4.181660	4.307220	3.931529	4.188883	4.223393	4.313806	5.275329	5.017705	5.064071	4.120296	4.230073	3.940235	5.409314	5.009721	5.164897	5.150345	5.154192	5.329694	3.825314	4.315607	4.112927	4.969897	3.487711	4.450965	3.958796	3.580319	3.772413	4.553238	4.308348	3.209109	3.760284	4.791432	4.080720	4.134718	4.779250	4.542417	5.331849	3.991131	4.806580	4.175536	3.957225	4.889825	4.951226	5.571259	4.447326	4.859838	4.911553	4.250835	4.919147

AC005229.4	9.137166	9.178776	9.457550	9.132911	9.503319	9.057198	9.568410	9.027513	7.523256	9.511361	9.613720	9.193621	9.639042	10.313708	9.604383	9.126584	8.682812	9.532146	8.752885	9.664693	9.590255	9.062914	8.554809	9.345116	9.302196	8.581959	7.658000	9.141971	7.946920	8.812340	8.985318	10.392057	10.409077	10.088368	9.566202	9.760564	9.863172	9.914232	9.787591	9.376678	9.164645	9.244855	9.458339	9.569197	9.636997	10.110653	8.790729	9.974679	9.926051	9.609822	9.624778	10.007529	9.944110	10.240599	9.685477	9.141665	10.096550	7.459959	10.543604	10.242440	10.733854	8.837687	9.672549	9.714868	10.224521	10.028112	10.402004	10.040996	9.830616	10.128236	9.793162	9.533221	8.964318	9.571519	9.788347	10.105095	10.093062	9.901638	9.491581	10.163693	9.984899	9.770001	9.912045	10.193710	10.159669	9.431743	9.939807	10.397912	9.611185	10.123062	9.576365	9.758277	9.672854	10.420556	9.881488	9.753457	9.549440	8.541337	9.605154	5.463454	8.752444	9.397486	9.301410	9.851210	9.917940	9.021575	9.089320	9.552229	8.114390	8.545522	9.564664	9.230297	9.917791	7.963288	9.322580	6.167736	9.512151	8.900824	7.772925	10.060756	9.425577	9.707507	9.020946	7.761592	9.163879	9.748719	8.478522	10.082245	9.509923	10.116828	10.201399	10.281096	9.096285	9.995272	8.687597	9.133380	10.034852	8.695277	9.990936	8.462927	9.240911	9.581310	8.658361	11.701015	9.507776	9.482720	9.499786	7.907702	9.488884	8.729147	9.567009	10.750993	10.538071	10.516678	10.884929	10.266295	9.702796	10.389876	9.626936	8.595469	10.173802	10.422850	10.641213	10.307712	10.717161	9.933222	10.319358	10.567716	9.612400	10.261790	10.807688	10.750537	10.202804	10.618351	9.361550	11.158336	8.127674	10.241662	10.659325	11.086626	9.639817	11.037001	9.380969	10.958111	11.221771	11.241501	9.819657	9.592749	10.322128	10.620851	9.983054	7.713603	9.374473	11.234364	10.552501	10.830911	11.077806	10.010197	9.889366	10.090978	10.971621	10.319973	10.370786	9.936190	10.329500	10.293094	10.804025	9.543140	9.316100	10.402900	9.589153	9.567637	9.504559	9.631081	8.753340	10.333120	8.375113	9.740870	6.987008	9.286114	9.533695	8.834349	9.897941	10.115138	8.637723	9.940405	9.466126	9.346035	8.568119	9.673965	8.652353	10.592600	7.570365	9.742434	8.900824	10.005361	9.284604	8.827765

LINC02019	6.204845	7.325321	8.356138	7.575743	8.764975	7.824779	8.591764	6.417161	7.965718	8.088325	8.175380	7.965553	7.907878	8.116849	8.820386	8.212526	7.648004	8.095831	7.945100	7.833385	7.023277	5.819727	3.629032	8.206480	8.028251	7.661530	6.956874	6.177466	8.227663	7.432820	9.020946	8.141476	7.617692	7.506792	8.003880	7.951067	8.197496	7.607143	6.927488	8.342909	7.871824	7.396943	7.476764	7.927895	7.992068	7.665283	6.902551	7.990967	8.893154	7.708229	7.506496	8.399403	8.498831	8.155151	8.871708	7.702383	8.685393	6.874986	7.233701	8.268392	8.293964	7.873357	7.106131	6.799110	7.157464	7.798155	6.998180	8.149462	6.477985	6.844206	7.629887	7.420547	8.180684	8.203624	7.582572	5.532567	7.672732	7.652204	7.581658	8.516857	8.512310	9.142892	8.332304	8.620028	8.318651	8.749866	8.629123	8.969607	7.406798	7.403812	7.136551	8.295598	7.579650	8.155934	7.551749	8.564352	8.640834	8.582697	7.691812	4.874437	8.281762	7.878104	7.680418	7.751051	7.263718	8.483559	7.686842	6.961817	7.432347	8.335081	6.928586	8.584534	8.414729	6.979716	9.029740	5.767752	6.747882	8.582980	7.898797	6.047635	6.974953	6.748483	7.383627	3.979779	6.162362	6.191307	5.789212	7.688106	7.414619	6.310605	6.824965	5.975338	6.264178	5.158305	6.479464	5.810203	6.234645	6.328231	7.392716	4.798317	6.803991	5.102586	5.263095	6.704142	7.381539	5.845645	5.036312	4.011621	6.491525	6.177136	7.165954	5.570880	7.284456	6.831962	6.875792	7.258388	6.297403	5.769200	7.481057	4.831864	5.931759	6.608681	6.411368	7.167633	6.619192	4.293345	7.643849	7.401308	7.404747	6.356611	7.021413	8.455966	7.721135	7.236888	5.809630	7.147390	6.354258	6.016047	6.927973	7.338317	7.336280	5.470251	5.751387	6.541874	6.553169	5.756267	6.586878	5.916922	5.725915	7.567185	6.088523	6.209840	6.407620	6.767805	5.626559	6.410697	6.299387	7.219180	7.336449	7.712658	7.169475	7.733473	7.758541	8.332609	5.200892	7.158379	9.042079	7.574659	6.462508	4.140390	6.968033	6.185209	7.731485	6.706060	7.082213	6.043810	6.771256	5.429045	4.751493	5.743916	6.598614	5.238860	6.677686	5.202236	7.221454	7.994267	7.293017	7.322640	5.377319	6.012483	5.437645	4.042997	6.227294	5.896175	7.254455	6.512602	9.543634	6.213743

LINC02458	3.363192	3.402652	3.291768	3.148035	3.083946	3.529043	3.427251	3.578380	3.551628	3.502944	3.672690	3.793394	3.604453	4.063079	3.777553	3.447280	3.823956	3.445260	4.868318	3.238603	5.285197	3.732755	5.891332	2.903558	4.873219	4.011825	3.606610	3.198153	3.090545	3.090860	3.744640	4.300892	4.715607	4.215317	5.859782	4.307689	4.350669	3.410052	3.235083	3.104462	3.431869	3.637869	3.592821	3.677001	6.130528	5.407524	3.933686	3.123088	4.763580	4.222766	4.153649	4.011437	5.827654	4.618879	4.080720	3.097736	6.255038	6.064868	3.910122	3.617825	3.192699	3.641891	5.618556	3.415696	4.097305	3.125920	2.975163	4.749126	3.098204	2.977966	3.930454	3.184892	3.159156	2.977873	3.196264	4.001688	3.409032	4.486379	3.704057	3.772413	4.386094	4.630254	3.508342	3.787161	5.937210	3.711134	4.737997	4.333986	3.717722	3.600679	3.900638	4.460059	5.287566	4.936406	3.687679	4.722166	4.598769	4.137598	3.333388	3.840068	4.217947	4.142802	3.124783	3.380500	4.449608	3.188619	5.004573	3.600306	3.003001	5.382123	2.835405	4.720656	3.548394	3.305531	3.420058	3.742589	3.221235	4.216647	3.162994	3.114254	3.624260	3.172804	3.097619	3.274345	3.610768	2.998519	3.601477	3.589579	3.239326	3.539190	3.231876	3.043405	3.318310	3.387908	3.156400	3.760284	3.138946	3.436524	4.070662	4.362190	2.991507	5.660681	3.147914	3.058619	3.414352	3.306975	3.441340	3.201820	3.472041	3.574995	3.517163	3.905507	3.487325	3.663264	3.829296	3.836911	3.556216	3.221947	3.974446	3.188468	3.153195	3.607655	5.198932	4.325277	4.966918	3.476161	3.122269	4.142605	4.505426	4.263555	3.888504	4.040146	4.599773	3.500497	3.048168	6.322725	3.947935	3.598158	3.514088	4.023218	4.469699	3.473810	3.806168	3.734015	5.934225	2.912576	4.079378	3.119124	2.935826	3.549478	3.660010	3.156400	3.398360	3.174332	3.520806	3.201427	3.599132	3.157765	3.714879	3.797572	4.575394	3.788448	3.554282	3.652214	3.079184	3.236242	3.916408	3.285045	3.659637	3.361618	3.478950	3.710670	3.605404	3.678427	3.794238	3.253865	3.358469	3.535542	3.286102	3.107369	3.481721	3.281352	3.476529	4.727660	3.127307	4.334841	4.678808	3.390681	3.215305	4.144295	4.280814	4.361519	3.209927	3.034896	3.069240	3.369248	3.189289	3.242655

LINC00390	3.512937	3.575932	4.422222	4.812916	5.226669	6.815085	4.943181	5.968805	5.746275	4.250189	4.782417	4.530956	4.250416	4.663487	4.238752	4.817923	4.856703	3.988826	6.185885	4.072967	4.510054	4.680730	8.641137	4.939947	4.612954	4.707447	3.758136	4.395213	4.328092	5.373984	3.812713	4.549312	3.152825	3.727139	4.899401	4.037989	4.301102	3.738525	4.863307	3.789308	4.402680	4.186196	3.474700	3.571891	3.456795	5.021985	6.448008	5.440059	4.504144	3.995086	4.758717	4.229003	4.279291	3.744640	3.650021	5.395778	3.745044	3.127205	3.786278	3.288016	3.411654	4.269404	3.475806	4.553466	3.866394	4.750429	3.995497	5.728468	4.557891	4.388677	4.453543	4.989560	4.749312	4.592522	3.972872	4.530309	5.195106	4.737997	4.579245	3.970758	5.003178	4.786621	3.873734	3.662430	3.044010	3.882986	5.891858	4.495125	4.880948	4.261553	3.358117	4.038891	3.544884	4.339197	4.709693	4.479959	3.698588	4.088287	5.219149	5.013631	6.851233	3.840068	4.224260	4.520187	3.285623	5.371076	5.454178	5.128988	6.174195	4.619695	6.780543	6.098553	6.274070	5.002123	4.513363	8.950129	5.319391	4.837655	5.056350	4.091186	4.279071	5.728276	4.529462	6.733538	5.425023	6.689329	3.969670	5.476909	5.460841	4.493308	5.545992	6.189601	6.478471	6.084054	6.056734	4.674939	5.589300	4.445656	5.714931	6.112982	5.463106	7.252773	5.502967	5.861398	4.957295	6.233086	5.905949	5.767752	5.249386	4.862293	5.236029	3.393483	5.526286	4.950838	4.599178	5.345157	5.107866	4.463946	5.969983	6.293193	4.983789	3.496532	3.372078	4.364149	4.846179	7.449204	4.169230	3.646626	4.695153	4.177867	3.750843	5.114965	3.721910	4.491346	5.625771	5.577407	3.021680	3.777746	3.291431	5.466753	5.951435	3.452949	3.487711	7.210504	4.861840	4.570485	5.889018	4.893955	3.828629	4.991612	4.861840	5.811119	4.974640	5.362720	4.753636	5.210230	4.253660	4.868526	5.446617	3.356209	4.189497	5.403667	5.122714	4.178750	5.180956	4.229251	3.892646	4.717502	4.538874	3.126623	4.973787	4.192585	3.842751	4.216015	5.020517	4.948324	5.593845	4.602914	4.533520	4.932634	4.753194	4.827825	5.813523	3.429708	5.297474	6.143072	4.269874	5.162860	5.932286	6.799110	6.907814	7.503062	6.497516	6.143754	6.204845	5.628592	4.659428	4.858360

AP000812.1	15.374032	13.802610	9.623524	12.531515	10.066011	11.269244	10.124118	10.889511	11.018364	11.554481	11.297790	13.442646	12.433076	10.200663	11.870853	12.920057	10.219870	11.326759	13.211099	12.494832	11.500918	10.556772	10.803437	12.793758	12.688794	13.123808	12.345812	14.452088	13.982398	12.384801	10.842875	13.576005	9.292820	12.589114	9.454180	12.318704	12.089977	12.038055	10.192816	10.391281	10.127268	15.027552	12.927977	13.159158	10.003957	10.745574	11.790004	14.882845	12.274566	11.365974	15.024101	9.989661	11.936235	11.396102	13.400373	10.972659	9.275465	10.246336	14.862987	11.814206	14.428612	10.119137	9.967417	14.264573	9.364801	13.933765	14.310316	10.549040	11.076776	14.098570	12.266082	10.019581	10.642391	12.146729	9.841226	9.660604	11.296731	12.694586	14.698837	9.402928	11.869049	12.458340	13.993598	12.961065	10.734915	13.894497	9.973403	9.591051	13.503228	14.538753	14.651063	12.642900	12.012961	9.606243	14.842135	9.866962	12.957538	10.133737	11.540693	10.568630	10.100136	11.970963	11.469421	9.792703	13.305996	10.476015	12.497494	10.239877	10.853742	11.464934	10.893147	12.922594	12.842983	10.813727	10.089927	11.610934	15.839623	12.046618	11.283702	10.445361	10.625346	10.037185	12.999330	10.783470	10.385173	10.371706	10.390475	9.610886	10.283384	13.869352	10.427019	10.161675	10.264251	11.807531	10.627787	9.825711	9.415037	10.465656	9.376503	10.461142	9.711248	9.777218	10.352051	9.854951	10.013258	11.476411	11.085434	11.568423	10.296166	10.404248	10.619249	10.318904	11.502476	9.878883	9.185019	9.959977	9.991732	9.818272	9.101177	10.674348	10.871301	9.382862	9.959383	9.408477	9.683095	10.903502	11.016776	9.627241	9.848007	11.167966	10.159975	9.062763	9.249360	10.208318	10.335694	11.597854	10.906300	10.791138	9.465964	10.163352	10.186968	10.618518	11.724539	9.953637	11.180812	10.846224	10.140756	9.709211	11.692156	11.244638	9.587330	12.619138	9.939807	9.912359	9.651348	9.632128	9.809361	11.065268	8.992256	9.701888	9.592440	10.361591	9.859596	9.649786	10.746942	9.435228	9.205341	10.919423	10.744115	13.225685	10.144630	9.762460	9.772051	10.915229	9.700189	10.018641	9.799359	10.322742	9.937753	9.670386	11.202068	10.158948	9.318499	10.029650	11.279887	10.478286	10.185274	9.660763	10.971457	10.007811	10.359776	11.024507	10.862048	9.511815	10.606387	10.267937	11.421486	10.276224

AL353681.1	4.490216	3.902722	3.725775	4.126791	4.125253	4.305947	3.618945	6.607708	5.499283	5.046672	4.560116	3.754841	4.439142	7.041278	3.205978	4.559007	5.648979	3.521682	4.979117	4.421071	5.271639	4.644930	4.128056	4.047589	4.273323	4.329672	4.695153	4.148555	4.655551	4.169649	4.807206	5.035696	5.457054	6.215426	5.259648	4.060851	3.863442	4.752528	5.334740	4.999837	4.034967	4.590157	4.655551	4.255890	4.666915	4.257644	4.177253	5.189186	4.453761	4.804325	4.280176	5.282173	3.571988	4.831232	4.457594	4.310605	3.452065	3.953338	5.110165	4.678180	4.140617	5.354706	5.285197	4.633723	4.915556	4.719803	4.174447	4.346971	4.723874	4.771384	3.924166	4.652372	4.777623	4.463946	4.478885	4.699871	4.306150	4.559658	4.173173	4.394991	4.242073	4.330775	5.080300	5.809426	4.485251	4.095169	4.547719	4.808479	5.932802	5.423847	4.911553	5.722705	5.077351	4.543555	4.670157	4.218585	3.382241	4.775942	4.255230	4.277079	4.521287	4.741289	4.235090	3.953753	5.330272	3.932604	5.519352	5.314416	5.788486	4.379733	6.114580	4.793938	5.833523	3.883367	4.338129	5.453218	5.118031	4.067541	4.410703	4.773688	4.184692	5.420746	4.472978	4.215112	4.018242	3.903359	6.363544	5.179750	4.186427	3.857911	4.805763	4.189728	4.887089	2.977135	4.408498	5.478043	5.207022	6.790236	5.598789	5.157266	4.864141	5.661421	4.809948	5.038906	3.807012	4.694948	3.381727	4.012032	3.868380	4.951226	4.674273	6.067163	5.027663	4.695972	4.807440	4.714961	5.704669	5.303420	5.383921	3.972872	4.292918	4.116148	3.612955	4.931984	3.753370	3.850353	5.853616	5.061210	4.281894	4.901952	4.354159	5.174952	5.356463	4.640113	4.221265	5.253153	3.768431	5.092686	4.687813	4.672062	3.859821	5.076314	5.150345	5.394962	4.486379	4.463269	4.961870	4.190822	5.077569	4.495974	4.851376	3.816516	4.219929	4.571161	4.551331	4.181660	4.534121	4.751901	4.999198	4.963948	4.955010	4.838497	5.217569	5.473683	5.137013	4.661814	4.552354	5.480817	5.324222	5.750655	5.677332	4.476926	4.676249	4.580573	4.677535	5.001085	5.527072	4.832927	4.965208	4.536948	7.525133	4.868318	4.596619	5.107426	4.050438	5.670843	5.966457	4.548146	3.619725	5.625588	5.916363	6.253010	4.022811	3.706468	3.950770	5.051969	4.268246	3.906869

AC097376.3	11.205234	10.534840	10.932033	10.415257	10.911326	10.398534	10.241203	10.110805	11.212440	10.470736	10.470433	10.411451	10.939212	10.489519	10.647894	10.755737	10.644883	10.962078	11.257536	11.245085	10.735480	11.397000	11.381887	10.187555	10.708399	10.740572	10.431723	9.870094	10.365154	10.068719	11.026466	10.703888	10.695729	10.987041	10.794980	11.099868	10.899971	10.561314	11.126639	11.171084	10.647743	11.377558	11.124577	11.305447	11.320099	11.123986	10.323820	11.240485	11.031779	11.063780	11.385706	11.081461	10.913094	10.760252	10.765528	10.159386	10.781020	11.196672	10.938612	10.858234	11.304114	10.311849	11.067334	11.360307	11.923414	11.094572	11.043725	10.933084	10.494224	11.166964	10.614968	10.602257	10.652206	10.658726	10.734462	10.678375	10.862604	10.509917	10.559019	10.359924	11.246940	10.809503	10.814170	10.948753	10.937605	11.013251	10.712868	11.104579	11.380384	11.022131	11.192799	11.028205	10.771657	10.737937	10.759701	10.993754	10.942565	11.095144	10.774190	11.014997	10.445813	10.563370	11.090033	10.700036	11.585156	10.258842	10.851359	10.923426	11.809762	10.608452	11.393682	11.333614	10.675830	10.961636	10.490251	10.285761	11.551818	10.753717	10.687204	11.867596	10.341850	11.071993	10.294312	10.980402	11.268724	11.755427	12.297752	11.385992	11.126783	10.843022	9.765569	11.170020	9.825243	10.258677	11.992461	10.920621	11.044439	12.232513	11.529216	10.717447	12.265421	11.811273	11.060858	10.536062	10.018949	10.095191	10.660933	11.647985	10.687817	12.175303	11.432854	11.455816	10.645900	11.871450	11.005839	11.251804	11.191198	11.471379	10.342153	11.244791	11.264849	11.755427	11.672076	12.397534	10.495855	10.827984	11.508193	11.234768	12.179253	11.667291	12.034708	11.588048	10.491566	11.246804	9.909868	11.748172	12.327798	11.594455	11.777620	11.988946	10.347504	12.771719	11.474995	12.568065	11.229765	10.834982	11.143394	10.366983	11.171084	10.651494	11.112307	9.475674	11.254856	11.619186	10.718671	11.161103	11.448683	10.468588	10.582172	11.794233	12.596981	11.107323	11.394876	12.207449	11.855631	11.222202	11.412947	11.789710	11.567573	11.385706	11.971606	10.974940	10.986432	10.946388	11.137872	11.292520	11.816438	11.790286	11.201144	10.151430	11.290218	10.022004	10.671047	11.934741	10.578433	11.935649	12.193831	11.648287	10.618976	11.077209	10.226672	11.116514	10.651913	11.112442	10.608914	11.055084	11.965535	10.158031

AC112484.1	11.130181	11.696089	11.287020	11.732727	11.715866	11.941813	10.926240	12.174985	12.209741	11.464337	11.501620	11.589071	11.025697	11.032397	10.863674	12.229570	12.196906	11.044439	12.640723	11.649756	12.339350	12.456129	12.576866	11.690625	11.787922	11.945301	12.144213	12.179702	12.008706	11.702389	11.990332	10.135303	10.192202	10.573635	10.563844	10.987336	10.560245	11.203158	11.509371	11.510541	11.851720	11.193556	10.901270	11.353017	10.570935	10.718371	12.229099	10.888932	10.873395	10.534405	10.891048	10.271020	10.282619	10.851957	10.787927	12.218048	10.195069	10.636673	10.837075	10.275033	10.966969	11.404558	11.372737	11.879689	11.392045	11.604162	11.919811	11.756489	11.423450	12.305259	11.876151	11.517806	12.015827	11.539677	11.444260	11.119415	11.425214	11.252662	11.421793	10.620851	10.293707	10.355061	10.121857	10.352202	10.495855	10.838131	10.365154	10.190552	10.951475	11.198236	11.063641	11.515630	10.832125	11.150393	10.677921	10.368356	10.723571	10.612294	11.226214	12.145607	11.485325	11.617285	11.472234	11.513041	12.272198	11.634790	12.241581	12.098604	12.610024	12.194924	12.497666	11.829878	11.599875	12.390623	11.567573	13.367661	11.267512	11.321849	11.831355	10.771657	11.870995	11.136100	11.500204	12.859001	11.689281	11.794233	12.046469	11.564634	11.312057	11.462482	11.313145	11.703267	11.137430	11.338491	12.295263	11.626596	11.323781	12.102545	10.599097	11.897652	12.087259	12.359115	12.124642	11.733781	11.216707	12.223028	12.269936	12.723310	11.570621	11.958138	11.106155	10.751903	11.011552	10.655933	10.985236	11.039164	11.197531	11.500644	11.333338	12.249289	11.412947	10.496003	11.550609	10.562917	10.942125	12.436133	11.087512	10.622632	10.572001	10.799503	10.238936	10.080716	10.430953	10.175307	11.513324	9.795034	10.212131	10.549645	10.062587	11.073280	11.391609	11.571927	11.462640	10.925632	10.880464	11.628067	12.001894	11.470932	11.708808	11.522478	11.284004	12.878188	11.190467	10.922099	11.351141	11.335396	11.091311	11.534947	10.161362	10.153888	10.264693	10.718205	10.128694	9.941976	11.110584	10.388806	10.013106	10.661925	10.755577	10.692859	11.335546	11.164944	11.054506	11.697551	11.241360	11.375164	11.433749	11.306805	11.081461	11.230487	11.534084	11.831805	10.910132	11.237555	11.941196	11.981061	11.281661	11.747596	12.507547	12.326688	12.150335	12.787176	12.740063	11.636925	11.500503	10.868295	10.813266	11.725566

LINC02413	9.732752	8.717816	9.077935	8.680362	9.790524	9.949060	8.951490	11.186572	11.443382	9.423613	10.457715	10.815810	10.977161	9.152637	8.427882	10.380607	9.932634	8.850610	9.405722	9.772221	8.709468	8.931776	6.520361	9.237341	8.880982	9.749888	12.170171	10.530395	11.014430	9.967099	11.336132	8.354756	8.995493	9.884098	9.359075	8.308548	9.005349	8.123563	8.301664	8.094026	10.574366	8.064574	7.962366	8.090942	7.379095	7.397106	11.664015	7.578705	8.518412	7.235637	7.920740	7.827764	7.470962	7.034226	6.762051	11.567272	6.626317	7.838143	7.938674	6.754906	7.199002	9.489658	7.265880	8.203158	7.537165	7.432657	8.195435	7.665283	7.812489	7.987496	8.359725	9.535972	10.057435	8.664392	8.056042	8.810595	8.555262	8.115323	9.501025	7.389191	7.083448	7.532225	7.708229	7.165039	7.195840	8.582420	7.537331	7.086134	8.092915	8.162350	7.834327	7.444912	8.969270	9.675550	10.229100	9.587009	8.401291	10.667558	11.197814	8.843566	11.179915	8.761524	10.850767	8.281286	7.588285	10.572294	9.430651	7.395552	8.506105	10.516399	7.957304	10.057120	8.684777	10.864843	11.004847	13.568953	8.608181	10.481536	10.638914	8.938682	9.345450	8.892522	8.908646	10.975234	10.251752	10.690215	12.493046	10.438091	10.690535	10.832422	11.026307	12.125666	9.057026	10.219240	12.189981	9.287393	9.809361	8.872170	9.492065	9.293321	8.547241	8.435395	11.653482	8.796854	8.774914	11.575527	11.642056	13.169214	10.701509	12.456287	9.805798	8.868537	9.863318	10.827379	8.799601	9.662803	9.063980	9.356220	8.711476	11.743482	8.752751	7.413675	7.769127	7.270940	7.533297	11.814644	7.315133	8.511835	7.448405	7.541435	8.438853	8.233570	7.413675	6.811299	10.440967	6.544521	8.282391	8.385428	6.703237	7.485047	8.682359	8.061379	7.769577	8.092170	7.672898	7.874285	7.783481	8.046403	8.878793	8.414587	9.986408	11.515630	8.230322	8.164882	8.705197	8.536680	8.665154	9.738061	7.692126	7.196002	7.666536	7.438844	7.634048	7.464195	8.880210	7.466203	7.159964	7.855309	7.798494	8.145183	7.420235	8.855472	9.696633	9.297087	9.897941	9.176514	11.014714	11.465404	8.191967	10.864387	9.196444	12.401299	7.675274	7.486340	11.976679	10.407694	7.679817	8.672339	11.873097	7.975388	10.791282	11.197104	11.940741	10.525656	10.993595	8.423466	10.629019	10.114821

AL162377.1	5.954371	5.870804	5.953089	5.747776	5.731934	5.532394	5.977433	6.236490	6.589328	5.565981	5.508567	4.852673	4.863103	5.060038	4.864141	5.689353	6.259130	6.051126	5.650491	5.800809	5.548054	6.614323	6.680462	5.659046	5.394390	4.828047	6.682427	5.078395	4.951026	5.340859	6.649358	5.282173	6.361514	5.899181	5.835886	5.720686	6.068409	6.298242	5.707790	5.979030	4.737156	6.413691	6.137188	5.726105	5.178571	6.144617	4.724937	5.669438	5.484613	5.907399	5.869386	6.603564	6.627098	5.997771	6.490004	5.076102	6.567597	6.744515	5.755704	5.864357	6.794787	4.705737	6.620183	6.608183	6.189430	6.539247	6.188764	6.240272	5.884057	6.477451	5.992878	5.964494	4.833748	6.176600	6.310763	6.496218	5.985916	6.195305	6.200170	6.237925	6.216975	6.360207	6.009291	6.327073	6.263018	5.771842	6.088675	6.586228	6.834845	6.955353	7.099426	7.187943	5.939326	6.185885	6.260479	6.152236	5.485941	4.986428	4.573081	5.120694	3.522634	5.653667	5.168280	5.399789	6.294060	4.583727	6.759287	5.819218	5.867047	5.377508	6.552999	6.657598	6.137019	5.448214	5.486353	3.804108	6.059027	5.412818	5.108708	6.548360	5.507986	5.476722	5.410318	4.999198	5.321288	4.146850	6.128820	6.357670	5.615740	4.815165	4.711587	4.000428	3.723702	3.299460	4.913039	6.379958	5.198528	5.594019	5.051393	5.247821	5.280527	5.424454	5.388935	5.501391	5.819218	4.838712	4.986033	3.815250	4.377383	6.207603	4.126337	4.314044	5.624269	5.683817	4.447569	5.449750	4.832062	5.678637	4.929514	4.717928	5.938973	7.182665	4.730409	4.571161	5.743377	4.798317	6.394353	5.171289	5.965900	4.462440	6.118370	5.289371	5.049796	6.876407	4.482648	6.103246	5.211625	5.021357	5.341799	6.485635	5.497551	5.592169	6.345907	6.390531	6.771077	6.230244	6.286020	5.273690	6.423176	5.572561	5.471801	4.200088	5.739181	5.762867	5.905278	5.995500	6.483897	6.300828	5.631886	5.687731	6.690120	5.804407	3.980200	5.791335	4.229251	5.822404	7.013910	6.274724	6.159072	6.189601	6.553084	5.179183	4.902149	5.333817	4.602105	3.978030	3.598158	3.431343	4.258087	3.578575	5.669795	3.827843	4.540205	6.256420	4.181660	6.421425	5.708658	5.495284	5.019934	6.157372	5.685095	4.835984	4.867232	5.183016	5.055944	6.262169	5.688595	4.929514

AC138965.1	4.106740	4.333762	4.306815	5.453218	5.746099	5.752847	4.287426	5.777617	5.507796	4.458439	4.452439	5.302829	4.405822	4.607549	4.709693	4.151676	5.072791	4.612707	3.120218	4.130630	4.361722	5.028096	6.472574	4.239916	4.115070	4.646404	4.927015	4.857743	5.177346	5.381153	3.983505	5.133823	4.055403	4.631763	4.318023	3.789104	3.554680	3.506641	4.256727	3.172503	5.039101	3.621479	4.442195	4.459269	3.546976	5.251176	5.663683	4.340039	4.378001	3.706865	3.746675	5.136441	5.274756	3.656424	3.419157	5.446414	3.652825	3.289377	3.445804	3.537427	3.372431	5.058371	4.232847	4.369342	4.393599	3.236796	3.739136	4.149177	4.239223	3.766295	5.032560	4.939531	4.691774	4.834368	4.148755	4.254797	5.264485	4.362400	4.054091	3.997680	4.729367	3.593240	3.081901	4.067317	3.199539	3.074008	5.465790	3.765302	3.090209	3.797157	4.266350	3.967025	3.532925	4.420222	4.832287	4.905873	4.444117	5.364627	5.255133	5.784124	7.051636	4.436050	4.531413	4.409145	5.620566	6.512439	3.813152	5.049595	5.926871	4.867232	6.430725	5.356651	5.920125	4.906492	4.534548	8.354284	4.986033	4.343285	4.567683	4.258947	4.503080	5.005774	5.234034	6.954068	5.262253	5.390655	4.512034	5.030342	5.429745	5.054726	6.000418	5.562589	5.740630	5.173917	5.507004	4.951226	6.215597	3.971793	3.476336	5.341978	6.320141	4.812916	5.476002	5.529728	5.222525	6.609828	7.111770	6.110246	4.631969	4.766147	5.775099	5.186207	5.431625	4.869381	3.550612	4.821475	4.747061	4.647475	5.102368	6.212521	3.660415	3.377394	3.255327	4.018026	4.853324	6.122524	4.429095	3.222403	3.739768	3.730665	4.864543	5.819906	3.617097	4.696606	4.830608	5.432006	4.832062	4.058871	3.536093	4.330993	5.011405	4.054525	4.448705	5.088396	4.034080	3.678826	3.990661	3.800090	4.586047	5.223904	4.979337	5.104831	5.019124	5.258688	4.323376	5.148135	4.887925	4.035847	5.338929	3.348845	3.690345	3.404556	4.265499	3.797353	5.808857	4.165107	3.869456	3.035084	4.635636	4.979942	4.362400	4.330557	4.273115	4.173575	4.830608	6.114220	5.468537	5.717993	4.385646	5.715850	5.447412	5.294445	5.958051	5.417191	5.989356	4.891666	4.296542	3.528850	5.015058	7.108364	5.897227	8.075328	5.719583	5.533876	6.104430	4.856921	5.102775	4.613988

AC140125.3	4.106740	4.333762	4.306815	5.453218	5.746099	5.752847	4.287426	5.777617	5.507796	4.458439	4.452439	5.302829	4.405822	4.607549	4.709693	4.151676	5.072791	4.612707	3.120218	4.130630	4.361722	5.028096	6.472574	4.239916	4.115070	4.646404	4.927015	4.857743	5.177346	5.381153	3.983505	5.133823	4.055403	4.631763	4.318023	3.789104	3.554680	3.506641	4.256727	3.172503	5.039101	3.621479	4.442195	4.459269	3.546976	5.251176	5.663683	4.340039	4.378001	3.706865	3.746675	5.136441	5.274756	3.656424	3.419157	5.446414	3.652825	3.289377	3.445804	3.537427	3.372431	5.058371	4.232847	4.369342	4.393599	3.236796	3.739136	4.149177	4.239223	3.766295	5.032560	4.939531	4.691774	4.834368	4.148755	4.254797	5.264485	4.362400	4.054091	3.997680	4.729367	3.593240	3.081901	4.067317	3.199539	3.074008	5.465790	3.765302	3.090209	3.797157	4.266350	3.967025	3.532925	4.420222	4.832287	4.905873	4.444117	5.364627	5.255133	5.784124	7.051636	4.436050	4.531413	4.409145	5.620566	6.512439	3.813152	5.049595	5.926871	4.867232	6.430725	5.356651	5.920125	4.906492	4.534548	8.354284	4.986033	4.343285	4.567683	4.258947	4.503080	5.005774	5.234034	6.954068	5.262253	5.390655	4.512034	5.030342	5.429745	5.054726	6.000418	5.562589	5.740630	5.173917	5.507004	4.951226	6.215597	3.971793	3.476336	5.341978	6.320141	4.812916	5.476002	5.529728	5.222525	6.609828	7.111770	6.110246	4.631969	4.766147	5.775099	5.186207	5.431625	4.869381	3.550612	4.821475	4.747061	4.647475	5.102368	6.212521	3.660415	3.377394	3.255327	4.018026	4.853324	6.122524	4.429095	3.222403	3.739768	3.730665	4.864543	5.819906	3.617097	4.696606	4.830608	5.432006	4.832062	4.058871	3.536093	4.330993	5.011405	4.054525	4.448705	5.088396	4.034080	3.678826	3.990661	3.800090	4.586047	5.223904	4.979337	5.104831	5.019124	5.258688	4.323376	5.148135	4.887925	4.035847	5.338929	3.348845	3.690345	3.404556	4.265499	3.797353	5.808857	4.165107	3.869456	3.035084	4.635636	4.979942	4.362400	4.330557	4.273115	4.173575	4.830608	6.114220	5.468537	5.717993	4.385646	5.715850	5.447412	5.294445	5.958051	5.417191	5.989356	4.891666	4.296542	3.528850	5.015058	7.108364	5.897227	8.075328	5.719583	5.533876	6.104430	4.856921	5.102775	4.613988

AL161757.5	9.439413	10.738523	8.709164	9.604210	9.226978	9.267666	9.068977	9.050843	9.006123	10.154668	9.280151	12.444832	10.102338	9.175876	10.283531	10.443810	8.802612	8.932990	11.517093	9.657496	10.100136	8.948718	9.503932	10.112915	10.359182	11.044307	12.971662	12.338259	13.243181	9.767395	8.867333	9.043309	8.742859	10.635345	8.500441	9.615752	9.371510	9.558784	8.783057	8.933459	9.113742	11.416462	10.174250	10.707919	8.715629	8.695418	9.792063	11.254561	9.395127	9.136846	10.992250	8.444090	9.268924	8.510113	10.040996	9.150640	8.466285	8.581371	12.037461	8.985318	10.368662	9.348012	8.672619	11.308100	8.244457	10.526564	12.141279	8.720864	8.798095	10.736232	10.254371	9.061101	8.975406	9.635213	8.780406	8.443933	9.098132	9.397158	11.963379	7.955865	8.081629	9.335876	9.744747	10.238782	8.206480	11.209956	7.945100	8.079016	9.232049	10.126629	11.531541	8.625298	9.064439	8.456424	11.417687	8.345680	9.545467	8.859114	9.717013	8.955627	9.096009	10.788398	10.131242	8.984412	11.245651	8.875260	9.094182	8.988823	9.478608	10.103112	8.380662	10.669868	11.171363	9.475820	8.717058	10.421303	9.096576	10.095497	10.863229	8.725877	9.104276	8.758075	9.400900	9.959522	9.256834	9.256070	8.827765	8.756219	9.475061	9.187848	10.254503	9.417175	9.077935	9.657496	8.752363	9.254350	9.796260	9.621976	8.102201	9.510547	9.309567	9.676753	10.294926	9.270748	9.307595	9.787767	9.893168	10.399733	9.261505	8.726647	8.944736	8.775501	9.165569	8.281448	9.052352	8.493834	9.454459	9.038018	9.067710	9.678618	9.095163	8.688827	9.268476	8.918918	8.839043	9.641913	9.446852	8.966033	9.082581	9.226345	8.914189	8.847228	8.674211	8.694827	8.909199	8.753032	8.893154	9.725448	8.518242	9.128284	9.431422	9.201379	9.983054	8.423009	8.803198	9.564348	8.452314	8.774143	9.141334	9.340437	9.157407	9.767677	8.824486	8.666083	8.308699	8.735642	9.131973	9.588576	7.991423	8.141476	8.498831	8.316068	8.335259	8.105747	9.247506	8.201668	8.564664	8.319415	8.490573	9.058310	8.274038	8.476639	8.601729	9.192582	8.772007	9.090786	9.276233	8.891290	9.292368	9.479495	9.112877	9.727298	8.927562	8.851981	9.759027	9.285515	8.343510	8.870231	9.477511	8.829820	9.539221	9.501173	9.908491	9.706104	9.208101	9.341317	9.227589	9.536430

GSEC	5.195497	5.925083	6.375840	6.052366	6.609496	6.299226	5.401255	6.646563	6.628549	5.532394	5.669624	5.571259	5.376740	5.858872	5.222327	6.259647	5.581896	5.912413	5.641209	6.183960	5.434367	6.005570	3.210733	6.509101	6.839990	6.259986	6.463039	5.434367	6.295600	6.265208	5.768316	5.635267	5.580774	6.483410	6.252502	6.467022	6.498737	6.191997	5.530479	6.420437	6.277988	5.725727	6.453805	5.976204	6.415011	5.964297	6.709596	5.922358	5.550523	5.660140	5.523280	6.306611	6.311949	6.071709	6.133974	6.661840	6.436473	6.256588	6.223992	5.937210	5.908486	7.022488	5.024042	5.568836	5.196697	5.381551	5.967706	5.212774	5.819564	5.240275	5.782736	5.879823	6.432086	6.116663	5.787010	5.652022	6.273716	6.092166	5.810751	5.115151	6.081461	6.105843	6.413054	6.176433	6.642422	6.155339	6.147950	6.071709	5.198932	5.300034	6.340247	5.851310	5.686601	5.833923	5.786653	6.087475	5.538448	6.164413	5.061210	6.942960	6.015887	7.646507	6.961340	6.373825	5.760509	6.591954	4.805149	5.680106	6.180653	6.646404	5.971051	5.554716	5.779447	6.574345	5.830114	7.923024	6.089748	6.074831	6.748483	5.689713	6.081971	6.497681	6.364065	7.208002	5.778189	5.927070	8.232535	7.201315	6.510415	4.571161	6.521864	6.090612	5.663324	5.789212	7.369988	7.859886	6.541874	6.229710	7.303825	5.395166	6.511935	5.673109	6.542050	7.297511	6.821189	6.891084	5.927937	7.289877	5.445055	5.776362	6.606716	6.393047	6.744003	6.767147	7.308960	6.792357	7.506496	6.445749	7.662814	6.891419	5.843704	7.180790	6.119556	6.269459	6.021175	6.384499	6.792669	6.501046	6.248293	6.116308	6.693760	7.553313	6.516905	6.842224	6.184684	7.207847	7.037634	6.476576	6.402954	6.073115	6.403278	5.730111	6.026255	5.769200	6.046399	5.784491	5.455327	5.825674	5.488194	6.191492	6.178658	5.646170	6.105503	6.604700	5.517999	6.223265	6.451151	6.412522	5.787562	6.508308	6.939832	6.726421	7.051636	6.948099	7.284738	6.077122	7.880784	6.339056	5.432219	7.693823	7.151068	5.322285	5.477861	5.500670	6.401461	6.374003	7.028768	5.716576	7.300476	6.561036	9.695858	7.808333	6.869820	7.083620	6.683406	6.208291	7.120267	5.669251	6.591465	6.443376	6.633056	7.155127	6.273564	7.467259	6.318237	6.521225	6.229889	6.490783

AC135586.2	11.063219	12.841260	13.373784	13.712190	13.371858	13.467530	13.423875	13.797469	13.845086	13.961604	14.276902	14.598641	14.312732	14.605414	14.474751	13.595594	14.421778	14.510324	13.343862	13.933244	12.431440	11.997786	11.995512	13.812477	14.244036	14.575995	10.532527	14.580994	13.494410	12.781892	14.310316	13.568039	13.612808	10.582900	12.900424	12.852304	12.816481	13.510627	12.529519	13.635217	13.621534	12.744556	11.369466	10.940687	9.985333	10.437361	12.906787	11.594455	13.411431	10.149344	12.062305	12.603915	12.806146	13.562433	13.657459	13.809650	13.276252	13.208477	12.851957	13.577816	13.470323	13.474637	9.507629	12.069772	12.232832	13.134732	12.650709	12.150502	12.312392	12.601747	13.310353	12.524622	13.784808	13.707475	14.160203	12.586128	13.378652	14.163659	13.368915	14.213984	12.555591	13.524608	12.760155	13.198035	13.704371	14.017264	14.006448	14.002435	12.217838	13.220404	13.105424	12.817679	12.218048	13.332466	11.836797	12.853171	14.409900	14.470819	13.465090	13.744601	14.217310	12.961780	13.769304	13.884089	12.379588	13.877418	12.401299	13.148247	12.141122	12.929387	12.393464	14.030557	14.280707	13.363482	14.345286	12.281925	12.008851	14.175838	13.990442	9.447310	11.989104	9.895350	10.051862	12.388237	12.848581	11.548736	8.315429	11.693642	11.127958	13.169407	10.624276	10.202804	10.426224	10.733110	11.150531	9.444057	11.889609	10.163352	9.611030	10.835737	9.062460	10.121103	10.684205	9.793310	9.999518	13.261024	12.706190	9.823307	11.239490	10.252933	11.170765	11.457426	13.084373	10.451395	11.402498	11.918344	12.527434	10.321983	11.050922	10.076365	11.264699	9.410650	9.577910	10.766421	10.551138	13.450944	10.113060	12.363068	10.074213	10.926394	10.251318	10.921049	10.927572	11.407693	9.238834	9.492065	10.353264	11.221164	11.122337	10.712574	9.410338	11.001938	11.280333	9.862557	10.469815	9.928472	12.400629	11.383209	10.939769	12.073426	12.252154	13.282824	11.470648	9.853064	12.470874	11.360018	10.573635	13.088879	9.826628	11.440486	12.065779	12.763614	11.303223	9.884994	11.282105	13.242002	10.795416	9.463307	10.782381	10.092469	11.536144	10.432156	9.699395	12.441914	10.359015	11.432403	10.444741	11.861116	9.876239	9.124740	9.846759	11.110303	10.952894	9.645810	11.621743	9.496268	10.839735	11.616843	12.621829	10.878852	9.671337	10.026399	13.108876	9.944548	12.574210	12.439334	11.140228	10.871585

AP000688.2	8.431370	9.036324	9.692997	8.964176	9.702032	9.789747	9.861920	9.119435	8.924735	10.000255	9.414400	9.412964	8.782749	9.826628	9.629531	9.412891	9.210370	9.978579	8.759174	9.821311	9.172036	9.140105	7.112068	9.410488	8.865514	9.126433	8.510590	10.101871	9.143958	9.639817	9.092833	9.911272	10.013689	9.144285	9.229567	9.773869	9.984899	9.901485	9.552071	9.761985	9.229567	10.019869	9.690233	9.754543	9.960750	10.059104	9.516988	10.049545	9.465179	10.095615	9.827819	9.964423	10.077903	9.902880	9.647512	8.969270	10.466534	10.521111	9.824324	9.916997	10.191897	9.534750	9.992805	9.806105	10.012445	10.135138	9.807001	9.615752	9.602855	10.023543	9.212673	9.307880	9.938377	10.089305	9.949662	9.932497	9.617155	9.665455	9.685927	10.265579	9.649786	9.700189	9.509444	9.837036	10.102182	9.280452	10.069492	9.764655	10.177038	9.420683	9.682776	9.813689	9.870677	9.881630	9.584223	9.995882	9.965900	9.502375	9.590114	9.386992	9.792860	9.506715	9.326595	9.959977	8.824643	9.545467	9.389452	9.417175	8.389280	9.316241	8.882195	9.620723	8.999951	9.387152	9.156761	10.412390	8.985166	9.167845	9.249936	9.203412	8.650870	8.131646	8.519771	9.072827	8.380007	8.291988	10.139689	8.537828	7.901974	9.314738	9.163879	9.224202	8.981195	9.727021	8.632197	8.568786	8.338549	8.590977	8.750348	7.025223	7.542035	7.235310	7.671322	9.294264	8.075160	9.415203	8.626099	10.217418	8.552881	7.669761	9.158035	9.944250	9.462714	10.332208	8.381284	9.230592	9.586230	8.485660	8.898560	10.820704	9.933712	11.366830	9.110950	8.952418	8.820086	7.783924	10.375141	8.027463	9.763248	9.305431	8.819866	9.072354	9.683738	9.082741	10.195828	11.981214	9.351997	9.793599	8.873102	9.404986	9.012062	9.079331	8.019824	9.380969	9.860824	8.212820	9.112570	8.919828	11.528173	8.775819	8.573812	8.422852	9.069131	9.074340	9.479802	8.347801	8.623592	9.002124	9.493938	9.066139	8.802316	8.696372	10.173663	9.696322	8.358778	9.562846	9.254029	8.161937	8.791374	9.285962	8.371282	8.807202	10.547178	9.407409	9.317616	9.338465	8.430558	9.522722	7.970369	8.951490	11.859791	9.034002	9.104509	8.295156	7.967258	7.220674	8.889622	7.300476	8.000418	8.072162	8.677427	10.666063	9.200620	8.021935	10.161998	8.795216	10.189657	7.646818

AP000688.1	8.431370	9.036324	9.692997	8.964176	9.702032	9.789747	9.861920	9.119435	8.924735	10.000255	9.414400	9.412964	8.782749	9.826628	9.629531	9.412891	9.210370	9.978579	8.759174	9.821311	9.172036	9.140105	7.112068	9.410488	8.865514	9.126433	8.510590	10.101871	9.143958	9.639817	9.092833	9.911272	10.013689	9.144285	9.229567	9.773869	9.984899	9.901485	9.552071	9.761985	9.229567	10.019869	9.690233	9.754543	9.960750	10.059104	9.516988	10.049545	9.465179	10.095615	9.827819	9.964423	10.077903	9.902880	9.647512	8.969270	10.466534	10.521111	9.824324	9.916997	10.191897	9.534750	9.992805	9.806105	10.012445	10.135138	9.807001	9.615752	9.602855	10.023543	9.212673	9.307880	9.938377	10.089305	9.949662	9.932497	9.617155	9.665455	9.685927	10.265579	9.649786	9.700189	9.509444	9.837036	10.102182	9.280452	10.069492	9.764655	10.177038	9.420683	9.682776	9.813689	9.870677	9.881630	9.584223	9.995882	9.965900	9.502375	9.590114	9.386992	9.792860	9.506715	9.326595	9.959977	8.824643	9.545467	9.389452	9.417175	8.389280	9.316241	8.882195	9.620723	8.999951	9.387152	9.156761	10.412390	8.985166	9.167845	9.249936	9.203412	8.650870	8.131646	8.519771	9.072827	8.380007	8.291988	10.139689	8.537828	7.901974	9.314738	9.163879	9.224202	8.981195	9.727021	8.632197	8.568786	8.338549	8.590977	8.750348	7.025223	7.542035	7.235310	7.671322	9.294264	8.075160	9.415203	8.626099	10.217418	8.552881	7.669761	9.158035	9.944250	9.462714	10.332208	8.381284	9.230592	9.586230	8.485660	8.898560	10.820704	9.933712	11.366830	9.110950	8.952418	8.820086	7.783924	10.375141	8.027463	9.763248	9.305431	8.819866	9.072354	9.683738	9.082741	10.195828	11.981214	9.351997	9.793599	8.873102	9.404986	9.012062	9.079331	8.019824	9.380969	9.860824	8.212820	9.112570	8.919828	11.528173	8.775819	8.573812	8.422852	9.069131	9.074340	9.479802	8.347801	8.623592	9.002124	9.493938	9.066139	8.802316	8.696372	10.173663	9.696322	8.358778	9.562846	9.254029	8.161937	8.791374	9.285962	8.371282	8.807202	10.547178	9.407409	9.317616	9.338465	8.430558	9.522722	7.970369	8.951490	11.859791	9.034002	9.104509	8.295156	7.967258	7.220674	8.889622	7.300476	8.000418	8.072162	8.677427	10.666063	9.200620	8.021935	10.161998	8.795216	10.189657	7.646818

AC080013.1	6.974792	8.093733	7.571434	7.712966	6.566553	6.211030	8.084927	5.025271	5.575751	7.267882	6.921619	7.088975	8.000277	7.312596	6.764246	7.348653	7.337844	8.138652	6.041239	7.238287	5.850428	5.179183	7.254931	6.509741	8.017553	7.857395	8.045582	7.067302	7.677137	7.601689	7.257882	8.326529	8.473263	7.636038	8.649523	8.501785	8.398520	7.950786	7.250407	6.705266	7.285979	7.983020	7.484133	7.549747	8.910390	7.970993	6.961500	7.780760	8.304376	8.139581	7.456825	8.272399	8.407880	8.615851	8.402822	7.438046	8.906269	7.351914	7.819194	8.920591	8.149782	7.502262	9.678618	7.195369	7.205220	7.824306	6.570524	7.127214	7.788217	7.269393	7.513578	7.373115	6.898358	8.026826	7.759138	7.894343	7.310875	8.274038	7.658000	8.594242	7.876859	8.103403	8.104169	8.856081	8.802612	8.365016	7.909883	7.796942	7.442516	7.427119	7.642764	6.995532	7.684886	7.934018	7.982864	7.938047	8.546108	7.572185	8.048929	7.707918	7.275773	7.712816	7.585017	6.963727	7.169643	6.948428	5.950898	6.773312	6.320848	7.255858	6.939515	5.729904	5.227496	6.070323	6.441853	4.564070	6.087306	8.205706	7.255549	8.559301	8.167657	10.191151	7.893273	8.183407	8.896716	8.555909	7.363567	7.742874	8.183260	7.797093	7.625464	7.679669	8.224937	4.458852	8.599122	9.083201	9.988728	7.003210	8.633832	6.276052	9.007159	9.205189	6.385776	6.405375	7.849325	7.344870	7.244686	6.491065	8.605884	8.450307	8.527057	8.181886	8.502324	8.559151	9.028564	8.375113	7.165525	7.240558	7.449527	8.647693	8.084927	8.465978	9.383323	9.585450	6.962614	6.533555	7.541435	8.458269	8.622822	7.465716	8.484922	10.016654	7.768234	9.318827	8.651181	9.915602	9.279051	8.731790	8.373268	7.711235	7.907702	7.705490	9.281062	6.968869	8.132536	7.676199	6.606716	8.533021	9.958930	8.481554	8.701068	7.402552	7.841406	8.434770	8.410456	8.777381	9.679857	7.811267	8.604996	9.960448	10.365940	7.908951	8.108499	8.960223	8.803036	7.659658	9.200481	9.306959	8.537260	5.960212	7.103764	8.320945	6.775273	7.418516	7.310410	7.437104	8.389429	7.951220	8.795062	7.104243	10.384715	8.900824	8.381590	7.133547	8.652809	7.001141	8.068471	5.329117	7.156534	7.319649	6.399129	6.739082	5.528583	8.907419	7.667897	8.713413	7.599534	6.840459

LINC00523	3.286535	3.195416	3.072259	2.987672	4.201853	3.722513	3.094426	3.648387	3.194590	4.575798	5.725727	4.493546	4.368291	4.004983	3.088427	3.062882	3.412735	2.977135	5.122508	3.087059	3.901874	3.222961	3.282097	2.920788	3.665884	4.172494	3.095417	3.656217	3.493155	3.001582	4.295235	3.371190	3.295748	3.014468	4.713285	3.744089	5.049796	3.076229	3.279275	3.037418	3.550612	4.579470	3.344639	3.150262	3.494089	5.144161	4.675173	3.127205	3.447475	3.553719	3.625598	3.427639	4.157168	4.051954	3.689939	4.426112	4.834572	2.887841	3.157765	3.304432	3.294060	3.067732	3.588616	3.318310	3.307903	3.278379	2.956356	3.199539	3.008075	2.991298	3.016992	2.916215	3.780400	2.933165	3.043405	3.540515	2.998995	3.016034	3.215814	3.524981	3.237557	3.809104	3.350047	4.690116	3.339291	3.461300	2.979944	3.713156	3.133090	3.353204	3.166776	3.511629	3.364854	3.141697	2.938701	2.960421	3.717722	3.174332	3.904201	2.922701	3.065121	3.144215	3.203211	2.914380	4.108500	3.437397	3.176261	3.196929	3.126399	4.674273	3.273434	4.198200	2.957078	3.940672	3.285471	3.117381	3.354007	3.346402	4.060178	3.239897	3.283480	3.008759	3.328510	4.723874	6.358684	4.083916	4.101263	3.212697	4.070060	5.678637	5.334554	4.618177	3.352877	3.395031	3.110843	4.381597	3.256882	4.045411	3.134377	3.436524	3.737108	3.180672	3.434709	3.731933	4.192341	3.019493	4.650040	4.239674	4.092696	3.892863	3.264238	3.288016	2.972326	4.534790	3.557966	4.739080	3.130994	3.260708	3.302377	5.938973	3.318632	3.429348	3.761124	3.795499	4.640113	4.903206	3.079971	3.689524	3.421705	3.472743	3.278379	3.915108	3.865362	3.346402	4.484384	4.101061	2.897589	2.969466	3.083840	2.954900	3.533846	3.654812	3.452771	3.474169	3.410757	2.946451	3.414881	3.001873	2.991298	2.990915	2.972619	4.811050	2.927701	2.978331	3.724930	3.159921	3.009356	3.133321	3.138221	3.500876	3.964673	3.300811	3.464678	3.433819	3.036549	3.287873	4.074510	3.025748	3.246230	3.656029	3.052890	3.158396	3.183693	3.114384	2.987672	3.186882	2.992210	3.647203	3.116393	3.739374	2.993084	3.070143	2.850232	4.519978	4.753418	3.213397	3.168395	3.371190	5.469134	3.637482	3.159792	2.887468	5.299668	4.963547	3.719129	2.985328	3.561567	5.965720

LINC02470	6.265389	4.619695	6.901263	5.279166	6.434442	6.305958	5.252360	4.409371	5.260874	7.203644	7.214830	5.641968	7.344269	6.032733	4.430487	5.371678	5.109773	7.440209	6.565927	7.732264	5.572941	6.582110	3.171558	4.253009	6.693087	6.347421	7.569085	6.750533	4.905025	5.672198	6.733827	7.540067	8.329486	6.238264	8.136662	4.566368	5.665156	3.962248	4.288770	7.745934	6.463039	2.818845	6.923207	6.767480	4.308819	6.965487	6.129664	6.418823	8.322775	4.198200	5.026259	6.427331	7.575127	6.484786	4.346108	5.800619	7.353569	7.294607	5.434751	4.135395	7.157612	5.981355	5.602452	6.502522	8.968056	4.407819	8.196705	5.256533	5.660140	6.553169	5.919921	6.969671	5.442356	6.683902	6.924019	4.476926	5.194478	5.321701	4.950017	4.153191	7.133076	4.428056	4.148555	6.185545	4.675173	7.695913	6.586060	6.695877	7.227500	7.284881	7.577914	6.874168	6.021366	7.091343	7.658623	6.670564	4.471916	6.472245	7.205507	5.531069	6.923867	5.164484	5.748864	4.603341	7.623778	6.450320	6.106563	5.452057	6.183047	5.825312	4.965410	5.317658	6.882766	5.648403	7.992704	8.595322	5.229535	5.057972	5.910976	6.878780	5.855085	8.261839	5.929013	7.383782	6.345056	7.182971	5.657575	5.356854	7.467854	7.956200	6.594072	7.725750	8.240832	5.889570	5.608542	5.455701	7.020298	9.033700	9.068977	7.528443	9.272401	6.896554	6.397288	9.105974	8.395144	6.862341	6.872894	6.779754	6.019182	6.907669	8.982132	7.810508	5.876206	7.743008	5.858684	7.334897	5.729904	5.880687	9.179081	8.064117	6.227804	8.044341	7.001141	4.495125	7.252773	7.079432	7.669912	7.639530	4.910501	6.150503	6.297553	7.768377	6.041748	5.987381	6.191997	6.618387	6.980510	6.184870	5.212870	7.729637	7.231492	5.677707	7.383297	9.871517	5.027874	8.372984	5.418161	6.295776	7.633323	6.158733	6.641748	8.028718	7.143465	7.295699	4.949812	5.495284	5.303048	5.614814	5.668327	7.423830	4.293345	5.996352	7.525133	5.641390	7.965252	5.834109	7.072119	8.431875	8.148523	7.039223	5.288139	6.294903	7.585017	7.088639	7.078154	6.474750	8.180535	7.524985	6.461669	7.281460	5.526286	6.500696	5.675825	8.186038	7.058232	8.618776	6.047304	6.109732	6.978944	7.371092	6.530971	12.651748	7.320886	7.724697	7.529332	4.878379	6.283794	6.862992

AP003128.1	3.344003	3.986951	3.626008	3.906869	3.799443	4.676681	3.814191	3.957225	4.398066	4.354817	3.585898	3.795069	4.005892	5.030546	4.768483	4.640321	3.689102	4.188655	4.931984	4.895390	4.520848	3.897372	2.891531	3.708838	3.947935	4.551957	3.796720	4.181237	4.231567	4.590570	3.592419	4.359584	3.519668	3.848851	4.015856	4.974843	3.613999	3.453144	3.835027	3.853814	4.459856	3.455487	3.768431	4.054525	3.656838	5.543158	4.448914	3.587872	3.726339	4.790580	3.870715	5.185200	4.210345	4.191471	4.047376	3.996401	4.090469	4.505663	4.019768	3.931741	4.374789	3.999159	3.826779	3.316203	3.577750	4.016290	4.526520	3.732967	3.621076	3.076788	4.095392	3.438594	4.410020	3.954599	3.752330	3.798589	3.922872	3.940235	3.765519	4.213645	3.914226	3.954387	3.568873	4.321835	3.903359	4.088961	4.596385	4.043422	4.371120	3.700152	4.263078	4.083044	3.438428	3.902531	3.981983	4.267189	3.688091	3.599926	3.651167	4.314992	4.597879	3.866608	3.769062	4.731657	4.481255	4.406247	5.155798	3.872015	4.145567	5.553381	4.096648	5.056559	4.110281	4.023218	3.958573	6.206594	4.201853	4.113765	6.056359	5.157073	4.388470	3.886347	4.270937	4.356986	6.656772	4.825211	4.324870	4.552801	4.684589	4.687185	5.012216	4.426112	4.932831	5.892964	4.967997	3.922224	4.656195	3.777942	4.922760	3.886152	4.498338	2.871971	5.506022	5.091025	5.705599	4.430721	5.077351	5.342558	5.054934	3.805556	3.691374	3.875056	4.676470	4.567453	4.465424	3.672869	3.707684	4.264885	4.441972	4.818129	3.289685	3.583598	4.714760	4.110047	4.903587	5.474833	3.214600	4.009340	4.403324	3.839648	4.643812	4.132553	4.329236	3.937514	5.735376	3.714449	4.415573	3.714654	3.893535	4.922988	4.491777	3.896611	3.956373	4.012899	3.757923	3.668671	3.707302	3.770710	2.844337	4.211644	4.405149	4.785361	4.003685	3.966608	4.292475	5.035490	4.335462	4.244327	3.725775	3.807201	4.295899	4.235090	4.665404	4.239223	4.522120	3.225561	3.426736	4.087434	4.667563	3.638690	3.910981	3.225561	3.833785	4.140617	5.216600	3.956373	5.277375	4.105442	3.746476	4.212963	4.461781	4.191252	5.350672	4.875897	5.822053	4.172286	3.764064	3.988380	5.201290	4.643607	5.322870	5.782546	5.613483	3.869015	4.437866	3.880019	4.167325	7.279886

LINC02640	6.086932	5.743559	5.699868	5.998669	5.522145	5.444298	5.463304	5.995839	6.076952	5.758326	6.432086	6.200479	4.983993	5.401078	5.570130	5.924919	6.210859	6.039123	6.981935	5.410703	6.311124	6.432597	7.380825	6.013848	5.567109	6.516569	5.495098	6.843037	5.319749	5.344963	6.006620	3.373077	5.753035	4.662858	5.499484	5.936863	5.744988	5.523672	5.979394	5.365588	5.849536	5.597613	5.120920	5.764306	4.692658	4.593406	5.672771	4.854156	5.657036	5.611787	5.817278	5.292942	5.209030	5.578727	5.469490	5.689540	4.054951	5.647068	5.345734	5.543334	5.694721	5.454755	5.554888	6.137696	5.595686	5.815647	5.610492	6.087137	5.951816	6.649670	5.824601	5.897938	5.503721	5.916363	5.862898	5.558862	6.091133	5.945313	5.803485	5.187613	5.276162	5.196697	5.394390	5.570130	5.235426	5.153997	5.644448	5.803115	5.114965	5.598387	5.639354	5.977611	5.628403	5.499091	5.798018	5.713273	4.871252	5.045882	5.594384	5.417383	5.352272	5.485941	5.598789	5.996198	6.560853	5.544106	6.269812	5.261427	6.639342	5.910976	7.130688	6.573541	5.839182	5.870430	5.405352	5.303625	6.149155	5.466753	5.794569	6.007549	6.029738	5.600431	6.214403	6.261499	6.160317	6.032733	5.766251	5.612184	5.816919	6.476576	6.296097	5.569403	5.268266	6.175069	5.341799	6.075192	5.633793	6.631873	5.387789	6.462173	6.822304	8.100252	6.117672	6.370064	6.511437	5.967524	6.763766	5.679386	5.774550	5.841877	5.349856	5.826762	5.657200	5.530310	5.877950	5.789407	6.424002	6.410697	6.317575	5.698946	6.369566	5.411498	6.435961	5.194478	5.650101	6.007362	5.795803	5.545624	5.696022	6.254554	5.553579	5.262870	5.874764	5.110970	5.473683	4.828247	5.804210	5.691739	5.832098	6.237135	5.846771	5.429045	6.142569	5.648403	5.931422	6.654673	6.171845	6.486942	6.744672	5.620741	5.884779	6.359193	5.962534	5.643100	5.762139	5.545078	6.349761	6.368368	5.428855	5.507191	5.918859	6.316559	4.643812	5.594202	5.958590	5.992335	5.813892	5.451663	5.598184	6.422546	6.170481	6.038416	6.246787	6.370064	6.354904	6.206911	5.620195	6.030252	5.384847	5.933526	5.254943	5.686968	5.598387	5.927070	5.732104	6.177466	5.913863	6.529490	6.343180	6.917090	5.784669	6.478471	6.099807	5.794931	5.540532	6.064519	5.971051	6.135673

LINC00376	6.388007	5.867214	5.976562	5.862898	5.594741	5.790268	5.301032	7.190316	6.020997	5.481833	5.523672	4.615553	3.983919	4.944576	4.444781	4.946937	7.999328	4.698939	6.383495	5.531069	6.105134	5.395571	6.354724	5.323238	5.792044	6.178147	4.631548	5.909697	4.669702	4.173385	6.597774	4.212963	5.936863	3.243811	5.584588	5.202855	5.602452	6.411029	6.933021	7.024588	5.128561	6.837524	4.566596	6.590303	4.835798	5.528391	4.858150	5.320125	5.141316	6.304777	5.707946	5.951816	4.903797	6.787106	4.943402	4.521287	4.904018	6.465546	6.423337	6.208471	6.975741	4.172286	6.115959	6.527163	5.884057	6.482390	6.050444	6.220905	5.844412	6.969980	5.482767	5.493884	4.235322	5.721228	5.805807	6.003941	6.632728	6.052025	6.293873	5.102368	5.750837	5.621849	5.748675	6.439892	6.332819	4.780742	6.577491	7.716139	6.565605	6.725575	7.823189	7.524374	5.510880	5.645803	5.077975	5.651060	5.549015	3.053850	3.560720	3.453504	4.094334	4.248186	3.939843	4.718328	6.482902	4.496646	6.629515	5.068602	6.364380	5.212013	6.731272	7.100637	6.895426	4.935777	6.464205	5.309663	6.864246	5.903090	5.035696	6.074503	6.106037	5.576839	5.729728	4.639063	6.146352	4.283814	6.279654	6.090960	4.230278	5.146754	4.446889	4.493107	4.032747	4.709048	5.249386	7.913863	4.094115	5.869021	5.552381	6.005203	4.294590	7.035762	5.239264	6.204695	6.007549	5.121307	6.087653	4.460269	4.367199	6.305436	4.179876	4.510054	3.031355	5.182826	5.160478	5.251390	6.182207	6.772023	6.562022	4.675590	6.975929	5.218154	7.408527	5.117619	5.705436	5.291967	5.643284	5.142355	6.596178	5.975338	5.570880	5.244018	7.121194	4.552175	4.445874	5.087993	6.446362	6.192527	5.950728	6.757650	3.743475	6.604700	6.299226	5.972718	6.154306	6.078651	6.066803	5.758326	6.651102	5.564486	5.544296	3.974446	5.421874	5.676369	6.033636	6.392523	6.319439	6.736122	5.235235	5.003583	5.467944	6.222275	5.968264	6.299387	4.187745	6.680626	7.274994	6.732375	6.726738	7.612789	7.849468	5.505239	5.826008	5.361536	5.744281	5.029939	4.032550	3.610373	3.912934	3.325316	3.643851	3.755901	4.268691	5.841340	4.192585	6.572093	5.733207	7.309782	4.843175	6.411686	6.230070	6.144967	5.403667	4.954166	3.994643	6.773975	5.689869	5.146547

AC025754.2	7.559365	7.525428	6.533555	5.717290	5.977257	5.532003	6.108169	7.096114	5.302426	6.289123	5.930572	6.700626	5.996352	5.629161	6.294723	6.644989	6.462173	7.315588	5.328489	6.998925	7.419924	7.289877	3.157430	6.661502	6.516230	6.776202	5.922358	6.553514	6.047817	6.815893	5.652761	7.197600	7.883516	7.112839	7.607580	6.729161	6.819738	7.069044	7.514341	6.763766	6.324566	7.133718	7.484743	6.992762	7.319649	6.501873	5.947042	8.395299	6.724980	7.975543	7.608977	7.250407	6.842576	6.591797	7.087059	6.437583	6.436969	7.930805	7.314004	7.477675	7.389333	6.209663	9.573979	6.525466	7.460716	7.093370	7.533833	7.198379	7.367347	7.245012	6.772836	6.617262	6.071530	7.195198	6.514226	7.762352	6.495744	7.881547	6.679132	7.542035	7.090101	7.812489	6.699791	7.441282	6.974792	7.023757	7.704063	7.628769	8.158230	7.302884	7.051012	7.427426	7.799277	7.106294	7.191824	6.293193	6.742581	6.243095	6.065578	6.424645	5.947585	6.415159	6.400484	6.682427	7.466031	6.622911	8.650119	5.827654	6.038087	5.925599	6.180988	6.165098	6.373468	5.656838	6.158733	6.087838	7.771578	6.043307	6.578148	7.163940	7.605001	7.430932	5.944073	6.259469	6.547423	5.719393	8.434296	6.360544	6.911310	7.177806	7.498515	6.929221	7.956793	8.464904	7.895579	7.817635	8.114718	7.426808	9.449677	7.292246	7.334577	7.070053	8.019387	7.663886	9.182199	7.170864	6.600064	6.470964	7.353283	7.457145	7.043338	8.053534	7.614214	8.130266	7.661235	7.392716	7.490716	8.081192	7.872133	7.375167	7.974770	8.750837	7.627512	7.738668	6.744337	5.981675	9.217596	7.888733	8.596576	8.119024	7.651747	7.300159	8.974817	8.320503	6.973815	7.332112	8.903845	8.146710	8.635528	9.557552	7.263718	9.263384	7.187472	8.278420	8.263689	8.607876	8.614503	7.633902	8.628667	7.280216	7.652974	5.889758	7.997958	7.409130	8.119024	7.887508	8.203158	7.193586	8.693250	7.633622	8.920452	6.982244	8.228867	9.297703	7.494871	8.265082	7.605001	9.003202	8.438131	8.614804	8.908317	8.104486	8.279940	7.733023	7.920740	7.460241	7.235637	6.599069	7.714215	8.182181	7.776998	6.733689	8.281921	8.651781	5.986628	9.049602	7.605162	8.229801	6.316736	7.364986	7.937895	7.222542	5.590046	7.290988	6.771561	8.467060	6.516401	7.670378

AC087386.1	8.673115	9.185181	9.347557	10.375445	11.429723	11.616406	10.698830	11.115623	12.126882	11.367114	11.247077	10.798914	11.211714	11.100303	10.316666	10.792768	11.133064	10.894605	10.952969	9.944691	9.215294	10.352202	9.594640	10.768349	11.094712	11.189047	9.111441	11.579668	10.469359	10.396441	11.354479	8.752444	9.200001	8.847714	9.499786	9.077347	10.374844	9.501326	9.876562	10.171241	10.957088	9.405140	9.468250	9.535347	9.217596	9.019569	11.360307	9.257737	10.158811	9.349719	8.977416	9.283859	8.819791	8.896861	9.445444	11.325112	9.210536	9.790792	8.932263	9.590890	10.116364	11.598873	8.740568	9.973403	8.366088	8.990766	9.131232	10.253361	9.310956	9.414552	10.106586	10.193091	12.238835	9.942884	9.694983	8.945820	10.175472	8.832102	9.057352	8.650718	9.836257	8.613447	8.493691	9.284015	9.190703	9.464554	9.434327	9.818886	9.080577	9.384364	9.290169	9.711867	9.502535	9.806528	9.783445	10.568922	10.000255	11.446009	11.550739	11.522052	11.471231	10.257113	10.567246	9.899900	10.119528	11.519525	10.809966	10.263119	10.674816	11.348487	10.470433	11.289319	10.852247	12.075438	12.085897	12.944064	10.040378	9.886402	11.889031	8.995493	8.692458	8.781348	8.850175	12.389993	10.198291	10.669127	9.785347	9.206285	10.094705	9.696322	10.297097	10.833585	10.249709	10.353871	10.142602	9.426037	10.625655	8.968056	8.414729	9.024321	9.538754	8.592859	10.488713	10.061353	9.119287	9.930780	10.606255	10.950587	9.373205	9.864980	9.290819	9.682193	9.213755	8.902638	8.881755	8.563207	8.622673	8.904614	8.972481	10.689027	8.485224	8.018603	8.328762	7.888433	8.209188	10.984493	8.551354	8.640510	8.049527	8.178407	8.469498	8.160386	7.954175	8.537828	9.377466	8.191012	8.454599	8.202112	8.000110	8.664994	10.096406	8.553172	8.120836	8.243401	8.790410	7.981758	8.016641	8.813200	8.460790	9.374150	8.622673	11.267954	8.775212	8.501060	8.310361	8.538157	8.232098	8.634259	8.818720	7.930949	8.073176	7.986373	8.566024	8.142105	9.899742	7.817143	8.780550	8.457045	8.163532	8.844016	8.997674	8.524014	8.653270	8.748411	8.917391	9.888932	10.381790	9.565422	8.718419	10.430011	10.265579	10.061198	9.447790	9.636846	9.729974	10.313989	9.214667	8.718897	9.621515	9.805954	10.391204	12.163592	12.057480	11.603886	11.268395	9.744273	9.572138	9.271815

FP700111.1	8.673115	9.185181	9.347557	10.375445	11.429723	11.616406	10.698830	11.115623	12.126882	11.367114	11.247077	10.798914	11.211714	11.100303	10.316666	10.792768	11.133064	10.894605	10.952969	9.944691	9.215294	10.352202	9.594640	10.768349	11.094712	11.189047	9.111441	11.579668	10.469359	10.396441	11.354479	8.752444	9.200001	8.847714	9.499786	9.077347	10.374844	9.501326	9.876562	10.171241	10.957088	9.405140	9.468250	9.535347	9.217596	9.019569	11.360307	9.257737	10.158811	9.349719	8.977416	9.283859	8.819791	8.896861	9.445444	11.325112	9.210536	9.790792	8.932263	9.590890	10.116364	11.598873	8.740568	9.973403	8.366088	8.990766	9.131232	10.253361	9.310956	9.414552	10.106586	10.193091	12.238835	9.942884	9.694983	8.945820	10.175472	8.832102	9.057352	8.650718	9.836257	8.613447	8.493691	9.284015	9.190703	9.464554	9.434327	9.818886	9.080577	9.384364	9.290169	9.711867	9.502535	9.806528	9.783445	10.568922	10.000255	11.446009	11.550739	11.522052	11.471231	10.257113	10.567246	9.899900	10.119528	11.519525	10.809966	10.263119	10.674816	11.348487	10.470433	11.289319	10.852247	12.075438	12.085897	12.944064	10.040378	9.886402	11.889031	8.995493	8.692458	8.781348	8.850175	12.389993	10.198291	10.669127	9.785347	9.206285	10.094705	9.696322	10.297097	10.833585	10.249709	10.353871	10.142602	9.426037	10.625655	8.968056	8.414729	9.024321	9.538754	8.592859	10.488713	10.061353	9.119287	9.930780	10.606255	10.950587	9.373205	9.864980	9.290819	9.682193	9.213755	8.902638	8.881755	8.563207	8.622673	8.904614	8.972481	10.689027	8.485224	8.018603	8.328762	7.888433	8.209188	10.984493	8.551354	8.640510	8.049527	8.178407	8.469498	8.160386	7.954175	8.537828	9.377466	8.191012	8.454599	8.202112	8.000110	8.664994	10.096406	8.553172	8.120836	8.243401	8.790410	7.981758	8.016641	8.813200	8.460790	9.374150	8.622673	11.267954	8.775212	8.501060	8.310361	8.538157	8.232098	8.634259	8.818720	7.930949	8.073176	7.986373	8.566024	8.142105	9.899742	7.817143	8.780550	8.457045	8.163532	8.844016	8.997674	8.524014	8.653270	8.748411	8.917391	9.888932	10.381790	9.565422	8.718419	10.430011	10.265579	10.061198	9.447790	9.636846	9.729974	10.313989	9.214667	8.718897	9.621515	9.805954	10.391204	12.163592	12.057480	11.603886	11.268395	9.744273	9.572138	9.271815

AC121757.2	3.940444	4.010157	3.414002	3.997446	4.002553	3.073107	3.315118	3.431525	3.410422	3.983505	3.611782	4.283174	5.336536	3.643851	3.190933	4.181013	3.642062	3.343649	4.634782	4.575394	4.156749	5.005573	6.411997	4.407592	4.327873	4.000224	5.360411	4.520848	4.824782	3.455487	3.627023	4.227886	3.617653	3.741157	4.766823	3.973522	3.487711	3.171175	3.452427	3.104231	3.366512	3.064605	3.525165	3.278205	3.659820	5.388534	3.997882	5.431041	3.836911	3.857911	3.639493	4.052798	3.863876	4.155826	5.116611	4.508113	4.044969	4.015184	4.725358	4.133608	2.833274	3.981754	4.360915	3.584146	4.215112	4.413089	3.211879	3.399682	4.633277	4.044741	3.289532	2.999469	3.443822	3.859401	3.371691	3.334861	3.147174	3.361618	3.390005	3.646235	3.547366	4.213192	3.723702	3.915108	4.555359	5.309024	2.890430	3.609596	3.999802	3.653400	4.887311	2.847893	4.125253	3.451172	3.970758	3.300811	3.707077	4.594913	3.452427	3.379996	2.985598	3.783146	3.682876	4.039525	4.406043	3.234379	4.585201	4.096439	3.301457	3.635673	2.812834	5.991295	4.101263	5.235030	3.525894	2.907490	3.345907	3.815038	3.992018	3.278037	3.436171	3.076658	3.218815	3.969455	3.518728	3.720576	3.262192	3.461300	4.104982	4.016060	3.290281	3.929346	4.391908	3.267491	3.396791	3.536283	3.148882	4.566165	3.469619	4.637809	3.663429	3.579178	5.167076	3.901467	2.960877	3.037128	3.416712	3.294523	4.166676	3.472563	3.491016	3.589954	3.318122	5.487292	3.947054	3.542469	3.252973	3.244699	3.031075	3.298831	2.935901	3.565350	3.269945	4.124633	4.679862	4.940938	2.808409	4.107435	3.796526	3.486521	5.779447	4.001688	4.292918	4.903797	3.268818	3.749398	5.028096	4.473849	3.356732	2.793161	4.321641	3.550424	3.775101	3.833985	5.158109	3.135892	3.075974	3.236658	2.813763	3.190933	3.105403	4.368922	3.036058	3.322496	3.307751	3.198002	3.304129	3.109451	3.189156	3.761533	4.482201	3.635440	3.533311	4.779910	4.651131	3.431869	4.700882	3.676604	3.360651	3.342665	2.808844	4.120097	3.347910	3.221947	3.249707	3.279275	3.261735	3.914887	4.631116	3.842751	4.685452	3.276202	3.143488	4.701513	3.685623	5.468124	3.952908	3.986286	4.483077	3.229455	4.575001	3.514088	3.782082	3.458581	3.337455	3.082998	3.173569	3.334212

AC092953.2	13.382399	12.974204	12.998952	13.572887	13.457219	13.319680	12.913978	13.884891	13.275436	13.270823	13.475325	12.365418	13.554828	13.341990	12.768875	13.324170	13.379067	13.060460	12.895646	13.304119	11.952744	12.478661	11.008580	13.327384	13.109074	13.456369	11.574797	12.956811	12.947549	12.670342	13.518195	12.990709	13.382821	12.362443	13.123620	13.011244	13.186638	13.408793	12.898302	13.005406	13.254784	12.708384	12.728453	12.895995	12.259110	12.707907	12.682648	12.636808	12.954789	12.695260	12.571330	12.825950	12.956427	13.603829	13.042069	13.401636	13.254196	11.881927	12.552169	12.289352	12.575735	13.060634	11.240771	12.915514	12.254686	12.977133	12.410313	12.832730	12.910727	12.629849	12.934369	12.874855	13.026553	13.037427	13.028031	12.898302	13.125775	12.184846	12.949809	13.317056	12.487990	13.216008	13.126907	13.451381	13.374445	13.474637	13.493039	13.186053	12.317007	12.946838	12.915163	12.412540	12.849996	13.427333	12.569043	13.311346	13.440127	13.084939	13.838739	12.658166	13.737346	12.834365	13.178579	12.601913	13.074158	12.998253	13.542327	13.164898	12.143436	13.294543	12.156344	13.662957	13.404202	13.243181	12.936720	13.888334	12.995839	13.419947	13.018455	12.272346	12.975830	12.460607	12.152049	13.013710	12.482465	13.624298	12.237106	13.003402	12.420436	13.824264	14.642705	12.458806	12.268074	10.785111	12.462402	12.413004	12.183450	10.374844	12.365559	11.032090	12.208851	11.753075	12.866895	12.231738	12.009009	12.419600	12.972030	11.916526	12.606727	12.886172	12.544889	12.210507	12.768522	12.876066	13.086475	12.695910	12.213629	11.942122	12.017038	12.599341	12.145157	11.590387	12.780199	12.915887	12.104230	13.369945	12.078357	13.201413	12.948421	12.220929	12.246399	12.773272	12.336360	12.033526	13.025833	12.096818	11.903572	11.766175	12.494832	11.699769	11.609488	11.980316	12.107738	11.753661	12.414781	12.605556	12.556219	12.072811	11.387935	12.610356	12.951286	14.348197	12.561872	12.309046	12.410614	12.377241	13.828942	13.107335	12.186187	13.163401	12.520730	13.393505	12.559114	12.046618	12.486817	12.980457	13.029905	11.215686	11.610188	11.161862	11.707755	11.938498	11.525317	13.783328	12.526938	12.751723	12.335103	13.388628	12.959381	12.664248	12.567417	13.363482	11.570035	11.234768	12.693222	13.056733	12.853171	12.766549	12.904642	12.297271	12.940803	11.987098	12.879408	13.896393	13.320905	12.170476	13.030100	12.185267

PTCHD1-AS	3.960968	4.441709	4.485941	3.810138	3.938324	3.640119	4.031073	5.172969	4.835798	5.054079	4.789101	3.075743	4.031479	5.010742	3.286382	4.363515	5.346739	4.617326	3.609188	4.379938	3.714025	5.035911	6.046399	4.594060	4.981730	4.842558	4.628254	5.466568	3.969226	3.679024	4.530309	3.104899	3.558141	3.483698	3.999159	4.089834	4.431806	4.715607	5.006189	5.016263	4.247095	4.758930	4.152302	3.561768	3.707485	3.959039	3.838235	3.237709	3.143153	4.382076	3.846532	4.150003	4.435637	4.489115	3.131467	4.328092	4.333078	4.387604	4.953764	4.453761	4.243420	4.261329	4.996288	5.170102	4.592310	4.758520	4.683975	4.701513	4.692443	4.729562	4.022173	3.931917	4.640721	4.275750	4.313402	4.283174	4.569162	4.466525	4.591620	4.045411	3.979593	4.171213	3.985840	4.379075	4.650482	3.481721	3.941921	4.987675	5.285388	4.959367	4.825211	5.117824	3.826564	3.751911	3.228359	3.931741	3.933686	3.932381	3.731709	4.600798	3.436352	4.253908	4.088961	4.369342	4.529657	4.291112	5.479850	4.887311	4.496646	4.957084	4.819605	4.982359	4.340922	3.771146	4.294590	3.036658	5.004365	4.707853	4.732932	3.931741	4.268246	3.649639	3.956800	3.436171	3.852056	3.193392	4.778450	4.959367	3.579359	3.608409	3.511834	3.918409	3.237557	3.520441	3.313984	4.990800	4.016698	4.381597	3.853571	3.356572	3.813595	5.257917	4.185545	4.186427	4.314992	3.284447	4.896431	3.941720	3.808277	4.560331	3.116070	3.366336	3.116629	3.571687	4.189270	4.236552	3.637684	4.507461	4.351778	3.747923	3.400545	3.960729	3.129094	3.463048	3.828431	3.513752	3.329348	3.093613	4.663683	3.988180	3.827419	3.770322	3.930648	3.566109	3.532733	3.437226	4.382536	4.421274	3.907267	3.921645	3.793794	5.008463	5.118860	4.733129	4.448486	4.591620	4.285445	4.274648	4.685015	3.758791	3.849937	4.756759	3.905736	3.637482	4.340694	4.373932	4.564910	4.846179	3.360502	4.003453	4.651571	4.154543	3.849489	4.520848	3.357933	4.421729	4.568743	4.802376	4.568743	4.721961	5.315869	3.666689	3.765519	3.382584	3.853359	3.363192	3.321225	3.496731	3.304924	3.106189	3.294523	3.471226	3.266021	3.524784	4.428265	5.454402	4.234619	4.521287	3.528312	4.117437	4.117207	3.986951	3.427441	3.303625	3.085290	4.985607	3.697351	3.508342

AC004470.1	3.960968	4.441709	4.485941	3.810138	3.938324	3.640119	4.031073	5.172969	4.835798	5.054079	4.789101	3.075743	4.031479	5.010742	3.286382	4.363515	5.346739	4.617326	3.609188	4.379938	3.714025	5.035911	6.046399	4.594060	4.981730	4.842558	4.628254	5.466568	3.969226	3.679024	4.530309	3.104899	3.558141	3.483698	3.999159	4.089834	4.431806	4.715607	5.006189	5.016263	4.247095	4.758930	4.152302	3.561768	3.707485	3.959039	3.838235	3.237709	3.143153	4.382076	3.846532	4.150003	4.435637	4.489115	3.131467	4.328092	4.333078	4.387604	4.953764	4.453761	4.243420	4.261329	4.996288	5.170102	4.592310	4.758520	4.683975	4.701513	4.692443	4.729562	4.022173	3.931917	4.640721	4.275750	4.313402	4.283174	4.569162	4.466525	4.591620	4.045411	3.979593	4.171213	3.985840	4.379075	4.650482	3.481721	3.941921	4.987675	5.285388	4.959367	4.825211	5.117824	3.826564	3.751911	3.228359	3.931741	3.933686	3.932381	3.731709	4.600798	3.436352	4.253908	4.088961	4.369342	4.529657	4.291112	5.479850	4.887311	4.496646	4.957084	4.819605	4.982359	4.340922	3.771146	4.294590	3.036658	5.004365	4.707853	4.732932	3.931741	4.268246	3.649639	3.956800	3.436171	3.852056	3.193392	4.778450	4.959367	3.579359	3.608409	3.511834	3.918409	3.237557	3.520441	3.313984	4.990800	4.016698	4.381597	3.853571	3.356572	3.813595	5.257917	4.185545	4.186427	4.314992	3.284447	4.896431	3.941720	3.808277	4.560331	3.116070	3.366336	3.116629	3.571687	4.189270	4.236552	3.637684	4.507461	4.351778	3.747923	3.400545	3.960729	3.129094	3.463048	3.828431	3.513752	3.329348	3.093613	4.663683	3.988180	3.827419	3.770322	3.930648	3.566109	3.532733	3.437226	4.382536	4.421274	3.907267	3.921645	3.793794	5.008463	5.118860	4.733129	4.448486	4.591620	4.285445	4.274648	4.685015	3.758791	3.849937	4.756759	3.905736	3.637482	4.340694	4.373932	4.564910	4.846179	3.360502	4.003453	4.651571	4.154543	3.849489	4.520848	3.357933	4.421729	4.568743	4.802376	4.568743	4.721961	5.315869	3.666689	3.765519	3.382584	3.853359	3.363192	3.321225	3.496731	3.304924	3.106189	3.294523	3.471226	3.266021	3.524784	4.428265	5.454402	4.234619	4.521287	3.528312	4.117437	4.117207	3.986951	3.427441	3.303625	3.085290	4.985607	3.697351	3.508342

HCG27	8.535166	7.910185	8.167229	8.757154	8.680959	8.199363	8.047693	8.880498	8.853796	7.925680	7.761907	8.169095	8.688984	7.554088	7.855309	8.727901	8.853642	8.787530	7.542633	8.187296	7.798982	8.279032	8.617543	8.445469	8.647979	8.128868	9.659506	8.462482	8.353838	8.234201	8.514294	8.273893	8.207850	8.942331	8.428517	8.448133	8.079921	7.850493	7.807713	8.545084	8.347074	8.036665	8.790410	8.719638	8.433072	8.319126	8.520666	8.372228	8.749624	8.139115	8.553764	8.482488	8.366367	7.972576	8.266186	7.264495	8.900672	6.618875	8.185093	8.332147	8.546108	7.864325	6.717341	8.304234	7.900849	8.638495	7.689797	8.390510	8.222383	8.598807	7.855309	8.380007	8.348833	8.422111	8.720713	8.196546	8.361723	8.210228	8.413538	8.215620	8.730470	8.421655	7.994877	8.241811	8.139429	8.479139	8.373268	8.670471	7.852039	8.491478	8.664235	8.570650	8.434442	8.243241	8.737947	8.717346	8.819040	9.112102	8.272535	7.185042	7.636038	8.474768	8.391772	8.398071	7.987342	8.416275	8.755929	8.124035	8.086448	8.207388	8.692954	8.704893	8.743457	8.306339	8.259246	7.750282	8.934723	8.576254	8.106649	8.664085	7.379253	7.613888	8.800210	7.968486	7.800367	7.838143	7.690710	7.976484	8.407117	7.509115	8.501568	7.578077	7.702525	8.168296	9.065038	8.761230	8.071834	8.385574	7.522964	7.607291	7.697302	8.937007	9.295979	7.290175	7.929278	8.253887	7.782573	8.559451	7.599534	9.191817	9.097671	7.574174	7.634654	8.524014	7.491660	8.035557	7.781356	7.853434	7.972915	8.063661	7.862315	8.179171	7.352823	7.881713	8.158230	9.301255	8.083795	8.050181	7.621041	8.188791	9.072984	7.842645	7.384091	8.528442	7.415371	7.437251	6.511268	8.138022	8.230249	8.256785	7.829277	7.431258	7.410915	8.192295	8.042970	7.216069	8.434296	7.935298	8.632336	7.442358	7.677784	7.965718	7.528812	8.460790	8.188791	7.884152	7.658623	7.426143	8.363165	8.234656	8.221156	8.039959	8.580126	7.918723	8.404354	8.053687	7.620447	7.121630	7.872457	8.558556	8.065034	7.677944	7.837640	8.453841	8.323674	8.747736	9.170919	8.598366	6.514561	7.838309	8.748492	8.008852	8.951813	6.979414	8.530777	7.980968	8.372378	8.098858	7.500400	7.871513	7.778846	8.274770	8.909943	7.597303	7.417135	7.865227	8.050463	7.454005

AL451105.2	6.549099	5.706630	6.162053	6.359193	7.170697	7.561665	6.571622	7.966334	7.625464	6.850135	7.037634	7.063488	6.496370	6.484247	5.369314	7.042080	6.724980	5.992161	5.824962	6.442684	6.524017	6.510260	6.728379	6.007165	6.070323	6.748483	7.265109	6.647547	7.042723	6.517718	6.821344	5.816007	5.173168	7.240235	5.967524	6.101888	5.689713	5.430290	5.988091	6.410859	7.148992	4.993938	6.455432	6.020997	5.813892	5.834109	7.264630	4.899606	5.684727	6.099807	6.045528	6.102226	5.660315	6.061533	5.246614	7.153231	5.817089	4.821688	4.703011	5.020329	4.503304	8.107575	5.145152	5.198116	5.081663	5.553982	4.482648	5.673307	5.404785	5.504676	5.834461	6.560853	7.298474	5.829913	6.171506	4.633927	6.287255	6.031651	5.950046	4.946291	4.856053	4.980326	4.865991	6.310763	5.443327	6.266553	6.829091	4.466966	5.109773	4.517005	4.963752	4.889393	6.239917	6.542716	6.640971	6.517550	6.277178	7.248850	7.044822	4.639479	7.742243	6.454936	5.683425	6.034559	4.260868	7.109818	6.671392	5.492415	7.022815	6.878619	5.877440	6.431244	6.898683	7.299545	7.155624	10.067980	4.806164	7.120267	7.264197	7.054425	6.819738	6.224501	7.232924	8.196072	7.033749	7.854689	7.793850	7.259489	8.401741	7.456987	7.496799	6.965325	6.872117	5.774175	7.999946	6.846929	6.310424	6.639018	6.713926	7.403345	6.140830	7.261987	6.622425	6.251818	6.392873	7.704547	7.509989	8.522011	6.628063	7.245923	7.158540	6.187221	7.584575	6.128820	6.822609	6.242444	6.450968	6.863771	6.382968	7.792182	5.949856	6.370900	6.576865	6.434799	6.442193	7.510460	6.022244	6.586545	6.561359	6.033636	7.284141	6.135515	7.278613	5.580219	6.519038	6.582457	3.127814	5.282574	5.716738	5.487095	6.723538	5.318061	5.505053	5.118456	5.631190	5.669251	5.265665	5.669061	5.582672	6.164262	6.842576	7.677784	6.567597	6.821189	5.261653	6.764402	6.537774	6.323204	4.807639	5.170899	6.330441	5.981675	6.379122	6.107488	7.503695	6.024627	5.660315	5.887451	5.310505	5.303420	5.594019	6.524348	7.423202	6.855573	7.099566	7.470794	7.707285	7.460397	4.989975	7.372217	7.771110	6.442361	5.952712	4.756117	7.145355	6.815250	6.579778	6.101011	7.783009	5.170495	6.352776	8.469344	7.497414	7.540820	8.527523	5.187613	7.907878	7.161729

AC108721.1	3.156795	3.100092	2.960337	2.919864	2.893066	3.033581	3.277250	3.035971	3.400545	3.257771	3.488475	3.561139	3.222539	3.852937	3.457694	3.093250	3.385511	3.242972	4.380378	3.200504	4.149594	3.014378	6.298576	3.396300	3.260431	3.031543	2.975923	2.973255	3.099977	3.023492	3.605404	4.704484	4.717502	3.097619	3.513752	4.023916	3.622668	3.243262	3.118062	3.127205	3.420660	3.254014	3.490098	4.008008	3.471047	3.866394	3.770137	3.870512	3.322813	3.927387	3.866193	3.487711	3.436845	3.900421	3.451172	3.358308	3.791187	4.138852	3.983713	3.615725	3.503315	2.981364	3.369092	3.058828	3.128093	3.058412	3.176261	3.106766	3.039515	3.031075	2.952851	2.772872	3.009947	3.073797	2.972138	3.687483	2.935480	2.947225	2.961862	3.623459	3.157896	3.583046	3.128217	3.246695	2.983345	3.271463	2.874047	3.059176	3.766295	4.631333	2.998611	2.891974	3.161074	3.207173	2.890825	2.962274	3.300075	3.081556	3.019944	2.976487	2.903204	3.190001	2.974081	2.944347	4.233519	4.391274	6.749427	3.089357	2.971185	3.541895	2.916310	4.054951	3.048272	4.334415	3.076552	2.857145	2.923214	3.000457	3.093250	3.049429	3.144788	2.933412	2.935557	2.838906	3.332583	2.956615	3.148764	3.374402	3.104899	3.275887	3.359154	2.995701	3.230761	3.160827	3.002449	3.312266	3.035361	3.318795	3.233635	3.664851	2.895812	5.715850	2.943661	3.088102	3.312266	2.973996	2.903654	2.997691	3.395581	3.336283	3.900187	3.430468	3.165304	3.457527	3.715250	3.651167	3.292959	3.103396	3.049958	3.186882	3.044137	3.489197	4.110948	3.672690	3.972341	3.220421	3.818633	3.443636	3.919983	4.078077	3.456247	3.319776	3.912495	3.208343	3.131365	3.450064	4.916169	3.868783	3.668479	3.237433	3.051115	3.336976	3.249291	3.223347	3.065028	3.160581	3.236658	3.190142	3.118879	3.127559	2.848679	3.561948	3.194860	2.911444	3.401932	2.931963	3.049767	3.020220	3.162394	3.355689	3.618361	3.229197	3.326154	3.870938	2.969108	3.434340	3.154663	3.554100	4.036922	3.044868	2.882521	3.848412	3.592078	2.952031	2.995029	2.896088	3.022998	3.079857	3.158396	2.987859	3.033387	3.016895	2.893983	4.491777	4.523234	7.051177	3.110252	3.277712	3.515144	2.875983	3.497840	3.412024	3.710670	2.987328	2.885392	2.948767	3.005456	3.145815

DLGAP4-AS1	10.525491	10.031468	10.697067	10.093823	10.609505	11.205099	10.383504	10.885843	11.351141	10.981301	11.034454	11.167684	10.403192	10.616435	9.158340	10.535606	10.622632	9.825091	9.688079	9.989661	9.879478	9.630443	10.281995	10.463030	9.852310	10.688294	11.625303	11.597121	11.443831	10.891777	10.719585	9.056876	9.221723	10.664303	9.417954	9.549440	9.466897	9.676294	9.759952	9.560659	11.006117	9.366294	9.693450	9.829671	9.759652	9.676599	11.804233	10.013832	9.962834	9.462413	9.496877	9.261505	9.061269	9.072203	9.174945	11.327922	9.111112	9.776270	9.049602	9.781923	9.278587	10.836911	9.904409	9.592749	9.136529	9.280309	9.567009	9.712195	9.623524	9.774780	10.305962	10.160447	11.302194	10.232060	10.274888	10.086530	10.005054	10.010461	9.983819	8.722060	8.874814	9.145926	8.566718	9.081958	8.793982	9.155395	8.896105	8.853466	8.906269	9.031263	9.276233	9.063380	9.681259	10.095615	9.898981	10.028430	9.247216	9.947489	10.373076	10.515780	10.807528	10.040543	10.475118	9.971850	9.447171	11.574932	11.467196	9.983819	10.091891	11.491448	9.334599	10.449467	10.520492	11.547691	10.677766	12.989969	10.001321	10.226672	11.650203	10.167666	10.187407	10.534840	10.881936	11.641926	11.269090	11.499775	10.838572	10.622929	11.255761	11.707306	11.288192	11.347275	11.385568	10.204297	11.924002	10.448409	10.505574	10.243674	9.870094	9.810408	9.970033	10.032414	11.406467	10.108827	10.826808	11.047654	11.563737	12.116974	11.474930	10.939064	9.912200	9.980904	10.884929	9.965297	9.770646	9.735468	10.456939	10.148232	9.910192	11.652576	9.892413	9.573538	9.625683	10.180258	9.491898	11.845386	9.932019	9.553280	9.414552	9.378994	9.691158	8.896560	9.400437	8.999804	10.985390	8.908475	9.257737	9.459105	9.084407	9.430794	11.331759	9.805321	9.654697	9.833636	9.273471	9.995433	9.707995	9.858336	10.003500	11.018066	10.734015	12.541262	10.865293	10.557223	10.302784	10.578284	10.402738	10.220026	9.353778	9.117427	9.001182	8.894686	9.218559	8.854082	10.102474	9.150322	8.795964	9.185335	9.265944	9.480728	9.686383	9.927841	10.288341	10.169807	10.369411	10.058014	11.592067	10.423303	10.283087	10.308933	10.934589	11.120569	9.829671	9.123526	12.113600	11.380054	9.971512	9.577910	11.916369	9.886092	10.437655	11.288902	12.204194	10.778996	11.131620	9.854297	10.887194	11.393514

C5orf66	13.971275	14.381702	15.238314	15.107375	15.158834	14.680965	14.845201	14.842680	15.129649	15.122829	15.120910	14.840176	15.193465	15.050698	14.933539	15.219810	14.938328	14.702993	15.496317	14.811620	15.467330	15.488676	14.708876	14.840176	14.758705	14.869310	14.030880	15.032714	14.596614	14.401522	15.236212	15.557764	15.157558	14.873940	14.606651	14.765753	15.112372	14.662761	14.714408	14.836534	14.784337	14.854342	14.105360	14.798922	14.344582	14.542602	14.778502	14.257993	15.064607	14.633014	14.478648	14.913617	14.653231	15.253250	14.065689	14.805656	14.739028	14.300589	15.025759	13.773961	14.689755	14.874444	13.680430	14.837049	13.667210	14.528702	14.710296	14.786318	14.689755	14.706865	15.052583	15.393740	14.908629	15.215752	15.367955	14.808644	15.357970	14.497164	14.602393	15.190719	15.205705	14.444149	14.856471	15.568457	15.101738	14.653231	15.075186	15.204490	15.067485	15.228590	13.961604	14.748776	14.747454	14.859978	14.719949	15.438162	15.219191	15.175273	14.957773	15.144888	15.253922	14.376307	15.217106	14.901289	15.075776	15.254640	15.040623	14.837049	14.509550	15.127111	15.524473	15.148768	14.705704	14.897610	15.564042	14.501441	13.562910	14.918365	15.312332	14.629113	14.901289	14.399625	14.306995	14.207485	15.045331	14.508030	14.551462	14.484052	14.443791	14.113444	14.841151	14.092821	14.705704	13.823470	14.724263	14.591979	14.266304	14.966246	14.934057	15.002418	14.463188	14.974820	14.778050	14.061195	14.224052	14.715882	14.267647	14.428612	14.217310	14.539639	14.443791	14.707518	14.127106	14.842135	14.429732	14.341372	14.313436	14.255152	14.150116	14.948503	14.016128	14.654144	14.474319	14.359381	14.163327	14.537935	14.567126	14.627259	14.254446	14.196229	14.072700	14.726960	14.465165	15.267864	14.801392	14.685534	14.753351	14.086526	14.768992	14.080347	14.239437	14.316430	14.408375	14.923336	14.108830	13.983511	14.334354	13.853818	14.057422	14.449433	14.526691	14.421008	14.545097	14.288622	14.710793	14.292020	14.935700	14.592389	14.602393	14.437151	14.765753	14.088628	14.405515	15.122829	14.855412	15.015687	15.427177	14.672364	14.196541	15.039342	15.035175	14.556030	14.679130	14.433838	14.173886	14.464001	14.733058	14.876538	14.198488	14.338926	14.632630	13.952379	13.805171	15.296733	14.056836	14.402866	15.044721	15.365568	14.611896	14.928143	14.199474	14.995622	14.747454	14.163659	14.792525	14.034368	14.898599	14.270558

AC100803.4	6.349412	6.630154	7.205220	6.150019	6.070817	3.877169	6.205388	3.930017	4.332057	5.989000	4.913698	6.587033	6.896050	4.920613	6.878132	6.302364	6.068594	6.101888	4.209072	5.576839	4.628254	3.600679	6.837524	4.422222	4.366775	5.627077	7.880642	5.662912	7.746562	5.745522	6.927797	7.042248	6.612157	6.148121	7.092774	5.062843	5.181765	4.077627	4.856053	5.351486	6.861077	6.414193	6.168430	5.895133	6.544521	4.262444	7.769734	5.264099	5.658330	5.381342	3.867730	4.892731	6.826732	4.800507	6.817963	6.186042	6.004097	4.928275	3.835458	5.214446	6.197911	8.301208	7.382525	5.364435	5.432219	5.857773	4.712842	5.042632	4.393599	5.010327	5.636578	5.497353	6.001274	6.364548	6.331981	6.288447	5.777437	5.964494	5.807238	6.387827	6.556302	7.569464	5.433754	4.726811	5.435125	6.915418	4.174012	5.559669	2.985121	5.054726	4.594913	5.326747	6.893978	5.735376	6.239096	6.772992	6.930818	7.001459	6.216975	6.896217	6.416995	5.715301	6.454610	5.301032	6.058527	7.671958	5.810576	6.666875	5.186596	6.479926	4.146850	4.387173	3.562130	6.087746	6.861854	5.248785	4.612707	7.204562	6.702584	5.786478	5.695854	5.148333	5.818870	3.715067	3.286969	5.179379	7.215915	5.354082	4.964786	6.028032	5.324768	6.534213	4.061501	5.919921	6.175069	5.962339	3.737108	5.327510	5.490169	4.009546	4.451832	5.555257	4.143246	4.006960	6.014010	4.136501	3.023492	5.515838	4.778843	7.272830	7.019841	6.655979	4.679862	6.673497	4.084550	4.494208	3.116275	4.265101	4.297673	4.823328	3.566891	5.134665	4.613988	4.698120	4.249061	6.003241	3.950770	4.438496	4.784747	3.942850	4.577985	4.336340	3.932046	4.589696	6.576181	4.119882	3.145372	4.531195	3.545984	5.070180	6.734308	3.320254	3.875659	5.712490	4.461136	2.864974	3.925461	4.462632	3.536689	3.960729	5.012009	4.382076	4.393824	5.840959	6.049248	4.508113	4.711587	4.617724	6.332819	4.737391	6.888402	4.154789	5.184212	4.083044	5.182603	4.996504	4.520611	2.907863	5.198932	5.584968	3.697132	6.030746	5.657391	6.159451	6.201557	5.174727	6.395482	3.596729	5.586609	4.890855	5.556569	5.296657	3.119124	3.977797	6.627260	4.018482	4.723440	4.424840	4.083916	3.771736	3.298231	3.049099	4.795822	4.573081	5.554888	4.716878	4.325069	5.198733

Z97056.1	5.699682	5.834109	5.615740	4.585625	5.674760	6.417972	7.066825	7.141433	6.735311	6.974953	7.174793	6.734147	6.176270	6.800254	4.955407	7.674015	6.091481	5.980467	5.000281	7.056979	5.600255	4.725144	4.461136	6.482390	4.920418	6.850610	5.167687	7.041908	6.411190	6.062445	6.604049	5.560215	6.051840	4.505883	6.052855	6.342020	6.689974	5.341799	5.192676	5.394390	7.131815	4.302412	6.399129	6.490919	6.050117	6.639174	6.321010	6.444900	6.929686	6.930178	6.354724	6.831311	6.307918	5.711196	6.679465	6.855086	5.236029	3.179263	4.740420	5.073843	4.067317	6.507989	3.817375	4.100404	4.298299	4.488884	5.428427	5.306575	5.643100	5.663868	7.099087	6.548360	7.592717	6.628233	7.146759	6.571007	6.551986	6.059900	6.390022	5.366204	5.671044	5.261427	5.051393	5.940063	6.079900	6.564660	3.669702	5.455513	3.051115	4.144750	3.763856	3.655456	6.637049	7.092294	5.714385	6.042958	6.478808	6.151749	5.689182	5.808321	4.588420	6.114764	6.424158	6.256420	4.568306	6.988909	7.118530	4.379733	3.383493	6.977531	3.322496	6.261164	4.781766	7.173996	7.465403	2.970179	4.169230	5.795999	6.367718	6.036861	5.194696	4.980126	4.315399	4.423983	6.824823	4.711587	5.121915	5.368759	6.456040	5.884779	5.840428	6.359358	5.893525	4.704262	4.799428	5.997433	4.971090	4.087871	6.279147	4.879022	4.248379	5.000281	4.570252	6.219571	6.854303	7.091977	4.792316	3.503137	6.929370	5.068602	3.792920	5.209030	7.007847	5.428855	6.212338	5.743559	4.749126	4.708061	4.117669	5.875675	3.242391	4.346322	7.217959	6.262169	6.872583	6.981481	7.335968	5.200081	6.651258	7.300970	6.434619	5.169098	6.831474	4.558533	5.521947	6.738926	2.988170	4.927218	3.452261	3.924386	6.079562	3.886152	3.915969	3.838007	5.090840	4.954166	5.601329	5.997605	5.116800	5.544684	7.065404	7.223425	6.312967	6.724009	6.349761	5.436282	4.868737	6.542050	3.315118	4.069644	6.460844	5.519185	4.988939	5.553982	4.532908	5.190131	4.486980	3.809313	3.622868	3.500336	4.409145	6.011945	5.861216	6.457563	5.505239	4.825841	3.404556	5.151558	7.118842	6.202793	6.052192	5.946886	4.516566	4.978895	5.781628	4.760599	3.987760	3.666279	7.537475	3.994643	5.204869	3.011063	6.318752	6.176433	5.378088	4.589046	6.159451	5.746482

LINC01626	6.325419	6.640307	6.750364	5.902912	6.137019	6.514561	6.076952	6.755073	6.649358	6.338556	6.180152	5.905949	5.383539	6.157021	5.481028	6.299534	6.506661	5.668159	6.487592	6.545806	6.292186	6.276841	4.288341	6.232414	5.994659	6.117316	5.979978	6.432597	6.208126	6.212025	6.613319	6.072935	6.202793	5.683817	5.900633	5.977433	6.425117	6.655174	6.489172	6.282784	6.565927	5.929339	5.812038	6.040904	6.024120	6.344224	6.189761	6.349761	6.212521	6.553341	5.829144	5.864523	6.603564	5.641968	5.829701	6.489172	5.741365	6.543218	5.668695	6.073647	6.095667	6.462676	6.529319	6.380125	6.377801	6.255725	7.023584	6.733538	6.652077	6.667856	6.423013	6.796536	6.424825	6.381820	6.647388	6.933931	6.598436	6.562847	6.536130	6.000919	5.831051	6.284639	5.644055	6.096168	5.875675	5.836054	5.404785	6.307251	6.548531	7.129868	6.878304	7.527432	5.949673	6.410549	5.932632	6.067709	6.205198	5.115151	5.213818	6.034196	5.506022	6.081461	6.308238	6.401461	6.449178	6.313497	6.957997	6.222763	6.000597	6.748935	5.886203	6.330944	6.303573	6.726070	6.122868	5.066749	6.203650	6.017781	6.075903	5.743559	7.118373	6.597860	6.319795	6.522506	6.640640	6.239426	5.547320	6.303573	6.243786	6.081607	5.861216	5.964122	5.520055	5.619275	5.983507	6.239250	5.994484	5.884422	6.132086	6.315037	6.207603	5.800993	6.821977	6.767639	6.032553	6.589655	6.805231	6.724009	6.844383	6.171172	5.242044	5.346143	6.268789	6.229015	6.696196	6.519214	7.130688	6.634360	6.652242	6.492034	7.193889	6.292519	5.983840	6.271383	5.831408	6.598436	6.550859	6.687933	6.362012	5.568071	5.671992	5.670662	6.672695	6.014010	6.695543	5.902746	5.993944	6.179672	5.966803	5.747384	6.447523	6.552518	6.810819	6.327073	6.619366	7.096876	6.922419	6.438065	6.464369	6.261339	6.693444	6.633547	6.569047	6.116663	6.663954	6.475930	6.575845	6.608356	5.487463	5.803115	6.422080	5.401078	5.476348	5.722134	5.907399	5.491679	5.450109	6.444202	6.251304	6.407708	6.776373	6.400990	6.406241	6.608518	6.049583	5.634913	4.662049	5.269469	6.666216	5.585889	6.187919	6.569361	6.457035	5.088396	6.242113	6.591797	5.506401	6.585224	7.095641	5.750104	6.672354	5.723468	6.639497	6.573358	5.278964	6.079562	5.940777	6.533555

AC112220.2	9.194034	10.744255	10.867997	11.449854	10.816269	10.965182	11.230487	12.357534	11.864535	11.322437	11.299253	11.970501	10.884011	11.408298	11.153776	11.486239	11.138009	11.555531	12.774471	11.541954	11.649619	12.487821	11.525317	12.200621	11.610629	11.086028	9.015766	12.123620	9.996752	9.538600	11.803667	10.715522	11.444132	9.083784	10.863380	10.684060	11.309400	11.264849	12.104074	11.784487	10.720354	12.130685	11.006838	10.850917	10.543883	10.465495	10.374263	12.009301	11.270124	11.213657	10.998620	11.344753	11.337754	12.285047	11.423001	10.857343	11.148846	12.310761	11.994581	11.531086	11.832712	10.695882	10.526109	12.252621	11.078407	12.171733	11.757533	11.630155	11.331890	11.998998	11.624862	11.563461	10.790561	11.634958	11.658499	11.188160	11.015142	11.097070	10.861451	10.472537	11.179135	11.186429	10.792489	11.467049	11.495782	11.347140	11.895183	11.926923	11.543025	12.125808	11.728087	11.050478	10.479922	11.440637	10.377106	11.194728	11.268796	11.480227	11.111448	11.226653	11.494211	11.190903	11.356819	11.898984	12.433552	10.453574	11.582266	11.902791	10.953197	11.139480	12.043495	11.943939	10.971908	11.289319	11.427955	9.296921	10.271785	10.536476	11.454892	8.748036	10.545363	9.768915	9.962834	10.165490	10.108827	10.037337	8.723880	10.160447	9.870596	10.037185	9.510392	10.254503	9.410032	10.711554	9.731680	9.847050	9.657496	9.854153	8.217444	10.586129	11.335546	11.490315	10.120339	10.291284	9.669110	10.403336	10.233994	8.794756	9.255278	9.777218	10.314899	10.472219	10.935945	9.681392	10.112741	10.506316	11.254561	11.882660	10.905092	10.153421	11.396845	9.213906	10.708068	9.992041	10.090224	9.705954	10.031960	9.436775	9.954586	10.676203	11.017053	9.766473	10.774046	9.733780	9.323814	8.756854	10.757949	10.472696	9.812449	12.142227	9.521307	11.666726	11.581259	10.552793	10.884458	11.820950	10.685395	11.455518	10.905092	10.286805	10.851206	10.951782	10.412994	10.368356	10.737617	10.437038	10.944920	10.070373	9.986541	11.338052	10.592327	11.280035	9.668298	10.930245	10.230524	10.622632	9.507924	11.367577	10.532841	9.773557	10.617007	10.560245	11.594299	10.465951	10.067207	10.507361	9.715026	10.371240	9.669719	9.792404	9.611353	11.047517	10.056961	10.621312	9.927064	11.114406	9.536127	10.263119	10.189346	9.436629	11.695921	8.578321	10.666667	8.262290	10.588511	11.192657	10.387620	10.831184

AC093157.1	3.627023	4.188223	4.271805	3.479650	3.221387	3.366167	3.924795	4.617086	3.709269	3.220057	3.781660	3.395212	3.161219	3.750843	3.124425	3.044660	3.341666	4.440482	5.610093	3.864506	4.757829	2.926892	3.754031	3.640310	3.743022	3.524404	3.097736	3.519668	3.746283	3.610154	4.892937	3.063467	3.161219	3.487711	3.226157	3.933031	3.813152	4.712642	4.553466	4.121401	3.721910	4.042312	3.270828	4.489115	3.312397	3.791654	3.295582	5.402259	3.107369	4.716241	3.632948	4.398484	3.503482	3.719129	3.297455	3.292205	3.596331	5.319749	3.830561	4.283596	3.335812	4.074510	4.524143	4.202921	4.922338	3.932381	4.065635	3.770710	3.894431	3.687679	3.283804	3.260431	3.346564	3.540515	3.429348	4.345899	3.947935	3.801618	3.568681	3.309540	3.855536	3.512749	3.740380	4.841044	4.428669	3.108164	3.155587	3.969226	3.468400	4.800062	4.082613	4.312513	3.047315	3.331917	2.885728	2.947986	3.249846	3.322172	2.982540	2.924028	2.848837	3.159437	3.540932	3.524206	4.662858	2.881617	4.400248	3.050475	2.997419	3.437038	3.661403	4.133401	4.124030	3.543818	4.194775	2.826793	3.373589	3.495782	5.286009	3.241347	5.130587	3.928494	3.596936	2.967997	3.069481	2.958724	3.895449	4.239223	3.074437	4.194119	3.079410	3.043133	3.126399	2.986558	2.986951	3.370455	3.721463	3.219672	3.335169	3.357588	2.960973	3.397663	3.083703	2.985787	3.614174	2.901598	3.392955	3.128450	4.423542	3.636471	3.228359	3.100934	4.066279	3.750622	3.647551	3.542870	3.122398	5.036945	4.105649	3.513752	2.935739	3.846532	5.206028	3.415696	3.598158	3.651989	3.661403	3.170910	4.819605	4.465643	3.372751	3.274802	4.752973	3.123311	3.135127	3.302535	3.556033	3.827843	3.581299	4.241852	3.579359	5.136803	4.558767	4.877177	4.259390	4.591411	5.224876	4.526954	3.101500	3.913358	3.509038	3.412024	3.891153	2.921280	4.698939	4.279959	4.617206	4.251723	3.615932	4.083676	4.694948	4.188883	3.918102	5.146547	3.040061	4.568093	4.177463	4.334201	4.483961	4.202047	6.163738	3.920194	4.208643	2.942255	2.969823	2.976228	2.882817	3.054972	3.112510	2.949946	3.224618	2.991917	3.361296	4.474278	3.271619	4.595326	4.343924	4.039525	3.925693	3.521873	3.273704	2.868195	3.642453	2.899003	2.990624	3.419157	3.541895	5.535095

TMCC1-AS1	5.796567	6.142058	5.596819	5.706289	5.402456	5.592538	6.239096	3.744836	3.370996	5.573354	6.743511	5.909357	5.664054	5.646170	5.497164	5.821306	4.063079	5.897757	5.213818	5.591754	5.999541	5.324768	3.618361	4.774499	6.082836	6.452268	6.661341	5.810403	6.154825	5.497928	4.537161	6.009128	7.029874	5.922559	6.584714	5.972886	6.297901	6.066083	6.045183	6.531278	5.488371	6.522347	5.705254	5.740812	5.933348	5.037736	5.488194	5.692261	4.516358	6.072935	6.319439	6.028906	6.577491	6.567597	5.365800	5.539594	7.447746	6.340567	6.635961	6.418492	6.766660	6.303742	6.737455	5.639734	7.087997	5.573354	5.973440	6.064179	6.680626	5.477492	5.301233	6.131717	4.763775	5.516058	6.066803	5.697684	5.914194	5.491117	6.236650	6.668981	6.910655	7.181888	6.758139	6.525818	6.733689	6.695389	6.632728	5.432219	5.715850	6.297553	6.888719	6.041931	6.228480	6.359193	6.739082	6.452605	5.247821	5.836962	6.218215	5.416020	5.197306	5.835193	5.264677	4.993108	5.334938	5.356278	3.787161	5.003789	4.395431	4.985191	4.135395	4.757390	4.037145	4.881804	4.472347	5.703743	6.171336	4.871035	4.511393	6.034380	5.632451	5.022770	5.886377	4.411994	6.601827	5.091838	7.456028	4.362400	5.413577	5.839699	6.255038	7.564103	5.495284	4.002553	6.603086	4.533330	6.816500	7.808778	6.818290	5.948272	4.854383	5.772748	5.708658	5.604154	11.349360	4.001936	5.978683	6.409686	5.998140	7.035624	5.338729	5.052568	4.284044	8.273134	5.122714	6.345390	3.840908	6.167379	6.535471	6.306442	5.489780	7.228734	3.991355	5.836808	4.314274	5.881412	7.826953	7.804638	4.633497	5.498320	6.681110	6.359019	4.490426	6.316736	7.283220	7.818572	7.497094	6.412522	6.827484	6.491222	6.087306	5.724374	4.557657	6.297901	5.375140	4.802168	6.121636	5.114771	6.255725	4.875476	4.504347	5.858142	5.860863	5.893709	5.135818	6.560227	4.926168	5.060038	6.559899	6.346393	7.039538	6.670249	5.951091	5.924919	6.606208	5.668508	8.579360	4.715396	5.752102	8.090173	5.881662	5.751191	4.767009	5.075261	6.053907	5.761773	6.941991	4.594060	7.026662	3.892199	7.938844	5.356854	4.446094	5.861398	5.843704	2.989405	6.088523	4.526954	5.134665	5.514453	4.052563	6.172693	4.873219	5.848095	4.554945	5.061015	6.794940	4.937893

AC136285.1	5.345539	6.001621	5.171385	6.062264	5.755895	6.724651	6.413860	5.301629	5.280737	6.688232	6.861393	6.883083	5.579462	5.081850	5.562964	6.777353	5.428231	6.244951	4.378669	6.365725	6.585567	4.769928	3.314806	5.059621	3.948370	6.229710	7.051488	6.174551	6.595852	6.403904	5.451257	5.075261	4.670157	5.797284	5.084460	5.149291	4.818987	4.208643	4.116995	4.668831	6.681424	2.921869	4.388910	5.176137	4.039309	3.806591	6.878132	3.565890	5.085724	3.164807	3.986286	4.742727	4.763361	3.525332	4.589046	6.907043	3.629032	3.787788	3.687063	3.431688	3.150753	6.351457	4.279291	4.837655	4.202274	3.968315	4.461347	5.165506	5.274525	3.344470	6.146017	6.089923	6.959726	5.844914	6.499883	5.650665	5.765566	5.284366	5.763409	4.020669	3.835458	4.502191	3.758136	3.671130	3.529594	6.276695	3.742020	4.526742	4.928648	3.233362	3.973973	4.626121	5.192676	5.513706	4.908622	5.317658	4.855023	5.169712	5.364627	5.443514	4.661386	5.201290	5.575947	5.336960	5.185394	6.580629	4.383610	5.239849	4.222766	6.819891	3.605586	4.504789	4.090941	7.125623	5.783088	4.497063	3.844991	6.108872	6.611143	5.114185	7.077988	5.976039	6.678964	6.173519	7.160265	6.372955	5.049984	5.139246	7.184736	7.442516	7.101269	6.476102	5.704288	6.925974	6.201890	5.443327	6.142756	3.695280	5.968439	6.182207	6.194626	3.664638	5.743733	5.207233	6.496048	7.114394	6.803481	6.544021	7.491020	5.697494	5.055751	5.910785	6.683564	5.096222	5.373801	5.285608	5.126754	4.926168	5.320125	7.365622	2.867173	5.005161	5.771240	3.587283	3.611973	8.247810	3.549833	5.285608	5.679002	4.927846	4.589267	4.810162	6.159451	5.348134	6.998483	3.346564	3.657866	3.558339	4.396141	3.046612	6.603879	3.297133	3.680841	4.570485	4.526322	4.871491	5.511823	5.705050	4.950416	6.460672	6.542552	8.069057	6.460347	6.318421	5.711196	6.138754	6.120281	6.255895	4.394772	4.411759	3.839112	3.999384	3.903970	4.027744	5.751747	4.745760	4.315840	5.076102	4.397856	4.256493	5.148519	5.591378	6.458352	6.257931	5.987923	5.711575	5.237869	5.949673	5.439680	6.355425	5.173168	6.206757	5.345539	4.546670	7.491173	5.070584	5.014462	4.004116	7.726369	4.918735	3.731092	3.466476	6.936660	6.455588	6.265713	4.229679	6.214073	7.818875

CASC15	3.760706	4.039945	3.838884	3.599926	4.304427	5.883889	5.084460	4.991612	5.137013	5.266871	5.177162	4.967357	4.988095	4.478041	4.805149	4.588420	3.336602	5.369136	4.699627	4.279071	4.097305	4.493308	4.021106	4.760783	3.905958	4.734603	6.509428	4.196467	5.229742	4.897234	3.693618	3.784906	6.391390	5.326937	3.168541	4.524788	6.528665	3.782941	3.942389	4.532673	5.053435	4.971901	4.069444	5.601705	3.534455	4.045616	5.051601	3.601673	4.370684	3.506434	3.480978	7.421974	5.794569	3.748721	4.329236	5.784311	3.739768	3.174479	3.605770	3.550424	3.456247	5.308286	3.907900	4.792691	3.107586	3.105510	4.040813	4.515737	3.403206	5.114965	4.481472	4.957517	4.798980	4.202921	5.058586	4.913698	4.776361	4.081762	4.323808	3.967464	4.655105	3.616132	3.754459	4.395908	3.825965	4.193897	4.901135	3.802663	3.330656	3.209242	4.201623	3.609188	3.215552	4.746858	4.487615	5.312635	4.513162	4.649422	5.498902	5.664256	5.846222	4.550664	4.648431	3.983919	4.077226	5.636578	4.645156	4.442859	5.607597	5.826559	5.111719	7.682592	4.738203	5.404268	4.899401	6.100310	4.374789	3.650427	4.748287	4.320790	5.036945	4.297673	4.210138	5.243412	5.069990	6.101704	5.824234	5.593845	5.429406	5.514046	5.957324	5.627077	5.445240	6.269643	4.967357	4.789101	5.508769	5.169309	4.691364	5.293505	5.071425	3.550026	5.237869	4.672733	6.187579	5.332656	5.147334	6.588530	4.003237	4.918512	5.581712	6.641604	5.447832	4.425470	4.426792	6.204845	5.178778	4.505883	4.681788	6.736780	5.245221	4.853324	5.772380	3.435272	3.913564	6.469202	5.872814	3.162507	3.497287	3.475980	6.804151	5.801382	3.815433	3.668288	5.476348	4.002780	3.183839	4.729562	3.504732	4.263766	5.221285	4.281578	5.042212	3.196648	4.227481	3.826564	4.662858	4.606924	5.777437	4.360707	5.130792	6.323720	4.797882	5.207233	5.463454	5.609336	4.785130	4.539078	4.622899	4.614662	3.829065	4.974640	4.935362	4.405822	4.992029	4.137797	3.742020	5.148333	4.452895	4.935362	4.788883	4.615121	5.492218	5.825674	6.008962	5.519715	5.684924	5.078395	4.663287	5.803834	5.761966	5.633793	5.336327	4.518062	6.039639	5.385211	4.784124	3.529594	6.037725	4.957084	7.653754	6.499728	5.725915	4.973579	5.083448	4.435400	5.840599	5.459435

LINC02294	4.235090	4.381390	4.937670	5.008283	5.074448	4.427225	5.393986	4.847873	5.089598	5.060442	5.435317	5.434949	4.996722	4.831651	4.014711	5.238461	4.865170	4.009340	5.272683	5.456085	4.988699	5.320716	4.461781	5.766090	5.420915	5.429568	5.311070	5.642899	5.674417	5.085506	4.915143	4.377383	4.113339	4.268916	4.939145	4.121401	4.714555	4.688438	4.762246	6.005022	5.339915	5.197508	4.270707	4.422440	4.197114	7.651747	5.589862	3.992680	3.212697	4.926387	3.952698	5.340859	3.990057	5.165506	4.331236	5.414157	3.842101	4.217517	4.705302	4.358088	3.307479	4.984172	4.769928	4.897894	4.422659	3.864320	4.923847	5.206824	5.013840	4.861638	5.297845	5.358339	5.606131	4.666915	4.902368	4.460477	4.781556	4.693307	4.837655	4.070841	5.039101	4.992029	4.264190	4.297429	3.824156	4.347401	4.211207	3.773677	3.023395	5.144750	4.399847	4.417533	4.892281	5.070383	4.818563	4.900045	3.798195	4.901741	5.207233	4.627574	5.253548	4.252568	4.772601	5.074842	5.368189	5.161854	4.798541	4.920613	5.185007	5.823480	4.130031	5.250790	5.175557	5.733728	4.530516	5.349462	4.488884	4.382733	5.499654	5.238860	4.962733	4.738203	5.210230	5.871685	6.043642	5.868676	5.367011	5.162655	6.545484	5.055139	5.060442	5.563547	4.204758	4.701302	5.294248	4.157373	3.665267	5.277938	5.809630	5.016059	6.200316	4.917266	5.721228	5.851489	7.632388	4.816461	5.436481	6.152236	5.787010	4.630905	5.307133	5.933698	4.085430	4.322056	6.279654	4.870222	5.604559	5.242255	5.909893	6.349235	4.857743	3.826564	5.154379	4.722391	7.656275	5.749561	5.386330	4.652372	5.150162	4.369123	7.276724	4.579245	4.776772	3.901062	6.286020	4.996722	5.293129	4.688859	4.843806	4.307910	5.460645	5.046672	4.747288	4.593852	7.091816	4.746622	5.102166	5.174727	4.491142	5.181372	5.209826	5.462213	4.875280	6.068929	4.396577	4.903797	4.544230	5.175953	4.280400	4.711373	4.469469	4.885618	3.786037	4.067541	4.170991	4.499842	3.998329	3.768431	5.657391	7.203493	4.291559	4.980126	4.686126	5.138445	4.664961	4.670370	4.899001	5.172120	4.794167	4.403745	5.141933	4.915972	4.339822	9.433276	6.046564	5.706129	4.847004	5.557563	6.017610	4.664293	6.945345	5.787944	6.159072	4.941572	4.771765	4.757390	5.315439	5.758523

LINC02293	4.235090	4.381390	4.937670	5.008283	5.074448	4.427225	5.393986	4.847873	5.089598	5.060442	5.435317	5.434949	4.996722	4.831651	4.014711	5.238461	4.865170	4.009340	5.272683	5.456085	4.988699	5.320716	4.461781	5.766090	5.420915	5.429568	5.311070	5.642899	5.674417	5.085506	4.915143	4.377383	4.113339	4.268916	4.939145	4.121401	4.714555	4.688438	4.762246	6.005022	5.339915	5.197508	4.270707	4.422440	4.197114	7.651747	5.589862	3.992680	3.212697	4.926387	3.952698	5.340859	3.990057	5.165506	4.331236	5.414157	3.842101	4.217517	4.705302	4.358088	3.307479	4.984172	4.769928	4.897894	4.422659	3.864320	4.923847	5.206824	5.013840	4.861638	5.297845	5.358339	5.606131	4.666915	4.902368	4.460477	4.781556	4.693307	4.837655	4.070841	5.039101	4.992029	4.264190	4.297429	3.824156	4.347401	4.211207	3.773677	3.023395	5.144750	4.399847	4.417533	4.892281	5.070383	4.818563	4.900045	3.798195	4.901741	5.207233	4.627574	5.253548	4.252568	4.772601	5.074842	5.368189	5.161854	4.798541	4.920613	5.185007	5.823480	4.130031	5.250790	5.175557	5.733728	4.530516	5.349462	4.488884	4.382733	5.499654	5.238860	4.962733	4.738203	5.210230	5.871685	6.043642	5.868676	5.367011	5.162655	6.545484	5.055139	5.060442	5.563547	4.204758	4.701302	5.294248	4.157373	3.665267	5.277938	5.809630	5.016059	6.200316	4.917266	5.721228	5.851489	7.632388	4.816461	5.436481	6.152236	5.787010	4.630905	5.307133	5.933698	4.085430	4.322056	6.279654	4.870222	5.604559	5.242255	5.909893	6.349235	4.857743	3.826564	5.154379	4.722391	7.656275	5.749561	5.386330	4.652372	5.150162	4.369123	7.276724	4.579245	4.776772	3.901062	6.286020	4.996722	5.293129	4.688859	4.843806	4.307910	5.460645	5.046672	4.747288	4.593852	7.091816	4.746622	5.102166	5.174727	4.491142	5.181372	5.209826	5.462213	4.875280	6.068929	4.396577	4.903797	4.544230	5.175953	4.280400	4.711373	4.469469	4.885618	3.786037	4.067541	4.170991	4.499842	3.998329	3.768431	5.657391	7.203493	4.291559	4.980126	4.686126	5.138445	4.664961	4.670370	4.899001	5.172120	4.794167	4.403745	5.141933	4.915972	4.339822	9.433276	6.046564	5.706129	4.847004	5.557563	6.017610	4.664293	6.945345	5.787944	6.159072	4.941572	4.771765	4.757390	5.315439	5.758523

AL110292.1	4.235090	4.381390	4.937670	5.008283	5.074448	4.427225	5.393986	4.847873	5.089598	5.060442	5.435317	5.434949	4.996722	4.831651	4.014711	5.238461	4.865170	4.009340	5.272683	5.456085	4.988699	5.320716	4.461781	5.766090	5.420915	5.429568	5.311070	5.642899	5.674417	5.085506	4.915143	4.377383	4.113339	4.268916	4.939145	4.121401	4.714555	4.688438	4.762246	6.005022	5.339915	5.197508	4.270707	4.422440	4.197114	7.651747	5.589862	3.992680	3.212697	4.926387	3.952698	5.340859	3.990057	5.165506	4.331236	5.414157	3.842101	4.217517	4.705302	4.358088	3.307479	4.984172	4.769928	4.897894	4.422659	3.864320	4.923847	5.206824	5.013840	4.861638	5.297845	5.358339	5.606131	4.666915	4.902368	4.460477	4.781556	4.693307	4.837655	4.070841	5.039101	4.992029	4.264190	4.297429	3.824156	4.347401	4.211207	3.773677	3.023395	5.144750	4.399847	4.417533	4.892281	5.070383	4.818563	4.900045	3.798195	4.901741	5.207233	4.627574	5.253548	4.252568	4.772601	5.074842	5.368189	5.161854	4.798541	4.920613	5.185007	5.823480	4.130031	5.250790	5.175557	5.733728	4.530516	5.349462	4.488884	4.382733	5.499654	5.238860	4.962733	4.738203	5.210230	5.871685	6.043642	5.868676	5.367011	5.162655	6.545484	5.055139	5.060442	5.563547	4.204758	4.701302	5.294248	4.157373	3.665267	5.277938	5.809630	5.016059	6.200316	4.917266	5.721228	5.851489	7.632388	4.816461	5.436481	6.152236	5.787010	4.630905	5.307133	5.933698	4.085430	4.322056	6.279654	4.870222	5.604559	5.242255	5.909893	6.349235	4.857743	3.826564	5.154379	4.722391	7.656275	5.749561	5.386330	4.652372	5.150162	4.369123	7.276724	4.579245	4.776772	3.901062	6.286020	4.996722	5.293129	4.688859	4.843806	4.307910	5.460645	5.046672	4.747288	4.593852	7.091816	4.746622	5.102166	5.174727	4.491142	5.181372	5.209826	5.462213	4.875280	6.068929	4.396577	4.903797	4.544230	5.175953	4.280400	4.711373	4.469469	4.885618	3.786037	4.067541	4.170991	4.499842	3.998329	3.768431	5.657391	7.203493	4.291559	4.980126	4.686126	5.138445	4.664961	4.670370	4.899001	5.172120	4.794167	4.403745	5.141933	4.915972	4.339822	9.433276	6.046564	5.706129	4.847004	5.557563	6.017610	4.664293	6.945345	5.787944	6.159072	4.941572	4.771765	4.757390	5.315439	5.758523

AC114498.1	9.368907	9.572872	9.092226	9.568103	9.515000	10.260570	10.472537	10.505574	10.052192	10.941113	10.629019	10.546111	10.200957	9.509606	8.722821	11.512308	10.186029	10.063481	11.200721	10.001482	10.909294	11.500773	10.373227	10.148869	10.803437	9.753292	10.033753	10.973802	9.886092	9.877347	10.256634	9.445145	10.795839	9.590587	9.522567	9.784276	9.755913	9.773401	10.671356	11.330601	10.050514	11.487573	9.207792	9.289113	8.849892	9.465805	10.968006	10.256634	9.986107	9.947489	9.325661	10.800245	9.155247	10.853314	10.239105	9.703107	9.790524	10.458804	10.427634	10.886712	10.556615	9.461985	7.954791	11.058929	9.435860	9.903641	10.559019	10.002883	10.379694	10.278197	10.741714	10.374121	11.154075	10.258997	10.265297	10.056350	9.367716	10.724471	10.568630	9.623840	9.560811	9.542052	8.772629	10.445527	10.947159	9.096576	10.331914	10.428662	9.841825	10.690215	9.990754	10.829909	9.598172	10.584342	10.032568	9.527248	10.017128	9.817990	10.137918	9.696933	10.292343	10.211224	9.497806	10.525956	10.016493	9.859745	9.791905	10.278521	9.965758	10.667256	11.210244	10.398534	10.222882	11.275733	10.994906	12.644874	9.462116	9.668780	11.331613	9.497175	9.041181	8.652972	9.653534	10.679894	10.053909	9.839846	8.373576	9.259991	9.665455	10.348093	9.321385	9.297087	9.050240	8.883274	9.268311	8.813200	9.971216	8.795349	9.155247	9.574283	9.458786	9.570274	9.126584	9.193891	8.642660	9.854153	9.168609	9.985649	9.997209	8.879106	8.932113	10.113777	11.700848	9.288485	9.341453	9.560327	9.221410	9.581469	9.941008	9.992195	9.794875	8.483106	9.205649	9.018527	9.654530	10.949809	8.893784	8.990467	8.745271	9.117571	8.525064	8.287349	9.868052	8.642371	9.067710	8.300189	8.925032	9.133821	8.840427	11.308373	9.500413	8.704437	9.970187	10.052635	10.163835	9.684992	10.204147	10.304016	10.365006	9.243968	9.136065	10.896881	9.336166	9.025867	9.578233	9.303246	9.592125	9.657050	8.758828	9.109422	9.298596	9.149261	9.027348	8.905380	8.664085	10.071474	8.532428	9.099970	8.465685	8.772484	9.411410	9.426987	9.036468	10.967834	8.478522	9.768131	9.027842	9.844727	8.993030	8.790410	8.647531	8.982441	8.893638	8.663627	9.840773	9.625053	9.756381	9.804238	10.904527	10.003500	8.935930	11.189332	10.504682	9.685927	11.689738	8.642211	10.326880	10.014606

AC144568.2	9.368907	9.572872	9.092226	9.568103	9.515000	10.260570	10.472537	10.505574	10.052192	10.941113	10.629019	10.546111	10.200957	9.509606	8.722821	11.512308	10.186029	10.063481	11.200721	10.001482	10.909294	11.500773	10.373227	10.148869	10.803437	9.753292	10.033753	10.973802	9.886092	9.877347	10.256634	9.445145	10.795839	9.590587	9.522567	9.784276	9.755913	9.773401	10.671356	11.330601	10.050514	11.487573	9.207792	9.289113	8.849892	9.465805	10.968006	10.256634	9.986107	9.947489	9.325661	10.800245	9.155247	10.853314	10.239105	9.703107	9.790524	10.458804	10.427634	10.886712	10.556615	9.461985	7.954791	11.058929	9.435860	9.903641	10.559019	10.002883	10.379694	10.278197	10.741714	10.374121	11.154075	10.258997	10.265297	10.056350	9.367716	10.724471	10.568630	9.623840	9.560811	9.542052	8.772629	10.445527	10.947159	9.096576	10.331914	10.428662	9.841825	10.690215	9.990754	10.829909	9.598172	10.584342	10.032568	9.527248	10.017128	9.817990	10.137918	9.696933	10.292343	10.211224	9.497806	10.525956	10.016493	9.859745	9.791905	10.278521	9.965758	10.667256	11.210244	10.398534	10.222882	11.275733	10.994906	12.644874	9.462116	9.668780	11.331613	9.497175	9.041181	8.652972	9.653534	10.679894	10.053909	9.839846	8.373576	9.259991	9.665455	10.348093	9.321385	9.297087	9.050240	8.883274	9.268311	8.813200	9.971216	8.795349	9.155247	9.574283	9.458786	9.570274	9.126584	9.193891	8.642660	9.854153	9.168609	9.985649	9.997209	8.879106	8.932113	10.113777	11.700848	9.288485	9.341453	9.560327	9.221410	9.581469	9.941008	9.992195	9.794875	8.483106	9.205649	9.018527	9.654530	10.949809	8.893784	8.990467	8.745271	9.117571	8.525064	8.287349	9.868052	8.642371	9.067710	8.300189	8.925032	9.133821	8.840427	11.308373	9.500413	8.704437	9.970187	10.052635	10.163835	9.684992	10.204147	10.304016	10.365006	9.243968	9.136065	10.896881	9.336166	9.025867	9.578233	9.303246	9.592125	9.657050	8.758828	9.109422	9.298596	9.149261	9.027348	8.905380	8.664085	10.071474	8.532428	9.099970	8.465685	8.772484	9.411410	9.426987	9.036468	10.967834	8.478522	9.768131	9.027842	9.844727	8.993030	8.790410	8.647531	8.982441	8.893638	8.663627	9.840773	9.625053	9.756381	9.804238	10.904527	10.003500	8.935930	11.189332	10.504682	9.685927	11.689738	8.642211	10.326880	10.014606

C5orf64	4.901741	3.189710	3.557581	4.113965	3.606610	4.844447	3.838432	4.019554	4.304213	3.370455	4.404666	4.539977	4.310159	3.508878	3.127424	3.512576	3.626812	4.437866	4.554733	3.790551	4.135166	3.820601	2.980874	3.324325	2.930172	4.327452	4.022811	3.787788	4.310159	5.584774	3.377394	3.527038	4.607765	4.526322	4.125253	3.888906	4.562063	4.291112	3.766295	3.999992	4.364615	3.047744	3.353669	4.346322	3.626008	5.081245	4.231140	2.933780	3.748541	4.517429	3.778359	3.610373	3.923719	3.926119	4.100404	4.056940	4.880948	3.256050	3.239897	3.365508	2.977408	4.137598	3.350916	3.748346	3.775550	4.136944	2.992717	3.894431	4.154991	3.191065	4.224260	4.444999	4.672505	3.896720	3.819912	4.820024	3.678626	4.531195	3.794458	3.611177	3.333065	3.919566	4.059769	4.045188	4.105649	4.162256	4.776982	4.295017	3.413828	4.578613	3.833320	3.615932	3.273704	4.090253	3.886748	4.137156	4.825841	4.212080	3.343324	3.884271	3.424717	3.243970	3.957225	4.124421	4.297007	4.580787	4.005674	3.156927	3.404038	5.091838	3.673284	3.354007	3.015866	4.486980	3.240032	4.036272	3.762370	3.818855	4.820465	4.301102	3.296065	3.303625	3.386728	3.352560	4.439142	3.198153	3.359512	3.979980	3.157660	4.158412	3.960098	3.944838	3.531287	5.481420	3.877169	4.122295	3.514626	3.355689	3.489710	3.554680	2.856555	2.969540	3.275739	2.850474	3.079298	3.753370	3.021553	3.588616	5.352484	3.265866	3.934702	3.139936	4.226166	3.188468	3.653225	4.156749	3.176969	3.180672	2.869857	4.011825	2.934625	3.419157	3.401420	3.792266	4.340694	4.370451	2.935901	3.792701	4.010802	3.699957	3.484451	3.759641	4.000622	3.365817	3.440139	3.743889	3.180263	2.966404	3.090589	2.903392	3.370101	3.373589	4.406483	4.292225	3.430280	2.963914	3.473087	3.066192	2.911537	3.075184	3.644257	5.112562	3.809533	3.538985	3.955463	3.414352	3.649639	3.666887	3.577750	3.666279	4.147485	3.475806	3.552011	3.458213	3.135255	3.911846	3.421184	3.242972	3.282097	3.596508	2.935901	3.087292	3.456601	3.195567	3.451366	3.393483	3.296519	3.512383	3.169872	3.072069	3.075743	3.283015	3.323316	4.643182	4.507907	3.204680	3.114613	3.138823	4.684175	3.567883	3.227892	2.934808	5.446840	3.517163	3.204801	3.631713	4.046238	4.713491

LINC02527	3.807012	3.896934	4.498788	4.245775	3.253117	3.425458	3.930246	3.660415	3.640693	4.119452	5.472341	4.880280	4.335291	4.626760	4.841900	3.716485	4.538874	3.761949	6.355059	4.810821	5.403869	5.022592	7.382687	4.800923	4.275750	4.939947	4.913698	5.206028	3.987558	3.424550	3.849064	5.030127	4.698722	6.169616	4.131875	4.308576	4.320571	4.131246	4.589492	3.992227	3.643851	4.768288	4.912630	3.973097	3.793589	5.907569	3.846944	4.108917	5.287944	4.137598	3.937294	4.460696	4.587990	4.528407	5.664600	4.117895	4.353548	2.971185	5.278342	4.648544	4.890642	4.625263	4.635636	5.239849	7.603129	3.800298	2.815447	3.953753	3.590928	4.470128	4.191920	4.516566	3.684817	4.036500	4.807440	3.617462	3.556975	3.846309	3.998329	3.922669	3.488662	4.389751	3.891573	4.284509	3.512383	4.319720	3.262344	4.466094	3.318632	4.340238	4.397856	4.677106	3.553917	4.361722	3.177342	4.193224	5.256134	4.236160	5.112562	4.389969	3.631713	4.341361	4.272236	5.399603	5.398042	3.330234	3.512576	4.939531	5.240060	3.899788	4.092057	3.576726	3.168670	3.777351	4.088287	3.092801	3.469431	4.765699	3.908793	5.092471	4.375223	4.756961	7.008016	3.223498	3.837678	4.293954	5.243037	3.798781	3.248412	4.255230	6.807255	3.018838	6.824026	3.888276	3.944424	6.293193	4.011621	5.080015	3.670528	4.569586	4.485251	5.148333	5.214034	7.587332	3.261735	3.394523	5.304215	3.693202	3.597725	4.478244	5.669981	5.642899	4.065006	4.351108	6.166148	4.208456	4.885618	6.141195	4.158864	3.463048	4.166893	6.129996	5.280957	5.390271	6.341221	3.299913	3.926712	6.600064	4.323129	5.189361	4.459073	4.373025	4.717502	5.176738	7.126590	3.871796	5.567854	7.767009	6.575670	8.039959	3.341822	4.239674	4.146850	8.809382	6.950590	5.406292	5.761196	6.580790	4.475603	3.110615	4.337231	3.583046	5.039903	4.545772	3.794028	3.432203	4.378864	3.896056	3.300530	5.938459	4.815381	4.045833	3.669497	3.948773	5.392589	7.165196	3.479108	5.297077	4.914529	4.017832	4.115709	5.124112	4.930410	5.696189	5.110560	5.991118	5.775099	5.178363	5.235426	4.545552	4.909483	4.441288	3.857006	7.687007	3.462345	4.771198	4.292692	5.294445	3.711334	3.579772	6.748935	3.010039	3.289973	3.472214	5.399411	5.979978	4.621851	3.433628

LINC00907	6.549699	6.767480	6.716549	6.589006	6.674294	6.975741	6.799271	7.726063	7.965553	7.071494	6.884396	6.974641	6.488062	6.862992	6.390363	6.379471	7.043199	6.725888	6.734661	6.509101	7.016162	7.160590	7.208002	6.975583	6.899672	7.017952	6.324927	7.056336	6.529154	6.726070	7.481352	6.701902	7.036542	6.131372	6.881513	6.741451	6.723049	6.757487	7.021413	7.203331	6.990471	6.662500	6.450000	6.570194	6.409342	6.247783	6.836129	7.077190	6.696964	7.194566	6.576684	6.745598	6.349235	6.527668	6.589006	6.994918	6.243095	7.201165	6.871129	6.785647	7.473087	6.475930	6.920814	7.037474	6.920499	6.995990	7.015004	6.924538	6.999995	7.493360	6.720973	7.030340	6.629051	6.736274	6.790070	6.742734	6.973051	6.963561	6.802210	6.569708	6.762051	6.457889	6.359531	6.681110	6.808558	6.255368	6.668503	6.870163	7.232778	6.815567	7.013009	7.543432	6.561694	6.566408	6.302543	6.450320	6.163654	6.352424	6.644025	6.668647	6.949641	6.314368	6.585060	6.750989	7.264952	6.491065	7.459357	6.815085	7.365622	6.937644	7.609761	7.616037	7.563478	7.248533	6.653213	7.483531	7.007190	6.684412	6.749255	6.750533	7.131970	6.805835	6.887252	7.138202	7.201468	7.455423	6.686460	7.275773	6.868569	6.960528	7.133547	6.712113	7.238594	7.115180	6.320511	6.919027	6.739567	7.544875	6.313672	7.087830	6.994459	7.611437	6.806159	6.852662	7.158379	6.994459	7.414619	7.366238	7.236888	7.154192	6.624362	6.562022	7.063169	6.776864	6.227975	6.719475	6.766184	7.176828	7.039223	7.090879	6.966758	6.487258	6.616420	6.954230	7.066199	7.456502	6.983822	6.437128	7.203644	6.707215	6.479137	6.152751	6.681267	6.111465	6.951856	6.340247	6.808714	6.811147	6.678786	7.763284	6.850296	7.271258	7.518351	7.244534	7.098777	7.289104	7.261055	7.514055	7.739929	7.186835	7.119495	7.434394	6.844034	6.541705	6.804151	7.027322	7.241803	6.875792	6.431404	6.240087	6.948257	6.834077	6.348452	6.539632	6.465373	6.826397	6.689803	7.091816	6.807413	6.921443	7.741021	6.815732	7.183775	7.058706	6.881192	6.441052	6.560227	6.734308	6.629353	6.888402	6.205756	7.040326	6.651431	6.729992	7.134838	7.086585	6.711800	7.248850	7.891377	7.127988	7.133895	8.532646	7.782857	7.085683	6.855890	7.109022	7.132283	7.257558

LINC01978	15.528080	12.486970	12.408855	13.310353	13.037820	13.207137	12.143103	12.914236	13.212044	13.211496	12.809469	12.681802	12.448476	12.309669	11.637393	12.972576	12.660693	11.944227	12.642076	12.530672	12.191034	12.213930	12.167732	12.660149	12.540468	12.737304	13.563621	13.165501	13.483216	13.024204	12.912699	11.930334	11.136974	12.279590	11.840838	11.841449	11.907737	12.026457	12.417994	12.191674	13.069852	12.252154	12.176242	11.976836	11.809331	11.795854	13.315405	12.042272	11.874610	11.935336	14.611416	11.340546	11.200580	11.372164	11.166369	12.998952	10.908532	11.511854	11.721674	11.166369	11.416326	13.063428	11.936861	12.178794	11.484271	11.565388	12.470629	12.201709	11.993060	12.237106	12.607232	12.593626	13.120243	12.596461	12.437588	12.307622	12.385620	12.135429	13.250652	10.878399	11.471947	10.922234	11.165803	11.093843	11.128092	11.464783	11.110584	11.206137	11.297513	11.646786	11.463337	11.557455	11.442790	12.049360	12.905530	11.741588	11.304260	12.075588	12.193991	13.367473	12.616315	12.381950	12.675868	12.638546	11.724680	13.182498	12.166337	12.311740	12.644874	13.546652	12.178954	12.281610	12.137705	13.612555	12.806490	14.647121	13.841373	12.348522	13.709682	12.571510	12.503046	12.691189	13.015726	13.891542	12.762553	13.066984	13.242390	12.819581	13.024204	12.823533	12.947738	12.895811	12.715418	12.314144	13.158966	12.401128	12.418964	12.259110	11.915924	12.150335	12.125254	12.057775	13.026028	12.522828	12.368300	12.840549	12.978958	13.855947	12.970251	12.520391	12.245314	12.144213	12.487141	12.129027	12.176872	11.931556	12.422459	12.642396	12.413338	13.322325	12.216837	12.214256	12.127814	11.837404	11.954390	13.407961	11.742298	11.670177	11.686355	12.904820	11.901889	11.272078	11.513628	11.241360	13.174180	11.252514	11.761777	11.635707	11.123509	11.610339	13.027278	12.141913	12.052666	11.728547	11.579668	12.398188	12.285989	12.012048	12.329087	12.548924	12.581318	13.397318	12.446254	12.306066	12.394097	12.447571	12.383384	12.397685	11.099443	11.252070	10.886126	10.969544	11.353164	10.969237	12.243145	11.131212	10.777392	11.469123	11.452540	11.651082	11.593433	11.653194	12.137094	12.174829	12.097568	12.370197	12.634617	12.177525	12.796078	12.250390	12.927582	13.171908	12.616955	12.099378	13.279450	12.357611	12.289665	12.122694	13.579444	12.140350	12.134816	13.152225	13.656748	12.736077	12.948751	11.695921	12.418655	13.013167

LINC01979	15.528080	12.486970	12.408855	13.310353	13.037820	13.207137	12.143103	12.914236	13.212044	13.211496	12.809469	12.681802	12.448476	12.309669	11.637393	12.972576	12.660693	11.944227	12.642076	12.530672	12.191034	12.213930	12.167732	12.660149	12.540468	12.737304	13.563621	13.165501	13.483216	13.024204	12.912699	11.930334	11.136974	12.279590	11.840838	11.841449	11.907737	12.026457	12.417994	12.191674	13.069852	12.252154	12.176242	11.976836	11.809331	11.795854	13.315405	12.042272	11.874610	11.935336	14.611416	11.340546	11.200580	11.372164	11.166369	12.998952	10.908532	11.511854	11.721674	11.166369	11.416326	13.063428	11.936861	12.178794	11.484271	11.565388	12.470629	12.201709	11.993060	12.237106	12.607232	12.593626	13.120243	12.596461	12.437588	12.307622	12.385620	12.135429	13.250652	10.878399	11.471947	10.922234	11.165803	11.093843	11.128092	11.464783	11.110584	11.206137	11.297513	11.646786	11.463337	11.557455	11.442790	12.049360	12.905530	11.741588	11.304260	12.075588	12.193991	13.367473	12.616315	12.381950	12.675868	12.638546	11.724680	13.182498	12.166337	12.311740	12.644874	13.546652	12.178954	12.281610	12.137705	13.612555	12.806490	14.647121	13.841373	12.348522	13.709682	12.571510	12.503046	12.691189	13.015726	13.891542	12.762553	13.066984	13.242390	12.819581	13.024204	12.823533	12.947738	12.895811	12.715418	12.314144	13.158966	12.401128	12.418964	12.259110	11.915924	12.150335	12.125254	12.057775	13.026028	12.522828	12.368300	12.840549	12.978958	13.855947	12.970251	12.520391	12.245314	12.144213	12.487141	12.129027	12.176872	11.931556	12.422459	12.642396	12.413338	13.322325	12.216837	12.214256	12.127814	11.837404	11.954390	13.407961	11.742298	11.670177	11.686355	12.904820	11.901889	11.272078	11.513628	11.241360	13.174180	11.252514	11.761777	11.635707	11.123509	11.610339	13.027278	12.141913	12.052666	11.728547	11.579668	12.398188	12.285989	12.012048	12.329087	12.548924	12.581318	13.397318	12.446254	12.306066	12.394097	12.447571	12.383384	12.397685	11.099443	11.252070	10.886126	10.969544	11.353164	10.969237	12.243145	11.131212	10.777392	11.469123	11.452540	11.651082	11.593433	11.653194	12.137094	12.174829	12.097568	12.370197	12.634617	12.177525	12.796078	12.250390	12.927582	13.171908	12.616955	12.099378	13.279450	12.357611	12.289665	12.122694	13.579444	12.140350	12.134816	13.152225	13.656748	12.736077	12.948751	11.695921	12.418655	13.013167

AC068888.1	9.208692	8.363935	8.610473	8.217284	8.460937	7.495997	8.712055	7.805246	9.060362	8.400184	8.318216	8.820230	8.835874	7.604670	9.546675	8.456739	8.842770	9.294904	8.547074	8.337276	7.539423	8.070476	10.411781	8.972042	9.557862	9.006263	9.252826	8.942008	8.762712	8.378160	8.250361	8.506585	7.880362	8.711476	9.048542	9.726052	8.789036	8.920452	8.738729	9.397648	7.675124	10.297710	9.508545	9.196602	10.230524	8.951490	7.880066	9.802412	9.474760	8.704893	9.311249	8.990170	9.056426	8.704285	9.878002	8.499634	9.185786	9.598341	9.827055	9.762301	9.417175	9.383032	10.061198	9.342359	8.932990	9.638262	9.155247	8.127977	8.372533	8.831940	8.372078	8.536401	7.899893	8.653728	8.385428	7.835968	8.352921	7.852497	8.490290	8.732552	9.265944	9.569197	8.833199	9.128119	8.680506	8.995493	8.395144	9.213597	8.655793	8.122129	9.604056	8.176794	8.672619	7.976790	8.721615	8.708855	9.547322	9.641485	8.375408	10.076034	9.539063	9.332303	9.163879	9.022477	9.516679	8.496424	9.727298	8.935020	9.736421	7.705631	7.841406	9.449194	9.113587	7.683968	8.373727	8.362863	9.955696	8.652353	8.186342	8.625761	7.495681	8.223133	7.865564	8.942331	8.059483	8.187900	9.132674	8.856378	9.173745	8.457938	9.074813	8.387874	7.027491	9.196762	8.715005	8.208762	9.840309	9.657640	8.361892	7.642151	8.490141	9.927545	9.407719	9.767533	8.397928	8.923868	9.659354	8.232693	8.065034	7.799610	8.964318	8.626389	8.453530	9.099347	10.839289	8.565249	8.472170	8.209938	9.060510	7.462771	9.507629	10.640300	8.919508	9.875439	9.211755	7.930519	9.428670	9.755164	8.472487	8.628231	9.536127	8.805298	8.304094	9.188155	8.755780	9.353627	10.034381	10.341850	10.348265	9.419138	9.297552	9.897656	9.910666	8.733640	9.321385	8.826140	7.853434	8.745271	8.482488	8.708574	8.875260	7.331016	7.814612	8.540706	7.459505	8.415496	7.912039	7.953412	8.595322	9.137166	9.331371	8.434770	8.505802	8.363017	8.954553	7.882321	10.090524	8.991194	8.091102	10.252933	8.152212	8.670956	7.812017	7.774290	8.567450	9.602218	9.893477	7.703146	9.334307	8.879581	9.828562	9.229567	8.527834	9.856672	9.352587	10.336597	8.634408	8.889622	6.883579	8.826297	8.179917	7.928181	6.812550	9.639817	8.075929	9.620412	7.618474	8.136495

AL591074.1	3.178289	4.673167	4.440018	3.455662	3.799869	4.207761	3.947691	4.596385	4.429565	4.042312	3.544693	3.522436	4.510736	4.146217	3.433628	4.318868	4.588651	3.165192	5.890109	3.472743	3.949084	4.191700	5.631552	3.521478	3.206112	4.045188	3.395581	2.989222	3.599742	4.295017	3.334212	5.974298	3.236957	4.002996	4.063276	3.719743	4.265308	4.083256	3.439414	3.260006	4.167541	3.756928	3.795711	4.920613	3.423840	7.135167	4.891867	4.061501	4.002996	3.545073	4.149594	4.819395	4.049324	4.834164	4.284044	6.057447	3.905077	5.283757	3.148514	4.234172	3.171175	3.608987	3.703446	3.630055	3.388600	3.390175	3.458955	4.626121	3.662035	3.087059	4.263974	3.888276	3.490484	3.452771	4.311047	4.958737	3.875266	3.569686	3.278696	4.841476	3.925247	4.912191	4.389553	5.048105	3.193659	4.726407	3.786037	3.229919	3.217861	3.319115	3.291272	3.861275	3.570038	3.589579	3.719743	3.547179	4.829317	2.943615	3.763184	5.335964	5.377881	4.016509	3.744268	3.844785	5.782186	3.915505	4.458238	3.893761	3.948370	3.695698	5.138648	3.735018	3.593435	4.427011	4.131671	7.058706	3.350047	4.230073	3.438428	3.369785	4.179211	3.941921	3.358117	5.684176	4.566806	4.193224	3.410422	3.102425	4.232008	3.901874	4.051722	4.899606	4.068386	3.531098	3.889387	4.743173	3.823735	3.477825	3.860875	4.038443	5.024042	3.512749	4.644930	3.065646	3.117943	4.297007	3.951398	5.258110	4.016509	3.119999	5.308286	4.190163	4.809096	4.130630	4.155180	4.453543	3.952698	3.365338	3.917040	3.774315	3.848851	4.110948	4.061967	4.322455	7.656558	5.853987	4.021106	3.406067	3.395399	4.921258	4.665614	4.000845	4.705113	4.728702	4.040595	4.375887	5.307327	3.099736	5.010327	3.921257	3.505344	3.780612	4.409608	4.212080	4.192585	3.000745	4.189963	3.997680	3.146302	4.354817	4.452895	4.315607	3.800298	4.311668	5.083066	4.217085	3.601673	3.180141	4.446299	4.252568	4.244102	4.791432	4.679456	4.343285	4.358758	3.812307	3.779991	4.175765	4.163148	3.363039	3.834204	3.927841	3.662430	3.553350	3.680841	3.827629	4.569356	5.394390	4.001936	4.841044	3.792471	3.762565	5.008463	6.657110	3.793159	3.265284	3.995744	4.031890	5.637531	4.466525	4.124030	5.839364	4.103439	4.225750	5.800232	3.376866	3.540932	3.561360

LINC01549	3.783575	3.560309	4.039525	3.616696	4.083468	4.233943	3.721688	3.697132	3.243676	3.245681	3.313790	3.080285	3.482786	3.556393	4.986033	3.563815	3.384652	3.663429	4.837448	4.311668	4.149407	3.461637	2.881013	3.494456	3.547366	3.545283	2.890735	3.051738	3.286231	3.301128	3.360967	3.928272	4.913698	4.636077	5.331246	3.842331	5.277375	4.662468	3.855094	3.497287	3.286678	3.721910	4.169649	5.170495	3.597342	5.389874	3.681040	5.339915	4.606057	4.947539	4.777623	5.014666	4.466966	5.387388	4.940761	3.074874	4.601214	6.039123	4.554107	5.528211	4.887311	3.079530	4.020460	4.091852	3.862165	3.693392	5.238653	4.220843	3.251420	3.816300	3.848646	4.416195	3.039726	3.297455	4.422881	3.812109	3.708262	4.267189	3.825314	5.676561	3.888699	4.492238	4.922128	4.798980	4.982161	4.474750	5.334186	4.703636	5.440059	4.512448	3.556216	3.730244	4.605023	3.619893	4.483077	3.577338	4.779673	3.781857	4.597046	3.364521	4.640321	3.171902	2.951964	4.046733	4.452047	3.544884	3.655248	3.832923	3.110054	3.179899	4.405372	3.822055	4.275750	3.622668	3.927387	6.527507	3.009356	3.782722	3.093147	6.529490	3.411476	5.400149	5.064275	4.734422	3.466290	4.135166	3.859999	3.224468	3.088772	3.137469	3.256478	3.692171	3.478413	3.568486	3.292658	3.321367	3.472743	3.418434	7.206289	3.649216	3.011939	2.875281	3.894213	3.535381	3.185015	3.681816	3.069020	3.698788	3.491351	3.174332	3.414533	4.565117	4.603341	6.678786	3.589579	4.800923	3.884029	3.567669	3.300392	3.115214	3.768431	3.801192	5.710123	3.949658	5.833177	3.551811	4.446299	3.909010	4.209698	3.604453	3.105510	5.721228	4.399610	3.721688	3.057265	6.592806	4.835195	4.117895	7.050689	5.293694	3.130149	4.138014	4.009758	3.954808	3.748119	4.202047	4.287894	3.406764	2.910452	3.397284	5.808321	2.984053	2.957334	5.061811	3.601673	3.087292	3.779991	3.542469	7.349096	3.742824	4.159936	5.282775	4.883487	4.245156	4.009340	3.593637	3.807616	4.715607	3.690955	3.440832	3.032967	4.036066	3.219832	3.322010	4.051084	3.327915	3.317112	4.358088	3.271149	3.678626	3.309540	4.235520	4.478041	4.838925	3.446532	2.947380	3.546976	3.227169	3.546976	3.686432	4.888552	4.602914	3.445453	5.468945	5.126372	3.120954	4.288341	3.149759

AL606760.1	7.661085	10.307427	10.429277	9.627096	9.284768	9.249208	8.767446	8.856529	9.668618	9.694363	9.152161	8.621584	9.573835	10.352051	10.169652	9.162455	10.009096	10.337200	10.369561	9.601111	9.616680	10.503462	6.664613	9.367251	10.225000	9.427430	8.925189	7.832150	9.140544	9.754226	10.014151	10.210629	9.746308	10.438390	9.523327	9.226210	9.196762	10.356463	10.261627	10.349330	9.484723	9.480888	9.287550	9.507924	9.317025	9.759499	8.912356	9.911427	10.024660	9.416569	9.458628	9.836571	9.957048	9.598172	10.797753	9.441595	9.765260	9.698167	9.954431	10.116521	9.729974	9.769058	9.326595	10.478878	10.506605	9.704296	10.166289	10.792335	11.344909	10.064516	8.991055	9.400437	8.724803	9.745680	9.344218	9.603010	9.696024	10.486410	9.394059	10.683331	10.301432	10.747396	10.841262	10.472372	9.941008	9.438039	9.989367	10.632606	10.947453	10.823092	9.507629	11.249845	10.391357	10.080716	9.115570	9.043484	10.358719	9.091908	9.918248	8.953796	10.116521	9.818886	10.396736	9.779375	12.103368	9.543634	9.549766	11.465697	10.683185	9.353931	8.605139	10.063932	11.218189	8.587540	10.279115	8.626562	7.914298	10.172908	9.551013	11.454602	11.650941	11.494352	11.211843	11.470789	12.108933	12.136054	10.235279	12.982574	12.434966	11.210981	10.828723	12.196471	10.304338	12.315273	9.464087	11.722134	12.325086	11.621431	11.901752	12.256329	13.782074	12.247673	11.184194	12.451713	12.445148	9.770646	12.378030	10.611842	11.916064	10.713301	12.953847	11.045432	10.993754	11.556839	11.704750	11.157147	10.753411	11.650359	12.600026	11.783740	11.760219	10.574229	11.934899	11.174736	11.777298	11.280035	9.794095	12.350062	12.203746	12.022078	11.308373	11.985408	11.578488	10.561618	12.011728	9.501025	12.566268	12.089192	11.875371	12.553785	10.901270	12.550390	12.986119	11.503209	10.658726	12.932422	10.893606	12.558326	12.837755	11.617713	11.664327	11.605367	11.094441	10.800245	10.964890	12.102387	11.346523	10.816269	10.090978	11.350698	12.414153	11.631664	10.684665	11.262427	10.845168	11.033754	10.655933	12.396485	11.662670	12.019013	11.702656	9.921845	11.343391	9.857725	10.251447	11.493752	10.976418	11.135818	12.165154	11.302476	10.896259	10.515170	11.909858	11.407065	12.578033	13.648695	12.343587	12.789606	11.019158	11.458006	11.885850	11.697702	8.469804	11.962445	11.680972	12.575087	12.748800	10.843773

PCDH9-AS2	4.262886	3.954187	3.294372	3.631713	3.667109	4.033413	3.326945	4.321019	4.826274	3.715067	3.210436	3.102425	3.231430	3.498889	4.508331	3.937922	4.358758	3.163655	4.186652	3.373442	4.035158	4.008667	3.645431	3.545984	4.044345	3.600306	4.459665	4.483077	3.879159	3.725135	4.616202	3.202692	3.342139	3.305388	4.388263	3.737871	3.417386	3.005824	3.684027	3.106094	3.313169	4.486379	3.817172	3.513752	3.586490	4.536102	6.471216	3.228359	3.206907	3.202948	3.175783	3.238603	3.633323	3.786732	4.069020	3.962928	3.947935	3.051529	3.245966	3.596936	4.250189	3.931093	4.233080	3.983919	3.209388	3.299620	2.990442	3.721463	3.576726	4.330993	3.786494	3.655820	3.630263	3.664851	3.684609	3.419157	4.443890	3.901467	3.953974	3.816090	3.835886	3.624461	3.237557	4.057579	3.198002	3.213524	3.577338	4.074067	3.382324	3.232555	3.940444	3.510009	3.623653	3.037030	3.580504	4.007174	3.421018	3.026034	3.277712	3.653830	3.867935	3.516064	4.145382	3.881073	3.988499	4.481977	3.749398	4.026036	4.629822	4.283174	3.979593	4.376322	3.982623	3.475634	3.404877	4.538206	4.192784	3.374079	3.598158	3.694253	3.207928	3.691374	3.655026	3.996830	3.380840	3.499062	3.922224	4.610371	4.656631	3.029478	2.943013	3.173183	3.682876	3.833118	4.389553	4.208850	2.987972	4.531615	3.107938	3.592616	4.168403	4.413993	4.264885	3.840275	4.179668	3.995086	4.559441	4.148555	3.380330	4.051282	3.768624	3.413453	2.997691	3.922669	3.516786	3.335963	2.970090	3.336976	3.571157	4.047376	3.155201	3.364693	3.213117	3.725335	4.348542	6.305436	3.112756	3.354171	3.086146	3.109994	3.077034	3.527209	3.951398	3.269432	3.487138	3.597934	3.064919	3.057068	3.517163	3.003614	3.339444	4.095823	3.313340	3.325791	4.013112	3.001873	3.522436	3.637482	3.089248	4.095823	4.115265	4.157781	4.121401	3.797157	3.343162	4.237860	4.071461	4.177253	3.344944	3.392134	3.791856	3.440139	3.526077	3.419322	3.208343	3.184892	3.588064	3.208736	3.236796	4.050438	4.102611	3.302676	3.069947	3.057796	3.925015	2.989405	4.285926	3.436697	3.250126	3.315547	3.517979	3.772832	4.188655	4.385878	4.246430	4.502632	3.599926	3.940444	4.281683	3.801618	4.552590	4.085680	3.801618	3.272546	3.567285	3.899129	3.852937	4.556230

AC093110.1	12.390314	11.055820	11.144712	11.365974	11.060277	10.541389	11.198103	10.423303	11.419262	11.053643	11.040745	11.192212	11.649159	10.589798	11.746252	11.024218	11.552116	11.375308	11.042851	10.471176	10.832125	10.586845	13.576247	10.698520	11.733626	11.922034	12.446584	11.638582	11.814644	11.071222	11.879689	11.390120	11.136230	11.373637	11.332182	10.883700	11.070628	11.089609	11.381596	11.046347	11.395640	12.205891	11.458769	11.272950	12.263695	11.345061	11.569154	8.129345	11.503353	10.906300	10.831803	11.297023	11.157897	11.034179	11.460244	11.313353	11.398216	11.858108	10.773596	11.789270	11.699008	12.098917	13.070763	11.085280	11.554922	11.867445	11.069585	11.091753	10.740196	11.794064	11.252662	10.918527	11.592678	11.296440	10.601523	10.809816	10.761296	10.631271	10.859242	11.053227	11.096621	12.233576	11.567413	11.582720	11.303833	11.463911	11.165668	11.188740	12.134342	11.366409	11.835114	11.791025	11.013535	10.690215	11.340546	11.248076	11.887512	11.824884	11.130181	11.666576	11.404241	11.264255	11.433898	11.156400	10.733574	11.544101	10.940687	10.521568	11.945921	11.038306	12.260507	11.181128	10.616136	11.350540	11.566982	10.934885	12.438343	11.339227	11.591454	11.557144	11.917597	11.287020	11.213966	12.131274	10.844403	12.067502	12.791839	11.913539	11.492028	12.480123	11.686785	11.896631	11.829878	12.646754	11.774769	12.082436	11.468075	11.641765	11.726750	11.245651	11.409205	11.356979	11.898984	11.758737	12.062616	11.941976	11.752614	11.606533	11.674147	12.697692	11.598004	11.809331	10.830328	10.591883	10.731036	11.393373	11.261832	11.453855	12.006559	11.995512	11.123230	11.075613	11.860703	10.588378	10.739768	11.413399	8.379692	10.377566	11.459334	11.694171	11.155939	10.705361	11.377558	11.563886	11.289617	11.347275	11.231436	11.501190	10.896259	11.739819	11.411961	12.054081	12.528252	10.911612	11.348027	11.733934	10.965940	11.743988	10.964890	10.291124	10.741574	11.894872	10.904667	11.181871	10.880317	10.945949	11.436941	11.137290	12.207449	11.517507	11.052815	12.024653	11.710591	11.980172	11.200874	11.715575	10.671047	12.455635	12.259890	12.686046	12.962524	11.824133	11.571034	11.705791	12.149284	11.779417	12.250390	11.431806	10.662071	12.683155	11.512761	12.466325	11.454602	13.128984	11.170466	12.535770	11.855782	11.296282	11.132065	12.012502	12.215941	11.839473	12.025558	11.747030	11.884206	11.604016	11.585590	11.201915

AL136369.2	7.217178	6.464876	7.005961	7.221773	6.920987	4.671026	7.183448	4.642400	3.781036	7.011554	5.779623	7.460560	6.867405	6.075352	7.064609	6.936015	6.947279	7.659658	6.138229	6.023946	5.295428	6.178514	9.545166	6.499570	8.039647	5.688595	7.851729	7.174321	8.124489	6.087306	6.743202	5.594019	6.443376	7.069552	7.006419	6.359193	6.552518	7.239061	7.174321	7.221302	6.860578	7.993945	7.522660	7.062210	8.132684	6.777517	7.998109	6.305609	5.846949	6.337715	5.791335	6.982081	7.311495	6.915418	7.672128	6.643535	7.840159	7.279568	7.810660	7.716582	7.402245	8.485660	9.056876	7.918723	6.097197	7.163940	5.460459	6.888075	4.909265	7.417135	6.831474	7.024588	7.259185	7.218267	6.444537	6.277687	6.134299	5.879995	6.359531	7.733926	7.597626	8.055425	7.511709	7.763447	7.772925	7.143310	6.443880	7.414306	7.199002	6.240272	7.359016	5.824601	6.318237	6.014177	5.318061	7.052716	7.289419	8.549523	6.956277	8.050619	7.021564	7.500244	7.896881	7.819680	7.055070	7.714929	7.235142	7.037167	8.746045	6.854156	7.083312	6.190787	5.188216	6.172188	5.744826	4.351543	6.392873	8.023620	7.786677	4.470128	4.576680	4.254350	5.486729	4.138852	3.478207	3.628398	4.227481	3.497998	3.226440	3.647986	3.408870	4.648544	3.652626	3.101726	7.053478	5.116998	3.556578	4.746389	3.769308	3.530322	3.693828	5.289371	3.939217	4.425470	3.327122	3.999802	3.534249	4.511172	3.468400	4.573962	4.368486	4.968621	3.481349	4.741069	3.370658	3.954808	4.401806	3.583962	4.379733	3.274197	4.322911	4.788469	3.452598	4.287666	4.253430	3.629032	3.269604	5.054312	4.881804	3.712529	3.231430	3.891367	4.435851	4.506771	6.330610	3.754031	3.251299	3.306493	3.904846	3.830958	6.606208	4.326770	4.366579	3.565350	5.390865	3.096137	3.669702	3.382241	3.612076	4.247095	3.703060	3.272701	4.509419	5.552989	4.586707	4.164884	4.247095	3.819056	7.116576	4.535232	4.883086	6.047988	5.721590	4.182761	3.671130	4.131875	5.001729	5.425814	5.542232	3.792701	4.670370	3.732140	3.241792	3.583046	4.399610	4.251723	5.376740	4.088500	4.737795	3.836911	3.786494	4.661156	4.885177	5.179750	4.468831	4.858150	4.329886	4.624415	3.165304	5.669624	5.103993	4.740420	3.961808	3.321367	4.628705	5.797284	4.562740	4.056272

LINC01771	4.556426	5.559465	4.907970	4.870826	5.220191	5.284565	4.815819	3.727139	3.632948	4.118599	3.090417	4.124217	3.691158	4.346523	5.097811	4.903206	3.988618	4.731233	4.540679	5.475801	6.325088	5.766833	4.974843	4.567244	4.430265	4.233080	6.375321	4.520187	4.964161	5.360800	3.523830	5.531616	5.140892	6.287577	5.772193	4.968621	4.242073	4.829317	4.303316	4.411106	3.866608	6.458537	6.379812	5.718496	5.611055	4.625919	4.028832	6.022897	4.423542	5.232404	6.226570	5.150345	4.825211	4.329236	4.993314	3.322641	5.413577	3.101045	4.695153	4.178992	5.416211	3.928272	4.888552	4.015184	4.845532	4.561847	6.012483	4.586047	6.998024	6.309603	5.391638	4.674733	4.728702	5.488941	5.147729	5.897574	4.187529	4.838091	6.730784	6.030587	4.391046	4.803256	5.702043	5.282991	5.503913	4.981730	4.774099	5.121307	3.497641	4.803466	5.778924	4.825841	6.180152	4.413089	6.359193	5.191700	4.407127	3.417386	4.710741	5.035062	3.174332	5.372660	4.893336	4.975473	5.112361	4.131246	5.086979	4.777623	3.840068	3.930246	5.332458	3.713806	3.761730	4.523906	3.339936	4.026873	4.029966	4.185118	4.518683	4.813320	5.601705	5.578508	4.925093	5.208018	4.535899	4.822102	4.214051	4.924665	4.784747	4.757390	4.835798	6.450636	4.085922	5.353882	4.026036	5.705784	4.502191	6.266222	5.062667	5.668602	6.241596	6.468844	3.979593	5.817278	7.159454	4.532673	7.431568	4.170551	5.785779	3.931917	5.602065	3.483337	5.292942	7.318079	5.348514	5.639555	4.858150	4.098850	5.874946	4.809948	5.933150	5.657036	5.725727	5.242646	4.331654	4.869580	5.380017	5.343992	5.003178	6.255725	5.055345	5.391433	3.983064	5.390271	4.992029	5.663324	5.332046	4.457358	5.783423	5.521763	5.163669	5.510469	3.911625	5.027874	5.115151	6.347604	6.028563	9.684031	7.131970	4.578189	4.708270	4.547511	6.614789	6.036011	5.261427	5.627262	5.639162	5.830469	5.135431	5.685865	5.894955	4.864751	6.059390	5.919744	4.372814	5.767564	5.946038	6.137188	5.073437	6.360044	5.512770	7.761907	5.108708	4.995442	5.059824	4.622273	3.292205	3.857219	5.991118	4.181446	5.556954	5.359108	6.151907	5.568644	4.901328	5.881571	5.910266	5.444097	4.122295	5.507386	4.099063	3.705674	4.925505	5.446414	5.360611	5.941954	4.440482	4.848888

DDX11-AS1	7.600778	6.626000	6.862022	6.920987	7.820271	7.632094	6.764402	8.261233	7.782109	6.593717	6.503812	6.538740	6.325780	6.846765	6.511268	6.732375	7.743500	6.712113	8.046561	6.735148	7.548082	7.944630	8.870889	6.908762	7.571280	7.349921	7.146759	7.714346	6.649508	7.136406	7.786851	6.678786	6.923712	6.613022	6.855086	6.979561	6.755724	6.866933	7.154341	7.635884	6.830874	7.497273	6.778627	7.147857	6.545166	5.847710	7.409741	6.363544	7.256941	6.328401	6.950590	6.266553	6.383826	6.971359	5.804920	6.638386	6.695231	7.234377	7.475368	7.151398	7.345331	6.778938	7.560294	7.608529	7.322331	7.369846	7.084910	7.137353	7.045786	7.470168	6.804768	7.139694	6.618714	6.828154	6.744168	6.884082	7.261822	7.279252	7.278463	6.739884	7.302884	6.581645	7.009963	7.079890	7.298624	6.708953	8.288718	7.430932	7.541274	7.509424	7.498515	7.811708	6.945345	6.700141	7.007190	6.952325	6.737942	7.365150	7.547904	7.702677	8.040277	6.805231	6.455262	6.507348	7.345331	6.530302	7.874285	7.585638	8.685237	6.575670	8.742859	8.591139	8.642660	7.243420	7.282895	10.888785	8.156894	7.107099	6.846171	7.794310	7.174163	7.774932	7.019995	9.967259	7.136255	7.426808	9.516845	8.824787	7.330723	7.507990	7.806014	9.081808	7.293973	7.888897	7.869842	8.356290	8.691670	8.820970	7.488204	7.835074	8.195255	8.978327	8.551804	8.510590	7.980968	8.424675	8.632538	8.699239	7.219504	7.567799	7.824617	8.968369	7.377685	8.379228	7.807111	7.057146	7.843275	7.910344	8.142105	8.833908	7.790524	8.470551	8.277390	6.838662	6.205198	7.598858	8.206187	7.744703	7.546612	7.305069	7.842645	7.566739	7.026340	7.766526	6.692925	9.101487	8.881298	7.970042	7.841406	7.938997	6.553514	7.818875	7.714645	7.513898	7.558907	7.612344	7.867093	7.464507	8.174583	7.003961	7.622688	7.046908	7.501958	8.203318	6.971967	7.704547	7.872765	7.686546	7.761745	7.318844	8.377828	7.240875	7.768842	7.783924	8.897149	7.915342	7.971630	8.301664	7.724392	9.894119	8.909661	7.466203	7.342373	7.299385	7.469565	8.633518	8.218549	7.189713	7.500244	7.720538	8.778712	7.594003	7.528881	8.346622	6.675083	8.232255	7.375318	7.929575	7.422439	9.121392	9.781037	11.505395	7.810345	8.304376	8.636910	8.265082	7.373429	7.165039

LINC01811	4.868526	5.035062	4.623340	4.347825	4.450065	5.066344	4.044969	5.525913	5.142772	4.215554	3.896720	4.212293	3.437547	4.279071	3.053145	5.188014	4.854383	2.877613	4.373262	4.720037	4.322056	5.191120	4.944987	5.378461	4.345246	4.773047	4.848293	4.710123	4.644274	4.776772	4.720858	5.272481	3.608409	5.021782	4.447792	4.663074	5.718496	3.838432	4.677535	4.288978	4.495974	4.632624	4.456047	4.637149	3.440673	4.842335	4.580993	4.857330	3.658843	6.509917	4.630905	3.225561	3.804516	4.598551	3.610768	4.594487	3.989853	3.190259	3.889150	4.726407	4.828464	4.429095	4.839153	5.106852	5.049984	4.466966	4.253430	4.968204	5.017906	4.988699	4.932634	5.102775	4.128056	5.147132	5.241252	5.490389	5.606848	5.533682	5.643100	5.010954	5.439113	4.243640	4.946094	4.454382	4.626760	5.637335	5.728468	5.197707	5.375342	5.614981	5.677332	6.339056	4.749752	4.344597	4.450279	5.231998	4.095169	3.420255	3.780612	4.011437	4.660922	4.048439	4.306150	4.379297	4.788883	4.623548	5.461042	5.112773	5.673843	4.700680	5.542788	5.833923	6.306611	4.744249	5.261427	6.854459	5.938120	4.142379	5.165298	4.570913	4.982161	4.944987	4.658582	4.470784	4.784334	3.902322	5.430663	5.263283	5.017503	3.996401	5.214034	4.172921	4.535899	3.176741	4.701513	5.412215	3.645431	5.835193	4.947124	4.946193	4.959144	6.486852	5.479634	5.345357	5.091838	5.765730	5.191700	5.294445	5.570335	5.126963	4.631116	4.629822	3.981754	4.875476	5.293315	5.848261	4.358295	5.069387	4.821263	5.105833	5.313012	4.168613	5.437272	5.103993	4.491346	5.385211	6.411836	4.644041	6.968555	4.957295	4.355705	3.791187	4.128953	3.352380	4.602311	4.775737	3.161949	5.320535	4.651356	5.493723	4.736346	5.109773	5.435714	4.760599	5.584968	5.797105	5.423847	5.251944	5.895664	5.529728	5.108505	5.107866	5.440614	5.371076	5.640661	5.863284	5.778924	5.628989	5.261427	3.976766	3.922669	4.582452	4.874048	5.446009	4.970485	5.604946	4.301736	5.188600	5.764499	9.189635	5.997605	4.572417	4.702563	4.745325	4.584561	4.015184	4.621637	4.379075	4.814969	3.827629	3.733620	5.284147	4.466966	4.711587	7.154507	5.791866	4.676014	5.774550	5.589095	6.231093	6.331636	7.322799	5.929695	7.041594	4.028609	5.810203	4.311668	4.962329

LINC01370	5.400334	5.082862	5.284771	5.262672	6.029738	5.610876	4.714127	5.986470	5.670490	5.798779	4.862293	5.146157	4.748940	4.922233	3.480782	5.232404	6.054615	3.466886	5.263473	5.126963	4.742152	5.515242	5.242646	5.239264	5.144534	5.438752	4.868117	5.648979	5.142956	5.739181	6.327896	6.391724	3.908360	5.653667	5.040093	4.987675	4.815381	4.495328	5.641019	4.517865	5.140077	4.813130	4.723015	5.482022	4.016060	5.593845	6.421107	4.271805	4.998379	4.875077	5.523836	4.446490	4.980732	5.579285	4.932634	5.517224	4.253009	5.484060	5.143570	4.905496	5.303625	5.600944	5.234617	5.881412	4.955624	5.073004	5.276568	4.913698	5.223134	5.584774	4.992029	5.672945	5.268846	4.844447	4.676249	5.309863	5.789578	5.801555	5.279964	5.508191	6.164105	5.018503	6.335522	5.088990	5.170495	4.815819	6.349083	5.153779	4.945220	5.709373	4.773047	5.696389	4.748729	5.331647	5.722134	4.792105	4.284260	4.870615	5.630433	5.537848	5.759620	4.571992	4.769928	4.788469	5.591192	4.941966	5.890109	5.930066	6.508768	5.678811	6.672197	6.376474	6.005942	5.464428	5.084680	7.168563	6.036696	4.850771	4.857954	5.293315	5.331246	5.286398	5.394390	6.922419	5.752653	5.703923	5.366014	6.082836	10.791138	5.671992	5.129394	5.856716	5.368946	5.750286	5.362552	6.025527	5.067971	5.334740	5.260039	5.696022	6.410859	6.441052	5.290382	5.863467	5.904540	6.099807	5.989536	6.521864	5.733015	6.633547	5.548432	4.683764	6.427847	5.029321	10.015727	4.796275	4.877984	5.037135	5.203864	6.318752	5.703743	5.133823	6.615109	4.886038	5.210833	6.260984	3.454372	7.228566	5.805975	5.954371	5.353882	6.627098	5.634528	4.920208	5.312841	5.330272	4.580993	5.943205	5.557747	5.974825	5.424454	5.644822	6.525307	5.056350	5.156247	5.938629	5.276363	5.111153	6.042288	5.041365	5.716929	5.997771	5.476530	5.341799	5.389488	6.121820	6.350277	5.560034	5.520989	4.909682	5.123314	5.702406	4.942614	5.636952	5.915666	5.771424	5.235426	6.118533	5.723098	5.306575	6.300676	4.937670	5.730493	5.250002	5.373011	5.045524	6.031651	5.231197	6.702249	5.787378	5.121711	5.512606	5.452837	5.334938	8.568786	5.865582	5.547131	6.202793	5.818870	6.601179	7.035453	7.954175	6.451330	5.730301	5.693593	6.129831	5.128768	5.473107

AC092640.1	7.661235	7.997674	7.668055	7.784942	8.097665	7.973828	7.158083	9.120056	9.403550	7.989271	7.784088	7.735034	6.903209	7.382525	7.304606	8.307762	8.722201	7.457145	8.647693	7.891070	8.032995	9.179234	9.610727	7.887659	7.809741	7.906169	8.810595	8.247528	7.514207	7.640322	7.925836	7.030189	7.632094	7.710796	7.563026	8.085241	8.216056	7.756185	8.072303	8.128868	7.819680	8.289196	8.638314	8.073351	8.552568	8.747882	8.348987	7.810799	7.915822	8.655375	7.192941	8.256188	7.988366	7.921791	7.901171	7.999175	8.212972	7.764204	8.063661	8.062420	7.673355	8.612986	9.158942	8.291830	7.822571	7.890928	8.340714	8.747415	8.403294	8.645143	8.433681	8.433072	7.582883	7.939788	7.941178	7.964325	8.308399	7.766091	7.861673	7.846711	7.298624	7.626091	7.082687	7.254931	7.821338	7.692886	7.839084	7.630529	7.801900	7.646673	7.847655	8.072444	8.110120	8.001687	7.321841	7.156988	8.104169	7.773688	7.489116	8.213459	8.051116	7.917531	7.786851	8.138187	8.109379	7.216069	8.897476	7.619230	8.781813	8.061379	8.920452	8.166286	7.892168	8.186673	7.866036	7.661235	7.764057	7.619091	7.925059	7.875964	7.789160	7.755009	8.295156	8.574419	7.718880	7.697764	7.805872	7.851415	7.826786	7.696224	7.490242	7.888433	8.009151	8.007160	8.377380	8.187296	7.030045	11.095424	7.095312	8.285318	8.624351	8.553172	7.539423	7.186986	7.141433	7.665421	7.885997	7.774461	7.894662	8.646197	7.409576	6.913084	7.172616	7.420891	6.873056	7.473087	8.118721	8.136815	7.989562	7.918723	7.871660	9.291287	7.204710	7.905856	8.930788	8.322308	7.419305	7.599995	7.838479	7.607725	7.698370	6.882930	6.986518	7.795089	7.658623	7.237820	6.912113	7.563478	7.261822	7.438669	8.417902	7.418369	8.267621	8.457861	7.766700	7.538769	8.239474	7.853114	7.820563	8.241141	7.497094	8.487552	7.232924	7.533611	7.320728	7.899733	7.568694	7.430775	7.145206	7.006750	6.826891	6.849484	7.133076	7.377032	7.886427	7.360429	6.889496	7.966799	7.669761	8.206780	7.889355	7.902878	7.876859	7.275617	7.392107	7.542477	7.864325	7.012349	7.162350	7.933412	7.614639	7.720677	7.910185	7.869407	7.713603	8.285627	7.781521	8.059955	7.778213	8.702595	8.286403	8.355695	9.114516	7.858931	7.176186	7.626412	7.779666	7.288452

AL513314.2	9.063380	8.290087	8.432259	9.266257	9.982756	9.736421	8.566293	10.338850	11.803092	9.660909	9.878591	9.489846	9.376353	9.512302	8.644089	9.538430	9.789131	8.468110	9.988254	9.324766	8.013862	9.700346	9.001514	9.355444	9.346191	9.766324	10.083729	10.287723	9.356658	8.519009	10.561928	8.701813	7.961155	8.791835	8.424087	8.929239	8.941567	8.347074	7.950014	8.400034	9.630159	8.148080	8.717976	8.836779	8.559612	8.279347	9.693584	7.739294	8.949507	8.694037	8.542119	8.357224	8.382372	8.433978	8.821128	9.041468	8.815760	7.951832	8.073673	8.078065	8.162515	8.970198	7.933412	9.065038	7.541602	8.208762	7.646673	7.935898	8.171446	7.552684	8.441197	8.656397	9.950421	8.139429	8.796272	8.633972	8.897149	8.226324	8.522011	8.346927	8.345535	8.018913	8.195435	8.171446	8.621747	8.698784	9.094947	8.482488	7.260304	8.415166	8.173625	7.680852	8.212228	8.377069	8.486125	9.147027	8.648567	10.466388	9.545767	9.304702	10.302633	8.063486	9.257879	9.074192	8.577424	9.920274	10.204611	8.910236	9.450783	9.644393	8.950273	9.788199	10.151883	10.319973	10.684971	11.604584	9.168008	9.907881	10.770016	10.100454	7.691660	8.271947	10.693165	10.650579	9.221723	9.383185	10.402284	10.064236	9.382710	9.144133	9.610292	9.143201	8.557313	8.656980	9.623228	9.660909	9.708289	8.929089	7.889831	8.606960	9.190087	7.770020	10.031468	8.469643	10.485177	9.320639	9.714868	10.245866	8.981195	10.572001	8.808284	8.759174	8.755477	8.309911	8.327291	8.284696	8.473863	8.295020	8.007770	9.655361	7.759759	7.894343	8.077123	8.231164	7.884152	8.940500	7.296211	7.900387	8.273458	8.081629	8.545954	8.168936	9.088211	8.490868	8.354442	8.680959	7.687340	8.604528	7.276110	8.494301	7.698822	9.823307	8.755929	8.257083	7.985002	8.187593	7.331642	7.819523	7.466791	8.051116	8.447805	10.023543	8.323519	8.573650	8.261975	8.224051	8.438131	8.279347	7.980345	7.969404	8.406973	8.536680	8.249737	8.044489	9.372113	8.209048	8.021345	7.410476	8.031490	8.553625	7.990815	7.863083	9.537676	8.092765	8.849419	9.347097	11.089762	9.506715	8.512612	10.616136	9.024005	9.583159	9.061578	9.146860	9.502845	10.396585	9.375129	8.583923	9.329390	8.214701	9.384364	10.638180	9.503174	9.092385	9.753292	9.280151	9.695275	9.020319

AC097511.1	3.670528	4.512448	4.477618	4.447792	3.854698	5.956091	5.232211	4.922988	4.301516	4.804933	5.788126	5.200488	3.949435	4.397228	4.836596	4.095392	3.747084	2.941574	4.657491	4.042090	4.896846	4.032337	4.917896	4.446889	3.773031	4.066903	3.860653	4.733992	4.995208	5.275538	3.710908	4.612707	4.264190	4.446299	5.052568	4.351327	3.937922	3.873734	4.173784	4.639479	5.657575	3.403206	3.567669	4.603754	3.843957	5.350035	5.396153	2.913137	3.835458	3.862377	3.944838	3.408177	4.044548	4.258522	4.516358	5.484266	4.077226	4.402233	4.598956	3.819707	5.066344	4.855023	4.348753	4.189728	5.388935	4.611640	3.421880	4.678180	4.598551	5.060442	4.619091	4.927846	5.271639	4.665178	3.930648	4.522120	5.421300	5.382508	4.974843	3.686638	3.394523	3.878287	3.823524	3.989853	4.050210	3.653830	4.677535	4.848293	4.302633	5.095423	4.267631	5.148333	3.793589	4.666471	4.420677	4.521910	3.636278	4.769928	3.812713	2.970723	5.183621	3.545643	4.514021	3.391947	4.880056	5.338103	4.872144	4.772601	4.560331	4.901328	4.920835	3.832712	4.261993	5.928111	4.231353	7.799143	4.037774	5.291576	4.809737	4.950838	5.706630	4.474088	5.472722	4.074510	6.591021	6.275725	4.130225	4.560560	5.668327	7.187166	5.500043	4.908396	4.663894	4.836596	4.976081	5.447613	4.973787	4.482874	4.348542	5.040958	4.553466	6.515220	4.652164	3.907475	6.220905	6.291862	5.828600	6.618065	6.590635	4.488254	3.774315	3.770910	5.582483	5.230559	4.447109	4.723874	5.036741	4.868967	4.805358	6.859944	4.521910	3.593829	5.511823	4.142173	4.704932	6.847580	2.831449	4.096860	4.035847	3.442401	4.307013	4.128728	4.408268	3.621281	5.462582	3.776160	4.304639	3.274197	3.751073	4.724322	5.742641	4.821475	4.895184	4.675819	4.313187	3.388949	4.441091	4.997369	4.889825	4.816890	5.403080	7.082687	5.929013	5.976204	4.639479	5.863653	5.216385	5.341623	3.550231	3.775945	4.260868	4.064578	4.188438	4.973787	4.301736	4.791873	4.816461	4.949812	5.272683	4.999198	5.143570	4.381162	4.852460	4.221265	4.467794	3.960098	5.795625	5.315869	3.551213	4.173575	4.008008	3.996626	3.647203	4.901741	6.060443	5.176738	6.963224	5.054532	6.139091	4.012684	4.424840	6.628714	7.376878	5.936863	4.659661	4.309062	7.561945	6.577491

LINC00616	3.670528	4.512448	4.477618	4.447792	3.854698	5.956091	5.232211	4.922988	4.301516	4.804933	5.788126	5.200488	3.949435	4.397228	4.836596	4.095392	3.747084	2.941574	4.657491	4.042090	4.896846	4.032337	4.917896	4.446889	3.773031	4.066903	3.860653	4.733992	4.995208	5.275538	3.710908	4.612707	4.264190	4.446299	5.052568	4.351327	3.937922	3.873734	4.173784	4.639479	5.657575	3.403206	3.567669	4.603754	3.843957	5.350035	5.396153	2.913137	3.835458	3.862377	3.944838	3.408177	4.044548	4.258522	4.516358	5.484266	4.077226	4.402233	4.598956	3.819707	5.066344	4.855023	4.348753	4.189728	5.388935	4.611640	3.421880	4.678180	4.598551	5.060442	4.619091	4.927846	5.271639	4.665178	3.930648	4.522120	5.421300	5.382508	4.974843	3.686638	3.394523	3.878287	3.823524	3.989853	4.050210	3.653830	4.677535	4.848293	4.302633	5.095423	4.267631	5.148333	3.793589	4.666471	4.420677	4.521910	3.636278	4.769928	3.812713	2.970723	5.183621	3.545643	4.514021	3.391947	4.880056	5.338103	4.872144	4.772601	4.560331	4.901328	4.920835	3.832712	4.261993	5.928111	4.231353	7.799143	4.037774	5.291576	4.809737	4.950838	5.706630	4.474088	5.472722	4.074510	6.591021	6.275725	4.130225	4.560560	5.668327	7.187166	5.500043	4.908396	4.663894	4.836596	4.976081	5.447613	4.973787	4.482874	4.348542	5.040958	4.553466	6.515220	4.652164	3.907475	6.220905	6.291862	5.828600	6.618065	6.590635	4.488254	3.774315	3.770910	5.582483	5.230559	4.447109	4.723874	5.036741	4.868967	4.805358	6.859944	4.521910	3.593829	5.511823	4.142173	4.704932	6.847580	2.831449	4.096860	4.035847	3.442401	4.307013	4.128728	4.408268	3.621281	5.462582	3.776160	4.304639	3.274197	3.751073	4.724322	5.742641	4.821475	4.895184	4.675819	4.313187	3.388949	4.441091	4.997369	4.889825	4.816890	5.403080	7.082687	5.929013	5.976204	4.639479	5.863653	5.216385	5.341623	3.550231	3.775945	4.260868	4.064578	4.188438	4.973787	4.301736	4.791873	4.816461	4.949812	5.272683	4.999198	5.143570	4.381162	4.852460	4.221265	4.467794	3.960098	5.795625	5.315869	3.551213	4.173575	4.008008	3.996626	3.647203	4.901741	6.060443	5.176738	6.963224	5.054532	6.139091	4.012684	4.424840	6.628714	7.376878	5.936863	4.659661	4.309062	7.561945	6.577491

TMEM132D-AS1	5.669795	4.356986	4.266147	4.640321	5.716222	6.098026	3.624461	3.729032	4.837448	5.215220	3.647551	4.725997	4.305530	4.636935	3.414002	4.512913	3.298831	3.581487	4.313402	4.024123	4.587990	4.797249	5.795625	4.130031	4.326548	3.562491	6.084236	4.755113	4.723440	6.766184	3.751073	4.751901	5.796753	5.094168	3.726752	5.783785	3.819912	3.941069	3.957460	3.953135	4.804715	4.526742	3.802024	3.684027	3.727937	5.759794	4.431388	3.986951	3.760284	3.880019	3.561360	5.038717	4.484599	4.255890	3.986069	5.245413	4.015856	3.901062	3.357243	3.964011	3.289829	4.889393	4.059980	4.427447	4.395908	3.552011	3.374551	4.075568	9.284768	4.653958	4.075160	4.787871	4.572849	4.655780	4.089190	5.553381	3.687880	3.761341	3.941921	3.846089	3.928494	3.855934	7.158083	3.943958	4.026036	3.935366	3.791187	3.470157	3.963800	4.433285	3.282239	4.188438	3.848646	5.471006	4.607149	3.864949	3.815250	4.110281	4.425695	4.989759	4.071461	4.005892	4.824782	4.838497	7.341300	4.694734	5.882835	3.818420	5.075669	3.980405	3.872654	5.062667	4.242727	4.475809	3.837575	5.396337	4.775112	4.908396	4.938930	5.940958	4.477155	3.567496	5.901681	5.583408	5.757968	4.003905	4.134951	4.250623	8.543841	4.175090	10.664886	3.665884	5.394569	3.565517	4.281251	3.241215	4.467583	4.166676	3.981517	4.039525	5.711575	7.453093	4.350467	5.865214	6.499409	4.879022	4.339822	4.252167	6.492192	4.014251	4.388072	5.039315	4.821263	3.496532	10.821920	3.655630	4.273784	4.347401	4.414432	5.430849	8.998880	7.731957	5.453409	4.152983	5.723468	4.524788	5.410898	4.012263	3.812109	4.698939	10.388663	4.287426	4.395213	3.516425	5.558123	8.365170	3.939843	7.842468	4.088092	4.407819	4.887505	4.194339	4.091186	4.004778	4.647912	3.192826	4.080021	9.574876	3.191743	4.689059	4.438927	4.952099	4.530746	5.919222	5.287566	6.172347	4.485503	3.365646	3.527760	3.979593	4.191252	5.157902	3.626417	6.645787	5.329881	3.093371	4.525013	3.659429	4.071884	3.895047	3.899988	3.443277	10.372281	4.527587	3.312266	3.993947	4.654176	4.256960	8.175531	3.389637	4.130822	5.266871	4.784935	13.117902	3.893314	5.742070	3.453504	5.534678	3.650983	3.903970	4.887696	4.453967	3.837575	5.597008	5.450109	4.460269	5.545245	5.845446

LINC00266-1	8.290408	7.532988	6.999824	7.558311	7.141928	8.303620	8.305897	7.164101	7.814319	8.214857	8.269821	7.491960	7.577303	7.601535	6.791208	7.677460	7.352207	7.170080	7.040326	7.382853	7.279413	6.374509	5.949005	7.596692	6.711800	6.901263	7.901974	6.302364	8.912802	8.466122	7.566262	7.772925	7.975388	8.394089	8.162073	7.936848	7.515705	6.858648	6.330772	6.895893	8.598366	6.429015	7.648891	6.795886	7.718421	7.012038	8.332937	8.088613	7.298925	7.636038	7.430775	7.479343	7.784243	7.804638	7.367347	8.819184	7.891543	7.321353	7.007040	7.521137	6.945652	8.529825	7.200082	5.981184	6.607883	7.192436	7.008016	6.767480	6.756046	6.413691	7.753792	7.426476	8.474166	7.604509	7.541891	7.880784	8.115905	7.265558	7.064140	7.998868	7.336449	7.377194	7.608060	7.151564	7.727157	7.118695	6.624196	8.424675	6.133795	6.437732	6.922566	6.299881	7.290668	8.006563	7.281951	7.707451	7.630838	7.348653	7.929878	8.101283	7.241967	7.974303	7.846866	6.587033	6.701586	8.417092	5.756267	6.103589	6.288447	8.895603	6.050288	5.283568	5.986093	8.870565	7.419464	7.608060	6.323720	7.930519	9.039461	8.639891	8.038201	6.489496	8.890976	6.355059	8.429598	7.482753	8.087711	9.319744	8.694336	7.885821	8.134218	7.374539	6.740220	6.121129	8.013862	8.203010	7.314510	5.884608	7.611437	6.920175	6.800931	6.354724	7.811870	6.465048	7.211984	7.801000	7.470633	10.306695	8.438280	8.271494	7.079432	7.274066	7.923024	8.343224	7.900849	7.614780	6.388514	5.047267	6.589501	9.479088	5.248606	7.296211	5.792044	7.507090	5.595130	8.346622	9.183450	8.078556	7.795863	7.248685	7.078154	8.202555	6.832940	7.119340	9.911047	8.582113	7.532690	6.577162	7.793850	5.218337	8.267121	6.821815	5.407741	7.165525	7.224648	6.119227	6.292836	6.798482	6.391724	8.362379	7.932955	8.184054	8.113311	8.301962	7.745146	8.006397	6.829747	7.077646	7.942647	7.240558	8.222383	7.302722	6.535471	7.603766	6.414848	6.620355	7.639852	5.673669	5.832637	5.867417	4.597655	6.790858	6.323720	6.767480	6.888860	7.042423	7.499450	7.654980	9.980403	6.215426	8.799440	7.741642	5.553777	6.489666	8.973768	5.493723	7.036232	7.469241	8.512310	5.757763	4.585201	5.557350	8.274770	8.622034	7.391479	5.740812	8.368619	8.218549

WWC2-AS2	9.969125	9.651799	9.774645	10.413303	10.608143	10.675689	9.925456	10.560792	11.618016	10.150520	10.637586	9.975417	10.251447	10.666968	9.846608	10.914318	10.379694	10.253789	9.630751	10.456786	9.447790	9.558329	10.985097	10.549347	10.794089	11.358851	11.103121	10.931888	10.114501	9.864701	11.492440	9.915602	10.346888	10.427336	10.289433	10.274723	10.367597	9.680619	10.158322	10.806779	10.156509	10.145963	10.595584	10.476787	10.462035	10.366240	10.689337	10.196439	9.812449	10.107149	9.959383	10.369561	9.875925	10.002425	9.504559	10.380154	10.329641	10.332682	9.693739	10.127268	10.338538	10.884165	10.105230	9.975738	9.285069	9.301255	10.447955	9.614388	9.523040	9.518073	9.978283	10.587598	10.897349	10.505273	10.632306	10.048802	10.461142	10.197653	10.114982	9.754870	10.670015	10.015553	10.054805	10.494077	10.699257	10.009096	11.192657	11.065268	10.216978	10.417667	10.126788	10.222241	10.353584	10.584051	10.731478	10.738093	9.903960	11.506409	10.570769	10.559179	11.167837	9.636997	10.154199	10.265433	9.400900	10.738234	11.638115	10.234797	8.744656	10.369561	10.569984	10.249835	10.436435	11.073138	9.791578	13.211668	10.167666	10.577397	11.067913	9.710638	10.252055	10.073145	10.400485	11.611963	9.720935	10.746036	10.667558	11.247492	10.134060	10.287574	10.466251	10.765528	10.523560	10.418738	11.528921	11.078552	10.573931	8.083328	10.013689	8.750837	10.422096	8.968219	11.158191	10.622041	11.099868	10.961636	10.662371	11.541661	9.743197	11.324835	10.222722	11.075319	10.210775	9.939807	10.291124	10.630981	9.781326	10.076034	10.145963	10.615704	9.376056	9.664693	9.641622	9.932019	9.739940	10.944920	9.616838	9.648868	9.855874	10.156215	9.490785	10.414788	10.070853	10.016345	10.044324	10.091582	9.391124	8.801425	9.127646	10.230524	10.506011	11.236266	9.786976	9.229858	9.888310	10.570283	8.979083	9.458948	9.194792	9.626779	10.253926	11.057484	10.355524	10.392503	10.525656	10.534840	10.595429	10.207052	10.528938	9.935902	9.139811	10.661777	10.563692	10.834204	10.728368	10.348265	9.737007	10.115138	10.550997	8.797307	9.719876	10.295232	10.114982	10.280645	11.041054	10.718205	11.946225	10.272596	9.395748	10.768349	10.989295	10.039141	9.978283	10.946082	10.853016	11.702238	10.147330	9.115115	10.540467	9.960286	10.292343	10.797454	11.404558	10.444741	11.730636	9.371429	10.521111	10.340966

BBOX1-AS1	6.719982	12.794657	12.856770	12.359732	13.016643	12.722295	12.597317	12.257409	11.667160	12.042411	12.126652	12.170632	12.142227	11.957101	12.342018	11.662670	11.872661	12.500922	13.743105	12.682992	13.643752	11.721525	9.310505	12.201396	11.395640	12.313325	7.994418	10.773444	10.801504	11.550891	11.558020	11.825487	13.160712	10.477090	12.013103	12.544554	12.945176	12.302166	12.486157	13.054184	12.328450	13.175184	12.430780	12.839995	11.748249	12.176095	11.578488	12.403382	12.422315	12.592823	12.075438	12.497666	13.236032	13.491499	12.207603	12.379891	12.156958	13.768548	12.844764	12.925070	13.112784	12.005028	8.076211	13.446919	12.090445	12.304038	13.056546	13.059891	12.297429	12.845626	12.816305	12.722972	11.833153	12.758922	12.314304	11.541661	12.227505	11.873246	11.976069	12.629207	12.560728	12.519372	11.980914	12.830991	12.144544	12.337925	12.589781	12.781401	13.530801	13.616838	13.000629	13.261613	12.950547	12.179253	10.963237	12.682137	12.692342	11.359582	12.727782	12.341032	11.705064	12.379287	11.969628	12.759589	12.952208	11.859491	11.737314	12.168200	13.664000	11.967485	13.857538	12.795892	12.374016	11.671790	12.711633	11.943639	7.172303	12.017344	10.933966	10.696190	10.268358	9.128602	9.718945	8.720555	10.426707	7.607580	5.883154	9.404526	8.975115	9.336166	7.187472	7.986373	9.774329	7.629887	10.073768	7.185534	9.245688	8.568049	9.462564	9.554494	9.265364	8.583613	8.445618	6.493471	8.162209	10.095497	8.356290	9.368751	8.500737	7.207080	8.140171	9.265792	8.531373	8.951021	7.700074	10.668858	8.051400	10.914011	7.952777	10.263119	10.006010	7.588731	7.662149	9.960135	9.889523	8.196546	8.731515	8.166768	9.628290	8.713136	7.511087	7.937139	8.490573	9.189054	11.184336	8.824787	8.888996	9.041327	9.740870	10.229417	7.421823	9.245501	10.896881	9.537522	12.623334	10.641916	11.403818	8.058580	12.813176	10.150370	9.430199	8.276190	9.083635	9.897040	10.609657	7.683194	7.285199	10.765971	9.555843	10.007983	9.571519	9.686383	8.488465	9.141158	8.402217	10.431723	11.283846	12.084016	10.404707	6.342674	11.701015	8.288581	9.675214	9.756549	10.347657	9.069273	7.708847	9.964730	9.816047	7.643684	11.061273	10.167973	7.995341	7.231968	9.141158	8.622962	9.377318	11.728547	9.652899	7.086443	9.302803	7.094174	11.742003	6.606374	8.387729	7.645874	11.466748	9.419441

KCNQ1OT1	13.090908	12.196906	11.993963	13.239764	13.525933	13.720180	12.383214	13.218581	14.631695	13.381772	13.551881	13.268434	13.342442	13.072450	12.503389	13.392668	13.476835	12.559575	13.210112	13.511061	12.427164	12.132968	12.227505	13.412516	13.566616	13.753345	14.739993	12.928662	13.951559	13.684300	14.085609	11.217885	11.570177	13.790706	13.075705	12.300641	12.514335	12.362592	12.513519	12.929565	13.508794	12.639389	13.039489	12.888611	12.507056	12.304038	14.205886	12.000568	12.183786	12.074208	12.255831	11.527615	11.846272	12.100593	11.567272	13.938772	11.575238	12.245455	12.650533	11.952432	12.099062	13.967702	12.219692	12.821251	11.887800	12.010041	12.378496	12.112840	11.554640	12.010940	12.420088	12.756165	14.076027	12.267455	12.268220	12.473114	12.645369	12.252460	12.243771	11.534802	12.120508	11.500644	11.430906	11.442182	11.904028	12.157725	13.327985	11.679352	11.755427	11.361662	11.486704	11.581538	12.168200	12.201546	12.501563	12.867443	11.697132	13.971275	13.381985	14.625993	14.116761	13.033565	13.455025	12.613373	11.838717	13.670083	13.535306	12.200941	13.671017	13.916607	11.604584	13.816046	13.307171	14.521454	13.155345	15.815434	13.635217	13.543949	14.693813	13.581820	12.456606	12.747457	13.362446	14.643124	12.978042	14.004154	15.345704	14.878115	14.029975	13.199300	13.599265	13.935214	13.440530	12.943369	14.783853	13.781822	13.447998	13.281802	12.843348	12.524622	12.115391	10.547330	14.050321	12.876249	12.060393	13.918583	13.445449	15.479687	12.831161	14.551853	13.830239	14.132353	13.054184	12.963458	12.899723	12.776199	12.680587	12.843869	12.897423	14.296316	12.411098	12.597984	12.950935	12.363880	11.905178	13.994194	12.533467	12.521236	11.858578	12.167248	12.438515	11.964028	12.158629	12.157725	13.531931	12.204667	12.203886	12.693563	11.648556	12.369200	13.402926	12.300959	12.462754	12.473611	12.096962	12.671375	12.210975	11.993647	11.763401	12.824533	13.040624	13.930021	13.132270	13.196531	12.547774	13.172295	12.797845	12.773792	12.709993	12.552337	11.658962	12.184541	13.144371	11.808281	13.709428	11.973905	12.302242	12.645722	12.699345	12.827872	11.993191	13.097119	12.323571	12.820920	13.535732	13.852202	14.707518	13.811158	13.512856	13.758124	14.356594	14.639680	13.249693	12.887380	14.243065	13.910783	12.298858	12.423432	14.208161	11.749074	14.096755	14.785821	14.226329	13.879304	13.906384	13.096912	13.455950	13.689737

AC025043.1	14.655931	13.716792	13.322111	13.284218	13.019365	12.996042	13.242607	12.405745	12.409843	13.011244	13.034689	13.066984	13.243546	13.059891	13.422596	12.901272	12.785225	12.935258	12.856045	12.839799	12.810376	12.606727	13.955750	12.855167	12.844915	12.861330	13.529678	13.136025	13.454810	13.469230	12.570522	13.963030	13.701651	14.311014	13.851451	13.536185	13.515355	13.216968	12.963458	12.882781	13.117902	13.982948	13.277634	13.372298	13.721765	13.179765	13.456831	13.554610	13.172483	12.976403	13.811158	13.410314	13.872088	13.398455	13.098061	12.738673	13.773464	13.518422	13.571674	13.630199	13.557270	13.470544	14.499877	13.263573	13.073519	13.099216	13.485295	13.398242	13.221977	13.541454	13.439251	13.271981	13.022526	13.574462	13.079675	13.787396	13.296189	13.730412	13.776673	13.105019	13.399301	13.988071	14.071794	13.472948	13.464002	13.658440	13.491941	13.688348	13.772438	13.337277	13.667422	13.815313	13.258606	13.291733	14.171915	13.599502	13.348676	13.204562	13.367473	12.573019	12.766196	13.221167	13.048973	12.607071	13.042766	13.172483	12.333192	12.358783	13.141663	12.841760	13.676917	12.437247	12.480968	12.542773	12.580816	12.646416	14.049746	13.409002	12.998073	13.478600	13.454810	13.280442	12.929211	13.078379	12.850884	13.841923	13.504598	13.812737	12.855862	13.321691	13.309538	13.419947	13.103890	12.519704	13.768298	13.684527	13.157107	13.315805	13.675535	12.932422	13.239764	13.769838	11.983055	12.695090	13.678503	13.328423	12.739016	13.296970	13.768777	13.198919	12.997305	13.320065	12.477677	13.239205	14.080659	13.921298	13.443765	12.561217	13.750320	13.126712	13.227872	13.480608	13.108685	13.421261	13.043715	12.085291	13.385597	13.685973	13.741572	13.534199	13.743347	13.841373	13.678008	14.425606	14.147617	14.179251	13.401636	12.763449	13.513091	13.369728	13.097119	13.620154	13.238446	13.521387	13.399301	13.002707	12.993637	12.716418	13.644259	13.599935	13.865143	13.022885	14.101917	14.199131	13.642031	14.150431	13.981326	13.715603	13.862600	13.549809	13.070948	12.846159	14.246947	13.892099	13.004306	13.381772	13.619696	13.960239	13.633485	13.192884	13.635217	14.086526	13.504804	14.000936	13.771628	13.912695	13.850447	12.845626	14.207819	13.348289	14.064771	13.488041	13.619935	13.685514	13.369328	12.875386	13.786118	14.259951	12.849653	13.949893	13.423212	13.327590	12.385139	13.972172	13.038207	13.439251	13.226650	12.304346

AC068205.2	3.633323	3.585700	3.326154	3.115700	2.991831	3.196504	3.388949	3.420058	3.513562	3.501795	3.627841	3.765098	3.294523	4.150401	4.028609	3.240314	3.759199	3.690137	4.575213	3.278205	5.575947	2.771095	4.651753	3.241215	3.138946	2.966116	3.058619	3.286231	3.236523	3.093751	3.720146	3.509038	4.744249	3.156556	3.734799	4.169428	3.784371	3.556216	3.422793	3.278993	3.502755	3.880019	3.674948	3.431343	3.833320	5.848095	3.579772	4.088500	3.822675	4.190383	4.149805	4.015628	4.770981	4.375223	4.578189	3.182569	4.242269	5.837488	3.615725	3.587089	3.090979	3.205070	3.905294	3.285045	3.476705	3.393483	2.792523	4.080720	3.212966	3.316944	3.101500	2.952203	4.085680	3.022175	3.281645	3.374079	3.030200	3.006446	3.020220	3.896283	3.513374	4.244526	3.548191	4.137156	3.435805	3.841530	2.999285	3.249584	2.934625	3.602660	3.258402	2.950032	3.007349	3.311774	3.004073	3.078754	3.971793	3.271787	3.080901	3.050905	2.895358	3.319115	3.071864	3.185015	4.469265	3.334035	3.453668	3.651167	3.058828	3.348845	3.205342	3.446902	3.167396	3.396300	3.586323	2.942434	3.022567	3.163259	3.270657	3.175549	3.782305	3.187544	3.184762	2.883803	3.831572	2.990174	3.267345	3.954187	3.230901	3.482975	3.217271	3.023596	3.369613	3.591339	2.982124	3.709665	3.247680	3.383293	3.459121	4.851376	2.757952	4.054728	3.041027	2.936439	3.292351	2.915804	3.026124	2.903392	3.591699	3.545984	3.367694	3.391393	3.357588	3.650619	3.864949	3.848646	3.784585	3.287565	3.074230	3.240931	3.461116	3.667908	3.504538	4.338539	5.395166	3.624828	3.916408	4.118354	4.428669	4.268916	3.892646	4.360707	4.579040	3.610373	3.099736	3.809313	2.889363	3.075410	3.317112	2.885220	3.312397	3.964011	3.779584	3.704057	3.451172	2.764973	3.985631	3.295582	3.219976	3.070885	3.034402	3.979150	3.065322	3.085938	3.347388	3.036164	3.191743	3.147783	3.314143	3.882548	4.781556	3.633884	3.620663	3.426570	3.128684	3.461815	3.405410	2.869142	3.476911	3.438735	2.868491	2.991917	3.434507	3.335465	3.156927	3.055709	2.934808	3.708456	3.260431	3.027789	3.084750	2.955162	2.985886	4.843175	3.237433	3.496731	3.661403	3.572272	3.236796	3.036549	2.969198	2.998688	3.288352	3.076552	2.958268	3.178836	3.243404	3.419322

AC068205.1	3.633323	3.585700	3.326154	3.115700	2.991831	3.196504	3.388949	3.420058	3.513562	3.501795	3.627841	3.765098	3.294523	4.150401	4.028609	3.240314	3.759199	3.690137	4.575213	3.278205	5.575947	2.771095	4.651753	3.241215	3.138946	2.966116	3.058619	3.286231	3.236523	3.093751	3.720146	3.509038	4.744249	3.156556	3.734799	4.169428	3.784371	3.556216	3.422793	3.278993	3.502755	3.880019	3.674948	3.431343	3.833320	5.848095	3.579772	4.088500	3.822675	4.190383	4.149805	4.015628	4.770981	4.375223	4.578189	3.182569	4.242269	5.837488	3.615725	3.587089	3.090979	3.205070	3.905294	3.285045	3.476705	3.393483	2.792523	4.080720	3.212966	3.316944	3.101500	2.952203	4.085680	3.022175	3.281645	3.374079	3.030200	3.006446	3.020220	3.896283	3.513374	4.244526	3.548191	4.137156	3.435805	3.841530	2.999285	3.249584	2.934625	3.602660	3.258402	2.950032	3.007349	3.311774	3.004073	3.078754	3.971793	3.271787	3.080901	3.050905	2.895358	3.319115	3.071864	3.185015	4.469265	3.334035	3.453668	3.651167	3.058828	3.348845	3.205342	3.446902	3.167396	3.396300	3.586323	2.942434	3.022567	3.163259	3.270657	3.175549	3.782305	3.187544	3.184762	2.883803	3.831572	2.990174	3.267345	3.954187	3.230901	3.482975	3.217271	3.023596	3.369613	3.591339	2.982124	3.709665	3.247680	3.383293	3.459121	4.851376	2.757952	4.054728	3.041027	2.936439	3.292351	2.915804	3.026124	2.903392	3.591699	3.545984	3.367694	3.391393	3.357588	3.650619	3.864949	3.848646	3.784585	3.287565	3.074230	3.240931	3.461116	3.667908	3.504538	4.338539	5.395166	3.624828	3.916408	4.118354	4.428669	4.268916	3.892646	4.360707	4.579040	3.610373	3.099736	3.809313	2.889363	3.075410	3.317112	2.885220	3.312397	3.964011	3.779584	3.704057	3.451172	2.764973	3.985631	3.295582	3.219976	3.070885	3.034402	3.979150	3.065322	3.085938	3.347388	3.036164	3.191743	3.147783	3.314143	3.882548	4.781556	3.633884	3.620663	3.426570	3.128684	3.461815	3.405410	2.869142	3.476911	3.438735	2.868491	2.991917	3.434507	3.335465	3.156927	3.055709	2.934808	3.708456	3.260431	3.027789	3.084750	2.955162	2.985886	4.843175	3.237433	3.496731	3.661403	3.572272	3.236796	3.036549	2.969198	2.998688	3.288352	3.076552	2.958268	3.178836	3.243404	3.419322

AC244213.1	5.424256	13.073967	13.242607	12.913034	13.262596	12.932954	12.843523	12.357997	13.268237	13.131724	12.939734	12.835422	12.575902	13.348898	13.235191	12.899883	13.443765	3.189710	12.384645	13.156712	12.499454	12.293733	12.455008	13.140875	13.331093	13.359085	13.118676	13.137385	13.190276	12.566429	13.261809	12.566268	13.151252	12.560728	12.819943	12.890515	4.646207	12.902509	13.130961	12.984259	13.155970	12.456606	3.469619	13.228465	13.346771	13.348067	12.922049	12.588301	12.950005	13.262182	12.722462	12.939558	5.491318	13.388411	4.740639	13.141252	3.662035	6.235310	13.216575	4.571161	12.712770	5.734828	11.811423	12.177525	12.180967	13.244726	12.354041	5.218337	4.882660	12.089510	13.015726	13.303685	5.118031	12.954237	13.174582	13.281221	5.207233	13.004475	13.137564	13.466605	13.288850	13.308155	13.618777	4.603961	13.236652	13.133191	13.442439	13.468361	12.321093	12.967881	13.324386	12.260195	12.972576	12.937259	12.927010	13.324601	4.544230	6.351290	13.180932	13.607100	13.354589	13.102943	5.021357	13.544379	11.991560	12.856216	13.642281	12.582617	12.724991	12.912866	5.333243	13.859679	13.505465	13.122136	6.830405	12.607880	4.007400	13.633485	13.198141	4.733129	10.689778	11.544514	11.302194	10.532527	11.030014	10.748747	15.148056	10.196594	9.672692	12.455951	13.879568	10.999696	10.872181	10.730905	11.573269	11.010354	3.313340	9.159846	12.888444	9.594008	10.841725	10.492998	13.433260	12.736959	10.221812	12.654999	9.764810	10.807688	10.695729	11.544514	11.790869	11.713327	10.459678	11.948824	11.892007	4.253009	10.704200	11.234364	11.127236	12.154339	10.771657	3.495403	10.498864	12.267902	11.693032	9.349409	10.261006	12.823360	10.688128	12.526303	10.929944	3.714654	10.600462	3.250993	10.963392	3.372751	4.469265	11.732422	3.864105	10.426856	6.263525	11.999153	11.489042	11.240198	12.167732	12.837080	4.858971	5.279336	10.445678	10.990195	11.104426	6.008962	11.051785	12.008552	11.981655	5.060442	10.954078	12.657829	12.891207	12.234198	10.778128	12.989260	4.164884	13.433260	10.985679	10.965351	10.665768	11.280614	11.836322	10.744707	9.851679	12.966913	10.979516	12.497024	13.061545	3.562130	4.388263	13.134359	12.278204	11.067626	13.151837	4.174447	11.980172	12.208551	12.967484	12.704827	11.456822	12.269936	9.424072	4.641771	13.585329	9.845032	12.560381	4.998160	9.549294	4.671443	10.246175	10.069314

AC100800.1	8.524156	6.613170	7.115823	8.163679	8.510905	7.285123	6.807413	7.737577	9.878449	7.953242	7.906928	6.932872	8.170198	7.613569	7.679669	8.069834	7.567942	7.828836	9.005162	7.535018	7.718880	7.526204	8.216056	8.239336	8.355695	8.147919	7.283831	7.932799	8.047520	6.143590	8.802470	6.416667	6.153773	7.332263	6.630766	6.346571	6.981009	6.813501	6.724651	7.068728	7.983020	6.214589	7.047385	7.302722	7.960849	6.446215	7.684592	6.965487	6.376310	6.671230	6.375503	6.009832	6.486440	6.590635	6.655174	8.527057	7.542035	7.421349	7.598858	6.924368	6.195305	7.618765	6.879088	7.405064	5.795803	6.804613	5.454582	7.005651	6.467703	5.778728	5.659580	6.640640	8.397774	6.108518	6.385776	5.981839	6.567898	6.214759	6.029573	7.090578	7.251854	6.598102	6.876549	6.909234	6.943923	7.207529	8.152684	6.604049	5.997961	5.843146	6.262353	5.916363	6.281101	5.973440	6.557781	7.751210	6.655174	9.482879	8.166444	8.912646	8.856845	7.404264	8.755638	7.900849	6.570194	8.342909	7.392271	7.150909	8.203908	7.833692	8.036507	9.074813	9.488725	8.295156	8.357843	8.984412	9.355444	8.313433	9.436174	7.768377	6.936495	5.818516	6.983822	7.338770	7.141280	6.572438	9.422970	8.811070	7.240393	6.846321	7.393365	7.426808	7.055431	6.995380	7.933723	6.398962	9.552380	6.604389	6.152405	9.240288	8.023925	5.671992	9.648272	5.584774	7.304277	8.129187	8.715790	10.217279	6.108169	7.804933	7.365307	8.293663	7.815906	6.828319	7.661530	7.165954	6.889664	6.681747	7.367347	7.777466	6.536288	6.864533	7.237820	8.167811	5.392017	8.020574	5.534285	7.526963	5.668508	5.819727	8.000418	7.852796	7.186039	7.809278	6.662662	6.730471	6.885519	7.052414	6.166510	6.522506	6.436473	7.159964	6.810006	6.421746	5.585157	6.026766	6.412706	6.600722	4.957935	6.338556	7.649962	7.051334	6.905732	5.872814	5.609716	5.900251	5.388347	6.774623	6.949641	7.389333	5.513884	7.079256	7.490387	6.654176	7.552070	6.810006	7.328515	5.388935	6.625344	6.466853	6.784675	6.405898	5.749929	6.980014	7.339085	7.633165	9.459105	6.749427	8.493981	7.777297	8.017407	8.631600	8.358470	7.400837	7.365458	9.536570	8.404680	7.439469	7.026662	8.198239	8.500123	8.421352	7.273894	6.196567	7.157299	8.021201	5.311819	6.829577

AC099535.2	10.826359	10.806201	11.121300	10.790007	10.025282	10.507824	10.653668	10.684350	11.590096	10.631884	10.560567	11.409515	10.559792	10.040378	10.664020	10.532527	10.400338	10.266446	9.993095	10.825164	10.239877	8.859422	10.372919	9.259535	9.089615	10.080427	10.490710	9.926337	11.050033	11.128092	10.752065	10.913714	10.682122	10.791885	10.921361	10.895336	10.776077	10.382728	9.676294	10.040236	10.878998	10.456003	10.649852	10.793806	10.891628	9.975129	10.245297	10.663435	11.039307	11.193393	11.043151	10.951630	10.996710	11.187721	11.381887	11.243882	10.754173	9.762143	9.932634	9.830156	9.920909	10.689027	10.813727	10.107923	9.882882	10.097780	9.319744	9.307273	9.455087	9.578233	10.561773	10.327034	10.408604	10.701664	10.630255	10.479922	10.719585	11.000467	11.051912	11.225924	10.911465	10.811375	10.498431	11.191787	10.742155	10.964018	10.851957	10.804455	10.033608	9.435389	9.306796	10.197958	10.414939	10.631731	10.867997	10.752372	11.242375	10.814754	11.424787	11.072990	11.551046	10.943897	10.631731	10.145201	10.963108	10.835128	9.901962	9.980403	10.392827	10.905092	9.821926	10.576772	9.452733	9.929996	11.441183	9.290023	9.394342	11.662078	9.981684	9.921529	10.478286	10.755416	9.925892	8.321873	10.775354	9.546391	10.759397	10.022143	10.731630	9.467510	10.175307	10.074063	9.219588	9.782381	10.080285	10.569382	10.563066	10.137149	10.417667	9.936498	9.373655	10.651913	9.234881	9.280309	11.031948	11.276298	8.620186	9.046541	10.686442	9.921681	10.621593	10.098577	10.376060	10.020947	10.889511	10.494961	9.352587	9.425426	10.202652	9.399848	9.601742	10.870211	9.840773	10.706106	10.072505	9.919552	9.869780	10.549196	11.250623	11.068070	11.136541	10.915990	11.626908	10.921515	11.217736	11.051636	9.799359	9.568254	9.430938	9.929233	9.864242	9.821765	9.763085	9.502704	9.817268	10.562074	9.151401	9.379298	10.169500	10.472068	10.881198	9.377152	10.902636	10.468311	10.559019	10.453118	10.747853	11.346391	10.443193	11.005405	10.312904	10.125527	10.944920	10.414630	9.594640	10.662816	10.369411	10.064990	9.524425	9.465521	10.491133	9.685477	10.532043	11.477701	10.871441	10.453118	10.444886	10.102182	10.474513	10.865608	10.686302	9.655028	10.710534	9.955381	11.155073	10.071631	9.644557	10.938063	9.230742	10.357076	10.189200	9.444674	8.911133	11.406029	10.358409	9.910192	10.964156	9.671337

AC009034.1	10.688294	10.773731	10.970303	11.249245	11.368160	10.638766	10.833152	9.424072	9.837660	10.319671	10.535450	11.046498	10.931745	10.821007	11.130492	10.542131	10.129307	10.972500	11.474995	11.043725	11.257082	10.192512	11.212440	11.099443	11.211433	11.137872	11.138461	10.583475	10.814911	10.508413	11.032245	10.667694	10.740051	10.475871	10.918527	10.704328	10.926394	10.686008	10.934885	10.581147	10.988997	11.228875	10.566794	10.427778	10.986739	10.684060	10.621312	10.805332	9.831828	10.606813	10.231014	10.804169	11.004703	10.734015	10.432465	10.903646	11.097482	11.589394	11.493018	10.885995	11.531829	11.271312	11.263943	11.131759	10.933966	11.365974	11.071366	10.748747	10.756632	11.057484	10.984205	10.890718	11.049296	10.975093	10.866981	10.841725	11.027041	10.596938	10.636673	10.926394	11.474995	11.403962	11.097929	11.519937	11.413548	10.807365	11.354479	11.830399	11.887650	11.486387	11.623005	11.774619	11.032834	10.808753	10.996398	11.281661	10.267937	11.182438	10.679894	10.292644	10.093662	10.689337	10.562644	10.971621	10.432922	10.629178	10.993881	10.040996	11.383354	10.469669	11.705791	10.955140	10.033753	9.956160	10.308167	10.820288	10.788704	11.041648	10.699257	10.446420	10.391357	11.466138	11.479798	10.085861	10.595584	10.608143	10.470433	10.820438	11.011977	10.490996	10.267489	11.040594	9.346806	11.073280	10.922541	9.825874	9.980586	10.775210	11.693331	10.197514	10.323048	10.417979	9.801645	10.308167	10.993001	10.022905	10.369106	10.365154	10.242440	10.055756	10.353124	10.218622	10.113319	10.117613	9.768766	10.650447	11.071672	10.979653	10.574366	11.436541	10.294779	11.525035	10.087619	10.069917	9.813386	10.139853	9.778165	9.972959	10.565474	10.497395	10.443501	10.228793	10.336744	10.563528	10.945211	10.381054	11.260928	10.350959	10.917489	10.285603	11.326759	10.699718	10.368958	10.763276	10.971621	10.748441	10.024169	10.111087	10.373518	10.194330	10.099670	10.115619	10.519232	10.600915	10.440659	10.266747	10.945949	10.337046	10.668143	11.402351	10.040996	11.017053	11.106894	10.579044	10.075427	10.765247	10.673442	11.401374	10.871747	11.434496	11.426361	10.375761	9.828267	10.598330	10.671047	9.801645	10.686008	10.484421	9.781775	9.471077	9.877034	9.793920	10.268677	10.255423	10.316237	11.362843	10.137745	11.041360	9.731236	10.829442	10.370335	9.830318	10.250420	9.440040	10.161066	10.643878	11.285190	9.808072

AC003986.1	4.451413	4.619283	4.552590	3.708077	4.895184	5.534285	3.929799	6.346221	7.695463	4.851592	5.586429	4.910284	4.788270	5.486138	3.148164	4.762713	6.116992	5.523280	5.431625	4.427875	4.536102	7.014074	6.594381	5.127943	5.003376	5.358537	5.493365	6.506661	3.956800	3.960098	5.910785	3.635857	4.779250	4.365717	4.494905	4.601214	4.932831	4.237634	5.308286	5.535274	4.094749	4.950017	5.884957	5.406292	5.157266	4.288874	3.877385	3.919983	5.086568	4.687185	4.181237	4.649613	4.035385	4.246430	4.018944	5.181765	5.357589	3.119247	4.565222	3.301128	4.303316	4.230677	4.021106	6.320848	3.550812	4.314044	4.525013	5.079803	4.581421	5.017705	4.733129	5.116998	4.933247	4.580148	4.940761	3.152335	3.815882	4.004562	4.290484	3.841099	3.783146	4.742152	3.656217	3.680636	3.949193	4.310828	3.412911	3.940235	3.042314	3.990914	3.650619	3.652825	5.167687	4.723015	4.456929	5.027663	5.089194	6.492516	5.839873	5.650665	4.779065	4.142276	5.713082	5.162091	5.389322	4.381821	7.710796	6.437280	5.219588	4.768875	4.442859	5.971051	3.955022	5.145729	5.362349	3.589954	4.708848	5.271047	4.707226	3.935574	4.277507	3.564394	3.594204	4.480424	5.499484	3.777553	5.648403	5.756463	3.759199	4.172921	4.046032	3.671371	4.253660	3.007845	4.646000	6.283964	4.787444	4.837238	3.829935	5.146157	4.716023	6.190268	4.724520	3.160050	4.212746	4.974416	6.276999	4.966042	4.022370	6.852320	3.862777	2.942527	4.191920	4.288341	3.609596	3.982184	3.257771	5.749744	3.789934	3.828051	3.307751	3.822877	5.393779	4.714961	4.081552	3.672132	4.462440	3.230192	4.017403	3.855323	3.478950	3.521478	3.651376	3.629852	4.194119	5.102166	3.126980	3.512576	3.200147	4.137598	3.293300	3.255912	5.218337	3.188619	3.152335	2.930079	3.848412	4.097105	3.740182	3.259588	4.515737	4.744674	3.835458	4.011238	3.132218	3.494456	3.702258	4.172921	3.615151	3.277400	4.010157	3.006162	3.426570	5.456853	3.808458	3.200504	3.647203	2.980874	4.647688	3.119010	4.870222	2.991009	3.759866	3.856357	4.324006	3.674735	5.599151	5.053435	4.274018	4.051084	4.293542	4.425043	4.471446	5.525734	3.523264	6.500213	5.544482	4.733340	5.109134	3.395031	4.439142	3.111885	5.709757	3.987558	4.037145	4.827617	4.742152	5.091838

AC097462.3	5.466381	5.897057	5.924182	6.500213	5.930750	6.211186	5.411498	7.246521	6.995829	5.617610	6.163228	5.916014	5.354296	5.478242	4.929972	5.701366	6.628714	5.937388	6.327567	5.823304	6.654503	6.664613	6.355577	5.958228	5.401645	5.437272	6.704797	5.671606	5.686221	5.533876	6.948905	5.090210	2.993084	5.710503	5.631732	5.412057	5.324597	6.043307	5.972718	5.964297	5.738433	6.116663	6.454936	6.750212	5.685095	6.104087	5.840959	5.224298	5.383723	5.587921	5.474270	5.389141	5.313797	5.984369	5.465597	6.128740	5.340859	3.524206	5.542603	5.030942	4.994580	5.629533	6.511935	6.625344	5.749929	5.683615	6.372612	6.500045	6.031288	6.352424	6.128168	5.615376	5.498320	5.843332	6.016224	5.651254	5.673483	5.106656	5.350251	4.463518	4.203616	4.818129	4.056940	4.483292	4.600798	5.576839	3.035479	4.941159	4.802838	6.060983	5.446009	5.178999	5.263283	5.261427	5.431625	4.582030	4.872144	5.095208	6.328905	6.671706	4.314773	5.820088	7.258046	5.966457	6.242601	5.612341	6.068237	6.567744	5.958962	5.989180	5.994659	5.292166	4.606924	6.587881	5.399012	4.081964	5.251390	5.201863	6.130528	6.128336	6.302189	6.051126	6.589328	6.602455	6.949322	6.203650	6.454610	6.843510	5.832274	6.175920	6.065393	5.981184	5.281755	5.892768	6.067528	6.781665	6.158213	7.212319	6.318421	6.908123	6.110971	7.258890	5.949351	5.791150	5.069592	6.555983	6.159627	5.855990	5.912785	6.866291	5.511627	5.305994	6.134299	5.956452	5.757968	5.695458	5.915291	6.893364	6.171677	6.889035	5.766833	6.805681	7.221302	6.434442	5.962877	6.521864	6.162208	5.267688	5.340097	5.324027	5.989536	5.349668	5.418743	5.489572	6.046918	5.983507	4.242073	5.849897	4.597449	5.238860	5.896175	6.630936	6.380948	6.236812	5.486905	6.628874	6.360544	8.541337	6.424158	6.260150	5.817622	6.633864	6.061533	5.809052	5.748312	6.287915	5.432797	5.646337	4.455432	4.637149	5.199473	3.921454	3.999992	4.523906	6.087137	3.735018	6.734147	4.512034	5.195106	6.201199	5.754253	6.386127	5.555640	5.801715	5.392017	5.895664	5.234417	6.064519	6.796372	5.747384	6.258264	8.278265	6.136700	6.205920	6.620355	6.505307	5.905278	6.273200	7.625771	6.291862	5.233026	4.034967	7.549912	6.030746	5.317249	5.841340	5.734458	6.633374

AC092053.5	3.859612	3.167149	3.173311	3.119124	2.959467	3.070040	3.574404	4.024737	5.223134	3.699776	4.446299	3.642657	3.183839	4.311047	3.348550	3.140672	5.705436	3.623459	5.404983	3.577338	5.004978	2.932255	5.137599	3.178836	3.206265	3.173183	2.946804	3.119247	3.179398	3.426736	5.841158	4.818759	3.012040	4.136501	3.338122	3.896720	3.655248	3.373733	3.479830	3.272701	3.348203	3.974642	2.965952	3.083438	5.530879	5.414539	3.543602	4.491993	3.147523	3.761341	3.430988	4.304639	4.088735	4.778843	3.502944	3.035361	3.786278	3.527760	3.488296	3.632530	3.535542	3.352560	3.578380	3.047416	3.980405	3.261430	4.565327	3.288923	3.162054	3.161219	2.929202	2.854366	2.956704	2.779067	2.899623	3.590928	3.017610	2.905401	2.923946	3.249455	3.087647	3.424550	5.776559	4.055179	3.150262	4.389354	3.346871	3.198532	3.143866	3.306029	3.182044	3.164945	2.982814	3.242816	3.135531	3.065217	3.653400	3.077141	2.995414	2.939146	2.920694	3.157660	3.004165	2.893176	2.896185	3.808277	3.181376	3.317763	3.064290	3.238467	2.952031	3.195295	3.043520	4.455844	2.989605	3.231727	3.007450	3.725577	2.842187	5.540532	3.236242	6.249386	3.017302	2.917603	3.435445	3.099402	3.689738	5.281972	3.597342	4.209072	3.168395	3.287730	4.730815	3.586121	3.423315	5.638273	3.209518	4.503938	4.223852	5.062460	2.877217	4.727241	3.248562	5.881927	6.605022	3.192046	3.360327	2.873486	3.457694	3.438428	4.414432	3.021771	3.315118	3.893314	4.584561	3.687262	4.383610	3.851412	3.460924	3.626008	4.111173	10.064373	3.984782	6.016900	5.030342	3.196648	5.287167	3.204137	4.912822	6.070667	4.882660	5.517603	5.175953	4.547293	6.326752	4.490665	5.178778	6.030746	4.442859	4.770332	3.070040	5.187401	4.901135	5.000064	4.349824	5.393779	4.502632	4.326548	4.455432	3.704887	3.529787	4.224260	7.184232	6.049071	4.180567	3.459121	3.503653	3.540932	4.118088	5.308659	5.190944	8.333829	4.433701	4.620375	3.674948	7.525891	4.178097	5.171289	4.410703	4.147890	4.136187	3.822254	4.011825	4.116561	3.901874	3.126876	3.306340	4.557457	5.916723	3.540335	3.004073	2.920622	3.452065	6.290979	3.775313	4.423983	3.271787	3.455153	3.536493	2.893899	8.610624	2.923853	4.567453	2.939745	2.897064	4.194544	8.628077	7.517578

AL133387.2	4.880724	4.489549	4.053659	4.648966	4.097537	4.511602	3.530520	5.246412	5.070805	4.015408	4.481723	3.462345	3.726533	3.527209	3.115578	3.903127	3.948147	4.970884	5.897057	4.048031	6.097863	4.803466	5.473683	3.995086	4.673167	4.452250	4.180105	4.834783	3.578575	3.552388	4.461136	3.307479	2.995316	3.361439	4.243870	4.205490	3.489376	4.752754	5.474461	5.217569	3.810138	4.970691	4.110743	4.998777	4.568093	4.139301	3.713806	3.849937	2.978793	4.700882	4.185118	3.480782	3.888276	3.696307	3.309708	3.552588	4.934074	3.729032	3.612379	3.386361	4.832287	4.104297	3.930648	4.686755	5.604946	4.512913	2.943756	4.651753	4.835984	4.139072	3.608987	3.957915	4.150618	3.776160	4.106299	4.017403	4.464171	4.340922	4.356767	3.315887	4.129176	3.603041	3.912072	3.852056	4.113121	4.425695	3.613168	4.462017	3.675983	4.689279	4.536530	5.357204	3.045673	4.727036	4.215317	3.794238	3.317763	3.679614	3.695929	3.782941	3.820601	3.529043	4.517223	4.428669	3.888906	2.927929	5.462213	4.549992	5.225884	4.082613	5.467740	4.794982	4.883281	3.723306	4.759964	5.049796	5.091641	4.000622	4.076809	4.163583	4.405822	4.111616	3.854056	4.366344	3.911207	3.905736	4.621238	4.941365	3.609384	4.026648	3.528663	3.362499	3.379837	3.884501	3.671942	4.735021	5.036741	4.048439	3.725976	5.694530	4.677535	6.136363	4.878379	4.311896	4.385189	4.413993	5.094583	3.322010	3.375281	4.781346	3.649432	2.990624	3.488837	4.147681	4.378669	4.049977	5.859782	4.911356	4.827617	3.293629	4.822102	4.293542	5.263473	5.142772	3.925015	4.240972	3.952483	4.508331	4.954568	3.735838	3.364044	3.487138	3.691777	3.108516	3.791856	4.476238	3.722702	3.495973	4.000224	4.210561	3.734186	4.520391	3.386728	6.004284	4.340922	3.146427	4.775532	4.848502	4.705302	4.266557	4.046961	4.339614	3.740380	3.566109	4.039945	4.318454	4.369342	4.821899	3.478586	4.562514	3.577965	3.040061	4.224051	3.400386	4.642400	4.030399	3.752527	3.089007	3.685428	4.195183	5.016059	3.773677	4.657710	4.630905	3.755044	4.468417	4.258314	3.286382	4.022591	3.710484	3.928272	4.700882	4.184692	4.334201	3.842101	5.333431	4.523456	5.843704	4.724322	6.001274	5.239632	5.575947	3.370825	4.723440	5.134458	4.773888	3.494456	3.722702

SAMMSON	12.567417	12.110845	12.099673	12.771719	13.102943	12.894925	12.713284	12.924711	13.220404	12.750339	13.358673	13.153629	12.894731	12.780199	12.717840	12.662746	13.160536	12.787861	12.413338	12.276968	11.933654	10.934125	12.230187	12.025944	12.714689	13.377996	13.970183	13.632044	13.139689	12.552012	12.976769	11.270443	11.486085	12.889319	11.958571	12.180653	12.161114	11.800745	11.894129	12.010792	13.162623	12.197990	12.312392	12.066090	12.796802	12.217913	13.727121	11.972822	11.729913	11.735541	11.701608	11.831805	11.338052	11.389403	11.723608	13.044826	11.639167	11.353164	11.209250	11.694552	11.478266	12.697859	12.284248	11.342184	11.438109	11.729303	11.538067	11.569456	11.394433	11.343861	12.485666	12.569208	13.412299	12.847185	12.602397	12.255993	12.142076	12.304191	12.226312	11.662821	11.696988	11.693331	11.294412	11.584392	11.729465	11.833153	12.016883	11.765403	11.136698	10.952431	11.228716	10.883094	11.954526	12.001025	12.542124	12.691189	11.828974	12.793186	12.503046	13.372507	12.516623	12.511287	13.138899	12.854219	10.120195	13.078847	12.683833	11.501766	11.473095	13.036697	10.578752	12.496355	12.417520	13.278200	12.796263	13.967702	12.389993	13.174367	13.700156	12.289514	12.295732	12.328136	12.939558	13.808354	12.949109	13.106206	13.124576	12.752950	12.641718	13.300869	13.196931	13.296386	12.903931	13.519354	13.088879	12.782224	12.882947	12.342178	12.193831	12.008552	11.231436	11.421630	12.455317	12.372749	12.952208	13.008664	13.465090	13.280056	12.577210	13.105616	11.915640	12.131737	12.770158	12.169426	12.111589	12.488929	11.821110	12.411264	12.389189	13.314543	12.084512	12.014628	12.372119	12.276073	12.293882	13.492381	12.022667	11.522184	11.832095	12.079262	12.007014	11.212440	11.486239	11.693642	12.855512	11.594601	11.189611	11.232126	11.380692	11.599324	13.268237	11.148846	11.484132	11.640106	11.570621	11.887800	11.527185	11.707159	11.373342	12.115695	12.735569	13.747333	12.815796	12.597148	12.439014	12.276225	12.820259	12.688641	11.960844	11.743921	12.024202	11.687959	11.699941	11.478556	12.315775	11.904904	11.735820	11.375164	11.493459	11.368020	11.107474	12.044706	12.398188	12.665284	12.876066	12.410460	12.992898	12.245147	12.389516	12.479650	12.732190	13.465968	12.673598	10.152638	13.263764	12.659816	11.238299	10.990910	13.294543	10.642239	12.471342	12.501102	13.719437	12.376294	12.830643	11.985255	12.739710	13.128443

AL391845.1	7.884605	8.918624	8.732710	8.963871	8.776752	8.963282	8.836181	9.464406	8.301521	8.901711	9.268924	8.756076	8.485826	8.502160	7.810799	9.054864	8.433072	8.023620	7.747653	8.963573	7.924598	7.922875	5.111153	8.288718	7.807410	8.652056	8.478229	9.467827	8.735164	8.630044	8.120075	8.317902	8.242495	8.210084	8.441936	8.244922	8.612810	8.531228	8.047984	7.626412	8.920911	7.637537	7.682404	7.539270	7.738513	7.845183	8.864614	8.373268	8.412918	8.287784	8.109085	7.990331	8.310979	9.038669	7.827891	8.810595	8.168776	8.031490	7.547904	7.732870	7.944780	8.741484	7.567799	7.888271	7.693823	7.540217	8.237377	7.992068	8.478824	7.909425	9.016215	8.896716	8.995047	8.860617	8.819635	8.493527	8.452168	8.329193	8.650279	7.617539	7.799754	7.882461	7.422117	8.069369	7.863243	8.141183	8.021935	7.949845	8.352610	8.065503	7.230565	7.192616	7.787922	9.036324	8.341870	8.532273	8.071381	8.145183	8.220853	8.477080	8.026668	8.562976	8.476639	8.742697	6.847723	8.833908	7.874891	7.640633	7.242763	9.188603	6.659694	8.021631	7.924442	9.243650	8.777533	8.729147	6.585387	8.559612	9.188455	7.821163	8.659450	8.505652	8.907201	8.664535	8.928332	8.680653	8.122926	7.938222	8.849892	8.719185	8.685838	8.981422	7.966199	7.920740	8.777673	7.793105	7.891543	7.137804	8.329650	7.658770	7.531887	7.681302	8.561461	7.804638	8.761974	8.926440	9.084561	8.822037	8.718743	7.807549	7.744557	7.455252	8.938979	8.137859	7.876859	8.455353	8.974670	8.441475	7.727325	8.884219	7.819359	7.379718	7.958992	7.589490	8.225862	9.023720	7.833385	7.787922	8.186038	8.071533	7.896420	8.360170	9.075141	7.629887	8.220554	8.018603	7.981300	7.683968	7.781521	8.305299	8.741333	7.645417	8.106204	7.677137	7.657214	8.429740	8.111766	8.542882	8.383906	8.626389	8.658207	9.257569	8.638802	8.247677	8.505509	8.164882	8.759006	8.812028	7.185369	7.639389	8.227358	7.585179	7.631767	7.970688	7.817308	7.960064	7.734581	7.859101	7.511385	7.298304	7.321996	7.685039	9.224202	8.597760	8.247528	8.007316	8.030875	7.989271	8.044812	8.005774	7.584419	8.584087	8.360634	7.389651	8.567907	7.800068	7.905055	7.330232	9.038018	6.761235	7.235957	5.996025	9.671026	7.974619	8.799150	7.229521	8.787346	8.879106

AC010273.2	3.973522	4.433919	4.972749	4.311668	4.214482	4.157373	4.706191	4.803039	5.390271	6.031651	4.747288	3.982184	3.933476	4.295235	3.245240	4.564465	5.138025	4.412213	4.643607	5.692261	4.374125	5.194263	6.823863	4.933461	4.217517	4.513567	4.201623	4.270081	4.713491	4.088500	4.801756	5.595302	3.238168	5.000471	4.834971	4.539078	4.971090	4.710518	3.982847	4.239453	5.097170	4.732932	4.455648	4.077019	4.476238	5.297845	4.781346	3.177764	4.468617	5.275538	5.804747	5.016903	6.880697	5.019124	3.760914	4.488254	5.148333	2.854242	5.510295	4.956452	5.261653	4.456699	5.721228	5.459435	5.747936	5.376539	4.637598	4.775323	4.964786	4.794982	4.833536	4.441972	4.586482	5.019124	6.451641	5.359285	4.773047	5.013417	5.141118	5.256331	3.875266	6.289787	4.968204	4.425695	5.474637	4.893555	4.334841	5.144352	5.571063	5.361935	4.935777	5.777077	4.458635	4.088092	3.739973	4.315840	5.321701	4.287894	3.549094	4.536102	3.123562	4.045833	4.071257	4.214482	4.345054	4.749752	5.123510	4.110948	5.366809	5.723646	5.060442	4.532673	4.193640	4.022173	4.774099	2.961473	4.851814	4.174641	4.862901	4.874651	5.278766	4.811884	4.748507	3.339777	4.369342	3.889150	5.522511	5.810403	5.033574	4.342640	4.560116	3.564573	3.565350	3.093751	4.661386	5.321701	3.239185	4.863307	5.818327	4.043227	3.985840	5.619849	3.815433	4.144750	5.807418	3.515313	4.481034	4.545552	4.825211	4.974843	4.637149	3.216802	5.504866	6.814127	4.762913	4.844242	3.858138	4.244327	4.556230	5.552010	5.607046	5.128371	5.401255	4.012263	4.758520	4.899185	3.494456	4.933036	5.026469	5.662741	4.779673	6.358855	4.125253	4.321835	5.172334	5.937761	6.653518	5.256331	4.669502	5.093749	4.645564	5.995839	5.601705	5.456085	5.492994	5.443704	4.767465	4.949392	4.712642	4.801357	4.516566	5.336536	5.207233	5.210432	5.277375	5.025466	5.416020	5.237472	5.025838	4.555132	5.614247	4.862901	3.773875	5.885856	4.161178	6.552849	6.688232	5.085506	4.949812	4.949392	5.889570	4.683764	4.202709	3.509805	3.783976	4.356986	3.571157	4.552354	5.271047	3.642453	3.578986	3.907084	3.473978	4.636303	4.514824	5.068410	5.420176	5.173736	5.241252	4.072108	3.948147	3.463557	4.409815	3.926528	3.549307	5.162860	4.944987	5.008684

AL096870.1	11.546206	11.758737	11.889910	11.802240	11.687807	10.975965	10.864681	10.978627	11.232005	10.788836	10.641778	10.768791	10.905411	11.559388	11.680837	10.064373	10.250420	11.286099	10.357811	11.595353	10.283384	10.642391	8.715458	11.223716	11.142960	11.042993	9.753457	10.667399	9.772051	11.102062	10.519370	12.395385	12.108482	11.072856	11.424197	11.234493	11.559648	11.222672	11.184811	12.099966	10.640618	11.573431	11.554325	11.483699	11.165084	11.674871	10.597699	11.769146	11.354634	11.714242	11.798796	11.713634	12.022968	11.847129	11.884633	10.648336	12.046914	12.495001	11.929527	11.625463	12.480968	10.325669	10.555064	11.559784	11.785232	11.895484	12.140350	12.555759	11.983055	11.976994	11.441033	11.536751	10.048197	11.547537	11.518252	11.763241	12.081214	11.484876	11.423001	11.187873	11.186151	11.696386	12.074060	11.816748	11.905325	11.418410	11.822299	11.742441	11.451147	12.327331	12.185716	11.614346	11.820662	11.768702	12.029310	11.715866	11.943486	10.761738	10.958405	11.073844	10.754495	11.129723	10.677496	11.343233	11.943044	10.444741	11.268516	10.697935	10.233407	10.611543	10.972801	11.639475	12.150924	9.933712	11.537331	9.256834	12.118005	10.995532	9.778003	11.400599	11.410967	10.984949	11.658803	9.616048	9.622911	12.147187	7.495847	7.670378	9.744273	11.247632	10.480213	9.170296	10.184366	8.899923	9.480728	9.381478	9.974195	8.773214	9.864082	7.893273	10.406224	9.155870	11.306661	10.545217	9.959522	10.452087	8.640199	7.733781	10.931591	9.048698	11.264849	10.966969	9.876239	10.087473	10.681521	11.019313	10.921361	10.386089	11.799242	7.005199	11.523082	10.502419	9.957986	9.944548	10.587164	10.552052	10.331612	10.325356	9.715949	11.393514	10.382558	10.536920	11.335254	11.805894	8.927706	9.279384	11.483101	11.337458	10.831653	12.324058	11.074426	9.530388	11.497278	12.058897	10.999997	9.736856	10.791439	9.824950	11.244028	10.399284	10.313397	9.108604	11.408599	11.482399	11.721853	9.880112	10.600166	11.197968	9.865449	10.648195	10.018488	11.803233	11.100738	10.512586	11.080441	10.159076	9.900361	11.785687	11.332335	10.254211	8.629744	11.501190	10.512879	10.560393	10.365006	10.543459	10.129307	10.007217	10.791885	8.664535	9.309261	10.034852	11.142960	10.019581	10.296166	9.480244	9.743493	9.831535	9.906344	9.844099	10.702258	7.088332	10.509324	9.009654	10.706580	10.195547	12.391607	9.262267

AC076968.1	10.567405	13.454152	12.581318	13.759150	12.508187	14.140523	14.311014	15.663417	13.695624	14.896544	14.097636	14.533026	13.359184	13.427110	12.950759	13.886198	13.356786	13.099956	13.501109	14.618611	12.954050	12.611352	10.996252	12.904820	13.863343	14.027387	11.679201	12.809121	12.780366	13.039489	13.704371	12.214094	12.609346	11.272804	13.085544	14.480925	13.842755	13.561295	13.805428	13.638755	13.723956	13.254196	13.100501	13.304746	12.631485	12.931521	12.419759	13.459822	12.163756	13.763964	11.876001	12.858637	13.001582	13.477694	13.780832	14.953863	13.170952	12.219989	12.932249	12.470387	12.811614	12.658166	10.784221	12.787176	13.223925	13.304746	12.697859	13.376481	12.738314	13.332466	13.383479	13.289866	12.866728	13.894360	13.154208	12.702790	12.798847	12.736077	12.942843	14.318185	12.046176	12.928492	12.460607	13.221977	14.050939	13.675309	12.916577	12.092910	12.322814	13.459166	13.667210	12.853507	12.982400	14.381001	11.322294	11.580850	12.935833	12.284727	12.984090	13.795660	14.101336	14.109446	12.548577	13.963570	14.115260	12.855167	13.831246	13.320270	12.907332	13.894764	12.309198	13.937690	13.480172	13.347873	13.359928	14.074236	11.148560	14.448331	12.687779	10.579352	13.748586	10.870510	11.971773	12.671548	13.163576	13.535732	11.852632	12.169723	12.317939	13.457435	11.697417	11.676108	11.192513	10.995680	12.463072	11.033609	11.458769	11.528321	11.081007	11.580709	11.742152	10.913411	12.040293	11.876001	11.107772	12.760155	11.946674	12.059052	11.836176	11.452540	12.094136	12.213629	13.323791	11.221164	13.439251	14.286571	11.820064	12.118493	11.838717	11.864087	12.517447	10.593778	11.688994	12.607714	12.945176	12.232358	10.989737	11.068971	11.839322	11.393373	10.740345	11.496833	11.584062	11.231267	11.624141	10.383969	10.850468	11.321148	11.099157	12.071732	11.590387	11.160511	11.830189	11.236126	10.728828	10.889218	13.725134	12.424072	11.802387	13.069482	12.664618	13.131339	12.577866	11.082761	13.249512	12.354682	11.214098	13.313756	10.934280	11.126352	11.017912	13.601026	11.039023	10.656544	12.006089	12.371489	11.379010	10.801939	11.910424	10.674816	11.799846	12.736077	11.332473	13.722489	11.126499	11.232861	11.133393	12.074516	11.911322	11.411961	11.760698	11.693331	11.116659	11.267512	11.914759	12.141913	11.413079	11.954526	13.626349	11.475276	11.302046	11.815094	13.421049	11.226941	12.760155	12.865330	11.323649	12.594269

AC076968.2	10.567405	13.454152	12.581318	13.759150	12.508187	14.140523	14.311014	15.663417	13.695624	14.896544	14.097636	14.533026	13.359184	13.427110	12.950759	13.886198	13.356786	13.099956	13.501109	14.618611	12.954050	12.611352	10.996252	12.904820	13.863343	14.027387	11.679201	12.809121	12.780366	13.039489	13.704371	12.214094	12.609346	11.272804	13.085544	14.480925	13.842755	13.561295	13.805428	13.638755	13.723956	13.254196	13.100501	13.304746	12.631485	12.931521	12.419759	13.459822	12.163756	13.763964	11.876001	12.858637	13.001582	13.477694	13.780832	14.953863	13.170952	12.219989	12.932249	12.470387	12.811614	12.658166	10.784221	12.787176	13.223925	13.304746	12.697859	13.376481	12.738314	13.332466	13.383479	13.289866	12.866728	13.894360	13.154208	12.702790	12.798847	12.736077	12.942843	14.318185	12.046176	12.928492	12.460607	13.221977	14.050939	13.675309	12.916577	12.092910	12.322814	13.459166	13.667210	12.853507	12.982400	14.381001	11.322294	11.580850	12.935833	12.284727	12.984090	13.795660	14.101336	14.109446	12.548577	13.963570	14.115260	12.855167	13.831246	13.320270	12.907332	13.894764	12.309198	13.937690	13.480172	13.347873	13.359928	14.074236	11.148560	14.448331	12.687779	10.579352	13.748586	10.870510	11.971773	12.671548	13.163576	13.535732	11.852632	12.169723	12.317939	13.457435	11.697417	11.676108	11.192513	10.995680	12.463072	11.033609	11.458769	11.528321	11.081007	11.580709	11.742152	10.913411	12.040293	11.876001	11.107772	12.760155	11.946674	12.059052	11.836176	11.452540	12.094136	12.213629	13.323791	11.221164	13.439251	14.286571	11.820064	12.118493	11.838717	11.864087	12.517447	10.593778	11.688994	12.607714	12.945176	12.232358	10.989737	11.068971	11.839322	11.393373	10.740345	11.496833	11.584062	11.231267	11.624141	10.383969	10.850468	11.321148	11.099157	12.071732	11.590387	11.160511	11.830189	11.236126	10.728828	10.889218	13.725134	12.424072	11.802387	13.069482	12.664618	13.131339	12.577866	11.082761	13.249512	12.354682	11.214098	13.313756	10.934280	11.126352	11.017912	13.601026	11.039023	10.656544	12.006089	12.371489	11.379010	10.801939	11.910424	10.674816	11.799846	12.736077	11.332473	13.722489	11.126499	11.232861	11.133393	12.074516	11.911322	11.411961	11.760698	11.693331	11.116659	11.267512	11.914759	12.141913	11.413079	11.954526	13.626349	11.475276	11.302046	11.815094	13.421049	11.226941	12.760155	12.865330	11.323649	12.594269

ZBTB20-AS3	8.313905	7.834327	7.484743	7.847036	7.955865	7.781356	8.045919	6.470882	7.781671	8.139115	8.161001	8.095669	8.592526	7.837050	8.076966	7.993011	7.044666	8.140905	7.619865	7.824001	6.535471	7.503220	8.594703	8.424530	8.180988	8.156599	8.156419	8.126601	8.507669	7.747952	7.765933	7.399457	7.768377	7.993011	7.921791	7.584575	7.687949	7.610841	7.832761	8.003407	8.076365	7.814030	8.489838	8.195255	8.882342	7.770961	8.243837	7.348334	7.561945	7.871229	7.706537	7.774143	8.237449	7.719642	7.838479	8.017102	8.298809	7.603766	7.602325	7.971157	7.154025	8.244922	7.270794	6.963224	7.607888	8.142539	6.926972	7.999946	7.009963	6.613022	7.314180	7.444912	8.342612	7.365458	7.493360	7.559819	7.408204	7.240875	7.352520	8.014777	8.083646	8.207388	7.857244	8.011566	7.936388	7.772925	7.721625	7.597303	6.584073	7.590884	7.887198	6.562847	7.518351	7.320886	7.782421	7.706992	7.585638	8.426298	7.543733	8.284855	7.802510	8.074715	8.367279	7.962833	6.110778	8.179776	8.314692	7.365307	7.619552	8.292913	6.602297	7.463738	7.642619	8.125103	7.928511	6.760447	8.727096	8.102201	8.554274	7.741340	7.024084	6.977391	7.658320	6.919542	8.075788	7.415219	7.506641	7.159168	7.973059	7.552537	7.988521	8.026381	7.097967	6.462508	8.038363	6.633215	8.116691	7.707918	7.245330	6.232252	7.599377	6.930178	8.092315	7.642002	7.520987	6.718962	7.161260	8.259547	7.651452	9.400733	7.598536	7.122273	7.270165	7.768842	7.702525	7.382853	6.841126	7.610220	6.982244	8.430887	6.700141	8.369704	7.388100	7.644624	6.612157	7.831872	6.573887	7.693362	7.036542	6.899330	7.771110	7.161260	6.326431	7.433286	8.343986	7.488204	6.515542	6.560227	7.620447	6.754571	7.834777	6.519531	6.047304	7.665739	7.221610	6.092872	7.209878	6.697442	6.704635	7.437104	7.396943	8.140171	7.124976	7.918723	7.499939	7.249782	6.687119	6.992106	8.257856	7.660314	7.302237	7.167473	7.481214	6.910328	6.586695	7.256013	7.729193	6.242941	7.344583	6.618875	5.503367	7.118530	6.718962	7.050231	7.183928	6.859944	8.307920	6.893978	8.956077	6.744976	7.838627	7.846866	7.450429	7.384691	7.667017	6.696046	7.822865	6.171845	8.102201	6.188082	6.582110	7.338770	8.366666	6.937142	6.655496	7.307573	7.811708	7.444912

AC015712.5	13.270630	13.355173	12.095201	12.721637	12.910356	13.216213	12.395385	13.028031	13.087036	13.081212	13.043339	13.361828	12.291860	11.749694	11.709829	13.588156	12.739710	11.292037	12.337925	13.126165	11.742598	11.784783	11.237147	12.588951	12.403076	13.387795	14.544276	13.618534	14.648021	13.428242	12.739710	11.676444	11.537179	12.324479	11.867133	12.181119	12.275923	12.266993	11.974649	12.194595	13.498914	12.548924	11.324685	12.105607	11.298381	11.215845	13.666938	12.360062	12.041947	11.525892	13.068904	11.070164	10.678967	11.378139	11.173730	13.490156	10.331914	10.753252	12.646567	10.696778	10.982204	13.039136	10.870667	12.007616	10.339746	11.524099	13.845633	11.847279	11.836322	12.203886	13.304746	12.707548	13.280648	12.897590	12.725309	12.321093	12.349434	12.384330	14.472293	10.817152	11.080729	10.990910	10.933681	11.442338	11.178222	12.348206	11.136391	10.954078	10.839735	11.783278	12.588785	10.787056	11.475989	11.783439	13.264773	11.664015	11.494786	11.319280	12.189499	12.867603	12.166484	12.888978	12.523811	12.257887	11.555799	13.167690	11.559918	11.382901	11.690176	13.672692	10.988091	12.053932	11.855631	13.654148	12.793389	13.604077	11.743921	12.826118	13.791466	12.083231	12.452958	11.556248	12.169111	13.204369	12.797845	12.546832	12.648312	12.399170	12.263096	12.573864	12.736612	12.277123	11.044022	11.603716	13.565725	12.190892	11.302476	11.744200	11.836026	11.234215	11.214237	10.883564	12.456916	11.950357	11.768702	13.074750	12.875210	13.583239	12.630012	12.849996	11.784334	11.925820	12.020782	11.751430	12.288317	11.839166	12.002196	11.673716	11.728472	13.305996	11.519246	10.786914	11.448683	11.036123	10.960769	13.081015	11.075744	12.112064	10.650301	11.741301	11.091172	10.450445	10.596191	10.555535	12.966563	11.161265	10.327943	10.864387	10.098273	10.268358	12.888793	10.981607	10.904376	10.267029	10.735347	11.678021	11.799525	11.459486	11.160805	12.453263	12.556362	13.395578	12.053618	12.012350	12.092137	11.881010	11.884206	12.457084	10.482463	10.546578	10.527453	10.286511	11.271009	10.602115	11.893812	10.854038	10.702585	10.570283	11.091462	11.859791	10.455244	10.946388	11.774016	11.966254	12.351495	11.424357	11.614495	12.127344	11.846574	11.631352	11.801933	12.365253	11.483837	11.023937	13.266805	11.572813	11.242685	11.200721	13.212044	10.787634	11.439106	11.664327	14.136689	11.810995	12.227351	10.855104	12.086801	12.722972

AC015712.6	13.270630	13.355173	12.095201	12.721637	12.910356	13.216213	12.395385	13.028031	13.087036	13.081212	13.043339	13.361828	12.291860	11.749694	11.709829	13.588156	12.739710	11.292037	12.337925	13.126165	11.742598	11.784783	11.237147	12.588951	12.403076	13.387795	14.544276	13.618534	14.648021	13.428242	12.739710	11.676444	11.537179	12.324479	11.867133	12.181119	12.275923	12.266993	11.974649	12.194595	13.498914	12.548924	11.324685	12.105607	11.298381	11.215845	13.666938	12.360062	12.041947	11.525892	13.068904	11.070164	10.678967	11.378139	11.173730	13.490156	10.331914	10.753252	12.646567	10.696778	10.982204	13.039136	10.870667	12.007616	10.339746	11.524099	13.845633	11.847279	11.836322	12.203886	13.304746	12.707548	13.280648	12.897590	12.725309	12.321093	12.349434	12.384330	14.472293	10.817152	11.080729	10.990910	10.933681	11.442338	11.178222	12.348206	11.136391	10.954078	10.839735	11.783278	12.588785	10.787056	11.475989	11.783439	13.264773	11.664015	11.494786	11.319280	12.189499	12.867603	12.166484	12.888978	12.523811	12.257887	11.555799	13.167690	11.559918	11.382901	11.690176	13.672692	10.988091	12.053932	11.855631	13.654148	12.793389	13.604077	11.743921	12.826118	13.791466	12.083231	12.452958	11.556248	12.169111	13.204369	12.797845	12.546832	12.648312	12.399170	12.263096	12.573864	12.736612	12.277123	11.044022	11.603716	13.565725	12.190892	11.302476	11.744200	11.836026	11.234215	11.214237	10.883564	12.456916	11.950357	11.768702	13.074750	12.875210	13.583239	12.630012	12.849996	11.784334	11.925820	12.020782	11.751430	12.288317	11.839166	12.002196	11.673716	11.728472	13.305996	11.519246	10.786914	11.448683	11.036123	10.960769	13.081015	11.075744	12.112064	10.650301	11.741301	11.091172	10.450445	10.596191	10.555535	12.966563	11.161265	10.327943	10.864387	10.098273	10.268358	12.888793	10.981607	10.904376	10.267029	10.735347	11.678021	11.799525	11.459486	11.160805	12.453263	12.556362	13.395578	12.053618	12.012350	12.092137	11.881010	11.884206	12.457084	10.482463	10.546578	10.527453	10.286511	11.271009	10.602115	11.893812	10.854038	10.702585	10.570283	11.091462	11.859791	10.455244	10.946388	11.774016	11.966254	12.351495	11.424357	11.614495	12.127344	11.846574	11.631352	11.801933	12.365253	11.483837	11.023937	13.266805	11.572813	11.242685	11.200721	13.212044	10.787634	11.439106	11.664327	14.136689	11.810995	12.227351	10.855104	12.086801	12.722972

AC015712.4	13.270630	13.355173	12.095201	12.721637	12.910356	13.216213	12.395385	13.028031	13.087036	13.081212	13.043339	13.361828	12.291860	11.749694	11.709829	13.588156	12.739710	11.292037	12.337925	13.126165	11.742598	11.784783	11.237147	12.588951	12.403076	13.387795	14.544276	13.618534	14.648021	13.428242	12.739710	11.676444	11.537179	12.324479	11.867133	12.181119	12.275923	12.266993	11.974649	12.194595	13.498914	12.548924	11.324685	12.105607	11.298381	11.215845	13.666938	12.360062	12.041947	11.525892	13.068904	11.070164	10.678967	11.378139	11.173730	13.490156	10.331914	10.753252	12.646567	10.696778	10.982204	13.039136	10.870667	12.007616	10.339746	11.524099	13.845633	11.847279	11.836322	12.203886	13.304746	12.707548	13.280648	12.897590	12.725309	12.321093	12.349434	12.384330	14.472293	10.817152	11.080729	10.990910	10.933681	11.442338	11.178222	12.348206	11.136391	10.954078	10.839735	11.783278	12.588785	10.787056	11.475989	11.783439	13.264773	11.664015	11.494786	11.319280	12.189499	12.867603	12.166484	12.888978	12.523811	12.257887	11.555799	13.167690	11.559918	11.382901	11.690176	13.672692	10.988091	12.053932	11.855631	13.654148	12.793389	13.604077	11.743921	12.826118	13.791466	12.083231	12.452958	11.556248	12.169111	13.204369	12.797845	12.546832	12.648312	12.399170	12.263096	12.573864	12.736612	12.277123	11.044022	11.603716	13.565725	12.190892	11.302476	11.744200	11.836026	11.234215	11.214237	10.883564	12.456916	11.950357	11.768702	13.074750	12.875210	13.583239	12.630012	12.849996	11.784334	11.925820	12.020782	11.751430	12.288317	11.839166	12.002196	11.673716	11.728472	13.305996	11.519246	10.786914	11.448683	11.036123	10.960769	13.081015	11.075744	12.112064	10.650301	11.741301	11.091172	10.450445	10.596191	10.555535	12.966563	11.161265	10.327943	10.864387	10.098273	10.268358	12.888793	10.981607	10.904376	10.267029	10.735347	11.678021	11.799525	11.459486	11.160805	12.453263	12.556362	13.395578	12.053618	12.012350	12.092137	11.881010	11.884206	12.457084	10.482463	10.546578	10.527453	10.286511	11.271009	10.602115	11.893812	10.854038	10.702585	10.570283	11.091462	11.859791	10.455244	10.946388	11.774016	11.966254	12.351495	11.424357	11.614495	12.127344	11.846574	11.631352	11.801933	12.365253	11.483837	11.023937	13.266805	11.572813	11.242685	11.200721	13.212044	10.787634	11.439106	11.664327	14.136689	11.810995	12.227351	10.855104	12.086801	12.722972

AC018616.1	9.943490	10.552353	9.560327	14.726520	5.643883	7.880784	9.851968	5.407524	5.788486	8.388820	7.480117	8.387729	11.098087	7.811708	9.354243	8.486435	7.610220	7.561801	9.882399	13.564364	6.304265	8.994735	7.587483	7.951667	7.658918	7.508474	9.192277	7.543116	9.981684	9.164862	7.774290	9.565885	9.366612	9.181558	12.747096	9.716088	10.109128	8.398835	6.662005	5.435125	8.562835	8.490431	10.188617	8.522763	10.602963	9.701427	9.021730	8.999804	8.985318	8.538625	10.253507	10.258842	10.939064	12.122694	9.965900	10.149488	10.924165	8.124646	11.926923	9.357110	7.380012	12.070827	10.214570	6.348452	8.132684	7.405064	7.233534	8.203318	8.570955	7.021281	10.924469	10.120339	8.601123	10.091114	10.385788	10.383504	10.741254	11.487129	11.100603	11.337754	10.309563	10.978923	10.214105	9.634319	10.087299	10.201710	11.518846	9.721686	8.352011	10.502569	7.749980	8.558709	9.001182	10.186206	8.902924	10.661313	9.721087	9.079184	8.060586	8.041321	8.682359	8.787017	9.685152	6.694243	7.132916	7.592559	6.341388	5.686968	6.891740	8.283450	7.128619	3.950770	5.273895	6.529995	6.808885	7.864173	6.067709	8.436316	9.636548	6.973815	10.205213	7.203950	9.240121	11.148990	4.875280	8.455510	4.817923	11.237555	6.293544	12.592493	5.792732	8.327120	3.412911	5.170899	10.988091	5.564109	4.009546	4.723874	9.452150	5.450480	6.398476	6.443210	6.767312	4.623340	6.725888	5.860123	4.101480	6.001621	7.501480	6.526356	8.698049	6.697122	9.463760	13.460482	7.976173	8.658361	6.273038	7.006419	5.562034	11.802547	6.493934	7.237038	7.042589	12.233426	8.207086	10.095931	6.645149	5.554537	10.650157	7.436945	9.274836	9.992352	8.522454	11.390861	15.705567	8.758681	10.041471	11.265170	11.142960	4.805358	9.223262	6.676570	3.776362	9.114516	7.041120	10.422096	7.609592	6.438240	3.744640	9.717806	7.725913	14.911437	8.098858	14.124934	15.189377	9.659506	12.109513	8.955928	11.038735	14.399253	7.688567	9.312325	15.278078	7.454147	6.677352	14.370460	14.408016	5.894592	12.625864	4.375223	10.173663	9.546241	14.286202	9.436929	8.258791	13.781822	5.164484	6.528481	7.781951	10.374263	10.726118	6.928586	6.099247	12.248657	5.323444	11.554781	6.734817	10.621011	7.809422	3.846089	9.435081	3.280580	6.855573	5.064275	14.009307	4.871491	12.299016	13.186438

AC068389.1	4.560331	6.051477	5.438936	5.547320	5.149598	5.435917	5.706129	5.233633	4.648966	5.415123	5.575751	4.747673	4.944783	5.338325	4.188883	6.491065	5.047267	5.767383	4.778450	5.397646	5.661798	5.518786	8.640041	5.307327	5.430096	5.691927	6.409505	6.163738	5.742070	6.292024	4.833748	5.772555	5.379059	4.907752	5.624864	5.740241	5.433353	5.237472	5.377508	4.937295	6.016723	4.389751	5.614049	5.331849	5.044934	4.353548	6.017427	4.807206	6.246099	5.321907	5.038528	5.667791	4.946094	5.084680	4.037774	6.191492	4.462440	3.102750	3.327915	3.523830	5.016475	6.124959	6.588047	4.773688	4.873648	4.554340	5.950379	5.826936	6.076077	5.257917	5.808857	5.582942	5.845087	5.590622	5.370703	5.710664	5.531616	5.674211	5.238653	4.751699	4.753636	4.810381	4.848293	3.912072	4.778032	4.856270	4.951888	4.181013	3.457527	4.777623	4.279490	5.374347	6.564660	5.554888	6.111319	5.458449	4.812278	4.644274	5.616676	5.102975	5.213361	5.392589	5.032759	5.342989	4.564070	5.772193	5.447613	5.881571	5.906838	6.118370	6.120803	5.471801	5.072791	5.470625	5.273109	5.746099	4.346762	4.874251	5.299668	5.192874	6.564009	6.476742	5.647068	6.397635	5.561665	5.666470	5.332255	5.283757	6.145501	5.955069	5.723267	6.189761	5.824779	5.351486	6.245437	5.538246	5.699307	5.806483	6.170824	6.260479	6.832128	6.919359	6.203297	6.859263	6.698868	6.722079	6.337381	5.803485	6.826397	4.481977	5.536264	5.355114	5.846583	5.794218	5.750467	6.034026	6.284798	5.694530	6.220262	6.320848	5.631552	5.122714	5.491509	5.135818	4.056076	6.430386	5.755531	5.773278	6.278983	4.837655	5.307133	5.138251	5.409120	5.230559	6.721446	4.304851	4.354817	4.746622	3.424029	5.596256	6.017781	5.289796	5.108098	5.071240	5.180737	6.031469	6.336858	6.358161	5.298266	5.987193	6.500369	6.351789	6.220262	5.893884	5.655195	6.457389	5.618176	5.831751	5.448989	5.111332	4.461136	5.167290	4.290484	5.347361	5.765200	4.509201	4.816027	4.847873	5.676369	6.056548	6.169451	6.428172	6.133795	5.809630	5.317463	5.535685	5.586055	5.057174	5.381923	5.927937	5.714931	5.509568	5.825842	6.175587	6.250151	5.337511	6.000418	5.797459	6.119757	6.511783	6.231257	5.690981	6.286581	5.690239	5.762139	5.203864	5.891163	5.927937

MUC20-OT1	7.415512	7.457620	8.015866	7.511240	8.858496	8.088027	7.670691	7.372371	7.603908	7.797998	7.728413	8.705827	8.543529	7.848701	8.423166	8.097528	7.788507	8.532566	6.511268	7.898519	6.375503	7.804315	6.635160	8.580581	8.422262	7.924758	7.769286	7.962989	8.038522	7.596093	8.388982	7.817308	7.414160	7.735340	8.287505	7.906019	7.559510	8.030083	7.000974	7.760531	7.859735	7.278463	7.841406	7.825526	8.709005	7.200082	7.408829	7.556289	8.017844	6.681267	8.159168	7.516167	7.331331	8.019230	7.899568	7.647559	7.811267	6.849180	8.414001	8.190857	7.557083	8.834648	7.055577	6.890454	7.115180	7.679669	7.687949	7.768086	7.828982	7.265109	7.558623	7.641842	7.393824	7.820127	7.217647	7.409893	7.708979	7.395991	7.640633	7.911114	7.914461	7.979109	7.992551	8.391312	7.617231	8.144078	8.252821	7.924267	6.625166	7.224338	8.330440	6.655338	7.894029	7.541435	7.923024	8.853185	7.472494	8.310361	7.915196	9.140691	8.084278	8.472487	7.463738	7.997958	7.077190	7.593028	7.994730	7.881547	6.828154	8.532874	7.172144	8.491790	8.519459	7.365938	7.974469	9.396827	8.890098	7.824155	7.502904	6.791693	7.006116	7.333066	7.466504	9.242420	7.081923	8.555423	7.479343	7.670222	9.058476	7.729193	8.494150	10.355676	8.391462	7.296877	7.700373	8.835874	10.458502	5.511256	7.297331	7.891377	9.154005	6.510260	10.518778	7.942176	7.870902	7.098929	8.248407	8.436002	8.011098	7.504612	7.877178	7.920144	7.965553	9.091908	8.874499	6.929852	6.849029	6.369034	7.749198	6.395964	7.100790	7.085071	6.606208	7.609761	7.018436	6.459852	6.895266	9.963969	6.758456	6.744337	7.045129	9.466126	6.838834	6.730314	8.013862	6.447691	7.310410	8.082112	7.500244	6.598774	7.790991	7.244060	7.040326	7.437104	6.636437	6.571931	6.942144	7.076686	6.372278	8.268392	8.458622	8.015092	6.950916	6.841609	7.520175	7.631767	6.615279	7.321996	7.420385	8.114390	7.097666	7.281951	7.840310	6.465048	7.927895	7.433914	7.425397	6.305762	6.746880	9.191505	6.226055	7.377364	7.044019	7.921791	7.352977	7.626091	8.747882	7.909425	9.675373	7.172461	8.097219	6.969180	8.027318	6.469350	8.423166	7.352662	8.473263	6.916498	7.417421	7.127214	7.716894	8.310071	7.576031	9.609476	8.581825	8.460023	7.217647	7.445391

AC124852.1	3.254505	3.551628	4.320571	4.642787	5.707221	4.847214	4.643812	4.979752	4.735884	4.745760	4.206614	4.792890	5.475801	4.299617	4.891666	4.520611	3.644045	5.643485	5.068188	4.369566	5.214034	4.774294	3.101167	4.948949	5.460233	5.351297	4.391274	4.802623	5.363098	5.439680	4.887089	4.432687	4.769494	4.732732	5.488756	4.136714	4.896431	4.168179	4.643391	5.528973	3.983281	4.788270	4.536749	4.279704	5.136622	5.543910	4.530956	5.230355	4.756537	4.289822	4.024321	5.356463	4.370011	4.354587	5.096968	4.276377	5.095208	5.415640	5.951984	4.947749	4.793710	5.239264	3.690345	4.694525	4.377152	4.438927	2.832589	4.494208	3.948370	4.751098	4.181446	4.069020	4.977646	4.737795	3.625598	4.761630	4.599394	4.074067	3.812920	5.413965	4.535021	5.796753	3.974198	4.375003	5.614981	5.032354	4.209072	4.590783	3.126980	4.141298	5.138648	3.939217	4.356986	4.265308	3.992227	3.532925	4.641771	5.223134	5.646696	5.473107	4.747061	4.942813	5.511823	5.195916	4.429095	5.058787	4.211644	4.522344	4.367199	5.216797	5.140454	5.677521	4.118816	4.148555	3.806591	6.618875	4.739080	4.646404	4.706397	3.566681	4.097759	4.741289	3.693828	6.042288	4.211644	5.416806	8.251460	4.493308	4.412414	4.537365	5.346337	6.408537	4.481255	4.394772	5.882107	3.758791	7.389033	4.806984	6.053040	4.502413	5.569226	3.019119	4.742152	5.644055	4.621044	4.162932	5.777263	5.949351	4.725569	4.365248	4.471226	5.383921	4.764426	5.541842	3.828431	4.438286	4.338757	4.627811	4.669702	4.518262	2.944727	3.665470	4.648966	4.322275	5.129796	5.070584	3.230761	4.439375	4.186897	4.296754	4.512247	4.771198	4.556647	4.177463	4.331849	6.888719	4.096230	3.811858	4.822317	2.911723	4.432013	3.690742	4.273784	5.135234	4.166469	2.819158	3.725976	3.527379	2.903558	4.614203	4.509641	4.352665	4.345246	4.026422	4.954568	4.912395	4.499005	4.500267	5.178778	3.826358	4.842115	3.864949	4.220612	4.219929	6.006620	4.282121	5.413577	2.984959	3.848412	5.084247	3.641701	4.128728	4.272457	3.899349	3.916184	5.179379	5.429745	4.024123	5.363470	4.974640	7.389191	4.911356	3.994432	4.850577	4.192784	4.631548	4.177040	3.553350	4.477618	5.363851	5.600779	5.972718	4.911789	5.653946	6.465215	5.526489	3.941069	4.042742

LINC00454	10.689778	9.487347	9.667624	10.747561	10.871895	10.855861	9.442516	10.732241	11.062597	10.241662	10.834204	10.365458	10.080121	9.570876	9.711552	10.672850	10.476151	9.722173	9.616048	10.004426	9.974195	9.137166	9.473227	10.053753	9.849770	10.556927	11.187721	10.507361	10.513902	10.519634	11.035057	9.490785	9.206427	10.418587	9.524595	9.630159	9.652120	9.330903	9.613640	9.453875	10.514677	9.062914	9.365664	9.407255	9.162133	9.747720	11.277024	9.239678	9.960286	9.326293	9.338465	9.030128	8.724803	8.704737	8.718581	10.990476	8.493691	8.762269	8.636459	8.536970	8.768535	10.544926	9.170296	9.051877	9.168609	8.653117	9.248124	9.643333	9.432358	9.204887	9.736856	9.911740	10.821608	9.671337	9.587971	9.535658	9.673010	9.488884	9.560811	8.385870	8.903553	8.449384	8.984120	8.588024	8.519918	9.101487	9.127646	8.876783	8.642371	8.456897	8.475262	8.475733	9.450937	9.727021	10.117613	9.564507	9.261051	10.043161	10.898488	10.561773	10.671515	9.524425	9.998272	9.968820	8.802155	10.781289	9.676141	10.085861	9.926337	10.889218	9.319212	9.784596	9.890283	11.824133	10.351285	12.772441	10.186206	9.854790	11.030014	10.697641	10.300662	10.640618	11.146494	12.674301	11.484876	11.323915	11.139782	11.299944	11.361662	11.581124	11.219673	11.476559	10.546889	10.863064	11.225630	11.018211	10.632606	11.016629	10.241972	10.712259	10.201859	9.567165	11.358396	10.750836	10.564137	11.267954	11.566391	12.296332	11.061419	10.879160	10.488263	10.623981	10.424856	10.100136	10.761158	10.566051	10.085100	10.688872	10.530093	12.256329	10.670884	10.471176	10.630112	10.282310	11.494936	12.103454	9.628464	10.128694	10.786013	10.547330	9.925456	9.848797	9.740417	9.667979	11.137872	9.365510	9.282612	9.562068	8.884660	9.874810	11.579070	9.599865	9.873486	9.620259	9.248587	10.579044	10.270147	10.538517	9.722314	11.045362	10.744560	12.365108	11.340846	10.689640	10.598011	10.322668	10.491566	10.358719	8.868843	9.408176	8.794756	9.745076	9.123078	9.086870	10.607990	10.004584	9.225119	9.087772	9.232220	9.644234	9.042970	9.736555	10.326880	10.162442	10.750389	10.817752	10.907795	10.717904	11.042557	10.862762	10.787634	11.140512	11.078117	9.212818	11.722608	10.527040	10.370938	9.315049	12.117551	9.769830	10.362545	11.250156	12.432907	11.436109	11.449438	9.959089	11.301442	11.747030

AC092669.1	3.521682	4.575394	5.292378	4.925747	5.003583	5.536264	4.700068	4.207761	5.562964	5.248606	5.677521	5.363098	6.066609	5.675664	4.628037	5.741001	4.802838	4.651943	5.390063	6.582110	4.903206	5.240060	6.781814	5.465020	4.554340	5.909011	5.492597	4.431806	4.732932	4.391908	5.724728	5.008684	5.344783	6.397635	5.822053	5.016475	5.715491	5.480462	5.013417	5.055139	5.402259	4.399610	5.799467	5.455127	5.612706	4.269404	6.368863	5.970151	4.672505	6.355059	5.022964	4.789720	4.944987	5.626357	4.312513	5.025654	4.595957	3.784371	5.343606	5.086146	5.300632	4.745112	5.237665	5.576123	4.573962	4.745539	4.908396	5.123104	5.019510	4.262444	4.933036	5.055546	5.533140	5.273109	5.136622	4.577328	4.584561	5.313012	4.192585	4.736955	4.636303	4.245365	4.862073	4.018026	4.993938	5.972524	5.077136	4.595108	4.076394	3.412911	4.240767	4.529657	5.591378	5.074842	5.266656	5.592538	5.707018	6.566720	5.837488	6.327073	4.577328	5.606848	5.165881	6.156847	4.957084	5.915847	5.189553	4.238752	5.135234	5.695458	4.051084	4.617724	3.778955	4.924665	3.953545	6.620034	2.829545	5.469895	6.293873	6.201713	4.811450	4.644693	6.318570	5.489978	4.992229	5.194263	7.765385	5.727722	6.865185	4.857743	4.324006	5.157902	5.890639	4.927846	8.383906	4.624831	6.927797	6.130691	7.977373	4.599773	5.748864	6.023946	6.077122	4.514214	5.290178	5.996543	5.033372	7.578395	3.931529	4.341361	4.896846	6.332486	5.373801	6.010539	5.247625	6.213909	6.115959	6.639342	5.339915	4.932634	5.416410	6.714928	4.735021	6.501553	3.858662	5.248221	8.313277	6.077446	6.084753	5.737157	7.013469	5.601705	5.591982	5.033991	4.776361	6.308579	5.733207	5.479850	5.403504	6.168600	5.686221	5.629161	6.128005	5.136622	5.389322	5.361935	4.958737	5.194263	5.390655	5.385758	5.699868	4.805960	5.251581	5.779803	4.951888	4.738203	4.619283	5.055751	4.755912	5.348890	5.263283	4.787036	5.446207	5.326937	5.134262	5.054312	4.512448	3.833571	4.975658	5.179925	4.703230	6.840955	5.603614	5.551813	5.054726	7.363410	7.542633	5.690981	7.655132	4.275288	8.346473	7.266636	6.316062	6.820707	4.517429	7.152007	5.969312	6.055659	4.270510	4.960598	4.805579	4.997755	5.127943	4.347207	4.745539	5.130200	5.471992	4.065006

LINC02058	5.932102	5.801873	5.879616	6.048901	6.073647	7.017952	5.304215	7.166260	7.248376	5.761966	6.122355	5.793485	5.592741	5.180956	5.122893	5.815094	6.958150	5.752290	6.658225	5.587157	5.681029	6.498235	8.783374	6.092366	6.521055	6.311781	5.629161	6.950916	5.565981	4.962733	6.775437	3.931917	4.995664	5.066111	5.994659	5.553182	5.693030	6.055134	6.410196	6.306926	6.167552	6.404233	5.763035	5.750467	5.503158	4.198200	5.680644	5.953430	5.197929	5.747936	5.679760	6.164593	4.911553	5.558298	4.754468	5.794931	5.862898	6.287915	5.755895	6.052855	6.564337	5.334014	6.710559	6.138578	5.716929	5.969815	6.382653	6.997719	6.037029	6.669632	5.793655	5.914942	6.059900	5.795803	6.052192	6.311124	6.518205	6.468844	6.409505	5.374742	6.087995	6.508146	5.850428	6.014345	6.342020	6.644989	6.900625	7.051488	6.148121	6.723049	6.481749	7.286929	5.852724	6.002904	5.627625	5.717116	5.823129	5.568836	4.767465	5.688249	5.664421	5.441793	5.347644	5.649908	6.677201	6.236650	7.291287	6.387497	6.951703	6.148479	7.089306	6.847240	6.847871	6.637692	6.340567	7.304277	6.192527	5.828600	5.893709	5.655947	6.180816	5.785945	5.928833	6.252502	10.151269	7.022655	6.661190	6.854940	5.732857	5.835368	8.771152	5.887073	5.497735	4.976700	5.779803	6.691609	8.095973	6.853335	5.764109	6.721767	6.297720	7.939788	6.488381	6.173182	6.424645	6.191829	8.281448	6.831026	5.450296	6.437425	6.053569	6.028563	7.433753	5.590809	5.679760	5.818327	6.240584	6.810976	6.657265	6.177991	6.934087	6.301513	5.536065	5.948272	3.940235	6.206757	6.070817	5.090658	6.512753	6.090782	5.886377	5.109773	6.113851	5.888143	5.611985	5.877609	7.253406	5.825674	8.398520	6.692425	5.612522	6.268440	6.577680	6.196190	6.197419	6.800425	7.069552	6.286218	6.843510	6.059390	6.917569	9.075596	6.254711	6.435793	6.141889	6.442889	6.477985	6.503174	5.800619	6.113504	6.183233	6.612494	6.309927	6.388174	6.909692	6.776202	6.665888	6.940887	6.721286	6.266890	7.318844	6.482234	6.253184	6.447523	6.160498	6.412181	5.910266	5.885495	5.346739	6.143417	6.107820	6.171172	5.813341	6.521703	6.059390	7.643053	6.370064	6.941664	6.503812	9.604857	6.848381	7.288783	6.280339	5.733728	8.011709	6.714928	6.016557	6.164413

AC132219.1	10.750389	10.320604	10.990910	10.096550	11.033295	10.531134	10.835583	10.986127	11.031483	10.693316	10.853314	10.139853	10.852098	10.805632	10.199467	10.759701	10.332815	10.871895	9.233328	11.024804	10.036868	10.082245	8.257368	10.289278	9.796404	10.058151	9.220198	10.380607	9.431088	9.939645	11.055538	10.277741	10.741714	9.690384	10.619838	10.613473	10.951404	10.667694	10.125091	10.103717	10.538968	9.439711	10.352666	10.932636	10.221670	10.701355	10.091421	9.698632	10.688872	10.795416	10.570769	10.946980	10.878560	11.074564	10.404707	10.582604	11.080729	10.087619	10.766127	10.104938	10.174250	9.866830	8.812340	9.376209	9.975574	10.187863	9.455713	10.449467	10.522363	8.844016	10.380008	10.497091	10.374979	10.522062	10.526564	10.152036	10.882791	11.160953	10.409534	10.829745	11.007562	10.973802	10.978333	11.184811	11.119269	10.724616	10.939911	11.222510	9.323513	10.456314	9.991246	9.709371	10.154668	10.819243	10.408443	10.882649	10.954993	10.900269	10.778275	10.704472	10.421018	10.745574	10.800888	11.164944	9.046377	10.880771	9.855121	10.366528	8.778417	10.497826	9.238262	9.932019	10.231920	10.714458	10.816269	9.531982	9.657788	10.432156	9.713877	10.335381	10.470433	10.029952	9.521307	8.806427	10.197218	9.391424	10.159669	10.070853	8.408361	9.512302	9.362773	8.137408	8.484300	9.746158	10.262095	8.910089	9.053617	9.103656	10.948753	10.005677	9.565725	8.047176	9.866387	10.711554	9.428985	10.528059	9.264430	8.979236	9.582846	8.932263	9.730434	9.032204	10.579502	11.424787	10.556615	10.527605	10.123658	9.370896	10.310359	8.907342	9.148150	11.261832	11.800745	10.096699	9.820421	9.990270	10.631433	10.432771	10.842557	10.684205	10.365940	11.183899	10.550253	10.892228	10.393410	11.350100	9.814640	10.389435	10.568013	9.528476	8.602666	9.218559	9.349094	9.627387	9.871071	8.868700	9.413487	9.636846	9.061877	9.757480	10.265579	9.057198	9.969878	10.289433	9.824794	9.950580	9.406477	10.481855	10.157125	9.925756	10.783006	10.705507	10.470736	12.035025	9.356076	10.416445	10.929328	9.374309	9.980249	10.529082	9.423469	9.257879	9.622602	10.734915	9.690691	9.675698	9.683246	9.219588	9.463454	8.831783	10.154373	9.999670	9.783615	8.599414	8.642817	8.374492	10.482608	9.386045	9.642543	8.474935	8.196996	10.241203	10.032116	10.422096	10.788244	9.345116	10.905252	8.933148

STX17-AS1	12.244222	12.179107	12.114778	6.882311	6.508471	5.342989	8.720086	5.967524	5.205618	7.935444	6.798331	8.131490	6.995380	6.504316	9.516518	6.672861	8.678828	8.789635	7.562125	5.501007	7.271866	7.697463	11.751278	6.164933	7.221939	5.190727	9.783445	6.422080	9.778612	11.469730	6.129010	9.462413	9.152937	9.290649	8.995337	9.100841	9.248587	9.211605	7.605306	6.905576	8.336979	9.709674	8.944285	7.505885	11.247077	8.904760	6.035624	11.089443	10.083729	10.231761	10.093506	9.694983	10.531004	8.532874	10.864681	8.633369	9.701888	9.969266	9.040540	9.780259	7.389802	10.851782	13.958770	8.571728	10.577397	10.944454	8.199678	8.861063	7.751051	8.173625	9.706262	7.792954	8.442390	9.521779	6.683902	9.849917	7.580698	11.178065	7.491173	11.057630	8.314539	10.970303	9.046541	9.834126	8.503715	8.953955	5.648403	8.982606	10.113319	8.971304	8.902324	9.107492	9.464238	7.821963	9.238600	8.542119	11.150678	8.716277	8.189423	8.801264	8.579823	8.968822	9.519473	9.105354	9.089471	8.227056	7.644929	7.991746	9.896747	7.270643	9.912507	7.087505	6.364856	5.572941	8.890098	5.416211	10.424393	9.796567	7.516020	9.160301	7.802216	8.696520	6.132918	4.368922	7.375490	5.393349	6.498576	3.197164	5.742641	5.999712	4.747061	8.786864	6.981009	6.640971	4.632624	6.815085	5.626914	6.872271	7.438669	7.389954	6.029573	9.863778	5.359285	5.632831	9.499648	7.673355	4.709048	4.154543	8.055259	6.683070	10.313989	7.514207	9.064588	7.734581	8.410456	9.634626	8.519918	6.241098	9.394200	4.363732	11.544514	7.830635	8.537994	9.738831	8.870231	4.691774	10.569842	7.127053	9.162292	8.567166	8.082867	9.154648	8.882036	10.090376	7.618175	7.675736	10.598160	8.661319	8.113311	9.961955	9.525550	9.251188	8.587856	8.794316	9.324307	7.830215	8.907026	8.410456	10.076034	8.099439	9.794237	4.102611	8.079617	8.679746	10.012145	7.112374	9.233800	7.729193	7.644012	7.858931	8.737797	8.615714	9.429446	8.532273	3.900869	8.454138	9.129389	8.725272	9.933222	7.695045	9.017297	5.987923	9.465354	9.260302	8.433816	8.179318	7.859428	7.276724	8.135264	7.138446	7.568409	8.708137	11.007280	7.703743	7.011554	5.278530	8.342466	8.366232	5.483876	8.493080	7.600485	2.844634	3.504352	6.985072	6.511109	9.146408	6.034881	7.217482

AC116351.1	7.284738	7.174480	7.426476	7.223425	7.230565	6.989994	7.526374	8.370503	7.770961	7.202252	6.793634	6.471755	7.498039	6.661502	6.990944	7.310106	7.992858	6.833773	7.705159	7.624339	7.905401	8.179637	11.097635	7.433592	7.764336	7.083159	8.197780	7.018100	7.421202	7.470321	7.084102	7.590442	8.187444	7.023584	7.754559	7.498818	7.741340	7.738827	7.931418	7.835387	7.127214	7.791899	8.251773	7.656558	8.226013	7.365622	7.489279	7.923339	7.802510	7.107418	7.922093	7.880362	8.192439	7.482584	7.434079	7.561200	7.664047	8.201668	7.873205	7.734581	8.226324	7.529936	10.322295	7.821807	7.868615	7.680701	8.309176	7.932955	8.031790	8.122441	7.549145	7.385610	6.918673	7.827120	8.027463	7.739616	7.794157	7.517578	7.886579	8.067722	7.631000	7.826786	7.525428	7.592412	7.865390	7.480444	8.653894	7.062528	8.864761	7.995502	9.628948	8.987164	8.160532	7.793394	7.289104	7.012518	7.409893	6.996153	6.779754	7.331485	7.623778	7.500092	7.184970	7.000133	7.813224	6.527992	7.728876	7.110328	8.335259	7.104714	8.395457	8.241734	8.423009	7.276110	7.162520	10.766421	8.558069	6.839827	7.035000	7.070710	7.532225	8.159780	7.413508	8.635686	7.002445	7.390553	8.390368	7.761745	7.967712	7.429167	7.334577	7.758694	7.072278	8.266186	7.885206	8.645888	7.510936	8.015395	8.062420	7.737739	9.298143	11.268237	8.357685	8.566024	7.182510	8.235738	7.648449	8.031790	7.518492	6.810338	8.089070	8.478664	7.490387	7.462448	7.617095	7.844523	8.151906	7.697916	8.684477	7.374236	8.403614	8.344769	8.090942	7.970525	7.378933	7.461032	8.086300	7.445236	7.874571	8.493527	8.171607	8.350523	7.759914	8.573236	7.701741	7.813075	8.095372	7.581658	7.712486	8.651781	7.826786	9.134778	7.893878	8.347801	8.643792	8.196392	8.343667	8.405306	8.226324	7.490073	7.887659	7.492285	8.342612	8.584972	7.852191	8.452630	7.855444	8.600192	8.372836	8.261839	7.583336	8.091424	8.316848	8.671970	8.304094	8.744205	7.087666	9.292688	8.562241	11.576476	8.668648	8.196235	7.765933	7.656886	7.297981	8.514597	8.564511	6.859119	6.905732	7.267588	7.792182	7.224648	7.469723	7.943715	6.696046	7.394907	6.970890	7.615409	7.721304	8.967268	8.878154	9.608075	7.751835	8.262136	9.221273	8.612161	6.974953	7.290501

SCAMP1-AS1	10.562074	10.052797	9.963969	9.941976	10.058310	9.418245	10.069025	9.202497	8.236065	9.162455	9.620873	9.838908	10.168590	10.077279	9.959832	9.558642	10.033478	10.013832	8.878485	10.011809	9.478457	9.616513	9.485482	9.805482	10.449011	9.552380	9.421602	9.609822	9.598172	10.306695	8.933305	10.803437	10.750692	10.497091	10.789285	10.375288	10.107468	10.013106	10.536920	10.644734	9.677983	10.858370	10.971305	10.402284	10.635345	10.853597	9.573371	10.647283	10.084367	10.097156	10.481855	10.525491	10.694662	10.167666	9.846139	9.363785	10.438091	11.166662	11.151852	11.343546	11.029412	9.539381	11.066743	10.948753	10.668429	10.412549	11.412637	11.308542	10.686620	10.468163	10.378185	10.399900	8.775280	10.508946	10.778275	11.082761	10.563986	10.445039	10.396441	10.585651	11.192657	10.535450	10.637424	10.725657	10.425753	10.607408	11.134182	10.743207	10.481102	11.099157	11.553592	10.041915	10.260726	10.327034	10.938471	10.550997	9.977259	9.064588	9.801200	9.556635	9.026744	9.953197	9.681866	10.208469	10.146257	9.325221	9.055814	9.269982	8.748190	9.613799	9.552380	10.199328	9.312170	9.050390	10.028112	9.345751	11.219066	9.841226	9.163229	9.345601	10.232963	9.832155	10.140329	10.098577	10.776975	10.537933	8.447805	8.670782	9.920116	9.376678	11.252362	10.979516	10.504080	10.338230	10.718371	11.261682	10.810677	9.186923	10.559792	10.315047	10.762044	10.422396	9.559692	11.347140	11.166662	9.551318	10.677354	9.109422	10.555843	10.549040	9.823607	9.833356	9.787132	11.704895	10.899253	9.959089	10.332961	11.214808	11.845084	10.660235	11.357963	10.519764	10.875904	11.365203	11.413703	8.982606	11.149750	11.584062	10.184971	10.381054	11.069731	11.601655	9.858664	10.692859	10.737617	10.509324	11.420870	11.209109	11.366119	11.390418	10.987934	11.185106	12.664433	10.291731	11.202548	11.217158	10.747238	10.945805	11.595768	10.849796	11.667440	8.336641	10.858655	11.209250	10.422850	11.668471	10.219691	10.086994	11.043290	10.950587	11.399407	10.711692	10.482307	11.934440	11.589394	10.643266	11.524887	10.177187	10.832721	10.701207	10.437199	10.906458	10.277608	10.186029	9.727298	10.335836	8.434134	10.636526	11.172234	9.864846	9.287393	10.561773	10.645484	9.335876	9.866241	10.101581	9.122924	10.776975	8.900076	10.424550	10.192349	8.908163	9.399396	12.657642	10.154986	11.811133	11.619649	11.215086

CRTC3-AS1	8.665791	7.788842	7.419464	8.476914	8.229507	9.489997	7.730863	10.001482	10.260726	9.228821	10.113060	9.374150	7.889831	9.487987	7.272982	9.832460	9.294119	8.658051	9.111441	9.354532	9.173899	9.304982	8.680959	8.645720	9.400900	9.114516	10.961939	10.472372	9.795507	7.799277	8.687758	6.570524	7.112679	8.093733	7.364986	6.825746	7.261987	7.383782	7.524985	7.533448	9.219434	7.738359	7.606691	7.463419	7.036155	6.956132	11.000620	6.800770	6.432761	6.740402	7.026498	6.319613	6.613481	6.772992	6.320331	10.569842	5.971232	6.535659	7.344870	6.976741	6.769290	9.072055	7.318527	8.431198	6.613022	6.864700	7.268030	7.622541	6.809699	7.499287	7.414619	7.825526	9.917640	7.375318	7.488051	7.895952	7.756185	7.355649	7.656711	6.823702	7.403178	7.157933	6.654673	6.937799	6.905093	7.313210	7.106453	6.361514	6.978170	7.028470	6.575845	6.808084	7.160265	7.188088	6.943280	7.611728	6.041748	7.905227	7.920886	8.820970	8.738410	7.268933	7.701453	7.869842	8.393633	8.676199	10.191897	8.655072	8.716906	9.446063	7.387618	8.635686	8.872639	11.161862	9.620873	8.614047	8.388820	9.069131	10.154986	8.631759	8.343986	7.078639	8.786239	8.911908	9.489187	8.755176	8.899769	8.990766	8.544468	8.186673	8.721185	9.380526	8.840733	8.261392	10.219095	7.992398	7.852796	8.409240	7.862157	8.431198	8.676360	8.532874	8.488914	8.364702	7.269844	9.597096	9.338318	10.127429	7.529471	9.853381	7.689797	7.538600	7.289562	7.355953	6.919186	6.845561	7.811267	8.462777	7.333524	9.751277	6.998180	7.054921	7.661085	6.437732	5.910976	10.532043	6.358513	7.239898	6.482902	6.986699	6.753389	6.161869	6.540849	6.131372	7.951832	5.434949	6.372612	7.236888	6.236971	6.648041	9.931101	6.269459	7.714780	6.992596	6.681577	6.853155	7.228566	6.906552	7.050534	7.319961	7.585490	10.635345	7.230114	7.323742	7.571715	7.699122	7.392419	7.422282	7.231664	6.448351	6.024627	6.066429	7.213260	6.915261	7.986058	6.976396	6.812716	7.408053	6.968033	6.829091	6.982716	7.531887	7.550974	7.499450	8.317157	7.569764	8.024983	8.565249	7.143793	7.735493	8.029503	8.578178	7.056646	7.274994	9.555370	8.987164	8.356138	7.323456	11.215086	7.404412	8.415166	8.677119	11.633139	8.381284	7.139538	8.272677	8.214857	8.654776

LINC01585	8.665791	7.788842	7.419464	8.476914	8.229507	9.489997	7.730863	10.001482	10.260726	9.228821	10.113060	9.374150	7.889831	9.487987	7.272982	9.832460	9.294119	8.658051	9.111441	9.354532	9.173899	9.304982	8.680959	8.645720	9.400900	9.114516	10.961939	10.472372	9.795507	7.799277	8.687758	6.570524	7.112679	8.093733	7.364986	6.825746	7.261987	7.383782	7.524985	7.533448	9.219434	7.738359	7.606691	7.463419	7.036155	6.956132	11.000620	6.800770	6.432761	6.740402	7.026498	6.319613	6.613481	6.772992	6.320331	10.569842	5.971232	6.535659	7.344870	6.976741	6.769290	9.072055	7.318527	8.431198	6.613022	6.864700	7.268030	7.622541	6.809699	7.499287	7.414619	7.825526	9.917640	7.375318	7.488051	7.895952	7.756185	7.355649	7.656711	6.823702	7.403178	7.157933	6.654673	6.937799	6.905093	7.313210	7.106453	6.361514	6.978170	7.028470	6.575845	6.808084	7.160265	7.188088	6.943280	7.611728	6.041748	7.905227	7.920886	8.820970	8.738410	7.268933	7.701453	7.869842	8.393633	8.676199	10.191897	8.655072	8.716906	9.446063	7.387618	8.635686	8.872639	11.161862	9.620873	8.614047	8.388820	9.069131	10.154986	8.631759	8.343986	7.078639	8.786239	8.911908	9.489187	8.755176	8.899769	8.990766	8.544468	8.186673	8.721185	9.380526	8.840733	8.261392	10.219095	7.992398	7.852796	8.409240	7.862157	8.431198	8.676360	8.532874	8.488914	8.364702	7.269844	9.597096	9.338318	10.127429	7.529471	9.853381	7.689797	7.538600	7.289562	7.355953	6.919186	6.845561	7.811267	8.462777	7.333524	9.751277	6.998180	7.054921	7.661085	6.437732	5.910976	10.532043	6.358513	7.239898	6.482902	6.986699	6.753389	6.161869	6.540849	6.131372	7.951832	5.434949	6.372612	7.236888	6.236971	6.648041	9.931101	6.269459	7.714780	6.992596	6.681577	6.853155	7.228566	6.906552	7.050534	7.319961	7.585490	10.635345	7.230114	7.323742	7.571715	7.699122	7.392419	7.422282	7.231664	6.448351	6.024627	6.066429	7.213260	6.915261	7.986058	6.976396	6.812716	7.408053	6.968033	6.829091	6.982716	7.531887	7.550974	7.499450	8.317157	7.569764	8.024983	8.565249	7.143793	7.735493	8.029503	8.578178	7.056646	7.274994	9.555370	8.987164	8.356138	7.323456	11.215086	7.404412	8.415166	8.677119	11.633139	8.381284	7.139538	8.272677	8.214857	8.654776

RB1-DT	6.122868	6.089390	6.033087	6.115959	6.245120	5.889206	6.200316	6.908123	6.041748	6.047635	6.219210	6.312121	5.907218	6.152405	4.971704	6.222924	7.170080	6.058707	6.499069	6.641604	5.912042	6.623080	6.567597	6.912288	6.374003	6.634026	5.516256	5.474270	6.117316	6.106391	7.042884	6.291862	6.128661	6.422858	6.940887	6.970286	6.874168	6.334855	6.155690	6.834688	6.233766	6.576510	7.408204	6.866291	7.237496	6.630632	5.991295	7.711867	6.564337	6.812716	6.985241	7.064609	6.705888	7.156379	6.259647	5.370132	7.148023	6.428351	6.381984	6.813340	7.028923	5.976903	6.356611	5.949856	6.741127	6.629515	6.699341	6.519531	6.087137	6.699791	6.526178	6.693929	5.727373	6.758456	6.702249	6.584231	6.529826	6.379471	6.381308	6.930818	5.994110	7.571434	6.771880	6.881346	7.059340	6.733827	7.424921	7.158083	6.764745	6.609006	6.828927	7.256173	6.820232	6.145501	6.739227	6.572438	6.819891	6.547740	6.025527	6.062774	5.529921	6.558456	6.006436	6.808558	6.290129	5.688775	7.136406	6.503324	6.692278	6.434967	6.807255	6.663632	6.872750	5.708658	5.674591	6.948257	5.838236	6.239917	5.592741	6.557965	5.662912	5.570508	5.715491	6.374509	5.289371	4.257422	6.894638	6.684412	5.702406	4.809510	6.655338	6.711499	5.295016	4.063721	6.250803	7.085989	6.314873	5.581341	6.501233	5.655559	5.391638	5.494505	5.920317	6.603086	6.054089	5.866135	5.342170	4.879622	4.857125	5.615740	4.927430	4.974640	6.129831	6.782927	5.621097	5.957686	5.184611	5.803302	5.514453	4.396577	5.933869	7.985161	6.422390	6.048741	6.183233	4.686755	7.141433	6.295600	6.870163	6.279486	6.385143	5.982024	6.348760	7.021281	5.788289	8.861351	6.145315	5.549584	6.535162	6.585732	6.005203	6.017427	5.667791	5.903276	6.735475	5.723098	6.102897	5.710312	6.442684	5.988481	5.815816	4.890032	6.350277	6.706701	6.001274	6.533240	6.872750	6.352264	6.878780	5.738626	8.389906	6.123031	5.944425	6.923052	6.068771	7.502758	7.931572	6.821977	6.098553	7.506496	6.282784	5.762867	5.594741	5.482767	5.725344	6.337715	6.663285	4.668831	6.178514	4.806984	7.945245	5.029742	6.266039	6.103756	4.469910	6.682588	6.313328	6.524506	4.242727	6.329228	5.783785	6.210188	5.246412	6.077284	5.615913	6.223644	6.372108	4.702139

DSG2-AS1	13.443082	11.959150	11.407356	12.658981	11.863499	11.940902	12.185267	12.351789	12.206044	12.405442	12.874685	11.970814	11.942440	11.875072	11.983502	12.547466	12.262785	11.856252	12.636992	11.952432	11.863352	12.500415	10.787337	12.121934	11.820354	12.935633	11.078407	12.532185	12.284103	11.906190	11.911179	12.986841	11.224748	12.489402	11.515630	11.653267	12.300173	12.372749	12.325086	11.848789	11.972983	12.564626	11.817176	12.466468	11.706874	11.900732	11.713495	13.002534	11.680518	12.250882	13.014108	12.245455	11.797058	11.978054	12.410313	11.720464	11.574486	11.754542	13.112985	11.881172	12.712283	11.437819	8.913424	11.973443	11.956209	12.132196	12.261028	11.540693	12.044549	12.323253	11.716739	11.317056	11.483412	11.381146	11.451301	11.151275	11.149750	11.426208	12.363568	11.418124	11.772119	11.545153	12.965441	11.893063	12.063188	12.446254	11.604734	11.981815	11.873849	12.381950	12.215055	11.694853	11.434198	11.757386	12.507056	11.502609	11.890938	11.526648	12.109069	11.252514	11.703267	12.052666	11.649323	12.504738	12.075117	11.890496	11.713495	12.074680	11.491172	11.880705	11.802693	12.599341	13.068499	11.560987	11.123670	10.793216	13.659596	12.249142	12.067983	13.683790	12.489492	13.469230	12.574922	12.277596	13.211293	12.688955	13.482826	12.964557	14.092524	13.528769	14.638867	14.115260	13.522760	15.196878	11.998395	13.241222	14.665858	13.793821	14.113158	12.198143	14.158986	12.734379	14.492765	14.422880	13.251043	12.809121	12.845274	11.257836	12.948751	13.340354	12.528560	13.829453	13.809161	13.636921	14.533864	12.924377	14.271565	14.181807	14.826972	13.057638	13.095783	15.160798	14.193699	13.115055	13.536412	13.407361	12.632959	14.100390	14.140212	13.147073	13.128244	13.041904	13.522292	12.952559	14.227945	12.924541	13.148076	13.106614	14.656836	13.687403	11.584856	13.237638	13.381550	12.722972	13.612309	13.103703	12.720078	13.735129	13.138696	13.000463	12.729813	13.251828	13.471189	13.171526	12.968209	13.991278	14.128591	13.400180	14.015842	13.652481	13.536847	13.303472	13.338109	13.948780	12.678360	13.130178	14.727457	12.953847	12.918970	13.733641	14.109446	12.964368	13.302266	12.034862	12.607232	13.798010	13.238041	12.555104	14.677419	14.331365	13.524143	13.927054	13.595594	14.081267	12.212235	13.132093	12.252315	12.824882	12.572773	13.379683	13.603336	13.859679	11.489500	14.232858	12.916404	13.024395	11.204945	13.500645

AC097625.1	3.272701	6.082328	6.217326	5.634913	4.956452	6.665251	8.602820	6.358855	4.907099	8.335713	7.605625	6.490462	6.899975	8.032389	3.658061	6.525818	6.355425	6.582607	6.687309	6.207080	8.312979	8.271201	6.552341	7.190930	6.407533	6.023065	3.460556	6.769936	5.089598	5.942491	6.841899	5.963776	8.016172	5.032949	7.447439	6.316905	5.828600	7.604831	7.311340	7.472799	5.392395	6.905252	6.179488	5.514823	4.089384	4.745112	8.708722	6.851706	7.284141	7.191688	6.532579	7.506792	6.552999	8.542271	7.833239	5.288139	6.482390	7.265109	6.432761	6.133974	8.278420	6.076260	3.701819	6.971518	5.618176	5.801186	7.213873	5.211443	6.799452	5.361147	7.382041	6.656465	7.457944	8.026527	6.593414	7.313538	5.797835	8.004991	6.273564	6.130163	5.543910	6.759442	5.899364	6.793483	8.474316	7.226699	7.366862	8.956668	6.464205	7.468145	6.528665	7.197133	6.451330	8.087867	5.725915	5.119275	6.076422	6.170659	5.712111	5.842965	5.079007	7.003520	5.525913	7.113000	7.022972	6.872117	6.896873	7.082529	3.895449	7.205967	5.155607	5.905080	5.957159	5.999377	8.879890	4.133608	3.994643	6.128661	6.526178	3.811402	3.607467	3.655820	5.987550	3.230494	5.819564	2.965658	3.894841	3.196648	5.402050	6.006620	4.011026	3.127307	5.843870	3.317445	4.944128	3.036164	2.881199	3.473243	3.505688	3.687483	3.629032	6.060983	4.088735	2.956356	3.328510	6.087137	5.067361	3.846089	4.498550	3.230901	3.057068	4.029752	6.161697	4.870222	3.915505	5.124300	3.394523	4.154082	3.466290	3.200504	4.918089	3.647766	3.461975	5.167486	4.506329	4.015184	2.879764	7.935298	4.379501	3.672321	3.026482	5.849536	4.642400	3.561360	5.333431	3.823109	4.340039	4.528611	3.991816	3.848646	3.447097	4.642591	6.702249	7.025384	6.015887	3.743889	4.576446	3.418265	8.351110	3.805962	4.573962	5.629161	4.347616	3.735615	4.570729	5.981021	3.314311	4.023461	3.191453	5.533876	6.081078	3.701159	3.379837	4.215554	3.121719	2.877511	4.700479	6.406241	3.430468	5.391260	3.533846	4.288556	4.031073	6.207946	4.956861	4.762051	3.684027	4.525222	3.174752	3.144562	3.333864	3.361975	4.263974	5.032949	4.139961	6.605446	7.801299	6.519531	3.592258	3.677597	4.123626	3.571157	5.617409	3.551628	6.756526	4.205490	3.629437	4.220124

AC005476.2	12.370837	11.302336	10.886269	11.640559	11.852935	12.275032	11.148846	12.153901	12.243919	11.304114	12.228964	11.866219	10.733854	10.905252	10.535767	11.755427	12.031827	10.792768	11.826393	11.109863	11.703716	11.075469	12.132196	11.214808	11.032397	12.040855	12.480800	12.262939	11.902027	11.729158	11.951841	10.123807	10.299864	11.209534	11.061419	11.165668	11.303833	10.900969	11.190467	11.455650	11.943486	11.277350	10.556299	11.360307	10.919103	11.107920	12.372913	11.061273	10.965940	10.831970	13.310550	10.603262	10.022905	10.330088	10.443573	12.056681	9.864242	10.079182	10.497236	10.266446	10.826511	11.682616	11.240198	11.360449	10.909986	11.202837	11.353017	11.021682	11.275334	11.934899	12.063494	11.970963	12.087860	12.011560	11.834673	11.755284	11.699008	11.792259	12.544041	9.937420	10.106287	10.049545	10.183168	10.058151	10.173198	10.759120	10.815810	10.322742	10.461731	10.621312	10.778689	11.125198	10.836162	11.022602	11.610492	11.049601	10.623981	11.001791	11.178683	11.355638	12.209422	11.390561	11.620096	11.522184	10.974940	11.857954	11.857830	10.958405	11.988788	11.979596	11.909265	11.675026	11.953330	12.307454	11.932456	14.873940	11.894274	11.512020	12.278819	11.096922	11.333902	10.985097	11.405580	12.992703	11.893211	11.980630	11.720318	11.500503	11.712047	12.210677	11.794980	12.061208	11.650051	12.916749	11.901010	11.495068	10.963108	11.519246	10.437507	11.527756	11.013967	11.856727	11.970814	11.350844	11.559784	12.442059	12.147509	12.463072	11.514222	12.045007	11.059685	10.693165	11.593285	10.906300	10.996548	11.280760	11.222825	11.352601	11.326759	12.208551	11.141929	10.090376	11.468687	10.771222	11.249397	12.483749	10.660235	10.769094	10.486270	11.807531	11.403818	9.932634	10.250874	10.550696	11.670899	10.216407	9.612400	10.187555	10.318748	10.776676	11.836629	11.020219	10.794399	10.879160	10.991933	10.987336	11.220409	11.176723	11.326901	11.581815	11.647091	12.467952	11.709110	11.557144	11.686489	11.577336	11.694853	12.089045	10.253361	9.739940	10.147476	10.440817	10.500010	10.196135	11.496207	10.765826	10.001946	10.518938	10.660096	10.508124	11.204945	11.013535	11.336873	11.701767	11.860081	11.506409	12.102820	11.460998	10.592897	11.451923	11.355638	11.814644	11.248076	10.749767	12.278662	11.639945	10.737488	11.424197	12.332394	11.526511	11.818688	13.476835	12.574210	12.074208	12.031682	10.598160	11.199093	11.914759

LINC01505	3.118879	4.058446	4.216443	3.665884	3.583778	4.334415	3.618011	3.593637	3.424029	3.872223	3.634474	3.737498	3.925461	4.128056	4.248186	3.120613	4.148655	3.567285	4.624205	3.656029	5.265445	2.909382	3.039031	3.967902	3.883170	4.012899	4.924478	3.346402	4.745760	3.259434	4.468199	4.554733	3.950770	5.525734	3.659820	4.113965	3.717910	3.349703	3.479469	3.343473	4.589267	3.435805	3.615523	3.360327	3.759199	5.615913	4.347616	3.027789	3.809313	3.880860	3.779768	3.646626	4.488254	4.338757	4.045188	3.836113	4.224260	3.507957	3.939217	3.960098	2.966934	4.490913	3.884029	3.322172	3.598390	3.534047	2.918405	3.828431	3.354861	2.976051	3.240744	3.553719	5.186019	3.556033	3.862994	3.539371	3.757509	3.636278	3.637261	3.866608	3.457163	4.113121	3.525510	4.393198	3.501629	3.751463	3.311947	3.667710	3.704471	3.583224	3.960518	3.023898	3.999159	3.230901	3.099280	3.320580	3.862377	4.040380	3.477470	3.165816	3.233635	3.686239	3.494265	3.622868	4.665614	3.483890	4.482201	3.618767	3.051738	4.166469	2.809993	3.203743	3.571157	5.473875	3.887853	3.187544	3.460369	4.128728	5.408923	3.275282	3.919131	3.953135	3.664050	3.364854	4.340039	3.166490	3.428989	3.435805	4.412646	3.445804	3.527928	3.565350	3.472563	3.639290	4.520611	4.588197	3.964222	3.371881	3.396948	4.525222	2.862605	2.936439	4.095392	3.718939	3.199768	3.365508	2.852987	4.154789	3.691158	4.312513	3.534047	3.893080	4.169878	3.544693	3.802663	3.760082	3.392489	3.587872	3.477470	4.403536	3.246230	3.663078	3.597725	4.258087	5.194696	4.517005	2.904037	4.110281	3.925693	3.758791	3.459491	4.318222	4.546670	3.595732	3.088427	3.850149	3.428828	3.687880	3.992473	2.864651	4.038230	3.938111	3.778157	3.896056	3.784156	2.891627	3.680636	3.441704	2.818522	3.206777	3.613999	4.326548	3.517979	2.986372	3.376527	3.644257	3.789513	3.638275	3.304280	3.861063	4.560966	3.610154	3.774917	3.592078	3.332891	3.472743	4.328317	3.315118	3.480211	3.747497	2.896185	4.158634	3.358117	3.146768	3.621281	3.059490	4.394513	4.155180	3.329990	3.015950	3.068050	3.097128	3.138701	5.046074	3.497287	4.766823	3.559932	3.356572	3.625812	3.272865	3.642062	3.207343	4.555796	3.279422	3.118161	4.036272	3.642272	4.059096

AL732323.1	3.118879	4.058446	4.216443	3.665884	3.583778	4.334415	3.618011	3.593637	3.424029	3.872223	3.634474	3.737498	3.925461	4.128056	4.248186	3.120613	4.148655	3.567285	4.624205	3.656029	5.265445	2.909382	3.039031	3.967902	3.883170	4.012899	4.924478	3.346402	4.745760	3.259434	4.468199	4.554733	3.950770	5.525734	3.659820	4.113965	3.717910	3.349703	3.479469	3.343473	4.589267	3.435805	3.615523	3.360327	3.759199	5.615913	4.347616	3.027789	3.809313	3.880860	3.779768	3.646626	4.488254	4.338757	4.045188	3.836113	4.224260	3.507957	3.939217	3.960098	2.966934	4.490913	3.884029	3.322172	3.598390	3.534047	2.918405	3.828431	3.354861	2.976051	3.240744	3.553719	5.186019	3.556033	3.862994	3.539371	3.757509	3.636278	3.637261	3.866608	3.457163	4.113121	3.525510	4.393198	3.501629	3.751463	3.311947	3.667710	3.704471	3.583224	3.960518	3.023898	3.999159	3.230901	3.099280	3.320580	3.862377	4.040380	3.477470	3.165816	3.233635	3.686239	3.494265	3.622868	4.665614	3.483890	4.482201	3.618767	3.051738	4.166469	2.809993	3.203743	3.571157	5.473875	3.887853	3.187544	3.460369	4.128728	5.408923	3.275282	3.919131	3.953135	3.664050	3.364854	4.340039	3.166490	3.428989	3.435805	4.412646	3.445804	3.527928	3.565350	3.472563	3.639290	4.520611	4.588197	3.964222	3.371881	3.396948	4.525222	2.862605	2.936439	4.095392	3.718939	3.199768	3.365508	2.852987	4.154789	3.691158	4.312513	3.534047	3.893080	4.169878	3.544693	3.802663	3.760082	3.392489	3.587872	3.477470	4.403536	3.246230	3.663078	3.597725	4.258087	5.194696	4.517005	2.904037	4.110281	3.925693	3.758791	3.459491	4.318222	4.546670	3.595732	3.088427	3.850149	3.428828	3.687880	3.992473	2.864651	4.038230	3.938111	3.778157	3.896056	3.784156	2.891627	3.680636	3.441704	2.818522	3.206777	3.613999	4.326548	3.517979	2.986372	3.376527	3.644257	3.789513	3.638275	3.304280	3.861063	4.560966	3.610154	3.774917	3.592078	3.332891	3.472743	4.328317	3.315118	3.480211	3.747497	2.896185	4.158634	3.358117	3.146768	3.621281	3.059490	4.394513	4.155180	3.329990	3.015950	3.068050	3.097128	3.138701	5.046074	3.497287	4.766823	3.559932	3.356572	3.625812	3.272865	3.642062	3.207343	4.555796	3.279422	3.118161	4.036272	3.642272	4.059096

AC077690.1	4.466739	5.962877	5.436108	5.672771	5.927937	5.662559	5.666871	6.423176	5.839699	5.816007	5.795108	5.054079	5.195106	5.404785	4.863721	6.115788	5.830666	5.899364	6.640971	5.255133	5.639932	5.263904	6.198084	6.386455	5.782546	6.338411	5.805626	6.349577	6.245620	5.508384	5.730301	4.645359	5.013631	5.801873	5.679203	4.761419	5.399987	5.270257	5.789012	6.254379	6.047108	4.981142	5.378859	5.683055	5.729728	5.182396	5.945313	5.647837	5.076726	5.715491	4.935777	5.334554	6.050117	5.703574	5.002315	6.137363	4.663894	3.519870	5.064681	4.443479	4.981328	5.954733	4.982359	5.219588	5.258891	4.994999	5.777437	6.361514	5.956634	5.719583	6.124641	5.660140	6.044821	5.636016	5.512606	5.418549	5.631732	5.466948	5.619454	5.080814	4.906706	4.792691	5.161670	3.344944	4.092275	5.286219	4.458852	4.688859	3.022567	5.217569	4.924879	4.605023	5.002927	5.274932	4.833111	5.515443	4.361307	5.872251	4.959576	5.876918	4.767870	5.834830	5.936504	5.982024	6.112317	6.241098	6.273370	5.809809	5.776362	6.269286	5.554716	5.910088	5.773628	6.189910	6.002723	4.733340	4.519978	4.955831	5.952712	5.361740	6.009651	6.219305	6.280339	6.294723	6.205388	5.702043	5.573704	5.084868	5.644266	5.274525	5.656461	5.186596	5.508567	5.419596	5.256728	5.607789	5.634528	6.933931	8.061523	5.255748	5.369930	6.052527	5.810937	5.820088	5.814413	5.607789	5.860123	5.477861	6.188243	4.676681	5.471489	4.802168	5.003956	5.690604	5.855085	5.270257	7.304606	5.920125	6.475405	5.843870	5.595889	6.296916	5.317249	5.088807	5.491876	6.133452	5.640502	5.075898	5.524779	5.149291	6.196023	6.637869	5.913302	4.353982	5.811871	5.404268	5.043266	5.068410	6.040904	5.163064	5.800993	4.353327	5.274315	5.491876	4.908200	5.732292	6.016224	6.018659	5.621291	5.918859	6.046736	6.057447	5.715491	6.770742	5.704106	5.476909	5.425023	5.765200	4.785559	4.791432	5.542439	4.911116	4.366344	4.418689	4.947941	4.690520	4.913908	5.437831	5.221285	5.448784	4.356325	5.291194	5.513884	5.469895	5.479634	4.563169	5.080404	5.685865	5.704106	5.465392	5.515655	5.747033	5.587720	6.079207	6.231589	5.849182	5.295428	5.524404	6.410196	5.504866	5.872251	5.304993	6.445076	5.472912	4.662255	5.641019	5.488546	5.357204

LINC01492	3.279590	3.415052	3.335311	3.433070	3.819912	4.137363	3.758336	3.698196	3.460556	3.454372	3.868380	3.742381	3.535381	4.034967	3.509805	3.255752	4.219685	3.735838	5.575751	3.600491	5.745747	3.940037	5.967890	3.658061	3.681816	3.789513	3.272404	3.430468	3.597156	3.394704	3.663842	4.667563	3.729236	3.125639	3.781857	4.246870	3.745473	3.698380	3.608791	3.621865	3.380664	4.227481	3.599539	3.316628	3.636766	5.398443	3.766295	4.767656	3.573734	4.211644	4.087037	3.822579	4.338539	4.758520	4.454188	3.420660	4.032121	5.078192	4.489766	4.093483	3.855740	3.481349	3.989667	3.669702	3.557169	4.169428	3.809313	3.363192	3.626812	3.069602	3.512749	3.821032	2.851120	3.614174	2.998429	3.322641	4.251497	3.803477	3.647986	3.812307	4.938279	3.837359	3.259434	4.269874	3.834808	3.945083	5.189361	4.437434	4.547719	3.823524	3.686042	4.306599	3.764711	3.110843	4.091630	4.038661	3.586706	4.152983	3.642453	4.026036	5.863088	3.499062	3.290950	3.807402	4.517644	3.912272	4.155180	4.110743	5.384096	3.235381	5.904897	5.262870	4.815819	3.185682	3.673900	7.616734	3.481721	3.237267	3.639699	3.559545	3.903786	3.979371	3.702258	4.446299	3.615054	4.928275	5.752847	3.735410	5.072402	5.097591	4.057800	5.630433	5.069990	5.088591	5.054726	3.877608	4.459665	5.375539	4.940144	4.907319	4.428875	6.203457	5.315660	4.746389	4.693307	5.467740	4.687813	4.998485	3.875266	4.395671	4.269156	3.554100	3.844161	3.814003	4.375003	4.072539	4.739775	4.447569	4.226166	5.262672	4.413763	4.231353	4.188438	4.034313	5.036945	5.221285	5.022964	3.833320	5.115570	4.614203	4.769299	5.005774	5.565981	4.704484	3.907267	5.203864	5.556382	5.255358	4.309458	4.875476	3.904662	4.502867	4.709048	4.384471	4.176606	4.923847	4.603341	4.067317	5.017104	4.517005	4.872994	4.860036	4.334644	4.807206	3.350047	5.007036	4.668615	3.999992	4.855423	3.803284	4.942999	4.847214	4.904018	5.546772	4.804325	4.322056	4.849690	5.290002	4.634571	3.906427	4.437434	4.896017	4.542656	4.553466	4.908396	4.849503	5.821680	4.867902	3.590513	4.862901	3.321552	6.005753	4.009758	4.882020	4.870615	4.287894	3.660415	3.876112	4.935151	4.617724	5.663106	5.198932	4.984779	4.163148	4.242727	3.560720	4.373474	3.921645

LINC00877	3.187544	5.764864	4.775112	3.790551	3.840068	4.011621	5.183216	4.124217	3.671942	3.416390	3.842951	3.885958	3.528663	4.189270	4.088092	3.638690	3.515884	4.094749	5.028491	3.138946	5.001521	5.030127	4.638845	3.805356	3.164876	3.630055	5.134458	3.496532	3.428299	5.455513	3.684609	5.047486	6.174889	5.977257	5.275128	5.873704	3.839470	4.500267	3.235659	3.854486	4.134951	4.500042	4.828672	4.105442	5.257917	4.578613	4.650918	3.324164	4.930190	5.139457	3.990057	3.760284	6.304937	3.952050	5.487095	4.313806	5.131397	4.671026	4.012899	6.674778	4.898566	3.038936	4.255890	3.061561	3.755267	4.457594	3.546357	4.403954	4.198394	4.485731	4.774499	4.462233	3.361794	4.661814	4.588197	5.725344	4.125253	5.241828	4.564465	5.164280	4.320370	4.859174	4.708061	4.318671	4.443479	4.704697	5.423616	3.734589	3.019585	3.249455	4.481255	3.599926	4.270510	4.428669	5.644055	5.936334	6.119404	4.716023	3.725775	3.789934	5.601135	4.229863	4.479749	3.242816	4.167786	3.089474	5.726105	3.602249	4.958542	3.525894	5.328489	5.126963	3.685044	3.760706	4.541317	6.793879	4.088500	4.379938	3.171791	3.356572	3.340297	3.803284	3.840908	4.919580	3.594204	3.525332	3.971363	3.411118	3.192046	3.445983	3.198895	3.776955	3.650619	3.673284	3.220421	3.483698	3.400057	6.137696	3.242391	4.300892	3.313017	5.920125	3.254505	2.986372	4.433701	3.738741	3.535725	3.166625	3.575527	3.430468	5.576839	3.901874	4.099296	3.542686	3.733181	3.775101	3.311611	3.171558	3.383146	3.184892	3.006528	3.956800	4.407357	4.140843	4.655105	3.638474	3.426736	3.780612	4.349824	4.053015	3.669264	4.423102	4.112439	4.064154	3.481721	5.478682	5.934947	4.478450	6.364380	4.527150	3.133914	3.362862	3.121240	3.223899	4.364843	2.850697	5.355591	3.460735	5.411498	4.259390	3.686432	3.074560	3.054044	4.642979	5.162860	3.198773	3.502367	3.065646	4.251298	3.603661	4.450279	3.556578	4.508331	3.514967	4.050878	3.417042	3.381371	2.945840	3.193392	3.312092	2.993540	3.163139	3.106541	4.430265	4.561427	3.576932	3.901680	3.329348	3.223091	4.532041	3.803896	3.335626	4.322686	4.532465	3.524981	5.753756	3.351071	3.272701	3.813152	4.453967	5.554340	5.033372	3.218550	4.062409	4.635852	3.798195	3.280580	3.294849

PRKG1-AS1	11.708338	11.191935	11.136541	10.431424	9.608994	9.545005	11.600742	7.651114	7.310728	10.067808	9.525379	9.974513	10.315777	10.356921	11.162013	10.262831	11.037133	10.632913	8.605139	9.571204	9.786258	9.381478	13.956584	9.904261	11.884935	10.788558	12.049360	10.680054	10.990030	11.152759	9.412599	11.790428	11.081888	11.319960	11.678914	11.854007	11.604734	11.600290	11.602116	11.475276	10.623514	12.307622	12.196619	11.691853	13.622440	11.822613	11.577922	11.636639	11.129584	11.424787	11.581405	12.268220	11.975594	11.730949	11.448235	10.562644	12.312233	12.075736	10.887492	12.557506	12.054398	11.663877	13.693813	11.094991	11.326303	11.576321	11.884048	11.442637	10.935332	11.201301	11.752750	11.278862	10.680668	11.811570	11.016496	11.460244	11.512020	11.729303	11.040159	11.613450	11.485624	11.888411	11.824292	11.595057	12.227205	11.392045	11.436255	11.486704	11.535996	10.288842	11.391895	11.411231	11.177479	10.962224	10.907071	11.027337	11.426361	11.244791	10.321065	11.946077	10.535450	11.772550	10.954367	11.089009	9.877686	10.312432	10.142602	10.028586	10.589937	10.295850	11.688705	9.486434	8.056362	9.827819	8.366526	8.508742	11.731240	11.216410	10.433508	10.074373	11.754542	11.626020	9.162455	10.041471	9.369059	10.373979	10.323671	11.120569	12.548577	10.757376	9.564348	11.668763	10.379400	10.333120	11.239056	11.720774	10.365612	9.841670	10.113060	9.444994	11.006254	11.661937	9.490951	11.825933	12.644060	9.940550	8.509059	9.478608	11.205541	10.781020	10.587164	11.683795	10.715970	10.694498	11.163491	12.189981	11.493170	9.887355	11.618460	8.833467	11.060858	11.339074	11.535996	11.491172	10.872641	8.087562	11.757533	10.972659	12.708953	12.900773	11.543325	11.934282	11.934117	12.641230	11.603290	11.973443	11.116825	10.774190	11.793767	11.878622	11.388656	11.157605	11.232005	11.176130	11.274607	10.998209	11.571999	11.767668	10.788244	11.203446	12.002347	8.232848	12.402762	11.877895	11.458309	12.418655	11.872205	11.909419	13.349953	12.131878	10.792186	11.718550	12.884205	12.583792	11.251436	12.142800	10.968006	11.990038	11.992764	10.888494	10.842267	12.664778	12.520217	12.041232	12.312544	12.208987	11.589394	10.190269	11.261074	11.387025	9.636701	11.767668	12.041676	11.309686	9.384500	11.535996	9.472171	10.182257	9.095706	12.323902	11.888248	9.835366	6.978944	10.139559	10.100782	11.780904	11.123080	11.993346

C9orf147	14.483652	14.130180	14.473933	14.147028	14.320207	14.160557	14.352362	14.516232	14.566322	14.293075	14.369760	14.477268	14.159585	14.764819	14.261678	14.080070	14.456521	14.555161	14.831507	14.543434	14.517028	14.451725	15.091311	14.308634	14.370844	14.466988	14.031176	14.057422	13.786622	13.984395	14.415465	14.467751	14.389681	13.522292	14.406566	14.642226	14.665858	14.386354	14.598641	14.604960	14.217619	14.511941	14.861942	14.753586	14.950618	14.818366	14.394046	14.890227	14.253427	14.775142	14.483264	14.813710	14.928143	14.751210	14.409528	14.377022	14.890760	14.384530	14.546394	14.771836	14.587687	14.028211	14.237167	14.444487	13.908273	14.634805	14.143957	14.618611	14.479847	14.468896	14.209486	14.327152	14.210459	14.144293	14.576819	14.539639	14.239134	14.255152	14.343514	14.443066	13.952936	14.299472	14.370460	14.644443	14.675960	14.314071	15.048970	14.671011	14.281068	14.498682	14.246286	14.411699	14.381702	14.043175	14.155124	14.504600	14.124934	14.347441	14.346007	14.711675	14.474751	14.365930	14.176803	14.587290	14.769473	14.341026	14.887600	14.330345	14.630796	14.258285	14.889685	14.669583	14.695696	14.066603	14.046714	14.091290	14.654622	14.101336	13.972743	13.139865	14.025054	13.440736	13.728107	13.682827	13.662485	13.197929	13.163201	13.702148	13.737835	13.828188	13.511757	13.936877	13.423212	14.312732	14.332054	13.490810	13.910238	13.775439	13.972172	13.796688	13.711167	14.787255	13.952936	13.629444	13.966043	13.531931	14.163659	13.490810	13.418619	14.144611	13.685268	13.876070	13.491941	13.817588	13.706089	13.629180	14.020402	13.894221	13.858073	13.228465	13.709428	12.820600	13.626821	14.050019	13.858854	13.301455	13.070042	14.474319	13.535732	13.315612	13.378652	13.875238	13.815048	13.719674	13.733881	12.852304	13.742058	13.352270	13.768030	13.289052	13.462463	14.092188	13.636433	13.586734	13.148784	13.393903	13.988680	13.729385	13.357219	13.672956	13.769304	13.857256	13.334586	13.165282	13.694028	14.126130	13.812477	13.863086	13.228263	13.661260	13.729901	13.189521	12.993823	13.881196	14.060641	12.922237	13.514012	13.313179	13.715852	13.359928	14.531467	13.730659	14.095820	13.236446	13.562221	14.167781	13.672956	14.147617	13.588156	13.456369	13.450346	13.658440	13.582741	12.735569	13.931643	14.508773	14.038510	14.632630	14.002435	13.964639	13.662485	13.329225	13.968272	13.976353	13.903911	14.002723	13.533746	13.686919

CERNA1	11.213047	11.924151	11.968105	12.087108	12.068594	12.212996	11.851720	12.073275	12.414625	11.957393	11.978531	11.750711	11.813591	12.008250	11.586469	12.127196	12.005939	11.485624	11.761456	11.733316	11.926448	11.154642	11.462482	12.044097	11.738648	11.874773	12.461907	12.013715	12.517283	12.362443	12.033678	11.092828	11.195637	11.773690	11.654834	11.613018	11.467049	11.832854	11.864833	11.697417	12.318376	11.501897	11.722441	11.579070	11.355342	11.078117	12.387919	11.527331	11.558489	11.254715	11.255007	11.079261	11.270728	11.231863	10.887345	12.560565	10.825316	11.565388	11.571480	10.775784	11.240485	12.342785	12.053932	11.735252	11.569303	11.373637	11.895835	12.013873	11.791206	11.563013	11.779706	11.792548	12.257254	11.690470	11.673410	11.809762	11.827397	11.413548	11.509371	10.693165	10.656678	10.389435	10.355373	10.681815	10.530253	10.923286	10.327627	10.373076	10.707338	10.906300	10.574366	10.556465	11.430298	11.299944	11.151275	11.100738	11.109722	11.490704	11.541804	12.489749	11.632987	12.198437	12.194140	12.043801	11.544982	12.226464	11.661180	11.885548	11.966878	12.531861	10.972053	12.023231	11.603886	12.621829	11.823531	12.927197	11.555942	11.603568	12.524312	11.556411	11.986674	12.141279	12.291073	12.809294	12.330024	12.227834	12.896328	12.330636	12.204356	11.913228	12.065315	12.119428	12.180653	11.996703	12.315592	12.038357	11.800003	11.904326	11.406614	12.078357	11.616406	11.615814	12.376771	12.100729	11.641331	11.954230	12.457691	13.019003	12.401457	11.925051	11.413991	11.666576	11.760219	11.933193	12.267749	11.431058	12.267304	12.109513	11.975098	12.645882	12.120977	12.023394	11.774477	11.425646	11.443831	12.534282	11.331326	11.818995	11.387935	11.344171	11.352031	11.788526	11.321148	10.744413	12.506563	10.865149	11.484876	11.797058	10.779419	11.598146	12.638368	11.870697	11.902647	11.736718	11.547842	11.942440	12.110845	12.068447	11.640863	11.894583	11.704750	12.715589	11.936536	11.557603	11.818688	11.856087	11.722280	11.675812	10.768939	10.710978	10.474973	10.575868	10.681815	10.656960	11.397315	10.704762	10.541250	10.951170	10.900969	11.062597	10.679116	11.515004	11.559918	11.229610	11.360018	11.379904	11.815237	11.522184	12.573503	11.775928	12.456916	12.405908	11.991560	11.470020	12.631637	12.031083	11.588472	11.726896	12.798496	11.191787	12.042106	12.331121	12.938874	12.038655	11.559648	11.299661	11.965086	12.423579

AC138627.1	7.448253	7.415795	7.196002	7.315270	7.558907	7.816968	7.393988	7.704712	7.542035	7.616362	7.689652	7.299864	7.282257	6.969819	7.333806	7.600626	7.294755	7.206128	7.644170	7.583649	7.548986	7.116576	6.764246	7.365150	7.523749	7.733926	7.906643	8.047520	7.754711	7.630212	7.686690	7.447919	7.029566	7.236268	7.145800	6.982537	7.169171	7.135615	7.632388	7.044339	7.919819	7.241188	7.091028	7.124348	6.986699	6.630154	7.905055	7.389491	6.947625	7.145206	6.709750	6.830244	6.889972	6.536130	6.942632	7.894343	6.659030	6.901907	6.916640	6.624196	6.768967	7.552070	7.104070	6.944562	7.009488	7.034373	7.439916	7.514932	7.340021	7.380528	7.657826	7.672732	7.934176	7.543264	7.620601	7.583021	7.688261	7.168868	7.354382	6.601510	6.794137	6.657929	6.500696	7.148992	6.746442	6.919882	6.685342	6.788922	7.200555	7.107731	7.054425	7.304121	7.154973	7.383462	7.425397	7.474601	6.836732	6.922885	7.190158	7.507090	7.233867	7.165039	7.376878	7.485516	7.291452	7.388100	7.764971	7.141596	7.237197	8.042008	7.388571	7.552843	7.456332	8.219027	7.283526	8.593171	7.536401	7.276724	7.890599	7.015163	7.409429	7.371092	7.492126	8.267621	8.019387	7.567340	7.267882	7.410189	7.786365	7.573258	7.553313	7.723151	7.250407	8.046247	7.664539	7.286616	7.658320	6.873056	7.405226	7.153563	6.824667	7.426143	7.651452	7.540669	7.713603	7.867408	8.189259	8.341455	7.631321	7.654980	7.137034	6.565927	7.759430	7.356249	7.384238	7.240235	7.506195	8.010496	7.295865	7.994730	7.556767	6.805835	7.400671	7.043338	6.906051	7.951354	7.225595	6.998626	7.040644	6.750842	7.349268	7.029874	7.254611	6.885991	7.783772	6.635475	7.054921	7.166091	6.522172	7.030340	8.026061	7.489591	7.355953	6.953765	6.936985	7.541435	7.662478	7.473992	7.629544	7.938997	7.783481	8.296548	7.604046	7.475990	7.552684	7.686690	7.379253	7.437104	6.714240	6.621298	7.057769	7.070868	6.568873	6.828610	7.057930	6.600722	6.895743	7.183928	7.169031	7.249927	7.670527	7.382041	7.620289	7.434700	7.435040	6.637380	6.973206	7.021413	7.336755	7.378634	7.304606	7.569464	7.284738	7.364834	7.306185	7.900697	7.154829	7.116904	8.341166	7.397543	7.660474	7.415795	8.634560	7.425554	7.861521	7.308803	7.332417	7.691660

LINC02211	3.616508	3.298546	5.240664	4.016060	3.488837	4.801541	3.863876	4.242269	3.832514	3.415206	4.040595	4.050878	3.135394	3.745674	3.480978	3.356906	3.521873	3.357764	3.515313	3.498544	4.587788	2.822427	5.756839	3.268254	2.898804	3.415052	4.106504	3.584146	3.709872	4.051282	3.669264	3.437904	3.383664	4.590982	3.482425	3.997882	4.354817	4.256727	4.167983	3.569686	4.454188	3.890045	4.001248	3.262043	3.749398	5.527261	3.915969	3.458409	3.580146	4.186652	4.035158	3.836113	4.495125	3.934099	3.908793	4.196670	3.946203	4.154312	3.317974	3.087392	4.218149	4.282121	4.028609	5.034412	4.066483	5.035490	3.998094	3.610969	3.814406	4.415762	4.065006	3.834808	4.321019	3.527209	7.166404	3.926528	4.481977	4.481723	3.878499	3.567285	4.523906	4.040380	3.261885	3.906634	4.492238	3.663078	4.882226	5.026050	2.888372	4.812486	4.863512	4.947124	3.082860	3.173699	3.492758	4.235959	3.875870	3.564969	3.217434	3.215429	4.973787	3.242816	4.250189	3.342824	3.418434	4.253908	4.293542	3.799007	4.237634	3.584353	4.288978	5.272049	4.631969	3.521682	3.512383	6.173696	4.059329	3.635673	3.671371	5.789012	3.770322	7.831401	3.457344	3.918499	3.692360	5.464037	4.917266	4.346108	3.618187	3.751278	4.657491	4.508979	3.623459	3.918798	4.219685	3.995497	3.372078	3.604642	3.623070	3.915969	2.805927	3.541683	3.486614	3.788669	4.915772	5.125769	4.117669	6.129664	4.957295	4.356325	3.267061	5.274525	4.466739	3.937075	4.041871	4.954364	3.530906	4.485024	3.563815	5.677332	3.092690	3.534991	4.134295	3.995744	5.057772	4.658804	3.520441	3.796132	4.138852	4.086354	3.747923	4.195839	5.041365	4.072332	3.758336	4.372155	2.858884	4.371963	4.311473	2.941487	3.637869	4.914945	4.370451	3.710908	3.997446	3.476529	3.866820	3.763620	6.323720	4.410703	4.162511	3.535179	3.993727	10.383504	4.112234	4.938279	4.646621	4.169230	3.579178	3.642453	4.326339	3.890274	4.484799	5.173917	4.832287	4.473434	3.826779	4.560966	3.311947	3.863667	4.562514	5.269249	3.235939	3.724930	4.967125	3.690559	4.514021	3.272988	3.175351	3.314806	3.208207	8.792916	3.212552	3.730244	3.847173	4.732302	3.795908	3.540335	3.463557	3.782082	5.082862	4.573081	4.137797	3.275450	4.518473	4.880724	3.416052	3.367892

AC009630.2	8.763855	10.520340	10.973089	10.142442	9.702347	9.600803	9.610570	7.921791	8.586509	9.744581	9.780111	9.575490	10.979225	9.823884	9.405273	9.307738	9.332770	9.640574	8.452314	10.208918	9.185181	8.691979	7.801299	9.876719	7.232439	9.273625	8.581533	7.076052	8.328445	10.053909	9.649319	11.558020	10.499007	11.164195	10.317267	10.347504	10.092047	10.023706	9.254187	9.471554	9.726052	8.538466	10.144161	9.755469	9.846915	10.477553	7.854375	9.342359	10.220177	10.044168	9.455567	10.098273	10.353124	10.344001	10.343107	9.563744	10.187258	10.024337	9.643171	9.967880	9.241660	9.255278	8.831940	8.253887	9.823468	9.234252	10.129455	9.511815	11.507445	8.314211	10.141648	10.316937	9.096148	10.274402	10.702431	10.314129	10.623370	10.519370	10.347055	10.556772	10.560714	10.431566	10.368062	10.799956	10.879893	10.135620	10.730905	10.677766	9.243504	9.952724	9.349567	9.088836	10.095036	11.038010	10.613754	10.345226	10.561160	9.587494	10.503158	9.284919	9.334014	9.685927	10.318307	9.781184	9.439413	9.905906	7.190009	8.619238	8.343060	8.908916	8.523224	9.268775	9.486588	8.610918	9.977545	10.223905	8.389753	10.114066	8.928172	11.213657	11.607855	11.446896	9.784102	10.322892	11.196086	11.436541	12.058239	12.255202	12.026020	11.164195	12.176095	12.127514	10.357811	10.858655	10.051862	11.476411	10.590818	11.107323	12.057480	11.384675	9.199210	10.328537	11.293530	9.591661	11.379300	10.590818	11.448972	11.934585	11.582863	10.372584	11.052815	12.223705	12.000077	11.612729	12.686212	11.390271	11.374333	9.463167	11.511108	10.869032	10.931134	12.682302	12.729547	11.744200	11.722749	11.216854	11.041360	12.020215	12.377553	11.837404	11.888108	11.985888	12.233736	11.987411	10.035315	11.515156	12.064848	10.917928	11.584062	10.908098	11.091024	11.830551	10.859103	10.036715	10.206147	10.392669	11.503797	12.465490	10.407254	11.898522	12.118005	10.955581	12.678900	11.806190	11.185257	11.562614	12.566429	11.519647	10.990476	11.307832	12.279737	11.479340	11.296440	11.607591	11.383354	12.692683	12.768875	9.748635	11.176269	11.793930	11.143830	11.303833	12.073275	11.433599	12.039839	11.319133	11.414999	12.273604	12.018090	11.451777	12.202678	9.304559	12.357687	10.190269	10.858370	11.750863	10.460269	10.686753	10.994463	10.458660	10.519634	12.386767	7.805559	11.436541	11.709538	12.356727	12.872501	11.716588

LINC02357	8.173166	7.693984	7.607580	7.983196	8.249737	8.650567	6.983656	9.001182	8.842443	7.727960	8.068759	8.145806	7.118214	7.569464	6.981168	8.265703	8.226755	8.104169	8.856845	7.819359	8.478664	8.768535	9.043309	7.781671	7.908340	8.149614	8.065503	8.729734	7.652378	7.329294	8.698199	7.272497	7.434394	7.081292	7.408829	7.551288	7.811416	7.917069	8.146260	8.143459	7.685796	8.159472	7.719509	7.754559	7.178293	7.537165	7.981300	7.762671	7.495681	7.592559	7.515856	7.569464	7.169475	7.457297	7.406172	7.885512	7.391165	8.033453	7.768978	7.672279	7.917531	7.197292	8.041861	8.626099	7.884438	8.363315	8.068615	8.347960	7.938047	8.556714	7.710796	7.985454	7.745467	7.837199	7.904431	7.867408	7.795703	7.774932	8.026381	7.528443	7.419618	7.406485	7.288615	7.540966	7.804638	7.604831	7.798320	7.989271	8.054325	8.773990	8.427711	8.793679	7.446825	7.505088	7.154829	7.407121	7.688871	7.434558	7.258561	7.596093	7.732264	7.310728	7.559979	7.753027	8.446086	7.661530	8.707227	8.212064	8.976956	7.984078	9.367879	8.785633	8.634726	8.536257	7.682251	9.293321	7.880066	7.695913	7.911412	7.708703	7.652521	7.434866	7.861216	8.360009	8.449060	7.608060	7.869699	8.109085	7.351763	7.538443	7.333971	7.447833	7.251999	7.021413	7.974303	7.878104	7.511538	8.431530	7.178765	8.004823	8.252222	8.973345	7.576995	7.889831	7.555653	7.974619	8.337740	8.235110	7.674175	8.300756	7.425076	7.110840	7.113147	7.169171	7.255711	7.449836	7.627977	8.119459	8.061379	7.605162	8.238086	7.633026	8.015237	7.085839	7.610979	8.024532	7.673196	7.247168	7.241639	7.556927	7.181722	6.593717	7.199608	6.806000	7.426808	6.810976	7.639852	7.658770	7.401008	8.188467	7.141116	8.488758	8.516062	7.883226	8.149308	7.864173	8.223604	7.730239	8.353996	7.572029	7.443943	7.829277	7.488051	7.648449	7.624107	7.865564	7.825526	7.963752	7.340021	6.993710	7.521441	7.507394	7.402070	7.515221	7.586605	7.753321	7.607725	8.172239	8.331848	8.073673	8.938239	7.239214	7.281001	7.404585	7.335828	7.573089	7.138598	7.173996	6.644025	7.133076	7.255390	7.375633	7.380528	7.501480	7.714215	8.266965	7.710796	8.652644	8.039647	9.211143	8.366088	8.654316	9.023274	7.349921	7.599022	7.763900	7.365785	7.862754

AC233976.1	9.968179	9.543634	9.683738	10.320132	9.990270	10.707919	10.062277	9.865136	11.175825	10.244280	10.784974	10.093062	10.890438	9.526187	9.829351	10.426073	10.214264	9.724538	9.883041	9.988100	9.254659	9.776906	10.540607	9.473843	9.681392	10.300503	11.784937	9.844876	10.732503	10.524141	11.026307	9.755913	9.926926	10.537651	10.578905	10.138664	9.950260	10.154199	10.595136	9.988100	10.355524	10.743052	9.673010	10.016803	9.935902	9.856190	10.779848	10.029952	9.962991	9.827675	9.377466	9.795661	9.877686	10.453118	9.930158	9.979650	9.803340	10.457252	9.374473	10.555535	10.126162	9.934782	11.653194	9.745076	10.799798	9.966054	10.189798	9.680791	10.365458	9.867722	10.111838	9.773103	10.976546	9.808072	10.291426	9.790950	9.877686	10.121388	9.842136	9.853854	9.347097	9.721686	9.449194	9.775229	9.739008	10.131092	10.364292	10.009427	10.390190	10.032710	9.893017	10.444886	9.609148	10.300168	10.094258	9.942574	9.846608	10.858794	10.109887	10.549791	11.004546	9.881777	10.195389	9.606408	10.484898	11.056421	8.838748	10.213038	10.076815	10.681237	10.378034	9.816203	9.563890	11.065562	10.438690	12.381004	9.983520	9.749266	10.284105	10.610796	10.609657	10.796728	10.640152	11.291891	10.840635	11.308833	12.116671	11.284886	11.996401	12.125096	11.476411	11.630608	10.629178	11.345331	11.844498	11.184194	9.548541	11.687068	12.287695	9.205341	10.757656	10.274568	9.730122	12.602064	10.948169	11.309686	11.424787	11.610786	10.219095	11.649619	11.751144	11.816173	10.299864	11.114713	12.000886	11.439898	10.626257	9.823738	10.519894	11.221474	11.806329	10.237139	9.834429	12.231738	11.477417	11.209250	12.934551	12.143436	10.986739	11.362553	10.395960	11.702959	11.405878	10.599248	10.534701	12.149134	12.330494	10.562355	13.946220	11.078117	10.069917	11.211573	9.324766	9.992511	9.774033	12.389829	9.854153	11.579070	9.309881	9.965758	10.580246	12.563656	10.441420	10.865449	9.961955	12.071894	10.111998	10.092624	10.425006	12.305259	9.103956	9.609476	10.768791	11.186293	10.659469	10.814470	12.311740	11.984692	10.599871	11.635559	11.522791	11.156096	10.939616	10.738234	10.528938	11.189047	11.508051	10.384855	11.222510	12.014918	11.190903	11.454144	11.274607	13.419287	12.643245	11.320390	8.823713	12.492085	10.772548	13.539712	12.311085	10.668429	11.153927	11.511560	11.053092	11.577041	10.232963	11.018523

AC078778.1	11.230943	10.414046	10.353725	11.304559	11.760698	11.380992	10.532370	10.531581	12.962912	11.106454	11.809762	11.275872	11.696988	10.849438	11.240771	11.793314	12.399170	10.606387	11.920101	11.694853	10.528799	10.405628	9.492544	10.580067	10.751755	11.396538	12.540317	10.840331	12.024202	10.673307	12.922424	10.196594	10.445527	11.708961	10.738234	10.530547	10.540467	9.495201	9.381628	9.639513	11.411098	9.783775	10.635038	10.646207	10.283247	10.826216	11.683795	9.606097	11.332767	10.356921	10.916730	10.372919	10.506605	10.319358	10.569055	11.639628	10.821308	9.619777	9.666547	9.669262	9.642543	11.441315	9.896439	10.081011	9.645810	9.396376	9.289113	10.491412	9.450135	9.833053	10.645194	11.106894	12.057936	10.499145	10.620285	11.044580	11.142070	10.798488	10.977739	10.666359	11.370215	11.579376	10.719109	10.814911	10.376671	10.952734	10.988377	10.592750	9.857887	9.572453	9.482879	9.956000	10.650012	10.584201	11.411822	12.425096	11.749834	12.677497	11.665514	13.316023	12.496861	10.657103	11.670622	10.941524	9.174045	12.525455	11.778669	10.449603	11.775050	11.722749	9.387333	12.686400	10.465346	12.104980	13.063428	11.030608	10.862604	12.191183	12.762381	11.832244	11.064219	11.006998	11.403392	11.839782	11.744798	11.727527	13.238041	12.495360	11.913228	11.595768	12.035950	11.413399	10.841568	10.832721	12.425904	12.020495	11.681866	12.180812	10.770306	10.817302	10.414788	8.404354	11.739966	9.955381	9.828267	10.571069	11.446299	12.814928	10.490251	13.311729	12.604579	11.707159	11.957688	11.615814	11.528321	10.583331	9.783775	10.201110	10.445678	12.378817	9.598799	10.619838	10.874430	10.331003	10.404862	12.580172	10.147930	11.109422	10.462340	10.692122	10.988232	10.740809	9.735468	10.547812	11.247632	10.691500	9.939497	9.772950	9.889840	10.533003	10.505273	10.455244	10.342962	10.474364	10.001482	10.749604	9.655960	10.344770	9.533072	11.210101	11.171084	12.313487	10.962648	11.563161	11.110584	11.006688	11.202677	11.468830	11.108364	11.520848	10.564580	10.838131	12.156344	11.042557	11.680837	11.029707	11.422093	10.354019	10.401393	10.758970	10.480956	11.830322	11.459949	11.840838	12.393793	12.164694	13.246936	12.491754	11.484725	12.496680	12.190585	12.596981	10.849717	8.408506	12.087998	13.296386	10.747238	10.660096	11.172831	9.270456	12.162952	12.088432	11.548287	11.365203	11.256626	10.291731	11.862307	11.145002

LINC00847	10.599565	10.832422	10.456003	10.538660	10.678806	11.207310	11.119269	10.766127	10.105679	10.682725	10.481536	10.594267	10.746036	10.346735	10.436435	10.794845	10.263794	10.724009	10.386560	10.946082	10.625655	11.162452	10.328381	10.663570	11.227093	10.552501	10.172908	11.518703	10.151430	11.231105	10.954214	10.497536	11.153618	11.201014	11.247214	10.861007	10.836326	10.798635	10.892843	10.939064	10.339585	11.317780	11.060277	10.923426	10.720490	10.626847	10.407254	11.083792	10.874275	11.139619	11.298834	11.249397	10.976265	11.639945	10.384236	10.465042	10.816269	11.130935	11.361048	11.378727	12.001749	10.311711	10.797295	10.876057	11.759762	10.587751	11.603716	11.094004	11.274739	11.016351	11.092539	11.078407	10.448409	10.892693	11.158776	11.061273	10.669542	10.857655	11.084401	9.902261	11.043433	10.674816	10.647743	11.140512	11.019767	10.606255	11.345196	11.245085	10.742594	11.336580	11.371885	11.101922	11.094860	10.804745	11.166523	10.935492	10.618518	9.785048	10.569914	10.659191	11.160666	10.712709	9.996027	11.051479	10.211657	10.646637	10.629335	10.506086	9.816653	10.353725	10.749604	10.604741	10.384541	10.808261	10.593619	13.923216	10.964294	9.990936	10.673881	11.432256	10.893881	11.732880	10.539109	10.866201	11.565093	11.006838	10.128997	10.282924	10.376811	9.754711	11.479943	10.965182	11.229444	10.622041	10.767431	10.861160	10.624904	10.481102	11.543468	10.678375	11.469123	10.785271	11.039598	11.246667	11.024507	10.750389	10.857810	10.515780	11.108364	10.650012	10.908696	10.835875	10.746482	11.626444	10.848877	10.656678	11.324962	11.212284	11.015894	11.071672	11.205984	11.156846	10.852730	11.354479	10.982204	10.526564	11.660885	11.451777	10.947592	10.778275	11.571034	11.505835	11.292520	11.316160	11.478556	11.577639	11.614961	11.301743	11.578488	11.758139	10.991775	11.355495	10.882081	12.482960	11.790869	11.410967	11.611382	11.357096	11.649619	11.352736	10.519634	9.979955	11.318667	11.505696	11.269542	11.244028	11.426649	10.618196	10.555685	10.412549	10.740051	11.125488	10.962648	11.301289	10.946980	11.146494	12.205414	11.017912	11.385568	12.161570	10.968909	11.055538	11.175825	10.330088	10.703034	10.832721	10.743802	10.561160	11.652130	11.047235	11.173601	10.472987	11.570177	10.993595	10.237708	9.949517	10.461430	11.392765	10.904667	10.846658	10.777970	12.898302	10.557368	11.570035	11.302774	11.175215	10.362213	10.910730

THAP7-AS1	6.251982	6.196190	6.566408	5.821306	6.502676	6.102897	6.385448	6.635639	7.101416	5.109966	6.633056	6.744976	7.485047	5.597613	6.315398	6.271705	6.818111	5.931921	5.798946	7.182036	5.042212	6.491854	6.473910	6.675937	6.297720	6.977531	7.362606	5.221697	5.432417	6.931281	5.857438	7.118055	7.059187	7.171477	7.611437	7.118373	7.121194	6.588047	6.895583	6.180988	5.476909	6.635796	7.464963	7.219043	7.140794	7.632388	5.148135	6.798807	5.912413	7.224175	7.100327	7.380184	7.235142	7.585179	7.829608	5.954546	8.240540	7.412597	6.153116	6.853977	6.928135	5.697300	7.442823	6.096514	6.594072	5.379836	7.304121	5.995352	6.496679	6.066254	6.287255	6.344224	5.184212	7.077988	7.533611	6.765874	6.861077	6.678173	6.688232	7.217647	6.927315	6.845047	7.547904	6.785321	6.970119	6.546804	7.314333	6.333836	7.002587	5.936504	6.833773	6.312471	6.176600	7.122892	6.801411	7.356091	6.382809	6.882462	6.278303	6.787415	6.805391	7.164719	6.721907	7.101416	6.503324	5.813523	6.442361	6.198084	6.125302	4.922562	6.679637	6.216124	5.028887	5.417383	6.077122	7.621653	6.591177	6.172347	6.126226	5.505053	6.442684	7.864018	5.052790	7.322331	5.710664	5.448593	7.906308	7.243901	5.584380	7.258046	8.453985	8.711913	5.975866	7.525133	5.660681	8.269676	7.215778	8.931289	8.686570	4.871916	7.579796	5.588122	7.244060	6.657757	8.214100	7.055726	5.953976	4.835195	6.417324	6.574822	6.905419	7.825526	7.447746	7.326749	7.345981	7.752415	5.987923	8.749240	6.926972	6.641471	6.216975	8.017102	7.762841	7.203798	7.952156	5.444097	8.261233	7.480444	8.146710	7.966199	7.375801	7.930369	8.924735	9.648405	6.558609	9.931696	9.263696	6.911143	8.148860	7.693051	6.526356	8.981195	7.783481	6.302007	6.943923	9.116189	6.498399	7.700541	5.509568	5.913302	6.564171	4.973376	8.289196	8.438280	7.477847	7.298304	8.443484	8.315925	8.197496	8.405771	8.275964	8.228265	7.721304	7.013009	6.128501	7.918723	6.943443	7.517738	7.663729	8.259851	7.747180	7.054770	8.727901	6.467195	8.187900	7.544535	6.564827	6.695877	6.406070	8.386650	8.300756	5.089403	8.914331	8.263544	5.212965	6.049418	6.441052	7.271405	5.232618	6.769612	7.476161	5.200277	6.377801	7.855897	7.030795	6.979111	6.474239	7.835236

TUBA3FP	6.251982	6.196190	6.566408	5.821306	6.502676	6.102897	6.385448	6.635639	7.101416	5.109966	6.633056	6.744976	7.485047	5.597613	6.315398	6.271705	6.818111	5.931921	5.798946	7.182036	5.042212	6.491854	6.473910	6.675937	6.297720	6.977531	7.362606	5.221697	5.432417	6.931281	5.857438	7.118055	7.059187	7.171477	7.611437	7.118373	7.121194	6.588047	6.895583	6.180988	5.476909	6.635796	7.464963	7.219043	7.140794	7.632388	5.148135	6.798807	5.912413	7.224175	7.100327	7.380184	7.235142	7.585179	7.829608	5.954546	8.240540	7.412597	6.153116	6.853977	6.928135	5.697300	7.442823	6.096514	6.594072	5.379836	7.304121	5.995352	6.496679	6.066254	6.287255	6.344224	5.184212	7.077988	7.533611	6.765874	6.861077	6.678173	6.688232	7.217647	6.927315	6.845047	7.547904	6.785321	6.970119	6.546804	7.314333	6.333836	7.002587	5.936504	6.833773	6.312471	6.176600	7.122892	6.801411	7.356091	6.382809	6.882462	6.278303	6.787415	6.805391	7.164719	6.721907	7.101416	6.503324	5.813523	6.442361	6.198084	6.125302	4.922562	6.679637	6.216124	5.028887	5.417383	6.077122	7.621653	6.591177	6.172347	6.126226	5.505053	6.442684	7.864018	5.052790	7.322331	5.710664	5.448593	7.906308	7.243901	5.584380	7.258046	8.453985	8.711913	5.975866	7.525133	5.660681	8.269676	7.215778	8.931289	8.686570	4.871916	7.579796	5.588122	7.244060	6.657757	8.214100	7.055726	5.953976	4.835195	6.417324	6.574822	6.905419	7.825526	7.447746	7.326749	7.345981	7.752415	5.987923	8.749240	6.926972	6.641471	6.216975	8.017102	7.762841	7.203798	7.952156	5.444097	8.261233	7.480444	8.146710	7.966199	7.375801	7.930369	8.924735	9.648405	6.558609	9.931696	9.263696	6.911143	8.148860	7.693051	6.526356	8.981195	7.783481	6.302007	6.943923	9.116189	6.498399	7.700541	5.509568	5.913302	6.564171	4.973376	8.289196	8.438280	7.477847	7.298304	8.443484	8.315925	8.197496	8.405771	8.275964	8.228265	7.721304	7.013009	6.128501	7.918723	6.943443	7.517738	7.663729	8.259851	7.747180	7.054770	8.727901	6.467195	8.187900	7.544535	6.564827	6.695877	6.406070	8.386650	8.300756	5.089403	8.914331	8.263544	5.212965	6.049418	6.441052	7.271405	5.232618	6.769612	7.476161	5.200277	6.377801	7.855897	7.030795	6.979111	6.474239	7.835236

AL022326.1	6.024457	6.715517	6.530971	6.439561	6.936495	6.646089	6.586060	6.935051	6.521703	6.842863	6.081971	5.742456	6.303063	6.388356	6.146192	7.028923	6.519214	5.944425	6.966591	6.859440	7.345011	7.554247	3.486956	7.127214	6.537774	6.805534	6.851861	7.053478	6.767480	6.864533	6.634360	5.924182	6.625166	5.874564	6.039824	6.289123	5.916177	6.822140	6.866291	6.568230	6.831962	6.030746	6.553679	6.923052	6.114220	6.460003	7.014840	6.442531	6.526670	6.656131	6.541196	6.544343	6.211523	6.463552	6.467545	7.273579	5.877950	6.596780	6.467861	5.915666	6.166510	6.809369	6.307918	6.751134	6.657433	6.487103	7.221148	6.903996	6.856737	7.139390	6.524815	6.262702	6.795557	6.137520	6.430386	6.735790	6.753389	6.339728	6.319966	6.472740	6.114404	6.229710	6.017258	6.065057	5.367778	6.235310	4.448244	5.688249	6.419962	6.863771	6.042616	6.702584	6.757186	6.809210	6.518050	6.025340	6.264325	5.765566	7.024084	6.345390	6.541524	6.592444	6.022738	6.381308	7.143875	6.634185	7.215915	7.171656	7.409893	6.955972	7.148341	7.033904	6.647877	7.035624	6.804768	7.306505	6.319966	6.485635	6.363544	5.967336	6.588047	6.443703	6.762515	7.842803	6.264502	6.372278	5.787010	5.709757	6.345747	6.223463	6.431244	6.579129	6.356807	8.582697	6.155339	5.887979	6.494605	6.876549	6.055326	7.229212	7.230711	6.966591	6.866291	6.651927	6.518878	6.751780	7.024249	6.831962	7.053478	5.757033	6.040731	6.528320	6.177655	5.925083	5.611389	5.751747	6.595852	6.355256	6.238082	7.194385	5.975696	5.563726	7.601689	6.132086	6.636437	7.668055	6.233243	6.144448	6.491525	6.591021	6.199146	6.112982	6.537292	5.526678	6.702584	4.986634	6.238082	6.002904	5.255945	5.944953	6.987163	6.511935	6.632208	6.241098	5.902022	7.064759	7.011725	6.942960	6.655496	5.978146	6.102541	7.491020	5.755895	5.213607	6.644356	6.153773	6.188929	6.489335	5.702228	5.790613	6.318925	6.201713	5.076314	4.670819	5.894955	5.215622	5.110970	6.051295	6.016047	6.457215	6.256093	6.395964	6.421573	6.340247	6.069980	5.981021	5.664777	6.683406	5.904197	6.870984	5.994297	6.436133	5.565784	6.259647	6.738430	6.511268	6.157372	6.314683	7.366705	6.523859	5.700984	6.321693	7.284141	7.648004	7.378490	6.623549	6.235819	7.003824

AC117383.1	10.411931	9.858186	10.038997	10.521739	10.377715	11.147807	10.151718	11.331326	11.156846	11.239635	10.964592	10.788074	10.117312	10.201399	9.761038	11.383500	10.149179	9.835054	10.202951	10.546111	9.600645	10.457105	9.991411	10.235895	10.306828	10.404707	11.096474	11.423905	10.615988	10.490556	10.471635	9.697251	9.608538	10.143544	9.786976	10.147330	9.909707	9.925599	9.950580	9.930637	11.029412	10.147476	10.056061	9.801959	9.925599	9.727298	11.335110	9.686981	9.912662	9.732885	9.756219	9.413187	9.513366	9.656882	9.490469	11.018364	9.489846	9.721843	10.056200	9.730269	10.031627	10.677354	10.482463	10.160730	9.721549	9.505969	10.061791	9.891185	9.696933	10.125839	10.670884	10.450036	11.684959	10.450933	10.594267	10.227905	10.369851	10.159823	10.669542	9.394510	9.001965	9.215117	9.563450	9.298143	9.474302	9.630914	9.661702	9.494887	9.760564	9.630751	9.689605	9.980586	9.621515	10.238620	9.988894	10.094875	9.665161	10.440659	10.593179	10.855244	10.344466	10.082692	10.458660	10.146257	9.881488	11.209812	10.102182	10.671515	10.281686	11.537619	10.070060	9.835818	10.200086	11.402055	11.209956	11.471379	10.579044	10.340199	11.816892	10.447656	9.970033	10.673442	10.354160	11.227093	10.548120	10.835128	11.227828	10.153421	10.600166	10.102793	10.663706	10.427336	11.033609	10.403949	10.731203	10.485177	10.903646	11.688705	11.069292	10.316666	11.578788	11.085135	11.808140	11.360307	9.782831	10.834345	11.608893	11.194590	10.356921	10.143844	9.942430	10.083894	9.957986	10.131242	11.741168	10.255569	10.344336	10.333451	11.129277	11.013967	10.751156	11.047378	10.346735	10.739148	10.230524	11.003077	10.836911	10.897614	9.762301	9.709999	9.910027	10.740962	9.485177	10.143702	10.453574	10.578752	11.146945	10.780289	11.129277	10.962810	10.335092	10.605030	10.211826	10.816868	10.483232	10.376347	10.035004	9.884098	10.427778	10.390190	10.194914	11.661642	10.026067	10.225601	10.213643	10.303868	10.020041	10.523398	9.792860	9.141334	9.778915	9.898981	10.265433	10.405008	10.195828	9.906054	11.717338	10.506605	9.808072	11.592521	10.769094	9.777687	10.166595	10.234797	10.314761	10.234963	10.539572	9.833356	11.637393	10.172605	11.354479	11.262567	10.267640	11.257232	11.033609	10.548595	11.242375	10.232060	11.049734	11.270124	11.072856	11.847451	11.620096	10.778275	10.087473	9.866241	10.237443	10.301888

AC013270.1	10.145963	10.448109	10.288200	10.646346	10.792032	10.880034	10.191626	11.028512	11.072990	10.502281	10.374844	10.330692	10.117613	10.089453	9.709048	11.295692	10.424856	9.734545	11.224021	11.370678	10.694805	10.478589	10.274087	10.462807	10.693316	10.529215	11.293672	11.163643	11.625759	10.999226	10.510355	9.738061	9.739328	9.616680	9.711867	9.472625	10.132441	10.190269	10.684205	10.022749	10.949809	10.741115	9.925044	10.253224	9.587971	10.162156	10.947592	10.908392	10.009271	10.423927	11.086028	9.634626	9.502704	10.037337	9.803650	11.130328	8.994888	9.431886	10.260405	9.202497	9.814480	10.814013	9.891491	10.594114	9.665009	9.883041	11.404986	10.454796	9.955381	10.641355	11.310412	11.403690	10.921361	11.195789	10.325821	10.027498	10.196594	10.611691	11.926602	9.246132	9.676753	9.815890	9.394972	10.152177	9.348642	10.189657	10.677766	10.424856	10.015553	10.312731	10.776077	10.177481	10.300344	10.347353	10.148869	9.903186	9.918999	9.670386	9.910805	10.520042	10.235895	10.161675	10.150370	10.079967	10.153421	10.754173	9.934023	10.294926	10.930535	11.450138	10.500604	10.442759	10.274723	11.668763	10.572001	12.437883	9.875439	10.211826	11.132929	9.590740	10.422850	10.126162	10.393559	11.769304	10.957229	10.368062	10.721646	10.627462	10.740809	10.472987	10.282147	11.255473	10.462499	10.456003	10.967395	10.364725	10.389118	10.529955	10.170273	10.609210	10.770741	10.318307	11.260634	10.740649	10.299419	10.796271	10.888785	11.655296	10.973802	10.373979	10.023218	10.008153	9.908194	10.006475	9.968968	10.050017	10.394473	10.848151	10.363564	11.241217	10.520042	9.695275	10.424856	9.729580	10.316237	11.435966	9.937420	9.731680	10.013258	10.192816	9.511205	9.333838	9.671026	9.043309	11.111588	8.790103	9.360960	9.687291	9.164187	9.662346	10.550696	10.214105	9.961668	9.823738	9.536303	10.691815	10.493142	10.112589	10.523247	10.529215	10.519091	11.417836	10.648195	9.986408	10.183454	10.212902	10.439142	11.307400	9.334439	9.668780	9.689137	9.471239	9.739008	9.180017	9.981215	10.686442	9.418245	9.778915	9.782381	9.763085	10.010612	10.016190	10.136546	10.128078	10.099200	9.991246	10.342962	9.984732	10.287089	10.009427	10.387783	10.354602	10.015875	10.201859	11.313224	10.383040	10.400485	10.526564	11.491895	10.376965	10.472696	10.763276	11.776832	10.462958	10.852403	9.607484	9.959667	10.646346

LINC01949	13.935762	13.709428	13.277634	14.729253	14.880110	15.188690	13.188396	14.051518	15.515150	14.141134	14.606262	14.267954	14.622046	14.306995	14.197180	14.228587	14.561327	13.990442	14.915189	14.452088	14.300954	14.633014	15.082324	14.566731	14.711236	15.431049	15.581893	14.295055	14.164900	14.959508	14.853259	12.504387	12.438515	15.065209	14.545097	13.514224	14.181178	14.273182	14.442351	14.653231	14.276547	14.530263	14.615742	14.527899	14.083435	14.029975	15.232746	13.565950	13.997582	13.840596	13.822921	13.070410	13.262596	13.888605	13.307926	14.570991	13.452027	12.990709	13.665236	13.970475	12.970971	15.138365	13.923492	14.714881	14.055678	13.682594	13.720921	13.859406	13.869088	14.624663	13.938513	14.042288	15.078176	13.427333	13.769304	13.998713	13.195318	13.021558	13.891542	12.696624	13.600599	13.039307	13.250652	13.154016	14.365566	12.753969	15.737267	14.256351	13.544622	12.934012	13.454586	13.994733	12.993251	13.983245	14.008461	13.887810	12.602739	14.784337	15.209711	15.259618	15.261637	13.654590	14.065689	14.128591	13.713891	14.626390	15.151242	15.089479	15.831819	14.704362	14.635230	14.795501	14.603726	15.537762	13.049719	16.045607	15.456675	14.531467	15.285115	15.372489	14.799393	14.987284	15.168793	16.131397	14.990649	15.544594	15.867496	15.732189	15.473917	15.329439	15.391393	15.479687	15.268645	15.278078	15.930069	15.530816	15.542881	15.455879	14.825958	15.048970	15.035758	14.652808	15.713696	15.152524	14.388223	15.180575	15.421067	16.175634	14.583170	15.808111	15.352487	15.665364	14.803797	14.547186	15.487850	14.948503	15.028728	15.224051	15.159483	15.581893	15.124619	14.758316	15.095711	15.289538	15.098818	15.449581	15.040623	15.008260	14.476349	14.922772	15.231361	14.438218	14.595757	14.826972	15.410836	14.936250	15.004809	14.975923	14.504993	15.096349	15.090125	14.993419	14.902407	15.002418	14.887600	15.198214	14.914667	14.649747	14.360826	15.026937	15.354914	15.272298	15.460905	15.248027	14.671445	15.023469	15.117212	14.843666	14.943193	14.871315	14.642226	14.851633	15.219810	14.828938	15.685230	14.697958	15.013920	15.301621	15.177955	15.053135	14.796877	14.940393	14.438218	14.892803	15.119035	15.724946	15.853383	15.382160	15.172005	15.391393	15.648843	15.881450	15.174615	14.806664	15.500573	15.682114	15.095132	14.648445	15.433373	15.287379	15.749943	15.859538	15.427861	15.737267	15.317503	15.107990	15.256065	15.345704

AP003396.5	9.673820	10.548595	10.077750	8.812917	7.260136	7.975701	9.249936	7.988091	7.960216	8.525979	8.240540	7.627834	9.103349	8.669568	8.923280	7.588888	8.853927	8.005443	9.207792	9.313949	9.333063	10.786914	12.552337	9.180782	8.931776	8.689568	11.940295	9.154648	9.311249	9.901485	8.475899	10.452240	11.153181	11.075319	11.150814	9.616680	9.956464	10.038559	10.913411	9.391895	8.016641	10.027195	11.186992	9.601437	10.392503	9.673965	9.405140	10.670295	9.241228	9.970033	10.227439	10.388103	10.464310	10.186968	9.958780	9.916212	10.196293	10.057740	10.309103	10.103273	10.736083	10.212902	14.761491	10.271020	11.832552	9.724689	11.261380	9.862229	11.292363	10.234963	9.720169	9.767982	8.681277	9.930780	9.323193	11.295839	9.653378	11.715728	9.615604	10.292188	10.370484	10.692551	10.407694	9.465964	9.806105	9.236583	11.171964	10.225137	12.154483	10.757808	10.189346	12.136334	11.167966	10.032414	10.260093	9.213597	9.240450	9.704886	8.625939	8.643630	9.678618	8.359575	8.740568	9.078876	9.929378	8.067428	9.081348	9.902411	10.071920	7.068565	10.603333	8.508449	7.776635	6.903040	7.714929	6.835808	9.870596	8.921058	7.764204	11.395949	12.679062	12.846490	11.875072	11.983809	11.521749	11.878180	13.084373	13.260449	11.619483	12.392356	11.234364	11.799097	11.318816	12.178954	11.369611	12.882408	12.192905	12.802671	13.483027	12.615190	14.405128	13.070042	11.142960	13.385185	12.533294	12.307454	10.330088	11.203302	12.391429	12.260855	13.019185	13.868836	12.362917	11.545455	12.649362	13.977501	13.714668	12.463227	13.896685	11.933654	13.230666	12.868144	12.933669	13.027671	11.436814	10.651778	12.522348	10.223174	13.563147	13.715358	12.462601	13.079309	13.232267	13.832307	11.364013	12.933838	13.485992	12.055738	13.058976	13.788457	12.310451	13.221167	12.958652	13.084018	13.656976	13.405029	13.339340	13.091080	13.755126	12.484062	13.539515	8.222845	14.242737	13.215627	12.635767	14.042006	13.520934	13.588376	13.348676	13.440327	11.735392	12.811445	14.573507	13.841137	13.127100	13.411431	13.067352	14.191449	14.189906	13.315805	13.685728	13.471411	13.820166	13.224930	13.784808	13.112784	14.128591	12.376451	11.499627	13.095970	11.458309	12.644395	13.461144	13.699890	9.328459	11.430746	11.507888	11.962317	10.354019	14.219249	14.000936	10.420096	7.412742	10.929640	11.891730	13.278412	12.023884	11.816438

USP2-AS1	9.673820	10.548595	10.077750	8.812917	7.260136	7.975701	9.249936	7.988091	7.960216	8.525979	8.240540	7.627834	9.103349	8.669568	8.923280	7.588888	8.853927	8.005443	9.207792	9.313949	9.333063	10.786914	12.552337	9.180782	8.931776	8.689568	11.940295	9.154648	9.311249	9.901485	8.475899	10.452240	11.153181	11.075319	11.150814	9.616680	9.956464	10.038559	10.913411	9.391895	8.016641	10.027195	11.186992	9.601437	10.392503	9.673965	9.405140	10.670295	9.241228	9.970033	10.227439	10.388103	10.464310	10.186968	9.958780	9.916212	10.196293	10.057740	10.309103	10.103273	10.736083	10.212902	14.761491	10.271020	11.832552	9.724689	11.261380	9.862229	11.292363	10.234963	9.720169	9.767982	8.681277	9.930780	9.323193	11.295839	9.653378	11.715728	9.615604	10.292188	10.370484	10.692551	10.407694	9.465964	9.806105	9.236583	11.171964	10.225137	12.154483	10.757808	10.189346	12.136334	11.167966	10.032414	10.260093	9.213597	9.240450	9.704886	8.625939	8.643630	9.678618	8.359575	8.740568	9.078876	9.929378	8.067428	9.081348	9.902411	10.071920	7.068565	10.603333	8.508449	7.776635	6.903040	7.714929	6.835808	9.870596	8.921058	7.764204	11.395949	12.679062	12.846490	11.875072	11.983809	11.521749	11.878180	13.084373	13.260449	11.619483	12.392356	11.234364	11.799097	11.318816	12.178954	11.369611	12.882408	12.192905	12.802671	13.483027	12.615190	14.405128	13.070042	11.142960	13.385185	12.533294	12.307454	10.330088	11.203302	12.391429	12.260855	13.019185	13.868836	12.362917	11.545455	12.649362	13.977501	13.714668	12.463227	13.896685	11.933654	13.230666	12.868144	12.933669	13.027671	11.436814	10.651778	12.522348	10.223174	13.563147	13.715358	12.462601	13.079309	13.232267	13.832307	11.364013	12.933838	13.485992	12.055738	13.058976	13.788457	12.310451	13.221167	12.958652	13.084018	13.656976	13.405029	13.339340	13.091080	13.755126	12.484062	13.539515	8.222845	14.242737	13.215627	12.635767	14.042006	13.520934	13.588376	13.348676	13.440327	11.735392	12.811445	14.573507	13.841137	13.127100	13.411431	13.067352	14.191449	14.189906	13.315805	13.685728	13.471411	13.820166	13.224930	13.784808	13.112784	14.128591	12.376451	11.499627	13.095970	11.458309	12.644395	13.461144	13.699890	9.328459	11.430746	11.507888	11.962317	10.354019	14.219249	14.000936	10.420096	7.412742	10.929640	11.891730	13.278412	12.023884	11.816438

LINC01706	3.194728	6.143072	4.803466	5.937761	4.449155	5.169505	5.829701	6.801570	4.975658	5.865057	6.206276	5.917767	4.921258	6.117316	3.506641	7.125453	5.664421	6.031113	6.118709	6.882160	5.811871	8.116083	2.896802	5.339915	5.069387	5.293694	3.397833	6.834077	4.522120	4.716878	6.217146	5.536657	5.934048	3.447097	4.982780	5.603232	6.091820	7.164273	6.022244	6.568705	6.005203	5.229742	6.585387	5.462398	4.368922	5.730301	3.211448	6.923712	6.874477	7.200242	5.811679	6.534041	4.570485	6.741956	6.299075	4.917266	5.157266	5.071019	6.848046	4.842558	6.158402	5.347540	4.005441	4.565553	4.287666	4.971901	6.784847	5.399206	6.179989	6.154988	6.961500	6.315037	5.465392	6.087137	6.882311	5.043683	5.442744	5.271224	5.520438	4.895390	3.842553	4.009758	3.580504	4.894172	4.864946	6.023065	3.431688	5.264877	4.959144	5.148725	4.294152	4.983002	4.481255	6.264006	4.775323	4.456263	5.919034	6.327414	4.956023	6.303405	5.046472	6.126600	6.528481	6.714928	6.132086	4.552354	7.038434	7.665583	4.292225	5.906156	5.233840	5.916177	3.294372	6.538118	5.714759	3.067732	3.596729	3.095909	5.316667	3.141816	4.122071	3.735018	3.183074	3.378974	3.627415	2.994217	4.173784	3.471558	3.589774	3.449712	3.167023	3.258402	3.593240	3.230351	3.539584	3.629032	3.036347	4.936406	3.586706	4.069444	3.935165	2.867083	3.227892	3.523830	3.245240	3.038936	3.770910	3.226736	3.545283	3.488107	3.549657	3.565704	3.984782	4.249268	3.729450	3.763005	4.029058	3.636278	3.367892	3.151102	4.246657	3.696741	3.521300	4.269404	5.147132	3.153417	4.024941	4.221682	4.754703	4.196022	6.011402	4.266974	4.630030	3.564191	3.132602	3.794458	4.687185	5.417778	5.131601	3.043028	3.285805	4.113965	4.194775	4.134500	3.985181	4.168179	3.753157	3.498710	2.996841	3.151510	3.092801	3.424029	4.480835	3.572883	3.330982	3.666279	3.185927	3.151248	3.276032	3.849489	4.389969	3.613168	3.680841	3.554492	3.321367	3.375999	3.445983	4.308819	3.704887	3.553350	3.028082	3.235526	3.452771	4.293542	3.604265	4.876743	5.125344	4.326548	3.332891	3.020820	3.462151	3.674117	3.053523	5.099511	3.188054	3.039726	5.695854	4.078077	3.408177	3.221947	3.567669	3.589211	4.541524	3.238168	3.237557	3.419704	3.508512	3.391764

AC104574.2	7.367028	7.731181	7.623177	7.987647	7.732409	7.490559	6.807572	8.979614	8.870392	7.475046	7.610841	7.275617	6.301153	6.899004	7.510154	7.865564	8.769584	7.281157	8.681590	7.898055	8.458106	8.336175	8.724655	7.720828	7.673686	7.924902	6.994765	8.560859	7.113612	6.823214	8.051562	6.255207	6.924368	5.649360	7.333524	7.539423	7.335363	7.818413	7.937295	8.034780	7.607725	7.825991	6.840811	7.789766	7.121790	7.328193	9.004857	7.467854	7.193101	7.458563	7.263718	7.437870	6.896873	7.300476	7.342696	7.654519	6.872894	7.474299	7.507556	7.630529	7.987940	6.890454	7.796488	8.388365	7.925376	8.142845	7.824779	8.550581	7.836741	8.760599	7.689026	7.701592	7.324066	7.493510	7.662956	7.629075	8.194463	7.879916	8.076511	7.369389	7.137190	7.678751	7.320594	7.493966	7.552843	7.432187	7.568557	7.879615	8.073510	8.376041	8.066258	8.983646	7.533758	7.627042	7.051177	7.358858	7.662002	6.251652	6.960356	7.266940	6.312471	7.307876	7.299081	7.309930	8.560859	7.366862	8.961298	8.199981	8.649047	7.671162	8.817289	8.299864	8.212972	7.639530	7.777297	7.022169	7.781521	7.406172	6.735952	7.201315	8.014164	7.331953	7.626880	8.081473	7.712175	6.602789	7.183775	7.901809	6.938118	7.015993	6.489172	6.739736	6.443376	7.127214	7.287232	8.117460	6.809699	8.158091	6.967570	7.945709	7.902721	8.950273	7.626412	7.748877	7.321205	7.941824	8.209188	7.410771	6.873859	7.568100	6.578980	6.775273	6.844547	6.885835	7.147559	7.158540	7.563777	7.944322	7.797093	7.617692	8.127674	6.418153	8.183407	6.938279	7.350691	9.097971	8.198553	7.101416	7.368431	7.727797	7.102506	6.632390	7.145969	6.261164	6.941502	6.243249	7.176042	7.270478	7.147710	7.893118	6.878619	8.138957	8.006102	7.734581	7.710952	7.665583	8.213942	7.853910	8.379228	7.643210	7.495213	7.508140	7.171812	7.026662	7.958043	7.888897	7.881547	8.207086	6.834077	6.775132	7.625172	7.552537	6.815409	7.422117	7.032657	7.643684	7.505088	7.678603	7.892168	7.735493	9.029665	7.543116	7.790524	7.530239	7.395552	7.365622	6.280180	6.709596	6.882462	6.399465	6.548531	6.819116	6.935199	7.554247	6.961969	8.124646	7.819040	8.776752	7.723151	8.593939	7.847939	7.717058	8.192912	7.605472	7.002808	8.001370	7.108059	6.745292

LINC00342	14.097093	13.076484	12.746911	13.734624	13.121579	13.673176	13.190645	13.036157	13.749588	13.769838	13.223528	13.637621	13.288631	13.391215	12.762924	13.310550	13.588156	13.411431	12.904102	13.647299	12.653093	13.499765	13.479048	13.460920	13.262983	13.503914	13.980804	13.754656	13.639425	13.113729	13.330495	12.727241	12.620992	13.363265	12.679396	12.777764	12.906948	12.217913	12.778119	13.075705	13.320668	12.828585	13.422596	12.646754	12.551723	12.486004	13.734624	12.656017	12.466153	12.353897	12.960120	12.159569	12.316396	12.245775	12.961230	13.189888	12.484861	12.634942	13.244123	13.384972	12.824716	13.699890	12.707385	13.492381	12.095335	12.837577	13.054557	13.053836	12.320002	12.993071	12.820415	13.227647	13.534199	13.089623	13.235399	13.273969	13.120033	13.067170	13.293157	12.347744	13.015183	12.278977	12.536567	12.402199	12.655512	12.592077	12.369546	12.464048	12.344564	13.080241	12.301709	12.449933	12.855684	13.054365	13.714163	13.581357	12.463883	13.772186	13.271981	13.741329	13.663761	13.256009	13.208294	13.372935	13.485529	13.501531	13.210542	12.868885	13.058207	13.460034	12.660501	13.489038	13.078555	13.616612	13.443307	13.228647	14.275877	13.640135	13.829200	12.639201	12.163903	12.514492	12.608186	12.956264	12.949809	13.084208	13.218764	13.359085	12.691855	12.542438	12.908398	12.989794	12.415626	12.081058	13.955482	12.749657	14.097093	12.321093	12.846834	12.760314	12.322207	12.077882	13.680430	12.673113	12.297752	12.788738	13.056178	14.113158	12.585159	13.988680	13.184093	13.282824	12.258058	12.754620	12.056973	12.268991	12.442059	12.396485	12.001437	12.669159	12.144382	12.657171	12.152993	12.290138	11.617713	12.907332	12.156031	13.013913	11.448837	11.579070	11.654537	12.816305	11.583468	11.898984	12.876931	12.281762	12.503046	12.221727	13.034495	11.688852	12.577537	12.041676	12.610190	12.210195	12.098604	12.174349	12.224905	11.282541	11.940741	12.873920	12.199376	12.893846	12.179402	12.801254	12.520730	11.969768	12.317939	12.555905	12.184698	12.394906	12.003696	11.332619	12.953654	12.573686	12.433882	11.701608	12.231416	12.374659	12.399784	14.641355	12.374349	12.548239	11.661180	12.173415	12.701928	12.718533	14.064771	12.711163	12.978958	12.500081	12.969511	14.399253	12.715740	11.336717	13.795129	12.306523	11.620399	12.579997	13.044646	12.829989	12.772064	13.463591	13.331093	12.532185	11.791350	13.095058	11.293081	12.667828

RAD21-AS1	11.628067	11.514381	11.380054	12.438343	12.880949	12.851777	10.872038	12.509156	13.552549	11.711018	12.187328	11.805746	11.555799	11.342331	11.225183	12.232513	12.261188	11.166369	12.306362	11.791508	11.574024	10.565474	11.570893	12.410794	12.290442	12.841760	13.369328	12.220472	12.687444	12.445481	12.568552	10.034381	10.496144	12.127984	11.515156	11.318232	11.251941	11.268237	11.412105	11.538822	12.476380	11.171500	11.758591	11.770488	11.463611	11.338491	13.052443	10.844879	11.332913	10.942125	10.999226	10.466097	10.542264	10.978333	10.372919	12.562670	10.320444	10.695129	10.688437	10.502860	10.527040	12.588951	11.205984	11.385252	10.802095	10.654131	11.205234	11.082337	10.860126	10.796577	11.761613	12.004567	12.907514	11.612729	11.439584	11.611675	11.969051	11.481782	11.503932	10.286213	11.021971	10.244280	10.193091	10.144458	10.640759	10.981465	12.157725	10.585380	10.445210	10.216690	10.294779	10.371089	10.793806	10.931134	11.214098	11.569749	10.825459	12.184238	11.856880	13.717719	13.132646	11.766326	12.502227	11.955591	11.143538	12.483254	11.526648	11.319628	12.968039	12.799200	11.325990	12.239149	12.133553	13.316023	12.024345	15.596409	11.843867	12.059200	13.323580	11.869049	11.505243	12.176716	12.576396	14.272867	11.984549	12.687272	13.570987	13.207325	12.493360	11.962317	12.372913	12.646567	12.318871	11.837854	13.607817	12.410794	12.144213	12.620500	11.578064	12.254351	11.122494	10.605180	13.105225	12.136649	11.469270	13.437980	12.837925	14.528293	11.404110	12.904820	12.378496	12.316396	11.758886	11.684658	11.559235	11.563013	11.824292	11.743759	11.846709	13.453723	11.107323	11.265020	11.753661	11.390561	10.956655	13.040426	11.396845	11.307098	11.039598	11.035981	11.195021	10.831500	11.020654	11.015753	12.410948	11.072990	10.937758	11.087667	10.368806	11.058656	11.944227	11.351141	10.923134	11.301140	10.831042	11.613750	10.967244	11.177770	10.705209	12.213183	12.239628	12.837577	12.322524	12.680421	11.606533	12.273123	12.011409	11.699314	11.270879	10.850333	10.512425	10.803737	11.419559	10.767583	12.755304	10.687519	10.867283	11.279433	11.403536	10.788836	10.535606	11.259576	10.950866	11.232861	12.117700	12.615979	13.260832	12.340191	12.225836	12.313487	13.149152	13.790706	12.973655	12.374518	12.922949	11.955891	11.607717	11.454294	13.020460	11.566261	13.130961	14.140523	13.302074	12.567904	12.805972	11.084833	12.082750	12.809469

LINC02372	7.640482	8.187149	7.734420	7.668657	7.753792	7.919036	7.630032	9.264903	8.657743	7.964652	7.234989	6.983494	6.682255	7.724392	6.468191	8.188180	8.279636	7.234377	8.166286	8.018756	8.629744	8.108184	6.982716	8.022388	8.187444	7.693051	8.129022	8.293805	7.934327	7.832466	7.972171	7.249008	7.759598	7.161880	7.720980	7.715374	7.794750	8.062273	8.192912	7.875662	8.081951	7.452118	7.498978	7.666698	7.190316	7.424453	8.040928	7.740704	7.369060	7.697463	7.215303	7.327546	7.443459	7.448561	6.953139	8.452928	6.257069	7.394443	7.285661	6.698868	7.276888	7.860319	7.712175	7.734581	7.268179	7.017144	8.481708	8.057438	7.926948	8.088765	8.058720	8.014321	7.975078	7.717365	7.837050	8.104985	7.986373	7.774785	7.778050	6.819280	7.232924	7.328040	8.192758	7.158850	6.827028	7.082365	6.659365	6.765059	7.765933	7.696834	7.512341	7.649794	7.771110	7.564875	7.131326	7.323456	6.883083	6.611684	7.477368	7.819973	7.498194	7.674327	7.658918	7.335363	8.115905	7.697463	9.148313	8.193679	8.160220	8.398224	7.602495	8.348256	8.136495	8.634259	7.682404	7.573718	7.264197	7.162193	8.039326	7.279720	8.181446	7.722516	7.971462	8.140306	8.403138	7.322331	7.649648	8.593623	7.209395	7.253866	6.845709	7.582731	7.308044	7.072747	8.083179	7.922567	6.915418	7.580544	7.579513	8.360934	7.547747	7.563944	7.771867	8.292913	7.351140	8.379382	7.914461	8.233737	8.391312	7.546612	7.228420	6.830092	7.602969	7.401924	7.287971	7.276888	8.084278	7.948319	7.405370	8.052786	7.648147	6.998483	7.960216	7.204562	7.550519	8.517642	7.617539	7.038584	7.508474	7.371577	7.093509	7.427426	7.764503	6.903996	7.890305	6.050621	7.145800	7.166559	6.736122	7.040480	7.799277	7.575291	7.632690	7.175395	7.014228	8.344769	8.081336	8.036983	8.008376	7.694279	7.530078	8.589904	7.561521	7.305394	7.903842	7.906928	8.039167	7.809278	7.005042	7.059340	7.565198	8.306046	6.520703	6.545014	7.062528	6.803168	6.508146	7.744413	7.482442	7.029717	7.744703	7.582572	7.722684	7.447140	7.487562	6.803833	7.066045	7.576031	7.541274	7.500547	7.540820	7.588731	7.303347	7.723151	8.323832	8.393345	7.565352	7.514634	8.867477	7.382525	8.156736	7.785598	9.225910	7.945391	7.536569	7.380184	7.241639	8.047031

AC044810.2	3.026761	3.903127	2.980038	3.596131	4.196900	4.548870	3.508878	4.935362	4.847214	3.306975	3.546167	4.373025	4.663894	3.803284	3.755476	5.037916	3.327407	3.572084	5.762690	4.096439	4.638845	4.609944	3.220421	3.539970	3.902722	4.388910	5.500266	4.116148	4.694331	3.154548	3.645431	3.441704	4.453325	3.995497	4.699871	3.823956	3.577153	4.075783	3.723903	3.822254	3.379675	2.978331	4.049098	3.167396	4.046961	5.148519	3.307599	2.975431	3.509610	3.322172	3.404877	3.442220	3.894646	3.860460	3.708949	3.483154	3.962248	3.005091	3.157304	6.176433	2.892982	3.095293	3.288642	4.516143	3.198666	3.846729	2.859822	4.036699	3.553719	2.872066	3.642657	4.051084	3.541683	3.250276	2.895725	3.520622	4.008667	3.788669	4.235322	3.517346	4.039945	3.820601	6.200170	4.227672	4.103439	3.804108	5.364627	4.037774	3.030691	3.172139	4.089608	4.266767	3.542870	3.246100	4.323129	3.841327	4.015856	3.909010	4.125890	4.797249	3.802024	3.962248	4.460696	3.637684	3.915302	3.634857	4.552590	3.820148	4.312513	3.927841	4.630254	5.083639	4.199237	3.233940	3.214885	4.847214	5.367401	4.153870	5.137404	3.371537	3.236796	3.179000	3.002049	4.742152	3.668288	3.248982	6.244622	4.140843	5.114965	3.437397	3.770527	2.993450	3.197164	3.402304	6.938569	3.677214	3.159038	2.956161	3.385360	4.021312	3.470503	3.379675	3.286535	2.847614	2.827972	4.787036	4.157992	5.442550	3.478770	3.987153	3.294060	4.963547	3.266295	4.703230	3.599338	3.549833	3.105154	5.297271	2.914185	3.133804	2.937913	3.468564	3.164186	4.016509	4.806984	3.426736	2.917403	3.695929	3.236383	3.333065	3.285045	3.784156	4.074510	3.403206	4.834572	3.955912	3.003614	2.956436	6.126600	2.850826	3.299325	4.356767	3.228185	3.251979	3.459121	2.855299	3.513146	3.296065	2.870336	3.003234	3.400891	4.381390	3.364521	3.137740	4.275090	3.554680	3.530135	3.633687	3.952908	3.613780	4.145567	6.343881	3.642657	4.032747	4.055845	3.183197	3.433974	3.679614	3.259139	3.357933	4.250623	3.334861	3.106189	5.204869	3.888075	4.557070	4.208229	4.360255	3.159671	5.371883	5.879995	4.623548	4.013337	3.463210	3.155801	5.026259	3.235381	3.789732	3.451883	4.288978	5.152347	5.897057	3.636278	3.165192	3.713806	5.718155	7.384537	3.422249

AC044810.3	3.026761	3.903127	2.980038	3.596131	4.196900	4.548870	3.508878	4.935362	4.847214	3.306975	3.546167	4.373025	4.663894	3.803284	3.755476	5.037916	3.327407	3.572084	5.762690	4.096439	4.638845	4.609944	3.220421	3.539970	3.902722	4.388910	5.500266	4.116148	4.694331	3.154548	3.645431	3.441704	4.453325	3.995497	4.699871	3.823956	3.577153	4.075783	3.723903	3.822254	3.379675	2.978331	4.049098	3.167396	4.046961	5.148519	3.307599	2.975431	3.509610	3.322172	3.404877	3.442220	3.894646	3.860460	3.708949	3.483154	3.962248	3.005091	3.157304	6.176433	2.892982	3.095293	3.288642	4.516143	3.198666	3.846729	2.859822	4.036699	3.553719	2.872066	3.642657	4.051084	3.541683	3.250276	2.895725	3.520622	4.008667	3.788669	4.235322	3.517346	4.039945	3.820601	6.200170	4.227672	4.103439	3.804108	5.364627	4.037774	3.030691	3.172139	4.089608	4.266767	3.542870	3.246100	4.323129	3.841327	4.015856	3.909010	4.125890	4.797249	3.802024	3.962248	4.460696	3.637684	3.915302	3.634857	4.552590	3.820148	4.312513	3.927841	4.630254	5.083639	4.199237	3.233940	3.214885	4.847214	5.367401	4.153870	5.137404	3.371537	3.236796	3.179000	3.002049	4.742152	3.668288	3.248982	6.244622	4.140843	5.114965	3.437397	3.770527	2.993450	3.197164	3.402304	6.938569	3.677214	3.159038	2.956161	3.385360	4.021312	3.470503	3.379675	3.286535	2.847614	2.827972	4.787036	4.157992	5.442550	3.478770	3.987153	3.294060	4.963547	3.266295	4.703230	3.599338	3.549833	3.105154	5.297271	2.914185	3.133804	2.937913	3.468564	3.164186	4.016509	4.806984	3.426736	2.917403	3.695929	3.236383	3.333065	3.285045	3.784156	4.074510	3.403206	4.834572	3.955912	3.003614	2.956436	6.126600	2.850826	3.299325	4.356767	3.228185	3.251979	3.459121	2.855299	3.513146	3.296065	2.870336	3.003234	3.400891	4.381390	3.364521	3.137740	4.275090	3.554680	3.530135	3.633687	3.952908	3.613780	4.145567	6.343881	3.642657	4.032747	4.055845	3.183197	3.433974	3.679614	3.259139	3.357933	4.250623	3.334861	3.106189	5.204869	3.888075	4.557070	4.208229	4.360255	3.159671	5.371883	5.879995	4.623548	4.013337	3.463210	3.155801	5.026259	3.235381	3.789732	3.451883	4.288978	5.152347	5.897057	3.636278	3.165192	3.713806	5.718155	7.384537	3.422249

LINC00870	4.035847	3.090209	3.465394	3.747084	4.183437	3.545643	3.951215	3.250993	3.167274	3.081673	3.190532	4.530746	3.696522	3.521300	3.321367	3.550026	3.191194	3.080594	3.487527	3.433819	3.965491	3.892646	3.495782	3.731092	3.646626	3.675983	3.769308	5.136022	3.830779	3.428828	3.594018	3.277138	3.495782	3.232555	4.745112	3.829515	3.400057	3.585700	3.232699	3.160450	4.390813	3.159792	3.983505	3.405718	3.828858	4.592701	3.599539	3.168395	3.266751	4.499424	3.491512	4.117437	3.631499	3.819912	3.488662	4.705969	4.237440	3.097255	3.141697	3.594399	3.064290	3.934099	3.479108	3.079857	3.233940	3.366868	4.520391	3.458955	3.458213	3.739547	3.716703	3.648387	3.815882	3.390353	3.393483	3.201160	3.569686	3.690345	5.145152	3.425458	3.373589	3.587474	3.268548	4.507002	4.530110	3.265284	4.132771	3.396948	3.427979	3.213675	4.132996	3.473978	3.589774	3.534047	4.100404	4.024737	3.421184	3.715868	3.661202	3.727364	3.631091	3.680841	3.943719	3.733411	3.996193	3.122165	2.910353	3.220421	3.219976	3.586323	3.227169	3.971571	3.563462	3.506434	3.271926	4.544458	4.445874	3.289377	3.954187	3.210436	4.559227	3.290448	3.264389	4.798541	3.389306	3.819707	3.841530	3.061561	4.371120	3.012867	3.664050	4.227030	4.054951	3.118062	3.391947	3.096907	3.559545	3.094765	4.241625	3.541120	3.410422	3.181516	3.606829	4.219035	4.801756	3.808070	6.099247	3.329178	4.421729	3.531098	3.257598	3.444894	4.442195	4.709478	5.116406	3.311317	2.982540	3.188883	3.505520	4.470334	3.155801	3.804309	3.589774	3.801192	4.392582	3.915505	3.029656	3.414002	3.199019	3.078385	3.062481	3.571157	3.978461	3.286535	4.555359	3.637261	3.105403	3.006617	3.512383	2.961673	3.997680	3.491185	3.227738	3.364521	3.490858	3.071765	3.451547	3.701159	3.019211	3.824627	3.968092	3.583415	4.115265	2.892149	3.409194	3.778157	3.602837	5.000281	3.091325	3.367536	3.778359	3.282404	3.482596	3.479469	3.259588	3.510558	5.754089	3.227451	3.181086	5.085506	3.477266	3.332891	3.458213	4.011437	3.464825	3.369785	3.474511	3.606289	4.712006	3.728166	3.421018	3.598775	3.995497	4.371120	4.467164	2.924280	3.668479	3.339936	4.422440	3.276820	3.453325	3.341985	3.558141	4.214253	4.051954	2.949520	3.861486	5.201486

SNHG7	8.146710	8.724496	8.692143	8.247245	9.031727	8.590205	7.903028	8.052321	10.321522	7.691812	8.973923	7.861363	8.626562	8.731515	9.302942	8.055096	9.003048	7.802954	8.694827	8.133329	7.082213	6.488381	4.260212	9.181558	9.215294	9.050240	8.663767	7.784553	7.259651	7.954640	7.383782	7.849748	7.351613	8.264935	8.753634	8.617997	8.146260	9.048228	6.689974	8.085691	7.842958	6.991455	8.174901	8.164571	8.529542	9.153375	7.008175	7.567050	8.745587	7.706992	7.778689	7.803857	8.924171	8.356438	9.246593	8.565560	8.254820	8.141802	7.621512	8.584839	7.811870	8.551510	7.061705	6.766347	7.311495	7.642619	7.128933	7.850049	8.078697	7.022655	7.740253	7.858470	8.692458	7.754268	8.161790	8.137701	7.996145	7.738048	7.598679	8.499325	9.258917	9.102439	8.294269	8.588507	7.799210	8.632743	8.900076	8.788596	6.836582	7.729485	7.781951	7.764815	7.848996	9.148150	8.391312	9.330000	8.957785	9.698934	9.030423	9.729350	9.431265	8.114888	8.754431	6.949322	7.389191	8.791995	9.883943	8.220385	8.574878	8.012294	5.540166	9.609972	9.124109	7.895767	9.448726	6.878780	7.757729	8.655960	8.796854	9.443592	10.104326	9.410809	8.694675	8.963282	9.570131	8.145645	8.841804	9.655511	8.501719	9.517769	8.809828	9.377152	8.465536	10.296016	7.664213	8.943397	7.909103	10.010612	8.191798	8.727734	6.765874	5.751933	9.335435	9.062320	8.802888	7.689945	9.659506	8.253582	8.367601	7.087505	9.154648	7.752553	9.168008	9.082279	9.438669	8.590668	10.063481	7.533448	9.232537	7.883682	8.357999	8.532874	9.222820	9.049602	9.286431	8.742249	7.064759	9.501800	8.714362	8.131953	8.183907	10.052026	9.146259	9.485965	8.374648	8.465833	9.000251	8.195107	9.055012	8.929239	8.370991	9.575947	8.227519	8.303462	8.431198	8.713717	8.223437	10.135460	6.291862	8.997674	8.353209	10.266295	8.895603	8.516378	8.916489	8.844962	8.709776	8.973768	8.907495	10.697351	9.713091	9.211454	9.460449	8.520218	8.737472	9.836571	9.908965	7.628930	9.688544	9.489187	8.562682	8.903060	10.141339	9.037545	9.895199	9.452905	9.267822	10.582028	9.597096	10.109280	8.560695	8.961744	7.027322	8.541182	9.175115	11.019767	9.786976	7.737895	9.121694	6.538740	9.365664	7.572029	7.878260	10.105829	7.890755	8.879890	8.771295	9.570274

AC090912.1	7.211509	5.900808	6.401942	6.946788	6.620976	6.505645	5.473470	7.331171	8.362543	6.445076	6.881192	6.242941	6.958624	7.144750	6.320511	6.740986	7.319485	6.590824	6.658065	7.557379	6.104430	6.398476	6.147950	7.416707	7.701899	7.916471	6.642551	6.784344	6.318752	5.194696	8.514907	5.302043	6.117144	7.062687	6.432261	6.432761	5.907765	6.541705	6.304777	6.658225	6.655000	6.520703	6.302007	5.940777	6.078138	8.106048	6.335031	6.659030	5.602065	6.730623	7.115031	6.680133	6.127483	5.925083	5.711017	5.968636	6.636437	5.925429	6.701746	7.488806	6.987946	6.512094	4.966720	5.976379	5.890305	5.073229	6.487592	6.667531	6.283128	6.509101	5.990959	6.243786	6.691766	5.859961	6.777825	6.389179	6.694563	6.562187	6.955198	6.643191	6.594520	6.237625	6.369198	6.616246	6.862022	6.559425	7.168868	7.475990	5.551244	5.967890	6.640806	6.098026	6.359193	6.730148	6.559425	7.845026	6.416166	7.520020	6.442016	6.964223	6.451788	6.459694	6.925831	6.123031	5.354909	6.977696	7.784088	6.740402	6.285854	6.143926	5.237472	7.182191	6.743835	6.423337	6.677045	4.441499	7.138598	6.599069	7.148188	7.551288	6.507348	6.995225	7.274704	5.610876	6.715863	5.637531	8.702278	8.057990	7.634821	6.854771	6.414041	7.115967	5.941126	6.054258	8.290571	7.621190	6.964669	6.294553	8.220715	6.574345	6.628874	7.185534	8.255721	7.308199	6.657433	6.462508	6.558456	7.353126	5.585889	8.098103	6.210351	6.294903	6.606716	6.339551	7.530239	6.689649	6.611312	7.389651	6.778153	7.286286	6.777676	7.643849	6.213909	6.412181	8.446869	7.157612	5.399789	7.207847	6.579129	8.511835	7.467106	6.729672	6.674449	6.227132	6.881674	8.314053	5.382123	6.496370	7.888113	6.783714	5.684727	7.001632	6.795248	6.903528	5.874764	6.898358	6.840459	7.141116	6.642091	6.809369	6.283455	7.352520	6.950755	6.427678	6.717965	7.322799	7.430775	8.632336	7.583498	7.900057	8.184054	7.241639	7.460875	7.480117	8.232255	7.471118	9.678457	6.039985	6.607883	7.991746	7.028923	6.794633	7.199772	6.965640	8.669426	6.757650	7.948001	6.626317	7.001632	7.823189	8.438720	6.789085	6.381984	5.439680	7.708553	8.492102	6.401307	7.019693	6.223992	5.486729	6.729672	6.705266	6.475089	6.857226	4.861224	7.339407	6.426975	7.512640

AL121796.1	9.296431	9.127501	9.062616	9.357110	10.001321	10.729560	9.732418	10.074846	11.226796	10.367747	10.885099	10.406956	9.741359	9.949662	8.666083	10.261006	9.911427	8.702129	8.733953	10.144304	7.588434	8.313513	7.672436	9.729733	8.882792	10.177187	11.233464	11.136838	10.193562	9.681108	10.473309	8.670782	8.024532	9.669890	8.847228	9.068474	9.121540	8.468844	7.860319	8.295598	10.501539	8.222845	8.060586	8.056204	7.780442	7.796019	10.870351	7.700231	9.141010	8.001687	8.365932	7.605472	7.704236	7.689652	7.734740	10.582745	7.376736	6.192675	7.126410	7.629233	7.213117	10.085395	6.718122	7.275147	7.427426	7.389651	7.970369	8.266810	8.098253	7.890305	9.601275	9.601742	10.942265	9.478293	9.731525	9.304141	9.565885	9.228039	9.260598	7.699442	7.880784	7.782109	7.707451	8.117615	7.924442	8.699834	8.106950	8.201220	7.199296	7.447592	7.158238	7.145206	8.555423	9.198923	8.889309	9.625854	8.496873	9.921216	9.754543	6.095860	9.862557	9.206573	9.978579	9.502704	7.281460	10.789717	9.428363	8.320945	8.021345	10.836029	7.482123	9.045190	9.792563	11.287619	10.804455	11.141203	8.303328	9.998125	11.437253	10.785111	9.008249	9.047757	10.275761	10.452536	10.858500	10.435219	10.630830	10.428979	10.772696	10.529215	10.791282	10.314761	9.573371	8.936855	10.918671	10.370179	8.942487	8.244137	9.563890	7.633475	8.304376	7.855444	9.736555	8.872320	10.068719	9.901001	10.830328	10.990476	9.512761	10.675241	9.283544	8.900672	9.897339	9.369517	9.580855	9.113587	9.160629	8.937952	8.610918	11.045293	8.784155	8.728842	8.493834	8.174746	8.257856	11.005994	8.195596	9.122611	8.170358	8.303620	8.464289	8.102045	7.894343	7.742712	10.112741	7.634654	6.345390	7.737577	8.200421	8.051259	10.333120	7.745934	7.936063	8.220237	7.895121	7.901171	8.152062	8.351410	8.137554	9.956316	9.644870	11.575684	9.631239	9.493154	9.562541	9.351391	9.703711	9.552380	7.512491	7.963140	8.336641	8.276915	8.315127	8.092448	9.785948	8.157192	8.200588	7.375955	7.395245	7.615888	7.902271	8.752281	9.235309	9.371346	9.666872	9.468105	10.826808	10.275187	7.161260	9.565121	10.094118	10.387783	9.294119	8.183907	10.558107	10.396283	8.887749	8.147768	10.727170	7.721135	8.748937	9.692841	11.192212	9.951078	9.848797	8.818569	10.717161	10.222096

FAM230I	5.892964	6.236971	6.128168	6.450151	6.645787	7.089448	5.940063	6.557617	6.878619	6.237925	5.993219	6.250316	5.692261	6.151907	5.244217	6.089390	6.611684	5.685594	6.230070	5.908291	6.566720	6.782610	7.376736	6.019899	6.203825	5.846583	6.344311	6.718634	6.109076	6.464543	6.264178	5.328291	5.287944	6.089923	5.702804	5.633382	5.668327	5.659956	6.317412	6.668503	6.514226	6.042288	6.331797	5.943205	5.755174	5.706824	6.307251	6.119556	5.873510	6.269643	5.534678	6.028563	5.562223	5.551433	5.310700	6.221600	5.037135	5.776362	6.494280	5.588122	5.714759	6.534213	6.634691	6.290302	6.578323	6.176773	6.338556	6.147073	6.299534	6.377471	6.312121	6.450636	5.915478	6.046399	6.042781	4.640929	6.288952	5.830277	6.131041	5.264099	5.614247	5.582847	4.975473	5.747776	5.083448	5.399012	6.367558	5.934593	6.732083	6.249133	6.071880	6.283637	5.792931	5.654640	5.761196	5.785415	5.768316	5.995014	6.324214	4.170075	7.395245	6.006099	6.056641	6.099988	6.599582	6.215255	6.778314	7.133718	7.608977	6.608020	7.568694	7.016654	7.188249	7.014074	6.130349	9.414244	6.815085	5.953623	5.865582	7.492126	7.196156	6.631099	6.632566	8.672031	7.010274	6.991111	7.583498	6.941664	7.056010	6.633215	7.087352	7.731332	7.113612	6.965162	6.439408	7.041594	6.293351	7.645269	5.940777	7.432040	7.761745	7.576995	7.025384	6.992596	6.507804	7.302237	7.297031	7.538121	6.658538	6.804305	6.741283	6.896050	6.871633	6.325780	6.178147	5.775818	6.535659	6.925012	7.140632	7.111620	6.622744	6.378320	6.901432	6.228660	5.795108	7.262980	7.398195	6.557617	6.908917	6.361202	6.255038	6.190787	6.873531	6.068771	6.283289	6.276052	5.840237	6.463039	5.933869	6.861393	7.012688	7.101874	6.860578	7.085071	6.707704	6.990803	6.906870	6.779754	7.012349	6.565605	6.712196	6.776864	6.668343	6.974149	5.284990	6.763766	6.666704	6.598436	5.948433	5.960936	6.080402	6.052025	6.267913	6.169784	6.907507	6.298576	5.908486	6.885035	6.284143	6.559759	6.662662	6.498895	6.830244	6.615435	7.041908	7.078474	7.213408	7.062837	4.760783	7.341605	6.584561	6.739567	6.981009	6.916950	7.061543	7.523415	7.218109	6.598929	8.267621	7.941504	7.803562	8.807664	7.207080	7.060597	7.129553	6.484961	6.560692	6.853796

Z93930.2	10.573790	14.282078	13.109074	14.419090	12.910540	14.640110	14.793559	16.478815	14.242737	15.211019	14.533492	15.114738	13.694716	14.188386	13.601738	14.205886	13.964639	13.484384	13.814571	15.135878	13.311729	12.380673	11.557743	13.084593	14.203084	14.873437	11.280614	13.220179	12.728453	13.217148	14.661796	12.978769	13.130178	11.895183	14.025995	15.403893	14.800437	14.119553	14.673717	14.203696	13.916872	14.162992	14.813710	14.491975	14.188986	14.656836	12.518430	14.927607	13.068294	15.316792	12.985188	14.362295	15.058545	15.150598	15.372489	15.157558	15.164670	12.975643	13.704371	13.700412	14.033777	12.697321	11.674871	13.451597	14.068933	14.278941	12.873210	14.171283	13.318278	13.769304	13.981851	13.753087	13.189322	14.473933	13.632044	13.380119	13.337899	12.972942	13.871263	15.749943	13.084593	14.225717	13.847752	14.831507	15.196878	14.879649	14.137614	13.180364	13.001190	14.327880	14.521454	13.582520	13.350574	15.198897	11.993505	12.241742	14.053306	13.170352	13.564116	13.725623	14.917362	14.828455	13.026368	14.637025	14.788228	13.147891	15.088208	14.255462	13.404601	14.116167	13.043520	14.218603	14.075445	13.376076	13.985572	14.605817	11.471087	15.344243	12.781728	10.681237	14.421008	10.953919	12.198750	12.409034	13.512208	13.807309	11.990332	12.611352	12.349904	13.918322	12.085444	12.484861	11.616406	11.881172	12.405908	10.878399	12.239471	11.439584	11.781211	12.277288	11.455214	11.857671	11.889031	12.000886	11.602546	13.162836	12.130980	11.566261	12.325086	11.872355	12.357184	12.517616	13.955482	11.631352	14.201164	14.950618	12.010648	12.499125	12.012663	11.827669	13.072064	11.030464	12.312060	14.154830	14.393289	12.409512	11.729004	12.204516	13.050446	12.262315	11.247922	12.788229	12.531002	12.397379	11.805894	10.877643	11.033754	11.732276	11.912357	13.087953	11.782660	12.326538	12.533294	11.964481	11.241501	11.439735	14.228894	12.830643	11.950357	13.402063	12.933838	13.262983	12.941926	11.298970	13.682355	12.497024	11.025411	13.996448	11.534664	12.101792	12.859356	14.766218	12.103454	11.351898	12.599183	13.417326	11.896481	11.977595	12.431923	11.214512	12.406661	12.936720	11.733781	14.439328	11.490236	11.884784	11.328057	12.606221	11.463264	11.395495	11.746705	11.449273	11.343861	10.820704	12.301709	12.889156	11.944663	12.508819	13.919105	12.207923	11.011088	10.609075	13.310948	11.466138	13.283038	13.623869	11.598426	12.971855

FAM27C	3.748966	5.299488	4.320790	5.734828	6.758456	6.417324	4.945436	5.875497	5.927937	4.141068	6.235468	6.251982	5.858872	4.619091	4.783894	5.127355	3.633884	4.189728	4.051084	5.780149	5.472341	3.688884	4.479959	5.390865	3.742824	6.430725	4.600200	3.073342	4.581826	4.360482	3.815433	4.571992	3.662649	4.626548	5.604559	5.298852	5.548054	3.507422	3.480782	3.173699	5.280349	3.848646	6.358329	5.594579	6.091820	5.263694	3.998522	4.985607	5.306753	5.606325	6.719475	6.119404	5.995187	6.896217	7.100327	6.052025	5.783785	3.536879	4.181870	4.513567	4.392359	5.132818	3.367694	4.171867	3.970758	3.650021	4.348753	3.512576	3.666461	3.486788	5.600779	5.853071	4.266350	5.840048	5.186794	5.324768	6.294223	5.491679	5.404608	5.732292	5.626357	7.429346	5.714005	5.880149	5.262064	5.182198	6.824504	6.031996	4.973155	4.084323	3.312397	5.288139	6.625846	6.351948	5.204460	6.258619	6.107970	6.814127	6.055991	6.266387	7.429685	5.919567	5.450296	4.368705	3.326468	5.582288	5.499654	3.829515	5.512606	4.423335	6.380125	6.243095	4.460921	4.513363	5.556382	9.456638	4.227886	5.766650	3.449361	4.644478	5.783785	4.852673	4.507907	7.594133	5.300411	5.895831	4.399395	4.095615	3.400891	5.147334	4.934074	6.546146	4.410020	4.809298	5.237066	3.751911	4.078269	3.251299	5.069990	4.741955	4.558533	3.594399	5.750104	3.114731	5.855263	6.395964	4.874867	6.221944	3.591339	3.551420	5.182198	5.056780	6.000261	5.631732	4.204989	5.353254	3.627023	3.395212	3.575730	4.932450	3.622038	6.699791	3.558339	4.302412	5.161575	3.647203	3.998746	4.900277	6.800254	4.768089	5.351677	6.050288	6.916362	6.693444	4.118088	6.452605	3.283615	4.035158	3.930648	3.302221	5.313797	3.260848	3.383664	3.300958	3.231727	2.838045	3.648387	3.298691	3.195567	6.572587	4.878379	3.188054	5.702804	5.045149	4.205947	5.546959	5.153391	5.226305	6.395482	4.756961	7.342373	5.083842	5.992878	6.010016	5.669624	5.391260	4.179668	4.335082	3.917257	3.428492	4.192585	7.563777	6.095321	4.151468	6.134154	6.079562	6.422692	4.650482	4.848086	6.799452	5.711575	5.052790	4.312966	3.360502	4.144088	5.252954	3.620663	3.435630	3.357076	6.354724	6.386626	4.154789	4.196022	6.880869	7.545653	3.454582	4.767656	3.544326

FAM182A	3.748966	5.299488	4.320790	5.734828	6.758456	6.417324	4.945436	5.875497	5.927937	4.141068	6.235468	6.251982	5.858872	4.619091	4.783894	5.127355	3.633884	4.189728	4.051084	5.780149	5.472341	3.688884	4.479959	5.390865	3.742824	6.430725	4.600200	3.073342	4.581826	4.360482	3.815433	4.571992	3.662649	4.626548	5.604559	5.298852	5.548054	3.507422	3.480782	3.173699	5.280349	3.848646	6.358329	5.594579	6.091820	5.263694	3.998522	4.985607	5.306753	5.606325	6.719475	6.119404	5.995187	6.896217	7.100327	6.052025	5.783785	3.536879	4.181870	4.513567	4.392359	5.132818	3.367694	4.171867	3.970758	3.650021	4.348753	3.512576	3.666461	3.486788	5.600779	5.853071	4.266350	5.840048	5.186794	5.324768	6.294223	5.491679	5.404608	5.732292	5.626357	7.429346	5.714005	5.880149	5.262064	5.182198	6.824504	6.031996	4.973155	4.084323	3.312397	5.288139	6.625846	6.351948	5.204460	6.258619	6.107970	6.814127	6.055991	6.266387	7.429685	5.919567	5.450296	4.368705	3.326468	5.582288	5.499654	3.829515	5.512606	4.423335	6.380125	6.243095	4.460921	4.513363	5.556382	9.456638	4.227886	5.766650	3.449361	4.644478	5.783785	4.852673	4.507907	7.594133	5.300411	5.895831	4.399395	4.095615	3.400891	5.147334	4.934074	6.546146	4.410020	4.809298	5.237066	3.751911	4.078269	3.251299	5.069990	4.741955	4.558533	3.594399	5.750104	3.114731	5.855263	6.395964	4.874867	6.221944	3.591339	3.551420	5.182198	5.056780	6.000261	5.631732	4.204989	5.353254	3.627023	3.395212	3.575730	4.932450	3.622038	6.699791	3.558339	4.302412	5.161575	3.647203	3.998746	4.900277	6.800254	4.768089	5.351677	6.050288	6.916362	6.693444	4.118088	6.452605	3.283615	4.035158	3.930648	3.302221	5.313797	3.260848	3.383664	3.300958	3.231727	2.838045	3.648387	3.298691	3.195567	6.572587	4.878379	3.188054	5.702804	5.045149	4.205947	5.546959	5.153391	5.226305	6.395482	4.756961	7.342373	5.083842	5.992878	6.010016	5.669624	5.391260	4.179668	4.335082	3.917257	3.428492	4.192585	7.563777	6.095321	4.151468	6.134154	6.079562	6.422692	4.650482	4.848086	6.799452	5.711575	5.052790	4.312966	3.360502	4.144088	5.252954	3.620663	3.435630	3.357076	6.354724	6.386626	4.154789	4.196022	6.880869	7.545653	3.454582	4.767656	3.544326

AL078587.1	3.748966	5.299488	4.320790	5.734828	6.758456	6.417324	4.945436	5.875497	5.927937	4.141068	6.235468	6.251982	5.858872	4.619091	4.783894	5.127355	3.633884	4.189728	4.051084	5.780149	5.472341	3.688884	4.479959	5.390865	3.742824	6.430725	4.600200	3.073342	4.581826	4.360482	3.815433	4.571992	3.662649	4.626548	5.604559	5.298852	5.548054	3.507422	3.480782	3.173699	5.280349	3.848646	6.358329	5.594579	6.091820	5.263694	3.998522	4.985607	5.306753	5.606325	6.719475	6.119404	5.995187	6.896217	7.100327	6.052025	5.783785	3.536879	4.181870	4.513567	4.392359	5.132818	3.367694	4.171867	3.970758	3.650021	4.348753	3.512576	3.666461	3.486788	5.600779	5.853071	4.266350	5.840048	5.186794	5.324768	6.294223	5.491679	5.404608	5.732292	5.626357	7.429346	5.714005	5.880149	5.262064	5.182198	6.824504	6.031996	4.973155	4.084323	3.312397	5.288139	6.625846	6.351948	5.204460	6.258619	6.107970	6.814127	6.055991	6.266387	7.429685	5.919567	5.450296	4.368705	3.326468	5.582288	5.499654	3.829515	5.512606	4.423335	6.380125	6.243095	4.460921	4.513363	5.556382	9.456638	4.227886	5.766650	3.449361	4.644478	5.783785	4.852673	4.507907	7.594133	5.300411	5.895831	4.399395	4.095615	3.400891	5.147334	4.934074	6.546146	4.410020	4.809298	5.237066	3.751911	4.078269	3.251299	5.069990	4.741955	4.558533	3.594399	5.750104	3.114731	5.855263	6.395964	4.874867	6.221944	3.591339	3.551420	5.182198	5.056780	6.000261	5.631732	4.204989	5.353254	3.627023	3.395212	3.575730	4.932450	3.622038	6.699791	3.558339	4.302412	5.161575	3.647203	3.998746	4.900277	6.800254	4.768089	5.351677	6.050288	6.916362	6.693444	4.118088	6.452605	3.283615	4.035158	3.930648	3.302221	5.313797	3.260848	3.383664	3.300958	3.231727	2.838045	3.648387	3.298691	3.195567	6.572587	4.878379	3.188054	5.702804	5.045149	4.205947	5.546959	5.153391	5.226305	6.395482	4.756961	7.342373	5.083842	5.992878	6.010016	5.669624	5.391260	4.179668	4.335082	3.917257	3.428492	4.192585	7.563777	6.095321	4.151468	6.134154	6.079562	6.422692	4.650482	4.848086	6.799452	5.711575	5.052790	4.312966	3.360502	4.144088	5.252954	3.620663	3.435630	3.357076	6.354724	6.386626	4.154789	4.196022	6.880869	7.545653	3.454582	4.767656	3.544326

LINC01703	8.240832	9.561747	9.833953	9.778612	9.991091	11.218772	10.534558	10.349010	8.527214	10.986127	11.370827	11.345061	9.537676	10.283668	10.017266	10.698830	9.499181	9.577144	10.548435	10.638627	10.085546	9.663577	10.191000	9.637784	9.487347	9.734386	7.747952	10.367747	10.213349	10.094402	9.493002	9.073136	10.553085	8.818720	9.189785	9.788977	9.851210	9.258777	9.879029	9.742594	11.338789	9.668932	9.091411	9.574138	9.594486	10.085395	10.455864	9.835965	10.667399	10.128392	9.593851	9.851816	9.149853	9.970502	9.821458	10.373979	9.395127	9.943787	9.485652	9.735158	9.925756	10.308167	9.345116	9.748309	9.751120	10.126629	10.251447	9.549607	9.985950	10.199050	11.295692	11.546206	10.963392	11.568715	10.420871	9.256976	10.157573	10.569293	10.609505	9.729812	9.669428	10.253789	9.547759	9.675373	11.029872	10.568476	11.222825	12.291548	9.832460	10.510632	10.573635	9.978744	10.250726	10.188159	9.465026	9.884692	10.582604	8.340857	8.617692	8.938096	8.394381	9.374625	9.293955	9.683095	9.243504	10.791738	9.332620	9.280309	9.451406	11.226506	10.598330	9.317827	8.942934	10.922691	10.174725	6.819593	8.376183	10.354444	10.479347	8.513701	8.416105	9.949211	8.562100	6.949322	10.553536	9.816987	9.157407	9.617475	10.648336	9.605617	10.175018	9.394200	9.736856	8.503865	8.753475	9.685927	10.899829	9.712336	11.026149	8.668823	10.785271	11.014118	8.471373	11.133855	9.861767	9.161680	8.284855	8.105589	9.950904	7.557235	8.235581	8.682359	10.176378	10.253507	10.707486	9.185019	8.254672	8.830404	10.048962	10.161839	10.150081	10.423927	9.184246	9.743643	11.267354	9.148458	8.224339	11.355934	9.008249	9.109422	9.263538	11.377843	8.011638	9.164484	10.289278	9.570720	11.415437	9.825553	9.555229	9.765726	10.175307	9.709211	10.274568	9.491735	9.534452	11.428860	8.889464	10.132299	8.591468	10.156215	10.430794	10.340199	10.062130	9.389757	9.602366	10.296948	10.378034	10.558564	9.384070	9.800580	10.511234	9.225443	9.561904	9.651648	9.281202	10.892545	11.059966	10.367141	9.414098	10.706880	8.506585	10.010197	8.886682	9.752532	10.032710	9.970033	8.991352	8.512159	10.391640	8.440424	8.893482	6.399819	8.631287	10.865293	9.398637	10.292343	10.972346	10.724311	9.137949	9.228368	7.866655	7.837640	11.426951	10.595877	7.925226	8.008376	9.756381	8.988078

AC025773.1	3.846089	4.426556	4.173173	4.328092	4.471226	4.202921	4.308576	4.717928	4.702350	3.774090	4.483961	3.772186	3.469235	4.689517	4.256493	4.842558	5.126754	4.209072	4.893555	4.350467	4.753418	4.802376	3.060451	4.689279	4.508331	4.585408	4.282734	4.741525	4.380153	4.093706	4.433285	3.218685	3.922002	4.334415	4.097105	4.306356	4.159720	4.523456	4.674518	4.001479	4.359375	3.965491	4.163148	3.746476	3.708643	4.816027	3.816516	4.618396	4.248186	5.002123	4.553466	4.499207	5.357204	3.915505	4.417754	4.694948	3.999159	5.735919	4.899185	4.929972	2.884827	4.715794	4.997962	4.617724	4.979337	4.370011	5.508966	4.886886	4.658804	4.969897	3.887853	3.683451	3.816718	4.351993	3.746476	4.228779	4.108500	4.008886	3.797760	4.657286	3.413828	4.162256	3.360810	5.312014	3.746085	5.492218	5.155213	3.774090	5.081245	4.427665	4.446094	4.567683	4.583521	3.704057	3.833118	3.672132	3.677001	3.454178	3.684027	4.411994	3.602660	3.817172	4.112673	3.837782	4.371560	4.375223	4.857330	4.620375	5.062667	4.525652	4.743795	4.794361	5.068188	4.622273	4.390178	3.982847	3.862377	4.161178	4.242269	4.370219	4.739080	4.522120	4.905873	5.195106	4.762246	4.553885	4.801966	4.698314	4.682249	4.791656	4.479330	4.425470	4.307220	4.867902	4.644693	5.424047	4.438927	5.292766	4.400248	5.443514	5.241438	4.123822	4.914337	5.095208	5.480817	4.804933	5.128131	4.494685	4.815165	5.314846	3.704272	6.279314	4.236770	4.573522	4.216443	4.708459	6.370733	5.036312	4.762246	4.682632	5.164280	5.088990	5.454582	6.474750	5.973236	5.232404	5.291773	4.965208	5.445240	5.454402	5.394781	4.946727	4.736547	4.705737	4.561205	4.867232	5.553982	3.673284	4.700680	4.820024	4.419593	4.313187	4.850771	5.313603	4.919813	5.667246	4.799201	4.595957	5.391069	4.244932	4.331236	5.292766	4.802623	3.598775	4.711587	4.672062	5.696022	4.558533	4.063954	4.630478	5.378859	4.075568	4.327221	4.681557	5.676561	4.764873	4.785984	4.987884	4.693712	4.592522	5.045712	4.552175	4.849906	4.690116	4.365248	4.353756	3.848851	6.704797	4.604611	4.935579	4.375223	4.499424	4.291994	4.724322	4.531413	5.859782	5.063633	5.103993	5.623704	5.455127	4.629125	6.909692	4.945653	5.157704	4.697660	5.164897	4.698314	5.057174

AP002989.1	3.905507	4.069444	4.247330	3.752527	3.184036	3.413453	4.590365	3.040595	3.357933	3.947935	3.489710	4.418223	3.557966	3.727738	6.395964	3.489018	3.338945	3.990057	4.447569	3.604453	4.177655	4.166469	3.272546	3.736901	4.032550	4.029966	4.267843	3.909259	4.296330	4.323129	3.485005	3.318473	4.375887	3.854056	3.416712	3.957014	3.523055	3.524206	3.706665	3.669497	4.137363	3.124671	4.011621	4.606700	4.378669	4.302168	3.925015	4.339822	3.294995	4.957935	3.994857	3.321058	4.973787	3.933266	4.365486	4.469699	3.693202	4.544458	4.133608	3.746476	4.074067	4.488884	4.502191	3.082345	3.227892	3.916408	3.668479	4.166676	3.976140	3.573430	3.937514	3.337955	4.514824	3.568681	3.723511	3.763395	3.428828	3.428299	3.655820	3.602066	3.692766	3.595367	3.070982	3.740582	3.936458	3.220706	2.919147	3.442756	4.132553	4.248186	3.051939	3.331129	3.205479	3.489890	3.345748	3.351555	4.010157	3.340818	3.460924	3.576116	3.038852	4.724734	3.954599	4.123822	4.117895	4.657286	3.794458	3.753370	3.244844	4.087647	3.348203	3.187793	3.066503	4.374789	4.214696	2.938626	3.289973	3.858770	3.398011	3.348203	3.877841	3.838658	4.444999	3.165689	4.100404	4.284044	3.125395	3.328072	4.535677	4.476026	3.861275	3.937075	3.626008	6.355425	3.093751	3.307751	3.557756	3.398675	3.366686	4.444552	3.145815	4.360033	3.593637	3.526294	4.699401	3.156400	4.154082	3.597156	4.988939	3.812518	3.262344	3.592419	3.856144	3.771146	3.654009	3.580893	3.626213	3.159437	3.511083	4.017832	2.991831	3.711334	4.166676	3.829744	4.036500	3.970521	4.287203	3.392489	4.259179	3.817172	3.314652	3.655630	3.942615	4.083468	4.630694	3.380840	4.409815	4.043422	3.796720	3.115819	4.597046	3.615725	3.336976	3.388778	3.221664	3.428989	3.763395	3.262940	3.091974	3.278696	3.279752	4.366995	3.572500	3.222815	3.659231	3.539970	3.121823	3.307751	3.559346	3.728592	3.650223	3.119999	3.482975	4.495328	3.174211	3.450610	3.248562	3.881295	3.812518	3.102534	2.899468	3.749777	3.797572	3.453325	3.215034	3.470703	3.288775	3.424633	3.866608	3.264674	3.196929	3.809905	3.864105	4.443890	4.345456	2.918762	3.074437	3.248562	4.327873	3.104231	3.113210	3.003983	3.785637	2.903914	2.986081	3.036937	3.446341	3.989003

AL133464.1	3.992680	4.062633	4.125253	4.495328	5.626914	4.399395	3.960098	4.869580	6.258961	4.030399	4.364149	3.994643	3.535725	4.038443	3.142180	3.680841	4.950017	3.596131	3.712125	4.219469	4.038230	3.621076	6.624196	3.956800	3.918899	4.258947	3.869665	4.078669	3.864105	3.737498	4.192341	3.924795	3.204261	3.672507	4.003905	3.892863	4.304851	4.637809	4.295457	4.017194	4.050210	3.084967	3.974446	5.048105	3.342139	4.703430	3.911846	5.178999	3.140544	4.403745	3.940865	3.643274	4.210774	4.482201	3.457163	4.481472	3.543056	5.282991	4.954780	4.106065	4.489115	4.005892	4.728053	4.472756	3.824156	4.315607	4.521287	4.343285	4.522553	5.632831	3.949881	4.481034	3.745044	4.019348	4.081964	4.371755	4.666915	4.567891	4.597046	4.589046	3.885739	3.665470	4.330775	4.809510	4.934923	4.884736	5.540532	4.132328	5.564296	4.699871	5.481638	5.471801	3.420255	3.965972	3.862994	4.319054	4.220843	3.301909	3.435272	5.029542	5.067971	3.535179	3.972667	3.911625	4.311473	3.986069	4.277739	4.891666	4.812278	4.120517	5.107866	5.295832	4.710741	4.367632	3.173569	6.086582	4.472031	3.615932	3.679614	3.608791	4.393599	4.061298	4.502632	6.201199	4.138630	4.104747	4.445874	5.525162	4.396141	4.234172	3.638275	4.909875	3.564761	4.013585	3.748966	4.466739	4.275963	4.998590	4.177463	3.900187	4.340694	5.542981	5.456853	4.832709	4.214051	4.307689	5.389322	4.889600	4.162019	4.202709	3.984979	5.125133	4.402464	3.883596	3.641503	3.643095	4.619283	4.609293	4.771198	4.524143	5.366629	3.898693	4.002351	3.485185	4.388072	5.307898	3.924576	3.250421	4.479552	3.956800	4.717502	3.746283	3.710670	4.495125	4.491142	4.574373	2.994298	3.439414	3.047106	4.307689	3.718335	4.965410	4.147890	3.143613	4.744249	2.934273	4.519116	4.390178	5.340097	4.772404	3.895047	4.789101	4.273115	4.768288	4.729367	4.882226	4.474088	5.262064	4.320790	3.718521	3.653225	4.496646	3.343001	4.657710	5.046472	3.995290	4.125060	5.264099	4.386748	4.717292	5.639555	4.098180	4.481034	3.958573	4.011437	4.642176	4.781346	4.631969	4.164473	5.012009	4.417533	4.792890	5.106656	5.001307	4.538643	4.486585	6.890130	4.708660	4.804508	5.649908	5.839699	7.421041	4.985607	4.148555	4.396577	4.735652	4.224467	4.717717

ENTPD1-AS1	8.577737	6.954541	7.467980	8.074418	7.780146	8.669568	7.738668	8.819333	10.081486	8.452479	9.172960	8.734707	8.346473	7.692731	7.735034	8.437092	8.628513	7.592260	7.180027	8.461720	7.431742	6.685006	7.442358	8.274926	7.907550	8.411708	9.284015	7.960216	9.558934	7.837808	8.782434	6.402954	6.632208	8.098103	7.548686	7.090101	7.671162	6.949175	7.363230	7.558478	8.885915	6.821014	7.098929	7.565801	7.391400	6.168263	9.466897	5.994834	7.239214	7.082859	7.372371	7.016162	6.750533	6.882160	6.883408	9.170762	7.265725	6.898200	7.529026	6.839336	6.790070	8.434607	7.167473	7.611728	6.700968	7.018436	6.821189	6.649508	6.336194	6.813820	7.131001	7.297649	9.500568	7.060427	7.137034	7.158083	7.614047	7.301614	7.280692	6.879880	7.656711	7.390711	7.246377	7.185203	7.882174	7.277201	8.048145	7.004570	7.253085	6.913240	7.257558	7.406172	6.906870	6.877809	7.142409	7.787922	7.227941	8.659306	8.341870	8.860617	8.656105	7.614780	8.085241	6.475765	6.689974	9.036933	8.996563	6.433281	7.147857	9.121694	6.978010	8.572191	8.037132	10.007217	8.789036	9.479495	7.807111	8.672031	10.530696	8.959155	7.014664	7.418833	8.651625	8.323980	7.503538	7.698229	9.628632	9.191651	8.011709	7.675589	8.124646	8.391945	7.338470	7.083939	8.866575	8.177531	6.806609	6.073115	7.029236	6.852320	5.050186	6.609329	6.849029	6.506176	6.327567	8.765747	7.372533	10.538517	7.362762	8.730470	8.030739	7.547429	7.512341	7.332577	6.734147	7.439160	6.993859	7.644771	6.776373	9.510217	6.065057	6.969671	8.205864	6.098553	5.152145	8.744503	6.553821	6.748333	6.606041	6.813820	6.733689	5.351297	7.234989	6.615749	8.042008	7.231968	6.036696	7.476764	6.131199	6.393714	8.261975	6.797498	7.303979	6.888860	7.418220	6.556152	6.197911	6.337219	6.649358	6.965797	7.287971	7.867408	7.518657	7.758541	7.239737	7.656711	7.509564	7.138922	6.998626	7.243086	8.091866	7.739616	8.336175	7.217334	7.444435	7.468472	7.234838	7.310568	7.515375	7.112068	7.964491	7.345011	6.525307	6.900625	7.273894	7.878566	9.139811	8.243691	7.698669	7.088332	7.748405	8.855177	6.365202	7.119808	8.581064	8.687456	6.768967	5.652204	8.014946	6.688548	8.711767	8.553021	9.566504	8.250361	7.792490	7.568409	7.812644	8.768833

AC004540.1	5.750655	5.675825	5.587720	5.978683	6.231428	6.691269	6.228838	6.537941	6.335357	6.334681	6.770091	6.704635	6.227804	6.454610	5.983672	6.302189	6.503324	5.681213	5.371678	6.344049	5.860123	5.521370	4.268466	5.726105	5.650297	6.799953	5.888841	7.738668	6.469852	6.366378	6.438750	5.122714	5.608157	5.979804	5.574273	5.937938	6.426975	6.105503	6.015535	6.002186	6.555983	6.016557	5.535095	5.917236	5.206236	4.709048	7.301928	6.810819	6.063646	6.143072	5.973440	6.205578	5.540710	5.818516	5.833177	6.375503	5.561114	6.413534	6.252821	5.637139	6.241596	6.144617	5.420358	6.150695	5.623364	5.611223	6.267913	5.883320	6.225380	6.374349	5.845843	5.683252	6.557965	5.609543	5.718155	6.077122	5.754253	6.012483	6.108872	5.449370	5.403273	5.724374	5.403667	5.663513	5.580774	5.783971	5.693220	5.594019	5.866856	5.990781	6.058707	6.527668	6.035818	6.141377	6.224688	5.875145	5.562770	6.022574	6.584561	5.893329	5.963587	5.434147	5.752475	5.870227	6.049418	6.309261	6.757330	5.945684	6.383495	6.602789	5.684361	6.662500	6.655979	6.503324	6.435274	8.130580	5.572745	6.251818	6.464205	6.368863	6.329072	5.649152	6.755724	6.888860	7.260440	6.752706	6.067343	6.591021	6.862341	7.146440	6.865024	6.904609	6.208650	6.598929	5.812805	6.051653	6.467545	6.323058	6.480576	6.059216	5.759442	6.986195	6.567744	6.528989	6.663789	6.538445	7.603445	6.794633	6.903528	5.704106	6.155871	5.872074	6.707042	5.801382	6.458196	6.110971	6.463376	6.430895	6.315207	6.987783	5.867966	6.066254	6.046918	5.884247	5.645988	7.624722	6.498399	6.217833	6.012970	6.262852	5.759442	5.375957	5.488546	5.562034	6.627577	5.835000	5.305796	5.634715	5.235030	5.687539	6.636280	5.769392	6.104430	6.030425	5.756267	6.078831	5.870430	6.113851	5.934593	5.621849	5.628403	7.472343	5.617223	5.614247	6.205920	5.198317	5.872074	6.118891	4.857125	5.314218	5.717290	5.416410	4.722578	5.385758	6.414041	5.253728	5.528583	5.753396	5.465392	6.477812	6.189761	5.839699	5.929876	6.174379	5.923827	6.030923	6.161322	6.322380	6.106910	6.308756	5.759620	6.550531	6.032173	5.449926	7.013182	6.306611	5.496067	6.192830	7.142089	5.984369	6.528989	7.525891	7.278325	7.016496	7.149441	5.907765	6.207781	6.700141

AL590705.2	4.767009	5.678263	5.270834	5.749031	5.425386	5.649152	5.404268	6.563191	6.355256	5.375957	4.638012	4.691364	4.365030	4.390178	3.547366	5.323047	6.430386	3.928725	5.015261	5.387789	7.333066	5.774175	6.191997	5.723098	5.031741	5.417979	4.684809	6.243249	4.808066	5.173357	7.064140	4.888552	5.066538	5.445240	4.657286	5.107256	4.662858	5.868488	5.618375	4.697031	5.221908	5.703743	5.067563	5.715301	4.956452	5.262672	4.333762	5.789578	5.054079	5.444298	5.271639	5.941126	5.375539	5.176137	4.876960	5.497164	4.120097	6.058868	5.750104	4.944128	5.736270	5.048505	5.965177	6.080235	5.533140	6.135334	5.950215	6.154122	5.670843	5.979585	5.445055	5.154379	5.227278	5.262870	5.377508	6.011402	5.669981	6.011588	6.003417	5.518583	5.132818	5.137404	5.399987	5.619100	5.426425	4.462632	5.158863	5.721949	6.698868	6.865815	6.425117	7.291614	5.316861	5.703923	4.856480	4.773047	5.370132	3.715469	3.998094	4.210561	3.627023	3.983281	4.613377	4.440018	5.811679	5.214626	6.467703	5.644627	4.729367	5.957324	6.547740	6.309261	5.798018	5.252360	5.665156	4.689059	5.565213	5.251581	4.896846	5.727541	6.163391	5.565213	6.131041	5.507796	6.416507	5.330272	5.867615	6.387666	5.950046	5.501991	4.365913	5.362905	5.467532	5.675316	4.946094	6.441853	5.081456	5.532003	5.945514	6.257247	5.296034	6.583114	6.051126	5.966803	5.966279	6.160979	6.465546	5.414539	6.050288	6.734147	4.817712	4.421729	6.437583	5.329500	5.548432	5.516058	5.870061	6.092872	5.653855	6.043958	6.291154	5.349077	6.543218	4.889190	4.958929	5.917588	6.061355	5.440433	6.783072	6.163054	5.549397	5.557935	5.856542	4.434540	5.965900	3.861486	6.181859	5.411685	5.728635	6.366226	5.857773	6.516905	6.719133	6.189239	6.136200	6.448187	6.205020	5.918522	6.866135	5.830666	5.639162	5.874232	5.539006	5.143974	6.492516	6.135515	6.286218	6.416667	5.459435	5.715491	6.186901	5.777077	5.170495	5.763590	4.279291	6.228660	5.746855	6.786300	6.549863	6.729161	7.484272	5.439863	6.287577	5.358725	5.247211	4.612282	3.785421	3.518201	5.200277	5.350035	4.772404	4.752528	4.581620	5.746855	6.025527	6.722079	6.157021	5.736270	6.606890	6.171845	6.289603	5.237665	5.493183	6.132434	5.106656	6.365363	5.477861	5.883320

AL590705.1	4.767009	5.678263	5.270834	5.749031	5.425386	5.649152	5.404268	6.563191	6.355256	5.375957	4.638012	4.691364	4.365030	4.390178	3.547366	5.323047	6.430386	3.928725	5.015261	5.387789	7.333066	5.774175	6.191997	5.723098	5.031741	5.417979	4.684809	6.243249	4.808066	5.173357	7.064140	4.888552	5.066538	5.445240	4.657286	5.107256	4.662858	5.868488	5.618375	4.697031	5.221908	5.703743	5.067563	5.715301	4.956452	5.262672	4.333762	5.789578	5.054079	5.444298	5.271639	5.941126	5.375539	5.176137	4.876960	5.497164	4.120097	6.058868	5.750104	4.944128	5.736270	5.048505	5.965177	6.080235	5.533140	6.135334	5.950215	6.154122	5.670843	5.979585	5.445055	5.154379	5.227278	5.262870	5.377508	6.011402	5.669981	6.011588	6.003417	5.518583	5.132818	5.137404	5.399987	5.619100	5.426425	4.462632	5.158863	5.721949	6.698868	6.865815	6.425117	7.291614	5.316861	5.703923	4.856480	4.773047	5.370132	3.715469	3.998094	4.210561	3.627023	3.983281	4.613377	4.440018	5.811679	5.214626	6.467703	5.644627	4.729367	5.957324	6.547740	6.309261	5.798018	5.252360	5.665156	4.689059	5.565213	5.251581	4.896846	5.727541	6.163391	5.565213	6.131041	5.507796	6.416507	5.330272	5.867615	6.387666	5.950046	5.501991	4.365913	5.362905	5.467532	5.675316	4.946094	6.441853	5.081456	5.532003	5.945514	6.257247	5.296034	6.583114	6.051126	5.966803	5.966279	6.160979	6.465546	5.414539	6.050288	6.734147	4.817712	4.421729	6.437583	5.329500	5.548432	5.516058	5.870061	6.092872	5.653855	6.043958	6.291154	5.349077	6.543218	4.889190	4.958929	5.917588	6.061355	5.440433	6.783072	6.163054	5.549397	5.557935	5.856542	4.434540	5.965900	3.861486	6.181859	5.411685	5.728635	6.366226	5.857773	6.516905	6.719133	6.189239	6.136200	6.448187	6.205020	5.918522	6.866135	5.830666	5.639162	5.874232	5.539006	5.143974	6.492516	6.135515	6.286218	6.416667	5.459435	5.715491	6.186901	5.777077	5.170495	5.763590	4.279291	6.228660	5.746855	6.786300	6.549863	6.729161	7.484272	5.439863	6.287577	5.358725	5.247211	4.612282	3.785421	3.518201	5.200277	5.350035	4.772404	4.752528	4.581620	5.746855	6.025527	6.722079	6.157021	5.736270	6.606890	6.171845	6.289603	5.237665	5.493183	6.132434	5.106656	6.365363	5.477861	5.883320

AL050341.2	10.438091	10.303719	10.265433	10.158664	10.476474	9.495049	10.638766	9.989806	9.087482	9.615452	9.958780	9.666389	9.877509	10.263794	10.792489	9.594166	9.856672	9.733340	9.349567	10.282465	8.810123	9.246593	10.468887	9.916366	10.103567	9.492722	8.538318	9.511023	9.602218	9.991091	8.763320	9.807593	10.898956	10.775784	11.036851	11.168397	11.095424	10.303719	10.438998	10.419200	9.718286	10.932488	11.007420	10.303868	11.495500	10.833585	9.059743	11.031779	10.883860	10.575116	10.800096	10.811946	11.171084	10.842709	10.756929	9.600027	10.922845	11.757848	11.031068	11.423761	11.680678	9.358026	11.205234	10.599097	11.991560	11.222342	11.498297	10.590818	11.349792	10.914464	10.719738	10.471332	9.417638	10.413585	10.613186	11.013825	10.810938	10.560245	10.659045	10.624589	9.980403	10.824136	10.510494	11.028132	11.125198	10.867283	10.813266	11.018523	10.189200	9.932911	11.431360	9.671482	10.232649	10.413447	10.596659	10.952091	10.638766	10.030230	10.345960	10.532370	8.883274	10.443646	10.035789	10.517131	9.994464	9.504734	8.742249	9.987919	9.145041	9.108604	10.567549	10.975539	8.688048	8.900672	10.083424	8.334787	11.093256	9.881018	8.874966	11.571927	10.181790	10.542688	9.890146	7.563944	9.377775	9.239370	10.152177	10.040077	9.137166	8.723730	10.489668	8.727572	13.306191	9.262763	9.972959	10.438390	8.644846	9.387152	9.946536	8.846939	9.212976	9.840456	8.995337	11.280333	10.425006	9.321212	9.517296	8.218720	9.767395	8.567308	9.174191	9.983209	10.693165	10.951630	10.895803	10.767132	11.092968	9.805482	10.502569	9.389452	13.716792	10.911326	11.206436	10.955883	10.736083	8.547528	13.512856	11.095709	10.881267	10.273491	11.285631	10.642239	10.156965	10.511848	9.643014	10.850468	11.635258	11.246940	11.623726	12.583952	9.145192	11.519246	11.286577	12.756522	11.739525	12.903931	10.425295	11.146945	11.464636	11.011088	11.162452	8.196546	11.232126	11.517367	10.672565	11.277646	11.023937	10.072985	10.966241	9.914535	11.641016	11.193556	10.627462	11.238920	9.980403	10.852247	11.740720	9.887683	9.250544	11.744952	9.699395	11.276880	10.806779	10.361744	10.221376	10.266879	8.534543	11.033609	11.949129	9.362308	11.742441	10.777670	10.894897	12.119428	9.100998	9.562068	10.204147	9.936352	8.917711	10.116680	11.140081	11.665355	7.701592	10.747087	10.769403	12.580816	12.105127	9.817990

AC022145.1	4.347616	3.450236	3.298831	3.873530	4.445425	4.252047	3.300958	5.230986	6.312121	3.715868	4.727844	4.023218	3.460015	4.258947	3.790147	3.834808	4.983403	4.523906	5.438752	3.903586	5.266067	4.733340	4.059769	4.248826	4.817310	4.744249	4.408498	5.244418	4.427875	4.523456	4.416874	3.529405	2.941020	4.388470	3.347210	3.934702	3.653225	3.181900	4.061298	3.708838	3.737108	2.913922	4.455228	4.170075	4.554733	4.749977	4.959791	3.406592	3.134992	3.639699	3.629852	3.682691	4.090033	3.710057	3.496532	4.410887	3.567103	3.042603	3.560924	3.298691	3.113539	3.956576	5.586787	5.373609	2.978709	3.629437	2.921236	3.570038	3.319776	2.919250	3.270828	3.391393	4.120097	3.089903	2.904417	3.157430	3.542870	3.432717	3.027358	3.385183	3.216236	3.838658	3.114145	4.249061	3.227610	4.597655	3.885958	3.422057	2.886294	3.035774	3.094660	3.034213	3.046203	3.478586	4.132553	3.513562	3.604836	5.275538	4.893555	5.599672	5.612706	3.552782	4.866428	4.892281	3.115214	4.429795	6.565450	5.300214	5.373984	4.249492	4.883487	5.515242	4.757595	5.873876	4.858971	6.619878	3.696123	4.363938	3.976335	3.803284	4.629357	3.671565	4.123402	4.796678	4.824555	4.526110	5.028289	4.624008	4.820657	5.319005	4.205490	4.871035	4.112234	4.502191	4.640113	4.278403	4.321413	5.129592	4.337686	4.350669	4.421071	3.600679	5.672408	4.560116	4.002553	5.193853	5.195497	4.795393	3.597536	4.742152	4.061055	2.996629	4.963547	4.045188	4.321207	4.408936	3.399850	5.020133	4.553015	4.710948	2.876759	3.365160	3.723702	4.037357	4.475377	5.248396	3.541317	3.218278	4.530516	5.202650	3.596729	3.642881	3.828629	4.134718	4.305090	4.356986	3.050364	4.921470	3.202692	2.990717	4.786821	5.226669	3.952698	4.477408	4.991007	5.094168	3.522057	3.994643	2.963837	3.564573	3.411297	5.102586	3.476705	3.113096	3.581694	3.601857	3.623070	3.263968	5.112145	3.525894	4.028609	3.186045	3.854271	3.617462	5.049361	4.115483	4.103651	2.861800	3.937075	3.199539	3.018838	3.448991	4.243420	4.081133	3.974642	3.572500	5.233633	5.392969	4.675590	4.641333	4.655105	4.892731	4.531822	3.231044	5.240856	5.755531	4.704262	4.870422	4.918935	4.624606	5.724934	5.060038	6.237788	5.187204	4.865575	4.539755	4.205947	4.562063

AC009478.1	5.746855	3.365817	3.240744	3.368594	3.869665	3.381552	3.848412	3.389306	3.523658	3.506641	4.063276	3.828629	4.066903	4.998590	4.218149	3.386728	3.728814	5.484266	5.172120	3.501419	5.595686	4.586482	3.818420	3.444542	4.401336	3.773677	3.054044	5.317069	4.176392	3.082998	3.952050	4.364402	3.339114	3.170245	3.756112	4.190163	3.829296	3.563042	3.578380	3.305708	4.353756	3.984336	3.640310	3.437732	3.739136	5.556382	3.572687	4.081358	3.724101	4.194339	4.161815	4.017403	4.384713	4.251723	4.522120	3.931093	4.225750	3.516786	4.290904	3.941720	3.775945	3.795304	3.800090	3.204801	5.899028	3.876112	2.863100	3.411476	3.201820	4.018944	3.262627	3.220568	5.469490	3.389306	3.015866	3.344161	3.107143	3.404213	3.241621	3.878499	3.431343	4.136501	5.437457	4.322455	3.368911	6.057108	3.225561	5.254349	3.153805	3.666689	3.367694	3.373733	3.210577	4.703861	3.193659	3.721688	3.915505	4.119663	3.749198	3.059490	3.323316	3.316355	3.525894	4.196900	4.456929	4.394991	6.120458	3.564761	3.453834	3.305388	3.150869	3.002910	4.459459	3.817794	3.253718	3.216664	3.284276	3.779584	5.348134	5.846583	3.515480	3.072446	5.056167	3.185015	3.799662	2.998611	3.278205	3.547366	3.236658	3.527760	3.233635	3.077141	4.431158	3.770910	2.998784	3.676812	3.965731	3.498362	3.474700	4.864543	2.820203	3.281645	3.341985	4.397643	3.027576	2.895525	2.921905	3.112229	3.589579	3.546557	4.106979	3.372751	3.321225	3.652214	3.873314	3.886347	3.738084	3.533846	4.000224	3.230761	3.745894	3.681816	3.525695	4.232008	5.285388	3.317112	4.136714	4.031890	4.456263	4.277507	3.877169	4.106065	4.420876	3.536493	3.082648	3.788448	3.261151	4.129377	4.100634	5.340278	3.555075	3.809533	3.716485	4.890032	3.578380	3.365508	3.778359	3.364370	3.126876	3.088208	2.923588	3.250569	5.413006	2.963837	3.326289	3.039394	3.189289	3.680036	5.480817	3.812518	4.524558	3.727738	3.745473	3.542686	5.225696	3.518911	4.176172	3.034513	3.565517	3.501981	2.897683	3.131467	3.231044	3.608409	3.062027	2.948932	3.143964	3.783146	3.316628	3.061561	3.077141	2.959195	3.013777	4.791432	3.858341	3.760284	3.643658	3.523055	4.698120	2.804194	3.101606	4.888354	3.277250	3.010039	3.036658	3.277400	4.709267	4.048661

SATB1-AS1	7.067141	7.806324	7.696379	7.086906	5.151120	6.750533	7.064609	6.397288	5.506211	6.032361	6.302543	7.079890	6.109076	4.894172	6.203825	7.265880	6.114580	6.739736	6.996619	6.742265	8.624195	8.957785	9.053917	9.006263	6.998180	4.813729	9.348168	6.132261	6.725888	8.782271	6.125147	7.964491	9.250707	8.573650	7.950786	8.082398	6.248637	6.626630	6.485132	7.680560	5.131601	8.062875	8.976069	7.608684	7.690085	7.537013	5.271639	7.922409	7.852039	7.056336	7.990967	6.764246	7.523112	7.136551	6.930985	5.531818	7.450429	7.614639	7.342696	7.078154	6.942471	5.411869	7.701453	6.991935	8.071679	7.166404	7.747797	6.047988	7.888585	7.701304	7.385917	6.364215	5.486729	7.044822	6.866463	8.454898	6.742581	7.965097	7.465403	7.231821	7.261055	7.453093	7.998563	7.880066	6.957844	7.607888	7.937139	7.035762	9.166648	8.092604	8.597328	8.972481	7.407740	7.232439	8.712662	7.499939	7.547904	5.151351	5.642536	6.106563	5.354518	7.411631	6.562022	5.911679	8.216812	7.015335	5.933150	7.880939	6.282784	5.917082	6.028736	5.922158	6.242444	6.186901	6.586228	6.297403	6.542716	6.431739	5.649152	7.912361	7.345011	8.080063	7.859886	6.229537	6.305958	7.188088	9.549294	6.127483	6.937304	7.283976	7.894196	5.440614	6.303916	5.702228	8.142105	7.436471	8.594088	9.149407	8.897149	7.809098	7.207529	9.742277	8.791209	6.459372	8.233737	6.718290	5.387388	6.200170	8.181745	6.890923	7.772768	8.706598	6.782999	6.774623	8.551354	8.601432	8.272535	6.032910	7.523591	8.482792	8.793679	8.480778	6.969980	7.971462	8.282080	5.083448	8.592859	8.460319	7.185203	8.285318	7.539578	7.806501	6.578148	7.439309	7.450737	9.160301	8.154688	7.884003	8.957459	8.646482	7.388571	8.023620	7.629708	8.054626	8.591616	7.388571	8.373422	7.193889	7.811708	8.468700	7.710499	6.549547	7.512950	8.570802	8.467264	6.713426	9.064439	7.881227	7.710952	6.979716	7.597944	8.016331	8.945362	8.658992	7.174938	7.832466	9.090210	9.066762	8.598194	8.629339	7.675124	8.263083	6.152579	7.518197	7.004570	6.583425	7.021874	5.855636	8.498529	4.579040	6.928920	6.540510	7.793105	7.732109	7.625464	6.866291	8.978787	8.849892	5.125562	8.421205	7.668657	8.812028	6.248466	8.137408	8.068471	7.183603	6.649192	7.204562

AC004083.1	7.014664	7.577303	7.770324	7.582246	8.547074	7.763140	7.390251	8.851059	9.389914	7.724062	8.123096	7.812644	8.005597	7.978468	7.693526	8.035557	8.131646	8.073029	8.326035	7.571876	7.894029	8.307762	7.821163	7.810799	8.158381	8.081629	8.046089	8.289941	7.742874	7.800683	8.445308	6.862821	6.796372	7.468615	7.237972	7.391479	7.307270	7.367980	7.780760	7.586300	8.062875	7.364365	7.446825	7.697302	7.572932	7.355023	8.464437	7.576995	7.651747	7.511385	6.991111	7.327894	6.714396	6.671061	7.027965	8.185578	6.725739	7.111288	7.105493	7.257719	7.095312	7.910344	7.651114	7.979416	7.011554	6.972117	7.439160	7.845933	7.166091	7.604670	7.423367	7.661681	7.934648	7.505232	7.481214	7.075764	7.338002	7.106590	7.218109	7.063971	7.387462	7.083787	6.769132	6.583114	6.892352	7.165196	7.588731	7.055577	7.217178	7.064609	6.976898	7.425554	7.099873	7.279252	7.594740	7.582572	7.304762	8.066972	8.608784	8.534845	8.515034	7.466031	8.163962	7.811267	7.730715	7.955709	8.855318	7.942332	8.943236	8.439490	8.618151	8.693709	8.422852	8.628513	7.893878	10.336136	7.707128	7.815753	8.394685	7.518421	7.902121	7.944780	7.975388	9.435860	8.106649	8.114097	8.504471	8.916335	7.970847	7.993626	8.583138	9.228821	7.728256	8.094770	8.111326	8.096450	8.601432	8.697899	7.405370	8.345535	8.066401	7.785295	8.776297	8.345381	7.787922	8.070641	8.513066	9.462564	8.048145	8.428664	8.610765	7.808778	8.056835	7.574659	7.667444	7.478884	7.949528	8.197327	7.889199	8.996563	7.767922	7.454918	7.587802	7.597626	7.348804	8.744959	7.437391	7.470633	7.978789	7.592104	7.617095	7.398195	7.173384	6.988738	7.838309	7.424608	7.364513	7.995191	6.982874	7.212144	7.943875	7.768537	7.933559	7.546796	7.034659	7.691976	7.706860	7.890305	7.717516	7.706860	7.804477	8.948426	7.920886	7.736809	7.424306	7.433448	7.646194	7.735034	7.068415	7.609761	7.214668	7.048505	7.005493	7.338924	7.895271	8.188953	7.473694	7.587193	7.535484	7.727960	7.557235	7.433129	7.358246	7.640482	7.759759	7.784873	8.685838	8.448133	7.929878	8.330105	8.108026	8.469643	7.942799	8.456268	8.160080	8.849892	7.723620	7.694438	8.835707	8.943397	8.855318	9.988254	9.251636	7.916120	9.073286	7.842303	7.873357	8.326365

LINC00535	4.443695	4.155600	3.726946	4.748729	4.105200	3.862777	3.443822	3.987976	4.389151	3.737108	3.436352	3.562676	3.155688	3.485375	3.053955	3.412420	3.834608	4.248379	5.803485	3.469063	3.668671	3.919983	3.610154	3.561567	2.937738	4.074943	4.226166	3.142064	3.381903	3.327729	6.125302	5.453616	4.474088	4.012684	3.521682	4.713705	4.166006	4.549553	4.422440	4.115928	3.236383	4.232008	4.323583	3.551024	3.964222	4.431388	3.493546	3.674319	3.396622	3.812518	4.886247	5.419596	4.441091	4.487836	3.271303	3.419704	4.636077	4.177655	3.764916	4.256960	4.844447	3.921454	7.225445	4.220612	5.259268	4.317809	3.281645	4.426556	5.161670	4.514824	4.219469	3.970758	3.369959	3.932805	4.040380	5.413006	3.934702	5.025654	4.065006	3.376527	4.435180	3.620077	4.580573	3.880019	4.578414	3.108051	4.984580	4.567453	3.900421	4.992675	4.695568	5.043462	5.035911	4.378001	3.788669	3.945317	3.617825	3.527928	3.048168	3.399529	3.428139	3.573430	3.247223	3.919566	3.575527	2.918334	4.595326	3.987153	3.403704	3.078635	4.681557	4.744005	4.786621	3.047512	3.114798	4.731657	3.853131	3.803896	3.414352	5.029542	4.228110	4.563629	4.638012	4.488050	3.941069	4.693921	4.055845	4.443074	3.769711	4.265308	4.264885	4.199007	3.347120	2.956087	4.452439	4.665824	4.333762	4.241406	4.245569	3.343324	3.853359	5.964657	3.465394	5.742261	4.152749	4.298115	4.684809	3.774707	3.406067	6.558766	3.710670	4.455432	3.330497	3.101167	4.734206	3.819283	4.470128	4.582030	4.728053	3.527038	2.909382	3.760508	3.972872	3.633884	3.763395	3.218112	3.541683	3.349197	3.855323	4.749521	4.856480	3.522236	4.015628	6.223823	4.124030	3.896720	4.115928	4.144522	4.521910	4.764632	4.020223	4.715794	4.392978	4.291335	5.186019	3.306029	4.770546	4.166893	3.803679	4.012684	4.516143	4.404426	4.685684	4.078461	4.192341	4.119254	5.219369	4.391694	5.116218	3.566109	3.709474	3.326289	3.471226	4.392582	4.500267	4.408699	4.478041	6.425977	5.785779	6.425977	5.713082	5.292766	3.925693	3.436697	4.023916	3.812920	3.782519	3.336436	3.772832	5.914194	3.948568	4.840860	4.823328	7.332417	3.359648	4.721070	3.589398	4.706397	3.323316	4.905251	5.058170	5.512770	3.797353	3.665678	4.486379	5.835368	4.292225	3.120613

AC018552.2	10.484421	11.293081	10.792489	11.327777	11.597568	11.742737	11.067181	12.086204	12.115229	11.444260	11.480227	11.280895	11.377558	11.059375	10.613340	11.699941	11.618460	10.907937	11.862005	11.316909	11.131496	12.016602	11.180060	11.208087	11.175685	11.459949	11.334799	11.917282	11.924735	11.809331	11.759040	10.283384	10.418587	10.711692	11.010198	10.511412	10.547330	11.025247	11.637244	11.169002	11.563886	11.055538	11.066449	11.323062	10.584768	11.141637	12.054081	11.634958	10.991194	11.642537	10.864090	10.844879	10.383504	10.638482	10.467258	11.778520	10.307130	10.880464	10.713717	10.241203	10.988232	12.082750	11.654083	11.433163	10.746036	11.007714	11.308100	11.232265	10.976418	11.588472	11.540839	11.360757	12.224905	11.050340	11.149918	10.836761	10.871747	10.919707	10.670015	10.008811	9.993380	10.052797	9.394660	10.001641	9.874226	10.302930	10.377424	10.258997	10.415709	10.679279	10.460708	11.011833	10.559632	10.680811	10.406639	10.406224	10.604165	11.097215	11.617879	12.015060	11.865925	11.296591	11.505545	11.173855	11.620096	11.962317	11.650051	11.467784	11.845695	11.977145	11.880421	11.658633	11.225924	12.215771	11.722901	12.979495	10.509472	10.768481	12.088432	10.393108	11.519102	11.694693	11.433004	13.247512	12.049502	12.022667	12.483120	12.188415	11.585010	11.814926	11.361808	11.990637	11.575684	11.743043	11.772261	11.368020	11.375308	11.852324	11.455375	11.575527	12.084324	11.293950	11.965821	11.729465	11.519102	12.013873	11.661797	12.548924	11.941351	11.482399	11.465093	11.272361	11.414710	10.621887	10.486117	11.029412	11.517507	11.301442	11.216559	12.553785	11.171227	10.249401	11.348487	10.588938	10.955581	12.989969	10.941674	10.091582	10.752793	11.167684	10.422396	10.362213	11.074858	10.992097	11.906665	9.699083	10.310211	10.720056	10.270574	11.188740	12.224905	11.015291	11.231267	11.040899	10.850468	11.617713	11.729158	11.240923	11.795854	11.217736	11.522478	12.282409	11.439262	11.108364	11.330888	11.520092	11.044439	11.066165	9.802412	9.928472	9.991246	10.227757	10.503777	10.147476	11.209534	10.442439	9.605154	10.602115	10.808104	10.612741	10.654587	10.712574	11.171659	11.089762	11.400756	11.118998	11.486995	11.873552	11.123825	11.716302	12.285680	12.018886	11.430579	11.348648	11.447635	11.522936	10.684060	10.815810	12.671216	11.537024	11.748029	12.234198	12.474915	11.710886	11.678446	10.899829	10.841861	11.938498

LINC02443	6.022574	6.465705	6.549386	6.543526	7.172144	6.624032	6.127120	7.650959	7.807549	6.460187	6.596780	5.779447	6.777993	7.452761	6.433787	6.849652	7.060280	6.828154	7.174793	6.672024	6.529826	8.236995	6.424977	7.359016	7.266775	7.510625	6.616765	6.543218	6.333836	6.876112	6.416667	6.200316	6.137696	6.633056	6.510765	6.212680	6.012785	6.650152	6.655496	6.871479	6.345747	6.574654	6.551327	6.677352	6.183618	6.891740	6.912442	6.397635	6.151391	6.625846	6.333155	6.911468	5.894247	6.944879	5.971960	6.554166	5.657036	6.309603	6.153773	5.577584	5.429406	6.485460	6.691111	6.175920	6.333671	6.008962	6.969499	7.013469	6.827484	6.186409	6.265036	7.044502	7.125777	6.311292	6.614473	6.375503	6.166148	6.486942	6.217146	5.576665	5.836808	5.635100	6.036011	6.312121	4.974843	5.409120	6.573050	5.870430	6.456040	5.670490	6.586545	5.959594	6.442193	6.670915	6.171845	6.323720	5.561304	6.708488	6.549020	7.085071	7.022026	6.529995	6.212338	6.429874	6.844707	6.865024	7.207379	7.696224	7.277515	7.364986	7.146440	7.432187	7.739929	6.640971	6.394353	8.990334	6.301153	6.605446	6.460844	6.594381	7.042589	7.002279	7.420235	8.369861	6.560378	7.056646	8.578764	8.566875	7.111288	6.482390	7.400837	7.180178	7.406634	6.785321	7.429995	7.324386	8.641591	8.593171	6.928432	6.999824	7.742243	7.210504	7.795559	7.797613	7.250720	6.767147	7.473387	7.517425	6.616082	6.838192	6.770246	7.367980	6.838834	6.327896	6.645475	6.277178	6.944724	7.172461	7.374539	7.897749	6.906051	8.215914	6.339228	6.327414	7.031715	7.330232	7.368131	5.987193	6.939673	6.365038	7.543264	6.243786	7.071494	6.691766	7.213117	6.921772	6.920025	7.150909	6.609956	6.923538	6.552518	7.108364	6.812101	6.614638	7.199296	7.337844	7.233231	6.930985	6.504481	6.126600	7.347217	7.415948	6.528837	6.959086	6.307403	5.981511	7.067906	5.948433	6.251304	6.565927	6.170659	6.110778	7.152494	5.569226	6.442193	7.121790	7.036077	7.665138	5.838236	6.310763	5.955394	6.955972	6.776202	6.953139	6.579946	7.292400	7.295865	7.004242	6.973517	6.146530	7.543264	6.753221	7.012038	6.819593	7.210504	7.600924	7.371267	6.806609	7.682746	7.730239	8.464437	12.799536	6.799110	6.938569	6.934574	6.773490	6.995380	6.805088

Z95115.2	11.278240	10.975234	10.553379	10.964156	11.486837	11.554325	10.965351	11.329825	11.860703	11.202995	11.358100	11.721993	11.379904	11.468967	11.206863	11.195173	11.639945	11.339525	10.284731	11.160233	10.337633	10.919567	10.500161	11.256626	11.151708	11.875511	11.518565	11.684367	11.624432	10.979516	11.613750	10.519634	10.444741	11.325406	10.467123	10.458181	10.490863	10.621163	10.479041	10.734602	11.384675	10.330389	10.689027	10.684350	10.394903	10.734310	11.802240	10.982623	10.571850	10.666359	10.835737	10.556465	10.544466	10.613340	10.534701	11.471231	10.476015	10.566794	10.889665	10.682588	10.978923	11.178849	11.208214	10.255734	10.379251	10.725060	11.105444	10.617160	10.920184	10.783768	11.022131	10.986432	11.546662	10.764768	10.950287	10.896734	10.938211	10.646057	10.974503	10.428215	10.583183	10.557368	10.477996	10.339450	10.265433	10.597699	10.482535	10.251752	10.307130	10.642844	10.180089	10.412086	10.489958	10.779258	10.920907	11.131212	10.756929	11.407214	11.428719	11.516358	11.837992	11.143394	11.410682	10.955140	10.303096	11.251658	10.377566	10.422993	10.721933	11.252802	10.230212	10.836621	11.487432	11.978054	11.202548	13.666222	11.101213	11.306499	11.689439	11.083792	10.468887	10.501830	11.107772	12.406352	11.370065	11.729608	11.892907	11.681120	11.500503	12.336360	11.715866	12.324058	12.044248	11.275334	11.177620	11.367280	11.788526	10.253086	10.379105	10.246175	11.013535	10.252933	11.821556	11.044169	11.499354	11.134006	12.037461	13.309752	10.731036	11.840676	11.029110	10.814170	11.164515	10.684350	10.131242	10.299569	10.784974	10.386560	10.610931	12.246555	10.461142	10.492203	10.341418	10.359924	10.325194	12.343274	10.672565	10.577828	10.225283	10.140602	10.245723	10.240898	10.078051	10.611210	11.140228	10.251178	10.173505	10.779258	10.046320	10.599248	11.230646	10.192202	9.863172	11.094441	11.286261	10.510212	10.567866	10.867134	10.960769	10.749455	10.464310	12.628852	10.584051	10.576915	10.688128	10.632749	10.587915	10.708399	10.165968	10.380607	10.541527	10.332062	10.190413	9.914396	11.183319	10.133149	11.061851	10.458181	10.691177	9.854464	10.158031	10.742311	10.638329	10.928007	11.379601	11.895619	11.961593	11.701015	10.759701	11.453283	11.318368	11.846272	10.820704	9.901485	12.043340	10.474667	10.204454	9.901303	12.928492	10.479789	10.808421	12.078825	12.003998	11.523246	11.846978	10.542264	11.410252	11.444132

SNHG28	6.503655	6.789740	7.063822	6.839672	7.101718	6.471755	7.437251	5.416994	5.494505	6.766039	7.438844	6.780704	7.304439	7.104070	7.409741	6.534525	5.572561	7.296370	4.502191	7.415219	5.577951	4.620607	4.213192	7.113452	6.783864	7.372217	6.049762	6.646901	6.880073	6.197741	5.657957	7.323456	7.268933	6.681903	7.101269	7.151068	7.334897	7.160446	6.041080	6.585387	6.796039	6.492516	6.608020	6.508622	7.548986	6.890614	6.499228	6.972302	6.871784	7.135167	7.195198	7.328659	7.067750	7.950478	7.835387	6.856901	7.448876	6.262702	5.337135	7.279413	5.894762	6.547258	4.876960	4.067119	4.975271	6.848878	6.375503	7.109658	6.562696	5.679570	6.755884	6.832128	7.000313	6.981330	6.740986	6.407869	6.430895	6.195129	6.838519	7.422754	7.649346	7.088478	7.710641	7.369686	7.313043	7.731029	6.916033	7.422597	3.767990	6.435793	6.426482	4.962329	7.332745	7.269546	6.921619	7.355023	7.851569	7.477847	6.708790	7.539896	6.049418	7.227793	7.048505	7.421823	4.114172	6.554786	6.144448	4.052563	4.980732	5.959854	3.745044	6.154648	6.022574	6.784020	5.926321	4.381162	5.917953	7.125950	6.236490	5.355303	5.446414	7.981607	6.278795	4.445100	8.105890	7.097815	6.724812	6.613816	8.114097	8.219476	6.353937	6.187221	6.091481	7.026662	7.106932	5.813341	7.457297	5.369930	9.176193	5.231292	5.799127	5.037327	6.651927	4.991830	7.183140	5.565981	5.343606	5.097811	6.824667	4.720456	7.344583	7.015993	8.409398	8.573379	7.249782	7.698975	6.740842	5.082253	6.946451	6.437425	5.516471	6.231918	6.106203	7.249927	7.136713	6.026766	6.639977	8.381126	6.734817	7.504924	6.702900	8.595167	7.139854	6.904609	7.187472	8.266356	7.608374	5.442550	9.178480	5.878879	5.304038	5.011793	4.693712	6.792669	5.824962	6.559099	6.161322	7.259489	6.422390	5.478477	7.187472	6.606041	6.751780	6.146352	6.543039	6.556302	6.722079	6.050621	7.312433	7.346434	8.201379	8.133618	6.605173	7.205658	7.061882	7.499773	8.303777	4.779910	7.165801	8.514437	5.331441	7.064759	6.053907	6.155871	7.174480	6.997868	6.417972	7.713269	8.068471	6.293544	7.754268	4.938930	7.462608	4.515053	5.421300	5.423414	4.950633	6.236971	6.721286	3.667908	3.670934	4.981524	5.712313	6.998626	7.202097	4.585625	7.229059	6.237472

LINC01293	6.376639	6.934258	6.262852	4.388263	6.879726	5.590242	5.000064	6.841899	6.240272	7.596093	5.622975	7.632856	7.922567	4.568306	5.969473	7.315893	4.479330	4.550441	6.068237	7.129387	5.919034	8.158860	6.866933	7.025548	2.924200	3.967902	8.324774	6.527333	6.934574	6.159072	7.096114	3.963800	5.299488	8.306636	6.724304	3.844991	6.601982	4.447569	3.524010	6.414848	4.671026	6.269812	6.918220	7.531887	3.425082	6.241098	6.255207	6.387151	3.068364	6.689974	7.293017	6.979258	6.471925	5.351677	5.591192	6.822140	6.776864	2.975609	7.984860	6.825119	6.422858	4.179668	5.838236	4.090469	6.889496	6.760754	7.009640	7.238138	6.037725	7.870132	7.077503	7.199937	7.327054	6.299881	6.732713	6.831026	4.148555	4.110743	6.223096	7.661235	7.262516	6.575512	6.998767	3.880253	4.043422	4.580993	7.592865	6.407533	6.479775	6.338886	7.507225	5.753589	6.771077	7.010112	4.398066	7.440361	7.087997	4.408014	6.331797	6.408201	3.707077	6.616937	4.203616	5.632279	7.375633	6.341221	6.489172	7.484898	8.542415	6.494605	4.622899	6.016224	3.477651	7.903197	5.886031	4.910919	5.799653	5.697116	7.223266	6.632032	7.413845	5.774012	5.683425	6.499228	5.584774	5.617970	8.150217	6.055741	8.056526	5.990072	9.346950	7.731181	6.976396	7.598536	7.274847	5.827305	3.458581	7.311670	7.467106	5.471006	8.163532	5.146157	7.144750	5.812805	4.094115	6.169121	5.848984	7.002445	5.754253	8.044341	4.712429	4.589492	7.291614	6.703693	6.081607	6.448187	4.758520	4.900698	6.243620	5.469490	7.263413	6.634850	6.841899	3.627415	6.149498	6.492368	7.384091	4.963356	4.368922	6.378971	6.809369	7.060125	9.676894	6.709910	6.006261	7.202252	6.554636	8.406665	6.375321	7.982864	4.058018	5.259448	5.056350	7.123519	5.374929	6.877809	6.720650	5.010742	6.861077	6.643535	6.961659	8.351557	6.032173	6.020461	5.596256	5.399206	4.004562	6.881513	7.492285	7.296877	6.754906	7.705490	3.446532	5.763933	4.148555	7.794622	6.758287	6.093905	6.476273	8.467060	6.886917	7.811708	9.282612	4.921690	6.865661	6.981609	3.786278	7.735934	6.357498	6.971663	6.546318	3.905507	3.503482	6.810006	7.966655	6.331290	9.396668	7.077503	6.170481	5.084680	5.880687	6.261339	6.868396	8.579521	3.788048	6.107118	5.468945	6.577340

AL138759.1	9.373205	10.111087	10.561160	10.159386	10.320751	10.039581	9.801046	8.355850	9.635352	10.012445	10.267489	9.581469	10.465042	10.290216	9.733340	10.462035	10.174725	9.383472	8.487706	10.414470	8.644551	10.207688	6.970730	10.348093	8.890904	10.126478	10.116364	7.978789	9.282148	10.266295	10.228061	11.352321	10.197070	10.860851	9.698474	10.169652	10.231313	10.026067	9.605311	9.103800	10.014741	8.579986	10.257704	9.691000	7.980027	9.046541	8.782271	10.169652	10.605030	10.215648	9.147183	9.667475	9.947176	10.197514	10.475430	10.076815	9.478766	9.791905	9.543140	9.333063	8.957952	9.631239	8.372685	8.273893	9.750672	8.947836	9.447007	9.795801	10.089160	8.353209	9.900225	10.159237	10.209210	10.164130	10.563986	10.006922	10.695129	9.894119	9.835965	10.494672	9.908039	10.425147	10.598478	11.408298	10.912200	10.330389	10.654274	10.526253	9.354834	9.154481	8.639114	8.959322	10.156965	10.709373	10.084968	10.479190	10.110042	10.692703	10.225458	9.339197	9.841825	9.181715	10.506011	8.493527	9.277081	10.786766	7.711542	8.743457	9.387152	9.797808	8.991194	8.063177	9.570876	10.003350	10.973234	8.700125	8.391462	10.419351	10.429581	11.070628	11.354771	10.804897	10.502419	11.356979	12.162058	10.170425	11.641765	11.768847	12.079107	11.816173	11.889910	12.052362	11.787010	10.202314	11.136698	12.071265	10.561160	11.523666	12.121293	10.419503	10.308026	11.093405	12.274878	11.167392	12.190143	10.124118	11.322149	12.225511	11.615402	11.766908	11.409053	11.520245	11.054075	11.184336	11.599324	10.474973	11.551356	10.210307	10.726118	12.075588	11.249397	11.716442	10.976864	9.804548	10.269429	11.852935	12.185267	11.619341	11.766772	10.355834	11.248503	11.155808	12.430104	11.441389	11.022434	10.555984	11.799242	11.084984	10.620123	10.187863	10.127429	10.118987	10.366684	9.871071	9.130166	10.989429	10.502146	11.222968	10.249835	11.348194	11.295692	11.298834	11.684797	11.026609	10.687045	10.257870	11.405580	11.093256	11.078407	11.243131	12.588140	9.860030	11.875232	11.628942	10.397036	12.229570	12.480288	9.545615	9.911816	10.321065	10.635641	11.039457	11.766908	10.738093	12.049973	10.472537	11.977595	11.328346	11.044877	12.772931	11.799679	9.219296	9.890739	10.941247	11.677730	11.048103	11.015587	9.825091	11.396538	10.482160	11.024075	12.076671	10.226065	12.791666	11.779565	11.224021	11.707892	12.723828

AC008127.1	3.137740	3.302676	2.971520	2.956161	3.087865	3.093751	3.139936	3.048798	3.306493	3.130356	3.318632	3.078861	3.170245	3.432543	3.738084	3.359154	3.232829	3.202823	4.174447	3.318958	3.903970	3.939843	2.835017	2.945757	2.874704	2.830175	2.890514	2.796100	3.270993	3.173949	3.400386	2.939913	5.171084	3.415541	3.928272	3.691777	3.292494	3.025748	3.161356	3.015950	3.351217	2.904784	3.458761	3.220706	3.615932	4.632197	5.782376	2.885220	3.673475	3.148640	3.085290	3.017781	3.513374	3.853814	3.725577	3.279422	4.070662	3.182313	3.326629	3.625812	2.825823	3.095909	3.604265	3.093751	3.321705	3.373442	3.104231	3.134492	3.131365	2.814697	3.053523	3.403206	3.172804	2.851618	2.874799	3.277138	3.160050	3.228185	3.485185	3.609188	3.236242	3.698196	3.460556	4.036272	3.358117	3.539190	3.334371	3.266440	3.836533	3.445079	3.195295	3.540932	4.016698	3.144918	3.163906	2.901076	3.553152	3.194728	3.088004	3.072259	3.107702	3.135531	2.962645	2.849469	4.428265	3.139187	2.910452	3.211573	3.338286	3.114254	3.247680	3.390175	3.776565	3.150988	3.170245	3.193531	2.946025	3.065646	3.192946	3.053432	3.240931	2.938878	2.995414	3.792920	3.569686	2.902115	3.043405	3.204019	2.999190	3.124029	2.950816	2.976586	3.060878	3.392134	2.932321	3.101726	2.995316	3.252820	3.092072	3.518541	3.146645	2.836268	3.779991	2.832736	3.362862	3.458025	2.775461	3.390353	3.391764	3.018195	3.084750	4.680510	3.078141	3.851853	3.469619	3.177960	3.041777	3.182173	3.011262	3.134888	2.856669	3.517346	3.234681	4.008667	4.393599	5.711383	2.847089	3.931093	2.947291	2.957685	2.950522	3.718335	4.089190	3.447839	3.349874	3.732755	3.069020	3.017496	3.513146	2.807495	3.222403	3.627415	3.238467	3.508161	3.540335	3.102314	3.295881	3.179000	2.823804	2.997794	3.045361	3.150003	2.880634	2.880815	3.250126	2.996944	3.156556	3.044226	3.154050	3.521300	3.906634	3.520038	3.564573	3.452598	3.082124	3.293467	3.409363	4.463060	3.309540	3.245966	3.377394	3.642062	3.183432	3.053145	2.908227	2.987048	2.972811	3.480782	3.111404	2.984795	2.966487	2.891531	2.932919	4.793308	3.154285	4.181446	3.211727	3.020128	3.389306	2.937467	3.463740	4.569356	3.803896	3.001582	2.976228	2.804194	3.102889	3.273570

AF131215.5	7.159454	6.564660	7.084910	7.230254	7.303187	6.467386	6.970435	5.759620	7.409576	7.115180	7.353569	6.582944	7.421974	7.589045	6.877000	6.905892	5.984867	6.992762	5.681975	7.468615	5.764109	6.060443	3.159671	7.092294	6.024627	6.608681	6.846765	6.012299	6.953765	6.733538	6.971198	7.457457	7.547276	7.070710	7.398827	7.226066	7.374716	7.013318	5.371479	5.293129	6.627907	5.594579	7.093070	6.873688	7.224502	7.289419	5.745522	6.467703	6.669308	7.411494	7.351914	7.258561	7.748264	7.656275	7.166993	6.857868	7.619865	5.352272	7.146288	7.202252	6.525640	6.248466	3.504538	5.486138	6.173342	5.715301	5.124112	6.459852	6.963394	5.156870	7.019693	6.986518	7.087059	6.925172	7.105361	7.028768	6.927315	6.946788	7.216069	7.934176	7.379401	7.357154	7.829918	7.738513	7.593826	7.612641	7.706238	7.433129	4.926830	6.591021	6.591021	5.268084	6.043810	6.975438	7.349764	7.614639	7.414462	8.080063	7.229212	7.112679	6.862666	7.460716	7.731957	7.364513	5.504676	7.101570	6.070817	6.679298	4.118088	6.935856	4.097537	7.014228	6.175920	6.756872	7.208783	4.414205	5.760333	7.009152	7.355349	6.391212	5.562223	5.572353	6.037203	4.176392	7.165525	4.810381	8.021791	6.218038	5.928833	6.398128	5.658509	5.234617	5.029542	2.968811	6.196023	3.631713	6.426143	4.940338	6.771409	5.617024	5.589666	3.046101	5.128988	3.174610	4.529040	6.597946	6.061708	6.768134	5.258688	5.175351	5.953780	4.869784	4.407357	7.548238	8.634560	7.365307	7.385917	4.192123	4.485251	8.191012	5.281557	7.858326	5.828774	8.305140	6.592623	7.235637	7.416992	7.590884	5.922158	5.899553	7.804315	7.638162	4.514021	7.619230	6.172347	7.205507	3.117004	4.959576	7.545653	4.865170	5.555427	3.830333	3.756928	3.695698	4.054293	4.296330	4.909483	5.740241	3.129814	7.084584	6.405375	6.170149	4.591620	6.178831	6.590303	3.994643	6.174195	5.673483	7.459357	7.432187	6.689329	7.573568	6.518205	6.502208	5.418743	6.570821	7.678284	3.350916	4.352893	6.790559	3.821455	5.092471	4.695972	7.520666	5.914039	3.366336	5.288957	4.107221	6.819436	3.785011	5.297271	3.992018	6.110433	4.781151	6.457035	6.557617	6.215255	6.135334	4.551538	4.312308	4.513800	3.254844	4.958149	4.981142	6.048901	5.476169	6.841294	3.557581

AP002748.4	11.979884	11.848510	11.629940	11.855782	11.739666	11.817784	12.056353	11.661180	11.915193	11.795267	12.493535	12.086801	12.156499	12.153600	12.056973	12.394751	11.963218	11.742737	12.190892	12.164377	12.138484	12.414781	13.455246	12.133553	12.460924	12.405908	12.975643	12.410313	12.336837	11.807078	12.582441	12.000733	11.762513	12.484062	11.803943	11.555531	11.671048	11.568120	11.961457	11.953330	11.942282	12.114935	11.953180	12.147660	12.697859	11.945301	12.369887	11.990484	11.589538	11.931707	11.846574	12.071578	11.751580	12.150786	11.596844	11.972520	12.192905	11.863656	11.821110	12.151398	12.227667	11.876001	12.265271	12.189336	12.087860	12.105607	12.104818	12.165154	12.291548	12.305577	12.294671	12.245147	12.172787	12.253114	12.257562	12.046469	12.233426	12.092283	12.284891	12.449101	12.074680	12.198437	12.327331	12.355477	12.513208	12.203746	12.569545	12.202831	11.743482	11.924735	12.277596	11.785972	12.320789	12.300012	12.251658	12.190585	11.819436	12.420436	12.478803	12.702108	11.974947	12.037148	11.494646	11.905178	11.831216	12.146258	12.053618	11.683795	12.116357	12.181582	12.114204	11.828693	11.800003	11.835276	11.690625	14.024445	11.786724	12.186990	12.262315	12.050737	12.525455	11.961736	12.269152	12.917840	12.423741	12.995633	13.604320	12.664096	13.049159	13.411431	13.008116	12.260507	12.795551	13.205336	12.750860	13.170547	12.383064	12.497333	12.536742	12.297429	13.858620	13.083849	12.966563	12.650533	12.367209	12.197990	12.404959	12.894731	12.477020	12.985188	12.334611	12.715232	11.512602	12.133274	13.037820	12.297271	11.236266	12.296782	12.369546	12.609195	12.274241	12.069772	11.962037	12.547000	12.034862	11.905478	11.690470	12.684200	11.541661	12.265765	12.426538	13.003402	11.463470	12.689295	13.326797	11.762942	12.857438	12.288785	12.217913	11.736869	12.614208	11.996132	12.177680	12.137705	12.622173	11.995348	11.956053	12.570995	13.247295	12.293129	12.498162	13.533746	12.228964	12.002950	12.382262	12.994539	12.611352	12.085596	12.621157	12.046618	12.020215	12.586128	12.356099	12.825581	13.179765	11.989871	12.585498	11.883896	11.943639	12.102241	12.269467	12.710821	11.956506	12.409034	13.317270	12.804049	13.164330	12.682817	13.293349	12.277123	13.519354	11.886002	12.106371	12.399323	12.739710	12.319676	11.823375	11.454433	12.411264	12.211265	12.032153	12.233576	11.892609	12.620818	12.984259	11.980172	12.112683	13.033913

AC073592.1	5.730853	4.987884	5.464840	5.784669	5.881412	5.344783	5.353060	6.156847	6.122524	5.560926	5.825842	5.585157	6.170659	5.923113	5.822952	5.783088	6.851861	5.882650	5.965900	6.202073	5.796753	6.032173	6.826234	5.777437	6.155871	6.422233	5.784124	6.427147	5.937761	4.661814	6.258264	4.754703	4.770332	5.921404	4.919813	4.888964	5.271843	5.573890	5.807238	5.547320	5.212965	5.464840	5.852553	5.325377	6.331636	4.831651	5.578508	5.122714	4.618396	5.240856	5.203669	5.034193	5.178999	4.902978	5.763409	5.574273	5.569758	4.832062	6.061895	5.408524	5.416020	6.179165	5.461230	6.022738	5.021357	5.182603	4.899606	5.615175	5.251390	5.561840	5.344783	5.322477	5.607979	5.333243	5.579462	5.044120	5.163064	4.704697	4.989759	5.303245	4.552801	4.789101	5.530879	5.467155	5.860307	5.772748	4.678590	6.121820	4.537806	5.327897	5.152764	5.391638	5.100918	4.987884	5.454402	5.707393	5.618740	7.129868	6.145662	6.062933	4.789101	6.013309	6.567597	5.633992	5.465597	5.411685	6.159072	5.632079	5.503367	5.686052	5.085724	5.775641	5.949351	5.668695	6.506176	3.043724	5.613677	6.027664	5.924008	6.413691	5.285197	5.107057	6.045357	4.845952	5.544296	5.515443	6.710714	5.776174	5.502582	6.197419	5.154379	6.034559	6.282784	6.091481	5.630433	6.234306	5.536264	6.889336	5.590622	5.629885	5.421654	5.900808	6.222763	5.346337	5.447613	4.660271	5.787010	4.812712	4.626946	5.724545	6.442889	6.505827	5.765908	5.126372	5.880687	5.687183	5.050781	5.587518	4.762713	5.573143	5.082253	6.202793	5.395965	5.415835	4.565553	5.199295	5.251390	4.250416	4.543780	5.451663	5.519352	5.015854	5.108923	5.669981	5.298266	5.758523	5.108923	5.792544	5.201083	4.691364	5.538448	5.107866	4.934923	5.302043	4.713491	4.443268	5.152764	5.515242	5.291392	4.722578	5.351860	5.951984	5.496995	4.983993	4.850148	4.815381	5.048303	5.170899	5.559054	5.789578	4.424630	6.042616	6.167031	5.447202	5.753934	5.775995	6.187579	4.983993	4.936406	4.908825	5.271428	5.895133	5.726273	5.694332	5.799305	6.491854	5.828040	6.573541	5.955561	5.815271	7.061705	6.871393	4.660922	6.108340	5.206028	5.984697	5.598184	5.587720	5.524971	4.874651	5.014252	3.470874	5.493884	4.849503	4.366995	5.549967	6.542863	5.392206

AC012447.1	7.919983	7.457145	7.131001	7.207847	7.625019	6.807572	7.252299	7.471256	8.527971	5.970151	7.394907	7.837337	7.254455	5.573354	7.753478	7.518041	7.612184	7.531584	7.588584	7.156534	7.392569	8.306046	7.339745	7.574816	6.795886	7.731029	8.007160	7.005042	7.107418	7.217647	7.077190	7.380361	7.556447	7.694121	7.363230	7.227793	7.151714	7.492895	7.234838	7.390405	7.312895	6.998626	7.329461	7.576848	7.160265	7.050231	7.149139	7.323595	7.366238	6.993859	6.963727	7.241025	7.175395	7.214181	7.239214	7.278325	7.335511	7.367980	7.224502	7.404585	7.504612	7.212644	8.749699	6.842711	7.106131	7.381539	7.352977	7.515856	7.224648	7.462608	7.323297	7.212810	7.277818	7.132283	7.580861	7.106131	7.068891	6.905093	7.181248	6.835648	7.204710	7.187166	7.007659	7.186189	6.928920	8.125778	7.115031	7.214181	7.348804	6.728379	6.735475	7.240719	7.333659	7.355349	9.055357	7.433286	7.701304	7.608834	7.212144	7.759138	7.765302	7.491333	7.597626	7.213117	7.377364	7.332112	8.774914	7.466956	7.796780	7.296044	7.246690	7.695315	7.997547	7.510625	7.411494	6.099068	7.434700	6.994310	7.200242	7.310260	7.969250	7.072893	7.629075	8.642986	7.416404	7.333226	8.320945	7.750438	6.882766	7.221148	8.840879	8.014321	5.944605	7.777904	7.041746	7.615127	8.610158	8.251460	7.210504	7.219352	8.334638	7.164573	8.207694	7.651747	8.018137	7.471256	7.652823	7.614047	7.461194	7.776635	7.647866	8.215479	7.802802	7.827616	7.859187	7.214976	7.879916	7.813075	7.587974	7.532374	7.887040	7.669912	8.080215	7.441282	7.237820	8.234201	8.212064	7.327054	7.532374	7.187316	8.006102	7.273736	7.476764	7.345496	7.366238	7.527128	7.765140	8.315925	7.586750	8.033303	7.077988	7.742387	7.413067	7.251051	8.548766	8.104486	7.717365	8.155472	7.716139	7.717814	7.420547	7.813738	7.907550	7.681453	7.084102	7.217178	7.458243	8.117928	7.339873	7.244382	7.766526	7.648612	7.701163	7.823335	8.817908	7.260304	7.724392	6.950312	7.375633	7.324386	7.301137	8.049384	7.952630	7.900387	7.683680	8.221008	7.899568	7.968034	7.540217	8.518412	8.080719	8.029333	7.512950	8.083795	7.706537	9.075596	7.792954	7.366550	7.943394	7.812489	8.029643	7.430775	7.114883	7.369224	6.586545	7.647109	7.657367	7.639694

AL356859.1	3.269432	3.254014	4.087434	3.722103	4.001688	4.980126	5.834643	4.375887	4.106065	3.972030	3.705487	4.107829	3.817172	4.157373	3.796132	4.317138	3.880860	4.886247	4.086354	4.009546	4.191920	3.103641	3.276820	3.597156	2.878713	2.856669	3.454769	4.565327	3.759641	4.325069	3.644431	3.529043	4.183837	3.004352	5.465211	3.836533	4.376736	4.002553	3.792701	4.016935	3.764064	3.069362	3.815038	3.274494	3.676220	4.849095	3.668862	2.868491	5.351297	4.011621	3.541494	3.908140	3.715868	4.436502	4.376322	3.917902	4.143246	3.451718	3.455153	3.779584	2.816359	3.748721	3.703237	3.571687	3.423315	3.455894	3.149886	3.114384	3.186761	2.827972	3.604642	4.270303	3.501061	4.496204	4.028609	4.047803	3.770910	3.639493	3.315118	3.683253	3.280717	6.922885	3.497463	4.022591	3.323316	3.638875	3.790356	4.128056	4.016935	3.467486	3.576333	3.040188	4.145382	3.517751	3.675783	4.433285	3.608025	5.330690	4.109148	3.280419	5.222327	4.338339	4.383150	4.932450	4.596619	3.477470	4.486379	4.666684	3.522634	3.767567	4.200088	4.004562	3.611397	3.536689	4.498125	6.547423	2.845063	3.745674	3.857219	3.716933	3.736479	3.489890	3.237851	3.790786	4.659010	4.318222	3.787788	3.877841	3.269270	3.877385	3.574795	3.496532	3.981076	3.898221	4.009340	4.098433	3.853571	3.229455	3.812307	3.689939	3.587872	5.406712	3.372751	2.841503	3.637869	3.845423	4.001479	3.441340	4.418457	3.766938	3.322974	4.036922	3.369785	5.139246	3.565350	3.925898	3.111180	3.981076	3.685860	3.790973	2.990536	3.580146	3.312397	4.121850	4.645359	4.188655	2.815060	5.444863	4.658123	2.971089	4.321019	4.702350	4.773047	4.026036	3.106431	3.803066	3.718939	3.124906	3.764916	4.734206	3.955022	4.193432	3.760706	3.695478	4.006530	3.728363	3.277564	3.633517	2.821982	3.298088	3.455894	3.664440	3.928494	3.498544	3.639080	3.174610	3.846309	3.338945	3.206525	4.364402	7.312129	3.838007	3.604642	3.972667	3.580146	3.975291	4.143859	3.655248	3.344003	4.037357	3.616696	3.729032	3.422423	3.182955	3.808070	4.308348	4.555796	5.465790	3.190794	4.913908	4.356767	3.942152	3.698588	4.943402	3.560924	5.182603	4.660504	3.609968	3.856357	5.101965	3.734799	3.434900	3.979980	3.740951	6.429547	2.938626	3.517979	3.738741

LINC01828	3.034792	7.022026	5.777617	5.229535	4.069444	5.869021	5.189553	5.240664	5.940611	5.390271	5.357782	5.891507	6.244951	3.988826	5.417778	6.825270	6.003241	4.328317	5.206236	5.688595	4.943641	6.358002	7.077031	5.385211	4.711800	3.497086	3.765704	6.889035	4.813320	4.646404	5.137599	4.549553	3.632310	4.025170	4.183639	5.727541	5.938459	6.155509	4.568743	7.340836	5.666470	4.458439	5.006417	4.555132	5.472341	5.568836	5.665725	3.600679	6.458196	6.303063	6.755884	5.331441	5.102368	4.317365	4.619695	4.130031	4.117669	5.443899	3.836745	4.734603	6.465215	5.353060	3.745252	4.558767	4.181660	5.801382	6.696363	6.113154	6.234645	6.896720	6.254020	6.651763	5.420746	6.105662	6.745450	4.375887	5.850948	4.905251	5.975168	4.027084	3.272250	3.767567	3.471047	4.186897	4.838497	3.590704	3.328676	3.506244	3.476529	4.295017	3.768196	5.434751	3.700564	5.041986	3.274494	3.619725	6.535017	3.557169	3.387061	3.698983	4.088287	6.026766	5.420746	6.759769	4.823740	5.291967	5.640130	4.703011	5.999906	5.751387	5.522145	3.139187	4.256083	5.921593	4.337686	3.322496	3.166776	4.174872	5.561471	3.019585	4.067119	3.324325	3.237267	3.097619	5.721042	4.365913	3.447665	3.209518	4.647912	4.129806	5.327707	4.030852	3.263680	4.495974	4.792105	3.513146	3.278379	3.009521	3.536493	4.230677	3.336283	5.996352	3.075635	3.089684	2.950032	3.410237	3.194035	3.859401	3.553152	4.192585	3.446902	3.654396	3.308901	3.398506	3.541895	3.694665	3.677597	3.509205	4.169230	4.712842	4.224467	3.591517	3.398852	4.277079	5.205831	4.153415	3.191611	5.192494	4.378001	4.099547	3.446341	4.006530	4.234838	3.466290	3.465216	3.778554	3.864506	3.413828	4.032550	3.931529	3.940865	3.780400	3.720796	3.715250	3.812518	3.274494	4.019348	3.544693	3.322329	3.722702	4.341124	4.181870	3.628214	3.723903	3.826990	3.208983	3.257771	4.796873	3.175288	3.639909	4.191920	3.548744	3.647203	3.545470	3.062027	3.313638	3.373274	3.259859	3.374079	3.480575	3.116070	3.399199	3.309540	3.103123	3.813804	3.391393	3.489197	3.907267	3.378618	3.220568	3.321225	3.543244	3.341666	3.160050	4.636303	3.369248	3.354520	3.397480	3.407994	3.635857	3.146189	3.818420	4.479749	3.300530	2.998519	3.111404	3.198773	3.666887

LINC01829	3.034792	7.022026	5.777617	5.229535	4.069444	5.869021	5.189553	5.240664	5.940611	5.390271	5.357782	5.891507	6.244951	3.988826	5.417778	6.825270	6.003241	4.328317	5.206236	5.688595	4.943641	6.358002	7.077031	5.385211	4.711800	3.497086	3.765704	6.889035	4.813320	4.646404	5.137599	4.549553	3.632310	4.025170	4.183639	5.727541	5.938459	6.155509	4.568743	7.340836	5.666470	4.458439	5.006417	4.555132	5.472341	5.568836	5.665725	3.600679	6.458196	6.303063	6.755884	5.331441	5.102368	4.317365	4.619695	4.130031	4.117669	5.443899	3.836745	4.734603	6.465215	5.353060	3.745252	4.558767	4.181660	5.801382	6.696363	6.113154	6.234645	6.896720	6.254020	6.651763	5.420746	6.105662	6.745450	4.375887	5.850948	4.905251	5.975168	4.027084	3.272250	3.767567	3.471047	4.186897	4.838497	3.590704	3.328676	3.506244	3.476529	4.295017	3.768196	5.434751	3.700564	5.041986	3.274494	3.619725	6.535017	3.557169	3.387061	3.698983	4.088287	6.026766	5.420746	6.759769	4.823740	5.291967	5.640130	4.703011	5.999906	5.751387	5.522145	3.139187	4.256083	5.921593	4.337686	3.322496	3.166776	4.174872	5.561471	3.019585	4.067119	3.324325	3.237267	3.097619	5.721042	4.365913	3.447665	3.209518	4.647912	4.129806	5.327707	4.030852	3.263680	4.495974	4.792105	3.513146	3.278379	3.009521	3.536493	4.230677	3.336283	5.996352	3.075635	3.089684	2.950032	3.410237	3.194035	3.859401	3.553152	4.192585	3.446902	3.654396	3.308901	3.398506	3.541895	3.694665	3.677597	3.509205	4.169230	4.712842	4.224467	3.591517	3.398852	4.277079	5.205831	4.153415	3.191611	5.192494	4.378001	4.099547	3.446341	4.006530	4.234838	3.466290	3.465216	3.778554	3.864506	3.413828	4.032550	3.931529	3.940865	3.780400	3.720796	3.715250	3.812518	3.274494	4.019348	3.544693	3.322329	3.722702	4.341124	4.181870	3.628214	3.723903	3.826990	3.208983	3.257771	4.796873	3.175288	3.639909	4.191920	3.548744	3.647203	3.545470	3.062027	3.313638	3.373274	3.259859	3.374079	3.480575	3.116070	3.399199	3.309540	3.103123	3.813804	3.391393	3.489197	3.907267	3.378618	3.220568	3.321225	3.543244	3.341666	3.160050	4.636303	3.369248	3.354520	3.397480	3.407994	3.635857	3.146189	3.818420	4.479749	3.300530	2.998519	3.111404	3.198773	3.666887

AC093714.1	3.290793	3.388422	3.161219	3.042823	2.915428	3.228359	3.470346	3.447665	3.338435	4.754468	3.447839	3.248706	3.924166	3.640480	3.265442	3.494846	6.097354	2.904591	4.145187	3.138221	4.386530	3.021136	3.615725	2.894327	2.893352	2.966574	2.956615	2.942843	3.421359	3.047512	3.585340	4.420677	3.803679	2.991009	3.497641	3.938998	5.390865	3.123684	3.376363	3.307751	3.405245	3.687262	3.756737	3.305708	5.574831	5.421874	3.235083	2.883185	4.931004	3.677597	3.590298	3.454769	4.233834	4.092474	3.931741	3.577965	4.124030	3.348203	3.252973	3.623869	2.934625	3.358816	3.732140	3.279422	3.502531	3.442756	3.957915	3.108051	3.131125	2.906249	3.129094	3.156556	3.236086	3.233108	3.984565	3.852937	3.032456	3.022890	3.060561	4.662255	3.378073	3.950106	3.545283	3.984132	3.400891	3.721463	3.649432	3.231305	4.843806	3.514626	3.230063	3.565517	3.822675	3.271787	2.960973	3.245376	3.779768	3.184361	3.137979	3.252098	3.631286	3.158019	2.979074	2.893708	4.589046	3.270526	3.314962	3.299913	2.969646	3.847173	2.860793	3.108874	3.045250	3.561768	4.067317	3.216108	2.975243	3.122398	3.198895	3.086948	3.494456	3.077475	3.091524	3.164945	3.631909	3.010039	3.235794	3.395399	3.059176	3.314652	3.025843	3.197053	3.136008	3.087647	3.819283	3.451547	2.850826	3.238603	3.176969	3.720796	2.901917	5.489572	2.858432	2.832589	2.922883	3.173828	2.883463	3.121101	3.415052	3.464122	3.472214	3.731296	4.058018	3.367066	3.676604	4.370219	3.213948	3.364693	3.609968	3.177091	3.544145	3.629437	3.347388	5.996025	4.900698	3.191453	2.835017	5.053865	3.535179	3.412420	3.303300	4.169649	4.940761	3.768093	3.567669	3.772596	3.216951	2.989506	3.472931	2.864326	3.281645	3.817794	3.828431	3.838432	4.107628	4.638630	3.246230	3.155076	2.863100	3.322172	3.154285	3.242972	3.102750	3.445260	3.721463	3.037724	3.313496	3.075075	3.866820	3.743475	4.314992	3.586323	4.043878	3.706865	3.108874	3.088208	3.378073	3.958573	3.385183	3.321848	3.637482	3.398360	3.384741	3.189408	3.023691	3.230192	3.572883	3.931529	3.356380	3.348845	3.197721	2.992210	3.191743	5.404785	3.205479	3.390005	3.609384	3.463740	3.153417	2.856077	3.241347	4.744674	3.405410	3.068474	2.936188	3.046403	3.164186	3.290793

AC100756.4	7.145053	7.139390	7.095474	7.493510	8.061804	7.887351	7.199296	7.265254	7.625172	7.259980	7.038741	7.183140	7.511861	7.647866	6.130858	7.546612	6.746276	6.843037	6.944078	7.461823	7.137353	7.515075	7.284304	7.424608	7.446013	6.985880	8.103552	6.941502	7.697302	7.560294	6.880073	6.933492	7.162865	7.049597	6.742879	6.936164	6.545014	6.988248	6.938890	6.706060	7.053011	6.868741	6.805534	6.575512	6.280919	6.080065	7.243253	7.014364	6.851233	6.855573	6.265543	6.734817	6.380781	6.382496	6.279828	7.582731	5.792544	6.310264	6.851861	6.244453	6.156680	7.525428	6.686786	6.649508	6.487258	6.520193	7.115335	6.957032	6.851861	6.847240	7.005355	7.047064	7.234377	6.894802	6.801570	6.475242	6.940592	6.749427	6.736274	6.604700	6.630632	6.304777	6.613481	6.720650	6.712952	6.711800	6.381039	6.712645	7.138283	6.651431	6.760754	7.021281	6.694089	6.815567	6.629353	6.513257	6.656310	7.152778	7.971306	5.782546	7.686252	6.766347	6.835477	6.858036	7.657668	7.231664	7.668348	7.857100	7.512022	7.974469	7.346140	7.607888	8.037881	7.883682	7.456182	9.471077	7.658152	6.781814	7.592104	6.883408	7.834327	7.462948	7.871824	9.247361	8.129950	7.192941	7.843121	8.059955	8.148080	7.934793	7.515856	8.689858	7.696994	7.637243	7.711542	7.375633	7.166260	7.649188	7.762049	7.211984	7.933559	7.793544	7.692886	7.594277	8.037285	7.232119	7.574494	8.286878	7.848245	6.991935	7.108059	7.449989	8.500281	7.620747	7.159016	6.975031	6.997572	7.800225	7.531277	7.558763	7.558478	7.504305	7.152154	6.499570	6.771409	7.650959	7.394907	7.521779	7.071013	7.762198	6.897530	7.183448	8.063486	6.421259	7.725434	6.384819	6.841126	6.738109	6.447013	6.842402	8.114888	7.499450	7.447746	6.655338	6.825270	7.571876	7.080977	7.872457	7.290824	7.294607	7.571715	7.925059	7.483685	6.969337	6.709100	7.335511	7.137353	6.922099	6.993072	6.878463	6.843699	7.065228	6.949028	7.095799	7.119635	6.660691	6.988569	7.398506	7.020942	6.911631	7.154341	7.227793	7.022334	7.281951	6.874986	8.451859	8.228550	7.942940	6.524017	8.247398	7.117723	7.125129	7.073401	7.703286	7.772318	8.670032	7.832466	7.595187	8.088168	7.506195	7.653147	9.354397	8.204349	8.561939	9.005162	7.783481	7.268785	7.818413

TTLL10-AS1	11.955891	12.779001	12.456759	12.666649	12.854406	12.751371	11.785826	12.800233	13.371671	12.260037	12.538617	12.275329	12.392815	12.181281	11.881927	12.629849	12.782063	11.650359	12.676204	12.460764	12.464370	12.734538	12.763935	12.177029	12.408694	12.720078	13.304338	12.849490	13.198527	13.193275	12.704515	11.612874	12.055738	12.808754	12.200007	11.633590	11.647091	12.048150	12.448174	12.225836	12.482634	12.331290	12.326612	12.078045	11.974211	11.773141	13.199119	12.048913	12.010792	11.777140	11.549007	11.332335	11.457857	11.719572	11.230646	13.086671	10.915527	11.486239	11.830768	11.386878	11.783740	12.805624	12.647798	12.467649	12.025400	11.987566	12.486643	12.350062	12.355628	12.612867	12.473611	12.355945	12.744389	12.152545	12.410460	12.981496	12.198437	12.655859	12.485843	11.360153	11.270879	11.179761	11.024661	11.092677	11.412504	11.683795	11.427239	11.212284	11.882660	11.751278	11.515926	12.118961	11.916369	11.813750	12.770516	11.634646	11.406029	12.235457	12.690509	12.850331	12.927769	12.100426	12.199224	11.941813	12.320299	12.427314	12.595226	12.848958	13.301047	13.048001	13.018455	12.823032	12.460438	13.196531	12.799850	15.002418	12.647084	12.296949	13.129969	12.038794	12.308719	12.941572	12.518587	13.916329	12.453112	12.804429	13.533515	12.861512	12.349742	12.291225	12.343747	12.808590	12.612002	12.780366	12.989626	12.118803	12.464195	12.628852	11.508343	12.867603	12.604915	12.426698	12.742146	12.417994	11.884206	12.632463	12.987601	13.502602	13.048001	12.843685	13.244529	12.364031	11.847595	13.130368	11.317927	12.774646	12.591825	12.863843	12.355628	12.949809	13.094857	11.899712	12.084512	11.655452	11.620849	13.137178	12.105743	11.532884	11.603716	11.584232	11.556248	11.184194	11.387167	11.202995	12.367059	11.069731	11.238299	11.660007	11.398515	11.616843	12.480452	12.618626	11.910150	12.177029	12.255831	12.029310	12.588621	12.302320	13.234006	12.698176	12.156187	13.401636	11.770058	13.709911	13.581357	12.099966	11.818235	11.889463	12.092910	11.128092	11.223716	11.363862	11.731685	11.053092	12.224905	11.025099	11.184648	11.888248	11.922470	12.535932	11.972074	11.636925	11.844639	12.227667	12.466646	12.293129	12.830477	12.208079	11.645189	12.786841	12.505911	12.573864	12.976037	12.383612	12.893846	12.790822	12.076671	12.488461	13.109444	12.696624	12.752265	14.242079	13.966624	12.918970	13.050251	11.883291	11.848932	12.615500

AL139353.2	5.846583	5.566556	5.915666	6.592623	7.218871	6.424645	6.538445	5.930416	6.722244	6.616593	6.510601	6.561359	7.228880	6.817477	5.634192	7.035923	5.666470	6.662321	7.150091	7.140023	6.748634	7.396612	6.947452	6.922731	6.966432	6.420945	6.425455	5.112562	5.634357	6.368863	6.660032	6.933776	6.447841	6.904304	6.668647	6.572093	7.355178	6.495088	6.586878	6.519373	5.540532	6.149868	6.772836	6.091481	6.511783	7.095799	6.073115	6.297403	5.858142	6.802515	7.105361	6.710243	7.449204	7.531277	6.469852	6.303405	6.995225	6.168963	6.696500	6.866773	6.729161	6.272537	5.496995	7.004242	5.869386	5.634715	6.277338	5.817445	5.666871	5.657575	6.720482	6.696046	6.695877	6.443034	7.115335	6.416338	6.583920	6.822609	6.620820	6.404233	6.549699	6.769450	6.771256	7.610523	7.289877	6.455878	7.499287	7.779827	6.845709	6.873056	6.405228	5.593845	6.891902	6.888237	6.584561	7.189713	6.410035	7.018269	5.935640	4.197968	6.416667	6.702584	6.513088	6.205020	6.806475	6.337546	6.101888	7.180027	6.661341	6.000919	7.258223	6.179488	6.639497	6.011588	6.838834	7.085535	5.082452	6.574201	7.138446	7.804774	6.127291	5.346739	7.112679	7.279087	6.038416	6.670097	6.211853	7.959450	7.560886	5.484426	7.397106	7.843121	6.520020	6.148979	6.931281	6.293873	7.773066	7.827120	6.388356	8.699378	7.917531	6.107118	7.208153	6.574201	5.316256	6.093905	5.880149	5.533140	6.137363	6.389356	6.656310	6.191829	6.992917	6.888567	7.038119	6.897378	7.329932	6.729161	7.689354	5.267688	6.124801	6.777177	5.763225	6.470026	7.396612	7.308348	5.968975	6.705266	6.595852	6.439408	6.970730	8.379542	7.385917	7.581800	5.437070	7.385917	5.558298	6.885363	5.266871	6.054615	5.687351	6.636123	7.448253	6.258790	5.671992	6.484071	6.021706	6.722898	5.738626	6.943280	6.659365	6.125492	7.028768	6.325780	5.805975	6.984290	6.171336	7.128141	6.292024	6.743835	7.599022	7.347870	7.158850	7.029236	7.236735	7.716894	6.882462	6.687772	6.577340	6.603253	5.937040	6.865500	6.708319	7.105361	6.821344	7.632094	7.296211	6.610455	4.943402	7.303657	7.249008	6.772836	6.483577	7.615127	6.820544	6.484247	6.947452	5.868676	6.410385	6.460844	6.560069	4.795182	5.974298	7.046755	4.682027	6.336018	6.919359	7.372217

LINC02060	5.218154	3.607835	4.167541	4.076394	4.833968	5.619645	5.042847	4.132996	5.116218	4.554733	6.531764	5.146547	4.761215	6.020286	5.598592	4.888552	4.050210	4.801756	5.340490	4.801966	5.260230	5.167486	6.188929	4.712429	5.184019	4.557070	5.077569	4.575611	5.042212	3.525894	5.374347	4.753849	4.819395	5.016682	4.549312	5.003956	4.734603	4.195839	3.898221	4.527383	4.781556	5.303048	4.306356	4.100404	4.654410	5.210637	4.816688	3.353835	3.332726	2.872176	3.322496	5.429568	4.254568	4.767009	5.689540	4.635852	4.830197	3.765098	3.781857	4.232240	4.863721	3.965297	3.765704	4.674733	4.093706	3.495403	3.069481	4.030399	3.844161	4.399847	3.913358	4.943181	4.890642	4.552354	5.335964	3.807616	4.936196	3.458213	4.043422	4.087871	3.863876	4.584149	5.469311	4.242727	4.860639	4.673167	5.584968	4.817515	3.791429	3.677808	4.429095	3.779151	4.824555	3.870296	4.445201	5.722134	5.066111	5.942139	5.556382	3.756737	5.153997	4.487189	5.030756	4.720037	4.308348	4.459269	5.340490	4.159074	4.644041	4.752754	4.569586	4.726601	5.448784	5.286988	3.597342	5.210432	4.334644	4.279959	5.567854	4.909040	3.461116	3.507957	4.550879	4.163148	6.030746	4.408936	6.170998	5.804920	4.710741	3.769711	5.398239	5.455701	3.870296	5.246614	4.980126	3.860245	5.212390	5.517224	5.862318	4.888751	4.660049	3.144458	4.096007	3.416712	3.074230	4.293735	4.619695	6.490004	3.850978	3.586490	5.051969	5.853436	3.686638	4.844447	4.464780	4.533107	5.205618	3.807012	5.024042	4.813536	4.286794	4.728936	4.612954	4.962329	4.938486	4.737156	5.481638	5.807043	6.387666	3.293467	5.415278	4.799646	4.397856	6.214917	4.248826	5.505847	3.781438	3.664440	4.013337	3.092690	4.386303	3.819283	4.222090	3.782941	3.726339	3.054254	3.594399	3.513374	3.035774	3.765098	5.535494	3.812518	4.341562	5.094583	3.329019	4.887311	3.769711	4.477618	3.814406	4.921258	4.659428	4.142379	4.460921	3.971363	5.360226	5.238860	5.063876	3.480575	4.198610	5.654829	3.538645	5.876566	4.335291	5.328913	5.305796	5.784669	5.668327	4.800062	4.089608	3.978243	4.984987	4.955624	5.423847	4.648966	4.985822	6.544343	3.554492	5.680106	4.546888	3.769711	3.733827	4.584561	4.459269	4.484384	4.632837	5.180737	4.986428	3.485005

AC068633.1	4.228324	5.723267	5.611787	5.722522	5.846222	5.633793	5.123314	6.187579	6.290806	5.301629	5.437070	4.540205	5.283182	6.211030	4.327452	5.479850	4.465860	5.275754	6.668343	5.438205	5.864357	6.729818	3.037323	5.947390	5.778728	5.247625	5.352068	5.454178	5.276568	5.125133	5.697116	5.355303	5.294827	4.303765	4.336340	5.830863	4.666013	4.441499	5.238860	5.173917	5.881412	5.245997	4.906706	4.776155	4.783483	5.103183	6.041416	4.948324	5.473470	5.339727	4.965827	5.718155	4.615751	5.222922	6.100152	5.738086	4.077019	4.394274	3.769513	4.739990	3.117943	5.890991	4.966918	5.650665	5.187008	4.425695	6.395964	4.939739	5.388158	4.656631	4.914730	4.957084	6.400484	4.828672	4.739775	5.056559	4.595326	4.420222	5.462754	5.485387	4.542417	5.184411	5.361935	6.210495	5.025654	4.654410	4.589267	4.290685	4.983584	4.639899	4.400675	4.869381	5.105634	4.930410	4.374567	4.543780	4.081552	5.201083	5.778924	5.396530	5.923488	5.022770	5.858327	5.855451	6.220727	5.924351	7.642764	6.337877	6.725278	5.998314	6.118533	6.716859	5.236233	5.813523	5.387168	6.932551	5.375140	6.022738	6.165625	4.721070	4.697230	5.265055	5.664974	6.718290	5.667601	4.597449	5.400520	5.564851	5.885687	4.900698	4.125060	5.105833	4.680289	3.960312	4.909040	4.146217	5.258688	5.001729	3.765302	5.514046	4.803678	2.920907	4.920835	5.728084	4.017194	5.140892	5.512606	5.302654	4.861840	3.588064	5.142148	4.573729	4.283381	6.461838	5.647262	3.957014	4.462868	3.698588	3.659820	5.728468	4.616662	4.785130	3.439414	4.201183	4.584996	6.179318	5.832274	4.110501	4.181870	3.573251	5.356854	4.527150	4.498125	5.574273	5.350251	4.321835	4.334201	3.600120	4.002147	2.944910	4.861046	5.195497	4.387604	6.286927	4.300245	5.712884	4.300454	5.145926	4.488884	4.338539	4.266557	6.135334	4.284260	4.417754	4.237860	4.462440	3.896502	5.256728	4.095823	4.067119	4.573305	3.651376	5.690239	3.841099	4.550441	4.192784	3.615725	4.958929	4.390400	5.038717	4.803917	3.994857	3.602837	4.455844	4.508542	4.651356	5.195916	4.659661	4.480635	4.489549	5.706824	6.660691	4.413993	5.442550	6.189761	6.530150	6.493471	5.840428	6.408537	5.844572	5.906509	7.838143	5.606325	6.260150	5.586609	4.075989	5.236428	4.707853

AP001150.1	8.043864	7.429685	7.491660	7.625612	8.112376	7.861216	7.169797	8.770850	8.819791	8.349433	6.964835	7.063971	7.155624	7.185698	7.114246	7.743172	8.359865	6.913856	8.703369	7.500400	8.302736	8.014164	8.526133	8.355988	7.849325	8.000720	7.423830	8.138502	7.542332	7.392271	7.975388	6.316233	6.871784	6.464205	6.990471	6.801251	7.102997	7.386684	7.529175	7.537165	7.546449	7.380528	6.971814	7.404121	6.813820	7.224648	7.700850	7.500244	7.534543	7.471425	7.079582	7.227500	6.524190	6.984122	6.598436	7.521137	6.121820	7.197600	7.380012	6.864533	7.252607	7.479343	8.235738	7.798320	7.390553	7.584112	7.888113	7.781203	7.565641	8.161001	7.582246	7.507990	7.508788	7.230565	7.364676	7.471877	7.792182	7.653754	7.786677	7.170547	7.286140	7.348503	6.904766	7.108192	7.222979	6.916190	7.100327	7.444598	7.687007	8.034493	7.760978	8.450156	7.224338	7.422893	7.417872	7.056172	7.077360	6.370064	7.408991	7.668203	7.873357	7.209237	6.574492	7.378793	7.437391	7.966655	8.816052	7.492583	8.846315	8.139429	8.591468	8.614211	8.736112	8.207850	7.792182	10.308167	8.299261	7.068227	7.738359	7.313859	7.818091	7.688422	7.562281	9.440040	7.626701	7.753478	8.225552	8.129022	8.218720	7.536246	7.079818	7.942940	7.988952	8.133472	7.700231	8.059636	7.006750	8.568954	7.389191	8.677280	7.879615	8.536093	8.575811	8.712812	8.234506	8.057573	8.169880	8.229646	7.659517	8.360934	7.256013	6.930178	7.158379	7.101416	6.928432	7.195369	7.866800	8.267621	7.749351	8.361249	7.811708	7.240393	8.482171	6.947279	7.212483	8.794460	7.676648	7.069707	7.892783	7.553011	7.089306	6.698868	7.255549	6.697923	7.666067	6.279654	7.439013	7.324214	7.085683	7.661085	7.652823	8.155006	7.928341	7.837640	7.670842	8.254205	7.888733	7.936532	8.378919	7.512793	7.510294	8.310673	7.507394	7.025077	7.599022	8.101742	8.014777	8.061672	7.541740	7.206919	7.671162	7.349921	7.048016	7.473087	7.999175	7.398195	7.345011	8.211887	8.092604	8.146874	8.997364	7.565494	7.897456	7.537331	7.455878	7.578853	7.120108	7.504456	6.881513	7.752279	7.787317	7.374236	7.222237	7.799910	7.967408	8.937800	7.533758	8.162350	8.650408	8.766838	8.731515	9.572872	8.705672	8.617997	8.488758	7.864481	7.515537	8.311463

AC013269.1	5.207022	6.383321	6.109896	6.298401	7.173384	7.249623	6.932551	7.732409	7.507556	7.017144	7.716427	7.432496	6.610626	6.915897	6.291515	6.519038	7.213561	7.081923	7.075905	5.923657	7.013182	7.291452	6.819738	6.569361	6.768134	6.955826	6.716203	7.916296	7.487101	6.732532	7.212483	5.420176	6.153453	6.289442	6.155509	6.100838	6.123900	6.267597	6.992259	6.732875	7.235310	6.238599	5.874405	5.732104	5.856542	6.352110	8.333537	5.625408	5.989180	6.086417	5.608330	5.892964	5.497353	5.615559	5.696567	7.840933	6.029076	6.039985	6.313142	6.021366	6.635321	6.803329	6.922566	7.380972	7.129705	6.519214	6.138229	6.740220	6.460518	6.826070	6.259130	6.495585	7.636038	6.749103	6.286409	6.333488	6.657433	6.878132	6.604389	6.069980	5.933698	5.844038	6.383999	5.981184	6.528665	6.438240	8.109811	6.547258	7.120747	6.794463	6.716023	7.460560	6.271383	6.545014	6.915103	6.300025	6.356984	6.049762	7.119181	6.749103	7.249008	5.994834	6.180491	6.497206	6.611312	6.900783	7.562281	6.662321	7.237496	7.563777	7.388257	7.656124	7.692436	8.630044	6.971518	8.930947	6.519682	6.689649	7.558311	6.187767	7.013910	5.936863	6.394997	8.141802	7.540366	7.620747	6.913999	7.279252	6.749255	7.351457	7.719779	7.868615	7.109022	7.656124	6.576510	6.920814	7.203012	6.599911	5.407122	6.936015	6.770091	7.810799	7.265109	7.062687	7.159168	7.235310	7.423532	8.061071	6.935199	7.223587	6.406553	5.973440	7.234838	5.875675	5.877282	5.968805	6.650301	7.390858	6.770246	7.906019	6.304777	5.535274	5.773799	5.664054	6.131199	8.907661	6.292672	6.005022	6.346221	5.888681	5.511627	4.605235	6.310954	5.529351	6.965325	5.838803	3.992878	5.870430	5.384096	6.725278	6.927637	6.679132	7.011235	6.945652	6.654503	7.173526	7.814319	6.752252	7.447746	5.945135	6.393213	8.677280	6.930656	6.219926	6.245934	6.296097	6.995990	7.045129	6.073647	6.055991	6.137868	7.453397	5.973787	6.174195	7.640962	9.477368	6.286020	6.934730	6.476422	6.190268	7.402552	6.490783	6.894955	8.578029	6.811147	7.164871	6.560069	7.316049	5.813892	7.140632	6.256911	6.494756	6.419326	5.343190	7.701453	6.932551	6.178514	6.825270	9.291751	7.512190	7.251697	7.919671	9.669262	7.570365	7.742941	7.052870	6.726070	7.406634

AC140481.2	5.207022	6.383321	6.109896	6.298401	7.173384	7.249623	6.932551	7.732409	7.507556	7.017144	7.716427	7.432496	6.610626	6.915897	6.291515	6.519038	7.213561	7.081923	7.075905	5.923657	7.013182	7.291452	6.819738	6.569361	6.768134	6.955826	6.716203	7.916296	7.487101	6.732532	7.212483	5.420176	6.153453	6.289442	6.155509	6.100838	6.123900	6.267597	6.992259	6.732875	7.235310	6.238599	5.874405	5.732104	5.856542	6.352110	8.333537	5.625408	5.989180	6.086417	5.608330	5.892964	5.497353	5.615559	5.696567	7.840933	6.029076	6.039985	6.313142	6.021366	6.635321	6.803329	6.922566	7.380972	7.129705	6.519214	6.138229	6.740220	6.460518	6.826070	6.259130	6.495585	7.636038	6.749103	6.286409	6.333488	6.657433	6.878132	6.604389	6.069980	5.933698	5.844038	6.383999	5.981184	6.528665	6.438240	8.109811	6.547258	7.120747	6.794463	6.716023	7.460560	6.271383	6.545014	6.915103	6.300025	6.356984	6.049762	7.119181	6.749103	7.249008	5.994834	6.180491	6.497206	6.611312	6.900783	7.562281	6.662321	7.237496	7.563777	7.388257	7.656124	7.692436	8.630044	6.971518	8.930947	6.519682	6.689649	7.558311	6.187767	7.013910	5.936863	6.394997	8.141802	7.540366	7.620747	6.913999	7.279252	6.749255	7.351457	7.719779	7.868615	7.109022	7.656124	6.576510	6.920814	7.203012	6.599911	5.407122	6.936015	6.770091	7.810799	7.265109	7.062687	7.159168	7.235310	7.423532	8.061071	6.935199	7.223587	6.406553	5.973440	7.234838	5.875675	5.877282	5.968805	6.650301	7.390858	6.770246	7.906019	6.304777	5.535274	5.773799	5.664054	6.131199	8.907661	6.292672	6.005022	6.346221	5.888681	5.511627	4.605235	6.310954	5.529351	6.965325	5.838803	3.992878	5.870430	5.384096	6.725278	6.927637	6.679132	7.011235	6.945652	6.654503	7.173526	7.814319	6.752252	7.447746	5.945135	6.393213	8.677280	6.930656	6.219926	6.245934	6.296097	6.995990	7.045129	6.073647	6.055991	6.137868	7.453397	5.973787	6.174195	7.640962	9.477368	6.286020	6.934730	6.476422	6.190268	7.402552	6.490783	6.894955	8.578029	6.811147	7.164871	6.560069	7.316049	5.813892	7.140632	6.256911	6.494756	6.419326	5.343190	7.701453	6.932551	6.178514	6.825270	9.291751	7.512190	7.251697	7.919671	9.669262	7.570365	7.742941	7.052870	6.726070	7.406634

AC104984.2	3.214885	3.188883	3.141547	2.986951	2.931158	3.013959	3.364994	3.347723	3.432717	3.945762	3.619327	3.719536	3.715868	3.866193	3.367892	3.117381	3.691777	3.203211	4.446094	3.365508	4.515925	2.893276	3.355689	3.193659	2.941808	2.962547	3.070452	3.058514	3.049958	3.136966	3.588616	3.770322	3.055709	3.343324	4.761419	5.468755	3.663659	3.211573	3.059490	2.866692	3.336602	3.120835	3.450995	4.625263	3.460735	4.837863	3.194161	2.978509	3.598578	4.070662	4.051496	3.845888	3.953135	3.960098	3.723306	3.042603	3.740951	4.958929	3.638690	3.235939	3.142565	3.035174	3.121823	3.087865	3.194444	3.054254	3.187406	3.056976	2.942255	2.937467	3.058828	3.398178	3.057495	3.056976	3.087392	4.254568	3.012499	3.056542	2.988170	3.563042	3.328510	4.751098	3.348203	3.631713	3.246695	3.544884	3.031543	3.129211	3.170770	3.289211	3.075743	3.864506	2.992607	4.951665	2.943219	3.003333	3.685428	3.867287	2.985231	3.095013	3.254178	3.223215	2.993540	3.795908	4.137363	3.048272	3.219377	3.147523	3.050804	3.345907	2.814093	3.158788	3.089248	3.221793	3.209656	3.958796	3.019853	3.001582	3.085489	3.329843	3.236523	3.067954	3.020820	2.860341	3.327407	3.421880	3.260848	3.496333	3.206648	3.492758	3.208873	3.551024	3.788884	3.524784	3.512383	3.655630	3.149759	3.332891	3.340106	3.818855	3.143041	3.153933	3.100328	2.852017	3.444725	3.296988	2.942101	3.092690	3.415387	4.370890	3.419704	3.054459	3.287419	5.019323	5.243412	3.746872	3.249291	3.055377	2.844483	3.133090	2.929714	3.472214	5.250184	3.763856	4.308576	3.156400	3.007845	3.808458	4.261553	4.115483	3.768624	3.709872	3.970080	3.339114	3.006794	3.431159	3.412735	3.288506	3.065854	2.985328	3.107248	3.084859	3.372751	3.301613	3.060451	2.889992	3.098076	2.929899	2.927073	3.225846	3.463925	3.060353	3.328072	3.256748	3.841327	3.024570	3.140290	3.076339	3.202692	4.054951	4.403324	3.427805	3.480386	3.298309	3.094426	3.403870	3.294995	3.057375	3.182695	3.108874	2.824801	2.949611	5.050976	3.364994	3.113778	3.136736	3.334035	3.144458	3.204019	3.195971	3.245524	3.087292	2.968264	4.381390	3.028768	3.208736	3.128807	3.504959	3.097255	3.084411	3.025532	2.942527	3.151248	2.981438	3.152335	3.154050	3.346402	3.176851

SNHG17	12.618274	12.453263	12.822839	12.522671	13.007936	12.296644	12.216837	11.926923	12.629032	12.165300	12.283028	12.322207	12.579838	12.754773	13.862105	12.468921	12.796802	13.035077	13.790457	13.068719	13.799817	13.185843	13.899912	13.691937	12.890515	12.698021	12.865155	11.637961	12.461907	13.450122	12.797127	12.900592	12.155866	12.580816	12.016602	12.136334	12.170015	12.665988	12.945900	13.585329	12.610356	12.923666	12.723671	12.767450	12.667306	12.857277	11.955891	12.465572	13.075705	12.298055	12.558326	12.025867	12.464195	12.601913	12.663564	12.845079	13.387795	13.944308	12.851230	12.794657	13.523010	13.168839	13.560165	13.729644	13.583453	13.105019	12.392060	13.388206	12.538957	13.135653	12.282255	12.740415	12.368300	12.829643	12.691855	12.750695	12.207292	12.606391	12.327958	13.727121	12.996574	12.924201	12.475057	13.377562	12.702108	12.591825	13.614204	12.579997	13.993914	13.919694	13.741572	13.167892	12.887037	12.992354	12.343108	13.036523	12.710332	13.157276	12.832376	12.206196	13.060802	12.483749	12.718459	12.493699	13.884615	12.930265	12.364031	14.234212	14.409900	12.600182	13.675077	13.671017	12.846651	12.572340	13.074945	12.731001	12.919527	12.866895	12.918772	12.611838	13.635698	12.882781	13.118676	13.822664	12.688477	12.781728	13.717240	13.389060	12.711470	13.160892	13.270823	13.586501	13.590518	13.860465	13.043520	13.174765	13.542327	15.067485	14.229857	14.240757	14.332054	14.229183	13.810879	13.331494	14.197180	13.058425	13.607572	13.499569	13.095423	13.302074	13.454810	13.511960	13.227239	13.317056	13.339924	12.731525	13.835602	13.802610	14.170650	13.854098	13.571924	13.655507	13.873927	13.837218	13.056546	13.423426	14.702993	13.424703	12.926827	12.759782	14.375530	13.161859	12.932422	13.069091	13.533066	13.927588	13.535095	13.775202	13.483665	14.010958	14.034368	14.086196	14.471558	13.459378	13.998449	13.746814	12.951111	13.550061	13.752566	13.613744	12.951111	13.455726	12.654474	13.249693	12.704515	13.391621	13.553021	12.966743	14.128868	13.101051	13.166504	13.335716	13.272382	13.540597	13.753859	14.314424	14.681870	13.691689	14.435274	14.223759	14.499076	13.140468	13.231052	13.213586	13.147891	13.325811	13.048388	13.479756	13.641034	13.644720	13.415034	13.498487	12.981496	15.029282	13.360372	14.485612	15.314555	14.416536	12.456759	14.881255	13.655038	13.732138	12.526938	13.531718	13.619271	13.052443	13.595345	13.362663

AC025171.1	9.047757	9.190561	9.086706	8.891594	9.365664	9.347097	10.233257	9.372650	9.522409	9.926926	9.996163	9.968179	9.123526	9.609972	9.354834	9.032961	9.117427	9.249650	9.628141	9.072354	9.589153	9.315341	10.323820	9.483634	9.131092	9.419441	9.425577	9.551464	9.107647	9.681546	9.743643	9.200778	9.231455	9.253595	8.963573	9.062914	8.954553	9.121392	9.087482	9.394342	9.319127	8.942629	8.721830	8.289941	8.683550	9.376353	9.410650	8.927706	9.335123	8.529542	8.660701	8.400640	8.720086	9.106123	8.856081	9.371967	9.007782	9.223414	9.325070	9.050550	9.222820	9.463454	9.193762	9.278587	9.280909	8.943397	9.108775	9.008088	9.126584	9.350045	9.308633	9.572453	9.331371	9.455263	9.599253	9.180782	9.438039	8.904760	8.861847	8.840427	9.104906	8.844331	8.727414	8.762269	9.108128	9.208246	9.011895	9.279993	9.577290	9.460288	9.219434	9.957686	9.248124	9.511361	9.172195	9.100559	9.247506	8.846787	9.019864	8.926121	8.414441	8.861523	9.213120	8.636910	9.557714	9.511205	9.809978	9.357110	9.494561	9.754543	9.919312	9.085823	9.226978	9.506119	9.289882	7.857542	8.909057	9.178776	9.190703	9.348642	9.469514	9.711706	9.527862	8.647850	9.628632	10.231014	9.098294	9.390348	10.067980	9.914081	10.410577	9.769058	10.451084	9.597258	9.411269	10.166289	10.074213	9.609390	9.554793	9.949803	9.602366	10.803737	10.357244	9.652899	9.433276	9.473843	10.572371	10.017871	10.454196	10.090978	9.255736	9.490626	10.248479	9.771440	9.261349	9.084407	10.412994	9.752062	9.936952	9.508226	9.798110	8.865208	9.118977	8.727734	10.136837	10.520191	9.603459	10.319223	9.530244	9.369831	9.395906	9.513068	9.393746	8.776752	9.893017	8.936547	9.602700	9.542983	9.279824	9.629098	9.698632	9.906208	9.819337	9.849598	9.666389	10.408443	9.649630	10.034063	9.988176	10.021710	9.809845	9.467958	9.879312	9.886562	9.546085	9.722467	9.460002	9.524275	9.216696	9.270915	9.833953	8.946909	8.848488	9.853064	10.086994	9.716397	9.539221	10.238466	9.410488	9.353931	10.508270	10.429431	10.871143	10.220637	8.864011	9.531300	9.309408	10.019135	9.437883	9.640875	10.004105	10.985236	9.010403	10.754004	9.531003	10.178593	9.834586	10.396131	9.527248	10.147784	8.894837	8.959927	9.413335	9.816047	9.437081	9.577144	9.387625	10.841725

AL136295.2	9.054717	9.456308	8.973345	9.562674	9.487184	9.214227	9.208246	8.877254	8.955627	9.271071	9.552530	8.995493	9.382862	9.594640	10.225137	8.640199	9.368318	9.946210	10.238620	9.558784	9.537522	9.065219	11.055820	9.477368	9.833053	9.206285	9.358941	8.882957	8.963724	9.146557	9.744429	9.850095	9.578383	9.975574	9.621823	9.426037	9.153845	9.488589	9.894872	9.390506	9.087482	10.013258	9.581758	9.275130	9.673342	10.027037	8.824932	9.593851	10.666510	9.412891	9.815723	9.979195	10.074213	10.084677	9.892861	9.028112	9.967259	10.422540	10.192680	10.468311	10.813727	8.988823	11.139480	10.622041	10.666667	10.170756	10.647894	10.430011	9.837036	9.910666	9.553140	9.339347	9.070503	9.416111	9.589501	9.748635	9.306796	9.800735	9.610727	10.146257	10.195988	10.303411	9.707507	10.985538	9.687619	9.808233	10.152177	10.525956	10.899103	10.473139	10.380008	11.136391	10.223316	9.723922	9.700800	9.936498	9.864397	9.363636	9.218710	9.487673	9.261505	8.989722	9.208101	9.420843	9.857581	9.566857	9.703711	10.628223	10.188320	8.717058	10.207514	10.622929	9.529282	9.296610	8.964030	8.923429	9.863624	9.336944	9.218055	10.360974	10.055903	10.185728	10.010197	9.504734	9.781037	10.285158	9.597336	8.987939	10.487203	9.587812	10.153581	9.925599	10.375600	9.902118	8.890668	10.768036	10.623839	11.247492	9.712939	10.111918	11.354634	10.553536	10.336891	10.352969	9.184093	9.459105	9.920274	9.253595	9.759952	9.540332	9.718945	9.930158	9.971673	10.422850	10.915384	9.424518	10.402738	10.474973	10.901113	9.623997	11.011088	10.718513	10.363870	10.310511	11.417687	10.179942	10.165569	10.506461	10.427634	10.347055	10.569692	11.893953	10.909711	10.372148	9.391424	10.456478	11.771398	11.350393	10.588804	11.252802	9.934782	11.369313	11.247077	11.379904	11.646786	11.126061	10.704762	11.009768	10.922388	10.638038	9.685477	9.033855	10.899381	10.084677	10.006475	9.865299	10.928144	10.513902	10.624433	10.063481	11.481075	10.107923	10.349902	10.479190	10.249984	11.616406	11.383057	11.692447	10.910897	11.271780	11.603148	10.579203	10.397036	9.457707	9.915602	10.652524	9.855275	9.752993	11.100452	10.791003	10.474364	9.881777	10.325356	11.482684	9.936352	11.520092	11.141637	10.845325	9.519152	11.462640	10.563066	10.823997	9.263696	11.056729	9.506865	10.432020	9.046541	10.193392

AC022137.4	11.437385	11.011088	11.599182	11.108811	11.199022	11.118406	11.596253	10.448550	11.250156	11.390271	11.095577	11.105444	10.978923	11.059685	11.227229	10.529955	10.443033	11.053352	11.154369	10.682874	10.947866	10.767277	10.793666	10.843477	10.460558	11.059824	10.599871	10.639211	11.178995	11.829273	9.887831	11.102976	11.531987	11.336132	11.367280	11.808140	11.511412	11.519647	11.627766	11.333902	11.367880	11.850196	11.719572	11.598146	10.979225	10.850767	10.875313	11.316909	11.279734	11.270443	11.022278	11.182158	11.423597	11.630301	11.241079	11.302046	11.411961	11.660588	11.716014	11.187132	12.143591	11.420288	11.287476	11.352461	11.495632	11.517229	11.848060	11.069731	11.053797	11.438372	11.237709	11.390418	10.955728	10.999617	11.318232	11.448381	11.516068	11.193556	11.626169	10.842709	11.436109	10.800888	11.253814	10.920907	11.532884	11.160511	11.249706	10.818491	11.628369	11.479023	11.692298	11.381737	11.036123	11.390120	11.706091	11.273543	11.242535	10.688872	11.277202	10.994322	11.093119	11.369466	10.799656	11.116825	11.327191	10.872332	10.952091	11.145288	11.534947	11.062006	12.076508	10.991775	10.848151	11.620096	11.047378	10.946980	11.912793	9.975417	10.804313	11.553894	11.313927	12.212996	11.660141	11.320686	10.634152	11.519647	11.101213	10.717011	11.506127	11.011245	11.805147	10.003957	11.629710	11.296282	10.698977	11.009906	11.097335	11.981214	9.928924	11.033441	9.912045	11.086931	11.352876	10.194166	9.976964	11.490704	11.571323	11.624141	11.275733	11.073280	11.110174	11.217005	11.259889	11.714384	12.355778	11.739666	12.318545	11.968244	11.346523	10.651494	12.010496	11.913837	12.647261	11.044022	10.623981	11.120843	11.769458	11.205984	11.377558	11.122951	12.323571	11.421867	11.946966	11.447786	11.444404	11.721853	11.852935	11.913837	11.427665	12.632959	10.736706	12.470221	11.830322	10.853597	11.879981	11.491308	10.953044	11.084833	11.470789	11.621139	11.695625	10.500604	10.918816	11.208500	11.296869	11.642235	10.982204	11.452083	11.563461	11.694244	11.627501	11.998716	11.104426	12.591825	11.098167	10.495118	11.599731	11.949912	11.814926	11.660731	12.001171	11.898984	11.718397	11.905620	12.236220	11.271780	10.832125	11.920531	11.629546	11.605367	10.745880	11.195935	11.023804	12.432090	11.740121	11.783113	11.461571	11.467630	11.106155	11.711161	11.553299	11.992298	11.024358	12.350513	11.091901	12.098917	10.658429	11.325112

AC073987.1	3.270657	6.207422	6.142058	4.589938	3.357764	4.710518	5.859422	5.767564	3.483154	5.985562	4.891666	6.362694	5.079210	4.634126	4.632197	5.137599	4.503304	5.794218	5.389322	4.298115	5.075898	6.494926	3.068806	4.846376	4.753849	5.251390	3.551811	4.200963	3.962928	5.368189	4.848086	4.939145	3.401420	4.942999	5.852724	5.708117	5.547864	6.088848	3.725976	5.293505	5.429045	4.745325	4.880502	4.291335	4.759750	6.602610	4.934923	4.856921	4.971090	5.383142	4.233943	4.760599	5.863827	4.676014	6.777825	4.509641	6.307251	4.870615	4.821899	4.475809	5.832098	4.421986	3.935366	4.561847	5.936160	5.916177	5.098046	3.858971	5.716738	6.159627	5.698946	4.130430	6.290979	5.967524	5.671418	4.855023	4.066279	5.087179	5.409314	5.943556	4.533715	5.601521	5.984182	6.434619	5.422259	5.741001	8.789036	4.472145	4.475809	5.042847	5.155213	4.133820	4.911356	4.685015	5.005774	5.049361	5.361740	4.277739	5.299488	3.751278	4.303076	4.971901	3.329019	6.050444	7.611123	4.875897	4.531195	6.136200	4.816230	5.253360	7.205049	3.881073	4.726601	5.359108	4.682440	4.425470	3.459121	4.761215	4.726203	4.003344	6.765217	4.873439	3.463925	3.360502	4.547719	5.139457	3.231430	3.529232	4.105200	4.090469	4.081964	3.254844	3.406592	3.534249	3.659429	3.706468	4.062633	4.866428	4.889825	4.300454	5.335131	2.983458	3.413645	4.692443	4.497281	4.400062	3.398178	3.093371	4.567244	3.558530	3.581102	3.379304	7.116576	6.698709	4.122955	6.643033	4.168613	3.994177	4.504347	3.591899	5.096222	4.611221	3.681816	4.619283	7.329779	5.539772	4.433285	4.043422	4.684175	4.724322	3.771535	4.556009	4.427665	4.771569	3.326783	7.520175	4.785559	4.622478	4.163837	5.844745	3.830142	4.012032	5.041771	4.324006	6.790070	4.682027	4.582669	5.386761	5.909516	5.189186	3.519105	6.063309	5.648788	4.426112	5.654640	3.275589	4.370890	5.700628	3.851185	3.957915	5.609716	8.007472	5.224298	4.798741	4.707226	10.320132	3.666066	4.192784	4.011437	3.544326	3.975082	3.663842	3.836319	4.715187	3.822483	3.222099	3.352062	4.289822	3.362151	3.285959	3.238005	3.158273	3.603220	5.127355	3.545984	3.097255	4.628705	5.264485	5.992513	8.560859	3.506053	3.498889	5.611389	3.303468	3.518541	3.483534	4.684394	3.447280

AL139815.1	9.794721	10.097471	10.517868	10.167973	10.347353	9.413487	10.439142	9.105974	9.271815	9.781478	10.321225	9.956160	9.889233	10.317582	10.046320	10.382728	10.622788	10.318027	9.442662	10.376347	9.799812	9.296610	10.230046	10.059401	10.903791	10.678664	10.592463	10.400485	10.060910	9.816653	9.428831	10.219095	10.447340	10.143394	10.685541	10.576772	10.470880	10.511412	10.185274	9.843353	10.371706	10.106999	10.240898	10.222414	10.896417	10.244280	9.956316	10.630554	10.201561	10.219240	10.069314	10.754933	10.698366	10.685697	10.495118	10.562074	10.971621	10.473914	10.017413	10.800245	10.340966	9.895820	10.623218	9.545166	9.674434	10.479505	10.333290	10.211657	10.031790	9.697544	10.400485	10.341990	9.870758	10.499861	10.345357	10.295548	10.531426	10.075573	10.012286	10.804897	10.387334	10.650579	10.510632	10.813727	11.110723	10.720621	10.601053	10.452826	9.960448	10.201561	9.996607	9.782680	10.725060	10.361904	10.357076	10.755886	10.606387	10.411451	10.388103	11.063055	9.767533	10.586129	10.308483	10.413733	8.959613	10.196135	10.645344	8.705827	9.364876	9.857414	9.036629	9.980743	9.525379	9.334599	9.510217	8.307119	9.400155	10.640152	10.108827	9.467958	8.774754	10.013986	8.839638	8.051562	8.302435	9.097822	10.100294	9.195778	8.885915	9.082741	8.743149	9.122924	9.418110	9.016068	10.455864	9.619777	8.772007	8.619099	9.663115	8.623592	8.830255	8.870392	9.422207	11.114258	9.136223	9.642543	7.446170	8.204198	9.818272	9.769677	9.726365	9.829351	8.959322	9.115266	9.700346	10.088700	9.293154	9.578383	9.486110	8.015866	8.548766	9.375451	10.020621	9.768131	9.387333	9.351702	10.387032	8.582420	9.734237	9.923681	9.742277	9.573979	10.090376	10.123198	9.687619	10.058151	9.843185	9.502078	9.602366	9.434621	9.631081	9.368622	8.715458	10.038692	9.188306	10.173663	9.811172	9.398142	11.219825	9.533834	10.032856	7.503538	9.854790	9.978283	10.062130	9.997062	10.491272	10.021565	10.562777	10.321065	9.999670	9.470011	10.511234	9.992041	9.301568	9.918248	9.810864	9.186600	10.136837	11.334188	8.842601	9.797194	9.684992	10.482160	9.902118	11.046498	9.914676	9.152161	10.098874	8.896265	10.405317	9.956316	9.427739	9.537362	8.528282	9.047293	8.526901	8.414001	9.342047	7.831082	10.532841	8.856221	8.105290	10.277128	9.897040	8.848488	8.634890	9.098736

NRAD1	3.370267	9.231906	3.280072	5.163669	3.214885	6.551153	5.130587	4.190383	4.693492	6.989679	6.398476	9.300176	9.065038	3.584748	6.134494	8.131027	3.377896	6.450151	7.861673	9.222670	7.307427	3.625205	4.664093	7.833074	6.722244	10.093356	8.488015	10.265433	9.427118	7.725750	3.660607	6.576865	4.941773	8.812183	3.676604	7.356091	9.597413	6.456215	4.660717	3.338945	3.339291	7.862157	8.586817	7.661530	6.185209	5.558672	8.603127	8.895603	7.984384	7.422754	7.808333	3.879570	7.179851	7.419618	8.763320	3.104462	3.997047	3.174610	9.469843	7.330395	7.881392	3.345907	3.816718	9.544116	3.298546	8.528920	8.047031	4.018242	6.153949	6.819436	6.544838	3.660815	3.089591	7.545035	3.499802	4.526110	6.432086	7.672128	9.663882	3.396948	3.152931	7.555801	9.337427	8.692290	6.298576	10.623981	3.537062	3.892646	6.776864	8.182181	8.744959	5.495098	7.104243	3.642881	9.814948	4.346108	9.479088	3.645219	7.988792	3.102889	3.030591	7.884003	5.993056	3.238890	7.608208	4.372594	6.849029	3.478013	4.215112	7.358111	3.505862	7.780442	5.614444	3.253582	3.995290	3.816090	4.057364	10.315629	6.350954	3.625390	5.393986	4.041017	4.505883	3.089786	4.532908	3.198410	4.350224	4.384713	3.665068	5.524600	3.625598	4.021995	3.536493	3.212842	3.863232	3.834416	3.143041	3.185682	4.624415	3.726139	3.260431	5.168494	3.416052	3.343473	5.727722	6.615919	4.983217	3.744836	5.413382	3.962484	6.476918	3.163139	6.968555	4.218149	4.490913	7.004729	6.132086	4.225973	3.781036	3.156795	6.424645	4.824555	5.389874	5.637531	5.121515	4.380795	5.444765	4.364615	5.444298	5.001521	4.247745	4.979337	4.534548	4.550664	3.318632	3.563462	3.185819	5.067563	5.284147	4.162256	3.158396	4.834971	7.599995	4.542193	5.971960	5.085724	5.148519	4.083044	7.375633	6.883240	3.533492	3.060451	4.330557	5.810937	4.257871	4.465643	4.709693	5.883154	4.362823	3.491185	4.891473	4.866626	4.738874	4.110281	5.318802	4.058241	4.201183	4.643391	4.794167	5.096023	4.190163	3.093869	3.538645	5.658878	3.337310	4.945885	3.116174	4.143033	3.709474	3.521300	3.466667	3.102889	3.495025	5.056780	6.157372	4.145567	3.570765	4.545332	3.597934	3.421532	4.983993	3.338781	3.204680	3.503315	3.932381	4.353327	5.435917	3.478013

STARD4-AS1	6.258790	6.637692	5.695854	9.110179	8.827765	11.048256	5.729540	9.771440	10.451084	6.226757	6.141377	7.141116	5.834109	6.055991	6.076422	6.604561	6.653693	6.167203	9.624150	7.504612	6.004648	7.314333	8.688827	6.425798	6.504161	7.191534	8.092170	5.922158	6.934887	6.845561	7.507225	7.723620	6.185885	8.012294	8.665154	8.626721	10.252933	7.816519	9.938052	8.527834	5.708658	7.294462	8.652353	7.854983	7.667600	8.163103	6.868396	6.738587	8.007472	6.113851	6.151907	7.990014	5.342558	7.950014	4.700479	6.753911	5.863653	6.343180	6.363544	7.106131	5.685292	6.070155	9.046851	10.030084	6.926148	6.417818	8.927404	7.096414	8.354912	8.799150	10.912644	12.847005	6.840313	11.449854	9.057198	8.764223	10.708553	10.936702	10.026576	6.778153	10.356772	6.062933	6.793483	6.196190	8.545801	6.808885	11.308969	9.541895	9.106435	8.258160	8.290571	9.085022	8.127337	7.967712	8.703369	10.037185	7.172461	7.668055	7.279886	9.583612	11.466748	7.115823	7.828691	8.792595	8.269676	7.490559	6.977696	7.515537	10.100294	6.550531	8.263850	10.513021	11.876727	9.208246	5.592356	16.533775	10.161998	6.830874	7.622087	7.925226	6.670564	8.581670	8.221444	12.289963	7.051636	9.162764	10.501376	11.845977	8.482171	8.208150	7.185534	8.780092	8.366367	7.489761	11.522184	9.413187	8.006706	8.993181	9.348949	10.301273	9.200778	9.743197	8.888686	10.274257	8.043558	13.931123	9.139031	11.295979	6.234806	10.698075	9.660909	9.019569	8.470095	9.938201	9.665161	11.244638	11.757848	10.715070	11.647837	9.137318	9.223893	9.004108	8.543056	7.777466	8.186673	8.519771	8.607415	7.498818	8.949039	7.743330	8.851683	7.496941	8.786239	8.851981	8.465536	9.885942	7.188552	9.761985	8.031326	8.690185	6.036861	7.818572	8.465383	7.856647	9.175570	9.838908	7.894980	8.212064	8.787683	11.569677	12.423741	6.000597	11.958284	13.937690	10.327332	11.746117	11.742899	9.910027	9.510060	7.446661	6.880550	8.725877	8.495523	9.045642	10.537371	10.002883	7.641532	10.872927	10.709515	6.990632	8.721903	9.251788	8.060250	8.507811	11.558653	11.001079	10.143394	9.249936	8.575191	9.302046	8.996270	11.328919	10.592327	8.046247	7.281623	10.504236	7.280850	6.677686	8.079763	9.014091	12.434731	15.195519	9.558482	8.725114	14.364077	8.075328	7.819973	7.866962

PSMG3-AS1	9.579952	8.611061	8.636014	8.337585	8.680362	9.194486	9.164933	8.652644	9.209941	8.414877	8.201533	8.236375	8.373422	8.696676	8.552439	8.558229	8.752751	9.047133	8.921058	8.777073	8.215758	9.963663	9.405722	8.943997	9.510547	8.212064	9.116026	9.264743	9.066922	9.683577	8.968219	8.995183	9.554047	8.938535	8.944146	8.943699	9.117571	9.214537	9.974049	10.033478	8.414146	9.697699	9.015152	8.744816	9.750211	9.260146	8.766232	9.665313	8.409398	9.898374	9.133527	9.271815	9.049437	8.531668	9.055814	8.130096	10.063627	9.656270	9.547923	9.932341	10.052486	8.892677	9.512302	10.029952	10.602549	10.169039	9.697699	9.175876	8.940945	9.630751	9.035284	9.210370	8.374648	8.937007	9.230147	8.871045	9.635632	9.480888	9.217322	10.054805	8.741484	8.559772	9.041013	9.610886	9.891332	8.851831	9.551013	9.547000	9.334439	9.054864	10.800421	9.021730	9.523811	9.221888	9.476585	8.937800	8.641436	9.121392	9.152637	8.958429	8.932263	9.285069	9.131973	8.613295	8.603917	8.318066	9.343609	8.746957	9.459862	8.511237	8.978174	9.292368	8.844814	8.310512	7.731029	10.335092	9.553744	8.576857	8.707834	9.020620	8.010496	8.970518	8.104486	9.404197	8.799601	8.337585	8.820838	9.904700	9.499970	7.907550	8.173935	8.421352	8.554202	8.255885	9.454768	8.746794	8.578625	8.843116	9.152161	8.034780	10.327627	9.054553	8.948426	10.546889	10.305812	9.014854	8.468400	9.256976	9.252371	8.528127	8.601432	8.656538	8.327120	9.082435	8.622183	8.945669	9.060051	8.781189	9.697083	8.858058	9.445907	8.540706	9.166044	8.338861	8.806738	8.601276	10.641916	8.105140	9.716701	8.536257	8.877552	8.907661	8.466285	9.135618	8.630992	12.385972	8.914940	8.648567	9.989516	8.967268	9.022798	9.879312	10.405164	9.544116	9.900517	9.688696	9.049275	8.417243	9.635632	8.880343	8.743149	8.307119	8.173625	9.326448	8.259851	9.063531	9.355134	9.031263	9.204729	8.887442	8.038522	8.416918	9.434546	9.468399	8.635856	8.523382	8.426610	8.939581	8.814119	10.697783	8.504625	8.468700	8.639417	8.742540	8.643311	8.538773	9.022623	8.692458	8.525385	8.983646	8.544468	8.180232	8.628077	8.055096	8.207388	8.546584	7.890456	8.737644	8.869138	9.091411	9.373655	10.026067	8.651781	8.960537	8.713136	8.749551	8.010042	8.479305

MIR4435-2HG	8.729734	8.679458	8.484010	8.434442	8.970518	8.788120	8.043864	9.037084	10.558269	8.438853	8.835465	8.375725	8.439638	8.753188	10.486410	9.020946	9.496268	8.001204	8.062875	8.867036	9.022327	8.896105	7.826473	8.703695	8.394832	8.522011	9.765411	9.206427	9.366774	8.900824	9.010820	8.291199	10.666968	9.289882	8.214394	8.464437	7.989420	9.679857	8.318066	8.315284	8.185892	8.085541	9.847836	8.372228	8.452011	9.020946	8.568189	10.206741	9.314441	7.917069	8.274612	8.402217	8.624991	7.922093	9.116948	9.171571	8.224051	8.500441	10.768642	8.777381	8.328762	8.257856	9.188306	8.233424	8.216505	8.481554	7.835387	8.373576	8.545378	7.705019	7.797700	8.074860	10.056200	8.033453	8.381126	11.414427	8.132684	8.611684	8.261975	8.850032	9.176193	9.012996	8.443325	8.167373	8.395457	9.976047	10.221095	9.148011	7.644473	8.449532	8.878315	7.895271	8.810123	8.567308	10.366240	8.941412	9.272995	8.694675	8.856984	9.874660	9.924138	9.434472	8.598366	8.074860	7.907878	9.485482	8.932840	8.413538	8.966798	8.597021	8.058438	9.148458	9.010403	9.176661	10.070853	10.826359	9.173899	8.629570	8.815901	9.539063	9.014405	9.028257	9.291136	9.204729	9.072827	8.431677	9.161517	10.430953	9.298003	8.789957	8.877552	8.824012	9.033700	10.398075	9.183769	9.195114	9.793920	9.011761	8.801264	8.815760	8.752885	8.229646	10.003654	8.495523	9.191651	9.305718	9.840623	10.490863	8.850472	9.299396	10.763875	10.596659	9.170158	8.907495	10.435833	8.399107	11.219825	8.850907	8.991632	9.037084	8.962835	11.667871	8.375408	8.865361	9.906961	9.171408	10.450613	9.497649	8.523382	9.091750	9.232852	9.346035	8.765277	8.921205	8.395299	8.600815	10.105829	11.461288	10.213349	9.292992	8.234201	10.631433	8.853796	8.482018	9.533834	8.551647	9.184707	9.266415	9.607021	8.723135	8.281921	10.407834	8.631128	9.031969	11.609621	8.558069	8.592063	8.406973	9.842744	9.383032	9.169378	9.153531	9.614684	9.922286	10.495855	10.168271	9.289571	8.103852	8.815901	9.229431	8.445017	9.111930	8.698484	10.601523	9.463027	10.106438	9.229700	8.740710	8.923868	9.705663	10.949054	9.134694	9.335876	8.306800	8.921804	9.873938	9.811665	8.260488	9.276071	8.781030	9.564664	9.987152	9.633683	9.395906	9.420843	9.274259	9.321385	8.304669

AC024361.2	4.419593	4.917062	4.508763	3.357764	3.356906	2.997603	4.800507	3.385183	3.212552	3.144331	4.166251	4.834783	3.930454	3.374551	4.584996	4.089384	3.235794	3.924386	4.478885	3.886551	3.940865	3.718118	2.942353	3.579575	3.987353	3.603661	3.416880	2.850107	3.526294	3.775550	4.701302	5.026830	5.177954	4.996288	4.858553	4.365486	4.227481	3.950770	4.258314	2.989605	3.268548	5.172533	4.420677	4.455015	3.407115	6.383651	3.165816	5.119073	5.296440	5.188600	5.189361	4.217298	5.705050	3.465394	3.437397	3.547179	5.528391	5.420176	4.437234	5.174138	5.043266	2.983971	5.950553	3.832923	4.738874	4.959791	5.173357	3.930876	4.621851	3.938998	4.601011	3.776565	3.604642	4.224260	4.520187	4.620607	3.888276	3.502944	4.195839	4.416652	3.155076	5.300411	4.687391	5.225509	3.783575	4.818563	3.309058	4.497929	5.199473	3.306975	5.311070	3.802439	4.472556	5.541080	5.480817	3.913138	4.256493	3.801408	4.460477	4.385189	2.945609	4.244727	4.173784	4.197114	4.808685	3.021235	2.915529	4.280614	2.922785	3.086040	3.698788	3.510886	3.100092	3.126294	3.826990	3.173051	4.866428	3.536879	3.092203	5.014462	4.294590	3.592616	3.455318	3.684214	4.120757	3.171558	5.127160	3.090417	3.416052	3.850777	5.327319	3.209927	3.091524	3.795069	3.210306	4.009963	5.155213	3.693392	3.937709	4.902149	3.670739	2.948609	3.464122	5.183621	3.634474	3.453325	3.451547	2.965075	4.150618	4.276840	3.840468	4.349409	4.796480	6.286409	5.107256	4.375003	4.883487	4.023916	4.233299	3.391201	4.898357	5.482205	3.607655	4.674518	6.496679	3.596331	5.814756	6.543526	5.198528	4.258714	6.760102	5.894592	5.582483	4.512683	3.469807	6.736422	7.315429	5.155798	5.258891	4.927218	4.160159	7.246849	4.887505	4.871491	5.448784	5.198528	4.726811	5.008061	5.768316	4.671238	3.724305	3.353040	4.421729	4.343091	4.699401	5.167687	3.645826	4.389751	5.034634	4.577568	6.181859	4.628037	4.213405	4.460269	3.368911	4.528611	5.997089	5.649152	4.129602	5.735376	4.176392	5.000684	5.255945	3.755678	3.469235	3.275122	3.346730	4.642176	5.906156	3.524981	7.067141	4.692874	4.301308	4.758717	4.353548	2.907677	5.412215	3.582650	3.485680	3.410757	3.053021	3.912272	3.358975	5.532394	4.360255	4.081358	4.257871	3.595009

LINC00278	3.002449	7.630529	8.346927	8.583467	9.850885	8.912802	8.225701	9.088689	9.230297	9.855874	9.417175	8.309320	8.435395	8.695139	8.673115	9.185481	7.834927	3.734186	9.772051	8.730911	8.637239	9.545320	9.479088	9.021121	8.813801	9.053286	7.672898	9.451098	8.101742	6.482715	9.469843	7.573718	8.415321	6.926316	7.927272	8.359426	3.568264	7.881227	8.594088	8.810440	9.054864	7.676347	3.312709	8.426298	8.374492	7.806501	8.773366	7.876282	8.168776	8.319558	8.161001	8.003407	4.038661	8.967589	3.697556	8.519607	5.948272	5.168280	8.027164	5.739181	7.982864	3.072161	5.090437	8.671909	5.974655	7.551749	7.805090	3.297290	3.126294	7.482906	7.721466	8.398224	3.053619	7.838479	8.039647	7.375955	3.109934	7.754107	7.675427	8.382675	7.694438	7.768537	7.975543	4.347616	8.707834	8.245241	8.325415	7.910633	7.612184	7.979579	7.172144	7.204873	7.711380	8.016641	7.117723	8.998577	3.679024	3.071974	8.994888	8.993639	8.424386	8.708574	2.972058	9.104428	8.019824	8.962369	9.381628	8.115323	7.714645	9.094484	3.417042	8.507514	8.918022	9.222218	3.009067	4.334841	3.277250	8.113934	9.330587	3.315269	4.455648	4.662858	5.257333	3.393995	5.297271	3.671942	4.207761	4.105841	3.933476	3.427251	5.876401	3.449554	8.086448	3.644431	6.279314	3.338286	3.256611	3.360160	5.552010	3.909010	5.582483	6.120967	3.646801	3.155467	4.071053	6.640481	3.292658	3.340980	3.666887	4.982161	3.939843	4.435851	3.662244	6.778153	5.071425	3.560111	6.616420	5.873510	4.513363	4.787036	3.533311	3.424717	3.862777	6.606540	3.999992	3.538047	3.334371	6.914636	4.643182	4.271174	4.156749	3.830779	5.175767	3.388600	4.869784	5.961446	2.928851	4.218384	6.056734	3.436352	3.164694	4.953562	6.531435	4.574373	4.927015	4.593146	3.603420	3.431869	4.196022	4.293345	4.529040	3.044660	4.105200	4.766823	5.744449	3.351386	3.996193	5.592917	5.715850	5.746855	6.050770	6.186042	3.698196	6.944407	3.167396	3.875266	3.521873	3.953338	3.812307	3.452949	3.539784	5.952342	4.083468	5.617409	5.278766	2.986165	3.313790	6.629353	5.517407	3.320743	3.648809	3.003522	5.234617	5.227918	5.027663	5.025271	6.174728	7.359095	3.912495	3.314962	4.259584	3.293300	8.434134	2.952659	2.981438	3.504732	3.895854	4.259179

AC139887.2	9.257737	8.773506	8.801192	9.090357	8.688827	8.094026	8.865208	8.642660	9.475061	8.715790	8.649523	8.795349	9.060211	9.324472	8.652353	8.550581	9.290980	9.655361	9.815584	8.564040	8.704737	9.689297	10.475718	9.687619	9.992805	9.718286	9.482261	10.035315	8.723730	8.465833	9.293796	8.183127	8.431677	9.581310	9.532146	9.018966	9.152019	9.254499	9.584146	9.803798	8.397030	10.060453	9.197647	8.976517	9.176949	9.134541	8.795349	9.541437	8.617319	8.661319	9.238262	8.802888	9.072827	8.849730	9.160920	8.312823	9.763401	9.946708	10.009587	9.319127	9.724389	8.923868	9.508842	10.115300	9.448726	9.631081	9.860520	9.357553	8.938386	9.463097	8.303462	8.838748	9.002437	8.756380	9.145331	8.773068	8.777381	8.463686	9.160920	9.097822	9.775382	9.237796	9.551318	9.223893	9.305277	8.902638	9.359075	9.838589	9.234733	9.425872	10.201561	10.263600	9.206866	9.082132	9.448419	10.262977	9.041919	9.879639	8.957459	9.845979	9.358192	9.095402	9.231455	9.058147	9.607774	8.561010	9.906054	9.435081	9.966653	8.818569	9.579165	10.258842	9.916212	9.427590	9.695275	7.683826	11.062006	9.431422	8.704893	9.669262	8.582845	8.941567	9.420843	8.938682	8.025420	9.762791	10.589350	9.348642	9.395272	8.256035	9.380526	9.230592	11.249397	8.771708	8.676820	9.779222	10.280189	10.153732	8.438572	8.931622	10.420096	9.655185	10.714317	9.175420	9.663731	8.424530	9.797967	9.200001	8.505967	9.877347	8.846176	9.282455	9.310505	9.800887	9.116348	9.358336	9.392669	10.104790	10.212412	9.287393	9.976964	8.835874	9.132278	8.853327	8.686278	8.916172	11.037267	8.434770	9.048542	9.352758	9.248587	9.721996	8.887920	9.497352	8.264324	10.551284	9.932341	10.900700	9.354991	9.993847	8.320503	10.722212	9.889997	10.780002	10.340663	9.794095	10.033321	10.341990	9.754226	8.658511	8.872478	9.801200	9.738374	9.537835	9.335876	9.456162	8.986242	9.273303	9.411123	10.185424	10.376671	10.515780	10.272760	9.829180	9.764347	9.977842	11.007562	10.292786	10.963108	12.158494	10.678215	10.215186	9.266572	9.361712	9.877830	9.484723	9.965611	8.959322	10.082394	9.396207	9.908340	10.155777	9.299396	11.640267	9.251636	9.423930	9.679997	9.358480	8.201821	9.310802	9.042653	9.842448	9.078242	9.395577	8.287784	10.248163	10.456649	8.542583

AF064860.2	5.054312	6.469852	3.332583	5.548626	3.791856	4.482420	3.090860	3.639080	3.729640	3.545643	3.295881	6.133265	3.753157	3.444194	3.722298	4.589046	3.248982	5.085272	4.647255	5.793485	3.990448	5.215622	6.076422	4.234172	4.315840	5.073638	4.479330	6.732532	4.352445	3.564573	3.392489	5.142956	5.261864	5.745334	4.684809	4.714760	3.783976	3.674117	3.124425	3.854271	3.297455	4.636303	3.777351	5.122099	3.323979	6.962923	4.208229	6.266890	3.519668	4.436050	6.086241	3.030788	3.436352	4.785559	5.492043	3.392134	7.818875	4.925093	6.512439	4.674518	4.624008	3.258079	4.947342	5.054532	6.082166	5.692261	5.099618	3.590125	5.307327	2.979842	3.384652	3.138348	3.202823	4.594487	2.830429	3.486788	3.617097	4.850372	6.556465	3.592419	3.217861	3.704674	5.115570	4.611640	3.632948	5.068793	4.574580	3.208598	3.749587	5.367011	6.706548	3.204548	5.412627	3.817794	6.195129	3.667908	6.171677	3.728166	4.877783	3.981290	3.793589	4.194544	5.265256	3.652414	4.391694	3.112133	4.266557	4.134067	3.587651	3.058514	4.405149	4.254797	5.860123	4.256960	3.540150	5.365022	4.651356	5.665330	4.508763	4.048439	5.242838	4.191920	5.688078	4.240349	6.101173	4.815819	3.716082	4.256493	4.713285	4.585851	4.884088	4.239453	4.416874	4.367632	4.464978	3.987976	5.244217	4.419593	8.276569	5.101759	4.720037	3.279422	4.802838	5.494903	3.911422	4.376947	4.921033	5.052375	4.518683	4.116995	4.688032	4.341562	5.216797	4.937082	5.701366	4.912630	5.175767	4.501617	4.782618	4.214253	4.304427	4.212963	4.899185	6.187399	7.066362	4.273115	3.066192	4.885836	4.000224	4.909040	4.334841	5.402456	5.018899	5.991459	4.344818	5.463106	7.881392	4.689716	5.331246	2.851394	6.722418	4.668615	4.365913	5.113938	4.307689	4.088092	4.541317	4.239453	6.231589	4.411319	3.567669	4.152302	4.420461	3.757114	3.970288	3.441340	3.671371	4.746622	4.779673	3.941517	3.875659	4.996504	5.367986	4.705737	4.415573	4.228532	6.251818	4.216015	4.688438	4.686349	3.783146	4.432464	4.074943	4.513800	4.273784	5.728084	4.365248	4.792691	4.168824	4.203381	4.144522	5.281972	4.501376	4.790580	4.158634	4.245365	4.812278	5.172120	4.476238	5.974996	4.531195	4.875476	5.770307	4.112008	4.367861	6.376000	4.792105	4.258087

PLSCR5-AS1	3.070681	4.057579	2.965658	3.789104	2.935655	3.104462	3.098313	3.073797	3.210865	2.942434	3.261007	3.379120	3.076339	3.696923	4.127199	3.129814	3.260431	3.519668	4.396359	3.158535	4.430721	3.200773	4.088961	3.237851	3.563042	3.488107	3.146072	4.183437	3.616879	3.128217	3.521682	3.825093	3.150377	3.663842	4.107221	3.703060	3.574404	3.286535	3.503982	3.292054	3.319425	5.436682	3.967702	3.000357	3.458409	5.492043	3.213260	4.185988	3.277250	4.880724	5.180533	3.688091	3.857663	3.718335	5.015459	4.093706	3.860875	5.434567	3.937075	3.636869	3.304432	3.047872	3.860460	3.118405	3.292959	5.594934	7.057146	3.909010	3.995290	3.072674	4.120757	3.576932	3.099853	3.778757	2.840883	4.411994	4.319054	3.630688	5.328100	3.335169	3.556216	3.578575	6.233583	6.226232	3.735228	3.246100	3.688293	3.427639	3.269945	3.716933	5.569576	3.254178	3.424387	3.121240	5.940777	3.240594	4.829094	3.039623	3.075184	3.059814	3.342665	4.109593	3.031355	3.160172	2.966317	3.112983	3.056767	3.303300	3.588616	3.370996	3.441340	3.139936	3.805556	3.145941	3.094765	4.018944	3.390353	3.590125	3.342665	3.420470	3.280580	3.478770	4.154991	3.571891	3.559932	3.607261	3.052385	3.156147	2.898622	3.079971	3.035971	5.176738	3.830958	3.543818	3.378256	3.213675	3.145941	3.613588	3.250569	3.867730	2.912392	3.465757	4.719590	3.863232	3.261007	3.145372	4.111173	3.605218	3.347388	3.322329	3.274345	3.218550	3.145815	4.692658	3.675584	3.545073	3.299180	3.446532	3.177091	4.126990	4.341985	3.394704	4.653042	3.768830	5.886902	3.490484	4.125452	3.373077	5.053865	3.748541	3.397833	3.553350	3.759754	4.164884	3.465570	5.966091	3.589021	3.282097	5.159865	3.058119	3.124552	5.106012	3.354345	3.417894	3.830142	6.643033	4.328092	3.509419	3.139069	3.775313	3.653830	3.046501	3.236957	6.663130	4.647912	3.934301	4.388263	4.175090	3.051529	3.387383	3.822675	5.258293	3.426395	3.478413	3.238603	4.287203	3.991816	3.185151	3.335311	3.497641	3.983919	3.643274	3.061222	3.224190	3.508701	3.510558	4.029752	3.413099	4.881587	3.253718	3.070262	3.411118	3.374235	2.990536	3.079184	3.117943	3.462494	3.433628	3.808655	3.026669	4.106979	5.820610	4.120757	3.277138	3.050254	3.918319	3.692972	3.592821

AL590556.3	12.408694	10.015875	9.716549	10.221812	11.038581	12.138038	10.569984	11.671500	12.041156	11.062165	11.808575	11.633139	11.315577	10.194330	9.478608	11.117822	11.477264	9.834126	10.403775	10.676287	9.711867	9.638574	10.313861	10.583183	10.039581	11.184811	12.055128	11.519395	11.505395	11.342479	11.522341	9.457241	9.357409	11.039457	10.327183	10.063036	10.483377	9.858664	10.302038	10.149656	10.986908	9.637166	9.587971	10.064236	9.285357	9.695275	12.240224	8.902785	9.963319	9.174484	11.600906	9.019430	8.355532	9.009028	8.879418	11.588775	8.576857	8.818873	8.851059	8.857898	8.954265	11.097335	9.048698	9.628290	9.397158	9.084689	9.385437	9.641761	9.300940	9.336631	10.248940	10.611842	11.665514	10.518615	10.428377	10.301273	10.355676	10.620204	10.822371	9.154648	9.663654	9.053286	9.307273	8.881902	9.249360	9.864242	11.293238	9.868678	9.023129	9.125977	9.097671	9.849294	9.740870	10.189798	11.642828	10.422252	9.936051	10.857343	11.084241	11.218485	11.697417	9.908340	10.441420	10.083133	9.412599	11.301874	11.109567	9.936051	10.734015	11.636481	9.971512	10.286654	10.324760	12.433552	11.191198	14.865570	11.204476	11.073997	12.240075	11.159639	11.018683	9.994619	10.010461	11.991408	11.417260	11.949129	12.220618	11.527331	11.188740	11.658340	11.587908	11.926448	10.315777	10.213349	11.897652	11.316160	10.305051	10.348720	10.434121	9.799644	9.607484	9.771286	11.227093	10.232495	10.163352	11.533482	11.362983	12.490065	11.068971	11.709994	10.244721	10.260870	11.631959	10.566051	10.373518	10.895803	10.421803	10.817596	10.278058	11.805598	9.818140	9.486434	10.435833	9.742594	10.191000	12.335719	9.149261	9.684512	9.707187	10.635198	9.241363	8.872006	9.313132	9.128435	10.758970	9.204887	8.940945	9.239831	8.818417	9.390059	10.642678	9.504085	9.491252	9.492722	9.010681	9.731377	10.136400	9.573979	9.052352	10.464739	10.604891	12.335719	10.876057	10.632606	10.255889	10.564425	10.910589	11.115175	9.583467	9.611030	9.348808	10.084052	9.651040	9.270142	10.847998	11.861844	9.663115	9.417469	9.484565	9.469514	9.946536	10.039741	10.228208	11.622683	10.975965	11.259576	11.582100	11.078266	10.249984	11.121598	10.739457	10.997004	10.099349	9.390348	11.422093	11.135665	9.626311	9.820554	12.362443	9.999048	10.532043	12.425739	12.887549	11.326454	12.107410	9.706727	10.844240	11.467339

AC097713.1	3.790551	4.831232	5.331849	5.021154	5.420915	4.264426	4.324006	5.905776	4.629125	4.637372	3.246954	4.021786	4.991405	3.544145	3.634068	5.457054	3.287873	3.188193	5.093551	5.675316	5.302426	5.523280	2.855829	6.360873	5.255945	4.681557	6.400824	4.644930	4.949181	5.590423	3.735228	3.135127	4.916406	5.919034	5.913302	5.605125	4.826069	4.939145	4.323129	4.747673	3.874606	5.082452	6.916498	6.359358	5.153174	6.253184	4.249061	3.612379	3.525165	5.165102	3.151870	2.996944	3.650983	4.751297	4.292475	4.713285	5.780899	4.112439	3.865362	3.825093	4.572849	5.019719	3.795304	4.479330	3.690137	4.065226	4.787444	5.251581	4.885836	4.854590	4.190613	4.679862	4.787643	4.960598	4.957517	5.257531	4.818129	4.630030	4.571394	5.027227	4.704057	4.039525	4.978895	4.162728	3.340297	4.515053	4.292918	3.683451	4.353327	4.525222	6.042449	3.815250	5.109134	4.616876	5.599151	6.003613	4.434340	4.909875	4.385418	3.992018	4.851592	6.180491	4.633927	4.093091	5.058787	5.442744	5.465597	5.695854	4.690116	5.035911	4.185118	5.065719	4.817515	4.432246	5.137833	5.377137	4.158634	4.584786	4.560764	4.642400	4.511172	4.635852	5.081029	5.774175	4.018026	3.852488	7.165039	4.309263	4.861224	4.375223	3.859821	5.058586	3.948976	3.253865	5.155213	3.319776	3.280717	8.189568	6.001794	4.710518	4.182119	2.858432	5.440059	4.424840	4.662653	4.596619	4.882858	5.264099	4.311668	3.666279	3.902531	3.927841	5.168494	4.824555	3.998329	4.155600	3.439792	3.592419	3.852717	4.000428	3.122398	5.353060	5.373609	4.131875	4.116995	4.569162	3.283331	3.730048	3.677808	3.025843	2.942182	3.710057	4.131034	3.360651	4.780099	6.607406	3.999159	3.678004	3.836533	2.893352	5.429568	3.871346	3.646626	3.650427	3.603420	3.721688	5.469704	4.822102	4.694734	3.890274	4.280400	4.494423	4.187106	4.344368	4.361966	3.855094	4.252367	4.505883	4.347616	4.945436	3.755044	4.489766	5.310505	3.360810	4.756759	3.760914	3.369092	4.052367	3.740951	8.221604	3.500685	3.900869	4.368291	4.920613	4.618177	4.515287	4.466328	3.924576	4.032121	5.169309	7.768537	4.245156	4.358295	4.799428	5.801186	5.135038	4.361307	4.228324	4.929972	5.020329	5.113751	3.773257	6.136363	6.263683	5.124915	4.339423	3.569466	4.385189

LINC01891	3.790551	4.831232	5.331849	5.021154	5.420915	4.264426	4.324006	5.905776	4.629125	4.637372	3.246954	4.021786	4.991405	3.544145	3.634068	5.457054	3.287873	3.188193	5.093551	5.675316	5.302426	5.523280	2.855829	6.360873	5.255945	4.681557	6.400824	4.644930	4.949181	5.590423	3.735228	3.135127	4.916406	5.919034	5.913302	5.605125	4.826069	4.939145	4.323129	4.747673	3.874606	5.082452	6.916498	6.359358	5.153174	6.253184	4.249061	3.612379	3.525165	5.165102	3.151870	2.996944	3.650983	4.751297	4.292475	4.713285	5.780899	4.112439	3.865362	3.825093	4.572849	5.019719	3.795304	4.479330	3.690137	4.065226	4.787444	5.251581	4.885836	4.854590	4.190613	4.679862	4.787643	4.960598	4.957517	5.257531	4.818129	4.630030	4.571394	5.027227	4.704057	4.039525	4.978895	4.162728	3.340297	4.515053	4.292918	3.683451	4.353327	4.525222	6.042449	3.815250	5.109134	4.616876	5.599151	6.003613	4.434340	4.909875	4.385418	3.992018	4.851592	6.180491	4.633927	4.093091	5.058787	5.442744	5.465597	5.695854	4.690116	5.035911	4.185118	5.065719	4.817515	4.432246	5.137833	5.377137	4.158634	4.584786	4.560764	4.642400	4.511172	4.635852	5.081029	5.774175	4.018026	3.852488	7.165039	4.309263	4.861224	4.375223	3.859821	5.058586	3.948976	3.253865	5.155213	3.319776	3.280717	8.189568	6.001794	4.710518	4.182119	2.858432	5.440059	4.424840	4.662653	4.596619	4.882858	5.264099	4.311668	3.666279	3.902531	3.927841	5.168494	4.824555	3.998329	4.155600	3.439792	3.592419	3.852717	4.000428	3.122398	5.353060	5.373609	4.131875	4.116995	4.569162	3.283331	3.730048	3.677808	3.025843	2.942182	3.710057	4.131034	3.360651	4.780099	6.607406	3.999159	3.678004	3.836533	2.893352	5.429568	3.871346	3.646626	3.650427	3.603420	3.721688	5.469704	4.822102	4.694734	3.890274	4.280400	4.494423	4.187106	4.344368	4.361966	3.855094	4.252367	4.505883	4.347616	4.945436	3.755044	4.489766	5.310505	3.360810	4.756759	3.760914	3.369092	4.052367	3.740951	8.221604	3.500685	3.900869	4.368291	4.920613	4.618177	4.515287	4.466328	3.924576	4.032121	5.169309	7.768537	4.245156	4.358295	4.799428	5.801186	5.135038	4.361307	4.228324	4.929972	5.020329	5.113751	3.773257	6.136363	6.263683	5.124915	4.339423	3.569466	4.385189

LINC01182	3.008858	3.065322	2.950634	2.990915	2.866588	2.969108	3.102750	3.066396	4.375667	3.070579	3.189289	2.992607	3.124150	3.350047	3.032852	3.033479	3.264982	2.910264	4.159720	3.068252	3.385855	3.048689	3.014839	2.900822	3.006886	2.941197	2.995910	2.897780	3.004804	2.977679	3.405245	3.023191	3.487527	2.979944	4.868117	3.732967	3.267648	3.002647	4.257644	3.054044	3.272546	3.029277	3.320580	3.076437	3.436171	7.639235	3.125273	3.088534	3.336976	3.331586	3.179647	3.137848	4.642591	3.821253	3.378618	2.993827	5.888681	3.556216	5.898668	3.538985	3.031650	3.014468	3.444542	3.099621	3.217985	3.223347	3.265866	3.077141	3.090979	4.285215	2.948609	2.895171	3.040703	2.880520	2.953929	3.195971	2.976959	2.888372	2.942527	3.377564	3.148514	3.502944	3.238603	3.600679	3.145662	3.186882	3.380664	3.479830	3.878719	3.335003	3.096657	3.024753	3.431525	3.068701	2.897589	2.924747	3.274034	3.146903	3.058954	3.000262	3.023793	3.361439	2.913710	2.807495	4.269404	2.952123	2.895077	3.284110	2.940141	3.011366	3.594809	3.103006	2.891009	3.084859	3.044948	3.471876	2.884158	2.991507	3.060771	2.985121	3.108739	2.927396	2.979637	3.050591	3.193103	2.884269	3.047416	4.649179	2.958067	3.032967	2.925521	2.941371	3.040061	2.945757	2.909069	3.174610	2.859593	3.255150	3.072349	3.167274	2.923946	2.978509	2.869515	2.918405	2.896885	2.869052	3.044868	3.035774	3.191743	3.178699	3.190259	3.416390	2.926721	4.638845	3.498544	3.243133	3.004073	4.238087	3.017403	3.087522	2.972768	3.367694	3.103265	3.663078	7.638786	3.054972	2.992607	3.456601	3.128951	3.069128	3.039394	4.444552	3.928910	3.233514	2.963216	5.765045	3.477651	5.374165	3.393995	2.947724	3.096798	3.473431	3.345270	3.363712	3.346402	3.307309	3.218685	3.138946	4.800722	2.924674	2.930332	2.997133	2.867589	2.907863	3.134992	2.963678	2.936817	2.990915	3.062236	3.357413	3.609968	3.260006	3.342302	3.273130	2.977408	3.119010	4.013112	3.664851	3.268108	3.127205	3.013456	3.193103	3.082761	2.997218	2.934112	3.731709	2.961557	3.343813	3.230761	3.148279	3.239897	2.984053	3.417042	4.583101	2.968562	2.874515	3.687262	3.116514	2.970441	3.229324	3.123904	3.120835	3.046612	2.946126	3.295748	2.870737	3.001185	3.083374

AC068196.1	8.375725	7.935134	8.142400	8.211273	7.782109	7.964652	8.009018	7.559365	8.773366	8.166444	8.098702	7.686842	8.443006	8.285774	8.004026	7.872457	7.938997	7.892325	9.046377	8.117780	8.807508	8.580126	7.612498	8.146260	8.232098	8.251602	8.122129	7.527128	7.847655	7.798822	8.593306	8.407575	7.922875	9.080099	8.656820	7.873506	7.792640	8.367279	8.429598	8.305738	7.764204	8.407722	7.841244	7.809422	7.528443	8.220554	8.204055	8.063486	8.434607	8.317000	7.820563	7.732109	8.360473	8.224479	7.879300	8.052945	9.088836	8.200920	8.561010	8.187149	8.280692	8.014946	8.254820	8.199201	8.900377	7.859273	8.177095	8.463830	8.538773	8.175683	7.909740	7.848400	8.269978	8.107430	8.387288	7.695178	8.015555	8.240081	7.890599	8.137259	8.129022	8.151760	9.223262	8.179776	8.051705	7.808494	8.062577	8.318066	7.779314	7.724697	7.543582	7.854375	8.075475	8.579659	7.793702	8.012895	7.860910	8.617543	8.701068	8.958268	8.627169	8.011263	8.382372	8.161466	8.513701	8.214100	8.342169	8.946600	8.666526	8.391617	8.164264	8.701371	8.199817	8.630992	8.045289	8.578764	8.607116	8.073812	8.263689	9.166801	10.340199	8.863223	8.953479	8.673596	8.990170	9.463454	9.508697	9.831389	9.179536	9.570274	9.153531	8.647397	8.580581	9.593539	8.759334	8.374187	8.722507	9.962091	7.850493	9.251188	9.390213	7.668497	8.624991	9.569977	9.665009	8.050181	8.898560	9.272474	8.592221	10.511571	10.676730	9.792229	10.298660	9.216398	9.304559	8.647531	10.021710	11.011977	10.033753	9.270142	11.300255	8.183260	9.045030	8.534543	8.946451	9.920576	9.879153	9.093582	11.146797	10.043594	8.719047	9.057993	10.113185	8.508601	8.739929	9.705173	9.717013	9.436174	10.046174	10.139408	7.977373	9.210851	9.401214	9.885624	8.934877	9.626779	9.914396	9.908813	9.276679	10.243674	9.633839	9.223120	9.633215	8.970668	8.868386	8.726802	9.702186	9.282941	8.963573	9.781775	9.082435	9.954431	8.508291	9.520363	9.447951	9.474607	9.749888	9.150168	8.696832	8.918022	9.138437	9.901962	8.990334	9.258188	8.441936	9.706262	10.141818	10.181167	8.532273	10.144911	9.129684	9.407255	8.774448	9.433582	8.107430	11.123509	10.661313	8.771295	9.077935	8.663627	9.346191	11.191061	8.491179	9.014694	8.837089	9.618091	9.712029	8.399260

AL357146.1	4.649613	4.145791	4.567453	3.948976	3.433628	4.620829	5.502762	5.040093	4.415122	5.826936	4.744674	4.635852	4.105442	4.336552	3.160172	4.774099	4.029277	3.004715	3.137235	4.543088	5.065915	2.984053	3.451366	4.630254	4.673384	4.586482	3.853131	4.696830	4.712642	4.664747	4.180324	3.800534	4.383150	3.634068	4.822917	3.688481	3.734186	4.540205	4.316930	3.742381	5.143570	4.144522	3.391764	3.744640	4.977248	5.501007	4.458635	3.307309	3.601290	5.203669	3.545643	3.551213	4.195183	3.825314	3.798781	5.245814	4.037989	6.228136	2.959467	4.356767	3.663659	4.668385	4.101480	3.822055	4.100404	3.965102	2.944437	3.830142	4.310605	3.238311	3.870512	4.353982	4.774099	4.606700	5.083066	4.200744	4.327221	4.482874	4.562740	4.472145	3.149648	5.759064	3.557169	3.597725	3.775945	3.612955	4.025364	3.705864	3.762166	4.199882	3.839470	4.821899	3.497998	3.655630	3.650223	3.610969	3.791654	3.757923	3.312876	3.410970	3.630688	3.783976	4.270081	4.303765	3.097128	5.364627	3.517551	3.791654	3.884898	5.883488	4.009546	5.094394	3.394158	5.231197	4.952305	4.349409	3.929346	4.015628	5.283372	3.624648	4.076394	3.339291	3.855934	3.341666	3.702258	4.390632	3.599338	3.707485	3.841899	4.002351	3.618767	3.985401	3.500497	3.030788	3.778554	3.885958	2.901684	4.261993	3.906188	3.662244	3.475268	3.249984	4.031703	4.850976	4.471446	3.928725	4.247330	3.661837	5.006848	3.344944	3.248706	3.487325	3.176969	4.387819	3.498362	3.413099	3.389306	3.980615	3.456247	4.564070	3.710670	3.478950	3.712529	5.271428	5.045343	5.191503	3.165069	3.799662	3.861705	3.344639	3.331129	3.995744	3.973302	3.400057	3.545643	3.689524	6.133084	2.920076	3.353040	3.999159	3.941517	3.886962	4.295457	4.891666	4.131450	2.948344	3.393833	4.179211	3.961596	4.219929	3.696923	4.691364	4.783690	4.772404	4.165795	4.259584	4.600410	4.567244	3.657453	3.357933	5.363287	3.349361	3.401932	3.997446	3.075410	3.938324	3.949193	3.797760	3.910981	3.942615	4.908825	3.236957	3.239051	3.177618	3.647766	3.160050	3.516064	3.538645	3.108516	3.377896	3.338435	4.210345	3.922669	5.191120	4.457149	3.233940	4.101929	4.435290	5.636384	3.907084	4.624415	4.028609	4.534121	3.409032	3.608603	3.751463	3.558530	4.767009

AC005908.3	3.034402	3.094210	2.934719	2.954241	3.696307	3.420255	3.084859	3.035276	3.201364	3.038936	3.171175	2.966404	3.133674	3.370267	3.086146	3.036058	3.209791	2.905501	4.002351	3.069362	3.381727	3.105287	2.951652	2.906828	2.959653	2.931963	2.931424	2.880718	3.041326	2.985413	3.450064	3.034680	3.753370	2.980773	4.326137	3.719129	3.234379	3.009174	3.120613	3.120954	4.221682	3.003614	3.382741	3.090545	3.511629	4.235959	3.334678	3.052050	4.062197	3.245376	3.087647	3.052262	5.621468	4.308576	4.680948	3.003001	3.913358	3.733411	3.140813	3.661202	2.931594	3.043520	3.508161	3.114865	3.733181	3.287257	3.332425	3.044868	3.125011	2.892414	3.293300	2.903033	3.105287	3.031471	2.929714	3.231186	2.973621	3.019669	2.943904	3.385685	3.153805	3.465935	5.018899	3.530135	3.141282	3.240314	3.365508	3.199916	4.036699	3.378974	3.316944	3.016261	3.618011	3.485680	2.895437	2.917323	3.262477	3.032852	3.743022	2.900459	2.958816	3.088004	2.923298	3.111066	4.307013	3.005371	2.865464	3.062992	3.165689	3.005943	2.916473	3.082345	3.524010	3.105727	3.174332	3.532537	2.856904	3.193913	3.110727	2.998262	3.137469	2.912392	2.980258	4.115709	3.335169	2.879209	3.033387	3.108874	3.232972	3.004535	2.900459	2.945514	3.046804	2.970513	2.942927	3.096273	2.938701	3.210577	3.064806	3.178160	2.940060	2.921570	3.393651	2.894087	2.884435	3.040703	2.840111	3.019493	3.216802	3.181086	3.160050	3.622237	2.939052	3.016157	3.471226	3.175025	3.019493	3.151643	3.374402	3.684214	2.949472	3.424550	3.116275	3.783976	3.882111	3.098313	2.931915	3.743475	3.020401	2.977873	2.974081	5.657575	4.533920	3.300811	2.979842	3.627023	3.612379	2.992717	3.470703	2.888990	3.142064	3.542277	3.335169	3.445079	3.406764	3.391578	3.162748	3.186045	2.886744	2.933891	2.934273	3.054459	2.868281	2.890430	3.176138	2.950634	2.939052	2.993450	3.566286	3.376363	3.615932	5.012617	3.324164	3.256343	3.697989	3.113096	3.295444	3.773875	3.297133	3.126980	2.995316	3.311317	3.135531	3.003120	2.924280	3.486788	3.279422	3.621479	3.065646	2.995228	3.337787	2.869225	3.006239	4.606485	3.021888	2.850474	3.034213	3.118642	2.999944	3.372751	3.109568	6.051653	3.054254	3.254990	3.841530	2.837022	3.058021	3.124671

AC108868.1	7.269844	6.600722	6.538445	6.579946	7.357647	7.720828	7.218267	7.646042	7.340176	7.072747	7.349431	7.506496	7.292562	6.887904	6.542552	6.823214	7.110988	6.079385	6.469512	6.486608	6.553514	6.272390	6.069272	6.132918	6.382809	6.594222	6.637692	6.759442	7.128141	7.224035	6.724149	5.321096	5.348134	6.712113	5.793128	5.817802	5.736270	5.601521	5.750286	6.009832	7.127841	5.828774	5.701880	5.674591	5.615740	4.787643	7.677630	5.248606	6.242444	5.718496	5.421112	5.866135	4.887696	5.141531	5.357397	7.362762	5.238860	5.276162	5.538641	5.971779	6.176773	7.281001	6.219753	6.110433	6.014517	6.122011	5.690981	6.068594	6.165098	6.393377	6.571789	6.684067	7.473087	6.500534	6.549177	6.465872	6.621633	6.452268	6.562696	5.720867	5.392395	5.558862	5.583037	5.837488	5.887979	5.674417	6.256420	6.287255	6.608356	6.426308	6.317412	6.854156	6.235642	6.341221	6.491699	6.566408	5.885856	6.540186	6.802353	7.291767	7.559652	6.323377	7.002889	6.385776	5.776362	7.646194	6.517718	6.062445	6.578487	7.273416	6.954382	6.709258	6.546146	7.436154	8.122441	10.537076	6.732375	7.211984	7.731485	6.903528	6.984610	6.799271	6.899505	8.574878	7.625019	8.249893	7.930670	7.341925	7.892325	7.935728	8.066831	8.359426	7.586605	7.766700	7.188552	6.985241	6.817169	7.827891	6.409686	6.796536	6.420945	7.430288	7.087505	6.928135	6.938425	7.610523	7.106932	8.401134	7.352977	6.793323	6.607708	6.518878	7.302722	6.317412	6.459196	6.362193	6.553169	6.610455	6.506176	8.240540	6.516754	7.022334	6.178147	5.896541	5.531069	8.430482	6.015713	5.981355	6.121304	5.726824	6.259290	5.039315	5.944953	5.588508	7.267734	5.461998	5.675995	5.632079	5.596446	6.524972	7.168099	6.436296	6.574492	6.255725	6.109076	6.423830	6.270170	6.619878	6.800600	7.112679	6.971359	8.184642	6.961659	6.689492	6.659694	6.854620	7.042248	6.949475	5.794931	5.542603	6.002904	6.133452	5.989536	6.186579	7.010888	6.327724	6.240427	6.775273	6.366226	6.856580	7.379401	6.729161	7.072893	6.951703	7.166993	6.899505	7.510625	7.011554	6.858036	7.169031	7.446170	7.412291	7.041441	5.937040	7.500244	6.911631	6.587534	6.572587	8.231949	7.271647	6.875163	7.317019	7.764815	7.810799	8.599881	6.813024	7.516320	8.362054

LINC01994	7.204095	6.538915	6.477985	6.578323	7.124012	7.296370	6.191997	7.086585	7.574967	7.248376	7.213117	7.622248	6.842863	6.952820	5.464634	7.222622	7.099873	6.511268	8.345680	7.471118	7.358246	7.650787	7.268030	7.603766	7.424763	7.572630	7.331171	7.855600	7.022655	5.894955	8.116235	5.345734	5.752847	5.569943	6.095860	6.135673	5.863088	6.349577	6.772519	6.318078	6.478808	7.081923	6.427678	6.452936	6.454137	6.183047	6.596637	6.854459	6.222924	6.708488	6.965487	6.355059	5.799127	5.763590	5.907765	6.192170	5.978520	6.980014	7.317019	6.466680	7.171971	5.698420	6.461838	7.596848	7.013318	7.317336	7.386840	6.735148	6.661658	7.338317	6.434967	6.591954	6.369386	6.492653	6.542716	6.657433	6.925659	6.747397	7.060597	6.504481	6.520879	6.828610	6.569361	7.215455	7.184736	6.032173	6.917243	7.173245	7.680267	7.705798	7.914461	7.935728	6.878463	6.414041	6.676101	6.654503	7.350077	6.937142	5.948086	6.193727	5.428855	6.235642	6.372108	6.750842	7.521137	6.118183	8.069057	6.811299	7.861831	6.535659	7.774461	7.863395	8.079921	6.780224	6.520193	8.050788	6.984911	7.030340	7.142089	6.917090	6.810976	6.502846	6.937142	7.808333	7.186675	5.972154	6.720315	7.317336	9.126433	8.302736	7.616951	5.229742	5.745334	5.410318	8.096125	7.645570	10.930535	7.443772	6.890763	7.471713	7.998718	8.656538	12.093979	6.970435	6.944724	6.779905	10.081486	9.885299	6.467022	7.157778	5.317845	7.585179	5.074842	5.598789	7.971157	6.320511	6.658065	6.805534	7.513578	6.261339	7.321681	6.569708	6.868396	7.179851	7.346294	6.721907	6.821498	7.455081	6.951549	7.292562	7.833239	6.619109	6.616246	5.605914	6.142569	6.110611	6.654176	8.120379	7.261516	7.319152	5.702406	7.355513	7.414920	7.194046	7.063661	7.061243	6.888719	6.753552	7.299864	7.948757	6.779905	6.268440	6.532414	6.505130	7.145511	7.216228	7.280850	7.351140	6.343881	8.010662	7.306346	6.521703	8.557469	7.034067	6.433444	7.490867	7.391023	7.608684	7.691976	8.357685	8.053687	6.857061	6.327073	7.591653	6.459196	7.273894	9.472171	5.481239	6.137363	4.655551	5.442160	5.673843	5.832460	6.924849	8.549357	8.354136	6.904150	7.372828	6.173342	9.792063	7.817635	9.424825	7.152154	8.389109	6.983822	7.527604	8.072573	8.357075

AC138904.3	4.119882	4.938708	6.226232	5.021357	4.404898	5.281972	7.497706	5.780149	5.707018	5.907938	6.124801	5.732857	4.719369	5.398443	5.604154	5.638981	5.337135	6.394030	5.438936	6.096683	4.838497	5.517224	5.034634	5.076919	5.287363	6.209840	6.299387	6.221767	5.631375	4.730815	5.780710	5.481239	4.350224	5.738273	5.821680	5.859422	4.741525	5.173168	5.300632	3.595732	5.621291	5.541080	6.440719	3.878061	3.800966	6.560853	6.208993	5.014666	5.734458	5.172969	4.706614	5.111719	6.495893	4.421274	4.734819	5.376539	6.705266	6.582283	3.595009	6.098553	4.101695	5.814591	3.866820	5.320912	4.601011	4.333762	4.925302	5.096023	4.305090	5.794031	5.191332	5.045524	6.889972	4.604611	5.707611	4.746622	4.715396	4.030852	4.521287	4.245156	3.206648	7.165039	3.976140	4.994999	4.908622	4.945653	3.367240	5.386761	3.751278	4.127424	6.700466	3.776955	3.989228	4.908622	3.625390	3.852056	5.367011	5.200277	4.744878	6.711800	3.103123	5.789735	5.669981	5.918696	6.588206	5.357589	6.499728	5.717648	4.790155	5.606500	4.497707	5.683615	6.363388	5.666470	6.927315	3.064806	4.092887	4.949604	5.346523	5.761388	5.659956	5.622231	4.231567	5.550523	6.256420	6.972302	7.698822	4.909265	6.914786	5.558298	5.287167	5.365800	4.389151	4.747061	4.322911	5.551244	6.867088	6.245934	6.155871	5.871338	6.138412	6.025527	5.657752	5.865382	4.114601	5.203474	6.928586	4.497063	4.379938	6.294223	4.802623	6.682909	5.824047	6.825424	6.949322	4.576446	5.145547	5.465020	5.269057	6.136200	5.671606	8.360934	4.103229	4.798317	6.636280	6.605173	6.520020	6.411997	4.502191	4.846579	5.518786	6.709910	4.196467	5.010327	4.679217	8.483274	7.557866	3.745674	6.012970	4.026036	4.703430	5.161480	6.185885	4.787036	4.301516	5.249169	4.260010	5.592917	4.713491	5.448214	4.985191	6.371417	4.234838	5.100721	4.083044	4.086570	4.011238	6.067883	3.636869	4.458238	8.519296	3.611605	6.395639	5.260230	5.154192	3.518728	7.227175	3.553917	3.991131	9.399848	5.722522	5.284565	4.779447	4.489992	3.965491	5.889206	4.556009	5.954174	5.757033	4.225973	7.395847	5.787378	4.011621	5.468945	7.190316	6.930487	5.454402	4.596165	6.077284	3.624461	5.051601	7.716894	6.611844	7.140632	3.768624	5.922745	5.068986	4.817515

AC138904.1	4.119882	4.938708	6.226232	5.021357	4.404898	5.281972	7.497706	5.780149	5.707018	5.907938	6.124801	5.732857	4.719369	5.398443	5.604154	5.638981	5.337135	6.394030	5.438936	6.096683	4.838497	5.517224	5.034634	5.076919	5.287363	6.209840	6.299387	6.221767	5.631375	4.730815	5.780710	5.481239	4.350224	5.738273	5.821680	5.859422	4.741525	5.173168	5.300632	3.595732	5.621291	5.541080	6.440719	3.878061	3.800966	6.560853	6.208993	5.014666	5.734458	5.172969	4.706614	5.111719	6.495893	4.421274	4.734819	5.376539	6.705266	6.582283	3.595009	6.098553	4.101695	5.814591	3.866820	5.320912	4.601011	4.333762	4.925302	5.096023	4.305090	5.794031	5.191332	5.045524	6.889972	4.604611	5.707611	4.746622	4.715396	4.030852	4.521287	4.245156	3.206648	7.165039	3.976140	4.994999	4.908622	4.945653	3.367240	5.386761	3.751278	4.127424	6.700466	3.776955	3.989228	4.908622	3.625390	3.852056	5.367011	5.200277	4.744878	6.711800	3.103123	5.789735	5.669981	5.918696	6.588206	5.357589	6.499728	5.717648	4.790155	5.606500	4.497707	5.683615	6.363388	5.666470	6.927315	3.064806	4.092887	4.949604	5.346523	5.761388	5.659956	5.622231	4.231567	5.550523	6.256420	6.972302	7.698822	4.909265	6.914786	5.558298	5.287167	5.365800	4.389151	4.747061	4.322911	5.551244	6.867088	6.245934	6.155871	5.871338	6.138412	6.025527	5.657752	5.865382	4.114601	5.203474	6.928586	4.497063	4.379938	6.294223	4.802623	6.682909	5.824047	6.825424	6.949322	4.576446	5.145547	5.465020	5.269057	6.136200	5.671606	8.360934	4.103229	4.798317	6.636280	6.605173	6.520020	6.411997	4.502191	4.846579	5.518786	6.709910	4.196467	5.010327	4.679217	8.483274	7.557866	3.745674	6.012970	4.026036	4.703430	5.161480	6.185885	4.787036	4.301516	5.249169	4.260010	5.592917	4.713491	5.448214	4.985191	6.371417	4.234838	5.100721	4.083044	4.086570	4.011238	6.067883	3.636869	4.458238	8.519296	3.611605	6.395639	5.260230	5.154192	3.518728	7.227175	3.553917	3.991131	9.399848	5.722522	5.284565	4.779447	4.489992	3.965491	5.889206	4.556009	5.954174	5.757033	4.225973	7.395847	5.787378	4.011621	5.468945	7.190316	6.930487	5.454402	4.596165	6.077284	3.624461	5.051601	7.716894	6.611844	7.140632	3.768624	5.922745	5.068986	4.817515

CASC20	3.515144	3.612955	3.361618	3.252661	4.046733	3.817794	3.416555	4.989560	4.313402	4.169230	3.591339	3.703237	3.801826	3.966608	3.804926	3.242102	3.779991	3.195416	4.987675	3.639909	5.482767	2.966746	3.500163	3.486614	3.915108	3.832712	3.498544	4.163837	3.260431	4.112234	3.789308	4.775737	4.099063	4.308819	3.740380	4.134295	3.679421	3.416207	3.558530	3.581900	3.474326	4.177040	3.837782	3.433070	3.799662	5.851124	3.838884	3.191888	3.911422	4.077019	3.950770	3.852937	4.573522	4.391274	4.132553	3.634068	4.270081	4.078669	3.725976	4.009758	2.917720	3.392796	4.044097	3.417042	4.208643	3.609968	3.365646	3.299913	3.510367	2.928427	3.529787	3.269757	3.537632	3.143488	3.094313	3.410052	3.521300	3.405718	3.423141	3.959460	3.539784	4.197968	3.535542	4.346762	3.518201	3.814815	3.968532	3.500163	4.740639	3.800747	3.370267	4.124421	3.999802	3.382241	3.425458	3.649022	3.895449	4.332682	4.655105	3.728814	4.381597	3.230494	3.225297	2.945275	4.843806	3.372905	3.559157	3.800534	4.216232	3.285045	4.968421	4.920208	4.191032	3.825314	3.966392	5.477091	3.264112	4.010356	3.176019	3.811201	6.366378	3.665068	4.883281	6.690628	5.088396	5.756267	5.212584	5.639555	5.320335	5.170899	4.413089	4.360033	5.537015	5.995014	3.652214	3.690137	5.073004	3.668095	4.380795	4.699164	4.125452	5.149493	4.601011	4.942614	4.264647	4.394991	5.369930	5.812964	3.917481	4.150003	6.121304	3.970970	4.170794	3.901276	4.797463	4.587333	3.713383	4.307910	3.936458	5.887816	4.895003	3.698588	3.507422	4.331849	5.357204	5.119896	2.947724	4.251056	4.216647	3.945317	4.073869	4.387604	4.798741	4.409145	4.983002	4.253660	3.921257	5.307713	4.014251	5.281372	3.468564	4.175765	5.501195	4.325528	5.216385	4.681366	4.144295	4.360482	3.065646	4.582230	3.569686	6.161322	3.899788	4.043878	3.824871	3.862994	3.547999	3.609384	3.552211	3.942389	4.729746	6.348609	3.729835	3.989853	4.456929	5.355688	3.435630	4.782417	5.001521	3.896502	4.502413	3.822055	3.612180	3.872015	3.878927	4.919147	5.191700	6.275222	5.688775	4.097105	4.112927	3.779584	4.297673	5.191894	3.750843	5.049595	4.747474	3.913564	5.068602	4.605023	6.615435	7.683036	4.005441	4.183193	5.545431	3.590513	5.386761	5.648576

LINC02346	6.214759	5.016903	5.429045	5.315660	5.886377	5.002927	4.805960	6.179672	6.987331	4.735652	5.426670	4.455015	4.540902	5.630077	4.345899	5.067171	6.210495	3.860653	5.237472	5.270834	5.883706	5.498902	5.275128	5.225089	5.446414	5.348514	4.636077	5.630815	4.990613	4.955624	6.271869	4.809510	4.312308	3.936016	4.747474	4.644930	5.425215	6.285141	5.886203	6.113851	4.869580	5.967171	3.559346	5.807596	4.502413	5.437272	5.267480	5.920857	5.552203	5.539969	5.388727	5.619645	4.868318	3.905958	4.540679	4.634571	4.389354	4.964590	5.217151	5.438752	6.397786	4.854811	7.390858	6.436771	6.332486	6.218710	5.305994	6.043307	5.260447	6.357152	5.442356	5.276751	5.442744	5.138251	4.862073	6.271536	5.958590	5.702043	5.962157	5.511256	4.854590	5.641968	5.246802	5.584380	5.753035	4.449155	5.613483	6.521545	6.686143	6.845709	6.460003	6.861854	5.052375	5.080195	5.179379	4.756329	5.110560	4.018242	4.571619	4.065226	4.709693	4.345679	4.488254	4.976700	5.412057	5.160478	6.215781	5.724728	5.743559	5.486905	6.361035	6.541196	6.608356	5.313797	5.813341	6.626161	6.670915	4.841265	5.053637	5.465597	5.360226	5.426425	5.138251	6.497516	5.871887	4.392128	5.885495	6.951856	5.091641	5.156870	5.023408	4.938930	5.173168	6.244622	5.259448	6.267078	4.568306	6.186736	5.575579	6.264678	4.905496	6.436133	5.706824	5.656838	5.790268	5.206824	6.264862	5.757389	5.355688	6.555493	4.794982	3.952050	5.123314	4.993108	5.625058	5.031543	6.581645	6.047635	6.276525	5.267883	6.919186	4.924291	7.157299	4.616662	5.113550	5.677707	6.057108	4.768089	5.902203	6.246624	5.860123	4.867689	5.535494	4.141298	5.091838	3.816090	6.261164	5.569943	5.800809	6.926316	5.000684	7.161420	6.861228	6.728047	6.907043	5.904725	6.258961	5.971415	6.847422	5.581535	5.655012	5.672603	5.494274	5.216032	6.440058	6.374837	6.034881	6.440869	5.012415	5.679946	6.043642	5.478043	5.506814	6.825424	4.647039	5.288957	6.770414	7.114731	6.928432	6.764402	7.664378	5.399789	5.842228	5.057772	4.530516	5.030942	3.773875	3.790356	4.316491	5.036116	4.120296	4.366132	4.100192	5.593845	5.871338	6.537460	6.476918	6.235139	5.901681	6.778153	6.274070	6.838192	5.680842	6.306114	5.703574	5.867966	5.566459	5.666470

SOX2-OT	3.146302	3.080285	2.987048	2.934273	2.890616	2.987779	3.223215	3.115938	3.335812	3.232441	3.441340	3.506244	3.205342	3.823735	3.276032	3.082015	4.181446	3.155331	4.200518	3.174211	5.649360	2.945840	4.739775	3.066929	3.297290	3.013376	2.976862	3.066396	3.007968	3.011858	3.622038	3.441518	3.095013	3.082069	3.524010	4.017832	3.641091	3.145815	3.182044	3.123904	3.387908	3.523658	3.382241	3.209656	3.360502	3.862586	3.234227	3.821840	3.147914	3.894213	3.911207	3.653830	3.593637	3.814815	3.372905	2.951652	5.130404	3.568486	3.880253	3.487527	3.808655	2.959101	3.265578	3.042314	3.629032	3.036058	3.006617	3.150377	3.003001	3.216802	2.931963	2.771095	2.988422	3.318310	2.997419	3.175288	2.933510	2.940658	3.151870	3.396622	3.117498	3.608987	3.789732	3.290588	2.997794	3.186180	3.142918	3.903036	3.280580	3.200255	2.999669	2.920166	2.977498	2.969388	2.897158	2.964287	3.362151	3.050060	2.992302	2.934452	3.034513	3.172650	3.304758	2.865157	4.167115	3.736479	2.999285	3.113539	3.156053	3.123193	3.193392	2.922522	3.067524	3.159156	3.067275	3.078754	3.206265	2.969466	3.050591	3.046304	3.129913	2.951849	2.950522	2.988814	3.205608	2.940819	3.114613	3.326629	3.079530	3.250276	3.125395	2.984859	3.213948	3.067172	2.935655	4.073634	3.002835	3.283950	3.182446	5.713823	2.875865	3.833571	2.970441	3.141052	2.971345	2.949339	2.954506	3.118529	3.325488	3.429179	3.303300	3.065121	3.116275	3.411297	3.721214	3.686239	3.134024	3.132846	3.025936	3.172503	3.361140	3.394330	3.274958	3.508342	3.988618	3.187273	3.886748	3.258402	3.956800	3.936123	3.570765	3.326629	3.803679	3.144215	3.005276	4.760157	3.397480	3.768431	3.531937	3.586121	3.016683	3.242251	3.249455	3.186339	3.058619	2.996944	3.335311	3.074974	3.497998	2.940422	2.854722	2.979346	2.977873	2.929989	3.159671	2.941371	3.034680	3.301457	3.153417	3.271303	3.608791	4.174641	3.339777	3.180141	2.928336	3.255752	3.183432	3.169763	3.190933	3.068578	2.926799	2.976959	2.993354	2.969823	2.994943	2.891362	3.165946	3.052172	3.147294	2.941487	3.012317	2.963579	2.987048	4.463946	3.302221	3.078141	3.131467	3.327268	3.022175	3.165689	3.108410	4.366579	3.106094	3.183197	3.010125	3.045148	2.965763	3.102425
[truncated: 17,774,581 more chars]
