# Supplementary material for: Immune-Related Long Non-coding RNA Signature and Clinical Nomogram to Evaluate Survival of Patients Suffering Esophageal Squamous Cell Carcinoma
Source: Front Cell Dev Biol. 2021 Mar 4;9:641960. doi: 10.3389/fcell.2021.641960 (PMC7969885; doi:10.3389/fcell.2021.641960)
Supplement: Supplementary Table 3 — mRNAs obtained by GSE53624 annotation. [file Table_3.docx]

id	GSM1297309	GSM1297307	GSM1297299	GSM1297297	GSM1297295	GSM1297239	GSM1297293	GSM1297139	GSM1297137	GSM1297291	GSM1297289	GSM1297287	GSM1297285	GSM1297283	GSM1297281	GSM1297305	GSM1297135	GSM1297279	GSM1297277	GSM1297303	GSM1297275	GSM1297273	GSM1297271	GSM1297269	GSM1297267	GSM1297265	GSM1297301	GSM1297263	GSM1297261	GSM1297259	GSM1297133	GSM1297237	GSM1297235	GSM1297131	GSM1297129	GSM1297127	GSM1297125	GSM1297123	GSM1297121	GSM1297119	GSM1297257	GSM1297117	GSM1297115	GSM1297113	GSM1297111	GSM1297109	GSM1297255	GSM1297107	GSM1297233	GSM1297105	GSM1297103	GSM1297101	GSM1297099	GSM1297097	GSM1297095	GSM1297253	GSM1297093	GSM1297091	GSM1297089	GSM1297087	GSM1297085	GSM1297251	GSM1297211	GSM1297209	GSM1297207	GSM1297205	GSM1297203	GSM1297201	GSM1297199	GSM1297197	GSM1297219	GSM1297217	GSM1297249	GSM1297215	GSM1297213	GSM1297083	GSM1297081	GSM1297079	GSM1297077	GSM1297195	GSM1297193	GSM1297191	GSM1297189	GSM1297187	GSM1297185	GSM1297231	GSM1297183	GSM1297181	GSM1297179	GSM1297177	GSM1297175	GSM1297173	GSM1297171	GSM1297169	GSM1297167	GSM1297165	GSM1297229	GSM1297159	GSM1297163	GSM1297157	GSM1297161	GSM1297155	GSM1297153	GSM1297151	GSM1297149	GSM1297247	GSM1297227	GSM1297147	GSM1297145	GSM1297245	GSM1297143	GSM1297225	GSM1297223	GSM1297243	GSM1297141	GSM1297313	GSM1297221	GSM1297311	GSM1297241	GSM1297308	GSM1297306	GSM1297298	GSM1297296	GSM1297294	GSM1297238	GSM1297292	GSM1297138	GSM1297136	GSM1297290	GSM1297288	GSM1297286	GSM1297284	GSM1297282	GSM1297280	GSM1297304	GSM1297134	GSM1297278	GSM1297276	GSM1297302	GSM1297274	GSM1297272	GSM1297270	GSM1297268	GSM1297266	GSM1297264	GSM1297300	GSM1297262	GSM1297260	GSM1297258	GSM1297132	GSM1297236	GSM1297234	GSM1297130	GSM1297128	GSM1297126	GSM1297124	GSM1297122	GSM1297120	GSM1297118	GSM1297256	GSM1297116	GSM1297114	GSM1297112	GSM1297110	GSM1297108	GSM1297254	GSM1297106	GSM1297232	GSM1297104	GSM1297102	GSM1297100	GSM1297098	GSM1297096	GSM1297094	GSM1297252	GSM1297092	GSM1297090	GSM1297088	GSM1297086	GSM1297084	GSM1297250	GSM1297210	GSM1297208	GSM1297206	GSM1297204	GSM1297202	GSM1297200	GSM1297198	GSM1297196	GSM1297218	GSM1297216	GSM1297248	GSM1297214	GSM1297212	GSM1297082	GSM1297080	GSM1297078	GSM1297076	GSM1297194	GSM1297192	GSM1297190	GSM1297188	GSM1297186	GSM1297184	GSM1297230	GSM1297182	GSM1297180	GSM1297178	GSM1297176	GSM1297174	GSM1297172	GSM1297170	GSM1297168	GSM1297166	GSM1297164	GSM1297228	GSM1297158	GSM1297162	GSM1297156	GSM1297160	GSM1297154	GSM1297152	GSM1297150	GSM1297148	GSM1297246	GSM1297226	GSM1297146	GSM1297144	GSM1297244	GSM1297142	GSM1297224	GSM1297222	GSM1297242	GSM1297140	GSM1297312	GSM1297220	GSM1297310	GSM1297240

CTNNA2	3.675983	3.546976	3.429179	3.257185	3.324164	3.643658	3.620077	3.375999	3.402821	3.806168	3.551811	3.738084	4.090469	3.593829	3.252236	3.349361	3.740764	3.478770	3.940136	3.683451	4.391694	3.748966	3.905077	3.791856	3.812518	3.764289	3.506879	3.476529	3.637482	3.563042	3.774707	3.992680	3.637869	3.733827	3.512937	3.753824	3.580694	3.638474	3.251299	3.904201	3.675388	3.465570	3.981983	5.068410	3.862994	5.123715	3.385360	3.298231	4.271585	4.120971	4.104747	4.469049	4.177867	3.824871	3.929574	3.760082	3.727937	4.020460	3.566891	3.598775	3.125920	4.767260	3.504732	3.467670	3.508512	3.531510	3.131700	3.382407	3.167396	3.125639	3.639290	3.281942	4.058446	3.412583	3.599742	3.523658	3.726533	3.634285	3.378073	4.083916	3.834416	3.933902	3.387908	3.819707	3.456795	3.899788	3.594399	3.542870	4.560966	3.339444	3.196384	3.829744	4.271174	3.595183	3.605586	3.694034	4.145791	3.976766	3.388249	3.731092	3.568074	3.384000	3.984336	3.732755	3.927387	3.655630	3.542277	3.476161	4.103884	4.025591	3.476529	3.591517	3.719948	4.224914	3.450800	4.139961	3.755901	3.288352	3.653400	3.294372	3.605046	3.218278	3.405574	3.631909	4.080922	3.616879	3.344161	3.465757	3.769308	3.418075	3.275739	4.065635	3.486956	3.645219	3.303802	3.783575	3.214463	3.308242	4.207295	3.757114	3.690559	4.560966	3.960098	3.000556	3.821651	3.466476	3.744089	3.963800	3.777746	3.406231	3.568074	3.136856	3.635265	3.485680	3.854880	3.860653	3.542469	3.671754	3.475442	4.801756	3.287873	3.789308	6.236812	3.807201	4.830410	4.008008	3.260560	4.111173	4.086810	4.527996	3.933476	4.659199	3.913138	3.608791	4.112927	3.572883	3.707684	3.201820	3.182569	3.160939	3.701392	3.418265	3.606829	3.543056	3.935165	2.994026	3.816090	3.446160	4.606057	3.370825	3.401766	4.790369	3.207803	3.416052	3.320254	3.264238	3.165554	3.157660	3.844785	3.631499	4.193008	3.384489	4.047589	3.496154	3.402652	3.154548	3.262477	3.570246	3.185285	3.266440	3.976766	3.806591	3.432044	3.304280	3.304758	3.205479	3.076108	3.598945	3.299180	3.123904	3.114979	3.340297	3.352721	4.127852	4.134500	3.296519	3.221793	4.026422	4.168613	3.665068	3.823524	5.917588	3.763620	3.386361	3.123193	3.354007	3.252973	3.776777

SNTG1	4.775737	5.317658	5.436682	5.637139	6.205920	7.088794	5.707946	6.878780	8.207548	5.895664	6.868396	6.032361	6.095149	6.353267	5.648207	6.060983	6.268096	6.364065	6.154472	5.497353	5.543334	6.381648	6.671556	6.176101	6.510415	6.563856	5.303245	6.906051	6.419326	5.901152	6.743037	5.124300	4.653285	5.242458	4.253009	4.490216	5.145152	4.742349	5.293315	5.348134	6.668179	4.880056	4.956023	4.752528	4.440018	3.761124	7.162865	5.164064	5.198528	5.226492	4.784935	4.976883	3.345270	4.674518	4.340694	7.229212	4.168824	5.400149	4.938708	5.036741	4.756961	6.146192	4.708459	5.990781	5.141316	5.128371	5.074237	5.419369	4.972338	5.027663	5.277375	5.768488	6.421917	5.168681	4.846579	5.259838	5.628989	5.467351	5.297271	4.542873	5.137208	4.406247	4.174641	5.060815	5.218747	4.448486	5.189553	5.600944	5.188407	5.260874	4.812916	5.433948	4.233943	5.276946	5.316472	4.989358	4.667563	6.510942	6.087995	6.488864	6.915418	5.024042	5.622781	5.181765	5.311070	6.232414	7.495997	5.967524	6.071709	6.797331	5.458276	6.354105	5.840777	7.632690	6.548856	7.855444	5.917082	6.643709	7.255095	5.913674	5.600604	4.978895	5.987742	6.735311	6.197075	7.069219	5.694907	6.026945	6.375674	5.855451	5.987381	7.566901	6.848795	6.846000	6.983026	5.217569	6.080902	5.589095	5.066749	6.954541	6.406070	6.548685	7.050084	6.566884	5.667420	6.961817	7.597626	8.025123	6.095497	6.830092	5.139865	4.042528	4.521910	4.534548	4.513800	5.148905	5.753589	5.481833	5.224094	7.096114	4.945220	4.119882	4.585625	3.910557	3.536283	7.118214	3.695478	5.069189	4.671850	4.645359	4.564465	3.369248	4.496423	5.178363	5.922924	3.220137	5.324027	4.828464	5.166894	5.532941	5.046472	5.147729	5.666470	5.512770	5.174952	5.285197	5.171084	5.236428	5.180737	5.438205	5.382508	7.445861	5.328489	5.473470	5.097591	5.508966	5.193853	5.277165	5.195106	4.294363	4.975271	4.254568	5.124915	5.195497	5.455701	5.116611	4.690520	5.262253	5.444666	4.588420	5.627262	4.958361	4.980532	4.596165	4.602914	5.302654	6.413054	6.350954	5.166273	6.160979	5.030942	6.399129	5.239632	4.161178	6.943923	7.298925	6.275222	5.249386	7.696224	5.178999	6.135673	6.857373	8.072573	7.083312	5.977257	5.318434	5.800031	6.758456

CCDC7	9.004857	8.583297	8.672031	8.606200	9.087336	8.714536	8.950273	8.592859	9.812289	8.825863	8.437250	8.935159	7.626880	8.216204	8.338549	8.436002	9.372889	8.857898	8.658511	8.762566	8.665154	8.184935	9.415515	8.857724	8.969110	8.424675	8.997674	8.794756	9.183297	8.687887	8.157192	8.684477	8.934257	8.784314	8.695889	8.737644	8.575356	8.011709	8.071679	8.933305	8.936392	8.704737	8.956821	9.450783	8.944434	8.677896	8.536970	8.621747	8.962525	8.667580	8.652644	8.995493	9.677053	9.037384	8.649201	8.620648	8.849730	9.204130	8.418559	8.508125	8.548924	8.918330	9.462264	8.345381	8.695584	8.785633	7.972576	7.995191	8.047520	8.982606	8.565719	8.618776	9.007782	8.627776	8.616784	9.031417	8.470262	8.590668	8.327291	7.985306	8.990766	9.192582	8.429598	8.628819	8.672619	8.755780	8.014777	8.561770	9.179536	8.693399	8.547074	8.831633	8.682202	8.908475	8.908163	9.354532	9.813229	8.819941	8.346622	8.744959	8.729147	8.580269	8.249596	9.522089	8.297298	9.098736	8.426765	8.294115	9.272265	9.070041	9.163075	9.235036	8.102799	8.452314	8.824787	10.303096	8.639114	8.632197	8.874344	8.817289	8.746513	8.450156	8.911908	9.645034	8.296244	8.126279	8.721472	9.453237	8.660064	8.660551	8.760599	8.104486	8.564352	8.234506	8.567308	9.450135	8.196392	8.888686	8.962835	8.291199	8.994252	9.501800	8.542730	9.100998	8.717816	8.970198	8.941567	8.976368	8.706443	8.743011	8.696520	8.721334	8.151277	8.532727	8.530929	8.680808	9.021885	8.757154	9.210536	8.519151	9.032649	8.534396	9.011761	8.286088	7.971157	8.272399	8.431794	9.108933	8.706305	8.801886	8.681103	8.920759	8.641739	8.420110	8.633073	9.709524	8.561316	8.145020	7.900849	8.157804	9.071428	8.411859	8.150964	8.728390	8.372984	8.223289	8.017553	7.925527	8.801264	8.480461	8.421352	9.574876	8.363776	8.888062	8.707044	8.376752	8.527834	8.545228	8.108784	8.373727	8.111607	8.500912	8.520379	8.471529	8.871211	8.310979	8.565560	8.945222	8.530152	8.713136	8.811877	8.736112	8.880498	8.964497	9.019430	10.288341	9.087772	8.371594	9.300335	8.712812	9.487522	8.688190	9.493614	8.728680	9.022179	7.609920	8.840427	9.710638	8.887600	9.052657	9.251933	9.333386	8.522603	8.868386	8.534075	8.247398	8.428823	8.839351

DNAJB1	13.803322	13.262797	14.384169	14.080973	14.052673	14.607572	14.836045	15.310201	14.633014	14.989019	14.946400	14.666375	13.975517	14.267351	14.898046	14.901840	15.318226	13.981851	13.920502	14.897610	13.662485	13.472730	12.533949	14.030880	14.561327	14.639680	13.232837	14.226329	14.240086	14.418012	14.511941	14.194332	14.308313	13.020807	14.267954	14.509168	14.683169	14.096755	13.547331	14.217310	14.597411	13.484858	14.317829	15.075776	14.394046	14.486353	14.109446	13.023662	16.115572	14.511568	14.227304	14.711236	14.439683	14.464405	15.245371	14.890760	15.000238	13.161477	13.698756	13.427333	13.410767	14.564707	13.318278	13.558652	13.341776	13.970725	12.745569	13.582053	12.971329	13.319284	14.360038	14.249198	14.679596	14.368698	14.764339	13.863343	14.886035	14.638391	13.884089	14.896544	14.371917	14.726960	13.815559	14.924899	14.507632	14.834088	14.992275	14.952794	13.292154	13.730412	13.031581	13.740837	14.013491	14.206497	14.045556	14.358378	15.295318	14.359066	14.811115	14.123055	15.055528	14.000936	14.060641	14.035874	13.024395	15.120250	14.317829	13.066780	13.537927	14.661796	13.577378	14.351258	14.767548	14.588093	15.146873	14.477842	12.669498	14.925433	14.402538	14.155728	14.026521	13.484139	13.823982	12.755993	13.572169	14.608380	12.003998	13.909712	13.441153	13.828722	13.394547	14.210459	13.411431	11.567130	13.287464	12.918772	12.984259	12.571856	13.359283	13.383910	12.657996	12.896328	12.891207	13.713659	12.493861	14.868226	13.406497	13.264151	13.497789	12.035025	13.979146	13.457435	14.787255	13.598139	14.232519	14.014363	12.798022	12.843523	12.948244	13.697330	13.022344	14.118926	13.884347	14.215329	14.232519	13.965167	13.931371	13.318676	14.521823	13.489934	13.434092	13.793821	14.025367	13.526138	14.485612	12.479812	12.413004	13.101051	12.849996	13.105804	13.974138	12.160484	12.863135	12.583952	12.983891	13.566385	12.873573	12.715589	12.830315	13.860465	14.282078	14.094001	13.571242	13.512420	14.213670	13.257008	13.578055	14.553109	12.168818	13.834052	13.710693	13.907439	13.615689	13.193873	13.492381	13.620619	13.853818	12.206509	12.554292	12.521728	13.533965	13.259393	14.349592	13.688585	13.681376	14.351612	12.741273	13.948475	13.577611	14.266304	12.342018	13.065305	12.546379	11.952588	13.422807	13.837964	12.403076	13.247295	13.302672	12.619316	13.179573	13.456831	14.234569	14.659090	13.881569	13.156342	13.788715	13.607817

SHANK1	11.150531	10.780444	10.879299	11.465983	11.711746	11.787010	10.809055	12.088732	12.437883	11.125488	11.510387	11.412815	10.803737	11.241360	10.284884	11.862452	12.375982	10.883700	11.097929	11.139043	10.870211	11.799679	11.751751	10.776676	11.323649	11.841289	12.765306	11.477417	11.764791	11.628794	11.970501	9.902880	10.659769	10.699257	10.518311	10.586252	10.791282	10.466097	10.773444	11.669857	11.648851	10.474667	11.096316	11.076192	10.606972	10.610648	12.211122	11.699459	10.981301	10.893753	10.846814	10.752225	9.972631	10.470586	10.413447	11.779993	9.992578	10.290059	10.422850	10.100633	10.715371	11.702072	10.442903	11.019615	10.390607	11.069292	11.134906	11.058092	10.938338	11.707306	11.598591	11.674446	12.265583	11.584062	11.110444	10.917038	11.120015	10.550104	11.001214	9.785196	10.056200	9.952724	9.524895	9.998736	10.288842	10.663131	9.903332	10.370938	10.838706	10.472696	10.482160	11.308691	11.239490	10.708724	10.665039	10.810113	10.708068	11.261529	11.634201	11.591601	11.537331	11.151411	11.553440	10.759261	11.420572	11.384675	11.825933	11.194590	12.113307	11.668471	12.151731	12.036847	11.586469	12.337312	12.217449	12.797127	10.997896	11.331890	12.150786	11.365654	10.891326	10.644734	11.424197	12.697151	11.688779	11.756489	11.879544	11.576165	11.689898	11.523392	11.476974	11.520245	10.850174	11.263797	11.710136	11.313224	11.120704	10.946082	10.423140	11.035559	11.073426	11.411822	11.037001	11.533937	11.149440	11.846434	11.915193	12.411884	11.581124	11.362843	10.807365	11.093119	11.124898	9.974513	9.803650	10.053909	10.224681	10.586401	10.463100	12.610190	10.248780	10.596659	11.054809	10.331462	10.362545	12.421355	10.361430	10.171241	10.756632	10.487353	11.014430	9.823738	11.300691	9.995272	11.361662	9.729974	9.401376	10.089002	9.594314	9.959230	11.846574	10.366828	10.241203	10.219536	10.399429	10.115901	10.257261	10.875163	10.831653	10.972346	11.050922	12.943369	10.921515	10.643734	10.617668	11.172387	10.955440	10.640900	9.574138	9.347399	10.087299	10.393559	9.934782	10.001641	10.912348	9.682921	9.601901	10.430794	10.362972	11.151138	11.059375	10.088368	11.355638	11.425504	12.269936	10.933822	11.265170	11.060427	10.399588	11.504954	11.080854	11.501471	10.506905	10.313564	11.895619	11.068828	10.504682	11.035057	12.244073	11.256480	11.528921	12.556219	13.001778	11.553894	11.133855	10.320444	11.424053	11.968413

OBSL1	12.152396	11.434496	11.297790	11.392615	11.585304	11.790869	11.368582	11.596253	12.104520	11.461880	11.824443	11.936861	11.281048	11.023655	11.087082	12.160178	11.616406	11.020795	11.413399	11.774311	11.265095	11.408446	12.439982	11.361662	11.783740	12.188852	13.098061	11.981214	12.559114	11.890496	12.006089	10.979372	10.920184	11.388349	11.132344	10.897043	10.924005	11.012272	11.389687	11.401374	11.898522	11.224167	11.129723	12.001304	11.522631	11.231436	12.415458	10.916730	10.918527	11.419997	11.256056	10.882081	10.622788	11.113033	10.738842	12.353242	10.562777	10.911762	11.108963	10.838572	10.944027	11.908802	11.939987	11.280760	11.554015	11.063780	10.987659	10.937294	10.790846	11.121444	11.757848	11.513041	12.153901	11.388220	11.257232	11.369916	11.536953	11.198236	11.273397	10.577397	10.490710	10.914011	10.573635	10.756471	10.584342	11.231105	10.656678	10.393559	10.729560	10.496144	10.063327	10.657698	11.047954	11.265890	11.287020	11.038306	11.063641	11.373920	11.436404	12.006852	11.540550	11.356089	11.691697	11.305148	10.915065	11.984838	11.831216	11.118254	11.751751	12.268379	11.012272	11.754990	11.364925	12.271073	11.592678	11.292363	11.852470	11.867596	12.318233	11.285190	11.468075	11.472951	11.405443	11.882216	11.250623	11.925820	13.662485	12.232669	11.496353	12.432585	12.174829	12.170015	12.242226	11.251510	13.222353	12.283174	11.611094	11.310266	12.012819	11.694097	12.528398	12.168505	12.283028	13.277438	12.639545	12.299354	11.796289	12.731681	11.905051	12.982246	11.250483	11.758300	11.660007	11.132929	11.950659	11.325694	11.910728	11.864688	11.876433	12.643905	11.952138	11.262567	11.841289	11.794551	11.516794	11.963379	12.416908	11.872355	11.971606	11.254400	12.203886	10.898488	11.491594	11.609049	12.672605	11.946535	11.086179	11.201144	11.542573	11.942720	12.071131	11.519937	10.917038	11.402351	11.391292	11.467492	11.441892	11.605506	12.072891	11.781075	12.039403	12.948591	11.744648	11.648993	11.776985	12.081058	11.602409	12.018571	11.613158	10.807205	10.986270	10.437199	11.575527	11.754080	12.408210	10.564580	11.915640	11.176130	11.237147	12.047379	11.386298	12.077573	11.848510	12.455635	12.285826	12.521236	12.467154	12.216524	12.214256	12.129935	12.739016	12.330329	11.098248	12.419759	12.197683	12.340685	11.944227	11.379601	12.370033	11.149750	12.302809	10.897614	12.978217	11.851720	11.549457	11.338924	11.835276	12.414938

FER	10.657248	10.819087	11.094004	11.323649	11.394806	11.457285	11.292669	11.617879	11.669088	11.619186	11.525035	11.399245	11.508929	11.961308	11.700684	11.671644	11.563737	11.589947	12.458956	11.229137	11.903261	12.282874	13.132450	11.395495	12.057480	11.703863	11.087512	11.600742	11.734231	10.885843	11.680972	11.209812	11.214660	11.225321	11.206137	11.283268	11.537179	11.628231	12.070082	12.156636	11.467049	12.286161	11.627214	11.632841	12.188415	11.232423	11.618460	11.323062	11.005117	11.603421	10.892228	11.809185	11.621605	11.537478	11.052063	11.213347	11.596704	12.016602	11.943044	11.597568	11.828401	11.394735	12.821603	12.449933	11.690932	11.808575	11.512897	11.412382	11.207310	11.821254	11.178535	11.209661	11.559784	11.189884	11.201594	11.136541	11.074564	11.214237	11.066310	11.429574	11.409661	11.436814	11.241079	11.351456	11.517507	11.402644	11.276449	11.349211	11.930928	11.612573	11.498297	11.765559	10.859529	11.033754	10.770016	11.210244	11.235078	12.072811	11.579070	11.486995	11.884048	11.368582	11.574347	11.626169	12.662239	11.289041	11.798061	12.193053	12.305725	11.545455	12.174666	12.332077	12.309198	11.472951	11.131351	11.974649	11.388081	11.622359	11.884633	10.104326	11.802693	11.218631	10.496489	11.082337	11.459334	11.212133	11.412382	11.547842	10.702123	11.164654	11.309839	11.406467	11.513924	11.437522	11.656893	10.620438	11.131212	12.152825	12.412694	11.858431	12.019601	12.622994	11.325694	11.689592	11.959150	10.614503	11.755741	11.402644	10.866362	12.154860	10.863944	11.573736	11.332042	11.398068	11.748779	11.540263	11.328346	12.521876	11.483264	12.741273	11.414134	10.988542	12.118961	11.777620	10.588511	11.601655	11.283416	10.203707	11.477556	10.865293	10.742155	10.972192	12.820600	11.547983	11.659417	11.560080	11.331759	10.720354	11.056896	11.420572	11.349211	12.125096	11.844337	11.737021	11.773690	11.301743	11.205541	10.837802	11.089609	10.411781	10.667979	12.306838	10.894745	11.244638	10.760404	11.213657	11.014430	11.132779	11.639167	11.575527	11.082337	11.569606	11.714992	11.245386	11.329537	11.514705	11.327340	11.818235	11.761777	11.101364	12.455008	11.040159	11.707755	11.244502	11.097335	10.717586	11.577336	12.606059	10.456157	11.357672	10.910445	10.847257	10.379694	10.498864	10.738376	11.793314	11.820206	12.003696	12.034131	11.446009	12.204814	11.314073	11.618620	9.992511	12.059200	11.336717	11.147376	11.396102

HOXB6	10.102793	7.807549	7.706238	9.276233	6.690947	7.631919	8.068471	7.631140	6.944562	7.274232	6.887904	7.226540	7.065228	6.002186	8.890904	7.610841	6.513566	7.786365	7.105179	8.401432	6.534041	7.086749	12.987390	6.926667	7.483367	7.385010	8.247677	8.604216	7.217178	7.200555	7.370309	10.542394	8.045755	6.774295	7.679960	7.684733	8.654920	8.002620	8.012294	7.546612	8.171288	9.228524	7.919331	9.556934	9.101326	9.552380	7.410333	9.230442	7.463106	8.633972	10.899971	8.848337	9.569642	8.607415	9.104428	6.744976	9.421747	8.105890	8.294418	8.926440	8.668025	7.917375	12.144699	7.480750	8.588673	8.497631	8.868700	7.043666	7.500547	8.612010	8.733797	7.810660	7.534392	8.753789	9.261051	7.533908	7.545344	8.775967	8.960071	9.127188	9.314441	8.425112	9.593851	8.839043	7.922409	8.833199	8.726505	8.311618	8.785471	9.334439	9.011895	8.505652	9.097041	7.613722	9.667624	8.319126	7.715071	6.345747	6.430725	6.368693	6.657433	8.266186	6.826564	6.905419	7.526204	8.235415	9.485482	6.849484	7.715979	7.104243	8.701371	7.421823	6.598280	7.691812	6.096514	7.224175	10.576472	7.415219	6.813024	7.371725	8.282999	9.973251	8.024983	7.862315	7.734091	7.629233	13.090908	9.669719	8.068615	8.557910	7.414306	9.651648	7.252455	6.930656	7.890305	9.586230	8.144547	7.181722	6.668503	7.119635	8.388057	9.289725	9.700644	9.268311	8.606356	8.240685	5.923488	7.811267	8.978474	9.851210	7.853910	10.244721	7.917823	7.291926	8.473114	9.589818	7.359642	6.457215	9.920116	6.987484	6.876248	7.291287	8.068315	10.590376	7.806972	7.283052	7.892947	10.074846	9.379453	8.833467	8.898403	8.732389	9.274416	10.012286	8.593623	8.941854	8.584250	6.724487	9.808233	8.499478	9.411560	8.920591	9.241363	8.142995	8.866879	8.034627	7.761133	7.577148	7.573412	8.522603	8.056204	7.735797	9.055670	8.966033	8.090942	9.372113	10.865897	9.515307	10.670427	9.036048	9.300335	9.752062	10.095036	9.472625	8.034780	10.081326	8.362863	9.014249	10.558709	7.180642	9.087175	10.559179	8.798699	10.106724	11.035706	9.183929	12.593626	9.356220	7.455252	8.208762	7.696073	8.621124	8.031179	9.519632	7.153231	9.279824	6.062618	6.862821	7.232277	9.750053	8.694514	6.887904	6.870984	9.004108	7.270004	8.286241	8.094336	6.805835

HOXB3	10.102793	7.807549	7.706238	9.276233	6.690947	7.631919	8.068471	7.631140	6.944562	7.274232	6.887904	7.226540	7.065228	6.002186	8.890904	7.610841	6.513566	7.786365	7.105179	8.401432	6.534041	7.086749	12.987390	6.926667	7.483367	7.385010	8.247677	8.604216	7.217178	7.200555	7.370309	10.542394	8.045755	6.774295	7.679960	7.684733	8.654920	8.002620	8.012294	7.546612	8.171288	9.228524	7.919331	9.556934	9.101326	9.552380	7.410333	9.230442	7.463106	8.633972	10.899971	8.848337	9.569642	8.607415	9.104428	6.744976	9.421747	8.105890	8.294418	8.926440	8.668025	7.917375	12.144699	7.480750	8.588673	8.497631	8.868700	7.043666	7.500547	8.612010	8.733797	7.810660	7.534392	8.753789	9.261051	7.533908	7.545344	8.775967	8.960071	9.127188	9.314441	8.425112	9.593851	8.839043	7.922409	8.833199	8.726505	8.311618	8.785471	9.334439	9.011895	8.505652	9.097041	7.613722	9.667624	8.319126	7.715071	6.345747	6.430725	6.368693	6.657433	8.266186	6.826564	6.905419	7.526204	8.235415	9.485482	6.849484	7.715979	7.104243	8.701371	7.421823	6.598280	7.691812	6.096514	7.224175	10.576472	7.415219	6.813024	7.371725	8.282999	9.973251	8.024983	7.862315	7.734091	7.629233	13.090908	9.669719	8.068615	8.557910	7.414306	9.651648	7.252455	6.930656	7.890305	9.586230	8.144547	7.181722	6.668503	7.119635	8.388057	9.289725	9.700644	9.268311	8.606356	8.240685	5.923488	7.811267	8.978474	9.851210	7.853910	10.244721	7.917823	7.291926	8.473114	9.589818	7.359642	6.457215	9.920116	6.987484	6.876248	7.291287	8.068315	10.590376	7.806972	7.283052	7.892947	10.074846	9.379453	8.833467	8.898403	8.732389	9.274416	10.012286	8.593623	8.941854	8.584250	6.724487	9.808233	8.499478	9.411560	8.920591	9.241363	8.142995	8.866879	8.034627	7.761133	7.577148	7.573412	8.522603	8.056204	7.735797	9.055670	8.966033	8.090942	9.372113	10.865897	9.515307	10.670427	9.036048	9.300335	9.752062	10.095036	9.472625	8.034780	10.081326	8.362863	9.014249	10.558709	7.180642	9.087175	10.559179	8.798699	10.106724	11.035706	9.183929	12.593626	9.356220	7.455252	8.208762	7.696073	8.621124	8.031179	9.519632	7.153231	9.279824	6.062618	6.862821	7.232277	9.750053	8.694514	6.887904	6.870984	9.004108	7.270004	8.286241	8.094336	6.805835

SULT1C4	7.345830	7.363230	7.566901	5.984369	5.111523	5.591192	7.531887	6.970435	4.085214	6.492368	5.782186	5.875848	5.379656	4.853109	6.671872	5.638273	5.523836	5.532567	5.419988	5.280957	5.365800	5.078395	6.114042	4.825402	4.975059	4.884088	6.116835	5.043266	6.338411	7.885047	4.529040	8.118397	7.696523	6.602610	8.502791	8.628077	8.460319	7.808333	6.892054	7.841996	5.751387	8.321113	7.340680	6.892201	8.677896	8.436316	6.026589	7.393988	7.770020	7.712486	7.775738	8.570802	8.751471	8.360634	7.759759	6.119556	8.600815	7.960535	5.808857	7.264952	7.314180	7.638786	8.465978	5.570700	7.143465	6.708142	7.078318	6.961184	6.565283	6.717651	7.738668	7.521618	6.002186	7.813738	7.865564	7.473225	7.809741	8.284523	7.149940	7.802510	5.984867	8.223604	7.221302	8.332304	8.053534	7.902878	6.969671	6.405727	6.814585	5.936160	6.709910	6.771711	7.494577	6.554476	6.953139	7.429837	8.629423	7.683194	5.369314	5.661605	6.642091	7.284456	6.715371	6.643191	5.461998	6.628714	5.577951	5.733015	5.439680	5.430849	6.903692	4.748507	4.894812	5.211052	4.967125	5.578913	6.342674	6.159273	4.949812	7.137662	6.392020	6.105662	4.844870	4.478658	5.336739	5.343606	10.331462	3.888699	4.981730	5.652572	4.992229	4.154312	4.184247	4.249931	5.358339	4.888964	3.832923	5.208422	6.350954	4.398066	4.777820	6.170824	3.380664	6.439708	5.299860	5.595490	3.700564	5.284990	5.758326	4.531615	6.948756	6.516905	6.252502	7.273894	7.608977	7.465716	5.228115	4.537597	6.924197	3.997237	6.984448	6.403278	5.516256	6.798331	8.056526	4.389151	10.169348	6.742734	7.060280	6.880697	6.977531	7.809278	7.355349	7.610220	6.040557	9.798602	7.739929	4.206388	7.928181	4.961870	6.868396	5.659046	5.227496	5.450480	6.110971	5.516058	6.175069	5.048938	5.216974	6.274392	6.933179	4.065006	6.338721	7.181888	6.641471	6.607242	6.686616	6.617107	7.119181	4.759526	6.955972	6.259647	6.749727	6.881833	5.727373	6.338886	5.641390	5.742641	6.046054	8.685393	5.185913	6.609006	6.645932	7.119635	6.362867	5.688249	5.356651	4.794770	4.463741	7.134512	8.275964	4.654618	6.075028	5.504117	4.891666	4.539755	5.885687	4.635636	4.680948	5.209227	5.074448	8.616164	5.002123	6.447691	4.383610	5.958962	4.203136	5.466753

LNPK	7.861985	7.763447	7.741944	8.039022	8.276257	8.223604	7.959294	9.523175	9.232049	8.443484	8.790729	7.915673	8.262784	8.626562	7.896560	8.929856	9.143350	8.552568	10.077590	8.616940	9.345894	8.759647	7.938524	8.646482	8.954868	8.600481	8.749699	9.666225	8.687527	7.388883	8.595641	7.976790	7.921791	7.001459	8.013377	7.830789	8.226166	8.175989	9.097364	8.279940	8.402822	8.503262	8.282080	8.653270	8.195435	7.987181	8.622673	8.666674	7.816672	8.353996	7.916768	8.310673	8.047984	8.300469	8.059955	8.783057	7.916296	8.693634	8.430243	8.179476	8.088613	8.306046	8.300189	9.235458	8.109664	8.105140	8.406229	8.380007	8.002620	8.162811	7.686388	7.855309	8.814119	7.830076	7.856943	7.914604	7.838309	7.701741	7.598858	7.747952	7.830932	7.850213	7.552226	7.539423	7.424921	7.623778	6.970435	7.925989	8.034047	7.955121	7.606389	7.596263	7.645097	7.521929	6.845709	7.246690	7.781951	8.536813	7.921218	8.150217	7.916296	7.834327	8.216505	8.768535	8.654623	8.190857	8.822507	8.688048	8.158381	8.932558	7.395991	8.561770	8.992872	8.863383	8.330258	3.248272	8.559772	8.055425	9.081035	7.409893	8.304531	7.493360	8.826297	8.430406	9.255278	7.876561	8.103097	8.614358	8.308845	8.691536	8.136815	8.932696	8.332461	8.871542	8.232995	8.126763	7.945557	8.942784	8.064267	9.250707	8.981350	8.404836	8.941247	8.826851	8.674365	8.222992	9.469192	8.675783	8.028251	8.057573	7.540966	7.311187	7.604046	7.815753	7.445711	7.634654	7.940095	8.771856	8.185578	8.299554	8.112072	8.601432	8.332937	8.108640	8.161613	9.056119	8.529384	7.613888	8.236836	7.745636	7.950478	8.216812	8.465219	8.166768	8.518092	8.307762	8.587856	8.259398	8.104486	8.498687	8.586509	8.861063	8.666821	8.566155	8.144389	8.583773	8.165502	8.110601	8.308399	7.742387	7.605777	9.975278	7.796019	7.411794	7.857244	7.639235	7.881392	7.961924	8.222845	7.824306	8.195917	7.867574	7.309440	7.769734	7.278770	7.983556	8.268231	8.051562	7.999017	7.680418	7.586901	7.275147	7.333226	7.456332	7.491660	7.646507	8.034188	8.313277	7.695615	7.786526	7.222846	7.602325	7.603602	7.917823	9.143799	9.080270	8.293064	8.058121	10.134533	6.817639	7.899893	5.182198	10.361744	7.180945	6.760102	8.046717	8.315429	9.060946

POPDC3	6.669775	3.674319	5.118031	3.825093	3.080594	5.187008	6.639977	3.591899	3.498362	5.070805	4.465860	4.475809	4.023916	4.954166	4.226825	5.450480	4.717717	5.323834	6.851706	4.948949	5.328715	3.030591	3.184101	3.270231	5.268266	4.806374	5.546374	3.957915	5.745927	4.894172	3.725135	6.142913	5.198116	5.163477	5.989903	6.554786	6.449178	3.935805	4.756117	4.200518	5.812964	5.113550	4.714555	4.843175	6.742879	5.521370	5.817089	5.749191	5.796567	6.143754	5.255748	7.090251	4.975882	6.622253	6.498895	5.172120	7.290335	4.564688	4.461559	5.760681	5.390271	4.758070	6.258264	4.110948	4.177253	4.869995	3.389835	4.692658	3.913757	3.043133	6.038597	6.248293	4.729367	6.184157	5.448989	5.556755	6.393213	5.036741	4.167541	4.464593	4.084550	6.317064	6.847580	6.312967	7.105179	6.276841	5.780149	5.695665	4.521491	3.846089	4.964988	4.122955	4.360482	6.546478	4.295457	5.839873	6.000597	5.951091	4.986843	5.151558	4.297673	5.486905	4.949181	4.665614	4.800293	5.262870	3.456247	3.621076	3.326468	5.730493	3.806591	3.521478	3.274958	4.217947	3.494089	3.458761	3.838235	6.070817	6.419483	3.976140	3.961168	4.034530	3.801618	3.067275	6.704456	3.749198	4.240573	4.883487	3.971363	4.051282	3.449181	3.605963	6.106391	3.790356	4.791432	4.701728	3.899988	4.127649	7.234377	4.615553	3.349197	5.287363	3.074560	5.125133	6.090105	3.470157	3.066192	5.195106	4.832709	5.765200	4.622687	4.684589	5.802945	6.638539	5.174952	4.187529	3.911207	3.571510	3.328333	5.079007	4.152983	4.455648	5.414924	4.378458	5.161854	4.177655	6.081607	4.169878	6.951067	4.465424	4.821475	4.357398	8.400492	5.172120	4.551105	5.206236	4.485731	4.412646	4.313602	4.023916	6.016047	4.849503	4.144522	4.061501	3.856793	3.407494	3.815250	3.853571	5.228700	4.044097	4.871252	4.039083	7.300797	4.045411	3.392300	5.044535	3.721463	3.779151	4.346108	3.866193	4.868318	7.616037	3.755476	6.904609	4.990613	5.442939	3.936458	4.168613	3.726139	3.540932	5.049796	4.170075	5.463844	4.052367	4.781151	5.609543	3.063094	4.307910	3.906634	4.074510	3.704057	3.186761	3.025146	5.187816	4.073634	4.299617	3.658061	3.725577	5.348134	3.554282	4.251497	3.010401	3.799232	3.315404	5.390865	3.639290	5.861943	6.845709

MAPKAP1	3.622237	3.132218	3.029872	3.335465	3.499062	4.230916	4.134500	3.261735	3.626008	3.318632	4.935362	3.531510	3.821840	3.628614	4.117895	4.117669	3.413828	4.892508	5.595490	3.342302	4.095169	3.947054	6.068409	4.245775	3.992473	3.804926	3.494456	3.960518	4.223185	3.334212	3.836319	3.870512	5.422817	3.758136	3.863876	3.976335	4.067541	3.173051	3.159156	5.420358	3.865968	3.302676	3.999802	3.893080	3.403206	4.871252	4.241852	3.862165	4.079820	3.905507	4.494905	3.681431	4.251723	3.774707	3.533103	4.565960	4.921258	3.037234	3.546768	3.277250	4.495125	3.865968	3.400057	4.682853	3.795304	4.352665	2.887371	4.014461	3.266581	3.107025	3.657866	3.687679	5.275956	3.288775	3.499242	3.229919	3.288016	3.760082	3.724101	4.139072	3.164562	3.772596	4.191471	3.731504	3.950106	4.618396	3.198666	5.187401	3.037621	3.232304	3.751073	3.463210	2.974439	3.387061	3.198153	4.010157	3.628398	5.298453	3.335465	3.242972	3.493366	3.722513	3.844785	4.000622	3.795908	4.754703	3.422423	3.906427	5.925599	4.273558	3.395581	4.586047	3.056542	3.478770	3.061222	3.063523	3.765098	4.162728	4.326137	3.406592	3.561948	3.310854	3.306340	3.161356	4.230073	3.206525	3.616696	3.734015	4.607765	3.602837	3.109568	3.321552	3.451718	3.128951	3.735018	3.403382	3.894431	3.822055	3.684027	3.580504	3.672507	6.023426	4.412646	3.598775	3.412024	3.098565	3.671754	3.775550	3.442941	3.862586	3.913358	4.448705	3.271926	3.246827	3.699196	3.654296	3.176503	3.097510	5.166894	3.947460	3.147783	3.452261	4.050438	3.581487	4.441709	4.239916	3.977212	3.323489	3.967228	4.598327	3.564761	4.652799	3.756112	3.987558	3.152825	3.415387	3.027480	3.384151	3.110353	4.744249	3.138221	4.685015	3.506879	3.283480	4.382303	2.884347	3.919566	3.680445	3.221387	2.980038	3.273434	4.377152	2.987779	3.176019	3.210733	3.295748	3.392629	3.039234	3.126045	4.346762	3.782194	3.129814	3.945762	3.443277	3.648603	3.244280	5.458449	2.967466	3.267214	3.155331	3.004804	2.968362	3.695929	3.244699	3.421705	3.558934	4.151676	4.211406	3.401604	5.287769	3.147640	3.187677	3.094537	4.256493	3.597156	4.858755	3.968532	5.964494	4.788469	3.224190	4.057171	3.074675	3.167023	3.151870	2.985985	4.093091	4.364402	3.903127

FGD6	7.877023	8.954704	9.277850	9.228967	9.242420	9.659354	9.664693	8.201973	9.267666	9.587330	9.877183	8.882644	9.505030	9.447951	8.919828	9.747100	9.035284	9.466593	9.328764	9.823884	9.633760	8.897627	9.833794	9.300486	9.224356	9.571519	9.457707	7.790093	8.898099	9.511361	9.926926	9.011895	9.293154	9.973551	9.477671	9.228821	9.722952	8.875260	9.390213	8.734707	9.997800	9.167413	8.359865	9.462714	9.065545	9.513820	9.618236	8.476502	8.856529	9.913924	8.541182	9.676141	9.003048	9.882726	9.044421	10.028734	9.800735	9.177420	9.091750	8.971009	9.419282	9.006556	9.385728	8.741333	9.627096	8.908005	8.648420	8.785047	8.883433	8.412310	9.219434	9.014854	9.579000	8.956668	9.276381	9.065685	9.618236	9.062320	8.852855	9.357409	9.450860	9.840152	9.848968	10.126326	10.317106	9.178174	10.090076	10.245152	9.956906	9.251636	9.138103	9.729505	9.245349	9.778317	8.717976	9.556322	8.891594	9.427739	9.787425	8.476502	8.708722	8.812340	8.708574	8.766072	8.975271	9.536888	9.993095	9.005162	9.264743	9.349252	9.643014	9.250083	8.734549	9.182046	9.142441	6.466356	7.109184	9.703711	9.402320	9.288170	10.630112	10.753717	9.885467	8.186182	10.324760	9.787591	10.505574	11.431058	10.680198	10.569204	9.407409	9.765127	9.881326	10.623687	10.312288	10.826511	10.609505	11.086931	11.602845	10.580549	10.640618	10.532043	11.027485	9.082279	8.854389	9.520834	8.972624	10.150663	10.700309	11.755140	9.984732	9.747411	10.508124	9.696322	9.793599	9.994317	9.014555	10.471779	9.643474	11.348648	9.228967	9.962991	11.533627	10.135460	10.255734	10.530844	8.257221	9.595531	10.822815	10.762198	10.220026	10.122918	11.002085	10.064373	11.161103	10.550537	9.538430	10.180089	10.094546	10.471779	9.886402	11.333338	10.185274	10.792335	9.434472	10.573204	9.541437	9.781037	9.629681	9.717177	10.554448	9.224522	9.642224	10.076651	10.340048	10.149179	10.675990	10.136988	10.549491	10.435219	10.449011	11.138604	10.754780	11.480931	9.418832	10.459101	10.190104	10.601053	10.486716	9.206719	10.951940	10.111838	10.012605	10.826946	10.330253	9.870914	10.737016	10.912497	8.640510	10.481102	8.091424	8.764687	9.012062	10.206592	10.583768	12.163903	11.028799	10.763416	10.430163	10.661622	10.377106	9.158942	8.630840	9.900076	10.817302	10.009271	12.291396	10.278824

RRM2B	9.625854	9.321704	9.602218	9.138750	8.868843	9.262594	9.817863	9.264276	7.126885	9.335876	9.220054	9.280598	8.089878	8.482338	8.529071	8.487552	8.519918	7.395399	7.980661	9.420683	8.633832	7.942332	7.311966	8.026217	6.751613	8.115622	7.447288	7.357154	8.490290	9.992644	7.246849	10.078848	10.223615	9.502704	10.455381	10.441560	10.432020	9.760255	9.247803	8.891290	9.077786	9.362915	9.150017	10.068558	10.202314	10.003818	8.253272	9.330903	10.416142	10.547499	10.264693	10.211375	10.273634	10.364292	10.094402	9.903960	10.087299	9.357266	9.411829	8.931776	9.418972	8.774603	9.598799	8.779634	9.038669	9.049134	8.803350	9.068809	9.190561	8.669568	9.513664	9.244855	8.523862	9.608538	9.159233	9.974821	9.732418	10.700452	9.868372	9.292061	9.670859	9.655028	10.029183	9.842888	9.748169	10.336597	10.071306	10.145061	8.897476	8.692789	8.667281	9.147686	8.927404	10.414788	9.459105	9.464087	10.411451	8.421040	9.718135	7.854212	8.320628	9.027048	8.146260	8.289196	8.705991	8.541337	8.071227	9.021121	7.322799	8.547074	8.432903	8.493981	7.190009	8.129662	9.392378	7.281784	8.008692	9.228039	7.375633	10.291731	9.633215	9.247803	9.067847	6.712799	9.454768	10.060453	8.266810	8.316232	8.275804	9.517441	9.448583	7.350077	8.490868	7.932799	8.466285	10.803584	6.709100	8.087256	10.514677	8.792288	8.185892	8.330105	7.445550	9.969266	10.108228	10.574079	8.036665	7.878989	10.137149	8.144863	9.837811	8.757154	10.450323	10.968475	11.026149	10.497236	9.478293	9.315965	9.773401	7.843764	9.866534	10.122789	11.027621	10.787337	11.156555	7.347692	8.812777	10.715522	11.008580	10.957378	10.394618	11.302336	11.035057	10.560091	10.011086	10.263944	10.230378	9.431743	9.827055	10.666968	8.387874	10.300662	9.890580	9.274979	8.899923	9.773557	9.390971	9.443592	9.394200	9.871673	9.935109	8.475899	10.032414	9.582066	10.417196	10.192512	10.478878	10.429431	7.624575	9.767248	9.514568	10.499425	9.688696	10.739457	9.604530	10.774902	10.583626	9.269079	8.763472	8.179171	9.375602	8.747097	10.921049	10.223028	9.317184	9.915448	7.847655	9.758720	10.426379	9.537835	8.112848	7.837337	8.898856	10.472219	7.598858	8.254971	10.209210	9.298930	8.205533	8.456424	9.822841	6.637380	7.865227	9.258344	9.362145	9.396668	11.159497	10.665611

ZNF7	9.679253	10.080716	10.140452	10.732799	11.071993	11.014277	9.827378	11.999458	11.884784	9.820866	10.017413	9.831828	9.647512	9.807143	9.474009	11.317187	10.425147	9.745680	11.250156	10.993300	10.373076	11.259576	11.464053	10.452666	11.103871	10.608452	11.715575	11.018990	11.362409	10.373076	11.208804	8.884828	9.652740	9.836416	10.440492	9.774329	9.756549	10.118219	10.338850	10.474513	10.963543	10.403336	10.506316	10.149344	10.359776	9.997359	10.885995	9.780402	9.562068	9.510701	9.808072	9.774780	9.792229	9.547157	9.433276	11.705942	9.459550	10.277301	10.056794	9.658740	10.141648	10.962507	10.138502	11.106894	9.618709	9.923196	10.123498	11.134324	9.612400	10.295850	10.707338	9.993847	10.861593	9.881165	9.889840	10.375141	10.978775	9.977119	10.025761	9.702796	9.538600	9.586539	9.437579	9.629836	9.599865	9.946870	10.049407	9.649935	9.882240	9.950580	9.780111	9.927396	9.622130	9.299554	9.576825	9.977259	8.977860	10.445813	10.070706	10.632446	10.421948	10.064373	10.741574	9.841075	10.551138	11.211433	10.860414	10.605932	11.433749	11.432854	10.717299	11.158476	10.132585	11.350100	10.467548	12.730491	10.509917	10.043446	11.406899	9.174045	9.440330	9.627540	10.615566	11.326134	9.896599	10.099349	12.096522	10.750692	10.030230	9.581469	10.014897	9.952136	9.863484	10.116055	11.081746	10.371390	10.413889	12.551234	9.258188	10.725359	11.369916	10.536771	10.640759	10.960176	10.551359	10.803437	11.057327	11.618460	9.731833	11.453138	9.545467	10.096699	9.562846	9.982301	9.078557	9.428363	9.396376	10.228938	10.164877	10.635777	9.587009	10.259940	10.147330	9.968341	9.614532	10.858937	10.187258	9.141505	9.280749	9.550699	10.602690	9.434778	9.554938	9.782831	9.979042	9.569197	10.315490	9.571818	8.954704	9.782381	10.115300	9.793162	10.520631	10.009427	9.820421	9.714221	9.347097	9.276523	9.909707	9.590114	9.649935	11.070312	9.633365	9.424676	9.823607	9.627387	9.842136	9.778165	9.923991	9.816653	9.434472	9.387940	9.871373	9.944985	10.589073	9.254946	9.799644	9.646902	10.053429	10.988542	10.303562	9.578531	9.041013	9.858186	9.918554	10.084818	11.547102	9.910357	9.326448	9.870258	10.397759	11.624141	9.673171	10.902185	10.946533	10.659325	10.094705	10.664468	10.601523	10.420871	11.010354	11.728087	11.679650	10.138805	10.588660	10.286370	9.481968	10.085231

ANKRD1	3.098454	4.152983	3.850353	3.171671	3.393310	4.091186	3.229611	4.203136	4.339614	3.286678	3.356906	4.285445	3.187925	4.594262	2.951652	4.445656	3.484795	3.605218	5.367986	4.056940	3.830561	3.800090	6.160658	4.294590	4.644274	3.836745	4.195624	5.436894	3.952908	3.640693	4.149407	3.248706	6.076952	5.530479	3.157189	3.989003	3.423141	3.788669	3.228916	3.962248	3.596729	3.128684	6.037029	3.475634	4.019348	4.645359	4.760783	6.652886	4.649826	3.026393	4.390178	3.332425	3.657270	3.004261	5.949174	3.016572	4.246657	3.381180	4.897894	5.955741	2.993084	3.226736	8.429740	4.947941	4.465207	5.768316	3.044010	3.077034	3.642657	4.795182	4.638630	3.536493	3.434709	3.763005	3.530906	3.218685	3.441874	6.350625	3.296369	3.483890	4.122295	3.776362	4.706826	5.429406	4.010356	4.752973	5.141531	6.156195	3.528850	4.420222	3.894646	4.186427	4.023218	3.258402	4.948949	6.536628	4.144088	3.476529	3.662649	3.045058	4.008227	3.173949	3.218416	4.433919	9.507031	3.651167	3.777746	4.170991	3.256611	3.121823	5.222525	3.764711	3.711134	3.011454	3.046894	4.472145	3.178836	4.169012	3.366868	3.482786	3.198532	3.513374	3.437226	3.603835	3.189850	3.316489	4.158864	4.298299	3.941921	6.164413	3.265442	3.282855	7.071494	8.337585	3.191611	3.450236	4.045616	3.930246	3.405718	3.385855	3.634285	5.417191	3.116275	3.021888	3.585700	4.375472	3.636278	3.467841	4.828464	3.324499	3.332425	3.742824	4.793710	3.095098	4.834368	5.198317	4.147052	3.227169	4.440247	3.251540	3.007067	4.709267	5.509568	5.461616	4.048031	3.602249	9.967099	4.044097	6.368043	8.030875	7.385610	5.970684	7.968486	4.530956	3.136856	7.433753	3.347539	3.288642	3.613588	6.498576	6.459372	5.912413	4.557891	3.943048	9.384665	3.042514	6.892054	5.934593	3.483890	3.912272	4.976700	4.256960	6.464703	3.407850	4.418223	7.142409	4.484599	4.840860	3.584353	3.668671	3.863667	3.935165	6.034881	6.234806	5.076102	6.099247	7.792182	3.445260	3.683640	6.684561	6.401157	4.293735	5.467944	5.787010	4.508113	3.548911	4.362400	3.521300	3.850978	5.170899	4.043227	6.380125	3.276507	4.944347	4.279490	4.808479	4.343705	3.823300	3.315269	5.463646	3.855934	4.273784	4.839385	3.171671	3.710908	3.010125	3.788669	3.940037

PACRG	4.543555	4.310159	4.855423	4.839616	4.431596	4.797060	4.869381	4.863937	5.545245	4.502191	3.662430	4.982161	3.975082	3.626008	4.371120	4.289621	5.202043	4.194339	6.204195	4.446299	5.422452	5.121915	7.576687	4.585201	4.474750	4.763775	4.212746	4.840860	4.307013	3.918319	4.404200	5.217151	4.731233	4.332682	4.975271	4.844447	3.582844	4.440018	4.737997	4.217085	4.843390	4.886038	4.142802	5.077351	4.245569	5.632831	4.353327	4.909875	6.263683	5.079803	4.515925	4.332259	4.521717	5.511627	5.390063	4.693712	6.055134	2.860134	4.200312	4.039083	4.626760	4.327649	4.688032	4.841265	4.187994	5.313196	4.507002	4.998969	3.874364	4.023017	4.106299	4.559227	4.324651	5.061015	5.131993	4.248379	4.385418	4.624831	4.643812	4.236552	5.579830	5.392969	4.601657	4.899001	4.532041	5.819037	4.135859	3.743889	4.695359	5.492043	4.782417	4.389151	4.284260	4.138852	4.635216	5.306383	6.054977	5.212182	4.573305	4.544678	4.459073	4.811675	4.716448	4.248826	4.663683	6.652077	5.059000	4.429565	5.579658	4.174447	4.577767	5.909011	4.328092	4.486379	5.659580	3.739374	5.254349	4.727241	3.482074	4.210345	4.044969	4.209292	3.896934	4.590157	4.584149	3.400891	4.652164	4.351993	4.621851	4.074943	3.660212	3.187406	3.144562	3.106895	3.661019	4.252787	3.912072	3.870938	3.527928	5.052157	4.079143	5.737715	4.704262	3.770710	3.725976	4.568525	4.238087	4.054728	4.207537	3.051004	3.873944	6.079900	4.337007	3.686638	4.302876	3.668479	4.373702	4.171654	4.857125	4.083468	4.776555	4.291335	4.911356	4.876507	5.103586	4.673856	5.016263	5.084247	4.273323	4.038230	3.639493	4.511393	4.681159	3.505862	3.348845	6.161502	4.860639	4.612954	3.049852	4.146648	3.884501	4.580787	4.861840	4.184469	4.771384	4.862073	4.489992	4.208643	3.054044	4.169878	4.991405	4.333544	4.616876	3.841899	3.872419	4.128508	4.463946	5.325377	3.933266	4.464978	4.725997	4.068163	4.496423	3.885110	4.705113	3.854486	4.973376	3.914443	4.224051	4.082186	4.114367	4.688438	4.186652	4.485251	4.548635	4.633927	4.651571	4.807861	4.088287	3.380170	5.511627	3.544326	3.420470	4.438066	5.540357	5.369930	4.141508	5.023856	4.992229	4.443890	4.168179	4.982161	5.063441	3.597725	3.515480	4.430721	3.926119	3.643851

SPEF2	12.239149	10.951475	9.469344	9.113294	6.691111	7.580396	9.673663	6.307251	6.306611	8.648892	7.111138	10.316237	8.308845	6.351290	9.087175	9.175570	8.675912	7.980180	9.914676	8.691230	8.273739	6.826891	9.868052	8.799286	9.421461	8.946451	10.511711	11.138009	12.564954	10.160896	8.101283	10.741574	9.967576	10.703580	10.596797	11.221910	11.167966	10.883700	9.458948	9.663882	8.172688	12.284727	8.757916	9.866241	9.169847	9.000419	8.655072	10.328234	11.943795	8.312374	12.062906	9.506564	9.999670	9.629681	10.305051	7.702215	10.249709	9.815273	11.716891	9.506420	11.464783	10.610931	11.437102	10.707486	9.242110	11.164048	12.050582	8.663457	8.707044	10.875313	12.087108	10.515170	8.267121	11.537024	10.283668	8.955338	9.700957	10.745154	13.688585	9.083635	9.143201	11.110723	11.205234	11.095577	10.224681	12.634097	9.492866	9.444057	10.112589	10.827827	12.471814	9.928924	9.768599	9.137471	12.934196	9.032501	12.266375	9.284296	8.568651	9.274979	8.909661	11.688994	9.641321	8.869138	8.981956	9.026892	7.688106	7.985002	9.278313	8.365932	9.245186	8.503105	9.569039	8.486586	7.069377	5.225884	11.982123	9.100099	9.087621	9.560659	9.939645	11.275733	8.450156	8.431370	8.073673	8.645888	9.363636	10.567405	7.821807	9.394200	7.347027	7.569307	6.772178	6.615435	9.113587	11.443078	7.923024	7.833540	8.259851	8.251460	9.032354	7.902121	6.575670	9.521604	10.415101	10.011809	7.092449	8.576254	9.341743	10.143394	10.847998	11.698725	10.292031	9.828267	10.435219	13.354589	12.040600	9.079626	12.209741	8.341166	9.890283	8.878315	9.660290	9.055523	7.478585	7.722083	9.065378	9.268924	10.034381	10.842407	9.258494	10.101871	10.377715	11.581405	8.893308	9.878591	9.948590	8.453070	9.748803	11.095577	9.704158	8.827010	9.218991	10.589937	11.261682	9.903042	10.532370	10.155465	10.114655	11.056896	11.532292	7.020771	12.854778	11.859791	9.957986	11.933805	12.083836	12.116834	9.785347	10.485950	8.420732	10.569842	12.016602	12.479650	10.695129	11.412382	10.446887	10.452087	11.097765	8.910839	9.825394	11.065422	11.466449	11.244791	11.522341	11.923566	11.974354	10.216690	9.304406	10.462499	7.957140	9.336024	12.117118	9.203559	7.070379	8.664994	7.372371	7.358246	7.781521	9.519152	10.934280	6.267597	6.623712	7.737577	9.688696	10.794534	10.295232	7.485352

NFATC1	10.593040	10.736232	10.667399	10.943468	11.112442	11.465404	10.417667	12.106988	12.095335	10.755416	11.063360	10.559179	10.520042	10.712868	10.610931	11.701300	11.771231	10.902185	12.733221	11.256626	11.897804	12.228465	12.584979	11.210560	11.382171	11.275177	11.589672	11.729465	11.413079	10.878852	11.581259	9.444674	10.231920	9.797808	10.288341	10.286213	10.137149	10.801353	11.319554	11.525892	11.296591	11.291891	10.766994	11.006117	10.553689	10.537786	11.384527	11.507746	10.666208	10.839132	10.867436	10.687817	10.319223	10.853896	10.649536	11.463053	10.183903	11.159344	11.273851	10.696336	11.125198	11.064664	12.184698	12.033377	11.443078	11.553299	11.634790	11.254129	11.057038	11.947881	10.855566	10.810938	11.415883	10.653995	10.710393	10.635777	10.764606	10.551729	10.622041	10.213194	10.177038	10.272441	10.345071	10.306414	10.212263	10.339585	10.097334	10.215042	11.163045	10.929185	10.713151	11.403962	10.401108	10.198884	9.838589	9.963816	10.359924	9.887037	9.868372	11.136838	10.222096	10.813266	11.037133	11.166369	12.017489	11.163914	12.015680	11.775503	12.187328	11.627501	12.219378	11.920674	11.693184	11.793767	11.198394	11.472082	11.397761	10.629643	11.533937	10.280033	11.342640	10.714458	10.993754	11.827250	11.372900	10.655784	11.740562	11.582863	10.892076	11.079261	10.276978	10.838984	10.715522	11.300978	11.362843	11.519525	10.856607	12.204037	10.536771	11.711892	12.160484	12.292654	11.686029	11.480227	10.991933	11.553156	11.373048	11.676518	11.147807	11.497855	10.020041	10.811801	10.443033	10.395504	10.302486	10.249259	10.942265	11.524269	11.348487	11.484271	11.266465	10.531275	11.246224	10.720056	10.834508	11.990173	11.167246	10.698075	10.960028	11.141047	10.824736	10.313861	11.054809	10.376060	11.219527	10.228505	11.057038	11.180978	10.583626	11.558489	11.246511	12.076837	12.059052	11.518846	11.638275	11.825777	11.566690	11.384387	11.774477	10.730465	10.850333	11.715866	10.944920	10.435072	10.777392	11.060572	10.773444	10.820861	10.085100	10.607548	10.480656	10.423140	10.220026	10.276374	10.728828	10.477389	10.019714	11.116077	10.928290	10.802540	11.503353	10.434589	10.579352	10.642678	10.232331	10.758970	10.396131	10.326735	10.782238	11.092378	11.081746	11.078407	10.799656	11.714912	11.234915	11.970071	11.214237	12.020495	11.990484	12.023112	11.900148	11.815555	11.904326	10.705811	11.395640	11.082917	10.851957	11.423905

MINAR2	3.520622	3.112510	4.036699	4.013112	3.087170	3.905507	3.146645	4.487836	3.715654	3.192046	5.182198	3.204801	3.553350	3.483698	3.016992	3.915721	4.879022	2.951204	6.452268	3.037621	3.835458	3.426570	6.347604	3.585137	3.188619	2.941644	5.145926	3.130036	3.219103	3.270828	3.492579	3.224468	3.871796	3.009843	3.145036	4.055403	4.457149	4.101263	3.755678	4.329886	3.294523	3.293775	3.288984	3.118161	3.410970	4.603961	4.853955	6.079043	4.486790	3.685428	6.044651	3.456601	3.800298	3.908140	3.635673	3.015866	3.856357	3.272988	3.512937	3.591339	3.510009	2.961673	5.313012	3.726533	3.234227	4.294797	3.080487	3.260848	3.321552	3.752961	3.623459	3.447665	2.991917	3.375800	3.021680	3.859401	3.507067	3.300958	3.258402	3.480386	3.634857	3.712023	3.130736	5.291773	4.140390	4.621044	4.274648	3.803284	3.752527	4.287203	3.758791	3.045250	3.200374	3.042193	3.527379	3.757923	3.446160	3.687063	3.093613	3.144788	3.627218	3.155467	3.087756	3.063410	4.353982	3.698588	4.056076	3.744089	3.148882	3.131700	3.371537	3.862586	4.359584	3.137360	3.653027	4.169878	3.735838	2.935992	3.034354	3.772186	3.380330	3.433246	3.301457	3.262043	4.136944	3.410757	5.865953	4.072539	3.414002	4.507693	3.265578	3.239051	3.084967	2.936995	4.194775	4.874867	2.883371	3.301128	4.129602	3.405807	2.875389	5.532003	3.313017	3.786732	3.707684	3.907267	2.924098	3.510558	3.623459	4.487427	3.303468	3.094426	2.955347	3.580694	3.984336	4.124421	3.523830	3.228505	3.544884	3.117381	3.617825	3.941720	3.169995	3.615151	4.158634	3.438239	6.404233	4.444117	3.630871	6.270865	3.289377	3.537427	3.961808	4.311896	3.148035	4.840449	3.281352	3.301288	3.459121	3.243536	3.826779	5.336960	3.576726	3.976560	4.229679	3.098204	3.496154	3.939637	3.128807	3.475806	3.559545	2.963216	3.430641	3.534991	4.161376	3.564969	3.391037	3.564761	3.789934	3.437547	3.773257	3.110843	4.539078	4.216443	4.099547	3.969874	4.213858	4.096439	4.187745	4.437866	4.342860	4.081358	3.484252	3.073342	3.311024	3.165689	2.997517	3.380840	3.148514	3.641891	3.431688	3.165437	3.091429	4.760994	3.400386	4.517223	3.534455	4.345246	2.981253	4.029058	3.791856	5.802051	3.096025	3.271787	3.328333	4.113765	3.222815	3.082447

PRUNE2	3.195700	3.543056	3.112756	3.824156	3.265284	3.138701	3.488475	3.533103	3.472214	3.894213	3.636278	3.636664	3.477470	3.985840	4.116356	3.505145	3.591699	3.563639	5.159455	3.598390	5.529351	3.619893	6.645475	3.202823	3.617097	3.078385	4.706826	3.423315	3.685044	3.709872	3.765519	4.598327	4.439596	3.172650	3.947248	4.325941	3.720576	3.832048	3.875056	3.867730	3.497086	3.918102	3.676220	3.418603	4.597879	5.761966	5.526489	3.828431	4.693081	4.054293	4.527792	3.638690	4.577141	4.623548	5.485556	4.146648	5.016682	4.591620	4.786621	4.440482	3.969226	3.712339	6.804468	4.202047	4.145567	3.618554	4.767260	3.739547	3.316203	3.062131	3.300811	3.435083	3.696522	3.474511	3.077588	3.415541	3.483154	3.297290	3.040276	4.686349	3.497086	4.491777	3.533669	5.338103	4.070841	6.768300	3.523830	3.835677	4.763580	3.872015	3.883596	3.182446	4.787643	4.336340	3.304432	4.801966	3.889613	4.001248	3.579772	5.050593	3.720576	4.137797	3.255631	3.753824	4.857125	4.922988	3.409715	4.200744	4.248186	3.550612	3.390523	2.978043	3.148164	3.355689	3.744640	3.328676	3.012127	4.307013	3.503982	3.084269	3.725335	3.049429	3.137360	2.892149	3.751073	3.044660	3.292658	3.467841	3.186882	3.444367	3.111657	3.309363	3.274197	3.680251	3.149886	3.513915	3.130356	3.522436	3.439792	4.635852	2.887027	5.571799	3.015552	3.098204	2.997517	3.106094	2.845610	2.900365	3.608603	3.534620	3.546976	4.119882	3.157896	3.580504	3.820601	3.770710	3.755267	3.605770	3.329682	3.238603	3.843957	3.729450	3.505520	4.368077	5.307713	3.832514	3.385183	4.211867	4.143246	3.951608	3.400891	4.383150	4.600410	4.283596	3.604265	3.895449	4.034530	4.133401	4.244932	2.915706	3.314483	4.340694	4.159936	4.130430	3.908140	3.285471	3.817172	3.509038	2.813126	3.112510	3.046710	3.278841	3.027576	2.917323	3.395752	3.072994	3.161484	3.093869	3.342475	3.907688	4.657931	3.588820	3.791654	3.625812	4.848293	4.727844	3.491351	4.035158	3.734015	3.582455	3.026669	3.739768	3.463925	3.195044	3.018930	3.017403	2.958353	4.309062	3.368271	3.104774	3.053021	3.024570	2.986951	5.238653	3.279275	3.465394	3.749998	3.460369	3.243404	3.703843	3.249142	2.960127	3.253442	3.082860	2.877138	3.116393	3.205608	3.411654

GPR137B	11.414563	10.817080	11.202677	10.854485	10.600317	10.076981	11.191787	10.631884	10.344001	10.965807	11.255007	10.629961	10.821007	11.078117	10.538357	10.799193	10.758681	10.375141	10.189508	10.637586	11.565808	10.818491	11.336873	10.418283	11.425080	10.766994	11.211143	10.874119	10.365302	11.465404	11.121300	11.793617	11.895030	11.239205	11.637244	11.470479	11.441183	11.703111	11.336278	11.014277	10.709207	11.569749	11.510541	11.436677	11.983657	11.394279	11.070312	11.047817	11.339525	11.466449	11.584232	11.235506	11.827250	11.809037	11.576321	10.957964	12.044097	10.992097	11.494786	11.514555	11.509371	10.633501	12.374199	11.396241	11.562313	11.258424	10.590541	10.943161	11.289617	11.347581	11.402498	10.969399	10.611842	11.220589	11.153776	11.488767	11.227693	12.210677	11.507007	11.806639	11.441607	11.507445	11.823222	11.394735	11.652576	11.283846	11.984250	11.597854	11.597121	11.110862	11.423597	11.791350	11.080729	11.429874	11.661180	11.813291	11.498377	10.662673	11.595221	10.090224	10.794845	10.665327	10.533962	10.783312	10.701804	10.972801	10.203849	11.104579	10.142906	10.571372	11.381447	9.713258	10.643878	10.430485	11.129277	8.789796	10.871441	10.685853	11.145435	12.006698	11.241217	12.431277	11.953033	11.607855	10.652524	11.777298	12.184698	11.561576	11.532717	10.975378	11.210560	11.059231	11.895030	10.024815	12.394906	11.209109	11.177032	12.679396	13.496693	11.849010	12.280654	11.628369	11.645473	13.302870	12.491934	11.638742	10.555535	11.370599	12.354358	12.274241	12.717840	12.066244	13.055794	12.971662	12.114935	12.901456	12.665646	12.111299	12.302809	11.778669	12.257562	13.293540	12.929922	12.815098	11.331478	11.331034	12.322660	12.565935	12.664946	12.448328	12.536272	12.587308	12.934196	12.808933	12.508357	12.783431	12.272198	11.771669	12.262939	12.715740	11.623447	12.170476	12.001171	12.022667	12.246874	12.290609	11.539526	11.237147	12.173565	11.809037	12.331584	10.825918	12.313639	12.744905	12.187328	12.406661	11.979284	11.743182	12.839276	13.159158	12.713635	11.613450	13.002534	12.867783	12.004877	12.625000	12.069772	12.781892	12.507380	11.938934	11.198394	11.783278	12.379731	11.832392	12.435481	12.534127	12.302965	11.630009	12.403886	11.747895	10.588378	11.838865	12.310761	12.689849	11.971606	11.535255	11.313927	12.191343	12.125403	12.138794	12.011560	11.460244	10.359472	11.138461	12.121934	12.541791	12.353897	10.928598

LMNTD1	15.100594	14.904566	15.499684	15.502988	15.763886	15.011645	15.832903	15.132734	14.405867	15.316021	15.649863	15.195519	15.303728	15.075776	15.116602	15.537762	15.192758	14.758705	14.718556	14.450216	14.707518	14.416187	14.213010	15.119035	15.095132	14.435274	14.611896	14.820339	15.545439	15.084166	15.372489	15.760765	14.991150	14.493157	15.514297	15.486206	15.771206	15.708514	14.850021	15.236212	15.512636	15.045993	14.134802	15.066902	14.202148	14.698837	15.382940	13.636675	15.758639	14.045844	15.017444	15.201679	14.381329	15.589004	15.264422	15.327968	14.953863	14.689299	14.693370	14.904024	15.017982	15.051945	14.391513	14.394753	14.844188	14.924443	14.298038	15.315239	14.803294	15.011645	14.803294	14.398487	15.434895	15.509129	15.277400	15.637542	15.078723	14.729253	14.484499	15.785756	15.832903	15.079901	15.819800	16.175634	15.565819	15.674965	15.222515	14.629537	14.856970	15.219810	14.868819	14.857509	15.603850	14.730705	14.715882	15.735297	15.829660	15.307946	15.482965	14.213324	15.370867	14.803294	15.355714	14.853259	14.506817	15.627072	14.648021	14.848968	14.578917	15.592691	14.800437	15.300250	14.824380	15.469767	15.296048	14.838073	14.494741	15.769072	15.069833	15.582778	15.146198	14.867676	15.232087	14.994487	15.973135	15.225383	15.416204	15.664400	15.580039	15.272298	15.700644	15.166041	14.546394	14.764339	15.263058	15.564042	14.951667	14.945774	15.654597	15.086549	14.555161	14.713935	14.584001	15.128463	15.608551	15.409272	15.528080	15.125800	15.367177	15.289538	15.003043	15.777495	15.560443	14.889685	15.153835	14.800886	14.833005	15.253922	14.882312	15.631882	14.857987	14.162045	15.115355	14.805224	14.778502	15.225383	14.388223	15.572722	14.951167	14.721418	14.734860	15.309448	15.175273	15.263058	15.584586	15.569300	14.231871	15.000238	14.633876	14.347082	15.936069	14.619411	15.240441	14.729702	14.917362	15.188078	14.652808	14.728340	14.510745	15.603850	15.970695	15.076369	15.265157	15.180575	15.001900	15.548789	15.655519	15.721897	14.984580	15.341190	15.784706	14.883369	15.101738	15.628950	15.599209	15.610464	15.564042	14.738602	14.440880	14.428990	14.182169	14.424884	15.488676	15.336747	14.906627	15.321092	15.008260	15.190719	15.159483	15.259618	14.817850	15.045993	14.239134	14.416536	15.148056	14.929175	15.362494	14.765245	15.253250	14.343152	15.003643	15.656594	15.350928	15.773226	15.327234	15.496317	15.150598	15.507347

CLEC7A	6.472402	7.906479	8.234802	5.814756	6.259986	7.226540	7.591348	6.355059	5.394196	6.565450	7.046406	7.311340	7.200694	7.332112	7.008904	6.957510	7.158083	6.969671	4.892281	8.482488	6.517061	7.059340	7.289259	5.411869	6.045883	4.833536	7.013620	4.774294	6.806000	8.633369	5.376333	8.684172	8.883274	9.214054	9.303847	8.854389	7.970525	8.028251	7.332745	6.705442	6.429367	7.212144	8.186825	8.190857	7.242922	7.847351	7.450429	7.636038	8.117009	8.818569	8.425436	8.932113	9.485652	8.844016	8.747097	7.985002	8.722060	7.259651	7.580544	7.414619	8.050788	7.349764	8.716113	6.962923	8.947993	7.604209	7.256173	7.637383	7.772016	7.728577	7.668974	8.581533	7.364203	8.267776	8.623129	9.533695	8.492740	9.306959	7.905856	8.741333	7.870451	9.187694	8.377828	8.868229	8.579521	7.994104	9.045341	8.321580	8.553764	8.312374	7.750438	8.633832	8.091424	9.263384	8.864142	7.928341	8.569898	7.090101	7.630374	6.050621	6.812241	6.842863	6.093031	6.348100	7.544535	6.880550	6.402768	7.238905	5.965532	7.214668	8.091102	5.281755	7.044502	6.698868	8.473263	3.943958	5.415835	7.000814	5.697884	9.856355	7.880226	9.847374	8.662549	4.642591	6.791693	8.149158	5.404268	6.459372	8.610624	7.493195	6.529319	5.500462	7.493195	4.986843	6.749904	8.585756	5.285608	8.609069	10.407402	6.026440	6.764576	7.421041	5.005994	5.155213	8.773832	8.159020	3.598578	5.645243	8.955928	6.474588	8.995967	8.904760	8.122441	8.972908	7.822263	8.291830	7.472645	7.345653	8.576254	6.514561	8.001042	11.865134	7.741944	8.499181	7.576179	6.239250	7.429530	7.428515	9.321845	10.254638	8.136815	9.449989	8.819184	9.248899	9.395577	7.936388	8.208150	9.438669	7.657521	9.460588	9.154319	8.632336	7.532852	10.079348	8.681429	8.381126	9.384665	8.069369	11.154213	7.817308	9.766324	7.960374	9.010977	9.621360	10.636237	8.315586	8.912217	8.688827	9.173435	8.948291	8.831783	8.620186	10.130623	9.365175	7.942332	9.950421	9.395272	9.357865	9.954586	7.501958	9.048867	8.617246	9.589654	10.457252	9.014555	7.797234	6.145315	9.968648	6.907980	6.776202	6.665888	6.565927	8.064117	7.278159	6.879266	5.650854	8.707378	7.717516	4.704932	7.299864	7.376122	6.968370	6.793634	5.506401	7.476921	7.735196	8.450307	9.607484

MMACHC	10.525341	8.836779	9.620412	9.242250	9.064290	9.119139	9.764810	8.157641	6.509590	9.241805	9.428192	9.058951	8.580730	8.649359	8.912050	8.984412	9.030689	8.800353	7.349096	9.990425	8.068901	8.136495	8.001687	7.914604	8.363315	8.783516	8.117780	8.109664	8.551058	8.809382	8.441781	10.331758	10.081326	9.916835	10.238329	10.144761	10.074685	10.070373	9.086870	8.066676	8.762269	8.236522	8.632336	9.980743	9.842603	10.034221	8.306486	8.595951	9.293634	10.357811	9.915448	10.001164	9.918392	10.469499	9.865593	8.679300	10.455244	8.016946	8.826560	9.188155	8.334787	8.823574	8.324774	7.844862	9.148150	8.439951	8.093562	8.462038	9.370301	7.662149	9.404032	9.656579	8.086143	9.289264	9.786686	9.409114	9.379147	9.457241	9.485807	8.851059	9.433276	9.955696	10.635198	10.341850	10.549196	10.222241	10.175781	9.906665	8.809523	9.261818	9.289264	8.300613	9.163408	9.861608	10.019305	9.866387	10.001482	8.866731	9.671636	7.634654	8.336479	8.402666	8.637389	8.580126	7.863083	8.359269	7.953560	8.738564	6.699964	8.300034	8.589222	8.260954	8.916018	8.214394	8.586387	7.380676	9.656882	9.056426	7.968783	11.469730	9.876085	9.968968	10.163835	8.262290	10.603402	8.724030	8.902924	9.552979	9.617799	9.987767	9.597882	7.667152	9.569197	8.640510	9.752062	9.975574	7.985454	7.553470	10.813574	8.297612	7.703584	7.621512	8.479139	10.066161	10.265579	9.032049	9.603315	8.268871	9.607484	9.455087	10.828444	10.349902	10.563066	10.920468	10.820704	10.852098	10.256634	10.448243	9.804238	9.747580	9.176514	10.438091	10.376210	10.274568	11.037558	9.323656	9.634319	10.240898	10.905852	10.348851	10.216978	10.529664	11.352876	10.411012	8.470262	11.552116	9.325363	10.205213	9.814480	8.919970	10.248940	8.991496	9.175115	9.198284	9.239831	8.922407	8.562532	9.212214	8.596881	9.924431	9.810719	8.386178	9.903960	10.174250	10.147018	9.769677	10.077440	9.575490	9.405722	9.290321	11.427955	10.544926	10.301888	11.495068	10.729560	11.072707	11.561147	9.033552	9.439413	10.522363	9.934336	9.942884	10.752942	9.853221	10.068114	9.521604	10.183168	10.804313	8.273592	10.090002	9.442955	9.093737	8.929545	9.459261	8.632606	9.691158	9.965611	6.507640	8.999654	8.456739	9.003351	11.049872	8.013547	8.915416	10.392215	9.913595	10.538517	11.377987

POU6F2	6.669632	5.174952	6.291515	5.546573	5.759977	5.788486	7.091343	7.181722	6.883240	6.881513	6.151554	5.473875	5.168280	5.264485	4.686349	6.500534	6.075531	4.679217	6.748333	5.850428	7.340680	8.103403	7.766091	6.576181	7.727960	5.632831	8.072573	8.150810	6.962923	7.883516	6.377625	7.154829	7.918437	8.997819	7.406634	6.843037	6.449494	6.200170	7.869232	6.369883	7.510460	6.236327	6.342020	6.377625	5.548432	7.935298	7.375318	7.055431	6.267418	7.981450	5.904725	6.573209	6.481906	7.016983	5.887073	6.013683	6.571789	7.880362	7.413508	5.723098	6.264502	6.444714	5.639555	6.628714	10.112153	5.857270	7.572107	5.998314	8.257083	6.533076	7.732264	6.182888	8.127752	5.865057	7.323122	6.639977	6.420618	7.566427	6.686295	5.210230	6.753057	7.340507	6.677352	7.589821	7.705798	6.854620	6.984753	7.634654	8.259547	6.196378	7.018752	7.078318	6.208126	7.788365	6.055326	5.857270	6.978010	7.411494	6.782450	7.737418	8.638171	5.357782	5.687539	5.319005	6.758965	5.990959	7.835968	6.938279	7.511709	6.193198	7.035000	9.602218	6.492830	6.159627	6.154306	9.443592	7.378933	7.492583	7.199296	10.778689	6.911143	7.749492	8.364546	10.211224	7.427903	7.478884	12.259420	12.618446	11.973127	6.491699	8.629256	7.790379	9.161365	8.311773	12.379588	10.904527	9.863624	10.965182	9.508697	10.192680	10.372426	11.010787	8.740568	11.692447	10.583183	7.745636	9.973251	10.644015	9.220659	5.843332	9.818575	10.145498	8.064734	8.604688	10.782238	10.128831	10.443347	9.330151	6.985072	11.300829	9.845979	10.984493	12.564800	10.232800	9.960892	6.634691	11.820064	9.384364	9.783775	9.852310	11.506720	7.169475	6.387306	8.743149	10.673442	10.712868	8.812340	10.237708	10.029183	10.267192	6.648363	10.613036	9.926775	9.648561	10.372584	12.019903	6.018321	9.472022	8.121469	7.121630	8.633073	10.549347	6.816998	9.782528	9.421896	7.396775	9.910511	7.944322	6.235819	10.848877	5.430849	9.579952	9.830915	10.734602	10.348851	9.758277	10.402284	9.118515	7.828982	8.359725	6.142756	7.432187	9.241070	8.850175	7.708979	10.584768	10.071306	11.610264	10.563528	6.370223	10.380450	10.126326	7.552537	12.308556	8.534684	12.141427	11.022602	9.887683	8.166768	8.631600	10.111087	10.872181	8.896861	9.573678	10.946388	10.969237	6.766833	10.605180

CAPN10	10.118063	7.104070	7.398827	9.280309	7.854983	8.466427	7.693984	8.067272	8.506270	8.144710	8.098103	7.946027	7.297331	8.143161	7.554414	7.758398	7.651278	7.890755	7.638944	7.845933	7.676983	8.296861	8.178250	7.382041	8.145645	7.875503	7.557235	8.753110	8.013217	7.207379	7.732409	9.455263	7.270940	7.256648	7.098777	7.592865	7.692886	7.385317	7.567942	7.851877	8.032995	7.253245	6.847079	7.652521	7.222979	7.463593	7.793850	6.964835	8.011860	7.664378	9.361876	7.566103	7.419464	7.421349	8.303938	7.857697	7.685180	7.933884	7.071013	7.678284	7.398506	7.488664	6.176101	7.370474	6.871479	7.673196	7.275617	7.220170	6.647718	7.387779	6.941991	7.401308	8.111918	7.465716	7.678105	7.146904	7.043666	7.015993	8.024223	7.319006	8.329950	7.550671	9.131804	7.435040	7.775580	7.709719	6.966758	6.966758	7.083787	7.513898	6.998024	7.153877	7.280692	7.654135	8.682676	7.616510	7.741944	8.330440	7.817308	7.705159	8.845719	7.291926	7.881547	8.428664	7.501657	7.950478	8.522159	8.025420	7.671486	8.168459	6.130691	7.971630	7.447919	8.907495	8.306186	7.464195	10.554766	8.132684	7.876711	6.872750	6.770577	6.287255	7.171971	7.878847	7.888271	7.335219	7.361373	7.334115	7.719509	7.623325	7.437251	6.988248	7.867250	6.801411	7.385465	7.343018	7.245330	7.224175	7.294297	6.901263	7.642917	7.548238	7.993790	7.891853	6.810006	7.368750	7.969250	8.189910	6.998925	7.145969	7.119495	7.013469	7.653463	7.285498	7.293485	7.002726	8.018756	6.807918	6.143072	7.823491	6.268096	6.137019	7.079100	6.950755	6.852662	7.905227	6.344739	7.541740	6.419156	6.688548	6.509428	6.914321	6.505645	6.637534	7.101115	6.966432	6.605173	6.875616	5.687890	6.406399	7.299240	5.600944	6.845217	6.366378	6.441853	6.083169	6.453116	6.492516	6.404890	6.841755	6.948905	8.567450	6.703693	6.473242	6.770091	6.338219	6.374349	7.063002	6.485635	7.212810	6.760754	6.882930	6.514561	6.296264	7.471713	6.466185	7.113000	5.865953	5.724728	7.258729	5.830277	6.268961	6.716859	7.389191	7.114246	6.850135	7.733473	7.209237	7.019538	7.293816	7.430474	7.440209	7.224035	6.061533	8.006102	7.075119	6.557446	6.989679	8.392568	5.717290	6.587033	6.612650	8.788596	7.572029	7.614214	8.211447	7.782109	7.485213

GPR35	10.118063	7.104070	7.398827	9.280309	7.854983	8.466427	7.693984	8.067272	8.506270	8.144710	8.098103	7.946027	7.297331	8.143161	7.554414	7.758398	7.651278	7.890755	7.638944	7.845933	7.676983	8.296861	8.178250	7.382041	8.145645	7.875503	7.557235	8.753110	8.013217	7.207379	7.732409	9.455263	7.270940	7.256648	7.098777	7.592865	7.692886	7.385317	7.567942	7.851877	8.032995	7.253245	6.847079	7.652521	7.222979	7.463593	7.793850	6.964835	8.011860	7.664378	9.361876	7.566103	7.419464	7.421349	8.303938	7.857697	7.685180	7.933884	7.071013	7.678284	7.398506	7.488664	6.176101	7.370474	6.871479	7.673196	7.275617	7.220170	6.647718	7.387779	6.941991	7.401308	8.111918	7.465716	7.678105	7.146904	7.043666	7.015993	8.024223	7.319006	8.329950	7.550671	9.131804	7.435040	7.775580	7.709719	6.966758	6.966758	7.083787	7.513898	6.998024	7.153877	7.280692	7.654135	8.682676	7.616510	7.741944	8.330440	7.817308	7.705159	8.845719	7.291926	7.881547	8.428664	7.501657	7.950478	8.522159	8.025420	7.671486	8.168459	6.130691	7.971630	7.447919	8.907495	8.306186	7.464195	10.554766	8.132684	7.876711	6.872750	6.770577	6.287255	7.171971	7.878847	7.888271	7.335219	7.361373	7.334115	7.719509	7.623325	7.437251	6.988248	7.867250	6.801411	7.385465	7.343018	7.245330	7.224175	7.294297	6.901263	7.642917	7.548238	7.993790	7.891853	6.810006	7.368750	7.969250	8.189910	6.998925	7.145969	7.119495	7.013469	7.653463	7.285498	7.293485	7.002726	8.018756	6.807918	6.143072	7.823491	6.268096	6.137019	7.079100	6.950755	6.852662	7.905227	6.344739	7.541740	6.419156	6.688548	6.509428	6.914321	6.505645	6.637534	7.101115	6.966432	6.605173	6.875616	5.687890	6.406399	7.299240	5.600944	6.845217	6.366378	6.441853	6.083169	6.453116	6.492516	6.404890	6.841755	6.948905	8.567450	6.703693	6.473242	6.770091	6.338219	6.374349	7.063002	6.485635	7.212810	6.760754	6.882930	6.514561	6.296264	7.471713	6.466185	7.113000	5.865953	5.724728	7.258729	5.830277	6.268961	6.716859	7.389191	7.114246	6.850135	7.733473	7.209237	7.019538	7.293816	7.430474	7.440209	7.224035	6.061533	8.006102	7.075119	6.557446	6.989679	8.392568	5.717290	6.587033	6.612650	8.788596	7.572029	7.614214	8.211447	7.782109	7.485213

GAL3ST1	9.107647	3.819476	4.413089	6.251818	3.794238	5.057772	4.755113	3.145506	3.263555	3.664851	3.292351	3.322010	3.466476	3.482074	4.084748	4.016698	3.341148	2.994379	5.511627	3.402304	5.726273	2.897780	8.226324	2.986165	2.967375	3.891965	5.126559	2.974981	3.702653	4.457594	4.356986	7.863555	5.178999	3.657644	4.669058	3.837146	5.147334	3.684817	4.366995	3.361439	3.355506	5.851310	3.369959	3.960518	6.084413	5.533321	4.615751	5.012811	5.034847	4.597879	7.259489	5.815094	4.885618	4.787871	4.831039	3.532333	5.913302	6.285328	3.034597	5.096781	5.911868	4.255446	7.115335	3.549094	5.027874	4.688438	3.846309	3.587872	3.241621	4.581198	4.314526	3.780400	5.088990	5.076919	4.396359	3.249455	3.340445	3.213675	5.076314	4.928464	5.144352	6.050621	7.569464	3.914887	4.996722	5.550338	3.372905	3.426919	4.818345	4.774707	4.429795	4.663683	3.318958	3.161356	6.396299	4.871916	3.795711	4.853550	3.228037	3.375999	3.097255	4.591859	3.391037	3.590928	3.262192	4.071461	4.058241	4.275090	3.439246	3.256210	3.182695	4.099547	2.950155	3.252362	3.170512	2.799671	8.882269	3.338122	4.168824	3.172503	3.385685	2.990915	3.035867	2.901417	4.043227	2.906581	3.074342	3.241215	3.034991	3.132602	3.019404	3.358975	3.074560	2.976679	3.139813	3.299460	3.687679	4.049098	3.302832	3.453834	3.091737	5.796753	4.526954	5.971232	6.144272	2.908227	2.901165	2.883640	3.501061	3.004431	4.324651	3.268982	3.189156	5.705436	3.609968	3.569686	3.332726	3.355506	4.025811	3.146903	4.106740	3.892199	3.300244	4.943891	5.032354	3.736280	7.330084	5.004978	3.770322	4.230677	4.112439	4.897059	4.108038	3.397663	3.036937	3.731296	5.864148	2.951204	4.716660	3.060451	5.571459	4.065226	3.558934	3.669702	3.608791	3.967702	3.510727	3.532151	3.167149	3.038635	2.991208	4.364402	3.130994	3.025349	3.218960	3.131467	3.056861	3.241215	3.580694	3.499242	4.721070	3.670112	3.479469	3.558339	3.244432	3.160321	3.877385	3.022890	3.328333	3.983919	3.182446	3.133914	3.200632	3.213260	3.039135	2.939052	2.933780	3.487711	3.958573	3.708262	3.011454	2.924830	2.835714	3.472743	4.983584	3.207343	3.272988	3.369785	3.126876	2.875475	3.011754	2.876455	3.196794	3.009067	2.866692	3.220989	3.115819	3.619143

ZNF521	3.316628	3.571337	3.354007	3.652626	3.876779	3.687483	3.787161	3.761949	3.560924	4.741069	3.644045	3.665678	4.893955	3.820601	3.666461	4.529240	3.856793	3.168171	5.190727	4.120097	5.242255	4.108703	6.865815	3.855536	4.023461	4.053659	4.725569	3.030991	5.200081	3.696123	5.530310	4.711800	3.495403	3.506641	3.710908	4.157992	3.727139	3.352062	3.276202	3.352560	3.940235	3.422336	3.654197	3.404378	4.214482	5.818870	6.190442	3.039623	4.991195	4.172494	4.046238	3.981076	4.578828	4.326548	4.069644	4.088287	4.942999	4.175765	4.875897	4.969038	2.869225	4.858360	3.787788	3.805356	3.645020	3.551811	3.065950	3.275589	3.294203	2.864974	3.498544	3.771953	4.799860	3.183972	3.352560	3.408870	3.418434	3.225710	3.284276	3.859401	3.506053	4.248611	4.363938	4.323808	4.031703	5.159668	4.722166	3.987353	4.121850	3.769513	3.411476	3.147640	4.092474	3.592821	3.337955	4.435400	3.872654	5.855821	3.719337	3.307903	3.583415	3.961808	4.731873	2.962817	4.621851	5.222149	3.936230	3.999802	3.878061	4.312966	3.373901	3.098565	3.222815	3.648603	3.531729	4.009963	3.072563	3.823956	5.257705	3.097128	3.714879	3.200908	4.110948	4.092474	3.799232	3.168786	4.379938	3.878719	3.211011	3.500336	3.301128	3.355506	3.191453	3.299325	4.170794	4.385646	2.972991	4.865575	3.525510	4.484799	5.729176	5.468537	3.143041	2.959467	4.011621	3.395212	2.915529	3.591899	3.494456	5.192874	4.450279	4.392582	3.736901	3.597536	3.780212	3.777351	3.447475	3.306029	3.413099	3.583598	3.047106	3.740764	3.448800	4.324006	5.245814	5.815647	2.947461	4.767260	4.381821	4.095169	3.800298	4.186897	4.433078	3.582100	3.527209	3.835886	4.031479	2.913137	3.752126	2.810698	3.342139	3.875478	4.102823	4.029277	3.787581	2.990263	3.492031	3.430804	2.823537	3.121823	3.540716	3.289685	3.525894	3.050591	3.541120	3.685623	3.177227	3.167023	3.679421	3.896720	4.664747	3.704057	4.246430	3.560924	5.271224	3.285202	3.597342	3.788884	3.613999	3.542469	3.021136	3.986510	3.470503	3.758336	4.384260	3.313790	5.885495	4.114172	3.313984	4.142802	3.371366	3.411297	3.249291	5.002123	3.162054	3.792920	3.657270	3.497840	3.659637	3.263834	4.113765	4.261080	3.228185	3.065121	3.150869	3.313340	4.077019	3.410579

SMC5	8.423320	6.586545	7.938524	7.688871	7.927895	8.535005	8.158705	8.885279	9.727298	8.843566	9.599253	9.337708	7.846086	7.924902	7.681002	8.666526	8.923429	7.395059	8.553021	8.219476	7.066362	6.958469	8.767930	7.189847	8.344300	8.400184	8.992256	7.958043	8.820838	8.139887	8.066831	6.957510	7.288783	8.394237	8.011263	8.217923	7.974136	6.604700	6.865500	7.787161	8.575648	6.961184	8.357224	8.272399	8.559151	7.974770	8.954704	6.946451	8.197934	7.988952	8.204349	7.786677	7.544702	7.567185	8.154551	8.641739	8.018913	7.097815	7.792043	6.869212	6.469512	8.985018	6.269643	7.315429	6.125852	7.066199	6.440547	7.429000	7.063002	7.340836	7.850793	8.367762	9.094797	8.036343	8.219328	7.934974	7.526038	7.426808	7.741021	7.664047	8.257083	8.021490	7.953099	8.014477	7.573718	8.304669	8.331700	8.413693	6.206911	6.464369	7.252455	6.810338	6.967877	7.555345	8.055749	8.729147	8.325256	9.560491	8.387288	8.242037	9.019286	7.744116	8.624827	7.551749	7.662956	9.241660	8.364848	7.432187	7.992398	8.865668	7.083448	8.036507	8.281921	9.289425	9.172960	9.302046	7.397543	8.659760	9.144597	7.852952	7.022655	7.758989	7.498661	7.098777	8.567166	8.771577	9.205975	8.896265	9.044566	9.564507	9.225751	8.447167	7.182971	8.127189	8.727249	8.550751	7.375490	7.911734	8.225701	6.892054	6.381130	7.570069	8.361560	8.001042	7.604831	8.446244	8.215311	9.803185	7.811870	8.325722	8.052321	8.358960	8.641356	8.323980	8.682951	8.132254	6.992259	7.765302	7.685946	9.007935	6.853497	7.852952	8.444544	8.557010	7.741642	9.188910	6.175920	8.059157	7.395847	7.796019	8.001992	7.710024	8.131953	7.992704	8.060923	7.774785	6.663954	7.708085	6.566884	5.739533	8.310071	6.069439	6.764402	6.267244	6.324053	6.934413	6.639174	6.650937	5.845087	8.221156	8.540372	10.116213	8.054773	7.732409	7.748405	7.900387	8.168936	8.613906	8.146260	9.089615	8.142033	8.808720	8.341166	8.314984	8.849419	7.894343	8.907661	6.609956	7.425855	7.349764	6.752554	7.246849	8.043711	8.433978	8.887442	9.139031	9.380804	8.683233	7.577303	8.955338	8.033614	8.569732	6.939673	7.392419	9.490469	8.309911	7.589821	6.904150	8.991352	6.584561	6.597460	5.882650	9.531300	9.108288	8.104652	7.206289	8.729876	8.770526

NDUFAF4	13.845086	12.723828	13.685728	13.190080	12.840179	12.649882	13.468573	12.150176	10.999696	12.809830	12.656017	12.622994	12.440789	12.146883	12.726202	12.445640	12.757890	12.687272	11.510387	12.562365	12.539305	12.078045	12.798496	12.266524	12.666171	12.380831	12.389189	11.603568	12.729457	13.047238	11.633888	13.706787	12.969135	13.100501	13.387343	13.145329	13.368506	13.196712	12.844401	13.509479	12.638546	12.399011	12.378643	13.120996	13.366449	13.058425	11.992622	12.857277	13.256816	12.803344	13.039307	12.988700	12.585807	12.954237	13.802610	12.758764	13.583239	12.479954	12.988128	12.411884	12.621674	13.175984	13.229444	12.311740	12.959744	13.474856	12.737637	12.920968	12.391607	12.583295	12.748800	12.815618	12.323739	13.386476	13.408374	13.233610	13.079487	12.946087	12.936361	13.652718	12.633304	13.888860	13.938227	13.856758	13.456140	13.337083	13.330066	12.967107	13.149340	12.898820	12.838265	12.934369	12.709644	13.448408	13.128443	13.265809	13.481700	12.658626	13.069091	11.874918	13.015899	13.000997	12.799704	12.298540	13.256009	12.448012	11.600163	12.883111	12.537248	12.847185	12.683155	12.362592	12.971662	12.030200	12.524939	12.385289	13.227058	13.161096	11.979884	13.535306	12.269302	12.729292	12.509321	13.110240	13.362873	13.697330	12.785061	12.943011	13.910783	12.875043	14.040253	13.062656	12.122850	13.117902	12.658466	13.338305	12.680421	12.839995	13.577611	12.726202	12.786494	13.297565	12.462928	14.065366	13.788167	12.951673	13.729644	12.960320	13.313561	12.020495	13.464415	13.402926	13.889403	12.416908	14.402866	13.166910	12.782063	12.569545	13.786622	13.351178	13.722010	13.054898	14.144293	14.181178	14.163659	11.521285	13.475103	14.330345	14.115861	13.806245	14.610567	13.859406	13.623609	13.223145	12.861696	13.517989	13.659354	13.474637	13.759150	12.532655	13.235816	13.036157	13.673865	13.166910	12.971855	13.148076	12.965989	13.796422	12.993251	13.911605	14.248543	12.884205	13.677364	13.197536	13.409465	14.020995	13.826360	13.381772	13.129561	12.897761	13.653197	13.470117	13.539933	14.020995	13.255583	13.508116	14.727873	13.311729	13.201795	13.788167	13.377996	12.632463	13.963304	13.390989	13.498914	13.637621	12.509651	12.642737	13.111759	13.522292	12.713993	12.422795	12.178309	12.947892	12.847857	13.010122	13.340998	13.680430	12.089045	14.043175	13.253022	13.890198	11.590688	14.195897	13.791466	13.389682	13.111379	12.765660

ARPC4	12.736267	12.732523	13.182307	13.338727	13.004475	12.696624	13.577816	13.670545	12.228317	13.261991	13.239764	13.469650	12.245922	12.862032	12.747792	12.715232	12.955360	12.700708	12.727604	13.081212	12.065157	12.512264	12.076202	12.687779	12.918224	12.818398	11.593866	12.698701	12.393629	13.021956	12.482634	13.501308	13.586257	12.664096	14.123055	13.901485	13.793282	12.958825	12.952366	13.252042	12.916049	12.928845	13.367886	13.193275	13.376481	13.484384	12.009598	12.861330	13.343460	13.382399	13.192485	13.575552	13.314147	14.063831	13.697560	13.038580	14.137944	13.152404	13.230081	13.115442	12.962142	12.793582	12.508519	12.737304	12.847364	13.108298	12.437588	12.618797	12.771890	12.623496	13.733398	13.414215	12.536567	13.309343	13.446264	13.444169	13.729644	13.384766	13.333079	12.910540	12.780366	13.304119	13.427110	13.754404	13.750799	13.562689	14.126434	13.666468	12.720078	13.189322	13.115807	13.023099	12.563831	13.403139	13.362873	13.795380	13.506342	12.672242	13.108298	11.495782	13.209096	12.745055	12.380353	12.893120	13.239992	12.425577	12.695910	12.788393	12.433076	12.534127	12.842271	12.696267	12.988128	12.756854	13.174008	12.108933	12.210195	12.953298	12.127196	12.130069	11.859202	12.941926	12.051330	11.280895	11.963054	12.235309	10.805632	11.707306	11.613018	11.869781	11.607591	11.479798	10.818334	11.458235	11.079978	12.322965	10.971013	11.315577	13.018544	11.385252	11.181727	12.215941	9.547611	12.115840	12.509156	12.318072	11.522052	9.888310	12.241919	10.802701	12.294510	12.314612	13.206127	12.656017	12.486817	13.037427	12.420264	12.481464	12.520550	11.357531	12.247353	12.299183	12.909480	12.707057	12.959381	10.965182	11.882525	12.269777	13.008116	12.910006	12.658466	12.219692	13.336430	13.041710	11.762372	12.898463	12.804235	12.508031	12.272798	13.036157	12.067188	11.988628	12.108321	12.017633	12.624160	13.754404	12.331584	12.312855	12.380518	12.671728	12.889667	11.349651	12.794117	13.189708	12.967586	12.474915	13.114296	13.061545	11.986208	12.052817	13.123808	12.991641	12.936361	13.371459	12.156187	13.135275	13.079309	12.665817	12.625513	12.574563	12.469565	12.130980	13.545519	12.607714	12.490917	12.570522	10.754495	11.951988	11.469574	12.629675	10.673716	10.507219	12.702108	12.157725	11.188740	11.113335	11.937773	11.727221	10.724009	12.138038	12.248657	11.274308	11.974061	11.669857	12.190892	12.803344	12.735217	11.908190

ARPC4-TTLL3	12.736267	12.732523	13.182307	13.338727	13.004475	12.696624	13.577816	13.670545	12.228317	13.261991	13.239764	13.469650	12.245922	12.862032	12.747792	12.715232	12.955360	12.700708	12.727604	13.081212	12.065157	12.512264	12.076202	12.687779	12.918224	12.818398	11.593866	12.698701	12.393629	13.021956	12.482634	13.501308	13.586257	12.664096	14.123055	13.901485	13.793282	12.958825	12.952366	13.252042	12.916049	12.928845	13.367886	13.193275	13.376481	13.484384	12.009598	12.861330	13.343460	13.382399	13.192485	13.575552	13.314147	14.063831	13.697560	13.038580	14.137944	13.152404	13.230081	13.115442	12.962142	12.793582	12.508519	12.737304	12.847364	13.108298	12.437588	12.618797	12.771890	12.623496	13.733398	13.414215	12.536567	13.309343	13.446264	13.444169	13.729644	13.384766	13.333079	12.910540	12.780366	13.304119	13.427110	13.754404	13.750799	13.562689	14.126434	13.666468	12.720078	13.189322	13.115807	13.023099	12.563831	13.403139	13.362873	13.795380	13.506342	12.672242	13.108298	11.495782	13.209096	12.745055	12.380353	12.893120	13.239992	12.425577	12.695910	12.788393	12.433076	12.534127	12.842271	12.696267	12.988128	12.756854	13.174008	12.108933	12.210195	12.953298	12.127196	12.130069	11.859202	12.941926	12.051330	11.280895	11.963054	12.235309	10.805632	11.707306	11.613018	11.869781	11.607591	11.479798	10.818334	11.458235	11.079978	12.322965	10.971013	11.315577	13.018544	11.385252	11.181727	12.215941	9.547611	12.115840	12.509156	12.318072	11.522052	9.888310	12.241919	10.802701	12.294510	12.314612	13.206127	12.656017	12.486817	13.037427	12.420264	12.481464	12.520550	11.357531	12.247353	12.299183	12.909480	12.707057	12.959381	10.965182	11.882525	12.269777	13.008116	12.910006	12.658466	12.219692	13.336430	13.041710	11.762372	12.898463	12.804235	12.508031	12.272798	13.036157	12.067188	11.988628	12.108321	12.017633	12.624160	13.754404	12.331584	12.312855	12.380518	12.671728	12.889667	11.349651	12.794117	13.189708	12.967586	12.474915	13.114296	13.061545	11.986208	12.052817	13.123808	12.991641	12.936361	13.371459	12.156187	13.135275	13.079309	12.665817	12.625513	12.574563	12.469565	12.130980	13.545519	12.607714	12.490917	12.570522	10.754495	11.951988	11.469574	12.629675	10.673716	10.507219	12.702108	12.157725	11.188740	11.113335	11.937773	11.727221	10.724009	12.138038	12.248657	11.274308	11.974061	11.669857	12.190892	12.803344	12.735217	11.908190

CRACR2A	10.345357	8.638314	8.632906	8.827612	9.194946	9.315341	8.561316	9.500568	9.483782	8.639891	8.602058	8.482719	8.027164	8.223289	7.830932	9.231455	9.026158	8.024223	9.384818	8.825236	8.740108	9.387777	9.107805	8.736877	9.141010	8.672782	9.781037	9.309567	9.385728	9.383918	9.169538	8.295296	8.675156	8.608629	8.369070	8.797307	8.159927	8.216662	8.507514	8.611061	9.026460	8.913424	8.476502	8.314845	8.118397	8.073351	9.430938	9.063831	8.605743	8.276739	9.207491	8.037132	7.879149	7.996760	8.012452	9.156761	7.658000	7.916120	7.923957	7.688422	8.227204	8.812028	8.787346	8.531668	8.075788	8.026061	9.099970	8.573979	8.572024	8.617543	9.065219	8.914940	9.145331	8.783516	8.672489	9.024766	8.639275	8.757464	9.854297	8.307442	8.068901	8.121626	8.506105	8.576106	7.993159	8.790577	8.264324	8.209622	8.375408	8.839797	8.960537	8.914784	8.335407	8.624351	9.492065	8.215620	8.609545	8.367126	8.670782	8.726505	9.294739	8.364085	8.575025	8.313905	8.447805	8.938682	9.546241	8.697583	8.973624	9.401696	8.902785	9.042373	8.676199	9.698334	8.928410	11.866983	8.963871	8.908005	9.268476	8.753634	8.795216	9.069131	9.330587	10.234170	9.177565	9.185181	9.095706	9.095402	8.957149	8.795216	8.681103	8.638171	8.470408	8.436796	9.416569	8.779006	8.114390	9.363051	8.762120	9.031888	9.460158	9.312799	9.267219	9.310358	9.021427	9.485807	9.503932	10.026067	9.688230	9.214386	9.166648	8.668344	8.517941	8.534543	8.422852	8.524310	8.889464	8.958885	9.177867	9.602855	8.851528	8.376183	8.659760	8.278265	8.196235	9.664852	8.529071	8.485224	8.414300	8.339965	8.209938	7.838143	8.229190	8.034047	9.376056	8.109811	8.473114	8.076799	8.004026	8.247106	9.503615	8.775967	8.641436	8.341747	8.324142	8.705513	8.925189	8.655522	9.348808	9.206285	9.136846	9.629238	8.843862	9.006998	9.178776	8.885766	8.982606	8.733342	8.506270	8.145183	8.353996	8.237618	8.553489	8.734707	8.795667	8.322623	9.310505	8.635686	8.813641	8.353066	9.080879	8.753956	8.942784	8.858496	8.739022	8.724336	9.164484	9.283708	8.792916	8.765121	8.922996	8.729568	8.880054	8.497774	9.477368	9.579308	9.020620	8.933933	9.916997	9.132598	9.047586	9.888161	10.132853	9.241513	9.915905	8.509059	8.872943	9.461453

D2HGDH	12.739166	12.381803	12.748800	13.105616	13.366020	12.991828	12.311085	12.654474	13.907695	12.801434	13.207720	13.396009	12.775662	13.578055	13.529454	12.642396	13.373356	13.177797	14.129877	13.084208	13.569407	13.661762	13.604320	13.110956	13.762178	13.791984	13.195507	13.036888	13.196931	12.192594	13.513091	12.556834	12.533788	12.386450	12.064261	12.161888	12.579997	12.181281	12.861330	12.913034	13.026553	12.837925	12.491562	12.528252	12.370359	12.667996	13.299825	12.365907	12.558642	12.618274	12.493206	12.720580	12.447571	12.720241	12.361470	12.974739	12.704827	13.270823	13.403996	13.165501	13.537477	12.489075	12.820775	13.796160	12.938338	12.821781	12.960120	12.642568	12.542124	13.095970	12.638003	12.252460	13.011946	12.230786	12.586128	12.611187	12.743377	12.050270	12.566429	12.974383	12.823209	12.990709	12.992898	12.696267	12.795726	12.871011	12.969318	12.672242	13.904184	13.525049	13.146133	13.345516	12.780366	12.547000	12.736077	13.237448	12.895811	13.574462	13.183703	13.261202	13.193477	12.129781	12.877659	12.894551	13.416705	12.925613	13.820961	13.097503	13.980518	12.880604	13.545741	14.048218	13.818903	13.321691	13.281221	11.934741	13.522760	12.882408	13.393700	12.904102	12.291225	11.506853	13.004678	12.801434	13.158966	12.388554	13.421261	13.395578	12.050582	12.842616	13.016643	13.857001	12.671548	12.359115	13.584643	13.206916	13.958500	13.033913	12.195580	12.924028	13.593289	13.732138	13.518872	13.100310	13.144764	12.415936	13.093911	13.762421	11.933193	13.782833	12.326538	12.945900	11.903261	11.986813	11.540693	11.779565	12.076036	12.563656	12.521575	13.819661	11.592521	12.913978	11.702518	11.753214	11.164797	12.926643	11.868033	12.459627	11.412504	11.073706	11.956809	11.972367	11.288046	12.381950	12.784340	12.476380	11.731995	13.061545	11.389403	12.830155	12.932588	13.021956	12.305101	13.446470	13.130961	12.372273	11.872661	12.640215	13.064543	11.354920	11.486837	13.895889	11.973605	11.573596	11.854155	11.912654	12.196008	12.676682	12.127984	12.246555	12.725852	12.223567	12.428334	11.344909	12.941926	12.426853	14.066903	12.903569	12.794117	11.787766	12.029467	12.879575	11.509940	12.564954	12.700378	13.401415	13.541252	13.047049	11.787922	12.891887	12.741273	13.707475	12.018886	13.155744	12.756522	13.603336	13.926780	12.743876	12.259110	13.214418	12.736784	12.410794	13.523456	11.337754	12.397227	12.841413	13.276462	12.286301

SPPL3	10.558425	9.958930	10.664611	10.520340	10.918390	10.517707	11.239490	10.455381	10.535296	10.708724	10.958111	11.055681	10.345071	10.172011	9.220963	10.343255	10.396585	10.439729	10.809966	11.036699	10.592463	10.462807	9.682637	10.476626	11.112153	10.624433	9.034796	9.837182	9.722628	10.523247	9.698789	10.275619	10.114208	10.709812	10.692859	10.830760	10.621593	10.201859	10.468163	9.689137	10.932488	9.537204	10.952265	10.789859	10.470586	11.222057	9.523175	10.048962	10.867134	10.965488	10.304766	10.672708	10.848591	10.970728	11.200158	10.533656	10.945211	10.687204	10.867436	10.519764	10.350375	10.846377	9.301096	10.144761	10.605791	10.467694	10.202314	10.203254	10.193562	10.118547	10.930669	10.915384	10.563219	10.838131	10.928290	10.396585	10.429581	10.760858	10.551729	10.126629	9.971850	11.361662	10.589350	10.697213	10.588660	10.650012	11.248954	11.069731	9.847836	10.299864	10.272441	10.392366	9.812607	10.636526	10.150370	11.106592	10.706880	10.553085	10.410131	9.998272	10.457252	10.177187	10.114351	10.667694	10.464170	10.297710	11.492591	10.768939	10.777462	10.368806	10.284582	11.270443	11.008142	9.821011	10.564137	10.031627	9.059743	10.697641	9.938377	11.750863	10.030393	10.211826	10.436142	10.403775	10.097156	10.913576	10.406956	10.107612	10.673881	10.389876	10.162442	10.016027	8.794756	9.144285	9.818886	9.857581	8.939739	10.840973	10.285761	10.363280	9.938052	10.651347	10.546727	10.973509	9.526340	9.418110	9.822841	10.908532	10.586252	9.260302	10.664468	9.696322	11.515156	10.784522	10.353725	10.405770	9.824794	9.674754	8.464904	10.879160	9.086111	11.028512	11.045017	10.662816	10.868295	9.845829	10.411931	10.488568	10.577682	10.047564	10.387474	10.386560	10.205059	10.867857	10.526253	11.121141	10.788987	10.646207	10.463100	9.392967	10.996788	9.346035	9.817268	10.810329	10.577682	10.446274	10.030230	10.074373	8.800353	10.907218	10.641355	9.656882	10.491272	10.485332	10.274402	10.096550	11.349211	10.664169	9.533987	9.843788	11.155808	10.605932	10.588660	10.270009	10.557223	10.882223	11.199316	9.459550	9.851372	9.794550	9.677379	9.334944	10.432310	10.385470	11.006117	10.356772	9.790211	10.354903	9.617155	9.591500	9.765260	9.425282	10.173953	9.017440	10.430794	11.163334	11.343087	10.412086	10.259775	9.775074	10.889830	9.786827	9.753457	10.370786	9.974679	9.317025	10.692859	10.046931

OR11G2	3.510558	3.601083	3.346730	3.165554	3.293629	3.300244	3.353367	3.585340	3.489710	3.460369	4.036922	3.399012	3.302053	3.896283	3.632948	3.235526	3.804926	3.111532	5.011405	3.253582	5.432219	4.992449	7.233701	2.979346	3.107369	2.957078	3.141816	3.091429	3.251818	3.078861	3.995086	3.844991	3.700762	3.122871	3.570423	4.447109	3.690345	3.351071	3.446532	3.272865	3.450610	3.820807	3.639290	3.389637	3.821032	5.886902	3.769308	3.336283	5.307327	4.028832	3.981983	3.900638	4.523234	4.318222	4.113121	3.201427	4.274425	3.240032	3.033076	3.818855	2.885303	3.202543	3.982847	3.389637	3.642062	3.564394	2.955347	3.351386	3.296210	2.971440	3.161074	3.103870	3.303158	3.052568	3.226873	3.383146	3.062674	2.977135	3.123088	3.929129	3.533492	4.213405	3.632126	4.356325	3.508878	4.957295	3.770527	3.290129	3.594018	3.806168	3.384322	3.248982	3.600306	3.368755	3.000837	3.049223	3.943958	3.223091	3.227738	2.990174	3.283480	3.257185	3.005456	3.206907	4.695359	3.671371	3.351386	3.723306	3.056767	3.374889	2.867977	3.465024	3.164186	3.364370	3.612180	3.449181	3.038434	3.114254	3.254505	3.141052	3.772413	3.200504	3.255327	4.163583	3.782941	3.001687	3.286828	3.485005	3.188761	3.417894	3.106766	3.379120	3.226440	3.299180	2.976862	3.717910	2.960516	3.849721	3.436697	4.695359	3.388059	5.242838	2.892787	3.203344	2.895725	3.438735	2.975703	3.120348	3.518728	3.537245	3.439246	3.620663	3.193461	3.516224	4.552354	3.739547	3.522634	3.427251	3.140672	3.193103	3.511629	3.673077	3.455894	4.347825	5.376539	3.649813	3.080594	5.208841	4.073434	3.964450	3.694034	4.299617	4.467366	3.620854	3.105977	3.859612	3.092914	2.840570	3.508342	2.854722	3.307903	4.116356	4.071686	4.021539	3.804926	2.899468	3.660607	3.432543	2.876167	3.089248	3.064605	3.279903	3.010483	3.122165	3.349518	3.064290	3.133205	3.153195	3.300958	3.927163	4.750862	3.691777	3.682236	3.522436	3.637058	3.222673	3.447839	3.279903	3.650619	3.568873	3.117943	3.270526	3.491185	3.337787	3.106541	3.037234	3.053740	4.073227	3.265442	3.434340	3.081556	3.544145	3.095195	5.076314	3.287111	3.382241	3.715868	3.546976	3.371881	3.164945	3.714449	3.517979	3.254014	4.219929	3.175288	3.192946	3.157049	3.370996

AKIRIN2	8.067272	7.650308	8.587856	6.485460	7.042589	7.667152	8.576857	8.858351	7.865564	8.673745	8.704125	7.766387	7.281623	7.119964	8.009296	8.411253	8.636158	7.987793	6.880224	7.878702	7.195520	7.332899	7.270165	7.292562	5.855263	6.227804	8.107279	6.357068	8.169880	9.004415	8.332937	8.154391	9.115115	7.685796	7.821163	8.471373	8.007472	7.718880	6.066254	6.647547	8.131342	6.354574	7.963288	8.725564	6.720650	7.395245	8.295916	7.380825	9.907428	8.490141	8.103097	7.456680	7.849325	6.371595	8.525064	9.314893	8.161001	6.384336	6.064179	6.878304	6.630936	8.310512	6.441853	6.254190	6.961011	7.235957	5.499091	7.643849	6.540849	6.248293	7.434558	7.759430	7.968189	8.181886	7.198837	8.119770	8.148860	8.518854	7.717365	7.711088	6.508471	7.696523	6.865661	7.056979	8.002934	9.457707	7.630212	8.413538	5.520232	5.739353	5.960403	6.767480	7.557379	8.272099	7.519245	7.762510	9.561136	8.062420	8.149614	7.126739	8.733797	7.659976	7.317019	7.198218	8.138807	9.710638	7.348953	6.726248	5.830114	9.018370	4.704932	6.911631	7.538600	7.753171	10.633662	6.022244	5.721228	9.357717	8.423320	8.292455	7.506496	7.418065	6.358684	7.965874	8.082112	7.928033	8.132097	7.829918	8.041063	7.885668	8.056978	7.028135	7.210504	7.406634	7.516478	7.910938	7.190458	6.524664	8.203624	7.144129	7.996300	5.627077	7.963443	4.323583	7.874427	9.839709	7.460397	7.373769	9.135468	8.162350	8.470480	8.409398	8.793845	7.471877	8.864761	7.754859	7.089612	5.175155	6.331112	8.524156	7.692436	7.814466	9.423138	7.551288	7.807549	8.678199	8.482018	8.327463	8.763780	8.807358	8.212064	7.788070	7.814030	7.243579	8.729427	8.401291	7.301137	6.896554	7.397106	7.205802	7.451363	7.911881	7.345011	6.365531	6.956132	4.838303	8.395299	8.123878	7.402873	8.212820	8.800353	8.043558	8.117316	6.918372	8.447639	8.654468	8.613148	8.249893	6.597629	6.702417	7.645097	7.271564	7.610523	8.429897	7.497555	8.173786	9.801200	4.771384	6.576008	5.899887	7.125777	6.868741	8.689294	8.352774	8.014777	8.566420	8.053099	7.230254	7.911412	7.608374	8.434134	7.085386	7.116127	6.080065	9.183929	8.806901	8.176794	7.475837	8.899620	5.008061	7.021100	3.774502	6.942632	8.406370	9.914982	6.412890	9.693450	7.206445

RNF6	10.471332	9.573039	10.018949	10.089927	10.642239	11.292037	11.083644	11.543325	11.224607	11.530149	11.383057	11.106894	10.545363	10.798488	10.835583	10.873250	10.919103	11.212744	11.563886	11.213821	11.080123	10.967395	10.962224	10.726580	10.056200	10.286654	9.881165	10.046039	10.705209	10.388663	11.299384	10.545217	10.059104	10.366087	10.753411	10.849149	10.794319	10.702585	11.101364	11.240485	11.111735	10.818184	10.659325	11.016629	11.169751	10.380008	10.525341	10.102793	10.640470	10.712259	10.296948	10.647435	10.300015	10.245014	10.627617	10.736390	10.787776	10.887194	10.564580	10.456157	10.481855	10.613340	10.631433	10.989151	10.188468	10.304169	10.055446	9.889840	9.907128	10.262380	10.279430	10.370179	10.814609	10.092323	10.311130	10.246336	10.371089	10.088845	10.110195	10.135934	9.932777	10.480213	10.340199	10.538810	10.914464	10.548890	10.569984	10.897742	10.297389	10.109280	9.932177	10.190858	9.698789	10.480506	9.878591	10.430163	10.382091	11.434353	10.935634	10.457252	11.345799	10.533504	10.454507	10.668998	11.347140	11.433898	11.049296	10.984035	10.687045	11.238007	10.751617	11.486837	11.135945	11.084401	11.078552	9.263062	10.588938	11.113335	11.004092	11.144115	10.460851	9.877686	10.611388	9.812907	10.241662	10.287723	10.353428	10.791138	10.827521	11.646361	11.167246	11.246511	10.186361	10.251906	10.391640	10.889665	10.500161	10.776676	10.256037	10.134060	11.004401	11.164944	8.914189	9.866534	10.319973	10.381054	10.143238	9.655185	10.572908	10.426856	10.149179	10.501376	11.223409	10.774758	10.704038	10.390339	10.347055	10.706272	10.471635	10.133737	10.336744	10.880917	11.055538	10.612741	10.252055	10.635777	9.392669	10.657842	11.253964	10.784522	10.592897	10.487023	10.690215	11.042247	11.067913	10.491566	10.563066	10.439142	10.479650	11.809331	10.347205	10.908236	11.342479	10.767583	10.373812	10.621887	9.840309	10.539572	10.026399	10.063481	10.470285	10.083424	10.685266	10.065121	10.544466	10.516841	10.463100	10.803291	9.600344	10.333744	10.555984	11.614495	10.220952	10.795565	10.365612	10.780871	10.736862	10.282310	10.437655	9.609659	10.103883	9.377318	10.497091	10.542543	9.943047	10.586129	11.163914	11.222825	10.593778	11.082337	10.119137	9.471239	10.029332	10.047564	10.829442	11.556839	10.400645	10.593479	9.982592	10.250874	10.384855	8.463989	10.247562	11.608755	10.779848	10.545676	10.840472	11.036553

LYPD3	10.560870	15.079901	16.279812	15.832903	15.784706	15.817557	15.784706	16.503819	16.115572	16.180048	15.726000	16.156164	16.166807	16.583150	16.230114	15.779491	16.180048	16.192130	16.365166	16.287200	15.908209	15.714733	14.348197	16.349770	16.290771	16.137062	12.630807	15.824202	15.570993	14.997957	16.114305	16.128614	15.342745	13.607100	15.932498	16.375050	15.936069	16.543736	16.361326	16.572650	15.849957	16.195154	16.409990	15.971905	15.733274	15.862906	15.152524	16.279812	15.723939	16.301700	15.633808	16.353525	16.559494	16.823927	16.730795	16.678432	16.847849	16.874939	16.714243	16.874939	15.735297	15.324219	11.878329	15.969476	16.422588	16.789194	15.583728	16.269830	16.220278	16.090084	16.271502	15.877039	15.403084	16.071028	16.248343	15.803838	16.648016	16.055100	15.054330	16.656647	16.005250	16.526136	15.066902	16.377052	16.456985	16.121338	15.763886	16.672020	15.707533	16.094324	16.065653	15.564042	16.079155	16.290771	14.470097	15.936069	16.478815	16.098515	15.921788	16.059162	16.228514	16.424706	16.430830	16.056486	17.002792	15.221141	16.319695	16.685246	16.094324	15.449581	15.417796	16.632960	16.428719	15.173276	16.632960	15.209711	12.769987	16.178602	15.872260	14.741814	15.312332	14.056836	14.152939	14.924899	14.902407	14.999084	11.126936	14.741814	12.498958	14.619813	12.987200	13.085901	12.100426	13.462700	13.784315	12.780199	13.789730	11.903881	11.611675	14.889167	15.438975	13.990142	13.660103	12.690337	10.555376	15.147454	14.082814	12.525614	14.433838	13.340771	13.880671	14.362295	15.437228	14.366636	13.487032	15.290231	13.284435	14.437151	14.715375	13.353546	14.478648	11.732727	14.934057	15.291679	15.049528	14.911437	11.639628	14.365218	14.184593	13.805428	12.484550	14.611021	13.744601	13.952379	12.559575	11.136838	13.455497	14.235912	13.514224	15.316021	14.773263	14.040253	15.384452	13.486625	14.591596	15.200938	14.600827	14.140840	13.330066	14.460072	15.241129	14.672364	14.473115	13.099019	14.931361	13.077428	12.940455	15.146198	13.607340	14.210111	14.635670	15.498917	14.216321	11.345331	13.840337	15.317503	12.977323	12.847005	13.196712	10.722360	13.113930	14.702993	12.764791	15.430200	14.612714	13.519354	11.508343	14.605817	14.587687	11.299108	13.381985	13.055072	13.679721	10.827241	14.620707	14.266657	14.582713	15.066902	15.166041	14.219589	14.001234	10.363564	15.385183	13.320270	14.522678	15.399178	14.903483	13.416505

MID1	3.792920	4.388263	4.681159	3.742196	3.941720	5.097372	5.558489	3.319596	3.538802	5.014462	4.531615	4.078904	4.207295	4.567244	4.979942	4.921917	3.358469	4.742727	4.357202	4.916624	5.111719	3.225038	6.371595	3.802243	3.602660	3.236242	3.784156	3.589021	5.323444	5.187401	3.522057	4.427875	4.355484	5.183621	4.868318	5.563726	4.706614	3.294849	3.316769	3.326289	5.372466	3.246890	4.074510	3.664440	5.603805	7.236268	5.035490	3.719845	5.177536	4.828672	3.318713	4.307689	3.730048	4.113121	4.891068	5.531069	4.058871	4.456929	3.664440	3.886152	2.932321	5.148905	3.860653	3.352560	3.483534	3.432363	3.607047	3.866608	3.587872	2.875865	5.287363	4.572417	4.591187	4.722578	5.093948	4.733792	4.605235	4.640721	4.118599	3.586323	3.314652	3.719337	3.367536	4.534121	3.295881	4.763986	2.941020	3.409531	4.458021	3.657270	3.206648	3.472563	4.318023	4.369342	3.126045	3.713585	4.153649	5.138648	4.141298	4.068386	4.869381	4.347825	4.617326	4.861840	4.398284	5.148333	4.573962	4.412414	3.377215	5.007256	3.099280	3.618945	2.947036	4.722818	4.333544	2.836829	2.924925	3.871553	4.719803	4.842115	4.351993	3.709060	3.749998	3.101983	3.898456	3.181767	3.912495	3.647373	4.765468	3.993947	4.380378	3.086146	3.827201	4.075989	3.942615	4.326137	3.826564	3.284905	4.723440	3.388249	2.976959	5.514823	4.907099	3.018736	3.957460	4.488884	2.866692	3.653400	5.847907	3.294060	3.367240	3.745252	4.379733	3.134492	6.339386	5.571259	3.457694	3.374235	3.192551	3.844372	3.124029	3.591699	3.229324	5.692478	6.807739	4.584149	3.185927	5.402259	5.202650	3.164186	4.427011	3.842751	4.630478	3.431159	5.898297	3.724930	4.394058	3.407660	3.831373	2.867173	5.816732	3.947248	3.773031	3.794679	3.642881	3.734589	4.332259	3.370101	2.846533	4.469699	4.402680	4.208643	4.500955	3.362499	4.168824	4.083916	3.570038	3.690345	3.149648	4.089190	3.970521	4.015856	3.498362	3.335963	3.577965	3.365983	3.366868	4.153415	3.553152	3.287419	2.905165	3.262792	3.608409	4.458635	5.313700	3.865362	4.104536	4.066061	3.851619	5.537848	3.570038	5.077136	3.619143	4.737997	4.607995	4.509641	4.221049	3.262344	3.627415	2.846808	3.176610	2.891716	4.008667	4.928464	3.115938	2.918243	5.114355	5.579658

EIF3B	13.127686	12.853171	13.615908	13.446715	13.413148	13.317163	13.320270	13.186053	12.557178	12.970251	12.974383	13.155145	13.311915	13.229053	12.989438	12.728950	12.998952	13.079117	12.821251	13.375655	12.981868	13.572169	12.671216	12.621991	12.800035	12.950361	12.895811	12.784870	12.679062	13.163768	12.979296	13.722010	12.905897	13.494170	13.263573	13.113538	13.461357	13.431930	13.501961	13.650065	12.985929	13.264960	13.570987	13.462242	13.711410	13.629444	12.600026	13.201991	13.274181	13.593519	13.519583	13.488803	13.594191	13.614714	13.616838	13.130569	13.847197	13.771343	13.832307	13.910238	13.286871	13.324601	13.319481	13.298414	13.823712	13.540808	13.115055	13.278803	13.080831	13.422986	13.031189	13.328625	12.647084	13.285876	13.352048	13.154391	13.311534	12.787348	13.071694	13.461144	13.050076	13.312545	13.460282	13.787649	13.488148	13.070763	13.232466	13.387976	13.292351	13.526542	13.226845	13.203403	13.087563	13.637384	13.209513	13.560400	13.284662	12.974204	13.362446	13.052245	13.106206	13.386902	13.271585	13.679001	13.882499	12.819943	13.386257	13.955482	13.750320	12.990890	13.281421	13.577170	13.841373	12.802478	13.167086	12.625000	13.467530	13.290718	12.614703	13.927588	13.767258	14.071478	14.267351	14.020694	13.756127	13.998713	13.479279	14.147617	14.408756	14.292381	14.212720	14.374258	13.919949	14.221539	13.395168	14.873437	13.817328	13.363673	13.508116	13.908831	14.130469	13.932204	13.335408	15.066359	14.798373	13.390537	14.303944	13.713891	13.986959	13.393505	13.854600	13.624536	14.915189	13.999261	14.516636	13.783587	14.438218	14.465165	14.271565	14.458572	14.190235	14.204958	14.730705	14.395843	13.790935	13.458723	14.123986	13.243734	14.176803	14.242737	14.472293	14.505707	14.386354	14.370460	13.734845	14.873940	13.525511	14.559663	14.258622	14.653700	14.131743	14.044056	14.418012	13.813000	13.959054	13.444821	13.388628	14.037892	13.903602	13.993598	13.587916	13.461577	13.792778	13.301664	13.835875	13.923216	14.700650	14.030557	12.905530	13.252042	14.101043	14.587290	13.416705	14.370844	14.720427	14.071794	14.956666	13.619020	13.512420	13.467091	14.565911	13.605717	14.357698	14.006174	13.858073	14.101917	12.941926	14.443066	14.375936	14.533864	13.391215	13.441594	13.770103	14.775599	12.445794	14.422502	14.211102	13.549579	13.694470	13.641034	14.101624	14.529893	12.920784	13.820166	14.070625	14.376307	14.193049	14.279316

HLA-A	15.892893	11.409205	14.072083	13.951559	14.723372	15.468133	13.438194	15.354914	14.776105	13.622196	14.225039	14.480925	14.756942	12.865155	14.725162	13.834324	14.017838	14.591596	13.624536	14.260943	14.095820	13.142467	14.241084	14.853812	14.814746	13.400373	14.488364	14.400018	15.100594	14.632630	14.030257	15.296048	14.558412	15.530816	14.304961	15.451967	14.434184	14.480189	13.487933	13.532839	15.117745	14.317829	14.640110	14.021565	14.016128	14.697958	15.077583	14.431588	14.498295	14.707518	14.352710	14.690192	14.689755	14.434526	13.714163	13.820961	14.909267	13.693113	14.590670	13.857256	13.652235	14.543000	14.042288	13.728857	12.630506	11.160233	13.079675	14.668191	14.434184	13.015361	15.085910	13.996448	14.378131	15.516865	14.823896	13.621305	14.010653	14.573507	14.204014	12.763449	13.149340	14.475985	14.128314	14.441229	13.713444	14.283430	14.884408	14.582713	14.138248	13.139308	13.880671	14.162992	13.883273	13.346348	14.088329	12.326856	13.583239	14.781328	14.566322	10.480071	14.529498	13.960239	14.299472	12.748645	12.869960	14.030557	14.761971	13.753345	13.243546	14.449826	14.507205	13.751062	14.768992	14.424884	16.009296	13.291349	14.639284	14.024445	14.409150	15.699656	13.129366	15.703652	15.442379	15.248690	15.105520	15.869850	14.887600	15.391393	15.569300	15.873355	15.157558	14.888617	16.202822	15.084166	16.048355	15.849957	14.905079	14.218284	15.300250	14.972000	14.907125	14.840176	14.957224	14.313436	15.414655	15.697564	15.028163	15.173970	15.431836	15.974511	15.505597	14.332725	13.879059	15.705567	14.484499	15.424949	14.351258	14.886035	14.898599	14.998489	14.226630	14.022985	15.047189	14.266657	14.757850	15.783769	14.100719	15.345704	15.270155	15.434895	15.161962	15.335238	15.253922	15.544594	16.071028	15.389194	14.427017	14.743665	14.208447	14.796433	15.822036	13.872610	15.362494	14.679130	11.395640	14.965697	14.459642	14.173200	14.405515	14.999084	14.400393	15.354130	15.193465	15.359530	14.999641	15.288862	15.186639	14.689299	15.461686	14.367353	15.767032	15.424214	16.218720	16.562284	15.620739	15.221141	15.014487	15.240441	15.689133	13.805968	14.316111	15.102359	15.620739	15.558628	13.025099	15.391393	15.788853	15.918070	10.764458	15.079318	14.608380	14.477088	14.630796	15.199621	15.553263	15.719891	13.596087	14.902955	15.827482	14.650177	15.789877	14.863545	16.040249	14.757850	16.253204	15.200291	16.294338	14.741363

NYAP1	7.287828	7.176989	7.165525	7.485516	7.725594	7.841244	7.354382	7.507849	7.372973	7.645719	7.259980	7.643684	7.598679	7.189847	7.045294	7.806014	7.229059	7.293658	7.135309	7.457620	7.500852	6.097354	7.386079	7.109984	7.200555	7.529175	8.598516	7.909425	8.572024	7.623618	7.400837	6.601343	6.084413	7.221773	6.998925	6.866291	7.042248	7.060933	6.613481	6.512439	7.706707	6.632896	6.744515	6.633547	6.844034	5.932286	7.995024	7.132591	7.185203	7.006419	6.891257	6.692278	6.568873	6.301153	6.405052	8.159168	6.214917	6.441696	6.260150	5.384096	5.030342	7.803715	6.441052	6.142404	5.632831	5.692843	6.559899	6.462173	6.401307	6.029573	7.227337	6.997424	8.024532	6.978170	6.844206	6.717502	6.709432	6.896217	6.775273	6.803009	6.225550	6.674620	6.318570	6.037899	5.705946	6.586695	5.537015	5.426425	4.837863	5.107866	5.322671	5.210637	6.453478	6.803833	7.193418	7.014840	6.775586	7.664983	7.234377	7.649188	7.778689	7.118842	7.311816	7.541274	7.162193	8.662390	6.999669	6.810338	6.669775	7.946920	4.423983	7.416867	7.353902	8.165811	8.059157	9.692997	7.512190	7.733926	7.759598	7.500400	7.347217	7.326749	7.713749	8.978023	7.891219	7.837199	8.402976	7.737739	8.089246	7.722684	8.064421	8.558069	8.072005	7.843121	8.159780	7.454763	7.313210	7.321353	7.580232	6.641921	6.577680	7.022169	8.611216	7.967552	7.627346	8.349595	8.432741	8.236065	7.691024	7.523920	7.032489	6.601510	7.645417	7.562869	7.276888	7.093070	6.961659	6.901263	7.229974	8.176328	6.313497	6.711048	6.790559	6.195488	6.385448	8.228550	7.178765	7.442823	7.160446	6.638539	7.320128	6.577680	6.912945	6.092366	7.950628	6.098380	6.409866	6.093207	4.932831	6.144784	7.852348	5.508191	5.988481	5.206643	5.633992	6.828319	6.750694	6.715371	5.888681	7.093995	7.249623	8.476374	7.209237	6.682588	6.945183	6.980170	6.884396	7.188088	7.065889	6.368368	6.632566	6.322725	7.326424	6.006261	6.756872	6.044295	5.671044	5.939326	5.323834	6.714097	4.923203	6.517896	7.201468	7.360104	7.230565	7.347870	8.087256	6.866135	8.001530	7.693362	7.223889	7.483367	7.108530	6.574042	8.496279	6.877175	6.879572	5.512009	8.973624	4.840025	7.342696	8.287640	8.562100	8.056042	8.402513	7.370785	7.730239	7.948163

MRPL50	12.965636	11.493891	12.389516	12.239319	12.262009	12.018734	12.557817	11.936396	11.340846	12.229570	12.118803	12.486568	11.759762	11.850968	12.031827	11.805147	11.769756	11.511108	11.558177	12.317768	11.099009	11.494063	11.391895	11.337301	11.282256	11.688416	11.968566	11.173303	11.581538	12.490737	11.910288	13.409904	11.842962	12.608186	12.295732	12.089045	12.384330	11.870697	11.928776	12.180812	12.136934	11.853395	11.483412	12.278977	11.963547	12.486970	11.619947	12.215941	12.709108	12.375118	12.541107	12.569861	12.046176	12.509805	12.540947	12.060702	12.799536	11.797058	12.044706	12.003852	12.059948	11.762225	11.628942	11.587163	12.560565	12.491398	11.642056	12.036697	12.054237	12.360207	12.219839	12.442059	11.303373	12.590794	12.819581	12.840359	12.472635	12.446937	12.696084	12.126733	11.737021	12.558642	13.122875	13.101809	12.403553	12.840549	12.907514	12.633137	12.100873	12.168674	12.030784	11.791206	11.868608	12.856932	13.011074	12.673786	12.739508	11.616116	12.281762	10.806633	11.760698	11.961308	11.843396	12.019305	12.669159	12.064261	10.998620	11.928618	11.753661	11.660731	12.213183	11.879689	11.552438	11.591314	12.088432	12.089348	11.915776	12.090811	11.257232	12.533949	12.780538	13.049325	12.014918	11.894872	12.931713	12.536567	11.473665	12.652080	13.144182	13.069296	12.417362	12.609852	11.885261	12.757381	12.819943	12.467788	12.028865	11.897513	13.208114	12.148694	13.144955	12.759589	12.178152	13.551649	13.400373	12.315928	13.544167	11.775216	12.724156	13.537927	12.279300	12.089977	12.516941	13.005778	14.206497	13.066403	12.538617	13.130760	12.884726	12.263250	13.261024	12.158042	12.968209	13.661031	13.911868	12.429483	11.864242	13.823177	13.269649	12.621510	12.673960	13.699452	13.540173	12.589947	12.866212	12.488312	12.300959	12.262939	12.686571	12.856045	11.827534	13.632998	13.219365	12.266524	11.792414	12.413808	12.591506	13.165282	12.690670	13.750073	12.952366	11.884784	13.301878	12.731164	12.980298	13.649857	13.424703	12.932065	12.447100	12.540317	13.814571	13.121958	12.814750	13.769045	12.996223	12.782775	13.481700	11.916216	12.599183	12.472313	13.128443	12.611187	14.014363	12.959381	12.743196	13.316425	12.435149	13.787649	12.172938	13.629938	11.069292	11.812582	12.461067	12.226312	12.587805	12.664618	11.894423	12.492884	13.450122	12.277288	12.639545	12.497186	11.618765	14.076340	13.109263	12.916404	12.778472	13.024565

UNC80	13.563867	12.273457	12.383691	13.549134	14.031741	14.548542	12.753452	13.623869	14.677877	13.469230	13.786622	13.791984	13.464192	13.020295	13.048590	13.370614	13.707475	12.804863	13.576247	13.386045	12.715232	13.517555	13.573817	13.254976	13.653197	14.001811	14.271254	13.087953	13.729644	14.199812	14.387857	11.881010	11.427813	14.215623	13.307696	12.914980	13.081778	12.374817	12.895102	13.306191	13.656487	13.152020	13.524608	13.281421	13.610599	12.860948	14.552678	12.161407	13.011074	12.515494	12.499786	12.330790	12.120508	12.719907	12.120080	14.035874	12.206651	12.428494	12.597492	12.382432	12.417662	14.275557	12.950759	13.504135	12.486004	12.521063	12.376451	12.124642	11.886208	12.641718	12.739710	13.302463	14.175520	12.720748	12.539812	12.917303	13.258986	12.716597	12.833655	11.853395	12.786993	12.079696	12.301709	11.969628	12.779855	12.304945	14.425228	12.645882	12.499629	11.666419	12.201865	12.587157	12.520217	12.852304	13.609219	13.458723	12.165154	14.193049	14.310666	14.726960	15.074615	12.851592	13.379474	12.913386	12.797496	14.111974	13.651246	13.481922	14.592810	13.957960	13.228076	14.038211	13.692656	14.407352	13.172483	16.206017	14.440880	13.847489	14.579342	13.797198	12.465162	13.193477	13.748094	15.547851	13.673412	14.924443	15.093809	14.801392	14.434912	13.938772	14.526691	14.465534	14.169373	13.808112	15.026937	14.155411	13.974396	14.042877	13.266014	13.525276	14.276234	12.303112	14.256351	13.758605	13.022344	14.696136	14.013491	15.657650	13.216968	14.735782	14.442714	14.125506	13.648940	13.450752	13.536634	13.431247	12.989794	13.036330	13.689045	14.880110	12.926482	13.106410	13.324170	13.357821	12.517113	14.477449	12.867783	12.911260	12.707907	12.801254	13.380119	12.422636	12.875731	13.279655	14.121021	13.614204	12.517769	13.101435	12.266302	12.999705	14.015006	12.915163	12.808933	12.947352	12.851957	12.773104	11.924594	12.359732	12.372577	13.370400	13.792498	14.355885	13.629686	14.047317	13.022344	13.711698	13.416905	13.302463	13.243546	12.867959	12.215771	13.173445	13.863086	12.891887	14.633014	12.844915	12.803851	13.376715	13.106997	12.770516	12.815098	13.400180	13.073039	13.466850	14.333069	14.630796	15.305145	14.265936	13.523456	14.667724	14.440521	14.940922	14.098263	13.454810	14.325045	14.119258	12.978391	12.597317	14.174885	13.335194	14.991770	15.692442	14.571778	14.720909	14.832004	13.517555	14.024760	14.221891

ELP2	13.687403	12.573686	13.069091	13.297753	13.500424	13.103890	13.325616	12.461245	13.250452	13.093696	13.322965	13.177797	12.972030	12.621342	12.467788	13.139865	13.310550	12.858133	12.953485	13.195507	12.974739	12.565784	13.137564	12.987788	13.444604	13.300682	13.770321	13.063428	13.506342	13.278412	13.500424	13.473987	12.387604	13.570340	13.304119	13.140068	13.107708	12.998439	12.640896	13.379894	13.419743	12.604080	12.978042	13.432385	13.612082	13.205336	13.562433	12.522051	13.258225	13.207720	13.377337	13.397792	13.434710	13.704144	13.358864	13.103703	13.432842	12.639201	13.223721	13.060802	12.782601	13.277835	12.908228	12.506563	13.080043	12.973483	12.771369	12.471196	12.434490	12.329087	13.134544	13.326420	13.154569	13.279753	13.400809	13.575781	12.954420	13.165282	13.648940	12.338888	12.286301	13.551649	13.494652	13.637843	13.383479	13.710171	13.559500	11.943639	12.673786	12.807870	12.926643	12.323739	12.512588	13.237448	13.701651	13.619696	13.394547	13.368915	13.318676	12.480642	13.198141	12.744556	13.197314	12.950547	12.191984	13.222745	12.607714	12.892258	12.861330	12.948421	12.931168	13.447352	12.755816	13.159931	13.285661	13.374219	13.464002	13.513328	13.158422	13.934939	12.117118	13.395578	13.775439	13.495548	13.726121	13.069852	14.026270	13.180750	13.538360	13.327179	14.221539	13.195709	12.351495	13.663761	13.882222	13.294745	13.607817	13.726121	13.562221	13.222566	13.486198	12.631160	13.209908	14.411699	14.519413	12.855167	13.597268	13.151837	13.450346	13.756127	13.331689	13.655271	13.483445	13.372065	13.612555	13.653197	13.437511	12.752073	13.492381	13.808908	12.823533	13.963030	13.571242	13.449721	13.948216	13.857256	12.810174	14.152666	13.395784	13.387555	13.775933	13.194918	12.971491	13.411645	13.609433	13.315405	12.514664	13.309969	13.027671	12.366707	13.693113	12.395067	13.540597	13.312545	13.265163	13.019185	12.989794	13.330272	12.257409	13.637621	13.577611	13.624759	13.276462	13.630199	13.500424	14.090418	13.681631	13.424295	13.893681	12.191829	13.651765	13.646825	13.745098	13.814571	13.293157	13.517555	15.436421	12.909315	13.242002	13.515121	13.443956	12.904297	13.537711	13.243734	13.462029	14.237480	14.091572	13.223145	13.292154	13.060802	13.617542	13.262182	13.056733	12.901798	13.638302	13.014996	13.123808	12.906948	13.600808	12.782063	13.583919	14.574333	13.612309	14.218603	13.541051	13.526138	13.672692	14.438961

LY6G6D	8.755318	8.680959	8.976368	9.300003	9.204578	9.257569	8.994576	9.839233	9.470780	9.373040	9.455263	9.323193	9.190397	8.953320	8.765277	9.008558	9.697470	9.001028	9.635213	9.597413	9.532449	9.598799	9.667475	8.485826	8.913049	8.986399	8.619557	8.800662	8.907026	8.943081	9.580581	9.438186	8.655658	8.917875	8.842770	9.007935	9.101177	9.195778	9.397486	9.533374	9.052042	8.920300	8.598194	8.988823	8.390510	9.310505	8.776297	8.869757	9.266257	9.090786	8.758395	9.369356	9.070041	9.586539	9.344831	9.147855	9.224813	9.210370	9.044724	9.510547	9.454607	9.010403	8.445923	9.229278	8.776457	9.149853	9.210159	8.941412	9.508068	9.431422	9.429273	9.474607	9.306959	9.075452	9.485652	9.324307	9.723922	8.877700	9.132598	9.294739	9.125977	9.274259	8.804154	9.594314	9.646149	9.225751	9.526340	9.581310	9.074813	9.510701	8.678364	9.428363	8.853327	9.104114	8.575811	8.925347	9.144754	9.042970	9.157875	8.733026	9.251342	8.469804	9.031727	9.055670	9.636066	9.411123	9.336631	9.605775	9.679406	8.936547	9.667023	9.841670	9.552530	9.371346	9.144754	9.578695	8.471529	9.210231	9.342971	8.351865	8.233737	8.251297	8.826851	9.158180	9.155870	8.732878	11.438226	8.962975	9.406477	10.024660	8.971304	8.187149	7.901019	8.552728	8.389906	8.965385	7.848848	9.437579	8.313592	9.163075	8.976517	9.440177	8.698784	8.842601	8.287945	9.004108	9.858664	9.540665	8.635856	8.401291	8.181745	9.050240	8.816989	8.483880	8.245543	8.342015	8.799440	8.926272	9.191345	8.853327	8.547997	7.679960	9.176949	8.403771	9.998570	9.199695	9.805321	9.286903	8.466122	8.580900	9.218055	8.854542	8.136323	10.907795	8.414001	9.359995	9.265207	8.355063	9.427118	9.159384	9.423469	8.829501	9.648561	7.986694	7.617539	8.391312	9.230742	9.526340	9.051294	9.439413	9.314441	9.557255	8.833608	8.404836	8.642515	9.584523	8.563436	9.399848	7.545653	9.854464	9.686093	8.694514	8.733342	7.759759	8.637867	8.784314	7.840776	8.069677	8.365337	11.057782	8.692623	8.849892	9.007324	9.188603	8.854158	9.432816	9.254659	9.586539	7.752415	8.567166	9.767982	8.403614	8.511664	8.204055	9.217443	8.984267	9.816047	9.083496	9.239678	8.939127	8.552114	8.545084	9.310802	10.250420	8.572024	9.176798	9.253052	9.081035

LY6G6F-LY6G6D	8.755318	8.680959	8.976368	9.300003	9.204578	9.257569	8.994576	9.839233	9.470780	9.373040	9.455263	9.323193	9.190397	8.953320	8.765277	9.008558	9.697470	9.001028	9.635213	9.597413	9.532449	9.598799	9.667475	8.485826	8.913049	8.986399	8.619557	8.800662	8.907026	8.943081	9.580581	9.438186	8.655658	8.917875	8.842770	9.007935	9.101177	9.195778	9.397486	9.533374	9.052042	8.920300	8.598194	8.988823	8.390510	9.310505	8.776297	8.869757	9.266257	9.090786	8.758395	9.369356	9.070041	9.586539	9.344831	9.147855	9.224813	9.210370	9.044724	9.510547	9.454607	9.010403	8.445923	9.229278	8.776457	9.149853	9.210159	8.941412	9.508068	9.431422	9.429273	9.474607	9.306959	9.075452	9.485652	9.324307	9.723922	8.877700	9.132598	9.294739	9.125977	9.274259	8.804154	9.594314	9.646149	9.225751	9.526340	9.581310	9.074813	9.510701	8.678364	9.428363	8.853327	9.104114	8.575811	8.925347	9.144754	9.042970	9.157875	8.733026	9.251342	8.469804	9.031727	9.055670	9.636066	9.411123	9.336631	9.605775	9.679406	8.936547	9.667023	9.841670	9.552530	9.371346	9.144754	9.578695	8.471529	9.210231	9.342971	8.351865	8.233737	8.251297	8.826851	9.158180	9.155870	8.732878	11.438226	8.962975	9.406477	10.024660	8.971304	8.187149	7.901019	8.552728	8.389906	8.965385	7.848848	9.437579	8.313592	9.163075	8.976517	9.440177	8.698784	8.842601	8.287945	9.004108	9.858664	9.540665	8.635856	8.401291	8.181745	9.050240	8.816989	8.483880	8.245543	8.342015	8.799440	8.926272	9.191345	8.853327	8.547997	7.679960	9.176949	8.403771	9.998570	9.199695	9.805321	9.286903	8.466122	8.580900	9.218055	8.854542	8.136323	10.907795	8.414001	9.359995	9.265207	8.355063	9.427118	9.159384	9.423469	8.829501	9.648561	7.986694	7.617539	8.391312	9.230742	9.526340	9.051294	9.439413	9.314441	9.557255	8.833608	8.404836	8.642515	9.584523	8.563436	9.399848	7.545653	9.854464	9.686093	8.694514	8.733342	7.759759	8.637867	8.784314	7.840776	8.069677	8.365337	11.057782	8.692623	8.849892	9.007324	9.188603	8.854158	9.432816	9.254659	9.586539	7.752415	8.567166	9.767982	8.403614	8.511664	8.204055	9.217443	8.984267	9.816047	9.083496	9.239678	8.939127	8.552114	8.545084	9.310802	10.250420	8.572024	9.176798	9.253052	9.081035

TPM2	15.531572	14.779929	15.003043	15.208375	14.679596	14.761491	16.052430	11.828254	13.013167	15.313805	14.702498	15.376978	14.601973	14.949574	15.140997	14.282413	16.122740	15.432586	14.317493	13.225685	15.325061	13.964639	17.021223	14.931911	16.130013	15.759701	16.541277	16.091387	15.895253	14.974306	14.906627	15.285921	14.016992	15.399178	15.918070	15.965762	15.541137	15.732189	15.621648	14.575515	15.394501	15.738421	15.079901	14.521823	16.441525	15.389194	17.105999	14.844188	16.357477	14.372995	14.467367	15.610464	16.154543	14.529893	15.382160	15.140997	16.166807	16.254772	15.577375	16.463480	16.063007	14.405128	17.422071	15.953297	15.269335	16.334866	15.488676	15.598287	14.013234	14.258950	15.908209	15.602028	15.546202	15.614975	14.950618	15.145529	15.190029	14.649747	14.251159	14.108513	15.250206	16.186008	15.716847	15.881450	15.809175	15.418602	15.399178	15.734237	15.185427	13.177997	15.643171	15.037486	14.319544	15.218536	14.244036	15.901055	15.847680	15.388004	15.448809	16.278273	14.591153	16.034860	14.759633	14.651938	14.851142	15.511748	15.427861	16.079155	15.971905	15.589004	14.971474	14.775142	13.683321	14.581385	14.186537	14.647558	14.878681	16.485350	16.034860	13.573342	14.243708	14.613543	14.119553	14.196229	14.008743	14.840176	14.932997	14.699277	13.793282	14.747454	13.769838	15.872260	14.598641	12.978577	15.795114	14.403650	14.047957	15.104927	14.834547	13.118268	14.073607	14.727873	13.325616	15.448809	15.195519	13.463136	13.011590	14.353744	14.214354	15.650841	14.182477	14.725162	13.674088	13.505003	13.258023	14.532252	13.735880	13.619696	14.625115	14.064420	13.797198	14.614415	13.978080	12.893120	12.809655	13.823177	13.447998	12.471030	14.697497	14.375936	14.222510	13.756127	15.341190	15.372489	15.634734	14.814746	13.323361	13.537270	13.699452	13.999261	16.546406	12.998952	13.533515	13.917771	14.128868	13.968540	13.391215	13.991278	14.868226	14.151380	14.560123	13.398875	14.807128	14.565911	14.144293	14.850021	14.788691	14.454664	15.351660	14.052401	13.718689	14.004466	14.566322	15.540294	14.531049	14.479005	14.933539	15.498075	15.611380	14.258622	14.653700	15.005364	14.587290	14.505307	14.558039	14.117673	14.975358	13.553239	13.909402	15.668159	13.938513	15.422546	14.033170	14.282749	14.044663	13.290718	12.039243	12.249289	14.270244	15.195519	14.527899	14.610103	12.642900	13.656014	13.621059	15.344956	13.417129	12.761009

AGA	12.989794	12.231738	11.124285	11.308542	12.205891	12.884558	13.260249	13.301878	12.170632	13.139505	13.198141	13.155970	12.246079	12.853341	12.107264	12.729457	12.764791	12.322660	11.824133	13.383693	11.564336	12.388554	9.923681	12.037461	12.182961	12.112540	10.666359	12.144213	12.370197	12.264187	12.410948	12.882781	12.372749	12.744026	12.940803	13.552076	13.782568	12.220618	12.152545	12.224164	13.109074	11.693488	12.933125	12.899003	12.616315	13.356199	12.248149	11.669696	13.355364	12.671375	13.133954	13.219767	12.655512	13.654792	13.166090	12.568720	13.287052	12.814409	12.061828	12.771890	12.259261	12.692008	11.052955	12.021228	11.978216	12.205277	12.661032	12.613560	12.420911	12.561046	13.130368	13.849377	12.608353	13.043905	12.940635	13.526786	13.651765	13.448635	13.683790	12.197056	12.361470	13.029353	13.587211	13.708928	13.237638	13.473987	13.509011	13.500645	12.384645	12.321603	12.124049	12.280366	12.339044	13.424295	12.789606	13.277438	13.343265	12.861151	12.721457	11.916369	13.083659	12.221092	11.972983	11.767960	12.499125	12.659650	13.229238	12.260195	11.460244	12.941572	11.963054	12.310609	11.440322	12.764108	13.631803	12.305725	11.137290	12.693740	11.693488	12.525104	11.715142	10.919423	10.906300	9.442662	12.240995	12.098604	11.344753	10.902906	12.484391	12.230037	11.557743	9.322286	10.833152	9.573538	11.982413	12.872501	11.046807	8.485660	12.074965	9.970923	10.485474	10.862048	7.294607	10.453877	12.003241	12.600886	11.243314	10.883700	12.067502	11.593593	12.023231	11.414427	12.372749	12.425254	11.724391	13.347004	10.796728	11.631808	10.376501	11.295692	11.288192	12.632134	12.396914	11.886002	11.959745	11.420720	10.016345	12.641908	11.599875	12.592493	12.345482	11.653194	12.328915	12.512588	11.511560	13.791726	11.254856	11.443675	11.344909	12.182585	12.014022	11.747895	10.642239	11.741732	11.629391	12.882033	12.085752	10.937143	12.279300	11.887800	12.313176	10.631587	13.075705	12.387097	12.896153	12.669662	12.258212	12.485499	11.492028	12.267146	13.780110	12.310302	12.772756	11.823375	12.650373	13.196531	13.017369	11.621059	11.570035	10.715522	11.556994	11.593433	11.758139	12.498162	13.033201	12.372577	11.744491	12.573339	11.365359	12.220618	10.699718	9.804238	11.373199	12.153901	10.802840	11.404390	12.027808	11.414134	11.246224	10.390756	10.355524	9.945139	11.972074	12.909480	12.683500	10.811946	12.401299	12.063646

CSDE1	5.924711	3.287257	4.461347	5.733728	4.726811	5.424256	4.349640	3.997047	6.101011	5.208231	4.884944	4.904203	5.499091	5.499484	6.429015	5.651254	5.903669	5.326544	6.085087	5.227060	4.646207	5.955741	5.162291	4.988308	5.076726	5.021985	6.089027	4.726407	5.174727	4.940338	6.075531	4.804508	5.223352	5.482940	4.828247	4.766823	4.340238	5.187008	4.357634	4.832062	4.847414	4.918512	5.670148	5.517603	5.673843	4.823944	5.679760	3.281037	5.815457	4.891263	4.600612	4.170991	4.587788	4.877385	5.720686	5.165102	6.118370	4.518683	5.394569	4.855630	4.716023	5.369503	4.757390	4.873439	3.531510	3.813152	3.581487	5.321096	4.438717	3.914658	4.448486	4.439142	5.298453	4.246198	4.287666	4.120971	4.826757	4.344134	4.037989	5.420358	5.741365	5.636758	5.910637	3.355179	5.101965	5.012811	5.480462	5.235426	4.098307	3.732755	3.803477	3.478950	4.530516	4.170794	5.375140	5.210833	6.116835	6.592623	5.981184	6.445408	6.379289	5.644822	5.557563	5.583037	5.305994	6.859944	5.208018	5.210021	5.602258	5.652572	6.162870	5.460841	4.868117	4.628705	7.435533	6.028032	5.987005	5.925599	6.595347	5.520641	5.085724	4.235959	6.107118	5.486138	4.180324	5.133404	5.595889	7.292400	5.772380	6.180152	6.819738	5.670662	5.850256	6.015887	6.232414	5.494698	6.901907	5.838803	5.026259	6.324566	6.300828	3.639290	6.621787	3.743475	4.653042	5.756654	5.367011	6.142913	4.661814	7.186189	6.622744	6.713105	5.932632	4.290904	5.391433	3.967902	4.794770	4.788270	4.578414	6.069439	3.895640	6.205920	5.611055	4.929972	4.859608	5.981021	4.844015	4.227481	4.650918	5.511434	5.449926	5.633193	8.525662	6.126600	4.796873	5.279748	5.104212	5.724200	4.438286	4.761835	5.358153	5.658509	4.472756	4.495328	4.787444	4.512034	4.977861	4.493107	4.635216	3.498710	4.536530	5.917767	4.780536	4.558767	5.173736	4.187106	4.499207	4.029752	5.928111	5.140676	4.888964	5.264297	6.010871	5.747211	5.464226	4.647688	5.447613	4.772404	4.388470	4.855836	4.300454	4.844015	5.019323	4.586047	5.116706	6.212161	6.129996	6.628549	5.602645	6.079720	6.479137	5.735558	5.878333	5.850948	5.924008	7.953706	6.496048	4.746622	6.958624	5.401078	6.566408	5.850948	4.624415	3.917481	4.870222	5.076919	6.326589	5.527261

NEK5	7.482442	8.572191	8.673115	8.214701	8.242770	10.104055	8.830706	10.513902	8.840579	9.005667	8.674683	9.270612	8.010797	8.447497	7.894497	9.311545	8.388680	7.668822	8.220237	9.058476	8.116770	7.961155	5.541177	7.806822	8.396552	7.541602	10.647743	8.742859	9.477984	10.230524	7.823644	7.758916	8.140306	9.265061	8.449532	8.798095	8.945222	8.501222	9.103509	8.364546	9.105197	8.791046	8.897149	8.981350	8.408361	8.796419	10.088845	9.027513	7.713269	9.706104	7.935444	8.720391	7.983341	8.078065	7.727632	9.493614	7.910185	8.442536	7.487725	7.426143	7.634962	9.353412	5.737361	7.447919	6.920987	7.450282	8.087562	8.026994	7.794009	7.529779	8.396721	8.518092	9.346503	8.715151	8.329950	8.731280	8.390213	8.562682	8.145645	7.775580	7.649509	7.779314	7.185534	7.913572	8.111174	7.939959	7.280850	8.474935	7.338317	7.650787	7.830502	7.158679	7.521779	8.098401	7.862916	7.850493	7.949845	7.579796	8.310979	8.193208	8.468400	8.478824	8.168133	8.441197	7.750734	8.813362	7.695615	8.196850	7.496300	9.716241	7.671796	8.141961	7.856647	10.453273	9.792229	10.585512	6.034196	9.135166	9.815723	8.219476	6.818463	6.813501	7.547429	7.332577	9.519152	8.800062	6.435274	6.663789	7.975388	7.838143	9.026892	7.437251	7.385917	7.560139	8.329805	6.622590	5.640329	7.069707	7.120267	6.688383	6.798482	6.561359	6.336018	6.678786	5.939883	8.437691	7.241188	9.258494	8.951490	7.090251	7.239214	6.269643	7.625019	6.919359	6.680307	8.064885	6.955826	7.297331	6.339228	8.784600	7.005493	6.781209	6.435793	6.370564	8.102799	9.081657	6.401157	6.442531	6.831645	5.816548	6.560378	5.899553	5.961446	5.953274	8.168296	5.330483	5.577775	6.487895	5.140257	5.424841	9.871517	5.470077	5.945514	5.880506	5.984697	5.761196	6.172693	5.234034	7.332577	7.069044	7.507225	8.933459	6.646404	7.741340	7.790379	6.668343	6.588530	7.049430	6.117316	5.092686	6.494280	5.953623	6.013483	5.635443	7.366550	5.854373	5.470077	5.639734	5.498320	5.123314	5.848644	6.752252	6.425455	7.856208	7.364676	7.175395	7.496799	6.604561	6.047481	7.470014	7.039382	8.522603	6.801570	5.826180	9.649786	5.539772	6.699791	6.309927	9.153845	7.376878	6.244951	5.594202	11.503797	8.848183	8.163833	5.939326	7.260136	8.790410

PGLYRP4	5.116998	8.075632	8.633972	7.605472	7.645417	9.965900	9.013781	11.495068	10.313397	9.865136	9.799962	9.218559	8.550127	8.983343	9.678618	8.858812	9.059425	8.735164	8.488914	10.659620	9.430651	10.814320	4.783045	7.868615	10.206147	8.191336	6.466021	9.848968	9.249360	7.783157	9.812137	9.018966	9.036791	8.306339	9.057993	10.132585	9.788049	8.806901	9.093582	9.510217	9.043649	7.765302	8.756380	9.087029	7.790991	10.322892	9.461669	7.647559	9.065378	9.049134	8.407426	9.665161	9.528969	10.180574	9.794095	9.521779	9.264136	9.679564	8.987939	8.890238	8.320195	7.938674	4.906706	8.454436	8.951490	9.421160	8.735952	7.925226	8.323250	8.141326	8.836925	8.689716	10.473914	9.085022	9.880559	8.367126	7.922093	10.102793	8.756076	9.105974	8.141040	9.433276	9.798436	9.996310	10.159237	9.825394	10.353428	9.505806	7.778050	8.612986	8.044812	8.574419	9.013468	8.991352	7.554725	8.967900	8.972042	9.906208	9.910027	7.262980	8.906422	8.264787	8.493691	10.129611	9.598172	9.792703	10.243835	10.850174	8.169398	9.620646	9.174191	8.585918	11.085876	8.350679	9.937420	6.924197	5.538448	9.848310	9.930158	6.340247	10.926544	7.479190	7.335219	8.466728	9.198592	7.636336	9.085355	12.158173	6.956719	11.202837	5.611588	4.191920	3.714025	4.259390	8.484598	6.248802	7.970525	7.141116	9.224038	8.715005	9.589014	4.140617	6.195854	7.006585	3.422057	8.823853	8.914022	7.174793	9.588120	9.376353	9.992511	10.490996	13.468361	11.269993	8.860183	11.436255	7.869699	8.649966	8.809523	7.837640	9.467350	9.654867	11.254129	10.043161	10.290520	12.274723	3.028768	9.175115	9.320329	10.713869	6.068056	8.535593	9.760100	11.303960	7.369389	9.100099	8.706598	10.114066	7.293816	7.417566	9.417469	8.474459	8.984549	9.237192	10.743665	8.264002	9.403086	7.128141	6.454454	8.828725	9.451718	10.977879	9.536303	10.505273	10.316666	9.612099	8.203475	9.451098	7.722994	10.351116	10.392965	11.517229	9.538754	8.588024	11.439898	11.493018	8.647237	5.672198	7.911574	3.594603	9.933372	9.746469	8.579079	10.014606	8.858205	9.796717	7.661829	11.086467	6.058191	3.091207	8.465219	5.862518	9.824950	5.029742	7.735493	8.444402	9.127357	7.336449	11.730636	5.945861	4.968204	7.299385	7.628292	5.449553	11.980630	7.317019	10.293094	9.015152

EDN1	13.772438	12.436293	13.158966	11.747338	12.579663	11.999293	12.803011	11.549457	13.151653	12.324479	12.312233	13.181502	12.179702	11.596704	11.984549	12.688122	13.378218	12.158494	12.563325	12.025867	12.589947	12.354988	12.849826	11.469421	11.995045	12.913386	13.413573	13.049159	13.700412	13.117173	13.403139	12.783261	12.515979	13.903326	12.741114	13.114296	12.689122	11.869930	12.360854	12.813513	13.710450	13.015539	14.170650	12.584643	14.420258	12.618964	11.747749	14.125846	12.673786	12.085596	12.200786	13.439251	12.375646	12.118316	13.679257	11.535109	12.799021	13.662723	13.250094	13.295341	12.665817	13.802610	12.728950	12.033841	13.345934	14.028776	12.464996	12.351650	11.268237	13.546180	13.236867	11.779706	12.978577	13.234224	12.271724	12.621510	11.160511	13.482590	12.921519	12.585159	14.757850	13.713659	12.251829	13.142299	13.190080	13.082732	13.408793	13.907998	13.943207	12.907514	13.372722	13.801374	12.720580	12.994903	12.783261	12.035336	12.948591	13.366847	12.479302	12.755118	12.507872	12.509156	12.736959	12.891724	12.937994	13.064543	12.929922	12.616658	12.495001	12.182658	12.533788	13.540597	11.310712	11.626309	12.386589	12.388737	12.487990	12.737304	12.625000	13.595345	12.362592	12.769413	12.614361	14.098570	12.041232	12.941758	12.751723	12.605556	12.593944	11.718826	14.518570	13.422986	12.875882	14.285845	12.671548	12.459458	14.601201	12.342322	9.836099	14.122158	13.798541	14.306647	14.847864	14.407674	11.363426	11.914277	11.932141	11.189187	13.071874	12.603238	12.290299	13.888066	13.216776	12.343913	12.796802	14.369760	12.941572	13.359928	13.775439	11.548135	14.165208	11.264699	12.409034	13.271222	12.494332	12.360369	12.594432	14.061195	13.164898	13.782334	13.785834	13.525511	11.932596	11.839322	11.207783	11.029412	13.026028	13.059142	14.425923	13.389682	13.416505	13.525511	13.927054	11.715283	13.033378	13.509011	13.209291	14.306995	12.087998	12.718533	14.801868	12.899003	12.586302	12.783797	13.576005	12.220306	11.723465	13.019739	13.328213	12.521728	12.226617	13.217987	14.002435	13.093498	13.015183	12.462402	12.327491	12.457385	12.520044	12.059510	12.300641	11.757998	13.080831	12.930265	14.568889	14.240420	13.531473	12.775662	12.724991	12.086064	12.418166	11.217302	11.513628	12.025076	14.242398	14.165841	12.106547	13.722979	13.759917	14.324402	13.554152	11.469574	10.661012	13.840878	13.830745	15.447299	12.953101	12.876579

PIPOX	6.455262	6.498399	7.148992	6.415472	6.859613	7.145969	6.451641	7.105962	6.864700	6.468342	6.472574	6.332486	6.132918	6.036353	6.396141	6.832287	7.546301	6.138578	6.802686	6.570356	7.983626	6.941340	6.234130	6.283289	6.520879	6.695231	6.971438	6.744003	6.632728	6.994160	6.932551	6.241098	6.757186	6.174195	6.364215	6.367040	6.170149	6.295088	6.556627	6.133452	6.699341	6.477635	6.415816	6.561513	5.953780	5.638630	7.486494	6.108872	6.584073	6.087306	6.545166	5.998849	5.829701	6.020286	5.545431	7.107883	5.637531	7.584112	6.553998	6.054615	6.155339	6.792357	6.732375	6.597629	6.192675	6.198588	5.948272	6.146530	6.269124	6.586878	6.532898	6.760608	6.671556	6.535312	6.487258	6.662807	6.613319	6.629353	6.431080	6.118370	6.169784	6.080402	5.778189	5.859070	5.600779	6.089390	5.819218	5.847321	6.139271	6.186042	5.771424	6.319268	6.182027	6.349577	6.767961	6.158888	6.435274	6.588358	6.826891	6.595852	6.865962	6.122011	6.344739	6.185718	6.867088	6.940287	7.106590	7.149790	6.848549	6.765721	6.592947	7.463901	6.468027	6.932401	6.910655	7.482123	6.739884	6.325609	6.858980	6.935856	6.890763	7.196457	7.183140	7.537331	7.531129	7.103141	6.954068	7.104562	7.334577	7.042589	7.090578	6.519214	6.679815	6.963224	6.909393	7.660474	6.430553	7.841700	6.617262	8.240832	7.427119	6.723225	7.315270	7.535780	6.709432	6.826891	7.496641	8.079480	7.060933	7.031715	6.577833	6.544184	6.531278	6.690459	6.793323	6.591465	7.341144	6.640971	7.058944	7.662478	6.782147	7.122273	7.057769	6.640971	5.895664	7.964652	7.034226	6.905252	6.287577	6.672197	6.776708	6.037899	6.075903	5.465983	7.209083	6.581476	7.681302	6.678964	6.354414	6.449017	6.944879	6.839336	6.512602	6.756046	6.238082	6.805835	6.462351	6.803168	6.884715	6.992259	6.926972	7.800830	6.573358	6.678466	6.743667	6.661840	6.680462	6.772337	5.877950	6.445919	5.862898	5.911336	6.153453	6.000418	6.815409	6.205388	6.202793	6.314683	6.163738	7.342224	6.340247	6.461507	6.918525	6.695069	6.524190	7.085231	6.972442	7.334421	7.258223	7.057617	6.868239	6.582944	5.987381	7.883516	7.214668	7.381252	7.500244	7.393824	7.639084	7.433286	7.063822	6.561036	8.151760	7.558311	7.431258	6.581952	6.710243	7.383462

JAK1	9.414865	8.924171	8.584250	9.115876	8.946272	8.794756	8.135425	8.874177	9.210851	8.781813	8.482338	8.693250	8.664994	8.503105	8.719507	9.257809	8.984549	8.940500	10.485037	8.879747	9.565422	11.111876	10.588073	9.399554	10.224053	9.336631	12.069478	10.015244	9.543634	8.874499	9.773259	8.315758	8.955338	9.587009	8.290722	8.125103	8.145806	7.626255	8.536545	9.182199	9.016869	8.765747	9.261665	8.775349	8.683845	8.520957	9.691617	8.306486	8.864614	8.115176	8.462777	8.011423	8.008230	7.912959	8.231309	9.143958	8.100567	8.728390	9.182351	8.668179	8.213459	8.492586	9.759333	10.347504	8.589904	8.714536	8.145806	8.493691	8.092765	8.516062	7.966334	7.962682	9.505192	8.023315	8.157042	8.854082	8.004643	8.642211	8.577014	8.381126	8.819478	8.630218	8.633684	7.857542	8.193053	8.662549	8.340415	8.698630	8.808882	8.417684	8.699095	9.387777	8.428351	8.251925	8.902003	8.775501	8.873841	9.137787	8.755638	8.887283	8.970518	8.343224	9.125660	8.696676	9.727598	9.713258	9.942430	10.362375	10.370017	8.973624	9.607630	8.954265	9.843788	9.267822	10.372584	9.373205	10.115451	9.424676	9.734386	9.622288	8.990614	8.390213	9.763871	9.735158	9.197188	9.634927	9.329557	9.113163	8.895309	8.882792	9.316409	9.365025	9.009491	10.174103	9.388862	9.366931	9.333214	10.439290	8.493527	10.167666	11.042557	10.561470	9.797967	9.207334	10.147930	9.921845	9.849917	10.050514	8.935611	10.630708	10.189346	9.501326	8.891442	8.319558	8.331048	8.487395	7.828229	9.555087	9.394342	10.219870	8.766990	9.432672	8.812483	8.596115	8.004026	10.905092	7.787922	8.513238	7.851877	8.643630	8.260003	8.391462	8.292913	8.483714	8.708855	7.927727	8.982750	9.869780	7.841996	9.288170	9.286431	8.861204	9.814948	8.837687	9.158340	8.973069	8.687318	8.147919	9.243504	7.998255	8.177966	10.657546	8.348118	8.395927	8.811877	8.337276	8.472170	8.471683	8.561173	8.625448	8.676661	9.148972	9.142441	8.904614	9.190561	8.402822	7.627977	9.438502	9.646315	8.337120	9.661397	8.485531	8.772484	8.685393	9.386518	9.497649	9.401376	9.374473	8.261557	9.555229	8.791209	9.722952	9.078076	9.650553	10.384855	10.702585	10.256188	9.956772	10.490413	9.896278	9.782831	9.320484	10.165185	8.501851	9.300176	9.262763	10.390190	9.476133

STIMATE-MUSTN1	13.267179	12.097725	12.916212	13.631382	11.941196	11.787619	12.980105	11.523666	10.499570	11.959299	11.688994	12.523476	12.481140	11.956956	12.221569	12.089667	13.440958	11.873387	12.022968	12.578859	11.972822	11.834822	13.282011	12.063188	14.080347	13.529898	13.806501	13.028594	13.386902	13.445662	12.651577	13.403996	12.486494	12.429938	13.561518	13.670785	13.225108	13.151653	14.066903	13.144577	12.924377	15.088816	14.836534	13.280841	15.589896	13.598384	12.455483	13.606881	11.529530	13.656487	13.368915	13.786878	13.812477	13.522531	13.171328	12.808222	14.656363	13.933523	12.092910	13.843499	13.429298	14.127106	14.675040	13.281802	13.133191	12.723489	13.207137	12.161724	12.754129	12.950935	14.218603	13.843499	12.772251	14.051810	13.336226	12.929387	13.952936	13.708445	13.186438	12.573686	12.944248	14.097636	12.734697	13.099406	13.330272	12.991245	13.450752	13.387343	13.174582	11.536144	12.938338	12.710992	12.828063	12.471970	12.225203	13.236652	13.447775	13.668871	12.595412	13.985291	12.854570	12.967688	12.874685	12.841260	11.426495	12.876415	13.024023	12.317621	13.199300	12.236952	13.083849	12.773964	11.647985	12.248149	11.409515	11.954903	13.366676	12.932954	13.141459	11.260634	11.556994	11.182712	12.441117	11.972367	10.782833	11.245386	11.274739	10.938063	10.884311	11.402055	10.738523	11.181128	11.068971	10.362375	12.005783	10.757808	9.913122	10.726741	10.982470	10.350219	10.895178	11.579521	10.584201	11.278409	10.778689	11.132427	11.150814	11.295540	12.045149	11.485786	11.348487	11.200158	11.973443	11.866837	11.522184	12.397227	11.801490	12.541107	12.450751	11.815094	11.708649	10.739298	11.599731	11.420870	11.614273	12.192292	11.362553	10.772988	11.685099	11.288046	11.100603	10.627143	10.243220	11.825339	11.318521	10.503929	10.841568	10.801353	10.513902	10.722072	11.940902	10.706580	10.809966	10.672285	10.680952	11.768259	11.029265	10.886712	10.591557	11.747749	12.015528	12.124499	12.172634	13.092393	11.903405	11.640559	12.105433	12.329402	10.888785	10.650579	10.741574	10.829909	10.361591	10.546423	10.752648	12.860767	10.600013	10.542264	11.230200	10.211657	10.219240	10.662229	11.299253	11.819577	11.335546	10.861451	10.760711	11.097215	10.429581	10.221951	10.355834	11.206863	10.746036	10.853896	11.560396	11.390271	10.390607	10.620713	12.036237	10.733261	11.081461	9.781184	11.665214	10.656389	10.372919	10.952734	10.778399	11.847771

AC006254.1	13.267179	12.097725	12.916212	13.631382	11.941196	11.787619	12.980105	11.523666	10.499570	11.959299	11.688994	12.523476	12.481140	11.956956	12.221569	12.089667	13.440958	11.873387	12.022968	12.578859	11.972822	11.834822	13.282011	12.063188	14.080347	13.529898	13.806501	13.028594	13.386902	13.445662	12.651577	13.403996	12.486494	12.429938	13.561518	13.670785	13.225108	13.151653	14.066903	13.144577	12.924377	15.088816	14.836534	13.280841	15.589896	13.598384	12.455483	13.606881	11.529530	13.656487	13.368915	13.786878	13.812477	13.522531	13.171328	12.808222	14.656363	13.933523	12.092910	13.843499	13.429298	14.127106	14.675040	13.281802	13.133191	12.723489	13.207137	12.161724	12.754129	12.950935	14.218603	13.843499	12.772251	14.051810	13.336226	12.929387	13.952936	13.708445	13.186438	12.573686	12.944248	14.097636	12.734697	13.099406	13.330272	12.991245	13.450752	13.387343	13.174582	11.536144	12.938338	12.710992	12.828063	12.471970	12.225203	13.236652	13.447775	13.668871	12.595412	13.985291	12.854570	12.967688	12.874685	12.841260	11.426495	12.876415	13.024023	12.317621	13.199300	12.236952	13.083849	12.773964	11.647985	12.248149	11.409515	11.954903	13.366676	12.932954	13.141459	11.260634	11.556994	11.182712	12.441117	11.972367	10.782833	11.245386	11.274739	10.938063	10.884311	11.402055	10.738523	11.181128	11.068971	10.362375	12.005783	10.757808	9.913122	10.726741	10.982470	10.350219	10.895178	11.579521	10.584201	11.278409	10.778689	11.132427	11.150814	11.295540	12.045149	11.485786	11.348487	11.200158	11.973443	11.866837	11.522184	12.397227	11.801490	12.541107	12.450751	11.815094	11.708649	10.739298	11.599731	11.420870	11.614273	12.192292	11.362553	10.772988	11.685099	11.288046	11.100603	10.627143	10.243220	11.825339	11.318521	10.503929	10.841568	10.801353	10.513902	10.722072	11.940902	10.706580	10.809966	10.672285	10.680952	11.768259	11.029265	10.886712	10.591557	11.747749	12.015528	12.124499	12.172634	13.092393	11.903405	11.640559	12.105433	12.329402	10.888785	10.650579	10.741574	10.829909	10.361591	10.546423	10.752648	12.860767	10.600013	10.542264	11.230200	10.211657	10.219240	10.662229	11.299253	11.819577	11.335546	10.861451	10.760711	11.097215	10.429581	10.221951	10.355834	11.206863	10.746036	10.853896	11.560396	11.390271	10.390607	10.620713	12.036237	10.733261	11.081461	9.781184	11.665214	10.656389	10.372919	10.952734	10.778399	11.847771

MUSTN1	13.267179	12.097725	12.916212	13.631382	11.941196	11.787619	12.980105	11.523666	10.499570	11.959299	11.688994	12.523476	12.481140	11.956956	12.221569	12.089667	13.440958	11.873387	12.022968	12.578859	11.972822	11.834822	13.282011	12.063188	14.080347	13.529898	13.806501	13.028594	13.386902	13.445662	12.651577	13.403996	12.486494	12.429938	13.561518	13.670785	13.225108	13.151653	14.066903	13.144577	12.924377	15.088816	14.836534	13.280841	15.589896	13.598384	12.455483	13.606881	11.529530	13.656487	13.368915	13.786878	13.812477	13.522531	13.171328	12.808222	14.656363	13.933523	12.092910	13.843499	13.429298	14.127106	14.675040	13.281802	13.133191	12.723489	13.207137	12.161724	12.754129	12.950935	14.218603	13.843499	12.772251	14.051810	13.336226	12.929387	13.952936	13.708445	13.186438	12.573686	12.944248	14.097636	12.734697	13.099406	13.330272	12.991245	13.450752	13.387343	13.174582	11.536144	12.938338	12.710992	12.828063	12.471970	12.225203	13.236652	13.447775	13.668871	12.595412	13.985291	12.854570	12.967688	12.874685	12.841260	11.426495	12.876415	13.024023	12.317621	13.199300	12.236952	13.083849	12.773964	11.647985	12.248149	11.409515	11.954903	13.366676	12.932954	13.141459	11.260634	11.556994	11.182712	12.441117	11.972367	10.782833	11.245386	11.274739	10.938063	10.884311	11.402055	10.738523	11.181128	11.068971	10.362375	12.005783	10.757808	9.913122	10.726741	10.982470	10.350219	10.895178	11.579521	10.584201	11.278409	10.778689	11.132427	11.150814	11.295540	12.045149	11.485786	11.348487	11.200158	11.973443	11.866837	11.522184	12.397227	11.801490	12.541107	12.450751	11.815094	11.708649	10.739298	11.599731	11.420870	11.614273	12.192292	11.362553	10.772988	11.685099	11.288046	11.100603	10.627143	10.243220	11.825339	11.318521	10.503929	10.841568	10.801353	10.513902	10.722072	11.940902	10.706580	10.809966	10.672285	10.680952	11.768259	11.029265	10.886712	10.591557	11.747749	12.015528	12.124499	12.172634	13.092393	11.903405	11.640559	12.105433	12.329402	10.888785	10.650579	10.741574	10.829909	10.361591	10.546423	10.752648	12.860767	10.600013	10.542264	11.230200	10.211657	10.219240	10.662229	11.299253	11.819577	11.335546	10.861451	10.760711	11.097215	10.429581	10.221951	10.355834	11.206863	10.746036	10.853896	11.560396	11.390271	10.390607	10.620713	12.036237	10.733261	11.081461	9.781184	11.665214	10.656389	10.372919	10.952734	10.778399	11.847771

HERC4	10.646958	9.248899	10.382091	10.027337	10.492850	10.694353	10.572908	10.757072	10.460851	10.982910	11.024661	10.528361	9.110950	9.670699	9.415816	10.618670	10.825019	9.502999	8.958589	10.484576	9.317754	9.133821	7.051334	9.117878	10.574965	11.263631	9.874356	10.562644	10.339746	10.415855	10.453727	10.537933	10.399900	10.697213	10.334644	10.782076	10.762958	9.587494	9.264276	9.697856	10.098273	8.564040	10.866362	10.616136	10.460118	10.894897	9.986408	9.467827	10.777532	10.676585	10.367907	10.627143	10.724311	10.987504	11.105734	10.517131	11.131914	9.074656	9.538134	9.747411	8.696372	10.071474	8.082867	8.532273	9.194792	9.727598	8.093260	8.967268	9.106595	8.059955	10.081967	10.515470	11.114868	10.279115	10.339746	10.527773	10.526418	10.742594	10.509472	9.310802	10.766421	10.588938	10.792913	10.889511	11.051341	10.430330	10.902843	11.220875	8.330105	9.173114	9.380048	8.602194	8.802888	10.696641	10.311562	10.550537	10.672137	10.968475	10.701207	8.453070	10.974650	8.946909	10.057120	10.447340	8.822037	10.780289	10.355971	9.848968	7.937295	10.446116	7.407121	10.116828	9.869932	10.275470	11.283702	9.377152	9.360960	10.836761	10.305518	10.447803	10.463567	10.136250	10.066907	10.350071	11.102976	10.424550	9.903802	10.522062	12.333973	10.884929	9.705663	9.285661	9.305277	9.066450	10.403336	10.913411	8.424820	8.269030	10.149948	9.705510	8.642817	7.283831	7.510936	10.960936	12.248817	10.108677	11.076192	9.761351	10.707761	10.926240	10.738093	10.648195	11.713634	10.255126	10.153421	10.616277	9.171885	9.932019	9.534452	10.426073	8.635856	10.863742	11.121598	10.644883	11.358695	11.144712	9.745544	10.570453	11.919091	10.767880	10.615988	11.105158	11.579521	11.044169	9.787289	10.809055	9.548072	9.765885	9.871673	9.425577	9.837036	8.339505	9.165409	9.445303	9.693739	8.366666	9.463027	9.377466	8.363460	10.326118	10.584768	10.996096	10.737016	10.216098	10.864538	10.639378	10.764174	10.985538	9.194034	10.993145	11.691853	11.687667	11.012108	11.816892	10.110954	10.975234	11.455650	8.686278	9.656270	9.207170	8.938682	9.249360	11.479023	10.521739	10.515623	10.583475	10.608452	11.352601	9.327661	10.625346	8.958885	9.632606	10.198291	8.422421	10.648499	10.825609	10.453727	7.541891	10.840331	6.880073	10.062899	8.240987	10.185886	10.319223	11.178849	9.550559	13.157657	10.372584

MMP16	5.285798	6.203297	5.751747	4.381597	4.236984	4.662049	6.179165	3.995086	4.806580	6.267913	5.106448	5.210230	4.743173	4.642176	5.444863	4.583521	4.070060	3.102534	5.837861	3.947691	4.745325	3.926712	8.422111	4.305530	4.370890	4.204758	6.038961	5.470251	6.096514	6.232414	4.212746	6.073470	5.354518	5.822776	5.955221	6.892688	6.827174	4.588857	4.585201	4.223185	4.408498	5.393349	6.636582	6.121304	7.659658	5.403504	5.374835	5.319005	5.839873	5.712688	5.671606	7.024588	7.472645	6.896554	6.698079	5.777437	7.558763	5.232404	4.334415	5.113938	4.972948	6.724651	6.686460	5.005774	5.946365	4.591187	4.618879	4.114601	4.279071	4.660049	5.838629	6.104605	5.151351	6.453116	5.634528	5.997961	6.007165	6.552341	5.293881	5.541842	6.696196	7.175232	6.168430	6.732083	6.889174	5.900433	6.679298	6.840313	5.776908	4.726407	4.780946	4.757829	5.392969	6.027838	5.478875	5.904725	6.078651	6.329072	4.736346	3.697556	5.665725	4.989560	4.844242	4.030642	3.708838	5.753589	4.095392	4.279704	4.113121	5.409725	4.547511	4.258522	3.754254	5.037736	4.418927	6.167031	4.831465	5.405155	5.081663	4.791656	6.705266	4.665824	4.571814	4.858971	5.757389	5.955069	5.880879	6.291154	5.799849	6.723847	5.875848	4.062409	4.461781	5.546959	5.352484	6.413691	3.606405	5.358339	6.424487	5.176961	4.981730	5.950379	3.554886	6.270338	8.394988	5.358907	4.017593	5.662165	5.789212	4.745959	5.624075	6.730314	6.535976	5.055345	6.878463	8.451235	5.607399	5.324768	4.362190	5.480719	5.244612	6.441696	7.342696	7.265558	5.024657	5.575579	6.383146	4.309458	6.941182	7.191984	6.069272	6.754244	8.572585	7.971306	6.564495	9.736169	3.555420	3.099402	4.918935	4.895184	6.184321	5.074448	5.682879	4.980732	4.723015	4.577328	5.675664	5.141316	3.023191	6.239749	7.361534	4.525222	8.179917	7.028306	6.808558	7.484272	7.516805	7.018100	6.281449	8.043261	6.788259	7.932799	8.511066	9.258777	5.784669	8.245543	6.699651	6.723381	6.575512	5.633010	4.733571	5.519352	10.036419	7.879463	8.234957	6.613170	6.308756	5.312218	4.637149	7.754107	3.540515	4.503503	4.806984	5.690981	5.028491	3.564969	3.762780	3.828431	5.540532	3.478950	6.116308	5.483876	5.767949	5.044120	6.071880	4.883281	6.699964	6.339228

AACS	12.443951	12.929387	13.252643	13.266411	13.223925	13.115807	12.551384	13.048973	12.799021	12.789606	12.640046	13.053474	12.918970	13.530568	12.995435	12.690828	12.485499	13.095970	13.943207	13.571242	13.727608	13.426653	12.942474	12.691020	12.892258	13.155145	11.447359	12.947003	12.207923	12.132820	12.785061	13.023662	12.959197	11.665355	11.974803	12.384180	12.768522	12.509651	12.847527	13.002707	12.885476	12.966743	12.087998	12.662746	11.966406	12.736077	12.598661	12.674839	12.486329	12.575563	12.728453	12.718385	12.453418	12.972212	12.673113	12.843523	12.720947	13.197729	13.266215	12.694911	13.117358	12.821429	11.939225	13.444403	13.124576	13.186246	13.004678	13.066780	12.741823	13.263961	12.652080	12.865869	12.600363	12.666814	12.936194	12.415458	12.741631	12.120667	12.795380	13.354589	12.670169	12.798847	12.886344	13.294543	13.222947	12.819943	13.021758	12.734044	13.741329	13.490156	13.670545	13.838475	12.844764	12.795892	12.857967	12.693740	12.820259	12.507056	12.583626	12.241742	12.751559	12.436907	12.432257	12.458806	13.787141	12.758075	13.087036	13.283038	14.003541	12.976584	14.404753	13.318676	13.041904	12.818205	12.925940	12.468679	12.095640	12.974569	12.399784	12.631817	12.875882	11.743625	12.772604	12.359732	12.253272	12.858133	10.774471	12.573019	12.790822	12.837755	11.745378	12.162501	11.963218	12.800035	11.992298	11.909110	12.272044	14.577676	11.391015	13.900992	12.937994	13.502602	13.331285	12.111007	12.101924	12.584643	12.928845	13.429094	12.624487	12.964739	12.456838	11.799097	12.462078	11.947446	12.325086	12.069926	12.550390	13.025833	12.156344	13.138306	12.456454	11.980630	12.121293	12.262160	11.904757	12.903231	10.764458	12.682475	11.898226	12.217765	12.072330	12.538079	11.469270	12.137705	12.470709	11.986513	12.436614	12.573864	11.969197	13.215627	12.453418	13.120033	12.773621	12.641390	12.346315	12.791666	11.962757	12.166633	12.792507	11.985888	12.537425	12.226768	12.485499	11.472792	12.030200	12.510646	12.583295	12.176716	12.262009	12.864781	12.188852	12.689849	12.082595	12.482801	12.269152	12.500081	13.832567	12.935069	13.027278	11.676370	13.203600	12.456759	11.953330	12.221727	11.921278	12.053155	10.843161	12.243919	11.341907	11.411534	11.705942	10.890293	11.956432	13.435992	12.596810	13.148076	14.071478	14.110109	12.499629	12.901113	12.406965	12.898820	12.858824	11.980914	12.264825	12.309839	12.768697	12.772064

SULT1A3	14.046714	13.509011	12.670521	13.725347	12.780366	12.833844	12.975099	12.252315	13.160712	12.857801	12.720078	13.164705	13.049159	12.959744	13.247129	12.732190	13.042766	12.834200	13.335001	13.151060	12.794117	13.102943	13.640364	13.272175	13.873927	13.574884	14.106916	13.008295	13.288240	13.396009	13.198919	13.941795	13.169781	14.588535	14.021820	13.484384	13.249121	13.025833	13.203792	13.228647	12.938700	13.424093	13.964894	13.275857	14.095820	13.433260	13.376076	13.063034	12.896421	13.197929	13.599502	13.407160	14.040527	13.227058	13.036697	13.011074	13.771343	13.128058	13.285244	12.761874	13.400373	13.457219	13.978080	13.375655	13.142467	13.038398	13.649396	13.040624	13.591444	13.087748	13.540385	13.540597	13.142638	13.377562	13.535967	13.427811	13.690456	13.355173	13.759917	13.574462	13.624991	14.162372	14.569736	13.037820	13.285244	13.263764	13.931123	13.297973	13.755126	12.707057	12.869225	13.389920	13.612555	13.189155	14.034970	13.410097	12.843171	13.607572	12.915887	13.161859	13.611109	12.677012	13.424093	12.353725	12.657996	12.866032	13.366676	13.003067	13.145329	12.726676	13.300869	13.259393	13.394330	13.264544	12.496680	14.108206	14.573111	13.143585	13.377337	14.298742	12.888793	13.347432	14.239437	13.849877	12.694078	14.365930	14.325742	13.608981	14.280056	13.477052	13.822664	14.384530	15.157558	13.763964	13.678771	13.578538	13.072450	13.487724	13.069482	12.996930	14.622502	13.671966	13.862105	13.405875	13.847489	13.473153	14.397321	14.117369	13.025652	13.348898	14.077463	14.060320	13.363265	13.379894	13.461577	12.878536	13.084018	13.364346	13.762967	14.012930	13.219365	13.264151	13.135653	12.910540	12.681285	13.160892	12.505911	13.274804	12.848771	13.359500	13.481256	13.225883	12.085444	14.224386	14.484873	13.791211	14.298393	13.253211	14.535929	13.893142	14.008743	13.286686	13.284435	13.717719	13.324170	13.286871	12.941926	13.628249	14.121601	13.731415	13.707724	13.939322	13.646113	13.932970	13.012290	13.440530	13.770321	13.327806	13.469871	14.073949	13.690456	13.518662	14.518957	12.916924	13.771628	13.039846	13.616612	13.810630	13.040624	14.408016	13.011946	14.108206	13.133954	13.483894	13.479517	13.953224	14.276902	13.524608	13.441594	13.948216	13.841137	14.513547	13.153806	13.239397	14.411699	14.235557	13.981851	13.938513	12.781052	13.531473	13.736836	14.232858	13.395578	14.167438	13.463591	13.986959	13.619020	13.385597

SULT1A4	14.046714	13.509011	12.670521	13.725347	12.780366	12.833844	12.975099	12.252315	13.160712	12.857801	12.720078	13.164705	13.049159	12.959744	13.247129	12.732190	13.042766	12.834200	13.335001	13.151060	12.794117	13.102943	13.640364	13.272175	13.873927	13.574884	14.106916	13.008295	13.288240	13.396009	13.198919	13.941795	13.169781	14.588535	14.021820	13.484384	13.249121	13.025833	13.203792	13.228647	12.938700	13.424093	13.964894	13.275857	14.095820	13.433260	13.376076	13.063034	12.896421	13.197929	13.599502	13.407160	14.040527	13.227058	13.036697	13.011074	13.771343	13.128058	13.285244	12.761874	13.400373	13.457219	13.978080	13.375655	13.142467	13.038398	13.649396	13.040624	13.591444	13.087748	13.540385	13.540597	13.142638	13.377562	13.535967	13.427811	13.690456	13.355173	13.759917	13.574462	13.624991	14.162372	14.569736	13.037820	13.285244	13.263764	13.931123	13.297973	13.755126	12.707057	12.869225	13.389920	13.612555	13.189155	14.034970	13.410097	12.843171	13.607572	12.915887	13.161859	13.611109	12.677012	13.424093	12.353725	12.657996	12.866032	13.366676	13.003067	13.145329	12.726676	13.300869	13.259393	13.394330	13.264544	12.496680	14.108206	14.573111	13.143585	13.377337	14.298742	12.888793	13.347432	14.239437	13.849877	12.694078	14.365930	14.325742	13.608981	14.280056	13.477052	13.822664	14.384530	15.157558	13.763964	13.678771	13.578538	13.072450	13.487724	13.069482	12.996930	14.622502	13.671966	13.862105	13.405875	13.847489	13.473153	14.397321	14.117369	13.025652	13.348898	14.077463	14.060320	13.363265	13.379894	13.461577	12.878536	13.084018	13.364346	13.762967	14.012930	13.219365	13.264151	13.135653	12.910540	12.681285	13.160892	12.505911	13.274804	12.848771	13.359500	13.481256	13.225883	12.085444	14.224386	14.484873	13.791211	14.298393	13.253211	14.535929	13.893142	14.008743	13.286686	13.284435	13.717719	13.324170	13.286871	12.941926	13.628249	14.121601	13.731415	13.707724	13.939322	13.646113	13.932970	13.012290	13.440530	13.770321	13.327806	13.469871	14.073949	13.690456	13.518662	14.518957	12.916924	13.771628	13.039846	13.616612	13.810630	13.040624	14.408016	13.011946	14.108206	13.133954	13.483894	13.479517	13.953224	14.276902	13.524608	13.441594	13.948216	13.841137	14.513547	13.153806	13.239397	14.411699	14.235557	13.981851	13.938513	12.781052	13.531473	13.736836	14.232858	13.395578	14.167438	13.463591	13.986959	13.619020	13.385597

ANKRD30A	4.250623	4.993108	4.626343	5.054726	4.786204	4.487427	4.255890	5.748675	6.334005	4.613576	5.449750	3.895246	3.918102	4.633723	4.327649	5.234225	5.768488	4.687813	5.411287	4.901741	5.175953	5.903669	3.412420	5.028096	4.223185	4.751699	5.741912	4.817310	3.769711	3.834416	5.746855	4.408268	4.373702	4.207295	4.418927	4.109814	4.018242	3.611177	4.407819	4.797060	3.824361	4.012459	5.552203	3.587283	5.321288	5.218532	4.612954	5.296034	5.468755	5.228917	4.788883	5.943205	4.483077	4.570729	4.515053	4.336552	4.152983	4.788062	4.650252	4.083044	4.908825	4.032550	4.049784	4.195839	4.016060	4.997369	4.885388	5.254943	4.710948	5.517407	4.715607	5.154789	4.182342	5.080015	5.255133	4.921033	5.106656	4.346322	5.190131	4.279490	4.020460	4.593852	4.215554	4.485251	4.527996	5.740064	4.562514	5.224298	4.540902	4.755521	4.313602	4.459665	4.561205	5.131805	4.040380	4.530956	4.857954	4.350224	3.596508	4.006327	4.189080	4.633497	4.813900	5.413965	5.386136	4.488050	6.281614	4.622478	4.467164	4.625263	3.749198	5.325747	5.378859	4.672289	6.072060	3.760914	4.691364	4.662468	4.523673	4.796275	4.700068	4.054728	4.924879	3.368431	4.825211	3.582650	4.459665	5.483503	4.209917	5.008684	4.452895	3.426395	4.018026	4.033222	4.334415	4.282734	4.241406	5.619645	4.082834	4.853109	5.808130	5.557935	4.311250	6.833773	5.611223	4.949812	4.513363	3.687063	5.860307	5.387168	3.697777	4.107628	4.394058	3.648809	4.209917	4.389751	4.052798	4.812075	4.742349	3.741588	3.961381	4.633927	4.740850	5.354518	5.030942	4.521048	4.664093	4.369790	5.496995	4.644274	5.649908	4.242269	5.442939	4.293954	4.129806	3.715469	4.637372	4.852673	4.252568	4.606924	3.695073	4.553885	4.023461	4.743378	5.063633	4.266767	5.022393	5.364627	5.356278	4.509641	4.738648	5.359285	5.020517	7.589045	4.546452	4.939145	4.575001	4.874048	3.667908	3.786951	4.346322	4.400675	3.892646	4.901535	4.756537	4.040380	4.255230	4.389553	4.549992	3.897142	5.124493	3.555075	8.662706	3.934506	4.384713	3.478413	3.731933	5.342170	4.321207	3.605404	4.651571	5.038331	3.612579	5.122893	5.286988	6.030923	4.782850	4.613988	4.817515	3.857006	4.280176	10.708553	5.193268	4.418689	7.642308	4.126791	4.818563	4.060633

BRD3	11.964481	11.377987	11.397914	11.364289	11.483101	11.217005	11.820802	10.843022	11.290061	11.327777	11.077209	11.506569	11.139782	11.240625	11.371423	11.188740	11.450569	11.426062	10.960936	11.572225	10.655009	10.912049	11.510826	11.336433	11.891870	11.569456	11.759762	11.293819	11.759915	11.392179	11.436941	11.480227	11.565239	11.301808	11.664174	11.318521	11.473235	11.460244	11.227093	12.164205	11.527480	11.374472	11.771822	11.818076	12.119923	11.647240	11.634348	11.182579	11.465697	11.537619	11.451631	11.634348	11.479943	11.773141	11.553739	11.303223	11.661027	11.265890	11.199598	11.846272	11.726448	11.447359	11.370678	11.191490	11.320848	11.325990	10.899668	10.928906	11.252514	10.757205	11.429272	11.520541	11.378571	11.428719	11.506127	11.392179	11.475562	11.646635	11.667871	10.411781	11.491752	11.509227	11.491895	11.513324	11.876288	11.541245	11.873246	11.863352	11.334950	10.978030	10.825918	11.051912	11.673410	11.729004	11.400599	11.593733	11.335983	11.446152	11.291724	11.530935	11.154642	11.472234	11.341907	11.436109	10.233407	11.194165	11.714541	11.500503	11.022434	11.256216	10.829442	11.639167	11.472498	11.555942	11.394579	10.451937	11.720774	11.442637	11.309686	11.713327	12.117551	12.209897	12.118493	11.264849	11.693184	11.873702	12.398015	12.359576	11.867133	11.650510	11.764352	12.537248	12.168033	11.833001	12.320151	11.994265	11.998237	12.731848	13.040990	11.343711	12.627817	11.932006	12.631160	13.065668	12.389516	11.032546	12.143905	11.513324	11.712614	12.666497	11.236568	11.524887	11.071672	12.029778	12.748286	11.541954	12.011560	11.549295	12.827872	11.392045	12.145773	12.276374	11.845543	12.202184	12.185867	11.480786	11.687372	12.746061	11.975098	11.178065	11.771231	12.875882	12.166192	11.826536	12.286918	11.778816	12.071265	11.410612	12.057936	11.558808	11.602546	11.438109	11.901752	11.482399	11.676832	11.428109	10.895257	11.772998	11.143689	12.232073	12.224005	11.801933	12.251829	11.939377	11.668318	12.283965	11.821409	11.701608	11.375308	12.417829	12.438343	11.778669	12.037911	12.307765	12.606727	12.281762	11.939225	12.022370	11.848510	12.300500	11.802387	12.294973	12.614703	11.877464	12.059052	12.212082	12.280960	12.334942	12.583626	12.379442	11.936083	11.628650	11.468687	11.844498	12.242834	13.050251	12.001025	11.800745	12.346154	11.699618	12.110693	12.708589	11.307971	12.621829	11.258424	12.320613	12.076837	12.496680

ADAM7	8.418410	7.327719	7.105493	7.745146	7.645874	7.769577	6.826234	8.949362	8.899620	7.503695	8.069834	7.582103	6.608840	7.197600	6.599911	7.531584	8.525979	7.190783	8.436002	7.570069	8.147603	7.688871	10.113777	7.369686	7.816357	7.931268	7.316850	8.440424	7.295535	6.731923	8.526736	7.100790	6.633215	6.008585	6.644679	6.992762	6.961659	7.415512	7.806171	7.966965	7.084263	7.528744	7.267588	7.163145	7.431568	7.320128	8.183733	7.354382	7.297804	7.386163	7.675274	7.717058	6.351290	7.074821	6.816661	7.983020	6.551642	7.335219	7.343519	6.949978	7.501316	6.676256	7.761592	8.106204	7.541274	7.933559	7.133895	7.493042	7.195198	8.180684	7.509249	7.270165	7.488204	7.284600	7.470794	7.162350	7.520818	7.468472	8.589431	6.913999	7.030942	6.984753	7.245475	7.157778	7.864481	7.435698	7.648147	7.743330	7.709719	7.944176	7.654830	8.474768	6.699964	7.067141	6.845868	6.865185	7.073401	6.402607	6.596013	6.832787	6.783375	6.665571	7.098623	6.749904	8.099931	7.091977	9.118833	7.930030	9.033855	7.946185	8.753789	8.417966	8.036665	7.866510	7.809741	7.582731	7.971783	7.506496	7.066825	7.484898	7.411794	6.950312	7.392107	7.772768	8.477734	7.483209	8.786705	8.162655	8.370333	8.224788	7.989106	8.507964	7.830357	8.929545	7.695913	8.066110	7.498515	8.041935	7.457145	8.577211	8.578321	9.782831	7.978000	8.304977	9.022179	8.531228	8.258005	8.507374	7.359788	8.725114	7.068891	6.984753	6.465048	6.733689	7.362910	6.702081	7.832609	8.115470	7.910633	7.694740	7.746079	6.692759	7.700541	7.479190	7.687949	8.519296	7.784088	7.701163	7.743798	7.646968	7.599022	6.257069	6.672024	6.558456	7.619865	6.805681	6.666704	7.070868	6.771256	7.712007	6.836278	7.865564	8.032995	7.594740	7.589345	7.207692	7.296044	8.282080	8.036191	7.383010	7.329932	8.430243	7.204710	7.209083	7.253866	7.842303	7.617095	7.937457	6.802029	6.561198	7.189234	7.254021	6.956277	7.671008	7.845512	7.256941	7.681782	7.760354	7.780442	7.711235	8.469173	7.936063	8.083490	7.450892	7.230254	7.762352	6.613816	7.259651	6.858980	7.891070	6.777825	7.149287	6.285328	7.467854	8.900970	8.692458	7.964169	8.545378	9.085192	8.382834	8.081052	7.927574	8.730609	8.276122	6.826070	7.779314	7.621512	8.505802

MOB3C	11.331759	11.071366	11.423308	11.177177	11.291145	11.305292	11.782962	10.825756	11.507158	11.392615	11.100303	11.182712	11.261380	11.388656	11.511703	10.622336	10.867717	11.346523	11.595221	11.206710	11.328919	10.565611	12.231416	10.901565	11.339227	11.586872	11.394279	10.831803	11.192799	11.745525	11.131496	11.338351	11.362843	11.482247	11.518972	11.309548	11.545901	11.713634	11.346662	11.727947	11.413548	11.647985	11.281195	11.691697	11.758886	11.448088	10.740809	11.593593	11.459639	11.745378	11.476974	11.725122	11.492740	11.721525	11.504805	11.503209	11.678021	11.258266	11.443831	11.181128	11.653773	11.700373	11.982268	11.569154	12.137705	11.836322	11.004546	10.988852	11.271480	11.190179	11.233924	11.095577	10.840049	11.101364	11.216854	11.081888	11.417391	11.094307	11.478113	11.793930	11.921595	11.722134	11.706874	12.061997	11.459795	11.520699	11.825339	12.134970	12.247505	11.415587	11.310412	12.068295	11.831355	11.851129	11.162899	11.234624	11.626444	10.941524	11.501190	10.496942	11.539526	11.404839	10.763276	10.993448	11.542723	10.852571	11.319427	11.104718	12.506563	11.089609	11.911633	11.522791	11.448837	10.690535	11.413548	9.725155	11.219974	11.278088	10.580549	11.524738	11.817939	11.975910	11.801490	11.276012	11.549582	12.156958	9.496425	11.589239	12.222189	11.810382	11.510541	12.094888	11.588926	11.474103	11.502905	11.192657	11.705505	10.658726	11.474995	12.640723	12.396914	12.583626	11.758591	11.778816	11.975737	12.205131	12.689295	11.252362	11.725272	11.982268	11.930619	11.261074	11.430826	11.816892	12.350370	11.939846	11.291291	12.285047	12.075588	10.390541	11.625759	10.468311	11.476126	12.465329	12.054237	10.491887	10.083133	12.873573	11.920253	12.015376	11.118254	11.945449	11.792548	11.429134	11.448381	11.237147	12.392501	11.712047	11.362553	12.273123	11.807364	12.393793	12.321742	11.846709	11.008883	12.300012	11.342331	11.370978	11.316005	11.981214	12.485326	11.206577	11.843554	11.776832	11.396538	12.064108	11.526648	11.622683	11.991699	12.028252	12.530838	12.431602	11.687522	12.289217	11.726896	11.666120	10.748141	12.134342	11.708199	10.682122	12.347281	11.926923	11.476828	11.275334	11.476411	11.669696	11.174295	12.161114	12.119288	11.983657	10.995378	11.790286	11.966551	11.450138	12.754466	11.549582	11.601794	12.818008	10.624433	12.344233	11.672529	6.711499	10.922691	11.553440	11.447496	12.840549	12.863843	11.434353

MKNK1	11.331759	11.071366	11.423308	11.177177	11.291145	11.305292	11.782962	10.825756	11.507158	11.392615	11.100303	11.182712	11.261380	11.388656	11.511703	10.622336	10.867717	11.346523	11.595221	11.206710	11.328919	10.565611	12.231416	10.901565	11.339227	11.586872	11.394279	10.831803	11.192799	11.745525	11.131496	11.338351	11.362843	11.482247	11.518972	11.309548	11.545901	11.713634	11.346662	11.727947	11.413548	11.647985	11.281195	11.691697	11.758886	11.448088	10.740809	11.593593	11.459639	11.745378	11.476974	11.725122	11.492740	11.721525	11.504805	11.503209	11.678021	11.258266	11.443831	11.181128	11.653773	11.700373	11.982268	11.569154	12.137705	11.836322	11.004546	10.988852	11.271480	11.190179	11.233924	11.095577	10.840049	11.101364	11.216854	11.081888	11.417391	11.094307	11.478113	11.793930	11.921595	11.722134	11.706874	12.061997	11.459795	11.520699	11.825339	12.134970	12.247505	11.415587	11.310412	12.068295	11.831355	11.851129	11.162899	11.234624	11.626444	10.941524	11.501190	10.496942	11.539526	11.404839	10.763276	10.993448	11.542723	10.852571	11.319427	11.104718	12.506563	11.089609	11.911633	11.522791	11.448837	10.690535	11.413548	9.725155	11.219974	11.278088	10.580549	11.524738	11.817939	11.975910	11.801490	11.276012	11.549582	12.156958	9.496425	11.589239	12.222189	11.810382	11.510541	12.094888	11.588926	11.474103	11.502905	11.192657	11.705505	10.658726	11.474995	12.640723	12.396914	12.583626	11.758591	11.778816	11.975737	12.205131	12.689295	11.252362	11.725272	11.982268	11.930619	11.261074	11.430826	11.816892	12.350370	11.939846	11.291291	12.285047	12.075588	10.390541	11.625759	10.468311	11.476126	12.465329	12.054237	10.491887	10.083133	12.873573	11.920253	12.015376	11.118254	11.945449	11.792548	11.429134	11.448381	11.237147	12.392501	11.712047	11.362553	12.273123	11.807364	12.393793	12.321742	11.846709	11.008883	12.300012	11.342331	11.370978	11.316005	11.981214	12.485326	11.206577	11.843554	11.776832	11.396538	12.064108	11.526648	11.622683	11.991699	12.028252	12.530838	12.431602	11.687522	12.289217	11.726896	11.666120	10.748141	12.134342	11.708199	10.682122	12.347281	11.926923	11.476828	11.275334	11.476411	11.669696	11.174295	12.161114	12.119288	11.983657	10.995378	11.790286	11.966551	11.450138	12.754466	11.549582	11.601794	12.818008	10.624433	12.344233	11.672529	6.711499	10.922691	11.553440	11.447496	12.840549	12.863843	11.434353

PILRB	12.309669	12.806311	12.482306	12.917840	13.378453	13.120800	12.383384	14.024760	13.928111	12.939734	13.576005	13.229053	12.015060	12.311428	12.583137	14.278587	14.277581	12.450587	13.293540	14.473115	13.163201	13.043520	13.776673	12.640385	13.235191	13.500208	13.354792	14.074236	13.659121	12.505236	13.566158	11.525463	13.175984	11.817333	13.504804	13.966043	13.535732	12.676204	13.118063	13.765216	14.017264	12.927379	13.341990	13.689045	14.230892	14.181178	13.183086	13.841638	13.297375	15.399178	14.280056	13.237448	12.000568	12.748989	12.552169	13.544167	12.536742	11.963379	12.239925	12.140193	12.550549	13.478600	12.185867	13.043339	12.718533	13.323155	12.224005	12.944614	12.527930	13.065502	15.170169	14.841605	13.923216	14.466988	14.640496	13.019185	13.384576	13.226062	13.806755	12.003241	12.173094	12.896716	11.699314	12.832557	12.566772	13.697560	12.817679	13.178579	12.635767	12.579011	12.541440	13.231650	12.513855	13.414830	12.914641	13.576005	13.039676	13.614835	12.852132	13.000997	12.966367	12.774471	13.699672	12.490571	12.698356	13.678503	13.258409	12.889837	13.642281	13.867543	13.254784	13.282824	13.102201	13.789730	13.830488	13.340559	12.781728	14.032887	13.754158	12.849310	11.726308	10.955728	12.257562	12.670521	12.229725	12.060393	14.363390	13.217768	11.801628	11.658803	12.337752	12.378817	11.165378	12.352896	13.602656	12.251209	12.830155	13.868320	11.651970	12.532019	12.728615	13.226454	13.013913	11.787766	11.725723	12.618797	12.670169	13.609930	11.486012	13.517555	12.518587	12.243629	11.218189	11.872045	11.686921	11.698867	11.674592	12.196619	12.445640	13.546876	12.103454	11.107323	11.748620	11.599731	13.277050	12.303273	11.263631	11.811570	11.519647	12.203746	11.578488	11.245651	10.999997	10.888785	12.034862	11.399245	11.790004	12.083231	11.478703	12.218928	12.206044	12.569545	12.122102	12.610699	11.715417	12.016430	11.562313	10.866526	12.543245	12.173415	11.977145	13.337700	12.109967	11.916369	11.832854	11.585010	12.152993	12.770845	11.223118	11.226796	11.882808	11.184811	12.172634	11.594141	13.001778	10.942871	11.899712	12.094888	11.824133	11.837100	13.094857	12.201396	11.698568	12.207449	12.507056	12.424576	13.097319	12.074208	11.308373	11.553592	12.280960	12.647433	10.830328	12.447724	12.575245	12.549091	13.608723	13.239009	12.183786	12.880782	12.461907	12.139582	13.718689	12.129027	11.824581	12.130685	12.611352	12.060100

PILRA	12.309669	12.806311	12.482306	12.917840	13.378453	13.120800	12.383384	14.024760	13.928111	12.939734	13.576005	13.229053	12.015060	12.311428	12.583137	14.278587	14.277581	12.450587	13.293540	14.473115	13.163201	13.043520	13.776673	12.640385	13.235191	13.500208	13.354792	14.074236	13.659121	12.505236	13.566158	11.525463	13.175984	11.817333	13.504804	13.966043	13.535732	12.676204	13.118063	13.765216	14.017264	12.927379	13.341990	13.689045	14.230892	14.181178	13.183086	13.841638	13.297375	15.399178	14.280056	13.237448	12.000568	12.748989	12.552169	13.544167	12.536742	11.963379	12.239925	12.140193	12.550549	13.478600	12.185867	13.043339	12.718533	13.323155	12.224005	12.944614	12.527930	13.065502	15.170169	14.841605	13.923216	14.466988	14.640496	13.019185	13.384576	13.226062	13.806755	12.003241	12.173094	12.896716	11.699314	12.832557	12.566772	13.697560	12.817679	13.178579	12.635767	12.579011	12.541440	13.231650	12.513855	13.414830	12.914641	13.576005	13.039676	13.614835	12.852132	13.000997	12.966367	12.774471	13.699672	12.490571	12.698356	13.678503	13.258409	12.889837	13.642281	13.867543	13.254784	13.282824	13.102201	13.789730	13.830488	13.340559	12.781728	14.032887	13.754158	12.849310	11.726308	10.955728	12.257562	12.670521	12.229725	12.060393	14.363390	13.217768	11.801628	11.658803	12.337752	12.378817	11.165378	12.352896	13.602656	12.251209	12.830155	13.868320	11.651970	12.532019	12.728615	13.226454	13.013913	11.787766	11.725723	12.618797	12.670169	13.609930	11.486012	13.517555	12.518587	12.243629	11.218189	11.872045	11.686921	11.698867	11.674592	12.196619	12.445640	13.546876	12.103454	11.107323	11.748620	11.599731	13.277050	12.303273	11.263631	11.811570	11.519647	12.203746	11.578488	11.245651	10.999997	10.888785	12.034862	11.399245	11.790004	12.083231	11.478703	12.218928	12.206044	12.569545	12.122102	12.610699	11.715417	12.016430	11.562313	10.866526	12.543245	12.173415	11.977145	13.337700	12.109967	11.916369	11.832854	11.585010	12.152993	12.770845	11.223118	11.226796	11.882808	11.184811	12.172634	11.594141	13.001778	10.942871	11.899712	12.094888	11.824133	11.837100	13.094857	12.201396	11.698568	12.207449	12.507056	12.424576	13.097319	12.074208	11.308373	11.553592	12.280960	12.647433	10.830328	12.447724	12.575245	12.549091	13.608723	13.239009	12.183786	12.880782	12.461907	12.139582	13.718689	12.129027	11.824581	12.130685	12.611352	12.060100

EFCAB11	10.617450	9.717013	10.925062	10.092624	10.483377	11.337604	11.324533	11.696817	11.194304	11.383763	11.451631	11.429574	11.045017	11.120430	10.047711	10.879299	11.403089	10.266879	10.609505	12.095947	11.259009	11.025247	9.876239	10.670884	10.189657	10.771942	9.334161	9.555229	10.074539	10.675830	10.750537	11.556411	11.142360	12.102545	11.258424	11.430579	11.463192	10.395361	10.460118	10.389435	11.222672	10.661622	11.305887	11.520985	11.283416	11.453428	10.127898	10.394618	11.470789	11.379762	11.063780	11.550016	11.050033	11.422252	11.166077	11.116371	11.368582	10.778852	11.103274	10.504682	11.015142	10.552353	10.070780	10.391640	10.886126	10.553085	10.992097	11.336278	11.096922	10.612584	11.345947	11.400910	10.632306	11.076058	11.548861	11.340546	11.793463	11.173601	11.398826	11.222057	11.282256	11.422252	11.369004	11.639628	11.549582	11.237278	11.278862	11.770488	10.824736	10.569692	10.222096	10.827110	11.146045	11.501471	11.718550	11.232575	11.107323	10.707633	11.212133	10.036868	11.252949	9.983054	10.670427	9.840309	10.516102	11.261380	10.974503	10.713869	10.448550	10.829909	11.397622	11.621293	10.218469	10.507667	11.303223	10.679432	9.102598	11.253964	10.508413	13.011590	10.218320	11.001368	11.790428	9.560659	11.025992	11.748029	9.333063	11.314528	11.723934	11.356089	12.188558	11.003790	11.824728	10.075427	10.903200	12.503725	10.100927	9.235309	11.080854	12.105743	11.223409	10.745281	11.051912	12.008706	11.908965	11.083497	9.173114	9.170296	11.495782	11.310881	11.651679	12.010940	10.963706	11.669383	11.786428	11.213657	10.424236	11.439735	10.826070	11.053493	10.891326	11.493601	11.902027	11.293819	12.218342	11.147376	9.978579	11.189473	11.907107	11.165084	11.832392	12.317479	12.606391	12.566593	11.599875	11.337458	11.691236	10.929785	10.209520	10.880620	12.161407	11.068070	11.617285	11.309113	10.596659	11.675894	11.081157	11.141784	11.537919	12.514335	12.212389	10.673716	12.196619	11.788973	11.803092	11.934585	12.380673	11.661642	12.095947	11.522184	12.301244	12.179402	11.392925	11.190758	11.403818	12.555263	12.190143	11.368020	10.694498	9.811338	11.076776	11.021682	12.424576	10.793950	11.245942	11.434496	10.655933	12.032298	10.630112	11.952893	10.404404	10.521111	10.537076	12.192594	10.354602	11.309113	10.906005	11.034454	11.864391	10.892382	11.984692	9.761826	11.063360	10.413149	11.753936	10.783006	11.518703	12.885289

GSTM5	14.800886	14.488728	13.561728	14.435670	13.502998	14.386005	13.416705	13.299434	12.773104	11.813442	13.093311	12.607553	14.317178	13.915290	14.026270	12.695570	13.232837	11.834043	13.494882	14.686942	11.793767	10.862762	13.244529	13.033736	13.072832	13.024565	11.286261	12.037292	12.180008	13.556369	12.719368	13.553239	12.221249	13.704613	14.247926	12.024501	13.741329	14.637945	14.071794	13.754901	14.249525	12.347129	12.654999	13.859930	12.421202	12.757381	11.999458	13.900462	12.062616	14.057422	12.786304	14.313436	10.643266	15.183376	13.611591	13.447775	14.205304	12.754129	14.122743	13.629812	13.872874	13.393094	13.959054	14.288296	13.750799	14.541751	14.680064	14.164273	13.068499	14.975358	11.953478	13.028398	11.615675	12.054398	14.497531	13.233234	12.779001	13.576961	12.334777	15.164050	13.930990	13.472274	14.705283	15.161962	14.367353	13.487933	14.997957	13.839011	14.490783	13.969061	13.938772	14.073025	13.682111	13.659354	13.766279	14.159585	13.162239	13.293957	14.302591	10.879160	13.781596	14.000102	13.225108	13.404827	15.005964	12.666171	13.387105	14.937805	12.729813	13.777164	13.694470	13.762967	13.591444	11.625759	11.743625	12.637159	14.375159	13.554828	12.533129	11.860241	12.185427	12.789055	14.193358	15.356364	13.403139	14.158638	11.302336	11.882981	15.565819	13.715358	15.383684	14.736202	12.163433	14.375530	12.435794	15.056697	15.400849	11.332182	12.028551	16.377052	16.559494	16.143053	14.680965	13.942960	14.621136	12.137555	15.521068	14.756024	14.880110	12.180333	13.967702	12.641230	13.239009	12.583952	15.918070	12.752609	13.888605	13.623383	15.648843	11.294668	15.927561	12.879072	11.442790	15.564042	15.909426	11.253526	12.913586	16.175634	15.122829	15.504733	15.060407	15.603850	11.650941	12.821251	14.591979	13.475325	13.697330	14.528702	12.639389	11.740862	13.676222	14.423235	14.556844	14.423617	12.703486	13.105804	14.478214	14.706637	13.295547	15.888240	15.628044	12.778119	15.399973	12.243771	13.570563	15.429349	12.703807	14.930806	12.779334	14.655931	14.696136	12.285526	12.849653	12.919720	14.976992	13.020460	12.833284	13.167086	12.767450	11.991699	11.612110	14.347809	13.214202	13.109649	12.963794	15.184064	14.097636	13.315612	15.618883	13.018269	12.536742	11.322149	14.907590	13.407160	15.244645	14.524343	13.425768	16.033556	13.558417	15.507347	12.449765	12.729457	11.128092	15.059149	12.913034	15.347177	14.243708	11.707448

GSTM3	14.800886	14.488728	13.561728	14.435670	13.502998	14.386005	13.416705	13.299434	12.773104	11.813442	13.093311	12.607553	14.317178	13.915290	14.026270	12.695570	13.232837	11.834043	13.494882	14.686942	11.793767	10.862762	13.244529	13.033736	13.072832	13.024565	11.286261	12.037292	12.180008	13.556369	12.719368	13.553239	12.221249	13.704613	14.247926	12.024501	13.741329	14.637945	14.071794	13.754901	14.249525	12.347129	12.654999	13.859930	12.421202	12.757381	11.999458	13.900462	12.062616	14.057422	12.786304	14.313436	10.643266	15.183376	13.611591	13.447775	14.205304	12.754129	14.122743	13.629812	13.872874	13.393094	13.959054	14.288296	13.750799	14.541751	14.680064	14.164273	13.068499	14.975358	11.953478	13.028398	11.615675	12.054398	14.497531	13.233234	12.779001	13.576961	12.334777	15.164050	13.930990	13.472274	14.705283	15.161962	14.367353	13.487933	14.997957	13.839011	14.490783	13.969061	13.938772	14.073025	13.682111	13.659354	13.766279	14.159585	13.162239	13.293957	14.302591	10.879160	13.781596	14.000102	13.225108	13.404827	15.005964	12.666171	13.387105	14.937805	12.729813	13.777164	13.694470	13.762967	13.591444	11.625759	11.743625	12.637159	14.375159	13.554828	12.533129	11.860241	12.185427	12.789055	14.193358	15.356364	13.403139	14.158638	11.302336	11.882981	15.565819	13.715358	15.383684	14.736202	12.163433	14.375530	12.435794	15.056697	15.400849	11.332182	12.028551	16.377052	16.559494	16.143053	14.680965	13.942960	14.621136	12.137555	15.521068	14.756024	14.880110	12.180333	13.967702	12.641230	13.239009	12.583952	15.918070	12.752609	13.888605	13.623383	15.648843	11.294668	15.927561	12.879072	11.442790	15.564042	15.909426	11.253526	12.913586	16.175634	15.122829	15.504733	15.060407	15.603850	11.650941	12.821251	14.591979	13.475325	13.697330	14.528702	12.639389	11.740862	13.676222	14.423235	14.556844	14.423617	12.703486	13.105804	14.478214	14.706637	13.295547	15.888240	15.628044	12.778119	15.399973	12.243771	13.570563	15.429349	12.703807	14.930806	12.779334	14.655931	14.696136	12.285526	12.849653	12.919720	14.976992	13.020460	12.833284	13.167086	12.767450	11.991699	11.612110	14.347809	13.214202	13.109649	12.963794	15.184064	14.097636	13.315612	15.618883	13.018269	12.536742	11.322149	14.907590	13.407160	15.244645	14.524343	13.425768	16.033556	13.558417	15.507347	12.449765	12.729457	11.128092	15.059149	12.913034	15.347177	14.243708	11.707448

KRTAP10-1	11.494211	12.305725	11.107920	11.879073	11.823375	12.507547	11.907737	13.430832	12.863485	12.639025	12.377877	13.070410	11.417099	11.327777	11.528614	12.534620	12.114497	11.166662	12.456916	12.293882	11.797645	11.641628	11.122188	11.706405	12.235592	12.374518	14.125236	12.762748	13.802855	12.354682	12.331442	10.763416	11.030164	12.038357	11.090592	11.997321	11.707306	11.497575	11.965086	11.733316	12.067983	12.285826	11.818688	12.500751	11.275035	11.439735	12.348967	12.413177	11.156708	11.859202	12.309356	11.109422	10.950433	11.406614	11.652891	12.656833	10.908098	10.511571	12.093060	10.492203	11.133393	11.893211	10.096264	11.673868	11.411534	11.728242	12.444034	11.530458	11.344451	11.703267	12.634942	11.747030	12.121584	12.213183	11.617144	11.440486	11.365974	11.687212	13.863595	11.160805	10.651347	10.789717	10.968006	11.267354	11.141493	12.411724	10.853314	10.377566	10.691661	11.476828	11.902791	10.932488	11.029265	11.786572	12.817160	10.994167	11.349651	11.685397	11.804392	12.175782	12.324058	12.456287	11.936536	11.823838	12.199062	12.026746	12.437588	11.818688	11.928915	12.573503	11.272950	12.114204	11.722280	12.566930	11.942584	14.339586	11.019905	12.298858	12.738146	11.143107	12.021368	11.221623	11.588472	12.901614	11.980914	12.200477	12.014841	12.309356	11.786118	12.208851	11.868313	11.694995	11.347880	11.661180	12.297906	11.676971	11.192946	11.142800	10.903357	11.443229	11.577639	11.033441	12.148257	11.717044	11.192513	12.587157	12.002950	12.970784	11.973763	11.855342	11.275872	11.536881	11.809631	11.217437	11.353164	12.261826	11.016629	11.801044	11.377558	12.387258	11.621743	10.654872	11.518100	11.510698	11.765253	12.612002	10.587598	10.568013	11.108364	11.258424	10.681966	10.586401	10.845477	10.284105	11.668608	9.939949	10.101089	10.678664	10.160981	10.887915	12.017633	11.537619	11.237350	10.716420	10.773291	11.151555	12.020215	11.458612	11.182301	11.960692	11.726597	12.798678	11.660289	11.857671	12.034420	11.379157	11.023071	12.121750	10.287089	10.229880	10.221516	11.225183	10.444886	9.944548	11.380384	10.696641	9.874660	9.867886	10.337200	10.135934	10.276978	10.867581	11.107632	12.099519	11.739966	11.443526	12.057611	11.667871	11.207310	11.586469	11.524099	12.068749	11.249094	10.241816	12.399952	12.036393	11.118254	11.207783	12.842437	11.035210	11.527756	11.858897	12.964557	11.849270	12.504062	11.029110	11.458459	12.178309

TSPEAR	11.494211	12.305725	11.107920	11.879073	11.823375	12.507547	11.907737	13.430832	12.863485	12.639025	12.377877	13.070410	11.417099	11.327777	11.528614	12.534620	12.114497	11.166662	12.456916	12.293882	11.797645	11.641628	11.122188	11.706405	12.235592	12.374518	14.125236	12.762748	13.802855	12.354682	12.331442	10.763416	11.030164	12.038357	11.090592	11.997321	11.707306	11.497575	11.965086	11.733316	12.067983	12.285826	11.818688	12.500751	11.275035	11.439735	12.348967	12.413177	11.156708	11.859202	12.309356	11.109422	10.950433	11.406614	11.652891	12.656833	10.908098	10.511571	12.093060	10.492203	11.133393	11.893211	10.096264	11.673868	11.411534	11.728242	12.444034	11.530458	11.344451	11.703267	12.634942	11.747030	12.121584	12.213183	11.617144	11.440486	11.365974	11.687212	13.863595	11.160805	10.651347	10.789717	10.968006	11.267354	11.141493	12.411724	10.853314	10.377566	10.691661	11.476828	11.902791	10.932488	11.029265	11.786572	12.817160	10.994167	11.349651	11.685397	11.804392	12.175782	12.324058	12.456287	11.936536	11.823838	12.199062	12.026746	12.437588	11.818688	11.928915	12.573503	11.272950	12.114204	11.722280	12.566930	11.942584	14.339586	11.019905	12.298858	12.738146	11.143107	12.021368	11.221623	11.588472	12.901614	11.980914	12.200477	12.014841	12.309356	11.786118	12.208851	11.868313	11.694995	11.347880	11.661180	12.297906	11.676971	11.192946	11.142800	10.903357	11.443229	11.577639	11.033441	12.148257	11.717044	11.192513	12.587157	12.002950	12.970784	11.973763	11.855342	11.275872	11.536881	11.809631	11.217437	11.353164	12.261826	11.016629	11.801044	11.377558	12.387258	11.621743	10.654872	11.518100	11.510698	11.765253	12.612002	10.587598	10.568013	11.108364	11.258424	10.681966	10.586401	10.845477	10.284105	11.668608	9.939949	10.101089	10.678664	10.160981	10.887915	12.017633	11.537619	11.237350	10.716420	10.773291	11.151555	12.020215	11.458612	11.182301	11.960692	11.726597	12.798678	11.660289	11.857671	12.034420	11.379157	11.023071	12.121750	10.287089	10.229880	10.221516	11.225183	10.444886	9.944548	11.380384	10.696641	9.874660	9.867886	10.337200	10.135934	10.276978	10.867581	11.107632	12.099519	11.739966	11.443526	12.057611	11.667871	11.207310	11.586469	11.524099	12.068749	11.249094	10.241816	12.399952	12.036393	11.118254	11.207783	12.842437	11.035210	11.527756	11.858897	12.964557	11.849270	12.504062	11.029110	11.458459	12.178309

EPB41L1	13.583919	12.130685	12.913386	12.431602	12.907514	12.842437	12.741273	13.401214	13.783587	12.939042	13.125984	12.748129	12.517113	13.311729	12.753969	12.614541	13.479048	12.598156	13.029169	13.305150	12.466325	13.110040	13.251828	12.298697	13.260832	13.180364	12.474915	13.023285	12.654649	12.207292	13.267397	13.473369	13.128611	12.794485	12.970060	13.306600	13.526786	12.864211	12.665284	12.678705	13.068091	12.616157	13.523456	13.420610	12.797845	13.082545	12.970971	12.601063	12.925434	13.581602	13.117173	12.840359	13.358864	13.516687	12.851230	13.465968	13.737085	12.617294	12.713635	12.930619	12.890172	12.437883	12.917840	12.962524	12.309669	12.537925	12.811770	13.050251	12.984441	12.919156	12.968039	13.182875	13.004475	12.962912	13.395578	13.561518	13.681631	13.456140	13.389482	12.400795	12.679547	12.382432	13.144764	13.226650	13.355364	12.997893	13.223325	13.072064	12.473452	12.206651	12.036533	12.505236	12.564954	13.354589	13.212415	13.298159	13.270823	13.167892	13.696085	12.869060	13.178579	12.421993	12.699016	12.638694	13.689281	13.320065	13.305996	13.109074	12.643076	13.214995	13.132646	13.393302	13.170952	13.153423	13.684034	11.883726	12.807201	13.014469	13.319062	11.452975	10.766421	11.861688	13.597930	11.425504	13.635468	12.294183	12.404662	11.336717	13.513209	11.743759	13.640135	12.875552	11.506409	11.798213	13.152801	10.855244	12.755658	12.015376	10.753717	13.382599	12.280813	12.493046	11.928470	11.449854	12.526786	13.142104	12.134970	12.413658	11.087512	12.656999	11.412947	11.466449	11.962614	12.398339	12.644473	12.377877	12.289665	10.731203	12.292501	11.014277	12.323414	11.899423	11.362553	13.405475	12.942474	12.742681	12.094734	12.891724	11.387475	12.502227	14.532252	12.609687	10.792032	13.739872	13.478817	12.453263	12.060534	12.680421	11.545312	11.694395	13.747333	11.486085	11.948669	12.277288	11.910576	9.932177	12.609195	11.243882	12.964557	13.140468	12.527775	11.999926	12.438191	11.800894	12.591170	12.537425	12.039995	12.753644	11.642983	10.958864	13.653439	11.808575	12.266993	11.463611	12.877478	11.829878	11.911476	11.105734	11.439898	10.647591	11.923703	12.480123	11.283702	11.475428	12.626503	12.244533	13.643032	11.849270	12.050582	11.503654	11.423761	12.177192	12.570678	11.093405	12.995435	11.427522	13.106997	12.632621	10.338376	12.834529	10.831970	11.164048	13.069852	11.755140	11.397622	13.030818	13.512631	10.607831

OPLAH	12.116196	11.643568	11.945613	11.868173	12.500415	11.999751	11.702072	11.444260	11.593285	12.200786	11.804233	12.043646	11.744648	12.338408	12.019013	11.640267	11.891244	11.753359	12.986841	12.093684	12.075438	12.277908	11.763401	11.703716	11.912357	12.040452	10.241816	11.373780	11.569749	11.046663	11.498901	11.735392	11.284447	11.408754	10.884458	11.519395	11.886135	11.831805	12.445298	12.501735	12.278662	12.138949	11.543325	11.988140	11.446601	11.959600	11.520699	12.137555	11.540550	12.092606	11.453711	11.935336	11.367114	12.254851	11.884500	12.197526	12.103782	12.502885	12.451862	12.231821	12.356099	11.678745	11.488461	12.605894	12.561217	12.154791	11.986356	11.833448	11.569154	12.382744	11.188596	11.850666	11.563161	11.252362	11.838285	11.267954	11.771822	11.437970	11.467196	11.912654	12.214094	11.849886	11.884500	12.002645	11.950357	11.976069	11.976679	12.061997	12.588140	12.261347	12.079547	12.261651	11.488318	11.908802	11.288336	11.638582	11.740562	11.799679	11.854007	11.471087	11.665066	12.001595	11.616550	12.217152	12.930440	11.787922	11.947588	12.779167	12.845812	12.154206	12.763935	12.902873	12.634617	12.014173	11.818545	11.261983	12.841587	11.957851	11.889463	10.152638	11.651970	10.364143	11.996843	12.268220	12.649190	12.552494	12.700895	11.025247	11.134006	12.071894	12.267455	11.450408	11.323214	10.641213	10.622929	12.280067	12.272956	13.355999	11.950357	12.830155	12.101924	12.202348	11.413703	11.887650	10.996398	11.455816	12.746061	12.324408	11.652428	10.810113	11.302046	10.766855	10.945347	11.736718	11.745819	10.639693	11.161103	12.734379	12.505236	11.272950	12.371963	12.361945	11.235828	11.724680	10.986270	11.126639	10.239265	12.838923	11.451923	11.863798	10.792768	12.398188	11.246667	11.941351	11.820802	9.804101	12.668989	12.405745	11.606677	11.448972	11.566982	12.243629	12.328915	11.769146	11.436677	11.537478	11.268796	11.272950	12.008706	11.689738	10.833302	12.165300	10.860414	9.481495	11.159344	11.291891	11.355342	11.627063	11.179915	11.153181	11.346980	11.678446	10.227905	11.368306	11.566690	10.912940	12.153901	11.911024	11.029556	11.094004	12.142364	11.125198	10.649682	11.649159	11.059536	11.803667	12.092757	10.730315	10.015392	10.563370	11.573736	10.770016	11.439262	12.091066	10.552938	11.285784	12.143436	12.728127	11.206137	10.852571	11.340239	11.228274	11.594907	11.030464	10.653521	11.308542	11.966406	10.964458

C16orf95	7.943394	6.216306	6.484961	8.417243	7.098929	8.350957	7.249782	7.494577	8.117316	7.827120	8.266965	8.068759	7.030503	7.042723	6.072743	7.695178	7.141433	7.314333	8.422572	7.041120	6.211523	6.421425	7.476312	6.785812	7.008826	7.663402	8.049234	7.472494	7.791899	7.695913	7.876139	8.428190	6.245437	6.963394	6.718452	6.005203	6.629353	6.010714	5.997605	6.135173	7.576358	5.715301	6.218038	6.311949	6.183960	4.877599	9.011278	5.431227	6.911310	6.962293	8.778111	6.971814	5.973067	6.387306	6.328572	8.428965	5.997605	3.726946	5.556954	5.385758	5.696189	7.869083	4.888964	5.280527	5.222725	5.710664	5.533140	6.195488	5.914578	5.566934	6.828774	7.140632	8.398520	6.472574	6.843360	6.630154	6.721907	6.503174	8.889943	5.449166	7.099426	5.971232	6.441376	5.969473	6.103756	7.248533	6.861228	6.296916	4.792316	6.694411	5.612522	5.237472	6.261339	6.759769	8.658361	7.166993	6.512753	7.505719	7.660947	7.059187	8.294571	6.307087	7.042248	6.301328	5.773447	7.697764	7.315588	6.417644	7.016496	8.269361	6.911932	7.378180	6.933021	9.139318	7.805090	9.713091	6.815567	7.888113	8.255721	7.255858	6.616937	6.485942	7.617381	8.523382	8.614955	8.438425	8.772629	6.839990	9.644234	8.703075	8.572461	8.321435	7.483367	7.756656	7.878260	7.122421	7.635566	7.860396	7.189063	7.050856	6.857373	7.608529	8.001370	7.460716	6.739736	8.153614	8.232255	9.296274	7.628292	7.808494	7.553470	7.231664	7.387317	6.422390	6.663789	6.710407	6.200170	6.501553	6.445408	8.930630	5.943035	6.010351	6.356807	6.499228	5.752653	9.701888	5.802751	6.938569	6.799271	6.957676	6.435628	5.789578	6.195305	6.068929	7.496149	6.259647	3.166490	5.302239	4.935579	5.171481	8.161158	5.272683	5.680644	5.688971	5.434147	5.718329	5.472171	6.285685	4.838497	6.911787	7.387934	10.069768	6.915595	6.685988	6.341865	6.746276	6.947794	7.381884	5.890991	6.472740	6.242941	6.236650	6.913553	6.198260	7.992068	6.493615	6.445578	5.249800	5.583037	6.047635	5.629710	6.913240	7.331642	7.774143	7.641687	7.621653	8.940500	7.837337	6.212025	8.101283	7.606249	7.402709	6.522506	5.872991	8.841648	7.337058	5.352484	5.971232	10.458181	7.117059	7.730554	7.013318	10.563986	8.506105	7.992228	6.451641	7.861216	8.805811

PBXIP1	8.287349	7.450282	7.409279	7.480444	7.381709	7.326110	8.328131	4.786621	6.358161	7.752415	7.456332	7.592559	8.037664	7.965409	8.250515	6.703546	7.280692	8.519296	7.662002	7.042080	7.786067	7.657214	10.424393	7.594003	8.763780	7.978951	8.450930	8.136495	7.859428	7.980345	7.261675	8.155472	8.818068	7.825370	7.710641	7.961924	8.101283	8.022679	7.948163	8.183733	7.535343	8.634259	7.850213	7.401924	8.626099	7.490716	8.612810	8.678364	8.346927	7.848996	7.828691	8.486435	8.242339	8.023315	8.647979	8.004026	8.524770	8.178250	8.848779	8.925514	8.697899	7.255549	9.361550	8.313127	8.498831	8.851059	7.791586	7.604831	7.542183	7.754559	7.355178	6.751613	7.557379	7.483531	7.049597	7.464507	7.181888	7.270643	7.243420	8.110742	7.754421	8.001370	8.157342	8.028572	8.166286	8.281762	7.401622	7.476921	8.015866	7.135477	7.807713	7.685796	7.482906	6.929686	7.377194	7.442212	7.930949	7.771110	7.332112	7.719199	7.075764	7.775738	7.278325	7.518657	6.555983	7.917227	7.936388	7.580861	7.631624	7.603296	6.969024	7.308493	7.261516	6.941502	6.955826	3.018650	8.972332	7.868615	7.889992	7.730715	7.101570	7.147559	8.689141	6.150695	7.469241	7.565198	6.369883	7.424763	6.935371	7.113612	7.012845	7.871513	8.379846	7.589821	7.114731	6.473593	8.603452	7.744247	7.364365	7.558311	7.939461	8.736423	8.127977	7.620747	8.113464	6.197419	6.680626	6.429547	8.265560	9.493614	6.828154	7.813075	7.099566	7.993945	6.819116	8.112376	6.907201	8.142694	7.836587	7.023919	7.575743	7.893878	7.198837	7.514341	6.279314	7.791421	7.983341	8.026217	7.964169	8.126763	7.981607	7.364834	8.130266	7.794622	8.483274	9.056728	7.660314	8.415166	7.703896	7.941663	8.663004	7.569912	7.432496	8.110900	8.445469	6.760278	7.000974	6.464876	7.865882	6.542210	6.820848	6.010714	6.937644	7.178923	7.026166	7.112523	6.524348	6.460844	8.363626	7.584728	7.661085	7.884438	7.340176	7.768086	7.328193	7.155289	6.700808	7.878989	7.462298	6.839336	6.708631	7.228105	6.749580	6.816835	7.079890	7.724392	7.644929	7.304121	8.114390	6.894802	6.834845	7.274066	7.417717	7.414462	8.097665	7.285348	6.370383	7.807549	7.088173	6.536628	7.794310	5.311259	6.741635	6.289442	5.361536	8.823713	7.579650	7.037782

ATXN7L1	11.744056	10.041323	9.965297	11.474238	10.722360	10.914915	10.702585	10.817302	11.285784	10.836621	11.087667	10.865608	10.433367	10.176535	9.648718	10.232190	10.588511	9.819337	10.601209	10.311562	9.857581	9.860030	10.502718	10.251318	10.532684	10.822441	10.487794	10.714913	11.023937	10.545676	10.983770	10.332362	9.738374	10.316793	10.211657	10.180574	10.205361	9.973251	10.315346	10.563370	10.966761	9.856355	9.793010	10.096550	10.175781	10.073625	10.995069	9.816506	9.959977	10.003058	10.351116	9.883470	9.511509	9.985804	9.580275	10.727633	9.903042	10.033478	9.875925	10.005847	9.954115	10.710268	9.564183	9.830470	9.974679	9.774780	9.921216	10.049864	10.092773	9.868678	10.526885	10.596344	10.932636	10.198139	10.787195	10.228061	10.347954	10.162595	11.127669	9.590255	10.197801	9.543140	10.102338	9.925044	10.125998	9.992041	10.040698	10.034063	9.713258	9.753141	9.747720	9.683095	10.085546	10.313861	10.612891	10.293707	9.780402	10.614231	10.665912	10.475430	11.073555	10.227130	10.313234	10.082544	10.305371	10.698977	10.511848	10.026868	10.296801	11.135945	10.208766	11.189187	10.694662	11.535407	10.463717	13.076263	11.067036	10.384236	11.144558	10.318904	10.429939	10.489668	10.222882	11.954825	10.341418	10.909711	10.434301	12.763614	10.613617	10.622189	10.269856	10.695729	9.579457	9.593696	11.526648	10.109727	9.811828	10.536771	10.687667	9.478766	9.429122	9.631529	11.147949	10.569539	10.385937	10.347205	11.028057	11.044439	10.543304	10.817596	11.385706	10.334644	10.535918	10.736083	9.882240	10.125091	10.000113	10.558564	10.363418	11.499049	10.325054	9.781184	10.135303	10.301273	10.363870	13.015183	9.688079	10.071163	10.051718	9.904261	9.543795	9.642396	9.821765	10.183009	10.848591	10.052486	9.561904	10.484576	9.444057	9.928472	10.749767	9.693154	9.733039	9.957526	9.947027	10.657396	9.895040	10.176378	9.275288	10.114066	10.443810	11.669538	10.380450	10.433959	10.138352	9.973551	10.632606	10.212263	9.898694	10.208469	10.111224	9.790792	10.047403	10.560714	10.978478	10.181458	10.399588	9.721234	9.404364	8.778712	9.480408	10.499570	10.308167	10.184839	10.711413	10.762508	11.185555	11.688705	9.900833	10.786329	10.333744	10.492998	9.987620	10.359626	10.619249	11.084092	9.737168	10.187555	11.202068	10.063627	11.410835	11.084241	11.344171	11.285928	11.333902	10.625962	10.261469	10.590228

TDO2	6.925494	9.179234	9.396827	7.214976	4.559441	7.326903	8.886682	6.703085	4.807861	7.336755	7.959604	6.541364	7.130688	5.451468	6.991455	7.555345	6.912945	7.592865	5.021782	7.714929	7.536246	8.146260	7.300641	6.634691	5.296253	6.321010	8.012742	6.867257	7.199002	9.775382	5.157073	8.625594	11.657028	11.373199	10.375909	9.186923	8.826560	7.021564	6.651258	5.297648	6.389857	7.069377	8.502465	8.812028	8.907835	9.513501	6.304424	8.073812	9.160000	9.604530	9.653700	8.839638	8.759647	9.405869	9.150489	7.247314	9.447171	7.674015	6.227132	7.881084	7.706992	6.946292	6.868396	6.036519	9.168451	7.476459	7.022655	7.627346	8.909943	6.654503	8.579823	8.093070	6.766039	8.624508	8.990170	11.221623	8.750517	11.208214	9.268311	9.030556	9.223579	9.308633	10.204454	8.444544	8.813641	8.515034	10.001321	8.442096	8.838748	8.798095	8.721472	9.543140	8.888851	9.817990	9.375129	8.812483	9.137318	7.558018	6.462351	5.152764	7.687645	7.233534	7.360270	6.407210	7.084910	6.868396	4.572211	7.276724	4.283381	7.034830	5.858327	5.404785	4.568951	6.174379	8.353838	7.073236	4.444333	7.405370	5.775099	10.548595	10.308324	10.546423	8.868537	7.650137	10.039141	8.581064	6.370383	9.449496	7.860630	10.160447	8.121469	7.048016	8.831633	10.678215	6.756526	11.901464	7.192295	6.352940	10.032710	9.014091	7.961776	8.284855	4.577328	6.029076	11.051205	10.700162	7.637847	6.107820	11.524427	8.031179	11.683647	11.997786	12.023884	10.693780	10.560091	11.314365	10.818042	7.914144	9.248587	9.674754	9.627387	8.071679	12.473452	10.758970	10.484345	8.924171	9.960448	7.591800	12.018886	12.198897	11.279290	10.223615	12.376294	13.032650	9.062616	9.469344	9.522722	8.478049	10.167210	10.185886	8.816517	9.852155	9.672092	8.800503	10.713301	10.093506	11.898984	11.235078	12.852645	11.080729	11.051341	6.667699	13.102943	11.904326	12.322814	11.610786	12.754773	10.277741	10.643734	11.838865	10.945949	12.996574	12.720748	13.675309	9.522722	12.481464	12.334126	10.301432	12.129478	8.810123	10.852730	10.343255	13.054008	11.854757	13.612808	10.257261	6.648197	10.543163	7.541435	11.001508	6.527835	7.135477	11.430160	7.905055	7.782275	4.383385	9.718945	8.014777	9.523327	8.822972	8.559931	3.575730	5.975168	7.978147	11.004703	10.321225	9.203107	10.191000

ZNF365	8.773990	13.486625	12.848215	13.881969	12.466727	13.575781	13.463591	14.914162	13.983511	13.880924	14.275219	13.773715	13.429511	13.024204	11.137430	14.545966	14.114654	13.242782	13.898972	14.318839	13.526138	13.493263	12.516941	13.641034	14.194332	13.241017	8.523862	14.369059	12.786674	11.571786	14.769473	12.272956	13.457219	8.540210	12.239628	13.098632	13.701651	12.990154	12.009153	12.704515	13.512856	13.223528	12.287847	13.083849	11.871132	13.257927	12.555591	12.117118	13.854876	14.084957	12.812473	14.176188	12.061208	13.446068	12.836529	12.848393	12.516941	13.448203	13.866495	12.617952	11.687212	12.231738	9.739940	12.905187	11.814644	12.364491	12.890172	13.254006	12.611838	12.400465	14.005012	13.713207	13.889925	13.295547	14.656363	12.748989	11.652428	12.546681	13.607340	12.750695	11.794389	13.094669	11.675300	13.548218	14.386005	13.297173	14.333069	14.096413	11.471518	13.314147	12.504062	12.460105	12.510296	13.905302	10.095036	12.449765	13.183285	14.440521	12.832557	13.951282	14.068626	12.765859	13.695182	13.583683	12.880949	13.195928	14.165841	14.025995	11.396394	13.902033	13.744601	12.992187	13.517777	14.030880	13.129172	11.880130	8.590817	11.887052	14.290687	8.862460	10.897614	10.656820	9.320905	9.644704	11.601212	9.659984	9.634169	11.033135	8.791209	11.124898	8.595641	11.419414	9.177266	8.922259	11.172697	10.171080	8.418259	8.941854	9.958930	9.301255	10.606321	9.245186	8.747097	11.417260	9.592273	11.276449	9.647368	9.004108	11.065101	9.537676	8.628667	10.570618	8.292759	9.185181	9.148011	10.834345	9.408019	10.115300	9.543317	9.197337	8.857582	8.215620	9.226978	9.733039	12.859356	10.885995	8.848183	9.995566	10.610648	10.023218	9.568749	9.961059	13.220765	11.158915	9.507776	10.082394	9.694205	9.894559	10.066763	9.114050	8.685237	8.685699	11.172387	9.259376	9.434621	10.849717	10.348574	9.633365	9.808072	9.802581	9.561136	11.635559	10.614503	10.948169	10.593040	11.062749	10.011528	12.747949	8.987649	10.859980	10.801645	9.596485	9.854790	9.890146	9.384818	10.487949	10.752512	10.424393	9.981975	8.585432	9.700346	9.399396	10.628861	8.859559	8.684477	9.621976	10.175472	8.871708	10.252055	10.985236	8.959155	9.996607	9.451553	9.767677	9.262432	9.991411	10.986598	10.709963	11.170020	9.952136	10.751156	8.530777	13.048973	8.680362	11.443526	8.953796	9.517921	10.525656

POU5F1	8.310071	7.197753	7.236582	7.515705	7.544535	7.880362	6.590635	8.104810	8.577147	7.044339	7.339745	7.456332	6.393714	6.560538	6.949978	7.371577	8.206040	7.695463	8.348256	7.360909	7.780760	7.992068	9.125313	7.752866	7.814612	7.778353	8.664392	7.991905	7.247314	6.922566	8.555588	6.884396	7.011554	6.986041	7.206128	7.254305	6.915752	7.134208	7.553771	7.900849	6.922265	7.819973	7.888897	7.641386	7.251051	7.506342	7.486173	7.503062	7.242261	7.452118	7.132916	7.234209	6.407869	7.012195	6.880869	7.203178	6.350277	7.216398	7.597944	7.373429	7.621653	6.843699	7.888897	8.307442	7.533908	7.825066	7.118530	7.535941	7.350547	8.190710	7.201165	7.297031	8.488606	7.514786	7.690876	7.800068	7.710341	7.666215	7.767780	6.976098	7.303657	7.098623	7.223728	6.939982	7.513578	7.717365	7.260304	7.712486	7.895271	7.861216	7.978147	8.578764	7.047856	7.369988	7.441756	7.494577	7.190930	7.335044	7.484898	7.189234	7.008344	6.852172	7.166846	7.199002	7.892947	7.182191	8.421803	7.855897	8.418725	7.169797	8.359426	8.419149	8.288718	7.770324	7.794622	7.841406	8.578178	7.558018	7.196156	7.636774	7.448561	7.229677	7.513898	7.701741	7.671162	6.851397	7.645874	8.190857	6.876695	6.974320	7.412455	6.488708	6.551816	7.486779	7.122273	8.035557	7.041120	8.284696	6.958927	7.657521	8.084927	8.907342	8.587096	7.822114	7.321681	7.469405	8.006397	7.760531	6.600872	8.426149	7.689354	6.858980	6.643709	7.105647	7.173838	7.023757	7.493966	7.707918	7.794465	6.973815	7.762198	7.190616	7.576687	7.115667	7.313699	7.780442	7.400216	7.573879	7.518041	7.066045	7.450146	6.861702	7.041746	6.467545	6.903040	6.287577	7.196624	7.604831	6.987331	7.849325	7.031101	7.828229	8.220853	7.775272	7.908800	7.560886	7.510460	7.522516	8.163679	7.245629	7.366705	8.206331	7.345496	7.465107	7.563186	7.788217	7.715526	7.915057	6.792834	7.036232	7.358723	7.469565	7.038741	7.380184	8.134737	7.475221	7.592559	8.027961	7.849606	7.810799	8.899769	7.344870	7.645719	7.824466	7.472188	7.758241	6.930332	8.003407	6.246435	7.263718	6.540510	7.691976	6.963727	7.595792	7.374236	8.453841	7.564260	8.317157	7.429995	8.448133	8.400970	7.939788	7.748877	7.477675	7.293816	7.978147	7.260903	7.020298

DDX19A	12.284248	11.908802	12.127025	11.964779	11.476270	12.112683	12.285047	11.683934	12.289514	12.137705	12.370197	12.220618	11.666419	11.755885	11.793617	11.701443	12.751039	11.498458	10.842407	12.145944	11.228140	10.814013	11.848352	11.209661	11.273685	12.192751	12.002055	10.584201	11.761456	12.546233	11.673261	11.824133	11.748029	12.690509	12.232669	12.383858	12.351035	11.638582	11.357531	11.538395	12.352896	11.596548	10.907937	11.845386	11.873246	11.997786	12.026155	11.238772	12.131274	11.991261	11.893812	12.067188	11.737491	12.479486	12.506387	12.476206	12.482306	11.462167	11.350393	11.500503	12.052224	12.653635	11.890938	11.581538	11.574486	11.734088	11.439898	11.659417	11.697270	11.713495	12.071265	12.283635	11.896055	12.133995	12.177525	12.064552	12.250230	12.223244	11.879850	11.411231	11.862156	12.411407	12.282409	12.115695	12.615500	11.690932	12.520730	12.118645	11.606533	11.299253	11.218485	11.530297	11.651535	12.226923	12.303112	12.145773	11.818403	11.744952	12.528710	11.976994	12.779001	11.496969	11.281507	11.611675	11.910004	11.978674	11.908569	11.815724	11.659417	12.010041	11.342184	12.088584	11.447635	11.690625	12.272493	12.997305	12.023884	12.520900	12.331442	12.614854	12.518010	12.689849	12.630986	12.584810	12.648843	13.099767	12.608022	12.478336	12.874855	12.922424	13.119650	12.688641	12.064690	12.273604	12.471493	13.212415	11.941052	11.625607	11.999293	11.463192	10.980857	11.623005	11.486239	11.551660	12.352431	12.009009	11.657747	12.198274	12.636117	12.116357	13.259393	12.927010	12.953485	11.965535	12.044097	12.474119	11.921437	11.818076	11.708338	13.541252	11.867133	10.871895	12.386589	11.959600	12.033988	12.365108	12.191183	12.395853	12.333973	12.430276	12.621991	11.888571	12.896328	12.895646	12.649718	12.688304	11.458920	11.753524	11.490551	12.887037	13.174008	11.384387	12.162658	12.162362	12.179561	12.117118	11.845084	12.857098	11.959150	12.740063	12.675695	12.520044	12.796960	12.448476	12.489908	12.856770	13.180160	12.456759	12.186990	12.360854	12.611352	12.995839	13.021352	12.799021	12.497186	12.641390	13.104834	12.295874	12.167248	11.674723	12.129331	12.611680	13.035629	13.207909	13.032476	12.800391	11.991560	13.124799	11.600742	13.386045	12.323571	11.603568	12.187947	12.168818	12.792841	12.326538	12.406965	11.335983	12.439669	11.829732	12.605068	11.542723	11.814644	13.237824	12.707057	12.257887	13.079309	13.313179

BRICD5	11.347880	10.553234	10.704200	10.551729	10.824581	10.977161	10.818334	11.301597	11.333338	11.357821	11.272950	11.213507	10.862192	10.947453	10.887345	10.682418	11.345799	11.134756	10.937605	11.266465	11.019313	10.799656	11.470178	10.766265	10.987504	11.232005	11.183899	10.743957	10.880179	10.977437	11.459334	11.083644	10.756929	11.260634	10.933084	11.047817	10.926976	10.578433	10.975234	10.781158	11.253098	9.918845	10.543883	10.968909	11.172697	11.320252	11.016201	10.921655	10.624904	11.025099	10.661777	10.935174	10.555843	10.673881	10.340048	11.127958	10.759975	10.530395	10.709659	10.749455	10.846814	11.069879	10.986908	10.800421	10.687667	10.478878	10.440817	10.200086	10.577682	10.496787	10.415417	10.428979	11.051912	10.586401	10.911045	10.603262	10.673572	10.378034	10.519091	10.132299	10.693912	10.579203	10.614651	10.658429	10.680668	10.749920	10.702431	10.895336	10.151883	10.405994	10.392965	10.694662	10.555984	11.011552	10.861911	11.079831	10.724471	10.935332	11.191490	10.591883	11.346523	10.261006	10.530253	10.598624	11.060112	10.906618	11.562171	11.248361	11.521285	11.110584	11.197241	10.916587	10.409222	10.663706	10.926976	10.758534	11.306046	11.210392	11.301597	12.382262	10.970001	11.003383	11.454144	11.811133	11.523246	11.328057	11.228716	12.071131	12.128287	11.929992	12.171733	11.944227	11.491308	11.904904	10.835737	11.568275	11.967186	12.131586	11.476828	11.419262	11.501471	12.589114	11.034889	12.295577	11.774016	10.842875	11.932596	11.733626	11.565808	10.986739	11.367114	11.451631	11.713779	11.015445	10.961939	10.826070	10.871585	11.408599	11.185403	12.243771	10.000412	11.157450	11.660588	11.671357	11.485014	11.554168	10.881641	11.223409	11.448524	11.027337	11.568120	11.266027	11.052955	10.856874	11.425803	11.427104	10.948169	11.179610	11.287619	11.179761	11.545901	10.692399	11.112884	10.954841	11.482953	11.029265	10.581882	11.456110	10.918229	10.936085	10.831184	12.072330	10.912497	10.944597	10.849578	11.056587	11.205234	10.909438	10.361591	11.013967	10.877643	10.874572	10.943606	10.871585	11.391756	10.959734	11.893953	10.595005	11.009628	11.571927	11.340088	10.902471	11.381737	11.527615	11.478556	11.186851	11.169751	11.231711	11.024507	12.099062	10.904059	11.013251	10.889665	11.494786	11.967186	11.967801	11.804233	12.054237	11.760858	12.087710	10.757949	10.451937	11.160805	11.364157	11.405727	11.632692	11.351751	12.356727

MLST8	11.347880	10.553234	10.704200	10.551729	10.824581	10.977161	10.818334	11.301597	11.333338	11.357821	11.272950	11.213507	10.862192	10.947453	10.887345	10.682418	11.345799	11.134756	10.937605	11.266465	11.019313	10.799656	11.470178	10.766265	10.987504	11.232005	11.183899	10.743957	10.880179	10.977437	11.459334	11.083644	10.756929	11.260634	10.933084	11.047817	10.926976	10.578433	10.975234	10.781158	11.253098	9.918845	10.543883	10.968909	11.172697	11.320252	11.016201	10.921655	10.624904	11.025099	10.661777	10.935174	10.555843	10.673881	10.340048	11.127958	10.759975	10.530395	10.709659	10.749455	10.846814	11.069879	10.986908	10.800421	10.687667	10.478878	10.440817	10.200086	10.577682	10.496787	10.415417	10.428979	11.051912	10.586401	10.911045	10.603262	10.673572	10.378034	10.519091	10.132299	10.693912	10.579203	10.614651	10.658429	10.680668	10.749920	10.702431	10.895336	10.151883	10.405994	10.392965	10.694662	10.555984	11.011552	10.861911	11.079831	10.724471	10.935332	11.191490	10.591883	11.346523	10.261006	10.530253	10.598624	11.060112	10.906618	11.562171	11.248361	11.521285	11.110584	11.197241	10.916587	10.409222	10.663706	10.926976	10.758534	11.306046	11.210392	11.301597	12.382262	10.970001	11.003383	11.454144	11.811133	11.523246	11.328057	11.228716	12.071131	12.128287	11.929992	12.171733	11.944227	11.491308	11.904904	10.835737	11.568275	11.967186	12.131586	11.476828	11.419262	11.501471	12.589114	11.034889	12.295577	11.774016	10.842875	11.932596	11.733626	11.565808	10.986739	11.367114	11.451631	11.713779	11.015445	10.961939	10.826070	10.871585	11.408599	11.185403	12.243771	10.000412	11.157450	11.660588	11.671357	11.485014	11.554168	10.881641	11.223409	11.448524	11.027337	11.568120	11.266027	11.052955	10.856874	11.425803	11.427104	10.948169	11.179610	11.287619	11.179761	11.545901	10.692399	11.112884	10.954841	11.482953	11.029265	10.581882	11.456110	10.918229	10.936085	10.831184	12.072330	10.912497	10.944597	10.849578	11.056587	11.205234	10.909438	10.361591	11.013967	10.877643	10.874572	10.943606	10.871585	11.391756	10.959734	11.893953	10.595005	11.009628	11.571927	11.340088	10.902471	11.381737	11.527615	11.478556	11.186851	11.169751	11.231711	11.024507	12.099062	10.904059	11.013251	10.889665	11.494786	11.967186	11.967801	11.804233	12.054237	11.760858	12.087710	10.757949	10.451937	11.160805	11.364157	11.405727	11.632692	11.351751	12.356727

CTC1	8.562835	10.266592	9.887831	9.735158	10.359015	10.983770	12.449101	10.575556	9.549294	11.211916	10.237139	9.859301	9.025867	9.400900	9.432816	11.979444	9.679857	9.516071	10.447955	11.127958	9.997492	10.698366	10.425899	10.189508	10.120667	10.028112	10.290374	10.550859	10.580846	9.667179	9.508697	8.982281	9.654697	9.075301	9.099653	9.267059	9.834744	9.607163	9.870258	10.094705	11.717338	9.727449	9.568410	10.261627	9.942884	10.942565	9.892413	9.832315	9.769058	10.672708	9.340883	9.206427	9.269391	9.893017	9.630914	10.450613	9.664186	9.945742	9.634765	9.588428	9.592749	10.958111	9.132598	10.094546	9.483039	10.022591	9.823884	9.812289	9.710316	10.335092	10.614085	11.913078	11.003790	10.900969	10.431883	9.811988	10.346434	9.409712	10.414470	8.958107	9.579952	9.458339	9.180782	9.815890	10.418283	9.723922	9.257116	9.969420	9.794095	9.798602	9.669565	9.714055	9.544844	10.129307	8.928332	9.711867	9.616838	9.168297	8.958268	9.692546	8.463077	9.995566	9.926621	9.846915	10.008945	10.423303	9.811338	10.121247	10.171699	12.008851	10.538071	9.845661	9.594008	11.909110	10.466097	8.505652	9.193762	10.292644	10.142149	8.721185	9.097512	8.259100	9.283708	9.665455	8.948886	9.944985	7.584112	7.770961	9.006123	8.822037	8.799005	8.574419	8.005597	8.792288	9.989367	8.072444	9.633519	9.190862	8.354284	9.637166	10.133901	10.014151	8.896413	7.959136	8.631915	8.921205	9.055161	8.701668	8.774914	8.862612	8.802752	8.306186	8.855027	8.892677	8.138502	8.589300	8.646046	9.196444	8.479812	9.539063	8.937335	9.118187	9.176364	8.627465	10.232800	9.288025	7.937295	8.488606	8.590205	8.667128	8.514294	8.741801	8.905075	8.615418	8.110742	9.427430	9.508397	9.241228	8.844814	8.811877	9.409712	8.171919	8.922699	9.481658	9.088836	8.082398	8.533473	8.537676	9.125660	8.777966	9.088211	9.591661	9.019117	8.628513	8.777234	8.376467	8.541658	9.215294	8.316530	8.707378	9.077786	8.632336	8.687887	8.861523	8.591616	9.081348	8.462482	8.911416	8.455824	8.256785	8.795216	9.049134	8.458444	8.360170	8.426610	8.769297	8.155622	8.249893	8.765596	8.092765	9.017912	8.539743	8.406973	8.372078	9.313619	9.500267	9.177565	9.442955	9.356962	8.873253	8.504625	8.086591	12.114054	7.811870	8.946123	8.640199	9.418385	9.244700

NNT	11.021387	9.736555	9.788049	9.913595	9.512448	9.346035	10.061198	9.063980	9.317184	9.483198	9.657788	9.537204	9.599406	9.301568	9.844563	9.774925	9.854153	9.620259	9.993987	10.070538	9.846139	10.261948	11.851271	9.598341	10.058151	10.046320	10.433959	9.722173	9.521928	9.843353	9.640574	10.265145	10.127098	10.631433	10.453574	10.382887	10.324283	9.822386	10.064516	10.193091	9.670545	10.704907	9.622457	10.457252	10.824286	10.479190	9.734386	9.936190	9.896278	10.371089	10.433224	10.496309	10.525793	10.484421	10.118987	9.784596	10.587164	10.786914	10.523853	10.761899	10.790142	9.725155	11.237709	10.460708	10.802701	10.300966	10.208000	10.039433	10.009427	10.411451	10.125679	10.156965	9.384364	10.208469	10.362213	10.171080	10.446580	10.144003	10.147640	10.090076	10.150370	10.482463	10.696036	10.487949	10.864236	10.237139	10.566949	10.668143	10.820570	10.383504	10.719260	10.733421	10.449732	10.148232	10.305371	10.082394	9.844727	9.996310	9.886402	10.193244	9.765569	9.748635	10.029801	9.766931	10.081641	9.402013	9.679564	9.740723	10.191897	9.572296	10.413447	9.899145	9.713258	9.380804	9.241228	8.590205	11.200874	9.650396	9.650895	9.279051	9.411694	10.646057	10.696919	9.056876	10.168122	9.344383	11.343861	9.152937	10.913094	9.965297	10.643415	9.860520	10.887915	10.269429	10.656389	10.638038	10.603714	10.195238	11.445862	10.304483	10.278521	11.426951	11.920823	10.758822	9.978579	10.263600	10.231313	10.683258	10.601053	10.521409	10.003213	10.678806	10.291731	11.368306	10.971013	10.534840	10.951630	10.091582	10.106142	9.692997	10.915674	9.143508	10.753411	10.060156	10.320132	8.831633	10.909294	10.840635	10.121103	10.241972	11.008580	10.902031	10.365777	11.015894	10.880179	11.305742	11.555370	10.606813	11.482953	11.062917	10.704907	11.585010	10.359626	11.580850	10.644156	10.650447	9.962091	10.352513	11.248223	10.178593	10.727937	9.816798	10.067514	10.655933	10.530093	10.781758	10.733574	10.824286	10.501539	10.704762	11.583331	10.539877	10.843773	11.858431	11.152759	10.628696	11.259294	11.525172	10.947309	12.190447	11.331478	11.013251	10.507667	10.507361	9.829982	10.754711	10.505710	10.162874	11.181871	11.170020	11.134182	11.863940	9.982906	12.365253	8.959927	10.585809	10.376060	10.398683	8.896560	10.931284	10.149179	9.698334	9.561303	11.050179	10.164877	10.345226	9.533298	11.030014

RPL8	16.815163	15.667217	15.359530	15.585396	16.697813	15.293858	16.496735	15.455098	15.086549	15.423337	15.910549	15.579100	16.292480	16.152946	16.487541	16.259687	15.650841	16.124210	15.971905	15.480428	16.137062	16.101458	16.409990	16.487541	15.066359	15.201679	15.081715	15.069269	16.181433	15.816498	16.088816	16.246616	16.321552	15.172005	15.572722	16.379042	16.383390	16.629840	15.637542	15.498917	16.387390	15.029900	15.392983	15.879245	15.678640	16.079155	15.642232	16.234936	16.024403	15.702691	16.290771	16.168373	14.581385	15.168107	15.571867	15.708514	16.178602	14.758705	16.315840	15.605713	16.329145	15.733274	16.264810	15.142982	15.874614	15.081140	15.598287	16.206017	15.156386	16.476725	14.384169	16.029650	16.064222	15.794025	16.036135	15.781709	15.066359	13.913967	14.359381	15.950994	16.187492	15.241129	15.583728	16.997234	15.628044	16.204400	15.610464	15.048970	16.435179	15.955729	15.813386	15.789877	15.916773	14.060010	15.410836	16.564795	16.433023	16.119818	16.130013	15.578257	15.999973	16.707941	16.536428	15.768020	15.571867	16.363316	16.312078	15.882641	16.210808	15.901055	16.420473	16.086016	16.165237	16.463480	14.776581	14.738602	16.105830	16.385298	14.614415	16.220278	16.659725	16.134222	16.740880	16.128614	16.807646	16.184460	16.313885	16.441525	16.585910	16.593935	16.707941	16.562284	16.391445	16.383390	15.919342	16.773335	16.551607	16.865778	16.175634	17.021223	16.489765	16.947470	16.281614	16.789194	16.681759	16.134222	16.591137	16.178602	16.467780	16.207472	16.264810	16.489765	16.917430	16.823927	16.441525	16.268140	16.234936	16.678432	16.723978	16.319695	16.685246	16.160600	16.605293	16.259687	16.678432	16.370743	15.968277	17.113525	16.361326	16.278273	16.107193	16.714243	16.556694	16.748238	16.551607	16.585910	16.405563	16.213991	16.401115	16.223639	16.629840	16.492130	16.650790	16.607860	16.580235	17.273415	16.492130	16.635635	15.930069	16.831787	16.730795	16.331210	16.727613	16.334866	15.891807	16.445545	16.531399	16.456985	15.846445	16.182924	16.481123	16.452541	16.023191	16.985483	16.815163	17.034716	16.874939	16.511406	16.418365	16.531399	16.701464	16.207472	16.546406	16.478815	16.059162	16.359390	16.226917	16.511406	16.348005	16.395147	16.002806	16.323317	15.836242	16.827810	16.041613	15.927561	16.835817	16.870018	16.253204	16.762688	16.412035	16.474625	15.976933	16.523640	16.287200	16.452541	16.902527	16.902527

CEBPA	12.996574	12.742681	12.628339	12.360524	12.418166	12.882408	12.980619	12.878188	13.022170	12.761697	12.720580	12.866895	12.391429	12.336035	12.727241	12.658790	13.172674	12.048596	12.530199	12.634450	11.811570	11.565952	11.580422	12.010648	12.229725	12.487307	13.134544	12.813342	13.095058	13.717240	12.798022	12.308229	12.182961	12.698531	12.663392	12.423121	12.506071	12.417211	12.314304	12.304945	12.743706	11.872205	12.004567	12.148544	12.318625	12.227205	13.228076	12.427666	13.336430	12.292362	12.020355	12.338408	12.612867	12.435481	12.796632	13.384972	12.246238	11.978531	11.588048	11.810382	11.686921	13.796688	12.451069	11.861116	12.369887	12.036393	11.854304	12.216379	11.997786	11.891870	13.049903	12.492085	13.034304	12.935163	12.565623	12.307156	12.673286	13.250280	12.957891	12.266082	12.123909	12.988336	11.599324	12.336677	11.747749	12.255523	11.812582	11.976069	11.448235	11.171227	10.938913	11.307098	12.415286	12.796960	12.625864	12.256329	13.157844	12.438343	12.676852	12.697321	12.929031	12.705525	12.532342	12.656999	11.542114	13.195709	12.419600	12.112840	11.885548	13.038781	11.360757	12.873920	12.558955	13.285244	13.113147	13.334586	12.206975	12.875731	12.973655	12.466808	12.492085	13.049159	12.873210	13.564577	14.011503	13.916329	12.744905	13.056733	13.531252	13.356586	13.614714	13.364551	12.676682	12.244533	13.857811	12.633304	12.672242	12.381631	12.507711	12.055881	12.309839	11.722280	12.895995	12.585646	12.863485	13.362873	13.384372	14.138248	13.479756	12.578352	13.280056	12.534127	13.757627	12.673598	13.174008	12.871760	12.366548	12.538424	12.657324	13.676917	13.478168	11.795725	13.148247	12.674134	12.497991	13.701386	11.896350	13.119840	12.701928	12.454528	12.026457	12.516782	11.965086	12.448174	13.605949	11.279887	12.183622	12.017038	11.547384	12.463731	13.991023	11.716588	12.647084	12.461401	12.820259	12.727241	12.571685	12.870321	11.790869	13.395784	13.684785	14.019002	13.137385	13.334586	13.502805	12.610024	13.225296	12.934551	11.588775	11.838131	12.595766	12.461245	12.517113	12.475535	12.899883	12.379588	12.412844	11.660731	11.815724	10.915826	11.629239	12.373219	13.765490	13.303085	13.238255	12.922949	13.045174	12.609687	13.361828	12.883847	12.612867	13.006514	13.150661	11.513485	13.616838	13.078187	11.825933	12.045007	13.946220	11.775784	13.424902	13.213390	13.680915	12.937259	13.377562	13.283632	13.130178	14.038211

ZEB2	13.682594	13.352048	13.619020	13.543518	12.697692	12.234983	13.800082	11.971127	11.458769	12.992530	12.356099	12.552169	12.986468	12.514995	13.393505	12.992354	13.170952	13.384372	12.539475	12.436293	12.869775	13.283833	15.194873	13.006158	14.512793	13.098440	14.187463	13.104067	13.801126	13.656748	12.929565	13.384372	13.152612	13.433879	14.559252	14.032590	13.826360	13.908273	14.162045	13.752811	13.356199	14.927607	14.906627	14.075445	15.397686	14.408016	13.123620	13.721765	12.950183	14.157670	13.579900	14.046420	14.800437	14.003265	13.277634	13.049719	14.749773	14.691959	13.484139	14.526310	14.448739	13.858854	15.372489	14.232213	13.924569	13.791984	13.909402	13.557270	13.318885	13.746570	14.046714	13.672469	13.145522	13.648013	13.147264	13.588156	13.978346	13.478600	13.407160	13.295547	13.040426	13.947918	13.647062	13.403139	14.237798	13.296569	13.536412	13.145329	14.084344	12.804235	13.917175	13.902528	13.079675	13.340998	13.285661	13.332700	13.510177	13.748849	12.937441	14.229504	13.003596	13.791726	13.492817	13.061923	12.656681	13.165894	13.467719	13.199300	14.034659	13.277050	14.066316	12.498635	12.041809	12.916404	11.922470	12.631817	13.958500	13.523692	13.592174	12.368752	12.262621	13.156161	13.088340	12.236220	12.557817	12.219537	12.705525	12.386292	11.947128	12.106547	11.766034	12.172013	12.382100	11.749526	13.250094	12.615500	12.082273	14.591979	13.124576	12.246555	13.435563	13.269247	11.840676	12.480123	14.119553	11.950501	11.738357	12.471970	12.848047	12.775154	13.099767	13.009396	11.794980	12.631485	11.798061	13.079675	12.719193	11.856573	13.754404	11.791350	13.129753	13.609219	12.622821	12.839626	12.109069	11.927550	12.404510	11.928150	12.700895	12.683325	12.566429	12.299522	12.733879	13.713891	12.382585	12.137705	12.577366	12.111007	12.417049	12.894551	13.258986	12.621510	12.871760	13.019185	13.181119	12.314612	12.746407	12.395385	12.780890	12.235907	12.539964	11.123080	12.672419	13.479517	12.862405	13.002160	12.842437	12.200477	12.761874	12.327798	11.526648	12.164845	13.598384	13.017369	12.416425	12.678191	12.137094	13.621985	13.498262	12.329402	12.613710	12.674301	13.000090	12.592652	13.400180	12.356888	13.131145	12.170476	11.651384	12.539475	11.540839	12.163756	13.367473	12.813784	12.284406	12.274399	11.859202	11.988461	11.880421	13.670083	13.125202	12.091525	12.373219	11.510541	11.370978	13.491281	10.979653	12.166942

GPR174	8.743900	8.060419	8.042970	8.764687	7.612184	8.382521	8.398367	7.952940	8.658677	7.198298	7.643516	7.728413	7.661380	6.298576	7.352063	8.632470	7.809278	8.029503	6.965162	8.974524	6.754571	9.895662	7.594883	8.330575	8.669282	6.699191	10.943468	6.164262	8.268700	9.904987	7.618023	8.957952	10.522517	9.862229	9.224967	9.018218	7.388729	7.875198	7.020771	8.723135	6.629846	8.347074	10.349010	9.262763	8.948154	9.613194	7.053640	7.982701	8.897960	8.945521	9.014694	8.385714	9.248281	8.769760	8.446387	6.635160	9.128435	8.734872	7.514055	8.836326	7.605001	6.581803	7.132134	8.414441	8.252957	7.649794	7.783772	8.359426	8.302103	8.010187	7.978147	8.337977	7.970993	8.979397	8.450156	9.726848	8.660064	9.002124	8.174113	7.804165	8.587856	8.609545	8.973481	8.188620	8.738106	9.177093	9.088375	8.027463	8.045439	8.189101	8.311463	8.251460	7.893878	8.414729	9.522255	9.864846	9.480095	7.422117	7.122578	7.640482	6.165098	8.415321	8.095215	7.734740	8.037734	8.055590	7.846238	8.640367	6.549863	7.254455	7.755464	7.013009	8.445308	8.191644	9.559999	4.891867	8.188314	7.463419	7.748566	9.406178	7.120438	8.887442	9.465805	5.471198	6.936495	8.771152	6.855251	7.698229	5.553982	5.464428	5.736968	3.627218	8.611216	5.122508	7.496941	7.591800	6.066254	8.109811	9.722952	5.652389	8.180838	8.400333	5.035276	5.252360	6.343881	7.867093	5.498320	6.942960	9.895971	8.838304	9.301890	7.294607	6.507640	9.493310	7.000313	7.867736	7.203798	6.087475	8.098253	4.069243	7.700373	9.312969	7.000485	7.747952	8.585291	7.258223	7.938844	7.443943	7.580071	8.361892	8.773068	9.448101	6.963071	8.688503	8.485224	8.063661	8.025265	8.224937	8.243837	6.894955	8.367601	7.091028	8.866433	9.335569	8.294418	7.552684	7.977373	7.095641	8.820691	8.341313	7.772016	5.666656	7.326903	9.799812	9.472328	7.577303	7.500092	7.083005	7.003383	7.490867	7.678371	7.061882	9.439867	8.531228	7.423979	7.981918	8.094336	8.412918	8.360170	6.493772	7.632856	6.591797	7.173677	7.513425	8.017257	6.960043	6.387306	4.997562	5.275956	7.562431	6.295933	6.384336	8.813954	7.020298	8.002620	7.249008	7.884890	7.245923	5.913125	8.402976	6.910328	6.245437	9.139031	6.137868	6.911468	7.248685	6.472574	7.179683

ICA1L	8.064117	7.169943	8.015555	7.733023	7.936532	6.769936	8.242495	5.409120	6.249967	7.755152	7.178605	7.615270	7.732580	7.422597	7.930949	7.010274	8.118397	7.771722	7.733023	7.515705	7.051012	7.045294	10.383504	7.288966	8.657895	7.696834	7.842803	7.352207	7.863716	7.752132	6.917722	8.273134	7.611728	8.303042	8.428664	8.413235	7.950628	8.443325	8.171607	8.150381	7.368431	8.020889	8.246658	7.946637	9.215763	8.073510	7.841085	7.735656	8.303938	8.197496	8.461565	8.458776	8.441329	8.211601	8.863223	8.126601	8.912646	8.102649	7.778689	8.259246	7.896881	8.156894	9.969266	7.781047	7.702677	8.442840	7.862606	7.489903	7.171656	7.753321	7.921218	7.719036	7.113933	7.718569	7.557379	7.606389	7.789766	7.693526	7.576516	8.109085	8.075632	8.764520	8.145020	8.752885	8.963871	8.170037	8.233424	8.559151	8.130266	7.530523	8.083179	7.795089	7.827449	7.977093	7.807549	8.342612	7.974469	8.386497	7.345155	8.329193	7.491660	8.087562	7.830932	7.392271	8.156082	7.521929	7.364513	7.891853	8.100710	7.570665	7.359312	7.760052	7.379718	6.844206	6.892837	5.165881	8.129022	7.937139	7.394443	7.491814	6.359358	7.206445	6.931597	5.416410	7.107577	7.659388	7.864325	5.017906	7.935579	7.029566	9.551464	9.218991	7.995024	6.275058	7.561665	7.269393	8.475419	10.645900	9.695127	6.817315	9.172647	9.301410	8.241603	9.053453	7.971462	7.477847	8.040118	9.127646	8.426298	6.202793	6.112145	7.352354	7.277044	7.214181	9.334439	7.291287	8.686278	5.004573	6.794294	5.833332	8.398367	7.711380	7.616815	8.778562	8.065658	5.937556	8.685533	9.612716	7.180642	7.551436	8.521422	9.258777	7.278770	8.807508	9.107183	8.586509	8.780705	8.259547	9.611512	6.329915	8.042316	7.293155	8.531825	7.702677	7.564103	8.729876	7.090725	6.225380	6.054977	8.700270	8.297767	6.798955	7.518967	7.573089	7.494731	7.414619	8.083021	7.459188	8.262615	8.220715	7.375318	7.584267	8.250212	9.277548	6.324927	6.918525	9.194946	7.988666	7.955121	8.816369	5.866298	7.621041	7.238826	6.992917	7.048804	8.297298	8.475419	5.766251	9.278447	8.239177	7.545491	7.434866	7.262516	9.350193	8.135109	6.723695	7.493510	9.047586	5.133626	9.208101	7.597468	8.059483	6.048901	8.668648	3.641701	8.630532	5.863467	5.865214

TMEM8B	10.994167	9.691309	9.617475	9.432029	9.432029	8.792916	10.026576	8.347074	8.712662	9.373989	9.749429	9.894722	8.953479	8.537385	9.459408	9.623069	9.656579	9.142130	10.030841	9.765726	9.307595	8.208900	11.477113	8.923429	10.501830	9.979337	10.070060	10.349760	9.881488	9.779222	9.506119	10.247122	10.162156	10.043446	9.843788	9.956160	10.162595	10.316666	9.655960	8.851059	9.597565	10.119137	9.431088	9.995272	10.603402	9.886873	9.834586	10.240898	10.407834	10.082987	10.589660	10.521409	10.344001	10.415417	10.450933	9.431886	10.401542	10.006922	10.318748	10.221095	10.354903	9.486434	11.279290	9.936051	9.699238	9.905763	9.701888	9.525226	8.969270	9.533695	9.849294	9.579952	9.295221	9.851679	9.505969	10.080427	9.805002	9.935758	10.199467	10.181167	10.073145	10.539729	10.794238	10.761000	10.541527	10.709373	10.413733	10.368508	10.691964	10.759120	10.657698	10.138805	9.514711	10.192816	10.498431	10.118547	10.497826	9.651799	9.493310	9.426499	9.052510	9.360960	8.728842	9.220963	9.041774	9.605311	7.945245	8.990467	9.512448	9.173114	9.672692	8.945521	8.705039	8.525523	9.560491	8.748329	10.442286	9.928779	9.091750	9.709371	9.424072	9.243810	9.574283	8.182493	7.752712	8.940339	10.069634	8.546108	8.772165	9.635073	9.744429	9.115417	8.300469	9.211143	9.593696	9.737762	8.800956	9.483198	11.097929	8.549523	8.723135	9.584068	9.034328	11.195935	11.039883	8.933630	9.005162	10.124419	9.754711	8.603917	9.542207	10.831970	8.742540	9.789131	9.582066	10.355061	9.176798	8.975910	9.540974	9.126126	8.718269	10.149488	9.292754	10.079015	9.700800	7.648147	9.565885	10.680518	9.400437	9.080721	10.422096	10.423303	9.789901	10.335694	10.069634	10.802991	9.994317	9.074979	9.511023	9.346648	10.275470	9.446852	8.975406	9.523327	9.469018	8.541974	8.924171	8.066258	11.625150	11.666726	9.580724	8.938682	9.847374	9.705663	9.222961	10.436727	10.320280	9.746469	10.630708	9.423296	8.903386	10.381196	10.310359	10.363564	9.544844	9.357865	10.992701	10.409077	9.906208	9.481049	10.067980	9.348012	9.574876	8.912971	8.957620	9.635491	9.903042	9.232049	10.218780	9.186753	9.478926	10.294312	9.108128	9.261349	8.799005	8.287945	9.835054	9.751741	8.346473	9.918999	8.563436	8.775967	7.406955	9.890580	9.350764	8.201973	9.064439	8.608029

RAB5IF	13.198527	14.071191	15.068646	14.435274	14.607132	14.636141	14.680064	14.297708	14.487150	14.776105	14.620267	14.655466	14.884408	14.634805	14.092821	14.808644	14.318504	14.697958	14.336137	14.667309	14.447934	14.398878	13.093127	14.752176	15.345704	14.366251	12.456129	14.952229	13.995849	14.052401	14.510745	14.395843	14.576819	13.772438	14.094594	14.160203	14.714408	14.629537	14.454290	14.773706	14.744545	13.860196	14.387085	14.284178	13.518422	14.157045	14.495949	13.586012	14.653700	14.092821	13.913198	13.653197	14.572654	14.764339	14.443066	14.394753	14.605414	14.393646	13.886730	14.173200	13.973052	14.636141	12.461548	14.503811	13.766757	14.215623	13.888605	14.329679	13.706318	13.961354	14.014100	14.513547	14.664987	11.807078	14.379181	14.323349	14.093399	13.970725	14.022985	14.075110	14.816832	13.954342	13.700662	15.154557	15.095711	14.168428	14.897059	14.983442	14.192430	14.446360	14.403999	14.198488	14.410971	14.640937	13.662485	14.115590	14.298393	14.354835	14.624663	14.034659	14.811115	13.901234	14.369760	14.080347	14.542200	14.548933	14.408756	14.298742	13.220952	14.794550	14.266988	14.581809	14.759178	14.763822	15.016274	13.985572	12.653975	14.284178	14.769473	14.146475	13.994733	13.496916	14.655036	14.251440	14.194016	13.867009	12.477354	13.514012	14.727873	13.582997	13.795129	13.897480	13.637147	13.910238	13.443956	13.903326	13.141902	13.351805	13.617769	14.446360	13.761927	14.710296	12.561046	13.872088	14.198163	14.475183	13.795903	13.851196	14.236540	13.253022	13.922688	13.691689	13.346771	13.600808	13.948216	13.642281	14.072083	13.501109	13.645181	14.096413	13.893951	12.936194	13.605717	14.234212	14.042877	13.911868	13.464192	14.125236	13.729901	13.876070	14.702065	13.883013	13.435155	13.598139	14.679596	13.329225	13.295754	13.742058	13.186053	13.637843	14.149795	14.069818	13.359500	13.722244	13.620619	13.501741	13.480608	13.194312	13.944829	13.492583	13.941517	13.911605	15.703652	13.266997	13.373992	13.910783	13.697330	13.478390	12.703486	13.918854	14.537158	14.499877	13.608041	14.383451	14.192430	14.257321	14.287951	13.204172	13.489728	13.211853	13.936877	12.944248	13.613516	13.762967	13.837460	14.345286	13.254196	13.582520	13.615908	13.612082	12.550390	12.485499	13.189322	13.280266	13.873413	13.210542	14.006174	14.492765	13.433260	13.492149	13.600149	13.589804	14.274531	14.396563	14.551021	14.277280	15.038723	13.564810

SLA2	13.198527	14.071191	15.068646	14.435274	14.607132	14.636141	14.680064	14.297708	14.487150	14.776105	14.620267	14.655466	14.884408	14.634805	14.092821	14.808644	14.318504	14.697958	14.336137	14.667309	14.447934	14.398878	13.093127	14.752176	15.345704	14.366251	12.456129	14.952229	13.995849	14.052401	14.510745	14.395843	14.576819	13.772438	14.094594	14.160203	14.714408	14.629537	14.454290	14.773706	14.744545	13.860196	14.387085	14.284178	13.518422	14.157045	14.495949	13.586012	14.653700	14.092821	13.913198	13.653197	14.572654	14.764339	14.443066	14.394753	14.605414	14.393646	13.886730	14.173200	13.973052	14.636141	12.461548	14.503811	13.766757	14.215623	13.888605	14.329679	13.706318	13.961354	14.014100	14.513547	14.664987	11.807078	14.379181	14.323349	14.093399	13.970725	14.022985	14.075110	14.816832	13.954342	13.700662	15.154557	15.095711	14.168428	14.897059	14.983442	14.192430	14.446360	14.403999	14.198488	14.410971	14.640937	13.662485	14.115590	14.298393	14.354835	14.624663	14.034659	14.811115	13.901234	14.369760	14.080347	14.542200	14.548933	14.408756	14.298742	13.220952	14.794550	14.266988	14.581809	14.759178	14.763822	15.016274	13.985572	12.653975	14.284178	14.769473	14.146475	13.994733	13.496916	14.655036	14.251440	14.194016	13.867009	12.477354	13.514012	14.727873	13.582997	13.795129	13.897480	13.637147	13.910238	13.443956	13.903326	13.141902	13.351805	13.617769	14.446360	13.761927	14.710296	12.561046	13.872088	14.198163	14.475183	13.795903	13.851196	14.236540	13.253022	13.922688	13.691689	13.346771	13.600808	13.948216	13.642281	14.072083	13.501109	13.645181	14.096413	13.893951	12.936194	13.605717	14.234212	14.042877	13.911868	13.464192	14.125236	13.729901	13.876070	14.702065	13.883013	13.435155	13.598139	14.679596	13.329225	13.295754	13.742058	13.186053	13.637843	14.149795	14.069818	13.359500	13.722244	13.620619	13.501741	13.480608	13.194312	13.944829	13.492583	13.941517	13.911605	15.703652	13.266997	13.373992	13.910783	13.697330	13.478390	12.703486	13.918854	14.537158	14.499877	13.608041	14.383451	14.192430	14.257321	14.287951	13.204172	13.489728	13.211853	13.936877	12.944248	13.613516	13.762967	13.837460	14.345286	13.254196	13.582520	13.615908	13.612082	12.550390	12.485499	13.189322	13.280266	13.873413	13.210542	14.006174	14.492765	13.433260	13.492149	13.600149	13.589804	14.274531	14.396563	14.551021	14.277280	15.038723	13.564810

MTPAP	12.175939	11.209109	12.440621	11.485014	11.949752	11.606238	12.085596	11.594601	11.591314	11.729757	11.559388	11.613750	11.042993	10.943897	11.138168	11.486562	11.758435	11.272222	10.211080	12.105127	11.048986	10.249259	10.961497	10.818648	11.719718	11.732276	11.275872	10.690865	11.250783	11.589947	10.809966	11.945613	11.933343	12.152049	12.149735	12.459627	12.357030	12.716965	11.159054	11.202837	11.637244	10.669700	11.331890	12.344708	12.443364	12.732190	10.829597	11.139619	12.349434	12.452025	12.128571	12.351946	12.315592	12.366707	12.230037	11.637393	12.504387	10.697641	11.003383	11.421024	10.669700	11.829273	10.627462	10.380154	11.426062	11.475428	10.499276	11.309982	11.360018	10.742742	12.018571	12.257719	11.385408	12.004288	12.185267	11.913228	12.434966	11.788817	11.977304	11.171363	12.581640	12.341032	12.427666	12.676852	12.607232	12.190585	12.421501	12.463731	11.604311	11.386601	11.513628	11.318368	11.142960	12.228964	12.076036	12.411884	12.020782	11.582863	12.167414	10.916445	11.840676	11.078266	11.379157	10.660852	11.148096	11.645473	11.028666	11.502190	11.108364	11.401919	11.459062	10.132585	11.531239	11.431202	11.930928	11.378139	11.149440	12.182658	11.287619	12.679396	11.037267	12.736267	11.665355	11.632395	12.054695	11.669088	12.435481	12.039995	11.397622	11.451777	11.313643	11.139480	10.633501	10.552052	11.900732	12.661199	10.703580	11.145288	12.253731	11.325255	9.984426	11.723465	10.141980	13.083849	11.665066	12.103282	12.799365	11.815724	11.801189	11.992915	12.002488	11.613158	12.679396	12.515666	12.417994	12.136934	13.058425	11.488021	11.389835	11.949288	11.894129	12.286301	12.525104	12.660501	12.972314	11.967332	12.120508	13.057276	12.908020	12.367209	12.432750	12.639025	12.818889	12.461245	11.136230	12.910540	10.316937	12.030339	11.104875	11.469270	12.000249	10.306115	10.142149	12.108644	11.428261	10.761738	11.571323	12.128287	12.104382	12.322965	12.032756	11.378421	12.469069	12.454210	12.086204	12.332724	12.973105	12.119577	10.906300	12.022667	13.201614	12.018090	12.068295	12.234514	12.124499	12.831338	13.309969	11.714992	11.704895	12.047981	11.940596	11.371738	11.961736	12.097869	11.906365	13.332262	11.625463	12.566268	12.099378	11.143986	11.357821	12.530199	10.467258	10.750836	12.279446	11.121444	12.657171	11.628942	11.643705	11.885697	10.611388	12.320789	10.982768	12.924541	12.346629	11.613605	12.445148	12.478336

YJU2	13.041710	13.016439	13.296569	13.045554	13.192104	12.837925	13.003243	12.565935	12.923310	12.663564	12.681285	12.883664	12.792841	13.006847	13.166910	12.414781	12.916212	13.097319	13.656014	13.201010	13.288631	13.258023	13.743835	12.848393	12.617792	13.036888	12.846651	12.493861	12.538424	12.737719	13.148247	13.322965	13.183703	13.006158	13.019003	12.946442	13.139308	13.250094	13.551187	13.413783	12.936194	13.701386	13.143196	13.195507	13.345835	13.405875	12.513855	13.383249	12.661720	13.069657	13.067352	13.247129	13.264960	13.136216	13.153806	12.835254	13.658895	14.078024	13.536847	13.763964	13.761156	12.842092	13.761927	13.568953	14.014363	13.846147	13.345516	13.480172	13.306191	13.932694	12.841082	13.007215	12.429140	13.118469	13.137385	12.870847	13.314970	12.701760	12.456916	13.081962	13.129172	13.420156	13.168083	13.286480	13.190645	13.126907	13.186829	13.158777	14.126769	13.793821	13.683790	13.545519	12.905001	12.908941	12.931002	13.212415	13.250280	12.979140	12.996042	12.960694	12.591989	12.913034	13.042069	13.030425	14.007596	12.825787	13.223145	13.203986	14.006716	12.847527	14.181178	13.491499	13.367661	12.425739	12.945804	11.671048	13.592616	13.122511	12.630807	12.942843	13.369536	13.269040	13.472274	12.591506	12.715054	12.395853	12.587474	13.106812	13.589326	13.314543	13.643515	13.544622	13.615908	12.310934	13.251828	13.259837	13.391621	13.716318	13.041546	13.341776	13.987492	14.390000	13.698230	14.594880	13.313561	12.659132	13.628936	12.758240	12.902330	12.828063	12.964739	13.274804	13.049903	13.365165	13.555264	12.860948	13.106997	13.593085	13.615689	12.699184	13.549344	13.978627	12.771018	13.130760	13.240382	12.786993	13.571462	13.102386	13.265367	12.661881	13.563867	12.844764	13.657220	13.243382	13.117902	12.827524	13.670785	13.459378	13.482826	13.551649	13.181119	14.161739	13.854876	13.905825	13.160892	14.233539	13.114296	13.121156	13.776673	13.332262	12.863135	13.050632	13.034689	13.253022	13.081962	12.943369	13.150110	13.090706	13.039676	13.417326	13.181711	13.334032	13.371459	13.532839	13.297973	13.329864	13.561060	14.392936	13.546876	13.735880	14.315445	12.896890	13.161685	12.769413	13.058616	12.328606	12.831161	12.884027	12.981496	13.174957	13.022170	13.375239	13.032650	14.579342	13.204562	13.728355	13.104067	13.900187	12.885289	14.503811	13.815048	12.685688	12.587308	13.053474	12.597651	13.608723	12.596273	13.798810

CAB39L	9.322876	7.539423	8.134068	8.572894	8.366088	8.392729	9.449989	7.895767	7.769577	8.852719	8.302103	8.158381	8.250361	7.934974	7.930519	8.389109	8.364848	7.972401	5.772193	8.717346	5.482022	6.431572	8.352774	7.060125	9.649786	8.684928	7.791129	8.339181	8.245391	8.270907	7.395714	9.265515	8.906121	8.072444	8.676509	8.869457	8.826140	9.276071	7.726369	7.445861	8.169880	6.880073	7.909883	8.450930	9.118833	8.587697	7.914533	7.914905	9.169996	8.874004	8.907661	9.351391	8.952566	9.241363	9.099498	8.339181	9.281361	8.088325	7.453397	8.198869	8.058438	8.696042	7.243420	6.005753	6.782610	7.596551	7.415219	7.684131	7.171017	5.964297	8.696832	8.428823	8.499478	8.680653	8.574419	8.802470	9.013927	8.642660	8.666821	8.311931	9.589818	9.259236	9.178480	9.469018	9.443280	9.102118	9.414865	9.620568	6.898842	7.573089	8.104486	7.040480	7.733172	9.122142	8.653728	8.809828	9.345116	8.305437	8.183127	7.575603	8.325722	7.443459	7.589979	7.912515	6.810647	8.949983	5.185394	6.260320	5.938459	8.212669	6.562187	6.522347	7.539578	7.816672	8.527674	5.741365	7.249175	8.355988	7.855309	8.557010	6.923538	7.678603	6.650152	6.336858	7.764971	8.322623	8.037443	7.472494	7.188718	8.050957	7.865090	7.476161	5.536065	6.901111	8.502947	8.322016	6.201199	6.107290	9.790059	6.471389	5.252144	6.434267	4.677535	8.916795	9.208546	7.760052	6.568059	7.052261	8.094770	7.063169	7.743966	8.074558	7.906169	8.278740	8.268871	8.388507	9.324307	6.242941	6.543039	5.930243	6.216641	8.354912	8.307119	7.959294	8.009151	6.671061	7.490867	8.025123	8.851386	9.682033	8.591468	8.357075	9.212818	8.763472	8.444252	9.201089	6.671392	6.619026	8.145645	7.621803	8.216812	6.149325	5.202236	6.537941	6.551642	6.511437	7.197292	7.442212	5.379836	8.408218	7.829277	6.233766	8.560387	8.267121	8.965706	8.408506	8.259100	8.877700	7.456332	9.302664	8.608929	9.581892	9.132278	9.805157	8.065802	8.994576	8.393192	6.365202	7.235310	7.776998	5.569576	6.869820	7.997230	8.337894	7.709122	8.294571	7.824617	8.109245	7.203493	6.717021	6.404890	5.947042	7.226066	5.801382	6.988104	4.705514	5.844230	6.414516	5.506607	5.362147	4.590982	6.242766	7.238138	7.548388	7.970688	6.700141	8.350208	6.567049

CAAP1	5.185007	4.147276	4.787036	4.277079	4.469049	4.571394	4.609944	3.525894	4.345246	4.639063	5.410515	4.880056	5.930750	4.307220	4.410264	5.091441	3.921645	3.520966	3.708456	5.618933	3.915108	3.013777	3.155201	4.404898	3.353367	4.002996	3.605586	3.851412	4.970294	4.335082	4.514021	3.134280	4.235322	5.499484	5.222922	4.742349	4.189728	3.983505	3.273434	3.986728	4.922338	2.976679	4.392582	4.330775	4.396359	3.673284	3.986510	3.157660	3.876965	4.338975	4.624008	3.907688	5.338929	4.020669	4.751493	4.575611	4.719369	3.931308	4.435180	3.954187	2.962180	4.906706	3.123684	3.386728	3.248829	3.648603	3.254014	4.133608	3.306340	3.637869	4.401594	4.913496	5.197508	4.896846	4.433078	4.554548	4.381162	4.115709	4.122295	4.452678	4.795182	4.221682	4.693307	4.489992	4.604190	4.567683	3.788669	4.156749	4.111173	3.214073	3.417042	3.600877	4.380980	4.597046	4.934698	6.126226	4.441499	6.042958	4.433285	5.305593	4.794770	5.723646	6.001979	4.662049	4.079820	4.988095	3.905294	3.338781	3.380840	4.829317	3.416207	3.573638	3.102636	5.106233	5.062258	3.318632	4.071686	5.556004	5.026469	3.466667	4.432246	3.271149	4.247095	3.015098	4.489115	3.436171	4.652799	3.901467	3.819056	3.746476	3.766938	3.344470	3.585898	3.369248	4.214253	3.408550	3.233219	3.471047	4.661814	3.507067	3.109333	3.108410	3.766086	2.977966	3.251818	3.068050	3.763620	3.592258	3.656622	4.031479	3.857219	3.915721	4.558102	4.989759	5.042427	4.541985	4.097968	2.969017	3.429708	4.290026	2.918966	4.644693	4.053433	4.368922	3.579178	4.678808	3.241347	4.949181	3.182569	3.266295	3.116393	4.621637	4.246430	4.046733	3.662430	3.890731	3.271303	4.149177	4.019991	3.183197	3.519870	3.097843	3.036756	3.142180	3.610373	3.152573	4.212746	3.776955	2.969281	4.379938	3.924795	4.639063	4.820657	4.217298	4.066903	4.262444	4.035385	3.749587	3.808277	5.556180	5.577407	4.073634	4.428477	4.542873	3.408550	4.319054	5.144352	3.237122	3.629437	3.936016	3.508701	4.174872	4.082613	4.467997	6.192675	3.961808	4.062633	4.282121	5.406110	4.698120	4.910284	5.162091	3.472743	3.641091	3.771953	3.748721	3.588270	3.704887	4.730815	3.097736	3.033804	2.861148	3.743022	3.722298	3.316047	3.471726	5.649730	4.213645

TPT1	8.697270	9.022623	9.619777	9.078242	9.459105	9.843353	9.148313	9.496425	9.400578	9.629098	9.098445	9.150795	9.396668	9.458488	9.974049	9.210997	9.449194	9.871673	9.826917	8.955928	9.389452	9.824177	9.260903	9.629098	9.605154	9.428831	8.620028	9.767533	9.483198	9.178174	9.110950	9.183929	9.098736	8.715309	8.508449	8.739630	8.788445	9.033855	9.272705	9.572296	9.596649	8.972481	8.559301	8.873253	9.081198	8.905804	9.330903	9.142892	9.962553	8.819184	8.655231	8.900227	9.256976	8.924017	9.491898	9.623678	9.506865	9.465672	8.848945	9.394972	8.899303	9.465354	9.310956	9.482720	9.109097	9.657050	8.831339	9.188306	8.750837	8.797307	8.291499	8.727901	9.549607	8.556080	8.690185	8.380007	9.359075	8.682812	8.533168	9.320484	9.452450	9.413800	8.942934	9.152637	9.266415	9.295062	9.073136	9.238911	8.836481	9.238911	9.051002	8.951490	8.842770	8.537994	8.722507	8.850175	9.509923	9.395272	9.508697	9.402607	10.070853	8.793845	9.577451	9.424518	9.577451	10.066907	10.449011	9.602061	10.454196	9.585450	9.219906	10.100136	10.201110	9.766931	10.523398	11.394433	9.328299	9.531003	9.553448	8.492896	9.207170	8.942008	8.748329	10.317872	8.551804	9.086559	7.804933	8.655793	8.924017	8.730911	9.417014	9.214227	8.755780	9.097671	8.908475	8.077123	9.392967	8.164723	7.376736	9.227124	7.900387	8.060419	8.547241	8.663767	9.089320	9.450937	8.858496	9.464698	8.506906	8.250361	8.764687	8.973768	9.000110	7.923957	8.319278	8.261557	8.749866	8.180684	8.269199	9.195936	8.322775	8.362692	7.520987	8.022388	8.161308	9.971364	9.049014	8.071227	9.659217	7.652204	8.267307	8.228114	9.152161	8.953012	8.276739	8.490868	8.101000	8.376467	8.976956	7.477368	8.842443	7.380361	9.051735	9.274698	8.343831	9.225591	8.235110	8.689294	7.371092	8.130729	8.241603	9.720345	8.440271	8.432432	7.914461	8.224339	8.374041	8.566875	8.324774	8.504777	8.653894	8.520817	8.334459	8.252821	8.604216	8.477080	8.451090	8.164882	8.258160	8.331700	7.278613	8.111326	8.745438	8.332609	8.834648	8.377983	9.037684	8.912500	7.987181	8.637723	8.560080	8.315586	8.542271	7.757279	9.112102	8.904760	9.959977	8.618458	9.022037	8.446244	9.349409	9.536731	9.707339	9.385728	9.336323	8.787831	8.360170	8.589431

DPF3	4.664961	5.188216	5.364435	4.989759	5.104607	4.658582	6.257762	5.518583	5.831582	5.089194	4.493992	5.096023	4.419800	5.564296	4.116148	5.205438	6.182378	5.462398	8.568501	4.776361	6.591177	6.339056	7.717516	4.232008	4.985394	5.789578	4.800062	5.648403	5.220560	4.275090	5.509370	6.084236	5.253728	4.321641	5.168494	5.820088	6.471925	5.951984	6.231093	5.763409	5.483145	6.218553	5.084041	4.864946	6.626000	5.286009	5.828417	4.957935	5.393349	5.887268	5.286009	4.780742	6.241436	4.059096	5.989180	5.929174	6.597629	6.944724	5.537433	6.207268	7.082213	4.893555	5.951435	6.065925	5.350464	7.312129	5.753756	4.837448	5.944776	6.004467	4.701728	5.479076	5.405537	6.421917	4.906706	5.155213	6.050444	6.173864	5.671044	5.264677	5.031937	6.468519	5.192099	5.887268	7.312743	6.447841	4.790580	6.703693	3.703237	5.457891	7.116435	5.947212	4.860639	5.779986	4.866428	5.775473	5.560215	5.570700	5.313603	6.486440	4.076809	4.993938	5.339320	5.987381	5.835534	5.300034	5.384096	6.242766	6.296759	5.683817	5.888681	4.584996	5.026830	4.832287	5.441005	4.581198	4.740639	6.046918	5.169098	4.718328	4.537806	4.389151	4.138630	5.038717	4.187293	4.399157	4.598956	4.505883	4.236552	4.239223	3.592258	4.818759	4.158864	4.255015	4.843806	4.238326	3.953338	7.195034	4.521491	4.346108	5.190332	7.232277	4.235959	4.628254	3.986286	4.554340	4.305090	4.375887	4.389151	4.348963	4.746162	4.593852	4.486585	3.890951	3.881295	5.570335	5.446617	5.515655	5.286792	4.590157	4.187529	4.363059	6.021706	4.564465	4.519978	5.086146	4.848293	5.192676	4.455648	5.316861	3.516786	4.694331	4.162511	3.853131	5.497928	4.826069	5.296253	2.987511	4.490665	5.005368	4.213645	4.941365	4.892077	5.186207	4.852882	4.830608	4.347207	4.776155	4.852673	4.782618	4.390813	4.786621	4.757595	4.038661	5.168072	4.653042	5.181177	5.189361	4.031273	3.740764	4.846579	4.022591	4.240767	4.683330	5.505443	4.266557	4.194339	3.720576	4.553674	4.397856	5.698228	5.039903	5.364244	5.355114	4.878379	5.066954	3.819056	4.012263	5.247413	4.002553	3.985631	4.705302	4.269645	4.819179	4.721287	4.289203	4.767656	5.274315	5.419147	5.039101	4.200744	4.796034	4.991830	4.478658	4.243870	5.580774	4.431596	4.747859

BSX	10.488104	9.281361	9.207170	9.556322	9.918845	9.954431	8.729876	10.064832	10.302486	9.228967	9.588576	9.232537	8.401432	8.636313	8.519918	9.757967	9.822841	8.590359	10.358566	9.372256	10.010197	9.841226	10.259465	9.312491	9.541895	9.425721	10.352360	10.033753	9.549766	9.479371	9.619017	8.583467	8.602194	8.953796	9.080648	9.245688	9.317754	9.332770	9.632923	9.868372	9.453395	9.699238	9.523811	9.710940	9.146259	9.459105	10.019448	9.386992	8.925347	9.251933	10.919103	9.045496	8.493691	8.535756	8.856984	9.916529	8.313592	9.029665	8.719946	8.585291	9.043804	9.336166	9.368473	9.914396	9.338015	9.138750	9.800112	9.985494	9.841511	10.325669	9.324472	9.477368	9.882077	9.491096	9.812907	9.772809	9.478293	9.320772	9.532146	8.920911	9.134109	8.763010	8.957149	8.469498	8.569732	8.700125	8.675912	9.333694	9.184855	9.443123	9.102892	9.513501	9.513664	9.429598	9.220054	9.041181	8.482792	8.863706	9.095875	9.651799	9.794875	8.924171	9.121085	9.699554	9.561747	9.521459	9.755013	9.537204	10.075883	9.816798	10.138215	9.605154	9.492065	10.463260	10.073289	11.652576	9.832315	8.997519	9.975129	9.461061	9.486588	9.338178	9.507629	10.397331	10.080716	9.733626	9.819206	10.018797	9.097041	10.364000	9.368829	9.213755	8.729734	9.006858	10.018641	9.689752	9.061877	9.767395	8.979537	9.485342	9.383472	9.894722	9.755013	9.834744	9.059909	10.027498	9.760564	10.145061	9.523040	9.760731	9.284604	9.284164	9.078876	8.940654	9.339347	9.030278	9.493782	9.889233	9.966216	10.388365	10.092624	9.088528	9.859150	9.458028	10.084367	12.007316	9.244700	8.965870	9.319744	9.897656	9.001514	8.405459	8.679458	8.623592	9.975574	8.212228	8.693709	8.959613	8.430243	9.013164	9.373508	9.514276	9.353198	9.232384	9.063980	9.670859	9.739498	9.752217	9.976964	9.563890	9.531481	10.947159	9.442348	9.376209	9.529282	9.279051	9.447171	9.851053	8.856845	8.783374	8.982750	9.231299	8.580435	8.189742	9.545166	8.739474	8.779928	9.160471	9.172647	8.663004	9.732418	9.345280	9.478926	9.176514	9.563592	10.118987	9.537362	9.858835	9.426808	9.342515	9.163229	9.309408	9.292820	9.174191	9.615752	9.856818	9.734237	9.416569	10.500604	9.941815	9.267666	10.701509	10.996548	9.431886	9.589654	9.295685	8.954868	9.540818

PCDHA1	4.201411	3.183197	3.170245	3.875478	3.813804	5.154607	3.078974	3.334678	3.302053	4.244526	5.018696	5.209030	4.058646	4.650040	5.752102	4.059980	3.268818	3.156147	5.022592	4.254568	3.879159	4.081964	6.973051	4.381390	5.547508	4.749521	4.488050	4.040813	3.736675	3.593435	3.477266	5.717822	4.838303	3.797572	4.127852	3.927163	3.368081	3.447280	3.164186	3.094889	5.121307	2.984795	3.817590	3.628614	4.364402	4.569356	5.404608	5.729904	4.730815	4.376523	3.191888	3.099031	3.442756	3.924166	3.581487	4.249700	5.152964	3.380500	4.466094	3.678826	2.933695	4.146217	3.830958	4.149805	4.324430	4.151468	3.178160	3.830779	3.328676	2.874426	3.962928	3.259296	3.981290	3.499640	4.045616	3.257039	3.797353	3.221235	3.537632	3.887853	3.418786	3.676812	3.852937	5.098644	3.268982	4.757829	3.359844	3.247533	3.938324	3.764711	3.515884	3.235234	3.715067	3.094426	3.442756	3.368755	3.409531	4.104098	5.194915	4.081133	4.444781	3.427979	3.994643	3.638690	4.781151	4.546670	3.522057	4.324870	3.293300	4.816027	3.888699	4.760385	4.133820	4.683764	4.757595	4.983217	4.209917	4.162932	4.392359	3.766938	3.776160	5.109345	4.135859	4.229455	4.868526	4.827825	4.646834	4.291773	5.457267	5.064071	6.660368	5.269057	4.993108	5.926133	4.284260	3.153544	6.031996	6.528481	4.439375	3.698196	4.198815	5.722134	4.278403	7.128619	6.136363	4.001248	5.440238	4.055403	4.739775	3.132846	3.843368	4.417754	3.186761	3.080901	5.626914	3.284599	4.185545	4.209292	3.516064	5.814756	2.929028	4.329472	4.184907	4.041347	4.290685	5.822590	5.434147	4.893127	3.211448	3.072674	4.436744	4.891263	4.085680	3.348689	6.141377	5.775818	5.338103	3.159921	4.415573	2.889637	4.693921	4.307910	3.506641	4.825841	3.567496	3.204941	3.469619	3.264389	2.881109	3.669264	3.117616	5.422259	3.635440	4.856921	3.416880	3.468724	3.649022	3.544326	3.735228	4.335916	3.832712	3.407494	3.476705	3.555846	4.884088	3.248982	3.375457	3.673475	3.859195	3.531287	3.225710	3.421532	3.497641	3.058619	3.281206	3.822877	4.290026	4.675819	4.547077	5.865582	4.600410	4.458238	3.302999	5.454938	5.153391	4.222533	4.394274	4.783045	6.213019	3.746476	4.471008	5.996352	5.723646	4.188438	4.226582	4.075160	3.983064	6.820544

PCDHA10	4.201411	3.183197	3.170245	3.875478	3.813804	5.154607	3.078974	3.334678	3.302053	4.244526	5.018696	5.209030	4.058646	4.650040	5.752102	4.059980	3.268818	3.156147	5.022592	4.254568	3.879159	4.081964	6.973051	4.381390	5.547508	4.749521	4.488050	4.040813	3.736675	3.593435	3.477266	5.717822	4.838303	3.797572	4.127852	3.927163	3.368081	3.447280	3.164186	3.094889	5.121307	2.984795	3.817590	3.628614	4.364402	4.569356	5.404608	5.729904	4.730815	4.376523	3.191888	3.099031	3.442756	3.924166	3.581487	4.249700	5.152964	3.380500	4.466094	3.678826	2.933695	4.146217	3.830958	4.149805	4.324430	4.151468	3.178160	3.830779	3.328676	2.874426	3.962928	3.259296	3.981290	3.499640	4.045616	3.257039	3.797353	3.221235	3.537632	3.887853	3.418786	3.676812	3.852937	5.098644	3.268982	4.757829	3.359844	3.247533	3.938324	3.764711	3.515884	3.235234	3.715067	3.094426	3.442756	3.368755	3.409531	4.104098	5.194915	4.081133	4.444781	3.427979	3.994643	3.638690	4.781151	4.546670	3.522057	4.324870	3.293300	4.816027	3.888699	4.760385	4.133820	4.683764	4.757595	4.983217	4.209917	4.162932	4.392359	3.766938	3.776160	5.109345	4.135859	4.229455	4.868526	4.827825	4.646834	4.291773	5.457267	5.064071	6.660368	5.269057	4.993108	5.926133	4.284260	3.153544	6.031996	6.528481	4.439375	3.698196	4.198815	5.722134	4.278403	7.128619	6.136363	4.001248	5.440238	4.055403	4.739775	3.132846	3.843368	4.417754	3.186761	3.080901	5.626914	3.284599	4.185545	4.209292	3.516064	5.814756	2.929028	4.329472	4.184907	4.041347	4.290685	5.822590	5.434147	4.893127	3.211448	3.072674	4.436744	4.891263	4.085680	3.348689	6.141377	5.775818	5.338103	3.159921	4.415573	2.889637	4.693921	4.307910	3.506641	4.825841	3.567496	3.204941	3.469619	3.264389	2.881109	3.669264	3.117616	5.422259	3.635440	4.856921	3.416880	3.468724	3.649022	3.544326	3.735228	4.335916	3.832712	3.407494	3.476705	3.555846	4.884088	3.248982	3.375457	3.673475	3.859195	3.531287	3.225710	3.421532	3.497641	3.058619	3.281206	3.822877	4.290026	4.675819	4.547077	5.865582	4.600410	4.458238	3.302999	5.454938	5.153391	4.222533	4.394274	4.783045	6.213019	3.746476	4.471008	5.996352	5.723646	4.188438	4.226582	4.075160	3.983064	6.820544

PCDHA3	4.201411	3.183197	3.170245	3.875478	3.813804	5.154607	3.078974	3.334678	3.302053	4.244526	5.018696	5.209030	4.058646	4.650040	5.752102	4.059980	3.268818	3.156147	5.022592	4.254568	3.879159	4.081964	6.973051	4.381390	5.547508	4.749521	4.488050	4.040813	3.736675	3.593435	3.477266	5.717822	4.838303	3.797572	4.127852	3.927163	3.368081	3.447280	3.164186	3.094889	5.121307	2.984795	3.817590	3.628614	4.364402	4.569356	5.404608	5.729904	4.730815	4.376523	3.191888	3.099031	3.442756	3.924166	3.581487	4.249700	5.152964	3.380500	4.466094	3.678826	2.933695	4.146217	3.830958	4.149805	4.324430	4.151468	3.178160	3.830779	3.328676	2.874426	3.962928	3.259296	3.981290	3.499640	4.045616	3.257039	3.797353	3.221235	3.537632	3.887853	3.418786	3.676812	3.852937	5.098644	3.268982	4.757829	3.359844	3.247533	3.938324	3.764711	3.515884	3.235234	3.715067	3.094426	3.442756	3.368755	3.409531	4.104098	5.194915	4.081133	4.444781	3.427979	3.994643	3.638690	4.781151	4.546670	3.522057	4.324870	3.293300	4.816027	3.888699	4.760385	4.133820	4.683764	4.757595	4.983217	4.209917	4.162932	4.392359	3.766938	3.776160	5.109345	4.135859	4.229455	4.868526	4.827825	4.646834	4.291773	5.457267	5.064071	6.660368	5.269057	4.993108	5.926133	4.284260	3.153544	6.031996	6.528481	4.439375	3.698196	4.198815	5.722134	4.278403	7.128619	6.136363	4.001248	5.440238	4.055403	4.739775	3.132846	3.843368	4.417754	3.186761	3.080901	5.626914	3.284599	4.185545	4.209292	3.516064	5.814756	2.929028	4.329472	4.184907	4.041347	4.290685	5.822590	5.434147	4.893127	3.211448	3.072674	4.436744	4.891263	4.085680	3.348689	6.141377	5.775818	5.338103	3.159921	4.415573	2.889637	4.693921	4.307910	3.506641	4.825841	3.567496	3.204941	3.469619	3.264389	2.881109	3.669264	3.117616	5.422259	3.635440	4.856921	3.416880	3.468724	3.649022	3.544326	3.735228	4.335916	3.832712	3.407494	3.476705	3.555846	4.884088	3.248982	3.375457	3.673475	3.859195	3.531287	3.225710	3.421532	3.497641	3.058619	3.281206	3.822877	4.290026	4.675819	4.547077	5.865582	4.600410	4.458238	3.302999	5.454938	5.153391	4.222533	4.394274	4.783045	6.213019	3.746476	4.471008	5.996352	5.723646	4.188438	4.226582	4.075160	3.983064	6.820544

PCDHA4	4.201411	3.183197	3.170245	3.875478	3.813804	5.154607	3.078974	3.334678	3.302053	4.244526	5.018696	5.209030	4.058646	4.650040	5.752102	4.059980	3.268818	3.156147	5.022592	4.254568	3.879159	4.081964	6.973051	4.381390	5.547508	4.749521	4.488050	4.040813	3.736675	3.593435	3.477266	5.717822	4.838303	3.797572	4.127852	3.927163	3.368081	3.447280	3.164186	3.094889	5.121307	2.984795	3.817590	3.628614	4.364402	4.569356	5.404608	5.729904	4.730815	4.376523	3.191888	3.099031	3.442756	3.924166	3.581487	4.249700	5.152964	3.380500	4.466094	3.678826	2.933695	4.146217	3.830958	4.149805	4.324430	4.151468	3.178160	3.830779	3.328676	2.874426	3.962928	3.259296	3.981290	3.499640	4.045616	3.257039	3.797353	3.221235	3.537632	3.887853	3.418786	3.676812	3.852937	5.098644	3.268982	4.757829	3.359844	3.247533	3.938324	3.764711	3.515884	3.235234	3.715067	3.094426	3.442756	3.368755	3.409531	4.104098	5.194915	4.081133	4.444781	3.427979	3.994643	3.638690	4.781151	4.546670	3.522057	4.324870	3.293300	4.816027	3.888699	4.760385	4.133820	4.683764	4.757595	4.983217	4.209917	4.162932	4.392359	3.766938	3.776160	5.109345	4.135859	4.229455	4.868526	4.827825	4.646834	4.291773	5.457267	5.064071	6.660368	5.269057	4.993108	5.926133	4.284260	3.153544	6.031996	6.528481	4.439375	3.698196	4.198815	5.722134	4.278403	7.128619	6.136363	4.001248	5.440238	4.055403	4.739775	3.132846	3.843368	4.417754	3.186761	3.080901	5.626914	3.284599	4.185545	4.209292	3.516064	5.814756	2.929028	4.329472	4.184907	4.041347	4.290685	5.822590	5.434147	4.893127	3.211448	3.072674	4.436744	4.891263	4.085680	3.348689	6.141377	5.775818	5.338103	3.159921	4.415573	2.889637	4.693921	4.307910	3.506641	4.825841	3.567496	3.204941	3.469619	3.264389	2.881109	3.669264	3.117616	5.422259	3.635440	4.856921	3.416880	3.468724	3.649022	3.544326	3.735228	4.335916	3.832712	3.407494	3.476705	3.555846	4.884088	3.248982	3.375457	3.673475	3.859195	3.531287	3.225710	3.421532	3.497641	3.058619	3.281206	3.822877	4.290026	4.675819	4.547077	5.865582	4.600410	4.458238	3.302999	5.454938	5.153391	4.222533	4.394274	4.783045	6.213019	3.746476	4.471008	5.996352	5.723646	4.188438	4.226582	4.075160	3.983064	6.820544

PCDHA6	4.201411	3.183197	3.170245	3.875478	3.813804	5.154607	3.078974	3.334678	3.302053	4.244526	5.018696	5.209030	4.058646	4.650040	5.752102	4.059980	3.268818	3.156147	5.022592	4.254568	3.879159	4.081964	6.973051	4.381390	5.547508	4.749521	4.488050	4.040813	3.736675	3.593435	3.477266	5.717822	4.838303	3.797572	4.127852	3.927163	3.368081	3.447280	3.164186	3.094889	5.121307	2.984795	3.817590	3.628614	4.364402	4.569356	5.404608	5.729904	4.730815	4.376523	3.191888	3.099031	3.442756	3.924166	3.581487	4.249700	5.152964	3.380500	4.466094	3.678826	2.933695	4.146217	3.830958	4.149805	4.324430	4.151468	3.178160	3.830779	3.328676	2.874426	3.962928	3.259296	3.981290	3.499640	4.045616	3.257039	3.797353	3.221235	3.537632	3.887853	3.418786	3.676812	3.852937	5.098644	3.268982	4.757829	3.359844	3.247533	3.938324	3.764711	3.515884	3.235234	3.715067	3.094426	3.442756	3.368755	3.409531	4.104098	5.194915	4.081133	4.444781	3.427979	3.994643	3.638690	4.781151	4.546670	3.522057	4.324870	3.293300	4.816027	3.888699	4.760385	4.133820	4.683764	4.757595	4.983217	4.209917	4.162932	4.392359	3.766938	3.776160	5.109345	4.135859	4.229455	4.868526	4.827825	4.646834	4.291773	5.457267	5.064071	6.660368	5.269057	4.993108	5.926133	4.284260	3.153544	6.031996	6.528481	4.439375	3.698196	4.198815	5.722134	4.278403	7.128619	6.136363	4.001248	5.440238	4.055403	4.739775	3.132846	3.843368	4.417754	3.186761	3.080901	5.626914	3.284599	4.185545	4.209292	3.516064	5.814756	2.929028	4.329472	4.184907	4.041347	4.290685	5.822590	5.434147	4.893127	3.211448	3.072674	4.436744	4.891263	4.085680	3.348689	6.141377	5.775818	5.338103	3.159921	4.415573	2.889637	4.693921	4.307910	3.506641	4.825841	3.567496	3.204941	3.469619	3.264389	2.881109	3.669264	3.117616	5.422259	3.635440	4.856921	3.416880	3.468724	3.649022	3.544326	3.735228	4.335916	3.832712	3.407494	3.476705	3.555846	4.884088	3.248982	3.375457	3.673475	3.859195	3.531287	3.225710	3.421532	3.497641	3.058619	3.281206	3.822877	4.290026	4.675819	4.547077	5.865582	4.600410	4.458238	3.302999	5.454938	5.153391	4.222533	4.394274	4.783045	6.213019	3.746476	4.471008	5.996352	5.723646	4.188438	4.226582	4.075160	3.983064	6.820544

PCDHA2	4.201411	3.183197	3.170245	3.875478	3.813804	5.154607	3.078974	3.334678	3.302053	4.244526	5.018696	5.209030	4.058646	4.650040	5.752102	4.059980	3.268818	3.156147	5.022592	4.254568	3.879159	4.081964	6.973051	4.381390	5.547508	4.749521	4.488050	4.040813	3.736675	3.593435	3.477266	5.717822	4.838303	3.797572	4.127852	3.927163	3.368081	3.447280	3.164186	3.094889	5.121307	2.984795	3.817590	3.628614	4.364402	4.569356	5.404608	5.729904	4.730815	4.376523	3.191888	3.099031	3.442756	3.924166	3.581487	4.249700	5.152964	3.380500	4.466094	3.678826	2.933695	4.146217	3.830958	4.149805	4.324430	4.151468	3.178160	3.830779	3.328676	2.874426	3.962928	3.259296	3.981290	3.499640	4.045616	3.257039	3.797353	3.221235	3.537632	3.887853	3.418786	3.676812	3.852937	5.098644	3.268982	4.757829	3.359844	3.247533	3.938324	3.764711	3.515884	3.235234	3.715067	3.094426	3.442756	3.368755	3.409531	4.104098	5.194915	4.081133	4.444781	3.427979	3.994643	3.638690	4.781151	4.546670	3.522057	4.324870	3.293300	4.816027	3.888699	4.760385	4.133820	4.683764	4.757595	4.983217	4.209917	4.162932	4.392359	3.766938	3.776160	5.109345	4.135859	4.229455	4.868526	4.827825	4.646834	4.291773	5.457267	5.064071	6.660368	5.269057	4.993108	5.926133	4.284260	3.153544	6.031996	6.528481	4.439375	3.698196	4.198815	5.722134	4.278403	7.128619	6.136363	4.001248	5.440238	4.055403	4.739775	3.132846	3.843368	4.417754	3.186761	3.080901	5.626914	3.284599	4.185545	4.209292	3.516064	5.814756	2.929028	4.329472	4.184907	4.041347	4.290685	5.822590	5.434147	4.893127	3.211448	3.072674	4.436744	4.891263	4.085680	3.348689	6.141377	5.775818	5.338103	3.159921	4.415573	2.889637	4.693921	4.307910	3.506641	4.825841	3.567496	3.204941	3.469619	3.264389	2.881109	3.669264	3.117616	5.422259	3.635440	4.856921	3.416880	3.468724	3.649022	3.544326	3.735228	4.335916	3.832712	3.407494	3.476705	3.555846	4.884088	3.248982	3.375457	3.673475	3.859195	3.531287	3.225710	3.421532	3.497641	3.058619	3.281206	3.822877	4.290026	4.675819	4.547077	5.865582	4.600410	4.458238	3.302999	5.454938	5.153391	4.222533	4.394274	4.783045	6.213019	3.746476	4.471008	5.996352	5.723646	4.188438	4.226582	4.075160	3.983064	6.820544

PCDHA8	4.201411	3.183197	3.170245	3.875478	3.813804	5.154607	3.078974	3.334678	3.302053	4.244526	5.018696	5.209030	4.058646	4.650040	5.752102	4.059980	3.268818	3.156147	5.022592	4.254568	3.879159	4.081964	6.973051	4.381390	5.547508	4.749521	4.488050	4.040813	3.736675	3.593435	3.477266	5.717822	4.838303	3.797572	4.127852	3.927163	3.368081	3.447280	3.164186	3.094889	5.121307	2.984795	3.817590	3.628614	4.364402	4.569356	5.404608	5.729904	4.730815	4.376523	3.191888	3.099031	3.442756	3.924166	3.581487	4.249700	5.152964	3.380500	4.466094	3.678826	2.933695	4.146217	3.830958	4.149805	4.324430	4.151468	3.178160	3.830779	3.328676	2.874426	3.962928	3.259296	3.981290	3.499640	4.045616	3.257039	3.797353	3.221235	3.537632	3.887853	3.418786	3.676812	3.852937	5.098644	3.268982	4.757829	3.359844	3.247533	3.938324	3.764711	3.515884	3.235234	3.715067	3.094426	3.442756	3.368755	3.409531	4.104098	5.194915	4.081133	4.444781	3.427979	3.994643	3.638690	4.781151	4.546670	3.522057	4.324870	3.293300	4.816027	3.888699	4.760385	4.133820	4.683764	4.757595	4.983217	4.209917	4.162932	4.392359	3.766938	3.776160	5.109345	4.135859	4.229455	4.868526	4.827825	4.646834	4.291773	5.457267	5.064071	6.660368	5.269057	4.993108	5.926133	4.284260	3.153544	6.031996	6.528481	4.439375	3.698196	4.198815	5.722134	4.278403	7.128619	6.136363	4.001248	5.440238	4.055403	4.739775	3.132846	3.843368	4.417754	3.186761	3.080901	5.626914	3.284599	4.185545	4.209292	3.516064	5.814756	2.929028	4.329472	4.184907	4.041347	4.290685	5.822590	5.434147	4.893127	3.211448	3.072674	4.436744	4.891263	4.085680	3.348689	6.141377	5.775818	5.338103	3.159921	4.415573	2.889637	4.693921	4.307910	3.506641	4.825841	3.567496	3.204941	3.469619	3.264389	2.881109	3.669264	3.117616	5.422259	3.635440	4.856921	3.416880	3.468724	3.649022	3.544326	3.735228	4.335916	3.832712	3.407494	3.476705	3.555846	4.884088	3.248982	3.375457	3.673475	3.859195	3.531287	3.225710	3.421532	3.497641	3.058619	3.281206	3.822877	4.290026	4.675819	4.547077	5.865582	4.600410	4.458238	3.302999	5.454938	5.153391	4.222533	4.394274	4.783045	6.213019	3.746476	4.471008	5.996352	5.723646	4.188438	4.226582	4.075160	3.983064	6.820544

PCDHA5	4.201411	3.183197	3.170245	3.875478	3.813804	5.154607	3.078974	3.334678	3.302053	4.244526	5.018696	5.209030	4.058646	4.650040	5.752102	4.059980	3.268818	3.156147	5.022592	4.254568	3.879159	4.081964	6.973051	4.381390	5.547508	4.749521	4.488050	4.040813	3.736675	3.593435	3.477266	5.717822	4.838303	3.797572	4.127852	3.927163	3.368081	3.447280	3.164186	3.094889	5.121307	2.984795	3.817590	3.628614	4.364402	4.569356	5.404608	5.729904	4.730815	4.376523	3.191888	3.099031	3.442756	3.924166	3.581487	4.249700	5.152964	3.380500	4.466094	3.678826	2.933695	4.146217	3.830958	4.149805	4.324430	4.151468	3.178160	3.830779	3.328676	2.874426	3.962928	3.259296	3.981290	3.499640	4.045616	3.257039	3.797353	3.221235	3.537632	3.887853	3.418786	3.676812	3.852937	5.098644	3.268982	4.757829	3.359844	3.247533	3.938324	3.764711	3.515884	3.235234	3.715067	3.094426	3.442756	3.368755	3.409531	4.104098	5.194915	4.081133	4.444781	3.427979	3.994643	3.638690	4.781151	4.546670	3.522057	4.324870	3.293300	4.816027	3.888699	4.760385	4.133820	4.683764	4.757595	4.983217	4.209917	4.162932	4.392359	3.766938	3.776160	5.109345	4.135859	4.229455	4.868526	4.827825	4.646834	4.291773	5.457267	5.064071	6.660368	5.269057	4.993108	5.926133	4.284260	3.153544	6.031996	6.528481	4.439375	3.698196	4.198815	5.722134	4.278403	7.128619	6.136363	4.001248	5.440238	4.055403	4.739775	3.132846	3.843368	4.417754	3.186761	3.080901	5.626914	3.284599	4.185545	4.209292	3.516064	5.814756	2.929028	4.329472	4.184907	4.041347	4.290685	5.822590	5.434147	4.893127	3.211448	3.072674	4.436744	4.891263	4.085680	3.348689	6.141377	5.775818	5.338103	3.159921	4.415573	2.889637	4.693921	4.307910	3.506641	4.825841	3.567496	3.204941	3.469619	3.264389	2.881109	3.669264	3.117616	5.422259	3.635440	4.856921	3.416880	3.468724	3.649022	3.544326	3.735228	4.335916	3.832712	3.407494	3.476705	3.555846	4.884088	3.248982	3.375457	3.673475	3.859195	3.531287	3.225710	3.421532	3.497641	3.058619	3.281206	3.822877	4.290026	4.675819	4.547077	5.865582	4.600410	4.458238	3.302999	5.454938	5.153391	4.222533	4.394274	4.783045	6.213019	3.746476	4.471008	5.996352	5.723646	4.188438	4.226582	4.075160	3.983064	6.820544

PCDHA11	4.201411	3.183197	3.170245	3.875478	3.813804	5.154607	3.078974	3.334678	3.302053	4.244526	5.018696	5.209030	4.058646	4.650040	5.752102	4.059980	3.268818	3.156147	5.022592	4.254568	3.879159	4.081964	6.973051	4.381390	5.547508	4.749521	4.488050	4.040813	3.736675	3.593435	3.477266	5.717822	4.838303	3.797572	4.127852	3.927163	3.368081	3.447280	3.164186	3.094889	5.121307	2.984795	3.817590	3.628614	4.364402	4.569356	5.404608	5.729904	4.730815	4.376523	3.191888	3.099031	3.442756	3.924166	3.581487	4.249700	5.152964	3.380500	4.466094	3.678826	2.933695	4.146217	3.830958	4.149805	4.324430	4.151468	3.178160	3.830779	3.328676	2.874426	3.962928	3.259296	3.981290	3.499640	4.045616	3.257039	3.797353	3.221235	3.537632	3.887853	3.418786	3.676812	3.852937	5.098644	3.268982	4.757829	3.359844	3.247533	3.938324	3.764711	3.515884	3.235234	3.715067	3.094426	3.442756	3.368755	3.409531	4.104098	5.194915	4.081133	4.444781	3.427979	3.994643	3.638690	4.781151	4.546670	3.522057	4.324870	3.293300	4.816027	3.888699	4.760385	4.133820	4.683764	4.757595	4.983217	4.209917	4.162932	4.392359	3.766938	3.776160	5.109345	4.135859	4.229455	4.868526	4.827825	4.646834	4.291773	5.457267	5.064071	6.660368	5.269057	4.993108	5.926133	4.284260	3.153544	6.031996	6.528481	4.439375	3.698196	4.198815	5.722134	4.278403	7.128619	6.136363	4.001248	5.440238	4.055403	4.739775	3.132846	3.843368	4.417754	3.186761	3.080901	5.626914	3.284599	4.185545	4.209292	3.516064	5.814756	2.929028	4.329472	4.184907	4.041347	4.290685	5.822590	5.434147	4.893127	3.211448	3.072674	4.436744	4.891263	4.085680	3.348689	6.141377	5.775818	5.338103	3.159921	4.415573	2.889637	4.693921	4.307910	3.506641	4.825841	3.567496	3.204941	3.469619	3.264389	2.881109	3.669264	3.117616	5.422259	3.635440	4.856921	3.416880	3.468724	3.649022	3.544326	3.735228	4.335916	3.832712	3.407494	3.476705	3.555846	4.884088	3.248982	3.375457	3.673475	3.859195	3.531287	3.225710	3.421532	3.497641	3.058619	3.281206	3.822877	4.290026	4.675819	4.547077	5.865582	4.600410	4.458238	3.302999	5.454938	5.153391	4.222533	4.394274	4.783045	6.213019	3.746476	4.471008	5.996352	5.723646	4.188438	4.226582	4.075160	3.983064	6.820544

PCDHA7	4.201411	3.183197	3.170245	3.875478	3.813804	5.154607	3.078974	3.334678	3.302053	4.244526	5.018696	5.209030	4.058646	4.650040	5.752102	4.059980	3.268818	3.156147	5.022592	4.254568	3.879159	4.081964	6.973051	4.381390	5.547508	4.749521	4.488050	4.040813	3.736675	3.593435	3.477266	5.717822	4.838303	3.797572	4.127852	3.927163	3.368081	3.447280	3.164186	3.094889	5.121307	2.984795	3.817590	3.628614	4.364402	4.569356	5.404608	5.729904	4.730815	4.376523	3.191888	3.099031	3.442756	3.924166	3.581487	4.249700	5.152964	3.380500	4.466094	3.678826	2.933695	4.146217	3.830958	4.149805	4.324430	4.151468	3.178160	3.830779	3.328676	2.874426	3.962928	3.259296	3.981290	3.499640	4.045616	3.257039	3.797353	3.221235	3.537632	3.887853	3.418786	3.676812	3.852937	5.098644	3.268982	4.757829	3.359844	3.247533	3.938324	3.764711	3.515884	3.235234	3.715067	3.094426	3.442756	3.368755	3.409531	4.104098	5.194915	4.081133	4.444781	3.427979	3.994643	3.638690	4.781151	4.546670	3.522057	4.324870	3.293300	4.816027	3.888699	4.760385	4.133820	4.683764	4.757595	4.983217	4.209917	4.162932	4.392359	3.766938	3.776160	5.109345	4.135859	4.229455	4.868526	4.827825	4.646834	4.291773	5.457267	5.064071	6.660368	5.269057	4.993108	5.926133	4.284260	3.153544	6.031996	6.528481	4.439375	3.698196	4.198815	5.722134	4.278403	7.128619	6.136363	4.001248	5.440238	4.055403	4.739775	3.132846	3.843368	4.417754	3.186761	3.080901	5.626914	3.284599	4.185545	4.209292	3.516064	5.814756	2.929028	4.329472	4.184907	4.041347	4.290685	5.822590	5.434147	4.893127	3.211448	3.072674	4.436744	4.891263	4.085680	3.348689	6.141377	5.775818	5.338103	3.159921	4.415573	2.889637	4.693921	4.307910	3.506641	4.825841	3.567496	3.204941	3.469619	3.264389	2.881109	3.669264	3.117616	5.422259	3.635440	4.856921	3.416880	3.468724	3.649022	3.544326	3.735228	4.335916	3.832712	3.407494	3.476705	3.555846	4.884088	3.248982	3.375457	3.673475	3.859195	3.531287	3.225710	3.421532	3.497641	3.058619	3.281206	3.822877	4.290026	4.675819	4.547077	5.865582	4.600410	4.458238	3.302999	5.454938	5.153391	4.222533	4.394274	4.783045	6.213019	3.746476	4.471008	5.996352	5.723646	4.188438	4.226582	4.075160	3.983064	6.820544

PCDHA9	4.201411	3.183197	3.170245	3.875478	3.813804	5.154607	3.078974	3.334678	3.302053	4.244526	5.018696	5.209030	4.058646	4.650040	5.752102	4.059980	3.268818	3.156147	5.022592	4.254568	3.879159	4.081964	6.973051	4.381390	5.547508	4.749521	4.488050	4.040813	3.736675	3.593435	3.477266	5.717822	4.838303	3.797572	4.127852	3.927163	3.368081	3.447280	3.164186	3.094889	5.121307	2.984795	3.817590	3.628614	4.364402	4.569356	5.404608	5.729904	4.730815	4.376523	3.191888	3.099031	3.442756	3.924166	3.581487	4.249700	5.152964	3.380500	4.466094	3.678826	2.933695	4.146217	3.830958	4.149805	4.324430	4.151468	3.178160	3.830779	3.328676	2.874426	3.962928	3.259296	3.981290	3.499640	4.045616	3.257039	3.797353	3.221235	3.537632	3.887853	3.418786	3.676812	3.852937	5.098644	3.268982	4.757829	3.359844	3.247533	3.938324	3.764711	3.515884	3.235234	3.715067	3.094426	3.442756	3.368755	3.409531	4.104098	5.194915	4.081133	4.444781	3.427979	3.994643	3.638690	4.781151	4.546670	3.522057	4.324870	3.293300	4.816027	3.888699	4.760385	4.133820	4.683764	4.757595	4.983217	4.209917	4.162932	4.392359	3.766938	3.776160	5.109345	4.135859	4.229455	4.868526	4.827825	4.646834	4.291773	5.457267	5.064071	6.660368	5.269057	4.993108	5.926133	4.284260	3.153544	6.031996	6.528481	4.439375	3.698196	4.198815	5.722134	4.278403	7.128619	6.136363	4.001248	5.440238	4.055403	4.739775	3.132846	3.843368	4.417754	3.186761	3.080901	5.626914	3.284599	4.185545	4.209292	3.516064	5.814756	2.929028	4.329472	4.184907	4.041347	4.290685	5.822590	5.434147	4.893127	3.211448	3.072674	4.436744	4.891263	4.085680	3.348689	6.141377	5.775818	5.338103	3.159921	4.415573	2.889637	4.693921	4.307910	3.506641	4.825841	3.567496	3.204941	3.469619	3.264389	2.881109	3.669264	3.117616	5.422259	3.635440	4.856921	3.416880	3.468724	3.649022	3.544326	3.735228	4.335916	3.832712	3.407494	3.476705	3.555846	4.884088	3.248982	3.375457	3.673475	3.859195	3.531287	3.225710	3.421532	3.497641	3.058619	3.281206	3.822877	4.290026	4.675819	4.547077	5.865582	4.600410	4.458238	3.302999	5.454938	5.153391	4.222533	4.394274	4.783045	6.213019	3.746476	4.471008	5.996352	5.723646	4.188438	4.226582	4.075160	3.983064	6.820544

SYNJ2	9.488153	9.564348	9.631239	10.073768	10.511993	10.590962	10.323048	10.478589	10.422993	10.200229	10.289433	10.397912	9.904851	10.337472	9.938201	10.320132	11.493891	10.101871	10.422396	9.499970	10.062277	10.103567	10.985815	9.747580	11.338628	10.674952	11.255609	10.767727	10.601825	10.010197	10.784668	8.919206	8.940804	9.534134	10.195547	10.225000	10.454651	10.519894	10.438690	10.538810	9.637621	10.984493	11.082917	10.229417	12.787176	10.247122	11.513170	10.646958	9.994317	10.609210	9.089930	10.450191	9.060510	8.867772	9.619482	10.021710	10.138805	10.327034	9.529282	10.338699	10.376060	10.306414	11.496833	10.307568	9.245349	9.858043	9.678618	9.576986	9.387474	9.925180	10.269288	9.879478	10.465797	10.187118	9.980403	9.829671	9.755164	9.190397	9.174945	9.356365	9.154157	9.376837	9.086422	9.151252	9.280749	9.028802	9.004108	9.665759	9.288170	8.685393	9.072055	9.318981	9.278447	9.892557	9.522089	9.462413	9.178480	10.227439	10.523247	11.839782	10.868738	9.491735	9.914676	10.267192	10.097946	10.602257	10.932033	10.617890	11.052955	11.045432	10.328691	10.641355	10.667558	10.742017	9.701277	12.114778	10.077279	10.939911	11.140081	9.380969	9.708289	9.471395	10.067057	11.104875	10.139254	10.141980	10.305051	9.888466	9.720169	10.247418	10.036419	10.522954	9.792229	10.149344	11.203885	10.134839	9.792063	10.219691	8.988078	9.752217	9.658575	9.799644	10.115756	9.951825	9.725903	9.582846	10.353428	10.468163	9.475674	10.527040	9.238600	9.644070	9.691617	9.174945	9.298596	9.212359	9.744581	9.775229	9.740092	9.968968	9.833499	9.448726	9.531481	9.559852	10.382254	10.397613	9.422207	9.293634	9.334014	9.242731	9.304559	8.632066	9.194946	9.535836	9.618091	8.997209	9.225751	9.332910	8.773366	9.435704	11.553024	9.586073	9.557255	9.267666	9.510547	9.533221	9.049602	9.140403	9.340284	9.299554	9.178624	10.911465	9.424349	9.180474	9.190243	9.170601	9.336777	9.078557	9.355134	8.526133	8.594242	9.296431	8.634112	8.577882	9.648405	8.664535	8.917711	9.162764	9.129684	9.132447	8.988227	8.981042	9.021885	9.099030	9.130010	9.767677	10.158322	9.567325	10.031008	9.838114	9.486271	10.044324	9.462413	9.126584	10.240163	11.574932	9.921992	9.594486	10.383188	10.436289	10.479789	11.379157	10.598330	10.608914	10.436289	9.638262	10.769710	9.503469

ACSL3	14.255787	11.659417	12.091525	12.599871	12.776199	12.262939	12.304637	12.197990	11.694552	12.409678	11.977757	12.330636	11.522631	11.398958	11.353312	12.099218	12.018432	11.491752	12.457861	12.484550	11.750711	11.739081	10.693912	11.646944	11.996843	12.796802	12.012208	12.409512	12.307305	11.962317	11.904904	12.596981	12.207764	11.250000	11.545607	11.725122	11.833741	11.289617	11.327922	11.050033	12.262785	11.509649	10.911326	12.031682	12.106371	11.756636	11.809631	11.436541	12.333040	12.343587	12.404042	12.165750	11.314216	12.269612	11.826829	12.059948	11.747749	11.367114	11.897652	11.319280	11.195021	12.358931	10.717447	11.063360	10.844098	11.414999	11.551514	11.272804	11.330459	10.955728	12.031083	11.866384	12.289665	12.037911	12.019601	11.576892	11.751580	11.696519	12.426698	11.272950	12.586817	12.395697	12.784340	12.813513	12.274399	12.658313	12.211122	12.286593	11.310881	11.799097	11.644748	11.362109	11.820802	12.139103	12.475368	11.884206	12.391429	11.707306	11.814058	10.898488	12.241433	11.045883	10.979653	10.732656	11.097335	12.263250	11.040450	11.300978	10.428215	12.453112	10.215321	11.043725	11.697551	12.034862	12.192131	12.196766	12.637159	12.561217	11.971127	12.141594	11.578788	10.967244	11.121141	10.946388	11.833448	11.369313	11.781211	12.152545	11.200300	11.173458	11.540263	11.528459	11.910004	12.045443	11.849423	10.871747	11.113492	11.306499	12.063188	11.140228	11.605953	10.723137	12.227505	11.450138	12.342322	11.714242	11.680386	12.558801	12.049360	12.297588	10.999386	10.861160	10.646346	12.173094	12.242226	11.185699	11.090456	11.018827	11.053227	12.768697	10.317728	10.535296	11.402055	11.483837	11.177479	11.587908	11.093692	11.957393	11.641331	11.191061	11.720935	10.691177	11.681708	11.652730	13.210340	12.248149	11.110444	10.980712	12.019305	10.137149	12.450751	10.397759	10.820438	10.034221	10.895178	11.589947	10.825756	10.992097	11.275177	10.801205	11.358851	11.300099	11.247355	11.214808	11.298525	11.368582	11.641331	11.822299	11.379762	12.041947	10.821920	11.440039	11.731380	11.952744	11.008580	11.365359	12.108789	10.602549	11.342795	10.717447	10.479041	10.422993	10.561470	11.020059	11.194864	11.405292	11.340397	10.387334	10.270147	11.149918	11.222273	10.345819	9.917330	10.385633	11.696386	8.625448	11.501620	11.611382	11.343546	10.285603	10.547653	9.616838	12.093514	12.648312	10.964890	10.456478	11.465697	10.671356

AFF3	9.611660	8.606504	8.612329	9.144893	9.367419	8.938979	7.823644	10.172770	10.392215	8.269978	8.615851	8.492740	7.881713	8.033614	7.948894	8.732552	9.563890	8.300756	9.932777	8.640834	9.071601	9.942122	10.596659	8.800815	8.991194	9.166648	9.357409	9.786390	8.660064	8.314984	9.780259	8.203755	8.295598	8.493080	8.426298	8.498198	8.534845	8.632606	9.009491	9.371801	8.457938	9.108604	8.555749	9.169996	8.646623	8.751640	8.653579	8.773664	8.616015	8.420244	8.803198	8.415946	7.823834	8.320195	8.333242	8.537994	8.129662	8.244304	8.468700	8.475093	8.884973	8.034047	8.801425	9.257879	8.747578	9.250237	9.009822	8.946272	8.526133	9.743643	9.085519	8.782434	8.451400	8.649681	8.760109	8.934257	9.026892	8.671577	9.385437	8.136662	8.329805	8.342315	8.527971	8.216204	8.542882	8.603763	8.719047	8.615851	8.886372	8.969436	9.089471	9.515156	8.379846	8.484152	9.108775	8.445923	8.598050	8.334148	9.106902	8.566420	9.064741	8.470727	8.947219	8.221769	9.300486	8.424232	9.844099	9.022798	10.462179	8.540524	9.940550	9.665455	9.154319	8.992872	9.054399	11.129440	9.581010	8.687456	8.495523	8.882269	8.628955	8.573812	8.900970	10.281995	8.753475	8.672782	9.416111	9.187084	8.370663	8.484449	8.270121	8.734401	8.069057	8.185578	8.806276	9.188155	8.137100	9.786097	8.226898	9.402607	9.722467	10.323048	9.391424	9.457319	8.759647	9.502229	9.580724	9.376837	8.069677	9.683888	8.702764	8.830099	8.288581	8.339807	8.615560	8.726647	9.430029	9.452733	9.906511	8.764087	9.553448	8.670340	9.318351	8.766397	8.898560	9.143350	8.594547	8.731790	8.878644	9.057352	8.523862	8.005597	8.275964	8.340415	8.163679	8.256475	8.342169	8.509365	8.224339	9.044254	8.337276	9.260146	8.972624	8.823124	9.005349	9.093884	9.137318	9.095089	9.411694	9.058951	8.996891	8.974817	8.848945	8.899923	8.718581	8.998880	9.001965	9.296431	7.972088	8.155313	8.376467	8.362192	8.527214	8.461720	9.349567	8.480622	8.272677	8.851683	8.802470	8.717665	9.643918	8.968822	9.223728	9.128931	9.340883	9.543317	9.469344	8.931622	8.125979	8.917875	8.507811	8.842290	8.614658	8.944736	8.567761	9.686521	8.953642	9.889997	9.161830	9.866689	9.840152	10.065869	9.611805	9.509606	9.812289	8.935765	8.521130	8.341022

ZNF563	7.233867	6.267757	6.916190	7.166404	7.390104	7.215303	7.336598	6.469674	7.400671	6.928293	7.291767	7.368750	7.218415	7.332745	6.615581	7.303511	6.546642	6.492653	7.673529	7.597778	6.750212	6.748483	8.525064	7.449989	7.796488	7.870285	7.326749	6.454936	6.941502	7.112523	7.005042	7.076359	7.673196	7.294607	8.111766	8.009296	7.968338	6.997719	7.514207	8.267453	7.061882	7.930519	7.966199	8.089878	7.862606	8.134218	6.756046	7.550222	7.192295	7.987647	7.848996	7.996300	7.447140	8.152684	7.070554	6.392190	8.349281	8.029041	7.869842	8.321580	8.009151	7.675427	6.610967	7.643516	7.260591	6.621787	7.571574	6.917722	6.782296	6.919027	7.402394	7.512640	7.100497	7.425397	7.824698	7.261987	7.762352	7.057440	7.506195	7.649962	7.810194	7.307573	7.638162	7.851569	7.984242	7.797378	8.181886	8.076511	7.604353	7.053815	8.049851	7.411070	7.054118	7.384091	7.570827	7.997674	6.925494	8.198869	7.272024	7.000313	6.945812	7.519719	7.351457	7.480895	6.826070	6.882766	6.602610	7.189713	6.790236	7.220840	6.891740	7.473850	6.742879	7.451641	7.123846	8.267918	6.692425	7.050689	6.939358	7.461972	5.470077	6.548037	6.743202	6.601827	6.525135	6.622590	6.324214	6.052366	6.522990	7.275464	6.393878	6.772178	6.289442	5.170694	7.249927	6.098553	6.130163	5.104607	6.677511	5.915113	6.015713	6.504965	7.188088	7.357804	6.086417	6.973977	5.956256	7.329134	6.710559	6.621633	6.873056	6.421998	7.097357	6.851096	7.666698	7.633902	6.321693	6.493156	7.261822	6.640806	7.000814	8.458939	7.507556	6.843037	6.996945	6.693087	6.865500	6.858648	7.025864	6.702417	7.802360	6.418492	7.564735	7.383930	6.470882	8.228419	7.536569	6.823702	7.883075	6.412706	7.067588	6.808084	6.428706	7.453866	7.019995	7.473694	6.108872	6.068409	6.255207	7.050689	7.005493	6.817820	7.210504	7.697156	6.925172	7.275147	6.544343	7.319239	8.071082	7.992068	7.421349	7.681302	7.827449	7.923024	7.138446	7.127841	8.007627	7.028923	6.573209	7.567050	7.191534	7.062687	6.772023	7.267272	6.858648	6.347604	7.358723	6.695231	6.282784	6.743202	6.958315	7.113452	6.772337	7.106131	6.689974	5.658330	5.666871	5.690793	6.535312	6.003417	6.360702	5.045882	7.074487	6.715863	7.048951	7.049913	6.648197	6.665078

CFAP65	3.841530	3.637058	3.319596	3.239051	3.217434	3.362499	3.326783	3.447475	4.366132	3.437226	3.521143	3.493546	3.279590	3.992473	3.758336	3.364994	3.713806	3.257039	4.645768	3.330169	5.416606	4.289203	5.993056	3.205978	3.070785	3.305388	3.027358	4.135395	3.217434	3.513374	3.700373	3.589774	4.860639	3.129580	3.607835	4.141298	3.737498	3.501795	3.552388	3.427979	3.495403	3.870938	3.945528	3.406231	3.874364	5.923113	3.770527	3.779768	5.153391	4.063954	3.991816	3.939428	4.603754	4.436995	4.157781	3.205204	4.312111	2.931158	2.990083	3.806389	3.455894	3.401235	4.090696	3.383848	3.613780	3.556975	2.851496	3.360651	3.310696	3.153805	3.121586	3.036842	3.321225	3.204137	3.275589	3.386208	3.166776	3.182826	3.120613	3.935366	3.533311	4.255890	3.618554	4.310605	3.510205	4.882433	3.694878	3.305865	3.227019	3.752527	3.362151	3.610768	3.428492	3.378618	3.073211	3.161721	4.227672	3.786951	3.148279	3.200037	3.847556	3.304432	3.063523	2.929503	4.589696	3.353521	3.386728	3.722513	3.615151	3.342665	3.357076	3.634857	3.056976	3.398360	3.675983	5.886031	3.402304	3.149389	3.281503	3.172255	3.864726	3.513752	3.356572	3.339936	3.857219	3.037944	3.236658	4.166469	3.174610	3.337147	3.114979	3.051939	3.481349	3.576521	3.158788	3.639909	3.016470	3.429348	3.445079	4.685684	3.848646	5.579285	3.307751	2.902397	3.299620	3.687880	3.559346	3.066084	3.594018	3.521300	3.990914	3.612759	3.227451	3.535907	3.843368	3.792266	3.712529	3.471876	3.189850	3.234542	4.803256	4.579245	3.480575	4.408268	5.434949	3.276507	3.288506	4.804508	4.119663	4.013337	3.763620	4.393405	4.658582	3.652626	3.113321	3.899548	2.862605	2.826317	3.502531	2.802833	3.340106	4.218585	4.015184	3.954599	3.773677	2.819679	3.678826	3.445983	2.966843	3.069362	3.065646	3.303158	3.277400	3.084157	3.352560	3.092203	3.178028	3.155201	3.613360	3.912272	4.830608	3.690559	3.663842	3.532151	4.819179	3.287419	3.463557	3.024958	3.593637	3.564191	3.835458	3.192314	3.493904	3.373901	3.142504	4.941966	2.992607	4.092474	3.249291	3.506244	3.354007	2.949165	2.842064	4.975658	3.351726	3.409363	3.690137	3.570038	3.231876	3.417386	3.239185	2.976679	3.287873	4.455015	4.240349	3.655026	3.194590	3.427086

PTPMT1	13.758124	13.175591	13.404202	13.107525	13.388628	12.941188	13.428458	12.420911	12.133995	12.811770	13.047049	13.120423	13.105991	12.906069	12.814228	12.821082	12.641908	13.177997	12.837080	12.960870	13.041374	12.837237	13.356398	13.121958	12.805972	12.879408	12.549730	12.595078	12.692858	13.201614	12.801434	13.377562	13.497789	13.199119	13.426884	13.099767	13.376917	13.113538	13.213390	13.451797	13.163401	13.002534	12.945351	12.994178	12.958452	13.273173	12.721637	13.567818	12.916049	13.058425	13.445036	13.436214	13.247713	12.569706	13.386476	12.776712	13.587916	13.357413	13.624065	13.465756	13.387105	13.057638	13.292760	13.118676	13.539515	13.240817	13.287464	12.919349	12.885836	13.123245	13.089814	13.114296	12.590626	13.139308	13.363673	13.391621	13.339732	13.521153	13.279019	13.543737	12.989794	13.560841	13.849077	13.647417	13.335613	13.361828	13.509947	13.450122	13.455497	13.514224	13.245524	13.238624	13.057638	13.219965	13.462242	13.473153	13.147685	12.649008	12.950005	12.327331	12.420264	12.988128	12.981688	12.834529	13.473788	12.905897	12.839438	13.366449	13.182307	12.840359	13.652033	12.997119	13.202977	12.468446	12.569861	12.126882	13.790457	13.133394	12.273604	13.310158	13.645181	13.523222	13.348676	12.838099	13.640816	12.894731	12.956981	12.704827	14.197518	13.411878	13.590518	12.891207	13.930021	13.485069	12.966194	13.600599	13.427110	13.513328	13.843499	13.264151	13.422986	14.126130	13.450944	14.373327	14.192138	12.993460	13.449081	12.831872	13.673176	13.426259	13.450752	13.559500	13.698230	13.045913	14.020108	12.952559	13.349515	13.804934	14.119553	12.603238	13.353762	13.802610	13.732138	13.774703	14.145227	13.057276	14.021820	13.916047	13.674334	13.385597	13.911868	13.976353	14.986168	13.786878	13.879568	14.145530	14.401146	14.151049	14.281068	13.436852	13.267179	14.201164	13.630442	13.697097	14.423999	13.630442	13.419743	14.122158	13.720180	13.698011	13.740367	12.743876	13.620619	12.987200	13.667672	13.355999	13.517555	13.335613	13.978627	13.189155	13.833551	14.252773	13.757132	14.690192	13.155345	13.973052	14.440521	13.674833	13.842996	14.573899	14.252773	13.104834	14.163952	13.588156	13.230081	13.571674	13.236446	13.833834	13.846147	13.783328	13.671278	12.165896	13.612309	13.549809	13.650975	13.714416	13.590518	13.634499	13.131537	13.845633	12.918024	14.318504	12.239149	12.462402	13.434297	13.621534	13.918583	13.803322

NDUFS3	13.758124	13.175591	13.404202	13.107525	13.388628	12.941188	13.428458	12.420911	12.133995	12.811770	13.047049	13.120423	13.105991	12.906069	12.814228	12.821082	12.641908	13.177997	12.837080	12.960870	13.041374	12.837237	13.356398	13.121958	12.805972	12.879408	12.549730	12.595078	12.692858	13.201614	12.801434	13.377562	13.497789	13.199119	13.426884	13.099767	13.376917	13.113538	13.213390	13.451797	13.163401	13.002534	12.945351	12.994178	12.958452	13.273173	12.721637	13.567818	12.916049	13.058425	13.445036	13.436214	13.247713	12.569706	13.386476	12.776712	13.587916	13.357413	13.624065	13.465756	13.387105	13.057638	13.292760	13.118676	13.539515	13.240817	13.287464	12.919349	12.885836	13.123245	13.089814	13.114296	12.590626	13.139308	13.363673	13.391621	13.339732	13.521153	13.279019	13.543737	12.989794	13.560841	13.849077	13.647417	13.335613	13.361828	13.509947	13.450122	13.455497	13.514224	13.245524	13.238624	13.057638	13.219965	13.462242	13.473153	13.147685	12.649008	12.950005	12.327331	12.420264	12.988128	12.981688	12.834529	13.473788	12.905897	12.839438	13.366449	13.182307	12.840359	13.652033	12.997119	13.202977	12.468446	12.569861	12.126882	13.790457	13.133394	12.273604	13.310158	13.645181	13.523222	13.348676	12.838099	13.640816	12.894731	12.956981	12.704827	14.197518	13.411878	13.590518	12.891207	13.930021	13.485069	12.966194	13.600599	13.427110	13.513328	13.843499	13.264151	13.422986	14.126130	13.450944	14.373327	14.192138	12.993460	13.449081	12.831872	13.673176	13.426259	13.450752	13.559500	13.698230	13.045913	14.020108	12.952559	13.349515	13.804934	14.119553	12.603238	13.353762	13.802610	13.732138	13.774703	14.145227	13.057276	14.021820	13.916047	13.674334	13.385597	13.911868	13.976353	14.986168	13.786878	13.879568	14.145530	14.401146	14.151049	14.281068	13.436852	13.267179	14.201164	13.630442	13.697097	14.423999	13.630442	13.419743	14.122158	13.720180	13.698011	13.740367	12.743876	13.620619	12.987200	13.667672	13.355999	13.517555	13.335613	13.978627	13.189155	13.833551	14.252773	13.757132	14.690192	13.155345	13.973052	14.440521	13.674833	13.842996	14.573899	14.252773	13.104834	14.163952	13.588156	13.230081	13.571674	13.236446	13.833834	13.846147	13.783328	13.671278	12.165896	13.612309	13.549809	13.650975	13.714416	13.590518	13.634499	13.131537	13.845633	12.918024	14.318504	12.239149	12.462402	13.434297	13.621534	13.918583	13.803322

PLPP3	14.332054	12.742146	13.220952	12.506387	12.391429	11.827966	13.483027	11.079395	9.992041	12.392195	12.085897	12.064108	11.875371	11.320099	12.665646	11.723321	12.353569	12.033841	10.922541	11.325112	11.623005	11.877315	13.127100	11.372309	12.994721	12.498330	12.654127	12.218928	13.023662	13.834843	11.562743	13.117513	13.242782	13.178784	13.931643	14.062109	13.792238	13.049719	12.886884	12.938530	12.675008	13.791984	13.491499	13.603336	15.005964	13.678771	12.213340	13.235191	13.364746	13.574884	13.778674	13.867543	14.617785	13.737085	13.099216	11.883896	14.097636	13.608281	12.619822	13.436852	12.965249	13.916872	14.580592	12.174829	13.208700	12.858461	13.009396	12.953847	12.773964	12.896716	13.793282	13.487933	12.131737	13.512420	12.966913	13.193873	13.610599	13.855412	13.545974	13.092572	12.850884	14.271254	13.757880	13.224516	13.802371	13.326991	13.263573	13.267817	13.406280	12.850331	13.261024	12.905001	12.786993	13.565499	13.268023	13.317056	13.629444	12.481977	12.455008	12.697692	11.862884	12.959007	12.421993	12.734538	12.476862	11.926602	11.838717	12.636300	12.875552	12.086504	13.314147	11.798654	11.416603	11.860555	11.357241	12.427314	13.705084	12.733709	11.963714	12.796078	11.194445	12.883664	11.623576	10.590086	11.278563	11.484132	12.424239	9.981361	11.632548	10.919103	11.072137	11.791350	11.392463	10.714609	12.147801	11.809631	10.553689	12.443196	13.713444	11.297951	11.983502	12.536742	11.200580	11.506720	11.753661	12.153751	11.398216	11.918948	11.879689	11.095577	12.336516	12.556219	11.379904	12.021923	12.480968	13.059891	12.038055	10.697494	12.542919	10.165968	11.789411	12.964739	11.980630	12.529181	11.941654	9.701744	13.458499	12.244688	12.151563	13.212614	12.434490	12.574744	11.907433	13.020807	11.884633	13.752310	12.530672	12.770845	12.094734	12.412212	13.291733	11.961308	11.131212	12.344088	12.582308	12.163433	12.165007	11.618969	13.070410	12.206351	12.329087	9.790373	12.672605	13.213195	12.943191	12.870847	12.335103	12.496861	12.178309	11.785073	12.327798	12.521876	14.015289	13.049541	11.788817	12.262315	12.130685	12.437247	12.473959	12.055881	11.554168	12.163756	12.362273	12.606892	12.524159	12.514165	11.125919	11.222672	12.782402	11.667024	13.521840	12.467649	13.396009	11.389264	11.084241	11.162307	12.604915	12.157396	10.550696	12.416734	12.333497	11.516950	11.072990	11.177177	10.945211	12.659132	12.028997	10.485474

AC119674.2	14.332054	12.742146	13.220952	12.506387	12.391429	11.827966	13.483027	11.079395	9.992041	12.392195	12.085897	12.064108	11.875371	11.320099	12.665646	11.723321	12.353569	12.033841	10.922541	11.325112	11.623005	11.877315	13.127100	11.372309	12.994721	12.498330	12.654127	12.218928	13.023662	13.834843	11.562743	13.117513	13.242782	13.178784	13.931643	14.062109	13.792238	13.049719	12.886884	12.938530	12.675008	13.791984	13.491499	13.603336	15.005964	13.678771	12.213340	13.235191	13.364746	13.574884	13.778674	13.867543	14.617785	13.737085	13.099216	11.883896	14.097636	13.608281	12.619822	13.436852	12.965249	13.916872	14.580592	12.174829	13.208700	12.858461	13.009396	12.953847	12.773964	12.896716	13.793282	13.487933	12.131737	13.512420	12.966913	13.193873	13.610599	13.855412	13.545974	13.092572	12.850884	14.271254	13.757880	13.224516	13.802371	13.326991	13.263573	13.267817	13.406280	12.850331	13.261024	12.905001	12.786993	13.565499	13.268023	13.317056	13.629444	12.481977	12.455008	12.697692	11.862884	12.959007	12.421993	12.734538	12.476862	11.926602	11.838717	12.636300	12.875552	12.086504	13.314147	11.798654	11.416603	11.860555	11.357241	12.427314	13.705084	12.733709	11.963714	12.796078	11.194445	12.883664	11.623576	10.590086	11.278563	11.484132	12.424239	9.981361	11.632548	10.919103	11.072137	11.791350	11.392463	10.714609	12.147801	11.809631	10.553689	12.443196	13.713444	11.297951	11.983502	12.536742	11.200580	11.506720	11.753661	12.153751	11.398216	11.918948	11.879689	11.095577	12.336516	12.556219	11.379904	12.021923	12.480968	13.059891	12.038055	10.697494	12.542919	10.165968	11.789411	12.964739	11.980630	12.529181	11.941654	9.701744	13.458499	12.244688	12.151563	13.212614	12.434490	12.574744	11.907433	13.020807	11.884633	13.752310	12.530672	12.770845	12.094734	12.412212	13.291733	11.961308	11.131212	12.344088	12.582308	12.163433	12.165007	11.618969	13.070410	12.206351	12.329087	9.790373	12.672605	13.213195	12.943191	12.870847	12.335103	12.496861	12.178309	11.785073	12.327798	12.521876	14.015289	13.049541	11.788817	12.262315	12.130685	12.437247	12.473959	12.055881	11.554168	12.163756	12.362273	12.606892	12.524159	12.514165	11.125919	11.222672	12.782402	11.667024	13.521840	12.467649	13.396009	11.389264	11.084241	11.162307	12.604915	12.157396	10.550696	12.416734	12.333497	11.516950	11.072990	11.177177	10.945211	12.659132	12.028997	10.485474

IFT22	8.553489	8.595641	8.833467	8.231949	8.079617	8.001992	8.588673	8.971157	7.159318	8.389906	8.714362	8.720086	7.986058	8.175989	8.513066	8.141183	8.213942	8.612010	8.454436	9.101829	8.580126	7.456182	9.863013	7.670062	8.756380	8.954400	7.521277	8.105589	7.916471	8.062875	7.460875	8.568954	8.701068	8.310979	8.983343	9.534452	9.271071	8.776128	8.689568	9.075896	8.135700	8.655522	8.839199	8.960071	9.383472	9.183143	7.794310	9.217443	8.878949	9.072055	9.106123	9.379298	9.137632	9.110791	9.317184	8.096125	9.704430	9.125500	9.103509	9.587642	9.081348	8.006259	8.099129	7.836137	8.835874	9.185942	8.408942	8.201145	8.044489	8.216356	8.590515	8.429598	7.684432	8.551354	8.608929	8.085541	8.399107	8.555588	8.409690	8.996119	8.100093	9.200620	8.956821	8.905075	9.203412	9.238440	8.794756	9.196444	8.241141	8.793982	8.970518	8.374816	7.907411	8.631600	8.670032	8.340131	8.729147	8.136969	8.418559	8.894543	8.150052	8.886833	8.232255	8.892677	9.268924	7.488965	7.993460	8.249148	8.599414	7.596848	8.256188	8.886993	8.285774	7.339085	8.005298	8.397632	8.577147	8.460319	7.629887	8.717058	8.788120	9.051002	7.697302	8.448899	7.905551	7.845347	9.307595	6.825589	8.051562	8.416609	8.547399	8.254672	7.249321	8.491331	8.104810	8.374973	7.930030	8.707526	10.040077	8.141326	7.744413	8.752751	8.460023	9.370141	9.012401	7.772925	8.067722	8.165502	8.273893	7.645417	8.506105	8.698630	8.668344	8.988978	9.754543	9.270748	8.624508	8.173786	8.711023	8.364702	8.606037	8.442840	8.946451	9.778769	9.401214	7.799277	9.884378	9.076223	8.841648	8.822360	9.407255	9.627839	9.490951	9.136846	8.196235	10.700036	9.641485	9.182199	10.139112	8.472816	8.074715	8.017257	7.830635	9.184246	8.073673	9.014694	8.302435	8.135851	7.973059	8.600815	8.346927	7.914144	8.467335	8.447167	8.018603	8.444544	9.384665	8.688353	9.345280	8.554054	9.668449	9.108933	8.765747	9.815273	9.371660	8.325878	9.600803	8.674991	8.197780	8.984120	8.532116	7.980345	8.426149	8.685982	8.661167	8.942784	7.859273	8.115905	9.270309	8.380355	9.630595	8.232098	8.428823	9.361550	7.735656	7.635106	8.704125	8.598050	8.108499	8.228709	9.562674	7.679523	7.613569	9.082279	7.832150	8.734549	7.984536	8.588347

SYNM	13.269841	12.952208	13.712695	13.118469	12.612867	13.401636	15.041266	8.086300	9.046071	14.018727	13.754158	14.018133	12.710332	13.485295	13.152020	12.809121	15.054906	13.380959	11.750863	11.584392	12.576866	12.313325	15.437228	12.523476	14.880110	15.155753	15.778533	14.410971	14.618223	13.879304	12.630807	13.601268	12.356727	14.191772	14.786789	14.780890	14.938328	13.731668	13.537063	12.834869	13.958770	13.427333	14.673717	13.211853	16.171220	13.831800	15.478014	13.388628	14.651063	13.905564	12.932755	14.616554	15.223282	14.113158	14.612308	13.914461	15.035758	13.414215	13.198336	14.487547	14.390000	13.717240	16.013271	13.494882	12.390154	14.260288	13.185843	13.782074	12.248657	13.303271	14.492765	14.651063	14.198163	15.178577	13.702414	14.524343	15.087640	14.002723	13.379282	13.394547	12.889667	14.870263	14.108513	14.910337	15.684238	14.227304	14.021288	14.736202	13.157107	11.639628	13.578055	13.478168	12.367843	14.851633	12.890688	14.594501	14.468143	13.957131	14.252485	14.996211	13.718916	13.942085	12.558326	13.924569	12.252952	13.663221	12.504062	12.944441	12.987200	14.180845	12.645563	12.881870	10.872478	12.643076	12.073126	12.333670	11.997321	15.159483	13.903911	10.809503	10.064236	11.641926	10.620713	10.034536	10.885551	12.861330	9.448263	12.029467	10.903502	10.928598	10.869173	14.189577	10.983350	8.741956	15.063409	11.321849	9.730122	13.118469	13.296189	8.959017	11.250623	13.358673	10.593946	11.535555	10.724616	10.007378	10.784668	12.190892	11.301442	13.598611	10.249099	9.008558	9.228667	11.118254	11.705354	11.240198	10.168890	9.136529	10.264537	10.922541	10.279430	12.137705	9.528337	11.065719	11.259741	9.888466	10.945211	10.941674	12.891887	10.939769	12.942642	11.443675	11.877895	13.687149	14.617366	13.761667	10.742155	11.031349	11.398826	10.334961	15.374826	9.785504	9.839846	9.404677	10.094875	10.180871	9.323989	8.248095	8.261839	11.415282	10.528219	10.558564	10.815220	12.044855	11.414848	11.578918	11.757998	9.923845	13.718425	11.257232	9.978283	10.678516	10.079182	11.528459	11.519102	11.692447	11.299108	13.042766	13.008295	11.657747	10.029478	11.394279	11.241501	11.252662	11.517367	10.653995	9.976964	9.633073	12.179853	10.546578	11.487573	13.556609	11.912931	10.277896	11.290392	9.984426	9.705031	9.990578	10.585975	13.228076	10.221516	9.667179	9.390971	11.386148	7.333384	13.190080	11.108664	10.754004

NQO2	15.082324	13.899116	14.690655	14.978704	15.057918	15.320436	14.291671	14.525087	15.995910	14.421778	14.842680	14.786318	14.234212	14.278941	14.047634	14.740482	15.370133	13.719437	14.262921	14.871778	13.826855	13.843499	14.035874	14.347441	14.634337	15.286692	15.830739	14.477449	14.800437	15.260285	14.928143	13.226252	13.363874	15.296048	15.143591	15.056697	14.987284	14.790142	14.397321	14.729253	14.757385	15.034636	15.860644	15.323382	15.643171	15.703652	15.192041	14.936250	14.645337	15.584586	15.124619	14.651063	14.768992	14.750726	14.382401	15.212349	14.605817	14.165208	14.325045	14.501067	14.246286	15.509129	14.156735	14.729702	13.985840	14.460448	14.248871	14.506441	13.770103	14.390763	14.955574	15.126455	15.248027	14.704765	14.944787	14.981620	14.388223	14.109446	14.558039	13.622440	13.389263	13.905825	13.585329	13.618534	14.289643	13.982674	15.075776	14.080070	13.093911	12.900424	13.270823	13.372935	13.756868	14.082814	14.543837	14.376679	13.616838	15.286692	14.916837	15.897567	15.310966	14.586050	15.117212	14.857987	13.904457	14.891747	15.360277	14.735321	15.254640	15.021686	13.689737	15.296733	14.746020	15.435694	14.388223	16.271502	15.330867	15.177955	15.496317	15.234869	14.751210	14.905079	15.203034	16.006630	15.113035	15.316021	16.100088	15.564859	15.164670	15.135260	15.346400	15.065808	14.721853	14.778940	15.632923	15.502234	14.360038	14.851142	15.013342	14.329679	14.244678	13.639891	15.346400	14.589339	14.240086	15.370133	15.095711	16.016830	14.712563	15.377722	14.985105	15.360997	15.327968	15.275187	15.495462	15.109227	15.043521	14.768542	15.226686	15.313805	14.910337	15.682114	15.686109	15.752097	15.394501	15.347923	15.226686	14.858502	15.639403	15.180575	15.088208	14.695696	14.918365	15.052583	15.213005	15.076957	14.403999	14.487547	14.442351	14.232858	15.317503	14.057693	14.517397	14.731754	14.569333	14.646275	14.570106	14.729702	13.938513	15.292522	15.451210	15.426445	15.386744	15.037486	15.017444	14.899137	15.038124	15.232087	14.519853	14.110411	14.284842	14.522236	14.998489	14.470819	15.561354	14.115590	14.649747	13.995291	14.105360	14.403650	13.693583	14.629537	14.750281	14.731220	14.982214	15.593684	15.787848	14.958920	15.455879	15.376978	15.781709	15.832903	15.114201	13.906384	15.467330	15.594598	14.487923	13.961100	15.467330	14.078902	15.564042	15.506499	15.475629	15.559524	15.477212	14.968533	15.415429	15.287379

DUSP3	13.298159	12.089045	12.570358	12.226312	12.615659	12.852132	13.254196	13.294952	12.762924	13.460482	13.002534	12.915163	12.240711	12.389993	11.955591	13.035807	13.142299	12.296044	12.803851	12.904469	12.417662	12.216524	13.362040	12.161973	12.830155	13.136025	13.482375	13.251437	13.025290	12.652080	13.070221	12.826480	11.634348	12.398695	13.057827	12.738146	13.002534	12.338106	12.799365	12.302965	13.150496	12.671548	12.633466	12.644060	13.810879	12.673598	13.399963	12.528093	13.054557	13.123808	13.005778	13.313179	12.980298	12.969318	12.985009	12.904297	13.531931	13.041546	12.389676	12.639389	12.587474	13.187428	13.763702	12.585646	11.987718	12.733048	12.301392	12.136181	12.096818	12.029158	12.941188	12.918597	12.753285	13.161096	12.948421	12.965249	12.936361	12.982246	12.964171	12.427016	12.207923	13.110956	12.976230	13.363072	13.552300	12.802136	13.399301	13.627987	12.655859	12.228786	12.662573	12.244222	12.214434	13.357219	12.840359	13.038010	12.839357	13.057827	12.835997	13.368915	13.001966	12.838609	12.457238	13.359714	12.571330	12.430939	12.409200	13.078763	12.209586	13.236867	12.010041	12.571685	12.087259	12.757036	12.278505	12.034420	12.837577	13.225296	13.184851	12.118005	11.383354	12.055502	11.353755	11.397000	11.439898	12.508749	11.238007	12.447571	12.222786	12.180494	12.437743	12.688477	11.806190	12.055422	12.904297	12.611187	12.257562	11.684959	11.787474	12.228964	12.058239	12.329711	11.683357	12.330636	12.876754	12.031230	12.117412	11.405878	12.672605	12.651078	12.592167	11.547384	13.270823	12.282874	13.147891	12.809121	11.663282	12.410313	12.222501	11.627063	11.968244	11.670766	13.056909	12.908941	12.390154	11.849423	11.748029	12.719045	13.140068	12.640723	12.837925	12.579166	13.092754	13.171908	12.950759	12.512753	12.360207	11.694693	11.538822	11.941497	13.600359	12.287369	11.817176	11.879689	12.188852	11.502190	11.655757	12.046914	12.098027	13.140268	12.980619	11.725723	12.880439	12.630185	12.521399	13.053301	12.564301	12.801254	12.558955	12.295577	12.336035	12.832911	12.805624	13.014830	12.747616	12.556362	12.628677	12.597317	12.197526	12.082135	12.172186	12.053694	12.441436	12.216677	12.262315	12.635424	12.203746	12.170325	12.734879	12.068749	11.465093	12.539475	12.441117	11.603716	12.247193	11.669233	11.569749	12.123320	11.722280	12.051034	11.961890	9.115876	12.167578	12.368604	11.897946	12.799704	12.196906	11.779417

CD4	12.246238	11.852324	11.527331	12.392060	12.342613	13.032306	11.716302	13.230859	14.425606	12.205727	12.895995	12.694750	11.915044	12.108933	11.873097	12.719723	13.778922	11.584062	12.621829	12.484713	12.175459	12.315273	12.661720	12.155713	12.552991	12.989626	14.587290	12.810731	12.416267	12.556996	13.295984	10.468311	10.637424	12.655512	12.761183	12.624672	12.091212	12.227205	12.659292	12.755118	12.520550	12.715143	13.940718	13.510832	13.793821	13.404827	13.192485	12.840899	12.093373	13.144955	12.924028	12.402606	11.819904	11.887212	11.851720	13.184481	11.576750	11.997634	12.237262	11.825645	11.927709	12.783797	12.798847	12.634771	11.667160	12.036088	11.969124	12.082595	11.412815	12.274399	12.409843	12.351330	13.203403	12.131274	12.304945	12.401128	11.984692	11.669088	11.967801	10.322128	11.085135	11.018066	10.451232	10.855244	10.822224	11.505395	11.276168	10.971305	10.614968	10.668285	10.668858	11.064379	11.417687	11.662821	12.011409	12.391754	11.426795	12.715589	12.580172	13.904754	12.971491	12.250568	12.773792	12.379731	11.868033	12.409034	13.601956	12.550893	13.293157	13.052613	11.539250	13.294148	12.939909	13.514680	12.327002	13.730904	12.886695	12.477677	13.231246	12.660869	12.456287	12.001437	12.779681	13.817064	12.975277	12.869416	14.308313	13.422807	12.970784	12.915514	13.141081	13.064722	12.402908	12.964368	13.300262	13.565264	12.167414	13.204762	12.008552	12.640385	12.567588	12.402123	12.951859	11.966551	11.439106	13.637147	13.144955	13.932970	11.997151	13.761409	12.330494	11.953784	12.518430	12.489402	12.128873	12.240857	12.291860	12.816305	12.969695	13.163201	12.494332	13.231839	13.495327	13.258606	13.059695	13.455726	13.086086	11.805598	12.806823	12.962718	12.579011	11.923119	11.848352	12.446416	12.564459	12.035336	12.019762	12.295263	11.683647	11.753524	12.603915	12.538254	12.431763	12.493360	12.110414	12.108933	12.284575	12.398510	11.851856	12.467154	12.454055	13.960526	12.535268	12.322965	12.583626	12.201865	12.283965	12.624487	11.040899	11.562888	11.281809	11.133393	12.124049	10.944165	12.861151	10.802247	11.509371	11.432097	11.886584	11.502905	11.443675	11.897073	12.135121	12.179402	13.303685	12.903569	13.420393	12.472957	12.373685	13.097503	13.226650	13.694245	12.710332	11.750117	13.470544	13.605949	11.989419	11.733781	13.882222	11.992156	13.443082	13.636198	14.168428	13.057276	11.752478	12.416908	12.425739	13.006688

SRPRB	13.131724	12.897590	12.609687	13.682827	13.166297	13.004306	13.676222	13.205336	11.738648	12.562670	12.679547	13.689281	12.863843	12.251052	12.668485	12.179253	12.575245	12.855512	12.473611	13.711698	12.472313	13.554152	11.704750	12.243919	11.638885	12.445946	11.857195	12.194595	12.627500	12.524622	11.875511	13.863595	12.807201	13.415659	13.585329	12.755816	13.083849	13.013350	12.837577	12.409358	13.122311	13.160146	12.550549	12.840811	12.489075	12.645205	12.222501	13.210340	13.197125	13.390336	13.363072	13.163401	12.521063	14.434184	12.990709	13.101611	13.117722	12.978042	13.194312	12.921144	12.911640	13.011779	12.519704	12.782402	12.999330	12.924028	12.922949	12.139723	12.919720	13.101611	13.430141	13.662957	12.392356	13.382399	12.905702	13.087748	13.489038	13.626137	13.049159	12.836529	12.523307	13.064722	13.521387	13.879568	13.016643	13.387976	14.029339	13.222745	13.480816	13.504598	12.844915	12.994008	12.929922	13.518422	13.502998	13.822206	13.311729	12.151239	13.625907	11.870697	12.006852	13.222745	12.427164	13.117358	13.277634	12.964739	12.626987	12.512914	13.181925	12.080437	13.756377	12.146258	12.593788	12.513855	12.137705	11.669538	13.109859	12.876931	12.298697	13.511061	13.079487	13.302463	13.887252	12.927582	13.085901	13.008116	12.341032	12.943191	13.895320	13.460684	13.362663	13.186829	13.377131	12.895811	12.656514	13.170169	13.358673	11.916369	13.054365	12.861151	13.696085	14.060010	12.441117	13.014830	13.953781	12.509485	13.903326	12.512428	13.518422	12.649882	13.206334	12.356099	13.171526	13.971862	13.884347	13.307295	12.859356	13.570764	12.895995	13.406964	13.312162	12.604409	13.532157	14.264949	13.892381	12.900071	12.892075	14.229183	13.334586	13.330689	13.026917	13.758124	13.581357	13.366449	13.936301	13.263961	13.841137	13.334586	13.669633	13.729117	13.302074	13.814789	12.790822	12.966367	12.526452	13.094296	12.938530	13.196334	13.438194	13.775933	13.165696	12.398858	13.644958	14.064771	12.802300	13.509232	13.471189	12.998253	12.593457	12.855684	14.521823	13.578538	13.516909	14.006716	13.381340	14.041399	13.965466	13.440530	13.079117	13.297173	13.778428	12.897761	13.868078	12.978577	12.929565	12.763759	12.219989	13.622196	13.470544	13.564364	12.618117	12.734213	12.884383	13.714163	12.805120	12.115391	12.939042	13.338514	12.497991	13.382599	11.996132	12.597984	12.435950	13.966887	12.975830	12.662914	13.638076	13.592392

MYNN	11.960368	10.503462	11.520394	11.354634	11.675812	11.195789	11.934282	11.992622	10.342962	11.601057	11.796594	11.825487	11.324835	10.607831	10.687204	11.086467	11.463911	10.668858	10.017871	11.581538	10.761296	10.494224	9.085022	10.602822	11.008142	11.383634	10.150081	11.439584	11.143986	11.713779	11.189473	11.745525	10.626401	11.749526	12.239925	11.968244	12.168975	11.494063	10.607687	10.850917	11.540839	10.600166	11.793165	11.954084	11.885091	11.971923	10.588938	11.014430	11.941052	11.862600	11.972367	12.174511	12.077573	12.247673	11.719874	11.528459	12.131274	10.936553	10.640470	10.736547	10.405317	11.213347	10.045720	10.443810	11.086931	10.889830	10.651347	10.682588	10.805332	9.937753	11.620849	11.581815	11.118709	11.711892	11.726896	11.744648	11.780301	11.937627	11.764495	10.869324	10.209666	11.984838	11.809488	11.979596	11.956809	12.125960	12.211933	12.277448	10.585064	11.093119	11.047378	10.256478	10.928144	11.833741	11.507297	11.902342	12.026746	11.584062	12.123320	9.971364	11.515630	11.092677	11.065101	11.775639	10.640300	10.941247	9.751120	10.383504	9.848474	11.230646	9.813834	10.368210	10.352360	11.189746	11.794551	10.373383	11.174436	11.512602	10.691177	12.253114	10.694207	12.461727	10.630708	10.872641	11.473964	11.763874	11.423160	11.298691	11.498901	11.904615	11.817489	11.151708	10.632170	8.918170	11.088131	11.786572	11.041788	9.872877	12.200477	10.782381	10.652361	9.977410	10.254371	11.096922	11.830901	11.671644	11.790568	10.826665	11.641173	11.560840	10.965182	10.361904	12.209130	12.747949	12.790143	12.364182	11.292669	11.215233	11.032834	10.888642	11.044735	12.579663	12.816563	13.083300	12.840549	10.602257	11.716588	12.552169	13.117358	12.329087	12.487821	11.921278	11.952744	11.709110	12.366707	12.107881	11.998716	10.884165	11.295540	11.015142	10.976695	11.351596	10.588938	10.848877	10.704200	11.611243	11.118854	11.356245	10.586401	12.249758	12.424744	9.885942	12.239319	12.683500	12.089192	12.455951	12.669327	11.749227	10.824581	11.308236	12.480123	12.532804	11.696519	12.297588	11.976836	12.868144	12.765477	10.613036	10.824736	10.959584	10.857655	10.899971	12.413658	12.446254	11.529600	12.110554	11.366830	12.297109	12.184541	12.028252	12.269612	10.824286	12.209586	11.087821	10.221095	10.857655	10.797454	11.147104	10.090224	10.162738	11.741301	11.656322	10.188468	12.891887	12.211933	11.466138	12.386589	10.980857

PPA1	14.240086	13.722489	14.503429	14.777106	15.038723	14.774671	14.880110	14.711236	14.002723	14.318504	14.676953	14.825958	14.264949	13.838475	13.968272	14.711675	14.625551	13.679487	14.107221	14.370128	14.215329	13.717944	11.906965	14.301287	14.100719	14.519413	13.355999	14.958920	14.290297	14.411336	14.130795	15.646016	14.148848	14.521454	14.792525	15.081140	15.090125	14.545545	14.015006	14.031741	14.782312	13.508579	14.752647	14.401854	14.111066	14.585219	14.053607	13.509232	15.263762	14.412071	15.088208	14.783853	14.442351	14.643124	14.905079	14.618223	15.606629	13.648255	13.921593	14.157364	13.291131	14.659974	13.004862	13.566616	13.804646	14.479847	13.717128	13.916607	13.993914	13.576961	14.771836	15.411549	14.467367	14.939862	15.079318	15.474785	15.396904	15.024101	15.283070	13.938513	15.209040	14.872324	15.424214	15.732189	14.614415	15.311610	14.927607	14.854342	13.160892	14.460851	14.099791	13.445036	14.019002	15.434895	15.143591	15.242555	15.482081	14.400393	14.509550	13.502195	14.700650	14.384169	14.608827	14.371917	14.286571	14.295055	13.514224	14.025054	12.668989	14.628138	13.084593	14.370460	14.121867	14.698837	14.726053	14.267954	13.716086	13.776922	14.095820	14.549326	13.622196	14.685534	13.564810	13.787896	14.230548	14.126130	13.425338	13.616405	15.172639	14.215329	14.260288	13.938513	13.824264	13.022170	13.883806	14.113444	12.209741	13.618534	14.796877	13.701136	13.229238	12.892258	12.584309	15.106780	14.502269	14.617366	14.452852	13.599935	14.266304	13.723478	13.840099	12.450587	14.822343	14.452088	14.157977	14.179251	14.774189	13.415034	13.627288	13.530341	13.587916	14.513890	14.681870	14.710296	14.891226	13.826605	13.522531	15.131490	14.796877	13.906117	14.583170	15.148768	14.974306	14.792525	13.845086	13.635468	13.765490	14.240420	14.043461	12.961780	13.609691	12.631817	14.126434	14.135776	13.747078	13.983800	13.529215	13.863343	13.204369	14.797410	14.630796	13.459822	14.770475	14.602856	14.671011	14.555161	14.922772	14.815284	14.651063	13.695182	15.318975	14.316814	14.452852	15.365568	14.201653	14.772763	15.303728	13.258800	13.578983	13.690683	13.287648	13.587916	14.927607	14.087732	14.584760	14.579771	13.140875	14.335789	13.802371	14.817306	13.418619	13.350971	13.994733	13.835602	13.632774	13.000997	14.235912	12.993460	14.137614	12.979897	14.388615	14.820855	14.279316	14.891747	14.540484	13.969621	15.723939	14.488364

EYA4	5.103993	3.232699	3.002835	4.554945	3.689524	4.059769	3.967902	5.233431	3.644257	5.199677	3.894841	5.534285	3.938758	3.941517	3.719337	3.810560	3.402108	3.478586	3.603661	5.019934	4.863721	3.215682	4.110948	3.348203	3.449361	3.701392	4.504144	4.161599	5.662559	4.300892	3.727139	4.758930	3.484067	5.212965	3.424199	3.996626	4.735652	3.183074	3.748721	3.064058	4.748287	3.102819	4.125060	4.390178	5.761020	5.436108	5.143159	2.997794	5.168280	3.418075	4.590982	4.331654	4.068163	4.063079	4.205714	3.871796	4.913496	3.111657	3.140934	6.137363	2.897338	5.587518	3.491861	3.716485	3.842951	3.783976	2.861591	3.855536	3.696522	3.911625	3.598578	4.341361	4.794167	4.124861	4.190163	3.301128	4.182980	3.686432	4.139072	3.964673	4.723229	3.918319	4.281251	4.484384	4.599394	3.598945	5.712884	4.387386	3.101295	3.869456	3.542277	3.486956	3.617097	3.947935	4.099547	4.623340	4.591411	4.875077	3.944424	3.921454	5.339727	3.710057	3.779373	3.992227	3.470157	5.171481	3.006446	3.735228	3.353040	4.320790	3.098313	3.795908	3.665470	5.749744	3.717910	5.922924	4.472031	4.299617	5.231386	4.174447	3.322974	3.004165	3.282404	4.868967	3.723702	5.521763	6.207080	4.411759	5.351486	5.058371	5.780525	4.686755	3.262792	3.352380	4.466739	3.306163	4.525882	3.027576	3.499062	4.196467	3.971168	5.681213	7.229521	3.017093	4.846179	5.278130	3.163139	6.277178	3.854698	3.695073	4.366995	5.013417	5.636952	3.286231	4.413326	4.393824	3.247824	3.263098	3.008166	6.041579	3.021888	3.813804	3.464678	4.308141	5.039903	6.548199	2.902673	6.492830	4.463269	5.054079	3.411831	4.669702	4.261771	4.035847	3.973302	3.804516	3.105616	2.943473	6.367040	2.840111	4.041460	3.445079	4.447326	3.475268	3.583778	2.853354	3.553350	3.394704	2.877511	4.266350	4.334644	6.588358	4.945653	3.847370	3.473243	4.026648	4.051084	4.306150	3.633517	3.720796	4.317809	3.629032	4.230278	3.854880	3.743241	4.138852	4.059980	2.975343	3.411476	3.394523	2.966317	4.064780	4.254568	3.532537	4.802623	4.152513	5.513884	3.833118	6.206276	4.752112	3.422423	3.815038	3.763856	3.699196	7.948461	4.532248	3.317112	3.309540	4.967357	3.201711	4.504347	3.970758	6.024815	5.968975	4.246870	3.024958	4.553674	3.875056

TGM2	10.289597	10.484745	10.249709	10.240599	9.155110	9.290023	10.818932	7.609592	7.742243	10.201859	9.672549	9.734090	10.338699	10.085861	10.139112	9.685927	10.190552	10.771657	9.305858	9.565885	9.706104	9.458628	13.649618	9.863922	11.324835	11.310712	12.486970	10.888061	10.582324	11.048690	9.697856	10.509773	10.839132	10.615103	11.032397	9.807001	9.967259	10.165338	10.876491	10.865149	10.430163	11.338628	11.131496	9.795801	11.247492	9.771907	11.269542	10.779119	10.418891	10.339310	10.504548	10.151883	10.670295	10.481389	10.458181	10.848448	10.780002	10.745421	10.246883	11.393682	10.903357	11.344909	13.009581	10.840635	10.196135	10.774902	9.975738	9.880112	10.624433	9.842744	9.785347	9.929378	9.946377	9.867722	9.499648	10.315931	10.077903	10.737168	9.733340	10.420249	10.077279	10.547330	10.258677	10.186361	10.570453	9.931241	10.242906	9.789292	10.572908	9.447951	9.870429	10.254638	10.336891	10.714168	9.777218	9.689001	10.205821	10.767583	9.780886	10.948753	9.954745	10.269429	10.752372	10.149023	10.643266	10.225283	10.776077	9.510547	10.569842	10.312288	9.923356	8.856221	8.916489	9.460752	9.111930	9.147530	10.652524	10.541677	10.344614	10.238329	10.273634	10.989295	10.689930	8.967435	8.766072	10.415855	10.282310	11.673261	9.637621	11.737021	10.401393	12.395993	13.692162	11.451631	10.916881	10.067980	9.302664	10.332961	10.044788	9.645490	10.939616	12.777955	8.262615	10.180089	10.503777	10.228365	8.616015	9.378542	11.399407	11.286728	10.861307	10.269429	9.598034	10.279891	8.413235	10.012286	9.268311	9.050390	11.680678	9.179371	11.005994	11.013094	9.791905	9.631674	10.407551	9.100559	10.992701	9.308789	10.965182	10.186361	11.274000	10.782076	11.279592	12.188558	11.724680	10.734749	11.122188	11.215686	10.610070	11.307971	12.256938	9.579165	10.749604	10.070208	10.990603	9.861920	9.854951	9.301568	11.473822	9.135166	9.643918	9.276833	8.923868	11.017510	10.394903	11.677730	10.065725	8.721472	13.114850	10.592463	9.513068	9.260739	9.867886	11.810536	10.463860	10.174868	10.173802	11.924594	12.270250	10.843161	10.595736	10.213349	10.860574	9.896747	10.619249	9.712939	8.674991	8.607116	9.787911	9.676141	9.501326	10.756778	11.208645	11.868173	10.239265	9.544579	9.598799	10.047564	9.385728	11.485173	11.023804	7.844214	9.088836	9.497649	8.009628	11.799242	8.295916	8.873698

SORD	9.270456	8.277666	8.786239	7.741489	9.258777	8.678828	8.822037	7.778984	7.802054	7.926641	8.370159	8.718121	8.315925	8.418410	8.609069	8.033614	8.238523	8.602983	9.405869	9.175876	9.210087	9.136379	10.158948	8.569732	8.864910	8.453530	8.011423	7.805090	7.729031	7.132916	9.083635	9.510217	9.246898	8.400034	8.485660	8.815133	9.272401	9.018370	10.012765	9.092833	8.490573	9.195450	8.221296	8.643311	8.947521	8.706907	8.208005	8.430558	9.038835	8.111766	8.269676	9.023720	8.849097	8.861847	9.224038	8.244922	9.326903	9.844727	9.093737	9.120947	9.757339	8.422262	9.819042	9.379881	9.130300	9.252684	9.164484	8.680202	9.085022	9.585450	8.599266	8.803984	8.557313	9.018370	8.518854	8.709005	8.995637	8.647531	8.511835	9.276833	9.403225	9.196095	9.446852	9.424676	9.455425	9.284449	9.141334	9.513501	10.188617	9.761509	9.743949	10.385019	9.159846	8.609684	8.859422	9.174793	9.019286	8.420888	9.140105	8.242770	8.850756	8.542583	8.012608	8.903998	9.666389	8.607116	8.591916	9.014249	9.665912	8.321720	10.396131	9.176661	8.868386	7.839369	8.707526	8.546419	9.900833	8.747415	8.192439	8.056978	9.953346	7.293973	9.006123	6.068771	8.313592	6.756207	8.584250	10.392827	6.626161	9.742277	8.538157	7.606999	6.773490	8.417392	8.598657	8.123421	8.737472	10.617597	9.493455	9.341602	7.557083	8.706747	7.020942	9.139490	9.442051	8.543056	9.076866	10.408604	6.554476	6.989522	7.425716	8.895958	9.431743	8.639746	10.198425	8.504326	9.687143	10.124118	9.702796	7.738668	9.565725	9.597413	9.510870	9.297246	8.056362	8.151610	9.686684	9.820128	9.373360	8.877113	8.115905	7.991593	9.021575	8.879418	9.893788	8.476914	8.798858	8.370333	10.933084	6.621138	8.768231	10.429431	10.761158	9.268924	8.986858	9.817990	7.019538	6.467703	8.196996	9.002579	8.834047	8.403138	8.915416	8.565872	8.066831	8.756854	9.388703	10.191000	9.502845	9.563002	8.230865	8.691979	9.531152	9.440330	8.929089	7.801900	8.354912	9.501613	7.905551	10.006922	11.212744	8.055425	7.125623	9.095089	7.816519	8.244304	8.368619	8.363626	7.099566	6.684249	7.015335	7.966199	8.376467	7.524374	7.966199	9.442348	9.728526	11.115020	8.310673	10.826070	8.091578	9.007159	7.027807	8.108945	9.075452	8.676059	9.177716	6.531435

GABRB3	10.358719	6.625003	7.205802	6.865500	5.412417	6.288774	8.003253	4.063954	3.801826	6.180152	4.181660	4.871916	8.532273	5.375758	5.780899	6.671872	6.514869	5.853436	6.642091	7.121194	5.257140	5.527072	9.943954	5.015459	5.523672	6.878780	7.693823	4.680068	6.525640	7.609277	5.757214	9.904261	7.926641	8.152839	8.313433	7.578853	8.919828	7.615580	7.615728	5.385758	6.692759	7.151398	6.730471	7.566575	8.147768	7.535183	6.767805	6.771880	8.396552	8.411859	8.984879	8.081052	8.195994	8.390510	7.787467	7.456825	8.700125	8.184797	6.197245	7.595792	6.368043	7.039059	8.784460	6.650608	7.986058	7.762671	7.568265	6.087306	8.667435	7.250091	7.270643	8.025420	6.692425	8.620966	7.306185	7.797700	8.208762	7.360909	8.618151	6.824177	6.685504	8.140905	9.527395	7.904130	8.194634	7.897456	7.983486	7.708553	7.711235	6.913700	8.277460	7.421675	6.298242	7.535484	9.302345	7.959450	7.947541	7.640791	7.663101	6.843510	5.207836	7.161880	7.088794	7.277515	7.663886	6.099247	6.678007	6.267597	6.038961	6.460672	5.716576	5.335333	3.487711	4.995664	6.007743	3.264389	10.158811	7.838778	6.019537	9.426808	4.126990	3.835245	3.967902	2.986651	11.312487	3.737108	3.369613	3.436524	3.138348	3.749587	5.066111	4.209499	3.303468	3.275122	8.612660	3.542076	2.872967	10.286370	4.904203	4.533715	3.208873	9.677053	2.882057	6.821344	8.671577	9.791905	2.801819	3.190142	3.981754	4.762051	4.921033	7.723928	3.544693	4.082186	7.709416	7.229974	7.428055	4.324651	5.946365	8.262136	5.823304	4.838497	4.594714	4.532248	5.265665	3.661019	6.858491	9.668618	6.064179	6.803833	10.304169	6.716023	6.001274	7.559652	9.187084	10.599565	3.322496	5.908843	6.423176	3.947460	7.588434	4.304427	4.181446	4.363515	4.437643	3.121348	3.837146	4.537597	5.879823	8.491028	4.987473	3.328676	6.520020	7.585809	8.490868	8.855919	4.692658	6.035071	6.121636	4.484799	5.296854	7.115823	4.705737	4.902554	4.597879	5.142569	4.167115	5.291773	5.735558	7.037948	3.703237	4.732932	4.195183	5.172334	5.455701	3.270087	3.772596	5.778924	4.893555	3.076437	5.565213	11.457125	5.295630	7.241967	3.341480	3.081335	3.747084	7.562281	3.251420	4.255446	3.398852	2.842344	3.290793	8.586817	2.981901	8.017407	3.924795	5.815816

DLG1	5.667791	4.668385	4.066688	4.557891	4.823328	5.977963	5.716576	7.135477	8.072872	7.243579	8.134813	6.204015	6.907201	6.132434	5.481833	7.474898	7.635404	5.358339	5.415123	6.977461	5.150938	3.573829	3.740182	5.204460	5.427255	5.543910	6.880697	4.566806	7.042884	4.411759	6.996790	4.741955	4.660049	6.012483	5.495885	4.372155	4.830820	4.079143	4.337686	4.559441	6.665571	3.639185	5.335551	5.177346	5.524779	4.978895	6.363388	3.206045	3.814191	4.658804	4.738648	4.271401	3.669057	4.931772	5.422062	6.911631	4.415573	3.625205	4.766370	4.629125	3.856144	6.405052	3.191611	3.746283	3.293629	4.174224	3.038852	6.273370	3.995497	3.262792	4.151892	5.827845	7.518351	4.068163	5.136227	4.346523	3.792920	3.787788	4.128056	4.552354	4.848706	4.972542	4.400888	4.408699	4.332682	6.153116	3.574228	3.698196	3.439974	3.224345	3.148383	2.947532	5.249579	4.610588	4.777623	6.430895	4.870422	7.659388	6.952479	7.716737	7.297804	6.208471	6.693760	5.069387	3.948773	7.456680	5.038528	3.711741	3.242391	6.834522	2.934273	5.703178	5.610093	7.731029	7.435209	3.285959	4.351435	6.980337	7.850213	6.253511	5.697494	3.806168	4.096007	5.003376	6.744515	6.242941	7.545980	8.852566	7.950478	6.997572	6.027480	7.619552	6.309443	3.933476	7.101874	6.945812	6.075903	3.662866	6.066083	4.867022	3.122398	3.257454	7.066045	3.352205	3.598158	5.884779	5.051969	7.038273	5.343795	8.334307	6.857547	7.316378	6.922419	6.405375	6.117316	4.770748	4.183639	5.675316	4.245365	7.972576	3.481721	4.909875	4.920835	6.130691	4.590982	8.710706	3.452771	6.024627	6.020286	4.681557	6.016723	6.449494	5.359485	4.158412	6.966132	3.502367	3.863232	5.186207	3.071333	5.979208	4.923203	4.096648	4.611640	3.578183	3.941921	4.736346	5.307530	5.264877	3.104560	5.887979	6.061172	7.534703	5.501195	4.447792	5.471380	4.226582	4.387819	5.558298	3.627415	6.483577	5.494080	5.232814	4.641771	4.070060	4.117669	5.536657	5.238255	3.280419	3.949881	4.129806	3.114731	5.996352	6.213909	6.847580	6.687456	6.151211	8.103236	8.552114	7.011725	8.119314	7.414619	7.481815	3.774090	4.186652	7.679817	6.533240	5.118253	5.073638	7.458563	2.879676	5.343992	3.310373	6.117672	6.767961	7.045786	4.943641	7.768086	6.524664

AC067752.1	11.232423	10.225000	10.289132	11.147515	10.650157	10.709659	9.274979	10.834204	11.548425	10.115756	10.732241	10.490251	9.562674	9.100841	8.969270	10.249401	11.330144	9.093285	10.025435	9.855417	9.752834	11.383057	11.339952	9.402162	8.636158	10.748889	11.264255	10.311282	11.218335	9.863922	11.465549	9.029197	8.796854	10.231920	9.010403	9.728056	9.484248	9.030556	9.588428	9.992195	10.379400	9.810118	9.737007	10.332520	9.731306	10.262244	12.117412	9.049134	10.373383	9.619017	10.233687	9.560158	8.625594	8.937156	9.062181	10.537076	8.531373	8.834785	8.803198	9.282941	9.507776	10.870211	10.144304	10.186664	9.839846	10.417829	10.233687	10.262380	10.010033	11.421630	10.774607	10.660852	10.892076	10.147640	10.375288	9.608075	10.264251	9.714221	10.345819	8.183407	8.588024	9.010820	8.548297	8.331048	9.111441	9.256070	9.170158	8.693560	8.832252	8.887920	9.342047	9.923356	9.558784	9.999518	10.313564	9.669262	9.410961	10.847104	10.739148	10.903646	11.318667	9.793162	10.212131	9.666724	10.356772	10.414186	10.387620	10.506605	12.145451	10.564425	10.823553	9.864082	9.782381	11.097765	10.472987	13.679951	10.278824	10.535147	11.499211	11.184501	11.096621	10.674034	11.368860	11.851720	10.188320	11.100018	11.634201	10.575868	10.620123	10.692263	10.736706	10.266295	9.868372	10.322892	10.741413	10.672708	10.116982	10.637586	9.188306	10.798780	11.463192	10.834982	10.598942	9.177565	9.564819	10.939212	10.578752	11.984395	10.090224	12.047831	10.404103	9.916055	10.021223	9.374150	9.867109	9.730122	10.140329	10.402145	10.566491	11.285190	10.138059	9.173279	10.516760	9.746613	10.255271	11.759472	9.830470	10.518615	9.800263	10.358877	10.142442	8.725722	9.344981	9.309102	9.759652	9.116348	8.887749	9.207632	8.949666	9.676141	9.758574	9.896599	9.923051	10.385633	10.192680	10.592327	10.349760	10.786914	10.938612	10.790437	10.624276	11.852470	10.520787	10.540607	9.576986	10.378348	10.158173	10.547030	8.598194	8.538157	8.881755	8.936392	8.982750	9.381799	10.689640	8.830099	8.813200	9.658419	9.499648	10.101264	10.058014	10.189059	10.891930	10.427634	10.656092	10.941674	11.782817	10.395504	9.665313	11.266027	10.654131	11.097635	9.708289	10.428377	10.622485	10.568476	10.317267	10.857955	10.985966	11.272804	10.330692	11.550311	12.250882	11.390636	10.961076	9.578383	10.505273	10.538071

ADH1A	14.133856	11.186851	11.603148	11.283702	9.877183	10.086530	13.671017	9.384220	9.219434	11.504652	10.393108	11.218772	11.080729	10.589937	11.704443	11.855782	11.830475	10.511848	8.097374	11.267076	9.134694	11.188016	10.176535	10.464310	11.434496	10.085713	12.482801	9.799359	12.601601	13.071521	11.513924	13.708928	12.487307	12.356334	14.469705	13.915290	13.916047	12.352576	11.885697	12.223705	12.735569	13.541913	12.948751	13.455497	14.509550	12.969318	11.604162	11.798936	11.668318	12.614208	13.800361	13.308940	13.016071	13.658895	12.330636	10.754780	13.882222	12.629207	10.841116	12.645369	12.447571	13.595594	12.760314	10.516841	12.147509	11.763401	11.834974	12.379442	11.653340	12.465654	13.898829	13.748094	11.420288	13.384972	13.332262	13.550257	14.473933	12.099825	13.488366	12.248323	12.895646	12.807024	13.043715	13.126344	13.403666	13.157459	12.793014	11.378727	12.282721	11.869781	12.439669	12.016135	11.913381	12.702108	13.792778	13.631592	13.700156	12.783965	12.606059	11.700684	12.182961	12.682905	11.199882	10.455701	9.649319	11.722280	10.838287	9.360960	11.471808	11.959745	12.708589	9.532146	9.325661	11.557603	10.494672	9.636219	13.383479	12.153901	12.463731	9.752834	5.814250	6.744672	10.525956	3.283480	6.321320	8.334148	4.320790	5.522145	7.729343	5.357029	6.624362	7.781951	4.803917	5.083639	11.074134	4.673856	7.333384	6.015887	7.473387	8.918022	5.879233	8.033453	5.221697	4.592086	9.862391	8.416105	5.399012	12.875552	11.141637	8.530152	8.213459	7.712340	7.193586	9.650235	8.614211	9.071262	7.660314	6.350954	7.663402	5.904725	9.894119	7.348804	7.603602	9.927841	8.994888	5.375140	8.518092	12.157725	7.466956	8.838748	11.697270	8.873398	8.112848	10.361120	11.122337	8.910839	8.359117	9.273789	8.263689	6.582771	11.637074	7.075119	5.257917	6.980510	8.833342	6.332486	8.249737	6.235310	6.777825	10.161530	7.864018	5.192494	7.830357	11.978674	10.389287	8.774143	6.746121	8.764831	9.388260	7.206614	7.925527	7.580232	7.113933	7.435209	9.265515	7.144129	6.668343	9.628464	8.506737	6.257597	6.224688	8.015709	6.854303	8.187296	8.244922	7.696675	7.163940	7.036077	10.416445	5.093749	6.243786	12.460261	9.633215	5.135616	10.741254	4.818987	8.755318	9.736095	6.060246	8.700270	4.562740	4.608201	8.837982	4.775737	6.532074	7.342525	6.807739	6.824346

ADH1B	14.133856	11.186851	11.603148	11.283702	9.877183	10.086530	13.671017	9.384220	9.219434	11.504652	10.393108	11.218772	11.080729	10.589937	11.704443	11.855782	11.830475	10.511848	8.097374	11.267076	9.134694	11.188016	10.176535	10.464310	11.434496	10.085713	12.482801	9.799359	12.601601	13.071521	11.513924	13.708928	12.487307	12.356334	14.469705	13.915290	13.916047	12.352576	11.885697	12.223705	12.735569	13.541913	12.948751	13.455497	14.509550	12.969318	11.604162	11.798936	11.668318	12.614208	13.800361	13.308940	13.016071	13.658895	12.330636	10.754780	13.882222	12.629207	10.841116	12.645369	12.447571	13.595594	12.760314	10.516841	12.147509	11.763401	11.834974	12.379442	11.653340	12.465654	13.898829	13.748094	11.420288	13.384972	13.332262	13.550257	14.473933	12.099825	13.488366	12.248323	12.895646	12.807024	13.043715	13.126344	13.403666	13.157459	12.793014	11.378727	12.282721	11.869781	12.439669	12.016135	11.913381	12.702108	13.792778	13.631592	13.700156	12.783965	12.606059	11.700684	12.182961	12.682905	11.199882	10.455701	9.649319	11.722280	10.838287	9.360960	11.471808	11.959745	12.708589	9.532146	9.325661	11.557603	10.494672	9.636219	13.383479	12.153901	12.463731	9.752834	5.814250	6.744672	10.525956	3.283480	6.321320	8.334148	4.320790	5.522145	7.729343	5.357029	6.624362	7.781951	4.803917	5.083639	11.074134	4.673856	7.333384	6.015887	7.473387	8.918022	5.879233	8.033453	5.221697	4.592086	9.862391	8.416105	5.399012	12.875552	11.141637	8.530152	8.213459	7.712340	7.193586	9.650235	8.614211	9.071262	7.660314	6.350954	7.663402	5.904725	9.894119	7.348804	7.603602	9.927841	8.994888	5.375140	8.518092	12.157725	7.466956	8.838748	11.697270	8.873398	8.112848	10.361120	11.122337	8.910839	8.359117	9.273789	8.263689	6.582771	11.637074	7.075119	5.257917	6.980510	8.833342	6.332486	8.249737	6.235310	6.777825	10.161530	7.864018	5.192494	7.830357	11.978674	10.389287	8.774143	6.746121	8.764831	9.388260	7.206614	7.925527	7.580232	7.113933	7.435209	9.265515	7.144129	6.668343	9.628464	8.506737	6.257597	6.224688	8.015709	6.854303	8.187296	8.244922	7.696675	7.163940	7.036077	10.416445	5.093749	6.243786	12.460261	9.633215	5.135616	10.741254	4.818987	8.755318	9.736095	6.060246	8.700270	4.562740	4.608201	8.837982	4.775737	6.532074	7.342525	6.807739	6.824346

TMTC2	7.233231	6.642551	6.843510	7.463265	7.521441	7.555503	6.497516	7.399610	9.581160	7.467717	7.733473	7.518657	8.006102	7.506049	7.770487	7.563478	7.516020	7.288615	6.757042	7.301614	6.079385	4.640113	4.464978	6.899823	6.009468	5.894955	7.705490	6.000919	7.532530	5.780338	7.273579	6.371084	5.961974	6.484623	6.020798	6.245286	6.508146	5.121711	5.064681	5.409725	7.463593	4.284744	6.596333	7.141280	6.713105	5.794218	6.926316	3.845423	5.293315	6.240427	6.548531	5.991631	7.283220	6.157021	6.161502	7.450892	6.539555	5.017705	5.031937	5.045524	4.846376	7.380825	3.425458	5.389141	3.461300	5.078395	3.478950	6.596178	4.412414	3.629234	5.632657	6.455262	8.020889	5.988821	6.592110	5.963587	6.078492	5.596642	6.148301	6.680053	7.331953	6.722244	6.237135	6.957191	5.567473	6.981330	6.018827	6.306114	3.486788	4.560116	4.324223	4.100634	6.358329	6.610125	6.494280	7.570962	6.428351	8.762856	7.172935	8.461565	8.433371	6.956874	8.000720	7.379718	4.794167	8.266504	6.726738	5.183016	6.078138	7.440515	3.530715	7.454005	5.985916	7.656558	7.807549	6.258790	5.914039	7.902271	7.913863	6.974641	6.247783	5.606131	6.563515	6.387497	6.034559	6.125302	6.466356	7.199608	6.176433	6.050444	5.915291	6.025340	4.298299	4.381390	8.568954	4.812916	5.946038	5.964494	6.561198	5.372466	3.486244	3.448612	7.657367	3.658626	3.080195	6.704456	5.021357	8.965706	3.993947	4.787871	7.774143	7.594883	7.301614	4.203849	6.492368	5.794762	4.827398	4.256493	3.524206	5.391638	4.092696	7.531887	6.255207	5.937938	5.490755	7.551436	3.436524	5.984697	4.628485	4.638413	6.831962	6.342505	4.884522	6.405727	6.306611	4.653042	3.790786	4.459856	3.664638	3.738525	6.096683	3.266440	3.621479	3.994432	3.919345	3.816718	5.457891	4.313402	3.244699	5.558862	6.335031	6.842224	5.715116	5.246802	6.022244	4.767009	5.836054	6.335357	5.879425	6.850460	5.188817	5.172533	6.073290	3.840908	6.581476	5.368759	6.119227	3.360327	4.327452	3.548911	4.193640	6.391724	4.876507	6.282954	5.619849	6.138578	7.855897	6.360376	6.146352	5.810751	5.917236	7.286286	6.318421	4.041676	6.970286	7.676199	5.619849	5.058586	6.379122	3.174211	4.566368	3.265127	7.008826	4.990182	5.826559	5.239632	5.376154	5.297474

FRMPD2	6.432761	12.285194	10.812838	12.092910	10.884850	14.633014	14.705704	15.475629	13.187814	15.370133	14.524706	15.034008	12.440145	12.340191	11.863352	13.917771	13.139505	12.065157	11.884500	14.755057	11.230338	10.842407	6.358855	11.358554	12.308719	13.294745	7.624722	11.270443	12.602220	13.116985	13.551649	11.587163	11.995974	10.459530	13.122511	15.011645	14.261678	12.588785	12.451862	12.336177	13.920234	11.899859	11.470648	12.154206	10.987659	11.601504	11.636032	12.603082	11.583181	12.555591	10.557083	11.588775	11.684367	12.292501	12.693740	15.333133	12.225511	10.373518	11.016056	10.715522	11.491308	12.453737	5.075463	11.034048	11.932141	12.241146	11.079978	12.113742	11.142960	11.780904	13.302870	12.859356	13.052443	13.936589	12.735749	12.464858	12.547929	12.197215	12.706541	13.737601	11.337604	12.112386	12.133853	12.584123	13.151837	13.504598	12.521728	10.945347	10.667694	12.106206	12.073275	11.505835	12.124642	14.580592	10.954841	10.638329	12.800391	11.923119	12.664248	12.544369	14.308984	13.443521	11.259294	12.875731	12.921963	12.986468	13.313756	11.508051	11.197531	13.981594	10.829303	12.430780	12.216995	13.381985	13.833309	14.105360	7.873058	15.131490	12.336360	7.296370	12.796802	6.890278	9.159233	9.463454	13.329436	13.574676	9.270142	10.938612	11.239635	13.803322	9.864082	7.680124	7.766387	6.515220	11.380054	7.354866	7.135477	4.943891	9.694363	8.236065	7.545035	5.744988	7.471256	7.484133	7.763447	12.371489	8.201668	8.378467	11.156239	8.980147	10.951940	11.332913	13.615908	10.180720	13.776922	14.639680	9.996607	10.278521	9.801346	10.068414	11.096621	7.113000	9.759333	11.324079	12.015060	11.029556	7.845933	9.818886	10.553689	9.996163	7.443772	10.385173	10.364143	9.639042	9.659051	7.396775	6.179488	8.813954	9.067847	10.915527	9.709848	8.257221	10.009271	9.004266	7.860780	8.040597	12.692683	10.993001	9.686684	12.840899	12.119748	13.122136	12.420749	9.206285	13.142835	11.921437	8.919357	13.191685	9.444994	9.890146	10.138502	13.471189	9.894559	8.801575	10.871301	12.112064	9.047586	6.356807	9.974365	7.054595	9.698632	11.795725	10.115138	14.151714	9.141010	9.525550	7.270643	11.463754	9.787767	8.720555	9.348168	8.628513	8.305738	6.778314	10.616277	10.543304	8.855318	9.972959	13.543949	8.967900	6.243095	3.958573	13.251437	8.842443	12.817846	11.683795	10.556927	12.480968

FBXO21	8.141476	7.468615	7.549912	7.295865	7.358246	6.778938	7.457779	6.499228	5.722326	7.824306	7.084584	7.600626	7.139390	6.908917	6.794940	7.152494	5.474055	6.930487	7.100178	7.873058	7.897027	8.237449	8.764975	7.106131	7.672436	7.241488	7.015498	6.662158	6.868239	6.974149	6.797851	7.934327	8.277825	7.696675	7.609439	8.045289	8.011860	7.369988	7.425076	7.666698	7.651114	7.317634	7.622688	7.801599	8.268065	7.729939	7.082687	7.593360	7.283976	7.744557	7.997816	8.182181	7.995024	8.157342	7.879149	6.987484	8.295378	8.278420	7.440209	7.931121	7.764057	7.535183	7.040034	7.057617	6.925659	7.810046	7.653601	6.679298	7.517263	7.856943	7.886135	7.733473	6.816835	7.722222	7.934018	7.890146	7.598679	7.267882	7.335219	7.773688	8.059794	7.412742	8.526133	8.179917	8.312070	7.842803	8.016946	8.563599	8.039167	7.914757	8.163833	7.312895	7.413067	7.459019	7.486494	7.738981	7.154829	7.480895	6.980510	6.334497	6.775132	7.522082	7.056979	7.733324	7.663558	6.625003	7.052414	7.453866	7.649346	7.374866	7.967867	6.610967	6.683406	5.964297	6.390022	4.578613	7.351457	7.841996	7.128781	7.869842	5.966279	7.425716	7.294911	5.811028	6.883408	5.814756	5.720302	6.176773	7.726063	7.131165	7.719199	5.995014	6.173182	6.008773	6.809210	5.650297	6.983026	7.582415	8.073673	6.992259	7.671322	8.317000	6.860106	7.812017	7.637084	6.377801	6.792518	6.441540	7.336755	6.207080	7.063346	7.288615	7.220497	6.798171	8.353996	7.170236	5.758523	6.908917	6.697122	6.389356	6.478984	7.450892	6.784020	8.193845	7.400216	6.097197	7.901488	7.733781	7.604831	7.664539	8.281448	7.788217	7.424131	7.456028	6.665888	8.872170	8.140459	7.395059	8.440271	6.859613	7.305394	7.046103	6.178514	6.709750	7.632540	5.520813	6.551327	7.378634	7.087059	7.677630	7.665283	5.204262	7.292400	7.237972	7.036396	7.625771	7.253406	6.826234	7.712007	7.461032	7.303825	7.241188	6.649044	6.963071	6.470376	7.746418	8.705361	7.426143	6.483410	8.206933	5.163477	6.562847	6.714240	6.786475	6.801084	6.072576	7.381709	5.552010	6.925831	6.484623	7.017952	6.764402	6.811929	7.637692	7.652378	6.506499	7.493042	8.547074	5.679946	7.753645	6.325088	7.470014	5.137833	8.762566	6.119929	6.889336	8.239336	6.902075

RFPL3	11.127076	9.562068	9.923196	10.264537	10.681376	11.617285	10.278824	11.181871	11.339525	10.435536	11.233464	11.110584	9.538430	9.792860	9.781775	11.035057	10.858794	8.753789	9.935441	11.398826	9.425426	9.728056	9.884535	9.442348	9.689137	10.325054	11.738943	10.606089	11.442042	10.782671	10.711692	9.218853	9.748976	10.235279	10.165028	10.321065	10.195069	9.920274	9.753921	9.913442	11.265020	9.658419	9.689437	10.143394	9.842136	10.039288	11.481782	9.351391	9.756700	9.679253	11.481500	9.611512	9.099653	9.563450	9.384665	11.099009	9.340740	8.829341	9.011278	9.027513	9.206573	10.809055	8.759794	9.222522	9.266894	9.453075	9.399848	9.505657	9.410961	9.471707	11.239944	11.204194	11.260184	11.407214	11.312487	10.568013	10.595584	10.768642	10.824430	8.701974	9.352587	9.224967	9.154950	9.400002	9.092054	10.229726	10.132992	11.153328	9.018822	9.024612	9.101177	9.110466	9.436477	10.155312	9.994941	10.203707	10.067808	9.986916	10.160896	10.003957	10.375445	10.143394	10.538660	10.506605	9.372574	11.318232	9.934782	9.519473	9.958780	11.259741	9.635782	10.084519	9.653378	11.760698	10.714458	13.329024	9.944399	10.522881	11.722608	10.632025	9.934336	9.727151	10.481536	11.737167	11.086931	10.947159	10.905252	10.487794	11.097070	10.982046	11.127958	10.218622	9.808891	9.947027	11.098087	10.507361	9.662498	10.089619	10.276834	9.406477	10.285158	9.641761	10.236834	9.980586	10.586706	11.311749	10.428979	12.393291	10.744255	10.656820	9.568410	9.966054	10.620713	10.483377	10.351898	10.290374	9.976348	10.057591	10.258179	11.569303	10.005518	9.593217	10.114982	10.074063	10.128694	11.633293	9.441595	9.915155	9.565725	10.459968	9.530543	9.211300	9.524595	9.187084	10.924627	9.187084	8.590817	9.136846	9.121085	9.105974	10.849578	9.181882	9.372889	9.429888	9.429742	9.641485	9.684838	9.575947	9.525717	10.922541	10.926688	11.772261	11.232575	10.777670	10.773731	10.328691	10.600462	11.104014	8.861351	9.328459	9.117099	9.662962	9.289425	9.307443	10.373383	10.147018	10.144761	8.911270	8.833199	9.621028	8.903060	9.386045	9.994941	10.537371	10.337340	10.400199	10.782238	10.100294	9.800263	9.917160	10.479041	10.790288	10.175472	10.129611	11.789710	10.013832	9.700494	10.556927	11.734816	9.488725	10.277608	10.265297	12.081360	10.852873	11.496207	9.501937	10.529955	11.224167

DDX19B	12.663072	12.341197	12.570017	11.977145	12.487478	12.534127	12.677174	12.528252	11.990173	12.412694	12.471493	12.461245	12.223401	12.390459	11.946535	12.205414	12.572856	12.036088	12.585969	12.926827	12.475857	13.141902	13.101051	12.174349	12.404959	12.196766	11.997634	12.014022	12.028406	12.233426	12.354202	12.653462	12.949621	12.542605	12.937259	12.878888	12.952935	12.526938	12.443867	12.659132	12.475368	12.397063	12.814569	12.616955	12.931002	12.949109	12.402275	12.262621	12.942109	12.600026	12.573686	12.829828	13.107173	12.809121	13.097680	12.320151	13.140468	13.004475	12.296489	12.460438	12.541791	12.405277	13.266014	12.623671	12.560055	12.418490	12.655686	12.353897	12.165300	12.679231	12.395853	12.661032	12.300500	12.472784	12.798338	7.737275	13.079117	12.465329	12.697692	12.354824	12.815287	12.856932	12.645369	12.791666	12.930265	12.680777	12.705525	12.942292	12.934012	12.652437	12.659816	12.840899	12.370681	12.470791	12.460764	12.653635	12.812473	12.486329	12.607071	12.081665	12.622342	12.479650	12.117262	12.195864	13.303085	12.466153	12.514830	13.083488	12.747282	12.264187	13.127100	12.882947	12.657484	12.259420	12.775824	11.876874	12.482306	12.521236	12.261188	11.692743	12.159115	12.834529	12.249142	12.257409	11.835718	12.022508	12.115695	11.788671	11.980765	12.145773	12.435636	12.474582	12.065157	11.789556	12.183277	13.103890	11.859791	11.899712	11.883291	11.986674	12.240857	12.644314	10.242906	11.510962	11.873246	12.273308	12.103936	12.025076	12.166484	12.418655	13.046106	12.477840	13.158034	12.308880	11.870252	12.427314	12.491754	12.567105	11.446009	12.330636	12.709456	11.213197	12.798496	11.782082	12.378030	12.443196	12.649008	12.600886	12.278204	12.468921	12.161570	12.076837	13.360143	12.478661	12.306362	12.079262	11.963218	11.933500	11.570758	13.553941	12.666497	12.208223	12.564954	12.587644	12.473611	12.824043	12.389042	12.261651	12.412694	12.819221	12.229877	11.906521	12.697692	12.192905	7.688422	12.171247	12.735923	12.493046	12.072497	12.493699	12.227834	13.244951	12.348967	12.757036	12.204037	13.157459	12.169111	13.391817	12.557657	11.946077	13.019562	12.691855	13.259193	12.955360	12.161273	12.380039	11.610629	12.389516	11.643282	13.016827	12.312060	11.371738	11.760077	12.971153	12.407294	12.098451	11.809488	12.220618	12.022667	12.895995	12.178954	11.750711	12.190585	12.754938	12.604915	12.978958	12.777583	12.954050

AC012184.2	12.663072	12.341197	12.570017	11.977145	12.487478	12.534127	12.677174	12.528252	11.990173	12.412694	12.471493	12.461245	12.223401	12.390459	11.946535	12.205414	12.572856	12.036088	12.585969	12.926827	12.475857	13.141902	13.101051	12.174349	12.404959	12.196766	11.997634	12.014022	12.028406	12.233426	12.354202	12.653462	12.949621	12.542605	12.937259	12.878888	12.952935	12.526938	12.443867	12.659132	12.475368	12.397063	12.814569	12.616955	12.931002	12.949109	12.402275	12.262621	12.942109	12.600026	12.573686	12.829828	13.107173	12.809121	13.097680	12.320151	13.140468	13.004475	12.296489	12.460438	12.541791	12.405277	13.266014	12.623671	12.560055	12.418490	12.655686	12.353897	12.165300	12.679231	12.395853	12.661032	12.300500	12.472784	12.798338	7.737275	13.079117	12.465329	12.697692	12.354824	12.815287	12.856932	12.645369	12.791666	12.930265	12.680777	12.705525	12.942292	12.934012	12.652437	12.659816	12.840899	12.370681	12.470791	12.460764	12.653635	12.812473	12.486329	12.607071	12.081665	12.622342	12.479650	12.117262	12.195864	13.303085	12.466153	12.514830	13.083488	12.747282	12.264187	13.127100	12.882947	12.657484	12.259420	12.775824	11.876874	12.482306	12.521236	12.261188	11.692743	12.159115	12.834529	12.249142	12.257409	11.835718	12.022508	12.115695	11.788671	11.980765	12.145773	12.435636	12.474582	12.065157	11.789556	12.183277	13.103890	11.859791	11.899712	11.883291	11.986674	12.240857	12.644314	10.242906	11.510962	11.873246	12.273308	12.103936	12.025076	12.166484	12.418655	13.046106	12.477840	13.158034	12.308880	11.870252	12.427314	12.491754	12.567105	11.446009	12.330636	12.709456	11.213197	12.798496	11.782082	12.378030	12.443196	12.649008	12.600886	12.278204	12.468921	12.161570	12.076837	13.360143	12.478661	12.306362	12.079262	11.963218	11.933500	11.570758	13.553941	12.666497	12.208223	12.564954	12.587644	12.473611	12.824043	12.389042	12.261651	12.412694	12.819221	12.229877	11.906521	12.697692	12.192905	7.688422	12.171247	12.735923	12.493046	12.072497	12.493699	12.227834	13.244951	12.348967	12.757036	12.204037	13.157459	12.169111	13.391817	12.557657	11.946077	13.019562	12.691855	13.259193	12.955360	12.161273	12.380039	11.610629	12.389516	11.643282	13.016827	12.312060	11.371738	11.760077	12.971153	12.407294	12.098451	11.809488	12.220618	12.022667	12.895995	12.178954	11.750711	12.190585	12.754938	12.604915	12.978958	12.777583	12.954050

PLCH1	4.557657	3.330731	2.996468	3.933686	4.036066	3.430280	3.108739	3.386208	3.301909	3.767775	3.303158	5.500266	3.313790	3.768196	3.268818	3.080690	3.275887	2.959013	3.561948	3.132846	4.494423	3.224050	4.295680	3.206648	3.180141	3.210048	4.010802	2.934535	3.405245	3.091863	3.768196	4.511823	3.689939	3.608603	3.543818	4.147485	3.559932	3.133459	3.644833	3.480386	3.471876	3.877169	3.894841	3.207343	3.652626	5.238461	4.663487	3.460924	3.488107	5.428855	3.317617	3.413645	4.162019	4.198815	3.761533	3.319425	4.144966	5.809994	3.438920	3.880658	4.401099	3.125011	3.808898	3.254844	3.457527	3.497840	3.086264	3.232699	3.261885	2.992905	3.521682	2.941729	3.690345	3.172014	2.904508	3.332726	3.005091	3.045578	3.425277	4.207063	3.262940	3.790356	3.355030	4.026422	3.865160	3.474326	4.309458	3.233782	3.825521	3.400545	3.597934	3.547577	4.072967	3.133321	3.773875	3.236086	3.567103	3.195700	3.157304	3.150130	3.122974	3.112756	3.415696	3.462494	4.451198	3.807012	3.328510	3.660212	3.235794	3.175414	3.016359	3.334861	2.927156	3.144562	3.970970	3.651791	3.883596	3.263273	3.204801	3.433819	3.442756	3.234227	3.068050	3.950551	3.538047	3.110727	3.504959	4.406483	4.104297	3.658626	4.677535	3.125138	3.356732	3.056221	3.449181	3.995744	4.169649	3.099621	3.152335	3.874839	3.712959	3.327729	3.854880	3.162507	3.525165	3.170245	3.981290	4.105841	3.448800	3.589398	3.480386	4.426112	3.307599	3.328676	4.881370	3.546768	3.112983	3.902531	3.601673	3.270526	4.771972	4.288978	3.275589	4.051282	4.915143	4.471916	3.073448	3.710908	4.735442	3.246827	3.175549	4.055179	4.345246	3.438735	3.468724	3.785421	5.879616	3.283173	3.901276	4.346762	3.245524	3.894646	3.711334	3.760914	3.726946	4.026873	3.534800	3.450800	4.685452	3.465935	3.166776	3.240178	3.309363	3.393144	3.392796	3.220285	3.471047	3.039843	4.036699	4.095392	4.115483	3.477825	3.597725	3.919566	3.315269	3.943276	3.591899	3.516425	3.559545	3.979593	3.057595	4.366344	3.895640	3.966608	3.725976	3.416052	3.370658	3.958573	3.801192	3.297612	3.393833	3.023793	3.274657	4.813130	3.313790	3.043913	4.191252	4.505232	3.105403	3.373442	3.911207	3.669702	5.341623	3.817375	3.207657	3.044761	3.144562	3.473087

POLR2M	11.368860	10.029183	10.184676	9.899439	9.160471	11.315857	11.432854	10.535296	10.169807	11.145736	10.921794	10.848877	9.871517	10.115756	10.382091	11.081746	10.652206	9.030689	9.417796	11.548425	9.435081	10.644299	10.206741	8.309320	9.840773	9.163879	11.381887	8.931119	11.155939	11.405292	10.565322	11.755427	11.823989	11.096779	11.376521	11.490551	11.473523	9.429888	9.722467	9.785797	11.306805	10.372281	10.575398	10.968006	11.065422	10.065121	11.018523	9.569977	11.710952	11.353895	11.473964	11.572679	11.596704	11.489358	10.767583	11.073555	11.159344	10.203404	9.557087	9.823884	10.353124	11.483101	10.580067	9.834894	9.809526	9.535972	9.669428	9.674434	9.338465	10.076201	11.515630	11.562614	10.801645	11.271637	11.203593	11.491308	11.601057	11.552438	11.388496	10.218469	10.355373	11.000920	10.691016	11.395196	11.201144	11.513170	10.864236	10.976265	10.259775	10.053753	9.724077	9.937249	10.359015	11.380054	11.661490	11.481643	11.691236	10.852173	11.413079	9.891491	10.909711	9.973102	9.745376	9.415816	10.497236	11.639628	9.264430	9.635632	9.816203	11.149750	10.633979	8.751640	8.652056	10.711830	11.240345	10.374549	9.380201	11.154369	10.903791	11.993346	10.942265	10.238936	9.897339	7.593028	11.828835	11.308542	11.106454	11.458309	11.816598	11.404110	11.190322	10.389876	10.085231	9.828875	11.914600	11.479943	9.310956	8.879581	11.772691	9.218401	10.719738	10.109887	7.911257	10.172148	9.873170	11.364925	9.389005	10.698366	11.806190	10.951170	10.887194	10.958566	10.880620	12.136054	12.245455	11.361808	9.485652	9.841825	10.046039	11.440486	10.167052	11.646361	11.539677	10.772548	10.130040	10.735347	10.129911	11.523534	11.864994	11.694552	12.079851	11.545607	11.613158	11.696386	12.400465	11.706559	9.975902	9.736421	9.996917	10.422850	11.076058	10.948319	10.345357	10.485177	9.798264	9.430199	9.214227	9.010532	9.969713	11.543468	11.271009	10.022295	11.391015	11.679201	11.547842	12.007014	11.391155	11.490704	10.695437	10.403336	10.496942	10.134688	11.359007	11.403962	11.033754	10.554766	10.957536	10.309414	9.730269	9.156761	10.170273	10.430630	11.005839	11.389264	11.128797	11.100018	10.925219	10.892228	10.497682	11.684658	10.570453	9.697396	9.347857	10.764606	10.777970	9.635213	10.445527	10.520951	10.841416	10.101718	9.245982	8.626099	10.594702	12.131878	11.804689	9.633683	11.826085	11.218772

OR4C6	8.833467	8.094921	8.500123	8.387288	8.902785	8.775967	7.643849	9.712784	9.757967	8.111454	8.454138	8.227815	7.923957	8.290087	8.029503	8.524310	9.594780	8.312531	9.477671	8.083646	8.952252	8.894400	10.028875	8.184642	8.844016	8.839199	7.991114	9.386992	8.109245	7.828982	9.794954	7.681453	7.856355	7.221610	7.732264	7.811267	8.156736	8.268392	8.382834	9.225119	8.721615	8.524310	7.855139	8.343060	7.619552	7.930030	9.050240	8.915861	8.313513	7.824466	8.209938	8.310361	7.702064	7.936848	8.054325	8.256475	7.538600	8.138337	8.213302	8.038832	8.654468	8.054325	8.899769	8.921659	8.467193	9.006858	8.576545	8.572191	8.448582	9.425577	8.511066	8.676360	8.676661	8.486586	8.245084	8.499181	8.609684	8.495364	8.760747	7.835236	8.063661	8.105290	7.906169	7.957608	8.425886	8.120696	7.923024	8.755318	9.118674	8.997519	8.825236	9.676141	8.633518	8.221296	7.911257	8.055425	8.573379	7.501815	8.500441	8.240832	8.234802	8.029783	8.012895	8.226468	9.054864	8.132391	9.561136	9.003202	9.750360	8.579823	9.836571	9.652257	9.311545	8.820970	8.988823	9.352291	9.033700	8.533770	8.127674	8.322153	8.349146	7.827616	8.041233	9.257116	8.892364	7.850360	8.686132	8.649966	7.991262	8.219933	7.788507	8.001992	7.661829	8.545801	8.220099	8.827175	8.365932	8.748190	7.888733	9.124887	8.852270	9.976520	8.475589	8.919067	8.258487	8.954400	9.400437	8.800956	8.335713	9.336245	8.018445	7.856494	7.836439	8.063024	8.162350	7.984384	8.490290	8.715941	8.679147	9.085022	8.717346	7.512640	8.811877	7.673841	8.365652	9.089780	7.967258	8.527834	8.350679	8.556399	8.302736	7.795248	8.366957	7.531887	7.991114	7.284881	7.902571	8.267776	7.934793	8.709313	7.829434	8.908163	8.807358	8.760915	8.860183	8.335713	8.428190	8.744816	9.142892	8.666821	8.365500	8.909199	8.093562	7.914757	8.360009	8.821587	8.671249	8.902003	7.558311	7.515856	8.298205	8.536257	7.700687	8.522913	8.614955	8.275089	8.328445	9.057198	8.786239	9.172804	10.134533	7.945865	8.715309	8.384354	8.989583	8.484922	7.751051	7.953099	7.898384	8.028874	7.732109	8.165664	7.822865	8.402822	8.329486	9.113163	8.562532	9.534594	8.602058	9.376209	8.952109	10.085395	9.789131	8.658828	8.431530	8.934567	8.148860	8.209188

SLC25A37	10.360069	10.413889	10.138215	10.697351	9.781037	9.886402	9.714868	10.507361	11.301743	9.826316	10.291124	10.345357	10.812551	10.172908	10.611076	10.388806	10.733421	10.215794	11.260318	10.483377	9.962834	11.620681	11.600580	10.141980	10.712709	10.815069	11.165530	10.631884	10.669264	9.951526	11.022131	10.198884	9.925756	10.960176	10.074213	9.826628	9.869932	10.403775	10.750993	10.366684	9.806855	11.154213	10.122161	10.355971	10.573635	10.170273	10.091582	10.683185	10.991323	10.129148	10.007811	10.019135	9.905450	10.255569	10.562644	10.385324	10.173198	10.312584	10.906300	10.236049	10.502419	10.085861	11.377091	10.710978	10.859529	10.737773	10.843943	9.803798	10.391918	10.340663	10.043315	9.828423	9.916692	9.926775	10.104055	9.929378	9.792404	10.669264	10.608914	9.846464	10.229572	10.531581	10.469964	10.307712	10.099983	10.568169	10.493289	10.518615	10.581595	10.590962	10.381790	10.492682	10.709659	10.762650	10.905411	10.193710	10.572446	10.580549	10.500314	10.098728	10.621011	9.663882	10.168737	9.884098	11.141047	10.371089	11.072707	10.688872	11.362553	9.689137	10.712574	10.866673	11.099868	9.899145	10.299569	10.870835	10.802095	10.713151	10.714168	11.466898	11.820662	9.982592	10.912497	10.363127	11.243601	10.391918	11.362109	11.647387	10.236515	11.435520	10.457869	10.548890	11.078697	11.958426	10.948753	11.354335	10.866038	10.424393	9.487184	11.339812	12.135755	11.155073	11.378286	10.664741	12.331584	10.963706	10.725518	11.152465	10.482608	11.959150	11.441183	10.722521	11.960063	11.046942	10.236834	10.829006	10.289278	11.574932	11.203446	12.220618	11.456523	10.675990	11.343711	11.956666	10.802840	12.625864	10.247991	10.733854	10.503620	10.694662	11.030306	11.105005	10.904224	12.083542	10.135620	10.433078	10.926394	11.396845	10.355061	12.018090	10.949054	12.322660	11.381737	11.538956	10.786766	11.670323	11.229137	11.244940	11.918799	10.616435	11.171659	11.472498	10.908772	10.603565	10.865449	10.677043	10.782076	10.486270	10.034536	10.616727	11.117676	11.526932	10.967694	11.271173	11.150393	11.413399	11.246940	10.768036	11.272078	11.819436	12.044855	11.254856	10.936085	11.506720	11.463192	10.889072	10.669700	10.921655	10.529807	11.651970	10.912200	9.652594	11.320543	11.277202	11.179135	12.418166	12.063646	11.313492	10.653379	10.723716	11.045153	11.648697	9.834586	10.965036	11.996542	11.108963	11.803532	10.907661

FMO5	11.895030	8.486125	8.037443	10.157733	7.073401	6.909858	7.640482	5.916723	4.580362	7.469405	6.377960	7.981607	7.725286	8.103712	7.460098	7.040480	5.795803	6.869059	7.679523	7.772629	6.949175	7.961466	7.217022	6.311949	6.975110	7.557866	6.412706	9.140544	7.479657	7.459810	5.752653	10.859380	8.682359	7.457145	7.712486	7.814935	8.247950	7.964325	7.645417	7.436017	6.405228	9.005015	8.168619	7.728577	8.399260	8.160080	6.437128	8.289941	8.404218	8.551354	10.963237	8.962369	8.653894	8.703369	8.450930	6.419799	8.209472	8.083646	8.390991	8.188953	8.940056	6.700968	8.373879	8.021345	7.984384	8.127189	8.224190	7.961466	7.439309	8.138337	7.689945	7.418684	6.469202	7.681937	7.336449	7.436945	8.251925	7.962682	9.535347	8.253119	9.302046	8.528920	10.789421	8.859264	9.160471	8.642515	8.787346	8.476639	8.700763	9.180314	9.022956	8.136005	8.285318	7.170697	10.555376	8.188055	8.601729	7.371577	7.128781	6.984290	6.709100	7.542183	6.899505	7.173384	6.539709	7.054266	7.807410	7.182354	7.563626	7.337348	8.438425	6.843699	6.002186	6.501873	7.125950	7.988227	12.035653	7.198684	6.754244	7.400060	5.002715	6.888860	7.971630	7.533297	5.226669	6.982081	4.889393	4.405609	7.086749	5.906509	7.214181	6.381308	6.153205	4.309458	7.821338	6.682753	6.556792	6.566408	5.947042	6.575152	7.060771	7.097357	5.696745	7.612965	7.363410	7.166091	5.840777	5.538246	7.126885	7.169643	7.007341	7.727157	6.980014	8.276122	6.055659	7.354382	5.612184	5.991295	6.422233	5.303245	7.461823	11.021387	5.324222	7.674955	7.438363	5.069790	6.622109	6.515048	7.237496	7.668822	7.304606	7.416992	7.418994	8.142400	7.309930	7.523256	7.677630	7.335828	6.844707	6.807413	6.851096	6.982716	5.870621	7.188249	7.103604	6.107118	6.054258	4.275963	7.165954	7.761745	6.149155	4.743795	7.386409	7.162193	6.949978	6.725575	5.974485	6.614638	7.811119	7.916384	6.909692	8.028572	7.527432	7.598098	7.701453	8.115024	8.486125	8.730171	7.277818	5.757968	6.221062	7.360104	6.324927	7.139220	6.617562	6.673975	6.561359	7.195034	8.834047	5.161670	5.654037	7.133232	6.844034	7.534238	5.697884	4.942399	6.484071	7.236106	3.743241	7.126261	7.554414	6.910655	5.257917	5.732672	8.431677	6.005203	8.951337	7.037948

C21orf58	7.976790	7.694279	7.556447	8.098702	8.570650	9.228667	8.259547	8.697420	9.112877	8.212820	8.404218	8.477815	7.570213	8.136323	7.436154	8.420110	8.462038	7.936999	9.375129	8.139887	7.858010	8.521564	7.854983	7.870451	8.455966	8.529222	9.295221	8.610473	8.400970	8.650119	8.681590	7.658000	7.714215	8.413828	7.983020	7.854059	7.574330	7.485352	7.702525	7.810194	8.337740	7.775738	7.911734	7.886427	7.835968	8.028572	9.920274	7.657214	7.962059	8.032084	7.766700	7.944941	7.875346	7.575291	7.191239	8.614503	7.543733	7.206614	7.443772	7.417872	7.429346	8.354284	7.362446	7.958214	7.574659	7.341925	7.754559	7.798320	7.721625	7.650959	8.072444	8.191798	8.689429	7.959604	8.175989	7.892026	8.013058	7.815753	8.011098	7.080201	7.542477	7.892947	7.345496	7.897027	7.196961	7.713749	7.520351	7.729485	7.406634	7.358559	7.298304	7.845347	7.804315	8.059318	8.131646	8.149308	7.508960	8.279188	8.522159	8.357685	9.069131	7.582246	8.116235	7.540966	7.710952	8.882957	8.085388	8.292608	8.638969	8.741333	8.449060	8.835707	8.200588	8.991194	8.200421	11.675164	7.950164	8.309320	8.547074	8.315586	8.121305	8.170959	8.822507	9.435389	9.363352	9.565121	9.494075	9.208388	9.108128	9.083496	9.761509	9.112425	8.599566	8.814414	8.991352	8.970518	9.178480	9.728056	8.674520	8.612493	9.098445	9.491096	8.871045	9.399848	9.030556	9.222522	9.266257	9.888002	8.763615	8.937504	8.583773	8.323100	8.651036	8.929856	8.356589	8.065970	8.681905	8.394237	8.649523	9.551619	8.476775	8.675628	8.586239	8.198081	8.447340	10.286511	8.517642	7.907241	8.380819	8.041408	8.543056	8.456897	7.743172	8.431370	9.285198	8.558556	9.274979	8.183260	8.258160	7.819523	9.047434	8.191175	8.172379	8.159020	8.286088	8.451544	7.797839	8.020288	8.589745	8.360324	8.395764	9.843644	8.444705	8.790577	8.231627	8.424530	8.726505	8.537260	7.919036	8.103552	8.401432	7.691511	7.975858	8.121305	9.224967	7.923817	8.343667	8.793982	7.696379	9.649168	8.345381	8.008376	8.322153	8.676509	8.845415	8.884508	8.998880	8.701974	8.107736	8.822972	8.601569	8.632197	7.943560	9.062460	9.043309	8.679595	9.493614	8.531075	9.244547	9.019286	9.123673	9.288170	9.785272	9.432029	9.725155	8.451235	8.653423	9.089930

LRCH1	12.904297	11.529388	11.799846	12.360678	12.337925	11.954526	12.477840	11.214237	11.817176	12.104683	12.073275	12.074353	12.162362	11.864994	12.232358	12.313325	12.517447	12.205727	12.678360	12.237262	12.549730	12.914157	14.648021	12.185716	13.388206	12.772064	13.699452	12.776712	12.870234	12.302965	12.468291	11.952744	11.856252	12.365730	11.803092	12.126285	11.916369	11.925820	12.122996	12.218793	12.110262	12.359576	12.853341	12.372119	13.462700	12.215618	13.079117	11.773852	12.075900	12.068749	11.976391	12.332224	12.510471	11.892907	12.156187	12.168818	12.196906	12.120220	11.873097	12.195094	12.071578	12.394906	13.459822	12.424072	11.845084	11.920396	11.487129	12.058084	11.148096	12.016745	11.759188	11.784633	12.408210	12.176872	11.637244	12.017633	11.919091	11.971293	11.593433	12.321603	11.882660	12.153901	11.938203	11.792414	12.536416	12.018249	11.857830	12.049820	12.615027	11.453138	11.909110	12.424744	11.977145	11.974354	11.926124	12.172787	12.076837	12.738673	12.004877	13.333079	12.364491	12.173415	12.450102	11.762657	11.920396	12.572340	12.412373	12.372273	13.585329	12.706715	12.555759	12.088432	12.411724	11.938053	12.016290	11.546807	12.820415	12.546069	13.040031	11.956809	12.170796	12.416734	12.175134	12.596810	11.366980	12.391089	13.138696	12.762748	12.569043	12.420594	12.953298	13.386677	12.264187	12.007474	13.496486	12.710332	12.332547	13.671278	11.717338	12.207764	12.991828	12.782402	11.973834	13.071328	12.739016	12.117700	12.203451	11.799525	11.992007	12.984441	12.546528	13.036888	12.328915	11.619186	11.696665	11.825777	11.774311	12.103623	11.973905	12.294023	11.770488	13.221376	12.375982	11.956053	11.648416	12.495176	12.325249	11.300099	13.023285	12.560565	12.533788	11.697270	12.595766	12.667306	12.752073	12.801968	11.798796	11.691529	11.859341	11.592214	13.253211	11.753359	12.036088	12.376294	12.281762	11.748620	11.515156	11.601655	12.273308	11.545153	12.069172	11.485325	12.022508	12.449765	12.160026	12.108644	11.923566	12.156958	12.488616	12.225836	11.989265	12.605068	12.450257	12.353569	12.617130	12.126428	12.045726	13.093498	12.514830	11.877752	12.369887	12.030200	12.262160	12.473785	13.002707	12.134342	13.514460	12.567417	11.795854	12.868885	12.779855	12.346154	11.996542	12.432257	12.108482	13.214995	11.725122	12.201396	11.306661	13.173050	12.553621	11.831507	11.537024	11.551948	12.462229	12.167732	12.800565	11.802080

PLA2G6	11.538224	10.042666	11.347140	11.156994	11.372164	12.255361	10.963865	11.949439	13.380525	11.617561	12.203746	12.013558	11.208356	11.017339	10.641500	11.828254	12.507380	10.572908	8.644551	11.516358	9.052042	9.109868	8.980580	11.094152	12.178954	12.358931	12.982760	12.573019	12.173896	11.267512	12.612002	10.148575	9.352445	11.787155	10.633501	10.835284	11.026307	10.061049	10.238329	10.138502	11.689738	9.529428	11.452234	11.436941	11.126783	11.360153	12.479302	10.168427	10.964294	10.917489	10.724471	10.362375	9.816653	10.236515	10.626550	12.041676	10.019135	8.913424	8.794902	9.191963	9.073438	11.670468	8.302103	8.891290	8.555588	9.792563	8.273592	9.303847	9.208692	8.876944	10.734749	10.883564	12.240559	10.791003	10.955728	10.868443	10.896337	10.638766	10.772850	10.484898	10.745016	10.374979	10.380154	10.229572	10.352666	10.722521	10.861911	10.956031	7.701304	8.310361	8.807970	8.441329	9.583773	10.602257	11.310412	11.338924	10.465656	12.301868	12.146729	11.733626	12.289811	10.420249	11.755885	10.672285	8.953320	11.864242	13.166504	10.511571	9.948590	12.363880	8.758395	11.044022	11.237709	12.702957	12.196008	14.262005	10.854176	12.324793	12.917840	12.235592	10.364000	11.162605	11.805220	12.511128	11.412637	12.074516	12.632803	12.623496	11.876727	12.076837	12.092283	12.218342	11.112307	10.972945	12.726676	12.247193	10.908849	9.140105	10.892989	9.613799	9.593381	9.625356	11.310266	11.402644	10.831500	11.975594	12.333192	12.788229	11.142502	12.952559	12.377241	10.966826	12.053932	10.741413	10.653102	11.629546	11.009325	10.449886	10.223453	12.894925	9.793310	10.935332	11.573114	10.982623	11.136541	12.281610	10.341850	10.659929	10.578433	11.005550	11.137722	10.128392	10.669700	10.665327	11.157605	10.481855	8.695277	9.085355	8.953170	9.526785	11.502905	8.366232	8.909516	9.112570	9.985950	8.654920	9.408176	9.873486	8.670956	10.862048	11.076192	12.945900	11.091311	11.077953	10.996866	10.936997	11.369916	11.008438	10.865608	10.489958	10.237443	10.837978	11.368441	10.175625	11.554640	10.868599	11.089291	8.097820	8.746649	9.846295	8.147026	9.808072	10.829442	11.355495	12.339191	11.830189	12.637480	12.080582	10.481102	11.812299	10.903646	12.096818	10.319358	8.401595	12.327331	13.675535	10.339310	9.300486	13.211496	8.692789	11.166523	12.342785	13.002534	11.950501	12.100729	10.303411	11.909419	11.893367

PARVG	7.756814	8.258005	8.368767	8.657895	8.999207	9.209005	8.296388	8.471218	9.120791	7.974619	8.345227	8.325088	8.231463	7.639852	8.513849	8.534543	8.334787	8.246193	8.413538	7.929575	7.772479	9.142892	8.706907	8.759174	8.700599	8.228550	10.516242	8.206633	8.760273	9.447475	8.477080	7.890928	9.060211	9.381799	8.550127	8.520528	7.753321	7.790829	7.951997	8.754570	8.278265	8.307442	9.293321	8.705827	8.329950	8.742249	8.731208	8.125709	8.657576	8.295020	8.082255	8.055892	8.299713	7.650787	8.151277	8.687013	8.213124	8.384965	8.137408	8.382675	7.692731	8.235110	8.212526	8.545084	7.915196	8.419942	7.709416	8.111607	8.173786	8.186038	7.863555	7.972255	8.498063	8.164571	8.193679	9.007782	8.139115	8.496726	8.128554	8.349747	8.721185	8.322944	7.917975	7.786365	8.007472	8.559301	8.320348	7.923339	7.919191	7.690876	7.954020	8.475093	8.578471	8.084126	9.035594	9.095402	8.244609	8.364386	8.617394	8.428965	8.975115	8.467193	8.170037	7.898958	8.573058	8.596272	9.065994	9.242731	9.007159	8.259398	8.682359	9.025237	9.060362	8.858952	8.803665	11.297023	8.353209	8.087401	8.098702	8.569732	7.940575	8.447340	9.467958	9.783941	8.294115	9.603315	8.652353	7.793259	8.164723	8.113605	8.527674	8.739022	8.867627	8.585432	8.279347	7.808028	7.869083	8.383127	7.693823	8.218403	8.664392	8.422713	7.975232	8.210084	8.835535	8.758540	8.697583	9.705173	8.663767	8.795507	9.195450	8.693097	8.315429	8.179009	7.412597	7.863243	7.750438	7.800830	8.989269	8.922259	7.773840	8.004643	8.150217	7.885668	7.889831	9.389757	7.356705	7.526823	7.332899	7.869842	7.682592	7.730715	7.414160	7.983196	8.696676	8.437844	7.621041	8.272998	7.886427	7.507090	9.066685	7.461972	7.997547	8.722201	8.329805	7.719347	8.102344	7.772768	8.243097	7.685039	8.017553	9.241660	8.162655	8.743457	8.743289	7.659822	7.969565	7.936228	8.303462	8.092915	7.747490	7.778213	8.544161	7.861216	8.792595	7.773066	8.191012	8.222690	8.320945	8.908772	8.024983	7.924902	7.879615	8.424386	8.966352	8.405306	8.820544	8.331995	7.604209	8.559772	7.936696	8.071679	8.960987	7.601689	8.509809	7.988366	8.563436	7.933104	8.760426	8.931119	8.656397	9.747100	9.819482	9.102118	9.238911	8.259100	7.689945	8.411545

BTRC	9.649472	9.237958	9.207016	9.206573	9.211755	9.293482	9.522409	8.798396	8.111607	9.491096	9.415666	9.301568	9.049014	9.733039	9.374964	9.391590	8.328299	9.441732	8.764687	9.541126	8.744959	9.370599	7.986529	8.795964	8.964176	8.647237	8.087711	8.910988	9.166648	8.974524	8.624195	9.624312	9.710473	8.917711	9.103509	9.338015	9.343442	9.210087	9.715472	9.651195	9.369356	9.751590	9.228821	9.254946	9.193891	9.279219	8.804775	9.694363	8.867772	9.644234	9.635213	9.728699	9.800887	9.738519	9.581469	9.077210	9.688696	10.283384	10.189200	10.007811	10.347504	8.980298	9.625998	10.224681	9.819206	9.624312	9.577775	9.144597	9.162133	9.679857	8.855620	9.012062	9.106435	8.989863	8.822673	9.213597	8.996430	9.146092	9.163554	9.396376	9.082132	9.123377	9.331691	9.517138	9.266894	9.715026	9.264136	9.426185	9.545767	9.646315	9.296921	9.304406	9.428363	9.342205	9.055012	9.145926	9.090357	8.928643	9.136379	8.804775	8.824932	9.195614	8.970668	9.324766	9.066292	9.050083	9.175728	9.465964	8.844814	9.322428	8.044654	9.524425	9.766625	9.750211	8.957303	7.643849	9.418972	9.239207	8.634112	9.514420	9.847836	8.504777	8.242770	6.962293	8.771295	8.878485	8.095669	7.806014	8.685237	9.609659	8.696219	8.695418	8.633832	8.298205	8.935020	7.942332	8.451400	8.486125	9.518530	8.380173	8.642055	7.839686	8.064421	8.958885	9.167413	7.907702	8.945362	8.628231	8.663004	8.703217	8.874966	8.576701	10.039141	9.361712	8.817434	9.120357	9.675852	9.210693	9.053125	8.773506	9.169538	7.976484	8.958429	9.152311	8.646914	9.380048	9.737612	9.627839	9.392037	8.850610	9.290321	9.661074	10.072985	9.746781	8.394381	9.899439	10.070060	9.659354	10.045253	9.379735	8.702764	9.042970	10.327775	9.753292	10.249099	9.503319	9.055357	8.665616	9.251933	8.761825	8.486125	9.091579	8.718743	8.250515	8.581825	8.985318	8.767930	8.759334	9.239831	8.966199	9.298766	8.740859	8.988823	8.929089	8.279347	9.376837	9.539381	8.775659	8.457490	8.322153	9.142743	9.140257	9.098132	8.780406	8.809969	8.413828	8.896413	9.525858	9.159694	8.824643	9.496589	9.068809	8.396721	7.966799	8.143761	8.454436	9.581615	8.739474	9.604857	7.805246	8.799907	7.815753	8.570494	8.513238	9.571043	9.232852	10.116521	8.618293

ZSWIM5	9.809208	8.772771	9.540332	9.068014	9.562249	9.491252	9.614087	9.445907	8.770850	9.668932	9.233645	9.069131	9.267666	9.678286	9.244700	9.576673	8.959613	9.339197	9.655676	10.075134	9.637621	9.694042	10.370484	8.746957	8.959322	9.417954	9.741509	9.606864	8.821277	8.952566	8.635686	9.755312	9.918845	9.923356	9.796086	10.179183	10.459258	9.425426	9.371967	9.055670	9.426185	9.863624	9.882399	9.628632	10.518184	9.821011	9.672549	9.193762	9.849917	9.896882	10.193562	10.230680	9.860199	9.379735	9.411410	9.109564	10.252055	9.097041	9.098736	9.479637	9.268924	9.361262	9.997647	9.142589	9.420243	8.856682	8.938386	9.775812	9.530388	9.213270	9.453555	9.183450	9.180938	9.695275	9.709371	9.790373	9.914396	9.437081	9.497504	9.490785	9.795507	9.719876	9.453237	10.032856	9.677830	9.790211	9.774645	10.040849	9.693309	9.372574	9.016533	9.881326	9.502845	9.657050	10.422396	10.256188	9.895350	9.637784	9.406773	9.606408	9.170762	8.788742	9.103656	9.518231	9.011126	8.922699	8.710861	9.369995	9.593696	9.445303	10.335529	9.663115	8.425591	9.015766	8.816206	8.742859	9.064741	9.544844	9.313309	10.965488	8.445017	7.855600	8.788445	8.291830	7.641386	8.037595	7.633475	9.616360	8.951021	8.013377	8.257688	6.871946	9.019864	6.287255	10.686620	9.032809	8.279490	8.617543	10.264395	9.095560	10.456157	9.092054	8.074558	8.870392	9.864242	8.106349	7.218267	9.017297	8.572191	9.864082	10.721226	8.942008	7.956035	11.345621	10.893753	8.739179	8.773366	8.400970	7.471425	9.475363	8.173625	8.325722	7.032798	9.288791	9.643778	7.802802	9.632285	10.195389	7.259651	7.648612	8.827010	9.753292	7.422439	10.352513	9.502999	9.727298	9.616211	9.115876	8.348833	8.511528	9.135312	8.066676	8.881137	10.496640	8.792439	8.526432	8.644846	6.530971	7.131165	9.654195	8.006998	8.581064	8.563436	9.464554	9.709211	9.616997	9.377318	7.990967	9.256834	8.579986	8.416275	8.638648	7.760978	8.268065	10.144003	9.668449	9.112570	8.200278	8.185578	11.109422	7.994730	9.050083	7.288783	7.556447	7.369389	6.581952	6.609648	6.633056	9.547157	9.037084	9.093137	9.818886	8.528767	10.032568	7.852191	8.374334	8.945066	9.659051	6.987331	8.856221	9.264575	7.931730	8.184935	7.996145	7.863243	10.904946	11.162156	8.507964

NSFL1C	11.018066	11.174591	11.640863	11.404110	11.799097	11.443382	11.843253	11.457426	10.782833	11.427955	11.559235	11.820662	11.442790	11.282105	11.399554	11.059824	11.777140	11.328346	11.201757	12.061039	11.223118	11.256056	11.644307	11.018066	11.782523	11.401062	10.805036	11.349360	11.113613	11.093256	11.058248	11.292520	11.738510	11.411376	11.281048	11.542272	11.680252	11.319133	11.787766	11.870252	11.636180	11.034889	12.029310	11.852176	11.981974	11.906965	10.867997	11.495353	11.591914	11.825192	11.843554	11.808653	11.625759	11.974500	11.792414	11.798936	12.000403	10.902906	11.896778	12.115840	11.670323	11.394433	10.979653	11.236856	11.469875	12.158950	10.724311	11.228419	11.136391	11.586749	11.548579	11.441464	11.084241	11.630454	11.403690	11.304114	11.589820	11.759915	11.436109	11.597121	11.702656	11.889609	11.538395	11.822002	12.121750	11.610492	11.773690	12.020355	11.132638	11.342060	11.441033	11.140512	11.525035	11.411822	10.969857	11.884784	11.566690	11.333206	11.855029	11.476411	11.521468	11.621293	11.381887	11.704895	11.369147	11.327191	11.509511	11.136541	11.273089	11.399554	11.414848	12.065779	11.929833	10.994601	11.503209	10.350451	11.458612	11.601504	10.853016	11.288046	11.781361	11.555942	11.326303	10.422993	11.471947	10.957964	10.910445	11.121888	11.898681	11.312057	11.329216	11.415733	11.662078	11.260634	10.974503	11.749074	10.531275	11.817489	11.746117	10.884165	10.980262	11.298525	11.274607	11.989104	11.601959	11.617561	11.077660	10.902781	11.137575	11.327777	12.053155	11.514222	11.202995	11.560987	10.873972	11.861688	10.479789	11.713929	10.717299	11.573269	10.135460	11.443382	12.383858	11.489042	11.508778	10.912348	10.846377	11.082183	11.475989	11.809331	11.316005	10.819847	11.651679	11.879073	11.942122	12.013264	11.219974	11.856727	11.761613	10.792626	12.663752	10.013258	11.418974	11.827669	11.704017	10.815069	10.612741	10.464013	10.239706	11.422716	10.707633	11.000467	10.779119	11.335983	11.235980	11.827814	11.774769	10.947159	11.899423	12.217449	11.904475	12.646567	10.914172	11.831216	12.465162	11.594141	11.640863	10.779119	11.091172	10.529215	11.643128	10.339746	11.263943	10.954078	11.252221	11.218485	10.962810	10.650874	11.429723	10.819699	10.584768	10.454796	10.944749	10.961776	10.502281	11.721370	11.595768	11.153254	11.183899	10.667979	12.250882	11.647691	11.247355	10.012286	12.186663	10.992855	11.413703	11.313065

RRBP1	15.604793	13.769838	13.743105	14.000102	13.717719	13.925407	13.820478	14.101917	14.091572	14.201495	13.880400	14.026521	14.005564	13.749340	13.343265	13.735356	13.913198	13.864888	13.197314	14.032887	13.514888	13.161096	12.837925	13.810378	13.992136	13.782568	14.489914	14.447111	14.100719	13.761409	14.028483	14.233212	13.896143	14.370460	13.874711	13.864108	13.551398	13.624298	13.845352	13.876872	13.926497	13.298159	14.627259	14.008169	13.845880	14.438961	14.023893	14.504993	14.052401	13.949083	14.749773	14.069209	14.075445	13.461357	14.141470	13.747078	14.361168	13.403370	14.289302	14.434184	13.658668	13.550257	13.610599	13.486415	13.400590	14.071794	13.931922	13.568702	13.930857	13.579444	13.795129	14.011503	13.735356	13.810158	13.763702	13.866239	13.671748	14.121601	14.292381	12.654999	14.150745	14.313722	13.467530	13.807016	13.600149	13.895320	13.639658	13.931643	13.016643	13.426259	13.684034	12.881870	14.295055	13.868320	14.054182	13.628713	13.900711	14.029339	14.289643	13.955482	14.186537	13.908544	13.841373	13.717490	13.673176	13.855680	14.372657	13.981851	13.249887	13.939322	12.939909	14.083132	14.769948	14.180225	14.718090	12.742146	15.090679	14.377416	14.009820	13.821965	14.575515	14.082507	14.579771	14.625993	14.307693	14.503811	14.200109	14.510324	14.516636	14.491542	14.500265	14.467367	14.735782	14.710296	14.327880	14.685039	14.448739	13.231052	13.410767	13.895620	14.078902	13.765490	14.746020	14.681870	14.747900	14.422502	14.301922	14.529101	14.329971	14.806121	14.379181	14.433838	14.356236	14.278587	14.229183	14.597411	13.963030	14.700188	14.062399	14.186189	13.147685	13.868320	14.610567	14.423235	14.228587	14.646275	13.405475	13.811931	14.456152	14.197180	14.613963	13.509011	14.652370	14.529101	14.736202	13.933765	13.066403	14.354129	14.002723	13.574249	14.714881	12.944441	13.332700	14.096118	14.041142	13.253022	13.870987	13.871812	13.236446	14.146475	14.208447	14.870263	14.006174	14.217951	14.266304	14.598641	13.902528	13.823470	13.840878	14.022116	13.521603	15.011645	14.451322	14.820339	14.581385	13.971862	14.154830	13.326797	13.697330	13.243181	13.732895	13.854373	14.303944	14.514695	14.289302	14.261678	15.212349	14.175520	14.074236	14.708876	13.644720	14.640496	14.465898	13.763209	14.588954	14.862987	13.727843	13.440958	14.629113	13.167086	14.864091	12.697151	14.334050	12.632134	14.210459	14.197832	13.937428	14.386354

C3orf22	10.191626	10.544311	10.206287	10.432626	9.984426	10.354444	10.454196	10.863229	10.495265	10.222882	10.297097	10.377715	10.207204	9.986541	9.752062	10.019581	10.552501	9.742743	9.649319	10.010929	9.717888	9.992644	9.837342	9.975738	10.096264	10.141048	10.734171	10.188617	10.473776	11.290392	10.320901	10.360509	10.689930	11.002085	11.215086	10.816421	10.665611	10.836761	10.941524	10.723137	10.309726	10.781910	10.685853	10.671652	10.823244	10.602822	10.263944	10.492998	10.283957	10.582324	10.860728	10.575699	10.536062	10.743207	10.441420	10.671652	10.471030	10.665039	10.638482	10.387032	10.653521	10.878852	11.414710	10.348093	10.690696	10.315198	10.467548	10.381790	10.654131	10.654274	10.679116	10.551588	10.333290	10.473776	10.462883	10.773291	10.437655	10.772249	10.521739	9.966953	9.792703	9.931696	9.672092	9.757177	9.998570	10.233687	9.775812	9.538754	9.751741	9.704158	9.584367	9.694983	10.052486	10.217133	10.281385	10.092624	10.208318	9.565280	10.159823	10.452240	9.937905	10.795256	10.560714	10.721360	10.216098	10.254503	10.233543	10.451232	9.921369	10.450613	9.696633	10.469201	10.405317	10.444422	10.015875	9.735326	10.430794	9.886715	10.411931	10.451937	11.046663	11.423450	11.283416	10.563066	10.912789	11.674302	12.135587	11.972520	10.810803	11.408754	10.781605	10.786172	10.644448	10.939339	10.776077	12.021923	10.281995	9.757177	11.604734	10.068558	10.602549	10.616577	10.327943	11.803532	11.565952	10.665768	10.534701	11.201757	11.977451	10.824286	11.132638	11.159921	11.364157	11.539677	11.203593	11.479023	10.784070	11.437522	11.230791	11.946461	11.035981	11.191490	11.736567	11.325406	10.783006	11.388349	11.114554	10.598330	11.441033	11.544678	11.084541	10.907937	11.717191	11.280760	11.520394	12.395993	10.975093	10.907218	10.945805	11.647387	11.665214	11.635098	11.104875	11.077366	11.040594	10.900409	11.003228	10.797295	11.445862	11.110014	11.216126	10.860126	11.294819	11.074858	11.349956	11.355068	11.445581	11.073706	10.601972	10.536195	10.780585	10.614651	10.892545	11.150678	11.111876	10.477695	10.684205	10.588938	10.622929	9.980249	10.882223	10.645194	11.474531	10.968609	10.856874	10.887033	10.432465	11.077806	11.553024	11.561576	11.933500	10.835128	11.338491	10.720354	10.738688	10.948607	10.526885	10.290374	11.493459	10.394903	11.166213	10.083729	10.801787	10.677766	10.467258	10.842267	10.668429	11.097070

CHST13	10.191626	10.544311	10.206287	10.432626	9.984426	10.354444	10.454196	10.863229	10.495265	10.222882	10.297097	10.377715	10.207204	9.986541	9.752062	10.019581	10.552501	9.742743	9.649319	10.010929	9.717888	9.992644	9.837342	9.975738	10.096264	10.141048	10.734171	10.188617	10.473776	11.290392	10.320901	10.360509	10.689930	11.002085	11.215086	10.816421	10.665611	10.836761	10.941524	10.723137	10.309726	10.781910	10.685853	10.671652	10.823244	10.602822	10.263944	10.492998	10.283957	10.582324	10.860728	10.575699	10.536062	10.743207	10.441420	10.671652	10.471030	10.665039	10.638482	10.387032	10.653521	10.878852	11.414710	10.348093	10.690696	10.315198	10.467548	10.381790	10.654131	10.654274	10.679116	10.551588	10.333290	10.473776	10.462883	10.773291	10.437655	10.772249	10.521739	9.966953	9.792703	9.931696	9.672092	9.757177	9.998570	10.233687	9.775812	9.538754	9.751741	9.704158	9.584367	9.694983	10.052486	10.217133	10.281385	10.092624	10.208318	9.565280	10.159823	10.452240	9.937905	10.795256	10.560714	10.721360	10.216098	10.254503	10.233543	10.451232	9.921369	10.450613	9.696633	10.469201	10.405317	10.444422	10.015875	9.735326	10.430794	9.886715	10.411931	10.451937	11.046663	11.423450	11.283416	10.563066	10.912789	11.674302	12.135587	11.972520	10.810803	11.408754	10.781605	10.786172	10.644448	10.939339	10.776077	12.021923	10.281995	9.757177	11.604734	10.068558	10.602549	10.616577	10.327943	11.803532	11.565952	10.665768	10.534701	11.201757	11.977451	10.824286	11.132638	11.159921	11.364157	11.539677	11.203593	11.479023	10.784070	11.437522	11.230791	11.946461	11.035981	11.191490	11.736567	11.325406	10.783006	11.388349	11.114554	10.598330	11.441033	11.544678	11.084541	10.907937	11.717191	11.280760	11.520394	12.395993	10.975093	10.907218	10.945805	11.647387	11.665214	11.635098	11.104875	11.077366	11.040594	10.900409	11.003228	10.797295	11.445862	11.110014	11.216126	10.860126	11.294819	11.074858	11.349956	11.355068	11.445581	11.073706	10.601972	10.536195	10.780585	10.614651	10.892545	11.150678	11.111876	10.477695	10.684205	10.588938	10.622929	9.980249	10.882223	10.645194	11.474531	10.968609	10.856874	10.887033	10.432465	11.077806	11.553024	11.561576	11.933500	10.835128	11.338491	10.720354	10.738688	10.948607	10.526885	10.290374	11.493459	10.394903	11.166213	10.083729	10.801787	10.677766	10.467258	10.842267	10.668429	11.097070

ADAM29	6.450151	7.086585	6.949475	7.223587	7.051334	7.612789	6.871479	7.195034	7.814612	6.925494	7.114394	6.952325	6.777825	6.514869	6.448680	7.966655	6.789740	6.313838	7.439309	7.099566	6.883914	7.855750	8.075475	7.497555	7.054595	6.379289	8.220385	6.894469	7.264630	7.278325	6.753221	6.781665	7.519393	7.933884	7.258890	6.978944	6.988738	6.910490	7.340356	6.990471	7.030795	7.431882	7.409741	7.580544	7.145206	7.448724	7.416992	7.049262	6.847240	7.372217	6.317250	6.291686	6.525221	6.918220	6.701120	7.266472	6.783375	6.822609	6.753552	6.226570	6.362193	7.518967	6.994918	7.498353	6.900141	6.845868	6.753057	7.044666	6.862022	7.183448	7.248685	7.356854	7.433753	7.170080	7.802802	7.422439	7.137190	7.243579	7.009152	6.440719	6.982244	6.481749	6.511935	6.405052	6.381648	7.302088	6.947452	6.541025	6.848549	6.600722	6.650301	6.938279	7.194888	6.961500	7.127512	7.497273	6.803009	7.307427	7.130033	7.557702	8.167960	7.046406	6.956429	6.734308	7.549145	7.351914	7.460716	7.770324	8.221915	7.323983	7.808333	7.654980	7.481512	7.479964	7.501316	9.485177	5.792044	6.792991	7.148023	6.987163	7.113147	7.046406	7.491660	7.373769	7.826149	7.197917	7.200242	7.331787	6.969819	7.022169	6.815250	6.711340	6.462508	6.675083	7.717365	6.861393	6.483079	7.566901	7.469565	7.073993	7.338470	8.083795	7.419775	7.303187	6.710407	7.867250	7.282413	7.710165	7.014840	6.819593	7.130525	7.182830	8.051848	7.188718	7.211984	7.178293	7.255858	7.964950	7.411494	7.570213	7.520520	7.476764	7.619230	7.104070	7.302722	7.285498	6.908604	7.099087	7.632094	6.991935	6.378641	6.394030	6.991290	6.750533	7.038434	7.319152	6.832287	6.884549	5.969312	7.032025	6.957032	7.507394	7.543116	7.224502	7.295535	7.178449	6.942793	6.979858	7.308960	7.333384	7.437870	7.564103	7.252773	7.949190	7.501815	7.047385	7.468145	7.244534	6.583759	6.626941	6.919713	6.851233	6.986699	6.511783	7.268627	6.770577	6.459041	7.062373	6.558935	6.861465	7.041441	7.108695	7.044666	7.405681	7.442212	7.049430	7.753478	7.166708	7.160938	7.504008	7.220170	6.901907	6.839508	7.085839	7.186039	7.961155	7.542183	7.778689	7.456502	7.741489	8.006846	6.622744	7.882013	7.037474	7.963140	6.576008	7.076686	6.900460

TMEM52	10.778996	8.930477	8.631915	8.995967	9.941496	9.263538	9.496731	8.423166	8.742697	8.828725	9.222067	9.205189	9.972796	8.754570	9.513820	8.599122	8.883123	9.015766	9.453395	8.720391	8.706598	8.961298	8.628955	8.849250	8.882957	9.059116	9.446213	8.918471	9.081509	9.536570	9.133075	9.639042	8.670629	9.494561	8.781030	8.348548	9.433874	9.206285	8.554054	9.338318	9.551167	9.091411	8.135851	8.689141	8.435835	9.179536	9.226054	9.164033	9.209476	9.105505	9.394342	9.325501	9.154648	9.917791	9.335281	9.342971	8.601123	9.707820	9.819657	9.109422	9.702796	8.822360	8.358620	9.437081	9.686383	8.659160	9.592908	9.361712	9.539221	9.436629	9.185335	9.134397	8.705039	8.668982	8.557313	8.233424	9.133220	8.999804	8.709313	9.168150	9.947336	9.682497	10.395504	9.255589	9.934782	9.589975	9.946044	9.291432	10.597404	10.321522	9.584523	8.790577	8.511528	9.394200	9.764963	9.166498	9.452450	9.065840	9.247052	8.868229	8.963724	8.310071	8.281921	8.002620	9.453727	8.920759	7.925527	9.109097	8.507964	9.246593	9.318827	9.433441	8.903060	8.963428	9.385126	9.652594	11.427104	9.299705	9.275616	10.121103	9.271815	9.010103	9.238440	9.388107	10.480362	11.043290	10.211080	10.265725	10.420249	9.582219	10.127429	9.085519	9.806528	10.189508	8.805667	9.556004	9.148011	11.582100	9.528969	9.617475	9.611185	10.046320	11.058524	12.206813	9.464554	9.417469	10.073625	10.686008	9.360643	9.708289	9.738677	8.504929	9.239986	10.683473	8.817908	9.458185	10.138502	9.063687	9.226526	10.327943	8.799907	8.549674	8.626865	9.287550	10.319510	10.719738	9.452905	9.943334	9.554047	8.922699	9.948435	10.440492	8.312374	9.112719	9.055670	8.965547	10.766855	11.045432	10.753090	10.209520	9.362458	9.601275	9.667979	10.943606	7.819973	10.131841	8.578764	11.802547	9.306635	9.882574	8.735164	11.362553	8.652644	8.682529	8.881137	9.129842	9.961344	8.358393	8.618151	9.430029	9.823884	10.061049	9.440805	8.718897	9.069737	9.336472	9.814319	7.811119	9.515750	12.300959	10.439142	8.379382	9.752834	8.973205	11.057327	9.044254	9.840773	9.569039	10.204002	9.101975	8.792595	8.423466	8.465536	11.152612	9.935596	9.384665	11.369761	9.798110	10.365154	10.047255	9.535194	10.445361	9.438186	10.127592	9.463454	8.829953	10.296166	9.772353

AFF2	7.238905	6.927637	6.850135	6.644513	7.063661	8.812917	7.944941	8.708137	10.055597	7.710499	8.356438	8.464743	7.227793	6.540336	5.082653	7.802954	7.884890	5.727009	4.778032	7.713749	4.843594	6.255368	6.107820	6.143072	6.113325	7.058536	9.558934	8.066831	8.640041	8.788596	8.358960	6.771880	8.236995	8.491946	7.622688	6.850930	7.856943	5.143570	5.394390	4.906492	7.993011	6.634691	6.289603	7.085839	7.515375	7.667017	8.830706	6.219571	8.189568	6.856249	6.959726	7.315739	6.941991	7.241488	7.235801	8.622034	7.463593	6.497206	5.412215	5.669981	4.875476	7.513109	4.167983	4.276840	7.503538	4.898779	4.956653	4.347616	5.827126	3.826149	6.834227	7.170864	8.779928	7.185534	7.185203	8.330105	6.970573	7.437546	7.525576	5.578136	6.802353	6.819738	7.369846	7.080037	6.881346	7.261207	7.922721	7.062210	5.156870	4.899606	7.233231	4.342419	6.154988	7.571121	7.627977	8.463830	7.688703	8.830099	8.475093	6.743344	9.018822	6.690296	7.398357	5.926480	3.303975	8.453070	3.959671	5.049361	4.114172	8.014014	4.465643	4.431158	5.444298	8.290722	9.170762	10.700036	5.259448	7.838143	9.454320	8.789796	5.703178	9.080099	8.381455	8.680959	10.792032	8.690507	8.874814	10.097946	9.240585	8.580900	9.842888	8.669872	7.429685	4.990613	8.424087	8.884058	7.001942	8.144547	11.030464	4.186897	8.269676	9.751904	7.123846	5.204460	6.376639	8.829820	9.644557	8.705513	8.050619	10.620438	8.557313	9.339959	7.994267	7.756185	7.962366	8.052945	4.909265	4.137156	5.935818	9.353198	6.374349	8.803665	5.932802	10.126788	5.739006	9.645490	9.185335	10.046469	7.584575	8.051116	8.302895	7.529026	7.052561	7.311340	9.274836	9.580581	7.414462	9.513501	8.922841	6.598436	8.160386	3.137360	5.351297	6.505307	8.181886	4.515737	5.641209	6.741283	4.302412	7.583649	9.644393	9.226978	7.303825	10.081787	7.497414	8.172072	10.028112	8.743149	5.253548	6.899823	6.873205	8.299864	7.684432	6.997868	8.363315	8.037595	9.447790	4.683975	5.862898	10.339163	3.544508	6.940592	6.939054	7.812324	8.463370	8.785633	10.411625	9.474159	8.967900	8.570650	8.310979	8.268871	7.665283	3.632948	9.794394	6.628714	8.123563	9.399241	9.130445	9.174330	6.887075	8.360473	10.122918	8.818241	9.310802	5.257917	8.650279	8.529384

OTUB1	12.166049	12.542279	12.654999	12.947179	12.667828	12.790989	12.580507	12.934196	12.223244	12.759782	12.798177	12.681971	12.710992	13.033201	13.100683	12.807530	12.896716	12.887549	13.675309	13.338305	13.123071	13.742612	13.287852	12.626022	12.616955	12.707907	12.282178	12.384801	12.097725	12.687630	12.824716	13.487724	13.215415	12.612867	12.823859	12.681457	12.911088	13.039489	13.191043	13.135275	12.608186	13.216008	12.873210	12.823032	12.799850	13.096345	12.255674	12.914484	13.044646	12.747792	12.910727	13.358673	13.197314	13.122311	13.128443	12.663564	13.500208	13.405475	13.316023	13.255784	13.310948	12.412056	13.375856	13.481700	13.614956	13.125598	13.364746	13.318089	13.376917	13.607817	13.218581	13.307696	12.428822	12.984259	13.541252	13.070948	13.158588	13.146687	12.860559	13.273969	13.301455	13.116206	12.992703	13.527900	13.432385	13.048973	13.429701	13.429701	13.902033	13.781086	13.501308	13.619482	13.475103	12.996574	12.989438	13.267397	13.031189	12.865672	12.959560	12.651411	12.749999	12.660149	12.916577	12.712283	14.368341	12.711633	12.841260	13.851570	13.473987	12.540801	13.940443	13.103364	13.243181	12.225203	12.948244	11.450723	12.626503	12.832557	12.457538	12.866399	13.945117	12.813176	13.267608	12.409678	13.070221	13.134359	12.923493	13.016643	12.879771	13.532613	12.587805	13.707475	13.524143	13.216968	12.640896	13.321094	13.311729	14.134512	13.108685	13.942362	13.948216	14.234874	12.737637	13.309969	13.411210	13.208114	13.045174	12.042883	13.102943	12.646251	13.524838	13.520034	13.496262	13.095058	13.522292	13.552549	12.839799	14.179542	13.714163	13.465090	13.636921	13.431706	13.287052	13.185843	13.678503	12.579997	13.227239	13.316216	13.689281	13.475946	13.712695	13.308547	14.099791	13.652033	13.100310	12.806823	13.539712	13.525511	13.257008	14.078621	12.797323	14.236540	13.873927	13.302463	13.790706	13.686701	14.212093	13.058425	13.407961	13.251240	13.161477	13.063231	14.529101	12.678705	13.671278	13.785834	13.791726	13.529215	13.202774	13.615689	13.586976	13.510396	13.468126	14.100719	13.604783	14.240086	14.131743	13.939322	13.770321	13.414830	14.673241	13.484618	13.744601	14.014363	13.433474	13.413783	12.360062	13.895060	12.958452	13.288240	12.998253	12.204516	12.982246	13.254976	12.303112	13.410994	13.537927	13.341580	12.811936	13.615437	13.277634	12.406200	12.435636	13.036523	13.225883	12.998253	13.262182	12.904102

RAP2B	9.052979	9.666547	10.631731	9.843038	9.698632	9.944250	10.463260	10.743513	11.077064	10.142294	10.265297	9.928924	10.017266	9.826316	10.258997	10.218622	10.432922	10.319057	11.142648	10.177187	10.340966	11.129440	10.464454	9.544271	9.133220	9.328916	9.131674	9.905450	9.422970	9.285739	10.323369	9.957686	10.702740	9.858186	10.260570	10.168737	10.830041	10.588938	10.444108	10.909567	10.032263	10.867134	9.585147	10.450323	9.931101	10.047711	9.343762	10.396585	10.027037	10.653521	10.161362	10.248333	10.938612	10.774902	10.623218	10.609923	10.571372	10.674499	10.349459	9.968492	10.631271	9.490469	10.566794	10.695882	10.842407	10.902327	10.230861	10.221095	10.266592	10.435536	10.250277	10.207204	9.486271	10.643569	10.142755	10.314277	10.549347	10.158496	9.654038	9.896439	9.731236	10.152177	10.334012	10.509324	10.352828	10.441702	10.720767	10.472987	11.141336	10.214731	10.026726	11.147515	10.354444	10.201859	9.802113	10.430557	10.433811	10.182714	10.710821	9.480888	10.736706	10.239553	9.924138	10.103422	11.220719	9.908813	10.276224	10.600603	11.108519	10.026399	11.250930	10.033912	10.556615	9.514420	11.043433	7.831559	9.320034	9.937098	9.907741	11.733934	11.152465	10.201561	10.393108	9.225591	9.294264	10.111224	9.346264	11.290061	9.067393	10.345960	8.773366	9.699554	8.648567	9.277385	10.067808	10.763720	9.977119	10.188320	9.338318	9.163229	11.637244	10.126478	10.212263	10.758681	8.747882	9.639191	7.119964	9.077210	9.841511	10.692703	8.858496	10.133592	10.116828	10.412390	10.617890	10.924469	10.011086	10.889511	10.866673	10.060602	10.438539	8.884356	10.691177	10.389594	10.191151	10.332362	9.249793	9.879153	11.835276	11.270275	10.102474	10.529082	11.076924	10.435394	10.411451	10.786329	11.913078	10.124282	10.979079	11.064379	9.353778	11.825487	11.263041	11.031633	10.461290	12.187471	10.749156	10.066907	10.070706	10.661925	10.506163	10.284425	11.092897	10.446420	10.222722	10.694051	11.488318	10.561470	9.448263	11.637826	10.275033	11.355068	10.408136	10.098273	9.489658	11.331759	9.658892	10.371545	9.607325	8.531228	10.149488	9.500882	10.091267	11.116961	9.426344	10.879160	9.640419	10.909986	10.824430	9.659506	10.250277	9.369059	9.663280	10.638627	9.287393	11.098696	10.459811	9.602855	9.781923	11.253380	10.453422	6.954382	8.849250	9.753141	9.485807	10.516841	10.403949	8.993336

NBPF11	7.825526	8.280995	8.551647	8.181745	8.799005	8.295296	8.403138	8.482018	9.657788	8.296996	8.112848	8.137859	8.224479	8.354284	8.175531	8.463224	8.577568	8.655231	9.517769	8.071082	8.426021	9.908813	9.412138	8.714674	8.625939	8.940500	8.360784	8.805073	8.589300	8.398367	9.362915	8.009910	8.117460	8.623429	8.161158	8.038029	8.127830	8.463989	8.852855	8.952566	8.505214	8.578764	8.217923	8.511528	8.563746	8.646482	8.585128	8.173935	8.569415	8.218403	7.794465	7.936228	7.962682	8.282551	8.614955	8.707526	8.241734	8.411859	8.564961	8.239474	8.428664	8.554647	8.720634	8.712662	8.711476	8.572726	8.167811	8.313750	8.331048	8.376752	8.179009	8.162959	8.651181	8.044166	8.157641	8.117928	8.224788	7.943875	7.995024	7.544535	8.136158	7.776055	7.606840	7.773840	7.326588	8.006397	7.663402	7.626091	7.617692	7.395399	7.496477	7.882760	8.351263	8.158091	8.229359	8.390673	8.109965	8.871377	9.148637	9.037084	9.341602	8.578321	8.772629	8.536093	9.124281	8.415496	10.027498	9.263538	10.349459	8.531075	9.304840	9.383781	9.934185	8.570802	9.087621	9.386992	8.839496	8.655793	8.475262	8.175531	9.161517	8.104010	8.428965	9.642697	8.093407	8.326818	8.485224	8.757384	8.344925	8.247106	8.251152	8.789635	8.416609	8.663004	8.027626	8.468844	9.197337	9.823884	8.800062	8.783833	9.985035	8.847714	9.207170	8.753956	8.460162	8.017407	9.075452	8.999362	8.585918	8.627169	8.552439	8.575356	9.260456	8.437092	8.406081	8.162073	8.378160	8.729427	9.108288	9.174641	8.785633	7.867887	8.851683	8.378761	8.624351	8.969436	8.766693	8.318066	8.439490	8.219027	8.480778	8.240081	8.953320	8.428517	8.915570	8.259547	8.440578	8.685393	8.394988	8.443781	7.973975	8.857133	8.837390	8.926766	8.585128	8.549357	8.269978	8.682359	8.772914	8.253119	8.230865	9.256070	8.150523	7.993011	7.955787	7.985306	8.242495	8.421352	7.272982	7.893878	8.626865	8.370333	8.274612	7.813386	8.633227	7.995341	8.517642	7.721466	7.976790	8.677746	8.022991	7.988952	8.309606	8.468252	8.504471	8.322775	9.052352	9.321704	8.105890	9.694983	8.211744	8.842601	8.228265	8.902170	8.685982	9.547475	8.785172	9.119435	8.426021	8.916489	9.379735	10.211826	8.561316	9.403879	8.617246	9.311249	8.570204	8.119173

DDX17	14.177726	13.184665	13.947085	13.884347	13.805428	13.534199	13.733641	13.399301	13.586501	13.909973	13.651504	13.769304	13.726879	13.410314	13.759150	13.555053	13.823712	13.320065	13.712951	13.435763	13.411878	14.005012	13.942362	13.392233	13.575112	13.743105	13.688112	13.838475	13.612555	13.774941	13.686919	13.437764	13.361393	13.751290	13.505253	13.621755	13.491941	13.383479	13.340998	13.405475	13.796422	13.559082	13.077040	14.065983	14.302959	13.660327	13.675309	13.214995	13.699890	13.290292	13.681855	13.502195	14.282413	13.396218	14.003878	13.637621	13.862600	13.373144	13.558417	13.763209	13.419066	13.630696	13.882760	13.593519	13.553691	13.711167	13.160146	14.013775	12.911088	13.409232	13.276462	13.248097	13.842755	13.373144	13.374839	13.523918	13.608723	13.515355	13.541252	12.945900	12.952737	13.772955	13.456831	13.924035	13.567818	13.472274	13.570123	13.511265	13.409232	13.155345	12.713819	13.338305	13.314543	13.067539	13.527257	13.658668	13.572645	13.875238	13.649618	13.599265	13.829979	13.119650	13.928111	13.587444	13.399078	13.739872	13.444604	13.568039	13.703179	13.677795	12.923860	13.728857	13.666222	13.631128	13.902821	12.981496	13.727608	14.051213	13.805428	13.620822	12.951111	13.702148	13.788993	13.594881	13.644958	13.740837	14.099196	13.518195	13.988071	13.566158	13.872610	14.087146	13.370400	13.040220	13.845633	13.679001	13.617064	13.213390	13.325998	13.348067	13.798810	13.442439	13.825287	13.239397	13.944308	13.453519	13.619482	13.156342	13.504598	14.025367	13.331494	13.793821	13.480608	13.111982	13.799344	13.361393	12.954789	13.239205	13.246114	13.829200	13.314762	12.677828	13.356199	13.349308	13.250452	13.663221	13.324170	13.410994	13.558417	13.694716	13.497141	13.452684	13.963570	13.463806	13.761927	13.903326	12.936194	13.082154	13.148421	13.234224	13.707475	13.385833	13.371459	13.181711	13.158422	13.364946	13.094478	13.182111	13.123808	13.509011	13.789231	13.637621	13.940718	13.789952	13.469010	13.582520	13.571462	13.820961	12.602739	12.892947	13.234224	13.062840	13.618283	13.435992	13.505253	13.690948	13.403139	13.065668	13.143196	13.041546	13.296970	13.373144	13.344471	13.510177	13.733138	13.612555	13.837460	13.508116	14.262283	13.488148	13.606881	13.531931	13.304119	12.769602	13.456369	12.811445	13.238812	13.189521	13.469010	12.939042	13.300869	13.665236	13.377562	13.826855	12.951859	13.380743	13.569183	13.325811

OTP	7.269393	7.332417	7.158083	7.161102	7.317927	7.762198	6.656465	8.510590	8.241293	7.318389	7.435851	7.140953	6.496218	6.781503	7.442078	7.321205	8.214857	7.314510	8.741333	7.346434	8.070641	7.489439	9.304406	7.478884	7.548238	6.939673	7.681782	7.943875	7.385465	7.084584	7.587974	6.140830	7.015663	5.712490	7.051808	6.899672	6.962442	7.294130	7.847801	7.616186	6.929077	7.807111	6.957676	7.226852	6.999519	7.673040	7.854689	7.546612	7.476921	7.444598	7.258890	7.396166	6.818290	7.186986	7.131001	7.304922	6.627577	7.604353	7.471425	7.399296	7.961466	7.336916	8.194308	8.309760	7.911881	7.901337	7.626701	7.717365	7.441933	8.165357	7.033591	7.063346	6.995380	7.032798	7.062210	7.486641	7.398965	7.376878	7.364203	6.926316	7.118055	7.178144	6.966132	7.172144	7.094982	7.363410	6.920814	7.390553	7.778689	8.156082	7.734420	8.392410	7.163317	7.154192	7.222979	6.771077	6.819738	5.714005	6.871479	6.603086	6.321010	6.517550	6.506176	6.664613	7.874285	7.243901	8.232255	7.763284	8.849419	7.522516	8.544315	8.530303	8.424087	7.722516	7.492285	8.125709	8.054162	7.163002	7.226852	7.172303	7.797839	7.102506	7.140794	7.384091	7.841996	6.707538	7.311045	7.760052	6.816661	7.147559	7.098777	6.752252	6.489829	7.450892	7.021564	7.902271	7.408829	8.271350	7.081142	8.128713	7.512640	9.413335	7.606544	7.833239	6.262524	7.608060	7.937592	7.796488	7.395245	7.622541	6.252157	7.008344	6.402270	6.973662	6.784180	6.766516	7.552537	7.860630	7.768978	7.300476	8.143298	7.129090	7.750438	7.464349	7.604831	8.143761	7.500852	7.306812	7.612025	7.800068	7.400837	7.150580	7.366100	6.938425	6.668816	6.610967	7.637084	7.680418	7.528117	8.030244	7.205372	8.247106	8.276569	7.971306	8.194463	7.735493	7.837050	7.706091	9.066450	7.087205	7.030942	7.759430	7.023584	6.928135	7.656275	7.411631	7.517884	7.764336	6.620976	7.011889	7.379095	7.317634	6.899672	7.385610	7.354552	7.288452	7.398827	7.767315	7.808938	7.822399	8.570204	6.788582	7.160111	7.180484	6.844875	6.805088	6.098380	7.057769	6.313497	6.211186	6.490919	6.436616	6.641126	7.765467	7.029566	8.327463	7.583190	8.097966	7.872609	8.466587	8.211137	8.548140	8.317902	7.593519	7.109184	7.921053	7.246690	7.352662

BRK1	14.686942	14.381001	14.806664	15.067485	14.505707	14.647558	14.829469	15.095132	14.318839	14.952794	14.905079	14.892803	14.503039	14.509168	14.025686	14.823396	14.873437	14.236540	14.268626	14.602393	14.398095	14.551021	13.510396	14.174885	14.272526	14.510745	13.944564	14.885495	14.467751	14.450216	14.864634	15.536016	15.303021	14.239134	15.116602	14.739028	14.781829	14.291337	14.536742	14.577469	14.614415	14.556424	14.833005	14.817850	15.279559	15.317503	14.577262	13.840099	15.336040	15.209040	14.633465	15.238314	15.607626	15.352487	14.826455	14.925975	15.407655	14.430074	14.269241	14.809104	14.054502	14.546394	14.236872	14.566322	14.224052	14.506817	13.817588	14.708876	14.507205	14.053306	15.293162	15.137104	14.420600	15.104224	15.005364	15.266520	15.473095	14.908102	14.637025	14.524706	14.790582	14.653700	15.113668	15.531572	15.248027	15.323382	15.688110	15.542881	13.977800	14.967376	14.122158	14.234874	14.508030	14.512377	14.904024	15.213664	15.109227	14.792525	14.977558	14.664987	14.872892	14.468143	14.634805	14.548089	14.126769	14.712563	14.327509	14.995622	13.493039	14.839682	13.844047	14.551853	14.642226	14.085269	15.168793	14.210781	13.872610	14.806664	14.554790	13.533281	13.736836	13.842996	13.966043	13.853253	14.526691	14.543000	13.392871	13.676917	13.836415	13.845880	14.103167	13.966624	13.290718	13.062840	13.929201	13.707724	13.289437	13.216405	15.026340	13.629444	13.186053	13.751062	13.070042	14.308634	14.565537	14.477449	14.235215	13.057446	13.832307	13.665236	14.139533	14.326431	14.517783	14.235557	14.260288	14.430074	14.196229	14.177726	13.809402	13.945929	13.679487	14.422502	14.299472	14.277280	14.651938	13.712440	13.148247	13.952379	14.121601	14.088628	14.213324	14.331005	14.615271	14.561327	13.780832	14.188386	14.186189	13.471830	13.826079	13.993914	14.557653	13.385185	13.617295	13.377996	13.886730	14.604561	13.750073	13.554152	13.545312	14.288296	14.316814	14.207819	14.278255	14.488728	14.341372	13.844292	14.400393	14.558412	13.376715	13.910238	14.527514	13.879568	14.180225	14.729253	14.121021	14.420258	14.540076	13.543949	13.688112	14.274191	13.522074	13.986378	14.746969	14.165527	14.054502	14.293414	13.109444	13.986668	14.032009	14.138571	13.764196	13.891542	13.739368	13.319680	13.902033	13.535306	13.823712	13.184093	13.691450	12.986468	14.271254	14.061504	14.258285	13.647535	14.324402	14.278255	14.068626	14.260288

F3	12.883111	10.875592	12.584475	10.705048	10.906146	10.763276	11.194590	12.716248	12.702790	12.831697	11.281661	12.576063	11.224748	11.092677	10.903646	10.983080	12.886514	10.803291	12.136181	10.452666	10.675241	11.410967	12.023231	11.406761	12.072811	13.022344	11.770623	12.315928	11.666726	11.611243	10.961939	11.372021	11.133688	10.732241	11.596844	11.271173	11.931845	12.090269	12.274241	12.417994	11.447359	12.195580	11.094004	11.694853	11.469421	10.916445	12.159874	12.666649	12.457691	11.687212	12.849137	12.492559	12.259736	10.621011	12.846314	11.921595	11.886584	12.407139	12.294834	11.703111	11.781504	11.794233	11.730047	11.375766	10.832721	12.671216	11.706258	10.860414	10.501830	11.989871	11.067626	10.610070	11.448683	11.171227	10.719432	11.328057	10.694805	12.737964	12.675358	11.418835	11.067036	12.419130	11.351596	11.652891	11.259889	11.530458	11.509057	10.672565	11.987250	12.145944	12.460924	10.700619	11.131759	11.184811	11.920674	10.250573	12.848771	11.030608	10.792626	11.258266	12.145607	13.059695	11.552728	12.091525	13.080831	11.841289	13.315612	12.584810	12.796448	12.023884	11.117822	12.255202	12.937800	10.984344	10.573931	10.125527	12.172186	13.092197	11.398216	9.310802	15.009958	11.624141	10.209520	10.465727	10.850917	12.651078	10.599709	9.992352	11.503490	13.251043	12.167414	12.348206	11.410105	14.283430	10.551138	10.013986	12.229099	11.125340	10.276978	11.457713	12.654306	12.012502	11.637677	10.045398	10.554299	12.726202	12.432421	8.536093	11.144256	11.565093	12.439669	12.253731	13.990442	11.157605	13.862105	12.876579	12.371333	13.490156	11.254561	10.008945	13.447352	10.297710	12.065779	11.926285	11.664174	10.799656	11.180515	11.661937	12.584475	13.877971	12.348522	11.969768	13.847489	10.407984	9.525717	10.569842	13.337083	12.033678	12.981868	13.167188	9.240450	13.419287	14.213010	9.239207	11.660141	12.606892	11.604438	12.447724	11.548135	11.321435	13.354977	13.407160	11.668911	9.118833	12.684724	11.738805	11.625884	12.011728	11.624718	12.298374	11.738805	13.792238	10.773877	14.689299	10.806918	12.295409	11.441033	11.271942	11.530935	11.259294	13.652481	12.296198	14.302275	12.041676	11.745095	11.512165	10.205213	13.266014	9.905906	15.022274	11.107037	8.157804	12.420436	10.830328	12.609687	13.965466	10.522806	11.716161	13.695872	13.086878	12.267146	7.378933	10.687817	12.255523	13.267608	11.573596	10.958405	13.405662

GINS2	11.997474	11.153776	11.524427	11.584312	10.936702	11.395949	11.142648	10.847257	10.889218	11.140636	11.500204	10.918229	11.361944	10.802397	9.897040	10.868443	11.405727	10.631271	11.074564	11.233769	9.809978	10.714015	11.069879	10.699421	10.441702	10.885995	11.158915	10.192949	10.323968	11.492740	11.374203	12.529347	11.127236	12.811111	11.502190	11.777298	11.471663	11.631664	11.161418	10.695882	11.092677	10.219095	11.426649	11.459795	11.492740	12.370681	10.905852	10.210629	11.402210	11.307178	11.436677	11.658499	11.676108	11.162971	11.622534	10.616870	11.712047	10.508413	10.646800	11.383057	10.387620	10.908996	10.018641	10.546889	11.740562	11.003790	11.471947	10.903938	12.081830	10.409375	11.392179	11.652891	10.543736	11.506274	11.991408	11.569749	12.016745	10.861307	11.535028	10.371998	10.578433	11.138902	11.581538	11.467339	12.115695	11.079831	11.555799	11.355934	11.052955	11.360602	10.452536	10.931888	11.633888	11.770058	11.781075	11.617879	11.541375	10.743052	11.283416	10.413447	10.533504	10.475286	11.058379	10.573060	11.093119	11.108963	10.562355	10.957667	11.332335	10.854176	11.226941	10.427471	11.028666	11.435658	10.954993	11.973290	10.817302	10.981607	11.086179	12.052362	12.415936	13.211099	11.689126	13.116395	12.572521	12.336986	13.379067	12.722462	13.670785	12.503556	13.740602	13.912401	12.587805	11.393514	11.624718	13.692656	12.197683	13.218984	12.912866	11.459639	11.427522	12.780366	11.816892	12.642396	13.258409	11.904475	13.195318	13.262398	13.340771	12.086504	12.580172	13.349953	12.751559	12.690828	13.031762	11.740121	13.567342	12.110554	11.777620	13.330272	12.183118	13.218764	13.508359	13.159158	13.790706	12.384018	12.584123	12.440469	13.646327	12.799021	13.902033	12.870847	13.597930	13.795903	12.632803	12.300012	12.480123	12.115391	12.749490	11.889157	11.982583	12.293428	12.931713	12.095489	11.873849	12.955360	12.363406	13.551398	11.730949	12.851064	13.421049	11.940136	13.295341	13.191685	12.896514	12.741631	14.021288	12.676354	11.474696	12.333823	13.531252	13.200662	12.670862	13.267179	12.621157	13.596087	14.251440	12.119428	12.760647	14.953342	13.078187	13.451597	13.352048	13.188004	12.702957	13.320065	11.578918	13.370400	12.936720	13.237824	13.245326	10.749767	12.856393	14.197180	12.343449	12.598661	13.163939	11.562313	12.100426	12.183786	12.443867	13.576482	12.077882	13.644259	12.968393	12.773964	11.811273	13.620619

KIF6	8.057711	8.050619	8.001370	7.959604	7.860319	8.598050	8.120911	8.543529	7.976636	7.919671	7.950014	7.902878	6.794137	7.069219	7.331787	7.899568	8.358315	6.958927	8.137701	7.575603	7.516962	6.975270	8.073176	7.227642	8.068315	7.949357	7.899114	8.301208	7.988366	8.251602	7.923664	7.324386	7.475221	7.423830	8.070314	7.981607	8.066258	8.100252	8.040118	8.009770	8.074558	7.882612	7.800068	8.104486	8.728390	7.709848	8.663767	7.906786	7.433914	8.123878	7.459660	7.803029	7.363410	8.109965	7.425999	8.479979	7.855600	7.834479	6.911787	7.870600	8.229359	8.234051	8.655960	7.697615	7.358858	7.409279	8.137859	7.683968	9.010103	8.132254	8.177531	8.020288	7.987496	8.010350	8.129662	8.142539	7.937457	8.098995	8.090493	7.201777	7.264041	7.847655	7.274704	6.934258	7.669290	7.549594	7.233534	7.446661	7.608060	7.759598	7.516167	7.654215	9.037684	7.921630	7.505885	7.427119	7.528278	6.849029	7.700850	7.225755	7.540067	7.673040	7.596692	7.241967	7.495997	7.968928	8.134662	7.362446	7.859428	8.265082	7.740395	7.874736	7.639530	8.305594	7.364513	10.139559	7.942799	7.803715	7.819359	7.449989	8.057282	7.931268	7.755300	8.798699	8.401134	8.392095	7.394443	7.554725	8.058438	8.555103	8.327809	7.869083	9.238440	8.860321	7.576516	7.920589	6.881674	7.079256	7.693984	8.150964	7.488204	7.922409	8.142249	7.961301	8.005774	8.135425	8.475262	8.824316	8.419769	7.539423	7.137190	7.457944	7.970525	7.840460	7.439013	7.815116	7.880939	7.622541	8.131825	8.759006	7.820563	6.577162	8.714025	7.562125	7.436323	9.022327	8.013217	7.261987	7.512022	7.484743	7.094174	6.831311	9.086706	9.816047	8.477734	8.364386	8.415946	6.936806	7.338770	7.615409	8.699834	6.946292	7.951354	6.821815	7.033428	7.755586	8.188314	10.997004	9.037684	8.387874	7.940740	8.939739	8.622673	7.859735	8.044166	8.118880	7.760531	7.768978	8.237926	8.488606	11.091901	7.132591	7.231027	6.878304	7.984242	7.478735	10.934442	7.966042	8.023315	7.565198	8.102949	10.604444	8.590063	7.849468	8.392568	8.588986	7.217334	7.610677	7.755300	7.540217	7.711088	8.488914	7.878104	7.318215	7.923339	7.139538	6.788259	8.090338	8.790410	7.956970	7.584871	7.914461	9.214963	8.473114	8.467956	7.428678	8.239920	8.541489

SLC7A14	4.116995	3.126623	4.979752	3.985631	4.440882	4.173784	4.233519	4.512034	4.885177	3.242251	3.353204	3.518201	3.783976	3.509419	4.839385	3.445453	4.304213	4.044345	4.557657	3.759199	7.683512	2.945757	6.237135	3.931093	3.113210	3.889387	5.109773	3.869456	3.412735	5.267883	3.930648	4.928275	5.392969	2.898904	5.320912	3.948773	3.454582	4.597046	4.428669	4.461559	3.265735	4.304851	4.191920	3.676604	4.619695	4.553674	5.549015	4.824132	4.537161	4.973787	4.937893	4.628254	5.183414	5.101759	4.495760	3.252362	3.765519	4.669058	4.599394	5.130200	3.327729	3.673696	5.947925	4.403536	4.812075	4.152302	3.054163	3.837359	3.848851	3.799662	4.842774	4.609078	3.676812	3.719536	4.654865	3.911422	4.138230	4.073227	4.014957	4.588420	4.797463	4.251929	4.296330	4.667960	4.780742	5.089403	5.267688	3.722103	5.205618	4.745959	4.960000	3.758791	3.320254	3.750187	5.051601	4.054091	4.519978	3.611397	4.301102	3.852056	4.801357	3.422057	3.718335	3.847370	5.608157	4.255230	4.710741	4.270937	3.966608	3.831827	3.857435	4.417080	5.003583	3.898456	3.200908	5.864523	3.918102	3.278841	3.716082	4.287894	3.668095	4.665614	3.833571	6.547740	4.723654	5.181177	4.616202	4.404426	4.107221	3.643274	4.631969	3.591699	3.716933	4.107221	3.879159	4.649613	4.165107	3.532537	3.793394	4.615968	3.496333	5.244418	3.717722	3.630055	3.805149	4.290484	3.986069	4.191920	5.100918	4.846179	4.510286	4.138630	4.241625	5.278342	4.191032	3.516425	4.041233	4.227672	3.926712	4.089834	3.823524	3.932174	4.006530	4.332885	4.025170	6.366062	8.107120	4.634126	5.111719	4.872144	3.509805	4.742727	4.186652	6.112494	4.263555	5.272683	4.098433	4.645359	4.780099	4.004116	6.296097	4.740192	4.837863	5.214034	3.384000	4.194119	4.535021	3.995950	4.163583	4.367861	4.309712	3.866608	4.204520	4.606257	4.148136	4.389751	4.462632	4.056940	4.670600	5.469895	4.947941	4.542193	3.725577	4.877599	5.431227	5.031937	4.436278	5.000064	4.922128	4.194339	4.648755	4.124217	3.968315	4.410887	4.069243	4.327452	4.784935	4.350669	4.014957	4.418457	6.210188	3.679614	4.210992	4.822917	4.907542	4.408014	4.763580	4.637149	4.236984	4.551331	6.216463	6.122011	3.870092	5.495683	5.578913	4.746389	4.149177	3.393995

AC026316.4	4.116995	3.126623	4.979752	3.985631	4.440882	4.173784	4.233519	4.512034	4.885177	3.242251	3.353204	3.518201	3.783976	3.509419	4.839385	3.445453	4.304213	4.044345	4.557657	3.759199	7.683512	2.945757	6.237135	3.931093	3.113210	3.889387	5.109773	3.869456	3.412735	5.267883	3.930648	4.928275	5.392969	2.898904	5.320912	3.948773	3.454582	4.597046	4.428669	4.461559	3.265735	4.304851	4.191920	3.676604	4.619695	4.553674	5.549015	4.824132	4.537161	4.973787	4.937893	4.628254	5.183414	5.101759	4.495760	3.252362	3.765519	4.669058	4.599394	5.130200	3.327729	3.673696	5.947925	4.403536	4.812075	4.152302	3.054163	3.837359	3.848851	3.799662	4.842774	4.609078	3.676812	3.719536	4.654865	3.911422	4.138230	4.073227	4.014957	4.588420	4.797463	4.251929	4.296330	4.667960	4.780742	5.089403	5.267688	3.722103	5.205618	4.745959	4.960000	3.758791	3.320254	3.750187	5.051601	4.054091	4.519978	3.611397	4.301102	3.852056	4.801357	3.422057	3.718335	3.847370	5.608157	4.255230	4.710741	4.270937	3.966608	3.831827	3.857435	4.417080	5.003583	3.898456	3.200908	5.864523	3.918102	3.278841	3.716082	4.287894	3.668095	4.665614	3.833571	6.547740	4.723654	5.181177	4.616202	4.404426	4.107221	3.643274	4.631969	3.591699	3.716933	4.107221	3.879159	4.649613	4.165107	3.532537	3.793394	4.615968	3.496333	5.244418	3.717722	3.630055	3.805149	4.290484	3.986069	4.191920	5.100918	4.846179	4.510286	4.138630	4.241625	5.278342	4.191032	3.516425	4.041233	4.227672	3.926712	4.089834	3.823524	3.932174	4.006530	4.332885	4.025170	6.366062	8.107120	4.634126	5.111719	4.872144	3.509805	4.742727	4.186652	6.112494	4.263555	5.272683	4.098433	4.645359	4.780099	4.004116	6.296097	4.740192	4.837863	5.214034	3.384000	4.194119	4.535021	3.995950	4.163583	4.367861	4.309712	3.866608	4.204520	4.606257	4.148136	4.389751	4.462632	4.056940	4.670600	5.469895	4.947941	4.542193	3.725577	4.877599	5.431227	5.031937	4.436278	5.000064	4.922128	4.194339	4.648755	4.124217	3.968315	4.410887	4.069243	4.327452	4.784935	4.350669	4.014957	4.418457	6.210188	3.679614	4.210992	4.822917	4.907542	4.408014	4.763580	4.637149	4.236984	4.551331	6.216463	6.122011	3.870092	5.495683	5.578913	4.746389	4.149177	3.393995

CLEC6A	3.137173	4.009758	3.833985	2.957515	2.871232	5.146754	3.202087	3.040276	3.275122	3.196504	3.358469	3.407300	3.677214	3.472214	3.230192	3.070262	3.294523	3.087865	5.952913	3.361794	3.825093	3.000837	3.188054	2.980425	3.212271	3.000262	3.586323	2.903815	2.993540	4.341124	3.411654	5.055345	5.289165	4.208229	4.257871	3.875659	3.297290	3.200255	3.138701	3.144662	3.341985	2.999567	4.906492	3.020517	3.731709	6.466356	3.146302	3.940235	5.330272	3.465394	3.072161	4.323808	4.820024	4.638630	3.433628	3.900187	3.824156	4.088092	3.799232	3.758566	3.028768	3.223215	3.683451	3.103265	3.296822	3.264112	3.345748	3.184101	4.082834	2.870546	3.549657	3.325633	3.632741	3.645431	3.104231	4.124030	3.555255	4.972948	3.527038	3.489559	3.931093	4.745959	3.687262	4.212963	3.706865	4.748940	4.294363	3.641091	4.184058	3.530135	3.128578	3.118161	3.828858	5.123104	3.832282	3.441180	3.280419	3.028960	3.079410	3.021347	3.744640	3.165554	2.944808	2.998995	4.326994	4.831465	2.908133	3.237122	3.446341	3.104058	2.883971	2.854366	3.296369	3.018736	3.192699	4.623792	2.864740	3.162994	3.061915	3.970758	3.722702	4.847414	3.126045	2.985514	4.539078	3.575186	3.070362	3.165689	3.052890	3.113441	4.172494	4.011437	3.479830	3.036937	3.011262	3.756526	2.957857	3.361794	5.885332	3.602066	3.046101	5.237270	2.917720	3.042314	2.940918	4.073634	3.207039	3.242519	6.247783	3.091863	6.062264	3.851619	3.302535	5.391638	3.588064	5.539969	3.262043	3.176969	5.304420	3.187925	2.932490	6.308393	4.615968	5.915113	6.640806	3.937294	3.773677	4.762713	4.993314	5.649730	5.441417	7.330723	6.065057	4.986843	3.566891	5.116611	4.015184	3.621076	3.703446	3.431343	3.848412	3.743677	4.197752	3.494647	3.395752	3.424550	4.421274	4.135859	4.469469	3.704674	4.736757	3.036658	3.378791	4.503720	5.493183	4.210774	4.820255	3.035971	4.513162	4.325528	5.693962	4.886886	7.385317	5.314846	3.520038	5.977794	6.259290	3.925015	3.752527	3.795499	3.103265	3.223746	5.055139	4.148341	5.113056	4.008667	3.096273	3.444894	3.160827	3.213675	3.204680	2.882057	6.230244	4.715794	3.882986	2.872066	3.106895	3.448991	3.610969	4.147890	3.102636	6.607057	4.758070	3.282710	3.044660	4.389553	3.408374	3.541120

MTPN	13.349515	14.868226	15.181269	15.451210	15.456675	15.197536	15.577375	16.013271	15.225383	15.857099	15.748884	15.417001	15.391393	15.627072	14.993988	16.121338	15.647882	15.347177	16.034860	15.469767	15.784706	16.152946	14.952794	15.383684	14.801868	15.416204	13.985572	15.279559	15.123478	14.803294	15.293858	15.889470	15.739503	14.768079	15.076957	15.323382	15.258866	15.914283	15.126455	15.356364	15.345704	15.851177	15.494689	15.093138	15.069833	15.114738	15.147454	15.321092	15.572722	15.507347	14.954990	15.377722	15.511748	15.588169	14.743665	15.282381	15.564042	15.369410	15.901055	15.152524	15.405445	15.125176	14.067493	15.616895	15.292522	15.241853	15.371654	15.568457	15.347177	15.478014	15.928804	15.781709	15.352487	15.265157	15.526235	15.278819	15.084722	14.933539	14.419877	16.218720	15.584586	15.140339	14.883849	15.859538	15.781709	15.525353	15.926414	16.221987	15.553263	15.945972	15.024650	15.709527	15.768020	15.455879	14.671011	15.608551	15.398455	15.520300	15.829660	14.810097	15.533380	15.342745	15.395261	15.182677	16.172846	15.438162	15.139006	16.024403	15.547851	15.580998	15.716847	15.626034	15.812427	15.183376	15.594598	15.056697	13.632300	15.602028	15.440770	15.307946	14.468143	13.993598	13.695390	13.921593	14.668191	14.560516	13.760910	14.876538	13.966887	14.119553	14.344223	14.570991	13.404827	14.448739	14.094594	14.255787	13.885163	13.812235	14.525087	14.543837	14.607960	14.530078	13.750799	14.430444	14.046144	15.094510	14.133255	14.178659	14.560516	14.769948	13.974684	14.539639	14.443791	14.650177	14.548933	14.753586	14.183082	14.713935	14.611896	14.302275	14.461603	14.102873	15.007673	14.655466	14.724698	14.746969	13.870987	14.686942	14.385259	14.678701	14.504993	14.390000	14.810589	14.950618	15.119706	14.383815	14.400018	13.932466	14.336460	14.440880	14.573507	15.137730	14.773706	14.446360	14.395843	14.389287	14.284842	14.222202	14.658149	14.625115	14.842135	14.087732	15.039342	14.637498	14.712131	14.742751	14.778050	15.268645	13.860992	14.650177	14.698400	14.722869	14.505707	14.731754	14.674591	14.616154	14.090137	13.992417	13.870710	14.038211	14.388223	13.970475	14.656836	14.834547	14.306647	14.798373	14.255787	14.226329	14.460072	14.589784	13.703898	13.775677	13.918854	14.343879	14.276055	14.616554	14.878681	14.963955	14.410783	14.415465	13.728857	13.828188	15.015687	14.684100	14.641807	14.687871	14.491152	13.845633

PAX9	9.661397	13.978080	13.696085	14.028776	14.351612	13.980518	14.031471	14.412450	14.748776	14.132353	14.508030	14.105067	14.018133	14.455753	13.928944	14.380267	14.358027	14.370844	15.032181	14.379907	14.333399	14.391136	12.778472	14.431588	14.969108	14.523150	12.197683	14.790142	13.422807	13.216213	14.645337	13.606202	13.682594	11.329687	13.466605	13.848828	14.381702	13.874185	14.309634	14.834088	13.911057	14.170650	14.221208	13.920502	13.963030	13.918854	13.560841	13.770321	13.792238	14.422133	13.678008	14.079765	14.431588	14.090994	13.991573	14.360826	14.123349	15.317503	14.745445	14.512377	13.787649	13.898284	9.767395	14.627259	13.116581	13.939322	14.243388	14.745445	14.382065	13.395371	13.905825	14.458184	14.360996	14.494741	14.044362	13.571674	13.790706	13.946220	13.944041	14.194648	13.944829	13.753859	13.101246	14.241406	14.361168	13.748342	13.886983	14.856471	13.041185	14.012650	13.747078	13.763467	14.065983	14.114980	13.011244	14.051810	13.898284	14.361946	13.680663	15.167431	14.298038	14.422502	14.405515	14.717187	14.120707	14.496768	14.054805	15.107990	13.504135	14.395843	14.433465	14.639680	15.008818	14.181495	15.085910	12.848393	9.910805	13.974138	14.610567	13.696837	10.783470	11.254856	12.630185	12.750339	12.933307	11.841136	11.782222	11.969197	12.708953	10.777827	10.516399	12.140350	11.125638	10.085100	12.696432	11.033754	11.185995	13.846147	9.232049	14.285533	10.601209	11.483264	12.734879	10.566794	10.044168	13.183285	13.549579	14.680064	10.962810	10.163352	10.097780	10.356921	10.118063	10.668429	11.900292	11.458006	13.314543	12.056040	11.634790	10.619400	11.206137	10.579352	9.121228	11.683069	13.047627	13.000815	9.729974	12.208851	10.132720	10.265297	13.541051	12.092757	10.516841	13.106410	10.254371	12.858824	11.458459	12.241919	10.919423	9.938847	12.398188	10.911465	13.402503	9.304982	9.065685	10.749317	11.972367	10.497960	9.755758	12.118961	10.528938	12.913209	11.654083	10.015875	11.518100	11.041788	11.598146	12.746061	11.765088	10.986127	11.821859	9.501474	10.482002	10.487794	11.148713	11.497713	10.101581	9.023129	10.226206	8.619728	10.728676	11.593593	11.040012	10.108228	10.245587	11.010354	11.616843	10.840472	11.233769	9.783281	12.544369	12.506563	12.516941	11.460712	12.222322	12.614541	13.076070	12.138794	12.556686	10.752793	11.403818	10.893304	13.809161	9.686241	8.972184	13.152404	9.294119	10.931888

RGMA	10.517131	12.357829	12.441436	13.016071	12.377877	12.830991	11.771962	13.441821	12.965249	12.523476	12.545893	12.441436	12.045877	12.522505	11.470020	13.332070	12.711633	11.566830	13.063978	13.122511	12.698837	12.667488	11.696817	12.640046	12.338722	12.914980	12.279446	13.336659	13.362040	12.893290	12.972030	11.507297	12.320613	11.384231	11.844802	11.665214	11.294540	12.059510	12.022667	12.065779	13.046106	11.419414	12.322965	12.348967	10.877054	11.812299	13.411645	12.446101	12.373219	12.376771	11.720028	11.380692	11.362983	12.205277	11.575684	13.497789	11.096474	11.188596	11.500773	11.354180	11.608315	12.996223	10.760858	12.026601	11.767362	12.018571	12.118316	11.938934	12.175939	12.207764	13.383249	12.584309	13.224320	12.681634	12.466325	11.849886	12.244533	12.399952	12.358636	10.852247	10.608316	10.486856	10.263119	10.859681	10.685853	12.383533	10.682874	10.353124	10.951782	11.419106	10.911612	11.206577	11.673868	12.021503	11.442042	11.176567	11.550311	12.005642	12.335244	12.776381	12.511596	12.623163	12.856573	12.269302	12.271548	12.736612	12.558482	12.542605	12.576557	13.049541	12.034131	12.318871	12.176872	13.089623	12.875386	13.594881	10.586706	12.486970	12.897068	10.726741	12.423121	11.695777	11.685252	13.066984	12.536567	12.079407	11.407356	12.021084	12.069172	12.178954	11.668608	11.792414	11.270879	11.347581	12.445481	11.067626	11.405580	11.001656	10.656544	12.104382	11.612874	11.546045	12.133414	11.502609	10.601358	13.313356	11.800003	12.683650	12.439820	11.359874	11.396538	11.691853	12.258361	11.134615	11.110723	11.685099	12.075900	11.465549	11.282541	12.529347	11.092378	9.978283	11.700848	11.438109	11.641765	13.536185	10.644448	12.246159	11.173601	11.208804	10.326429	10.301596	11.019457	10.486716	11.846709	9.734545	10.418587	10.591713	10.486637	11.016351	12.049502	11.603421	11.538395	10.936702	10.829442	11.727527	12.260347	11.525172	11.141929	12.181582	12.081981	13.331903	11.633293	11.274000	11.903572	11.663120	11.356089	12.160822	10.124118	10.577682	10.771942	10.702258	10.324612	9.708602	11.718826	10.302930	9.457860	10.037784	10.613473	9.909707	10.430011	11.589071	11.305292	12.078677	11.089903	11.764203	11.676832	11.827113	11.575684	11.014582	11.769146	11.643417	11.443229	10.390190	12.442059	11.405143	11.149750	11.860833	13.572645	11.374753	11.520394	12.006559	13.551881	11.566261	12.360369	11.311900	11.214808	12.376134

C9orf92	4.372155	4.551105	4.677314	4.195624	5.003956	4.956231	5.084247	5.994297	5.436682	5.691561	5.466753	5.344189	5.615913	5.525352	5.767564	5.201486	5.515242	5.695458	6.534701	6.618065	5.648576	5.664974	6.758287	5.409314	6.033450	6.149155	6.146352	5.299282	5.948816	4.819179	6.056359	4.931589	5.068410	4.669502	5.171907	4.888552	4.513162	5.459635	6.144617	5.415640	5.228700	5.611055	5.602452	5.766833	4.793308	5.294248	5.924351	6.058191	5.114771	5.855085	5.067778	4.641333	6.041579	5.532003	5.196697	4.892077	5.257531	4.833328	5.580405	4.463946	5.696567	4.916406	5.051601	5.848814	5.064275	4.912822	4.912630	4.579040	4.845326	5.209621	4.654410	4.623113	6.202073	4.541317	5.152145	4.656406	5.126754	5.041771	5.586055	4.996722	5.560034	5.225089	4.705302	5.376333	5.211443	5.918522	5.000471	5.080195	5.468945	5.274525	4.202921	6.982537	4.979117	5.029542	4.114601	5.753934	4.984987	6.270338	5.378461	5.737715	6.649508	5.163875	5.939707	4.896655	5.109773	5.496995	3.657453	5.783423	5.860683	5.183621	5.996543	6.041579	5.332656	5.123314	4.796873	6.201016	5.199473	5.350035	5.820088	5.646170	4.947941	5.072202	4.060633	4.861046	4.667368	4.952305	7.196156	4.615121	3.865968	4.356767	5.853816	4.928648	5.737715	4.968821	5.009514	6.126791	6.600064	5.483145	5.634357	4.648115	6.065057	5.349668	4.463741	5.272049	4.144750	5.073638	4.758311	4.845739	4.511172	6.298242	4.286794	5.706450	5.388727	5.577407	4.721070	5.100522	5.273296	5.015654	4.633723	4.578613	5.415278	6.223096	5.846222	4.929751	4.837238	5.356081	5.981184	5.666110	5.232404	5.501195	4.983584	5.555072	5.493530	5.101551	4.504144	4.358758	4.814772	4.553674	5.109541	4.678180	4.612954	5.072013	5.662559	4.730815	5.357204	4.689905	5.120920	4.835984	5.263473	5.030127	5.067361	4.464171	4.868318	5.978683	4.810821	5.071631	4.787242	5.778189	5.048938	5.284147	5.855451	6.243620	5.087179	5.390655	5.181177	5.360611	4.628705	5.465211	4.894812	4.324651	6.089564	3.994643	4.324870	4.135166	5.336327	4.811675	5.642352	5.160478	4.982359	6.636745	6.273808	5.239632	4.826975	5.470448	4.320370	4.401336	4.752528	4.163148	4.630030	6.094972	5.553982	5.973067	4.853324	4.842335	4.598551	4.436744	4.355484	4.722818

HS1BP3	10.457568	9.829507	9.879478	10.199612	9.803798	10.108076	10.471176	9.567325	9.259535	10.155465	10.357533	10.245440	10.086994	10.126326	9.873016	9.695713	10.197218	10.169039	10.710978	10.122478	10.350819	9.936952	12.303903	9.955223	11.039307	10.581306	10.034458	10.366240	10.238936	10.283087	10.051559	10.129759	9.942884	9.802726	10.416896	10.360974	10.533504	10.518184	10.639993	10.153888	10.297862	10.551588	9.801046	10.142906	10.798780	9.908194	10.352051	9.943637	9.941008	10.121706	10.034692	10.349760	10.378959	10.387951	10.586401	10.477389	10.593778	10.507508	10.494672	10.724616	10.881053	9.930473	11.248655	10.201561	10.202314	10.417364	10.041611	9.836740	10.015553	10.046174	10.135777	10.099503	10.297862	10.125390	10.012145	10.562777	10.131841	10.189346	10.064516	9.823607	9.677525	10.436289	10.306828	10.369106	10.761738	10.054131	10.456939	10.365006	10.402738	10.329641	10.274568	10.640152	9.755312	10.103717	9.985035	10.208766	9.896882	9.954745	9.921216	9.987305	9.483198	9.844400	9.610886	9.985333	9.748309	9.921992	10.046174	9.685152	10.380918	10.122304	10.585651	9.902411	10.535918	9.789901	9.333694	7.494117	10.640618	10.096988	9.815273	10.300168	10.019448	10.411625	10.354297	9.815108	10.465192	10.395201	9.774406	11.060277	10.919103	10.540309	11.066165	10.802095	9.897339	10.804455	10.255734	10.383667	10.437805	11.096025	10.985679	10.377566	10.482608	11.479340	11.554922	11.460104	11.681573	9.468870	10.329641	10.198425	10.562355	9.912200	10.053753	10.115451	10.093965	10.050200	10.283531	10.050200	10.154986	10.491721	10.879746	11.424787	10.042381	10.427019	10.277741	10.303562	9.811828	9.898374	9.891644	10.406224	10.625804	10.163693	10.141818	9.972796	10.975378	10.730315	10.820288	10.583331	10.366388	10.910589	10.165812	11.009483	10.605340	10.780289	10.201710	10.161675	10.293540	10.038559	10.251318	10.097334	10.036268	10.185274	10.105985	11.033441	10.335529	10.372281	10.299127	10.323820	10.624726	10.875592	10.212581	10.278824	10.937605	10.251030	10.314435	10.113777	10.304338	10.695437	10.558868	10.700036	10.450323	10.141818	11.142070	10.023543	10.308628	10.132720	10.290059	10.810536	10.236834	10.236834	11.068537	10.376060	9.841075	10.134987	9.921529	10.324445	9.861608	10.012286	10.627617	10.568169	10.516242	10.710117	9.896439	9.540026	9.573194	10.818491	9.138600	11.014997	10.358566	10.475430

AL138752.2	10.915990	11.124128	11.273685	11.750265	11.361202	11.583331	11.228274	12.198594	10.735347	11.240923	11.785073	11.329687	11.389264	11.330738	11.200874	11.618016	11.170020	11.668608	12.102073	11.755741	11.310134	10.209061	10.486856	11.342795	11.682317	11.459795	10.538212	11.774016	10.810402	10.738376	11.781940	11.185995	11.202837	10.677496	10.954523	11.197968	11.299661	11.508778	11.706559	11.494063	11.251941	11.665514	11.106317	11.165530	11.077366	11.317187	10.826511	11.233156	11.613311	11.480091	11.008580	11.429723	11.341475	11.661937	11.340088	11.204776	11.394116	11.415282	11.763874	11.611675	11.330888	11.085434	10.862314	11.335396	10.805632	11.036851	10.664886	11.089762	10.934442	10.584914	10.819699	10.716117	11.409349	10.948018	10.920468	10.854786	10.855861	10.987504	11.092378	11.006838	10.887634	11.563602	11.267197	11.603009	11.468535	11.207480	11.799097	12.079407	11.410967	11.981504	11.230338	11.611382	11.133855	11.356819	10.735064	10.971775	11.299253	11.400756	10.989882	11.135202	11.427955	10.973509	11.015142	11.642694	11.118110	11.448837	11.571480	11.905888	10.532370	11.619186	11.017510	11.357096	11.670323	11.424357	11.424939	10.870510	11.116659	11.109863	10.968609	10.677496	12.202831	11.465248	11.077366	10.510068	11.557743	11.207480	10.496309	10.808421	11.490551	11.517806	10.516841	10.943897	11.704750	10.714913	11.673108	10.607548	11.383634	11.020938	11.341314	11.105584	10.846071	10.416445	11.359874	11.898369	11.910004	11.038581	12.151088	10.434589	11.109107	10.912348	10.728368	11.691368	11.399961	11.703565	11.611675	11.533035	10.896111	12.144058	11.543468	10.724139	11.440893	11.627357	11.556994	12.156031	12.118316	11.930334	10.967556	11.612729	11.024804	11.183767	10.771383	12.145005	11.897222	11.191935	10.573345	11.016201	11.609910	11.651970	11.050033	12.322054	10.770448	10.984649	11.963714	10.402900	10.929328	10.902636	10.667399	11.656322	12.788576	11.167392	11.333338	10.992556	11.340239	10.606255	10.816113	11.268237	10.858500	11.038451	10.884458	10.539109	12.466981	11.523246	10.652809	12.781728	11.028968	11.725426	11.571323	11.425348	11.502051	11.529830	12.431923	10.751755	11.414272	10.736232	10.769243	10.822224	10.156215	10.754495	12.286760	11.203302	10.034221	10.670884	10.987504	11.773690	10.461142	11.896350	11.940741	11.450408	11.654387	10.798914	10.427471	11.352876	11.172697	11.918344	11.213821	11.474781	11.283115	10.883700

DCAF10	10.915990	11.124128	11.273685	11.750265	11.361202	11.583331	11.228274	12.198594	10.735347	11.240923	11.785073	11.329687	11.389264	11.330738	11.200874	11.618016	11.170020	11.668608	12.102073	11.755741	11.310134	10.209061	10.486856	11.342795	11.682317	11.459795	10.538212	11.774016	10.810402	10.738376	11.781940	11.185995	11.202837	10.677496	10.954523	11.197968	11.299661	11.508778	11.706559	11.494063	11.251941	11.665514	11.106317	11.165530	11.077366	11.317187	10.826511	11.233156	11.613311	11.480091	11.008580	11.429723	11.341475	11.661937	11.340088	11.204776	11.394116	11.415282	11.763874	11.611675	11.330888	11.085434	10.862314	11.335396	10.805632	11.036851	10.664886	11.089762	10.934442	10.584914	10.819699	10.716117	11.409349	10.948018	10.920468	10.854786	10.855861	10.987504	11.092378	11.006838	10.887634	11.563602	11.267197	11.603009	11.468535	11.207480	11.799097	12.079407	11.410967	11.981504	11.230338	11.611382	11.133855	11.356819	10.735064	10.971775	11.299253	11.400756	10.989882	11.135202	11.427955	10.973509	11.015142	11.642694	11.118110	11.448837	11.571480	11.905888	10.532370	11.619186	11.017510	11.357096	11.670323	11.424357	11.424939	10.870510	11.116659	11.109863	10.968609	10.677496	12.202831	11.465248	11.077366	10.510068	11.557743	11.207480	10.496309	10.808421	11.490551	11.517806	10.516841	10.943897	11.704750	10.714913	11.673108	10.607548	11.383634	11.020938	11.341314	11.105584	10.846071	10.416445	11.359874	11.898369	11.910004	11.038581	12.151088	10.434589	11.109107	10.912348	10.728368	11.691368	11.399961	11.703565	11.611675	11.533035	10.896111	12.144058	11.543468	10.724139	11.440893	11.627357	11.556994	12.156031	12.118316	11.930334	10.967556	11.612729	11.024804	11.183767	10.771383	12.145005	11.897222	11.191935	10.573345	11.016201	11.609910	11.651970	11.050033	12.322054	10.770448	10.984649	11.963714	10.402900	10.929328	10.902636	10.667399	11.656322	12.788576	11.167392	11.333338	10.992556	11.340239	10.606255	10.816113	11.268237	10.858500	11.038451	10.884458	10.539109	12.466981	11.523246	10.652809	12.781728	11.028968	11.725426	11.571323	11.425348	11.502051	11.529830	12.431923	10.751755	11.414272	10.736232	10.769243	10.822224	10.156215	10.754495	12.286760	11.203302	10.034221	10.670884	10.987504	11.773690	10.461142	11.896350	11.940741	11.450408	11.654387	10.798914	10.427471	11.352876	11.172697	11.918344	11.213821	11.474781	11.283115	10.883700

C11orf54	12.308719	10.853016	10.671196	10.670427	10.554299	11.131914	10.789568	11.455518	10.116680	10.962371	10.793359	10.624433	10.500761	10.592028	10.416749	10.563370	10.832422	10.836326	9.547157	11.375308	9.791422	10.881198	9.449194	10.035789	10.130777	9.813834	9.841075	10.243220	10.189798	10.781020	10.021710	11.320543	10.972346	10.631587	11.597121	11.772400	12.063952	11.829273	11.189473	11.023352	10.932934	10.872332	10.344336	11.179454	11.666726	10.424711	9.908491	10.397613	11.157291	11.741732	12.033678	11.733026	11.771669	11.848932	11.338197	10.763571	11.609049	11.057484	10.656678	10.597077	11.424500	10.255271	11.092828	10.581000	10.658275	10.925940	11.212284	11.257836	10.644734	10.629485	11.526648	11.404685	10.256793	11.129277	11.625303	11.348487	11.648993	11.394433	11.710281	10.714609	11.549154	11.092677	11.634506	11.637961	11.451301	11.546356	11.566546	11.479340	10.575699	10.458181	10.264104	10.388806	11.413548	11.078839	12.061357	11.250930	11.354029	11.220719	11.338197	10.520492	11.045577	10.547330	10.563370	10.755416	10.936997	10.183600	9.904543	11.046663	9.265061	10.437507	10.372744	10.116521	10.929057	10.786621	11.404390	9.500882	11.603421	11.035057	10.685853	11.132065	9.704430	9.890146	9.842288	8.712662	10.640300	10.880464	9.939172	9.689297	8.613447	8.682047	10.268677	8.781030	8.333986	10.391204	10.374121	10.099670	10.720204	8.261557	11.248503	10.293094	9.771605	8.939581	9.064900	9.689437	10.072673	10.405628	9.144754	8.233292	10.282310	8.887749	10.045559	9.929996	11.515464	11.647837	10.898956	12.020926	11.015291	10.277741	11.170321	9.573979	10.519232	10.009895	11.013251	11.531390	9.764655	9.180624	10.683914	11.490395	10.918671	11.852176	11.203158	11.069585	11.049601	11.097215	9.485028	11.283115	10.845168	10.404862	10.227583	10.913714	9.736421	10.278971	9.952136	10.531882	11.216854	9.089471	10.603105	10.884165	10.675830	11.322595	11.384675	7.791586	10.462179	11.217885	11.603568	11.373920	10.246644	11.098557	9.872877	10.621163	11.090033	11.091024	10.943161	11.276728	11.751430	11.595353	11.928776	10.470130	9.515156	9.789131	10.673572	10.656544	10.370938	11.486387	10.649682	10.638038	10.157433	10.564580	10.373518	9.460752	10.003957	9.445145	10.697641	9.423469	9.448879	9.239068	10.310967	9.483039	8.562976	9.549766	8.245874	9.791905	9.953637	11.444544	10.141487	10.448109	12.596981	10.408604

AP001273.2	12.308719	10.853016	10.671196	10.670427	10.554299	11.131914	10.789568	11.455518	10.116680	10.962371	10.793359	10.624433	10.500761	10.592028	10.416749	10.563370	10.832422	10.836326	9.547157	11.375308	9.791422	10.881198	9.449194	10.035789	10.130777	9.813834	9.841075	10.243220	10.189798	10.781020	10.021710	11.320543	10.972346	10.631587	11.597121	11.772400	12.063952	11.829273	11.189473	11.023352	10.932934	10.872332	10.344336	11.179454	11.666726	10.424711	9.908491	10.397613	11.157291	11.741732	12.033678	11.733026	11.771669	11.848932	11.338197	10.763571	11.609049	11.057484	10.656678	10.597077	11.424500	10.255271	11.092828	10.581000	10.658275	10.925940	11.212284	11.257836	10.644734	10.629485	11.526648	11.404685	10.256793	11.129277	11.625303	11.348487	11.648993	11.394433	11.710281	10.714609	11.549154	11.092677	11.634506	11.637961	11.451301	11.546356	11.566546	11.479340	10.575699	10.458181	10.264104	10.388806	11.413548	11.078839	12.061357	11.250930	11.354029	11.220719	11.338197	10.520492	11.045577	10.547330	10.563370	10.755416	10.936997	10.183600	9.904543	11.046663	9.265061	10.437507	10.372744	10.116521	10.929057	10.786621	11.404390	9.500882	11.603421	11.035057	10.685853	11.132065	9.704430	9.890146	9.842288	8.712662	10.640300	10.880464	9.939172	9.689297	8.613447	8.682047	10.268677	8.781030	8.333986	10.391204	10.374121	10.099670	10.720204	8.261557	11.248503	10.293094	9.771605	8.939581	9.064900	9.689437	10.072673	10.405628	9.144754	8.233292	10.282310	8.887749	10.045559	9.929996	11.515464	11.647837	10.898956	12.020926	11.015291	10.277741	11.170321	9.573979	10.519232	10.009895	11.013251	11.531390	9.764655	9.180624	10.683914	11.490395	10.918671	11.852176	11.203158	11.069585	11.049601	11.097215	9.485028	11.283115	10.845168	10.404862	10.227583	10.913714	9.736421	10.278971	9.952136	10.531882	11.216854	9.089471	10.603105	10.884165	10.675830	11.322595	11.384675	7.791586	10.462179	11.217885	11.603568	11.373920	10.246644	11.098557	9.872877	10.621163	11.090033	11.091024	10.943161	11.276728	11.751430	11.595353	11.928776	10.470130	9.515156	9.789131	10.673572	10.656544	10.370938	11.486387	10.649682	10.638038	10.157433	10.564580	10.373518	9.460752	10.003957	9.445145	10.697641	9.423469	9.448879	9.239068	10.310967	9.483039	8.562976	9.549766	8.245874	9.791905	9.953637	11.444544	10.141487	10.448109	12.596981	10.408604

TAF1D	12.308719	10.853016	10.671196	10.670427	10.554299	11.131914	10.789568	11.455518	10.116680	10.962371	10.793359	10.624433	10.500761	10.592028	10.416749	10.563370	10.832422	10.836326	9.547157	11.375308	9.791422	10.881198	9.449194	10.035789	10.130777	9.813834	9.841075	10.243220	10.189798	10.781020	10.021710	11.320543	10.972346	10.631587	11.597121	11.772400	12.063952	11.829273	11.189473	11.023352	10.932934	10.872332	10.344336	11.179454	11.666726	10.424711	9.908491	10.397613	11.157291	11.741732	12.033678	11.733026	11.771669	11.848932	11.338197	10.763571	11.609049	11.057484	10.656678	10.597077	11.424500	10.255271	11.092828	10.581000	10.658275	10.925940	11.212284	11.257836	10.644734	10.629485	11.526648	11.404685	10.256793	11.129277	11.625303	11.348487	11.648993	11.394433	11.710281	10.714609	11.549154	11.092677	11.634506	11.637961	11.451301	11.546356	11.566546	11.479340	10.575699	10.458181	10.264104	10.388806	11.413548	11.078839	12.061357	11.250930	11.354029	11.220719	11.338197	10.520492	11.045577	10.547330	10.563370	10.755416	10.936997	10.183600	9.904543	11.046663	9.265061	10.437507	10.372744	10.116521	10.929057	10.786621	11.404390	9.500882	11.603421	11.035057	10.685853	11.132065	9.704430	9.890146	9.842288	8.712662	10.640300	10.880464	9.939172	9.689297	8.613447	8.682047	10.268677	8.781030	8.333986	10.391204	10.374121	10.099670	10.720204	8.261557	11.248503	10.293094	9.771605	8.939581	9.064900	9.689437	10.072673	10.405628	9.144754	8.233292	10.282310	8.887749	10.045559	9.929996	11.515464	11.647837	10.898956	12.020926	11.015291	10.277741	11.170321	9.573979	10.519232	10.009895	11.013251	11.531390	9.764655	9.180624	10.683914	11.490395	10.918671	11.852176	11.203158	11.069585	11.049601	11.097215	9.485028	11.283115	10.845168	10.404862	10.227583	10.913714	9.736421	10.278971	9.952136	10.531882	11.216854	9.089471	10.603105	10.884165	10.675830	11.322595	11.384675	7.791586	10.462179	11.217885	11.603568	11.373920	10.246644	11.098557	9.872877	10.621163	11.090033	11.091024	10.943161	11.276728	11.751430	11.595353	11.928776	10.470130	9.515156	9.789131	10.673572	10.656544	10.370938	11.486387	10.649682	10.638038	10.157433	10.564580	10.373518	9.460752	10.003957	9.445145	10.697641	9.423469	9.448879	9.239068	10.310967	9.483039	8.562976	9.549766	8.245874	9.791905	9.953637	11.444544	10.141487	10.448109	12.596981	10.408604

TGFB2	10.830911	10.223453	9.557087	9.880559	8.597760	9.661238	10.373076	8.844490	9.496731	9.812137	9.789131	10.643569	10.726410	10.106859	9.839992	10.996710	11.541528	9.227888	9.559999	10.136837	8.604216	8.950413	10.839735	9.630159	10.955728	10.335240	11.392765	11.423597	11.704443	11.925971	9.033552	10.276978	9.760421	10.605574	10.549938	10.733727	10.687204	10.121857	9.639191	8.949983	9.791905	10.101264	10.030841	10.721789	11.605953	9.896130	11.117105	10.972659	10.345672	10.699718	11.093256	10.577397	10.190858	10.871441	11.096172	10.445527	11.229016	9.443912	9.349094	9.935109	9.726052	10.426707	12.016745	10.025602	9.634473	9.461373	9.447171	9.497955	8.847553	9.717339	10.329013	10.131679	9.980249	10.507987	9.698021	9.665455	10.039288	10.813866	10.230524	9.330289	10.521258	10.944749	11.301597	10.679279	10.880034	10.345226	10.426379	10.818785	10.056200	9.904261	9.885299	9.158942	9.220517	10.072505	10.941674	10.051261	10.745154	10.827827	9.897496	10.601825	10.424393	9.655511	9.447475	9.403709	9.487673	10.097624	9.837507	8.622673	8.649681	9.977545	8.724496	10.255734	9.242180	8.934723	9.348168	8.910839	10.109727	10.523700	10.521909	9.211454	12.103782	10.869610	9.708142	9.399554	10.951332	9.987919	12.297906	12.774646	10.741574	12.368300	10.710978	11.796904	12.927010	11.141929	13.088513	12.415626	9.914396	9.295525	12.134816	8.811217	10.826808	9.618556	10.565611	11.837689	11.120843	11.581666	14.004726	10.146709	11.575684	10.376671	10.465495	11.621743	11.639628	10.580246	13.771102	11.945155	10.587003	11.777298	9.785797	11.428109	10.020330	10.214264	10.937444	11.205984	10.680668	10.748602	12.804778	11.448972	12.868513	11.846125	11.339812	10.743513	13.514680	10.630708	12.326190	11.666726	9.984109	9.512448	10.945054	11.002234	12.142942	11.191198	10.835737	9.603604	10.705664	11.544101	10.559954	10.375909	10.441420	10.330540	11.605367	9.708602	12.059948	10.307273	11.284747	12.308229	10.986127	11.062443	10.597246	11.871756	10.528515	12.217913	12.726021	11.749694	11.002796	12.345318	12.013264	10.806633	10.366983	12.434806	9.887831	10.222567	12.742336	11.816438	12.109069	12.102387	12.150030	10.570453	10.796271	13.482826	9.971512	9.411694	11.065268	11.215987	12.058084	11.121300	8.440901	9.085022	12.404662	10.590818	12.438036	9.353198	7.319325	11.623379	11.078552	10.149179	10.660235	12.348814

CKMT1A	14.068082	13.918854	15.151876	14.224386	14.388973	14.906118	14.055121	14.830972	13.719437	14.531467	14.260639	14.352710	14.593639	15.341190	14.361168	13.695624	14.707094	14.595757	14.117033	14.995049	13.937151	13.734372	11.855631	14.120707	13.757132	14.319186	12.114497	13.337083	13.839284	13.248097	14.092524	15.628950	14.622502	13.197929	14.420600	14.220551	15.350177	13.409002	14.948503	15.227905	14.473115	14.232519	14.247926	14.769948	14.548089	15.164670	14.240086	15.107990	14.180845	14.953863	14.163659	14.901840	15.271483	14.900158	14.939387	13.211496	15.607626	14.768542	14.239751	14.273856	14.761042	14.119859	10.727311	14.761491	12.819581	15.328703	14.786553	14.997389	14.771390	14.340345	14.094001	15.012809	14.062649	14.887119	15.037486	14.116481	14.929736	14.305307	14.045556	13.190080	14.958920	14.513547	14.686004	15.143591	15.200938	14.579771	14.914162	14.347809	13.533965	15.339008	14.680520	13.436214	14.640110	15.175273	14.434184	14.982214	13.463806	14.597809	14.830972	13.858854	14.781829	14.533864	14.199812	14.540937	14.362657	14.683638	14.411699	14.251159	13.474198	14.580207	13.655750	14.718090	15.149924	14.357312	14.641807	14.267351	14.070098	13.277233	14.106283	12.349742	14.039946	12.658313	13.964639	13.898284	14.305623	14.552270	11.192946	14.678701	13.149931	14.173886	14.375936	13.317270	11.365203	13.372722	12.639889	13.154939	14.384530	10.973367	11.634790	12.128733	13.274601	13.925700	13.320668	14.243708	9.125182	13.696837	13.825824	13.629938	12.335561	13.304945	12.771018	12.777077	13.597687	12.418964	15.342745	13.294952	14.565911	12.983713	14.202755	14.462773	14.012930	12.874105	14.813710	14.713029	14.369059	14.102873	12.535440	14.274191	13.448871	15.028728	12.867055	13.558417	12.673286	13.393302	13.386476	10.356294	13.522760	14.125236	13.498035	13.589326	13.927054	13.417980	13.576005	14.262283	13.233810	14.261678	12.431763	14.019562	11.521903	13.399078	12.962718	14.404753	13.444403	13.883806	14.351258	13.680430	13.837460	14.199474	10.985236	14.341372	13.234411	13.429094	13.976899	13.487496	13.649857	13.497565	14.237167	12.706190	12.748286	11.908802	12.000077	13.133191	12.803344	13.701386	13.020993	14.386005	13.747078	13.405029	13.807309	11.910728	12.632959	13.496693	10.739617	12.099966	13.784808	14.022985	13.098440	13.659121	13.834567	13.835602	13.821463	12.822839	14.403650	14.022408	13.551649	12.148979	14.713469	13.380119

MYCBP	9.540026	9.476414	10.285158	9.659506	10.302633	9.643333	10.376965	10.494370	9.428521	10.257870	10.223316	9.769520	9.422970	10.384236	9.520226	9.733039	10.027337	10.319358	10.777970	10.062587	10.674816	10.046779	9.799644	9.839071	8.395689	8.462629	8.571866	8.950273	8.180535	9.126584	9.713091	9.950580	10.659191	9.984899	9.835522	10.020621	10.420096	9.924735	10.222567	10.657698	9.336631	9.861767	9.400283	9.973102	9.770486	10.028734	9.533834	10.006772	10.377715	9.734545	10.096988	9.881488	10.013557	10.360365	10.554613	9.919633	10.754335	10.347657	10.570126	9.966500	9.956464	8.838597	8.811217	10.334961	10.396283	9.904543	9.483198	10.501072	10.236196	10.905697	9.992041	10.245297	9.662346	10.062130	10.554299	10.042830	10.566197	10.310064	10.316078	10.347353	10.108529	10.256330	10.130357	10.221812	10.270009	10.266163	10.558269	10.596035	10.213953	10.727937	9.939807	10.829597	10.016345	10.102793	9.750518	10.530253	10.351729	9.563299	9.819984	9.616680	9.741675	10.074063	9.852983	10.424393	11.703716	9.463307	8.313127	11.041360	10.624589	9.912662	10.528668	9.803650	9.985333	9.253295	9.748309	9.112263	9.666547	9.708602	9.674912	11.057038	9.600803	8.977562	9.638262	7.350547	8.932113	8.735642	7.949190	10.052026	8.829501	9.495797	9.096736	7.375167	8.456594	8.613295	9.130300	8.858205	8.154551	8.700270	8.878011	9.779527	9.417469	10.075003	8.854389	7.463738	7.840460	9.423296	8.568954	7.430775	9.267822	9.584671	9.202497	9.023558	9.766473	9.619482	9.623390	10.102954	9.736856	9.902880	9.790662	8.172546	9.240585	8.469947	9.759027	9.723440	9.829822	9.284296	8.913424	9.698334	9.511660	9.417469	8.899923	9.328916	9.472171	10.026868	8.972624	9.557406	10.335836	9.439413	9.773259	9.807767	8.764223	9.663731	10.738688	10.466251	9.556482	9.951526	9.764810	9.373655	10.796577	9.662803	9.553903	8.017690	9.785948	9.570720	9.731377	9.583773	10.246336	9.832460	9.384818	9.788660	10.228793	10.138967	8.991496	9.301568	9.661397	9.836740	9.550393	8.791835	8.984267	8.844175	9.729654	9.294739	9.177266	9.485177	9.213676	8.801121	7.353126	10.050017	8.920759	9.149407	8.669426	9.030423	9.200174	9.435228	8.521130	8.049077	10.780147	10.266592	9.637621	9.083635	9.282941	6.522990	8.384808	7.722222	9.782304	10.858937	10.438091	9.384070

AC093155.3	7.637243	7.194888	7.160752	7.731181	8.810910	8.028111	7.040480	8.443781	8.050788	7.635884	7.247314	7.864173	7.009806	6.950590	6.554476	7.576516	8.008692	7.094649	7.907241	7.198061	7.107418	8.128115	9.023720	7.580232	7.662149	7.913282	7.932034	8.034188	8.071679	7.661829	8.280091	7.018269	7.591030	7.150580	7.016496	6.977046	7.169340	6.935051	7.445236	7.726686	7.707451	7.486779	7.049113	7.430138	7.265404	7.177969	7.855309	7.119964	7.267429	7.263271	7.391638	6.873688	6.710407	7.028306	6.652886	7.710341	7.306185	7.008175	7.374236	7.072588	7.133895	7.311816	7.378933	7.543582	7.245629	7.354382	7.222702	7.260136	7.311187	7.673040	7.467854	7.396166	7.733473	7.423685	7.189847	7.483209	7.319649	7.160752	7.747797	6.875616	6.894955	7.116904	7.031545	7.196961	7.379718	7.100637	7.112679	7.388420	7.203012	7.588584	7.502904	8.008852	7.128470	7.149622	7.535343	7.188088	6.907507	7.297185	8.011995	7.319795	7.547747	6.966591	7.568409	7.335828	8.454290	7.511709	8.150964	7.460098	8.559151	7.837337	8.209472	8.000110	7.641687	8.378612	7.626412	9.837182	8.036507	7.564875	7.879300	7.346294	7.897749	7.504456	7.960849	9.139811	8.371749	7.929719	8.596272	8.033141	7.701304	7.732580	7.897307	8.004347	7.290824	8.779155	7.563777	8.261392	7.418220	8.206480	7.249623	8.129345	8.703369	8.992256	8.567616	8.433371	7.494577	8.215914	8.478049	8.565719	7.625464	8.489514	7.617855	8.182032	7.443000	7.233060	7.155127	7.399762	7.861216	8.545378	8.110900	8.159168	7.731029	7.242596	7.820127	7.019054	7.088478	8.151277	7.620128	7.580861	7.429995	7.239557	7.074170	6.694902	7.118530	6.880073	7.463901	6.943118	6.954382	7.376276	7.029566	7.438046	7.579940	7.707451	7.899568	7.698822	7.653754	7.412915	7.592104	7.657521	8.221604	7.434700	7.661681	8.510749	7.644473	7.443459	7.507394	7.742874	7.634962	7.885997	7.290824	6.909077	7.172935	7.476608	7.282895	7.420736	8.239020	7.466791	7.225755	7.526374	7.601380	7.805727	8.561316	7.474745	8.083795	7.855600	8.095372	8.358156	8.385284	8.563599	7.354709	8.330258	7.935728	8.083021	7.552684	8.508449	8.096280	8.261557	7.778689	8.051562	8.482018	8.638314	8.147768	9.452602	8.715151	8.205382	8.142249	7.889355	7.547904	7.710641

KHDC4	16.306908	15.954463	15.782778	16.228514	15.878115	16.536428	15.831819	15.211019	16.962425	15.797434	16.249977	16.063007	16.312078	16.117096	16.100088	16.075022	16.685246	16.195154	16.312078	16.175634	16.632960	15.537762	16.559494	15.914283	16.306908	16.399202	16.648016	15.971905	15.780590	16.751734	16.526136	15.130850	14.743665	15.997211	16.359390	16.727613	16.405563	16.564795	17.076555	16.662863	16.274878	16.467780	16.937351	16.947470	16.533775	17.027876	16.405563	16.602299	16.346048	16.397385	16.152946	16.249977	16.273273	16.068270	15.950994	16.342113	16.212290	15.189377	16.104387	15.743790	15.689133	16.839687	16.445545	16.186008	15.440770	15.990738	16.045607	16.215284	14.894936	16.361326	16.403395	16.435179	16.751734	16.049744	16.355654	16.114305	16.105830	15.279559	15.084722	14.861942	14.824380	15.958303	15.108611	15.964494	16.145748	15.434117	16.759174	16.038921	15.450314	14.607960	14.783853	15.739503	15.648843	15.310201	15.627072	16.401115	14.216655	16.492130	16.892439	16.629840	16.659725	15.743790	16.190578	15.949780	16.414122	16.065653	16.599646	16.744583	16.781100	16.140018	15.910549	16.264810	16.238386	17.042023	15.550613	16.678432	16.281614	16.567554	16.308700	16.737719	16.769750	16.248343	16.585910	16.737719	16.499240	16.759174	16.907711	16.815163	16.800018	16.659725	16.730795	16.865778	16.815163	16.751734	16.800018	16.907711	16.620426	16.847849	16.618881	16.874939	16.580235	16.377052	16.656647	16.487541	16.065653	16.533775	16.523640	16.851732	16.192130	16.796364	16.827810	16.902527	16.803816	16.523640	16.762688	16.621970	16.501423	17.090717	16.932890	16.674913	16.593935	16.839687	16.997234	16.990930	16.932890	16.688349	16.902527	16.580235	16.784906	16.694520	16.572650	16.678432	16.737719	16.714243	16.602299	16.691628	16.130013	16.283537	16.094324	15.975682	16.665968	16.629840	16.343924	16.312078	16.263142	16.478815	16.365166	16.445545	15.805918	17.021223	16.997234	16.596872	16.843250	16.499240	16.274878	16.523640	16.559494	16.465580	16.223639	16.147173	16.450253	16.287200	17.105999	16.494444	16.823927	16.157751	16.549084	16.372711	16.243477	16.310500	16.204400	16.624262	16.359390	16.241684	16.717316	16.734486	16.851732	16.819885	16.734486	16.952421	17.002792	16.947470	16.125498	16.503819	16.717316	16.839687	16.762688	16.178602	16.796364	16.433023	16.734486	16.831787	16.685246	17.105999	16.659725	16.618881	16.811523	16.704885

CIRBP	5.595490	6.431080	6.227132	5.859782	6.165625	6.102054	5.554888	6.379471	6.775273	5.955221	5.735745	5.743377	5.342778	5.422452	5.369930	6.666704	6.277838	5.914039	7.242119	5.877609	7.151398	8.034780	6.861077	6.342674	6.074670	5.427467	6.635961	5.486729	5.961974	6.843510	5.510681	6.118533	6.740842	6.369386	5.694332	5.745747	5.386761	5.860307	6.075714	6.649192	5.455127	6.114220	6.382968	6.805835	6.465048	5.950379	6.394833	6.749727	6.515723	6.322563	5.805110	6.175069	6.220581	6.225380	6.465215	6.204520	5.508966	6.635160	5.864148	5.853247	6.527992	5.935107	6.416667	6.830707	5.949856	6.654176	6.428351	6.506176	5.908486	6.334497	6.362535	6.134823	5.476348	5.774910	6.436296	6.356437	6.093031	6.136363	6.008585	5.754786	6.096861	6.140472	5.569576	5.965532	5.714931	6.823372	4.126990	5.417383	5.697300	5.885138	6.011588	6.267913	6.144617	6.173342	5.635849	5.940611	5.705599	6.324566	6.205756	6.111319	6.265036	6.653693	5.946038	6.099807	6.640640	6.018144	6.902882	6.834978	5.745334	6.461669	6.031825	6.666388	5.767949	6.514720	6.411526	4.942813	5.491117	6.095321	5.486729	5.602258	6.158888	5.465020	5.699132	5.415835	6.095497	5.286219	5.094789	5.211443	5.159455	5.805451	4.976700	5.551044	5.757389	5.262459	5.441005	4.812075	5.089598	6.079207	5.777077	6.393714	7.189713	7.497273	4.838712	5.478477	5.110367	5.050781	5.671606	5.721590	6.762363	4.563852	7.206614	6.206594	6.146884	5.283757	5.485751	5.382123	5.653855	6.289123	6.134823	5.850256	5.868862	4.593852	7.016333	5.947758	6.050444	6.664613	5.619849	6.454454	5.759442	5.805807	6.322896	6.602297	6.064343	5.251581	5.742641	4.363732	6.478640	5.889206	5.209621	6.005570	5.584179	6.871129	6.540510	5.690061	5.955921	5.647837	6.333155	5.946205	6.195488	5.835886	5.269469	5.673109	4.925961	4.938930	5.934048	5.448214	5.177346	5.569032	4.733571	5.600779	6.144967	5.211443	5.362349	4.617724	6.339551	3.807616	6.795886	5.251776	5.224298	6.594072	5.837676	5.577951	6.054977	5.934593	5.708822	6.322563	5.433001	6.490462	5.543334	6.232076	5.736968	6.324405	5.550691	6.336858	5.845843	5.109134	5.965720	5.969815	6.005381	6.083882	5.575213	5.012216	6.532898	6.506831	5.091237	6.092703	5.466568	6.545166

MGST1	13.365580	12.859886	13.789730	13.154939	13.675077	13.873120	14.283789	14.400780	13.150845	13.379474	13.844544	14.601201	13.277634	13.535533	13.270023	13.885163	13.106206	13.377791	12.654306	14.757385	12.248323	11.859051	10.263119	11.794841	11.626752	12.578033	11.257393	12.349742	13.151060	12.812303	13.241222	14.522236	14.051518	13.631592	13.887531	13.204947	12.577366	13.379474	12.027939	11.853542	13.774703	12.020641	11.269542	13.725868	13.761156	13.916872	12.753969	12.728127	13.923778	14.970250	13.985572	13.775677	10.334168	14.931911	14.397680	13.804646	13.949622	12.815455	11.850350	13.191901	11.546045	13.873120	12.321742	11.873849	12.344233	12.924903	12.149134	12.672242	12.086204	12.061828	14.546813	13.654386	13.056546	13.141252	14.308634	13.920502	13.219174	13.676465	13.431034	13.290718	12.033377	13.025833	15.106780	14.905079	14.665437	13.949622	13.978346	13.912944	12.689122	12.657484	12.905530	12.764791	13.049541	13.896143	13.417980	14.366251	13.596807	13.774477	13.896393	12.067188	13.666468	12.705875	13.609930	12.238050	13.052245	14.285224	11.825645	12.410794	11.318368	14.173200	12.735048	11.729757	12.840359	13.592841	12.373685	12.849826	11.586749	13.646573	13.411645	14.409150	13.010316	12.491075	12.168200	13.354386	14.355177	13.954619	11.523953	8.258160	14.987786	13.387105	11.869488	14.225039	11.121444	12.871176	12.920413	14.331365	12.495694	11.194032	9.031888	13.563376	5.607597	8.443632	13.320270	11.937329	14.832004	13.665236	13.332882	12.515153	14.007596	13.040220	10.457252	13.994733	14.414735	13.624298	15.061588	13.376280	11.061851	12.217765	9.997359	14.218284	12.831161	10.527605	14.194016	14.051518	13.481922	13.277050	10.678072	14.509938	12.679396	13.675535	11.237147	14.026521	13.634245	13.245719	14.295679	14.348562	10.774902	12.500587	12.073577	12.883472	11.407911	12.519704	10.536920	12.836345	12.196315	7.826149	13.832809	13.888605	13.901790	13.912401	13.282220	9.667624	15.147454	13.729117	14.062947	14.562573	14.273182	14.530652	10.142602	13.118676	15.522760	10.465797	12.095947	13.784315	14.422502	14.598641	13.995849	10.492850	10.685395	10.819699	13.290718	12.362917	9.522887	13.584890	10.305962	14.562171	7.755300	13.575781	13.394547	8.558556	11.469574	12.732865	12.034278	10.029332	13.043520	12.488771	13.087748	13.658440	13.922688	11.319702	7.644333	11.712895	13.204762	14.894400	14.518159	13.029169	14.573507	13.450346

FAM78B	6.208471	5.646170	6.175417	6.087306	6.590635	6.426143	5.693794	7.482286	7.514932	6.163391	6.299075	6.081971	5.655361	6.266387	5.712313	6.336018	7.154341	6.425798	7.217959	6.149325	6.906552	6.922885	8.201070	6.831169	6.766039	6.871306	6.787415	6.935528	6.018144	5.590046	7.241333	5.156673	5.740241	5.386970	5.582288	5.604154	6.105503	6.363544	6.761235	6.827338	6.134823	6.637380	6.035071	5.795280	5.797284	4.351778	6.468683	5.819564	6.125675	5.612522	5.728993	6.098380	5.234826	5.251944	5.577775	6.164105	5.042847	5.887621	6.050288	5.997605	6.465373	6.164413	7.109503	7.005961	6.230070	6.142058	6.105843	7.056807	6.219571	6.735952	6.932401	6.261678	6.626777	6.073647	6.355910	6.128005	6.583114	6.191997	6.339805	5.714005	5.891684	5.769392	5.870227	6.077620	6.206085	5.944605	6.548685	6.404576	6.941991	6.617746	6.498895	7.114883	5.612184	5.841522	5.780338	6.131041	6.101173	6.032553	6.492034	6.858036	6.220905	6.042288	6.540186	6.346731	6.532898	5.844572	7.124824	6.494605	7.186349	6.387151	6.968033	7.035624	6.548685	6.785321	6.443034	6.172016	6.783072	6.120281	6.587705	6.142404	6.028219	6.201199	6.104268	6.886996	6.620820	5.688595	7.561058	7.049113	5.868676	5.984867	5.438752	5.767383	5.278766	6.465705	6.284297	7.013318	6.135173	6.967719	5.858142	6.762363	6.657598	7.463738	6.727092	6.636745	6.539709	6.410859	6.895743	6.420945	5.876918	7.335511	6.117854	6.271383	5.767949	5.717822	5.901681	5.711575	6.455432	7.429346	6.426645	6.095497	6.501718	5.886377	5.227712	5.524971	5.294445	6.626475	6.273716	6.281449	6.185384	5.807238	5.887979	5.360977	5.751387	5.299860	5.854373	5.988091	6.244301	6.111618	5.971594	6.686786	5.710503	6.656465	6.947452	6.494441	6.305762	6.330260	6.335850	6.630766	6.802029	6.278795	6.054447	6.558766	6.065925	6.126791	6.177466	6.520193	6.531435	6.577162	5.803658	5.792732	6.248637	5.976204	5.896338	6.238775	6.577680	6.284798	6.447013	7.125777	6.586378	7.104871	7.299240	6.248466	6.358513	6.516230	6.406726	6.513895	6.244301	5.909516	5.534087	6.316233	5.894762	6.084925	6.031996	6.572895	5.788486	6.912288	6.431080	6.904447	6.354904	7.019693	7.074654	7.718727	6.708142	7.094332	6.053569	6.852320	6.053040	5.992161

PLSCR4	12.088879	11.130031	10.541527	10.901875	10.001946	10.924165	11.774311	10.745730	11.152612	11.281354	10.650579	11.027915	11.293672	10.227300	11.351751	11.222672	11.547842	10.281686	10.622485	11.160511	10.364867	11.204630	12.559720	9.992352	10.711127	10.720621	12.215618	10.875011	12.102073	12.150176	11.288046	12.346785	12.114358	12.825230	12.482141	12.568243	12.280813	10.785414	11.127076	10.979653	11.691697	12.309198	11.328200	11.892308	12.955722	11.521131	11.410967	11.614795	11.869200	11.956956	12.056520	12.492884	11.502609	12.233736	11.753936	11.996703	12.660693	11.288902	10.856320	11.291891	11.610188	12.432421	12.442520	11.375615	11.733316	11.041054	11.327922	11.560528	10.683631	11.432696	12.395544	11.949587	11.392463	12.211933	11.543627	12.292501	12.357184	12.925780	12.162658	11.261380	11.115623	12.247505	12.093684	12.229428	12.169426	12.238354	12.312544	11.886280	11.776985	10.889218	11.215086	11.459639	11.409053	11.814345	12.356248	12.130529	12.174985	12.268220	12.038515	11.612417	11.641016	11.454008	10.970001	10.315629	10.264251	11.915776	11.228419	11.158052	11.231564	11.398826	12.213629	9.584367	9.292820	10.243835	11.473822	10.050349	11.239056	11.659709	11.619033	11.428109	10.727045	10.117312	10.955581	7.836896	9.257270	12.571167	11.380538	11.938498	10.930808	11.536600	9.601584	11.325112	11.595768	8.432586	13.164705	12.162807	9.926926	10.380918	11.381737	10.783617	11.576750	11.098087	11.494352	10.428662	11.158776	11.643705	7.401622	11.155666	11.745095	12.800745	11.758139	11.054661	10.169807	12.746742	11.999597	12.167877	9.861442	9.535499	10.857955	9.663577	10.711692	10.251178	10.377270	11.629083	11.037869	9.935596	10.445678	11.726597	12.124642	11.812084	11.500503	11.341014	10.472537	12.108644	12.272420	11.777772	11.001508	10.685541	10.850767	11.812873	11.985717	11.209534	10.803437	11.676245	10.555843	10.910589	11.544982	9.798436	11.553156	11.606822	11.505545	10.071306	11.595353	12.177989	11.972822	12.233426	11.284447	11.522791	11.642828	11.618169	9.918702	11.419106	12.217765	11.598426	11.423905	11.820802	10.378509	11.555799	10.988689	9.281202	11.086467	12.089045	11.363426	11.448837	11.257232	12.052996	12.025867	12.173255	10.641355	11.563461	10.785271	10.359626	10.313050	11.143689	10.012286	11.340687	11.332042	9.474760	11.007714	11.669857	11.268796	7.600485	9.202802	9.120646	11.195473	10.905092	12.309669	11.177479

PON2	11.914277	12.283028	12.510794	11.942282	11.621431	11.594601	11.935813	11.423308	11.272515	11.628067	11.799242	11.904028	11.575856	11.886889	12.167877	10.946533	11.701300	11.851856	11.008289	11.457713	11.068537	11.890634	11.854304	11.524738	11.826393	11.612573	11.717191	11.662957	11.710136	11.945767	11.063780	12.120362	12.525969	12.386589	12.542279	12.504904	12.134671	12.496182	12.444271	12.363568	11.520699	12.793758	12.460924	12.124933	12.550217	12.710821	11.063641	12.996396	11.939846	12.399011	12.863135	12.648668	12.290299	12.344394	12.714534	11.568275	12.749999	13.040220	12.781216	13.150661	12.834013	11.582100	13.067170	11.615675	13.272175	12.804049	12.169723	12.074060	12.487990	12.014764	12.174666	11.835114	11.348941	12.060702	11.919811	12.318233	12.481303	12.461401	12.295874	12.571685	12.079547	12.327002	12.852132	12.503725	12.220778	11.886280	12.825950	12.410460	12.253893	12.180967	12.280654	12.098308	12.483254	12.165750	12.449933	12.370681	12.196161	11.104875	11.855631	11.390120	11.574486	12.040781	11.687807	11.865618	11.467920	11.539103	11.115623	11.992622	11.401919	11.137430	11.403962	11.589071	12.093373	11.226506	11.347140	11.055820	12.354541	11.861116	11.600013	13.007761	12.737964	13.034304	12.533294	12.780366	11.782962	12.491075	13.327985	11.977757	11.901464	12.457691	12.457238	12.620336	11.903881	12.562209	13.033027	12.730652	12.892947	11.990038	13.168839	11.507746	12.099673	12.144382	11.958426	13.107335	13.948475	12.303595	12.319186	12.137865	12.898820	12.671038	13.093498	12.619661	13.074553	13.153220	12.890348	13.196137	12.502227	13.202580	13.075888	13.326991	14.145530	13.121375	14.352710	14.054805	12.795726	12.329402	12.870321	13.225685	13.405662	12.997893	12.522828	13.134732	13.670545	12.548754	12.788393	13.020993	13.562910	13.295754	13.660103	13.828450	11.727596	13.211099	11.811860	13.316023	13.367473	13.779388	12.350370	12.729457	12.691020	12.537925	12.994351	12.268515	12.917840	14.029975	12.970595	12.631485	13.772698	12.835615	13.139114	13.302672	13.448101	12.833456	13.144182	13.874448	13.864620	13.201614	13.321475	12.652437	12.240396	12.403382	13.189521	13.530341	13.158422	12.408357	12.205131	13.040990	12.202348	12.060534	13.252439	12.497333	12.483576	13.028594	12.682475	12.964557	11.479340	11.587012	12.771543	13.084780	12.459940	11.449854	13.382599	13.316841	11.383909	12.288154	12.404510	13.709169	11.593285	12.526938

NFKBIA	13.986959	14.565537	14.485961	14.336805	13.550946	13.948216	14.473115	14.639680	14.882312	14.402538	14.446004	13.949622	14.300589	13.999805	14.655036	15.007673	14.771836	14.017838	14.390763	14.153617	13.278412	15.014487	14.515850	14.525087	15.289538	13.733881	14.164594	14.847864	14.085609	14.591596	15.170771	14.662761	15.085293	14.594501	14.480594	14.182770	14.074808	14.043461	14.322632	14.618223	14.871778	14.774671	13.890198	14.764339	14.738102	14.199474	13.955896	14.853259	15.969476	14.712563	13.818348	14.518159	14.461989	14.133856	14.923889	14.746490	14.168734	14.216655	14.413643	14.570106	13.366847	14.090418	16.163437	13.709169	14.773706	15.119706	14.130180	15.533380	14.179251	14.893861	14.622502	13.900711	14.440081	14.258950	14.989606	14.629537	14.468520	14.912538	14.686498	14.754551	15.091311	14.841605	14.027670	14.405515	14.156066	14.930806	13.791726	14.800886	14.218945	14.448739	14.470465	13.654590	15.533380	12.559435	14.260639	14.673717	15.741560	14.746020	14.467367	15.157558	14.841151	12.686750	14.514288	13.496262	15.412346	14.334703	14.951667	15.736354	14.176188	14.217310	13.466396	14.051810	14.765753	14.305307	15.896385	14.144611	13.972172	15.028163	15.170169	14.764339	14.158986	13.492149	14.442351	15.653688	14.722386	13.879868	14.633876	14.407674	14.051518	14.212093	14.929736	14.243388	14.185875	13.579900	14.126769	14.584760	13.895320	14.318504	14.421398	15.373253	14.680520	13.569183	13.813787	13.175390	14.691512	15.017444	13.985291	12.914484	14.031741	14.086526	14.719474	14.590670	14.723372	14.531467	14.761971	14.717654	14.616972	14.679130	14.432364	14.477449	14.519413	14.404365	14.320921	14.732623	14.403650	15.050118	13.678503	13.692162	13.970725	14.549736	14.345644	14.215030	14.904566	14.700188	13.720921	15.004214	14.359066	14.443791	13.775202	14.725162	14.574718	13.916872	15.250968	13.922148	14.403999	14.308634	14.082814	14.141470	13.984682	14.563854	15.133349	14.805656	14.877613	14.523537	14.703436	14.102873	14.252485	14.711236	13.773715	13.952936	15.047833	14.535541	14.981028	14.514695	14.038793	15.188690	14.333706	13.666938	13.844830	13.149535	14.758316	13.688112	15.734237	14.979898	14.591596	14.464777	13.836415	14.556030	13.827929	15.058545	15.275879	14.229857	15.017444	13.933765	14.155728	14.820855	13.747078	13.857538	14.575515	13.906117	14.944787	14.085905	14.064771	14.131436	14.853259	14.263241	14.249198	14.235912

ILDR2	6.307584	6.623882	6.945031	7.213260	7.131658	7.249473	7.171812	6.825589	6.692115	6.843699	6.485786	6.696363	6.314446	5.804583	5.711383	7.047214	6.477635	6.006436	6.929686	6.793634	6.282450	5.739181	7.785744	5.942682	6.415011	6.377121	7.136114	6.778767	7.465716	7.034226	6.629202	7.030045	6.660520	6.446077	7.000814	7.085839	7.209572	6.485635	6.096022	6.411526	7.170405	6.107658	4.902554	5.811119	7.243086	4.564465	7.512950	6.078138	6.625344	6.132261	6.121636	6.687309	6.726070	6.676879	6.481906	7.369389	6.607556	6.691435	5.871685	5.957866	5.686601	7.087830	7.855139	6.123720	5.914194	5.895664	6.428009	6.542210	6.248637	5.304420	6.738587	6.920499	7.180484	6.907669	6.828774	6.916792	6.849029	6.776864	6.136200	6.692925	6.106037	6.552999	7.360750	6.013848	6.764402	6.610125	6.551642	5.945313	5.671418	5.641209	5.415278	5.274932	6.001794	6.548531	6.245437	6.323541	6.371595	7.362606	6.609828	7.010112	7.323456	6.932401	6.864858	6.906051	5.486535	7.088332	5.582483	6.208471	6.398476	7.404412	5.844038	6.932551	6.460844	7.389802	6.413860	8.007770	7.278325	6.722418	7.016822	8.305140	6.600383	7.105724	7.089306	7.886288	7.211328	7.322955	10.072223	6.769612	6.817169	6.513257	5.995187	6.747557	7.353126	4.896017	7.486494	5.968264	5.840959	5.771842	6.885991	6.110433	6.390861	5.350672	6.675596	7.470477	8.056362	7.244210	6.800931	7.332263	7.057617	6.632208	6.139974	6.790314	6.443376	8.353838	7.294297	7.885668	6.549547	6.069980	6.412706	6.759287	6.263356	6.581133	6.233399	6.624362	4.230480	6.718122	8.076799	6.703693	7.337058	5.940063	7.531584	6.924680	6.645787	7.063169	7.892168	8.659450	7.204235	6.734661	6.460003	6.246267	7.684131	5.490169	5.832460	6.705732	5.873876	6.138754	6.532738	6.458196	5.522331	7.393516	7.433129	6.938731	7.093818	7.376584	6.655000	7.657826	6.929370	6.824026	7.855750	7.355649	6.591954	8.121793	5.531069	7.037328	6.585387	6.691609	7.399457	6.538274	5.969983	8.472017	4.834572	6.418823	6.560538	6.415816	5.984006	7.058080	8.310673	6.307744	7.693051	6.633056	8.335081	7.083620	6.750989	7.027807	6.658538	5.913482	6.487103	5.298852	7.124976	6.677686	7.104562	8.513380	7.093070	6.675596	6.583920	6.382809	6.985880	6.784522

NAALADL1	8.335713	9.282762	9.497649	9.234252	8.680653	8.668025	9.481968	8.848488	8.755176	9.032204	8.935611	8.748036	8.554809	8.791995	9.314274	8.801575	9.109712	8.964176	8.810440	8.575191	8.643945	9.131538	10.588804	8.845719	9.491898	9.143799	8.827314	9.032649	9.088528	9.093430	8.698049	9.489658	9.660684	8.971882	9.799495	9.642697	9.605311	9.498874	9.771440	9.590890	9.270612	10.282310	9.224813	9.364566	10.057435	9.539381	9.119586	9.709211	8.891594	9.659506	9.093582	9.863922	9.817117	9.839846	9.321212	9.063687	9.934185	10.466097	9.035284	10.144911	10.567716	9.153080	11.518972	9.358192	10.243523	9.997954	9.828267	9.558026	9.517138	9.871071	9.569977	9.348480	9.040866	9.411694	9.330748	9.571204	9.552669	9.254946	9.156149	9.768766	9.233008	9.820128	9.495797	9.461985	9.730122	9.437579	9.513366	9.176514	10.195238	9.670859	10.330253	10.130189	9.381878	9.439260	9.237493	9.339959	9.213906	8.706158	8.790103	9.044869	8.230395	9.309881	8.850756	8.913738	8.853327	8.657576	8.972768	8.363315	8.544600	8.959458	9.538277	8.312823	8.339181	8.896265	8.680053	7.941504	9.027197	9.041919	8.813057	8.469643	8.372533	9.743197	8.722060	8.881137	9.060946	8.982750	8.581219	8.802019	9.064900	9.097971	8.835707	9.408658	8.121159	8.478374	8.363315	8.983047	8.001042	8.737163	8.248863	7.615580	8.669282	9.295377	8.016483	8.668025	9.707037	9.223414	9.195936	8.496726	9.283708	9.049602	8.613148	9.138103	8.731936	8.523862	8.554484	9.547611	8.872478	8.353209	9.761985	9.192277	9.434472	8.793679	8.897794	9.007159	8.642817	9.494730	8.722507	8.424232	9.728056	9.495797	9.032354	9.071115	9.513820	9.993987	8.815760	10.116680	9.543959	8.453689	9.525226	9.926337	9.466743	8.953955	8.700916	9.831229	10.048025	8.811703	9.320329	9.120791	9.589654	8.734549	9.224202	9.788049	9.462868	9.421896	9.392037	9.863778	9.494730	9.095875	9.973866	8.925815	9.132278	9.052817	9.846759	9.975417	8.746205	9.506420	8.710550	10.400199	10.212131	9.781478	11.133064	9.220054	9.688079	9.408658	9.328621	8.656980	8.709164	8.370008	7.865882	8.794153	7.804933	9.024321	9.127357	8.763615	8.952859	7.835744	7.345653	7.404585	9.502845	9.069737	8.373123	8.299127	9.209780	8.769760	8.825386	9.503932	8.826707	9.013781

LRRC4C	4.881587	3.614770	3.362151	3.173311	3.011754	3.481909	3.365646	3.602249	3.500876	3.471558	3.565704	3.425277	3.327122	3.904846	3.683253	3.233514	3.819283	3.159156	5.002508	3.259725	6.623882	2.809301	3.588270	2.995591	3.165816	2.970632	3.350916	3.114865	3.254990	3.280717	5.248966	5.653855	6.073804	3.127814	3.586905	5.168280	3.702441	3.365983	3.444725	3.275450	3.452427	3.838007	3.640900	3.395399	3.823735	5.893525	5.514046	5.368387	6.024815	4.057171	3.999384	3.925693	4.527383	4.327221	4.222293	3.201021	4.278403	3.336436	3.064517	3.852717	2.894780	3.203454	3.987976	3.783976	3.652414	3.572883	2.961058	3.364693	3.310230	2.974624	3.472563	3.091098	3.304758	3.005824	3.239740	3.841099	3.064290	3.004804	3.022471	3.941921	3.542686	4.222293	3.632530	4.369790	3.514967	5.146754	3.124552	3.292054	3.662430	3.827201	3.394158	3.270368	3.583962	3.372078	3.003234	3.048798	3.940865	3.453504	3.251420	2.964816	2.954241	3.262192	3.030280	2.954042	4.704057	3.839112	3.352062	3.755476	3.063753	3.323817	2.865368	3.000046	3.154548	3.366868	4.362190	3.250126	3.100092	3.489890	3.253718	3.106648	3.787161	3.208598	3.267061	2.894419	4.380795	2.999469	3.294203	3.495025	3.197053	3.424029	3.117004	3.024851	3.230901	3.337623	3.109116	3.734589	2.981538	3.469235	3.445453	6.070980	2.792523	5.468124	2.898151	2.934273	2.906163	3.169872	2.952851	2.972619	3.534800	4.884522	5.206236	3.917481	3.206265	3.539584	4.230278	3.752126	3.538985	3.425996	3.145506	3.193659	3.524010	3.676220	3.463210	4.349824	5.384644	5.280349	5.346739	5.843515	4.130430	3.999384	3.720146	4.296116	4.472556	3.622868	3.105015	3.860875	3.160172	2.847233	3.556393	2.806319	3.305233	4.125890	4.117207	4.035385	3.816090	2.903558	3.682236	3.454979	2.878344	3.101983	3.067732	3.278696	3.010748	3.059490	4.222533	3.066834	3.144788	3.154285	3.306834	3.936856	4.763986	3.691579	3.688674	3.524588	3.682236	3.361296	3.448612	3.340980	3.669057	3.581900	3.170136	3.263098	3.494647	3.345400	3.429526	3.563232	3.009424	4.087037	3.271463	3.161721	3.052385	2.931424	2.935480	5.086779	4.408268	3.377394	3.756526	3.566891	3.199916	2.831066	3.095565	2.963837	3.254844	3.065217	2.958816	3.232699	3.169155	3.367066

CRYBG2	12.125254	14.482061	14.746490	14.354835	14.481669	14.552270	14.673717	15.246670	15.357170	15.075776	14.848417	14.672821	14.775599	15.040623	14.903483	14.648824	15.173276	15.015383	15.460009	14.833005	14.785315	15.301621	14.112273	14.827462	15.764986	15.081140	12.667488	15.084722	14.250206	13.916607	14.979310	14.326431	14.304961	12.759782	14.191138	14.604151	14.882312	14.680064	14.979310	15.955729	14.637498	15.015078	14.434912	14.514695	14.171613	14.046420	14.094594	14.797410	14.698400	15.037486	13.825546	14.864634	15.529882	14.631248	15.290231	14.814210	15.110454	15.971905	15.158834	15.396904	15.042927	14.445242	11.509789	15.682114	14.847864	15.720880	14.947964	15.325061	14.793559	15.457513	14.360460	14.852694	14.353021	14.771390	14.527899	14.280056	14.996793	14.444840	14.260943	15.700644	15.385183	15.325830	14.603726	15.374032	14.707518	14.279673	14.237480	15.237652	14.690192	15.582778	15.007098	15.585396	14.812693	14.937229	13.524143	14.630796	14.876067	14.886035	14.462773	15.432586	15.300977	14.793559	14.810097	14.750281	16.130013	14.401146	15.283070	16.115572	15.356364	14.626808	14.916232	15.362494	15.272298	14.443429	15.763886	14.538753	11.735820	15.127801	14.768079	12.743377	12.970784	12.707385	13.182498	13.594434	13.302266	13.924299	12.056828	13.655038	13.275630	13.444403	12.918597	11.814780	11.327340	12.818735	13.082921	11.656735	13.329864	11.696238	11.441752	13.298159	12.844209	12.299354	13.820961	11.408142	10.790007	13.987791	14.137944	12.856770	12.720947	12.966563	12.643245	12.882781	13.791984	12.539305	12.067832	13.400373	12.520550	13.261613	12.236364	13.104067	12.133853	11.218189	13.007761	12.555422	13.298825	13.843499	11.094307	13.046106	12.112386	12.364182	11.589538	12.095201	12.859886	12.568878	12.001304	11.186293	12.141913	13.162423	12.364975	14.733058	12.486004	12.965824	13.330495	13.076836	12.836168	13.319864	12.909129	12.485843	12.097117	12.819043	12.510794	13.820478	12.676354	12.214583	13.011244	12.530838	12.592334	13.772955	11.711327	13.401636	13.191901	13.932970	11.990332	11.440893	12.939212	12.546832	11.588775	12.254851	12.393629	10.664886	12.994539	12.624160	12.157109	13.078763	12.768169	13.043715	12.336360	13.103703	12.413004	12.028997	12.166337	13.672692	11.821859	11.317780	13.464192	13.539515	13.891289	14.094892	14.128591	12.559435	13.086292	12.235457	14.413234	12.422315	13.203792	12.699345	13.003243	12.910910

MANBA	12.669837	12.701760	12.430615	12.403076	12.981325	12.735217	13.277634	12.802300	12.486643	12.771018	12.886172	12.987601	12.281273	12.589947	12.503895	12.627164	12.882692	12.304038	12.715895	12.620992	12.805972	12.636300	13.359928	12.059200	12.507056	12.747536	12.655512	12.535616	12.534781	12.764272	12.623824	13.028986	13.013710	12.885289	13.348067	13.051845	13.187814	12.726510	12.587644	12.455008	12.878888	12.427164	13.125775	12.796448	13.221572	13.315805	12.333973	12.709826	12.757215	13.115266	12.722802	12.864041	13.465756	13.364746	13.237061	13.061734	13.619271	12.646416	12.932065	12.755304	12.786993	12.692514	13.181502	12.608353	12.918970	12.720405	12.418807	12.505071	12.643905	12.673286	13.091080	12.969318	12.467154	13.008116	12.950005	13.012290	13.081595	12.942843	12.775319	12.665988	12.530368	13.070763	12.884205	13.491043	13.217768	13.143585	13.147466	13.134544	13.072243	12.702618	12.776712	13.033736	12.697518	13.340354	12.963290	13.216776	12.739166	12.646251	13.103890	12.251209	12.783797	12.482465	11.898839	12.635955	12.948332	12.396312	12.385620	12.555759	13.100683	12.510471	13.367256	12.355312	12.211418	12.687630	12.632959	12.258361	11.972983	13.017023	12.214739	12.309519	12.991245	12.545063	12.567417	12.443196	12.331121	12.387757	12.828585	12.968955	13.243734	12.885836	12.843523	12.164377	11.926448	12.637320	12.572187	13.445862	11.123360	12.724316	12.488312	12.416103	12.779517	13.268628	11.832095	12.605717	13.332700	13.219965	12.698356	12.750695	12.569043	12.868696	12.564301	13.250652	12.883285	13.308155	13.394330	13.443082	13.344471	12.932588	12.322814	12.877478	12.331442	12.726676	13.997282	13.283833	13.214797	11.952291	12.791666	13.394129	13.542577	13.278020	14.106283	12.437077	13.029169	13.619271	13.234224	12.890856	12.190143	12.605894	12.771018	12.504738	13.505253	13.342212	12.278977	12.318072	12.967881	12.790499	12.343449	12.434966	13.048388	13.357413	13.260736	12.410614	13.120996	12.779681	12.670692	13.807016	13.515121	12.995079	12.699016	12.442847	13.297173	13.659596	12.584643	13.410314	13.685268	12.868513	13.542120	13.102386	12.593788	12.030200	13.317056	13.087563	13.526328	12.941572	12.892420	13.549134	12.354824	12.513519	12.501413	13.236652	12.521728	12.255993	12.861151	13.241017	12.340875	12.335876	12.774962	13.039676	12.324191	13.268023	12.634450	11.734381	12.829461	12.884894	12.789606	12.461245	13.412931	13.060802

SGSM2	10.492998	10.061953	10.316523	10.686890	11.208804	10.897742	10.673148	10.711692	11.740420	10.865747	10.768791	11.060112	10.894897	10.984649	10.703580	10.870667	10.981148	11.400278	11.531239	11.040450	10.985966	11.242229	12.049360	11.035380	11.797346	11.465697	11.187573	11.464636	10.810536	10.166289	10.818932	10.306254	10.354764	10.590679	10.171391	10.589798	10.827827	10.431267	10.939339	11.092677	11.004977	10.853314	10.677043	10.836467	10.993754	10.698669	10.997896	10.659325	10.100782	10.600754	10.815810	10.727937	10.495118	10.174725	10.576915	10.865293	10.594702	10.951940	11.666120	11.230487	10.768349	10.893147	11.066595	11.351456	10.578433	10.864236	10.293394	10.147476	10.133901	10.565322	9.922889	10.317872	11.082044	10.044933	9.973866	10.230046	10.375445	10.154056	10.232649	10.557223	10.128997	10.331462	10.752372	10.719738	10.770448	10.907795	10.699115	10.835583	10.729132	10.736862	10.784070	10.737937	10.215794	10.201258	10.392669	10.709373	10.164597	11.611531	10.752065	11.105734	11.300255	10.735785	11.034179	11.006550	11.063641	10.569382	11.435387	11.150075	11.252221	10.623514	10.864843	11.918799	11.954390	11.089903	10.948753	9.746781	11.892168	10.901565	11.059966	11.371738	9.960448	9.871973	11.074134	10.685266	9.862391	11.068971	10.820704	10.633662	9.631529	10.348851	11.010491	10.757072	10.082846	10.417045	10.987180	10.834038	11.404110	11.032090	10.035789	11.232265	11.057934	11.312768	11.889910	9.919633	11.360018	10.245152	10.759701	10.149811	10.013832	11.245386	10.926240	11.094152	10.668858	10.574229	10.758822	10.183009	10.722821	11.108364	10.988852	10.943468	10.103567	10.102182	11.220719	9.923681	10.111224	11.175962	9.949803	10.518462	10.212131	10.066454	10.398387	9.955532	10.614358	9.959230	9.743792	10.775932	10.629178	11.114554	10.372426	10.255423	11.197814	9.794721	10.544020	10.285603	11.487280	9.347711	10.110195	9.985494	10.566491	9.552530	9.998895	10.208989	10.309563	9.709524	9.770486	10.158322	9.806105	10.245866	10.682588	9.802271	10.538212	10.529524	10.399429	10.592175	11.708961	11.026466	10.719908	10.491412	10.632749	10.384099	10.690069	10.930535	10.046779	10.051261	9.920576	10.807528	10.687045	10.292500	10.304016	10.690696	10.971305	11.273397	10.226206	9.836571	10.491887	11.195637	10.501830	10.411154	10.442006	11.044735	10.537933	10.533808	10.436590	9.413487	10.031960	11.357821	11.109422	10.536195

ST8SIA4	4.529888	4.805763	4.661156	3.990914	2.985514	4.209698	5.227496	4.303540	5.188014	3.371190	4.151044	3.639699	4.121850	4.042997	3.862777	4.368486	4.774499	3.669702	5.199110	4.604611	5.063052	2.895437	7.427752	3.687063	2.995228	3.684027	4.172494	3.550812	4.119034	6.353603	3.975942	4.487615	5.183216	5.374545	5.000890	4.367199	5.113550	4.123402	4.075989	4.413541	3.815038	4.403105	3.494846	3.268403	3.691947	5.549397	3.975291	3.136966	6.282611	4.747474	3.719743	4.471008	4.071257	4.070662	4.923203	3.372078	4.144750	3.165554	3.469619	3.734799	2.966219	4.272646	4.615968	4.245365	4.288106	4.578414	2.870842	3.824627	3.673900	3.720796	4.810162	4.565758	4.699164	4.515053	4.286131	5.434751	4.132328	4.858553	4.505232	3.691947	3.856567	4.849289	3.519492	4.300892	4.424224	4.927430	3.259588	3.659637	4.933247	4.495328	3.986069	5.011176	3.527576	4.416195	4.975059	4.140191	5.046472	3.624461	3.106094	3.283173	2.998429	3.974872	3.514792	3.528850	3.358212	5.583994	4.135395	3.779768	3.212271	4.278181	3.151974	4.208850	3.355340	3.285623	5.844572	3.036164	4.247541	4.602712	3.781438	5.049157	4.820657	4.600612	3.588064	2.947291	3.784585	4.761835	4.758520	4.951447	3.136966	3.366336	3.147523	3.018363	3.306029	3.463925	3.565159	4.861224	3.196929	4.175765	5.374742	4.359819	2.956356	6.667856	3.450064	2.881304	3.937709	4.403745	2.910828	3.887189	6.599582	4.495974	5.186019	4.034313	4.915556	4.668169	3.732140	4.924879	4.379297	4.056495	4.175090	3.176741	3.268548	3.572687	3.308743	4.162019	5.170694	3.847973	3.001390	5.138025	3.878719	3.644257	4.955407	3.949769	4.270510	3.565350	6.909549	3.779991	3.140934	3.101983	3.707485	2.861800	3.698983	4.752528	4.243640	4.894172	4.806580	2.862041	3.938536	3.958573	4.796034	4.481472	4.561205	3.251299	3.984565	4.508113	5.553182	4.282318	4.534790	4.341985	3.215429	3.738525	4.602523	3.622868	3.692972	3.886152	3.529594	3.780400	4.233943	4.881152	4.515053	4.075989	4.692874	3.657644	4.131034	4.237440	4.374789	3.227019	2.936817	3.600306	3.720796	3.146189	3.380664	3.038635	3.696923	3.485680	5.307327	5.165102	4.222293	4.777623	3.210577	3.627841	3.819056	3.356572	3.847173	4.970691	3.693618	4.950633	3.796526	3.445983

FILIP1L	4.250416	3.362672	3.804108	4.334841	4.100634	3.933266	3.960518	4.001936	6.159627	4.386530	5.081029	4.581198	4.645564	4.352665	3.920614	4.209292	3.517979	5.116218	6.400824	4.226387	4.021539	5.116800	3.318310	4.569162	5.033779	4.912191	4.991195	3.466886	5.221489	4.143646	5.531616	4.438066	4.468831	4.247745	3.254330	3.721214	3.908140	3.697351	3.860460	3.967902	3.719536	3.103544	4.169012	3.772186	3.816090	4.517429	4.109148	3.054972	3.343813	3.512576	3.486788	3.615340	3.686239	3.954808	4.736346	5.563155	3.906869	3.556773	3.753370	3.366686	2.898622	5.302043	3.572500	4.209072	3.470157	4.328766	3.066929	3.758791	3.118529	3.032657	3.307599	3.567285	4.633927	3.264238	3.311179	3.200908	3.075184	3.448178	3.057265	4.101263	4.225750	4.466966	4.270510	4.488660	3.752726	4.562740	5.498120	3.298231	3.063410	3.296519	3.264817	2.944347	3.445636	3.186180	3.473810	4.087871	4.456263	5.971594	4.318222	5.198528	6.637534	4.284044	4.703636	3.975729	5.281557	5.772380	4.083916	4.672733	6.475765	4.929303	5.587157	5.230761	5.136441	3.961808	5.841877	6.541025	4.224051	5.259448	5.360226	4.450747	5.035062	3.651989	4.874251	4.980732	5.556569	3.549478	6.969980	6.579778	5.711196	4.515287	4.663487	6.338411	5.312635	4.598956	5.455327	5.007648	7.465403	7.092774	4.194544	4.776361	4.852235	4.134718	6.546478	5.572187	5.664600	4.658359	7.319961	6.249627	4.249492	7.534238	6.902230	6.100838	5.737892	5.367227	5.017318	3.826358	3.131125	5.867615	5.025838	6.640143	3.044421	6.051126	5.040502	4.674273	4.415349	7.172303	3.098076	4.561847	3.768830	3.819056	5.441215	5.884247	3.841327	4.336124	4.632405	4.273323	5.197508	4.204989	3.428989	6.477985	3.807201	6.068409	4.476026	4.110743	3.976140	3.360651	4.286343	4.344368	3.369434	3.766740	4.008227	5.474833	4.822317	4.423335	3.649216	3.954808	3.988618	3.925693	5.121515	5.144750	5.925962	5.310700	4.825610	4.847648	4.962938	4.968621	5.710503	4.545122	4.866199	6.302543	5.114185	4.370011	4.830820	4.145382	5.737715	5.210230	6.666216	6.331457	5.761388	6.472916	6.627417	5.937040	5.220191	7.116127	6.360044	7.916471	6.053214	3.749398	6.157532	6.725435	6.757487	8.081336	3.997680	4.278634	4.139542	3.873314	4.948142	6.380948

CMSS1	4.250416	3.362672	3.804108	4.334841	4.100634	3.933266	3.960518	4.001936	6.159627	4.386530	5.081029	4.581198	4.645564	4.352665	3.920614	4.209292	3.517979	5.116218	6.400824	4.226387	4.021539	5.116800	3.318310	4.569162	5.033779	4.912191	4.991195	3.466886	5.221489	4.143646	5.531616	4.438066	4.468831	4.247745	3.254330	3.721214	3.908140	3.697351	3.860460	3.967902	3.719536	3.103544	4.169012	3.772186	3.816090	4.517429	4.109148	3.054972	3.343813	3.512576	3.486788	3.615340	3.686239	3.954808	4.736346	5.563155	3.906869	3.556773	3.753370	3.366686	2.898622	5.302043	3.572500	4.209072	3.470157	4.328766	3.066929	3.758791	3.118529	3.032657	3.307599	3.567285	4.633927	3.264238	3.311179	3.200908	3.075184	3.448178	3.057265	4.101263	4.225750	4.466966	4.270510	4.488660	3.752726	4.562740	5.498120	3.298231	3.063410	3.296519	3.264817	2.944347	3.445636	3.186180	3.473810	4.087871	4.456263	5.971594	4.318222	5.198528	6.637534	4.284044	4.703636	3.975729	5.281557	5.772380	4.083916	4.672733	6.475765	4.929303	5.587157	5.230761	5.136441	3.961808	5.841877	6.541025	4.224051	5.259448	5.360226	4.450747	5.035062	3.651989	4.874251	4.980732	5.556569	3.549478	6.969980	6.579778	5.711196	4.515287	4.663487	6.338411	5.312635	4.598956	5.455327	5.007648	7.465403	7.092774	4.194544	4.776361	4.852235	4.134718	6.546478	5.572187	5.664600	4.658359	7.319961	6.249627	4.249492	7.534238	6.902230	6.100838	5.737892	5.367227	5.017318	3.826358	3.131125	5.867615	5.025838	6.640143	3.044421	6.051126	5.040502	4.674273	4.415349	7.172303	3.098076	4.561847	3.768830	3.819056	5.441215	5.884247	3.841327	4.336124	4.632405	4.273323	5.197508	4.204989	3.428989	6.477985	3.807201	6.068409	4.476026	4.110743	3.976140	3.360651	4.286343	4.344368	3.369434	3.766740	4.008227	5.474833	4.822317	4.423335	3.649216	3.954808	3.988618	3.925693	5.121515	5.144750	5.925962	5.310700	4.825610	4.847648	4.962938	4.968621	5.710503	4.545122	4.866199	6.302543	5.114185	4.370011	4.830820	4.145382	5.737715	5.210230	6.666216	6.331457	5.761388	6.472916	6.627417	5.937040	5.220191	7.116127	6.360044	7.916471	6.053214	3.749398	6.157532	6.725435	6.757487	8.081336	3.997680	4.278634	4.139542	3.873314	4.948142	6.380948

OR7D2	7.034659	7.460560	7.543432	6.915418	7.683194	6.790559	5.825674	7.861831	7.923817	6.026766	5.841158	6.153773	5.664974	6.158733	5.953780	7.360598	7.779974	6.094089	8.085541	7.116904	7.018436	8.497483	8.715941	6.222433	6.870163	6.896554	6.411836	7.367178	7.398506	6.567229	7.132134	7.043506	7.156379	4.888354	6.249967	6.575152	6.606374	7.064609	7.588888	7.263271	6.905252	7.370940	6.071530	6.673180	5.765045	6.394997	6.845868	7.128470	7.976325	6.622590	6.664453	6.992762	6.017427	7.217959	6.556302	7.519556	6.123720	7.449204	7.228420	6.968033	7.345011	7.175395	7.087352	8.238226	6.704299	8.006998	8.408805	7.895767	7.268489	8.209472	7.867408	7.595483	6.365895	7.317777	7.122734	7.758694	7.917667	7.529026	7.694438	6.828319	6.668017	6.974953	6.642422	7.257106	7.261987	7.182665	7.033252	7.156534	7.490387	7.365150	7.250091	7.766387	6.682255	6.883083	6.317742	6.373468	6.938890	6.126045	6.475585	7.011065	7.076523	7.100327	7.150257	7.166708	8.349595	6.317742	7.711380	7.980661	7.942940	6.359889	8.240081	8.375725	7.120888	7.383159	7.486938	7.185698	7.127214	6.170824	6.586695	6.334005	6.354258	6.220905	6.016723	7.426808	6.315570	5.981675	7.482123	6.096346	5.416606	5.745163	5.717993	5.929174	5.862518	5.731388	6.199294	6.998024	5.415450	7.002445	6.042288	6.992106	6.740044	7.791586	7.226699	6.881987	6.487895	7.317927	7.182036	6.704456	5.741546	6.598774	6.297403	6.626777	5.815816	6.162208	6.715222	5.999712	6.919713	7.511861	7.155289	5.923113	7.015163	5.182198	6.987484	5.712313	6.018827	6.484623	5.951435	6.971359	5.946365	6.734308	6.210351	5.903452	6.407210	5.721394	6.394506	5.990959	6.419156	6.393714	6.166312	6.681747	5.582483	6.386290	7.097815	6.687119	6.483079	6.486761	7.000133	6.308928	7.372685	6.975583	6.504965	5.749561	6.248137	6.087838	7.018269	6.820232	6.908917	7.548238	5.801555	5.877440	6.943596	6.788763	5.940611	6.821815	7.257558	7.193734	6.682588	6.806159	6.778627	6.850135	7.572932	6.557284	6.660520	6.689974	6.549863	7.157933	7.038434	6.122172	5.339320	6.708488	6.325946	6.834373	5.844412	7.210823	5.723468	7.127371	6.672861	7.469565	6.207080	7.871229	7.355513	7.566103	6.844547	6.611844	6.080402	7.174480	5.839528	5.973624

TUBE1	9.986251	7.688261	7.634654	8.402370	8.426454	8.764520	8.474459	9.351997	9.891491	9.570876	9.186923	8.601432	8.264324	8.037132	7.738048	9.362773	10.064236	8.392729	8.358778	9.259835	8.484766	8.274926	8.974670	8.306186	7.574816	9.270915	8.825090	8.551804	9.705813	7.702525	9.775518	8.622962	7.528744	8.814849	8.395299	8.392872	8.443484	7.521618	7.824386	8.074127	9.077935	7.564418	8.013058	9.496268	9.167255	8.872478	9.102439	7.571715	9.129543	8.890098	9.072827	8.778562	8.727734	8.620028	8.487395	8.743011	8.917711	7.424921	7.766091	7.933104	7.528278	9.090642	6.989522	8.030739	6.766039	7.508140	7.203644	8.064421	7.582883	7.455878	8.166604	8.772007	9.531481	8.184186	8.183127	8.003253	8.537260	8.117460	8.476201	7.662149	7.991746	8.450626	8.774143	8.641739	8.812633	9.002906	8.469344	8.763170	7.212319	7.356705	7.492583	7.217178	7.638944	8.232693	8.767930	9.405722	9.165262	10.237571	9.284015	8.993798	9.460752	8.749866	8.616310	8.113605	8.012155	10.090835	9.764038	8.909057	8.326656	9.549607	7.435533	8.356290	8.076511	9.559382	10.209367	7.301217	8.471373	9.621028	9.502375	9.597725	7.598247	7.563478	8.569098	6.997868	8.358620	8.589300	9.959832	9.693309	9.205823	7.965718	8.249596	7.066362	7.408991	6.571789	9.482879	9.363636	8.076365	8.197934	8.956217	8.136495	8.013058	7.854983	8.029783	7.020447	8.234201	8.509664	8.254529	9.590255	7.878260	9.853543	9.587971	7.974923	8.735320	8.308699	8.664235	8.053391	7.208002	7.792043	7.888433	8.961589	7.073993	8.356756	9.452017	8.737472	8.231463	8.705513	7.935444	8.824316	8.471373	8.795349	9.124740	8.512472	8.333986	8.660064	8.399403	9.303246	7.286449	7.714215	7.390251	7.013620	7.970688	6.992259	7.553313	7.450146	7.028135	6.900783	7.583021	8.279940	7.389491	7.813224	8.030578	9.090210	8.272677	8.074860	8.213459	8.327120	8.485224	8.220237	7.565641	7.749822	7.981758	8.603917	9.011895	8.314211	8.622183	8.316848	8.928486	7.150091	7.183140	8.480144	7.174793	7.962531	8.343986	8.517642	9.258494	9.231906	9.444057	8.413828	8.540060	9.108933	8.866879	8.516685	7.296044	6.997719	8.958885	9.981684	8.640199	7.890755	7.654830	7.510154	7.558907	6.863914	9.003351	8.698484	8.208599	7.492895	10.164877	7.610841

JAKMIP3	10.121247	10.974369	10.557368	11.186712	10.472068	11.064957	10.499570	10.960475	10.321983	10.285603	11.339369	10.757205	10.720490	9.966653	9.623390	10.407108	10.096114	9.163554	9.072507	10.405770	9.789980	8.458622	8.778562	9.671636	8.817585	10.196594	10.832274	9.987620	11.004546	11.211287	10.499861	9.035594	9.417175	10.239105	9.990112	10.215321	9.885467	10.088070	9.341453	9.156011	10.898647	8.811217	10.238329	9.899439	9.447007	10.005054	11.107920	9.851210	9.689297	10.370017	9.533834	9.464857	9.070196	9.960135	9.133220	11.096779	8.922996	8.906121	8.742249	8.983952	8.436936	10.716709	8.525662	9.035125	9.395906	9.259535	9.875925	9.771440	10.205670	9.213597	11.206436	10.979079	10.553536	10.842407	10.853597	10.818042	10.565746	10.395504	10.579925	9.394200	9.285069	9.094042	8.795820	9.434921	9.292212	9.541747	10.138215	9.342359	8.206780	9.101829	8.877113	8.641591	9.939340	9.900361	10.268218	9.781478	9.105050	9.505969	10.462035	10.468732	10.266020	10.754642	10.691661	10.328083	9.358480	10.461290	8.748632	9.344530	9.146860	10.773444	8.729734	8.724655	9.144893	10.672285	10.081787	13.030251	8.642515	10.279891	10.888350	9.926621	10.840331	10.749920	11.126783	11.757848	11.488607	10.937909	10.490556	9.948125	11.128957	12.624160	11.692880	11.022755	10.465495	10.831500	10.651778	10.180089	9.719718	8.807970	10.120815	9.956772	8.978023	9.439711	10.358409	9.842603	10.377106	11.305148	10.167052	11.650941	11.081007	10.196916	9.775960	9.796886	10.736706	9.979195	10.263444	10.311711	10.545676	9.532917	9.209941	11.375308	9.304559	10.465797	9.960135	9.485482	10.368210	11.429874	9.863172	9.467194	10.185886	9.692841	9.278313	9.265650	10.226980	8.841178	11.075319	8.958589	8.547528	8.537113	8.838748	8.445618	11.277646	8.678364	8.934567	9.406773	9.402461	10.326118	10.070538	10.613473	8.853185	10.997288	10.997454	13.115807	10.836911	10.728368	10.710117	10.671356	10.820211	11.337604	8.986858	9.215763	9.321704	9.877034	9.014091	9.077786	10.245866	9.366459	9.488302	8.191644	8.777808	8.647979	8.596115	10.038406	10.682122	10.756043	10.472987	9.795351	10.505273	10.972500	10.867581	10.499570	10.682725	12.139723	10.656092	9.590429	11.249094	9.231754	9.167554	8.913738	11.748779	8.758828	8.866122	9.934023	11.490236	11.202677	11.416046	8.510590	10.012765	12.045877

FRZB	10.874572	10.317267	10.417667	9.908965	8.004643	8.140171	10.558425	6.696813	5.210021	8.634408	7.552843	9.851679	9.827055	7.290668	9.396668	9.218853	8.806901	8.990467	5.704866	9.136846	6.457563	8.147919	12.158335	8.871377	8.884660	8.818873	8.808284	11.110444	10.201710	11.127384	7.969250	11.449438	9.715792	11.337458	10.843477	11.132344	11.257836	9.980743	9.403550	9.269982	9.166952	10.184062	12.136934	11.457125	13.921875	11.690625	10.201859	11.726896	10.193874	11.586607	11.452975	12.054695	11.534230	11.254265	10.951630	8.242770	11.340846	9.974679	9.158942	10.568312	10.411296	10.697641	10.746326	8.605447	9.978283	9.052657	9.206719	9.502229	9.893328	9.583001	10.795715	10.451655	9.055670	10.845627	10.847104	11.203810	10.753860	10.823997	10.632170	10.332062	10.587003	11.584703	11.182859	11.239056	11.007141	10.860851	11.186293	11.212599	10.125998	9.197036	10.071016	9.930320	8.881603	9.917330	11.273397	10.626550	11.079261	10.140756	9.042653	9.016697	8.868700	9.577451	9.698474	9.367716	7.792490	8.989583	6.842711	7.464647	6.876695	7.708553	9.002906	7.005812	6.246267	7.971306	7.483828	7.022972	10.515332	8.832102	8.542271	10.886421	7.478298	10.531581	10.642988	7.454307	8.679908	10.301596	10.094402	6.656632	7.957608	8.598657	8.902638	7.440662	9.001182	7.642764	10.762198	7.796324	7.970688	7.718273	10.582172	6.457889	6.854940	8.999042	6.190615	7.919191	9.251788	8.493527	6.638708	8.734085	11.363568	8.843711	10.221670	6.933336	9.765411	12.276968	10.029650	11.503654	10.031163	6.229015	8.060250	6.618235	9.406640	11.398826	11.222202	11.133064	9.857414	6.608020	11.630754	10.440353	10.247271	10.165028	11.997786	10.528799	9.645490	11.294105	10.790007	13.300042	10.057591	9.289264	10.465656	9.681866	12.354682	7.572185	7.708979	10.441855	9.913768	7.103604	10.196293	11.863352	9.982906	10.037185	10.340199	6.757487	10.293394	13.042069	10.810402	11.826829	10.263600	9.528632	11.831216	10.933379	11.229137	9.801504	12.365730	10.148869	9.407409	10.904224	10.631884	11.921278	9.460905	8.763472	6.842576	9.556170	10.629178	10.332682	10.737016	9.594780	10.613473	7.302722	9.778003	9.074979	10.028875	10.113060	11.345331	8.257528	8.244922	5.574617	8.182981	7.795703	6.599421	10.672422	8.740108	6.914786	6.871784	9.879782	8.074860	10.065056	9.078406	8.892991

INKA2	12.313639	11.466592	11.559235	11.723321	11.953330	11.894583	11.594907	12.157886	11.720935	11.668763	11.598287	11.490850	11.026307	11.496066	10.838984	11.780301	11.947588	10.991471	11.582266	11.623726	11.425504	11.793022	11.850666	11.067181	11.453283	11.937174	12.172013	11.816961	11.997634	12.024501	11.594299	10.810113	10.786172	11.478113	11.909419	11.745676	11.744200	12.030784	12.158335	11.739525	12.216095	11.809037	11.979284	11.499775	11.907107	11.738062	12.329087	11.594141	11.542573	11.246511	11.147376	11.172088	10.816719	11.012108	10.739768	12.012961	11.011088	11.318087	11.017053	11.532579	11.240771	12.009153	11.824581	11.602276	11.299815	11.371589	11.347880	11.395035	11.257232	11.538395	12.428156	12.818568	12.123755	12.662746	12.176389	11.584392	11.695305	11.890634	11.716161	11.303373	10.981607	11.104149	10.772548	10.871585	11.032688	11.068220	12.146584	11.123080	10.937143	10.731036	10.789859	10.935332	11.221164	11.398068	10.983494	11.147949	10.999386	11.229765	11.529216	11.829878	11.585740	11.802240	11.609049	11.574932	11.571927	11.729303	11.405443	11.569303	11.936536	12.166484	11.572071	11.416326	11.369147	11.998857	11.402792	13.676917	11.255473	11.692743	12.224164	11.012108	11.978216	11.275334	11.830475	12.825581	11.863352	11.659709	11.587908	11.240625	11.308542	11.553739	11.562464	11.231105	10.861307	11.263486	11.877032	11.221025	10.839132	11.299661	10.819554	11.260782	11.382171	11.715417	11.581815	11.303088	11.270443	11.905178	11.912931	12.439504	11.722134	11.365203	11.360307	10.884165	11.878622	11.468687	11.698725	11.755885	12.156499	11.977145	11.500773	12.304424	11.515464	11.122188	11.480091	11.384387	11.671790	12.310451	11.409205	11.203885	11.122188	11.379300	10.896259	10.280486	10.849149	10.615103	11.582567	10.175472	11.128520	11.022755	11.138009	10.648782	12.357030	11.168109	11.188446	11.217158	10.740494	11.331759	11.596548	11.236722	11.164944	11.667871	12.302166	12.336177	12.210054	11.674446	11.564634	11.681708	11.685252	12.105900	10.844240	11.201442	11.116825	11.070018	10.432310	10.990603	11.396845	12.682992	10.834667	10.653668	10.513616	10.178885	10.861911	11.563161	11.368441	12.020926	11.247285	11.098557	11.297951	11.501326	10.825609	11.122494	11.577041	12.114358	11.219527	11.199162	11.847451	11.167392	11.160666	11.297653	12.123909	11.403818	11.450138	11.911774	12.266993	11.335396	11.850817	11.119269	11.421024	12.062769

DNASE2B	7.543733	3.617653	3.108516	3.008249	2.951849	3.090209	3.954187	3.270828	3.316944	3.199434	3.342302	3.052684	3.496333	3.655456	3.120467	4.440882	3.391578	2.973074	4.691774	3.145506	4.309062	3.384997	3.372431	3.435272	3.472563	3.179899	2.980968	4.013112	3.137360	3.630688	3.581900	4.849095	4.771569	3.612379	3.888699	3.968532	4.250416	3.508878	3.529787	3.629641	3.421532	4.196670	3.774707	3.210733	3.645826	5.523280	3.684027	4.435851	7.628930	4.172286	5.244217	3.129913	6.031469	4.174641	4.280400	3.112376	4.146850	4.902554	4.127649	4.059096	3.066396	3.119999	4.686559	4.103884	3.536493	4.859838	4.550441	3.336118	3.255150	2.889192	3.767567	3.224190	3.164434	3.443822	3.767372	3.674117	3.432044	3.355877	4.756759	4.762486	3.514264	4.689279	6.854156	4.257422	3.874606	5.149087	4.492437	3.148882	4.697895	4.289822	5.497353	5.115354	3.743241	3.274345	7.235637	5.644055	3.517979	3.462866	3.864320	2.962547	3.603041	3.178423	3.093488	3.025843	4.564910	3.506244	3.099402	3.206407	3.190532	3.200147	3.295141	3.179770	3.131365	3.476911	3.413645	4.087647	5.521947	3.108164	3.176557	5.574273	3.516425	3.990914	3.760284	2.947532	3.558934	3.396300	3.189850	3.296210	3.051529	3.801826	2.944347	3.056861	3.215034	2.981253	5.593460	3.279275	2.884539	3.364854	4.387386	4.290484	3.023793	5.111153	2.876858	3.306834	3.150988	3.026305	2.904966	3.233514	8.731651	3.390353	5.897408	5.859607	3.239740	8.350354	3.673077	7.459019	3.829296	3.577153	4.566165	3.180813	3.924166	3.641293	3.280419	5.171289	4.959791	3.174752	3.937294	7.067452	8.351557	3.686870	5.752290	6.465872	5.362720	7.172616	3.241074	3.959247	4.872994	3.982623	4.687586	2.908499	3.432717	4.208229	4.977646	6.739884	4.970485	4.129806	7.065074	5.228700	2.955254	6.439561	4.391046	3.119866	7.716296	7.406798	5.023623	3.784585	4.354159	3.099736	6.930818	7.622541	4.875692	3.712339	4.305328	3.623869	5.584179	9.316873	8.087401	6.644356	4.849095	3.492956	2.984327	3.743475	3.306029	4.916169	3.632530	3.331764	3.313638	4.320147	4.227672	3.153195	3.088322	3.053243	7.208301	4.933854	3.137979	3.873944	3.910773	3.409888	3.169763	7.695615	3.302832	3.820148	3.159038	3.039135	2.971345	4.927015	3.143153	3.233219

ACBD5	12.739892	11.638885	12.054081	11.905748	12.335561	12.233736	12.113307	12.404662	12.186019	12.189499	12.270901	12.479650	11.408754	12.063042	11.145577	12.067345	12.277596	11.181427	11.649756	12.488312	11.599023	11.928150	10.426073	11.344019	10.994601	11.636790	11.488900	11.596704	11.352601	11.858108	12.245147	12.693903	12.184238	12.154791	12.357829	12.420911	12.526140	11.714992	11.907737	11.414999	11.974061	11.897804	11.921278	12.019762	12.014471	12.170171	12.198437	11.093405	12.490737	12.275625	12.306676	12.553295	12.355778	12.325731	11.774477	12.176389	12.649882	11.650785	11.294105	11.503209	12.046618	11.958426	11.693331	11.676832	11.772998	11.711161	11.725872	11.515780	11.732422	11.362688	12.250390	12.512428	12.154339	12.510646	12.522505	12.348814	12.788229	12.368300	12.652080	11.625150	11.925051	12.380187	12.745569	12.379891	12.605717	12.676682	12.675008	12.692342	11.927388	11.979596	11.869488	12.136934	11.656322	12.683833	13.005589	12.858461	12.242693	12.203594	12.249142	11.496969	12.059357	11.748172	11.568275	11.829413	11.523534	12.518735	11.757091	11.500644	11.116514	11.999751	11.583468	12.084324	11.409964	12.032600	12.210827	11.086931	12.062451	12.026155	11.876433	12.726510	11.230061	11.272804	11.762657	10.750537	11.906809	11.936083	11.197390	12.074680	11.173855	11.145141	11.157147	10.587915	11.263347	10.764768	12.383064	12.641089	10.183317	11.413079	12.406965	11.235219	11.515630	11.410105	10.603870	11.513170	11.387935	12.617952	11.908646	11.765088	11.936536	12.604730	12.192292	10.659769	12.746572	12.382432	11.735392	12.243771	12.392195	11.651970	12.230786	11.944525	11.804534	11.769304	12.514995	12.085596	12.160822	11.598873	11.707755	12.947179	12.005180	12.167097	11.754375	12.305725	12.367990	12.180812	11.581405	11.680103	11.484725	11.843867	11.076625	11.759472	11.868173	11.402210	11.068828	12.486817	11.809331	11.351898	11.327628	11.023804	12.634771	12.058084	11.926777	11.470932	12.001171	12.060393	12.254045	11.795854	12.474435	11.990173	11.575856	11.173855	11.947128	11.881590	11.995512	12.011096	11.757241	12.068126	12.114778	11.848200	11.387025	10.828444	11.654083	11.397159	11.329825	12.065618	11.955741	13.238812	11.694693	12.190289	11.887512	11.038581	11.305148	11.233464	11.008883	11.485624	12.133274	10.830474	12.510963	12.418807	11.863940	10.977016	11.581666	11.017912	11.252514	11.971127	12.637652	11.333467	12.185267	11.914892

LCE1B	6.150503	6.998024	7.031249	6.825270	7.179683	6.734308	6.759131	7.005493	7.127371	6.968555	6.914949	6.813657	6.587371	6.855573	6.331112	7.121630	7.119808	5.945313	6.183233	7.306812	5.796567	8.553172	6.276382	5.824416	6.189239	6.622253	5.670490	6.891582	6.935856	6.952973	7.417421	7.300310	6.494926	6.492830	9.136529	6.641748	6.667380	7.000648	7.834927	6.440058	7.081775	6.001621	6.011036	5.778004	5.077136	5.849360	7.054770	5.786478	7.533297	6.313328	5.232211	6.928747	5.501796	7.827616	6.445578	6.918525	6.040904	6.789904	6.824026	6.329383	6.919542	7.034830	4.315196	6.734661	6.471925	8.370663	7.521618	7.440209	6.712279	8.327972	7.973521	8.162811	6.800254	8.684777	8.048638	7.137804	7.402709	8.951168	7.368917	6.922265	6.708631	7.125129	8.650567	7.184551	7.175887	7.083005	9.393593	7.480582	6.524506	6.796677	7.283526	7.253406	7.237349	7.166260	6.767480	6.467022	6.924368	6.593887	7.074317	6.971663	6.936985	6.683070	6.812101	6.757330	7.750282	7.135167	6.522018	7.625330	7.702215	7.074170	8.247528	6.652077	7.508140	6.965797	7.036232	8.206933	5.495284	6.869820	6.823702	6.808714	7.239214	5.710123	4.744878	7.808494	6.580451	7.070379	6.848224	7.115823	8.174746	7.670378	6.819891	5.891163	6.207422	5.412417	5.907218	6.149155	5.522694	6.817169	8.902638	5.621468	6.591465	6.212680	6.612157	6.093905	4.932634	6.750842	7.994104	6.699191	8.015395	5.537848	7.449679	6.685988	9.369517	9.039784	6.233766	9.158634	6.395803	5.719936	5.590242	7.816357	5.569032	3.814003	5.049361	8.817145	5.730111	8.234802	3.920397	8.304834	8.143761	5.787010	4.577141	5.511434	10.650579	6.386290	6.305257	7.417566	9.750518	5.733911	7.729031	6.688707	6.719642	5.761388	5.365588	4.596826	11.534230	6.168963	10.130935	6.825914	6.094796	6.903209	6.402607	7.871513	12.666497	9.162916	7.289419	8.129817	8.669426	7.535018	5.922158	9.275769	10.788836	7.015663	6.682909	5.383723	7.147710	13.128798	9.562674	5.844230	6.289603	4.375887	6.844383	5.446414	5.815094	6.876407	6.945183	6.569199	6.803481	8.101283	9.148011	6.852662	7.089152	5.820610	6.382153	5.677876	7.838778	6.655338	7.311816	7.872765	7.819523	9.662014	6.487895	8.041861	7.214668	7.665583	9.467827	5.489978	6.502846	7.183928

CD99	6.951549	7.628292	8.072573	7.575603	8.425261	8.073812	8.260812	8.499478	8.988978	8.130266	9.209157	8.128713	8.377672	7.569307	7.631000	8.992404	8.318796	7.771110	8.572331	7.810194	7.859101	7.977678	8.852413	8.149782	8.680202	8.295296	8.507669	9.005015	8.439638	7.952475	8.211601	7.460716	7.433286	7.812489	7.837979	7.821338	7.724062	7.207847	7.536868	7.407585	7.955563	7.800830	8.393633	7.775580	8.061955	8.017982	8.452168	7.934793	8.074715	7.784553	7.545980	7.984691	7.780442	8.152212	8.453070	8.285162	8.107736	7.351140	8.024837	7.295217	7.309930	8.173323	8.050181	7.982864	7.375004	8.177095	7.582883	7.488516	7.409893	7.353569	7.930519	8.495987	8.469344	8.201973	7.989562	8.051400	7.743798	7.837979	7.790686	8.105747	8.490431	8.338060	8.300034	7.959604	7.952475	8.255295	8.170358	8.909057	7.412455	7.476161	7.956793	7.696675	7.870285	8.062875	7.891070	8.770850	8.100252	9.157556	8.381126	8.758828	8.607415	8.482018	8.549073	8.190541	7.625464	8.316232	8.181446	8.636910	8.270267	8.106048	8.287784	8.915416	8.327120	8.453220	8.799005	8.128115	7.621512	8.913281	8.317902	7.117230	7.571574	7.484133	7.221454	8.006706	7.321508	7.767009	8.818873	7.525289	7.130837	8.032680	8.495049	7.448876	8.175833	7.231189	8.150655	8.061672	6.621465	7.767159	8.059483	7.427752	7.426886	8.274332	7.520020	7.595034	8.665379	7.981758	7.767619	8.355063	7.331171	7.288120	7.574659	7.239898	7.433592	7.464042	8.316693	8.055425	7.516637	7.128619	7.256786	7.748119	6.921619	8.217923	7.762671	7.813386	8.004347	8.052166	7.144281	7.648147	7.188552	7.335828	7.569009	7.908184	7.628611	8.016331	7.954020	7.276724	7.383930	6.950590	6.895583	6.792834	7.586605	7.841085	7.469723	7.022655	7.203644	7.513747	7.163145	7.730554	7.156988	7.743966	7.595034	7.909264	7.837640	7.502904	7.715669	7.821338	7.464195	7.754268	7.819822	8.385284	7.314980	8.739325	8.003253	8.274038	7.650959	7.615270	8.383906	7.385155	7.085839	6.424158	7.716894	7.280216	8.052166	8.108325	8.406665	7.928816	9.809526	8.102045	7.683351	7.357647	7.859591	8.293521	7.406955	8.008230	7.733926	8.166936	8.319872	8.055259	7.683680	7.674779	7.760531	7.228105	8.020116	7.733473	7.407740	7.741197	7.513425	7.379718

ABI3BP	11.833609	11.809935	11.397622	9.783445	8.721615	9.735326	12.154483	7.382198	8.021048	11.049127	9.712641	10.236834	10.138059	9.731833	11.269090	10.358105	10.205821	9.927064	9.805002	10.644015	8.678046	10.586252	13.265809	10.257870	9.711706	8.607729	12.038203	9.160920	11.824884	12.431763	9.159549	12.623334	12.102545	11.658962	12.979897	12.729292	12.532804	11.174591	11.550739	11.544514	11.203593	12.375466	12.333192	11.915640	13.587211	11.886584	11.725723	11.349651	12.085120	11.535555	12.027808	12.926482	12.862405	12.098451	11.785542	10.493600	13.048973	11.635258	10.688294	11.885330	11.536600	12.897927	14.366997	10.398231	12.136503	10.880620	11.448524	11.460396	11.430579	11.678021	12.557978	12.170476	10.601972	12.353242	11.411680	13.035891	12.615027	12.983891	12.246079	12.247983	12.800233	12.774471	11.835114	12.029467	12.267902	11.931391	12.084016	11.458920	12.147040	11.163491	11.751007	12.437883	11.458006	11.973290	12.521575	12.088432	12.680777	12.105127	10.754642	10.345226	10.431267	11.380054	10.499570	9.973551	10.664539	11.038878	8.980451	10.228649	10.758389	10.394017	12.008090	8.804313	7.708085	9.309567	10.044641	8.424967	10.401702	11.350698	10.229572	11.897222	8.381455	8.873698	9.178174	3.025644	6.545014	8.340714	9.525717	7.184736	7.097508	6.472740	6.906870	8.337740	6.647388	6.209504	11.302927	9.105197	5.389141	8.858351	10.073916	5.765045	6.743037	10.718671	5.075064	6.163570	9.189635	8.344605	4.496204	8.269978	10.185424	9.294119	9.893623	7.531887	7.839084	10.375445	7.944475	10.264693	8.215018	8.078556	9.464087	7.071814	10.494672	9.444994	7.920456	9.549766	9.195450	6.486761	10.047403	9.278587	9.409559	8.613295	10.998209	8.978636	9.694834	12.676040	10.839735	10.195682	9.862062	8.884828	10.174432	8.089724	12.933669	8.789635	7.548238	8.693399	12.103454	9.315204	10.042063	6.382653	8.966033	11.169880	8.624195	4.935151	10.188320	12.735217	10.782381	10.748747	12.036088	8.772771	10.951940	9.179371	9.103197	8.695584	9.424676	9.421896	8.381748	9.715179	10.354444	12.124933	11.667567	9.022327	9.786390	9.817564	8.179476	8.632336	11.235828	8.015555	7.924442	7.741021	8.464904	7.311816	7.779974	8.413828	10.993145	8.408648	9.138891	5.885856	7.912515	8.060923	6.376000	11.918508	7.188088	6.697923	8.089246	7.878702	6.205483	10.604302	7.806972	8.233142

PLN	11.149918	11.000770	10.838572	10.319223	7.950786	9.146860	11.778372	4.582882	4.261329	10.455568	9.658419	10.409829	9.417954	10.306695	10.930388	9.600027	11.299253	10.310967	8.949507	8.561316	9.899145	9.652899	14.527106	8.775349	11.578338	10.150370	12.386142	9.782680	11.174007	10.983634	8.481414	11.524269	10.702585	10.908849	12.140517	12.001595	11.740562	11.460104	12.009598	11.246804	10.372426	13.380327	11.699769	11.286577	13.741329	10.962078	12.246707	11.535109	11.667160	11.261074	11.103415	12.952737	11.518703	11.699008	11.620096	11.107474	12.353897	12.698701	10.790007	12.493535	12.314784	11.392765	15.510860	11.976069	11.844337	12.319032	11.430440	11.306355	10.867283	11.589239	11.935187	11.586188	10.639993	11.643868	10.823997	11.994409	11.659862	12.032600	10.889665	11.193097	11.743759	11.654387	11.250783	11.700848	11.935047	11.193702	10.986598	11.562028	12.524939	10.789568	11.883726	12.131737	11.786724	11.107920	10.577397	11.531829	11.973290	11.003228	10.627927	11.866837	10.090376	11.283702	10.203707	10.511086	10.662816	9.685311	9.186600	10.845325	10.828294	9.232049	12.381309	9.203559	5.977257	8.836326	8.297298	6.842224	11.465404	11.562313	10.068558	9.227589	8.202867	9.794237	7.650640	2.973255	6.893530	8.615560	7.006419	3.579949	5.842614	5.201863	6.708319	9.871373	7.798494	4.449155	11.543954	8.161466	6.712279	9.277238	9.894275	4.357398	7.508297	11.832712	4.716241	7.668657	3.500685	6.661840	3.082345	6.715079	8.057849	9.612400	8.271654	6.202793	7.074654	9.470176	8.113151	10.282465	8.317157	7.774143	11.042851	4.560331	9.458948	10.233543	6.565450	8.104486	8.478374	4.931984	9.299705	7.754559	8.555909	9.180624	10.666510	8.766546	10.024017	11.196525	11.163197	10.939064	9.721234	9.530388	9.265364	8.981042	11.848352	9.022623	6.966758	9.514568	10.463260	8.580581	9.076060	6.759287	8.132391	9.457550	8.785314	4.307910	9.181391	11.230791	10.599565	10.264251	9.904851	7.732109	13.018455	9.036468	7.641842	8.331048	7.865390	8.582697	7.264495	9.202802	9.250394	12.498635	12.458656	9.634473	9.304141	11.173855	8.899769	9.109255	9.954281	7.488965	4.522785	5.187401	9.696489	5.715491	7.196156	11.748325	9.783281	8.087401	6.322725	5.574989	6.026255	8.406665	4.491993	12.286593	5.141933	5.204460	3.693202	7.124348	3.406592	10.808421	7.961301	5.867047

CEP85L	11.149918	11.000770	10.838572	10.319223	7.950786	9.146860	11.778372	4.582882	4.261329	10.455568	9.658419	10.409829	9.417954	10.306695	10.930388	9.600027	11.299253	10.310967	8.949507	8.561316	9.899145	9.652899	14.527106	8.775349	11.578338	10.150370	12.386142	9.782680	11.174007	10.983634	8.481414	11.524269	10.702585	10.908849	12.140517	12.001595	11.740562	11.460104	12.009598	11.246804	10.372426	13.380327	11.699769	11.286577	13.741329	10.962078	12.246707	11.535109	11.667160	11.261074	11.103415	12.952737	11.518703	11.699008	11.620096	11.107474	12.353897	12.698701	10.790007	12.493535	12.314784	11.392765	15.510860	11.976069	11.844337	12.319032	11.430440	11.306355	10.867283	11.589239	11.935187	11.586188	10.639993	11.643868	10.823997	11.994409	11.659862	12.032600	10.889665	11.193097	11.743759	11.654387	11.250783	11.700848	11.935047	11.193702	10.986598	11.562028	12.524939	10.789568	11.883726	12.131737	11.786724	11.107920	10.577397	11.531829	11.973290	11.003228	10.627927	11.866837	10.090376	11.283702	10.203707	10.511086	10.662816	9.685311	9.186600	10.845325	10.828294	9.232049	12.381309	9.203559	5.977257	8.836326	8.297298	6.842224	11.465404	11.562313	10.068558	9.227589	8.202867	9.794237	7.650640	2.973255	6.893530	8.615560	7.006419	3.579949	5.842614	5.201863	6.708319	9.871373	7.798494	4.449155	11.543954	8.161466	6.712279	9.277238	9.894275	4.357398	7.508297	11.832712	4.716241	7.668657	3.500685	6.661840	3.082345	6.715079	8.057849	9.612400	8.271654	6.202793	7.074654	9.470176	8.113151	10.282465	8.317157	7.774143	11.042851	4.560331	9.458948	10.233543	6.565450	8.104486	8.478374	4.931984	9.299705	7.754559	8.555909	9.180624	10.666510	8.766546	10.024017	11.196525	11.163197	10.939064	9.721234	9.530388	9.265364	8.981042	11.848352	9.022623	6.966758	9.514568	10.463260	8.580581	9.076060	6.759287	8.132391	9.457550	8.785314	4.307910	9.181391	11.230791	10.599565	10.264251	9.904851	7.732109	13.018455	9.036468	7.641842	8.331048	7.865390	8.582697	7.264495	9.202802	9.250394	12.498635	12.458656	9.634473	9.304141	11.173855	8.899769	9.109255	9.954281	7.488965	4.522785	5.187401	9.696489	5.715491	7.196156	11.748325	9.783281	8.087401	6.322725	5.574989	6.026255	8.406665	4.491993	12.286593	5.141933	5.204460	3.693202	7.124348	3.406592	10.808421	7.961301	5.867047

NFAM1	8.428823	9.669719	10.312904	8.868076	8.783057	8.243401	9.120196	7.378490	7.094332	8.457783	8.195435	8.132536	8.887920	8.227815	8.394237	8.298973	7.111288	8.767446	8.678199	9.017297	9.238262	10.223767	9.438812	9.153531	9.099498	8.706747	11.107323	8.379067	8.754115	10.907526	7.975701	10.087299	10.771060	9.825394	10.309103	9.542207	9.296921	9.497806	9.827819	9.551464	8.726020	9.380804	9.918845	9.116670	9.509159	9.943637	8.476914	9.723922	9.713565	9.589818	9.470474	9.605026	10.522517	9.094797	9.713877	8.162350	10.398387	9.986541	9.133380	9.647063	10.072223	9.326153	11.888876	9.408800	10.295548	9.668618	10.170916	8.795820	10.558564	9.808233	8.991632	9.148150	8.426021	9.904409	9.669719	10.216258	9.909707	10.552353	9.695713	10.408918	9.894119	10.019305	9.753620	9.737007	9.851053	9.061269	10.510212	10.306695	11.387625	10.269126	10.673148	11.890634	10.490101	10.735629	9.920576	9.604702	9.860824	8.009628	8.460023	6.855086	8.198382	8.478664	8.903386	7.972255	9.662174	8.295459	8.583467	8.646623	9.143958	8.150655	10.333120	8.034047	7.834777	7.501958	8.575504	8.820544	8.142249	8.723582	7.892473	10.136400	8.866122	10.823553	10.791738	9.160153	8.625939	9.485342	8.849419	8.206933	8.844016	8.568501	8.036983	8.242934	10.696778	8.605743	9.131973	7.685796	7.974619	12.221727	11.598732	10.957667	11.710136	11.947446	8.187296	8.869457	11.530788	9.215117	8.748329	9.665759	11.631959	9.614826	10.526418	10.000704	8.748937	10.230212	8.377215	9.831983	9.808233	8.214394	10.687519	8.259246	10.317582	11.009906	9.054238	10.059401	9.558934	8.565872	9.740870	8.905662	9.743348	9.846464	10.082245	10.320901	9.814948	10.371998	9.639817	10.166289	9.821458	10.147930	9.993695	10.083270	11.175825	9.333838	11.001656	10.960321	10.308167	9.870758	9.989516	9.937420	11.741588	9.311872	9.509159	7.646673	9.541580	10.377270	10.029952	9.531652	9.818140	8.412310	10.386880	10.151718	9.833636	9.499476	11.070312	10.635198	9.505499	10.637586	10.902031	12.222189	11.095144	10.109727	10.406784	9.055012	9.458339	9.789443	10.131841	9.403709	8.736729	7.778984	7.963752	8.444854	8.948426	8.080719	10.600603	11.550311	9.559382	7.853434	10.642536	10.850018	7.957304	12.152396	9.053766	8.004183	8.821587	8.352011	9.100559	10.074063	8.532727	9.810864

COPS5	13.310550	12.837755	13.399078	13.104248	13.663221	13.372935	13.804110	13.591911	13.135275	13.571674	13.633254	13.649164	13.072631	13.013350	13.001399	13.379474	13.555911	12.920598	12.604409	13.640603	13.100874	13.516009	12.500081	12.775662	12.398858	13.156712	12.487141	12.918404	13.086292	13.040220	13.450944	14.221539	14.054502	13.678503	13.745098	13.822206	13.905302	13.347004	13.001399	12.556507	13.578983	12.687630	13.422370	13.381550	13.344260	13.494170	13.247713	12.566107	13.422596	13.407961	13.494410	13.894764	13.136216	13.548445	13.591911	13.498487	14.138911	12.846159	12.824533	13.001966	13.067170	13.374641	12.899183	12.400795	13.022526	13.091627	12.819043	12.961230	13.113355	12.971153	13.483445	13.458278	13.264773	13.474198	13.678246	13.632774	13.723726	13.837218	13.419502	12.819043	13.727121	13.406964	13.584890	13.737346	13.619271	13.699672	13.851943	13.653197	13.096345	13.133751	12.609687	13.054557	13.228843	13.504360	13.333719	13.778222	13.851451	13.617542	13.998449	12.570995	13.624759	13.306600	13.184851	13.450944	13.414215	13.667422	13.895620	13.291733	12.658790	13.394129	13.283038	12.802136	13.017706	13.295754	13.315405	12.787861	12.976403	13.119075	12.660869	14.124606	13.076484	13.191901	13.366676	12.793014	13.963304	13.791211	13.338927	12.945900	13.866741	13.768030	14.155124	12.918772	13.225883	12.550390	13.249887	14.203355	12.739710	13.117358	13.706546	13.566875	13.152612	13.391817	12.356420	13.974396	13.843254	13.719166	13.347432	13.672469	13.560841	13.672215	13.293349	14.000355	14.248223	14.212720	14.204014	13.954934	13.113538	12.946630	13.042247	13.690456	13.236032	13.859679	14.440521	14.049457	14.585623	13.516454	13.416505	14.316814	14.235215	14.160557	13.823982	13.606413	14.269241	14.320921	14.056836	13.679001	12.805285	13.087953	13.491043	12.894925	13.647299	13.326217	13.858357	13.533746	13.176594	12.977674	13.173253	13.549134	12.724498	13.685040	14.035241	13.160892	14.177402	14.067206	13.734120	13.860196	13.564810	14.019845	12.808222	13.577936	14.015578	14.355177	14.101917	14.822949	14.204014	14.277909	14.695696	12.816305	13.413997	14.203855	13.738603	12.932249	14.280707	14.118926	13.637384	13.671966	13.383029	13.732652	12.573864	14.231500	12.628339	12.867443	13.446470	13.336858	13.102201	14.019562	13.921875	13.251638	13.414830	13.301878	12.778119	12.682137	13.077040	14.307986	14.401522	13.138117	14.871315	14.422133

PPP1R42	13.310550	12.837755	13.399078	13.104248	13.663221	13.372935	13.804110	13.591911	13.135275	13.571674	13.633254	13.649164	13.072631	13.013350	13.001399	13.379474	13.555911	12.920598	12.604409	13.640603	13.100874	13.516009	12.500081	12.775662	12.398858	13.156712	12.487141	12.918404	13.086292	13.040220	13.450944	14.221539	14.054502	13.678503	13.745098	13.822206	13.905302	13.347004	13.001399	12.556507	13.578983	12.687630	13.422370	13.381550	13.344260	13.494170	13.247713	12.566107	13.422596	13.407961	13.494410	13.894764	13.136216	13.548445	13.591911	13.498487	14.138911	12.846159	12.824533	13.001966	13.067170	13.374641	12.899183	12.400795	13.022526	13.091627	12.819043	12.961230	13.113355	12.971153	13.483445	13.458278	13.264773	13.474198	13.678246	13.632774	13.723726	13.837218	13.419502	12.819043	13.727121	13.406964	13.584890	13.737346	13.619271	13.699672	13.851943	13.653197	13.096345	13.133751	12.609687	13.054557	13.228843	13.504360	13.333719	13.778222	13.851451	13.617542	13.998449	12.570995	13.624759	13.306600	13.184851	13.450944	13.414215	13.667422	13.895620	13.291733	12.658790	13.394129	13.283038	12.802136	13.017706	13.295754	13.315405	12.787861	12.976403	13.119075	12.660869	14.124606	13.076484	13.191901	13.366676	12.793014	13.963304	13.791211	13.338927	12.945900	13.866741	13.768030	14.155124	12.918772	13.225883	12.550390	13.249887	14.203355	12.739710	13.117358	13.706546	13.566875	13.152612	13.391817	12.356420	13.974396	13.843254	13.719166	13.347432	13.672469	13.560841	13.672215	13.293349	14.000355	14.248223	14.212720	14.204014	13.954934	13.113538	12.946630	13.042247	13.690456	13.236032	13.859679	14.440521	14.049457	14.585623	13.516454	13.416505	14.316814	14.235215	14.160557	13.823982	13.606413	14.269241	14.320921	14.056836	13.679001	12.805285	13.087953	13.491043	12.894925	13.647299	13.326217	13.858357	13.533746	13.176594	12.977674	13.173253	13.549134	12.724498	13.685040	14.035241	13.160892	14.177402	14.067206	13.734120	13.860196	13.564810	14.019845	12.808222	13.577936	14.015578	14.355177	14.101917	14.822949	14.204014	14.277909	14.695696	12.816305	13.413997	14.203855	13.738603	12.932249	14.280707	14.118926	13.637384	13.671966	13.383029	13.732652	12.573864	14.231500	12.628339	12.867443	13.446470	13.336858	13.102201	14.019562	13.921875	13.251638	13.414830	13.301878	12.778119	12.682137	13.077040	14.307986	14.401522	13.138117	14.871315	14.422133

MEF2C	8.003253	7.654357	7.682592	8.156599	8.371908	8.186673	7.330879	9.210851	9.145331	7.991593	8.074267	7.731957	7.565945	7.919036	7.425716	8.049234	8.774914	7.754859	8.946451	7.521441	8.383127	8.847553	9.392378	8.202867	8.330575	8.402976	8.378322	8.874499	8.055259	7.363727	8.814849	7.291767	7.379556	7.685331	7.616815	7.671958	7.672732	7.674327	8.256475	8.557313	7.818572	8.406518	7.849606	8.151610	7.902121	7.830789	8.034188	7.849325	7.862606	7.596093	7.697302	7.446170	7.181248	7.375955	7.354215	7.900849	7.417566	7.872765	7.880362	7.562431	7.971157	7.513425	8.240396	8.843862	7.890146	8.211447	7.901642	8.037881	7.619865	8.573058	7.621041	7.731332	8.056835	7.492285	7.758072	7.787317	7.889501	7.459810	7.926812	7.533448	7.417279	7.411359	7.326749	7.420235	7.956970	7.502758	7.457297	7.641095	8.192758	7.973373	8.007160	8.693560	7.651592	7.477534	7.567799	7.750438	7.595187	8.129345	7.922875	8.218245	8.266671	7.610677	7.918129	7.728256	8.211137	7.856943	9.086559	8.162655	8.916955	8.365500	8.853927	9.017440	8.437092	8.436936	7.952630	8.477251	8.519296	7.930369	8.032533	7.800225	8.049077	7.596093	8.348987	8.768535	8.320792	8.178706	8.823713	8.478049	8.318216	8.119314	8.015092	8.301664	7.968034	8.213302	7.902121	8.573379	7.901019	9.223120	7.389033	8.693709	8.754570	9.365664	8.560242	8.791593	8.572726	8.276739	9.097364	8.698484	7.819973	8.576857	8.599566	7.996613	7.287828	7.509989	7.569604	7.321353	8.246522	8.712812	8.600963	8.412762	8.249440	7.671958	8.102045	7.607291	7.698370	8.436154	8.096900	8.087256	7.755152	7.618931	7.870600	7.242922	7.241488	7.303979	7.898958	7.125129	7.638944	7.864791	7.218710	8.096450	7.599534	8.521422	8.324630	8.108945	8.039489	7.910185	7.749652	8.056835	8.236995	7.777904	7.775580	8.622183	7.625612	7.594133	7.706238	7.906169	7.774785	7.984242	7.311670	7.280042	7.386548	7.290668	7.323456	7.673686	8.247810	7.384238	7.378793	8.149015	7.997816	8.191012	8.510590	7.897027	8.063486	8.124953	8.074267	8.195596	8.453070	8.697502	7.834160	8.287784	7.418994	8.117615	7.658152	7.901809	8.429432	9.336323	7.898958	8.566155	8.733026	8.910236	8.480778	8.771440	9.120947	8.140171	7.584728	8.234355	7.633766	8.249440

NPIPA1	13.606413	13.064342	13.237448	13.518872	13.804379	13.606633	13.262797	12.791497	14.321619	13.491043	13.852979	13.660103	13.295148	13.372722	13.476158	13.787896	14.244036	13.147264	14.057105	13.815048	13.494170	13.971570	14.537158	13.482826	14.076889	13.974684	15.375547	13.421709	14.282413	13.798810	14.381702	12.982246	13.133954	14.307693	13.920234	13.744082	14.069516	13.396671	13.562221	13.671534	13.770103	14.329679	14.570568	13.993914	14.622046	13.784808	13.918056	13.388838	13.374445	13.571462	13.499122	13.491043	13.400590	13.344050	13.587671	13.741097	13.497353	13.366020	13.358446	13.748586	13.299825	14.123682	14.073949	14.043175	13.217987	13.476158	13.122311	13.382177	12.667488	13.620619	13.137385	13.428889	13.932694	13.335408	13.568953	13.579444	13.271026	12.920413	13.280266	13.095970	13.073039	13.669377	13.130368	13.042957	13.675309	13.293540	13.490156	13.568148	13.490810	12.792507	13.136216	13.401016	13.111379	13.441153	13.498262	13.875803	13.243546	14.675960	13.834843	14.558412	14.354835	13.210340	14.020402	13.553941	13.507906	14.019284	14.087427	14.003265	14.393289	13.775933	13.736836	14.163659	13.783328	14.050019	14.049746	13.962183	14.346007	13.865399	14.846797	14.117673	12.911640	13.113147	13.798541	14.296633	13.373992	14.178034	15.201679	14.834547	13.640135	13.587444	13.905027	13.950183	13.700662	13.971570	14.662761	14.378512	13.107335	13.855412	13.806501	13.339127	14.418012	13.539515	13.378218	12.864593	13.007394	13.472047	13.762421	14.427017	12.939392	14.719949	14.460851	14.744972	13.566385	13.690948	13.527900	13.618534	13.484618	13.840878	13.729117	14.266304	13.564577	14.811620	14.347082	13.919949	13.170352	13.916872	12.830315	13.731149	13.217360	13.499569	14.481276	14.199131	13.068904	13.566616	13.722489	14.253766	13.575552	13.356586	13.233033	13.536185	14.063237	13.507223	13.395168	13.336659	13.404202	13.045554	12.799365	13.609219	13.103890	13.270211	13.450541	14.030880	13.542120	13.695872	13.344260	13.497789	13.620404	13.232466	13.206710	13.118063	12.867959	13.716792	13.953485	13.655750	14.053881	13.036330	14.164594	13.581602	13.319864	13.744601	13.465295	13.605486	13.192303	13.818086	13.967990	14.197832	15.081715	14.068367	13.859137	14.452476	14.283430	14.119553	13.291530	13.958500	14.468143	14.669062	13.753345	13.492817	13.529678	13.868320	13.722725	14.503811	14.066316	13.550486	13.345088	14.150745	14.341372	13.373784

AL109810.2	9.806391	12.202831	13.100310	12.568720	12.459940	12.604080	11.345799	12.676852	13.816046	11.612110	12.039403	11.665355	10.689190	12.352099	10.937909	12.406051	13.828722	11.665214	13.244322	12.583626	12.651235	13.213784	12.850331	11.974803	12.010940	11.687667	10.341269	12.974204	11.829273	11.345061	12.570358	11.435387	11.720774	9.726848	11.689126	11.670622	11.476270	11.799846	12.355628	12.623163	12.047677	11.882216	11.563602	11.791508	12.378969	12.724156	11.887512	11.907279	12.271548	12.420436	12.678191	12.733709	11.171363	12.775154	11.834822	11.858897	12.236364	12.045589	12.529347	11.893812	12.215771	11.786724	11.136391	12.603413	11.797346	13.230464	12.600886	12.404198	12.361792	13.756377	13.798810	13.466850	12.277123	14.081267	13.394726	12.175939	13.624536	12.542279	13.812235	11.436677	11.545153	11.304855	11.250623	11.956506	13.235615	12.761874	12.083836	12.647613	12.103782	12.466153	13.043145	12.499929	11.895619	12.351330	10.922691	12.233358	12.012048	13.303085	12.153901	12.478661	11.831962	12.189021	12.839438	12.183450	13.043520	11.985558	12.291548	12.578352	12.905355	12.004288	13.359500	12.240396	12.873573	12.165896	13.091080	12.064848	9.634169	12.230037	12.251658	9.185786	9.590740	9.296135	9.445444	10.430953	10.839445	9.125977	8.619393	8.996729	9.365510	9.097512	8.962369	8.889464	8.976804	8.937007	9.981518	9.046071	8.317157	9.387940	9.055814	9.516071	9.449194	9.816798	9.057845	9.245031	9.077037	11.519937	9.716701	9.834429	9.928630	8.748036	9.105505	9.033855	10.091582	9.272858	8.654920	9.732123	9.204578	10.314618	9.198592	9.898827	10.584613	9.020771	9.732418	9.334765	11.942873	10.610379	9.578695	9.481658	9.461985	9.597882	9.316571	8.680362	9.169228	8.867036	9.377152	8.758828	8.851230	8.828074	9.354397	8.983047	9.282941	9.487831	9.440177	9.285962	9.294119	9.325070	10.259317	9.556781	10.573479	10.282619	9.851210	10.838841	9.821765	9.535347	10.453118	9.601584	9.229567	10.951033	8.473263	8.532727	9.504396	9.078406	8.438425	8.807358	9.061269	9.281361	8.409398	8.502624	8.738564	9.071900	9.242578	8.946451	9.342816	9.350940	9.231455	8.835535	9.056272	8.935453	8.556080	8.856682	8.779006	9.104114	9.003351	8.896861	10.043315	8.746346	10.006922	11.462019	10.640900	10.062755	9.191027	10.080427	11.957101	9.558026	9.396207	9.064900	8.801264	9.821926

TRIM67	9.806391	12.202831	13.100310	12.568720	12.459940	12.604080	11.345799	12.676852	13.816046	11.612110	12.039403	11.665355	10.689190	12.352099	10.937909	12.406051	13.828722	11.665214	13.244322	12.583626	12.651235	13.213784	12.850331	11.974803	12.010940	11.687667	10.341269	12.974204	11.829273	11.345061	12.570358	11.435387	11.720774	9.726848	11.689126	11.670622	11.476270	11.799846	12.355628	12.623163	12.047677	11.882216	11.563602	11.791508	12.378969	12.724156	11.887512	11.907279	12.271548	12.420436	12.678191	12.733709	11.171363	12.775154	11.834822	11.858897	12.236364	12.045589	12.529347	11.893812	12.215771	11.786724	11.136391	12.603413	11.797346	13.230464	12.600886	12.404198	12.361792	13.756377	13.798810	13.466850	12.277123	14.081267	13.394726	12.175939	13.624536	12.542279	13.812235	11.436677	11.545153	11.304855	11.250623	11.956506	13.235615	12.761874	12.083836	12.647613	12.103782	12.466153	13.043145	12.499929	11.895619	12.351330	10.922691	12.233358	12.012048	13.303085	12.153901	12.478661	11.831962	12.189021	12.839438	12.183450	13.043520	11.985558	12.291548	12.578352	12.905355	12.004288	13.359500	12.240396	12.873573	12.165896	13.091080	12.064848	9.634169	12.230037	12.251658	9.185786	9.590740	9.296135	9.445444	10.430953	10.839445	9.125977	8.619393	8.996729	9.365510	9.097512	8.962369	8.889464	8.976804	8.937007	9.981518	9.046071	8.317157	9.387940	9.055814	9.516071	9.449194	9.816798	9.057845	9.245031	9.077037	11.519937	9.716701	9.834429	9.928630	8.748036	9.105505	9.033855	10.091582	9.272858	8.654920	9.732123	9.204578	10.314618	9.198592	9.898827	10.584613	9.020771	9.732418	9.334765	11.942873	10.610379	9.578695	9.481658	9.461985	9.597882	9.316571	8.680362	9.169228	8.867036	9.377152	8.758828	8.851230	8.828074	9.354397	8.983047	9.282941	9.487831	9.440177	9.285962	9.294119	9.325070	10.259317	9.556781	10.573479	10.282619	9.851210	10.838841	9.821765	9.535347	10.453118	9.601584	9.229567	10.951033	8.473263	8.532727	9.504396	9.078406	8.438425	8.807358	9.061269	9.281361	8.409398	8.502624	8.738564	9.071900	9.242578	8.946451	9.342816	9.350940	9.231455	8.835535	9.056272	8.935453	8.556080	8.856682	8.779006	9.104114	9.003351	8.896861	10.043315	8.746346	10.006922	11.462019	10.640900	10.062755	9.191027	10.080427	11.957101	9.558026	9.396207	9.064900	8.801264	9.821926

EIF4E	13.155345	13.619482	13.388628	13.891833	13.390537	13.411645	13.801126	13.952110	12.801968	13.714416	13.649396	13.479279	13.420393	13.624759	13.075520	13.815559	13.449721	13.215415	13.336226	13.704144	13.440127	13.817826	11.569606	13.421709	13.525049	13.534199	12.072185	13.577378	12.721275	13.235191	13.191256	13.918056	13.960811	13.584387	13.635698	13.653913	13.600149	13.384372	12.906948	13.053836	13.650975	12.616477	13.846147	13.845352	13.381340	13.844830	12.817593	12.986294	14.129877	14.105943	13.625467	14.158638	14.028483	13.906117	14.005012	13.390157	13.896143	13.272567	12.759589	13.347873	12.800391	13.318278	12.014173	12.716248	12.717320	13.324998	12.698701	13.103175	13.061923	13.005778	13.646573	13.700412	13.496693	13.594881	13.660327	13.372722	13.837713	13.581115	13.570563	14.587290	14.090692	14.021820	13.936877	14.084808	14.129527	13.806245	13.818636	13.996150	13.042576	13.667931	13.246311	13.390537	13.825287	13.939615	13.328213	13.745828	13.943207	13.263764	13.525933	13.025290	13.607340	13.489501	13.361622	13.057276	13.299046	13.243546	12.822489	13.265367	13.032476	13.582997	12.992898	13.120423	13.017536	13.408151	14.114029	13.098245	12.142942	13.570340	12.985374	13.337481	13.805968	13.071874	13.074158	12.729636	13.780832	12.533949	11.783668	12.233426	12.734879	12.767083	12.462754	12.621674	12.600707	12.988891	12.460438	12.773448	11.889157	11.789411	13.341166	13.151837	13.023099	12.371021	11.763724	13.012474	13.330689	13.819403	13.316023	11.841289	13.362235	12.146729	12.480123	12.647613	12.831161	13.015361	12.978391	13.484618	12.711326	12.572521	12.664778	12.925613	12.447268	13.196137	13.688814	13.877971	13.904321	12.590461	11.879384	12.793186	13.840878	13.421261	13.365165	13.482140	13.623383	13.373992	13.239205	13.465295	12.548754	12.752782	13.236652	12.597317	11.949752	12.977506	12.805624	11.898226	13.054711	12.636992	12.608022	12.851777	12.266993	12.884726	13.117722	12.485499	13.277634	12.919349	13.133751	12.965824	13.432385	13.362235	12.695910	12.974908	13.855947	13.915790	13.733881	13.243931	12.596981	13.800082	13.383910	12.379442	13.153006	12.818568	13.076654	13.272175	13.466605	13.331689	12.810934	12.903047	11.290392	13.211099	12.409200	12.969318	11.909265	12.352259	12.678191	12.384330	12.032445	11.930776	13.977501	13.110780	13.175795	12.423432	12.052072	12.691189	13.166715	13.196931	13.731668	12.598323	13.312746	13.209096

TRPA1	7.326266	5.909697	5.681420	5.965177	3.293126	5.568836	5.960936	5.157073	4.511393	5.158207	5.252732	4.678180	5.779623	5.148333	5.289592	5.587157	5.577218	4.940761	4.920011	5.904540	6.464876	5.737715	6.604561	4.236984	5.275128	4.721961	5.207639	5.432612	5.033779	6.895583	4.227258	7.617855	7.679669	5.983328	7.255858	6.971967	6.732375	5.759262	6.045528	6.169288	5.635849	7.019054	6.748634	6.221248	7.631140	5.786478	4.947749	6.857061	6.850296	6.807095	6.242601	7.848996	7.492285	7.202097	6.820386	5.500266	7.377194	6.821189	4.395671	6.098896	6.106037	6.621465	8.408805	5.805451	6.927488	5.690430	6.170824	6.091820	5.991118	6.419648	6.874986	6.348934	4.888751	7.090413	6.227975	6.950755	6.270170	6.901754	6.172347	7.483367	6.987783	7.406172	7.157612	7.145355	7.028135	7.237820	6.436473	6.664782	6.262852	5.655559	5.423011	5.883154	6.620820	6.350277	6.381308	6.539082	6.984911	6.422546	5.116218	4.392978	4.310828	5.965720	5.332847	4.766823	5.124300	6.085087	5.423414	5.944425	5.336327	4.927430	6.427847	5.202650	3.406943	4.791656	4.871916	3.116275	5.461798	5.701366	4.955831	6.475585	6.973815	6.317926	4.717292	3.231565	5.414727	4.120296	6.409018	8.339807	7.568694	5.134841	4.294363	8.546755	5.329287	6.344566	4.579470	9.111930	6.021175	6.927637	8.747736	5.821481	8.096768	7.029717	3.178423	5.421874	8.822972	4.605637	3.197721	3.852056	7.915342	4.765269	7.662314	7.003666	7.865090	6.390022	7.467417	8.070641	5.815647	6.846929	6.442193	6.899004	5.859961	6.494441	7.956793	6.873056	7.761430	4.217947	7.646194	7.544076	9.516216	8.636910	7.657521	6.843510	9.231299	7.916120	8.009296	8.136815	5.849536	4.734603	5.867788	7.238594	5.411287	6.762196	7.718880	4.998777	6.621633	6.571789	5.670490	7.794928	5.864523	6.094796	7.957304	4.768671	8.189259	8.385284	6.489496	8.259705	7.375490	6.806316	8.263388	7.777145	6.526178	8.997674	8.174901	8.203624	5.856204	8.559772	8.484300	6.867088	6.222275	5.283372	7.016822	6.824667	8.003583	6.438660	6.809369	6.711340	5.451663	6.435274	7.217178	7.730239	5.663683	4.018026	6.893841	7.152494	6.246624	6.921935	4.052798	8.109811	5.632451	6.481906	5.691365	3.114384	4.847214	6.636437	6.214917	5.606325	8.821736	5.257333

CYTH1	13.905027	14.262592	14.528702	14.015289	14.398095	13.944564	14.084661	14.120452	14.326082	14.179838	14.064122	14.003878	14.087732	13.420156	14.006174	14.351612	13.902528	14.094892	14.884961	13.652481	14.456976	14.910864	14.628138	14.453914	14.290297	14.103167	14.350592	14.433099	13.735356	14.165841	14.182169	14.265614	14.482460	14.254446	13.960239	14.142986	14.357698	14.458915	14.637945	14.591596	13.921032	15.030442	14.827462	14.657265	14.621589	14.485225	14.025367	14.724698	14.419877	14.240420	14.102560	14.291337	14.027670	14.025686	14.422502	14.132353	14.044975	14.713935	14.675489	14.405515	14.942582	13.847489	14.327509	14.793038	14.616154	15.060999	14.672821	14.245669	14.585219	14.981620	14.173886	14.226329	14.103780	14.440880	14.182477	14.980454	14.642705	14.898046	14.018727	14.738602	14.711236	14.133559	14.208161	14.396205	14.032009	14.185875	14.644025	15.111191	15.254640	14.951167	14.215030	15.414655	14.386005	14.663179	14.300589	14.367691	14.027938	14.187776	14.239134	14.183722	14.364475	14.021820	14.219932	14.301287	15.122219	14.009820	14.450565	14.678701	14.949574	14.152064	15.246007	14.578479	14.785315	13.970183	14.487547	13.548218	14.143957	14.312732	14.199812	13.267179	13.593289	13.446470	13.612808	13.439251	13.695624	13.793026	13.983245	13.691689	13.592841	13.361622	14.003878	14.237480	15.668159	14.051810	13.688814	14.102560	14.403276	14.868819	14.796433	15.313805	14.899137	15.195519	14.451725	14.671925	13.770612	13.868078	13.909128	13.916468	13.685268	13.870987	13.519100	13.953781	13.788715	14.133559	14.135776	13.665736	13.414215	14.423999	14.767548	13.639658	14.332725	13.960811	13.590983	14.342816	13.972172	13.361000	13.813787	13.634499	13.864620	13.779882	13.773464	14.580994	13.711946	13.578284	13.574051	14.834547	14.719474	14.455021	14.057693	14.219589	14.055678	14.774189	14.830006	13.685040	13.845880	13.795660	14.303599	13.888334	14.637025	13.931123	14.041399	13.125984	13.776922	14.010076	13.840337	13.556139	12.984259	14.178943	14.181807	13.936589	13.964894	13.882499	13.996994	14.135776	13.947375	13.792498	13.319284	14.415816	13.995849	15.189377	14.607572	13.689737	13.303685	13.490810	13.612555	14.308634	13.738340	13.279019	13.741803	13.487496	14.484499	13.863595	14.316814	14.265286	13.693359	13.828188	15.587282	15.437228	13.460920	15.107990	13.903911	13.243931	13.916607	13.357004	13.566158	14.441603	13.771628	12.888269

RPL37A	4.638413	6.075714	5.490389	5.630528	4.837031	5.971594	6.163570	3.922669	5.435317	4.570913	5.108098	5.328291	5.002927	5.826762	6.367881	4.600612	5.144534	4.953764	4.575611	6.655174	5.807596	4.253225	5.771842	5.073004	4.704262	5.090437	3.932174	4.234619	4.528407	5.385942	4.724520	6.932252	6.352600	5.472341	6.802846	6.356611	6.203825	5.688775	5.306383	5.223543	5.817089	5.066538	5.376154	5.867615	6.486608	7.060280	5.359285	6.205756	6.826070	6.368195	6.155509	7.361534	6.607057	6.519038	7.051808	5.390458	6.799271	6.363201	5.553777	5.855636	6.524017	6.070817	5.743007	5.353445	5.578727	5.840599	3.263680	5.351089	6.555305	5.780338	6.604224	6.028736	4.558533	6.058191	6.159981	6.322563	6.361035	6.460347	5.499091	7.319649	6.521385	7.023584	6.585732	7.180178	6.324214	7.273579	6.667531	7.353126	5.923827	5.677707	6.203825	6.965797	6.522817	6.655174	5.564851	5.968264	6.852479	5.104831	5.219974	5.167871	6.164262	5.207233	5.491679	6.207603	5.802945	6.535312	4.197524	6.391043	5.050186	4.154543	6.198084	5.509173	5.134458	4.364615	5.827479	7.095967	4.342189	4.940144	4.811675	5.437272	5.885332	5.284771	6.631873	4.447326	6.288089	4.531822	5.165881	6.990315	6.172854	6.018512	6.033365	6.803168	5.867214	5.356278	4.216869	5.642899	4.670157	4.048031	5.848095	6.741451	6.484435	5.602850	7.485863	8.283918	5.808857	6.104430	8.935765	4.985191	5.799653	3.694665	4.890032	5.599867	7.286616	6.537120	6.449656	6.654673	5.830277	5.422062	5.768830	5.622592	3.716933	6.104430	7.438363	6.991935	8.023775	3.550026	7.242922	7.066501	6.601827	6.548531	6.782296	7.061705	8.340131	7.026662	4.109148	6.420618	5.084868	4.904637	7.170236	5.121515	6.242941	8.334638	5.778004	6.163391	6.071333	6.643535	4.800293	6.549699	6.320331	6.825119	6.231093	3.929129	6.214589	5.321096	6.251304	7.341144	6.459694	8.036507	7.464195	7.655283	6.741451	6.224175	7.040480	6.618387	6.121636	6.550364	7.085839	3.016261	5.682156	4.950218	6.062264	5.836646	9.264276	6.173182	5.350251	6.185384	6.798331	5.801186	5.548626	6.627577	6.278158	5.273109	5.006627	4.820845	5.482205	6.477288	6.938279	6.984122	3.444542	6.686786	5.639932	4.824555	3.807012	5.632451	5.533682	5.878510	5.142956	7.576358

WASF2	5.487827	6.166868	6.747055	6.314683	6.611312	6.551642	6.172526	6.447349	8.692623	6.060619	6.719642	6.075192	6.921142	6.985241	7.003068	7.468910	7.548986	6.487592	7.037167	6.744822	5.479850	7.575443	6.851233	7.502112	7.725913	7.272024	6.751780	6.520703	6.421425	5.910976	7.006585	5.066538	6.253010	6.446533	6.004842	6.168083	6.158574	5.451468	6.070817	6.526516	7.212319	5.614619	6.852835	7.150257	6.894802	6.659030	6.398128	6.664453	6.886607	6.282111	6.029915	6.931129	6.540849	6.474428	7.218415	6.915261	6.769936	6.470026	6.393377	6.016723	6.735311	6.276203	5.666656	6.824504	5.909357	6.867906	6.001274	6.906870	5.367011	6.247965	5.706450	6.532898	6.744672	5.897574	6.207268	6.207080	6.230070	5.796922	6.359193	6.170311	7.345830	7.752132	6.405228	7.287083	6.008773	6.953139	6.666542	6.905093	5.635267	6.396973	5.825842	5.812964	7.269393	6.532242	6.499570	7.781671	7.607143	7.593670	6.524815	7.634486	7.418369	6.625506	7.247168	6.398962	6.185545	8.348688	8.774603	6.380621	8.068901	6.622590	6.246787	7.683826	7.474299	6.949814	8.495845	6.575343	5.980847	7.442823	6.911143	6.087137	6.197075	5.517407	6.072243	5.795999	6.134397	5.340097	5.957495	6.925659	5.869888	5.723468	5.612341	5.126754	4.838712	4.447326	6.403278	6.473080	5.921404	6.076077	5.554888	5.865953	7.066825	7.329461	7.250091	4.372155	5.185007	5.846583	6.194961	6.103756	5.434949	5.641771	6.298082	6.518720	6.459852	6.053214	5.994110	6.369725	6.168430	5.628989	6.185885	5.970151	5.783423	5.693962	7.072893	6.202255	5.873185	6.917722	5.299668	5.891163	5.664054	6.345907	6.018512	6.568873	5.823480	6.351623	5.375758	5.456287	5.824779	5.755351	5.684361	5.858872	5.161480	6.445749	5.755174	6.302900	6.007362	5.248396	5.633193	6.245766	5.765045	5.530479	6.030587	6.891902	6.309443	5.273296	6.033450	5.653503	5.890305	6.756373	5.317069	7.016654	6.580451	6.860415	6.620976	5.922559	6.330944	6.186901	5.935459	4.572417	6.090782	5.331054	5.325377	7.176828	6.093207	6.885035	7.604831	6.520543	6.877000	6.829091	5.569943	5.940438	6.012970	6.076077	5.449166	4.834971	6.807739	7.925680	6.451641	6.333671	7.071340	5.959156	5.195497	4.047376	6.168963	5.410127	5.922924	6.227294	6.244796	5.276363

C9orf153	3.314652	3.189850	3.173949	3.176851	2.952291	4.787036	4.003685	3.242102	4.175090	3.342665	3.749998	3.755901	4.043645	4.448914	4.397228	3.706665	3.736070	3.394523	3.240314	3.344944	4.533520	2.909221	5.765200	3.162940	3.348550	3.204137	3.700762	3.148514	3.491351	3.051939	3.559737	6.881033	4.562063	4.054525	4.408268	4.038661	4.515053	3.433628	3.418075	3.254330	3.434147	3.981517	3.351555	3.432203	3.456601	5.352964	3.376866	4.480424	4.710123	6.190968	4.045411	4.754255	5.275538	3.855934	4.612491	3.355030	3.873314	3.548911	3.760508	3.549657	3.717333	4.295457	3.607835	3.094660	3.226298	3.229455	2.887760	3.264112	3.114254	3.229042	3.528850	3.436697	3.521300	3.665068	3.526294	3.207803	3.601477	3.429876	3.058412	4.318671	3.119866	3.709872	3.717529	3.588820	3.552978	4.693712	3.469431	3.153309	3.065950	3.321367	3.126876	3.010582	2.883371	4.273115	3.462151	3.410757	4.680068	6.099449	4.375887	3.336976	4.194119	3.844372	3.507239	3.241487	4.990613	4.195839	3.158535	3.343001	3.036347	3.271463	3.000046	3.033387	3.081779	3.204801	4.375887	4.320147	3.008759	4.178750	3.722702	3.585898	3.841899	2.998519	3.140290	2.939146	4.984172	3.576932	3.884029	4.097759	3.557756	4.622899	3.518728	3.422793	3.613360	4.356533	3.987558	3.543602	3.093250	4.321413	4.110743	3.919345	3.055377	5.584588	3.031075	3.178536	3.187010	3.065121	2.984511	3.488662	3.466290	3.332238	6.168263	4.153870	4.343285	4.493992	4.135166	4.478658	3.523264	3.305708	3.121463	3.191611	3.993947	4.444117	3.789104	3.723107	5.031937	3.246386	4.586266	4.287894	6.494280	4.122740	3.952698	4.920418	3.773257	3.666689	3.517346	4.038661	3.533103	3.730048	3.609384	3.461116	3.814191	4.238326	3.364044	3.297133	3.227169	2.894780	3.528663	3.251540	4.700680	3.745674	3.284447	3.730876	3.375800	3.216520	3.190532	3.386535	3.606173	3.198666	4.245989	3.316355	3.772596	3.146189	3.999384	3.417042	4.153649	4.052167	3.985840	3.005002	3.306163	3.277400	2.978709	3.324325	4.053879	3.090007	3.233575	4.040813	5.081029	3.477825	3.867507	4.479106	3.319776	3.127814	2.996090	5.872251	4.512683	4.286794	3.330300	3.516964	3.269757	2.907112	3.006239	3.091325	4.096230	4.333544	6.083169	3.121240	3.542469	3.979980

METTL23	12.227505	12.140193	11.842350	11.489358	11.366409	10.997597	11.913683	10.967694	9.479495	11.200874	11.377843	11.320848	11.531829	11.765403	12.129781	10.691016	11.039307	11.644166	10.143844	11.336580	10.417045	11.091311	10.703034	11.178535	11.242985	10.980857	10.324901	10.335611	10.852730	11.958138	9.961955	12.172470	12.403382	12.081360	11.945155	11.930928	11.954677	11.967950	11.725122	12.183277	11.025697	12.293733	11.960220	11.916216	11.710430	12.152825	10.935634	12.229877	11.985888	11.959745	12.242226	12.167414	12.498162	12.493206	12.365253	10.993881	12.425406	12.662414	12.158494	12.550217	12.930440	11.063219	11.550159	11.586607	12.575563	12.497831	12.228465	11.704017	12.414938	11.962037	11.639091	11.682461	10.473776	11.785826	11.869488	12.339191	11.920674	12.202831	11.931707	12.159708	12.071265	12.156789	12.181119	12.276660	12.062305	12.147660	12.249599	12.008402	11.809631	12.071265	12.056520	12.121750	12.331927	12.006852	12.150502	12.043045	12.012583	10.615426	11.621605	10.724919	10.811654	11.745819	10.934125	11.370520	11.357672	10.405628	10.836621	11.330601	10.500761	10.486856	11.591454	11.328200	11.754833	10.228365	11.360449	10.326880	12.564954	11.209393	9.627691	11.891870	12.279139	11.879226	11.429874	9.975574	11.603009	11.354479	10.703580	10.744255	11.754080	11.387625	11.493459	12.100593	13.465295	12.142518	10.731932	12.275467	11.476974	10.553536	11.953180	11.697842	11.017912	11.766326	10.785414	12.849310	12.940276	11.567979	11.832854	9.951687	12.008851	10.859242	11.926923	12.104818	12.805972	12.476535	12.482801	12.045443	11.787766	12.557978	12.821603	11.854155	12.879950	12.500587	12.291073	12.092606	12.936361	10.989882	12.816808	12.555263	13.223145	12.375118	12.276523	12.870487	12.851957	12.330956	11.518565	12.885476	12.942292	12.936720	12.542919	12.746572	12.079696	12.247833	12.542773	12.548405	13.002534	11.863047	12.161888	12.886000	12.713284	12.552169	12.293733	9.889233	11.977595	12.728294	12.502885	12.592493	11.912654	12.383384	12.236647	12.081513	12.969511	12.313810	12.215332	12.467321	11.868608	12.731336	13.018633	12.175782	11.949912	12.876066	12.398695	12.914157	12.426698	11.756331	11.774910	12.762381	10.353871	11.954825	12.100117	12.255523	12.050418	10.959032	12.426853	11.640106	9.583919	11.174150	11.689898	11.634958	9.975417	11.413399	11.903405	12.530023	9.981215	12.671548	12.350062	12.786494	12.129781	11.520092

AC005837.2	12.227505	12.140193	11.842350	11.489358	11.366409	10.997597	11.913683	10.967694	9.479495	11.200874	11.377843	11.320848	11.531829	11.765403	12.129781	10.691016	11.039307	11.644166	10.143844	11.336580	10.417045	11.091311	10.703034	11.178535	11.242985	10.980857	10.324901	10.335611	10.852730	11.958138	9.961955	12.172470	12.403382	12.081360	11.945155	11.930928	11.954677	11.967950	11.725122	12.183277	11.025697	12.293733	11.960220	11.916216	11.710430	12.152825	10.935634	12.229877	11.985888	11.959745	12.242226	12.167414	12.498162	12.493206	12.365253	10.993881	12.425406	12.662414	12.158494	12.550217	12.930440	11.063219	11.550159	11.586607	12.575563	12.497831	12.228465	11.704017	12.414938	11.962037	11.639091	11.682461	10.473776	11.785826	11.869488	12.339191	11.920674	12.202831	11.931707	12.159708	12.071265	12.156789	12.181119	12.276660	12.062305	12.147660	12.249599	12.008402	11.809631	12.071265	12.056520	12.121750	12.331927	12.006852	12.150502	12.043045	12.012583	10.615426	11.621605	10.724919	10.811654	11.745819	10.934125	11.370520	11.357672	10.405628	10.836621	11.330601	10.500761	10.486856	11.591454	11.328200	11.754833	10.228365	11.360449	10.326880	12.564954	11.209393	9.627691	11.891870	12.279139	11.879226	11.429874	9.975574	11.603009	11.354479	10.703580	10.744255	11.754080	11.387625	11.493459	12.100593	13.465295	12.142518	10.731932	12.275467	11.476974	10.553536	11.953180	11.697842	11.017912	11.766326	10.785414	12.849310	12.940276	11.567979	11.832854	9.951687	12.008851	10.859242	11.926923	12.104818	12.805972	12.476535	12.482801	12.045443	11.787766	12.557978	12.821603	11.854155	12.879950	12.500587	12.291073	12.092606	12.936361	10.989882	12.816808	12.555263	13.223145	12.375118	12.276523	12.870487	12.851957	12.330956	11.518565	12.885476	12.942292	12.936720	12.542919	12.746572	12.079696	12.247833	12.542773	12.548405	13.002534	11.863047	12.161888	12.886000	12.713284	12.552169	12.293733	9.889233	11.977595	12.728294	12.502885	12.592493	11.912654	12.383384	12.236647	12.081513	12.969511	12.313810	12.215332	12.467321	11.868608	12.731336	13.018633	12.175782	11.949912	12.876066	12.398695	12.914157	12.426698	11.756331	11.774910	12.762381	10.353871	11.954825	12.100117	12.255523	12.050418	10.959032	12.426853	11.640106	9.583919	11.174150	11.689898	11.634958	9.975417	11.413399	11.903405	12.530023	9.981215	12.671548	12.350062	12.786494	12.129781	11.520092

DCAF4	10.217133	10.092047	10.199765	10.008303	10.161362	10.442595	10.240459	10.586845	10.554613	10.367597	10.628696	10.524883	10.292031	10.652063	9.991882	10.094875	10.300015	10.377270	10.228061	10.300812	10.169500	10.505710	9.836416	10.211984	10.358105	10.430485	10.161362	10.586252	10.497536	10.312584	10.055148	9.734163	9.805321	10.154986	10.164279	10.280806	10.403638	10.328234	10.870211	10.525656	10.400960	10.532207	10.194166	10.156673	10.030230	10.162442	10.594267	10.044788	9.446373	10.061198	10.200806	10.162292	10.250726	10.295232	9.867886	10.454651	10.384099	10.526564	10.775210	10.596938	10.900542	10.351574	10.722674	10.843330	11.259576	10.203254	10.271935	10.185728	10.165185	10.694662	10.158664	9.937905	10.814170	10.154515	10.086358	10.079519	10.335381	10.350959	10.091891	9.780111	9.799359	9.838908	10.388103	10.414470	9.660909	10.100633	10.013557	9.953492	10.256037	10.175472	9.913595	9.681546	9.839071	10.118700	9.708602	10.126478	9.437744	10.137149	10.434992	9.994150	10.129611	10.046625	10.089619	10.356463	10.401393	10.271167	10.681815	10.391769	10.431883	10.401239	9.735326	10.867857	10.582745	10.475718	10.076365	11.194728	10.557654	10.162156	10.508876	11.290061	10.757072	10.220637	11.605506	10.375909	11.214379	10.333451	10.270574	10.428516	10.474206	11.095294	10.554929	10.391204	11.735962	10.885099	10.325669	11.189332	10.444108	10.478137	9.834894	10.027644	9.898527	10.086677	10.776676	12.377241	10.816113	10.240017	11.240198	10.364867	10.401855	10.200086	10.162874	10.123969	10.178435	10.360509	10.696778	10.176535	10.505842	11.844802	11.623576	10.181016	11.412382	10.828444	10.666510	10.398075	10.787337	11.154075	10.170585	10.320444	10.215948	10.499425	10.436727	10.808753	11.494211	10.480213	10.405472	10.300966	10.490101	12.667996	10.024815	12.287695	11.023215	11.446009	11.016275	12.106846	11.671048	10.850018	10.652206	10.621312	11.439106	10.244867	9.832758	11.106317	10.925491	9.801811	10.216258	10.857655	10.254063	10.380759	10.630830	9.964114	11.360449	10.872332	10.034381	10.306414	10.727937	11.063780	10.909294	11.077660	10.353428	10.159823	10.310064	10.357076	11.017781	10.340199	10.794238	9.852765	10.652524	10.883860	10.354903	11.088277	10.494961	10.752065	10.706580	12.156031	10.615704	11.953632	10.211984	9.997800	11.153776	9.773557	11.288746	10.834508	10.835583	10.596868	10.885415	10.950728	11.529981	10.968317

ICE2	9.191027	8.015237	8.536257	8.245391	8.676820	8.470727	8.845859	7.844360	8.442536	8.146104	8.163962	8.314845	8.328604	7.758541	7.787161	8.806276	8.189101	8.308999	7.570962	8.959155	8.390991	6.884238	8.601123	8.647089	8.609391	8.316693	8.994416	7.455878	8.329486	7.932481	9.095402	8.517795	9.050550	8.890976	8.766397	8.876306	8.984712	8.236836	8.536680	8.865668	8.299127	7.983626	8.873253	8.537385	8.742107	8.809674	8.163103	7.666698	8.545228	8.584387	8.604053	8.925347	9.032809	9.002579	8.632906	7.554088	8.981350	8.507070	8.299127	8.548140	7.972255	8.521708	8.226603	7.887351	7.930949	8.309760	7.697615	7.660474	7.974770	7.856797	8.739777	8.494612	8.367279	8.611378	8.541825	8.365788	8.843566	8.498198	8.688827	8.983646	8.594242	8.906121	8.991632	8.796272	9.371967	9.091750	9.077037	9.253295	8.177241	8.328299	8.506906	8.277230	8.572331	8.679746	8.286715	8.576857	8.850472	8.949507	8.328445	8.302736	7.999328	8.743289	7.914014	7.082859	7.734740	8.158860	7.880784	8.712055	7.720231	8.380007	8.117928	8.124176	8.031021	8.465055	7.965718	6.518205	8.072872	8.504929	8.410617	8.701813	7.679523	8.077583	7.910938	6.488546	7.763447	6.842863	9.259079	9.645183	8.026668	7.543733	7.294755	7.394907	5.980140	6.828610	9.088528	8.103236	8.401291	7.369988	9.175728	7.161420	7.012195	8.159780	6.827338	8.053534	7.531737	7.281460	7.524985	7.997958	7.919191	9.868052	7.147559	8.100093	8.191485	8.566718	8.866575	8.105890	8.114718	7.684733	7.807872	6.876248	7.349431	8.367601	8.964936	8.046247	8.123878	7.059340	7.187619	9.032649	8.395144	8.290087	9.154005	8.187593	8.653728	8.592063	9.001965	8.965087	7.608834	7.580396	8.742397	6.936660	6.838192	7.668348	7.559979	7.980661	8.244304	7.251854	7.235637	7.341925	7.397106	7.931572	7.717516	6.824667	8.151117	8.389280	7.846866	8.627465	8.548766	8.409838	9.295979	8.283150	8.313433	7.684432	8.344141	8.734549	7.993159	8.026994	8.489362	7.801746	7.794465	8.566155	8.421655	8.123421	7.175395	7.649346	7.845347	7.930670	9.358630	7.730715	7.754421	8.180838	8.707526	7.929278	6.117492	6.997424	9.134241	8.420888	9.090065	8.810595	7.941663	7.820563	7.771423	8.732552	7.659517	8.181595	7.128302	8.317461	8.008230	7.755586

SMAD4	12.778842	12.734379	12.692683	12.478803	12.498799	11.938498	12.350849	11.581405	11.680103	11.948186	12.068447	11.738510	11.644595	12.062451	12.262315	12.091377	12.070827	11.956809	12.326538	12.065925	12.144544	11.765403	12.580980	11.997151	12.288317	12.062451	11.925351	12.009153	11.988015	12.065618	11.516794	12.606221	12.903231	12.513855	12.486494	12.377553	12.723985	12.325086	12.398188	12.570198	12.070991	12.577537	12.399784	12.831872	12.920223	12.526615	11.684658	12.543722	12.685364	12.471493	12.636634	12.705163	12.809563	12.804601	12.539123	11.977595	12.670692	12.951673	12.582141	12.646567	12.636300	12.063806	12.710821	12.266375	12.716418	12.669159	12.502567	12.548405	12.405117	12.172470	12.245455	12.401781	11.713634	12.569208	12.169111	12.428658	12.449933	12.419759	12.413338	12.490403	12.653292	12.746572	12.712441	12.930094	12.610356	12.816808	12.452481	12.669837	12.395544	12.437247	12.004219	12.364654	12.653292	12.619822	12.740585	12.690183	12.792190	11.876288	12.218206	12.224164	12.097725	12.271228	12.259420	12.336035	12.134507	12.221727	12.187163	12.439175	12.245922	11.985255	12.876931	12.270578	11.739081	11.595942	12.294834	11.379300	12.570017	12.427314	11.490236	12.347744	10.871441	12.429308	11.860081	11.139340	12.230488	11.351898	12.270250	11.016776	11.615814	11.138761	12.132968	11.683357	11.765559	11.246940	12.357997	12.086801	11.789556	12.863311	12.987965	12.401619	12.098166	11.989265	11.594008	13.000090	12.522671	12.237890	11.464934	10.304626	11.984395	11.359717	11.697702	12.122102	12.063042	12.467788	13.118676	12.521236	13.331285	11.956809	12.758584	11.223716	12.418490	13.075147	12.979495	12.902330	12.714173	11.373780	12.606221	13.027092	12.832557	12.587968	13.102386	12.967314	12.292196	12.901272	12.653635	13.158966	12.897927	12.578859	12.919886	12.075117	11.617879	12.158335	11.810826	12.482634	12.991245	11.524738	12.237422	12.744026	12.292981	12.556996	12.511451	10.877054	12.198750	12.561872	12.535268	13.082545	12.539123	12.366220	12.486817	12.458956	11.778966	11.884935	12.404662	12.665111	11.992156	12.194289	12.807363	12.268991	12.351789	13.139308	11.744798	12.442693	12.076353	12.305898	12.233290	12.514492	11.748493	11.758300	13.091997	11.467339	13.006158	11.822299	12.500415	13.287852	10.694051	11.442637	12.739334	12.664618	11.411376	12.642737	12.307156	13.913198	11.209661	13.042069	12.071894	12.452958	11.553739	12.159708

SHANK2	11.932456	11.954526	11.767509	12.215771	12.425577	13.070763	12.298858	12.815796	13.526328	12.816972	13.042247	12.696624	12.112840	11.807225	11.268654	12.776712	12.746911	11.435314	12.280654	12.679736	11.329368	12.076353	11.280469	11.356381	12.487141	12.829828	13.407961	12.912866	13.035259	12.860767	12.940455	11.498901	11.466898	12.034420	11.698422	11.779259	11.944663	11.398216	11.866700	11.733781	12.966194	11.594299	11.904326	11.971923	11.641765	11.515780	13.228465	11.250930	11.704017	11.584703	11.393514	11.204194	11.345621	11.340687	11.275177	13.065305	11.147664	11.175962	11.130778	10.920323	11.038010	12.976952	10.870351	11.157291	10.963237	11.015291	11.436941	11.692880	11.400440	11.497575	12.093060	12.355628	13.098440	12.184989	12.187471	12.110262	11.997151	11.730489	11.989104	11.409205	11.349651	11.416927	10.900269	11.456523	11.500773	11.665066	11.561285	11.663282	11.016056	11.113613	10.852098	10.859380	11.557455	11.839782	11.912357	11.964779	11.425646	12.396312	12.201865	13.058112	12.539305	11.758591	12.457385	11.863798	11.757691	13.110956	12.398188	11.331478	11.731098	13.192203	11.051636	12.340875	11.935497	13.436646	12.928845	13.944041	11.455375	12.646754	13.681146	11.957851	12.320942	11.869641	12.430104	13.254784	12.913792	13.025099	13.301878	12.858461	13.190080	13.121579	12.921144	12.613710	11.803385	11.674592	13.065502	12.388886	12.011560	11.861688	12.366380	11.251658	12.057308	11.363862	12.166942	12.228465	12.197526	12.677497	13.006514	13.635217	12.741631	13.051634	11.923703	12.084324	12.347129	12.124642	12.226464	11.992461	11.599324	12.018571	12.069478	13.411878	12.156958	11.746557	12.053155	11.603568	11.622042	13.399078	11.615814	11.621743	11.340239	11.409809	11.322149	11.501545	11.161862	11.768414	12.579338	11.028357	11.290706	11.000620	11.047235	11.348194	12.998952	11.079395	11.752033	11.152318	11.067334	11.291432	11.816748	11.341907	11.422716	12.141427	12.445794	13.680191	11.988788	12.397685	12.375821	11.932304	11.956666	12.073577	11.347581	11.508193	11.359302	11.263347	11.656596	11.587606	12.294973	11.463192	11.456977	10.982470	11.099443	10.806201	10.856734	11.890041	11.930928	12.200621	12.420008	12.109069	12.802671	12.250568	11.813291	12.181736	12.386142	12.599699	11.741301	11.433898	13.143030	12.929922	11.240345	11.099868	13.448408	11.115175	12.175303	12.838440	13.585542	12.269467	12.657171	11.165378	12.805624	13.018633

PATJ	12.080437	12.361628	12.603082	12.141594	12.348689	12.775824	12.619822	12.655686	12.599341	12.585807	13.089999	12.609852	12.028551	12.686212	12.227205	12.411407	12.539964	12.218048	11.866384	12.717145	11.460712	11.499049	10.646484	11.972222	12.394906	12.422795	10.366240	11.836941	11.872045	11.851720	11.974947	11.953478	12.113742	12.065779	12.282255	12.768169	12.630986	12.419759	12.058559	12.240224	12.490403	12.151088	12.224164	12.391429	11.782962	12.467321	11.772998	11.534802	12.530023	12.514664	12.167248	12.619661	12.658981	12.551868	12.585328	12.427164	12.654649	12.224164	11.936861	11.933343	12.367368	12.393629	10.731478	12.326361	11.412105	12.036533	11.872045	12.042883	11.842797	12.248323	12.052224	12.703807	12.760647	12.531515	12.696982	12.217152	12.829989	12.260677	12.234346	12.436133	11.868608	12.713119	12.546681	12.602220	12.896514	12.416578	12.730311	12.981325	12.277746	12.015680	12.123620	12.791340	12.178468	12.602912	12.397379	12.596810	12.103782	12.516134	12.456606	12.486817	12.767266	12.232073	12.242069	12.131737	11.896778	12.355628	12.401128	12.518430	11.732727	12.515324	11.792259	12.681285	12.324793	12.727955	12.808222	12.163282	11.949288	12.684911	12.083083	12.362753	12.128873	11.964932	11.503490	11.866700	12.311740	12.138334	11.404241	11.614652	11.703267	11.843716	11.813001	11.570758	11.252949	11.730949	11.795586	11.821409	11.359155	11.154939	12.208987	11.159203	11.191935	10.377270	11.577492	11.776541	9.839846	11.091753	10.929185	11.464200	11.218631	11.273089	12.322054	11.410252	12.830827	11.514381	12.335719	12.020215	12.082750	11.862600	11.710741	11.550159	11.811860	10.805196	12.166049	11.239356	11.575095	11.125638	10.603105	12.234514	11.825487	11.874610	11.650646	11.990332	12.465654	11.937016	11.189611	12.040929	11.633734	11.408892	11.610339	12.066401	11.573431	11.359439	12.067502	11.746705	11.637244	11.544982	11.561147	11.539378	11.846125	11.920396	12.448961	11.888725	12.165444	11.985717	12.031230	11.980478	12.027045	12.372119	12.029621	11.262129	12.332876	12.648312	11.767815	12.573019	11.834369	11.987859	12.042106	12.357534	11.328775	12.256151	12.480123	11.414134	11.712047	11.685099	11.805894	11.727665	11.433004	11.840838	11.433444	11.216126	12.232669	11.679053	11.390120	10.569692	11.456523	11.845235	11.992298	11.123825	12.055881	11.248076	11.841912	11.886732	12.368752	12.358931	11.293819	11.887212	12.797127	11.587606

PDLIM3	10.031468	10.699257	10.302329	9.437418	9.443592	8.556557	10.068275	7.939959	8.535593	9.381310	9.136846	9.633365	9.401696	9.383472	10.188009	9.783445	10.161362	10.254371	9.545467	8.858058	9.346035	8.955928	13.454810	9.522255	11.759188	9.607630	11.180812	10.363720	10.967395	9.790950	9.425721	10.437805	10.054204	9.857725	10.115756	10.243674	10.204002	10.257870	10.641061	10.152488	9.941496	11.355638	10.632749	9.671636	12.126882	9.893328	10.958864	11.395788	10.276541	9.937420	9.912962	10.361904	10.416749	9.584839	10.690375	9.656426	10.791003	11.081888	10.726580	11.017339	11.327497	9.959832	13.897223	10.830760	10.267338	11.456110	10.195828	10.342797	9.725305	10.627143	10.143238	9.773557	9.944399	10.331462	9.432202	10.445210	9.950904	9.989046	9.095706	10.857188	10.285020	10.643266	10.058310	10.126478	10.774758	10.156965	9.596328	11.286728	11.382463	9.944473	11.242229	10.464170	10.266020	9.675852	9.239370	10.131679	10.645044	10.823244	9.363785	10.711413	9.395428	10.222882	10.136837	10.425147	9.654530	9.850742	9.642224	10.122012	10.667979	9.947650	10.871143	9.243051	8.809052	9.312325	8.873102	9.275130	10.276978	10.656820	10.341850	9.560491	9.593851	10.550397	8.870889	9.030278	7.839529	8.652056	9.170919	9.584999	8.573236	8.496581	8.201070	10.739148	9.052200	8.823574	10.536920	9.834429	9.290980	9.217179	9.582699	8.661006	10.529524	11.325694	8.529384	10.320901	9.205043	8.718121	7.928954	9.310802	8.565560	10.580067	9.098294	9.772221	9.081958	8.913738	9.085355	10.338850	9.519781	9.489187	11.496969	8.545657	10.275619	10.857034	8.545522	9.807001	9.043957	8.315284	10.183600	8.754570	9.910357	9.946536	9.774181	9.139811	9.929378	11.181871	9.951687	10.218469	10.206741	9.733183	9.582540	10.432626	11.456523	9.071978	8.849097	8.898099	10.428065	9.098445	9.518530	9.802581	9.655028	9.506715	9.724538	7.911881	9.786686	10.350819	9.192429	10.606972	9.867261	9.390797	11.715575	9.402320	8.435395	9.486874	9.492544	10.255569	9.549005	9.519313	9.618091	11.664472	11.561717	10.462035	9.641321	9.972631	9.900225	9.141158	9.806528	9.361712	11.281969	9.024321	9.235785	9.337859	8.266671	10.043161	11.121750	10.215486	8.975406	9.038327	8.478374	9.560158	9.142589	11.915487	10.295232	8.215914	8.421040	8.599881	7.453233	11.031948	9.189473	7.880939

SH2D7	5.433948	3.445983	2.962105	3.233940	4.384959	4.208850	3.627841	3.522634	4.271401	3.631286	4.084103	3.791187	3.221235	3.971571	3.588616	3.174752	3.422966	5.187401	5.260230	3.216375	6.355577	4.402007	5.624075	3.175025	3.255752	2.940574	3.326154	3.650223	3.098313	3.546167	3.805556	3.937294	2.942671	3.073107	3.472373	3.999159	3.718738	3.369959	3.331586	3.053955	3.409194	3.538456	3.501981	3.161949	3.438428	5.261653	3.603661	4.336124	5.584380	3.914773	3.963170	5.222922	4.151259	3.825737	3.570423	2.998161	3.796319	3.530135	3.732517	3.585340	3.555420	3.206112	3.381371	3.019669	3.550424	3.208207	2.853988	3.295298	3.465394	3.099621	2.933330	2.824801	4.273115	2.977791	2.989405	3.178423	2.944259	3.089903	2.938528	3.349874	3.179899	3.773677	3.089007	4.098630	3.177227	5.362552	2.962547	3.209791	2.990263	3.202402	3.150513	3.076788	2.896251	2.972247	5.234417	2.957515	5.580041	3.242655	4.501617	3.032050	3.373442	3.187677	3.199539	2.916007	2.991831	3.949435	3.086827	3.341315	3.878927	3.210733	3.259859	3.712529	3.031650	3.223899	3.202823	4.605418	6.323541	2.992814	3.162277	2.858664	3.203211	3.276674	3.000556	5.131015	5.049157	4.010802	3.526294	4.722166	3.163529	3.797572	3.933031	3.312876	3.656029	3.334035	3.111995	3.418603	4.995208	4.065635	3.353835	6.238948	3.915302	5.786822	3.632310	3.764711	3.527760	2.956909	3.998746	3.092443	3.530906	3.462151	3.341822	2.973908	3.095565	3.371366	3.711741	3.757923	3.358117	3.223347	3.013169	3.616696	3.453144	3.885512	3.193531	3.673696	4.947539	3.722298	4.432464	6.362694	4.066279	4.029509	4.451610	3.613588	3.810335	3.210733	3.766740	3.441704	3.576932	3.643095	3.615725	3.236957	3.826564	3.334035	3.142064	3.183197	3.203211	2.858140	3.607261	3.366336	3.224468	2.956356	2.913243	4.549553	2.967466	2.923401	3.149886	2.951744	3.089357	2.988170	3.468909	3.478413	3.966177	3.156556	3.593240	3.441000	4.961642	3.475806	3.317974	2.951397	3.215955	3.332425	3.048272	2.979165	2.997691	4.795822	2.971520	3.976335	3.900421	3.495973	3.196264	3.261885	3.223626	2.981438	2.844634	3.032233	4.606485	3.136611	3.288191	3.469807	4.038891	4.775532	3.448178	4.634571	3.739136	4.870826	4.441288	4.323376	2.967652	3.230901

TRIM29	10.330253	15.416204	15.599209	15.406237	15.541137	15.625168	15.994548	15.815434	15.101143	15.638552	15.387598	15.606629	15.417796	15.761752	15.593684	15.256065	15.287379	15.309448	15.363249	16.092758	15.374032	15.260285	13.169593	14.985629	14.847864	15.706649	13.016439	14.898046	14.819862	15.156386	15.117212	16.073627	15.512636	14.428245	15.424214	15.243311	15.528894	15.190029	15.543645	15.625168	15.469767	15.236952	15.888240	15.691311	15.095711	15.558628	14.831507	15.153250	15.628044	15.748884	15.023469	15.799508	16.225319	16.416212	16.221987	16.127121	16.478815	15.177280	15.490401	15.661481	15.138365	15.436421	10.588511	15.217824	14.784337	15.198897	15.032181	15.573696	15.406237	15.599209	15.973135	15.786742	15.009362	16.051180	15.724946	15.840619	15.942235	15.608551	14.948503	16.258072	16.008000	16.137062	14.586497	16.223639	16.201225	15.649863	15.918070	16.271502	15.212349	15.724946	15.200291	14.879649	15.901055	16.112915	14.368341	15.942235	15.843040	15.283711	15.671141	15.475629	15.700644	15.729053	15.414655	15.315239	15.461686	15.317503	15.458388	15.340435	15.492990	15.562229	15.081140	15.934855	15.686109	14.926494	16.013271	14.779447	11.743482	15.389194	14.828455	13.724916	13.828188	14.084344	14.137614	14.263241	14.597809	14.245947	13.307488	13.954061	13.988980	14.169058	14.101917	13.570563	12.178309	12.111007	14.748776	14.363735	13.675754	12.420911	13.217148	14.012342	14.228894	14.147923	14.268626	13.752310	9.468250	14.922772	14.440880	13.620154	13.876318	13.552300	13.976074	14.183722	14.558412	14.628630	14.938328	14.631695	14.127386	14.011503	13.847752	13.859137	13.469871	13.839284	14.594501	15.286692	14.554379	14.129209	11.957393	15.042927	14.478648	14.915674	14.528293	14.417635	13.647299	14.247611	13.528336	13.012808	14.100719	14.092188	13.316425	13.773464	14.413234	14.350592	14.201495	13.664000	14.531879	13.195507	14.174222	13.321286	12.017489	14.370460	15.071019	14.147348	13.762711	14.335070	14.154223	14.223479	14.088015	14.944787	13.248301	14.582286	14.101917	15.212349	14.248223	13.424093	14.897610	14.431588	14.317493	13.238812	13.080428	12.242693	14.195248	13.795660	13.390157	15.198214	13.468361	13.964639	13.423875	13.867009	14.140212	13.000629	14.168428	13.731668	13.146687	12.158629	14.326082	14.009820	14.637025	14.524343	13.684300	13.491711	13.412516	10.807688	14.886609	13.914755	15.005364	13.995561	15.196878	14.528702

ZNF506	9.874083	9.295221	9.265650	9.117278	9.187084	9.289571	9.452450	8.455195	9.144456	9.073438	9.169228	9.073896	9.207491	8.925972	9.675373	9.287859	8.886372	9.333538	9.778460	9.445444	9.341170	9.595374	10.445678	9.698934	9.614989	9.364092	9.510060	8.597021	8.958743	9.309408	8.672339	9.375451	9.938052	9.660142	9.981518	9.820554	9.817863	9.925756	10.186829	10.289908	8.699378	10.512586	10.293860	9.809677	10.145498	9.799359	8.432106	9.759952	9.474607	9.809845	9.743643	9.953197	9.544712	9.834281	9.531817	8.654155	9.981518	10.241044	9.712489	10.034063	10.407984	9.166801	10.677766	9.793310	10.006166	10.068275	9.759333	9.541580	9.238102	9.966054	9.474903	9.626458	8.793066	9.551777	9.423772	9.629238	9.505342	9.738061	9.565725	9.756057	9.829507	9.515307	9.759952	9.698789	9.752686	10.022905	9.707187	9.725305	10.292343	10.033608	10.027195	10.100633	10.255126	9.563148	9.925892	10.112665	9.818886	9.633215	9.078706	9.536570	9.443123	9.683738	9.137787	9.484419	10.427336	8.578625	9.564183	10.138059	10.332520	8.860469	10.383504	9.774181	9.887831	8.769925	8.622673	6.937460	10.260726	9.069888	8.892214	10.874430	9.492544	9.348642	9.242889	8.257856	8.727096	8.229190	9.296921	7.659976	8.362192	9.252538	9.157407	8.525523	8.393345	8.616164	9.371195	8.856378	9.365340	9.989367	9.274698	8.721757	8.868076	9.392967	9.116516	9.456955	8.125564	8.641137	8.710395	8.507514	8.317753	8.519771	9.696489	9.549294	9.674273	9.894559	9.844876	9.953492	9.754059	9.806528	9.886715	8.730911	9.971512	10.302784	10.296016	9.568254	8.847874	8.297469	9.265061	9.160629	9.250083	9.073136	9.967099	8.850610	10.105829	9.662174	8.800503	9.345894	8.988657	9.220517	9.919156	9.848007	9.123078	11.178995	9.457860	9.529591	10.602257	8.745107	9.541895	9.353931	10.086994	9.499181	9.394200	8.552439	9.443912	9.422051	9.057198	9.351997	9.098890	9.810719	11.086626	9.807593	9.272705	10.270875	9.873016	9.332164	9.984732	8.857274	9.986838	10.605791	9.741831	10.513902	10.554299	10.635038	9.723772	9.739786	9.773557	9.190561	9.351090	9.782680	9.241660	9.186105	9.365980	9.703107	9.349567	9.936797	8.789321	9.312647	9.129842	8.848031	8.272535	9.958130	9.249360	9.663115	8.731651	9.079331	6.793483	9.457860	8.891903	8.778562

SPDYE17	11.911322	12.108644	12.135906	12.463562	12.690337	12.400969	11.817639	11.964028	13.063805	12.353569	12.755993	12.364182	12.548754	12.486329	12.245455	12.148094	12.772931	12.171247	12.626503	12.589445	12.412540	12.518430	12.744389	12.105127	12.820415	12.735923	13.614204	12.487990	12.484713	12.286918	13.247713	11.929992	12.180812	12.676682	12.116974	11.961457	11.959745	11.876433	12.705000	12.465490	12.372913	12.499786	12.321255	12.131436	12.214434	12.277746	12.810174	11.931845	12.171102	12.134671	11.929392	12.057308	11.896631	12.247046	11.999926	12.505236	12.100426	12.344088	12.304346	12.420088	12.186842	12.486157	12.842437	12.556219	12.646567	12.086064	12.427016	12.320942	12.327331	12.640723	12.203451	12.262160	12.718533	12.029310	12.369200	12.356573	12.012961	11.841289	11.764040	11.740264	12.447724	12.395993	12.041521	12.119577	12.353897	11.997933	12.278351	12.352896	12.674301	12.480968	12.419436	12.153299	12.146584	12.156789	12.151088	12.701417	11.888571	12.800035	12.614041	12.956616	12.379731	11.968715	12.723135	12.349580	12.642737	12.497024	12.929753	12.913978	12.723135	12.763272	12.547624	13.183285	12.691020	12.819748	12.652596	12.977133	12.539475	12.254686	13.394129	12.793186	12.259261	12.452025	13.306600	13.601268	12.993251	13.119075	14.755534	13.798010	13.354386	12.783797	13.727121	13.932970	12.150335	12.595766	13.807309	13.165501	13.891542	14.036663	13.164898	12.736959	13.469871	13.555677	13.645878	12.718016	12.876249	12.448476	13.260449	13.644259	12.620642	13.838218	12.935833	13.254784	11.881927	12.584309	12.777426	12.259736	12.299354	13.344050	13.335408	13.692656	13.608723	13.090537	12.798847	12.479954	12.093684	13.254395	11.804981	12.759782	12.031682	12.327640	13.134732	12.764791	12.057775	12.696084	13.399078	12.533129	12.874855	12.903931	12.592167	12.905355	13.530568	13.028809	12.934196	12.784513	12.626503	13.257008	12.310934	12.515826	13.162623	13.178186	13.066209	13.216008	12.442202	12.817505	12.686046	12.140678	13.286686	12.415626	12.279300	12.811445	12.888611	12.662414	12.609346	12.383214	13.638949	12.099378	12.940276	12.740227	12.226923	12.451389	13.215415	13.297753	11.969922	12.322207	12.820415	12.964739	13.713891	12.318233	12.887748	12.618117	13.326420	13.249121	12.626987	12.577039	13.420393	13.580155	13.040220	12.910540	12.873402	12.914980	12.854570	12.549566	13.240382	12.533788	12.546069	13.446264	13.268822	12.949809

SPDYE11	11.911322	12.108644	12.135906	12.463562	12.690337	12.400969	11.817639	11.964028	13.063805	12.353569	12.755993	12.364182	12.548754	12.486329	12.245455	12.148094	12.772931	12.171247	12.626503	12.589445	12.412540	12.518430	12.744389	12.105127	12.820415	12.735923	13.614204	12.487990	12.484713	12.286918	13.247713	11.929992	12.180812	12.676682	12.116974	11.961457	11.959745	11.876433	12.705000	12.465490	12.372913	12.499786	12.321255	12.131436	12.214434	12.277746	12.810174	11.931845	12.171102	12.134671	11.929392	12.057308	11.896631	12.247046	11.999926	12.505236	12.100426	12.344088	12.304346	12.420088	12.186842	12.486157	12.842437	12.556219	12.646567	12.086064	12.427016	12.320942	12.327331	12.640723	12.203451	12.262160	12.718533	12.029310	12.369200	12.356573	12.012961	11.841289	11.764040	11.740264	12.447724	12.395993	12.041521	12.119577	12.353897	11.997933	12.278351	12.352896	12.674301	12.480968	12.419436	12.153299	12.146584	12.156789	12.151088	12.701417	11.888571	12.800035	12.614041	12.956616	12.379731	11.968715	12.723135	12.349580	12.642737	12.497024	12.929753	12.913978	12.723135	12.763272	12.547624	13.183285	12.691020	12.819748	12.652596	12.977133	12.539475	12.254686	13.394129	12.793186	12.259261	12.452025	13.306600	13.601268	12.993251	13.119075	14.755534	13.798010	13.354386	12.783797	13.727121	13.932970	12.150335	12.595766	13.807309	13.165501	13.891542	14.036663	13.164898	12.736959	13.469871	13.555677	13.645878	12.718016	12.876249	12.448476	13.260449	13.644259	12.620642	13.838218	12.935833	13.254784	11.881927	12.584309	12.777426	12.259736	12.299354	13.344050	13.335408	13.692656	13.608723	13.090537	12.798847	12.479954	12.093684	13.254395	11.804981	12.759782	12.031682	12.327640	13.134732	12.764791	12.057775	12.696084	13.399078	12.533129	12.874855	12.903931	12.592167	12.905355	13.530568	13.028809	12.934196	12.784513	12.626503	13.257008	12.310934	12.515826	13.162623	13.178186	13.066209	13.216008	12.442202	12.817505	12.686046	12.140678	13.286686	12.415626	12.279300	12.811445	12.888611	12.662414	12.609346	12.383214	13.638949	12.099378	12.940276	12.740227	12.226923	12.451389	13.215415	13.297753	11.969922	12.322207	12.820415	12.964739	13.713891	12.318233	12.887748	12.618117	13.326420	13.249121	12.626987	12.577039	13.420393	13.580155	13.040220	12.910540	12.873402	12.914980	12.854570	12.549566	13.240382	12.533788	12.546069	13.446264	13.268822	12.949809

ASTN1	8.633073	4.792890	4.850577	6.195129	3.463740	5.344585	5.418942	4.417292	3.714025	3.331917	3.494647	5.316472	3.768431	4.296330	7.161578	4.939531	3.361975	3.720796	5.459435	6.259130	4.526110	5.690430	8.147603	4.955407	3.088772	3.568681	6.008773	4.676470	4.411994	6.147242	3.635857	8.086591	5.591000	7.404121	6.213374	4.835195	7.093995	3.886551	4.553238	4.330333	3.374551	6.861702	5.893884	7.304922	9.449040	6.491699	8.593939	6.120458	6.349235	6.842863	7.758989	7.994104	7.197753	7.358559	6.312471	3.997237	7.524675	5.415835	4.231803	6.190268	5.376740	6.691111	8.659609	5.050781	6.370223	4.856270	5.304215	4.892508	4.347401	5.914578	6.496679	6.542210	3.795069	6.333155	6.137363	4.902368	5.727541	6.004648	6.866463	5.939883	5.088990	6.791839	7.532225	6.970730	6.215952	6.628714	6.800111	4.084550	5.621849	5.809052	5.599867	3.117138	4.816230	4.629125	7.135949	5.187008	7.475689	5.584774	4.107435	4.082186	6.046918	5.050976	4.383610	4.024123	3.293467	4.302633	2.986472	4.073227	4.519116	3.420660	6.058191	3.894213	4.032747	5.368387	3.086361	7.838778	6.225380	4.743570	3.316628	4.179211	3.196384	6.040557	3.585700	4.090469	3.289973	6.034026	4.876507	3.182569	3.707874	3.593015	4.335916	4.608664	4.612282	4.667764	8.448133	6.692115	3.704471	7.006419	6.102725	3.937294	5.455513	7.608208	3.400057	4.225572	4.057579	5.859241	3.913993	5.738086	3.544884	3.871134	4.645564	4.683975	6.217833	7.116576	4.568743	5.221107	3.125639	3.043133	5.491318	3.591699	3.808277	4.751901	3.709665	5.960034	5.423011	3.410757	9.750053	5.711575	4.594913	4.515053	5.079600	5.480262	5.150938	7.540366	7.225900	10.702431	3.256882	5.177346	4.303986	3.381552	9.013164	4.631333	3.268548	3.328072	4.130630	2.901076	3.612379	3.312397	4.834971	4.194339	3.635857	3.052568	5.023856	5.904025	3.912495	6.526001	5.571259	3.569686	5.526489	3.454010	4.804508	4.464593	4.376947	4.981929	6.322725	5.283372	5.526489	8.148380	4.715187	8.317305	3.146189	4.753194	3.073566	5.427255	3.994643	5.210021	6.679815	3.348550	6.542050	5.887621	8.168936	7.522372	5.162860	9.957686	3.447280	3.009843	3.238168	5.294623	3.318795	7.827120	6.244622	7.289104	4.096648	3.803896	4.668831	5.680842	3.662430	3.201577

DACH1	9.879782	8.963871	9.492933	8.261233	5.512606	6.277687	9.009654	3.733827	3.325633	7.394285	6.171336	6.206085	7.755152	6.099449	8.717976	6.509917	4.595957	7.225595	6.463552	7.194566	6.537617	6.122011	7.336280	5.955394	7.166993	5.593652	7.139220	6.397635	7.240393	9.188455	5.065508	10.271317	9.826469	8.437250	10.242742	9.665912	9.558784	8.687887	9.118025	9.107332	7.506934	9.936352	8.644401	9.480244	9.364876	9.532294	5.925429	9.754226	8.314845	9.522887	9.646619	9.424518	9.878883	9.624312	8.823574	6.259647	9.877686	9.656037	7.494271	8.885766	9.535194	9.805321	10.905852	7.123846	9.908813	9.093137	9.138253	8.675628	8.573520	8.837687	9.221577	8.665616	6.718806	9.273303	8.907026	9.496124	8.867627	9.508842	8.947993	9.246741	9.033855	9.732123	9.493002	9.593851	9.221888	8.859114	9.318827	9.722952	9.901001	9.010681	9.060211	10.273930	8.937007	9.058786	9.664360	8.834192	9.339791	8.396872	6.610455	7.001459	6.716702	8.590817	7.582883	8.073029	7.012038	7.583190	5.308084	5.534285	8.086004	6.929686	10.307427	6.840632	3.112756	5.736449	5.925083	4.599773	9.191817	7.168868	6.224688	8.413538	8.196392	6.579946	7.090251	4.017593	5.174952	6.524815	10.297710	3.351900	7.753171	5.308477	9.955696	6.698541	4.242508	3.910122	9.166801	3.973522	9.508697	8.074267	5.792732	5.478242	8.129662	10.937605	7.415371	5.465020	6.477812	5.402259	3.881506	5.567109	8.487865	6.101888	7.838627	8.611061	7.290668	9.656579	10.605498	8.666821	7.449365	5.747563	8.593939	5.451468	10.942718	7.100946	7.349599	8.312218	7.954640	3.467086	11.360602	9.561904	8.840278	8.564511	8.676199	9.022037	8.089070	8.723582	10.474973	10.936234	8.582113	6.909858	9.742900	8.205533	8.085388	8.347801	7.653310	7.161880	8.416275	6.899004	8.051705	6.982874	7.929108	9.378088	9.124740	3.266021	7.760761	8.000883	9.438350	7.822571	8.397169	9.075141	8.278420	7.458563	8.929545	7.869548	9.458185	7.985002	6.005942	8.433222	7.319649	8.157804	8.438720	8.743748	6.919359	8.249440	8.661774	8.756688	7.507849	6.667203	3.970970	7.272024	10.855861	5.633193	10.362833	3.933902	7.832150	5.918859	6.329072	3.359328	7.444598	9.495345	4.628705	8.442390	5.432219	7.257106	3.515313	10.810536	8.869457	7.163769	5.396732	5.273296

CRLS1	14.197518	12.163756	12.676354	12.983527	12.955541	12.401457	12.819748	12.026746	11.910004	12.332876	12.563831	12.491562	12.733547	11.907107	11.900004	12.434966	12.221727	12.148544	11.578338	12.340685	12.172634	11.086782	11.077953	11.838207	11.157147	12.616315	12.494332	11.672678	12.454375	12.529678	11.911924	12.774141	12.701221	12.973483	12.705699	12.552494	12.629517	12.267304	11.723465	11.706874	12.514995	11.579215	10.982910	12.531515	12.433717	12.489908	12.323101	11.662670	12.539123	12.425254	12.797845	12.262315	12.723489	12.681457	12.502885	12.825230	12.203451	11.199882	12.145451	11.945155	11.661349	12.298219	11.860703	11.348194	11.976069	11.961308	11.506569	11.627501	12.054832	11.514222	12.515666	12.582441	12.215055	12.621342	12.500751	12.316075	12.157396	12.680252	12.557657	11.443675	12.446937	12.679547	13.019003	13.092197	12.739334	12.721110	12.962142	12.199062	11.880705	12.225678	11.786118	11.596548	12.626671	12.378353	12.968566	12.726341	12.557332	11.928470	12.785766	11.742737	12.255202	11.715283	12.144058	11.989871	11.454294	12.449933	11.795267	11.892007	11.645041	12.316396	11.678914	12.311900	11.990484	12.161407	11.866837	11.965245	13.224119	12.775483	11.944663	13.133580	13.056178	12.992703	13.369328	12.994008	13.042247	12.937441	13.155145	13.472510	13.549579	12.881502	13.417523	13.369945	12.395853	12.507872	12.792841	13.399963	12.634942	12.715054	13.824511	12.214094	12.219378	11.918508	13.456599	13.446470	13.299219	13.490594	12.554292	12.902705	12.674134	11.965086	12.849137	12.847005	12.793758	12.174197	13.011244	12.949458	12.324058	12.540801	11.820950	13.407961	11.703267	12.096818	13.356786	12.680949	13.087953	12.982957	11.487873	12.786841	12.744741	13.009749	13.795380	12.941572	13.422986	13.269040	13.618777	14.018727	12.307454	12.553137	12.445640	11.305887	13.462926	11.365974	11.686785	12.121934	11.892609	11.807078	12.290138	12.060393	11.928150	12.905001	12.978042	12.739892	12.633466	12.920784	13.199727	13.533281	13.455025	13.094296	12.105127	12.668989	13.229238	13.547331	13.064161	13.730157	13.411210	13.212614	14.557653	11.916369	12.503725	12.268074	12.517769	12.182961	13.376481	13.410314	13.163401	13.172295	12.842271	12.236813	12.886000	13.813000	12.626987	12.509651	12.179561	12.431923	12.907332	12.638003	12.701928	12.228152	12.155866	12.266375	12.751886	14.133559	12.181736	13.650755	13.249693	12.227205	12.942109	13.000629

PLEKHA7	12.770158	13.135455	13.707475	13.183897	13.876596	13.549134	13.081778	13.818348	13.684785	13.764461	13.366449	12.744556	12.695415	13.686701	12.985738	13.424902	12.971662	13.240605	13.132093	13.517555	13.038580	13.065843	11.713188	13.603336	13.394547	12.969695	10.249259	13.311729	12.342018	12.106371	12.885289	13.456831	13.489038	10.832721	12.493861	13.522292	13.671966	13.082545	12.865491	13.987492	13.197536	13.075520	12.507212	13.434297	12.617631	13.464641	12.658313	12.489908	12.989626	13.267608	13.059334	12.969511	13.516235	13.230859	13.014469	13.111379	13.572169	13.662003	13.084939	13.222947	13.415659	12.969318	9.762791	13.132270	11.989496	12.926114	13.200848	13.246703	13.081015	13.028594	12.903569	13.619482	13.199727	13.351397	13.431930	13.264151	14.191138	13.204369	13.334132	12.727782	13.924035	12.894925	13.186438	13.597474	13.710171	13.397551	13.463591	13.664989	12.756697	13.038959	12.584979	13.146323	13.616612	13.666468	12.682992	13.424487	13.426884	13.453292	13.186438	13.240179	13.632998	12.899372	13.002332	13.220952	13.459822	13.202291	14.236540	13.003777	13.227432	13.621755	13.403761	13.760666	13.643272	13.411210	13.533066	12.688794	12.598484	13.272567	12.896890	12.374817	9.030423	11.397159	11.737771	9.985035	11.663120	10.531426	10.517413	9.905601	10.826070	10.077590	10.674499	9.025080	10.396893	7.758989	11.905478	9.592908	11.726750	10.468439	10.611691	11.388349	9.252981	11.097929	10.704907	10.020041	8.027961	11.325990	11.674007	8.522454	10.170916	9.038502	10.268837	10.446116	10.692703	10.258038	10.501072	11.108070	11.534802	10.628223	11.434496	10.388663	11.505395	10.254371	11.710136	11.338052	11.392179	10.646637	9.872877	11.899786	10.175931	9.258344	10.587449	10.539877	8.529985	10.440195	9.301890	12.534620	12.065467	11.553739	10.913714	10.649536	9.761826	9.277385	10.958864	10.268218	11.559235	10.734602	11.071222	9.567958	9.838908	11.167543	11.435658	10.208000	9.986994	10.616435	11.363426	9.276523	9.853381	11.846272	9.728056	10.933223	10.935020	9.172491	10.442006	11.295400	10.660719	11.224321	10.322892	11.096922	9.573039	10.947309	10.281995	10.765971	9.051294	9.442348	10.664886	11.335682	9.768599	10.034221	10.438091	8.204777	9.864082	10.812838	10.894029	8.630532	10.417196	11.221025	12.176546	11.486562	11.283846	11.268947	8.764379	12.084016	12.994351	10.210936	11.736567	11.998560	11.552884	7.702215

TUBB2A	11.349956	13.643272	13.455950	13.036157	12.197683	12.564954	13.101809	13.521153	12.197370	12.391894	12.851402	12.819943	12.685861	12.308719	13.121958	12.886514	13.064903	12.816481	13.353964	13.127862	12.354682	12.532342	11.931556	12.870847	12.281273	12.805120	11.614961	12.738673	12.516623	12.966913	12.845970	12.913034	12.619480	12.338106	13.165092	12.212996	12.960320	13.227647	13.765490	12.869416	12.520044	12.682817	12.448632	13.437764	13.004678	13.495548	12.342322	13.708445	13.689979	13.602656	12.666334	13.063805	12.676204	13.229238	14.128314	13.707475	12.364339	12.947352	12.665471	12.264825	12.938700	12.299854	12.698701	12.615346	13.289437	14.049746	12.779681	13.146687	12.605717	12.720947	13.792498	13.182111	12.291548	13.079487	12.932755	12.407767	12.130364	12.844209	12.929387	12.508996	13.007579	13.620154	12.922594	13.224721	13.179151	13.214797	13.567342	13.180932	12.611680	12.952737	12.718866	12.745380	12.399784	12.188852	12.403727	12.169864	13.650755	12.303753	12.833844	12.684030	13.298825	13.062098	12.640385	12.855334	13.750320	12.525969	12.594761	13.967420	13.342212	12.905355	12.565935	12.734213	12.757036	12.432907	13.652950	11.692608	10.797174	13.520479	11.771231	11.345331	14.130795	13.042766	12.031230	12.157886	12.826480	12.475857	12.342322	11.717496	12.235309	12.556996	12.498799	12.118005	12.010792	12.837925	11.914124	12.447100	11.983954	12.442059	12.160336	11.840239	11.696519	11.813291	12.288938	12.366548	12.249758	13.468361	12.683325	11.482684	12.517283	11.737167	13.254395	12.417049	13.785359	13.323361	12.798678	13.609930	12.964368	13.549134	12.891724	11.982583	12.980298	12.238197	14.058288	13.446919	13.805428	13.149739	13.910543	13.523456	13.889925	13.311346	12.779517	12.240711	13.530801	13.121772	12.262315	11.762074	12.907135	12.461067	12.690828	14.036135	11.823838	12.524622	13.216968	12.727604	13.161096	13.750540	14.000102	12.989794	12.518735	13.208899	13.342212	12.956055	13.600599	12.018734	12.820259	12.537757	12.843523	13.489501	11.959150	13.026028	13.371134	14.886035	12.609852	13.110410	12.633137	13.905564	12.613560	12.638003	12.823859	15.007098	12.821251	12.563325	12.774962	13.954619	13.058018	12.818008	11.627636	13.489728	12.719723	12.469727	12.290442	12.014628	12.550058	12.760481	12.637159	10.940377	12.371808	12.508519	12.914641	11.954230	12.409678	11.737167	12.177372	13.810879	12.458016	12.230037	12.255020	12.815796

NBPF9	7.933723	6.666053	7.284881	7.816357	7.917975	8.513701	7.312275	8.228867	9.035428	7.233383	7.690559	8.088467	8.174583	7.250566	7.404747	7.685180	8.900227	6.714097	7.945557	8.041562	6.780884	7.901642	8.166936	7.468305	7.225595	8.326656	8.101596	6.700141	7.161102	7.842303	8.003733	7.900697	7.372973	8.441329	8.298501	8.194144	8.114560	7.674467	6.926148	7.392419	7.307573	7.710341	7.605001	8.238226	8.409551	8.950569	7.784873	7.107099	8.569098	7.981300	8.208900	8.041233	8.069369	8.098858	8.518715	7.394285	8.296861	7.363410	7.102676	7.077031	7.189713	7.605625	7.948757	7.538600	7.081450	7.568100	6.713749	7.087059	6.934574	7.416992	7.419464	7.810799	7.209572	7.664213	7.773530	7.498515	7.915975	7.856208	7.695615	7.327546	7.550974	8.018445	8.086448	7.929278	7.768978	8.545378	8.833772	8.285931	6.982081	6.211683	6.972602	6.965325	6.667380	7.717365	8.190071	8.382372	8.611378	9.127188	8.412020	8.900970	9.462264	7.606999	7.979109	7.734091	8.273458	8.807046	7.454005	8.188620	9.030128	7.510460	9.004704	7.758241	7.854534	7.424306	9.310505	11.602116	8.027463	8.276415	8.005443	7.772629	7.619230	8.237768	8.109085	9.340883	8.329054	8.220099	9.590429	8.536401	7.972915	8.136323	8.837234	8.567616	7.933723	8.489226	8.789321	8.929707	7.750282	8.582980	9.281202	8.058996	8.970198	7.974469	8.815433	8.547074	8.557010	9.030556	8.362692	8.468400	7.453550	9.090713	9.522409	9.034328	9.035284	8.970362	9.000251	8.686866	7.801900	7.806972	8.137100	8.526901	8.177095	9.726365	8.681750	8.875412	8.995047	8.367433	8.294418	8.488758	8.522913	8.847080	8.512159	9.056578	9.317184	8.935611	7.816519	9.110639	8.472968	8.056123	7.670378	7.997387	7.223889	8.257528	7.513425	8.191967	7.639530	7.811572	7.106590	7.870743	7.396310	7.838061	8.051400	7.974770	8.803515	8.581959	7.997816	8.621889	8.425886	8.881755	8.121961	7.987719	8.584839	8.947364	9.041181	9.015016	9.330000	8.749699	9.522409	8.287195	7.474299	9.233946	8.077423	7.622388	8.732389	8.403138	8.954553	9.799812	9.997954	9.191505	9.325829	9.633683	8.773664	8.720391	8.404218	9.059909	8.463077	9.316100	8.172072	8.065503	7.374716	9.224202	9.029501	9.296921	7.635736	8.806820	9.073598	7.780146	8.826430	8.370828

SRRD	10.595429	8.520064	10.265297	10.237708	9.912200	10.445039	10.747853	10.180413	7.949691	10.369851	10.276070	10.298003	10.783168	8.712212	8.168776	10.012765	9.941340	8.636014	8.941854	10.910296	7.680701	10.176239	8.606818	7.761283	7.114246	9.638433	8.966798	8.859884	9.599094	10.491566	8.974964	11.270275	10.051862	10.863944	10.623218	10.547030	10.762650	9.470923	8.792916	8.407880	10.072068	8.512310	9.147855	10.593040	10.486716	10.806353	9.277081	8.767930	10.717011	10.841725	11.064514	10.834345	10.980998	11.310881	10.947735	10.893456	11.095294	8.742540	8.576623	9.166351	10.033912	9.818140	8.802752	8.696676	9.481968	9.385126	8.536093	8.550896	8.789174	8.887749	10.688128	10.691661	9.646463	10.589350	10.506163	10.915990	11.044877	11.426951	10.942718	9.300486	9.505499	10.895178	11.494211	11.374203	10.975234	10.910730	11.520699	11.126783	8.849250	8.616784	8.498063	8.904141	8.844016	10.746795	10.759828	10.690535	10.750069	9.600803	10.879299	8.924017	9.761826	9.185181	9.022327	9.058786	10.190858	9.700030	8.534236	9.432816	7.747797	9.751904	8.655658	7.648612	8.800503	9.145331	10.629794	9.098736	8.390673	10.725060	8.999495	10.413149	9.665009	10.579788	10.277457	8.862310	11.047654	10.305204	8.978787	10.007378	10.695437	11.059685	10.601825	10.634437	8.956529	8.736423	9.667179	10.558269	8.878793	7.696224	10.578592	8.728842	9.229700	9.677830	8.158381	9.209780	11.396845	11.547983	10.552353	8.780870	11.236126	9.964578	10.538517	10.170916	11.208214	11.504078	11.426795	11.644595	9.809845	9.778317	8.888686	10.447955	9.636548	9.486874	11.157147	11.211573	11.745676	10.423303	9.082741	11.865455	12.423579	12.073426	11.124285	11.681278	12.584979	11.433444	10.791597	11.602692	8.759174	9.310045	9.946536	10.307568	10.502419	9.430349	9.564507	8.935159	9.350468	9.526785	9.495630	9.639042	9.782073	11.323214	11.217158	9.953197	11.841289	11.249094	11.650646	11.280469	11.010640	11.746705	9.761038	9.970187	10.936085	11.505545	11.004546	12.222786	10.941674	12.109069	12.742511	9.081808	8.725272	9.359687	9.843038	9.024766	11.929674	11.567130	11.112153	11.206137	9.088211	10.799193	9.985804	12.174040	9.216247	9.389608	9.749266	9.018966	9.638103	8.225089	9.070964	8.672782	10.030393	8.883274	8.846939	8.194802	9.102118	10.981465	11.105158	10.430485	10.982623	11.038010

AC058822.1	6.932722	7.470168	7.362150	7.568265	7.512190	7.286449	7.171477	7.821338	8.404836	7.212644	6.934574	7.457297	6.441376	6.789423	6.857697	7.401008	7.949357	6.823074	7.638162	7.155766	7.200694	8.010797	8.775659	6.840159	6.886288	7.080037	7.494731	7.755863	7.356249	7.316850	7.139220	7.707128	7.343817	7.030503	6.874168	7.146614	7.171971	7.052716	7.165350	7.956511	6.891740	7.293658	7.294755	7.641687	6.767312	7.231189	7.631767	7.648004	7.787607	7.053011	7.147559	7.390858	6.863914	7.318215	7.355649	7.504008	6.967877	6.925012	7.342853	6.817315	7.278325	6.997719	7.179231	7.515075	7.742097	8.025893	7.372973	7.534064	7.540513	8.297149	7.968631	7.351296	6.872117	7.691812	7.365150	7.195034	7.698370	7.611602	7.754107	6.871946	6.441853	6.571007	6.563011	7.105647	7.235310	7.324386	7.024588	7.135167	7.408367	7.643053	7.449040	7.980345	7.471713	7.474601	7.097508	7.238138	7.049913	7.272673	7.779666	6.964378	7.432187	7.328515	7.520351	7.198684	7.597003	7.696523	7.311340	7.411794	8.091722	7.499773	8.555423	7.338470	7.670842	7.068063	7.412291	8.117460	6.706548	7.015993	7.463901	7.303657	7.355513	7.312275	7.452599	8.012452	8.249893	7.376736	7.230254	7.799277	7.761745	7.602495	7.430775	7.447592	7.250881	6.913084	7.023670	7.356705	6.194067	7.813738	7.472343	8.086004	8.586509	9.772809	7.706238	8.188467	7.619230	7.652974	8.190541	7.794009	7.652204	6.913700	7.182510	7.317492	7.456987	7.175395	7.585179	7.417566	8.518715	8.250672	8.217923	7.657826	8.582268	7.384091	8.360170	7.132283	7.875964	7.894421	7.444435	8.607569	7.678751	7.796942	7.142879	7.214181	7.823644	6.828154	7.569464	7.035923	7.668497	7.375801	7.321681	8.248700	7.069552	8.286088	8.295916	7.714215	7.047549	8.156894	8.127337	7.894811	8.456424	8.148380	7.914461	7.598679	7.806324	7.623325	7.526204	7.456680	7.506342	7.742097	6.443703	7.126885	7.714929	6.993221	6.529490	7.405370	7.583649	6.941340	6.817820	7.598679	7.489761	7.892633	8.090338	7.956793	8.443154	8.045127	7.499603	7.926641	7.353422	8.147447	7.980180	7.577148	7.522227	7.153877	7.776635	8.275513	7.936999	6.683564	8.341166	9.237341	8.010350	8.755929	7.587974	7.872919	7.978951	7.967408	7.528443	7.564260	7.056172	7.473850

DLX4	4.345054	5.938629	4.899606	5.772748	5.379258	5.531069	5.914377	4.970485	3.920809	6.597774	6.411526	5.629885	7.464507	6.108677	6.054977	5.705599	6.078492	4.941773	7.147061	6.716702	5.207836	6.905252	7.229677	4.691573	5.406110	5.626721	4.711587	4.255667	6.124461	6.725435	5.960936	5.505652	5.192099	6.470376	6.283289	4.737391	3.926325	5.477091	6.440058	3.948147	6.057108	5.047909	4.935579	5.528973	4.341124	6.430041	5.159455	4.774907	4.529240	5.247821	4.732732	5.440433	6.122524	6.044821	5.180956	6.689329	4.374567	2.906315	6.589823	4.615751	4.224914	5.152556	3.675584	5.991827	5.944073	5.883154	4.193224	4.680948	5.657575	3.950770	5.710664	5.052157	6.455588	5.558123	5.548626	4.788690	4.180105	5.121307	5.424047	5.739181	3.422249	5.511823	6.344898	4.352225	3.892199	6.507804	6.290462	4.905025	5.741185	6.898683	4.749977	4.346762	5.231593	4.040146	4.738648	5.375758	4.389751	5.895320	6.055823	4.511602	4.114269	5.625058	4.897234	5.244418	4.707853	6.299881	3.943719	5.309215	6.687772	6.253184	5.158109	5.742456	3.774315	5.126754	5.084868	4.422440	4.955010	5.894419	6.003417	6.713250	6.406893	6.761235	6.465705	6.890614	8.039489	7.531887	8.644089	8.427882	8.852566	8.644089	8.055749	6.414695	8.549674	8.246033	6.062774	6.547423	8.377069	8.357075	8.382213	8.196546	9.014405	9.203704	9.710156	8.615278	5.826762	4.701513	5.945684	7.129705	6.966936	7.045129	4.851173	6.280180	6.256588	7.040034	8.858058	5.982617	5.959156	7.174638	7.845347	7.664213	6.894469	7.475837	5.915666	7.341925	7.446500	7.694879	8.608029	7.511538	7.260136	8.116083	7.542633	7.652378	8.113934	6.062445	8.965466	8.946123	7.854689	7.402552	8.336175	4.940527	6.999519	9.459408	9.038502	5.987742	7.041441	6.982395	7.032182	7.166846	4.616425	7.488051	7.286616	6.773160	7.206445	6.775273	6.722579	6.045183	6.479620	7.033749	7.721928	7.999017	8.146104	8.253439	9.761038	4.970884	6.621955	8.714844	8.386331	6.980014	8.242495	10.672137	7.926641	7.100790	7.027322	7.659976	7.909583	7.069044	8.039326	7.241803	8.773366	5.161854	8.671094	6.792518	4.699627	7.707128	6.961011	4.735884	6.368368	8.725272	6.311781	7.055070	5.478043	3.766938	5.561304	8.209784	5.910637	6.420124	8.023465	8.278584

FCGR1A	6.282450	8.674050	8.858656	7.307270	8.077123	8.993798	8.997042	9.735468	8.176634	9.649021	8.882792	8.494150	8.499968	7.955709	7.046103	9.819818	8.845259	8.353066	6.885035	8.300613	8.271201	7.732409	7.185698	8.058274	9.367419	7.670062	9.182351	9.923526	8.773214	8.820970	8.224937	7.312433	8.209188	8.648420	7.379556	8.060100	9.137471	8.721757	7.955404	9.039921	9.209476	6.615109	8.049234	7.688261	7.231189	8.061229	8.696219	7.846394	9.209005	8.207086	7.883682	8.301521	7.850360	7.567498	8.287784	8.493834	8.150217	7.948894	7.648004	7.462608	8.090173	9.618091	7.878566	6.990944	7.570519	7.824001	7.676648	7.578705	8.279787	6.835143	7.983773	8.991352	10.554140	9.166351	8.704285	8.508291	8.124489	8.728390	7.909425	6.958927	8.003733	7.658623	6.673327	8.324142	8.562835	7.331331	8.106503	9.072354	6.809854	7.145511	7.035624	7.440064	8.327809	9.632445	7.003296	7.538600	8.144078	8.108325	7.799277	8.867477	9.327210	8.803198	8.704285	9.440482	7.591499	8.943551	7.904280	8.289335	6.779417	10.450191	7.872609	8.269199	8.507964	10.329335	9.608538	10.256330	6.771711	7.333659	9.015152	7.625933	8.197159	8.274471	7.943093	8.746513	8.549830	8.129345	7.265880	7.736809	8.414001	8.181304	7.286286	7.716582	8.786399	6.550996	8.069216	7.020447	7.532374	7.518492	7.481352	7.302884	7.940095	7.830789	6.711199	7.437251	8.889773	8.391312	7.677784	9.133220	8.504041	8.930947	8.292455	7.959604	8.329193	7.758398	7.055726	7.951667	7.460397	7.191824	8.698630	8.089558	6.328070	8.647850	7.674467	7.691660	8.148380	8.651479	6.444714	7.549594	7.663263	7.852348	7.381539	7.630212	8.232255	8.917875	8.313277	7.343187	6.868741	7.341925	7.512491	7.432496	9.017157	6.183800	7.151855	8.717346	7.546796	7.664378	7.691976	7.687797	7.696834	7.656711	7.891377	9.546391	8.130422	7.709416	8.298501	7.804933	7.717365	7.171315	7.344870	7.636336	7.523920	8.072162	8.189259	8.203010	8.312823	7.549145	7.156988	7.810345	8.907495	6.487592	6.409505	7.717814	7.703743	7.442358	8.181222	7.748566	8.341022	7.491489	7.558311	7.898797	7.404121	7.653601	9.319744	8.059483	7.874427	5.890991	7.740092	6.592272	9.172195	7.750734	9.081198	10.797016	10.463100	7.728256	8.341022	8.568119	6.856901	8.230249

MUC6	17.761057	7.604353	7.971938	17.335857	9.302345	8.688827	7.866357	7.956035	7.636038	7.393203	8.072719	8.096125	7.875964	7.013910	13.014469	7.773688	7.773066	7.309440	6.801722	7.354552	7.581658	7.643358	10.822815	6.607242	7.268627	8.273134	8.011263	8.637389	7.991746	8.102649	8.671577	17.469225	11.015587	7.422893	6.815732	6.830707	7.200694	6.865024	7.141596	6.122524	7.698975	6.750842	7.675274	8.942331	7.506342	7.166993	8.933148	7.005042	7.568483	6.552682	17.907698	13.347208	6.322896	6.496048	6.047817	8.352148	6.637206	6.125852	7.460241	6.457389	6.207781	7.731485	7.104243	8.311618	5.571063	6.778153	5.929339	6.453958	5.899028	8.226755	8.254672	7.244686	8.211601	6.526846	7.008506	6.657929	8.175989	6.399129	17.055536	6.276841	17.209463	6.621787	17.487844	8.074558	6.750212	7.740253	7.684733	6.961500	6.521385	15.926414	7.040480	8.175531	7.319649	8.509059	17.229824	7.322799	6.779417	8.330882	9.470011	7.253085	9.242250	8.181595	7.164423	6.757650	7.017782	8.241293	6.355910	8.026826	8.418870	7.809422	7.912200	8.286403	8.656689	15.757552	9.683409	12.229428	17.761057	8.244137	8.026381	15.012809	7.306185	7.092916	14.649251	9.577618	8.387288	7.606249	8.449532	7.061387	7.634200	7.409893	7.822714	7.401008	7.103438	10.251178	8.101596	7.058384	7.801599	8.763472	6.656465	8.245391	7.674327	8.326365	7.170547	7.232439	7.323456	8.293216	9.224038	7.803715	7.666363	7.677137	14.060010	14.525499	7.694121	6.739392	6.930002	6.654503	6.569361	6.664294	6.354258	8.424386	6.774623	6.899975	7.069044	6.643033	6.952973	9.206130	7.167473	6.830092	6.980337	15.093809	10.372744	6.041748	6.747397	6.062618	7.438669	7.247454	7.009640	7.311045	6.228307	5.809994	8.847228	6.320331	7.270794	5.526878	6.308928	7.066199	6.124461	6.189601	10.531882	7.093070	6.813975	9.258494	6.497681	6.627417	6.278646	6.929077	5.909893	13.759418	6.711199	13.551398	6.256911	14.079493	11.037133	6.611997	7.592717	6.652886	6.609496	6.687456	12.513519	6.916950	7.000648	6.619026	7.146904	14.225717	7.553153	7.341925	8.076799	8.262784	6.486275	8.861679	11.006550	7.332745	6.176773	5.867966	8.345381	7.112374	7.183140	8.192295	7.987181	7.226066	7.869842	11.030902	14.201809	9.151539	10.233845	14.561753	8.684172	7.657040

AC004687.2	12.194595	12.043801	11.773277	12.652080	12.903734	12.354202	12.385972	12.592652	12.456759	12.734044	12.795726	12.197990	12.875552	12.354202	12.011096	12.801613	12.635955	12.207137	13.045174	12.468921	12.920968	13.357004	11.284152	12.268379	12.653975	12.783624	11.294819	12.853682	12.133995	11.915341	13.113930	11.965086	11.880861	11.967332	12.497024	12.181582	12.707057	12.254206	12.896716	12.600363	12.629032	12.723310	12.083542	13.254784	11.919972	12.861151	12.076202	12.128415	12.413808	12.996930	12.650904	12.805449	12.313009	12.759968	11.718080	12.125545	11.685541	12.567752	12.587805	12.686750	12.849490	11.934741	8.215914	12.782775	12.317479	11.495210	12.684030	12.934891	12.622511	12.730151	12.252315	12.813513	12.669498	12.601063	12.694078	12.008851	12.513371	12.084810	12.376451	12.236505	12.163120	12.594923	12.295732	12.777426	12.624672	12.775154	12.826118	12.909649	12.623334	12.988128	11.913381	13.193477	12.643905	12.218342	12.302166	12.629207	12.405594	12.345161	12.568720	12.741631	12.017633	11.698277	12.340373	12.777426	13.431034	12.874685	13.100874	13.210340	12.821781	12.706040	13.614714	13.092940	13.167086	12.583137	12.165300	11.676245	12.435325	12.082273	12.435950	12.192751	12.210352	13.232466	12.215771	12.136334	11.938632	11.468535	13.645649	11.620399	11.641173	11.841598	12.464195	12.042551	12.752950	11.476974	11.912208	12.218206	12.847695	13.474856	13.680915	12.506071	13.739103	13.233234	13.422807	12.822669	7.331331	11.914759	11.865618	11.980765	12.030634	12.770845	11.508193	12.232669	11.466592	12.326361	12.971329	11.869930	11.189611	13.049903	12.491754	12.211598	12.694911	13.009202	10.085546	13.073797	12.838773	12.084512	12.820920	12.566593	12.704327	12.258961	11.795418	12.406661	12.619822	11.050179	12.936720	14.588535	13.243931	12.463227	12.773792	13.240382	11.320099	13.699890	12.641718	12.177372	10.957831	12.806823	12.456759	12.168975	10.810803	13.177598	12.185131	11.759040	11.594455	12.065618	12.065925	11.250318	12.298858	12.975277	11.953709	12.051190	12.396166	12.463883	11.973443	11.968566	12.354041	12.369546	12.442847	11.331034	11.535555	14.063237	12.296782	12.760481	12.504387	11.756948	11.975261	12.555263	11.918646	12.649190	12.599699	11.514083	14.401146	13.248301	11.186992	11.903261	11.795725	12.188415	12.033077	13.304537	11.604311	13.709428	12.508819	12.571167	12.305725	13.907695	12.059357	11.936694	11.669538	12.485326

RNF43	12.194595	12.043801	11.773277	12.652080	12.903734	12.354202	12.385972	12.592652	12.456759	12.734044	12.795726	12.197990	12.875552	12.354202	12.011096	12.801613	12.635955	12.207137	13.045174	12.468921	12.920968	13.357004	11.284152	12.268379	12.653975	12.783624	11.294819	12.853682	12.133995	11.915341	13.113930	11.965086	11.880861	11.967332	12.497024	12.181582	12.707057	12.254206	12.896716	12.600363	12.629032	12.723310	12.083542	13.254784	11.919972	12.861151	12.076202	12.128415	12.413808	12.996930	12.650904	12.805449	12.313009	12.759968	11.718080	12.125545	11.685541	12.567752	12.587805	12.686750	12.849490	11.934741	8.215914	12.782775	12.317479	11.495210	12.684030	12.934891	12.622511	12.730151	12.252315	12.813513	12.669498	12.601063	12.694078	12.008851	12.513371	12.084810	12.376451	12.236505	12.163120	12.594923	12.295732	12.777426	12.624672	12.775154	12.826118	12.909649	12.623334	12.988128	11.913381	13.193477	12.643905	12.218342	12.302166	12.629207	12.405594	12.345161	12.568720	12.741631	12.017633	11.698277	12.340373	12.777426	13.431034	12.874685	13.100874	13.210340	12.821781	12.706040	13.614714	13.092940	13.167086	12.583137	12.165300	11.676245	12.435325	12.082273	12.435950	12.192751	12.210352	13.232466	12.215771	12.136334	11.938632	11.468535	13.645649	11.620399	11.641173	11.841598	12.464195	12.042551	12.752950	11.476974	11.912208	12.218206	12.847695	13.474856	13.680915	12.506071	13.739103	13.233234	13.422807	12.822669	7.331331	11.914759	11.865618	11.980765	12.030634	12.770845	11.508193	12.232669	11.466592	12.326361	12.971329	11.869930	11.189611	13.049903	12.491754	12.211598	12.694911	13.009202	10.085546	13.073797	12.838773	12.084512	12.820920	12.566593	12.704327	12.258961	11.795418	12.406661	12.619822	11.050179	12.936720	14.588535	13.243931	12.463227	12.773792	13.240382	11.320099	13.699890	12.641718	12.177372	10.957831	12.806823	12.456759	12.168975	10.810803	13.177598	12.185131	11.759040	11.594455	12.065618	12.065925	11.250318	12.298858	12.975277	11.953709	12.051190	12.396166	12.463883	11.973443	11.968566	12.354041	12.369546	12.442847	11.331034	11.535555	14.063237	12.296782	12.760481	12.504387	11.756948	11.975261	12.555263	11.918646	12.649190	12.599699	11.514083	14.401146	13.248301	11.186992	11.903261	11.795725	12.188415	12.033077	13.304537	11.604311	13.709428	12.508819	12.571167	12.305725	13.907695	12.059357	11.936694	11.669538	12.485326

TXNRD2	6.801722	7.094820	7.144590	7.671653	7.258388	7.874571	7.415219	7.585336	7.752866	7.857542	7.778846	7.916768	7.158238	7.428367	6.930332	7.700541	7.468145	7.423039	8.768990	7.660947	7.797234	6.927797	6.761400	7.110328	7.288615	7.698975	7.384691	8.166604	7.488051	7.648891	7.330084	7.396612	7.585638	7.035624	7.451958	7.219180	7.318215	7.126261	7.235637	7.396775	7.792043	6.870310	7.292079	7.270643	7.362150	7.544535	7.432347	7.171477	7.937457	7.427588	7.159016	7.162350	7.337198	7.847219	7.448405	8.132838	7.343668	7.155624	6.688232	6.727389	7.196156	7.965409	6.252648	6.989840	7.509710	7.586605	7.106294	6.795557	6.919027	7.353981	7.663729	7.446661	7.564260	7.432496	7.556289	7.268030	7.223425	7.335661	7.115967	7.018100	7.098777	7.390553	6.984753	7.163616	7.059962	8.095669	5.672771	6.417161	6.508471	6.993546	7.084741	7.157612	6.838038	7.250091	6.693444	7.013620	7.863243	7.630529	7.624187	7.211670	7.658770	7.275303	7.036868	7.367807	7.659976	7.603602	6.983979	7.706860	6.521225	7.785744	6.647062	6.752087	6.907201	8.022827	8.447340	7.320728	6.480417	7.652823	7.407121	6.401780	7.565037	6.533076	7.076523	7.975078	7.893570	7.379253	6.304265	7.581800	7.486779	7.848245	7.468910	7.414306	6.913398	6.977696	6.628549	7.350831	6.448842	6.449821	6.140472	6.945974	5.785023	6.663954	6.681903	6.954382	7.820856	7.658918	7.631919	7.152628	7.499142	7.476608	7.087059	7.666536	7.733023	6.900141	6.951242	7.606389	7.146440	7.243086	7.130688	7.657367	7.204562	6.272390	6.958781	7.306185	7.371092	7.908644	6.453805	7.772629	7.256648	7.128933	6.762196	6.661502	7.530523	6.871633	7.079582	6.410035	5.670662	5.319191	5.556180	7.049913	7.515075	6.664453	6.348100	5.848814	6.634850	6.318752	7.342853	7.123062	6.667041	7.723476	7.470168	8.278869	7.492437	6.629051	7.498818	7.533611	7.317019	7.617381	6.041931	7.231664	6.882620	7.477847	7.062210	7.619699	7.510936	6.674131	6.261678	5.126754	5.864523	5.497551	6.237316	7.019371	7.104871	7.957449	7.359946	6.879088	7.403345	7.834020	6.577340	7.640322	6.406553	6.694902	6.398962	5.408723	7.551749	6.070817	5.907032	6.329383	8.191798	5.390655	5.795108	5.688971	8.254672	7.187166	7.499450	7.436154	7.156827	7.662478

MUC2	4.665614	5.685865	5.502582	8.537113	5.465211	5.553381	5.523836	6.883757	4.947124	5.805451	5.465020	4.771198	5.666656	5.551044	4.587333	6.004467	6.295776	4.761419	5.485210	6.005942	5.589862	6.164766	4.674518	5.416211	5.830277	5.713273	6.158049	6.645787	6.637380	5.691927	5.344374	5.314416	5.195106	4.825402	5.231998	4.591411	5.313408	5.733911	6.060443	5.226669	5.892040	4.942399	5.271639	4.986843	4.994580	4.195839	6.043307	5.424454	5.373984	4.979752	5.242838	4.323376	4.586266	4.629125	4.694111	6.573541	3.879159	3.538047	5.226859	4.346971	4.767009	6.013848	5.937761	5.625217	4.908622	4.572417	5.605327	5.683252	5.268084	5.677876	5.566361	4.900484	6.216641	5.873510	5.294827	5.565981	5.553381	5.283942	5.739713	4.713921	4.312966	5.410127	5.944605	4.314274	4.479330	5.531417	4.115265	4.620829	3.802855	5.366014	5.052375	5.768488	5.074842	5.352869	4.834971	5.247211	4.436502	4.961642	5.503913	5.241641	4.309712	5.267688	5.269057	5.510880	5.545992	6.021366	6.157700	5.933526	5.957495	6.556792	5.301629	5.592356	5.229742	6.268440	5.502197	3.802855	5.463106	5.159455	5.776731	6.466680	7.297331	5.473107	6.849813	6.111142	7.450429	6.545166	5.943721	4.780742	6.069439	6.704797	5.268846	6.483741	5.702406	6.550026	5.996352	5.957159	4.746622	5.447832	5.862703	6.120458	5.857773	6.477451	9.360471	6.490919	6.074144	6.105134	6.582771	5.831929	6.791035	5.563335	5.051783	4.297007	7.528582	5.439863	4.911356	6.575845	5.737539	6.287752	5.603982	7.084584	4.885388	5.109541	5.636952	5.446840	3.909692	6.573722	8.001841	5.135234	4.944347	5.985562	5.242044	3.379996	4.963752	3.614582	6.673895	3.493904	4.874437	5.470251	4.546237	3.206648	8.649820	5.020938	5.316075	4.887696	5.033779	7.033109	5.962707	5.544106	5.606848	5.142569	5.401645	6.479464	6.196567	5.074237	5.830863	6.775740	5.747776	5.943900	4.529040	5.505652	4.570033	3.914443	3.630688	4.915357	6.054615	5.538641	4.126337	4.354587	4.736547	5.249579	5.990425	5.454938	6.538445	6.525818	5.216196	4.714337	5.355901	5.834643	5.322870	5.989356	4.566806	7.124976	5.349273	4.837448	6.252335	4.946094	5.609146	6.751932	8.205864	4.987675	5.083639	4.121617	7.040034	5.507386	5.083066	6.707390	5.774175	6.132754

ATMIN	13.245524	13.469871	13.711167	13.371671	13.537270	13.595842	13.576715	13.781596	13.204762	13.698011	13.706787	13.395784	13.629686	14.009020	13.543949	13.459608	13.633254	13.941795	13.777688	13.769541	13.682355	13.007761	12.778288	13.747333	13.707975	13.274601	12.726202	13.457435	13.279019	13.221376	13.461577	13.509723	13.727608	13.351397	14.084957	13.918854	14.165841	13.950998	14.052673	14.029660	13.557035	14.002723	13.817588	13.769304	13.739368	13.961884	13.365165	13.192104	13.308940	13.676917	13.727608	13.999805	13.853818	14.091290	13.310948	13.464002	13.937690	14.108206	14.124606	13.814571	13.778222	13.434092	13.136216	13.734845	13.360816	13.704846	13.589804	13.706787	13.544622	13.724675	13.643515	13.701861	13.368506	13.779388	13.551398	13.611591	13.898577	13.391621	13.440530	13.675535	13.605717	13.885964	13.486415	13.855137	14.057105	13.632044	13.762967	14.108206	14.110759	13.915790	13.749950	13.661762	13.521387	13.847752	13.238812	13.668155	13.385402	13.925407	13.812477	13.436214	13.585329	13.365784	13.496693	13.627523	13.354191	13.558417	13.499351	13.423426	13.459378	13.817588	13.387795	13.878229	13.610144	13.449301	13.568702	13.029714	13.285446	13.697796	13.525049	12.972212	12.771214	12.803692	12.975455	12.758764	12.772251	12.971329	12.965086	13.859930	13.074158	12.391894	13.269841	12.692683	12.455784	11.973127	13.403761	13.127862	13.052959	12.955360	12.999145	12.168975	12.500751	12.449269	11.924880	12.424576	12.549566	13.170169	12.501897	12.055582	12.965249	12.218642	12.188102	12.726851	12.275765	12.297429	13.252250	13.055989	12.910006	12.618274	13.232466	12.913386	13.289639	13.085147	13.170749	13.238041	13.017706	12.682992	13.145522	13.336858	13.309136	12.917111	13.186053	13.233610	12.670010	13.109444	13.005039	13.063428	13.066597	12.416908	13.068294	12.886884	12.628507	13.730904	13.294952	13.478600	12.939558	13.291948	13.169964	12.314612	12.715418	12.908941	13.194078	12.560874	12.857438	13.306393	13.030425	13.062656	13.426884	13.128984	12.569208	13.311534	12.495694	13.122311	13.364346	13.213993	12.565935	13.295984	13.340354	13.643272	12.860948	13.165092	13.015899	12.988336	12.404042	13.629180	12.746911	13.066024	12.610190	12.398695	12.839276	12.734879	13.285050	12.148544	12.716965	13.037820	12.858824	13.156342	13.319864	13.245152	12.600707	12.635424	13.149152	12.466325	13.154391	13.296766	13.078763	13.540173	12.804601	12.489749

AC092718.8	13.245524	13.469871	13.711167	13.371671	13.537270	13.595842	13.576715	13.781596	13.204762	13.698011	13.706787	13.395784	13.629686	14.009020	13.543949	13.459608	13.633254	13.941795	13.777688	13.769541	13.682355	13.007761	12.778288	13.747333	13.707975	13.274601	12.726202	13.457435	13.279019	13.221376	13.461577	13.509723	13.727608	13.351397	14.084957	13.918854	14.165841	13.950998	14.052673	14.029660	13.557035	14.002723	13.817588	13.769304	13.739368	13.961884	13.365165	13.192104	13.308940	13.676917	13.727608	13.999805	13.853818	14.091290	13.310948	13.464002	13.937690	14.108206	14.124606	13.814571	13.778222	13.434092	13.136216	13.734845	13.360816	13.704846	13.589804	13.706787	13.544622	13.724675	13.643515	13.701861	13.368506	13.779388	13.551398	13.611591	13.898577	13.391621	13.440530	13.675535	13.605717	13.885964	13.486415	13.855137	14.057105	13.632044	13.762967	14.108206	14.110759	13.915790	13.749950	13.661762	13.521387	13.847752	13.238812	13.668155	13.385402	13.925407	13.812477	13.436214	13.585329	13.365784	13.496693	13.627523	13.354191	13.558417	13.499351	13.423426	13.459378	13.817588	13.387795	13.878229	13.610144	13.449301	13.568702	13.029714	13.285446	13.697796	13.525049	12.972212	12.771214	12.803692	12.975455	12.758764	12.772251	12.971329	12.965086	13.859930	13.074158	12.391894	13.269841	12.692683	12.455784	11.973127	13.403761	13.127862	13.052959	12.955360	12.999145	12.168975	12.500751	12.449269	11.924880	12.424576	12.549566	13.170169	12.501897	12.055582	12.965249	12.218642	12.188102	12.726851	12.275765	12.297429	13.252250	13.055989	12.910006	12.618274	13.232466	12.913386	13.289639	13.085147	13.170749	13.238041	13.017706	12.682992	13.145522	13.336858	13.309136	12.917111	13.186053	13.233610	12.670010	13.109444	13.005039	13.063428	13.066597	12.416908	13.068294	12.886884	12.628507	13.730904	13.294952	13.478600	12.939558	13.291948	13.169964	12.314612	12.715418	12.908941	13.194078	12.560874	12.857438	13.306393	13.030425	13.062656	13.426884	13.128984	12.569208	13.311534	12.495694	13.122311	13.364346	13.213993	12.565935	13.295984	13.340354	13.643272	12.860948	13.165092	13.015899	12.988336	12.404042	13.629180	12.746911	13.066024	12.610190	12.398695	12.839276	12.734879	13.285050	12.148544	12.716965	13.037820	12.858824	13.156342	13.319864	13.245152	12.600707	12.635424	13.149152	12.466325	13.154391	13.296766	13.078763	13.540173	12.804601	12.489749

AKT3	10.349176	9.145192	9.409114	8.463529	7.191824	8.828725	10.954523	6.478155	5.440059	9.801200	9.182199	9.081198	8.987489	8.701516	9.241513	9.679997	10.392965	9.256667	7.271730	8.765904	8.453985	8.035245	11.478857	8.089558	10.019305	8.887749	10.574229	8.746346	10.521568	10.835875	7.966334	10.832274	10.218622	10.381054	11.099443	10.850174	10.810402	10.453727	10.228208	9.957845	9.509606	10.804597	9.631239	9.958305	11.121750	10.001021	10.889218	9.666389	10.767132	10.520951	10.228061	11.111876	10.699257	9.919961	10.465797	9.871071	10.810258	10.479789	9.229123	10.434911	10.377715	10.564279	12.500081	9.776906	10.167052	9.906344	9.423613	9.421747	9.077935	9.818426	10.510212	10.194166	9.552825	10.939339	9.873790	10.674499	10.727937	10.905252	9.905132	10.178735	10.569842	10.600317	10.183600	10.709659	11.053643	10.439584	10.188159	10.343685	10.523853	9.061877	9.617944	10.136250	9.319298	10.165649	10.118840	10.394903	10.950587	10.397759	10.014305	10.306828	9.521307	9.930637	9.169683	9.318351	9.034473	9.803340	8.989121	9.505030	9.047586	9.797029	9.370447	7.919983	6.694563	8.492896	8.516857	7.089929	9.736095	10.414939	9.721996	9.816506	8.981350	10.071631	8.261975	7.720377	8.318960	10.563066	10.891628	9.496425	11.373199	9.736421	11.593733	11.793930	11.167966	8.250515	10.715371	10.245440	9.979195	12.534282	12.828929	8.404997	10.986270	10.231761	5.633010	10.760858	11.132344	8.941247	6.486608	7.772173	10.724139	10.498125	10.152342	10.720767	9.499476	10.825918	12.246079	11.141336	10.104632	9.401854	10.051261	11.779120	11.581124	12.069615	9.631529	9.882882	10.764929	7.093070	11.092206	11.913837	11.352601	10.799193	10.831042	11.654537	10.574514	11.501897	12.465830	11.308833	10.163187	8.899150	9.927224	10.029801	11.516647	10.234480	9.509006	9.278174	9.845343	9.594939	9.278447	9.318197	9.934938	10.522954	11.636481	7.841085	10.529215	11.761777	10.557516	11.406029	10.903791	10.183168	10.950121	10.435682	9.992195	9.874226	11.074288	11.335396	9.737449	10.397036	11.647387	10.879596	10.469201	11.985558	10.411012	9.781037	11.934741	10.521258	10.601825	9.735158	9.616360	9.884227	11.800284	11.627636	7.597778	9.232220	10.108677	11.730195	10.538968	7.957140	8.263240	10.738984	7.681937	10.432156	11.014997	11.359874	7.084263	12.939042	9.322428	10.652524	9.208246	8.873253

KIFC3	9.269228	9.671026	9.785347	9.639736	9.874083	10.011379	10.258677	10.560245	10.740196	10.438091	10.452087	9.821458	9.912359	10.425899	9.929996	10.118987	10.337200	10.167524	10.451084	9.707662	10.179636	10.656092	10.075727	10.177956	10.321065	9.964578	9.761826	10.779119	10.173505	9.787591	10.242137	9.522255	9.513967	9.612257	9.461669	9.560158	9.613954	9.980586	10.101264	10.496942	9.999518	9.645339	9.498106	9.526012	9.086706	9.368622	10.165649	9.935902	9.593851	9.577290	9.436629	9.299232	9.527706	9.497175	9.662014	10.357533	9.246431	9.760100	9.702651	9.535194	9.771907	10.288034	9.914232	9.960286	9.773869	10.055148	9.862229	9.978895	9.495049	9.961516	9.704430	9.782986	10.409375	9.612400	9.709848	9.564664	9.807143	9.601901	9.628948	9.087336	9.554047	9.332910	9.244109	9.481495	9.289113	9.268924	9.260456	9.447790	9.737449	9.268775	9.304272	9.952432	9.493154	9.686383	8.850610	9.371033	9.422970	10.131092	9.864701	9.951825	10.352360	9.813834	9.710940	9.806391	10.415101	9.992195	11.236266	10.624433	10.120036	10.407984	9.780722	10.329926	10.668559	10.470130	10.175931	10.321225	9.796886	9.868222	10.101264	9.496124	10.463717	9.750360	9.739328	10.469499	10.101871	10.660096	10.035315	10.674348	10.310511	10.643734	9.929861	10.522062	10.905549	10.635038	9.882882	10.544617	9.798923	10.203254	9.411410	10.166289	10.206147	9.741022	10.201710	10.657103	10.526109	9.682033	9.932911	10.593778	10.058619	10.427471	9.984109	10.082846	10.372584	9.331538	9.098736	9.514851	10.140152	10.429581	9.918999	10.767727	9.688544	9.504879	10.217724	9.166648	9.680141	11.137575	9.902118	9.643918	10.066161	9.898527	9.172491	9.294739	10.196749	9.841226	10.012286	8.685982	9.656115	9.790662	9.252826	10.747087	10.311130	9.754388	10.090671	10.191000	9.992951	10.383819	10.033608	9.869932	10.535450	9.714055	9.777687	10.991053	9.940846	9.335435	9.883041	9.852904	10.056961	9.897040	9.304406	9.837182	9.960448	10.055756	9.706727	9.680791	9.742434	9.972959	9.162133	9.777366	10.025435	9.809361	10.584914	9.702953	10.326281	10.304626	10.116982	10.175625	10.192202	10.404707	9.501326	10.348720	9.670699	9.537522	9.811988	10.370335	10.090835	10.875592	10.184839	10.014459	11.385844	9.991411	10.539729	9.655185	10.466388	9.519313	9.808735	10.437655	9.764038	10.465495

HNRNPK	14.511123	14.280056	14.297014	14.630796	14.854894	14.623346	14.639680	14.582286	14.222202	14.394753	14.640496	14.553109	14.704362	14.254782	14.535929	14.751698	14.653231	14.453914	14.592810	13.966887	14.263585	14.213670	13.933244	14.441229	13.939913	14.321619	14.286571	13.956288	14.464001	14.532252	14.684603	15.336040	14.885495	14.754551	14.755057	14.643584	15.064046	15.098171	14.504600	14.309339	14.711236	14.596200	14.732223	14.531879	14.571778	14.619027	14.430826	14.056836	14.851142	14.248543	14.648445	14.648445	13.937428	14.002993	14.816832	14.332395	15.252474	14.260639	14.160203	14.261313	13.871517	14.808644	14.406946	13.888860	14.525087	14.466988	14.066316	14.630361	14.136689	14.342281	14.292726	14.568889	14.591596	14.632175	14.640110	14.656836	14.563854	14.159585	14.370128	14.552678	14.638391	14.121021	14.742751	15.298804	14.859978	14.659533	14.880110	15.054906	14.544632	14.251440	13.922148	14.394753	14.255152	14.055969	14.472714	15.005364	14.423617	14.814210	14.855908	13.561728	14.694246	14.369059	14.739993	14.521823	14.422133	14.831507	14.241406	14.579342	14.552270	14.580994	14.045286	14.160048	13.896959	14.671445	14.291337	13.680430	14.057422	15.019773	14.443429	15.406237	14.929736	14.942038	14.634337	14.589784	14.954990	14.897610	14.761971	14.924443	15.022274	14.854342	14.983442	14.740963	14.469284	14.513547	14.944787	15.302315	14.546813	14.992275	15.369410	14.724698	14.438602	14.360460	14.652808	14.781328	14.672364	14.947964	14.800437	14.528702	14.821872	14.993988	14.570991	15.083595	14.951667	15.180575	15.659589	15.061588	15.188078	14.830506	15.000238	14.778050	15.028163	15.010559	15.418602	15.054906	15.101143	14.626390	14.405128	15.344956	15.211019	15.047833	14.973725	15.168793	15.397686	15.139712	14.939862	15.293162	14.775142	14.560123	14.853259	14.850021	14.372657	14.802845	14.961694	14.164594	14.248543	14.651534	14.667724	14.902955	14.506070	15.045993	15.162646	14.324402	15.335238	14.950618	14.916837	15.032181	15.188690	14.999084	15.087640	15.143591	15.515150	14.992275	14.720427	15.903390	15.176581	15.473917	15.515150	14.857509	14.590224	14.996793	15.230037	14.637025	15.201679	14.512793	14.750281	15.341190	14.937805	14.993419	15.271483	15.226686	14.759178	14.352710	14.692440	15.034636	14.581385	14.505307	14.932454	14.781829	14.820855	14.023591	14.682292	14.978704	14.513890	15.343482	14.963394	14.989606	15.291679	14.923336

RHOH	9.803798	10.651913	10.097471	8.935020	7.391638	8.743011	9.533072	9.058310	6.920499	7.275940	7.961466	8.861351	8.303938	7.646341	8.982606	9.050843	7.249321	9.563450	7.462298	9.170158	7.594133	10.754173	7.399610	10.609923	9.123220	6.840632	9.843946	7.960849	8.894400	11.599182	5.788677	10.141048	11.961160	11.482100	11.293672	10.489800	9.240121	9.913281	8.364235	10.023706	7.306812	10.704472	11.427239	10.081967	10.309414	10.956934	7.903686	10.822063	10.719432	9.815273	10.677043	10.027644	10.892076	10.045720	11.010198	7.777904	10.388663	10.280952	9.174793	10.452381	10.496144	7.633622	10.415101	9.015924	10.894182	10.582604	9.659835	9.417325	10.333876	10.077279	9.680465	8.962062	7.393203	10.020783	9.689137	11.455058	9.446852	10.459392	10.626257	10.502860	10.938471	11.009325	10.557792	10.007983	9.810118	10.134987	10.246336	9.621823	10.494828	10.039433	11.485624	10.773291	10.033478	10.144761	11.376800	10.656389	11.331034	7.106932	8.437983	7.385317	7.088478	10.269126	8.337509	8.204349	9.698934	8.114390	9.063070	9.909114	7.205802	6.499728	8.897794	7.471570	9.171092	7.367653	9.609476	6.010714	10.496942	8.575648	6.583114	10.184839	8.513380	10.502419	9.400733	7.088173	7.693984	8.433072	7.108530	6.062086	6.321320	6.565605	7.331642	6.154648	9.125660	8.197934	7.707918	8.001687	6.925341	8.323674	9.812767	7.382198	7.816672	8.762712	6.172347	5.834109	9.360643	8.626562	6.852172	7.211820	10.056502	7.797527	10.957378	9.467827	8.549225	10.512879	8.208005	10.306414	9.891957	7.365307	10.103112	6.250803	11.467492	10.196135	9.485652	10.341850	10.212263	7.016496	10.622485	8.841648	9.908340	11.367280	10.619400	10.696778	9.957526	10.160447	8.282714	9.587971	11.576750	10.451538	10.880179	11.353895	8.995637	10.291124	10.483671	10.813415	11.151708	9.312647	11.150234	11.109254	11.535407	9.976663	9.379298	6.413357	8.916955	11.294819	11.240625	9.118833	9.197036	8.816989	10.437361	9.533374	9.818575	9.700957	11.343233	10.483985	7.716582	10.095615	9.523175	10.501539	10.423303	10.144761	10.311282	9.020172	9.937420	9.905450	10.002883	8.226898	7.256173	7.364676	7.346731	9.348642	8.376902	7.626091	11.260184	9.074656	8.674991	9.962244	8.963724	8.720713	6.708953	10.132585	8.056835	7.530985	8.949362	8.345999	8.092006	9.026744	6.998180	7.803104

ABHD8	14.432364	13.795903	13.794078	14.086526	14.094287	13.797198	13.876318	13.315612	12.678006	13.777971	13.621305	13.725868	13.658895	14.101917	13.363482	13.841638	13.511061	13.635217	12.885665	13.475544	13.064903	13.419287	14.052986	13.883550	13.708445	13.892647	13.948780	13.335818	13.823982	13.678008	13.112784	14.709387	14.019284	14.006448	14.219932	13.685728	14.153274	14.201495	14.232858	13.954619	13.755369	13.661260	13.897480	14.315445	14.519853	14.578917	13.709169	13.701386	13.686701	14.202755	14.186189	14.248223	14.016722	14.289961	14.068082	13.567588	14.840681	14.172267	14.273182	14.445611	14.319544	13.593974	14.336460	13.836415	13.737346	14.172567	13.959373	14.196229	13.724916	13.532157	14.069516	14.393289	13.499987	14.113158	14.386005	14.536366	14.667724	13.928944	14.361946	13.171710	13.910238	14.345286	14.334354	14.680520	14.497531	14.128591	14.192726	14.022116	13.699452	13.752566	13.772698	13.393903	14.124606	14.051213	14.569736	14.578479	13.956042	13.732385	14.214704	13.636675	13.456369	13.974684	13.628249	14.022116	14.114980	13.873927	13.217568	13.568257	13.065113	13.832809	13.486198	13.597930	13.878453	13.449507	13.426460	13.443765	14.520255	13.836415	13.497353	14.741363	13.743347	13.964108	14.308634	13.723956	14.109446	14.109446	13.347208	13.660103	14.061504	13.750799	14.149163	14.083435	14.035571	12.933669	14.276547	13.655271	13.707475	13.413997	15.010559	13.256816	14.018427	14.062649	13.853253	14.847322	13.778222	13.920234	14.264253	13.952110	13.669861	12.822141	13.660327	14.011814	13.927324	13.933523	14.676463	13.717240	14.105067	13.846933	14.215623	14.133255	13.632774	15.122829	13.365784	14.736202	14.800437	13.304945	13.990142	14.688427	14.920608	13.818348	14.644443	14.769473	14.088933	14.289643	13.969061	14.002993	14.157670	14.088628	14.314071	13.483894	14.668633	13.597054	13.668609	14.041713	13.902528	14.107221	13.381340	14.893360	13.452250	14.742275	14.178943	13.410994	13.939322	14.611021	14.323349	13.919949	14.465898	14.465534	13.239992	14.707518	14.251440	14.212720	14.391513	14.390763	14.019562	14.279673	14.700650	14.043788	13.676917	13.929201	13.458052	14.206838	14.696136	13.991871	13.927054	13.850953	14.037892	14.078329	14.363735	14.062649	13.393700	14.162992	13.799572	13.742058	14.028483	13.082921	14.282413	13.625907	13.398875	13.966624	13.720180	14.368698	13.199503	14.750726	13.214418	14.267954	14.038793	14.432364

MRPL34	14.432364	13.795903	13.794078	14.086526	14.094287	13.797198	13.876318	13.315612	12.678006	13.777971	13.621305	13.725868	13.658895	14.101917	13.363482	13.841638	13.511061	13.635217	12.885665	13.475544	13.064903	13.419287	14.052986	13.883550	13.708445	13.892647	13.948780	13.335818	13.823982	13.678008	13.112784	14.709387	14.019284	14.006448	14.219932	13.685728	14.153274	14.201495	14.232858	13.954619	13.755369	13.661260	13.897480	14.315445	14.519853	14.578917	13.709169	13.701386	13.686701	14.202755	14.186189	14.248223	14.016722	14.289961	14.068082	13.567588	14.840681	14.172267	14.273182	14.445611	14.319544	13.593974	14.336460	13.836415	13.737346	14.172567	13.959373	14.196229	13.724916	13.532157	14.069516	14.393289	13.499987	14.113158	14.386005	14.536366	14.667724	13.928944	14.361946	13.171710	13.910238	14.345286	14.334354	14.680520	14.497531	14.128591	14.192726	14.022116	13.699452	13.752566	13.772698	13.393903	14.124606	14.051213	14.569736	14.578479	13.956042	13.732385	14.214704	13.636675	13.456369	13.974684	13.628249	14.022116	14.114980	13.873927	13.217568	13.568257	13.065113	13.832809	13.486198	13.597930	13.878453	13.449507	13.426460	13.443765	14.520255	13.836415	13.497353	14.741363	13.743347	13.964108	14.308634	13.723956	14.109446	14.109446	13.347208	13.660103	14.061504	13.750799	14.149163	14.083435	14.035571	12.933669	14.276547	13.655271	13.707475	13.413997	15.010559	13.256816	14.018427	14.062649	13.853253	14.847322	13.778222	13.920234	14.264253	13.952110	13.669861	12.822141	13.660327	14.011814	13.927324	13.933523	14.676463	13.717240	14.105067	13.846933	14.215623	14.133255	13.632774	15.122829	13.365784	14.736202	14.800437	13.304945	13.990142	14.688427	14.920608	13.818348	14.644443	14.769473	14.088933	14.289643	13.969061	14.002993	14.157670	14.088628	14.314071	13.483894	14.668633	13.597054	13.668609	14.041713	13.902528	14.107221	13.381340	14.893360	13.452250	14.742275	14.178943	13.410994	13.939322	14.611021	14.323349	13.919949	14.465898	14.465534	13.239992	14.707518	14.251440	14.212720	14.391513	14.390763	14.019562	14.279673	14.700650	14.043788	13.676917	13.929201	13.458052	14.206838	14.696136	13.991871	13.927054	13.850953	14.037892	14.078329	14.363735	14.062649	13.393700	14.162992	13.799572	13.742058	14.028483	13.082921	14.282413	13.625907	13.398875	13.966624	13.720180	14.368698	13.199503	14.750726	13.214418	14.267954	14.038793	14.432364

CC2D2A	6.798652	7.078639	7.008175	7.474299	7.684279	7.073059	5.298266	8.098858	8.320030	7.039538	7.352063	6.628549	6.188929	7.402873	5.852202	7.335363	7.334732	6.447013	8.743289	7.585179	7.189713	8.235415	9.894559	7.063971	7.996760	7.753478	8.106503	7.075460	7.620289	6.513895	7.552537	6.077122	6.250630	6.999376	6.472574	6.179488	6.004648	6.765217	7.569307	7.186349	6.752706	7.003383	7.323595	7.931730	7.129553	6.776708	7.750734	6.596013	7.273894	6.605592	6.498576	5.787010	5.980847	7.128470	5.709192	7.660137	6.334340	6.733689	7.293485	6.994310	6.527333	7.237972	7.115180	8.312218	6.766833	6.804468	6.452101	7.220674	6.444900	7.138283	6.661840	7.378793	7.301462	6.096168	6.554476	6.744672	6.774130	6.381820	6.571622	6.404576	6.727719	8.616473	6.205756	6.254379	6.611997	6.669475	6.683902	6.314526	6.881346	6.805088	6.742430	6.625846	6.400990	6.862173	6.119929	7.017298	6.292836	7.799425	7.231027	7.892783	7.451641	6.399465	7.456502	6.742734	7.280541	7.041278	8.275804	7.990014	8.604688	7.196791	8.144547	7.939959	7.220994	7.953706	7.625933	7.967867	7.478298	7.428515	7.328812	6.491854	6.512277	6.687309	6.930487	7.933412	6.953139	6.730314	8.205080	7.438512	7.754859	6.403904	6.463746	6.840632	6.637049	6.115466	7.195686	7.212966	7.768234	8.081336	6.555125	7.908033	8.455966	9.212520	7.709559	7.436017	6.256911	7.786930	8.058720	8.288718	6.248137	8.348548	7.251537	7.572480	6.453116	6.435961	6.991611	6.549020	7.245330	8.528767	7.739134	6.926316	7.719509	7.124170	8.403459	6.846321	6.475930	6.973662	7.243086	7.410476	6.402115	6.517061	6.863771	6.292519	6.883083	6.208832	6.271028	6.422546	6.569361	7.480444	5.700818	6.095149	6.454306	6.812404	7.739616	6.949978	6.794463	6.872894	6.730953	6.870813	6.640481	6.447349	7.078944	7.032798	6.816500	6.592272	6.473753	6.967719	6.570194	6.752706	6.477451	5.942139	7.875198	6.722726	6.521545	6.362193	7.957449	6.416995	6.372450	7.214830	7.596551	7.883516	6.891740	7.151068	7.343967	6.484786	6.969337	7.719199	7.939959	7.194046	7.046406	7.592559	7.650640	7.808494	6.166312	7.160752	7.395552	8.306636	7.582572	7.832466	7.345981	7.540820	7.148828	8.242339	7.877023	7.584267	6.848549	7.546449	7.164273	6.365038

AHNAK	3.108304	3.919566	4.003237	3.819283	3.411831	4.053659	4.135625	3.208073	3.205732	3.857435	4.600200	3.291907	3.560111	3.532151	3.911207	3.839470	3.968532	3.856357	4.463946	3.575186	3.613360	3.007561	4.274425	3.491669	3.086948	3.610550	4.734206	2.953929	4.713705	4.247541	3.468038	4.816890	4.700271	3.656217	3.053243	3.895854	3.397480	3.384997	3.163323	3.046612	4.248379	3.086146	3.642453	4.144088	3.446341	4.484911	4.277970	3.394330	4.231140	3.600306	3.441340	3.314311	3.485520	3.937075	5.037135	4.900919	3.886551	3.626618	3.371691	3.607655	3.192444	4.082384	3.516064	3.121463	3.244569	3.823109	3.206907	3.793794	3.641091	2.942606	4.013112	3.779151	4.509641	3.236242	3.749198	3.815038	4.245775	3.488837	3.836113	3.445079	3.611782	3.641293	3.621479	3.793159	3.196384	4.741525	2.931868	3.174752	3.859612	3.410757	3.122628	3.037323	3.266150	3.685428	3.470157	3.569066	3.908793	4.179211	3.762565	3.076658	2.980038	3.693392	4.115928	3.423840	4.329236	4.731233	3.972872	3.759866	3.194161	4.825610	3.140408	3.415206	3.333864	3.664440	4.762713	2.909302	3.069947	4.940761	4.009546	3.712959	3.559157	3.197591	3.889613	3.298831	3.855536	3.506053	3.583962	3.593015	4.489992	4.666915	3.865362	3.684027	3.190142	2.930172	3.881295	3.296369	3.495782	3.330982	3.271303	3.286382	3.605963	5.930924	3.411654	3.716082	3.566480	3.170910	3.457694	4.031479	4.780099	3.818855	4.011437	3.225976	3.625812	3.007349	3.621076	3.420470	3.075859	3.187793	3.010582	5.100522	2.998889	3.418265	4.141719	3.672869	3.959773	4.845952	3.273130	3.442756	3.473810	3.338435	3.412735	3.407494	4.013825	3.260153	4.432246	3.532925	3.585898	3.145941	3.450610	3.028271	4.551752	3.561567	3.459121	3.410579	3.400891	3.237122	3.500497	3.770910	2.948429	3.335465	3.797760	3.917683	3.914887	3.098313	3.998211	3.745894	3.061684	3.857911	3.094889	3.460369	3.741358	3.211164	3.433246	3.331586	3.345907	3.364521	4.036922	3.655630	3.343649	3.181230	3.128807	3.095680	4.009758	3.461116	3.481157	3.218416	3.359328	3.609596	3.445079	3.594809	3.305531	5.996901	3.022786	4.689279	5.683817	3.248706	3.695478	3.242816	5.087580	2.939146	3.005456	2.875281	3.809313	4.207063	3.706665	2.920622	4.124861	5.131805

ADAMTS18	4.705969	8.919067	6.484435	3.940865	5.320335	5.223707	4.824325	8.736729	3.264817	7.240235	5.403080	5.139018	6.135334	4.034749	4.550441	3.015552	3.413453	4.982161	5.046672	5.351486	6.262169	3.684424	6.456884	3.884501	3.660415	3.595732	5.156673	5.152764	5.792732	6.601017	4.409815	6.673975	7.881227	7.152007	5.917082	4.368705	6.021175	5.501007	6.550859	2.945757	5.590809	4.678389	6.328231	4.264426	3.905507	5.167290	6.495744	7.278927	4.893955	4.059534	6.647877	5.447613	7.724989	4.906492	6.277687	6.337219	8.862151	2.908776	6.260320	6.068594	5.323834	5.954371	9.369059	4.402892	7.052870	5.169309	2.830622	3.859999	6.313672	4.584365	5.965720	4.304427	5.821481	5.217960	4.446690	7.940884	5.319391	9.598799	6.860269	6.566408	6.834227	7.183306	6.136867	4.900919	4.947941	5.690981	7.153391	5.523836	7.551749	4.105442	6.080902	5.552010	7.451050	7.121335	5.673483	4.813536	4.514603	5.011599	4.465643	3.049958	8.602194	7.141116	4.556426	4.621238	4.016509	4.276612	6.482902	4.214482	3.230636	4.784747	3.237433	5.085724	4.395213	3.857435	4.144966	3.578380	4.448244	6.363201	4.503720	6.552682	6.185046	5.579830	5.590622	7.380012	4.740639	5.377137	14.073307	3.922224	6.203297	6.139271	8.771295	5.263694	3.884687	3.385360	6.443034	4.994379	9.822386	7.088975	10.727782	4.123822	6.111319	7.343018	7.298474	6.148479	7.465257	4.728936	3.079184	5.013631	6.417818	6.583920	8.140022	9.049275	5.401078	7.277818	6.112642	8.443325	8.021935	6.449821	6.289289	4.567032	7.600924	6.579309	5.321096	7.494871	6.247463	4.323583	8.915570	6.476576	6.939832	6.045528	7.400216	8.883738	6.442684	7.706091	8.128554	12.703807	6.607708	5.658509	6.978487	5.199677	6.673327	5.250586	6.007743	7.994267	8.513238	5.769010	5.464428	5.173357	5.752653	7.508140	7.017144	4.088735	6.162716	8.375886	8.967589	7.740395	6.431572	7.413210	7.272830	7.522082	6.957350	5.800809	7.465716	6.495585	5.965177	6.981481	7.707918	7.938385	7.571715	11.430746	4.620829	6.380781	6.323204	5.896175	5.917588	6.806475	8.073965	4.356325	6.350625	5.922559	12.784513	4.839385	7.268785	5.615376	5.272049	3.074230	4.915972	5.413577	4.602712	6.421107	5.079407	3.915505	4.538874	3.939637	4.109814	6.319104	5.411685	7.147857

ANKRD36C	15.161343	14.290297	13.606202	14.620707	13.644025	13.869088	13.376076	12.513691	14.491975	14.166466	13.795660	14.022689	13.848547	13.823712	13.513328	13.692881	13.887252	13.741329	13.921032	13.885964	13.066780	13.952644	13.982141	13.973052	14.273182	14.389287	15.455098	14.297708	14.513178	14.771836	13.956042	13.577170	13.747588	15.271483	13.670545	13.763964	13.724455	13.156342	13.386677	14.112869	13.883806	14.217310	13.901234	14.099196	13.615689	13.561518	14.535115	13.254784	13.556139	13.289437	14.044975	12.762553	13.333300	13.528769	14.014703	14.020995	13.165282	13.420156	13.755369	13.870167	13.888066	14.342484	14.084036	14.380635	13.796422	13.869352	13.770612	14.077200	13.518872	13.790203	13.311534	13.696085	14.185238	13.320065	13.592841	14.287271	13.444604	13.979690	14.395124	12.748989	13.774210	13.465527	14.005290	12.939212	13.386045	14.025995	13.609219	12.862032	13.967167	13.463806	12.328136	13.310550	13.435992	13.485992	14.659533	14.057105	13.597687	14.254446	13.980804	14.243708	14.242398	13.655271	13.459378	13.589555	13.535095	13.967420	14.589339	14.119553	14.385259	14.026521	13.207531	14.193049	13.698230	14.728340	13.542577	14.218945	15.109821	14.831507	14.991150	14.320921	13.698484	14.324060	14.009539	14.325742	14.289643	14.446360	15.618883	14.954990	14.285845	14.243065	14.305307	14.245367	13.831800	13.722244	15.248690	15.129649	15.267207	14.556030	14.048831	13.829453	13.587916	12.966367	14.759178	13.957666	13.553941	13.510396	14.294729	15.627072	14.370844	15.269335	15.069269	14.448739	13.048973	14.762371	14.143957	14.465165	14.335789	13.853253	13.919694	14.376679	14.580592	13.847752	13.221376	13.601738	12.957704	13.643272	14.212720	14.746490	12.770340	13.629180	14.038510	14.085269	12.891375	13.414830	14.149795	13.973310	13.985572	14.269241	14.277909	13.372935	14.223156	14.378131	13.928691	14.010352	13.739368	13.650539	13.865143	13.291948	14.817850	14.307332	13.539058	14.309339	13.516909	14.675960	14.721418	13.304338	13.430141	13.324386	14.756024	14.141134	13.448635	12.984620	14.877613	14.091290	14.275219	13.331689	13.726632	14.046144	13.665482	15.615956	13.769304	13.662485	13.365784	13.394726	14.518957	14.633240	15.403084	14.456521	14.533864	14.438602	14.780447	15.511748	14.587687	13.706546	15.085910	14.034659	13.385185	14.012930	13.856222	14.076027	14.425606	15.514297	14.352005	14.655036	12.608850	14.233212	12.910727	13.988680

ANKRD36B	15.161343	14.290297	13.606202	14.620707	13.644025	13.869088	13.376076	12.513691	14.491975	14.166466	13.795660	14.022689	13.848547	13.823712	13.513328	13.692881	13.887252	13.741329	13.921032	13.885964	13.066780	13.952644	13.982141	13.973052	14.273182	14.389287	15.455098	14.297708	14.513178	14.771836	13.956042	13.577170	13.747588	15.271483	13.670545	13.763964	13.724455	13.156342	13.386677	14.112869	13.883806	14.217310	13.901234	14.099196	13.615689	13.561518	14.535115	13.254784	13.556139	13.289437	14.044975	12.762553	13.333300	13.528769	14.014703	14.020995	13.165282	13.420156	13.755369	13.870167	13.888066	14.342484	14.084036	14.380635	13.796422	13.869352	13.770612	14.077200	13.518872	13.790203	13.311534	13.696085	14.185238	13.320065	13.592841	14.287271	13.444604	13.979690	14.395124	12.748989	13.774210	13.465527	14.005290	12.939212	13.386045	14.025995	13.609219	12.862032	13.967167	13.463806	12.328136	13.310550	13.435992	13.485992	14.659533	14.057105	13.597687	14.254446	13.980804	14.243708	14.242398	13.655271	13.459378	13.589555	13.535095	13.967420	14.589339	14.119553	14.385259	14.026521	13.207531	14.193049	13.698230	14.728340	13.542577	14.218945	15.109821	14.831507	14.991150	14.320921	13.698484	14.324060	14.009539	14.325742	14.289643	14.446360	15.618883	14.954990	14.285845	14.243065	14.305307	14.245367	13.831800	13.722244	15.248690	15.129649	15.267207	14.556030	14.048831	13.829453	13.587916	12.966367	14.759178	13.957666	13.553941	13.510396	14.294729	15.627072	14.370844	15.269335	15.069269	14.448739	13.048973	14.762371	14.143957	14.465165	14.335789	13.853253	13.919694	14.376679	14.580592	13.847752	13.221376	13.601738	12.957704	13.643272	14.212720	14.746490	12.770340	13.629180	14.038510	14.085269	12.891375	13.414830	14.149795	13.973310	13.985572	14.269241	14.277909	13.372935	14.223156	14.378131	13.928691	14.010352	13.739368	13.650539	13.865143	13.291948	14.817850	14.307332	13.539058	14.309339	13.516909	14.675960	14.721418	13.304338	13.430141	13.324386	14.756024	14.141134	13.448635	12.984620	14.877613	14.091290	14.275219	13.331689	13.726632	14.046144	13.665482	15.615956	13.769304	13.662485	13.365784	13.394726	14.518957	14.633240	15.403084	14.456521	14.533864	14.438602	14.780447	15.511748	14.587687	13.706546	15.085910	14.034659	13.385185	14.012930	13.856222	14.076027	14.425606	15.514297	14.352005	14.655036	12.608850	14.233212	12.910727	13.988680

UPRT	13.060993	11.056587	11.822002	11.148560	11.931845	12.444671	12.158335	12.443196	12.775824	12.215485	12.004288	12.050127	11.232575	11.214808	11.006117	12.621829	12.160336	11.340088	11.800445	12.248149	10.813866	11.086028	10.186029	11.295264	11.279734	11.752181	10.834038	11.869641	11.552884	11.506274	12.466468	12.139271	12.071894	10.910897	11.687068	12.155411	12.428156	10.886421	10.400485	10.958253	12.654306	11.019457	10.118219	11.565093	11.413399	10.860851	12.325410	9.978579	12.318545	12.073426	12.010184	12.280226	10.859835	11.938785	11.836797	11.723934	11.967332	10.801045	10.365940	10.633662	10.661622	11.835426	10.396585	10.733854	10.032856	10.832583	10.745421	11.016201	10.162156	10.889511	12.238682	11.981504	12.002196	12.146111	12.033077	12.048678	12.299016	11.756782	12.129478	12.250568	12.185061	12.045443	11.920971	12.369716	12.225203	12.146258	11.864994	11.926448	10.787634	11.521612	10.945805	11.006838	10.860728	11.796151	12.185427	12.015212	12.348206	11.997004	11.931223	11.666419	12.226923	10.939911	11.394433	11.821409	11.379157	12.289665	11.281195	10.593619	9.992352	12.641230	10.487647	11.065879	10.386413	12.344394	12.691189	11.003077	11.214098	12.050881	12.765660	9.715179	10.851641	9.944548	10.291426	8.735793	10.990768	9.615149	10.680054	9.184855	10.710821	11.185699	11.464783	8.825863	8.595641	10.240459	10.858937	10.289000	10.417364	10.722212	12.565935	7.650787	10.214731	9.764810	10.672137	9.315818	11.429874	11.801784	9.187538	8.875702	11.624303	12.021503	10.877948	11.177620	10.811523	10.368062	11.737491	10.901565	9.753921	9.598476	9.636066	8.719507	9.531152	8.778865	11.088131	10.062899	10.519232	8.813362	8.878315	9.885624	10.036715	10.701056	10.731478	10.609210	11.721853	11.040159	9.929996	11.356381	9.908340	9.033262	9.896439	9.596161	10.145661	10.520492	9.785196	9.624463	9.864846	8.302895	9.218559	9.671410	10.228793	11.155073	11.504954	10.471779	11.131496	10.860574	11.375453	11.353455	10.208918	10.911326	11.585304	11.883139	11.922470	10.896111	11.464636	12.255523	10.292644	10.399138	12.138794	10.479789	9.902734	9.929695	10.372584	9.944399	10.995378	11.561285	11.180812	10.451232	10.468163	11.411822	10.453422	11.608175	9.652120	10.167210	10.310810	12.050582	10.902031	11.179610	8.835535	9.402771	10.165490	9.125977	10.077750	7.833692	11.503654	10.643878	9.352445	10.179183	10.520951	11.275035

BAX	9.939497	9.859885	9.680298	9.248587	9.607163	10.267937	10.015392	9.948125	10.345819	10.047564	10.297389	10.191000	9.688544	9.654364	9.808233	9.893017	10.583475	9.395577	9.109712	10.700036	8.836481	9.966653	8.826851	10.007378	10.507219	10.354444	10.139408	10.173953	9.196762	10.699572	9.772221	10.434301	10.137297	10.836621	10.728981	10.442286	10.205821	9.613488	10.108989	9.847374	9.973251	9.078242	10.024169	10.845782	10.625655	10.759701	9.387940	10.515623	10.715826	10.704907	10.178593	10.560714	10.159669	10.953621	11.071058	10.101581	10.476626	9.726052	9.845829	10.589798	10.301888	10.195389	9.126898	9.217742	9.159846	9.797503	9.937098	9.856956	10.189346	9.627096	10.409077	10.459101	9.499181	10.469815	10.668143	10.900123	9.814786	10.188159	10.196916	10.686442	10.527453	11.160666	10.796271	10.566346	10.851782	10.116364	11.068070	10.884311	9.451718	9.335710	9.654364	10.179803	9.667979	12.258508	10.281837	10.361744	10.335092	9.499786	9.782528	10.822224	10.648499	9.603604	9.762628	10.041471	10.170273	10.398683	10.053909	9.838268	9.173899	9.587009	9.083038	8.677593	9.991882	8.857582	10.508560	10.201561	10.419038	9.875282	10.545676	10.201258	10.468163	10.066310	9.849917	10.517262	10.705048	9.944548	9.624073	10.603402	10.686890	10.170130	10.977437	10.560091	10.235895	11.199882	9.527706	11.201442	9.441732	10.192816	10.495265	10.318307	10.575699	11.409515	9.954745	11.483264	10.825918	9.997954	11.566546	10.673716	11.186572	9.937098	11.244199	10.657396	11.969922	11.087821	10.723571	10.696336	9.853854	10.786621	10.772696	11.432097	10.790846	10.687045	12.073894	11.475989	11.163914	9.889840	11.427955	10.819847	11.482399	10.831500	11.458920	10.572740	11.637393	11.151275	10.579203	10.551138	10.137598	10.602690	10.517707	10.822955	10.302486	9.975129	10.922099	9.957211	10.024169	10.695729	10.312584	11.126061	10.573060	10.833447	11.156239	9.964730	10.770306	10.613340	11.362688	9.894424	10.874572	10.980402	10.751156	11.006998	11.800445	11.264402	11.148096	11.063499	10.175160	11.459197	11.512020	9.991246	10.080121	10.640618	10.870510	9.907273	12.071131	10.719585	10.581882	10.530253	9.505030	10.376210	11.119415	11.430015	9.659835	10.115138	10.542840	10.476787	11.429945	10.914172	10.664303	9.581892	10.305204	10.057120	8.594404	9.082889	9.180624	11.284747	10.220796	11.530935	9.934023	11.756172

GFRAL	4.083256	3.609596	5.299282	4.151468	4.190613	3.820601	3.420255	5.604354	4.199007	3.479469	3.549657	3.963586	3.282097	3.930246	3.710276	3.977012	4.820845	3.284905	5.172533	3.722103	5.527072	4.632624	7.020771	3.694253	4.458439	3.732140	3.785011	6.230595	3.399012	3.306834	4.292692	3.705487	5.191894	3.240594	3.563815	4.125253	3.708838	4.309946	4.522120	4.801148	3.765704	4.823740	4.159720	3.940444	3.829744	5.899553	4.732932	3.875659	4.606485	4.022811	4.023218	4.372814	4.579040	4.328766	4.119034	3.569686	4.270510	3.095293	3.562491	3.783976	4.647039	4.016060	4.876104	5.286398	4.530110	5.269469	3.482425	4.524788	4.444333	4.927430	4.325528	4.385418	3.730048	4.367861	4.412855	4.191920	5.155213	5.088396	5.125562	4.481723	3.891573	4.235728	4.111378	4.647688	4.202709	5.082049	4.455432	4.861840	4.820845	5.134262	5.733207	5.929525	3.442756	3.941310	3.635673	3.749998	4.344818	3.920397	3.239051	3.563462	4.311896	3.272865	4.059096	3.452598	5.102775	3.576726	4.619091	4.666013	5.385758	3.487711	5.106656	4.921258	5.077569	3.763184	3.542277	5.105453	4.894812	4.417754	3.782082	4.317596	4.011825	4.774907	4.482648	3.855740	3.962484	3.221793	4.864946	4.640524	3.559545	3.404213	3.934099	3.745044	4.115070	3.357243	5.063876	4.679009	3.235083	4.753194	3.497287	4.814569	4.407819	6.824177	4.303765	4.969897	4.217728	3.982401	5.747936	4.342419	3.652825	3.933902	3.872419	4.297220	3.246827	3.524010	3.925015	3.768431	4.472347	4.076198	4.641771	4.005892	3.719743	3.684817	4.427665	4.348963	5.401078	5.254943	3.563042	4.295017	4.797060	4.053433	3.742196	4.337446	4.501376	3.618767	3.199539	4.642979	2.976407	3.266751	4.466966	5.011793	3.322641	5.077975	4.808066	4.742543	5.304038	2.872264	4.170991	4.668169	5.206733	4.243640	4.246198	3.427805	4.707853	4.560764	4.772404	4.890226	5.024042	5.171712	3.843368	3.938998	4.779910	4.300454	3.862994	4.366775	4.866626	5.306921	4.391694	5.024855	4.431596	5.245997	5.956452	4.675590	3.718118	3.845888	4.036699	4.049977	3.435989	4.050878	3.267061	3.522436	3.538456	3.725976	3.697556	5.102975	3.856144	4.648544	4.786204	5.071019	5.050186	5.180956	5.453616	6.420618	4.114367	3.283480	3.569686	4.855423	3.948147	3.576932

MRPL42	11.943639	11.153927	12.043801	11.591754	11.857671	11.683207	11.843554	11.773277	10.134374	11.432540	11.764040	11.629546	11.461422	10.995821	11.123360	10.982333	11.557301	11.046942	10.414630	11.577776	11.104718	11.109107	10.894605	10.759553	10.581000	11.258854	10.138967	10.644592	10.830474	11.514083	10.861451	12.114204	12.321897	11.868313	11.997634	11.996703	12.146584	11.272662	10.954214	10.897349	11.398216	10.845627	10.931888	12.020782	11.831058	12.043340	10.822656	10.906618	12.070678	12.046176	12.046176	12.145607	11.325406	12.449603	12.204037	11.627766	12.361470	10.955581	10.926688	11.532441	10.719585	11.436109	10.726580	10.641061	11.376800	11.183614	10.924165	11.150531	11.341779	10.769710	11.780607	11.719572	10.867581	11.774311	11.735252	12.119577	12.026913	12.206196	11.864391	12.102972	11.186293	12.303440	12.216095	12.302476	12.053308	12.076202	12.238682	11.904757	11.128957	11.361048	11.247077	10.846519	10.764768	12.030495	11.828693	12.054398	12.173094	11.350540	12.027658	11.117676	11.423160	11.375615	11.448235	11.876151	11.453283	11.555942	10.200663	10.926096	10.565193	11.366409	11.424787	10.178735	10.759975	10.944454	12.046469	10.910730	11.187573	11.762074	10.417196	12.188852	11.346092	11.981815	11.643417	11.002938	12.197215	11.475847	11.071366	11.483981	11.734522	11.551660	10.559019	10.662229	10.795256	10.367456	11.044735	11.951260	9.980403	10.817456	12.660869	11.427955	10.877054	11.810691	10.717011	11.631959	11.869488	11.772261	11.787619	11.141929	11.829413	11.330888	11.331187	11.244350	12.150924	12.216837	12.579338	11.880421	11.142648	10.956359	11.025697	11.643868	11.010491	12.052510	12.667306	12.509485	12.711078	10.776235	11.599023	12.452166	12.531172	11.958138	12.249142	11.438954	12.367529	12.199376	11.844021	12.262939	11.498458	11.292209	12.254851	10.994906	11.003228	11.654682	10.941388	11.532717	11.032397	11.564187	11.023071	11.533482	11.085876	11.845834	12.025076	10.258997	11.771398	11.936235	12.107738	12.046618	12.730828	11.700081	11.643568	10.630554	12.393945	11.748029	11.883430	12.267596	10.917928	12.416425	13.427333	10.696190	11.066875	11.454602	11.228419	10.769710	11.993647	11.940454	11.803803	11.495210	10.893147	12.007474	12.377241	11.472082	10.636826	11.180670	11.802387	11.427239	11.115772	10.827110	11.726896	11.684797	11.156994	11.930928	11.048103	12.626022	10.518030	12.499288	12.425096	11.650203	12.944441	11.632395

EVL	13.550061	13.580404	13.384142	13.161859	13.583919	13.196137	13.205915	12.289811	13.180160	12.701417	13.206916	13.183285	13.296970	13.086086	13.667672	12.994721	13.284863	13.629686	13.661031	13.171328	13.477273	14.447515	13.614956	13.627746	13.969621	13.250187	14.683638	13.335001	13.466188	13.823470	13.301878	13.565499	14.165841	14.468143	13.817064	13.970725	13.261202	13.475103	14.078902	13.879868	13.158034	14.606651	14.616154	14.038510	14.259287	14.039659	13.387795	13.986959	13.159931	13.781596	13.769541	13.814296	13.785590	13.414432	13.029353	13.254976	13.908273	14.261678	13.947375	14.418363	14.108513	12.925613	15.302315	14.299472	13.928691	13.411878	13.567112	14.109785	13.969908	14.135457	13.172863	13.233400	13.147891	13.535095	13.185635	14.148532	13.225296	13.838739	13.238624	13.439251	13.366847	13.632044	13.671966	12.929211	13.233033	13.312162	13.637147	13.007936	14.601201	13.739872	14.189906	14.519413	13.560400	13.135653	13.924569	13.846933	13.493480	13.560841	13.197729	13.572645	13.347657	13.461144	13.375462	13.442860	13.500882	13.229650	13.669377	14.242398	13.915790	12.987200	13.203792	13.577816	13.711410	12.962718	13.384576	12.694078	14.370460	13.385597	13.279851	13.584643	13.296569	13.727369	14.006448	13.567112	13.126528	13.600359	13.847197	13.381772	13.529454	13.372722	13.473369	13.026917	14.046420	13.522531	13.678246	13.949083	13.741329	13.861539	13.595594	13.447998	14.602393	13.432385	14.746969	14.221891	13.622665	12.638694	12.732190	13.269439	13.543518	14.039659	13.988680	14.036411	13.140268	14.271885	13.068719	13.952936	13.353325	13.548218	14.477088	12.588301	13.976353	14.414735	13.418183	13.853818	13.344471	13.044264	14.479847	13.121579	13.193698	13.364551	13.835875	13.551649	13.319284	14.484873	13.691937	14.133856	14.483264	13.760666	13.773961	14.064420	13.935762	14.278255	13.993914	14.412450	14.408756	14.145840	13.626349	12.928662	14.150116	13.016643	13.365580	13.093696	13.669109	14.205886	13.855412	13.793282	13.692881	13.583239	14.050643	14.162992	13.035259	13.187040	14.296316	14.074554	14.051810	13.380525	14.615271	14.802845	14.507205	13.916872	14.046144	13.540279	13.353095	13.402704	13.693359	13.455950	14.421778	13.258409	14.016405	14.069818	13.668378	14.610567	14.215030	14.019284	13.826855	14.097636	14.262005	13.714879	12.844764	13.827131	14.252773	13.919105	13.387105	13.189322	12.836701	14.307693	13.529678	13.412516

DEGS2	13.550061	13.580404	13.384142	13.161859	13.583919	13.196137	13.205915	12.289811	13.180160	12.701417	13.206916	13.183285	13.296970	13.086086	13.667672	12.994721	13.284863	13.629686	13.661031	13.171328	13.477273	14.447515	13.614956	13.627746	13.969621	13.250187	14.683638	13.335001	13.466188	13.823470	13.301878	13.565499	14.165841	14.468143	13.817064	13.970725	13.261202	13.475103	14.078902	13.879868	13.158034	14.606651	14.616154	14.038510	14.259287	14.039659	13.387795	13.986959	13.159931	13.781596	13.769541	13.814296	13.785590	13.414432	13.029353	13.254976	13.908273	14.261678	13.947375	14.418363	14.108513	12.925613	15.302315	14.299472	13.928691	13.411878	13.567112	14.109785	13.969908	14.135457	13.172863	13.233400	13.147891	13.535095	13.185635	14.148532	13.225296	13.838739	13.238624	13.439251	13.366847	13.632044	13.671966	12.929211	13.233033	13.312162	13.637147	13.007936	14.601201	13.739872	14.189906	14.519413	13.560400	13.135653	13.924569	13.846933	13.493480	13.560841	13.197729	13.572645	13.347657	13.461144	13.375462	13.442860	13.500882	13.229650	13.669377	14.242398	13.915790	12.987200	13.203792	13.577816	13.711410	12.962718	13.384576	12.694078	14.370460	13.385597	13.279851	13.584643	13.296569	13.727369	14.006448	13.567112	13.126528	13.600359	13.847197	13.381772	13.529454	13.372722	13.473369	13.026917	14.046420	13.522531	13.678246	13.949083	13.741329	13.861539	13.595594	13.447998	14.602393	13.432385	14.746969	14.221891	13.622665	12.638694	12.732190	13.269439	13.543518	14.039659	13.988680	14.036411	13.140268	14.271885	13.068719	13.952936	13.353325	13.548218	14.477088	12.588301	13.976353	14.414735	13.418183	13.853818	13.344471	13.044264	14.479847	13.121579	13.193698	13.364551	13.835875	13.551649	13.319284	14.484873	13.691937	14.133856	14.483264	13.760666	13.773961	14.064420	13.935762	14.278255	13.993914	14.412450	14.408756	14.145840	13.626349	12.928662	14.150116	13.016643	13.365580	13.093696	13.669109	14.205886	13.855412	13.793282	13.692881	13.583239	14.050643	14.162992	13.035259	13.187040	14.296316	14.074554	14.051810	13.380525	14.615271	14.802845	14.507205	13.916872	14.046144	13.540279	13.353095	13.402704	13.693359	13.455950	14.421778	13.258409	14.016405	14.069818	13.668378	14.610567	14.215030	14.019284	13.826855	14.097636	14.262005	13.714879	12.844764	13.827131	14.252773	13.919105	13.387105	13.189322	12.836701	14.307693	13.529678	13.412516

CMTM1	7.183928	7.081923	6.872894	7.701304	8.157947	7.983020	6.816661	10.744634	9.142279	7.687797	8.232693	6.822459	6.514226	7.076359	5.222149	7.641386	7.833692	7.245764	8.520817	8.226898	7.245923	8.034188	7.994267	8.179009	7.989848	7.793544	6.798807	8.148523	7.518351	6.667856	8.159168	6.262852	7.245173	6.401461	8.591289	8.255576	7.512640	7.927574	7.630529	7.754107	8.285931	6.496858	7.391321	7.026996	6.907980	6.538584	7.491960	7.460098	7.159016	6.986518	7.018597	7.385155	6.918047	7.066988	7.090101	9.638574	7.536719	7.580071	6.547569	6.821650	7.063661	8.124953	5.736114	7.753171	6.024120	7.373429	7.088794	8.260954	6.806475	7.560139	8.122765	6.989357	7.601986	7.375490	7.642481	6.962293	8.335081	7.164719	7.143631	7.027965	6.394030	7.291767	6.336194	6.865336	7.316534	7.364203	8.201973	6.869212	7.047385	6.901432	7.032959	7.202252	6.631711	7.090879	6.680462	7.512950	6.321010	7.516962	7.592260	6.839672	7.248533	7.569912	8.474316	7.454763	7.204398	8.945066	8.999804	7.432820	6.826564	9.073896	7.228105	6.746276	6.588358	8.491179	7.784873	5.162860	7.631919	7.019995	7.952475	6.137188	5.425215	5.318802	6.415472	6.568387	7.559979	5.401460	6.937962	6.637206	6.107820	5.950215	6.454610	4.710123	4.264885	4.372814	6.491065	6.960043	6.232252	6.813191	6.060096	5.265055	6.244453	6.657265	6.606540	6.233086	6.072404	6.200316	6.535162	6.562355	5.794931	6.017258	6.553679	6.333488	5.586429	5.709572	6.293693	5.781818	6.616082	6.322896	6.568544	6.610626	6.049248	6.272050	5.793310	5.218947	5.987742	5.473286	6.106037	6.458037	5.649152	6.138412	6.930002	5.729084	5.386761	6.434442	6.043810	4.087434	6.154122	5.733015	5.940438	5.833923	6.457563	5.608542	5.878151	5.873704	6.129010	6.011036	6.064688	6.524972	6.447841	6.440547	5.949174	7.014515	5.653855	5.629161	6.480249	5.953623	5.860863	6.480576	6.249790	6.125302	5.905442	6.029427	6.247965	6.291154	5.562964	5.941126	6.388847	6.133084	5.838053	5.707221	6.379122	6.102386	5.794762	6.023946	5.790780	5.524600	5.500842	5.404608	4.994999	5.354518	6.182888	6.013309	5.617024	5.484266	7.044822	5.873876	6.818962	6.688383	6.568059	6.117854	5.476909	4.793938	7.755586	5.288749	5.134051	6.200659	6.500369	6.737455

CKLF-CMTM1	7.183928	7.081923	6.872894	7.701304	8.157947	7.983020	6.816661	10.744634	9.142279	7.687797	8.232693	6.822459	6.514226	7.076359	5.222149	7.641386	7.833692	7.245764	8.520817	8.226898	7.245923	8.034188	7.994267	8.179009	7.989848	7.793544	6.798807	8.148523	7.518351	6.667856	8.159168	6.262852	7.245173	6.401461	8.591289	8.255576	7.512640	7.927574	7.630529	7.754107	8.285931	6.496858	7.391321	7.026996	6.907980	6.538584	7.491960	7.460098	7.159016	6.986518	7.018597	7.385155	6.918047	7.066988	7.090101	9.638574	7.536719	7.580071	6.547569	6.821650	7.063661	8.124953	5.736114	7.753171	6.024120	7.373429	7.088794	8.260954	6.806475	7.560139	8.122765	6.989357	7.601986	7.375490	7.642481	6.962293	8.335081	7.164719	7.143631	7.027965	6.394030	7.291767	6.336194	6.865336	7.316534	7.364203	8.201973	6.869212	7.047385	6.901432	7.032959	7.202252	6.631711	7.090879	6.680462	7.512950	6.321010	7.516962	7.592260	6.839672	7.248533	7.569912	8.474316	7.454763	7.204398	8.945066	8.999804	7.432820	6.826564	9.073896	7.228105	6.746276	6.588358	8.491179	7.784873	5.162860	7.631919	7.019995	7.952475	6.137188	5.425215	5.318802	6.415472	6.568387	7.559979	5.401460	6.937962	6.637206	6.107820	5.950215	6.454610	4.710123	4.264885	4.372814	6.491065	6.960043	6.232252	6.813191	6.060096	5.265055	6.244453	6.657265	6.606540	6.233086	6.072404	6.200316	6.535162	6.562355	5.794931	6.017258	6.553679	6.333488	5.586429	5.709572	6.293693	5.781818	6.616082	6.322896	6.568544	6.610626	6.049248	6.272050	5.793310	5.218947	5.987742	5.473286	6.106037	6.458037	5.649152	6.138412	6.930002	5.729084	5.386761	6.434442	6.043810	4.087434	6.154122	5.733015	5.940438	5.833923	6.457563	5.608542	5.878151	5.873704	6.129010	6.011036	6.064688	6.524972	6.447841	6.440547	5.949174	7.014515	5.653855	5.629161	6.480249	5.953623	5.860863	6.480576	6.249790	6.125302	5.905442	6.029427	6.247965	6.291154	5.562964	5.941126	6.388847	6.133084	5.838053	5.707221	6.379122	6.102386	5.794762	6.023946	5.790780	5.524600	5.500842	5.404608	4.994999	5.354518	6.182888	6.013309	5.617024	5.484266	7.044822	5.873876	6.818962	6.688383	6.568059	6.117854	5.476909	4.793938	7.755586	5.288749	5.134051	6.200659	6.500369	6.737455

SPATA5	8.340131	8.174901	8.453383	8.375265	7.432820	7.412915	8.336800	5.982795	5.707790	7.842803	7.686546	7.758541	7.658770	7.910344	8.238086	7.629708	6.919027	7.946185	8.216812	8.160080	8.138957	9.279219	10.130482	7.247008	7.709559	7.397399	7.517109	6.691766	7.201627	7.968034	7.334421	9.341317	8.942487	9.016697	8.406081	8.664838	8.831497	8.140611	8.959765	8.535593	7.704547	8.736112	8.402822	8.020116	8.191798	8.451090	7.036396	8.673745	8.457624	8.878793	8.644996	8.561461	8.686278	8.675912	8.301962	7.589649	8.807202	8.946272	8.995798	8.823574	9.190087	7.951832	9.151110	8.298205	9.327055	8.720229	8.987020	8.076511	8.878793	8.877254	8.371594	8.241141	7.794157	8.526581	8.400970	8.531825	8.703695	8.911908	8.455824	8.609236	8.793229	8.853327	9.192737	8.691670	8.749240	8.853327	8.165029	8.886537	9.758117	9.006707	8.708855	9.445303	8.561461	8.560535	8.271654	8.411404	8.628819	8.165029	8.115470	7.476764	7.131001	8.144710	7.640962	8.200278	9.307116	7.550222	7.474745	8.789485	8.846787	7.326588	9.175262	7.108192	7.324386	6.717965	7.789160	5.831051	8.101596	8.053853	7.102025	9.421302	8.929545	8.848031	8.415017	7.749822	9.681546	9.376209	9.020620	8.959765	8.575356	8.749240	8.428035	7.796324	8.418107	9.342359	8.149158	9.029665	8.485224	9.104114	10.231313	9.493782	9.524595	9.824794	7.882321	10.281537	9.739498	7.691350	8.024380	8.107889	9.152780	9.626627	8.416918	8.562532	8.821277	9.081509	9.308789	9.145041	8.483880	9.569642	8.533923	9.389155	9.222067	9.445907	9.368622	8.850610	9.072984	7.664047	9.847836	8.687758	9.697396	8.945669	9.085355	9.034328	9.438812	8.860321	7.786221	9.622288	9.703711	9.549938	9.698789	9.682352	8.020288	10.371545	9.478608	9.684363	8.975737	9.902734	8.378322	9.145493	9.896599	8.814706	8.513380	6.877809	8.650718	9.054717	8.634726	8.491632	9.883183	9.035594	8.666821	9.490144	9.505969	9.186600	9.013927	9.454320	8.719946	9.511023	9.456638	9.922286	9.143508	9.463167	10.405917	9.166801	8.975406	8.018603	8.229359	8.083490	7.478298	8.918022	8.551354	8.226324	8.989863	6.545166	8.412161	9.986107	7.223728	8.544468	10.776525	9.070196	8.288103	9.842888	8.704737	7.860910	6.678786	9.292992	9.547475	9.295221	9.234102	9.483345

SCAMP1	11.183614	9.924291	10.271625	9.759499	10.068719	9.386668	10.445361	10.100927	10.203254	9.962091	10.040698	10.057740	10.048197	9.730607	10.519232	10.075427	9.918248	10.741115	10.719908	10.276224	10.475566	9.910192	12.121431	9.826469	10.621738	9.948283	10.136988	9.871373	9.952574	9.656882	10.065569	10.619105	10.618351	10.348574	10.617890	11.058929	10.662229	10.564279	10.654274	10.648782	10.077590	11.384818	10.741413	10.968475	11.488767	10.713006	9.737911	11.041512	10.691815	11.075613	10.930388	11.224957	10.689778	10.947592	10.978923	9.732263	10.981607	11.152318	11.075007	11.115474	11.250930	9.974980	11.480510	10.988232	10.938762	11.040594	10.665912	10.674952	10.235585	10.737937	10.400338	10.376501	9.862701	10.570453	10.450933	10.183600	10.319223	10.385633	10.408136	10.571850	10.570618	10.965351	10.681664	11.036263	10.940232	10.821164	10.594114	11.166369	11.615814	10.978923	11.196818	11.380384	10.482463	10.435072	10.538071	10.377566	10.963543	10.674348	10.238329	10.378655	10.409534	10.332208	10.274087	10.613473	10.873395	10.020041	10.307427	10.310810	10.912348	10.072985	11.391015	10.165185	10.586129	9.517921	9.961059	9.809058	11.653194	10.287417	9.902411	9.517296	9.055357	9.584068	9.319744	8.910089	9.828267	9.983675	10.043738	9.998125	9.086706	8.204198	9.977682	10.441855	9.402607	9.668449	10.853896	10.571850	10.129307	10.519634	11.352601	10.459968	10.710978	10.715371	7.962059	11.145002	9.466126	9.128931	8.917093	10.136400	9.505806	10.819847	10.349330	9.561452	10.604302	11.135202	10.034063	10.215648	9.880559	9.698021	10.589210	10.711967	10.154056	11.010941	11.858108	11.085135	10.717904	9.629836	12.134970	11.630454	10.821608	10.342317	11.246511	10.920769	10.251906	10.733854	10.916152	11.076625	11.547537	10.474364	11.269993	10.306695	9.637166	10.223453	11.030608	11.127669	11.124577	11.113773	9.724995	9.904108	10.387620	10.042830	10.144161	8.710249	10.507667	10.144458	9.522409	10.869173	10.435219	9.739940	10.778128	10.301888	9.875282	10.573931	10.885843	11.564033	10.045398	10.583475	11.915776	11.258115	10.582460	11.173730	10.804897	9.531982	8.968056	9.511815	9.854153	9.664693	9.829982	10.774607	10.552645	9.805482	9.033552	9.778612	10.113185	10.607831	9.947027	10.948753	10.501539	12.858133	8.330440	11.930619	10.001641	9.604702	8.953642	11.102683	10.531134	10.615704	10.835583	9.129229

PRRT3	9.030556	10.189657	10.219691	9.970361	9.782227	10.841416	10.504838	9.792563	10.010197	10.892228	11.319824	10.812246	9.982592	10.673716	9.821311	10.636826	10.139254	11.334799	11.619033	10.601523	11.766908	12.617294	11.374333	11.710281	11.708808	10.583183	9.884378	11.747596	9.235309	9.896599	11.165378	10.894469	11.239797	9.466897	10.315047	10.086020	10.282228	10.310810	11.427665	11.661027	9.803036	11.082183	10.179183	9.449677	9.995882	10.177785	11.946386	10.770741	10.791597	10.253086	10.151718	10.597851	10.284105	11.004401	10.909438	9.858043	10.686302	11.586330	10.884011	11.117968	11.443229	11.167684	11.081461	11.106454	10.464310	10.792489	11.235675	10.169963	10.781158	10.701664	10.619838	10.577067	11.372164	10.821308	10.406956	10.277301	10.338538	10.228061	10.429581	10.766855	10.280486	10.499425	10.348720	10.918816	11.324303	10.305675	11.078552	11.302336	11.613311	11.255761	11.120569	12.400969	10.457568	10.601358	10.495424	10.479922	10.271625	10.003058	10.134213	10.613473	9.881018	10.363564	10.350819	11.096172	11.191061	11.190179	10.468311	10.899971	9.705031	11.059231	12.006559	10.773135	11.393969	10.422252	11.522791	8.971157	9.513068	9.612400	10.795256	8.330258	8.822360	9.954745	10.071016	9.729974	10.893881	10.746795	8.159780	9.545767	9.534452	9.770646	9.904851	8.910839	9.529282	9.797029	9.190397	9.188910	10.339163	10.401855	11.535848	10.750389	11.392925	11.800594	9.379147	10.531581	10.880317	9.404197	10.063036	7.705322	9.687929	9.408176	9.148972	9.540332	9.294119	9.392213	9.062460	9.642697	9.741203	10.178885	9.711867	10.284105	10.091731	10.367597	9.391590	9.763085	10.312904	8.959155	10.536625	9.606243	9.127037	9.507924	10.172297	9.843946	9.359390	9.563148	9.881018	10.946082	11.184951	11.003790	10.002111	10.993881	10.537371	10.237289	10.380607	9.739008	10.436289	11.101634	8.983047	10.541677	10.462340	9.430794	9.499970	10.103273	8.845415	9.958620	9.350764	9.406640	9.596485	9.399396	9.758277	9.507629	10.527040	10.475118	10.082245	10.054204	9.639954	9.758720	11.240198	11.463470	10.212729	12.762748	11.374894	10.206592	10.058151	9.079184	9.648561	8.805524	8.507070	8.736423	10.011226	9.332454	9.759952	8.756380	9.541126	11.701015	10.671515	9.292212	10.164449	9.941340	8.014477	10.299569	9.974679	9.943637	10.093199	9.145041	9.668618	9.787289	10.025914	10.381941

TRAF3IP2	13.191043	12.417829	12.558955	12.863311	12.674839	12.565285	12.724498	13.112985	12.922594	12.958452	12.585646	12.537757	12.718193	12.937994	13.051023	12.847364	12.807870	12.785598	13.025652	12.878010	12.486329	13.761667	11.802240	12.507380	12.545714	12.764108	11.820064	12.942292	12.161570	12.239319	12.858461	12.967107	13.001582	12.741823	12.730151	12.798338	12.710992	12.776543	13.378878	13.348898	12.472784	13.458952	13.086878	12.774646	12.504387	12.731681	12.460607	13.271981	12.845626	13.136216	13.054008	13.039489	12.909315	12.769062	13.161296	12.855512	12.847695	13.650975	13.451174	13.633991	13.479517	12.452025	12.879237	13.502602	13.230666	13.263573	13.086086	13.093911	12.708762	13.377337	12.620010	12.579838	12.505754	12.624672	12.750339	12.619661	12.832199	13.008849	12.716063	12.822839	12.563000	12.557817	12.876415	12.993251	12.664096	12.795036	12.872130	13.281038	13.023845	13.266591	13.015726	13.208477	12.958452	12.910006	12.698531	12.743377	12.710821	12.580335	12.889319	12.945707	12.794317	12.665284	12.658166	13.004678	13.255784	12.681634	13.367062	13.593974	12.467788	12.581823	12.542438	13.359500	13.390336	12.488616	12.956811	11.985558	13.488586	12.704678	12.549408	11.697997	12.363568	12.351789	11.642694	12.392060	12.100873	13.124799	12.660869	13.580404	12.214434	13.220585	12.556362	12.723310	12.980619	13.040804	12.067345	12.964557	12.231416	12.606059	13.286686	12.194595	13.303685	11.755885	12.885092	13.539286	12.812654	12.960501	12.650060	12.333192	12.419287	12.565127	13.173253	13.468126	13.763467	12.332724	12.366707	13.287464	13.014650	13.040624	13.574462	12.695090	13.101611	12.907514	13.731149	13.697560	12.519877	12.833655	12.901614	12.745055	13.138899	13.362235	12.384984	13.225108	13.832809	13.098245	11.765403	13.887810	12.976403	13.134359	13.610375	14.021288	12.380673	13.913715	14.107553	12.945001	12.608516	13.167086	13.153220	13.587916	13.528551	12.465995	12.685364	12.956811	13.153629	12.348967	12.623334	12.949621	12.919349	12.881322	12.718690	12.672419	12.682475	12.902509	12.594107	12.685861	12.702451	13.132807	12.606221	13.145143	13.048184	11.468830	13.180932	12.793936	13.241017	13.004678	12.691524	13.157459	12.033841	12.316396	12.333192	12.602739	13.654386	12.618117	12.650533	13.038580	12.018090	13.689737	12.929922	13.052245	13.177797	12.694911	13.555053	11.676671	12.469069	11.728547	13.509723	13.034495	12.400465	12.624672

COPZ2	10.560091	10.913411	11.337754	11.078117	10.766855	10.249259	11.196939	10.223174	8.955481	10.475118	10.446274	10.702889	11.897375	10.706106	10.600317	10.570618	11.050179	10.349760	10.520492	11.003790	10.289433	10.835128	13.324998	9.991091	12.151731	11.516497	12.259890	11.198550	10.980120	11.005550	10.322742	11.567130	11.554325	12.360524	12.093373	11.210981	11.620681	11.214237	11.604162	10.991323	11.108811	11.609621	11.818854	11.620252	12.586624	11.897652	12.048425	11.886135	11.740420	11.167966	11.831507	12.205573	11.403089	11.477113	11.516358	11.640559	12.757381	11.485624	12.142364	11.953784	12.056353	11.839166	13.558188	11.059091	11.757691	11.781211	11.897513	10.934280	11.008289	11.520394	11.981214	11.420427	10.375288	11.602409	11.261983	12.191343	11.814345	11.405953	11.086782	12.288638	10.559328	11.767202	11.575457	11.608755	12.188852	11.626020	12.148979	11.842648	11.938934	11.006550	11.860555	11.942720	11.302927	11.717638	11.516497	11.887359	11.468967	10.604165	11.771669	11.252949	10.660556	11.853082	10.506316	11.192657	10.818932	10.383667	10.625962	10.440659	11.208087	10.358105	11.544838	10.651183	10.529082	8.948580	9.729812	9.019117	11.088131	11.382315	10.426707	10.153109	12.211933	13.139114	10.963543	12.628339	12.183622	13.289866	9.482261	12.647613	11.663576	12.781892	13.325421	13.726879	11.250783	12.378969	12.254351	12.780022	12.863485	10.340966	10.641644	12.209422	13.031189	12.881870	12.668810	13.002160	13.721765	13.015539	10.683042	10.387334	12.944807	13.440530	12.410460	12.955722	12.605068	12.301392	13.730157	13.948780	12.050127	13.020993	12.495360	12.969695	11.901010	11.314216	14.163952	13.734624	12.982957	11.774769	11.136230	12.871377	14.133559	13.750540	11.895484	14.013491	13.394547	14.028776	12.688641	12.185267	13.381550	12.454055	12.703486	13.078004	13.017023	13.304119	12.766737	12.139582	12.521236	12.930801	12.652596	12.190585	11.355342	13.249512	13.418619	11.524581	13.112197	12.961953	12.326538	13.971012	14.106565	13.530801	13.447998	13.143196	14.031741	14.150745	14.234212	13.737601	12.621342	13.440327	12.704167	13.377337	13.208114	10.811801	13.215196	12.950547	12.843348	13.452889	13.264346	13.105616	12.588140	13.604077	12.487307	12.354041	11.250783	11.634646	13.189708	12.862405	10.806353	10.222241	11.860081	11.205234	10.556615	12.712948	12.941188	7.379857	8.729568	12.141427	12.596981	12.620500	12.492245	11.711473

USP6NL	11.828974	12.401781	12.975277	12.417662	13.114655	13.228647	13.422370	14.017548	14.203355	13.963822	14.115590	13.456831	12.670342	13.101809	12.041809	13.774703	13.710693	12.932422	12.725309	13.575112	12.573019	13.339924	11.271637	13.144955	13.040990	13.075888	11.521749	14.058590	12.963458	12.997678	14.149163	12.899883	13.527474	11.732880	13.069296	13.512420	13.789486	13.240605	13.057638	13.108685	13.350971	12.405277	13.515121	13.118872	12.270085	12.892420	13.412516	12.195580	13.439680	13.496025	12.857277	13.425768	14.050643	13.615689	13.344692	13.028398	13.366676	12.478001	12.038942	11.927240	12.373531	13.681146	10.813574	12.728769	11.556994	12.164845	12.050270	12.084810	11.649898	12.290138	13.205135	13.260048	13.629686	13.646825	13.419947	13.510177	13.533515	13.420156	13.136798	13.194312	11.528614	13.195928	12.775662	13.757000	13.394547	12.986841	13.062098	13.833834	11.710281	12.245455	11.746705	12.339191	12.066571	13.471411	12.577537	13.300495	13.584643	13.846933	13.565264	13.024204	14.221891	12.534781	12.821251	13.284435	12.562827	13.526542	13.846403	13.104067	11.733026	13.815313	12.120362	12.686571	13.039846	13.555911	13.813787	13.018455	10.659929	13.738842	13.947375	12.010648	10.266020	11.262567	10.344466	10.523700	11.285190	11.694995	11.730489	11.784783	10.757072	10.770741	10.750692	10.193392	10.056660	9.306796	11.735541	11.849732	10.549491	10.179803	11.617427	10.941674	10.199765	10.764768	8.663321	11.087082	10.583893	12.030069	11.448524	10.286805	11.591754	11.636925	10.911326	10.970303	12.565935	12.081360	11.558653	11.723465	11.373637	10.599709	10.829442	11.001791	10.803437	11.673557	11.332619	11.938053	12.049502	12.071131	10.843773	11.865774	11.532884	11.190758	11.512897	11.434639	11.749227	11.165084	13.131339	11.435242	10.402738	10.792913	10.179942	11.032245	10.674034	9.763561	10.400338	11.218031	10.561618	9.806697	11.130633	10.451538	11.611963	11.996703	11.771536	11.580132	11.252949	11.369313	12.198437	10.495716	11.843396	12.050270	10.449011	9.298003	11.915341	11.162013	11.320686	10.745352	10.916152	11.998395	11.594455	9.928321	9.832615	10.341418	10.867436	9.782227	9.862701	11.305602	10.713869	11.071058	11.307706	11.336278	11.405443	9.497806	10.957667	10.649241	10.650157	10.381941	11.364444	11.814644	11.668177	10.993145	11.924880	10.962078	10.059698	11.215086	12.897068	12.331768	12.700895	10.793359	11.646067	11.017053

RCOR3	11.098557	10.829672	10.911045	11.434046	11.651970	11.215382	11.477556	10.986270	11.491895	11.450569	11.585439	11.250623	11.393682	11.341014	11.386878	11.438510	11.880269	11.268395	11.507297	11.280760	11.211714	11.517093	12.204356	11.084541	11.360757	11.762513	11.821556	11.360602	11.490069	11.135665	11.593866	10.923853	11.022434	11.583022	10.961222	11.009906	11.194032	10.708230	11.051912	11.274456	11.362109	11.232725	11.096621	11.425080	11.433163	11.129871	11.418410	10.468439	11.395949	11.233924	10.933966	11.164797	11.250930	11.337082	11.184648	11.387935	11.362257	11.148990	11.699618	11.154213	11.648556	11.135510	11.426649	11.482247	11.346391	11.028205	11.099443	11.440893	11.109722	11.115474	10.809338	11.319824	11.667440	10.886421	11.286424	11.108664	11.326901	10.954214	11.455650	11.304412	10.634740	11.246940	11.328775	11.344308	11.274000	11.289917	11.166369	11.727061	11.116961	10.822512	10.795127	11.622188	10.653521	11.153181	11.003511	11.385568	11.151852	11.594601	11.102824	12.174829	11.297194	11.369761	11.481367	11.542114	11.665514	11.637393	11.299253	11.846574	11.508929	11.098087	11.770207	11.885091	11.763874	11.517947	11.576321	10.637424	11.686191	11.240198	11.177918	11.465248	10.785111	11.073426	11.711327	11.216707	12.420436	11.413399	12.070249	11.655296	12.257254	10.598624	11.499049	12.234909	11.296130	11.021387	11.507158	11.588326	11.599023	12.160822	12.355477	11.508636	12.318376	11.605218	11.762794	11.330144	11.687807	11.046807	11.641173	11.497421	11.128382	11.907894	11.885261	11.518416	12.040600	11.394279	11.106454	10.745574	10.385324	10.976546	11.085590	11.870089	10.889381	12.138659	11.423001	11.377693	11.541528	11.492591	11.406467	12.077573	11.768702	11.592214	10.871143	11.427665	11.629710	11.190322	12.023884	11.306661	11.561877	12.150030	10.932329	11.742003	11.180670	11.154369	11.729913	11.299529	11.185106	11.078117	10.651028	10.955883	11.093843	10.915990	11.161265	10.804745	11.157753	11.135510	11.219825	11.199316	11.321148	11.383209	11.457578	10.793216	11.693488	11.183029	11.105584	11.497121	11.457578	11.713033	11.501057	11.519937	10.678967	12.342480	10.539572	10.792626	11.404558	10.923568	11.505545	12.201709	12.134970	11.694693	12.028865	11.689898	11.352876	11.655452	11.345061	11.366540	11.199598	11.836322	11.528459	11.553592	10.733727	11.080123	12.154206	12.623334	11.003940	12.565127	11.090592	11.787155	11.636639	10.907373

FAM193B	13.152612	12.739710	12.679396	13.578983	12.978769	13.184481	12.512086	13.572645	13.479517	12.762216	12.502721	12.389516	12.543410	12.712110	11.778071	13.337700	13.073229	11.855029	13.390537	13.352500	12.807530	12.794657	12.898463	12.718193	13.002534	12.630807	13.421049	12.882781	13.557508	13.373992	13.027092	12.259890	12.082923	12.004288	12.734697	12.474271	12.552826	12.795639	13.287852	12.970060	13.104067	12.993823	13.248522	13.035807	12.649882	12.513066	13.311157	13.118268	12.512588	13.273969	12.661032	12.247833	12.034420	12.025867	11.573736	13.520034	11.503209	12.495360	12.325410	11.748325	11.999458	13.410314	12.831516	12.972030	12.204037	12.175459	12.778842	12.940803	12.440469	12.713119	13.156161	13.032134	13.228843	12.736784	12.497186	12.828063	13.056363	12.726510	12.661032	11.840239	11.708338	11.506720	11.802693	11.880705	11.702959	11.890041	11.521131	12.257803	12.493861	12.196008	11.882381	12.247983	12.355945	12.215199	11.969628	11.998089	11.741301	12.186990	12.234834	13.276051	12.568878	12.808590	12.741631	12.590988	12.860230	13.075888	13.171908	13.159158	13.051444	13.666705	12.855862	13.301878	12.866399	13.690456	13.232053	13.939322	13.809161	12.481464	13.392233	11.935813	12.599183	12.503556	12.816305	13.454152	12.893664	12.938874	13.206710	13.199503	13.073229	12.341549	12.186019	12.762748	12.946838	12.185267	13.301249	12.598836	12.049673	13.122875	12.328915	12.627500	12.881690	12.634942	12.989969	12.966563	12.019903	12.804235	12.657324	13.675309	13.118872	12.832911	12.098761	12.518890	12.137094	12.319524	11.933805	12.024345	12.662746	12.925070	12.608672	13.219568	12.363731	12.461907	12.615027	12.163756	12.247193	13.302870	12.773448	12.104520	12.366062	12.287223	12.894372	11.816892	11.957688	11.475722	13.309752	11.153927	12.126882	12.194431	11.483412	11.824728	13.419502	12.642231	12.642396	12.271073	12.027347	12.720580	12.630185	12.254686	12.482306	12.416425	12.620500	13.350135	12.174666	12.136054	12.740585	12.730491	12.358457	12.487141	11.617427	11.316305	11.312923	11.480091	11.720464	11.413847	12.054237	11.221474	11.338052	12.448796	12.068594	12.333343	12.187163	12.004721	12.348206	12.253427	12.529519	12.266082	12.452816	11.980914	12.630986	12.264028	12.732017	12.799365	12.213340	12.611021	13.073430	13.309343	12.608850	12.481140	13.584890	12.789412	13.042576	13.386476	13.874711	12.557506	12.589781	12.225511	12.135276	12.782402

KCNN2	4.139757	3.308901	3.160172	3.042065	2.929615	3.186339	3.295748	3.810138	3.384000	4.905025	3.502755	3.349874	3.995950	3.624260	3.135127	3.160172	5.135431	2.899544	4.409608	3.556033	4.259390	2.945091	6.845709	2.837600	2.860793	3.320743	3.160827	2.931766	2.991100	3.038936	3.567496	3.884271	3.444194	3.072887	4.368922	3.983064	3.499640	3.102534	4.949604	3.023191	3.344161	3.159671	3.506434	3.272404	3.651573	5.453616	5.597418	2.852017	4.719369	3.786494	3.689102	3.617653	3.937075	4.115070	4.162728	3.138823	4.093293	3.431869	2.927229	3.512007	2.867688	3.123684	4.080249	3.709269	3.465757	3.382741	3.049852	3.090739	3.132846	2.861494	3.117845	3.836319	3.179263	2.946251	3.089786	3.325791	3.071207	2.992607	3.051529	3.686042	3.392134	3.975291	3.551420	3.990448	3.546557	4.879022	3.879377	3.570961	3.672321	3.524206	3.241215	3.442401	3.559545	4.018242	2.988422	2.988075	3.765704	3.703060	3.102113	2.942527	3.301772	3.453834	2.975343	2.911012	4.468831	4.739537	3.130253	3.253865	4.305738	3.151974	2.828626	3.335963	3.026482	3.245376	3.550812	3.461300	3.261306	3.090209	3.169028	3.033479	3.399199	3.077588	3.088534	3.104774	3.534620	3.118529	3.224618	3.418786	4.582669	3.366686	3.260006	3.001002	3.127559	3.000661	3.069827	4.688212	2.849093	3.303802	3.341666	3.640480	2.874515	5.764109	3.155331	2.782823	2.895634	3.172650	3.198410	3.455153	3.338286	3.422423	3.439595	3.456795	3.243262	4.134295	3.707302	3.561360	3.149389	4.988939	2.979165	3.120348	2.982002	3.558530	3.286678	4.112673	4.784543	4.588651	2.823537	4.491573	3.689738	3.531098	3.435805	3.953753	4.239674	3.503137	3.057796	3.746872	3.300392	2.837022	3.167396	2.823537	3.234824	3.610969	3.806591	3.741358	4.346971	2.999190	3.195044	3.134492	2.827972	3.054044	3.033076	3.167650	2.954774	3.000357	3.291907	3.060878	3.058119	3.091863	3.196929	3.754254	4.338975	3.605963	3.552978	3.445804	3.941921	3.143738	3.424387	3.396455	3.986510	3.328860	2.868281	3.218416	3.366167	3.181516	3.139452	2.993540	3.241947	3.741157	3.194444	3.082015	3.011063	2.976959	3.182446	4.788062	5.584588	3.123088	3.185819	5.153573	3.088427	3.313790	3.303468	2.935217	3.155076	3.083503	3.023395	3.061684	3.124029	3.232441

TMEM61	10.188009	9.985333	10.443193	10.226509	9.508226	9.598341	11.082337	9.929076	8.808720	10.148232	10.779119	11.089762	10.339163	11.095709	10.574965	10.424711	11.055538	10.650874	9.090933	10.471779	9.601111	8.018603	9.908039	10.302178	11.675026	11.396845	9.491411	11.141336	11.166523	9.989516	10.209666	10.528219	10.563986	9.771440	11.133549	10.888350	11.185858	11.367280	10.943468	10.251447	9.785948	10.958864	10.697783	10.677766	9.786827	10.852247	9.781184	11.072707	10.629019	10.794089	10.697351	10.910445	10.979805	10.609210	11.250783	11.296440	11.605367	11.782962	11.880130	11.471518	12.397227	10.061953	10.699115	10.429581	9.860973	11.913683	10.684823	10.128078	10.142906	10.295699	9.937098	10.376671	10.407254	10.573635	10.019714	10.041915	10.523247	10.850917	10.560567	11.040450	9.811172	11.228875	11.183899	11.278409	11.436814	11.070472	10.869324	11.303088	10.856734	11.017339	10.882649	9.993095	10.316379	10.760858	10.601523	10.925790	11.102534	9.956906	11.223409	10.850468	10.296801	11.311317	9.968029	10.204906	9.054152	10.449177	11.247077	10.377715	9.726215	10.236667	9.218710	10.525043	10.722674	9.701109	9.528337	10.236049	10.941833	11.158629	9.802880	7.668974	8.164723	8.613447	7.868330	8.237926	7.330232	8.084751	6.521703	8.156894	7.517109	8.890238	8.167657	9.187379	10.357076	7.831235	8.429123	9.216553	7.276417	8.494761	8.166131	8.435693	7.461972	8.425591	7.031545	9.278447	7.225445	8.633518	7.425554	8.342015	10.011809	7.726838	9.718286	7.966042	10.248333	10.583331	9.372726	9.057026	8.666965	8.732710	7.060771	7.694121	8.367279	7.612498	9.429598	8.049851	9.175115	8.953479	8.995798	9.700800	7.171971	8.247810	8.587096	10.126478	9.253123	9.521928	7.939154	8.412310	11.696817	10.743665	9.067710	10.204762	9.294119	9.006263	8.877700	8.791835	10.300344	8.448899	8.312531	6.967400	10.989737	8.400184	7.446975	9.452150	8.353209	8.961134	8.575963	9.203412	8.717201	9.619319	10.992097	6.853497	10.358877	9.299232	8.573979	7.563186	8.262447	10.495573	8.431198	9.696933	9.371510	11.158629	7.672436	8.129662	7.928511	8.487060	8.930315	9.750518	7.550222	8.910988	10.402145	8.038673	8.926766	8.340263	7.645097	8.460937	8.627031	6.996619	8.584972	8.154862	8.356589	7.166559	8.995047	7.200694	8.744057	8.635856	8.998577	6.352776	9.651348	7.934793

TRIP12	9.616680	8.676820	9.067393	9.325501	10.340507	9.516071	8.847228	10.678215	12.347575	9.785504	10.315777	9.761038	9.704158	9.666076	9.327985	10.373383	10.691661	9.733183	11.318667	9.569803	10.003500	11.173138	11.650203	9.898827	10.610220	10.443646	11.786268	10.364292	10.665188	8.534543	11.853844	8.175531	8.240987	9.548072	9.023558	8.806427	9.170601	8.356909	9.361712	9.468105	9.857725	9.395428	10.065869	9.795034	10.035467	9.344218	10.474667	8.859264	8.909199	8.990911	9.311708	8.854719	8.849250	8.396872	9.148812	9.937905	9.087175	8.847553	9.278174	8.849419	8.307442	9.708902	9.473089	9.951978	8.050957	9.070354	8.061672	9.035284	7.979734	8.807664	8.376613	8.739325	10.657698	8.667580	8.641436	8.713136	8.594088	8.487233	8.410136	8.955928	9.081035	9.395272	9.073756	8.636910	8.977562	9.244246	9.323193	9.330587	8.510749	8.437404	8.377983	8.554809	8.549674	8.461246	8.935159	9.989046	8.739777	11.562313	9.857107	10.343685	10.909438	9.201379	10.303562	9.526630	9.827521	10.728085	10.354602	9.268147	11.628942	10.166289	10.185886	10.593946	10.740051	10.438251	11.055538	9.665313	10.199926	10.447955	11.700541	9.834429	9.463454	7.464195	9.209617	9.919156	9.134541	8.731061	11.707017	11.406467	8.826002	9.400155	9.335710	9.385728	9.148458	8.958743	10.870835	10.351285	10.171549	9.702953	9.185181	9.932911	11.162013	9.925756	10.554613	8.294418	8.998256	9.788816	9.837507	11.470648	7.883516	12.565285	10.136988	10.370639	9.421896	9.177867	9.489658	8.799601	8.195255	9.301410	8.995047	11.164195	8.125849	9.534300	9.685622	9.588862	8.465978	11.183186	8.680202	8.768231	8.454436	9.453237	9.547475	8.748785	8.955775	9.017297	8.991496	9.109422	8.576254	9.394814	7.934176	9.372574	8.763320	9.955223	9.454926	8.849419	8.780550	8.247677	8.804313	8.558709	8.833772	8.176949	9.032961	10.838706	9.570579	8.424386	9.099030	8.039647	8.230545	9.038669	9.137949	9.484092	9.393593	9.363352	10.059845	9.253885	9.644557	9.098294	9.679564	8.580126	9.611185	8.269821	9.092540	8.943081	8.249596	9.850095	10.125390	9.917640	11.639475	10.673572	8.962687	10.355676	9.859885	10.057740	9.536303	9.708602	10.576472	11.317631	9.535972	10.531733	10.477090	10.023074	10.090835	8.109965	10.035315	8.598050	9.714536	9.548379	10.464884	9.111762

NF1	6.981481	5.494505	6.659365	6.980170	7.847939	7.403178	7.533758	7.171812	7.373586	7.959136	7.936388	7.731800	7.410915	6.971814	5.469134	7.307876	6.635796	6.077284	6.164933	7.970993	6.441376	7.343187	6.460672	6.933634	6.399293	7.624722	7.877641	7.584871	8.248548	7.353283	7.368431	7.451050	6.486761	8.235110	7.472188	7.497706	7.281951	6.255725	6.243953	6.124461	7.719347	5.994297	7.933245	7.057287	7.635884	6.741635	7.261207	4.979117	6.946292	7.057146	7.219504	7.030189	7.251999	7.739134	7.562281	7.124348	7.654215	5.791150	5.680644	6.475765	5.496448	7.881227	4.584365	5.339320	5.640661	5.604749	5.540894	6.177822	6.294553	5.887979	7.193586	7.134995	7.620289	7.588434	7.493678	6.953906	7.568694	7.210174	7.176186	6.860916	7.054118	7.132591	7.700687	6.996153	7.679960	7.987940	8.172546	7.984691	3.795711	5.917588	6.305436	5.491117	5.728084	6.846321	7.027322	7.839084	6.887075	8.455510	7.191239	6.469852	7.847939	7.173384	7.356249	7.026011	5.630261	7.471425	7.072893	6.881192	5.305994	7.981450	5.625771	5.974118	5.812237	8.021345	7.224338	8.305299	6.944078	7.209083	8.070168	7.648283	6.248977	6.863771	6.891257	7.009322	7.510294	7.152007	8.742697	8.634259	8.039959	7.041120	8.012608	6.999376	6.805758	5.720686	7.438844	7.605952	5.981021	7.635247	9.045341	6.794463	7.838479	6.617422	7.139071	6.750364	8.846008	7.024084	7.470168	8.688353	8.186673	9.394342	7.686388	6.406726	8.623129	9.417014	8.676661	7.970369	6.411836	6.599911	6.204845	8.488165	5.916014	8.269526	8.450307	7.896267	6.368043	7.595792	6.856341	7.876561	9.154319	8.153757	7.668822	7.716737	8.640980	7.714346	7.919671	8.915240	6.610967	5.424841	7.297331	5.781067	8.163532	5.366408	5.732672	5.988481	5.493884	6.454774	5.801715	6.779094	6.206276	8.711618	7.799143	8.193517	7.875662	8.727096	6.951703	7.781951	9.084407	8.133618	7.260136	8.378919	7.976790	8.368071	7.875043	8.709936	7.755300	8.263850	8.104010	4.312308	5.893884	6.859613	6.030252	5.240060	6.548938	7.279720	8.032856	7.626701	8.775967	6.802846	7.275464	7.583967	7.650787	7.712816	7.191688	5.609543	7.785744	7.462771	7.185369	6.518553	7.733324	5.528763	5.728635	5.019323	7.627977	6.913700	7.074487	7.553771	6.747226	7.339085

ZIM2	15.796320	12.874491	14.251813	13.487496	13.833047	14.096118	14.989019	13.321286	14.302959	14.788691	13.637384	14.674118	14.378131	12.731001	13.307488	14.670049	15.116030	14.277280	10.727937	14.764819	10.290216	10.992855	12.580656	13.715603	14.197180	14.940922	15.762943	15.625168	15.350928	14.501441	15.104927	14.309634	10.862314	14.464777	15.502234	14.651534	14.143339	13.134732	14.107221	13.596087	15.377722	14.528293	15.489513	15.480428	16.013271	16.044226	15.529882	13.790706	14.867085	14.318185	14.070098	14.658149	15.524473	12.502721	14.280559	14.426320	15.988198	12.506387	12.474915	12.352576	13.266411	16.245035	13.281221	11.099443	11.353312	11.885850	12.713284	11.125198	10.789859	12.194431	15.982133	13.904457	15.090679	14.573899	15.527160	13.768548	13.696837	15.095132	13.165282	12.673786	12.995633	16.092758	13.635468	12.800565	14.313722	15.157558	14.451322	15.435694	12.383858	11.506409	12.135587	11.852005	11.816438	13.067904	13.040624	14.118926	13.559500	14.543837	15.047189	15.306516	14.546394	15.230680	14.964550	14.738102	10.314618	14.670049	14.010352	10.844098	12.347575	15.029282	11.573596	11.933343	12.248492	14.958920	14.551021	14.808146	14.770475	15.265827	15.826441	14.477842	12.239925	15.165299	14.293075	15.899823	14.845201	15.135878	16.298008	15.153835	14.475985	15.697564	14.775142	15.247395	14.557264	15.384452	15.357170	15.087640	14.433465	11.419997	13.313356	11.227828	11.895030	12.327002	14.164900	13.761927	13.933244	15.003643	14.257035	15.571867	14.713469	16.253204	15.801695	15.041784	15.979533	15.239718	15.950994	16.751734	17.434795	13.779642	13.211006	15.064607	13.761156	16.174278	16.292480	16.377052	14.806121	15.213664	16.819885	14.764819	16.233280	16.202822	15.729053	15.205421	16.051180	15.676954	15.370867	15.511748	14.012342	12.871579	14.135776	14.459642	14.981620	10.189657	11.368860	13.449931	13.855680	12.896716	14.202755	13.459166	12.328915	15.013920	16.543736	14.553963	16.210808	15.414655	15.593684	15.826441	15.068646	15.919342	15.573696	15.758639	14.573111	15.416204	16.648016	16.720589	14.900158	15.502988	15.434895	12.957891	13.282220	13.984682	12.259261	15.795114	15.785756	16.052430	15.271483	16.226917	16.907711	14.701092	14.768542	16.199785	14.300251	15.491291	15.031025	11.187573	16.769750	12.556686	10.310511	10.981607	14.576434	12.202831	14.298038	13.327985	14.581385	14.715375	15.074047	14.824917	16.134222	14.164594

LHPP	6.625003	7.096262	6.962923	7.340021	7.586300	7.890146	6.828610	7.328659	7.158379	7.272360	7.220674	7.600158	7.120590	7.082213	6.775893	7.194566	7.154829	7.052109	7.687645	6.991111	7.245330	7.565801	7.758541	7.232119	7.458563	7.551749	7.793850	7.647109	7.636038	7.876419	7.342696	6.476918	6.362694	7.336280	6.676256	6.749580	6.605022	6.684864	7.144281	7.139390	7.437704	6.803991	7.143020	7.274232	6.954882	7.054118	8.093260	6.743667	6.664134	6.728047	6.738109	6.360207	6.443880	6.540675	6.290632	7.411070	6.102725	6.454454	6.774469	6.259469	6.639497	7.493042	7.080486	7.191984	6.799110	6.896554	7.072893	7.023757	6.971814	7.268179	7.043666	7.082071	7.663729	6.775893	6.964378	7.060427	6.809210	6.834978	6.868741	6.317575	6.106746	6.262353	6.504481	6.183960	6.211523	6.303246	5.893884	6.387497	6.346571	6.340056	6.382325	6.858189	6.877490	6.773975	7.029396	6.868069	6.235139	6.938279	7.611728	7.226387	7.336916	7.032329	7.101115	7.124824	7.079582	7.569161	7.278325	7.543904	8.164882	7.475837	7.518351	7.228105	7.223131	7.911114	7.165039	8.276415	7.193101	7.218571	7.507394	6.891902	7.184381	7.893118	7.624107	8.513548	7.729031	7.749652	7.939788	6.857061	7.377194	7.431568	7.540820	7.884003	7.729637	7.601067	7.023420	7.220994	7.158379	8.356290	7.088639	7.547276	7.667152	7.809278	8.002152	7.629544	7.127683	7.345653	8.196996	8.393936	7.474601	7.679233	7.213408	7.051488	7.292246	7.124511	6.874005	6.828610	7.205049	7.538276	7.587483	8.349281	7.097967	7.331331	8.009628	6.748483	6.936806	8.050313	7.266030	6.887904	7.008344	7.173384	6.703390	6.522990	6.473080	6.379471	7.503062	6.156523	6.691940	6.917243	6.236812	6.762969	7.806171	6.499570	6.829419	6.962614	8.290087	7.231821	6.885035	7.045294	7.300476	6.962768	7.144129	8.150052	7.150750	6.976239	6.923207	6.889664	7.026662	6.887904	6.416338	6.338721	6.978816	6.487433	6.694902	6.196904	7.607291	6.178996	6.308073	6.930818	6.741283	6.811147	6.848046	7.290988	7.266030	7.061705	7.446170	7.608374	7.505719	7.573568	7.456028	7.698370	8.202867	7.848092	7.149622	7.138446	8.010496	7.685481	7.660474	7.177471	8.358315	7.735493	7.300970	7.552226	8.375265	7.733172	7.348503	7.011725	7.497273	7.712966

PRIMA1	10.318464	8.632470	8.965547	9.494887	9.850250	9.179848	10.586560	7.948606	7.668974	10.306828	9.643171	9.899145	9.449194	9.287550	9.339487	10.214264	10.558564	9.815723	9.203256	9.327210	9.645034	9.680298	12.377075	8.669426	10.617007	10.393108	11.818235	10.811728	10.460269	9.981518	10.737773	10.440195	8.910390	9.771286	10.216690	9.921681	9.760421	9.334439	9.861294	8.455824	10.694662	9.462868	10.125091	9.482720	10.805632	10.284261	11.455058	8.799766	9.763723	9.723120	9.608373	10.396283	10.140452	9.963157	9.795661	11.032546	10.418738	9.264136	9.816506	10.148575	9.134862	10.289132	11.156708	9.440040	9.184093	9.316241	8.275241	9.068319	8.638648	8.469008	9.566857	9.744273	10.289132	9.947800	9.380804	9.360471	9.819818	10.005054	9.814069	9.674122	8.975271	9.986838	10.073289	10.333876	10.615426	10.167812	9.846464	9.529917	9.881630	8.527057	9.301890	8.653579	8.921659	10.075003	9.068977	10.336136	9.397984	11.011245	10.169807	10.286213	10.366983	9.399554	9.548541	9.504559	8.214100	10.577237	9.935273	9.300176	9.359995	10.691964	9.199065	8.818417	7.178923	9.199838	8.341313	6.222113	8.914492	10.812401	10.841725	11.969197	8.095215	7.812489	8.239920	8.593306	11.911179	10.338376	10.349459	12.149882	4.158193	10.051261	9.414552	7.966655	8.309248	3.049660	10.401108	11.442486	8.903697	9.541747	11.105301	8.846625	6.979414	9.655511	10.474206	8.198081	4.908396	8.181140	4.312308	7.863555	8.072162	8.659760	9.459550	8.529222	6.039453	9.700189	9.305858	8.430079	8.663941	6.816500	4.876960	6.901432	5.739533	8.137259	6.395639	9.214386	8.711767	10.806779	7.696523	8.555103	6.430895	4.326137	9.188155	9.302046	8.028572	11.335254	10.075573	9.550248	8.064117	9.107492	8.181886	8.194308	11.438372	5.343992	6.712799	7.020447	7.832314	8.746045	7.985306	7.476764	5.448411	8.489838	7.559064	8.053241	8.870565	10.164279	7.991423	8.428664	8.348402	8.468700	9.028410	8.158860	9.829180	6.719816	9.428048	8.448899	9.004415	9.753457	11.432696	8.987320	8.398520	5.547131	9.519152	8.039326	8.419307	6.794463	7.994571	7.371267	7.441605	9.146408	8.503865	6.521055	9.185786	8.911573	8.420888	9.625524	8.805380	10.039581	11.121300	6.343517	8.992404	8.901108	8.500912	3.909692	7.514207	5.798581	4.833748	9.175876	7.210174	9.749429

MB21D2	8.902924	8.902638	8.639417	7.637084	7.008175	6.835143	8.538925	7.472188	5.471992	7.662314	7.758398	7.804315	7.946027	6.829577	7.584419	8.094336	7.024421	7.361373	6.868239	8.730171	6.485635	7.556615	7.621803	7.054595	7.259651	7.207379	6.962124	6.827028	6.927150	9.013634	7.592717	9.556781	8.342315	8.545378	8.726647	8.839951	8.675912	8.078556	7.269844	6.855251	7.298925	7.252607	8.325568	8.290887	9.061427	8.627922	6.600216	8.104010	9.153673	8.732236	8.480461	9.220659	9.510217	9.459408	9.675059	7.771578	9.255892	8.758395	7.889992	8.548924	8.342909	7.552226	9.198592	6.803168	7.842803	7.715979	7.693362	7.942495	8.147603	7.384238	8.943397	8.568786	7.342076	8.730027	8.736729	8.723284	8.379846	9.568254	8.717201	8.453220	9.096148	9.904987	9.471707	9.143350	8.822822	8.941097	9.096895	9.160765	8.170358	8.332304	7.719036	8.133618	8.354284	8.806276	8.949507	7.977233	9.724389	7.360429	7.714346	6.014517	6.514720	7.669595	7.389033	7.579650	8.128713	7.015663	5.636952	6.651258	6.001274	6.231918	7.338770	6.464703	6.540849	7.351914	8.208900	3.652626	7.127988	8.215914	7.186189	7.306970	9.547475	9.462264	6.486608	8.479979	8.223133	8.515034	7.961776	8.568119	9.128602	9.490626	8.325568	8.230545	8.237449	7.434079	7.107731	8.331700	7.958214	5.167687	9.029335	6.402954	7.575743	8.163833	9.234563	9.060211	10.421018	7.406172	6.596333	4.369342	9.143799	8.913424	9.620873	8.311618	10.120961	9.281202	10.162738	9.990425	7.298925	8.917249	7.506641	8.830255	8.202867	8.711767	10.207843	10.352828	9.238600	7.859735	9.284768	9.518373	10.583183	9.907128	9.243504	10.264251	11.090314	9.941815	9.223728	10.066454	9.706727	9.090513	9.737303	10.614358	7.975078	8.612161	9.704430	7.445391	9.156924	10.524431	8.329805	9.204578	8.071082	8.764223	10.576019	7.499142	9.568899	10.109280	9.708142	9.041626	9.642396	9.785196	9.032354	10.790437	9.718616	10.156215	10.188617	10.806779	8.622520	10.826216	9.571519	9.227275	8.640510	8.683089	9.213755	8.855027	10.357076	10.603565	9.510392	8.549225	6.551816	7.595624	8.558069	10.385324	9.499476	6.921278	9.442516	9.197498	6.456884	6.342844	6.307403	8.223899	7.153076	8.369861	10.707032	5.498320	5.738273	8.822507	9.571668	7.724844	8.802316	8.867772

PRKAR2A	11.229444	10.610513	11.567413	11.568275	12.080437	12.023550	11.562028	12.214094	12.100593	12.136054	12.097117	12.097254	11.808140	11.793617	10.951782	11.626309	12.150644	12.032600	11.521612	11.840095	11.108070	11.221315	11.352601	11.992156	12.029778	12.009598	10.972192	11.790869	11.929227	11.044022	12.393291	11.449854	10.696036	10.755416	11.370215	11.433599	11.380538	11.013401	11.401062	11.342331	12.121131	11.276728	11.394433	11.482813	11.307629	11.527756	11.946821	11.608893	11.211433	11.673108	11.238457	11.485786	11.472640	12.068126	11.704602	11.890346	11.678294	10.454651	10.944749	11.519395	11.913381	11.575387	10.120036	10.547030	10.267776	11.127532	10.492371	10.432156	10.712574	10.565193	11.156239	11.050340	11.880421	11.042557	11.190615	10.913860	11.547102	10.973089	11.190615	10.581306	11.562028	11.245386	11.428987	11.436404	11.696238	11.461422	11.743340	11.834521	10.250726	11.031483	10.794980	10.496787	10.215948	11.171811	10.629019	11.308100	11.044877	12.070678	11.751430	11.414427	11.940295	11.428860	11.852324	12.049973	11.170321	11.898369	11.089903	10.858234	10.888642	12.104683	10.478435	12.035025	12.212996	12.007316	11.731685	10.837527	11.576165	12.034708	12.306838	10.213349	9.828115	10.362213	11.080729	11.482813	11.301743	10.887033	10.556299	11.644307	12.261188	11.305742	11.257970	10.917928	10.142602	10.898327	10.952579	10.503015	11.435387	9.945429	10.384236	10.618976	10.598160	11.496969	10.959158	11.998089	11.644012	10.594267	11.500204	10.115451	11.089903	10.949348	11.085280	10.183009	11.659417	10.405008	10.784818	10.973659	11.084241	10.611076	10.063772	11.094991	10.709373	10.520492	10.902031	10.846377	10.780147	12.118961	10.493289	10.652809	10.882081	10.688872	10.629178	10.823244	11.610188	10.701804	10.438251	10.409375	9.906665	10.518938	10.549196	11.518846	11.564187	9.605026	10.061353	9.452298	10.745574	10.002558	9.829351	10.501539	10.100782	10.586706	10.388365	10.962810	10.312904	9.969572	10.569055	10.331142	10.679584	10.659045	9.516518	11.019767	10.960626	10.977879	10.707761	11.071521	11.084092	11.080441	11.194445	9.947336	10.085231	10.364867	10.244125	9.817268	11.090745	10.837243	10.659045	10.587449	11.213507	11.082761	10.592028	11.032397	10.213194	10.799798	10.899668	10.249984	12.038203	10.674348	10.200663	10.086189	11.078697	10.323968	10.774607	10.266879	11.581259	9.990270	11.109107	11.446896	11.252514	11.753661

PRAM1	10.244125	10.983494	10.988997	10.723716	10.891326	10.726580	10.673572	10.818491	10.812246	10.742742	10.532370	10.547330	9.728056	10.271475	10.136690	10.667829	10.532684	10.060006	10.466973	10.305675	10.899253	10.944749	9.956000	10.911612	10.531426	10.799059	10.983900	10.780871	10.885690	11.250783	10.825459	10.226065	10.571684	10.982623	11.016776	10.878106	10.615256	10.846519	11.040450	11.068828	11.232005	10.687667	10.943322	10.696336	10.526885	10.665327	11.070164	10.550997	10.644015	10.732367	10.476787	10.441113	10.697641	10.739457	10.317267	10.975539	10.241662	10.861593	10.358409	10.398387	10.773596	10.951940	11.528321	10.804897	11.026892	10.902471	11.317325	10.803143	10.987504	11.275601	11.138168	11.080590	11.018364	10.989295	11.114713	10.963392	10.683914	10.630554	10.647743	9.885624	9.742900	9.730269	9.526785	9.782986	9.833953	10.347802	9.927396	9.775812	10.309563	10.141487	9.926926	11.023655	10.278664	10.392215	10.274087	10.044497	10.326281	9.955223	10.137297	10.853742	10.282924	11.064664	10.999997	11.070312	11.039457	10.310810	10.716420	10.991621	10.635345	11.054809	10.345960	10.293860	10.253644	11.202837	10.195238	11.219527	9.800887	10.161228	10.806201	10.492682	10.941833	11.413232	11.204945	11.616116	10.986270	10.680198	10.618670	10.552501	11.266314	10.859681	10.725060	10.144458	10.441113	10.472372	10.452536	10.752942	10.013832	11.025411	10.334339	10.979805	10.987041	10.927572	11.085135	11.102385	10.791738	10.996398	10.922983	11.315857	11.510243	10.943606	10.596035	10.718823	11.282541	10.779258	10.712709	10.439290	10.980402	11.328644	11.353604	11.633888	11.274308	11.477840	11.077806	10.527453	11.105870	11.418410	11.328499	10.542394	10.903200	10.591883	10.351285	10.610070	11.046663	11.006254	10.971457	10.317728	10.840635	10.648046	10.668285	10.731351	11.452975	11.229016	11.044439	11.675976	11.081007	11.435520	10.826216	11.045017	11.307552	11.235506	11.154369	11.780148	11.018211	11.126352	10.845325	11.090592	11.366409	10.845477	9.969572	9.923196	9.746781	9.539708	9.553280	9.753292	10.467402	10.004264	10.122304	11.107920	10.850468	10.168271	10.073916	10.465951	10.607990	10.254975	10.282619	10.040543	10.477090	10.388806	10.956934	10.474513	11.333206	11.127236	11.201594	10.906005	11.226360	10.815965	10.985390	10.970442	11.442182	11.480371	10.482307	10.289132	11.261983	11.147664	10.164279	11.062298	10.182411	11.379443

DIS3L2	12.187328	12.113307	11.868033	11.846574	12.435794	11.902491	12.584810	13.097119	12.855512	12.859886	12.538788	11.837541	11.705791	12.208223	12.193531	12.195730	12.762553	12.625698	12.108644	12.365418	12.364339	12.206044	11.864535	12.217605	12.236059	12.523015	11.559388	11.967645	11.817176	11.549716	13.104645	11.840095	11.663282	11.125059	11.686355	11.898522	12.075279	12.438685	12.694911	12.938874	12.495856	12.103782	12.104818	12.401128	11.894129	11.983809	11.677583	12.521399	12.161407	12.866728	12.095201	12.364031	12.456606	11.940741	12.503725	11.931707	12.473452	12.699870	12.482634	12.500257	12.167248	11.947128	11.803092	12.270749	12.155866	12.682817	12.389993	12.097725	11.954084	12.653292	11.640863	12.073577	11.945449	11.767960	12.092606	11.801189	11.933343	11.667871	12.035185	12.168505	12.215771	12.484238	11.894274	11.880861	12.496680	12.115840	11.442486	11.887878	12.085752	12.306676	12.141122	12.166049	11.805746	12.182366	11.263206	11.926285	12.490403	12.086801	11.984250	12.840179	12.480642	12.208851	12.470874	12.405442	12.644233	12.310761	13.580155	12.415936	12.644551	12.594107	12.092137	13.086671	12.934196	12.662414	12.585969	11.770344	12.729457	12.579166	12.007161	11.760532	11.428576	11.286577	11.375164	11.700373	11.308969	11.243470	12.147801	11.771669	11.243882	11.517367	11.571034	11.567694	11.399961	11.557144	11.750421	11.996262	11.765403	11.459197	11.468830	11.614795	11.400756	11.578064	11.839322	11.694552	11.629239	11.583468	11.670022	11.415733	11.238920	12.362753	11.028057	11.715575	11.269855	10.879893	11.472951	11.075168	11.695468	11.685883	11.583753	12.065157	11.822002	11.333338	11.552884	11.366119	11.494352	11.781361	12.255202	11.364925	11.616550	11.838717	11.717044	11.196939	11.565952	11.577194	11.696238	11.776387	11.448524	11.734816	11.685541	11.559519	11.402055	11.541954	11.648993	11.600906	11.994731	11.617427	11.648129	11.691529	11.935946	10.998047	11.498297	12.088584	11.796594	11.095709	11.527756	11.412815	11.494063	11.652130	11.292037	11.365055	11.706559	11.263631	11.389403	11.286577	11.894274	11.153181	11.628650	11.438510	11.627766	11.402498	11.729158	11.322294	11.309548	11.559388	11.423308	11.653632	11.659265	11.361357	11.423905	11.323781	11.836176	12.132655	11.320099	11.511560	11.943486	12.336677	11.401374	11.611094	11.928618	11.669383	11.897375	12.167097	12.393464	11.752181	11.399700	12.053155	11.437522	11.662957

GYPC	10.652947	11.702812	11.650941	11.011833	10.931441	10.721226	11.376521	9.826170	9.258639	10.469045	10.480506	10.651028	10.492517	10.843330	10.850618	10.081326	10.674816	10.848448	9.239207	10.408299	9.863484	10.522954	10.363564	11.107037	11.717496	10.984949	11.856087	10.285020	10.641213	11.511412	9.810719	11.247632	11.700373	11.214379	11.638582	11.981214	11.871450	11.666120	11.614652	11.655606	10.817456	11.832552	12.415458	11.587606	12.871579	11.993346	11.102534	11.895619	11.419559	11.783740	11.788373	12.438685	11.995974	12.229570	11.828974	10.513312	12.120508	12.276523	11.650051	12.179402	12.468757	11.291724	12.503202	10.877490	11.620849	11.859940	11.365654	11.284596	10.895631	12.323414	10.998047	11.479176	10.474815	11.193097	11.074288	11.521468	11.184194	11.092539	11.000137	11.408446	11.821556	11.725872	11.603886	11.511703	12.077128	10.950121	11.298834	11.555370	11.448524	11.757091	11.925820	11.136698	11.813142	10.946388	11.436677	11.187132	11.906190	10.962371	11.293383	10.891184	10.544020	11.211573	10.605340	10.617890	10.289278	10.565746	10.478589	10.447031	10.743352	10.521909	10.027498	11.032546	10.263119	10.004749	10.885551	10.762198	11.056421	10.762198	10.732799	10.292343	10.254638	11.451147	10.495265	9.774780	9.140403	9.774645	11.031068	9.058786	9.122293	9.673663	12.579997	10.578359	11.330317	9.655511	10.428979	10.241044	9.156445	8.787683	11.479176	8.835535	9.669262	9.202653	8.872320	10.071474	10.841416	10.053753	9.089471	9.771907	10.661160	9.683095	10.988689	10.279731	9.821458	10.286654	10.306414	10.974650	10.341118	9.435543	11.181871	9.188009	11.062006	10.933966	10.136988	10.697213	10.120667	9.242578	12.034862	9.694983	10.895043	10.943468	11.487432	10.901414	11.016629	12.106371	10.919423	13.023480	10.879442	10.731478	11.101785	11.200439	11.669383	9.549938	9.925892	11.047086	11.970814	9.936352	11.075168	10.062755	13.016253	10.161839	10.372281	9.525379	10.281231	11.277350	10.935174	10.873089	10.494529	10.137297	11.685883	10.607264	10.007217	10.480362	11.419262	11.135818	9.711248	10.537786	10.338850	11.567979	12.187471	10.710534	9.738519	10.819554	10.613754	10.695590	10.649097	9.746469	10.405628	9.445145	9.275616	10.292343	11.471087	10.256188	11.342479	10.250420	10.002883	8.975271	9.525379	9.441732	9.475363	9.582540	10.523247	9.362773	9.973866	9.697396	9.511977	11.396684	9.210997	9.425282

RDM1	7.699442	7.294755	7.589490	7.807263	7.422893	8.309760	7.819194	7.869975	8.235894	8.304669	8.397632	8.102949	7.821338	7.701899	7.621653	8.292608	7.453866	8.027797	7.998868	8.058274	8.996729	8.824170	8.540872	8.015709	7.914144	8.251460	8.371426	8.097219	8.454436	8.025737	7.964950	9.259236	8.173935	8.803984	7.788679	7.703584	7.685039	7.664983	7.001307	8.015092	8.411545	8.176167	8.198869	7.733172	7.943093	7.555981	8.802155	7.355513	7.724392	7.835809	7.324066	7.968338	7.710641	7.465403	7.479964	8.305738	7.934176	7.259036	7.645719	8.509809	7.424306	8.288718	6.860106	7.680418	7.999789	7.606999	7.708085	7.235637	7.380361	7.342224	7.470962	7.757898	8.542730	7.584728	8.078394	7.672577	7.476161	7.290668	7.594003	7.547276	8.545084	7.281300	7.526963	8.313905	8.251460	7.555981	7.734091	8.135563	7.389802	7.270478	7.782857	7.356854	7.675274	7.871982	7.845512	7.924598	6.844383	8.037132	7.698822	7.788842	7.277515	7.977519	7.974619	7.432040	7.347217	8.852855	8.522763	8.704737	7.764336	8.430243	8.089070	8.059636	6.915897	8.716431	7.950164	7.649041	7.844214	8.133618	9.039311	9.089149	7.732264	9.184546	7.891543	7.433129	8.595469	8.267918	8.798699	8.217118	9.182973	8.592063	9.473089	8.871377	8.858656	7.684432	7.814763	9.764963	9.554494	10.126162	9.985187	8.597021	8.875260	8.699971	8.193053	8.604996	9.661852	8.378761	9.214667	8.963282	8.797014	8.463224	8.404680	8.975575	7.044183	9.372574	8.975271	8.466285	7.260903	6.829091	8.663180	9.582379	9.681392	10.737488	7.190458	7.853434	8.322623	9.693584	8.987805	8.395457	7.522516	7.649188	9.241228	8.059157	6.960528	8.588507	9.141010	9.365816	9.376678	9.191963	10.338376	8.598657	8.947836	7.454005	8.336800	8.280995	8.041233	8.502160	7.585017	7.407903	7.804477	8.431370	8.120696	8.920759	8.495049	7.968928	7.882321	8.649201	9.288791	7.992398	8.338549	9.057198	7.418833	8.424232	9.006998	9.196896	8.928643	8.781813	10.216548	7.641235	7.765302	9.556635	9.195778	7.762049	8.258005	8.003253	8.037285	7.819523	8.479305	9.374964	8.336641	7.777610	9.886243	8.491478	7.856797	8.096048	9.283248	8.040118	11.466592	8.001992	8.918918	7.857857	8.361560	8.456424	9.456308	9.237796	8.977700	8.889622	8.279188	8.566293

C16orf54	10.403488	11.201301	11.838131	10.046469	9.197498	9.738519	10.266295	8.468540	8.683845	8.877393	8.970829	9.446373	8.938979	8.119314	9.321212	10.211826	6.906222	10.357244	9.880559	10.439729	9.977410	11.404839	9.051148	11.418269	10.037337	8.823411	11.794841	7.535941	9.985035	12.455784	7.471713	12.251983	12.993251	11.743340	11.623796	11.466592	10.155626	9.950580	9.091908	10.702431	9.564819	11.216854	11.435658	10.625962	11.111015	11.404110	8.817585	11.447213	11.023352	10.928007	11.310712	10.634588	11.311032	10.293540	10.799798	7.638623	11.203446	11.384527	9.951078	10.354019	10.315047	8.805380	10.836162	9.819657	11.247766	11.179135	10.150520	10.034692	10.937143	11.243314	11.107474	10.396736	8.760747	11.383763	10.901414	12.626839	10.811801	11.340163	10.482931	11.078978	11.116514	10.874713	11.754223	10.720767	11.066449	11.438954	11.111146	10.485784	12.155713	10.702740	11.822458	11.724977	10.597545	10.822371	11.742298	11.062443	11.530458	9.757807	8.743149	7.610523	8.089724	11.024075	9.979650	9.505806	10.350959	9.148313	9.389155	10.008474	7.968631	8.271350	10.850767	8.562532	8.942169	8.589904	9.842136	9.053125	10.407551	10.321672	8.941097	10.309726	10.131679	11.565239	10.576089	7.703423	7.825991	8.809828	7.846551	10.933153	7.464042	10.610931	7.159784	6.926148	9.854297	8.454436	9.313949	6.377960	7.579940	9.267666	10.739904	8.541658	9.223893	9.888772	7.896712	6.509590	9.288025	8.945669	5.374742	7.978633	10.859980	7.735934	11.259156	11.037133	9.684838	11.037396	9.239207	10.600317	9.357110	9.247361	10.475118	6.839827	11.678161	10.325669	9.554189	10.219536	10.128547	7.193263	10.569984	9.288170	9.790792	11.106019	10.480656	10.182257	9.701109	10.635777	9.001818	9.911891	10.487794	10.130357	10.471930	10.055298	10.812551	9.248755	9.873647	11.048552	11.003077	9.306026	10.454947	9.714536	11.758591	10.500528	9.529591	5.451062	9.881018	12.118803	11.270579	9.707187	9.928704	8.715629	11.274456	11.066875	9.538430	10.006313	10.957536	10.004264	9.513366	11.189473	10.039917	11.746705	10.550696	9.706727	10.520787	9.743043	10.748889	10.364435	9.989046	9.063225	8.130422	8.913583	6.752554	7.601380	10.146257	7.892783	11.376076	9.513967	10.777392	6.631873	9.388562	9.326595	8.098858	11.224748	9.972959	5.606500	9.496124	8.203475	8.657283	9.162292	10.024497	8.646623

ASB3	11.884206	12.201396	11.955591	12.417994	12.475857	12.193677	12.419436	12.385972	12.153600	12.203451	12.153299	12.311900	12.451389	12.241146	12.041372	12.643905	12.558148	11.867283	11.384602	12.687272	11.597854	12.396914	11.126639	11.681866	12.633795	12.168505	12.124203	12.521575	12.308556	12.213629	12.470874	12.191499	12.543561	12.493360	12.432750	12.558955	12.623824	11.960844	11.659709	12.091212	12.219537	11.979131	12.651577	12.729292	12.677340	12.438515	11.973763	11.526366	12.622821	12.858637	12.863485	12.789606	12.566772	12.747282	12.151731	11.918508	12.614361	11.771398	11.826672	12.174349	11.928150	11.867445	11.547537	11.516950	11.567413	12.044415	11.412815	11.917597	12.065467	11.317631	12.392964	12.253272	12.036533	12.227834	12.407456	12.589114	12.537757	12.290609	12.390314	12.819581	12.459117	12.437883	12.414625	12.519526	12.618274	12.747949	12.703315	12.677497	11.443990	11.986208	11.662223	11.443306	12.289811	12.348370	12.510471	12.989260	12.707214	12.914157	12.376451	12.386142	12.401299	12.424399	12.318233	12.304791	11.233156	12.183450	11.332042	11.463754	11.026892	12.286760	11.746557	12.190447	12.307622	12.262939	12.093212	11.193252	12.207292	11.935946	12.335244	13.220404	12.193376	12.189499	12.618117	12.294023	12.135429	12.237727	13.231839	12.426215	12.180653	11.907737	12.050881	11.895762	12.202988	11.761456	12.394267	13.284032	11.671790	11.128520	13.189322	11.463911	12.256329	10.955440	12.452958	12.534282	12.497991	12.180653	12.223567	12.372577	12.156187	13.402704	12.625338	13.098245	12.335719	12.954985	13.066780	12.580816	11.929674	11.676971	12.325249	12.842983	11.940454	13.292154	13.042957	12.992354	12.341032	11.694395	13.042069	12.685200	12.765306	12.782224	12.924201	12.988517	13.265575	12.546069	12.666497	14.124290	11.472366	12.070249	12.855862	12.604579	11.541101	12.022821	11.513041	12.285365	12.098027	11.457426	11.751580	12.539305	11.874773	12.699870	12.662414	10.723137	12.677012	12.946087	12.522671	12.426059	12.939558	12.890012	12.728615	12.902873	13.096155	12.351946	12.399170	12.725852	12.558801	12.854778	13.006688	11.384387	11.834521	12.455784	11.876727	13.446264	12.725694	12.416103	12.528252	12.690509	12.854978	12.385289	13.230081	12.127984	12.698531	12.347575	12.449933	11.671048	11.426495	11.838426	12.174829	12.043959	11.701608	11.611821	12.258812	12.991641	11.780465	12.877825	11.906365	12.607232	12.802822	11.688416

TOGARAM2	8.022679	7.369389	7.132134	7.659388	7.924267	8.484449	8.119314	7.549912	7.971630	8.164571	8.523071	8.281286	7.690392	7.697916	7.068063	7.353422	7.559819	7.428367	7.100637	7.254764	6.801570	7.709277	7.848701	7.316049	7.737275	7.734887	7.837979	7.809896	8.693881	8.297469	7.356553	6.915595	7.281951	8.053099	7.587974	7.317777	7.028306	7.436471	7.488051	7.643849	8.480944	7.175887	7.314510	7.513578	7.600626	6.912113	8.738106	7.281623	7.274556	6.867088	7.015663	7.401771	7.195369	7.083159	7.121335	8.627031	6.995675	7.333659	7.691812	7.016983	7.039223	8.393485	7.774143	7.250720	7.311816	7.386991	7.353569	7.459959	7.480117	7.450892	7.717516	7.635884	8.694514	7.447592	7.668348	7.098469	7.255095	7.277975	7.404412	6.760102	6.737298	6.865815	6.678326	6.579946	6.309603	7.227642	6.728379	6.602156	6.267913	6.594690	6.562527	6.797498	7.180945	7.311966	7.285048	7.289562	6.781047	7.767463	7.925059	7.730715	8.064421	7.460875	7.347692	7.477063	7.318079	8.794153	7.673686	7.702064	8.030083	8.530634	7.417566	7.772173	7.800516	8.955481	7.651747	8.875412	8.336333	7.733625	8.454517	7.521779	8.344459	7.799910	7.874891	8.904922	8.505652	9.085519	7.602803	8.534075	8.576401	9.051442	8.604216	8.398520	8.019387	8.343667	7.356999	7.964169	7.522516	7.898223	7.009806	7.626701	8.537994	8.532646	8.393192	7.881547	7.683194	7.626880	8.459105	9.198437	9.203996	7.923817	7.596408	7.662149	8.489064	7.706992	7.766700	7.314004	7.962682	7.751682	8.231793	9.457860	7.646042	7.797234	8.605743	7.557083	7.068891	9.341898	7.248850	7.482906	7.903842	7.641532	7.444119	7.094649	7.732870	7.030651	8.844175	6.732875	7.354709	7.935444	6.947452	7.625330	8.530634	7.613107	8.297612	7.705631	7.531129	7.987020	7.821500	8.146104	7.771423	8.167373	7.955563	9.409559	7.805246	7.531584	7.186521	7.743500	8.298658	7.644473	6.788922	6.958781	7.127371	6.919882	7.255711	6.638386	8.108784	7.005355	6.778627	6.887904	7.093070	8.534396	7.115335	7.966042	8.107889	7.905856	7.868761	8.135264	8.058996	8.265560	7.942647	8.389753	7.842803	8.218871	7.641235	7.302884	8.990766	8.399557	8.050181	7.426641	10.990540	8.128270	8.167075	8.178859	8.754279	8.407575	8.887283	7.710952	8.344605	8.684777

RHBDL2	12.443951	12.621991	13.055240	12.715054	12.963290	13.264773	12.972416	13.365580	13.426006	13.350761	13.397092	13.101246	12.879950	13.382399	12.356573	13.258986	13.334586	12.580980	11.837404	13.705820	12.182810	12.960870	9.834744	13.118469	13.166910	13.169593	10.874430	13.427811	12.137255	11.491594	12.989626	13.151060	12.983333	12.363880	12.640215	13.498262	13.485295	12.815287	12.530368	13.011412	13.142638	11.916216	13.073039	12.765660	12.338554	13.190080	13.045554	11.951108	13.552300	13.063428	12.902160	13.224516	13.499569	13.086086	13.122511	12.748989	13.593744	12.128571	12.088584	12.724156	12.998253	12.666814	8.584250	12.172013	11.837992	12.254206	12.419130	12.533467	12.523149	12.560874	12.653462	13.340998	13.525049	13.015726	13.454586	12.706541	13.544058	12.817505	12.875731	13.242189	12.913209	12.945707	12.686750	13.123245	13.529678	13.045174	13.391215	13.261991	11.463754	12.076981	12.103782	11.939692	12.562827	13.246497	12.777955	12.996930	12.939734	13.169964	13.181502	13.223925	13.301455	12.636117	12.901113	13.000815	12.041676	13.205915	13.456831	12.041521	11.618765	13.084018	12.162658	13.337481	13.110603	13.445662	13.516009	13.167290	11.884633	13.129969	12.962718	12.722462	12.157886	12.500257	12.142076	12.168818	11.818995	12.283635	11.492880	11.767049	11.868900	12.758401	10.930976	12.498162	10.959158	11.726020	12.555263	11.810691	10.871441	8.859264	11.766473	10.899829	10.924932	8.613766	12.121584	10.746795	11.132638	12.302476	11.688272	11.758737	11.079687	11.819577	12.785412	12.226002	13.059891	12.072185	11.687807	13.144764	11.744798	12.042883	11.557603	11.678446	11.263631	10.489958	12.594269	11.505101	12.232073	12.552826	9.609148	12.681634	12.512914	12.528710	11.712469	11.892007	12.191499	12.250230	11.551514	11.843253	10.905852	11.717792	11.110174	12.395385	11.641926	10.099503	11.330601	11.563161	10.875163	12.435636	11.970649	12.010496	11.873849	11.413548	12.699345	12.819581	12.597148	11.911476	12.304503	12.748286	12.071894	12.792028	11.606533	12.975455	12.691020	12.730311	12.172341	11.654083	11.960220	12.130980	11.271637	10.497395	11.059010	10.527605	11.084984	12.169723	11.941351	11.909709	11.962614	12.259890	11.771669	12.919720	10.547962	11.137136	11.474696	11.654225	12.161037	8.824170	12.077723	12.010041	11.865774	10.806779	13.091272	9.153531	12.048425	11.242685	13.049541	11.133235	12.637828	12.412212	14.102234	10.968006

LRWD1	12.048596	12.199376	12.105433	11.945155	11.829126	11.771398	11.830901	11.561147	12.024036	11.901608	11.711746	11.779847	11.816598	12.133414	11.923854	11.848431	11.964164	12.197370	12.307924	11.822914	12.509651	12.828063	12.456916	12.071423	12.140038	11.763874	11.953033	11.637543	12.008250	11.846125	11.934899	12.004151	11.792854	12.232212	11.776541	11.504214	11.560840	12.261651	12.219378	12.178309	11.810536	12.166633	11.921879	11.740264	11.504954	11.653482	12.016745	11.887052	11.601655	11.479340	11.750711	11.612110	11.756948	11.841745	11.995202	12.079696	11.733626	11.979596	12.241297	12.066882	12.168818	11.754080	12.504224	12.419287	12.730311	12.640533	12.340685	12.356727	12.151563	12.497024	11.736262	12.055280	11.838426	12.042721	12.076036	12.039701	11.916526	11.876001	11.778520	12.225064	12.023394	11.897222	12.076508	12.046769	11.987025	11.826393	11.829273	11.726750	12.552826	12.474435	12.233142	12.257254	11.989265	12.204814	11.869641	11.690176	11.680972	11.589071	11.993060	11.743625	12.086352	11.854007	12.223101	11.807837	13.167086	11.802080	12.233892	12.980977	12.807703	12.058084	12.498479	12.384018	12.222032	12.064108	11.981655	12.387757	11.985255	11.940295	11.962757	12.300335	12.707548	12.609521	12.564626	13.149739	12.352431	12.388737	13.852424	12.942843	12.429308	12.322814	12.998772	12.490571	12.133853	13.177598	12.314784	12.497105	12.560381	13.633730	12.818008	12.677340	12.626022	13.082921	12.804601	13.679721	13.043339	11.967801	12.310451	12.564800	12.437883	12.373219	13.322965	12.680777	12.591334	12.617456	12.470874	12.324191	12.882226	12.858287	13.135275	13.633254	13.743347	12.511911	12.862948	12.378496	11.872045	12.734379	13.403370	12.315928	12.478491	12.556362	12.265421	12.399470	12.694078	12.695736	12.202348	12.782601	13.551187	13.256009	13.164133	13.313561	12.145944	13.189888	13.103364	13.366218	13.086086	13.371459	12.586980	12.849653	13.497141	12.654306	12.813692	12.558326	12.539305	12.488145	12.845626	12.710821	13.402063	12.633633	12.582617	12.571330	13.066024	12.465329	12.780022	12.976230	13.497353	12.522348	13.548671	12.989073	12.705525	12.747096	13.839834	12.959381	12.864593	12.868885	13.015361	12.532655	12.622511	12.408210	12.723828	13.311915	13.325811	12.500922	12.166192	13.597268	12.378643	12.963290	14.190534	12.848581	12.356420	13.129753	13.011779	13.016827	12.530838	12.638870	12.603082	13.028398	12.512086	12.978577

HOXA3	6.814438	7.775107	8.214240	7.396166	8.482792	7.959136	7.831401	7.363410	8.974964	8.126601	7.934974	7.606840	7.728109	7.901171	7.620128	8.317000	7.714780	7.157299	8.062120	8.066401	8.429897	8.085541	8.895958	7.964491	8.567450	7.825691	7.651747	7.364043	7.882013	7.645417	8.616473	7.937457	8.298658	7.820271	8.443484	8.286878	8.595787	7.532374	7.776496	7.763447	8.264458	8.056204	8.041861	7.908800	8.256188	8.601729	7.990331	7.918129	7.541740	8.572331	8.413384	8.249737	8.877113	8.438572	8.029503	7.762049	8.644401	8.582268	8.101896	8.145954	8.826140	8.203010	8.949195	8.360170	7.981300	7.646673	8.252222	8.150217	7.584112	8.373879	7.927727	7.995341	8.073351	7.748264	8.185252	7.738513	8.328445	7.460397	7.731332	8.182981	7.993319	8.132684	7.843275	8.294269	8.410780	8.123251	8.023141	7.795703	9.177420	8.032533	8.177391	8.292455	8.223437	8.340131	7.326110	8.437550	7.712966	8.208900	7.489903	7.779491	7.229369	7.703286	7.347389	7.832020	7.923817	7.441605	8.202701	8.216356	8.212373	8.026668	8.893784	8.372378	7.406331	8.256329	7.207080	7.446500	6.128501	7.693526	8.030875	7.455423	5.959677	7.287679	8.511528	5.609716	6.657433	7.432657	7.449204	8.714844	7.421349	7.133232	5.986093	6.431908	6.527507	5.692261	9.011607	7.214668	5.915847	6.439892	7.005961	6.824965	7.156379	8.030875	6.230244	7.591653	5.519715	7.127053	5.077569	7.164273	7.693823	8.853927	5.520641	6.450968	7.768377	6.822918	6.683070	7.470633	6.583596	6.576349	7.326424	6.711340	6.977224	7.142720	6.621138	6.812101	6.592444	6.825914	6.658538	6.360207	6.471216	7.582415	6.870984	7.080815	6.584561	8.423166	7.678459	7.428055	5.804747	7.827291	5.963776	7.224175	8.254529	7.436471	7.312895	8.482646	6.830405	6.687772	6.693760	6.213566	7.856647	6.832940	6.683902	6.716023	6.772178	7.285048	7.022815	7.459810	7.926479	7.263271	8.459576	6.840811	6.951549	6.750073	7.232778	6.917410	7.334577	7.112679	6.605022	8.175380	7.631624	7.075602	7.743966	8.190228	6.887252	6.730784	6.403278	6.749904	7.461972	6.018512	6.780543	6.789577	6.509590	6.837369	6.756046	7.485047	6.806000	7.778213	9.875126	6.746121	7.004729	6.766987	6.008419	5.276946	8.501568	6.832615	6.682427	6.163228	6.639497	6.674131

EPS15L1	10.421474	10.860728	11.100158	10.706418	10.953197	10.487497	10.647283	11.446152	11.581405	10.746036	10.498125	10.522806	10.662816	11.207935	11.218189	10.611388	11.091753	10.933966	11.241786	11.022905	10.824581	11.608893	10.510494	10.559481	10.898182	10.564279	10.320901	10.305518	9.723922	10.148077	11.213966	11.273225	11.001791	10.284582	10.813141	10.954523	11.176130	11.181727	11.557743	11.558489	10.377715	11.189332	10.880179	11.238152	11.226653	11.169437	9.973251	10.933529	11.013094	11.068537	11.055964	11.319280	11.156096	10.841416	11.437970	10.348429	11.352182	11.465248	11.236722	11.440893	11.175825	10.090671	10.880464	10.610379	11.240073	11.058929	11.346523	11.058929	10.942265	10.645484	10.656389	11.000137	10.149811	11.405143	11.075613	10.942718	11.150980	11.054661	10.781758	10.585228	11.103871	11.429417	11.062165	11.292037	11.235752	10.934589	10.926394	11.652428	11.188894	11.603886	10.877350	11.171579	11.419997	11.081007	10.647591	10.948465	11.431058	10.925632	10.999997	11.109254	11.247632	10.776525	11.282980	11.210101	11.740420	11.211143	10.873702	11.157605	11.177479	10.632306	11.004248	11.349360	11.938053	10.009271	11.281048	9.576225	10.723285	10.800421	10.090224	10.510787	10.759120	10.453574	10.480506	10.600754	11.568715	10.507219	9.610292	11.708649	9.974679	10.539400	10.098728	11.028512	10.492371	10.100136	10.112741	10.479347	10.837243	10.978775	11.592816	10.682588	11.454433	11.381737	10.940842	10.919263	11.073997	10.544466	11.338351	9.525717	9.778003	10.107612	10.935174	10.698977	10.933379	11.230646	10.799656	10.960321	10.901875	10.599871	11.288615	11.205234	10.684205	12.463072	11.666120	11.848060	11.437676	10.529382	11.039598	10.815666	11.348648	11.019767	11.096172	11.174591	11.138902	10.990910	10.484745	10.822815	11.868313	10.699115	11.510541	11.284152	10.477996	11.557873	10.716860	10.352051	10.806048	11.874610	11.009325	11.712895	11.222202	10.473139	10.512139	10.599097	10.992556	10.394903	10.762198	10.977879	11.327191	10.839445	10.347657	11.097482	11.158776	11.297023	10.750836	11.710886	10.476015	11.003940	11.681120	10.960475	11.147241	11.973605	11.451479	11.117536	11.258731	11.086179	10.582460	10.442286	11.217573	10.268511	10.517413	10.267776	9.958930	10.596344	10.632749	11.087385	10.302329	11.590838	12.395697	11.932141	9.936952	11.615965	9.783445	11.666726	9.822386	9.561136	10.272099	11.730335	10.706272	9.371801

ANKRD40CL	5.310072	6.094972	5.649548	6.236327	4.803361	5.186207	6.130349	5.970859	5.669624	6.251304	6.354258	6.274392	4.202480	5.412215	6.395639	6.033087	5.386761	4.980532	5.316667	5.345357	4.814353	4.975473	5.568462	4.996090	5.145352	5.066749	5.637885	5.700463	6.039453	6.155690	5.107256	5.253360	6.080402	5.927599	5.561304	5.453218	5.432219	5.691927	4.954780	5.190944	6.636582	4.763151	6.075028	5.048723	5.084680	5.377508	6.037725	4.889393	5.448593	6.049071	6.174551	6.293010	3.991131	5.267480	3.572687	6.227132	3.907084	3.172804	3.926528	4.156749	4.587788	6.250316	5.216797	4.047589	5.222922	5.273895	5.524027	5.699132	5.945514	5.579658	6.462837	6.612852	6.508146	6.582283	6.481749	5.990248	5.565981	5.806305	6.389695	3.596729	4.053433	4.454595	5.332656	4.223606	3.925015	5.605914	4.159720	4.546011	3.109116	4.876507	4.121617	4.429329	4.702801	5.179925	4.786821	5.880506	5.160090	4.832287	4.928275	5.160676	3.887402	5.705050	5.505239	5.532941	5.343606	7.192436	5.007858	5.178778	5.210432	6.915752	5.644627	4.283596	3.854698	6.851706	6.069439	4.495328	5.081850	5.296253	6.456375	5.038113	6.422233	5.709757	5.829333	4.672505	6.254868	5.714385	5.006848	4.780946	5.598387	6.016394	5.746275	4.350669	4.540205	6.115297	4.762913	5.298058	3.481349	4.373474	4.870222	5.585889	4.195183	7.328659	4.817712	4.698314	5.172533	5.405155	5.284771	5.899181	6.754404	4.915556	4.904637	5.615913	6.526356	5.821960	5.853816	5.457488	6.040358	5.646519	5.479452	6.860415	5.609902	5.934753	5.995187	5.747563	6.035454	6.957997	3.658843	4.894601	6.173696	5.331849	6.095321	4.572211	4.921917	4.284509	6.249133	3.496154	3.070262	3.740380	4.664522	4.807639	6.176773	6.099633	4.833968	5.243216	5.015654	5.750467	5.814929	6.170659	5.825489	6.678634	6.472740	6.826070	6.120458	6.023595	6.021366	5.894247	6.527025	6.079720	3.565704	3.571337	4.179448	4.839385	8.134813	4.529462	5.229116	4.379297	4.710123	3.009174	4.248186	4.458635	5.170694	4.941572	6.287915	5.317845	5.805266	4.379075	4.106979	3.561567	5.659956	4.543995	5.093948	5.262064	5.230986	4.735021	6.802846	4.869580	5.465020	5.735376	6.320511	5.687890	4.403954	3.198002	6.214838	4.817923	4.787242	4.592522	5.904725	6.701746

BRS3	6.793483	9.565885	9.264430	9.237958	9.817117	10.341418	9.244700	12.389361	10.439290	9.465805	9.506715	9.027513	8.288718	9.242889	8.237449	11.377843	10.270009	8.809674	10.028875	10.596344	10.006922	10.535918	9.001818	9.327512	9.491735	8.982281	6.885835	10.443347	10.240313	9.435704	9.076408	8.319872	8.839951	6.779417	8.834785	9.582066	9.481820	9.692546	10.420096	10.115901	10.763571	9.357553	10.928007	11.236428	10.763720	11.583602	9.922889	12.386292	9.367576	13.527257	11.219527	11.778669	8.845259	9.831229	8.998256	10.541527	8.678987	9.735326	9.435228	8.679300	9.477832	10.101871	7.961616	9.729812	9.001028	9.737007	10.492517	10.129911	9.803798	10.212581	10.579788	10.588511	10.835583	10.264395	10.139559	9.659984	9.946210	9.603904	9.463760	9.132447	8.685982	8.645888	8.026381	8.800662	8.920452	8.944895	8.491632	9.406930	8.995637	9.372405	8.946909	9.296274	8.999495	9.372889	7.582572	8.248095	9.017912	9.051002	8.686419	9.582219	9.139252	9.859596	9.515599	9.759184	9.627241	10.422850	9.981684	9.621360	8.804775	11.941813	9.822544	9.623997	9.475527	11.142206	10.287254	9.237493	7.296370	9.109255	9.810408	6.897209	8.500912	6.964056	6.952175	8.227663	8.890238	7.576995	7.693051	7.271258	7.599203	7.243086	6.946617	7.866800	6.889972	7.512341	8.924312	7.180027	7.246079	7.522964	7.223425	7.956511	7.636629	7.926479	6.854459	7.769428	7.600322	9.246741	7.556447	8.184054	7.402709	7.029236	6.673180	6.873370	6.947625	7.053478	8.014946	7.406485	7.164101	8.155791	7.651592	8.681429	7.327894	8.589745	8.011098	7.708229	10.892382	7.913711	8.897010	7.542477	9.423930	8.045582	8.900824	7.021874	7.410189	7.139220	8.577277	6.657757	7.996613	6.850460	6.998024	7.419924	7.135167	7.269393	7.192941	7.290668	7.552380	7.411359	7.817936	7.790686	7.667305	8.026527	9.456470	9.234252	8.018756	7.978000	7.577303	7.935134	8.170037	8.319126	6.488546	6.257247	7.618627	7.201945	6.350954	7.265254	7.412132	6.376950	7.567050	7.497273	7.581501	8.515903	8.453220	6.910490	8.312823	7.082213	7.860319	7.245764	7.664983	7.435375	7.659517	7.595187	7.794009	6.907043	7.016496	7.613266	8.234201	7.092294	7.736514	8.062577	9.240450	8.156255	7.699912	9.353484	10.698200	8.963871	7.143957	7.368591	8.056685	7.266940

NADK	12.682137	11.598873	11.340239	11.676518	11.541804	11.710741	11.444132	12.076671	11.937627	11.823989	11.512453	11.599731	11.247214	11.732727	11.345196	11.826393	11.620849	11.623726	12.059805	11.822002	11.775784	12.337443	12.462078	11.608755	11.782962	11.730804	12.114778	11.891560	11.772261	11.808575	11.923268	11.269090	11.643705	11.994265	11.440039	11.100018	11.207310	11.692608	12.047379	11.965821	11.803233	11.816173	11.733781	11.507297	11.214808	11.325549	11.699459	11.468386	11.237709	11.385252	11.673108	11.288746	11.710136	11.775366	11.632098	11.687212	11.551514	11.946821	11.953632	11.987859	11.939692	11.747596	12.296332	11.951702	12.118803	11.845543	12.120362	11.624862	11.974354	12.072497	12.520550	12.382744	11.627357	12.090445	12.123468	11.786428	11.503353	11.924880	12.115534	11.185995	11.245085	11.308691	11.715417	11.217005	11.286577	11.120843	11.230061	11.381737	11.777916	11.587908	11.473523	11.779565	11.784633	11.795267	11.482399	11.228419	10.895803	11.078266	11.174436	11.402644	11.341633	11.626444	11.449273	11.478418	12.305259	11.342331	11.813001	12.206975	12.071578	11.914449	12.033841	11.937909	12.071578	11.973905	11.209534	11.757386	13.024751	11.401656	11.664771	11.562464	11.629391	11.703111	12.396775	12.198594	11.958999	12.241919	11.465248	12.529854	12.282874	11.727947	11.404390	11.788671	12.341379	12.336360	11.567130	11.745962	12.400629	12.417520	11.000920	11.792414	12.565935	12.741114	11.892609	12.678534	12.218793	11.477264	12.341859	12.023884	12.053155	12.496680	11.614201	11.600906	11.684959	11.670899	10.786621	11.388799	11.658189	12.372577	12.024345	11.987250	12.043045	11.729608	11.639945	10.891628	11.450138	11.983212	11.189611	11.347723	12.025727	11.457578	11.538395	11.709110	11.533180	11.941654	11.691236	11.260483	11.658962	12.266375	12.129027	12.546233	12.234346	11.942584	12.280067	12.900238	12.213488	12.101494	11.767668	12.066244	12.497186	11.613311	11.213047	12.878888	11.619033	11.461422	11.882660	11.405878	12.033841	11.725566	11.444695	11.187721	11.685397	11.648416	11.477840	11.694552	11.566546	11.517806	11.245651	12.066401	11.791975	11.551660	11.917597	11.955287	11.720627	11.556248	12.306066	11.086467	11.826972	12.073126	11.479650	11.129112	11.195021	11.648993	11.665969	11.815395	12.253731	12.354541	11.735541	12.145157	12.610190	12.146584	12.155713	12.163756	12.425096	11.227093	11.033295	12.274566	11.095144	12.035185

TBC1D8	6.432425	6.598280	6.755073	6.177822	6.886446	6.563856	6.944724	6.797677	6.638539	6.394679	6.281779	6.571479	6.018321	6.488546	6.518878	6.692595	6.294903	6.563011	6.729672	6.429707	6.300025	6.443034	6.551496	6.638849	6.228480	6.317575	6.411526	6.416995	6.715863	6.374667	6.224856	7.121194	7.143171	6.106391	6.600558	6.198084	6.631246	6.821189	6.761889	6.854620	6.493315	6.404392	6.377801	6.436969	6.247463	6.775132	6.460003	7.000648	7.185042	6.279828	6.744822	6.410697	6.454306	6.535471	6.738587	6.572093	6.246949	6.616593	6.583920	5.779280	6.232927	6.106910	6.857868	6.445076	6.140472	5.641771	7.274232	6.706217	6.930818	7.011889	7.347692	6.957510	6.554958	6.779578	6.979414	6.983822	6.985720	6.800425	6.354414	6.229710	5.870621	5.979030	5.976204	6.430211	5.577584	6.575152	6.557965	5.865214	5.792377	5.967336	5.957495	5.745522	6.926825	6.605173	6.075714	6.288952	7.057930	6.509428	6.526178	6.708488	7.015993	6.624661	6.581476	7.009322	6.993221	6.503324	5.913482	6.064005	8.019387	6.220262	6.973815	7.477368	7.692731	6.509917	6.507348	8.203908	6.518720	5.887621	6.245766	6.511437	6.704931	6.931449	6.481071	7.470321	6.993859	6.849029	6.964378	6.165625	6.680626	6.387827	6.964524	6.501233	6.374349	6.694243	6.546953	6.730471	6.759769	7.040480	6.126425	6.224856	6.701746	7.866510	7.433592	6.305436	6.355910	6.576349	6.564827	7.380361	6.748333	6.410035	6.436616	6.493934	6.616679	6.921278	6.803991	6.563191	7.442823	7.362150	7.539896	6.739227	7.584112	6.669775	7.029077	6.624833	7.083939	6.955662	7.132134	7.101570	6.315037	6.964378	5.919567	6.584073	6.778458	6.513566	7.020447	6.895893	6.583596	6.218553	6.448842	6.068771	6.393047	7.354060	7.391638	6.442684	5.846771	7.555205	7.004242	6.630313	7.450589	7.355178	7.164955	6.989840	6.878463	6.900460	7.236106	6.757487	6.794787	6.581803	6.372955	6.216641	5.773122	6.405559	6.203297	6.011945	6.984290	6.226055	5.709005	5.867214	5.983840	6.634185	5.879233	7.101874	6.811147	6.475089	6.202255	6.751438	6.983822	6.512277	7.225135	6.761889	7.100327	6.856249	7.234989	7.038584	6.893180	5.846222	6.264178	8.652204	7.036077	7.068891	7.562721	7.486938	7.300159	7.121945	6.987163	6.751134	6.174889	6.431244

MAP4	14.135457	13.395784	13.559500	13.752566	13.512856	13.354792	13.929475	13.310550	13.419947	13.450346	13.440736	13.793556	13.470117	13.773715	13.738081	13.487273	13.859930	13.862600	13.868567	13.538596	13.896685	14.138248	15.104224	13.717490	13.925119	13.571674	14.699677	13.614714	13.804646	13.365580	14.120170	13.867818	13.869905	13.593519	14.034659	13.858620	13.704144	13.686442	14.569333	14.116167	13.653197	14.961694	14.280707	14.041142	15.057310	14.114324	14.083435	13.925700	13.924569	13.682827	13.427811	14.152064	14.521823	13.784079	13.803086	13.616405	14.356236	14.474751	13.953485	14.241406	14.388223	13.668155	15.403084	14.285845	14.331727	14.114324	13.584890	14.084661	13.564116	14.356954	14.307986	14.004466	13.615437	13.951559	13.907439	13.779642	13.816316	13.282011	13.560165	13.187239	13.430597	14.156066	13.123245	13.844544	14.054502	14.057105	14.025054	14.076340	13.815786	13.508579	13.967990	13.905564	13.693583	13.254784	13.598384	14.129877	13.392871	14.101624	13.973052	14.651534	13.896143	14.146755	13.912944	13.860465	14.809575	13.652481	14.248543	14.873940	14.582713	13.544622	14.165527	14.171283	14.008169	13.399764	13.572169	12.610544	14.066603	13.876318	13.837964	12.486494	13.753087	13.242607	13.492149	13.541051	12.879950	13.628249	13.559941	14.599964	13.704371	13.642517	12.926643	14.169373	13.307295	14.441603	13.744082	13.952379	13.376917	14.419090	13.385597	13.652481	14.202148	14.019284	12.754298	14.096118	14.310666	13.085733	13.222566	13.288240	13.540173	14.487923	13.101611	13.791726	13.922935	13.381985	12.913209	13.916047	13.873120	14.077200	14.088628	13.773212	13.413997	13.653913	14.232519	13.008295	12.738484	13.864620	13.066597	12.755118	14.015842	14.038793	13.664721	12.625513	14.066603	13.827679	13.754656	13.036523	12.990890	13.264544	13.311729	13.789952	14.341026	13.688585	14.008461	13.645181	14.076027	14.330345	13.493953	13.421709	13.757880	13.180750	13.705084	13.292351	13.955482	13.864108	13.121772	13.722244	14.092524	13.902033	13.777164	13.853253	12.978391	13.813787	13.778222	13.705084	13.517777	13.064722	12.940635	13.990741	13.554388	12.954420	13.510627	13.713891	13.975250	13.968817	14.125506	12.092283	14.162045	13.884615	12.889319	13.894360	12.593788	13.743597	13.406733	12.915341	13.363072	14.279316	12.779001	13.446068	13.838739	13.663221	14.011814	13.152020	13.272984	11.639016	13.186246	14.047957	13.578538	13.354792

AKAP9	3.096907	3.065121	2.981438	2.999867	3.044226	3.567285	3.065646	3.466476	3.258402	4.282532	4.693492	3.526862	4.154991	3.419157	3.367536	3.865160	3.315118	3.380500	3.003614	5.334554	3.790786	3.323651	4.479106	3.107143	3.078263	3.058954	3.299325	3.406764	4.027084	3.164020	3.437397	3.897785	3.436611	3.261151	3.262043	3.392955	3.990254	3.107248	3.166213	3.119247	3.733181	3.348380	3.176503	3.052485	3.200773	4.208004	3.799007	3.642881	3.143382	3.331266	3.381180	3.152825	3.732755	3.360327	3.306659	3.321367	3.372751	3.478950	3.568681	3.270368	3.187677	2.966404	3.248829	2.892787	3.063753	3.090114	3.019493	3.070681	3.002910	3.052262	2.977135	3.091524	3.971793	2.908133	3.122398	3.067954	2.897502	2.975807	2.949946	3.140544	3.040703	3.299022	3.067628	3.591899	3.092690	3.236383	2.895171	3.084859	3.239599	3.046710	2.995910	2.976586	3.254679	2.985231	2.900722	2.994658	3.295748	4.399847	3.162394	3.420470	3.027183	3.467841	3.343813	3.203604	3.182173	4.712842	3.086732	3.159554	2.931158	3.725775	2.935557	2.949681	3.049223	3.921050	3.088102	3.098565	2.925007	3.573638	4.490665	3.546167	3.440139	3.056221	3.229455	2.902569	3.246230	3.110154	6.205920	4.570913	3.711546	3.056020	3.629032	3.220831	4.485503	3.145152	4.025170	3.559157	3.098204	3.161949	3.432871	3.408870	2.939052	4.945220	2.971185	3.022567	2.935655	2.912944	3.103750	4.173784	3.729640	3.829515	4.265722	3.356906	3.022268	3.133459	3.134142	3.334371	3.097510	3.090860	3.140408	3.916408	3.494089	3.093869	2.992006	3.348845	3.998094	3.799443	3.557381	3.140934	3.294696	3.383848	3.109814	3.531510	3.343162	3.052385	4.034749	3.203901	3.503137	3.502944	3.329019	3.550424	3.040276	3.201820	2.997794	3.096657	3.344003	3.024366	3.082124	3.543818	3.176741	2.941487	3.085489	3.070040	3.067954	2.947900	3.258539	3.040379	2.915428	3.163794	3.038936	3.175549	3.347910	3.124286	3.360502	3.389637	2.968683	3.248706	3.169283	3.098565	3.039947	3.285805	3.017403	3.237122	3.041680	2.974439	2.966934	2.925438	3.998094	3.058514	3.483337	3.117138	4.154789	3.153805	3.079184	3.258846	3.763005	3.222255	3.113539	3.237267	3.229755	2.911101	2.948429	2.952942	3.378791	2.988516	3.026669	3.225437	3.806168	3.327268

NAPA	14.045286	14.539639	14.240086	14.464777	14.356594	14.316814	14.386005	14.484052	14.550161	14.429371	14.547186	14.447515	14.198488	14.404753	14.156390	14.565911	14.679130	14.447515	14.543000	15.004214	14.332395	14.833576	14.205591	14.157364	14.326082	14.405128	13.430372	14.354516	14.254105	14.095205	14.371589	14.066603	14.203084	13.774477	14.739993	14.649747	14.822343	14.649747	14.984580	15.088208	14.563436	14.947411	14.729253	14.407352	14.517397	14.632175	14.269241	14.439683	14.435274	14.724698	14.495949	14.779929	14.726520	15.000238	14.517783	14.491542	14.661345	14.960579	14.887119	14.565911	14.893861	14.445611	14.285845	14.946400	14.642226	15.224770	14.792018	14.822949	14.655466	15.270155	15.848852	15.579100	14.501857	15.103033	14.942582	14.331365	14.877613	14.693813	14.853259	14.867085	14.657723	14.484873	13.954619	14.539165	14.484873	14.396205	14.735782	15.107990	14.881255	14.830506	14.821872	15.064607	14.658618	15.204490	13.828722	14.697497	14.045844	14.311014	14.186537	14.553109	14.303277	14.528293	14.423617	14.480594	14.583170	14.403999	14.575515	14.487923	14.517028	14.355522	14.477842	14.876067	14.795023	14.347441	14.880110	14.225335	13.976899	14.976992	14.319544	13.399078	13.688348	13.625907	13.871812	13.673865	13.932204	13.803853	13.068719	13.425542	13.706318	13.777435	13.700412	12.968955	13.800361	13.384972	13.460920	13.393094	12.996223	13.316216	12.773448	13.143030	13.994473	13.773464	12.991245	12.939392	12.939042	13.896143	13.423212	13.622196	13.669109	13.610850	12.979696	12.957359	13.468573	13.931643	13.405475	13.833047	13.597268	13.713891	13.834567	13.929742	13.993914	12.983527	13.681376	13.713891	13.857811	14.025686	13.213993	13.319284	13.300682	13.753859	13.576247	13.156520	12.892075	13.364346	13.913967	13.613971	13.710918	13.680663	13.665978	14.171915	13.704846	13.776435	14.311709	13.922935	13.801661	13.530801	14.121601	13.078931	14.270558	13.542577	13.641034	13.940443	13.785834	13.574462	13.854876	13.314762	13.751062	14.000643	13.530801	13.293957	13.907439	14.412818	13.351805	14.194648	13.231246	13.780832	13.310353	14.253766	13.977800	13.627288	14.316430	13.567112	13.498487	14.216003	13.559834	13.153006	12.855773	13.302463	13.595345	13.313356	12.909129	12.795726	13.106410	13.579444	13.941234	13.040426	13.384372	14.227650	13.950442	13.028031	13.646573	13.029905	14.308984	13.375462	13.028031	13.721501	13.450944	13.620154

TYROBP	13.385833	14.384169	13.633730	12.793389	13.119650	13.103364	12.979140	12.369546	12.625160	12.705163	12.894731	13.095058	13.226650	12.378496	13.213784	12.640896	12.942109	13.402063	12.972757	12.606564	13.309136	13.570123	14.250206	13.196137	13.747078	12.281273	14.307986	12.971855	13.499122	13.926780	12.645882	13.961604	14.629930	14.068626	14.012650	14.524343	13.430141	13.884891	14.524706	13.266215	13.106614	14.023893	14.368698	13.429701	13.446068	14.370460	13.732385	13.976899	14.228894	13.493039	14.096755	14.015578	14.169373	13.497565	13.147073	12.811936	14.463605	14.005012	13.489934	13.266591	14.117990	13.217568	16.310500	12.949293	13.623148	13.352270	14.453914	13.085733	14.524343	13.426884	13.704846	13.628490	13.323580	13.801661	13.762421	13.653197	13.475544	14.127106	14.123055	13.358864	13.807016	14.316430	13.905825	13.518195	13.442236	13.255583	14.372657	13.845880	14.014703	13.053474	14.445242	15.111816	13.674833	14.524706	14.272867	13.184851	14.059166	13.132646	13.154208	12.504570	13.145329	13.216575	13.255784	12.213183	13.478390	13.027483	13.471189	13.249512	13.454152	12.904820	13.865942	12.763935	12.735217	12.930801	13.441364	13.145143	12.839105	13.356199	12.920223	14.039072	14.029339	15.327968	14.335070	13.589804	13.574249	14.412818	13.663761	13.852202	13.353762	13.287261	13.276051	13.327590	14.911983	13.842996	14.376307	13.665236	12.979296	15.318226	14.798922	14.849518	15.205705	15.186033	12.748470	13.219365	14.997389	13.996150	13.311157	14.365218	14.836045	14.818366	14.984013	14.860478	13.605717	14.862987	13.041374	14.504600	14.817850	12.934722	15.470625	13.442646	14.902407	14.986168	14.700650	14.923336	14.264253	13.606413	14.824917	13.605255	14.729702	14.789144	14.851142	14.913079	14.382401	15.546202	14.543434	14.578102	15.016274	14.518957	14.601575	14.891226	14.869812	14.719474	15.473917	15.499684	15.045993	14.648445	15.056095	14.429732	15.126455	14.286202	14.327152	13.008664	14.847864	15.137730	15.008260	14.647558	14.674591	13.712951	15.042367	14.591153	14.457804	14.230209	15.348633	15.247395	14.466988	14.943708	15.192041	16.134222	15.739503	14.726053	15.321092	14.848968	14.432364	14.567539	15.143591	14.208447	14.257659	14.084661	13.858073	13.828942	13.141663	13.801374	15.159483	15.510860	13.931371	13.736365	14.857509	14.884408	13.247129	15.623471	14.370844	14.110109	13.895060	13.167290	13.676465	15.209040	13.243382	13.836415

HCST	13.385833	14.384169	13.633730	12.793389	13.119650	13.103364	12.979140	12.369546	12.625160	12.705163	12.894731	13.095058	13.226650	12.378496	13.213784	12.640896	12.942109	13.402063	12.972757	12.606564	13.309136	13.570123	14.250206	13.196137	13.747078	12.281273	14.307986	12.971855	13.499122	13.926780	12.645882	13.961604	14.629930	14.068626	14.012650	14.524343	13.430141	13.884891	14.524706	13.266215	13.106614	14.023893	14.368698	13.429701	13.446068	14.370460	13.732385	13.976899	14.228894	13.493039	14.096755	14.015578	14.169373	13.497565	13.147073	12.811936	14.463605	14.005012	13.489934	13.266591	14.117990	13.217568	16.310500	12.949293	13.623148	13.352270	14.453914	13.085733	14.524343	13.426884	13.704846	13.628490	13.323580	13.801661	13.762421	13.653197	13.475544	14.127106	14.123055	13.358864	13.807016	14.316430	13.905825	13.518195	13.442236	13.255583	14.372657	13.845880	14.014703	13.053474	14.445242	15.111816	13.674833	14.524706	14.272867	13.184851	14.059166	13.132646	13.154208	12.504570	13.145329	13.216575	13.255784	12.213183	13.478390	13.027483	13.471189	13.249512	13.454152	12.904820	13.865942	12.763935	12.735217	12.930801	13.441364	13.145143	12.839105	13.356199	12.920223	14.039072	14.029339	15.327968	14.335070	13.589804	13.574249	14.412818	13.663761	13.852202	13.353762	13.287261	13.276051	13.327590	14.911983	13.842996	14.376307	13.665236	12.979296	15.318226	14.798922	14.849518	15.205705	15.186033	12.748470	13.219365	14.997389	13.996150	13.311157	14.365218	14.836045	14.818366	14.984013	14.860478	13.605717	14.862987	13.041374	14.504600	14.817850	12.934722	15.470625	13.442646	14.902407	14.986168	14.700650	14.923336	14.264253	13.606413	14.824917	13.605255	14.729702	14.789144	14.851142	14.913079	14.382401	15.546202	14.543434	14.578102	15.016274	14.518957	14.601575	14.891226	14.869812	14.719474	15.473917	15.499684	15.045993	14.648445	15.056095	14.429732	15.126455	14.286202	14.327152	13.008664	14.847864	15.137730	15.008260	14.647558	14.674591	13.712951	15.042367	14.591153	14.457804	14.230209	15.348633	15.247395	14.466988	14.943708	15.192041	16.134222	15.739503	14.726053	15.321092	14.848968	14.432364	14.567539	15.143591	14.208447	14.257659	14.084661	13.858073	13.828942	13.141663	13.801374	15.159483	15.510860	13.931371	13.736365	14.857509	14.884408	13.247129	15.623471	14.370844	14.110109	13.895060	13.167290	13.676465	15.209040	13.243382	13.836415

RC3H2	11.781211	11.278563	11.614961	11.780148	11.680252	11.992156	12.015528	12.045877	10.831342	11.855029	11.735680	11.622359	11.747895	11.484132	10.859835	11.555058	11.599594	11.402937	10.199050	11.791807	10.727045	10.438251	9.797342	10.986908	11.002234	11.334950	10.778128	11.647837	11.210560	11.809185	11.070628	11.848789	12.025642	11.634043	11.788222	11.803233	11.840239	11.184811	11.222968	11.629083	11.569154	10.650874	11.909858	11.964932	11.739819	11.968868	11.729913	10.936702	11.873097	12.118005	11.990332	12.050881	11.942122	12.188415	12.053768	11.848655	12.115229	10.038839	10.486410	11.450408	10.939064	11.494646	10.081486	10.177628	10.105679	10.779419	9.700346	10.564878	11.041512	9.805482	11.594771	11.609343	11.567979	11.833001	11.712748	11.627501	11.704602	11.398958	11.680678	12.342944	11.852470	11.809935	11.708507	12.070082	11.944525	11.966551	12.302965	12.455635	10.286511	10.306115	10.729132	10.777248	11.813909	12.142076	11.593733	11.795120	11.825487	11.476689	11.881010	11.309262	11.862307	11.781075	11.387167	11.657747	10.340353	11.286261	10.415417	11.298239	10.229267	11.651535	9.829180	10.857343	11.778520	11.647091	11.820206	11.683934	10.768036	11.512165	10.987180	11.805147	12.411724	12.119288	11.824292	10.943027	12.121934	12.196161	11.256480	11.783439	11.594839	12.148979	11.853542	12.056205	11.217885	11.048406	11.272804	12.200007	11.237421	9.931241	12.411724	11.409809	11.019158	11.256626	11.709994	11.655296	12.306523	11.572374	12.479954	11.414272	12.285047	12.063188	11.771962	12.379588	11.976530	12.230786	12.920223	12.209897	11.605808	11.778372	11.521749	11.597708	10.864681	12.168746	12.618117	12.691855	12.750174	11.524427	10.894314	12.496861	12.617792	11.907279	11.851418	12.965824	12.615659	12.088137	11.889910	11.759915	11.359874	10.584613	12.040781	11.354029	11.707448	11.734231	11.506853	10.318748	10.926544	10.498431	11.081888	11.462167	10.249099	12.402606	12.410948	11.675491	12.211122	12.007772	12.168818	12.348522	13.599502	12.225678	12.401781	12.928662	12.797496	12.703649	12.196161	12.793186	12.532342	12.303595	12.196471	10.663570	10.629178	11.253964	11.569303	11.838285	12.912009	11.863047	11.526214	12.322054	10.474667	12.705525	12.570522	12.673113	11.153465	10.931284	11.699314	10.430330	10.831970	11.105005	11.851129	11.521468	12.197990	10.654131	11.773277	11.428987	12.377553	12.378969	12.083083	11.275032	11.793314	12.421829

SPTB	3.793794	3.269270	3.040931	2.992814	3.276359	4.579470	3.375281	3.305084	3.458761	3.460556	3.641701	3.727738	3.330807	3.998746	3.890951	3.178423	3.621865	3.658246	7.397106	3.201820	5.072591	2.823310	3.291768	3.024753	2.994026	2.858664	2.945941	2.866297	3.133205	3.164807	3.682876	3.285471	3.551213	3.255631	3.480575	4.130031	3.736479	3.315269	3.268108	4.641552	3.455318	2.953586	3.571337	3.345113	3.724305	6.632390	3.299770	3.150753	3.950322	4.005892	3.998329	4.678180	4.376736	4.201623	3.963800	3.130149	4.185118	3.039234	3.803284	3.733620	5.489780	3.104231	3.724305	3.131949	3.392489	3.376704	2.843764	3.342665	3.284276	2.843334	3.021235	2.876455	3.134888	3.314483	2.922785	3.354345	3.108164	3.014178	3.314806	3.808277	3.413453	4.107829	3.428672	4.352445	3.467670	5.560926	3.580893	3.299022	3.252513	3.580998	3.274034	3.119753	3.498184	3.146539	3.788048	2.972058	3.861275	3.176969	3.085408	3.294060	4.253225	3.284599	3.143964	2.875281	4.554107	3.779151	3.085603	3.512749	3.324325	3.306029	3.125920	3.734799	3.731296	3.323165	3.265284	5.654829	2.930867	3.048168	3.212271	3.064806	3.404710	2.996541	3.043316	3.843761	4.817310	3.225182	3.220137	3.438594	3.202543	3.457869	3.182695	3.228916	3.537834	3.458761	2.971964	3.560522	3.145662	4.056715	3.371881	4.362823	2.809656	5.298852	3.840275	2.859124	2.807495	3.505145	3.027480	3.050591	3.577338	3.357076	3.378618	3.502944	3.186474	3.418972	3.821651	3.781036	3.314483	3.243133	3.527379	3.198002	2.846421	3.642272	3.409888	4.190163	6.487433	3.454178	3.128578	3.957460	4.078077	4.028172	3.639909	4.127649	4.385878	3.547577	3.068474	3.774315	2.981829	3.435805	3.571510	5.445240	3.227451	3.738939	3.465394	3.591699	3.525695	2.839365	3.663078	3.262627	2.836657	3.236383	2.973908	3.135255	3.231044	2.933780	3.326629	3.046004	3.139069	3.400545	3.272404	3.796720	4.448486	3.520441	3.734389	3.581900	5.083448	3.475634	3.471558	3.050142	3.452427	3.420255	3.010039	3.312266	3.198410	4.292225	2.994379	3.204548	3.192444	3.565704	3.277400	3.592419	3.242251	3.000143	3.711920	4.981524	3.249984	3.064058	3.523055	3.390175	3.203211	3.182044	4.883487	5.021154	3.247223	3.547366	4.214482	2.922961	3.082761	3.356052

USP26	3.810138	3.391201	3.007349	3.126980	3.329178	4.229003	3.770910	3.416880	3.384997	3.393833	3.508342	4.099296	3.811402	4.071053	3.705674	3.214073	3.494647	4.923433	5.251176	3.240744	5.133206	3.905507	5.587720	3.903586	3.506879	3.603420	3.583598	3.560720	3.582844	3.503137	4.252568	4.383150	3.006528	3.639493	3.813350	5.181974	3.756326	3.850978	3.625008	3.434709	3.403206	5.429045	4.104982	4.194339	3.783146	5.598592	3.867507	5.682879	4.092696	4.023461	5.754614	5.573528	4.151044	3.971571	4.343924	3.465394	4.043645	4.005218	4.036500	3.921050	3.891965	3.426570	5.148135	3.290448	3.555255	4.505009	3.438067	3.384652	3.552388	5.015058	3.586121	3.358816	3.572500	3.875659	4.114172	3.824361	3.539371	3.781036	5.417579	3.530322	3.357588	4.427011	3.984979	4.238537	3.215162	5.609336	3.087059	3.478013	3.284757	3.980405	6.277687	4.335291	2.956250	3.291100	4.093091	3.473431	4.309263	4.033858	3.314483	3.070681	3.013777	3.788048	3.841530	3.341148	2.946451	4.432464	3.262344	3.422630	4.294797	4.423542	4.234408	4.220367	3.183839	3.481536	3.511629	3.410579	4.255015	3.581487	3.280419	3.486788	3.344944	5.862703	3.196135	3.405895	5.180318	4.263344	3.682876	3.449712	3.810335	4.780324	4.979117	3.638474	4.903797	4.705113	3.054547	3.492212	4.026648	3.703060	4.360033	4.430487	3.278379	5.691739	3.355689	4.279291	3.896283	3.506053	3.361975	3.883596	3.690559	4.023017	3.739768	3.048905	3.951215	4.147276	4.452439	4.453325	4.588197	3.625598	4.249492	4.490665	4.943181	3.939843	4.301736	3.862586	5.250586	3.980405	4.788062	3.436171	4.214051	4.893762	4.677961	3.638875	3.962484	4.083256	4.795393	3.597156	4.112439	3.999992	4.087434	3.584949	3.533492	3.904846	3.607467	3.513374	3.632310	2.995228	3.754647	3.492579	3.929346	3.246695	3.627611	6.345056	3.610768	3.521478	3.146427	4.227481	4.336552	4.796480	3.170136	3.533311	4.219685	4.033222	4.175304	3.862994	3.812920	3.660212	3.323316	3.242519	3.990914	3.630688	5.104212	3.554100	4.613988	4.193008	4.322455	3.643095	4.503938	4.636935	3.230901	3.334861	3.146427	4.238326	3.100092	2.955347	4.909875	3.655026	3.511083	3.990057	4.823549	3.269270	3.386886	3.514626	3.656424	4.116356	3.183432	3.845666	3.764496	4.464780

EYS	3.028669	3.333551	3.144458	2.988612	2.982627	3.846532	3.414352	3.433819	3.440483	3.465216	3.804108	3.680445	3.372263	3.807402	3.289377	3.208343	3.692360	4.438066	4.925093	3.540150	4.677314	3.003820	3.652214	2.889447	3.477093	3.162748	3.010961	3.182826	3.196929	3.047315	4.288341	4.377580	4.859174	3.165816	3.979779	4.086354	3.694034	3.552011	3.335465	3.257598	3.349197	3.403382	3.884271	3.281785	3.594399	5.392395	3.774090	5.923827	4.107829	4.201183	4.063079	4.765269	4.338757	4.197524	3.813595	3.422793	3.934932	4.714961	4.230073	3.895854	3.297133	3.055187	3.744836	3.255631	3.498544	3.340106	3.424029	3.285623	3.759641	2.914475	3.280717	2.934808	3.198002	2.984959	3.090114	3.324325	3.024958	3.095417	2.994658	3.716485	3.407494	3.959875	3.337455	4.220612	3.416052	5.000471	2.903654	3.198153	4.231140	3.663842	3.223091	2.863100	3.038755	3.176969	2.949946	2.984597	3.653830	3.302676	3.453834	2.978889	2.883803	3.253277	3.010672	3.014557	4.388263	3.900869	4.166676	3.202402	2.980773	3.256882	2.805927	3.107248	3.228505	3.245109	3.790973	2.841503	3.065950	3.041027	3.076229	3.009272	3.438239	3.075075	3.027882	2.886653	4.619476	2.996541	3.282097	3.489376	3.218815	3.799662	3.194161	3.063198	3.208983	3.074675	2.971703	3.655026	3.636869	3.448991	3.501419	3.965491	2.916215	5.721228	2.851394	3.277712	3.136503	2.996090	2.987972	2.886744	3.519668	3.654009	3.532925	3.251979	3.249846	3.920614	3.779991	3.778359	3.830142	3.398675	3.128951	3.143153	3.164945	3.946856	3.624461	3.963170	4.743378	3.936856	4.284744	3.810995	4.492677	4.150618	4.363938	4.605637	4.291335	3.395752	3.200147	3.545283	4.619929	4.052563	3.877169	3.036447	3.103006	3.765704	3.851619	3.832712	3.528663	3.421705	3.568074	3.272103	2.889727	3.091624	3.282097	3.404378	3.017698	2.987230	3.291768	3.139452	3.351900	3.077475	3.240314	3.791856	4.219250	3.366512	3.648387	3.417570	3.667710	3.505145	3.360810	3.967025	3.554886	3.350383	2.835405	2.968562	3.198773	3.208207	3.019944	2.923214	2.927497	3.917902	3.315547	3.025052	3.041440	2.928675	2.893450	4.808066	3.699776	3.023990	3.119753	3.288775	3.168060	2.791795	2.902316	2.992717	3.176019	2.981829	2.916669	3.303468	3.051422	3.173051

AC005324.3	8.648119	8.741172	9.987460	9.042224	8.972624	9.559249	8.808590	9.705510	10.962078	9.647967	10.199926	9.055357	9.758866	9.631817	9.099030	9.661238	9.563520	9.671188	10.678215	9.148972	9.678129	10.323671	8.544161	10.463030	10.447194	9.403225	9.053125	10.170916	9.285357	7.770796	10.348093	9.349094	8.812183	7.680418	8.203010	7.877792	8.625448	8.785931	9.638262	10.613754	9.107647	8.453070	8.673441	8.460633	7.995191	8.740568	10.117613	8.205533	9.429598	8.591616	8.212972	8.904922	8.640041	8.596736	9.470707	9.039461	8.557469	8.188620	8.819941	9.092226	8.979691	9.472328	5.866135	9.173435	8.103097	9.192582	8.208762	9.128284	8.918330	8.400970	7.938524	8.748190	10.037937	9.402162	8.446695	8.468110	9.380201	8.596881	9.003202	8.336479	9.431578	9.548379	9.160765	8.336025	9.582219	9.170762	9.055978	10.195238	7.467717	7.807410	7.919491	9.489846	8.374648	9.176514	7.822399	9.209617	9.278901	9.648405	9.565280	9.734237	9.866534	8.989269	9.506259	9.455567	10.144458	9.909249	9.918845	9.148458	10.029478	9.297552	8.542271	9.929861	11.176723	10.054805	10.806498	8.531825	9.319449	9.449989	9.536303	6.964223	9.755913	8.783670	7.649509	9.605026	8.562976	10.266879	6.613170	9.894559	9.152780	8.946600	7.604353	10.380306	6.674294	9.234252	8.585756	8.118880	9.408658	9.115876	7.597778	10.333610	10.605340	9.114516	8.459410	7.345830	9.570876	8.711618	10.817302	8.997042	8.276257	9.096736	8.489838	9.152937	10.455244	7.036695	8.138337	9.662014	7.252149	9.925180	8.556557	7.754268	8.882494	7.393988	10.180871	8.732552	9.012852	9.178624	6.093732	9.267059	9.488884	8.479979	7.260440	9.641025	8.150052	8.393485	8.301810	6.117672	6.542379	7.781047	6.982537	10.552793	7.100637	7.227941	9.010532	7.214504	8.157042	7.635884	8.943551	8.269676	7.737577	9.240121	8.980732	10.935020	8.758540	6.333320	8.974964	8.784460	8.846939	8.313127	6.701746	9.688696	8.777234	10.349902	8.064574	8.318216	9.529428	8.848945	7.232439	7.181406	7.319006	6.121304	8.678364	8.193995	8.211887	9.562846	7.338317	9.769058	8.607569	10.191897	8.438425	7.580396	6.810819	6.601982	8.301065	5.842228	9.713091	10.563692	8.450156	9.190397	10.070538	8.912356	7.576848	7.264197	8.555588	7.728876	9.675373	9.856526	7.857100	8.086750

CDRT1	8.648119	8.741172	9.987460	9.042224	8.972624	9.559249	8.808590	9.705510	10.962078	9.647967	10.199926	9.055357	9.758866	9.631817	9.099030	9.661238	9.563520	9.671188	10.678215	9.148972	9.678129	10.323671	8.544161	10.463030	10.447194	9.403225	9.053125	10.170916	9.285357	7.770796	10.348093	9.349094	8.812183	7.680418	8.203010	7.877792	8.625448	8.785931	9.638262	10.613754	9.107647	8.453070	8.673441	8.460633	7.995191	8.740568	10.117613	8.205533	9.429598	8.591616	8.212972	8.904922	8.640041	8.596736	9.470707	9.039461	8.557469	8.188620	8.819941	9.092226	8.979691	9.472328	5.866135	9.173435	8.103097	9.192582	8.208762	9.128284	8.918330	8.400970	7.938524	8.748190	10.037937	9.402162	8.446695	8.468110	9.380201	8.596881	9.003202	8.336479	9.431578	9.548379	9.160765	8.336025	9.582219	9.170762	9.055978	10.195238	7.467717	7.807410	7.919491	9.489846	8.374648	9.176514	7.822399	9.209617	9.278901	9.648405	9.565280	9.734237	9.866534	8.989269	9.506259	9.455567	10.144458	9.909249	9.918845	9.148458	10.029478	9.297552	8.542271	9.929861	11.176723	10.054805	10.806498	8.531825	9.319449	9.449989	9.536303	6.964223	9.755913	8.783670	7.649509	9.605026	8.562976	10.266879	6.613170	9.894559	9.152780	8.946600	7.604353	10.380306	6.674294	9.234252	8.585756	8.118880	9.408658	9.115876	7.597778	10.333610	10.605340	9.114516	8.459410	7.345830	9.570876	8.711618	10.817302	8.997042	8.276257	9.096736	8.489838	9.152937	10.455244	7.036695	8.138337	9.662014	7.252149	9.925180	8.556557	7.754268	8.882494	7.393988	10.180871	8.732552	9.012852	9.178624	6.093732	9.267059	9.488884	8.479979	7.260440	9.641025	8.150052	8.393485	8.301810	6.117672	6.542379	7.781047	6.982537	10.552793	7.100637	7.227941	9.010532	7.214504	8.157042	7.635884	8.943551	8.269676	7.737577	9.240121	8.980732	10.935020	8.758540	6.333320	8.974964	8.784460	8.846939	8.313127	6.701746	9.688696	8.777234	10.349902	8.064574	8.318216	9.529428	8.848945	7.232439	7.181406	7.319006	6.121304	8.678364	8.193995	8.211887	9.562846	7.338317	9.769058	8.607569	10.191897	8.438425	7.580396	6.810819	6.601982	8.301065	5.842228	9.713091	10.563692	8.450156	9.190397	10.070538	8.912356	7.576848	7.264197	8.555588	7.728876	9.675373	9.856526	7.857100	8.086750

UTP14A	12.455483	12.184698	12.137865	11.704750	11.930334	11.837992	12.294671	11.904757	11.507746	12.243307	11.939528	11.975261	11.900292	11.995512	12.220778	11.896778	12.036237	12.428003	12.364975	12.252154	12.476025	12.546681	12.850884	12.127196	12.012819	12.128733	12.012048	11.527903	12.084672	12.051649	11.752925	12.482141	12.192450	12.086652	12.479302	12.403076	12.352099	12.758584	12.395544	12.571330	12.262939	12.438036	12.299016	12.459117	12.441586	12.321432	11.671357	12.184541	12.136334	12.106371	12.199524	12.363731	12.082273	12.536272	12.522051	11.857830	12.345161	12.288317	12.221427	12.442360	12.567588	12.129935	12.854406	12.306362	12.086204	12.120220	11.982123	12.227667	11.995974	12.525777	11.940136	11.971773	11.749834	11.959299	12.019013	11.951988	12.247505	11.881010	12.085897	11.815237	12.297752	12.029310	12.142076	12.406051	12.495360	12.198594	11.958709	11.818235	12.722115	12.208079	12.023112	12.326361	11.901464	12.268220	11.851556	12.224317	12.335876	11.874773	12.067502	11.773425	12.075117	12.298055	12.014764	11.857195	13.537270	11.934117	12.372433	12.710821	13.095248	12.044248	12.956616	12.249599	12.265583	12.006089	11.986356	10.924627	12.236952	12.132348	11.697417	12.585328	13.047238	12.561379	12.464531	11.693803	12.932954	12.013103	12.475857	13.064722	12.609687	12.706190	12.534911	12.666963	12.199062	12.243629	11.995202	12.957359	12.752429	13.432385	13.240382	12.647084	12.685200	13.068904	11.852632	12.115080	12.730491	11.572225	12.383214	11.778816	12.678360	12.265122	13.051023	12.765130	12.887037	12.696084	12.445002	12.465329	12.812829	13.625684	12.799200	13.102010	13.090158	12.791666	13.176594	12.690509	12.915887	12.393629	12.922049	12.364491	13.216575	12.975455	12.958825	12.668162	12.626987	12.547774	12.131138	12.822839	12.277908	13.017369	12.407615	12.322207	12.911260	12.726851	13.764461	12.600707	12.019164	12.984090	12.284891	12.272493	12.737801	12.286918	13.064722	12.346629	12.382100	12.497991	12.414483	12.905001	13.524838	12.537583	12.028718	12.369370	13.305359	11.825192	12.203451	13.144955	12.833284	13.232837	12.734379	12.756348	12.604249	12.611187	12.906255	12.244853	13.295754	12.836529	12.052224	12.432090	11.913228	12.980105	12.471030	13.129561	12.360678	11.822914	12.228631	13.418183	11.869930	13.391215	12.920784	12.710992	11.733178	13.292760	12.762553	13.186829	11.663877	13.181319	11.776688	13.023845	12.275923	12.373685

DNAH7	5.342348	5.187401	5.021154	5.361536	5.235030	4.691364	4.494208	6.117144	5.396732	4.744005	4.898779	6.318570	4.487836	3.662035	4.453761	4.922338	5.336143	5.224298	6.758139	4.113339	4.665824	4.757162	5.083639	4.808066	4.587091	5.206028	5.945135	4.951026	5.487827	4.119254	4.914730	6.107488	5.875848	5.505652	5.236860	4.494208	4.712006	3.901467	5.048723	2.916572	5.691168	4.640721	5.545431	4.958542	5.903669	4.341788	4.960000	5.297077	5.760333	4.852235	5.479452	4.994155	5.507599	5.270257	5.413965	4.059980	4.476238	3.922002	5.506814	5.244018	3.094426	4.528825	5.446617	4.563169	4.960000	5.762324	3.069717	5.059621	4.576680	5.066538	5.305796	4.214051	4.441288	4.312729	4.331456	5.692843	4.724520	5.213361	4.467997	4.838091	5.258688	5.715491	4.769494	5.227496	4.796480	6.271536	6.573209	6.845561	4.831039	4.977646	5.350672	5.165881	5.311070	4.596165	5.231386	5.263283	5.583037	6.242113	5.240856	5.657752	5.365800	5.072591	5.991996	5.773628	4.973155	5.661046	4.376322	4.870222	4.853109	4.689716	5.094789	5.541274	5.546573	4.366775	4.609293	6.257597	5.970326	6.288269	5.583408	5.287167	3.877169	4.354159	4.433701	4.702801	5.430849	4.476479	4.580993	4.422881	3.964886	5.117824	4.173575	4.223185	4.711800	4.215554	5.190727	4.710741	4.113339	7.644333	6.495402	4.704484	5.070383	5.654433	4.211207	4.341985	5.150526	5.432219	4.624008	5.904380	4.323583	5.127753	4.709048	5.596446	6.046054	5.189959	4.487427	4.748940	4.248379	5.242044	4.615553	5.694332	2.905987	5.684541	4.311250	4.715794	3.757114	4.703861	5.468334	4.547077	4.451610	4.282734	6.407708	5.240856	5.249800	4.623548	4.119254	7.410189	6.423652	5.392589	4.696606	4.408498	4.432687	4.348075	3.518201	4.727455	4.276612	3.939428	4.515287	3.884501	4.934923	4.490426	4.264426	4.589492	5.182603	4.435637	4.202480	4.667563	4.571394	4.413326	5.443704	5.323834	4.577141	4.759964	5.485387	5.659219	5.583037	5.090437	4.672289	5.046074	4.512247	5.528211	5.533682	5.072013	3.769513	5.221285	4.267843	4.881370	5.847710	4.500267	5.829701	4.650703	6.406893	4.977248	6.558111	5.468537	5.324966	4.829317	4.081762	4.150204	4.116788	5.198932	6.998483	5.340006	4.511602	3.871346	4.845532	4.261329	4.552175	3.761124

XRRA1	7.551749	7.882174	8.546584	8.695889	9.280452	8.938239	8.552439	9.360161	10.474667	9.989210	9.364727	8.726647	9.703851	10.031008	8.930159	9.447171	9.238757	9.379735	10.009427	8.854542	8.999654	11.103577	9.331691	10.390607	9.288325	9.340284	9.274979	10.305371	9.290484	7.486340	9.824324	6.906393	7.694121	7.656275	8.000110	7.668822	8.531966	8.567616	8.915570	9.338896	9.056272	7.675736	8.379846	8.731790	8.349747	8.635528	9.992951	7.990498	8.248700	7.875662	7.354709	8.215311	7.860047	8.542119	8.283150	8.542583	8.821587	8.244777	8.946451	8.409240	8.654155	9.260456	6.985241	9.476275	6.951856	7.909583	7.831559	8.342758	7.814104	7.692126	7.653147	8.602058	9.463167	8.671736	8.769760	7.593360	8.240081	7.491960	7.455423	8.363460	8.373879	7.460875	8.444090	8.781030	9.014405	8.703980	9.106746	8.681429	6.445919	8.476775	7.844066	7.208002	8.145020	8.381748	7.372217	9.315654	7.841244	10.270147	9.589975	9.772353	9.174945	9.128435	9.462868	8.801264	9.023413	9.360471	9.535499	9.484723	8.817145	9.885942	9.254946	8.397632	9.646315	9.931696	8.496424	7.707285	7.727325	9.028564	10.969237	7.453233	6.479926	6.357152	8.760109	6.914636	7.883075	8.135264	7.735340	8.177241	8.637389	8.803198	8.192439	7.531429	7.135477	7.747653	9.095706	8.588347	9.591971	7.331171	7.037167	7.842468	8.876541	6.668981	8.621266	7.240071	6.635961	7.704877	10.054496	9.171092	5.806483	9.036185	6.918525	7.602148	8.108945	6.555657	7.784553	7.109503	6.895743	7.422754	7.218710	7.367347	6.515220	6.773160	7.187472	8.166444	7.659080	7.615728	6.384167	7.770177	5.745522	6.577340	7.747797	7.375004	7.246079	7.194717	6.723695	7.507849	6.299075	8.457490	6.628874	6.599750	7.360909	5.886723	7.559819	6.731107	6.137520	6.183411	7.251999	7.263413	5.188216	6.865500	7.440064	7.928816	6.347604	6.365202	6.816661	6.807095	5.708658	6.292672	6.909234	7.213117	7.192697	7.384238	7.147061	7.533448	8.068471	6.826891	7.538600	5.502582	6.538445	7.230565	5.422633	6.712113	5.607399	8.182340	7.478135	7.085386	9.128602	8.303620	9.010977	7.826149	8.864614	8.836612	7.213408	6.461838	9.343124	7.758072	8.506270	6.857868	7.698975	7.066045	6.757978	7.489903	9.496425	7.594740	6.716392	7.740862	7.769881	8.889622

KCNMB1	12.687444	13.835331	13.479980	13.713891	12.536416	13.681631	14.781829	15.172639	12.988891	14.640496	13.851689	14.479473	12.831697	13.441153	13.267179	13.842204	13.878229	13.164705	13.459822	13.950442	13.321286	12.852480	15.527160	12.643076	14.757850	14.143610	14.197832	13.374445	13.241587	12.942843	13.364346	13.141663	13.166715	12.891207	13.912401	14.928143	14.542602	14.123055	14.369411	13.938513	13.451797	14.485612	13.945117	13.563621	15.618883	13.559725	13.821463	13.830239	13.326618	14.110109	12.990154	14.454664	14.092188	14.324060	14.506070	14.666375	14.700650	14.039946	13.899912	14.863545	14.822949	12.898820	15.914283	14.076340	14.154830	15.011117	14.289643	14.261678	13.826605	14.444149	14.563854	14.377022	13.132093	14.629930	13.685040	13.655750	13.968407	13.644259	13.441821	13.855680	13.557035	14.178659	13.783848	14.443791	15.197536	14.542200	14.066316	13.414432	13.437764	13.376715	14.795501	13.946486	13.700893	14.898599	12.952935	13.504135	13.754158	13.168263	13.180561	14.820855	13.750320	14.357312	13.174180	13.633730	14.039502	12.760647	13.723478	13.220404	13.640364	13.884089	13.493953	13.275630	12.952366	12.836529	12.171410	13.718167	13.529678	14.741814	13.130569	10.904224	13.388838	12.685364	11.576750	12.033988	12.428658	13.680430	12.035653	11.174150	11.652730	13.047627	11.535407	13.351805	11.768702	10.801045	13.811158	11.998395	11.020654	12.473114	11.807837	11.038306	11.333467	13.407769	11.172234	11.916216	10.321225	12.082135	11.372379	12.108789	11.554015	12.588458	11.767815	12.061357	13.150496	11.587606	13.287464	14.602393	11.504078	12.041676	12.162219	11.554168	12.659650	12.495360	11.469123	12.451227	12.891375	11.678294	11.457713	10.783168	11.795120	11.909858	12.459458	11.420135	12.490240	13.307926	13.239992	13.024751	11.107188	11.974803	12.021368	12.511911	14.073025	11.213657	11.759614	12.011096	11.914600	11.382171	13.572418	12.504387	12.170015	13.167290	12.692172	12.676204	12.792028	12.288154	13.257008	12.451069	12.082923	13.022170	14.334050	11.205836	11.333902	13.811679	11.771231	11.619649	12.124933	12.924711	11.385102	13.808112	13.852712	12.417662	12.269936	13.834567	12.348967	13.968540	12.351197	11.173730	10.830197	11.277646	12.340875	10.780871	11.560528	13.416905	12.127196	10.991053	11.680252	11.440893	10.633979	11.942122	13.385597	13.440736	10.733574	12.392060	12.468291	10.859529	12.127984	13.811158	10.995960	12.168975

KCNIP1	12.687444	13.835331	13.479980	13.713891	12.536416	13.681631	14.781829	15.172639	12.988891	14.640496	13.851689	14.479473	12.831697	13.441153	13.267179	13.842204	13.878229	13.164705	13.459822	13.950442	13.321286	12.852480	15.527160	12.643076	14.757850	14.143610	14.197832	13.374445	13.241587	12.942843	13.364346	13.141663	13.166715	12.891207	13.912401	14.928143	14.542602	14.123055	14.369411	13.938513	13.451797	14.485612	13.945117	13.563621	15.618883	13.559725	13.821463	13.830239	13.326618	14.110109	12.990154	14.454664	14.092188	14.324060	14.506070	14.666375	14.700650	14.039946	13.899912	14.863545	14.822949	12.898820	15.914283	14.076340	14.154830	15.011117	14.289643	14.261678	13.826605	14.444149	14.563854	14.377022	13.132093	14.629930	13.685040	13.655750	13.968407	13.644259	13.441821	13.855680	13.557035	14.178659	13.783848	14.443791	15.197536	14.542200	14.066316	13.414432	13.437764	13.376715	14.795501	13.946486	13.700893	14.898599	12.952935	13.504135	13.754158	13.168263	13.180561	14.820855	13.750320	14.357312	13.174180	13.633730	14.039502	12.760647	13.723478	13.220404	13.640364	13.884089	13.493953	13.275630	12.952366	12.836529	12.171410	13.718167	13.529678	14.741814	13.130569	10.904224	13.388838	12.685364	11.576750	12.033988	12.428658	13.680430	12.035653	11.174150	11.652730	13.047627	11.535407	13.351805	11.768702	10.801045	13.811158	11.998395	11.020654	12.473114	11.807837	11.038306	11.333467	13.407769	11.172234	11.916216	10.321225	12.082135	11.372379	12.108789	11.554015	12.588458	11.767815	12.061357	13.150496	11.587606	13.287464	14.602393	11.504078	12.041676	12.162219	11.554168	12.659650	12.495360	11.469123	12.451227	12.891375	11.678294	11.457713	10.783168	11.795120	11.909858	12.459458	11.420135	12.490240	13.307926	13.239992	13.024751	11.107188	11.974803	12.021368	12.511911	14.073025	11.213657	11.759614	12.011096	11.914600	11.382171	13.572418	12.504387	12.170015	13.167290	12.692172	12.676204	12.792028	12.288154	13.257008	12.451069	12.082923	13.022170	14.334050	11.205836	11.333902	13.811679	11.771231	11.619649	12.124933	12.924711	11.385102	13.808112	13.852712	12.417662	12.269936	13.834567	12.348967	13.968540	12.351197	11.173730	10.830197	11.277646	12.340875	10.780871	11.560528	13.416905	12.127196	10.991053	11.680252	11.440893	10.633979	11.942122	13.385597	13.440736	10.733574	12.392060	12.468291	10.859529	12.127984	13.811158	10.995960	12.168975

PTS	5.877950	5.539594	5.650297	6.341705	6.833280	6.773490	6.149155	5.226859	6.826070	6.452765	6.673975	6.867906	6.401780	5.823680	5.293315	6.389356	5.758146	6.976098	5.858142	7.123215	5.896338	5.271047	5.826762	6.835648	7.046406	6.634185	7.169943	6.361699	6.649358	5.867417	7.256173	5.319005	6.548360	6.491699	6.461507	5.513154	6.126045	5.730853	5.851850	6.059027	6.988104	5.689182	6.670097	6.376000	6.792357	5.172120	6.941340	5.913302	5.985034	5.701366	6.027838	5.432417	6.615919	6.276695	6.497681	6.456040	6.330441	4.041233	6.752706	5.929695	4.900484	6.727241	5.037736	6.203650	5.363851	5.543334	4.672936	5.459048	5.232814	4.885618	5.900980	5.895320	6.727719	6.012132	6.420618	6.174728	5.574617	6.265036	5.918522	6.294553	6.064005	6.311949	5.671418	6.089390	6.541196	6.818290	6.572737	6.282611	3.670528	5.559465	5.425023	4.893955	5.994297	5.553777	6.079385	6.376795	5.416410	7.884753	6.485132	7.057617	7.015827	6.510415	6.924368	6.304265	5.447832	6.701426	5.575395	6.371084	5.487997	6.849972	3.947248	7.507990	6.549863	7.040172	6.583596	5.965347	6.482556	7.061882	7.783009	4.193008	5.489978	4.817515	5.931422	5.417778	5.315439	5.900633	6.948756	6.470712	4.283596	5.416994	6.888567	5.008172	4.698520	2.974731	6.331112	5.740431	6.217326	4.283381	6.026440	5.597810	4.279959	5.634528	7.031875	5.044934	4.874651	6.400316	5.561304	5.053206	4.958737	6.786940	5.936334	6.235988	5.249579	6.355577	5.486905	6.049248	3.674319	4.795599	5.235235	4.892077	3.006794	5.516844	5.306198	5.575947	4.043878	5.140676	3.278841	5.825842	4.536530	4.232847	6.379471	5.193075	4.690748	6.054977	4.720037	3.530322	3.927841	5.522331	4.907752	4.651131	5.604559	4.530110	4.850148	4.622478	5.817445	3.084646	5.129592	4.454595	2.933891	5.141118	5.857270	5.003178	4.815587	5.620566	5.730301	5.594019	4.582452	5.491318	5.640130	5.635849	6.405559	5.807238	6.218038	5.761196	6.832287	5.067778	6.579465	3.454582	4.434540	4.427447	4.084103	5.116611	4.581620	5.800432	5.292942	4.956861	7.715526	5.824779	6.030587	5.115354	6.024120	6.814127	5.324768	4.591620	5.818516	4.151044	4.979117	4.452439	5.985373	3.615523	5.910637	5.482022	5.614444	5.233633	4.837655	5.599318	6.272225	6.014517

PLET1	5.877950	5.539594	5.650297	6.341705	6.833280	6.773490	6.149155	5.226859	6.826070	6.452765	6.673975	6.867906	6.401780	5.823680	5.293315	6.389356	5.758146	6.976098	5.858142	7.123215	5.896338	5.271047	5.826762	6.835648	7.046406	6.634185	7.169943	6.361699	6.649358	5.867417	7.256173	5.319005	6.548360	6.491699	6.461507	5.513154	6.126045	5.730853	5.851850	6.059027	6.988104	5.689182	6.670097	6.376000	6.792357	5.172120	6.941340	5.913302	5.985034	5.701366	6.027838	5.432417	6.615919	6.276695	6.497681	6.456040	6.330441	4.041233	6.752706	5.929695	4.900484	6.727241	5.037736	6.203650	5.363851	5.543334	4.672936	5.459048	5.232814	4.885618	5.900980	5.895320	6.727719	6.012132	6.420618	6.174728	5.574617	6.265036	5.918522	6.294553	6.064005	6.311949	5.671418	6.089390	6.541196	6.818290	6.572737	6.282611	3.670528	5.559465	5.425023	4.893955	5.994297	5.553777	6.079385	6.376795	5.416410	7.884753	6.485132	7.057617	7.015827	6.510415	6.924368	6.304265	5.447832	6.701426	5.575395	6.371084	5.487997	6.849972	3.947248	7.507990	6.549863	7.040172	6.583596	5.965347	6.482556	7.061882	7.783009	4.193008	5.489978	4.817515	5.931422	5.417778	5.315439	5.900633	6.948756	6.470712	4.283596	5.416994	6.888567	5.008172	4.698520	2.974731	6.331112	5.740431	6.217326	4.283381	6.026440	5.597810	4.279959	5.634528	7.031875	5.044934	4.874651	6.400316	5.561304	5.053206	4.958737	6.786940	5.936334	6.235988	5.249579	6.355577	5.486905	6.049248	3.674319	4.795599	5.235235	4.892077	3.006794	5.516844	5.306198	5.575947	4.043878	5.140676	3.278841	5.825842	4.536530	4.232847	6.379471	5.193075	4.690748	6.054977	4.720037	3.530322	3.927841	5.522331	4.907752	4.651131	5.604559	4.530110	4.850148	4.622478	5.817445	3.084646	5.129592	4.454595	2.933891	5.141118	5.857270	5.003178	4.815587	5.620566	5.730301	5.594019	4.582452	5.491318	5.640130	5.635849	6.405559	5.807238	6.218038	5.761196	6.832287	5.067778	6.579465	3.454582	4.434540	4.427447	4.084103	5.116611	4.581620	5.800432	5.292942	4.956861	7.715526	5.824779	6.030587	5.115354	6.024120	6.814127	5.324768	4.591620	5.818516	4.151044	4.979117	4.452439	5.985373	3.615523	5.910637	5.482022	5.614444	5.233633	4.837655	5.599318	6.272225	6.014517

NSF	11.019615	11.044877	10.904224	11.030608	11.686630	11.073706	10.818648	11.989871	11.721084	11.327340	11.835276	11.368020	11.918166	11.894727	10.664886	11.789270	11.654834	12.244688	12.297429	11.198394	11.580709	12.614854	11.344019	11.726020	11.981061	11.604016	10.300662	11.235506	10.499570	10.870667	11.201014	11.137430	11.140636	10.605180	10.479190	10.978478	11.246511	11.874166	11.407693	12.212531	10.941674	11.283268	11.749526	10.438539	10.966093	11.358100	11.498757	10.886421	11.042091	10.724919	11.556994	11.752478	11.250623	11.508778	11.370215	11.257689	11.718244	11.561285	11.370065	12.044248	11.306805	11.254400	10.985679	11.698422	11.347140	11.318667	11.291724	11.544678	11.392765	11.034179	10.762808	11.288046	11.730949	11.153618	10.803737	10.524581	11.167110	10.544617	11.293238	10.662532	11.237555	11.540263	10.960626	11.206436	11.409205	11.071058	10.810402	11.449438	11.117393	11.249397	11.321435	11.052217	11.368860	10.875451	10.914603	11.100018	10.887345	11.613605	11.074134	10.289597	11.987098	11.242610	11.583022	11.134324	11.957393	11.051912	11.471663	12.052224	9.890283	11.553739	10.671515	12.238197	12.654649	11.360018	12.377241	9.736555	11.383209	10.387783	10.742311	10.959584	10.766265	10.353725	10.727311	11.160367	10.798335	10.025602	9.970923	11.942873	10.430953	10.748889	11.281661	11.420870	10.440195	10.002425	10.403949	11.136698	12.175134	11.437522	9.921369	10.955728	11.886002	11.275334	10.331142	10.650579	11.690932	10.780444	9.606560	8.746649	10.825756	11.036408	10.385633	10.685541	11.054370	10.518311	10.792032	10.949662	10.852571	10.529215	11.279887	9.993251	10.868882	11.015291	10.591883	10.966826	11.369611	11.544838	10.684665	10.614968	10.405917	11.146945	11.052955	10.516102	10.745281	10.581882	10.969060	10.643123	11.274882	11.015291	11.224462	10.902327	11.002085	11.419106	11.382901	11.074134	11.019905	10.682418	10.702889	10.918671	11.073706	11.081746	10.791738	9.957369	10.873822	10.348429	10.378655	10.288521	10.254638	10.820288	10.105230	10.494224	11.243470	11.460104	11.133235	10.653251	10.860414	10.891777	10.849297	10.681237	10.813574	10.942565	10.342641	11.271780	10.516020	10.290520	10.132299	10.240163	10.316078	10.763121	10.341712	10.519091	10.271475	10.858234	10.531733	11.163643	10.504406	9.406773	11.383209	10.518778	11.199453	9.843353	11.368306	11.375921	10.918947	11.198701	11.070018	11.534377	10.285158	10.330692

AC011462.1	9.554494	10.029650	10.107468	9.528007	9.360643	8.853185	9.678789	9.192737	8.330882	9.713091	9.136379	9.472022	9.318197	10.045253	9.525550	9.142743	8.958743	9.718772	7.794750	9.488725	8.871852	9.482879	7.560582	9.144456	9.271660	9.239068	8.345999	9.280909	8.983483	9.441911	8.519009	10.380918	9.883787	9.491252	9.564183	9.407719	9.541895	9.720786	9.928156	9.799077	9.476275	9.839071	9.335876	9.141819	9.402928	9.706104	9.124887	10.035315	9.529282	9.768131	9.807143	9.919470	10.136988	9.954924	9.669110	9.243051	9.807593	10.515937	10.575116	10.113185	10.615988	8.997674	10.843773	10.230861	10.508876	10.530844	9.848310	9.563744	10.056061	10.254638	9.560659	9.382397	9.049602	9.303419	9.396057	9.787767	9.365816	9.455087	9.415347	9.500267	9.880394	9.856672	9.758574	10.021565	9.622752	9.959383	9.483345	9.681866	10.894182	10.246494	10.006010	10.353725	9.771605	9.583001	9.479088	9.454459	9.559249	8.988078	9.842448	9.043484	8.861204	9.420087	9.171408	9.983675	9.356737	9.115417	8.523862	8.964318	8.402822	8.668179	7.899893	9.655361	9.559089	8.897960	9.690530	9.711100	9.547475	9.491252	9.224668	9.696489	10.275761	10.117613	9.946044	10.057941	9.556781	8.727096	7.980493	8.203755	9.210231	8.940056	8.819941	9.021427	10.203707	8.873398	8.657576	9.335435	8.637723	9.504231	10.011667	9.197647	9.676141	8.229507	9.627540	9.798602	10.414630	9.235458	9.474903	9.090933	9.659051	8.215620	9.170296	9.172195	9.853684	9.559692	9.252826	9.722173	9.990754	10.186500	10.483671	10.426540	10.604302	10.468588	9.900225	9.711552	9.512302	8.709313	9.801346	9.765569	10.227905	9.873016	10.177956	9.771286	10.189798	9.844563	9.826780	10.104182	10.170756	9.759652	10.727633	10.872782	10.327332	10.053117	10.544466	10.560393	9.894722	11.043725	9.517921	9.383323	10.448550	9.134694	9.519931	9.073286	9.125034	9.131538	9.353052	9.380364	9.510701	9.050390	9.682776	9.676753	9.133674	10.108827	9.790373	10.246175	9.268775	9.600803	9.864846	10.683331	10.154056	10.970158	10.440817	9.775681	9.543140	9.254946	9.281525	8.658992	7.750734	9.187848	9.128435	9.302664	8.790410	8.886993	9.648868	9.315049	9.290023	9.020466	9.730269	10.669868	8.910236	8.174113	10.270415	9.117427	8.905538	9.852612	9.657205	10.419803	9.728056	9.569197

B9D2	9.554494	10.029650	10.107468	9.528007	9.360643	8.853185	9.678789	9.192737	8.330882	9.713091	9.136379	9.472022	9.318197	10.045253	9.525550	9.142743	8.958743	9.718772	7.794750	9.488725	8.871852	9.482879	7.560582	9.144456	9.271660	9.239068	8.345999	9.280909	8.983483	9.441911	8.519009	10.380918	9.883787	9.491252	9.564183	9.407719	9.541895	9.720786	9.928156	9.799077	9.476275	9.839071	9.335876	9.141819	9.402928	9.706104	9.124887	10.035315	9.529282	9.768131	9.807143	9.919470	10.136988	9.954924	9.669110	9.243051	9.807593	10.515937	10.575116	10.113185	10.615988	8.997674	10.843773	10.230861	10.508876	10.530844	9.848310	9.563744	10.056061	10.254638	9.560659	9.382397	9.049602	9.303419	9.396057	9.787767	9.365816	9.455087	9.415347	9.500267	9.880394	9.856672	9.758574	10.021565	9.622752	9.959383	9.483345	9.681866	10.894182	10.246494	10.006010	10.353725	9.771605	9.583001	9.479088	9.454459	9.559249	8.988078	9.842448	9.043484	8.861204	9.420087	9.171408	9.983675	9.356737	9.115417	8.523862	8.964318	8.402822	8.668179	7.899893	9.655361	9.559089	8.897960	9.690530	9.711100	9.547475	9.491252	9.224668	9.696489	10.275761	10.117613	9.946044	10.057941	9.556781	8.727096	7.980493	8.203755	9.210231	8.940056	8.819941	9.021427	10.203707	8.873398	8.657576	9.335435	8.637723	9.504231	10.011667	9.197647	9.676141	8.229507	9.627540	9.798602	10.414630	9.235458	9.474903	9.090933	9.659051	8.215620	9.170296	9.172195	9.853684	9.559692	9.252826	9.722173	9.990754	10.186500	10.483671	10.426540	10.604302	10.468588	9.900225	9.711552	9.512302	8.709313	9.801346	9.765569	10.227905	9.873016	10.177956	9.771286	10.189798	9.844563	9.826780	10.104182	10.170756	9.759652	10.727633	10.872782	10.327332	10.053117	10.544466	10.560393	9.894722	11.043725	9.517921	9.383323	10.448550	9.134694	9.519931	9.073286	9.125034	9.131538	9.353052	9.380364	9.510701	9.050390	9.682776	9.676753	9.133674	10.108827	9.790373	10.246175	9.268775	9.600803	9.864846	10.683331	10.154056	10.970158	10.440817	9.775681	9.543140	9.254946	9.281525	8.658992	7.750734	9.187848	9.128435	9.302664	8.790410	8.886993	9.648868	9.315049	9.290023	9.020466	9.730269	10.669868	8.910236	8.174113	10.270415	9.117427	8.905538	9.852612	9.657205	10.419803	9.728056	9.569197

TMEM91	9.554494	10.029650	10.107468	9.528007	9.360643	8.853185	9.678789	9.192737	8.330882	9.713091	9.136379	9.472022	9.318197	10.045253	9.525550	9.142743	8.958743	9.718772	7.794750	9.488725	8.871852	9.482879	7.560582	9.144456	9.271660	9.239068	8.345999	9.280909	8.983483	9.441911	8.519009	10.380918	9.883787	9.491252	9.564183	9.407719	9.541895	9.720786	9.928156	9.799077	9.476275	9.839071	9.335876	9.141819	9.402928	9.706104	9.124887	10.035315	9.529282	9.768131	9.807143	9.919470	10.136988	9.954924	9.669110	9.243051	9.807593	10.515937	10.575116	10.113185	10.615988	8.997674	10.843773	10.230861	10.508876	10.530844	9.848310	9.563744	10.056061	10.254638	9.560659	9.382397	9.049602	9.303419	9.396057	9.787767	9.365816	9.455087	9.415347	9.500267	9.880394	9.856672	9.758574	10.021565	9.622752	9.959383	9.483345	9.681866	10.894182	10.246494	10.006010	10.353725	9.771605	9.583001	9.479088	9.454459	9.559249	8.988078	9.842448	9.043484	8.861204	9.420087	9.171408	9.983675	9.356737	9.115417	8.523862	8.964318	8.402822	8.668179	7.899893	9.655361	9.559089	8.897960	9.690530	9.711100	9.547475	9.491252	9.224668	9.696489	10.275761	10.117613	9.946044	10.057941	9.556781	8.727096	7.980493	8.203755	9.210231	8.940056	8.819941	9.021427	10.203707	8.873398	8.657576	9.335435	8.637723	9.504231	10.011667	9.197647	9.676141	8.229507	9.627540	9.798602	10.414630	9.235458	9.474903	9.090933	9.659051	8.215620	9.170296	9.172195	9.853684	9.559692	9.252826	9.722173	9.990754	10.186500	10.483671	10.426540	10.604302	10.468588	9.900225	9.711552	9.512302	8.709313	9.801346	9.765569	10.227905	9.873016	10.177956	9.771286	10.189798	9.844563	9.826780	10.104182	10.170756	9.759652	10.727633	10.872782	10.327332	10.053117	10.544466	10.560393	9.894722	11.043725	9.517921	9.383323	10.448550	9.134694	9.519931	9.073286	9.125034	9.131538	9.353052	9.380364	9.510701	9.050390	9.682776	9.676753	9.133674	10.108827	9.790373	10.246175	9.268775	9.600803	9.864846	10.683331	10.154056	10.970158	10.440817	9.775681	9.543140	9.254946	9.281525	8.658992	7.750734	9.187848	9.128435	9.302664	8.790410	8.886993	9.648868	9.315049	9.290023	9.020466	9.730269	10.669868	8.910236	8.174113	10.270415	9.117427	8.905538	9.852612	9.657205	10.419803	9.728056	9.569197

ATP5MD	14.966837	15.044189	15.694552	15.495462	15.446499	14.992829	15.367177	15.395261	15.069269	15.264422	15.009362	15.117745	15.317503	15.498917	15.637542	15.043521	15.117212	15.624294	15.165299	14.675040	15.337535	15.377722	15.638552	15.718870	14.915674	14.932454	14.173886	14.685039	14.710296	14.539165	15.194140	15.523618	15.393740	14.570568	15.170169	15.242555	15.338251	15.464870	15.709527	15.676063	14.935419	15.347177	14.800886	14.840176	14.874444	15.512636	14.843171	14.757385	15.044189	14.799393	14.931361	15.288862	14.978704	15.209040	15.145529	15.141653	15.385183	14.498295	15.544594	14.804759	15.018571	14.960579	15.413146	15.181269	14.848968	15.151876	14.529893	15.497254	14.947411	15.471498	15.276640	15.895253	14.816832	15.496317	15.756462	14.794023	15.288168	14.616972	14.484052	15.239037	15.305847	15.533380	15.118321	15.598287	15.571867	15.676954	15.561354	15.232746	15.560443	15.431836	15.275879	15.098171	15.298176	15.389194	14.487547	15.398455	15.462495	15.181938	15.313805	14.866612	15.134599	15.244645	15.708514	15.415429	15.844203	15.421791	15.416204	15.165299	15.327968	15.074047	15.385183	15.695521	15.628044	14.923336	15.125800	14.101917	15.332400	15.183376	15.001364	14.969696	15.539462	14.496768	14.856471	14.650647	15.078176	14.776105	14.378512	14.892803	14.726960	14.961694	14.768542	14.741814	14.948503	14.640937	14.828938	14.730211	15.003643	15.591703	15.086549	15.498075	15.074047	15.576365	14.505307	15.181938	14.943708	14.914162	15.304417	14.797866	14.713935	14.865080	14.751698	14.521080	15.109821	14.783853	14.886609	14.630361	15.012809	15.357970	15.028728	15.383684	15.029282	14.873437	15.293858	15.009958	14.850021	15.159483	14.763321	15.258866	14.632175	14.384877	14.622906	14.844188	15.168793	15.133349	14.651063	15.361722	15.168107	14.802357	14.831507	15.062228	14.800437	15.012247	15.548789	15.074047	14.925975	15.403893	14.932454	14.645337	14.937229	14.834547	14.705704	14.756024	14.988450	14.538753	14.777592	14.685534	14.935138	14.889167	14.688427	14.724263	14.392936	14.802845	15.114201	15.191395	14.159585	15.165299	15.711627	14.760083	14.992829	15.029900	15.219191	14.828455	14.523924	14.854894	14.742751	14.537158	14.806121	14.694707	14.886609	14.902407	15.039969	14.531049	14.591596	15.123478	14.344919	15.674067	15.739503	15.421791	14.795970	15.601071	14.999084	14.990649	14.761971	15.047833	15.354914	14.859475	15.178577	14.913079

TAF5	14.966837	15.044189	15.694552	15.495462	15.446499	14.992829	15.367177	15.395261	15.069269	15.264422	15.009362	15.117745	15.317503	15.498917	15.637542	15.043521	15.117212	15.624294	15.165299	14.675040	15.337535	15.377722	15.638552	15.718870	14.915674	14.932454	14.173886	14.685039	14.710296	14.539165	15.194140	15.523618	15.393740	14.570568	15.170169	15.242555	15.338251	15.464870	15.709527	15.676063	14.935419	15.347177	14.800886	14.840176	14.874444	15.512636	14.843171	14.757385	15.044189	14.799393	14.931361	15.288862	14.978704	15.209040	15.145529	15.141653	15.385183	14.498295	15.544594	14.804759	15.018571	14.960579	15.413146	15.181269	14.848968	15.151876	14.529893	15.497254	14.947411	15.471498	15.276640	15.895253	14.816832	15.496317	15.756462	14.794023	15.288168	14.616972	14.484052	15.239037	15.305847	15.533380	15.118321	15.598287	15.571867	15.676954	15.561354	15.232746	15.560443	15.431836	15.275879	15.098171	15.298176	15.389194	14.487547	15.398455	15.462495	15.181938	15.313805	14.866612	15.134599	15.244645	15.708514	15.415429	15.844203	15.421791	15.416204	15.165299	15.327968	15.074047	15.385183	15.695521	15.628044	14.923336	15.125800	14.101917	15.332400	15.183376	15.001364	14.969696	15.539462	14.496768	14.856471	14.650647	15.078176	14.776105	14.378512	14.892803	14.726960	14.961694	14.768542	14.741814	14.948503	14.640937	14.828938	14.730211	15.003643	15.591703	15.086549	15.498075	15.074047	15.576365	14.505307	15.181938	14.943708	14.914162	15.304417	14.797866	14.713935	14.865080	14.751698	14.521080	15.109821	14.783853	14.886609	14.630361	15.012809	15.357970	15.028728	15.383684	15.029282	14.873437	15.293858	15.009958	14.850021	15.159483	14.763321	15.258866	14.632175	14.384877	14.622906	14.844188	15.168793	15.133349	14.651063	15.361722	15.168107	14.802357	14.831507	15.062228	14.800437	15.012247	15.548789	15.074047	14.925975	15.403893	14.932454	14.645337	14.937229	14.834547	14.705704	14.756024	14.988450	14.538753	14.777592	14.685534	14.935138	14.889167	14.688427	14.724263	14.392936	14.802845	15.114201	15.191395	14.159585	15.165299	15.711627	14.760083	14.992829	15.029900	15.219191	14.828455	14.523924	14.854894	14.742751	14.537158	14.806121	14.694707	14.886609	14.902407	15.039969	14.531049	14.591596	15.123478	14.344919	15.674067	15.739503	15.421791	14.795970	15.601071	14.999084	14.990649	14.761971	15.047833	15.354914	14.859475	15.178577	14.913079

CCNL1	13.305996	12.985929	13.439032	13.183086	13.213390	12.771543	12.985009	13.507449	14.567967	13.192705	13.347873	13.443956	13.336659	12.901272	13.190080	12.862774	14.074808	13.422596	14.208831	12.857438	13.241017	13.073229	14.008743	13.333719	13.795903	13.734845	14.451725	13.320065	14.002435	13.602656	13.763209	12.360524	13.133751	13.113930	12.999330	12.809469	12.941572	12.767631	12.288317	12.803851	13.302672	12.626022	13.275630	13.392871	12.755481	13.191256	12.918772	12.889319	14.213324	12.898097	12.771890	12.833655	12.933669	12.684378	13.870167	14.118926	12.838773	12.512753	12.822839	12.994351	12.155089	13.437764	13.348898	12.929565	12.957538	13.768777	12.039083	12.717840	12.262621	11.944381	12.372749	12.214256	13.366847	12.340373	12.487478	12.927977	12.667122	12.949621	12.576557	12.690670	12.437418	13.375056	12.597817	12.725852	12.502391	13.729901	12.863135	12.806311	12.472142	12.285680	12.288476	12.821962	12.798338	12.442202	12.487478	12.777583	13.610144	13.671278	13.594660	13.196137	13.778222	12.663235	13.228647	13.464002	13.633730	13.555911	14.142986	13.317644	14.512377	13.075705	13.032134	13.542120	13.797469	13.520479	13.934635	12.417362	13.870710	13.644259	13.881196	13.640135	13.391427	13.696586	13.330689	14.305307	13.196334	13.542577	14.529498	13.875238	13.335194	13.259620	12.623671	14.050019	13.605255	12.721457	13.755126	14.235557	14.214704	14.062947	12.729124	13.829200	13.730904	14.153935	14.377416	12.817345	12.881137	13.262797	14.312048	13.454152	12.991054	14.898599	13.335818	13.120996	14.354516	13.200662	13.476158	12.847857	13.040426	13.088340	13.411645	13.734372	12.934722	12.747457	13.800613	14.017838	13.557270	14.081267	12.582966	13.653439	13.478390	13.373144	13.239009	12.994351	12.646567	12.614541	12.790989	12.508819	13.649164	13.516235	12.695910	13.472510	12.904642	13.132093	13.125775	12.605717	13.097867	13.287261	12.912347	13.593744	12.353482	13.375056	13.039489	13.735356	12.883847	13.014282	13.246497	12.784870	13.503447	12.873035	12.120667	13.884347	13.713444	13.234805	13.536412	13.234318	12.999705	13.492381	13.560621	12.807363	12.922772	13.000090	13.534858	13.250452	13.389482	13.956868	13.573817	14.320048	14.604960	14.249198	14.447515	14.229183	14.795501	14.149163	13.545519	13.681146	13.876872	14.884408	13.690683	13.017369	13.579215	13.520934	14.002151	14.386354	13.112597	14.363390	13.557508	13.910543	14.089539	12.631817

EXO1	9.948751	11.135665	11.296440	11.128092	10.709963	10.878399	10.989882	10.283531	10.485177	11.276168	11.018364	10.621312	11.045432	10.998047	10.255889	10.419946	10.719260	11.141929	10.775641	10.787337	10.639852	11.275872	9.328138	10.789138	10.243220	10.390190	9.676894	9.589501	10.360811	11.034586	10.428065	11.572961	10.654734	12.224769	10.970442	11.281969	11.185403	11.128382	11.353090	11.178535	11.076330	10.500604	10.797454	10.635922	10.481250	11.539821	10.108989	10.406371	10.785111	10.782381	10.365006	11.089291	10.956204	10.721496	10.613340	10.819087	10.882791	10.735629	10.860274	11.074704	11.018990	10.944454	9.873170	10.592175	11.873849	10.551729	11.594455	10.576317	12.165600	10.601523	10.741115	11.142502	10.775064	10.610513	11.275872	10.636526	11.593733	10.908849	10.699115	10.298160	10.766127	10.798635	11.766175	11.493459	11.499049	10.780289	11.006254	11.147241	11.201594	10.725657	10.686620	11.108664	11.267076	11.724680	10.783692	10.672565	10.738234	10.432156	10.868443	10.106999	10.426224	10.500604	10.821920	10.862314	11.259889	10.798635	10.636091	11.131082	11.050033	10.876491	11.551660	10.791597	11.000620	11.174436	10.565322	11.034738	9.913768	10.610648	10.561928	12.873920	12.599871	12.987965	12.015212	12.726021	13.034884	11.963218	12.701417	12.626987	12.781728	12.779334	13.058018	13.311729	12.878352	12.334611	10.739904	12.829989	12.516782	14.089838	13.492266	12.280960	12.649538	13.238255	12.365907	13.306600	12.703137	11.198550	12.560381	11.993346	12.784157	11.683795	12.430276	12.304346	12.999330	13.074553	12.923493	11.708961	13.110240	12.333343	12.734879	12.962142	13.512631	13.854876	13.050076	12.265502	13.319481	12.620818	13.361828	12.855167	13.529678	12.954420	12.312544	13.515560	13.545857	12.220618	12.141594	13.206916	13.233033	12.981868	13.183703	12.601240	12.111901	12.392195	13.396009	12.429637	11.604016	13.219568	11.672678	13.157276	12.768697	12.283635	13.357611	12.511756	12.496355	12.522828	12.011267	12.220150	13.157276	12.423432	11.483101	12.154206	13.194312	12.336677	12.039701	12.889486	12.121293	13.392668	13.205525	12.878707	12.379731	14.132353	12.945900	12.431602	13.086671	11.514925	12.142518	12.606727	11.303960	12.903231	11.993060	12.394589	12.111299	10.275619	12.519704	13.428889	12.487307	12.839995	12.687096	12.720580	12.369370	12.700895	12.994178	14.603726	11.473523	13.607100	12.463731	13.168641	11.863656	12.821251

GMDS	10.156816	8.231949	8.121961	9.504879	7.839230	8.009296	7.373769	9.419138	9.330151	8.318066	7.584728	8.168459	8.326972	7.992551	9.487035	8.782434	10.360069	8.112229	9.489658	8.239474	7.853763	9.150795	8.839043	8.510274	9.112719	8.563905	9.254946	8.904922	8.456739	7.099087	8.413828	7.401924	6.775893	7.431258	7.026340	7.213561	7.758072	8.492586	7.822399	8.581825	7.997816	8.109811	7.751835	7.906786	7.829918	8.193679	8.434296	7.912959	9.217742	8.175218	8.523545	7.946493	7.293017	7.104871	9.121979	7.941974	8.210991	7.926319	8.219775	7.374539	7.737895	8.068615	8.155622	8.813801	7.120590	8.281448	7.501815	9.128435	7.354215	7.810799	7.294755	7.131970	8.568189	7.325643	7.424306	7.124511	7.677784	7.069219	7.780597	7.776338	8.470262	8.928486	8.304234	8.546419	7.415219	8.068759	7.451502	7.313859	7.439764	8.110438	8.030420	8.292913	8.172997	7.639389	8.826430	8.349595	9.195936	7.841700	7.761745	9.512914	8.854235	7.661380	8.182340	8.157493	9.015613	9.623524	9.900361	8.943847	9.943787	8.961744	8.615418	10.156509	9.876239	9.722952	10.993001	6.663130	10.869324	8.215620	8.640670	7.080341	7.792954	6.835648	8.142400	8.241887	8.884973	7.221939	7.691350	7.552684	8.057573	7.261207	7.697156	8.339505	8.481866	8.438853	8.153159	7.413356	8.351110	9.246431	6.900947	8.695418	8.417966	9.179371	8.789036	7.632094	7.628292	9.211300	9.009109	7.916620	7.232119	7.972088	8.322944	6.875957	7.632856	7.169031	6.711048	7.117230	8.164882	7.819359	7.411070	8.612986	7.551900	6.838192	7.235637	7.213873	7.807263	8.573812	7.644170	6.889972	7.125950	7.365150	7.267272	6.957032	6.723695	7.064451	7.646042	7.044822	7.285048	8.118721	6.771880	7.107099	7.567050	7.830215	8.393936	7.488051	7.282114	7.180328	7.275940	7.654056	8.043407	7.354382	6.912288	10.927572	6.445749	5.770307	8.295598	6.685342	6.909549	7.636483	6.900947	6.934258	8.093070	7.649188	7.736225	6.754244	7.288966	7.235142	7.126261	7.358246	7.798320	9.527548	8.376613	7.375318	7.691812	7.484133	7.770020	7.833692	7.528443	8.289633	7.122734	7.484743	8.267776	7.967867	6.800111	7.642002	9.016215	8.840579	8.605139	7.887956	8.382834	7.554247	8.368619	7.783317	9.198437	8.266186	7.521277	7.693823	7.614356	7.682251

LYRM1	10.861749	9.623524	9.742900	10.243835	10.309726	10.772399	9.516071	11.037869	11.632245	10.244867	10.875592	10.632606	9.872579	10.095497	10.022004	10.596344	11.038306	10.016027	11.470020	10.220494	10.945054	10.511412	11.641478	10.585651	10.765826	10.997597	11.763241	11.444132	11.045505	9.775960	11.251225	8.863223	8.917711	9.436024	9.268147	9.426808	9.543634	9.642543	10.170273	10.390024	10.379694	10.253086	9.542810	10.232190	9.682033	9.720011	11.306962	9.702953	9.445303	9.430794	9.637950	9.200778	8.945966	9.120357	9.362308	10.725946	8.930630	9.652120	9.626936	9.485177	9.769225	10.395504	10.151576	10.484898	9.766026	9.992352	10.175307	10.128547	9.867722	10.567246	9.770646	9.956624	10.858655	9.613345	9.764510	9.961344	10.075727	9.695713	10.022004	9.215920	9.357865	9.356365	9.225591	9.202202	9.227275	9.394200	9.165096	9.285817	9.905450	9.808072	9.637784	10.408443	9.740417	9.678789	9.828423	9.989516	9.247361	10.181309	10.166289	10.695882	10.583475	9.505499	10.056794	10.034381	10.343107	10.674193	11.047517	10.370786	11.382463	10.906618	11.162013	10.898647	10.459968	11.479798	10.827110	12.227205	10.985097	10.530253	11.465248	10.047483	9.890879	9.486588	9.998125	11.144558	10.209367	10.276834	10.423780	10.566794	10.141339	10.402586	9.941496	10.341418	10.115451	10.486270	10.860126	10.635038	10.129611	10.796121	9.248899	10.694207	10.322128	11.090874	10.923426	10.362213	9.702796	10.588938	11.371885	11.483549	9.548217	11.322294	9.334439	9.922132	9.430494	9.370141	9.684031	9.200778	10.211224	10.262677	10.137297	10.547653	10.064686	9.184400	10.217279	9.866086	9.833499	11.138604	9.600494	9.628290	9.373824	9.521928	9.388107	8.902924	9.138750	9.200933	9.895500	8.790890	9.332620	9.574876	9.137166	9.760895	9.902734	10.037937	9.920576	9.749732	9.708142	9.871517	9.754870	10.063175	10.102022	9.850250	9.685152	11.339812	9.242250	9.380526	9.637322	9.660604	9.881929	9.837036	8.925514	8.904922	9.168755	9.151857	9.237958	9.059743	10.110348	9.018527	8.912217	9.786258	9.526785	9.829982	10.468311	9.711248	9.683409	9.771286	10.313989	9.987152	10.286961	10.898327	9.470633	9.917640	9.655185	10.599709	9.398469	9.816203	10.834820	11.015894	9.862391	10.505428	11.360757	10.692399	10.392669	11.569154	11.561285	10.099200	9.911127	9.928321	10.230524	10.050816

C3orf67	4.453967	4.938930	5.010145	5.574989	6.525307	5.326747	5.344963	5.096023	6.541524	6.272225	5.908115	5.304420	5.826376	6.159981	3.904201	6.423013	5.296034	5.094789	7.201165	5.583994	4.880724	4.884522	2.965270	6.262169	6.372612	5.774720	4.133199	5.163875	4.818759	3.643475	6.635160	5.889206	6.246267	5.166466	4.422440	5.691168	6.399293	5.621468	5.779280	4.497707	5.562034	5.256331	4.621436	5.555640	4.964988	6.180816	4.477837	5.934593	6.187767	5.097170	4.532041	5.821866	5.981675	7.392865	5.415123	5.694907	6.233399	5.390063	4.891666	4.287426	5.381734	4.596385	3.792471	5.206236	4.587990	5.323047	3.085603	5.048938	5.653667	4.175969	5.978848	5.741912	5.913482	5.014666	6.342674	4.320370	4.435637	4.687813	4.769077	5.083258	5.490389	5.140077	5.412215	6.369034	6.736274	6.568230	6.378320	6.296759	4.225144	5.037529	4.931589	3.679837	3.970288	5.913125	4.243870	5.598789	5.307133	7.764971	5.340859	6.271536	5.413197	5.371258	6.138928	6.481749	5.626357	5.741546	4.436278	5.895493	3.855934	5.938973	4.637598	5.623704	5.158863	5.871338	5.450109	3.496154	5.138251	5.246614	6.156523	4.809948	5.789407	4.190822	5.608330	5.200892	4.579245	4.955831	4.909875	8.710395	8.517165	6.609956	5.910785	4.922128	4.320370	5.566361	7.377364	7.476459	7.120590	6.518383	3.530520	4.874251	5.246412	5.289592	4.316078	5.909011	5.083639	3.572883	5.687539	5.301233	6.463376	3.051640	5.963956	5.681420	6.592110	3.395031	7.592559	3.604836	7.085386	6.237472	5.416994	5.482205	5.681788	6.101704	6.706548	6.475089	7.508788	4.326339	3.620461	6.135334	6.149325	7.387462	6.799452	7.074487	8.213302	6.248466	6.216124	3.776362	6.386290	5.301434	5.887073	6.000261	3.272988	6.490154	7.023919	5.892964	4.732302	6.420124	3.662649	6.364856	5.132818	7.163616	7.094491	5.582087	5.631732	5.706630	6.116835	5.742830	6.600558	5.135818	4.985394	8.320195	4.550879	6.049071	3.655456	3.742824	6.379812	6.586228	3.474511	4.752973	4.900045	3.955259	3.604028	6.311292	3.899349	6.416842	4.265722	7.202097	5.751747	8.196705	7.124170	6.334681	6.579465	4.594060	5.213169	7.849325	5.363664	7.264041	5.863994	5.219764	4.989358	4.891473	5.110367	3.095098	5.403504	4.737391	7.305703	7.376122	7.713675	7.199937

VRK2	4.156948	4.145985	5.552989	4.894601	4.556647	6.102226	5.424841	3.885958	4.316930	5.464634	5.992513	4.321019	5.283942	3.713383	3.990057	4.885177	4.118816	5.087383	5.395778	5.496995	4.695972	4.507461	6.489335	5.102166	4.958737	4.694331	6.677045	5.994110	5.637531	5.104607	4.179876	4.645156	5.910785	6.358513	5.149291	4.466966	5.009721	4.748287	5.065069	5.539006	5.367401	4.914529	6.178333	5.166273	4.919580	5.564660	5.756839	3.755267	4.939531	5.029113	4.917896	4.480835	4.297220	4.081964	3.810767	5.547320	5.274315	4.488254	4.553674	4.951447	4.383150	6.593107	5.037529	4.430265	4.389354	3.839470	3.377215	4.268049	4.264426	4.572639	5.163258	4.610996	5.977794	5.018899	4.586266	4.775112	4.629357	4.703230	5.138025	4.301102	5.582087	4.269404	5.358907	4.657491	4.427665	5.600604	5.718496	5.048303	4.232008	4.318454	4.787871	4.766823	5.116611	5.259268	5.366204	5.417383	4.006760	5.183016	5.274097	5.772555	6.079720	5.616676	5.511627	4.846579	4.466328	5.451257	5.641390	5.638630	5.227918	5.830863	4.904841	4.855836	4.904429	5.335964	4.644930	6.755724	5.227496	5.182396	5.873704	4.915556	4.359584	4.609293	4.912822	5.460233	4.620607	5.706450	5.939707	4.765699	4.967357	5.292942	5.008061	5.195916	5.272267	4.769713	5.788289	4.387173	5.952712	5.015058	4.631969	4.662255	5.466948	6.289971	5.958051	5.042427	4.842335	6.069980	5.308286	5.917422	4.770748	5.237066	5.672603	5.763765	4.331654	4.467366	4.350022	4.940938	5.038906	4.663683	5.221489	4.845079	3.623653	5.753206	5.582847	4.661386	5.450109	4.754919	3.503137	5.747384	5.208841	5.019934	5.021985	4.779447	5.645243	5.726824	5.191120	6.151554	4.406483	5.096409	3.910773	4.456263	4.871491	4.686970	4.595557	4.695775	4.874251	3.363362	4.540468	4.387819	3.116514	5.089092	4.817515	5.723098	4.872994	5.260447	4.793308	4.799201	5.021985	5.329694	4.482874	5.242646	4.810821	5.680644	5.388935	5.185584	5.732672	5.612899	4.749521	5.112958	4.961439	5.626160	4.929514	5.216974	4.160365	4.957084	6.013483	4.980126	6.067528	5.272267	5.423110	5.528763	4.696830	5.523096	6.179165	4.875280	7.123846	5.310505	5.016475	5.298660	5.114355	5.466171	5.652931	7.944014	5.445822	5.520989	4.663894	4.934698	4.664961	4.869173

LGI2	7.165662	7.350547	7.432040	6.084236	3.120117	6.580287	7.863243	4.031890	3.307479	6.176433	6.317064	6.068594	5.682541	3.977405	5.253728	6.244951	6.616246	3.952908	4.247330	7.683036	5.056780	5.534897	6.907507	4.251723	5.172969	4.704484	10.103717	5.535095	6.962124	8.364235	4.003685	8.871708	9.686383	8.775061	8.947836	7.452274	7.846086	6.995064	5.546959	3.923504	6.080745	5.372063	7.978633	7.047698	8.836481	7.669761	8.269030	5.489143	7.264789	7.765302	7.787161	8.664235	8.742249	7.440662	7.587048	8.165664	8.531966	4.475158	3.837146	6.060619	6.610287	7.355349	9.230442	5.003789	6.155690	5.330860	4.548399	4.767009	6.244796	4.288556	8.188467	7.469869	5.667601	8.596576	7.027648	9.726365	7.955563	9.456794	7.676043	7.102997	8.622673	8.222067	7.994730	7.907550	7.907550	7.315893	8.051400	7.169031	6.658703	5.026469	6.051840	5.686968	7.341605	7.542183	8.409838	7.038434	7.096262	6.823702	6.930656	6.334340	5.426024	5.848448	6.006099	5.166666	5.626914	6.065925	4.944347	4.083916	3.517346	5.238653	3.024084	3.063869	3.097510	5.289371	6.386807	4.004337	4.668615	6.790391	5.713463	8.716747	5.737715	8.267121	7.207529	3.068701	4.570033	8.078855	6.981481	3.284110	6.089206	4.243640	6.962442	9.033552	3.862586	4.539543	8.233737	5.911153	3.392300	6.447841	12.387424	4.366775	7.343519	6.231428	3.035361	4.406483	6.948600	5.877609	3.475268	5.137404	7.386991	6.996945	8.075788	6.833435	5.011599	7.009322	5.327707	8.281921	6.567597	4.084103	4.867022	5.643284	5.736968	10.204002	3.809313	6.717651	7.087505	3.601290	6.415816	6.259647	7.018269	6.953765	8.910390	7.403034	8.537676	10.741254	8.565393	9.519473	3.769513	4.631333	4.679671	5.558123	10.108529	4.055403	3.751073	4.334415	7.781671	6.074315	5.422452	3.539970	3.322974	7.874141	6.771077	3.180533	8.753188	9.811988	7.948461	8.925032	8.921659	6.197075	8.416754	7.574659	7.933559	6.954882	7.344269	7.099426	7.251697	8.257528	7.950478	8.129022	7.940418	6.842050	4.014356	7.260903	6.570356	7.618175	7.786221	4.123402	4.059329	6.878780	7.874736	4.339197	3.866394	7.934499	7.470168	3.082648	6.344739	4.575611	3.819476	6.129831	4.976495	5.876019	3.250849	4.526742	5.285798	5.666871	3.130253	8.177095	6.069272	3.528663

TSHZ3	10.573345	10.518184	10.848877	10.299127	9.786097	9.043649	11.252802	7.783924	7.784873	10.233407	10.009895	9.386045	10.432020	10.197801	11.346523	9.941651	10.628696	10.824136	10.252055	9.538134	10.395050	9.416569	13.151461	9.594314	11.651679	10.925355	11.331613	10.228365	10.926240	10.661160	9.886243	10.532684	10.415257	10.754933	11.234768	11.107553	11.116223	11.459197	11.863499	11.101922	10.302930	12.469727	11.289319	11.009167	12.470550	10.866824	10.953197	11.599731	10.780585	10.863810	10.637121	11.322916	11.185699	10.868295	11.175522	10.334799	11.684367	11.895183	11.186851	11.890346	11.817784	10.788398	13.653913	11.354029	11.962182	11.731240	10.900123	11.408446	10.491887	11.244350	10.736547	10.446116	10.317267	11.020512	10.297097	10.745730	10.962371	10.982470	10.045253	11.317473	10.673148	11.220264	11.047378	10.853742	11.318521	10.818648	11.119415	11.374203	12.438515	10.834345	11.625884	12.046769	10.600533	10.571850	10.385788	10.809338	10.767132	10.847257	10.405472	11.428860	10.660403	10.810402	10.484421	10.686620	10.584613	10.255734	9.839071	10.851206	11.555370	10.524734	10.936398	9.627096	9.270309	8.865668	9.207334	8.158705	11.079831	10.872641	10.597699	10.847549	11.613605	11.614495	9.831389	11.089903	9.122142	11.598287	10.925491	11.375843	9.404364	10.513463	9.311096	12.414781	11.475847	12.108321	10.770448	11.142206	10.861911	10.325669	11.409661	10.018641	10.783470	10.843161	11.151852	12.967881	12.212531	10.685266	8.573650	9.285962	10.587598	11.154075	10.753860	12.148694	11.074426	11.238920	10.201258	12.184406	11.218911	11.770940	12.380039	9.190243	11.253380	11.491019	11.620849	11.977757	10.128831	9.330587	12.399170	9.751904	12.567588	12.057936	11.279136	11.004401	12.696267	12.183786	11.192374	12.023394	11.431058	10.364143	11.008736	12.616955	11.852470	12.131737	11.341173	11.149918	11.989104	11.495500	11.186293	11.370366	11.774619	10.860414	11.718397	6.457389	11.686630	11.660429	11.570893	11.613450	11.753359	11.828974	12.664096	11.416773	11.679502	11.679960	12.106702	13.147466	11.173458	11.753075	12.051190	12.899561	12.440302	10.936398	12.648668	11.394876	11.919358	11.032989	11.194590	11.277024	11.998716	10.675389	10.760858	11.876727	11.622359	11.398667	11.685955	12.522348	9.157717	9.085022	9.408800	10.959888	9.004857	11.359007	12.462229	8.247528	7.543733	9.839071	9.793460	11.623576	10.930245	10.746942

SENP1	5.402864	5.255945	5.437457	6.232252	5.566167	5.373424	5.280957	5.747384	6.043307	5.380192	5.307713	5.524971	6.015357	5.748485	5.973236	5.956634	5.572010	6.177655	6.044295	6.062618	5.104433	6.840313	4.836376	6.318925	5.990248	6.544521	5.966279	5.952913	5.504866	5.537646	5.620566	4.867443	6.038250	6.410196	6.174379	6.213207	5.742456	5.939707	5.781628	5.972340	5.590242	6.286409	6.561858	6.202971	6.141020	3.993727	5.593652	5.151943	5.895493	5.898668	6.002904	5.205438	5.927231	6.214225	5.244018	5.590622	6.376310	6.227804	6.363201	6.060443	5.861398	5.794569	5.833923	6.133795	6.175250	5.767752	6.376639	6.011588	5.862518	5.934225	5.762867	6.288952	5.412627	5.932445	6.090277	6.185384	5.947390	5.809809	5.793485	6.109896	6.372787	6.054977	6.682427	5.890109	6.006099	5.073638	6.773662	6.328750	5.571799	5.840959	6.495585	5.752102	6.535813	5.887451	6.386290	7.060597	5.670314	6.168779	5.481420	6.177822	6.382153	6.031288	6.145662	5.831929	5.696389	5.464428	6.448008	6.315398	6.576349	5.570508	6.317250	6.201557	6.560853	5.968636	6.445076	7.421974	6.240087	5.863653	5.459435	5.917588	5.799127	6.127825	5.554537	6.363201	5.151558	5.991459	5.979804	6.273370	6.152236	5.661798	5.962157	6.598614	6.315037	6.815085	6.104775	6.286927	6.015713	6.864533	5.899181	6.223644	6.377960	6.262353	6.788582	6.517061	6.304098	6.008773	6.395482	6.241098	5.378461	6.063646	6.376157	6.815250	6.346731	6.260320	6.393213	5.918696	6.246099	6.024627	6.520543	6.556302	5.718855	6.499228	7.100178	6.214759	6.222924	6.444202	6.500881	5.522145	5.723098	6.086241	6.298401	6.191997	6.179820	6.546642	5.589480	6.934574	6.319795	6.183047	6.643191	5.783088	5.659956	6.227132	6.119404	7.046103	6.737942	7.034521	6.005753	6.518383	6.029427	5.933869	6.096346	5.818516	6.076952	6.256740	5.608760	6.125492	6.581803	5.671044	6.764576	6.987946	6.716203	6.556465	6.852010	6.508622	5.984867	6.279486	6.322725	6.293351	6.577162	7.229974	6.219210	6.808244	6.346221	6.318570	6.152938	6.604877	6.576349	6.589984	6.057813	6.243095	7.040326	6.077953	6.194626	6.319613	5.991118	6.888719	6.816039	5.806483	6.345229	6.274724	7.055864	7.184381	6.458037	6.160498	5.035062	6.095149	6.152579	5.474637

ARHGDIA	14.300589	14.466587	14.495138	14.563436	14.509938	14.299113	14.273526	14.051810	14.231500	14.463188	14.371917	14.236872	14.303599	14.368027	14.159585	14.246286	14.608380	14.508773	14.972581	14.154529	14.512377	14.320552	14.743181	14.189577	14.301922	14.347441	14.653700	14.483264	14.100097	14.059470	14.485225	14.885495	14.184313	14.333706	14.177090	13.763467	14.324726	14.508030	14.611896	14.596200	14.239437	14.316111	14.594880	14.353021	14.650647	14.186537	14.207485	14.166159	14.189577	14.147617	14.091572	14.356236	13.995849	13.664721	14.302959	14.289961	14.918365	14.687432	14.625993	14.859475	14.131743	14.493157	13.880400	14.202755	14.316814	14.704362	14.164594	14.629113	14.173886	14.269566	13.829453	13.971862	14.229504	14.274191	14.160557	14.115590	14.255462	14.507205	13.521387	14.285224	14.590670	14.513178	14.239437	14.267351	14.295373	14.064122	14.062109	14.628138	14.274531	14.286202	13.897223	14.224386	14.093399	13.876318	13.794844	14.455390	14.188094	14.510745	14.209806	14.581809	14.148848	14.617785	14.531879	14.664523	14.858502	14.533492	13.933765	14.855412	14.742275	14.304308	14.636601	14.405515	15.041784	14.104742	14.756024	13.105616	14.262592	14.791560	14.455390	14.070910	14.960579	14.180553	14.436781	14.297379	14.434912	14.610103	14.476349	15.190719	14.461603	14.848968	15.001364	15.133964	14.956666	14.971474	14.569736	14.981620	14.961694	14.825429	14.766663	14.985105	14.828938	15.427861	15.021020	15.469004	14.668633	14.533864	15.072836	14.362657	14.659090	14.316430	14.498295	15.244645	14.508030	13.976074	14.711236	14.458184	14.086526	15.505597	15.056697	14.668191	14.140212	15.185427	14.934577	14.699677	14.725604	14.199474	14.097959	14.158638	14.989606	14.422502	14.543837	15.036921	15.241129	14.926494	14.661796	14.087427	14.553562	14.822343	14.229183	14.616554	14.807649	15.260285	15.344956	13.810158	14.316111	14.430074	14.328965	14.459642	14.233539	14.170304	14.406946	14.819862	14.344919	14.193049	14.582286	14.419519	13.870453	14.713935	14.680064	15.246670	14.743665	15.092451	14.716286	14.933539	14.759633	14.367353	14.608827	14.642226	14.616554	14.731220	14.899137	14.406566	14.786789	14.640110	14.463188	14.278587	14.813710	14.722869	14.612714	15.321822	13.818086	14.887119	14.511123	14.915189	14.409900	14.812187	14.985629	15.036300	14.449082	14.993988	14.752647	13.744349	14.167781	13.741572	14.355885	15.062228	14.387857	14.707989

ATP1A1	9.351391	8.649523	8.406806	7.985912	7.783924	7.456680	8.261839	6.491380	6.369034	7.689945	7.536401	6.924197	7.191824	7.994418	8.121159	7.473087	6.960860	7.747653	6.564337	7.944176	6.643709	7.016496	7.739775	7.919491	7.854689	7.216709	7.264789	7.441756	7.828229	8.115905	6.577340	9.000419	8.839797	8.196996	8.610302	8.589143	8.681429	8.396241	8.343224	8.006259	7.686546	8.583613	7.903028	8.429598	8.787017	8.357526	6.466356	7.612965	8.036665	8.170358	8.765747	8.471683	8.899150	8.793982	7.912361	7.485213	9.226836	9.154319	8.324297	8.907201	9.301890	7.945557	7.946342	7.559365	9.124574	7.893570	9.052042	7.921791	9.035594	8.156255	8.735320	8.471849	7.004242	7.760697	8.285627	8.043864	8.890530	7.758541	8.836326	7.962366	7.929278	8.145338	8.701221	9.110179	8.903213	8.575963	8.691536	8.385123	8.049687	8.556238	8.481554	7.696523	8.482967	7.940255	9.319298	8.759794	8.400640	7.090251	7.224502	7.753171	5.703574	8.417966	7.816672	7.994730	6.403439	7.012195	6.112642	6.609496	6.428172	7.406634	8.633684	6.334340	6.934887	7.597303	7.715979	4.162019	9.123673	7.060771	6.718122	8.310673	7.938385	8.195435	6.995990	5.948433	6.126600	6.068771	6.477812	6.169288	6.968714	7.360429	8.340131	5.928111	6.580287	7.366862	7.140632	7.683036	7.335968	6.647222	7.714346	6.994310	7.004886	7.184970	7.184084	7.250091	7.944322	6.816191	7.234047	6.640806	8.067133	6.354414	7.234209	8.926272	7.997674	7.895767	9.598034	7.579177	8.469804	8.318651	7.935898	6.620820	8.067133	7.645719	7.566103	7.693526	7.608684	6.470376	7.755300	8.107736	8.096450	8.305299	8.878154	8.928332	11.123080	8.572331	7.218710	9.319298	8.778417	8.493243	8.743748	7.401308	7.966042	7.906308	7.329623	8.397333	8.569732	7.789614	7.628611	7.916296	8.860469	8.182820	8.033303	6.534701	7.681937	8.347200	7.883365	9.286431	8.146426	7.772318	7.696675	7.194888	7.130189	7.380361	7.870902	9.340437	7.596408	7.914144	9.362614	7.804933	7.813075	7.478884	8.129950	9.489997	7.626255	7.339873	7.837808	8.232255	5.672408	6.724651	8.608784	7.015993	9.350323	7.144750	8.886372	8.611857	6.323377	5.582847	7.558763	5.829913	6.762196	6.748935	6.151907	9.174484	6.580113	7.627673	5.921773	8.015395	7.143465	8.318216

NEURL2	9.427279	9.153531	9.125660	8.981810	9.119892	8.442390	9.539221	8.533168	8.305299	9.161365	8.706305	8.978023	9.244397	9.330903	9.138750	8.892827	8.565249	9.329705	8.904760	9.222961	8.861523	10.181309	10.709963	9.056876	9.422207	9.226526	9.316409	9.620723	9.365816	8.832406	9.039461	9.563450	9.876719	9.450783	9.508068	9.472171	9.405869	9.659984	9.831229	9.717806	9.056426	10.146396	9.131232	9.285198	9.952136	9.298596	9.211908	9.568899	9.351391	9.589014	9.360332	9.663882	9.983355	9.927990	9.535658	9.063980	10.105985	10.001482	10.261948	10.114066	10.385173	10.804169	11.854007	9.809978	9.955081	9.315654	9.759184	9.581758	9.550699	10.142755	9.444994	9.507486	9.041774	9.343442	9.689297	9.443437	9.454459	9.721549	9.220517	9.922734	9.828718	9.682637	9.657788	9.843644	9.796567	9.589330	10.051718	9.785948	10.092624	9.852455	9.792703	9.843185	9.368173	9.488884	9.307116	9.636701	9.245031	8.935930	9.349896	9.288025	8.381284	9.263538	9.197337	9.657205	10.315629	8.353369	9.695127	10.116680	9.750053	8.837089	10.207688	9.136846	9.509444	8.732710	8.552114	8.308699	9.582846	9.112102	8.746045	9.241954	8.809052	9.334014	9.075596	10.067514	9.041468	9.008558	8.323388	8.329193	8.777966	8.301521	9.337708	8.546108	9.144285	8.934877	8.830706	9.602855	8.232389	8.986560	8.497631	9.819206	10.564878	10.966093	7.896112	9.092385	10.394763	9.458786	10.188320	8.803515	9.077210	10.034381	9.275288	9.728056	9.154319	9.825553	9.049275	9.414244	10.600317	9.811665	9.801811	10.137918	9.595374	9.550699	10.310511	9.989046	9.202802	8.405606	10.062587	9.526012	9.353778	9.546241	10.651183	9.671780	9.035125	9.694834	9.877509	10.991775	10.547653	10.381639	9.591500	9.841825	10.782833	10.330253	8.776457	10.202479	11.067626	9.231754	9.406773	9.247506	10.528515	10.012286	9.331216	8.921964	9.112102	9.862391	9.290649	9.661852	9.973251	8.969110	9.785658	9.209476	10.206741	9.702032	8.818873	10.181458	10.073446	9.651648	10.254503	9.909568	10.164449	9.092226	10.063327	9.776123	9.224668	8.537113	8.993639	9.160153	7.871513	8.510434	10.820134	9.701427	9.697251	9.751277	9.431743	11.452234	8.149462	10.534114	9.996310	10.108827	8.700916	10.673307	8.484598	9.876085	9.475820	9.193891	8.985166	10.120036	9.700189	10.120036

CFAP100	4.791873	4.445656	5.227712	5.860863	5.422062	6.490623	6.186736	4.373262	5.155607	5.857270	6.609006	6.056359	5.476002	4.853550	4.657491	5.458649	4.718543	4.870826	5.138445	5.142772	6.635796	5.867047	6.586695	4.878603	4.855214	5.082253	4.971901	4.422440	5.523836	5.249169	5.604749	5.644448	5.437457	4.888117	5.197099	5.420176	5.031543	5.104607	4.544458	4.865575	5.565213	4.241406	5.164897	5.379836	4.594060	4.629125	5.620017	4.103333	5.299282	5.773447	5.395778	4.310605	3.611397	5.053206	5.184611	5.212584	5.766090	3.250993	5.540710	4.429565	4.389151	5.494698	4.311668	4.525882	4.694525	4.122740	4.796480	4.666013	5.073437	5.036534	4.844670	4.754703	5.224480	5.141118	5.690239	4.567891	5.688775	3.933031	4.586707	4.641133	4.530516	4.436744	5.101129	4.978711	5.240664	5.527261	4.589938	5.029542	3.616696	4.145382	4.097537	4.889600	4.899185	4.887505	5.318615	4.720858	4.832287	5.269249	6.154306	4.360482	4.832709	4.757829	5.259448	5.361536	4.843594	6.728047	5.100522	5.255133	5.142148	5.843515	4.119882	4.634782	4.712212	6.206085	4.416652	5.540894	4.462868	5.691168	5.424841	4.777623	5.132818	5.286009	6.447013	5.706289	10.062277	6.288952	5.894592	5.192286	6.713587	6.741283	6.598929	5.824601	4.844015	4.891867	5.497164	5.163064	5.610093	5.754253	5.353882	5.442356	7.093370	6.169616	6.208650	5.283182	4.570033	4.414658	8.046247	6.167379	5.730301	6.082664	5.089403	5.570700	5.109773	5.611389	6.035624	5.341422	4.975271	5.296440	5.154789	6.392020	5.227496	5.307530	5.561840	5.759620	4.164664	6.416842	5.137833	6.354414	5.468334	4.576680	5.739006	5.316861	5.233026	5.167687	5.282991	5.786290	3.249455	5.537848	4.804325	3.286102	5.430096	4.866199	4.924079	5.157481	4.041233	4.490426	4.307220	4.990394	3.264238	4.580362	4.642591	5.837861	4.934074	5.253548	4.461347	5.422259	4.495760	4.510054	3.908575	5.334373	6.144010	5.725727	4.075783	5.599867	5.016059	4.871701	6.073976	4.378241	4.047589	5.567281	5.467351	5.043056	4.691573	5.110970	4.779065	4.812486	6.040358	6.199832	5.215840	5.104607	5.782376	5.677521	4.492437	4.779065	7.606691	4.676470	4.938486	5.545907	6.032733	4.779250	3.604836	4.525652	6.488546	5.943721	5.632451	4.555132	5.964122	5.698228

OSBPL11	11.201301	11.762942	12.183277	12.169723	12.188253	11.654083	12.400465	12.152049	12.070991	12.138659	12.279737	12.124801	12.450751	11.873996	11.721370	11.934117	11.958855	11.677302	12.012961	12.334431	11.828693	12.010792	11.141203	11.656178	11.825645	11.737771	11.269993	11.873849	11.431661	11.921278	12.107264	12.448328	12.371644	12.349434	12.550058	12.067670	12.182211	11.804534	11.903405	11.779120	11.918019	12.137398	11.402644	11.982268	11.964028	12.113904	11.540408	11.435966	12.425406	12.287532	12.143742	12.275329	12.164553	12.849996	12.077882	12.132348	12.642568	11.911322	12.478173	11.849886	12.245455	11.392925	11.975910	12.038942	12.435794	12.046618	11.963547	11.921879	12.151881	12.147801	12.710992	12.302809	11.918344	12.314450	12.312855	12.336177	12.181281	12.416103	12.663909	12.683325	12.176546	12.468291	12.321432	12.654306	12.484550	12.650533	12.740585	12.616802	12.124933	11.766034	12.019305	12.340685	12.315775	12.413496	12.154206	12.404510	12.382891	11.887359	12.417994	11.939987	12.025400	11.992298	11.885850	12.360369	12.138484	12.208851	12.066729	12.273604	11.258731	11.821859	11.872954	11.376944	11.851556	11.660289	12.369716	11.160666	11.042391	12.177680	11.694244	12.610544	11.973127	12.346315	12.315273	11.015670	12.782601	11.454602	11.628794	11.815555	12.082273	11.767049	12.462601	12.544369	11.558653	10.862604	11.867596	11.525597	11.149599	11.028512	12.060100	12.289514	12.132045	12.045149	12.025558	12.249922	11.990798	11.640106	12.290916	10.905411	12.149599	12.626987	12.142942	11.318816	12.284727	13.278803	12.637159	12.542773	11.762074	11.941351	12.191829	12.238354	11.981061	11.712614	12.555263	12.964557	12.188253	11.938498	11.647837	12.890172	12.205573	12.261651	12.478001	12.278662	12.497024	12.231903	12.089977	12.114358	12.380831	12.110693	12.309519	12.429784	12.091066	12.581982	12.440469	12.140517	11.842350	11.563013	12.257887	11.880861	12.630345	12.258961	11.925662	10.993595	12.617130	12.620336	12.146258	12.143742	12.482306	12.378643	12.373219	12.263250	12.893290	13.171328	12.296489	12.886695	11.393682	12.859001	13.236652	12.018734	11.764642	12.205891	12.475368	12.163592	12.390314	12.143103	11.709994	11.958999	11.952893	13.021758	13.083488	11.813909	11.943939	11.848655	11.901010	12.393945	11.384967	11.874166	12.516134	12.469727	11.938632	12.375118	10.355834	11.582567	11.246224	12.261347	12.218928	11.752750	12.920968	11.631509

RAD51D	4.077019	3.926936	4.190163	5.059621	4.841044	4.435400	4.626343	4.975059	5.639354	3.982184	5.050781	4.763361	4.579470	4.631333	4.608201	4.694734	4.421071	3.420660	5.720302	4.914136	4.017194	4.217947	6.162545	4.861046	4.319502	4.861638	5.487095	5.423011	4.888751	4.862684	3.852056	4.941365	5.062843	5.676369	4.004337	3.883367	3.564009	3.774707	3.520038	3.095811	4.864340	3.434507	4.392978	4.277507	5.874232	5.417191	6.076952	3.363712	4.963118	3.675168	3.729835	4.113121	4.284509	3.819283	4.236984	5.278530	4.231353	2.879860	3.043233	4.286998	3.013959	5.499857	3.718335	3.130470	3.349874	3.639909	2.912842	3.993522	3.889613	3.021136	4.413993	4.453325	5.080814	4.203616	4.784935	4.683975	4.239453	3.983713	3.970970	4.067119	3.544884	3.715868	4.344597	4.754703	4.370219	5.261653	3.866820	4.490665	3.076658	3.896502	3.297766	3.088004	4.467366	4.771569	4.982359	5.579658	4.193008	5.527261	4.649179	5.207233	4.894812	4.130430	4.548635	4.314773	3.709872	5.225089	4.631969	4.322455	3.081216	5.282991	3.312876	3.341666	3.673379	5.044730	5.225301	5.660140	4.412646	5.131601	5.268846	4.979337	4.012899	4.497496	5.085272	5.558862	5.057174	4.718963	4.758930	5.029321	5.449370	4.952935	5.954546	5.184212	3.769308	4.630254	5.040958	5.016263	4.019151	3.767990	4.688642	4.717928	3.725976	5.719018	5.595686	3.665884	5.276946	4.689059	5.203864	5.428638	4.707226	4.184692	5.003583	6.069272	5.887268	5.736616	4.874344	4.725997	3.632741	3.882327	3.000927	6.107820	3.305388	4.732302	4.019151	5.634528	4.902978	6.112145	3.271787	4.984580	3.529405	3.585898	5.504676	5.107256	3.947935	4.751098	4.578414	3.706050	2.893527	4.046032	3.131589	2.931424	4.982563	3.798589	3.458409	3.508878	3.473087	2.906924	4.402464	4.455432	3.008488	5.150526	4.783690	4.895003	4.805579	4.282532	4.490665	4.106504	4.102158	4.230916	4.528825	4.587091	5.124493	4.115265	5.112361	3.611973	5.298852	5.041578	5.572745	2.980607	4.223852	4.319275	3.032337	5.049361	4.064578	5.306753	5.148519	5.432006	5.136227	5.181765	5.580041	5.048723	4.426343	4.832062	3.826358	4.131034	5.181372	4.837655	4.377797	3.441874	4.565553	3.052890	3.541895	3.285202	4.859174	4.869784	4.941159	3.624828	5.080814	4.389354

AC004223.3	4.077019	3.926936	4.190163	5.059621	4.841044	4.435400	4.626343	4.975059	5.639354	3.982184	5.050781	4.763361	4.579470	4.631333	4.608201	4.694734	4.421071	3.420660	5.720302	4.914136	4.017194	4.217947	6.162545	4.861046	4.319502	4.861638	5.487095	5.423011	4.888751	4.862684	3.852056	4.941365	5.062843	5.676369	4.004337	3.883367	3.564009	3.774707	3.520038	3.095811	4.864340	3.434507	4.392978	4.277507	5.874232	5.417191	6.076952	3.363712	4.963118	3.675168	3.729835	4.113121	4.284509	3.819283	4.236984	5.278530	4.231353	2.879860	3.043233	4.286998	3.013959	5.499857	3.718335	3.130470	3.349874	3.639909	2.912842	3.993522	3.889613	3.021136	4.413993	4.453325	5.080814	4.203616	4.784935	4.683975	4.239453	3.983713	3.970970	4.067119	3.544884	3.715868	4.344597	4.754703	4.370219	5.261653	3.866820	4.490665	3.076658	3.896502	3.297766	3.088004	4.467366	4.771569	4.982359	5.579658	4.193008	5.527261	4.649179	5.207233	4.894812	4.130430	4.548635	4.314773	3.709872	5.225089	4.631969	4.322455	3.081216	5.282991	3.312876	3.341666	3.673379	5.044730	5.225301	5.660140	4.412646	5.131601	5.268846	4.979337	4.012899	4.497496	5.085272	5.558862	5.057174	4.718963	4.758930	5.029321	5.449370	4.952935	5.954546	5.184212	3.769308	4.630254	5.040958	5.016263	4.019151	3.767990	4.688642	4.717928	3.725976	5.719018	5.595686	3.665884	5.276946	4.689059	5.203864	5.428638	4.707226	4.184692	5.003583	6.069272	5.887268	5.736616	4.874344	4.725997	3.632741	3.882327	3.000927	6.107820	3.305388	4.732302	4.019151	5.634528	4.902978	6.112145	3.271787	4.984580	3.529405	3.585898	5.504676	5.107256	3.947935	4.751098	4.578414	3.706050	2.893527	4.046032	3.131589	2.931424	4.982563	3.798589	3.458409	3.508878	3.473087	2.906924	4.402464	4.455432	3.008488	5.150526	4.783690	4.895003	4.805579	4.282532	4.490665	4.106504	4.102158	4.230916	4.528825	4.587091	5.124493	4.115265	5.112361	3.611973	5.298852	5.041578	5.572745	2.980607	4.223852	4.319275	3.032337	5.049361	4.064578	5.306753	5.148519	5.432006	5.136227	5.181765	5.580041	5.048723	4.426343	4.832062	3.826358	4.131034	5.181372	4.837655	4.377797	3.441874	4.565553	3.052890	3.541895	3.285202	4.859174	4.869784	4.941159	3.624828	5.080814	4.389354

CAPN14	9.772646	15.117212	15.849957	13.450346	15.091311	14.985105	15.482081	15.988198	15.569300	15.252474	15.034636	15.178577	13.939322	15.365568	14.785315	15.275879	15.174615	15.473095	14.435670	14.867676	15.008818	15.544594	13.703179	15.661481	16.057790	15.440770	11.325255	15.614087	14.657723	13.664249	14.839682	14.846797	14.828938	11.452378	15.789877	15.398455	15.390720	14.789144	15.271483	15.499684	14.966837	12.611506	15.272298	14.752647	15.370133	14.910337	14.970250	14.338926	14.882845	15.138365	14.475985	15.003643	16.290771	15.119706	15.840619	15.269335	16.372711	15.872260	14.624254	15.810180	15.183376	15.547001	12.224317	15.438975	13.940978	15.982133	14.865570	15.238314	15.250968	15.374032	14.630796	15.794025	15.095711	14.878115	15.091311	15.418602	16.140018	15.209040	15.673080	15.503877	15.924163	16.124210	14.455021	16.032263	15.775426	14.094001	14.539639	15.536844	13.988980	15.025759	15.272991	15.517756	14.600827	15.460009	15.387598	15.578257	15.531572	14.801868	14.906118	15.684238	15.404616	15.197536	15.830739	15.469767	15.744893	15.156386	15.359530	15.304417	14.571778	15.127801	14.910337	15.029900	15.352487	15.387598	16.138567	14.874444	10.499276	15.751009	15.039969	13.565950	9.685152	10.232963	12.394751	10.673148	11.705064	10.211080	10.637273	10.016345	10.083729	10.290059	9.617475	8.858496	9.677830	9.030556	12.932588	9.859596	10.609789	8.221296	9.561747	10.995221	9.061877	9.508842	10.938211	10.316078	9.308633	13.759150	10.350375	10.140602	9.399697	9.981518	10.095346	11.414272	8.985166	9.795661	10.602257	11.647387	10.352828	11.405292	10.259317	10.138805	8.631915	9.099970	9.670545	9.606560	12.222955	10.037784	9.812137	11.760698	9.400155	9.755913	10.347205	9.621823	9.562541	10.299569	9.677525	9.972475	9.639654	12.642900	10.615704	9.522488	10.686442	8.581371	10.133149	9.488884	10.025602	9.397486	10.261006	9.557255	10.769403	11.177479	10.113467	13.218764	9.545767	9.726699	12.694586	10.152036	9.660763	12.949293	9.441911	9.453727	12.062616	9.284768	9.908813	9.703268	9.553140	9.220659	9.808398	8.862151	9.536570	9.799077	9.820128	10.452966	9.576517	10.034692	10.134374	11.044735	10.436590	9.789901	9.940247	10.152488	10.405917	10.432020	10.395808	8.627316	12.720947	10.393410	12.631325	13.650975	10.180871	8.107575	9.586073	10.858086	14.601201	9.872579	8.567907	11.324685	10.269567	9.296274

KMT2D	8.703539	8.354284	8.408648	8.361249	8.638171	8.781813	8.316232	9.253455	9.777842	8.463686	8.423936	8.258160	8.027626	8.780092	8.211447	8.405459	8.792140	8.763472	9.479371	8.422852	9.006123	9.924735	9.759499	8.753634	8.946763	8.795349	8.757464	9.031565	8.242037	8.200757	8.889309	7.799910	8.132684	8.116384	7.981607	8.154551	8.512472	8.375725	9.067710	8.821587	8.419013	8.589431	8.393936	8.923429	8.664994	8.309176	8.686570	8.352459	7.898797	8.642371	8.203475	8.309911	8.537536	8.397333	8.535943	8.724336	8.720391	8.762856	8.898856	8.485531	8.833908	8.513995	8.529542	9.378232	8.508291	8.835394	8.625761	8.388680	8.352534	8.481554	8.071082	8.223754	8.512472	8.262928	8.150217	8.187593	8.401902	7.953099	8.353996	8.460937	8.639114	8.574581	8.667046	8.761524	8.746794	8.202701	8.420732	8.968517	8.632336	8.591139	8.597607	8.945521	8.242186	8.547842	8.109965	8.249009	7.793394	8.104652	8.200421	8.637389	8.482171	8.172688	8.320503	8.686866	9.242731	8.381284	9.531982	9.238757	9.312017	8.754570	9.067393	8.790577	8.819184	9.160471	8.628955	9.145493	8.877700	8.450004	8.539248	8.864465	8.490868	8.073510	8.863054	9.229431	9.014249	8.622673	8.278869	9.024612	8.781813	8.503865	8.436649	8.274332	8.708432	8.532116	8.453689	8.843418	8.357685	9.323193	8.035876	9.665759	10.033321	10.157573	8.838141	9.249501	8.984120	8.630218	9.394660	8.785471	8.648567	9.335123	7.811119	7.597778	8.299261	7.778050	8.339505	7.865564	8.516857	9.057198	8.281598	9.135773	8.170037	8.474316	9.094797	8.784001	8.269030	9.160471	8.563436	7.970993	8.513701	8.078065	8.230865	8.170198	8.511237	8.470408	8.706907	8.664838	8.797467	8.784884	8.227663	9.049275	8.862460	9.334944	9.075596	8.851528	8.102799	8.750986	8.063973	8.368767	8.463686	8.191644	8.409992	9.282762	7.999328	7.906928	7.934721	7.905227	8.376041	8.200421	7.979579	8.423788	8.550581	8.529542	7.843927	8.900377	8.005145	8.431794	8.098858	8.726355	8.219177	8.724169	8.743149	8.068759	8.701974	8.316693	8.388982	7.702677	8.021345	8.400184	8.087091	7.885206	7.911257	8.338702	7.908951	8.761230	8.756532	9.216398	8.975406	8.749391	9.237341	9.063980	8.452314	7.964950	9.572872	7.760354	7.845183	8.649966	8.330258	8.931939

TULP4	11.633888	10.979225	10.851641	10.925940	11.623866	11.515780	11.282391	11.787766	11.338924	11.760698	11.260782	11.270124	10.584342	11.033609	10.663570	11.605218	11.147376	10.126629	11.138902	11.882808	11.070164	11.948032	10.575398	9.999366	10.551284	10.898647	11.136391	10.947866	10.942565	11.332619	10.813866	11.161418	11.920396	11.510541	11.421337	11.158052	11.408892	10.887194	11.143394	10.761158	11.228419	10.997755	10.694051	11.489199	11.310266	11.163197	11.014277	10.873702	11.152612	11.910288	11.414272	11.542114	10.797454	11.326605	11.418269	11.728087	11.212133	10.936835	11.170020	10.785583	11.276728	11.135665	11.238152	11.620979	11.281507	11.019158	11.074564	10.951940	10.974503	11.355638	11.687372	11.941813	11.408142	11.698568	11.639475	11.664174	11.610934	11.580289	11.404110	11.150393	10.792489	11.487573	11.620252	11.532717	11.663435	11.620681	11.903572	11.958855	11.923854	11.246667	11.053643	11.906190	11.402792	11.691529	11.409349	11.488607	11.107474	10.669264	11.780904	10.925355	11.597708	10.774607	10.495716	11.024804	11.478113	11.085434	10.956204	11.503051	11.377252	11.727061	11.418682	10.400485	10.712709	11.112588	11.420572	10.437507	10.461430	11.624862	10.881053	11.298239	10.667256	11.215382	11.724086	11.040012	11.452975	11.995669	11.101785	11.846125	11.363862	11.511108	11.819746	10.740494	10.498431	11.222202	11.806941	11.176723	10.333876	11.307769	12.458340	11.107188	11.779993	10.817007	10.318904	11.605071	10.940980	11.569895	11.775503	10.570935	11.793314	11.731098	11.237278	11.717338	12.140517	11.258585	11.316765	11.742298	10.920907	10.721789	11.084833	11.978824	10.784522	10.367456	12.543561	12.292654	11.199453	11.192072	11.264551	11.568275	12.498958	11.986208	11.326454	11.018990	12.723985	11.261983	11.033295	13.090908	11.870252	11.223409	12.019762	10.741115	11.389403	11.444835	11.540976	11.663576	11.167966	10.037185	11.290218	11.091753	11.839633	12.157396	12.039083	10.970303	11.799846	11.632098	11.441892	11.650785	12.168351	11.663435	11.071993	10.927410	11.219673	11.824133	11.189611	11.566261	11.414272	11.790568	11.478113	11.708961	10.900269	10.874859	11.545765	11.106592	11.248361	11.254400	11.617713	11.700241	11.067181	10.663570	11.363720	11.790568	12.151239	11.066875	10.734171	11.007999	10.696778	10.298160	10.731351	11.654387	12.318871	11.978216	10.856607	10.057591	11.654463	10.615256	12.089816	10.986598	12.465830	11.463911

COTL1	13.759418	12.929753	12.800745	12.872501	11.603290	12.318871	12.247046	12.184238	12.347415	11.729004	12.268515	12.048760	12.512264	11.795854	11.712177	11.995045	12.356248	11.982268	11.977145	12.468446	12.269467	14.334354	12.562827	13.108505	12.326361	11.701767	13.988680	11.518972	12.153151	13.567342	12.295577	13.466396	13.968817	13.708220	14.157364	13.765216	12.263096	12.690027	12.879575	12.563162	11.869200	12.721811	13.559500	12.906787	12.965086	13.057638	12.608516	12.665817	13.077040	12.478001	13.001778	12.549730	13.007394	12.335719	12.167578	12.051034	12.886344	12.348060	11.803943	12.316558	12.067502	12.352099	13.322740	12.089977	12.856770	12.433390	12.305577	11.820662	12.689676	12.360524	12.651078	12.313980	11.917136	12.799536	12.593944	13.371239	12.681123	12.848047	12.996223	12.263250	12.647798	12.917932	13.154762	12.076981	12.903931	12.516623	12.964171	12.117118	13.126344	12.647798	12.536416	13.380525	13.020295	12.562209	13.397792	12.473286	12.730491	11.683647	12.605068	11.019767	12.418807	12.278977	12.125096	10.947309	12.696084	11.963218	12.837755	12.550714	12.504570	11.683795	12.800233	11.651082	11.924735	11.448235	12.315592	11.734962	13.425768	12.412694	12.055280	13.324601	13.460920	13.746814	13.782074	13.702414	13.153629	13.320471	12.524939	13.639425	13.616144	13.415442	13.074158	13.394129	14.341372	14.512377	12.804235	13.601268	12.889486	13.961604	13.011946	13.832809	13.537477	14.038510	12.571330	13.064543	14.550582	13.228465	12.756165	12.723985	13.761667	14.226969	13.693113	13.568039	12.653292	13.054184	11.930143	13.234006	12.376614	13.739103	13.409232	13.690206	13.391215	12.970971	12.784157	13.353325	12.691524	13.992685	13.214995	12.306066	13.689737	13.297565	13.787896	12.440954	13.697796	13.704613	13.537711	12.120080	12.663235	13.099592	12.551384	13.524838	14.039072	13.885693	13.605004	13.357004	13.178579	13.502998	13.517989	13.276051	13.661762	12.837755	13.265575	12.509805	13.295547	13.696085	13.590725	13.033565	13.665736	12.692172	13.077040	12.687444	13.115266	12.762381	14.179542	13.471598	13.109649	13.605004	14.056836	14.097093	13.817328	13.960811	13.971275	12.771543	14.058288	13.792498	14.332725	13.573121	12.961589	12.815455	11.673716	14.934577	12.399170	12.132820	13.571924	13.671748	13.640364	13.024751	13.763964	13.067718	14.347082	13.877148	13.723726	13.084018	13.338109	12.859356	13.319680	13.576715	12.531002	14.560951

ZNF451	9.489846	8.553764	9.289113	8.890976	8.870721	8.588347	8.668648	8.490431	8.309468	9.118833	9.088985	8.439951	8.914784	8.738729	8.922106	8.821898	9.130300	9.113896	9.147686	8.980884	8.190710	9.255589	10.431883	8.983194	9.128119	8.830099	8.495987	8.551354	8.720229	8.368071	8.641137	8.834785	9.280452	9.428831	9.161517	9.251027	9.298143	9.183297	9.160471	9.841226	8.826707	9.131804	8.824012	9.409873	9.580855	8.814990	8.591916	8.874344	8.836779	9.169683	8.858351	8.842290	9.148011	9.381310	9.077935	8.510905	9.629378	8.946600	8.990467	9.212818	8.834349	8.699239	9.474903	9.126584	8.773664	8.748036	8.011423	8.478522	8.620493	7.944941	8.408942	8.400184	8.668344	8.375113	8.259246	8.488758	8.666526	8.682359	8.598516	9.011895	8.531531	9.023558	9.224038	9.069592	9.098736	9.213597	8.998406	8.743011	8.817585	8.388820	8.269676	7.911412	7.999482	8.949983	8.588177	8.881755	9.017754	9.425721	9.360471	8.879418	9.088836	9.372405	9.215763	9.254029	8.932696	9.114964	8.196235	9.149548	9.306478	9.039625	8.778712	8.336025	9.443437	8.438131	9.160471	6.420437	9.985333	9.063831	9.052657	10.271317	9.940101	9.039156	9.490626	9.674434	9.475967	9.340437	10.791138	10.187407	9.870258	10.325356	9.399554	8.813641	9.853854	9.572138	9.845661	9.695569	9.540332	9.985804	9.143799	8.862612	10.149023	10.071920	9.858664	10.089305	9.602061	7.567050	10.238936	9.129684	9.368173	10.101264	10.083133	10.711127	10.201710	10.096114	10.566197	9.930637	9.400733	10.135620	10.276374	9.832913	10.211511	10.760858	9.681546	9.881929	9.300643	8.880210	10.205361	10.183317	10.339585	10.055446	10.198291	9.953948	10.508876	9.435081	8.919206	10.683185	10.728676	9.693309	10.995221	10.080285	8.363935	10.738376	10.282924	9.031417	8.632743	10.251030	8.983483	9.426656	9.048228	9.140691	9.757177	8.682202	9.654195	8.675471	9.319298	9.329557	9.562541	9.461531	9.861442	9.814145	9.838268	9.405722	9.916212	10.536920	9.620873	9.526012	9.931382	9.118674	9.139186	10.035467	8.909057	10.089002	9.894559	10.531275	9.729350	9.825243	10.114655	10.704200	9.915905	10.755577	10.360365	9.590255	9.448101	9.638720	9.722314	10.451084	9.041774	10.157433	9.090210	9.068474	10.341118	10.700162	8.111766	9.927545	10.385173	11.141047	9.346035	9.359075

CDH4	14.274870	9.338896	9.500267	11.734962	10.743513	11.400278	10.147476	11.451020	12.079851	11.738062	12.244853	11.500204	10.679279	10.242296	9.600027	10.493458	11.346391	9.731236	10.780444	9.985187	10.041915	10.471332	11.828835	10.015244	10.230861	11.174150	12.028718	11.060999	11.446009	10.770306	11.828693	11.248361	9.109712	10.469201	9.235309	9.487673	9.415037	9.330587	9.884840	10.055298	11.206863	9.892101	9.670699	9.970631	9.498106	9.818272	12.507547	9.276071	9.900076	8.969436	11.480786	9.057993	8.288418	8.798544	8.782909	11.643705	8.534684	9.111762	9.121540	9.068474	9.352074	11.443990	10.080574	10.069492	9.361550	9.675852	9.494561	9.336944	9.242578	10.213349	9.703107	9.864543	11.549154	9.341898	9.548845	9.918845	9.771131	9.541747	12.017489	8.870565	10.048802	8.827314	10.476151	9.018966	9.004704	9.377318	9.323513	9.205823	9.771440	9.738831	9.384220	10.364578	9.585307	9.760100	11.932141	9.783615	9.366774	10.352828	11.178065	10.701056	11.082044	9.468725	10.216834	9.848968	10.183009	11.102824	10.930245	10.216098	11.752925	11.616246	11.292805	10.637586	10.398387	12.431277	11.074065	13.334349	11.565671	10.748441	12.193531	11.210101	10.125679	9.991411	10.440967	11.988461	11.417391	11.715283	11.747192	10.958719	11.315577	11.582863	11.897073	11.272361	10.796728	11.042718	11.327340	10.641061	10.512425	11.277646	10.211657	10.871143	10.627292	11.398667	11.069585	10.635345	10.121103	11.321288	11.424939	12.949293	10.953768	12.342480	10.348265	10.148077	10.202804	9.468105	9.770001	9.685777	10.342153	10.614651	10.503462	12.253114	10.215186	9.538600	10.342477	9.478608	9.878741	12.400465	9.631239	9.754711	9.282455	10.054496	9.479088	8.687013	9.095560	9.130166	10.553234	8.849578	9.151110	9.529145	9.744747	9.736555	10.488104	9.920741	9.718135	9.957845	9.835686	10.043446	9.520834	10.030841	10.132147	10.070208	10.150520	13.101611	9.761660	10.140152	9.689137	9.925599	10.132853	10.362545	9.546391	8.947669	9.092981	9.441911	9.711552	9.080577	10.828444	9.078242	9.093582	9.937579	9.679086	9.711552	10.566491	10.163031	10.367141	10.680054	11.057484	11.037724	11.207013	10.697935	9.558934	11.372021	10.467548	11.510826	9.902118	10.985236	11.830475	11.182859	10.008647	10.735064	11.712469	11.474238	10.759120	11.816015	13.576005	11.550609	11.013094	9.962244	10.550696	11.474531

SLC7A11	13.318885	9.810408	9.356658	10.707919	8.947836	10.079690	10.626098	11.672364	10.269126	9.851210	10.155626	9.955081	10.843616	11.251075	9.057352	10.134839	10.635198	8.975115	10.963392	11.074426	9.039784	9.650087	8.667128	8.889622	8.537828	10.016803	8.157342	9.545767	10.418738	11.765719	10.308628	11.927081	10.764174	11.865618	9.591500	10.387951	9.659667	10.650301	11.029707	10.594114	9.578851	8.810595	9.041919	10.021399	9.142130	10.588224	9.280909	10.295091	10.219095	10.193562	10.491887	10.356463	9.542983	10.641644	9.740576	10.899668	9.930473	9.057511	8.837234	9.628003	8.640670	12.030784	8.501222	9.761826	9.850577	9.674122	9.599253	9.543140	11.022434	9.430494	10.016190	11.899287	8.506105	10.819087	10.199765	10.134060	10.239706	11.442042	10.547653	10.893147	11.076776	11.564336	12.302631	11.072990	10.616277	10.671967	12.160336	11.544514	10.234797	10.043446	9.115721	9.740576	9.919633	10.517413	10.776828	10.122625	9.836740	10.478589	11.115020	8.354442	11.613605	11.320390	9.768766	8.834349	12.438515	10.604444	7.413356	11.893367	10.420414	10.557083	9.036324	8.289633	11.036408	10.016345	9.202358	9.801046	10.849009	11.031948	10.383040	11.488900	11.856087	9.758574	9.168451	13.461357	13.847197	13.758124	9.002437	12.990709	14.773263	12.623496	13.198527	13.650755	9.182666	13.686442	9.161231	14.957224	13.358864	7.048016	9.816653	14.321264	14.539639	14.472293	9.899900	10.352666	15.172005	11.291291	14.169692	13.189888	14.342816	12.467952	12.910727	14.007596	11.651970	10.569539	14.034970	11.993963	11.722134	12.007616	12.360524	11.145141	13.408151	13.360596	10.175472	13.978627	14.528293	8.925660	8.421655	14.413643	14.099791	14.462773	14.464405	13.088702	11.511259	10.508722	13.981326	10.516242	11.424053	10.986127	11.031349	10.893753	8.882342	12.497991	10.953197	10.255734	8.832900	11.451147	12.591670	14.144293	10.980998	14.570106	14.019284	11.585439	14.269900	12.954599	12.148544	14.229857	12.060702	13.429298	8.851059	12.624833	11.859940	13.216575	12.676354	11.680103	12.320466	12.208987	9.636374	9.157875	12.026746	10.862604	11.275464	10.505710	13.158777	12.766737	11.768847	14.621136	13.432615	13.931123	14.414362	10.363418	8.562976	7.842803	12.565935	9.633519	12.366882	11.145736	13.260641	14.176188	9.355751	11.479023	9.802880	8.670956	7.988227	13.893951	12.926482	13.248301	14.029080	12.378188

SLC16A1	15.574596	9.324472	9.976348	12.667306	9.909395	9.582699	8.891290	9.472022	10.201399	9.638720	9.811665	9.382262	8.819478	9.760564	9.226526	9.111274	10.010461	9.479371	9.167845	10.418130	9.318053	9.714385	7.778353	9.698021	8.840879	9.033700	8.282551	8.862460	8.745884	8.147026	10.510937	13.066780	9.158340	9.605154	8.699691	9.417796	9.375602	9.749429	10.101418	10.796271	9.505969	9.416569	9.244700	9.371195	9.067710	9.558859	8.855027	8.802752	9.701277	9.493614	11.708338	9.653882	9.121085	9.884227	9.620568	9.021885	9.548379	10.352202	9.837182	10.020621	9.528187	9.151857	8.061955	9.524895	10.348265	9.838268	10.080574	9.410650	10.270574	9.254659	9.244547	9.829671	8.857133	9.635491	9.927990	9.383618	9.623997	9.481049	11.119269	9.366138	11.924735	9.651972	13.670308	10.069634	10.174575	9.321704	9.217026	10.289433	9.489187	10.201258	9.373360	9.823738	8.858205	9.811011	13.208899	9.653700	9.634169	9.430349	8.964652	9.441911	8.061672	9.516071	9.774645	9.921216	10.193874	9.613799	10.373227	9.598956	9.357266	9.202802	8.356138	10.269288	9.726699	9.971216	9.520679	8.578029	15.624294	9.535194	9.671636	11.175380	9.076408	8.370663	12.771214	9.502375	8.560242	10.529382	8.846008	10.251178	9.381478	10.120507	9.000858	7.935728	8.610918	8.282080	9.241070	9.363934	8.274038	8.968369	8.450004	8.873841	8.747882	7.670062	10.037488	11.055379	8.523382	10.056961	10.996252	9.307443	7.961776	9.132993	10.759975	9.228667	9.859150	9.339197	8.068471	8.799440	11.496833	10.735480	8.640367	8.616784	9.494075	10.375600	10.895475	8.794460	9.384220	10.780803	7.447746	8.395144	8.653270	9.589501	9.771605	8.409099	8.249596	9.264743	7.906169	8.661167	8.956987	11.857954	9.376353	9.008722	12.605230	8.950728	9.311096	8.868990	9.921216	9.524595	8.775349	11.344451	8.161613	8.547842	8.454898	11.674871	9.229700	8.792916	10.642844	8.725114	9.023860	8.953955	9.049938	8.595167	9.409712	9.747720	9.288485	10.016493	10.119312	9.764347	10.242742	9.079028	8.977700	7.642917	9.131092	9.365980	9.065219	9.096895	9.869171	9.168451	8.737472	9.631529	8.124489	6.735790	9.732263	10.911171	9.754870	10.538660	9.359075	11.542870	9.021275	8.488914	9.469018	7.980027	8.707378	8.853015	9.024005	7.849325	10.745730	9.878002	11.496673	9.030278

CYP21A2	14.457804	13.743597	13.530568	13.561518	12.418807	11.673261	14.395124	8.685533	9.797808	13.295754	11.475989	13.061185	13.872353	12.680421	13.960811	12.068126	11.898681	13.213390	12.173896	12.192594	11.933500	12.129331	15.790919	12.174040	14.391513	12.663752	14.055678	13.683082	14.627259	13.875509	12.746061	14.204014	13.826855	13.739368	14.083132	14.105943	14.411699	14.225335	14.447111	14.497531	13.537063	14.821349	14.433465	13.884891	16.213991	14.047003	14.156066	13.860465	13.613276	14.155124	13.330272	14.744972	15.124619	14.045844	14.341372	12.580980	15.357170	14.986168	14.882845	15.285921	14.683169	15.919342	16.006630	14.248223	15.082962	14.285845	14.207485	14.041399	13.695624	14.819862	14.731754	14.558412	12.714355	13.825046	13.452684	13.227872	14.578102	14.192430	13.917771	15.513476	14.695210	14.933539	14.217619	13.963822	14.216321	14.197180	13.854373	13.902528	14.260639	12.866399	14.430444	13.473153	13.712440	13.319864	13.423212	13.412931	14.263585	14.764819	13.001966	13.948780	13.699452	14.227945	13.975517	13.697330	12.374518	13.353095	12.868696	12.976037	15.250968	12.326030	15.017444	11.395035	11.322149	12.549882	11.033754	11.887800	14.126769	14.158986	13.703179	11.617285	9.366459	9.986994	12.941926	10.155777	9.338616	10.832876	9.481968	8.026668	10.994601	10.660096	9.725903	12.319852	10.654734	9.972329	13.185047	8.312070	9.857414	11.981815	10.698520	9.763723	10.553379	13.719674	9.669110	10.551729	10.610796	9.673171	9.563890	10.534701	10.646958	11.977451	10.472987	10.369411	9.293482	10.607264	9.504396	10.956934	10.167666	10.396893	10.707338	9.785504	12.216379	10.023858	9.540026	10.810677	10.753411	9.674434	11.396241	11.456254	10.099670	9.418972	10.932033	9.952136	9.569490	11.595630	13.612082	10.477695	11.129112	11.052955	10.690215	9.282612	14.131436	10.160284	9.829822	10.753252	13.398242	10.055148	10.687976	9.366294	10.219536	11.187272	10.204906	10.172297	9.909249	12.811936	10.722674	10.053753	10.457869	10.163031	11.948336	9.766473	9.807917	9.987620	10.579659	9.829982	10.260726	9.401532	9.923356	12.835615	11.931556	10.333290	8.718121	9.807767	9.715792	9.984426	10.036868	9.685927	10.357076	9.589330	10.236515	9.726848	9.625356	11.687212	10.881487	9.710793	11.645767	8.540524	9.678129	10.693316	9.698632	13.034689	9.795661	10.231595	10.885415	9.213906	8.532273	12.657484	10.045094	10.546727

TNXB	14.457804	13.743597	13.530568	13.561518	12.418807	11.673261	14.395124	8.685533	9.797808	13.295754	11.475989	13.061185	13.872353	12.680421	13.960811	12.068126	11.898681	13.213390	12.173896	12.192594	11.933500	12.129331	15.790919	12.174040	14.391513	12.663752	14.055678	13.683082	14.627259	13.875509	12.746061	14.204014	13.826855	13.739368	14.083132	14.105943	14.411699	14.225335	14.447111	14.497531	13.537063	14.821349	14.433465	13.884891	16.213991	14.047003	14.156066	13.860465	13.613276	14.155124	13.330272	14.744972	15.124619	14.045844	14.341372	12.580980	15.357170	14.986168	14.882845	15.285921	14.683169	15.919342	16.006630	14.248223	15.082962	14.285845	14.207485	14.041399	13.695624	14.819862	14.731754	14.558412	12.714355	13.825046	13.452684	13.227872	14.578102	14.192430	13.917771	15.513476	14.695210	14.933539	14.217619	13.963822	14.216321	14.197180	13.854373	13.902528	14.260639	12.866399	14.430444	13.473153	13.712440	13.319864	13.423212	13.412931	14.263585	14.764819	13.001966	13.948780	13.699452	14.227945	13.975517	13.697330	12.374518	13.353095	12.868696	12.976037	15.250968	12.326030	15.017444	11.395035	11.322149	12.549882	11.033754	11.887800	14.126769	14.158986	13.703179	11.617285	9.366459	9.986994	12.941926	10.155777	9.338616	10.832876	9.481968	8.026668	10.994601	10.660096	9.725903	12.319852	10.654734	9.972329	13.185047	8.312070	9.857414	11.981815	10.698520	9.763723	10.553379	13.719674	9.669110	10.551729	10.610796	9.673171	9.563890	10.534701	10.646958	11.977451	10.472987	10.369411	9.293482	10.607264	9.504396	10.956934	10.167666	10.396893	10.707338	9.785504	12.216379	10.023858	9.540026	10.810677	10.753411	9.674434	11.396241	11.456254	10.099670	9.418972	10.932033	9.952136	9.569490	11.595630	13.612082	10.477695	11.129112	11.052955	10.690215	9.282612	14.131436	10.160284	9.829822	10.753252	13.398242	10.055148	10.687976	9.366294	10.219536	11.187272	10.204906	10.172297	9.909249	12.811936	10.722674	10.053753	10.457869	10.163031	11.948336	9.766473	9.807917	9.987620	10.579659	9.829982	10.260726	9.401532	9.923356	12.835615	11.931556	10.333290	8.718121	9.807767	9.715792	9.984426	10.036868	9.685927	10.357076	9.589330	10.236515	9.726848	9.625356	11.687212	10.881487	9.710793	11.645767	8.540524	9.678129	10.693316	9.698632	13.034689	9.795661	10.231595	10.885415	9.213906	8.532273	12.657484	10.045094	10.546727

ABI2	15.873355	15.706649	15.422546	15.433373	15.389194	15.282381	15.448809	15.748884	15.740567	15.462495	15.624294	15.299532	15.359530	15.582778	15.280236	15.915538	15.823135	15.392156	16.254772	15.904568	16.037601	15.573696	16.051180	15.734237	15.945972	15.697564	16.011919	15.757552	15.867496	15.307206	15.671141	15.666334	15.615956	15.384452	15.933725	15.770228	15.873355	15.823135	16.414122	16.412035	15.741560	16.439291	15.768020	15.908209	16.289097	15.999973	15.663417	15.837350	16.057790	16.032263	15.507347	15.824202	16.372711	15.998443	15.682114	15.851177	15.826441	16.308700	16.519186	16.233280	16.119818	15.602028	16.397385	16.638676	16.496735	16.418365	16.171220	16.523640	15.960718	16.048355	16.163437	15.978326	15.377722	15.288168	15.544594	16.572650	16.327143	15.678135	15.815434	16.019400	15.926414	15.978326	15.630940	15.898712	15.309448	15.789877	15.127111	15.042927	15.740567	15.887107	15.559524	15.751009	15.926414	15.983265	15.321092	15.823135	16.026974	15.469004	15.440770	15.979533	15.374032	16.014400	15.740567	15.597310	16.256382	15.224051	15.783769	16.727613	16.312078	15.489513	16.154543	16.094324	16.032263	15.490401	15.953297	15.463984	16.049744	15.723003	15.637542	15.674067	15.790919	14.962238	15.549763	15.741560	15.711627	15.653688	15.631882	15.763886	15.606629	15.518640	15.781709	15.813386	15.650841	15.922928	15.544594	15.433373	15.468133	16.048355	15.359530	15.928804	16.029650	16.115572	16.003998	16.258880	15.939590	15.480428	15.979533	15.933725	15.416204	15.719891	14.992275	15.825349	15.428569	15.188690	15.602028	15.133349	15.376257	16.015596	15.993114	16.047049	15.658565	15.564859	15.548789	15.845299	15.558628	15.676954	15.552451	15.625168	15.804881	15.217824	15.482081	15.817557	16.006630	15.603038	15.767032	15.604793	15.756462	15.858328	15.589004	15.974511	15.718870	16.015596	16.104387	15.753148	15.901055	15.719891	15.581893	15.801695	15.258165	15.589004	15.482965	16.032263	15.191395	14.925433	15.354914	15.676063	15.827482	15.746888	15.262346	15.491291	15.423337	15.349378	15.262346	15.048391	15.696546	14.857509	14.974306	15.521068	15.561354	15.624294	15.592691	15.584586	15.683269	15.429349	15.454339	15.960718	15.326543	15.474785	15.854635	15.396904	15.553263	15.679147	14.822949	15.847680	15.681084	15.728021	16.125498	15.968277	16.140018	15.695521	15.888240	15.377722	15.928804	15.839623	15.612324	15.817557	15.726974	15.672165

IGBP1	14.200109	13.065843	13.344050	13.277050	13.711410	13.676465	14.087427	13.602422	12.862205	13.669633	13.652718	13.655038	13.150110	13.316619	13.326991	13.704371	13.252643	12.989260	12.908228	13.833047	13.157844	13.644958	13.035807	12.980105	12.321255	13.010512	12.864977	12.930440	13.439680	13.692656	13.200284	14.202473	14.315126	13.484139	14.088933	13.865942	14.006716	13.115807	12.828063	13.080043	13.731903	13.127862	13.946797	13.831246	13.769304	13.985840	13.337700	12.974908	13.988680	14.067777	13.920769	14.115260	13.743347	13.942085	13.817826	13.386677	14.082814	13.376917	12.814228	13.216575	13.540385	13.344260	13.002332	13.060802	13.056546	13.079117	13.347004	13.401844	13.522531	13.615204	14.105943	13.872353	13.501741	13.913440	14.196229	14.144912	13.903071	14.025995	14.042877	13.942960	12.968566	13.672469	13.860196	14.303277	14.070625	14.199474	14.061795	13.892381	13.375462	13.227647	13.435563	13.702414	13.556834	13.959054	14.073025	14.104742	13.977193	13.403761	13.751547	12.457385	13.464002	13.236032	13.016071	13.705820	13.142299	13.620404	13.645649	13.472510	13.096345	13.469650	13.880924	12.954420	12.844401	13.401415	13.455950	13.005406	12.952208	13.528125	13.154939	13.418183	12.938338	12.994903	12.545219	12.474582	13.236867	13.105225	12.966743	13.020807	13.557035	13.094857	13.868836	13.095970	12.564459	12.089816	13.329436	13.631592	13.057446	12.668485	13.567588	13.361828	13.325811	13.184851	12.798338	11.648129	13.278020	13.202977	12.574744	12.271724	13.597687	12.939734	13.415858	13.520267	13.918056	13.507700	13.857538	13.662723	12.219839	13.212236	12.924903	13.270823	13.011779	13.223925	13.498487	13.593974	13.834843	12.886514	13.173253	13.918322	13.511265	13.782074	14.009820	13.857538	13.639425	13.585102	13.237824	13.634745	12.369546	12.600363	12.575902	12.683650	14.288989	12.795726	12.159708	13.091440	13.079675	12.538424	13.300495	12.729124	12.994539	13.512208	14.237798	12.629849	14.049129	13.878755	13.618017	13.953485	13.362663	13.860733	13.705582	12.584309	13.950442	13.188004	13.385833	13.751062	13.024565	13.652950	14.265286	12.768697	12.570198	12.417994	12.818889	13.023099	13.490363	13.627987	13.376481	13.426884	12.392356	12.947179	13.707232	13.514224	12.756854	13.104454	13.254976	13.553021	13.003596	13.175491	13.039676	12.961230	12.779001	13.420156	12.389676	12.639201	12.989438	13.861539	12.837080	13.122690	13.143981	13.208899

LIPG	8.419149	8.890668	7.902414	7.185203	3.940235	5.802411	7.468305	6.882620	6.532579	6.241268	6.239250	5.618740	5.971051	6.663467	7.285199	4.908396	5.778189	3.558934	7.518657	7.233867	5.606325	7.383010	7.738827	6.157883	5.112145	5.233431	8.035245	4.382956	6.554166	9.144893	4.087037	9.486434	9.479802	9.211755	8.449060	7.287679	6.552341	7.775107	7.620747	5.313196	4.572849	6.941502	8.663004	6.892688	6.905732	7.005812	6.701426	8.653579	9.851968	8.072573	9.005667	8.087867	8.197780	7.967552	8.743900	7.930805	7.223728	7.016496	6.080902	7.061088	6.740701	6.385280	9.249046	6.843510	8.547241	7.439916	6.651258	5.922924	8.407880	5.547706	7.339873	6.761889	6.062933	6.958150	6.202438	9.121694	7.210174	9.962091	8.130096	8.433681	6.809524	8.800353	8.460319	6.868069	6.439708	6.925831	8.334638	7.535484	8.662549	7.656711	6.228480	7.619091	8.676972	10.023706	8.785047	6.727719	8.353066	4.536948	6.111465	4.144750	7.632690	5.913482	6.191492	6.315909	9.612559	6.472574	6.453805	8.026061	7.415371	4.609944	4.181237	5.030756	6.733538	4.998590	7.999482	6.190268	8.324932	7.861673	6.499228	8.794460	10.331296	8.825386	6.223463	7.571280	8.791046	9.236897	7.548532	7.835968	6.385280	8.909057	7.421202	9.266572	10.520787	11.633139	6.432425	7.195198	6.806940	7.939461	11.079831	8.182665	8.582697	7.547904	7.047385	11.079540	9.050240	9.526785	7.042248	4.128277	8.320195	6.949175	11.393682	10.172908	10.667399	10.357679	7.809741	9.278901	10.219536	10.951033	8.079480	5.932286	9.555843	9.379147	10.265297	9.241363	9.677215	10.595736	8.752584	8.044166	9.442348	8.190857	9.720502	9.198763	11.859791	10.111838	8.147026	10.538968	9.910027	8.449060	8.754431	6.955662	8.039167	8.834942	9.518692	5.887816	10.300662	11.884352	10.951332	8.167075	7.765933	8.236375	10.279584	10.266163	7.608684	9.408477	10.013412	11.068392	9.036468	9.736095	9.524595	7.975232	10.412390	8.858656	9.417469	10.806201	8.473581	9.786827	9.431578	10.016190	10.130935	7.910787	8.292759	8.600192	10.449011	10.251318	10.892545	8.362863	6.805231	10.477236	6.877809	11.899574	9.472625	7.326903	8.257528	11.501766	7.992858	9.771440	6.673180	9.262432	10.554613	8.572191	9.105354	5.093749	5.189746	7.205134	9.645183	8.733026	6.148301	11.871889

RCAN3	11.084541	11.013401	11.053092	10.873702	11.916831	11.887052	11.664015	12.653093	12.120820	12.294973	11.881590	11.865618	11.617561	11.665819	11.189884	11.901166	12.068901	11.491172	11.787922	11.961308	11.037869	11.945613	10.616727	11.540128	11.320543	11.663120	10.491133	12.147944	11.223559	11.296869	11.839013	11.567979	11.596548	10.810258	11.325255	11.795586	11.830768	11.405292	11.587764	11.744491	11.711892	11.027041	12.265271	11.967801	11.763724	11.964164	11.561422	10.841568	11.861406	12.103782	11.853542	11.826085	11.865134	11.820512	11.717338	11.319280	11.807225	11.225775	11.822149	11.439584	11.709538	11.615530	9.466126	11.384967	10.890139	10.701056	11.188301	11.056421	11.109254	11.005699	11.282690	11.680103	11.824728	11.428860	11.517507	11.217736	11.487873	11.219233	11.560243	11.667871	10.966241	11.464200	11.583181	11.736404	11.909419	11.472366	11.869049	12.185585	11.072280	11.094441	10.868295	11.050179	11.131914	11.405292	11.101364	11.422789	11.672977	11.845386	11.449574	11.486387	12.117551	11.465624	11.280614	12.046769	11.948669	11.714683	12.147040	11.656178	10.754335	11.945301	11.397159	12.189898	11.899712	11.877610	11.759614	9.871373	10.496640	11.684367	11.930467	11.213047	10.850333	10.518248	10.780444	10.890438	11.743340	11.449574	10.831970	11.789270	10.885995	11.607591	10.802397	10.033478	10.062899	10.750836	11.085280	10.385937	10.721789	10.119603	11.675026	10.536771	10.296641	10.120507	11.799679	11.145141	9.947962	10.943027	11.471231	11.498149	10.979949	11.025555	11.694395	10.788558	11.869930	11.240073	11.127958	11.811705	9.797194	11.459486	10.589350	10.601053	10.453118	11.118406	12.018249	11.727140	11.557144	12.006245	9.694363	11.529079	11.691697	11.266027	10.943897	11.172234	12.516941	10.934442	10.112915	11.190903	10.070853	11.700373	11.516227	12.084672	11.142502	11.365508	10.412994	10.463260	9.962834	10.887634	10.406956	10.782381	9.717177	10.797454	10.623514	11.478703	11.308100	11.279663	11.272662	10.912644	10.555535	11.021387	10.828572	10.864387	12.189815	11.894727	10.432020	11.951260	11.420572	11.570621	10.890583	10.883860	9.701277	10.079182	10.981739	9.966953	11.166077	9.922286	10.434445	11.027915	11.613311	11.693032	10.839445	10.182714	10.195069	10.926544	10.810258	10.653521	11.246365	11.455650	10.377424	10.240163	12.325410	10.659325	10.888785	8.057141	11.449574	10.686008	10.978923	10.715070	10.859380	11.241643

ZNF773	9.693450	9.273471	9.380969	9.285661	10.020783	10.171080	9.830616	10.068275	10.323820	9.939017	9.830616	9.589153	9.564966	9.852612	9.956624	9.384220	9.333386	9.731833	9.419599	9.473557	9.092678	8.876306	9.372889	9.681392	9.966054	9.788490	9.126741	9.676005	9.415203	9.569197	9.728526	9.600803	9.926775	9.618393	9.802726	10.040543	9.689916	9.869000	10.070373	10.193392	9.923681	10.151576	9.837507	9.850250	9.840309	9.775812	10.055148	10.222882	9.932634	10.323820	9.705173	10.104790	9.833499	10.159386	9.358941	9.726052	9.997062	9.813541	9.908340	10.385633	10.531134	9.631378	9.112719	9.744936	9.676005	9.560968	9.607484	9.718286	9.553280	9.932777	9.937905	9.912045	9.577618	9.700030	9.890146	9.699873	9.763871	9.506715	9.968648	10.044788	9.228210	9.457707	9.919961	9.883323	9.946708	9.927990	9.886092	9.934485	9.058600	9.894275	10.358566	9.192120	9.559999	9.939645	9.782986	9.755013	9.440040	9.670216	9.763871	9.805798	9.871223	9.581541	9.409873	9.463923	9.387474	9.725608	10.255271	9.623678	9.588428	10.075134	9.426987	10.522062	9.986408	10.434742	9.706564	10.991621	10.060602	9.682033	9.863624	10.074539	9.736242	10.123342	9.676894	10.627143	10.260870	10.045559	9.669565	8.748492	9.502375	9.486737	9.292688	9.468250	9.377930	9.867560	9.112719	9.097971	9.588120	8.417837	10.877792	8.826140	9.011126	9.136672	10.169200	9.561747	9.352150	9.840929	10.163187	10.032568	9.288636	9.892861	8.618776	9.599562	9.461985	9.479239	10.226206	9.850577	10.570769	10.526253	9.973102	9.872421	10.634588	8.292913	10.496787	9.032809	9.315654	10.495716	10.418435	9.898224	8.988657	9.675698	9.866086	9.950260	9.825091	9.278587	10.349330	9.893788	9.676450	9.864980	10.152342	11.116223	9.371195	9.621028	9.982906	8.811217	10.180871	8.948426	8.999362	9.991732	9.743792	9.735623	9.688696	9.918999	8.881603	9.438812	8.846787	9.370447	9.172804	9.492217	10.130935	9.003048	9.989046	10.637121	9.823307	10.745574	9.956464	9.110950	10.150520	9.322141	9.515307	10.736547	9.344981	10.113907	9.764347	10.441702	10.291572	9.625854	9.335281	9.813689	10.136988	9.612257	8.951958	9.286592	9.308487	9.478457	9.199695	10.063772	8.745271	9.815723	10.152488	8.911735	10.378655	10.558425	10.391918	10.272760	9.573678	9.673010	8.882195	9.492544

AC003005.1	9.693450	9.273471	9.380969	9.285661	10.020783	10.171080	9.830616	10.068275	10.323820	9.939017	9.830616	9.589153	9.564966	9.852612	9.956624	9.384220	9.333386	9.731833	9.419599	9.473557	9.092678	8.876306	9.372889	9.681392	9.966054	9.788490	9.126741	9.676005	9.415203	9.569197	9.728526	9.600803	9.926775	9.618393	9.802726	10.040543	9.689916	9.869000	10.070373	10.193392	9.923681	10.151576	9.837507	9.850250	9.840309	9.775812	10.055148	10.222882	9.932634	10.323820	9.705173	10.104790	9.833499	10.159386	9.358941	9.726052	9.997062	9.813541	9.908340	10.385633	10.531134	9.631378	9.112719	9.744936	9.676005	9.560968	9.607484	9.718286	9.553280	9.932777	9.937905	9.912045	9.577618	9.700030	9.890146	9.699873	9.763871	9.506715	9.968648	10.044788	9.228210	9.457707	9.919961	9.883323	9.946708	9.927990	9.886092	9.934485	9.058600	9.894275	10.358566	9.192120	9.559999	9.939645	9.782986	9.755013	9.440040	9.670216	9.763871	9.805798	9.871223	9.581541	9.409873	9.463923	9.387474	9.725608	10.255271	9.623678	9.588428	10.075134	9.426987	10.522062	9.986408	10.434742	9.706564	10.991621	10.060602	9.682033	9.863624	10.074539	9.736242	10.123342	9.676894	10.627143	10.260870	10.045559	9.669565	8.748492	9.502375	9.486737	9.292688	9.468250	9.377930	9.867560	9.112719	9.097971	9.588120	8.417837	10.877792	8.826140	9.011126	9.136672	10.169200	9.561747	9.352150	9.840929	10.163187	10.032568	9.288636	9.892861	8.618776	9.599562	9.461985	9.479239	10.226206	9.850577	10.570769	10.526253	9.973102	9.872421	10.634588	8.292913	10.496787	9.032809	9.315654	10.495716	10.418435	9.898224	8.988657	9.675698	9.866086	9.950260	9.825091	9.278587	10.349330	9.893788	9.676450	9.864980	10.152342	11.116223	9.371195	9.621028	9.982906	8.811217	10.180871	8.948426	8.999362	9.991732	9.743792	9.735623	9.688696	9.918999	8.881603	9.438812	8.846787	9.370447	9.172804	9.492217	10.130935	9.003048	9.989046	10.637121	9.823307	10.745574	9.956464	9.110950	10.150520	9.322141	9.515307	10.736547	9.344981	10.113907	9.764347	10.441702	10.291572	9.625854	9.335281	9.813689	10.136988	9.612257	8.951958	9.286592	9.308487	9.478457	9.199695	10.063772	8.745271	9.815723	10.152488	8.911735	10.378655	10.558425	10.391918	10.272760	9.573678	9.673010	8.882195	9.492544

KDM4D	6.568705	6.323058	6.632208	6.479137	6.379636	6.129505	6.646729	5.764109	4.565758	6.188929	5.971960	6.198966	6.697278	6.160317	5.874946	6.529154	5.900251	6.445578	5.135234	7.140953	5.855085	6.402768	5.475412	5.743559	5.532003	5.815647	5.534285	5.567473	5.600944	6.275725	5.205831	7.738048	7.146288	6.806770	7.429530	7.113763	7.491173	6.508768	5.900808	6.107658	6.184321	6.949978	7.737739	7.636181	7.786221	7.775272	6.492034	5.724728	6.471389	7.993319	7.794928	7.962531	7.736955	7.674175	6.432086	6.020286	7.808494	6.693444	7.594582	7.605777	7.020611	6.218892	5.246802	5.704866	6.656772	6.513727	6.283794	6.289442	7.362150	6.422858	7.136255	6.848549	5.743733	6.852662	7.469565	7.061243	7.053173	6.705266	6.850776	7.245475	6.028736	7.165350	7.243253	7.665283	7.754711	7.672279	7.441605	7.710165	6.870163	6.869212	7.205658	5.590423	6.715222	7.398195	7.283052	6.891740	6.669632	6.224688	6.882620	5.927772	5.308477	6.592806	6.193564	7.122273	4.880724	5.849706	6.202438	5.596070	5.008885	5.309215	6.094448	6.012629	5.695854	4.858755	5.690061	4.726203	6.538915	5.966091	5.140077	7.647866	6.813975	7.325012	6.935199	5.799467	8.135563	6.981009	7.382687	6.462351	7.001459	6.194249	5.894762	6.987331	7.737418	7.382041	7.048951	7.646673	6.628063	6.567744	8.596881	7.104714	6.414193	6.646089	7.613569	8.398520	7.895767	6.630766	6.347762	5.266469	6.887904	7.055251	6.629051	7.393665	7.641386	8.698949	9.266894	8.546108	7.024922	7.210325	7.024084	6.579617	6.548360	10.231761	8.820970	8.574878	8.357368	6.563191	8.287505	8.582697	8.448278	8.724169	9.361712	8.779155	8.560695	8.175833	7.318215	8.955017	8.197780	8.918170	7.841561	7.033591	7.378793	7.957883	6.902230	5.737715	7.250566	6.806770	6.303573	7.577460	6.593414	7.232439	7.774461	5.492994	7.651452	8.301810	8.007472	8.363626	7.771867	7.324214	8.574739	6.861228	9.231455	8.102649	7.927574	9.746613	8.251602	8.634112	9.750053	6.916498	7.417717	7.885821	7.671322	6.700626	7.595034	7.387618	7.170622	6.832940	6.688383	6.977224	7.720677	6.939832	9.324766	4.838925	7.623470	7.548986	6.729992	7.535627	8.396872	5.985916	5.899364	5.843332	6.678964	5.880149	4.938930	7.681782	6.889035	8.192912	7.540217	8.743149

MAML3	5.504312	4.448486	5.034847	5.069790	6.096683	5.540357	5.334740	5.631017	6.549177	6.343011	6.286409	6.388356	5.736449	4.542873	5.724374	6.545166	5.620741	4.247945	6.757330	5.727373	4.801541	4.417754	6.609648	4.793518	4.542873	5.909516	5.155009	5.269469	6.017083	4.216869	7.315588	5.467351	4.781346	5.079600	5.049796	4.714337	4.746622	3.750622	4.309062	4.257644	5.359672	3.808777	4.681366	5.512606	5.334938	5.585717	6.407375	3.684027	4.775532	5.044321	5.045882	4.646621	5.549767	5.198932	4.626946	6.240763	5.782186	3.449554	3.217434	3.933902	3.054459	6.116129	4.755730	4.114367	4.132093	4.223185	2.948429	3.748346	3.292959	3.756112	4.290685	4.704932	6.532898	4.006114	4.529888	5.760167	5.001729	4.330775	4.787643	4.223185	4.732510	5.130200	3.394704	4.915772	5.108098	6.197419	5.135038	4.906273	3.262792	4.381597	3.549833	3.658626	4.479552	5.261254	5.019719	6.723381	5.748485	7.064140	6.000418	5.676185	6.005570	4.579696	5.159668	4.401336	3.051231	6.944078	6.812550	3.689939	3.756112	6.058707	3.773257	5.270257	3.702258	6.617262	6.786131	6.501553	3.514967	6.247965	5.956634	5.846393	4.338129	3.306163	3.937709	3.955912	5.700066	5.252954	5.775473	4.480424	5.666294	4.434129	4.525013	4.979942	3.547366	4.983789	6.123548	3.822254	3.087392	4.758070	4.345246	4.152983	3.809721	6.035454	4.004337	3.463557	4.565327	5.007256	3.745473	5.925599	3.785223	6.048206	5.528391	3.986728	3.399349	3.260153	4.554340	3.663078	3.329178	3.470346	3.387383	5.217569	3.907900	5.339727	3.623869	4.411549	5.189553	6.539247	3.327268	5.077351	3.825965	3.693618	4.254130	3.870092	4.285690	4.472756	4.046498	4.332454	3.529405	3.060657	3.828244	3.505145	4.658123	3.780212	3.558731	3.596508	3.696522	2.951119	3.639699	3.466667	2.984859	3.680036	4.440018	4.647688	4.030176	4.519569	5.947042	3.900638	3.852717	4.291112	3.565517	3.627841	4.576019	4.451198	4.855423	3.921822	4.924291	4.395431	6.030923	3.125532	3.502367	3.489376	4.078077	4.434340	4.468617	4.778450	4.375887	4.732302	5.920125	5.021357	3.998746	4.159518	3.395581	3.425458	3.306659	3.077828	5.361935	5.235030	4.389354	3.733620	4.110743	4.001936	3.409194	3.181086	5.579658	3.810560	3.869875	3.809721	5.929013	4.567032

L3MBTL3	3.399199	3.418603	3.227738	4.072967	3.543244	3.749777	3.363531	5.091838	4.544458	5.388347	4.666915	4.212746	3.600877	3.719129	4.725997	3.595938	3.747700	2.934452	4.879225	4.263766	4.570033	2.967997	3.076229	2.907763	2.890514	4.024123	4.305090	4.518683	4.900919	3.049958	3.701577	4.322455	3.546167	3.550612	3.604453	4.057171	4.017832	3.231044	3.240314	3.131467	3.935574	3.154548	3.562491	3.326945	3.724528	5.630261	4.678590	2.857028	5.006417	4.002147	3.831373	3.764496	4.087871	4.240767	3.975942	3.569066	4.143455	4.044741	3.039234	3.696123	2.834424	3.665884	3.612180	3.313984	3.559346	3.481536	3.144788	3.787161	3.206407	2.835714	3.261430	3.242816	4.137363	3.086586	3.120954	3.642272	3.053145	3.075527	3.007968	3.739973	3.444014	4.039945	3.593015	4.091630	3.448800	4.540468	2.897780	3.271619	4.014061	3.612759	3.270828	2.879764	3.637482	3.315404	3.434147	3.115700	3.796132	3.766514	3.152573	3.684817	2.888014	3.214600	3.266295	3.393651	4.497929	4.355913	3.052568	3.349011	2.978709	3.805556	3.263968	3.404213	3.018838	4.908825	5.113154	2.958164	3.510727	3.109208	3.897142	4.617086	3.529405	3.123779	5.389322	3.585898	5.405155	2.971440	4.570729	4.495328	6.110971	4.392978	4.757162	3.237122	3.157765	5.867214	3.139187	3.638875	3.022682	4.867022	4.148755	3.862586	2.964561	5.402259	3.260153	3.607655	3.344161	3.048905	6.387151	5.702610	3.393483	3.534800	3.524588	3.540716	3.314143	4.391694	3.754647	3.666461	3.317278	3.283615	3.054254	4.738421	2.960127	3.608791	3.344796	4.217728	4.970105	4.581421	2.812056	4.983217	4.048215	3.735018	3.582650	4.100404	4.371341	3.550026	3.063753	3.798389	3.879790	2.857028	3.395399	2.784658	4.176172	3.647551	3.886748	3.905077	3.690955	3.062481	3.237709	3.251669	2.786208	3.228037	3.056020	5.957686	3.006886	3.017496	3.332078	3.055089	3.118766	3.121463	3.228916	3.826990	4.457149	3.658843	3.588064	3.464825	3.586121	3.456418	3.430468	3.678004	3.460369	3.839112	2.837022	3.251299	3.397833	3.725775	3.044226	2.945840	3.024181	3.930454	3.325488	3.036756	3.021136	3.414352	3.062576	4.832509	5.328291	4.131034	3.735018	3.367066	3.952698	3.109116	3.042715	2.923134	5.060815	4.252787	3.645219	3.176261	3.337310	3.481909

GLG1	5.785023	5.523672	5.616676	6.243620	6.428535	6.686616	6.469202	6.435961	7.721928	6.834077	6.456712	5.606684	6.295260	6.979561	4.844870	7.065889	6.323541	5.712490	5.502582	6.538740	4.619476	7.035147	6.981481	5.982617	5.874232	6.832787	6.041239	6.033636	6.393047	5.576665	6.928293	6.376000	5.037736	6.032553	5.504312	5.417579	5.474637	6.013848	5.460459	6.251818	6.134299	5.229116	6.441853	5.720686	6.394353	5.674591	6.203825	5.132613	6.780224	6.497516	5.863284	6.200479	6.374667	6.372612	6.722079	6.038416	6.222924	6.185885	5.369930	5.169712	4.268049	6.590635	4.869173	5.455701	4.477408	5.455701	5.234417	5.698420	5.273895	5.178999	5.997771	6.255725	7.029396	5.948634	6.378810	5.639354	5.803115	5.268846	5.792377	5.936863	7.122734	6.897530	5.976562	6.915595	6.940443	6.497040	6.214225	6.461005	5.151558	5.631552	4.962329	5.683055	5.598592	5.848261	5.876918	6.379289	6.032173	6.561198	5.620195	6.848381	6.796536	6.053907	6.618387	6.133974	6.259647	7.708979	6.592444	6.700466	5.661605	6.869358	5.111523	6.232414	5.708310	6.434799	7.326266	5.964494	4.776982	6.646089	6.385953	5.547131	5.683252	4.891263	5.308477	6.574654	6.382809	6.162053	5.788486	6.783550	5.242838	5.635267	6.129996	5.206028	5.436282	4.913496	6.455107	6.446691	6.093560	5.014666	4.787444	5.213361	5.660681	6.201016	5.072402	4.302876	5.980467	6.128005	5.889018	5.282574	4.836806	6.474906	7.100790	5.546772	5.946205	4.912191	5.031543	5.222525	4.950633	4.688212	4.621851	6.689492	5.477861	5.226859	5.960308	6.590303	5.877609	7.233060	5.457488	6.397130	5.851664	5.904897	6.161502	6.298741	5.873704	6.302728	5.834461	5.133206	6.314368	5.372272	5.429406	4.736955	5.292942	5.579658	5.774720	5.147545	5.180956	5.283757	5.174521	4.903414	4.977861	5.282363	6.004284	6.411686	5.906596	5.037135	5.719583	5.261064	5.528973	5.858504	5.061015	6.733827	6.040557	6.450151	6.628397	5.612184	5.984697	5.944425	6.477105	5.261064	5.027449	4.709267	4.721510	5.087179	6.192830	6.603879	7.134672	5.908486	6.579129	5.505239	5.274525	7.036542	6.194799	5.244823	5.155009	5.544296	6.906051	6.557781	6.125147	4.652799	6.450151	5.010742	5.436682	3.353835	6.325088	5.883488	6.352264	4.464978	6.564009	5.598387

MYT1L	10.461885	9.505499	9.566857	9.607774	9.830772	10.224836	9.285962	10.513768	10.665327	9.770486	9.866689	9.626458	9.165262	9.616997	9.331216	9.819482	10.427634	9.619017	11.201594	9.714221	10.584051	9.992041	11.420135	9.801200	10.002558	10.229726	9.943490	10.655009	9.768599	9.667023	10.502419	8.642660	9.094633	8.754861	9.136223	9.259079	9.357553	9.589501	10.085861	10.289000	9.863484	10.132441	9.312325	10.002270	9.411694	9.710638	9.912200	9.742594	9.483782	9.344218	9.663577	9.403399	9.010977	9.399241	9.421602	9.867109	8.956821	9.753292	9.766182	9.504879	9.959230	9.550248	10.801787	10.551284	10.190104	10.233845	10.273491	9.971364	9.798602	10.799798	9.607163	9.739328	9.756057	9.447632	9.604383	9.747254	9.876085	9.789600	9.952574	9.342047	9.415969	9.493455	9.451098	9.418832	9.346950	9.304702	9.396978	9.553448	10.308483	10.112915	10.013258	10.688872	9.470011	9.545005	9.497806	9.456162	9.375129	8.587856	9.035284	9.314738	9.191651	9.109712	9.262911	9.574283	10.528361	9.667023	10.577542	10.277457	11.047235	9.974821	11.029872	10.653668	10.486856	10.399900	9.857107	10.640900	10.768187	9.530244	9.753292	9.635491	10.057277	9.726699	10.246964	10.789138	10.187258	9.525226	9.944847	10.506461	9.940550	10.122918	9.628464	10.077125	9.521160	10.188617	9.687929	10.340663	9.391590	11.097335	9.293796	10.553981	10.232331	11.338197	10.040543	10.323048	10.081162	9.944110	10.674952	10.541837	9.954431	10.488419	9.132751	9.508068	9.435860	9.358941	9.431886	9.633365	10.064832	10.632606	10.496787	10.524141	10.746326	9.540974	10.650157	9.574724	10.011226	10.693482	9.639042	9.589330	9.580110	9.896130	9.436929	9.220054	9.666547	9.335710	9.778460	9.021121	9.677525	9.906961	9.473843	10.225000	9.694205	10.704472	10.697494	10.590679	10.388515	10.533808	10.286654	10.163352	11.073280	9.815427	9.854464	10.587598	9.765726	9.484491	9.959383	10.027644	10.093965	10.091891	9.235176	9.382862	9.589501	9.687778	9.318981	9.554494	9.912045	9.674754	9.369517	10.438091	10.129611	10.082987	10.945054	9.778003	10.212263	10.014305	9.905132	10.284884	9.369517	9.491096	9.283708	10.147018	9.184093	9.820866	9.600803	10.431267	10.099038	10.524581	10.269714	10.798060	11.069134	10.990768	10.567716	10.823244	10.907937	9.689297	9.584671	10.008153	9.667023	10.162874

HGD	7.617231	8.456268	9.051442	8.429897	9.216247	9.304141	9.039461	8.721472	8.738106	9.007476	8.459105	9.011126	8.605587	9.172195	8.732010	8.352921	7.520818	8.872170	10.416445	8.972908	9.474447	9.883634	8.660064	8.458269	8.583297	7.794928	7.195520	8.975406	7.224175	8.002934	8.705672	9.242420	8.546755	8.234051	7.693362	8.735642	8.047520	8.675628	8.177241	8.874004	9.561303	8.223604	7.811708	8.568786	7.714505	7.266636	7.845650	8.578029	9.307738	7.902571	8.061379	8.153907	8.171114	7.986373	8.004502	8.865812	8.395613	8.541974	8.517165	8.316848	8.673745	8.289335	8.019671	8.229943	8.822507	8.953479	8.295296	7.764204	8.295598	9.247657	9.179536	9.092678	8.729734	8.235894	8.914492	7.854534	8.554956	8.289790	8.041562	8.012155	7.848550	7.148023	7.781047	8.314211	8.392095	9.393437	7.755863	8.342169	8.959017	8.362863	8.593010	8.890098	8.224643	8.482171	6.850930	7.737577	9.289425	8.934723	8.679746	8.730609	8.522159	8.716580	8.584684	8.250672	9.751590	8.862310	9.897656	9.017607	9.733183	8.998406	9.625524	8.644996	9.304559	8.939739	9.182973	7.157464	8.301810	8.499816	7.491960	9.142279	8.829188	7.099707	9.044254	7.023584	8.207229	7.114883	6.268605	8.378160	7.584267	9.478766	6.148819	7.434558	6.537617	7.421675	7.238454	8.697747	6.788922	6.603564	7.213117	8.474628	8.006706	8.485224	8.048145	6.526178	8.968517	8.195107	7.924267	9.107332	7.482286	7.578233	8.923580	8.402976	8.223289	7.722684	6.630936	7.701592	8.175380	9.224522	8.221915	8.008852	8.103552	6.365038	8.362054	7.289419	7.633475	9.444517	6.606374	9.084561	7.846711	8.089401	7.492583	7.518967	8.472319	8.043558	7.430625	5.029113	7.125300	7.979109	6.964056	8.250672	8.453383	9.119139	8.789036	7.057287	7.667017	8.177835	8.793229	8.018603	7.749037	8.109516	8.318216	8.307282	8.616473	7.781047	7.957608	7.764815	7.437546	8.082554	7.795409	7.995817	9.003802	8.217284	7.869083	7.846711	8.470262	8.072162	7.186039	7.665739	8.328762	7.334115	9.302942	7.999017	8.899150	9.007159	7.554247	8.413076	8.051848	9.446549	7.114394	9.658575	6.920499	6.756872	8.037881	7.407585	7.426963	8.265248	9.245828	7.941031	8.531373	7.556615	7.360270	6.830405	8.832570	6.737942	8.458269	8.146710	7.756016	8.921205

GPR82	7.754268	7.466203	7.846238	6.894955	7.691660	7.785158	7.933559	7.875503	8.272677	6.820707	8.172546	8.093070	7.632243	8.196392	7.457457	8.124489	7.106590	7.106932	8.073351	8.772323	7.710796	8.694983	6.817315	7.844066	7.178605	6.723381	7.910787	5.291194	7.175395	8.298973	7.038119	8.567027	9.048698	9.041468	9.852155	9.556004	8.445159	8.365932	7.681453	8.223604	7.522660	8.262784	9.230883	8.649681	8.563599	8.905230	6.385280	7.131326	7.816813	8.705991	8.913281	8.505967	9.095402	8.697270	8.086920	7.368131	9.039001	8.298431	7.385610	7.946637	8.534075	7.790686	6.668981	7.801000	8.068759	7.306655	7.767780	7.618765	8.080886	8.006706	7.901974	8.206933	8.250993	8.711169	9.035892	9.252231	8.617088	8.154551	8.837843	8.557608	9.417796	8.692954	8.911908	8.832252	8.768535	8.092448	8.977263	7.982237	8.521276	8.408361	8.651625	8.321435	8.482018	9.240911	9.289571	9.484565	8.092604	9.029335	7.743008	7.695762	8.203010	8.189101	8.100252	7.950786	10.392503	7.370150	8.819791	8.232693	7.336449	6.996308	7.678909	7.808028	7.059187	7.015827	8.285627	4.925302	6.976098	7.945100	7.218109	8.974964	7.291452	8.154391	7.166846	7.424453	8.218549	7.538600	9.291914	7.488806	7.592104	7.448724	8.336333	6.323720	7.800367	6.540849	8.560387	7.757729	6.022244	9.293955	9.533987	6.680932	7.278770	6.734817	8.330440	6.132261	4.832927	7.240393	5.907938	7.418684	7.545491	8.370745	7.948894	8.194144	8.335568	9.734237	8.185737	8.646046	7.876561	6.385448	8.570650	7.263561	8.233292	8.386497	9.045496	9.019286	8.111326	5.843704	7.021874	8.170959	8.717058	9.169074	8.266021	8.990614	8.688503	9.323193	8.150810	9.226210	8.110900	7.743660	7.995659	8.373576	7.227642	7.804165	8.020574	9.370141	7.713903	7.553470	8.518854	7.802054	8.299261	8.207388	8.344605	7.013769	8.899150	8.946763	10.159237	8.501719	8.434296	8.591916	9.509006	10.025121	9.327512	9.449354	10.088543	10.184839	7.830635	9.017440	9.143799	10.196594	9.613640	7.650473	8.564664	9.072667	8.900227	9.661074	10.018641	8.312374	8.848945	7.446170	7.284304	8.457783	8.301065	8.239639	8.919658	10.263600	7.751360	10.159386	8.743457	8.175833	7.014515	7.593028	7.397106	3.063626	6.686460	7.159318	5.835708	7.953706	7.235310	7.228420

CASK	7.754268	7.466203	7.846238	6.894955	7.691660	7.785158	7.933559	7.875503	8.272677	6.820707	8.172546	8.093070	7.632243	8.196392	7.457457	8.124489	7.106590	7.106932	8.073351	8.772323	7.710796	8.694983	6.817315	7.844066	7.178605	6.723381	7.910787	5.291194	7.175395	8.298973	7.038119	8.567027	9.048698	9.041468	9.852155	9.556004	8.445159	8.365932	7.681453	8.223604	7.522660	8.262784	9.230883	8.649681	8.563599	8.905230	6.385280	7.131326	7.816813	8.705991	8.913281	8.505967	9.095402	8.697270	8.086920	7.368131	9.039001	8.298431	7.385610	7.946637	8.534075	7.790686	6.668981	7.801000	8.068759	7.306655	7.767780	7.618765	8.080886	8.006706	7.901974	8.206933	8.250993	8.711169	9.035892	9.252231	8.617088	8.154551	8.837843	8.557608	9.417796	8.692954	8.911908	8.832252	8.768535	8.092448	8.977263	7.982237	8.521276	8.408361	8.651625	8.321435	8.482018	9.240911	9.289571	9.484565	8.092604	9.029335	7.743008	7.695762	8.203010	8.189101	8.100252	7.950786	10.392503	7.370150	8.819791	8.232693	7.336449	6.996308	7.678909	7.808028	7.059187	7.015827	8.285627	4.925302	6.976098	7.945100	7.218109	8.974964	7.291452	8.154391	7.166846	7.424453	8.218549	7.538600	9.291914	7.488806	7.592104	7.448724	8.336333	6.323720	7.800367	6.540849	8.560387	7.757729	6.022244	9.293955	9.533987	6.680932	7.278770	6.734817	8.330440	6.132261	4.832927	7.240393	5.907938	7.418684	7.545491	8.370745	7.948894	8.194144	8.335568	9.734237	8.185737	8.646046	7.876561	6.385448	8.570650	7.263561	8.233292	8.386497	9.045496	9.019286	8.111326	5.843704	7.021874	8.170959	8.717058	9.169074	8.266021	8.990614	8.688503	9.323193	8.150810	9.226210	8.110900	7.743660	7.995659	8.373576	7.227642	7.804165	8.020574	9.370141	7.713903	7.553470	8.518854	7.802054	8.299261	8.207388	8.344605	7.013769	8.899150	8.946763	10.159237	8.501719	8.434296	8.591916	9.509006	10.025121	9.327512	9.449354	10.088543	10.184839	7.830635	9.017440	9.143799	10.196594	9.613640	7.650473	8.564664	9.072667	8.900227	9.661074	10.018641	8.312374	8.848945	7.446170	7.284304	8.457783	8.301065	8.239639	8.919658	10.263600	7.751360	10.159386	8.743457	8.175833	7.014515	7.593028	7.397106	3.063626	6.686460	7.159318	5.835708	7.953706	7.235310	7.228420

PRODH	10.850333	12.228786	12.217765	12.475368	12.581640	13.664000	13.378878	13.177402	13.159717	13.534431	13.647299	13.727369	12.474582	12.797679	12.066882	13.181925	13.356199	12.268825	11.562464	13.196931	11.908348	11.537478	10.844098	12.294023	11.971293	12.653635	12.197526	12.771292	13.020460	12.893478	13.323361	11.860081	11.293819	12.252952	12.566930	12.572187	12.739508	12.199376	12.136503	11.987566	13.440327	11.688272	12.109213	11.948495	11.632245	12.043646	13.403996	11.593285	12.487141	11.850117	11.709393	11.979884	11.876433	12.358306	11.799525	13.375856	11.464636	11.746705	11.390561	11.903261	11.729004	13.443521	10.023706	11.648287	11.085135	11.746117	11.611531	11.946225	12.166779	11.624141	13.012290	13.220404	13.637147	13.156342	13.307696	12.535770	13.132547	12.697859	12.859172	11.190043	11.510698	11.543325	11.018683	11.658340	11.487721	12.136934	11.362409	11.556994	10.685853	11.230487	11.077064	10.943897	11.670468	12.650060	12.319524	12.849996	11.815871	12.581161	12.636458	12.958452	12.968955	12.792507	12.750174	12.023720	11.590096	13.384142	12.322365	12.642396	11.406761	13.474856	11.569749	12.522505	12.198594	13.614482	13.607572	14.317493	10.393410	13.031373	13.516454	11.024955	11.718826	11.835877	12.238354	13.240179	13.399963	13.045174	12.344394	12.784870	13.317859	13.315805	13.686701	12.869225	11.637961	11.407526	12.769791	11.594455	13.844830	11.199598	11.916672	11.526788	11.376659	12.053308	13.949893	11.672364	11.507888	12.812654	13.222154	13.090158	12.050737	12.122102	11.486085	12.352576	12.441914	11.840994	12.713819	11.504652	11.909709	11.683069	12.089348	12.734697	12.424929	12.602064	11.681708	12.756854	12.233892	12.800233	10.735207	13.031672	11.639945	12.960694	12.068749	11.403962	11.286099	11.356819	13.138306	10.587915	12.187947	12.057308	12.448796	11.546511	12.265271	10.806498	11.557455	10.957378	12.224317	11.107995	11.330459	11.309839	10.517707	13.197314	12.624833	13.452455	11.595768	12.798678	12.298374	11.579822	11.830189	12.513208	10.847257	11.824292	11.617713	11.062006	10.919423	10.290216	12.283965	10.982046	10.512425	10.518184	10.392057	10.776828	11.157897	11.361662	11.506409	11.631352	11.552438	12.556686	12.546832	11.729608	13.194518	11.346810	12.405908	12.705000	11.395949	9.818886	12.788393	12.232073	11.376221	12.267146	13.166910	12.282409	11.909419	11.886889	13.384142	12.955178	12.669159	11.413399	13.085733	12.957359

AC007326.4	10.850333	12.228786	12.217765	12.475368	12.581640	13.664000	13.378878	13.177402	13.159717	13.534431	13.647299	13.727369	12.474582	12.797679	12.066882	13.181925	13.356199	12.268825	11.562464	13.196931	11.908348	11.537478	10.844098	12.294023	11.971293	12.653635	12.197526	12.771292	13.020460	12.893478	13.323361	11.860081	11.293819	12.252952	12.566930	12.572187	12.739508	12.199376	12.136503	11.987566	13.440327	11.688272	12.109213	11.948495	11.632245	12.043646	13.403996	11.593285	12.487141	11.850117	11.709393	11.979884	11.876433	12.358306	11.799525	13.375856	11.464636	11.746705	11.390561	11.903261	11.729004	13.443521	10.023706	11.648287	11.085135	11.746117	11.611531	11.946225	12.166779	11.624141	13.012290	13.220404	13.637147	13.156342	13.307696	12.535770	13.132547	12.697859	12.859172	11.190043	11.510698	11.543325	11.018683	11.658340	11.487721	12.136934	11.362409	11.556994	10.685853	11.230487	11.077064	10.943897	11.670468	12.650060	12.319524	12.849996	11.815871	12.581161	12.636458	12.958452	12.968955	12.792507	12.750174	12.023720	11.590096	13.384142	12.322365	12.642396	11.406761	13.474856	11.569749	12.522505	12.198594	13.614482	13.607572	14.317493	10.393410	13.031373	13.516454	11.024955	11.718826	11.835877	12.238354	13.240179	13.399963	13.045174	12.344394	12.784870	13.317859	13.315805	13.686701	12.869225	11.637961	11.407526	12.769791	11.594455	13.844830	11.199598	11.916672	11.526788	11.376659	12.053308	13.949893	11.672364	11.507888	12.812654	13.222154	13.090158	12.050737	12.122102	11.486085	12.352576	12.441914	11.840994	12.713819	11.504652	11.909709	11.683069	12.089348	12.734697	12.424929	12.602064	11.681708	12.756854	12.233892	12.800233	10.735207	13.031672	11.639945	12.960694	12.068749	11.403962	11.286099	11.356819	13.138306	10.587915	12.187947	12.057308	12.448796	11.546511	12.265271	10.806498	11.557455	10.957378	12.224317	11.107995	11.330459	11.309839	10.517707	13.197314	12.624833	13.452455	11.595768	12.798678	12.298374	11.579822	11.830189	12.513208	10.847257	11.824292	11.617713	11.062006	10.919423	10.290216	12.283965	10.982046	10.512425	10.518184	10.392057	10.776828	11.157897	11.361662	11.506409	11.631352	11.552438	12.556686	12.546832	11.729608	13.194518	11.346810	12.405908	12.705000	11.395949	9.818886	12.788393	12.232073	11.376221	12.267146	13.166910	12.282409	11.909419	11.886889	13.384142	12.955178	12.669159	11.413399	13.085733	12.957359

DGCR6	10.850333	12.228786	12.217765	12.475368	12.581640	13.664000	13.378878	13.177402	13.159717	13.534431	13.647299	13.727369	12.474582	12.797679	12.066882	13.181925	13.356199	12.268825	11.562464	13.196931	11.908348	11.537478	10.844098	12.294023	11.971293	12.653635	12.197526	12.771292	13.020460	12.893478	13.323361	11.860081	11.293819	12.252952	12.566930	12.572187	12.739508	12.199376	12.136503	11.987566	13.440327	11.688272	12.109213	11.948495	11.632245	12.043646	13.403996	11.593285	12.487141	11.850117	11.709393	11.979884	11.876433	12.358306	11.799525	13.375856	11.464636	11.746705	11.390561	11.903261	11.729004	13.443521	10.023706	11.648287	11.085135	11.746117	11.611531	11.946225	12.166779	11.624141	13.012290	13.220404	13.637147	13.156342	13.307696	12.535770	13.132547	12.697859	12.859172	11.190043	11.510698	11.543325	11.018683	11.658340	11.487721	12.136934	11.362409	11.556994	10.685853	11.230487	11.077064	10.943897	11.670468	12.650060	12.319524	12.849996	11.815871	12.581161	12.636458	12.958452	12.968955	12.792507	12.750174	12.023720	11.590096	13.384142	12.322365	12.642396	11.406761	13.474856	11.569749	12.522505	12.198594	13.614482	13.607572	14.317493	10.393410	13.031373	13.516454	11.024955	11.718826	11.835877	12.238354	13.240179	13.399963	13.045174	12.344394	12.784870	13.317859	13.315805	13.686701	12.869225	11.637961	11.407526	12.769791	11.594455	13.844830	11.199598	11.916672	11.526788	11.376659	12.053308	13.949893	11.672364	11.507888	12.812654	13.222154	13.090158	12.050737	12.122102	11.486085	12.352576	12.441914	11.840994	12.713819	11.504652	11.909709	11.683069	12.089348	12.734697	12.424929	12.602064	11.681708	12.756854	12.233892	12.800233	10.735207	13.031672	11.639945	12.960694	12.068749	11.403962	11.286099	11.356819	13.138306	10.587915	12.187947	12.057308	12.448796	11.546511	12.265271	10.806498	11.557455	10.957378	12.224317	11.107995	11.330459	11.309839	10.517707	13.197314	12.624833	13.452455	11.595768	12.798678	12.298374	11.579822	11.830189	12.513208	10.847257	11.824292	11.617713	11.062006	10.919423	10.290216	12.283965	10.982046	10.512425	10.518184	10.392057	10.776828	11.157897	11.361662	11.506409	11.631352	11.552438	12.556686	12.546832	11.729608	13.194518	11.346810	12.405908	12.705000	11.395949	9.818886	12.788393	12.232073	11.376221	12.267146	13.166910	12.282409	11.909419	11.886889	13.384142	12.955178	12.669159	11.413399	13.085733	12.957359

MLIP	5.429406	4.921258	3.701159	4.839829	6.159451	7.521618	4.759750	6.201016	6.792834	6.746568	7.252607	5.570880	5.609716	6.441219	5.420358	7.553313	6.185718	5.971051	5.823680	7.314813	4.564910	6.843037	6.088324	5.276162	5.746855	5.859241	6.218892	5.643883	6.263018	4.409815	7.840933	4.748507	4.495551	5.868488	4.803039	4.739775	5.448014	3.482229	4.124217	4.319720	7.299385	4.467997	5.076919	4.842335	5.199887	5.679760	7.348503	3.661837	5.904380	4.616662	3.663429	4.092057	5.517407	5.420915	5.265665	6.297553	6.327896	4.759341	4.714555	4.101263	4.903414	6.717021	3.949658	5.954371	4.447326	4.255015	4.266557	5.373011	4.421986	4.138630	5.055139	5.914578	6.904609	5.660863	5.912590	4.618177	5.331849	5.411287	5.416994	5.085506	6.166148	4.457789	5.095809	5.946702	6.137696	5.501991	6.366865	5.702228	5.229330	4.384260	4.156749	4.169012	4.763361	5.000281	5.779803	6.839012	3.690742	7.734420	6.653518	6.199473	5.467155	4.917062	6.035254	5.426425	4.594913	7.128302	6.248137	5.835886	4.798741	7.127841	4.756537	5.427467	3.797353	7.015163	6.086091	7.728256	3.310230	6.679815	7.044822	8.425886	4.877177	3.930246	6.615435	4.524351	5.280527	6.128661	3.582276	7.097035	6.250316	8.037595	5.306198	3.318958	3.943958	3.822254	10.912200	5.336143	6.060246	3.317974	3.613168	3.871796	6.253184	5.418549	4.459856	3.331917	3.893761	6.581645	5.052375	6.180491	3.722894	5.698420	6.182027	5.285388	4.049098	5.056167	4.136501	4.973376	4.584786	3.578788	4.214482	5.074237	3.793159	3.684027	4.957517	6.340247	5.083639	6.278158	3.066288	5.912590	4.648966	4.656406	4.066903	4.272029	4.561847	5.498523	4.125660	3.839304	4.680948	3.763184	4.051282	5.622407	3.735838	4.915556	5.841340	4.502191	4.196239	3.836533	4.605023	4.193432	2.843630	6.219753	5.670148	6.069439	5.410127	6.176270	5.127160	4.888117	5.995662	5.382725	4.444999	6.442361	4.478041	4.502191	7.033252	4.534790	4.521910	5.475033	4.034313	4.256960	4.397228	3.687679	4.610790	4.907970	3.436697	6.421425	5.702980	4.939947	5.441215	5.328489	5.005573	3.316628	3.915721	4.642787	3.227317	4.963547	6.702249	5.749031	4.934698	5.057972	7.147061	4.353327	3.926325	3.837575	6.549699	4.579914	6.932551	4.117895	7.545980	5.831751

IGF2BP1	5.146342	6.554958	6.544343	5.910459	5.726105	6.529826	6.307584	6.513088	6.205388	6.043642	5.890458	6.101011	5.341041	6.089748	5.989717	5.964122	6.317926	5.555427	6.744822	6.560069	7.077190	7.375318	6.360544	6.055495	5.404608	5.777617	4.793710	5.987381	5.166666	5.310882	6.123900	5.919744	5.850428	5.884247	5.867417	6.502846	6.329550	6.823214	6.811607	6.700968	6.291686	6.583270	5.818327	6.497040	6.747557	5.090840	5.475801	6.244951	6.178147	6.492988	5.746099	6.059561	5.820796	6.114042	5.908486	5.893148	5.751568	6.364695	6.012785	6.015713	6.418647	5.805626	6.849324	6.474750	7.060597	6.584561	6.728667	6.389179	6.676570	7.453233	6.920646	6.518720	5.770690	6.571166	6.975929	5.844914	6.192830	6.378971	6.456884	4.934074	4.684394	4.946094	4.476675	5.649152	5.455701	5.925785	4.414432	5.774175	5.774910	5.885856	5.669251	5.821866	5.815816	5.838236	5.208422	5.714200	6.114220	5.172769	5.287363	5.474833	5.431807	6.382496	6.030425	6.779905	7.139694	6.056359	6.079207	6.406070	6.756207	5.869712	7.370474	5.464037	5.557350	6.105134	5.602258	5.538051	4.147485	5.967171	6.037378	6.080065	6.769612	5.243216	6.435793	3.857911	5.793485	5.093109	6.426308	6.053907	6.015887	6.764402	6.354724	5.987923	6.764910	5.432417	5.112145	6.494280	4.885618	5.561665	6.841126	5.622037	5.056780	6.390194	5.533140	4.530516	6.766660	5.936160	5.471598	5.980467	5.384847	5.281372	5.427467	4.344597	6.152751	5.710664	6.359531	5.743007	5.501796	7.148490	5.777617	5.464226	6.092548	4.193008	5.672603	6.077787	6.461336	6.400484	5.582483	5.348514	6.278158	5.516844	5.206028	6.247611	6.649508	5.659219	4.747474	3.922444	5.448014	6.050770	6.106746	5.790446	6.001274	7.345011	6.904927	5.627077	5.636210	6.364065	6.959558	6.402954	6.572895	5.968636	6.003768	5.823129	6.723847	5.963234	5.740064	5.361935	5.708117	6.701120	4.453114	6.751613	5.977611	4.394513	5.173553	4.390178	4.620607	5.533682	6.799271	4.946505	5.314008	7.684886	6.366865	5.822053	6.300993	5.689540	6.363905	5.403504	4.187529	6.106203	4.848888	6.189430	7.779314	4.827193	6.219571	6.680307	5.943556	6.983656	6.540849	5.981511	5.628214	5.219974	4.813729	3.386208	5.968264	4.920613	6.667041	5.171481	5.648788	6.762969

AP3M2	10.868738	10.453574	11.237555	10.976695	11.347880	10.030841	11.012953	9.615149	10.035941	10.679584	10.979516	10.165028	10.945949	10.678215	10.918671	10.554140	10.715826	10.972192	10.110501	10.605030	9.955081	10.583893	10.512879	10.636237	11.251658	11.256343	11.318974	10.750537	10.698520	10.557083	11.249706	10.946219	10.745880	10.797295	11.157450	10.851359	10.974650	10.815666	10.649536	10.675830	10.312432	11.100894	11.062917	11.095294	11.384387	10.854176	10.323671	10.688592	11.328775	10.816421	10.722360	10.871143	10.991471	11.318368	10.286805	10.201399	11.385706	10.845477	10.931284	10.869610	11.955591	10.574514	10.793498	10.297542	11.002085	10.786621	11.069879	10.669403	10.801939	10.587449	10.877350	10.992250	10.668712	10.971908	10.991933	10.713717	10.910296	10.912348	10.907373	10.332520	11.305602	11.184951	11.166213	11.246365	11.000137	11.305447	11.421486	11.530935	11.102824	10.797295	10.728368	10.469964	10.586706	10.847846	11.270728	11.265170	11.391155	10.747087	10.990341	10.586129	10.661012	10.445039	10.669542	10.842709	10.383188	10.946833	9.556322	10.339310	10.431097	10.066310	10.413149	11.223716	10.009427	10.072505	10.846377	10.802991	11.153465	10.728676	10.485950	11.667024	11.323357	12.051034	10.741863	10.152807	10.947735	10.787634	12.836884	11.954825	11.972983	11.939528	12.371808	12.121750	11.678745	11.964932	11.176723	11.826245	12.401128	12.672242	13.069852	11.917442	12.508519	12.108031	12.846834	11.939377	12.040293	10.649376	12.125960	13.141459	11.918799	11.508778	11.159054	11.701015	11.010491	11.673410	12.844764	11.796904	11.624718	10.798780	12.150030	11.584232	12.252154	12.390314	12.373219	12.454210	11.676245	11.435242	12.296782	12.558801	11.403818	11.405580	11.708961	12.344233	11.134465	12.080906	11.344171	11.980914	12.362917	10.981148	12.580656	11.560987	11.537331	11.640417	11.755284	11.759188	12.085897	11.366119	10.783617	11.297358	10.940377	11.566546	12.224905	12.073894	12.084016	11.843554	10.797174	12.086652	12.459294	11.681120	11.448837	11.639796	11.384231	11.827966	11.857355	11.575095	12.389993	11.381146	12.484861	11.089291	11.613311	12.613037	11.558489	11.137722	11.279136	11.626444	12.466808	11.767202	12.047677	11.757691	12.266082	11.178065	12.090269	11.605660	11.812156	10.925790	12.168674	11.639316	11.525172	11.973605	10.316937	11.861844	11.649619	13.143383	9.740870	12.315106	11.853082	12.666814	12.412844	10.879746

EPHA7	8.930003	7.145206	6.778627	7.792348	6.027116	8.648726	8.250672	5.751191	7.487562	8.557910	8.783833	8.599566	7.766526	7.353733	8.019077	7.395059	6.787106	7.298624	7.094332	7.241639	6.931281	6.844383	13.049903	7.589979	8.183127	7.232778	12.376916	8.787683	9.265650	9.080879	7.293017	9.254187	6.750842	10.290812	7.522817	8.094476	7.263889	7.029874	6.369725	8.167960	8.365932	8.526432	7.364365	8.502160	11.797931	7.923024	11.812728	7.998718	7.301137	7.500547	9.056272	8.458622	8.518570	8.109085	7.558311	8.689568	8.584250	7.722840	7.795248	8.117009	8.626389	8.720086	12.994721	6.963071	9.636997	7.363878	9.091258	6.860916	8.247677	7.925059	8.213302	7.609439	8.756076	7.242596	6.673327	6.526846	7.210823	8.247677	8.536813	7.557531	9.436174	8.567616	8.966496	8.157641	8.168459	7.868615	7.833074	7.225900	7.739134	7.488362	8.089724	7.095641	8.142249	6.744672	8.949983	7.103764	8.295020	7.721928	6.475765	7.021564	7.083312	8.058996	7.478448	6.387306	6.560227	9.488302	7.138598	6.832447	7.845788	8.696042	7.278927	6.686143	6.514720	10.003654	6.579129	7.757898	8.843269	7.209733	9.945139	8.759488	5.731757	7.486025	7.837808	5.521947	8.374973	10.208469	8.995047	12.749657	9.091411	8.227204	9.760731	10.450445	7.209083	6.250630	11.351898	7.347533	8.088467	9.761038	11.267512	8.017690	8.903553	10.756929	10.326281	9.621515	7.861521	7.107267	11.757241	10.362375	8.315758	10.414186	6.854771	8.221296	6.827174	5.836646	10.550253	6.763766	6.881192	6.853796	6.818791	10.617450	9.895820	10.172011	6.353267	10.707761	7.347027	9.785196	10.582460	9.351240	7.164573	7.582731	11.336132	8.335259	8.476502	10.406784	10.852247	13.650975	8.731790	12.709993	10.244280	7.808635	11.505545	6.466680	7.830502	6.006436	7.784873	5.524971	8.379067	7.115031	6.138229	8.827765	8.606504	9.771758	7.959450	7.232439	5.698593	8.638648	8.115622	7.324066	10.515001	5.362905	5.554340	6.928293	6.796536	6.789577	8.806738	10.931284	8.241455	10.774339	10.671196	10.830760	6.929852	11.842648	7.165196	8.077123	8.800210	7.464806	7.237820	7.388729	9.284015	7.181406	8.312823	10.062277	8.972184	10.649536	9.693739	9.663280	9.512761	10.441702	10.281837	10.117464	6.494926	9.725305	9.869780	5.969157	7.220994	10.955728	8.714674	7.295374

HS6ST3	3.996401	3.427441	3.467086	3.602837	3.558141	4.755113	4.034080	3.706665	4.141298	3.938998	4.669502	4.271401	4.475377	3.829065	4.089608	3.400726	3.664851	3.537427	4.174012	3.433628	4.088287	3.516425	3.045889	3.291907	3.311317	3.828244	3.939217	3.810767	3.804926	4.513363	3.715250	3.934099	3.893535	3.800298	4.085922	3.996193	3.953545	3.172139	3.703060	4.275527	3.849284	3.802663	3.605404	3.892863	4.155406	4.748287	4.757595	3.400057	3.933476	3.725577	4.074728	3.104004	3.661019	3.852717	3.483534	4.019554	3.795069	5.274315	3.984979	3.956373	3.762370	3.967464	4.459073	3.521300	3.896283	3.695698	3.901276	3.755678	3.855934	4.869173	4.002553	3.863876	4.182119	3.713806	3.905736	3.398852	4.365486	3.796132	3.791654	3.593829	3.700373	3.937514	3.159437	3.646429	3.868783	3.196929	5.696022	3.556393	4.408014	3.358308	3.218278	3.439595	3.886748	3.686638	4.065635	3.786278	3.554680	4.244727	3.612759	4.605235	5.230355	3.812109	3.997446	3.665678	4.322275	3.962694	3.331586	3.628614	3.976335	3.536283	3.878927	3.179520	3.517979	4.198815	3.669919	7.348953	3.444367	3.561768	3.964222	5.369821	3.859999	3.812518	3.434340	4.002996	4.262886	5.006189	3.793159	4.474945	4.535232	4.904203	5.527680	5.142569	4.171654	4.316078	4.117207	4.206158	4.203616	3.694034	3.223091	4.252367	3.562864	3.155076	3.609803	3.314962	3.679024	4.867022	3.557581	5.179925	3.937075	4.208643	3.796319	4.324651	4.697230	3.778157	3.932805	4.191471	3.900638	3.885958	4.020669	4.608853	4.734206	4.398284	3.975516	4.314526	4.124633	4.722818	5.192286	4.355913	4.770981	4.795393	4.538427	4.902368	4.831651	4.530309	4.141068	4.369123	4.249931	4.472347	3.670528	4.251298	3.893990	4.091630	4.419800	4.712842	4.469049	4.447792	3.899129	4.238991	4.772813	3.918319	4.175090	4.865788	4.356986	4.459073	3.911846	4.501376	4.317138	4.426343	4.198610	4.383385	4.485731	4.379938	4.189728	4.462233	5.221107	3.322813	3.685860	5.030756	4.023461	3.937294	3.891965	4.051282	4.072332	4.044969	4.268916	5.375758	5.318434	4.571814	3.859195	4.289411	5.001307	3.958117	4.104747	4.528407	4.134067	3.716703	3.610969	4.145791	5.009311	3.956373	3.380840	3.226440	5.014666	4.261553	4.779447	3.414177	4.744878	4.134067

PLXNB2	14.703436	13.582290	13.749826	14.155124	14.163659	13.984682	13.773212	14.060010	14.089539	14.105943	13.779388	13.869905	14.238141	14.336460	13.978080	13.995849	14.152064	14.257659	14.324402	14.038793	14.331005	14.591153	14.375159	14.159893	14.610567	14.350592	13.846674	14.255152	13.608281	13.573817	14.153935	14.738602	13.707975	13.926227	14.015842	13.913198	14.304308	14.118589	14.348197	14.675960	13.875238	14.873437	14.563854	14.217619	14.521454	14.291671	14.029080	14.110411	13.486814	14.126434	14.116761	14.250487	14.084344	13.573817	13.850714	13.775439	14.364475	14.730211	14.241084	14.628630	14.496768	13.836966	13.675077	14.713029	13.939615	14.028211	14.042877	14.371917	14.287271	14.358378	13.808908	13.774703	14.015289	13.914755	14.004466	13.748849	14.278941	13.884347	14.165208	13.956868	13.769304	13.636675	13.979424	13.958770	14.159585	13.816557	13.844292	14.517783	13.756638	14.132353	14.023893	14.035241	13.917771	13.826605	14.001811	14.025367	13.689737	14.375936	14.126130	14.225039	13.913967	13.905825	14.056258	14.165208	14.299472	13.836694	14.501067	14.989019	14.070375	13.843499	14.405128	14.268271	14.678334	13.865143	14.216003	12.692342	15.036300	14.139239	14.084661	12.947352	13.736365	14.165208	14.116167	14.178338	13.277438	14.236872	14.266657	14.880110	14.129527	14.300589	14.211747	14.551853	14.872324	13.936301	14.393289	14.070910	14.339964	13.597474	14.078902	14.189262	14.466587	14.070375	14.747454	14.231871	14.338198	14.195897	14.134512	13.872874	13.930021	14.082814	13.962742	14.957773	14.155411	14.291025	14.556424	14.142096	14.125506	14.448331	14.491542	13.973052	14.046144	14.695210	14.421778	14.654144	13.867259	14.241084	14.656363	14.162045	14.747454	14.233212	14.520691	14.025367	14.739028	14.587687	14.497164	14.372657	13.701651	14.089539	14.295055	14.504993	14.019845	14.275557	14.219249	13.988393	14.028776	14.487923	13.971862	13.809402	13.681855	13.878229	14.272202	14.185875	14.022985	14.441603	13.805428	13.868320	14.379907	14.638391	14.072700	14.646701	14.331365	14.581809	14.178943	14.179251	14.454664	14.162045	13.840596	14.046420	13.995561	13.061923	14.172267	14.252773	14.184052	14.102560	14.486353	14.174222	14.198488	13.775677	14.274870	14.336805	14.446741	14.760560	14.247254	14.237167	13.500424	14.509550	14.509168	13.861260	12.273457	14.270938	14.323669	13.270630	13.814789	13.288240	13.453090	14.779929	13.851196	13.842996

TPST1	8.494761	7.779314	7.622541	8.722201	8.930315	8.570650	8.164882	8.581371	9.086111	8.311463	9.014249	8.412020	8.378322	8.676661	8.124646	8.729427	8.804918	8.024837	9.990754	8.874966	9.317184	9.743792	8.549357	8.073965	8.222227	9.164789	9.008722	9.016068	8.280091	8.161158	10.169039	8.347074	8.862905	9.009822	7.854983	8.063817	8.343986	7.428367	8.568189	8.120379	8.063661	8.587856	8.278120	8.046403	8.000110	8.126763	8.662549	7.615127	8.290249	8.516062	8.146260	8.343986	8.285627	8.973923	8.092915	8.547074	8.317753	8.196850	8.444854	8.275673	8.685393	7.889199	7.891690	8.606818	8.533318	8.327809	7.696675	7.745467	7.553011	7.565945	7.794157	8.037595	8.431198	8.209472	8.390063	8.107430	7.766387	7.935134	8.322623	7.935134	8.306636	8.022249	8.181886	8.703075	8.329322	8.512760	8.508449	8.937952	9.157090	8.545954	8.121159	8.996563	8.526736	8.431198	8.699378	9.055670	8.242770	9.069592	8.593456	8.693560	8.574264	7.769734	8.618776	8.471373	8.407253	9.115266	9.464238	8.746205	8.552881	8.558851	9.326153	8.899620	8.797944	8.215914	8.736262	6.373651	9.335710	8.736112	9.371801	9.101487	8.741172	6.955501	9.110179	8.079921	8.073176	10.202314	10.911465	11.243746	8.272834	11.433749	9.063687	11.193953	8.374334	10.334168	10.989151	9.395272	8.918624	8.996270	9.034153	9.492065	10.873089	8.057990	7.595483	8.085541	9.209780	8.639584	10.356139	10.663706	11.068675	10.131092	8.630044	8.470408	8.158525	7.862754	7.915502	7.631455	7.665421	10.913576	9.803956	8.337894	9.293482	8.498349	9.028257	9.876562	8.517642	8.800662	7.184381	7.511709	11.047235	10.950287	8.764379	7.921935	12.075438	7.941974	9.417014	9.350620	7.960216	9.122611	7.903984	8.755176	9.623069	11.715283	8.057282	9.306026	9.168451	10.628223	8.044341	7.608529	8.063661	7.422439	8.296548	8.763780	9.985950	8.246193	7.788507	9.334765	8.559612	10.122304	7.921791	8.369861	8.841804	8.728213	8.595951	8.305140	8.937335	8.145338	9.233328	9.497504	9.013164	8.921205	10.202155	9.816047	9.642061	11.007562	10.775641	8.394685	9.607163	9.302664	7.883682	8.314984	9.884840	9.004108	7.423039	10.248163	8.328604	11.022131	10.624726	7.544875	8.143298	8.039489	7.107267	6.051477	8.462629	6.969499	8.289633	10.825019	10.679584	10.065569

ASB11	10.217724	7.112523	6.664782	7.041908	8.070916	8.066110	7.618627	7.595034	7.492895	8.141644	7.504305	7.868475	7.097035	7.337505	6.647547	7.568557	7.220008	7.409429	7.075602	7.142250	6.896050	7.581800	7.745146	7.359946	6.799796	7.510154	7.948894	8.463529	8.066676	8.158860	8.092915	6.859944	7.345011	7.325643	7.587802	7.147559	6.844707	6.692425	6.754404	7.079100	8.038363	7.104405	7.166708	7.290335	7.276110	8.594879	8.828392	7.335828	7.557235	7.317336	7.099707	6.959873	6.546478	6.766660	6.871129	7.850948	6.287255	6.053214	6.378810	6.463376	6.312644	8.099298	6.609648	6.780704	6.468683	6.414695	6.724980	6.762817	6.759442	7.115031	7.306107	7.235310	7.877792	7.099873	7.271258	7.366550	6.985241	7.101269	7.209237	6.867559	6.824346	6.485460	7.841085	6.429182	6.629846	6.699651	6.336858	6.805681	6.390861	6.210351	6.348609	6.696813	6.972755	7.112374	7.558478	6.888075	6.896873	7.459019	8.634259	7.285048	8.172546	6.952479	7.187166	7.132283	7.230711	8.938239	7.997387	7.708703	7.311495	8.175989	7.711542	7.231664	6.841442	8.186342	8.044966	10.655784	9.968341	7.444912	7.954949	8.384200	7.812244	7.674327	7.946342	9.226054	8.307762	8.824932	7.942940	7.654357	8.946600	8.527674	8.514154	8.195107	8.170959	8.020288	8.129022	7.340176	8.031633	7.877349	7.623954	7.724062	7.949845	7.104714	7.931730	8.134516	7.875503	8.227519	8.546267	8.782749	8.567616	7.867408	8.317157	8.175380	7.825066	8.169398	7.696523	7.563944	7.976636	8.138022	7.817308	8.688665	7.803104	7.356249	7.607430	7.382198	8.932696	9.526012	7.977373	7.890928	7.476608	7.755152	7.541602	6.885672	7.255390	7.243750	8.546584	7.122109	7.322799	7.070554	7.020771	7.527936	8.858058	6.693444	7.137804	7.487725	7.149287	7.973059	7.247314	7.354709	7.943715	7.787317	7.775107	8.700599	7.661380	7.688567	7.610523	7.548388	8.206780	7.506195	7.341605	7.193734	7.032798	7.103141	7.486938	7.193586	7.848092	7.100946	7.483531	7.632690	6.908452	7.332899	7.390711	7.397868	7.930670	7.971157	8.178859	7.889831	8.463077	8.175380	7.539738	8.170810	7.292562	7.876282	7.619865	7.339407	9.402928	8.320503	7.334421	6.806159	9.043152	8.069994	7.589490	7.551590	8.736112	9.209780	8.978925	7.183448	7.879916	8.535756

IL13	7.120590	7.802054	9.016942	3.006886	3.485185	4.326994	3.204548	3.190259	3.818633	3.417386	4.271805	3.350579	3.718738	3.671565	5.746099	3.465394	3.246827	2.984243	5.018300	4.385189	4.262444	4.234619	3.575730	3.837575	3.088004	2.994863	4.955010	2.960243	3.554492	5.794218	3.678826	5.470448	4.813130	5.599318	8.396552	3.973745	3.347388	3.268982	3.366167	3.502755	3.468909	2.965270	4.764207	3.194444	5.373801	5.190727	4.369123	8.572024	5.626914	5.610093	2.860671	4.386959	5.286594	4.148755	8.119314	3.124783	8.305897	5.214626	3.832712	3.975516	3.325163	5.089194	12.429308	3.619528	6.030085	5.356651	3.434507	3.494265	3.614384	2.876167	3.082761	3.191743	3.223626	4.230073	3.648190	6.209329	3.905736	4.448013	3.653615	7.223728	3.325488	6.219210	3.447839	5.113938	5.748312	3.610373	3.069240	5.655195	5.669251	4.104747	4.571992	5.076314	4.482874	5.861216	6.747722	5.945684	9.035125	3.581900	3.838432	3.067954	3.011939	4.109814	3.524981	3.536283	4.632083	4.689517	3.625812	3.282710	3.427251	3.138106	5.048505	3.473087	2.937314	3.234074	4.692443	3.094426	3.993280	3.984979	3.389637	6.386455	5.462582	5.484060	4.088092	3.256478	6.173023	3.239740	3.152335	3.949658	3.605770	4.061298	4.231567	4.437866	4.019991	3.231727	3.378073	3.444894	2.878713	7.223425	5.022964	4.464978	5.996198	6.885835	3.755267	2.942671	4.200518	4.249061	3.358469	3.098076	5.472912	3.913757	6.883579	4.126337	3.848851	5.059824	4.484173	4.593634	3.418786	3.382584	5.816919	3.673284	6.520703	5.251944	4.358295	4.374352	4.801148	4.743795	7.217959	4.146648	5.388935	6.337033	5.531254	5.899028	5.254943	5.664974	3.440832	4.325528	8.144245	3.652825	4.023692	5.338729	4.278634	4.845532	4.732302	5.929339	4.381821	5.599151	5.877095	4.603540	4.950017	3.242519	5.419794	4.036699	3.660010	4.593634	5.821680	5.118456	3.600877	3.467279	4.130630	4.846376	4.990613	3.840468	5.809052	6.660184	3.303625	5.092275	6.502026	5.798946	4.983584	4.248826	4.839616	3.656838	5.032949	5.591589	4.203616	4.627160	3.925461	4.141946	3.888075	3.984336	3.189156	4.749126	3.969874	5.231197	5.749370	4.630030	5.916363	5.470077	3.690559	5.435714	3.126509	2.886566	3.516064	3.723107	4.478041	4.602914	3.853814	3.803066

TICRR	6.211381	7.441605	8.684010	8.436316	8.258487	8.516533	8.076511	7.351914	8.685838	8.340263	8.080215	8.334148	8.150655	8.811703	7.825209	7.521137	8.115759	8.130096	8.081192	9.197337	7.529626	8.013547	6.132918	7.398506	7.972576	8.372685	7.313859	6.442684	7.232277	7.376415	8.508449	8.497923	8.057849	8.161308	8.444402	8.935159	8.290249	8.197159	8.909943	8.159927	8.584839	7.453233	8.696372	8.259705	7.885206	9.107805	7.822865	7.478735	7.830357	8.183407	7.966965	8.438720	8.135109	8.454138	7.225827	8.481866	8.293216	8.078556	8.292759	8.512159	8.091263	8.228709	6.732226	8.288257	8.147162	7.197133	8.717346	7.270940	8.567166	7.358559	7.675589	7.828836	8.246193	7.678105	8.286715	7.792182	8.205229	7.333384	8.545084	7.407121	8.367762	8.853185	8.527214	8.792140	8.518570	7.634654	7.935298	8.556858	7.916296	8.197327	7.140467	7.376584	7.606999	9.023413	8.162959	7.980027	8.892055	7.957304	8.241887	8.042654	8.353532	8.382834	8.278420	7.547593	8.043864	8.221915	8.502791	8.250212	7.817478	8.420244	8.226166	8.806738	8.262136	8.789635	7.942176	7.368131	6.650152	8.096900	7.915057	9.115266	8.117316	9.819984	8.260645	8.905662	9.241363	9.002579	9.420683	10.371545	9.534899	8.940204	9.620259	7.898055	8.987020	7.887351	7.570519	9.629681	9.463923	8.948718	9.447475	8.797797	8.527523	9.678939	8.443781	9.117099	8.701974	7.488362	8.942487	9.518843	8.890976	8.021201	8.975575	9.674122	8.990911	10.405917	10.405917	7.553935	9.496877	8.743011	8.781348	9.199378	8.721334	10.187555	10.380918	9.715628	9.566202	11.130031	9.757480	8.746513	9.786827	9.893623	9.835522	9.055523	9.228039	8.691086	9.073136	8.339181	8.985474	8.659450	10.306115	9.071115	11.223559	10.270720	8.414441	8.361249	7.741021	10.219377	8.596272	9.646315	8.418107	8.943847	9.022037	9.322286	8.835236	8.330440	8.596272	8.518854	9.415969	9.421602	7.327208	9.226210	10.022905	8.940654	8.654920	10.285451	9.186435	8.840579	10.215042	8.694336	8.272834	7.532056	8.924459	8.453383	9.021121	8.628231	8.776601	9.689437	9.640265	8.589431	8.289790	9.780402	10.948912	7.190316	7.542332	9.376353	8.613906	11.291005	10.469359	8.253736	9.194184	8.425261	9.285069	8.533021	7.625330	8.436936	9.095402	8.408039	7.564569	9.420526

KIF7	6.211381	7.441605	8.684010	8.436316	8.258487	8.516533	8.076511	7.351914	8.685838	8.340263	8.080215	8.334148	8.150655	8.811703	7.825209	7.521137	8.115759	8.130096	8.081192	9.197337	7.529626	8.013547	6.132918	7.398506	7.972576	8.372685	7.313859	6.442684	7.232277	7.376415	8.508449	8.497923	8.057849	8.161308	8.444402	8.935159	8.290249	8.197159	8.909943	8.159927	8.584839	7.453233	8.696372	8.259705	7.885206	9.107805	7.822865	7.478735	7.830357	8.183407	7.966965	8.438720	8.135109	8.454138	7.225827	8.481866	8.293216	8.078556	8.292759	8.512159	8.091263	8.228709	6.732226	8.288257	8.147162	7.197133	8.717346	7.270940	8.567166	7.358559	7.675589	7.828836	8.246193	7.678105	8.286715	7.792182	8.205229	7.333384	8.545084	7.407121	8.367762	8.853185	8.527214	8.792140	8.518570	7.634654	7.935298	8.556858	7.916296	8.197327	7.140467	7.376584	7.606999	9.023413	8.162959	7.980027	8.892055	7.957304	8.241887	8.042654	8.353532	8.382834	8.278420	7.547593	8.043864	8.221915	8.502791	8.250212	7.817478	8.420244	8.226166	8.806738	8.262136	8.789635	7.942176	7.368131	6.650152	8.096900	7.915057	9.115266	8.117316	9.819984	8.260645	8.905662	9.241363	9.002579	9.420683	10.371545	9.534899	8.940204	9.620259	7.898055	8.987020	7.887351	7.570519	9.629681	9.463923	8.948718	9.447475	8.797797	8.527523	9.678939	8.443781	9.117099	8.701974	7.488362	8.942487	9.518843	8.890976	8.021201	8.975575	9.674122	8.990911	10.405917	10.405917	7.553935	9.496877	8.743011	8.781348	9.199378	8.721334	10.187555	10.380918	9.715628	9.566202	11.130031	9.757480	8.746513	9.786827	9.893623	9.835522	9.055523	9.228039	8.691086	9.073136	8.339181	8.985474	8.659450	10.306115	9.071115	11.223559	10.270720	8.414441	8.361249	7.741021	10.219377	8.596272	9.646315	8.418107	8.943847	9.022037	9.322286	8.835236	8.330440	8.596272	8.518854	9.415969	9.421602	7.327208	9.226210	10.022905	8.940654	8.654920	10.285451	9.186435	8.840579	10.215042	8.694336	8.272834	7.532056	8.924459	8.453383	9.021121	8.628231	8.776601	9.689437	9.640265	8.589431	8.289790	9.780402	10.948912	7.190316	7.542332	9.376353	8.613906	11.291005	10.469359	8.253736	9.194184	8.425261	9.285069	8.533021	7.625330	8.436936	9.095402	8.408039	7.564569	9.420526

ARHGEF16	11.784937	10.907526	11.334799	11.219233	11.337151	11.302774	10.728981	11.225024	10.701804	11.025555	11.381146	11.454294	11.364157	11.968413	10.927267	10.593479	11.286424	11.597416	10.936398	11.178849	11.343233	11.106894	9.706727	11.194728	11.248223	11.878918	9.931531	11.071993	10.389743	10.476474	11.286099	11.366409	10.905549	11.432097	10.560870	11.010941	11.119136	10.937605	11.455518	11.407214	11.267354	11.288902	10.476950	10.830911	10.546111	11.348793	10.579925	11.587163	11.059091	11.279592	11.117248	11.290706	10.563692	10.911326	11.207480	10.961776	10.908696	10.361904	11.359007	11.689592	11.376076	10.316237	9.281361	11.548287	11.806792	11.925820	11.813291	10.821454	11.643493	11.075469	10.812246	10.799656	10.965182	10.647591	11.205234	10.655171	11.302774	10.534405	11.444404	11.289319	11.410105	11.408298	11.824443	11.477701	11.136838	11.246511	11.450138	11.317325	11.031633	11.520848	11.927550	11.581405	11.618620	11.346810	11.083497	10.811375	10.533656	10.855104	11.084092	10.028586	11.195473	10.603262	10.575556	10.365458	11.084241	10.466534	12.425406	11.703565	11.129723	10.803437	10.906146	12.325562	12.290138	11.130181	11.172547	9.498874	12.417994	11.140921	10.376671	10.725359	10.675241	10.895178	11.000467	11.498149	10.971457	11.164195	10.473914	10.074685	11.324962	11.480371	10.508722	11.497855	10.620851	12.245922	9.815427	12.008706	10.820438	10.539729	11.087512	11.029110	10.122918	10.793806	12.326190	11.190179	9.884613	10.987934	11.512897	10.429132	10.083270	11.948032	10.722521	12.553295	10.659191	10.167210	9.982592	9.854621	10.927410	10.959734	12.277746	10.165338	11.624862	9.731977	10.300344	10.986908	12.298697	10.193392	10.881053	11.808281	11.512020	10.847257	10.489519	11.857830	11.078552	10.647591	9.994317	11.342331	10.029801	11.191787	10.674348	11.959449	10.570769	11.981061	11.337604	12.591170	10.783617	10.774830	10.802701	10.780585	9.979804	10.186361	9.931101	10.809338	11.598287	8.761378	9.551318	9.347857	10.747238	12.013873	9.590587	11.321435	11.696238	11.418410	11.417687	10.968006	12.580980	10.755416	9.772353	10.064516	9.953197	11.179288	11.008580	10.897486	11.261529	9.271214	10.276224	10.804897	10.766574	11.315577	10.284731	10.800421	10.116680	10.316793	9.755758	9.320329	10.258038	11.356674	12.244222	11.383354	11.563461	9.316100	12.546528	9.959089	10.437507	11.118854	9.934938	11.339812	11.464200	10.334644

RET	8.714198	7.251697	6.804922	6.752888	6.641302	7.197292	7.042723	7.930670	6.826891	7.618474	7.709277	7.216228	6.884082	6.885519	6.452936	7.379718	7.362762	6.442684	7.713118	7.118214	5.837134	6.748634	9.589153	7.694590	7.071013	7.156988	6.890763	7.263889	6.947121	6.930332	7.067588	7.530678	7.395059	6.831799	7.327400	6.808714	7.394607	6.369386	6.941182	7.076205	6.890454	7.458863	6.778938	6.084753	7.212144	6.726070	7.537013	6.646901	7.277201	6.365363	7.592412	7.515537	7.783157	7.369686	6.648197	6.949978	7.187943	7.269702	7.063971	6.269124	7.535941	7.458563	9.294591	7.218571	7.463106	6.979258	8.029783	6.850930	6.760608	6.932872	7.047214	6.903209	7.458563	7.507556	7.101416	7.019693	7.036232	7.636928	7.819040	7.222406	7.233534	7.776826	8.120236	7.144913	6.996467	7.124824	6.863627	7.185369	7.985002	7.393665	8.066831	7.587483	7.045129	6.792518	6.864858	6.569361	6.593107	6.689803	7.354709	6.617262	6.334164	6.243620	6.392873	6.749427	7.135309	7.107267	7.774461	7.176647	6.604561	7.476608	7.342373	6.867257	6.276695	6.914480	7.124664	8.114718	8.056204	7.257882	7.494271	6.810647	8.087867	6.654347	6.547740	6.631395	6.670564	7.310410	6.348278	6.284973	6.661341	6.916498	7.542183	5.899713	7.341925	7.239898	6.826234	7.593195	5.399206	7.062837	8.172842	6.090277	8.529222	8.367911	7.022169	8.954868	9.193041	6.207946	5.646696	6.728667	6.185384	7.719927	8.141476	6.181161	7.211670	8.279636	6.339728	8.054016	6.959726	6.710559	6.655979	6.587534	6.968370	5.554340	6.291862	7.409576	7.286286	7.091816	7.822865	8.630218	6.743667	7.542797	7.749492	7.850493	7.354709	7.166559	6.531764	7.443772	9.125977	7.017298	7.695913	10.773291	7.361058	6.790858	7.281623	7.916768	7.246849	9.630301	6.934413	6.454306	6.803991	7.520020	6.665395	9.028257	7.549434	7.284141	6.743835	7.908479	7.201468	7.006419	9.105505	7.721466	7.649509	9.355444	8.587391	8.408218	6.388007	8.365016	6.599421	9.750211	8.466427	11.466592	8.555262	6.812404	8.071082	7.005651	9.102118	6.616765	6.103928	7.049113	6.806475	6.345229	5.714005	7.835236	6.111142	9.618556	6.892352	8.045289	7.113933	7.030189	7.270643	10.097946	7.536099	5.476169	6.631246	7.706860	7.499939	6.909549	9.403086	5.902022

C5AR1	8.248548	9.874083	9.511815	6.611997	6.240938	6.341563	8.052496	3.748721	5.335758	7.102025	6.288952	6.263683	7.247454	7.275303	7.737739	5.884779	6.054258	6.606041	6.393047	6.580113	5.451663	8.060100	7.659241	6.683070	8.472666	9.558482	8.888851	6.955198	7.964808	11.398667	5.854171	9.088689	9.083496	8.734549	8.922699	8.443781	8.900076	8.470095	7.462140	7.665900	6.783375	7.978468	8.309911	7.058536	8.735952	8.519918	7.874141	8.795667	9.607630	8.685533	8.628077	9.298453	10.018023	8.374334	10.237289	8.172842	10.146866	8.391140	7.164423	8.837390	8.186986	8.908646	11.871295	7.099426	9.786613	9.333386	7.590584	8.137408	8.590359	8.070314	8.240396	7.726063	6.799110	8.799601	8.069834	9.769058	8.619099	10.530395	8.104985	10.164279	8.999654	10.362213	8.501222	8.734085	8.706747	7.991593	9.230147	8.995798	8.667886	8.381126	7.766856	9.792563	9.406930	9.755615	9.207632	8.338861	9.855874	7.561362	7.652974	6.678634	7.413675	7.406331	7.531584	7.536246	8.092006	7.617095	6.617562	7.327894	7.661235	6.052691	8.024380	5.143570	5.883706	5.267688	6.877809	4.321207	7.846711	7.065228	6.294060	9.098445	9.873647	10.278824	8.652644	8.572461	8.671736	8.923868	6.001794	7.578077	8.576701	8.196235	7.825370	8.795507	10.792913	9.600956	7.368131	8.199029	7.980493	9.693584	8.820691	11.241643	10.884311	10.459530	9.592440	9.110639	12.451069	8.518854	8.622822	8.028251	11.360757	9.681108	11.201757	10.144003	9.509159	9.549146	8.516771	9.997492	9.823607	8.115905	10.760711	8.068759	10.387032	9.318499	9.384364	10.062755	9.284296	8.258639	9.088082	8.397333	9.956464	10.161998	9.586696	10.482307	10.318616	11.044022	10.306254	9.817268	9.620568	9.381478	9.881018	10.417667	10.652524	10.018488	10.480956	10.561314	11.063499	9.464087	10.327627	10.234170	11.459062	9.238600	9.896130	7.644771	9.989806	9.738216	11.103722	9.945591	10.469045	8.999362	10.421173	9.720786	10.337633	9.377622	10.873702	10.362833	8.473263	10.398977	10.067514	10.242137	11.177032	9.173279	10.498577	9.317025	10.216098	10.120815	10.147476	9.078557	8.514597	9.173745	8.767446	9.088082	7.458863	8.013058	10.939339	9.867722	9.499476	7.274556	10.242137	10.283384	8.528442	10.525043	9.315818	5.584588	6.533555	7.817798	8.860765	10.348574	7.809583	9.365510

NUP153	11.740264	12.614854	12.219378	12.232993	12.157572	11.900148	12.358457	12.184698	11.783278	11.892609	11.766473	11.730335	12.012048	12.126571	12.310761	12.065315	11.562888	12.162658	12.198897	12.253272	12.026457	12.290442	11.990038	11.993346	12.244688	11.829732	11.147308	11.673108	11.276591	12.285365	11.720169	12.036847	12.242384	12.565127	12.213629	12.217449	12.202831	11.959600	11.761613	11.898984	11.959299	11.235980	12.505403	12.559575	12.535268	12.477840	11.423308	12.112064	12.383064	12.692858	12.241581	12.618117	12.508357	12.348370	12.616658	12.307156	12.412844	12.315273	12.109351	12.216235	12.154206	11.916216	11.659862	11.875072	12.251209	12.809469	12.264825	12.130685	11.974947	12.026746	12.155411	11.999458	11.561877	12.346785	12.147801	12.377877	12.466325	12.135121	12.043959	12.426853	12.834696	13.060802	12.705525	13.073608	12.499786	12.212707	12.437743	13.019562	12.707731	12.495176	11.961308	12.502885	12.584123	12.662573	12.095201	12.338888	12.362103	11.486562	11.880269	12.091683	11.984395	12.122102	12.043045	12.061828	12.396622	12.074208	11.602546	11.882808	12.440302	12.127344	12.516304	10.891184	11.832552	11.340687	12.226464	11.408892	11.269542	12.338259	11.472082	12.530023	12.751206	12.046024	12.743876	11.833609	12.455171	12.430448	12.753644	11.296591	12.685688	11.204776	12.829284	11.980630	12.632301	12.706715	11.715575	11.836176	11.921595	12.937622	11.684797	12.851592	13.152020	13.420156	12.021646	12.282101	12.422968	11.929077	12.171876	10.302784	12.508357	11.768847	12.346315	11.378139	12.676852	12.971329	12.561379	12.169723	12.097725	11.929077	12.031376	12.744026	11.331326	12.483899	13.191256	12.657484	12.889319	11.859940	12.435325	12.195094	13.000815	13.081212	13.475946	12.582791	12.676682	11.748921	11.728395	13.057096	13.002534	12.139271	13.232466	12.181582	11.434198	12.370033	12.397534	12.299854	13.022716	12.319676	11.826672	12.407767	12.462229	12.301709	13.004475	11.985408	12.457084	11.873097	12.513691	12.115840	13.133580	12.484062	12.082750	12.254526	12.880949	12.775824	12.132968	12.776381	11.696238	12.607071	12.632463	12.103128	12.178954	13.268237	12.346785	12.278351	12.815618	13.298159	12.356573	12.229570	11.208645	12.374199	12.311242	11.508051	12.948751	11.634043	11.726750	12.435325	12.336035	11.497278	12.339350	12.087710	11.804689	12.483749	10.809187	12.900773	11.526932	12.752950	13.005956	11.869049	12.577039	12.072035

CCN6	4.551538	6.859613	3.861486	5.194061	4.030852	6.462351	3.607835	3.783771	3.946856	4.024123	3.597156	6.230070	8.419769	4.401336	4.861046	5.020938	3.546357	4.354817	6.672528	5.761773	4.894383	5.370703	5.792377	4.847873	4.766823	4.144295	5.459635	6.477105	5.840599	4.347616	3.703237	4.998590	5.495472	7.743500	5.603614	6.001621	5.004978	6.002186	6.085604	3.848186	3.738525	7.761133	6.016723	4.498125	3.893990	5.057355	4.045411	7.443000	6.026589	5.876566	7.259735	5.252144	5.739713	4.830410	5.992161	3.835027	5.339320	3.060259	6.942632	5.008684	6.098721	3.795069	4.255667	5.595490	9.082889	6.843859	7.357647	4.876299	7.251051	4.948729	5.506814	4.702563	4.101695	5.605914	4.251723	3.897785	4.432464	8.022991	7.210041	3.858138	3.859401	2.845345	7.327208	6.284143	3.969874	6.549020	3.072674	3.889150	6.332154	7.019207	7.557235	4.240972	4.992449	7.974469	7.835236	3.685623	6.542379	4.186652	5.140892	3.745894	3.470503	5.167290	4.708061	4.416652	5.399603	5.377702	5.590242	3.397284	3.306975	4.711373	4.890226	5.940958	3.986069	4.339197	4.482420	5.759262	5.135616	4.599394	4.183639	4.774499	4.730610	6.866291	8.386497	4.404200	5.948433	7.818413	4.560560	7.234047	3.180263	6.404233	3.747497	3.121463	3.273130	3.604453	8.809382	3.463379	3.450610	8.786567	5.911679	4.218585	3.133555	5.703574	3.801826	7.411494	2.892982	9.095402	6.676729	7.871375	4.048661	7.539270	7.691812	4.386303	6.692925	4.194980	4.274425	7.057440	8.676360	4.691187	3.640310	7.103286	7.752712	4.649179	11.093554	4.942813	4.845952	9.032354	4.701302	4.856270	4.460696	7.096414	9.435543	5.452837	4.343705	7.563478	3.736675	11.278862	7.717814	9.988415	5.240060	2.967728	6.204695	5.113751	9.632756	6.120803	4.718124	10.550253	7.148490	8.621889	8.184487	4.976495	6.196904	7.441605	10.396131	5.006627	3.633323	3.676812	3.203743	4.129377	9.130166	5.735202	8.829658	9.720646	8.595641	7.201315	3.697132	3.953135	3.975516	11.128092	6.712466	4.014061	9.844876	4.922760	5.216032	3.808070	8.635528	3.475268	3.768624	5.291194	3.244699	2.968811	7.046103	9.473694	3.565517	11.575238	5.460459	8.875559	3.435989	3.286382	9.269079	3.561948	6.929852	3.018930	6.005381	2.984859	10.020947	8.550896	8.701371	3.409363

ATXN3	10.310657	10.658893	11.061273	10.652947	11.097335	10.351116	10.800743	10.085546	9.747720	10.220346	10.172908	10.233109	9.919156	10.431723	10.615704	11.438806	9.919470	10.626987	10.403949	10.879596	11.112884	10.864986	11.854007	10.679432	11.299661	10.631731	9.880868	10.727170	10.219377	10.285451	10.003818	10.663706	11.106454	10.529664	10.483377	10.695129	10.630981	11.033609	10.757072	10.961076	10.718513	11.162013	10.250726	10.479650	10.700919	10.807528	10.682874	10.369707	10.944920	10.680668	10.286961	10.791439	10.604589	11.064957	10.215794	10.405008	10.761000	11.123360	10.895043	10.724471	11.274882	10.748441	11.014430	11.025099	10.837527	11.082183	10.967244	10.698520	10.595877	10.690696	11.001368	11.126207	11.358695	11.256056	11.123230	10.846519	10.572908	10.555984	10.638038	10.646346	10.524883	10.676585	10.497682	10.947309	10.943027	10.851641	10.854485	10.936835	11.022131	10.639852	10.776975	10.548435	10.664468	10.467258	10.317425	10.767880	10.821608	10.468588	10.161839	10.275187	10.004105	10.635198	10.465042	10.709515	10.584201	10.296016	10.630402	10.202155	10.855244	11.049127	11.825777	10.401702	10.049101	10.978192	10.457422	9.431578	9.992805	10.389118	10.382254	11.091598	10.870211	10.456786	10.827110	9.478136	9.893168	10.102022	9.704017	9.726052	9.424072	10.076508	9.572739	9.503319	9.113587	9.990578	10.495424	10.326429	9.750518	10.171241	9.948435	10.214105	9.742594	10.122304	11.191198	10.560245	10.364435	9.659506	9.915155	9.106435	10.156673	9.816348	10.264395	10.587449	10.206147	10.779848	10.620438	10.576019	10.771942	11.067181	11.003511	9.050390	10.674499	10.034692	10.380759	9.922889	10.820288	11.095294	10.154668	10.799656	10.434121	10.300015	10.726741	9.966953	11.485786	10.812838	9.957369	10.835875	10.301733	10.698830	10.140152	9.932497	10.847257	9.624150	10.434742	10.565322	10.904224	10.559632	10.760252	9.958930	10.497395	10.864236	10.786766	9.485177	11.883726	10.617007	10.320132	10.822656	10.910445	10.690535	11.033295	10.193874	10.843477	10.257400	10.336744	10.732241	10.411931	10.571684	10.723285	10.926544	10.184062	10.223453	9.908039	10.157573	10.034852	10.137745	10.383339	10.300168	9.700346	9.908813	10.001946	9.912200	10.492517	10.599413	10.459258	9.905282	9.793010	9.771286	9.979650	9.617944	10.239265	10.908098	9.677379	8.483412	10.358719	10.049407	9.432202	10.245587	11.018683	9.778769

PPFIA4	9.841975	8.841504	8.821736	9.404526	10.038406	9.734090	8.549357	10.583626	10.663131	8.863223	9.584523	9.494236	9.766931	8.887442	8.847228	8.796126	10.177628	8.965087	9.872421	8.510905	9.787289	9.301968	10.605180	9.175115	9.259376	10.111998	9.454320	10.085231	8.949362	9.484419	10.327332	8.101896	8.318352	8.289633	8.306486	8.540210	8.637239	8.775212	9.146408	9.283859	9.243810	9.246132	8.561770	9.093582	8.536401	8.791520	10.458339	8.735320	8.777234	8.337585	9.023274	8.448278	7.957732	8.390673	8.376183	9.873938	7.976636	8.655960	8.777381	8.656246	9.214667	9.262594	9.414400	9.385126	9.060362	9.239986	9.104276	9.096009	9.023274	9.797808	8.702430	8.683701	9.323358	8.527674	8.612329	8.989269	9.044099	8.898403	9.248899	8.203158	8.414001	8.567308	8.478982	8.401595	8.574500	8.537385	8.722201	8.955928	9.295062	9.345366	9.201856	9.973866	8.740710	8.998256	9.983675	8.714025	8.534396	8.384497	10.058476	8.873102	9.819337	8.305437	8.638802	8.956987	9.215611	8.913424	9.937905	9.254659	10.301888	9.198113	10.358105	10.143394	9.897496	10.104790	9.759333	12.493360	10.072505	9.418110	9.539221	9.200174	9.666389	9.534750	9.447310	11.554015	10.277457	10.103567	10.010461	9.642850	9.783615	10.132299	10.138352	9.651799	9.331061	10.302038	9.247657	9.789131	9.520066	10.075727	8.568651	10.270009	9.668144	10.638180	10.113907	9.837964	9.517769	10.033321	10.880034	10.786473	10.278058	10.176378	9.027842	8.729147	9.161830	8.637074	8.780167	8.713880	9.641171	9.621515	9.627096	10.803584	9.597725	8.478049	9.813065	8.674683	9.206719	10.912940	8.696372	8.742397	8.833772	9.208246	8.643945	8.282999	8.645568	8.426929	9.138437	8.221296	8.553172	9.014694	8.717495	9.499324	9.051075	9.546241	9.503777	9.405273	9.312017	9.595225	9.406320	9.688696	10.072673	9.097041	9.110466	11.877752	8.959927	8.858496	9.085022	9.215920	9.290484	9.440966	8.349433	8.395927	8.908646	8.853185	8.541489	8.809674	9.518073	8.992086	8.824012	9.407719	9.304406	9.269539	10.364578	9.123809	9.479371	9.676894	9.754870	9.668932	9.824636	9.529145	8.490290	10.117147	9.106902	9.317338	9.249046	9.150640	9.474607	10.011226	9.259991	9.830772	9.898827	10.401855	10.184516	11.736404	12.549408	10.797753	10.579659	9.384220	9.500568	10.209367

TNKS1BP1	7.379095	7.475528	7.854375	7.635247	8.409992	8.109379	7.520987	8.686736	9.073896	7.671486	7.583498	8.120075	7.776338	7.398827	7.969565	8.112533	8.507811	7.833385	8.374648	7.797234	7.807549	8.120236	9.408176	8.213942	8.343224	8.109664	8.797797	8.960680	9.029335	7.866800	8.592526	7.321996	7.018597	7.602325	7.405681	7.514494	7.568557	7.887812	8.025123	7.913282	8.180074	7.819523	8.024072	7.959136	8.026217	7.212810	8.437550	7.717058	7.992704	7.398506	7.189556	7.077646	7.084584	6.699191	7.000485	8.692954	6.702584	7.635566	7.449679	7.052261	7.321681	9.903186	8.451859	8.170959	7.331016	7.552684	7.540067	7.749351	7.360429	8.053534	7.739464	7.652521	8.551804	7.633475	7.365622	7.728577	7.875819	7.679960	7.984078	7.717220	7.723319	7.479497	6.922885	7.481512	7.326749	7.651452	7.278770	7.391165	7.725434	7.340836	7.246690	7.851877	7.948757	7.498661	7.567340	7.624575	7.393824	8.721757	8.526432	8.930315	9.159846	7.747337	8.257856	7.985306	7.632243	8.477574	9.059743	8.089558	9.002437	8.363460	8.511237	8.747736	8.485531	8.042654	8.594242	10.622929	8.782749	8.310361	8.376183	7.736225	7.728256	7.581340	7.766700	9.049602	7.535343	8.157947	8.836612	8.155622	7.588888	7.470168	7.699122	8.406370	7.961924	8.627316	8.635686	8.046717	7.744557	8.350817	7.980968	8.026061	8.130096	8.439018	8.919828	8.360784	7.743798	8.475262	8.352459	8.884828	7.267272	8.278584	7.974303	7.883516	7.713749	7.346434	7.718880	7.268627	7.865090	8.057711	7.991423	8.576545	7.814466	8.022249	7.997547	7.338621	7.094982	8.199201	7.733473	7.754268	7.406955	7.271730	7.215778	6.903372	6.828774	6.742430	8.363165	6.559425	7.443608	7.486025	6.913240	7.674175	7.838309	8.094184	7.824466	7.540217	7.720075	7.832761	7.477368	7.699442	7.789160	7.584419	7.723774	8.447805	7.415080	7.623470	7.833855	7.708703	7.833074	7.836741	7.756499	6.891419	7.233701	7.142250	7.698822	7.316219	8.259547	7.161880	7.517425	7.840776	7.639852	7.676043	8.160459	7.633323	7.558623	7.578233	7.712340	8.549073	8.570494	8.474935	7.965252	8.020708	8.043407	8.348402	7.652041	7.567340	7.842468	8.766546	7.618627	7.990179	8.234802	8.616473	8.534543	10.086530	8.121793	8.879106	8.833772	8.090649	8.181304	7.416551

RARB	4.858755	3.117261	3.028867	3.667710	4.060633	4.204520	4.340238	4.386303	4.021786	5.434567	5.250989	3.645431	4.256960	5.021782	3.601477	5.319749	5.631375	4.291335	4.745112	4.107628	4.139072	3.876342	7.077988	2.952746	4.902149	2.981438	7.227002	4.300892	4.451413	3.223626	5.811871	3.582455	5.383335	3.913564	4.496646	4.318868	3.625008	3.156291	5.100721	3.849489	3.366336	3.304924	3.405410	3.227451	5.558862	4.369566	7.833692	3.318310	4.473629	3.982847	3.988180	4.973787	5.026469	3.897372	4.854383	4.567032	5.158488	3.397123	3.728166	3.346951	3.742196	2.871436	6.832287	3.252362	3.141417	3.865160	2.953226	5.061593	2.953586	2.937218	2.958067	3.212131	6.094448	4.251056	3.035580	3.238535	3.557966	3.292658	3.116393	4.110281	3.209518	3.788448	3.919566	3.546976	5.283942	4.624415	4.684589	5.091025	6.133265	3.265578	3.332078	2.877038	2.946451	3.899788	3.172384	6.058527	3.541683	6.716549	4.972338	6.162716	5.271639	4.631333	4.209698	3.985840	4.288341	5.901312	4.492437	4.067541	4.808685	5.833523	2.896339	3.821840	3.665884	3.728592	5.291194	4.365913	3.375457	5.044730	7.283670	2.969894	3.168541	2.987328	2.980607	2.871339	5.449166	2.959195	6.592110	3.353669	3.125771	3.386361	3.541494	3.601477	3.612759	3.048798	6.460518	3.490858	4.672936	4.582669	6.523680	3.579359	3.475634	5.693962	3.865160	3.063094	2.934452	3.652626	2.947624	4.806984	3.404213	4.031273	3.354861	4.257186	3.345748	3.366167	3.736675	3.704674	3.161074	3.133459	2.907199	3.160172	3.077373	3.447475	3.273704	4.025170	4.087871	7.260591	5.720507	4.339614	4.096648	4.021995	4.791656	3.619143	3.879790	3.239599	4.869580	3.435630	3.247376	3.560522	3.221664	3.340980	5.344783	3.548744	3.364370	3.238005	3.111066	2.936539	3.423646	2.973807	6.034881	2.973344	2.916877	3.181900	3.036347	2.963760	3.219103	3.004165	3.120954	3.170245	3.652414	3.443447	3.822483	3.220421	4.223852	3.448612	3.588064	3.068147	4.972338	5.494698	3.212552	3.248143	2.872761	2.939310	3.109008	3.055278	3.067524	4.502413	4.148341	3.569850	3.892448	3.028082	5.852724	3.074342	3.094426	4.639899	3.724732	3.080285	3.139936	3.336784	3.136389	3.281645	4.445425	4.736547	3.155938	3.012671	2.993084	3.071437	3.098676	3.407115

NPHP4	5.790613	6.322198	6.354105	7.059481	6.712952	6.916950	6.467861	5.923488	6.866291	6.604389	7.128933	7.247314	7.330879	7.402709	6.852172	6.677511	6.735475	7.272982	7.152007	7.445861	6.991455	7.020447	7.173526	6.832940	7.328812	7.306812	8.226013	6.596333	6.822754	5.950046	7.576848	7.023420	7.082529	6.907507	6.624032	5.966457	6.334681	6.296590	6.319966	6.022574	6.932089	5.958051	7.262980	6.945652	7.066362	6.733213	6.793634	6.269124	7.921494	7.046248	6.548685	7.071647	6.999213	6.868741	7.064918	7.277975	6.653041	5.551813	6.836582	7.011725	5.426425	6.845047	6.482902	6.265208	6.570356	6.677201	6.186223	6.043958	6.428172	5.421462	5.997249	5.868488	7.315739	6.236812	6.131041	6.148301	6.039985	6.328401	6.084413	6.571789	6.372450	6.521703	6.749904	6.424977	6.398800	7.029566	6.097863	6.224856	5.373609	5.745747	5.380962	4.862293	6.115466	5.988291	5.833177	6.472402	7.473087	7.662002	6.962442	7.148023	6.987163	6.317575	7.630032	6.453958	6.903996	7.526963	6.920646	7.028768	6.330096	7.405226	4.649613	6.353937	6.600216	6.257421	6.750989	3.848646	6.006261	7.404121	7.415795	7.009963	7.578395	6.500369	7.510625	7.473850	8.739630	7.085071	8.408942	9.263062	8.031937	8.289036	7.059020	7.431423	7.865090	8.252377	7.446975	8.713286	8.022991	8.337432	7.765933	7.625933	8.153907	7.674955	8.435693	8.863852	8.782749	7.015498	9.344218	7.000313	7.625612	8.721757	7.423532	8.059483	7.668348	7.112523	6.924019	7.056495	6.271204	7.153391	7.174793	8.994252	5.285608	8.295459	7.940255	7.488965	7.586901	8.249148	7.191984	8.573379	8.255576	7.491173	8.262784	7.592865	7.911412	8.012452	7.444912	7.798320	7.411494	7.494577	7.834160	6.921278	7.704712	7.275617	5.905278	8.186038	7.158540	7.331787	6.472245	7.592260	6.631873	6.074144	6.481237	8.837390	7.022169	6.272537	6.537941	7.027648	7.003961	6.355910	7.426963	7.170697	7.594133	8.123251	7.459810	7.348804	7.569464	7.013318	7.911881	6.238948	6.436616	6.962768	5.539213	7.007190	6.959086	7.145511	8.262615	8.065802	8.386497	8.331848	7.508788	7.094332	8.591616	7.345011	6.207781	7.106590	7.900697	8.806108	7.344870	6.096683	8.295020	5.209826	6.956719	4.814969	6.582110	5.642536	7.183306	6.821815	7.706992	8.071533

IQSEC1	5.985749	5.130200	4.946291	4.991830	5.709925	7.225755	5.184019	7.094820	7.652674	5.789735	6.677045	6.805391	5.769200	5.824962	5.991996	5.687183	6.527992	5.383539	6.808398	5.139865	5.563155	6.139619	6.286409	5.791150	5.049984	6.791521	6.299075	7.035147	5.735919	6.431080	6.860578	5.090840	5.509568	5.585717	5.070584	4.801148	5.098442	4.427665	6.503995	5.446840	5.475801	5.681420	5.851489	5.520813	5.371479	5.787562	7.943875	5.157481	6.225720	4.770748	5.401078	5.523476	4.591187	4.384260	5.054934	6.943280	4.925302	5.008463	5.418355	4.103651	5.534897	5.693412	5.436108	6.439253	5.118860	5.452837	5.297845	5.296854	5.027227	5.140454	4.376122	5.004156	6.173864	5.060038	5.075669	5.692082	5.012415	5.366809	5.276946	5.113550	3.611177	4.539755	4.511393	4.370219	5.897057	5.891332	3.965297	5.814756	4.505009	5.411498	5.281972	5.387590	5.342348	5.656282	6.762668	5.640502	5.463454	6.318237	7.409279	5.681975	6.881513	4.928648	5.747563	5.576123	5.414539	6.014848	7.228566	6.089923	5.727722	5.830469	6.068771	6.266553	5.025838	7.166260	7.081619	8.644551	5.890639	6.137782	5.872991	5.478477	5.152764	4.668169	5.438382	6.479620	7.543904	7.126261	6.563677	6.165458	7.266940	6.931748	7.471570	5.389322	5.114561	7.204022	5.598950	5.538833	6.115959	6.439708	5.950379	6.383321	5.745334	6.677849	6.781340	5.099724	6.017966	5.800993	6.933634	7.705631	6.916498	6.760754	5.930066	5.987742	6.237625	5.248966	5.092892	5.330483	5.584179	7.011235	5.733728	7.502262	5.262064	5.208635	5.739533	5.730853	5.304609	8.470095	5.226305	5.509757	4.729367	4.665824	5.145352	4.346971	4.505663	4.337007	5.900068	5.210637	4.870222	4.316287	4.201623	5.452453	6.070817	5.495683	6.141553	5.168072	5.212584	4.189728	5.662165	5.512948	4.923626	5.157704	5.332458	8.572024	5.183621	5.328291	5.496448	4.701728	5.557350	5.217771	3.901874	4.367399	4.660504	3.706865	4.635852	5.991118	6.563191	4.112234	5.436481	5.043056	5.722705	5.118456	5.128131	5.294827	5.670148	6.206594	6.538915	6.531278	6.614638	6.898513	4.834164	7.030045	5.505847	5.034847	5.195705	5.289371	5.884057	6.861393	6.507348	5.857270	6.611684	5.499091	5.123104	6.339551	9.018822	7.783924	7.739464	5.182396	6.350954	6.918220

NAAA	11.432403	12.281925	12.278057	11.714384	12.167877	12.240224	11.911476	11.949288	10.852098	11.825046	11.398515	11.902027	11.122655	12.663072	11.920971	10.716263	11.958426	12.182512	11.175052	11.975098	11.016907	12.589616	9.809058	11.419106	11.525172	11.349792	9.910805	12.261188	11.107037	11.443078	10.890139	11.677730	12.091837	10.716566	12.151563	12.378496	12.517447	12.152825	12.154206	12.829114	11.323357	13.184851	12.679547	12.308073	12.067502	12.273762	10.263119	13.111379	11.302476	12.627500	12.882226	12.506907	12.719045	12.090726	12.517283	10.866981	12.865155	13.055072	13.103175	12.989969	13.405029	11.514222	11.966551	12.727955	12.141761	12.801434	13.541051	12.617631	12.670692	13.017023	11.687212	11.983055	11.499480	12.345482	12.413974	12.367529	12.226002	11.993346	13.332466	12.187793	12.378188	12.328136	12.174829	12.419130	12.416267	12.224905	12.068749	12.352576	12.682648	12.816808	13.429298	12.393291	11.849732	12.642737	13.196712	11.929992	12.180812	11.723766	11.452975	11.170020	11.529530	11.993963	11.694244	12.342613	11.675812	11.086467	11.300543	12.001304	10.843773	10.876634	12.449765	12.712770	12.199224	11.016907	11.472082	9.581758	11.785687	11.423450	10.346123	11.180515	10.226672	11.809935	11.471947	9.616211	10.357533	10.767508	8.050181	8.217593	9.531817	9.572872	8.451859	8.722971	10.486716	10.323968	9.159846	9.714536	9.316873	9.478926	10.794980	8.086920	10.388365	9.745544	8.409690	10.395050	10.459101	10.705209	10.272900	8.874004	11.108811	8.872320	11.193252	10.219536	10.686753	10.860851	9.694670	11.270728	10.606972	9.894722	11.893668	8.596736	11.454294	11.151555	12.255674	10.664303	11.138168	8.116849	10.979372	9.778769	11.046049	10.552501	10.595429	11.049446	10.479922	11.841598	9.991411	11.754990	11.463911	11.386742	11.828107	12.215485	10.741863	11.391015	11.240625	11.710886	12.429784	11.771822	11.126639	11.376659	12.041809	10.406371	10.316937	9.861294	11.820802	11.365804	11.500644	10.436142	10.131092	11.301442	11.082337	11.237000	11.170466	11.591454	11.417994	11.574932	10.261790	10.825019	10.557516	12.406051	11.450569	10.691661	11.477417	10.684205	11.876001	10.786914	10.441420	10.664611	9.784891	8.934723	9.185335	9.581160	9.408730	10.739617	11.870552	10.449732	8.712212	9.372113	11.224891	9.718135	8.837534	10.742155	11.265456	7.432820	10.673716	9.383618	9.891801	10.845782	10.797753	9.173435

RXFP2	6.875163	5.900980	5.588508	6.091481	6.231093	8.445759	6.700295	6.739736	7.033591	6.720973	6.852479	6.658391	6.009832	5.342558	4.442432	6.111791	7.589490	5.778189	5.328715	5.925258	5.632831	7.493042	6.939673	5.474055	5.719750	6.224175	6.757487	7.307427	6.359531	7.358723	6.170824	5.209227	6.079720	5.928833	6.188416	7.186521	6.639497	5.681788	6.309443	5.747936	7.140632	5.763765	6.363905	5.743183	5.950046	7.426641	7.803857	6.360702	6.276382	6.204520	5.979208	6.399654	4.999198	5.418743	5.373011	7.758241	4.735442	5.933348	5.676369	4.953147	5.584380	6.604389	5.994834	6.215597	5.955221	5.434367	5.622975	6.669308	6.386980	6.888075	6.023770	6.558111	7.926641	6.914321	6.533555	6.261678	6.581803	5.930066	6.210859	4.771569	5.402456	5.903276	5.255748	4.548399	5.692478	6.397635	4.836806	5.268667	5.505652	5.830863	5.775818	6.819116	6.084413	5.984697	6.558456	6.298401	5.977433	5.690239	7.049913	6.379289	8.386331	5.536657	6.491699	5.731574	5.817802	6.926316	5.825139	5.728818	5.970151	7.219504	6.538274	6.500213	5.807949	8.697899	7.040810	11.457125	5.831929	7.823834	6.862992	7.149790	6.274070	6.204845	6.562696	7.502262	7.993460	8.069369	6.486608	6.697597	7.500852	7.443314	7.318693	6.902882	6.569708	5.873185	6.619535	7.405528	5.618375	6.216306	6.241596	6.203143	6.757650	7.883226	6.306611	6.622911	6.361514	7.482286	7.750575	7.488051	7.484272	6.656632	5.956256	6.099449	6.506499	6.951703	6.999213	6.866463	6.524664	6.639819	6.161502	8.607116	6.225380	6.931281	6.727719	6.661341	7.925527	8.152526	6.159981	5.883320	6.444537	6.326589	6.433086	5.267688	5.738626	5.657036	7.560294	5.228700	6.124083	5.744656	4.662468	5.372660	7.392716	5.742830	6.305257	6.101534	5.788289	5.895320	6.586228	6.876112	6.709432	6.236327	6.719133	8.389906	6.417161	6.345568	6.583114	6.499728	6.245934	6.452423	5.093948	5.485008	6.208993	5.610876	4.650252	6.174728	6.889035	5.302043	5.614049	5.670662	5.605914	7.555205	6.976898	6.152067	6.631099	6.518383	7.271730	6.306611	6.457889	6.512094	6.366543	7.267114	6.199995	7.229369	5.946365	5.935640	7.284304	5.983328	6.015713	6.049248	8.581670	6.696813	6.078831	7.159604	10.100633	8.040277	9.613640	5.884422	8.795964	7.349599

LRRC73	8.834942	8.233897	7.942176	8.270740	8.506737	8.029783	8.246938	8.157804	8.321873	7.520351	7.685331	8.255885	7.648147	7.296707	8.074860	7.931730	7.961155	8.662231	7.930670	7.750438	8.173935	8.433681	8.437983	7.910483	8.041562	8.297612	8.175380	8.273306	7.975388	7.632388	8.239920	8.714674	7.922409	7.689026	7.764336	7.636336	7.828691	8.050019	8.559772	8.128270	8.164571	8.251773	7.979734	8.444090	7.517578	7.755863	7.537619	8.838597	8.029186	8.426765	8.490431	8.313592	8.186986	8.509515	8.684928	7.819973	7.786365	8.437844	8.845719	8.745587	8.913424	7.767159	8.189568	8.462482	8.317000	8.960537	8.562835	7.968631	8.114241	8.893154	8.149158	7.797527	7.568853	7.720377	8.434442	7.714780	7.900697	7.794622	8.476775	8.637723	7.984691	8.526275	8.196235	8.979236	8.306636	8.836326	8.075788	8.532428	8.594879	8.631128	8.352459	8.717976	8.445308	8.242934	8.164264	7.682746	7.877023	7.363230	8.319558	7.709559	7.602148	8.120986	7.750506	7.936532	8.026668	7.431568	7.751835	8.178250	8.431955	7.993790	8.213302	8.398977	8.458444	7.548532	7.427588	8.049077	8.953012	7.958836	7.197600	8.561173	8.471373	8.444705	8.317305	9.539708	7.927106	8.058580	7.871982	7.941031	7.604046	8.222227	8.621747	8.380819	8.801121	8.944434	7.592104	8.149308	8.249596	7.927727	7.745783	9.049602	10.510068	9.061578	8.241887	10.053117	10.374695	9.801645	9.851816	7.463419	7.761283	7.796171	7.741197	7.840159	8.933782	7.944176	9.248899	7.755300	7.892783	8.883738	8.571281	8.703217	8.814563	8.922996	9.228039	8.775819	9.271660	8.233570	9.361423	9.972182	8.207694	8.497341	8.878315	9.439413	8.734401	8.120379	7.414462	10.328856	9.179081	8.449681	10.316078	9.028725	8.284696	7.845933	8.306963	8.489686	8.942487	8.695889	7.790524	7.816968	9.415666	8.180988	7.791742	8.397632	7.578233	9.046701	7.715979	9.564183	9.580275	8.279032	8.161308	8.126922	9.245501	9.623524	8.177095	9.667979	9.193762	7.844066	9.053125	8.453985	8.212228	9.440640	8.903998	8.942487	8.919067	7.710874	8.603609	8.599728	7.483685	8.118091	9.087621	9.000858	8.781959	7.421202	7.932799	8.071381	7.706091	7.699442	7.421823	8.769925	7.796942	8.992569	7.771110	9.105197	7.753171	8.846787	8.715458	8.115622	8.464289	7.297511

TRAPPC10	12.008706	11.882981	12.196471	11.782817	12.193376	11.687372	11.864391	11.856252	11.389687	11.993647	11.725872	11.658340	11.792854	11.992915	11.742737	11.654834	11.100738	12.233290	11.806639	11.731240	11.980914	12.057480	11.709829	12.155249	12.125545	11.528459	11.283416	11.762443	11.495068	11.782523	11.496969	12.028252	12.485843	11.719282	11.887052	12.041676	11.967950	11.811133	12.293428	12.385620	11.735680	12.123909	12.166049	11.949912	11.755741	11.957393	11.409515	11.795267	11.551046	11.796594	11.933805	11.893367	12.250568	11.953632	11.996542	11.394279	12.086352	11.808140	12.059200	12.242991	12.061682	11.676518	10.905697	11.779120	11.788222	12.124933	11.543954	11.602276	11.808438	11.854007	11.431058	11.805448	11.737771	11.892308	11.653340	12.074353	12.154631	12.051785	11.877315	12.310451	12.261502	12.134671	12.118645	12.273123	12.195094	11.902791	12.145773	12.535440	12.045726	11.958709	12.039083	12.120362	11.906965	11.902491	11.617285	11.891244	11.657322	11.815395	11.677730	11.470648	11.909709	11.976994	11.767960	11.990332	12.209897	11.475562	11.813442	12.295263	11.818995	11.724680	12.128571	11.960999	11.974803	11.624718	11.681708	11.351141	12.288938	11.811273	11.413399	11.694552	11.340397	11.075744	11.969628	9.553903	11.376659	11.213507	11.560680	11.155593	10.809338	11.376371	10.829303	11.395035	11.630454	10.952265	11.588775	10.841116	11.350987	11.216261	12.325731	11.473380	11.468687	12.335719	10.633662	11.971451	11.596096	11.136838	10.951706	11.060705	11.253526	12.009153	11.585010	11.289771	11.601351	12.119288	11.421486	11.333902	11.943188	11.634348	11.797645	11.591754	11.583331	12.095640	12.248492	11.876433	11.555942	11.485467	11.680103	11.906037	11.410835	11.508343	11.700848	11.730949	11.624432	11.649471	11.969051	12.242384	11.705354	11.394433	11.486562	11.391155	11.139619	10.359472	11.375766	11.454602	12.418166	11.196525	11.304710	11.165084	11.525892	11.215686	10.989737	10.752225	11.515004	11.925351	11.619186	11.342940	11.679502	11.451147	12.324191	12.108933	11.288046	11.832854	11.925195	11.974500	11.246804	11.826085	12.392815	12.124203	11.586042	12.094424	11.851556	11.306962	11.083368	11.623866	11.479176	10.799338	10.732241	11.037396	10.968909	11.200158	10.699421	10.993001	11.511108	11.911179	11.072990	11.247214	11.707604	12.226768	11.601794	12.452481	11.651384	11.776985	11.312627	10.949961	11.350698	12.087108	11.878463	10.856734

FP565260.6	12.008706	11.882981	12.196471	11.782817	12.193376	11.687372	11.864391	11.856252	11.389687	11.993647	11.725872	11.658340	11.792854	11.992915	11.742737	11.654834	11.100738	12.233290	11.806639	11.731240	11.980914	12.057480	11.709829	12.155249	12.125545	11.528459	11.283416	11.762443	11.495068	11.782523	11.496969	12.028252	12.485843	11.719282	11.887052	12.041676	11.967950	11.811133	12.293428	12.385620	11.735680	12.123909	12.166049	11.949912	11.755741	11.957393	11.409515	11.795267	11.551046	11.796594	11.933805	11.893367	12.250568	11.953632	11.996542	11.394279	12.086352	11.808140	12.059200	12.242991	12.061682	11.676518	10.905697	11.779120	11.788222	12.124933	11.543954	11.602276	11.808438	11.854007	11.431058	11.805448	11.737771	11.892308	11.653340	12.074353	12.154631	12.051785	11.877315	12.310451	12.261502	12.134671	12.118645	12.273123	12.195094	11.902791	12.145773	12.535440	12.045726	11.958709	12.039083	12.120362	11.906965	11.902491	11.617285	11.891244	11.657322	11.815395	11.677730	11.470648	11.909709	11.976994	11.767960	11.990332	12.209897	11.475562	11.813442	12.295263	11.818995	11.724680	12.128571	11.960999	11.974803	11.624718	11.681708	11.351141	12.288938	11.811273	11.413399	11.694552	11.340397	11.075744	11.969628	9.553903	11.376659	11.213507	11.560680	11.155593	10.809338	11.376371	10.829303	11.395035	11.630454	10.952265	11.588775	10.841116	11.350987	11.216261	12.325731	11.473380	11.468687	12.335719	10.633662	11.971451	11.596096	11.136838	10.951706	11.060705	11.253526	12.009153	11.585010	11.289771	11.601351	12.119288	11.421486	11.333902	11.943188	11.634348	11.797645	11.591754	11.583331	12.095640	12.248492	11.876433	11.555942	11.485467	11.680103	11.906037	11.410835	11.508343	11.700848	11.730949	11.624432	11.649471	11.969051	12.242384	11.705354	11.394433	11.486562	11.391155	11.139619	10.359472	11.375766	11.454602	12.418166	11.196525	11.304710	11.165084	11.525892	11.215686	10.989737	10.752225	11.515004	11.925351	11.619186	11.342940	11.679502	11.451147	12.324191	12.108933	11.288046	11.832854	11.925195	11.974500	11.246804	11.826085	12.392815	12.124203	11.586042	12.094424	11.851556	11.306962	11.083368	11.623866	11.479176	10.799338	10.732241	11.037396	10.968909	11.200158	10.699421	10.993001	11.511108	11.911179	11.072990	11.247214	11.707604	12.226768	11.601794	12.452481	11.651384	11.776985	11.312627	10.949961	11.350698	12.087108	11.878463	10.856734

CPEB4	12.338408	11.913683	11.971606	11.787766	11.937909	12.791154	12.507547	13.447560	13.214589	12.753644	13.020637	12.488616	12.177680	11.913539	11.523534	13.021352	12.196008	11.245235	12.056828	12.776543	11.798796	11.339952	10.626401	11.697132	11.547537	11.467492	11.697132	12.207923	11.989419	12.300959	12.644233	11.599594	11.567979	11.341173	11.537179	11.960063	12.025558	11.489500	11.753936	11.807837	12.313176	11.806047	11.149440	11.809331	11.125638	11.424500	12.884558	11.902791	12.656514	12.221569	11.481223	11.944822	11.783439	11.589538	12.156789	12.935431	11.576018	10.906618	11.215686	11.005117	11.515630	12.147944	11.813591	11.688994	11.391683	12.149882	11.518972	11.327628	11.230200	11.446009	11.946821	12.094888	12.993251	12.468757	12.141122	12.373531	12.543245	12.579166	12.129172	12.370681	11.909419	12.286760	11.404390	11.956358	11.867283	11.979884	11.911322	12.222032	11.644892	11.949752	11.476689	12.490737	11.902342	12.390623	11.872045	11.850968	12.209586	12.258961	12.023550	11.941052	13.273354	11.995974	11.801784	12.152396	11.707017	13.260832	12.647084	11.443990	11.193252	13.065668	11.436404	11.859051	12.248657	12.689676	13.009396	11.944227	11.139340	12.698837	13.091804	12.725309	12.142800	10.594114	10.295850	9.426037	11.338628	10.635641	8.914640	11.401790	9.406320	10.663859	10.003350	8.815582	10.016654	8.818873	11.379157	9.464698	8.558383	7.804017	10.338538	10.898787	8.677746	9.270456	10.456649	8.844814	10.227300	12.176546	8.939739	10.541990	10.684506	10.052192	11.051785	9.446710	11.933654	11.674007	10.140390	11.058929	9.962991	10.283668	10.493458	10.678215	10.773291	9.956906	10.755101	10.834982	10.812838	11.565388	9.989661	10.474364	10.991621	10.913253	10.853180	11.031349	10.831970	10.940377	11.343233	9.721087	10.157881	10.428215	10.283957	11.098248	10.574965	10.217279	10.101089	10.621887	10.711413	10.208469	11.136230	10.217133	11.111146	11.089443	10.877193	11.864833	10.876767	10.762352	11.805598	10.598790	10.703174	11.664327	11.035380	10.939911	10.666359	10.774471	10.928442	10.596486	9.198923	11.757241	10.883564	10.437805	10.804313	10.520492	10.592531	10.049254	11.163491	11.565093	11.058379	9.735623	9.658419	10.652361	10.268837	10.478737	9.735774	9.659835	10.667114	10.119603	10.786473	10.426224	10.517131	11.089149	11.702959	9.731525	10.213496	8.660931	12.046769	9.307595	12.157252	10.042994	11.004977	10.376671

SLC9A1	7.535484	7.187472	7.195034	7.258890	7.175161	7.650308	6.593107	8.551058	8.040436	7.016162	7.106770	6.854156	5.940063	6.888860	6.651763	7.302326	8.015866	7.082859	8.990467	7.149139	7.599534	7.633165	8.578625	7.109658	7.572932	7.383930	7.574019	7.607430	7.303037	6.639342	7.960535	6.719133	6.655496	6.173342	6.543376	6.791208	6.971967	6.944237	7.570519	7.774290	7.165525	7.243579	6.591465	7.025384	6.613481	6.731272	7.539270	7.336449	6.841899	7.048951	7.008983	7.219180	6.527333	7.752866	6.780884	7.118055	6.421746	7.246849	7.331485	7.133718	7.584728	6.841442	7.587483	7.876711	7.643053	7.913282	7.430775	7.383010	7.155921	8.142105	7.101115	7.040810	7.332112	7.080648	7.086585	7.257882	7.518657	7.305703	7.579023	6.853335	6.945031	7.234377	7.049597	7.475990	7.711542	7.421202	7.121335	7.566575	8.143761	7.853763	7.830635	8.110438	6.712113	7.032959	6.142756	6.831474	6.705888	6.095667	5.813148	6.597946	6.553679	6.720482	6.747722	7.017480	7.506342	7.359312	8.558069	7.524066	8.013058	7.733781	8.378160	7.974619	7.719642	7.749492	7.426641	7.664829	7.890146	7.279413	7.636181	7.547120	7.467564	6.854620	7.507849	7.629708	7.581800	6.508308	7.917375	7.683968	6.738272	6.975438	6.395482	6.359193	6.129831	6.786777	7.350396	8.036821	6.803168	7.908033	6.751613	8.009151	7.261516	9.020771	7.553313	7.507225	7.297031	7.204235	7.491333	7.443239	6.804151	7.771423	6.689001	7.298148	6.612494	6.737298	6.508471	6.851553	7.576687	7.974923	7.651747	6.851861	7.748723	7.025384	7.922875	6.815732	7.254455	7.600626	7.223266	7.208936	7.324693	7.334115	7.316219	6.390861	7.659822	6.701586	7.058536	6.288620	7.135949	7.465403	6.999519	7.943394	7.166260	8.022090	8.091578	7.918584	7.966655	7.740543	7.569604	7.679669	8.458622	7.057930	6.870163	6.996153	7.136879	6.813024	7.321681	7.550671	7.620601	7.850793	6.620183	6.969337	7.437546	7.280692	6.827338	7.801746	7.461194	7.612344	7.347389	8.216356	7.434223	7.631696	8.672180	7.444765	7.467564	7.229677	6.986195	7.097967	6.395163	6.505130	6.440058	6.569199	6.641302	6.399974	6.542050	7.558018	7.266775	8.471683	8.035393	8.117460	7.541671	8.416275	7.818572	7.333384	7.591191	6.800254	7.172303	7.958214	7.145800	6.967094

PRDX1	14.162706	16.152946	15.930069	15.352487	16.036135	15.719891	15.733274	16.023191	14.732623	15.415429	15.974511	15.173276	15.785756	15.968277	15.340435	16.141552	15.594598	15.548789	15.016274	15.965762	14.099791	14.430444	13.622665	15.953297	15.086549	14.954990	13.519354	14.970250	15.122829	14.739028	15.387598	16.112915	15.852280	15.488676	15.533380	15.238314	15.691311	15.529882	16.029650	15.603850	15.922928	14.906118	15.855890	15.395261	15.474785	14.867676	15.354914	15.396107	15.001900	15.007098	15.252474	15.854635	15.054330	16.218720	15.697564	15.606629	16.521294	15.065209	15.364729	15.848852	14.854894	15.350928	13.498487	14.867676	15.483826	15.899823	15.142270	15.889470	15.975682	14.926494	15.122829	15.562229	15.663417	15.635602	16.349770	16.011919	15.552451	15.606629	15.439830	16.289097	16.258072	15.991977	15.633808	16.665968	16.157751	16.157751	15.911833	16.467780	15.442379	15.674067	15.215025	15.086549	16.570086	15.810180	16.107193	16.391445	15.674965	15.630940	15.783769	14.287271	15.742683	16.323317	16.393332	14.300251	14.911437	15.354130	15.260952	14.949574	14.140523	15.627072	14.607132	15.570171	16.182924	15.558628	15.674965	14.841151	13.297565	15.469004	14.842135	16.395147	15.646933	15.201679	15.961903	15.155753	15.417796	15.619793	14.457804	15.439830	15.909426	15.123478	16.029650	15.658565	15.408414	15.283711	15.031025	15.473917	15.593684	14.453201	15.173276	15.704556	15.664400	15.498075	15.287379	15.885839	15.875766	15.913059	15.740567	15.578257	15.908209	14.750726	15.575476	15.545439	15.824202	15.473095	16.056486	15.523618	15.292522	15.489513	15.799508	15.150598	14.970861	15.005364	15.785756	15.522760	15.961903	15.047833	14.855412	16.621970	15.781709	15.660615	15.368664	15.752097	15.747854	15.860644	15.685230	14.981620	15.794025	15.800609	15.512636	14.956666	15.937305	14.746490	15.598287	15.196878	15.357970	16.192130	15.589004	15.952203	15.036921	16.397385	16.492130	14.921174	16.212290	15.283070	15.609450	15.910549	15.819800	15.773226	14.838073	15.520300	16.059162	15.820930	15.229335	15.613151	15.624294	15.576365	15.127801	14.710296	14.841605	15.068646	14.223479	15.537762	15.809175	15.085910	15.195519	15.701693	15.479687	15.525353	16.189003	15.300977	15.122829	14.595344	15.074615	13.972172	15.419437	14.817850	15.410054	15.184064	15.589004	14.338198	15.082962	14.641807	15.263058	15.878115	15.873355	16.148537	16.213991	15.822036

PIK3C2B	5.408149	5.564851	5.144534	5.671418	5.949516	5.883706	4.830608	5.165688	5.946365	4.784124	5.357397	4.761215	4.441499	4.571619	4.258522	4.947124	5.236860	5.305994	5.327510	6.131717	5.558672	4.987884	6.059561	4.917690	5.413006	5.613847	5.542037	5.439113	5.729540	5.486729	5.651842	4.514603	5.368759	5.252954	5.320125	5.104607	5.608760	4.714337	5.406292	5.169309	5.533510	4.882226	5.310272	4.660922	5.455882	5.795625	5.138025	5.318242	5.438752	4.594262	5.377702	6.369566	5.328489	5.378088	5.275030	5.678067	5.540532	4.167541	5.383921	5.450652	5.347361	5.381551	5.135616	4.763580	5.467351	5.214446	5.701700	5.177346	5.447832	5.619454	5.790446	5.545624	5.879995	5.712111	6.036696	5.461998	5.569576	5.436282	5.170283	4.772601	5.268266	5.764682	5.302043	4.989154	5.798204	5.883320	3.898693	5.527848	5.899713	5.755531	5.938629	5.446414	5.216196	5.348318	5.367584	6.138229	5.017705	4.725774	5.228917	5.429045	4.176172	5.622975	5.144352	5.368578	4.600410	6.140472	5.416994	5.318802	5.214240	5.500670	4.785361	5.208422	5.027663	5.529351	5.363851	4.878379	5.970151	4.776982	5.081029	5.252550	5.325189	5.280737	5.730493	5.709757	6.388174	5.081029	5.083066	5.957324	4.984172	5.619849	4.949181	4.133401	4.557457	7.102997	4.873219	5.504117	5.893709	5.468755	5.747384	5.203062	4.902149	6.052855	5.162091	5.537848	5.532739	5.148333	5.675664	5.038113	5.496448	5.402259	4.813900	4.438927	5.856901	4.791226	5.284565	5.074044	4.257422	5.388534	4.465207	5.482575	5.455327	5.339128	4.914945	5.032354	5.241057	6.342169	4.731657	5.253153	4.986634	5.032759	5.746482	4.518683	5.053206	4.110281	5.694332	6.216805	3.972872	4.723229	4.657710	4.445874	5.694530	4.651753	5.236233	6.014010	5.319391	5.707946	5.398631	5.649548	5.419794	5.726273	5.565029	6.174195	5.462398	5.552587	5.398443	5.455327	5.577407	5.561665	3.516786	5.649908	5.170694	5.456085	4.473849	5.248966	5.971232	4.876507	5.140077	5.164897	5.135234	5.734458	6.023946	4.737607	5.743183	5.113751	4.961235	4.591620	3.993727	4.850976	5.592538	6.077787	5.215220	5.549197	4.864340	4.991405	5.910266	5.510469	4.846376	5.326747	5.582288	4.875897	5.055546	4.187994	5.622407	6.039453	5.391638	5.073004	5.635631	5.544106

CENPH	9.269690	11.460396	11.699459	10.750993	10.413447	10.765528	11.430160	10.170756	10.231448	10.699718	11.139199	11.089903	11.480371	11.016056	10.877490	10.538968	10.983080	10.928751	10.144003	11.650941	10.918527	11.612417	9.320772	10.205978	9.406930	10.280033	9.964114	8.679147	9.738519	11.832712	9.472022	12.262009	11.573269	13.106997	12.360369	12.191984	11.850666	11.485939	11.682169	11.117676	11.054075	11.073280	11.968566	11.836322	11.704316	12.321174	9.090210	11.317631	11.931707	11.885548	11.412637	12.059805	11.178995	11.613605	12.085897	11.001656	11.380384	11.209250	11.380054	11.434353	11.593733	11.318974	11.099868	10.789285	13.076836	11.418124	12.074811	11.301743	11.864994	11.432097	11.630904	11.804392	9.957986	11.744648	12.107738	11.946077	11.828693	11.761613	11.444695	11.413232	11.659709	11.742598	11.141047	11.906365	11.877895	11.453567	11.628487	11.843867	12.076036	11.147807	11.627357	12.051649	11.226506	12.147509	11.499480	11.672977	11.587012	10.630255	11.579521	10.791885	10.513902	10.929057	10.871143	10.564716	12.264187	10.548745	9.809058	11.790719	11.387025	9.501937	12.413338	10.738234	10.443810	9.839709	11.369313	10.876767	9.715792	10.970158	10.176239	11.118254	11.620252	12.429483	10.966697	10.466673	12.306838	11.561147	12.691705	12.208987	11.719718	11.485786	12.899372	12.447416	12.370837	11.765559	10.660852	12.140193	10.740649	13.042413	13.739368	12.749170	12.046618	12.245147	10.257704	12.565127	11.920971	11.301874	12.120667	12.351946	12.254526	11.800284	11.671644	11.590838	12.996223	12.994903	12.845274	11.753359	12.775824	12.075588	12.240075	12.641550	13.063978	13.061923	13.937428	12.440469	13.099592	11.404685	13.718167	12.982246	13.526786	12.588458	13.128984	12.739710	12.706190	12.362917	13.015539	12.622342	13.632044	12.309198	13.149931	11.836629	11.535255	12.761009	12.977133	12.202516	11.883139	12.947003	12.229877	13.076070	12.542773	12.710169	12.953298	10.894182	12.797127	13.006847	11.945613	12.586128	12.926643	12.784966	11.884633	12.638185	13.223325	12.026913	11.914600	13.376481	11.931223	12.841587	13.467530	12.748989	11.987411	13.040990	12.477509	11.916369	12.651577	11.461422	11.697702	12.563162	10.903502	12.864398	12.437883	12.629517	11.457285	10.712709	11.597854	12.660869	10.819554	12.222786	13.178186	13.038580	10.893881	12.891207	12.101182	12.227834	9.963969	13.744082	12.228465	12.289963	11.858897	12.755816

SH3GL3	7.145800	9.150795	9.117878	9.082279	8.785782	9.345280	10.050349	9.584671	9.948435	9.197036	9.511977	9.529428	8.860765	9.658575	9.083496	9.496425	9.353339	9.420243	10.512879	9.698789	9.624616	10.403336	8.894837	8.904141	8.862000	8.519771	7.606389	8.981956	8.737163	8.717816	9.934023	9.232694	9.181558	8.928486	9.473089	8.845719	9.292992	9.731377	10.131679	9.673663	9.427118	9.511660	9.078406	9.484876	9.411410	9.350620	8.581064	9.730932	8.932414	9.911577	8.241455	9.494730	8.913127	10.107303	9.477832	9.362614	9.036324	9.748635	9.105505	9.182813	9.444674	8.867036	9.062763	9.567009	9.735774	9.921529	9.457550	9.355444	9.296135	9.691158	9.892861	9.720935	9.123078	9.360960	9.878591	9.131674	9.205492	8.841026	8.801264	9.476741	8.510434	8.814414	8.613295	9.342664	9.165262	9.750518	9.652120	9.595082	9.344530	9.184093	8.975575	9.456955	8.748632	8.983194	7.630212	8.811217	9.127188	9.619777	9.348642	9.444211	9.856025	9.387625	9.109097	9.541126	10.319973	9.481820	9.464406	10.730465	10.378509	9.778165	10.496942	9.503319	9.110639	9.594314	9.682921	8.925815	7.232119	8.628373	8.696042	9.029816	9.374150	8.157042	9.261966	8.390832	8.165963	7.637243	7.211007	8.908772	8.511066	8.767611	7.337198	7.568694	7.719642	8.510590	8.218720	9.124887	7.817143	7.660627	7.506641	9.367419	8.555262	8.325722	8.036191	7.227500	9.042804	7.769428	7.382525	9.021427	7.730402	7.697302	8.362192	8.184935	9.037545	8.767446	7.445391	8.511392	9.039784	9.694670	8.350208	8.328299	8.797307	6.933336	8.356756	9.343762	9.460905	8.884828	9.541580	8.304531	9.249501	8.690660	8.228265	7.990331	9.194647	8.732552	8.452011	6.321167	8.364235	9.081509	8.411859	8.376183	8.064574	9.841825	9.159233	8.393785	7.713749	8.554054	9.549294	8.583923	7.971938	8.219177	8.157192	7.795089	9.013468	8.261839	8.755176	8.646197	7.833540	8.531668	8.438853	9.124887	9.288170	8.342015	7.873506	8.565560	8.316530	9.335123	9.629238	7.882612	8.853327	7.185369	9.535194	8.858351	8.805963	8.826140	7.651887	8.904469	8.306046	9.423930	8.250672	9.824324	9.228524	7.091636	8.853466	8.470551	8.222992	8.942487	9.896278	9.951825	8.380173	7.783317	8.300189	7.175887	8.707526	8.791835	8.448422	7.550222	7.997547	8.930630

SRSF5	10.804455	10.978333	10.739904	11.662509	11.562888	11.399821	10.355061	12.157396	13.045728	11.138761	12.113151	11.319960	11.197531	10.730598	9.696933	11.615530	12.459458	10.867134	12.002645	11.964318	11.852005	11.447213	11.960220	11.548579	11.159921	11.671357	11.779565	12.415458	11.504214	10.672137	12.263250	8.887131	10.898327	9.854012	11.695625	12.052996	11.452687	11.456674	12.038357	12.523982	11.826536	11.012393	11.017912	11.887957	12.104074	11.254400	12.225064	11.477264	10.983900	11.379300	10.398534	10.813141	9.804390	10.573931	10.655009	11.639475	10.486558	10.269429	9.945279	10.101089	11.141047	11.613311	9.938052	11.720627	10.471635	11.933193	11.139043	10.747987	10.898787	12.471647	12.925070	11.178995	11.471518	11.850196	12.023231	10.714913	12.499786	11.174150	11.557873	9.979804	9.444517	10.450191	9.153845	9.778612	10.395201	11.491019	10.125998	10.178120	10.578433	10.586252	10.891480	11.463192	10.293707	10.667256	11.344171	10.935769	10.449011	10.885258	11.539821	11.114554	11.538067	10.980712	11.832854	11.204194	10.952091	11.865774	11.916981	11.106019	12.081981	11.540976	11.564033	11.251075	10.726118	11.994103	11.899859	13.075325	10.531275	11.468239	11.832095	10.223905	10.090835	10.024169	10.261165	11.494500	10.965807	11.290218	11.237000	10.541101	10.891628	11.315857	11.428109	11.491019	10.306414	10.419658	10.976418	10.472987	10.246964	10.820704	9.796886	10.990341	10.146709	11.154075	10.701962	10.155465	10.017709	11.320686	11.132510	12.464195	10.556465	11.460998	9.745999	10.272441	9.435704	9.531652	9.693584	9.975574	10.791003	10.905092	10.517262	11.952138	10.166595	9.399697	10.485784	10.172011	11.005550	12.040929	9.682921	10.016962	9.732752	9.788977	9.694205	8.861847	9.165740	9.204130	10.245723	9.407255	8.791835	9.265207	9.147183	9.915602	9.454768	9.788049	9.854464	9.763401	10.101581	9.734545	9.674912	10.332520	10.349010	10.422540	9.818426	12.597317	10.074213	10.075573	10.195389	10.351729	10.020187	10.985097	8.816660	8.844016	9.739631	9.092678	9.171237	9.452017	10.683473	9.475674	9.363051	9.763401	9.730607	9.837036	10.498280	10.645484	10.650579	10.684060	10.874430	10.898488	10.847846	10.593179	10.327627	11.194304	10.806918	11.088865	9.821011	9.891801	11.389403	10.845325	10.294926	11.210844	10.996866	10.875163	10.314129	10.941388	13.246936	10.903938	10.884311	9.493154	10.197070	10.921361

TTC4	11.034586	11.159344	11.279887	11.006254	10.781605	10.470285	10.939339	10.342641	9.511977	10.372919	10.745730	10.782238	10.735064	11.051341	11.460547	9.923991	10.346580	10.802095	10.284425	10.843773	9.766625	10.355524	10.214105	10.507508	10.306254	10.382091	9.370447	10.218179	10.055446	10.727474	10.169500	11.341633	11.409349	10.881641	11.273543	11.224021	11.280895	11.328644	10.514215	11.012393	10.344466	10.604444	10.768036	11.308100	11.194728	11.411680	9.577618	10.645344	10.886562	11.143986	11.169307	11.452975	11.378421	11.518252	11.454144	10.652524	11.486995	11.217005	11.553156	11.765719	11.130778	10.479922	10.249835	10.405472	11.009628	11.338351	10.919103	10.924165	11.544514	10.327480	10.737617	10.931284	9.921069	10.884165	10.878998	10.810536	11.059231	11.038163	10.813866	11.245512	11.393217	11.250000	11.579980	11.674147	11.274882	11.374064	11.306661	11.435387	10.736706	11.380615	10.713575	10.831803	11.144712	11.193702	10.995821	11.125198	11.355780	10.071781	11.002514	9.763401	10.378811	10.785414	10.008647	10.140907	11.614961	9.772809	9.562846	10.657842	10.535450	10.074373	11.255319	9.313787	11.122025	9.677687	10.505113	9.294264	10.885258	10.687204	9.525226	11.505998	11.967332	11.905478	11.321148	10.450191	11.538822	11.246804	10.043315	11.190758	10.878998	11.543787	10.443501	10.991323	10.908392	12.260195	10.440659	11.776688	10.208624	10.389118	11.714992	11.273685	10.678375	11.538395	11.060705	12.080010	12.416578	11.066743	11.733178	9.778460	11.946386	11.000137	12.441586	11.254129	11.915487	11.852935	11.779259	12.116025	11.751278	11.492591	12.110414	10.929944	10.910296	11.252802	12.353569	11.732276	11.723179	10.513312	11.537179	12.101029	12.324058	12.142227	11.317473	11.707892	12.593292	11.536751	10.778275	12.181896	11.983954	12.418490	12.128571	12.461067	11.180812	11.864688	12.063646	11.257232	11.346253	12.717655	11.367880	12.051938	11.159497	11.281195	11.276880	10.764929	11.606677	11.226360	11.625303	11.811705	12.067502	11.014277	11.858431	11.549154	12.460105	12.524939	11.122792	12.048150	11.440322	12.360369	11.642537	11.817176	11.467339	11.433898	11.721223	11.662361	12.218793	10.930535	11.347880	11.733178	8.967765	12.445002	11.453855	11.446896	10.798335	9.889071	10.999386	11.993647	10.239105	10.928598	11.092539	11.428860	11.527756	11.348194	11.120430	11.622534	9.768915	11.019457	11.403089	12.329869	12.380353	11.777772

MROH7-TTC4	11.034586	11.159344	11.279887	11.006254	10.781605	10.470285	10.939339	10.342641	9.511977	10.372919	10.745730	10.782238	10.735064	11.051341	11.460547	9.923991	10.346580	10.802095	10.284425	10.843773	9.766625	10.355524	10.214105	10.507508	10.306254	10.382091	9.370447	10.218179	10.055446	10.727474	10.169500	11.341633	11.409349	10.881641	11.273543	11.224021	11.280895	11.328644	10.514215	11.012393	10.344466	10.604444	10.768036	11.308100	11.194728	11.411680	9.577618	10.645344	10.886562	11.143986	11.169307	11.452975	11.378421	11.518252	11.454144	10.652524	11.486995	11.217005	11.553156	11.765719	11.130778	10.479922	10.249835	10.405472	11.009628	11.338351	10.919103	10.924165	11.544514	10.327480	10.737617	10.931284	9.921069	10.884165	10.878998	10.810536	11.059231	11.038163	10.813866	11.245512	11.393217	11.250000	11.579980	11.674147	11.274882	11.374064	11.306661	11.435387	10.736706	11.380615	10.713575	10.831803	11.144712	11.193702	10.995821	11.125198	11.355780	10.071781	11.002514	9.763401	10.378811	10.785414	10.008647	10.140907	11.614961	9.772809	9.562846	10.657842	10.535450	10.074373	11.255319	9.313787	11.122025	9.677687	10.505113	9.294264	10.885258	10.687204	9.525226	11.505998	11.967332	11.905478	11.321148	10.450191	11.538822	11.246804	10.043315	11.190758	10.878998	11.543787	10.443501	10.991323	10.908392	12.260195	10.440659	11.776688	10.208624	10.389118	11.714992	11.273685	10.678375	11.538395	11.060705	12.080010	12.416578	11.066743	11.733178	9.778460	11.946386	11.000137	12.441586	11.254129	11.915487	11.852935	11.779259	12.116025	11.751278	11.492591	12.110414	10.929944	10.910296	11.252802	12.353569	11.732276	11.723179	10.513312	11.537179	12.101029	12.324058	12.142227	11.317473	11.707892	12.593292	11.536751	10.778275	12.181896	11.983954	12.418490	12.128571	12.461067	11.180812	11.864688	12.063646	11.257232	11.346253	12.717655	11.367880	12.051938	11.159497	11.281195	11.276880	10.764929	11.606677	11.226360	11.625303	11.811705	12.067502	11.014277	11.858431	11.549154	12.460105	12.524939	11.122792	12.048150	11.440322	12.360369	11.642537	11.817176	11.467339	11.433898	11.721223	11.662361	12.218793	10.930535	11.347880	11.733178	8.967765	12.445002	11.453855	11.446896	10.798335	9.889071	10.999386	11.993647	10.239105	10.928598	11.092539	11.428860	11.527756	11.348194	11.120430	11.622534	9.768915	11.019457	11.403089	12.329869	12.380353	11.777772

CABYR	6.978944	8.560535	7.615580	8.702129	8.025737	8.147919	8.528767	6.638849	7.046103	8.453841	8.955627	8.532566	8.381590	7.859735	8.555749	8.395927	8.264623	8.211744	8.956821	8.479665	7.808938	7.290668	9.550248	8.202269	9.269835	8.984549	8.543841	9.114516	8.909799	8.836181	7.871375	8.816206	8.978474	9.775229	8.950129	8.321580	8.159168	8.295916	9.099806	8.791374	7.889992	8.897010	8.690507	8.517004	8.933305	10.056502	8.490573	9.236434	8.814706	8.883900	9.145041	8.997042	8.537994	8.806574	9.326153	8.669568	8.588986	8.172842	9.380364	9.321044	8.509971	6.571326	10.224521	8.609851	10.805904	8.982281	9.959667	8.050619	10.443501	7.794465	8.729568	7.400060	7.949190	8.153614	8.418725	8.695277	7.525289	9.117571	8.666526	8.797307	8.167075	8.477408	9.413800	8.657423	8.698199	8.924735	9.766473	8.996270	8.891290	9.227888	9.700346	7.728725	9.205492	8.086300	9.061269	8.156255	8.090338	7.311187	7.274387	8.373422	7.628930	8.527834	8.494612	8.443484	7.279413	7.764659	7.599995	7.942495	7.717220	7.619865	6.945652	8.361560	7.911734	7.943394	8.596115	5.708822	8.773506	8.357685	8.047832	9.320484	9.421008	9.510217	10.565474	9.843038	8.833908	9.034002	9.912507	8.345080	14.182770	10.228793	12.324636	13.578284	10.314435	11.969628	9.125034	12.297906	12.034131	11.938203	10.308933	10.934589	12.594761	12.857801	10.103273	12.584309	12.717320	9.768599	11.041054	9.860824	11.612874	9.772646	9.405273	11.012393	9.986683	8.885915	11.572813	10.201859	9.386205	10.738523	12.073275	9.855121	13.360143	11.896778	10.127429	11.409053	14.280410	7.847503	10.845927	14.208831	10.839289	10.891628	11.771398	13.096345	10.222414	9.752370	13.433260	11.850666	11.104718	12.131737	10.641355	10.284731	9.194647	10.420715	11.393969	9.466743	11.514845	12.225366	9.541747	10.433508	10.657248	11.866219	10.384381	8.706443	10.686008	10.004749	9.151110	9.931101	9.979042	12.442059	9.704430	10.225742	10.184839	10.131679	9.761509	10.298003	12.273123	9.540665	11.212599	9.938377	10.791738	11.585010	8.851230	10.196916	9.228524	9.066139	9.436336	12.454528	10.403638	9.696322	13.818903	10.900123	12.007014	9.098599	11.633293	11.111735	12.915341	9.429598	10.329500	13.740121	8.488318	10.042222	11.188446	10.973947	7.154341	14.378131	11.284886	11.664624	10.820134	10.190703

TRDMT1	9.151857	9.039001	9.114050	8.606504	8.367126	8.856221	9.205043	8.156736	7.861985	8.496726	8.824932	8.723582	8.342758	8.510749	8.663767	8.606037	8.456739	8.529985	9.332620	9.033855	8.451235	9.165740	10.382887	8.588347	8.473581	8.739022	9.295850	7.840605	8.601729	8.955338	7.900057	9.505030	9.465672	9.334944	9.313309	9.249936	9.358630	8.826140	9.045190	8.805524	8.474316	9.240739	9.312491	9.320194	9.441911	9.848160	8.393785	9.104114	9.358480	9.362915	9.519781	9.550393	9.869327	9.609972	9.693309	8.419013	9.515907	9.075596	9.171740	8.903697	9.108288	8.616164	10.028430	9.292368	9.030848	9.008722	9.039001	9.184707	8.880982	9.012546	8.996119	9.141819	8.459410	9.401214	9.218055	9.654195	9.211300	9.280151	9.268476	9.082279	9.410488	9.507341	9.449496	9.403550	9.771907	9.397486	9.644234	9.581469	10.097780	9.346806	9.355284	9.542207	8.864614	9.220819	9.538277	9.657952	9.629238	9.142743	8.890530	8.807358	8.520957	8.443325	8.514294	8.460633	9.261505	8.905970	8.191485	9.285515	9.258494	8.670956	9.903186	8.176472	8.039647	7.954175	8.780406	8.362692	8.499968	8.834502	8.605139	9.226696	8.821736	9.497175	8.602983	7.204562	8.577277	7.830789	9.637621	9.268621	7.364365	8.598963	8.473429	7.687340	8.934257	8.599881	8.498349	9.422644	7.994104	9.807001	9.486737	8.483274	8.129345	9.812137	7.923024	10.546727	9.803650	9.531003	8.929089	8.154078	8.986084	8.272099	9.529145	9.146716	10.100136	9.675852	9.301410	9.296761	9.375753	9.149548	10.248629	8.553172	9.183450	9.943637	9.538904	9.708289	9.719259	8.494911	10.233109	9.192889	10.281096	9.846295	9.503319	9.800580	10.740809	9.886402	8.824316	10.128078	9.163554	9.643333	9.292061	9.609148	8.002934	8.864614	9.594939	9.302664	9.029665	9.608075	10.013689	9.479952	11.035981	9.183769	8.922996	8.338060	10.146709	9.524743	9.887206	9.479637	9.844254	9.546391	9.439413	9.298143	9.604210	9.034002	9.560968	9.784423	9.258567	10.526564	10.412232	9.983054	9.620568	9.376503	10.291731	8.875412	9.721843	9.126898	10.066011	11.007999	8.392258	9.135312	10.120339	8.187744	9.919156	9.454768	8.358620	10.725800	8.807664	9.525056	8.494911	8.946763	8.594404	9.921845	9.178024	8.789174	8.794756	9.747411	8.952716	8.471218	7.356091	9.204729

FGFR1	10.748441	10.496942	10.452666	10.916291	11.257836	11.132205	10.188009	11.477417	11.958138	10.849438	10.962648	10.980712	10.751302	10.533349	10.475286	10.943746	11.705224	10.601671	11.568120	10.391503	11.258115	11.578488	12.417049	10.855104	11.173138	11.347723	11.796000	11.786118	11.509057	10.641355	11.650785	9.758866	9.732752	10.392215	10.083133	10.174250	10.056660	10.359776	10.885551	11.009167	10.876331	10.884769	10.642239	10.792032	10.529524	10.540036	11.630009	10.497091	10.403949	10.085395	10.373661	9.931382	9.597725	9.835965	9.928156	11.151555	9.615893	10.169963	10.343107	9.990936	10.476626	11.503051	11.257393	11.294819	10.474206	10.773731	10.527913	10.545217	10.433508	11.384967	10.735480	10.664169	11.413079	10.406371	10.472372	10.477843	10.450323	10.316793	10.620438	9.760421	9.803650	9.885790	9.526630	9.660455	9.894424	9.968820	9.895500	9.850414	10.468732	10.268511	10.263285	10.940842	10.195238	10.179032	10.324127	10.224836	10.110501	11.305148	11.234768	11.412245	11.543025	10.457869	10.858234	10.609075	10.919263	11.133855	11.680386	11.096025	12.207292	11.158776	11.898092	11.365889	11.071222	11.601959	11.011245	13.296386	11.012801	10.826359	11.448683	10.361591	10.643734	10.710534	10.893753	12.376451	10.939212	11.144256	12.113151	11.309982	10.982470	10.870667	11.486837	11.834043	11.019905	11.254265	11.135202	10.986127	10.917038	12.037766	9.995098	11.153927	11.535695	11.848060	11.510541	11.305887	10.709515	11.049296	11.635098	12.129935	10.505842	11.571927	10.707633	10.648046	10.266879	10.065419	10.208469	10.122625	10.781158	11.116825	11.102683	11.481075	10.940687	10.304338	10.887345	10.307568	10.624904	11.572071	10.674348	10.512879	10.010322	10.540179	10.210775	9.594166	9.769830	9.654697	10.710669	9.540818	9.937249	10.360509	9.511205	10.380759	11.408142	10.808596	10.886126	10.706106	10.422993	10.869477	10.622041	10.847998	11.030608	10.657842	10.764768	11.968566	10.433959	10.444263	10.537076	10.470433	10.643123	10.641500	9.660455	9.579457	9.695127	9.798775	9.980109	9.617636	10.929057	9.957845	9.664186	10.552645	10.410277	11.289319	10.998209	10.314435	10.804597	10.601825	11.058784	11.175052	11.802547	11.073138	10.553379	11.199453	11.236428	11.314836	10.573345	10.740196	11.593733	11.224321	10.608759	11.255007	11.745676	11.924435	11.324835	12.515979	12.155249	11.343546	11.165530	10.521739	10.509017	10.952431

MOG	6.736274	6.447841	6.216463	6.693598	6.376474	6.994160	6.675596	6.961011	6.811147	7.218710	6.467386	6.455878	6.174728	6.479137	6.117316	6.452936	6.443539	6.079562	6.935856	6.617422	6.289289	7.724989	7.104070	6.220415	5.635267	6.412347	6.762363	6.680462	6.682588	6.816998	6.442889	6.372955	5.783606	6.428535	6.031288	6.468191	5.885495	5.981675	6.211853	6.346221	6.726070	6.012483	6.739567	6.406241	6.269286	7.335828	6.680785	6.726738	5.960034	6.419799	6.254711	6.479304	6.154122	6.567898	5.902563	7.165525	5.679570	5.423847	5.805626	5.764109	6.257421	6.842711	7.418369	6.690628	6.716549	6.452936	6.400824	5.973067	6.267757	6.910016	6.996153	6.528320	6.948099	6.371935	6.375158	6.043120	6.646258	6.629051	6.482715	5.648403	5.404785	5.916177	5.477492	5.491117	5.367011	6.152751	5.786478	5.225696	5.589095	5.984867	5.681420	6.178996	6.224856	6.139619	6.013309	5.574831	5.639162	5.899553	6.709258	6.514720	6.245286	6.128168	6.370223	6.317412	6.549863	7.013009	6.397876	6.903827	6.842050	7.206767	6.793403	6.403750	5.785779	6.767961	6.001621	6.992596	6.416842	6.676729	6.504316	6.917410	7.388729	6.244135	7.005355	7.327400	7.465716	7.439764	6.241098	6.643191	7.672732	7.601535	7.069377	7.118055	6.700808	7.148490	6.388685	6.754244	6.426645	6.936164	6.643860	6.756707	7.765140	7.566262	6.931449	7.019054	6.951703	7.326266	6.867405	7.298474	7.746708	6.317742	6.784847	6.486942	6.949322	5.705599	6.333488	6.724487	6.874812	7.539578	6.728667	7.328659	6.416507	6.513895	7.060280	6.630313	8.127189	8.107120	7.282257	6.307087	7.340021	7.342525	6.747722	6.312295	7.315588	6.350111	7.293155	5.225509	5.901312	6.232252	5.657752	6.072576	7.404264	7.144444	7.135309	6.310763	6.509590	7.165350	7.146759	6.686616	6.916640	6.710407	6.860496	7.852497	7.092916	6.402270	6.541364	7.100790	6.738587	6.970286	6.572093	5.790268	6.367376	6.194961	5.355114	6.542379	6.735311	6.692425	4.914136	6.425612	6.094448	6.313672	6.318570	6.213566	7.282733	6.935856	7.026662	6.377121	6.483577	7.158540	6.026440	7.184551	6.589006	6.669308	6.517896	5.976039	7.261359	6.049762	6.371255	6.213207	8.632606	6.776864	6.107488	5.777806	7.562281	6.982395	6.852835	5.766251	6.841755	7.824466

HSPA8	8.698484	8.627922	8.783833	9.183929	8.555909	9.296274	9.325501	8.576545	8.230097	8.665616	8.479139	8.764687	9.272995	8.871377	12.811770	8.386650	13.701861	9.266257	10.559792	7.973216	9.570131	10.328856	10.721646	9.514420	9.625854	10.073446	11.934741	9.286747	9.045341	8.853927	9.306308	9.264743	8.660064	8.373727	8.175833	8.320628	8.262784	8.995493	8.732878	8.826707	9.045789	8.384200	8.408218	8.340415	8.169560	8.850756	9.006707	7.979256	10.467844	8.235581	7.829434	8.447805	8.246339	8.409099	8.704594	9.308789	9.246132	8.956077	8.680506	8.455668	8.749391	9.327364	10.657103	9.392525	8.844331	9.003955	8.167229	8.844814	8.428035	9.214227	7.959450	7.949691	8.563436	7.843927	8.341593	8.026826	9.091579	7.267734	7.930949	8.563284	9.349896	8.846008	8.893784	8.940204	8.133925	8.667128	8.118240	8.675315	8.748785	8.892677	8.607266	8.985620	8.969436	8.399403	9.403879	9.128602	10.053263	9.019286	9.817990	10.284425	9.660455	9.400578	8.512159	9.174191	9.731680	10.360365	9.316571	10.258347	11.189473	9.215117	9.902566	8.975115	10.172908	9.398637	11.115474	6.366378	9.380667	8.959458	10.009587	8.868537	10.732079	9.197337	10.261006	9.865593	10.253224	11.370366	9.304982	10.940842	9.510392	9.156011	9.947027	10.184227	9.594640	10.888202	9.166498	11.245791	11.231863	11.969197	8.457624	9.951526	10.968609	11.031483	10.823092	10.394618	10.755101	11.059375	11.437676	10.234648	9.796886	10.241363	11.209661	8.489977	9.407255	8.674365	9.011126	8.453070	8.529692	9.317338	9.188155	11.946821	9.021730	9.634626	9.492217	9.020620	9.041013	11.603886	9.335710	9.586073	9.558482	9.860677	9.554938	9.815723	9.334439	9.493614	10.715522	9.071115	10.085231	9.548541	9.043309	9.562398	9.666225	11.017510	10.020187	9.284296	10.046039	8.833199	9.530075	8.883433	9.349409	8.174272	8.769297	12.008250	8.455824	8.492438	8.345680	9.009822	7.996300	9.585922	8.647397	9.474009	10.117756	9.246431	10.697067	8.903213	10.034536	8.794316	10.408299	9.235949	10.719432	9.264903	11.007280	10.627617	9.528187	10.487647	10.690069	11.741732	9.649319	10.077279	9.521160	12.413974	10.312135	7.961616	9.235176	10.404404	10.540179	12.671548	11.979131	10.194330	9.328138	10.379552	11.066743	12.213629	9.039625	11.956506	9.673171	8.879581	9.727086	9.795202

TRPS1	4.181870	6.649826	6.801865	6.233928	7.400364	8.046717	6.979858	7.063822	8.197496	8.770989	7.916768	7.102676	7.582415	7.780597	7.473087	8.146426	7.490559	8.339505	10.641213	7.726369	8.621266	9.400002	8.430079	7.819523	7.637243	7.091491	6.343517	6.415636	7.441605	6.339386	7.990643	4.916406	6.977046	6.391555	5.942139	7.425716	5.556755	5.779280	6.270504	7.155766	8.352011	5.260658	6.375840	6.181859	6.514053	7.402873	8.254205	6.331457	6.327414	5.479076	5.994297	5.685865	7.247604	5.816371	6.438750	8.598366	6.816835	6.122355	7.829608	5.444863	5.561114	6.766039	6.177655	7.381709	4.941773	6.769450	5.581535	6.383321	5.359485	5.966637	6.000081	5.625771	8.406665	4.635852	5.679002	5.430487	4.633497	5.758717	4.962329	5.868862	5.585889	6.696046	5.195317	6.630936	6.735790	7.560752	4.534351	5.075669	5.815816	5.589300	5.182396	5.062460	5.435125	5.995839	5.033150	6.759287	6.077284	8.232693	7.402394	7.776338	7.106590	7.336449	7.808179	7.115667	7.715669	7.967408	8.142324	7.565494	8.352610	8.254820	6.946788	8.655793	7.640962	7.403034	7.954640	3.019669	3.229197	8.510113	7.404121	7.223131	4.158864	4.300245	5.406712	5.296854	7.043839	5.964494	5.908672	6.760913	5.671044	6.346731	6.208832	4.131246	5.837861	3.576116	4.704484	5.104212	6.398294	7.114581	6.135173	6.864858	8.477080	6.649508	4.769494	4.336552	6.667699	5.449750	5.271224	6.079720	5.636210	4.782618	5.348890	6.190615	6.170311	5.435317	6.277503	4.669502	4.429565	4.625919	5.531616	6.615279	4.786402	6.254711	5.398239	4.240767	7.091636	7.761283	5.376740	5.283757	4.561847	6.144617	4.599993	6.012629	6.202793	6.511614	5.864523	6.073976	3.029190	5.637885	3.742020	4.370890	6.285328	5.043462	6.359019	4.887089	4.343091	2.989605	4.630694	4.102375	5.338729	4.911356	5.586258	6.093732	5.224094	3.938998	4.335082	3.895246	3.926528	5.434751	4.479552	5.457696	4.843175	6.169784	6.054797	4.366132	3.755476	4.969897	5.476002	5.638981	5.361740	8.152993	4.365030	4.950017	5.487827	6.994459	7.622541	3.843761	4.504789	6.170481	5.490582	4.449838	6.008962	5.197306	4.191032	5.805451	6.515893	6.356984	6.115466	5.896541	7.088332	4.620829	6.081807	4.148555	6.487592	5.670490	3.633884	5.833722	6.556627	7.396310

LAT2	14.473933	14.152064	14.348562	14.930234	15.101143	14.853259	14.150116	14.378847	15.905774	14.344582	14.691512	14.637945	14.517783	14.601575	14.487923	14.620267	15.053135	14.553562	14.982214	14.367353	14.648824	14.615742	14.668633	14.658149	15.009362	15.331635	15.591703	14.526691	14.761491	14.928661	14.908102	13.028398	13.239009	15.101738	14.580592	14.369411	14.195562	14.525087	14.696565	14.778940	14.832004	14.726520	15.084166	14.767089	14.757850	14.881255	15.190029	14.032009	14.178659	14.385642	14.194648	13.949622	14.037892	14.274870	14.040527	15.011645	13.981326	14.233212	14.709387	14.186189	14.345644	15.212349	14.604151	15.101143	14.145227	14.464777	14.329971	14.525499	13.898577	14.769948	14.458572	14.757850	15.009958	14.189906	14.516636	14.348197	14.403999	13.805428	13.870987	13.146687	13.516909	13.445036	13.121579	13.255365	13.647535	13.900992	14.500649	13.362663	13.508359	13.191043	13.458952	13.740602	13.837713	13.807016	14.352005	14.325742	13.331903	14.894936	14.796433	15.642232	15.246670	14.711236	15.074615	14.748776	14.370460	14.677877	15.427177	15.467330	15.966981	14.801868	14.331365	15.253250	15.011645	15.207719	14.353395	16.240034	15.240441	14.870791	15.303021	14.639680	14.504194	14.558039	15.063409	15.979533	14.531049	15.074615	15.849957	15.265157	14.997957	14.861441	15.095711	15.303728	14.997389	15.137104	15.314555	15.037486	15.128463	15.429349	14.052986	14.958920	15.069269	14.139239	15.506499	14.781829	14.037568	14.980454	15.023469	15.926414	14.299113	15.186033	14.789655	15.036921	14.925975	14.381329	14.259621	14.237798	14.565137	14.888152	14.834547	15.321092	14.416904	14.328965	14.746490	14.392613	14.177402	15.241853	14.265614	14.132353	14.084036	14.186189	14.158320	13.685268	14.195562	14.452476	14.684603	14.253427	14.244999	14.591596	14.037892	14.143957	14.965697	14.528702	14.769473	14.790582	14.524343	14.691512	14.400018	14.662329	14.070910	14.528702	14.866612	15.156386	14.692440	14.563854	14.267647	14.498295	14.502269	14.572654	13.790706	13.898829	13.525717	13.831246	14.353021	13.484384	15.272991	13.497141	13.740367	14.252158	14.233886	14.344582	13.966334	14.575995	14.194016	14.376307	14.765753	15.140997	15.492147	14.829469	14.925433	15.213005	15.719891	15.723003	14.896544	14.183082	15.229335	15.601071	14.920091	14.541332	15.161962	14.660894	15.435694	15.714733	15.229335	15.408414	15.308698	14.739537	14.881255	14.999641

KIAA1257	8.998406	8.662549	8.558556	9.008394	9.478293	9.136672	9.523040	8.793388	8.345680	8.897149	9.283390	9.302196	8.504041	9.498106	9.666389	8.715790	8.825863	9.083784	8.170810	9.053125	7.995024	8.217444	8.594404	8.970198	10.449603	9.343762	9.081657	9.394660	9.099347	9.537972	9.027197	8.987320	8.763170	9.076408	9.017157	9.220198	9.236097	9.354834	9.959667	9.384665	9.392378	10.574514	9.229858	8.454436	9.340123	9.394660	9.283390	8.699239	9.070503	8.846315	8.916645	9.341170	9.055814	9.182973	9.070354	9.396668	9.603754	9.561303	9.212673	10.229267	9.944399	9.099806	9.401696	9.149696	9.402162	9.718135	9.440040	9.447310	8.387874	8.956217	9.070964	8.901711	9.310956	9.099191	9.067393	8.834942	8.631287	8.724336	8.640834	9.480095	9.833499	9.415816	9.007159	9.863922	10.382558	8.753634	9.497175	9.325501	9.897040	9.223262	9.664041	8.678046	8.628373	9.250707	8.373576	8.746957	9.135312	9.450783	8.713136	9.223893	9.000251	9.438669	8.746205	9.151110	6.904447	9.171885	9.939340	8.342758	8.422111	9.620873	7.660474	8.825536	9.484723	10.199765	8.394237	9.732263	11.451923	9.258917	9.760255	9.674122	9.402928	8.756076	9.516988	11.873996	12.347415	11.238618	9.041626	9.474159	11.538542	10.553379	10.442148	11.261231	9.908491	8.866879	8.791374	8.652502	11.387625	7.744557	7.995817	7.834927	11.520848	9.103656	8.850175	12.667306	8.761068	8.400034	11.848789	9.474903	9.828562	10.072829	9.363051	8.356756	8.898856	10.448243	10.538212	8.181304	8.987164	8.319872	9.939340	11.434353	13.106206	12.671375	7.859735	10.486117	9.883323	10.110954	10.287089	10.875592	9.225751	8.579360	9.210370	9.997209	8.638171	9.298930	9.486434	9.425135	11.530149	9.539708	12.021794	9.785948	9.472923	9.189785	11.014997	8.601432	7.981450	10.755261	8.444402	8.082112	11.108364	12.539646	9.250394	12.054832	8.687887	9.733780	8.180838	9.052657	8.983047	8.170198	8.840733	11.616116	10.910589	9.480728	11.610188	9.246431	9.044254	9.435704	12.053932	10.628531	9.403550	13.787649	9.154798	9.005667	8.540060	9.207491	8.803515	8.971445	9.671188	9.188155	10.974369	8.618293	8.551354	9.743493	8.148242	7.932481	10.708399	9.787132	8.326818	9.028725	11.190322	9.989367	8.099129	10.617450	11.441892	10.968164	10.421948	12.316075	10.672708	8.776752

EPOR	9.535347	8.760109	8.628373	9.499476	8.703217	8.827175	8.865361	9.081958	9.729350	8.690507	8.939279	8.831497	8.704437	8.449215	8.821898	8.764223	8.861351	8.630992	8.698949	8.619099	8.205229	9.387152	9.478926	8.453070	9.110021	9.303557	9.332770	9.061877	9.409712	9.327512	9.151857	8.911416	8.760273	8.852566	8.870081	8.665949	8.503865	8.422572	8.859559	8.673596	8.989121	9.042804	8.704737	8.685838	9.147686	8.842121	9.344831	8.712506	8.553764	8.794316	8.711476	8.467335	9.102118	8.733797	8.437844	8.905804	8.989583	9.268476	8.669872	8.767146	9.265792	9.172491	9.388260	9.019117	9.164033	8.494911	8.913127	8.663627	8.683845	9.044254	8.803665	8.566024	8.808284	8.671407	8.832570	8.767777	8.776297	8.817289	8.727414	8.863054	8.805667	8.890976	9.029036	8.656689	9.036629	8.461565	9.045030	8.635277	8.878011	8.768231	8.921354	8.903697	8.621124	8.632743	8.890098	8.516533	8.452479	8.880667	8.730322	8.643945	8.909199	8.279636	8.712362	8.502947	8.062273	9.047434	8.517165	8.642986	9.312647	8.895467	9.077347	8.820691	8.567450	9.297703	8.713413	10.271020	10.856320	8.632743	9.300643	8.712812	10.207204	9.207170	9.318351	10.230861	9.537676	9.875601	9.449820	9.885467	9.197647	10.152342	9.462868	10.524284	9.921069	10.446732	9.174793	10.005677	8.881755	9.481049	9.179848	9.328916	10.027037	9.624150	10.379863	11.101364	10.944454	9.267376	9.411987	9.868678	9.702032	9.456638	9.607163	9.653700	10.121103	9.695127	9.530828	8.973205	9.645183	10.350959	9.904851	10.301596	9.310505	9.248587	9.209005	8.991055	9.397313	10.120339	9.941169	8.987320	10.313050	9.233800	9.163075	9.433716	10.974077	9.603754	9.395906	9.403086	10.177956	9.607325	9.248124	10.090524	9.923356	9.972959	9.945591	9.224967	9.236434	10.393108	9.021575	9.545615	9.620723	9.031263	9.294119	10.416445	9.489336	9.700189	9.329390	9.668144	9.562068	9.270915	9.460752	9.753620	9.539221	9.726052	9.863624	10.428662	9.697856	10.296948	9.315818	9.924735	9.751590	10.163693	10.158322	9.872723	10.700309	9.916997	10.011086	9.733932	9.522567	10.006631	8.942487	10.365006	9.318981	9.856355	9.705954	10.234797	9.321044	9.510701	9.652899	9.153531	10.038241	9.753777	9.995098	9.414708	9.322141	9.558934	9.372113	9.317465	9.140544	10.354602

FAM174C	13.487933	13.514224	13.887252	13.727369	13.690456	13.219767	13.309343	12.894185	12.294671	13.215196	13.082921	13.297973	13.231839	13.857256	13.795129	12.989626	13.063231	13.705582	14.238141	13.071874	14.178338	13.662485	13.592616	13.565950	13.356786	13.113538	13.308756	12.950361	13.069296	13.413573	12.989438	14.056836	13.614714	13.376715	13.442439	13.162239	13.332262	13.497789	13.800613	13.482375	13.264151	13.707724	13.617064	13.469871	13.197314	13.795903	13.026194	13.778428	12.959007	13.265809	13.473788	13.453519	13.686919	13.755645	13.426460	13.058976	13.541684	13.851324	14.105067	13.842996	14.131266	13.336858	13.978346	14.331727	14.085609	13.802120	13.736123	14.324726	13.755645	14.181495	13.293756	13.558188	13.007761	13.524396	13.560400	13.726121	13.588156	13.564577	13.164898	14.329679	13.960526	13.693813	13.867818	14.384877	13.643752	13.490810	14.036411	13.527900	14.625993	14.488728	14.173886	14.439328	14.114980	13.927849	13.537270	13.915536	13.502805	12.969511	13.414830	12.624672	12.939042	13.189155	12.940096	13.092393	14.586863	12.967484	13.349308	14.515850	14.453564	13.142104	14.536742	13.567112	13.863595	13.093911	13.089449	12.750860	13.695182	13.683082	12.873920	13.915004	14.309339	13.742058	14.382065	13.971862	14.269566	13.763964	12.661376	13.726369	14.490355	14.180845	14.356594	14.821872	14.467367	14.350242	13.345293	13.712695	14.328233	14.898046	14.446741	15.129649	15.493839	15.782778	13.922688	14.836534	14.606651	13.528125	14.492360	13.955482	14.219589	12.900773	13.799817	14.094287	13.613044	13.799817	13.879868	13.618777	13.975805	15.085910	14.403276	14.446004	14.579342	13.748586	13.710918	14.073607	14.364816	13.596345	13.044463	14.126434	14.192726	13.376280	13.666468	14.111684	13.946797	13.625684	14.506441	13.896959	14.548542	14.132654	14.433838	14.266304	14.156390	15.122829	14.471172	14.333069	14.231871	14.661796	13.891028	14.430444	14.402538	14.201164	13.983800	14.013234	13.485992	13.980804	14.200109	13.806107	14.098263	14.077463	13.868320	14.291671	14.110411	14.486353	14.051213	14.691055	14.151049	13.934369	14.869310	15.026340	14.305307	14.712563	15.206355	13.942085	13.825287	13.794844	13.228465	13.355999	13.004126	13.415034	13.910543	13.463806	12.897761	13.718425	13.396889	15.515150	13.892647	13.954934	15.232746	15.225383	13.441594	15.348633	13.900711	13.280841	13.520934	14.203696	13.689737	14.262005	13.666222	14.437151

FBXO41	8.589904	9.377622	9.030689	9.323027	9.210693	8.974670	8.958107	8.532874	8.090338	8.760109	9.193041	8.905538	9.814145	9.105197	7.661380	9.443280	8.084435	9.263235	9.269690	9.878591	8.349146	9.167845	8.050463	9.398310	9.310215	9.566857	10.947159	8.573520	8.882036	10.527304	9.417796	9.913595	10.417667	10.808596	9.717648	9.434019	9.007782	9.677983	9.380804	8.388218	9.312647	8.619557	9.827223	8.954400	9.279384	9.574283	9.243504	8.360324	9.185942	9.246281	8.925972	9.450783	9.481198	9.453727	9.395127	9.079184	9.328621	8.557469	8.948291	9.657347	8.608929	8.649201	8.171919	8.020116	8.975575	9.222218	8.804918	7.718273	9.633073	8.289036	9.200778	8.912646	8.608629	9.494887	9.465354	9.800580	9.479239	9.440805	9.060051	9.763723	8.460937	9.455567	9.996752	9.592273	9.829982	9.702953	10.275761	10.131533	8.572585	8.564352	8.708574	8.740267	9.138103	9.780722	9.675059	9.164033	9.193041	9.253455	9.535972	8.885279	8.380173	8.952109	9.380969	8.997519	9.711706	8.743289	8.057141	8.857582	7.765302	8.777234	8.058274	7.932329	9.200481	8.843418	8.587391	6.282954	8.814254	9.369356	9.295525	10.451395	9.264430	10.071163	10.288521	9.842448	10.156816	10.803437	11.353164	8.121305	11.008736	9.391747	10.645044	10.909438	9.334765	9.385126	8.533620	9.270915	11.842797	10.462340	11.686029	9.389298	10.051718	10.041323	11.275334	11.170020	10.582460	9.760421	11.109567	9.587244	10.264251	11.842350	10.371706	10.988852	10.115901	10.530093	11.586469	9.571360	11.756172	10.788704	9.621360	11.181275	9.820866	12.024345	8.246339	11.134465	10.301888	11.415883	10.048197	11.661027	9.158502	10.501376	10.134213	11.963054	9.523327	10.090835	10.889218	11.016629	9.985950	10.594702	11.208087	9.477056	10.246028	7.889051	8.965385	8.828725	10.706418	10.423140	8.855773	10.288341	10.124928	10.668712	10.403192	10.251030	9.910805	9.952136	9.607163	10.172453	10.166289	10.354602	10.455568	9.444057	11.432696	10.853448	10.906300	11.268947	10.784221	10.514527	11.802821	8.959155	8.388365	10.077903	10.151883	8.558556	9.621515	9.491581	9.401854	10.686158	10.819554	9.408330	10.892989	10.614358	9.513967	10.902031	9.835054	12.066882	11.210560	10.276978	10.223028	9.760895	10.066606	9.892267	7.777904	10.047711	9.399554	11.368860	9.821458	10.798060	10.801787	11.739222

EIF1AY	3.343473	10.402738	11.158476	10.899523	10.829161	10.722821	11.345196	10.537786	9.897040	10.822224	10.679279	10.456157	10.277457	10.065869	10.145661	10.853742	10.544790	3.044421	9.321704	11.222510	10.488419	10.170585	10.716117	10.056961	9.432508	9.993987	10.810113	9.406773	10.388515	11.013401	10.433959	11.702812	11.881927	9.980586	11.486995	11.460244	3.498362	11.321996	10.626987	10.044933	11.119567	10.468588	3.361794	10.938211	11.072137	11.071672	10.506163	10.336453	11.675812	11.262567	10.951475	11.587764	4.370219	11.575527	3.757923	11.047654	3.939843	2.892414	9.915448	3.275739	10.534334	3.441340	11.164361	9.909707	9.752686	10.125091	9.960135	3.445453	3.015950	10.537517	11.389096	11.201915	3.074974	11.209393	11.198236	11.281195	3.387383	11.148990	10.999696	10.486410	10.445210	11.364289	11.353164	3.990661	11.730195	11.751278	11.433444	11.292363	10.247122	10.036097	10.516678	10.823396	10.650579	11.227093	11.190758	11.508343	3.775101	4.077825	11.171084	10.165968	10.775064	10.266592	3.128332	9.371801	10.800421	10.566949	10.307862	10.024017	9.608075	10.226206	3.497641	9.400002	9.717013	9.959383	4.624831	9.505192	3.857219	11.324220	10.358877	3.466108	7.791899	9.393270	8.419769	4.994800	8.825386	8.113605	9.663115	6.982716	7.406010	9.423613	11.495915	8.031490	6.979258	6.153607	9.626163	8.128554	3.373274	6.866463	11.540408	6.742265	8.222537	7.992551	8.762566	8.657128	7.823032	10.514527	5.429568	7.172144	9.412745	8.232255	9.726052	9.035125	7.895271	11.168835	10.195828	3.583962	9.120947	7.833855	8.951168	9.988254	10.258347	3.883367	8.166768	10.979516	9.647662	5.693593	7.989106	11.102534	9.334765	10.642988	9.762301	4.049324	9.333694	3.360502	9.255278	3.522236	2.898333	7.524675	3.045148	7.865730	3.623070	10.108529	9.580581	8.370159	9.306959	10.013258	3.780612	3.785421	9.104590	9.296610	8.893482	3.566109	9.234563	10.151883	10.226672	3.583224	9.323656	10.410429	10.152036	9.493455	9.313619	11.496207	3.549478	11.308691	8.552568	9.272123	8.847714	9.151110	8.994888	7.359946	7.776496	9.523949	9.229997	10.945054	10.860728	3.405895	3.051004	7.832150	8.289036	8.094476	9.272549	3.132715	8.073812	7.643210	7.476459	9.081035	9.228039	11.290061	6.286581	3.286231	9.026158	3.351071	9.583467	3.335812	6.601343	4.492677	7.731957	7.456332

SLC14A2	6.811147	5.284565	4.993108	6.380296	5.601135	7.865730	6.606374	6.231727	6.141731	7.744995	8.491478	7.898223	6.139091	5.363851	4.453967	7.330158	7.448253	4.530956	5.405925	7.666067	4.784124	5.243809	5.835886	4.355913	5.257917	5.339320	7.417421	6.535312	8.328604	6.664613	6.362867	4.917266	4.829094	7.289104	6.791693	6.085920	6.321505	4.872775	4.957295	3.837782	7.262822	2.952581	5.214034	4.814353	5.472171	5.450854	9.154798	5.859241	5.825139	4.716023	3.745044	4.235959	4.139301	4.277307	4.449838	7.871982	5.133404	3.027480	3.586905	3.484067	3.093041	8.228867	4.708660	3.869015	4.183437	4.654410	4.235322	4.616425	4.370890	4.372594	5.930416	6.166868	8.120696	6.387306	5.828960	5.834109	6.049762	5.342558	5.337890	4.842988	4.180567	3.884898	4.318023	4.319720	4.009963	5.374929	3.950551	3.463210	2.977056	3.156400	3.490484	3.392134	4.084981	5.490389	5.787748	6.417972	5.114185	8.006998	7.491660	6.606716	7.822263	6.187067	6.097512	5.358537	5.251581	7.831559	4.411549	5.454755	4.538427	7.964808	4.669934	4.143033	4.844870	8.115024	7.080977	8.568189	5.131397	8.086300	7.960849	6.670249	5.372063	4.639269	5.490755	5.792044	6.190787	7.689797	8.620345	6.232414	6.702081	7.656711	7.837337	7.292246	5.219369	4.869784	7.616951	5.737892	4.999402	4.981142	5.614814	4.133820	4.854590	5.777617	4.950416	5.635631	5.391069	7.196961	5.817089	7.724844	5.650854	6.962124	6.132434	5.635100	7.339745	5.601705	4.862492	6.625166	4.519116	4.850976	4.372155	7.053976	2.910533	4.493107	3.757114	4.137363	5.053865	7.547120	5.125133	5.465211	4.011026	3.780400	3.512208	3.744836	4.023017	4.333078	7.117230	4.725774	3.060046	3.302053	3.371691	2.968734	7.120267	3.289532	3.127814	3.215305	4.170551	2.849361	4.021539	3.599742	5.562034	5.196697	5.881412	6.266387	5.559256	6.128501	5.069189	6.134823	4.914337	6.150503	4.252568	3.614582	4.113339	3.364197	3.967464	3.540150	6.261843	3.388949	5.110367	2.923134	3.973097	3.328510	3.158396	4.689517	6.188929	6.855733	7.191688	6.764101	8.633518	7.680701	5.251176	6.703693	7.298148	7.017952	4.960214	3.745674	7.089306	3.108051	4.131246	3.748721	7.228420	4.809737	4.701302	6.032733	6.674449	7.148828	7.695913	5.821481	7.583967	6.189910

SHOX2	15.501368	12.665284	12.465329	12.397534	12.161724	13.952644	12.676040	13.571674	12.940186	12.878536	13.512631	13.085147	11.960999	10.461430	11.060858	13.786622	12.816808	10.616435	12.078200	14.346713	11.887800	11.939692	11.631199	11.344607	11.098087	12.043646	12.263695	12.631485	13.450122	13.641524	12.566593	11.052514	12.456129	12.165750	13.453519	12.259890	13.996150	13.824511	12.275177	12.421993	13.765490	12.095039	11.064802	12.360678	11.304114	11.814486	13.114655	11.560680	13.300495	12.851592	14.033477	10.199193	10.783312	12.234514	10.831653	13.639188	10.538212	10.530844	11.231267	10.745574	10.696036	13.166297	11.074858	11.776075	11.442486	11.676245	12.375982	11.900872	12.284727	12.193218	14.706637	14.550582	13.574051	14.400393	13.625684	12.063806	12.551234	13.728607	13.769045	9.544116	9.294739	10.837243	11.301442	10.353193	12.548754	13.409232	13.831246	13.759418	10.382403	11.047517	10.866362	10.929944	11.665214	12.592652	11.843716	11.794551	12.394267	11.400133	11.762794	12.470062	11.932456	12.026746	12.503725	11.898839	11.253671	13.051444	11.443078	11.056587	11.740862	13.339340	11.649323	11.189473	11.313224	13.489283	12.997893	13.785091	11.643705	13.184093	13.285050	12.277123	13.087953	11.958709	12.121431	12.423912	13.443956	13.294357	11.393088	12.235761	12.640215	13.410531	11.952291	12.124642	11.376076	11.511560	13.117358	12.084965	11.931845	11.762513	12.227998	12.856573	12.063806	12.726341	12.738673	11.177335	11.467920	13.127862	11.860414	13.085901	13.240817	13.253211	11.545153	12.000886	13.350761	12.686046	12.465490	13.499569	12.916404	12.404662	12.870487	13.232053	12.024653	10.016345	11.968868	11.483699	12.114204	13.554388	11.870252	12.236220	12.171565	13.221771	10.502860	10.970588	11.385844	10.535450	13.065113	9.604210	10.806498	11.464636	10.955581	11.171811	12.121431	12.672419	11.507158	12.207449	10.907937	12.523015	12.370197	12.055582	11.230338	12.963629	13.242002	13.672692	13.755369	12.150644	12.487990	12.475535	13.291948	13.859930	10.948753	9.759952	10.953197	12.089510	11.487721	12.412540	12.196315	14.236540	11.834974	11.064802	11.488607	9.907428	11.959299	12.053308	12.369200	13.380525	12.594923	12.622994	13.185843	12.612171	11.723321	11.655452	11.888108	12.989626	11.531987	10.752942	12.674493	11.338491	11.218557	12.265583	13.466188	11.560840	11.559519	12.229099	13.919694	11.851856	12.359421	10.941524	12.683650	12.834200

KAT6B	12.630506	11.834673	11.700081	12.258508	12.547624	12.037292	12.507711	11.727061	12.350213	11.968715	12.210827	12.085897	12.372433	12.091377	11.948495	12.330329	12.428983	12.310003	12.549091	12.184541	12.374349	12.351946	13.857001	12.417049	12.711326	12.665284	12.591825	12.381950	12.321603	11.996401	12.571330	11.713634	11.854007	11.501897	11.884633	11.932902	12.228786	12.022508	11.867888	11.751899	12.401299	11.912793	12.408210	12.266375	12.923493	12.181896	12.283820	10.885551	11.726448	11.970213	12.022968	12.162952	12.119748	12.020355	11.809631	12.329562	12.374659	11.899125	11.890346	12.205573	11.885400	12.268991	12.406352	11.853082	11.544374	11.874773	11.504078	11.726750	11.419414	11.551660	11.794841	11.981974	12.274566	11.898522	11.863499	11.660289	11.844183	11.375921	11.541954	11.138761	11.816173	11.989265	11.842192	11.842962	12.609021	11.992461	11.848932	12.205573	11.951566	11.213347	11.920036	11.526511	11.642235	11.480371	11.555669	12.144699	11.939377	12.756165	12.075117	13.110240	11.999751	12.546528	12.505754	12.491562	11.523666	12.573503	12.767631	11.955591	12.566107	12.452642	12.375646	12.255831	12.075117	12.190143	12.088732	9.996752	12.695090	12.432090	12.770516	11.401220	11.272804	11.445714	11.945155	11.872506	12.075117	11.628369	12.698837	12.157886	13.601026	11.394876	11.892007	12.505071	10.769710	9.997492	12.503895	11.129277	12.395544	12.677340	11.896778	11.776985	12.459294	12.579011	12.116671	12.787861	12.247983	10.840331	12.250568	11.641628	11.200022	12.602572	11.345947	11.549868	11.547691	11.054370	11.530297	11.233156	11.452083	10.473776	11.001079	11.801628	10.887345	12.197215	12.123620	12.045443	11.682169	11.485173	11.651679	11.703111	11.359717	11.032397	12.064410	11.795725	11.326759	11.397914	11.758435	11.943044	11.486562	11.076330	11.590838	11.114554	11.987566	11.024075	10.664169	11.578488	11.305742	10.889830	10.986908	10.841568	10.617741	11.285928	11.331326	10.801045	11.404390	11.547102	11.031779	10.923426	11.009483	11.073280	11.180670	11.162013	12.147040	11.015587	12.290138	11.884206	13.248522	11.318368	12.244999	12.203133	11.156708	12.182658	11.260034	11.331478	11.235350	11.322437	11.725496	10.824581	12.532342	11.280035	12.672938	11.048835	12.579838	12.699705	11.372737	11.666419	12.102545	13.072243	11.541661	11.763874	11.331759	12.478001	10.939616	11.580422	11.332767	11.867888	10.873539	11.340687	11.798796	11.077660

UROS	12.728615	12.450102	12.416103	12.255523	11.908965	12.053445	12.544205	12.158335	10.705507	12.143273	12.020215	12.491398	12.060393	12.124346	12.109351	11.740121	12.084173	12.079107	11.976069	12.456287	12.204037	12.776543	13.294357	12.005028	11.652891	11.743759	11.862307	10.935634	11.567694	12.118493	11.546807	13.106614	12.963098	12.666171	13.087953	13.100683	12.780199	12.924711	12.864398	12.502885	12.120508	12.702618	13.061734	12.541791	12.796078	13.282417	11.672529	12.623334	12.284248	12.850513	13.073797	13.279450	12.536900	13.386045	12.904642	11.811423	13.353095	12.608850	12.753644	13.096725	12.958825	11.932902	13.962742	12.369546	12.590988	12.522051	12.628507	12.543561	12.738673	13.157657	12.868885	13.050446	10.850917	12.813692	13.040220	12.776712	12.869060	12.442520	12.780022	12.251658	12.667306	12.453418	12.938338	13.058425	13.218764	12.985549	13.129561	12.457385	12.953298	12.710652	12.685688	13.061361	12.565127	12.818205	12.876579	12.823533	12.656999	11.851418	12.260677	11.717938	12.045877	12.622994	11.541804	12.118153	13.377337	11.526788	12.285826	12.705525	13.092572	11.478703	13.011590	11.964164	12.533949	11.121598	11.373920	10.966697	12.909480	12.106371	11.031948	12.478661	12.218642	13.142638	11.947881	10.290812	12.488461	11.400440	11.652286	12.103128	12.741981	11.964164	11.887212	12.462601	12.047831	10.335092	11.618460	12.491754	11.891870	12.256329	13.320668	11.437522	12.248149	13.462926	10.666510	12.186019	12.266082	11.511854	11.104149	10.314899	12.009153	11.801189	12.787522	12.345006	12.353242	12.877121	12.447863	12.718866	12.455317	12.144544	12.386292	11.620252	12.976403	13.332070	13.225499	13.168263	13.084018	10.849149	12.747949	12.835422	13.723726	12.415781	12.352259	13.605486	13.163576	13.055601	11.839166	12.763272	13.430141	12.629207	13.117722	12.610356	12.177525	12.120820	12.802300	12.631160	12.808590	13.570563	12.587968	11.970501	13.165501	12.770516	12.687955	10.822063	12.874491	12.954985	12.759589	12.482634	13.290292	12.616477	12.231738	12.769062	13.521840	12.310609	12.890856	13.201208	12.645722	12.816972	13.608723	13.443765	12.603413	12.596810	13.350761	12.757215	13.274388	12.077435	13.088513	12.254351	12.066729	11.810382	13.245910	12.573686	12.702273	10.959734	12.701928	13.271585	10.881487	12.578683	13.048778	12.769413	11.082044	13.186053	12.120667	12.628852	10.809966	13.171124	11.826829	12.634617	12.703568	12.609021

ZNF677	6.824026	6.000081	6.781503	6.544184	6.405559	6.261678	7.455252	5.021985	4.941159	6.597946	6.062774	6.035624	6.064343	6.512439	6.983979	6.783714	4.700479	6.935371	5.285388	6.742734	5.055546	6.499570	7.626412	5.892386	6.649826	5.699307	6.538915	5.956634	6.660691	6.134494	4.356108	7.867250	7.532056	6.459372	7.570365	7.831717	7.866357	7.314660	6.750842	7.664378	6.556627	7.625933	7.183140	7.432187	8.069994	6.683734	5.790077	6.540186	6.527992	7.636483	7.502589	7.856208	8.049851	7.365622	6.807739	5.787748	8.342015	7.648004	6.390194	7.601535	8.115024	6.991611	7.426317	6.472740	6.646089	6.639819	7.500244	6.952016	6.087475	7.168868	7.443000	7.523112	6.251495	7.336916	7.354060	6.655806	7.344722	6.240763	7.215915	7.034373	7.776496	6.947279	7.710165	8.176328	7.903984	7.745146	7.066362	6.927973	7.069874	6.085754	7.114093	6.624833	7.142089	7.331331	7.542183	7.259036	6.736422	7.036396	6.194799	6.680932	5.925962	7.305240	6.247129	6.286409	6.505130	5.641390	3.627023	5.473107	5.231998	6.058527	6.789423	4.694111	4.860848	6.092703	4.963547	6.707704	7.096562	6.500045	6.409866	4.771765	5.012811	6.000597	6.598102	4.516358	4.517223	5.923488	4.798980	4.034080	4.573305	4.882858	4.913039	5.163875	6.102226	6.498576	5.468124	4.217085	3.999159	4.007805	5.304215	4.360707	5.348703	5.856716	3.668288	5.350035	4.084103	6.413054	6.093905	5.268480	5.621670	5.429568	3.928494	4.801966	6.102386	6.036353	6.113696	7.244534	6.888567	4.405372	5.182603	5.137833	5.161480	5.681975	4.950017	5.334373	5.258110	4.708270	5.221908	5.322477	5.921773	6.662158	6.417972	5.990781	5.194061	7.545653	5.752653	6.858980	4.282532	4.445874	5.415640	5.518976	6.084236	7.335661	4.324223	4.153649	5.341799	3.642657	5.111332	4.419593	5.518976	5.815816	6.211381	4.260010	6.249294	6.689803	5.645034	6.623549	5.454938	5.528763	7.025077	6.629515	5.421300	6.346221	6.738587	7.014515	5.831224	6.385280	4.605847	5.506607	5.227060	7.129868	6.734661	6.240584	6.495243	5.828960	5.724545	5.342558	5.618176	4.150003	5.055139	4.701302	4.373932	5.276162	4.462440	3.045780	5.255554	3.340106	3.591517	3.531937	4.347825	5.312014	4.773254	6.379471	4.644274	4.913496	4.609078	5.377319	5.040280	4.498788

TK2	11.658189	11.387625	11.744798	11.713327	11.630454	10.924005	11.845235	10.989151	10.635496	11.547236	11.168397	11.207783	10.987180	11.243314	11.383634	11.491594	10.929712	11.130031	12.456759	11.618765	11.694395	11.711018	9.200336	11.683496	11.783598	11.768847	11.417099	11.175522	11.241643	11.853395	11.405143	11.920823	11.768560	11.041648	11.925051	11.647837	11.810068	11.749984	12.218494	12.378188	11.562313	12.676852	11.391292	11.434496	11.741869	11.165803	11.217158	12.065618	11.058784	11.454008	11.649619	11.800745	11.902027	11.581405	11.127076	11.248361	11.353017	12.351197	11.864994	11.982413	12.197990	11.705942	13.209700	12.583137	12.341379	11.706405	12.240559	11.493891	11.734381	12.264028	11.662821	11.284747	10.888202	11.339525	11.545607	11.860555	11.774016	11.704316	11.615241	11.939846	11.737635	11.872355	11.683496	11.549648	11.777620	11.966878	11.812156	12.089816	12.505403	11.993963	12.248989	12.578521	11.613311	11.442790	11.642537	11.462914	11.531239	11.659107	11.539677	10.994167	11.038163	11.380992	11.226506	11.414563	11.745095	11.079831	11.870252	11.200580	12.548924	11.605506	9.521307	12.066571	11.690040	10.771383	11.080441	9.948751	12.384330	11.440637	10.994463	10.410869	10.591557	11.606677	11.729465	9.650553	10.029478	10.785727	10.765383	11.670899	10.780734	10.402284	9.882574	10.680952	11.246940	10.818491	11.538956	11.859653	10.013258	11.302336	11.037267	10.890897	11.459486	8.280832	9.700030	11.579980	12.327958	10.607687	9.273471	9.796331	11.395640	11.543627	11.564784	11.230487	10.346580	10.950587	10.733421	11.612110	11.929527	12.087259	12.280813	10.336136	11.964481	10.653995	10.550253	10.706272	10.203707	8.075788	10.915384	10.684506	10.918229	10.905697	11.575856	11.141784	11.228558	11.782660	11.608622	10.606089	11.724539	11.048256	10.837075	12.036393	11.768414	11.696386	12.088732	12.052072	12.226768	11.678161	11.001214	10.967834	12.441914	11.285485	10.954078	9.743493	10.932934	11.586749	11.454008	11.750117	11.931067	11.093843	11.990637	11.633293	10.399429	11.054506	11.433898	11.590688	10.999696	11.077953	11.653482	12.480642	11.875072	11.195021	12.135587	11.393682	11.122494	11.051785	11.420572	11.302634	11.257082	11.311460	10.886269	11.475562	10.517868	10.143844	11.345947	12.326688	10.632306	10.554929	10.855244	11.315434	9.660763	8.986399	11.786428	10.134688	10.691815	10.207843	10.826359	11.596981	11.540550	11.817489

AC010542.3	11.658189	11.387625	11.744798	11.713327	11.630454	10.924005	11.845235	10.989151	10.635496	11.547236	11.168397	11.207783	10.987180	11.243314	11.383634	11.491594	10.929712	11.130031	12.456759	11.618765	11.694395	11.711018	9.200336	11.683496	11.783598	11.768847	11.417099	11.175522	11.241643	11.853395	11.405143	11.920823	11.768560	11.041648	11.925051	11.647837	11.810068	11.749984	12.218494	12.378188	11.562313	12.676852	11.391292	11.434496	11.741869	11.165803	11.217158	12.065618	11.058784	11.454008	11.649619	11.800745	11.902027	11.581405	11.127076	11.248361	11.353017	12.351197	11.864994	11.982413	12.197990	11.705942	13.209700	12.583137	12.341379	11.706405	12.240559	11.493891	11.734381	12.264028	11.662821	11.284747	10.888202	11.339525	11.545607	11.860555	11.774016	11.704316	11.615241	11.939846	11.737635	11.872355	11.683496	11.549648	11.777620	11.966878	11.812156	12.089816	12.505403	11.993963	12.248989	12.578521	11.613311	11.442790	11.642537	11.462914	11.531239	11.659107	11.539677	10.994167	11.038163	11.380992	11.226506	11.414563	11.745095	11.079831	11.870252	11.200580	12.548924	11.605506	9.521307	12.066571	11.690040	10.771383	11.080441	9.948751	12.384330	11.440637	10.994463	10.410869	10.591557	11.606677	11.729465	9.650553	10.029478	10.785727	10.765383	11.670899	10.780734	10.402284	9.882574	10.680952	11.246940	10.818491	11.538956	11.859653	10.013258	11.302336	11.037267	10.890897	11.459486	8.280832	9.700030	11.579980	12.327958	10.607687	9.273471	9.796331	11.395640	11.543627	11.564784	11.230487	10.346580	10.950587	10.733421	11.612110	11.929527	12.087259	12.280813	10.336136	11.964481	10.653995	10.550253	10.706272	10.203707	8.075788	10.915384	10.684506	10.918229	10.905697	11.575856	11.141784	11.228558	11.782660	11.608622	10.606089	11.724539	11.048256	10.837075	12.036393	11.768414	11.696386	12.088732	12.052072	12.226768	11.678161	11.001214	10.967834	12.441914	11.285485	10.954078	9.743493	10.932934	11.586749	11.454008	11.750117	11.931067	11.093843	11.990637	11.633293	10.399429	11.054506	11.433898	11.590688	10.999696	11.077953	11.653482	12.480642	11.875072	11.195021	12.135587	11.393682	11.122494	11.051785	11.420572	11.302634	11.257082	11.311460	10.886269	11.475562	10.517868	10.143844	11.345947	12.326688	10.632306	10.554929	10.855244	11.315434	9.660763	8.986399	11.786428	10.134688	10.691815	10.207843	10.826359	11.596981	11.540550	11.817489

PTER	11.341314	5.794031	9.958305	9.831229	9.227124	8.818241	9.060660	7.674955	7.603129	8.938834	6.543856	9.531652	8.865812	9.373824	5.873704	9.066762	9.572739	6.103928	5.971960	7.464647	6.474239	4.320790	9.109422	5.709192	7.641095	8.164723	8.674991	8.502624	6.370064	6.047481	8.655522	8.013862	8.186342	9.269079	9.388260	7.229677	7.114394	10.327480	8.174272	9.731833	8.974524	8.117780	7.441605	9.537676	9.495957	10.293540	8.501395	5.282173	9.289571	9.435704	10.544020	9.359225	7.077503	9.610124	10.022749	9.274698	9.523489	8.230395	5.546573	8.602527	9.205649	8.786239	8.084927	8.328762	8.273134	8.011566	8.979397	9.414708	9.531152	7.863395	6.315207	9.128766	8.729291	9.596649	9.090933	9.533072	6.652567	9.387333	6.008962	6.566103	10.199328	9.621515	10.414939	6.725278	10.687045	9.860350	9.672854	9.959667	4.871701	8.713413	8.516857	5.106852	8.663180	10.085100	11.058379	10.381639	6.607406	7.450429	9.096736	5.110765	8.357368	9.973251	8.516237	9.050843	4.932831	9.028112	7.459188	7.916925	3.980200	8.897627	7.980661	4.494208	6.905892	8.267453	9.611512	7.054118	10.100136	8.626865	9.439711	9.791905	4.474750	9.915905	9.245688	6.732875	8.351557	8.040770	8.290571	5.880879	5.869547	5.855821	9.198923	7.992858	9.650396	3.856567	9.400900	10.684823	4.761630	5.102166	7.552684	5.151120	3.439974	9.446063	3.317763	9.310654	7.565641	9.102439	9.829180	5.533682	5.560405	9.525858	5.666871	6.072060	9.373655	9.121839	5.964297	6.170481	10.744560	7.962833	9.030848	8.949507	6.399974	7.132134	9.797194	8.968219	9.750979	7.651278	3.086040	8.611378	9.608227	9.637950	9.164033	5.775641	9.994464	10.351574	10.398534	9.641913	8.192912	3.599132	7.205372	9.057352	8.159617	8.475733	8.181304	8.111766	7.949528	8.472170	8.443325	9.421896	10.059546	6.015535	9.001028	9.236434	9.704296	8.821898	9.169683	5.499283	10.208162	4.209072	6.085920	9.896882	10.047403	9.593696	5.821680	10.198139	8.333986	9.524595	10.761158	4.928275	8.121469	7.536868	4.816230	7.812489	8.859422	9.851548	9.172647	6.137188	5.959677	9.458948	6.163570	6.087995	8.894686	9.434327	8.509216	4.775323	7.696994	7.487395	9.168906	4.414432	9.082279	7.416992	3.679837	6.135673	7.927106	9.047434	10.967244	8.591916	6.628874	9.892557

HEY1	10.007529	9.881630	9.118977	9.233328	8.141326	9.245688	10.179480	9.251027	8.354136	9.746922	9.169683	9.889840	9.921529	8.497023	9.873170	9.290484	9.062320	9.065840	9.084561	10.344001	8.647237	10.144161	10.669542	8.304977	9.096895	9.428985	10.727474	9.357553	10.419946	12.118961	9.381956	10.656678	10.337046	11.949752	10.981465	10.291284	10.443033	10.181016	10.746326	9.877686	9.459862	10.893304	10.861451	10.451803	10.256188	9.932497	9.616513	9.637166	10.338538	10.070706	10.664611	10.742742	10.424856	10.609789	9.453727	10.277896	10.130482	9.903332	10.413303	10.197218	10.549645	10.551284	12.551069	10.325194	11.262129	9.902566	10.770448	10.138967	10.362972	10.674193	10.339585	10.299127	9.533695	10.527159	10.055903	10.612140	10.261006	11.151411	10.712259	9.716549	10.122789	10.537371	10.306695	10.485631	10.509173	10.799503	10.821920	10.123969	11.318974	10.185134	10.162442	10.961497	10.933223	10.100782	10.885099	10.323206	10.454947	10.279731	9.999208	9.222218	9.644393	9.310654	9.366931	9.181882	9.405418	9.667475	9.064900	9.857253	10.022143	9.464857	10.475118	9.124740	8.465978	8.679300	10.152177	8.541974	10.018641	9.596161	9.630751	10.423614	11.754833	12.469402	9.150640	11.932141	13.387976	13.543949	14.262921	12.894185	12.167732	10.693165	14.549326	13.659596	12.643586	10.773444	10.621887	11.038735	13.309343	14.284842	13.680191	12.404510	14.731220	14.884408	13.098061	14.506817	11.809037	12.950935	11.113773	14.104388	13.176379	11.612874	10.865293	12.512428	11.093554	11.746871	12.998952	11.487873	10.866981	10.783470	12.480968	12.570995	13.062656	11.108364	10.433661	13.205135	11.945613	9.399241	13.706546	13.885163	12.778842	11.428860	11.313643	12.837080	12.166779	14.086854	14.123682	13.814043	13.649618	12.738484	13.263764	11.243314	13.073229	11.269993	12.494508	10.698669	11.164195	11.561717	12.158042	11.137430	10.808753	13.463136	13.073967	9.265944	12.151398	12.335244	11.792707	12.473785	12.681802	12.701417	11.811860	10.663570	10.485177	10.563219	11.843106	11.781075	12.862774	11.527903	10.653668	11.562313	11.773141	12.686212	11.897946	13.931643	12.776909	11.214237	13.012808	12.502567	12.515666	11.479023	14.573899	12.579166	14.812693	13.894497	12.410173	14.769948	12.166484	13.230666	12.105127	14.836534	11.111876	13.880400	13.138899	11.985255	9.871973	14.257993	10.509549	12.314947	10.079690	11.232005

TBX18	7.251697	9.545933	9.334014	10.035941	9.344383	8.424087	10.440492	9.141505	7.380184	9.378381	8.310361	8.363315	11.065101	9.116948	8.769448	8.555423	9.695713	9.831229	9.528476	8.877393	8.780550	10.412690	11.144404	8.756076	10.100633	10.291572	9.987460	9.371195	9.666225	10.069025	9.302196	9.541279	9.209157	10.783906	10.177785	10.349613	10.212902	10.624904	10.994906	10.517131	9.634765	11.179135	10.562917	9.510217	11.246940	9.715343	10.294926	10.499861	9.269982	10.192057	9.085192	10.361120	10.606387	10.085546	10.280645	9.483039	10.917038	10.704200	10.042063	10.767880	10.425603	10.806498	11.837541	10.300966	10.714015	10.338850	10.378959	10.007811	10.655314	9.936271	10.023858	9.329853	9.311872	9.993380	9.394059	9.424072	10.205978	9.245828	9.037850	10.600915	9.503174	10.722821	10.142755	10.053753	10.613036	10.309103	10.036268	10.516102	10.720925	9.228967	10.434589	10.437655	9.540026	10.486117	8.359725	9.531481	10.435536	10.197801	10.247418	10.385788	9.706727	10.227583	9.603459	9.680465	8.856221	8.892677	9.598639	9.614989	9.677525	9.222961	10.496787	8.794902	8.242037	7.788842	8.560859	9.416264	8.585918	9.221577	9.086559	8.165357	11.775366	9.381628	11.157291	10.707189	8.335259	8.954400	8.191012	9.200336	7.919983	8.567308	11.025992	11.989574	12.246555	8.382521	11.458769	9.268476	9.575790	8.221769	11.772400	8.590359	12.568065	11.696386	11.852935	9.759792	12.204194	9.019569	7.654678	8.458106	11.597121	8.792595	11.726597	11.747030	12.443548	10.394320	11.978981	11.362843	11.852005	8.879418	10.844879	10.159975	10.787337	9.050550	8.376183	9.183297	8.667128	12.401619	10.920184	11.053092	9.965154	11.728708	12.766912	9.821619	10.004887	12.408069	9.504879	9.634927	12.441117	10.072829	11.955591	9.441268	11.362257	12.733048	13.220952	12.014319	10.735064	11.712177	9.769520	11.644166	8.951813	8.890098	12.655175	12.552169	11.433599	10.042994	8.871211	11.272804	11.619033	10.781447	10.628531	12.546379	10.410131	12.717320	10.966697	11.484876	9.544433	9.777523	11.646944	12.071732	11.576750	9.297852	9.105657	11.124739	11.767362	8.991632	11.568841	9.516845	11.385706	11.684076	8.452630	11.657028	10.906005	10.809966	9.498268	9.072203	10.820134	12.334611	10.774471	10.806201	10.385937	11.723042	9.979955	8.991632	7.289259	11.057630	8.457339	12.428983	8.176794	7.841835

PSMB9	10.099038	10.344614	10.832125	9.569803	9.514568	9.913122	10.087473	8.431048	8.086750	8.812917	9.443739	9.354397	10.011528	8.446086	9.476585	9.965758	8.688665	9.787132	7.695762	10.415564	8.048473	10.275916	8.626721	10.558269	9.072667	8.437404	10.578592	7.810194	9.319298	11.682169	7.404585	11.206863	12.454055	12.151881	11.575095	11.252070	10.422850	9.686684	9.275993	9.932019	9.107183	10.183009	11.057782	10.778555	10.825316	11.227693	8.633227	10.541677	10.614231	10.738842	11.243131	11.034179	11.300099	11.053352	10.882223	8.815901	11.460712	10.495855	9.539381	10.050349	10.355208	9.431265	11.096172	9.555843	10.291882	9.861608	9.979195	10.171549	10.588804	9.924291	10.659469	10.336891	8.812483	10.829597	10.816113	11.860414	10.552052	11.405727	10.767431	11.108217	11.312627	11.346092	11.072137	10.638914	10.743513	10.633501	11.254400	10.949961	10.761158	10.005677	10.967106	10.821454	10.263600	11.430440	11.767362	11.221910	11.043725	9.476893	9.324307	9.277385	9.024467	10.097624	9.375129	9.025237	9.469668	9.065545	8.327291	9.959832	8.336641	8.203908	9.963485	8.095973	8.029041	8.614047	10.253644	8.162350	9.378542	9.604056	7.985454	10.572908	10.516533	11.764203	10.166750	8.565719	8.680653	10.615426	7.762198	9.156011	10.588378	10.015244	8.951656	8.673896	10.997288	8.275513	11.147807	11.111735	8.844175	8.119024	10.955728	8.421040	10.493743	9.835818	7.700373	6.566263	10.730762	9.779969	7.778353	8.244058	10.674193	10.287723	11.221910	11.135362	9.892413	11.941196	9.387333	10.993145	9.574876	9.266894	10.274257	8.194144	10.053753	10.584613	11.010198	10.540309	11.275177	8.511392	10.300015	11.171363	10.887345	11.701916	11.133688	11.622846	11.379443	12.024345	10.130482	10.875011	10.746036	10.057867	10.371998	10.721933	10.270720	10.250420	11.030306	11.109254	10.619249	10.114821	10.690069	9.880715	11.439584	10.819554	10.897742	7.778353	10.416284	12.090445	11.969922	11.070018	10.717586	9.891957	12.256489	11.288336	11.866384	11.424641	12.048425	12.067039	10.336136	11.442637	10.652063	11.218031	10.917191	9.873790	11.286577	10.517868	11.513170	11.324962	11.161265	10.969318	9.981518	11.116077	9.758430	10.903200	9.893477	8.303777	11.359717	11.306661	9.520997	9.138103	10.069768	9.642697	8.707044	10.188159	9.645966	5.796753	9.798110	8.898560	11.589947	10.223028	11.233924	10.772988

TAP1	10.099038	10.344614	10.832125	9.569803	9.514568	9.913122	10.087473	8.431048	8.086750	8.812917	9.443739	9.354397	10.011528	8.446086	9.476585	9.965758	8.688665	9.787132	7.695762	10.415564	8.048473	10.275916	8.626721	10.558269	9.072667	8.437404	10.578592	7.810194	9.319298	11.682169	7.404585	11.206863	12.454055	12.151881	11.575095	11.252070	10.422850	9.686684	9.275993	9.932019	9.107183	10.183009	11.057782	10.778555	10.825316	11.227693	8.633227	10.541677	10.614231	10.738842	11.243131	11.034179	11.300099	11.053352	10.882223	8.815901	11.460712	10.495855	9.539381	10.050349	10.355208	9.431265	11.096172	9.555843	10.291882	9.861608	9.979195	10.171549	10.588804	9.924291	10.659469	10.336891	8.812483	10.829597	10.816113	11.860414	10.552052	11.405727	10.767431	11.108217	11.312627	11.346092	11.072137	10.638914	10.743513	10.633501	11.254400	10.949961	10.761158	10.005677	10.967106	10.821454	10.263600	11.430440	11.767362	11.221910	11.043725	9.476893	9.324307	9.277385	9.024467	10.097624	9.375129	9.025237	9.469668	9.065545	8.327291	9.959832	8.336641	8.203908	9.963485	8.095973	8.029041	8.614047	10.253644	8.162350	9.378542	9.604056	7.985454	10.572908	10.516533	11.764203	10.166750	8.565719	8.680653	10.615426	7.762198	9.156011	10.588378	10.015244	8.951656	8.673896	10.997288	8.275513	11.147807	11.111735	8.844175	8.119024	10.955728	8.421040	10.493743	9.835818	7.700373	6.566263	10.730762	9.779969	7.778353	8.244058	10.674193	10.287723	11.221910	11.135362	9.892413	11.941196	9.387333	10.993145	9.574876	9.266894	10.274257	8.194144	10.053753	10.584613	11.010198	10.540309	11.275177	8.511392	10.300015	11.171363	10.887345	11.701916	11.133688	11.622846	11.379443	12.024345	10.130482	10.875011	10.746036	10.057867	10.371998	10.721933	10.270720	10.250420	11.030306	11.109254	10.619249	10.114821	10.690069	9.880715	11.439584	10.819554	10.897742	7.778353	10.416284	12.090445	11.969922	11.070018	10.717586	9.891957	12.256489	11.288336	11.866384	11.424641	12.048425	12.067039	10.336136	11.442637	10.652063	11.218031	10.917191	9.873790	11.286577	10.517868	11.513170	11.324962	11.161265	10.969318	9.981518	11.116077	9.758430	10.903200	9.893477	8.303777	11.359717	11.306661	9.520997	9.138103	10.069768	9.642697	8.707044	10.188159	9.645966	5.796753	9.798110	8.898560	11.589947	10.223028	11.233924	10.772988

MPP3	7.521929	7.090725	7.469644	6.784522	7.123687	6.478640	6.853000	5.910088	7.260304	7.362910	7.304922	7.070868	6.695543	7.306812	7.412455	6.995532	7.052414	7.585179	8.611216	7.417717	6.925659	7.773210	7.764659	6.715371	7.841561	6.876549	6.114764	7.137662	7.195369	6.636123	7.129553	7.607291	7.128933	7.132916	6.990944	7.152928	7.095641	7.165954	7.751835	7.207245	7.081142	7.426476	6.591797	6.970730	7.124348	7.298304	7.458563	6.988909	7.389191	7.309930	7.071013	7.820703	7.668348	7.102506	7.491814	7.053173	7.694121	8.103712	7.749980	8.189423	7.507708	7.098929	7.763900	8.073029	7.691024	7.771578	7.385610	8.126124	7.343187	7.437870	6.747055	7.201945	6.969180	7.215915	6.942312	6.386290	7.153231	6.031469	6.671556	7.862157	7.607143	8.019529	7.669290	8.034780	7.633622	7.978305	7.129090	7.531129	7.951067	8.161466	7.578077	7.728413	6.767312	7.151068	7.014228	7.478298	7.281951	7.699612	7.321841	7.880784	7.226540	7.401622	7.541740	7.418994	8.355223	7.094491	7.003824	8.117928	8.765747	7.520666	7.931883	7.285498	7.050534	6.653041	8.169560	5.386970	7.770961	7.817936	7.666536	8.908772	7.599706	6.941664	7.796780	6.454610	5.865953	7.846866	6.950590	7.936696	8.856845	7.254931	8.517941	8.562682	6.476918	8.061071	6.241268	8.408726	10.052635	9.646902	7.135477	8.909516	11.100738	9.486737	7.887956	7.931572	7.813882	5.401645	8.697270	7.683512	8.068175	7.298474	8.596576	8.277230	8.241887	8.254529	10.114501	7.284881	7.086749	9.156924	8.931776	7.397708	9.071601	8.268231	7.111620	8.333675	7.937895	7.632856	6.324405	9.933372	7.713118	7.508788	7.553935	9.660909	7.753947	7.745146	6.760102	6.813657	9.149548	8.124953	8.018913	8.989269	6.519531	8.698784	8.916489	8.392568	7.587974	8.001042	6.650457	8.523382	8.921205	9.074813	8.051116	6.522506	7.519870	6.698868	6.473593	8.147447	7.888585	7.452274	6.993859	8.427711	7.933412	8.070916	7.940884	7.639852	8.054626	7.617855	7.384537	7.871824	7.697302	9.970033	8.774754	7.635566	6.912595	6.280526	7.478884	7.855139	8.634890	7.812644	8.591764	6.570672	7.130189	7.755464	7.198061	8.267918	8.936855	8.234051	8.579659	10.378959	5.286009	9.878591	6.668179	3.494265	5.065280	9.975574	7.231968	9.201691	8.122926	6.958781

ATF6	11.958003	11.188740	11.087385	10.915384	11.497575	11.130492	11.350844	10.897742	11.360449	11.346391	11.424787	11.257393	11.315135	11.640863	11.679352	10.880695	11.026609	11.550159	12.509321	11.251658	12.029158	12.011560	13.056546	11.239205	11.750711	11.421793	11.255761	11.648129	11.581259	10.816113	11.649898	11.187573	11.443229	11.136230	11.295540	11.147376	11.350258	11.110862	11.573431	11.400910	11.280614	12.001894	10.983494	11.260184	11.776075	10.912348	11.354771	11.261231	11.286728	10.969237	11.034889	11.093843	11.297358	11.540839	11.104718	11.136100	11.458006	12.115229	11.449715	11.547384	11.491019	11.524581	12.547929	11.996542	11.485325	11.115929	11.088725	11.388799	11.089443	11.884500	10.853448	10.774758	11.261832	10.783312	10.659045	11.317325	11.263347	11.104014	11.006688	11.048690	11.291891	11.652286	11.024804	11.245085	11.364771	11.371953	11.309839	11.291576	11.636925	11.494063	11.389687	11.986356	11.309982	10.831653	11.195021	11.150980	11.437970	11.302476	11.263797	11.352601	11.098696	11.376371	11.730949	11.585439	12.621829	11.585889	12.042721	12.469069	12.314144	11.383354	12.367134	11.819287	11.403818	11.173601	11.425348	9.970502	12.313009	11.495353	11.626444	11.902176	11.020512	10.990476	11.328057	10.811229	11.655296	11.333751	11.333751	11.317187	11.026609	11.638115	11.074288	11.324079	11.542870	11.867133	11.326303	10.882369	11.637543	12.973999	11.843867	11.634122	12.450905	12.508519	11.260928	11.312338	12.115695	10.786621	11.033754	11.099443	11.305008	12.437743	11.081007	11.448837	11.211714	11.528777	11.048406	11.059685	10.965351	11.355206	10.977437	11.768414	11.019313	11.275733	10.659325	10.842709	10.207688	11.520394	11.621431	11.313065	10.887492	10.541837	10.953350	10.615103	10.878700	10.974225	11.953784	11.635707	11.247766	10.891480	11.014430	10.891048	12.019305	11.375766	11.375024	11.803532	11.380054	11.190043	10.545972	10.515623	11.771822	10.792626	10.465346	10.537933	10.550696	11.123360	11.263041	11.280760	11.283268	10.724139	11.530622	11.037001	10.730021	10.738842	11.282105	11.179915	10.780871	10.961939	10.946533	11.555669	11.567130	11.604584	11.358100	10.526253	10.497236	10.853742	11.199453	11.012272	11.583753	11.267660	10.984649	10.460558	11.328644	11.244502	11.216707	11.861844	11.357096	11.385102	12.008250	12.234983	11.214098	11.933974	11.690176	10.976116	10.928290	11.401220	11.175215	11.277795	11.228140	10.511848

ERCC2	9.172036	12.257562	12.993460	13.146687	12.930265	13.273550	13.112597	13.484384	12.706360	12.281444	13.320471	13.506799	13.416705	13.871263	13.652235	13.119075	13.474422	13.801126	13.269649	13.817826	13.125984	13.455246	10.911326	13.530801	13.107525	12.945900	8.923712	12.458490	11.978903	12.340685	13.055240	12.980792	12.590988	11.724977	12.497186	13.026368	13.672692	13.905825	14.033627	13.816557	12.730151	13.471830	12.792841	13.121375	12.437077	13.431471	12.097725	13.191901	12.708077	12.579011	12.069926	13.380119	12.427834	13.824782	14.039072	13.102386	13.330907	13.747333	13.706318	13.591911	13.722725	13.047627	8.736262	13.441821	13.738842	13.945117	13.102943	13.740367	13.150306	13.655750	13.382399	13.718916	12.227351	13.340559	13.763467	12.408519	13.212823	12.514165	12.867959	14.128314	13.031762	13.310755	12.739710	14.015006	13.612808	13.306855	13.521387	13.579900	13.014282	13.392461	13.569407	13.037820	13.269649	13.584172	11.547691	13.169214	13.596345	13.251437	12.929922	12.700204	13.306600	13.784553	13.198336	13.683082	14.296316	12.610190	12.978577	14.001519	13.908273	12.758401	13.232267	13.914229	14.236872	13.028986	12.957704	13.287464	10.105095	13.203403	12.607880	11.295540	13.183505	12.649190	12.563000	13.532613	11.968868	13.292951	10.563370	12.909129	11.512897	12.726021	13.101435	9.763723	12.774962	14.083758	9.999518	12.257409	12.160336	8.799601	11.121598	13.906117	13.645878	12.593944	12.866728	13.318461	8.919828	12.814569	13.569877	11.732575	12.387258	12.069615	13.926497	12.528560	14.849518	13.539286	13.574676	13.430832	14.399253	13.808908	14.147923	11.639796	14.001519	8.624195	14.479473	14.166159	13.343654	13.079117	12.221648	13.099592	12.467488	13.577611	13.097319	11.870412	14.156066	12.637480	10.549040	11.613605	14.110759	13.478600	13.686919	14.145530	10.998620	13.293957	13.694028	13.022170	13.265575	14.376679	13.255784	13.159327	11.664472	13.466396	13.407769	13.122690	11.994581	12.486157	12.661376	12.687096	13.324800	12.894372	11.647091	13.118872	13.076654	14.727457	12.717320	14.485961	13.195507	13.665236	13.018455	13.193873	12.537248	10.150237	13.439032	12.104980	12.668989	13.344260	12.860559	12.221872	10.633501	13.312545	13.658201	12.120820	10.688872	11.282541	10.521409	12.782948	11.910728	12.260855	11.412637	13.095970	13.167086	13.420393	12.868513	10.477553	12.360207	12.252315	13.016071	12.451545	13.906384	12.781728

KLC3	9.172036	12.257562	12.993460	13.146687	12.930265	13.273550	13.112597	13.484384	12.706360	12.281444	13.320471	13.506799	13.416705	13.871263	13.652235	13.119075	13.474422	13.801126	13.269649	13.817826	13.125984	13.455246	10.911326	13.530801	13.107525	12.945900	8.923712	12.458490	11.978903	12.340685	13.055240	12.980792	12.590988	11.724977	12.497186	13.026368	13.672692	13.905825	14.033627	13.816557	12.730151	13.471830	12.792841	13.121375	12.437077	13.431471	12.097725	13.191901	12.708077	12.579011	12.069926	13.380119	12.427834	13.824782	14.039072	13.102386	13.330907	13.747333	13.706318	13.591911	13.722725	13.047627	8.736262	13.441821	13.738842	13.945117	13.102943	13.740367	13.150306	13.655750	13.382399	13.718916	12.227351	13.340559	13.763467	12.408519	13.212823	12.514165	12.867959	14.128314	13.031762	13.310755	12.739710	14.015006	13.612808	13.306855	13.521387	13.579900	13.014282	13.392461	13.569407	13.037820	13.269649	13.584172	11.547691	13.169214	13.596345	13.251437	12.929922	12.700204	13.306600	13.784553	13.198336	13.683082	14.296316	12.610190	12.978577	14.001519	13.908273	12.758401	13.232267	13.914229	14.236872	13.028986	12.957704	13.287464	10.105095	13.203403	12.607880	11.295540	13.183505	12.649190	12.563000	13.532613	11.968868	13.292951	10.563370	12.909129	11.512897	12.726021	13.101435	9.763723	12.774962	14.083758	9.999518	12.257409	12.160336	8.799601	11.121598	13.906117	13.645878	12.593944	12.866728	13.318461	8.919828	12.814569	13.569877	11.732575	12.387258	12.069615	13.926497	12.528560	14.849518	13.539286	13.574676	13.430832	14.399253	13.808908	14.147923	11.639796	14.001519	8.624195	14.479473	14.166159	13.343654	13.079117	12.221648	13.099592	12.467488	13.577611	13.097319	11.870412	14.156066	12.637480	10.549040	11.613605	14.110759	13.478600	13.686919	14.145530	10.998620	13.293957	13.694028	13.022170	13.265575	14.376679	13.255784	13.159327	11.664472	13.466396	13.407769	13.122690	11.994581	12.486157	12.661376	12.687096	13.324800	12.894372	11.647091	13.118872	13.076654	14.727457	12.717320	14.485961	13.195507	13.665236	13.018455	13.193873	12.537248	10.150237	13.439032	12.104980	12.668989	13.344260	12.860559	12.221872	10.633501	13.312545	13.658201	12.120820	10.688872	11.282541	10.521409	12.782948	11.910728	12.260855	11.412637	13.095970	13.167086	13.420393	12.868513	10.477553	12.360207	12.252315	13.016071	12.451545	13.906384	12.781728

POLR3G	9.742743	9.793162	9.956464	10.124928	9.919156	10.089927	9.725903	10.970868	10.419200	10.008153	10.014459	9.776425	9.873315	10.260405	9.380804	10.204454	10.627462	10.128831	10.428516	10.590228	9.808891	10.451655	10.144911	9.963157	10.033023	9.620568	8.919970	10.113185	9.351702	9.249046	10.871585	10.065268	10.027037	9.276833	9.442955	9.794550	9.670859	9.891644	10.041915	9.938201	9.635352	9.381956	9.448419	10.292786	9.607021	10.477843	9.147183	9.819206	9.894559	10.275470	10.104326	10.208918	9.763723	10.365154	10.339585	9.822073	10.062437	9.905763	9.935109	10.134374	9.891185	9.403399	9.148011	9.882726	9.761351	9.854012	10.154056	9.858336	10.112450	9.902261	10.063481	9.835686	9.939017	9.976047	10.064373	10.099200	9.878156	10.557223	10.300344	10.588938	9.609659	10.338538	10.101581	10.042666	10.270147	9.676894	10.575116	10.409985	9.497955	10.332362	9.946708	10.184516	9.622457	10.252352	9.290023	10.051862	9.939172	9.799495	10.267029	9.126584	10.266592	9.648561	9.689605	9.865926	10.482002	9.575947	10.352969	10.142294	10.148077	10.142442	10.440033	9.643474	10.588804	9.839560	10.145061	10.141339	9.354834	10.359338	9.793010	9.883470	9.609304	9.259079	10.395504	10.584473	11.385102	11.097635	9.475363	11.227693	9.737168	10.090524	9.594486	8.683701	8.903998	9.722952	9.575176	9.727598	9.608994	9.901485	10.912940	10.011976	9.705954	10.124419	9.455425	11.648697	10.110042	10.359015	10.847400	8.770226	9.072354	9.749266	11.177918	10.380759	11.502609	10.783768	10.522363	10.469201	10.566794	10.883860	9.972329	11.348793	9.822999	10.387179	11.892308	10.186500	11.503353	11.573192	10.064516	10.288842	11.314678	10.465042	9.899439	10.666063	9.914081	10.597404	8.988823	10.122161	9.992195	10.383819	10.049705	10.590086	9.339034	9.627691	10.695437	9.263847	10.063327	11.262723	9.656115	11.285343	9.666547	11.167246	10.341990	10.669403	9.514711	10.089619	10.903938	10.632606	11.174007	10.987180	9.369356	10.026726	10.586706	10.603402	9.968648	10.860851	10.299271	12.018432	11.987098	9.541895	10.096550	10.167052	10.299419	9.536303	10.342797	10.783906	10.642074	9.347399	8.527674	10.824581	9.212673	9.023558	8.802155	9.264903	10.052797	8.923429	9.175570	10.119603	9.507341	10.103717	10.621593	10.182107	9.055161	10.966241	9.848631	11.526788	10.977016	10.542543	10.030540	11.507746

PPP1R3F	9.845504	10.024017	10.258179	10.104182	10.534405	10.335529	9.906961	10.754495	10.983350	10.037185	10.134688	9.858664	9.903802	9.892861	9.297852	10.481102	10.630112	10.223905	10.680359	10.514677	10.419038	11.105444	10.989882	10.125091	10.407694	10.704328	11.350540	10.683914	10.242602	9.959383	10.704907	9.877509	9.679564	9.999048	9.765411	9.801959	9.753620	9.569977	10.206592	10.336744	10.225898	10.266879	10.199328	10.453574	10.411451	10.428662	10.381054	10.148077	9.745228	10.294008	10.004887	10.253361	9.819482	9.750053	9.728056	10.250420	10.002111	10.062587	10.212581	9.870758	10.106999	9.978433	10.359182	10.393702	10.504548	10.385470	10.226672	10.050969	10.110042	10.452381	9.835965	10.128392	10.472987	9.872579	10.174432	10.014606	10.140152	9.912045	10.048648	10.047255	9.943197	10.485037	9.973251	9.932911	10.010322	9.869487	9.805002	9.808072	10.160447	10.079690	9.974195	10.464884	9.912200	10.506461	9.989661	10.004887	9.881018	10.112589	10.126478	10.543736	10.454796	9.887037	10.429132	10.115619	10.264537	10.161675	10.940232	10.380918	11.303373	10.621887	10.872478	10.677043	10.824581	10.768349	10.331296	11.419262	10.374121	10.286511	10.476950	10.398075	9.803036	10.256634	10.518938	11.179610	10.455701	10.439434	10.344614	10.296166	11.981061	9.882399	9.975417	9.916692	10.211511	9.650396	11.234915	10.634740	9.755913	11.033295	11.784186	10.232060	11.299384	11.613910	10.266446	11.841136	11.003228	10.577989	10.256330	11.075319	10.631884	10.589210	9.991577	9.733780	9.334599	10.137297	9.446213	9.651972	9.787132	10.299127	10.509173	10.809338	10.430163	10.990195	11.187721	10.620713	10.701056	10.621312	10.204297	9.812607	9.783615	9.710638	10.853896	10.034692	9.140105	10.167973	10.218950	10.683473	11.502190	10.806201	10.209061	10.613340	10.104477	10.084677	10.388978	11.400440	10.747087	11.276591	10.024497	9.867722	10.230212	9.977992	9.773557	10.884458	10.312135	10.015244	10.329013	9.915155	10.289278	9.948590	10.183903	10.167210	9.802271	9.775812	10.087299	10.704907	11.317927	9.655185	10.600603	10.163983	10.258677	9.508397	10.464170	10.016962	10.536345	9.716549	9.951825	10.036097	10.177339	11.188016	11.460104	11.491594	10.743513	10.253789	9.474760	11.661797	10.588938	10.444582	10.584342	10.610220	11.029556	10.985679	10.782381	11.522052	11.204630	10.367294	9.973693	9.999518	9.714695	10.737331

KCNJ9	6.058191	5.036534	5.762324	5.254163	5.863994	5.086979	4.293146	6.019899	6.025527	4.515287	5.162468	4.749977	4.389751	4.568525	4.195839	5.238653	5.655749	4.834783	6.075352	4.992892	4.698314	6.556627	7.245173	5.690061	5.634715	5.701700	5.637139	4.822497	4.776155	4.761835	6.210859	5.639932	5.495885	4.737795	4.600612	5.607789	4.361519	5.373984	5.476348	5.499857	5.102975	5.429406	5.055139	5.962707	4.485731	5.035276	5.088591	4.161815	5.761773	5.509932	4.917690	4.972119	4.839385	4.963752	5.043462	4.955407	5.367227	3.509805	5.197929	5.470077	5.220735	5.401645	5.730301	5.677332	5.611223	5.528391	5.462213	5.814413	5.760333	6.013683	5.195106	5.176324	4.917478	4.863937	4.998160	5.216600	5.861035	5.371678	5.648020	5.376333	4.088961	4.489992	4.494685	4.780324	5.248785	5.637531	4.199007	5.328715	6.297082	6.075192	6.089206	6.101888	5.261254	5.073229	5.010742	5.119688	3.504352	5.566361	5.575395	3.824627	4.811255	4.607348	5.238255	5.145547	5.700066	4.520391	5.938799	5.850948	6.326589	5.334186	6.172347	6.064005	6.105503	4.910082	5.688078	6.194067	6.838662	5.109345	5.485008	4.705737	5.074842	5.347540	5.151735	6.947942	5.210021	4.663287	5.638458	5.491679	4.573081	5.627262	4.023916	4.394274	4.522996	4.955624	5.473470	5.757587	5.488941	6.231257	5.164064	4.915357	6.164105	6.836582	5.925962	5.579462	5.040958	5.318615	5.351486	4.655780	5.437070	6.207781	4.937670	4.741069	5.371479	5.221489	5.396732	4.503503	5.870621	5.557935	5.777806	4.984172	5.914377	4.818759	6.815893	5.328100	4.676909	5.815094	3.379304	5.831224	5.592917	5.277165	5.193853	3.675388	4.128277	4.286998	4.673618	3.619725	5.505053	5.009093	5.436682	5.188994	5.044321	6.174889	6.109076	6.519844	5.611985	5.561665	5.596642	5.430290	6.367040	5.347143	5.630077	5.397296	5.046074	4.399157	5.558123	5.491679	5.416410	5.724200	5.160880	5.700066	4.175765	5.336960	3.587089	5.520989	5.457696	5.212390	5.581896	5.902022	5.414539	6.317926	6.505307	5.414924	5.396530	5.402050	4.891068	5.006417	5.295428	4.514214	4.158864	5.240664	4.581826	4.376523	4.873648	5.580219	5.464634	6.501553	5.752290	6.285328	5.599318	5.981511	5.271843	7.146288	5.771842	6.612852	6.265543	6.259821	4.706826	5.025466

RBM8A	12.975455	14.412818	14.499076	14.378512	14.389681	14.131436	14.424443	14.667724	14.486754	14.461603	14.295055	14.427017	14.568485	14.628630	14.486754	14.519413	14.521823	15.037486	13.920234	14.441978	14.408756	15.012809	13.103703	14.740482	14.580207	14.310316	12.462754	14.632175	14.034063	13.868078	14.397680	14.295679	14.109785	14.053607	14.124606	14.338926	14.498682	14.849518	14.842135	15.238314	14.372657	14.233886	13.989848	14.158986	14.169373	13.891833	14.065689	14.036411	14.470097	14.274191	13.977193	14.398878	14.111684	14.217619	14.772763	14.769948	14.421008	14.242737	14.379907	14.391852	14.262283	14.395502	13.480172	14.375159	14.355177	15.248690	14.133255	14.994487	14.127386	14.509938	14.563014	14.756024	14.216003	14.464777	14.532252	14.544276	14.611021	14.110109	14.016722	14.679596	14.618223	14.267351	14.252485	14.946400	14.623346	14.081566	14.110759	14.455753	14.005898	14.334050	13.790706	14.047317	14.540484	14.687432	13.916607	14.289961	14.262921	14.335789	14.419090	13.986378	14.945774	14.525087	14.696136	14.770908	14.675960	13.931371	14.724698	14.814746	13.923778	14.633876	13.924299	14.396205	14.518159	14.475583	14.378131	13.848547	13.056733	14.488728	13.689979	14.506817	14.127698	14.075110	14.334050	14.497164	14.554790	14.463605	13.179765	13.129753	14.790582	14.230209	14.432746	14.553562	14.984013	14.517028	13.320905	14.232519	14.694707	13.900462	14.182477	14.386005	14.833005	14.465165	14.116167	14.923889	14.295991	14.262592	14.358027	13.186246	14.489554	13.972172	14.038793	14.594501	14.464777	14.039346	14.001519	13.569877	14.051213	14.467751	14.257993	14.460851	14.826972	14.519853	14.538340	14.303277	14.796877	13.759659	14.071191	14.298393	13.931123	14.069209	13.884891	14.527899	14.851142	13.959373	13.780621	14.505307	14.718090	14.402538	14.254105	14.588954	14.284842	14.141470	14.409150	14.269566	14.149487	14.601973	13.912140	14.545545	14.163327	14.044975	14.245947	13.926497	14.135616	14.015578	13.961100	13.881969	14.312386	14.183396	13.798810	13.834843	14.425606	14.094001	13.995003	14.375159	14.115590	14.535115	14.307693	14.189906	13.914879	14.648824	13.643752	14.401522	14.219589	14.037568	14.203355	14.504600	13.004678	14.338926	14.712563	14.151714	14.224052	13.200478	13.924569	14.053881	13.819661	13.740367	13.593085	13.804646	14.126130	13.676687	14.501067	14.817306	14.430826	14.647121	14.133559	14.995049	13.956042	15.026340

C14orf39	12.975455	14.412818	14.499076	14.378512	14.389681	14.131436	14.424443	14.667724	14.486754	14.461603	14.295055	14.427017	14.568485	14.628630	14.486754	14.519413	14.521823	15.037486	13.920234	14.441978	14.408756	15.012809	13.103703	14.740482	14.580207	14.310316	12.462754	14.632175	14.034063	13.868078	14.397680	14.295679	14.109785	14.053607	14.124606	14.338926	14.498682	14.849518	14.842135	15.238314	14.372657	14.233886	13.989848	14.158986	14.169373	13.891833	14.065689	14.036411	14.470097	14.274191	13.977193	14.398878	14.111684	14.217619	14.772763	14.769948	14.421008	14.242737	14.379907	14.391852	14.262283	14.395502	13.480172	14.375159	14.355177	15.248690	14.133255	14.994487	14.127386	14.509938	14.563014	14.756024	14.216003	14.464777	14.532252	14.544276	14.611021	14.110109	14.016722	14.679596	14.618223	14.267351	14.252485	14.946400	14.623346	14.081566	14.110759	14.455753	14.005898	14.334050	13.790706	14.047317	14.540484	14.687432	13.916607	14.289961	14.262921	14.335789	14.419090	13.986378	14.945774	14.525087	14.696136	14.770908	14.675960	13.931371	14.724698	14.814746	13.923778	14.633876	13.924299	14.396205	14.518159	14.475583	14.378131	13.848547	13.056733	14.488728	13.689979	14.506817	14.127698	14.075110	14.334050	14.497164	14.554790	14.463605	13.179765	13.129753	14.790582	14.230209	14.432746	14.553562	14.984013	14.517028	13.320905	14.232519	14.694707	13.900462	14.182477	14.386005	14.833005	14.465165	14.116167	14.923889	14.295991	14.262592	14.358027	13.186246	14.489554	13.972172	14.038793	14.594501	14.464777	14.039346	14.001519	13.569877	14.051213	14.467751	14.257993	14.460851	14.826972	14.519853	14.538340	14.303277	14.796877	13.759659	14.071191	14.298393	13.931123	14.069209	13.884891	14.527899	14.851142	13.959373	13.780621	14.505307	14.718090	14.402538	14.254105	14.588954	14.284842	14.141470	14.409150	14.269566	14.149487	14.601973	13.912140	14.545545	14.163327	14.044975	14.245947	13.926497	14.135616	14.015578	13.961100	13.881969	14.312386	14.183396	13.798810	13.834843	14.425606	14.094001	13.995003	14.375159	14.115590	14.535115	14.307693	14.189906	13.914879	14.648824	13.643752	14.401522	14.219589	14.037568	14.203355	14.504600	13.004678	14.338926	14.712563	14.151714	14.224052	13.200478	13.924569	14.053881	13.819661	13.740367	13.593085	13.804646	14.126130	13.676687	14.501067	14.817306	14.430826	14.647121	14.133559	14.995049	13.956042	15.026340

TRIM46	7.493825	7.095149	7.621347	7.692283	8.287044	8.654155	8.244457	8.060250	8.553489	8.750190	8.916795	8.677427	8.044166	8.144863	7.669450	7.676500	8.195596	7.712658	8.651326	7.643684	8.334937	8.527361	10.570769	7.956970	8.151277	8.286545	8.311618	8.843862	8.411404	8.266504	8.441197	7.643516	7.527604	7.692436	7.159964	7.384537	7.708229	7.514341	7.975701	8.065970	8.497631	7.995817	7.228566	7.393516	7.489439	7.067588	9.308487	7.109658	8.512159	7.166708	7.022972	6.860751	6.945974	6.842863	7.199772	8.128410	7.113452	7.413845	7.339253	7.596093	7.512640	8.695746	7.911257	8.116235	7.390104	7.573412	7.418369	7.276110	7.346731	8.059318	7.499287	7.582103	8.533620	7.742874	7.652823	7.757729	8.174113	7.657826	7.726369	7.150415	7.320728	7.052414	6.881033	7.053976	7.461345	7.675124	7.216069	7.611287	7.487887	7.230565	7.318693	8.344605	7.366238	7.687007	7.969565	7.746562	7.297649	8.021490	8.527214	7.839529	6.324741	7.347217	7.741944	7.985002	7.726063	8.712055	8.432741	8.119607	8.954400	8.561316	8.500592	8.036343	8.394685	8.841352	8.344141	9.882574	8.573812	7.998255	8.917711	7.857100	7.440987	7.449040	7.907241	9.347399	8.090493	9.210997	8.356138	8.588830	8.589431	8.777533	9.057352	8.781189	8.169095	8.421803	8.113934	8.347349	8.304531	8.756532	7.437704	8.529825	8.820970	9.080270	8.236689	8.122926	7.781356	8.024380	8.505652	9.241805	7.943235	8.756380	8.232535	8.555342	7.645269	7.119340	8.000550	7.589345	8.140171	8.406973	8.349890	9.795351	7.966199	7.417421	7.795409	7.368591	7.113308	9.240450	7.406634	8.439018	7.331331	7.336449	7.015663	6.861077	7.011388	6.976741	7.943715	7.134838	7.153877	7.423367	6.972892	7.619552	8.419013	7.417872	7.642619	7.895121	7.747797	7.203331	7.251051	7.532988	8.024837	7.609133	7.654830	9.385879	7.484898	7.624722	7.461823	7.605625	7.722369	7.924112	7.177806	7.181093	7.094332	7.063488	7.461507	7.288966	8.474459	7.140023	7.183306	7.609761	7.488516	8.590063	8.411859	7.994730	8.251152	7.997674	8.324932	8.674520	8.159780	8.485373	7.439610	6.316062	7.510294	8.161613	7.753792	8.003733	8.798858	9.091908	7.879300	8.523071	8.706747	8.950129	8.077423	8.935765	9.338762	8.498529	8.730322	8.144078	8.010942	8.051848

AL713999.1	7.493825	7.095149	7.621347	7.692283	8.287044	8.654155	8.244457	8.060250	8.553489	8.750190	8.916795	8.677427	8.044166	8.144863	7.669450	7.676500	8.195596	7.712658	8.651326	7.643684	8.334937	8.527361	10.570769	7.956970	8.151277	8.286545	8.311618	8.843862	8.411404	8.266504	8.441197	7.643516	7.527604	7.692436	7.159964	7.384537	7.708229	7.514341	7.975701	8.065970	8.497631	7.995817	7.228566	7.393516	7.489439	7.067588	9.308487	7.109658	8.512159	7.166708	7.022972	6.860751	6.945974	6.842863	7.199772	8.128410	7.113452	7.413845	7.339253	7.596093	7.512640	8.695746	7.911257	8.116235	7.390104	7.573412	7.418369	7.276110	7.346731	8.059318	7.499287	7.582103	8.533620	7.742874	7.652823	7.757729	8.174113	7.657826	7.726369	7.150415	7.320728	7.052414	6.881033	7.053976	7.461345	7.675124	7.216069	7.611287	7.487887	7.230565	7.318693	8.344605	7.366238	7.687007	7.969565	7.746562	7.297649	8.021490	8.527214	7.839529	6.324741	7.347217	7.741944	7.985002	7.726063	8.712055	8.432741	8.119607	8.954400	8.561316	8.500592	8.036343	8.394685	8.841352	8.344141	9.882574	8.573812	7.998255	8.917711	7.857100	7.440987	7.449040	7.907241	9.347399	8.090493	9.210997	8.356138	8.588830	8.589431	8.777533	9.057352	8.781189	8.169095	8.421803	8.113934	8.347349	8.304531	8.756532	7.437704	8.529825	8.820970	9.080270	8.236689	8.122926	7.781356	8.024380	8.505652	9.241805	7.943235	8.756380	8.232535	8.555342	7.645269	7.119340	8.000550	7.589345	8.140171	8.406973	8.349890	9.795351	7.966199	7.417421	7.795409	7.368591	7.113308	9.240450	7.406634	8.439018	7.331331	7.336449	7.015663	6.861077	7.011388	6.976741	7.943715	7.134838	7.153877	7.423367	6.972892	7.619552	8.419013	7.417872	7.642619	7.895121	7.747797	7.203331	7.251051	7.532988	8.024837	7.609133	7.654830	9.385879	7.484898	7.624722	7.461823	7.605625	7.722369	7.924112	7.177806	7.181093	7.094332	7.063488	7.461507	7.288966	8.474459	7.140023	7.183306	7.609761	7.488516	8.590063	8.411859	7.994730	8.251152	7.997674	8.324932	8.674520	8.159780	8.485373	7.439610	6.316062	7.510294	8.161613	7.753792	8.003733	8.798858	9.091908	7.879300	8.523071	8.706747	8.950129	8.077423	8.935765	9.338762	8.498529	8.730322	8.144078	8.010942	8.051848

IGF2R	13.030999	12.537248	12.776004	12.671548	12.863135	12.513371	12.709644	12.287695	12.093373	12.532494	12.606221	12.604915	12.582791	13.078931	12.174197	12.508819	12.230187	12.483405	13.310948	13.046847	12.847857	12.887748	13.197314	12.713819	12.356888	12.589947	12.188102	12.229877	11.977451	12.509321	12.234198	12.653635	12.705699	13.412299	12.763935	12.836345	12.734044	12.909649	13.016071	12.626178	12.529519	13.038207	12.920784	12.749833	12.732190	12.945707	12.165750	12.517113	12.213183	12.622994	12.618964	12.759968	12.837237	12.724156	12.576396	12.502721	13.198527	13.053642	13.234411	12.772756	12.857801	12.440145	13.124995	12.953654	13.224721	13.032476	12.794117	12.553621	12.599183	12.623334	12.712441	12.911260	12.241297	12.740585	12.923860	12.740063	12.837577	12.676682	12.769062	12.956811	12.570017	12.723135	12.894731	13.075888	12.987200	12.884383	13.135842	13.116581	13.322325	12.576234	12.678705	13.163401	12.787348	12.993637	12.895995	12.973655	12.226312	12.595078	12.887748	12.590794	12.649538	12.753795	12.690509	12.983430	12.931331	12.086352	12.331290	13.122136	13.237448	12.270578	13.622440	13.145143	12.796632	12.297429	12.230643	11.927550	13.049159	12.708589	11.792707	13.452027	12.408357	13.178954	13.126344	12.835422	12.056040	12.893120	12.937441	13.310353	12.906787	12.715589	12.498330	12.074353	12.884383	13.206430	12.716779	13.089999	12.887748	12.826480	13.231451	12.865491	13.562689	13.042957	13.295148	12.757381	13.039489	12.579997	12.805120	11.839166	12.916749	12.987200	13.183505	13.570123	13.403996	12.730828	12.872324	13.527900	12.983141	13.107173	13.332262	12.449269	12.765477	13.364346	13.344050	13.550061	12.734879	11.863499	12.467649	13.217148	13.771102	13.412299	12.687272	13.566616	13.876872	13.077813	12.313810	13.439032	13.693583	13.183285	13.892381	13.825287	11.323214	13.957409	13.516687	13.013167	12.938530	13.518662	13.472047	13.634745	13.291131	13.548445	13.112985	11.781361	13.639425	12.838440	12.860065	13.504804	13.379067	12.985374	13.180561	13.557717	13.073797	13.964639	13.188586	13.239591	13.128984	13.104454	13.330272	13.469230	13.296970	12.649362	13.553456	13.156520	13.417129	12.958825	13.129561	13.384576	13.070589	12.533294	13.862600	13.881196	13.570340	13.466396	13.107335	13.672692	11.976069	12.767631	13.162623	14.017548	12.033841	14.043788	13.574462	12.380673	11.883726	12.716063	13.634990	13.714416	12.979495	12.554944

KCNA6	9.347857	10.853180	9.858997	11.008580	10.376671	12.555422	11.939377	11.498901	10.943322	13.000090	12.307924	12.626178	10.720056	11.145577	10.980120	10.845782	10.640470	10.459258	12.157396	11.359439	11.214237	11.016496	10.659620	10.406224	11.186151	11.390861	9.719099	10.436727	11.493313	11.527331	11.332913	10.294312	10.739457	9.279672	9.844727	10.867581	10.783168	10.767880	11.178222	11.144404	12.202018	10.767880	9.616997	9.993540	9.424212	9.429122	11.359717	9.933063	10.561928	9.599698	9.376837	9.814480	10.395050	11.141047	11.494500	12.804949	10.825756	10.132720	10.712259	10.354903	10.941833	11.167246	9.225751	10.990603	11.390418	11.583022	10.430163	11.079687	10.491272	11.525740	11.079831	10.785414	11.624718	11.196818	10.507987	10.224206	10.313708	10.248629	10.847688	11.100303	9.963157	10.719260	10.390607	11.413991	11.853844	11.733178	11.001508	9.997954	10.734915	11.678602	11.774619	11.668837	11.181128	11.872954	10.046469	9.657205	10.950121	10.037337	10.692551	10.547653	11.410252	10.850174	9.915301	11.002085	12.209741	11.532717	11.539250	11.394876	11.455650	12.005783	11.319960	11.197531	10.516533	12.287067	10.861007	13.177797	8.879418	11.442933	11.200580	9.336631	11.518252	9.130629	9.533530	11.550016	11.076058	11.261231	11.267354	10.149023	10.081486	11.716302	10.058151	10.001321	9.708142	10.179032	9.703426	11.858578	8.986560	8.780406	8.448422	9.363205	9.577775	9.288170	9.595856	9.017015	8.921205	11.258115	10.155158	10.707032	10.119753	10.067514	10.425603	10.995221	11.384067	9.263984	10.660719	11.860241	10.122012	10.589516	10.108989	10.620123	10.896881	8.233897	9.143958	9.613799	10.029332	11.472498	9.661702	10.361430	9.275465	9.502704	8.529985	9.610570	9.989959	9.454180	9.358480	9.362011	8.002306	9.261349	9.255589	10.662229	10.070706	9.673342	10.294615	9.285515	8.807202	9.141971	11.956506	10.637273	9.873790	11.264092	10.741115	11.853395	10.595136	9.091411	11.089443	10.476626	9.449677	11.339952	8.710706	9.167845	9.337859	11.621139	9.413036	8.965870	10.885415	10.861593	8.937007	8.838597	9.970187	11.842962	10.126326	11.592678	9.906511	12.825230	10.441113	10.310359	10.385788	10.577237	9.440640	10.205213	9.975902	9.811172	9.415515	9.685477	10.372919	10.299864	9.671636	10.520042	11.705224	10.756176	9.830156	13.083113	12.051190	10.604302	11.187873	10.680952	9.555843	11.305008

GALNT8	9.347857	10.853180	9.858997	11.008580	10.376671	12.555422	11.939377	11.498901	10.943322	13.000090	12.307924	12.626178	10.720056	11.145577	10.980120	10.845782	10.640470	10.459258	12.157396	11.359439	11.214237	11.016496	10.659620	10.406224	11.186151	11.390861	9.719099	10.436727	11.493313	11.527331	11.332913	10.294312	10.739457	9.279672	9.844727	10.867581	10.783168	10.767880	11.178222	11.144404	12.202018	10.767880	9.616997	9.993540	9.424212	9.429122	11.359717	9.933063	10.561928	9.599698	9.376837	9.814480	10.395050	11.141047	11.494500	12.804949	10.825756	10.132720	10.712259	10.354903	10.941833	11.167246	9.225751	10.990603	11.390418	11.583022	10.430163	11.079687	10.491272	11.525740	11.079831	10.785414	11.624718	11.196818	10.507987	10.224206	10.313708	10.248629	10.847688	11.100303	9.963157	10.719260	10.390607	11.413991	11.853844	11.733178	11.001508	9.997954	10.734915	11.678602	11.774619	11.668837	11.181128	11.872954	10.046469	9.657205	10.950121	10.037337	10.692551	10.547653	11.410252	10.850174	9.915301	11.002085	12.209741	11.532717	11.539250	11.394876	11.455650	12.005783	11.319960	11.197531	10.516533	12.287067	10.861007	13.177797	8.879418	11.442933	11.200580	9.336631	11.518252	9.130629	9.533530	11.550016	11.076058	11.261231	11.267354	10.149023	10.081486	11.716302	10.058151	10.001321	9.708142	10.179032	9.703426	11.858578	8.986560	8.780406	8.448422	9.363205	9.577775	9.288170	9.595856	9.017015	8.921205	11.258115	10.155158	10.707032	10.119753	10.067514	10.425603	10.995221	11.384067	9.263984	10.660719	11.860241	10.122012	10.589516	10.108989	10.620123	10.896881	8.233897	9.143958	9.613799	10.029332	11.472498	9.661702	10.361430	9.275465	9.502704	8.529985	9.610570	9.989959	9.454180	9.358480	9.362011	8.002306	9.261349	9.255589	10.662229	10.070706	9.673342	10.294615	9.285515	8.807202	9.141971	11.956506	10.637273	9.873790	11.264092	10.741115	11.853395	10.595136	9.091411	11.089443	10.476626	9.449677	11.339952	8.710706	9.167845	9.337859	11.621139	9.413036	8.965870	10.885415	10.861593	8.937007	8.838597	9.970187	11.842962	10.126326	11.592678	9.906511	12.825230	10.441113	10.310359	10.385788	10.577237	9.440640	10.205213	9.975902	9.811172	9.415515	9.685477	10.372919	10.299864	9.671636	10.520042	11.705224	10.756176	9.830156	13.083113	12.051190	10.604302	11.187873	10.680952	9.555843	11.305008

VPS8	12.020355	11.166077	11.857035	11.900447	11.615675	11.555942	11.786870	12.434039	11.941052	11.673410	11.789411	11.836629	11.926448	11.762372	11.508343	11.382618	12.047525	11.315712	12.074208	11.997151	11.561422	11.926777	11.750421	11.834974	11.506853	11.947731	11.085434	11.373485	11.273851	11.476689	11.793314	11.934440	12.071732	11.816748	12.425406	12.222501	12.167877	11.892609	11.743340	11.935336	11.582567	11.997786	12.370197	12.045443	12.026311	12.151563	11.321595	11.836026	11.806485	12.121750	11.928915	12.271395	12.108482	12.582141	12.019762	11.708649	12.123909	12.054551	12.504387	11.993191	12.161724	11.288336	11.891078	11.790869	11.321435	11.478857	11.609766	11.447213	11.752478	11.498609	11.852324	11.535848	11.370827	11.667024	11.783278	11.555370	11.937909	11.887512	11.721853	11.675812	10.955728	11.819746	11.787320	11.995974	12.206975	12.140038	12.211418	11.948967	11.711327	11.619341	11.460998	11.771536	11.635559	11.735252	11.524581	11.812156	12.022370	11.805894	12.338259	11.443229	11.776075	11.871450	11.236126	11.527331	12.430276	11.425646	11.977757	11.969485	12.009009	11.147241	12.010792	12.060534	12.009735	11.502609	11.749526	10.277128	11.900732	11.764203	11.137872	12.158785	11.701015	12.188253	11.849270	11.373637	11.834822	11.007141	11.990950	12.630506	11.676518	11.363426	11.270275	11.611963	12.181896	10.283247	11.603009	12.228786	11.049601	11.376221	12.135121	11.720464	11.708808	11.826829	11.675647	11.555223	11.550461	11.200158	11.748172	10.851782	11.553299	12.716418	12.046469	12.277123	12.564626	12.600182	12.434656	12.059948	11.523246	11.978054	11.945301	11.530622	11.893668	12.124801	12.200477	12.688641	12.076981	11.544514	12.420911	12.481303	12.974569	11.681278	12.261028	12.211598	12.090445	11.793463	11.717938	11.813750	12.457385	12.287223	12.077882	12.126121	11.677143	12.180967	12.035185	11.486995	11.251510	11.589239	11.833298	11.998998	11.488318	12.137555	11.882660	11.058524	11.986052	11.783886	11.993346	11.982268	11.881010	11.931067	11.568120	11.870853	12.371808	12.432090	11.826972	11.742598	12.103454	12.614703	12.570358	11.759614	11.714683	11.271637	12.251510	11.763092	12.458340	12.433552	11.788056	11.915776	12.267146	12.602912	13.177402	11.742298	12.488312	11.843396	12.008090	12.775824	11.496833	13.524396	12.395231	12.482465	11.173601	12.264028	12.585969	12.058084	10.990030	12.996930	11.620399	12.457691	12.364654	11.191638

PCDHB15	8.235265	8.341870	8.185093	7.817478	7.018436	6.305609	8.075929	6.878304	7.529779	7.469565	7.729031	7.508788	8.037664	7.419775	8.277666	7.384238	7.681453	7.473087	7.796780	7.688871	7.284600	7.584871	10.302038	7.094649	7.494871	8.308074	8.478522	7.718880	8.066831	7.921791	8.184797	8.933148	8.820838	7.797234	8.128713	8.267121	8.372984	7.773375	8.060586	7.429837	8.010350	8.729147	8.452479	8.472319	9.526785	8.222227	8.206633	8.192295	8.380355	8.919508	8.140611	8.822972	8.789174	8.553489	9.164033	8.237926	9.034002	8.533546	8.322623	8.167075	8.605303	8.680362	10.956031	8.470875	8.429897	7.943715	7.952630	8.360009	7.861831	7.856494	8.191485	8.060923	7.507990	7.996760	8.022249	8.282551	8.108325	8.801121	8.010187	8.723582	8.249893	9.354243	8.823853	8.468700	8.562976	8.523703	8.108325	8.402976	8.531531	7.744116	7.862157	8.211137	8.294571	8.127337	8.363460	7.956793	9.000251	8.756219	8.202269	8.362692	8.238226	7.685039	7.845512	8.097062	8.435543	7.706992	8.129492	8.388507	8.371426	7.709559	9.121392	7.497414	7.272360	6.912751	7.133718	8.917548	8.620966	7.847503	7.642151	6.962923	8.583467	10.083270	6.776373	8.188620	6.902230	9.528187	7.051177	8.892827	6.934887	6.529072	8.001687	7.756185	6.820386	6.904447	9.277692	8.833772	9.274698	6.581476	9.363051	8.309760	8.356589	8.524461	6.911932	7.034830	7.600626	6.792176	7.068063	7.660627	7.129243	9.596007	8.795349	9.377622	7.763738	7.712658	7.670378	9.111274	7.870743	7.962059	7.467564	7.338470	7.388257	8.486745	8.793229	9.554047	7.753478	9.388107	7.562571	7.422893	8.433816	8.806901	9.191345	8.403294	8.683773	9.992352	9.188306	8.512001	8.143607	8.102045	7.820271	10.037937	8.698348	8.341747	7.199452	9.621823	7.866036	8.094336	9.157875	9.812907	7.367980	7.854534	8.266186	9.716865	8.486274	8.349146	8.006563	9.288325	8.642371	8.003733	9.319298	8.654623	8.093733	9.430349	9.391747	8.241603	9.925599	9.218710	8.187593	8.328131	9.007632	7.507556	8.219624	8.778865	8.715790	9.189936	8.232535	8.573058	8.202413	10.883094	9.125500	8.032389	7.739616	8.143925	8.422262	10.033912	6.421425	9.808570	8.051848	9.679709	8.323980	9.594314	9.266572	7.685039	6.283128	6.759935	8.033141	8.866122	7.441933	7.651114

MAP3K20	5.052568	5.872251	5.725140	5.456085	5.440816	4.105649	5.141316	6.236149	5.641209	5.188994	5.549584	4.897894	5.684924	4.763361	5.052375	6.074503	6.201713	5.674028	6.189096	5.538448	5.541648	5.734630	7.085989	4.974843	4.560560	5.645429	5.742456	5.425023	5.583801	4.618177	5.649548	6.607708	6.293693	6.737455	5.626914	5.445240	4.633497	4.706826	4.466525	5.818676	6.016557	5.166894	6.257931	5.706450	6.314181	5.927937	6.295776	5.825674	5.832637	5.263694	8.179776	5.965177	6.707995	5.789893	6.403278	6.246624	5.694139	5.184611	6.304098	5.205618	6.123900	4.862073	7.158238	4.911789	5.877786	5.621097	5.018503	5.809052	5.850080	5.395166	5.317069	4.842558	5.514243	5.712313	5.655195	6.210188	5.451468	6.071153	6.521385	6.265036	6.413357	6.492368	5.612899	6.483577	6.167203	6.653041	5.802411	5.816007	5.193075	5.526878	5.765566	5.549967	4.982161	5.174138	7.282571	6.288089	5.870430	6.854303	5.597810	6.882160	6.366712	6.037539	6.454774	5.566361	6.505475	6.424825	7.326424	6.252335	5.766251	4.439375	4.657286	6.666704	4.900277	5.384847	7.097815	4.503720	4.332259	6.912595	4.439818	6.208291	6.592110	4.204989	3.943719	4.232648	4.694948	6.092872	7.036542	6.549386	4.718124	5.217960	4.733792	5.455513	3.950770	3.835886	5.487657	7.220329	4.569828	7.807111	5.441793	4.831039	5.672771	6.137188	5.342778	4.549075	7.505232	5.261427	4.178524	5.668508	4.868967	5.260874	7.834020	6.608356	6.535312	5.732104	4.334841	5.642899	3.714240	5.975338	6.221944	6.853155	5.497353	5.563547	5.700818	6.238438	5.431807	7.014515	4.810612	5.654640	5.815183	6.057447	5.981511	6.311621	7.211007	6.486761	5.676760	4.927218	5.832460	6.100488	5.625771	9.021575	4.270937	7.046248	7.232924	5.393177	5.425023	6.122684	6.133974	6.080567	4.726407	6.059900	6.095497	5.806134	7.033749	5.092471	6.119929	4.948324	5.852202	5.851399	6.393878	6.232581	6.051295	6.882311	6.673024	6.050621	5.271843	7.018752	5.564851	5.306575	5.778374	4.159936	7.107731	5.635631	6.762817	7.448724	7.069707	5.908291	5.640661	7.196961	6.062264	7.150091	7.106131	4.187745	5.373011	5.158305	4.869580	8.316384	5.370703	5.486353	6.707390	4.596619	5.674946	5.202453	5.063876	4.392359	7.325321	5.821680	8.023925	6.034380

SMCO4	11.030608	10.050200	9.697251	9.289264	8.479305	8.524918	9.627540	9.255442	7.183928	9.425426	9.005015	9.459710	9.115721	9.761038	9.775382	8.489362	9.209476	9.357717	9.076060	9.748034	8.840427	9.987305	8.244922	8.827765	7.489903	8.956821	7.166404	8.695277	8.326818	9.298766	7.821338	10.341556	9.745999	8.828392	9.390643	9.841361	9.490144	9.835686	9.349409	9.263384	7.966799	10.269567	9.242889	9.482879	8.769138	9.757807	8.496139	9.865593	9.926775	9.869932	10.121706	9.807438	9.957986	9.732418	9.912825	8.041723	9.723277	10.049705	9.682033	9.711100	9.986251	8.155006	9.308633	9.580724	9.308948	9.844727	10.181016	9.411829	10.145498	9.776507	9.709524	9.417014	8.807202	9.907428	9.838908	9.628948	9.379881	9.854012	10.697641	9.647063	9.741022	10.144458	10.494370	10.025121	9.862557	9.912507	9.494561	9.740417	9.700644	10.342317	10.107923	10.115619	9.753921	10.188320	10.398815	9.386364	10.023382	7.853910	8.635047	7.644333	9.548541	9.276953	8.275804	9.609148	9.715026	8.665460	9.491096	9.554189	9.525717	8.622520	9.515750	9.766182	9.678286	8.656689	9.262763	6.090782	11.072430	9.528476	6.924197	10.083894	8.531531	10.272596	8.752751	7.309276	8.214857	9.539873	7.658320	7.294607	9.007324	7.900239	9.898527	8.124176	8.699691	9.726547	6.371935	8.170037	9.785658	8.627031	9.137787	9.175728	8.786705	10.481389	7.051808	8.432741	9.723772	8.392872	8.271201	6.480089	9.495797	6.825589	9.254946	9.441911	9.198437	9.719403	9.571519	9.968968	9.690233	7.579796	10.003500	7.601067	9.869171	10.388365	9.935596	9.988728	9.209476	7.452925	9.150322	10.242137	9.553280	9.743348	9.459408	10.711127	9.295062	10.477090	8.480944	9.518530	10.894029	9.266095	10.245152	10.061791	8.073510	8.984549	8.826560	9.649630	10.700036	9.887037	9.556635	9.908039	10.014459	9.372405	10.138502	6.592947	9.926202	10.795565	9.742594	10.132720	9.906961	9.533834	9.573039	9.436336	10.850917	9.693891	10.276541	10.784367	9.951231	10.062277	11.383763	10.576317	10.216098	9.414400	10.752512	9.324916	9.972475	10.737488	9.514128	9.003955	8.042154	7.476459	9.481198	8.009018	8.703369	7.432187	10.173198	9.874507	8.486586	6.958150	10.249984	9.619319	7.287522	9.593381	8.602983	8.126601	8.234802	9.968648	7.922567	9.699709	8.487706	6.827174

KIF16B	10.960475	9.527395	12.003696	11.435520	12.035653	11.592521	11.704169	11.255761	11.059824	11.497855	11.808731	11.383133	11.729757	10.874430	10.596659	11.327497	11.326303	10.123498	10.476151	11.674723	10.502718	9.351391	8.253736	10.749455	10.165968	10.597404	10.255569	9.659051	10.998779	11.441892	11.536144	11.832244	11.794389	11.464053	11.920971	11.826536	11.824728	11.020795	10.044641	9.548379	11.258585	9.697856	11.567573	11.827113	11.562313	11.967186	10.924932	10.052486	11.486387	11.887512	11.632841	12.120080	11.948186	11.709681	11.355780	11.730636	11.916064	11.191490	11.491752	10.887194	10.543163	11.193393	9.298453	9.253455	10.093199	10.489033	10.545524	10.352666	10.534701	9.740241	11.406614	11.484580	11.255319	11.929077	11.569154	11.477980	11.892168	12.305419	11.464783	12.106044	10.338699	11.920674	11.740862	11.978054	11.990484	11.875845	12.114778	12.020641	10.002111	10.731932	10.001946	10.166750	10.617450	11.544245	11.145141	11.683207	11.685252	11.425929	12.059652	11.025411	11.421793	11.505835	10.914464	10.612294	10.128547	11.648416	9.542129	9.983355	8.966033	11.291432	8.976221	10.946082	10.858794	10.852403	11.546662	10.532207	9.063980	11.796425	11.281809	12.220618	10.609210	11.009628	11.248223	11.792112	12.292196	12.143742	10.376210	12.583463	11.819147	11.385844	11.322595	12.258361	9.437081	10.845031	11.109254	12.628172	10.245866	10.034221	11.632098	10.919423	10.202804	8.570955	10.931441	11.030164	10.688437	12.068901	11.024804	10.734310	11.815237	11.138168	11.944227	11.238618	12.280226	12.284103	11.493018	12.215199	10.925355	10.720354	10.439875	12.091837	9.654867	10.585064	11.949752	12.235309	11.188301	11.782962	8.951813	12.170476	12.388886	12.798496	11.469875	12.048284	12.731525	12.717320	11.698867	11.567694	11.794980	11.197241	10.969544	10.783470	11.807225	9.224356	9.971364	10.164279	10.003350	10.894745	10.356625	10.674952	9.160471	12.547466	12.194924	10.968475	12.016745	12.190447	12.329246	12.708762	11.573596	12.274723	12.801968	10.769559	11.808438	13.014469	11.606677	12.046914	11.822002	12.519526	13.485749	10.218780	10.900409	8.796272	11.092539	9.719403	11.862156	12.065467	11.581124	12.707731	11.963714	11.558808	12.738314	9.615291	11.225024	11.001508	10.254638	10.471490	9.961955	11.553739	9.931531	9.578078	10.816719	9.992352	10.733110	7.863861	10.321819	11.493313	12.583463	10.583768	12.249758	11.678446

CLASRP	11.010491	11.040745	10.880620	11.399095	11.543954	11.372900	11.017781	11.389551	11.990798	11.344308	11.104280	11.181578	11.158336	11.360307	11.097482	11.218485	11.662957	11.489042	11.774619	10.924780	11.828107	12.152049	11.957688	11.472082	11.559388	11.531541	12.409200	11.336433	11.885400	11.454008	11.650051	10.628861	10.704472	10.731203	10.518615	10.465656	10.411931	10.815291	11.183899	11.303960	11.632395	10.979079	10.552353	10.787337	10.364725	10.471779	12.118493	10.650012	11.429417	10.423780	10.426224	10.240898	10.334799	10.466973	10.596035	11.752315	10.193392	10.851957	10.984949	10.664020	10.818184	11.497713	11.650203	11.666726	11.006117	11.224748	11.150814	11.147807	10.695590	11.467492	11.232423	11.124285	11.657173	10.512139	10.785111	10.902906	11.130633	10.496144	10.829006	10.369411	10.589937	10.524883	10.323369	10.395504	10.430330	10.529082	10.406224	10.169963	10.848591	10.591883	10.551729	10.925062	10.678215	10.757376	10.617160	10.615988	10.472987	11.018990	11.205099	11.469270	11.468239	10.710117	10.982623	10.944454	11.349516	11.425348	12.261188	11.836629	12.265271	12.006559	11.369313	12.003998	11.728547	12.467788	11.273543	12.256799	11.758591	11.255761	11.660885	10.860274	11.273685	11.154075	11.702238	12.610699	11.790004	11.805894	12.076837	11.764791	11.619649	11.406899	11.719425	12.257089	11.987098	11.740720	11.494936	11.464934	11.963873	12.526140	11.000137	12.169111	12.337599	12.239471	12.358457	11.759472	11.007420	11.388656	12.092283	12.915341	11.817176	12.384801	11.315857	11.168701	11.148560	10.435394	10.654587	10.195988	11.126061	11.486837	11.366540	12.506730	11.331478	10.628531	11.393088	10.331758	10.577067	12.350213	10.977591	11.293383	10.898647	10.830911	10.686620	10.518615	10.840472	10.329013	11.555370	10.114351	10.529807	11.466898	10.326579	11.071366	11.615814	11.041512	11.436677	11.200158	11.161265	11.407356	11.136100	11.286424	11.277646	11.010941	11.198394	12.493699	10.803737	10.722072	10.835284	11.101364	10.828572	10.793216	10.425147	10.415564	10.770306	10.592463	10.527040	10.572446	11.435520	10.531204	10.434742	11.180344	11.078978	10.912049	11.186992	11.234364	11.392615	11.329687	11.310412	11.335396	11.848510	11.677583	11.049127	11.602845	11.094925	11.501190	10.700919	11.213821	12.231738	12.340526	11.378421	11.319554	12.561543	11.840529	12.072185	12.530838	12.502885	11.906190	11.055235	11.352736	11.181275	11.844950

SLBP	12.723671	12.226312	13.654590	13.002332	12.411407	12.627995	12.846314	11.988788	11.684658	12.296782	12.439175	12.525777	12.573503	12.146410	12.337312	11.994409	12.404198	12.517447	11.459062	12.041521	11.719874	12.037461	11.158336	11.820802	11.633293	12.084173	11.627063	11.610934	11.883896	12.867055	11.965964	13.470117	12.794117	13.722489	12.810001	13.094478	13.011590	12.738314	12.197370	12.317768	12.563972	11.929833	12.831697	12.828755	13.144371	13.129561	11.669538	11.926777	13.232053	12.567588	12.825230	13.074945	13.121772	13.162423	13.177184	12.346315	13.413573	11.964318	12.521728	13.114121	13.001778	12.693398	11.863047	11.572225	11.821859	12.163433	11.774910	12.333192	12.485499	12.129781	12.811111	12.887922	11.994581	13.022344	12.790651	13.017899	13.410767	13.137564	12.807703	13.369728	12.224905	12.908664	13.053301	13.435992	13.080241	13.135455	13.121375	13.233810	12.143103	12.386767	12.280067	12.293428	12.288785	13.123620	13.083488	13.047049	13.139689	12.357368	12.892947	12.209273	12.068447	12.798022	12.606564	12.920598	12.206509	12.804235	11.099298	12.184093	11.541245	12.345006	12.474582	12.287067	11.593285	12.018886	13.093498	11.963379	12.317479	12.569374	11.596704	13.333079	12.440302	13.201208	12.803190	11.117968	12.732865	13.398018	13.003596	12.758922	13.667931	12.668333	12.731848	13.011590	12.837925	11.740562	12.079547	13.786622	12.153299	12.206509	14.050321	12.182810	12.329246	12.839626	13.754404	12.921878	13.193086	13.089999	13.326217	12.477354	13.537270	12.912347	12.748470	12.586302	12.829284	13.862600	13.608281	13.445449	13.024924	12.882226	13.059334	13.017899	12.541107	13.835083	13.679001	13.063805	13.178186	11.868033	13.208899	13.032839	14.050939	13.247211	13.632300	14.111384	13.695872	13.624991	13.131924	14.079493	12.811936	13.553691	13.352695	13.410767	12.762924	12.953485	12.762553	12.503046	12.723135	12.495360	12.394906	13.261991	12.637828	13.022344	13.435346	11.308542	13.493480	13.788993	13.390989	13.226454	13.690948	13.398875	13.132450	12.278977	13.991573	13.961354	13.006688	13.847752	12.720078	13.409904	14.391852	12.415089	12.296198	13.190470	12.956264	12.138794	13.511960	12.853171	13.038959	12.996396	12.095640	13.081212	13.579444	13.623609	13.166297	12.294973	13.816557	12.925253	12.337443	12.681457	12.947738	12.705699	11.943939	12.354988	13.046278	12.883285	11.695777	13.299825	13.578284	12.870487	14.218945	13.449081

C1orf167	8.607415	8.742697	8.772484	8.721334	8.931119	9.333214	9.100099	9.115417	9.356076	9.160153	9.044421	8.873398	8.768392	8.956821	8.464590	8.775212	8.721472	8.479473	9.439413	9.224813	9.146092	9.913281	9.378827	8.583467	8.524310	8.770683	8.973481	8.793982	8.753475	9.033700	9.255278	8.989121	8.226898	9.022623	8.811877	8.817738	8.911735	8.610765	8.840278	8.721185	9.169847	8.304531	8.546584	8.598366	8.377828	8.515175	8.964652	8.464590	8.755477	8.641739	8.543056	8.703695	8.723431	8.869921	8.802888	9.284015	8.579360	8.658828	8.436154	8.369384	8.468400	8.847080	8.467193	8.997364	8.935930	9.028257	8.690022	8.595167	8.698348	8.660551	8.859264	8.963428	8.890668	8.878949	8.740859	8.737644	8.926440	8.470875	8.386650	8.399403	9.189192	8.704125	8.891290	8.889464	8.632066	8.651036	8.272099	8.620648	8.882036	8.422421	8.213942	8.819040	8.760273	8.987020	8.500281	8.718581	8.525979	8.964936	9.073286	8.823574	9.257879	8.489064	8.700125	8.450156	9.259991	9.069737	9.485177	9.443280	9.473843	9.317338	9.001352	8.811217	8.877254	9.539063	9.256201	9.470011	8.276739	9.111602	9.318981	9.383185	9.119892	8.628819	9.353052	9.627241	9.998570	9.564819	8.828881	10.062587	10.054345	9.477056	9.549440	9.808233	9.721234	9.275915	8.973481	9.374309	9.095706	9.917640	9.674912	9.560890	10.283087	10.113627	8.967268	9.361114	9.333838	8.963871	9.263235	8.904922	9.490307	9.913281	9.403086	8.769760	9.571980	9.368473	9.222522	8.673896	8.541825	9.340740	9.040712	10.114655	8.829188	9.657952	9.408330	8.743457	9.318827	9.811513	9.334439	9.350468	9.335710	9.379735	9.295221	9.564819	9.832155	9.065545	9.280452	9.301568	9.085022	8.996891	8.532273	9.429888	9.254350	9.410961	9.591810	9.195272	8.854885	9.459862	8.412762	9.216076	8.788742	9.171571	9.141334	9.655511	8.991055	8.698348	8.934093	8.805811	9.188155	8.712055	8.636910	9.173745	9.368907	8.763780	8.843566	9.298766	9.067544	9.432508	9.031888	8.875113	8.744503	9.457860	9.256590	8.832570	9.218991	9.477511	9.568588	9.126741	8.854719	9.409404	9.286592	9.568410	8.686866	8.609391	8.246522	9.017912	9.660763	9.504085	8.981494	9.122293	10.135303	9.330151	9.132278	9.355609	9.519781	9.455087	8.995798	9.236097	8.807508	10.461430

SEMA6D	8.546108	7.614945	7.072417	6.885195	6.271383	6.394506	8.254820	4.234838	4.907319	7.219504	6.165780	7.124664	7.381393	5.779280	8.045289	7.138120	7.199452	6.662005	5.843515	7.345981	5.766833	6.196023	10.072829	6.184321	6.422692	7.401008	7.930212	5.222149	8.165029	8.794533	7.601067	8.631759	7.303037	8.620804	8.420395	8.633073	8.476775	7.876282	7.480117	7.220329	8.171114	8.099775	8.717816	8.359117	9.391747	8.675783	7.998868	7.215915	7.788999	8.730763	8.442390	8.550275	9.008878	8.836326	7.943394	7.861831	8.988657	7.618627	7.685039	7.945865	7.787317	8.469947	9.412891	7.137501	7.827291	6.551642	7.312275	7.207379	6.666216	6.262169	8.402217	7.745467	7.264789	7.992068	8.035245	8.389753	7.787010	8.704285	7.793394	8.270594	8.107575	8.546584	9.009654	7.923498	8.756854	8.190228	8.802752	8.193845	8.042654	7.046406	7.112839	7.375167	8.101742	7.581181	8.339181	7.821338	8.183260	8.170810	6.826397	7.589649	7.588584	7.759759	6.889496	7.665583	6.962614	8.171766	6.058527	5.914039	7.246377	6.938731	7.521277	5.832460	5.004978	5.628989	5.947212	6.429182	7.621653	7.382348	6.807413	7.614356	8.121626	7.468615	6.607406	10.218469	7.578395	10.146709	9.680791	9.068319	8.941567	10.366087	10.073145	8.450156	6.321505	5.798946	7.172144	8.226755	10.669195	9.396514	11.805448	4.599993	10.210139	11.072280	8.793229	9.012701	9.046988	6.757487	8.272535	7.533448	8.132254	7.295071	7.884438	9.184093	7.064300	8.592859	12.536108	9.451553	7.959136	4.710518	8.263083	8.513701	8.774288	7.855600	7.348953	10.033181	9.128284	5.981355	7.934176	9.996163	9.101649	8.962369	7.765933	8.840733	9.746078	8.857582	11.163914	9.241660	8.417837	7.615888	11.299108	7.511385	9.186923	6.673497	6.611997	6.448842	6.897032	5.576665	6.912113	5.307713	4.791226	10.581595	9.675059	3.971168	9.343298	9.976192	8.073351	9.222361	8.153907	10.093356	8.524608	9.338616	8.504041	7.748566	8.633972	8.572585	9.779375	7.929878	8.564352	7.543904	7.881864	6.732532	5.411091	8.037734	8.502000	10.261165	8.252222	9.831229	10.054204	5.557935	10.616435	8.447805	8.580730	5.706630	7.593826	8.260488	10.541677	7.909264	5.254349	7.986868	6.107290	12.054237	8.474935	8.637555	4.613988	10.547962	5.566167	9.508697	8.495694	6.664134

TWIST1	10.753860	11.972669	11.668318	10.248333	10.330389	10.477695	12.098166	10.026226	10.130040	10.698669	10.785583	10.898647	11.125488	10.885258	11.104014	10.701962	11.603716	11.132997	10.542840	10.744869	10.519091	11.344019	11.836176	10.676287	12.121131	11.716442	11.697270	10.901270	11.334043	12.116506	11.020059	10.840635	11.658803	12.110108	12.473785	12.112223	12.188253	12.376614	12.121584	12.054832	11.009906	12.804601	12.110693	10.900269	12.515153	12.212235	11.018827	12.052072	12.361158	11.408142	10.966557	11.877752	12.674301	11.934899	12.569545	10.859103	13.080043	12.933125	11.422093	12.742860	12.905702	12.072811	13.862600	11.933343	13.246936	12.619822	12.332724	12.154051	11.893063	12.549566	11.923566	11.811423	11.114713	12.356099	12.107128	11.953632	12.133719	13.093911	11.186712	12.104230	11.080590	12.635599	11.478857	11.871602	12.394267	11.732422	11.933193	11.851720	12.730311	11.317325	12.641390	12.846159	12.223856	11.843253	10.947453	12.068126	12.542605	11.874610	11.249845	12.051330	11.343391	12.076353	11.518703	11.702389	11.630009	10.961364	11.292363	11.641553	11.966551	10.869744	12.275923	10.876634	9.809361	10.405628	9.842744	10.522668	10.572446	11.379762	11.549154	11.218335	12.144544	12.893120	11.007861	12.184541	10.435394	12.236505	11.923566	12.578180	10.627292	12.201103	11.837247	11.654988	12.829461	12.204516	11.329537	12.703486	11.784487	11.205984	12.471814	11.324079	12.840179	12.143436	11.106019	12.536272	13.849377	11.858431	10.860274	11.539821	11.837100	10.454346	12.610024	12.545533	12.386932	11.349792	12.165444	13.272984	12.653975	11.062006	12.672419	11.662670	13.084593	11.455214	11.981504	12.381233	11.779706	11.342184	14.785315	11.220589	12.989260	13.022885	11.822149	12.576723	13.388411	13.080831	11.738510	13.129753	12.711956	11.922622	12.467321	12.645369	11.792707	12.841413	13.098814	12.691189	13.420610	12.609687	12.875043	12.143591	13.571674	11.993505	12.970784	11.276298	12.936720	13.173253	12.683833	12.798022	12.622511	12.814569	12.949809	12.467154	11.685883	12.936017	14.578479	13.614204	11.559784	13.135842	13.089623	13.557035	13.130368	12.064994	13.173253	12.263250	13.719166	13.045174	13.251240	12.672776	12.639296	12.014173	11.392045	13.457857	12.148094	11.412105	13.601268	14.337170	11.534230	12.557332	11.074564	11.845543	11.451923	12.574563	13.449507	10.008945	10.065725	11.348194	11.303223	12.346315	11.378571	12.292819

CTDSP2	13.991278	12.704515	13.363482	13.833309	13.872353	13.655038	13.702916	13.159158	13.369123	13.595124	13.583919	13.613971	13.832042	13.054184	12.591989	13.622915	13.199727	12.950547	12.446101	13.669377	12.861696	12.689849	11.488767	12.844915	12.599699	13.635940	14.264949	13.649857	13.727369	13.676917	13.974684	14.280056	13.292154	14.042288	14.100719	13.773212	14.257035	13.333932	12.879072	12.681457	13.686919	13.138899	13.762421	13.793026	14.052673	13.647062	13.833047	11.947881	13.828722	13.694245	13.606633	13.849877	13.307926	13.615437	13.482375	13.611868	13.611109	12.372749	12.446101	13.046106	12.841082	13.731668	12.631637	12.623163	12.805285	12.528398	12.631485	12.617456	13.229650	12.562057	13.770853	13.890483	13.780110	14.350910	13.919400	14.580994	14.344582	14.094001	13.742612	12.564626	13.526786	13.776435	14.142986	13.940978	13.922148	13.983511	14.112576	14.267351	12.685688	12.839438	12.563485	12.737126	13.002707	14.012079	14.296316	14.073307	14.010958	14.019845	14.069516	12.989794	13.626349	13.234611	13.485992	13.670083	11.654083	13.698011	12.098604	12.634617	12.196008	13.742058	11.701767	13.538596	13.043715	13.376280	13.869088	13.437511	13.271787	13.695390	13.869088	13.929742	12.392657	13.491711	13.413997	13.901790	13.394129	14.010653	14.163327	14.203084	13.948345	13.851689	13.627746	13.331689	13.515560	12.955887	14.362657	14.103476	13.055240	12.404042	13.802371	13.195507	12.792190	11.111300	13.352270	13.504360	13.852424	13.800082	13.598139	13.709682	13.640013	14.412818	13.753345	12.974569	13.779388	13.747078	14.296633	13.935487	13.304537	12.818889	12.879575	13.281038	12.923310	14.114324	13.830239	13.976639	13.149931	13.847489	12.875552	13.965466	13.602862	13.700156	13.728607	14.575995	13.955750	14.165208	13.863595	14.401854	12.404804	12.553472	13.071874	12.779167	14.350910	11.918166	12.526303	12.511451	13.277050	12.478001	13.040624	12.661199	12.458956	13.881196	13.965167	12.609521	13.884615	14.278587	13.573121	14.079765	14.110759	13.965466	12.863135	13.887252	13.229650	13.528769	14.185875	14.052986	14.020108	13.698984	13.968272	12.398510	12.752265	13.150110	12.025251	13.229650	13.749340	13.405475	13.769045	13.723478	14.145530	13.817588	13.740837	14.164900	14.175228	14.086526	13.727121	12.811770	13.659354	11.721084	12.691189	12.451713	13.389060	11.501471	13.982141	12.948055	13.245326	13.601738	13.602191	13.173630	13.831022	13.259620

AQP6	9.493782	8.278740	9.135166	8.383585	8.892055	8.342466	8.780241	8.868990	8.126279	8.437404	8.772629	9.033552	7.911654	8.279490	8.827483	8.991496	8.839043	7.728109	7.510294	8.116545	8.053099	7.963592	8.209329	7.774143	8.011098	8.768156	9.491898	8.975575	9.198763	9.733183	8.462629	7.375633	7.473694	8.715151	8.227815	8.004347	8.423788	7.866800	7.645269	7.259331	9.358026	8.376902	7.989708	9.547759	8.758395	8.428965	9.316241	9.045030	9.329853	9.169996	8.064574	8.588673	8.874004	7.871513	8.794606	8.941854	8.285459	8.550275	7.023277	7.862606	7.679069	9.130166	10.477090	7.771423	9.432816	8.477574	7.793394	8.869757	7.905227	8.047984	8.437550	8.344769	9.334014	8.345999	8.956987	8.393192	8.822673	8.359725	7.759279	8.706158	7.524523	8.582555	8.040597	6.842711	7.184898	8.733493	6.923374	7.177313	7.491660	7.928954	7.353902	8.811877	8.139429	8.437983	8.739474	8.223899	8.653193	8.203318	8.309176	8.683550	8.566590	9.157407	9.100686	9.272705	7.913119	9.188603	8.624032	7.760209	9.120196	9.174641	8.055892	8.097966	8.433072	9.007324	8.965547	10.154056	8.441329	8.735793	8.872639	9.059425	8.511392	8.638314	9.026613	10.027037	9.371967	9.292992	8.459880	8.249737	8.489838	9.173899	10.711692	8.472816	8.285318	8.555588	8.904760	8.136495	8.800503	8.394542	8.645143	8.217766	8.590817	8.155006	8.451400	8.495213	9.534300	9.102892	8.748785	9.362011	9.500267	8.326529	8.118091	8.350047	8.889309	8.386026	7.996760	7.879463	8.426929	8.158091	8.411708	9.766782	9.063380	7.768086	8.925032	8.809828	8.070314	9.138103	8.205229	8.131825	8.553625	8.170810	8.467335	8.353690	8.525385	7.919331	8.931622	8.079159	8.717976	7.720980	7.174480	9.154950	8.850472	8.670956	8.022991	8.118880	7.918437	8.752132	8.590817	8.188953	7.955709	8.373576	9.336024	9.775382	8.292304	8.240832	8.310809	8.357224	8.766232	8.512159	7.524374	8.208437	8.922106	7.563478	7.858326	7.828229	8.238086	7.342525	9.190087	6.926825	7.792954	7.573412	8.060419	7.807549	8.739929	8.270907	8.463224	9.341453	8.733493	8.255576	8.707227	8.683089	8.431955	8.396721	8.104810	7.364513	9.279525	7.933245	7.594883	8.419610	9.413800	8.136323	8.441936	8.472487	9.267981	9.346950	9.093582	7.979416	9.043957	8.975115

TET3	6.697442	5.931261	6.555824	7.116576	7.873506	7.100497	6.527835	7.999639	9.166648	7.648004	7.698520	7.199772	7.270794	7.038119	7.023127	7.616186	7.731029	7.199153	8.089558	7.294911	5.263904	8.288418	5.522694	7.573089	8.131027	8.250047	8.465219	8.064421	8.201379	6.949028	9.299554	5.901849	5.709757	7.322720	6.127483	5.552203	6.210495	5.851664	6.335031	6.250956	7.548238	5.922924	7.175232	7.103764	7.118842	6.043307	7.582246	5.471198	7.238746	6.460672	5.985191	5.766438	5.984182	5.816187	6.609648	7.182036	6.255725	5.752653	6.183047	6.276999	5.665156	7.332577	4.516782	6.910016	5.066538	6.319104	5.609146	6.391892	5.548626	5.694139	4.913908	6.378641	8.065802	6.058868	5.613847	6.129182	6.133974	5.326544	5.608760	5.556954	6.153294	6.398294	6.211030	5.751933	5.876401	6.656942	6.170659	6.777825	4.814140	5.081029	5.448214	5.560750	5.739006	5.990613	6.764402	7.268785	6.544021	8.957952	7.899893	7.272190	7.741489	6.568544	7.950478	6.834845	7.531584	7.820127	8.403138	7.214035	7.749198	8.121159	5.566754	7.611881	8.075328	8.343831	8.370159	6.182544	7.871375	7.793702	8.887442	7.644771	5.976379	5.995662	8.150964	8.873841	7.258046	7.373586	8.248548	8.397928	7.707598	6.647718	7.981300	7.432657	6.606041	6.606540	7.062373	7.508788	8.558851	7.563337	6.455739	6.758456	8.432741	6.623712	8.730171	6.388514	6.043958	7.074170	8.982441	8.382213	6.763922	8.861523	7.474444	7.765467	6.888075	6.167552	7.193263	5.455513	6.481906	7.287971	6.504643	8.511664	5.759064	7.351914	6.505307	7.220497	5.844038	8.083490	5.877950	7.019538	5.712688	5.936160	6.279486	5.904197	6.015535	6.463884	6.582607	6.167909	5.387590	6.442193	5.218747	6.120635	7.235957	6.010194	7.256648	5.760509	5.421300	5.682156	6.003768	6.469350	5.789578	5.768830	6.555983	7.725142	5.283942	5.617787	5.605540	5.077975	5.979208	5.859422	5.394962	7.169943	6.606716	6.309772	6.999824	5.622781	7.295374	6.863627	6.274562	5.201290	5.477682	6.687309	6.082328	5.620917	5.865382	6.505130	6.930002	7.404264	8.718581	8.090942	6.921935	7.521137	6.549177	8.495364	6.286409	6.531604	8.808720	8.196705	7.379718	6.623549	8.122129	6.272050	6.974641	7.786067	8.073351	7.621803	6.925012	7.746418	7.592717	7.128781

GET4	12.374199	12.414938	12.497991	12.754773	12.974569	12.524939	12.471647	12.799850	13.132270	12.900773	12.777955	12.535770	12.563325	13.089449	12.727048	12.708589	12.517616	12.752073	13.828942	12.841260	13.319284	14.010352	13.835602	12.988128	13.104834	13.095423	13.066984	13.208114	12.616658	12.053445	13.202580	12.658466	13.053836	12.380673	12.481464	12.511128	12.602064	12.636992	13.477694	13.639188	12.620500	13.609433	12.886172	12.879575	12.954237	12.892947	12.941572	12.549882	12.683325	12.887549	12.855512	12.553951	12.376614	12.941758	11.961160	12.530672	12.724156	13.426460	13.184851	13.214589	13.454346	12.621342	13.516009	13.907439	13.254596	12.830827	13.229868	12.990154	12.983713	13.116985	12.423912	12.786103	12.839438	12.551558	12.899183	12.681634	12.717840	12.179107	12.809655	12.903734	12.981325	12.371333	12.646754	13.126165	12.659292	12.492884	13.278803	13.223721	13.875803	13.489283	13.309538	13.608041	13.251828	12.920057	12.797845	13.147466	12.109967	12.807363	12.943369	12.692858	12.938338	12.570522	12.627335	12.891887	13.620619	12.618797	13.353762	13.598611	13.880924	12.801434	13.751547	13.573121	13.379282	12.845274	12.648312	11.942282	13.726879	12.451069	13.003596	13.690683	12.850168	12.491398	13.437764	13.288740	12.391754	13.161296	12.934551	13.605949	13.140875	13.032306	12.824716	13.064342	12.619480	13.703898	13.414830	13.569407	13.149535	14.009020	12.570017	13.316841	14.085609	13.864888	12.874855	13.750320	13.567112	12.750860	13.506587	13.329654	12.710169	13.670545	12.629358	12.869060	13.269247	12.820259	12.792507	12.611187	13.172863	13.259193	13.306191	13.626349	13.218984	12.734879	13.253798	13.486198	12.121584	13.106812	13.606633	12.411407	12.518587	12.690337	13.470952	12.450905	11.891870	13.097119	12.616658	13.413997	13.075147	13.277050	13.192884	13.727369	13.233033	13.843499	13.264544	13.189708	13.560621	12.532019	12.574210	12.639389	13.809402	12.960320	12.638003	12.673960	12.550390	12.862205	12.470550	12.977674	13.486625	12.826659	12.658166	12.617130	12.879771	13.190276	12.468921	13.031373	13.718689	12.565127	13.594660	13.791726	13.139865	13.213003	14.475985	13.167086	12.542124	12.408069	12.784513	13.242189	13.694470	13.014650	13.185434	12.913792	12.500081	13.094478	12.257887	14.143339	12.343274	13.723956	14.267647	13.604783	12.742146	13.815559	12.896514	13.574462	12.747949	12.727428	13.378652	12.924711	13.763702	12.776712

SUN1	12.374199	12.414938	12.497991	12.754773	12.974569	12.524939	12.471647	12.799850	13.132270	12.900773	12.777955	12.535770	12.563325	13.089449	12.727048	12.708589	12.517616	12.752073	13.828942	12.841260	13.319284	14.010352	13.835602	12.988128	13.104834	13.095423	13.066984	13.208114	12.616658	12.053445	13.202580	12.658466	13.053836	12.380673	12.481464	12.511128	12.602064	12.636992	13.477694	13.639188	12.620500	13.609433	12.886172	12.879575	12.954237	12.892947	12.941572	12.549882	12.683325	12.887549	12.855512	12.553951	12.376614	12.941758	11.961160	12.530672	12.724156	13.426460	13.184851	13.214589	13.454346	12.621342	13.516009	13.907439	13.254596	12.830827	13.229868	12.990154	12.983713	13.116985	12.423912	12.786103	12.839438	12.551558	12.899183	12.681634	12.717840	12.179107	12.809655	12.903734	12.981325	12.371333	12.646754	13.126165	12.659292	12.492884	13.278803	13.223721	13.875803	13.489283	13.309538	13.608041	13.251828	12.920057	12.797845	13.147466	12.109967	12.807363	12.943369	12.692858	12.938338	12.570522	12.627335	12.891887	13.620619	12.618797	13.353762	13.598611	13.880924	12.801434	13.751547	13.573121	13.379282	12.845274	12.648312	11.942282	13.726879	12.451069	13.003596	13.690683	12.850168	12.491398	13.437764	13.288740	12.391754	13.161296	12.934551	13.605949	13.140875	13.032306	12.824716	13.064342	12.619480	13.703898	13.414830	13.569407	13.149535	14.009020	12.570017	13.316841	14.085609	13.864888	12.874855	13.750320	13.567112	12.750860	13.506587	13.329654	12.710169	13.670545	12.629358	12.869060	13.269247	12.820259	12.792507	12.611187	13.172863	13.259193	13.306191	13.626349	13.218984	12.734879	13.253798	13.486198	12.121584	13.106812	13.606633	12.411407	12.518587	12.690337	13.470952	12.450905	11.891870	13.097119	12.616658	13.413997	13.075147	13.277050	13.192884	13.727369	13.233033	13.843499	13.264544	13.189708	13.560621	12.532019	12.574210	12.639389	13.809402	12.960320	12.638003	12.673960	12.550390	12.862205	12.470550	12.977674	13.486625	12.826659	12.658166	12.617130	12.879771	13.190276	12.468921	13.031373	13.718689	12.565127	13.594660	13.791726	13.139865	13.213003	14.475985	13.167086	12.542124	12.408069	12.784513	13.242189	13.694470	13.014650	13.185434	12.913792	12.500081	13.094478	12.257887	14.143339	12.343274	13.723956	14.267647	13.604783	12.742146	13.815559	12.896514	13.574462	12.747949	12.727428	13.378652	12.924711	13.763702	12.776712

REC8	12.990324	10.327627	10.374399	10.608759	10.411625	9.517602	9.672854	9.490144	9.784596	9.451553	10.157573	9.933530	10.190858	10.768481	10.317582	9.559249	9.683888	10.577682	11.456392	10.600754	10.679735	11.097070	11.886732	10.372281	11.121819	10.741254	11.511412	10.368806	10.264852	10.561160	10.484898	11.251075	11.386452	10.643123	10.043161	10.011976	9.726848	9.597413	10.022449	9.680465	9.794875	10.996252	10.510632	9.779375	9.930936	10.004887	10.183600	10.269714	9.907881	9.681546	10.086189	10.016345	10.220346	9.600344	10.181167	10.032263	9.902880	10.616136	10.619249	10.642678	10.643266	9.397811	11.660429	11.529530	10.514527	10.731351	10.749920	10.541101	10.204762	10.434742	9.352897	9.323193	10.017128	9.953637	9.672393	10.870510	9.443739	9.360643	9.576825	10.612294	10.156362	10.286511	10.953768	9.860350	10.373076	10.202804	10.665460	10.054496	11.521749	10.983211	11.269399	12.059200	10.344916	10.270279	11.149599	10.252352	10.331003	10.240737	9.917791	9.711397	10.136546	9.515307	10.020621	9.413187	10.713575	9.490626	10.648649	10.588224	11.317631	10.145800	12.110108	10.447955	10.232060	9.499324	11.011833	7.051964	13.509479	10.451395	10.029952	11.142648	10.883417	10.516678	11.222968	10.143394	10.181458	10.725946	8.840879	10.383339	10.980712	10.349459	10.260093	10.182411	12.126285	10.230046	10.604891	11.428416	10.583044	12.710821	10.372919	11.690176	13.335194	11.172387	12.336837	9.285198	9.259682	9.940405	12.934196	10.033023	10.333290	12.045149	10.819998	10.930096	9.999208	10.691500	9.890879	10.013689	9.788977	11.146045	11.562614	9.581310	11.169165	10.079519	10.289000	10.103567	10.032263	11.531829	9.111112	9.636846	9.254350	10.143067	9.866689	10.934589	10.025602	10.641778	10.029478	9.569642	10.414788	11.335110	9.743120	11.504506	11.244502	10.975965	11.987411	11.533329	10.827521	11.779847	10.200086	10.451395	11.132929	9.904987	10.894745	10.122161	10.004887	9.953346	10.458339	8.865068	9.412599	9.672092	11.282541	11.051785	11.335110	11.452830	11.563303	10.861749	11.525892	11.330144	9.597413	12.087259	11.852632	9.572739	11.843716	10.713006	10.000862	10.073446	10.957536	10.795256	11.471518	11.680678	9.386834	11.343711	9.079789	12.788897	10.542394	12.362753	11.201301	12.333192	11.114868	11.670177	11.332913	11.462167	11.471231	6.777993	10.773596	8.647237	9.790950	11.484580	9.830772	11.726175

IPO4	12.990324	10.327627	10.374399	10.608759	10.411625	9.517602	9.672854	9.490144	9.784596	9.451553	10.157573	9.933530	10.190858	10.768481	10.317582	9.559249	9.683888	10.577682	11.456392	10.600754	10.679735	11.097070	11.886732	10.372281	11.121819	10.741254	11.511412	10.368806	10.264852	10.561160	10.484898	11.251075	11.386452	10.643123	10.043161	10.011976	9.726848	9.597413	10.022449	9.680465	9.794875	10.996252	10.510632	9.779375	9.930936	10.004887	10.183600	10.269714	9.907881	9.681546	10.086189	10.016345	10.220346	9.600344	10.181167	10.032263	9.902880	10.616136	10.619249	10.642678	10.643266	9.397811	11.660429	11.529530	10.514527	10.731351	10.749920	10.541101	10.204762	10.434742	9.352897	9.323193	10.017128	9.953637	9.672393	10.870510	9.443739	9.360643	9.576825	10.612294	10.156362	10.286511	10.953768	9.860350	10.373076	10.202804	10.665460	10.054496	11.521749	10.983211	11.269399	12.059200	10.344916	10.270279	11.149599	10.252352	10.331003	10.240737	9.917791	9.711397	10.136546	9.515307	10.020621	9.413187	10.713575	9.490626	10.648649	10.588224	11.317631	10.145800	12.110108	10.447955	10.232060	9.499324	11.011833	7.051964	13.509479	10.451395	10.029952	11.142648	10.883417	10.516678	11.222968	10.143394	10.181458	10.725946	8.840879	10.383339	10.980712	10.349459	10.260093	10.182411	12.126285	10.230046	10.604891	11.428416	10.583044	12.710821	10.372919	11.690176	13.335194	11.172387	12.336837	9.285198	9.259682	9.940405	12.934196	10.033023	10.333290	12.045149	10.819998	10.930096	9.999208	10.691500	9.890879	10.013689	9.788977	11.146045	11.562614	9.581310	11.169165	10.079519	10.289000	10.103567	10.032263	11.531829	9.111112	9.636846	9.254350	10.143067	9.866689	10.934589	10.025602	10.641778	10.029478	9.569642	10.414788	11.335110	9.743120	11.504506	11.244502	10.975965	11.987411	11.533329	10.827521	11.779847	10.200086	10.451395	11.132929	9.904987	10.894745	10.122161	10.004887	9.953346	10.458339	8.865068	9.412599	9.672092	11.282541	11.051785	11.335110	11.452830	11.563303	10.861749	11.525892	11.330144	9.597413	12.087259	11.852632	9.572739	11.843716	10.713006	10.000862	10.073446	10.957536	10.795256	11.471518	11.680678	9.386834	11.343711	9.079789	12.788897	10.542394	12.362753	11.201301	12.333192	11.114868	11.670177	11.332913	11.462167	11.471231	6.777993	10.773596	8.647237	9.790950	11.484580	9.830772	11.726175

SETBP1	6.465373	4.281894	4.703011	6.105329	6.028906	6.639659	5.507004	5.568462	6.331981	6.344739	6.644989	6.618714	6.967254	6.006099	6.149498	6.042958	5.746482	6.897684	4.941966	5.510295	4.682249	6.225550	6.784344	6.599750	6.925494	6.248466	6.635796	7.548532	7.095474	5.718684	6.382325	4.687391	5.522145	6.608356	5.958051	5.069790	5.306575	4.749521	4.602712	5.618375	6.592110	4.916860	6.424977	5.692666	7.319006	5.624269	6.942144	2.842787	4.302876	5.782546	5.896885	4.410887	5.272049	4.863512	5.096409	6.220727	6.318078	3.624461	5.493183	5.163064	5.089809	6.563515	4.666471	4.409371	3.604453	4.858360	3.061454	4.758930	4.102823	4.568525	5.169712	4.837448	6.350795	5.431432	5.220376	5.518195	4.975271	4.177867	4.712006	5.594019	5.444473	5.399012	5.905776	4.659010	5.148725	5.866135	4.240573	5.092892	3.808898	4.578828	4.638196	4.510948	4.597046	4.970884	5.631017	5.959156	6.014686	7.814319	5.767194	6.304424	5.782005	5.654829	6.600872	6.440058	4.948729	6.965004	6.055823	5.275329	5.194696	6.481071	5.029542	4.756759	5.189746	6.109572	5.844230	4.610371	6.470376	5.899887	6.979258	6.104775	4.992892	3.668862	6.207080	5.413006	6.501233	5.426219	7.625172	4.976700	6.461005	6.616765	6.948600	6.801411	6.233583	7.112068	5.937556	6.589501	7.543432	5.637885	5.854373	4.820657	5.461798	5.878333	6.415159	5.677332	5.651640	3.772186	6.989522	5.584968	5.846583	6.608681	6.045183	6.764101	5.683615	5.011793	5.706129	4.325747	3.330497	4.569356	4.536749	6.106746	3.446812	5.417579	5.489572	6.537460	5.026259	6.871306	4.221265	4.928060	4.396359	5.133404	6.335031	5.725538	4.437434	4.837031	5.766650	6.969671	3.465305	5.338325	3.586905	4.123822	6.445919	3.814191	4.008468	4.068163	5.698775	2.990809	4.759341	4.937893	2.853354	4.651571	4.835798	6.471216	4.335462	4.981328	5.175155	4.951447	4.244102	5.193652	5.622037	5.056780	4.781766	5.900633	5.337511	5.356651	5.843704	4.611855	5.800031	3.471558	5.190944	4.827193	3.659062	4.433495	5.433001	5.832808	5.020938	5.804583	7.817143	6.530462	5.892964	6.209329	6.459372	6.194626	4.891263	5.924182	6.716549	4.908825	3.955912	4.064364	6.612157	5.037736	4.097968	4.663683	6.433787	5.174727	4.184247	6.180653	5.320335	6.913856

CCDC85B	12.227834	12.732358	12.118961	12.294356	12.098308	12.126121	13.130368	11.961736	11.496066	12.207603	12.744905	12.607071	12.854034	12.917574	13.070763	11.654682	12.663072	12.871377	12.519372	12.268074	12.519046	12.759089	13.187239	12.137255	12.617294	12.540947	12.238507	12.242834	11.876727	12.142800	12.216379	12.635273	12.728453	13.147891	13.278020	12.688641	12.834696	13.018087	13.288850	13.075520	12.041232	13.312162	13.001399	12.701599	13.457219	13.166504	11.797192	13.246936	12.832730	12.729989	12.491754	13.221771	12.839105	13.048590	13.230280	12.239149	13.211293	13.530568	12.787690	13.743835	13.510832	12.226002	14.684603	13.070948	13.872353	13.544379	12.857438	12.587805	12.831516	13.213784	12.520730	12.690027	11.884352	12.440145	12.685200	12.602912	12.582141	12.311242	12.231261	12.793758	12.514664	12.811936	12.880949	12.715418	12.729989	12.829461	12.596461	12.361628	13.337481	12.958452	13.308155	13.667422	12.862405	12.680777	12.456454	12.620177	13.299630	12.665111	12.330790	12.467321	12.356888	12.525777	12.095797	12.249922	13.246703	11.819147	12.715589	13.335194	12.986119	11.800745	12.593126	12.788040	13.053301	11.848060	12.151563	11.872661	12.884726	12.389189	11.786118	13.325811	13.449081	12.641089	12.966913	12.366220	12.722462	13.040804	12.345649	13.215196	13.117513	13.829979	13.084593	13.789730	13.502404	13.404601	11.701767	13.152020	13.141252	13.172674	12.417520	13.078763	13.012640	12.949458	11.940741	13.849877	14.289961	12.112986	13.250452	11.828401	12.445640	12.502069	13.016827	13.596807	13.022526	12.396622	12.268379	12.716779	12.882033	13.634245	13.295754	12.696084	13.479980	13.253798	13.134732	12.946442	14.266988	12.146729	13.436430	12.590988	13.098245	12.881690	13.546180	13.228843	13.579444	13.809161	12.481636	13.027853	13.213586	14.121867	13.052959	13.653439	12.742511	13.358673	13.771343	13.552782	13.691450	12.764108	13.109263	13.999805	12.890688	12.578683	12.441914	12.886695	13.196531	12.444353	13.117513	13.209908	13.310550	12.651411	12.868052	12.451389	13.569652	12.552991	12.541440	12.758922	13.539515	13.076654	13.833047	13.800613	13.533281	13.392871	13.966624	13.396889	13.579215	13.166090	13.214797	12.906787	12.239471	13.256221	12.582441	13.501741	12.309356	12.152049	13.461357	13.085901	12.403553	13.354589	12.885289	12.424744	12.399952	12.750518	12.664946	13.352048	12.185867	12.979140	13.228076	13.930857	12.496861	13.742612

ARNTL	6.015357	5.224094	6.283128	3.835886	5.897574	5.962707	6.051653	5.472537	7.124976	5.824416	6.047481	7.103141	4.471916	5.175351	5.484613	5.117824	6.129505	4.841693	4.078077	5.648403	4.620375	4.453967	6.329550	4.822317	2.941644	5.143372	5.649360	2.903112	5.611389	7.236106	4.457149	7.035286	5.812805	6.957032	7.176647	7.010571	5.611389	4.466328	3.271787	4.659849	5.458858	4.300684	6.102054	5.454178	6.331290	4.972338	4.920418	2.998343	7.144444	6.539247	6.255038	4.587555	7.186675	6.090277	7.849325	5.248221	6.738109	4.048439	3.173699	4.641133	4.390632	6.638032	4.344818	3.249291	3.417570	6.385448	3.348380	4.347401	3.866820	4.051954	5.619645	5.448014	7.055070	6.005022	6.174035	7.053640	4.987082	7.062373	6.680462	5.841522	7.165196	7.448405	5.887268	6.273200	5.050399	6.303742	5.841340	6.119063	4.189497	3.471394	4.122955	4.763361	5.306921	6.343705	6.910655	7.500092	7.349599	6.517718	6.288774	4.670600	7.235637	5.771660	6.375503	6.901432	4.968204	6.663954	7.870132	5.699868	3.741358	4.455228	2.918874	5.161272	3.473431	5.049361	7.808028	5.435917	4.077226	7.649041	4.772813	6.678007	6.247129	6.179165	4.229251	4.753418	5.870061	5.269656	4.659428	5.628592	5.526086	6.045528	5.761388	3.975516	4.150401	5.305197	4.929067	6.289289	4.025364	3.219832	5.311819	4.644041	3.323165	3.078033	4.662858	2.868595	3.418603	4.884308	3.491351	4.913496	6.326589	3.480211	7.513425	5.829507	7.251366	6.815732	6.776038	6.470026	3.894213	3.285959	4.497929	4.420461	3.251540	5.616107	5.345357	6.221944	4.434754	5.339523	2.867173	6.370223	6.967570	6.782927	5.329287	7.282571	6.515723	7.061543	5.653503	6.532242	3.959460	3.011063	4.431596	4.096230	5.977257	4.777623	3.714654	4.326137	5.665514	3.915505	5.214446	4.471916	2.886842	5.659219	5.931261	5.758717	6.572093	6.141889	7.339873	5.070383	6.896720	7.403508	6.174551	7.156070	6.825270	6.484786	7.507225	5.814075	5.645243	6.428009	7.032182	3.875870	5.137833	3.848851	5.333630	5.179750	6.319104	7.311045	7.687797	7.071013	6.199995	6.362012	4.787643	6.866135	5.168072	4.499207	6.551642	4.884088	5.231593	6.716549	5.574273	4.478244	3.403704	3.000046	3.015024	2.847758	4.651131	6.086091	6.204520	4.647475	8.131646	5.224298

HNRNPA1P48	14.541751	14.533492	14.565537	14.816215	14.863545	14.426644	15.184749	14.088329	14.114654	14.294067	14.765245	14.542200	14.498295	14.307693	14.802357	14.683638	14.443429	14.415465	14.388223	13.791726	14.865570	14.575995	14.731220	14.337170	13.872088	14.479005	14.768542	13.460482	14.616972	14.767089	14.875498	15.748884	15.246670	14.779447	15.250206	14.993988	15.014487	14.754551	14.649747	14.590670	14.737586	14.580207	13.612808	14.384530	14.621136	14.623346	14.502269	13.824264	14.910337	14.232519	14.972000	14.743181	15.145529	14.304625	14.866053	14.365218	14.439683	14.056836	13.624759	13.976899	14.294396	15.201679	14.529498	14.037568	14.659533	14.752647	14.235912	14.311014	14.347809	14.646275	14.572654	14.805656	14.723372	14.894400	14.867676	15.140997	14.710296	13.791726	14.751698	14.183722	14.808644	14.928143	14.669062	15.397686	14.704765	15.400849	14.737145	14.197518	14.718998	14.585219	14.211417	14.938834	14.322632	14.013234	15.173970	15.320436	15.487065	14.837526	14.841605	13.522760	14.576819	14.220551	14.526310	14.405515	14.798922	14.623346	14.455021	14.548089	14.830006	14.520691	14.835529	14.319186	13.612808	14.842680	14.297379	14.334354	14.045844	15.129649	14.295373	15.304417	14.771390	14.863545	14.670593	14.859010	15.233438	14.592389	15.262346	14.822343	15.070405	14.893861	15.206355	14.458184	14.806664	14.402538	14.804293	15.261637	14.349592	15.197536	15.079318	15.021020	14.888152	14.963955	14.675960	14.790582	14.651938	14.910337	15.029900	14.746969	14.671445	14.896544	14.419519	14.716286	14.928661	14.680064	14.931361	14.725162	14.570106	14.392249	14.674591	15.142982	14.575995	14.303599	15.179287	14.510324	14.785821	14.680064	14.766218	15.152524	14.646275	14.566322	14.830972	14.900158	14.988450	14.851142	14.747900	15.237652	14.262592	14.344582	14.580994	14.449826	14.276902	14.422502	14.790142	14.706135	14.638391	14.680520	14.503429	14.604151	14.315778	14.967925	14.958920	14.678334	14.972581	15.002418	14.756024	14.906118	15.120910	14.972000	14.218284	14.488364	14.890760	14.727873	14.695210	15.270868	14.591979	15.093138	15.491291	14.596200	14.650647	14.994487	14.906118	14.852694	15.314555	14.648824	14.663619	15.001364	14.866612	15.086549	14.746490	15.262346	14.640110	14.779929	14.447934	14.635670	14.798922	15.273761	14.820339	14.689299	14.874444	14.820339	15.258165	15.353308	14.592389	15.344243	14.781829	14.847322	14.477842	15.084166

OR8H1	3.284447	3.923084	3.385685	3.931529	3.464489	4.610371	4.181870	3.835245	3.313790	3.109333	3.730876	3.838235	3.662035	3.727364	3.758791	3.897142	3.380170	2.887669	3.252587	3.890045	4.311047	3.607655	5.224094	4.108038	3.289685	3.581299	3.762565	2.957334	3.316355	3.850353	3.671942	4.637598	3.742589	3.782722	4.801966	3.963386	5.065069	3.359648	3.494647	3.492401	3.775313	4.012684	3.525894	3.239740	3.664440	5.570700	3.368431	3.466108	3.645219	3.660010	3.540335	3.534047	4.377152	4.587788	3.881910	4.549992	4.109365	3.413828	3.185819	3.744640	3.061684	3.620854	3.890045	3.420058	3.502150	3.506641	3.022175	3.346871	3.633517	4.006114	4.074510	4.751493	3.399529	3.610768	3.473978	3.776565	3.860653	3.943719	3.764496	3.514792	4.165107	3.786037	3.392489	3.853359	3.684609	3.634857	4.425910	3.631713	3.567103	3.435805	3.223498	4.000622	4.102375	3.348056	3.635440	3.800534	3.740764	3.041132	3.502150	3.359512	5.711750	3.762370	3.996193	3.730665	5.833177	3.903359	3.736280	4.733571	4.743378	4.274425	3.761124	4.113558	4.618652	4.527587	3.574404	6.980675	3.993074	3.238168	3.542076	3.265442	4.036066	3.827843	3.960098	4.016935	4.225973	4.365248	3.120218	3.775752	4.203136	3.583046	3.592821	4.253430	3.922444	2.990083	4.460269	3.182569	2.835200	4.272029	3.385183	4.535677	3.234681	3.368594	3.811858	3.604836	3.620461	4.995208	3.595938	4.276612	4.188655	3.499422	3.478950	3.681431	3.970080	4.223606	3.672321	4.855214	3.311947	3.422793	3.538456	4.198610	4.185988	3.582276	3.293775	4.101929	5.127355	4.187529	3.084967	3.881506	3.452427	3.393651	4.332885	4.193640	4.311668	3.475268	4.349640	4.962329	3.430988	3.100328	3.718521	4.095823	3.559157	3.988826	3.771146	3.798389	3.716485	3.014323	3.988826	3.587283	3.034991	4.415573	4.383833	3.848412	3.785223	4.189270	3.534047	4.090696	3.947935	4.108917	3.645826	3.595732	4.096007	3.391201	4.212293	3.500336	3.763856	4.447326	3.382407	3.261577	3.371537	3.353521	2.952703	3.347210	3.725135	3.716284	3.946203	3.890951	5.263473	4.002147	3.906427	3.526862	3.672321	4.258714	3.996830	5.498120	3.772186	4.017593	3.358117	3.449885	4.519569	3.991571	4.682440	5.960569	4.947941	3.684027	4.082384	3.065217	4.008008	4.450279

TARM1	8.522763	7.065228	7.299864	7.537165	8.021631	9.526925	8.557608	9.571519	9.662962	8.638802	9.227275	9.726215	8.444252	7.807410	7.306970	8.125849	9.612878	7.231821	8.717495	7.655630	8.565110	7.719642	10.413447	7.382348	7.515375	8.988507	9.162455	8.519151	8.645281	9.871517	9.469344	7.457145	7.511087	8.431048	7.267272	7.493510	7.442676	7.414000	7.778689	8.095372	8.925972	7.745299	7.076686	7.581955	6.980014	7.281300	11.078117	7.346579	8.157493	6.680932	7.309782	7.384091	6.586878	6.929221	6.497857	9.729350	6.588530	6.159810	7.166846	7.217178	7.594432	8.890098	7.569604	7.904431	7.682889	7.868761	7.600924	7.445550	7.623954	8.282391	7.850948	7.737739	8.953170	7.569307	7.709277	8.557152	8.044166	7.929878	8.593939	7.033109	7.398357	7.330879	7.461032	7.182354	7.385010	7.724547	7.627977	7.726996	7.921630	7.936848	8.142694	8.412918	7.873818	8.021631	10.151269	7.896712	7.976016	7.953412	10.092469	4.954364	9.427430	7.005812	7.193586	6.980675	7.737107	8.869138	7.914014	8.511664	9.061578	8.608784	8.715941	8.659306	8.384354	9.997209	9.144893	12.793389	8.350047	9.015924	9.418832	9.092054	7.779974	7.622087	7.810345	9.284164	9.655028	10.201399	10.257553	8.729568	9.727449	10.420556	10.213038	9.574283	8.189742	9.329390	8.981042	8.888851	8.150381	9.323513	7.883365	9.004108	8.058121	9.519931	8.998256	8.285931	8.443154	10.094546	9.152937	9.352445	9.983209	9.681392	8.549674	8.376902	8.807664	8.098702	7.697916	8.077123	8.641436	9.524895	8.637389	10.638914	8.226166	7.668974	8.652353	7.320440	7.741489	11.709994	7.575127	8.026826	7.164273	7.860474	9.232220	6.652403	7.444912	6.857547	8.498198	6.963224	6.295088	7.432347	7.248533	8.111174	8.838597	8.096606	8.051705	8.049687	8.098548	8.073510	7.684279	8.416609	8.604996	8.103097	8.001204	10.436435	9.092540	8.730171	8.183407	8.206331	8.572331	9.435704	7.205220	7.606544	7.542948	7.792043	7.340836	8.054626	9.284768	7.928511	8.010662	8.139429	8.099931	8.617692	9.101487	8.306486	9.887206	9.197647	9.182515	10.859980	10.096114	9.807438	5.430487	10.989882	8.076211	9.142892	7.292867	8.154232	9.178624	8.992404	8.481554	8.918759	9.345280	8.911573	8.956987	9.038186	12.322660	10.316666	10.383504	8.099775	9.332000	9.792703

CRAMP1	10.796422	9.601111	10.014305	9.797029	10.017128	10.639993	10.909844	9.938052	10.247418	10.501072	10.877643	10.721496	10.105829	10.209812	9.004108	10.324760	9.657205	9.074052	10.306695	10.742155	9.743949	10.039741	10.091421	9.295377	9.350764	9.770967	10.130040	9.374798	10.101089	10.531004	9.836257	10.324445	10.449326	11.087667	10.369851	10.595736	10.645414	9.397313	9.691617	9.561747	10.543736	9.801200	9.681699	9.959089	9.635352	9.375295	10.323369	9.071428	10.293707	10.217418	9.713091	10.310064	9.907273	10.093356	9.839396	10.610379	9.767677	9.822386	9.516988	9.498731	9.988575	10.754004	9.510701	9.900361	9.861294	9.098890	9.427739	8.687758	9.110466	9.822073	10.223767	10.656820	10.645194	10.509472	10.431267	10.169039	10.601671	10.416006	10.133149	10.117756	9.799962	9.670216	10.022295	10.148232	10.103883	10.487647	10.161839	10.255889	10.376501	9.256667	9.610292	10.592600	9.781923	10.621011	10.150237	10.197070	9.968179	10.599871	10.563219	9.693584	10.369561	9.921992	9.568254	10.171845	10.121388	10.168890	9.462264	10.074539	10.363127	10.489958	11.135510	9.422799	9.320329	10.305204	10.502146	9.299554	8.845259	10.796121	10.003654	12.606564	10.061488	10.226509	10.703888	8.763472	10.086677	10.593946	11.454433	10.829161	10.718958	10.993754	10.865293	10.158173	10.034536	9.544579	10.573479	10.160131	8.727572	11.136698	10.851497	9.980403	9.715949	10.662532	8.398367	10.548745	10.047255	10.143394	9.590114	9.939497	10.332362	9.187379	10.935020	10.755886	11.752315	10.233845	10.464170	10.440967	10.251447	10.491566	9.783775	10.484268	10.104326	9.772353	11.018990	10.175307	9.401854	10.112741	8.888525	10.897486	10.238398	10.416142	10.956655	10.305962	10.577067	9.913442	10.531004	11.044439	9.692692	9.554494	9.795351	10.223615	10.907937	9.586230	9.743043	9.356076	10.084209	9.475061	9.126433	9.447310	10.369851	10.584768	10.950866	9.875439	10.664886	10.388365	10.177628	10.535296	11.071058	10.363870	10.103422	9.988575	10.017871	10.445361	9.636846	10.215948	10.360974	10.379105	10.289433	10.298160	9.531481	10.128997	10.292031	9.934185	10.933223	10.375761	10.137297	10.222882	9.689437	10.586706	10.579788	11.044022	10.572740	8.553021	9.558482	11.509789	10.349330	9.705954	10.979516	11.079687	10.477996	10.905411	8.701371	8.102649	10.302486	10.093965	10.911465	10.044168	11.578064	10.785111

AL031708.1	10.796422	9.601111	10.014305	9.797029	10.017128	10.639993	10.909844	9.938052	10.247418	10.501072	10.877643	10.721496	10.105829	10.209812	9.004108	10.324760	9.657205	9.074052	10.306695	10.742155	9.743949	10.039741	10.091421	9.295377	9.350764	9.770967	10.130040	9.374798	10.101089	10.531004	9.836257	10.324445	10.449326	11.087667	10.369851	10.595736	10.645414	9.397313	9.691617	9.561747	10.543736	9.801200	9.681699	9.959089	9.635352	9.375295	10.323369	9.071428	10.293707	10.217418	9.713091	10.310064	9.907273	10.093356	9.839396	10.610379	9.767677	9.822386	9.516988	9.498731	9.988575	10.754004	9.510701	9.900361	9.861294	9.098890	9.427739	8.687758	9.110466	9.822073	10.223767	10.656820	10.645194	10.509472	10.431267	10.169039	10.601671	10.416006	10.133149	10.117756	9.799962	9.670216	10.022295	10.148232	10.103883	10.487647	10.161839	10.255889	10.376501	9.256667	9.610292	10.592600	9.781923	10.621011	10.150237	10.197070	9.968179	10.599871	10.563219	9.693584	10.369561	9.921992	9.568254	10.171845	10.121388	10.168890	9.462264	10.074539	10.363127	10.489958	11.135510	9.422799	9.320329	10.305204	10.502146	9.299554	8.845259	10.796121	10.003654	12.606564	10.061488	10.226509	10.703888	8.763472	10.086677	10.593946	11.454433	10.829161	10.718958	10.993754	10.865293	10.158173	10.034536	9.544579	10.573479	10.160131	8.727572	11.136698	10.851497	9.980403	9.715949	10.662532	8.398367	10.548745	10.047255	10.143394	9.590114	9.939497	10.332362	9.187379	10.935020	10.755886	11.752315	10.233845	10.464170	10.440967	10.251447	10.491566	9.783775	10.484268	10.104326	9.772353	11.018990	10.175307	9.401854	10.112741	8.888525	10.897486	10.238398	10.416142	10.956655	10.305962	10.577067	9.913442	10.531004	11.044439	9.692692	9.554494	9.795351	10.223615	10.907937	9.586230	9.743043	9.356076	10.084209	9.475061	9.126433	9.447310	10.369851	10.584768	10.950866	9.875439	10.664886	10.388365	10.177628	10.535296	11.071058	10.363870	10.103422	9.988575	10.017871	10.445361	9.636846	10.215948	10.360974	10.379105	10.289433	10.298160	9.531481	10.128997	10.292031	9.934185	10.933223	10.375761	10.137297	10.222882	9.689437	10.586706	10.579788	11.044022	10.572740	8.553021	9.558482	11.509789	10.349330	9.705954	10.979516	11.079687	10.477996	10.905411	8.701371	8.102649	10.302486	10.093965	10.911465	10.044168	11.578064	10.785111

GFPT1	12.866728	11.363130	11.120300	10.736547	10.184516	10.365302	10.688128	10.268511	9.446213	10.202804	10.407551	10.492998	10.325965	9.844727	10.062277	10.463860	9.648129	10.422706	10.031316	10.760404	9.985035	10.269126	9.774645	10.029183	10.360811	10.408524	10.681966	10.411451	10.472068	10.476787	9.108288	11.442182	10.587003	10.886871	10.975539	11.408142	11.185699	11.181275	9.921681	10.412232	9.771907	11.483101	11.248655	11.489500	11.230646	11.347723	9.188910	11.123986	10.997597	11.100018	12.424239	10.991194	11.270275	11.113182	11.592964	10.065725	11.357672	10.557950	10.865149	10.752793	11.007714	10.158948	11.407693	10.569539	10.543604	10.953197	11.685397	10.604741	10.610648	10.926688	10.915990	10.838423	10.061049	11.285928	10.975234	10.786473	11.214379	11.409515	12.133414	11.241079	11.518416	11.542723	12.186508	11.548861	10.846377	11.787320	10.809966	11.157753	10.991194	10.963237	11.475562	10.916152	11.359007	11.218631	12.599341	11.044580	11.572679	9.816653	10.232060	10.661622	10.414788	11.486704	10.791885	10.552052	11.142360	9.931382	10.679116	10.854955	10.976341	9.607630	10.675689	10.343850	10.601053	9.827378	10.436289	9.754870	12.158173	10.319814	9.020172	11.295839	10.821007	10.826216	10.031468	10.495265	10.027037	10.473776	11.646786	9.787591	10.959298	10.090076	9.708902	10.315198	9.958930	10.762508	9.654697	10.537222	9.555843	10.774339	11.563013	9.849132	9.553744	10.698830	9.731680	10.601053	10.437954	10.844098	10.352666	9.619482	10.067514	9.939017	10.718063	9.981055	10.996252	11.277350	11.079687	11.007714	11.485467	10.326579	10.907218	9.576365	10.938612	11.626020	11.132779	10.765528	10.497091	8.768463	12.061208	11.094712	10.780871	11.020371	11.228274	11.492740	11.032397	11.401220	10.698977	12.131878	11.153618	10.506461	11.565520	10.901270	9.177867	10.920769	10.118388	10.136837	10.795127	10.485784	10.707338	11.202548	11.357963	10.681521	11.503654	8.939900	10.730905	11.118110	10.606523	11.104149	11.622359	10.990195	11.348648	11.068220	11.203735	11.047954	11.120300	11.094152	10.479650	10.636826	11.474379	10.875592	10.415101	11.785826	10.847257	11.534947	10.811946	11.427665	11.239635	10.998939	10.063175	9.759792	11.742003	10.198291	11.582720	10.407834	10.765971	10.672422	9.320034	8.688827	11.091172	11.491308	8.262136	10.728524	9.851372	10.334644	8.828725	11.270879	9.857107	11.140774	10.501539	8.935930

WFDC11	3.400227	3.797572	3.714449	4.159074	4.850577	5.067563	3.832923	3.491351	6.143926	4.233943	4.401099	3.759641	4.184907	5.401255	4.691364	3.613588	4.320370	5.052568	5.458276	4.992449	4.945436	6.478984	3.983064	4.774907	5.086568	5.237665	3.196264	4.944783	3.406943	3.203743	5.710849	4.690299	4.336340	4.391274	4.149805	4.140390	3.868783	3.651376	3.368431	4.487427	4.408936	3.717722	4.410703	4.425470	4.510736	5.408333	4.444552	5.594934	4.970691	4.253225	4.140617	5.209227	4.323129	4.507461	4.185338	4.182980	4.031890	4.756759	4.274648	3.893080	3.459278	4.680068	3.683640	4.511602	3.864949	3.342824	3.351900	4.438066	3.559346	4.859389	4.150204	3.722298	4.086354	3.828431	4.069444	3.337310	3.520242	3.232441	3.215552	3.759199	3.737290	4.010802	3.394158	4.132996	3.345113	4.674065	3.206907	3.147523	4.121187	3.735410	3.463210	4.018708	3.031355	3.711920	3.349518	4.528201	4.155180	5.902366	5.544482	4.795599	4.763151	3.866820	4.659010	4.497281	4.484599	4.576680	5.319191	4.103439	5.647837	4.525441	3.980864	4.536948	3.395752	5.220735	4.849503	3.734186	3.082015	4.401336	4.933652	4.462632	3.788669	4.782417	3.438735	4.482648	4.490665	3.872862	3.310033	3.725135	3.540335	3.859821	3.231727	3.078263	3.285623	3.215682	3.384000	3.706050	5.124112	5.412818	3.557966	4.231140	4.585408	5.558489	4.312111	3.851412	3.495403	3.297937	4.646000	3.586490	3.538047	4.448705	5.252732	3.278841	3.949193	4.516782	4.163148	3.829296	3.871134	3.412911	4.239916	3.792266	3.385685	4.334415	3.499422	5.721394	4.906492	5.041986	4.066903	5.698054	4.567453	4.248379	4.860231	4.118088	4.347616	3.451883	3.153056	3.625390	4.576019	4.094334	3.912710	3.096025	3.723306	3.681040	4.008227	3.948147	3.503482	3.288352	4.541524	3.871134	3.049099	5.793310	5.250184	3.490098	4.087037	3.151510	3.316489	3.283173	3.181376	3.479271	4.155180	5.086979	4.307013	4.306356	4.672289	3.638474	4.292225	4.244327	3.310373	3.854698	4.493786	3.354520	4.258714	3.780830	3.256478	4.251929	4.743795	4.784438	5.733015	5.977963	4.690963	3.648387	3.658061	5.140077	3.319596	4.853109	5.031335	5.015058	4.907970	5.978683	4.221462	4.068386	3.130994	3.349518	4.438717	3.454178	4.319275	3.369434	5.456085	3.376176

RPL31	15.265157	15.426445	15.830739	15.936069	16.199785	15.659589	16.405563	15.290979	15.516865	15.644037	16.014400	15.420214	16.331210	16.401115	16.223639	15.938416	15.534337	15.419437	15.889470	14.906627	16.290771	15.993114	16.041613	15.898712	15.219810	14.962802	15.036921	14.934057	15.993114	15.707533	15.974511	15.984477	15.743790	15.006579	15.969476	15.792922	16.196709	15.901055	15.764986	15.720880	15.961903	15.268645	13.600359	15.235562	14.842680	14.485612	15.701693	14.365566	15.947333	14.637025	15.167431	15.598287	14.565537	15.465655	15.700644	15.762943	14.815737	15.345704	15.035175	15.347923	15.654597	15.878115	15.594598	15.626034	15.275879	15.288862	15.066902	15.459167	14.775599	15.887107	14.920091	16.032263	15.897567	15.175273	15.748884	15.921788	15.997211	14.328233	14.567126	15.851177	16.108530	15.497254	15.969476	16.494444	16.433023	16.234936	16.215284	15.313043	15.794025	16.040249	15.820930	15.723939	15.495462	14.957773	15.296048	15.455879	16.059162	15.957029	15.897567	14.835035	15.758639	15.549763	16.199785	15.480428	15.843040	16.177217	15.861722	15.744893	16.122740	16.147173	15.740567	15.745908	15.302315	16.047049	14.808644	15.290979	14.889685	15.959440	15.433373	15.930069	15.681084	15.337535	15.312332	15.205705	15.746888	15.351660	15.674067	15.801695	15.657650	15.376978	15.743790	15.173276	15.417796	15.452790	15.392156	16.009296	16.055100	15.987023	15.753148	15.944816	15.546202	15.892893	15.662431	15.646933	15.638552	15.536016	15.636638	15.520300	15.093138	15.492990	15.456675	15.695521	15.498075	15.149334	15.352487	14.974306	15.543645	15.413146	15.676954	15.604793	15.541988	14.075731	14.586050	13.979146	14.519413	15.392983	14.624254	16.090084	13.976353	14.570568	14.493157	15.671141	15.624294	15.497254	15.671141	15.772150	15.208375	15.412346	15.057310	15.500573	15.582778	15.372489	15.431836	15.406237	15.345704	15.335238	15.023469	15.358743	14.970861	15.635602	15.472304	15.448809	15.859538	15.160128	14.858502	14.911983	15.623471	15.543645	15.370133	15.182677	15.463234	15.299532	15.495462	15.933725	15.591703	15.697564	16.213991	15.702691	15.614975	15.427177	15.802772	15.460905	15.486206	15.352487	15.443248	15.440770	15.425683	15.491291	15.544594	15.660615	15.339763	15.181269	14.759633	15.628950	15.356364	15.795114	16.010487	15.764986	15.296048	15.863999	15.617837	16.073627	15.439830	15.887107	15.534337	15.901055	15.457513	15.694552

ZNF613	9.868052	9.891185	10.089002	9.803956	10.072985	9.719550	10.115138	9.411987	8.729427	9.839992	9.835965	9.804849	9.427118	9.817564	9.462264	9.693450	8.737472	9.804390	8.530634	10.082692	9.540818	9.702186	8.999804	9.770821	9.557714	9.727598	7.927895	9.363934	8.859884	9.123809	9.315204	10.349902	10.563528	9.815584	10.254975	10.435536	10.589350	10.097624	9.326153	10.093356	9.816348	9.356816	9.970033	10.165028	10.204611	10.235895	9.286747	9.261194	10.245014	10.242137	10.482767	10.363870	10.365612	10.697351	10.156215	9.123526	10.795715	10.138502	9.531982	10.419200	9.944847	9.700644	8.910390	9.152161	9.549607	9.626458	9.385584	9.461061	9.999670	9.171408	10.202951	10.168122	9.224202	10.291882	10.148232	9.986251	10.422252	10.017266	10.260405	10.102338	10.413585	10.112589	10.254815	10.731781	10.334496	10.276374	10.538517	10.806048	9.449194	9.517138	9.874961	9.309261	10.263944	10.570935	10.083424	9.981215	10.101581	9.723772	9.813992	9.511509	9.608835	10.245440	9.671482	9.822841	9.325661	9.202802	9.760100	9.592603	8.784600	9.472923	9.443739	9.192429	9.599253	8.917711	9.545320	8.030083	9.534594	9.525550	8.531966	9.935758	9.911427	10.542996	10.412690	7.767009	8.720229	9.336944	8.541489	6.253010	9.015314	9.002579	8.814849	7.794750	9.252981	8.873841	9.192737	8.519009	8.826140	9.106280	9.371195	8.831339	9.098294	9.451098	9.167255	8.733797	7.584267	9.796086	4.771765	6.767312	9.191817	10.201399	9.451258	8.422572	9.927841	10.467402	10.583626	10.530844	10.096114	8.844962	9.520524	5.589862	7.641386	9.546085	10.389876	9.353484	8.907342	8.609851	9.729192	9.840623	9.344530	9.445303	10.811946	10.414186	10.325194	10.141487	7.632243	10.830613	8.076365	9.236897	10.443810	10.374399	9.411829	8.394685	8.755318	8.818720	9.034964	7.953242	9.214054	9.659667	9.592356	8.852118	9.792404	7.458563	10.111838	10.070373	9.151857	9.884692	9.518373	9.784891	10.483232	10.147175	8.810289	10.847257	9.902880	11.327497	9.558784	9.938539	10.308167	9.791261	9.234881	9.389298	9.429598	10.183009	9.776425	9.871673	10.178885	9.336024	8.441197	9.819657	10.196439	9.251933	8.827175	8.207086	8.975406	9.778612	8.838141	8.919658	9.639347	8.015709	8.335568	7.746868	8.858058	9.827223	8.481554	10.247700	9.283080	8.438720	9.222961	8.556080

TMEM47	12.917303	11.893367	11.419106	11.280469	9.914232	10.392366	12.327491	6.594072	6.458537	11.621293	10.373812	11.031068	11.183186	10.307568	11.558177	11.048986	12.662746	10.996096	9.381799	10.411296	9.766182	9.914396	13.776922	10.427919	12.877478	12.783797	12.826984	12.450751	12.426371	11.644819	9.808570	12.573686	11.343546	11.651384	12.052996	12.753285	12.544739	11.712047	11.621431	10.797295	11.224748	11.584548	12.430448	12.670521	14.454664	12.115229	12.423121	11.825339	11.643568	12.224476	12.530524	13.403370	12.574386	12.238197	12.622342	11.978531	13.189521	11.664624	11.308236	12.985374	12.106547	12.319186	13.332882	11.123509	10.785414	11.512761	10.421803	11.445581	10.745154	10.458181	12.190585	11.904475	11.678745	12.325086	11.256626	12.047084	11.733934	12.156499	11.456977	12.228317	13.139865	12.621342	12.274878	11.883291	12.916049	12.463227	11.905178	12.573339	11.358851	10.176874	10.687976	11.096621	11.987098	11.581967	11.805894	12.185427	12.335244	12.385468	11.284447	12.832911	11.059536	12.211265	11.394116	10.654872	9.833499	11.005994	9.185942	9.894424	10.497826	11.477556	10.775784	10.281995	8.113151	10.524431	9.357717	9.772353	11.878180	12.098761	11.235078	10.233109	10.755101	11.373920	9.433716	8.331848	8.654155	10.288200	10.354019	9.061578	9.282762	9.692401	9.463167	11.319280	9.509923	8.648119	11.958138	11.081157	9.393746	8.929545	10.382403	8.279636	9.768915	11.003511	7.187316	11.131496	12.096234	10.030230	7.261516	8.742540	10.974369	10.134533	11.242685	11.234364	10.446732	10.484898	11.329537	11.999153	10.625496	9.049134	10.659469	8.590668	9.797657	11.880130	10.967834	10.792032	10.676888	6.491222	10.296801	10.708891	11.793314	12.054398	11.845977	11.488607	11.468075	13.040220	12.332224	12.272346	10.542996	9.892267	11.140081	11.097215	12.562827	9.696322	9.012401	9.690691	10.754642	9.542810	10.511711	10.552645	9.159384	10.788558	11.849886	7.228105	11.802954	11.706405	11.525317	12.550549	11.509227	11.301597	13.266591	11.751899	10.486558	11.025099	11.992298	11.837854	10.483523	11.398667	11.039457	11.087821	11.411098	10.093823	9.992805	11.810536	11.366830	11.275464	11.730489	11.392045	10.676434	9.757480	10.768791	10.543604	9.825553	12.030069	11.184648	10.580549	8.808590	6.315570	7.861073	9.773103	7.938222	11.491308	10.524000	8.443325	5.695665	9.896599	9.095560	10.782381	9.887355	8.560387

TMEM191C	9.410193	11.479650	11.903720	12.089192	11.318667	11.905478	11.414563	12.024202	11.486562	11.704316	11.915776	12.578180	12.147040	12.482634	12.207449	11.125774	11.668911	11.954677	13.261613	11.850039	11.964553	12.698021	11.894274	12.019013	11.846709	11.401790	9.949359	11.741588	10.484268	11.288046	12.049360	12.070398	12.079547	11.542414	11.601057	11.376371	11.700373	11.664472	12.431440	12.432750	11.805293	11.986356	11.431806	11.189473	11.399961	11.750265	11.423597	12.080906	11.373920	11.648287	11.408892	11.738510	11.708507	11.941654	11.621293	11.279887	12.086204	12.561718	12.515324	12.215485	12.762553	11.063360	11.338052	12.758240	12.247353	12.244533	12.517113	11.943044	12.175459	12.618274	11.628067	11.955591	11.091172	11.691086	12.191984	11.539976	11.946386	11.211990	11.717191	12.543722	11.937329	11.621139	12.062305	12.351650	11.757998	11.982747	11.609910	11.753661	12.853171	12.747457	12.257254	12.796802	11.709538	11.502333	11.480931	11.613910	11.492440	11.436541	11.366540	11.569606	11.617879	11.405727	11.340687	11.734522	12.676682	11.444544	12.027507	12.740227	12.652254	11.156708	13.461357	12.930265	12.180008	11.203302	11.367114	10.825459	9.889997	11.500503	10.910897	10.334799	11.481643	9.927841	11.198103	10.597545	10.651347	11.653773	10.047255	11.667871	10.713301	11.935187	12.445794	11.476689	9.910666	11.964779	9.827819	10.796867	12.451545	12.329087	10.387701	12.419130	12.420436	12.118493	11.186992	12.703315	9.719259	11.995045	12.095797	9.951526	11.353164	10.393847	11.463470	12.030495	11.268796	11.293950	11.738217	10.961776	11.752925	12.323571	12.129935	10.759975	11.953033	10.377270	11.088572	11.720774	11.362688	12.493699	10.867134	12.274566	11.981364	11.402792	11.963054	11.367280	11.862307	12.659466	10.794845	10.932636	13.618777	13.023099	12.692172	11.865618	11.234364	12.578521	12.272798	12.138038	14.526691	12.275467	11.579668	11.776075	12.254686	12.031376	11.556552	12.447724	10.985679	11.635875	10.889665	11.180978	11.727379	11.563886	11.388081	12.364491	12.150924	11.670899	11.208214	10.794845	11.808281	11.662821	13.372722	13.066209	11.412637	10.496309	12.897761	10.913094	10.909438	10.902781	10.120507	11.024218	11.071993	10.349176	10.600013	10.531134	11.227828	10.941388	11.294265	10.795839	11.128662	11.157450	12.342322	12.415936	11.473235	13.268822	10.956934	9.079331	10.871585	10.622336	11.913837	12.282874	11.628231	10.865747

TMEM191B	9.410193	11.479650	11.903720	12.089192	11.318667	11.905478	11.414563	12.024202	11.486562	11.704316	11.915776	12.578180	12.147040	12.482634	12.207449	11.125774	11.668911	11.954677	13.261613	11.850039	11.964553	12.698021	11.894274	12.019013	11.846709	11.401790	9.949359	11.741588	10.484268	11.288046	12.049360	12.070398	12.079547	11.542414	11.601057	11.376371	11.700373	11.664472	12.431440	12.432750	11.805293	11.986356	11.431806	11.189473	11.399961	11.750265	11.423597	12.080906	11.373920	11.648287	11.408892	11.738510	11.708507	11.941654	11.621293	11.279887	12.086204	12.561718	12.515324	12.215485	12.762553	11.063360	11.338052	12.758240	12.247353	12.244533	12.517113	11.943044	12.175459	12.618274	11.628067	11.955591	11.091172	11.691086	12.191984	11.539976	11.946386	11.211990	11.717191	12.543722	11.937329	11.621139	12.062305	12.351650	11.757998	11.982747	11.609910	11.753661	12.853171	12.747457	12.257254	12.796802	11.709538	11.502333	11.480931	11.613910	11.492440	11.436541	11.366540	11.569606	11.617879	11.405727	11.340687	11.734522	12.676682	11.444544	12.027507	12.740227	12.652254	11.156708	13.461357	12.930265	12.180008	11.203302	11.367114	10.825459	9.889997	11.500503	10.910897	10.334799	11.481643	9.927841	11.198103	10.597545	10.651347	11.653773	10.047255	11.667871	10.713301	11.935187	12.445794	11.476689	9.910666	11.964779	9.827819	10.796867	12.451545	12.329087	10.387701	12.419130	12.420436	12.118493	11.186992	12.703315	9.719259	11.995045	12.095797	9.951526	11.353164	10.393847	11.463470	12.030495	11.268796	11.293950	11.738217	10.961776	11.752925	12.323571	12.129935	10.759975	11.953033	10.377270	11.088572	11.720774	11.362688	12.493699	10.867134	12.274566	11.981364	11.402792	11.963054	11.367280	11.862307	12.659466	10.794845	10.932636	13.618777	13.023099	12.692172	11.865618	11.234364	12.578521	12.272798	12.138038	14.526691	12.275467	11.579668	11.776075	12.254686	12.031376	11.556552	12.447724	10.985679	11.635875	10.889665	11.180978	11.727379	11.563886	11.388081	12.364491	12.150924	11.670899	11.208214	10.794845	11.808281	11.662821	13.372722	13.066209	11.412637	10.496309	12.897761	10.913094	10.909438	10.902781	10.120507	11.024218	11.071993	10.349176	10.600013	10.531134	11.227828	10.941388	11.294265	10.795839	11.128662	11.157450	12.342322	12.415936	11.473235	13.268822	10.956934	9.079331	10.871585	10.622336	11.913837	12.282874	11.628231	10.865747

TMEM18	10.175625	9.722785	9.766182	9.416569	9.432672	9.539536	9.587642	8.977416	8.655960	9.296431	9.046071	9.332620	9.416410	9.489036	9.589818	9.347399	9.002124	9.395428	8.476043	9.682776	8.544600	10.064074	9.277850	9.108455	9.655511	9.810864	8.872478	9.216398	8.801575	9.480244	8.909057	9.594939	10.076365	9.820866	10.126788	10.102022	10.184062	9.498564	9.836416	9.625356	9.408658	9.625053	10.326880	10.337046	10.473452	10.084818	8.577277	10.430011	9.966054	10.058014	10.147784	10.415855	10.599565	10.247562	9.948751	9.081198	10.423303	10.350375	9.785048	10.517131	10.143238	9.628948	9.780402	9.282612	9.682193	9.976964	9.809526	9.797194	9.377466	9.523040	10.026868	9.959089	9.513967	10.102182	10.034063	9.988575	10.383969	9.997359	10.047104	9.988894	10.417045	10.222567	10.095036	10.505273	10.287574	10.011086	10.351729	10.546111	9.731085	9.736095	9.805636	9.414098	9.957369	9.912200	10.454196	10.313708	9.766182	9.263696	9.733489	9.568410	9.214227	9.653700	9.082279	9.606097	9.506119	8.633832	8.476201	9.137166	9.427430	9.113447	10.200086	9.135166	9.032961	8.650567	9.433716	9.691309	10.099818	9.281986	8.139719	9.891491	10.292953	9.937098	9.263384	8.972481	9.892413	9.107805	10.608143	9.310215	9.548217	8.813954	9.831309	10.363870	9.180169	9.596795	9.625356	9.861128	9.230592	9.674605	10.421638	8.708574	8.953955	9.580110	8.348402	9.709599	10.885843	9.424349	9.236897	9.218228	9.450295	9.736704	9.336472	10.280486	9.277238	10.531004	9.993695	9.788347	9.752993	9.842288	9.803798	9.946870	8.934423	10.542840	10.074539	9.926775	9.241228	7.951067	11.731098	10.328691	10.497536	9.580724	10.311130	9.943047	10.011528	10.715970	9.403879	11.726020	8.896560	10.027337	10.293094	9.507186	9.482261	9.453875	9.593851	9.215763	10.247700	9.149853	9.163229	9.195936	9.131387	10.061049	9.947336	8.147603	9.996472	10.439875	9.634626	10.465656	10.343107	10.154515	10.852873	10.131679	9.904700	9.836571	9.892707	10.449886	9.842603	9.811338	10.922388	9.434019	9.389298	10.283384	10.214570	10.046174	10.156509	9.377622	9.826018	9.791905	8.756688	9.603010	9.800887	9.599094	10.669542	9.034473	9.722785	8.658361	8.576401	8.253119	9.876416	9.373824	8.476914	9.544271	9.182515	11.436814	8.799005	10.957536	8.463529	9.966653	10.179032	8.411859

MAEL	8.517165	4.436502	4.444333	6.235310	3.314483	4.306150	5.475033	3.691579	3.436171	5.347947	3.631286	3.731504	4.172695	4.893555	3.238005	3.654812	5.452837	3.167932	4.582030	3.695280	4.411549	2.903033	7.907702	3.641701	2.793161	3.023596	5.843515	3.479830	4.481977	3.249984	3.582844	5.969157	4.922338	6.184495	5.457891	5.752102	6.236149	4.760385	2.998611	3.715067	4.988095	4.627372	4.075568	4.294797	7.840310	5.044934	6.567898	6.028219	4.696394	4.893127	4.210774	6.806770	6.136534	4.577568	6.425117	4.120296	5.973440	4.137598	5.729728	5.852553	6.804922	4.075783	8.296861	4.212512	12.525614	5.151943	4.834783	5.277562	3.290129	2.920076	4.984987	4.924079	4.685015	6.571479	4.851814	4.739775	5.110560	4.116148	3.811201	6.748634	5.485941	5.305197	7.876711	3.850978	5.186403	5.356854	3.610768	5.757587	5.067361	4.049977	5.087179	4.118816	4.618879	4.407592	5.342170	5.828040	5.316075	5.977963	4.102375	5.438205	3.141282	4.923433	4.594714	4.393599	7.287828	4.758311	3.154285	5.324966	4.070266	5.418743	3.898221	3.254178	3.108164	3.219976	3.765884	3.044010	3.014468	4.818563	3.419492	13.895620	3.783575	4.059534	12.781216	8.877700	4.842115	6.801411	5.775099	3.486614	3.368911	3.491861	3.331129	3.778955	5.597810	3.045889	4.693081	4.409608	5.574273	3.359512	3.933686	3.739374	5.226669	5.952342	2.826095	10.057591	5.150162	3.074874	2.922785	3.313638	5.160478	3.588616	5.774363	3.224618	3.522436	8.750837	6.244622	6.216463	4.125890	3.018195	2.830429	3.130865	9.873790	5.448989	3.607261	5.486905	4.266147	3.218550	6.008773	4.332259	5.007256	4.120296	6.302900	4.853955	4.148977	5.904380	4.646621	6.703996	5.317845	6.550996	5.031543	12.933125	6.023770	3.505344	3.605770	6.694089	3.316628	9.097199	4.281894	4.186196	2.867977	4.071884	5.557935	3.461815	5.246614	5.282775	3.700373	6.391212	3.508161	4.897059	6.950590	6.251304	4.626343	14.046144	3.530135	3.618767	4.012459	4.185988	5.217960	5.177954	5.044120	5.405352	2.838906	5.496273	3.243811	4.816461	3.485520	4.732732	6.275386	3.585898	9.193891	3.113096	6.888719	4.454595	3.441000	14.117673	3.266021	5.986093	4.809096	4.020669	3.171175	5.102975	3.189408	3.579949	3.148383	3.962248	2.940730	3.459278	8.522011	3.203604

TAF4B	9.789131	10.158322	10.384236	9.355444	9.871517	9.534750	10.160131	8.785633	10.221095	9.390059	9.639347	9.590429	10.145661	9.875126	10.143394	9.573538	9.386518	10.190858	8.282853	10.052192	9.212818	8.760599	7.639235	9.864082	9.313787	9.258639	9.271368	8.184054	9.121085	9.951825	9.420243	9.719259	10.465797	10.173198	9.887513	9.903802	10.162292	10.023382	9.961205	9.922286	9.608835	9.400283	10.198884	10.046779	9.934635	10.530696	8.539908	10.264537	10.318027	10.189200	9.837507	10.487949	10.318027	10.252055	10.424393	9.710793	9.960286	9.935441	10.181458	10.111224	9.986541	9.248899	8.560859	8.729734	9.938052	10.893147	9.421461	9.560327	9.894119	8.919658	9.648405	9.916366	9.287550	9.949211	9.954924	10.320444	9.903960	9.987767	9.697083	9.515750	10.548745	10.656092	10.377424	10.846956	10.247700	10.215321	10.094875	10.580396	9.336166	9.715628	9.550559	9.026744	10.368662	10.634289	9.970923	10.193392	10.373383	9.620412	10.094546	9.676141	9.538904	10.038997	9.577910	10.019448	9.548379	9.616360	9.474009	8.699378	9.001182	9.223579	8.807202	9.735158	9.631378	8.844175	10.405770	7.384386	9.289571	10.159975	9.121228	10.823396	10.061353	10.458959	9.591195	7.392569	10.253644	8.426765	10.208162	10.588804	10.557223	9.365510	9.792563	9.765260	8.636158	10.854645	9.419282	9.896882	10.030393	8.671577	11.013672	8.730027	9.477671	8.289335	9.501025	11.332473	10.552501	9.133674	10.291124	7.321508	9.521604	10.235279	9.988415	10.153732	10.554766	10.108076	10.610796	10.164737	10.149811	10.486856	10.520492	10.169200	9.951825	11.661490	10.298660	10.694662	11.911179	10.022749	9.876085	11.033609	10.598011	10.183903	10.971013	10.621011	11.488607	9.769520	10.013986	10.276541	9.966351	10.969060	11.205099	8.908916	9.470474	8.625298	10.384855	10.031960	11.013094	10.289433	9.867411	11.081305	8.458269	10.471030	10.349760	8.780092	10.339011	10.506163	10.651628	10.711692	10.912348	9.540026	9.599562	10.480506	11.090745	10.076034	9.956624	10.838984	11.035559	10.136988	11.511108	8.887749	9.365816	9.063831	10.234797	9.649630	9.734090	10.199193	10.298660	9.763723	10.310511	10.069634	10.728828	10.495118	11.031779	8.343986	10.190552	9.822386	10.106586	10.665768	10.038097	9.394972	8.999495	9.324612	9.357266	12.130069	8.528442	10.752793	10.173198	9.950904	9.547923	10.101581

CASC4	13.519798	11.652428	11.506569	11.890346	11.674723	12.298858	12.219989	11.457713	12.213488	12.427016	12.201865	12.434341	11.882981	11.170321	11.455375	12.257409	12.010940	11.456523	9.562541	12.190746	10.718063	9.494236	10.528515	11.432403	10.406511	11.197814	13.083113	11.807078	13.077225	12.517769	11.211433	12.330024	11.367280	12.901113	12.360062	12.183935	12.027347	10.834820	10.610513	10.561618	12.538788	10.889665	11.476559	11.281354	11.771074	11.290862	13.006339	11.302476	11.769756	11.312923	12.191183	11.734522	11.557873	11.181727	10.899381	12.098917	11.136698	10.136250	10.385937	10.268677	10.246175	12.850513	11.135945	9.809677	9.961826	10.328234	9.995433	10.832721	10.351116	10.285304	12.233576	12.038942	12.551723	12.069172	11.909110	12.344564	12.079262	12.157725	12.588301	9.469668	11.300255	11.411534	11.184036	11.082761	11.290546	12.104074	10.997454	11.017053	10.584051	10.138215	10.559481	10.593778	11.213047	11.647837	12.219074	11.815395	11.709538	12.121750	12.153299	11.445862	12.044415	11.218631	11.516497	11.000920	9.320639	12.556507	10.966241	9.730269	10.086825	12.537925	9.400733	11.368582	10.601825	12.471647	11.550891	11.268516	11.316160	12.391754	12.873210	12.198143	11.481500	12.043801	11.927081	12.243145	12.165896	12.989073	12.874491	13.268628	12.107738	12.797679	12.396485	12.410173	11.306661	10.664303	13.021352	12.871579	11.840529	9.921992	12.367693	10.843161	10.399429	10.584342	10.271785	10.693780	11.818854	12.263096	11.519525	13.059142	13.306600	11.952744	12.159874	11.592521	11.488767	11.858897	12.655686	12.423741	11.122494	10.860126	11.218772	12.589286	10.951033	11.075896	11.355638	11.157753	11.399407	12.058559	11.199022	12.470221	12.043646	11.924435	12.174666	11.374614	11.708649	11.726175	13.274601	11.312057	10.267192	10.094546	10.505273	10.700452	12.649008	10.377270	10.432310	10.768642	10.769710	10.201859	10.994601	10.370017	10.673148	12.594432	12.665111	12.368451	12.844839	12.999705	12.373381	13.096527	12.870661	12.486817	10.268677	11.444404	10.959032	10.597246	11.853688	11.632692	11.800284	11.271942	10.844098	11.246804	10.867134	10.405472	10.730021	11.821698	12.083836	12.754298	12.234198	12.582141	13.261408	11.824728	12.590461	12.454859	11.873849	12.001749	11.777916	9.978579	13.281605	11.246511	11.093554	10.983770	12.551069	9.967099	12.203594	10.353264	11.804392	13.387105	11.737314	11.213347	11.924594	11.702238

TOR1AIP2	13.317056	13.043339	13.220179	13.273550	13.184093	12.919720	13.299046	13.410767	13.775202	13.501961	13.157459	13.302870	13.439251	13.595345	13.127299	13.723956	13.364551	13.554388	14.347809	13.417727	14.096118	14.355522	13.939054	13.635217	13.906928	13.260449	12.880439	13.874973	12.781558	12.962912	13.341166	13.016071	13.379067	12.779334	12.740415	13.180561	13.407563	13.660327	13.612808	14.099512	13.213586	13.615689	13.371239	13.351178	13.137178	13.113930	13.262398	13.162064	13.747078	13.175591	12.870487	13.338927	13.341580	13.323155	13.502805	13.084373	13.062840	13.574884	13.457219	13.344888	13.520707	13.363265	13.485069	13.903911	13.485749	13.701651	13.416299	13.783328	13.121772	13.802120	13.063034	13.548900	13.442036	13.354792	13.447138	13.153423	13.310755	13.100126	12.984802	13.332466	13.184851	12.976584	12.889837	13.481027	13.558886	13.119251	13.607340	13.780832	13.687645	13.562689	13.488366	14.205886	13.222947	13.289052	12.815963	13.172389	13.374839	13.399301	13.206710	13.558417	13.733881	13.317859	13.341776	13.349750	14.249198	13.321286	14.221539	14.676463	14.296633	13.436430	14.486353	13.605949	13.712695	13.414830	13.557717	12.897245	13.387555	13.153423	13.690456	12.679062	13.359283	13.239009	12.845445	13.850953	13.379894	13.005316	13.009941	13.056178	13.363482	13.527257	12.536416	13.653913	13.728607	13.734120	13.271410	12.816481	13.343862	14.340345	12.765477	14.029660	14.282413	14.299871	13.344692	12.995435	13.466605	13.019365	13.109263	12.797323	13.149152	14.081267	13.304945	13.398875	13.522292	13.459608	12.736450	13.067539	13.170169	13.823470	13.298414	14.019562	13.586976	13.072064	13.148784	12.737304	12.852480	13.513091	13.451174	13.046467	13.000629	13.297375	12.701760	12.454709	12.949621	12.615659	13.179765	13.409664	13.064161	13.024924	12.920057	13.558652	12.672938	13.969621	13.467091	13.531252	13.409465	13.187239	12.753548	13.143383	13.223528	12.629517	12.934369	13.033027	13.057096	12.933307	13.017899	12.913586	12.956616	12.889319	12.763614	13.047238	13.086957	13.128058	13.094096	13.322740	12.798678	13.246497	12.887380	13.719555	13.105804	13.437980	13.055240	13.250652	13.042957	13.185434	13.406084	13.452889	13.872874	13.623869	12.511287	12.754938	12.877478	12.800233	12.488145	14.086854	12.978769	13.571242	13.829200	13.883273	13.445036	13.475946	13.514224	13.228263	13.275630	13.587671	13.427333	13.204562	12.509485	13.306191

CNPPD1	10.227583	10.984344	11.038581	11.135510	11.276449	11.781504	11.372021	11.881320	12.172470	11.989727	11.572813	11.443078	11.147376	11.270728	10.691500	11.655296	11.546807	11.289617	11.433163	11.346391	11.048552	11.432097	10.742594	11.333338	11.179761	11.381737	10.964294	11.947291	11.307706	11.183319	11.708199	10.709963	11.173303	10.752225	10.702740	10.692859	10.972053	10.839132	11.600742	11.305742	11.608315	10.851641	10.722212	10.675689	10.668858	10.613997	11.558653	10.341990	11.253814	10.720354	10.640900	10.944749	10.781447	10.878399	10.878560	11.657473	10.714609	11.295839	10.631587	11.112884	10.791738	11.548135	10.000412	10.731932	10.806048	11.000620	11.108664	11.031779	10.829597	10.729876	11.136391	11.163914	11.665819	11.427239	11.275035	11.020059	11.081305	11.001368	11.107474	10.439290	10.991933	10.717743	10.329926	10.955440	10.903646	10.894605	10.969060	11.452234	10.788704	10.923134	10.708068	10.628531	10.818648	11.006117	10.602963	11.251804	10.777392	11.142360	11.168835	11.572374	11.769458	11.063219	11.377558	11.192513	10.876491	11.848352	11.489652	11.042718	10.863944	11.869930	11.070628	11.749393	11.202068	11.768560	12.144699	12.726510	10.170916	11.279887	12.015376	10.437655	10.826665	10.581882	10.715070	11.538688	11.482538	11.427104	10.505428	11.456392	10.997004	11.356537	10.690069	11.238920	10.440817	10.297862	11.282850	11.019905	10.181943	9.861767	10.421474	10.733110	10.198139	10.139689	11.029707	10.663570	10.270279	11.144404	11.394876	11.407214	10.949194	11.422716	10.592463	10.823846	11.319702	11.002514	10.744255	10.756929	10.876634	11.003940	10.589798	11.638582	10.567246	10.502281	11.155073	10.806779	10.583768	11.944381	10.185582	10.890438	10.640152	11.009768	10.552132	10.297097	10.898327	10.541250	10.950587	10.187118	10.345517	10.567716	10.175307	10.564137	10.827984	10.009096	10.271167	10.168427	10.284261	10.758086	10.812104	10.511086	10.416591	10.817887	10.947866	11.778217	11.148846	10.305204	10.865747	10.546423	10.670580	11.038735	9.891032	10.702123	10.734915	10.319814	10.141980	10.352969	10.914603	10.809187	10.645900	9.961516	10.024497	9.910511	10.120815	10.349010	10.760252	11.153328	10.767880	11.518416	12.014471	11.143538	10.896111	10.621452	10.935020	10.713006	10.522062	9.854621	11.354771	10.892076	10.428662	10.777248	11.698867	10.090224	11.457426	10.002270	11.702656	10.974650	11.186572	10.673572	11.137872	10.687354

CYP1A2	8.919658	8.842770	8.608029	9.113587	9.663115	10.599413	9.215448	10.424711	11.314983	10.156509	10.253789	9.752993	9.480888	9.211755	8.663180	10.329335	9.705954	8.667580	8.883590	9.455870	7.976325	9.227738	7.679233	9.754543	9.213597	9.314118	10.434911	9.083038	11.111300	10.228365	10.545363	7.801000	8.389600	9.386045	8.808590	8.546419	8.758828	8.886682	8.906727	8.978925	10.420249	8.711023	8.548297	8.984879	8.777381	8.183907	11.006400	8.242934	8.920759	8.408942	8.251297	8.387874	8.395299	8.702764	8.523862	10.909151	8.415946	8.569561	8.779483	8.357075	8.130869	10.318027	8.422421	8.778865	8.038115	8.091722	8.431370	9.005828	8.283150	8.586817	9.058310	9.006707	11.022434	8.903553	8.862310	8.935453	9.065219	8.670340	8.418559	8.207850	8.156419	8.192758	7.556289	7.524066	8.160080	9.112102	8.504929	7.652974	7.133895	7.347870	7.656275	6.529653	8.064574	8.438131	8.617394	9.067544	8.593782	10.388978	9.633215	10.536625	10.488104	9.401532	10.045253	9.152161	8.327809	10.881936	10.281385	8.726505	8.778712	11.043151	7.489903	9.665161	9.744273	11.513924	10.225283	12.516134	8.615278	9.713877	11.542723	9.131538	8.845415	8.103403	8.940204	10.350675	9.590587	10.137918	10.828572	10.651628	10.167812	9.549146	9.856956	9.345751	9.172960	8.395299	10.742902	9.251788	9.029972	8.077583	8.895157	8.011860	8.863706	7.054266	9.949956	8.255885	8.199678	10.275619	9.352150	12.368145	9.247052	10.462958	9.084861	9.013634	9.397811	8.595787	8.711913	8.475733	8.580126	8.424820	8.579234	10.849578	7.565641	8.068615	8.687318	8.325878	7.779666	11.100603	8.036191	8.545084	8.128410	8.156082	8.187444	8.301664	8.664994	8.484598	10.322450	8.470875	8.571281	8.559612	7.800830	8.107575	9.662962	8.439798	8.281598	8.235110	8.258639	8.420558	8.469947	8.017690	8.065034	9.105974	8.899303	10.411625	8.957303	8.772914	8.867263	8.913281	8.670471	8.763615	8.221156	8.048638	7.993011	7.618765	7.965252	7.756656	9.105810	7.737418	7.686094	7.114246	7.301775	7.409741	6.682255	8.318651	8.286241	8.765277	8.796555	8.900970	10.286654	9.791422	9.420683	9.672692	9.971065	10.546111	9.281688	7.400216	10.838131	10.272441	8.319278	7.711380	11.284747	7.272024	9.889071	10.075275	11.094507	9.717339	9.831681	8.209784	9.380364	10.257553

NPB	12.432257	10.937294	11.046498	11.418410	11.845084	11.863352	10.899103	12.007938	12.356420	11.422861	11.944525	11.743340	11.087082	11.496506	11.283268	12.064690	12.218793	11.671644	12.455635	11.445581	11.843253	11.930143	11.855196	12.039995	12.361945	11.744798	13.254596	12.354041	11.998237	11.437385	12.106206	10.236196	10.937294	11.123080	10.761899	10.658429	10.831803	10.847400	11.335546	11.368582	11.671500	11.422716	11.420720	11.113773	11.334799	11.408754	12.299854	10.889218	10.750836	10.717161	10.562917	10.747561	10.340199	10.487949	10.414046	11.782817	10.873089	11.145002	11.010787	10.991471	11.045432	11.563461	11.008580	11.831962	11.161727	10.801205	11.193393	11.001079	11.008142	11.457426	11.136838	11.312487	11.956809	11.102534	11.297653	10.948018	11.067913	10.723137	10.825459	10.501072	10.723137	10.774339	10.778852	10.462665	10.561773	10.696919	10.548435	10.407694	11.387310	10.885551	11.221315	11.336278	10.682588	10.702123	11.167684	11.161862	10.702258	11.883430	11.176414	11.808140	11.583331	10.837802	11.646786	11.321723	11.458612	11.797500	11.593135	11.528614	12.037009	12.318072	11.756331	11.953478	12.031230	12.614854	11.862307	12.950183	11.833001	11.593433	12.622511	11.342640	11.448088	12.035185	11.871132	12.747616	11.894423	13.005316	13.670083	12.402762	11.614652	12.447863	12.207923	11.893515	11.691697	11.951702	12.229570	12.045301	11.511703	15.235562	14.166778	12.591506	12.516782	11.893668	12.519216	12.577866	11.753359	12.580507	12.731681	12.679231	11.271780	12.379588	11.257836	10.663131	11.240485	11.211843	11.474238	11.260928	11.472640	12.475535	11.706091	12.689122	11.620543	13.205135	12.209741	11.730335	11.623005	12.551069	13.152225	10.646958	11.633590	11.412947	10.889665	10.573931	11.141047	10.417045	11.185106	14.403650	11.722901	11.935946	10.709053	11.625463	11.565520	13.541913	12.149599	10.661777	11.366119	12.265271	11.184951	11.530788	13.414830	11.397622	11.071819	12.433552	11.225183	11.134465	10.899971	11.409053	10.988377	11.497575	10.891048	11.427665	11.342940	11.761011	10.969060	10.184676	11.781211	10.676118	10.723716	13.052443	10.849297	12.566772	12.605390	11.364013	11.221025	11.270879	12.593788	10.871143	11.996843	11.167966	10.982768	11.336433	12.361312	11.863798	11.564033	12.450418	12.300335	12.256329	11.073555	11.865925	12.665471	12.401128	11.447943	12.364339	13.031581	11.373780	12.015060	11.437819	12.978391	11.649898

PCYT2	12.432257	10.937294	11.046498	11.418410	11.845084	11.863352	10.899103	12.007938	12.356420	11.422861	11.944525	11.743340	11.087082	11.496506	11.283268	12.064690	12.218793	11.671644	12.455635	11.445581	11.843253	11.930143	11.855196	12.039995	12.361945	11.744798	13.254596	12.354041	11.998237	11.437385	12.106206	10.236196	10.937294	11.123080	10.761899	10.658429	10.831803	10.847400	11.335546	11.368582	11.671500	11.422716	11.420720	11.113773	11.334799	11.408754	12.299854	10.889218	10.750836	10.717161	10.562917	10.747561	10.340199	10.487949	10.414046	11.782817	10.873089	11.145002	11.010787	10.991471	11.045432	11.563461	11.008580	11.831962	11.161727	10.801205	11.193393	11.001079	11.008142	11.457426	11.136838	11.312487	11.956809	11.102534	11.297653	10.948018	11.067913	10.723137	10.825459	10.501072	10.723137	10.774339	10.778852	10.462665	10.561773	10.696919	10.548435	10.407694	11.387310	10.885551	11.221315	11.336278	10.682588	10.702123	11.167684	11.161862	10.702258	11.883430	11.176414	11.808140	11.583331	10.837802	11.646786	11.321723	11.458612	11.797500	11.593135	11.528614	12.037009	12.318072	11.756331	11.953478	12.031230	12.614854	11.862307	12.950183	11.833001	11.593433	12.622511	11.342640	11.448088	12.035185	11.871132	12.747616	11.894423	13.005316	13.670083	12.402762	11.614652	12.447863	12.207923	11.893515	11.691697	11.951702	12.229570	12.045301	11.511703	15.235562	14.166778	12.591506	12.516782	11.893668	12.519216	12.577866	11.753359	12.580507	12.731681	12.679231	11.271780	12.379588	11.257836	10.663131	11.240485	11.211843	11.474238	11.260928	11.472640	12.475535	11.706091	12.689122	11.620543	13.205135	12.209741	11.730335	11.623005	12.551069	13.152225	10.646958	11.633590	11.412947	10.889665	10.573931	11.141047	10.417045	11.185106	14.403650	11.722901	11.935946	10.709053	11.625463	11.565520	13.541913	12.149599	10.661777	11.366119	12.265271	11.184951	11.530788	13.414830	11.397622	11.071819	12.433552	11.225183	11.134465	10.899971	11.409053	10.988377	11.497575	10.891048	11.427665	11.342940	11.761011	10.969060	10.184676	11.781211	10.676118	10.723716	13.052443	10.849297	12.566772	12.605390	11.364013	11.221025	11.270879	12.593788	10.871143	11.996843	11.167966	10.982768	11.336433	12.361312	11.863798	11.564033	12.450418	12.300335	12.256329	11.073555	11.865925	12.665471	12.401128	11.447943	12.364339	13.031581	11.373780	12.015060	11.437819	12.978391	11.649898

CA3	6.749427	5.294069	5.101965	5.844745	4.923626	4.713705	4.796678	5.567109	5.412818	4.638196	4.288106	4.413541	3.872015	4.174641	3.940672	5.004156	4.968821	5.335551	6.032910	5.066749	5.180129	5.010542	4.775532	4.903414	4.432882	5.031741	4.011026	5.394196	4.549553	4.455844	5.963956	4.953347	5.481833	3.955463	6.265874	5.602850	4.648318	5.032949	5.628214	5.204262	4.598956	5.847907	4.366995	6.200659	4.258314	5.065280	4.091186	5.737539	4.824782	5.536065	6.234806	5.389488	5.287566	6.379636	5.041986	4.647688	5.142569	4.013825	5.588697	4.883876	5.769576	4.362400	5.473107	6.127660	5.188014	5.906325	4.628485	5.164280	5.029113	4.438286	5.459635	4.721070	4.368291	5.332046	5.480262	5.536065	5.465983	6.009291	5.796922	5.586258	5.393349	5.474055	5.703743	6.223644	5.568462	5.967890	5.757214	6.143417	5.920125	7.031101	5.976903	5.634192	4.730610	5.318242	5.478477	5.507386	5.162860	4.691364	4.212080	3.756112	2.922883	4.322056	4.725358	4.481723	5.425814	3.812109	5.438382	4.969692	4.911553	4.312513	5.350672	5.811491	5.082452	4.094115	4.399395	3.214073	5.362552	4.905873	4.120971	4.475158	5.369136	4.259179	4.438717	3.653027	4.469699	3.258539	4.643607	6.060096	3.203454	4.125890	3.269432	3.353521	3.508878	3.034680	3.784156	4.817923	4.304851	5.720507	5.415640	4.642591	3.973522	6.908123	4.443479	4.268916	4.655316	5.375342	4.022591	3.330982	5.321484	5.204669	3.366868	4.125660	4.122295	5.846583	5.017503	6.472574	4.293735	6.064343	4.629125	3.568681	5.435917	6.596178	6.352110	3.899548	4.346322	4.704057	4.671850	4.506555	5.967336	6.061355	4.938091	6.207268	6.395163	5.636210	4.736547	3.500685	5.289796	5.104607	4.703430	6.704456	4.611221	6.882311	6.325088	6.114764	5.765566	5.718684	5.533321	5.210833	5.530310	4.554340	3.842101	3.524784	5.709192	4.265308	6.105329	6.529826	6.341388	6.254020	4.517005	6.808084	5.958405	7.639389	4.283596	5.851489	4.274873	6.948756	6.182710	6.095497	7.107099	6.141195	6.247965	4.260648	5.434147	5.202650	3.929129	3.779151	2.964653	3.633323	3.202692	3.095195	3.035084	3.280580	3.514792	5.839528	3.418265	6.284973	5.353675	5.716576	4.698722	5.052996	5.092471	4.408936	3.936016	3.539970	4.848293	4.945653	6.442684	4.514021

CNGB3	7.505232	6.640640	6.568873	6.928747	6.396141	6.828774	5.813148	7.755009	7.482906	6.412347	6.796847	6.712113	4.845326	5.781067	5.501391	6.207603	7.550671	6.425977	7.271564	6.481071	7.547276	5.907399	8.104810	6.991935	6.844875	6.394997	6.927150	6.718962	5.352272	6.738430	7.473225	5.770307	6.060246	7.375801	6.484247	6.295600	6.546478	6.685661	7.094332	6.440230	5.334186	6.862666	5.938459	6.535017	6.352424	6.855733	6.422080	7.022334	6.662158	6.596333	6.478315	7.337058	6.186223	6.186409	5.137833	6.078831	5.826180	6.836278	7.256786	7.221610	7.182354	5.729350	6.620518	7.120108	9.102439	7.108059	6.643535	6.962124	8.450307	7.244686	6.601671	6.500881	5.982412	6.417818	6.692595	6.625846	7.039382	8.258639	7.198218	6.211683	6.963727	6.514720	8.897010	6.478640	7.076052	6.306442	7.723476	6.925659	7.796171	7.424453	7.440987	7.690710	7.094982	6.987163	6.118709	6.715696	5.895831	5.391837	4.955624	5.925083	6.233086	5.722522	5.207022	6.026766	6.095497	6.260652	7.732264	6.374667	7.716296	6.170824	7.799910	7.966965	7.347870	6.390022	7.221302	8.444544	7.645097	7.157148	6.590824	10.305812	10.108529	8.628231	10.654274	9.933222	7.962205	7.747653	7.150091	9.238440	8.871045	9.987460	10.020621	8.271947	5.541274	6.080902	8.566590	8.757916	9.617155	7.311187	8.165502	8.419942	7.185698	9.838114	10.161530	9.663577	7.845788	9.138253	9.068164	8.484598	8.327809	11.214808	7.966495	8.388365	9.374798	5.815457	10.203707	8.895467	9.214807	9.290819	7.540217	7.741340	10.220637	9.788490	8.136495	9.305431	10.058151	11.099157	7.013182	9.854621	8.951656	8.883900	8.272248	9.088082	6.971038	7.939959	9.257737	5.869386	9.471707	9.672249	9.028112	10.532527	7.770796	8.958885	8.918918	8.901410	9.856355	8.915240	9.716241	7.458863	7.685796	8.469344	9.619941	7.982237	9.133976	8.620493	8.700125	8.520528	10.126629	9.560491	6.057290	9.392820	6.819116	9.350045	9.041774	9.865768	9.260302	10.631125	10.038241	10.902906	9.594780	7.601535	8.800062	9.948590	7.740395	10.657698	9.331216	7.830502	8.275513	8.693709	7.824617	9.560327	5.961292	8.107889	7.316049	7.573258	8.865812	9.149261	8.115176	9.577290	9.379298	8.448278	9.403086	11.440039	7.185534	10.827682	10.603714	9.659354	11.147664	10.839888

TMEM178A	8.147603	8.830404	8.334148	8.785633	8.706305	9.433441	8.866731	9.404833	8.115470	9.236267	8.974670	8.856845	7.794310	8.369704	6.968869	9.773259	8.664235	7.526669	7.540820	8.843862	7.462771	8.775967	9.325221	7.934018	8.065034	8.631600	7.911881	8.831175	8.890530	8.818068	8.472666	8.471849	7.976016	8.173625	7.817936	8.368477	7.978789	8.238226	7.968783	8.019529	9.863624	7.655466	7.128302	8.040436	7.994104	8.212526	8.960537	8.189259	8.652972	7.770487	8.295020	8.185093	7.471425	7.738048	8.072719	9.131387	7.605001	7.511240	8.139429	7.593195	7.443164	8.953170	8.257221	7.481352	7.372828	7.762998	7.835236	7.955250	7.950164	8.163833	9.380048	9.497352	9.557862	8.987164	9.220517	8.669282	8.947521	8.462038	8.849892	7.566103	7.571434	7.880784	7.348334	7.981450	7.700231	7.964491	6.922566	7.914905	7.520987	8.217923	7.957304	7.775417	7.802510	8.836612	8.262136	8.167229	7.804165	7.750575	7.914905	8.539575	7.907089	8.235581	8.366526	7.750125	7.972915	9.219906	8.444705	7.760354	8.127063	10.328083	8.176634	7.784389	7.518041	9.995272	8.826560	6.589655	7.942332	8.582697	9.181882	8.986399	9.661702	9.281837	8.847714	8.780870	9.421896	8.939900	7.892325	8.586667	9.346648	9.275288	9.952895	9.301410	7.078474	6.911143	8.421352	9.282762	7.946027	7.985002	9.614826	7.863243	8.384047	8.888996	8.207229	7.516478	9.746469	9.828423	9.003636	9.304141	10.281231	7.308044	7.629708	8.527674	9.445907	8.215758	8.991795	7.503062	8.345535	8.488318	8.064421	11.032397	8.031937	7.474299	9.299232	7.869232	8.815133	10.389118	7.376415	8.744503	8.724169	8.806427	8.247106	8.899923	8.228709	7.492437	9.874226	8.180232	7.574019	7.266472	7.374069	7.281157	9.580724	7.694740	7.564569	7.693207	7.645570	7.649346	8.797014	8.324774	8.516685	9.228039	10.370786	10.110042	9.686684	9.047133	9.125313	9.607774	9.981835	9.824324	6.954068	7.415948	8.114241	7.287083	6.843859	7.743500	8.576701	7.437104	7.946637	7.070868	6.958150	7.844862	7.482906	7.634048	9.728386	8.911416	8.303462	8.407880	6.885991	7.974303	8.719785	8.103712	7.716737	8.008532	7.121043	7.005493	9.208546	8.394089	7.879149	7.842958	10.614085	7.786365	6.674131	7.320294	10.098577	9.245828	9.249650	7.225755	8.633832	10.220952

AL353588.1	4.795822	3.862165	4.321207	4.090941	4.277970	4.739775	3.160705	5.989717	5.995662	3.435805	4.212512	4.458439	3.409715	3.590513	3.165946	3.708456	5.168882	3.699591	5.434567	4.130822	5.049048	3.745674	5.592356	4.295457	3.963800	4.279071	3.748119	4.292225	2.935217	4.072108	5.004761	3.791856	4.872994	3.738525	5.196495	3.965491	4.339197	5.330483	4.829983	4.480635	3.824871	5.102368	3.832048	3.667489	4.778843	5.489345	3.893535	3.920397	4.104098	3.150262	4.323129	4.042997	4.326770	3.703843	3.681611	3.111066	3.785421	3.250849	3.236796	4.566368	4.667764	3.465935	4.981524	5.122714	5.499091	4.294363	4.086354	4.391046	4.513800	4.028832	4.296330	4.684394	3.652825	3.739768	4.167325	4.055403	4.853955	4.896218	4.647475	4.599993	4.053879	4.294797	5.146342	4.131875	4.828889	3.448800	5.555427	5.194061	5.291392	5.846393	5.594579	5.689713	3.439595	4.759750	4.130031	3.429526	4.610588	2.968910	3.279590	3.611397	4.247945	3.201711	3.728592	4.088287	4.694331	3.743022	4.044969	4.304427	5.283372	4.143646	5.914039	5.586258	4.114828	3.715868	4.780536	6.305958	5.629161	3.680636	4.031703	4.428477	5.955741	6.162545	4.217517	5.497551	5.053435	4.983217	5.856901	5.882835	4.107435	3.965102	3.486039	3.411297	5.716222	5.194263	4.558305	6.486107	3.709269	5.281372	6.353769	5.329500	5.027033	7.069707	4.461781	3.918319	4.198815	5.020329	4.803256	4.421986	5.181765	6.011945	3.749398	5.421654	4.511602	6.555305	5.138445	4.493308	5.849360	5.561471	6.054977	4.363059	5.831582	5.659389	5.515242	5.235636	5.000684	4.929751	4.016509	5.415123	6.419326	5.522511	4.826757	6.430553	5.336536	6.210188	3.619528	6.432597	5.143570	5.053206	5.930750	6.135673	3.461975	6.180491	6.780059	6.664928	5.550148	5.816371	5.343606	6.313497	6.294060	4.939531	4.469049	3.133674	5.136441	4.709907	5.664974	6.038597	5.683425	5.580405	6.176101	5.398042	6.464038	6.089923	6.850930	8.234051	5.244217	6.062774	5.874232	7.815434	7.273273	6.166510	7.100031	5.047697	5.204262	5.377702	5.373200	5.433754	4.170305	4.453325	4.062858	4.978070	4.064154	3.771736	5.482940	6.864246	3.965297	4.612282	6.276841	5.961628	3.916834	5.880506	7.296536	4.331849	3.510009	4.783894	5.436481	6.031113	4.662468	5.248396

SPATS1	4.795822	3.862165	4.321207	4.090941	4.277970	4.739775	3.160705	5.989717	5.995662	3.435805	4.212512	4.458439	3.409715	3.590513	3.165946	3.708456	5.168882	3.699591	5.434567	4.130822	5.049048	3.745674	5.592356	4.295457	3.963800	4.279071	3.748119	4.292225	2.935217	4.072108	5.004761	3.791856	4.872994	3.738525	5.196495	3.965491	4.339197	5.330483	4.829983	4.480635	3.824871	5.102368	3.832048	3.667489	4.778843	5.489345	3.893535	3.920397	4.104098	3.150262	4.323129	4.042997	4.326770	3.703843	3.681611	3.111066	3.785421	3.250849	3.236796	4.566368	4.667764	3.465935	4.981524	5.122714	5.499091	4.294363	4.086354	4.391046	4.513800	4.028832	4.296330	4.684394	3.652825	3.739768	4.167325	4.055403	4.853955	4.896218	4.647475	4.599993	4.053879	4.294797	5.146342	4.131875	4.828889	3.448800	5.555427	5.194061	5.291392	5.846393	5.594579	5.689713	3.439595	4.759750	4.130031	3.429526	4.610588	2.968910	3.279590	3.611397	4.247945	3.201711	3.728592	4.088287	4.694331	3.743022	4.044969	4.304427	5.283372	4.143646	5.914039	5.586258	4.114828	3.715868	4.780536	6.305958	5.629161	3.680636	4.031703	4.428477	5.955741	6.162545	4.217517	5.497551	5.053435	4.983217	5.856901	5.882835	4.107435	3.965102	3.486039	3.411297	5.716222	5.194263	4.558305	6.486107	3.709269	5.281372	6.353769	5.329500	5.027033	7.069707	4.461781	3.918319	4.198815	5.020329	4.803256	4.421986	5.181765	6.011945	3.749398	5.421654	4.511602	6.555305	5.138445	4.493308	5.849360	5.561471	6.054977	4.363059	5.831582	5.659389	5.515242	5.235636	5.000684	4.929751	4.016509	5.415123	6.419326	5.522511	4.826757	6.430553	5.336536	6.210188	3.619528	6.432597	5.143570	5.053206	5.930750	6.135673	3.461975	6.180491	6.780059	6.664928	5.550148	5.816371	5.343606	6.313497	6.294060	4.939531	4.469049	3.133674	5.136441	4.709907	5.664974	6.038597	5.683425	5.580405	6.176101	5.398042	6.464038	6.089923	6.850930	8.234051	5.244217	6.062774	5.874232	7.815434	7.273273	6.166510	7.100031	5.047697	5.204262	5.377702	5.373200	5.433754	4.170305	4.453325	4.062858	4.978070	4.064154	3.771736	5.482940	6.864246	3.965297	4.612282	6.276841	5.961628	3.916834	5.880506	7.296536	4.331849	3.510009	4.783894	5.436481	6.031113	4.662468	5.248396

SLC19A1	11.022755	11.534802	10.735064	11.116961	10.547962	10.592251	11.371135	10.173198	10.267029	10.764037	10.828294	10.900827	11.239635	10.612741	11.319702	10.499145	11.119136	11.301442	11.524738	10.659469	11.256343	11.527480	12.472957	11.021537	11.606112	11.879384	11.449199	11.324533	11.264402	11.466748	11.018211	11.329984	11.524887	11.939987	11.586188	11.313065	11.323781	11.266616	12.232073	11.820662	11.408142	12.307924	11.885697	11.049734	11.844802	11.500352	10.974369	12.331290	10.805036	11.508491	11.631199	11.672076	11.678446	11.636790	11.605367	11.059824	11.671048	11.907279	11.671357	12.034131	12.110108	11.810826	13.882222	12.243771	12.606059	11.869342	11.761011	11.121598	11.496353	11.900872	11.301442	10.982768	11.081157	11.023352	11.306355	11.466449	10.870351	11.942873	11.108519	11.596390	11.467049	11.880861	11.357241	11.105734	11.392765	11.230791	11.603421	11.466296	12.680949	11.447062	11.536600	12.337925	11.607297	11.145435	10.984790	10.847846	11.008438	11.374542	10.469669	10.871747	10.991053	11.143689	10.795839	11.289617	11.464859	10.818785	11.804392	11.427955	11.936861	10.420096	11.367880	11.530935	10.620713	10.351422	9.651648	10.176090	11.605660	10.803437	10.658003	11.319702	12.211265	11.767960	12.296949	11.590536	11.002085	12.330956	12.676354	12.076202	11.806792	12.078825	12.185427	12.801968	12.038055	12.468601	11.267354	12.545714	12.228152	12.169111	11.982413	11.669538	13.032306	12.855334	12.151398	13.424703	12.065467	11.304710	10.901715	11.507445	12.175621	12.119110	11.788671	12.350062	11.712327	11.369147	11.797645	12.431602	11.561717	12.435149	12.574744	11.519525	12.310003	11.473822	11.512308	11.966716	11.756331	11.231436	12.161724	10.737168	12.310934	12.709644	12.028718	11.212902	12.739508	12.308401	11.849270	13.353095	12.159268	11.873387	12.169864	12.609852	12.211265	12.511128	12.735398	12.366707	12.556362	12.133115	12.392815	11.845543	12.419600	11.311032	11.855342	11.349516	12.183622	12.001304	11.740562	12.585328	12.194744	12.184541	13.221977	12.555104	11.706258	12.421658	12.762216	12.101792	12.044549	12.250724	11.124128	13.576961	12.832911	13.102010	12.539475	11.907585	13.226650	12.982400	13.411878	12.458016	12.671038	11.404316	11.174295	12.664096	11.737167	11.862307	11.980027	12.076671	11.469730	11.320686	10.820288	11.941813	12.330956	12.255361	13.356199	9.387474	10.518184	10.954993	10.590679	12.771214	12.236364	12.141913

COL18A1	11.022755	11.534802	10.735064	11.116961	10.547962	10.592251	11.371135	10.173198	10.267029	10.764037	10.828294	10.900827	11.239635	10.612741	11.319702	10.499145	11.119136	11.301442	11.524738	10.659469	11.256343	11.527480	12.472957	11.021537	11.606112	11.879384	11.449199	11.324533	11.264402	11.466748	11.018211	11.329984	11.524887	11.939987	11.586188	11.313065	11.323781	11.266616	12.232073	11.820662	11.408142	12.307924	11.885697	11.049734	11.844802	11.500352	10.974369	12.331290	10.805036	11.508491	11.631199	11.672076	11.678446	11.636790	11.605367	11.059824	11.671048	11.907279	11.671357	12.034131	12.110108	11.810826	13.882222	12.243771	12.606059	11.869342	11.761011	11.121598	11.496353	11.900872	11.301442	10.982768	11.081157	11.023352	11.306355	11.466449	10.870351	11.942873	11.108519	11.596390	11.467049	11.880861	11.357241	11.105734	11.392765	11.230791	11.603421	11.466296	12.680949	11.447062	11.536600	12.337925	11.607297	11.145435	10.984790	10.847846	11.008438	11.374542	10.469669	10.871747	10.991053	11.143689	10.795839	11.289617	11.464859	10.818785	11.804392	11.427955	11.936861	10.420096	11.367880	11.530935	10.620713	10.351422	9.651648	10.176090	11.605660	10.803437	10.658003	11.319702	12.211265	11.767960	12.296949	11.590536	11.002085	12.330956	12.676354	12.076202	11.806792	12.078825	12.185427	12.801968	12.038055	12.468601	11.267354	12.545714	12.228152	12.169111	11.982413	11.669538	13.032306	12.855334	12.151398	13.424703	12.065467	11.304710	10.901715	11.507445	12.175621	12.119110	11.788671	12.350062	11.712327	11.369147	11.797645	12.431602	11.561717	12.435149	12.574744	11.519525	12.310003	11.473822	11.512308	11.966716	11.756331	11.231436	12.161724	10.737168	12.310934	12.709644	12.028718	11.212902	12.739508	12.308401	11.849270	13.353095	12.159268	11.873387	12.169864	12.609852	12.211265	12.511128	12.735398	12.366707	12.556362	12.133115	12.392815	11.845543	12.419600	11.311032	11.855342	11.349516	12.183622	12.001304	11.740562	12.585328	12.194744	12.184541	13.221977	12.555104	11.706258	12.421658	12.762216	12.101792	12.044549	12.250724	11.124128	13.576961	12.832911	13.102010	12.539475	11.907585	13.226650	12.982400	13.411878	12.458016	12.671038	11.404316	11.174295	12.664096	11.737167	11.862307	11.980027	12.076671	11.469730	11.320686	10.820288	11.941813	12.330956	12.255361	13.356199	9.387474	10.518184	10.954993	10.590679	12.771214	12.236364	12.141913

IZUMO1R	6.387497	4.967577	4.978711	4.599587	4.687185	4.937670	5.063876	5.475224	5.059000	4.663683	4.783257	4.122526	3.648387	4.557457	3.712529	5.004573	5.492799	3.726533	5.248966	4.342419	4.089190	4.526742	5.853436	4.550228	4.758717	4.511602	5.108098	5.111719	5.414350	4.310159	4.269156	2.949012	4.996288	4.329006	4.259390	4.513162	4.162511	4.660717	4.886461	3.930876	4.845532	4.431806	4.933652	3.624828	4.589046	4.216015	5.358725	4.797249	4.523906	3.587474	3.425458	4.315399	3.564969	3.881910	4.143246	4.985394	3.806168	3.766086	3.695929	4.606057	4.660504	5.174727	5.339523	5.368387	4.325941	5.153174	5.009311	5.229535	4.713285	5.267082	4.834368	5.057355	4.894172	5.325747	5.214446	5.308841	4.822497	4.812712	4.910501	4.626946	3.179263	4.908200	4.024941	4.452895	4.908200	4.541102	3.767990	4.104982	5.738814	4.285690	4.983403	4.854590	3.388778	4.441288	4.867232	4.413326	5.761196	5.409922	4.393824	4.770142	3.394704	4.396577	4.896431	4.574580	4.659428	4.892281	5.291194	4.725774	4.374567	5.338509	4.036500	4.582452	4.578189	5.014462	4.419593	3.172255	5.958405	5.281755	5.349668	5.036741	4.935876	4.275750	4.959144	3.984336	5.290575	3.748346	5.073638	4.404898	4.476675	4.657286	4.066688	3.742381	3.529232	3.201160	4.491573	5.432219	4.054293	5.154192	4.768089	4.779065	4.238087	5.637046	4.907319	3.936856	5.054532	4.287203	5.228322	4.708660	4.591859	4.704262	3.493904	4.473434	3.918319	4.454595	4.307220	3.815433	4.529240	4.864543	4.503720	4.715187	4.827398	4.907542	3.197292	3.899788	3.895246	4.927632	3.580893	4.290244	3.436697	3.560720	4.827398	4.384471	3.955259	3.234074	5.053637	4.273558	5.282363	3.516964	3.647203	4.154789	5.399411	5.319749	5.786822	4.012032	4.816027	4.739299	5.596446	5.389322	5.742456	4.855630	4.778843	5.215622	4.646000	4.182980	5.503913	4.692874	5.149493	5.065719	3.252513	3.881073	5.243609	3.955463	3.466476	4.462017	4.314274	3.780830	4.599587	5.286398	4.512913	4.854383	4.515508	3.307599	4.814140	4.986428	4.308819	5.292942	3.855740	3.802024	4.540679	3.636278	3.858138	4.657491	3.928045	4.701109	5.154192	5.418743	5.107426	5.160478	5.868125	5.014252	4.111378	4.210774	4.722391	4.755521	4.363298	5.530479	4.541747	5.098442

BOC	10.462499	12.233290	12.168975	12.858824	12.874301	12.306362	13.232466	10.591268	11.412245	12.305725	13.078004	13.069091	12.726676	12.711326	13.154016	12.536108	12.856932	12.907514	13.598139	12.546379	12.977506	12.050582	13.562221	12.814228	13.590299	13.600149	12.411574	12.526303	13.024395	12.357997	12.983891	11.874918	12.163120	11.006688	12.754938	11.903881	12.442520	12.196161	13.422986	12.982246	13.182307	13.392233	12.568243	12.835058	12.989438	12.369370	12.184698	12.479302	11.914759	13.102386	12.080733	12.864781	12.566107	13.299825	11.960220	12.734879	12.385620	13.116395	13.234224	13.073608	13.782568	12.511596	14.626808	13.393094	12.777955	13.044993	12.677174	12.346950	12.206351	12.899723	12.725852	12.353897	12.387258	12.095039	12.329087	11.996262	11.947446	12.426698	12.192751	12.559114	12.231096	13.401844	11.826245	12.202678	13.021558	12.734379	12.731848	12.637652	13.131145	12.936910	13.049325	12.865869	11.877752	12.144058	10.984493	12.565623	12.022667	13.037619	12.376771	13.279655	11.327051	13.387343	12.836884	12.956981	12.057480	12.260037	12.483749	12.590117	13.301249	12.486157	13.183086	13.304338	11.721370	12.213629	11.275035	11.871295	11.497121	12.396622	12.737637	11.258585	9.852904	11.664174	11.364013	10.900969	10.946688	12.285680	12.168674	10.922691	9.711552	10.049705	10.847257	12.159115	10.497236	10.164737	11.860833	9.972475	11.559088	11.358851	10.652361	9.699395	11.178065	12.762029	11.846709	11.833741	9.441108	10.410577	9.441595	11.260928	10.840331	11.569606	10.580246	10.434301	9.166648	10.940064	9.629378	11.429723	10.393108	10.476950	11.499775	10.299127	11.311166	12.479650	10.718671	9.830470	10.176874	9.293796	14.250802	9.903802	9.970033	10.580246	11.023215	9.822386	10.694498	11.876585	12.282101	12.188558	11.551198	11.100603	11.402055	11.051205	13.146323	9.972329	11.693803	11.638425	11.419262	11.353895	10.509773	10.200229	12.004430	10.119452	10.371775	10.398683	10.364000	12.568720	10.689640	10.871441	10.445527	10.764309	13.321286	10.204002	11.759915	10.214105	11.113919	11.160953	11.547236	10.706734	10.162156	13.278020	12.555984	10.077750	10.467258	11.467920	10.447340	9.930158	11.492028	10.162292	11.102224	10.252493	10.773877	10.054641	12.620336	12.316848	11.126207	10.689474	10.411012	9.181095	9.229700	10.376965	10.090076	12.629675	10.800096	10.084367	11.790869	9.451406	9.459710	11.839166	10.275619	10.509626

ZNF529	10.843477	10.347802	11.232575	10.432465	10.697351	10.224053	10.690215	9.852310	10.750537	9.990578	10.041323	10.132299	9.967576	9.639654	10.473309	10.254503	9.964867	9.642850	9.767842	10.555843	9.748635	9.388862	10.656544	9.895662	10.390475	10.373227	10.558107	9.914232	10.319814	10.552938	9.712641	10.651494	10.961636	10.433811	10.786914	10.944749	11.015142	11.313065	9.945894	10.134533	10.243374	10.231920	10.651778	11.191198	11.226796	10.825459	9.495049	10.441249	11.518100	10.884929	10.975378	10.970442	11.066017	10.975673	11.288336	10.399588	11.195935	10.774046	9.492866	10.732503	10.271020	10.756632	10.445210	9.639954	10.307130	10.905172	10.049407	10.388663	9.942430	10.063627	10.900542	10.808753	9.836099	10.966826	10.724139	10.868882	10.985815	10.791439	10.784818	10.438690	10.445361	11.347880	10.976265	11.379601	11.237555	11.027337	10.975823	11.346523	10.372919	10.472219	10.346434	10.510787	10.401542	10.828868	10.878399	10.895336	11.434802	10.822512	10.490101	10.812696	10.691016	10.630554	10.427778	10.701207	10.174103	10.956504	9.477984	9.767842	10.663706	10.388230	10.520340	9.307273	9.687778	9.940550	11.399554	10.540607	10.242906	10.763875	10.310657	10.665327	10.685697	11.466592	10.689640	9.671482	9.858664	10.586706	11.013094	8.102649	9.975902	9.429742	10.467123	8.625298	9.651348	10.563844	10.395960	8.497774	9.914081	11.082761	10.928598	9.952278	8.818720	10.121388	9.986541	12.049973	9.848474	10.348720	10.519506	9.946377	9.593381	11.126207	10.473914	10.465951	10.492682	11.598426	11.782082	11.307971	11.711018	10.132441	9.936636	11.208214	10.021856	10.430485	11.749227	11.004248	10.581147	9.607325	11.349074	11.217005	10.654587	10.869610	11.879689	11.143689	10.686620	10.851206	10.723285	11.374064	10.706734	8.627031	11.201301	10.119312	10.316078	9.696776	9.742434	9.616048	10.657396	10.927713	10.023858	10.688732	9.859150	10.840635	11.020795	9.164645	10.741115	10.996866	10.213496	10.948465	10.164597	10.838841	10.808891	10.392965	10.940458	11.235078	11.319554	12.683833	10.386256	9.893788	11.162156	10.849578	10.252193	10.765247	10.885551	10.639852	11.398352	10.473914	11.239056	10.702889	10.479190	10.661012	10.848448	10.223453	10.226354	9.922286	10.558107	10.724009	10.582900	10.185134	8.137859	9.714055	9.171885	12.141427	9.507629	10.131992	9.034964	11.709393	10.033321	10.563528	10.447194	9.628464

EMP3	5.850774	6.139271	6.260984	6.245766	6.390194	6.319439	6.378320	5.959510	5.244418	6.040169	5.849897	6.251495	6.394353	6.031825	6.148121	6.233928	6.042449	6.198966	5.673109	6.981935	5.040502	6.675238	5.341799	5.976903	5.796387	6.328905	6.126955	5.499857	5.363851	6.327724	6.118370	5.579285	6.583596	7.014840	7.222084	6.713749	6.909858	6.332819	6.137868	6.292672	5.712313	6.234964	6.731107	6.897952	6.292342	7.319325	5.309863	6.976898	6.900625	7.476459	6.378641	6.753389	7.293155	7.484743	7.486641	5.787010	7.014840	6.899823	6.587033	6.705597	7.177313	5.665156	6.753221	6.697442	7.100637	7.162520	7.107577	7.265880	6.333488	6.949641	7.023919	6.970286	6.157883	6.804305	7.309440	6.752407	6.930487	6.296420	5.898487	7.323742	6.754571	7.672128	6.788096	7.842645	7.172616	6.633547	6.694089	7.379556	7.259980	6.842576	7.010888	7.226852	7.165954	7.264630	7.315588	7.097191	6.761235	6.596178	7.161102	5.939707	6.027116	6.148121	5.356463	6.000758	7.223728	6.202255	6.727092	7.134995	6.505827	5.896721	7.109984	5.943205	5.559669	5.822404	6.393714	4.745112	5.475412	6.154472	4.836806	5.892040	6.639977	6.770414	6.552176	7.559064	6.002530	6.902716	5.598592	6.872429	6.292024	6.060803	6.762817	6.351290	5.758326	6.428535	5.445439	7.163145	6.737455	6.847723	6.885519	6.278983	7.295374	7.537089	7.354709	7.163616	7.443459	5.870804	5.839528	4.576019	6.629691	5.779447	4.631969	5.779986	7.027491	6.957032	7.394443	6.189096	6.449333	6.990140	7.403812	6.033087	6.289123	7.282413	6.431908	6.101534	7.659241	6.481749	6.186901	6.521864	9.053617	6.577011	6.538740	7.579023	8.214701	7.633323	4.946727	7.355178	6.934887	6.139446	7.018597	7.356705	6.716702	7.191384	8.127337	6.001794	6.496370	7.268933	7.408053	6.610967	6.071530	7.139538	7.581658	6.068771	7.289724	7.137983	6.321693	7.246079	8.481092	6.799452	7.316219	7.808494	7.883682	6.986518	6.950916	8.019529	6.388847	7.568557	7.141280	7.442516	6.697278	7.843587	7.324535	8.384808	7.567799	7.398357	7.354866	6.528481	5.571259	5.474461	6.638539	7.582103	6.242113	6.146192	5.921968	7.013769	7.276559	5.642352	7.812489	7.006896	6.865024	6.821650	5.904197	3.293775	6.034728	7.406485	6.603879	5.921041	6.748786	6.680932

CCDC114	5.850774	6.139271	6.260984	6.245766	6.390194	6.319439	6.378320	5.959510	5.244418	6.040169	5.849897	6.251495	6.394353	6.031825	6.148121	6.233928	6.042449	6.198966	5.673109	6.981935	5.040502	6.675238	5.341799	5.976903	5.796387	6.328905	6.126955	5.499857	5.363851	6.327724	6.118370	5.579285	6.583596	7.014840	7.222084	6.713749	6.909858	6.332819	6.137868	6.292672	5.712313	6.234964	6.731107	6.897952	6.292342	7.319325	5.309863	6.976898	6.900625	7.476459	6.378641	6.753389	7.293155	7.484743	7.486641	5.787010	7.014840	6.899823	6.587033	6.705597	7.177313	5.665156	6.753221	6.697442	7.100637	7.162520	7.107577	7.265880	6.333488	6.949641	7.023919	6.970286	6.157883	6.804305	7.309440	6.752407	6.930487	6.296420	5.898487	7.323742	6.754571	7.672128	6.788096	7.842645	7.172616	6.633547	6.694089	7.379556	7.259980	6.842576	7.010888	7.226852	7.165954	7.264630	7.315588	7.097191	6.761235	6.596178	7.161102	5.939707	6.027116	6.148121	5.356463	6.000758	7.223728	6.202255	6.727092	7.134995	6.505827	5.896721	7.109984	5.943205	5.559669	5.822404	6.393714	4.745112	5.475412	6.154472	4.836806	5.892040	6.639977	6.770414	6.552176	7.559064	6.002530	6.902716	5.598592	6.872429	6.292024	6.060803	6.762817	6.351290	5.758326	6.428535	5.445439	7.163145	6.737455	6.847723	6.885519	6.278983	7.295374	7.537089	7.354709	7.163616	7.443459	5.870804	5.839528	4.576019	6.629691	5.779447	4.631969	5.779986	7.027491	6.957032	7.394443	6.189096	6.449333	6.990140	7.403812	6.033087	6.289123	7.282413	6.431908	6.101534	7.659241	6.481749	6.186901	6.521864	9.053617	6.577011	6.538740	7.579023	8.214701	7.633323	4.946727	7.355178	6.934887	6.139446	7.018597	7.356705	6.716702	7.191384	8.127337	6.001794	6.496370	7.268933	7.408053	6.610967	6.071530	7.139538	7.581658	6.068771	7.289724	7.137983	6.321693	7.246079	8.481092	6.799452	7.316219	7.808494	7.883682	6.986518	6.950916	8.019529	6.388847	7.568557	7.141280	7.442516	6.697278	7.843587	7.324535	8.384808	7.567799	7.398357	7.354866	6.528481	5.571259	5.474461	6.638539	7.582103	6.242113	6.146192	5.921968	7.013769	7.276559	5.642352	7.812489	7.006896	6.865024	6.821650	5.904197	3.293775	6.034728	7.406485	6.603879	5.921041	6.748786	6.680932

PADI2	8.242624	8.742107	8.566293	8.742397	8.243097	8.202867	9.298286	8.831019	7.818572	8.444854	8.497483	9.806528	8.825536	8.319558	8.739474	10.231158	8.331848	8.597328	8.010942	9.445750	8.342612	7.950929	8.035070	9.224813	9.092678	8.415646	8.061804	11.257232	11.103871	10.077590	8.959765	9.344383	9.165409	8.826430	9.896599	8.939900	8.704594	9.070196	8.515034	7.911114	8.326529	8.497023	10.217724	9.192889	8.708432	9.255442	8.635194	9.072055	9.444839	8.991935	10.610931	9.474760	9.393123	8.891767	10.054204	8.316530	11.994265	8.450472	10.082394	9.258188	8.971304	8.865812	7.646968	7.626701	8.165963	8.621124	8.591139	8.283450	8.523224	9.046541	8.795667	8.666374	8.429897	8.557469	8.561316	9.302504	8.385123	8.799766	8.894256	8.693881	9.272858	9.385728	8.597896	8.877113	8.978636	8.926440	8.699834	8.673745	8.190378	8.594404	8.324932	8.214240	8.760747	8.592374	9.746613	9.141010	9.266257	8.014321	8.566293	8.248407	7.872609	9.724231	8.479812	7.857244	7.937895	8.218076	9.359842	7.595483	7.929278	8.636313	7.699122	8.584762	8.728680	8.220099	9.114675	7.210664	7.841406	9.524105	8.426021	8.760747	7.762198	8.170666	9.645183	7.830932	8.056685	8.179476	8.948154	6.993221	7.578233	7.752866	7.578853	7.713749	9.470176	7.915057	8.249009	7.022815	6.820707	9.720011	9.052979	8.166131	7.323297	8.356138	7.385610	7.558763	7.246216	7.960849	8.305140	8.989583	9.835965	8.169095	9.029335	8.282714	8.925032	9.866534	7.773530	8.299713	8.983343	7.416551	7.520666	7.717058	7.865227	11.460396	8.506737	8.607415	8.735484	7.916620	9.290649	8.325722	8.761068	9.721996	9.333063	8.624032	8.742249	10.154668	8.718581	14.667309	8.193995	10.059104	8.680362	8.474316	9.966216	6.568230	7.350077	9.146716	8.266965	7.774932	8.037881	7.486494	11.098696	8.054016	7.676043	7.893570	8.163833	8.352774	9.791103	7.777466	8.469344	8.681277	8.337894	9.310802	8.824486	8.660218	8.758540	9.436336	8.061379	8.833342	8.164423	8.912217	8.121793	8.243979	7.311816	8.353532	8.390673	10.075883	9.656714	8.191012	7.537475	8.083795	7.807549	7.503220	11.684797	7.683680	8.588347	7.119635	8.057711	7.435698	7.775107	7.802054	7.535780	7.571121	7.487562	9.242110	8.056204	7.774614	7.132916	8.124646	11.008289	7.671322

C2orf73	15.522760	14.189577	14.118589	14.293761	14.099196	14.599964	14.030116	15.149924	14.701092	14.121021	14.663619	14.061195	13.622196	13.487273	12.672938	14.303277	14.607572	12.765477	13.608509	15.377722	13.516235	13.740121	12.786304	13.602191	13.498714	14.036663	14.533492	14.436030	14.672821	14.733058	13.924850	13.409232	13.994194	14.137016	15.566607	14.692917	14.727873	14.881255	14.428990	13.940443	14.677877	13.521387	12.835422	13.652235	13.652033	13.344692	14.415078	13.765216	13.852712	14.095514	14.575995	13.053474	12.261651	12.570198	13.073430	15.414655	12.470550	12.649190	12.879771	12.510140	12.757215	14.904024	11.952432	13.396455	13.389263	13.478817	13.769304	13.927849	13.079117	13.543266	15.631882	15.097561	14.365566	14.920608	14.739993	13.503677	13.986959	14.346364	14.142398	12.440469	13.267817	13.440958	13.021352	12.929753	14.825958	13.966043	15.676954	14.891747	12.794117	12.948751	12.473452	13.096527	13.352886	13.972453	13.151461	14.077463	13.357004	13.381340	14.111684	14.607572	14.187463	13.621985	14.070910	13.487032	12.777955	14.766218	13.739103	13.012640	13.364946	14.732623	13.031581	13.267179	12.690183	14.591153	14.244331	15.517756	13.183285	14.558412	14.797410	13.458499	14.813167	13.761156	13.656487	14.582713	14.318504	14.536366	14.580592	13.634245	13.766508	14.407352	13.648940	13.467314	12.870661	12.974204	14.069516	13.991278	12.503202	12.377241	13.728355	13.071328	12.839995	12.243145	14.051810	13.053130	12.361792	14.244331	13.739368	14.641807	14.453564	13.546180	13.704613	13.892882	14.840176	14.358378	13.918322	14.782854	14.300589	14.232858	13.440958	14.312732	13.303472	11.751580	13.474637	13.280841	13.675309	14.502659	12.455951	13.741097	14.162372	14.178659	12.185267	11.902934	12.199524	12.242384	14.099196	11.545153	11.941976	12.498635	12.406503	12.237422	14.186189	12.592823	12.728950	13.230280	12.685861	13.644958	13.761409	13.073608	12.423279	14.058874	14.430074	14.607572	14.703436	13.819912	13.760910	13.649164	14.222510	14.498295	12.235761	13.557508	13.067352	13.756377	12.694911	13.678503	13.644259	15.580998	13.595124	12.357030	12.804429	12.520217	12.751886	13.418399	13.763964	14.251440	13.691189	13.689737	14.120452	13.634990	13.619271	12.867603	13.526328	13.839834	12.829643	11.646496	14.473115	12.731681	12.328915	13.004475	14.671445	12.762029	13.063978	14.153935	14.618223	13.549134	14.523150	12.330636	13.789730	14.279673

CCDC9	16.501423	15.659589	16.063007	16.409990	16.010487	16.685246	15.149924	15.955729	16.874939	16.218720	16.730795	16.367000	16.577604	15.940843	15.928804	16.138567	16.927813	15.985841	16.494444	16.508802	16.551607	16.266495	16.823927	16.040249	16.437172	17.034716	17.135902	16.420473	16.195154	16.593935	16.819885	15.373253	15.088208	16.346048	16.831787	16.315840	16.409990	16.433023	16.843250	16.393332	16.266495	16.662863	17.262291	16.942750	16.744583	17.144930	17.042023	16.827810	16.811523	16.819885	16.531399	16.496735	16.073627	16.041613	16.206017	16.887432	16.391445	16.061651	16.283537	16.672020	16.187492	17.189676	16.632960	16.927813	16.264810	16.541277	16.513720	16.737719	15.990738	17.061989	16.564795	16.492130	16.201225	16.065653	16.163437	16.865778	16.161910	15.701693	15.985841	15.409272	16.020683	16.369009	15.791820	15.690182	16.168373	15.399973	16.541277	16.025583	15.925276	15.565819	15.423337	16.225319	16.112915	15.952203	16.567554	16.379042	15.250968	16.397385	17.262291	16.773335	16.599646	15.957029	15.915538	16.202822	16.653630	16.128614	16.883200	17.144930	17.434795	16.125498	16.577604	16.401115	16.523640	16.428719	15.619793	17.055536	16.973803	17.090717	16.827810	16.800018	16.973803	16.361326	16.711084	16.815163	16.610744	17.048853	16.927813	16.855860	17.021223	16.800018	16.855860	17.069256	17.021223	17.021223	16.870018	16.789194	16.968008	16.835817	16.572650	17.098194	16.851732	16.711084	16.907711	16.847849	16.519186	16.762688	16.947470	17.027876	16.997234	17.034716	16.638676	16.827810	16.521294	16.461214	16.437172	16.246616	16.469988	16.744583	16.577604	17.179789	16.213991	16.264810	16.656647	16.800018	16.672020	16.865778	16.835817	16.469988	16.701464	16.605293	16.704885	16.501423	16.734486	16.648016	16.653630	16.450253	16.124210	16.508802	16.226917	16.348005	16.572650	16.292480	16.401115	16.474625	16.469988	16.409990	16.391445	16.792711	15.963250	16.762688	16.962425	17.152637	16.624262	16.234936	16.104387	16.650790	16.635635	16.564795	16.238386	16.258072	16.132820	16.215284	16.632960	16.261246	16.860748	15.879245	16.087400	16.430830	16.377052	16.526136	16.315840	16.526136	16.559494	16.258072	16.762688	17.042023	16.823927	16.874939	16.599646	16.957850	17.055536	17.042023	15.925276	16.459030	16.759174	16.769750	16.800018	16.575146	16.947470	16.704885	16.902527	16.997234	17.322495	17.285107	16.648016	16.585910	16.847849	16.751734

TRPV6	9.726365	10.106438	10.167524	9.821311	11.226506	10.262095	9.807767	8.664535	9.445303	9.572296	9.689437	10.028875	9.748976	10.571684	10.242602	9.884378	9.587494	10.509017	10.222722	10.390024	11.000920	11.004977	8.148380	10.487203	10.214105	10.092047	7.387168	9.504085	8.806574	8.977416	9.958780	10.040236	9.721686	8.765596	9.131973	9.942884	10.753090	10.100454	9.863484	10.362696	10.118987	9.356962	8.773214	10.097334	9.644870	10.529382	8.555423	9.595856	10.596191	9.768599	9.404677	10.388978	10.125235	9.869000	10.142149	9.507924	10.099038	10.930096	10.772399	10.108529	10.771942	9.328299	6.625344	10.322128	10.261948	10.833585	9.913768	10.708724	9.486434	10.621887	9.821765	10.422252	9.562674	9.687929	10.554929	9.714868	10.512279	8.700270	9.767395	10.046469	10.046320	10.197070	10.177628	10.839888	10.478286	10.518938	9.736421	10.086189	10.884458	10.565322	10.998939	10.802540	9.654195	9.208388	9.729192	10.691348	10.326579	9.981975	9.812907	9.956624	8.507070	10.219095	9.870758	10.716117	10.090835	9.714868	10.850618	10.499570	11.622042	9.701277	12.349742	11.281048	11.233156	10.353124	10.252493	8.194634	9.969420	9.253295	9.466897	11.480931	5.748675	8.947521	10.918527	9.762628	7.065228	5.928643	9.066292	5.519533	8.849250	7.246377	8.043711	5.171907	6.120635	6.278303	6.689649	5.340859	7.254305	8.563746	5.597008	5.985562	9.155560	8.868537	10.586845	6.448523	5.221107	7.285979	6.905576	7.570213	9.334161	5.807783	6.454936	6.926825	6.067709	9.508068	9.034964	7.521929	6.738272	7.106590	6.822304	6.157700	10.374979	9.667179	5.922158	10.322295	7.705945	6.753389	8.422421	10.883417	5.965177	6.571166	7.301462	7.299385	5.639354	8.307442	8.033745	9.919470	11.467630	9.277238	8.775212	6.147424	7.222542	6.329550	8.270594	9.777842	6.963394	6.889496	8.641898	6.807918	12.334942	9.044869	8.412310	6.903372	6.038087	7.796324	7.952309	6.293010	6.695389	7.649794	5.679760	9.894722	8.321720	5.652389	9.707507	5.907765	7.434558	6.603253	9.961516	10.726252	6.316408	6.949641	6.669931	5.593845	5.143159	7.424921	6.056359	7.321039	9.244855	5.057568	9.356076	5.187008	9.711397	8.194308	6.290462	6.576510	8.647850	5.661798	8.705991	10.709812	7.000974	8.603281	6.156847	7.216561	9.342664	7.127512	6.092703	6.046222	7.760978	5.536657

ATP6V1B2	13.143981	13.618534	13.587671	13.766016	13.619935	13.434092	13.291948	13.340998	13.523456	13.382599	13.588156	13.584172	13.671278	13.843768	13.368506	13.581357	13.353095	13.568486	13.750320	13.962461	13.539286	13.539515	12.435950	13.702665	13.817588	13.650539	13.594434	13.659596	13.371858	13.292760	13.904184	13.755126	13.871812	12.812473	13.222566	13.313356	13.336430	13.697796	14.256071	13.628936	13.531718	13.928111	13.241587	13.506342	13.178372	13.762178	13.492381	13.719437	13.247295	13.664472	13.149739	13.274804	13.450944	13.635940	12.585159	13.878453	13.739103	13.553456	13.855680	13.632774	13.553691	13.586976	13.249512	13.569183	13.770853	13.763964	13.614956	13.353325	13.679487	13.387795	13.585102	13.364746	13.520707	13.405475	13.513556	13.066209	13.557508	13.631592	13.340998	13.812737	13.833834	13.619696	13.420393	14.012342	13.574676	13.546652	13.849377	13.627059	13.729901	13.843254	13.631803	13.676222	13.698484	13.863343	13.306393	13.955482	13.358268	13.482140	13.962183	12.963629	13.622196	13.185843	13.367256	12.981496	13.366020	13.562221	13.767473	13.597930	13.726369	13.645649	12.951859	13.660789	13.802610	13.368506	13.602862	13.147685	13.160536	13.852424	13.710693	13.772955	13.544167	13.576715	13.632774	12.976037	13.352886	12.837755	13.500208	13.375462	12.746572	13.131724	12.735569	12.933125	13.140268	13.286871	13.126528	13.087211	13.275857	13.490810	12.959197	13.978346	13.291131	12.684911	13.985840	12.984620	13.353095	13.680191	13.347873	13.358045	13.706994	13.422172	13.439894	13.279655	13.529215	13.640135	13.226454	13.706787	13.434092	13.557717	14.108206	13.641034	13.830488	13.143196	13.446470	13.729644	12.985188	14.168090	13.236349	13.489934	13.222154	13.087563	13.362040	13.367473	13.160536	13.957409	13.067718	12.750695	13.348289	13.295984	13.314970	13.290940	13.394547	13.489934	13.114121	13.669861	13.275436	13.348289	13.950717	12.906428	13.318461	13.077040	13.447560	13.714879	13.251043	13.077428	12.738484	13.109263	13.093127	13.417129	13.608281	13.349515	13.056733	13.467530	13.657459	13.606202	13.405662	13.767258	13.859679	13.832567	13.788457	14.102873	13.426653	13.207909	12.857438	13.717490	13.628713	13.292760	13.437980	13.000278	13.190645	12.873035	12.879575	12.609021	13.327985	12.956055	13.113355	13.867818	13.567112	13.662003	13.157276	12.819581	13.371239	13.483216	13.430597	13.392871	14.280707	13.775202	13.207137	13.511504

IL7R	11.737635	12.171410	12.718385	11.772261	10.719432	11.939692	11.321148	9.664852	10.967106	10.343395	10.796121	11.911024	11.014714	10.694051	10.660096	11.340239	10.083571	12.534781	10.365006	11.997933	10.966697	12.230643	9.608227	13.314762	11.821254	10.104477	13.459608	10.171549	11.794064	13.587444	10.734915	13.031189	13.254196	13.440736	12.864211	13.349750	10.684428	11.634348	11.349074	12.783114	10.888494	11.686785	13.297973	11.893367	11.652730	12.430448	11.075007	11.971923	12.667828	11.023522	11.851556	11.352461	11.978981	11.650359	11.525463	9.774329	12.083688	11.882981	11.003228	12.039083	10.883700	11.242985	10.587164	10.924780	11.920396	11.599731	10.533962	10.582745	11.977595	11.766034	11.119708	10.970728	11.610052	11.598732	11.363720	12.618964	11.014430	11.595057	11.973763	12.358306	13.171124	12.616157	11.996542	10.937909	10.916730	12.121934	11.991026	11.564033	12.986646	11.194165	12.535101	13.199119	11.908348	11.278409	13.055794	11.397914	12.303753	10.539729	11.154213	10.198715	10.680811	12.381803	11.896202	10.282778	12.063188	11.816748	11.562888	12.333040	9.998736	10.330088	10.409534	9.809677	11.392925	10.934885	12.245314	11.757091	11.793463	12.303112	10.693013	12.535268	13.190645	13.304945	12.738484	11.925051	11.427813	11.800594	10.855861	11.160233	10.738376	12.976952	12.798338	9.890283	11.743043	14.116761	10.946219	13.723478	11.378286	11.052217	10.981465	11.289771	12.017489	11.048406	9.725752	10.701509	13.507700	10.939769	10.418587	10.760108	12.552675	12.536742	12.790989	13.117358	12.060702	11.720464	10.732799	12.729636	12.898626	12.485843	13.199727	12.468757	12.036088	11.669088	12.454528	12.445298	11.831658	11.652130	12.484550	11.171084	12.462928	13.507906	12.488616	12.166942	12.689295	12.999705	10.649852	10.398683	12.127814	12.250070	12.496355	13.375239	13.517989	12.631986	11.791508	11.376521	13.481484	13.340126	12.772064	12.138334	12.894185	11.637677	12.207449	11.344753	13.062840	12.951479	12.323739	13.227239	13.112784	12.344564	12.827524	12.476380	11.816892	13.066209	14.450917	15.032714	11.829732	13.376715	12.234346	13.548218	13.703179	12.063494	13.333079	12.071423	13.789730	12.750860	12.953101	12.509485	12.038942	12.938338	9.936352	14.291025	10.886871	11.561576	12.810001	12.651078	12.816972	10.963108	12.045726	12.084324	11.997786	12.610699	13.512631	10.750069	11.209393	10.776077	11.913381	12.643905	11.907737	13.820478

COX6B2	6.079900	7.647714	8.990170	7.487101	8.687887	7.929428	8.594879	9.866241	11.087385	7.076205	8.757464	8.954265	8.063973	8.654920	8.463077	8.412020	8.854389	7.847219	8.136815	9.173745	8.353838	8.683020	6.080902	8.415946	8.682359	8.703075	9.268924	9.450937	7.528744	7.119635	8.205080	9.164187	8.307920	10.000704	8.888062	7.625933	9.268147	8.921804	9.059279	8.737163	9.019712	8.614804	7.535343	9.085355	7.229059	7.948163	8.345999	8.931119	9.021121	8.297149	8.128713	8.179171	7.950786	8.220385	8.728213	7.804315	8.628667	8.493080	8.844331	9.070196	9.993847	7.276724	8.496726	10.011667	8.888996	9.011126	8.780870	8.761974	8.527523	9.129684	7.648612	8.502324	8.932840	7.395552	8.132391	8.677119	8.879418	9.220351	7.866036	8.466884	8.598050	7.988792	9.601742	8.631915	8.646046	8.292304	8.771295	8.471849	8.941716	8.533021	8.188467	9.818731	8.470551	8.849578	8.231309	8.448740	8.746205	7.481352	8.216812	7.628292	8.456268	7.589345	9.307273	7.796324	10.241816	7.757279	9.772646	9.010532	8.506270	8.154688	9.226054	8.896861	8.735164	7.097815	8.440271	9.999048	7.251537	10.097156	8.736571	6.728047	8.489977	10.492682	9.492722	11.428860	7.412915	11.508343	12.359115	12.630345	7.627977	10.047564	8.441936	8.865514	9.894872	11.311460	9.743643	11.021971	6.484786	8.964318	10.359015	10.818785	10.351116	7.043666	9.739786	9.991411	7.789614	11.006838	11.505998	7.751360	8.527674	11.063641	9.090859	7.900849	11.653773	10.898647	8.461405	11.067036	10.464884	11.402055	10.626401	12.620177	9.472328	8.786567	11.665355	7.919983	8.920911	9.304141	9.162455	8.899150	10.139254	10.233407	9.483198	7.868615	9.803036	8.814563	6.841442	9.628948	9.503174	9.733340	10.311282	12.130364	8.422262	10.976418	12.203451	9.975417	9.813992	10.800584	8.671094	9.979042	7.940418	8.345227	9.782227	12.627650	6.708142	9.332770	9.050843	10.550537	10.803584	8.976517	10.256330	9.602061	7.618175	12.089192	9.752993	10.664611	8.669134	9.463760	8.617394	10.707032	10.745281	7.528744	10.588378	10.025914	9.805482	8.321580	10.802840	9.216076	8.491478	9.468870	8.102045	8.713880	8.724968	12.395544	8.877113	10.747707	9.836892	11.288477	10.069025	10.075134	11.062443	10.764929	10.584201	7.542035	7.676347	8.519771	9.770486	8.603452	12.539475	10.614358

RFNG	10.759120	10.477236	10.315346	10.581882	10.621738	10.847688	10.717586	11.204776	11.144404	10.954993	11.143538	11.195935	10.712259	11.252949	11.145002	10.492517	11.020654	11.198550	11.464783	10.679116	11.188894	11.210101	10.516533	10.816421	11.000920	11.157897	10.996710	11.376221	10.396585	10.106142	10.968909	10.912348	10.708230	10.585809	10.606813	10.896568	10.903357	10.968756	11.282256	11.451020	10.749920	11.540839	10.764309	10.752372	10.861307	11.093692	10.697067	11.182712	10.374263	10.967556	10.838913	10.967834	10.644592	10.917489	10.886269	10.508560	10.829909	11.493891	11.456977	11.456392	11.818235	10.529382	11.727061	11.451777	10.871585	11.094004	10.776676	10.385633	10.521568	11.065719	10.410277	10.466534	10.521111	10.339746	10.568476	10.376671	10.492371	10.436289	10.493743	10.724311	10.632913	10.591557	10.576772	10.708399	10.597246	10.857955	10.581306	10.873539	11.396102	11.130935	11.089609	11.656322	10.672137	10.381341	10.332362	10.758086	10.674657	10.988852	11.050779	10.622485	11.100603	10.566641	10.656544	10.820134	11.336278	10.644734	11.247492	11.824581	11.412947	10.671356	10.634152	11.397000	11.616116	10.941833	10.651347	11.515310	11.369147	10.843330	10.763571	10.456157	10.724544	10.348574	10.627292	11.033295	10.756632	10.735347	10.747987	10.889072	10.514677	11.107188	11.296731	11.189473	11.062006	11.886135	10.532527	11.343233	10.960398	11.111588	10.288521	11.808575	10.883094	10.734915	10.873539	11.441892	10.987934	10.766127	11.393373	10.544617	10.477090	10.987934	10.868295	10.795256	10.863522	10.620285	10.419038	10.725203	10.861749	11.705650	11.390271	11.163045	11.291576	10.831184	10.795715	11.078552	11.339812	10.999696	11.128382	10.625804	11.101213	10.611210	10.576472	10.767583	10.672137	10.985390	10.387951	10.967556	11.352736	11.500069	10.699115	11.485939	10.634588	12.001749	11.430160	10.527304	11.395344	10.299569	10.576915	10.544466	11.081461	10.300168	10.334012	11.184648	10.198884	10.265145	10.330692	10.734602	10.258347	10.790437	10.628696	10.848151	11.045739	11.665969	10.499861	10.775210	10.975823	10.756043	10.750537	11.615402	11.060858	11.058524	12.120820	10.942405	10.442903	10.792186	10.850917	10.632606	10.941987	11.072990	11.012393	10.556927	10.078545	10.790007	10.872782	11.425929	10.580396	10.978775	12.056973	10.879596	10.917038	10.356294	11.065562	11.123080	11.155666	10.632913	10.552793	11.258731	10.889830	10.806353

IFT122	9.959089	9.405140	9.466435	9.881929	9.612878	9.557087	9.413036	9.180938	9.226210	10.010197	9.554189	9.947489	9.953492	10.047711	10.173505	9.310505	9.407409	10.547178	10.713446	9.736421	10.038839	9.702347	11.041788	10.050816	10.206287	10.212412	9.875601	10.026726	9.781775	9.317901	9.668618	9.470474	9.137471	9.491581	9.354834	9.324472	9.316241	9.816987	10.135934	10.133901	9.832155	10.252933	9.287715	9.505030	9.929076	9.487184	9.818140	9.289571	9.253295	9.730434	9.652120	9.389608	9.605775	9.899900	9.472775	9.793599	9.527395	10.175472	10.519232	10.015727	9.740417	9.650087	9.730856	9.967576	10.196594	9.714868	9.478926	9.492217	9.306478	9.223262	9.028410	8.846939	9.675059	8.821432	9.003955	8.803036	8.877700	8.813801	9.238911	9.243051	9.449354	9.556781	9.382710	9.399697	9.603904	9.336777	9.369995	9.393908	9.994619	9.658740	9.739498	9.642061	9.147027	8.973624	9.068014	9.312799	9.353484	9.649319	9.703268	9.443280	9.390213	9.395906	9.746158	9.766782	9.481198	9.647967	9.434778	10.082093	10.433811	9.700189	10.048505	10.315629	10.161362	9.489846	9.317025	8.414146	10.519764	9.864980	9.929996	9.911577	9.678129	10.120961	10.770609	11.355342	10.361120	10.104055	10.857810	10.249099	10.645044	10.156215	10.357958	11.265593	10.596938	10.407402	10.011667	10.463392	11.453855	11.927388	9.912045	10.956031	10.637882	11.375921	11.385568	11.325112	10.996096	9.424212	11.284296	10.581752	10.223453	10.820704	10.037185	9.501474	9.985494	11.013672	10.170756	9.779075	10.134374	10.845031	10.708553	11.923854	10.168122	10.300344	10.487203	10.537651	9.481658	10.881936	10.461731	9.479088	9.887037	9.773259	10.064516	9.752834	10.177481	10.074685	10.306994	10.723285	11.966106	10.506011	10.991775	10.819395	9.964264	10.210474	10.275470	10.215948	9.852310	9.581010	9.952278	9.338616	11.277927	9.279993	8.596881	10.156362	9.322003	9.418245	8.749240	9.419282	9.517769	8.823411	9.803798	9.632923	10.406224	9.849294	9.689916	10.229572	10.341712	10.150237	10.666208	10.729414	10.477389	11.292947	10.918671	9.912507	9.226054	9.500568	9.521160	9.916366	10.210139	9.733932	10.490413	9.784423	11.397914	9.557862	9.508397	10.810113	10.473914	10.258677	10.924932	10.280645	10.074063	10.477996	10.318027	11.560987	9.564893	9.856672	9.307595	10.309258	10.242742	10.273200

BMP8A	12.046313	11.616692	11.719718	12.029925	12.771214	12.939212	11.909858	12.450257	13.028031	12.185716	12.215941	12.231096	12.398510	13.520479	12.182043	12.816972	13.261202	12.724316	12.725694	12.114497	12.033678	12.521963	13.555459	12.149284	12.659975	13.109263	12.511451	12.156187	12.332724	12.219537	12.908398	11.579668	11.811570	12.174197	12.230488	12.268074	12.873920	12.129781	12.217765	13.566875	12.369716	12.437743	12.885289	12.187793	12.933477	12.151239	13.260048	13.728857	11.927240	11.828107	11.765559	12.402275	12.124499	12.096962	11.990173	12.231577	12.696982	12.206044	12.033377	12.278977	12.830477	12.992187	13.120033	12.384801	12.318545	12.247353	12.188852	12.435149	12.098166	12.604249	12.542773	12.458016	13.595842	13.057276	12.050270	12.029310	12.517931	11.509371	12.109661	11.392463	12.251209	11.970071	11.641173	11.955287	12.693563	12.293428	11.806639	12.978577	13.548218	12.773448	12.692172	13.738081	13.525276	12.029621	12.085897	12.815287	12.010496	13.592616	12.849490	12.693903	12.805797	12.399952	12.635273	12.046469	12.662746	12.024202	12.578033	12.775483	13.396889	12.219378	13.463136	13.653197	12.843685	12.788393	13.626349	14.458572	13.029169	12.346006	12.820920	13.028210	11.845543	11.720935	12.388237	13.573817	12.038515	12.509156	12.559435	11.799373	12.468921	12.283028	12.222786	12.675868	13.796160	11.932304	12.706360	12.478661	12.191984	12.892768	11.828254	12.164047	12.827695	12.103936	11.947128	12.456916	12.215332	12.309046	12.195864	12.873750	11.837247	12.974383	12.843348	12.811770	12.255831	11.534230	11.308969	11.807225	11.664327	11.431952	12.062451	12.342480	13.441364	11.128662	11.284152	11.486562	11.884935	12.406661	11.658633	11.786268	12.233142	11.483981	12.513208	12.053768	12.552494	11.764948	11.575387	12.141761	12.087108	12.284575	11.593866	12.143742	12.679736	11.919811	11.665969	12.248492	11.972074	11.494063	11.393969	11.267076	11.308373	12.166942	11.694097	13.037427	11.969197	11.934899	11.550891	12.549408	11.328775	11.454144	12.065467	11.004248	11.966716	13.065305	12.427666	12.450418	12.187947	11.579521	11.159203	13.286686	13.031373	13.931643	13.736592	12.209422	12.763272	12.588785	13.778922	12.333823	13.187239	11.794389	11.042247	11.999153	12.067502	11.831355	11.944525	11.436941	12.007161	12.358457	12.335719	11.487129	11.870412	12.814569	12.896890	14.369411	13.182111	12.683833	12.319355	12.834869	13.181925	11.604311

COA6	13.378453	13.078004	13.490156	13.312746	13.299434	12.950005	13.272567	13.265809	13.026732	12.475535	13.205718	12.731164	13.635698	13.143196	12.787176	12.759418	13.110410	12.911640	12.553951	12.600886	12.299354	11.415282	10.838572	12.728769	11.822149	12.489402	12.136649	11.808438	12.733395	12.948591	13.044993	13.480608	13.231839	13.754404	13.107335	12.860767	13.547537	13.686701	13.148247	12.415781	13.136420	11.201144	12.610699	13.384766	12.631986	12.606059	12.779517	11.697997	12.338554	12.219537	13.066403	13.608509	12.437418	13.084373	13.320471	12.596273	13.627523	11.941654	12.338106	12.982957	12.315592	13.037427	11.616996	11.509789	12.460924	12.950183	12.292024	12.613710	12.340685	12.054832	12.471196	12.484062	12.782402	12.818568	13.543058	13.155744	12.557506	13.064161	12.732697	12.649362	11.718550	13.004126	13.800876	13.448635	13.498487	13.250845	13.243181	13.549344	11.994103	12.617631	11.952138	12.106206	12.836884	13.191256	12.999520	13.193477	12.812829	13.200284	13.446068	12.111589	13.282417	13.128984	13.537477	12.925070	12.500081	13.026194	13.085251	12.498330	11.400278	13.292951	11.640106	12.245613	13.261202	13.061734	11.825046	12.358931	12.271228	12.918024	12.780022	13.575329	13.915004	13.782334	13.278622	14.163327	14.616554	13.944041	13.866239	14.010958	15.317503	14.083132	15.007098	14.205886	13.328213	13.597687	13.481922	14.358378	14.753586	13.767473	14.619411	13.993598	14.223759	13.899912	13.468792	13.670308	14.425228	13.360372	13.831022	13.952936	14.500649	13.656487	14.114980	14.192726	13.919949	14.127698	14.931911	13.873927	14.264573	13.741803	13.869352	14.057105	14.021288	13.626349	14.100097	14.282413	14.836045	13.684300	12.502227	15.101738	14.811620	14.518159	13.883273	14.931361	14.269900	13.973837	14.197518	14.189577	13.508116	13.095970	13.851943	13.084593	13.283038	12.940455	13.610850	13.256614	13.014282	13.193477	13.092754	14.115260	12.595934	14.357312	15.173276	13.210340	14.849518	13.924035	14.015006	14.372290	14.054182	14.208447	12.948055	13.198725	14.370128	13.645181	14.717187	14.465165	13.912140	14.428612	14.303277	12.465162	13.467091	13.306393	12.709826	13.573121	14.085269	14.206838	13.694956	14.956666	14.544276	14.091290	14.816832	13.855680	13.070948	13.167690	14.195897	11.877172	14.725604	14.614852	12.865330	13.691450	13.685268	12.795036	13.208700	14.306647	12.667828	15.502234	14.243708	14.425923	14.435670	13.728355

DHRS4	14.450917	15.048391	14.702498	14.634337	14.452088	14.454290	14.947964	14.248223	14.234569	14.637945	14.324726	14.610103	14.584001	14.676953	14.144611	14.471558	14.406227	14.293761	14.530263	14.759178	14.690192	14.563014	14.529498	14.479473	14.760083	14.887600	14.717187	14.396941	14.751210	14.649747	14.487150	14.461989	14.413643	14.580207	14.697958	14.664070	14.708876	14.832004	15.009362	14.870791	14.716286	14.945774	14.478648	14.504600	14.686942	14.395124	14.808644	14.714881	14.544632	15.093809	14.185875	14.582286	15.071599	14.992275	14.744131	14.911437	14.659533	15.143286	15.063409	15.017982	15.047833	14.867085	15.045993	14.992829	15.037486	15.234869	15.098818	15.160798	14.735321	15.038723	14.999084	14.800437	14.537158	14.418012	14.589784	14.917362	15.230680	14.487923	14.602856	15.072836	14.426320	14.453564	14.493555	14.729702	14.480594	14.477088	14.014363	13.818636	14.428245	14.907125	14.564295	14.779929	14.361581	14.946885	14.222846	14.484499	14.789144	14.055969	14.327509	15.040623	14.305307	15.053711	14.532252	14.556844	14.450917	14.286202	14.756942	14.828938	15.171380	14.649251	14.607960	14.416904	14.530652	14.743665	14.463605	14.551021	14.407352	14.839161	14.451322	14.335070	14.340690	14.289302	14.270558	14.494741	14.484052	14.821349	14.229183	14.167438	14.630361	14.264573	14.566322	14.707094	14.477088	14.223156	14.334703	14.256071	14.184052	14.451725	13.854600	14.378512	15.587282	14.338926	14.465165	14.560516	14.267351	14.275877	14.475985	14.731220	14.514288	14.385259	14.013491	14.105943	14.257993	13.844544	13.995849	13.800876	14.518159	14.471172	14.470097	14.600408	14.584760	13.630442	14.087427	14.494365	14.545966	14.637498	13.902528	14.498295	14.076889	14.064771	14.188678	15.028728	14.378131	14.475183	14.733504	13.742612	14.484873	14.471966	13.949622	14.369411	14.784831	14.508368	14.456976	14.411336	14.500265	14.673241	14.415465	14.680520	14.124606	14.454664	14.346713	14.532252	14.321264	13.765490	14.110411	14.384530	14.365930	14.225335	13.680915	13.652033	14.452476	13.672469	13.576005	13.607817	14.324060	13.944829	13.588617	14.519413	14.104742	13.888334	14.658149	14.390000	14.271885	13.916329	13.944308	14.491542	14.033170	14.283789	14.799897	14.421778	14.396941	14.440081	13.861539	15.584586	14.533864	14.497164	15.701693	14.636601	14.839682	14.260943	14.162992	14.035571	14.669062	14.863545	14.104048	14.758705	14.343514	14.684603

BMP1	9.123377	10.301596	10.279115	10.411296	10.462958	10.959584	10.166750	10.365006	10.712709	10.765247	10.284884	10.400199	10.830911	11.233464	10.283800	10.137446	10.901875	10.742742	10.180720	10.609505	9.495630	10.417045	9.707820	10.824286	11.067181	10.908696	10.455381	10.883700	10.574665	10.226833	10.909844	10.397912	9.900685	11.084401	10.080574	9.897794	9.838745	10.204762	10.550104	10.809338	10.419658	9.752370	9.953867	9.988575	10.134688	10.090835	10.524734	10.080574	10.698669	10.010612	9.781184	10.358232	9.977545	10.278197	10.464739	11.069445	10.406956	10.596035	10.788987	10.549645	11.163491	10.881780	11.219382	9.955532	10.801045	10.653315	10.039741	9.935273	10.047255	9.606097	9.707995	10.005054	10.336292	10.044497	9.823307	10.203707	10.002425	10.456157	9.779375	10.599248	10.350675	10.488263	9.982125	10.441631	10.314435	10.208918	10.537371	10.956204	9.861920	9.773557	9.576825	9.596007	10.467694	9.985035	9.454459	10.127898	10.273634	10.663292	10.318464	10.518311	11.122188	10.126943	10.302038	10.411012	9.748976	10.846519	10.755416	9.566353	9.336777	10.131242	8.923712	10.385937	11.112724	10.254063	10.764174	8.418259	9.583919	10.682418	10.561314	10.272270	12.654999	12.215618	11.472234	11.552884	11.228274	12.071423	11.820950	12.274399	11.523666	12.753644	11.262723	12.574563	12.247983	12.752265	11.514555	12.785412	11.774311	9.969266	10.722674	11.687667	12.412540	10.967395	13.096912	12.665817	12.323414	11.792707	11.322595	11.956809	12.357997	12.335103	12.149735	12.963290	11.998716	11.376076	11.931707	13.046106	12.542279	12.149134	13.125598	11.907433	11.089903	11.202229	12.863843	12.557506	11.590838	12.621510	10.323369	12.012961	13.109074	12.598661	11.909709	12.260507	13.486625	12.843348	11.129723	10.783312	12.080733	11.452234	12.045301	12.997483	11.404685	11.665355	11.471808	12.127344	12.426538	12.984441	11.920396	11.634958	10.912940	11.478703	12.324793	12.215332	12.723907	11.415587	11.708808	13.180932	12.592167	12.417662	12.195864	13.161477	11.271942	12.819581	13.600808	13.321286	12.561046	12.793582	11.608020	11.382463	12.670521	11.440178	11.813591	12.245455	12.980298	12.689122	13.163020	12.786494	12.752073	12.327168	11.557455	12.881502	11.142648	11.631509	12.474435	10.703735	10.955440	12.317402	9.705813	10.274087	11.282105	10.921655	12.821082	9.155247	10.254638	10.273342	11.834673	12.761874	11.951988	12.428494

ITLN2	6.770915	7.331953	7.402070	7.057930	6.373651	7.362303	7.347692	7.642308	7.791273	7.436471	6.274562	7.064759	6.563191	7.341765	6.420945	6.971038	7.464963	7.069219	7.051964	7.405370	6.264006	7.335828	9.134397	6.720650	6.472574	7.081450	5.996901	7.155127	6.384666	6.832615	7.713118	7.325797	7.309112	7.530378	7.054425	6.556152	6.759769	7.049262	7.502112	7.543264	7.022026	7.421041	6.293351	6.445919	6.464543	6.314526	6.794137	7.322955	7.072747	6.911631	7.033252	7.213260	6.945183	7.662002	6.799796	6.899169	7.227941	7.357964	7.490559	7.576995	7.543582	6.650152	6.351290	7.188718	7.114883	7.439160	7.638327	7.240719	7.197133	7.091816	8.082112	7.703146	6.990632	7.774614	7.979874	7.361219	7.349431	7.321353	7.648612	7.594432	7.389651	7.229059	7.479657	7.178765	7.156379	7.354709	7.371092	7.641842	7.478884	8.129817	7.542183	7.586605	6.830244	7.197133	6.892054	6.691766	7.097035	6.208126	6.493471	6.659030	5.733384	7.293485	7.115667	7.291140	6.907201	6.310424	7.041120	6.395639	7.333659	6.926505	7.642619	7.449836	7.121945	6.706548	7.368917	6.518383	6.720650	7.188552	6.440719	8.044489	7.231492	8.219328	6.531604	7.247752	7.218109	6.555824	7.037167	8.034780	7.721625	7.062047	7.308803	5.902563	5.852907	5.438382	5.814929	7.231821	7.651278	8.009151	7.104405	6.570672	6.917878	8.171919	7.046908	6.727870	6.986699	6.660184	8.706305	6.524506	7.293816	7.811119	6.760754	7.303511	7.712340	7.695913	7.541740	6.703844	7.139538	6.858817	7.428515	7.732409	7.668348	6.825914	7.276265	7.825691	6.494756	7.568409	7.026811	7.079746	6.512277	7.014074	6.011759	7.344115	6.068056	5.451468	6.891740	7.128302	7.363230	7.673529	10.167524	8.217923	6.602455	6.915261	7.315429	6.767961	7.043839	7.499287	7.729193	7.446500	7.370150	8.418870	7.823644	7.407269	7.997230	7.609920	7.548238	7.178923	8.024983	7.491173	5.826559	7.972915	7.186349	7.741489	7.341605	6.614323	6.745598	7.771269	8.583923	7.364043	6.920025	8.620648	7.796780	6.381466	6.855890	7.691350	7.541740	7.461507	6.409176	7.151068	7.541891	5.553381	10.460420	7.371092	6.662005	6.258112	6.787592	7.935298	6.295933	7.730863	6.918372	7.161880	8.013058	7.468910	6.914786	7.556139	6.156030	6.631873	6.855086	6.734990

ARID4A	11.879981	11.220719	11.441607	11.038010	11.335254	10.902471	11.704017	11.188596	11.685541	11.167246	11.346391	11.226506	11.009483	10.781910	10.709515	11.391015	11.175962	11.228558	10.113060	11.365654	10.682266	11.349211	10.592028	10.753860	10.643123	10.973659	10.597404	11.167684	10.691661	11.249706	10.903646	11.437253	12.174197	11.918508	11.831805	12.005642	11.956956	11.016496	10.617741	10.754173	10.933529	10.894605	11.217573	11.655140	11.903720	11.842489	10.701207	10.785872	12.315273	12.130834	11.849886	11.923119	11.433305	12.057936	12.118153	10.626098	12.169284	11.194445	10.703735	11.383634	11.420288	10.967244	11.321288	10.784221	11.255609	11.173458	10.841116	11.326759	10.970728	11.037869	11.863798	11.964779	11.127384	11.919358	11.793767	12.109967	12.111007	11.839473	11.979284	12.097568	11.276880	12.039403	11.912283	12.183786	11.957237	12.002055	11.986674	12.422163	11.166523	10.746193	11.169307	11.870995	11.357963	12.089977	12.056040	12.047677	12.134816	11.241786	11.180344	11.462325	11.340397	11.388799	10.904224	11.864994	10.414788	10.750836	11.407356	11.200721	10.369561	10.553981	11.504214	10.218950	10.924932	11.257082	11.626444	9.782227	10.735207	11.268796	10.379251	11.496673	10.440967	11.387769	10.464310	9.354532	9.704886	10.313708	10.049864	10.362060	9.779667	10.049864	10.040996	9.470328	9.467958	9.007782	10.337200	11.742737	9.535347	9.573039	10.129307	10.343850	9.370301	9.527248	10.181458	10.105381	10.225742	11.139480	9.512608	9.063380	10.149023	10.957229	10.895043	10.250277	11.139782	11.459062	11.142502	11.219527	11.783598	9.757177	9.926775	8.626257	10.246028	9.745228	11.385252	11.017200	11.558409	10.685116	10.166289	11.465248	11.119136	11.342479	11.733626	10.697213	11.805598	11.852935	9.783615	11.583181	10.349010	10.895631	10.388103	10.953478	10.582028	10.118219	9.991732	10.667979	11.211714	10.227300	11.191935	10.251612	11.040450	11.415883	11.331478	9.009189	11.849270	11.768560	11.416773	11.181727	11.789411	11.360307	11.969485	10.222096	11.873849	11.805147	11.345479	11.823688	11.130328	12.415286	11.498609	11.143240	10.400645	10.463860	10.789138	11.158476	11.579668	11.273685	11.174436	11.228419	10.465951	11.129440	11.473095	11.317927	10.741254	10.917191	11.669538	10.380918	10.003957	9.886243	10.597324	10.373661	10.043315	10.421303	9.744936	9.307738	9.993695	11.939528	10.663131	10.765247	11.805147	10.269288

GSTA3	10.858500	8.149308	8.157947	9.094797	9.533987	9.531300	7.921494	9.118025	9.182515	8.172997	8.949830	8.449681	9.332910	8.382997	7.213408	8.468700	9.279219	7.440807	6.189601	8.562532	6.581301	7.147212	6.345747	8.045289	8.262615	8.800062	8.996430	9.205189	9.370750	8.155934	8.700599	7.877349	7.532690	8.214240	10.034381	8.072005	8.343367	9.073286	6.433444	7.452446	8.994888	5.876566	6.657598	7.508474	7.712486	7.783924	8.146710	6.945974	8.025420	7.715979	8.345227	7.072417	6.436473	7.676199	7.894343	8.894686	7.761907	7.507708	9.492217	7.493360	7.894980	8.576401	5.783606	6.149868	9.121392	7.042248	7.801599	8.028251	8.360784	7.144750	8.039022	8.649359	8.835394	7.924112	8.340131	8.036191	8.724655	8.024072	8.200757	8.350208	7.958836	7.727797	8.698949	8.047832	7.922875	8.172842	8.477574	7.633026	6.449821	6.564337	7.126739	6.371417	8.275089	8.258487	8.644846	8.907026	8.668823	8.632906	8.977416	8.485965	9.304406	8.255721	7.886885	7.451363	6.251495	8.375568	7.697463	6.329550	6.664134	8.868386	6.147424	6.695543	8.252222	9.703584	8.290571	9.737449	10.432626	8.908317	8.955928	8.710861	7.246690	7.703584	9.682921	9.906054	9.496124	9.343124	9.504085	8.509809	10.716420	8.807202	12.192594	8.448133	7.705798	8.033901	10.100782	8.699691	9.105050	7.298624	7.731181	6.434967	7.021100	8.460633	8.754115	7.821163	7.858326	10.430330	10.692399	9.309567	10.479190	8.658992	8.583297	9.746781	8.838896	8.039647	8.985620	7.370940	7.165801	6.964378	7.579023	8.318216	9.006263	8.093070	7.601380	8.592681	7.277363	8.336025	6.815732	10.571218	7.356854	7.237820	9.092981	8.077747	7.663729	7.407740	11.137430	6.982537	10.757072	8.277230	10.523247	8.427087	8.248548	6.267913	6.996945	8.752751	8.951813	6.944879	9.276071	6.938731	7.136713	11.093843	9.292212	9.087336	8.602363	7.605001	7.648449	9.728526	7.865959	8.003407	8.236065	8.528920	8.857274	7.558623	9.856818	7.326749	10.152342	9.408800	8.704594	6.436133	7.755464	6.109732	6.622590	9.526785	8.351557	9.111112	10.089453	8.718419	8.855620	8.476639	9.409114	8.136158	8.610765	9.329078	8.389906	6.416507	10.168890	8.051400	7.584728	8.947219	8.841648	7.751835	7.570665	8.397030	8.812028	9.636548	8.803665	7.897170	8.674365	8.780550

RPS9	10.114982	11.504078	11.475722	9.588428	9.281361	9.308181	10.286060	9.410266	9.584839	9.531481	8.876622	9.347097	9.807001	9.318660	10.192816	9.103050	9.324766	9.865299	10.252193	9.545320	9.968029	10.150370	11.258424	9.760731	9.885139	9.796404	12.660501	10.301733	10.604020	11.610786	9.730122	11.097335	11.886135	11.572071	11.836941	11.101364	10.797753	10.973367	11.473235	11.373199	9.574876	10.489183	11.631199	9.879312	10.896881	11.330601	10.601053	11.903261	11.919662	10.886269	11.511560	11.697702	11.722134	10.346434	11.827966	8.946763	12.068749	11.437970	11.022131	11.303088	12.138484	11.052661	13.717944	10.663435	12.179702	11.898681	11.484410	10.743665	12.027658	10.945347	10.644015	10.626987	9.449118	11.092828	10.428516	11.935813	10.740494	12.565285	11.067474	11.791025	11.884935	12.214583	10.946388	11.104280	11.020938	10.153581	11.801784	11.757533	11.912931	11.122792	11.350844	12.823360	11.904475	12.566429	11.412245	10.593778	11.458769	9.392820	9.538277	9.380526	10.194475	10.022143	10.262677	10.360069	11.482813	9.395577	10.455087	11.064219	10.880771	8.795667	10.670732	9.275616	10.310211	8.729427	9.689605	10.626098	11.052661	10.050200	9.078706	11.281969	11.399700	12.689500	11.660007	10.756471	9.717969	11.146644	9.562249	10.852571	10.244125	9.804548	10.710821	10.098577	12.624487	11.817939	10.041611	10.961636	10.057740	12.069027	11.631046	11.719425	12.322814	12.368752	9.153845	10.134213	13.246114	11.020938	10.658582	10.577542	12.266230	11.798508	13.088141	12.610024	11.671195	11.622042	10.069492	11.983502	11.854455	11.047378	13.405475	10.031316	12.248657	12.251052	11.578338	11.989727	11.984838	11.212599	12.523982	10.462665	12.146729	12.610699	12.332394	12.412844	12.031230	13.216968	10.586845	11.484876	12.333192	12.328285	12.419600	13.119650	12.833106	12.065467	12.653635	13.027853	13.297173	12.333973	12.801434	12.337925	13.064161	11.354771	12.110414	10.036268	11.442637	12.004877	13.014469	11.750421	12.080159	10.760552	12.613710	11.774016	11.519246	11.944984	13.253798	12.259577	11.389966	12.434806	12.491229	13.239205	13.808908	12.638368	13.006847	11.449438	12.031303	12.024797	13.051634	11.211287	10.505428	10.639378	10.289597	11.052063	9.882882	10.870979	13.634499	12.695910	11.100452	11.066595	12.005336	11.838717	9.752686	12.660501	11.434353	10.652524	9.998272	9.888310	10.683631	13.313179	9.439711	11.465248

LILRB3	10.114982	11.504078	11.475722	9.588428	9.281361	9.308181	10.286060	9.410266	9.584839	9.531481	8.876622	9.347097	9.807001	9.318660	10.192816	9.103050	9.324766	9.865299	10.252193	9.545320	9.968029	10.150370	11.258424	9.760731	9.885139	9.796404	12.660501	10.301733	10.604020	11.610786	9.730122	11.097335	11.886135	11.572071	11.836941	11.101364	10.797753	10.973367	11.473235	11.373199	9.574876	10.489183	11.631199	9.879312	10.896881	11.330601	10.601053	11.903261	11.919662	10.886269	11.511560	11.697702	11.722134	10.346434	11.827966	8.946763	12.068749	11.437970	11.022131	11.303088	12.138484	11.052661	13.717944	10.663435	12.179702	11.898681	11.484410	10.743665	12.027658	10.945347	10.644015	10.626987	9.449118	11.092828	10.428516	11.935813	10.740494	12.565285	11.067474	11.791025	11.884935	12.214583	10.946388	11.104280	11.020938	10.153581	11.801784	11.757533	11.912931	11.122792	11.350844	12.823360	11.904475	12.566429	11.412245	10.593778	11.458769	9.392820	9.538277	9.380526	10.194475	10.022143	10.262677	10.360069	11.482813	9.395577	10.455087	11.064219	10.880771	8.795667	10.670732	9.275616	10.310211	8.729427	9.689605	10.626098	11.052661	10.050200	9.078706	11.281969	11.399700	12.689500	11.660007	10.756471	9.717969	11.146644	9.562249	10.852571	10.244125	9.804548	10.710821	10.098577	12.624487	11.817939	10.041611	10.961636	10.057740	12.069027	11.631046	11.719425	12.322814	12.368752	9.153845	10.134213	13.246114	11.020938	10.658582	10.577542	12.266230	11.798508	13.088141	12.610024	11.671195	11.622042	10.069492	11.983502	11.854455	11.047378	13.405475	10.031316	12.248657	12.251052	11.578338	11.989727	11.984838	11.212599	12.523982	10.462665	12.146729	12.610699	12.332394	12.412844	12.031230	13.216968	10.586845	11.484876	12.333192	12.328285	12.419600	13.119650	12.833106	12.065467	12.653635	13.027853	13.297173	12.333973	12.801434	12.337925	13.064161	11.354771	12.110414	10.036268	11.442637	12.004877	13.014469	11.750421	12.080159	10.760552	12.613710	11.774016	11.519246	11.944984	13.253798	12.259577	11.389966	12.434806	12.491229	13.239205	13.808908	12.638368	13.006847	11.449438	12.031303	12.024797	13.051634	11.211287	10.505428	10.639378	10.289597	11.052063	9.882882	10.870979	13.634499	12.695910	11.100452	11.066595	12.005336	11.838717	9.752686	12.660501	11.434353	10.652524	9.998272	9.888310	10.683631	13.313179	9.439711	11.465248

TAC3	5.669624	4.805579	4.376947	4.300684	3.544326	4.148555	3.242251	3.755901	3.354345	3.542076	3.892646	3.411476	4.134067	3.499964	3.771953	3.315690	3.285623	4.868318	5.598387	4.654865	4.309458	5.845265	8.398071	4.531822	4.253430	3.742824	4.486980	2.902480	4.023692	3.555846	4.699871	7.178449	7.809896	4.831232	5.159865	5.374929	3.365160	3.802663	4.438717	3.305084	3.393833	3.139813	4.829094	4.361966	4.553238	5.108708	3.652214	5.328913	4.704484	4.022370	5.736968	5.238653	7.042589	5.052790	3.828431	3.865767	4.107628	4.361109	3.974642	4.873845	5.216032	3.927387	7.152628	3.776362	5.261653	3.450425	4.118816	4.268246	5.639354	4.281894	4.816027	3.988826	3.714240	4.303765	4.307910	6.231918	4.180769	5.354296	4.953147	5.563547	5.869712	6.049762	5.007648	5.774363	3.898693	5.019510	5.023623	4.559007	5.983840	6.041416	5.786290	5.054079	4.741955	4.373932	6.814127	4.305738	3.556675	3.806168	4.445201	4.085922	3.431343	4.108038	4.401594	3.631909	4.555359	4.267843	3.017302	4.001479	3.751278	3.402821	4.296754	3.310696	3.000556	4.633497	4.509641	3.641293	6.761400	4.489549	3.401604	2.925007	3.454979	2.960600	3.036756	3.736479	5.096968	3.178289	4.331654	3.272103	5.587720	3.180933	4.197524	3.272701	3.095013	3.398011	3.064058	3.180263	3.876112	10.104182	7.998255	3.810335	4.213405	5.118253	5.126372	3.523465	3.229197	2.976228	3.531287	3.176503	3.533103	4.560560	3.876965	4.771972	3.893761	4.388470	5.239264	3.893314	3.690345	3.394875	3.196504	3.169155	4.222533	7.138922	3.412583	4.178097	4.735238	3.506434	3.377564	4.010157	3.353204	3.721214	3.013376	5.731934	4.323253	3.465935	3.074008	4.298299	4.198610	4.681557	6.120116	2.899169	4.123626	4.773688	4.355265	3.919566	3.950770	3.565517	3.608791	3.426570	2.861260	4.728936	3.606610	3.183197	3.006446	3.873314	4.051282	3.031543	3.139813	3.190001	3.432871	4.211644	4.723874	3.589398	4.715187	3.504352	3.711546	3.140290	4.148555	4.089190	3.800534	8.543841	3.294372	4.024321	3.313638	3.150003	3.017781	3.178699	3.675388	4.741289	3.228776	6.113154	8.651036	2.982814	3.330807	4.940938	3.527576	3.546557	4.993721	3.744268	3.183197	4.347616	3.141186	3.009424	4.569586	3.567285	3.977588	3.154801	4.356767	3.299180

SCMH1	10.959584	10.256188	9.689752	10.053263	9.559089	9.771440	10.320901	8.949507	9.580581	9.640875	10.115619	10.113777	9.917640	9.822386	10.863064	10.096264	10.181309	10.211080	8.236065	10.121857	8.868076	9.545767	10.843943	9.841226	10.703444	10.595136	10.867134	10.099670	9.941340	9.852455	9.942734	10.678516	10.658275	10.899381	10.386880	10.246336	10.227300	10.254638	10.899103	10.890897	9.725448	11.083077	10.516399	10.582460	11.049872	10.021565	10.528361	11.104875	10.095615	10.488713	10.822224	10.510787	10.504838	10.625496	10.568780	9.742743	10.687519	10.065419	11.107474	11.445862	11.382315	9.777842	11.059536	10.820861	11.450945	11.150980	10.591407	10.485631	10.569055	10.709812	9.798436	9.784276	9.356515	10.071631	10.005200	10.085231	10.078202	10.307130	10.337472	10.849717	10.578592	10.517560	10.816113	10.668712	10.684971	10.577828	10.642678	10.625804	9.966500	11.113773	11.311749	9.930780	10.529955	10.228061	10.536920	10.465042	10.308026	10.242906	9.961344	10.704762	9.881488	10.377566	10.248333	10.271317	9.176798	9.962834	10.137918	10.244867	9.492217	9.554494	8.292455	10.371390	10.156509	8.944584	9.579308	9.915301	11.602845	10.292188	9.983355	11.991869	10.085861	9.830616	9.915155	9.290484	9.619319	9.760421	10.796271	9.800887	9.533530	9.916997	10.265433	9.960892	11.622683	11.098696	10.844240	10.521739	9.527862	8.004823	10.973802	9.275288	9.826316	8.775819	9.534899	10.861911	11.037558	9.804101	9.049938	9.604702	9.359225	11.047517	10.934280	10.711339	8.860183	10.421303	10.179183	10.812246	10.182714	11.338491	10.761158	9.757967	11.704017	10.119905	10.912644	10.198291	10.135620	10.004584	11.337903	9.979491	9.923526	10.376347	10.900123	10.111382	10.569382	10.863522	9.962553	11.225487	10.404404	10.997755	11.412033	11.682616	10.728524	11.110584	11.134615	11.014277	10.889218	12.533788	10.244867	10.569692	11.746252	9.568899	10.319973	9.736555	10.150237	10.515780	9.979195	10.749604	10.075883	10.380154	10.897742	10.710821	10.980857	10.823846	10.443810	11.062597	10.342797	9.995566	10.436890	10.148575	11.023522	11.166369	10.249259	10.408443	9.995098	9.535972	10.231014	10.412994	10.173953	9.991577	10.947735	9.867261	11.030608	10.757656	10.418587	8.851683	9.471554	9.046071	10.521568	9.202653	9.092226	8.296702	11.190758	10.568476	9.428670	9.688392	9.660604	12.193677	10.868443	9.308341

WIPI1	11.434944	9.993095	10.672285	10.584201	10.989882	10.790007	10.887915	11.243882	12.091066	11.021537	10.925491	11.120500	11.418124	10.760711	9.552071	10.683473	10.410869	10.540467	11.026609	11.236266	10.682874	11.689738	10.788558	11.161418	11.233464	11.981504	11.385992	11.500573	10.223453	10.738842	10.669403	10.001021	10.921361	10.735629	11.185858	11.503353	11.470479	10.762958	10.924932	10.726580	10.796577	11.244028	10.747396	10.852403	11.770623	11.213821	10.189346	10.845325	11.059966	11.140921	11.237000	10.962648	10.404404	11.132065	10.978030	10.057740	11.359302	11.071222	10.876767	10.935769	11.199385	11.279433	11.147104	11.000920	10.220026	10.487949	11.053797	9.466593	10.271167	10.975823	10.926836	11.202837	10.663706	11.403089	11.317631	10.784668	10.873395	11.078407	11.009906	10.363720	10.295699	10.297542	11.239356	10.857343	11.105870	10.537651	11.687667	11.129723	11.047954	10.146257	10.960321	10.693316	10.945509	10.743352	11.407356	11.188894	10.541837	11.575095	10.793806	11.436541	11.016629	11.020512	10.976116	11.070628	11.371135	9.756549	11.685720	10.692399	11.572071	10.511571	11.128877	10.799059	10.734462	10.703174	9.919633	8.691384	11.352031	10.713717	10.717904	11.705650	9.336777	11.072990	10.893606	11.677143	10.163518	10.385019	10.801045	11.085876	10.552645	10.317425	11.365974	11.785972	9.622288	9.334014	10.352051	9.896747	11.173855	9.806528	10.397475	9.679709	11.811570	10.225137	11.174150	10.863674	10.850333	10.360509	11.870089	10.888932	9.873486	10.380306	10.873250	11.763241	10.612891	10.605498	11.539250	11.759188	10.148077	10.384099	10.729414	10.768036	10.857505	10.363280	10.983770	12.371190	10.788244	9.996027	10.493458	11.585304	11.336873	11.434639	11.077953	9.977682	11.355206	11.222510	9.274416	10.895475	11.567272	10.148575	11.550461	10.752648	11.020795	9.873938	10.056961	10.002558	10.223453	10.471490	10.977161	10.334961	10.660403	11.165803	11.711327	9.765411	11.618317	11.882808	10.630554	11.346523	11.212902	11.184811	10.725060	10.851497	10.273634	11.541101	11.888108	10.788704	10.691964	11.617427	10.948319	11.676671	10.328234	9.855744	9.869000	10.928007	11.029412	11.623999	11.722134	11.706718	12.024501	9.737007	10.766265	10.536920	11.149440	11.331187	11.132510	10.636673	10.200957	10.100633	9.567473	11.117105	10.422706	11.934741	10.630981	8.539743	10.547178	10.252193	8.137701	11.021084	10.680952	9.915602

ARSG	11.434944	9.993095	10.672285	10.584201	10.989882	10.790007	10.887915	11.243882	12.091066	11.021537	10.925491	11.120500	11.418124	10.760711	9.552071	10.683473	10.410869	10.540467	11.026609	11.236266	10.682874	11.689738	10.788558	11.161418	11.233464	11.981504	11.385992	11.500573	10.223453	10.738842	10.669403	10.001021	10.921361	10.735629	11.185858	11.503353	11.470479	10.762958	10.924932	10.726580	10.796577	11.244028	10.747396	10.852403	11.770623	11.213821	10.189346	10.845325	11.059966	11.140921	11.237000	10.962648	10.404404	11.132065	10.978030	10.057740	11.359302	11.071222	10.876767	10.935769	11.199385	11.279433	11.147104	11.000920	10.220026	10.487949	11.053797	9.466593	10.271167	10.975823	10.926836	11.202837	10.663706	11.403089	11.317631	10.784668	10.873395	11.078407	11.009906	10.363720	10.295699	10.297542	11.239356	10.857343	11.105870	10.537651	11.687667	11.129723	11.047954	10.146257	10.960321	10.693316	10.945509	10.743352	11.407356	11.188894	10.541837	11.575095	10.793806	11.436541	11.016629	11.020512	10.976116	11.070628	11.371135	9.756549	11.685720	10.692399	11.572071	10.511571	11.128877	10.799059	10.734462	10.703174	9.919633	8.691384	11.352031	10.713717	10.717904	11.705650	9.336777	11.072990	10.893606	11.677143	10.163518	10.385019	10.801045	11.085876	10.552645	10.317425	11.365974	11.785972	9.622288	9.334014	10.352051	9.896747	11.173855	9.806528	10.397475	9.679709	11.811570	10.225137	11.174150	10.863674	10.850333	10.360509	11.870089	10.888932	9.873486	10.380306	10.873250	11.763241	10.612891	10.605498	11.539250	11.759188	10.148077	10.384099	10.729414	10.768036	10.857505	10.363280	10.983770	12.371190	10.788244	9.996027	10.493458	11.585304	11.336873	11.434639	11.077953	9.977682	11.355206	11.222510	9.274416	10.895475	11.567272	10.148575	11.550461	10.752648	11.020795	9.873938	10.056961	10.002558	10.223453	10.471490	10.977161	10.334961	10.660403	11.165803	11.711327	9.765411	11.618317	11.882808	10.630554	11.346523	11.212902	11.184811	10.725060	10.851497	10.273634	11.541101	11.888108	10.788704	10.691964	11.617427	10.948319	11.676671	10.328234	9.855744	9.869000	10.928007	11.029412	11.623999	11.722134	11.706718	12.024501	9.737007	10.766265	10.536920	11.149440	11.331187	11.132510	10.636673	10.200957	10.100633	9.567473	11.117105	10.422706	11.934741	10.630981	8.539743	10.547178	10.252193	8.137701	11.021084	10.680952	9.915602

PLGLB1	9.488725	9.211755	10.635345	10.501830	11.106728	10.183454	9.440805	9.307595	11.094441	10.369707	9.812137	10.098273	11.237709	10.465192	10.050200	10.747087	9.871517	9.866387	11.739525	10.701509	10.369411	11.052514	10.899381	11.456822	11.222672	11.402937	11.268395	11.186851	11.290218	9.584367	11.637074	9.916212	8.837687	10.162595	9.544712	10.208000	9.814640	8.865208	10.239706	10.508560	10.719260	10.047403	9.409404	9.739940	8.760273	9.562398	10.890718	7.730402	8.363165	9.948125	8.367279	8.560242	9.787767	9.116189	7.672436	9.924583	9.798264	9.879782	9.923051	9.667801	9.906813	9.357865	9.695415	10.439729	8.447016	9.863624	8.185578	10.203098	8.237305	9.046377	9.929695	8.881137	10.911612	9.625998	8.548924	9.391590	9.524743	9.665009	9.735774	9.188910	10.305051	10.329500	9.940550	9.566857	10.177038	10.269288	10.865293	8.840579	9.024321	7.908033	9.992352	10.512139	8.745438	9.017754	10.461731	10.932934	9.197647	11.258345	11.001791	10.569055	9.880559	10.287089	10.925219	10.319814	10.074213	9.305858	10.800421	10.641916	11.701015	10.468163	10.580549	11.157897	10.952265	11.177177	8.756380	10.376811	10.222241	9.923196	10.707032	9.532449	9.282612	9.927691	10.656544	10.049864	8.999495	9.619941	11.909265	10.433367	10.463567	9.370750	10.140329	10.901113	9.672393	9.880868	10.572446	10.214424	11.502333	12.066244	10.483985	9.374309	11.772261	10.133737	11.570621	10.911904	10.648649	9.428192	10.395201	10.798200	8.872478	11.091172	10.124758	9.993251	9.021575	9.517138	10.310211	9.355609	8.412020	9.764191	10.585975	9.577775	9.711706	9.886402	8.587540	8.316384	8.978327	9.608075	7.542948	9.297552	9.556934	8.287044	8.921804	10.400199	8.717665	8.591289	9.969572	9.914535	9.658248	9.556004	9.686981	9.375129	8.546267	9.748976	9.781553	9.180624	9.882961	9.874810	9.538600	8.630840	9.021885	9.769830	9.385879	8.578029	9.684672	9.125034	8.934567	9.385879	9.836740	9.605466	9.073896	9.813541	9.928630	10.185728	10.118388	10.045253	10.072673	9.706262	9.111441	10.006922	9.086706	11.148713	10.312904	8.599414	9.306026	10.299127	10.209061	10.079690	11.216261	10.224206	9.438812	10.274257	10.343395	10.199193	9.872421	10.009427	9.657050	10.784974	10.315346	10.472696	8.627169	10.510494	9.799495	11.871450	10.519370	10.363127	9.409559	10.162874	10.020621	9.532686

RGPD1	9.488725	9.211755	10.635345	10.501830	11.106728	10.183454	9.440805	9.307595	11.094441	10.369707	9.812137	10.098273	11.237709	10.465192	10.050200	10.747087	9.871517	9.866387	11.739525	10.701509	10.369411	11.052514	10.899381	11.456822	11.222672	11.402937	11.268395	11.186851	11.290218	9.584367	11.637074	9.916212	8.837687	10.162595	9.544712	10.208000	9.814640	8.865208	10.239706	10.508560	10.719260	10.047403	9.409404	9.739940	8.760273	9.562398	10.890718	7.730402	8.363165	9.948125	8.367279	8.560242	9.787767	9.116189	7.672436	9.924583	9.798264	9.879782	9.923051	9.667801	9.906813	9.357865	9.695415	10.439729	8.447016	9.863624	8.185578	10.203098	8.237305	9.046377	9.929695	8.881137	10.911612	9.625998	8.548924	9.391590	9.524743	9.665009	9.735774	9.188910	10.305051	10.329500	9.940550	9.566857	10.177038	10.269288	10.865293	8.840579	9.024321	7.908033	9.992352	10.512139	8.745438	9.017754	10.461731	10.932934	9.197647	11.258345	11.001791	10.569055	9.880559	10.287089	10.925219	10.319814	10.074213	9.305858	10.800421	10.641916	11.701015	10.468163	10.580549	11.157897	10.952265	11.177177	8.756380	10.376811	10.222241	9.923196	10.707032	9.532449	9.282612	9.927691	10.656544	10.049864	8.999495	9.619941	11.909265	10.433367	10.463567	9.370750	10.140329	10.901113	9.672393	9.880868	10.572446	10.214424	11.502333	12.066244	10.483985	9.374309	11.772261	10.133737	11.570621	10.911904	10.648649	9.428192	10.395201	10.798200	8.872478	11.091172	10.124758	9.993251	9.021575	9.517138	10.310211	9.355609	8.412020	9.764191	10.585975	9.577775	9.711706	9.886402	8.587540	8.316384	8.978327	9.608075	7.542948	9.297552	9.556934	8.287044	8.921804	10.400199	8.717665	8.591289	9.969572	9.914535	9.658248	9.556004	9.686981	9.375129	8.546267	9.748976	9.781553	9.180624	9.882961	9.874810	9.538600	8.630840	9.021885	9.769830	9.385879	8.578029	9.684672	9.125034	8.934567	9.385879	9.836740	9.605466	9.073896	9.813541	9.928630	10.185728	10.118388	10.045253	10.072673	9.706262	9.111441	10.006922	9.086706	11.148713	10.312904	8.599414	9.306026	10.299127	10.209061	10.079690	11.216261	10.224206	9.438812	10.274257	10.343395	10.199193	9.872421	10.009427	9.657050	10.784974	10.315346	10.472696	8.627169	10.510494	9.799495	11.871450	10.519370	10.363127	9.409559	10.162874	10.020621	9.532686

RGPD2	9.488725	9.211755	10.635345	10.501830	11.106728	10.183454	9.440805	9.307595	11.094441	10.369707	9.812137	10.098273	11.237709	10.465192	10.050200	10.747087	9.871517	9.866387	11.739525	10.701509	10.369411	11.052514	10.899381	11.456822	11.222672	11.402937	11.268395	11.186851	11.290218	9.584367	11.637074	9.916212	8.837687	10.162595	9.544712	10.208000	9.814640	8.865208	10.239706	10.508560	10.719260	10.047403	9.409404	9.739940	8.760273	9.562398	10.890718	7.730402	8.363165	9.948125	8.367279	8.560242	9.787767	9.116189	7.672436	9.924583	9.798264	9.879782	9.923051	9.667801	9.906813	9.357865	9.695415	10.439729	8.447016	9.863624	8.185578	10.203098	8.237305	9.046377	9.929695	8.881137	10.911612	9.625998	8.548924	9.391590	9.524743	9.665009	9.735774	9.188910	10.305051	10.329500	9.940550	9.566857	10.177038	10.269288	10.865293	8.840579	9.024321	7.908033	9.992352	10.512139	8.745438	9.017754	10.461731	10.932934	9.197647	11.258345	11.001791	10.569055	9.880559	10.287089	10.925219	10.319814	10.074213	9.305858	10.800421	10.641916	11.701015	10.468163	10.580549	11.157897	10.952265	11.177177	8.756380	10.376811	10.222241	9.923196	10.707032	9.532449	9.282612	9.927691	10.656544	10.049864	8.999495	9.619941	11.909265	10.433367	10.463567	9.370750	10.140329	10.901113	9.672393	9.880868	10.572446	10.214424	11.502333	12.066244	10.483985	9.374309	11.772261	10.133737	11.570621	10.911904	10.648649	9.428192	10.395201	10.798200	8.872478	11.091172	10.124758	9.993251	9.021575	9.517138	10.310211	9.355609	8.412020	9.764191	10.585975	9.577775	9.711706	9.886402	8.587540	8.316384	8.978327	9.608075	7.542948	9.297552	9.556934	8.287044	8.921804	10.400199	8.717665	8.591289	9.969572	9.914535	9.658248	9.556004	9.686981	9.375129	8.546267	9.748976	9.781553	9.180624	9.882961	9.874810	9.538600	8.630840	9.021885	9.769830	9.385879	8.578029	9.684672	9.125034	8.934567	9.385879	9.836740	9.605466	9.073896	9.813541	9.928630	10.185728	10.118388	10.045253	10.072673	9.706262	9.111441	10.006922	9.086706	11.148713	10.312904	8.599414	9.306026	10.299127	10.209061	10.079690	11.216261	10.224206	9.438812	10.274257	10.343395	10.199193	9.872421	10.009427	9.657050	10.784974	10.315346	10.472696	8.627169	10.510494	9.799495	11.871450	10.519370	10.363127	9.409559	10.162874	10.020621	9.532686

USP27X	10.137745	8.547997	9.455713	9.410650	9.366774	9.332000	9.745999	9.295062	9.217322	9.880868	9.435081	9.418537	8.866433	9.421896	8.493381	9.742594	9.333538	9.606560	9.241660	10.055298	8.575648	9.399241	10.481689	8.856081	9.117427	9.019286	8.957303	8.675628	9.235949	9.290169	9.814319	10.512728	9.744581	9.493938	10.013986	10.324127	10.340353	9.171740	9.292527	9.798775	9.667801	9.150640	8.899150	10.128392	10.566197	10.001321	9.424212	9.580275	10.305675	10.443501	10.160896	10.350528	10.316078	10.378655	10.292953	8.826707	10.551284	9.512911	8.969436	9.694983	9.712489	9.579308	9.452150	9.070964	9.107183	8.930159	8.915570	8.776297	8.808590	8.952418	9.781326	10.029478	9.135312	10.244280	9.898827	9.998736	10.570126	10.161066	10.163835	10.018340	9.526340	10.204611	10.368662	10.797174	10.475430	10.016493	10.445678	11.148250	10.127592	9.395577	9.372256	9.917791	8.538625	10.139254	9.731236	9.702651	10.237571	9.739328	9.831068	9.536049	9.325661	9.560491	9.481353	9.249793	8.764831	9.372726	8.856221	9.085022	9.162292	9.275915	9.586230	8.963724	8.779928	8.719185	9.822386	8.960537	9.720011	10.000113	9.218401	9.913281	8.102799	9.615149	9.105050	8.515175	9.168609	9.313787	9.969420	8.924312	9.298596	8.901410	9.974513	7.703743	9.631239	8.193370	9.662014	10.117917	8.803515	9.499023	10.861307	8.386026	9.301890	10.152342	8.989863	9.762791	9.105354	10.143702	8.756380	8.693399	9.217895	9.835965	8.768535	8.724496	8.604528	10.368508	11.223858	10.158496	8.214394	8.655793	10.015875	8.076966	8.118880	9.147855	9.649021	10.838572	9.803484	7.513747	10.255569	10.346580	10.214880	9.575018	10.460851	10.334961	10.087772	9.869000	9.490951	10.867436	9.554494	10.290216	10.207334	9.171408	8.752751	9.124887	8.695277	8.865361	8.887442	8.831339	8.251925	8.491331	8.816835	9.580110	9.395906	8.730609	9.857107	9.917160	9.699083	10.651183	9.795584	9.541580	10.037784	9.216398	9.880559	10.110042	10.006631	10.632306	9.491411	9.780259	10.849875	9.857253	9.085823	9.120357	9.406178	8.126124	9.100248	9.834281	9.549005	9.079028	9.363205	9.766931	11.431505	10.004584	10.305371	9.681259	8.902489	10.108827	9.530388	9.399396	9.754388	10.096406	8.831497	8.942487	9.292688	8.600815	8.255576	10.356625	9.022798	9.230742	9.383918	8.927562

CHP2	9.403879	7.315739	7.573568	9.185335	8.601893	8.665154	8.068615	8.394542	8.417392	7.806653	7.801900	8.650567	10.511848	8.496424	6.321010	7.245764	8.766072	7.606544	8.168619	8.774143	8.017690	10.017413	8.623894	7.971630	7.948606	7.726369	8.148860	7.694279	7.268335	9.115115	8.342758	7.757576	7.904130	9.273149	10.798488	7.675589	8.454290	9.878449	8.337740	7.983341	8.806574	7.760209	6.519373	7.208783	8.035475	8.374041	6.849652	7.027965	9.158035	7.714929	7.866800	8.528920	8.177241	7.017952	7.891219	9.024612	7.988521	8.423936	10.609075	7.476459	8.274612	8.060250	7.451958	7.348334	10.332961	8.505509	8.485826	7.860319	9.913768	7.875662	8.120457	8.770383	7.585638	7.947076	8.593456	7.367653	8.359725	10.414939	7.513109	9.008878	7.433592	8.083021	11.199022	8.870081	8.654776	8.980298	8.648119	8.600639	8.405606	7.768537	7.823834	7.671008	8.867627	10.340966	7.733172	7.362910	7.749492	7.433129	9.036791	8.599122	7.595187	8.438853	7.131326	8.321113	7.734091	8.139429	8.131342	8.338386	8.666374	7.869699	8.403771	8.561173	8.125256	7.557235	8.014946	9.789443	8.408506	9.949359	7.372828	13.188973	8.566024	10.071920	13.542327	13.162836	7.672279	10.215486	7.826953	7.929575	11.642694	10.813574	13.401415	12.331584	9.913768	7.233701	8.128410	7.629887	12.481464	8.895603	6.290632	11.348793	10.882926	10.032856	8.676972	11.763565	7.169797	12.168975	8.766990	12.288785	9.585147	7.911257	9.943490	12.041372	10.016962	10.834204	10.363720	8.471683	7.751360	7.799754	9.971512	7.350988	12.207449	7.339407	9.841975	11.733781	11.509057	6.327236	7.839230	13.304945	8.410935	11.334799	8.148697	13.159158	5.874564	10.432310	10.127268	6.269643	13.521840	10.416749	10.083894	12.011560	12.265924	8.244609	9.226836	11.046205	11.306661	10.173953	11.525317	9.562674	7.604209	12.007316	12.686571	11.649323	8.493691	6.603879	6.722726	11.955137	9.174191	8.117460	9.183450	10.790561	6.980675	11.266314	12.911451	8.567761	10.855244	8.680362	9.516216	9.473387	10.423140	6.989679	8.688984	9.743348	7.087352	11.536302	8.076966	9.099653	7.906308	8.882792	13.488148	6.414848	6.367718	8.770383	12.207292	11.256930	12.601326	9.132911	10.168122	11.426495	8.588024	8.431530	10.117147	9.166648	7.548532	11.971606	12.233576	9.625683	14.326431	7.413845

POLDIP3	13.686442	12.940455	13.105616	13.350346	13.518662	13.129561	13.196137	12.587805	12.613560	13.245910	13.227872	13.103703	13.235191	13.245152	12.798678	13.209096	12.734538	13.204369	13.163020	13.276051	13.357219	13.736365	11.444835	13.392020	13.625234	13.444821	13.022526	13.531931	13.068091	12.810376	13.312545	13.165092	13.118063	12.829461	12.801254	12.849137	13.480608	13.065843	13.387555	13.438408	13.261613	13.511265	12.713284	13.022716	12.838440	12.828929	13.101246	12.651748	12.619661	12.798678	12.816646	13.046467	12.745204	12.739334	12.482801	13.158236	12.566268	13.198725	13.267179	13.194078	13.280442	13.126344	12.735048	13.222566	12.967484	13.100501	13.263764	12.619316	13.093498	13.088513	12.957538	13.034120	13.316425	12.799365	12.891207	12.959932	13.311346	12.954789	12.864211	13.315405	13.212823	12.942474	13.769838	13.644259	13.386902	13.098061	13.255365	13.413783	13.231650	13.108685	13.164516	13.084373	12.947003	13.164705	13.214995	13.310158	12.747457	13.026732	13.173050	12.841760	12.951479	12.805797	13.029169	13.053301	13.218764	12.833456	13.114121	13.108685	13.244123	13.281221	11.712327	13.142104	13.267608	13.051634	13.349098	12.811111	13.494410	13.105804	13.007936	13.001966	13.213195	13.209096	13.225108	13.107335	13.457857	12.850884	13.081595	13.629686	13.623148	13.081015	13.360596	13.410314	13.006847	12.594598	12.983713	12.994351	13.417727	13.078931	13.462463	12.798338	13.503228	11.442338	13.714668	13.744349	14.032590	13.300682	13.564577	12.996930	13.072243	13.116206	12.746742	13.744349	12.581982	13.020637	13.613276	12.671897	13.031373	12.956616	13.543058	12.975643	13.146881	12.290609	12.564124	12.725151	12.566429	12.816481	13.108505	13.373564	13.255784	12.767450	12.681802	13.190846	13.189238	12.974204	13.450122	13.564577	12.666814	12.963098	13.144955	12.716597	13.163201	13.204369	12.904820	12.971855	12.451389	13.248097	13.076654	12.730828	13.149931	13.105804	13.257609	12.801793	13.174765	13.157107	12.391825	12.824043	12.913386	13.202193	13.286282	13.390989	13.031373	12.786103	13.352500	13.617064	13.585776	13.107708	13.182875	13.303085	12.722802	13.184851	13.231650	12.659650	13.118063	12.689500	13.108685	13.352048	12.763759	12.860767	12.991054	13.384142	13.222353	12.513371	12.807024	13.095058	12.472313	12.849653	13.558188	12.716965	12.755993	11.571034	13.143030	13.154016	12.805797	13.240605	13.007394	13.136216	13.604320	13.132093

NT5DC2	11.559784	13.999261	14.101336	14.797410	13.819912	14.313722	14.737586	13.671534	12.049063	14.527514	14.191138	14.556030	13.591683	14.369059	13.506587	13.837218	13.266014	14.427017	14.223156	14.872324	14.170650	14.958920	12.987965	14.747900	13.624991	14.126769	12.171410	13.755645	13.366020	13.419947	13.467905	14.290297	13.960526	13.082154	14.329971	14.332395	14.497164	14.338926	15.457513	15.082962	14.193049	15.321822	13.862105	13.706318	14.297708	14.383074	13.508359	14.333069	12.240224	14.194648	13.378878	14.258950	14.182477	14.762870	13.592841	13.577170	14.579771	15.269335	15.199621	14.961694	14.975358	14.371236	13.576482	15.111816	14.241749	14.339964	14.831507	13.147891	14.222202	14.622906	14.694707	14.203355	13.408595	14.012650	14.607960	13.861843	14.271885	13.607817	13.830488	15.151242	13.677133	12.902330	14.223479	14.625115	14.953863	14.447934	14.449082	14.233886	14.518957	14.520691	13.974396	14.101336	13.595124	14.197180	13.098814	14.227650	12.650904	13.973310	13.945929	13.157657	13.629938	14.599064	14.075731	14.508368	14.368698	12.239785	13.192485	15.296048	14.577676	13.899912	14.702993	14.015842	14.188386	13.470117	11.947731	12.991245	11.900148	13.888066	13.477273	12.805797	12.256151	13.274388	12.858287	12.745569	12.678360	12.227351	13.911324	13.629938	14.283430	12.937622	13.315405	14.332054	12.934196	14.352362	11.688416	13.201795	13.506342	13.839834	11.923119	12.888611	13.167290	13.962183	11.733934	14.813710	14.010653	11.848352	13.208899	10.954523	12.592334	11.052359	13.626821	14.603287	14.294067	13.298634	13.957409	14.000643	14.521080	14.118926	14.247254	12.692342	14.414362	14.422133	13.789231	13.844047	13.399078	11.268237	14.221208	13.472948	14.114029	13.254395	12.884027	13.314762	14.881789	13.898577	11.802547	13.820478	14.247254	13.399078	14.255462	14.402181	13.039676	13.683790	12.902330	12.396312	13.928404	13.300869	13.813249	14.807649	13.916047	13.229650	13.392461	11.209393	13.352048	12.959007	12.562365	13.625684	14.093713	13.774703	12.483749	13.442439	14.036135	12.719907	13.336430	13.104454	13.559500	13.611591	14.018727	14.142712	13.157459	15.523618	13.864620	13.986668	14.251159	13.194518	13.563147	13.532379	12.070991	12.280654	13.450122	13.294357	13.476835	12.580335	13.462700	15.401561	11.355780	12.096962	12.903734	13.115055	11.419997	13.780110	12.969891	14.743665	12.602739	13.753087	12.751206	14.749773	12.768875	14.090137

ADAMTS7	7.735196	7.704877	7.964491	7.810948	7.964030	7.633165	7.894811	8.292455	8.686278	7.464507	7.513578	7.249782	7.542035	7.338770	7.658152	7.949190	7.669595	7.599534	8.354587	7.623618	7.867887	8.543056	8.949666	7.313043	7.618931	7.711088	7.567799	8.192912	7.476921	7.541740	7.897456	7.015004	7.814319	7.472799	7.216561	7.704236	7.466956	7.507225	8.097665	7.744413	7.729485	7.594277	7.531129	7.543116	7.749822	7.557531	7.529026	7.544875	7.539423	7.891853	7.614639	7.360750	7.259489	7.543432	7.468615	7.794157	7.396463	7.319795	7.824617	7.330232	7.875503	7.600485	8.563129	7.989708	7.818875	7.497273	7.803562	7.693984	7.832466	8.025893	7.562869	8.196546	8.045755	7.544702	7.982069	7.648147	7.283831	7.509989	7.844066	7.109503	7.228270	7.178449	6.937962	7.423830	7.019538	7.882013	7.020447	7.217816	7.669761	7.374716	7.119808	8.108945	7.941031	7.186349	7.713749	7.520818	7.272673	7.590442	7.836741	7.855309	8.096280	7.628930	7.581501	7.624575	7.733023	7.419618	8.504326	8.044341	8.498529	7.875503	7.970847	8.073029	7.173677	7.783924	7.724989	7.730554	8.051037	7.490242	7.601986	7.913119	8.531228	8.223604	8.177531	8.923868	8.401595	8.280399	8.670782	9.071262	8.229033	8.312070	8.414441	8.457339	8.113761	8.432106	7.896881	8.250993	8.246033	8.942331	8.254049	8.289790	9.982125	8.988078	8.530929	8.686278	9.420526	7.866962	8.484152	8.556557	8.227519	8.097297	7.616510	9.026010	8.134382	7.434700	8.678046	8.285459	8.584534	9.344664	9.204268	8.206633	7.817478	7.800068	8.055892	8.448278	8.183907	8.408942	8.304234	8.570955	8.892522	8.440099	8.470262	7.909883	8.611061	8.268392	8.064653	8.287640	7.839230	8.015709	7.999639	8.638495	7.752415	8.628513	8.488606	8.108325	7.897027	8.317753	8.098253	8.791835	8.205382	7.836137	8.706907	8.522913	8.321435	8.428664	8.281286	8.494150	8.267621	8.503865	7.882612	8.361415	8.325878	7.706387	8.056526	8.518715	9.156597	8.112997	7.329461	8.377672	8.338861	8.348402	8.790260	8.378761	8.419149	9.035428	8.130580	8.471373	9.049134	8.471036	8.660064	8.851230	7.944322	8.165963	7.844679	8.401741	7.728109	8.496279	7.943235	8.616015	8.655522	8.885915	8.643792	8.039022	8.389280	8.499478	8.006563	8.976069	7.796324	8.236522

ZNF718	8.788742	7.906169	7.633622	7.726838	8.226898	8.193845	8.547528	7.851729	8.401741	8.400640	8.455966	8.379692	8.207086	8.020288	8.516237	8.289196	7.456680	8.220715	8.254820	8.729427	7.068415	8.131027	8.994252	7.553313	7.159016	8.014777	8.233292	7.347533	8.211744	8.668179	8.729876	8.963573	8.739325	8.823713	9.317616	9.002124	8.707378	7.646673	7.301297	8.314367	7.695178	8.366367	7.998255	8.459410	8.069057	8.244304	7.960064	7.334421	9.472775	8.788596	8.739777	8.352011	8.738729	9.099347	8.813954	7.897593	8.283150	8.398835	7.270940	8.195435	8.004502	8.515332	7.970369	8.223133	7.731332	7.906169	7.819523	7.119181	7.923664	7.483209	8.026826	8.547399	8.272834	8.923280	8.321580	8.597328	8.790103	8.990614	8.732236	8.325256	8.420395	8.833467	8.630365	8.673195	8.937007	9.226526	8.539908	9.101829	7.945100	7.968486	7.802954	7.903509	8.329805	8.479473	8.751131	8.912217	9.074192	9.295062	8.099775	8.081052	8.498687	8.449215	7.749351	7.557866	8.190378	9.004704	7.839230	7.668055	8.055590	8.594404	7.313210	9.560158	6.779094	7.877178	8.394988	5.082253	7.763447	8.742859	8.415646	9.029665	7.115967	6.905892	7.315056	3.853359	7.176186	8.427711	9.427118	7.309610	8.592221	6.826732	7.895121	7.111288	7.685180	8.229801	8.904141	7.744866	8.618458	8.025580	9.205492	5.930066	8.417540	8.471529	8.595322	5.943721	6.657929	7.647714	7.784088	7.816205	7.921791	9.482879	8.878485	8.653270	7.310728	9.105197	9.517921	8.659902	7.001784	6.913700	8.226603	7.449989	8.435543	8.499181	8.385284	7.601380	8.070168	7.611287	8.149308	8.702430	8.600192	7.542035	9.141819	9.359995	9.638887	8.731061	6.992917	8.876306	8.839797	6.888402	8.184487	7.758241	7.704236	8.801425	8.337740	7.716059	8.213459	7.516805	6.118183	8.616310	7.557531	7.188718	8.492586	6.191829	8.920132	8.210366	8.616015	8.325878	8.897794	8.563436	7.992704	8.638969	9.335435	8.939279	8.448133	8.995337	8.620493	8.000720	7.134838	8.153907	8.034047	8.865361	8.349433	8.851831	7.930030	7.956657	8.215914	8.445469	8.086920	8.284696	8.009018	9.026306	10.628531	7.338317	7.127841	8.778417	8.409099	8.180379	7.986694	7.331787	6.788096	5.143372	8.404071	4.115709	7.099247	3.773481	7.624410	8.290887	9.481049	8.046561

CAGE1	14.865080	16.088816	15.933725	15.837350	16.541277	15.463984	16.014400	15.168793	13.904754	15.298176	14.705283	15.605713	15.490401	16.238386	15.614975	15.566607	15.032714	15.444784	15.074047	14.560951	15.789877	16.025583	15.476405	15.553263	14.673241	14.884408	14.637498	13.541051	15.528894	16.243477	15.093809	16.685246	16.334866	14.527514	15.928804	15.940843	16.259687	16.065653	15.256065	15.413146	15.889470	15.658565	15.232087	15.735297	15.452790	15.163378	15.403893	15.268645	15.800609	15.644970	15.573696	15.814383	14.431995	15.963250	16.064222	15.230680	14.307986	16.177217	14.847322	15.739503	15.746888	15.772150	15.325830	15.227905	15.395261	14.817850	15.654597	15.758639	13.636433	15.710626	14.773706	16.189003	15.408414	16.546406	16.285236	15.931238	14.769473	15.219191	14.506817	16.057790	15.425683	15.560443	16.090084	16.773335	15.908209	15.932498	16.184460	14.868226	16.251585	15.991977	15.701693	15.578257	15.738421	15.028163	15.451210	16.357477	16.381342	15.409272	15.983265	11.762794	15.350177	15.210334	15.964494	15.636638	15.724946	15.467330	15.003643	15.509129	16.124210	15.529882	16.131397	15.011645	15.553263	15.250206	13.971275	14.823896	15.344243	15.571867	14.042006	16.377052	15.417001	15.267864	16.059162	15.044721	16.040249	15.659589	15.596409	15.035175	15.655519	16.389378	15.788853	15.644037	14.993419	16.010487	15.321822	15.983265	15.602028	15.425683	15.714733	16.157751	15.366350	15.474785	15.550613	15.872260	15.773226	15.497254	16.163437	15.040623	14.871778	14.866612	14.975358	15.573696	16.245035	15.300977	15.636638	15.382940	15.343482	15.526235	15.818695	15.314555	15.520300	15.449581	15.344243	15.528080	15.529882	14.484052	15.042367	16.154543	14.865080	15.383684	15.163378	15.768020	15.966981	15.801695	15.835010	16.469988	15.516865	15.958303	15.463984	15.661481	15.660615	15.691311	15.841896	15.325830	15.772150	16.015596	15.283711	16.624262	15.400849	15.959440	16.036135	14.857987	15.631882	15.523618	15.628044	16.148537	15.955729	16.102827	15.248027	15.605713	15.901055	15.651828	15.158834	16.481123	15.854635	15.804881	16.385298	15.459167	15.339008	15.621648	15.973135	15.119035	15.908209	15.566607	14.878681	14.982833	14.626808	15.045993	16.026974	15.375547	16.086016	15.537762	15.264422	15.463984	14.988450	15.406952	16.206017	15.481254	14.638867	15.762943	15.215025	15.711627	15.448092	16.124210	15.581893	15.569300	15.872260	16.140018

SSR1	14.865080	16.088816	15.933725	15.837350	16.541277	15.463984	16.014400	15.168793	13.904754	15.298176	14.705283	15.605713	15.490401	16.238386	15.614975	15.566607	15.032714	15.444784	15.074047	14.560951	15.789877	16.025583	15.476405	15.553263	14.673241	14.884408	14.637498	13.541051	15.528894	16.243477	15.093809	16.685246	16.334866	14.527514	15.928804	15.940843	16.259687	16.065653	15.256065	15.413146	15.889470	15.658565	15.232087	15.735297	15.452790	15.163378	15.403893	15.268645	15.800609	15.644970	15.573696	15.814383	14.431995	15.963250	16.064222	15.230680	14.307986	16.177217	14.847322	15.739503	15.746888	15.772150	15.325830	15.227905	15.395261	14.817850	15.654597	15.758639	13.636433	15.710626	14.773706	16.189003	15.408414	16.546406	16.285236	15.931238	14.769473	15.219191	14.506817	16.057790	15.425683	15.560443	16.090084	16.773335	15.908209	15.932498	16.184460	14.868226	16.251585	15.991977	15.701693	15.578257	15.738421	15.028163	15.451210	16.357477	16.381342	15.409272	15.983265	11.762794	15.350177	15.210334	15.964494	15.636638	15.724946	15.467330	15.003643	15.509129	16.124210	15.529882	16.131397	15.011645	15.553263	15.250206	13.971275	14.823896	15.344243	15.571867	14.042006	16.377052	15.417001	15.267864	16.059162	15.044721	16.040249	15.659589	15.596409	15.035175	15.655519	16.389378	15.788853	15.644037	14.993419	16.010487	15.321822	15.983265	15.602028	15.425683	15.714733	16.157751	15.366350	15.474785	15.550613	15.872260	15.773226	15.497254	16.163437	15.040623	14.871778	14.866612	14.975358	15.573696	16.245035	15.300977	15.636638	15.382940	15.343482	15.526235	15.818695	15.314555	15.520300	15.449581	15.344243	15.528080	15.529882	14.484052	15.042367	16.154543	14.865080	15.383684	15.163378	15.768020	15.966981	15.801695	15.835010	16.469988	15.516865	15.958303	15.463984	15.661481	15.660615	15.691311	15.841896	15.325830	15.772150	16.015596	15.283711	16.624262	15.400849	15.959440	16.036135	14.857987	15.631882	15.523618	15.628044	16.148537	15.955729	16.102827	15.248027	15.605713	15.901055	15.651828	15.158834	16.481123	15.854635	15.804881	16.385298	15.459167	15.339008	15.621648	15.973135	15.119035	15.908209	15.566607	14.878681	14.982833	14.626808	15.045993	16.026974	15.375547	16.086016	15.537762	15.264422	15.463984	14.988450	15.406952	16.206017	15.481254	14.638867	15.762943	15.215025	15.711627	15.448092	16.124210	15.581893	15.569300	15.872260	16.140018

PARD6G	6.792176	8.105589	8.656538	8.309468	8.514437	8.794153	8.255721	8.209622	8.152212	8.183583	7.645097	7.830357	7.906479	7.783157	7.428055	8.404680	7.386840	7.861363	8.337740	8.624652	8.066831	8.419689	7.964950	7.893570	7.873058	7.596263	7.199772	7.648612	8.724336	8.635194	8.193679	6.896050	7.182036	7.406010	7.130033	7.177471	7.341605	7.026662	7.838627	7.160590	8.645720	7.345981	7.422754	7.289877	7.643684	6.951703	8.160532	7.488806	7.062687	7.933559	7.095312	7.263271	8.006102	8.213942	7.462608	9.159233	7.414000	8.511237	7.500092	7.622248	7.734887	8.163962	7.533908	7.895952	7.397708	7.882612	8.548297	8.184337	8.204629	7.873982	8.344769	8.439347	8.402370	8.202269	7.878989	7.632540	8.023465	7.728256	7.788507	7.648283	7.736955	7.644929	7.735196	8.149015	7.792804	7.554088	7.821662	7.776496	7.796780	7.708553	7.587332	7.235310	7.873205	7.936532	7.270940	7.574494	6.968204	7.299081	7.639389	7.922567	7.429167	7.574330	7.943235	8.247398	7.758398	8.026527	7.545191	8.175070	8.632606	8.157042	7.543733	7.691024	7.690392	8.079308	8.019077	9.170158	7.341765	7.722369	8.097820	6.672354	8.427236	8.070314	7.977373	8.942629	9.007324	8.684477	7.503538	7.410915	8.479979	7.917227	7.480582	7.940255	7.932184	7.242261	7.592260	7.003210	8.027463	8.522603	8.477734	8.299261	9.145633	7.728876	8.348987	7.974303	7.643210	7.456502	8.604528	8.519918	8.942008	7.026811	6.560227	7.190316	8.947669	7.164101	7.224035	7.025864	8.202413	7.958836	6.953594	9.018670	7.110988	6.557284	7.014228	7.944941	6.805534	8.917548	9.576986	7.694590	7.771110	7.240719	6.492653	8.157641	7.888733	7.023420	9.006123	8.546912	8.930788	7.089929	7.664047	7.497555	7.873506	8.067722	7.487887	7.255549	7.464195	8.841178	8.492264	8.006998	7.068891	8.418259	8.684928	8.954868	8.284855	7.099087	7.827616	8.052786	8.031021	8.671249	7.340356	8.181140	7.690876	8.157342	7.175395	7.705159	7.455252	8.548453	9.579000	7.314004	7.002445	7.954949	7.250091	7.849468	6.820232	7.808333	7.636629	7.787467	6.999064	7.473850	7.655962	6.978010	8.353066	7.216228	7.918883	6.549699	7.617231	7.167160	7.827449	8.259246	8.239474	7.119181	7.126261	8.887600	8.585918	8.337120	8.224937	7.982540	8.246938	8.052014

BAP1	9.577775	9.489508	9.957526	10.042521	10.147784	9.978744	10.275333	9.767982	9.573371	10.051403	10.250874	10.412690	10.069314	10.540036	9.492065	10.081787	9.868222	9.990112	10.811801	10.588511	9.770155	10.785583	10.160447	10.139408	9.695713	10.137297	9.897656	9.035428	9.383323	9.733932	9.818886	10.263365	10.181943	10.040378	9.958459	9.866387	10.009096	9.993251	10.529215	10.512279	9.875764	10.267338	9.645339	9.718772	9.856818	9.681866	9.392669	9.292527	9.680298	9.785658	9.535836	9.937829	10.008945	10.418435	10.220177	10.014151	10.304626	10.157733	9.973551	10.261311	10.417196	9.840456	10.159534	10.564878	10.117147	10.084209	9.781629	9.425721	10.085395	9.853221	9.796260	9.610727	9.689001	9.593381	9.775074	9.550248	9.955223	9.701109	9.571980	10.117613	9.227888	9.562541	9.815273	10.096406	10.242296	10.084818	10.272270	10.091421	9.976192	9.710556	9.665912	10.021710	9.496268	9.888002	9.565422	10.270279	9.663731	10.325523	10.234170	9.709371	9.750825	10.367456	10.249099	10.144161	10.772249	9.586378	10.298660	10.645194	10.820288	9.555843	9.941815	10.453877	10.797595	9.167255	10.063627	6.943443	9.819042	10.092323	9.472923	9.468250	9.920909	9.428985	10.023382	9.877686	9.932341	9.737762	10.228505	10.283531	11.974500	10.675526	10.184227	10.691661	9.632756	10.214105	9.003802	10.823092	10.108388	9.932777	10.296948	10.261311	10.816421	11.156096	10.121706	10.403488	10.340199	9.574876	9.549938	8.440578	9.996310	9.381628	9.948435	11.213347	10.811086	9.677215	10.505710	9.800263	10.908098	10.438251	11.252362	9.017015	10.890139	10.266446	8.594879	10.401542	10.261165	9.266894	8.835394	10.480071	10.244125	10.188617	10.205978	10.885843	11.569895	10.373227	9.632128	10.031790	10.287574	10.287878	10.083270	10.259465	10.229100	11.612573	10.581882	9.759499	9.430938	10.278971	9.839560	10.869032	10.015244	9.703107	10.399900	8.532273	10.294615	9.557862	9.741203	9.598034	10.115756	10.457869	9.950260	10.003957	10.559954	9.968648	10.450323	10.316793	10.201561	9.905601	9.752532	10.333610	10.160611	9.880394	10.232190	9.961668	10.622632	10.980998	10.352202	10.290059	9.328916	10.584914	10.875904	10.573204	9.727598	9.572872	9.867109	11.107632	9.419599	10.379694	10.543883	10.733261	8.663457	10.131679	10.228365	8.922407	8.552881	10.024960	9.634319	11.203158	10.640470	11.216410

BCAS3	12.030634	12.127514	12.085752	12.186508	12.045726	11.655606	12.177029	12.007161	11.394579	11.948032	12.262785	12.101029	12.396312	12.651910	12.632134	11.851418	12.142518	12.196161	12.928312	12.228152	12.870929	13.139689	13.151653	12.190447	12.368451	12.059052	11.899125	12.240224	11.681278	11.725122	12.035653	12.461907	12.581982	12.406200	12.327798	11.920823	12.278662	12.301868	13.036157	12.621510	11.841449	13.292351	12.248989	12.438343	12.406816	12.389516	11.909709	12.682475	12.224619	12.537757	12.489908	12.554292	12.671548	12.817345	12.475368	11.860241	12.210507	13.108876	13.375462	12.974569	13.531718	11.488318	13.172095	13.348067	13.111379	13.069852	12.926298	13.044091	12.772756	13.160892	12.472957	12.193053	11.881320	12.340875	12.510794	12.768875	12.393464	12.633304	12.516134	12.607315	12.250724	12.439820	12.246874	12.490240	12.252154	12.428658	12.760647	12.731336	13.413997	13.113355	13.024023	13.434297	12.886514	12.137705	12.451069	12.025251	12.125254	12.126428	12.615979	12.150924	12.147660	12.183450	11.836176	12.561046	12.732358	11.941813	12.243629	13.120033	12.682302	11.682920	13.305150	12.769602	12.360207	11.288746	12.234041	10.729132	12.746742	11.976069	11.673716	12.318871	10.948982	12.063646	12.224317	11.562614	12.104382	11.455967	11.778520	12.240396	12.494832	11.210844	13.015726	12.674493	12.708589	11.733316	11.939528	12.669662	13.196931	13.162064	11.922034	13.557270	13.493480	13.421932	12.462229	13.137935	12.736450	11.348194	12.244688	12.040452	12.089045	12.233736	11.960220	12.843348	11.947588	12.286447	12.883111	12.221092	11.543325	12.785412	13.105424	12.139103	13.213195	13.464002	12.196906	12.531515	12.369546	10.772100	13.295984	12.156344	11.973443	11.824884	12.548071	11.703111	11.797346	11.507746	12.798496	13.307696	12.933477	12.756522	12.680421	12.077128	12.745728	13.446068	12.895455	13.242782	12.737801	12.534620	12.709993	12.333192	13.053301	12.340685	12.504387	10.964294	12.373685	12.706881	12.233142	11.928470	11.882981	12.707907	12.553295	11.925662	11.794841	12.117118	12.078970	11.854889	12.554619	12.253114	12.539646	12.971153	12.275032	12.781558	13.045554	13.109074	11.955287	11.761931	14.097093	12.468601	11.952744	11.981504	12.917665	11.802080	12.494508	12.768610	12.141761	13.325998	11.294819	11.547236	12.393629	13.417523	11.127669	13.287052	12.687955	12.043199	11.528321	11.807531	11.620849	13.228263	12.151563	12.017489

AC087721.2	13.580621	13.571674	14.049746	14.106283	14.152378	13.893395	13.951850	13.701861	13.772698	14.005012	14.099512	13.821463	14.185875	13.953485	13.311915	13.711946	13.869352	13.744601	13.048590	13.932970	13.459378	13.050819	12.851230	13.869642	13.410994	14.238833	13.940443	13.406964	13.677133	13.634745	12.905897	13.933523	13.693359	14.048218	14.384169	13.869088	14.384169	13.995849	13.767473	13.862844	13.866741	13.727608	13.851451	14.260288	14.477449	14.168090	13.650975	12.738673	13.653439	14.271565	13.819912	14.407674	14.544632	13.995849	13.477273	14.010352	14.411336	14.172567	14.464001	13.834843	13.653197	13.692412	12.809121	13.270823	13.185843	13.794844	13.389060	13.544622	14.063237	12.885092	14.118279	14.330345	13.869642	14.328965	14.174543	14.146475	14.371589	14.193699	13.937151	14.226329	13.229868	14.189262	14.292726	14.091572	14.543837	14.372657	14.536366	14.620267	13.199903	13.876596	13.580155	13.354977	13.330272	14.102234	13.852424	14.145530	13.852712	14.212093	14.346364	14.101917	13.832307	13.709428	14.009020	14.243388	12.495001	14.103780	13.686701	13.314147	12.969511	13.782568	12.674576	14.192430	13.761409	13.693813	13.411645	14.086854	13.329024	14.142398	13.766016	13.402704	13.229238	12.826659	12.840899	13.314543	13.371030	13.150661	14.058874	14.241406	13.125419	13.602656	13.563376	13.721765	12.675008	12.198594	14.330677	13.556609	13.548009	12.173896	13.742334	12.051938	12.705875	12.408357	13.202193	12.634450	13.134908	13.060802	13.703898	13.674334	13.408793	13.055794	13.093911	12.579166	13.104248	13.273969	14.405515	13.779166	13.541684	12.901113	13.462926	13.289639	12.941369	14.233539	14.192430	13.713659	13.459822	13.183505	12.437743	14.042006	13.987492	13.661499	13.384142	13.239764	14.060320	13.616838	13.469650	13.927324	13.144764	12.607714	14.192726	12.907332	13.113147	12.838923	12.629849	13.174008	13.307696	13.197314	12.282721	12.390623	12.237565	13.677795	13.439894	13.783328	13.940978	13.862844	13.535095	13.720180	13.596345	14.095514	13.941517	12.903734	12.850884	12.946630	13.827679	13.132988	13.156161	13.667672	13.487724	12.579663	12.742681	13.033913	12.396485	13.055429	12.930094	14.043175	13.315612	13.862600	14.039072	13.134908	13.648695	13.478168	14.389287	13.640816	13.360372	11.978826	13.285244	13.355364	13.450541	13.081212	14.006448	12.620818	13.570340	13.823712	13.553691	14.120707	13.237061	13.610599	13.386677	12.116671

FGF14	9.922132	10.039741	10.212581	10.276070	10.636673	10.128997	10.034536	10.707633	11.899574	10.530395	10.172148	10.271475	10.771504	10.133149	10.665912	10.899523	11.120569	10.872332	10.934442	10.733727	10.617450	11.027621	12.250724	10.969060	11.532142	10.826511	11.916216	10.960176	11.625463	10.233845	11.422554	9.647967	9.736242	10.363720	10.256188	10.227905	10.062437	10.309103	10.637424	11.059966	10.939769	10.678516	10.549938	10.698200	10.507987	10.260405	10.878106	10.383969	10.386413	10.311421	9.659506	10.196916	9.641913	9.653882	9.964264	10.551138	9.639954	10.387620	10.586129	10.394473	10.074846	11.359874	10.263444	11.015587	9.994941	9.903491	10.134533	10.437038	9.848968	10.336453	10.099670	10.288034	10.830760	10.030084	10.479650	10.240898	10.115300	9.993095	10.144003	10.058476	10.017413	10.105230	9.480408	10.078545	9.811011	10.240459	9.824324	10.420556	10.386712	10.088219	10.202155	10.411781	9.966801	10.067514	9.611353	10.290960	10.560714	11.741007	10.212131	11.158915	10.835284	10.241662	10.787634	10.593179	10.378811	10.980402	11.540408	10.974225	12.173415	10.762650	10.980998	10.942125	10.709053	11.222672	11.323062	10.808261	10.098577	10.711830	11.644595	10.486558	9.917481	10.323671	10.572001	11.149440	9.925180	9.682497	11.202068	10.655784	9.834586	9.870758	11.440486	10.704834	9.846759	10.166750	10.671196	10.388978	12.558326	11.257536	9.880394	10.317106	10.495855	10.294779	10.218179	9.910027	10.287723	10.375288	11.043290	10.833010	10.204611	11.086028	10.821454	10.217279	9.926337	9.809978	9.841825	9.817117	10.522517	10.342641	10.453273	10.458181	10.672285	9.622130	10.113907	10.020621	9.886243	11.073555	10.254211	9.758430	9.590971	9.981215	10.093965	9.001352	9.439413	9.680465	10.114982	9.071736	9.915301	10.122012	9.538754	9.974365	10.536345	9.841361	9.675852	10.162292	10.039288	10.084818	10.444422	10.203557	10.596486	9.853064	10.288842	10.927267	10.322295	10.319358	10.252352	10.130623	10.135138	10.100927	9.466897	9.596649	9.697396	9.998417	10.254503	9.632445	10.007656	9.824482	11.303833	10.492371	10.421303	9.832913	10.154056	10.184227	10.003213	10.474815	10.259615	10.870510	11.688994	10.533349	10.064990	10.425753	10.366528	10.778852	10.402432	10.079015	10.695882	12.185427	10.425147	10.396736	10.382403	10.370179	10.587309	10.453877	10.756778	10.138352	9.830915	10.537076	10.852247	9.984270

BCR	12.543561	12.328136	12.493360	11.865618	12.541971	12.206509	12.379588	11.986513	12.698176	12.298219	12.109069	12.686212	12.584979	12.640046	12.219074	12.132348	11.937329	12.447571	12.898097	11.802821	12.350213	12.949293	12.856932	12.107582	13.077603	12.929922	12.313009	12.689122	12.087405	11.841136	12.306523	12.472142	12.345318	12.567262	12.508996	12.133274	12.513691	12.615979	13.185434	13.050251	12.136181	13.106997	12.700378	11.969197	12.813342	12.620500	11.982413	13.222566	11.618169	12.159708	12.226768	12.544369	11.870252	12.396775	12.357829	12.554462	12.669327	12.993460	13.466850	12.488461	13.092754	12.462402	12.127673	13.113147	12.921703	12.947352	12.996930	11.774311	12.696432	13.048590	12.095947	11.929527	12.248657	12.518270	12.254526	11.895762	12.533788	12.255831	11.780148	12.717655	12.264825	12.319852	12.910006	12.313639	12.586624	12.564459	12.540801	12.860065	13.336430	12.189173	12.714861	13.385597	12.773792	12.208987	12.352576	12.086204	11.906809	12.691342	12.521399	11.718698	12.685364	12.231903	12.288154	12.665284	13.391215	11.896350	12.586128	13.188396	12.776909	11.898226	12.830643	12.889667	12.958652	11.825046	11.687372	11.532441	13.475103	12.546528	11.856727	12.230643	12.932065	11.665969	12.686920	12.420436	11.983809	13.216213	12.658626	13.793821	12.329087	12.814928	12.546832	14.195248	12.487141	13.212236	13.117173	12.045007	12.811770	13.435155	12.438849	12.205727	13.157107	12.851777	12.769413	13.614956	13.114466	12.583137	13.187612	12.912347	11.813142	12.513519	12.458956	13.911868	13.005589	12.314304	12.716597	12.874491	12.392501	13.992989	13.297565	12.950361	12.541971	12.994008	12.987390	13.259837	12.313009	12.814055	13.458052	12.487643	13.547770	12.691524	12.803011	12.039543	13.356586	13.162836	12.253731	12.161570	13.196931	12.580172	12.740770	12.644874	13.481922	12.506730	13.625684	12.827524	12.758584	13.627288	12.282409	12.661720	13.298159	12.564301	12.411574	13.273354	12.590461	12.686750	11.327051	12.337599	13.363482	12.541791	12.557817	13.154208	11.787155	12.841587	11.683795	12.541791	13.020460	12.427314	13.247903	12.646251	11.839322	12.376916	13.727369	11.529459	13.023099	12.378353	11.213047	12.405594	12.909129	12.372119	11.580422	12.734044	12.284727	12.831872	12.228317	12.105900	12.687444	12.766549	13.543058	12.606892	12.042272	13.204762	12.596096	11.995822	12.207603	11.079978	12.868341	13.098061	11.811570	12.212707

LRRC8C	9.720502	9.781923	9.674122	9.322428	8.388218	8.423466	9.528476	7.362150	7.428825	8.707526	8.775349	8.692143	8.930477	8.260812	9.460905	8.788742	8.344925	9.611938	7.043199	8.905230	7.821338	9.968492	9.267666	9.010681	9.672249	9.166801	10.662371	8.723135	9.497175	10.422396	8.101742	10.120339	10.189954	10.505574	10.003957	10.000255	9.635632	9.529428	9.856190	10.163518	8.825090	9.708602	11.047378	9.805636	11.046205	10.273491	8.386178	10.315198	9.761195	10.110042	9.961344	10.045559	10.456786	9.670216	10.323968	8.322469	10.340353	9.413335	10.130189	10.643415	9.677525	9.740417	10.748747	9.106123	10.184366	10.326281	8.592374	9.928779	10.051718	9.223120	9.513068	9.513366	8.742859	9.676141	9.437498	10.432310	9.408477	10.073768	9.437744	10.106586	10.142442	10.403059	10.163835	9.927990	9.973102	9.710473	10.144458	10.110653	9.522722	9.178624	9.544844	8.988823	9.545005	9.946377	9.898827	9.572138	10.151269	9.567325	8.658511	8.699378	8.482488	9.691000	9.542207	9.390797	8.784155	9.246898	7.815587	8.995967	8.079308	8.135264	8.537994	7.970847	8.908475	7.544378	9.165409	7.244534	9.676599	9.040866	8.711767	9.807001	11.624581	10.498125	10.370179	9.888002	9.189635	11.353017	8.739777	11.615241	9.627540	11.879073	9.786976	11.574642	10.897349	11.936536	9.765569	11.716588	10.473309	8.686278	10.899523	10.194030	10.886269	9.664186	12.388237	11.769458	12.001304	11.251225	9.291287	8.820230	11.190322	10.003818	11.143240	10.851641	10.902031	10.469815	11.126639	10.516981	10.300966	12.166192	11.657322	10.451803	9.937905	10.502569	10.806779	10.856607	10.690215	9.980904	10.706734	10.169039	11.624432	11.137722	10.407694	10.814470	12.895284	11.220589	10.452381	10.025121	10.453422	11.118854	11.613018	11.182438	11.023071	10.680054	11.217158	10.617300	10.682588	10.553379	10.395201	11.158476	10.686158	9.676294	10.642239	9.996917	10.764606	10.445678	10.989582	10.936398	11.066875	10.787493	11.172088	11.447213	11.076058	10.856734	11.167246	11.649898	10.576772	11.475428	10.432626	10.141187	10.614085	9.993251	11.077209	9.909249	11.738510	10.736706	12.110693	10.838706	9.541126	10.594267	10.041471	11.760858	10.623370	10.552793	11.207310	9.734702	9.544579	10.879746	9.271368	8.968219	10.960626	9.893168	10.977016	9.154950	7.945557	8.639584	10.372426	10.851957	9.300643	11.005550

AC093423.3	9.720502	9.781923	9.674122	9.322428	8.388218	8.423466	9.528476	7.362150	7.428825	8.707526	8.775349	8.692143	8.930477	8.260812	9.460905	8.788742	8.344925	9.611938	7.043199	8.905230	7.821338	9.968492	9.267666	9.010681	9.672249	9.166801	10.662371	8.723135	9.497175	10.422396	8.101742	10.120339	10.189954	10.505574	10.003957	10.000255	9.635632	9.529428	9.856190	10.163518	8.825090	9.708602	11.047378	9.805636	11.046205	10.273491	8.386178	10.315198	9.761195	10.110042	9.961344	10.045559	10.456786	9.670216	10.323968	8.322469	10.340353	9.413335	10.130189	10.643415	9.677525	9.740417	10.748747	9.106123	10.184366	10.326281	8.592374	9.928779	10.051718	9.223120	9.513068	9.513366	8.742859	9.676141	9.437498	10.432310	9.408477	10.073768	9.437744	10.106586	10.142442	10.403059	10.163835	9.927990	9.973102	9.710473	10.144458	10.110653	9.522722	9.178624	9.544844	8.988823	9.545005	9.946377	9.898827	9.572138	10.151269	9.567325	8.658511	8.699378	8.482488	9.691000	9.542207	9.390797	8.784155	9.246898	7.815587	8.995967	8.079308	8.135264	8.537994	7.970847	8.908475	7.544378	9.165409	7.244534	9.676599	9.040866	8.711767	9.807001	11.624581	10.498125	10.370179	9.888002	9.189635	11.353017	8.739777	11.615241	9.627540	11.879073	9.786976	11.574642	10.897349	11.936536	9.765569	11.716588	10.473309	8.686278	10.899523	10.194030	10.886269	9.664186	12.388237	11.769458	12.001304	11.251225	9.291287	8.820230	11.190322	10.003818	11.143240	10.851641	10.902031	10.469815	11.126639	10.516981	10.300966	12.166192	11.657322	10.451803	9.937905	10.502569	10.806779	10.856607	10.690215	9.980904	10.706734	10.169039	11.624432	11.137722	10.407694	10.814470	12.895284	11.220589	10.452381	10.025121	10.453422	11.118854	11.613018	11.182438	11.023071	10.680054	11.217158	10.617300	10.682588	10.553379	10.395201	11.158476	10.686158	9.676294	10.642239	9.996917	10.764606	10.445678	10.989582	10.936398	11.066875	10.787493	11.172088	11.447213	11.076058	10.856734	11.167246	11.649898	10.576772	11.475428	10.432626	10.141187	10.614085	9.993251	11.077209	9.909249	11.738510	10.736706	12.110693	10.838706	9.541126	10.594267	10.041471	11.760858	10.623370	10.552793	11.207310	9.734702	9.544579	10.879746	9.271368	8.968219	10.960626	9.893168	10.977016	9.154950	7.945557	8.639584	10.372426	10.851957	9.300643	11.005550

GRAP	8.593171	9.533072	9.010532	9.111762	8.912500	9.441595	9.095089	9.780402	9.299705	8.962062	8.957149	8.924735	8.504929	8.711023	8.768990	9.490307	9.520524	8.743289	9.995433	9.081035	9.384220	10.802840	9.857253	9.963485	9.392820	9.707507	10.506316	9.667331	9.330587	9.824636	9.495493	8.736112	9.972329	9.701109	9.449496	9.240739	8.924312	9.467673	9.481495	9.712641	9.196095	9.767842	10.001641	9.436336	9.596161	9.387152	9.745376	9.118977	8.926611	9.288791	9.339197	9.213906	9.395906	9.468725	8.782434	9.233328	9.110791	9.711248	8.919658	9.279384	9.573371	9.104114	9.957211	9.642543	9.879153	9.354397	9.662962	9.375753	9.625356	10.037644	9.609822	9.219434	9.289032	9.248755	9.289113	10.010322	9.349567	9.557714	9.482261	9.164645	9.234252	9.033700	9.177266	8.734401	8.788881	8.762269	9.226054	8.662549	9.541895	9.641025	10.102338	9.280598	9.287715	9.126741	9.409114	9.414708	8.989269	8.367762	8.372228	8.822202	8.065802	9.281986	8.900076	8.748785	9.527862	8.902638	10.376347	9.568254	9.723772	9.571818	9.784739	9.380048	9.358192	9.819042	9.126433	7.409741	9.123809	8.854885	9.193891	9.075141	9.739008	9.256514	9.664186	9.329705	9.101649	8.745884	8.929089	8.733026	8.998406	9.130781	8.685533	9.141505	8.806738	8.791374	8.962835	9.254659	8.487552	10.056061	8.483714	9.048076	9.767533	10.231014	8.983047	9.219741	10.030841	8.929089	9.168008	9.237796	9.328459	9.952136	8.600815	9.204729	8.725564	8.953642	8.515903	9.430029	9.587812	9.709048	9.964578	9.221273	9.906511	9.107805	9.534899	9.083496	8.845571	9.039461	8.884660	8.733172	9.314893	9.357553	9.129389	9.136672	9.523489	9.616513	8.994888	9.118187	9.406024	9.056876	9.701277	9.138437	9.433874	9.292820	9.551619	9.664693	9.995882	9.814319	9.907128	9.552380	9.896130	9.392213	9.164789	9.433441	9.131804	9.763871	9.681392	9.250544	9.446852	9.141334	9.261194	9.000251	9.136379	9.404364	9.342816	9.423772	8.718581	9.332910	8.549674	9.776123	9.911272	10.550537	9.009654	9.329853	10.099349	9.588576	9.146408	8.364085	8.227815	8.635528	8.564796	9.334439	8.796696	9.166121	9.249501	8.814849	9.171885	9.171408	8.998256	9.214807	9.729654	9.345751	8.724030	8.686570	9.950580	8.523703	8.284696	9.553140	8.166444	9.024766

GRAPL	8.593171	9.533072	9.010532	9.111762	8.912500	9.441595	9.095089	9.780402	9.299705	8.962062	8.957149	8.924735	8.504929	8.711023	8.768990	9.490307	9.520524	8.743289	9.995433	9.081035	9.384220	10.802840	9.857253	9.963485	9.392820	9.707507	10.506316	9.667331	9.330587	9.824636	9.495493	8.736112	9.972329	9.701109	9.449496	9.240739	8.924312	9.467673	9.481495	9.712641	9.196095	9.767842	10.001641	9.436336	9.596161	9.387152	9.745376	9.118977	8.926611	9.288791	9.339197	9.213906	9.395906	9.468725	8.782434	9.233328	9.110791	9.711248	8.919658	9.279384	9.573371	9.104114	9.957211	9.642543	9.879153	9.354397	9.662962	9.375753	9.625356	10.037644	9.609822	9.219434	9.289032	9.248755	9.289113	10.010322	9.349567	9.557714	9.482261	9.164645	9.234252	9.033700	9.177266	8.734401	8.788881	8.762269	9.226054	8.662549	9.541895	9.641025	10.102338	9.280598	9.287715	9.126741	9.409114	9.414708	8.989269	8.367762	8.372228	8.822202	8.065802	9.281986	8.900076	8.748785	9.527862	8.902638	10.376347	9.568254	9.723772	9.571818	9.784739	9.380048	9.358192	9.819042	9.126433	7.409741	9.123809	8.854885	9.193891	9.075141	9.739008	9.256514	9.664186	9.329705	9.101649	8.745884	8.929089	8.733026	8.998406	9.130781	8.685533	9.141505	8.806738	8.791374	8.962835	9.254659	8.487552	10.056061	8.483714	9.048076	9.767533	10.231014	8.983047	9.219741	10.030841	8.929089	9.168008	9.237796	9.328459	9.952136	8.600815	9.204729	8.725564	8.953642	8.515903	9.430029	9.587812	9.709048	9.964578	9.221273	9.906511	9.107805	9.534899	9.083496	8.845571	9.039461	8.884660	8.733172	9.314893	9.357553	9.129389	9.136672	9.523489	9.616513	8.994888	9.118187	9.406024	9.056876	9.701277	9.138437	9.433874	9.292820	9.551619	9.664693	9.995882	9.814319	9.907128	9.552380	9.896130	9.392213	9.164789	9.433441	9.131804	9.763871	9.681392	9.250544	9.446852	9.141334	9.261194	9.000251	9.136379	9.404364	9.342816	9.423772	8.718581	9.332910	8.549674	9.776123	9.911272	10.550537	9.009654	9.329853	10.099349	9.588576	9.146408	8.364085	8.227815	8.635528	8.564796	9.334439	8.796696	9.166121	9.249501	8.814849	9.171885	9.171408	8.998256	9.214807	9.729654	9.345751	8.724030	8.686570	9.950580	8.523703	8.284696	9.553140	8.166444	9.024766

TCEANC2	9.176661	10.356463	10.150237	10.072673	10.766127	10.611210	10.306115	11.937174	10.890718	10.788704	10.289132	10.299127	10.029801	10.435072	9.544844	11.069879	10.385173	10.246644	10.571531	10.656244	10.890139	10.527304	10.327943	9.924735	10.547962	10.378509	10.061641	10.959584	10.450613	9.947176	10.820438	10.204454	10.479789	9.544433	10.606089	10.532684	11.062749	10.738688	11.177770	11.207167	10.904224	11.396241	11.508778	10.409686	10.466810	10.909711	10.858937	11.614495	10.233543	11.907737	10.857955	11.033441	10.376501	10.238166	10.339746	10.938612	10.022905	10.995821	10.733421	10.530547	11.013094	10.675830	10.249543	11.431952	10.812246	10.613473	10.598624	10.789568	10.950728	11.174736	10.304910	10.463717	10.951033	10.634437	10.753717	10.557368	10.509324	10.466810	10.185728	10.325965	10.403488	9.692242	10.173663	10.374695	10.547812	10.382728	10.097156	10.902636	10.260093	10.891184	10.751156	10.984649	10.413303	10.531275	9.585599	10.133459	10.031627	10.240898	10.376060	10.601671	10.787056	10.299345	10.091582	10.587164	10.421474	10.498280	11.271173	10.666510	9.591195	10.861307	10.365302	11.226360	10.590679	10.960321	11.451301	10.776676	9.839992	10.155777	11.038010	9.691940	10.011667	9.498564	9.903042	10.396736	10.159076	10.036097	9.282306	10.027337	9.721996	9.853854	9.690384	9.466743	9.632606	9.839396	10.196749	10.090978	9.218559	8.971604	9.659354	9.786097	9.832315	9.412599	9.570720	9.873790	9.548845	9.974049	9.973551	12.046769	9.748169	9.833356	10.898956	9.869000	9.993987	9.904987	9.909568	10.128392	10.186664	10.685697	10.756176	10.525956	10.356625	10.015244	10.203404	9.869932	10.371240	10.443347	10.677921	9.981835	10.545972	10.249835	10.088368	9.725305	10.184062	9.836571	11.530788	9.781629	10.910132	10.670884	10.584051	10.971621	9.822544	10.681815	10.698669	10.593778	10.252352	10.547812	10.095191	10.472068	10.592600	9.716088	9.853684	11.661490	9.909568	9.588502	9.983209	9.777218	9.757177	9.822841	9.804101	9.679709	10.306414	10.380306	9.918845	9.942272	9.818575	10.019135	10.085231	10.693912	10.200229	10.687976	10.731630	9.912825	9.788490	9.433716	9.365664	9.724995	9.168609	10.334168	9.507776	11.321148	9.714536	9.719718	9.744273	10.254503	9.915301	10.632913	10.537786	9.532764	10.472219	9.305277	9.838589	10.251612	11.072137	9.054553	9.839560	10.582604	10.559792	10.891628

SLC25A48	5.409725	6.288089	6.131199	6.798807	7.605952	7.709416	7.717665	5.349077	7.230410	7.411494	8.317902	7.666067	7.865882	7.355953	8.044341	7.372685	6.553821	7.564735	7.481663	7.574967	6.167552	6.324566	5.562964	6.610796	7.466956	7.485213	5.860480	6.620661	8.257083	7.274066	7.959450	6.080235	6.299387	6.705732	7.168260	6.487753	6.276841	6.433787	7.099873	6.025527	7.816055	6.325254	6.213743	6.228660	6.165625	5.169895	7.170697	6.588358	7.127683	7.029566	5.389689	6.208471	7.576179	7.241803	6.382496	7.747014	6.610125	6.492034	7.688703	6.240938	6.522172	7.833239	4.683975	6.567229	7.268030	7.301775	6.137868	5.981355	5.807043	5.513333	6.853796	6.619366	8.318352	6.632032	6.846482	6.416995	5.852014	6.051295	5.984867	6.752087	5.757389	7.518492	6.529826	6.315909	6.164262	6.743344	6.672024	5.621097	7.372060	7.423039	6.003613	5.900808	5.657391	6.267597	5.697116	6.786777	6.925341	7.850049	7.362910	7.071494	7.644170	7.010274	6.951067	6.473421	5.620195	7.756185	6.738763	6.768300	6.970435	7.847801	5.691927	7.089448	5.697116	7.687007	7.735493	7.503220	5.066749	7.298474	7.844523	5.862898	5.869213	6.097691	6.380125	7.289259	8.724336	7.499939	6.634691	6.550026	7.298304	7.595034	7.620747	7.662002	6.469202	6.085431	6.339386	6.814751	6.608020	6.242601	6.762969	6.959726	5.744281	6.801722	6.945974	9.439867	5.903831	6.692759	8.352610	7.731957	6.282271	6.097197	5.727373	7.726063	6.283128	6.785647	6.010016	7.170697	5.456085	6.373651	6.314012	7.543116	6.321882	6.716203	5.726450	6.942471	4.820024	7.632540	5.817445	7.528881	6.334497	6.489666	6.123379	6.324053	6.053381	5.869021	6.503995	4.054293	6.972602	6.383495	6.113851	4.085922	7.471118	5.081663	5.364627	5.912042	4.997962	6.859440	6.405727	5.390655	5.052790	6.876549	6.284639	8.411708	6.867559	5.274315	6.395639	7.071013	5.306198	6.974953	7.964169	6.030587	7.709559	8.117928	6.488220	5.591589	6.576349	6.662005	5.946702	6.400484	5.390655	8.569732	5.375957	6.101332	6.007165	6.542552	6.476422	7.509115	7.981127	7.052414	6.892352	6.699341	6.881513	7.478735	5.870621	4.601657	7.603908	6.392873	5.884247	7.804477	7.891543	8.448582	5.844038	7.441933	7.866197	7.711380	6.977855	9.027513	8.005145	9.169378

ATP6V0E2	7.179851	6.603417	6.846321	7.077031	7.214504	7.563944	7.338924	7.424453	7.875198	7.700231	7.556767	7.357154	7.127683	7.428825	6.255895	8.084927	7.172144	6.596333	6.648533	7.555653	6.853335	7.595034	6.699341	7.256941	7.525891	7.466791	7.993460	7.326424	7.958691	8.261975	8.012742	6.190787	6.296264	7.199937	7.130525	6.483410	6.438911	6.397966	7.145355	7.481057	7.570962	7.099873	6.382153	6.611684	6.244951	5.860307	8.099606	6.194799	7.192436	6.474078	5.899713	5.658509	5.969983	5.712490	5.391260	8.216812	5.389874	5.667420	6.166312	5.698593	6.321505	8.196996	6.441696	6.664928	5.975338	5.681788	6.284973	6.152579	6.261843	6.686616	7.044339	7.022815	8.387874	6.938118	6.676256	7.412132	7.188396	6.860578	6.681577	5.462213	6.123195	5.951091	6.067163	5.980467	6.115466	6.059216	5.525913	6.188764	6.350795	6.213566	6.169288	6.381984	6.559257	7.039538	6.881346	7.391321	6.048392	7.320440	7.400837	6.915418	6.513895	6.439561	6.851706	6.480733	6.975438	7.665421	7.856797	7.117559	7.136114	8.448740	6.167031	7.268335	6.995829	8.478049	7.105801	7.661530	7.485700	7.469241	8.504326	7.330879	6.965640	7.022655	7.763738	8.050019	8.029783	7.652204	8.098548	7.672577	8.246658	7.622838	7.963443	7.649509	7.225135	6.927973	8.283450	7.507556	7.067906	8.156082	7.410915	7.102355	7.751051	6.807413	7.827891	7.761283	7.092916	7.500852	7.524066	8.836181	8.321274	8.103403	6.876549	6.535017	6.822304	7.247314	6.760608	7.027807	6.840955	7.528278	7.361058	8.108499	6.766516	6.587705	6.871784	6.436133	6.177655	8.760109	6.955501	7.111460	6.897032	6.615109	6.674294	6.090612	5.863994	5.927772	7.927415	5.714759	6.576684	6.156680	5.761196	6.017427	8.256475	6.509428	6.535162	6.214225	6.370223	7.033904	6.707704	6.803991	6.730471	7.181722	7.270312	8.545522	7.363410	6.746568	7.260754	7.707128	7.270794	7.033904	6.069808	6.261678	6.284143	6.044295	6.329072	5.945313	6.694724	5.901504	6.235988	6.724651	5.993944	7.606249	6.414193	6.561036	7.245475	7.089781	7.351457	6.465215	7.398827	6.627260	6.887904	7.120888	6.961817	7.174638	6.212680	6.526670	8.414146	8.037595	6.545484	7.278613	8.772484	5.934593	7.199296	6.754070	9.050550	8.197159	7.325643	7.086749	7.825370	8.156419

KLRD1	5.636016	4.849289	4.817310	5.140257	3.421359	5.765566	6.764101	3.444367	4.397453	4.388910	4.803466	4.291335	4.460696	3.695698	5.257705	5.669795	3.924386	3.947248	4.194119	4.527383	4.627372	4.395431	3.641503	3.739136	3.189710	3.979980	4.812486	2.908590	5.540532	6.237472	4.085922	4.459364	4.310387	5.181563	5.910266	6.219399	6.080235	3.605404	5.109966	5.165506	3.711741	6.575670	5.446009	5.431807	7.064140	5.375342	3.366512	6.000919	4.898566	6.675083	5.649548	6.946617	5.596256	6.027838	6.253339	4.080720	6.009832	6.588698	3.004431	4.823740	6.740540	5.694332	6.383495	5.400880	5.264677	6.532738	4.475377	4.466094	3.907688	5.875675	6.032910	6.224688	5.033574	5.726450	5.961292	4.926387	4.798741	4.972542	5.311259	5.755174	4.850577	5.975696	5.930066	5.879425	6.209157	5.379258	5.536460	5.011599	5.388935	5.410318	5.394962	5.096222	4.950416	5.761196	5.242838	6.158213	5.910266	4.731011	4.670157	4.510948	4.000622	5.597206	5.341422	5.514823	4.590365	5.257917	4.773254	4.797249	4.819605	3.787788	4.893336	4.103229	5.245814	4.094535	3.839470	4.797882	3.869015	5.044120	3.502150	6.180324	4.641333	4.743173	4.601011	3.188619	5.206824	4.755730	3.343473	3.395942	3.079857	4.513162	3.315690	3.875056	3.530715	3.007147	5.674760	4.011825	3.630688	3.243676	4.546011	3.949435	4.527383	6.457563	3.344003	2.774163	3.100092	3.750622	2.861366	5.392969	5.870061	4.738421	3.956373	4.663894	4.896655	4.327221	4.829761	6.102811	3.205608	3.308743	4.866626	4.327649	4.981730	4.468617	4.603341	5.541080	4.698722	3.168541	2.817234	4.852235	5.903831	5.100098	5.712884	4.544678	5.229116	7.005812	4.434754	6.113325	3.885512	2.873384	4.490216	5.087383	4.232240	3.817172	3.978243	6.422858	5.314846	4.644041	5.273109	4.182761	5.842795	5.204082	6.132434	3.273130	5.811491	5.544106	5.039315	5.003583	4.921690	5.792732	5.953623	5.159455	4.797060	6.560069	6.343011	5.202043	4.147052	5.286009	5.077136	5.494274	5.115151	3.349518	3.890951	4.676909	4.747673	5.250989	5.351089	4.780946	4.567683	4.123626	5.089194	3.466290	3.337623	4.068580	5.305404	4.804715	4.419362	3.083703	4.680289	4.971704	3.661019	3.746283	4.989560	7.016983	4.395431	3.180672	3.861945	4.816890	4.747061	3.295141

VPS53	6.254711	5.953430	6.249294	6.204695	7.342853	7.522372	6.296097	6.735475	8.324774	6.388514	7.063971	6.572093	6.514380	6.339386	7.030340	6.951703	7.047385	7.087505	7.094820	6.854459	6.841294	7.484272	7.082071	6.364215	6.909077	6.997424	7.224803	6.976396	6.585224	6.150337	7.528744	5.860307	6.291686	6.238775	6.016224	5.775473	5.630622	5.935986	6.514869	6.255368	6.604561	6.536469	6.590464	5.362905	7.073059	6.595678	8.051400	4.540468	6.009128	6.271869	6.542050	6.290806	6.573209	4.817096	6.961011	6.616593	6.315909	6.677849	5.888319	6.293693	6.775740	6.732713	6.547258	6.679465	5.941446	6.094274	5.891684	6.930002	5.534285	6.265874	5.613082	6.240584	7.187785	6.001274	5.727904	6.306280	5.616498	6.054797	5.647837	6.386127	6.673180	6.899330	6.001621	6.093031	5.886377	6.207422	6.312471	6.220905	6.164933	5.896007	5.780525	6.337877	6.199995	5.973067	6.485942	6.954382	5.767564	7.872292	6.701426	7.590734	7.564418	6.354258	7.539270	6.919713	6.948600	7.990014	7.466791	6.814751	7.277975	6.904150	7.711088	7.524066	7.540966	6.559257	7.989271	5.899553	6.855890	6.999669	7.189847	6.533725	7.044339	6.067343	6.214759	7.414000	7.322163	6.015023	7.173677	6.955026	6.039824	6.697122	6.427678	6.686295	6.055991	6.950916	6.580113	7.050375	7.050084	6.581133	6.414516	6.709596	7.306346	6.643191	7.345155	5.857438	7.435851	6.951067	7.168420	6.441696	6.211030	7.130688	7.707451	7.421041	6.887409	6.594222	6.306114	6.322380	5.958405	6.608840	6.497516	7.827891	5.934593	6.346891	7.080815	6.994765	6.599750	8.752751	5.561471	5.681611	6.317575	6.945502	6.402270	6.680133	6.259130	6.026440	6.383146	5.695665	6.989049	6.168779	5.729176	6.674449	6.415636	6.671556	6.539864	6.415636	6.178996	6.153116	5.924919	5.662352	6.241930	5.608760	6.231727	6.905252	6.448008	5.704486	6.597629	5.833722	5.946541	6.356807	6.566103	6.942312	6.272853	7.377860	6.952820	7.208630	6.604224	6.808714	7.076870	6.295088	6.717651	6.158049	6.640640	6.667699	6.455878	6.893364	6.832787	6.778314	6.874639	6.912595	7.156070	7.321353	7.558311	6.615435	5.914377	6.389695	7.030045	8.274471	6.877972	7.006419	7.083939	7.311816	6.454774	5.245413	6.340567	5.733911	6.440869	6.376474	7.869975	6.576349

RTKN2	8.247810	10.295699	10.375835	9.947176	11.512020	10.976265	10.677202	10.997150	12.046618	11.623999	10.722360	9.736555	10.475118	11.059091	10.442286	11.043570	9.703268	10.919103	10.705811	11.183899	10.957536	11.034048	8.772914	11.066743	10.764929	10.702740	9.016215	11.133393	9.910192	9.729350	10.981148	10.731478	11.143240	9.411410	9.807767	10.752372	10.990195	10.033753	10.139559	11.285190	10.813415	9.728699	10.481536	10.282147	9.545005	10.660096	10.732799	9.673663	10.211511	10.834667	10.555685	10.325356	10.480801	9.911127	9.295685	10.022143	11.182012	10.762958	9.684838	10.160447	10.936085	10.423140	7.491020	10.863229	9.859459	9.772502	10.533504	10.471030	10.402738	10.024169	9.729812	10.638038	10.965807	10.491721	10.046625	10.061049	11.196236	10.200528	10.446887	10.067057	11.060705	9.987305	10.266446	11.049296	10.706734	10.309258	10.424393	11.092539	10.766574	10.609657	10.041611	11.645924	10.985679	11.275733	10.471635	11.037267	10.480506	11.206137	10.482160	9.811828	11.107474	10.080574	10.722674	10.372744	10.639852	10.463260	11.904904	10.878852	10.999853	11.069879	11.514705	10.673572	10.623839	11.562171	10.716860	9.223120	7.941031	10.515470	10.331758	10.632749	8.725420	11.250930	10.525043	7.227941	10.186968	9.361876	10.875592	11.167837	9.159694	9.072507	9.962553	9.689605	10.341556	6.951549	9.500267	8.912971	9.783941	12.497666	11.272078	9.672854	10.466973	9.640101	10.020041	10.910730	7.850948	9.631239	10.547962	9.230442	10.917038	8.610624	8.865668	9.833953	8.025265	10.474513	10.044168	9.810561	10.902471	7.853595	9.587009	6.827484	9.698474	10.182257	7.856943	9.720502	9.690384	7.725142	11.464477	10.739904	10.070538	8.960680	10.910730	10.271020	12.416425	10.656092	9.352445	10.121247	10.783906	9.479802	11.669696	8.861847	8.733172	7.838143	11.240485	10.020041	9.551167	10.139254	9.474447	8.767446	9.846608	10.207514	9.658740	8.426149	8.980298	10.639141	10.100136	9.628141	9.401532	10.607264	9.628003	11.095577	9.131538	9.404833	10.454947	10.365458	8.951656	10.016654	10.548595	11.121300	9.566857	12.214911	8.848779	9.785196	8.858058	8.738106	8.822507	9.507924	10.929640	7.694590	9.148458	9.607163	10.775641	8.447805	10.510632	9.109564	10.568312	9.916529	13.005224	11.059231	10.638482	11.782817	11.036699	11.587012	10.772249	9.583467	7.421349	11.099157	10.293394	8.996270

DHX8	5.517224	5.931759	6.286581	6.169784	6.547107	6.375674	6.063823	6.306114	6.049071	6.287255	6.103246	5.944776	6.075352	6.199566	6.078138	6.208471	6.455432	6.206594	6.826732	6.094796	6.547889	7.321508	5.990248	6.368863	6.799452	6.234645	6.410549	6.485942	5.781452	6.173864	5.640329	5.835193	6.243786	6.204695	6.180653	6.161322	6.485635	6.424487	6.534213	6.656310	5.955394	6.174889	6.410859	6.341049	6.012629	5.599318	5.769929	6.273200	6.315207	6.245620	6.262169	6.196904	6.464876	6.508622	6.837708	6.228480	6.346571	6.446864	6.249967	6.691111	6.304098	6.321882	6.667699	6.535312	6.620183	6.342844	6.735790	6.477105	6.770091	6.836278	6.224501	6.444202	6.315398	6.376310	6.240087	6.411526	6.595347	6.376795	6.140293	6.308393	6.327724	6.122172	6.328572	6.209504	6.447183	5.505053	6.035818	6.812241	6.454774	6.431739	6.198588	6.161144	6.517234	6.630936	6.271869	6.336359	6.392707	6.212161	6.579465	6.627260	6.775273	6.118017	6.041416	6.401610	6.851553	6.413860	6.382153	6.642091	6.861702	6.313328	7.138598	6.656310	6.584714	6.363037	6.690947	7.783772	6.695389	5.972718	6.025164	5.814591	6.607242	6.618235	6.510260	7.348653	6.805681	6.429015	6.709432	6.298401	6.157021	6.789577	6.285685	6.655000	6.714097	6.867727	6.682255	6.517397	6.807739	6.932252	6.227294	7.278770	8.015237	7.281157	7.134838	7.044339	6.703390	6.914949	7.087505	7.286213	6.959726	6.460187	6.388007	6.938731	6.674778	6.503174	6.620183	6.162053	6.735790	6.745941	6.837856	6.777010	6.329228	6.627907	7.476764	7.017298	7.079746	6.924019	6.607883	6.504316	6.657929	6.566263	6.317250	6.595180	6.479775	6.238775	6.539247	6.193889	6.533884	6.607057	6.295437	6.566103	6.297553	7.101718	7.348015	6.711646	6.741635	7.158083	6.410196	7.132134	7.166404	6.728379	6.409505	6.795886	6.423013	6.128168	6.411526	6.396973	6.746725	6.146352	6.256740	6.499228	6.926825	6.643535	6.221767	6.045883	6.402270	6.162716	6.466524	6.308238	6.397051	6.351948	6.413534	6.628063	6.329072	6.392523	6.249790	7.011235	6.727555	7.414000	6.779578	6.576349	6.684249	6.522655	6.588854	7.201777	6.540026	7.585809	6.941182	7.150257	6.756526	7.229677	7.154664	7.786851	6.729309	6.779754	7.082529	7.008826	6.524972	6.619878

ANKRD34B	3.218112	4.090253	3.473810	3.006352	2.863100	3.764289	3.068364	3.732140	3.369613	3.195700	4.785984	6.215255	3.294849	4.569586	3.675983	2.939913	3.321705	3.329019	4.255230	4.182342	4.763580	2.964816	3.457344	3.066288	2.950032	2.860229	3.783771	2.871536	3.150003	3.412583	3.757923	5.013223	4.594487	4.518915	4.559007	3.971793	3.597156	3.074342	3.347388	3.261885	3.460186	3.289829	3.470157	3.213948	5.186794	5.277165	4.710948	4.754703	4.415573	3.276507	3.337623	3.414352	3.905294	4.148341	3.823109	3.115938	4.106065	4.426112	3.346564	3.766514	2.964561	3.133555	3.612379	3.700564	3.760284	3.356209	2.959101	3.207511	3.316944	2.898242	3.220137	2.931044	3.225561	3.095293	4.201853	4.308819	2.996944	6.251304	3.284905	4.342189	4.149177	3.884271	4.570913	4.338339	3.113539	3.490659	3.033479	3.224190	3.414177	3.857219	3.225437	3.038319	3.804926	3.136114	3.608025	3.191888	3.670314	3.251299	3.051939	2.950816	2.953042	3.151974	2.965002	2.818522	4.152088	4.517223	3.023691	3.392955	2.988422	3.201160	2.899721	2.841712	2.886566	3.291272	3.544884	2.934010	2.910724	3.654613	3.617257	4.657066	3.414881	5.694530	3.050364	4.243640	7.847503	6.847871	8.207548	6.310605	3.038852	4.490913	11.900447	3.175876	7.767315	3.328333	6.795407	4.419157	5.587720	5.552381	10.459968	4.100192	5.098046	3.087059	2.949423	2.873923	3.565350	2.898242	6.675083	3.109451	4.341985	3.561567	3.901276	6.668816	3.354861	6.292519	3.689939	3.584949	4.896017	3.282855	3.131365	3.198895	3.245109	3.540932	3.496154	6.321320	4.937893	4.657491	6.949814	5.473470	4.822102	3.284757	3.220831	7.192616	4.338539	3.477266	3.415696	4.063079	6.013309	3.842751	3.743677	3.159554	3.257598	3.612379	4.091852	5.783252	6.170998	4.307220	4.306356	3.412911	7.180328	5.123919	5.047697	3.214073	3.416712	7.776635	3.914887	3.102636	5.389322	3.159296	3.414881	7.592559	4.260648	5.052996	7.964950	5.585356	5.595889	4.296116	6.059216	3.151870	4.921690	3.372431	2.982978	4.409608	3.228654	4.943891	3.266751	3.104231	2.992905	5.941779	5.238461	3.006528	3.154426	3.006794	3.244844	4.501376	5.675129	4.370011	6.029738	3.296822	3.158019	4.541985	3.328510	4.074067	3.167274	4.782194	2.923489	2.936539	9.641171	4.193008

TFEC	8.448206	8.206780	8.084126	7.641095	6.739082	7.518657	8.070168	6.129664	5.473107	7.069044	7.326266	7.298780	7.815434	5.931422	7.053173	7.848848	6.543218	7.425716	5.236029	8.045919	4.966918	6.470026	4.616425	7.328976	7.230565	6.325088	8.923143	6.790858	8.266356	9.941815	5.712111	9.691158	11.094307	9.531817	9.487347	9.231033	8.832406	7.972401	6.441540	6.937304	7.227642	6.926148	9.857414	8.856378	8.931776	9.285515	8.042008	8.671407	9.258777	8.839496	8.968967	8.966352	9.697083	8.467193	8.951337	6.964835	9.180169	7.518492	7.150257	7.879916	7.887198	7.734740	7.577303	5.914578	7.472494	7.427119	6.956874	7.522817	8.076073	6.078232	8.302279	7.692731	6.739884	8.789036	8.256188	9.897339	8.295598	9.493002	8.794316	8.089070	9.957526	9.549005	9.333694	8.567450	8.781813	8.755929	9.489997	8.990467	7.293323	7.306185	8.079016	8.005443	7.593519	9.934185	9.657496	8.520817	9.479952	7.483059	7.799143	5.766438	6.509590	8.147768	7.379556	6.009468	6.992420	6.982244	6.175587	6.089748	4.227886	5.832274	5.681213	5.241057	4.840654	6.396973	7.687797	4.209698	7.248376	7.623002	6.579309	10.568169	8.617246	10.066606	9.776906	5.773122	7.930805	9.623997	6.812404	8.366232	7.693207	7.698669	7.272190	6.428009	9.781037	8.212972	7.591030	9.070964	5.693030	8.273739	10.309414	7.525428	6.665571	6.816998	5.518195	6.267757	10.679116	8.549225	7.367653	8.221156	11.077366	10.305204	10.665327	10.047403	8.424967	10.903200	8.610624	9.790373	9.230883	5.305197	9.524595	6.472916	8.017982	13.185843	9.625356	9.540184	9.428192	7.268179	8.963871	8.992872	9.842603	10.122478	10.182867	11.059536	9.442200	10.817152	10.194758	9.636701	8.652204	8.722657	9.112425	9.236434	10.256634	6.985556	8.012742	9.206866	9.447632	8.324774	8.960834	8.094921	8.953320	9.238440	8.812777	5.235235	9.791103	10.598790	10.361284	9.320639	9.365340	7.933412	9.004857	10.230524	10.233257	9.830318	11.038581	10.933223	9.384500	10.623839	10.885551	9.280909	10.094118	7.835809	8.552276	8.041723	9.616680	9.576986	10.596344	9.193041	7.959294	8.661634	8.401741	9.432816	7.364043	7.611287	10.453422	8.860321	7.423830	9.186600	7.530523	7.504305	6.551642	9.230592	7.760354	4.790369	8.203755	7.310568	8.812340	10.197958	8.036507	8.773366

SRSF7	12.503895	11.173303	12.050418	11.931067	12.140350	11.409661	11.854889	11.676245	11.878463	11.889299	11.673716	11.607297	11.419559	11.019158	11.647985	11.947731	11.860081	11.883726	10.972192	11.695305	11.811133	11.858266	11.755427	10.942718	10.803872	12.120220	12.299016	11.319824	12.069615	11.919811	12.079262	12.191343	12.450257	11.901010	11.222202	11.881010	11.699941	11.041648	10.646207	10.581438	12.024653	10.962961	11.489042	12.176095	12.244367	11.924735	11.545607	12.319186	12.582308	12.286447	11.920101	12.057480	11.834043	12.227834	12.168674	11.920036	11.763092	10.441560	10.732079	11.251364	11.180344	12.183277	11.456254	10.781910	11.139043	11.713929	10.611543	11.570455	11.213047	11.297023	11.812873	12.047084	11.752033	11.802387	11.965674	11.522478	11.872205	11.533035	11.859791	11.235078	12.031827	12.376916	12.444353	12.277448	12.162501	12.145311	11.629239	11.566261	11.051054	11.312487	11.081888	10.712414	11.206137	11.807837	11.988461	12.051649	12.524779	12.105433	12.003100	6.623549	12.017344	11.360153	11.742298	11.737771	11.676971	12.011560	11.684076	11.961736	11.373637	11.970071	11.357531	11.248655	10.949961	11.789710	12.242226	11.486704	11.291145	12.228631	11.968244	12.378353	10.774046	11.683207	12.038203	12.151731	12.632463	11.285928	13.050446	11.287767	12.188102	11.557873	12.737637	12.003998	11.924735	10.865149	12.273308	12.003998	12.247353	11.496207	13.074553	11.764203	12.426215	12.339350	12.063806	11.317927	12.297906	11.982902	11.836941	12.282557	12.258961	12.643245	11.726750	12.042551	10.964592	12.000886	12.076671	11.671934	10.576915	10.876700	10.506316	12.945001	10.600603	12.506563	11.990173	11.881927	11.560987	11.687959	12.882226	12.061682	11.719572	11.420720	12.252621	12.357997	11.684367	11.938785	12.969511	12.679547	11.140228	10.757205	11.703716	11.153776	11.729608	11.074426	10.772399	10.999226	10.789138	11.282256	10.614651	11.068971	11.209661	11.308236	11.896631	11.299661	11.454008	11.967485	11.347880	11.677583	12.263096	11.703111	11.121750	11.946674	11.871132	11.808438	11.645767	12.095489	11.406029	11.682317	13.122875	10.665611	11.162013	11.792854	10.960176	10.842557	11.194445	11.305887	11.734663	11.454144	11.776227	11.503932	7.235637	12.108031	12.148694	12.881137	11.343711	11.701443	12.645882	11.641478	11.850039	11.293672	11.953478	11.615530	11.731240	12.030069	12.081830	13.352695	11.764203	11.543167	12.011905	12.251510

MMP14	10.627787	10.996710	11.150531	11.532884	12.520900	12.781401	11.232265	12.151239	12.506730	11.669233	12.112223	11.653632	12.313639	12.971662	11.194864	12.519877	12.396622	11.940136	11.464200	11.842962	11.527185	10.654872	10.827984	11.042993	10.965807	12.736077	11.525740	11.653482	11.345061	12.012502	12.482801	10.964294	11.954825	11.495782	10.870211	11.968715	12.933669	10.814013	12.254045	12.452025	11.980172	11.766772	11.595353	11.266180	10.918947	11.175522	12.847527	12.939558	11.956053	11.372309	10.957831	11.099298	11.027621	11.131351	10.971457	12.469908	11.317473	10.489348	10.529955	11.269993	11.376944	12.576723	10.996398	11.359582	11.742298	11.149137	10.695514	11.181128	11.377693	11.058379	12.658466	12.391255	13.472510	13.488366	11.297194	11.093256	11.968868	12.359421	12.064108	10.789859	11.808875	10.833585	11.029872	11.103121	12.122850	11.216261	12.772441	13.065305	11.989104	11.935497	11.985888	12.158494	13.056546	11.648851	11.729303	12.659466	11.023655	12.588621	12.768875	6.642422	12.631485	11.143240	11.443526	11.146945	11.150234	11.691236	11.377401	11.680103	11.572521	12.038055	11.527903	12.839276	12.233426	12.446584	13.478600	14.814746	11.828556	11.960368	12.396312	11.984966	12.399011	12.415286	12.439014	14.121867	12.215332	13.412516	12.433717	12.936720	12.544041	12.827182	12.486329	12.980457	13.451381	13.195507	12.449765	13.486814	12.641718	11.604734	11.142206	12.412373	12.109213	12.101494	12.226617	13.285446	12.802136	12.900071	12.475223	12.593788	13.064722	13.487724	12.900424	13.905564	12.431102	11.263943	12.610024	13.080142	12.435325	12.739508	13.019903	13.051243	12.109351	11.567838	12.082135	12.130834	11.719718	13.143772	11.747596	11.980316	13.438194	12.686212	12.568552	12.132968	13.608281	12.646059	12.034566	11.459639	11.661642	12.044248	11.418124	13.277634	11.723321	11.970213	12.227351	12.017200	12.618626	12.364809	11.829126	12.063042	12.124346	12.076353	12.534911	13.143772	12.897245	12.194924	11.762942	13.528125	12.774471	12.953101	12.062769	12.069772	12.639389	13.516009	12.989438	13.497565	13.219568	13.179765	11.805293	12.787176	13.321475	13.745342	13.005224	12.328136	13.639425	13.505253	14.648824	13.268434	13.598139	13.264773	7.484585	13.787141	12.373531	12.611506	12.467154	13.113538	11.830627	11.996843	11.663877	11.646786	12.833284	11.868900	13.823712	14.299113	13.005956	13.122511	13.037247	13.101051	12.419287	12.793758

NCSTN	13.152225	12.558326	11.722280	13.062098	12.635424	12.501102	13.100126	12.673286	12.071423	12.899003	12.983891	12.751206	12.401128	12.992530	11.717044	12.434656	12.644060	11.852324	11.491308	12.546233	11.563161	12.407294	11.666271	11.828556	12.045589	12.145451	11.807225	12.062305	12.777077	12.911451	12.117412	12.994008	11.763565	13.252042	12.634097	12.681802	12.789606	11.374894	11.456110	11.840095	12.730652	11.537766	12.854570	12.872680	12.969511	12.369887	12.511451	10.902781	12.692172	12.829828	12.892258	12.612527	13.202580	12.982957	12.861696	12.700708	12.974908	11.646207	11.670177	11.848510	11.903405	12.733221	11.697270	11.421190	11.597416	11.670766	11.696089	10.923568	11.605218	11.692880	12.729813	12.650533	12.512264	12.286918	12.351330	12.738146	12.920784	12.805285	12.857277	12.802136	12.662065	13.052959	12.746236	12.874685	12.826984	12.722641	12.908020	12.905702	11.689898	11.463911	11.076058	11.650646	11.976069	12.674493	12.958452	12.758922	12.381631	12.643586	12.739710	11.995045	12.688955	12.912009	12.474582	11.468386	11.728708	12.412373	11.684367	11.286099	11.339227	12.601747	11.670022	12.197990	12.325249	12.515494	12.725694	11.705064	11.793165	12.719524	11.721674	12.930440	12.734044	12.500081	13.416299	11.932006	13.724916	13.458499	11.889157	12.375646	13.047442	13.341166	12.751039	12.509156	13.265809	12.216379	12.225678	12.600182	11.745525	11.607855	13.475756	12.129478	12.848215	12.250390	11.623310	12.278662	12.819396	12.487990	11.516227	11.980630	13.488803	12.540137	13.322740	12.769791	12.918404	13.558417	13.124576	13.389920	11.375164	12.002950	11.722749	12.699870	11.429272	13.672956	13.446919	12.830155	12.356420	12.496355	11.011245	13.309969	12.912009	13.463363	12.786494	13.080428	13.259837	12.957359	13.605717	13.201614	11.738648	11.645924	12.103623	12.555263	13.513556	11.826829	11.815237	11.998560	12.020053	11.992007	10.601358	11.516497	11.750421	12.607398	12.704678	12.247046	12.860065	12.316234	13.131145	13.156712	13.096527	12.605556	13.237448	13.054365	12.760314	13.349750	12.415936	13.244529	13.179374	12.635086	12.496521	12.012663	11.559388	11.597416	11.767668	12.343587	12.844050	12.813259	13.232267	13.497565	12.158335	12.914314	12.747282	12.361945	12.760155	12.450905	12.131436	11.703426	11.945767	11.243470	11.099868	11.949129	12.126571	11.555531	12.827695	12.136503	12.345318	12.906069	12.959560	12.501246	12.640896	11.886584

ROBO1	4.061967	3.242102	3.442050	3.474890	4.019348	3.998329	3.639909	3.874160	4.376736	3.878719	4.840025	3.489018	4.575394	4.690748	4.239453	4.595765	3.894841	5.716738	5.025466	4.298529	4.166676	5.122714	3.563639	4.923626	5.073004	5.025838	4.762713	3.726533	4.729138	3.147032	4.993534	3.580146	4.928648	5.093749	3.803896	4.158634	3.454769	3.269432	3.847741	3.909010	4.867443	4.032747	4.509419	3.711546	4.348542	4.394772	4.339423	4.041871	4.202709	4.236984	3.454979	4.458021	4.668615	4.038891	3.987353	5.058371	4.102611	3.358117	3.916620	3.174479	3.159554	4.285215	3.674530	4.363298	3.276977	3.292494	3.116629	3.747923	3.406231	3.756526	3.246230	3.197591	5.545245	3.154159	3.309708	3.556033	3.370267	3.148992	3.263555	4.112008	3.817794	3.569066	3.703446	3.624260	3.678427	3.823735	3.562676	3.663264	3.474169	3.118879	3.046501	3.435445	3.269945	3.341985	3.396300	4.417292	3.856793	5.627077	4.463946	4.177253	3.779373	4.143859	5.047267	4.184469	3.984782	5.064862	5.070584	4.150618	3.699196	4.659010	3.435445	3.531729	3.682876	4.975882	4.497707	3.039726	3.153195	4.786621	5.275754	3.490659	3.256210	3.216236	4.112234	3.417203	4.755912	3.197053	4.751493	3.703843	3.418972	3.766295	4.181870	3.408177	3.002248	3.798781	3.296988	3.938998	6.227294	6.547889	4.217085	3.348845	3.635265	3.163655	4.951888	3.606610	4.310605	3.457163	3.313017	3.727139	3.233108	3.747923	3.572500	6.077787	4.100634	3.150130	3.618361	3.266295	4.323129	3.678427	3.210306	7.056010	3.496532	5.777437	3.776160	5.401255	4.196467	3.373733	3.110054	4.175969	3.335311	3.517751	4.202047	4.281894	3.623869	3.650021	4.030852	3.774707	3.672321	4.026422	3.109691	3.179000	3.323979	3.302221	3.643274	3.327268	3.670112	3.800298	3.006239	3.016683	3.443277	3.153417	3.200773	4.434540	3.143613	3.141417	3.420470	3.133914	4.083044	3.334035	3.525165	3.925247	3.656029	4.070266	3.953974	3.688293	3.503315	3.633128	3.448389	3.305531	3.206777	3.404877	3.099853	3.313790	3.147032	3.701819	3.888276	3.982184	4.894383	6.487433	3.138823	3.792048	4.646621	3.921822	3.422249	4.445201	5.225696	5.059192	3.160827	3.093751	3.396622	3.142794	3.147783	5.251390	3.374235	3.123088	2.998995	3.965102	3.400726	3.850978

OST4	14.227945	14.634337	14.583563	14.742751	14.810589	14.517783	14.568485	14.450917	14.002435	14.528702	14.565537	14.471558	14.686498	14.951667	14.594880	14.484499	14.424884	14.524343	14.718998	14.297708	14.794023	15.056095	15.253922	14.322301	14.241406	14.368698	14.007288	14.479847	14.238833	14.415078	14.476727	14.884408	14.750281	14.650647	14.726053	14.477088	14.571778	14.563014	14.573899	14.554790	14.246947	14.750281	14.782312	14.734860	14.727457	14.854894	14.443791	14.001519	14.739537	14.443791	14.949574	15.203034	14.773263	14.182477	14.441229	14.670593	14.786789	15.211659	15.088816	14.877086	14.425923	14.237167	14.772763	15.170771	14.843666	14.530652	14.652370	14.948503	14.709387	14.729702	14.336805	14.781328	14.243708	14.602856	14.747900	14.494741	14.774189	14.396941	14.000936	14.551462	14.509168	14.531879	15.007673	15.053135	14.744131	14.759633	14.867085	14.763822	14.885495	15.068093	14.565911	14.843666	14.451725	14.180225	14.365218	14.654144	14.560951	14.568485	14.672364	14.080973	14.495544	14.231871	14.451322	14.347082	14.434526	14.674118	14.587687	14.984580	14.804759	14.525897	15.297462	14.812187	14.825958	14.330677	14.101336	13.909402	14.384877	14.887600	14.089238	14.706135	14.458915	14.201809	14.168428	14.267351	14.165841	14.153935	14.568889	14.487150	14.297379	14.162992	14.452088	14.008461	14.525087	14.126434	14.412818	14.765753	14.043788	13.907177	14.974306	14.497164	14.517028	14.774671	14.702065	14.763321	14.531049	14.786789	14.511568	13.637147	14.002723	13.985840	14.597809	14.789655	14.424884	14.356954	14.631695	14.444840	14.541332	14.793038	14.788228	14.766218	14.443429	14.847864	14.772271	14.980454	14.836534	14.065366	14.741363	14.514288	14.703881	14.169692	14.680965	14.566731	15.102359	14.973125	13.992989	14.967925	14.519413	14.480189	14.591153	14.962802	14.310666	14.874974	14.323669	14.438961	14.652808	14.533864	14.381001	14.366997	14.526310	14.721853	14.612308	13.915290	14.750281	14.687432	14.666890	14.415816	14.900723	14.995622	14.466224	14.630361	14.433838	14.561753	14.785315	14.976470	14.511123	14.929736	14.998489	14.560123	14.752176	14.808146	14.525897	14.872324	14.661796	14.540937	14.425228	14.689755	14.184313	14.224052	14.725604	14.846246	14.623791	14.881789	14.288296	14.900723	13.753087	14.524706	14.893861	14.852976	13.945117	14.914667	14.855412	14.766663	14.108513	14.828938	14.339249	14.961131	14.413643	14.354129

AGBL5	14.227945	14.634337	14.583563	14.742751	14.810589	14.517783	14.568485	14.450917	14.002435	14.528702	14.565537	14.471558	14.686498	14.951667	14.594880	14.484499	14.424884	14.524343	14.718998	14.297708	14.794023	15.056095	15.253922	14.322301	14.241406	14.368698	14.007288	14.479847	14.238833	14.415078	14.476727	14.884408	14.750281	14.650647	14.726053	14.477088	14.571778	14.563014	14.573899	14.554790	14.246947	14.750281	14.782312	14.734860	14.727457	14.854894	14.443791	14.001519	14.739537	14.443791	14.949574	15.203034	14.773263	14.182477	14.441229	14.670593	14.786789	15.211659	15.088816	14.877086	14.425923	14.237167	14.772763	15.170771	14.843666	14.530652	14.652370	14.948503	14.709387	14.729702	14.336805	14.781328	14.243708	14.602856	14.747900	14.494741	14.774189	14.396941	14.000936	14.551462	14.509168	14.531879	15.007673	15.053135	14.744131	14.759633	14.867085	14.763822	14.885495	15.068093	14.565911	14.843666	14.451725	14.180225	14.365218	14.654144	14.560951	14.568485	14.672364	14.080973	14.495544	14.231871	14.451322	14.347082	14.434526	14.674118	14.587687	14.984580	14.804759	14.525897	15.297462	14.812187	14.825958	14.330677	14.101336	13.909402	14.384877	14.887600	14.089238	14.706135	14.458915	14.201809	14.168428	14.267351	14.165841	14.153935	14.568889	14.487150	14.297379	14.162992	14.452088	14.008461	14.525087	14.126434	14.412818	14.765753	14.043788	13.907177	14.974306	14.497164	14.517028	14.774671	14.702065	14.763321	14.531049	14.786789	14.511568	13.637147	14.002723	13.985840	14.597809	14.789655	14.424884	14.356954	14.631695	14.444840	14.541332	14.793038	14.788228	14.766218	14.443429	14.847864	14.772271	14.980454	14.836534	14.065366	14.741363	14.514288	14.703881	14.169692	14.680965	14.566731	15.102359	14.973125	13.992989	14.967925	14.519413	14.480189	14.591153	14.962802	14.310666	14.874974	14.323669	14.438961	14.652808	14.533864	14.381001	14.366997	14.526310	14.721853	14.612308	13.915290	14.750281	14.687432	14.666890	14.415816	14.900723	14.995622	14.466224	14.630361	14.433838	14.561753	14.785315	14.976470	14.511123	14.929736	14.998489	14.560123	14.752176	14.808146	14.525897	14.872324	14.661796	14.540937	14.425228	14.689755	14.184313	14.224052	14.725604	14.846246	14.623791	14.881789	14.288296	14.900723	13.753087	14.524706	14.893861	14.852976	13.945117	14.914667	14.855412	14.766663	14.108513	14.828938	14.339249	14.961131	14.413643	14.354129

RAP2A	9.962703	11.381887	11.722441	11.679201	11.113182	11.761312	11.536881	12.599699	10.809658	11.775928	11.576321	11.279290	11.206938	11.562171	10.891480	11.728242	11.364013	11.537478	11.097635	11.807531	11.327051	11.907433	9.906054	11.507746	10.994322	10.886269	9.287550	11.591914	10.627617	11.200439	11.012272	11.540263	12.020495	10.621312	11.404685	12.140678	11.802887	11.725872	11.109863	11.187721	11.429134	11.198394	11.492028	11.414134	11.103121	11.484580	11.300099	10.469964	11.518416	11.917282	11.336433	11.759472	11.840676	12.018432	11.287321	11.395196	11.393088	11.171964	10.514372	11.165668	11.355934	11.325990	10.831653	11.362409	10.657698	10.893881	11.130633	10.923853	11.067036	11.146191	11.511259	11.605506	11.417534	11.763874	11.911774	11.595942	11.538956	11.580566	11.642235	11.752750	11.507445	11.496969	11.514222	11.852324	11.970071	11.631046	11.711018	11.887512	11.153927	11.452975	10.467694	11.393514	11.669383	11.980765	11.351141	11.263347	11.505243	11.789270	11.584392	11.178995	12.166192	11.098557	11.435100	11.305292	11.898839	11.629239	11.953632	12.041080	10.618828	12.074965	11.549007	11.162307	10.894182	11.917442	11.433444	10.355061	9.189192	11.181578	11.061124	10.263119	10.814013	11.403962	9.912962	9.329078	11.390710	10.542840	9.442516	9.436553	10.246336	10.504682	10.692122	10.363280	9.152473	10.809503	10.318172	11.001079	10.247700	11.830475	10.636387	10.489183	10.379552	9.924291	8.222227	10.219691	11.052514	10.881936	8.974079	8.204471	10.952894	8.360324	10.386256	10.610220	11.410682	11.473822	10.316078	10.893753	10.258038	9.963663	10.451937	10.038097	10.286654	10.060453	10.893304	10.831653	11.427104	10.862192	10.261165	10.380008	11.254715	11.579980	10.829303	11.457713	11.510962	11.385706	11.118560	13.245524	10.887345	9.787132	10.941524	10.315777	9.274836	9.676450	10.226354	10.936997	10.627143	10.335529	10.311711	10.767727	10.408443	10.501539	10.965351	9.124109	11.608622	10.855721	11.326759	11.378421	11.412245	11.897222	10.654587	11.550609	10.987659	11.628067	11.127809	11.213657	10.723866	11.557301	11.748172	10.864387	10.668285	12.102695	11.394116	10.514677	11.149599	11.229444	10.727474	10.535606	9.075755	10.397613	10.144911	10.313234	10.298003	9.484419	10.591635	10.748602	8.923580	9.416708	11.314983	10.836162	9.567009	10.553981	9.677215	10.304338	11.175522	10.975965	11.186429	10.627927	11.992764	9.646149

GTF2B	12.973272	12.948591	13.446715	12.602572	12.858637	12.874301	13.180160	13.659596	13.007394	13.309752	13.132547	13.146133	13.072064	13.286282	12.946442	12.887549	13.307488	13.026194	13.157276	12.964171	13.223721	13.707232	13.484618	12.976037	13.328423	12.672069	11.757998	13.157459	12.549566	13.048184	12.990709	13.289866	13.496693	12.451545	13.425338	13.446919	13.657694	13.497141	13.234006	13.668871	12.914314	13.610850	13.937151	13.610375	13.750540	13.718916	12.465329	13.594434	13.730412	13.726632	13.742874	13.925407	13.810879	13.419743	13.752566	12.776199	13.790935	13.675535	13.280266	13.334586	13.441153	12.738848	13.747333	13.282417	13.285050	13.574884	13.189708	13.554388	13.102010	13.425542	12.761009	13.076836	12.929753	13.279234	13.213586	13.155145	13.382821	13.116206	12.947003	13.820478	13.404601	13.410314	13.038580	13.401636	13.007215	13.348898	13.162423	13.492266	13.337083	13.338305	13.114850	13.512420	13.028031	13.300262	13.031581	12.455951	13.706994	13.560621	13.068091	13.197125	13.558417	13.288424	13.302870	12.861330	13.677586	13.099956	13.537063	13.589099	13.233400	13.115266	13.847197	13.284435	13.309343	12.871377	13.980518	12.131138	13.275229	12.848771	12.724991	12.931002	12.982957	13.107904	12.438685	12.512428	12.552012	12.924028	12.300805	12.497333	12.331768	12.800391	12.094424	11.947731	12.566268	12.476206	12.540317	12.719524	12.082595	12.447100	13.375856	13.013710	13.397551	13.065502	12.855862	12.663564	12.833456	12.905530	12.790822	11.854455	12.702790	12.723135	13.175390	12.639889	13.818903	13.143772	13.215627	12.774471	12.862948	13.125598	12.856932	12.133853	13.134908	13.358446	13.958500	13.569877	13.470952	12.692514	13.591797	13.144955	13.613744	13.588156	13.663476	13.313561	13.386476	13.259837	12.251358	13.634245	13.956868	13.346348	13.913198	14.089539	12.429784	13.652718	13.287052	13.472948	13.340126	13.218179	13.056178	13.127100	13.389060	13.381772	13.024751	12.237890	12.874855	13.088702	13.239591	12.831338	13.162836	12.796802	13.031373	12.887922	13.093311	13.599051	13.149739	13.702148	12.797496	13.130961	13.185047	13.037427	12.840359	13.035259	13.467091	13.106206	13.119650	12.714689	14.306995	12.913298	12.082436	13.395578	12.938700	12.966367	13.149535	12.299016	12.967688	13.359928	11.857830	13.301664	13.481256	13.363482	12.762029	13.620822	13.162064	13.630916	12.539812	12.571167	13.604544	12.995256	12.945530	13.087953

UGDH	14.365566	14.170650	14.368027	13.597474	14.286202	13.734624	14.089539	13.686919	13.498262	12.896716	13.875238	13.394726	13.429701	13.544167	12.878536	13.368069	13.357611	12.687779	13.561973	13.878229	13.003777	13.154569	12.766382	12.974908	13.336226	12.739508	13.218984	12.838099	12.945176	13.297753	13.064722	13.689737	13.888860	13.837964	14.298742	13.756638	14.251440	13.680663	13.418183	13.469230	14.253427	13.388838	13.634990	14.239437	13.768164	14.138248	13.135842	12.965249	13.862600	14.634337	13.800876	13.776673	12.792190	14.190235	13.190846	13.560165	14.154830	13.719674	12.842803	12.787861	13.111167	14.054182	13.842481	13.345736	13.596345	13.670308	13.314762	13.998713	13.308547	13.554610	13.271585	14.278587	13.644259	13.828450	13.878453	13.869088	14.064771	13.252643	13.691689	14.254782	14.336805	14.666890	14.312048	14.021565	14.284178	13.982398	14.513178	14.761042	13.945117	13.765754	13.588874	13.781822	13.552782	13.519798	13.253414	14.143957	14.170304	13.213993	14.132353	13.281802	13.753483	13.963822	13.236251	12.467154	13.163768	13.454810	13.134908	13.549809	13.273354	13.995561	13.240817	13.788457	13.351178	13.689494	13.860733	13.959054	12.938530	13.304338	13.793556	13.162836	12.456129	13.165092	14.439683	13.119251	13.582997	13.074553	13.487496	13.047798	15.021020	12.398188	14.079765	14.528702	13.384142	12.997678	13.025099	14.663179	14.160557	12.926114	12.956264	14.616972	14.141781	15.111816	12.811614	13.509723	14.734860	14.241084	14.168734	14.224052	13.769838	13.204762	12.747282	13.257421	12.336516	12.180008	13.939054	13.138696	12.618964	13.108876	13.463806	12.663909	12.640046	13.281802	12.890348	14.231500	13.680915	12.165600	12.875210	14.149163	13.090706	13.361000	13.913198	14.131096	12.904732	13.524143	14.734860	13.434297	12.035812	13.324601	12.047981	12.529016	13.127100	12.927582	13.088879	12.391089	13.121156	12.485169	12.833284	12.279590	12.976769	14.037568	13.633730	12.031518	13.876070	12.742511	12.894031	14.056836	14.076608	13.529678	13.266805	13.017536	12.160822	13.498714	13.675974	13.753621	13.246936	12.545386	12.340373	13.020101	13.325208	12.544739	13.390157	13.789231	14.047634	14.184313	13.167478	13.240817	14.406946	12.566772	13.419287	14.141781	12.553951	13.185249	13.196137	12.720580	14.392936	13.126907	12.523307	14.859010	12.011267	13.821718	13.499351	12.475535	13.286082	13.958253	13.273173	13.634990	14.556844	13.050076

PTK2	4.856270	3.967025	3.767567	4.659661	4.091404	4.222970	4.137156	3.986951	4.660922	4.680948	4.777820	4.934501	5.434367	4.537994	4.845532	4.965827	4.118354	4.099063	4.818345	4.875077	4.848502	4.195183	4.197310	4.107628	4.261329	4.472756	5.371479	4.567244	5.204460	4.141298	4.539755	4.525882	3.822483	4.996504	4.191920	4.156749	4.096439	3.759199	3.880253	3.457694	3.976560	3.746675	4.587333	3.854056	4.697330	5.308659	4.174012	3.407300	3.715868	4.129176	4.004983	4.822102	4.589938	4.286569	4.629125	5.372466	4.379075	4.429795	4.787242	4.093706	3.086264	4.688859	3.820807	3.909010	3.851185	3.600877	3.261430	3.902531	3.199539	3.486614	3.276674	3.273130	4.747673	3.314806	3.917257	3.891965	3.680251	3.422423	3.395212	4.056272	4.853109	4.370684	4.580993	4.300684	4.397017	4.365030	4.025170	3.817172	3.999587	3.558339	3.335812	3.380330	4.291994	3.575355	4.069243	4.897059	3.924795	5.672771	4.036500	5.354518	4.493992	4.842988	5.053206	4.143246	3.999384	4.850771	3.891965	4.039945	4.060633	3.804926	3.495025	3.983281	3.382407	4.502005	4.778032	4.170794	3.729640	5.423208	5.422259	4.619091	4.898566	3.399682	5.325189	4.130225	3.690137	4.417080	6.625166	5.411685	5.377881	5.507004	5.002123	5.491318	5.558862	4.826757	5.312635	4.879417	4.496646	5.275329	3.990057	4.339822	3.801618	4.942813	4.690116	4.421729	4.552175	3.876112	4.932210	5.604559	3.864320	5.474833	4.531822	5.150526	5.095617	4.872577	3.737290	3.921454	4.174641	4.185545	3.559545	4.324870	3.892646	4.354159	3.713156	4.394991	4.998777	5.805110	3.300244	3.881690	4.333078	4.348963	4.477155	5.255748	5.086568	4.896655	5.520438	3.751463	4.219929	4.871035	3.890274	2.947036	4.292225	4.128953	4.247745	3.758136	3.836745	4.204989	3.564573	3.452598	2.940060	4.059980	4.962329	4.660271	4.599394	3.673475	4.248826	3.676604	4.750862	4.931589	4.360915	5.393779	4.433285	5.604946	5.534678	5.741744	5.097591	4.052563	4.032992	3.507618	4.036066	3.874364	3.373901	4.598956	4.887089	4.004562	4.769077	4.750654	6.947279	5.046672	3.951827	5.651451	5.020732	5.555822	3.463210	4.308348	5.487463	4.560560	4.602523	4.336340	4.209499	3.552388	3.889854	3.159296	4.694331	3.691374	3.802243	3.755678	5.542232	4.739775

AIFM3	9.080099	8.911416	8.772007	8.918918	9.263696	9.165740	8.621124	9.428521	9.309714	8.767146	9.364092	9.264136	8.537536	9.075755	8.925189	9.442200	9.746158	8.740710	9.345116	8.860035	9.334161	9.086111	10.840472	8.716906	8.800503	9.046071	8.856378	9.557255	8.675156	8.371132	9.657640	8.800815	8.768833	9.054399	8.606664	8.808882	8.881902	9.195114	9.056426	9.493614	8.928787	9.101487	8.224643	8.833199	8.429598	8.875702	8.949039	8.540060	8.618626	8.562976	8.739179	8.767146	8.410935	8.803036	8.753188	8.736423	8.886993	8.756854	9.003048	8.953955	9.557714	8.529384	8.973069	9.113163	9.521779	9.932177	9.317465	8.871852	9.336472	9.691309	9.036933	8.975115	8.876783	9.042516	9.227589	8.857724	9.163554	9.293796	9.560158	8.517477	8.248407	8.835236	9.918392	8.896265	8.929239	8.946123	8.875260	9.059743	9.026158	9.073286	9.210693	9.713408	9.259079	8.868386	9.142130	8.812917	9.072055	8.150655	8.963573	8.456594	8.073351	8.485965	8.657576	8.628513	9.200336	8.852566	9.048228	9.073756	9.581615	8.840124	10.254815	9.206130	9.080577	8.864761	8.685237	9.197498	8.862310	9.050240	8.963428	9.240288	9.053125	8.710706	10.070853	9.533221	9.278749	9.211300	9.303246	9.844876	9.376209	9.892101	10.102954	9.412451	8.740267	10.524883	9.965013	9.552229	9.749575	9.060510	8.981956	9.546675	9.787767	10.912789	9.640265	9.706401	9.250083	9.567165	9.989210	10.082245	9.393593	10.061791	8.950728	9.272858	9.298453	9.455567	9.791578	9.294436	9.078706	10.093823	10.113777	9.892413	9.540332	8.440424	9.802880	8.973923	9.121839	9.830156	8.907026	9.615291	9.005162	9.675214	8.985474	9.295525	9.096009	9.368622	9.696933	9.328916	9.798436	10.195547	9.323513	10.441420	10.084367	10.027337	9.393908	9.676294	10.695882	9.593696	9.337427	10.791282	9.620259	9.313619	9.823468	9.990754	9.452733	8.888996	9.084561	10.036268	9.308487	10.050200	8.191336	8.756532	10.749026	11.027185	8.511664	8.516857	9.688696	9.356658	10.849009	9.059573	9.063831	9.358630	9.736704	10.544926	9.597096	10.035391	9.246132	9.747254	9.024612	9.151401	10.212729	8.895157	8.985166	8.875113	8.827483	9.224038	9.829671	10.143394	10.133901	9.938377	9.330587	10.137918	8.830255	8.635361	9.459261	9.922889	9.492544	9.378827	9.638262	9.346335

SOS2	12.116671	12.473114	12.712770	12.552675	12.562209	12.168505	12.644718	12.212707	12.050582	12.497024	12.516134	12.355945	12.538424	12.904469	12.211418	12.705699	12.066401	12.305419	12.813176	12.650373	12.739166	12.648312	12.621342	12.671728	12.491075	12.629675	11.588775	12.910540	12.171733	12.205573	12.182961	12.167097	12.723671	12.673598	12.630661	12.673786	12.871011	12.232358	12.359421	12.560874	12.324636	12.565451	12.702108	12.734879	12.771719	12.556996	12.528252	12.493046	11.755140	12.637480	12.389829	12.568065	12.806823	12.618274	12.268675	12.552012	12.786674	13.213784	12.541618	12.784685	12.389189	12.371963	12.476535	12.440789	12.397534	12.450418	11.984094	12.014173	12.118153	11.929833	12.539305	12.404510	12.624672	12.566930	12.325410	12.695910	12.656757	12.741823	12.272798	12.870847	12.475535	12.237727	12.598156	12.990154	12.970060	12.437588	12.717483	13.044646	12.643737	12.699705	12.317479	12.730491	12.524939	12.624160	12.089348	12.570198	12.024036	12.391255	12.477840	12.437418	12.456606	12.576063	12.284727	12.774471	12.248323	12.085291	11.962903	12.613373	12.081830	12.549882	13.201991	12.603569	12.398015	12.359732	12.034862	11.475642	12.295409	12.092910	12.136795	13.449081	12.009009	12.645369	12.527930	11.208956	11.524581	12.168033	10.720767	12.774808	11.291291	12.171247	11.586607	11.393088	11.911633	11.216559	12.572340	11.543954	11.365654	11.637244	11.308833	12.167248	11.419106	11.452378	12.112064	12.329711	11.868033	12.116196	10.858500	9.811988	11.772119	11.431202	12.250070	11.851129	12.272044	12.358783	12.122772	12.389042	13.385185	12.214256	11.967950	10.481250	11.376076	11.034048	12.742336	11.843716	11.659417	12.260855	11.049872	11.769146	12.387424	12.228631	12.371808	12.157886	13.099956	12.407139	11.285343	12.747616	12.182961	12.000077	11.790004	12.800035	12.219214	12.026155	11.934440	11.956506	12.356420	11.718971	11.541661	11.752033	12.448174	11.555669	12.284727	10.625655	12.104230	12.339692	12.299522	12.318233	12.693032	12.543410	12.618274	12.420088	12.870487	12.783797	11.935649	12.225836	12.075279	13.221167	11.785397	12.592167	11.864833	11.685252	12.098308	12.245314	12.172787	12.923860	11.859051	11.535695	11.494786	11.986674	12.243919	12.337443	11.354920	12.538957	12.108933	12.394097	11.051205	11.051912	12.483120	11.716014	11.513777	12.188253	12.510794	10.879299	11.527615	11.318974	11.812728	12.377877	13.123245	12.331927

SH3GLB2	11.210244	11.993191	12.218642	12.371021	12.577210	12.505580	12.136334	13.358673	12.704515	12.879771	12.670862	12.381004	12.185716	13.066403	11.732727	12.206651	12.372577	12.549882	13.309343	12.616658	12.315775	13.696837	12.686920	12.682302	13.004475	13.150306	11.846125	13.158588	11.911322	11.274000	12.966194	11.627766	11.742152	10.700919	11.997004	12.178152	12.811445	12.593788	13.266411	12.956427	12.610869	12.328606	12.166192	12.287369	12.245147	12.428156	12.086352	12.380518	11.611531	12.284575	11.637961	12.215485	12.325086	12.035185	12.400126	12.777077	12.187947	12.761697	12.656514	12.404804	12.289352	12.017772	11.226653	12.457084	11.996542	12.551868	12.431102	11.710741	11.753359	12.501246	11.943044	12.023720	12.315273	11.930467	11.916369	11.834209	11.767202	10.986127	11.702656	11.783598	12.170796	11.809331	11.711473	12.100117	12.379287	12.031987	12.387097	12.811936	11.569303	12.573019	12.289078	12.110693	12.184093	11.934899	11.221315	11.708649	11.594771	12.319676	12.056520	12.211265	12.577702	12.112386	12.295874	11.978054	12.921519	12.285194	13.532157	12.962142	12.828237	12.556507	12.550714	13.329225	13.210738	12.776909	12.218928	10.981607	12.320002	12.028718	12.714861	10.324283	10.851782	10.835284	11.361048	11.364771	11.844950	11.898681	10.707486	10.842267	11.251658	11.504506	11.227693	11.316160	11.984549	11.893953	11.411534	10.672999	11.887512	11.572679	10.358409	12.193376	12.533294	12.471814	11.908049	11.911476	10.936997	11.296440	12.826830	11.529388	10.716263	12.367368	10.407108	10.272900	10.740494	11.067913	11.269399	10.982910	12.241297	11.700081	11.042557	11.206577	10.906458	10.427919	10.832583	11.481643	11.567979	12.220778	10.580846	11.254715	10.441560	10.238166	10.430630	11.435808	10.744560	11.024955	11.118709	9.968648	11.507007	11.430160	11.269244	10.601053	10.970001	11.674007	11.239056	11.327922	11.306962	10.689640	11.059231	10.570935	11.241643	10.885995	10.287878	13.056363	10.044641	9.893168	10.861160	10.619400	10.266592	10.968164	10.730598	11.606379	11.110014	10.993145	10.585512	11.107188	11.030902	10.348574	10.904059	10.862460	11.094860	11.219974	11.480786	10.428816	10.549196	10.599871	10.367141	10.504406	11.053352	11.771398	11.012953	10.615426	10.221236	11.349792	10.170585	10.117756	11.936396	11.046942	11.495782	12.009448	13.138696	10.904808	11.399245	10.262095	13.073430	11.372309	9.906665	11.400278	10.787776	11.375024

KCTD9	10.595736	11.360153	11.799373	11.631959	11.754223	11.329687	11.513324	11.804534	11.978824	11.534084	11.494137	11.485173	11.672678	11.571323	11.658962	11.836797	11.199316	11.752614	11.166964	11.824292	11.167684	10.883860	10.636387	11.649159	11.350258	11.356245	10.684665	11.592964	11.379762	10.991194	11.341633	11.471087	11.380212	10.563986	11.170618	11.317187	11.142648	11.233156	11.650785	11.295839	11.514222	10.855721	11.058929	11.119269	11.199162	11.198394	11.267660	11.038451	11.719282	11.251658	10.760858	11.402644	11.267954	11.553156	11.670323	11.885261	11.117676	10.813866	11.033754	11.428261	10.879893	11.332767	10.039829	10.516102	10.663435	11.656040	10.293540	10.810536	10.936702	10.638038	11.170321	11.138461	11.405443	11.222342	11.396684	11.081007	11.209393	11.076776	11.169002	12.016430	11.874773	11.790869	11.196818	11.807983	11.714313	11.373199	11.995822	12.102972	10.453727	10.758970	11.240923	11.216854	11.717638	11.632245	11.022905	11.323062	11.481940	11.145435	11.457713	11.104718	11.496833	11.045153	11.174007	11.428109	10.645194	11.880130	11.478266	11.338351	10.968475	11.436814	10.331296	11.797346	11.437385	11.394735	12.067345	11.069585	10.374844	11.837854	11.679800	10.328537	11.685397	10.238166	10.761456	9.736421	11.109722	10.051559	10.627927	10.769403	10.602690	11.198236	9.755469	11.517806	10.045877	10.483523	10.693627	8.816206	10.268511	9.744273	10.379552	11.512453	10.345672	10.437507	11.624998	9.914396	10.778128	11.718826	10.391503	11.437253	11.359302	10.745421	10.632913	11.487721	11.817639	10.878560	11.031948	11.071058	10.681664	11.599182	10.945949	11.494352	10.637737	10.742155	11.769756	11.209109	10.902781	12.302476	9.451553	11.421190	11.689281	10.790288	11.090314	11.150234	11.532142	11.546511	11.061851	9.966351	10.467694	10.346735	10.900827	10.472844	10.740962	11.021387	10.542840	10.603565	10.381639	10.735785	11.013401	10.384099	9.839396	10.488419	11.892007	11.556552	11.103274	10.205978	10.778399	11.423597	10.766421	11.448837	11.134182	11.577639	11.100894	12.078897	11.048406	11.406029	10.335092	11.847451	11.135510	9.796404	10.375909	11.263041	10.985679	10.663131	10.406371	11.185555	10.278350	10.658143	10.165028	10.639211	10.116828	10.465192	9.732580	8.803828	10.812246	9.218228	10.974077	11.056896	11.601281	11.386452	10.880464	9.449194	10.082846	9.561597	10.818184	11.260483	12.318233	10.976418	11.382901	10.859380

KRTAP12-1	5.338103	5.101129	4.488884	4.496646	4.986033	5.953780	4.470128	5.102166	4.379938	4.182761	3.657270	3.798389	5.131199	3.959247	3.703237	4.347401	4.255667	5.552778	5.718496	4.404898	5.368387	5.654240	4.778450	4.318222	4.198610	4.393599	4.838712	4.980732	4.630905	5.041365	4.799428	4.052563	5.234034	3.897785	5.263473	4.960000	3.837146	4.037774	4.673856	4.558305	3.969455	3.386886	5.569758	4.687813	4.858150	5.704669	4.863103	5.721394	6.131898	5.201290	4.145791	5.384096	4.474278	4.838925	4.891666	4.929514	4.130630	4.062858	4.582030	4.097759	3.012127	5.047697	4.820024	4.341562	4.944128	4.305947	5.632451	5.320335	4.764873	5.525162	4.588420	4.308576	4.584786	4.593634	4.629357	4.666013	4.696606	4.406043	4.377580	4.228779	3.545812	4.436050	3.430641	4.252787	3.469619	5.750104	3.876112	3.904201	3.345556	3.627841	4.245989	4.579040	4.823549	4.702563	5.020938	4.197968	4.814772	5.058170	5.886203	5.361351	5.136441	4.765468	4.794361	5.064275	4.544678	5.223543	5.522886	5.719936	5.833177	4.862684	5.501195	5.759262	4.913908	4.481977	5.029321	5.593652	5.114355	4.265101	3.491016	5.885856	6.187767	4.910082	4.401336	5.699682	5.929339	4.492677	8.861847	3.674948	3.958573	4.024123	3.921050	4.129806	4.499842	4.928648	4.262444	3.830779	5.862518	6.570524	7.685180	4.617724	5.155411	6.332486	4.669058	4.808685	4.018482	4.609716	4.714555	5.035696	4.904429	3.729032	3.937709	4.161815	4.689517	4.628254	4.256282	3.812713	4.277970	5.351089	4.151892	4.398284	3.003614	5.567659	5.202855	4.639063	5.147334	5.108923	5.865214	4.427875	5.133206	4.235322	5.066111	4.156948	4.352893	4.349824	4.604801	3.692171	3.446902	3.817794	3.302676	3.890731	5.325555	5.700984	3.958573	4.421729	4.752754	5.358537	5.137404	5.205070	5.473107	4.426556	4.609293	5.026650	4.462440	4.461781	4.535468	4.211406	3.993074	4.488455	3.432044	4.691987	4.671238	4.710123	3.653615	3.470703	5.851850	3.331586	3.971793	3.138594	4.293146	5.710503	4.306599	4.647039	4.478450	4.854156	3.886962	4.759750	4.380590	4.072332	4.553238	4.956231	5.622407	4.475603	5.474833	4.934074	5.169309	4.415573	5.208018	5.029939	5.163669	6.044476	5.629358	4.880948	4.892077	5.464428	5.778189	3.979150	4.527996	4.241625

ADGRE5	7.342525	6.950590	6.814585	7.519107	7.774461	8.608029	7.569307	8.802470	8.712506	8.128554	7.904280	8.269676	7.930805	7.706091	7.650787	7.483531	7.939788	7.887508	7.963882	7.761592	7.359016	7.804477	7.779974	7.657826	7.389191	8.429897	10.972659	8.292304	8.182181	7.768978	8.481866	7.319485	6.647062	8.496279	7.396166	7.304439	7.452274	6.724304	7.165954	7.338621	7.925059	7.316378	7.956793	7.420891	7.001459	7.019995	8.278420	7.014515	7.465403	6.282450	7.066825	7.092449	6.864398	6.481419	6.943280	8.139275	6.687456	6.908917	6.663954	6.506499	6.436771	7.801445	6.701902	6.886607	6.679132	6.551327	6.771711	7.127841	6.664613	7.208936	6.945812	7.340993	8.507964	7.045950	7.059340	7.766526	7.473087	7.446335	7.621942	6.486440	7.064759	6.838347	6.514720	6.447013	6.119063	7.767315	6.804468	6.790559	6.373825	6.067883	6.247783	6.361360	6.801084	7.238905	7.991905	7.622838	7.316049	8.233897	8.763170	8.032389	9.188603	6.880224	7.650640	7.386991	7.417717	8.343224	8.139581	7.885997	8.360634	8.304234	7.324066	7.868615	7.202847	8.830569	8.850756	10.614358	7.943715	8.393039	8.436316	8.087256	6.939358	7.019538	7.668203	8.542119	8.348987	8.374492	9.140691	8.031490	8.707378	8.237768	9.197337	8.719638	8.364085	8.296702	8.604996	8.305058	7.936228	7.619699	7.749351	7.515856	7.650473	7.937295	7.756974	7.556927	8.456103	9.505657	8.482646	8.595641	7.585809	9.214807	8.494468	7.655283	8.512310	7.892325	7.413508	7.280042	7.922721	7.886427	7.381102	8.812340	7.256013	7.378933	7.609277	7.156070	6.120281	8.784001	6.804922	7.284738	6.783375	7.546796	7.507225	6.918220	6.790708	7.353126	7.405852	6.718290	6.725575	7.324535	5.872074	7.249175	7.236582	7.383627	7.185203	7.175887	6.750073	7.114731	6.733213	7.466365	7.154973	7.403508	7.406955	9.208692	7.160590	7.565945	7.395552	7.186521	7.789294	7.323742	6.782296	6.627260	6.902075	7.343967	7.463419	7.055251	8.369224	6.720650	7.457944	6.875616	6.723303	6.978653	7.425716	7.487725	8.186342	7.721928	8.165811	8.688048	9.115721	9.004415	7.584871	8.767777	7.452446	7.505395	7.267882	8.066676	9.383032	9.028410	8.122926	7.205220	9.292820	7.775272	7.623325	6.479926	9.020466	8.569415	7.982701	7.260304	8.012608	7.375955

MAGI2	3.806805	3.246230	3.164186	3.162886	2.974624	3.202239	3.450425	3.339291	3.349518	3.403027	3.550231	3.574795	3.210436	3.656424	3.463740	3.351071	4.078269	3.379490	3.505520	3.145036	4.124217	2.889090	4.531822	3.375457	3.302053	3.021968	3.329019	3.113210	3.343473	3.224345	3.868591	4.006760	4.628254	3.257039	3.751463	3.702441	3.570961	3.217271	3.164694	3.115462	3.304924	3.486872	3.542277	3.215552	3.512383	5.871520	3.275589	4.525652	4.105841	4.115265	3.852056	3.786037	3.870512	3.723306	3.568681	3.413453	3.632741	3.940672	3.578986	3.424717	3.173569	3.219976	3.183693	3.227019	3.265866	3.329682	3.303625	3.361794	3.212131	3.058828	3.248143	3.218112	3.263834	3.251540	3.116393	3.298831	3.079410	3.132846	3.077475	4.022591	3.387908	4.247330	3.477470	4.505663	3.517346	3.294849	3.663842	3.252362	3.610154	3.607655	3.128332	3.014268	3.648603	3.529405	3.045058	3.240032	3.505344	3.550026	3.200147	3.390681	3.258230	3.269270	3.125639	3.082447	3.148764	3.075245	3.101295	3.417742	3.031075	3.395752	2.971853	3.246827	3.114384	3.378073	3.335812	3.217589	3.047744	3.026761	3.105403	3.478770	3.276202	3.061684	3.080285	2.952475	3.160172	2.968632	3.173949	3.418434	3.672507	3.550026	3.174211	3.235939	3.436845	3.802663	3.227169	4.041233	3.551024	3.225561	3.235659	3.917257	3.066288	3.637058	3.085822	3.151248	3.171433	2.976312	3.192826	3.158788	3.601290	3.889854	3.424717	3.025146	3.493366	3.548191	3.495403	3.552211	3.387383	3.124783	3.032744	3.386728	3.333065	3.201300	3.201300	3.794881	5.771071	3.018838	3.565517	4.261993	4.079820	4.354587	3.642881	4.493786	4.702139	3.623070	3.439246	3.427251	3.950770	3.443277	3.300671	3.133674	3.558339	3.443822	3.701819	3.408711	3.285202	3.042065	3.257039	3.296210	3.071095	3.272250	3.165689	3.051842	3.166091	3.185682	3.358308	3.153933	3.419896	3.269945	3.504732	3.987976	4.027084	3.320580	4.072762	3.608409	3.311774	3.799007	3.139813	3.335465	3.294372	3.436524	3.404038	3.236242	3.375457	3.326468	3.112756	3.064058	3.662866	3.295444	3.403532	3.311774	3.108304	3.055089	3.140544	3.377017	3.047106	3.313169	3.351555	3.471394	3.316355	3.029060	3.092072	3.085290	3.550424	3.012867	2.956909	3.302676	3.184101	3.055476

SNX22	7.682404	8.494468	7.373269	7.546964	7.088639	8.640670	7.861673	8.427398	6.993221	7.616037	7.820703	8.167229	7.224502	7.335968	7.420891	8.197159	6.845217	8.527361	7.927895	7.835680	7.543733	10.194758	7.320294	9.072203	8.103552	7.491020	9.526925	8.288257	8.079921	8.735003	6.834373	7.845183	9.998570	9.528007	8.400034	8.448582	7.113612	7.808635	7.529779	8.433978	6.917722	9.148458	9.240288	8.318066	8.687456	9.067544	8.348688	8.323832	7.728109	7.801000	7.485047	8.162350	8.540060	7.723151	8.167960	7.156225	8.653270	9.148458	7.538942	8.233292	9.082132	6.853155	6.422233	8.284696	8.742859	7.618328	7.547429	7.638623	7.781951	8.556399	8.172239	7.321996	7.848092	8.453841	8.254820	9.542676	7.115967	8.032389	7.822399	7.503538	8.153907	7.681614	8.413538	7.125453	7.674955	8.447497	7.497555	6.755395	8.636313	8.455824	9.431088	8.129817	7.687645	7.000974	8.851230	8.436796	8.455824	7.408204	7.801000	6.728831	7.256173	8.173786	7.183928	6.948257	8.321435	7.600626	7.985161	8.624827	7.224338	7.690392	6.995064	7.590734	7.738827	8.398835	7.416551	7.278613	8.400184	6.769290	7.419305	7.946920	6.205920	6.854940	8.251152	7.159964	8.382675	7.481352	8.782589	6.023247	7.348334	7.100327	7.877938	7.305240	6.999824	7.468757	7.131970	6.317575	8.348987	7.296044	6.946451	6.922885	7.907878	8.271947	7.428055	6.980014	6.760278	7.157612	8.509809	8.429432	7.263561	6.360702	7.512190	7.282571	7.434866	7.325492	7.715374	6.798807	6.626475	7.145206	7.187619	7.235475	9.118833	9.516845	6.811769	7.512793	7.586750	7.777145	7.637537	7.127371	6.575152	6.880869	7.610841	7.401461	6.670564	7.142720	6.690628	12.968209	8.362192	7.927574	8.568651	8.360170	7.371424	6.605303	6.768134	7.713435	8.596115	7.287232	8.381126	6.236327	8.040277	8.122600	6.235642	8.316068	6.223992	9.193762	8.217766	5.574445	6.378641	6.241098	5.951984	5.816371	6.776373	6.919359	8.104330	6.225550	7.913572	5.634913	6.653213	7.567799	6.992762	8.114241	6.462837	6.458196	6.388356	6.402115	7.273736	6.882930	7.098777	6.922099	6.887566	7.021564	6.400824	7.309930	8.353532	6.720482	8.707973	7.056336	7.004729	6.877972	6.910490	7.443164	6.961969	10.654274	9.351240	7.940095	6.889820	6.940592	6.111142	7.185875

ZNF254	10.648046	9.218228	8.560859	9.081657	8.370503	10.128547	10.766711	10.420249	9.226345	10.700309	11.161418	10.024660	9.296761	9.227425	9.290169	10.836621	10.062130	8.934800	6.934574	10.575699	7.218571	6.595347	6.110611	9.496425	7.689354	9.018966	8.277230	9.107052	9.911740	9.987919	9.724995	10.374121	10.713869	9.872723	10.187258	10.491721	10.527040	8.520528	8.380355	8.478824	11.122655	8.482171	7.772173	9.938052	9.283390	9.285198	9.364876	8.887283	10.346274	10.156215	9.743348	10.110195	10.060006	10.480801	9.446549	9.960750	9.950260	8.346622	7.838941	8.275089	8.095669	10.177339	6.632390	7.853280	8.630840	7.806324	7.903028	9.051585	8.112997	7.731485	10.521258	9.930473	10.236049	10.416445	10.532527	10.396131	10.432310	10.146257	10.413733	9.261505	9.896599	10.233994	10.424550	10.852247	10.736390	10.800743	9.794550	10.501539	8.088325	8.449850	8.239639	7.819194	9.337273	10.431097	10.380154	10.280332	10.591557	10.706734	10.584914	9.644557	10.698830	9.134109	8.940945	8.514154	7.356249	9.706262	8.116083	7.675589	6.562022	10.511848	7.239214	8.680362	6.913240	10.249984	10.478589	8.862000	7.898958	10.498864	9.885299	10.566949	8.996430	7.090725	9.404986	3.273434	7.833855	8.126124	8.662231	6.645317	8.121159	8.912971	10.310967	6.060096	8.187593	7.510460	9.577910	7.866197	7.671322	5.484805	9.908194	6.168430	6.385280	6.982537	6.659189	7.614495	8.282080	9.677830	8.426149	7.083448	7.884296	7.741802	8.592063	9.464554	7.750575	9.394059	10.274257	10.560393	7.747653	4.904203	7.512640	8.671407	6.779254	7.096715	10.395504	8.660218	7.872919	6.954704	9.111602	9.832460	8.753188	9.128766	10.410131	8.717058	10.004105	9.282306	9.810261	11.462640	7.925527	6.066609	8.280543	8.109085	8.386026	7.681453	6.570524	6.907669	8.522315	4.964988	7.992398	7.035624	8.425261	9.263984	9.776589	8.607415	9.967099	9.783775	9.304982	9.935596	8.574739	10.397036	10.357811	9.639191	9.794875	10.254211	10.438091	10.423303	9.077347	8.005145	9.997062	7.866197	6.579465	8.552568	7.198379	9.884692	9.936797	9.966216	9.812607	8.487865	9.253295	8.733026	9.899742	8.470020	8.243837	8.000720	6.603086	7.858176	7.855309	4.388263	5.600944	7.815434	8.577882	4.577985	5.385023	7.358404	9.225751	8.513380	7.539578	8.266810	10.029019	8.266810

BMP7	9.559852	10.310064	10.932488	10.940377	11.013967	11.039457	10.947592	9.118977	9.394510	10.016345	10.915065	10.878106	11.769915	11.028357	11.028968	9.878883	10.571069	11.314528	9.916529	10.936702	10.792186	10.289433	7.097666	11.039164	9.725903	10.614231	9.372113	7.772479	8.982281	10.353264	10.434445	11.268395	10.301733	11.619341	11.005839	11.281195	11.429272	10.917626	11.104875	11.011398	11.156846	10.287089	10.366087	10.462807	10.868599	11.102224	8.384656	10.371706	11.098087	10.867857	10.252781	11.226360	11.112959	11.628067	11.378139	10.892382	11.256343	10.989429	11.615965	11.418124	11.266465	10.214731	7.679371	9.979337	11.427522	11.254400	10.387179	10.349010	11.375453	10.955140	10.697641	10.438539	10.218950	10.426379	10.961222	10.072505	11.168397	10.539729	10.042666	11.126936	10.505113	11.080287	11.490551	11.529079	11.293383	11.230646	11.056587	11.300829	10.224836	10.450191	10.628696	10.591713	10.636091	10.776676	10.205361	11.023937	10.728828	10.854176	11.059091	10.318616	9.780558	11.119415	10.872927	10.710821	10.362545	10.888932	9.296135	10.517560	10.483072	10.321225	11.140636	11.455058	11.040450	9.426344	9.904851	8.216505	9.982592	10.220026	9.355284	12.666171	10.063327	10.890897	12.463562	10.369561	11.463053	10.581000	12.241742	11.593733	11.608315	10.711692	11.880130	10.948169	10.209666	6.868741	9.085355	10.633055	11.791975	11.170765	12.094734	11.551514	12.403727	11.602276	11.200300	10.975965	7.358858	9.017754	11.242229	12.211122	11.293530	5.048723	10.093062	11.085280	10.267937	9.576517	11.904904	10.857505	12.484391	9.111274	11.972822	10.478435	11.975910	8.814990	7.831401	12.721275	12.205131	10.213194	11.705064	12.354541	10.748889	11.666875	13.221572	12.114497	11.183466	11.190043	12.633633	12.891724	12.513208	12.645722	12.255831	10.332961	12.192751	10.236834	9.450439	9.959230	8.098401	9.843946	11.232575	11.188894	6.802353	10.774339	10.814911	9.381310	10.360811	10.271167	9.559089	10.568476	10.185424	10.973947	9.999670	11.226360	12.105285	10.559328	10.403638	7.783772	11.732575	11.283702	11.829126	9.171740	9.601437	12.470874	10.103567	11.679650	10.116521	11.044580	9.839560	12.151398	10.833585	8.966496	12.730652	8.771708	12.701928	10.300662	10.662816	10.127748	11.531390	10.027803	9.298003	11.715417	8.929384	11.080441	10.507987	10.815965	8.521422	12.770516	9.466435	12.285989	11.927240	7.937592

ANO5	4.493308	4.205714	3.694665	3.729835	3.528850	4.589492	4.127852	4.658582	4.792523	4.098433	4.086810	3.960729	3.609968	4.008008	4.426792	4.084103	4.131246	3.966392	4.457358	4.753849	4.448705	4.163347	4.753194	3.647373	4.470128	4.332682	4.847214	4.529888	4.563852	4.749752	5.153997	3.647986	5.636016	4.049098	5.016475	5.759794	4.130225	3.183972	4.306356	3.835027	5.075463	4.424419	4.803256	4.002780	4.520611	6.359358	4.630254	5.955394	4.329236	3.912495	3.960312	4.378001	4.368077	3.896934	3.784156	4.258522	4.313602	2.939236	3.475806	3.866394	3.957460	4.187994	4.964389	4.292692	4.313187	4.492437	2.888289	3.832712	5.302239	4.001688	4.097759	4.034080	5.081663	4.736757	4.435851	4.910284	4.916406	4.071461	4.424840	3.636076	3.468209	4.048215	4.419157	4.419157	4.111801	3.490484	3.769513	4.071686	4.082384	4.984987	4.909265	5.050399	3.806591	3.817590	3.935805	3.952698	3.777351	3.669264	4.383610	4.271805	3.495973	3.959039	4.180324	3.674319	4.017929	5.052996	3.869240	4.223852	4.647475	4.553238	4.674273	5.142355	3.509805	4.607995	4.313602	3.359154	4.779250	4.068163	4.698520	4.230073	3.897372	3.376866	4.316491	3.249142	4.978711	3.739136	6.071530	4.566806	4.581620	3.682035	3.926119	3.330300	3.573251	3.922002	4.350669	4.353117	3.174332	3.752726	4.802623	3.796720	3.872223	6.268096	3.510367	3.837146	3.320580	4.301102	4.041676	3.966177	4.749977	4.938486	3.820377	4.553238	3.695698	4.112008	6.350795	5.153779	3.247680	4.400675	4.310387	4.721287	5.939145	4.552354	3.732517	4.007400	6.501392	4.600612	5.655749	4.243194	3.995497	3.961808	4.559658	4.038443	3.970521	3.373589	4.278859	6.186579	2.924747	3.121240	6.463552	2.996841	4.638012	3.653400	4.520611	4.285445	3.662649	2.871232	3.773875	6.842402	4.920613	3.692171	4.089834	5.809052	4.620143	5.929174	4.967792	5.028096	3.822483	4.383150	3.614957	3.658626	4.200518	3.744640	3.567285	4.326137	3.389306	3.662649	5.304993	2.968910	4.186196	4.831864	5.467740	3.684817	3.840679	4.415762	4.700068	3.342302	3.415541	4.423102	3.573059	3.516786	3.248272	5.117409	3.139069	6.691940	4.714127	3.928272	3.275887	4.128953	4.817923	4.606057	4.368291	3.323489	4.625263	3.131365	3.201711	4.762246	4.173385	4.413089

PVR	9.798264	8.829025	9.099653	9.130300	8.799286	8.367911	8.905230	8.489514	8.380662	8.769584	8.794756	8.765596	8.463529	8.358315	8.837687	8.139115	9.219434	8.681429	7.721779	8.818873	8.292455	8.579234	8.249737	8.126124	7.557702	8.646914	8.454436	8.737163	8.635528	8.692954	9.239370	9.310505	9.256834	9.166498	8.699095	8.500281	8.698199	8.453530	8.196392	8.255129	8.858058	8.046717	8.036507	8.587856	8.966199	8.404521	7.972255	8.180232	9.743348	8.797307	8.933148	8.745438	9.242889	8.960834	9.505657	9.075755	9.051002	7.817936	8.247106	8.492438	7.936999	9.237192	9.403399	8.057141	8.411096	8.918918	7.635884	8.111766	8.258639	7.657521	8.223754	8.524461	8.468844	8.573650	8.369224	7.834020	8.425886	8.952418	8.618776	8.653894	9.212359	9.502229	9.376353	9.168150	9.150322	9.018822	8.836027	9.202653	7.693051	7.701453	7.633902	7.776998	8.495364	9.297552	8.266021	8.650408	9.558482	8.887600	8.785314	8.607116	9.226978	8.602527	8.517004	8.932263	9.339197	8.866879	8.098401	8.942331	8.370991	8.544600	6.671872	7.471425	8.492438	8.014946	8.906422	6.517896	8.899923	9.337708	8.036983	8.869289	10.500161	9.740576	9.396514	9.980904	9.930936	10.334799	8.310979	9.657347	9.781478	9.501025	9.973251	9.583467	10.273930	10.734015	8.085844	10.350375	8.907201	7.939314	10.648931	9.829507	9.712029	8.950728	9.562068	8.988078	10.167666	9.933222	9.811172	7.867093	10.093823	10.802991	11.300099	9.690083	10.371545	9.788490	10.073446	9.897941	8.975271	10.080574	9.477056	10.085713	8.923712	9.365664	10.102338	10.018797	9.630595	9.540974	8.199678	9.635632	10.370938	10.258842	10.332208	9.912045	10.383819	9.724538	9.411269	9.363051	9.253455	9.399241	9.790059	10.297862	9.772502	9.500882	10.380306	8.625761	8.424675	10.306560	8.964176	9.906665	8.816052	8.845859	9.769058	9.620259	9.465964	8.484010	9.623678	10.231920	9.929695	9.649935	9.084407	9.593060	10.047711	10.977437	9.782227	10.919567	9.690233	10.222722	9.696322	8.712212	8.766232	9.709211	9.926476	9.664041	11.103722	10.575116	9.766026	9.353052	8.207086	10.220796	9.834281	11.857035	9.032049	9.122460	9.318351	10.309258	9.535836	9.348480	9.197337	9.125034	9.346035	7.544378	9.367576	7.147061	7.427903	9.440177	10.154056	9.712939	9.542983	10.704038

CLEC4D	5.930243	7.074821	7.869975	5.633382	5.485751	5.635849	6.106910	6.524190	6.703996	5.806134	5.290575	4.768483	4.961439	5.195106	4.974843	5.501796	6.270170	5.138648	5.785779	5.046472	7.092916	6.099807	6.402607	5.841522	5.866298	5.360226	6.235468	6.042958	5.491509	7.292246	5.948816	7.815256	7.755464	6.246435	6.989994	5.899364	7.120747	6.894802	7.190009	7.160752	5.662352	6.420776	6.789085	5.516471	5.644627	6.999519	5.328715	6.864246	9.679406	6.422080	6.297901	7.333659	7.791273	5.553982	8.660218	6.070667	7.825370	6.594520	5.244612	6.690120	7.139071	5.404785	10.057591	5.992513	7.924758	7.554725	6.760102	6.162716	7.268030	5.907399	5.936504	6.240763	5.448989	6.768134	5.882835	7.717814	6.402431	8.706907	7.047214	8.867333	7.391023	8.133846	5.871520	6.823214	6.677849	5.248396	7.456332	8.191485	7.895952	7.450589	6.751438	9.162292	7.987940	8.459105	6.084236	6.512277	7.936388	4.816027	5.524404	5.526678	5.297845	5.542232	5.137599	5.665514	6.685504	5.272910	6.248977	6.393047	6.754571	5.560750	6.492192	5.859241	6.086091	5.574617	5.834109	6.434267	5.927937	5.519876	4.646000	6.000261	6.896050	6.627098	5.903090	5.825674	6.177466	5.539969	6.294903	6.660032	5.611787	5.632831	5.317069	5.654240	6.265543	6.012132	5.481420	6.759287	5.100918	6.240938	6.256420	6.970435	7.234690	8.097062	6.070155	5.749744	6.601179	6.066973	5.412818	5.416606	6.878780	6.271028	8.191967	6.803329	5.922559	6.380453	5.749031	7.910483	7.337348	6.602156	8.952418	6.160836	7.807713	7.603766	6.879088	7.621803	7.625172	6.204015	7.464042	5.949351	7.420235	7.791742	8.054626	7.807713	7.826786	8.072303	5.778728	7.560294	7.390104	6.418325	8.156255	8.459410	6.274392	8.580900	7.204710	6.508946	8.441475	7.987496	9.360161	7.535183	9.098599	6.821650	7.108364	5.521563	6.291862	6.716392	8.514154	6.469202	7.573258	6.703844	8.182181	6.246949	7.513898	7.560886	8.582697	7.203493	5.016263	8.103097	7.015163	8.157493	8.677427	7.869699	7.656711	6.651258	7.425236	7.415795	7.917375	5.814413	5.241252	5.985916	4.998777	5.833523	5.200081	5.045524	8.187444	6.280339	5.384096	6.353603	6.392355	7.593028	5.929525	7.963592	6.282784	8.024072	5.565388	5.642738	5.770690	7.562869	5.288542	5.588508

FOXC1	10.313708	11.523534	11.043290	9.720786	10.197958	10.265145	11.321435	10.701962	10.614358	10.758230	10.506765	11.417099	11.461288	9.587642	10.741254	12.162807	10.848877	9.817990	9.336323	12.028097	9.735002	8.397333	7.214830	9.979955	9.874356	11.011088	11.275177	12.088432	12.798678	11.314528	9.368318	10.718513	10.100927	11.440637	10.204147	10.621312	11.409053	9.557552	9.569343	9.572587	10.944027	11.005405	10.340663	10.974225	10.779848	10.070706	11.842489	10.188771	11.378571	10.463567	10.728368	10.630255	11.388220	10.888202	11.418835	10.446580	10.752372	9.808891	10.430330	10.560870	10.473452	11.563737	11.753524	11.189746	10.233845	9.908194	9.977410	10.098874	9.658892	10.913411	11.164654	10.450933	10.145351	11.172831	10.496003	10.465042	10.845168	11.897375	11.645767	9.553140	9.921992	11.699459	11.133688	10.980998	10.496489	12.522828	10.084519	10.198291	10.107923	10.474513	10.113060	9.805954	10.575868	10.860574	11.989104	10.384381	12.316234	10.102474	11.576321	10.118700	10.795839	10.054057	9.958130	10.425753	9.468725	10.925219	9.572739	9.037850	9.190243	10.751617	8.171919	10.639535	9.054399	9.984899	11.242685	8.263388	7.705019	11.564033	10.789717	10.567100	10.492998	10.412549	8.638314	9.400578	8.372378	9.501173	12.114644	10.005054	10.359776	10.252193	9.934185	9.718454	10.751617	10.413889	11.344909	9.590429	7.796324	10.282310	10.798488	9.647063	8.726355	7.869548	10.263444	9.831389	9.613345	12.314612	9.593539	8.670782	11.033895	9.401376	11.899423	9.814319	12.082135	10.922983	10.311282	12.144846	9.921529	9.863624	10.745574	8.458939	9.715949	10.625346	11.820354	9.638887	9.668932	10.148232	12.234346	10.341269	10.379863	10.742902	11.185995	10.640900	10.891628	10.795127	10.482002	12.726021	10.026576	8.782909	9.103349	9.529758	10.773877	9.656115	10.668858	9.721996	10.404553	10.457422	9.795507	10.176239	11.976069	11.600742	11.365804	10.147175	9.895500	11.580566	11.533627	10.832422	10.993001	11.654988	10.176239	10.153274	10.399900	10.424091	9.502375	10.625346	10.873822	10.489958	10.819087	11.167837	9.591500	8.531668	9.515156	9.713721	10.950433	11.530149	11.536442	10.936398	9.673663	11.103274	10.984493	9.974049	13.465756	10.700619	10.209812	9.721843	10.404553	8.167229	8.620186	9.504879	12.153600	6.780059	10.232963	11.713495	9.511361	10.108827	12.125808	9.275288	9.883634	9.523489

WFDC9	4.833328	5.560405	5.297845	5.325377	4.874437	4.730184	4.358969	6.562022	5.876206	4.459459	4.876743	4.185118	3.608409	3.510558	3.346871	5.182826	6.222275	4.634782	5.957159	4.424419	3.888504	5.495885	4.201183	4.013337	4.477837	4.647039	4.361519	6.295776	3.682691	3.980405	5.481638	5.671606	3.547999	3.808898	4.911116	4.451832	4.218793	4.568525	5.356463	5.529921	4.326548	4.479330	5.215422	4.810821	3.430280	5.406292	5.779623	5.127355	5.712884	5.015654	5.781271	5.679002	3.521300	5.265256	3.929346	4.131246	3.790786	3.516064	4.686349	4.183639	5.703574	3.699591	5.511627	6.047988	5.565581	5.764864	5.129993	5.216032	4.996090	6.362012	5.223352	5.867788	4.897460	5.064681	5.628020	5.374929	5.554537	5.620917	6.260824	4.701728	5.065719	5.284771	5.248396	5.147545	6.064868	4.611640	4.701109	6.038416	5.707790	5.738273	5.521370	6.257069	4.993108	5.189959	5.068793	4.300684	4.725569	3.886748	4.221265	4.421506	3.956800	4.974640	4.649422	4.669058	4.785361	5.648403	6.276052	5.066538	5.731388	4.948142	5.900433	5.469704	6.342844	5.178778	5.531254	4.193008	5.605327	5.355688	4.555132	4.621851	5.179925	4.509641	4.980326	5.849360	4.762913	4.160365	5.148519	4.984377	4.285445	4.865170	4.299199	3.839470	4.207761	4.092275	4.315607	5.688078	4.221682	5.050781	4.427225	3.500685	4.157168	6.266222	4.546670	4.826274	4.882858	4.964590	4.787871	4.577985	4.275288	4.502632	3.655248	3.419322	4.973155	4.509201	4.310828	4.175536	4.198815	4.901535	4.546011	4.707226	4.888751	4.525652	5.559465	3.611397	5.830114	5.939145	3.558141	4.489115	5.037916	4.930806	4.507002	4.818987	4.179448	3.828244	4.503080	4.613162	3.872015	4.916406	4.352225	4.745539	4.443479	5.560405	5.887073	5.499857	5.515838	5.050186	4.990394	4.976700	5.911515	4.872775	4.969254	5.912785	4.955407	4.581620	5.087179	4.868526	5.954733	5.614619	3.679225	4.039945	5.147545	4.728702	4.178097	5.507796	4.363938	4.930625	5.179925	5.383539	4.913039	4.726203	5.313603	3.985631	4.936617	5.047064	4.563408	4.190822	4.141068	4.123822	3.883794	4.521048	4.259179	4.324651	4.299199	5.145352	4.856480	6.223823	5.269249	5.797835	4.785361	5.033779	5.542439	5.646696	5.184212	4.857330	5.609146	5.862898	4.657931	4.917896

CPM	12.226148	12.804949	12.893478	12.613560	12.968566	12.566268	12.516941	12.909129	12.415626	12.323253	12.771214	12.449603	12.612348	12.797127	12.405117	12.763103	12.527775	12.419436	12.258675	12.816305	12.422459	12.160822	11.243314	12.506071	11.972222	12.357184	12.217765	11.987250	12.156499	12.611352	12.304791	12.432421	12.481977	12.611352	12.734538	12.672605	12.691855	12.365907	12.298540	12.171733	12.511128	12.386589	12.813004	12.937089	12.769062	12.344233	12.601063	12.435325	12.459772	13.131924	12.663909	12.675008	12.619138	12.906787	12.603413	12.708953	12.671375	12.299183	12.169579	12.300012	12.045877	12.344853	12.260195	12.419759	12.394267	12.462928	12.285680	12.595412	12.415458	12.435481	12.673286	12.729124	12.473114	12.911640	12.833456	13.155545	13.030100	12.418490	12.588951	13.077603	12.587968	12.650060	12.916404	12.892609	12.596273	12.624672	12.685514	12.919527	12.546681	12.506387	12.371489	12.735398	12.613710	13.132988	12.823360	12.940096	12.671375	12.354541	12.425577	12.373685	12.389829	12.735048	12.707214	12.834869	12.502069	12.539305	12.854034	12.397858	12.243771	12.583137	11.938785	11.995822	12.625160	12.313325	12.972757	11.857513	11.917886	13.006339	12.378969	14.223156	12.247353	12.933125	13.383693	11.949587	12.681971	11.816305	13.922935	11.949752	13.044646	11.988788	12.630986	11.782817	12.270578	11.616843	12.241433	11.848352	12.161407	12.281444	12.838923	12.281277	12.473611	12.262315	12.630185	11.578205	12.244367	12.948910	12.437743	12.530838	12.491562	12.369546	12.467321	11.898369	13.352991	12.867268	12.954789	12.483749	11.970367	11.983055	11.840994	12.269467	12.744026	13.130760	12.558326	12.621674	12.386932	12.096818	12.697321	12.833106	12.855334	12.645369	12.481303	13.675974	12.060534	12.374349	12.613037	13.202580	12.796960	12.396914	13.353095	13.881710	12.098604	12.440954	12.211771	12.395067	12.837405	12.563972	12.559720	12.269936	12.595587	12.699016	12.964368	12.111742	12.304038	12.806146	12.918224	12.263397	12.824882	12.854978	12.232212	12.445298	13.043715	12.090726	12.943738	12.378643	11.641173	12.473611	12.882781	12.089348	11.892168	12.601913	12.087562	12.508187	12.203451	12.512588	12.380187	12.294510	12.061997	11.819362	12.847695	12.016883	12.655512	12.240857	12.854219	11.913837	12.615346	12.901976	12.712524	12.810731	12.576234	11.987098	12.020355	12.346785	12.477509	13.174367	13.068499	11.996401	12.447416	11.988461

MDM2	12.226148	12.804949	12.893478	12.613560	12.968566	12.566268	12.516941	12.909129	12.415626	12.323253	12.771214	12.449603	12.612348	12.797127	12.405117	12.763103	12.527775	12.419436	12.258675	12.816305	12.422459	12.160822	11.243314	12.506071	11.972222	12.357184	12.217765	11.987250	12.156499	12.611352	12.304791	12.432421	12.481977	12.611352	12.734538	12.672605	12.691855	12.365907	12.298540	12.171733	12.511128	12.386589	12.813004	12.937089	12.769062	12.344233	12.601063	12.435325	12.459772	13.131924	12.663909	12.675008	12.619138	12.906787	12.603413	12.708953	12.671375	12.299183	12.169579	12.300012	12.045877	12.344853	12.260195	12.419759	12.394267	12.462928	12.285680	12.595412	12.415458	12.435481	12.673286	12.729124	12.473114	12.911640	12.833456	13.155545	13.030100	12.418490	12.588951	13.077603	12.587968	12.650060	12.916404	12.892609	12.596273	12.624672	12.685514	12.919527	12.546681	12.506387	12.371489	12.735398	12.613710	13.132988	12.823360	12.940096	12.671375	12.354541	12.425577	12.373685	12.389829	12.735048	12.707214	12.834869	12.502069	12.539305	12.854034	12.397858	12.243771	12.583137	11.938785	11.995822	12.625160	12.313325	12.972757	11.857513	11.917886	13.006339	12.378969	14.223156	12.247353	12.933125	13.383693	11.949587	12.681971	11.816305	13.922935	11.949752	13.044646	11.988788	12.630986	11.782817	12.270578	11.616843	12.241433	11.848352	12.161407	12.281444	12.838923	12.281277	12.473611	12.262315	12.630185	11.578205	12.244367	12.948910	12.437743	12.530838	12.491562	12.369546	12.467321	11.898369	13.352991	12.867268	12.954789	12.483749	11.970367	11.983055	11.840994	12.269467	12.744026	13.130760	12.558326	12.621674	12.386932	12.096818	12.697321	12.833106	12.855334	12.645369	12.481303	13.675974	12.060534	12.374349	12.613037	13.202580	12.796960	12.396914	13.353095	13.881710	12.098604	12.440954	12.211771	12.395067	12.837405	12.563972	12.559720	12.269936	12.595587	12.699016	12.964368	12.111742	12.304038	12.806146	12.918224	12.263397	12.824882	12.854978	12.232212	12.445298	13.043715	12.090726	12.943738	12.378643	11.641173	12.473611	12.882781	12.089348	11.892168	12.601913	12.087562	12.508187	12.203451	12.512588	12.380187	12.294510	12.061997	11.819362	12.847695	12.016883	12.655512	12.240857	12.854219	11.913837	12.615346	12.901976	12.712524	12.810731	12.576234	11.987098	12.020355	12.346785	12.477509	13.174367	13.068499	11.996401	12.447416	11.988461

AC010422.8	12.235143	12.124933	11.727527	11.687667	11.740420	11.313492	12.000568	11.784487	11.080590	11.759472	11.580132	11.727061	11.513485	12.033841	12.317939	11.336132	11.744351	12.154483	11.917442	11.720169	11.909110	12.601240	12.298055	11.728087	11.848200	11.686785	10.731203	11.208087	11.138316	11.602409	11.411376	12.357687	12.062616	12.015212	12.109069	12.064410	11.967186	12.288638	12.502227	12.400126	11.441892	12.603082	11.911179	11.920823	11.967645	12.391754	11.253671	12.542605	12.111299	12.242384	12.174349	12.395993	12.447724	12.498162	12.519046	11.454008	12.482634	12.955887	12.749833	12.685053	13.174367	11.524269	12.962142	12.916212	12.955541	12.926643	12.694586	12.554118	12.480547	12.761183	11.916216	12.065467	11.106454	11.851856	12.004567	12.044248	12.050418	12.058410	12.123620	12.091066	12.063042	12.284575	12.071894	12.348060	12.001025	12.238997	11.880566	11.922181	12.677669	12.611352	12.505580	12.717320	12.389189	12.030069	12.168505	12.180168	12.432907	11.434046	11.648697	11.525740	11.651535	12.210352	11.855029	11.828107	13.233810	10.947453	12.426215	13.035974	12.448012	11.199882	12.667996	12.412694	12.595226	11.081157	11.716442	10.657546	12.793936	11.749834	10.683914	12.639025	11.948495	11.940454	12.592167	10.771657	12.544205	12.215055	11.051479	11.482684	11.431661	11.705064	12.042551	11.908802	11.637074	11.572521	11.056273	11.930334	12.181582	11.902791	11.860414	12.214434	12.786841	12.615027	11.661349	13.066780	11.892168	11.472640	11.468687	10.766574	11.379157	10.899829	12.167877	12.117118	12.586624	12.287067	12.053768	11.724977	12.283820	12.725496	12.557817	11.701443	12.863843	13.255166	12.413004	11.769458	12.655859	11.240073	12.261651	12.032756	12.683650	12.273308	12.471647	12.813692	12.966913	12.511287	11.417391	12.032756	12.930801	12.456454	13.048184	12.806146	12.162501	13.677133	13.190846	12.972942	13.073797	12.833844	12.076508	12.391894	12.963290	12.116671	12.035185	11.410682	11.735820	12.245147	12.175621	12.301244	12.245455	12.212996	11.860703	12.115840	12.022508	12.180653	11.834521	12.351946	12.485666	11.890938	12.123468	12.745204	12.214583	13.425768	13.512208	12.351197	12.619822	12.204037	11.509511	11.516794	11.599182	11.922181	12.347415	11.922034	11.553440	11.489358	11.705791	13.091997	11.034454	12.051649	11.722749	12.756348	11.454602	12.774646	12.348522	11.834209	11.116825	12.052666	11.935649	12.757381	11.856727	12.145451

DHPS	12.235143	12.124933	11.727527	11.687667	11.740420	11.313492	12.000568	11.784487	11.080590	11.759472	11.580132	11.727061	11.513485	12.033841	12.317939	11.336132	11.744351	12.154483	11.917442	11.720169	11.909110	12.601240	12.298055	11.728087	11.848200	11.686785	10.731203	11.208087	11.138316	11.602409	11.411376	12.357687	12.062616	12.015212	12.109069	12.064410	11.967186	12.288638	12.502227	12.400126	11.441892	12.603082	11.911179	11.920823	11.967645	12.391754	11.253671	12.542605	12.111299	12.242384	12.174349	12.395993	12.447724	12.498162	12.519046	11.454008	12.482634	12.955887	12.749833	12.685053	13.174367	11.524269	12.962142	12.916212	12.955541	12.926643	12.694586	12.554118	12.480547	12.761183	11.916216	12.065467	11.106454	11.851856	12.004567	12.044248	12.050418	12.058410	12.123620	12.091066	12.063042	12.284575	12.071894	12.348060	12.001025	12.238997	11.880566	11.922181	12.677669	12.611352	12.505580	12.717320	12.389189	12.030069	12.168505	12.180168	12.432907	11.434046	11.648697	11.525740	11.651535	12.210352	11.855029	11.828107	13.233810	10.947453	12.426215	13.035974	12.448012	11.199882	12.667996	12.412694	12.595226	11.081157	11.716442	10.657546	12.793936	11.749834	10.683914	12.639025	11.948495	11.940454	12.592167	10.771657	12.544205	12.215055	11.051479	11.482684	11.431661	11.705064	12.042551	11.908802	11.637074	11.572521	11.056273	11.930334	12.181582	11.902791	11.860414	12.214434	12.786841	12.615027	11.661349	13.066780	11.892168	11.472640	11.468687	10.766574	11.379157	10.899829	12.167877	12.117118	12.586624	12.287067	12.053768	11.724977	12.283820	12.725496	12.557817	11.701443	12.863843	13.255166	12.413004	11.769458	12.655859	11.240073	12.261651	12.032756	12.683650	12.273308	12.471647	12.813692	12.966913	12.511287	11.417391	12.032756	12.930801	12.456454	13.048184	12.806146	12.162501	13.677133	13.190846	12.972942	13.073797	12.833844	12.076508	12.391894	12.963290	12.116671	12.035185	11.410682	11.735820	12.245147	12.175621	12.301244	12.245455	12.212996	11.860703	12.115840	12.022508	12.180653	11.834521	12.351946	12.485666	11.890938	12.123468	12.745204	12.214583	13.425768	13.512208	12.351197	12.619822	12.204037	11.509511	11.516794	11.599182	11.922181	12.347415	11.922034	11.553440	11.489358	11.705791	13.091997	11.034454	12.051649	11.722749	12.756348	11.454602	12.774646	12.348522	11.834209	11.116825	12.052666	11.935649	12.757381	11.856727	12.145451

FDFT1	9.135468	8.911908	9.001514	9.559249	10.073916	9.837036	8.867920	9.841075	11.009167	9.320194	9.904108	9.586073	9.499786	9.561747	10.030393	9.692841	10.544020	9.898694	11.394116	9.303419	10.223615	10.178271	11.763565	9.923526	10.374399	10.559328	10.405917	10.870351	9.747411	8.826560	10.451313	8.353209	8.468844	9.213755	8.677119	8.893784	8.926942	8.902785	9.555676	9.760895	9.386364	9.819818	9.569039	9.570429	9.564026	9.662014	9.895199	9.406320	8.924017	9.141158	9.254659	8.895309	8.571418	8.642515	9.033411	9.596161	8.915240	9.278587	9.421461	9.206130	9.240739	9.359075	9.840623	10.199612	9.354991	9.256667	9.204423	9.159694	8.988978	9.770486	8.816369	8.977700	9.810561	8.881755	9.072203	8.858812	8.974964	8.791374	8.990911	8.903998	9.122460	9.191651	9.054553	8.667435	8.399882	8.893638	9.115266	8.806574	9.552669	8.915861	8.930003	9.766473	9.018218	8.669134	9.117878	9.431886	8.858812	10.261790	9.678618	9.977119	10.024017	8.938834	9.806105	9.813229	10.004426	10.239265	10.376965	10.113185	11.400599	9.731680	10.994463	10.691500	10.900700	10.119905	10.415257	11.472792	10.355834	9.763561	10.170916	9.363934	9.476414	9.141665	9.924431	11.055379	10.349902	9.702186	10.556772	10.067808	10.035467	9.786686	10.132441	10.676730	9.986408	10.622485	9.799495	9.731085	10.630112	11.425214	8.876155	10.539400	10.776828	11.167543	11.107188	10.138967	10.207334	9.865593	11.171227	10.389118	9.307738	10.663859	9.857414	10.061353	9.631817	9.007935	9.573678	9.113027	9.534750	10.052342	10.415564	10.324901	9.754059	9.496268	9.897040	9.736856	9.733489	10.762352	9.528632	8.911270	9.327364	9.336166	9.605311	9.096148	9.218401	9.298286	9.326153	8.933782	9.443739	9.626311	8.931939	9.849598	9.449820	10.200882	10.225283	10.158664	9.517441	9.729026	9.038502	9.432672	9.698632	9.073136	9.191817	10.781910	9.215294	9.047293	8.953955	9.161830	9.231299	9.486271	9.067393	9.667179	9.273471	9.679709	9.311708	8.894109	9.847374	8.928787	9.109712	9.755469	9.665455	9.709371	10.016654	9.382710	9.299086	9.483634	9.705173	9.668780	10.626257	9.863922	9.231754	9.968820	9.979042	9.960604	9.793310	9.910666	10.432156	11.630155	10.364000	10.569984	10.559792	11.110584	10.571850	11.104280	10.476474	9.594640	9.867886	9.922578	10.146111	9.796886

TRIB3	9.472625	11.399821	11.357821	10.999538	11.501897	11.236568	10.907071	12.634617	11.953180	11.372593	11.311166	10.842875	10.341118	10.975673	10.563844	11.726750	11.458006	10.965488	12.775154	11.296130	12.011728	12.412540	11.542870	11.701015	11.423450	11.355342	9.682776	11.511335	10.902031	10.470880	11.015142	10.429717	11.407985	9.551013	10.887345	11.157147	11.244940	12.161114	12.043959	11.804534	10.708068	11.674723	11.396684	11.207783	10.421803	11.455058	11.126936	11.436941	11.519937	11.678021	11.420572	11.446601	11.187873	11.656322	11.425929	11.439430	11.073706	12.045372	11.562313	11.151138	11.758300	10.734015	9.419138	12.490240	11.430298	11.577041	11.873552	11.668608	11.100452	12.123909	11.371423	11.255319	11.298834	11.136541	11.300099	10.536771	10.879893	10.932934	10.984790	10.432922	10.246964	10.257113	9.627839	10.697067	10.720354	11.164944	10.214570	10.650579	10.902636	11.539378	10.864843	11.148713	10.630255	10.700452	9.922734	10.129307	11.484580	11.081888	10.621011	11.452759	11.279734	11.363130	11.495915	11.792414	12.478971	11.367280	11.720627	12.228964	12.318233	11.435100	12.721275	12.097254	12.012208	11.566391	11.687667	10.551435	9.365980	11.002796	11.343546	10.097624	9.597413	9.372405	10.169807	9.434778	10.173663	10.123807	8.994576	10.380607	9.575634	9.636997	10.018797	10.550537	8.916172	9.499181	9.739786	9.372726	8.915570	10.017413	8.868537	10.501376	9.347557	9.761509	9.960448	9.332303	9.765127	10.570935	10.585651	10.162738	9.892267	9.088528	8.798858	9.281202	10.555843	8.860617	9.318053	10.243523	10.580695	10.563986	9.416264	9.764038	10.506163	9.731377	9.813229	9.974049	10.710978	11.155939	9.539708	9.612559	10.099038	9.735158	10.483232	9.182973	10.671652	9.418245	9.449194	8.959613	9.674754	10.242602	9.857107	9.941496	9.881326	11.016056	10.931745	9.655028	9.127501	10.022591	11.051636	10.281231	10.934731	9.708449	9.263235	11.754682	9.538600	8.827612	9.885467	9.072667	9.272995	10.470433	8.573650	8.853466	9.393270	9.252981	8.476201	8.510749	8.700125	9.548217	8.468700	9.231754	9.359995	9.116189	8.797307	8.837089	9.220351	8.622822	8.715790	9.523644	8.739929	8.954704	8.889622	8.916335	9.143958	9.162611	9.085022	8.288718	9.793920	9.760895	10.981739	11.303833	10.722821	9.580855	9.340577	9.234409	11.793930	8.855027	9.755312	8.595641	9.469843	9.598639

ERC1	12.101182	11.866700	11.842192	12.020641	12.147944	12.626178	12.807870	13.023845	12.282557	12.606564	13.063978	12.634097	12.337443	12.626354	11.674007	12.913586	12.393291	11.754223	10.691016	12.434806	10.982470	10.050969	9.875282	11.869200	12.192751	12.213340	13.075520	12.031083	12.603413	12.603238	13.017706	12.914641	12.344564	12.297906	12.579997	12.461907	12.676040	12.412056	12.304791	11.749227	12.871579	12.016290	11.901889	12.501413	12.664096	12.393793	13.074750	11.589394	12.247833	12.709993	12.084324	12.767266	12.310761	12.197841	12.050418	12.877302	12.384018	11.894583	11.978386	12.146410	12.272044	12.847185	11.780904	11.093405	11.888725	11.750863	11.683795	11.533937	11.621743	11.708338	12.563485	12.244073	12.688122	12.496017	12.412844	12.551723	12.634617	12.144058	12.040929	12.146410	12.614361	12.208851	12.322524	12.841934	12.809294	12.632301	12.427834	12.371333	12.129935	12.018571	11.872205	12.054081	12.270412	12.578683	12.198897	12.722972	12.588785	12.687096	12.813513	12.547624	12.541971	11.887052	12.076036	11.823531	10.511086	12.652437	12.100593	9.916529	10.621011	12.511756	10.017560	11.536144	12.106846	12.631160	12.646251	11.860081	11.060572	12.899723	12.684724	13.166504	12.441748	12.205414	11.854155	11.777916	12.111153	13.024751	12.569861	13.292154	13.654386	12.627995	12.905355	12.637159	10.953197	11.435808	12.540631	12.415286	11.978981	10.724311	13.932466	10.829597	9.764510	9.999670	11.566103	11.438954	13.031189	12.476862	11.952432	11.783278	12.360678	12.506232	12.361009	12.958279	12.968393	12.574386	12.504062	12.552991	12.244073	12.775154	12.002196	13.592392	12.175621	13.582053	11.934741	12.013408	11.644307	12.618446	11.278715	12.170171	12.879408	12.473286	12.091212	12.753122	12.600707	12.123151	12.934196	12.869585	12.042883	11.886135	12.169723	12.462229	13.142638	10.875592	11.392925	11.839166	12.137398	11.694097	11.936694	11.490395	12.015827	11.791025	13.273969	12.620642	12.453263	12.359115	12.691189	12.380187	13.193698	12.871943	12.215771	12.773448	13.021758	12.289514	12.556062	12.638368	12.758401	12.421501	13.378878	12.068447	11.938632	11.922181	12.740063	11.791508	12.813342	12.764987	12.750695	12.262621	12.189336	13.108685	11.862884	11.928304	11.679650	11.527756	11.181727	10.378348	12.210352	11.915640	10.719109	10.722975	13.399301	9.964867	11.158191	12.117118	12.578683	11.867445	13.040990	11.644307	12.653816	13.111982

TSPYL4	12.262939	11.656735	12.063494	11.798061	12.125960	11.510541	12.640046	11.788526	12.701928	12.382891	12.649190	12.311242	11.834043	12.281762	11.717496	11.857355	12.455784	12.179107	12.112064	11.686921	11.486239	12.102073	12.860230	12.265765	11.615402	11.589538	11.546206	11.291291	12.154206	11.509057	12.869060	12.293591	12.560216	11.906665	12.528252	12.161114	12.105900	12.723135	12.411884	12.684558	12.013873	13.209700	11.649323	11.772550	12.254686	12.146883	11.730636	11.482538	12.900592	12.152049	11.991102	12.041676	12.389829	12.716418	12.276073	11.649323	12.339350	12.696432	11.641628	11.750265	12.035336	11.991699	12.834696	12.391089	12.098917	12.395993	11.980914	11.744798	11.798936	11.803943	12.291860	11.798654	11.757091	12.322365	12.140193	12.150335	11.607591	11.878180	11.731839	11.568275	11.923703	11.939692	11.903096	12.518587	12.111589	12.489402	11.949912	12.338259	12.131586	12.010184	11.906965	12.267749	11.312487	11.625759	12.009598	12.170796	12.590461	12.759589	12.387604	12.189655	12.632621	12.343274	11.948967	11.840239	13.235399	12.396914	12.183935	11.969768	12.142076	12.379122	12.317621	12.220306	11.619483	11.513324	12.106846	10.917350	12.496017	12.107264	12.777583	10.618828	10.482160	10.845782	9.975738	10.027195	10.089619	11.181578	12.692683	11.027337	10.803437	10.563692	12.170171	10.756043	11.831058	11.303223	11.694244	11.840994	11.427378	12.057936	10.632170	9.904409	11.463470	11.201014	10.630112	11.636925	11.307257	11.590688	10.362060	10.311988	11.150814	12.404042	10.985097	11.816438	11.197814	11.284886	11.656322	11.882981	11.801628	10.422540	11.571631	11.022434	11.746117	10.817887	11.636790	11.318087	11.148096	10.025602	12.695090	10.921794	10.806918	11.312057	11.503051	10.993300	12.316696	11.680972	11.475428	12.955722	11.661027	11.090314	11.447635	11.832854	11.159497	11.507607	11.121444	11.977451	11.404390	11.250318	11.315712	11.230061	12.382585	10.610070	11.141493	10.070373	11.991560	11.390710	11.323781	11.861844	11.812299	11.255473	11.307971	11.484580	11.010640	11.380842	11.558020	11.577492	11.611821	11.534802	12.309046	11.728242	11.074858	11.678294	10.616277	11.130935	11.533775	11.582424	11.617427	12.012048	12.032911	11.379443	11.584232	12.956981	12.590117	11.788526	11.096025	12.881137	11.020938	11.569154	10.522222	11.546045	10.823997	11.940741	11.847279	12.638185	10.763416	11.178065	11.943795	11.720318	11.191418	10.526253

FOXD3	9.653534	9.509295	9.296135	9.779075	10.160447	10.173348	9.789292	10.108388	10.437507	9.646619	9.693450	10.235279	9.854297	9.217443	9.142279	9.582379	10.119603	9.214386	9.882077	9.535836	9.711706	10.090224	10.576472	9.582540	9.871973	10.333610	10.762352	10.633212	10.805904	10.204611	10.063772	9.195778	8.933305	10.159669	9.636374	9.521779	9.792860	9.827819	9.620259	9.695415	10.215186	9.617475	9.521604	9.603010	9.797967	9.186600	10.629019	9.561136	9.579000	9.243810	9.042653	9.033552	9.220963	9.608994	9.325070	10.398977	9.127188	9.296921	9.371195	9.091411	9.294436	10.145351	10.074685	9.509923	9.150322	9.189785	9.532764	9.166351	9.226054	9.636997	9.914396	9.735774	10.267489	9.644704	9.574876	9.317184	9.649472	9.642543	9.737303	8.400970	9.020172	9.003490	8.734401	8.588177	8.895467	9.421160	9.068319	8.825536	9.162611	8.690809	8.844490	8.843862	9.081657	9.359075	9.754388	9.360161	9.224202	9.598956	10.529664	10.061049	10.995221	9.830915	9.726848	9.417638	9.551318	9.807593	10.490710	9.772809	10.416284	10.220177	9.899900	10.349613	10.372281	10.610379	9.605154	13.339537	10.172908	9.755013	10.347055	9.276833	10.080285	10.089453	10.009096	11.717191	10.249835	10.964018	10.713869	10.717299	10.772249	10.495855	11.039733	11.085733	11.970814	10.838841	10.612584	10.652524	10.354160	10.394618	10.304338	10.639852	11.380538	10.466534	10.893881	11.241360	11.314073	10.716709	11.053643	11.673108	10.379694	10.787634	10.136690	9.640875	10.122789	10.128997	10.757526	10.026576	10.258997	10.099349	10.216690	10.865149	9.881326	9.841511	9.782831	10.283957	9.777068	10.867997	10.825019	10.700036	9.920741	9.794394	9.958780	10.285304	10.742017	10.300503	10.708068	9.661074	10.218028	9.361114	9.611805	9.769677	10.913094	10.018949	10.307712	9.310045	9.341602	10.425295	9.456011	9.564183	9.929695	9.952895	10.046625	11.534802	9.731680	10.258842	9.593851	10.020621	10.045559	9.865768	9.449194	9.689001	9.949060	9.235620	10.297240	10.373979	10.051261	9.764963	9.970187	9.833794	9.419441	11.427665	9.863624	9.482261	10.113777	10.326281	10.555843	10.175472	11.182712	10.525956	10.365006	11.410967	10.750219	10.607118	10.058151	10.715670	11.165939	10.420556	9.269228	9.965900	11.032989	10.464310	11.185995	12.467649	10.945949	11.289617	10.668285	10.475566	9.368032	10.715826

USP3	7.264197	6.800425	6.001274	6.884082	7.007495	7.468615	7.165525	6.999064	6.891902	7.462140	7.365622	7.712340	7.003666	6.942144	6.462837	6.956277	6.491065	7.022972	6.638708	7.149622	6.034881	6.775740	7.255095	6.433951	6.755245	7.110988	7.334421	7.724547	6.946970	7.459505	6.807413	7.078154	6.626941	7.214347	6.758965	6.885195	6.848712	7.109984	6.612650	6.637869	7.139390	6.697597	6.881987	6.757487	7.589821	6.874986	7.825209	7.131970	6.739392	6.871129	7.685331	6.496858	6.659868	6.420618	6.476273	7.583021	6.084753	6.195129	6.597115	6.060096	6.322896	7.277658	6.326752	6.258790	6.577833	6.548685	6.715079	6.411686	6.575670	6.596013	6.858817	6.459041	7.274994	6.723225	6.707215	6.504481	6.493156	6.470203	6.965487	5.609146	5.836243	5.702406	6.334340	5.823851	5.563547	6.613816	5.415278	5.834461	5.511627	5.510117	5.774363	5.902203	6.297403	6.585224	7.267272	6.322896	6.331457	6.735632	7.467717	6.615279	7.088639	6.588358	6.719475	6.320684	6.550364	7.241188	7.606544	6.860578	6.128336	7.394443	6.289289	6.472916	6.760447	7.737107	6.824026	8.673896	6.992762	6.863627	7.257558	7.293973	7.067906	7.010422	7.904280	7.953242	8.157342	8.269526	6.642091	7.169031	8.277666	8.232995	7.907241	8.036665	7.482123	7.736081	6.904609	7.176042	7.355811	7.124012	7.415512	7.065889	6.790391	7.565494	7.294297	7.768537	8.178250	7.418684	8.314053	7.297804	8.298363	7.221454	7.512491	6.936985	7.562125	6.939832	7.338924	6.883757	7.261516	7.911574	7.418994	8.577014	7.467980	6.993221	7.592717	7.804477	7.838309	8.561939	6.863627	6.956132	8.079617	7.581800	6.951549	7.003961	7.102676	6.838662	7.915673	6.573722	6.309261	6.674294	6.386807	6.952175	7.946185	6.749580	6.657433	7.046406	6.781209	7.151564	6.979716	7.363230	6.452936	7.436323	7.031249	8.816369	7.361534	7.590734	7.049913	6.929531	7.254611	6.589655	5.662559	6.588047	6.597629	7.109184	5.817964	6.042449	7.340680	6.467861	5.829333	5.837134	5.794218	6.261843	6.367217	6.706548	7.193734	6.960860	7.048016	7.102025	7.531584	7.794157	7.609277	7.461194	6.967719	7.392419	7.001459	6.213566	8.216975	8.040597	6.762668	6.354724	8.646337	6.495744	7.229521	7.235142	8.263850	7.938997	7.578705	7.156225	7.589045	8.497023

FAM155B	7.134672	6.249627	6.191130	7.607580	7.229677	8.388057	6.448842	7.123846	7.401308	6.600064	6.830092	6.728831	6.199294	6.596780	6.512277	7.164423	6.702584	6.485292	7.607430	6.741283	6.674449	7.253866	8.090493	6.434799	6.356073	7.030942	7.297649	7.864481	6.730953	7.160938	6.608356	6.533076	6.410196	7.611728	6.582944	6.418988	7.142561	6.145843	6.982874	6.273200	6.809524	5.733207	6.361035	6.401610	6.434267	6.231428	7.883766	6.113325	6.954068	5.603033	5.928289	6.018512	5.080814	5.492799	5.442356	7.115335	6.053723	3.724305	5.170899	5.946205	5.119499	6.731428	6.322896	7.019693	6.585387	6.015357	6.360044	7.104070	6.719816	6.741283	7.143793	7.860047	7.076359	7.340836	6.599069	7.202097	7.308044	6.957676	6.984290	5.661228	6.913398	6.241098	6.191665	5.659956	5.814756	6.455107	7.596093	8.260812	5.975866	5.307327	6.263683	6.664453	6.323541	6.924680	7.213873	6.992762	6.483741	6.852479	6.867257	8.475589	7.532056	6.197582	6.536628	7.583498	6.086932	7.141596	6.802515	6.812241	6.970730	7.009322	6.194433	7.033591	6.965797	7.687159	7.271730	9.484876	7.736514	6.979561	7.223728	7.196961	6.085604	6.988248	7.194717	8.646197	8.106503	7.413508	6.885035	7.312129	7.241639	6.587881	7.464042	6.974792	6.460003	8.041562	7.734581	6.672861	8.147603	7.109022	7.189713	6.937644	8.350817	7.990815	7.085683	7.489116	6.879572	8.173166	8.190071	7.414306	6.795248	7.240719	7.162520	7.685796	7.748405	7.167633	7.121630	7.697156	7.369988	7.211820	7.818572	7.437704	6.219571	6.623549	6.112494	6.898513	6.755073	8.792916	6.084236	7.220994	6.286409	6.839990	6.376157	5.981021	5.698593	6.708953	7.102823	6.505645	6.339228	7.111138	6.247611	6.233766	7.885206	5.960936	6.157700	6.805534	6.726738	6.329383	7.056646	6.905093	6.640307	7.307127	7.713118	7.701592	7.467106	8.621889	7.456680	7.575743	7.314813	6.865962	6.652886	5.816548	7.228880	6.504965	6.255529	6.200479	8.087256	7.039698	7.146133	6.598929	6.501046	5.797835	6.831474	6.382968	6.406893	7.443314	7.277818	7.837050	7.127371	7.278325	7.555055	6.193889	6.703237	7.302088	8.288581	6.736422	6.976898	6.571931	6.394833	6.339386	7.332417	7.514055	6.994459	9.550248	7.739294	7.277658	7.464042	6.954882	7.301462	6.958315

PP2D1	4.939531	4.212080	2.935394	3.774917	3.394158	4.015184	4.288341	4.340922	3.225297	3.169645	3.297290	3.344305	3.642881	3.462494	4.363732	4.338539	3.285045	3.585898	4.760994	3.794458	4.004116	3.810767	3.631091	4.532465	4.100634	3.705864	5.220931	4.123175	4.119254	6.621955	4.251929	6.029259	3.857663	4.514021	3.807012	3.853131	3.447097	3.432363	3.564394	4.087434	3.653400	5.022202	3.689939	3.853571	3.957709	4.261771	4.556426	4.872994	4.643182	3.436524	3.341666	3.253865	4.607149	3.865362	3.423646	4.043227	3.934099	4.744674	3.615151	3.667489	4.546011	4.148136	4.753418	5.029542	3.933031	4.340922	3.981290	3.766514	4.000845	4.122955	3.960729	3.473087	4.291335	3.508701	3.774315	4.428056	4.213405	3.470874	3.921822	4.745760	3.853131	4.617724	4.350467	5.062027	4.277970	4.911992	3.362333	3.967464	5.247821	4.566165	3.331129	3.270828	4.092696	4.881370	3.940865	3.708838	3.363362	3.871553	4.525222	3.553917	3.459121	4.244526	3.340818	3.156147	4.356986	3.736901	4.774294	4.941159	4.195183	4.185338	2.911012	4.326339	4.061501	4.535468	4.401099	3.764064	3.904662	3.891965	4.276377	4.375667	3.201942	3.766740	4.090941	3.189017	4.240349	3.759435	3.701819	3.620461	3.350741	3.390523	3.880460	4.375887	3.077588	3.880658	3.335963	3.211573	4.144750	4.309062	3.269432	3.574592	4.104536	5.270656	3.843155	3.088208	3.898693	3.584548	3.596936	4.164264	6.276841	3.216520	4.336552	3.380170	3.438067	3.091325	3.594603	3.397833	3.022268	3.197854	3.732517	3.533669	5.110560	3.971363	4.525013	3.743241	4.093091	4.354587	3.450800	4.322056	3.305531	3.274657	3.122513	3.456978	3.993727	3.805149	3.931093	3.951827	3.976335	3.720995	3.606173	3.723511	3.464489	4.232008	4.793518	4.252787	4.065417	3.111180	3.548911	3.872015	3.051640	3.558339	3.725775	4.154082	3.235526	3.595009	3.785850	3.459278	3.462669	4.294363	3.129814	3.420058	4.177463	4.427011	3.499062	4.037145	3.370996	3.277138	4.035158	3.492956	3.606405	3.474890	3.524981	3.792471	3.800534	3.650619	3.482596	3.279903	3.132218	3.711920	3.586490	3.247376	3.486039	3.652214	3.192551	4.773254	4.048661	4.023218	4.030642	4.007805	4.756329	3.472743	3.874364	3.399012	5.001521	3.790973	3.500685	3.607655	3.667489	3.615932

AGAP2	10.717904	10.366828	10.136400	10.146709	9.789292	10.297240	10.022449	10.286370	10.462665	9.750979	9.859885	9.636548	9.559249	10.123807	9.463454	10.588938	9.979195	10.066454	10.715070	10.685697	10.574079	11.078552	11.010043	10.430485	10.302930	9.958930	12.525614	10.391918	10.449603	11.162452	10.745880	10.241816	11.104579	10.778128	10.379400	10.010461	9.551013	10.124928	10.150370	10.394763	10.164130	10.345071	10.907373	10.220177	10.070538	10.412390	10.533656	10.420556	10.134839	10.350959	10.076651	10.145498	10.347657	9.964578	10.025121	10.374399	10.058310	10.273491	10.181016	10.515780	10.334012	10.445527	11.303088	10.786621	10.756778	10.442006	10.392503	10.443960	10.460118	10.841568	10.154373	10.061791	10.083270	10.140602	10.388663	11.147241	10.339585	10.146396	10.051862	10.526564	10.504548	10.293860	10.389743	10.194330	10.260093	10.072356	10.121247	9.811172	10.576159	10.126943	10.016190	10.757072	9.761038	10.144630	10.723422	10.393559	10.117147	9.223262	9.309881	9.813541	9.308789	10.216548	10.002425	9.726052	10.831184	9.860030	10.709515	11.072632	10.754642	10.238002	10.770016	10.327183	10.057120	10.552938	10.279891	10.179636	10.329171	9.851679	10.406639	11.264402	10.394903	10.478589	11.181275	11.136100	10.247122	10.606813	9.227275	10.139408	9.663882	9.948283	9.776270	9.874810	10.809966	9.919961	10.414333	9.816348	9.826469	10.845927	10.244125	10.445361	10.885843	11.148401	9.737911	10.340199	10.783617	10.724139	10.294008	10.742902	11.232725	11.126783	10.613754	10.225458	10.582324	10.140152	8.996891	9.639817	10.073145	10.055903	10.137446	10.820861	10.301432	10.592327	10.049705	10.144003	10.239105	10.705664	10.403638	9.747100	10.150520	10.262095	10.159534	10.234797	9.944250	10.114501	10.635641	9.948283	10.030540	10.395432	10.057435	10.489183	11.466296	10.418587	10.677921	11.072990	10.563066	10.625496	10.429717	10.011379	11.071672	9.987620	10.130777	11.367432	9.876085	10.786329	10.718205	10.139993	10.068414	9.616211	10.583331	10.155158	10.104632	9.985333	11.314216	10.305051	9.929861	10.447955	9.989046	10.860574	10.389118	9.627096	10.325194	9.554189	10.390475	10.493142	10.504838	9.406640	9.104428	10.291124	9.344082	9.833636	9.774033	10.646958	10.339897	10.743052	10.982333	10.470736	11.295540	10.465495	10.608759	11.220589	10.534263	11.003511	11.476559	9.475200	9.566691	10.036868	10.078202	10.933966

SLC25A35	12.917482	12.096669	12.116196	12.273762	11.698277	11.452378	12.055738	10.771222	10.458339	11.473095	11.541954	11.608459	11.867888	11.831058	11.901537	11.717338	11.649159	12.020782	11.476828	11.930467	11.734962	12.382891	13.114466	11.699769	12.301392	11.785826	12.624160	11.723321	11.898369	12.268515	11.816598	12.854034	12.713455	12.929031	12.228786	12.026384	11.747895	12.287695	12.771890	12.395544	11.607717	12.767083	12.375982	12.191829	13.054184	12.443867	12.068594	12.103623	11.800745	11.963714	12.111299	12.295732	12.435481	12.436907	12.107738	11.515004	12.421993	12.087405	12.281762	12.417049	12.850702	11.912061	13.830239	12.431602	12.598836	12.467952	12.456759	12.439669	12.388554	12.624833	12.446416	12.110845	11.350258	12.147660	12.317007	12.695570	11.985558	12.368752	12.127025	11.923703	12.181736	12.376614	12.581640	12.158785	12.116671	12.372749	12.397063	11.845977	12.330494	12.288317	12.372119	12.525283	12.075117	12.170015	12.547624	12.445002	12.197056	11.517367	11.831216	11.822299	11.547983	11.873097	11.798356	11.731098	12.706715	11.659564	10.967694	12.487307	12.188253	11.394876	12.252621	11.798356	11.947291	11.008289	11.492028	10.743665	13.327179	11.654537	11.396102	12.340029	13.173050	13.008116	12.020355	13.236251	11.974211	12.688477	11.990637	11.447062	12.967314	13.206525	13.409664	13.128984	13.261809	13.306600	12.089816	12.955360	13.417129	12.758584	12.867959	12.099966	13.091080	13.109444	12.460438	13.041185	13.890198	12.185716	12.219074	10.630830	13.068091	12.573503	12.728615	12.865672	12.678006	13.221977	12.792190	12.377714	13.107708	13.242782	13.334586	13.070221	12.934551	12.678360	12.283820	12.268825	12.887214	12.303112	12.228964	12.472142	13.094478	12.727241	11.600906	13.063231	13.061185	12.925070	12.288638	13.396671	13.362040	12.464048	13.533066	12.726851	13.227872	12.600544	12.939734	13.061185	12.983527	12.999520	12.424072	12.809655	13.347657	12.130980	12.709456	12.679909	12.752950	12.816305	12.691342	12.732358	12.831697	13.057276	12.644718	13.531473	13.163576	13.286480	13.001778	13.413997	12.933669	13.240382	13.218764	13.066403	13.168083	13.278020	13.128798	12.987020	13.181119	13.024751	12.590461	12.957538	12.079407	13.420156	13.421932	13.562689	13.409232	11.976530	12.606391	12.638694	12.578521	12.152682	12.955178	12.539123	12.032153	12.543078	13.492583	12.298055	11.185403	12.943897	13.087953	12.035653	13.255365	13.684785

RANGRF	12.917482	12.096669	12.116196	12.273762	11.698277	11.452378	12.055738	10.771222	10.458339	11.473095	11.541954	11.608459	11.867888	11.831058	11.901537	11.717338	11.649159	12.020782	11.476828	11.930467	11.734962	12.382891	13.114466	11.699769	12.301392	11.785826	12.624160	11.723321	11.898369	12.268515	11.816598	12.854034	12.713455	12.929031	12.228786	12.026384	11.747895	12.287695	12.771890	12.395544	11.607717	12.767083	12.375982	12.191829	13.054184	12.443867	12.068594	12.103623	11.800745	11.963714	12.111299	12.295732	12.435481	12.436907	12.107738	11.515004	12.421993	12.087405	12.281762	12.417049	12.850702	11.912061	13.830239	12.431602	12.598836	12.467952	12.456759	12.439669	12.388554	12.624833	12.446416	12.110845	11.350258	12.147660	12.317007	12.695570	11.985558	12.368752	12.127025	11.923703	12.181736	12.376614	12.581640	12.158785	12.116671	12.372749	12.397063	11.845977	12.330494	12.288317	12.372119	12.525283	12.075117	12.170015	12.547624	12.445002	12.197056	11.517367	11.831216	11.822299	11.547983	11.873097	11.798356	11.731098	12.706715	11.659564	10.967694	12.487307	12.188253	11.394876	12.252621	11.798356	11.947291	11.008289	11.492028	10.743665	13.327179	11.654537	11.396102	12.340029	13.173050	13.008116	12.020355	13.236251	11.974211	12.688477	11.990637	11.447062	12.967314	13.206525	13.409664	13.128984	13.261809	13.306600	12.089816	12.955360	13.417129	12.758584	12.867959	12.099966	13.091080	13.109444	12.460438	13.041185	13.890198	12.185716	12.219074	10.630830	13.068091	12.573503	12.728615	12.865672	12.678006	13.221977	12.792190	12.377714	13.107708	13.242782	13.334586	13.070221	12.934551	12.678360	12.283820	12.268825	12.887214	12.303112	12.228964	12.472142	13.094478	12.727241	11.600906	13.063231	13.061185	12.925070	12.288638	13.396671	13.362040	12.464048	13.533066	12.726851	13.227872	12.600544	12.939734	13.061185	12.983527	12.999520	12.424072	12.809655	13.347657	12.130980	12.709456	12.679909	12.752950	12.816305	12.691342	12.732358	12.831697	13.057276	12.644718	13.531473	13.163576	13.286480	13.001778	13.413997	12.933669	13.240382	13.218764	13.066403	13.168083	13.278020	13.128798	12.987020	13.181119	13.024751	12.590461	12.957538	12.079407	13.420156	13.421932	13.562689	13.409232	11.976530	12.606391	12.638694	12.578521	12.152682	12.955178	12.539123	12.032153	12.543078	13.492583	12.298055	11.185403	12.943897	13.087953	12.035653	13.255365	13.684785

MBNL1	7.339085	7.514494	7.510460	7.643210	8.278584	7.462140	7.410189	8.689294	10.046174	8.192912	8.980451	8.785314	8.543841	7.488051	7.208630	8.418559	8.509059	7.896712	8.487060	7.732726	7.496149	6.752252	7.169475	7.999175	7.917667	8.122441	8.503865	6.965162	9.482879	8.216975	9.145493	7.150580	7.968631	8.168133	7.564260	7.405852	6.918844	6.854940	7.055431	6.823863	8.660218	7.028638	8.424675	7.598247	8.321435	7.309440	8.298809	5.289165	7.763140	7.294297	7.496799	7.120747	7.814935	6.277178	7.913711	8.844331	7.492895	7.072278	7.434558	7.312275	6.002186	8.168936	7.287366	6.933931	5.731934	7.270478	5.389141	7.179231	6.250956	6.318752	6.927637	6.946788	9.277850	6.903692	6.887904	7.794465	6.898200	7.344115	6.773823	6.024457	7.640322	7.828059	7.140953	7.263561	7.397106	7.961776	7.095641	7.009152	5.718329	6.548037	6.383321	5.512770	6.790236	6.325088	7.022026	7.900057	7.715526	9.981215	8.442536	8.795820	8.791995	7.630529	8.683550	8.230545	7.011388	10.115138	7.377364	6.985880	6.592623	8.208762	5.133206	8.549674	7.718273	8.532727	10.163518	6.124461	7.154192	8.837982	8.837982	7.931572	7.820703	6.992596	7.400216	7.448724	8.051705	7.777466	8.689716	9.278313	8.262136	8.516062	7.524523	7.977233	7.126739	5.513706	8.119607	8.926440	8.489226	6.931597	7.755863	6.829927	6.538915	6.052192	8.256628	6.286927	7.453093	6.573050	6.653361	8.437691	7.486641	9.410488	8.806574	8.260645	8.300469	7.909264	7.255858	6.989197	6.509428	6.940443	6.695069	9.032049	5.670314	7.858776	7.891853	8.117928	6.865962	9.363051	5.065069	7.875503	7.406798	7.429167	7.473087	7.585179	7.317336	7.319795	8.530152	7.095474	7.040480	7.014228	6.397786	6.334681	7.315270	7.088975	6.483741	6.399293	6.724812	6.190615	6.336359	6.414360	5.620378	6.931924	6.900296	7.806171	7.092126	7.040034	7.650959	6.412890	7.154025	6.939054	5.849706	8.960537	7.307573	7.663558	8.052166	7.508474	7.009640	8.086750	7.722516	4.935151	7.013182	5.482205	6.328231	6.289971	7.289877	7.624107	8.039489	7.645874	9.428521	9.650895	8.036343	8.646197	8.163833	7.571434	7.964652	7.339873	7.532056	8.210366	7.280042	5.835886	7.831559	4.844670	6.836885	6.008773	7.416254	7.910344	7.627042	6.492368	9.064136	7.504456

NOX1	8.285162	8.834502	9.600027	8.663004	8.934423	8.878011	9.042373	8.764831	7.426143	8.583773	8.396077	8.008230	8.877254	9.071601	8.313592	7.813567	8.332609	8.912287	8.194634	9.560491	8.922699	8.173935	8.413384	8.364085	7.801746	8.313750	7.113308	7.082859	6.977224	7.763602	7.589490	8.905380	9.259835	8.307442	9.493455	10.176535	9.893962	9.830915	8.742540	8.966798	8.534684	8.338549	8.874344	9.454926	9.148972	9.409873	7.289724	8.413538	9.231906	9.577618	9.018370	9.563450	9.655812	9.676599	9.411269	8.461720	10.423140	9.099970	8.134971	9.546827	9.169996	8.084751	7.640962	8.137859	8.813954	9.132911	8.696520	9.089471	9.566057	8.160840	8.909370	9.336166	8.055892	8.955775	9.553744	9.055161	9.907881	9.092226	9.197498	8.919658	8.661915	9.731085	9.507341	9.604383	9.839071	9.373989	9.362458	9.793460	9.206573	9.472328	9.239068	9.405273	8.989583	9.459408	8.937156	9.748976	9.318499	8.392729	8.485660	8.300322	8.271046	8.770989	8.809969	9.027048	8.695418	8.676972	7.841244	8.134971	8.375408	8.441475	9.296431	7.217959	8.560859	8.166131	8.815582	6.379636	8.127337	8.329805	7.266319	8.386331	8.764687	8.321720	7.438844	5.970326	9.551928	6.979258	7.335828	8.374648	7.869083	6.976239	7.037167	7.978951	7.366100	6.635639	7.695315	8.658992	8.136005	7.523749	10.835583	8.368340	7.791899	8.339643	6.661658	6.612494	9.332770	7.169171	7.344583	6.418153	8.610765	7.313859	7.445550	9.335876	8.680053	9.315341	9.574584	8.863383	9.029816	7.905709	8.540706	8.169560	7.878104	9.181248	9.803340	9.698334	9.135166	6.350954	9.067393	9.814145	9.751437	8.627465	9.383472	9.833053	9.125660	9.118187	8.612329	10.381639	9.001665	7.678603	9.937098	7.828542	8.525662	8.380819	7.920456	8.353690	8.469498	8.832900	7.971938	8.092765	7.778846	8.992872	9.393908	6.298082	8.402217	9.193621	8.690507	9.089149	8.620804	9.114355	8.452479	8.906569	9.790059	8.844962	8.875260	9.387940	7.954250	9.219741	9.953948	8.565719	8.335866	10.339450	8.343986	7.650787	7.701741	8.559931	8.012895	8.103097	6.991290	7.761745	8.598657	7.663729	7.864325	7.379253	8.370333	7.563478	7.068565	7.974303	9.289425	9.238102	6.669144	8.222690	5.588901	7.349096	7.335511	8.707227	6.993072	9.544116	8.013547	6.740986

KSR2	6.923538	5.886203	6.462837	6.090105	6.238438	6.368195	6.641126	7.515375	7.010741	6.291686	6.698079	6.939673	6.561198	6.722418	5.832637	6.780059	6.102386	6.658225	6.079720	7.612641	5.896885	7.733625	6.689803	6.571007	6.470882	6.730471	6.328905	6.959726	6.385607	6.801865	6.453805	6.752407	6.080235	6.418823	6.248637	6.411029	6.373313	6.598280	6.707842	6.664782	6.649192	6.646404	6.557284	6.559099	6.776038	4.905496	6.452765	7.385010	6.239749	6.848224	7.277363	6.625166	6.407375	6.784020	6.092703	6.426975	6.351290	6.610626	6.553821	6.437128	6.570672	6.589006	5.221107	6.968033	5.828214	6.742265	6.727389	5.778924	6.537774	6.009291	6.446533	6.555125	6.354181	6.956719	6.676729	6.112145	6.475585	6.611844	6.970890	6.941837	6.681747	5.826008	6.885035	7.191688	6.820058	7.328360	6.671061	7.173526	6.349577	6.136700	7.095641	6.608356	6.391555	6.300351	6.973051	5.993753	6.027116	6.066254	6.501553	6.493471	6.956874	6.506499	6.457035	6.741794	6.849972	6.015023	6.322198	6.716392	7.480750	6.830549	7.753027	6.469852	7.027491	6.450320	6.551327	8.721757	6.815409	6.283964	6.631099	7.727475	6.039824	7.580396	5.686778	7.100946	6.479620	6.832128	7.931730	7.487256	6.777825	6.792834	7.142561	7.030189	6.390531	6.513727	5.998502	6.012483	6.635961	9.300643	10.136546	7.502112	7.690876	7.523591	6.737942	8.136495	6.824177	6.815567	6.762817	7.018894	6.543690	5.712490	6.620355	6.142404	6.447349	6.493156	6.855573	7.015004	6.008773	5.907218	7.019538	6.965004	6.524664	8.732236	5.995839	6.997868	4.567891	7.420547	8.665949	6.216805	6.250956	6.615919	8.241293	6.283964	4.774099	5.800232	6.749727	7.163769	8.106793	6.707704	5.775290	5.873185	7.939314	4.733992	6.109732	6.236650	5.609902	5.854373	6.444900	6.007890	5.778189	6.676256	7.236735	7.137662	6.536628	7.262516	6.165625	7.459959	7.526204	7.376878	6.145662	6.519214	6.238775	5.280957	6.395482	6.911310	7.065558	6.050288	9.122924	5.360034	5.519352	7.504008	6.395316	6.197419	6.359531	6.662662	6.049071	6.800600	7.703743	6.193018	6.990315	6.745598	6.842050	6.366226	6.635796	7.158379	7.008826	6.766184	5.643691	8.102799	7.322799	8.060419	6.817477	9.926926	6.956277	7.120590	6.960210	5.990613	6.724980	6.553998

RHCG	8.634408	12.498635	12.436907	12.390154	13.275229	12.492404	12.581823	13.489283	14.975923	12.765218	12.920223	11.940902	12.703315	12.982574	14.037255	13.402926	13.634245	12.963629	13.949345	12.548071	12.664618	14.404753	12.820259	13.413148	13.686442	13.465295	11.897946	13.569652	12.292654	11.477113	14.062109	11.126207	10.559019	9.994705	10.711554	11.327191	11.782370	11.390561	12.527089	12.640046	12.798338	11.649323	11.276168	12.752265	12.162807	12.338888	12.926482	11.675026	12.349580	11.787766	11.324220	11.567413	11.681866	11.190322	12.538424	12.877121	11.178375	12.038055	11.900292	11.131351	12.203746	12.856393	9.686521	13.294952	11.619033	13.262797	12.117700	14.195562	11.531086	12.835997	11.160805	12.514830	12.921878	11.410967	12.337443	10.931284	11.536302	10.363870	11.934282	12.089348	12.999705	13.007579	11.187573	12.436448	11.084984	12.826118	11.774477	11.758300	11.527480	12.110414	11.379904	12.886172	13.022885	11.878180	11.522936	13.635698	13.040220	13.573342	12.361792	14.521454	13.828942	11.807225	13.128984	12.306362	12.421202	13.459166	15.141653	12.775154	15.084722	12.997119	14.102873	14.791560	14.347082	13.559309	14.080659	12.750695	9.736555	13.005589	13.621755	8.986242	11.553739	9.748309	10.045559	13.622915	11.036851	10.557516	9.859459	10.224206	9.684672	11.052359	9.624312	9.907128	9.633519	10.186829	11.868313	9.265364	11.262723	10.378034	8.712055	11.182012	10.353264	9.536888	10.414046	9.936498	9.535658	11.187272	11.140228	10.575261	9.953492	10.171241	11.518846	10.642074	10.590541	10.060602	9.705173	10.060756	9.656882	10.802247	10.391769	11.158052	10.603402	9.004704	10.747396	9.932497	10.642074	12.116834	9.140257	9.758866	9.632606	9.937420	9.108128	8.976956	9.465026	9.889523	9.175876	8.591468	9.424825	10.455568	9.167255	12.335561	12.239785	10.486856	11.461880	10.725203	9.959522	10.128078	11.254129	10.834508	9.997359	10.110805	10.679659	13.831800	9.657050	8.775212	11.053493	9.209941	9.241228	11.500503	8.755780	10.774190	10.735785	9.389608	11.164515	8.870565	10.091582	11.075007	10.634437	9.053453	10.414630	8.939423	11.153927	10.489519	10.077750	11.991102	11.035981	10.812401	11.008438	11.155211	9.380667	9.531300	9.899599	11.047378	9.515436	9.783615	11.550461	12.509321	11.729303	11.644012	12.693903	11.238457	10.809658	11.448381	13.214589	10.199467	10.780147	9.505030	9.873170	10.308097

UPP1	9.497175	13.607572	13.010512	14.386354	13.519354	13.272382	14.280056	14.869310	12.671897	13.868836	14.148848	13.171328	13.936877	14.117673	13.171526	14.847322	14.019002	14.295055	13.800082	14.900158	13.968817	14.740963	12.291225	13.969061	14.121867	13.974138	9.465026	14.599518	12.848958	12.681802	13.885693	13.090158	12.480642	11.299253	12.534282	13.321094	13.215801	14.131096	12.861867	13.192705	13.736365	13.566875	13.354386	13.250094	11.061124	12.936542	11.817939	13.717719	13.223145	13.610144	12.643586	14.026801	13.533281	14.535541	13.487032	12.859527	14.418363	14.764819	13.984082	13.003243	13.189322	12.860392	11.159639	14.359381	13.179963	13.763467	13.643752	13.074750	13.282417	13.290292	13.558652	13.820478	13.753087	13.345516	14.780447	12.446758	12.513208	13.229053	13.654792	15.151876	12.678191	13.035435	12.374659	14.761042	14.777592	13.323791	14.894936	14.065689	12.579338	14.042288	13.678771	13.408595	14.006716	14.470097	11.583468	12.978042	13.191466	14.137614	13.403761	13.219767	14.175228	13.870453	13.208700	13.803853	14.963394	12.671897	14.343152	14.065689	12.427164	13.960239	13.590299	14.054502	13.915790	14.075445	13.751801	12.552494	9.514568	13.234991	13.276852	9.151110	13.208294	11.064514	10.202012	11.446009	11.221315	12.979296	9.146716	11.676971	11.534084	12.132348	10.566491	11.848510	11.203158	13.271787	11.710591	12.288476	13.469871	8.567761	9.618866	11.280035	11.369147	10.898036	10.813866	12.634771	11.184811	11.935336	11.281969	9.020466	12.552494	9.759333	12.119577	12.147357	12.963098	10.857034	11.375164	12.278977	11.402351	12.556219	12.651910	11.742298	12.608186	10.112296	11.497121	10.860851	13.487032	11.873387	9.876562	9.937098	12.107881	13.083488	11.133393	11.853082	13.231052	12.942109	9.671026	10.086994	11.623447	12.412056	11.427955	13.281421	11.123230	13.034689	12.646251	10.376347	11.916064	13.069296	13.239205	13.832042	11.322437	11.848655	12.983713	12.919886	13.680430	11.087512	13.042957	13.098245	11.564033	13.894497	11.766175	11.731533	12.739334	13.362873	11.204776	11.472234	12.149284	12.880604	10.947309	11.319280	12.277746	10.551284	13.482375	11.320686	13.029529	13.834843	11.532142	12.074965	9.039625	12.272044	10.228505	14.208831	9.299855	9.626163	12.269936	11.588196	10.105381	11.953033	11.625884	11.554481	12.369200	11.724977	10.857034	8.615560	13.638514	9.827965	13.535967	12.434656	10.587309	12.577702

USP6	15.461686	9.856672	10.389118	13.390157	11.265456	11.591754	10.881053	11.313353	11.631352	11.324079	11.192072	11.176414	10.657031	11.688416	10.253926	10.815965	10.375141	10.794399	11.752478	10.724311	11.126061	11.579521	10.373518	10.812696	10.937294	10.915065	10.959158	10.905549	10.742594	10.181016	11.517947	12.348206	9.972017	9.796717	9.944985	10.435682	10.552938	10.497960	10.909438	11.246940	11.248361	10.641500	10.327332	10.515001	10.350889	10.334168	11.069585	10.809966	10.156509	10.415101	15.198897	10.384236	9.846608	9.795507	10.281537	10.884311	10.242296	10.644156	10.740809	10.312135	10.564580	11.039598	9.931382	11.352321	10.123498	10.355061	10.588511	9.816348	10.237289	11.145141	10.109582	10.374979	11.168249	10.188920	10.293540	10.188920	10.551898	10.200086	12.025251	10.413889	11.584062	9.641171	11.772998	10.154056	9.797967	9.860030	9.860677	10.383969	10.612438	10.508722	10.125839	10.652947	9.981835	10.347657	12.366220	10.024337	9.701109	10.902906	10.914603	10.835430	10.955140	10.429581	10.717586	11.007562	11.455518	10.857810	11.821698	11.706874	11.596390	11.695777	11.338052	11.368020	11.493891	12.111901	10.871143	12.016290	15.552451	10.786766	11.724086	11.673108	11.215532	9.876877	11.899574	11.610786	9.734702	11.132929	9.875126	10.990768	10.924627	12.379891	10.703174	11.120015	10.247848	11.535996	10.848296	11.384387	11.201014	10.398075	9.174945	10.480801	10.829597	10.471635	11.894872	10.682588	9.390643	10.505113	11.745378	11.350393	10.001321	10.566197	11.187132	10.635038	11.001079	9.628799	10.810402	9.966351	10.410869	10.870667	10.614503	10.495716	10.786013	9.459105	10.329926	10.664468	10.426073	10.877948	9.614243	11.208645	9.912045	12.761354	9.705954	10.151576	10.239105	10.778996	9.919470	8.967900	10.194616	10.539877	9.728056	10.148232	10.479922	10.673572	10.699884	10.034852	10.245297	10.617450	10.238466	10.206432	10.212902	10.436590	10.115300	11.422861	10.740345	9.494403	10.015392	10.387951	10.033753	10.632170	10.215486	10.069768	9.566202	10.264537	9.640574	10.015078	10.694498	10.198572	9.227425	9.954115	10.052797	9.477368	11.048406	9.622602	10.075003	10.590228	9.738831	10.470433	10.550253	9.820277	11.016201	10.315931	9.908194	10.996548	10.145061	10.215321	11.143107	11.538822	11.041360	10.887915	10.760552	11.024507	10.784668	11.175685	11.956358	10.795715	10.597077	11.656893	10.781605	10.016027

ZNF232	15.461686	9.856672	10.389118	13.390157	11.265456	11.591754	10.881053	11.313353	11.631352	11.324079	11.192072	11.176414	10.657031	11.688416	10.253926	10.815965	10.375141	10.794399	11.752478	10.724311	11.126061	11.579521	10.373518	10.812696	10.937294	10.915065	10.959158	10.905549	10.742594	10.181016	11.517947	12.348206	9.972017	9.796717	9.944985	10.435682	10.552938	10.497960	10.909438	11.246940	11.248361	10.641500	10.327332	10.515001	10.350889	10.334168	11.069585	10.809966	10.156509	10.415101	15.198897	10.384236	9.846608	9.795507	10.281537	10.884311	10.242296	10.644156	10.740809	10.312135	10.564580	11.039598	9.931382	11.352321	10.123498	10.355061	10.588511	9.816348	10.237289	11.145141	10.109582	10.374979	11.168249	10.188920	10.293540	10.188920	10.551898	10.200086	12.025251	10.413889	11.584062	9.641171	11.772998	10.154056	9.797967	9.860030	9.860677	10.383969	10.612438	10.508722	10.125839	10.652947	9.981835	10.347657	12.366220	10.024337	9.701109	10.902906	10.914603	10.835430	10.955140	10.429581	10.717586	11.007562	11.455518	10.857810	11.821698	11.706874	11.596390	11.695777	11.338052	11.368020	11.493891	12.111901	10.871143	12.016290	15.552451	10.786766	11.724086	11.673108	11.215532	9.876877	11.899574	11.610786	9.734702	11.132929	9.875126	10.990768	10.924627	12.379891	10.703174	11.120015	10.247848	11.535996	10.848296	11.384387	11.201014	10.398075	9.174945	10.480801	10.829597	10.471635	11.894872	10.682588	9.390643	10.505113	11.745378	11.350393	10.001321	10.566197	11.187132	10.635038	11.001079	9.628799	10.810402	9.966351	10.410869	10.870667	10.614503	10.495716	10.786013	9.459105	10.329926	10.664468	10.426073	10.877948	9.614243	11.208645	9.912045	12.761354	9.705954	10.151576	10.239105	10.778996	9.919470	8.967900	10.194616	10.539877	9.728056	10.148232	10.479922	10.673572	10.699884	10.034852	10.245297	10.617450	10.238466	10.206432	10.212902	10.436590	10.115300	11.422861	10.740345	9.494403	10.015392	10.387951	10.033753	10.632170	10.215486	10.069768	9.566202	10.264537	9.640574	10.015078	10.694498	10.198572	9.227425	9.954115	10.052797	9.477368	11.048406	9.622602	10.075003	10.590228	9.738831	10.470433	10.550253	9.820277	11.016201	10.315931	9.908194	10.996548	10.145061	10.215321	11.143107	11.538822	11.041360	10.887915	10.760552	11.024507	10.784668	11.175685	11.956358	10.795715	10.597077	11.656893	10.781605	10.016027

FBF1	8.385123	8.719507	8.920591	8.659306	8.940500	8.947669	8.734872	8.595167	8.907661	8.921659	9.208246	8.888374	8.701068	9.168609	8.807202	8.613599	9.047293	9.054399	8.959927	9.006556	8.531966	9.943637	8.583923	8.923143	8.944146	9.040378	9.038835	8.911270	8.312979	9.073598	8.899150	9.223120	9.097199	9.371510	8.970362	9.028257	9.006556	8.618939	9.225274	9.252231	8.902324	8.844490	8.725564	8.724803	8.701516	8.834942	8.549674	8.878315	8.834942	8.429740	8.554956	8.932263	8.846470	9.272549	9.243504	9.058310	8.923868	9.347711	9.396668	9.245186	9.610292	8.610009	8.870565	9.209476	9.073286	9.256358	9.209005	8.648726	9.193621	9.316873	8.497483	9.000251	8.648726	8.690185	9.106902	9.074656	8.894991	8.916955	8.616164	8.627776	8.671249	8.929239	8.736571	8.920300	8.856221	8.841352	8.540210	8.768392	9.151857	9.029501	8.929089	9.003048	8.853642	9.207016	8.857898	9.012401	8.731353	8.890833	9.024321	8.759950	8.703539	8.721025	9.083038	9.123673	9.260903	9.038835	9.611938	9.225119	9.127037	8.788276	8.824643	9.360161	9.408176	8.703695	8.616473	8.925972	8.403771	8.609069	8.927404	9.487831	9.205492	8.999951	9.136065	9.180169	9.406930	9.483634	8.582980	9.131387	9.954924	9.678618	9.530075	9.301890	10.828572	10.322742	8.450472	9.752217	9.126299	9.385879	9.378088	9.340577	10.301596	9.899145	9.624463	9.937579	9.505806	9.167255	9.702186	9.190862	9.727754	9.362145	9.468552	9.000251	9.148972	9.748976	8.951958	8.792439	8.500441	10.218028	9.563148	9.460158	9.283248	9.553744	9.145493	9.031727	9.808233	9.183297	8.960071	9.105050	8.832252	9.422496	9.224522	9.505969	11.082337	8.854885	9.171885	9.191817	10.051559	9.873647	8.978174	10.230046	9.633073	10.773877	10.234963	9.799812	9.192737	10.145661	8.774914	9.758117	9.642061	8.522315	9.298453	10.068558	8.983194	9.629836	9.631674	9.130010	8.904469	8.847714	8.822822	9.330748	9.721087	9.592440	8.987649	9.518373	9.087336	9.537972	9.476133	9.601584	9.262432	8.864910	9.682352	9.249208	10.214880	9.796717	9.534594	9.376209	8.799907	9.686093	9.067242	9.681108	9.131387	9.524105	9.252684	10.010461	9.210087	9.382397	9.120357	9.134241	9.731977	8.988368	8.879747	9.360471	9.347097	8.827612	9.173578	8.847553	8.901108	10.584914

AC087289.3	8.385123	8.719507	8.920591	8.659306	8.940500	8.947669	8.734872	8.595167	8.907661	8.921659	9.208246	8.888374	8.701068	9.168609	8.807202	8.613599	9.047293	9.054399	8.959927	9.006556	8.531966	9.943637	8.583923	8.923143	8.944146	9.040378	9.038835	8.911270	8.312979	9.073598	8.899150	9.223120	9.097199	9.371510	8.970362	9.028257	9.006556	8.618939	9.225274	9.252231	8.902324	8.844490	8.725564	8.724803	8.701516	8.834942	8.549674	8.878315	8.834942	8.429740	8.554956	8.932263	8.846470	9.272549	9.243504	9.058310	8.923868	9.347711	9.396668	9.245186	9.610292	8.610009	8.870565	9.209476	9.073286	9.256358	9.209005	8.648726	9.193621	9.316873	8.497483	9.000251	8.648726	8.690185	9.106902	9.074656	8.894991	8.916955	8.616164	8.627776	8.671249	8.929239	8.736571	8.920300	8.856221	8.841352	8.540210	8.768392	9.151857	9.029501	8.929089	9.003048	8.853642	9.207016	8.857898	9.012401	8.731353	8.890833	9.024321	8.759950	8.703539	8.721025	9.083038	9.123673	9.260903	9.038835	9.611938	9.225119	9.127037	8.788276	8.824643	9.360161	9.408176	8.703695	8.616473	8.925972	8.403771	8.609069	8.927404	9.487831	9.205492	8.999951	9.136065	9.180169	9.406930	9.483634	8.582980	9.131387	9.954924	9.678618	9.530075	9.301890	10.828572	10.322742	8.450472	9.752217	9.126299	9.385879	9.378088	9.340577	10.301596	9.899145	9.624463	9.937579	9.505806	9.167255	9.702186	9.190862	9.727754	9.362145	9.468552	9.000251	9.148972	9.748976	8.951958	8.792439	8.500441	10.218028	9.563148	9.460158	9.283248	9.553744	9.145493	9.031727	9.808233	9.183297	8.960071	9.105050	8.832252	9.422496	9.224522	9.505969	11.082337	8.854885	9.171885	9.191817	10.051559	9.873647	8.978174	10.230046	9.633073	10.773877	10.234963	9.799812	9.192737	10.145661	8.774914	9.758117	9.642061	8.522315	9.298453	10.068558	8.983194	9.629836	9.631674	9.130010	8.904469	8.847714	8.822822	9.330748	9.721087	9.592440	8.987649	9.518373	9.087336	9.537972	9.476133	9.601584	9.262432	8.864910	9.682352	9.249208	10.214880	9.796717	9.534594	9.376209	8.799907	9.686093	9.067242	9.681108	9.131387	9.524105	9.252684	10.010461	9.210087	9.382397	9.120357	9.134241	9.731977	8.988368	8.879747	9.360471	9.347097	8.827612	9.173578	8.847553	8.901108	10.584914

ARRDC1	15.011117	12.619480	12.671728	13.705084	12.741114	13.020101	12.689500	13.635217	13.548671	13.230666	13.169042	13.045360	12.998253	13.338514	12.781401	13.338927	13.324601	13.146881	13.845086	13.424595	13.081778	13.455025	12.380353	13.145522	13.184093	12.896514	12.524939	13.403996	12.720405	12.346154	13.413148	13.467905	12.952208	12.217300	12.353897	12.470221	12.892947	12.595766	13.144182	13.401844	12.906069	13.342644	12.896890	12.718866	12.204037	12.573686	12.533294	12.909649	12.793758	13.151837	13.203403	12.950547	12.788576	13.136608	12.999705	12.961424	13.010893	13.562910	13.155345	13.372298	13.276852	12.631160	11.615814	13.604544	12.951479	13.246114	13.169781	13.379067	12.856573	13.043339	12.870661	13.053130	13.074158	13.025476	13.106812	12.710821	12.860559	12.828585	13.442970	13.206916	12.975455	12.829284	13.006339	13.132807	13.085733	12.742511	13.234991	13.417980	12.874685	13.770103	13.242980	13.026732	12.814928	12.994351	13.010893	12.939042	12.894551	13.329225	12.675008	13.497141	13.333079	13.034884	12.994539	13.189155	13.502602	12.817505	13.290515	13.652718	12.829643	13.130961	13.390989	13.384576	13.327179	13.344888	13.436104	12.944248	13.864108	12.735048	13.360372	12.757890	12.720241	11.735680	12.324479	12.313639	12.146258	13.064342	11.585439	12.765130	12.395544	12.435950	12.088137	12.611506	12.701417	12.092606	12.687096	12.523307	12.715054	11.944381	11.631275	12.230037	12.662746	12.651078	12.661199	12.317479	11.094004	12.091212	12.959560	12.158950	11.600580	12.633940	12.326190	12.456759	12.086064	12.330636	12.878536	12.049063	13.214995	13.372065	12.780538	12.231096	12.781892	12.126571	11.925662	12.412844	12.408069	12.528398	11.068828	13.204369	11.897513	11.935946	12.435325	12.594761	12.581640	12.211265	11.721853	10.824286	12.996223	12.586980	12.319355	13.099956	12.047677	13.388838	13.045360	11.031212	12.508357	12.579011	12.727428	12.590461	12.898463	12.741273	12.622342	12.851064	12.154928	11.757998	12.683500	12.248989	12.017038	12.516464	12.180168	13.121375	12.822489	12.337752	12.080582	12.637652	12.555759	12.965086	12.560874	12.972030	12.383064	12.158335	13.362446	12.304945	12.409200	12.420749	12.223705	12.789960	12.656681	12.829284	12.969511	12.309519	11.626169	11.896202	12.162219	11.739081	12.501735	13.047442	12.964557	12.561379	12.968759	12.200007	12.069926	11.421630	13.025833	12.627164	11.870995	12.836884	12.950547	12.209897

SLC26A8	3.395212	3.613588	3.461815	3.665678	3.155688	3.134024	3.417042	3.666887	4.856270	3.507239	3.641091	3.658433	3.356052	3.885110	3.845423	3.236523	5.241252	3.295881	5.121307	3.349518	5.523280	4.351543	3.574795	3.316047	3.271149	3.021553	3.684609	3.114613	3.310854	3.291616	5.152764	4.002147	4.206614	3.972872	3.689322	4.155180	3.945083	3.373901	3.322496	3.205978	3.450236	3.674117	3.715654	3.387739	3.796720	5.874232	3.622038	4.957731	5.317658	4.172921	4.069020	4.007615	4.468417	4.350224	5.754786	3.180263	4.260212	4.037570	3.182044	3.912934	2.899544	3.181767	3.888906	3.417203	3.665068	3.953338	3.003001	3.391201	3.922224	2.909221	3.281785	3.003120	3.294849	4.149594	3.248272	3.458581	3.249707	4.055179	4.212963	4.967997	3.567285	4.219469	3.903786	4.365717	3.522436	5.003789	3.026581	3.783345	4.041676	3.833785	3.399349	4.651356	3.516964	4.071053	3.355877	3.372595	4.835798	3.656622	3.228916	2.973164	3.015866	3.495222	3.400057	6.562847	4.678389	3.603835	3.239897	3.812713	3.442401	3.308242	2.852384	3.705864	3.227610	3.347723	3.542686	2.978982	3.696522	3.864320	3.232167	3.112756	3.778757	3.951215	3.584949	3.201711	3.920397	3.019669	3.338619	4.872775	3.224190	3.497641	3.334861	3.036447	3.350916	3.426736	3.308406	5.048723	3.043520	3.493904	3.483698	4.795393	3.872654	5.415123	3.036549	2.982540	2.947225	3.059943	2.953130	3.181230	3.488837	4.702801	3.524784	3.481349	4.151892	3.611177	3.835677	3.794679	3.480978	3.324164	3.129814	3.184762	3.213948	3.663842	4.226825	4.317365	5.342778	3.854880	3.217985	4.280614	4.388072	4.136714	3.823300	4.222970	4.448486	4.472145	3.084509	3.842331	3.881506	2.879088	3.681816	2.822427	3.481349	4.005218	4.124633	4.058871	3.826779	2.941574	3.629032	3.507789	2.848569	3.116732	3.077828	3.259139	3.067628	3.058954	3.517551	3.284599	3.199019	3.389455	3.302221	3.933476	4.741069	3.966608	3.684609	3.535381	3.579178	3.452427	3.457163	3.721688	3.678004	3.591699	3.340980	3.218815	3.504352	3.376866	3.249142	2.950436	2.940495	4.062858	3.492031	3.024181	3.084269	4.691364	7.305553	5.059824	4.214696	3.222403	3.832923	3.589579	3.189850	3.298388	3.001687	2.993254	3.426736	3.138823	3.587474	3.395752	3.151974	3.306659

KRT73	9.820554	9.841075	9.830772	10.319671	10.711967	11.086626	10.143394	11.482684	11.800445	10.619535	11.076625	10.558709	10.307568	10.281995	9.891644	10.986598	10.952734	9.949359	10.944920	10.532684	9.878300	10.622485	9.879639	10.283247	10.304338	10.820288	11.082761	11.022755	11.374203	10.465346	11.577639	9.270309	9.323358	9.895662	9.462714	9.558026	9.779527	9.483497	10.218950	9.873016	11.077209	9.783941	9.599698	9.966216	9.722467	9.493002	11.484580	9.733183	9.501613	9.687451	9.187848	9.599698	9.058600	9.554189	9.319592	11.333467	9.243650	9.654867	9.609304	9.432358	9.298596	10.701664	9.340284	10.183317	9.467510	9.436174	9.540818	9.790211	9.566202	9.717969	9.829507	9.905450	11.260034	9.645034	9.682352	9.659217	9.674912	9.592125	9.640574	9.506420	9.445750	9.140691	9.137318	9.418245	9.498564	9.616838	9.641321	9.756549	9.314274	9.596951	9.292212	9.561904	9.820128	9.706564	9.791261	9.941496	9.389608	10.607990	10.677043	11.065719	11.045883	9.805157	10.454947	10.191313	9.782227	11.341014	10.737331	10.074846	10.800096	11.211843	9.695569	10.947159	11.103121	11.522791	10.935332	13.021558	10.259615	10.543163	12.042411	9.864242	10.293236	9.669428	10.536625	11.769600	10.357076	10.805904	10.410131	10.905852	10.462807	10.904808	10.280486	10.804313	10.120195	10.281686	10.895631	10.633662	10.542688	9.965013	9.583324	10.356772	10.806201	9.332454	10.939212	10.022143	9.734702	10.640759	10.853314	11.809331	10.143844	11.057630	10.279430	10.219240	10.391055	9.448419	9.578383	9.721087	9.587330	10.549491	9.879639	11.588326	9.618556	8.922566	9.785797	9.811011	9.603163	12.222032	8.798246	9.387777	9.518231	9.645490	9.474447	9.026892	9.579457	9.663577	10.811801	8.700428	9.378682	9.588735	8.975737	9.812767	10.589073	9.749266	9.776425	9.316571	9.523949	9.497029	9.845979	9.723277	9.636066	9.782831	9.819984	12.224164	10.096699	9.246431	9.871673	9.713091	9.665912	9.838114	9.662648	9.272858	9.222961	9.316873	9.215448	9.260302	10.265725	9.584367	8.937648	8.994735	9.206130	9.049134	9.591343	9.864242	9.772353	10.024815	10.158948	10.107303	11.055538	10.850333	10.016962	10.137446	10.098434	10.776378	9.974679	8.727249	11.086626	10.945347	9.761351	9.769225	11.822299	9.624616	10.612438	12.410614	11.770058	10.816421	11.475722	9.883041	10.634740	10.648046

CUX1	8.551354	8.702430	8.992569	8.779318	8.635856	8.932558	8.783516	9.124740	8.684010	8.521276	8.624351	8.428351	9.395577	9.244700	8.536093	8.606200	8.311931	8.673896	8.578920	8.914940	8.836326	9.053617	9.957048	8.725564	8.548140	8.568786	8.347960	8.414729	7.909425	8.442536	8.503715	9.163075	9.580426	8.807508	8.829658	8.722201	8.980884	8.922106	9.088528	8.661634	8.345999	8.621889	8.880054	8.545522	9.375753	8.638648	8.607876	8.776297	8.847714	8.994100	8.887920	9.062763	9.168297	8.933305	9.144456	8.639746	9.499786	9.428521	9.454180	9.484723	9.224522	8.453220	9.583001	8.917711	9.026460	8.820386	8.785633	8.511392	9.311872	8.569261	8.672031	8.704437	8.224190	8.873398	8.793388	8.922259	8.982441	9.385285	8.575191	9.276381	8.932990	8.895756	9.080099	9.144456	9.259079	8.987649	9.518231	9.490144	8.995183	8.811217	8.833199	8.667886	9.416111	9.149853	8.645568	8.753475	8.871542	9.055012	8.893945	8.698199	8.406973	8.774143	8.909057	9.276833	9.271969	8.758241	8.553489	8.909370	8.156419	8.583773	8.590668	8.335568	8.499004	7.869083	8.310979	9.035125	8.516149	8.759174	8.667128	8.819791	9.336944	9.548072	9.845661	10.504236	9.747720	9.005510	9.876239	10.916587	9.440330	9.346648	9.843644	8.696372	7.998868	8.808590	10.014459	9.502229	10.655479	9.295979	9.600344	9.619017	10.103273	9.931241	9.897496	9.892413	9.734090	8.886993	9.407099	8.837390	8.463686	8.654623	9.984579	9.657205	9.745376	9.130445	10.959888	10.016493	10.239265	9.640101	10.257113	10.035161	9.858496	9.708602	10.013258	11.359717	9.587157	8.850175	9.438812	9.953346	10.189059	10.057591	10.219691	10.348093	10.153109	9.867886	9.159694	9.858997	10.603565	10.242137	10.784221	9.817117	8.825236	9.514851	9.645810	9.441980	9.612400	9.651348	9.879312	9.724389	8.801121	10.038559	10.355061	8.583138	9.436629	9.835522	9.384220	9.446852	10.100454	9.683577	9.910357	9.761195	9.029816	10.036715	9.878156	9.488884	10.007072	9.765411	9.525056	9.060660	9.304141	9.298286	9.969266	10.168427	9.234563	9.450783	9.584523	9.365175	9.813541	8.539403	10.134688	8.572191	9.706401	9.188155	9.528632	9.706401	8.737472	10.472219	9.787289	10.020783	8.956370	10.199328	9.231170	8.143761	7.412132	8.232995	9.244109	10.752793	10.558709	9.460752

TNIK	10.459968	9.248755	9.461531	9.912200	9.768915	9.512608	9.780402	9.635849	9.516988	9.628632	9.290321	9.722785	9.408330	7.804477	9.443739	9.752062	9.135924	9.008878	9.684197	9.409404	9.344218	8.996270	9.130010	9.328062	8.547997	9.032961	10.904946	8.291988	10.114351	10.447500	9.579792	9.771907	9.545320	9.829671	10.330389	9.672854	9.556482	9.570876	9.248356	8.751640	10.128236	8.927562	9.663731	9.247361	9.617155	9.051148	10.129911	8.530152	9.042373	9.428670	9.670386	9.454320	9.163408	9.598034	9.499023	10.380759	9.273303	9.470633	9.557255	9.014249	8.746045	9.679564	9.396827	8.201533	8.995337	9.400733	8.204198	8.920300	9.175728	7.883850	10.665768	9.641025	9.832913	9.808570	10.054496	10.180258	9.737303	10.191151	9.742594	10.041156	9.670699	9.592603	8.942784	9.373655	9.495957	9.460449	9.972329	10.172770	8.968517	9.837342	8.814706	9.692401	9.134241	9.328916	9.881929	9.311545	9.265944	9.314738	8.933782	10.511711	8.895157	9.869171	9.960750	9.943197	7.559064	10.128078	8.874344	9.165894	8.285459	10.147175	8.434770	8.935159	8.134382	8.783374	8.630840	8.027626	9.262594	9.377466	9.592908	9.451258	9.005828	9.902118	9.034473	8.642371	8.097665	8.772771	9.308024	8.687758	8.557910	7.799425	8.955928	7.978468	9.388703	7.025077	9.702186	8.404438	9.449040	7.738981	9.467827	8.783210	8.057849	8.940804	11.436255	9.488884	8.440424	8.378322	7.086134	11.234364	9.605617	9.364727	8.991055	9.250237	8.223899	10.603402	9.819337	8.978925	8.376183	7.752132	9.059116	10.310359	8.188620	11.158776	8.285162	9.055978	8.954109	9.037545	8.475262	9.617944	9.367576	8.778562	8.704437	8.974079	8.824932	9.939017	10.042666	8.704125	9.062460	8.437844	9.266572	9.714868	9.733626	8.486745	7.503062	8.138502	9.201856	10.431883	8.871377	8.090649	8.188180	9.934336	9.107492	7.633165	9.965758	10.937605	9.884535	9.931382	9.329557	9.299705	9.941651	10.956031	8.684477	7.932633	9.645183	9.372113	9.033552	11.112588	8.823280	9.884535	9.770821	9.432029	9.498874	8.393485	10.211984	9.334599	9.300335	9.186600	8.954400	6.954068	9.983054	11.209812	9.360332	8.491790	10.736706	8.461880	9.964867	8.593939	9.406773	8.709164	10.399588	8.772165	12.078200	9.966801	8.620648	8.439951	7.618175	8.677119	7.371092	8.279940

ASCC1	10.957229	10.998620	11.505998	11.551198	10.592897	11.020371	12.262621	9.331216	10.451232	11.764948	11.190615	11.317325	10.514841	10.878560	11.684212	11.228419	12.741823	11.558935	10.816719	10.231595	11.761312	9.787289	13.435763	11.126783	12.501563	12.078677	13.216213	12.259736	12.355155	10.707189	12.161273	11.465549	10.934125	11.938934	12.241919	12.385620	12.286593	12.565784	13.103548	12.674576	11.783598	13.593859	12.812113	12.138484	13.732895	12.070249	13.146323	11.752478	11.913539	12.022968	11.637543	12.472784	12.130529	12.019903	11.803667	11.013094	12.352896	13.219965	12.040141	11.928915	13.093911	11.411376	14.663619	12.015827	12.260347	12.690027	12.561379	12.667996	11.408142	11.912061	12.152235	12.038055	12.065925	12.353897	11.520985	11.725426	9.236267	11.404986	11.295839	10.804025	10.345226	10.779419	10.558107	11.137290	10.535450	11.565671	9.828115	10.764929	11.464934	10.250726	11.489358	10.552353	11.117822	10.706580	10.574079	11.773425	11.784041	12.058897	11.853542	13.900711	11.103722	13.077225	12.000077	12.620500	11.542272	12.088732	12.274074	12.657484	12.816143	12.467321	11.347275	11.592964	10.635777	11.274308	10.119603	10.537933	12.096083	11.943486	12.588140	9.420087	9.461202	10.462179	10.312904	9.797029	9.496425	10.474973	10.901715	10.507667	10.742902	9.383032	9.671636	10.902185	9.518998	8.905075	12.775824	9.798602	10.597699	11.572813	10.338072	9.424212	10.423303	10.904808	10.270574	9.978895	8.922407	8.940945	9.649319	10.718671	9.259079	12.352576	9.854464	9.903186	9.472171	10.090076	9.345894	10.254815	9.752834	9.888466	10.931591	9.606560	10.440817	10.943468	9.460158	9.207632	9.423772	9.766324	9.813229	9.125034	9.857253	10.059259	10.728368	9.673342	10.032710	11.240073	11.376076	10.618976	10.295232	10.701509	9.558784	9.622602	12.994539	9.644393	9.103656	10.693482	10.388806	9.789600	9.933222	9.970923	9.393908	10.039141	9.924291	9.581758	9.830915	10.879442	10.427019	8.456897	10.178271	9.477368	11.258854	9.315049	9.385879	8.820086	9.754226	8.569415	9.974365	8.934567	9.682637	11.645767	11.278088	9.479495	8.759006	10.926688	8.878315	9.628141	10.165649	9.315503	10.845627	9.260598	11.119567	9.084561	10.653251	12.216095	11.215086	9.823884	10.716420	11.026742	9.978579	9.591661	9.828423	11.509649	10.247848	9.912662	9.810261	9.313309	8.669872	11.878918	9.659835	8.952859

ANAPC16	10.957229	10.998620	11.505998	11.551198	10.592897	11.020371	12.262621	9.331216	10.451232	11.764948	11.190615	11.317325	10.514841	10.878560	11.684212	11.228419	12.741823	11.558935	10.816719	10.231595	11.761312	9.787289	13.435763	11.126783	12.501563	12.078677	13.216213	12.259736	12.355155	10.707189	12.161273	11.465549	10.934125	11.938934	12.241919	12.385620	12.286593	12.565784	13.103548	12.674576	11.783598	13.593859	12.812113	12.138484	13.732895	12.070249	13.146323	11.752478	11.913539	12.022968	11.637543	12.472784	12.130529	12.019903	11.803667	11.013094	12.352896	13.219965	12.040141	11.928915	13.093911	11.411376	14.663619	12.015827	12.260347	12.690027	12.561379	12.667996	11.408142	11.912061	12.152235	12.038055	12.065925	12.353897	11.520985	11.725426	9.236267	11.404986	11.295839	10.804025	10.345226	10.779419	10.558107	11.137290	10.535450	11.565671	9.828115	10.764929	11.464934	10.250726	11.489358	10.552353	11.117822	10.706580	10.574079	11.773425	11.784041	12.058897	11.853542	13.900711	11.103722	13.077225	12.000077	12.620500	11.542272	12.088732	12.274074	12.657484	12.816143	12.467321	11.347275	11.592964	10.635777	11.274308	10.119603	10.537933	12.096083	11.943486	12.588140	9.420087	9.461202	10.462179	10.312904	9.797029	9.496425	10.474973	10.901715	10.507667	10.742902	9.383032	9.671636	10.902185	9.518998	8.905075	12.775824	9.798602	10.597699	11.572813	10.338072	9.424212	10.423303	10.904808	10.270574	9.978895	8.922407	8.940945	9.649319	10.718671	9.259079	12.352576	9.854464	9.903186	9.472171	10.090076	9.345894	10.254815	9.752834	9.888466	10.931591	9.606560	10.440817	10.943468	9.460158	9.207632	9.423772	9.766324	9.813229	9.125034	9.857253	10.059259	10.728368	9.673342	10.032710	11.240073	11.376076	10.618976	10.295232	10.701509	9.558784	9.622602	12.994539	9.644393	9.103656	10.693482	10.388806	9.789600	9.933222	9.970923	9.393908	10.039141	9.924291	9.581758	9.830915	10.879442	10.427019	8.456897	10.178271	9.477368	11.258854	9.315049	9.385879	8.820086	9.754226	8.569415	9.974365	8.934567	9.682637	11.645767	11.278088	9.479495	8.759006	10.926688	8.878315	9.628141	10.165649	9.315503	10.845627	9.260598	11.119567	9.084561	10.653251	12.216095	11.215086	9.823884	10.716420	11.026742	9.978579	9.591661	9.828423	11.509649	10.247848	9.912662	9.810261	9.313309	8.669872	11.878918	9.659835	8.952859

GPATCH8	10.948018	10.889985	11.457285	11.180670	11.757533	11.298381	11.460855	11.577194	11.432854	11.313492	11.359582	11.001214	11.433599	10.931591	9.660763	11.299384	11.631352	9.640419	10.771222	11.527615	9.992511	10.200663	8.790410	10.568922	10.494529	10.781758	9.935109	10.667558	11.052661	11.213347	11.098248	11.643282	11.918019	11.268395	11.533775	11.708961	11.659709	10.818785	10.434589	11.017912	11.094441	10.205670	11.714834	11.789556	11.666271	11.655606	10.516242	10.091267	11.664926	11.813909	11.618620	11.889759	11.614652	11.558808	11.617214	10.960769	11.862748	10.698075	10.917489	11.025846	10.164597	11.022755	9.292527	10.063327	10.571218	10.597077	10.594561	11.357672	10.622632	9.630443	11.309686	11.443526	11.306203	11.656735	11.438109	11.568999	11.707448	11.691697	11.534802	11.954390	11.373920	11.865377	11.691697	11.835114	11.828107	11.761312	11.858266	12.218494	9.997359	10.939616	10.252055	9.952895	10.625496	11.433163	11.248223	11.532884	11.796745	11.816305	11.508778	11.461731	11.654537	11.429723	11.275601	10.883564	10.730156	11.539526	9.430199	10.249543	9.156761	11.259009	8.963282	10.285020	10.899103	11.163772	11.615402	10.027644	10.189200	11.352031	10.911465	11.387025	11.054075	11.084241	10.510212	9.968179	11.119415	11.205099	11.109422	11.266775	10.908849	10.787776	10.901715	10.863380	9.988894	8.524014	10.756176	11.875072	9.513967	10.599097	11.760698	9.975129	10.422252	9.072667	10.437954	10.811654	11.415437	10.449177	10.030393	10.617741	11.237421	11.205836	11.147807	11.636180	11.665819	11.743625	12.098451	11.391895	10.407402	10.594561	11.232005	11.047086	10.044933	12.155249	12.097254	11.651535	11.616116	10.519438	10.701664	11.632841	11.966106	11.618317	11.619033	11.822772	11.767362	11.418835	11.339525	12.113904	10.968164	10.694353	11.175522	10.092047	11.155666	9.965460	10.396585	10.714168	11.160511	10.808104	10.579925	10.665188	9.571980	11.731685	11.499627	9.646619	11.673868	11.703111	11.650359	11.543025	11.991261	11.893211	11.926602	11.283416	11.857035	11.812582	11.991408	11.986208	11.194864	11.960692	12.252460	9.767842	10.493142	10.819847	9.991577	10.765528	11.073555	11.214660	11.030014	11.282850	11.189047	11.330317	11.676245	10.334961	11.392045	10.885415	10.570453	10.712115	10.576019	9.704158	10.455864	9.885942	10.427471	9.025867	10.052635	9.947800	10.294926	10.955883	11.440322	11.054661	11.467049	10.689337

MKI67	8.885604	9.895820	10.282465	10.106859	10.275619	10.573635	9.818886	9.456638	9.758117	10.415564	9.835212	10.019714	10.543304	10.235279	9.656882	9.945894	10.045094	10.740649	8.707227	10.671356	9.067393	9.930320	8.157804	10.261469	9.378232	10.068558	9.210231	8.072444	9.024914	9.904543	10.214424	10.960475	9.831229	11.338491	10.118547	10.719738	10.458181	10.127429	10.032263	10.735940	10.047403	9.242578	10.491272	10.235585	10.535147	10.902185	9.156149	9.084407	10.355971	10.678806	9.964423	10.510494	10.519232	10.130935	10.259158	9.266415	10.108989	9.177093	9.412599	10.425295	9.500105	9.760421	7.251697	8.657576	9.606097	9.240121	8.725722	9.744936	10.253224	8.372533	9.540818	9.775229	9.636846	9.713408	10.277896	9.758430	10.078545	9.580426	9.579000	9.933882	9.980743	10.132147	9.930936	10.478737	10.683631	9.792860	9.832155	10.293094	8.106793	9.048542	9.454607	8.622034	8.717201	10.124419	9.662498	9.920741	10.174432	10.488104	10.548263	9.761195	9.842288	9.597096	10.438848	10.451084	8.671736	10.404862	9.212818	9.795943	8.372078	10.069314	8.272998	9.481820	10.381790	9.956000	10.303562	9.595856	9.403399	10.123807	9.701109	12.131737	11.727947	12.150644	11.036553	11.095144	12.652934	11.737167	12.470874	12.313487	12.247193	12.186019	12.320789	12.795726	12.212531	11.060705	10.124282	12.488145	11.580132	12.028551	12.622342	10.247848	10.593329	10.312288	11.748620	12.064848	12.158173	9.519313	11.712469	12.030069	11.956809	11.892168	12.089348	11.832095	11.685397	11.991869	11.992764	11.142960	12.347281	11.179288	11.941052	12.458340	10.872927	12.920968	12.789228	11.690932	11.821859	11.901311	11.752181	11.474696	12.757036	11.666120	11.905620	12.895811	13.016827	12.175621	10.928442	11.255159	11.449715	10.983634	11.764948	10.549040	10.827379	10.026399	11.007714	10.885415	10.088845	10.228061	10.837802	11.211843	8.910390	10.714913	10.912940	11.583468	11.188301	11.457857	10.741254	11.165084	12.735217	11.263347	11.742598	11.616246	12.926643	10.600754	11.511108	12.286161	12.082923	11.940596	12.506232	9.628003	10.665327	12.033526	10.433367	10.269429	12.393464	10.801504	11.433163	12.878188	12.151563	12.681802	12.388237	12.816808	12.715895	9.277385	11.471379	10.579659	11.005839	11.422554	12.185267	9.215920	11.562313	8.595322	11.431952	13.025833	10.036868	11.569895	11.265020	11.370678	11.561717	12.150786

ZNF500	7.043839	6.336540	6.247129	6.059216	6.257597	5.915291	6.552682	7.349764	6.447841	6.868396	6.742430	6.613319	6.360873	5.217371	4.845952	6.696500	6.392707	5.721042	6.453304	6.678786	5.948433	6.768807	7.724547	5.889945	5.749191	6.492653	6.599750	6.035454	6.110433	6.349577	6.769612	7.338621	7.899733	6.406726	6.606716	6.910016	6.632208	6.462351	6.626777	5.872814	6.164766	6.493156	7.197133	6.305436	6.608518	6.266222	5.917767	7.295374	7.313210	6.846929	6.771880	7.013769	6.483249	6.764910	6.532242	6.170998	7.032959	6.582607	6.052855	6.245286	6.101173	6.149498	6.864398	6.513413	6.303063	6.382653	6.542210	5.993219	5.860480	5.879823	6.938731	6.156847	6.264678	6.701586	6.782927	6.527507	6.399293	6.213566	6.644989	7.032798	6.109395	6.908452	7.338002	7.101874	7.507090	7.314004	6.940443	7.158379	6.458853	6.203297	6.293873	6.269984	6.471578	6.924368	6.837708	6.901263	6.518553	6.535813	7.437391	6.231093	5.677876	6.294553	6.937644	6.247129	6.489031	6.300025	6.368195	6.567229	6.057447	6.436771	5.490755	5.859782	5.695283	6.472916	5.702406	3.475806	6.475242	6.409866	6.710559	6.443376	6.138928	6.197911	5.994297	5.580219	5.882107	5.782921	6.654828	6.598280	6.125147	6.187221	6.201016	5.237270	4.308576	4.646207	6.394679	6.747557	5.634192	6.653361	7.006750	5.153573	6.512094	7.429995	5.649730	5.335758	7.677137	5.963234	5.745522	5.777806	6.098721	7.129090	6.076577	6.946970	6.489335	6.507989	6.794137	6.608356	5.514243	6.233766	6.071333	5.715301	6.190787	7.046566	5.894762	6.514869	6.333488	5.651640	6.040557	7.183448	6.706548	6.101173	6.759287	7.813386	6.581133	6.526516	6.161869	7.711380	7.156070	5.975696	6.445240	5.961797	6.086417	7.537785	6.101534	5.501007	5.930416	5.635100	6.206911	5.802237	6.241268	6.805391	6.251495	6.289971	6.755554	6.564827	6.408030	6.759287	6.281963	6.249479	6.678786	6.513257	6.175069	7.596093	6.577011	7.638162	6.488381	6.354258	7.265880	6.477635	5.910637	6.421746	6.164413	6.118621	6.296097	6.515723	6.702745	5.931261	5.841877	6.752554	6.342844	5.895133	6.230595	5.779623	5.367401	7.247008	6.369883	6.482390	6.381820	7.378634	5.825842	5.131199	4.856053	4.625504	6.549177	5.224480	5.121711	6.035454	6.176433	6.110611

C16orf71	7.043839	6.336540	6.247129	6.059216	6.257597	5.915291	6.552682	7.349764	6.447841	6.868396	6.742430	6.613319	6.360873	5.217371	4.845952	6.696500	6.392707	5.721042	6.453304	6.678786	5.948433	6.768807	7.724547	5.889945	5.749191	6.492653	6.599750	6.035454	6.110433	6.349577	6.769612	7.338621	7.899733	6.406726	6.606716	6.910016	6.632208	6.462351	6.626777	5.872814	6.164766	6.493156	7.197133	6.305436	6.608518	6.266222	5.917767	7.295374	7.313210	6.846929	6.771880	7.013769	6.483249	6.764910	6.532242	6.170998	7.032959	6.582607	6.052855	6.245286	6.101173	6.149498	6.864398	6.513413	6.303063	6.382653	6.542210	5.993219	5.860480	5.879823	6.938731	6.156847	6.264678	6.701586	6.782927	6.527507	6.399293	6.213566	6.644989	7.032798	6.109395	6.908452	7.338002	7.101874	7.507090	7.314004	6.940443	7.158379	6.458853	6.203297	6.293873	6.269984	6.471578	6.924368	6.837708	6.901263	6.518553	6.535813	7.437391	6.231093	5.677876	6.294553	6.937644	6.247129	6.489031	6.300025	6.368195	6.567229	6.057447	6.436771	5.490755	5.859782	5.695283	6.472916	5.702406	3.475806	6.475242	6.409866	6.710559	6.443376	6.138928	6.197911	5.994297	5.580219	5.882107	5.782921	6.654828	6.598280	6.125147	6.187221	6.201016	5.237270	4.308576	4.646207	6.394679	6.747557	5.634192	6.653361	7.006750	5.153573	6.512094	7.429995	5.649730	5.335758	7.677137	5.963234	5.745522	5.777806	6.098721	7.129090	6.076577	6.946970	6.489335	6.507989	6.794137	6.608356	5.514243	6.233766	6.071333	5.715301	6.190787	7.046566	5.894762	6.514869	6.333488	5.651640	6.040557	7.183448	6.706548	6.101173	6.759287	7.813386	6.581133	6.526516	6.161869	7.711380	7.156070	5.975696	6.445240	5.961797	6.086417	7.537785	6.101534	5.501007	5.930416	5.635100	6.206911	5.802237	6.241268	6.805391	6.251495	6.289971	6.755554	6.564827	6.408030	6.759287	6.281963	6.249479	6.678786	6.513257	6.175069	7.596093	6.577011	7.638162	6.488381	6.354258	7.265880	6.477635	5.910637	6.421746	6.164413	6.118621	6.296097	6.515723	6.702745	5.931261	5.841877	6.752554	6.342844	5.895133	6.230595	5.779623	5.367401	7.247008	6.369883	6.482390	6.381820	7.378634	5.825842	5.131199	4.856053	4.625504	6.549177	5.224480	5.121711	6.035454	6.176433	6.110611

EDA2R	7.328193	7.298474	7.218571	7.652041	5.960936	6.320848	7.572330	3.990057	4.932450	6.072404	7.312129	6.356259	7.006419	6.809369	7.909883	7.286929	5.675129	6.892352	7.255240	7.150257	7.618328	6.509917	8.307762	7.004886	6.952479	7.971157	7.197441	7.627042	7.789294	7.176989	5.611985	8.401432	7.795703	6.465546	7.504612	7.327719	7.082859	7.470962	7.460716	7.033749	6.922731	7.832020	6.913553	7.551900	7.885512	6.798482	7.325171	7.886728	6.641748	7.571121	7.371424	7.744866	7.907089	7.851729	7.635884	6.595678	8.181140	7.884152	7.275303	7.856208	8.574739	6.647062	9.470011	7.685946	7.765779	7.640962	7.786365	6.902716	7.164871	7.208227	7.609277	6.549386	7.574967	7.814319	7.513898	7.740395	7.096188	7.698822	7.619402	8.367279	7.429995	8.452630	7.962682	7.849905	8.117928	8.560080	8.664392	8.098702	8.648726	8.430243	7.573879	7.620601	6.715371	7.002279	7.528278	7.519556	7.422117	7.031249	5.562403	6.198084	5.881051	6.655496	6.604561	6.506661	6.575152	6.533555	5.176541	5.824416	6.965964	6.263525	7.094332	6.832447	4.211867	6.728532	6.662807	3.174065	6.558766	6.594520	6.392355	7.764659	7.743008	7.514207	6.159627	4.384043	6.327724	6.077284	6.069980	6.799796	5.031335	6.822140	4.353756	6.763607	7.270478	5.982957	6.171845	7.182830	4.739080	5.481420	5.280737	5.684727	6.567413	7.059187	4.471226	8.463989	9.275616	6.145843	7.016983	4.760157	6.449017	6.445749	7.466203	8.190071	7.020611	6.746880	7.014664	8.564352	7.375004	6.797331	8.706907	7.231345	7.998255	6.512277	7.138922	7.631624	5.008283	4.589492	6.328401	5.325555	8.516378	7.156534	7.123519	7.718880	7.551900	9.153845	6.947121	8.639417	7.737577	6.057813	7.739616	8.776601	7.072278	6.791354	7.244857	6.963224	7.990643	7.298780	7.222979	7.557702	7.002726	6.780704	7.409130	4.317138	7.864018	7.561362	7.505232	8.019387	8.429897	7.678459	8.901863	8.018445	6.893530	8.055259	9.030985	9.425135	7.579023	8.870231	9.923845	8.919970	9.282306	8.646197	8.061804	7.659658	7.815256	7.727475	7.932955	7.145800	6.989840	6.574345	5.491117	8.070641	6.039284	6.660368	6.725130	8.452011	4.692874	4.031273	4.453114	6.389695	4.462868	7.561200	7.946027	3.026669	5.337710	4.930410	6.208126	7.696994	5.379258	5.459844

TRIM15	9.257270	5.878879	6.890923	7.425999	8.816206	8.092315	7.505232	7.846866	7.997816	7.836587	7.901337	7.829434	7.277201	6.476273	7.009806	6.811443	7.287232	6.355256	7.611602	6.685166	5.941296	6.350446	7.249321	6.652734	6.293351	8.039489	8.350957	7.521277	7.819973	7.392716	8.238367	7.489591	6.505130	7.691350	6.942960	6.828774	6.388007	6.230595	5.680935	5.915291	7.038119	5.908115	6.373825	6.022066	6.334681	5.991996	8.399557	5.479634	7.727475	6.284297	6.310605	5.685095	5.832274	5.473683	6.002723	7.879463	5.820965	4.739775	5.999377	5.785223	5.291576	7.439160	6.051477	6.145501	5.287566	5.893148	5.338509	5.548432	5.095000	6.038087	6.411836	6.706701	8.001042	6.629202	6.623405	6.436969	6.527992	6.600216	6.552341	5.866650	5.787748	6.262524	6.326915	5.804210	6.027116	7.112679	7.468145	6.644356	6.302728	5.129196	5.686601	5.727541	6.045714	7.904584	8.221156	7.348804	6.424158	8.109379	9.053125	7.681151	8.952859	6.586695	6.906870	6.655496	6.510260	7.113308	5.953623	6.428351	7.191384	7.033109	6.491699	7.375167	7.507394	8.414587	7.742387	11.693331	7.173838	7.569464	7.747797	7.762049	6.554476	7.984242	6.920025	10.381474	7.476312	8.564352	7.288297	7.836137	8.353066	8.350047	8.524014	8.095831	7.403964	8.344459	7.744247	7.370785	7.300159	7.167633	6.777177	5.890818	6.532738	7.364676	7.835387	6.641302	7.972255	7.988792	8.198553	8.808590	7.053316	8.537385	7.551900	7.739775	8.171607	7.575743	6.846765	6.980510	6.952663	6.699191	6.781814	8.275673	6.615109	9.660142	6.787106	6.453478	5.958590	9.029501	5.736616	7.063661	6.574822	6.493615	6.090782	6.292186	6.274724	6.641302	6.971518	6.605446	6.564982	6.685342	5.194478	6.216641	7.701899	6.065057	6.228480	6.057813	6.746276	6.133795	5.830469	5.872991	6.255038	6.445919	6.928432	9.150489	7.229369	6.907814	6.411029	6.468844	7.513425	6.961969	6.329383	6.038250	6.548360	6.404890	7.461663	5.911336	8.340557	6.483741	9.537204	6.273900	6.195674	6.728999	6.772178	6.957350	7.743660	7.801145	8.284375	8.460790	8.946909	8.369070	7.312895	9.067083	7.214976	7.248376	7.345496	7.907089	7.461823	6.782450	7.714346	6.323058	8.579986	7.081142	7.815906	8.261392	9.077642	9.923051	10.142602	6.444714	8.068315	7.688567

FGF13	6.557111	4.419362	4.312729	3.609596	3.151510	4.991405	6.285328	3.358469	3.236658	3.635265	3.752961	4.079378	4.921917	3.449554	3.815667	3.973522	4.009963	2.891716	3.383146	4.309712	3.806389	2.920166	4.528201	3.954187	3.194728	3.980405	5.553982	3.744268	4.867689	4.504789	3.296369	5.830277	6.136200	5.022592	5.442744	5.814075	6.321693	4.958361	3.801192	3.789104	5.387590	3.447280	5.493723	3.993280	7.022488	5.256728	6.549699	6.115466	6.019724	5.658878	4.835984	6.007165	6.473753	5.820088	6.159273	4.639479	6.814751	2.916669	4.501816	4.654865	4.210561	5.715301	5.644055	3.146302	3.356209	3.743241	2.969982	4.406925	4.266147	3.006528	5.170694	4.770546	4.589492	6.293544	4.658582	4.148755	5.469311	4.824132	4.884944	6.391555	4.584786	5.929525	5.589300	5.881051	7.248072	5.627454	4.168179	5.544296	4.348297	4.044548	4.414893	3.868138	4.047803	5.802411	5.006417	4.697031	5.277375	5.708310	6.121129	6.016224	3.875266	6.055823	4.411759	5.681611	5.051193	3.746872	3.079184	3.261151	3.039843	4.943181	2.917227	3.110615	2.923059	3.216375	3.197591	3.332238	3.651167	5.476002	4.191700	4.978895	3.358619	3.067732	3.616879	3.041132	5.389488	4.363732	3.941069	4.173784	3.471558	3.092914	4.812486	3.665678	3.062481	2.952386	4.814969	3.248982	2.877613	3.047001	3.406231	3.386886	2.930729	5.950046	2.883463	2.896701	3.879159	3.151248	3.675584	3.014178	3.448178	3.127950	5.609716	5.387388	4.759526	3.099176	5.620017	6.415011	3.764711	3.290129	3.040595	4.035385	3.364854	3.571510	3.287257	4.515925	4.775532	3.654812	6.106563	5.823680	5.267268	3.584353	5.074630	4.676014	4.293542	4.673618	4.703430	5.099511	2.940658	2.916788	3.264112	2.941729	6.145843	3.885512	3.471876	3.530135	4.293542	2.963995	3.478207	3.177227	2.987672	5.943205	5.613287	3.220831	3.552211	3.594809	3.610373	3.501981	3.596331	4.339197	4.531195	3.495782	4.680948	3.590125	3.949435	3.905507	3.634285	3.697132	3.838007	3.056655	3.271463	6.627731	3.991571	3.190001	3.748119	3.181376	3.477266	3.269121	3.565517	4.942813	5.308659	3.531937	3.483337	5.252144	3.334035	4.438717	3.312876	3.100427	3.195700	3.280866	3.135664	3.071974	3.090632	3.166490	3.159921	3.016359	5.768648	4.172089	3.171558	3.986728

ZBP1	8.595322	9.759952	8.823853	8.721903	8.169880	8.813362	8.046403	8.467335	8.628231	7.511861	8.064117	8.058580	7.997958	7.760826	8.408218	8.729568	8.004026	8.848031	8.742540	8.629901	8.490141	9.984967	9.401214	9.258777	9.180938	7.819680	11.336132	8.817585	8.344605	10.845325	8.078697	9.330440	10.826359	11.068392	9.243968	9.200336	8.444544	8.595787	7.934176	9.686684	8.050463	9.648129	10.372744	8.960987	8.695418	9.659586	8.415017	9.966351	9.060792	8.876306	9.865926	9.278313	9.454320	8.565719	9.681108	8.032995	8.606200	9.703107	8.999207	9.525226	9.630914	7.812790	9.015314	9.583159	10.611543	9.288325	9.786976	9.479952	10.426707	9.966216	8.645720	8.690507	7.987342	9.272123	9.198437	11.453711	8.712812	9.671482	9.422970	9.329557	9.587157	9.379147	9.686241	8.758828	8.879418	9.285962	9.312969	8.837687	10.399588	9.690233	9.759184	10.744115	9.521928	10.610648	10.181016	9.722785	9.197188	7.644333	8.079617	7.798655	7.712340	8.891290	8.103236	8.079763	9.542515	8.119607	8.785782	9.155395	8.854082	8.081792	9.357110	8.296548	8.498687	8.463686	8.346473	9.798602	9.773869	8.254672	8.036343	10.629335	9.761509	10.577682	10.678375	8.626865	9.169996	8.895309	7.486340	7.821662	7.685796	8.578625	7.746256	7.035286	11.441315	9.243810	7.865730	10.882791	8.231463	9.854951	10.031316	9.091908	10.945211	10.348720	7.537785	7.306655	7.535627	11.043725	8.258639	8.750022	10.444886	9.358788	11.355495	8.587856	8.799766	10.479190	8.952566	9.764347	10.054057	9.326153	10.353264	7.434223	11.087667	10.473309	8.950569	9.392820	10.800096	9.353339	10.269714	8.615111	8.745107	10.635641	10.116521	10.254503	7.711380	9.408330	9.096736	8.412161	10.491887	12.124933	10.706880	10.303868	8.821736	11.001079	11.409053	11.622188	11.115929	10.241816	11.092828	10.666063	12.701760	9.395906	9.719876	8.180988	8.495987	10.694498	11.641765	8.706443	8.340415	8.565393	10.961636	9.370141	9.597882	10.730762	10.867436	11.187873	8.785931	9.568103	8.759006	10.483815	11.081007	9.766931	11.742152	8.460937	9.407719	10.178593	11.164515	8.730171	8.515759	10.430630	7.683680	9.048076	8.916335	8.505652	11.383209	10.883564	9.749266	8.009628	9.712029	9.739328	9.255589	11.061124	9.852155	9.098890	9.971512	8.182340	8.516533	9.952278	7.320128	9.339487

ZSCAN5A	8.514294	8.099931	8.113605	8.851981	8.964497	9.057352	7.720980	9.451098	10.036097	8.753634	8.840427	8.361019	8.406370	8.289790	8.060923	8.663627	8.887283	8.181304	9.580724	9.397811	8.668179	9.362614	9.778460	8.715941	9.212520	8.815760	9.930780	9.402771	9.171571	8.529692	9.041013	7.860047	7.963592	8.251602	7.867736	7.921935	7.946342	8.131197	8.900377	8.908916	8.759794	8.410456	8.088765	8.358778	7.963882	8.052014	10.011379	7.751210	8.955174	7.871982	7.979416	7.852651	7.302565	7.471256	7.229677	8.946272	7.226699	8.157947	8.145020	8.238226	8.251460	8.847714	9.181558	8.864465	7.777297	7.976484	8.545228	8.514907	7.888271	8.618151	8.338861	8.431048	9.060792	8.235415	8.301208	8.280399	8.507514	8.221008	8.307282	7.726532	7.960692	7.825691	7.694438	7.746079	7.931121	7.930949	8.399882	7.832609	8.273134	8.027463	7.927415	8.522315	8.142995	8.134382	8.182181	8.159168	7.530523	8.610473	8.833467	9.403709	9.417796	8.133618	8.547842	8.302895	8.403771	8.873698	9.661397	8.632470	9.799495	9.322876	9.082279	9.218559	8.866731	9.737762	8.975575	10.892076	9.058786	8.352011	9.496877	9.013468	9.270309	9.102283	9.299086	10.498864	8.967268	8.892827	9.882077	9.810261	9.514711	9.523040	8.953796	10.093965	9.168297	9.702347	9.634319	9.482720	8.772914	10.256478	11.994581	9.527248	10.392057	9.300940	9.543795	10.097334	9.440640	9.383185	9.562674	10.262831	8.900522	9.750211	8.887600	9.014694	8.584972	8.344459	8.647089	8.945066	9.258917	9.380804	10.011379	9.890580	9.214054	8.702920	9.069131	8.601276	8.357999	9.717013	8.270421	8.711476	8.803350	8.836481	8.520957	8.406806	8.722971	8.475899	9.048698	8.170037	8.363165	8.616784	8.218076	8.698949	9.148150	8.202413	8.732710	8.485224	8.614211	9.299554	9.125500	8.995637	9.444365	8.994888	9.349409	9.218710	9.274098	9.061578	8.682676	9.744106	9.284919	9.141158	8.468110	8.331213	7.990643	8.234506	8.453689	8.442536	8.876016	8.327809	7.997816	8.871542	8.888374	8.691819	8.845859	8.896105	9.029036	8.926611	8.823411	9.045642	9.530244	9.383918	8.753475	9.336323	8.638648	9.488445	9.247361	8.908317	9.015459	10.163983	8.444854	9.084861	9.743643	9.991577	9.913924	9.970033	10.340663	9.417175	9.221118	9.521160	8.452479	9.158180

C1orf52	12.256799	11.479023	12.354358	11.992461	12.195580	12.026746	12.481303	11.965674	12.453112	12.007014	12.024942	12.183935	12.083231	11.577776	11.627063	11.764203	12.259420	12.168818	11.055538	11.973763	11.443078	11.409205	11.583468	11.404685	11.125488	11.942440	11.969628	11.574347	11.993963	11.889463	11.229444	12.083083	12.259577	11.734088	12.020053	12.054551	12.102820	11.895338	10.996866	11.862748	12.008090	11.328200	11.923268	12.156958	12.227505	11.965245	11.592678	11.510243	12.721811	12.173565	12.138038	12.301244	12.434341	12.336516	12.932755	12.253427	12.096669	10.998939	11.017781	11.913381	11.662361	11.927240	11.311032	10.770306	11.842350	12.235592	10.652524	11.559388	11.418682	11.431202	11.587012	11.940295	12.167732	12.212389	12.098917	12.353897	11.830627	12.303753	12.139420	12.303273	11.050033	12.285680	12.426538	12.299854	11.989265	12.332077	12.181896	12.606892	11.163772	11.551948	11.555370	11.715866	11.335546	12.267146	12.011267	12.221092	12.115840	12.119577	11.942720	12.080906	12.065618	11.735106	12.022222	12.070991	11.432403	12.088271	12.101333	11.661797	11.735541	12.003100	11.827534	11.162013	12.234041	12.221092	12.874855	11.733461	11.938632	12.277746	12.291942	12.299522	12.163756	12.385139	11.931391	12.875210	12.348060	12.567262	12.308556	12.460607	12.079547	12.508357	11.680386	11.871756	11.907433	12.022370	11.933974	12.333973	11.589071	10.913253	12.316234	11.871295	11.836470	11.499922	11.830322	11.323357	12.534620	12.645882	12.414483	12.332394	12.235761	11.812013	12.463389	12.255523	13.021758	12.386292	12.820600	12.493046	12.454210	11.709393	12.203451	12.217300	11.613018	12.055422	12.887380	12.360854	12.336516	12.369200	12.716779	12.556062	12.908579	12.961953	12.293428	12.343747	12.622994	12.332724	11.720464	12.979495	12.071894	11.892007	13.088340	12.427164	12.236813	11.660007	10.992701	12.353897	12.069478	11.661027	11.978054	12.219378	11.731240	12.507872	12.376451	11.944822	13.115055	12.473452	12.811936	12.175303	12.776712	12.436754	12.261651	11.339952	13.118872	13.241017	12.909649	13.465090	12.261502	12.703315	12.393629	11.134324	11.962445	11.838426	12.040600	11.926777	13.012290	12.327958	12.194431	12.473611	12.056973	12.967688	12.385139	12.632301	12.512086	11.945767	12.331290	11.792112	11.887650	13.094669	11.963873	11.073555	12.625338	12.029778	11.643417	13.019739	11.860975	12.414781	13.028594	11.909566	12.659292	13.205336

RHOT2	11.359007	11.326901	11.591454	12.106702	11.764040	11.540550	10.920468	11.323214	11.509057	11.327497	11.627063	11.512453	11.515630	11.810536	11.543468	11.448683	11.564497	11.476270	13.004475	11.724236	12.278819	12.486817	13.226252	11.816748	12.201865	12.061208	12.153445	11.838865	11.558808	11.410542	11.674592	11.688705	11.547983	10.889218	10.743052	11.154213	11.235506	11.200874	11.384067	11.520245	11.497713	11.822458	11.077064	11.491172	11.127809	11.223409	11.833001	11.291145	11.532292	11.292037	10.930535	11.291145	10.897043	11.530935	11.120300	11.658059	10.947309	11.855496	11.567838	11.580850	11.331326	11.338351	11.981655	11.984549	12.087259	11.535407	11.641173	11.576892	11.354180	11.407214	11.047517	11.122188	11.321595	11.047306	11.234364	11.259576	11.157291	10.847846	11.174436	11.196939	11.673557	11.529388	11.380538	11.578338	11.623310	11.308691	11.614054	11.761011	12.739166	12.269302	11.781361	12.520217	11.424939	11.021233	11.263347	11.625463	11.307706	11.285784	11.429574	10.874430	11.356979	10.704762	11.285928	11.207013	12.077723	11.865618	12.168818	12.076508	12.557817	11.662670	12.699705	12.083083	11.926777	11.660731	12.180333	10.094258	12.202678	11.411680	12.021794	13.400373	12.100275	11.555370	12.687272	12.600544	11.669857	12.422163	12.426215	12.259110	11.976836	12.033377	12.644874	12.477840	12.090269	12.508031	11.892609	12.041080	12.690337	13.548900	11.622042	13.074945	12.387097	13.314357	12.355477	12.904469	12.824882	11.774910	12.841413	11.993505	12.091683	11.357241	11.836026	12.663392	11.802387	10.887194	11.325406	11.312203	11.585156	12.428334	12.161407	13.641785	11.727061	10.909151	12.294023	11.356245	10.632606	12.552675	11.064514	11.750421	11.458006	11.478113	11.615965	11.367114	11.650646	11.734522	11.906809	11.385252	11.113773	11.552438	11.611675	12.172013	12.301709	11.992915	11.984395	11.799525	11.835877	11.762372	11.443831	11.795854	11.802821	11.282541	11.571480	12.980457	11.520394	11.151411	11.411822	11.277202	11.606112	11.524269	11.563886	11.936536	11.691368	11.991102	11.670899	11.761312	11.789411	11.794064	12.605556	12.525777	12.341859	12.171565	12.885665	11.812728	11.624718	11.481223	11.630608	11.769458	11.346810	11.703426	11.096025	11.954979	11.716014	11.368020	11.581259	13.000090	12.031682	12.816972	13.210112	12.586457	12.124642	12.905355	11.545153	11.219066	12.480968	11.062165	12.434341	12.264668	12.866581	12.285826

VNN2	7.470321	7.061705	7.389491	4.895390	3.046203	5.394962	6.985556	6.029076	4.246430	3.571687	3.411831	5.309863	4.387386	3.798589	5.943556	6.364380	4.618652	3.267949	3.566681	5.899887	6.274896	6.946451	6.513566	4.718124	3.018838	3.836319	8.054935	4.750862	5.340859	9.327210	3.658061	9.136846	7.805727	7.679817	6.598929	6.464205	6.125675	5.804920	4.026422	5.512770	4.453967	6.724304	7.923181	5.853071	6.088324	8.044489	6.938731	5.670490	9.298003	7.058706	8.602666	7.917823	8.281598	6.549386	8.504777	4.796480	8.444854	5.713082	2.949076	5.616498	5.228700	5.248785	7.611287	5.592356	7.601689	6.489829	4.445425	4.771384	7.320294	4.140191	7.134208	5.210432	4.829094	7.099247	6.151391	8.180535	6.340247	8.692143	8.692789	8.921354	6.713587	7.259980	7.785439	7.714929	7.435533	7.760697	7.709122	7.934793	5.719583	6.392190	7.493042	6.971967	7.288615	9.152019	8.691536	7.515705	9.045496	3.595367	6.210017	2.933598	3.096137	5.447016	3.444725	3.315547	4.677535	6.463206	4.002553	4.081552	3.018650	3.656217	3.645020	3.224749	3.374402	4.935976	5.859070	4.401099	3.944424	4.914136	4.637149	6.832615	4.608853	6.167203	5.809809	2.895077	5.489780	4.231567	2.832736	3.134142	3.345400	4.609495	4.849095	3.187677	5.499654	4.942999	5.659046	3.185015	3.016359	5.920679	6.496679	5.723368	3.541494	6.319104	2.933891	2.917403	6.785321	6.649670	3.544884	3.898920	8.577737	3.449361	8.005597	6.001621	7.538600	7.911881	4.286343	7.353126	5.028096	3.349011	5.355495	3.206112	6.942312	6.385607	6.394679	7.785744	6.817963	4.901535	3.262344	5.923488	6.562022	7.658770	8.536545	7.445550	7.648749	8.447805	5.546573	6.476422	5.599491	4.880724	6.659868	5.200081	6.519682	5.138445	5.827479	6.781503	6.967719	2.943219	7.304277	5.305197	6.457889	7.419464	5.915113	4.618177	5.002123	7.361534	8.037595	6.500213	6.405375	6.870310	8.030739	5.312841	6.835648	7.164871	8.923868	5.555822	5.339523	8.037881	6.644513	4.639678	4.192784	6.280716	3.874364	5.587518	7.143465	7.591800	8.322469	4.078461	3.220989	4.009963	3.146903	4.777820	3.478950	3.196929	5.637335	3.200255	7.862455	3.017093	3.882327	3.349703	3.406943	3.915108	3.668095	2.793797	6.468191	4.944128	6.769612	4.724734	3.650223	4.918935

BABAM2	12.551069	12.851957	12.748129	12.667996	12.730311	12.671728	12.871377	12.975455	11.534230	12.570995	12.706715	12.726341	12.866212	13.016253	12.698531	12.628507	12.832911	12.568243	12.905897	12.927769	12.720947	13.435763	13.162423	12.770845	12.548577	12.461401	12.218928	12.329087	12.134970	12.688794	12.272044	13.183285	12.681285	12.828755	13.225499	13.161296	12.959560	13.047627	13.206525	13.046662	12.294671	13.244322	13.229053	13.116581	13.223145	13.543518	12.280067	13.157459	12.899561	13.263573	13.125598	13.343862	13.295547	13.489283	13.026917	12.452334	13.280841	13.690456	13.246703	13.255166	13.152020	12.235761	13.792778	13.240382	13.054008	13.160352	13.020637	12.721275	12.904469	12.991998	12.736267	12.734213	12.200941	12.706881	12.943559	12.964911	12.748800	12.708589	12.878707	12.937800	12.592334	13.158588	12.956616	13.318885	13.473153	13.043905	13.256418	13.403569	13.539712	13.319864	13.157459	13.416905	12.911451	13.032306	12.938173	12.819043	12.845626	12.498330	12.728950	12.377877	12.758584	12.746236	12.546233	12.660693	13.382821	12.448476	12.424239	13.328625	12.987965	12.387757	13.635940	13.131537	13.143585	12.130217	12.753969	11.386298	12.931168	12.394097	12.238997	13.250280	12.103936	12.942292	12.251209	11.924435	12.485499	12.345812	12.354988	12.617631	12.530199	12.139420	12.544889	12.212235	12.320151	12.027045	12.344853	12.908579	11.861844	12.971855	13.555677	12.627164	12.381157	12.992703	12.167578	12.383691	13.308756	12.759589	12.301091	11.303223	12.162219	12.010792	13.140875	13.118268	12.575902	12.634450	13.005224	13.341361	13.226252	12.678191	13.361622	12.959744	13.299825	13.866741	13.124002	13.494410	13.160892	10.826070	13.732652	12.968566	13.608509	13.179963	13.309752	12.919527	12.915163	13.635940	12.263695	13.407563	13.121156	13.243931	13.384972	13.600808	12.862774	12.866212	12.758584	13.368069	13.051023	13.313966	13.232656	13.007579	13.367256	13.140875	13.010316	11.462482	13.416505	13.327590	13.088702	13.079851	13.918583	12.627164	13.787896	12.816305	12.858133	13.232656	13.362446	12.813176	12.239925	13.894764	13.787649	13.322965	13.368304	13.479756	13.696085	13.053474	12.983713	12.600544	13.664721	13.592616	11.881927	12.586457	13.288044	12.136934	12.773792	11.909858	13.303085	13.266805	11.349516	12.579997	13.577611	13.589804	11.524738	13.548218	12.681634	12.902160	11.729004	12.339044	12.458656	13.115624	13.679951	12.382891

TRIM3	9.793162	9.324612	9.189333	9.989661	9.475527	9.195936	9.258034	8.868229	8.420395	9.158035	9.284919	9.018966	9.118515	9.479637	9.220351	8.944736	9.501173	8.852566	8.975271	8.908646	7.780295	9.447790	9.020771	9.315965	9.841975	9.218853	9.573371	9.734090	9.757639	9.504396	9.009334	9.301410	8.640510	8.911416	9.062181	9.107183	9.077037	9.208692	9.238102	8.993798	9.411410	9.104826	8.879106	9.160765	9.339197	9.099653	9.627691	8.461880	8.982916	8.800353	9.282941	8.746957	9.110950	9.129082	8.907661	9.792703	9.000709	9.292527	9.577775	9.199378	8.814849	9.477056	8.881902	9.218055	9.314738	9.296135	9.307116	8.894543	8.973768	8.332461	9.142589	9.217322	9.367879	9.023274	8.953012	9.019286	9.179371	8.840579	8.896105	8.561461	8.652056	8.735320	9.097822	8.644089	8.839043	9.120056	8.638648	8.561316	8.181745	8.884058	8.963120	7.289724	8.636014	8.718581	8.751808	8.872790	8.688827	9.139811	8.897010	8.986560	8.568954	9.374150	8.991935	9.003048	8.644401	9.219146	9.005015	8.684010	7.832150	9.475200	6.853000	9.224356	9.223728	9.336024	9.435860	6.986518	10.561470	9.006998	9.373824	9.189054	10.079823	9.478293	10.118700	9.041919	10.322594	9.269982	9.120492	9.110179	9.037239	9.114675	9.371660	9.416708	9.824019	10.112296	8.935314	9.882077	8.815433	8.840879	9.074979	8.542583	9.574584	8.791520	9.146716	10.116982	10.436289	9.319592	9.487347	9.240450	9.796404	9.300486	8.876306	8.713413	9.491581	9.172346	9.081958	8.935453	9.640101	9.585307	9.726547	9.415203	8.875260	8.921659	9.390059	9.026613	8.761974	9.822688	8.635856	9.116189	9.155247	9.246281	8.762712	9.149117	10.009587	9.302504	9.985950	9.066450	9.580855	9.714536	9.200174	8.843711	9.780886	8.593456	8.837534	9.396514	9.276071	9.451861	8.903697	9.402928	8.525662	9.332910	9.171408	9.898694	9.335569	8.783374	9.163075	9.344664	9.278447	9.181715	9.122460	8.985318	8.918330	9.454180	9.050390	9.771758	9.109255	9.125817	8.946123	9.038186	9.409267	9.311249	8.666239	8.674211	9.444057	8.855620	8.967435	8.977263	9.140257	9.678286	9.427739	9.526187	8.671736	9.130010	9.170762	9.098890	8.865668	8.844410	8.450307	8.087711	9.603163	7.098929	9.351702	8.641436	9.190561	8.912802	8.335259	9.388107	9.451098	10.292953

SDR42E1	11.441033	10.795127	11.159921	11.157147	11.466296	11.250156	11.251075	11.410252	10.559792	11.152165	11.291576	11.291432	11.401374	11.220719	10.121857	10.922234	10.947735	10.674034	10.521568	11.255473	11.159203	10.600754	10.120507	10.021223	9.611030	10.461579	9.491735	10.094705	10.598011	10.922099	10.871895	11.619483	10.799656	11.366540	11.107188	11.377091	11.371423	11.134615	10.219095	10.846658	11.034048	10.375909	11.011245	11.296282	10.441855	11.185699	10.023074	10.656389	11.761931	11.532579	11.314365	11.234364	11.549457	11.613311	11.174007	10.941113	11.327777	10.970158	10.690865	10.735207	10.917350	10.958405	8.770850	10.520191	10.469499	10.302329	10.538517	11.025099	11.093554	10.633361	11.134756	11.458920	10.830197	11.355934	11.640106	11.423597	11.750863	11.571480	11.881010	11.281195	10.862314	11.431360	11.444260	11.760698	11.170466	11.586042	11.416462	11.591914	10.976546	11.075168	10.674499	10.890897	10.956204	11.863196	10.886871	11.469421	11.854007	10.211511	11.300543	10.540943	10.534405	11.252362	10.744869	10.838131	11.109863	11.017053	10.541389	10.717586	10.668712	10.775784	11.152465	10.708399	11.007420	11.048835	11.299815	9.709211	10.725060	11.020654	10.263119	11.998998	10.953350	11.631352	11.889157	12.340373	12.290916	11.584703	8.039167	10.537933	12.764108	11.340687	11.498901	10.727782	11.323649	10.178735	10.977879	11.820354	6.485635	10.493600	8.030875	11.061554	10.945347	10.593040	10.335998	10.102629	7.270478	11.881172	11.778372	10.774902	11.556994	11.745239	6.787106	7.645269	12.418964	11.704750	11.524427	11.864242	10.858655	9.975902	10.585064	12.125666	10.456786	11.353017	11.818403	11.783598	11.785232	7.217647	10.587751	12.018090	9.596328	12.061828	11.991869	8.763320	7.891853	10.364725	11.803092	12.257719	10.681966	10.667829	10.460708	11.684367	11.164195	11.755284	11.643417	9.191027	9.908813	11.285343	10.061953	10.759975	10.342317	10.336597	11.760077	10.647435	11.444132	8.802316	11.795725	12.007772	12.193376	10.578127	10.955290	7.235310	12.727428	12.421829	11.357390	11.692743	11.407985	11.178683	11.653921	10.497091	10.087473	10.670884	11.387475	10.587003	10.645760	11.830768	10.896881	7.231189	10.237139	9.428831	12.133719	11.312768	11.456254	10.501981	10.399733	11.372450	11.391756	10.724767	9.639042	11.223858	11.594299	10.191151	10.568780	9.658892	10.534263	12.104074	11.980316	11.099729	11.834521	12.012208

CPNE2	11.115623	12.112683	11.972367	11.503209	12.233576	11.544101	11.965674	11.552728	11.620849	12.360524	12.190447	11.668018	11.549582	11.516950	12.155557	11.776075	12.087860	12.050582	12.130980	11.568999	12.173726	12.601601	12.268991	12.289963	12.529519	12.585328	11.705650	12.187793	11.598287	11.709994	11.920531	12.144058	12.193991	12.467569	12.283484	12.025867	12.701599	12.322814	12.845812	12.820920	11.979284	12.762029	12.652437	12.383533	12.862774	12.238997	11.831507	12.885092	11.370520	12.312855	12.605894	12.461067	12.294671	11.846434	11.920674	11.856406	12.607232	12.920968	12.864977	12.954599	12.927197	12.048760	13.486814	12.796960	13.131537	12.602572	12.783431	12.077573	12.821603	12.874105	11.947446	12.200157	11.686785	11.822458	11.951988	12.091525	12.239319	12.134671	11.939225	11.962445	11.947128	12.060393	12.042883	12.098917	12.600707	12.216524	12.106988	12.084173	13.127492	12.885836	12.923860	12.848958	12.883472	12.221249	11.507607	12.226312	11.772691	12.097568	12.291396	11.658633	11.784487	12.037614	12.103282	12.306995	12.235457	11.306203	13.156712	11.768105	12.248149	11.621605	12.078970	12.386142	12.731001	11.989419	11.544514	9.520834	11.606379	11.697417	11.749393	12.261028	12.329087	12.668810	12.368451	12.432750	12.231903	12.759168	12.541440	13.022170	12.491562	12.569706	13.112597	13.290071	13.272984	12.832911	12.293428	12.757557	13.259620	12.358156	12.698837	12.404198	12.813176	12.629032	12.449603	13.749588	13.473987	12.216379	11.473095	13.045913	12.314304	12.235592	12.992187	13.906117	12.661032	12.721275	13.396455	12.795036	12.681285	13.700662	13.483665	12.192292	13.856222	12.567417	13.490156	13.481922	12.700895	11.920101	13.343055	13.143585	13.189708	12.782402	13.206916	12.397858	13.899912	13.329654	11.673261	12.514008	12.615979	12.835058	12.700708	13.656241	12.363406	12.802478	13.401844	13.305150	12.761009	13.630916	12.866212	12.831338	13.290718	12.344708	12.611352	11.459334	13.006514	12.446416	12.250568	13.048388	12.890172	12.934012	12.749170	12.867603	12.847527	12.313980	13.018087	12.779167	13.067539	12.967484	11.839473	13.606202	13.179765	12.856045	13.768777	13.541252	12.526140	12.879950	12.583626	13.436430	12.434341	12.136649	13.529215	12.497024	13.148076	12.385972	12.771543	12.996930	12.774646	11.885400	12.829114	11.994409	11.938934	12.536567	13.109444	11.227537	11.572521	12.813342	11.906965	13.997851	12.262009	12.521876

STOML2	14.099512	14.226969	14.571403	14.174543	13.825046	13.563147	14.292381	13.250452	12.085291	13.614482	13.509723	13.849077	13.783587	14.041142	14.377416	12.825950	13.359500	14.124290	13.519798	13.422370	13.932466	13.594660	14.085609	13.678771	12.570678	13.242607	12.285047	12.427666	12.764600	13.689494	12.782601	14.861441	13.983800	13.963030	14.286917	14.156735	14.540484	14.381001	14.338582	14.562171	13.500882	14.395502	13.814043	13.714416	13.623148	13.963570	12.729292	14.323349	13.757132	14.063831	14.107553	14.433099	13.918056	13.973576	14.552678	13.273354	14.929736	14.303599	14.326793	14.845201	14.539165	13.705336	14.455753	13.966887	14.633465	14.755534	14.329679	14.518159	13.771343	13.329225	12.713635	14.189906	12.732358	14.092821	14.465165	14.327509	14.040527	13.980251	14.172567	14.301287	14.615271	13.532379	14.328600	14.994487	14.026270	14.287951	14.289643	14.337881	14.609701	14.764819	14.430826	14.167101	14.654622	14.379907	14.280056	14.528293	14.278941	12.916749	13.682111	12.372749	13.142835	14.471558	13.504804	14.012079	14.962802	13.000463	13.195709	14.456152	13.798541	12.841934	14.787255	13.762421	14.244999	12.894551	12.569706	13.828722	14.002435	13.678246	12.451713	13.410531	15.151242	14.156066	13.636921	12.927010	13.934635	13.778222	13.011074	13.118676	13.954619	14.092188	13.709911	13.699672	14.392936	14.401854	12.409200	13.954342	13.967167	14.339249	14.295373	14.287271	14.355885	14.604151	13.289866	15.533380	14.434526	13.357413	14.531049	13.071521	14.031471	12.782948	13.595345	14.313722	14.199474	14.339964	15.039342	14.097376	14.887119	14.702498	14.662761	13.646327	14.633014	14.780447	14.382758	14.945286	14.906118	13.595842	14.529893	15.049528	14.067206	13.953781	13.958253	15.108611	15.088208	14.167438	13.201413	14.329679	15.111191	14.393646	14.436030	14.860927	13.929475	14.097797	15.050698	14.201809	14.404365	14.904024	14.309634	15.430200	15.502988	15.738421	14.579342	13.492583	14.755057	14.175228	14.236540	14.740482	14.590224	14.268966	14.649747	14.070625	15.420214	14.418742	13.651765	15.718870	13.990442	14.894400	15.394501	14.944249	14.578917	15.382940	15.799508	14.497531	14.963394	13.838739	13.698984	13.886730	11.802240	13.391215	15.114738	14.653700	13.535533	13.821463	14.074808	15.198897	13.112784	13.780110	14.897610	14.894400	13.697330	15.140997	13.868567	14.356954	12.859701	15.362494	13.683790	14.782854	13.643032	13.926227

PCDH11Y	5.531417	5.605125	5.023856	6.804468	4.628705	4.822317	6.042449	4.566596	3.823735	6.166868	5.722134	5.920495	5.317069	4.517223	6.831169	6.167031	6.719302	4.666013	3.248272	6.461336	3.865160	6.906870	9.659354	5.975531	5.175351	4.786204	6.477105	4.768089	6.269984	5.916723	6.823863	7.069044	5.176961	6.297246	6.904447	5.362552	3.419704	5.628214	6.014010	4.306150	5.416211	6.931924	4.606924	6.557617	6.769775	5.105634	3.975516	5.050186	6.362694	5.448411	5.991118	6.145144	5.141118	6.197245	4.648115	4.440018	5.211625	3.537245	5.558862	3.455662	4.405372	4.981929	6.854771	5.339915	5.483145	5.399411	5.507796	4.310605	4.070841	5.194478	5.719936	6.463552	5.474637	5.973953	6.437732	5.851489	5.662456	5.405155	6.107290	6.037203	7.009963	7.016983	6.676570	3.830333	5.759620	6.788410	3.975516	5.217771	6.330260	5.927599	5.296253	5.965720	6.066083	4.322455	7.187166	5.952913	4.590365	3.381552	5.581535	4.190822	5.232211	4.605847	5.071425	5.689869	7.145660	5.814591	4.242269	4.068580	6.079207	3.950977	4.338539	5.073437	4.630030	6.857373	5.100098	3.577750	4.492238	6.223992	5.769010	3.452065	4.199007	3.261430	4.381390	4.733340	4.077627	5.208018	5.687183	3.120954	5.110367	5.314846	4.034313	3.212842	3.091098	5.004573	6.124083	7.377032	2.829545	3.002140	4.284744	3.449361	4.578828	7.127988	3.394330	3.564969	6.637380	5.552587	3.358975	3.268690	6.047481	8.321435	4.099063	7.003961	5.207233	5.686415	4.282734	3.379996	4.779910	6.313838	4.209917	3.504538	6.144784	3.534047	3.458761	5.148333	4.714961	3.465757	3.096025	5.086779	3.873944	4.801541	4.651943	3.849064	4.392359	3.353204	4.658123	4.328766	5.184019	5.296657	5.138445	4.874048	3.267793	6.346068	4.573962	6.266553	4.841476	4.075989	4.601214	4.093091	3.265127	5.202043	4.787871	4.133401	3.847556	5.916177	4.521910	4.992229	4.208004	5.836443	3.962248	4.164047	5.042632	5.009514	4.456263	4.900045	4.173173	4.064780	3.898456	4.957084	4.602712	4.112008	4.811255	5.707221	3.238755	4.337231	5.789735	3.237851	3.472743	4.008227	3.492579	4.445425	2.972619	4.471446	3.976335	8.420395	6.639018	4.094946	3.841327	5.780710	3.588820	3.697132	4.129806	3.768830	7.078318	4.501816	3.595732	4.398066	3.898920	4.072539

PCDH11X	5.531417	5.605125	5.023856	6.804468	4.628705	4.822317	6.042449	4.566596	3.823735	6.166868	5.722134	5.920495	5.317069	4.517223	6.831169	6.167031	6.719302	4.666013	3.248272	6.461336	3.865160	6.906870	9.659354	5.975531	5.175351	4.786204	6.477105	4.768089	6.269984	5.916723	6.823863	7.069044	5.176961	6.297246	6.904447	5.362552	3.419704	5.628214	6.014010	4.306150	5.416211	6.931924	4.606924	6.557617	6.769775	5.105634	3.975516	5.050186	6.362694	5.448411	5.991118	6.145144	5.141118	6.197245	4.648115	4.440018	5.211625	3.537245	5.558862	3.455662	4.405372	4.981929	6.854771	5.339915	5.483145	5.399411	5.507796	4.310605	4.070841	5.194478	5.719936	6.463552	5.474637	5.973953	6.437732	5.851489	5.662456	5.405155	6.107290	6.037203	7.009963	7.016983	6.676570	3.830333	5.759620	6.788410	3.975516	5.217771	6.330260	5.927599	5.296253	5.965720	6.066083	4.322455	7.187166	5.952913	4.590365	3.381552	5.581535	4.190822	5.232211	4.605847	5.071425	5.689869	7.145660	5.814591	4.242269	4.068580	6.079207	3.950977	4.338539	5.073437	4.630030	6.857373	5.100098	3.577750	4.492238	6.223992	5.769010	3.452065	4.199007	3.261430	4.381390	4.733340	4.077627	5.208018	5.687183	3.120954	5.110367	5.314846	4.034313	3.212842	3.091098	5.004573	6.124083	7.377032	2.829545	3.002140	4.284744	3.449361	4.578828	7.127988	3.394330	3.564969	6.637380	5.552587	3.358975	3.268690	6.047481	8.321435	4.099063	7.003961	5.207233	5.686415	4.282734	3.379996	4.779910	6.313838	4.209917	3.504538	6.144784	3.534047	3.458761	5.148333	4.714961	3.465757	3.096025	5.086779	3.873944	4.801541	4.651943	3.849064	4.392359	3.353204	4.658123	4.328766	5.184019	5.296657	5.138445	4.874048	3.267793	6.346068	4.573962	6.266553	4.841476	4.075989	4.601214	4.093091	3.265127	5.202043	4.787871	4.133401	3.847556	5.916177	4.521910	4.992229	4.208004	5.836443	3.962248	4.164047	5.042632	5.009514	4.456263	4.900045	4.173173	4.064780	3.898456	4.957084	4.602712	4.112008	4.811255	5.707221	3.238755	4.337231	5.789735	3.237851	3.472743	4.008227	3.492579	4.445425	2.972619	4.471446	3.976335	8.420395	6.639018	4.094946	3.841327	5.780710	3.588820	3.697132	4.129806	3.768830	7.078318	4.501816	3.595732	4.398066	3.898920	4.072539

TRIM56	11.674302	11.421190	11.360907	11.993505	12.471970	12.312713	11.744200	12.147509	13.158236	12.248817	12.253272	12.030634	12.273308	12.005861	11.472951	12.555905	12.402762	12.010330	13.141252	12.398188	12.603082	13.115442	13.602862	12.522201	12.910176	12.887214	13.518195	12.745897	12.840723	11.975261	12.846159	10.744707	10.657396	11.729465	11.506409	11.646361	11.544245	11.738943	12.208223	12.033526	12.409843	12.118645	12.335719	11.897652	12.207292	11.977757	13.071141	11.769600	11.328346	11.638275	11.388799	11.358851	11.174736	11.440893	10.911045	12.462754	11.304559	11.634506	11.696238	11.363266	11.770058	12.306066	12.441117	12.587157	11.425646	11.429574	11.893515	11.498901	11.353604	11.967332	11.769304	11.682031	12.840899	11.617561	11.685883	11.519647	11.668763	11.377558	11.554325	11.153618	11.146352	11.055538	11.011398	11.048406	11.280760	11.247766	11.161418	11.238152	11.673716	11.153465	11.460855	11.703716	11.490704	11.624432	11.544101	11.614961	10.848296	12.744556	11.859202	12.952034	12.346315	11.671357	12.335103	11.972222	12.375118	12.601601	13.280648	12.632301	12.785930	12.853341	12.550893	12.690670	12.145311	13.058207	11.926923	13.685514	12.425406	12.021646	13.276661	11.680252	11.404839	11.534664	12.284103	13.225108	11.639016	12.076671	13.058207	12.622173	12.404343	11.695921	12.195260	12.741273	12.062136	11.776985	13.455950	12.101792	12.358457	13.698011	11.921595	11.781075	13.216575	12.537425	12.779167	12.107881	11.796425	12.037009	12.255020	13.465090	11.633293	12.934012	11.570621	11.204194	11.250156	11.499627	11.185699	11.219382	11.680518	11.941052	11.961736	12.674839	11.548287	11.733781	11.555223	11.497713	11.433749	12.604579	11.379601	11.177770	11.256480	11.167543	11.466449	10.927713	11.243882	10.929640	12.219378	11.449854	11.061273	11.345196	10.985236	11.256783	12.457238	11.665819	12.094888	12.016745	10.976265	11.791508	11.253380	11.134615	11.696817	11.403536	11.337754	12.575563	11.434496	11.583914	11.394735	11.558328	11.556691	11.536442	11.521612	11.126061	10.926394	10.729560	11.216261	10.864090	11.915487	10.611388	10.601209	11.881590	11.333751	11.251364	11.683647	11.488767	11.390561	11.353017	11.698867	11.593733	13.200284	11.401656	11.685541	11.615530	12.015060	12.719723	11.445125	12.482801	13.012808	13.015899	12.292981	11.870089	12.766912	12.144544	12.296782	12.629517	13.099956	11.966106	11.773425	11.837689	11.673410	12.102695

PAGE2	5.070584	6.262524	5.986093	5.982024	5.890991	6.837708	7.957140	6.452423	5.837861	6.240584	5.996025	6.007743	5.987005	6.507804	4.837863	6.382968	5.517407	5.834643	5.660315	6.543039	6.001097	5.299668	4.233299	6.643709	8.296996	5.493183	5.362905	5.484266	5.711196	6.389695	6.023247	6.130528	6.437732	7.799610	5.898668	5.980670	5.228700	6.008053	5.793655	5.897408	6.358855	6.126791	6.484623	6.322896	5.668695	5.950553	6.088150	6.236812	7.294607	6.688080	5.995839	6.697278	5.606325	5.788486	6.782610	6.043958	6.362535	3.428492	5.302654	5.908291	5.653127	6.110246	4.836806	6.012132	5.208231	6.098553	6.232076	6.356073	6.418325	5.973236	6.137363	6.940135	6.442016	7.826301	6.023770	8.002152	6.550859	6.070155	6.118891	5.809630	6.379958	6.188588	5.591000	6.107488	6.646729	6.111465	5.697884	6.068594	4.728936	5.501796	6.122524	5.849182	5.721773	6.891257	6.302728	6.104605	6.771711	6.079900	5.457696	5.936160	6.757487	6.126045	5.816919	5.800031	6.156355	6.274392	7.386684	5.733384	4.387604	6.066803	5.236233	5.726273	6.011402	6.327724	7.269702	10.167052	4.966720	6.066429	5.440614	6.247297	5.826376	6.527025	8.100093	6.190968	6.360207	14.452852	5.258688	5.407299	7.544535	5.967524	6.561359	6.141553	5.997605	4.788270	6.634515	5.254163	5.305994	5.480262	7.746868	5.380017	5.066111	5.600779	6.122868	14.834088	5.214446	6.231727	5.741185	6.557111	7.035762	5.865057	5.834287	4.879022	6.138754	6.353603	4.940144	5.769200	3.967902	5.585717	4.197524	7.934327	11.757848	5.831051	6.070155	4.873648	5.648576	6.436616	5.970151	6.639659	6.591797	6.008773	4.442859	5.813523	5.765908	5.846949	6.520543	5.601329	4.014461	5.204669	8.612010	5.556954	7.877641	4.092696	4.442432	4.718747	11.574797	4.981730	5.609716	10.619400	5.422817	6.555824	6.298576	8.044021	13.851943	5.583994	13.875803	6.916792	6.103589	4.985394	6.107820	5.867214	5.602452	5.040737	6.051477	5.945861	5.316472	5.983672	6.463376	3.516786	4.595957	4.713069	5.217771	4.456263	5.256331	6.016224	6.109395	6.819436	6.881674	5.813341	9.670057	5.819218	5.653503	8.690660	4.765468	4.919371	7.812790	7.108862	6.379636	6.662005	6.579309	4.972542	3.934932	4.815819	6.477105	6.283637	14.790582	3.388949	8.310213	5.784855

SPATA46	7.248072	8.798396	9.048076	8.764831	9.467350	9.688230	8.857724	9.842448	9.628003	9.265944	8.959017	8.843566	8.321873	9.126898	7.973665	9.480408	9.086111	8.282853	9.276953	9.032049	8.799907	9.792703	8.467956	8.778111	8.998577	8.878644	8.601123	9.216162	9.004415	9.031124	9.380364	7.999946	8.018913	8.269978	8.194463	8.250361	8.375408	8.865812	9.009334	8.939581	9.681108	8.679300	9.047586	8.786239	8.078230	8.555749	9.460588	8.727249	8.851683	8.931939	8.150381	8.512001	8.119173	8.230249	8.089878	9.904543	8.017982	8.281921	8.314984	7.758072	8.164115	9.348168	7.800683	9.020172	8.739474	8.656246	9.189936	9.019286	8.974230	9.301255	8.731651	9.040866	9.894119	8.863706	9.073286	8.617692	8.902489	8.443632	8.497631	7.675589	7.603766	7.651452	7.480282	7.911734	7.551288	7.858621	7.269237	7.351613	7.756656	7.987793	7.585977	7.932034	8.148080	8.547241	7.936388	7.925989	7.983486	8.167506	8.742540	9.103050	9.208388	8.814706	8.972042	9.144456	9.121085	9.489658	9.509295	9.391747	9.118344	10.392215	8.951168	9.182973	8.950873	10.079690	9.522089	9.792860	7.783481	8.211447	9.727298	8.254529	8.886682	8.337894	9.111602	10.203707	9.398637	8.597328	7.981300	8.531373	8.860469	8.948291	8.497774	8.577737	8.365788	8.784884	8.942331	8.425261	8.092006	9.303709	7.954492	9.309714	10.100927	9.324472	9.327512	8.563284	8.069531	9.449496	9.597096	10.251906	9.436775	8.659450	8.064117	8.301521	8.919206	8.133472	8.306046	8.043558	8.507374	9.202358	8.639584	9.684672	9.131973	8.879581	9.166801	8.989121	8.755929	10.094118	8.039959	8.774914	8.953320	8.517317	8.759174	8.246522	8.475899	8.394542	9.149696	7.789766	8.310809	8.269978	7.820703	8.197159	8.950728	9.228210	8.597607	8.666526	8.133019	8.934423	9.131538	9.396207	8.865208	8.991496	9.257270	10.762198	8.351704	7.868615	8.540706	8.734872	8.473729	8.687456	7.265254	8.094921	7.887812	7.804774	7.903197	7.357319	8.446086	7.288120	7.706238	7.516805	7.666067	7.736364	8.444705	8.140459	8.386331	8.279940	7.981607	8.423936	8.388057	8.603452	8.766397	8.515759	9.024766	8.834942	8.183733	8.820838	9.373655	8.821736	8.995798	9.217596	10.167524	8.805380	8.809828	9.451718	10.483523	9.208692	9.072055	8.778272	8.424967	9.489036

AL512785.2	7.248072	8.798396	9.048076	8.764831	9.467350	9.688230	8.857724	9.842448	9.628003	9.265944	8.959017	8.843566	8.321873	9.126898	7.973665	9.480408	9.086111	8.282853	9.276953	9.032049	8.799907	9.792703	8.467956	8.778111	8.998577	8.878644	8.601123	9.216162	9.004415	9.031124	9.380364	7.999946	8.018913	8.269978	8.194463	8.250361	8.375408	8.865812	9.009334	8.939581	9.681108	8.679300	9.047586	8.786239	8.078230	8.555749	9.460588	8.727249	8.851683	8.931939	8.150381	8.512001	8.119173	8.230249	8.089878	9.904543	8.017982	8.281921	8.314984	7.758072	8.164115	9.348168	7.800683	9.020172	8.739474	8.656246	9.189936	9.019286	8.974230	9.301255	8.731651	9.040866	9.894119	8.863706	9.073286	8.617692	8.902489	8.443632	8.497631	7.675589	7.603766	7.651452	7.480282	7.911734	7.551288	7.858621	7.269237	7.351613	7.756656	7.987793	7.585977	7.932034	8.148080	8.547241	7.936388	7.925989	7.983486	8.167506	8.742540	9.103050	9.208388	8.814706	8.972042	9.144456	9.121085	9.489658	9.509295	9.391747	9.118344	10.392215	8.951168	9.182973	8.950873	10.079690	9.522089	9.792860	7.783481	8.211447	9.727298	8.254529	8.886682	8.337894	9.111602	10.203707	9.398637	8.597328	7.981300	8.531373	8.860469	8.948291	8.497774	8.577737	8.365788	8.784884	8.942331	8.425261	8.092006	9.303709	7.954492	9.309714	10.100927	9.324472	9.327512	8.563284	8.069531	9.449496	9.597096	10.251906	9.436775	8.659450	8.064117	8.301521	8.919206	8.133472	8.306046	8.043558	8.507374	9.202358	8.639584	9.684672	9.131973	8.879581	9.166801	8.989121	8.755929	10.094118	8.039959	8.774914	8.953320	8.517317	8.759174	8.246522	8.475899	8.394542	9.149696	7.789766	8.310809	8.269978	7.820703	8.197159	8.950728	9.228210	8.597607	8.666526	8.133019	8.934423	9.131538	9.396207	8.865208	8.991496	9.257270	10.762198	8.351704	7.868615	8.540706	8.734872	8.473729	8.687456	7.265254	8.094921	7.887812	7.804774	7.903197	7.357319	8.446086	7.288120	7.706238	7.516805	7.666067	7.736364	8.444705	8.140459	8.386331	8.279940	7.981607	8.423936	8.388057	8.603452	8.766397	8.515759	9.024766	8.834942	8.183733	8.820838	9.373655	8.821736	8.995798	9.217596	10.167524	8.805380	8.809828	9.451718	10.483523	9.208692	9.072055	8.778272	8.424967	9.489036

GRM8	6.132434	3.728166	3.298088	3.804108	3.662430	3.554492	3.666887	3.589211	3.290518	3.141052	3.288506	3.694253	4.012899	3.802855	4.484384	3.629032	3.254990	4.130630	4.698314	4.659428	4.445874	4.315399	6.283455	3.661837	4.346108	3.582844	4.068163	3.231565	3.643475	3.530520	3.684817	4.663894	3.504538	5.127555	5.895831	3.935574	3.808070	3.518728	3.463379	3.372431	3.645219	3.862994	4.274648	3.002248	4.098850	5.811775	4.533520	3.718521	4.822317	3.378256	4.097105	3.542686	4.022591	3.966797	4.384713	3.364044	4.247330	3.474700	3.015183	3.845666	3.320743	3.981517	4.220124	3.132846	3.779584	3.810995	2.980038	3.194161	3.656424	2.984053	3.742196	3.236957	3.377215	3.682876	2.944347	3.260291	4.038230	3.380664	3.440673	3.376866	3.166776	3.193913	4.282734	4.017403	3.054254	3.777146	3.869015	4.618652	3.392955	3.751911	3.529594	3.096907	3.545984	3.239326	3.959247	3.262043	3.888276	4.071461	3.996626	3.609188	4.493786	3.722702	3.749198	3.815038	3.066288	4.708660	3.018838	3.499242	4.338975	3.735228	3.760082	3.594809	3.821840	4.025170	4.384713	3.647002	6.042449	3.261151	3.134643	4.328543	5.285388	4.707226	3.617462	3.421705	4.536102	4.255446	6.132434	3.769062	3.948370	3.660815	5.934947	3.928272	4.399395	4.339614	3.293775	3.161356	4.516143	4.624415	4.625504	3.870938	3.708838	5.631732	3.797157	4.348075	4.533107	3.568873	3.980405	4.080922	5.207233	3.878499	4.694111	4.709478	4.144522	5.911153	5.157902	5.105634	4.138630	4.704484	3.373442	3.268548	4.016509	5.295630	5.238060	5.096222	6.222589	4.808260	4.724520	6.144272	4.614203	4.820845	5.604946	4.588197	4.571161	4.723654	3.697989	6.469512	5.087383	3.248982	5.560572	4.655780	4.138401	3.829065	5.091025	4.106065	3.773875	2.990442	5.074842	3.811402	4.639269	4.541102	4.686349	3.760284	4.728053	3.548191	3.670314	5.088396	4.026329	5.366629	4.551331	5.959677	6.505130	4.267843	3.978716	5.304609	5.068793	3.944654	4.834783	3.195847	4.527383	4.683975	3.698196	4.530110	3.808898	4.493308	5.023196	5.388347	4.821899	4.319054	4.148341	5.630433	5.162468	3.764916	3.901276	3.102215	4.453325	3.069128	4.289822	4.042528	3.594809	4.556647	4.053015	6.790070	3.918697	6.343011	3.691947	4.351108	3.412420	5.209227

SDF2	13.133394	12.916049	13.125419	13.257830	13.002891	12.833106	13.109859	13.037427	12.863135	13.068719	13.282011	12.993251	13.302672	13.360143	13.335818	12.994008	12.971491	12.976769	13.894497	13.151461	13.658895	13.964108	13.732652	12.876754	13.061923	12.829284	12.630012	13.070763	12.841260	12.856770	13.243931	13.520934	13.198336	12.565784	12.658626	12.638870	12.751039	12.944441	13.056363	13.067904	12.900943	13.448635	12.637159	12.864041	12.677669	12.772756	12.814928	12.736077	13.083300	12.786674	13.050251	13.169407	13.150496	13.038398	12.863485	12.850702	13.100310	13.140268	13.337481	13.289639	13.164516	12.815287	13.838218	13.544379	13.494652	13.334795	13.314970	13.373992	13.115807	13.290515	12.639545	12.808043	12.948751	13.006688	13.051444	13.046278	13.131825	12.871760	12.964911	13.213586	13.037820	12.834013	13.010316	13.236652	13.446919	13.192303	13.489934	13.487496	14.010076	13.856222	13.198141	13.793282	13.746068	13.145911	13.372065	13.157459	13.136025	12.836884	13.169593	12.623334	13.015361	12.645205	12.587157	12.566268	14.074554	13.038398	13.180750	13.702148	13.590078	12.956616	14.051518	13.078555	13.350574	12.750695	13.153423	12.360524	13.175591	12.908579	12.784340	12.955887	13.543949	13.038781	13.063428	12.815618	13.617295	13.241587	12.331121	12.947003	13.063624	12.656189	13.253022	13.222745	13.734372	13.317859	13.241797	12.780716	13.559309	13.845086	13.150496	14.104388	14.205591	14.239134	13.734845	13.573817	13.735356	13.544870	13.149152	12.674660	13.443521	13.636921	13.102201	13.731415	12.940803	13.595345	13.538127	13.541252	12.892075	13.878755	13.705582	13.742612	13.686919	12.719524	13.384372	13.137178	12.897590	13.261408	13.377337	13.351592	13.163939	13.107525	12.847857	13.107173	13.634990	13.107904	13.200478	13.525717	14.007880	13.358673	13.322965	13.937559	13.389920	13.704846	13.934635	13.444403	13.482375	13.411210	13.029353	13.481027	13.614714	13.455950	13.239992	12.921327	13.390157	13.262398	13.335001	13.351592	13.484858	13.460034	13.439680	13.817588	13.973837	13.558886	13.877148	13.701136	13.381772	13.936589	13.817588	14.088933	13.823982	14.674118	14.427855	13.655271	13.431247	13.485992	13.752811	13.408595	12.859356	13.100501	12.952935	13.083113	13.084593	13.382599	12.908398	14.161160	13.110603	13.610850	13.986102	14.114980	12.950361	14.053607	13.608723	12.870146	12.802671	13.496025	13.392871	13.609930	12.998439	13.276462

GALNT2	11.605660	11.731533	11.595221	11.879544	11.649159	11.352321	11.536751	12.082750	11.430746	11.897073	11.942282	11.868313	11.655906	12.613710	11.172547	11.381887	11.181128	11.616116	11.717044	11.309686	11.641173	12.771018	12.173726	11.807983	11.764203	11.432696	11.316005	12.018090	11.248076	11.994731	11.164048	11.794698	11.739222	12.243771	11.733781	11.464934	11.412105	11.637961	12.241742	12.145311	11.588472	11.928915	11.829413	11.780148	11.932456	11.562614	11.455816	11.799525	10.992250	11.608893	11.769304	11.741168	12.091683	11.277350	11.892007	11.689592	11.691697	11.701916	11.816892	12.046313	11.588775	11.528777	12.342480	12.156958	12.476535	12.011409	11.735962	11.285928	12.181281	11.938632	11.721223	11.233924	11.533775	11.383209	11.446601	11.616246	11.783598	12.234198	11.802240	11.214660	11.600906	11.363862	12.117551	11.412105	11.951428	11.410682	11.635707	11.833001	11.438510	11.764948	11.664327	11.639395	11.325112	11.713495	11.565388	11.333751	10.791885	10.783168	11.727818	11.365804	11.557455	11.296731	11.660141	11.568715	11.788973	10.643415	12.041232	12.424072	11.703035	11.169002	11.939528	11.447943	11.758591	11.392179	10.951475	10.837243	11.668608	11.785826	10.914915	11.842489	12.191674	12.494508	12.493861	11.851418	11.503051	12.607398	12.413808	12.457538	11.396538	12.531515	12.224619	13.182111	13.148784	12.733879	11.488900	12.952366	12.010940	11.878463	12.884027	12.382744	12.755658	12.015376	12.723985	13.266997	13.578768	11.957393	10.821767	11.042557	12.852304	11.721674	12.152396	12.801093	11.926124	12.300500	11.826829	12.486970	12.381309	13.541913	12.563485	12.463072	12.522828	11.818076	12.098917	12.182961	12.144699	11.695468	11.967950	11.879689	13.297173	12.833106	11.920531	11.929674	13.937428	12.310609	12.771543	13.137749	11.979444	12.485843	12.311740	13.333932	12.523476	11.989871	12.771214	11.668018	12.406200	12.965441	11.931707	12.558955	12.300335	11.215233	12.085596	11.266930	12.948751	12.251358	12.715418	12.471342	12.432907	12.391894	12.503389	12.562827	12.636458	12.828929	12.433717	12.565935	12.284575	13.297375	12.471970	12.399011	12.814055	13.709428	12.068749	12.701417	13.576247	12.134671	13.170169	12.933669	11.238299	12.561718	11.981434	13.531252	11.162452	12.325086	12.910176	13.124995	11.227229	12.375982	12.070678	12.051330	11.538542	11.923414	13.941795	12.673286	11.330738	11.290392	11.405580	13.206525	11.401523	12.604080

AMZ1	7.813738	8.474166	8.222067	8.380007	8.610624	8.909057	8.216204	9.495493	9.933063	8.280832	8.218871	8.329193	8.454436	8.439798	8.176949	8.890098	8.749391	8.223604	9.449820	8.657576	8.777073	9.365025	8.988657	9.056578	9.070503	8.939127	9.514568	9.048228	8.908916	9.099347	8.831940	7.452274	7.551132	8.542271	8.601569	8.440424	8.467956	8.460633	8.912217	8.749551	8.603763	8.689716	9.130445	8.804154	8.601893	8.512472	8.973624	8.848779	7.940884	8.529985	8.252679	7.978305	8.209048	8.316530	8.290887	9.125660	7.814763	8.637239	8.730911	8.004347	8.401741	8.832406	9.518692	9.451406	8.442536	8.564796	9.339197	8.909943	8.640670	9.323656	8.401595	8.411253	9.093582	8.380819	8.461565	8.607876	8.537828	8.415646	8.417092	8.123096	8.006102	8.095215	7.878566	7.507990	7.204710	7.632094	7.619699	7.321039	7.997081	7.695615	7.911574	7.903028	8.374973	8.309320	8.436154	8.461085	7.397868	8.253272	8.774448	9.317465	9.117571	8.691536	8.944285	8.894109	9.430199	8.690185	10.134060	9.312170	9.571204	9.242889	8.431048	9.591661	9.290980	9.433276	8.506270	10.125091	8.894256	8.094631	8.960379	8.092604	8.744503	8.391312	8.757304	9.803484	8.924312	8.750837	9.330748	9.175341	8.730322	8.499478	8.487395	9.121540	8.773214	8.995493	8.916489	8.840579	8.737317	9.812289	8.457339	9.226836	9.725305	8.829501	9.569642	9.193041	8.655375	9.241660	9.451553	9.836416	8.584972	8.731936	8.243691	8.322944	8.812633	8.162811	8.221604	8.048145	8.357999	8.897476	8.605884	9.427590	8.400492	8.732389	8.992872	8.555423	8.453689	9.431422	8.712506	7.673355	8.601893	8.267307	8.138022	8.340263	8.661167	8.313750	8.728985	8.155006	8.715458	8.829025	7.909103	8.899452	8.816660	9.588862	9.289571	8.934567	8.584684	9.458786	8.989121	9.157249	9.043484	8.669134	8.527214	9.641247	8.509515	8.177095	8.519459	8.494761	8.661489	8.661319	8.214556	8.233142	8.395613	8.381284	8.047984	7.455252	8.170810	7.418516	7.832927	8.621584	8.298363	8.648567	8.478982	8.550581	8.695277	8.452314	8.701813	8.032084	8.782589	8.855773	8.742697	9.105657	9.060792	9.252981	9.247506	9.142130	9.030278	10.206899	8.872639	8.623284	9.914081	8.798095	9.720169	9.970502	9.978433	9.055670	8.644846	8.790729	8.385574	9.175115

GNA12	7.813738	8.474166	8.222067	8.380007	8.610624	8.909057	8.216204	9.495493	9.933063	8.280832	8.218871	8.329193	8.454436	8.439798	8.176949	8.890098	8.749391	8.223604	9.449820	8.657576	8.777073	9.365025	8.988657	9.056578	9.070503	8.939127	9.514568	9.048228	8.908916	9.099347	8.831940	7.452274	7.551132	8.542271	8.601569	8.440424	8.467956	8.460633	8.912217	8.749551	8.603763	8.689716	9.130445	8.804154	8.601893	8.512472	8.973624	8.848779	7.940884	8.529985	8.252679	7.978305	8.209048	8.316530	8.290887	9.125660	7.814763	8.637239	8.730911	8.004347	8.401741	8.832406	9.518692	9.451406	8.442536	8.564796	9.339197	8.909943	8.640670	9.323656	8.401595	8.411253	9.093582	8.380819	8.461565	8.607876	8.537828	8.415646	8.417092	8.123096	8.006102	8.095215	7.878566	7.507990	7.204710	7.632094	7.619699	7.321039	7.997081	7.695615	7.911574	7.903028	8.374973	8.309320	8.436154	8.461085	7.397868	8.253272	8.774448	9.317465	9.117571	8.691536	8.944285	8.894109	9.430199	8.690185	10.134060	9.312170	9.571204	9.242889	8.431048	9.591661	9.290980	9.433276	8.506270	10.125091	8.894256	8.094631	8.960379	8.092604	8.744503	8.391312	8.757304	9.803484	8.924312	8.750837	9.330748	9.175341	8.730322	8.499478	8.487395	9.121540	8.773214	8.995493	8.916489	8.840579	8.737317	9.812289	8.457339	9.226836	9.725305	8.829501	9.569642	9.193041	8.655375	9.241660	9.451553	9.836416	8.584972	8.731936	8.243691	8.322944	8.812633	8.162811	8.221604	8.048145	8.357999	8.897476	8.605884	9.427590	8.400492	8.732389	8.992872	8.555423	8.453689	9.431422	8.712506	7.673355	8.601893	8.267307	8.138022	8.340263	8.661167	8.313750	8.728985	8.155006	8.715458	8.829025	7.909103	8.899452	8.816660	9.588862	9.289571	8.934567	8.584684	9.458786	8.989121	9.157249	9.043484	8.669134	8.527214	9.641247	8.509515	8.177095	8.519459	8.494761	8.661489	8.661319	8.214556	8.233142	8.395613	8.381284	8.047984	7.455252	8.170810	7.418516	7.832927	8.621584	8.298363	8.648567	8.478982	8.550581	8.695277	8.452314	8.701813	8.032084	8.782589	8.855773	8.742697	9.105657	9.060792	9.252981	9.247506	9.142130	9.030278	10.206899	8.872639	8.623284	9.914081	8.798095	9.720169	9.970502	9.978433	9.055670	8.644846	8.790729	8.385574	9.175115

SYT6	5.598387	5.919567	5.595889	5.184212	5.461798	4.939531	5.340097	5.581341	5.166894	5.786123	4.844015	5.064681	5.461798	6.162208	5.480262	6.061895	4.458021	5.691927	3.576726	5.993753	5.885138	6.425798	6.242113	5.667601	4.981328	5.799467	6.169451	5.485941	6.078831	5.491679	4.976495	5.668159	4.597879	5.024042	5.724019	5.904897	4.335462	5.333431	5.619645	5.059000	4.886667	4.900919	6.769211	6.750073	6.695712	6.562355	7.183775	7.053316	6.162208	8.181304	6.377471	6.363388	5.661046	5.402864	5.072013	5.285388	4.603341	4.985394	5.819037	5.090210	4.771569	5.096023	5.693962	6.139446	5.633992	4.791016	5.076102	5.776362	5.710503	6.580629	5.573890	5.663106	5.879616	4.919371	5.510880	5.466568	5.289796	5.524212	4.952305	5.413382	6.214917	5.135234	5.748126	3.902945	4.975473	5.307898	4.679217	4.302412	5.242646	6.082664	5.709925	5.811119	5.299488	5.655012	5.923657	6.052192	4.967357	4.925093	6.136867	5.046472	5.928111	5.496608	4.871491	5.296440	6.042781	5.839528	6.150337	6.341388	5.515655	5.319191	6.976396	4.961870	5.544684	5.196121	5.762324	6.157021	5.671418	4.055845	4.813130	4.400062	5.495683	6.724980	5.325747	6.502846	4.674273	5.617787	4.003685	4.917896	4.901741	4.126565	4.564070	5.295016	6.306280	5.846949	5.828040	4.016060	4.445874	6.117854	6.509428	4.698939	6.561198	5.959156	4.977248	5.022770	5.677876	5.410318	5.919744	5.912785	5.279166	5.857965	5.243037	4.323583	4.709907	6.334855	4.269874	4.460269	4.509419	5.457696	5.768648	4.644478	4.703230	7.286140	6.662321	6.776373	7.236582	7.263561	7.399457	5.324966	7.640322	6.299709	6.116835	6.084753	4.852673	5.546160	6.136200	5.237869	3.312092	4.618879	5.984182	4.670370	5.726273	5.594934	6.778767	7.436323	5.198116	5.612341	5.488371	6.010016	7.435698	4.519767	4.956452	6.325088	4.338339	5.515655	5.929013	5.737715	4.992675	4.438496	6.128820	5.157073	5.551244	5.934225	4.678389	5.116218	5.371678	3.646035	3.444894	6.860269	6.899672	5.054079	5.650101	5.773278	5.485556	5.927937	5.352484	4.727455	4.957295	4.857954	4.679671	5.186596	5.196697	4.744467	5.678811	6.677352	6.183800	5.396153	5.632279	5.019323	5.399206	6.911143	5.837310	6.649826	6.009291	5.785223	5.931085	4.515925	4.220367	5.108299

TMEM64	3.162277	4.172921	3.689939	3.258402	3.227169	3.711546	3.520038	3.638275	3.356732	3.234379	4.091852	3.641091	3.862377	3.702855	3.082761	3.555846	3.555255	2.870014	4.383833	3.388778	4.652164	3.894213	2.963837	3.190259	2.858432	3.260560	3.333065	2.922883	4.169878	3.044226	3.668288	4.402680	3.711920	3.494456	4.049098	4.148555	3.468909	3.113210	3.288506	3.244844	3.412735	3.360160	3.509205	3.575527	3.642881	5.316861	3.836745	2.856424	3.812109	3.723702	3.523465	3.434709	3.843761	4.200088	4.795822	3.133674	4.067954	3.828629	3.063523	3.658433	2.901917	3.495782	3.613588	3.275122	3.478950	3.438067	3.193392	3.294203	3.493734	2.866692	3.563639	3.684214	3.204421	3.891153	4.225364	3.776955	3.718335	3.187273	3.385360	3.657270	4.231353	3.890511	3.526678	3.813595	4.273784	4.050645	4.390632	3.838007	3.951398	3.542076	3.204137	2.841922	3.718521	3.254330	3.579178	3.593435	3.677408	4.438066	3.865565	3.467841	3.246954	3.651989	3.450064	3.124425	4.539977	4.393824	3.121586	3.207511	3.652825	3.114865	3.908360	3.163794	2.986272	3.295582	4.726203	4.966918	3.176610	3.613588	3.038227	3.232304	3.438920	3.047106	3.676604	4.198394	3.591899	3.320580	4.647912	3.911207	3.606173	3.552978	5.662165	3.150988	3.752726	2.968562	3.631286	3.448991	4.679217	3.824627	3.661635	3.497086	3.423315	2.893899	3.828431	2.938528	3.427639	3.975291	3.388778	4.089608	3.389637	3.513374	3.493155	4.655551	3.203085	4.702350	4.956023	3.535542	3.163139	3.303468	3.113210	3.770137	3.155467	4.500504	3.316489	4.443268	4.688212	3.902531	2.805152	4.072220	3.583778	3.358816	4.361722	5.381551	4.344818	3.978030	3.529232	4.737997	3.706665	2.911822	3.461975	2.846128	3.269432	3.671565	3.802663	3.789513	3.637684	3.128578	3.125395	3.777746	2.828839	4.286998	4.031890	3.201160	3.255752	3.706468	3.619327	3.251979	3.065747	3.620077	3.259296	3.746283	4.256083	3.587089	3.547999	4.931984	5.427041	4.063502	6.797851	3.628398	3.368594	3.257943	2.808409	3.303300	3.343324	3.192046	3.019853	3.694034	3.628019	4.058646	4.025591	3.634285	4.055845	3.455153	3.014925	5.278766	3.404378	3.214600	3.843155	3.218416	3.096025	3.541683	3.565350	4.807206	3.291431	3.808655	3.917257	3.416555	3.463557	4.991612

AC012531.3	6.388847	5.692478	5.070805	6.471389	8.049851	6.925831	3.969670	6.531764	5.896007	5.202453	6.029738	6.605173	8.434442	5.730301	5.397296	5.315232	6.280919	4.441972	7.870600	7.819523	6.269286	6.752554	2.950339	5.957324	5.611588	6.288269	5.954174	4.849906	4.588651	7.301614	5.638981	6.241436	5.033574	10.031316	7.855750	6.380781	5.093331	5.986628	6.646089	5.021357	5.015058	5.880328	6.909393	5.404785	5.141933	6.813975	4.999402	6.000418	4.446490	5.506401	6.588047	6.518878	7.724392	9.294264	6.678634	7.249008	6.112982	4.352225	7.324066	4.619695	6.064868	4.812486	4.520611	5.528763	8.654468	4.612282	6.214225	4.724322	6.844206	5.028289	5.243412	4.932210	5.107646	5.384847	5.199473	4.888354	5.762690	8.298501	7.219504	5.271843	6.299075	6.362376	9.287715	5.474637	6.926316	6.408698	7.904130	7.293658	8.413384	5.908486	5.217151	5.594019	5.966803	8.462198	6.130163	4.536530	5.653303	4.192341	6.636745	5.077136	6.507348	5.746680	5.035911	5.208018	6.236971	5.259268	5.146754	6.962442	5.105042	4.609078	5.387388	6.203143	5.654640	4.125253	6.678326	8.470408	4.910706	5.872632	6.023946	10.743802	9.394342	8.512159	6.823537	9.999208	12.197683	7.125950	10.737016	9.831229	5.039717	10.582460	10.645194	5.744092	11.125198	9.961059	9.479637	11.281661	8.829025	6.339228	10.608316	10.396585	10.577828	2.880411	10.515780	11.933974	11.457125	6.778153	4.834368	8.621584	10.175307	9.624463	9.637166	8.832252	7.999946	9.167413	10.796577	8.524461	10.003818	8.506585	9.047586	10.655638	8.114560	10.686008	5.544882	10.217279	10.102022	10.384381	10.281837	7.054921	10.309414	9.734545	11.166213	10.769710	11.068828	10.938612	6.760447	9.993540	9.356515	8.490573	9.942884	7.727960	10.037024	8.426765	9.522255	10.227905	5.392017	10.344614	8.812633	8.263850	7.405528	7.206445	5.738433	10.823692	9.640574	8.519918	9.667179	9.116516	7.808778	9.619017	10.010033	11.231564	11.296591	10.455244	10.489668	11.752925	9.595082	11.110584	12.278057	10.324978	8.905380	8.201668	10.621738	9.953047	10.910730	10.077750	9.742594	9.941008	4.821475	10.722674	10.714609	11.997321	10.525185	9.006410	9.467958	11.711327	9.627387	9.535972	11.113182	6.808084	9.015459	9.251636	11.147807	9.996607	6.492368	11.511854	9.048867	5.948816	9.600494	11.092058

HOXC10	6.388847	5.692478	5.070805	6.471389	8.049851	6.925831	3.969670	6.531764	5.896007	5.202453	6.029738	6.605173	8.434442	5.730301	5.397296	5.315232	6.280919	4.441972	7.870600	7.819523	6.269286	6.752554	2.950339	5.957324	5.611588	6.288269	5.954174	4.849906	4.588651	7.301614	5.638981	6.241436	5.033574	10.031316	7.855750	6.380781	5.093331	5.986628	6.646089	5.021357	5.015058	5.880328	6.909393	5.404785	5.141933	6.813975	4.999402	6.000418	4.446490	5.506401	6.588047	6.518878	7.724392	9.294264	6.678634	7.249008	6.112982	4.352225	7.324066	4.619695	6.064868	4.812486	4.520611	5.528763	8.654468	4.612282	6.214225	4.724322	6.844206	5.028289	5.243412	4.932210	5.107646	5.384847	5.199473	4.888354	5.762690	8.298501	7.219504	5.271843	6.299075	6.362376	9.287715	5.474637	6.926316	6.408698	7.904130	7.293658	8.413384	5.908486	5.217151	5.594019	5.966803	8.462198	6.130163	4.536530	5.653303	4.192341	6.636745	5.077136	6.507348	5.746680	5.035911	5.208018	6.236971	5.259268	5.146754	6.962442	5.105042	4.609078	5.387388	6.203143	5.654640	4.125253	6.678326	8.470408	4.910706	5.872632	6.023946	10.743802	9.394342	8.512159	6.823537	9.999208	12.197683	7.125950	10.737016	9.831229	5.039717	10.582460	10.645194	5.744092	11.125198	9.961059	9.479637	11.281661	8.829025	6.339228	10.608316	10.396585	10.577828	2.880411	10.515780	11.933974	11.457125	6.778153	4.834368	8.621584	10.175307	9.624463	9.637166	8.832252	7.999946	9.167413	10.796577	8.524461	10.003818	8.506585	9.047586	10.655638	8.114560	10.686008	5.544882	10.217279	10.102022	10.384381	10.281837	7.054921	10.309414	9.734545	11.166213	10.769710	11.068828	10.938612	6.760447	9.993540	9.356515	8.490573	9.942884	7.727960	10.037024	8.426765	9.522255	10.227905	5.392017	10.344614	8.812633	8.263850	7.405528	7.206445	5.738433	10.823692	9.640574	8.519918	9.667179	9.116516	7.808778	9.619017	10.010033	11.231564	11.296591	10.455244	10.489668	11.752925	9.595082	11.110584	12.278057	10.324978	8.905380	8.201668	10.621738	9.953047	10.910730	10.077750	9.742594	9.941008	4.821475	10.722674	10.714609	11.997321	10.525185	9.006410	9.467958	11.711327	9.627387	9.535972	11.113182	6.808084	9.015459	9.251636	11.147807	9.996607	6.492368	11.511854	9.048867	5.948816	9.600494	11.092058

CCDC62	3.141935	3.326289	4.996504	3.293126	4.612954	5.275754	4.177655	3.406067	3.895246	5.319391	4.006530	3.999587	4.929067	3.913993	3.804108	4.335082	3.346402	4.204054	4.252787	3.757923	4.768483	3.665470	3.064058	4.937670	4.556426	3.556393	4.063502	3.659820	4.283596	4.403536	3.915302	6.020286	5.293129	4.616876	5.380410	4.502867	4.262886	4.258087	5.863827	4.329672	3.796319	4.867902	5.540532	4.131875	5.223707	5.233026	4.307440	6.147073	3.694034	4.382536	3.274802	4.850771	5.128131	5.243809	4.923433	4.001479	4.745539	3.269757	5.226305	3.829065	4.861224	4.061298	3.774707	4.858971	4.227672	3.492579	4.636077	4.521048	3.545984	3.620663	3.940672	3.509610	3.762166	4.001936	3.976766	4.596385	4.450747	3.768431	3.450064	4.937893	3.783976	4.406925	4.866626	5.167290	4.382303	3.620854	3.808458	4.030852	5.392589	4.807861	4.713069	4.548870	4.983217	3.469235	4.889393	4.356533	4.841693	3.817794	4.987473	3.906869	3.088772	3.933686	4.690520	4.690520	4.603754	4.837863	3.051422	4.618177	3.304280	3.156053	3.831179	4.341361	4.916860	3.279590	3.553536	3.551420	3.475076	3.548744	3.943958	3.421880	3.578380	4.215112	3.445079	3.861486	4.621851	3.338122	3.547366	3.257771	5.555257	4.072108	2.978612	3.577965	3.856567	4.685922	3.418265	3.223626	3.463210	3.346402	4.627574	4.094946	3.180009	2.948685	4.798102	5.126372	3.270368	3.083140	3.314652	3.359512	3.801408	4.693921	4.647475	4.680289	4.141068	4.816027	3.831572	3.573829	3.169645	4.343091	3.852937	3.207173	3.516964	6.332318	4.636935	4.611221	4.938279	3.256050	6.559759	3.965972	3.421880	3.195700	3.166091	4.882226	5.244418	3.822877	3.332425	4.244526	6.006099	3.465216	5.062667	4.387604	3.983505	3.825965	4.000224	3.815038	5.533140	7.053976	3.612955	3.910981	2.866975	3.181767	3.304924	4.051282	3.017983	4.116995	4.226387	3.083503	3.281352	3.049338	4.441499	4.851592	4.410703	3.657453	4.047183	5.278766	3.345270	3.287730	3.881910	3.470503	4.233080	4.432882	3.458955	4.240972	5.341041	3.510727	5.293694	3.965731	3.143269	4.778032	3.620269	4.028365	3.916184	5.126754	3.759199	4.974843	4.536948	4.501170	4.342189	4.065635	4.366995	3.120954	4.327873	4.036500	3.437732	4.232648	4.041460	3.575932	3.167932	4.106979

PLEKHM2	11.228274	11.421337	11.654988	12.315273	12.292362	12.199524	12.261347	12.550390	12.256799	12.110554	12.344708	12.256489	12.408357	12.837237	11.989871	12.067188	12.145157	12.281444	13.102010	12.473611	12.597651	12.794485	12.862774	12.486157	12.324636	12.140840	11.713327	12.116357	11.566391	11.654225	12.525969	11.838717	11.976391	12.221872	11.659417	11.899287	12.089667	12.583792	12.712606	12.737126	11.984838	12.679231	12.111299	12.042272	12.197056	12.102695	11.579668	12.118645	11.759353	11.852632	11.429272	11.939377	11.788973	12.121584	11.977595	12.038942	12.037911	12.219214	12.175134	12.362443	12.321897	11.937174	12.380518	12.708077	12.346950	12.017200	11.933193	11.735962	11.971773	12.016290	11.421630	11.413991	11.923566	11.653340	11.944085	11.543468	11.694395	12.028865	11.602692	11.941813	11.632245	11.623005	11.467784	11.869781	11.807364	11.900148	12.236059	12.369887	12.012208	11.739666	11.624718	12.093373	11.621293	11.661642	11.126061	11.777140	11.672829	12.413496	12.183450	12.021503	12.366548	12.247673	12.079262	12.510794	13.126712	12.077128	12.670010	13.182307	12.933307	11.929992	12.840179	12.812113	13.286480	11.686355	12.120977	10.671652	12.011560	12.068295	11.889157	12.024345	11.820206	11.798936	12.593292	11.966406	11.271942	12.586624	12.093826	13.267817	12.518430	12.589286	11.401220	11.788222	12.407615	12.549244	11.513041	12.140678	11.854007	12.699345	11.751007	12.840179	12.580172	12.551868	12.135587	12.399323	12.762553	11.688416	12.326856	11.301743	12.004721	12.519526	12.197990	12.598661	12.780199	11.837992	11.213047	12.547774	11.990950	12.707057	12.068126	12.046914	11.706718	12.007474	12.547310	12.369200	12.085120	12.711163	11.943350	11.956053	12.719524	12.160178	12.086064	11.749227	12.170949	12.155089	11.205541	11.828835	11.894727	12.141279	11.990173	12.927977	12.236505	12.586980	12.255020	11.936861	12.354824	12.576234	12.378030	12.027045	12.166779	11.367880	11.488318	11.664624	12.177989	11.936777	11.576476	11.857954	11.988461	11.862748	11.640267	12.265271	12.593292	12.723489	11.994409	12.222032	12.287067	12.056828	11.518252	13.266215	12.109661	11.159782	11.912061	11.126207	11.876433	12.001025	11.885697	11.856880	12.281925	12.594107	12.247353	11.675164	12.110108	12.108644	12.274074	12.789412	11.666726	12.767982	12.647433	12.472784	12.190289	12.687272	12.306362	11.398826	11.297513	10.739457	11.739966	12.688955	11.389966	12.120667

TMEM185B	9.926926	10.215321	10.803291	10.527605	10.760328	10.637121	10.651628	10.436000	9.997209	10.566949	10.597246	10.220637	11.563602	11.039733	11.041197	10.193244	10.513021	10.943606	10.098874	10.793060	10.425006	9.807438	10.298003	10.241044	10.569539	10.865608	10.670732	10.370938	10.201399	10.385324	10.675689	10.918816	10.808104	11.327191	10.239553	10.678967	10.904224	10.671810	10.994776	10.921206	10.496787	10.508722	11.049601	11.087667	11.110174	10.907373	9.906427	11.057327	10.465495	10.803437	10.807528	10.707761	10.757526	10.794399	10.841568	10.614802	10.990030	10.336292	10.422096	11.286577	10.492203	10.418435	10.700757	10.575261	11.335682	11.546807	10.310967	10.785271	10.905092	10.415417	10.026067	10.145351	10.129455	10.340663	10.304338	10.417364	10.704907	10.867857	10.314761	10.872641	10.721360	10.774758	11.208214	10.807967	10.753252	10.265145	10.738093	10.721085	10.688872	11.262723	10.760552	9.772950	10.338072	10.824136	10.554766	10.781289	10.571372	10.772696	10.893881	9.471239	10.894469	10.554140	10.651778	10.381639	10.717447	10.654425	10.656244	10.679116	10.318307	10.713446	9.789901	11.242375	11.243746	9.796567	10.361904	9.814786	10.820704	10.618196	10.525415	10.700452	11.609766	10.880771	11.433599	11.460547	11.725426	11.743043	11.719874	12.100873	11.910872	12.052294	12.495856	11.863656	12.253893	12.170796	11.491308	11.935047	13.269649	10.614085	11.484876	12.109661	10.681815	10.636526	12.407767	13.682355	12.678191	10.609923	11.709681	11.717938	11.192212	11.681573	11.031948	13.166504	11.333064	11.595630	12.051034	11.142648	11.329062	12.287695	12.206509	11.465093	11.874610	12.514995	11.789270	11.841912	12.795036	11.202995	12.665111	11.395196	12.142659	12.192450	12.038055	12.017489	12.635086	11.771822	11.653921	12.092137	11.490395	11.877315	11.494646	12.537248	11.332473	11.318087	11.432403	11.434353	11.808438	11.974875	12.091683	12.014918	11.393514	11.496506	11.651679	11.192374	12.403229	11.468535	11.316464	11.227229	11.899574	11.675976	11.654537	12.019762	12.880604	12.000886	11.761011	12.334777	13.441594	12.302320	12.482465	10.862913	12.224317	11.775784	11.749984	12.129781	11.737635	11.934899	11.280333	11.394279	11.508778	12.292196	10.987180	11.949129	12.137255	12.031083	11.442637	11.519246	11.547691	13.238812	11.674007	10.785111	11.439584	10.475286	12.041080	11.814926	9.956624	11.235980	11.306661	13.063034	12.630986	11.863940

ZFP1	10.549938	10.554299	10.237443	10.338699	10.294312	10.189798	10.880464	9.932019	10.035004	10.386637	10.152807	10.073446	10.564279	10.383339	10.382558	10.157433	9.896278	10.390024	8.502947	10.610931	10.007529	8.436936	9.317616	10.113627	10.555231	10.110653	9.442516	9.447632	9.819042	10.244867	9.735158	11.076924	11.177770	11.070312	11.142648	10.883094	11.119269	10.658143	10.205213	10.314899	10.371545	10.061198	10.837978	10.888785	10.985390	10.865149	9.926621	10.050200	11.086467	11.018990	10.987791	11.069879	11.092378	11.348194	10.821164	9.780259	11.144712	10.698669	10.451803	11.235506	10.465656	10.498280	10.026399	9.255892	10.268977	10.711692	10.487353	9.967880	10.559481	9.489036	10.827379	10.839735	9.625053	10.545363	10.820438	10.795715	11.170163	10.800096	10.742902	10.724009	10.934885	10.864387	10.910445	11.319702	11.015753	11.018211	10.932033	11.112018	10.280952	10.300344	10.078202	9.808398	10.966697	10.895336	10.499276	10.653521	10.852247	10.419803	10.585228	10.453727	10.167812	10.710393	10.541677	9.785948	9.308341	10.096406	7.659658	9.876719	8.810289	9.726365	9.025867	10.636091	9.911427	9.582219	10.228365	8.490868	9.941651	10.321819	9.747411	10.456478	10.986270	10.861307	10.298333	10.343107	10.608759	9.577144	11.152895	10.975093	10.551588	10.421803	10.704762	10.397475	10.376347	10.065725	10.717161	11.745095	9.942884	8.913738	10.904224	9.629681	8.251460	8.639275	9.751741	11.303833	11.458159	10.256188	9.995882	9.140105	10.340353	10.496942	11.263347	11.381146	11.282038	11.348941	10.984205	11.328346	10.395361	10.614231	10.528668	10.177481	10.524000	11.615965	11.539378	10.621312	10.956031	9.880715	11.543167	11.438954	11.638742	11.331187	11.890777	10.999226	12.017633	11.370827	10.440492	11.702389	10.808104	10.883094	11.127384	11.064664	10.530696	9.559999	9.957845	10.352828	11.023804	11.214098	10.285020	11.403962	9.881488	10.406073	11.220409	8.597607	11.071366	11.281809	10.782157	11.572887	11.432256	10.816583	11.141929	11.317927	11.640714	11.358396	11.195021	11.469123	10.766855	11.608020	11.670899	10.416591	10.743352	10.925355	10.754495	11.335110	11.663120	11.421793	10.822955	10.991053	10.614802	11.107920	11.205099	11.144404	11.991560	10.129759	10.222567	10.737488	10.257870	8.985318	11.041788	9.584068	9.762949	9.135994	11.538542	11.353455	8.848945	11.433599	11.397315	11.028357	10.957229	11.245235

ST3GAL3	9.518998	9.493782	9.004415	8.585756	8.098103	8.073029	9.580275	7.503840	7.272497	8.095669	7.946776	8.307282	8.947364	8.122287	8.941247	8.340714	8.834502	8.366957	7.529471	8.430406	8.052945	8.127189	10.904376	7.357496	9.036933	8.858812	9.178624	8.451544	8.327291	9.057026	8.061229	9.709524	9.334765	9.754543	9.641913	9.516216	9.705031	9.343609	9.025712	9.111441	8.614047	9.383323	9.322003	9.327661	10.555231	9.432508	8.668344	8.665616	9.478293	9.537972	9.356816	9.957211	9.949060	9.897193	10.060308	8.218720	10.379863	9.567790	9.016869	10.005054	9.975417	9.103956	10.857034	8.443006	9.610570	9.821011	8.915082	8.897010	9.484248	8.962525	9.462714	9.348642	7.689500	9.582379	9.249208	9.402013	9.568410	9.530244	9.356220	9.870758	9.510870	9.935273	9.854464	9.939645	10.058310	9.594486	9.511815	9.602855	9.278174	8.787346	9.446063	9.086559	9.283544	9.507776	9.288791	9.635073	9.557255	8.287640	8.512924	9.437081	7.866357	9.771605	8.816052	9.139490	8.538925	7.753171	7.849748	8.742107	8.925189	7.853114	9.460002	8.312531	7.984860	7.272190	7.899257	6.478984	9.378381	8.983047	7.444765	9.976192	9.820277	9.819482	9.022327	7.240393	9.580110	9.234102	9.023558	9.531003	10.571069	8.870889	10.529664	9.864701	9.499023	9.646463	8.651181	9.697544	9.383032	8.267453	10.119137	8.510905	10.670427	10.652063	9.126898	10.361284	9.732418	8.502160	10.597699	7.521779	9.628948	9.217026	10.432020	9.791757	10.210474	10.401702	11.110303	10.367597	9.551928	9.848007	11.473380	8.434134	10.806201	9.335435	10.272441	10.573204	9.569977	7.293155	9.611938	11.010640	10.272441	10.289000	9.559169	11.558020	10.449467	10.320604	8.687456	11.162307	10.549645	9.871223	11.525597	9.769365	9.281986	9.912962	9.435389	8.554809	9.669262	11.095709	8.758395	9.081348	9.207016	9.975738	11.037558	6.905892	10.273342	9.763723	9.898374	10.919263	10.187407	9.602218	10.472219	10.456786	10.652524	10.422096	10.034063	10.247562	9.907581	9.993540	9.401532	9.400578	9.174793	9.911891	9.455567	9.711100	10.565746	8.882342	9.928472	10.819847	12.317939	9.498874	11.749694	9.990425	10.366087	8.447497	10.567549	9.300797	7.941663	8.533770	9.425577	10.529382	7.549594	9.983675	9.366294	8.723135	7.100497	9.468250	8.893308	11.381887	9.862391	9.481968

OR6S1	4.187994	4.273115	5.020938	5.009514	6.811929	7.071340	6.541364	6.053723	5.755531	5.314416	6.272537	5.719393	5.614049	4.396789	4.608664	4.208456	6.193564	4.228779	3.454979	4.223852	4.833328	5.009920	7.146614	4.460696	4.394772	5.141316	4.433495	5.743733	5.196697	6.449178	5.661986	4.524351	6.422692	5.350897	5.323637	4.636303	5.099302	5.432006	6.183960	5.621468	4.536530	5.309024	3.645616	4.816688	3.734186	5.546573	5.430849	5.754786	5.597418	4.930625	4.642979	5.948634	4.857954	5.007858	4.698314	5.949174	4.582452	5.489143	5.343992	4.766605	5.517999	3.735410	6.101173	5.371678	5.882992	5.661605	4.786204	4.693712	4.937670	5.368759	5.498902	4.665614	4.019991	5.279336	5.698054	4.863937	5.479266	5.621097	5.469895	4.314992	5.119688	3.714449	4.322686	4.821688	5.378642	5.776731	6.065235	6.037725	5.802237	5.542603	5.618740	6.444367	4.197968	5.095809	5.356463	4.995442	5.405537	4.105442	6.235468	3.517751	5.710664	3.915505	4.929303	4.807639	4.803466	5.487997	4.758717	5.093331	5.907938	5.151558	5.970502	5.961118	6.037899	4.512913	6.289787	7.811870	4.961642	5.418355	5.408524	5.515443	4.807206	4.714555	4.744249	6.556947	7.594883	5.262253	4.942813	5.400149	5.434367	6.338721	5.654640	4.939739	4.503304	4.904018	4.080720	6.383651	3.681611	4.159518	4.711800	4.967792	4.911116	7.274556	5.137208	5.208231	5.048105	5.452248	5.141933	5.608542	5.974118	5.263095	4.371341	6.076952	4.665614	4.325747	4.913908	6.017610	5.330083	5.469311	5.909182	5.022770	5.753206	3.585340	5.493530	3.885512	5.211825	6.098553	4.718328	5.985373	6.053907	5.732104	5.832995	5.120920	5.166466	5.675129	6.030923	5.530879	6.000261	5.371479	5.214034	5.650491	5.572561	6.643860	6.059390	6.099633	5.431041	4.532248	5.112773	5.034847	5.806670	5.379656	4.706826	5.081850	5.611055	5.195407	4.821475	5.945514	5.783423	5.614981	5.372272	5.678263	4.956231	4.379297	4.677740	4.761215	6.478808	6.005203	5.594384	5.625588	5.555427	6.255368	7.161880	5.565981	5.122099	6.577999	4.680068	6.488546	4.654865	5.435714	4.070662	5.655361	3.782305	4.102158	4.587555	5.422452	6.403904	5.509568	5.301233	6.489666	5.528973	6.080402	5.433948	5.743377	5.751747	7.121194	7.486340	5.573143	5.560034	5.109541

SEM1	4.735238	4.351327	4.240135	5.642738	5.687351	7.074317	5.403504	4.837863	4.669934	5.089403	5.095423	4.710318	6.479926	4.763775	4.824555	5.289796	4.964389	5.925083	5.795280	5.554340	6.699341	5.030942	7.283387	5.160090	5.920495	4.875692	6.049248	5.617610	5.131397	4.050210	4.818129	4.529240	5.334554	6.219399	6.009128	6.213566	4.841044	4.462233	5.307530	5.317069	5.364627	5.430290	6.135673	5.099097	5.888319	6.101534	4.748729	5.764682	5.329117	4.676470	4.207295	5.394569	5.879425	5.939522	4.644693	4.900045	5.502582	3.361975	3.809905	4.907542	4.974843	5.906683	4.259390	5.098234	4.140843	4.305738	5.360977	4.493786	3.802855	4.741718	3.677001	4.841796	6.347250	5.120920	5.387388	3.950977	4.818759	4.370890	5.021985	3.855094	5.818676	3.678826	5.386136	5.468124	5.748485	6.076260	5.339320	5.144750	5.509757	4.480835	4.884308	5.007036	4.894172	5.122315	4.458635	4.966720	4.548146	7.197133	4.985607	5.414727	5.061210	5.359863	6.250956	5.156870	4.633277	6.547569	5.401078	5.721042	5.721949	5.964122	4.927218	5.232814	5.327707	5.846771	4.509851	5.064071	4.858553	5.533876	5.679002	5.772555	4.466966	4.773477	6.822459	5.466381	6.354258	6.658391	8.864761	7.584112	5.740064	5.066954	5.880879	4.625705	3.533492	3.765302	6.643191	5.451257	8.233570	7.634821	5.041771	6.027838	6.434113	6.320141	4.067751	4.524558	5.485556	5.123314	4.523456	6.466524	4.798317	5.231792	6.256420	6.286409	4.984779	6.059561	5.587157	4.813320	4.122526	4.639269	5.014044	6.811929	4.928060	5.995014	5.008061	4.652372	5.861573	5.441596	5.427660	5.147334	5.854373	5.034193	5.627077	6.529154	5.693962	6.423337	4.800923	6.161697	3.276202	3.718521	5.675497	4.973155	5.081850	4.570485	4.816027	6.539555	5.345937	5.507796	4.468831	4.025364	6.003768	4.712006	5.518396	5.247211	5.955221	5.298852	5.071831	5.237066	4.942189	4.550879	4.415978	6.041080	3.696123	6.007165	7.463593	5.562964	6.491525	5.001307	5.222149	5.129394	4.130630	5.319749	5.019934	4.790580	3.370658	5.014852	4.580993	5.488194	7.248072	5.780149	4.941159	5.014462	6.778314	5.963587	4.196239	5.000471	5.782005	5.816187	7.600924	4.933036	4.835195	4.820845	5.279537	5.801555	5.149291	4.538643	5.535863	5.506022	6.888402	4.227672

RERE	6.021706	5.729350	5.248033	6.283455	5.215622	6.385607	6.157198	5.694139	4.983002	5.929339	5.781271	5.066344	5.112562	5.720686	5.129196	6.281963	5.072013	5.079007	5.297474	5.903090	5.618176	6.091650	5.347540	5.508384	5.600062	5.779106	5.894955	6.677045	5.672945	8.408218	5.110165	5.710123	5.690793	5.949351	5.685292	5.324966	5.348318	5.148519	5.478242	5.446009	6.668343	5.642899	5.316472	5.178157	5.439113	5.191700	6.306611	5.780899	5.243809	5.448784	4.700680	5.697684	5.497928	4.623792	4.955209	6.177466	4.686755	2.966669	5.282775	4.913908	5.236860	6.049418	5.988291	5.605914	5.379059	5.041771	6.064688	5.570700	6.144617	5.277938	5.993219	5.755174	6.198428	6.055134	6.182888	5.937040	5.377702	5.818516	5.645803	5.018300	4.317138	4.945220	4.570485	4.877984	4.207537	5.951091	3.418075	4.839153	4.605637	7.166993	5.100918	5.155607	5.477861	5.480462	5.629358	5.197306	4.837863	4.875476	5.835886	5.295016	4.311250	5.649908	5.696567	5.272910	4.829094	6.321505	5.337890	5.478682	4.626121	5.956984	4.245989	5.512606	4.012899	5.967171	5.288542	3.618945	4.397017	5.073638	6.119227	5.430663	6.119404	5.929876	6.671556	5.257917	7.331171	6.041579	5.239632	5.470448	6.282611	6.772992	5.724374	5.404435	6.023946	6.083169	5.206440	5.554340	5.251390	5.116800	5.924711	5.490933	6.569047	5.936696	5.216974	6.595678	6.537617	6.223265	6.302189	6.060803	8.748329	4.775532	4.582669	5.210833	6.401307	5.764499	6.542552	5.925962	5.677134	6.506499	6.158402	7.176042	5.566556	5.299069	5.948433	5.004573	4.841900	7.050084	5.560405	5.895664	6.227294	5.972886	5.411287	5.268266	6.421107	5.221107	6.555125	4.368486	3.027576	5.464037	6.093378	4.388677	6.761061	5.152964	5.626721	5.898757	5.079600	6.222433	6.505475	6.325946	5.956091	6.557781	6.308928	7.057769	6.499570	5.979585	5.982617	6.029915	6.155339	6.053214	4.337446	4.846579	5.490169	4.874651	3.414352	4.361307	5.474270	4.744005	4.131450	4.208229	6.693087	5.751191	4.779065	5.413965	5.995352	5.689713	5.582288	4.713705	4.758311	5.722134	5.897227	5.885138	5.621468	5.947758	4.721961	4.377580	6.475585	5.348703	5.388158	5.286594	6.812550	4.090469	5.473470	3.553350	6.561198	5.700984	5.131397	4.554945	6.003941	6.975270

NR1H3	12.195260	11.371284	10.269856	11.422716	10.062587	10.306994	10.817752	9.390506	8.971744	10.633979	10.926836	10.512139	10.496640	10.676287	10.908996	10.273782	10.114982	10.771060	9.623069	10.681664	9.933530	11.821698	11.637543	10.737168	10.814609	10.715970	11.650510	10.905852	10.691815	11.415128	10.153109	11.850039	11.658962	11.649619	11.166077	10.945662	10.919103	11.190322	10.813141	10.966969	10.386256	11.275733	11.427378	10.785111	11.597416	11.107474	10.847688	11.131496	10.726741	11.097070	11.439262	11.125919	11.486387	11.306499	10.749920	9.814640	11.288746	11.381737	11.133393	11.451479	11.600742	10.711413	11.859051	10.569204	11.549295	11.154213	11.463911	10.303257	11.263347	10.968609	11.365804	10.734462	10.229100	10.966697	11.088131	11.789856	10.928906	11.213047	11.294959	11.160511	11.175522	11.162307	11.605506	10.876057	11.040899	11.127669	11.189047	10.416006	11.024804	10.873822	11.074704	10.982046	11.177620	11.203735	11.979596	10.928442	11.196086	10.592175	10.938338	10.228061	10.304169	11.090592	10.665039	10.366828	10.385470	10.010322	10.218622	10.300812	10.533349	9.589153	11.154369	10.550253	10.507508	10.002721	10.069634	10.240163	12.199224	10.445527	10.068875	11.892457	11.486387	11.857830	12.432750	9.852765	11.646496	11.953033	10.512279	10.388663	11.578064	11.261231	11.901889	12.206196	12.851777	11.372450	10.718671	11.796151	11.659107	12.328915	11.997151	11.901311	12.186187	12.543561	9.676599	11.977757	12.734697	11.089443	11.356381	12.210352	12.984090	12.096522	12.072662	11.828556	11.821254	12.312855	10.846519	11.212599	11.128240	10.383504	12.284103	11.206863	11.943639	12.333040	10.618351	11.305447	11.029556	11.472082	12.052362	11.037558	11.409809	10.806918	11.794064	12.320299	12.605230	12.012208	11.474696	11.704895	11.457125	11.925971	11.584856	11.670468	11.692298	10.483671	11.788671	12.180812	12.267596	11.483981	11.859491	11.737635	12.666649	11.678161	11.671934	10.666510	12.126882	11.881927	11.500773	11.183186	10.732656	10.479650	12.044549	10.846658	11.279362	10.784522	12.175782	11.569303	11.932596	12.016430	12.664248	12.428822	11.866384	11.293950	11.863940	11.475722	11.826829	11.167837	12.046769	11.057038	10.931745	12.006559	10.880317	11.537024	11.566391	10.893881	12.998952	11.334950	11.681430	10.259775	11.047954	10.539400	10.201258	12.863666	11.385706	10.934442	11.422861	10.854645	11.000137	11.943188	10.909844	12.449451

TAT	5.448989	6.495893	5.919402	6.562847	6.857697	6.568544	5.487657	7.685039	7.093370	5.913482	7.589045	6.374003	6.955026	6.389179	5.700263	6.224337	7.186835	5.929695	6.457563	6.107290	6.533725	7.138446	6.892054	5.759064	6.400824	6.601510	5.999204	6.124801	6.534041	6.716859	6.914480	4.965410	6.590464	6.279486	5.782921	5.792931	5.161670	5.764109	5.740812	5.924711	5.194263	7.199452	6.498235	5.586055	5.036945	6.169451	7.093223	6.882462	7.106294	5.577038	6.811443	5.914578	5.244612	5.571799	5.622975	7.145969	4.783690	5.852202	7.151564	5.406712	6.340567	6.233766	6.581803	6.639819	5.868125	6.620976	6.539396	5.815094	5.452837	6.205920	5.834830	5.766251	5.612184	5.506401	5.542603	5.967336	6.103589	5.900433	6.467861	5.779106	5.534897	5.827126	5.846583	5.831408	4.979752	6.689163	6.877175	5.552203	7.368591	7.101269	7.310728	6.707842	5.892040	5.792377	7.792348	5.109134	5.602065	5.311259	7.181567	6.788922	6.646901	5.112361	5.535863	5.808677	6.854156	5.591378	6.718634	7.016162	6.947121	5.977433	7.295535	7.666363	7.129553	6.870984	5.765566	7.890599	6.526356	6.050444	6.019002	5.942682	7.733172	6.577340	6.038961	8.264324	7.288615	7.267114	6.568544	6.453644	6.931597	9.317616	7.389491	6.821344	7.776338	7.321353	6.059726	6.780884	7.165801	7.071647	5.551433	7.010888	7.415795	8.039489	7.234838	7.099873	7.092603	6.527992	7.144913	7.061088	6.558111	7.410771	6.721286	5.569403	6.558766	6.532738	6.266890	6.490623	6.127483	7.017144	6.244135	6.842402	6.703546	6.496048	5.955069	5.337890	6.534372	7.556139	7.478298	6.521385	6.259130	6.308238	6.445240	5.174138	5.602645	5.274756	6.323541	5.423414	5.947925	6.234806	5.255554	6.095667	6.196567	7.401924	6.497681	6.097354	5.691739	7.159318	6.086091	7.265558	7.152928	5.756654	5.965347	10.302329	6.025877	6.581645	5.448784	6.454610	5.833177	7.265725	6.445408	6.213566	6.429015	6.940887	6.797498	5.796183	6.263844	5.566934	7.326749	7.187785	6.321505	6.203825	6.924197	5.959510	5.892040	6.057447	6.034026	6.405375	6.050288	8.001992	6.976741	7.013620	6.715863	8.144245	6.377960	8.856984	6.564495	7.052261	7.074965	7.507849	8.498198	7.447439	7.797093	8.011709	8.123563	7.344722	7.271730	6.156355	6.182888	7.911734

ATG16L1	11.043892	10.225137	10.445527	10.287574	10.462499	10.937444	10.294459	10.180258	10.300812	10.930976	10.809503	11.032688	10.672285	10.664886	10.694969	10.474064	10.662816	10.862604	9.908965	10.537651	10.056794	10.508876	10.145351	10.732079	10.873250	10.675389	10.914172	10.599413	10.903502	10.473139	10.582604	10.170273	10.241363	10.421638	10.169807	10.353264	10.216834	10.455244	10.622041	10.822371	10.669700	10.726410	10.177785	10.146866	10.189059	10.148722	11.254400	10.358566	10.099349	10.109582	10.456314	10.264852	10.401239	10.200806	10.762958	10.745574	10.339011	10.680198	11.107920	10.749317	11.018827	10.225137	10.730465	10.658429	10.864538	10.836029	10.650951	10.209982	9.931531	10.314435	10.138502	10.018488	10.910296	10.064990	9.932177	10.272596	10.006010	10.180871	10.493600	10.205978	10.248479	10.196749	10.411154	10.431097	10.084519	10.284425	10.201258	10.065419	9.941496	10.344336	10.537933	9.988575	10.168737	10.217724	10.535767	10.466251	10.326429	10.523105	10.447340	10.439434	11.308236	10.548595	10.198572	9.989210	10.844572	11.155520	9.849132	10.409829	10.578752	10.601523	9.795034	11.280895	11.421024	10.569984	10.803584	14.123055	11.393682	11.028666	10.838287	11.028057	11.175380	9.889523	10.244867	10.991621	10.060756	10.961636	10.500604	10.443033	10.631271	10.847998	10.886269	11.886732	11.100303	11.297194	10.225742	10.585809	10.509773	9.696933	10.141648	10.530093	10.866673	10.227757	11.004248	11.320252	11.006254	11.063499	10.908532	11.183466	10.292031	10.660235	10.180413	10.888061	10.383819	10.473776	10.932329	10.128694	11.126499	10.772175	11.043725	10.655479	11.270879	9.970502	10.444886	10.662983	10.610796	10.927410	10.747561	10.466251	10.882791	10.773877	10.438848	10.713575	11.918508	10.534114	10.319510	10.018340	11.223858	11.315292	10.888202	11.180812	10.271475	11.072556	11.026609	10.131679	10.313708	10.455244	10.516533	10.419038	10.461885	10.077903	10.223174	10.424550	10.680359	9.886873	10.094546	9.966054	10.174725	10.266020	10.409985	10.623687	10.914603	9.977842	9.955081	10.563692	10.643266	10.912200	10.104182	9.985804	10.731932	10.246644	10.344466	10.449603	10.646637	10.409829	10.357381	11.008142	10.822955	10.811086	10.639071	10.831803	10.346888	10.551284	10.248780	10.735480	10.939339	9.694363	10.017413	10.074373	10.684205	10.057740	11.223716	12.168975	10.587449	11.082183	11.111588	11.392925	10.539572	10.717904

SVIL	8.234201	7.724844	7.871660	8.222067	8.637239	8.402370	8.319415	8.267121	9.677379	8.341747	8.661319	8.893308	8.454290	8.638314	8.547842	8.189259	8.419307	8.812633	8.834785	7.956793	8.195435	8.644694	9.428670	8.968369	9.264136	9.129229	8.585918	9.204729	9.022956	8.377983	9.129229	7.358404	8.144389	8.542415	8.572024	7.924598	8.160080	8.162811	8.394685	8.910089	8.541182	8.758395	7.987496	8.135700	8.665616	7.804638	9.159384	7.594003	7.766526	7.629708	7.974923	7.603129	7.946776	7.502262	8.152839	8.334307	8.393785	8.074127	8.719185	8.020116	8.448278	9.327512	9.159233	8.542271	7.993011	8.335866	7.607291	7.447439	7.532374	7.860047	7.495361	7.753792	9.114675	7.692731	7.785598	7.726369	7.808494	8.145954	7.700687	7.894029	8.303938	8.058121	8.132684	8.051116	7.972401	7.992551	8.121793	8.053241	7.960849	7.306970	8.230865	8.465536	7.865730	7.958540	8.073510	8.381455	8.015092	9.451406	8.481092	8.790410	8.877254	8.229801	8.760109	8.088613	7.805396	8.684172	9.755758	8.747097	9.194184	8.612161	8.114241	9.248899	8.711169	9.287859	8.626562	8.731790	9.453395	8.461720	8.910390	7.820563	7.592865	7.887508	8.319126	8.862760	8.024380	8.321873	9.533530	8.254529	7.847939	8.596272	8.415946	8.585756	8.458269	8.561461	8.243546	8.919970	8.486586	8.783057	7.423830	8.010042	8.692458	8.554346	9.362458	8.949039	9.641761	8.147768	8.947063	9.477511	8.441625	8.656397	8.272248	8.274186	8.493243	8.216812	7.223587	8.012608	8.322469	8.329054	9.111762	8.572024	7.738668	7.168714	8.353996	8.096768	6.996467	9.228039	7.814763	7.821807	6.940135	7.690085	7.095967	7.281001	7.503220	7.947387	7.984860	8.381126	7.201165	8.549674	7.575127	8.237768	8.826560	7.344722	7.799610	8.401291	8.791520	7.214504	7.198837	7.523920	8.492264	7.173677	7.428367	9.600956	7.828383	7.476921	7.770647	7.467717	7.868761	7.748877	8.381126	8.089246	8.050019	8.384808	8.557313	8.360634	8.697420	8.028874	8.266671	8.770226	8.583613	8.688048	8.189910	7.914298	7.683512	8.357224	8.334307	9.531652	9.815427	8.791665	7.957732	8.150964	8.798699	9.913924	8.089401	7.500244	8.847714	8.555103	8.907201	8.059794	8.761825	8.561010	8.663321	8.239783	9.247506	8.228114	7.748877	8.607116	7.942799	8.406370

CCDC106	8.363776	9.003490	8.550275	8.332937	8.285318	8.187593	8.770072	8.451859	7.406172	8.206780	8.621889	8.557775	8.129662	8.701221	8.554202	8.348548	8.358470	9.032809	8.531531	8.585432	8.432903	9.028410	9.132447	8.666239	8.392410	8.545228	8.677280	8.033901	8.063024	8.807046	8.053241	9.339034	9.275465	9.068319	9.200174	8.952252	9.127037	9.217322	9.261505	9.011455	8.465685	9.132113	8.689141	9.216696	8.843269	8.250361	8.309911	8.963724	8.545657	8.840124	8.779155	8.963573	9.270309	9.109097	8.943081	8.811877	8.990766	9.402607	9.112102	9.325829	9.870258	8.114097	9.799359	8.668648	9.741509	9.375295	9.438948	8.837390	9.418692	9.172491	9.064588	8.778417	8.055425	8.625298	8.865668	9.480095	8.984879	8.880667	9.011761	9.112877	8.692789	8.949195	9.478457	8.967589	9.179692	9.369517	9.036324	8.894837	9.576365	9.133976	9.038502	8.945362	9.261818	9.095875	9.065994	8.916489	8.456103	7.769286	8.865812	8.181595	7.978951	8.852566	8.382675	8.268231	8.707378	7.875043	8.385123	9.069737	8.402513	8.160678	8.763780	8.690185	8.834942	8.261557	7.882760	7.858010	8.897010	8.170358	7.540366	7.587634	8.096280	8.768535	8.501222	8.304669	8.867333	7.923957	7.851254	7.918584	8.572331	8.079016	7.939314	8.141183	8.289502	7.910938	7.523112	8.708279	8.270267	8.187744	8.711767	9.155247	9.205189	9.973866	7.831717	9.164323	9.270142	8.530777	9.247506	7.821500	8.886682	8.115470	8.481092	8.233424	9.289113	9.106280	8.273592	9.028879	8.536401	8.690809	9.773709	9.338465	9.399396	9.163229	9.030985	8.729876	7.422754	8.435835	9.589501	8.928003	8.940654	8.941567	9.018966	8.396241	8.129817	8.789485	8.876306	9.770314	8.534236	9.431088	9.577775	10.189346	9.004561	9.047586	8.023465	9.502229	9.612716	9.099030	8.675156	8.721903	9.621194	9.388260	8.607876	7.597626	8.394089	8.544161	9.316873	8.667281	8.832732	8.649681	8.576401	7.994730	9.752993	9.260598	8.465055	8.748937	8.514294	8.089878	9.044099	9.137949	8.673745	9.107052	8.365652	9.006998	8.911908	8.426610	9.370599	8.334937	7.647273	7.899114	9.222670	7.813386	8.019965	8.194964	8.669872	7.822865	8.504777	6.757042	7.770177	8.951021	8.258160	8.589431	8.458776	8.792439	8.472170	8.841178	7.975232	9.860350	7.889501	7.646507

SCNN1B	7.192295	7.384386	7.649648	7.778213	8.362192	7.955563	7.420070	8.614358	10.171845	7.810948	8.064574	7.416084	8.141476	8.413235	8.343667	8.319126	7.585809	8.461565	9.756381	7.715071	7.089306	9.424518	8.330575	8.320195	8.356756	8.130869	8.281762	9.331216	7.352977	7.375318	8.205533	6.895266	7.620447	7.613414	7.703286	7.582415	7.045129	7.197917	8.104486	8.935453	7.466365	8.117928	7.848400	7.423532	7.583336	7.595034	7.445236	7.732726	7.666215	7.156225	7.223728	7.557866	7.661681	7.318844	8.150381	7.470962	6.967719	7.913282	8.075328	6.921443	7.664213	8.182820	6.748017	8.428823	7.156070	7.716737	7.573258	7.853280	7.552684	7.775738	7.106131	7.577914	8.285459	7.024084	7.766526	7.088975	6.824346	6.785973	7.485047	8.100858	8.415496	6.999995	7.076686	7.887508	7.241333	7.616362	7.685180	8.571129	6.937304	7.441282	6.936015	6.596483	7.832609	7.367028	6.885835	8.072573	7.512793	8.377380	7.462948	8.208150	8.392258	7.493195	7.742874	7.792182	7.711867	8.276569	9.061877	8.681277	8.032680	8.074558	7.594582	9.149853	8.756532	8.347659	9.193041	8.371749	8.921205	7.480282	8.028417	6.688845	6.635006	6.757042	7.591348	8.332304	7.445711	7.789160	7.169943	7.392107	7.061543	6.566408	7.124976	7.305703	7.418220	7.081923	7.225295	6.735790	7.361534	7.396775	6.076577	6.497206	8.851683	7.786067	6.973517	7.055577	6.581301	7.413356	7.300159	8.247245	6.791208	7.444598	7.898384	7.087830	7.616186	7.250720	6.778938	6.565762	6.446077	7.378933	7.144750	7.180328	6.877000	6.844547	6.823214	7.164101	6.854459	8.251773	7.569464	7.167473	6.359889	6.248802	7.283670	6.613022	6.382653	6.864078	6.805231	6.522655	6.947452	7.308803	5.897227	7.256786	7.116435	6.176773	7.463265	7.105962	6.845047	7.116127	7.962205	7.010888	8.484922	6.872750	7.114394	7.784088	6.818634	7.134672	6.801411	6.450803	6.368863	7.088478	6.337033	6.846765	6.318421	6.693760	7.518657	5.210637	7.939314	8.254971	6.618875	6.612157	6.313672	6.247129	6.094796	6.790559	6.827484	6.736946	7.213260	7.168260	7.366100	7.137804	7.435375	6.989357	7.036232	7.011725	7.436323	7.235475	7.587193	7.618328	7.640322	8.009910	8.131953	7.311495	7.675897	8.227663	8.174583	7.220994	7.306655	7.453397	6.909692	6.642700

ZPR1	5.671044	4.058018	5.455701	4.390632	5.718496	6.055991	6.612313	3.671130	5.919744	6.089564	5.703923	5.124710	5.380192	5.431807	6.510765	4.590570	5.136022	6.015713	5.506814	5.627077	5.260658	5.309863	5.960034	5.174333	5.936160	5.724374	5.560405	4.796678	5.151735	6.029259	4.149177	6.034881	6.673024	5.230355	6.471047	5.625960	5.906683	6.949322	5.820610	6.365202	5.485751	5.033574	6.337381	5.151120	7.702832	5.664256	6.326915	6.714240	6.110971	5.542037	5.375539	7.436945	5.956091	5.503367	5.630077	6.080402	5.557143	3.738084	6.244301	6.248466	6.162208	5.700263	5.672198	4.588420	4.742349	5.234617	5.797284	5.702980	5.360611	5.032560	5.485941	5.711017	5.343386	5.883154	5.668880	4.933247	5.278342	5.351089	4.742543	6.892517	5.643485	5.067971	4.792523	5.659751	5.582483	6.591797	6.459852	6.764101	4.511172	4.984779	4.565553	5.263904	5.175557	6.649508	5.069790	6.311444	5.796387	6.057108	6.697923	6.403592	6.274724	7.086585	6.463376	6.847240	6.161144	4.797882	4.065006	5.498120	6.234806	5.870227	7.082859	5.474637	6.553514	4.536102	4.808066	7.949845	5.804747	5.623704	5.131199	4.254797	3.594809	4.389354	4.213405	5.571612	4.948324	4.896017	4.559658	4.823117	3.811858	4.540902	4.817712	4.772813	4.894812	5.755531	5.169309	5.192676	5.691739	3.989228	6.492988	4.509201	5.734630	5.960749	5.042212	5.772748	4.160965	4.986428	4.572417	5.124710	5.369713	3.662866	4.506771	5.458858	5.343992	5.966637	4.752528	5.162091	5.134051	4.008667	5.940777	5.259838	3.279063	5.918522	4.516566	7.026166	5.141118	4.599587	5.566754	6.338043	5.397296	4.293345	7.226387	5.805110	4.421506	5.210432	5.647262	4.100634	3.028480	5.369713	4.617086	4.900919	6.131898	4.345246	3.887630	3.766514	4.951888	5.775473	3.625812	4.452047	3.135255	4.084323	4.667368	4.608201	4.062197	4.573522	3.766514	4.002780	4.753636	4.813900	6.434799	4.891263	4.764632	5.572353	5.080015	3.652825	6.218710	5.304215	7.057930	4.899001	4.170794	3.444894	5.338103	4.491573	4.886667	4.552175	4.413326	5.409120	5.441793	4.540679	5.241828	5.524404	5.575213	6.075352	6.521225	5.003956	5.411091	3.479469	4.130031	4.368922	3.655456	7.101269	4.993721	7.163769	4.919813	6.192351	6.257421	5.239849	4.972948	3.572272

KRTAP1-3	15.817557	14.764339	15.533380	15.500573	15.186033	15.552451	15.833929	16.174278	15.138365	15.729053	15.472304	15.848852	16.294338	15.848852	15.288168	15.453625	15.476405	15.265157	14.665858	15.299532	14.683638	15.150598	14.452476	15.032714	14.893360	15.261637	14.652370	15.291679	15.648843	15.417001	15.388411	15.156920	14.522236	15.017444	15.636638	15.221865	15.281689	15.165299	15.196216	15.211659	15.894116	14.722386	15.583728	14.814746	14.784831	15.207719	15.350177	15.446499	14.846246	15.251620	14.669583	15.072836	15.103652	15.411549	15.262346	15.792922	15.355714	14.598235	14.864091	14.923889	14.625993	15.496317	13.836141	14.374090	14.919494	14.176803	14.216975	14.134158	14.503811	14.837049	15.628950	15.690182	15.710626	15.211659	15.689133	15.081715	15.192041	15.278078	14.662329	15.536844	13.988680	14.496348	14.392613	15.541137	15.307206	15.175273	14.947411	14.813167	14.111066	13.884089	14.653231	14.591979	14.097636	15.149334	14.082814	14.692440	15.306516	15.536016	15.485427	15.605713	15.459167	15.733274	15.407655	15.424949	14.938834	15.764986	15.534337	15.084166	14.712131	15.541988	14.591596	15.322593	15.523618	15.836242	15.500573	15.267207	14.760083	15.503877	15.508198	15.238314	15.908209	14.954990	15.135260	15.802772	15.386012	15.592691	14.879151	15.135260	15.234130	15.440770	15.374826	15.313043	15.234869	14.659974	15.045993	14.835529	14.835529	13.986668	14.775142	14.231500	14.397680	14.184899	15.234869	14.842135	14.946400	15.144888	15.149924	15.541988	15.196878	15.200938	14.975923	14.835035	15.932498	16.100088	15.857099	15.627072	14.737586	14.760560	14.680965	15.254640	14.835035	14.717187	14.477449	15.599209	15.488676	15.505597	14.519853	15.324219	14.900158	14.845201	14.090137	13.936042	14.352005	13.996707	15.499684	13.654148	13.969061	14.097959	14.166466	14.471172	15.486206	13.668871	14.133856	13.832809	14.938328	14.132934	14.588954	14.458184	13.924299	15.540294	15.602028	15.630940	15.915538	14.921174	15.144888	14.918365	15.130285	15.502234	14.597411	14.860927	14.583170	15.069269	14.698400	14.141781	15.246670	16.331210	14.497900	13.968272	14.143957	13.800613	14.306647	14.836534	14.604561	15.219810	15.146873	14.965129	15.194873	15.528894	15.428569	14.606262	15.042367	15.371654	14.605414	13.442646	15.281689	14.532680	13.853253	14.479847	15.965762	14.908629	14.569736	14.994487	15.365568	15.100594	15.676063	15.022882	16.506136	15.246007

C3	13.229650	14.618611	13.271884	12.691342	11.136698	12.183786	13.831532	9.176047	9.125817	12.497666	11.324220	12.782402	12.919527	12.818568	13.336016	12.716965	11.394735	13.642031	10.538968	12.976403	11.960844	14.163952	15.055528	13.747829	13.474637	12.679062	14.785821	13.229650	14.391852	14.230892	11.858108	13.914461	15.069833	14.862987	15.735297	14.479847	13.921875	13.514460	14.268966	13.880137	13.144182	14.072083	15.496317	14.625993	16.428719	15.133349	14.098263	14.388615	13.836966	14.238141	13.652718	14.472714	15.627072	14.443791	13.353325	12.392964	15.019171	13.696837	13.804379	14.276547	14.720427	14.872892	16.441525	13.285244	13.779882	12.968759	14.458184	13.460920	14.130469	13.540173	13.905302	14.058590	11.816305	13.365370	12.582141	14.846797	14.279316	15.098171	14.554790	13.994473	14.388973	15.248690	13.787896	13.457023	13.919949	13.781822	13.863343	12.732523	13.118676	12.187471	14.056836	14.222846	13.233610	13.707975	14.870263	13.456831	14.255462	12.562827	13.008849	12.130685	13.900187	13.781822	13.583683	12.405117	13.591229	12.371333	13.321094	13.354792	13.835083	11.994895	13.872088	10.601972	12.240396	11.688105	10.962810	11.131351	12.904820	13.508359	12.420749	14.320379	11.013251	14.398487	14.272867	10.832422	10.205978	12.389042	10.244422	10.079690	11.802954	12.237106	11.174295	13.049903	14.298038	11.143107	13.785834	11.603290	12.238835	12.651910	11.574642	11.620681	13.419502	14.560123	10.212581	10.157881	12.395231	12.752265	10.758822	11.681573	13.366218	11.894274	14.116761	12.914314	12.950759	12.964739	10.277457	12.641550	12.434490	13.292351	14.438602	11.485173	13.546180	13.330495	13.013913	13.260048	11.642235	11.668608	13.779388	12.568405	13.045913	12.722641	14.143957	11.889031	12.286161	15.258165	13.354386	13.675077	13.231451	12.583463	12.559435	13.091272	14.846246	10.475718	11.896055	13.772438	14.517397	12.056973	13.058207	12.513208	14.447111	12.932755	12.969511	11.632692	13.780832	15.122219	12.942843	13.791211	12.956427	11.386601	13.737601	12.718385	12.596631	12.213780	13.954061	12.586624	12.377714	12.495694	12.439982	13.892882	14.205886	10.442759	12.753795	13.861843	12.973272	12.377877	13.760666	12.900773	13.055429	11.474103	11.614201	12.813342	13.421709	13.852313	14.644025	11.112153	13.105991	10.274568	9.449677	11.045017	9.378232	14.817306	13.593744	9.732263	11.751580	11.694552	11.912931	13.201614	10.832583	13.602422

CCZ1	13.099767	13.729117	13.392461	13.718916	13.841137	13.452455	13.465527	13.367256	13.084018	13.388628	13.552782	13.385833	13.683550	13.754901	13.908544	13.242980	13.089250	13.844544	12.251209	13.516909	12.236505	13.917175	12.490065	13.643515	13.642031	13.252439	12.671897	13.382599	12.957538	13.156520	13.043715	13.441594	13.513091	13.083488	13.537270	13.527030	13.666468	13.839284	14.100390	14.096755	13.218581	14.068367	13.386045	13.395371	13.525276	13.671966	13.254976	13.239205	13.346151	13.364551	13.328213	13.436852	13.683082	13.533746	13.682355	13.397092	13.849877	13.718167	13.917771	13.867259	13.557508	13.128443	13.609691	12.774306	13.934369	14.023284	13.654792	13.876318	13.717944	14.024760	12.985009	13.171124	13.370614	13.092940	13.394935	13.366847	13.609433	13.050632	13.198725	13.667931	13.666705	13.363673	13.429298	13.833047	13.393094	13.278622	13.451797	13.664472	13.335818	13.969908	13.873413	13.729644	13.387555	13.316841	13.275229	13.380525	13.216776	13.341580	13.438408	13.128058	13.295148	13.595124	13.561973	13.310353	12.562670	13.710450	13.285876	12.525283	11.890346	13.409232	12.375286	13.816316	13.891028	13.284032	13.433055	12.752429	13.584643	13.459166	13.246936	13.453090	13.184481	13.221376	13.981851	13.391817	13.078555	13.335001	12.922049	13.907439	13.731668	13.541252	13.457642	13.507449	13.213390	13.723726	13.535095	13.801661	13.858620	12.117262	13.395784	12.105900	13.959948	12.728950	13.402261	14.270244	13.829200	13.358268	13.641034	13.180160	13.392233	13.061734	12.998253	13.181119	13.906117	13.223528	13.670545	13.271787	13.670083	13.720681	14.052401	14.012342	14.292381	13.478390	14.177090	13.829200	13.333932	13.668609	14.241084	12.728769	13.527474	13.471189	13.813249	13.598854	13.938772	13.767728	13.483665	14.204014	13.010122	13.910783	13.906657	14.615742	13.126907	13.658895	13.027853	13.942362	13.757880	13.430832	13.367661	13.894497	14.106916	13.401214	13.235615	12.732697	13.308940	13.252848	13.197729	13.181119	14.024445	13.333932	13.353325	13.603593	13.670308	13.654148	13.233400	13.909973	13.376280	13.515355	13.642031	13.714163	13.782568	14.048540	14.563854	13.466850	13.351397	13.181925	13.155744	13.469871	13.367256	13.065668	13.930857	13.853561	12.735569	13.076836	12.989969	13.286480	12.480123	13.991023	12.395544	12.191829	13.154016	12.396914	13.624298	14.341372	12.927197	13.484139	13.504598	14.071794	13.901485	13.257830

CCZ1B	13.099767	13.729117	13.392461	13.718916	13.841137	13.452455	13.465527	13.367256	13.084018	13.388628	13.552782	13.385833	13.683550	13.754901	13.908544	13.242980	13.089250	13.844544	12.251209	13.516909	12.236505	13.917175	12.490065	13.643515	13.642031	13.252439	12.671897	13.382599	12.957538	13.156520	13.043715	13.441594	13.513091	13.083488	13.537270	13.527030	13.666468	13.839284	14.100390	14.096755	13.218581	14.068367	13.386045	13.395371	13.525276	13.671966	13.254976	13.239205	13.346151	13.364551	13.328213	13.436852	13.683082	13.533746	13.682355	13.397092	13.849877	13.718167	13.917771	13.867259	13.557508	13.128443	13.609691	12.774306	13.934369	14.023284	13.654792	13.876318	13.717944	14.024760	12.985009	13.171124	13.370614	13.092940	13.394935	13.366847	13.609433	13.050632	13.198725	13.667931	13.666705	13.363673	13.429298	13.833047	13.393094	13.278622	13.451797	13.664472	13.335818	13.969908	13.873413	13.729644	13.387555	13.316841	13.275229	13.380525	13.216776	13.341580	13.438408	13.128058	13.295148	13.595124	13.561973	13.310353	12.562670	13.710450	13.285876	12.525283	11.890346	13.409232	12.375286	13.816316	13.891028	13.284032	13.433055	12.752429	13.584643	13.459166	13.246936	13.453090	13.184481	13.221376	13.981851	13.391817	13.078555	13.335001	12.922049	13.907439	13.731668	13.541252	13.457642	13.507449	13.213390	13.723726	13.535095	13.801661	13.858620	12.117262	13.395784	12.105900	13.959948	12.728950	13.402261	14.270244	13.829200	13.358268	13.641034	13.180160	13.392233	13.061734	12.998253	13.181119	13.906117	13.223528	13.670545	13.271787	13.670083	13.720681	14.052401	14.012342	14.292381	13.478390	14.177090	13.829200	13.333932	13.668609	14.241084	12.728769	13.527474	13.471189	13.813249	13.598854	13.938772	13.767728	13.483665	14.204014	13.010122	13.910783	13.906657	14.615742	13.126907	13.658895	13.027853	13.942362	13.757880	13.430832	13.367661	13.894497	14.106916	13.401214	13.235615	12.732697	13.308940	13.252848	13.197729	13.181119	14.024445	13.333932	13.353325	13.603593	13.670308	13.654148	13.233400	13.909973	13.376280	13.515355	13.642031	13.714163	13.782568	14.048540	14.563854	13.466850	13.351397	13.181925	13.155744	13.469871	13.367256	13.065668	13.930857	13.853561	12.735569	13.076836	12.989969	13.286480	12.480123	13.991023	12.395544	12.191829	13.154016	12.396914	13.624298	14.341372	12.927197	13.484139	13.504598	14.071794	13.901485	13.257830

PACSIN3	13.670785	13.721200	14.201495	13.981996	14.460448	14.227945	13.891289	14.068367	14.313051	13.728355	14.040253	14.056258	14.005898	14.622502	13.943775	14.186537	14.281752	13.878755	15.462495	14.421778	14.804293	14.630361	15.333793	13.889403	14.014100	14.765753	14.000102	14.078902	13.957960	13.682355	14.420600	13.947918	14.097959	13.518872	13.686919	13.633254	14.294729	14.134802	14.199131	14.381001	14.193358	14.322301	14.385642	14.381001	14.241406	14.506817	14.042288	14.408375	13.722489	14.136088	13.816812	14.270938	13.665736	13.352500	13.903326	14.032887	14.331727	14.141470	14.174885	14.038510	14.318504	14.053607	13.615204	15.079318	14.030557	14.414735	14.534643	14.479473	13.624759	14.655466	13.746814	13.944564	14.087146	13.855680	14.256071	13.877707	14.334050	13.989252	13.699229	14.520255	14.363390	14.206838	14.094001	14.613163	14.392249	14.064771	14.352362	15.043521	15.154557	14.820855	14.031471	14.485961	14.102560	14.290687	13.827679	14.202755	13.717719	14.161452	13.959373	13.911057	14.093117	13.632044	14.043788	14.484873	15.096970	14.084344	14.562573	14.445611	14.873940	14.285224	15.626034	14.868226	14.604561	14.161739	14.206207	14.017264	14.177090	13.762178	14.152378	13.822440	14.128019	13.637147	14.577262	13.952644	14.171915	14.028776	13.924850	13.924035	14.604151	14.418363	14.359688	14.208447	13.937428	14.573111	14.354835	13.960239	14.685039	14.754065	13.622440	14.815284	15.213664	15.538559	14.765753	14.951667	13.738842	13.912401	14.357312	14.262283	13.558188	14.436403	14.313436	13.863086	14.141470	13.359500	14.617366	13.236867	14.217310	15.007673	14.480594	13.948475	14.705283	13.472510	13.964894	14.150431	14.155728	14.303599	14.032280	14.364816	14.230209	14.264949	14.252158	13.943207	14.647558	14.061195	14.704362	13.563376	14.750726	14.551021	13.992685	14.348562	13.826855	14.881255	14.259621	14.567967	13.992989	13.959054	13.852712	14.311389	14.179251	14.008743	14.365566	14.653231	13.880137	13.041904	13.831022	13.412114	13.794332	13.963822	13.637147	13.647062	13.402063	13.789486	13.454346	14.009539	14.196229	13.935214	13.342212	14.265286	14.166466	14.495544	15.005964	13.682111	13.507906	13.517109	13.480172	14.362657	13.968678	14.401522	14.233212	13.354792	14.027670	13.635217	13.190276	14.521080	14.394753	14.666375	14.596200	15.131490	14.292726	14.635230	13.700156	13.988980	14.471558	13.541454	13.913198	13.724675	14.772271	14.607572

UBA1	14.064122	14.534260	14.272867	14.644889	14.301287	14.555161	14.247254	14.678334	14.951667	14.906118	14.462369	14.318839	14.628138	14.712131	14.154529	14.622502	14.660429	14.723372	14.406566	14.414735	14.341026	15.059742	13.736123	14.733504	14.553963	14.970861	13.741572	14.553963	13.956288	14.121021	14.528702	14.561327	14.172876	14.180845	14.419519	14.090994	14.845201	14.595757	15.339763	14.969696	14.766218	14.671445	14.740482	14.879151	15.133964	14.721853	14.444487	14.164273	14.005012	14.404753	13.930021	14.452088	14.802845	14.315445	14.157045	14.757850	14.619411	15.036300	14.874974	14.738602	14.088933	14.753116	13.876318	14.923889	14.611021	14.628630	14.739028	15.170771	14.801868	14.213670	14.461989	14.477449	14.680520	14.224715	14.398878	14.560951	14.642226	14.501857	14.443429	14.142712	14.404365	14.408016	14.021288	14.672364	14.381329	14.200109	13.990442	14.156066	14.183082	14.654622	14.244036	13.941517	14.520255	14.471558	13.825046	14.548542	14.077463	14.704765	14.412071	14.329679	14.448331	14.569736	14.753116	14.349592	14.651938	14.354129	14.493555	14.548542	14.371236	14.505707	13.811158	14.721418	14.972000	14.582713	14.557653	14.669583	14.334050	14.813167	15.005964	13.868836	14.352362	13.972743	14.182477	14.232213	14.220551	14.131743	14.491975	13.931371	14.547186	14.369059	14.540937	14.220880	14.307693	14.478214	14.740963	14.392936	14.204014	13.993914	14.832004	13.640816	14.201164	13.914755	14.587290	15.013342	14.244999	14.352362	14.517028	14.578102	14.117033	14.562171	13.820166	14.211417	13.601956	14.215623	14.422502	14.325416	14.142712	14.385259	15.368664	13.999805	14.168428	14.510324	14.611416	14.692917	13.889152	13.810158	14.271254	14.087732	14.588093	14.066903	13.916329	13.719938	14.313722	14.503429	14.208831	14.116761	14.009307	14.346364	14.466988	13.816557	14.346007	14.305307	14.657265	14.378847	14.097636	14.259287	14.555161	14.289961	13.526786	13.785834	13.995561	15.024650	14.340690	13.842755	14.242398	14.796433	13.957666	14.245367	13.932204	14.965129	13.836694	14.267351	14.159585	14.522678	14.566731	13.570340	14.588954	14.058590	14.295679	14.450565	13.940443	14.055121	14.204316	14.652370	14.557653	14.096118	14.734860	14.387085	15.892893	14.753586	14.692440	14.577676	14.034368	14.529498	14.025367	14.612308	14.421778	13.913198	14.199812	13.656748	14.441978	13.939322	14.582713	13.715145	13.945384	14.388223	14.335789	14.469284

NR1H2	13.329225	13.290292	13.496693	13.167478	13.089623	13.210914	13.317056	12.515666	13.139865	13.197929	13.168839	13.238624	13.242390	13.355173	13.333079	12.986468	12.797127	13.425104	13.295148	13.337083	13.329225	12.913209	13.180750	13.142299	13.240382	13.046106	13.483216	12.835997	13.071521	13.330066	13.306191	13.305996	13.194078	12.730311	13.023480	12.888444	12.978958	13.053301	13.178579	13.266411	13.246936	13.355999	12.629675	12.775483	13.352048	13.212823	13.325208	13.145329	13.185434	12.837237	12.764108	13.211099	13.087211	13.247295	13.227872	13.197729	13.346151	13.557270	13.577170	13.229444	13.361828	13.177402	14.258285	13.358268	13.261024	13.493480	12.925613	13.180364	12.934551	13.397318	12.947738	12.982400	13.060802	13.047627	12.920968	12.709456	13.032476	12.862948	12.658466	13.165282	13.238812	13.581115	12.950183	13.638302	13.301455	13.396218	13.345088	13.503914	13.845086	13.601956	13.543058	14.005564	13.164705	13.090908	12.729292	13.176786	13.576482	12.937441	13.054557	12.748286	13.012808	12.936720	12.746911	12.864781	13.569407	13.280841	13.198336	13.555459	13.641300	13.199119	12.823360	13.224721	13.122511	13.079117	13.144577	12.782948	12.933125	13.394129	13.229650	13.258225	13.184288	13.386045	13.488366	13.185047	13.260048	13.369536	12.407767	13.259620	13.669861	13.073229	13.353095	13.285050	13.415442	12.713119	13.056909	12.809830	13.016253	13.148076	12.716779	13.069482	13.647795	13.039489	13.393302	12.888611	13.104645	12.971491	13.192104	13.264346	13.595842	13.813525	13.027483	13.060802	13.114466	13.110956	13.222353	13.206127	13.286480	13.438194	13.645649	14.034063	13.757393	12.967314	13.652235	13.004862	12.658981	13.133394	12.936194	13.502602	12.993637	13.010316	13.129366	12.820920	12.813004	12.775662	13.539712	12.952737	12.734379	12.586302	12.699345	13.554828	13.535967	12.949458	13.594434	13.336226	13.068719	12.829828	12.588140	12.347744	13.107904	12.703807	13.216405	13.157276	12.514830	12.594107	12.858133	12.850513	13.049159	12.941188	13.511960	12.798847	12.834869	13.653197	13.165894	13.555677	13.195118	13.124799	12.956616	13.893681	13.413573	13.219965	13.677795	13.025652	13.091080	13.427333	13.043339	12.794859	12.846834	12.759782	13.132093	13.204172	12.049063	12.348522	12.552494	13.845352	13.955201	13.307048	13.813787	14.188986	13.258409	12.691705	12.971153	12.053618	12.912524	13.457023	13.032839	13.169781	13.376280	13.445237

GPR26	7.655796	6.749580	6.681110	6.840955	7.466791	7.087997	5.924711	8.089246	7.951354	6.292342	6.810819	6.428351	5.008885	5.984549	6.286581	6.534213	8.164264	6.494605	6.978487	6.603417	7.759914	7.099873	8.700763	7.027648	6.702900	6.813501	6.510942	7.316378	5.233026	5.902366	8.066110	6.411836	6.225380	4.961642	6.452605	6.718962	6.439708	6.860269	7.319961	6.912945	5.070383	7.214976	6.180816	7.588120	6.553514	6.774623	6.640971	6.829927	6.856249	6.465872	6.886135	7.525735	6.013483	6.512602	6.211683	5.739353	5.400334	7.193889	6.996467	7.036695	7.426476	5.584968	7.475368	7.602658	7.296044	7.712816	6.965487	7.165662	6.965640	7.830076	6.539709	6.662807	5.861398	6.528989	6.770091	7.061543	7.283670	7.143020	7.408527	6.796847	7.084263	6.741451	6.886288	6.528320	7.250091	7.617855	7.656558	7.445391	7.738827	7.894196	7.726838	8.702595	6.873531	6.715371	6.778938	6.612313	7.257257	4.830197	6.185046	6.430386	6.542050	5.746275	5.524404	6.352424	6.900296	6.179989	7.898055	6.962614	7.491333	6.507490	8.395299	7.684886	8.137554	6.392873	7.128933	9.390971	7.946185	6.424310	5.974825	7.479964	7.072588	7.313043	6.378130	7.996917	7.078396	5.929013	7.573718	7.665900	8.451693	6.290302	6.169288	6.532898	6.598102	6.752407	6.703390	8.060923	6.936015	7.410189	6.667380	8.172072	6.693760	9.841226	6.963394	7.464806	6.733689	7.612184	7.046660	6.066083	5.616310	8.270740	6.733993	6.429707	6.165285	6.460518	8.113605	6.734147	7.327546	7.565641	7.542477	5.521370	7.604353	6.693598	8.271494	7.038119	7.185534	6.710714	7.358404	7.296707	7.375955	7.514494	7.282895	6.136867	6.682255	6.656310	5.990781	6.314181	7.157933	7.230114	7.780597	7.741489	5.836243	8.054016	7.978305	7.510785	7.903984	7.349096	7.283052	7.138922	7.912515	6.925974	6.920646	5.697300	6.931924	6.844034	7.334897	7.417872	7.642619	7.677630	7.194222	6.770742	7.132283	7.072119	6.910016	6.967400	7.854059	7.241803	8.406518	7.992551	7.814935	7.765616	9.246132	6.995990	6.844875	6.759611	6.603417	7.796942	7.092449	5.246614	9.723277	6.524190	6.309603	5.785415	6.366226	7.107577	6.105843	8.155791	7.312596	8.025580	6.463039	8.431794	7.407740	9.137787	6.829577	7.273736	7.909883	7.842468	6.915103	6.077284

GPSM1	12.755118	13.236652	13.153006	13.422986	13.453519	13.739368	12.966367	14.202473	14.257659	13.465193	13.572887	13.337899	12.940803	13.117902	12.965636	13.748094	13.896143	12.865491	13.680663	13.607340	13.098245	12.664946	12.708384	13.341361	13.207531	13.316619	13.512208	13.591444	13.852979	13.360816	12.793936	12.145607	12.192292	12.421993	12.804429	13.075888	13.262983	13.401415	13.899912	13.429298	13.550061	13.453292	13.654792	13.913198	13.535095	13.505905	13.686701	13.649396	12.840723	14.402866	13.193275	13.260449	12.623163	12.509969	12.437743	13.632774	12.421501	13.058425	12.499454	12.462402	12.275177	13.364346	12.009301	13.113355	12.344394	12.409358	13.270823	13.304119	12.967688	12.808933	13.737346	13.573121	13.675309	13.605717	13.422370	12.896328	13.110410	12.889319	12.959381	12.232358	12.056973	11.773277	11.345799	12.005336	12.128873	12.343913	12.172470	12.393945	12.052362	12.277448	11.877464	11.759472	12.570017	12.730491	12.202678	12.439334	12.550549	13.095058	12.917111	14.158986	13.411878	13.481700	13.766757	13.918056	12.677828	13.760910	12.787176	13.270023	13.053130	14.062109	12.306362	13.545741	13.255583	13.972453	13.531473	14.982214	12.299183	13.138306	14.001519	12.272198	12.663909	12.678360	12.826984	13.950183	13.054711	13.163939	13.683321	13.213003	12.792672	12.826118	12.695090	12.825581	12.639389	12.617294	13.448871	12.666814	12.364491	12.751206	12.154206	12.500257	12.380353	12.309198	13.263199	12.575245	12.443027	13.451797	13.258606	13.931371	13.015899	12.091525	12.145157	12.196619	12.901113	12.596631	12.127025	12.516304	12.879771	12.848958	12.572689	13.382177	12.234198	12.228075	13.193873	12.395993	12.995256	13.569407	12.278977	11.605660	12.754938	12.512588	12.010648	11.345479	11.891560	11.745676	12.959560	11.038451	11.539103	11.754542	11.387475	11.947291	12.957538	12.090269	12.222322	11.912793	11.829878	12.469727	12.569545	12.643737	12.016135	12.767631	12.698837	13.806755	12.878010	12.571685	12.648312	12.614854	12.707907	13.019903	11.480371	11.690777	11.183466	11.337903	11.560396	11.182301	12.578033	11.763565	11.425080	11.527049	11.480786	11.897222	11.295112	12.111443	12.194744	12.359732	12.477840	12.390759	12.941572	12.370681	12.689122	12.490065	13.287648	13.539515	12.774646	11.854155	13.278803	12.559575	12.149882	11.978054	13.725134	12.050418	12.714689	13.101809	14.094594	12.528093	13.464192	11.820662	12.311428	13.318461

SP140	10.833152	11.495782	10.931888	11.048690	11.185995	11.656735	10.740649	11.213507	11.876585	10.817752	10.761456	11.072556	11.616692	10.693013	10.711554	11.342795	10.738093	11.313492	11.329687	11.142360	11.063780	11.633590	10.769243	12.139271	11.665969	10.903357	13.661762	10.792335	11.380212	12.450751	11.024804	11.068828	12.259420	12.527930	11.466898	11.418974	10.927713	10.956031	10.653826	11.413079	10.894469	11.748779	12.226923	11.589820	11.593593	11.885850	11.728242	11.428109	11.247077	11.117676	11.314678	11.280187	11.329062	11.007999	11.074704	10.750993	11.135945	11.393373	11.206291	11.409515	11.166662	10.294779	11.031483	11.508343	12.261826	10.936997	10.547330	10.727474	11.267512	10.766574	10.658275	10.266446	10.827984	10.722975	10.516981	11.838566	10.574366	11.050779	10.953621	11.041360	11.380538	11.051341	11.330317	10.610513	10.978627	11.079540	11.070913	10.558425	11.765719	11.062443	11.994265	11.366980	11.097215	11.262723	11.560680	11.429272	11.261231	11.104014	11.230061	11.004847	11.294265	11.245942	10.720925	10.320280	10.357076	11.195637	12.197370	11.212744	11.218031	10.887915	10.601671	11.247632	11.735392	11.241643	11.625607	11.974211	11.517507	10.911171	11.554640	12.338888	11.789710	11.547102	12.393464	11.692298	11.450869	12.055881	11.628650	13.098061	12.098451	12.017038	10.807817	11.754542	12.747096	11.796904	12.495856	12.483405	11.982747	11.769756	11.779847	12.269936	11.680678	11.737635	12.274399	10.464170	12.351035	11.322149	12.359576	13.413783	12.099378	12.927977	12.526615	12.379731	11.129112	11.842962	11.696817	11.460104	11.390710	11.227986	11.466138	12.283820	12.012819	11.814644	11.832552	11.522936	11.378727	12.328915	11.418124	11.303668	11.112153	12.110554	12.574922	11.828254	11.093405	11.765559	12.731001	10.785111	11.550891	12.496182	11.578995	11.704750	11.729158	11.331478	11.981655	12.185267	11.924002	11.192513	11.486562	11.218772	11.849886	11.056273	11.006400	12.223567	11.168397	11.834209	11.859051	10.623514	10.806353	10.070706	12.378496	12.207449	11.156096	11.734816	12.471814	12.562827	12.095947	11.797931	11.323062	12.178794	12.348370	9.534750	12.016745	11.689281	11.103415	11.776688	11.707448	12.414938	11.608175	12.597317	11.212902	12.509321	11.106894	11.782660	12.343449	12.208079	12.422163	12.690337	11.964481	12.415089	12.071131	11.878918	11.915487	11.258585	12.469238	10.602822	12.213629	12.856770	12.078677	12.206813

AIFM2	8.867627	9.890739	10.293094	9.737303	10.249401	10.799503	10.964747	10.289759	10.385470	10.472987	10.822371	10.779563	9.789747	10.170425	10.320604	10.243674	10.721646	10.012145	9.695127	10.954078	10.131679	9.252684	9.717969	9.226696	8.542730	9.506865	9.207632	8.942331	9.503932	10.452666	10.938338	11.046049	10.256961	11.226506	11.008142	11.293672	11.111735	10.656820	10.274402	10.017128	10.679116	9.938052	10.073768	10.895043	10.400044	11.076924	9.879639	9.861294	10.618518	11.177770	10.849009	11.263943	11.055235	11.118406	10.766711	10.292031	10.797174	10.468004	10.378348	10.313050	10.262533	10.195238	10.091421	9.905450	10.190858	9.983355	10.103567	10.086358	10.078372	10.140152	10.703318	10.952091	9.836257	10.839888	10.984205	11.111448	11.041944	10.988542	10.765692	9.598956	10.902781	10.479650	10.648336	10.995532	11.125919	10.507824	10.580549	10.438320	10.644883	10.466534	9.962553	10.294779	9.679253	10.643878	10.423614	11.013535	10.710821	10.281995	10.880464	10.016345	10.313564	9.778317	10.308784	10.352202	10.241203	10.665188	9.363051	10.314435	10.034852	10.537651	10.869477	9.628290	9.957048	9.893788	10.647283	8.341166	7.526521	10.635345	9.935273	10.176378	9.525379	10.674952	10.457422	9.965611	11.681708	7.649346	11.981655	11.968715	13.166910	11.011977	11.874918	10.693165	11.074288	9.050550	11.019313	12.551384	10.544466	10.975093	11.069134	9.738374	10.173198	10.601523	8.880054	10.637273	10.824882	9.345894	10.804169	7.318079	11.079395	11.451020	10.561618	9.955696	11.326605	10.621738	11.287321	10.100782	9.958620	10.040849	10.123342	11.660731	10.593479	11.817784	11.656040	11.270579	12.072330	10.757072	12.268074	11.304260	11.418543	10.877792	11.662821	11.576892	12.158950	11.110444	10.520340	11.443078	10.346123	11.268947	10.818648	10.736862	10.987791	9.602700	10.462499	10.436142	8.908646	10.757656	10.121857	10.167666	10.225137	11.243601	11.058929	10.299864	11.555669	11.235219	10.562917	10.875313	10.748141	11.620399	10.520042	10.997150	12.033077	10.692936	11.238299	10.016027	10.909986	11.463337	11.476270	11.000920	10.171391	11.169002	10.218320	10.494224	10.918671	10.907795	11.749310	10.774339	10.826359	11.245942	11.734381	9.361550	11.743340	10.707338	10.984649	11.553024	12.080582	9.707187	11.403962	8.845859	11.278088	10.205821	11.204776	12.084965	9.627691	12.130529	11.222057	11.231564	12.686400	11.657173

TNFSF12-TNFSF13	12.535101	11.787766	12.091986	11.699156	10.978775	10.650579	12.286161	8.161790	8.743748	11.202677	10.771942	11.186151	11.470648	10.784221	11.340687	11.311032	10.963865	11.498757	9.845032	10.879160	10.983634	10.343685	11.342331	11.275035	12.499786	11.667717	13.093498	11.785542	11.987566	12.223567	11.478857	12.142227	12.138949	11.653049	12.170325	12.063952	11.678446	11.916526	11.576476	11.526648	11.821698	11.998395	11.474238	11.229016	12.545533	11.663731	12.385620	10.949499	11.635875	11.410105	11.530622	12.093684	12.261028	11.881462	11.733626	11.344451	12.460105	11.986052	11.270443	12.271395	11.940596	12.034862	13.175795	11.495210	12.028865	11.890777	11.566982	11.361662	11.355342	11.283569	12.001437	11.821627	11.538688	12.192131	11.635559	11.864242	11.780607	11.630904	11.704169	11.708507	12.152682	12.387757	12.314450	12.246555	12.574922	11.844802	12.189499	11.870697	11.915924	11.338197	12.381475	11.913984	11.599023	11.739362	12.063646	12.059200	11.946386	11.494352	11.156239	11.952744	10.999088	11.558020	11.342184	10.372148	10.171845	11.581538	10.113060	10.759397	11.407214	11.614795	10.152488	9.727021	9.369204	10.622336	10.335836	9.423296	11.865300	11.958426	12.037614	11.090592	10.960176	12.472635	11.432256	9.673342	9.771758	11.656735	10.721933	10.988232	10.128392	10.422850	10.577828	11.488021	11.109107	9.927545	12.309519	10.669542	10.626696	11.482247	11.442637	10.425753	11.201594	9.740241	8.704594	10.897349	12.336360	10.311282	8.894109	10.818491	11.901752	12.162658	11.493313	11.316613	10.096699	11.573114	10.531733	11.589394	11.046049	9.333838	11.721525	10.168271	10.747853	10.911687	10.512657	10.765826	10.129148	9.794550	9.947176	10.892843	11.000920	11.300978	11.262723	11.515004	10.800888	12.264668	12.144058	12.009009	11.140921	10.366240	11.397159	11.035981	12.901456	10.057591	10.683473	11.941196	11.585590	10.641213	10.900409	10.888061	11.342940	10.968756	11.336433	8.618626	11.472951	12.192594	11.681278	11.897804	11.436404	10.886421	12.453577	11.834209	11.030608	11.221474	12.553621	12.365559	11.049601	11.694395	11.423450	12.599341	12.307454	10.949194	11.460244	11.204945	11.184036	11.341779	11.676971	11.009768	12.010041	10.379694	11.779120	10.535606	10.288200	11.014430	11.301743	11.664472	11.004703	8.175070	10.903502	10.601358	9.455567	10.979225	10.762958	7.403812	9.778003	10.717586	10.042521	11.341633	11.293672	10.616727

TNFSF12	12.535101	11.787766	12.091986	11.699156	10.978775	10.650579	12.286161	8.161790	8.743748	11.202677	10.771942	11.186151	11.470648	10.784221	11.340687	11.311032	10.963865	11.498757	9.845032	10.879160	10.983634	10.343685	11.342331	11.275035	12.499786	11.667717	13.093498	11.785542	11.987566	12.223567	11.478857	12.142227	12.138949	11.653049	12.170325	12.063952	11.678446	11.916526	11.576476	11.526648	11.821698	11.998395	11.474238	11.229016	12.545533	11.663731	12.385620	10.949499	11.635875	11.410105	11.530622	12.093684	12.261028	11.881462	11.733626	11.344451	12.460105	11.986052	11.270443	12.271395	11.940596	12.034862	13.175795	11.495210	12.028865	11.890777	11.566982	11.361662	11.355342	11.283569	12.001437	11.821627	11.538688	12.192131	11.635559	11.864242	11.780607	11.630904	11.704169	11.708507	12.152682	12.387757	12.314450	12.246555	12.574922	11.844802	12.189499	11.870697	11.915924	11.338197	12.381475	11.913984	11.599023	11.739362	12.063646	12.059200	11.946386	11.494352	11.156239	11.952744	10.999088	11.558020	11.342184	10.372148	10.171845	11.581538	10.113060	10.759397	11.407214	11.614795	10.152488	9.727021	9.369204	10.622336	10.335836	9.423296	11.865300	11.958426	12.037614	11.090592	10.960176	12.472635	11.432256	9.673342	9.771758	11.656735	10.721933	10.988232	10.128392	10.422850	10.577828	11.488021	11.109107	9.927545	12.309519	10.669542	10.626696	11.482247	11.442637	10.425753	11.201594	9.740241	8.704594	10.897349	12.336360	10.311282	8.894109	10.818491	11.901752	12.162658	11.493313	11.316613	10.096699	11.573114	10.531733	11.589394	11.046049	9.333838	11.721525	10.168271	10.747853	10.911687	10.512657	10.765826	10.129148	9.794550	9.947176	10.892843	11.000920	11.300978	11.262723	11.515004	10.800888	12.264668	12.144058	12.009009	11.140921	10.366240	11.397159	11.035981	12.901456	10.057591	10.683473	11.941196	11.585590	10.641213	10.900409	10.888061	11.342940	10.968756	11.336433	8.618626	11.472951	12.192594	11.681278	11.897804	11.436404	10.886421	12.453577	11.834209	11.030608	11.221474	12.553621	12.365559	11.049601	11.694395	11.423450	12.599341	12.307454	10.949194	11.460244	11.204945	11.184036	11.341779	11.676971	11.009768	12.010041	10.379694	11.779120	10.535606	10.288200	11.014430	11.301743	11.664472	11.004703	8.175070	10.903502	10.601358	9.455567	10.979225	10.762958	7.403812	9.778003	10.717586	10.042521	11.341633	11.293672	10.616727

AL662884.4	9.090065	8.944434	9.178174	9.301890	9.187848	9.315818	8.965087	9.949803	8.187593	8.723730	9.191963	8.720391	8.800353	8.973768	9.162455	9.006858	8.744205	8.847874	8.867627	9.555526	8.876155	9.133976	9.507186	8.384354	8.680506	9.447790	7.811267	8.713286	8.228550	8.568786	8.739630	9.059743	8.227971	8.963871	8.902324	8.997042	9.224668	8.879747	9.259835	8.462629	8.957459	8.949039	8.474628	8.811548	8.753188	9.000858	8.590817	8.680808	8.395764	9.191181	8.721903	9.075896	9.053453	9.388107	8.837982	9.112719	9.120492	9.354834	9.314604	9.353778	9.394660	8.628955	8.866575	9.207939	9.225274	8.898245	9.521459	9.325501	9.278587	8.700763	9.129684	8.901410	8.829188	9.119435	9.308181	8.724655	9.002906	9.027348	9.264136	9.151252	9.048313	9.003955	8.949507	9.183143	9.002733	8.553322	9.045924	9.595708	8.800353	9.523811	9.109255	9.135312	8.613447	8.729568	8.925660	8.815582	8.727096	8.781189	8.648119	8.981494	8.277093	8.829341	8.352774	8.569415	8.760191	9.142130	8.116545	9.144893	8.427882	8.804313	9.195614	8.961744	8.639891	8.870081	8.234051	6.508308	9.705663	8.826430	8.309911	9.028564	8.630690	8.010187	8.720086	8.379542	8.297298	8.304531	9.516679	7.872133	8.402513	9.583919	9.573194	8.060923	8.581371	8.776128	8.769138	8.158230	8.470262	8.344300	8.067949	9.414865	9.316100	9.741359	8.142539	8.090022	10.358566	8.458269	9.494403	8.338549	8.267453	7.721135	7.692126	8.425886	8.596115	8.734085	8.624991	8.028572	7.908184	8.932414	8.551354	9.549146	7.738981	8.924735	8.408648	7.973975	8.958885	8.622822	8.218549	8.837234	7.937295	7.379401	8.808720	8.457045	9.375295	9.385584	7.675274	9.533072	8.560242	9.426344	9.136529	7.946342	8.739022	10.505574	9.714868	8.879747	10.001641	8.638314	8.818569	8.660551	9.452017	8.004026	7.905856	9.137318	8.401291	8.254205	8.323250	9.753457	8.903845	9.212359	8.519607	8.446548	9.156286	8.919658	8.562976	8.069057	8.945066	8.761068	9.320034	7.802652	8.286878	9.781629	8.954400	8.416754	8.885279	9.458185	8.127189	7.591191	8.060586	9.413487	8.155006	8.432106	8.905804	9.665161	8.029643	8.984120	8.802888	7.700850	10.009427	7.782109	8.515903	8.207086	7.405370	8.448422	8.958268	8.599566	8.818873	7.767009	8.834648	8.339965

PRRT1	9.090065	8.944434	9.178174	9.301890	9.187848	9.315818	8.965087	9.949803	8.187593	8.723730	9.191963	8.720391	8.800353	8.973768	9.162455	9.006858	8.744205	8.847874	8.867627	9.555526	8.876155	9.133976	9.507186	8.384354	8.680506	9.447790	7.811267	8.713286	8.228550	8.568786	8.739630	9.059743	8.227971	8.963871	8.902324	8.997042	9.224668	8.879747	9.259835	8.462629	8.957459	8.949039	8.474628	8.811548	8.753188	9.000858	8.590817	8.680808	8.395764	9.191181	8.721903	9.075896	9.053453	9.388107	8.837982	9.112719	9.120492	9.354834	9.314604	9.353778	9.394660	8.628955	8.866575	9.207939	9.225274	8.898245	9.521459	9.325501	9.278587	8.700763	9.129684	8.901410	8.829188	9.119435	9.308181	8.724655	9.002906	9.027348	9.264136	9.151252	9.048313	9.003955	8.949507	9.183143	9.002733	8.553322	9.045924	9.595708	8.800353	9.523811	9.109255	9.135312	8.613447	8.729568	8.925660	8.815582	8.727096	8.781189	8.648119	8.981494	8.277093	8.829341	8.352774	8.569415	8.760191	9.142130	8.116545	9.144893	8.427882	8.804313	9.195614	8.961744	8.639891	8.870081	8.234051	6.508308	9.705663	8.826430	8.309911	9.028564	8.630690	8.010187	8.720086	8.379542	8.297298	8.304531	9.516679	7.872133	8.402513	9.583919	9.573194	8.060923	8.581371	8.776128	8.769138	8.158230	8.470262	8.344300	8.067949	9.414865	9.316100	9.741359	8.142539	8.090022	10.358566	8.458269	9.494403	8.338549	8.267453	7.721135	7.692126	8.425886	8.596115	8.734085	8.624991	8.028572	7.908184	8.932414	8.551354	9.549146	7.738981	8.924735	8.408648	7.973975	8.958885	8.622822	8.218549	8.837234	7.937295	7.379401	8.808720	8.457045	9.375295	9.385584	7.675274	9.533072	8.560242	9.426344	9.136529	7.946342	8.739022	10.505574	9.714868	8.879747	10.001641	8.638314	8.818569	8.660551	9.452017	8.004026	7.905856	9.137318	8.401291	8.254205	8.323250	9.753457	8.903845	9.212359	8.519607	8.446548	9.156286	8.919658	8.562976	8.069057	8.945066	8.761068	9.320034	7.802652	8.286878	9.781629	8.954400	8.416754	8.885279	9.458185	8.127189	7.591191	8.060586	9.413487	8.155006	8.432106	8.905804	9.665161	8.029643	8.984120	8.802888	7.700850	10.009427	7.782109	8.515903	8.207086	7.405370	8.448422	8.958268	8.599566	8.818873	7.767009	8.834648	8.339965

CCDC144NL	3.365983	3.234542	2.953408	3.094765	3.089684	4.381390	3.228359	3.369613	3.363531	3.293775	3.482074	3.570246	3.300244	3.538645	3.723511	3.235381	3.305708	3.524981	4.813900	3.160172	4.371755	3.967902	3.000262	3.109691	3.263834	4.105442	3.242251	2.898804	2.810370	3.841707	3.391393	3.584353	4.178312	5.196495	4.533920	4.007615	3.428299	3.494846	3.263418	3.245524	3.352560	3.134769	3.751278	3.229042	3.548744	4.858360	3.913358	4.751297	3.958573	3.803896	4.186427	3.037944	4.175304	4.016290	3.709872	4.236552	3.984336	4.311896	4.171654	3.905958	2.809301	3.063293	3.879377	3.615523	4.053659	3.148882	3.428672	3.290281	3.498184	3.864320	2.937825	3.775101	3.382741	3.769910	3.600120	3.318473	4.330993	4.070266	3.741588	3.680251	4.009111	3.762565	4.038891	4.244526	3.575730	3.279903	4.618652	4.040595	4.364402	3.714240	3.206907	3.174065	3.709269	3.537632	3.269757	3.732967	3.434507	3.867061	3.122747	3.351900	3.665068	3.557169	3.212415	2.923298	4.176825	3.215682	3.024753	4.172921	3.192444	3.225846	2.898151	3.072674	3.125138	4.116788	3.109568	6.271028	3.386361	4.321413	3.435272	6.328905	3.322329	3.842553	4.334415	6.897032	3.977012	6.011218	4.613162	3.989003	3.633884	3.623258	4.279959	3.300392	3.877169	3.364693	3.835458	3.251132	3.157189	3.689102	3.249142	3.852056	3.800090	5.035696	2.970992	4.381162	8.787190	4.294363	3.388249	7.393516	3.551024	3.044544	9.437883	3.921257	3.428672	4.569828	7.138598	3.435805	3.568074	3.300244	3.159156	5.303625	3.020517	3.562491	3.280580	3.839470	4.482420	4.104297	4.156948	3.699196	3.742196	4.782850	6.858491	3.960098	4.111801	3.869015	9.875126	3.590125	4.229679	4.062633	4.256727	8.876016	3.132715	3.955721	3.629641	6.227975	7.150091	3.515313	3.580146	3.320901	2.888372	4.958737	3.813350	7.097815	5.405155	6.894638	3.284599	4.022591	6.501553	3.608025	3.212415	3.669497	3.869240	4.444999	4.134067	3.917683	4.180769	3.216664	3.807402	4.063502	3.558141	3.321367	3.223899	3.442756	3.196264	3.046203	4.586903	3.371366	4.023916	3.701159	7.580232	3.181086	5.064478	4.070060	3.621667	9.065840	5.651842	6.118709	9.376056	3.215552	3.734186	2.814093	5.832808	5.250184	4.386530	2.967375	4.452439	4.457149	9.952136	7.135790

RYR1	12.679909	13.892381	13.486415	14.018727	13.939913	14.553109	14.686942	14.489157	14.749262	15.116602	14.697055	14.799897	14.255152	14.661796	13.630916	14.652370	14.617366	14.229504	15.117745	14.517028	14.443066	15.761752	14.262592	14.539165	14.652808	14.759178	13.097319	14.994487	14.822343	14.250206	14.664523	13.332070	13.428685	12.273762	13.540808	13.596087	13.771894	13.845086	13.917771	14.226969	14.843171	13.912401	14.034970	13.815559	13.209096	13.528125	14.755534	13.633991	14.032887	14.111684	13.450346	13.834843	14.011230	14.327509	13.410994	14.908102	13.761409	14.032887	13.847197	13.974396	13.695872	14.942582	12.341549	13.891542	13.308547	13.638755	14.188386	14.096755	13.679257	14.301613	14.219589	14.314071	14.789898	13.994733	14.073949	13.939322	14.096413	13.386902	13.833551	13.666705	13.086878	13.326797	12.895455	13.714163	13.553021	13.521387	12.837577	13.304746	13.476603	13.216213	13.481484	13.486814	13.316425	13.530801	12.827344	13.053642	13.935762	14.192726	14.165527	14.901289	14.306294	14.169058	14.267351	14.166466	14.533864	14.363002	15.060999	14.930806	14.382401	14.922259	13.835875	14.220551	14.601973	14.963955	14.867676	13.772186	12.363880	14.212381	14.985629	11.694395	11.845084	11.597854	12.502567	12.581318	13.118063	12.875043	11.918948	12.428822	12.501563	12.514165	12.280067	12.392657	11.744351	12.268074	12.805797	11.612729	12.147801	12.152049	11.143107	12.445481	12.751465	12.746742	12.292501	11.775216	11.560080	13.238624	12.571510	12.803011	12.443196	12.332394	11.828974	11.782660	12.757890	10.882369	10.822371	11.363266	11.673410	12.045007	11.639167	12.651910	11.920253	10.787927	11.991869	11.605218	12.451227	13.824511	11.555942	11.742737	11.496506	11.611821	11.733461	10.874119	11.485624	11.434353	12.194289	10.556299	11.143394	11.539378	11.426208	12.059948	12.357368	12.175134	11.757241	11.853688	11.775503	11.714992	12.329562	12.122532	11.837100	11.892308	11.948495	13.925700	11.574932	10.944920	11.991560	11.356747	11.776387	12.285047	10.474667	10.877643	11.399700	11.213507	10.939911	10.450933	11.822613	10.988997	10.238002	11.341314	11.160233	10.835284	11.486995	11.623159	11.543025	12.142800	11.769600	11.633888	11.775216	12.435149	11.488021	11.351751	11.862884	12.126882	11.131082	11.097635	13.148581	12.534620	12.265583	12.663909	13.655038	11.928776	11.713495	11.279136	14.630361	11.662670	12.135429	12.435636	11.826536	12.202348

CIT	7.819822	7.919331	7.887351	7.862606	8.205382	8.686419	8.149015	9.293321	9.514851	8.712812	8.408648	8.282080	7.856208	8.339807	7.919819	8.916335	8.531599	8.379382	9.186923	8.514597	8.879418	9.597096	9.173279	8.645143	8.581825	8.636313	9.205975	9.358941	8.674834	8.443781	9.033700	7.122273	7.499773	7.950317	7.839230	7.805246	7.820127	8.206187	8.958885	8.703695	8.968369	8.142249	8.185892	8.374816	7.952309	8.136969	9.513967	8.886537	8.249893	8.717816	8.199817	8.404680	8.170037	8.391140	8.290249	8.752281	7.809896	8.419769	8.464289	8.062577	8.470408	9.097041	9.077786	9.190243	8.453985	8.771856	9.108288	8.709005	8.587540	9.291751	8.439638	8.496581	8.973069	8.217444	8.302736	8.107120	8.101146	7.702983	7.955563	7.307573	7.368917	7.363410	7.182036	7.360270	7.318527	7.825526	6.957191	6.969499	7.704877	7.516478	7.229814	7.873982	7.643849	7.656275	7.247454	7.388420	7.835809	7.822865	8.159472	8.956668	8.410456	8.335713	8.666374	8.732878	9.282612	8.558383	10.194475	9.386045	9.634319	9.230442	9.272858	9.038669	9.204130	9.394059	8.783833	9.458185	8.127830	7.952630	8.814563	7.711088	8.054325	7.831559	8.094336	9.221723	8.692143	7.997387	8.583467	8.469344	8.296996	8.173625	8.109245	8.528611	8.081336	8.699378	8.326529	8.109245	8.306963	9.227275	7.936063	8.617088	9.525858	9.186268	8.841352	8.572024	8.062577	8.915725	8.944895	9.234563	8.290722	8.483559	7.345830	8.054016	8.415017	7.351613	7.240235	7.213726	8.121159	8.494468	7.973975	9.223262	7.978789	7.822865	8.321113	8.230097	8.245241	9.482103	8.737472	7.605306	8.402822	8.368201	8.390673	8.032084	8.521130	8.194802	8.156736	7.681151	8.198239	8.298809	8.144389	8.361892	8.594547	9.118977	8.854082	8.694037	8.490290	8.997819	8.505802	8.892827	8.914331	7.993945	8.207086	9.909707	7.699122	7.506641	7.828691	7.944322	8.110742	8.041408	7.159016	7.265254	7.392271	7.369988	7.055577	7.035000	7.915502	6.878780	6.801251	7.735934	7.373769	7.450737	7.674467	7.396612	7.610841	7.548238	7.585336	8.013862	8.055096	8.583773	7.970042	8.362863	8.612161	8.799005	8.214701	8.118549	9.118674	9.575634	8.377069	8.489686	9.399085	9.116948	9.201089	9.678129	9.570503	8.914492	8.400034	8.006397	7.820703	8.739777

ARHGEF18	12.450257	11.347581	11.948032	11.723934	12.401128	12.620500	12.616879	13.915290	12.859172	13.065843	13.127100	12.646567	12.611838	12.757890	11.414134	13.129969	12.459117	12.410173	12.851230	12.839105	11.959600	13.044646	11.746705	12.432585	12.061039	12.568720	12.532804	12.848215	12.252952	12.310154	13.127492	12.897590	13.016827	12.451069	12.932422	12.586302	12.775483	13.032650	12.435325	12.355778	12.816646	12.307454	12.950759	12.985738	12.454375	12.581161	12.779334	11.630454	12.036533	12.678006	12.468921	12.694239	12.950547	12.494508	12.548577	12.629207	13.272175	12.255112	12.540947	12.409034	12.168505	11.766772	11.601794	12.402908	11.818854	11.809762	11.633293	11.398515	12.250230	12.114644	12.318545	12.356420	12.876415	12.668810	12.628172	12.954420	12.834200	12.809294	12.338259	12.056828	12.462402	12.096818	12.122371	12.594107	12.419928	12.748800	12.784870	12.856573	11.873387	11.776688	11.383354	12.074516	12.190892	12.507547	12.604249	12.454210	12.355945	13.341990	13.304945	12.619138	13.057638	12.390154	12.549882	12.322660	12.947738	12.026020	12.956616	13.014282	11.129277	12.832557	11.934899	12.130685	12.928492	12.751559	12.486970	11.538688	12.066090	12.508679	12.857615	12.243629	11.901889	11.218911	12.043959	12.002055	12.101029	12.174349	11.039733	12.908020	12.372119	12.591989	12.212082	12.669327	12.763614	12.454709	12.185867	12.901976	11.688994	11.565388	11.899287	12.021646	12.676204	12.238354	12.073126	12.464531	12.119577	12.651577	12.226002	11.601504	12.009009	12.278977	12.193531	12.171410	12.978769	12.536567	12.839105	12.691189	12.577210	12.509805	12.296044	12.021084	11.921278	11.749074	12.421501	12.725852	12.441276	12.562057	10.881198	12.104382	12.604730	12.385468	12.474435	12.228152	13.193698	12.352099	12.233290	11.598287	11.900872	11.914277	12.114778	12.210975	12.983713	12.144846	12.040293	11.521131	11.610934	12.043340	11.898369	11.623576	12.003696	12.858133	12.453737	12.168975	11.993191	12.388554	12.461907	12.096377	12.494832	12.555422	11.339369	12.247046	11.780465	12.870661	12.110108	12.587644	12.356248	12.340685	12.170796	11.519525	11.257836	10.967556	12.172013	11.122655	12.662065	11.656735	11.611243	11.816173	11.722608	12.457538	12.462601	13.585542	11.293672	12.770680	11.764352	12.295118	12.070082	12.072972	11.236856	12.058239	12.771018	12.013408	12.334126	10.300662	12.998253	11.072430	12.294834	12.240559	12.556219	12.803522

BOLL	3.679024	3.859401	5.186207	3.320901	3.892863	5.728635	4.781987	3.606405	3.796319	5.067971	3.179124	5.525913	4.995885	4.756961	3.013864	3.933476	3.291431	4.066279	5.398239	4.502632	3.984132	2.899623	5.755174	3.823956	3.033186	2.855056	3.817590	4.504144	3.780400	5.187613	3.692171	6.184870	6.428009	6.660520	5.091740	5.092275	4.064780	4.026422	3.136611	3.104774	3.672507	3.030788	5.828417	4.623340	5.062667	6.365725	3.082998	5.690430	4.951888	3.326945	3.149648	5.542439	5.187613	6.549020	3.354007	3.754031	4.774707	4.641771	2.914185	3.081216	4.196900	3.271463	5.550338	4.246430	7.143793	4.937295	3.224190	3.743889	6.653693	4.808685	5.368759	4.647255	4.514021	4.620829	4.758717	4.896655	4.913908	5.990425	4.450965	4.493107	5.517799	4.601657	4.305530	4.158412	6.239250	4.728053	5.127355	5.855451	3.780400	4.773688	4.990182	4.848706	4.788469	6.660691	4.882226	4.662255	4.967577	4.492238	5.773799	3.071765	5.149087	4.622273	3.116871	3.326289	3.138701	3.975516	4.105200	3.655248	4.575001	3.385855	3.146903	4.031073	4.480424	3.650223	3.210577	8.300613	4.552801	5.397296	3.798781	7.215612	7.532690	7.005651	6.932089	5.999906	7.658152	6.906870	7.182036	8.225251	8.386497	7.804774	5.973953	6.597303	5.926670	6.821014	9.807438	8.506737	6.968555	7.843927	8.625448	5.346143	7.079746	7.028638	4.775323	4.844447	4.660717	6.911310	7.237972	8.535756	6.687619	8.681103	7.464647	6.214073	6.889820	7.822714	7.119340	7.485863	8.016331	7.958043	6.230434	8.288718	5.901504	7.820856	7.050231	8.259547	7.971462	6.534701	6.267418	8.284375	6.438911	6.243437	8.590668	7.507225	6.698233	6.235819	7.247454	6.707215	9.163879	7.371577	6.395163	8.147296	6.817639	8.789321	7.904584	6.704299	5.355303	7.926948	5.523836	6.222275	7.187472	8.037595	7.277818	6.821498	6.762051	6.676101	6.805088	6.438750	8.698784	4.657066	6.386626	7.701001	6.706060	7.761133	7.323742	8.928934	5.892225	6.102725	7.762352	6.995064	7.061705	5.901152	9.103050	7.662149	7.343361	5.889945	6.270170	7.485863	6.997719	8.412605	7.612641	6.143590	6.093905	6.202073	6.668017	6.611312	6.400824	9.740576	9.190561	7.959136	6.668017	4.322056	7.057930	7.684279	6.815893	7.620601	7.981450	8.347960	8.137408	8.370663

GSTCD	12.443548	11.794841	12.322524	12.554944	12.893120	13.307048	13.200478	12.375982	13.136025	13.464002	13.565950	13.005039	12.547310	11.596096	11.362257	13.312973	12.617952	11.641016	10.602115	13.015539	11.999751	10.715070	11.185995	11.068392	10.460708	12.440469	13.774941	12.057775	12.754620	13.045457	13.248301	11.694853	11.775928	12.747282	12.448012	12.428156	12.410460	12.580172	9.981518	10.220177	13.760666	9.650396	10.470130	11.143538	10.889830	11.261832	13.424295	9.414865	11.468687	10.930096	10.933529	10.972192	10.547962	10.972053	10.680518	13.386476	10.590679	9.899439	10.043446	10.102954	9.917791	12.901976	9.720011	9.708602	9.965900	9.896882	9.542052	9.778915	10.290216	9.793310	13.484384	13.233033	13.584172	13.160536	12.697151	13.003777	12.855684	12.401619	12.975455	11.270275	11.192374	11.259009	10.728225	11.601959	11.343711	12.121431	11.428860	11.492740	9.621194	9.824636	9.457707	9.733932	11.180978	12.434341	12.248323	12.525455	11.999597	12.193677	12.139103	12.129478	11.979284	11.562614	12.291714	12.210975	9.870258	13.261809	11.917886	10.222096	10.394147	13.568039	10.421474	9.687929	10.897486	13.780359	12.629358	12.530838	9.623840	13.184481	13.790935	12.704515	12.011267	12.615190	12.434181	12.707057	13.426460	12.727428	13.127492	13.039307	12.860767	13.459166	12.646059	12.046024	11.275464	11.635559	13.447775	12.535101	11.391756	10.634437	12.825069	11.639628	10.761738	10.977591	10.156062	10.819847	11.791738	12.989438	11.910288	12.703984	13.049541	13.476158	11.799525	11.859791	12.419759	11.987250	12.835058	12.280960	12.839995	10.397759	10.627462	13.239205	10.285158	11.183767	11.912793	11.133855	11.631352	13.187040	9.733340	11.634043	11.510826	11.366257	11.132638	10.826511	11.285784	10.907661	13.151653	11.232126	10.522222	10.489183	10.719908	10.355524	12.679231	10.816719	10.207688	9.894872	10.278197	10.342477	9.948904	10.697213	10.289759	13.185843	12.949458	12.969695	13.380327	12.613037	12.968039	13.042247	12.891375	12.949809	11.246224	11.444695	11.432256	10.998348	11.704316	11.354920	11.930619	11.526214	11.370978	9.995433	10.175307	10.280332	10.351574	11.687667	12.657171	12.982246	12.523811	12.091212	12.634942	12.435481	12.031987	12.102972	11.729158	12.332394	11.970213	10.467258	13.172483	12.294671	11.491308	10.388978	13.066780	10.957964	9.735468	11.273851	13.257195	13.637621	12.811614	10.693780	12.686750	13.032650

INTS12	12.443548	11.794841	12.322524	12.554944	12.893120	13.307048	13.200478	12.375982	13.136025	13.464002	13.565950	13.005039	12.547310	11.596096	11.362257	13.312973	12.617952	11.641016	10.602115	13.015539	11.999751	10.715070	11.185995	11.068392	10.460708	12.440469	13.774941	12.057775	12.754620	13.045457	13.248301	11.694853	11.775928	12.747282	12.448012	12.428156	12.410460	12.580172	9.981518	10.220177	13.760666	9.650396	10.470130	11.143538	10.889830	11.261832	13.424295	9.414865	11.468687	10.930096	10.933529	10.972192	10.547962	10.972053	10.680518	13.386476	10.590679	9.899439	10.043446	10.102954	9.917791	12.901976	9.720011	9.708602	9.965900	9.896882	9.542052	9.778915	10.290216	9.793310	13.484384	13.233033	13.584172	13.160536	12.697151	13.003777	12.855684	12.401619	12.975455	11.270275	11.192374	11.259009	10.728225	11.601959	11.343711	12.121431	11.428860	11.492740	9.621194	9.824636	9.457707	9.733932	11.180978	12.434341	12.248323	12.525455	11.999597	12.193677	12.139103	12.129478	11.979284	11.562614	12.291714	12.210975	9.870258	13.261809	11.917886	10.222096	10.394147	13.568039	10.421474	9.687929	10.897486	13.780359	12.629358	12.530838	9.623840	13.184481	13.790935	12.704515	12.011267	12.615190	12.434181	12.707057	13.426460	12.727428	13.127492	13.039307	12.860767	13.459166	12.646059	12.046024	11.275464	11.635559	13.447775	12.535101	11.391756	10.634437	12.825069	11.639628	10.761738	10.977591	10.156062	10.819847	11.791738	12.989438	11.910288	12.703984	13.049541	13.476158	11.799525	11.859791	12.419759	11.987250	12.835058	12.280960	12.839995	10.397759	10.627462	13.239205	10.285158	11.183767	11.912793	11.133855	11.631352	13.187040	9.733340	11.634043	11.510826	11.366257	11.132638	10.826511	11.285784	10.907661	13.151653	11.232126	10.522222	10.489183	10.719908	10.355524	12.679231	10.816719	10.207688	9.894872	10.278197	10.342477	9.948904	10.697213	10.289759	13.185843	12.949458	12.969695	13.380327	12.613037	12.968039	13.042247	12.891375	12.949809	11.246224	11.444695	11.432256	10.998348	11.704316	11.354920	11.930619	11.526214	11.370978	9.995433	10.175307	10.280332	10.351574	11.687667	12.657171	12.982246	12.523811	12.091212	12.634942	12.435481	12.031987	12.102972	11.729158	12.332394	11.970213	10.467258	13.172483	12.294671	11.491308	10.388978	13.066780	10.957964	9.735468	11.273851	13.257195	13.637621	12.811614	10.693780	12.686750	13.032650

NT5C3A	12.858824	12.705875	13.240817	12.810376	12.958825	12.467321	12.655686	12.876931	11.758591	12.851402	13.018455	12.884027	12.896514	12.676682	12.412212	12.489749	11.797058	12.981496	11.171659	12.575005	12.182211	12.416734	10.206432	12.475057	12.075358	11.744952	11.466138	11.379157	11.943939	13.153629	11.670622	13.424703	13.605949	13.154939	12.882226	13.140676	12.888444	13.103890	11.897222	11.992156	12.513208	11.620399	13.471830	12.549244	12.747949	13.163939	12.711326	12.135906	13.326991	13.074750	13.174180	13.159717	13.302672	12.907135	13.414215	12.204971	13.646113	11.631509	11.996542	13.419743	12.240224	12.176716	11.634506	11.220875	12.134342	12.562057	11.788526	12.599871	12.630185	12.271894	12.669159	12.920784	12.885836	13.497141	13.285661	13.056909	13.392020	13.556834	13.207720	13.341776	13.495327	12.965824	13.124799	13.488586	12.597817	12.843348	13.113147	14.011814	11.823989	12.283174	12.392964	12.226464	13.220952	14.047634	13.666705	13.102773	13.034689	12.752429	12.637320	11.862884	12.239319	12.791154	12.836345	12.058239	11.724539	13.078379	12.255674	12.325731	10.769403	11.962614	11.696386	11.576165	12.452025	12.374817	13.458723	11.937773	12.076508	12.361792	12.372749	14.080070	13.717240	13.418183	13.243734	12.490737	12.952034	13.690456	12.123468	12.576866	13.194518	13.071874	12.432907	12.203291	13.661762	12.334942	13.000278	14.212381	11.484580	11.050635	13.216776	12.104520	12.425577	11.696988	12.713635	12.897068	13.013913	13.991278	11.934117	12.505236	13.478817	12.262315	13.110240	12.638003	13.994194	14.360038	13.552300	13.268822	13.932694	12.551723	12.309356	12.268074	12.250390	13.662003	14.020995	13.580864	13.872610	13.031189	12.729813	12.824203	13.425104	13.440127	13.724210	13.910783	13.871263	13.379683	13.392020	13.343265	12.834696	13.708684	13.384372	13.593974	12.716418	12.175782	13.161685	13.464002	13.560400	13.081015	13.642784	13.625234	13.150496	13.822440	14.127698	11.647546	13.365580	13.485069	13.717015	12.980977	13.787396	13.091080	13.884615	13.481256	13.912401	14.349898	13.622665	15.012247	13.311729	14.646275	13.507906	12.035653	12.826284	12.944614	13.433055	13.263369	14.057995	12.827872	12.939392	12.923860	11.662223	14.025995	13.783848	13.912944	12.147801	11.707448	12.282409	12.622342	12.225366	12.569706	13.448635	12.005783	13.412299	11.901311	12.798847	12.912347	12.794485	13.292557	13.927054	12.692858	13.231451	13.670083

ERAP2	12.365730	12.003998	12.922772	11.892609	11.944822	12.514165	9.016068	10.912348	8.905538	11.896910	12.631817	9.152161	12.277596	11.531829	11.840994	9.605775	8.981810	11.955891	12.056973	11.767362	9.355751	9.690083	12.999705	12.064410	9.671636	11.914124	9.634626	10.852730	9.172804	10.427471	11.217158	10.000862	13.902821	13.495100	10.289759	9.723120	12.106206	13.064722	11.939225	9.716865	11.743625	12.495694	13.050076	12.532185	9.867886	12.547466	11.203593	11.612110	12.084672	9.547000	12.401619	12.520900	13.077040	12.673960	9.253123	11.822002	9.924735	12.712606	11.930467	12.799536	12.474915	11.781211	13.221572	12.149284	12.754938	12.268825	9.009334	9.769520	12.464048	12.301868	13.121579	12.172787	11.362843	9.807917	12.168975	10.648195	13.096155	13.163768	12.461067	10.047564	12.772251	12.766912	12.684911	11.998716	12.221569	12.242226	13.073430	12.509321	13.452455	12.017927	9.936498	10.509173	9.679997	13.574462	13.439251	13.456369	12.670692	9.774925	11.840994	12.118961	8.716431	12.186990	11.844337	11.449124	12.492245	11.743625	10.430794	11.568275	11.578788	10.985679	10.649241	11.627501	11.147104	11.744798	10.018340	11.496673	12.135755	11.867445	10.813574	12.585498	14.079189	13.756638	11.663282	11.019158	12.904642	9.735326	11.506274	8.909057	12.759968	12.300335	8.545228	10.860728	13.247129	12.390459	10.275187	10.539729	11.494786	12.705338	11.658340	8.610302	9.783615	13.180561	9.471077	6.564660	13.492817	9.352587	9.406773	9.359995	9.965154	13.532157	10.674348	12.894731	11.438654	10.930669	8.440271	13.093911	13.411431	12.742336	9.817117	11.629710	12.277746	11.942282	12.037148	9.161081	12.003241	11.452540	11.187721	12.084810	9.411694	12.637320	11.841289	13.927588	10.849009	10.315629	14.221891	9.556635	13.144955	12.696267	13.079851	12.493861	12.040600	11.652576	13.346566	12.310302	12.007014	9.509444	9.452450	11.761613	12.620010	12.583792	12.230037	11.992622	9.117878	13.193698	10.127592	13.139865	13.174008	12.455008	10.930535	13.220585	12.513208	12.550714	13.647299	13.285446	10.750069	13.655038	12.371021	13.839834	13.072064	8.261557	11.299529	9.564183	13.627523	13.296189	13.382177	11.298834	9.515750	13.705084	11.898226	10.473776	10.287723	11.498007	13.225108	13.807854	11.415437	12.992354	13.851943	14.088628	12.718016	10.502146	13.734845	11.887512	12.810174	7.141928	13.435563	12.204356	10.449177	12.905702

RUNX2	10.340353	11.142206	10.542131	9.855275	10.210307	9.871223	9.986408	8.955174	8.805380	9.783775	10.142755	10.502718	10.596938	10.069917	10.632446	10.449326	8.834648	10.421803	9.684363	10.611076	10.269429	10.058619	10.035161	10.725359	10.716860	10.343107	11.116961	11.745095	10.941987	11.523246	8.766693	10.987791	11.149440	11.441315	11.181427	11.409349	10.266592	10.326118	10.072068	11.187418	9.341170	10.676888	11.132510	11.343087	10.125527	11.111300	10.901270	10.964018	10.668285	10.231595	11.115020	10.167973	11.127384	10.165338	10.771383	9.518073	10.947453	10.341850	11.249550	11.077209	11.482100	9.385285	9.914081	10.534114	10.671196	10.670580	10.423303	10.430953	10.893456	10.568630	10.409686	9.976520	10.019448	10.613473	10.169652	11.423761	9.981361	11.493313	11.411822	10.928290	11.307257	10.921049	11.578205	10.268677	10.204611	11.318816	10.830760	10.367141	11.038010	10.923426	11.408754	11.258424	10.285761	10.452966	11.739362	10.872332	10.632170	9.200336	10.475118	8.464904	8.608181	10.366684	9.771907	9.581010	10.135460	9.958930	10.400485	9.867886	9.282941	9.735326	10.156673	9.265650	10.019869	9.664041	9.733039	7.603602	10.748279	10.433224	9.690691	12.112540	12.535616	12.627817	11.644012	12.105206	11.337458	12.267455	13.992417	11.606981	10.634740	12.064994	11.360449	11.281809	12.499454	12.212531	11.896055	12.845274	11.485014	11.088431	11.932141	11.346523	11.980914	11.256783	10.429431	13.150306	12.883111	11.464783	10.375288	10.802991	12.105127	10.974503	12.447100	13.254196	11.595630	11.092206	11.601504	12.931713	12.230950	12.426698	13.090537	12.146584	11.145896	12.775154	11.400756	11.723465	11.163045	10.428516	13.258800	10.382887	12.482141	12.936542	11.347880	11.909110	13.217768	12.726510	11.425504	11.562313	11.630608	11.663120	11.946225	12.965249	10.733854	11.819746	11.692743	12.126882	12.941758	12.841499	11.950074	12.080298	11.815724	11.534520	12.049973	9.642061	12.834200	12.900943	12.950183	13.660327	13.269439	12.824360	13.254395	12.880782	10.744560	12.304637	13.710171	13.871812	12.354202	12.848771	11.711612	12.175459	12.800565	12.723671	12.130217	13.770612	13.154939	12.487141	12.767801	12.553785	12.967484	11.782660	11.867133	12.448328	12.965441	11.373485	12.729636	12.482306	10.388515	12.539812	11.856573	11.224891	11.068070	12.262009	12.848215	11.929227	9.970187	11.956809	11.707017	11.893211	11.338789	11.589071

TRABD2B	8.694827	8.941854	8.493527	8.344925	8.377672	8.789635	8.609236	8.296702	7.649346	8.448422	8.374041	8.492264	7.944475	7.518197	8.930630	8.194802	8.458106	8.090493	8.339021	7.822714	7.271564	6.874812	9.424072	7.530378	8.099298	8.931622	9.459408	8.574739	9.027842	9.116948	8.491946	8.781348	8.274471	8.443484	8.215018	8.484449	8.113605	8.258942	9.168008	8.836481	8.353209	9.391269	8.366232	8.198553	9.215611	8.003880	9.021575	9.320194	8.420244	8.868076	8.695584	8.502000	8.596576	8.476043	8.751131	8.397030	9.045190	8.475733	8.855620	9.364566	8.930003	9.770967	10.093356	9.095706	9.005015	8.772323	8.703369	8.422852	8.735484	9.087336	8.851386	8.325568	8.758075	8.249596	8.395927	8.331213	8.400333	8.997519	8.658207	8.509809	8.432432	9.595708	8.456103	8.665298	8.506270	8.380819	8.364546	8.644846	8.878315	8.638969	8.785172	9.178776	8.721185	8.227358	8.296388	8.055590	8.310512	8.193845	8.037881	7.751682	8.280091	8.166444	8.568786	8.036191	7.681453	8.582555	8.090942	7.839686	8.789635	8.385123	8.040770	7.697764	8.109516	7.894029	8.269821	10.040236	8.533923	8.509365	9.210693	8.257368	7.936063	8.139887	8.418725	9.397486	8.382521	8.970668	8.727096	8.250993	8.448278	8.436002	8.759794	8.285627	8.136158	9.037384	8.672180	8.583773	7.205372	8.045439	8.135700	8.397333	7.237038	9.013781	7.821163	8.780705	8.367126	9.437249	8.379692	9.422970	8.387588	8.505072	8.878315	7.913119	8.042486	8.167229	7.609592	8.035707	8.068759	8.805216	8.485224	8.928172	8.554647	7.717058	8.649681	7.803857	7.442212	9.187229	8.733797	8.471529	8.517004	8.662706	9.045924	7.411631	9.922427	8.591616	9.144597	8.996270	7.947387	7.830076	8.274186	8.875260	9.832913	7.998255	7.707764	8.232389	9.021275	9.624150	8.352921	8.204471	8.817738	8.250993	8.173323	9.826316	8.554809	9.456308	8.049851	8.801886	8.469804	8.260003	8.653728	7.931572	8.470727	7.942940	8.296702	8.428965	8.258160	7.828059	7.286616	8.998406	8.884219	8.142539	8.456268	8.143607	8.276257	8.499816	8.113934	7.932184	8.059483	7.411494	8.190710	8.173786	7.670378	7.988366	8.040436	7.572630	8.378467	7.356854	7.353283	8.092006	8.961298	8.193845	7.995191	8.255576	8.801575	8.674834	8.474459	8.370828	7.909264	8.752444

TYMS	10.965940	9.901001	9.944399	9.379298	11.084763	10.099038	9.569197	10.718063	12.109513	9.761351	10.627143	10.100136	10.772850	10.261311	9.802412	11.261832	11.381887	9.850414	12.312060	10.599248	11.226214	10.655171	12.616157	10.684060	11.360449	11.179610	10.959888	10.472696	11.068070	9.597413	11.401919	9.997954	9.849770	10.114982	9.533834	9.720935	9.733183	10.023074	10.195988	9.054399	10.534840	10.665768	9.600803	10.675241	10.645900	10.058014	10.713575	9.986541	9.983675	10.180720	10.294779	9.863172	9.729812	9.626936	9.837342	10.778852	9.863484	10.252493	10.485950	9.918554	9.933063	10.329500	11.151852	11.029707	10.484421	9.329390	9.569490	10.705507	9.740417	9.227275	9.735326	9.920576	10.984344	9.441108	9.134109	9.772950	9.990425	9.662962	10.011226	10.118547	10.268677	10.281686	10.188009	10.361430	9.800735	10.258347	9.882726	9.656115	10.992097	10.232963	10.498577	9.521307	10.317582	9.666547	10.606972	11.006254	10.207052	11.876727	10.601825	11.080791	10.504080	9.824177	10.201258	10.284884	11.063055	10.430163	10.977591	11.172387	12.204971	10.723571	11.123360	11.442637	10.839132	11.100738	10.387032	8.255721	11.385568	10.352202	11.578064	10.811654	10.965351	9.271815	9.859885	9.775518	9.887206	9.126126	11.997474	11.610492	10.761456	9.484565	9.478608	10.648046	8.892055	8.978787	11.770207	12.633466	10.457422	12.011728	9.534899	11.364604	11.355934	11.337604	11.583602	9.995882	10.599871	8.759334	10.872038	10.376347	9.590255	11.593135	11.056273	11.703267	10.971621	10.227130	10.018949	9.576153	10.213194	11.431058	9.360332	10.968006	10.372584	10.032568	10.646484	10.538810	10.326118	11.302634	9.495345	10.089002	9.607926	9.495957	10.265002	9.239370	9.434327	9.410650	10.296336	10.229100	11.404558	9.943334	9.511361	10.132853	10.051862	11.355206	10.600462	11.808731	9.999518	9.296761	10.290374	10.100294	9.006707	10.442006	10.066011	10.610796	10.171080	9.174191	9.793920	9.386834	10.065268	9.978130	9.744936	11.482100	10.484268	9.588428	10.623514	9.458185	11.499049	9.968968	10.222096	10.853016	10.273342	10.147476	10.009271	11.019158	9.254029	10.562217	10.951033	9.576517	10.818042	9.702796	11.501897	9.949956	11.465697	9.846759	9.406773	11.178849	10.447340	13.001966	13.136985	11.492740	8.078697	10.656244	10.723716	11.687807	9.979491	10.084052	10.227300	11.220409	9.608994	12.005028

ENOSF1	10.965940	9.901001	9.944399	9.379298	11.084763	10.099038	9.569197	10.718063	12.109513	9.761351	10.627143	10.100136	10.772850	10.261311	9.802412	11.261832	11.381887	9.850414	12.312060	10.599248	11.226214	10.655171	12.616157	10.684060	11.360449	11.179610	10.959888	10.472696	11.068070	9.597413	11.401919	9.997954	9.849770	10.114982	9.533834	9.720935	9.733183	10.023074	10.195988	9.054399	10.534840	10.665768	9.600803	10.675241	10.645900	10.058014	10.713575	9.986541	9.983675	10.180720	10.294779	9.863172	9.729812	9.626936	9.837342	10.778852	9.863484	10.252493	10.485950	9.918554	9.933063	10.329500	11.151852	11.029707	10.484421	9.329390	9.569490	10.705507	9.740417	9.227275	9.735326	9.920576	10.984344	9.441108	9.134109	9.772950	9.990425	9.662962	10.011226	10.118547	10.268677	10.281686	10.188009	10.361430	9.800735	10.258347	9.882726	9.656115	10.992097	10.232963	10.498577	9.521307	10.317582	9.666547	10.606972	11.006254	10.207052	11.876727	10.601825	11.080791	10.504080	9.824177	10.201258	10.284884	11.063055	10.430163	10.977591	11.172387	12.204971	10.723571	11.123360	11.442637	10.839132	11.100738	10.387032	8.255721	11.385568	10.352202	11.578064	10.811654	10.965351	9.271815	9.859885	9.775518	9.887206	9.126126	11.997474	11.610492	10.761456	9.484565	9.478608	10.648046	8.892055	8.978787	11.770207	12.633466	10.457422	12.011728	9.534899	11.364604	11.355934	11.337604	11.583602	9.995882	10.599871	8.759334	10.872038	10.376347	9.590255	11.593135	11.056273	11.703267	10.971621	10.227130	10.018949	9.576153	10.213194	11.431058	9.360332	10.968006	10.372584	10.032568	10.646484	10.538810	10.326118	11.302634	9.495345	10.089002	9.607926	9.495957	10.265002	9.239370	9.434327	9.410650	10.296336	10.229100	11.404558	9.943334	9.511361	10.132853	10.051862	11.355206	10.600462	11.808731	9.999518	9.296761	10.290374	10.100294	9.006707	10.442006	10.066011	10.610796	10.171080	9.174191	9.793920	9.386834	10.065268	9.978130	9.744936	11.482100	10.484268	9.588428	10.623514	9.458185	11.499049	9.968968	10.222096	10.853016	10.273342	10.147476	10.009271	11.019158	9.254029	10.562217	10.951033	9.576517	10.818042	9.702796	11.501897	9.949956	11.465697	9.846759	9.406773	11.178849	10.447340	13.001966	13.136985	11.492740	8.078697	10.656244	10.723716	11.687807	9.979491	10.084052	10.227300	11.220409	9.608994	12.005028

RUNX1	3.300075	5.162291	4.508113	3.895449	3.368271	5.044934	4.389151	5.576665	4.107628	4.000224	3.616132	4.200518	3.374551	3.980615	3.656838	4.816027	3.674530	3.309058	5.148333	4.113965	5.192874	2.983458	5.648403	3.810995	3.145941	3.478770	3.538985	2.877725	4.131450	4.080720	3.704471	3.458025	4.967997	3.465757	3.644431	4.949812	3.739768	5.100918	4.375223	4.205490	4.517865	3.112376	5.899028	4.195419	3.839648	5.785779	4.668615	6.250956	4.151259	4.405822	3.948568	3.815250	4.466094	4.320147	4.288106	4.862901	6.282784	3.018540	3.679614	4.103026	2.942671	4.281473	4.006530	3.280580	3.568264	3.823300	3.834204	3.426919	3.803679	2.986951	5.001085	3.910981	4.202047	4.140191	4.396577	3.572272	3.899349	3.602466	3.969670	3.898920	3.477825	4.171654	3.870296	4.431596	3.526678	4.656195	3.159792	4.882226	4.265308	3.790551	3.387578	3.285326	3.890951	3.744640	3.142180	3.439246	3.925461	3.388778	4.606924	3.475806	3.639909	4.949604	4.835606	3.993522	4.680948	4.385189	3.089357	3.676812	3.124958	5.273690	3.188332	3.272250	2.989012	3.919345	3.652214	4.253430	3.138594	3.695280	3.306975	3.832923	4.379733	3.820807	3.281352	2.992407	5.359672	3.339114	3.030894	3.366167	3.720350	3.624648	3.230351	3.050591	3.261885	3.282551	3.563042	3.597536	3.016034	3.530906	3.516786	4.487836	3.173699	5.462668	3.229755	2.912656	3.479650	4.294590	2.830429	3.063198	4.490426	3.400057	3.432363	3.734015	4.039309	3.546768	3.830779	4.128277	3.544145	4.035605	3.561567	3.500876	2.943316	5.456476	4.304427	4.312111	5.277562	5.170694	6.392020	4.067541	4.313402	3.933476	3.562864	4.243194	4.519978	3.589021	4.430265	5.385211	3.031075	3.190658	3.841707	2.851247	3.612379	4.121850	3.882760	3.909259	4.028609	2.892332	4.029058	3.746476	2.816668	4.419800	3.842751	3.869456	3.760082	3.152702	3.342139	3.369434	3.278993	3.494456	3.290793	3.879570	4.610371	3.615340	3.777942	3.625205	3.354520	3.511629	4.695972	3.312266	3.640693	3.560111	3.230351	3.397123	3.322496	3.269945	3.329682	2.951568	3.030894	5.254349	4.996504	3.372431	3.879790	3.261306	3.392629	5.106012	3.965731	3.054547	3.663429	3.506641	5.033991	3.014839	2.997419	3.268690	3.787581	3.583598	3.856357	3.297290	3.436524	3.804108

CHD1L	9.031263	8.522159	8.463529	8.719047	8.475262	8.628955	8.829820	8.182981	9.091411	8.785471	8.994888	8.909661	8.781959	9.032809	8.901561	8.740411	8.906121	9.516845	9.641913	8.697270	8.213942	8.627031	8.701516	8.996563	9.154648	8.500281	8.771856	8.580435	9.343762	8.211447	8.841178	8.904614	8.538466	8.837390	8.615560	8.749391	8.862310	9.180782	9.107963	8.500737	8.711476	8.591139	8.643630	8.614358	8.865812	8.640510	8.384808	8.423166	8.564352	8.418107	8.505802	8.590515	8.798544	9.107805	9.139490	9.012701	9.063687	9.131674	9.298286	9.362011	8.750190	8.525385	8.040770	8.430715	8.304834	8.786090	8.477896	8.634726	8.368619	7.640791	7.888271	8.221444	8.812183	8.208005	7.969404	8.198553	8.395764	8.520064	8.222690	8.051259	8.450767	8.740108	8.737644	8.852719	8.926272	8.578104	8.445923	8.728075	7.949691	8.406229	8.400640	6.923712	8.286088	8.457624	8.234802	8.573979	8.734872	8.940204	8.686736	8.589745	8.890098	8.805667	9.190243	8.537113	7.905856	9.051585	8.200588	9.044566	7.872919	9.147027	6.992259	8.734707	9.314274	8.739179	8.964176	7.647273	9.873938	8.985474	9.258494	8.924876	9.005162	8.149782	8.858952	9.424518	9.720345	9.137949	10.527605	9.598172	9.953948	9.547475	9.675373	9.486874	9.655812	9.874810	8.355063	10.045253	11.042851	8.593456	10.508560	8.700763	9.761660	8.958885	9.637621	9.502999	10.480506	8.663627	9.669110	9.073438	9.657496	10.242440	9.302803	9.877347	9.538277	9.365025	9.950260	8.998099	9.538600	9.774181	9.824636	10.227130	9.354532	12.132348	9.002437	9.034153	9.729026	9.333214	9.072507	9.489336	9.407868	9.603010	8.965870	10.570618	9.704576	9.516374	9.052657	9.837811	10.369561	9.866387	9.244109	8.901561	8.662072	7.921053	9.305574	9.013781	8.959765	9.113896	8.031021	8.789796	8.149924	8.176634	8.614358	9.633073	8.536545	8.043558	8.405459	8.928003	8.684630	8.588347	8.082255	9.069131	9.406930	8.839797	8.954704	9.454030	8.909057	9.166952	10.065121	7.942647	8.594242	9.470328	7.431568	8.525064	8.633369	8.618939	9.178480	9.904409	9.568588	9.846464	9.872421	8.872478	9.148637	9.117730	8.696676	7.781203	9.702796	10.821920	9.251483	7.973373	9.317754	7.072747	9.671482	10.857343	9.246132	9.278749	9.416264	10.257553	8.739022	9.967099

IL4R	12.746236	13.430372	13.925700	13.377562	13.914755	13.568953	13.412516	13.959373	13.694028	13.599502	13.548445	13.361393	13.441153	13.899912	13.404202	13.383029	13.714416	13.689979	13.877707	13.600149	14.032280	14.565911	14.333399	13.829200	13.698230	13.696325	14.270558	13.536847	13.640364	13.550257	13.498914	13.198725	13.698984	13.567818	13.435155	13.854098	13.303085	13.023845	13.152801	13.651246	13.588376	13.516454	13.682355	13.630199	13.773715	13.524143	13.694245	13.656487	13.855412	13.420610	13.168458	13.410994	14.053607	13.043339	13.548009	14.025995	13.878755	13.154391	13.475946	14.233886	13.371671	13.691189	13.994194	13.704846	13.254784	13.581602	13.126165	13.817064	13.372722	14.006448	13.528769	13.519354	13.607100	13.465090	13.625234	13.684300	13.798279	13.680663	13.375462	13.826855	13.601956	13.608041	12.908228	13.872610	13.291530	13.342212	13.511960	13.967167	13.563376	13.386476	13.435992	13.491711	13.781330	13.955750	13.099216	13.690206	13.507906	13.484384	13.873413	13.591911	13.776673	13.397551	13.305570	13.184851	14.771836	13.546426	14.365930	14.383451	14.169692	13.462242	13.982141	13.282626	14.188678	13.445449	14.185238	13.116985	12.648312	13.913715	13.427333	14.403276	13.763702	13.541051	14.132934	13.896143	12.856393	13.671278	12.191984	13.414635	12.558642	13.785359	12.666649	13.773715	13.200848	13.766757	14.164900	12.884894	11.951566	12.652934	13.230280	13.120243	14.339249	13.370614	12.394097	13.109649	13.306600	13.934066	13.021956	12.771214	13.138696	13.726121	13.805171	13.849877	14.068933	13.044091	11.532441	13.409904	13.133954	13.940199	12.696624	13.668871	12.740063	12.108177	13.819912	13.002332	12.807703	14.365218	11.964028	12.594761	13.007579	13.640135	12.903734	12.558955	14.155124	13.498487	13.061923	11.681120	12.976769	13.310550	12.303112	13.759659	14.292726	13.688348	13.693813	13.909712	13.781822	13.585102	13.501531	13.363274	13.353095	12.735923	13.252848	14.124934	13.444604	13.167892	13.813525	13.326797	13.956042	13.690456	13.532379	13.705084	13.828188	14.504600	13.798279	13.773464	13.321691	14.193358	12.809469	13.306600	13.809161	12.474915	14.175838	12.839995	13.559725	13.775439	13.228076	13.064903	12.911451	13.800082	12.442847	14.217951	12.115534	13.047798	13.025833	13.765216	13.434710	13.273776	14.362295	13.628490	13.833047	13.099019	13.745342	10.830474	13.755369	12.226464	14.412450	13.607572	13.927324	13.484384

PDLIM7	10.692122	11.174150	10.988377	11.068220	10.577237	10.275033	11.818403	9.043804	10.199926	11.006400	11.040594	11.194032	11.301140	11.529216	11.673716	10.630981	12.357534	11.952893	11.564187	10.015244	11.144256	11.062298	14.254782	10.916881	12.997305	12.129935	12.341379	12.261826	11.594907	10.875011	11.372900	11.106317	10.667979	11.888876	11.555058	11.531829	11.460998	11.841745	12.306066	11.501620	10.940842	12.516304	11.590536	10.623687	12.665646	11.081888	12.405117	11.878043	10.535918	10.990195	10.776525	12.155089	11.478113	11.417915	11.717496	11.815555	12.346006	12.915717	11.962317	13.068091	12.738484	11.366830	15.225383	12.835615	12.284103	12.726021	12.101333	11.847595	11.156708	12.069027	11.473235	11.155520	11.460712	11.300829	10.611691	11.048986	11.281195	11.003383	10.263444	11.766908	11.311611	11.549154	11.281969	11.415733	11.984250	11.045577	11.093554	10.942125	12.677012	11.035380	12.127673	11.970649	11.180206	10.907373	10.486270	11.683934	11.083214	11.473235	11.190322	12.903569	11.387310	11.713929	11.231105	11.339369	11.221025	10.986432	12.096522	11.725872	11.758737	11.114868	11.042557	11.178995	11.206291	10.496640	10.054975	9.011761	11.850968	11.944822	11.766034	10.557083	11.691853	11.679800	10.793666	11.572374	10.481855	11.854007	11.038735	11.186712	10.771504	11.516497	10.971908	12.745380	12.750695	12.184846	11.574024	11.878180	11.377091	11.322149	10.516678	11.656322	12.443196	12.120977	11.284886	12.538424	12.218206	10.147640	10.758086	10.391769	10.904808	12.942474	11.441607	11.944381	11.522184	11.286099	10.221812	11.929992	11.781799	12.913792	12.473785	11.628231	11.697842	11.371423	11.695468	10.928598	10.437805	11.658340	10.920769	9.784596	11.904615	11.849090	11.630009	11.164361	12.373381	12.773621	11.888876	11.830627	12.639201	11.618169	11.725272	13.214995	12.515324	12.211933	11.873702	12.262454	12.492085	13.293957	12.096818	11.506409	11.793767	10.550397	11.264699	11.974354	11.901166	11.249845	11.524887	12.276810	11.790286	11.044580	12.821429	11.519246	10.971168	11.859202	11.610052	12.281925	11.404685	11.694995	11.285784	13.286082	13.308940	11.959600	12.874491	12.149438	13.028210	11.725426	11.855631	11.359582	12.022508	11.470335	11.065879	13.671534	11.074704	12.216379	11.785397	12.139271	10.192680	12.057611	10.857188	10.836621	11.321288	11.626752	13.234006	10.044168	8.984267	9.144893	10.371842	13.030425	10.900269	12.414483

CACNA1G	9.568254	10.408918	10.280806	10.266747	10.747707	10.809658	10.723422	11.294412	11.664472	10.655933	10.761000	10.476474	10.301596	10.777392	10.015078	10.970442	11.028057	10.278824	11.600441	10.507824	11.056896	10.781910	10.366684	10.870038	10.568630	10.420249	10.699572	10.675689	11.207639	10.633822	12.307156	9.591195	9.700030	9.506259	10.022591	10.132147	10.300812	10.574079	11.410835	10.975673	11.200721	10.682874	9.930158	10.471779	9.800421	10.049407	10.858655	10.468163	10.075275	10.064832	9.959977	9.918554	9.994317	10.009742	9.889689	11.021233	9.686521	10.367294	9.966953	10.078051	10.206147	10.954078	9.976047	10.704617	10.359776	10.276541	10.764037	10.520340	10.500604	11.426062	10.476474	10.313564	11.257536	9.992352	10.330389	10.231920	10.398075	9.744106	10.080574	9.814145	9.741203	10.078202	9.540184	9.865136	9.609148	10.088219	9.429598	9.486434	10.238329	10.256961	9.961668	10.189059	9.607926	9.812137	9.031263	9.577534	10.023543	10.176874	9.655676	10.866981	9.986107	10.431097	10.779419	10.677496	10.964592	10.901875	12.132348	11.120150	10.984649	11.659709	10.743957	11.262129	11.331478	11.962614	10.756778	9.370896	9.607021	10.214731	11.040311	9.590890	10.018340	10.211224	10.291124	9.966801	10.245866	9.954281	10.038097	11.048552	10.501693	10.109727	9.229997	9.701427	9.444839	9.651500	10.216548	10.069634	9.440482	9.977259	9.464554	10.639211	10.340199	10.453877	10.368210	9.954745	9.865299	10.130482	10.345960	10.824286	10.460851	10.328381	9.364876	9.524895	9.979650	9.557714	9.673342	9.677215	10.268083	10.526564	10.252781	10.396441	10.052954	10.041471	10.515937	9.714536	10.023543	10.901875	10.597246	9.642850	9.631081	10.195238	9.564348	9.658419	9.872579	8.914189	10.264395	9.051148	10.302178	9.425721	9.663882	10.063481	10.457422	10.292500	10.344916	9.736555	9.112570	10.673307	10.134688	10.212581	10.233407	9.843644	9.768915	10.925355	9.455263	9.710473	9.845032	9.799359	9.804703	9.779375	8.940204	9.923681	9.614087	10.570935	10.586706	9.321044	9.414098	9.633365	8.224788	9.831681	9.431743	9.589654	9.317184	9.406024	9.690691	9.672692	9.663577	9.261194	9.547611	9.373205	9.917481	9.546391	9.936797	9.994941	9.698474	9.384500	10.762808	11.223716	10.183168	9.969878	11.212744	10.043887	10.455087	8.713570	11.379904	9.808072	9.856818	9.953492	9.979650	10.225283

UNC5C	8.508291	5.406483	5.734828	5.788863	3.422057	6.164933	6.592272	4.578189	3.275739	5.447832	4.423102	5.385942	5.057355	3.955259	5.536657	5.145547	3.304598	4.920418	4.367199	4.969038	3.730048	3.979150	6.128168	3.677001	3.169995	3.248562	6.258961	4.416874	6.520879	6.606540	3.727937	8.230395	7.107883	6.118709	6.862173	7.257719	6.954882	4.741525	4.158864	4.186427	5.061811	5.964657	5.714385	6.744337	7.622388	4.355705	5.427467	5.030942	6.007890	6.756707	7.182354	7.169643	6.584073	7.245173	6.498235	4.493992	7.396775	4.042997	3.372431	5.290575	4.534351	7.291926	5.338103	3.962928	5.469704	3.734015	4.745325	4.875077	4.689059	4.430487	6.946617	6.618387	4.933461	6.861854	5.944605	6.221944	6.193727	6.622744	6.589328	6.227975	6.047988	7.432820	7.070219	7.156827	6.334005	7.012518	5.688415	5.288139	3.812518	3.379837	3.920397	3.894431	4.817310	5.783088	7.158679	5.575213	7.542332	6.599069	4.991830	5.316861	5.052157	5.393558	5.482022	5.196314	4.376523	6.424158	2.978982	4.441091	3.095417	4.432882	4.119452	2.846952	2.904417	4.753849	4.366344	5.382123	5.052790	6.181677	5.280957	5.502384	5.003376	6.312811	4.379733	3.718738	5.369930	5.793655	5.899181	6.374667	4.341985	5.053435	4.476675	3.608791	6.005753	4.316491	5.463304	6.634360	4.404426	3.311024	6.771561	4.672936	5.016903	5.855085	3.296210	3.646626	3.494265	5.809240	3.168920	4.555796	6.120116	4.371560	5.319191	6.628874	5.595302	5.876206	6.694243	8.004183	4.504570	3.233219	3.067061	4.970485	3.737498	4.776361	4.800923	6.279314	4.062633	4.272457	8.919357	6.030587	7.554247	6.726248	6.222113	6.384819	7.032959	7.767922	5.828960	6.916190	4.901535	3.167149	3.761124	4.781151	5.678637	3.749198	4.242269	4.629822	4.688642	3.342388	5.187401	4.125452	3.404378	6.094972	7.160590	3.118766	7.763284	7.393824	6.069104	7.372973	7.390405	6.778938	4.816890	6.280180	5.077975	6.238264	8.172842	7.932955	5.852907	6.908452	5.883706	3.609384	3.709060	3.902531	4.478244	5.057355	7.062047	6.616420	5.759620	6.695543	6.759611	5.583408	4.173385	6.491222	4.061055	3.567285	6.770577	4.757829	4.493546	2.884916	4.357398	3.417894	3.318310	3.294203	4.284044	2.868281	3.350741	4.252167	5.098874	4.920835	6.317250	5.739006

ZNF846	6.739392	7.299697	7.348503	7.429995	7.096562	6.392523	7.637692	6.522018	6.760447	7.078944	7.399914	7.422282	7.599840	7.662653	7.376878	7.159604	6.713749	7.600322	8.466728	6.883579	6.576684	6.665725	8.220099	7.627346	7.928181	7.940095	6.447349	7.623177	7.140322	7.393665	6.412347	7.363727	7.581955	6.793323	7.709559	7.800225	7.894811	7.452761	7.467106	7.810046	6.667380	7.730402	7.498661	7.567340	8.068315	7.531584	7.325492	7.382348	7.386991	7.816672	8.138022	8.102344	7.984078	8.030875	7.606544	7.123687	8.340857	8.330575	7.739775	8.345831	8.405306	7.104070	7.301928	7.204562	7.478298	7.754107	7.410476	7.251051	7.020942	6.309927	7.096414	7.000313	7.137190	7.469869	7.166559	7.175574	7.213726	6.834077	7.352662	7.884605	7.617855	7.676983	7.492437	7.980968	7.934793	7.789614	7.187316	7.615409	6.536469	7.696379	7.210823	6.795726	7.242261	6.977696	6.951549	7.218109	7.348953	7.760354	7.062373	7.538443	5.559669	7.386163	7.334897	7.031402	6.696046	6.709910	7.953242	6.456712	6.504813	7.021100	6.954882	6.788410	5.971960	6.987331	6.852172	4.293735	7.212144	7.419618	6.943280	7.860910	6.297246	6.881033	7.191688	4.781766	5.355901	6.404576	5.519185	4.885177	6.355910	6.155871	6.938731	6.256249	6.074831	5.144534	6.967719	4.831039	7.661085	5.138833	6.500534	6.935051	5.981675	6.760913	7.477997	6.535471	5.454402	6.055823	6.104430	6.097512	6.704142	5.628592	6.590635	7.345011	5.951984	6.583596	7.004242	7.503220	6.558290	7.262670	7.102676	4.708848	6.707390	6.673649	6.039284	7.041278	7.051012	6.533076	6.033822	6.968869	6.741794	6.846765	6.981330	6.930178	7.771269	7.414920	6.717810	7.354866	7.269078	6.827174	7.512341	7.628448	6.394997	7.101570	5.910088	7.349096	7.314180	6.852662	6.482390	5.541466	5.947042	6.630766	6.126045	4.985191	6.891257	6.884873	6.664453	6.470538	5.577775	7.162049	7.664213	8.445159	7.201019	7.959912	6.948428	6.865962	5.819906	7.269237	6.540026	6.484623	7.263127	6.381984	6.442361	7.106294	6.934087	6.594381	5.836646	6.299709	7.087059	6.079720	6.568873	4.326548	6.422390	7.303657	6.288620	5.201486	5.818870	6.344049	6.013683	6.606041	5.234225	5.618740	4.700680	3.127307	5.684176	4.923847	6.146530	8.089878	7.158850	5.801873

PRELID2	6.422546	6.970730	7.112679	6.953906	7.018894	7.132916	8.053853	7.648749	6.949641	7.473850	7.476459	7.166993	6.987484	7.374069	7.297331	7.553771	7.073059	7.502904	6.842711	7.129090	7.107883	6.795726	6.578654	7.056336	7.009640	7.103764	7.061882	7.294297	7.284456	7.008016	6.803833	6.924368	7.251697	7.158083	7.254021	7.356553	7.276265	7.639694	7.340176	7.247168	7.606691	6.825746	6.651431	6.626777	7.019371	6.134823	7.491489	6.910160	7.286286	6.579778	6.638032	6.691609	6.966132	7.122421	6.666388	7.490242	6.412181	6.865500	6.458537	6.453304	6.577680	7.130189	7.061387	6.629051	6.841609	6.782147	7.448405	7.504008	7.464349	7.035147	7.737739	7.815753	7.254021	7.634337	7.646968	7.659080	7.468757	7.343018	7.109658	6.109395	6.207080	6.194626	6.029573	6.271204	6.369386	6.996153	6.214073	6.247965	5.985373	6.004097	5.864148	5.937761	7.050375	7.110988	6.729309	7.000648	6.847422	7.113763	7.044019	7.399762	7.082365	7.661235	7.637383	7.622087	6.708631	7.277658	7.342224	7.358111	6.728379	7.597944	6.427331	6.929077	7.252299	7.437104	6.958781	6.408201	6.763922	6.619366	7.223889	7.004395	7.322955	7.207245	7.071013	6.694243	7.826786	7.114093	6.959401	6.824346	7.512341	7.298304	7.201777	6.867088	6.913553	7.127683	7.101416	7.407426	7.236423	6.589006	7.004100	7.486641	6.606890	6.788922	7.622541	6.998626	7.657826	7.284304	7.502758	7.007190	7.209878	6.396973	6.443880	6.443034	7.767463	6.942471	7.232277	6.794787	7.399457	7.393665	7.382525	7.601986	7.368285	6.852662	6.992259	6.622744	6.826234	7.502112	6.761061	7.112523	6.959726	6.832287	6.965487	7.020298	7.204562	6.867727	7.429685	6.952175	7.120747	7.027491	6.533404	6.898358	7.519393	6.811607	6.663632	7.212644	6.882462	7.650640	7.865730	7.538942	6.978944	7.645719	7.737895	7.459019	7.560439	7.074317	7.407740	7.185875	7.499450	7.390405	6.111142	6.028736	6.732375	6.317926	6.107290	6.474239	6.216306	6.318078	6.272537	5.904197	6.131717	5.972718	6.078831	6.901599	7.272673	6.679637	6.789577	6.505645	6.746442	7.282114	7.562571	7.090101	7.035147	7.241333	7.400525	6.282611	6.886607	7.472951	7.304439	6.326915	7.792640	6.463376	7.298474	6.422080	7.509850	6.477288	6.442016	6.907043	6.806770	7.676500

BMP15	3.436697	3.817172	3.197053	3.442756	4.112234	3.921050	3.616508	3.710908	4.292475	3.847370	3.830142	3.578575	3.392134	3.596131	3.278993	3.575932	3.838658	3.033387	6.283637	3.821032	4.648544	4.415349	6.883914	3.488837	4.421729	4.206158	4.171213	4.113965	4.014061	3.446722	5.241438	3.506434	3.410970	3.788448	3.319115	3.960968	3.521682	3.126399	3.641091	3.840068	3.296065	3.425996	4.007805	3.290448	3.650983	5.574831	4.282734	3.023096	3.717333	3.817375	3.829744	3.679225	4.303316	4.044345	3.902945	3.951827	4.612491	2.992210	3.146768	3.496154	2.945091	3.458213	3.684027	3.901467	3.792471	3.796720	2.963837	4.184469	3.473243	3.025242	3.639080	3.621865	4.074067	3.998522	3.680251	3.676812	3.932805	3.563639	3.794881	3.771535	3.696741	4.286343	3.529405	4.191700	3.450995	5.141118	3.633128	3.879790	3.304598	3.825737	3.514792	4.160965	5.434567	3.456418	3.412583	3.752961	3.831572	4.120517	3.465757	3.610768	3.364521	3.389455	3.695280	3.710276	4.498788	3.838007	4.153415	3.471726	4.162728	4.793518	4.687586	3.690345	3.425458	3.858138	4.051084	3.576116	4.899185	3.688091	3.598578	3.810138	3.492579	3.126045	4.161815	4.201853	4.187745	3.804926	3.669702	4.347401	3.459846	3.929129	4.661156	3.195162	3.119999	3.404877	3.818855	4.085430	2.929359	5.052375	3.537427	3.950322	4.401099	5.913863	3.805753	4.607765	3.788048	4.131246	4.362190	4.020669	3.452949	5.396922	3.369613	3.410579	4.356325	3.558934	3.708643	3.596729	4.426792	3.866193	4.298299	3.456247	3.171433	4.308576	3.329538	4.091852	5.009514	4.442432	2.973996	4.012684	3.781236	3.723306	3.497463	4.096007	4.172494	4.300454	3.660415	4.188223	2.959373	2.900004	3.538645	2.867688	4.358969	4.501376	3.842751	3.765519	4.215317	2.931594	3.543818	3.147411	2.894419	3.189710	3.441340	5.033372	3.532537	3.326468	3.635265	3.744268	3.735228	3.832712	3.231565	3.776362	4.456478	3.546167	3.606610	3.479830	4.715794	3.180407	4.016060	4.455015	3.440673	4.302633	4.915972	5.226305	3.618187	3.322329	3.923504	3.654009	4.018708	5.193075	3.295444	3.555255	3.365646	3.794238	3.457527	4.885618	4.122071	4.778641	4.514824	5.066111	4.525013	4.736955	3.817590	4.362606	4.360707	4.032337	3.639080	4.303076	4.511823	4.301308

GABRD	7.692126	8.364546	8.196392	7.837050	8.857724	8.348833	8.329650	9.141010	9.031565	7.983196	8.290571	7.880066	7.988952	7.856355	7.234543	8.607876	8.695418	7.763140	8.981195	8.102344	8.279787	8.593623	9.701744	8.214857	8.278869	8.570802	8.284075	8.613906	8.556714	8.465685	8.804706	7.840605	8.232848	8.147768	7.892947	8.263850	8.022679	8.274471	8.481554	8.190710	8.457202	7.838143	7.785913	8.244777	7.642151	7.775891	9.108455	8.304094	8.355223	7.999017	7.796324	7.844523	7.765616	8.238691	7.685946	8.644694	7.610076	8.198382	8.203318	8.014946	8.291346	9.391424	8.885604	8.449532	8.245543	8.249440	8.410780	8.417243	8.250993	8.638018	8.259246	8.397333	8.460937	8.101146	8.161613	8.362379	8.370159	8.202269	8.281762	7.895579	8.042970	7.994104	7.695315	8.060586	8.085844	7.742874	8.141802	8.358778	8.475093	8.411859	8.568049	8.618776	8.019387	8.231014	7.904431	7.694279	7.866962	7.752279	8.688048	8.164423	8.253119	7.762998	7.494271	7.545191	8.588024	8.054935	8.839496	8.764379	8.958743	8.670340	8.878011	9.026010	8.631915	9.558026	7.868475	8.681590	8.146572	7.985621	8.285459	9.017015	8.756854	9.518843	8.532874	9.714385	8.586387	9.279672	8.438853	8.766546	8.447968	8.895467	8.359117	8.543841	8.623894	8.296996	8.214556	8.495845	7.767009	8.804154	7.941332	8.472487	8.545522	9.566504	9.392597	9.649021	9.385285	8.445017	8.873398	9.978283	9.081657	8.869598	8.159316	8.172842	8.333675	10.725203	8.019387	8.043711	8.849730	8.623284	8.740411	9.097199	8.676360	8.000883	9.062181	8.046717	8.014777	9.958780	9.404677	8.319558	8.596881	8.279940	8.459259	8.008078	8.512924	8.467956	8.413235	8.949039	9.406178	8.344300	8.925347	8.724655	8.904614	8.457938	9.070041	8.540524	9.404833	9.216696	8.908475	8.772484	9.002733	8.336333	8.619728	9.408958	8.527057	8.728680	8.337120	8.998577	9.279993	8.575811	8.155472	8.613766	8.805963	8.131490	8.483274	8.413693	8.967268	8.141326	8.424386	8.733026	8.466884	8.611857	9.182973	9.047293	9.297852	8.725272	8.645568	8.245699	8.629123	8.450307	8.882195	8.716580	8.094184	8.436936	8.145645	8.961134	8.440578	8.802888	8.941412	8.531373	9.417796	9.332910	9.228368	8.875412	10.462340	8.749240	8.119770	9.045030	8.465536	9.030848

IMPG2	7.113147	7.543733	7.596551	6.729487	6.769290	6.931597	6.951067	7.862606	7.760978	7.659976	6.985720	7.463738	6.708319	7.577914	6.753389	6.960356	7.166260	7.379556	8.570053	7.080341	7.945865	8.366666	8.787977	7.529175	7.589205	8.055590	7.285048	8.439638	7.582246	6.709258	8.026668	6.788763	6.697122	6.643191	7.005812	6.807739	6.987783	7.322799	7.451050	7.398027	7.180790	7.219180	6.235642	6.596178	6.314683	6.103589	6.135173	6.937799	7.039875	7.225900	6.657929	7.014364	6.950755	7.217178	7.333659	7.233701	6.855086	7.452118	7.433129	7.026662	7.568265	6.995829	7.359788	7.535018	6.648533	6.924849	7.317019	6.877000	6.858491	7.225755	6.708631	6.601017	7.317019	6.632390	6.745598	6.517896	6.713587	6.103246	6.713926	6.627907	6.931449	7.193586	6.697122	7.213117	7.047064	7.034830	5.786123	6.337033	7.191239	6.910016	6.530621	7.298304	6.415636	6.305094	6.167031	6.177466	6.869820	6.444034	7.364513	7.339873	6.910490	6.885519	7.153563	7.541891	8.924600	6.829253	8.719507	8.601569	7.964169	7.152928	7.731332	7.753321	7.847036	7.277363	7.613266	5.762505	7.165350	7.223728	7.378490	6.809369	7.851254	7.080977	7.165525	7.345653	7.235957	7.374236	6.491854	7.789934	7.263561	6.578148	7.140023	7.778050	7.591800	9.059116	6.507182	7.325012	7.413508	8.131490	6.109395	8.504326	8.017982	9.396057	7.351914	8.121159	7.617539	7.201627	8.177241	7.058080	7.370785	8.664680	6.006099	5.611055	7.456332	6.414041	6.469852	6.730784	6.615109	7.894029	6.822140	7.915502	6.835477	6.557446	7.472951	6.552849	6.296420	8.659760	6.941991	6.584073	7.063488	7.160752	7.184232	6.870984	7.574967	7.586901	7.049913	6.462173	6.955353	7.391797	7.067302	7.235957	7.369846	7.308348	7.141928	6.761400	7.041594	7.220840	6.554636	6.880224	6.825746	6.171172	6.319268	8.540210	6.362867	6.261499	6.288952	6.622109	6.721446	7.101115	6.582771	6.525135	6.915752	7.203798	6.458695	6.974953	6.389695	7.585179	6.947794	6.984610	6.967877	6.545978	7.405681	6.176600	6.825270	7.006116	6.848878	6.213743	6.610125	6.793969	6.766184	6.865962	6.707042	7.384238	6.969499	7.283220	8.009296	7.966199	7.910344	7.551132	8.925514	7.932481	7.679960	7.389802	8.863852	7.213726	6.970435	6.794137	7.552684	7.884296

BRF1	7.669450	7.250091	7.141928	7.892783	7.742537	8.173166	7.766091	7.418369	6.930002	8.167075	8.365170	7.935579	7.723476	8.218245	7.480750	7.525735	7.403034	7.499603	7.109818	7.455878	7.460716	8.058580	7.523112	7.504612	7.071814	7.707598	7.994571	7.930670	8.042486	7.671796	7.334732	7.334421	7.333384	7.979579	7.037474	7.257257	7.006585	6.846929	7.121945	6.908452	8.138957	6.941664	7.273736	6.958927	7.216398	7.130688	8.092915	7.430775	6.864078	7.339745	7.343817	7.298148	7.008826	7.314980	6.523325	7.969250	7.252922	7.263127	7.209572	7.508297	7.075288	7.719779	6.636280	6.984122	7.755464	6.854459	7.054425	6.941182	7.471877	7.624410	7.119635	7.258890	8.046247	7.092916	7.314980	7.113000	7.064759	7.035762	7.249782	6.947942	6.635475	6.804922	7.374539	6.933776	6.868239	7.212144	6.674131	6.617262	6.567898	6.830092	6.921443	6.550996	6.885991	7.095799	7.369988	7.532988	6.430386	7.514207	7.421202	7.034373	7.322640	6.895266	7.571280	7.468145	6.630936	8.141183	7.564103	7.236106	6.701120	8.268871	6.563515	7.219180	7.733324	8.179476	7.705798	8.026527	7.314980	7.459660	7.952630	8.663457	8.397632	7.587802	9.718616	8.663627	9.100841	8.601569	8.473429	8.260003	9.478136	9.595082	9.223120	9.059425	8.622673	8.678364	7.336449	9.177420	8.572726	8.592526	7.744116	8.136969	8.550127	8.062120	9.535499	8.178108	8.265248	7.903984	9.457098	8.387874	8.695418	8.154862	7.776496	8.441936	7.652974	8.260488	7.792640	7.423367	6.959245	8.276915	7.772016	9.102735	7.343018	7.586750	7.449040	7.535484	7.504760	9.097822	8.516533	7.682746	7.850793	7.941031	8.360324	7.848550	8.546267	8.403294	8.732552	8.509365	8.156255	8.927404	7.759138	7.933412	8.712662	8.427711	7.922875	8.247245	8.802316	7.723151	7.567799	7.578551	8.269199	7.430474	7.704236	9.186268	7.940418	6.996467	7.549747	7.976636	7.841996	7.778846	7.595944	7.794310	8.976665	7.888271	7.282413	7.779314	8.603763	7.697615	8.168296	7.157299	7.702525	7.819523	7.502427	8.045439	8.344769	7.767851	8.215479	7.759279	7.668348	7.986212	7.710499	9.122142	8.443781	8.360170	8.213459	7.694590	9.224522	8.304094	7.912200	6.972302	9.564507	6.922566	7.935134	6.990944	8.699691	8.636601	8.354587	8.006563	8.663321	9.461669

UBAP1	12.200157	12.645722	13.123245	13.180932	13.348898	13.562910	13.413573	14.141781	13.996707	13.753621	13.769045	13.476603	13.353546	13.189155	13.010122	13.733398	13.684527	13.077428	13.446470	13.925119	13.458952	13.737346	12.823209	12.986119	12.921327	13.080428	11.992764	13.386045	12.893290	12.810174	14.032009	13.420820	13.227058	12.077573	12.845274	13.004862	13.300495	12.802478	13.002332	13.325208	13.378218	12.998952	12.913792	12.918024	12.818889	12.224164	13.151252	12.731336	13.856222	13.361622	13.155970	13.479279	12.953485	13.031373	13.715852	13.582741	13.313356	13.090908	12.397227	12.787522	12.827695	13.274601	13.070221	13.093311	12.538424	13.437980	12.729636	12.830315	12.219214	12.496680	13.083488	12.813004	13.580864	13.291131	13.254006	13.145143	13.069482	13.385597	12.982246	13.634245	12.864593	13.192884	12.875731	13.580404	13.442646	13.276462	13.504360	13.892099	13.328825	13.057276	12.637159	13.499987	12.690183	13.445237	12.576866	13.277233	13.676687	13.782074	13.326618	13.491941	13.751290	13.040031	13.098440	13.639891	13.754158	13.605486	13.148247	13.584890	12.541618	13.572418	12.909649	13.106410	13.373784	13.490594	14.135457	12.240396	11.311032	13.417523	13.869642	11.357241	12.936910	11.691236	11.984838	12.024036	12.688122	12.682817	11.496673	12.712441	12.847005	12.921519	11.621139	12.104382	12.218206	11.972669	13.559725	12.412540	12.344853	11.623310	12.294356	12.901976	13.605486	12.107881	12.604915	12.700204	12.302320	13.284662	13.098061	11.971127	12.498162	12.367767	12.414309	13.332700	12.765859	12.718016	13.357821	13.125419	12.820920	12.893846	13.056733	11.579822	12.562670	11.910424	13.080043	13.238812	12.810548	13.341580	11.095424	13.242607	12.509969	12.779681	12.614208	12.832376	13.537270	12.554292	12.161273	11.700684	12.184989	11.642537	11.515156	13.190846	12.343108	12.470387	13.051845	11.704602	11.672529	12.375466	12.184989	12.293733	13.648940	12.364491	13.160712	12.829461	13.135934	12.269936	12.587157	12.761183	12.369370	12.946630	12.707907	11.887359	12.934551	12.615346	12.108933	13.551398	12.244222	12.971153	12.685861	12.721811	11.749834	11.725566	13.403996	11.667160	12.553785	12.801434	12.083542	12.452095	11.253814	11.895030	12.923129	13.314357	11.496066	12.619480	12.395231	12.006089	12.746061	13.405875	12.743020	12.720748	13.381340	12.024345	11.744351	10.558868	13.159717	13.079851	12.818398	12.282101	12.760647	12.399323

CRYAB	12.911640	15.097561	14.430444	15.344956	14.684603	14.763822	14.776581	15.621648	15.370867	15.354130	15.276640	14.974820	15.635602	15.266520	14.648021	14.765753	15.592691	15.443248	15.620739	15.733274	14.359381	16.383390	15.913059	14.433465	15.245371	15.593684	14.033170	14.935700	14.596200	13.900711	15.184749	14.242398	14.956666	13.747078	15.103652	14.905079	14.484873	15.179965	16.353525	15.430200	14.954990	15.845299	15.569300	14.553109	15.410836	15.959440	14.817306	15.943548	15.196878	15.868600	13.966887	15.590850	15.090125	15.704556	14.036962	15.059742	15.170771	15.878115	15.779491	15.194140	15.360997	14.442351	15.656594	15.198897	14.810589	15.969476	15.590850	14.795501	14.782854	15.290231	14.668191	14.935138	14.754065	15.250206	14.990123	14.443066	14.915189	15.573696	14.173545	15.553263	16.095846	15.030442	14.526691	14.232213	15.221141	15.497254	15.449581	15.820930	14.685534	15.088816	15.373253	15.260285	15.021020	15.296733	12.996741	14.479847	15.072183	15.285115	14.931911	15.612324	15.223282	15.000238	14.850021	14.973725	15.407655	15.350177	15.368664	16.095846	15.300250	14.784337	14.938834	15.764986	15.013342	14.438602	14.814746	13.769541	13.601504	15.682114	15.320436	12.804949	11.957851	13.029714	13.490594	13.545312	15.060407	13.807016	12.280508	12.792190	13.125598	13.068294	12.244073	13.190080	12.215941	12.025558	13.617295	12.332547	12.555759	12.552012	11.385992	15.120910	13.311915	14.609256	12.777077	14.189906	11.205541	15.027552	15.182677	12.900238	14.016128	12.956811	11.620681	12.060534	13.575781	13.098440	11.356245	13.889403	12.446254	14.317829	13.692412	12.908228	13.516235	11.908049	13.869642	15.716847	14.904566	13.435563	12.933838	12.196008	12.989794	13.081778	12.474915	11.798061	12.441117	15.631882	14.463188	12.440954	13.108876	13.927588	13.276462	14.009307	13.888066	13.798010	14.684603	12.271724	12.162362	12.741358	14.198163	14.433099	12.172186	13.647299	12.331121	14.583563	12.397227	14.320921	13.520479	12.650373	12.089667	13.945117	13.023845	12.628172	13.890768	12.131138	14.854342	13.161096	12.267902	12.285047	14.716286	14.391136	14.511123	12.078357	13.323580	12.413004	11.818076	12.535268	12.232993	12.438343	14.079189	12.722972	12.679396	11.577336	12.021368	14.041713	12.223101	14.460851	12.963098	12.791666	12.932755	14.234874	13.585102	13.189888	12.474271	11.342184	14.625993	12.267304	13.515121	13.762421	12.351035	12.208079

RHPN2	13.729117	12.608022	11.738510	13.082921	10.622189	14.497164	14.230892	14.565137	12.464370	14.855412	14.241749	14.761971	12.034708	12.332876	11.794233	12.924711	12.007014	11.135510	12.334126	13.635940	11.727947	11.394279	9.258777	11.043290	12.052666	12.746742	9.888161	11.681708	12.543078	12.749999	12.751039	13.217360	11.536600	11.177918	11.484410	13.490363	13.088141	11.878918	12.218928	12.277123	13.671017	12.525777	11.915044	12.054832	11.298691	11.730804	11.606238	12.333343	11.743759	12.150502	12.300959	11.477417	11.499211	11.988293	12.865155	14.689299	11.899423	11.442933	12.188253	11.642537	12.314450	12.158494	10.470736	12.089510	12.384801	12.721457	11.957237	11.806485	11.414710	12.397858	12.309839	12.138794	12.571510	12.880604	11.777298	11.319427	11.437102	11.399961	12.548924	12.783431	11.931223	11.812582	13.254976	12.164845	12.860065	12.990154	11.841449	11.186851	11.617144	12.503389	12.933669	12.377553	11.866983	13.213586	12.816808	10.354764	12.170015	10.802991	11.305148	11.717044	12.819748	12.093060	10.491133	11.988946	13.110780	12.643905	12.773792	11.939377	11.678161	13.717944	11.297951	12.070541	12.105743	12.985009	12.751206	13.528984	14.056836	14.147028	12.093060	12.467154	12.967688	12.176950	10.918390	11.008289	13.263369	13.681376	10.670295	11.171084	11.616996	13.574884	11.798213	11.662957	12.427314	11.680518	10.711127	10.416142	10.832876	12.672938	10.746326	11.243882	11.834043	9.762460	12.830643	12.514335	10.914464	12.243771	11.417762	10.845782	11.519647	11.036263	11.781211	11.126352	13.409664	11.783740	12.940635	13.551649	11.568715	11.321595	11.567573	11.706874	12.188558	12.236220	11.597121	11.827113	12.313176	11.664015	12.091525	12.290609	11.194165	11.529216	11.714384	11.471087	10.697783	10.986432	10.494077	11.932304	12.786304	11.448837	12.565127	12.897590	11.136974	11.399407	11.988788	11.820064	11.852324	11.434802	12.433552	11.972983	12.645205	12.180008	12.056973	12.901113	11.859051	10.173056	12.053618	11.230487	11.388955	12.446254	10.951170	10.971908	10.947592	12.860559	10.947735	12.799536	11.608459	11.761931	11.619186	11.636032	11.358243	12.617456	13.148247	11.710136	11.834209	13.141902	10.691815	11.511854	10.474206	10.659325	11.468687	10.845782	10.904224	10.927116	10.413149	11.386148	12.116671	12.332547	11.388081	11.932902	13.562910	11.534084	12.471814	12.857615	12.945351	11.903720	12.479486	12.137398	11.423001	12.467649

SIPA1L1	11.974061	12.500587	12.813176	12.991828	13.142104	13.083113	13.132646	13.209096	12.709644	13.276661	13.238812	13.106512	13.238255	12.880782	12.592493	13.019903	13.140068	12.293428	12.938700	13.190080	12.449603	13.552300	12.532655	12.662914	12.078200	12.968759	11.965964	12.632803	12.443548	12.507056	13.180932	13.344888	13.324800	12.589286	13.208899	13.272175	13.239009	13.019185	12.790316	12.619480	12.893120	12.438849	13.455950	13.083300	12.818568	13.377791	12.412373	12.639718	13.309538	13.551187	13.244726	13.632300	13.579215	13.627746	13.154208	12.683325	13.595345	12.941188	12.966913	12.733221	12.978769	12.470874	12.684724	12.816972	12.652080	12.805285	12.840359	12.918772	12.981688	12.871760	13.490156	13.376076	12.633633	13.230081	13.563867	13.110603	13.267397	13.383910	13.343654	12.563972	12.600182	12.763759	13.293157	13.601738	13.341990	13.298825	13.390989	13.493953	12.962524	13.171526	12.774141	13.066780	13.105804	13.391427	12.963999	13.436852	13.173253	13.289866	13.320270	12.889486	13.166910	12.835615	12.917482	13.326420	13.232053	13.122690	12.730652	13.017023	12.750174	12.978391	13.278020	13.512631	12.825950	12.760816	13.251828	12.375982	12.131586	13.157657	12.701221	12.622342	12.665284	12.469565	11.960692	12.244533	12.804049	12.532655	11.999153	13.370832	12.711326	12.563325	12.422459	12.442202	12.021923	12.247673	12.596273	12.947738	11.706091	12.262009	13.926227	12.456916	12.723671	12.889156	11.874773	11.828974	13.091272	13.068294	12.506387	11.835574	13.126344	13.158777	13.346151	13.393903	13.579444	13.311157	13.072631	13.132988	12.372577	13.343862	12.401619	13.064342	12.649718	13.019003	13.928944	13.701651	13.381550	12.524779	12.937441	12.895284	13.753345	13.761156	13.583683	13.320065	13.449721	13.273550	12.232513	13.341361	12.858133	13.185249	12.381631	12.758240	13.205816	13.250452	12.485326	12.744741	12.574744	12.586817	12.977868	12.619661	13.088879	12.722462	13.662957	11.926777	13.180932	13.194518	13.329436	13.603593	13.126165	13.184288	12.254526	12.626839	14.255462	13.259393	13.081212	14.038211	12.599503	13.481484	13.845633	12.721953	12.944807	12.455483	13.112784	12.607398	13.232837	13.104645	12.581161	13.363482	12.279139	12.799850	12.911451	13.362235	12.590461	12.298697	12.845079	13.764196	11.943044	12.432090	12.765130	12.922772	12.075438	12.900773	12.988891	12.676682	12.335244	12.584810	13.067539	13.020993	12.485005	12.397685

DCP1A	11.372450	11.371738	11.970649	11.961890	11.613018	11.056896	11.870995	11.509940	11.070018	11.332335	11.781653	11.791350	11.325406	11.605808	11.308373	11.256480	11.395495	11.324962	11.803233	11.406982	11.534520	12.465654	11.664771	11.623726	11.148401	11.509789	10.830911	11.294105	11.329984	11.725426	11.403089	11.868313	12.051190	11.435808	11.706718	11.608459	11.758591	11.600013	11.812299	11.502476	11.368582	11.929392	11.614201	11.885548	12.096818	11.709994	11.018683	11.376371	11.974649	11.983360	11.516068	11.869049	12.051330	12.109351	11.984094	11.363720	12.080159	12.127196	11.692447	11.718971	11.914449	11.407065	12.162952	11.654682	11.963547	12.302631	11.130181	11.426951	11.376076	11.046663	11.713779	11.441892	11.186992	11.654988	11.600906	11.824884	11.968244	11.713634	11.533937	12.417362	11.438372	12.178617	11.872954	11.996542	11.750421	12.075438	12.311242	12.513691	12.113600	11.919222	11.770782	12.252783	11.190467	11.834369	11.739525	11.629546	11.884935	11.377693	11.498297	11.175685	11.353604	11.798796	11.540408	12.081360	12.179253	10.979653	11.122188	12.111901	11.635875	11.222342	12.244533	11.050779	12.050737	10.798060	11.645189	10.681103	11.701015	11.472792	10.822815	11.181427	11.022278	10.942871	11.184951	10.360069	11.066017	10.314129	10.786473	10.402738	12.159708	10.528668	10.782671	11.100303	10.904946	10.634437	10.257870	11.163045	10.558709	11.434639	11.202548	11.599023	11.967030	11.920531	10.702431	11.362257	11.821859	11.296591	10.680359	9.074813	11.136974	10.962961	10.864236	11.328499	11.415733	11.038735	11.483981	11.276728	11.513924	10.855398	11.168109	10.172148	11.137575	11.751278	11.593733	11.458769	11.819746	9.630301	11.165378	10.997972	11.192374	11.523534	11.724236	12.057480	11.298239	11.420870	10.731036	12.238197	11.941497	11.739362	11.468687	12.180168	11.352876	11.498458	11.521131	11.259294	11.916831	11.305148	10.939911	11.367577	11.540976	11.399821	11.522184	9.475527	11.227693	11.306661	11.380212	10.906146	11.404685	11.543787	11.466898	10.814013	11.724086	11.274000	11.567413	12.016883	10.603870	11.271480	11.807364	11.651679	11.167966	11.605506	11.532142	10.857955	11.390710	11.178222	11.309548	11.075007	10.025602	10.986127	11.385844	10.849717	11.182859	10.383188	11.729913	12.503202	9.967099	11.358100	12.295118	11.536751	9.739786	11.946966	10.766855	11.929674	10.166595	11.074134	11.383763	11.993793	11.827669	10.958253

TTLL11	6.553341	6.852010	7.145800	6.839180	6.395639	6.567597	7.119015	6.149155	5.642738	7.161420	6.541025	6.289289	6.358855	6.758456	6.852010	6.591465	6.535312	6.521385	6.590824	6.936578	7.138283	6.951402	8.274332	6.227804	5.957159	6.415636	6.510415	6.335357	6.746121	6.976396	5.863994	7.238454	7.128781	6.208993	6.966278	6.829253	6.677849	6.913240	6.845217	6.512753	7.014664	6.849180	6.404233	6.534701	6.910328	5.932102	6.565762	6.744976	7.142409	6.923374	6.602156	7.155766	7.295217	7.180790	6.666704	7.417717	7.611602	7.430775	6.876248	7.015163	7.228105	6.969819	8.207850	7.082213	7.347389	7.303825	7.134995	6.829747	6.690296	6.776202	6.554786	6.507640	6.841368	6.440058	6.527992	6.526001	6.944724	6.647062	6.563011	6.975270	7.062528	7.529471	6.853155	7.240875	6.964524	7.145206	5.776559	6.600722	7.112374	7.050231	6.713250	6.812241	6.666704	7.214504	6.211030	6.432261	7.202536	5.986093	6.639659	7.039382	6.717810	7.146614	6.593887	6.837058	7.399130	6.852172	7.449679	7.335968	6.925341	6.815250	7.633165	6.186409	6.551642	6.804922	7.037328	5.865057	5.954174	6.881346	6.614323	7.237496	8.705197	6.728831	6.405228	6.330441	6.567898	7.747337	4.700271	5.626160	5.815271	7.551288	6.320848	6.178333	7.492437	7.548388	6.197582	6.748786	6.390861	5.361935	6.513088	7.328515	7.635247	7.442676	6.352940	6.549547	7.462298	6.931523	5.318802	5.556755	7.225295	5.537433	6.941182	6.792991	6.892054	6.004467	6.894955	7.425236	6.701280	8.985018	5.419596	6.428172	6.265543	5.954888	6.252335	6.603879	4.904018	6.588358	6.097354	6.588530	7.848996	7.400837	6.360376	7.974469	10.134213	7.250407	6.646729	6.019899	7.261516	7.326266	6.865185	8.276915	7.008983	9.803956	10.829909	7.234209	6.596935	8.669426	6.845392	6.537120	7.255390	6.505827	6.803329	7.458243	6.689001	5.789735	7.856943	7.740704	7.451641	6.544021	8.165963	7.422282	5.843870	8.627776	6.377288	9.790059	5.807238	7.743330	6.698868	7.449365	7.945709	5.108299	9.009028	6.137868	9.844563	6.931281	6.111319	5.586972	5.396732	9.070196	6.821498	9.442516	5.942682	5.740064	6.242601	8.165188	6.274562	6.017610	6.792669	6.342339	7.061243	6.661502	7.339579	5.014252	6.807739	6.962293	7.185875	6.294553	6.004284	10.036419

MCTS1	9.468725	8.467193	9.160000	9.133821	9.970361	8.910543	8.924171	8.510749	9.325501	8.993942	9.047586	9.006263	9.757639	9.328299	8.803665	9.552071	7.882174	9.369995	9.501326	9.789292	8.827175	8.863852	9.229858	9.952895	9.470176	9.673663	9.918999	9.022798	9.273149	8.749240	9.433117	8.829025	9.099806	9.450135	9.479239	9.437249	9.272995	9.151110	8.711169	9.654038	9.199378	9.491096	9.741203	9.468105	9.789600	9.760895	8.875412	9.049757	8.711618	9.108455	9.368751	9.303995	9.212976	9.145795	8.815760	8.617692	9.749429	9.712641	9.572138	9.544271	9.304272	9.223414	8.799766	8.826002	9.275769	9.219434	8.668648	8.737644	8.825863	8.754570	8.834942	9.313949	9.343298	9.211300	9.251027	9.135773	8.733026	9.034002	9.092833	9.619777	9.408019	9.111682	9.520679	9.276953	9.308341	9.383185	9.712939	9.589818	9.315654	9.264430	9.778003	9.295377	9.255128	9.288791	9.526630	10.138805	8.746649	10.102182	9.100841	9.286747	9.386668	9.384364	9.435081	9.256976	8.533318	8.577737	9.796717	9.277850	9.494236	9.085673	8.763010	10.044933	9.551167	9.265944	8.080215	8.176634	10.365612	9.184855	9.330000	8.830569	8.734549	8.636601	9.097671	8.791520	9.709371	8.016172	10.200663	10.254211	9.059116	8.744816	9.239370	8.967435	8.303938	8.115470	9.813541	9.182973	9.800580	10.809503	10.172908	8.897794	9.316241	8.430887	9.301255	8.258005	9.303246	8.002620	8.529985	9.324612	8.285931	9.238911	9.470011	10.898956	9.276233	9.328459	9.217179	9.400155	9.130781	9.059743	9.770314	9.456794	9.226526	9.296610	9.734237	9.377318	9.015766	7.809896	9.449194	9.169538	9.270309	9.018966	9.642396	8.915725	8.411404	9.176949	8.791835	9.696173	9.187379	9.230297	9.609476	8.347493	9.229567	8.867772	8.584972	10.079182	9.104114	9.023413	8.511237	8.803515	8.642515	9.031888	9.581758	8.620186	8.862460	9.461202	8.825236	8.731353	9.055012	9.193324	9.996027	9.810561	9.616680	9.686981	10.280806	9.239986	9.547322	9.688849	10.062277	10.013557	9.475061	10.852098	8.435395	8.578321	8.775212	9.610124	9.486110	9.184707	10.339163	9.068319	8.766072	8.136969	9.603904	10.054345	8.911735	9.609659	9.202010	9.603754	10.008153	9.607630	7.976173	8.581371	9.093884	9.493310	8.760599	8.207850	7.329932	9.999817	8.376324	8.269526

CLEC12B	7.337058	10.444263	9.672692	10.926976	9.092540	10.636826	10.509324	13.140875	10.011809	11.283846	10.478137	10.872782	9.345116	10.266747	9.946536	10.006010	9.857253	9.804238	11.516358	10.711967	10.713575	9.907741	7.996465	9.240288	10.244125	9.868372	7.891070	9.184546	8.532874	8.751471	10.593479	9.396668	9.906054	8.486586	9.418110	11.156096	10.575699	10.371390	11.107920	10.798335	9.629836	10.762044	10.369246	11.059685	10.155465	10.860126	8.302435	11.420720	9.452298	11.682169	9.758720	10.757808	10.497091	10.692703	11.383209	10.703174	10.836326	9.755615	10.415417	10.004105	10.555685	8.396396	8.138502	10.726741	11.082627	10.950728	9.884994	10.690375	9.918999	10.766421	9.764810	9.728699	8.757154	10.285451	9.451258	9.301410	9.201691	9.028112	9.696173	11.262567	9.757807	10.219240	10.383504	10.657248	11.009768	11.092058	10.127429	9.452602	9.957369	11.358554	11.462325	10.749317	9.716701	10.667694	8.218549	8.005145	10.473309	9.271660	9.354991	9.625854	11.340239	10.536625	8.778111	10.613186	12.302809	8.557010	11.791206	11.179915	10.328691	9.904700	10.032710	11.226653	10.700036	9.077935	9.922286	9.893168	8.109085	10.602394	8.300756	9.140257	10.952015	8.537536	9.214667	9.587330	9.612257	10.145201	8.084126	9.034328	8.848183	9.868052	8.023315	9.025237	8.261557	8.207388	8.410935	8.851981	7.384537	7.882461	8.138337	8.675783	8.429897	8.220237	7.960064	8.368619	8.572585	9.128602	8.524156	8.754431	8.685533	8.262784	9.513820	10.428979	10.062587	7.935134	10.170585	11.048256	8.739022	9.682776	9.461985	8.498687	10.105679	8.367126	9.443123	10.505842	11.256930	8.615851	8.264787	8.300756	10.060006	9.381628	7.917069	9.614087	9.212057	9.179371	8.174901	7.412291	7.676983	9.268311	8.570349	10.599871	8.273893	8.553322	9.721686	9.187084	8.194802	8.861204	11.229765	9.994790	9.229567	9.648718	9.316571	9.424825	9.729505	8.304234	9.821458	9.498268	8.670782	9.936797	8.306800	8.985776	8.721185	11.370366	8.659160	8.055096	8.826140	10.171699	8.014014	7.937457	9.801811	8.077276	9.204729	9.569977	8.483274	10.217581	8.103552	8.124953	7.778846	8.874814	8.888374	7.649648	7.938524	7.887508	8.525523	7.542183	8.246033	9.863624	8.868843	9.483198	10.122304	8.859422	9.696633	10.567716	9.340740	7.812644	9.965154	10.442006	9.094042	9.411829

DAO	10.581000	11.283268	10.966969	11.502905	11.185699	11.235828	10.528799	11.833609	11.045017	10.887784	10.352202	10.173198	10.009427	10.344173	9.334765	11.471379	11.183767	9.732123	11.844639	11.650646	11.614346	11.193097	10.756929	10.944165	10.270720	10.504977	9.908491	10.803584	11.410105	11.162307	10.229267	10.216258	9.881630	9.713721	10.632025	10.547330	10.718205	11.292805	11.549582	10.812104	11.557873	10.681815	10.861160	11.217736	10.312584	10.857955	10.917626	10.738376	10.435536	11.225487	10.116364	10.196135	9.659506	9.871673	9.440040	11.937329	8.968673	10.049254	10.122161	9.260598	9.614684	11.163772	9.658106	10.775932	9.914982	10.221812	11.228875	10.946082	10.656544	10.949499	11.731685	11.839944	11.357821	11.259576	11.049296	10.665768	11.171084	10.970001	11.327340	9.585307	9.789292	9.406178	9.289113	10.016493	9.989367	9.886402	9.611185	9.983355	10.044031	10.261790	9.629238	10.117312	10.032116	10.663131	9.630372	9.619941	9.857107	9.411694	10.061641	10.747707	10.133901	10.959032	10.601358	10.440817	10.551588	10.666208	10.707919	10.858794	11.074134	11.778966	11.178375	10.713869	10.272441	11.742298	10.837385	11.189746	10.083270	10.593329	10.892989	9.656714	11.415128	10.464310	10.668143	11.441315	10.724919	10.378655	9.834586	10.316793	10.652947	10.432465	9.769830	10.187555	9.377152	9.960604	10.591268	10.470736	9.360799	10.543736	10.306994	10.589210	10.240599	10.409222	11.022755	10.052635	10.116982	11.082337	10.545824	11.182579	11.286261	9.593539	9.744273	9.496589	10.421948	9.982125	10.398387	10.451937	11.116659	11.485014	10.149948	11.294412	10.465656	9.243051	11.064957	10.435833	11.054941	11.495915	9.315818	9.987620	10.975673	10.183759	9.549766	9.194486	9.684031	8.806276	11.170618	8.251773	9.298766	9.714868	8.850472	9.317754	10.702740	10.114821	10.071631	9.851053	9.424072	10.601523	10.658726	10.033753	9.613488	10.271475	10.721646	11.560243	10.403059	9.823607	10.475718	10.781020	10.595584	10.928007	9.000566	9.403086	9.134241	9.237341	9.016386	9.390506	9.611030	9.794721	8.910839	9.461531	9.651500	8.862612	9.688696	9.705173	10.501693	10.388663	9.818426	9.701888	9.212746	9.928156	9.698789	9.739008	9.887037	9.685152	9.515000	9.431578	10.645044	10.243523	10.166113	10.548890	11.835426	10.223028	10.284261	10.460269	11.579521	10.378348	10.428662	9.298930	10.481462	11.413991

SPX	10.706734	9.314274	8.040118	7.605625	4.769928	5.077136	8.291041	4.064780	4.803039	6.763922	5.556569	8.847714	6.475585	5.949174	7.867093	7.896420	7.806014	6.638207	8.901252	6.943118	7.123062	5.828960	10.169039	7.429530	7.738827	7.722840	10.012605	9.867560	11.090456	8.736423	6.607883	8.785314	8.806427	8.866731	9.700644	10.968317	9.417325	9.887513	9.081958	8.521866	5.996198	11.311749	8.848031	10.177187	10.600013	10.179032	6.931281	11.656735	11.070472	9.841670	11.424053	9.185481	8.313905	7.984691	8.509809	5.654829	8.293663	8.498063	9.350045	8.755638	10.184676	7.990014	11.069879	10.117147	9.060792	9.835686	10.947159	8.517795	8.484598	10.949348	11.348194	9.669110	6.304098	10.404103	9.668144	8.158230	8.657423	9.821458	11.523246	7.589979	6.819891	9.029036	9.069131	8.499634	7.953706	11.765559	7.261822	7.815434	8.693399	8.882494	10.751903	8.805216	8.913127	8.102799	11.002938	7.474146	10.325054	6.710714	7.434223	7.767780	7.057930	10.495855	8.801264	7.168714	8.230545	7.182036	5.738433	7.199452	8.382051	6.214073	8.203908	6.350277	8.070476	6.686952	5.636210	3.508342	9.902118	7.596408	7.136879	8.818720	8.505802	10.238701	7.004886	5.642352	5.800809	6.484961	7.232778	9.062320	5.151351	7.664378	6.335850	4.982563	4.271585	5.394781	7.790524	10.497395	5.879233	6.538118	7.033591	7.253406	8.233142	7.364203	6.329228	8.011423	9.664360	9.615528	6.248802	6.965004	8.026217	8.036022	8.655072	10.182867	8.812340	9.032649	10.338538	11.394433	10.973659	8.716906	11.174893	6.459196	9.552979	8.821432	9.958780	10.352969	8.611536	5.347947	10.759975	8.627776	11.584062	10.440659	8.677119	8.383274	8.485531	9.833356	6.760278	8.245874	8.939279	6.275725	9.068014	10.266163	7.532988	8.725420	9.135618	10.509773	10.335836	9.781629	10.553981	10.073768	10.659769	10.583475	10.957229	3.851619	12.052666	11.416927	9.083496	11.081305	11.308236	11.023655	7.750125	8.067272	6.439064	8.665154	9.114516	9.563002	10.054641	9.425872	8.846176	9.022477	8.916489	7.696834	9.099191	10.343850	10.506461	9.413335	9.785948	10.224681	8.446548	8.816052	7.998868	8.791046	6.862821	8.534684	10.262380	8.558851	4.876299	7.054425	6.432425	6.800111	4.802623	8.864465	8.737947	5.570880	4.344368	5.722134	7.368285	9.591343	9.071601	6.137019

RASA2	10.451655	10.071163	11.220719	10.881198	11.131351	10.747238	10.995821	11.179610	11.940741	11.300099	11.332473	11.054506	11.616843	10.898647	10.366087	11.248808	10.722674	10.580846	10.096699	11.090033	9.321556	9.869632	8.397774	11.412637	10.728085	10.728828	9.412745	11.797931	10.476319	10.831803	11.452083	10.582604	10.535918	10.917765	10.928598	10.924005	11.014118	10.887915	10.005847	10.877350	10.811801	10.234963	11.080931	10.960321	10.662673	10.735940	10.108228	10.754933	11.125774	10.897742	10.671967	10.776525	11.379904	11.410252	11.187573	10.682874	11.330738	10.863064	10.602549	10.566197	10.486716	10.716117	9.225443	10.196293	9.943490	10.936835	9.816798	10.235279	10.040378	9.763085	10.802540	10.590679	11.089903	11.145141	10.640900	11.150814	11.017653	10.939769	10.739148	11.284747	10.814320	11.039457	11.241501	11.254400	11.223249	11.298525	11.353604	11.413079	10.118388	10.624726	10.272099	10.020947	10.141648	10.925790	10.678967	10.867509	11.225487	11.505243	11.051636	11.229765	11.445862	11.191935	11.088131	11.432540	10.009271	10.766711	9.881777	10.062587	8.812028	11.251075	9.389757	10.715070	10.607408	11.028968	11.061419	9.416410	9.619319	10.932033	11.093843	10.763720	9.994619	10.451232	10.114821	9.249936	9.553589	10.118219	10.871143	10.296641	10.159534	10.188009	9.611660	9.988100	10.553981	8.684172	10.499145	11.006550	9.976520	9.113447	10.436142	9.111930	9.225910	8.520528	10.025914	9.947176	10.427919	9.854621	10.686158	9.394342	10.395361	11.081746	10.538660	8.590977	10.897742	10.791003	10.936997	10.907071	10.138805	10.053909	10.416142	9.901303	9.499648	10.627462	11.312627	10.867997	10.546267	10.565474	10.272099	11.255007	10.023218	10.720925	10.726410	10.927116	10.986908	10.641355	9.766782	10.612140	10.960626	9.987919	10.762958	10.975823	9.448726	9.983209	9.514568	9.463307	10.172770	10.640759	9.832460	9.156011	9.616997	10.457869	10.244721	9.260903	10.239706	10.845325	10.445678	10.479347	10.321391	10.357679	10.932636	10.501072	11.267954	11.077366	10.714458	11.040159	9.658419	10.977016	10.412690	9.693309	9.376353	10.080574	9.344831	9.148637	9.421008	10.730021	10.079015	10.585064	11.066875	10.347657	11.300691	9.689001	10.973802	10.431097	10.679116	9.971065	10.273342	9.661074	9.745841	9.415203	10.169963	9.117878	10.654274	10.520787	10.079348	10.429431	10.670580	10.075275	11.095577	9.645339

B3GNT10	8.589300	6.841294	6.816039	7.459505	7.517578	8.028718	7.959136	6.770091	6.307918	6.711646	6.989679	7.338924	7.207529	7.535484	6.585901	7.499142	7.637692	7.882760	6.263525	7.553935	6.133618	6.447013	6.640971	6.636745	6.592806	7.282114	7.865390	7.177313	6.780059	7.573412	6.602156	8.769448	7.202847	7.834020	8.108499	8.448740	8.304234	7.447439	7.030651	6.316408	7.126107	7.021874	7.920886	8.125564	8.121626	7.468757	6.515220	7.311187	8.006846	8.412310	8.461246	8.701974	8.193679	8.606818	8.144547	7.272190	8.969270	6.796847	7.060125	8.507231	7.832314	6.746121	7.014228	6.344566	6.600558	6.732532	6.955198	7.158679	8.184797	6.074503	8.135851	8.254367	6.433618	8.318352	8.155934	8.470727	8.239177	8.200920	8.095669	8.376324	7.449040	7.776998	8.160386	8.771708	8.477408	8.670032	8.860321	8.724655	7.067588	7.080201	7.464963	6.829419	7.542035	8.276122	8.390213	8.275673	7.262146	7.377032	7.670062	7.176647	6.609828	7.483531	6.639977	7.740862	6.415636	7.063971	6.028736	6.229383	4.938279	6.849324	4.899838	5.650297	7.015827	6.329228	7.322163	6.532738	7.317019	7.469405	7.224972	8.172842	7.611728	6.910160	6.620820	5.917588	8.565110	7.442212	6.772519	8.731353	7.326749	7.433753	7.694121	7.454918	7.825691	7.090101	8.751471	8.802155	8.718419	6.435126	7.735797	6.790236	6.704456	7.828982	6.850460	8.570053	10.054641	7.100637	9.181391	6.748634	8.692789	9.140257	6.799452	5.291967	7.429837	8.861063	9.640419	8.688665	7.940255	8.094770	5.808321	6.916498	7.710165	9.262911	8.649359	8.957620	8.107120	7.915975	7.025077	9.573039	9.149117	8.421803	8.374334	10.036268	8.955174	8.607116	8.166131	8.797307	7.500852	7.881864	8.506906	9.230812	7.652521	9.307273	7.827616	6.176949	6.016047	7.179077	7.018752	9.549440	6.076077	9.289425	8.600639	7.661085	8.862000	8.878485	8.474628	8.069057	9.671337	8.219476	9.032809	8.513701	10.071631	7.559214	8.704594	9.067393	9.016215	8.733640	9.447310	7.611728	6.995675	7.794009	8.314845	8.359725	9.439086	7.676500	7.220840	7.720538	7.735934	8.670956	8.705039	6.512094	7.223266	6.392190	7.267114	7.537475	6.893689	5.774720	6.031469	6.269643	8.763320	4.638845	6.265036	4.562514	6.956429	8.651036	7.099873	8.011566	8.082255	8.924876

FXYD4	10.891480	5.424652	5.632279	6.598614	6.734479	7.020298	6.273200	6.680462	7.066988	6.667041	6.864533	7.421974	6.462351	6.673327	5.733207	7.259651	6.069272	5.705784	6.328905	6.732375	5.398817	5.925785	6.418647	6.911932	6.354105	6.532738	6.430041	7.422282	7.871375	7.451786	6.616593	7.326588	5.117203	6.553341	5.569226	5.518396	5.600604	5.339523	5.615559	5.595686	6.952973	5.896721	5.982617	7.397259	5.240664	6.034026	7.547429	7.082071	7.584419	6.000261	9.894559	5.973440	5.400520	5.696389	5.773122	7.153563	5.023196	5.968070	6.011218	5.799127	5.422062	6.534841	5.022770	6.538118	5.814591	5.809630	7.501152	5.678811	5.870227	7.876859	5.896721	5.893148	7.101416	5.936504	5.820610	5.442356	5.784124	6.117316	6.853977	5.827305	7.494117	6.120281	8.851683	5.963776	5.213361	7.335968	5.813892	6.098026	5.733728	7.171812	6.571789	6.631395	5.724200	6.103246	8.020574	5.922358	5.877440	6.263188	7.048804	6.078138	7.160938	5.785597	5.920857	5.798398	6.235988	6.336194	7.272830	6.029738	6.638539	6.856249	6.014345	6.425798	6.873531	8.731515	6.771256	9.236583	12.342178	6.541874	6.912442	7.532852	6.221062	5.843704	5.918331	8.081052	6.987946	7.589490	6.613481	7.073540	8.233142	7.486025	7.723151	7.341300	6.353603	6.535162	6.303573	5.331054	6.352940	6.512753	6.635006	6.316233	6.285141	6.482715	7.362762	9.356515	6.046399	6.556627	7.409279	7.804638	6.810338	6.243095	6.369034	5.782921	7.066825	6.746568	5.558298	5.245997	5.504312	6.035624	5.589095	8.306800	5.812591	7.070053	6.309927	7.175574	6.648884	8.295020	5.542439	8.450004	6.240087	6.454936	5.824601	5.703574	5.168494	5.530879	6.307584	5.195497	6.784522	5.434567	7.585638	5.959347	7.134672	6.020084	6.666216	5.987005	5.667791	6.689163	5.668508	6.237625	6.488864	6.114404	6.125675	8.802316	5.882107	5.690061	5.344374	6.250316	6.320511	6.225189	5.006417	6.244951	6.858345	5.583614	5.966803	5.597810	7.329623	7.395399	6.442016	4.620143	5.746680	9.436024	6.593414	6.036519	7.070219	6.676101	7.151068	6.759442	6.910160	6.717651	7.310410	7.142250	6.414193	6.128168	5.649360	6.296759	7.144913	7.204562	6.404392	6.212025	7.940740	6.728532	6.472574	7.035147	8.296702	8.239474	8.456897	6.170998	7.735797	7.032489

FANCL	12.609521	12.457238	12.539646	12.731164	12.969135	12.941572	12.790143	13.275857	13.272778	12.820259	12.975830	12.734213	12.761009	13.058018	13.111982	13.048184	13.259620	12.860559	13.003936	12.689676	12.505403	13.056909	13.142299	12.460261	12.255202	12.385139	12.879575	12.165444	12.715895	12.835615	13.438408	13.394330	12.776004	13.302672	12.859356	12.890172	12.957704	12.884558	12.699345	12.860559	12.847527	12.756348	12.820259	12.956055	12.641390	12.692342	12.862948	12.072497	12.977228	12.547624	12.726341	12.934012	13.072832	12.708762	12.913386	12.716597	12.992703	12.462402	12.526786	12.638546	12.820920	12.851402	12.635273	12.810548	12.740063	12.448174	12.676523	13.020295	12.387604	12.827872	12.194140	12.840723	12.691855	13.180561	12.830315	13.029529	13.226454	12.762553	12.745569	12.713819	13.102943	12.641230	13.082347	13.234224	13.041374	12.990498	12.927379	13.010122	13.139689	12.800391	12.537086	13.145720	12.771018	13.252643	13.038207	12.957891	13.237448	13.082732	13.213390	12.486004	13.389482	12.617631	12.690027	12.666334	13.928944	13.099767	12.455317	13.373144	13.417326	12.835806	13.513783	13.012474	12.718866	12.904297	12.944614	12.416103	12.386589	13.399764	12.974383	13.377337	12.631637	13.455025	12.445794	13.193275	13.315805	13.524608	13.639658	13.913440	13.603098	12.922049	13.103548	12.732865	13.002707	12.834869	13.175184	13.963030	13.455726	14.307986	14.032590	12.625338	13.216405	13.541684	12.881690	12.493861	12.645722	13.103703	12.779167	12.760816	12.917482	13.789952	13.551881	13.038959	13.011244	13.264773	13.737835	12.985374	12.951859	12.783431	13.324998	13.290515	13.152225	13.784553	13.060993	12.987390	13.177797	13.097119	12.783965	13.581357	13.043905	13.035077	13.109263	13.281421	13.183897	12.690183	13.275436	13.530801	13.592616	12.691020	12.901456	13.108685	12.395993	13.333719	13.459608	12.759782	12.437743	13.093498	12.670010	13.329654	13.180932	13.377791	13.277438	12.648843	12.735048	12.978577	13.069482	12.757711	13.630199	13.349953	12.682302	13.495100	13.576005	13.146687	12.608516	13.358446	12.792190	13.298159	13.222353	13.256418	12.870661	13.097867	13.786622	12.961065	12.996574	12.763272	13.042413	13.163939	13.189708	13.595124	12.849137	13.231839	12.534127	12.388737	12.936017	13.363874	13.120243	13.285446	13.397792	13.734624	12.835997	13.819403	13.270023	14.205886	12.604080	13.842755	12.918404	13.011074	13.156520	12.696432

ARMC8	7.573089	5.704486	6.587534	7.362762	8.177391	7.070554	6.351623	7.131970	7.882612	7.188396	7.490242	7.575887	7.902878	7.005199	6.315207	7.943560	6.276203	6.601017	8.070785	7.687797	5.836962	8.269030	6.822918	7.953412	7.103438	7.511861	8.218245	7.679523	7.930030	6.093732	8.267776	6.465705	5.813703	8.031790	7.290335	6.683406	6.832447	6.172016	6.002186	6.167379	7.697463	6.044821	7.724222	6.981481	7.162692	7.091636	7.843927	5.441417	6.457563	6.676570	6.534525	6.738763	6.809032	7.118055	7.530678	7.225755	7.605306	6.114764	6.874477	5.922924	6.109572	7.634337	6.011945	7.222542	5.695092	5.876019	5.902912	5.647068	5.407122	6.109395	6.566884	6.668017	8.183583	6.524815	6.660846	6.764576	6.555125	6.326261	6.568230	6.034196	7.252922	6.078831	7.119964	7.090725	7.442358	6.770246	7.306655	6.978816	5.809426	5.066749	5.848984	5.548432	6.616246	6.737629	6.729161	7.773210	6.264502	8.673275	7.708085	7.381884	7.832314	7.282413	7.513898	6.824667	6.783864	6.991611	6.504643	7.555345	7.480895	7.780295	5.698684	6.682753	6.374509	8.072162	6.657757	6.190615	6.820544	8.153614	8.117615	7.188718	5.849360	6.452101	7.705322	7.086906	6.466680	6.795557	8.486274	8.428351	7.158850	7.030795	7.256941	7.101718	6.281614	5.802051	8.217284	7.753478	7.630838	7.496477	6.663632	7.161729	8.226898	7.191384	8.463077	6.399654	5.936863	6.742107	7.820563	7.920886	6.111791	9.557255	6.914786	5.925429	7.930030	7.826473	7.417717	6.500881	5.837488	6.419326	6.082499	8.039326	5.528031	7.141433	7.044339	7.195198	6.590824	7.447140	2.901598	7.725594	6.522990	6.108340	7.545980	6.892201	6.678786	7.136879	6.581476	6.989679	6.193889	6.722898	5.899364	6.269124	6.911787	6.580967	6.560853	6.008053	5.348134	5.101364	5.744281	5.565388	5.898297	6.545484	6.510765	6.920175	6.766516	6.687772	5.967890	6.405559	5.947585	6.459694	6.481071	6.986852	7.308803	7.728725	7.320886	7.454763	6.920025	7.568853	7.035624	5.972154	5.777617	6.483079	5.997771	6.397288	5.636210	7.234838	7.205049	6.894802	8.835707	7.691187	7.131326	7.228566	7.215129	7.607430	6.093560	7.098301	7.568853	5.396922	7.402070	6.549020	7.226852	5.567473	7.146759	6.499883	6.965004	6.352776	5.266656	6.116129	8.117460	6.975741

NME9	7.573089	5.704486	6.587534	7.362762	8.177391	7.070554	6.351623	7.131970	7.882612	7.188396	7.490242	7.575887	7.902878	7.005199	6.315207	7.943560	6.276203	6.601017	8.070785	7.687797	5.836962	8.269030	6.822918	7.953412	7.103438	7.511861	8.218245	7.679523	7.930030	6.093732	8.267776	6.465705	5.813703	8.031790	7.290335	6.683406	6.832447	6.172016	6.002186	6.167379	7.697463	6.044821	7.724222	6.981481	7.162692	7.091636	7.843927	5.441417	6.457563	6.676570	6.534525	6.738763	6.809032	7.118055	7.530678	7.225755	7.605306	6.114764	6.874477	5.922924	6.109572	7.634337	6.011945	7.222542	5.695092	5.876019	5.902912	5.647068	5.407122	6.109395	6.566884	6.668017	8.183583	6.524815	6.660846	6.764576	6.555125	6.326261	6.568230	6.034196	7.252922	6.078831	7.119964	7.090725	7.442358	6.770246	7.306655	6.978816	5.809426	5.066749	5.848984	5.548432	6.616246	6.737629	6.729161	7.773210	6.264502	8.673275	7.708085	7.381884	7.832314	7.282413	7.513898	6.824667	6.783864	6.991611	6.504643	7.555345	7.480895	7.780295	5.698684	6.682753	6.374509	8.072162	6.657757	6.190615	6.820544	8.153614	8.117615	7.188718	5.849360	6.452101	7.705322	7.086906	6.466680	6.795557	8.486274	8.428351	7.158850	7.030795	7.256941	7.101718	6.281614	5.802051	8.217284	7.753478	7.630838	7.496477	6.663632	7.161729	8.226898	7.191384	8.463077	6.399654	5.936863	6.742107	7.820563	7.920886	6.111791	9.557255	6.914786	5.925429	7.930030	7.826473	7.417717	6.500881	5.837488	6.419326	6.082499	8.039326	5.528031	7.141433	7.044339	7.195198	6.590824	7.447140	2.901598	7.725594	6.522990	6.108340	7.545980	6.892201	6.678786	7.136879	6.581476	6.989679	6.193889	6.722898	5.899364	6.269124	6.911787	6.580967	6.560853	6.008053	5.348134	5.101364	5.744281	5.565388	5.898297	6.545484	6.510765	6.920175	6.766516	6.687772	5.967890	6.405559	5.947585	6.459694	6.481071	6.986852	7.308803	7.728725	7.320886	7.454763	6.920025	7.568853	7.035624	5.972154	5.777617	6.483079	5.997771	6.397288	5.636210	7.234838	7.205049	6.894802	8.835707	7.691187	7.131326	7.228566	7.215129	7.607430	6.093560	7.098301	7.568853	5.396922	7.402070	6.549020	7.226852	5.567473	7.146759	6.499883	6.965004	6.352776	5.266656	6.116129	8.117460	6.975741

ZNF391	7.471425	7.467564	7.367028	8.707834	7.766700	7.127683	8.621435	6.964378	7.025548	8.104486	8.198008	7.670062	8.650119	8.498687	8.673275	7.989562	7.772925	7.884753	9.798923	8.083179	8.402370	9.351550	10.722360	7.925059	8.411708	8.321873	7.714645	7.467106	7.821500	8.142539	7.511385	8.409099	8.122287	8.367911	9.071601	8.750517	8.812340	8.706443	8.879252	8.931776	8.284696	9.684031	8.211137	9.083635	8.876155	7.967552	7.917667	8.897010	8.554202	8.899452	8.453220	8.744816	9.247657	9.260739	7.932184	8.045289	9.180782	9.711552	9.303847	9.430494	9.841226	8.195751	10.020041	9.589818	9.660909	8.221604	8.693881	8.515175	8.767777	9.105197	8.101000	8.387729	7.413356	8.056362	8.245241	8.184935	8.412020	8.150381	8.408218	8.834502	8.892677	8.495523	9.137949	8.583297	9.070196	9.370599	8.916018	8.758395	9.863172	8.887283	9.184400	8.933933	8.342612	8.503412	8.361723	8.063817	8.488606	8.278342	8.846470	8.374973	7.471877	8.525979	7.685331	8.283918	8.785782	7.800516	7.408367	8.259398	7.495527	7.498661	9.045190	7.671162	6.975929	7.910633	6.910490	7.920144	8.530152	8.012452	8.026994	8.106503	7.467417	8.196850	8.685838	6.762515	6.841899	7.825839	8.745587	8.298062	8.806108	8.340714	9.049014	7.159318	9.689752	9.443280	9.097971	7.641095	8.051400	10.705209	6.578840	8.999207	9.146716	9.670699	8.399260	9.264903	7.827449	7.856494	7.593360	6.621787	8.712662	9.278587	9.848007	8.001992	8.177531	10.211826	9.636219	9.280598	8.960223	8.933630	9.036048	7.872133	9.380048	9.634765	8.963871	8.681429	8.761524	8.828074	10.674499	9.312325	8.664392	8.714025	9.551777	9.049602	9.323027	9.584367	7.989106	9.832913	10.392366	9.398469	9.821151	9.929695	8.964176	10.555685	9.395748	10.598160	8.590063	9.284768	7.478135	9.151110	9.395748	7.240393	8.094921	8.102201	9.051294	9.075596	8.726020	9.702796	9.017157	8.028111	9.451406	9.958620	9.538134	9.673499	8.606200	9.877183	9.503932	9.224202	9.477984	9.690836	9.114833	9.894275	9.873315	9.800263	9.590587	9.021730	8.173166	8.785782	8.328762	9.937579	9.227888	8.245391	9.377622	8.935453	7.468910	10.508722	6.967094	7.477675	8.379067	7.582572	7.970847	8.052014	9.041468	9.681392	7.782421	8.901108	9.735326	8.777533	9.112570	8.780092

ARSJ	8.702278	8.328445	8.826297	7.494420	7.088332	7.265404	8.560535	6.147424	6.807255	7.849606	7.316534	6.878304	7.879916	7.642002	8.954704	6.612852	7.396943	8.766838	4.420003	7.088478	7.053316	5.582288	6.904447	6.972117	7.486173	7.404747	6.363722	6.584714	6.977391	7.819359	5.965177	8.772323	8.654155	8.396396	8.036343	8.622183	8.600049	9.021885	7.769286	8.140905	7.093818	8.010662	7.906479	8.123421	8.696372	7.915342	6.440869	9.037684	9.079184	8.341166	8.736262	8.635047	9.311708	8.035393	9.565885	7.201468	9.298766	8.677280	7.612184	9.275055	8.515034	7.109022	8.097820	6.965162	8.278265	9.658419	7.678105	8.379846	7.806014	6.910328	7.438046	7.411211	7.153231	8.237618	7.761592	8.050463	8.422262	9.074052	7.867574	9.209005	8.748329	9.453727	8.986560	8.907026	8.267453	8.491632	8.269978	9.236897	8.022249	8.474768	8.128713	8.354587	8.052321	8.614211	7.740253	8.145020	9.355898	7.648749	8.120836	7.754711	8.065177	8.305594	8.375408	7.572029	8.295296	7.744703	5.616848	8.113934	7.003210	6.833435	6.847871	7.209083	8.051848	5.603805	8.778712	5.675825	8.163242	8.121626	6.692595	6.898513	9.894424	8.725272	5.444473	8.379067	7.242922	8.164723	9.449040	10.501539	6.788410	9.617155	6.615279	6.987163	9.640875	9.965460	7.831235	9.893788	7.975858	4.656631	10.085713	8.409099	7.020447	5.684727	8.718581	10.481102	10.500010	7.916620	3.278544	7.132134	8.911133	7.054595	8.513380	9.625195	9.094947	7.572185	9.170448	10.626847	8.859721	9.929861	9.561747	8.135109	9.080413	7.634486	9.929378	9.826780	8.482018	8.237449	9.499648	8.858952	11.072280	9.312325	8.407253	10.276374	10.692122	9.574876	8.108640	10.463567	8.489362	8.040436	8.809828	9.671337	6.957191	10.120507	9.872723	7.109022	8.330258	10.950287	8.498349	10.350071	6.208471	7.570519	8.727572	7.148490	9.516216	9.098294	9.528969	9.083496	10.360811	9.481820	8.790103	10.818858	10.556615	9.660290	10.686158	9.521928	8.205864	10.357244	10.493142	8.887442	9.355898	9.833499	9.469018	8.573449	10.043738	9.749888	8.988368	8.949666	8.698049	8.694514	8.624508	9.631239	8.049851	8.478049	8.363315	10.444741	5.826376	8.700599	8.064421	8.536813	8.208900	7.796171	9.883470	4.285445	5.430663	7.191094	9.418972	10.013689	6.632032	8.916955

PLA1A	11.355495	10.279891	8.742397	7.510936	6.619366	6.604700	8.509059	6.941991	5.414727	7.986529	6.565762	6.616937	6.945812	6.257931	8.731061	6.822754	6.219571	7.083312	6.620518	7.944322	7.253549	7.949038	8.536093	7.028768	7.075764	6.168263	8.618776	7.120108	7.928678	9.404833	5.962534	9.000709	10.179636	9.381478	9.001514	8.306800	7.977233	8.111607	8.888525	8.818873	7.414306	9.058476	10.655933	8.858205	9.235309	8.454138	7.383010	10.117917	8.084751	8.841949	10.257870	8.897010	9.760255	9.651348	9.254946	6.629202	10.007656	8.811548	7.963752	9.256201	8.453985	7.997745	10.091114	7.889199	9.619777	8.640980	8.078556	7.895579	9.664041	9.293321	9.327985	8.325722	6.716023	8.236218	8.298363	8.796696	8.709468	10.162738	8.282714	9.473557	9.186268	9.647967	9.198113	8.347200	7.768234	8.520528	9.181558	8.670629	10.157573	8.933782	8.346766	10.314761	10.550859	9.660142	9.480573	8.189259	8.279347	7.249008	6.804151	6.209840	6.124801	8.293064	7.496941	6.985720	8.654016	7.607143	6.827989	8.629256	7.144281	6.584231	8.439638	6.627098	7.165662	6.053040	6.985880	4.421729	11.100894	7.782421	5.815647	9.028112	8.001687	9.598476	7.997547	5.547706	6.548199	7.547747	9.137632	6.611312	6.707842	6.152938	6.782147	5.541274	8.474166	6.068929	6.881987	6.566103	5.439113	9.865593	9.739786	6.638032	7.765933	8.026381	5.800232	7.216857	6.895266	7.688422	5.981355	6.957350	9.253295	6.265036	8.811877	7.649962	6.911143	9.716701	7.614639	8.017553	7.431096	7.350247	9.598034	5.882992	10.129455	10.789717	7.795089	8.785931	9.160920	7.271730	10.890438	8.686570	8.456739	7.567799	10.761899	9.376353	7.876859	8.784884	8.835394	11.264699	10.213496	9.417325	9.822688	7.149287	8.540706	8.169398	8.698199	9.966953	9.037545	7.862606	9.056119	7.438363	9.151539	8.674211	8.265560	6.138053	7.096262	10.058476	8.882036	8.291041	7.988792	7.524374	9.301890	7.433129	8.992569	7.014515	8.056835	7.718421	7.287828	7.529626	8.320030	10.349010	9.492065	9.213906	8.158705	7.801599	8.132684	7.310260	7.877938	6.532738	6.756373	5.697300	7.912658	7.568409	7.839230	6.808398	8.289335	9.525858	7.996917	7.022488	8.012742	9.099191	6.612494	10.731630	6.475089	6.110060	7.522227	8.412468	5.330690	7.459959	5.661798	7.498661

AKAP8	7.584575	6.822918	7.060771	7.442516	7.419618	6.891419	7.586459	7.887508	7.627346	7.609133	7.797378	7.360429	7.442358	7.745299	7.375633	6.930985	7.293485	8.173786	8.036022	7.417421	7.670527	8.024532	8.487865	7.119635	7.613414	7.661085	7.997816	7.682592	7.446975	6.892837	8.014946	7.662814	7.645874	7.672436	7.526823	7.247454	7.061705	7.107577	7.433286	7.188088	7.263889	7.648283	7.867887	7.583021	7.993159	7.711542	7.004886	7.257558	7.274847	7.498661	7.807549	7.914905	7.797234	7.681614	7.340993	7.153391	7.740253	6.599911	7.194046	7.813075	7.766700	7.153231	7.392569	7.891070	7.074654	7.033749	6.210859	7.423532	6.578840	6.547258	6.528665	6.354904	7.385917	6.611997	6.840159	4.770546	6.473421	6.651431	7.078318	6.817639	7.656886	7.508788	7.853763	7.722083	6.702081	7.623470	6.739227	7.296211	7.090413	6.784847	6.868569	6.324053	6.734479	7.065715	7.073401	7.271564	7.507708	7.934793	7.417135	7.391165	7.492437	7.254171	7.559510	6.668503	7.709277	7.436945	8.302736	8.123563	7.457145	7.225900	7.346868	8.413693	7.561665	7.387779	7.507090	5.411287	8.352921	7.389802	7.611805	8.405148	6.997121	7.390251	9.094182	6.967400	7.859735	7.881713	7.325012	7.597003	7.350077	7.269393	8.045582	7.987020	7.705159	6.914321	7.668348	8.014321	8.617246	8.406806	7.181406	7.589649	9.210693	8.013547	8.211447	7.740253	7.122273	6.594072	7.471877	7.140794	7.185698	7.913863	7.558478	7.676347	7.709277	7.720538	8.032995	7.256173	7.335363	8.138807	7.561801	7.601841	7.378634	9.396978	7.842468	7.506049	7.584575	7.452599	6.714755	7.674779	7.235310	7.063488	8.094921	8.434134	7.586605	7.296044	7.591191	7.115503	6.806000	6.965162	7.664983	7.335661	8.169880	7.491489	7.272673	7.435375	7.455081	6.646258	6.268789	8.041408	6.779905	6.956874	6.713105	7.841244	6.467195	6.787592	4.975882	6.236650	6.708790	6.501553	7.032489	7.926948	7.323742	8.371426	7.127371	7.563626	7.578395	6.980014	7.284456	6.975110	7.075460	7.876139	6.701746	7.588120	7.021564	7.013769	7.209878	6.961184	8.539575	7.808858	7.493510	7.254611	6.927488	8.345831	6.398800	7.758843	7.673529	8.944146	7.757135	7.417135	7.602495	7.900057	7.504456	6.097197	7.336916	7.517263	6.415011	8.314692	7.848550	7.306812

CNTNAP5	5.384644	4.894383	4.783483	5.744449	6.879572	5.837134	5.487095	5.798398	5.132404	5.520813	5.658509	6.143926	5.686415	5.565581	4.854156	5.786123	5.182603	5.483876	6.061708	5.049595	5.542603	5.638981	5.408923	5.348318	5.050186	5.569226	6.106746	5.507004	6.048575	7.101718	5.704486	6.972442	5.841522	6.029915	5.548243	7.079256	5.346523	4.670819	4.943181	5.374347	5.284147	5.617787	5.765908	5.511256	6.221600	6.156847	6.127660	3.548569	5.905949	5.317069	5.814250	5.696189	4.813536	5.959854	5.274097	7.004242	6.398128	4.061501	4.048661	4.404666	5.186596	5.462582	5.112361	5.765730	5.262870	5.303420	5.626914	5.607219	5.303625	5.323541	6.102054	6.082328	5.647464	5.604154	6.054447	5.098874	5.404608	5.110165	5.636016	4.761630	5.599867	5.210432	6.283964	4.727455	4.607995	5.427255	6.478984	4.709693	6.025164	4.784543	4.798317	4.824555	5.670314	5.793655	5.652204	6.082328	5.232211	6.107820	5.489780	4.020460	6.273370	5.436481	5.861573	5.458649	6.293873	5.632657	4.646834	5.628214	5.183414	5.521563	5.892040	5.415278	5.064071	6.535162	7.027169	7.069707	5.214830	5.331054	5.854925	6.090612	6.100488	5.491509	6.497206	6.541874	5.811119	6.526178	7.805727	6.109732	5.985916	6.345390	9.671337	5.591378	5.397646	4.678180	6.613816	6.160979	5.984867	6.417324	5.107057	5.410127	6.114764	5.922745	6.338886	6.579465	6.314012	6.379958	5.826559	7.060933	5.740630	7.108364	7.326110	7.060280	6.518205	5.857773	10.209210	6.756707	6.183233	6.847240	6.410385	6.370383	6.755554	6.944078	5.930924	6.805391	8.527674	6.764402	6.450968	7.899568	6.215781	6.409176	5.597206	7.004729	6.141377	6.229889	9.251933	9.139637	5.255945	5.311617	5.881243	5.626357	7.618765	4.609078	5.666871	6.423013	5.066344	5.668327	6.181161	5.861774	5.382725	6.601179	6.868239	5.387168	6.726905	6.481419	5.763590	6.386290	6.215952	6.222113	6.440547	5.525913	5.136622	5.970859	5.891507	5.494080	6.746121	5.902746	4.937082	5.088591	5.821306	5.736968	5.498722	6.331981	6.942312	6.771077	7.325797	6.774130	7.604831	6.699191	4.654410	7.040810	6.434799	6.858980	5.523836	5.715491	5.527848	4.443695	5.940438	5.875319	5.453987	5.863827	6.907669	7.768842	5.581896	10.120036	5.427255	5.182826	5.633793	6.706701

AC005670.2	8.355532	8.397928	8.041723	7.917531	9.245982	8.673115	8.577882	8.719354	8.421040	9.160920	8.697124	9.309714	6.951242	8.044812	7.846711	8.842601	8.868990	8.318651	8.101896	8.467638	8.072005	9.379453	8.273458	8.652353	9.313619	7.372685	7.828383	8.452479	8.521130	7.900239	9.487987	7.301928	6.921772	6.681577	6.838662	8.078697	8.304531	8.791995	8.957785	9.027348	9.211755	8.057711	7.705945	7.910344	9.504396	8.685237	7.964169	7.758398	9.082889	7.538769	8.107889	8.682529	6.987783	8.488318	8.872790	8.323832	7.749198	9.344082	7.636629	7.585179	7.588285	9.196896	7.372371	7.572330	7.553771	8.278584	8.376752	7.574967	7.583190	8.184054	7.850360	8.986709	8.766546	9.417014	9.211755	7.057930	7.694590	7.609277	8.930630	7.830076	9.770821	9.062616	8.386026	8.441781	8.944434	8.239177	7.039538	9.042516	9.481353	9.725608	9.819337	8.441625	6.934087	8.361249	6.951936	8.351704	8.684172	7.432040	7.069044	8.582697	8.308845	8.842290	8.021048	8.169560	8.574419	9.285198	9.370750	8.788276	9.448263	9.643014	8.500912	8.888374	8.829501	9.171092	7.649509	7.673686	7.661235	7.853763	8.816206	7.012845	7.467259	6.951242	7.280216	8.625939	7.880784	7.094649	6.955662	7.267882	7.507990	7.431742	8.161001	7.660137	9.322724	7.683680	7.473694	7.195369	7.196961	7.610841	6.985072	7.123062	8.716277	7.917375	7.766091	8.977116	9.045496	7.708085	7.732109	8.302279	7.769881	7.596692	6.269643	6.599421	7.332417	6.677201	6.934730	6.576181	6.934730	6.995532	7.416254	8.017844	6.890763	8.376613	6.876549	6.926972	6.927973	8.367126	10.778399	6.693087	6.589328	6.836732	6.997572	6.081607	6.828927	7.384386	7.582731	7.373908	6.639977	5.680307	6.534372	6.782927	7.845650	6.482390	6.559257	6.608681	7.214830	7.058232	6.909858	7.236268	7.506049	7.561058	7.153877	8.343986	7.323297	7.255711	6.711646	6.909858	7.210823	7.196301	6.409342	6.818463	6.870163	6.513895	6.378641	6.631099	6.820232	6.814438	7.386840	7.762352	7.609920	8.507231	7.350247	6.202438	7.518657	6.878937	7.006969	6.697923	6.515893	6.964524	6.394030	8.878485	7.026011	7.173677	6.637049	8.375408	8.529825	7.261822	7.010422	7.337505	9.111602	7.461507	7.525358	8.088168	8.644996	7.844066	7.715828	7.052716	6.922731	9.075452

RPRML	8.355532	8.397928	8.041723	7.917531	9.245982	8.673115	8.577882	8.719354	8.421040	9.160920	8.697124	9.309714	6.951242	8.044812	7.846711	8.842601	8.868990	8.318651	8.101896	8.467638	8.072005	9.379453	8.273458	8.652353	9.313619	7.372685	7.828383	8.452479	8.521130	7.900239	9.487987	7.301928	6.921772	6.681577	6.838662	8.078697	8.304531	8.791995	8.957785	9.027348	9.211755	8.057711	7.705945	7.910344	9.504396	8.685237	7.964169	7.758398	9.082889	7.538769	8.107889	8.682529	6.987783	8.488318	8.872790	8.323832	7.749198	9.344082	7.636629	7.585179	7.588285	9.196896	7.372371	7.572330	7.553771	8.278584	8.376752	7.574967	7.583190	8.184054	7.850360	8.986709	8.766546	9.417014	9.211755	7.057930	7.694590	7.609277	8.930630	7.830076	9.770821	9.062616	8.386026	8.441781	8.944434	8.239177	7.039538	9.042516	9.481353	9.725608	9.819337	8.441625	6.934087	8.361249	6.951936	8.351704	8.684172	7.432040	7.069044	8.582697	8.308845	8.842290	8.021048	8.169560	8.574419	9.285198	9.370750	8.788276	9.448263	9.643014	8.500912	8.888374	8.829501	9.171092	7.649509	7.673686	7.661235	7.853763	8.816206	7.012845	7.467259	6.951242	7.280216	8.625939	7.880784	7.094649	6.955662	7.267882	7.507990	7.431742	8.161001	7.660137	9.322724	7.683680	7.473694	7.195369	7.196961	7.610841	6.985072	7.123062	8.716277	7.917375	7.766091	8.977116	9.045496	7.708085	7.732109	8.302279	7.769881	7.596692	6.269643	6.599421	7.332417	6.677201	6.934730	6.576181	6.934730	6.995532	7.416254	8.017844	6.890763	8.376613	6.876549	6.926972	6.927973	8.367126	10.778399	6.693087	6.589328	6.836732	6.997572	6.081607	6.828927	7.384386	7.582731	7.373908	6.639977	5.680307	6.534372	6.782927	7.845650	6.482390	6.559257	6.608681	7.214830	7.058232	6.909858	7.236268	7.506049	7.561058	7.153877	8.343986	7.323297	7.255711	6.711646	6.909858	7.210823	7.196301	6.409342	6.818463	6.870163	6.513895	6.378641	6.631099	6.820232	6.814438	7.386840	7.762352	7.609920	8.507231	7.350247	6.202438	7.518657	6.878937	7.006969	6.697923	6.515893	6.964524	6.394030	8.878485	7.026011	7.173677	6.637049	8.375408	8.529825	7.261822	7.010422	7.337505	9.111602	7.461507	7.525358	8.088168	8.644996	7.844066	7.715828	7.052716	6.922731	9.075452

SAMD14	6.649670	7.062687	6.450968	6.726582	6.703085	7.189556	7.190009	6.138578	6.717180	6.599241	6.434442	5.930243	6.892994	6.757487	6.557446	6.410859	6.721286	6.509278	6.625668	6.334164	6.363388	6.857373	8.094476	6.646258	7.778213	7.084263	7.418684	7.507556	6.858345	7.186835	6.424825	7.356390	7.145660	7.217647	7.143171	6.712466	6.728532	6.592806	6.821498	6.901432	6.824667	6.783072	7.052716	6.407052	6.837217	5.357975	7.280216	6.601179	6.662321	6.533884	6.761558	7.178605	7.173996	6.668503	6.722244	7.221773	7.285348	7.216069	7.340507	7.395245	7.285348	7.122109	8.414441	7.296211	7.283387	6.834688	7.001784	6.600216	6.973051	6.648197	6.627731	6.441696	6.928920	6.587371	6.461336	6.650608	6.690459	6.758287	6.337546	7.152007	6.940887	6.966936	7.182971	6.243786	6.789577	7.514207	6.513727	6.401461	7.466203	6.104775	6.980337	7.063169	7.350831	6.772992	6.920335	6.839336	6.436296	7.080977	7.852039	6.983656	7.450429	6.720973	6.641921	6.004284	6.584561	6.915103	7.432040	7.029077	7.229814	6.196731	6.990315	7.154664	6.484435	6.536782	6.629051	9.198113	7.148341	6.412181	6.573887	6.667380	8.182665	7.714645	7.399457	8.531668	7.051177	7.892473	7.557083	7.307427	7.294911	8.817145	7.599022	8.062729	8.425261	8.333675	6.638708	7.756334	7.262670	7.404910	7.353569	6.934887	8.115905	8.059794	7.270478	8.026061	8.578764	7.086749	7.804165	7.637084	7.793394	7.121790	7.639389	7.926147	7.857542	7.065404	7.208936	7.737418	7.756656	7.699262	7.956035	7.796780	7.415795	7.782708	7.345011	7.312596	6.156847	7.655132	7.925989	6.985241	7.715071	7.586605	7.389802	7.274556	8.395613	8.289633	7.396166	8.377380	7.761907	7.468615	8.115759	8.337432	7.492126	8.462629	7.314180	7.578077	7.810948	7.670691	7.965252	7.535627	7.587802	6.963071	7.098469	7.723928	7.612025	7.221939	7.202384	8.266021	7.504760	7.255095	8.281921	8.028111	7.341765	7.910633	8.064421	7.719347	7.983020	7.616510	6.573358	8.707677	8.243979	9.003955	7.780146	7.962366	8.369704	8.221444	7.704386	7.319485	8.112072	7.897456	7.414462	8.449532	7.018894	7.394285	7.302884	7.879916	6.980337	6.810181	7.036542	6.603564	7.827891	7.737895	7.942332	8.426021	7.210664	7.943394	7.979256	8.195751	7.220170	7.023127

DLGAP4	10.683331	11.491448	11.269855	11.842192	11.494500	12.300641	11.650203	12.238354	12.298540	12.251052	12.441036	12.313639	11.614346	11.855496	11.288192	12.021368	11.878774	11.551948	12.748800	12.091377	12.214583	11.709110	11.749074	11.883139	11.914892	12.310609	11.657605	12.085897	11.933500	11.743759	13.270211	10.946082	11.090456	10.821454	10.900969	11.057038	10.948753	11.322149	11.939089	11.745819	12.366707	11.413399	11.014714	11.287476	10.729718	10.875592	12.079696	11.037558	11.070312	10.833152	10.708068	10.747987	10.782533	11.222057	11.333467	12.471493	10.951940	10.926544	11.193097	11.167543	11.440039	11.790719	10.876331	11.552884	11.531684	11.708338	11.311900	11.386601	11.238152	12.278662	11.855342	11.601212	12.290765	11.571034	11.735962	11.233464	11.375615	11.170765	11.472640	11.138009	10.533962	10.887634	10.550253	10.990603	11.078978	11.800133	10.541837	10.337771	11.061554	11.383763	11.551046	11.688272	11.038735	11.586749	10.411451	10.909567	11.504652	11.573736	11.455375	12.071894	11.868033	11.674302	11.822772	11.962317	12.278977	11.896055	12.262160	11.886441	12.157109	12.251052	12.056828	11.705942	11.631808	12.317939	11.721674	12.058239	10.787776	11.798936	11.988293	10.851497	11.781211	10.842709	11.421793	12.064994	11.780760	11.727818	11.570035	11.568423	11.659709	11.944984	11.519525	11.537619	11.144404	11.259428	11.216707	11.106728	10.991933	11.572961	10.257400	11.358100	11.293950	11.456110	11.748921	11.180670	11.001938	11.737021	11.824443	12.155866	11.432696	11.779993	11.307098	11.357241	11.565671	10.539729	10.815810	11.311166	11.082627	11.424641	11.131914	12.058897	11.441315	10.444582	11.360602	10.868738	11.190615	12.251658	10.852098	10.824581	10.920023	11.016776	10.535918	10.230524	10.603870	10.187407	11.257082	9.858043	10.314129	10.542688	10.317425	11.218335	11.590688	11.213047	11.184194	10.803437	10.614231	11.138902	11.825339	11.368582	11.268089	11.547842	11.348487	12.464996	11.614961	10.778689	11.410105	11.160953	10.917928	11.609621	9.958459	10.351574	10.355208	11.341314	10.437805	9.895500	11.215382	10.642391	9.968820	10.495716	10.639071	10.462958	11.094712	11.163197	10.971168	11.990484	11.505998	11.054941	11.474696	11.506409	11.113033	11.261529	11.413079	11.768414	10.752648	10.601671	12.112986	11.635875	10.827827	11.468687	12.452816	11.325694	10.989295	11.295400	12.392195	10.952431	11.208087	11.294540	11.179454	11.940741

ERGIC1	15.041784	13.572887	13.337700	13.829200	13.667422	13.686442	13.559725	13.335001	13.432385	13.488148	13.662485	13.641300	13.740602	13.868320	13.789231	13.432385	13.371459	13.696837	13.464002	13.402704	12.950183	14.374090	13.426653	13.608281	13.748094	13.546426	13.826079	13.795380	13.504598	13.414432	13.216968	13.757393	13.239205	13.590078	13.484139	13.653679	13.538596	13.485295	13.846147	13.984981	13.234611	14.286917	13.634245	13.559725	14.020108	13.605255	13.317056	13.850447	13.138117	13.450346	14.376679	13.497141	13.248097	13.521153	13.731903	13.609691	13.758124	13.884347	14.550582	13.650975	14.211747	13.556369	14.405867	14.141470	13.958500	14.114980	14.046714	13.283833	13.379474	14.039946	13.718916	13.580404	13.447998	13.324170	13.628490	13.232466	13.415442	13.490594	13.857256	13.293957	13.450944	13.718689	13.754404	13.592392	13.609930	13.593519	13.203179	13.823982	14.194332	13.930857	13.792778	14.016405	14.052986	13.351805	13.983800	13.844047	13.459822	13.601268	13.418399	13.307926	13.436214	13.659861	13.594881	13.421049	13.054365	13.435346	13.713207	13.237061	13.284863	13.410422	12.347744	13.846933	14.170650	13.581357	13.663761	13.210914	15.198897	13.912695	13.626821	13.677795	13.680915	13.789952	13.132988	13.312746	13.118872	13.123620	13.475756	13.367661	13.457435	13.273776	13.752811	13.674088	13.785590	13.401016	13.583453	13.439032	13.770321	12.864977	13.763702	13.989848	14.011814	12.621157	14.146140	13.773715	14.751698	12.852480	13.377337	13.733138	13.270630	13.646113	13.672956	13.808354	13.630442	13.933765	13.020460	13.306191	13.511960	14.324402	13.829710	13.689979	14.135457	14.482061	13.894497	13.776185	13.248737	13.619696	13.615908	14.238833	13.673646	13.196334	13.820166	13.823177	13.904606	13.854098	14.079189	13.787649	14.062649	13.725347	13.911605	13.997851	13.629686	13.753087	13.441153	14.443429	13.599265	13.690456	14.097636	13.393505	13.896959	13.295148	13.111982	13.792778	13.745098	13.569877	13.536847	13.536847	13.431471	13.078931	13.754656	13.824264	12.944248	13.073330	13.964108	13.599935	13.849377	13.926497	14.034368	14.392249	14.223479	14.975923	14.713935	13.805428	13.956584	13.427110	13.312746	13.133394	13.451381	12.876579	13.712440	13.433474	13.669861	13.364551	13.535732	12.650533	13.156520	13.475946	13.345088	13.708684	13.140676	12.755118	14.016992	13.154391	13.568039	12.844580	13.158236	13.842755	13.109263	13.364946

EFNB1	10.904224	11.119708	11.718397	11.740720	11.155520	11.620681	11.459062	10.608914	12.002196	11.348027	11.617427	10.937909	11.925051	11.412382	12.288938	10.774607	11.386452	10.530093	11.153181	11.351898	9.855275	10.009427	9.723120	10.668712	11.054941	11.334188	11.665355	10.213800	11.135945	11.656596	11.299661	11.510243	10.847998	12.119923	11.115020	10.634740	10.664020	11.480931	11.803943	11.558177	11.168249	10.696336	11.191787	11.766620	11.486239	11.237147	11.002368	10.484745	11.276012	11.559088	11.345621	10.967395	11.491594	11.586188	12.191034	11.898226	11.171659	10.856734	11.607591	11.615241	11.152318	11.261074	10.389876	11.043570	11.962317	12.220929	11.409053	11.387475	11.173601	10.059845	10.958253	11.009483	11.100158	11.229016	11.064219	10.871895	11.347723	11.423597	10.740494	11.920253	11.312203	11.902647	11.701443	11.473523	10.805904	10.808104	11.745239	11.693642	10.368662	11.213657	9.979804	10.281995	11.727665	11.979749	10.998939	11.237278	10.855566	10.867857	11.118406	10.944165	11.597416	10.791003	11.331613	10.912497	11.263206	11.780607	10.646346	10.830041	10.633822	11.277499	9.567473	10.972192	11.721853	10.142294	12.011728	11.154939	10.869894	11.934585	11.779259	10.516533	13.337899	12.783261	12.536108	13.304537	13.569652	13.347432	12.452958	14.149795	13.644498	14.110759	12.306066	13.562433	12.105127	14.233539	12.785598	13.076263	11.947291	11.559918	12.913978	11.893367	12.469238	11.163197	13.487724	12.723671	11.746252	12.157886	12.363232	12.269777	13.962183	12.041232	12.607880	12.870487	13.054711	12.155557	11.533775	12.597984	11.586469	14.207819	13.073039	13.636675	12.207764	11.417836	13.449301	13.955482	13.118268	13.738603	10.010197	11.974803	13.182875	13.682827	11.883139	13.119650	13.594660	12.432585	12.957160	11.392765	12.249599	13.381550	11.945301	12.548405	13.538596	13.190276	12.611506	12.266082	11.823222	14.122447	12.773792	12.822312	9.739008	12.028865	13.786622	13.626349	12.694415	12.781052	12.866399	13.284662	12.641159	12.631325	12.412373	13.763209	14.718998	13.170749	13.026553	12.727241	12.067188	13.442646	12.054237	10.390024	12.847857	10.176704	11.195637	12.106988	12.989794	14.114324	13.503677	12.294671	13.156914	13.078379	12.571856	12.907332	12.637828	11.524099	12.006245	12.707731	13.509723	13.513783	11.807364	11.418543	13.136025	11.032688	12.827524	9.956624	11.460855	10.835430	12.967881	11.767362	12.353725	13.825046

CELA2B	14.654144	7.702832	8.514437	11.335546	8.267307	9.778915	9.723922	9.021275	8.473729	9.450603	9.717339	9.530075	8.474013	8.421352	8.758395	9.603010	9.163554	8.204349	6.667531	9.399697	8.712506	7.735493	8.007316	7.942940	7.567498	8.418410	9.493614	8.388680	9.740723	9.609148	8.630992	11.658059	8.371908	9.471870	8.832252	8.989722	9.096576	7.384537	7.155127	7.496149	9.976964	6.935528	8.163679	7.880642	8.059636	7.845650	10.060602	7.866357	8.383906	8.178108	8.003583	8.414729	7.652204	7.726686	7.870285	10.136250	7.804017	7.327719	6.834077	7.270643	7.620747	9.577290	8.223899	7.316378	7.788365	7.499939	7.391797	7.497414	8.011423	7.505395	9.391895	9.485028	9.869705	9.472328	9.442051	9.457550	9.254659	9.182515	10.167812	7.867093	14.160557	7.895767	7.547276	7.722516	7.585809	8.227663	8.066532	7.963443	7.643053	11.082627	7.401622	8.237449	8.783210	8.888686	10.130482	8.970668	8.060923	8.356909	8.981956	8.631915	8.807970	8.426765	8.606664	8.762712	7.328659	9.702347	7.169171	7.296211	8.105890	10.229267	8.099298	7.753027	8.105890	9.805321	9.332000	10.308324	9.867560	9.612257	9.684197	10.156362	7.263413	8.322308	9.415347	9.173279	10.800245	8.902170	8.198709	8.593782	9.729974	9.392669	10.074685	9.192277	8.097528	8.743457	9.391590	8.624991	8.371282	7.313859	9.444517	9.395906	7.606999	7.860474	8.730322	8.533318	8.655231	9.153845	9.022623	9.536888	9.824019	8.315758	8.300034	7.589821	9.444365	8.648119	9.274098	8.362379	6.993221	7.284600	6.939832	10.406073	7.346868	8.484449	7.300159	7.739616	8.252070	9.793010	7.204562	8.499478	8.101283	7.496941	8.234355	7.687645	7.773530	7.809422	10.051862	7.745146	7.300797	7.186349	7.343519	8.053241	10.644592	7.606389	7.129868	7.164573	7.832020	7.604209	7.557531	8.152367	7.635566	9.575790	9.269228	9.781184	9.114964	9.188910	9.020018	8.809969	8.923712	9.576986	7.402873	8.402822	8.017102	7.303347	7.290335	6.923867	8.118721	7.468145	7.667444	7.234989	7.165350	6.254711	7.631321	7.680560	8.647397	9.691787	8.300907	7.804638	8.400640	8.440743	8.719354	8.168619	8.431677	8.524461	8.473263	6.459852	9.810719	7.246216	7.460098	7.023127	9.595708	7.800683	7.993626	7.630032	9.731525	9.648272	9.344906	6.944724	9.256201	9.969266

CELA2A	14.654144	7.702832	8.514437	11.335546	8.267307	9.778915	9.723922	9.021275	8.473729	9.450603	9.717339	9.530075	8.474013	8.421352	8.758395	9.603010	9.163554	8.204349	6.667531	9.399697	8.712506	7.735493	8.007316	7.942940	7.567498	8.418410	9.493614	8.388680	9.740723	9.609148	8.630992	11.658059	8.371908	9.471870	8.832252	8.989722	9.096576	7.384537	7.155127	7.496149	9.976964	6.935528	8.163679	7.880642	8.059636	7.845650	10.060602	7.866357	8.383906	8.178108	8.003583	8.414729	7.652204	7.726686	7.870285	10.136250	7.804017	7.327719	6.834077	7.270643	7.620747	9.577290	8.223899	7.316378	7.788365	7.499939	7.391797	7.497414	8.011423	7.505395	9.391895	9.485028	9.869705	9.472328	9.442051	9.457550	9.254659	9.182515	10.167812	7.867093	14.160557	7.895767	7.547276	7.722516	7.585809	8.227663	8.066532	7.963443	7.643053	11.082627	7.401622	8.237449	8.783210	8.888686	10.130482	8.970668	8.060923	8.356909	8.981956	8.631915	8.807970	8.426765	8.606664	8.762712	7.328659	9.702347	7.169171	7.296211	8.105890	10.229267	8.099298	7.753027	8.105890	9.805321	9.332000	10.308324	9.867560	9.612257	9.684197	10.156362	7.263413	8.322308	9.415347	9.173279	10.800245	8.902170	8.198709	8.593782	9.729974	9.392669	10.074685	9.192277	8.097528	8.743457	9.391590	8.624991	8.371282	7.313859	9.444517	9.395906	7.606999	7.860474	8.730322	8.533318	8.655231	9.153845	9.022623	9.536888	9.824019	8.315758	8.300034	7.589821	9.444365	8.648119	9.274098	8.362379	6.993221	7.284600	6.939832	10.406073	7.346868	8.484449	7.300159	7.739616	8.252070	9.793010	7.204562	8.499478	8.101283	7.496941	8.234355	7.687645	7.773530	7.809422	10.051862	7.745146	7.300797	7.186349	7.343519	8.053241	10.644592	7.606389	7.129868	7.164573	7.832020	7.604209	7.557531	8.152367	7.635566	9.575790	9.269228	9.781184	9.114964	9.188910	9.020018	8.809969	8.923712	9.576986	7.402873	8.402822	8.017102	7.303347	7.290335	6.923867	8.118721	7.468145	7.667444	7.234989	7.165350	6.254711	7.631321	7.680560	8.647397	9.691787	8.300907	7.804638	8.400640	8.440743	8.719354	8.168619	8.431677	8.524461	8.473263	6.459852	9.810719	7.246216	7.460098	7.023127	9.595708	7.800683	7.993626	7.630032	9.731525	9.648272	9.344906	6.944724	9.256201	9.969266

DDR1	11.339369	11.262723	11.541954	12.312392	12.196471	12.224769	11.827814	11.739525	12.258961	12.001595	12.270578	12.137705	11.958709	12.894551	12.296644	11.619947	11.913837	12.144544	13.502805	12.218642	12.164047	12.357030	11.325549	12.388554	12.314612	12.980105	11.773141	12.337925	12.016430	11.598591	12.226464	11.905178	11.650646	11.999458	11.334799	11.064379	11.744351	11.873552	12.708077	12.209897	11.863352	11.848510	11.382032	11.960368	11.108664	11.473665	11.531987	11.534664	11.916216	11.839633	11.545765	11.666419	11.579668	11.948186	11.684959	12.273762	11.205984	11.779993	12.822141	11.772998	12.112540	11.500204	9.319592	12.537086	12.115614	12.136649	11.807837	12.032298	11.738062	12.092137	11.327191	11.097482	12.042106	11.130328	11.211287	11.097635	11.473523	11.380384	11.386742	11.759472	11.472366	11.675026	11.591754	11.801189	11.544838	12.089192	11.605808	11.839473	11.852324	12.355628	11.627766	11.423308	11.634958	10.922388	11.130181	11.816598	11.466592	11.867888	12.233576	11.940295	11.687959	11.351596	11.713033	12.026311	12.110262	11.634958	12.286760	12.008402	12.237106	12.248149	12.764108	12.691189	12.900592	11.878918	12.103128	11.152318	11.891078	11.916672	11.680972	12.555422	11.520848	11.958571	13.726632	12.414938	12.525283	13.280266	12.399011	12.046024	13.227647	13.835331	13.871812	12.899723	13.141081	13.481484	12.353395	11.635098	13.625907	12.901798	11.596096	12.893846	14.181178	13.945929	13.939913	12.764425	11.427104	12.472784	13.569183	13.262182	12.771719	12.239785	11.598287	13.164330	11.994731	12.171733	12.748989	11.602116	11.987859	13.337481	12.420749	12.783431	11.775216	12.190892	12.329246	11.874166	12.105285	11.864535	11.284747	12.543887	11.560243	12.318704	12.323902	12.276374	12.310302	11.653921	12.814409	11.833609	12.564301	13.126165	12.718385	12.450102	12.900943	12.719193	12.816972	12.452958	12.261028	12.201396	11.613450	11.662821	12.031827	11.503353	11.745525	12.655175	11.997786	11.326303	11.463053	12.705163	11.806485	12.199224	11.623005	12.194595	12.402908	11.966551	11.523246	10.809966	12.701928	11.333902	11.344909	11.285190	11.989104	11.275177	11.765088	12.146584	12.315775	12.780890	11.999926	12.380831	12.244073	11.952744	12.798496	10.930388	13.040624	13.304746	11.413548	11.502750	12.902705	12.414938	12.613560	12.361945	12.065157	12.336986	12.895284	11.338491	12.642482	12.196619	12.255202	12.417662	13.230666	11.763401

ADAM15	13.262182	14.788691	14.934057	14.287592	14.754065	14.581385	14.249525	14.239751	14.375159	14.460072	14.251440	14.508773	14.548089	14.854894	14.274531	14.279316	14.379014	14.839161	15.660615	14.694246	15.764986	14.999084	15.314555	14.599518	14.592389	14.773263	14.733504	14.512377	14.566731	13.917477	14.591153	14.509168	14.180553	14.386698	14.212720	14.417282	14.499877	14.562573	15.120250	15.223282	14.599518	15.083595	14.654622	14.599064	14.507632	14.529893	14.270558	14.605414	13.365370	14.442714	14.068367	14.494365	14.039072	14.820339	14.419877	14.629113	15.188690	14.176188	14.620267	14.416904	14.817306	14.642226	14.839682	14.974820	14.600827	14.911983	14.767548	14.939387	14.822949	15.126455	14.567967	15.121584	14.444840	14.833576	14.776581	14.463188	15.020432	14.585623	14.433838	14.662761	14.341736	14.284842	14.602856	15.179287	14.839161	14.464001	14.678701	14.938328	15.124619	14.952794	14.455390	15.404616	14.742275	14.822343	14.305938	14.484873	13.918322	14.739993	14.582286	14.403999	14.838073	14.682727	14.668633	14.697055	15.678135	14.247926	14.896010	15.402282	15.666334	14.349898	15.871113	14.744131	14.997957	14.493958	14.395843	14.651063	14.246947	14.699677	14.635670	15.307206	14.204626	13.907177	14.985105	14.830506	14.188094	14.689299	13.619020	14.817306	14.303599	14.685534	14.316111	14.141470	14.933539	15.177955	14.641807	13.501308	14.599518	15.179287	13.832809	15.317503	15.253922	15.614087	14.739028	14.724263	14.100390	14.199812	14.367353	14.225717	14.487150	15.035175	14.429371	14.197180	14.097636	14.363390	13.504135	13.810158	14.701092	14.409150	13.473153	14.594880	14.254782	13.496693	13.851196	14.177726	14.080347	15.096349	13.042576	14.469284	13.298634	13.888605	13.645878	14.227945	13.734624	14.035874	14.369760	12.841760	14.165527	14.249863	13.806755	13.737601	14.698837	14.627696	14.841605	14.615271	13.786622	14.147923	14.011503	13.839011	14.272202	14.123682	13.986959	14.981620	13.396218	13.892381	13.920502	13.789952	13.991573	14.007880	13.726121	14.068933	14.063565	13.819161	13.757393	13.805698	14.394753	14.252158	13.568257	14.608827	14.097636	14.438961	14.011230	13.980804	13.698756	13.860992	14.661345	14.411699	14.038793	14.402538	13.922935	13.375056	13.579900	14.148848	13.701861	14.769473	14.590670	14.649251	15.400849	15.270868	15.211659	14.723372	14.440081	13.938227	14.671925	13.846933	14.554379	14.343514	14.453914	14.452852

IPO7	11.096316	10.548263	10.859242	11.707604	11.559235	12.001304	11.181427	12.051785	12.394751	11.891078	12.191984	11.848352	11.806639	12.046618	11.872205	11.597256	12.115534	11.423160	11.809037	12.039995	11.176723	13.072631	10.879596	11.883430	11.672206	11.748620	12.446758	12.244222	12.237262	11.483699	12.041372	10.993881	11.547691	10.873539	11.073706	11.356979	11.208087	11.150234	11.324685	11.404110	11.945301	11.002368	11.314836	11.668177	11.479798	11.716014	11.700241	11.071058	11.491172	11.327922	11.319554	11.726020	11.425348	11.709538	11.575856	11.672678	11.291005	11.211287	10.774607	10.783617	11.090033	11.403089	10.583475	11.204334	11.079540	11.113613	11.134615	10.642391	10.909711	10.952894	11.212744	11.346523	12.100275	11.357096	11.754542	11.391609	10.919423	11.033754	11.110174	11.284152	11.572374	11.609839	11.394433	11.274456	11.592521	11.355780	11.668318	11.671048	11.077953	11.082839	10.964890	10.952734	11.705505	11.658499	11.599023	11.621431	11.604438	12.086955	11.796425	11.827250	11.775366	10.765826	11.838717	10.314129	11.032834	12.055881	12.397858	11.316005	10.505574	12.404042	10.971621	11.713327	11.208804	11.825192	11.713495	10.375835	11.289041	11.295264	12.725151	11.747596	11.697417	11.610052	12.124346	11.876288	12.484550	11.379300	12.056353	11.309839	12.324058	11.353312	12.116357	12.113904	11.914449	12.074516	12.269152	12.447571	12.772756	11.725272	11.876001	11.651226	13.256614	11.982413	11.736869	11.802387	12.514830	12.189655	12.524939	12.549244	12.022968	11.900584	10.913714	11.428109	11.360018	10.850018	11.552276	11.000137	10.451803	11.373920	11.019905	12.765306	10.579788	12.147357	12.149599	12.429637	12.117412	12.479812	11.555799	11.904174	10.923286	11.661180	12.555591	11.816173	11.585590	12.264187	12.684378	12.021794	11.811348	11.658962	11.333064	10.688592	11.767960	11.121598	11.141784	11.498901	11.514705	11.253671	10.584768	10.887784	10.891048	11.260034	11.661349	12.232212	11.755284	11.564336	11.312627	11.882067	11.644166	12.033841	11.751430	11.177918	11.853235	11.933654	12.139271	12.406661	11.474531	12.022078	12.637652	11.646786	11.384527	11.387475	11.598004	11.220264	11.711892	12.408210	12.909823	11.501897	12.600886	12.085596	11.416188	11.682461	11.477556	12.141279	10.320132	10.634437	12.588301	12.631325	10.943161	10.911465	12.460261	10.866201	10.840794	13.577378	12.063042	11.596253	10.946082	11.344607	12.208698	12.059200

MCF2L	6.229015	5.908843	6.308928	6.610967	6.967719	6.261843	5.677134	4.793308	5.996352	6.892517	6.414193	6.617422	6.889820	6.617562	6.780059	7.223728	5.664777	6.961184	7.978951	6.853000	5.627262	8.006259	8.570955	6.628874	8.357685	6.379958	6.808885	7.237038	7.024421	5.721042	6.982081	4.165107	5.487292	5.864523	6.406070	5.822776	5.768488	5.563155	5.490389	6.661658	6.233086	6.329736	6.515542	6.704456	6.679637	7.215778	6.339551	5.240275	6.710407	5.323238	5.772006	7.254455	6.105843	5.998849	5.786290	6.644836	6.711965	7.114731	8.100093	5.697684	6.516401	6.587371	6.326589	8.089246	5.167076	6.763449	5.759977	6.084054	5.400880	5.225089	4.854156	5.148725	6.299881	4.752112	5.630622	5.731934	6.852010	5.149493	5.347947	5.552587	5.633578	6.755554	6.546478	4.780536	6.393714	6.562022	5.557350	6.392190	5.466171	4.987082	6.364215	5.691365	6.601671	5.189186	6.028219	6.304614	5.815816	7.695615	6.650152	6.754571	6.050770	6.619366	7.138283	7.025623	6.423830	6.329228	6.431080	7.111620	8.413538	6.771880	6.104775	7.084910	7.151398	6.643374	6.650608	5.271428	7.332899	6.848712	7.656124	5.366809	5.499091	6.801084	5.790077	6.600216	6.455107	5.830666	7.835968	4.653729	7.525289	6.139619	6.648704	6.343881	4.553466	5.005994	5.632451	4.980930	8.065970	7.424608	5.586787	5.204669	7.651114	7.195686	6.614965	5.970859	4.901328	5.884957	6.623246	6.257762	5.203864	4.953347	4.791226	6.493772	4.837031	6.303246	5.848095	4.759964	5.129993	5.059000	5.837861	5.454582	4.636935	4.766147	6.573050	5.139018	6.858648	6.191997	5.200892	4.792523	4.459665	5.019124	8.696832	4.224051	4.584149	5.436481	5.912042	6.533404	5.761577	5.836962	5.116800	5.077351	6.818111	5.300823	5.617409	5.357589	4.991830	4.739775	4.788270	4.378864	4.404898	5.110970	5.761577	5.287944	4.583521	4.980532	4.963118	4.674733	4.632197	5.659580	4.292225	7.594133	5.627815	4.776772	5.708481	4.721961	6.428706	4.502632	5.827305	3.967902	4.995208	7.769127	4.497496	5.096968	4.869173	5.579462	5.257140	9.378232	8.066831	5.308659	6.247129	5.167076	6.824667	6.754726	4.765079	5.221285	6.812404	4.812712	7.072747	6.681577	5.342989	5.881051	5.321907	5.034847	6.796039	6.549863	5.120694	5.820260	6.197741	5.634715

GNAO1	7.086292	7.892783	7.983773	8.087256	8.631600	8.404218	7.856647	8.820086	8.740639	8.054480	8.229359	7.993790	7.968631	8.164882	7.893878	7.997230	8.225089	8.082867	8.500281	7.766700	8.505967	8.739630	8.649359	8.180074	8.092765	8.249893	7.958540	8.434689	8.164723	8.168296	8.162959	7.376878	7.479657	7.678105	7.969093	7.850793	7.923817	8.014321	8.436316	8.165664	8.211137	7.897027	7.919671	7.795409	7.795703	7.595483	8.406518	8.128270	7.639530	7.859886	7.395245	7.701899	7.823491	7.974923	7.753321	8.405932	7.591348	8.277093	8.016016	7.734887	8.097665	8.175380	8.254529	8.505509	8.313277	8.157042	8.479473	8.183583	8.405932	8.656980	7.943394	7.921935	8.243691	7.832314	7.843927	8.072005	7.981918	7.897749	7.887508	7.789934	7.741340	7.699122	7.481352	7.377685	7.469405	7.321681	7.534238	7.673841	8.011995	7.888271	7.646507	8.251297	8.136158	7.974770	7.600158	7.756499	7.357964	7.931268	8.152839	8.262784	8.420888	7.985002	8.332937	8.347960	8.685838	8.270740	8.263240	8.360473	8.867036	8.435835	8.501568	8.923429	8.639746	8.399403	8.105589	10.230680	7.979734	7.772016	8.326365	7.457297	8.244457	7.882612	8.065802	9.977682	8.581219	8.434607	7.623177	10.279731	8.402059	8.097062	8.165963	8.254205	8.607415	8.809207	7.721304	7.644333	8.472816	8.199201	7.498194	8.576106	9.063070	8.424386	9.426185	8.369555	8.276257	8.336175	8.804918	8.787017	8.557010	8.050313	8.291499	7.743330	8.215620	8.153907	7.549287	7.556447	7.708847	7.988792	7.811416	9.126741	7.811267	7.450282	7.973373	7.778050	7.857857	10.884165	8.214240	7.028306	7.850493	7.388883	8.020116	7.818091	7.979579	7.717665	8.136815	7.376878	7.756185	7.921053	7.618023	8.193053	8.292608	8.327636	8.356909	8.367762	7.757729	8.111766	8.261704	8.274038	9.021575	7.875198	7.777904	9.248587	7.709719	7.583967	8.080215	7.655132	7.868192	7.813224	8.200278	7.494420	7.802054	7.182830	7.498661	7.815256	8.029333	7.170405	7.040810	7.887956	7.587634	7.420891	7.639694	7.427752	7.761133	7.721779	8.106793	7.691187	7.960374	8.863706	7.720538	7.997547	7.930030	8.335568	8.600192	8.533620	8.643164	8.635686	8.308074	8.031633	9.268924	8.150381	9.065219	8.840124	8.840124	9.247960	9.056272	7.715979	7.658918	8.506105

C14orf119	7.627192	5.523280	7.464647	6.259821	4.514214	5.416020	7.532852	4.116561	4.074728	6.526670	6.434113	6.484623	6.293544	5.973953	5.870227	6.209329	5.192286	4.846179	5.044321	7.243901	4.393198	3.745473	5.864709	4.568525	4.541524	4.857954	5.081850	4.462868	5.141933	5.905442	3.858138	7.187943	6.714755	6.763922	7.890928	7.170864	6.801570	5.947758	4.321835	3.008358	5.417191	3.336784	6.043120	7.234047	5.937040	6.323377	5.257140	4.156948	4.907319	7.410771	7.082859	7.241333	8.190228	8.354912	7.667897	5.909357	6.989197	5.661421	5.799849	4.512683	5.571459	6.010351	3.281352	5.024458	5.013840	5.015654	5.791671	4.355265	4.923626	3.724305	7.141433	5.982617	5.809809	7.732409	7.287083	6.394353	7.058080	6.664453	6.931597	7.077646	6.873531	7.247604	7.490387	7.522817	7.530078	6.729818	7.266176	6.033450	4.500504	4.883686	5.136227	4.621436	7.474444	7.815587	6.779905	7.422597	6.199473	4.359819	5.297077	6.370223	5.691739	6.579778	6.300828	5.941446	4.977448	5.182396	4.245365	4.071884	3.261577	4.305947	3.332238	3.707485	2.955892	4.634359	5.544482	3.833320	3.747084	6.724487	5.242044	7.572630	5.942139	7.123062	6.241762	3.337787	5.387388	4.741289	4.690299	3.262866	6.149155	6.045183	5.498902	5.664054	4.682632	4.586707	5.637695	5.382725	3.882760	3.324325	6.822609	3.824871	3.309363	6.003613	3.474890	3.696522	5.302239	5.027227	2.925521	3.306834	5.584968	3.414692	5.304993	5.719393	7.611728	7.580232	6.728831	6.849813	5.119275	3.974446	3.643851	3.183074	3.215552	5.380962	7.340507	5.692082	6.201016	5.789407	4.255446	5.235636	7.128781	7.033428	7.181093	8.227519	8.857582	7.979109	5.880687	7.391321	5.240664	5.653127	4.214051	4.044345	5.822053	3.237851	4.173784	5.339128	4.876104	6.083687	4.907319	4.736955	3.510367	6.954541	5.172120	4.693492	8.763929	6.761727	6.720804	7.004729	7.024922	7.322955	7.439309	7.583336	7.942940	7.726996	7.008506	7.742243	5.234417	8.287784	5.947758	4.693081	4.874651	5.296253	4.168179	7.348503	7.681782	6.347920	6.674131	4.315399	3.102215	5.051601	6.721128	6.981781	6.201380	5.213607	5.007858	5.366629	4.697895	3.015098	4.153415	3.429348	4.785984	3.255474	3.332078	2.880225	4.491142	5.987550	5.296440	3.486428	7.830789	5.892768

LMLN2	7.627192	5.523280	7.464647	6.259821	4.514214	5.416020	7.532852	4.116561	4.074728	6.526670	6.434113	6.484623	6.293544	5.973953	5.870227	6.209329	5.192286	4.846179	5.044321	7.243901	4.393198	3.745473	5.864709	4.568525	4.541524	4.857954	5.081850	4.462868	5.141933	5.905442	3.858138	7.187943	6.714755	6.763922	7.890928	7.170864	6.801570	5.947758	4.321835	3.008358	5.417191	3.336784	6.043120	7.234047	5.937040	6.323377	5.257140	4.156948	4.907319	7.410771	7.082859	7.241333	8.190228	8.354912	7.667897	5.909357	6.989197	5.661421	5.799849	4.512683	5.571459	6.010351	3.281352	5.024458	5.013840	5.015654	5.791671	4.355265	4.923626	3.724305	7.141433	5.982617	5.809809	7.732409	7.287083	6.394353	7.058080	6.664453	6.931597	7.077646	6.873531	7.247604	7.490387	7.522817	7.530078	6.729818	7.266176	6.033450	4.500504	4.883686	5.136227	4.621436	7.474444	7.815587	6.779905	7.422597	6.199473	4.359819	5.297077	6.370223	5.691739	6.579778	6.300828	5.941446	4.977448	5.182396	4.245365	4.071884	3.261577	4.305947	3.332238	3.707485	2.955892	4.634359	5.544482	3.833320	3.747084	6.724487	5.242044	7.572630	5.942139	7.123062	6.241762	3.337787	5.387388	4.741289	4.690299	3.262866	6.149155	6.045183	5.498902	5.664054	4.682632	4.586707	5.637695	5.382725	3.882760	3.324325	6.822609	3.824871	3.309363	6.003613	3.474890	3.696522	5.302239	5.027227	2.925521	3.306834	5.584968	3.414692	5.304993	5.719393	7.611728	7.580232	6.728831	6.849813	5.119275	3.974446	3.643851	3.183074	3.215552	5.380962	7.340507	5.692082	6.201016	5.789407	4.255446	5.235636	7.128781	7.033428	7.181093	8.227519	8.857582	7.979109	5.880687	7.391321	5.240664	5.653127	4.214051	4.044345	5.822053	3.237851	4.173784	5.339128	4.876104	6.083687	4.907319	4.736955	3.510367	6.954541	5.172120	4.693492	8.763929	6.761727	6.720804	7.004729	7.024922	7.322955	7.439309	7.583336	7.942940	7.726996	7.008506	7.742243	5.234417	8.287784	5.947758	4.693081	4.874651	5.296253	4.168179	7.348503	7.681782	6.347920	6.674131	4.315399	3.102215	5.051601	6.721128	6.981781	6.201380	5.213607	5.007858	5.366629	4.697895	3.015098	4.153415	3.429348	4.785984	3.255474	3.332078	2.880225	4.491142	5.987550	5.296440	3.486428	7.830789	5.892768

ZUP1	10.326281	10.718958	11.074564	9.919803	9.993251	9.831983	10.626696	10.377106	9.597096	9.913924	10.202804	9.908194	9.753620	10.210139	10.399588	9.320484	9.907581	10.836911	9.829507	10.223767	10.219691	10.106859	9.827675	10.297240	10.118388	9.626627	8.841504	9.390213	9.479239	10.279265	9.801811	10.765826	10.879893	10.395050	10.851497	10.894469	10.869032	11.329984	10.799193	11.229137	9.953787	10.624904	10.518462	10.855398	10.408836	10.902031	9.205975	10.789285	10.838706	10.787776	10.626847	10.846814	10.878852	10.948465	11.294959	9.770486	11.086333	11.528035	11.066310	10.987934	11.626169	9.632756	10.062899	10.268218	11.266616	11.525597	10.927410	10.593479	10.809816	10.278971	10.424236	10.321391	9.532764	10.436890	10.385019	10.554140	10.941524	10.880464	10.780734	11.107323	11.077366	11.018364	10.997288	11.099157	10.982768	10.589516	10.910897	11.501057	11.161862	11.051785	11.126061	11.170163	10.956031	11.284152	10.366684	10.499425	10.682588	9.480573	10.157733	9.351997	9.973693	10.469964	9.700800	10.506011	10.406371	9.788816	10.520787	9.989210	10.104790	9.780722	10.642239	10.535147	10.705361	9.915301	10.662983	9.223414	10.527913	10.235895	9.145041	10.524431	10.158173	10.723866	9.622752	8.561316	9.158783	9.601584	9.550699	9.911127	9.373989	9.504734	9.701109	8.948154	10.232331	9.733489	8.564352	10.280952	10.002111	9.641485	10.556772	9.796404	10.224206	10.023382	9.688696	11.304114	9.950421	10.602394	10.377106	9.183929	10.343540	9.854951	9.502078	9.740241	11.332767	10.645044	11.205234	10.867134	11.526366	11.563303	11.387025	9.425135	11.165084	10.736706	12.075736	11.177177	11.012108	9.272265	11.283115	11.266775	10.987336	11.415733	11.144558	11.185555	11.408446	10.598330	9.194486	11.064957	12.262009	11.356089	12.325249	11.772998	8.965385	10.846071	11.115324	10.926976	11.069292	11.190467	11.104718	11.275334	11.017912	10.640900	10.948018	9.566691	10.858794	10.083729	10.396283	11.040159	11.283702	10.677496	11.297653	11.205541	10.922691	11.466898	10.604891	11.831658	9.010820	11.155520	11.395420	11.535996	11.103415	11.571323	10.401108	10.894745	11.273397	10.434911	10.041156	10.632446	9.472923	9.514128	10.281096	10.559179	9.756864	9.223120	10.461142	11.183899	9.428831	10.799956	10.658893	11.421337	9.550559	11.201144	10.404553	10.669403	9.527548	12.259261	11.083497	11.035981	10.529382	8.901711

DDIT4L	8.875412	8.414146	8.214556	8.584534	7.075764	7.898958	8.006397	8.043558	7.847939	7.642764	7.497273	7.361534	7.378933	7.475528	7.962059	7.941332	8.233292	7.903197	7.798494	8.456897	8.018603	8.204934	10.991471	7.500997	8.269821	9.145192	8.753340	8.277531	7.630032	7.376122	8.200588	8.406518	8.538157	8.378919	8.418107	8.211601	8.813200	8.549357	8.613906	8.722201	7.473225	8.855773	8.330575	8.813641	9.517769	8.117780	8.859884	8.098253	8.269361	8.363626	7.698370	8.155006	8.313750	8.699543	7.864173	7.530985	9.054717	8.558383	8.533318	8.974079	9.433276	8.245241	10.168890	8.909799	9.132911	8.341313	8.992404	8.243097	8.320195	8.557775	8.095531	8.404680	8.040928	8.431048	8.107889	8.335259	9.018370	8.080719	8.516685	7.541435	7.489279	8.722201	8.975575	8.698049	9.062320	8.838141	8.718121	8.659450	8.438853	8.370008	8.403138	9.080270	8.305437	8.218549	8.512924	7.401157	8.930003	8.052786	6.977696	7.739616	6.613319	8.570955	7.692436	7.351296	8.761524	7.162350	8.279636	8.154688	8.393936	7.885047	9.786097	7.756016	7.391479	8.150655	7.041278	6.834845	8.604053	7.831082	7.381252	7.905709	8.090338	8.949362	7.871229	6.957997	6.787415	7.671322	9.306635	7.908951	6.839827	7.255711	7.546964	7.657826	7.130837	6.437583	8.201379	8.627776	8.137701	8.457490	9.519473	8.134218	8.598963	9.613042	7.987793	7.716582	8.316068	7.966334	7.575743	7.252773	7.739464	8.805667	7.400525	7.714645	7.426476	7.458563	7.361839	8.745739	7.838309	7.838941	8.769760	6.779754	8.273458	7.233231	7.406485	7.434394	7.064451	7.008344	10.310211	7.027965	7.457779	7.991114	8.336333	7.447746	7.893724	9.685927	9.137632	8.978327	7.616951	8.058121	7.709719	7.767463	8.402059	8.263689	8.511528	8.245391	8.431370	7.648891	8.370663	7.410632	8.106793	7.956035	7.497414	7.192295	8.333829	8.541337	8.009224	9.245688	8.680959	7.904584	9.526785	7.485352	7.166260	7.867093	7.738209	8.300189	7.698069	9.069273	7.614495	8.582845	9.272401	8.212064	8.552439	9.677525	8.398665	7.376346	8.632066	7.949691	7.567185	6.893364	7.865090	6.736274	10.564580	8.381455	7.743798	7.567498	7.240875	8.123096	8.111607	8.334307	7.716582	9.384070	7.960849	7.353569	7.714215	7.393203	6.028736	8.562532	7.365458	6.949322

SPATA1	6.404576	5.555072	6.174889	6.298082	6.938425	6.933336	6.433281	5.067971	5.458089	7.069377	6.814915	6.518878	7.242596	6.985720	6.737942	6.668503	5.050399	6.671706	5.646889	6.854940	6.352776	4.221883	3.587089	6.041416	6.167736	6.239917	6.646901	5.742641	6.807572	5.715666	6.511935	6.636745	7.010741	7.522372	6.944237	6.956719	6.812716	6.142404	5.461042	6.582944	6.344898	5.574989	7.061243	6.456542	7.118530	6.143417	6.103756	6.003768	5.902912	6.318925	6.386980	6.534841	7.290501	6.767312	6.847723	6.097354	7.139220	6.197582	6.005381	6.887409	5.304215	7.149139	5.045343	5.300411	4.901741	5.295630	5.105234	5.566754	5.494903	5.044730	5.779106	6.123900	6.897032	6.516230	5.987193	6.162362	5.986628	6.282611	5.941779	7.085989	6.523509	6.195305	7.126590	6.251818	7.292400	6.540336	6.631395	7.016162	5.184411	4.950218	5.707790	4.811884	6.421917	6.274070	5.747563	6.750842	5.976379	8.101896	6.595180	6.743835	5.994659	7.418516	7.374392	6.566408	4.666915	6.615749	4.503080	5.376740	4.360255	6.998925	3.630688	3.858770	6.017258	6.579946	6.771409	3.458409	5.631552	6.285141	6.799796	5.655749	4.864946	5.386549	6.119757	3.224190	4.638845	5.518396	5.869547	6.617262	3.890045	5.513333	4.215778	4.352445	5.844914	4.470784	7.742537	5.634192	6.258961	5.106233	7.076205	4.465860	3.912495	3.175677	5.467944	3.561768	5.347947	4.262886	3.346402	4.993314	4.086810	7.393824	7.047064	6.310605	7.142561	6.522817	5.226669	6.106037	4.619929	4.419800	4.088735	5.328100	4.142802	6.908604	6.298082	5.218747	4.692443	4.953147	5.146157	5.781452	5.047064	5.489978	6.256911	5.880687	5.737892	6.434267	5.590423	6.617107	5.261653	5.477492	5.027663	4.484173	7.176828	4.038891	4.088961	5.362349	4.675819	5.422817	4.769713	4.171454	4.696606	5.029742	5.078192	3.374402	5.365392	5.767383	5.401255	5.191894	5.514243	5.397471	7.220008	7.127512	6.312967	7.020142	6.948600	6.313838	5.093331	5.787010	5.434367	4.891666	4.860848	4.328092	3.775101	4.887505	3.984782	5.256331	6.170311	5.329117	6.900296	6.099633	6.198966	5.194696	6.606374	6.224501	5.534087	3.951215	5.193853	5.072202	5.086364	4.332259	5.106656	3.141052	3.739973	3.251669	4.834368	3.432203	3.956140	5.583994	5.990959	5.067563

GGT5	10.356921	10.728828	10.361284	9.763723	9.607926	9.342816	10.143544	8.478824	8.902324	9.519632	8.997966	9.558934	10.035004	9.118674	10.350375	9.501326	9.426037	9.779816	9.554332	9.118025	9.317338	10.343540	11.440486	9.581310	9.893168	10.020621	10.614085	10.225601	9.968648	10.503158	9.707507	10.980712	10.890139	10.382254	11.229920	10.640618	10.241044	9.855565	11.180670	11.440486	9.390059	11.552884	11.113033	10.415417	11.523534	10.061488	9.454320	10.749026	10.472296	10.413447	10.793806	10.249099	11.035981	10.714317	10.269567	9.243810	11.173138	10.739298	10.270720	10.876767	10.866981	10.524734	13.080831	10.523560	11.301442	10.520787	10.591114	10.430794	10.344770	10.004105	9.955696	9.716701	9.227738	9.942734	9.966801	10.747707	10.188468	11.085434	10.225898	10.601523	10.398075	11.021682	10.171845	10.243523	10.113777	10.037784	10.298333	10.399138	10.897486	10.676730	10.406784	10.618976	10.660235	9.951687	10.332961	9.950580	10.583475	9.760255	9.296921	9.521779	10.336597	9.980403	10.099503	9.977682	9.705338	9.792229	9.532146	10.019869	10.751156	9.139963	10.474815	9.759027	9.445444	9.367576	8.926942	10.315931	10.902636	9.282306	9.372256	10.217279	11.352736	11.591169	10.695729	10.557083	9.657788	10.400199	10.997004	11.081461	10.194330	10.411931	10.770741	11.010354	11.592359	11.464200	10.399733	11.680518	10.393559	10.833447	9.425426	10.237063	12.193531	11.437253	9.697251	11.872045	12.169426	10.294779	10.213349	10.091891	11.069134	10.308167	11.673716	11.888876	10.327943	10.744255	10.188320	11.666576	10.929185	11.079978	13.078931	10.119452	12.374199	11.037869	11.120569	11.248076	9.927990	9.938539	11.195637	10.646484	11.647387	11.490236	11.118406	11.011977	11.563461	11.791671	10.207514	11.063499	11.183899	10.647119	11.274151	11.582720	10.912348	11.266180	11.490236	11.311166	11.653340	11.238920	11.490850	11.341014	11.135510	10.215321	11.067626	10.027958	10.854645	11.190758	11.124739	11.266314	11.192374	10.695729	11.863940	11.295839	10.445039	11.053092	12.174430	11.997321	11.342331	11.035981	10.265002	11.710430	12.250230	11.237000	11.220719	11.413991	11.989871	11.443526	12.031682	11.568999	10.521909	10.883564	9.725752	12.133853	10.151430	10.721360	11.639475	11.077806	10.139853	10.219240	9.789901	10.300015	10.186361	11.558177	11.998857	10.724471	9.744747	9.854464	9.374473	12.025727	9.265515	10.539254

ZNF74	8.652204	8.503262	8.384656	8.892055	9.051442	9.230442	9.109422	8.084927	8.662390	8.994416	9.272549	9.421747	9.461373	9.720646	8.903998	8.665616	8.779775	9.692841	9.163075	8.974230	8.969270	9.032204	8.856221	8.987320	9.589654	9.092054	8.838141	8.943997	8.622365	8.564961	8.998099	9.016869	8.567166	9.083635	8.545657	8.622673	8.846470	8.921205	9.303094	9.085673	8.633227	8.587540	8.369704	8.784155	8.576701	8.818720	8.903998	8.484010	8.564511	8.706598	8.367911	8.919067	8.883900	9.016215	8.485965	8.997519	9.022956	9.247361	9.566691	9.298766	8.769584	8.602527	8.751131	9.152473	9.634169	8.596736	8.846939	8.978787	8.660064	8.834502	8.224643	8.339643	8.826707	8.440271	8.432106	8.203624	8.681590	8.596272	8.460937	8.644401	8.628077	8.803350	9.489658	8.937335	8.973345	8.810289	8.762856	8.790260	9.413036	8.806427	8.502465	7.993011	8.463529	8.663004	8.561173	8.725722	8.246658	8.966798	9.371801	8.902924	9.169683	8.676059	8.853327	8.750837	8.368909	8.841504	9.431265	9.097671	8.977263	9.023274	8.929856	9.526496	9.459710	9.092054	8.488015	10.039433	9.656270	8.777073	8.911573	8.942331	9.366138	8.450767	10.078051	10.218179	9.755469	9.776589	10.038406	9.802581	10.261790	9.734237	11.007861	10.245723	10.507824	9.748034	9.028112	9.300335	10.509626	9.900076	8.959765	9.652740	9.683888	9.979804	9.681699	11.118110	9.587971	9.293482	10.258512	9.608373	9.277692	9.340284	9.348012	9.929695	9.278313	8.614047	9.883041	9.042373	9.404986	10.134213	9.812137	9.380048	9.022179	9.278749	9.469018	9.199695	9.146860	9.308181	10.228365	9.947800	9.481658	8.910390	9.809845	9.856818	10.315047	10.266163	9.547611	10.031627	10.472987	10.884769	10.176090	9.148313	9.594486	9.103800	9.845032	9.993987	10.550696	10.021856	9.004108	9.706104	9.755312	8.885604	8.680202	9.629836	9.251636	8.967900	8.376467	9.046071	9.004108	9.106123	9.022477	9.355444	9.755013	9.585147	9.294904	9.740870	9.753921	9.394660	11.123825	9.338465	9.123673	10.242296	8.365170	9.317338	9.791103	9.174793	9.191181	9.372405	9.461985	9.743643	9.852612	10.268083	9.890739	10.107768	8.851683	8.602666	9.865449	9.942574	9.247657	8.626562	9.327364	9.274549	9.790950	11.135818	9.449989	9.761985	9.271071	10.297240	9.176193	10.392965

PROK1	4.206388	3.806389	3.274657	2.969108	3.512383	4.128728	3.372905	3.286678	3.640900	3.388422	3.524206	3.558339	3.379304	3.722298	3.299620	3.161484	3.468400	3.495782	4.980930	3.436845	4.600612	3.158149	3.512749	3.082345	3.624039	3.361140	3.207343	3.236957	3.267949	3.425458	3.473243	4.134718	5.113352	3.508701	4.425257	4.019348	4.571992	3.617097	4.384713	3.455153	3.936458	3.760082	3.735018	3.209109	3.543981	5.212774	3.141697	5.101129	3.296210	4.142605	3.954187	5.671238	4.343091	4.150003	3.678209	3.154930	5.366204	4.993314	4.418223	4.042528	3.677597	3.495403	6.799452	3.295582	3.497641	3.435989	3.763184	4.270510	3.801618	2.965075	3.274802	3.033479	2.994477	2.956161	2.994026	3.638474	3.242519	4.060633	3.029478	3.714449	3.358117	3.799007	3.116070	4.268916	3.479650	5.165298	4.464384	3.424029	4.592522	3.776565	3.547577	3.832048	5.881927	3.497287	3.115819	2.956909	3.404877	3.192946	4.038230	2.970823	2.952475	3.224618	2.971440	2.916877	4.421071	3.480575	3.089786	4.208643	3.297455	3.268982	2.856302	3.502944	3.259725	3.462866	3.830333	3.285805	3.694253	2.981829	2.988814	3.425827	3.412024	3.512576	3.167274	3.660010	4.996722	3.256210	3.562130	3.335626	3.187010	3.319425	3.528312	3.132332	3.189289	3.081673	3.267793	3.467279	3.630489	3.461300	3.417894	3.977405	2.965377	5.508966	4.515737	3.505520	3.258697	3.156291	3.792920	3.245966	3.561139	3.274657	3.442220	3.608791	3.282239	4.230278	3.720995	4.474088	3.947691	3.514626	3.396300	3.458761	3.721214	4.114601	3.283950	3.803477	4.686559	4.281683	4.806984	3.447839	4.378669	3.995744	7.902414	4.663894	4.212080	3.295748	3.242102	4.657710	4.963356	4.353117	4.159074	3.350218	3.941921	4.906899	3.896934	3.867507	3.596508	4.257186	4.551538	3.990914	2.976862	3.351900	3.072161	3.764064	3.088322	2.918334	3.950770	3.125395	3.355689	3.576521	4.181013	3.751463	3.949881	3.101859	3.662866	4.087434	3.809313	3.247223	3.398675	4.324870	3.688884	3.865968	3.520622	3.045474	3.158535	3.050591	2.969894	2.968362	2.988244	4.078669	3.304598	3.015866	3.094313	3.013169	3.128332	4.837863	3.575730	3.004535	4.029966	4.038891	3.171902	3.210048	3.392134	3.064605	3.587089	3.600306	3.454372	3.416712	3.261885	3.925461

IMMT	12.612171	11.828401	11.953478	12.127344	12.266082	12.355477	12.171733	12.229260	12.258508	12.428822	12.575087	12.281610	12.365418	12.400126	12.362443	12.334279	12.390154	12.186187	12.811111	12.281444	12.617952	13.240605	13.393505	12.074680	11.706718	12.475223	12.619480	11.845543	12.450102	12.242384	12.083384	12.400294	11.925502	12.043495	12.369716	12.188852	12.513519	12.182366	12.572689	12.273123	12.607071	12.146729	12.604730	12.394589	12.653292	12.255993	12.394751	11.708808	12.223705	12.550893	12.113448	12.576396	11.841912	11.912793	12.358783	12.197370	12.114358	12.001171	12.108321	12.039701	12.102972	12.200477	12.862587	12.652767	12.128125	11.904615	11.932456	11.770940	11.908190	12.135906	11.621897	11.775366	12.389361	11.730804	11.841136	11.848510	12.253427	11.840239	11.834043	11.473964	12.093826	12.227834	12.114571	11.846434	12.240559	11.983657	12.054398	12.041080	12.382585	11.908646	11.701300	12.125403	11.826393	11.740862	11.835877	12.272346	12.029621	12.656189	12.441276	11.923119	12.346468	12.061997	12.075279	11.759188	12.682137	12.581318	12.428822	12.503556	13.158034	12.526615	12.888091	12.377553	12.493046	12.454709	12.081665	11.655296	12.390923	12.400126	12.435481	12.744209	12.624160	12.455951	12.974908	12.275329	12.933477	12.874685	13.341990	12.808222	13.352500	12.372749	13.389482	13.266591	12.709269	12.986119	12.783797	13.614714	13.256418	13.703179	13.091627	13.198141	13.211293	13.699452	12.961589	13.402503	13.185843	12.338888	12.883472	13.028986	12.928312	12.607232	13.154762	12.943559	12.432750	12.857615	13.220585	12.312544	12.417662	12.854406	13.227647	12.932065	12.817846	13.633991	13.023099	12.967314	13.023099	12.638870	12.986468	12.884027	13.162836	12.384330	12.722802	12.721811	12.819043	12.790316	12.911640	12.307156	12.984090	12.633137	12.405442	12.663564	12.779681	13.094478	13.079487	12.688304	12.027939	12.275765	11.933343	12.646916	12.285365	12.549730	12.170796	12.621157	12.810001	12.539646	12.071131	11.867888	12.748989	12.424576	11.795120	12.719045	12.365907	12.384180	12.676682	12.294510	12.683833	12.850884	13.081595	13.124397	12.558801	12.813513	13.258800	12.165444	12.652437	12.357030	12.645037	12.617456	12.697518	12.544554	12.923860	12.829114	12.376134	12.400969	12.013408	13.283632	13.034495	13.346771	13.095579	13.109649	12.617130	13.583239	12.824533	13.022170	12.418807	13.022344	11.922337	12.405908	12.810731	13.530568

BLOC1S5-TXNDC5	3.730665	3.284757	3.140672	3.173828	3.136966	3.656622	3.303468	3.318473	3.364044	3.419704	3.543818	3.671565	4.036922	4.355265	3.332891	3.323979	3.592419	3.892547	4.068163	3.729835	4.640321	4.300454	4.833536	3.363039	4.279959	3.092443	3.894646	3.059176	3.644431	3.479271	3.300671	3.519105	4.223393	3.889387	4.075989	3.998940	3.789513	3.234379	3.188883	2.909736	3.548744	3.252362	4.036272	4.964786	3.579575	5.411685	4.376122	3.365338	3.520038	3.985181	4.029509	5.123314	4.463269	3.937709	4.887925	3.138221	3.943276	2.899623	3.455487	3.163655	3.063626	5.338929	3.535179	3.793159	3.857219	3.556033	2.843198	3.227317	3.671130	3.117321	3.032967	3.754031	5.489978	3.699384	3.119618	3.293629	3.570765	3.222815	3.395031	3.639290	3.491512	4.033222	3.410052	3.962030	3.391393	4.798317	3.938536	3.210048	2.929714	3.359512	3.783575	2.936081	3.084037	3.483154	3.561768	4.009111	3.838884	3.862586	3.253117	3.527928	3.407994	3.520038	3.498184	3.406231	4.106504	4.468199	3.348056	3.352560	3.962694	3.693202	3.841899	3.211011	3.305084	3.589021	3.211573	4.915556	3.379304	3.756526	3.547999	3.422630	3.393483	3.058514	3.408711	3.092443	3.533311	3.069362	3.353204	3.399682	3.312092	3.411831	3.462151	3.682691	4.587555	3.128807	3.603220	3.575730	3.180813	3.416880	3.407494	3.954808	4.246657	6.008773	3.741804	2.900629	2.859593	4.048439	2.941644	3.071974	3.529043	3.163529	3.312092	5.019719	3.355179	3.788448	3.734799	3.703635	4.110281	3.130356	2.863598	3.297766	3.027576	3.541120	5.019510	3.967702	4.896655	4.331654	3.411831	3.769910	4.101480	4.068386	5.109134	4.141719	4.036699	3.435989	3.206648	3.517551	2.879575	3.089474	2.966530	2.950032	4.707020	3.544145	3.478413	3.438428	3.920809	2.825823	3.418075	2.978509	3.024459	3.024654	3.282477	4.185545	3.330300	3.026669	3.276359	3.034513	3.227610	3.770910	3.212842	3.681040	4.292475	3.662866	3.559932	3.447839	3.701577	3.183972	3.374079	2.880225	3.235083	3.243676	2.900459	3.017610	3.246827	3.201577	3.583598	3.193531	3.706468	5.336143	3.195044	3.401766	4.088961	3.354520	2.928427	4.551538	4.026235	3.412735	3.521143	3.341985	4.044741	4.233080	3.549657	4.874867	3.368755	3.021136	3.318958	3.773677	3.705674	3.329843

KCNIP3	3.680445	4.253430	4.360915	4.672289	5.884779	6.211381	4.885618	5.183414	5.787748	4.821688	5.566556	5.286792	5.145729	4.711587	4.931589	5.465790	6.090612	5.528211	5.034847	5.040958	5.416211	5.555257	4.481977	5.188407	6.011036	5.872251	4.863937	5.113938	5.428030	5.193075	5.210432	3.601857	4.593406	5.087783	5.237472	4.728053	5.781067	5.012009	5.540532	5.987193	5.468945	5.278530	5.764109	5.907218	5.382322	4.393599	5.408723	2.928005	4.613787	4.184692	5.318242	5.564486	5.665725	4.987884	5.385402	5.245221	5.457488	4.052367	5.337890	5.095208	5.385211	4.934289	5.540166	5.456657	5.341422	4.527587	5.182603	5.067361	5.478682	5.427843	5.286398	4.471699	5.960403	4.928275	5.610876	4.849503	4.541524	3.873734	4.049098	5.777806	4.640929	4.895184	3.726139	5.932102	4.757829	5.367778	4.132996	5.000064	3.708262	4.699627	4.959367	5.111332	5.226103	5.112145	4.811884	4.961018	4.613377	5.763933	6.276203	5.709572	5.277938	5.035062	5.390063	5.848095	4.056495	5.412417	5.492043	5.015058	5.380962	5.231998	5.429568	6.124641	4.759750	4.407127	5.864878	6.207422	5.579462	5.053865	5.261653	4.927430	5.291773	5.313012	4.950416	6.867559	6.189096	5.244823	6.365725	5.101551	5.367011	5.097372	4.993534	6.753057	3.936016	5.887979	4.271401	5.818676	5.573354	6.802029	5.411498	5.008885	6.469202	6.354414	5.396153	5.601135	4.224260	4.828672	5.696389	5.870975	5.278766	4.826274	4.083468	4.917062	4.909265	6.853335	5.067778	4.974843	4.004983	4.187994	5.472341	4.517429	4.769494	6.008246	5.520232	5.477861	3.764916	4.929972	7.908951	4.700882	4.055620	4.108703	4.822497	5.116800	4.109365	4.270081	5.309024	5.906683	3.626812	3.889854	5.229116	4.839616	5.506401	4.638012	5.140676	5.332458	4.388072	5.894762	4.987675	4.562740	3.074230	5.431227	4.397453	5.508966	4.695568	3.732140	3.926325	4.561637	4.512683	4.101480	4.987279	5.110560	4.286569	3.213524	5.248221	3.417742	5.492799	4.229455	4.638845	4.989560	4.356108	8.280091	4.344134	5.103791	4.708061	4.374789	4.527383	5.930416	5.384847	5.784855	5.217569	4.877385	6.006620	6.482902	3.634068	5.210637	5.315660	5.106012	4.587333	6.910490	5.046672	5.518195	4.755730	6.851553	5.562223	6.283964	5.957686	4.802838	5.057972	4.600200

NSD3	7.410476	8.056362	8.165502	7.010422	7.056010	6.628874	7.454005	7.501815	7.863395	7.080341	6.832128	6.646089	6.811769	7.045467	7.568409	7.277658	6.863457	7.636774	7.440209	7.547904	6.571931	7.905401	7.050084	7.187316	6.757487	6.767805	6.478640	7.250091	6.456215	7.426143	7.206128	8.139275	8.227519	8.008078	7.845026	8.301065	7.838143	7.918437	7.981607	7.925226	6.602297	7.925989	8.089401	8.102949	7.758398	8.905075	6.621787	8.628667	8.872478	8.178706	8.051705	8.540372	8.771577	7.961924	8.846008	6.835965	8.148080	8.702430	8.084278	8.231164	8.473429	6.696196	7.827120	7.882612	9.144754	8.652809	7.849606	8.258942	8.242770	7.685481	7.473547	7.674175	6.897684	7.780597	7.537785	8.136969	8.012895	8.278120	7.859886	8.530464	7.614356	8.808590	7.945865	8.291667	8.117316	8.008230	8.101896	8.909799	8.079308	8.163384	8.187149	8.743149	8.024380	8.603917	7.856797	7.732109	8.457490	7.119181	7.156225	7.830635	6.692759	7.552843	7.204235	8.101596	7.865227	7.762049	7.760209	7.999789	7.367028	6.484435	7.703286	7.969404	7.737275	6.846765	8.007627	5.326937	7.360909	7.752553	6.517397	7.829918	8.308548	8.184337	6.944562	5.551813	6.967570	6.241436	7.391638	9.106902	8.584972	6.587534	7.243420	6.663285	5.960212	6.451641	7.076052	7.082365	6.900947	7.276417	8.641739	7.288615	8.395927	8.249148	6.985241	6.468844	7.254764	7.469869	10.107923	7.171017	7.090251	6.491222	6.908762	5.951816	8.055096	7.639694	9.136379	8.248407	8.574581	8.002152	8.627169	6.242269	9.174641	8.984712	9.925324	9.112425	11.365974	6.461996	8.875260	8.624351	8.636758	9.965013	9.369675	9.530244	8.416609	8.739179	6.610626	7.168714	10.247991	10.448243	9.150322	9.378381	6.287413	8.857724	8.855773	8.584387	9.578383	8.144863	8.447016	9.028410	8.027318	8.688827	8.739777	6.447523	7.933245	8.176794	8.185409	8.088027	8.734549	8.350523	7.630374	7.740862	8.278869	8.637389	7.925376	8.670782	6.824667	8.271789	8.897010	7.333524	8.709164	8.628231	8.560387	7.625330	9.772221	8.483559	9.235949	7.733781	6.649508	7.901488	9.667475	7.220170	7.964491	7.424921	8.532874	7.518806	7.183775	8.474166	8.530777	8.608629	6.004842	8.815433	6.669775	9.036791	6.326915	9.256514	7.932329	7.679233	9.720646	6.504316

ZCCHC7	6.883914	6.306442	6.226940	6.857226	7.245330	6.855573	6.005942	7.104871	6.911932	6.972602	7.002587	6.466356	6.711800	7.220497	5.152764	6.951242	7.229974	6.500534	6.872894	6.779094	6.121129	6.750694	6.652886	7.039382	6.537460	6.289289	6.701586	6.967877	6.821815	5.431227	6.903450	5.318802	6.187067	6.422546	6.410385	5.832995	5.560034	6.244301	6.270689	6.992106	6.742581	5.879233	6.157198	6.908604	6.394833	6.533404	6.411686	6.283964	6.822140	6.572895	6.280919	6.671872	6.257597	5.961628	6.420124	6.845392	6.424002	5.562589	6.822918	7.313376	6.297553	6.418988	5.552587	6.837058	5.970502	6.687619	6.298401	6.647547	6.340875	6.640481	6.041579	6.165285	6.842863	6.393549	6.032361	6.399974	6.639018	6.290462	6.386626	6.384336	6.497681	6.293193	6.060619	4.715396	6.503324	6.234471	6.110778	6.142242	5.861216	6.509278	6.844547	6.434967	5.825139	6.090105	5.759620	6.277838	6.529653	6.836732	6.079207	6.520703	6.685825	6.918844	6.864078	6.808714	6.534041	7.352520	6.897684	6.684561	6.006989	7.160111	6.440230	7.193263	6.588854	6.601017	7.576179	5.210230	7.122109	7.032489	6.680307	6.384499	7.016496	5.640130	7.162520	6.467195	6.862821	6.369034	6.392873	7.299240	7.200399	7.053316	6.425977	6.029738	6.454306	5.217960	6.097512	6.900460	7.209572	6.160498	6.844034	5.324597	6.569550	7.308644	7.409576	5.778374	7.043338	6.147599	8.323674	6.890923	5.755174	6.643033	6.254711	6.903996	5.966457	6.029259	6.754244	5.834830	6.092548	6.628397	6.286020	6.815409	5.731037	6.941340	6.869059	6.445578	6.296916	7.228270	4.998379	7.055251	5.968070	6.067343	6.729672	6.955972	6.840459	5.701700	6.562022	4.860639	5.979585	6.590464	6.821498	6.230434	5.723646	6.142404	6.974953	5.803658	6.040557	6.451474	6.298082	6.609828	6.646901	7.212483	6.197582	6.908295	6.244135	5.802237	6.406070	6.171336	6.269643	6.459196	5.938282	6.666388	6.934087	6.521055	5.736795	6.863627	6.182888	5.934404	6.563515	5.913674	6.027309	6.346068	6.068929	5.554151	6.120281	6.184495	6.878132	6.094796	5.932632	6.715371	7.187472	7.192697	6.174379	7.200242	6.019537	6.391555	7.173526	7.117059	6.749580	5.601329	7.556289	5.650101	6.564495	6.740701	6.628233	7.272360	6.005203	6.773312	7.625464	7.033749

CNOT3	8.977860	9.208692	8.926121	9.356962	9.919312	9.912662	9.352445	9.892267	10.112153	9.823884	9.667475	9.604702	9.127794	9.297552	8.690022	9.736555	9.581469	9.061578	9.667023	9.313132	9.538754	9.444839	9.883041	9.535054	9.645648	9.522722	10.199467	10.234963	10.387179	9.614826	9.786539	8.333380	8.623284	9.332164	9.102118	9.033112	9.064136	9.205189	9.339034	9.279993	9.935273	9.209941	9.055670	9.426499	9.134862	8.568954	10.447500	9.153845	8.709164	8.832252	8.838597	8.577147	8.647979	8.523224	8.475419	10.193244	8.204629	8.787977	8.883274	8.537536	8.539743	9.734777	9.348480	9.408176	8.841352	8.798095	9.302345	9.225910	8.998880	9.298453	9.339624	9.363485	10.037784	9.059279	9.142279	9.074192	9.332770	8.944434	9.298003	8.442240	8.350047	8.210651	8.159168	8.167811	8.167506	8.674050	7.975701	8.084588	8.108784	8.096606	8.182665	8.129022	8.747882	8.832900	8.969980	8.929239	8.222690	9.322003	9.536303	9.880715	9.718945	9.504085	9.633365	9.416264	9.310956	9.779969	9.505806	9.733932	9.934635	10.243981	9.283080	10.041611	9.588735	10.425458	9.498874	11.021084	9.491581	8.917711	10.335529	8.903060	9.596485	9.148011	9.699709	10.852571	9.761351	9.827819	10.409077	9.778612	9.896278	9.650553	9.785797	9.867261	9.567790	9.518073	9.869780	9.497175	9.491898	9.903042	9.022477	9.344082	9.780402	9.737303	10.137446	9.608075	9.292212	9.770001	9.907741	10.674193	9.711867	10.118388	9.053125	9.180782	9.486874	9.204729	9.038186	9.008878	9.422644	9.803798	9.409559	10.262244	9.084243	9.039156	9.639954	9.011895	8.615278	10.494529	9.158502	8.819635	9.013927	8.853466	8.785471	8.762856	8.970518	8.745438	10.012145	8.650870	8.845259	8.926272	8.310673	8.921205	10.011667	9.308487	9.254788	9.207632	8.856845	9.552979	9.172804	9.103956	9.376503	9.397158	9.310358	10.455568	9.188155	8.994252	9.100411	9.382113	9.214227	9.190243	8.567616	8.443325	8.445159	8.551510	8.353838	8.439798	9.249360	8.170810	8.103712	8.607116	8.482792	8.791520	8.389280	8.968517	9.302942	9.182666	9.234409	9.113587	9.606716	9.544579	9.373040	9.698934	9.839846	9.916212	9.259079	8.914640	10.118700	9.774780	9.308633	9.157717	10.340507	9.302942	10.077125	9.977119	10.663706	9.567958	9.670386	9.237341	9.023274	9.774181

METTL3	12.844050	13.079117	13.184093	13.095579	13.338827	12.610356	12.876754	12.509969	12.536108	12.862032	12.873750	12.852985	13.008477	12.994721	12.931521	12.792190	12.840179	13.082154	13.512208	13.012290	13.119251	13.516687	13.899396	12.897590	13.305150	13.218764	12.927197	12.931521	13.041185	12.769791	12.966743	12.807870	13.188209	13.274601	12.843523	12.888978	12.937441	12.937259	13.359085	13.219365	12.994903	13.308756	13.077603	13.179573	13.475325	13.186053	13.000629	12.719368	12.927977	12.956055	12.700204	12.999520	13.029529	12.827344	12.985374	12.774141	13.060993	13.273173	13.071141	13.206916	13.324998	12.616658	13.657220	13.244322	13.072631	13.129969	13.100310	13.250452	13.097867	13.343265	12.960870	12.867959	12.818398	12.816972	12.969135	12.987020	13.016253	12.705699	12.877121	13.124002	13.142835	12.934012	12.838440	13.246114	13.135842	13.052959	12.992703	13.175984	13.442036	13.148076	13.197125	13.446919	12.919720	12.867959	13.094857	13.117722	13.016439	12.953847	12.832730	13.122311	12.791340	12.827182	12.940276	12.977133	13.451597	12.814750	13.198919	13.603336	13.457435	12.917303	13.472047	13.168458	12.896890	12.710652	12.625338	12.276374	13.124002	12.874301	12.784685	13.116011	13.202774	13.170169	13.158588	12.816972	12.694415	12.784870	12.541107	11.821698	12.646567	12.550549	12.932335	13.241587	12.978217	12.741114	13.061923	13.003777	13.327590	13.497565	12.284248	12.870487	12.632301	13.452250	13.305570	12.752609	12.764272	12.519046	12.479130	12.214094	12.523307	12.953485	13.026732	13.103703	12.675008	12.994008	12.915341	12.602397	12.431923	12.532342	12.826984	12.977674	12.846159	12.398264	12.876249	12.919886	13.365165	12.582308	12.344637	13.171124	13.170547	12.770158	13.325421	12.996741	13.425338	13.115266	12.417520	12.980792	12.888091	13.083488	12.423912	12.479650	13.197314	13.007936	13.015899	13.026368	13.255784	13.010698	12.744556	12.674660	12.872130	12.507547	12.661720	12.698701	13.441364	12.696624	12.532185	13.018269	13.199727	12.997893	13.188004	12.996396	13.291349	13.010122	12.825950	13.125775	13.329024	13.751801	13.121156	13.377562	12.965086	13.040804	13.365165	13.075147	13.309538	12.562209	13.093696	13.109263	12.766196	13.189322	13.102943	13.400180	13.178186	13.182498	12.568552	13.518872	12.703984	12.909129	13.014469	13.112389	12.586302	13.406497	12.812303	13.739872	12.684378	13.815313	12.638368	12.958825	13.505905	13.221376

TOX4	12.844050	13.079117	13.184093	13.095579	13.338827	12.610356	12.876754	12.509969	12.536108	12.862032	12.873750	12.852985	13.008477	12.994721	12.931521	12.792190	12.840179	13.082154	13.512208	13.012290	13.119251	13.516687	13.899396	12.897590	13.305150	13.218764	12.927197	12.931521	13.041185	12.769791	12.966743	12.807870	13.188209	13.274601	12.843523	12.888978	12.937441	12.937259	13.359085	13.219365	12.994903	13.308756	13.077603	13.179573	13.475325	13.186053	13.000629	12.719368	12.927977	12.956055	12.700204	12.999520	13.029529	12.827344	12.985374	12.774141	13.060993	13.273173	13.071141	13.206916	13.324998	12.616658	13.657220	13.244322	13.072631	13.129969	13.100310	13.250452	13.097867	13.343265	12.960870	12.867959	12.818398	12.816972	12.969135	12.987020	13.016253	12.705699	12.877121	13.124002	13.142835	12.934012	12.838440	13.246114	13.135842	13.052959	12.992703	13.175984	13.442036	13.148076	13.197125	13.446919	12.919720	12.867959	13.094857	13.117722	13.016439	12.953847	12.832730	13.122311	12.791340	12.827182	12.940276	12.977133	13.451597	12.814750	13.198919	13.603336	13.457435	12.917303	13.472047	13.168458	12.896890	12.710652	12.625338	12.276374	13.124002	12.874301	12.784685	13.116011	13.202774	13.170169	13.158588	12.816972	12.694415	12.784870	12.541107	11.821698	12.646567	12.550549	12.932335	13.241587	12.978217	12.741114	13.061923	13.003777	13.327590	13.497565	12.284248	12.870487	12.632301	13.452250	13.305570	12.752609	12.764272	12.519046	12.479130	12.214094	12.523307	12.953485	13.026732	13.103703	12.675008	12.994008	12.915341	12.602397	12.431923	12.532342	12.826984	12.977674	12.846159	12.398264	12.876249	12.919886	13.365165	12.582308	12.344637	13.171124	13.170547	12.770158	13.325421	12.996741	13.425338	13.115266	12.417520	12.980792	12.888091	13.083488	12.423912	12.479650	13.197314	13.007936	13.015899	13.026368	13.255784	13.010698	12.744556	12.674660	12.872130	12.507547	12.661720	12.698701	13.441364	12.696624	12.532185	13.018269	13.199727	12.997893	13.188004	12.996396	13.291349	13.010122	12.825950	13.125775	13.329024	13.751801	13.121156	13.377562	12.965086	13.040804	13.365165	13.075147	13.309538	12.562209	13.093696	13.109263	12.766196	13.189322	13.102943	13.400180	13.178186	13.182498	12.568552	13.518872	12.703984	12.909129	13.014469	13.112389	12.586302	13.406497	12.812303	13.739872	12.684378	13.815313	12.638368	12.958825	13.505905	13.221376

FNBP1	11.812728	12.558148	12.554462	12.425904	12.285365	12.210054	12.900773	11.737922	11.554922	12.286161	12.346154	12.218642	12.150502	12.623334	12.230950	12.245922	12.700204	12.456287	11.854007	12.185867	11.768414	12.540137	13.670785	12.562057	13.123808	12.640723	13.601268	12.599341	12.639201	12.663072	12.254526	12.487643	13.340559	12.813876	12.997893	13.037071	12.766030	12.664778	12.550217	12.110262	12.295874	12.009153	13.132988	12.469402	14.201495	12.879950	13.073608	11.944822	12.695090	12.523982	12.329711	13.001778	13.180364	12.954599	12.811274	12.344708	13.522074	13.451381	12.672938	13.769838	12.483405	11.769756	13.329864	11.826393	11.878774	12.846651	12.511451	12.212707	12.184541	11.382901	12.236220	12.332394	12.445946	12.895995	12.122241	12.933477	12.830643	12.458016	12.055128	12.857967	12.532019	12.576723	12.718866	12.711956	13.214589	12.591334	12.773448	12.900238	11.719874	11.873552	12.496182	11.952432	12.436614	12.838265	12.572023	12.821082	12.816646	12.664433	12.443364	13.677586	12.331442	13.209513	12.624487	12.644395	11.369313	12.373685	11.047378	12.391754	11.519102	12.607714	11.824133	11.792112	11.792259	11.642056	11.742598	10.270279	11.305602	13.052245	12.669498	11.874918	10.872641	11.712469	11.263797	8.793066	10.695729	11.160511	11.264551	11.056421	10.332682	10.584914	11.052063	12.047224	11.072137	10.362696	12.838265	10.365612	11.450002	12.354988	11.642537	10.917350	11.153776	11.836026	11.026742	10.581306	11.481940	10.338376	9.857253	10.395884	11.236266	12.308556	11.440322	10.964747	10.886871	11.909110	11.428576	11.806639	11.317056	10.037488	11.296282	8.514597	11.056587	12.668647	10.935332	11.200022	11.375921	10.489519	10.816421	11.754375	11.079831	11.230200	12.051785	12.148694	11.586749	12.659975	12.411098	12.109661	12.050737	11.628942	11.884500	10.690069	13.038398	9.592603	10.822224	11.293530	11.568275	10.778128	11.246365	11.102062	11.053797	11.788222	11.219825	9.788347	10.929640	12.138484	11.920101	11.352031	11.089903	11.079127	12.726851	11.876585	10.747087	11.280187	12.111299	11.128957	11.453855	11.174150	11.153328	11.416462	12.066244	11.349360	10.011667	11.587908	10.671810	10.999538	11.279734	10.978030	11.110303	11.172831	11.746557	10.230524	10.734462	12.045149	12.204516	10.370484	11.362983	9.480728	11.280035	10.691815	11.458159	11.845386	10.770741	11.841289	11.019905	10.076651	9.994317	11.416046	10.653251	10.512586

CCM2	11.708507	12.257887	12.235143	11.976530	12.508819	12.168033	12.149735	11.454736	11.236266	11.929527	11.911476	12.116196	12.353725	12.166484	12.172787	12.345812	11.739666	12.273123	13.005778	12.422636	12.648482	13.191901	12.183450	12.582617	12.028097	12.423432	13.415241	11.922034	12.445148	12.593944	12.147040	12.353725	12.864398	13.177598	12.294973	11.952291	11.842049	12.138334	12.368451	12.297752	12.242384	12.670521	12.196906	11.934440	12.095335	12.288638	12.342018	12.215618	11.888108	11.808875	11.904615	12.138038	12.152235	11.509057	11.823222	12.228152	12.146883	12.464531	12.528560	12.651411	12.599699	11.974803	13.468126	12.586457	13.577816	12.224769	12.573864	12.239785	12.502567	12.795380	12.289078	12.063353	12.025558	12.111299	12.330024	12.543245	12.099519	11.943639	11.779706	12.564301	12.126571	12.016290	12.156958	12.370033	12.210352	12.152049	12.630345	12.272641	13.221167	12.675358	13.038580	13.022716	12.318871	12.114644	12.211771	12.256640	11.776227	11.784937	12.097254	11.639167	11.354771	11.869488	11.927863	11.608175	12.503389	12.003535	12.262785	12.650060	13.124799	12.106547	12.207764	12.464048	12.688794	11.968105	11.818403	10.541677	12.640046	11.539250	11.968868	14.468896	13.354792	12.928845	14.517783	12.683500	13.002332	13.357219	12.296044	14.042877	13.653913	13.603336	12.658466	13.631382	13.671017	13.924569	13.230081	13.426653	12.938700	13.366218	12.853682	13.009941	13.974138	13.537063	13.708684	14.534643	13.645878	12.362917	13.342844	12.678191	13.552076	13.770612	13.399537	12.887549	12.848581	12.847364	12.700895	13.151653	13.507015	14.044056	13.873413	13.891542	13.812737	12.159874	13.401844	12.322660	12.711633	13.413997	12.490403	12.148397	13.074750	13.747829	13.002332	12.768169	13.810378	13.158236	12.966913	12.851230	13.335408	13.632774	12.848215	14.050643	13.980251	13.868078	14.153935	13.426259	13.531007	13.802855	13.221376	13.119251	13.940199	12.514995	12.945707	13.443956	12.959007	12.972576	13.037427	13.155145	13.504360	13.196712	13.569652	13.462029	13.421932	14.048540	13.295547	13.716537	13.549809	13.340998	12.436293	14.254446	13.825824	12.993637	14.335422	13.066403	13.592616	13.456831	13.488803	13.296970	12.385620	13.315174	12.339855	14.382758	11.727947	12.443548	12.537757	14.314071	12.731001	13.272778	13.141252	13.021758	13.038398	12.738848	13.390157	12.032911	12.362917	12.529854	13.265809	13.218380	14.159282	13.973837

PICALM	11.426795	11.320390	11.676671	11.305887	11.358243	11.453711	11.330888	11.736567	12.068901	11.516068	11.309400	11.290061	11.448524	11.236568	10.769710	11.758435	11.503490	10.800096	9.938693	11.425646	9.821926	10.299864	10.079015	11.337903	11.073280	11.372737	11.689898	11.738805	11.370827	11.574642	11.753936	11.754375	11.816892	10.947309	11.576165	11.438510	11.492440	11.100738	10.522222	10.915826	11.823222	10.715070	11.020059	11.488900	11.333614	11.382171	11.751899	11.151708	11.956666	11.423905	11.243314	11.489358	11.568715	11.552438	11.671934	11.687522	11.791671	11.012515	10.698977	11.463470	10.688732	11.796425	11.400756	10.590086	10.799059	10.938211	10.971908	10.734602	10.886712	10.809187	11.378139	11.377987	11.836470	11.598426	11.342479	11.435520	11.557301	11.701300	11.230200	10.216834	11.608755	11.787155	11.477556	11.847129	11.444404	11.348648	11.423597	11.581815	10.702889	10.823692	10.423303	10.668712	11.293950	11.551948	11.366409	11.562464	11.879226	11.474531	11.482247	11.397315	11.570758	10.896568	11.249706	10.831500	10.603714	11.723465	11.459486	9.983976	9.755164	11.818688	9.863624	11.511412	11.198858	11.483981	11.896350	10.341418	10.150081	11.289771	11.681430	10.937444	11.452687	11.342060	11.077589	10.684350	11.756028	11.858897	10.904808	11.470178	11.554168	12.665471	11.288746	11.258115	10.624119	12.122694	11.521131	11.961308	11.040311	8.819791	11.568423	10.407984	10.303719	10.059401	11.639628	11.319133	11.462914	11.867888	11.017781	10.046320	11.255159	11.523953	11.183899	11.308969	11.800133	11.533035	11.298091	11.326303	10.424550	10.595005	10.921794	11.406029	9.903186	10.795839	11.449574	11.432540	11.004401	11.483699	9.567637	11.380992	11.914124	11.939846	11.548861	11.543325	12.015985	11.961736	11.403536	11.163643	10.818648	11.233001	10.869477	10.826511	11.332182	11.204630	10.958111	10.549645	11.428109	10.642844	10.515470	10.955440	10.728676	11.571034	11.722280	10.750389	11.303833	11.464783	11.986356	12.068295	11.584856	11.603148	10.478589	11.628369	11.626169	11.671195	11.990798	12.458016	11.229920	11.339952	11.834822	11.185106	11.025555	10.436289	11.241501	10.573790	11.505998	12.156499	11.735962	10.862604	11.786870	10.934125	10.745016	11.918019	11.037396	11.117968	10.815965	9.620873	11.214660	10.845927	10.128694	9.791261	11.270124	9.842288	10.938762	9.673171	11.621743	11.392463	12.081830	10.560091	12.378188	11.271637

MACF1	6.307403	5.310700	5.698775	5.161272	5.370515	7.577303	4.955624	6.276999	6.057290	4.677961	5.354082	4.964161	4.820465	5.261864	6.381648	5.096222	5.837134	5.580219	4.890032	5.508966	4.242727	5.797651	4.400888	4.940144	5.060815	4.878171	5.096409	5.885332	6.331290	5.182826	6.319966	5.044934	5.794569	4.940338	5.843870	5.644266	4.875692	5.214830	5.753035	5.719583	5.299282	5.716738	5.629358	6.037203	5.474461	7.207245	6.464369	5.865783	5.712111	5.121711	6.085262	5.124493	5.702228	4.463741	5.350897	6.079385	5.361740	5.108923	4.410020	5.103993	5.225696	6.552682	6.309443	5.832808	5.991631	5.203062	4.993534	5.560034	5.191894	6.669775	5.472912	5.384460	5.864148	5.355495	5.810203	5.826008	5.223904	5.189959	5.459435	5.970684	4.886247	5.514046	6.043642	6.252502	5.090210	4.417533	4.855214	4.938279	5.956812	5.379836	5.495098	5.269469	5.212013	5.324406	5.263095	5.082862	5.785023	5.618556	6.387306	6.142756	5.260874	4.908622	5.261864	5.595490	5.770307	6.452101	5.816919	5.958962	6.413357	6.095667	5.793655	6.342505	5.041578	6.195854	5.321907	5.541648	5.687183	5.694721	6.134299	5.872632	4.977248	5.071019	4.908200	5.643284	7.101416	5.784669	5.392395	6.007549	5.195916	4.662255	4.434966	5.535685	5.046074	4.424419	5.011793	5.786290	4.433285	4.835798	5.323238	5.241438	5.278766	5.642536	5.631190	4.658123	4.231140	5.113352	4.796480	5.953089	5.568071	5.965017	4.987473	5.075463	5.194061	5.818145	5.758717	4.895184	5.049984	5.559256	5.887073	5.980847	5.140257	5.571063	6.586378	5.437457	7.424763	6.301676	5.677707	5.030546	4.851376	6.413054	4.219035	4.342860	4.797463	3.963800	5.659219	5.461399	4.849503	4.982780	4.647039	5.646519	5.474833	5.982957	5.486905	5.238060	4.381597	5.291576	4.957517	5.075261	4.406925	6.324927	5.193075	5.404785	5.440238	5.768136	5.536460	4.961439	5.507004	5.323444	5.133206	4.947749	5.778728	5.965177	5.789012	5.409725	4.937893	5.688078	5.011793	6.033087	5.334740	5.735558	5.008061	4.982780	5.190727	5.805975	5.573143	5.272683	5.350035	6.039123	5.165506	4.976495	4.938486	4.678389	4.807639	5.247211	6.234471	5.615175	5.663106	5.914942	5.548830	5.617970	5.593845	5.580595	6.933021	4.681557	5.646696	5.742261	5.519876	5.194478

TENT4A	11.863352	11.937329	12.152545	11.691529	11.866384	11.594141	12.085444	11.190758	12.409678	11.775216	11.864994	11.683934	11.835574	11.499211	11.612573	11.618620	11.876001	11.798213	12.405745	11.374333	11.926124	12.388886	12.505911	11.399407	11.988293	11.789113	11.826672	11.286728	11.975261	11.966878	12.116974	11.620399	11.785542	12.898463	11.616246	11.742152	11.932456	12.016602	11.793314	11.823531	11.838285	11.965674	12.030069	12.313487	12.299854	12.106846	11.493891	11.650646	11.938934	11.720028	11.594907	11.924735	12.004430	11.839782	12.427314	12.071265	12.114935	11.440322	12.113600	11.700373	11.747749	12.031827	12.045726	11.934282	12.326538	12.101182	11.463470	11.708338	11.609982	11.710430	11.620979	11.790428	12.007161	12.061519	11.882067	11.929392	11.857035	11.678914	11.476411	12.461727	12.110262	12.137555	12.292819	12.317621	11.985107	11.855782	12.098451	12.512086	12.551234	11.372593	11.579668	12.449765	11.899859	12.683650	11.826245	11.809331	11.935497	12.333823	11.842350	12.005783	12.230488	11.901464	12.019762	12.296489	12.530838	11.796151	12.075438	12.643905	12.812473	11.941351	12.756854	11.810536	12.186842	11.926777	12.338408	10.756778	11.712748	12.037292	11.935187	12.145005	12.271073	12.437743	11.921741	11.721084	12.187328	11.669383	13.426006	12.405908	12.427016	12.471493	12.049063	12.495001	11.922337	12.704515	12.843171	12.803522	12.489749	13.392020	13.986102	12.618274	12.596461	12.920598	13.100501	12.338106	12.269467	12.864977	12.691855	13.002332	12.665471	12.563831	12.560874	12.681634	12.528867	13.336226	12.679231	12.393945	12.643905	12.724156	12.238197	12.449933	12.623496	12.149284	12.991641	12.622173	12.816481	11.676671	12.318545	12.884383	12.471493	12.253579	12.022222	12.983713	12.785225	12.098604	11.940596	12.407139	13.036888	12.688122	12.791154	12.641550	12.627164	13.281421	12.352431	13.350574	12.645205	12.690183	12.320299	12.899372	12.689676	12.740770	12.644233	11.732575	11.881590	12.466981	12.390923	13.435155	13.101246	12.645563	11.860081	12.997119	12.966194	12.853341	12.406965	12.965086	12.749916	12.391429	12.885665	12.901113	11.684076	12.482465	12.619138	12.142942	12.813004	12.082923	12.724991	12.736077	12.663072	12.686212	12.568878	12.086801	13.090343	12.619138	12.298697	13.871812	12.702451	13.640603	13.318885	13.658895	11.454008	13.461357	12.252952	13.285446	10.919423	12.656189	12.926643	11.982583	13.469871	12.478173

FCAR	6.344049	8.719785	9.479802	6.211030	7.465866	6.284471	7.204710	6.874324	6.737298	6.649192	6.823537	6.786777	5.845645	6.437732	6.544021	6.329736	6.999519	6.104087	6.143590	6.193564	6.375158	6.077122	6.453304	6.198588	5.730301	7.050689	7.731485	6.702745	6.703844	9.369831	6.422390	8.383274	8.224339	7.580861	8.186182	7.290988	8.528127	8.900824	7.266940	8.093733	6.823863	7.661530	8.125256	6.621138	8.578625	8.234051	6.962293	8.327291	9.817412	8.010350	8.532428	8.513238	9.713258	8.535166	10.153109	6.742734	9.407099	8.599122	6.844383	8.615418	9.724231	7.341449	11.498007	6.186901	8.036022	9.553589	7.654980	9.306026	9.661551	7.835680	7.198532	7.788217	6.570356	7.644624	7.249623	9.542515	7.391321	9.580855	8.861134	9.084861	8.184935	10.022449	7.651887	8.525817	7.383297	6.706217	9.048076	8.613599	7.605625	8.077423	8.013547	9.441268	9.338015	9.698632	8.206780	8.667742	9.011607	6.284973	6.496218	6.525307	7.443608	6.323875	6.364215	8.488606	7.105179	6.703546	6.496048	6.095321	7.265254	6.662960	7.539896	7.037008	7.479807	6.787922	6.635961	8.602194	8.232255	6.025685	6.831474	6.616420	8.596576	9.203107	6.246099	8.630840	7.055577	7.111620	6.751613	6.216975	7.187316	7.246216	7.521929	6.764745	6.868239	7.513109	6.438911	7.809741	6.279828	7.525133	6.701902	7.192137	6.658391	7.492126	7.085231	6.596780	7.982237	7.888897	7.023584	7.199153	8.614955	6.595007	9.955696	9.041626	8.557469	7.649188	7.524985	9.318827	9.611805	7.783317	9.268311	7.495997	9.064900	8.976804	8.669282	10.134374	8.606037	7.417135	8.550127	8.602666	9.275769	10.226509	9.064588	9.626779	10.003213	10.325356	6.966758	9.219741	9.281986	7.903686	9.504231	10.837802	8.169725	10.348646	6.832787	7.545653	10.365302	8.104169	10.924780	11.026149	9.280452	7.748723	9.068649	7.432187	7.831082	7.843425	10.261469	8.142249	9.425426	9.159384	8.519009	8.525385	9.848631	8.897794	10.229572	7.656711	7.221939	9.655812	8.547842	7.866036	9.171571	9.787425	10.011976	8.714674	9.811665	9.178776	10.057120	8.471036	6.512277	6.794137	5.999541	7.887956	6.539247	6.442193	10.071306	7.471118	7.325954	6.923207	6.781814	6.596483	7.813075	8.269978	7.414765	7.728256	7.264495	7.377032	7.854983	9.548217	6.270170	7.671008

NANP	7.691350	7.702677	9.100841	6.877654	7.530523	7.267882	8.112533	6.711800	5.415640	7.680124	7.186189	7.695178	7.531277	7.897749	8.054162	7.172616	6.346731	7.116904	7.230565	7.807263	7.857857	6.640971	7.421041	6.488708	6.288774	6.623712	5.361935	4.586047	6.207946	6.897378	5.851489	8.630532	8.836779	7.820703	8.251460	8.587697	8.843418	8.626865	8.216056	8.037881	7.626255	8.082255	7.672279	8.019077	8.080215	8.301521	6.397786	7.870743	8.553625	8.358315	7.952940	8.642515	8.573236	8.967900	9.049275	7.243579	8.843862	8.796555	8.606200	8.098548	8.409240	7.635566	8.468252	7.711088	9.240450	8.507811	7.783157	8.041233	8.407426	7.988366	7.878989	7.726532	6.288620	7.990967	8.224190	7.958043	8.098253	8.483559	7.727475	8.008376	7.659822	8.681103	8.423629	8.485373	8.369555	8.880343	8.933305	9.084689	8.565560	7.976484	7.968034	8.652056	8.629256	8.690354	7.520818	7.780295	8.581670	7.257719	7.943093	6.649044	7.122892	7.957304	7.436154	7.570365	8.635528	7.217334	6.491065	8.086591	7.941824	6.458352	8.253439	6.931281	6.892994	6.563677	7.930949	4.997562	7.460098	8.016016	5.396530	7.685796	8.233142	8.123563	6.808885	4.846179	7.339085	6.437425	6.827028	7.504612	8.340131	7.172764	8.164423	6.875957	6.451946	7.514207	6.939358	7.569912	7.156225	7.592717	8.413076	6.387666	7.175574	7.830635	6.923207	6.830549	7.985774	6.958781	5.578136	5.593090	6.481749	4.845532	7.801000	8.336025	8.615560	8.733493	9.584523	9.054064	8.596115	8.152839	8.591916	6.546953	9.020620	7.754711	8.221296	8.759794	8.590063	4.983993	7.743966	8.994576	9.078557	8.942934	9.822224	8.801886	9.339791	8.831497	7.194717	9.046701	9.234733	8.818417	8.392095	8.437250	7.870600	7.068891	8.003733	8.813200	8.288103	7.648749	8.044021	8.187744	7.708703	8.630218	8.317305	5.938282	8.375408	8.305299	8.386809	8.727572	8.326972	8.218720	8.672031	7.835968	9.209005	9.515156	8.104486	9.410488	8.717201	9.292688	8.682202	8.528282	7.736660	8.492102	8.893482	7.925680	8.176949	9.161517	7.858176	8.097219	7.147212	7.387317	8.717346	7.937895	7.758694	6.550026	7.994877	9.166648	6.387666	7.082071	8.280091	9.047921	4.002351	8.645281	7.513268	6.424645	4.493992	7.812165	8.034188	8.738265	9.258639	7.013769

SNRPB2	13.674594	12.822312	13.424487	13.258986	13.329654	12.991440	13.380119	12.857277	13.036330	12.944807	13.256009	12.965824	13.464002	13.102386	13.192104	13.025290	13.218581	13.383029	13.232656	13.142104	13.450752	13.251043	13.771343	13.396009	13.367473	13.219568	13.287052	12.929753	13.248737	13.222353	13.317859	13.503914	13.131339	13.341990	13.451174	13.222745	13.216213	13.677795	13.348676	13.276252	13.178372	13.393094	13.343654	13.089053	13.147685	13.529898	13.183285	12.877825	13.229238	12.985009	13.262797	13.108685	13.284863	13.091080	12.999520	13.077813	13.329864	13.159717	13.225685	13.344260	13.468361	13.146881	14.097959	13.281421	13.450122	13.361828	13.251043	13.370400	13.100683	13.473575	12.905355	13.226062	13.209700	13.347657	13.196334	13.037247	12.872130	13.144577	13.007394	12.807201	13.425768	13.299219	12.932422	13.344888	13.234006	13.078187	13.479279	12.892075	13.805968	12.972757	13.093127	13.120996	13.186638	13.186638	12.966743	13.203600	13.301249	13.378218	13.517989	12.870146	13.143383	13.270630	13.532157	13.116395	13.466396	13.405875	13.690456	13.507906	13.613276	13.362663	13.250280	13.253211	13.380743	13.231246	13.108505	12.215199	13.550061	13.664721	13.379067	13.527474	14.427447	13.875803	14.044975	14.456976	14.355885	13.580404	14.058288	14.100097	14.497531	13.881969	14.301613	14.049129	14.426320	14.281401	13.603829	14.235912	14.295055	14.599518	13.940443	14.169985	14.299113	14.369411	14.440880	14.633014	14.287951	13.916607	14.241749	14.043175	13.800876	13.932970	13.998449	13.864108	13.675077	13.691689	14.138571	13.883013	13.749075	13.859930	13.951282	14.310316	13.743597	13.764461	14.322988	14.005898	13.844292	14.170650	13.924569	13.597474	14.005012	13.979146	14.243065	13.707232	14.215030	14.401854	14.276902	14.266988	14.143957	14.052986	13.997851	13.544167	14.275219	13.746570	14.019002	14.048218	13.602191	13.826079	13.899396	13.799344	13.754901	14.216321	13.804379	13.979146	13.457023	13.829200	13.857811	14.363735	14.229857	13.734845	14.068082	13.909712	14.121601	14.522678	14.348910	14.808146	13.980518	13.961354	15.351660	14.229504	14.427447	14.545545	14.823896	13.371459	13.947918	14.174543	14.087146	13.729644	14.434184	13.630442	14.169373	14.197180	13.957131	13.868320	13.645649	14.540484	14.052067	15.283711	14.000102	14.143957	13.690206	14.044362	13.547770	14.073307	13.390157	14.260943	14.271885	13.835602	13.470952	14.183722

TRIM55	8.739929	9.276833	9.036629	9.612716	9.691000	9.335281	8.887442	10.394147	10.597851	9.610435	9.738519	9.404986	8.807358	9.094182	8.408942	9.698632	9.945429	9.039784	9.588428	9.081657	9.857887	10.851359	9.643632	9.432508	9.138891	9.622911	9.018822	10.312904	9.964423	9.067083	10.368806	9.150489	9.460002	8.896105	8.704594	9.070964	8.540372	9.029036	8.770850	9.390213	9.651195	8.339333	8.856081	9.148313	8.538466	8.361560	10.378509	9.152019	10.150370	8.418410	8.851683	8.162209	8.374816	8.651326	8.322775	9.549766	8.010042	8.136005	8.440743	8.230249	8.985474	9.335569	7.946185	8.948154	8.916645	9.400002	9.284919	8.855318	9.092054	9.918999	10.324445	10.424550	9.452905	9.505969	9.889689	9.222218	9.814480	9.292992	9.609659	7.945709	7.843275	8.067428	8.005443	8.253582	8.524918	9.713408	7.888733	7.766700	8.580435	8.374187	8.509059	9.125034	9.420843	9.625854	9.089149	9.243185	9.312170	9.217026	9.660909	9.664360	9.603904	9.494887	9.926337	9.087336	9.347711	9.112877	10.915826	9.279525	9.780111	9.768599	9.549766	9.674434	9.909249	9.895040	9.610124	10.953919	8.317157	9.281525	9.372574	8.713570	9.051877	8.783516	8.957149	9.831389	8.983646	8.886073	8.098401	8.421205	8.645143	8.793229	8.493981	8.863852	8.881603	8.735320	8.627169	8.711325	8.410303	8.478049	8.364085	8.968517	8.718581	8.890098	8.894837	9.133821	8.861847	10.542688	9.251636	9.498106	9.019864	8.746346	8.406081	8.309911	8.717665	8.642515	8.307762	8.389429	9.015613	9.019430	8.720864	9.164933	8.826778	7.872609	9.005162	8.136495	8.576254	9.879029	8.326656	8.980147	8.351263	8.711618	7.843121	7.950786	8.282999	7.916471	8.920132	7.526374	7.719509	8.073351	7.784088	8.166936	8.953170	7.851415	8.459105	8.520528	8.435100	8.724803	8.905970	8.705991	8.809523	9.038835	9.121979	9.548072	8.932558	8.718419	8.965087	8.755638	8.778712	9.204729	7.691350	7.469081	8.098401	7.843927	7.651278	8.223604	8.753475	7.714052	7.122578	8.298205	8.086300	7.663729	8.355850	8.760273	8.731651	9.052657	8.680959	8.483274	8.431530	9.125817	8.437092	8.821587	8.431370	8.676360	9.008878	7.926147	8.848779	8.476775	8.701221	9.176193	9.542983	8.381906	9.114675	9.626163	10.532684	8.985620	9.124887	8.372378	8.456739	9.289113

CENPI	6.957676	7.710952	8.115323	7.588584	7.014664	7.796488	7.447919	5.461998	7.715374	7.648147	7.705322	6.767639	8.102344	7.992228	8.038201	6.778314	7.843121	7.212483	7.005493	8.382213	6.313497	7.044502	6.579055	7.621512	7.500244	7.422597	7.593195	5.643485	6.401157	8.272998	7.007040	8.703369	8.366232	8.726647	7.961155	8.403614	7.957449	8.535943	7.222979	7.440361	7.116576	6.966758	7.851095	7.387317	8.010187	8.868537	5.961446	5.631190	8.164264	8.157042	7.645719	8.358315	8.577147	8.302895	8.350523	7.164101	8.281598	7.701304	8.245241	8.783374	6.955501	7.237677	5.939707	6.119929	8.399403	7.903342	8.798544	7.912515	8.851059	6.568387	7.562721	8.183907	6.573209	7.548082	8.452789	7.822399	8.067133	8.198709	7.533908	8.276122	8.599122	8.953320	8.319558	8.251152	8.792439	7.416404	8.432903	8.831940	6.599421	7.198532	7.102676	5.301839	8.518242	8.483880	7.743172	8.019965	8.092006	7.907089	7.627834	7.982383	6.861536	7.464647	7.751210	7.088478	7.952309	7.436471	6.216975	7.007495	5.453616	6.774963	5.573890	6.841442	7.443608	6.712279	7.868330	4.702350	6.805231	7.157778	7.283831	9.262112	9.277692	9.235785	8.426765	7.999175	10.378034	8.776297	9.434778	10.740494	9.994150	9.488153	9.522409	9.532764	9.287550	10.313050	7.042884	10.585228	9.188009	9.756700	10.695882	8.282714	8.436002	8.909943	9.878002	9.941340	9.971673	7.451206	9.142892	8.948580	10.176378	8.851230	9.772353	10.760552	9.712195	9.674912	10.137918	8.983646	10.125235	9.197337	9.403709	9.007632	9.107183	9.119892	10.186968	9.538277	10.052635	8.713413	8.301962	9.262432	10.201399	9.900361	10.327183	10.216548	10.330850	9.521604	8.895756	9.116670	10.238002	10.243220	10.457105	8.785782	9.112570	9.438350	8.696984	9.114833	9.746613	11.059685	9.646619	10.095931	9.136529	9.902261	10.275033	8.337120	9.548217	9.844254	9.252538	9.168755	10.280806	9.239370	9.575634	10.486410	11.236126	9.530828	9.436629	10.832583	9.603459	10.709053	10.527304	9.098445	9.105197	9.543317	8.408218	9.084561	9.706564	8.582268	8.666965	10.148575	9.937098	9.825091	9.877830	10.455864	9.215117	7.500852	8.298363	11.110444	8.198709	9.859301	9.606097	7.687491	8.092765	6.826234	8.685838	10.208000	7.781203	9.154798	8.392729	9.361876	7.870902	9.621194

GATAD2B	7.232778	6.286218	5.885495	6.184495	6.151391	6.469674	6.476918	6.285685	6.139091	6.239587	6.447349	6.526178	6.104775	6.174035	6.881674	6.063823	7.986373	5.918331	5.817278	6.056179	4.762246	5.760681	5.827654	5.641390	5.514046	6.459041	6.500881	6.202073	7.034659	7.071967	6.643033	5.847321	5.484613	6.505130	6.053723	5.606684	5.803302	5.945861	5.754614	5.737157	6.129831	5.510295	5.878692	6.034026	5.690793	5.100918	7.024588	6.328750	6.093560	6.190615	5.696745	5.949351	5.666656	5.086146	6.026766	7.018100	5.129592	5.350035	6.644989	5.423208	5.250586	6.382325	5.996025	6.022574	5.546573	5.938282	5.914039	6.377960	5.635100	5.744656	6.318570	5.897057	6.269124	5.976204	5.891684	5.684176	6.025527	5.698228	6.384973	5.491876	5.516654	6.156523	5.981184	4.958361	5.027227	5.398631	4.912395	5.389322	5.428231	5.510295	5.018110	6.140293	5.779280	5.964827	6.133452	5.992878	6.371084	7.026166	6.974477	7.193586	6.879726	6.228307	6.219047	5.848644	5.886723	7.443459	6.411029	6.179165	7.062373	7.004242	6.231093	7.153563	5.766833	6.813820	6.807413	6.737298	7.727157	6.346891	6.335031	6.315909	6.449017	5.773447	7.170236	6.773160	6.672024	6.826234	5.702228	6.199659	6.668981	6.834077	6.198966	6.968869	6.774963	6.417479	6.285328	6.310111	6.165098	6.242941	5.762505	6.104087	6.187579	7.072278	7.000814	6.682427	6.158049	5.943205	6.608518	7.123687	7.067906	6.968033	6.774295	5.874052	6.515220	5.927411	5.639734	5.729728	6.047304	6.205756	5.890818	6.937304	5.716401	5.078395	6.318570	5.664421	4.688438	7.716427	5.982957	5.859961	6.609186	6.238082	5.729904	5.090437	5.870227	5.291773	6.470026	4.595957	3.728166	6.781047	5.591754	5.744281	6.445408	6.412181	5.908291	5.379059	5.561665	5.935284	6.224501	6.393878	6.049418	5.896885	6.127291	8.330105	6.388685	5.500266	5.909357	6.086738	6.111142	6.695231	5.379059	6.054977	5.459048	5.876019	5.349462	5.441793	5.936696	5.690793	5.171084	5.555427	5.244018	5.223352	6.008419	6.074503	6.580967	6.629202	6.874477	6.601179	6.471925	7.186835	5.879616	6.914148	6.425281	6.078831	6.084925	5.097372	6.957844	6.243786	5.946038	5.260230	8.259246	6.613319	6.548199	6.909393	6.809210	6.524664	6.400990	5.893709	6.129010	7.240875

APBA3	7.752415	7.537165	8.082112	7.437870	7.364043	7.533448	7.281784	7.475990	7.567942	7.858326	6.885991	7.551590	6.822459	7.555503	6.551153	7.652041	7.093223	7.078474	8.340557	7.458098	7.830502	7.488516	5.968439	7.778530	7.780146	7.182971	6.932401	7.854212	7.566427	7.071340	7.468910	7.770487	7.592559	6.853000	7.479190	7.478448	7.553313	7.824155	8.359269	8.314053	7.560582	7.727960	7.563478	7.365785	7.296707	7.512950	7.710641	7.732580	7.663101	7.681002	7.582103	7.875346	7.916768	7.644473	7.401157	7.521441	7.501316	8.123491	7.814612	8.042008	7.995024	7.597778	7.098130	8.057711	7.601380	7.761592	7.925680	7.859591	7.834020	8.020889	7.312275	7.579650	7.715828	7.501958	7.589045	7.414000	7.499287	7.143793	7.519870	7.201165	7.339873	6.950916	7.229212	7.448092	7.102025	7.254171	6.576684	7.332577	7.610220	7.858326	7.512950	7.112679	7.573089	7.589045	7.043338	6.998626	7.202252	6.910985	7.619402	7.409130	7.422754	7.472951	7.260754	7.934018	8.047832	7.495029	8.216204	7.830932	7.425855	7.612789	7.152778	7.314180	8.027164	7.672128	7.882917	6.923207	7.564875	7.014074	7.812790	7.428678	7.613888	7.678284	8.139275	7.323901	7.725594	7.547429	6.507989	7.228880	7.420235	7.329779	7.116904	7.588434	7.620128	6.683070	7.122734	6.851233	6.801084	7.814935	7.755300	7.754559	8.183733	7.220008	7.669912	7.440209	7.605625	7.165039	6.794787	7.380012	7.665583	6.517234	7.403660	7.310728	7.605625	7.506792	6.986699	7.156534	7.669761	7.968783	7.771269	7.706992	7.707598	7.391949	7.212966	7.037474	7.296536	7.836587	7.518197	7.325492	7.584728	7.255390	7.418684	7.254455	8.149158	7.374866	7.576358	7.557702	7.760826	7.883850	7.373908	8.073812	7.582883	7.739616	7.966655	8.040770	7.537785	7.777297	7.544235	7.351613	8.444252	7.192436	7.569464	7.932481	7.411631	6.940443	7.218415	7.377540	7.191534	7.771269	6.952820	7.093070	7.313043	7.589490	7.169797	7.736364	7.149622	7.593826	7.472188	7.505232	7.280692	7.802054	7.985306	7.057287	7.669450	7.171812	6.892517	6.777993	6.839180	7.781203	7.043039	7.543733	6.603564	7.427269	7.301462	7.034830	7.288783	7.112679	7.423202	7.813567	7.899733	6.868239	6.770246	7.745299	7.569307	6.823537	7.146133	7.160590	7.273273	8.055590

TJP3	7.752415	7.537165	8.082112	7.437870	7.364043	7.533448	7.281784	7.475990	7.567942	7.858326	6.885991	7.551590	6.822459	7.555503	6.551153	7.652041	7.093223	7.078474	8.340557	7.458098	7.830502	7.488516	5.968439	7.778530	7.780146	7.182971	6.932401	7.854212	7.566427	7.071340	7.468910	7.770487	7.592559	6.853000	7.479190	7.478448	7.553313	7.824155	8.359269	8.314053	7.560582	7.727960	7.563478	7.365785	7.296707	7.512950	7.710641	7.732580	7.663101	7.681002	7.582103	7.875346	7.916768	7.644473	7.401157	7.521441	7.501316	8.123491	7.814612	8.042008	7.995024	7.597778	7.098130	8.057711	7.601380	7.761592	7.925680	7.859591	7.834020	8.020889	7.312275	7.579650	7.715828	7.501958	7.589045	7.414000	7.499287	7.143793	7.519870	7.201165	7.339873	6.950916	7.229212	7.448092	7.102025	7.254171	6.576684	7.332577	7.610220	7.858326	7.512950	7.112679	7.573089	7.589045	7.043338	6.998626	7.202252	6.910985	7.619402	7.409130	7.422754	7.472951	7.260754	7.934018	8.047832	7.495029	8.216204	7.830932	7.425855	7.612789	7.152778	7.314180	8.027164	7.672128	7.882917	6.923207	7.564875	7.014074	7.812790	7.428678	7.613888	7.678284	8.139275	7.323901	7.725594	7.547429	6.507989	7.228880	7.420235	7.329779	7.116904	7.588434	7.620128	6.683070	7.122734	6.851233	6.801084	7.814935	7.755300	7.754559	8.183733	7.220008	7.669912	7.440209	7.605625	7.165039	6.794787	7.380012	7.665583	6.517234	7.403660	7.310728	7.605625	7.506792	6.986699	7.156534	7.669761	7.968783	7.771269	7.706992	7.707598	7.391949	7.212966	7.037474	7.296536	7.836587	7.518197	7.325492	7.584728	7.255390	7.418684	7.254455	8.149158	7.374866	7.576358	7.557702	7.760826	7.883850	7.373908	8.073812	7.582883	7.739616	7.966655	8.040770	7.537785	7.777297	7.544235	7.351613	8.444252	7.192436	7.569464	7.932481	7.411631	6.940443	7.218415	7.377540	7.191534	7.771269	6.952820	7.093070	7.313043	7.589490	7.169797	7.736364	7.149622	7.593826	7.472188	7.505232	7.280692	7.802054	7.985306	7.057287	7.669450	7.171812	6.892517	6.777993	6.839180	7.781203	7.043039	7.543733	6.603564	7.427269	7.301462	7.034830	7.288783	7.112679	7.423202	7.813567	7.899733	6.868239	6.770246	7.745299	7.569307	6.823537	7.146133	7.160590	7.273273	8.055590

MCM3AP	9.529428	9.734237	9.819657	9.513223	10.283247	9.957369	9.434019	9.401532	10.679279	9.645490	9.854464	9.583001	9.362011	9.573371	9.883787	10.043161	9.717177	10.511086	10.430630	9.718454	9.445907	10.096550	9.472171	9.924291	10.146709	9.826170	9.625683	9.615893	9.455087	9.554494	10.183759	9.508545	9.667023	9.596485	9.999817	9.740092	10.012922	9.738519	10.065121	10.615566	9.992041	9.996607	9.784423	10.211224	10.136837	9.637322	9.171571	9.479637	9.837660	9.855744	9.415969	9.690611	9.908965	9.983976	10.112741	9.757807	10.023382	10.213496	10.047564	10.155465	10.012605	9.577451	9.667979	9.873486	10.213038	9.791103	9.603315	9.686521	9.961059	9.989210	9.183450	9.575634	9.632445	9.755615	9.497029	9.448101	9.482720	9.273625	9.145493	9.382710	9.910666	9.822688	9.667979	9.997492	9.864543	9.709999	10.075134	9.941976	10.139993	10.368062	9.808891	9.997062	9.922734	9.705031	9.297246	9.744936	10.060453	9.983819	10.186968	9.951078	10.054805	9.576986	10.214264	9.974195	10.314129	9.919156	9.767080	10.607990	10.492998	9.947962	10.159823	10.083270	10.096550	9.940701	10.172297	11.086028	9.754543	9.795351	10.177038	10.700757	10.040077	9.393908	10.335529	9.854621	11.365359	10.120339	12.210507	11.348487	11.166077	10.348429	10.532527	11.068392	10.066011	10.596659	11.089762	10.336453	11.670022	11.250318	10.179942	10.524431	10.110805	10.761000	10.207514	10.482002	11.098860	9.328138	10.809338	10.738842	10.012145	11.839473	10.380008	10.491272	10.017871	11.536302	10.993001	10.333876	10.981465	10.832876	10.904667	10.584613	10.720056	11.297951	11.053092	11.276728	11.047378	11.440893	10.752793	11.080441	9.766473	10.489183	10.621738	10.751617	10.170130	10.028430	10.848448	10.790142	10.872038	10.863810	10.018340	10.542688	9.835366	11.215233	10.448550	9.569642	10.521739	9.767842	9.891491	10.586129	10.644156	10.079519	9.089471	10.346123	10.134533	10.104632	9.871071	10.332062	9.823607	10.016803	9.205189	9.993251	9.786390	9.743949	9.862854	10.246494	11.331759	10.698977	11.393088	10.315490	10.299419	11.655757	11.098411	10.724009	9.485807	10.360069	10.516841	11.112724	10.773291	10.747853	10.849009	9.460158	11.128092	10.641778	9.886562	10.957536	10.374695	11.409661	10.801504	11.015753	10.494224	10.803737	9.525858	11.809935	9.949211	11.440039	10.676888	11.608893	9.914676	10.825609

RPL13	14.803797	14.352005	14.601973	14.734860	14.570568	14.570568	14.720427	13.912140	13.401415	14.194016	14.346713	14.380267	14.189577	14.905079	14.844672	14.182477	14.314792	14.597809	14.237167	14.749262	14.755057	14.479847	14.446360	14.680965	13.990442	13.903071	14.061504	13.745592	14.267647	14.291671	14.161739	14.792018	14.225335	13.775202	15.071019	14.800437	14.789144	14.672821	14.782312	14.737145	14.487547	14.294396	14.443429	14.461603	14.979898	14.446004	14.005564	14.226329	14.591979	14.508773	14.726960	14.419090	15.660615	14.972581	14.434912	14.403650	14.680520	15.170771	14.490355	15.168793	14.264573	14.457391	14.406566	14.231871	14.191449	14.387470	14.499485	14.310666	14.109446	14.596998	14.730211	14.503429	14.258950	14.221208	14.809104	14.843666	14.336137	13.590983	14.400018	14.042006	14.031176	14.878681	14.343514	15.086549	15.127801	14.877613	14.925433	13.987492	14.303944	14.658618	14.891747	14.256694	14.144611	13.547770	14.201164	14.453914	14.704362	14.195248	14.486754	14.035106	14.362295	14.069818	14.671925	14.255152	14.630361	14.523537	14.269566	14.672821	14.735321	14.179251	14.694246	14.216655	14.410595	14.071191	14.124606	13.915536	14.494365	14.352362	13.617295	14.565911	14.645337	13.680430	14.390000	14.380635	14.419877	14.203696	14.375530	14.388223	14.578102	14.016722	14.370128	14.819862	13.801661	14.857987	13.965167	14.456976	14.531049	13.944829	13.094857	14.650647	13.429094	13.931123	13.830488	14.150745	14.978704	13.883013	14.940922	13.834843	14.095205	13.452455	13.740967	14.248871	14.832510	13.986959	12.962524	13.916872	14.040527	14.422880	14.233886	14.836534	13.898829	14.472293	14.509550	13.658895	14.029975	13.850447	15.099414	14.183722	13.892099	13.918583	14.169692	13.342844	14.735782	14.565537	13.811422	14.070625	13.744082	13.999535	13.517315	14.530263	14.421398	13.654148	13.903602	14.653231	14.682727	14.155728	14.203084	14.535115	13.698484	13.856758	13.776435	14.292381	14.587687	14.188386	13.801126	14.461232	14.815737	13.925407	13.146881	12.776909	13.704371	13.987492	14.050019	13.659354	13.872353	14.242079	15.028728	13.533746	14.263896	12.892947	13.958770	14.303944	14.490783	14.499877	13.589099	14.086196	13.741329	14.110411	14.316430	14.377022	13.944308	14.086196	14.814746	13.859406	14.117369	14.550582	14.761491	13.332882	13.913198	13.836415	14.114029	13.794332	13.991573	14.392249	14.304961	14.312386	14.355177	14.786789

ARHGAP21	10.555231	9.707037	9.706564	10.363564	10.518462	10.508270	9.765569	10.322742	11.129112	10.302178	10.727170	10.189200	10.346434	10.254638	9.587157	10.409222	10.368806	9.907428	11.031779	10.053753	10.254975	10.522222	11.337014	10.228208	10.669264	10.451232	10.809816	11.056421	11.042247	10.065569	10.584201	9.274259	8.999654	9.878883	9.311708	9.267981	9.407719	9.676141	10.078682	10.154515	10.354444	9.935758	9.815723	9.822841	9.702651	9.330587	10.853314	9.670216	9.517441	9.240450	10.732656	8.901711	8.943397	9.102283	9.383781	10.636387	8.925815	9.377622	9.746922	9.343124	9.291432	10.497395	10.280952	10.222567	9.426808	9.537972	9.990578	9.895971	9.551167	10.247418	9.850577	9.800421	10.694281	9.707662	9.695569	9.755013	9.721234	9.560968	9.989959	8.911270	9.342971	8.959765	8.998099	8.962835	8.960680	9.130166	9.173435	9.587812	9.262112	9.165409	9.743643	9.680950	9.442955	9.553903	9.594640	9.599406	9.040712	10.130623	10.147476	10.471332	10.547962	9.752993	10.162442	10.105381	10.038559	10.530253	10.627617	10.358719	10.837978	10.859835	10.395808	10.423456	10.555685	11.320848	10.559632	11.898681	11.309548	9.925756	11.377693	9.995882	10.319057	9.933712	10.326735	11.650203	10.507987	10.800743	11.181578	11.075319	10.695590	10.605498	10.423927	10.862192	10.686620	10.718513	10.820134	11.022905	10.378509	11.724680	9.668298	10.516981	10.922541	10.886126	11.158915	10.816113	10.307273	10.644156	11.036699	12.127984	10.296016	11.194590	10.386089	10.381341	10.304766	9.853684	9.680791	9.694042	10.626987	11.056729	10.911045	11.110014	10.313564	9.503615	10.394017	9.690083	10.058476	11.614495	9.632756	9.829671	9.722467	10.633055	9.570876	9.286747	9.635352	9.347711	10.557516	9.178931	9.449820	10.149656	8.949983	10.197365	10.642239	10.355373	10.317582	10.181309	9.629836	10.290059	9.997359	10.153109	10.360365	9.993987	10.065869	11.519102	10.059104	9.747100	9.939017	10.080121	10.094258	10.106724	9.133220	9.341028	9.350940	9.297552	9.499786	9.352758	10.316523	9.305788	9.444211	9.701888	10.057591	10.319223	10.383667	9.965758	10.241203	10.368662	10.442148	10.818334	11.156994	10.750993	10.261006	10.550253	10.331296	11.297513	10.142294	10.354297	11.158629	11.794064	10.437199	10.556615	11.287767	10.623687	10.953919	11.146494	11.570035	10.209061	10.653379	10.142149	10.299864	10.835284

FEZF1	7.787317	6.915418	6.468342	7.281300	5.727722	5.963410	6.221944	7.389491	6.338219	5.971415	6.011402	6.081078	6.210351	6.260824	5.440816	7.039875	6.647388	5.922924	6.093378	6.541524	6.550188	6.786300	6.937644	6.532659	6.585387	5.989903	7.246849	6.405052	6.771711	7.007040	6.549386	6.308756	5.331647	6.669931	6.307251	6.667856	6.029259	6.136700	6.408860	5.877609	6.579778	6.736946	6.711965	5.771071	6.012299	5.964494	6.700808	6.502522	6.038597	6.418492	6.757186	5.776559	6.336540	6.335850	5.934753	7.158083	6.487433	6.841442	6.288089	6.173519	6.103756	6.779417	7.086585	6.462351	6.076077	6.228136	6.997868	6.826732	7.197600	7.013469	6.190787	6.023065	6.869358	6.341563	6.190968	6.667041	6.339728	6.398633	6.524664	6.417644	5.866650	6.496048	6.141195	5.795280	5.199295	5.976562	4.958361	5.067778	6.444900	6.047988	6.290129	6.058868	6.262169	6.073470	6.111465	5.919744	5.545245	6.606374	6.368693	6.627907	5.215220	6.538445	6.388514	6.572093	7.318215	6.744003	6.727870	6.971198	6.829577	6.900783	6.147242	6.467545	5.978848	6.853977	6.312121	6.567413	9.362915	6.794633	6.057290	7.203493	8.113761	6.679637	7.967867	6.297553	8.249009	6.645317	6.994160	6.365895	6.667856	7.295535	9.721234	6.500534	7.131491	6.672197	6.926825	6.711646	9.011455	6.634515	6.221062	6.731754	7.258388	7.372371	6.760102	7.745299	6.589655	7.266472	7.328193	7.070379	7.308960	6.648363	6.229015	6.323377	7.371725	5.950046	9.839560	6.086417	6.078138	7.003666	5.829701	7.626701	9.097199	6.490623	6.508946	6.582457	6.913084	7.124976	9.510547	6.279654	6.738926	6.494756	5.935284	6.811929	7.178605	6.865962	7.597944	6.579309	8.207388	7.874427	8.923712	6.919882	6.915103	7.919983	6.833280	7.035286	6.814915	7.401622	7.425716	7.180790	6.898842	5.915113	6.275386	7.716582	6.743202	7.968034	6.814751	6.832615	6.554636	6.652567	6.256588	7.261055	6.410697	6.110971	5.398042	6.623549	6.068594	6.122355	5.758146	6.655979	6.147073	6.403118	6.334681	6.003417	6.964524	6.947942	5.923657	5.984182	9.716701	7.141280	7.389033	5.848095	6.975741	7.460875	6.465048	7.523112	7.373429	8.201070	8.230865	9.770486	7.459505	7.485700	6.210682	6.999824	7.311045	6.916033	7.044666	7.073401	6.314683	7.167935

DEDD2	13.222154	12.874685	12.841260	13.112597	12.804049	12.813876	13.036888	13.185249	13.049541	13.286871	13.141081	12.913792	13.101246	13.315072	13.223925	13.304338	13.468361	13.384766	14.111684	13.262596	13.835602	13.809161	14.172876	13.641785	13.745592	13.218179	12.897927	13.902033	12.869225	13.064903	13.468126	12.962331	13.380959	12.471196	12.586980	12.553785	12.788576	12.995256	13.361622	13.533746	13.020101	13.271222	12.558482	12.598836	12.142076	12.650060	13.093127	12.679231	13.922935	12.699870	12.743377	12.806490	12.639201	13.182307	13.215627	12.863666	12.998772	13.227872	13.350971	12.961953	13.323155	12.906948	13.905564	13.566385	13.428458	13.234224	13.009581	13.222745	12.956811	13.653439	12.785412	12.782948	13.356199	13.054184	13.097503	12.916049	12.907680	13.117358	12.966563	13.514224	13.268023	13.287464	12.931882	13.565264	13.076070	12.950183	13.309343	13.589326	13.981326	13.752811	13.465968	14.209486	13.326797	13.359085	13.164705	12.877302	13.192485	12.838440	12.962718	12.871176	13.048973	13.054898	12.892947	13.419066	13.535533	13.349750	13.819403	13.898018	13.385402	13.262182	14.196229	13.305786	13.657694	13.267608	13.771343	12.221569	13.142104	12.750339	13.189888	13.378218	14.126434	14.003541	13.307926	14.020108	12.928147	13.209513	12.937994	12.975643	13.511265	12.441436	13.278803	13.103548	13.626588	13.515355	12.221727	12.889486	12.848393	14.854894	11.905178	14.166778	14.210111	14.525087	13.313179	13.479980	12.787861	13.928111	14.069818	13.217360	13.122311	13.038398	12.718866	12.747457	13.480386	13.019903	13.466396	13.087563	13.061274	13.054365	13.832042	13.158034	14.070098	12.278819	13.504135	13.293957	13.212236	13.459166	13.498914	13.158777	13.170169	13.412720	13.285876	13.403996	12.836884	13.301047	13.627746	13.108876	13.570340	13.555053	13.641524	13.944308	13.693359	13.492149	14.503429	13.605949	13.511265	13.911324	13.705820	13.165092	13.548900	12.972212	13.719166	13.448635	12.845812	12.743540	12.982400	12.993460	13.124397	13.228647	13.152612	13.476391	13.217360	14.199812	13.854600	14.014100	13.315805	13.509479	13.601738	14.218603	13.622915	13.933244	14.700418	12.782948	13.013167	12.993251	13.527900	13.174582	12.546379	13.138306	13.029169	13.648940	12.313009	12.136795	12.798847	14.479005	13.142467	13.789952	14.850633	14.907590	13.244951	14.420258	13.409232	13.357219	12.787176	14.084661	13.609219	13.365267	14.022689	13.079851

ZDHHC11B	14.906118	12.771214	13.480172	14.186189	14.289961	13.302672	13.759150	12.980977	15.534337	12.856045	13.996448	14.298742	13.574676	13.368069	14.746020	13.055601	15.305847	14.777592	14.658149	13.504804	14.173886	13.587211	15.065808	13.646825	15.400849	14.225335	13.569183	13.404401	14.478214	12.002488	14.631248	12.859356	12.077573	13.686701	13.675535	13.420156	13.469230	13.132988	13.733641	13.254784	14.218284	13.661762	12.096234	12.907332	14.546394	12.794317	13.722725	14.128591	13.601026	12.983527	12.477677	13.181711	13.404401	13.101809	14.078902	13.985840	13.644498	13.743835	14.607572	14.517028	14.095514	13.862356	14.128868	15.125176	14.170650	15.469004	14.126434	13.988980	13.172483	14.844188	13.060460	14.238479	13.247903	12.720947	13.798010	13.694028	13.500424	13.301878	14.384877	13.410531	14.392936	14.879151	13.635217	13.859679	14.068933	13.675754	14.739993	14.842135	14.171915	14.370844	13.776435	14.768079	13.974945	14.360460	13.149535	13.731668	13.877971	14.905079	14.287951	15.073466	15.065808	13.328625	13.755126	14.477088	14.480189	13.974684	13.435992	15.491291	14.615271	13.660327	13.802855	14.252773	13.921032	13.567342	14.684100	14.242398	14.925975	15.463984	13.839552	12.178309	10.596938	11.224607	12.271395	12.558482	11.063360	13.851689	13.551881	12.924377	13.624536	13.299825	12.723489	13.123071	12.257887	11.703426	14.164273	11.559235	14.698179	11.751144	13.188396	13.318885	13.634745	13.533066	15.121584	12.965249	10.189059	13.836966	14.453564	14.732223	12.119110	15.025759	12.580980	10.361744	12.721457	13.545741	13.801661	13.451597	12.996042	13.486520	13.145911	11.068971	13.567818	10.430953	10.690865	12.487643	12.973272	10.382403	11.183029	14.214704	11.638582	12.250724	12.605556	13.081212	11.026892	11.108963	12.806146	10.181383	11.648416	12.774306	13.302074	13.469871	12.913209	12.840899	12.722295	14.031176	13.467091	10.861911	11.626596	12.578683	11.615814	13.858073	13.444403	12.758075	10.494077	11.572374	11.851556	13.075888	11.743921	14.046144	11.878622	12.565127	12.024345	11.627636	12.064261	12.014764	14.165841	10.680668	11.405580	12.731336	12.684378	11.868608	12.132820	12.976584	11.841745	11.490704	11.047654	12.422636	13.844830	11.235219	14.493157	12.125666	11.771398	15.203034	11.741588	11.997933	15.183376	14.487923	12.420264	14.303277	10.795565	13.601738	11.684959	12.765306	12.835615	13.391817	11.047954	14.166466	11.343087	13.454346

CCDC25	12.508357	11.946386	12.099218	12.222322	12.340029	11.812156	11.903881	11.145141	10.712868	11.905051	11.880130	11.807837	11.902491	12.122996	11.723321	11.798508	11.745962	11.998560	11.483981	12.458016	11.613910	11.004703	10.947309	12.255523	12.537583	12.313810	11.869200	11.835426	11.998998	11.732727	11.791025	12.196161	12.197990	11.530788	11.893367	11.824884	11.809762	12.064108	12.277746	11.888108	12.248989	11.953033	11.564033	11.635559	11.873552	11.954825	11.911179	11.982123	11.908646	11.862748	11.611675	11.929077	11.718244	12.052817	11.627214	12.206351	11.864688	12.090589	12.414625	12.027939	11.887650	12.036088	11.688416	11.841912	11.912793	12.028865	12.194595	11.731995	11.944381	11.268654	11.823688	11.639167	11.909419	11.722280	11.741588	11.560528	11.795854	11.703111	11.775784	12.502885	12.342480	12.350849	12.194595	12.467321	12.535770	12.204194	12.457084	12.486643	11.695305	12.672605	12.577702	11.745525	11.903572	11.642694	11.701443	11.983360	11.945301	11.359439	11.990637	11.305602	11.430746	11.337082	11.112018	11.387167	10.731203	12.011905	11.749984	11.118560	11.802240	11.848200	10.128831	11.911633	11.924151	11.816748	11.842489	11.514555	12.389189	12.092137	11.967950	11.859051	12.423579	12.140038	12.249758	10.377715	11.854889	10.776378	11.761159	10.918816	11.284886	11.684367	12.004567	11.881462	10.612741	11.464636	11.526932	11.544982	10.912789	11.850968	11.324079	12.080159	10.595005	10.705664	12.107264	11.348487	12.268675	11.772691	11.170020	12.262939	11.680518	11.294959	11.099157	11.847904	12.015985	11.317854	11.927863	11.937016	11.721674	12.238050	12.289963	12.572856	12.304346	11.778520	12.137398	11.962757	11.438954	12.149134	12.313810	12.156958	11.944525	11.190322	12.092757	11.198701	11.366830	12.526615	11.446732	11.012108	11.689126	11.448683	12.012048	10.909711	12.675868	11.847129	11.679053	12.480800	11.710591	11.731685	11.576321	11.217736	10.410277	11.863196	12.279737	12.099673	11.719874	11.330144	11.224167	12.087259	11.939225	11.966716	12.146883	11.913837	11.201442	12.380353	12.297752	11.855782	12.091683	12.087710	11.664926	11.453855	12.277596	12.986119	11.838566	11.295979	10.711413	11.346092	11.209250	11.736718	10.621887	11.396845	10.816719	11.255473	11.309839	10.230378	11.049734	10.627787	11.071521	12.388554	11.375164	11.999153	10.871747	10.631731	11.030014	11.881010	11.479943	11.678294	12.715418	12.162362	11.923414	11.953180

TIAF1	8.687013	10.733854	10.783312	10.762352	11.110014	11.568568	11.308691	11.874166	11.705791	11.477556	11.354771	11.083497	11.317780	11.574024	11.237709	10.743802	11.214237	11.641331	10.506163	10.988232	10.777970	10.478137	9.818272	11.281195	11.347140	10.999853	9.744429	11.310881	10.813266	10.894182	11.615814	10.579044	10.577237	9.969420	10.333610	10.825459	11.009483	10.926394	11.538395	11.891078	11.136391	11.313643	11.174295	11.232265	11.044877	11.204776	10.745574	11.369313	10.441249	11.276012	10.874275	11.165668	10.752942	10.706272	10.960321	11.126639	10.926688	11.614795	11.134324	11.060427	11.349792	11.086028	10.288842	11.472234	10.974793	11.243470	10.780444	10.726741	10.554613	10.953478	10.015875	10.272760	11.172697	10.317425	10.289433	10.065569	10.491721	10.151883	10.164877	10.904376	10.956655	10.885415	10.718063	10.902781	10.979225	10.402738	10.690215	11.290218	11.202068	11.359582	11.123360	11.078839	10.663292	10.429277	9.758720	10.255569	10.684060	11.397471	10.692122	11.048256	11.211714	10.869894	10.995960	11.188894	11.015142	11.282256	11.961890	11.611531	10.763720	11.478556	10.058950	12.098761	11.901464	11.278240	11.207935	9.871071	10.881936	10.912049	11.082627	8.433072	10.703174	9.858664	10.357679	9.561747	10.921361	11.225321	9.178174	10.373076	11.389096	10.549938	10.616577	10.497960	10.278521	11.316464	10.017709	9.817723	10.816269	8.687165	9.798110	10.187555	9.484248	9.720935	10.272900	10.266446	10.200663	9.573678	9.959832	10.216906	11.049872	10.467548	10.292953	10.548890	10.539400	9.916692	10.117613	10.381196	10.045720	11.283268	10.807967	11.356381	10.501830	10.274257	11.106317	10.729414	10.690535	10.704200	10.006010	10.035004	10.402432	10.560870	10.153581	10.346888	10.815220	9.976348	10.580846	10.003500	10.348574	10.517868	9.924291	11.424500	11.042091	10.001795	11.548425	10.936398	10.710978	9.914676	10.142906	10.569204	11.008289	10.340048	9.907273	11.275872	9.935758	9.499786	10.015078	9.887355	10.025282	9.947336	10.156362	10.678806	10.733110	11.112884	10.169652	10.099349	10.069634	10.761158	9.824019	10.900123	10.709659	11.503051	10.776975	9.821011	9.748034	10.145061	10.305675	9.829822	9.960286	10.192816	9.884227	9.170158	9.698632	9.653700	9.991882	9.655960	10.188159	10.387783	10.997755	10.703444	11.332042	9.501937	10.396736	8.142249	11.308100	8.656538	10.879160	10.237708	10.499145	10.974650

MYO18A	8.687013	10.733854	10.783312	10.762352	11.110014	11.568568	11.308691	11.874166	11.705791	11.477556	11.354771	11.083497	11.317780	11.574024	11.237709	10.743802	11.214237	11.641331	10.506163	10.988232	10.777970	10.478137	9.818272	11.281195	11.347140	10.999853	9.744429	11.310881	10.813266	10.894182	11.615814	10.579044	10.577237	9.969420	10.333610	10.825459	11.009483	10.926394	11.538395	11.891078	11.136391	11.313643	11.174295	11.232265	11.044877	11.204776	10.745574	11.369313	10.441249	11.276012	10.874275	11.165668	10.752942	10.706272	10.960321	11.126639	10.926688	11.614795	11.134324	11.060427	11.349792	11.086028	10.288842	11.472234	10.974793	11.243470	10.780444	10.726741	10.554613	10.953478	10.015875	10.272760	11.172697	10.317425	10.289433	10.065569	10.491721	10.151883	10.164877	10.904376	10.956655	10.885415	10.718063	10.902781	10.979225	10.402738	10.690215	11.290218	11.202068	11.359582	11.123360	11.078839	10.663292	10.429277	9.758720	10.255569	10.684060	11.397471	10.692122	11.048256	11.211714	10.869894	10.995960	11.188894	11.015142	11.282256	11.961890	11.611531	10.763720	11.478556	10.058950	12.098761	11.901464	11.278240	11.207935	9.871071	10.881936	10.912049	11.082627	8.433072	10.703174	9.858664	10.357679	9.561747	10.921361	11.225321	9.178174	10.373076	11.389096	10.549938	10.616577	10.497960	10.278521	11.316464	10.017709	9.817723	10.816269	8.687165	9.798110	10.187555	9.484248	9.720935	10.272900	10.266446	10.200663	9.573678	9.959832	10.216906	11.049872	10.467548	10.292953	10.548890	10.539400	9.916692	10.117613	10.381196	10.045720	11.283268	10.807967	11.356381	10.501830	10.274257	11.106317	10.729414	10.690535	10.704200	10.006010	10.035004	10.402432	10.560870	10.153581	10.346888	10.815220	9.976348	10.580846	10.003500	10.348574	10.517868	9.924291	11.424500	11.042091	10.001795	11.548425	10.936398	10.710978	9.914676	10.142906	10.569204	11.008289	10.340048	9.907273	11.275872	9.935758	9.499786	10.015078	9.887355	10.025282	9.947336	10.156362	10.678806	10.733110	11.112884	10.169652	10.099349	10.069634	10.761158	9.824019	10.900123	10.709659	11.503051	10.776975	9.821011	9.748034	10.145061	10.305675	9.829822	9.960286	10.192816	9.884227	9.170158	9.698632	9.653700	9.991882	9.655960	10.188159	10.387783	10.997755	10.703444	11.332042	9.501937	10.396736	8.142249	11.308100	8.656538	10.879160	10.237708	10.499145	10.974650

MROH7	8.677119	7.174321	7.175887	7.648004	8.048777	7.951832	7.392107	8.113464	8.361415	10.215186	8.050019	8.618458	8.047984	7.370785	7.838778	7.982383	8.078697	7.604831	9.003802	8.132536	8.016331	7.723476	10.341418	7.777466	8.024680	8.086750	8.821277	8.682047	7.782275	7.023919	8.122129	7.578233	7.234838	7.466031	7.294607	8.968517	8.565393	7.761133	7.921494	8.015395	7.369531	7.963592	7.946637	7.878416	8.382834	7.238454	8.227358	8.244609	7.855897	7.586901	8.119173	7.538769	7.175887	7.397543	8.352774	7.724062	8.333675	7.859886	8.766838	8.489600	7.742243	7.584419	9.288485	8.407426	7.361695	8.268231	7.624881	7.477997	6.982537	8.339181	6.933931	7.259489	7.890755	7.190930	6.851861	7.010741	7.429530	6.859263	7.481976	7.826301	7.422439	7.534238	8.001530	7.678105	7.726996	8.065034	7.610076	7.739616	7.409130	8.516062	7.907241	7.669132	7.663101	7.819822	7.755300	7.832609	7.867574	8.726355	7.696224	9.228210	7.565801	8.841504	7.641687	7.914757	8.554809	7.863716	8.054480	8.382521	8.230249	7.711235	7.265109	8.122926	8.635528	7.735797	7.395847	5.677134	9.784596	8.263083	8.219933	6.396462	7.195369	6.714097	6.388174	6.890763	7.786067	7.422282	8.125849	8.298658	7.988792	8.691384	6.419799	8.024680	6.399465	5.632279	9.199535	7.375955	6.770246	6.985880	10.790007	7.339579	8.406081	7.889831	9.097199	6.614473	6.847079	6.501873	7.072417	7.028470	6.841294	8.538318	7.277818	7.192137	7.468910	7.701453	8.079016	8.602058	5.770307	6.688232	7.493195	6.418492	6.287413	7.514932	6.777676	7.212319	6.555983	7.114093	6.839012	7.303037	6.330096	7.278927	5.929013	5.983328	6.112982	6.926148	7.489279	5.777077	7.199937	7.134512	7.198218	7.392419	7.737577	6.902716	6.901754	6.718122	6.427512	6.319613	7.168714	8.150964	7.575743	6.715371	6.489031	7.914905	6.830092	6.718962	7.052716	7.426808	6.898358	7.595624	7.797093	7.954949	6.360044	7.805727	7.570069	6.734903	7.540217	8.556714	5.764864	8.004183	7.960064	5.577038	6.910490	9.472775	7.061882	7.050534	7.393365	7.119495	7.418684	6.930818	8.021561	5.743733	8.210651	6.464369	7.517425	5.745334	7.181093	8.296996	6.625506	6.612157	8.308699	7.330232	6.062264	5.740431	6.939212	6.207422	6.221767	7.887351	10.632025	7.482584

ALDH6A1	14.552678	11.297358	12.000403	12.328606	12.460105	12.292819	12.538254	12.432090	12.569374	12.702108	12.487990	12.638003	12.365730	12.042883	11.862005	12.339692	12.504387	12.005488	12.131586	12.773272	12.128571	12.699016	12.813004	11.822458	11.575238	12.579663	11.776387	12.337312	12.126428	11.848352	12.339692	12.683833	12.747616	12.919720	12.463072	13.041710	13.192884	11.677583	11.923703	12.261028	12.563000	12.353395	12.576723	12.800745	13.080241	12.596631	12.074353	12.343747	12.934891	13.045913	13.213390	13.145522	12.807201	13.218380	12.610544	12.150644	12.822312	12.803190	12.773621	12.918024	12.378353	12.264825	12.184541	12.168818	12.064994	12.063353	12.409034	12.388237	12.344394	12.386589	12.863843	12.820259	12.191343	12.557817	12.473785	12.541791	12.873750	12.581640	12.988517	12.714534	12.573864	12.698531	12.984259	13.118063	13.216776	12.849490	12.579489	12.948421	12.315433	12.349904	12.286593	12.231738	12.240857	12.536416	13.168839	12.929387	12.824203	12.797323	12.567105	13.067539	12.447724	12.580980	12.254686	12.468921	12.304346	12.490737	11.899125	11.957393	12.006089	12.358636	12.807870	12.050881	11.903646	12.195094	12.686046	11.927240	13.867543	12.783624	12.314612	13.563621	9.604383	11.095709	12.156958	11.009768	12.138182	11.291291	12.136795	11.813291	12.246874	11.361944	12.486494	11.014997	11.006688	9.971065	12.455784	12.088271	11.553592	10.479789	11.393826	12.238682	10.902636	11.363130	11.578854	10.154515	12.119110	11.835574	11.235506	10.940980	11.818688	12.212235	12.043045	12.285365	11.703565	12.608672	11.887359	12.415626	10.225601	12.033678	11.335546	11.527049	11.170163	12.245314	11.145141	12.044549	12.302631	11.962317	11.791508	12.303903	11.634348	11.494211	12.683325	11.298525	11.311317	12.236952	12.065467	13.137385	11.633888	12.623999	11.407356	11.342184	13.087036	11.234915	11.151708	11.607591	11.231105	11.319554	11.951841	11.013251	11.854757	12.075900	11.763401	10.287417	12.276660	11.892609	11.514705	12.273913	12.096818	11.760698	12.271724	10.841416	12.464996	11.554481	11.695468	11.289771	11.782222	11.789270	12.877302	11.267660	11.128662	11.385568	11.188740	12.009892	11.894872	12.091837	11.514845	12.063353	11.745378	11.204776	12.230950	11.075744	12.509156	12.259577	11.975436	11.846709	11.508778	10.569204	11.515310	11.050922	11.466138	10.763720	11.096474	10.489958	12.015680	12.248657	11.893953	11.731995	12.169426	12.164205

BBOF1	14.552678	11.297358	12.000403	12.328606	12.460105	12.292819	12.538254	12.432090	12.569374	12.702108	12.487990	12.638003	12.365730	12.042883	11.862005	12.339692	12.504387	12.005488	12.131586	12.773272	12.128571	12.699016	12.813004	11.822458	11.575238	12.579663	11.776387	12.337312	12.126428	11.848352	12.339692	12.683833	12.747616	12.919720	12.463072	13.041710	13.192884	11.677583	11.923703	12.261028	12.563000	12.353395	12.576723	12.800745	13.080241	12.596631	12.074353	12.343747	12.934891	13.045913	13.213390	13.145522	12.807201	13.218380	12.610544	12.150644	12.822312	12.803190	12.773621	12.918024	12.378353	12.264825	12.184541	12.168818	12.064994	12.063353	12.409034	12.388237	12.344394	12.386589	12.863843	12.820259	12.191343	12.557817	12.473785	12.541791	12.873750	12.581640	12.988517	12.714534	12.573864	12.698531	12.984259	13.118063	13.216776	12.849490	12.579489	12.948421	12.315433	12.349904	12.286593	12.231738	12.240857	12.536416	13.168839	12.929387	12.824203	12.797323	12.567105	13.067539	12.447724	12.580980	12.254686	12.468921	12.304346	12.490737	11.899125	11.957393	12.006089	12.358636	12.807870	12.050881	11.903646	12.195094	12.686046	11.927240	13.867543	12.783624	12.314612	13.563621	9.604383	11.095709	12.156958	11.009768	12.138182	11.291291	12.136795	11.813291	12.246874	11.361944	12.486494	11.014997	11.006688	9.971065	12.455784	12.088271	11.553592	10.479789	11.393826	12.238682	10.902636	11.363130	11.578854	10.154515	12.119110	11.835574	11.235506	10.940980	11.818688	12.212235	12.043045	12.285365	11.703565	12.608672	11.887359	12.415626	10.225601	12.033678	11.335546	11.527049	11.170163	12.245314	11.145141	12.044549	12.302631	11.962317	11.791508	12.303903	11.634348	11.494211	12.683325	11.298525	11.311317	12.236952	12.065467	13.137385	11.633888	12.623999	11.407356	11.342184	13.087036	11.234915	11.151708	11.607591	11.231105	11.319554	11.951841	11.013251	11.854757	12.075900	11.763401	10.287417	12.276660	11.892609	11.514705	12.273913	12.096818	11.760698	12.271724	10.841416	12.464996	11.554481	11.695468	11.289771	11.782222	11.789270	12.877302	11.267660	11.128662	11.385568	11.188740	12.009892	11.894872	12.091837	11.514845	12.063353	11.745378	11.204776	12.230950	11.075744	12.509156	12.259577	11.975436	11.846709	11.508778	10.569204	11.515310	11.050922	11.466138	10.763720	11.096474	10.489958	12.015680	12.248657	11.893953	11.731995	12.169426	12.164205

AC244197.3	9.991732	10.813866	11.262129	10.719908	10.914915	11.298091	11.216410	11.727221	11.306203	11.403818	11.188301	11.356089	10.769710	11.085590	9.989959	11.586749	11.264551	10.837243	11.060112	11.229444	10.791003	10.587164	9.287241	10.318027	10.406371	10.645760	9.878449	11.526511	10.510787	11.061124	10.639211	11.388081	12.101333	11.185257	11.069134	10.996866	11.200158	10.601209	10.439290	10.893881	11.395788	9.628632	10.942871	10.806918	11.005994	10.757808	11.295979	9.954586	11.712614	11.352182	10.639993	11.232725	10.974793	11.278715	11.664174	11.157605	11.004401	10.357381	10.576159	10.669264	10.233687	10.770306	9.966653	10.010197	10.034536	10.881053	10.099349	10.210936	10.421474	9.912507	11.094991	11.119269	11.474696	11.292805	11.135510	11.280760	11.093405	10.998047	10.995680	11.484410	10.772988	11.430298	11.103415	11.357096	11.569154	11.309839	11.312203	11.812013	10.020483	10.719109	10.139408	10.131533	10.752225	11.399891	10.856459	11.102683	11.737314	10.833302	11.006998	10.757526	11.491594	10.975823	10.627787	11.388799	9.978283	11.246224	10.270009	10.644015	9.034153	11.450002	10.140152	10.344770	11.307098	11.397315	12.026020	9.833953	8.935020	11.112307	11.080123	10.905697	11.129871	11.436404	10.667829	8.954868	11.432256	10.774190	8.854235	11.623005	9.740241	10.914603	10.560091	10.915674	10.477090	10.435072	9.383918	10.733854	10.110042	9.710473	11.591601	10.511848	9.191027	9.218991	8.531825	9.169378	12.151881	10.690865	10.907373	9.727909	11.218031	8.650870	10.653521	10.912348	11.328057	11.136974	9.943954	10.927267	9.895199	11.576476	10.377874	11.119708	9.413934	10.596344	10.545002	11.132929	10.505842	11.391292	8.640199	10.756043	11.557743	11.062006	10.393702	10.056502	10.465042	10.909294	11.322437	10.323671	10.056794	10.281686	10.354764	9.705338	11.855342	9.196602	11.113613	9.551464	10.786473	11.042247	10.665039	9.392525	9.544116	10.618670	11.303373	10.330389	10.321391	12.310154	10.722821	11.105584	12.550549	11.544838	10.995680	10.940232	11.526932	11.816015	11.013825	10.869032	9.927691	11.156555	10.492047	10.327480	9.899439	8.883900	9.458628	9.507924	10.865897	11.193393	10.086677	10.453273	10.424091	9.513068	10.111696	10.715970	9.009654	9.775812	11.118254	9.338178	9.854297	9.147855	9.772353	10.252055	10.371545	9.286903	9.484248	8.087401	10.987659	9.646463	9.912662	10.935945	10.360216	8.771856

IDS	9.991732	10.813866	11.262129	10.719908	10.914915	11.298091	11.216410	11.727221	11.306203	11.403818	11.188301	11.356089	10.769710	11.085590	9.989959	11.586749	11.264551	10.837243	11.060112	11.229444	10.791003	10.587164	9.287241	10.318027	10.406371	10.645760	9.878449	11.526511	10.510787	11.061124	10.639211	11.388081	12.101333	11.185257	11.069134	10.996866	11.200158	10.601209	10.439290	10.893881	11.395788	9.628632	10.942871	10.806918	11.005994	10.757808	11.295979	9.954586	11.712614	11.352182	10.639993	11.232725	10.974793	11.278715	11.664174	11.157605	11.004401	10.357381	10.576159	10.669264	10.233687	10.770306	9.966653	10.010197	10.034536	10.881053	10.099349	10.210936	10.421474	9.912507	11.094991	11.119269	11.474696	11.292805	11.135510	11.280760	11.093405	10.998047	10.995680	11.484410	10.772988	11.430298	11.103415	11.357096	11.569154	11.309839	11.312203	11.812013	10.020483	10.719109	10.139408	10.131533	10.752225	11.399891	10.856459	11.102683	11.737314	10.833302	11.006998	10.757526	11.491594	10.975823	10.627787	11.388799	9.978283	11.246224	10.270009	10.644015	9.034153	11.450002	10.140152	10.344770	11.307098	11.397315	12.026020	9.833953	8.935020	11.112307	11.080123	10.905697	11.129871	11.436404	10.667829	8.954868	11.432256	10.774190	8.854235	11.623005	9.740241	10.914603	10.560091	10.915674	10.477090	10.435072	9.383918	10.733854	10.110042	9.710473	11.591601	10.511848	9.191027	9.218991	8.531825	9.169378	12.151881	10.690865	10.907373	9.727909	11.218031	8.650870	10.653521	10.912348	11.328057	11.136974	9.943954	10.927267	9.895199	11.576476	10.377874	11.119708	9.413934	10.596344	10.545002	11.132929	10.505842	11.391292	8.640199	10.756043	11.557743	11.062006	10.393702	10.056502	10.465042	10.909294	11.322437	10.323671	10.056794	10.281686	10.354764	9.705338	11.855342	9.196602	11.113613	9.551464	10.786473	11.042247	10.665039	9.392525	9.544116	10.618670	11.303373	10.330389	10.321391	12.310154	10.722821	11.105584	12.550549	11.544838	10.995680	10.940232	11.526932	11.816015	11.013825	10.869032	9.927691	11.156555	10.492047	10.327480	9.899439	8.883900	9.458628	9.507924	10.865897	11.193393	10.086677	10.453273	10.424091	9.513068	10.111696	10.715970	9.009654	9.775812	11.118254	9.338178	9.854297	9.147855	9.772353	10.252055	10.371545	9.286903	9.484248	8.087401	10.987659	9.646463	9.912662	10.935945	10.360216	8.771856

BNC2	4.243870	3.944654	3.686042	3.079184	3.103641	4.198394	4.981142	6.487103	3.392489	5.365217	4.661814	5.753589	4.379075	4.369342	3.352062	4.384959	4.970105	4.672289	5.152145	3.989853	4.864946	4.698722	7.592559	3.926936	4.786621	3.495025	5.112145	5.275128	5.208841	4.643607	3.881910	5.059192	5.014044	5.300214	5.568644	4.233943	4.858755	4.081964	3.343324	5.088990	5.322477	5.109966	4.687813	4.312111	5.434751	5.669624	5.997961	2.968734	5.572941	4.348542	3.763395	4.001936	4.320790	4.112673	4.884944	4.199007	5.475033	3.041879	3.032456	4.391494	4.258087	6.499883	6.520361	4.939145	3.919774	3.510727	2.970360	3.919983	3.570961	3.908360	3.538645	4.041017	5.429406	4.235520	3.743475	4.022811	4.403745	3.605046	4.104297	4.837863	3.937922	4.086810	3.651791	4.315840	5.468537	6.095149	3.342139	3.669919	3.393310	3.602249	4.177040	4.260648	3.577338	3.274576	4.160768	5.883488	4.564070	6.314873	4.387386	6.066254	4.964786	5.546573	5.245997	3.796319	4.949392	6.024457	4.030852	4.612954	5.289165	5.520232	4.373025	4.793308	3.282404	4.447792	4.842988	4.333544	3.366167	5.482394	4.950633	4.297904	3.867507	4.070060	3.190794	3.008683	3.698788	3.936016	9.063380	3.413453	3.136856	3.387237	3.270993	3.700564	3.677808	3.141697	4.574160	4.404666	3.527760	7.157612	7.639694	6.346068	5.410703	5.349273	2.856669	2.977135	4.935579	3.288191	3.473431	4.865575	3.513915	3.357243	5.480462	5.919567	4.569828	3.342302	3.722298	6.358329	3.326629	3.340445	5.278964	3.148882	3.480211	6.254554	3.439246	4.323583	5.108098	3.292054	2.908866	4.929067	3.860460	4.496204	3.514088	4.144750	4.247745	5.057174	5.938282	3.791429	2.978612	2.857822	3.288506	3.931741	4.548399	4.014957	3.990057	3.847973	3.661403	2.929714	3.516224	3.204261	2.889363	3.080777	4.589046	3.287111	5.086146	3.968315	4.282532	5.339523	3.865968	4.334644	5.636210	5.030756	4.495760	3.618554	5.935284	4.887311	4.233519	4.120971	3.433070	3.167274	5.345734	3.472214	3.617825	5.027449	4.110501	4.840237	6.053569	5.000684	6.470712	5.235235	4.023461	5.398817	4.413089	4.747288	4.096007	5.165102	3.501629	3.349874	3.389835	3.341666	3.196135	4.483726	5.299668	3.253277	3.238603	3.024270	2.999285	5.154192	4.195624	3.412911

RALGAPA1	8.342466	7.501316	7.997547	7.955121	8.688984	7.914461	7.514055	8.447016	9.822544	8.100408	8.587391	8.081951	8.324630	8.466285	8.710249	8.633369	8.934877	8.135700	8.785931	8.073965	7.478135	7.844679	8.781348	8.564511	8.489064	8.397483	8.308242	8.388507	8.707677	7.012195	8.772323	7.229521	7.413210	8.336479	7.674467	7.654056	7.735196	7.511385	7.305394	7.584267	7.948757	7.132283	8.265248	8.167657	8.544002	7.927574	8.031633	7.124664	7.914604	7.722840	7.574659	7.381884	7.881084	7.268489	8.093070	8.389600	8.163962	7.472022	7.916925	7.880939	7.228880	8.003253	7.089612	7.573258	6.622425	7.462948	6.814585	8.123421	7.061882	6.573541	7.099426	7.701899	8.736262	7.593360	7.392569	7.268933	7.208301	7.375955	7.109339	8.279347	8.391945	7.941974	8.118880	7.447919	7.874285	8.119607	7.913711	7.970993	6.487103	6.742581	7.130525	6.572438	7.792954	7.591653	7.466365	8.659306	8.553021	10.046779	8.106048	9.399554	9.266732	8.685097	9.307738	8.681590	7.511240	9.599406	8.610009	7.803104	8.715941	8.284523	8.301367	9.129082	8.781511	8.535462	10.285761	8.559151	7.826301	8.943997	8.956370	9.656579	7.389491	7.077031	8.921804	10.494529	7.206767	7.300641	9.188306	9.218401	7.914144	7.820563	8.410456	7.626412	7.884438	7.428825	8.657423	8.547074	8.242770	8.622822	7.098469	9.594939	7.843275	7.526521	9.923051	7.587802	7.008344	8.396241	8.305897	8.161937	6.556627	8.121469	8.602983	8.604996	7.755724	7.968486	8.592221	7.511087	8.985166	7.836896	8.133925	7.014074	6.518050	7.534238	7.651114	7.945709	8.350957	8.693097	6.976898	7.533758	7.014515	7.122578	7.913119	8.710395	7.778353	8.378919	7.305069	8.001687	7.127053	7.715237	7.267734	7.038741	7.489761	7.033591	8.519296	7.044183	7.480895	6.883757	7.525428	7.811572	6.496218	7.397708	7.558763	8.740267	8.112848	7.161880	7.545491	7.228420	7.483209	7.943394	8.063661	8.921659	8.085541	8.369861	8.124795	7.905055	8.641591	8.595167	8.333986	7.180945	7.365458	8.123251	7.117559	8.040928	7.536569	8.456424	8.716580	8.626721	9.391747	8.517941	8.773990	8.894686	9.353627	8.462482	8.329193	7.690876	8.471683	8.148080	7.925989	7.289877	8.397774	7.839529	8.637555	6.762363	8.631915	8.099775	7.198379	7.355953	8.761230	8.721334

DTNBP1	12.546528	12.022370	11.936694	11.423450	11.301874	10.777248	11.929674	10.453727	9.878002	11.340687	11.197390	11.564187	11.018523	11.495782	11.595057	10.856607	11.136391	11.633734	10.612891	11.486239	11.019457	12.030069	11.694853	11.259156	11.225924	11.583331	11.602276	10.442759	11.135818	11.909566	9.962991	12.302009	12.790499	12.316396	12.508519	12.506907	12.376294	12.604409	11.730804	11.713033	11.021817	12.149438	12.121584	11.694995	12.656189	12.232212	10.656544	11.893953	12.002645	11.846574	12.221569	12.439504	12.434656	12.452958	12.228964	10.783312	12.508519	12.127514	11.950501	12.212707	12.779681	11.260634	12.907135	11.460998	12.272641	12.327491	12.128125	11.992915	12.111742	12.293428	12.317621	12.355312	10.620123	12.431923	12.528093	12.701221	12.687444	12.695260	12.590988	11.788222	12.112064	12.408855	12.594598	12.350513	12.285194	12.367209	12.352576	12.227059	12.097568	12.077128	12.240857	12.116506	12.003535	12.495176	12.728769	12.486643	12.258675	10.486716	11.505101	11.275733	10.746193	12.031083	11.039164	11.603642	12.089510	10.265297	10.694498	11.620681	11.204040	10.255569	11.605506	11.028512	11.569303	10.390607	10.904808	9.929076	12.448174	11.241932	10.106586	12.532342	11.626020	12.664946	12.247673	10.777970	11.675812	10.681664	11.099157	10.101089	11.421793	10.313708	11.966106	11.048256	12.295874	11.290546	10.971621	11.134324	10.823244	11.803532	11.852632	11.487129	11.961457	12.046024	11.655606	11.147807	11.242375	11.255159	11.584548	9.977682	11.509649	11.519937	12.113904	11.226941	11.896202	13.118268	12.353725	12.272956	12.311085	11.250156	11.915341	10.784818	11.960368	11.292669	11.837541	11.741301	11.819147	10.353124	12.464370	12.452816	12.048150	12.317162	12.035499	11.909858	11.776832	12.067502	10.978478	12.547310	12.965441	11.935336	13.443956	11.852935	11.655606	11.781361	11.467339	12.577537	12.465654	12.191034	12.167414	12.114644	12.761354	12.376614	12.358636	10.480506	12.249289	12.590988	12.776543	12.193991	12.739166	12.208987	12.187947	11.566546	12.361312	12.373381	11.879384	12.785412	11.536600	12.274074	12.237890	12.523632	12.352739	13.495327	12.250568	12.163433	12.708077	12.144699	12.031376	11.876288	10.669403	11.916981	12.567105	10.532527	11.860555	11.200022	11.679650	12.097117	10.976116	10.333876	11.341633	11.347275	10.174725	11.955591	11.808875	11.628067	10.699421	12.334279	11.207639	12.503725	12.184989	10.337472

ZNF804B	3.823300	3.660815	3.646801	3.598390	3.447097	3.547366	3.329019	3.518911	3.546976	3.449361	3.512383	3.393995	3.262940	3.986951	3.773875	3.452261	3.938536	3.185015	5.047909	3.260560	5.534285	2.786208	3.807616	3.337310	3.057495	2.904508	3.230761	3.114521	3.187010	3.368431	4.735652	5.081456	3.822675	3.130470	3.604642	4.132553	3.716933	3.450236	3.959247	3.365817	3.540335	3.851185	3.709474	3.402821	3.881506	5.928289	3.593829	3.674948	5.170283	4.016509	3.974198	3.896056	4.601214	4.397017	4.678808	3.211164	4.311250	2.975073	2.929503	3.799662	2.856904	3.281206	4.069243	3.391037	3.638690	3.680251	2.873704	3.684214	3.320420	3.097736	3.362862	3.614957	3.333388	3.222673	3.245109	3.391764	3.558530	3.609188	3.260291	3.956576	3.555255	4.161815	3.644257	4.347401	3.503315	4.531822	3.886152	3.814406	3.275739	4.084748	3.532733	3.870938	3.543602	3.393483	3.145506	3.239326	3.977797	3.098565	3.614582	3.110154	4.354817	3.285045	3.332238	3.170910	4.676681	3.602249	4.306150	3.953135	3.143738	3.340445	3.759641	3.916620	4.052167	3.397284	3.655630	4.920011	3.375800	3.170136	3.284110	3.169414	3.872862	3.204801	3.262792	3.860460	3.858971	2.990809	3.318473	3.617653	3.175876	3.344639	3.094426	3.731504	3.268690	3.394875	3.507789	3.666279	2.987859	5.137013	3.439974	4.826975	3.244280	5.164675	3.128807	2.893708	3.472214	3.861275	3.801408	4.015184	3.582455	4.690116	4.716660	3.711134	3.234379	3.546557	3.833985	3.766740	3.661019	3.433819	3.166907	3.382241	3.548744	3.699384	3.495604	4.413993	5.435317	3.501981	3.162054	5.016682	4.021539	3.968532	3.707585	4.388263	4.608431	3.754254	3.223899	3.898920	2.885033	2.899911	3.432044	2.769141	3.338619	4.198394	4.023017	3.998329	3.798781	2.837184	3.700762	3.463557	2.925111	3.645020	3.999802	3.313340	3.140672	3.417894	3.360160	3.869875	4.141508	3.526862	3.313017	3.944222	4.658804	3.712709	3.671565	3.631499	4.368705	3.217271	3.462345	3.037234	3.630263	3.564009	3.579359	3.255752	3.507239	3.381180	4.003685	3.509610	4.647255	4.160365	3.254178	3.015024	3.063753	3.319115	3.364044	5.068793	3.555846	4.290244	3.751278	3.637684	3.226873	2.875281	4.538874	7.706537	3.282710	3.418265	3.907084	3.747301	3.192163	3.425651

OLFM1	15.982133	16.036135	16.053839	16.180048	16.052430	16.526136	16.040249	15.556779	16.538902	16.329145	16.016830	16.230114	15.840619	16.258072	15.360277	16.674913	16.551607	15.814383	13.990442	16.399202	14.162372	15.711627	14.333706	16.190578	15.952203	16.361326	16.094324	16.644905	16.201225	15.991977	16.213991	15.579100	15.117212	15.521068	16.081863	16.531399	16.274878	15.388411	15.838499	16.678432	16.538902	15.726974	16.487541	16.077730	16.519186	16.874939	16.740880	15.753148	16.327143	16.585910	15.798440	16.281614	16.310500	15.061588	15.773226	15.911833	16.704885	16.287200	15.294538	16.124210	15.242555	16.759174	15.784706	15.424949	14.557653	15.802772	15.927561	15.550613	15.336747	15.223282	16.748238	16.701464	16.489765	16.377052	16.879013	16.118448	16.064222	16.172846	15.627072	16.002806	15.279559	16.851732	15.910549	15.547001	16.744583	15.960718	16.395147	16.564795	14.328965	16.061651	14.806664	15.362494	15.100018	16.087400	15.236952	15.333793	15.004214	16.055100	16.217060	16.769750	16.283537	16.105830	16.351585	16.827810	14.097093	16.607860	16.907711	15.535143	15.428569	16.694520	13.914229	15.578257	15.913059	16.641548	15.866357	16.819885	15.697564	16.627037	17.008331	15.692442	15.053711	15.166759	15.521068	16.171220	15.373253	15.752097	16.338520	15.812427	15.490401	15.767032	15.701693	15.801695	15.684238	15.641330	16.015596	15.700644	15.153250	13.854373	14.888152	13.543518	13.858357	13.240605	15.582778	14.688427	14.785821	15.625168	15.521877	16.038921	14.949574	16.234936	15.413146	16.094324	15.861722	15.434117	15.894116	16.198362	16.707941	14.733935	14.834088	15.762943	14.422880	15.909426	15.731087	16.097256	15.782778	15.707533	16.319695	15.248027	15.903390	15.800609	15.769072	14.815737	16.061651	15.510860	15.576365	15.236952	14.545966	14.150745	15.283390	14.495949	15.412346	12.900592	13.690948	15.084166	14.911983	13.778922	14.832510	14.989019	13.469871	15.224051	16.143053	15.777495	15.877039	15.254640	15.443248	15.630002	15.473917	15.974511	15.454339	15.755343	15.030442	15.369410	16.209127	15.888240	15.749943	15.269335	15.325061	13.924035	14.978704	15.463234	13.762711	16.076368	15.555037	15.639403	15.429349	16.131397	16.357477	15.492990	15.332400	15.862906	15.669151	16.091387	15.012809	13.114296	16.226917	15.381398	14.396205	13.676222	15.554200	13.547770	15.490401	15.882641	15.743790	15.885839	15.751009	15.230680	16.125498	15.184749

PARVB	7.518967	7.634200	7.422439	7.358111	7.521207	7.214504	7.045786	8.769584	8.250672	7.305394	7.317777	6.809854	6.621633	7.131658	6.500696	7.034521	8.038832	6.766039	8.296702	7.061543	7.803104	8.108499	9.220198	7.419775	7.317492	7.876711	7.008175	7.939154	7.076359	6.959558	7.642917	6.713250	6.724487	6.475585	7.090879	7.140953	6.912945	7.295217	7.795559	7.424306	7.531277	7.643053	7.017782	7.306505	7.781356	6.997424	7.280042	7.127988	7.492285	7.275773	6.732083	7.021726	6.578323	7.023919	7.298780	7.643210	6.644513	7.243750	7.038273	7.179851	7.739616	7.011554	7.497414	7.662478	7.681782	7.702525	7.226387	7.479807	7.612641	8.031179	7.069874	7.287232	7.105647	7.285048	7.301462	7.395245	7.603296	7.576687	7.639694	7.021874	6.981609	7.080977	7.045467	7.261675	7.045619	7.075905	7.098301	7.278463	7.662653	7.752712	7.712486	8.335568	6.946970	7.192436	6.705103	6.798482	6.810647	6.457215	7.174638	6.878937	6.715371	6.748786	6.865661	6.877175	7.460560	7.148656	8.026061	7.743966	8.365337	7.400837	8.286545	8.073742	7.466651	7.447288	7.193101	6.186736	7.989271	7.133232	7.113308	7.044822	8.133767	7.345155	7.434394	8.135851	8.018756	7.270794	7.482442	7.854689	7.192616	7.668203	6.963071	7.223131	6.685661	7.143465	6.826564	7.862916	6.883757	8.141183	7.077360	7.699912	7.970201	9.102598	7.636774	7.900239	8.126601	7.430625	8.333675	7.237820	7.654980	7.627346	6.588047	6.739227	7.169943	7.101874	7.134995	6.945974	7.258046	8.194634	7.820856	7.961776	8.096606	7.343817	7.922721	8.169238	7.474745	8.338225	7.284141	7.324066	7.778846	6.977224	6.864700	6.847240	7.300970	7.087059	7.312129	6.527163	7.214504	7.499142	7.225295	7.872609	7.350547	8.090805	8.064117	7.632540	7.948163	7.743172	7.816968	7.727960	8.151277	7.187166	7.359312	8.163962	7.306346	7.047064	7.651747	7.720075	7.882760	7.946493	6.613976	7.128470	7.308348	7.437546	6.714928	7.041594	7.192137	7.574967	7.061387	7.627673	7.575887	7.674628	8.478049	6.841755	7.537955	7.499142	7.279720	7.013910	6.750073	6.910830	7.166846	7.190616	6.492516	7.032329	6.782927	7.081450	7.593028	7.896560	7.358723	8.336800	8.384965	8.171114	7.907878	6.993391	8.016483	7.449527	7.246521	7.893118	7.205372	7.798982

FAT4	5.341623	3.727546	5.162091	4.424840	3.560111	3.213524	5.230559	4.347616	4.516358	3.957225	4.526954	4.033858	5.252360	3.175783	4.521491	4.690520	4.086354	4.325277	3.540515	4.874651	3.371537	4.464384	4.628705	4.264647	4.896846	4.265308	4.937295	4.382303	5.324966	4.657066	3.814815	4.891263	4.394513	5.659751	5.812591	5.648979	5.334373	4.636077	4.286343	4.775737	3.849721	5.071631	5.269846	4.662653	4.855214	3.776565	4.472145	3.704057	4.463946	4.360033	3.876342	4.222766	4.870422	4.244327	5.060442	4.536303	4.584786	4.538643	4.509641	4.282734	3.329178	6.474078	4.893127	4.316930	4.037570	4.421071	3.992473	4.141508	3.777146	3.912272	4.080493	4.330333	5.124710	4.784124	4.777405	4.005441	4.379501	4.504789	3.910122	4.388263	4.612282	4.889825	5.192494	3.974446	5.145926	4.028172	3.167529	4.993938	3.957915	3.673077	4.274225	4.539755	4.290244	4.229003	4.329472	4.888751	4.736102	6.119063	4.235520	5.334373	4.700882	5.447832	5.033991	4.930410	4.120517	4.114828	4.701302	4.347616	4.898117	3.911846	4.175090	3.878499	3.438594	4.514410	4.039083	3.459121	4.440247	5.427660	5.271639	3.840068	3.172384	4.075568	3.471726	3.016572	3.110154	3.417742	5.930066	3.889613	3.130036	3.520038	3.225846	3.134280	3.118161	3.145266	4.149594	3.802024	2.963914	3.115109	3.683070	3.140189	3.450236	3.232699	4.699164	3.143613	3.186882	3.387739	3.318310	3.781660	3.229919	3.884029	3.960729	4.792691	4.530309	3.872419	3.170910	5.487997	4.472145	3.699384	3.961381	3.242391	3.439414	4.154312	3.385360	5.016263	3.748119	3.095098	3.061454	3.421018	3.516964	3.911207	3.597934	3.958573	4.273115	3.732755	3.510558	3.418434	3.931308	3.573251	4.169428	4.151044	4.184469	3.516786	3.660607	4.298299	3.489890	3.537834	3.987353	3.540150	3.040188	3.328676	4.181870	3.168920	3.599742	4.204054	4.111173	4.181870	3.873944	3.574228	4.078904	4.402464	4.085922	4.845079	5.200488	3.858138	2.974981	4.015408	4.016509	3.747301	3.351555	4.044548	3.499062	5.069189	3.727364	4.720656	4.425470	3.754647	5.915291	5.040958	3.889150	3.852056	3.696741	4.262886	3.195295	4.369790	3.372263	4.304427	3.246540	3.716933	3.123088	3.649432	3.167529	4.275750	3.295881	2.954608	3.204941	4.829317	3.850353	3.622868

TUBB6	8.090173	9.136379	10.100294	9.625524	9.481968	10.292188	10.689474	11.670468	11.697132	11.128662	10.864986	10.455864	10.276070	10.611997	10.060910	10.648195	11.009906	10.123342	11.622534	9.816125	10.207843	10.420715	10.922388	10.312904	10.329335	10.021101	8.818569	11.363130	9.329557	9.635782	10.724311	9.723772	9.869327	9.089930	10.398387	9.855874	10.721933	10.077279	10.662816	11.033441	9.810261	10.613473	10.261006	10.536062	10.404404	9.811338	9.848160	10.540036	10.771222	10.304483	10.597545	10.825019	10.098874	10.861007	11.232575	10.072223	10.350959	11.241932	10.191475	10.714756	10.876057	10.078545	10.900123	10.283800	9.833356	10.089927	10.035789	10.074685	9.905450	9.702032	9.810719	9.729654	10.475118	10.189657	9.430199	10.147930	9.770001	10.183317	9.732263	10.293394	10.082987	10.294779	9.661702	9.707187	10.864986	9.832913	10.602822	11.932006	9.611660	10.061953	9.755913	10.110954	9.611660	10.290059	8.572191	10.062277	10.791138	10.629961	9.575790	11.016776	10.921942	10.081486	9.483497	10.420414	10.214570	10.571372	10.886126	10.104790	9.532146	10.018340	9.733183	10.702585	10.949662	9.851053	10.940064	8.184642	8.428035	10.404553	10.345226	8.390991	9.734237	9.052979	7.418994	9.414708	9.951231	9.130781	7.974619	9.958459	8.640834	9.396057	7.825691	9.267511	9.351997	10.119905	9.459105	10.412232	7.906786	8.811070	8.844331	8.884219	10.297097	9.650087	9.402320	8.578625	8.381284	9.165569	7.803407	7.180484	9.342359	9.314738	9.549005	9.473843	11.668018	10.198291	8.946451	10.670427	9.231455	10.853448	9.832460	8.742249	9.522567	8.714362	11.154213	10.328234	10.541250	10.165968	8.857582	9.777218	10.255423	10.853742	9.263062	9.780259	11.514381	9.829822	9.068977	9.308487	10.522806	9.733039	9.578308	12.367843	9.539873	10.801045	10.015078	9.483634	9.709048	9.571204	9.440040	10.225137	8.658051	8.590359	9.249501	9.623390	9.443123	8.643311	9.822688	9.636997	9.839846	9.926051	9.698632	10.296948	10.768939	10.428377	8.634726	10.075883	9.082889	10.823244	10.299569	9.133976	9.751120	8.053391	9.600645	9.586696	10.413447	10.198715	9.460449	9.821458	9.761985	9.142441	10.979805	10.295850	8.466427	8.236995	8.836181	9.665912	9.300940	12.280067	8.953479	9.376837	9.575018	8.399557	10.407551	6.746121	8.903998	8.606818	9.048698	9.838114	8.380972	10.690069

BCL2L14	15.333793	14.869812	15.380594	15.595469	15.610464	15.022274	15.495462	14.222846	14.570568	15.071599	15.132734	15.044189	15.205705	15.258866	15.627072	15.015078	14.797410	15.343482	14.827984	13.945929	15.487850	14.950618	15.679147	14.684603	14.293761	14.861942	14.852694	14.059470	14.900158	15.000238	15.241853	16.095846	15.437228	15.221865	15.381398	15.278078	15.370867	15.405445	15.768020	15.417796	15.085293	15.269335	15.088208	15.404616	14.855908	15.296048	14.943193	14.685039	15.594598	14.867676	14.929175	14.645337	15.140997	14.767548	14.927607	14.855908	14.934057	15.069833	14.680520	14.706135	14.989606	15.344956	15.243980	15.116030	15.565819	15.371654	14.709849	14.691055	14.713029	14.653231	15.173276	15.143591	15.401561	14.896544	15.046534	15.052583	15.376978	14.395843	14.707094	14.690192	15.145529	14.588535	15.418602	15.700644	15.336747	15.557764	15.180575	14.961694	15.815434	14.848417	15.115355	15.236212	15.192758	14.865570	14.721418	14.869812	15.360277	15.408414	15.248027	14.603726	14.930234	14.361581	15.515982	14.607572	14.581385	15.241853	15.324219	14.748776	15.718870	15.049528	15.734237	15.179965	14.681437	15.421791	14.258622	14.580994	15.764986	15.738421	14.921174	15.840619	15.782778	15.328703	15.484605	15.491291	16.010487	15.446499	16.002806	15.937305	15.631882	15.632923	15.582778	15.908209	15.825349	15.541988	15.521068	16.019400	15.516865	15.785756	15.632923	16.063007	15.619793	15.802772	15.812427	15.763886	15.293858	15.443248	15.595469	15.799508	15.569300	15.676063	15.009362	15.416204	15.285115	15.394501	15.695521	15.193465	15.486206	15.445672	15.649863	15.938416	15.492990	15.370867	15.709527	15.482081	15.222515	15.758639	15.502234	15.775426	15.679147	15.523618	15.527160	15.296733	15.755343	15.632923	15.927561	15.847680	15.345704	15.419437	15.353308	15.296048	15.865084	15.565819	15.674965	15.128463	15.107375	15.546202	15.315239	15.495462	15.257473	15.234130	15.599209	15.285115	15.685230	15.318226	14.987786	15.179965	15.630940	15.519489	15.485427	15.328703	16.088816	15.347177	15.681084	16.226917	15.652837	15.921788	16.111440	15.887107	15.718870	15.769072	15.817557	15.532455	15.370867	15.313805	14.985105	15.544594	15.589896	15.768020	15.487065	15.847680	15.497254	15.217106	15.042367	15.545439	15.734237	15.921788	16.393332	15.971905	15.744893	15.808111	15.848852	16.091387	15.301621	15.960718	15.542881	15.961903	15.746888	15.965762

PTMA	15.333793	14.869812	15.380594	15.595469	15.610464	15.022274	15.495462	14.222846	14.570568	15.071599	15.132734	15.044189	15.205705	15.258866	15.627072	15.015078	14.797410	15.343482	14.827984	13.945929	15.487850	14.950618	15.679147	14.684603	14.293761	14.861942	14.852694	14.059470	14.900158	15.000238	15.241853	16.095846	15.437228	15.221865	15.381398	15.278078	15.370867	15.405445	15.768020	15.417796	15.085293	15.269335	15.088208	15.404616	14.855908	15.296048	14.943193	14.685039	15.594598	14.867676	14.929175	14.645337	15.140997	14.767548	14.927607	14.855908	14.934057	15.069833	14.680520	14.706135	14.989606	15.344956	15.243980	15.116030	15.565819	15.371654	14.709849	14.691055	14.713029	14.653231	15.173276	15.143591	15.401561	14.896544	15.046534	15.052583	15.376978	14.395843	14.707094	14.690192	15.145529	14.588535	15.418602	15.700644	15.336747	15.557764	15.180575	14.961694	15.815434	14.848417	15.115355	15.236212	15.192758	14.865570	14.721418	14.869812	15.360277	15.408414	15.248027	14.603726	14.930234	14.361581	15.515982	14.607572	14.581385	15.241853	15.324219	14.748776	15.718870	15.049528	15.734237	15.179965	14.681437	15.421791	14.258622	14.580994	15.764986	15.738421	14.921174	15.840619	15.782778	15.328703	15.484605	15.491291	16.010487	15.446499	16.002806	15.937305	15.631882	15.632923	15.582778	15.908209	15.825349	15.541988	15.521068	16.019400	15.516865	15.785756	15.632923	16.063007	15.619793	15.802772	15.812427	15.763886	15.293858	15.443248	15.595469	15.799508	15.569300	15.676063	15.009362	15.416204	15.285115	15.394501	15.695521	15.193465	15.486206	15.445672	15.649863	15.938416	15.492990	15.370867	15.709527	15.482081	15.222515	15.758639	15.502234	15.775426	15.679147	15.523618	15.527160	15.296733	15.755343	15.632923	15.927561	15.847680	15.345704	15.419437	15.353308	15.296048	15.865084	15.565819	15.674965	15.128463	15.107375	15.546202	15.315239	15.495462	15.257473	15.234130	15.599209	15.285115	15.685230	15.318226	14.987786	15.179965	15.630940	15.519489	15.485427	15.328703	16.088816	15.347177	15.681084	16.226917	15.652837	15.921788	16.111440	15.887107	15.718870	15.769072	15.817557	15.532455	15.370867	15.313805	14.985105	15.544594	15.589896	15.768020	15.487065	15.847680	15.497254	15.217106	15.042367	15.545439	15.734237	15.921788	16.393332	15.971905	15.744893	15.808111	15.848852	16.091387	15.301621	15.960718	15.542881	15.961903	15.746888	15.965762

DTWD2	15.333793	14.869812	15.380594	15.595469	15.610464	15.022274	15.495462	14.222846	14.570568	15.071599	15.132734	15.044189	15.205705	15.258866	15.627072	15.015078	14.797410	15.343482	14.827984	13.945929	15.487850	14.950618	15.679147	14.684603	14.293761	14.861942	14.852694	14.059470	14.900158	15.000238	15.241853	16.095846	15.437228	15.221865	15.381398	15.278078	15.370867	15.405445	15.768020	15.417796	15.085293	15.269335	15.088208	15.404616	14.855908	15.296048	14.943193	14.685039	15.594598	14.867676	14.929175	14.645337	15.140997	14.767548	14.927607	14.855908	14.934057	15.069833	14.680520	14.706135	14.989606	15.344956	15.243980	15.116030	15.565819	15.371654	14.709849	14.691055	14.713029	14.653231	15.173276	15.143591	15.401561	14.896544	15.046534	15.052583	15.376978	14.395843	14.707094	14.690192	15.145529	14.588535	15.418602	15.700644	15.336747	15.557764	15.180575	14.961694	15.815434	14.848417	15.115355	15.236212	15.192758	14.865570	14.721418	14.869812	15.360277	15.408414	15.248027	14.603726	14.930234	14.361581	15.515982	14.607572	14.581385	15.241853	15.324219	14.748776	15.718870	15.049528	15.734237	15.179965	14.681437	15.421791	14.258622	14.580994	15.764986	15.738421	14.921174	15.840619	15.782778	15.328703	15.484605	15.491291	16.010487	15.446499	16.002806	15.937305	15.631882	15.632923	15.582778	15.908209	15.825349	15.541988	15.521068	16.019400	15.516865	15.785756	15.632923	16.063007	15.619793	15.802772	15.812427	15.763886	15.293858	15.443248	15.595469	15.799508	15.569300	15.676063	15.009362	15.416204	15.285115	15.394501	15.695521	15.193465	15.486206	15.445672	15.649863	15.938416	15.492990	15.370867	15.709527	15.482081	15.222515	15.758639	15.502234	15.775426	15.679147	15.523618	15.527160	15.296733	15.755343	15.632923	15.927561	15.847680	15.345704	15.419437	15.353308	15.296048	15.865084	15.565819	15.674965	15.128463	15.107375	15.546202	15.315239	15.495462	15.257473	15.234130	15.599209	15.285115	15.685230	15.318226	14.987786	15.179965	15.630940	15.519489	15.485427	15.328703	16.088816	15.347177	15.681084	16.226917	15.652837	15.921788	16.111440	15.887107	15.718870	15.769072	15.817557	15.532455	15.370867	15.313805	14.985105	15.544594	15.589896	15.768020	15.487065	15.847680	15.497254	15.217106	15.042367	15.545439	15.734237	15.921788	16.393332	15.971905	15.744893	15.808111	15.848852	16.091387	15.301621	15.960718	15.542881	15.961903	15.746888	15.965762

ITGA9	15.246007	14.168734	13.900711	14.201164	13.709428	14.082208	14.448739	12.802136	14.157364	14.616154	13.647062	14.412450	14.051518	13.224119	13.488148	14.511568	14.678334	13.964639	12.327002	14.465534	12.962142	12.101494	13.556139	13.693359	13.949893	14.517397	15.736354	15.171380	15.085910	14.414735	14.696136	13.581357	11.806190	14.082507	15.025188	14.374427	13.815559	12.927582	14.135120	14.098263	15.161343	14.388615	14.907125	14.428245	15.503877	15.344243	15.331635	13.473788	14.263241	13.873120	13.522074	14.149795	14.466061	12.831697	13.352500	14.350910	14.847322	13.094857	12.495176	12.937441	12.930619	16.033556	15.250206	12.868242	12.351330	12.107582	12.787522	12.739334	11.740562	13.234805	15.224770	13.511061	14.917864	14.045286	14.861942	13.393700	13.598139	14.678334	13.209908	12.635767	13.182875	15.407655	13.404601	12.494420	13.479279	14.595757	13.757880	14.392936	12.420436	11.923414	12.976230	12.512753	11.873996	13.108685	13.099406	13.781086	13.399301	14.027387	14.505707	15.190029	14.017548	14.630796	14.732223	14.575995	11.629391	14.448911	14.692440	13.739103	14.196861	14.917362	12.710502	12.697518	12.586302	14.968533	14.059748	14.878115	14.641807	14.996793	15.672165	14.183722	13.585102	14.892277	14.884408	15.726974	14.672821	14.938328	15.922928	14.894400	14.348197	15.438162	14.578917	15.091892	14.913079	15.552451	15.248027	14.763822	14.188986	13.045174	13.036330	13.639891	12.856932	13.013710	14.242737	13.515355	13.473369	14.803294	14.058288	15.502988	14.573507	15.926414	15.386744	15.008260	15.527160	14.784831	15.607626	16.169798	16.897220	13.841923	13.704846	14.965129	13.692881	15.601071	15.543645	15.805918	13.925119	15.080534	16.572650	14.324402	15.763886	15.681084	15.234130	14.128314	15.683269	14.640937	15.116602	14.431229	13.510177	12.853832	14.826455	13.934369	14.850021	11.846434	12.970971	14.422880	14.064122	12.788738	14.515058	14.657723	13.072631	14.489554	15.987023	14.627696	15.745908	14.890760	15.291679	15.454339	14.586497	15.787848	15.281689	15.553263	13.597687	14.730705	16.143053	15.763886	14.521823	14.689299	14.479473	12.997893	13.791984	15.048970	12.909129	16.026974	15.609450	15.779491	14.969696	15.975682	16.538902	14.572654	14.614415	15.705567	14.111066	15.367177	14.854894	12.256151	16.474625	14.113158	12.814750	12.491754	14.659974	13.121375	14.957224	13.693359	14.742751	14.382065	14.583563	14.601973	15.632923	14.129209

BARD1	10.781605	12.002645	12.559575	11.462482	11.880861	11.419699	12.253272	12.225836	13.081015	12.084672	11.958138	12.269777	11.746871	11.776688	11.785073	12.137865	12.527256	12.360854	12.282874	11.587383	12.386767	11.926923	12.353569	11.562171	11.695305	11.602409	11.037396	11.485786	11.590536	11.946077	12.271395	12.481813	12.049213	11.976530	11.711473	12.109069	11.916216	11.570035	11.062917	11.954230	12.169579	11.079540	12.334279	11.618620	12.238997	11.983954	11.286424	12.328606	12.623334	12.160484	11.710591	11.896631	12.567262	11.523811	12.686046	12.063494	11.986052	11.568423	11.728087	11.928618	11.906037	11.695625	11.446299	11.441607	12.000886	12.739710	11.295979	11.806329	11.782082	11.360907	11.852176	12.032153	11.995822	12.071423	12.079547	12.470221	12.298374	12.057308	11.792548	11.970814	11.656449	12.318072	11.730047	12.735569	12.363232	12.245922	11.694552	12.899561	12.076671	11.508193	12.029925	12.174197	11.418124	12.146410	11.720318	12.082436	12.555905	12.508187	11.881462	12.650373	11.564336	12.017489	12.327331	12.884027	11.841136	12.313325	12.615659	11.746557	11.850817	11.437253	12.450102	12.333497	12.200157	11.914277	12.596981	11.232575	10.604165	12.446584	11.306203	11.758139	10.961076	11.456523	11.462325	10.307568	11.933343	11.208356	12.535770	11.812156	11.775216	11.521285	11.104875	11.854611	11.609621	11.028512	11.188016	12.280508	11.119708	11.937329	11.792548	11.325845	11.813591	12.410614	12.301709	11.941052	11.168109	11.567838	11.618016	12.216095	12.010184	12.018249	11.915776	10.795839	11.784937	12.477191	12.623999	11.559519	12.402275	10.204611	10.969695	11.049872	11.848352	11.986953	11.486995	11.733026	11.515004	11.329825	12.610024	12.046618	11.245085	12.013408	12.436448	11.163491	12.595226	11.992461	12.013103	11.742152	11.776387	11.865618	12.090895	11.427104	11.532884	11.362553	11.170765	11.043433	11.628487	11.593866	11.371589	11.819436	11.126352	11.325990	11.724977	10.703174	11.970367	12.064994	11.798508	10.947866	11.386298	11.618317	11.821254	11.029556	13.146515	11.454892	11.306661	12.330329	11.985888	11.839166	11.872205	11.825339	11.006254	11.972074	11.934585	11.202548	11.409515	11.277499	10.854330	12.221092	11.283846	12.330024	11.943795	11.293238	12.373862	10.692122	11.743043	11.838717	11.718550	11.723042	12.478001	12.068749	10.965488	11.369313	11.326605	12.615979	11.135202	12.540468	11.704895	12.095039	11.505998	11.623726

CCM2L	16.456985	15.379850	15.706649	15.815434	15.882641	16.234936	16.048355	15.883639	15.760765	16.076368	15.915538	16.163437	16.001371	15.781709	15.450314	15.873355	15.924163	15.547001	15.476405	15.779491	15.340435	14.736679	15.160128	15.404616	15.499684	15.616895	15.911833	16.036135	16.443550	16.111440	15.954463	15.871113	15.059742	15.536844	15.715793	15.558628	15.988198	15.887107	15.729053	15.219191	16.412035	15.731087	15.813386	15.663417	15.890581	15.934855	16.182924	15.729053	15.978326	15.781709	15.726974	15.637542	15.682114	15.249434	15.531572	15.726000	15.717865	15.308698	15.256065	15.451967	15.131490	16.152946	15.598287	15.224051	15.368664	15.507347	15.368664	15.589896	15.315239	15.195519	16.127121	16.115572	16.259687	15.998443	15.994548	15.997211	15.837350	15.482081	15.604793	15.390024	14.993419	15.419437	14.887600	15.655519	15.517756	15.790919	15.430200	15.589896	15.044721	15.191395	15.128463	14.913617	15.333793	15.186033	15.278819	15.682114	15.630940	15.756462	15.827482	15.728021	15.854635	16.020683	16.057790	15.730084	15.082962	16.430830	15.420214	15.979533	15.310201	16.355654	14.926494	15.481254	15.756462	16.369009	15.964494	15.613151	15.810180	16.472279	16.156164	16.175634	15.903390	15.478014	15.847680	15.916773	16.261246	16.086016	16.010487	16.184460	16.131397	16.259687	16.171220	15.966981	16.011919	15.570171	15.983265	15.817557	15.462495	15.385183	16.064222	15.205705	15.124619	14.888617	15.836242	15.784706	15.897567	15.724946	16.010487	16.207472	16.308700	15.809175	15.380594	15.745908	16.213991	16.115572	16.370743	15.985841	15.985841	15.984477	15.942235	16.161910	15.656594	15.948483	15.874614	15.970695	15.932498	16.249977	15.448092	15.934855	15.953297	15.455879	15.418602	15.526235	15.738421	15.458388	16.562284	15.484605	15.412346	15.043521	15.160798	15.304417	16.483130	15.113035	15.173276	15.078176	15.189377	15.455879	15.379121	15.523618	14.817306	16.263142	16.228514	16.178602	15.998443	15.613151	16.005250	15.958303	15.989529	15.979533	15.227331	15.371654	15.505597	15.343482	15.268645	15.487850	15.889470	15.437228	15.363968	15.059742	15.153250	15.222515	14.938834	15.607626	15.854635	15.852280	15.521068	15.875766	15.789877	15.903390	16.433023	15.819800	15.852280	15.924163	15.204490	15.168793	16.066984	15.626034	15.966981	15.186033	16.358433	14.856471	15.411549	15.292522	16.125498	15.978326	16.160600	15.732189	16.196709	16.228514

PMP22	14.229183	13.590078	13.465090	12.992354	11.814058	11.369916	13.789952	9.743949	9.763871	12.644233	11.518100	12.124346	12.795211	12.042551	13.488803	12.119428	12.591334	12.811445	10.881487	11.400440	11.623159	12.051649	14.817850	11.806639	13.488148	12.598836	14.019562	12.714861	13.436646	13.745592	12.295874	13.648255	13.600359	13.525717	14.447934	13.682111	13.583919	13.643515	13.969621	13.432842	12.932755	14.679130	13.940443	13.460920	15.493839	13.871517	13.336016	14.006174	13.756638	13.459608	13.459378	14.028776	14.522236	13.163201	13.642281	12.671038	14.794023	14.380635	13.441364	14.335070	13.959054	14.274870	15.731087	13.083849	14.602856	13.994733	13.601738	13.339732	13.369945	13.633991	13.637147	13.324800	12.417049	13.597930	13.094857	13.529678	13.335613	13.987492	13.387976	13.579900	13.703414	14.750281	13.828188	13.318089	13.503447	13.265809	13.617542	13.754656	13.508794	13.068091	14.146755	13.507015	13.676465	13.151060	13.595345	13.432385	13.865399	13.333079	12.287847	13.319481	12.707385	13.631382	13.134359	12.830827	12.231738	12.583792	12.748645	12.637320	13.509479	12.196619	13.669109	11.554325	10.929328	11.596253	11.081007	11.627063	14.097959	13.222566	12.697518	12.642231	13.693359	13.927324	13.098632	13.747588	11.869488	13.282220	12.075117	12.401457	12.767982	13.004475	12.536900	13.377996	13.874448	14.071191	13.214202	13.483216	12.905897	12.630986	12.969695	13.153629	13.865399	13.343055	12.958279	13.483216	14.767548	12.753795	11.180344	12.315928	13.435346	12.584123	13.436214	13.837460	12.434656	13.094857	12.860065	13.934369	13.392020	11.977304	14.468520	12.037292	13.460920	13.158588	13.052053	13.463363	12.764791	11.186151	13.547770	12.076508	13.766508	14.077463	13.547331	13.519798	14.212720	14.297014	13.564577	13.671534	13.896959	12.728615	13.905302	13.533515	14.491152	12.656999	13.322545	13.598854	14.570106	13.514460	13.392668	13.578983	13.178579	12.808400	13.708445	11.484725	13.813000	14.127698	13.510832	14.166778	13.616612	13.660327	14.287592	13.532839	12.423121	13.159158	14.671925	14.348910	13.248925	13.541913	12.954985	14.081267	14.697055	13.537063	13.026368	13.458052	13.955201	13.478168	14.057693	13.640135	13.804379	12.436133	12.898302	13.345516	12.219692	12.968955	13.970725	13.514012	12.457861	11.521612	11.979444	12.297906	13.558188	13.942665	14.213670	12.100593	10.539572	12.202831	11.729465	13.999261	12.102820	13.368069

PBX3	11.374064	11.075469	11.324685	11.624432	11.587012	10.963392	11.390271	11.228274	10.736547	11.198550	11.032989	10.966399	11.219233	11.090745	11.211990	11.296440	11.242229	11.392179	11.799242	11.331326	11.529671	11.818545	12.844401	11.090745	11.419997	11.556839	11.679960	11.055379	11.115175	11.111735	11.513324	11.805448	11.732880	10.818334	11.456110	11.620849	11.752614	11.359582	11.585304	11.317187	11.436814	11.899287	11.084833	11.755741	12.176389	11.591754	11.764642	11.176414	11.747895	11.806190	11.369761	12.207292	11.907737	11.579980	11.234915	10.898787	11.961890	11.936396	11.160805	11.774172	12.175134	11.566103	13.085544	11.868456	11.820950	11.599182	11.894274	11.610339	11.459639	11.853844	11.376944	11.481782	11.110174	11.656449	11.370366	11.697842	11.341984	11.309839	11.274151	11.356089	11.656322	11.801784	11.405292	11.777140	11.800445	11.654988	11.663576	12.045443	11.957237	11.423450	11.856406	11.930619	11.610492	11.488767	11.180978	11.570455	11.708507	11.493752	11.601212	11.609910	11.203593	11.302927	11.278862	11.211143	11.616843	11.059824	11.958709	11.440486	11.745095	11.044307	12.154791	11.301140	11.265170	10.907661	10.785271	10.315346	11.765719	11.268395	10.911762	11.661349	10.353428	11.368733	11.689898	10.554766	9.712489	11.253964	10.110195	10.553379	10.700309	10.878246	10.484576	11.451631	11.854304	10.766855	11.440178	10.939616	10.791282	11.546206	11.567694	11.366980	12.784685	11.943486	12.384645	12.968759	11.454736	10.657698	10.756471	9.826469	10.832876	12.382100	10.947592	10.838423	9.456955	11.203158	12.352896	11.638425	11.747596	11.114868	11.071222	11.862307	11.545765	13.453926	11.324386	11.547102	10.697935	9.400002	12.238997	10.592897	11.450138	10.960626	10.926544	11.794980	12.666497	11.852324	11.747192	11.568999	12.228465	10.583893	11.166523	11.628942	11.890634	12.601411	11.674007	11.694693	11.760077	11.811133	10.736390	10.972500	11.564859	11.209661	11.322595	9.292527	11.296130	11.945767	11.021971	11.766620	11.324962	11.615402	11.870853	11.820206	10.729271	11.234364	11.481782	12.259261	11.017653	11.019905	10.582460	12.091212	11.301597	10.299864	11.594771	10.862460	11.060705	10.655479	11.087082	11.144860	11.633734	10.958719	11.187798	12.403382	9.827223	11.308901	11.311611	12.473452	10.558564	11.325845	10.390756	11.493601	11.405443	11.559918	12.030634	13.273173	10.856734	10.752372	10.236196	11.248361	9.841975	10.937294

GID4	3.645431	4.233299	3.944654	3.403206	4.065006	4.505663	4.724322	3.988380	5.846047	4.473849	5.378088	5.878151	4.493308	4.203849	5.054934	4.852673	4.736547	3.624648	4.665178	4.843806	5.465020	2.823537	3.802243	3.875056	3.560522	4.454821	4.213192	4.328317	4.459269	4.831864	4.341562	6.120116	5.073004	4.547077	5.271224	5.948086	5.088396	4.220367	3.813804	3.753157	4.947539	3.876342	5.755174	3.509419	4.553885	5.909357	5.411869	3.270657	6.602789	3.975942	3.936458	5.372063	5.831408	5.853436	7.415795	4.899001	5.156870	5.406712	4.266557	5.215220	4.730409	5.244612	4.041233	4.132771	3.653400	4.654618	4.534351	4.484599	3.665267	3.826358	4.755912	4.778641	4.027744	5.082253	4.956861	5.699307	4.131671	5.331849	3.821253	6.177136	5.732486	6.402431	4.289203	5.161480	5.329881	5.684727	4.893955	7.131001	3.412911	4.606257	4.317365	3.327915	4.806984	4.851814	4.909483	4.967357	5.481833	5.470823	4.132328	5.459435	4.690299	4.494905	4.826757	5.112361	4.704262	5.209429	5.266656	3.943502	3.055709	3.896502	3.225561	3.460556	3.054863	4.398284	6.348609	3.396622	3.460015	4.775737	3.468209	3.989667	5.823851	3.939637	3.420255	3.404378	4.560116	3.407850	3.285959	3.914658	4.290904	5.218747	5.302426	4.021539	3.252973	4.459459	3.379120	4.073434	2.929714	3.457869	4.887089	4.733992	3.560720	5.562034	3.331764	2.867885	4.622687	3.737290	4.441288	3.453668	3.949881	3.614770	5.131397	4.580993	5.506022	5.229951	6.225380	5.417383	3.641091	3.895854	3.624039	4.099972	3.610373	4.662468	5.297845	5.979208	5.415640	5.988641	2.963416	5.971051	3.923290	3.876965	4.904203	4.397228	6.026064	5.482767	4.175969	3.901874	2.956250	2.812834	5.518786	4.378669	4.917062	4.165795	4.095169	4.022591	3.848186	3.366512	4.563629	3.670112	2.898436	5.250184	4.035847	3.467670	4.763580	3.661837	5.104433	4.631763	4.617724	4.145187	5.320912	5.932632	6.470538	6.215597	4.402007	6.359697	4.452047	5.187008	6.350795	3.102636	3.622668	3.565063	3.903127	4.572849	5.231593	5.600944	4.605023	4.627811	3.859999	4.230278	6.169288	4.941966	4.275288	3.556216	3.827629	5.084247	4.116148	5.577775	4.332885	4.428056	4.751901	3.107476	3.217589	2.927701	3.864105	5.209826	4.961235	4.177655	4.420876	4.299398

IFI44L	8.893482	9.628141	9.592273	7.922721	7.238287	7.612184	8.133176	6.327414	4.676909	7.727325	7.651452	7.553392	8.623592	9.218853	8.448740	7.157299	8.222227	9.583773	6.015023	9.238911	7.185534	7.003068	9.714221	6.581952	7.853595	6.838519	9.855275	7.321841	8.120535	10.538071	7.851569	9.641485	12.288938	11.547236	10.635198	9.559382	9.535972	9.894119	9.249208	8.990614	6.935528	10.462807	11.624432	9.793010	10.731478	10.556615	8.168296	9.125313	9.057670	10.067207	10.994776	12.771719	10.692122	9.737303	9.554646	7.903342	9.705663	9.446852	9.612878	9.980743	9.512608	9.431422	9.449989	8.853466	10.118840	9.800112	9.744936	12.078970	10.285603	8.424820	8.864761	8.734085	6.945345	9.229700	8.766397	11.256930	9.103509	9.206573	9.881165	11.369761	10.111998	9.615893	10.316793	10.381941	9.648272	8.813801	10.814170	10.247700	10.249401	9.581010	10.387032	11.855342	10.500924	12.436614	11.866080	9.317184	10.490413	9.508226	9.081657	7.959757	8.222992	8.960223	8.392568	8.309760	7.399762	7.435040	7.078318	7.401924	7.914461	7.061387	9.158783	8.328299	6.393878	6.043958	8.652972	5.501391	9.968492	9.137166	7.134995	12.749319	12.496521	12.637320	11.330601	7.239737	12.027045	12.287847	6.337381	8.310512	11.531239	9.296610	6.761558	7.748723	13.972743	9.808735	10.602963	13.535306	8.297298	10.187407	11.810826	11.626444	13.208114	11.274882	10.170756	6.757650	11.768847	12.664096	9.528969	9.566353	11.685099	13.377234	10.746193	9.477056	10.443810	13.318278	9.208692	10.874119	11.644012	12.263096	12.022222	8.128713	11.330317	12.503046	12.615190	11.595630	12.775662	8.809207	10.398534	10.014741	10.896881	13.312162	12.277596	13.690948	9.521604	12.060100	12.327168	13.131537	11.295400	12.802478	11.231105	12.472957	11.626444	9.904108	12.977674	13.228843	11.936536	11.406467	11.446009	10.603402	13.048184	9.656882	12.212235	7.597778	9.917330	10.857505	12.646754	9.924735	9.882240	9.359225	13.196334	11.069134	12.839438	14.263896	12.381475	14.421398	11.320848	11.449273	10.665188	11.610052	13.255166	8.939581	12.891207	10.791885	11.803667	11.253814	12.837755	10.757808	8.887920	13.452889	10.862314	11.769756	8.138337	7.881227	11.850350	11.762225	9.956464	7.822571	10.844403	10.446116	11.705064	10.684971	12.959560	5.754786	12.371644	7.289419	11.568841	13.257008	10.234334	11.911024

BAALC	5.770865	5.882462	6.407375	5.632657	5.436282	5.636758	6.563191	5.730674	4.504570	5.063633	5.445634	4.528611	6.073804	5.525162	4.481255	5.656461	5.826762	5.224480	4.655551	4.932210	4.725774	4.979337	6.624362	5.034193	6.389008	5.478875	5.247625	5.455327	5.466568	7.932799	4.842115	7.511240	6.649826	6.590464	7.180328	6.648363	6.662807	6.634026	6.110778	6.214589	5.426670	5.855821	6.434267	6.529490	7.041594	6.712952	6.683406	5.497551	6.804151	6.573209	6.555305	6.941033	7.469241	6.632390	7.143310	5.536657	7.181093	6.346068	6.277988	6.556302	6.923207	5.676185	6.354574	4.626946	6.851397	6.935199	6.248137	5.980140	5.960936	5.430487	6.901907	5.874564	5.133626	6.547740	6.692278	6.491222	6.470026	7.207847	6.022066	6.599421	6.574042	7.132761	6.763766	6.572587	6.663954	6.570038	6.615109	6.707042	5.355495	6.276052	6.696813	4.892508	6.141377	7.010422	6.537120	6.308073	5.765200	5.754614	5.972154	5.185806	4.842115	5.896721	5.471198	5.601521	5.204082	5.649908	5.826180	5.342989	4.650482	4.972338	5.006627	4.363732	5.445634	4.664093	4.776555	5.547864	6.418823	5.824779	4.677314	9.458185	6.913999	8.493381	8.228867	7.015663	6.228136	7.771423	6.979258	6.343011	5.681611	7.934327	6.844383	6.567413	7.427119	5.208018	7.512022	7.541891	6.126955	5.206440	6.913084	5.988481	7.341300	6.474750	7.727797	9.498874	8.427882	5.318061	5.326168	6.617903	7.724844	5.762867	6.788410	7.951667	6.636123	9.872275	7.786851	8.944434	7.320594	6.423337	7.155127	6.029738	6.394997	7.923181	9.241805	8.071381	7.248226	6.551816	6.574654	8.875113	8.633972	7.874285	6.447523	8.842290	8.256628	10.738376	6.625668	7.890755	8.649201	6.500534	8.465219	7.084584	7.753645	6.767805	6.264678	7.527432	6.890614	9.657788	7.116904	6.324405	5.782376	6.853497	7.743008	6.131372	8.147919	9.766182	7.807263	8.879252	8.343367	7.533448	8.716906	9.645648	8.944895	8.387588	7.702983	9.383323	8.771708	8.981956	7.636774	8.734872	7.994267	6.472740	4.970294	7.615728	9.086870	6.939212	6.491525	7.141928	6.271536	5.903276	8.817289	6.133795	6.930656	5.773278	7.252455	7.522817	5.634528	6.691940	6.328750	5.147729	8.325088	5.600062	7.742387	6.191665	5.591000	6.246099	6.384819	7.310728	9.514851	8.876622

BCKDHB	12.073426	11.199742	11.204040	11.115929	10.965182	10.959888	11.256480	10.785872	10.503317	11.075744	10.937909	11.342479	10.593479	10.787634	11.242375	10.748141	10.737488	11.089685	10.878323	11.069134	11.051912	10.077440	10.864538	10.922845	10.905549	11.177918	10.463260	11.183466	11.122655	10.807060	10.580067	11.027337	10.860126	10.642678	11.093405	11.197663	11.224021	11.374203	11.597568	11.626675	10.952431	12.514995	10.714168	11.051912	11.166662	10.976546	10.722072	12.113742	11.198858	11.343233	11.508343	11.220719	11.427665	11.523082	11.381596	10.815965	11.465983	12.224619	12.197056	11.706258	12.242226	10.778399	12.399627	11.914124	11.645624	11.957101	12.326361	11.427665	11.320252	11.943188	11.505696	11.290862	10.612584	11.111735	11.024218	11.047378	11.208087	11.102824	12.041372	11.339952	11.354634	11.303088	11.448837	11.448972	11.256343	11.258424	11.186429	11.268947	12.061519	11.984692	12.185867	11.642694	11.385706	11.009768	11.733934	11.179288	11.324220	10.680668	11.048690	11.142648	10.809658	11.297790	10.968756	11.588472	11.101499	10.788074	10.814754	11.407985	11.384387	10.746942	10.689337	11.564784	10.853180	11.141637	10.604891	11.048256	12.733879	10.859529	10.689337	10.712574	9.730269	10.663292	9.878156	9.598172	9.643632	9.854153	11.274607	10.020947	10.059845	9.929695	10.102629	10.272760	9.214537	9.890432	11.055610	10.100294	10.076365	10.639993	11.471808	9.988728	9.354991	9.844400	10.666811	9.766782	9.890283	10.907795	10.254815	10.831500	10.062899	10.779119	10.147330	10.961222	10.238782	10.079690	10.029019	10.755577	10.658429	11.109422	11.016201	10.515332	11.526788	10.628861	9.878156	10.315931	10.279115	10.584914	11.924880	10.111224	10.547030	10.270009	11.699618	9.894872	10.046320	10.525185	10.955440	11.908493	11.886002	11.191935	11.737021	10.924325	10.262977	12.169864	10.880464	11.764495	11.300543	10.536625	10.415564	10.616577	11.803385	10.224681	10.370335	9.179692	11.039307	10.618196	10.358566	10.522363	10.459258	10.786172	10.557368	10.316523	10.537651	10.280486	10.728828	10.259775	10.259775	9.745999	11.665969	11.021233	10.831970	11.497855	11.636180	10.696036	9.875282	10.100927	10.087772	10.410429	10.517560	9.673499	10.847998	9.601437	10.833447	10.602690	10.626550	9.574584	9.419757	10.827682	11.765719	11.809762	9.488589	9.696024	10.377874	11.837541	10.613910	9.851816	10.617300	10.582028	10.875751	9.331538

NUB1	11.508193	11.017510	10.804597	10.375761	10.218028	10.088219	10.929785	9.651648	8.262290	10.281995	10.106142	10.630402	9.916835	10.587164	10.551284	10.345672	9.757967	10.060156	10.138059	10.810803	10.435219	11.506853	11.030464	9.965013	10.377715	10.262380	11.015894	9.614684	9.921992	11.276012	9.181882	11.131496	11.126352	11.429495	11.382315	11.268089	11.196525	10.273782	10.663131	10.293540	10.462340	10.999386	11.503490	11.011398	11.363862	11.016056	9.732123	10.480362	10.895631	11.295839	11.043433	11.354920	11.726308	11.611094	11.023071	10.366240	11.435242	11.009021	11.080590	11.256343	11.767049	10.642239	11.587454	10.997004	11.264255	11.099443	11.152465	11.037869	11.052217	11.215987	11.347723	11.150075	9.821765	11.457125	11.260928	11.548736	11.532884	11.179288	11.474995	10.881487	11.465404	11.269244	11.481223	11.565520	11.479176	10.856159	11.328644	11.431202	11.703267	11.288046	11.441607	11.720028	11.260318	11.935336	11.855631	11.660731	10.730021	9.245349	10.532841	10.002270	9.579952	10.741115	9.733932	10.220346	10.954841	9.687619	9.726547	10.620579	10.942125	9.790059	11.882525	9.800580	9.557714	8.844331	10.590086	6.976098	10.407108	10.560393	8.834502	10.785414	11.544678	11.842350	10.346735	7.649041	10.691348	11.012953	8.417837	9.674122	9.039311	9.120056	10.100927	10.007983	10.566197	10.740962	9.358480	10.108529	8.974524	11.448235	11.813001	10.020947	11.525597	10.883417	9.635491	10.708068	10.523398	11.453138	10.127592	9.783941	11.534230	11.044735	10.458339	9.850885	10.592028	11.516794	11.119708	10.926907	9.794550	10.569539	10.646637	8.749091	10.884458	11.324835	11.450138	10.880771	10.815069	8.662390	10.104632	10.782238	10.989151	11.202548	11.314836	12.312060	11.442338	11.524887	10.972801	11.528459	11.083644	11.288477	11.254400	10.938211	11.414134	10.983080	11.950217	12.155557	11.003511	11.744056	10.329500	10.305962	11.594008	11.086931	11.387025	8.261392	11.002938	11.512897	11.484271	11.216410	11.310560	11.004248	11.110014	11.430298	11.083077	11.620252	11.245235	11.474696	10.257261	11.030164	10.701355	11.843716	11.325845	10.647970	11.465983	10.497091	11.861688	11.156096	11.060572	10.075501	8.234051	10.477996	10.493142	10.909711	9.612878	9.324472	10.954367	11.385568	9.271368	9.234563	10.334496	10.978030	9.825874	11.787155	10.142906	8.636910	9.460002	11.105301	8.465055	10.437880	10.511993	9.771758

WDR86	11.508193	11.017510	10.804597	10.375761	10.218028	10.088219	10.929785	9.651648	8.262290	10.281995	10.106142	10.630402	9.916835	10.587164	10.551284	10.345672	9.757967	10.060156	10.138059	10.810803	10.435219	11.506853	11.030464	9.965013	10.377715	10.262380	11.015894	9.614684	9.921992	11.276012	9.181882	11.131496	11.126352	11.429495	11.382315	11.268089	11.196525	10.273782	10.663131	10.293540	10.462340	10.999386	11.503490	11.011398	11.363862	11.016056	9.732123	10.480362	10.895631	11.295839	11.043433	11.354920	11.726308	11.611094	11.023071	10.366240	11.435242	11.009021	11.080590	11.256343	11.767049	10.642239	11.587454	10.997004	11.264255	11.099443	11.152465	11.037869	11.052217	11.215987	11.347723	11.150075	9.821765	11.457125	11.260928	11.548736	11.532884	11.179288	11.474995	10.881487	11.465404	11.269244	11.481223	11.565520	11.479176	10.856159	11.328644	11.431202	11.703267	11.288046	11.441607	11.720028	11.260318	11.935336	11.855631	11.660731	10.730021	9.245349	10.532841	10.002270	9.579952	10.741115	9.733932	10.220346	10.954841	9.687619	9.726547	10.620579	10.942125	9.790059	11.882525	9.800580	9.557714	8.844331	10.590086	6.976098	10.407108	10.560393	8.834502	10.785414	11.544678	11.842350	10.346735	7.649041	10.691348	11.012953	8.417837	9.674122	9.039311	9.120056	10.100927	10.007983	10.566197	10.740962	9.358480	10.108529	8.974524	11.448235	11.813001	10.020947	11.525597	10.883417	9.635491	10.708068	10.523398	11.453138	10.127592	9.783941	11.534230	11.044735	10.458339	9.850885	10.592028	11.516794	11.119708	10.926907	9.794550	10.569539	10.646637	8.749091	10.884458	11.324835	11.450138	10.880771	10.815069	8.662390	10.104632	10.782238	10.989151	11.202548	11.314836	12.312060	11.442338	11.524887	10.972801	11.528459	11.083644	11.288477	11.254400	10.938211	11.414134	10.983080	11.950217	12.155557	11.003511	11.744056	10.329500	10.305962	11.594008	11.086931	11.387025	8.261392	11.002938	11.512897	11.484271	11.216410	11.310560	11.004248	11.110014	11.430298	11.083077	11.620252	11.245235	11.474696	10.257261	11.030164	10.701355	11.843716	11.325845	10.647970	11.465983	10.497091	11.861688	11.156096	11.060572	10.075501	8.234051	10.477996	10.493142	10.909711	9.612878	9.324472	10.954367	11.385568	9.271368	9.234563	10.334496	10.978030	9.825874	11.787155	10.142906	8.636910	9.460002	11.105301	8.465055	10.437880	10.511993	9.771758

LMOD3	5.589095	7.061088	7.759279	7.367807	6.469852	5.511064	8.566718	3.410970	3.913993	7.512022	6.762817	7.958214	7.295374	7.439916	7.244382	7.278770	5.708822	8.146710	7.955404	6.572269	6.814127	6.281449	11.940902	7.668822	8.647237	8.350679	8.350817	5.850774	7.445391	6.949475	6.696196	6.890454	8.129817	7.481214	8.376613	8.535166	8.857133	8.717346	6.729818	5.608956	8.199817	8.228867	6.843699	8.057438	7.154664	8.554202	8.651479	8.064734	8.091424	8.581219	7.561058	7.726229	8.398520	7.864791	8.731515	6.674131	8.849097	9.163879	8.522603	8.555103	9.241363	6.923374	11.365359	7.727157	8.352921	9.152019	8.750986	8.646337	7.514494	8.178706	8.988227	7.957304	5.286594	8.562100	7.931883	7.319961	8.400492	7.290175	8.203908	9.144456	5.737361	7.213408	8.882342	8.765121	9.067710	7.586605	8.721185	7.013620	10.002425	8.640510	9.402928	9.641622	7.896560	7.863555	7.247752	7.366400	8.700270	7.379253	7.211176	5.241252	7.900541	7.868615	7.755300	8.207388	7.107418	5.356854	6.118891	5.991631	7.556615	7.298474	9.393123	5.752653	5.691739	6.322563	5.671803	8.708137	5.136441	7.178293	7.058536	3.673475	3.740582	6.094614	4.835984	5.541080	3.866394	5.646519	3.243404	3.633128	4.419800	3.747301	4.988095	6.701586	3.913757	3.727738	7.518967	3.459671	3.212415	4.742152	5.310072	4.615751	4.479106	8.424087	4.522553	5.005161	4.620375	6.338886	2.854722	5.155213	4.498338	5.811119	5.022964	3.656029	5.921593	6.079900	5.761020	6.637534	4.532248	3.614957	3.491351	4.720037	4.695153	5.275128	5.086364	4.451198	5.142569	5.629161	3.327729	5.524404	6.201016	5.511064	6.110778	6.102897	6.234645	7.597003	5.749191	7.278927	3.806168	3.955259	5.241828	4.490426	7.039698	4.896655	4.041017	4.930410	5.436894	4.929751	4.701513	3.733411	4.499207	6.283794	4.869580	3.303468	6.030587	6.296264	5.282173	5.820965	5.570700	5.469490	7.880784	4.058018	4.982780	6.780704	5.802582	6.244796	4.794770	6.297246	4.506771	8.113151	7.758843	4.394058	4.987279	5.456853	4.922128	4.615553	4.998969	5.976903	5.853071	4.298741	4.572639	6.019002	5.178157	6.265389	6.343517	6.066083	4.145187	3.340445	5.055139	3.919345	3.460186	8.040928	5.527477	4.620375	3.903970	3.957915	5.565784	6.274237	5.105042	4.594714

IQCM	3.772596	3.787788	4.242269	4.751699	5.214240	4.300684	3.722298	5.420176	4.404666	4.972542	5.040958	4.434966	4.321019	4.624008	4.358758	4.720656	4.292225	3.075635	5.089194	4.309062	5.306753	5.330483	6.323377	4.940761	5.065508	4.565327	4.807440	4.630694	5.135431	4.923847	4.553885	4.470334	4.635852	4.140617	4.635636	5.096594	4.053659	4.157781	3.979593	4.366132	5.064681	3.951215	4.300892	3.641293	3.956373	5.906838	7.777768	3.179770	5.339523	4.327452	4.177040	3.840908	4.598769	4.414893	4.939947	5.876019	4.299617	2.988170	3.893761	4.639899	4.694111	5.848448	4.048661	4.200312	4.222293	4.192585	2.867782	3.919131	3.499964	4.395671	5.306921	5.118253	4.672733	4.600410	5.120292	4.058646	4.299398	3.567883	4.161376	3.910557	3.799869	4.923433	3.988826	4.232240	3.776565	4.153649	4.440882	4.026648	3.302535	4.173575	3.554282	3.428299	4.275527	4.249268	4.391494	4.142379	4.765904	5.195916	5.196121	5.027227	4.670370	4.206614	4.925093	4.804715	4.621436	4.551331	5.136803	5.311445	4.951665	4.077627	8.266671	3.438428	4.208456	4.617086	4.301308	3.196135	4.335462	4.201411	4.659428	4.703430	4.607765	4.451198	5.067778	5.711383	6.147599	4.298115	4.045411	4.973579	5.278342	5.241252	4.933854	4.031890	4.662653	6.154825	4.614203	4.741069	3.956800	5.462754	3.512576	4.565327	5.052996	6.841442	5.225509	3.873314	4.559227	4.714555	5.066344	5.532003	6.839180	5.432417	4.497063	4.262655	4.614662	4.413089	4.402680	3.904426	3.694034	4.928060	4.074275	6.191829	3.754841	4.280176	3.971793	4.414658	5.420915	8.135109	2.911167	4.842335	3.986728	4.415573	3.662866	4.411106	4.636935	4.428669	5.131805	3.909914	3.519105	4.481472	4.658804	4.208456	6.091481	4.980126	4.097305	4.862901	4.587788	3.243970	4.470128	4.705113	4.255015	4.597449	4.825841	6.366062	4.902149	4.371755	4.426792	4.226387	4.097759	4.573305	3.865968	4.003685	4.879417	4.231803	3.993280	3.532733	4.831651	4.198394	3.878287	3.031966	4.394513	4.224914	4.703430	3.918102	4.868967	4.707020	4.786821	4.909875	4.828247	4.904203	4.837031	5.030756	4.268246	5.227712	4.701513	4.988511	5.986093	5.122893	5.610876	5.071240	5.923827	8.216812	3.894431	3.439246	6.318421	4.353117	3.674948	3.715067	5.039504	4.864946

GIMAP8	11.052063	9.670699	9.638433	9.586230	9.178931	9.566353	11.011088	7.460241	7.608977	10.010322	9.608835	9.089149	9.353339	7.702215	8.267307	10.412549	8.688984	8.576254	7.709122	10.405164	8.605587	10.359472	9.751590	8.634112	8.338702	8.727734	11.310881	8.743457	10.148412	11.396538	9.467194	11.306355	11.474379	11.627636	12.220929	11.612417	11.249397	9.660455	9.947489	10.230212	9.782831	10.844403	11.703267	11.060040	11.658962	11.234624	10.059698	9.838745	10.478435	11.445424	11.131620	11.323062	11.597256	11.609910	10.182107	8.668344	11.905620	9.849435	8.989121	9.910511	10.591268	11.727818	10.855104	9.282148	9.961205	8.983194	9.612716	9.410032	10.011226	9.934336	11.463337	11.243601	9.608835	11.530935	10.876767	11.560243	11.297358	11.579215	11.441892	9.750825	11.300829	11.554015	10.902906	11.447943	11.391895	10.578592	11.745525	11.603421	10.332682	9.462714	9.806697	10.831653	10.156673	11.986953	11.690932	10.886126	10.651183	10.984790	9.448263	8.632197	10.003500	10.320751	9.128766	8.272399	9.271660	9.933882	8.245874	8.657423	8.321274	8.914784	10.476474	7.472645	6.740044	8.995047	9.217179	7.924112	8.983483	9.608075	9.622911	10.483671	9.991882	10.654872	9.795801	7.561801	8.755019	9.771605	8.270907	8.290722	8.958885	8.939739	8.412468	8.088467	9.536570	6.787415	10.555231	9.282941	5.643691	8.426610	10.831042	7.940418	9.614684	9.698093	5.272049	6.236812	10.369411	10.009587	6.873688	10.170425	11.240198	11.037396	11.354479	9.749888	10.360216	11.804233	10.006010	11.580709	8.420732	7.814612	9.934023	7.621803	9.670386	10.501693	10.130935	10.135934	10.284261	7.839844	9.260903	10.999997	10.614231	10.704907	11.393217	11.065719	10.538517	11.396394	9.966801	10.484898	8.731061	8.289633	9.024914	8.970055	11.125774	8.271494	9.371346	9.528337	9.421896	8.779775	9.444057	8.436480	9.221888	10.724009	10.043887	6.166148	10.623514	11.874316	11.716161	10.773135	10.488419	9.772221	9.276381	9.998895	10.332062	10.356625	11.501897	11.422093	9.665009	10.751156	10.199050	10.850468	9.997800	8.660701	9.114050	8.867920	10.831500	10.801939	11.168249	9.161517	9.151539	9.554189	8.529542	10.606813	7.919819	8.166444	9.496268	9.649786	9.276381	6.027309	8.387288	8.351865	7.504157	10.191897	7.667444	4.891867	8.827688	8.985166	9.022623	9.492544	8.330575	9.371195

AL049697.1	3.751463	5.212965	4.068580	4.312111	3.175288	3.480211	6.051840	4.897059	3.458409	4.369123	5.643883	5.322477	4.981524	4.953562	4.628705	4.096439	3.960312	5.259448	4.604801	5.407122	5.009721	5.639162	4.587990	3.372905	4.713491	4.556840	4.006760	3.606405	5.564296	4.778245	4.857330	6.729309	6.763607	5.800232	5.969657	6.467386	5.334186	5.313797	6.188588	4.877599	5.025271	5.774550	6.442193	5.015459	6.000418	5.873876	4.036699	6.630154	3.892448	6.664533	6.904447	6.822918	6.260652	6.346068	6.701746	4.830410	6.269984	5.274932	5.049157	6.247463	6.237472	5.026469	6.832447	4.411994	5.527072	5.018696	6.450484	4.516566	5.543158	5.787562	6.206911	4.695972	4.685452	5.281557	5.765730	5.866298	5.373424	5.770690	6.300188	5.829144	5.394390	6.453304	6.498895	6.302900	5.798779	6.620661	6.038250	6.524348	4.887696	4.465860	5.690981	4.543312	5.324027	5.919921	5.979978	5.723098	5.297648	5.067971	5.659580	4.389553	3.745252	5.434751	5.433353	5.790780	4.420461	4.015628	4.023218	4.251298	4.257186	3.580146	5.303048	5.208841	3.996626	3.379120	4.844670	3.457694	3.625812	4.215317	3.253117	4.795599	5.461042	5.059000	3.174332	3.124029	3.856793	4.303076	7.236582	5.227712	4.721287	4.896017	5.737157	3.290448	6.017781	4.960000	4.548146	5.499283	3.593829	6.452423	7.108364	4.266557	4.824132	6.367558	3.531729	3.847741	6.824504	4.462632	3.610373	3.878061	5.354518	6.336540	5.420358	6.743037	5.112773	7.178765	7.260903	6.898683	4.493546	6.202971	6.173519	3.492758	5.796753	7.177806	5.422062	6.276052	5.395778	3.421880	7.468145	5.029321	7.184232	6.759287	6.798171	6.671872	6.254190	7.095149	5.757214	9.419599	6.567049	5.067971	7.582731	5.729540	4.448914	6.521545	5.422259	4.691774	5.311070	4.273115	6.296916	4.598327	9.471554	5.410127	5.479634	4.721961	6.089923	5.638630	5.631190	6.486761	6.225189	5.591982	5.744281	6.600064	5.628798	7.277658	7.429530	7.267429	5.765730	6.865815	5.889758	5.606500	5.533321	5.474833	5.355114	6.152938	6.589006	7.014364	6.399465	6.482234	5.253951	4.968204	5.082653	6.132591	7.340176	4.638196	5.516058	5.314218	3.524588	4.409608	4.835606	6.416667	3.235083	5.375957	5.935640	4.594913	3.271619	3.859612	4.282734	4.580148	6.962768	4.445656

CFAP206	3.751463	5.212965	4.068580	4.312111	3.175288	3.480211	6.051840	4.897059	3.458409	4.369123	5.643883	5.322477	4.981524	4.953562	4.628705	4.096439	3.960312	5.259448	4.604801	5.407122	5.009721	5.639162	4.587990	3.372905	4.713491	4.556840	4.006760	3.606405	5.564296	4.778245	4.857330	6.729309	6.763607	5.800232	5.969657	6.467386	5.334186	5.313797	6.188588	4.877599	5.025271	5.774550	6.442193	5.015459	6.000418	5.873876	4.036699	6.630154	3.892448	6.664533	6.904447	6.822918	6.260652	6.346068	6.701746	4.830410	6.269984	5.274932	5.049157	6.247463	6.237472	5.026469	6.832447	4.411994	5.527072	5.018696	6.450484	4.516566	5.543158	5.787562	6.206911	4.695972	4.685452	5.281557	5.765730	5.866298	5.373424	5.770690	6.300188	5.829144	5.394390	6.453304	6.498895	6.302900	5.798779	6.620661	6.038250	6.524348	4.887696	4.465860	5.690981	4.543312	5.324027	5.919921	5.979978	5.723098	5.297648	5.067971	5.659580	4.389553	3.745252	5.434751	5.433353	5.790780	4.420461	4.015628	4.023218	4.251298	4.257186	3.580146	5.303048	5.208841	3.996626	3.379120	4.844670	3.457694	3.625812	4.215317	3.253117	4.795599	5.461042	5.059000	3.174332	3.124029	3.856793	4.303076	7.236582	5.227712	4.721287	4.896017	5.737157	3.290448	6.017781	4.960000	4.548146	5.499283	3.593829	6.452423	7.108364	4.266557	4.824132	6.367558	3.531729	3.847741	6.824504	4.462632	3.610373	3.878061	5.354518	6.336540	5.420358	6.743037	5.112773	7.178765	7.260903	6.898683	4.493546	6.202971	6.173519	3.492758	5.796753	7.177806	5.422062	6.276052	5.395778	3.421880	7.468145	5.029321	7.184232	6.759287	6.798171	6.671872	6.254190	7.095149	5.757214	9.419599	6.567049	5.067971	7.582731	5.729540	4.448914	6.521545	5.422259	4.691774	5.311070	4.273115	6.296916	4.598327	9.471554	5.410127	5.479634	4.721961	6.089923	5.638630	5.631190	6.486761	6.225189	5.591982	5.744281	6.600064	5.628798	7.277658	7.429530	7.267429	5.765730	6.865815	5.889758	5.606500	5.533321	5.474833	5.355114	6.152938	6.589006	7.014364	6.399465	6.482234	5.253951	4.968204	5.082653	6.132591	7.340176	4.638196	5.516058	5.314218	3.524588	4.409608	4.835606	6.416667	3.235083	5.375957	5.935640	4.594913	3.271619	3.859612	4.282734	4.580148	6.962768	4.445656

FAM153A	8.830873	6.400824	6.389695	7.290175	6.468342	7.612965	6.297553	5.717116	8.250515	5.889394	6.766039	6.039824	6.178831	5.197099	6.713105	7.163317	6.320511	5.624475	7.381393	7.739929	6.053723	7.171164	10.893304	6.993072	6.480417	7.353422	7.839084	5.041771	5.995187	6.869510	4.391694	6.748017	7.646194	6.716023	7.425397	7.001784	7.946637	6.657757	6.501473	7.534703	6.261678	9.011126	8.046561	7.852796	8.694827	7.127988	6.927637	7.483685	7.236106	7.360598	7.940095	8.275673	6.791521	6.576349	7.188088	5.374929	6.969980	6.005203	6.835477	6.559257	6.917090	5.394569	7.467564	7.030651	8.657743	7.044666	6.844206	5.859070	5.437457	6.685504	6.598102	6.019537	5.375957	6.241268	7.205967	7.024748	6.619878	6.576684	6.020626	7.731029	7.502589	6.858648	7.516962	6.688080	6.852479	7.985454	6.492192	6.137520	7.230410	7.700687	6.801251	7.049913	7.384386	6.327724	7.552684	7.678603	7.623778	7.795703	7.392569	6.687456	7.384238	7.172303	7.015663	6.192830	7.341144	7.039059	6.593717	7.578853	7.557702	6.775740	6.809854	7.344870	6.814915	6.538445	6.623080	8.152212	8.959613	6.714591	6.937962	7.000648	6.470538	5.756839	4.808479	6.132261	8.834047	4.541102	4.352445	6.398476	4.663894	5.516844	5.140676	6.286581	4.836172	3.721463	7.067906	4.083676	4.974843	4.873219	9.487831	8.136815	10.332682	9.819818	4.927218	4.926830	3.304280	5.875675	3.801826	5.420746	4.783045	7.576358	8.625298	6.629515	5.491679	8.105747	9.730932	5.470251	4.837448	4.364149	6.202971	5.299668	8.759006	5.497164	5.204869	5.862703	6.751438	4.765079	6.523680	8.943551	4.730184	4.307910	4.699401	9.320034	4.712429	5.093109	5.839364	6.504481	5.393986	5.169098	7.898958	4.975882	5.269057	7.590292	4.770332	6.237472	6.171506	4.096860	4.742727	3.497287	2.923401	7.309440	4.675355	4.343091	5.286398	6.566720	5.920679	4.704484	5.198116	5.059621	5.362147	5.496273	4.947749	6.051477	6.023426	3.594204	5.354706	4.088287	4.083676	5.112562	5.604154	4.801966	5.604354	4.791873	4.633497	4.165577	4.635852	5.203062	9.462714	5.168280	7.996613	5.426425	5.629533	4.492888	5.407524	6.430895	6.876407	10.012765	5.345937	11.061124	5.305593	10.171845	7.308348	7.553771	5.337135	4.748507	7.157856	5.725344	6.302364	4.488660

RBMS3	3.846532	3.410052	3.068578	3.031650	2.899082	3.838884	3.301909	3.412735	3.406231	3.298231	3.561139	3.449712	3.371881	3.910352	3.926528	3.155331	3.489197	3.235526	4.651131	3.252513	4.983403	2.935072	2.897683	3.019025	2.926540	3.159671	4.137797	2.831266	3.469965	3.183307	3.753157	4.221682	4.024123	3.731092	3.541683	4.074510	3.652825	3.219514	3.472214	3.322172	3.477093	3.276359	3.609803	3.348056	3.778554	5.585157	3.703237	2.898333	3.842751	4.308819	3.472373	3.428989	4.330993	4.300684	4.030852	3.179398	4.250189	3.215955	3.627023	3.937514	2.871110	3.938111	3.916834	3.278696	3.551628	3.555652	2.974245	3.301288	3.266295	2.819928	3.131949	3.706254	3.735410	2.885639	2.880520	3.381727	3.046101	2.946711	3.232555	3.853131	3.433070	4.067751	3.718118	4.362190	3.489559	4.132771	3.393310	3.308061	3.841899	3.628214	3.284757	3.104774	4.124861	3.260153	3.274958	3.280419	3.847973	4.266767	3.171433	3.220568	3.678826	3.229755	3.076788	3.093488	4.706614	3.742824	3.263418	4.070060	3.261885	3.391947	2.821356	3.241487	2.894688	3.330807	3.519290	3.428299	2.911822	3.473978	3.250849	3.072349	3.559545	3.010748	3.087865	2.955517	4.209072	2.955694	3.227738	3.376704	3.097619	3.336784	3.039031	3.047969	3.233940	3.506053	3.151777	3.351217	2.985701	3.375800	3.249584	4.277079	2.935739	2.861148	2.912304	2.839514	2.836657	3.161604	2.795482	3.270993	3.568074	3.520806	3.477093	3.959039	3.256611	3.407660	3.769711	3.648809	3.241215	3.451172	3.177342	3.216375	3.112229	3.662430	3.419492	4.288556	5.167076	3.261735	2.833451	4.151468	3.530322	3.328676	3.207343	4.260010	4.521287	3.602837	3.096273	3.875478	3.145266	3.190794	3.914226	2.815060	3.320901	3.999587	3.779768	3.911207	3.816718	2.948513	3.623869	3.423315	2.791795	3.069717	3.033387	3.276820	2.950938	3.165437	3.361618	3.047744	3.052485	3.108516	3.285326	3.830142	4.504789	3.663264	3.746085	3.584748	3.678004	3.423141	3.472041	3.445453	3.498362	7.150750	2.972991	3.705075	3.364854	3.166490	3.567883	2.992100	4.547511	4.089608	3.290129	3.015183	3.085714	2.923298	2.955040	5.081663	3.232829	3.294372	3.529594	3.297766	3.211011	2.805927	3.093488	2.855496	3.229197	3.078033	2.976862	2.910452	3.188332	3.384830

AC098650.1	3.846532	3.410052	3.068578	3.031650	2.899082	3.838884	3.301909	3.412735	3.406231	3.298231	3.561139	3.449712	3.371881	3.910352	3.926528	3.155331	3.489197	3.235526	4.651131	3.252513	4.983403	2.935072	2.897683	3.019025	2.926540	3.159671	4.137797	2.831266	3.469965	3.183307	3.753157	4.221682	4.024123	3.731092	3.541683	4.074510	3.652825	3.219514	3.472214	3.322172	3.477093	3.276359	3.609803	3.348056	3.778554	5.585157	3.703237	2.898333	3.842751	4.308819	3.472373	3.428989	4.330993	4.300684	4.030852	3.179398	4.250189	3.215955	3.627023	3.937514	2.871110	3.938111	3.916834	3.278696	3.551628	3.555652	2.974245	3.301288	3.266295	2.819928	3.131949	3.706254	3.735410	2.885639	2.880520	3.381727	3.046101	2.946711	3.232555	3.853131	3.433070	4.067751	3.718118	4.362190	3.489559	4.132771	3.393310	3.308061	3.841899	3.628214	3.284757	3.104774	4.124861	3.260153	3.274958	3.280419	3.847973	4.266767	3.171433	3.220568	3.678826	3.229755	3.076788	3.093488	4.706614	3.742824	3.263418	4.070060	3.261885	3.391947	2.821356	3.241487	2.894688	3.330807	3.519290	3.428299	2.911822	3.473978	3.250849	3.072349	3.559545	3.010748	3.087865	2.955517	4.209072	2.955694	3.227738	3.376704	3.097619	3.336784	3.039031	3.047969	3.233940	3.506053	3.151777	3.351217	2.985701	3.375800	3.249584	4.277079	2.935739	2.861148	2.912304	2.839514	2.836657	3.161604	2.795482	3.270993	3.568074	3.520806	3.477093	3.959039	3.256611	3.407660	3.769711	3.648809	3.241215	3.451172	3.177342	3.216375	3.112229	3.662430	3.419492	4.288556	5.167076	3.261735	2.833451	4.151468	3.530322	3.328676	3.207343	4.260010	4.521287	3.602837	3.096273	3.875478	3.145266	3.190794	3.914226	2.815060	3.320901	3.999587	3.779768	3.911207	3.816718	2.948513	3.623869	3.423315	2.791795	3.069717	3.033387	3.276820	2.950938	3.165437	3.361618	3.047744	3.052485	3.108516	3.285326	3.830142	4.504789	3.663264	3.746085	3.584748	3.678004	3.423141	3.472041	3.445453	3.498362	7.150750	2.972991	3.705075	3.364854	3.166490	3.567883	2.992100	4.547511	4.089608	3.290129	3.015183	3.085714	2.923298	2.955040	5.081663	3.232829	3.294372	3.529594	3.297766	3.211011	2.805927	3.093488	2.855496	3.229197	3.078033	2.976862	2.910452	3.188332	3.384830

AGBL1	3.330807	3.655026	3.294060	3.529405	3.779373	4.589046	4.768875	3.793159	4.575611	4.772404	4.191471	3.804710	3.876965	4.126990	3.984565	3.970758	3.592258	3.097510	4.375472	3.526862	6.177991	4.860231	2.840406	4.423102	3.929129	3.973097	3.891965	4.164884	3.884271	4.567453	3.746085	4.651356	5.075261	4.205246	4.753194	4.048215	3.542686	3.776777	3.823300	4.237224	4.061298	3.163529	3.840275	3.713156	3.792471	5.544106	4.852882	2.846290	3.874364	3.523055	3.105727	4.012459	4.269874	4.291335	4.453114	3.881910	4.252167	3.615151	5.181372	3.889613	2.792523	4.204289	4.610996	4.570485	5.176961	4.014251	3.911207	3.939428	3.604265	3.275887	4.068386	4.474088	4.366995	3.709474	3.558731	4.130031	3.922002	3.852717	3.686239	4.464384	3.664638	4.015408	3.576726	4.519116	3.687483	3.798195	4.690963	3.330982	5.192099	4.481723	3.689524	4.301736	4.611640	4.310159	4.248826	3.596508	4.198200	3.418434	3.671754	3.401046	4.400675	4.196900	3.282710	5.199473	4.719153	4.116788	3.789104	4.075568	3.576726	4.014957	3.619725	4.304639	4.459269	3.878287	4.079820	5.046472	3.826149	3.148640	3.808070	3.547366	4.384959	3.814815	3.879570	4.608853	4.570252	3.795069	4.335082	4.029752	4.382536	4.438717	4.158412	4.160569	4.143859	3.561139	4.037774	3.214200	3.691947	3.289685	5.119499	4.268049	4.654410	2.801819	3.853131	3.990254	5.920125	3.857435	3.983505	4.058871	5.206236	3.981290	5.183414	4.191471	4.336552	5.062027	4.123626	4.085214	4.258087	4.123175	4.535468	3.936856	5.111332	4.294152	4.281683	4.416195	5.145547	5.022393	4.987884	5.448989	3.178160	4.971305	4.807639	4.807639	4.796275	4.503080	3.978243	3.944424	5.447513	5.140077	5.320716	4.691187	3.697556	4.505426	4.655780	5.092686	4.771972	4.809948	4.424630	4.328317	5.808857	3.759435	4.722818	3.708456	3.518376	4.089384	4.217298	3.693618	3.889387	3.617097	3.528312	4.318671	4.441288	4.529888	4.512683	4.072967	4.291994	3.989853	3.831827	5.133206	4.387604	4.518062	3.961808	3.864506	5.147334	4.618177	4.332454	4.937893	4.164473	4.200744	4.290685	4.344818	4.581421	3.478207	4.752754	5.887979	5.245026	3.312397	3.861705	5.281154	4.074067	4.701940	4.976883	6.801411	3.539584	4.457149	4.147276	4.086810	3.352721	3.658246

GPATCH2L	6.462508	6.403439	6.330096	5.940611	6.014848	5.978520	5.827654	6.962442	6.819436	5.724374	6.159981	6.004467	5.297271	5.624670	5.857096	5.847321	7.315893	5.929013	7.454763	5.910266	6.918673	6.368520	8.041063	6.642700	6.794137	6.771077	6.320684	7.113308	5.242044	5.483503	7.258388	5.523096	5.719583	5.647464	5.924711	6.071709	6.126226	6.536952	6.685988	6.998767	5.814250	6.872750	6.127291	6.697597	5.987005	5.328913	5.964827	6.126791	5.814929	5.808677	6.444202	6.067883	5.819218	6.307584	6.235310	5.486905	6.484071	6.619535	6.778458	6.706394	7.105493	5.963410	7.619552	7.121945	7.163145	7.086134	6.879880	6.584561	6.618387	7.165801	6.320848	5.892386	6.099988	6.110246	6.189096	6.475089	6.761727	6.829747	7.016162	6.502676	6.327073	6.521864	6.438065	6.806940	6.713749	6.074831	6.276203	6.957676	7.477847	7.429837	7.297185	7.633475	6.333488	6.423337	5.941126	6.266890	6.246267	5.418355	5.680106	5.021154	4.517005	5.614619	5.686221	5.754089	6.773823	5.542603	6.982874	6.314526	6.619878	5.823680	7.321205	7.168563	7.426641	5.450652	6.225380	3.626008	6.667531	6.316233	5.741365	6.522347	6.707042	6.023247	6.155509	5.824416	6.433086	4.630694	6.204695	6.854459	5.367584	6.665078	6.942144	4.991830	5.577407	6.910016	5.986282	7.675124	6.300676	6.947794	6.140137	7.088794	6.682255	7.911114	6.435459	7.042248	6.868239	5.900068	6.667203	4.605235	5.979585	7.011065	5.655195	5.818676	5.831751	6.319268	6.542716	6.161697	5.970326	7.125129	7.177645	5.004573	7.138922	6.337715	7.863083	5.780525	5.801382	6.414516	6.077284	5.508567	6.435793	7.050084	5.972154	6.173519	6.800931	6.226232	5.395571	5.952550	6.742265	7.023919	6.624661	7.208301	5.868125	7.258046	7.552843	7.278613	7.101416	7.183603	6.790559	7.089448	7.602658	6.239917	6.480576	6.473421	6.491380	6.136867	6.710407	6.839508	7.070554	7.191984	6.457035	6.918673	6.955662	6.839827	6.606041	7.021564	5.642738	7.369224	6.755724	7.586137	7.124348	7.007040	8.709776	6.189910	6.835477	6.838834	6.351290	5.750286	6.062086	5.570335	4.993314	5.794384	5.256134	5.958590	5.859782	6.850296	5.801873	7.160446	7.196002	7.166846	5.991459	7.003383	6.891257	4.797674	5.037327	6.001794	5.625960	7.369846	6.327567	6.609496

HEY2	9.311408	8.762047	9.631976	9.602700	8.434134	7.638327	9.486271	6.000081	6.358684	8.335866	7.410333	8.124795	8.305437	7.408367	9.506715	8.504471	7.673841	8.673115	7.392107	8.806901	8.641739	7.135309	9.502229	7.942495	7.615409	8.698949	8.449060	8.498198	9.016697	9.180017	6.874005	9.691940	9.471239	9.155560	9.515599	9.560491	9.752062	8.842770	8.939581	8.754431	8.431370	10.251752	9.933712	10.034063	10.668998	9.352150	8.007160	8.913738	8.654016	9.686521	9.331061	9.811988	10.332815	10.264693	9.407409	7.549145	10.434121	10.162292	8.607116	9.988415	9.088375	9.699238	9.638262	9.683409	9.654867	8.733172	9.185181	9.474903	8.943551	8.859422	9.558171	9.641171	7.593360	9.757339	9.613954	9.123377	9.864980	9.183598	9.133075	9.378381	9.414708	10.228365	10.169500	9.748470	10.158031	9.776745	9.941976	9.373655	10.312584	9.464554	9.855275	9.839709	9.461373	9.307880	9.321704	9.164484	9.699395	9.065994	8.720864	5.010327	8.292913	9.153531	8.960223	7.819194	8.105442	8.209329	6.804613	7.957449	8.994416	7.763602	10.324612	8.106048	8.770383	7.252455	7.618627	7.062047	8.471683	8.907495	8.831783	9.496877	9.605311	8.748036	8.017102	6.788096	7.625464	8.812633	8.636758	9.329705	7.686388	8.695746	7.501152	7.354382	8.613295	8.596115	8.182340	9.475200	7.225295	7.203012	9.427118	8.205382	6.913553	8.066401	10.305962	9.330587	7.572480	7.840776	7.581800	9.134541	8.816369	7.579940	9.571204	10.313989	8.221769	8.634259	9.064290	9.861128	8.763929	7.647414	9.466593	7.086443	8.822673	8.322944	9.271368	9.360643	8.520957	7.609439	12.507711	8.384965	9.834744	9.903491	8.783516	9.278015	10.204454	9.854297	8.124646	9.812449	9.972959	8.795349	9.584839	9.110021	7.683826	9.043649	7.983020	9.787591	8.486745	8.548924	9.709999	8.678987	7.772925	9.384220	9.691787	5.476530	9.889233	10.146552	9.642396	9.890739	9.625998	9.258639	10.131533	10.094705	9.713565	10.204762	11.253380	11.170163	9.469192	10.635922	10.437954	10.449326	10.149656	9.339487	9.336631	9.830318	10.541527	10.277301	10.524581	9.679086	8.842290	9.545615	5.723468	9.815108	8.818569	7.941974	8.065503	9.877830	5.684176	8.165502	8.745438	7.753947	7.074821	9.959230	9.409873	6.709258	6.756373	8.064885	9.085519	8.889943	8.424386	9.518998

MCPH1	14.371917	14.898599	14.898046	14.963955	14.524343	15.040623	15.102359	16.016830	14.778050	15.718870	15.160798	15.498917	15.133964	15.237652	14.619813	15.644970	15.472304	14.975358	14.952229	15.465655	15.219191	15.258165	13.898577	15.112372	15.113668	15.513476	13.192104	15.274457	14.736202	14.891226	15.373253	14.739993	14.534260	12.869416	14.601973	15.062814	14.575515	15.007673	14.557264	15.510860	15.480428	14.439683	14.726960	14.294396	13.791726	14.460851	14.830506	15.148056	15.438975	14.952794	14.249198	14.769473	15.617837	15.384452	15.493839	15.482965	14.773706	15.083595	14.625551	14.681870	14.708876	15.304417	13.192705	15.148056	14.521080	15.033365	14.914667	14.682292	14.721418	15.168107	15.527160	15.490401	15.279559	15.242555	15.399178	14.541332	15.244645	15.071599	14.871315	15.674067	15.009958	14.729253	13.349308	15.301621	15.300250	14.838593	13.986959	14.811620	14.110109	14.844672	14.601973	14.704362	14.788691	15.674965	14.061195	14.730211	15.775426	15.123478	15.056095	15.638552	15.335238	15.452790	14.731220	15.260285	15.849957	15.155102	15.465655	16.029650	14.800437	14.953863	14.746490	15.250968	15.068646	15.443248	15.740567	14.965129	12.833456	15.247395	15.253922	13.209291	14.874974	13.595124	13.287261	14.966246	14.313436	14.590224	12.962142	13.403370	12.696084	13.577611	13.003243	12.573019	12.578521	12.423279	13.595345	12.260677	12.419600	12.531002	12.258508	13.363482	13.027092	12.632463	12.887748	12.434656	12.715418	14.746969	13.270023	13.833309	13.930296	13.832307	13.954342	13.270211	15.251620	14.801392	12.928492	14.731220	13.186246	12.996042	13.138899	13.984981	13.556834	11.933974	13.052613	13.426653	14.359381	14.826972	12.420594	13.048001	12.945351	13.253022	11.632395	12.618964	12.464996	12.502391	13.335613	10.781910	11.637826	12.378643	12.371190	13.366847	13.740602	12.570358	13.418509	12.510140	13.694716	12.864398	14.051810	13.363274	13.495100	14.123349	13.828942	14.869310	14.043788	13.574051	14.576819	12.950547	13.356786	14.729253	12.047379	13.055989	13.864108	14.902407	13.886983	11.924594	13.896685	14.905582	11.874918	12.713284	12.926643	11.256216	12.476380	13.502404	12.447268	13.953224	13.341361	12.868885	12.608516	14.364077	12.539305	12.434341	13.273776	13.148784	12.726021	12.225366	14.042604	12.733547	12.868696	13.854876	15.273761	14.480925	12.558955	13.168641	14.878115	13.185635	14.557653	12.875731	14.319186	13.728857

CHM	7.493510	7.503695	8.163103	7.200082	7.213260	7.020611	7.269702	7.184232	7.647109	7.475689	7.152928	7.536246	7.669290	6.368863	6.563856	7.180790	7.545980	6.976396	6.134823	7.663886	6.376639	6.328750	7.512341	6.049418	6.662321	6.972442	5.914751	7.018597	6.051653	7.303511	7.649041	8.436796	7.817478	7.629887	7.341925	7.687491	7.821963	7.116904	7.092449	6.639174	6.963561	6.655496	7.504157	7.191384	7.398027	7.462771	6.137019	7.333806	7.296877	7.486340	7.164273	7.570962	8.274926	7.745467	7.531584	7.378933	8.048777	6.478155	5.650665	6.807918	7.178605	7.264789	6.949322	5.742261	7.159016	6.867257	6.853796	7.270312	7.459505	5.810751	7.305394	7.151855	6.952973	7.572330	7.481815	6.857697	7.692283	7.600158	7.220674	7.586901	8.283307	7.874891	7.988666	8.200920	8.032084	7.559652	7.675897	8.332786	7.204398	6.839672	6.978653	6.428172	6.644025	8.044812	7.446975	7.519870	8.134662	7.697615	7.580544	6.044821	7.378180	7.366550	7.000485	6.851233	7.731800	7.581181	7.478585	7.804017	6.184157	6.724304	6.196731	6.709100	7.423202	7.005042	8.359426	4.271174	6.865185	7.436154	7.165801	8.136005	8.537113	8.307282	7.298624	8.043864	9.733183	8.194964	6.903372	9.423930	8.759006	8.783374	8.579360	7.658461	7.385010	7.406798	7.660790	8.480778	7.383930	7.214181	9.396978	7.749980	6.733368	7.333384	7.049597	7.797378	9.764038	6.960210	9.024766	6.808714	8.896105	8.501222	8.541489	7.910344	9.175262	7.506195	9.360161	9.602061	6.143926	7.929575	7.475990	8.622365	7.798655	8.390368	8.695139	9.450135	8.390213	7.734740	8.269361	8.183583	9.407719	8.448278	8.908163	8.602820	8.867627	8.502465	8.874177	7.910028	7.559214	6.978944	8.975575	8.615851	7.951997	8.342758	7.796488	7.164273	7.322482	7.967408	7.770961	8.558229	6.935528	9.894119	8.754115	9.034153	8.405148	8.606356	7.931883	8.321873	8.040597	8.212373	7.520987	9.544844	10.204611	8.447016	9.374473	9.886715	7.770487	9.113587	9.127037	7.596551	7.372828	8.563436	7.879463	6.260150	8.167657	8.321873	8.660394	8.723431	7.918437	9.046235	8.197780	9.748635	7.624881	6.973662	7.285348	9.158502	8.208681	10.540036	8.311931	8.136005	8.483559	7.333066	8.437983	7.465563	6.043810	8.483274	8.726647	8.768990	7.823491	9.092540

GTPBP10	12.664946	12.214739	12.194744	12.341379	12.334431	11.854304	12.009892	11.460712	11.702959	11.750421	11.889910	11.671934	11.849423	12.188708	12.358457	11.719874	11.857830	12.083083	11.748921	12.372749	11.948336	11.657823	12.215941	11.934117	12.112840	11.942720	11.660588	11.977145	11.403962	11.907279	11.913984	12.072811	12.529678	11.955741	11.945767	12.080906	12.139723	12.315592	12.498958	12.785930	11.643705	12.657996	12.162501	12.079262	11.851720	12.309046	11.440322	11.844950	12.470221	12.126733	12.337752	12.244999	12.426853	12.256640	12.334126	11.579980	12.690828	12.861330	12.458656	12.791666	12.910910	11.582424	12.066882	12.631986	13.091804	12.459458	12.702273	12.830643	12.445148	12.735048	12.166633	12.574922	11.718550	12.351197	12.468111	12.067502	12.634275	12.173726	12.641813	12.366220	12.777955	12.324942	12.420911	12.501563	12.471970	12.253427	12.476025	12.613373	13.126907	13.049541	12.847185	12.602064	12.291548	12.532494	12.561872	12.342178	12.401781	11.624718	11.873702	11.837992	11.711612	12.209348	11.810995	11.878329	11.765559	11.871602	11.509789	11.762942	12.223244	11.761456	12.572023	12.312544	12.285989	11.639551	12.069926	11.869049	12.632621	11.847451	11.572679	12.812113	12.758075	12.326190	12.294671	11.817639	11.723608	11.765876	13.509947	12.572340	11.984838	11.496833	12.168033	12.143273	11.560528	12.325731	12.974569	12.686750	12.301244	12.548577	12.247505	11.835114	11.137290	11.868456	11.856252	12.407456	12.562827	12.057936	12.614703	12.256489	12.082135	11.816748	13.183897	12.171733	12.069615	12.449269	12.493699	12.163282	12.773104	12.782224	12.748989	12.449765	13.330907	12.555104	12.699016	11.948495	12.438343	11.933193	12.583952	12.613037	12.259420	12.240995	12.648482	12.873210	12.727955	12.566107	12.060393	12.347129	12.927769	13.448635	12.616157	13.214589	12.061357	12.680421	13.160892	13.343862	13.080428	13.339732	12.480288	12.890172	12.750695	12.905355	12.515324	11.734816	12.093060	12.757036	12.507547	12.607398	13.153220	12.471342	13.171328	12.733547	12.951859	12.439175	12.338722	13.358673	13.617769	12.370529	13.428685	13.062656	12.677669	13.006847	14.191138	13.102201	12.448012	11.953478	12.008250	12.813176	12.037009	11.848352	12.842983	12.143905	12.492404	12.173094	12.017489	12.381803	11.861260	12.408210	12.987788	12.417049	11.505101	12.001171	12.436754	13.018816	11.418543	12.583295	12.520730	13.157107	12.955178	12.251983

ZNF33B	9.140861	8.161613	8.122926	8.332786	8.755780	8.596272	8.296548	8.617088	8.068175	8.533318	8.052786	8.295296	8.021791	8.184186	8.125709	8.307442	7.665739	8.188180	7.480582	8.321720	7.703423	7.757576	8.497631	8.717346	8.833056	8.363460	8.079921	8.614658	8.216204	7.817798	7.647559	8.030420	8.601569	8.429740	8.034780	8.395764	8.450626	8.191336	8.274471	8.682359	8.050019	8.427236	8.595951	8.758681	8.298205	8.752444	8.105589	7.497706	8.276569	8.386977	8.786705	8.681429	8.314539	8.095531	7.473087	7.549594	8.767146	8.257528	8.986858	8.233424	9.270142	7.915975	7.215303	7.722369	7.866510	8.428035	8.627031	8.184487	8.298205	8.097219	8.064885	8.263544	8.571129	8.085241	8.277093	7.856870	8.423320	8.031326	8.634259	7.851877	8.486745	8.215620	8.617088	8.371282	8.408805	8.877254	7.899568	8.540060	8.128713	8.460319	8.841504	8.376902	8.553489	8.270121	8.843711	8.431370	7.956035	8.536680	8.117009	7.715237	7.882917	8.163384	8.242186	8.489064	7.573718	7.502427	8.365652	7.653147	8.298062	8.233737	8.863543	8.315586	8.818873	8.578625	7.602803	9.364247	9.239068	7.705945	8.295156	10.032263	8.720555	7.356091	9.426344	8.087711	7.582246	8.098103	9.653238	8.332304	8.543369	8.133176	10.109128	8.712506	8.096125	7.603445	8.795667	7.924902	10.023218	9.537039	6.226570	7.427903	9.332454	8.465978	8.469643	10.191000	9.301255	7.502262	8.818068	10.728828	7.852651	8.174583	7.568100	7.618765	7.959294	8.845415	9.827378	8.148523	7.855897	8.456424	9.447951	7.380972	8.475733	10.204454	7.919331	8.800662	8.516378	7.818413	8.803350	9.560327	8.772629	7.500701	9.039784	9.209941	9.322141	9.200174	7.334115	8.511835	8.665460	8.264002	9.106902	9.371033	7.638944	7.413675	8.868076	9.637784	9.467827	7.128302	8.304234	7.197753	7.678603	9.306478	9.187694	8.245699	7.685331	8.904760	8.485531	9.929861	8.400034	8.167229	8.687165	8.484598	7.629708	7.848550	9.067710	8.103852	7.994267	8.180535	8.660855	8.754719	7.826301	7.695315	8.514437	9.211143	8.831940	8.425112	7.819973	7.643210	8.581825	7.185875	8.273458	8.807046	8.472017	8.890833	7.492744	7.431882	9.014555	8.562976	7.539104	7.592559	7.990498	8.908772	9.552380	10.592175	8.473581	10.595584	7.976173	8.694983	8.257221	9.908039

PRAMEF14	4.894383	5.054079	3.878287	4.786621	6.516060	5.572561	4.539078	6.212025	6.343881	5.169098	5.055751	5.445439	4.154082	5.327707	5.005573	4.126565	5.373984	4.884308	7.991262	5.052157	5.522145	5.375957	4.305738	4.671443	4.765699	5.209227	3.794238	4.767870	3.936230	5.394962	4.773477	4.777193	4.484384	4.743378	5.035276	4.084981	4.591411	4.026648	8.118091	3.480575	4.388910	3.925015	5.090008	4.929751	4.741955	5.083258	5.541842	5.481239	6.640640	4.244102	4.648966	5.532003	4.705113	4.828047	4.625039	5.465983	3.842553	4.983403	4.411549	4.010356	3.722894	4.497281	4.530309	4.601911	5.629885	3.891153	4.129602	4.302876	4.311473	4.073634	4.394274	4.451198	4.467366	4.052367	4.085214	3.906634	4.734819	4.732932	5.430290	5.908672	4.741955	4.305738	5.033991	4.165795	3.599926	5.996543	6.258790	4.823944	4.472145	4.432464	4.489549	4.316491	3.472743	3.827629	5.568071	5.037916	4.785361	5.947390	6.224337	5.971415	7.565198	4.077019	4.357202	4.989560	4.433919	4.960214	4.210138	4.574791	6.156680	5.096409	6.864858	6.706060	6.783375	5.316861	5.266871	10.663131	6.123900	4.293146	4.465860	4.309946	4.348542	5.162091	3.770137	7.787161	4.595557	6.446077	3.731092	5.057772	4.934289	4.874251	5.319749	5.982795	6.613637	6.625846	5.617610	4.685218	5.558123	4.846791	3.931741	5.028694	5.329881	6.054089	5.675664	6.006820	4.858755	6.389356	6.172693	5.742070	4.768671	4.721070	5.599672	4.678180	6.062618	4.825015	3.768196	5.323444	4.251056	4.472756	4.228532	5.128561	3.857327	4.455432	5.812591	4.105200	4.727241	5.448014	4.743570	5.792931	5.640329	4.258314	4.670819	4.811255	4.941572	5.685865	4.555796	6.046399	4.888964	4.367861	4.135625	4.323583	4.873439	4.001936	4.232008	4.596619	4.252568	4.208643	4.044741	3.681040	3.156400	4.440882	4.329236	5.659751	4.215317	5.084460	4.086354	4.498125	4.866428	5.204082	6.277503	4.010356	4.066903	5.072791	5.559669	3.723903	6.925341	4.378001	3.565159	4.204289	3.995290	5.590046	4.555359	4.005892	3.856144	4.149805	4.770546	5.901681	6.582607	6.160151	3.809905	6.824177	4.963547	4.690520	5.988291	4.790369	4.380378	4.233943	4.642176	4.521491	5.729904	6.862341	7.787607	8.404680	6.139789	6.697122	8.566293	4.861046	4.343285	4.480424

RNF114	12.307622	11.781799	11.888876	11.693642	11.547691	11.538067	11.994409	11.379010	10.510937	11.454736	11.581666	11.721525	11.742598	11.479798	11.604438	11.479650	11.067181	11.415437	11.061273	11.733934	11.620543	11.503932	11.051341	10.872782	10.814911	11.217437	10.999997	10.705209	10.972801	12.025400	10.536476	12.138659	12.534620	12.205277	12.022508	12.278057	12.274241	11.703565	11.252662	11.406317	11.328775	11.879384	12.051938	12.067502	11.874773	11.788056	10.976265	10.987041	12.070398	12.187328	12.164047	11.995822	12.252154	12.516782	12.033154	11.457285	11.806329	11.536600	11.485624	11.550311	11.991261	11.527480	11.825339	11.391015	11.779565	10.969857	11.180206	11.134006	10.960769	11.202068	11.950810	12.015680	11.126936	11.785232	11.859202	11.979749	11.896350	12.075117	12.003535	11.867743	11.921125	11.654083	12.102695	12.115534	11.993647	12.186990	11.986953	11.819577	12.133995	11.466449	11.496673	12.020782	12.035025	12.316396	12.150644	12.123909	12.164845	11.151138	12.149438	11.010787	11.475276	11.737167	11.209109	11.221164	11.428719	11.410967	10.463567	11.151708	11.937016	11.227986	11.943188	10.719109	11.165530	11.046049	11.689360	10.273200	11.468239	11.462019	10.403336	13.208899	12.495856	12.659975	11.974354	11.079687	11.912654	11.285485	11.859940	11.769756	11.338924	11.232861	11.417391	11.614961	12.605390	11.630454	11.408446	12.625513	10.338376	12.504387	13.225883	12.103623	11.932141	12.108321	9.640719	11.117536	12.556062	12.726851	11.239205	11.737167	12.111007	11.801345	12.207764	11.632548	12.196836	13.131924	12.492884	12.632134	12.344708	11.292805	11.692007	11.694244	11.915850	12.316396	12.772251	12.398510	12.322207	11.201915	11.933500	12.597651	12.157725	12.578683	13.064543	12.544369	12.368898	12.743540	13.037619	14.163659	11.988015	12.092606	12.119288	11.917748	11.900292	12.167732	12.028718	11.787155	11.284596	11.433444	11.380992	11.313791	13.216213	11.802954	11.871602	10.873395	12.467154	11.872661	11.787922	12.576557	12.030784	11.602116	12.641230	11.569154	12.001749	12.290765	12.029310	12.676852	12.546069	12.171410	12.673960	12.345482	11.722608	11.661180	12.710502	11.881172	12.571330	11.981974	11.985107	12.568720	11.647546	12.414625	12.502885	12.652080	13.219568	12.051330	12.097568	12.613867	11.320848	11.244028	12.511451	13.080612	10.836326	11.914449	11.182712	11.396102	11.122188	12.260855	12.334942	11.770344	13.100310	11.546662

PTPRG	3.945083	3.954808	3.408711	4.834572	3.741358	4.116356	4.448486	3.707077	4.232847	4.244727	3.896283	3.898000	5.442744	4.224051	4.894383	4.260868	5.305404	5.905278	6.168600	4.220124	4.483519	5.356278	8.028718	4.084103	4.395671	5.341041	5.184019	3.636664	5.701366	3.923950	5.316075	4.624415	4.882433	5.109966	5.682349	4.097537	4.900919	5.125769	5.075463	5.363098	4.170551	6.157700	5.157266	4.481723	5.629710	4.967792	4.177463	5.464226	4.758717	4.187529	4.861840	5.100098	5.072402	4.707853	5.152556	4.807639	5.031937	4.434754	4.576446	5.165688	4.701302	4.717292	6.816500	5.585889	5.185394	4.347207	3.121463	4.861224	3.645431	4.656406	3.040931	3.732326	5.341137	3.639699	3.914443	4.032747	3.793589	4.159518	4.000224	5.822053	5.608542	4.509851	4.731233	4.478885	3.703446	5.699682	5.702406	4.421986	3.850777	4.397453	4.084550	5.165688	4.441709	3.774917	3.496154	5.093331	4.750429	6.133084	4.184469	5.434949	5.301629	5.303625	4.919813	4.464171	5.847521	5.722326	3.607047	5.459635	6.390022	4.442195	6.383651	5.921968	4.630905	3.673077	4.923203	5.323834	5.197929	4.548399	5.549967	3.488475	4.191032	3.364370	4.047589	3.479830	4.547511	3.893535	6.495088	5.753206	4.131875	4.286569	4.312966	4.081133	4.098630	3.566286	5.532394	4.175765	5.637139	5.695092	3.938998	3.806591	6.871479	5.827845	4.465207	4.083256	5.661986	3.741804	3.586490	3.896720	3.927841	5.300214	6.078831	6.691940	4.089190	4.259773	3.983064	5.148725	3.671942	4.855023	6.090277	4.378241	3.456073	6.303742	3.683070	5.287944	4.399395	4.254568	4.607348	3.915721	4.412855	4.221049	5.159865	4.302633	4.218384	3.823300	4.700680	3.782082	4.181870	4.028172	3.661837	5.621097	3.899788	6.138053	6.297403	5.557563	5.811119	3.057796	4.105649	3.849284	4.504789	3.360006	4.063276	4.447326	4.585408	4.198815	4.051722	4.343480	4.097105	4.527587	5.872074	5.721590	4.281683	4.896017	5.371076	5.064071	4.282318	4.156749	4.511393	5.283568	6.143926	6.203650	4.427011	4.367199	5.346337	5.599491	5.386330	5.198116	6.774963	5.671044	3.944222	5.635267	5.470251	5.206824	5.286988	7.201165	4.464384	6.799632	3.432543	5.577218	3.173828	5.342989	5.969657	5.088591	3.177091	2.943661	4.178992	5.171084	4.581198	5.198932

SRP72	11.679960	11.433305	11.947881	11.413626	11.503654	11.139340	11.452830	10.927858	10.496144	11.335836	11.353017	11.167966	11.113335	11.302476	11.144404	10.685853	10.976546	10.823553	11.138009	11.523534	11.021817	11.216126	11.465549	10.946219	10.640152	10.719738	10.473139	10.731203	10.869610	11.599731	10.370639	11.829413	11.916064	11.409205	11.462325	11.692447	11.425080	11.453711	11.430298	11.294959	11.614961	11.471518	10.756471	11.427104	10.801205	11.159054	11.087667	10.667256	11.842192	11.299661	11.496066	11.604311	11.729158	11.705791	11.618765	11.368020	11.497421	11.532142	11.105158	11.069134	11.495068	11.429574	11.707755	11.054370	11.530458	11.223118	11.268395	11.347581	11.099009	11.229610	11.438372	11.502750	10.909986	11.659265	11.620681	11.735962	11.666576	11.488174	11.551356	11.016351	11.922622	11.988628	11.973290	12.005488	11.871756	11.904028	11.748620	11.971923	11.823531	11.638275	11.252949	11.727665	11.633139	11.591914	11.778669	11.419262	11.731380	10.690535	11.471379	10.285908	11.206710	11.264849	10.929185	11.001079	12.265765	11.271173	10.800245	11.112442	11.305292	11.106728	11.789556	10.577989	11.145288	10.928598	11.500503	11.234624	10.954685	11.782660	10.896259	12.081058	12.061208	11.407838	12.138659	10.577397	12.003535	11.089009	10.570769	11.221771	11.860081	11.872812	10.505574	10.983494	11.249245	11.690625	10.805761	11.370827	10.391769	11.087082	11.899574	11.312203	11.583022	11.354180	10.095346	10.948465	11.616692	11.529216	11.276728	10.709659	11.667871	10.913860	11.401656	11.038581	11.081888	11.255007	11.337604	11.686785	11.455214	11.406761	11.423160	11.897804	11.180344	10.701509	11.340846	11.035706	12.073050	11.136391	10.790288	10.991933	11.787320	11.243131	11.073997	11.307832	11.812013	11.455518	11.521468	11.414427	10.804169	11.046807	11.223249	11.126352	11.327051	11.828107	11.779706	10.928007	11.408754	11.702518	11.045017	11.017510	11.337903	11.601655	11.524099	9.599253	11.408142	11.901608	11.480371	11.623796	12.369200	11.512308	10.779419	12.131586	11.719874	12.114358	11.802080	11.902176	11.641765	11.848789	11.499627	11.893812	11.288192	10.575699	11.619033	11.206436	11.543787	11.843867	11.377401	10.524141	10.718823	11.784937	10.464739	11.427665	10.405008	10.832274	10.971305	11.465404	11.532292	10.726876	11.868033	11.481500	11.476828	11.481643	10.933379	9.772646	10.533808	10.962224	12.141761	10.746795	11.658962	11.972222

KIF9	7.471877	7.943715	7.716737	7.877938	7.164573	6.629051	7.897027	7.542035	7.330723	8.313592	7.270004	7.633766	7.300797	7.708085	7.782421	6.827028	7.662478	7.843587	7.578853	7.383159	9.170919	7.392865	8.799005	7.337058	7.817798	7.522372	7.555055	7.302565	7.332577	7.214976	7.313210	8.860035	7.956035	6.846171	8.248548	7.780597	7.710024	7.586750	8.081951	8.494761	7.706238	6.617262	8.320792	7.301614	7.840159	7.486025	7.229814	7.860474	7.681302	8.044021	8.236218	8.163384	8.125256	7.830789	8.549674	7.359312	8.249893	8.708432	7.910028	8.541182	7.539738	7.678105	8.781348	7.785158	7.241025	8.181815	7.511861	6.936806	7.862916	6.824177	7.676648	7.377194	6.611684	7.944322	7.469723	7.726532	7.561521	7.881864	7.705490	8.266504	8.593456	8.559301	8.291041	8.374816	8.295756	8.206480	7.736081	8.889162	7.634821	7.457779	7.575291	7.220329	7.645719	7.631000	7.468910	7.507708	7.573412	7.447140	7.561521	7.142250	7.258388	7.854375	7.545191	7.441130	8.495049	7.084102	6.818111	7.581501	7.313210	7.313376	6.520543	6.858648	6.751276	5.919402	6.643191	3.828629	6.989049	8.221444	8.591764	6.307251	6.925172	7.121335	6.310264	5.435917	6.180988	5.140892	6.113851	5.902366	8.039022	6.636437	6.517896	6.001621	6.316559	7.421349	6.303742	6.066254	6.949978	7.578077	7.374392	10.428662	6.311781	8.555103	5.846047	8.333242	7.985161	5.723098	6.466853	4.832709	6.592272	6.221429	6.902551	7.600158	8.558992	7.039875	7.020447	7.054770	6.848878	6.988104	7.248226	6.668179	6.605446	8.984412	6.644989	6.880224	7.553935	7.781810	5.947390	7.223266	7.085535	7.372973	7.347217	8.349747	8.096606	7.173245	6.257931	8.390063	7.082071	7.281001	7.755863	6.203650	8.847553	7.085535	6.450484	6.713926	7.189234	6.472094	6.869995	6.966591	6.811443	7.792348	6.870310	7.572932	6.385143	6.793483	7.487395	7.206919	6.965487	7.289259	7.638327	7.249927	7.397106	6.944724	7.424306	8.379382	7.284456	7.139390	8.026668	6.895743	6.626475	7.545653	7.549287	6.496218	6.586695	6.637049	6.484786	6.457727	5.331647	6.862821	6.819593	6.102054	7.642151	5.337710	5.520438	6.222113	7.640177	6.113325	5.898668	7.162193	6.508146	6.720151	5.914751	6.606890	4.986634	5.848448	6.012299	6.905732	6.487592	9.309261

WDR5B	10.207052	8.923868	9.713565	9.587157	9.621679	9.571818	10.158811	9.737168	9.313619	9.634002	9.840773	9.817564	9.846837	8.945521	9.268311	9.948904	9.354991	8.953955	9.629378	10.912049	8.790890	9.134862	10.036097	9.348320	8.654920	9.568254	9.652594	8.415166	9.334599	9.355444	9.762143	9.009822	9.474159	9.396668	10.467402	10.450036	10.319510	9.292368	8.453841	8.127977	9.980249	8.656105	9.425577	10.086020	10.510355	9.886562	9.200620	8.042154	10.129611	10.410131	9.645490	10.346434	10.112741	10.586560	10.040849	10.296336	10.261627	9.160301	8.355850	9.194792	9.294119	9.997284	9.309261	8.397928	8.205533	8.717976	8.723431	8.906121	8.697270	8.757756	10.433661	10.108388	9.774780	10.135620	10.324760	9.339347	9.949803	9.495201	9.934485	10.057591	10.035789	10.224836	10.115138	10.080285	10.394147	10.058151	10.666208	10.237289	9.202358	8.818873	9.149261	8.782122	9.869000	9.824794	9.714385	10.126326	9.616360	9.907881	9.941496	9.986683	9.559852	9.398310	9.485965	9.295685	8.251297	9.212214	7.606101	8.999654	9.015152	9.530075	8.790103	8.986084	7.161578	9.534300	9.134694	9.034964	8.007472	10.092469	9.148150	9.195114	9.172804	9.879153	8.789485	8.332147	9.209005	8.886223	9.819042	8.418410	8.764379	9.284604	8.696372	9.713258	7.212144	7.669450	9.658248	9.414552	8.004643	9.134862	10.984035	7.811708	9.143060	8.595469	8.780550	6.754906	8.298501	9.135018	8.868537	9.860677	8.978787	9.475674	8.636158	8.890238	9.622288	10.581000	10.074373	10.230861	8.297149	7.583967	7.189847	9.649786	7.522660	8.902638	10.406224	10.726118	9.529428	8.500912	7.631000	10.471490	10.083424	9.301890	9.974049	9.528187	9.591195	9.606864	10.319057	10.165968	8.250515	6.816351	9.145041	7.659976	9.781478	8.721472	7.289724	7.560886	8.531966	7.320886	8.139429	7.959757	8.073510	9.311408	9.405556	8.691536	10.121103	9.963157	8.795964	9.992644	9.385285	9.786827	9.881326	9.411123	9.937420	10.044031	9.766182	9.514851	9.656270	9.591051	10.094118	7.698975	8.037595	8.702920	8.032680	8.971604	8.629423	9.081657	9.170919	9.510547	9.879153	9.333063	9.989367	8.167373	8.975910	9.589975	8.103712	6.792669	8.949830	6.586378	8.549674	10.189954	8.446548	7.828836	6.824026	4.970884	9.434921	9.108775	8.796419	6.952175	10.404103	8.031633

FAM162A	10.207052	8.923868	9.713565	9.587157	9.621679	9.571818	10.158811	9.737168	9.313619	9.634002	9.840773	9.817564	9.846837	8.945521	9.268311	9.948904	9.354991	8.953955	9.629378	10.912049	8.790890	9.134862	10.036097	9.348320	8.654920	9.568254	9.652594	8.415166	9.334599	9.355444	9.762143	9.009822	9.474159	9.396668	10.467402	10.450036	10.319510	9.292368	8.453841	8.127977	9.980249	8.656105	9.425577	10.086020	10.510355	9.886562	9.200620	8.042154	10.129611	10.410131	9.645490	10.346434	10.112741	10.586560	10.040849	10.296336	10.261627	9.160301	8.355850	9.194792	9.294119	9.997284	9.309261	8.397928	8.205533	8.717976	8.723431	8.906121	8.697270	8.757756	10.433661	10.108388	9.774780	10.135620	10.324760	9.339347	9.949803	9.495201	9.934485	10.057591	10.035789	10.224836	10.115138	10.080285	10.394147	10.058151	10.666208	10.237289	9.202358	8.818873	9.149261	8.782122	9.869000	9.824794	9.714385	10.126326	9.616360	9.907881	9.941496	9.986683	9.559852	9.398310	9.485965	9.295685	8.251297	9.212214	7.606101	8.999654	9.015152	9.530075	8.790103	8.986084	7.161578	9.534300	9.134694	9.034964	8.007472	10.092469	9.148150	9.195114	9.172804	9.879153	8.789485	8.332147	9.209005	8.886223	9.819042	8.418410	8.764379	9.284604	8.696372	9.713258	7.212144	7.669450	9.658248	9.414552	8.004643	9.134862	10.984035	7.811708	9.143060	8.595469	8.780550	6.754906	8.298501	9.135018	8.868537	9.860677	8.978787	9.475674	8.636158	8.890238	9.622288	10.581000	10.074373	10.230861	8.297149	7.583967	7.189847	9.649786	7.522660	8.902638	10.406224	10.726118	9.529428	8.500912	7.631000	10.471490	10.083424	9.301890	9.974049	9.528187	9.591195	9.606864	10.319057	10.165968	8.250515	6.816351	9.145041	7.659976	9.781478	8.721472	7.289724	7.560886	8.531966	7.320886	8.139429	7.959757	8.073510	9.311408	9.405556	8.691536	10.121103	9.963157	8.795964	9.992644	9.385285	9.786827	9.881326	9.411123	9.937420	10.044031	9.766182	9.514851	9.656270	9.591051	10.094118	7.698975	8.037595	8.702920	8.032680	8.971604	8.629423	9.081657	9.170919	9.510547	9.879153	9.333063	9.989367	8.167373	8.975910	9.589975	8.103712	6.792669	8.949830	6.586378	8.549674	10.189954	8.446548	7.828836	6.824026	4.970884	9.434921	9.108775	8.796419	6.952175	10.404103	8.031633

CHRFAM7A	11.094712	10.364725	10.538071	10.725203	10.489958	10.214424	10.514215	10.586706	9.769520	10.190552	10.266592	9.999670	9.577290	10.341269	10.102629	10.471030	10.183009	10.405008	8.517642	10.321672	9.751590	10.247700	9.281361	10.180413	10.082692	10.011379	9.793460	10.745016	9.777218	10.128694	10.056660	10.655638	10.806498	10.286654	10.274888	10.844098	11.076058	10.698520	10.600915	10.801205	10.558564	10.386880	10.332062	10.541990	9.319127	9.925456	9.968492	10.308167	10.956934	9.898224	11.167543	10.243674	11.017912	10.777532	11.148846	10.381054	11.036408	11.872954	10.355061	11.105005	10.466097	10.103422	10.120507	9.957686	10.685116	10.427471	10.866981	11.497575	11.320998	10.728981	10.931134	11.025411	10.339163	11.168701	11.215845	10.999226	11.272662	10.892228	10.794845	9.743043	9.743949	9.907128	9.796567	10.571531	9.867560	10.943897	9.841075	9.854297	10.006922	10.513021	9.844563	9.293796	10.457869	10.422540	10.582324	10.372919	11.124128	9.623390	9.696322	9.934336	9.722628	10.560245	10.728981	10.862913	9.147686	10.000412	10.903646	9.449989	8.994252	10.463717	8.861351	9.966216	10.444263	10.530395	10.655638	9.620086	11.782082	9.793010	9.792229	10.097334	10.398231	10.905092	10.074373	10.063327	10.988997	9.823607	10.240163	9.520363	10.250420	10.227583	11.024075	9.955381	10.122625	10.250420	9.831229	9.945742	10.071631	9.895500	10.604589	9.969878	10.135460	10.140907	10.029183	10.306695	10.836467	10.918082	10.582604	10.196135	10.563528	9.439576	10.048346	10.153274	10.775932	10.518615	10.560870	11.015894	10.842709	11.063922	11.025411	10.261627	11.301874	11.743482	10.488877	9.997492	10.824136	10.918816	10.934125	10.891628	9.838589	10.604302	10.649536	11.492440	10.620438	11.387769	10.098728	11.433163	11.491895	11.063499	11.412245	10.139853	10.722360	10.280332	10.157433	11.370366	10.915229	10.983494	11.477417	11.134182	10.862762	10.975673	11.520245	11.607717	11.255473	11.285784	10.963543	11.450002	11.046942	10.985236	9.908340	9.469514	10.290059	9.083201	10.010322	9.803340	9.850742	10.131533	10.365302	9.842288	10.004105	10.106142	9.243810	9.834894	10.535147	10.345517	9.890739	9.972631	9.002124	9.787425	11.186429	9.654195	10.306695	10.808891	10.869173	10.003350	10.128547	10.039741	10.003818	11.066595	10.742902	9.540818	10.174103	10.937294	10.884769	10.446420	9.637950	10.569055	9.353778	10.364435

CHRNA7	11.094712	10.364725	10.538071	10.725203	10.489958	10.214424	10.514215	10.586706	9.769520	10.190552	10.266592	9.999670	9.577290	10.341269	10.102629	10.471030	10.183009	10.405008	8.517642	10.321672	9.751590	10.247700	9.281361	10.180413	10.082692	10.011379	9.793460	10.745016	9.777218	10.128694	10.056660	10.655638	10.806498	10.286654	10.274888	10.844098	11.076058	10.698520	10.600915	10.801205	10.558564	10.386880	10.332062	10.541990	9.319127	9.925456	9.968492	10.308167	10.956934	9.898224	11.167543	10.243674	11.017912	10.777532	11.148846	10.381054	11.036408	11.872954	10.355061	11.105005	10.466097	10.103422	10.120507	9.957686	10.685116	10.427471	10.866981	11.497575	11.320998	10.728981	10.931134	11.025411	10.339163	11.168701	11.215845	10.999226	11.272662	10.892228	10.794845	9.743043	9.743949	9.907128	9.796567	10.571531	9.867560	10.943897	9.841075	9.854297	10.006922	10.513021	9.844563	9.293796	10.457869	10.422540	10.582324	10.372919	11.124128	9.623390	9.696322	9.934336	9.722628	10.560245	10.728981	10.862913	9.147686	10.000412	10.903646	9.449989	8.994252	10.463717	8.861351	9.966216	10.444263	10.530395	10.655638	9.620086	11.782082	9.793010	9.792229	10.097334	10.398231	10.905092	10.074373	10.063327	10.988997	9.823607	10.240163	9.520363	10.250420	10.227583	11.024075	9.955381	10.122625	10.250420	9.831229	9.945742	10.071631	9.895500	10.604589	9.969878	10.135460	10.140907	10.029183	10.306695	10.836467	10.918082	10.582604	10.196135	10.563528	9.439576	10.048346	10.153274	10.775932	10.518615	10.560870	11.015894	10.842709	11.063922	11.025411	10.261627	11.301874	11.743482	10.488877	9.997492	10.824136	10.918816	10.934125	10.891628	9.838589	10.604302	10.649536	11.492440	10.620438	11.387769	10.098728	11.433163	11.491895	11.063499	11.412245	10.139853	10.722360	10.280332	10.157433	11.370366	10.915229	10.983494	11.477417	11.134182	10.862762	10.975673	11.520245	11.607717	11.255473	11.285784	10.963543	11.450002	11.046942	10.985236	9.908340	9.469514	10.290059	9.083201	10.010322	9.803340	9.850742	10.131533	10.365302	9.842288	10.004105	10.106142	9.243810	9.834894	10.535147	10.345517	9.890739	9.972631	9.002124	9.787425	11.186429	9.654195	10.306695	10.808891	10.869173	10.003350	10.128547	10.039741	10.003818	11.066595	10.742902	9.540818	10.174103	10.937294	10.884769	10.446420	9.637950	10.569055	9.353778	10.364435

CTBP1	8.073029	7.745934	8.393936	9.066762	9.537676	8.231627	7.854837	9.312969	10.351574	8.176949	8.286715	9.017440	8.746957	9.448726	8.655072	8.622520	8.592063	9.188603	9.118515	8.458106	8.290249	10.128831	8.586667	9.945591	9.783445	9.112102	9.822073	8.398835	8.214701	7.803407	8.474628	7.676806	8.062420	9.300797	8.624991	8.162350	8.466427	8.444544	9.165894	9.161517	8.326818	9.173745	9.603163	9.207170	9.236267	9.087175	7.883226	8.907201	8.193517	8.449681	8.400184	8.347801	8.181595	8.055590	8.644254	7.843275	8.527523	8.908163	9.413934	8.482018	8.930159	8.557608	8.906269	9.606560	8.803350	8.992714	8.660218	8.524014	8.003583	8.934423	7.884152	8.514597	8.883433	8.286403	8.577014	8.325256	8.279347	7.807263	8.020288	8.464743	8.601276	8.038673	8.478374	8.189101	8.009151	8.456424	8.464437	8.557010	8.414001	8.284855	9.216398	8.105290	8.955627	8.207850	8.540706	9.395428	8.308242	11.028799	8.663767	9.169847	9.361262	8.920300	9.288485	8.940056	9.146557	8.077583	10.555984	9.644234	9.607484	8.352921	8.820544	9.697083	9.694834	8.550896	8.787190	10.465042	9.717806	8.789635	8.210366	8.144863	7.749492	7.946637	9.333386	9.747720	7.887508	7.691511	9.081509	8.804313	8.555423	8.034493	8.868700	8.711169	8.661319	8.969110	8.360934	8.554809	8.527674	8.773366	8.190228	8.264002	9.933882	8.570650	10.938762	8.673745	7.768842	8.680959	8.427087	8.759794	7.571121	8.388820	8.900522	8.584534	9.101487	8.295459	7.744413	8.491179	8.823411	9.444517	9.098890	7.755724	8.899150	8.864465	9.207632	8.754861	8.564352	7.478448	8.743900	7.839230	8.263544	8.374973	8.090493	8.345080	7.810508	8.718581	7.001632	8.358315	8.268065	9.356365	8.153471	8.551058	8.221444	9.159093	9.068474	9.685622	8.675628	8.507811	8.741801	8.607876	8.567308	7.569912	8.101742	8.492586	8.293964	8.233424	8.028874	7.966655	7.870600	8.352309	8.600815	8.654468	8.303042	9.086706	8.881463	7.946185	8.882036	8.076365	8.776297	8.703369	8.855773	9.876877	8.336479	8.846939	8.549674	8.856984	9.226526	8.924459	11.177770	8.257528	8.062120	9.215448	8.775819	9.459408	8.885766	8.570802	8.403921	9.904851	9.323193	8.522454	7.726996	8.573979	9.067393	9.229431	8.657423	8.695584	9.101177	9.057511	8.622365	8.331532

PPARGC1B	11.064379	10.784668	11.514381	10.296801	11.752033	11.503797	11.293530	10.998487	11.087821	11.542870	11.501897	11.687959	11.987250	11.880130	10.332362	11.402498	10.919263	10.638038	9.841075	11.263347	9.649935	11.180670	11.267812	10.496003	10.988091	11.622683	10.684971	10.610070	11.055538	10.551284	10.883417	11.525317	11.027485	11.148401	11.064802	11.511854	11.945921	10.874713	10.839735	10.676287	11.697270	10.199612	11.199453	11.212284	11.072556	11.782817	11.579822	10.079015	10.250726	11.693032	11.034738	11.247077	11.321723	11.322916	10.614231	10.980857	10.966969	10.291572	10.926394	11.290862	10.385019	11.090456	9.860520	10.116680	9.851679	9.114355	9.629531	9.429598	9.524275	9.981835	11.150234	11.289771	11.773546	11.897513	11.344607	11.243746	12.110693	10.973367	11.417687	10.313708	11.519785	10.640300	11.570177	11.720774	11.806941	11.522341	11.806190	11.280035	10.245587	10.159237	10.183454	10.704328	11.035380	11.620681	10.682122	11.444544	10.309563	11.790286	11.829413	11.538688	11.097929	11.047954	11.425348	11.063055	11.140774	10.992855	10.356294	10.122478	10.179480	12.001749	10.760108	11.509940	10.911465	11.819577	10.667256	11.304855	9.939172	11.366830	12.190447	10.723285	10.667558	10.263285	10.327775	10.043887	11.146352	10.386712	8.146260	11.230791	11.214237	10.124758	10.885690	11.350987	9.015924	8.297298	11.251510	9.469668	11.101922	9.939340	11.945921	9.874226	10.497091	10.303719	10.788398	10.567405	10.313708	9.038835	11.335254	11.432097	10.132992	9.844099	10.999696	10.468163	10.953768	10.341269	11.239056	10.190269	9.581310	9.804390	9.338465	12.008552	9.597882	11.897946	10.489958	11.030164	11.428860	10.877643	9.082889	11.821859	11.274882	10.716860	10.931441	11.379300	10.820570	10.884769	10.660719	9.451406	10.080121	10.604741	10.104477	8.890833	11.284747	8.233737	9.069737	9.747720	8.621124	7.788365	9.490626	9.288485	7.593826	11.596844	10.556615	9.717013	10.185424	10.389594	10.654872	10.509324	10.030084	10.604020	9.936636	11.009167	11.290862	10.181309	10.693912	10.640300	11.328644	11.644166	11.420870	9.528007	9.215294	7.366862	9.884994	9.728526	10.132585	9.121979	9.937905	9.594640	10.749156	9.734090	10.436000	9.797342	9.459550	9.645810	9.599698	8.978174	11.321148	9.223262	10.010612	11.163197	10.071016	10.674952	10.202155	10.021101	11.339525	9.949517	10.702740	9.442955	11.290061	11.091311

MS4A5	3.254178	3.412024	3.293629	3.304758	3.303802	4.360482	4.041460	3.720576	3.984979	3.512937	3.682453	3.787581	3.334861	3.901467	3.647203	3.626417	5.140676	3.390353	4.950017	3.559157	5.039903	4.207537	4.012459	2.910264	3.606610	3.610373	4.040487	3.097370	3.022998	3.634676	3.739973	4.310387	5.401645	3.406943	4.605847	4.135625	3.911846	3.421184	3.097255	2.965270	3.405895	3.122269	4.033222	3.363362	5.048938	5.521184	3.696522	3.183432	4.378669	4.229679	4.133820	6.079043	4.351778	4.431388	3.949658	3.079971	4.014061	4.346971	3.840679	3.656217	2.871646	3.198773	4.878603	4.318023	5.344963	3.339291	4.442638	3.515884	4.040380	4.354362	3.641701	3.848186	3.313340	3.517979	3.808070	4.665404	4.220612	3.799007	3.841530	3.738525	3.707874	4.102823	3.464678	4.118088	3.372905	4.232847	3.382584	4.927846	3.768830	3.649432	3.621076	4.237440	4.007615	4.333078	4.007805	4.199007	3.783146	2.999867	3.945762	3.281942	4.263344	3.371881	3.144215	3.346871	4.389553	3.729236	4.215778	4.159936	4.252367	4.527383	3.581900	4.614433	3.438735	3.556033	4.485503	5.724545	3.802024	3.333551	3.287419	3.455487	3.521873	3.151361	3.100195	3.454769	3.938536	4.530110	3.325163	3.863442	3.243676	3.535725	3.226440	3.619528	3.231727	3.287730	4.631969	5.374165	3.693618	3.885512	3.460735	4.270510	4.128728	5.750467	3.208736	3.122747	3.024181	4.015184	3.588432	3.698380	3.526678	3.570590	3.547776	4.533107	3.388249	4.415349	3.823524	3.815882	3.550424	3.124286	3.280717	3.755678	2.910452	4.533715	3.792048	5.563335	4.883086	3.631713	3.150513	4.057800	4.534121	4.241625	5.619275	4.092887	4.304851	3.478013	3.405410	3.634285	4.157168	3.465757	3.433819	5.966988	3.378618	4.526520	4.637598	4.704697	3.440314	4.183437	3.327268	3.595009	2.891158	3.169155	3.523264	3.137740	4.030852	3.511629	3.799869	4.092887	3.719536	3.636664	3.519492	3.828244	4.450509	3.526077	3.613780	4.544230	3.718939	3.429179	4.573729	3.482786	4.255667	3.717333	3.439792	3.975516	3.651573	5.041986	3.311611	3.391201	3.414533	4.931589	3.325977	2.958067	3.388422	3.177091	3.455153	5.160090	3.450610	4.108280	3.862994	3.570590	4.134718	3.322974	4.750429	4.690963	4.304213	3.530520	4.501617	3.383848	3.345748	3.831827

EDNRA	10.731932	10.998779	11.131914	10.590376	10.277128	9.314118	10.640152	9.075896	8.795216	9.834744	9.814232	9.640574	10.587598	9.667331	11.204194	10.141818	9.350323	10.679735	10.961776	10.038097	11.229292	10.138664	13.405875	10.035315	10.538071	10.545217	9.432358	10.307568	10.592897	10.692122	10.002558	11.370520	11.379300	10.307273	10.699421	10.692551	10.595288	10.757526	11.307832	11.032834	10.665327	12.052817	11.154075	10.801645	11.630608	11.034738	9.801811	11.400440	10.570453	11.030014	10.661622	10.877948	11.221771	10.802397	10.667399	9.817723	11.056587	11.473235	10.666359	11.432097	11.565808	10.670159	12.493206	11.215845	12.037911	11.426795	11.266930	11.402937	10.763276	11.858266	10.992250	10.639211	9.932911	10.752512	10.917191	10.516242	10.674034	11.017912	10.287089	10.908996	10.772696	11.658633	10.852247	11.098087	11.128382	11.026609	11.456110	11.450002	12.267902	11.465549	11.434802	12.404804	11.258266	10.371089	10.313708	10.328381	11.252662	10.052342	9.927064	9.994941	10.182411	10.305518	10.552212	10.579996	11.490395	10.381341	10.159823	11.268089	11.741869	9.249360	12.663072	10.177038	9.618866	9.352445	9.276679	9.740241	10.916152	10.271317	9.850095	10.158948	12.273913	11.467492	10.018949	10.796867	9.873315	10.727633	11.535996	11.226064	10.199926	11.653049	10.278058	10.951332	11.275334	12.144382	10.104055	11.685397	10.798488	11.662821	11.272804	11.470648	11.942122	11.412947	10.974503	12.350670	10.537786	11.411534	9.099191	10.201561	10.775784	10.135460	11.011686	11.875232	10.573204	10.503158	10.689930	12.060100	11.602692	12.190143	12.461067	10.166449	11.789556	10.381341	10.839888	11.315434	10.332682	9.698474	11.729303	9.901303	11.774172	11.709255	11.031948	10.809503	13.341990	11.653194	10.965807	11.976069	11.286882	10.425458	11.398068	11.782660	10.380306	12.762748	12.356099	11.911476	11.506569	10.978030	11.631808	11.498458	11.482399	10.482307	11.134006	8.371426	11.300400	10.903200	10.838287	11.486837	11.691236	11.519246	12.485843	11.584062	10.985538	11.967332	12.645205	12.544205	11.255900	12.225203	10.810803	12.544739	12.717145	11.937329	12.521876	11.480091	12.562670	11.875845	12.270085	11.586330	11.254561	11.237555	9.667331	12.515826	10.775501	10.235585	11.145736	12.872324	9.203412	10.686890	11.019767	11.594008	9.799208	12.949621	11.932596	9.926337	8.281286	9.781326	11.298834	11.235506	10.194758	11.718971

BDNF	4.720456	4.643812	3.903586	3.804516	3.737290	4.488884	4.821688	3.411831	3.279133	3.900187	4.042742	3.963170	4.305090	3.657644	4.539543	4.271174	3.389637	5.462933	5.511064	3.982623	5.924182	2.885929	7.760826	4.461136	4.842558	4.389751	4.155826	4.077825	3.463379	4.133608	3.444894	4.854383	5.480262	4.892731	5.334554	5.038906	4.921033	4.937082	4.464593	4.659199	4.109365	4.855023	5.138833	4.479552	5.743559	4.561427	3.294372	5.710503	5.342778	4.711373	4.721070	6.735475	4.574160	4.882020	4.244102	3.791856	5.005774	5.462213	4.680068	5.070805	5.191503	4.596826	5.898129	3.800090	5.831929	4.853765	4.251056	4.155826	4.055845	4.037570	4.630694	3.618767	4.178750	4.135859	4.217517	3.872862	3.873530	4.412414	4.103651	5.099896	3.908140	5.382123	5.287944	5.215840	5.739713	5.938973	4.047803	4.584561	4.892281	4.705113	5.251176	4.738874	4.727036	3.852263	5.206643	4.555570	4.974416	5.505053	4.266557	3.580146	4.538874	4.805763	4.398938	4.475377	3.806389	3.659637	3.083140	3.186612	4.404666	3.877608	3.633687	4.225973	3.907475	3.210577	3.217115	5.382725	5.142772	4.144522	3.525332	3.425651	3.520441	3.387237	3.304432	4.051722	4.635636	3.312092	4.493107	3.804108	3.201711	3.257316	5.547131	3.222815	5.183016	2.982627	4.871035	3.378073	7.350691	4.637149	5.085272	5.128561	5.227278	6.493615	5.616848	3.182826	5.715666	3.908575	3.399682	5.951984	3.474511	3.264238	3.279133	4.144295	3.412420	4.803678	6.240584	4.129806	3.233782	4.166006	6.300025	3.153417	6.681267	3.688674	4.563852	6.573050	4.240573	3.166776	4.421986	6.854771	4.756117	4.046733	4.831039	4.792890	3.885739	4.569162	3.724930	6.013134	6.723847	4.000622	7.296707	3.495025	3.720995	4.977248	3.303300	4.174872	4.347207	2.913243	4.452047	3.143613	3.406067	6.356984	6.078831	3.095417	3.087392	3.407300	3.435630	4.210992	3.136389	3.383293	4.614662	5.046672	4.429565	3.700948	6.915103	4.942999	4.083468	3.622038	3.344470	4.893762	4.643812	6.438400	3.088322	3.431688	3.255912	3.431159	3.133459	3.769062	5.074630	3.392955	5.077569	5.516654	5.294827	3.699196	3.952698	4.238991	4.848293	3.087170	3.167529	5.684361	3.537632	3.062769	4.415573	6.937962	3.462669	5.552010	3.090545	4.020223	3.464292	3.198895

YPEL2	12.207603	11.406614	11.370366	11.339812	11.261682	11.538822	11.699769	12.380831	11.813001	12.080733	12.089348	11.329537	11.670323	12.064848	10.863810	12.035336	11.264849	11.127669	12.543245	11.689592	11.236266	12.052362	12.860559	11.328200	11.521468	11.336873	11.580987	11.749526	11.076482	11.578918	12.192450	11.659107	12.281444	11.404390	11.724086	12.026020	11.914277	11.435520	12.051330	11.695625	11.306355	12.537925	11.798796	11.745962	11.896055	11.785397	12.105607	11.335682	12.375982	11.975594	11.620849	11.745676	11.510541	12.060393	11.756331	11.271942	12.202348	12.317768	11.818403	12.040781	12.506387	11.613450	13.045554	12.473286	11.616246	11.503209	12.051649	11.417687	11.260034	12.015680	11.925502	12.111299	12.294356	11.919514	11.928150	12.015212	11.995430	12.576063	12.800565	11.465833	11.781799	11.812013	11.351456	11.736262	11.972520	11.962317	11.825487	12.666497	12.288476	11.658633	11.997004	12.692342	11.818854	11.962614	12.191422	11.943939	12.511911	11.888571	11.643705	12.233736	11.644012	11.463470	11.173138	11.695305	11.878463	12.138794	12.623671	12.822312	11.326605	11.351965	12.883285	11.968244	11.468075	11.847279	11.276012	10.311562	11.826972	11.566103	11.177032	11.199598	11.084541	10.687045	10.808261	9.363785	9.856025	9.720646	10.201110	10.520787	9.562398	10.387620	10.216548	9.698632	10.246336	8.609684	11.437522	10.145498	9.849598	10.186206	10.367294	10.664468	10.928007	10.775210	7.959450	10.066011	10.345226	10.962224	9.133821	8.730609	10.888642	11.279290	11.440486	10.477996	12.113904	11.322294	11.242082	11.400910	10.900409	10.600915	10.950866	9.381799	11.643128	10.280645	10.170425	11.368020	10.737331	11.379601	10.779258	11.572225	11.159497	10.737016	11.028205	10.490251	10.581882	11.335396	10.745016	11.131914	11.092378	10.551284	10.791282	11.233464	10.644015	11.470648	11.305447	11.729158	12.132968	10.162595	11.394279	10.272270	11.586469	11.278862	11.517367	10.220637	11.356381	11.528035	11.696238	11.232005	10.603714	11.801189	11.300099	10.708399	11.123509	11.084984	11.418543	10.704617	10.218179	11.521285	10.928598	11.919091	10.931591	10.598160	11.309839	11.574797	11.565952	11.601212	11.184501	10.613617	10.909711	9.876562	11.908965	10.853896	10.913253	10.925790	10.518938	11.360449	9.972329	11.407838	11.772550	12.007161	11.608893	11.793165	10.311130	9.017607	10.589073	10.368806	11.550739	11.394657	10.782833	9.914535

MEF2A	12.096818	11.824213	12.018090	11.809331	11.787922	11.449574	12.074680	11.285190	11.173730	11.588775	11.769146	11.610629	12.239628	11.951260	12.258508	11.699459	11.611382	11.835114	11.264849	11.532717	11.492740	12.016430	12.581823	11.716014	12.480452	12.045443	12.393291	11.873702	12.155557	11.952432	11.883896	12.003100	12.556834	12.227205	12.205727	12.056520	12.030784	12.007772	12.501735	12.256640	11.961160	12.161114	12.255674	12.189336	12.789606	11.856573	12.168033	12.280226	12.121934	11.615675	11.699618	12.278977	12.087259	12.190585	11.996401	11.867283	12.425904	11.811570	12.418964	12.388096	12.338259	11.802387	12.973655	11.863940	12.404198	12.394906	11.840529	11.989574	11.661180	11.651970	11.764791	11.784186	11.509649	11.932006	11.606238	12.158785	11.899574	11.882381	11.728873	11.990798	12.015827	12.286447	12.197990	12.090895	12.103936	12.360369	12.067832	12.004877	11.651535	11.753524	12.185585	11.995974	12.089816	11.915924	11.838717	12.129781	12.074965	11.790150	11.732727	12.012663	12.096818	12.015680	11.618460	12.129478	11.747480	11.712895	12.146729	11.806941	11.796151	11.520985	11.571034	11.802693	12.015212	11.142960	10.939911	10.702889	12.418655	11.884935	11.762372	11.844802	12.221872	11.903405	11.580422	12.067345	11.388496	12.286161	12.069926	12.897927	12.560381	12.042721	12.028718	12.574210	11.955137	12.069326	12.081058	12.443027	12.699345	11.802954	11.582100	11.862884	13.228647	11.878329	11.802080	12.355155	11.770940	11.447213	11.759040	11.988461	12.241297	12.564954	11.607297	11.524738	11.448972	12.441117	11.942584	11.947881	12.565784	12.515494	12.582966	11.992156	12.043570	11.765876	12.099062	11.851856	11.866080	11.886002	12.518587	12.257719	13.165092	12.154051	11.733781	11.739362	12.231096	12.396775	12.522051	12.253731	12.212840	12.015528	12.329402	12.045443	12.272798	11.876874	11.813442	12.128873	12.305898	11.541804	11.664926	11.435242	11.321435	11.272515	11.642385	11.438510	11.623999	12.001171	11.944227	11.949439	12.014319	11.850666	11.926124	12.389042	11.607717	11.858431	12.061357	12.654127	11.879544	11.976069	11.638742	11.622683	12.334279	11.826536	11.718080	12.034708	11.377558	12.319032	11.931556	11.829273	12.850513	12.187637	11.966254	11.520699	12.432421	11.702518	11.867445	11.005117	11.888876	12.065779	12.372119	12.669498	11.861688	11.441183	11.874475	12.182043	11.003664	12.041232	11.604897	12.055280	11.760698	10.998487

LYSMD4	12.096818	11.824213	12.018090	11.809331	11.787922	11.449574	12.074680	11.285190	11.173730	11.588775	11.769146	11.610629	12.239628	11.951260	12.258508	11.699459	11.611382	11.835114	11.264849	11.532717	11.492740	12.016430	12.581823	11.716014	12.480452	12.045443	12.393291	11.873702	12.155557	11.952432	11.883896	12.003100	12.556834	12.227205	12.205727	12.056520	12.030784	12.007772	12.501735	12.256640	11.961160	12.161114	12.255674	12.189336	12.789606	11.856573	12.168033	12.280226	12.121934	11.615675	11.699618	12.278977	12.087259	12.190585	11.996401	11.867283	12.425904	11.811570	12.418964	12.388096	12.338259	11.802387	12.973655	11.863940	12.404198	12.394906	11.840529	11.989574	11.661180	11.651970	11.764791	11.784186	11.509649	11.932006	11.606238	12.158785	11.899574	11.882381	11.728873	11.990798	12.015827	12.286447	12.197990	12.090895	12.103936	12.360369	12.067832	12.004877	11.651535	11.753524	12.185585	11.995974	12.089816	11.915924	11.838717	12.129781	12.074965	11.790150	11.732727	12.012663	12.096818	12.015680	11.618460	12.129478	11.747480	11.712895	12.146729	11.806941	11.796151	11.520985	11.571034	11.802693	12.015212	11.142960	10.939911	10.702889	12.418655	11.884935	11.762372	11.844802	12.221872	11.903405	11.580422	12.067345	11.388496	12.286161	12.069926	12.897927	12.560381	12.042721	12.028718	12.574210	11.955137	12.069326	12.081058	12.443027	12.699345	11.802954	11.582100	11.862884	13.228647	11.878329	11.802080	12.355155	11.770940	11.447213	11.759040	11.988461	12.241297	12.564954	11.607297	11.524738	11.448972	12.441117	11.942584	11.947881	12.565784	12.515494	12.582966	11.992156	12.043570	11.765876	12.099062	11.851856	11.866080	11.886002	12.518587	12.257719	13.165092	12.154051	11.733781	11.739362	12.231096	12.396775	12.522051	12.253731	12.212840	12.015528	12.329402	12.045443	12.272798	11.876874	11.813442	12.128873	12.305898	11.541804	11.664926	11.435242	11.321435	11.272515	11.642385	11.438510	11.623999	12.001171	11.944227	11.949439	12.014319	11.850666	11.926124	12.389042	11.607717	11.858431	12.061357	12.654127	11.879544	11.976069	11.638742	11.622683	12.334279	11.826536	11.718080	12.034708	11.377558	12.319032	11.931556	11.829273	12.850513	12.187637	11.966254	11.520699	12.432421	11.702518	11.867445	11.005117	11.888876	12.065779	12.372119	12.669498	11.861688	11.441183	11.874475	12.182043	11.003664	12.041232	11.604897	12.055280	11.760698	10.998487

CDH23	8.073176	8.192912	7.975155	8.416754	8.302736	8.918471	8.663627	8.370663	7.997387	8.629423	8.603127	8.655072	7.964030	8.000883	7.976946	8.184935	8.116083	8.554346	7.412915	7.915822	7.859735	8.459641	8.803350	7.990179	8.154391	8.671577	9.256667	9.266095	9.122611	8.850472	7.992398	7.689500	8.256188	8.381906	8.315586	8.239177	8.158860	8.006563	8.291499	7.969867	8.732710	8.321873	8.366526	8.345535	8.997209	8.137554	9.362773	8.378919	7.531129	8.014477	7.904130	8.151442	8.562976	8.111918	8.077423	8.921804	8.706443	8.332147	7.828229	9.080879	8.387588	8.796854	9.445145	8.259246	8.862151	7.936696	8.158230	7.982540	8.022551	8.009468	8.396241	8.164882	9.015766	8.175833	8.145020	8.024837	8.115622	8.265861	8.056042	8.081629	8.162811	8.166286	7.602658	7.501480	7.573568	8.009296	7.959450	7.590442	8.326529	7.359489	7.856647	8.653423	7.981450	8.085388	8.055892	7.870600	7.774143	8.272099	8.351034	8.237305	8.777966	8.290887	8.177966	8.115470	7.907411	8.562100	7.996145	8.135563	8.363776	8.828551	7.866036	8.025123	7.324693	9.817990	7.990815	10.893304	7.945865	8.216505	9.032204	8.157641	8.208437	9.009189	8.692789	9.631976	8.932840	9.304840	8.468400	8.618151	9.153845	9.005828	9.172036	9.353778	9.578851	8.569261	8.725564	9.392037	8.005774	10.718958	8.207850	8.372533	8.845571	8.894109	8.742859	8.669568	10.057867	9.181715	9.488153	9.590587	8.768535	9.099653	7.987793	8.248407	8.495523	8.635194	7.947234	8.640199	8.599881	8.242037	8.797467	9.645490	8.157804	8.163103	8.737317	8.202112	7.849748	9.626627	8.736571	7.797527	8.532116	8.443325	8.395764	8.013547	8.639114	9.460752	8.965466	9.966054	8.541337	7.965718	9.188455	8.219624	9.461373	7.809583	7.660627	8.079480	8.897010	8.302435	8.338861	8.086004	8.024680	8.290571	8.174901	10.388515	8.527523	8.966651	8.008852	8.887600	8.902785	8.544315	8.993798	8.185737	7.773375	8.233570	8.971157	8.668648	8.715790	8.459259	7.913711	10.037937	9.108288	7.524834	8.750348	8.531228	9.249046	8.655522	9.457396	8.812183	9.027677	8.823713	7.941663	9.005980	8.696676	8.727734	9.377318	8.126434	9.012996	7.864481	7.901642	7.545035	10.050349	8.595016	8.513548	9.967725	10.667694	8.761068	9.013634	8.246033	8.752751	9.196602

FAM178B	8.873398	8.300189	8.573058	9.410032	9.932634	10.377566	9.167845	10.568312	10.697641	9.822544	10.614651	10.606972	9.809677	9.468399	9.145493	9.551619	10.295091	8.882342	9.311096	9.174330	8.702920	9.408958	9.416708	9.243051	8.744816	9.419921	9.777218	10.070538	10.271935	9.594486	10.684665	8.084278	8.008078	9.345894	8.742697	8.235738	8.513701	8.955017	9.279824	8.656246	9.794721	9.094797	8.570802	8.319872	8.529985	8.299864	10.579352	8.295296	8.902324	8.137554	7.501958	8.568786	8.072719	8.735320	8.280091	10.298003	8.208762	8.722060	8.876016	8.692458	8.574264	9.742594	9.117571	9.016697	9.397648	8.764520	8.493834	8.519607	8.218549	8.283150	8.567616	8.861351	10.710117	9.153531	9.311096	8.313592	8.402217	7.939959	8.470262	9.128931	8.129345	8.429740	8.539092	8.500592	8.718121	8.891594	9.037545	9.251933	8.463077	9.246898	9.539063	8.361723	8.423788	8.992714	9.682921	8.745587	8.362543	9.342205	10.658275	9.486737	9.933063	8.141961	9.258777	8.900076	9.128766	10.133737	9.547475	9.511023	9.269982	10.355676	8.940056	9.874961	9.489187	11.254561	10.078202	12.469565	8.555262	9.598341	10.494961	10.317582	8.427563	8.433222	10.077440	11.256480	9.920909	10.117756	10.369561	9.953637	9.781629	10.303257	10.109727	9.941651	10.060308	9.648405	9.913281	9.389452	8.909370	9.107332	7.976484	8.246033	8.226013	8.460790	10.750836	8.276257	8.068023	9.544116	9.726547	11.432403	9.205043	10.232190	8.553625	8.349281	9.025080	8.183733	8.308699	8.388218	8.623129	9.735002	8.449850	10.467694	8.152839	8.457202	7.870285	8.167373	8.101742	10.884929	7.634486	7.824155	7.368131	7.752132	7.811708	7.281623	8.151277	8.141326	8.968369	9.949956	7.873667	10.115138	7.834927	9.525379	9.966500	8.046717	7.909740	10.096334	8.201220	8.040770	8.113151	8.909370	9.412891	8.067428	8.335259	11.154939	9.591810	8.296861	7.972255	8.057711	7.826473	8.555262	8.110120	8.482171	7.597166	9.635782	7.715669	7.666860	9.872275	7.624575	8.815901	7.962531	9.658575	12.778642	7.702383	8.597896	9.071262	9.062763	9.256667	9.191027	9.906054	9.936352	8.112229	9.654530	8.778111	10.095615	8.537676	9.592908	10.063700	9.667475	9.006998	8.523224	10.726118	8.727096	10.050200	9.964730	11.748029	10.575699	11.343861	8.147603	10.518184	9.802412

GPAT2	11.534664	10.636673	11.079687	10.712115	10.755261	11.494936	10.912940	10.815522	10.919423	11.001938	11.324533	11.218631	10.198715	10.136250	9.755758	10.802991	10.454947	10.286511	10.768036	10.644299	10.306695	12.219692	11.674446	10.374979	10.153109	11.229292	11.519246	11.584856	11.338628	11.264092	11.063055	11.317927	10.950866	10.729271	10.207334	10.129759	9.467827	9.575490	10.092469	10.190552	11.353455	9.887206	10.375761	10.828144	10.093506	10.240313	11.595221	9.614989	10.896568	10.211224	9.958930	9.748803	9.241954	9.755913	9.844563	11.313643	10.229267	9.229858	10.007656	10.456478	11.506127	10.880179	12.384645	10.242906	10.419503	11.028666	10.638038	9.808891	10.247271	10.235895	11.224462	10.745016	11.164654	10.211375	10.308324	10.592175	10.249984	10.416284	10.510212	10.140756	9.952136	10.574819	9.300869	10.056961	10.244280	10.318464	10.241816	10.293860	10.679432	10.497682	11.558489	10.403638	10.964747	10.065268	11.036851	10.797295	10.409985	10.549938	10.707486	10.922099	10.974077	10.080427	9.899145	10.081087	10.440817	11.153927	11.518100	10.794399	11.450002	11.176414	10.836029	10.504548	10.736390	11.781504	10.911326	13.266997	11.515156	10.718063	11.500204	10.202804	10.247418	10.945805	11.711612	12.323571	11.125340	11.483412	11.421630	10.616870	11.065101	11.460547	11.400133	11.058248	10.574229	10.471332	11.043290	10.577828	10.503620	11.110303	10.006166	10.602257	11.256056	11.318667	11.154369	10.870211	11.142360	11.002796	11.329537	12.080582	11.165228	10.948607	11.227537	11.276298	10.681815	10.134374	10.256188	9.937905	10.560393	10.860274	10.893753	11.651679	10.405164	9.964264	10.800421	9.763248	10.208918	11.809935	9.985035	10.520340	10.786473	10.108827	9.961059	9.079331	10.044324	10.646484	10.955140	9.836099	9.655361	13.077428	10.064236	11.101213	11.324386	10.354160	10.102474	10.606255	10.691016	10.537786	10.952734	10.693013	10.451395	10.862192	10.839735	11.853844	10.660556	10.612140	12.304791	10.544311	10.726118	10.501693	10.345071	9.322876	10.326429	9.302664	10.497826	10.125839	12.758584	10.271167	10.118547	11.156400	10.802095	11.404390	10.228505	11.468386	11.075613	11.127532	12.204667	11.371738	11.661797	10.803291	10.284582	11.083497	10.669127	10.802701	10.569842	10.150959	11.417836	11.129112	10.411451	10.983350	11.555942	11.415437	10.873972	12.548071	11.836322	11.404685	11.081461	10.571684	10.263444	11.274151

EVI5	12.213340	11.278240	11.362843	11.237709	10.935492	10.976546	11.752478	11.249397	9.746158	11.140371	11.355068	11.550739	10.989151	11.351456	11.479650	10.773731	11.092206	11.158915	11.677583	11.182579	11.581405	11.158191	12.342613	10.764309	10.988997	10.601523	10.456478	10.260249	10.840331	11.189884	9.921845	11.863047	11.981655	11.334652	12.036237	11.838285	11.978531	11.538542	11.788817	11.636481	11.066449	12.307156	11.411961	11.500773	11.666419	11.246081	10.944920	11.505395	11.273225	11.387310	11.539526	11.645189	11.963054	11.719282	11.275733	11.096621	11.943795	12.220618	11.839013	11.792414	12.151731	11.143538	12.833284	11.995202	11.979444	11.679502	11.733461	11.519647	11.267660	12.264187	11.633434	11.688705	10.522222	11.958138	11.673868	11.924002	11.910728	12.294973	11.627766	11.999751	12.061357	11.854455	11.995822	11.912512	11.900447	11.779565	11.908802	12.291396	12.389042	11.956506	11.876288	12.503202	11.643282	11.636331	11.983055	11.889609	11.497855	10.973367	11.353312	10.803291	11.212133	11.516497	11.068675	11.451923	12.414309	10.612438	11.059685	12.615979	11.848352	10.794845	12.604730	11.097765	11.395196	10.573931	10.491566	10.263285	12.200314	11.191198	9.896882	10.919103	11.631664	11.118560	10.283247	8.866731	9.665912	11.024804	10.553234	10.497236	9.982457	11.022278	10.565474	10.359338	10.778399	11.679502	10.711692	11.253526	9.926127	10.357381	11.692880	11.235350	11.291891	11.521612	10.136400	11.148096	11.601351	10.772850	9.613488	9.224356	10.925355	10.527773	11.201594	11.123986	12.127984	11.388220	11.895619	11.647691	11.367577	11.502333	11.754682	9.745076	11.381447	10.491721	11.274456	10.899523	10.368356	9.978744	11.638115	10.833302	11.918646	11.437676	11.193097	11.573877	12.432090	11.913539	10.896259	11.549716	12.108177	11.239490	11.547102	11.407838	10.580695	12.399952	11.434496	11.635098	12.169111	11.587012	11.051912	11.239635	11.935336	11.526057	11.668911	8.973205	11.684959	11.871295	11.730195	11.965821	12.091377	11.727818	12.255202	11.842049	10.933084	11.945449	11.657028	12.089977	10.793216	11.477417	11.320099	12.430276	11.635875	11.237709	12.261028	11.317780	11.146797	11.529530	11.469270	11.134465	9.763871	11.453711	11.009768	12.205277	10.714168	10.811946	11.474103	12.034420	9.132911	10.488263	11.834974	11.944085	9.970033	12.439820	11.386878	10.367747	9.775812	10.169200	11.023804	11.677874	10.983080	10.365458

ZNF582	6.770414	6.796847	6.876846	6.226940	6.384666	6.281449	6.769132	5.041986	5.351677	6.966132	6.424310	6.518205	5.973787	6.509101	6.505645	6.760447	5.077975	7.679371	6.326915	6.583920	7.110154	6.208993	7.743172	6.698233	6.871479	6.049931	6.129505	6.741794	6.691940	6.976564	4.922988	7.188899	7.702677	6.567898	7.207847	7.243901	7.474146	7.325954	6.488546	7.057440	6.989994	6.939673	6.752554	6.742879	6.858189	6.096514	6.107820	6.244301	6.520020	6.727719	6.926825	7.316378	7.729343	7.537165	6.789423	6.201557	7.413508	7.415948	6.525818	7.229521	7.167160	6.693444	6.889972	5.870621	6.630936	7.034067	6.757978	6.713105	6.588047	6.541025	7.165039	6.810491	6.381466	6.762196	6.891740	6.626317	6.905892	6.377121	6.503012	7.092603	6.849813	6.640143	7.029077	6.975583	7.190458	7.570962	5.972154	5.914751	6.241436	6.696196	7.138598	6.029738	6.468191	6.380296	6.566408	6.526846	6.759287	6.342169	6.302364	6.190787	5.953623	7.060771	6.609006	6.928293	6.251652	6.320848	5.568253	6.308928	5.901681	7.010571	6.081461	5.591192	5.624269	6.547423	5.853816	5.480817	6.234130	6.335522	6.104268	6.111965	5.740241	7.011235	7.172764	5.227918	6.114404	5.749744	4.599587	4.750429	5.719393	5.955561	5.953780	5.075669	5.914377	6.836732	5.696745	4.354817	6.578840	5.874405	6.076260	6.283128	5.338929	8.027463	5.420915	5.997249	5.649360	6.283455	6.144617	5.707018	6.528665	4.904637	5.742070	6.366865	5.837488	6.451151	7.082213	7.185698	7.277658	6.158574	6.664453	5.146958	6.633056	6.047108	6.569361	5.445822	5.427041	4.903797	4.977646	6.358684	5.621097	6.069272	6.887075	7.238454	6.803833	7.146759	6.095860	6.802029	5.829507	5.694907	6.157372	5.816548	6.329550	5.545822	5.795108	5.946038	6.550364	5.962877	6.208993	6.278303	6.612157	6.758139	6.359531	5.111153	6.378971	6.988909	6.304098	6.844383	6.105662	6.145843	7.222406	6.110611	6.009832	5.780710	6.729487	6.993546	6.519531	5.690604	5.010954	6.275725	6.378971	7.578077	5.314218	6.606716	6.222589	6.211523	6.880869	5.778924	6.066083	6.255725	6.038250	6.217668	5.402050	6.281779	6.566103	5.743183	5.764682	4.011825	4.663683	5.886551	6.274896	6.367040	5.502762	6.493315	6.410035	5.150526	5.284565	5.914578	5.491679	6.460844

NAA50	16.744583	15.011645	14.909806	15.146873	15.065808	15.084722	15.113035	15.111191	15.327234	14.876067	15.200291	15.667217	15.129069	14.843666	14.432746	15.302315	14.785315	14.638391	14.515058	15.122219	14.518957	14.569736	14.096118	14.708876	14.714881	15.142982	15.764986	15.099414	15.861722	14.855908	15.027552	15.031602	15.061588	15.137104	15.101143	15.156920	15.330867	14.961131	14.532680	14.751210	15.044189	14.828938	14.529101	14.840681	14.559663	15.038124	15.270868	15.060407	14.742751	14.908102	16.264810	14.460072	14.160203	14.795970	14.591979	15.278078	14.395124	13.740837	14.504194	13.574462	14.129527	15.227331	13.186053	14.599964	14.410971	15.177280	14.482061	14.381329	14.174543	14.734398	16.144373	15.137730	15.075186	15.194140	14.880110	14.907125	15.073466	14.674118	15.932498	14.845201	14.940922	13.982398	14.898046	15.760765	14.871315	15.667217	15.032714	14.136689	14.382401	14.737145	14.480189	14.300589	14.796877	15.016274	15.780590	14.940922	14.968533	14.934057	15.362494	14.129527	15.133349	14.925433	14.819361	14.106565	14.375159	15.074047	14.516636	14.378847	14.232689	15.181269	14.259951	14.970250	14.293075	15.307946	14.818887	15.895253	14.563436	15.327968	15.333133	15.468133	15.101738	14.396205	14.995049	15.108611	15.219810	15.250206	15.214315	15.122219	15.386012	15.051945	15.158183	15.413926	14.866612	14.400780	15.125800	15.040623	14.770475	14.940922	14.686498	15.134599	15.060999	15.446499	15.202396	15.055528	15.005364	15.199621	15.455098	15.595469	15.190029	15.564042	14.441603	14.622502	14.945286	14.852694	15.114201	14.603726	14.756024	14.836534	14.738102	15.557764	14.308313	14.072391	15.075186	14.920091	14.928661	15.336747	13.888470	15.232746	14.415078	14.942038	14.708439	14.459315	14.462369	14.617785	15.455098	14.096118	13.776185	14.396563	14.215329	14.310316	15.081140	14.456152	14.427855	14.200109	14.356954	13.804934	14.470465	14.317829	14.068082	14.953342	15.026340	15.113668	14.967925	14.836045	14.386005	14.602393	14.847864	14.929736	14.436781	14.645337	15.332400	14.904024	14.525087	15.173276	15.025759	15.124619	15.364729	14.276234	14.678701	15.022274	14.815737	14.882312	14.714881	14.976470	14.785315	15.187361	15.140997	15.265827	14.917864	14.921685	14.908629	15.012809	13.816316	14.447934	15.010559	14.491152	15.333133	14.871315	15.219191	14.659533	14.410971	15.385183	15.276640	15.448809	15.445672	14.432746	15.452790	15.195519

SEC14L6	8.147768	7.066825	7.132761	6.965964	7.077988	7.434558	7.049113	6.858491	6.597115	7.102997	6.731428	7.200399	6.380948	6.616246	6.249967	7.098929	6.918525	6.512277	5.906683	6.836439	6.464543	6.622911	6.618545	6.674294	7.018597	6.603879	6.562187	7.406010	7.146759	7.270478	6.638032	7.196961	6.786777	6.674930	6.664134	6.588206	6.328231	6.461005	6.564827	6.750989	7.111138	7.115823	6.642260	6.572093	8.076073	6.350277	7.256941	6.713749	6.464205	6.392523	8.054773	6.798171	7.021413	6.675596	6.055495	7.548986	6.475242	6.148301	6.434967	6.771880	6.555657	7.081775	8.006706	6.402270	6.643535	6.234806	6.450000	6.478315	6.346393	6.938731	6.890454	6.342020	7.319649	6.445408	6.879572	6.221600	6.349412	6.596178	7.818572	7.477675	6.605303	6.776533	6.969980	6.241596	6.529995	6.862173	6.586228	6.238948	6.408366	6.920814	6.534372	6.632728	6.690459	6.643374	7.778984	6.576865	6.634691	6.300025	7.295535	6.359889	7.245012	6.274724	6.534701	6.585387	5.738086	7.078154	6.790559	6.584073	7.094820	7.474601	6.409686	6.725435	6.619878	7.828982	6.938890	8.464122	8.041861	6.556792	7.246216	6.543856	7.397543	7.333384	7.011554	8.444402	7.479657	7.976173	6.217326	6.706701	7.237677	7.434079	6.869358	7.485700	8.742397	6.996153	6.904766	6.723695	6.751932	6.536469	6.351115	6.929221	6.824346	7.218710	6.970435	8.459707	7.520666	7.249927	7.749980	8.940339	7.718569	6.920987	6.194067	6.988248	7.514341	7.169797	5.878333	6.850296	6.077787	6.919359	6.888860	8.338060	6.538118	5.607399	6.952973	6.173342	6.146192	8.368909	6.410549	5.867966	6.522506	7.203012	5.659580	5.874564	7.032025	6.264862	7.111063	5.339523	5.459844	6.305094	5.923657	6.556947	7.363727	6.420124	6.311781	6.513566	7.842151	6.599421	6.601343	6.578487	6.539396	6.215952	6.372612	8.212669	7.197133	6.600722	6.476273	6.424158	6.595852	7.689026	7.827891	6.602610	6.218038	6.563191	5.815094	6.903528	7.297649	8.465536	6.329383	6.071333	6.866463	5.880879	6.729992	6.619878	7.191534	6.914949	6.922099	7.468145	6.837217	7.560752	6.031113	7.414160	6.454774	6.410549	7.064300	7.036232	7.221939	6.782296	6.399974	5.830277	9.346191	6.711965	7.324693	7.851877	8.690022	7.314004	8.385284	6.739884	6.650779	7.522082

DCDC1	3.206112	3.730048	3.071333	3.594603	2.886653	4.033222	3.569850	5.197306	3.238168	3.319425	3.443277	3.485680	4.359819	3.504352	3.088534	3.330169	4.117669	3.040188	6.502846	3.136503	4.070662	2.886653	3.496532	3.372751	3.801408	2.900528	3.515685	3.029872	3.090114	3.116732	3.309058	3.357413	3.090209	3.047969	4.143455	5.077975	3.474326	3.171791	3.195162	2.932919	3.230494	3.206265	3.685044	3.186339	3.412583	4.927846	3.110154	3.196384	3.335963	3.797157	4.310387	3.585340	4.105200	5.302426	4.708848	3.085063	5.153573	3.734389	3.462866	3.258402	4.173173	3.034123	3.377896	3.100685	3.249142	3.109568	2.957685	3.202948	3.405245	2.972991	3.904846	3.327563	3.021888	2.932169	3.386728	3.236242	3.099517	3.006045	4.554733	3.847741	3.273434	3.865565	3.060259	3.968740	3.324164	3.305233	3.973745	3.171311	3.213805	3.343324	3.378791	3.694034	3.175677	3.274034	3.872223	3.031966	3.656622	3.544508	3.609384	2.984419	2.896185	4.284260	3.011159	3.235234	4.288770	2.980968	3.202087	3.168541	3.462669	3.198532	3.574031	3.375281	3.020720	3.178289	3.099176	2.969540	2.976407	2.974907	3.055187	3.480782	4.748070	3.012411	3.028669	3.351900	4.097759	2.939146	4.827398	3.279133	3.126980	3.300392	3.597342	3.611605	3.089903	2.987048	3.972456	3.836745	3.924166	3.508512	3.200773	3.525510	3.306163	4.185785	3.172804	2.932490	3.845423	3.245376	2.932649	2.928336	3.344796	3.188054	3.331917	3.087170	3.011642	4.298115	4.776155	3.858971	6.792003	3.191322	2.902397	3.083503	3.057068	3.419322	3.208598	3.657043	4.333307	3.081779	3.240460	4.041017	3.835027	3.839885	3.461116	3.792701	4.492888	3.784585	3.633128	7.537619	3.031164	3.153195	4.748940	3.079298	3.099402	4.936617	3.458581	3.408374	4.592086	2.944259	3.410052	3.008683	2.982814	3.385685	3.138221	3.014748	3.113651	2.981085	3.205978	3.358117	3.057912	3.599132	3.287111	3.564191	3.995744	5.471598	3.537245	3.572687	3.696307	3.131266	3.980615	3.100685	3.881690	3.220568	3.003001	3.588432	3.146189	3.137979	4.057800	3.244432	3.038227	3.499964	3.158914	3.085822	6.591954	3.006617	3.175783	4.691364	2.979842	3.217115	3.141282	3.236086	3.034123	3.869665	3.407300	3.065950	3.134643	3.261306	2.911614	3.033804	2.983258	3.139128

TMOD4	9.590429	9.037850	9.031417	9.031417	9.371195	10.745880	10.315931	9.302942	9.535658	9.968968	10.499425	10.975823	9.662014	9.459183	9.721381	8.861204	9.397984	9.624312	9.726052	9.266572	9.172491	9.617155	11.237863	9.222670	9.601901	9.285661	9.631976	9.010103	11.445714	9.855275	9.597565	8.964176	9.074979	9.727298	8.951490	8.930788	8.925032	9.231754	9.525550	9.096736	10.625496	9.546524	8.568501	8.454599	9.491411	8.906121	10.474064	8.794460	9.128119	8.560859	8.456268	8.808720	8.813362	8.875559	9.151857	10.683631	9.149696	9.408176	9.426808	9.403225	9.419757	10.995680	10.846956	9.876719	9.463307	9.494403	9.406024	9.308487	9.053766	9.847208	9.229997	9.380048	10.197653	9.160765	9.208246	9.086111	9.105810	8.858812	9.172960	9.085673	9.038186	9.139318	9.208388	8.776752	9.332620	9.219588	9.480408	9.217895	9.993987	9.257116	9.435228	10.025121	9.333063	9.445303	9.296431	9.632128	9.147027	9.932911	9.647368	9.364412	10.039581	8.948426	9.229997	8.991055	9.757807	10.140602	9.237341	9.860677	10.160284	10.492682	9.916055	9.202202	9.289264	10.193710	9.416111	12.450905	9.484092	9.748976	11.014714	10.595736	9.858186	9.834126	9.800735	10.430794	10.453574	11.495782	10.078202	9.489187	11.630608	11.167966	11.333751	10.427778	10.336292	10.347055	10.076651	9.697396	10.416749	10.939911	10.614968	9.551928	11.359874	10.412086	10.470130	9.459261	9.502455	9.914982	10.384855	12.368451	10.703888	10.555685	9.380804	9.718772	10.261469	9.504231	9.999817	9.688079	10.146396	10.813141	9.800735	11.016496	10.423140	8.708279	8.697124	9.036791	9.327985	10.539572	8.823713	10.771383	9.432358	8.877700	8.982833	9.844727	9.683577	9.018370	10.685116	8.981810	9.708902	9.248587	10.459258	10.214264	10.948607	10.149023	9.909568	9.875126	9.258917	10.266446	9.117427	9.601275	9.526630	10.667558	10.305675	11.373199	9.974679	9.575634	9.367251	9.747411	10.121388	9.861767	9.511023	9.712195	9.278174	9.131387	9.245828	9.872723	10.678215	8.786239	9.119139	10.675082	9.805002	10.245866	10.369851	10.442286	10.356921	10.263444	10.193562	10.187863	10.620438	9.488884	9.681699	9.745999	9.388703	10.179942	9.654195	9.482562	12.194289	10.863674	9.671337	10.024815	10.737937	10.341418	9.339624	10.384381	10.549938	10.618976	10.434742	9.755469	9.526187	12.183450

VPS72	9.590429	9.037850	9.031417	9.031417	9.371195	10.745880	10.315931	9.302942	9.535658	9.968968	10.499425	10.975823	9.662014	9.459183	9.721381	8.861204	9.397984	9.624312	9.726052	9.266572	9.172491	9.617155	11.237863	9.222670	9.601901	9.285661	9.631976	9.010103	11.445714	9.855275	9.597565	8.964176	9.074979	9.727298	8.951490	8.930788	8.925032	9.231754	9.525550	9.096736	10.625496	9.546524	8.568501	8.454599	9.491411	8.906121	10.474064	8.794460	9.128119	8.560859	8.456268	8.808720	8.813362	8.875559	9.151857	10.683631	9.149696	9.408176	9.426808	9.403225	9.419757	10.995680	10.846956	9.876719	9.463307	9.494403	9.406024	9.308487	9.053766	9.847208	9.229997	9.380048	10.197653	9.160765	9.208246	9.086111	9.105810	8.858812	9.172960	9.085673	9.038186	9.139318	9.208388	8.776752	9.332620	9.219588	9.480408	9.217895	9.993987	9.257116	9.435228	10.025121	9.333063	9.445303	9.296431	9.632128	9.147027	9.932911	9.647368	9.364412	10.039581	8.948426	9.229997	8.991055	9.757807	10.140602	9.237341	9.860677	10.160284	10.492682	9.916055	9.202202	9.289264	10.193710	9.416111	12.450905	9.484092	9.748976	11.014714	10.595736	9.858186	9.834126	9.800735	10.430794	10.453574	11.495782	10.078202	9.489187	11.630608	11.167966	11.333751	10.427778	10.336292	10.347055	10.076651	9.697396	10.416749	10.939911	10.614968	9.551928	11.359874	10.412086	10.470130	9.459261	9.502455	9.914982	10.384855	12.368451	10.703888	10.555685	9.380804	9.718772	10.261469	9.504231	9.999817	9.688079	10.146396	10.813141	9.800735	11.016496	10.423140	8.708279	8.697124	9.036791	9.327985	10.539572	8.823713	10.771383	9.432358	8.877700	8.982833	9.844727	9.683577	9.018370	10.685116	8.981810	9.708902	9.248587	10.459258	10.214264	10.948607	10.149023	9.909568	9.875126	9.258917	10.266446	9.117427	9.601275	9.526630	10.667558	10.305675	11.373199	9.974679	9.575634	9.367251	9.747411	10.121388	9.861767	9.511023	9.712195	9.278174	9.131387	9.245828	9.872723	10.678215	8.786239	9.119139	10.675082	9.805002	10.245866	10.369851	10.442286	10.356921	10.263444	10.193562	10.187863	10.620438	9.488884	9.681699	9.745999	9.388703	10.179942	9.654195	9.482562	12.194289	10.863674	9.671337	10.024815	10.737937	10.341418	9.339624	10.384381	10.549938	10.618976	10.434742	9.755469	9.526187	12.183450

SCNM1	9.590429	9.037850	9.031417	9.031417	9.371195	10.745880	10.315931	9.302942	9.535658	9.968968	10.499425	10.975823	9.662014	9.459183	9.721381	8.861204	9.397984	9.624312	9.726052	9.266572	9.172491	9.617155	11.237863	9.222670	9.601901	9.285661	9.631976	9.010103	11.445714	9.855275	9.597565	8.964176	9.074979	9.727298	8.951490	8.930788	8.925032	9.231754	9.525550	9.096736	10.625496	9.546524	8.568501	8.454599	9.491411	8.906121	10.474064	8.794460	9.128119	8.560859	8.456268	8.808720	8.813362	8.875559	9.151857	10.683631	9.149696	9.408176	9.426808	9.403225	9.419757	10.995680	10.846956	9.876719	9.463307	9.494403	9.406024	9.308487	9.053766	9.847208	9.229997	9.380048	10.197653	9.160765	9.208246	9.086111	9.105810	8.858812	9.172960	9.085673	9.038186	9.139318	9.208388	8.776752	9.332620	9.219588	9.480408	9.217895	9.993987	9.257116	9.435228	10.025121	9.333063	9.445303	9.296431	9.632128	9.147027	9.932911	9.647368	9.364412	10.039581	8.948426	9.229997	8.991055	9.757807	10.140602	9.237341	9.860677	10.160284	10.492682	9.916055	9.202202	9.289264	10.193710	9.416111	12.450905	9.484092	9.748976	11.014714	10.595736	9.858186	9.834126	9.800735	10.430794	10.453574	11.495782	10.078202	9.489187	11.630608	11.167966	11.333751	10.427778	10.336292	10.347055	10.076651	9.697396	10.416749	10.939911	10.614968	9.551928	11.359874	10.412086	10.470130	9.459261	9.502455	9.914982	10.384855	12.368451	10.703888	10.555685	9.380804	9.718772	10.261469	9.504231	9.999817	9.688079	10.146396	10.813141	9.800735	11.016496	10.423140	8.708279	8.697124	9.036791	9.327985	10.539572	8.823713	10.771383	9.432358	8.877700	8.982833	9.844727	9.683577	9.018370	10.685116	8.981810	9.708902	9.248587	10.459258	10.214264	10.948607	10.149023	9.909568	9.875126	9.258917	10.266446	9.117427	9.601275	9.526630	10.667558	10.305675	11.373199	9.974679	9.575634	9.367251	9.747411	10.121388	9.861767	9.511023	9.712195	9.278174	9.131387	9.245828	9.872723	10.678215	8.786239	9.119139	10.675082	9.805002	10.245866	10.369851	10.442286	10.356921	10.263444	10.193562	10.187863	10.620438	9.488884	9.681699	9.745999	9.388703	10.179942	9.654195	9.482562	12.194289	10.863674	9.671337	10.024815	10.737937	10.341418	9.339624	10.384381	10.549938	10.618976	10.434742	9.755469	9.526187	12.183450

GP1BB	10.292644	12.601913	12.690183	12.890172	13.081420	12.944807	12.542124	13.103060	12.836345	12.871943	13.020295	12.741823	12.859886	13.254006	12.969891	12.714355	12.609852	13.001582	14.529893	13.099216	13.561728	14.107221	14.524706	13.027853	13.101809	13.189322	11.268796	13.385833	12.062451	11.873849	13.020295	12.514165	12.589947	10.909711	11.676971	12.126882	12.378969	12.810001	13.416705	13.264544	12.711470	12.832035	12.585969	12.659132	12.605230	12.658313	12.367767	12.538254	12.324942	12.419287	12.102695	12.408357	12.596273	12.988517	12.682137	13.181925	12.827182	13.534637	13.194918	12.751559	13.288631	12.480288	13.200096	13.880671	12.810934	12.994008	13.064903	13.042413	12.733221	13.451174	12.508031	12.941369	12.880949	12.905897	13.084018	12.355477	12.542773	12.761526	12.574563	13.064161	12.881502	12.973272	12.323571	12.636458	13.149739	12.379442	13.193477	13.266805	13.497789	13.516235	13.219174	13.953224	13.285244	12.559887	11.443526	12.222032	12.666334	12.653975	12.642231	13.118676	12.971329	12.750174	13.038398	13.097680	14.535115	12.801793	13.516235	14.437499	14.268271	12.765306	14.148532	13.447560	13.583239	12.748129	13.026194	11.743482	11.014430	12.891724	13.075705	9.415347	11.760375	10.537933	10.086530	13.568257	12.201103	12.053445	12.169426	11.815237	11.550016	12.359732	12.428494	13.896393	12.727782	13.270411	11.138316	11.723766	13.759917	12.101029	10.497826	13.778674	13.015899	13.780621	12.804949	12.749657	12.132968	12.412056	11.833448	11.191638	11.840676	11.871132	11.718826	11.655906	11.788373	11.199022	12.564459	11.829878	10.688437	12.373531	12.871943	11.176868	12.655859	9.755758	11.391015	11.688994	12.396622	13.316425	12.258812	13.107904	11.817489	12.111589	10.670884	11.809037	12.333192	12.189815	11.072137	11.322595	12.622664	11.929392	13.017536	12.043646	11.143107	13.761927	12.533467	10.280806	12.075736	12.248492	12.936361	11.620543	11.326303	11.062597	11.826245	12.930619	12.447268	10.687204	11.070018	12.073275	10.675389	12.806823	11.169880	11.673410	11.134182	12.244367	12.028252	11.149750	11.660289	12.832376	11.113919	12.020355	11.843554	12.552991	12.675523	11.534802	11.617144	11.037724	11.544838	12.439669	12.414781	11.740562	11.545153	11.240771	12.261347	11.692447	10.834345	12.336035	12.055582	11.303088	11.578064	12.906787	12.274566	12.698701	12.435636	11.124128	12.787522	11.898984	11.389966	11.267812	11.193393	12.296782

SEPTIN5	10.292644	12.601913	12.690183	12.890172	13.081420	12.944807	12.542124	13.103060	12.836345	12.871943	13.020295	12.741823	12.859886	13.254006	12.969891	12.714355	12.609852	13.001582	14.529893	13.099216	13.561728	14.107221	14.524706	13.027853	13.101809	13.189322	11.268796	13.385833	12.062451	11.873849	13.020295	12.514165	12.589947	10.909711	11.676971	12.126882	12.378969	12.810001	13.416705	13.264544	12.711470	12.832035	12.585969	12.659132	12.605230	12.658313	12.367767	12.538254	12.324942	12.419287	12.102695	12.408357	12.596273	12.988517	12.682137	13.181925	12.827182	13.534637	13.194918	12.751559	13.288631	12.480288	13.200096	13.880671	12.810934	12.994008	13.064903	13.042413	12.733221	13.451174	12.508031	12.941369	12.880949	12.905897	13.084018	12.355477	12.542773	12.761526	12.574563	13.064161	12.881502	12.973272	12.323571	12.636458	13.149739	12.379442	13.193477	13.266805	13.497789	13.516235	13.219174	13.953224	13.285244	12.559887	11.443526	12.222032	12.666334	12.653975	12.642231	13.118676	12.971329	12.750174	13.038398	13.097680	14.535115	12.801793	13.516235	14.437499	14.268271	12.765306	14.148532	13.447560	13.583239	12.748129	13.026194	11.743482	11.014430	12.891724	13.075705	9.415347	11.760375	10.537933	10.086530	13.568257	12.201103	12.053445	12.169426	11.815237	11.550016	12.359732	12.428494	13.896393	12.727782	13.270411	11.138316	11.723766	13.759917	12.101029	10.497826	13.778674	13.015899	13.780621	12.804949	12.749657	12.132968	12.412056	11.833448	11.191638	11.840676	11.871132	11.718826	11.655906	11.788373	11.199022	12.564459	11.829878	10.688437	12.373531	12.871943	11.176868	12.655859	9.755758	11.391015	11.688994	12.396622	13.316425	12.258812	13.107904	11.817489	12.111589	10.670884	11.809037	12.333192	12.189815	11.072137	11.322595	12.622664	11.929392	13.017536	12.043646	11.143107	13.761927	12.533467	10.280806	12.075736	12.248492	12.936361	11.620543	11.326303	11.062597	11.826245	12.930619	12.447268	10.687204	11.070018	12.073275	10.675389	12.806823	11.169880	11.673410	11.134182	12.244367	12.028252	11.149750	11.660289	12.832376	11.113919	12.020355	11.843554	12.552991	12.675523	11.534802	11.617144	11.037724	11.544838	12.439669	12.414781	11.740562	11.545153	11.240771	12.261347	11.692447	10.834345	12.336035	12.055582	11.303088	11.578064	12.906787	12.274566	12.698701	12.435636	11.124128	12.787522	11.898984	11.389966	11.267812	11.193393	12.296782

AC000093.1	10.292644	12.601913	12.690183	12.890172	13.081420	12.944807	12.542124	13.103060	12.836345	12.871943	13.020295	12.741823	12.859886	13.254006	12.969891	12.714355	12.609852	13.001582	14.529893	13.099216	13.561728	14.107221	14.524706	13.027853	13.101809	13.189322	11.268796	13.385833	12.062451	11.873849	13.020295	12.514165	12.589947	10.909711	11.676971	12.126882	12.378969	12.810001	13.416705	13.264544	12.711470	12.832035	12.585969	12.659132	12.605230	12.658313	12.367767	12.538254	12.324942	12.419287	12.102695	12.408357	12.596273	12.988517	12.682137	13.181925	12.827182	13.534637	13.194918	12.751559	13.288631	12.480288	13.200096	13.880671	12.810934	12.994008	13.064903	13.042413	12.733221	13.451174	12.508031	12.941369	12.880949	12.905897	13.084018	12.355477	12.542773	12.761526	12.574563	13.064161	12.881502	12.973272	12.323571	12.636458	13.149739	12.379442	13.193477	13.266805	13.497789	13.516235	13.219174	13.953224	13.285244	12.559887	11.443526	12.222032	12.666334	12.653975	12.642231	13.118676	12.971329	12.750174	13.038398	13.097680	14.535115	12.801793	13.516235	14.437499	14.268271	12.765306	14.148532	13.447560	13.583239	12.748129	13.026194	11.743482	11.014430	12.891724	13.075705	9.415347	11.760375	10.537933	10.086530	13.568257	12.201103	12.053445	12.169426	11.815237	11.550016	12.359732	12.428494	13.896393	12.727782	13.270411	11.138316	11.723766	13.759917	12.101029	10.497826	13.778674	13.015899	13.780621	12.804949	12.749657	12.132968	12.412056	11.833448	11.191638	11.840676	11.871132	11.718826	11.655906	11.788373	11.199022	12.564459	11.829878	10.688437	12.373531	12.871943	11.176868	12.655859	9.755758	11.391015	11.688994	12.396622	13.316425	12.258812	13.107904	11.817489	12.111589	10.670884	11.809037	12.333192	12.189815	11.072137	11.322595	12.622664	11.929392	13.017536	12.043646	11.143107	13.761927	12.533467	10.280806	12.075736	12.248492	12.936361	11.620543	11.326303	11.062597	11.826245	12.930619	12.447268	10.687204	11.070018	12.073275	10.675389	12.806823	11.169880	11.673410	11.134182	12.244367	12.028252	11.149750	11.660289	12.832376	11.113919	12.020355	11.843554	12.552991	12.675523	11.534802	11.617144	11.037724	11.544838	12.439669	12.414781	11.740562	11.545153	11.240771	12.261347	11.692447	10.834345	12.336035	12.055582	11.303088	11.578064	12.906787	12.274566	12.698701	12.435636	11.124128	12.787522	11.898984	11.389966	11.267812	11.193393	12.296782

DEXI	11.097635	11.411376	11.041512	10.887194	10.738688	10.713301	11.125488	11.073630	10.768036	10.868882	10.748889	10.926240	10.678967	11.094860	11.062833	10.741413	10.872641	11.133549	11.553440	11.087082	11.422716	12.584810	12.780538	11.124739	11.416046	11.408446	11.589239	10.984344	10.598330	10.911465	10.628379	10.836162	11.067036	11.236428	11.423761	11.313643	11.393373	11.778217	11.941813	11.604162	10.683914	11.896055	11.330459	11.253671	11.639796	11.496066	11.012953	11.682779	10.783168	11.261231	11.032989	11.363130	11.586872	11.905178	11.719282	10.985390	11.679053	12.169579	11.904174	11.935336	12.466808	10.888061	12.978769	12.161888	12.175459	11.975261	12.323101	11.546955	11.831355	12.506071	11.458920	11.438109	10.385173	11.495632	11.534520	11.415587	11.271780	11.534084	11.484580	11.522341	11.237421	11.325406	11.545312	11.678914	11.598948	10.921361	11.544245	11.611094	12.563000	11.924002	12.002645	12.721811	11.766034	11.534230	11.347581	11.633139	10.950866	10.095773	10.725518	10.634289	10.474815	11.266616	10.661160	10.884011	12.507212	10.238466	11.497855	12.117118	12.280366	10.514841	12.471342	11.063499	11.322916	10.912348	10.142906	10.333610	11.605367	10.877948	10.273491	10.825756	11.169880	11.518846	11.502333	11.088725	10.798914	11.183186	10.129455	11.473380	11.100452	11.284004	11.497421	11.694853	11.375615	12.086352	11.024075	11.088277	10.990768	11.254561	11.051054	11.455518	12.445298	13.233400	10.334496	11.915193	11.847451	11.093554	11.456110	10.506316	10.953350	10.193710	10.980120	11.112442	11.959600	10.805196	10.471779	11.270124	11.688852	12.222189	11.635258	11.253964	11.928470	11.681866	12.105900	11.443831	11.353017	11.002796	10.953621	11.050033	11.632548	11.324533	11.442338	12.288938	12.018432	11.976229	11.077518	12.341549	12.376294	11.845235	11.919811	12.192905	11.414427	12.905897	12.166337	11.833153	12.565285	12.039701	12.001304	12.150786	12.358156	11.313643	11.084833	11.339682	11.630155	11.139619	11.568999	11.806792	12.279446	11.477701	11.610188	11.319427	11.385408	11.821698	11.133393	11.370065	10.862762	11.816438	11.997151	12.471970	11.976679	11.854007	12.480288	12.147357	12.282101	12.190892	12.021794	10.829006	10.376347	10.919864	10.960769	11.591314	11.293383	10.835875	11.413399	12.265924	11.076192	11.157897	11.499480	11.806485	11.349651	12.441914	11.495068	11.475722	11.529671	11.751007	10.742017	12.229032	10.988689	11.171227

CIITA	11.097635	11.411376	11.041512	10.887194	10.738688	10.713301	11.125488	11.073630	10.768036	10.868882	10.748889	10.926240	10.678967	11.094860	11.062833	10.741413	10.872641	11.133549	11.553440	11.087082	11.422716	12.584810	12.780538	11.124739	11.416046	11.408446	11.589239	10.984344	10.598330	10.911465	10.628379	10.836162	11.067036	11.236428	11.423761	11.313643	11.393373	11.778217	11.941813	11.604162	10.683914	11.896055	11.330459	11.253671	11.639796	11.496066	11.012953	11.682779	10.783168	11.261231	11.032989	11.363130	11.586872	11.905178	11.719282	10.985390	11.679053	12.169579	11.904174	11.935336	12.466808	10.888061	12.978769	12.161888	12.175459	11.975261	12.323101	11.546955	11.831355	12.506071	11.458920	11.438109	10.385173	11.495632	11.534520	11.415587	11.271780	11.534084	11.484580	11.522341	11.237421	11.325406	11.545312	11.678914	11.598948	10.921361	11.544245	11.611094	12.563000	11.924002	12.002645	12.721811	11.766034	11.534230	11.347581	11.633139	10.950866	10.095773	10.725518	10.634289	10.474815	11.266616	10.661160	10.884011	12.507212	10.238466	11.497855	12.117118	12.280366	10.514841	12.471342	11.063499	11.322916	10.912348	10.142906	10.333610	11.605367	10.877948	10.273491	10.825756	11.169880	11.518846	11.502333	11.088725	10.798914	11.183186	10.129455	11.473380	11.100452	11.284004	11.497421	11.694853	11.375615	12.086352	11.024075	11.088277	10.990768	11.254561	11.051054	11.455518	12.445298	13.233400	10.334496	11.915193	11.847451	11.093554	11.456110	10.506316	10.953350	10.193710	10.980120	11.112442	11.959600	10.805196	10.471779	11.270124	11.688852	12.222189	11.635258	11.253964	11.928470	11.681866	12.105900	11.443831	11.353017	11.002796	10.953621	11.050033	11.632548	11.324533	11.442338	12.288938	12.018432	11.976229	11.077518	12.341549	12.376294	11.845235	11.919811	12.192905	11.414427	12.905897	12.166337	11.833153	12.565285	12.039701	12.001304	12.150786	12.358156	11.313643	11.084833	11.339682	11.630155	11.139619	11.568999	11.806792	12.279446	11.477701	11.610188	11.319427	11.385408	11.821698	11.133393	11.370065	10.862762	11.816438	11.997151	12.471970	11.976679	11.854007	12.480288	12.147357	12.282101	12.190892	12.021794	10.829006	10.376347	10.919864	10.960769	11.591314	11.293383	10.835875	11.413399	12.265924	11.076192	11.157897	11.499480	11.806485	11.349651	12.441914	11.495068	11.475722	11.529671	11.751007	10.742017	12.229032	10.988689	11.171227

RBKS	11.158336	11.982123	12.270412	12.139103	12.624487	12.041947	12.140193	12.832035	12.173415	12.592977	12.343913	12.494020	12.576866	12.593788	12.243145	12.048150	12.619480	12.513208	12.792672	12.555591	12.319355	12.831338	11.233307	12.130980	12.151398	12.688477	10.878560	12.191499	11.487873	11.794064	12.377714	11.673716	12.397063	11.563737	12.190289	12.091377	12.360062	12.502391	12.933669	12.745728	11.992764	12.741981	12.163903	12.149134	12.030200	12.283635	12.157109	12.704167	12.359421	12.329402	12.252460	12.648312	12.356727	13.047798	11.801784	12.199524	12.297752	13.022885	13.117513	12.728127	12.776712	11.618620	11.993425	12.799200	12.458806	12.637159	12.536567	12.445002	12.361628	12.807024	12.409998	12.277596	11.901889	12.137555	12.489402	12.205727	12.294671	12.226923	12.237890	12.235457	12.649362	12.284103	11.994265	12.474749	12.730491	12.392195	12.700378	12.802671	12.782224	13.092940	12.919886	12.779001	12.350370	12.074208	12.220150	12.086801	12.112540	12.175782	12.689295	12.201238	12.686571	12.159431	12.028406	12.516941	13.112784	11.692447	12.790316	12.981325	12.591506	12.009448	12.738848	12.913386	12.700047	11.678602	12.399470	13.626821	12.097405	12.120080	11.788973	11.635875	10.802991	11.429574	11.230646	13.145143	10.947309	11.436404	11.536302	11.887957	11.512453	11.363862	11.304559	11.816892	11.780301	12.137705	11.017510	12.098308	11.573269	12.324336	12.790143	12.094734	11.227828	11.383763	11.768414	11.243131	12.240559	12.188708	11.264551	11.292947	11.252949	11.577336	11.797787	11.894727	11.998560	11.822914	10.887194	11.587012	11.329216	12.503389	12.224769	12.627650	11.368306	10.850018	11.530788	10.990476	11.667291	12.300012	12.358306	11.304855	11.717792	11.604016	11.633293	11.434353	11.971923	11.653632	10.133309	12.141122	11.470932	12.378353	11.785073	13.371671	11.965535	11.886135	11.614346	12.182810	12.075279	13.321898	12.576557	10.782833	13.124002	10.915527	11.322149	12.228786	11.904615	11.366830	11.808575	11.054370	12.127514	11.630454	12.618446	11.152465	12.264495	12.575245	11.199022	11.019158	11.735106	12.231261	10.906934	12.063952	12.165896	12.135276	13.186638	11.704017	11.785542	11.642694	11.880861	12.652596	11.869781	11.500069	11.573269	12.186990	12.252460	11.401656	11.761159	12.770680	10.764037	12.231416	12.365730	11.814058	11.724977	11.159054	12.642900	13.254395	12.184698	12.182658	12.240559	11.568423	12.530023	11.636180

MRPL33	11.158336	11.982123	12.270412	12.139103	12.624487	12.041947	12.140193	12.832035	12.173415	12.592977	12.343913	12.494020	12.576866	12.593788	12.243145	12.048150	12.619480	12.513208	12.792672	12.555591	12.319355	12.831338	11.233307	12.130980	12.151398	12.688477	10.878560	12.191499	11.487873	11.794064	12.377714	11.673716	12.397063	11.563737	12.190289	12.091377	12.360062	12.502391	12.933669	12.745728	11.992764	12.741981	12.163903	12.149134	12.030200	12.283635	12.157109	12.704167	12.359421	12.329402	12.252460	12.648312	12.356727	13.047798	11.801784	12.199524	12.297752	13.022885	13.117513	12.728127	12.776712	11.618620	11.993425	12.799200	12.458806	12.637159	12.536567	12.445002	12.361628	12.807024	12.409998	12.277596	11.901889	12.137555	12.489402	12.205727	12.294671	12.226923	12.237890	12.235457	12.649362	12.284103	11.994265	12.474749	12.730491	12.392195	12.700378	12.802671	12.782224	13.092940	12.919886	12.779001	12.350370	12.074208	12.220150	12.086801	12.112540	12.175782	12.689295	12.201238	12.686571	12.159431	12.028406	12.516941	13.112784	11.692447	12.790316	12.981325	12.591506	12.009448	12.738848	12.913386	12.700047	11.678602	12.399470	13.626821	12.097405	12.120080	11.788973	11.635875	10.802991	11.429574	11.230646	13.145143	10.947309	11.436404	11.536302	11.887957	11.512453	11.363862	11.304559	11.816892	11.780301	12.137705	11.017510	12.098308	11.573269	12.324336	12.790143	12.094734	11.227828	11.383763	11.768414	11.243131	12.240559	12.188708	11.264551	11.292947	11.252949	11.577336	11.797787	11.894727	11.998560	11.822914	10.887194	11.587012	11.329216	12.503389	12.224769	12.627650	11.368306	10.850018	11.530788	10.990476	11.667291	12.300012	12.358306	11.304855	11.717792	11.604016	11.633293	11.434353	11.971923	11.653632	10.133309	12.141122	11.470932	12.378353	11.785073	13.371671	11.965535	11.886135	11.614346	12.182810	12.075279	13.321898	12.576557	10.782833	13.124002	10.915527	11.322149	12.228786	11.904615	11.366830	11.808575	11.054370	12.127514	11.630454	12.618446	11.152465	12.264495	12.575245	11.199022	11.019158	11.735106	12.231261	10.906934	12.063952	12.165896	12.135276	13.186638	11.704017	11.785542	11.642694	11.880861	12.652596	11.869781	11.500069	11.573269	12.186990	12.252460	11.401656	11.761159	12.770680	10.764037	12.231416	12.365730	11.814058	11.724977	11.159054	12.642900	13.254395	12.184698	12.182658	12.240559	11.568423	12.530023	11.636180

PALMD	12.247353	14.285533	14.489157	13.801661	14.103476	13.692656	14.076889	14.517397	14.311389	14.289961	13.888066	14.434912	13.950442	13.800613	13.791466	14.205591	14.143339	14.376679	15.421791	14.371917	14.826972	14.221539	14.134512	14.119859	14.036663	14.120707	12.955887	13.849656	14.408016	13.662003	13.856506	14.241914	14.446741	12.865672	14.131436	14.210781	13.904457	13.884089	14.051810	14.204014	14.219589	14.557653	15.179287	13.629180	14.697958	14.281068	13.493953	15.073466	14.612714	14.226969	13.757880	14.531467	15.303021	14.272867	14.545545	14.370128	14.462773	15.252474	15.155102	14.354835	14.025995	13.700893	14.235912	14.680965	13.940443	14.868226	14.161160	14.291025	13.708928	14.369760	14.533864	14.421008	13.612309	14.002723	13.820727	14.528702	13.635468	14.862987	14.197832	14.388615	15.307946	14.587290	13.341990	14.501441	14.313051	14.577262	14.136088	15.254640	14.802845	14.467367	14.940922	14.356954	14.487547	13.698484	13.150306	14.082208	14.289961	14.424443	14.076027	14.144611	14.140212	13.957409	14.001519	14.221891	15.145529	14.018727	13.885163	14.909267	14.527106	13.753345	14.872892	14.543434	14.298393	13.687885	14.497531	13.815313	12.120820	14.985629	13.494652	11.569303	12.977133	12.245314	11.460104	13.053301	12.603569	12.480800	10.793498	13.004862	10.590962	12.525283	10.172908	12.014918	13.101435	11.627063	13.113930	11.131351	10.887114	11.949439	11.968244	13.194078	13.050819	12.976230	12.089192	12.544554	10.810677	13.678246	13.282011	11.093843	12.222955	12.287695	12.798177	12.457084	13.610144	13.628713	12.424072	13.757627	12.047677	13.747829	13.053836	12.368451	12.866399	11.854611	14.701564	12.887214	13.025833	12.371333	12.043646	11.880566	13.367256	13.520034	11.953632	11.821254	12.686750	12.632134	11.314983	11.321849	12.235457	12.665471	11.992007	13.588617	12.613211	14.145840	13.597930	13.030100	12.342944	12.931521	13.040426	13.523456	12.885665	12.475688	12.488461	12.860948	12.771369	13.360372	13.373356	13.445036	11.882525	13.017536	13.066780	12.150176	13.364346	14.400780	13.048973	13.211099	11.933343	12.890515	12.692342	14.277909	13.159931	11.495500	13.934635	12.479486	13.658895	13.591229	12.306995	12.696084	11.257836	13.008849	11.600906	12.463731	12.241433	12.276225	11.623005	12.111443	11.518972	13.351397	11.882216	13.122690	12.931521	13.078004	12.796802	10.675389	13.445449	10.786013	13.161096	12.440469	12.209741	13.319864

ZNF106	12.786674	12.316696	12.185267	11.730489	12.630345	12.495360	12.838440	12.282255	11.820206	12.661032	12.612171	12.492245	12.691855	12.548577	11.650941	12.410948	12.368604	12.387919	11.954677	12.446101	11.840676	12.290609	13.002160	12.361312	12.561718	12.697321	13.128611	12.469069	12.653816	12.596461	12.281925	12.950005	12.881502	12.802478	13.034884	12.845626	12.984620	13.091997	12.385468	12.272641	12.530524	12.350370	12.774646	12.720947	13.361000	12.923666	12.996574	12.075588	12.360524	12.914314	12.949458	13.019739	13.199903	12.668810	12.820415	12.289217	13.085147	12.150644	12.668162	12.920413	12.994351	12.492245	13.360143	12.384180	11.816748	12.249840	12.372749	12.001171	12.484550	12.456129	12.528867	12.850702	12.549566	12.949621	12.568065	12.986119	12.932755	12.573503	12.615500	12.163282	12.408855	12.110554	12.672776	12.876066	12.954050	12.893846	12.667122	12.888269	11.945613	11.405580	11.862452	11.912208	12.109967	12.684558	12.612527	12.874105	12.606892	12.800938	12.737801	12.974908	12.386450	12.795036	12.605894	12.626022	12.481140	12.295874	11.834822	12.224905	12.051785	12.638546	12.255020	11.474995	11.913078	12.170632	11.989727	11.866547	12.295577	12.613560	12.478661	12.504062	12.092606	11.563013	11.609621	12.178309	12.497333	11.922622	12.462402	13.176594	12.346154	12.804235	12.382100	12.505236	11.375921	11.824292	12.996223	12.099966	12.126121	11.818854	13.010316	11.040745	11.851418	12.274723	11.552438	11.857035	12.449933	11.430160	12.270578	11.813142	12.462928	12.154483	12.219989	12.376916	12.239319	12.176546	12.680079	12.166484	12.469402	11.796904	11.684658	12.309046	11.997474	12.956981	12.853832	12.283484	12.395544	12.056205	11.919091	12.721110	12.795211	12.601601	12.257409	12.166049	12.963999	12.713284	12.474582	13.085901	11.755284	12.060100	12.488616	11.939089	13.072832	12.218048	11.902176	11.991869	11.668911	12.067502	11.684658	11.855782	11.910004	12.506387	12.205573	11.424053	12.242226	12.836884	12.243145	12.549566	12.907514	12.344394	11.814486	12.253114	12.576234	11.583331	12.130529	12.038203	12.299522	12.024942	12.318072	11.755741	11.087667	11.146352	11.656178	11.769600	12.193531	12.157252	11.788526	12.659132	12.083384	11.892609	12.576723	12.560565	12.753969	12.667828	12.236952	11.567413	12.007616	11.958284	12.515666	12.054975	11.879689	12.130980	11.500069	12.734213	11.483264	12.748286	12.435794	11.821110	12.377075	11.560680

CBWD5	14.191138	14.016722	14.524706	14.324402	14.724698	14.320921	14.387470	14.028211	14.422880	14.199474	14.320921	14.268271	14.555161	14.201809	14.492360	14.231187	14.119258	14.165208	14.702498	14.401146	14.634805	14.304625	14.439683	14.295373	13.909712	14.347082	14.108830	13.401415	14.625115	14.132353	14.375530	14.648824	14.185552	14.531879	14.278587	13.831022	14.130469	14.666890	14.091572	14.243388	14.662329	14.137944	13.801126	14.258622	13.339537	13.978080	14.172876	13.940978	13.937428	14.003541	13.891833	13.862844	13.860465	14.030880	14.411699	14.615271	14.667724	13.979424	14.153935	14.003265	13.814789	14.408756	13.819912	13.897223	14.270244	14.903483	13.870710	14.311389	13.566385	13.830488	13.810378	14.275219	14.520691	13.971275	14.538753	14.793559	14.029080	13.778222	14.009539	13.751290	13.557270	13.640135	13.998982	14.754551	14.629113	14.140523	14.779447	14.481276	14.452852	14.203084	13.977501	14.267647	14.331365	14.024155	14.273856	14.424884	14.167781	14.633876	14.433465	13.402261	14.410595	14.250206	14.233539	14.191449	15.067485	14.528702	14.466224	14.600827	14.753586	14.291025	14.746020	14.150116	14.312048	14.549736	13.618534	14.129527	13.782074	14.742275	14.359066	14.330345	14.590224	14.255152	14.434912	14.521454	14.444149	14.598641	14.118926	14.239751	14.698400	14.280056	14.361581	14.463605	14.415465	13.443082	14.410971	14.578917	14.428245	14.727457	14.549326	14.260288	14.978163	14.744972	14.913079	14.553562	14.136402	14.043461	15.039342	14.601201	14.239751	15.001364	14.318185	14.559663	13.638076	14.177402	14.722386	14.012079	13.839011	14.592810	14.580994	14.226969	14.531879	13.674594	13.962183	14.314792	14.121322	14.559663	13.722244	14.816832	13.654792	13.862844	14.105657	14.317178	14.331005	14.030257	13.989252	13.660327	14.243065	13.790935	14.315445	14.321619	14.371236	14.743181	14.717187	14.009820	13.950442	14.539165	14.145840	14.569736	14.913617	14.722386	14.606262	14.348910	14.817850	14.453201	13.936042	14.636141	14.603726	14.202755	14.135776	14.042604	14.691055	14.325742	13.733398	14.830506	14.651534	14.465898	14.449082	14.356594	14.275219	14.265936	14.776105	14.571778	14.536742	13.985572	14.479473	14.749773	14.436030	14.290297	14.415465	15.009362	13.557508	14.158638	13.684034	14.204958	14.598235	14.749262	14.699677	14.876067	14.444487	14.639284	14.635670	14.697958	14.326793	15.258866	14.376679	14.502659	14.490355	14.451725

ZNF28	10.758970	9.819984	9.738519	10.170425	10.118063	9.986683	10.327627	9.611185	9.474760	9.749732	9.835522	9.828562	9.801346	9.591051	9.661702	10.058014	9.419599	10.037937	9.764038	9.961059	9.042373	9.738598	9.474009	9.789600	9.760731	10.039433	10.116680	9.782986	9.401070	9.906208	8.900672	10.313564	10.274402	10.214264	10.838984	10.870351	10.762352	10.695283	9.850095	10.077125	9.748169	10.056200	10.382728	10.668712	10.265297	10.359182	9.088836	10.406073	10.072223	10.800421	10.604302	10.563528	10.734015	10.790142	9.982592	9.335281	10.918390	10.780871	10.307130	10.630112	10.542394	10.234480	9.709848	9.992511	9.873647	10.001482	10.454947	10.205670	10.103422	10.046625	10.640618	10.751903	9.772051	10.456649	10.671967	10.530696	10.947735	10.215648	10.608759	9.557714	10.541250	10.061198	10.544926	10.911612	10.677631	10.430011	10.565322	10.605791	10.286370	10.190413	10.251612	10.010929	10.253644	10.663859	11.107920	11.076924	10.479922	9.787425	10.162156	10.053909	9.696173	10.243220	9.870914	9.687451	9.842888	9.278901	9.901303	9.646619	9.916997	9.393746	10.602822	9.438669	9.595374	10.046469	9.891957	11.108217	10.514372	9.663882	9.826628	10.802840	9.556635	10.262831	10.636091	10.042830	9.189785	10.152953	10.301733	8.831940	9.978130	9.759792	10.817887	8.785471	10.136250	9.823884	9.979955	9.523327	10.259775	9.869932	9.033482	9.021275	8.912646	9.656115	10.430485	10.084130	8.995967	10.273491	10.791439	9.710473	9.256976	9.791757	10.045877	9.743792	9.795351	11.391155	11.835718	11.190467	11.735541	9.669262	10.385470	8.524770	9.761195	10.686753	11.838426	10.219870	9.625998	9.534750	10.983634	9.804849	10.251612	10.561314	11.766034	10.749156	11.066449	10.762650	9.045190	11.061554	11.560680	10.497395	11.184951	11.118709	8.976221	11.365654	10.181623	9.159233	10.819395	10.264395	10.104055	10.118547	9.935902	10.864843	10.911465	9.073756	10.305204	10.369561	10.069492	10.766127	9.890283	10.341269	10.127898	10.968006	10.111382	11.362109	10.954367	11.744200	10.251318	9.635213	11.189473	10.383819	10.195389	10.589660	10.665460	11.195637	10.578752	11.296440	11.757241	10.430330	9.413934	10.685697	10.885099	10.270574	9.803650	9.890146	9.411123	10.979516	9.767677	9.943197	9.526012	9.105810	9.080099	9.692841	10.188159	11.360153	9.406024	10.510068	9.269539	10.705507	10.474667	9.935596

ZNF468	10.758970	9.819984	9.738519	10.170425	10.118063	9.986683	10.327627	9.611185	9.474760	9.749732	9.835522	9.828562	9.801346	9.591051	9.661702	10.058014	9.419599	10.037937	9.764038	9.961059	9.042373	9.738598	9.474009	9.789600	9.760731	10.039433	10.116680	9.782986	9.401070	9.906208	8.900672	10.313564	10.274402	10.214264	10.838984	10.870351	10.762352	10.695283	9.850095	10.077125	9.748169	10.056200	10.382728	10.668712	10.265297	10.359182	9.088836	10.406073	10.072223	10.800421	10.604302	10.563528	10.734015	10.790142	9.982592	9.335281	10.918390	10.780871	10.307130	10.630112	10.542394	10.234480	9.709848	9.992511	9.873647	10.001482	10.454947	10.205670	10.103422	10.046625	10.640618	10.751903	9.772051	10.456649	10.671967	10.530696	10.947735	10.215648	10.608759	9.557714	10.541250	10.061198	10.544926	10.911612	10.677631	10.430011	10.565322	10.605791	10.286370	10.190413	10.251612	10.010929	10.253644	10.663859	11.107920	11.076924	10.479922	9.787425	10.162156	10.053909	9.696173	10.243220	9.870914	9.687451	9.842888	9.278901	9.901303	9.646619	9.916997	9.393746	10.602822	9.438669	9.595374	10.046469	9.891957	11.108217	10.514372	9.663882	9.826628	10.802840	9.556635	10.262831	10.636091	10.042830	9.189785	10.152953	10.301733	8.831940	9.978130	9.759792	10.817887	8.785471	10.136250	9.823884	9.979955	9.523327	10.259775	9.869932	9.033482	9.021275	8.912646	9.656115	10.430485	10.084130	8.995967	10.273491	10.791439	9.710473	9.256976	9.791757	10.045877	9.743792	9.795351	11.391155	11.835718	11.190467	11.735541	9.669262	10.385470	8.524770	9.761195	10.686753	11.838426	10.219870	9.625998	9.534750	10.983634	9.804849	10.251612	10.561314	11.766034	10.749156	11.066449	10.762650	9.045190	11.061554	11.560680	10.497395	11.184951	11.118709	8.976221	11.365654	10.181623	9.159233	10.819395	10.264395	10.104055	10.118547	9.935902	10.864843	10.911465	9.073756	10.305204	10.369561	10.069492	10.766127	9.890283	10.341269	10.127898	10.968006	10.111382	11.362109	10.954367	11.744200	10.251318	9.635213	11.189473	10.383819	10.195389	10.589660	10.665460	11.195637	10.578752	11.296440	11.757241	10.430330	9.413934	10.685697	10.885099	10.270574	9.803650	9.890146	9.411123	10.979516	9.767677	9.943197	9.526012	9.105810	9.080099	9.692841	10.188159	11.360153	9.406024	10.510068	9.269539	10.705507	10.474667	9.935596

ZNF320	10.758970	9.819984	9.738519	10.170425	10.118063	9.986683	10.327627	9.611185	9.474760	9.749732	9.835522	9.828562	9.801346	9.591051	9.661702	10.058014	9.419599	10.037937	9.764038	9.961059	9.042373	9.738598	9.474009	9.789600	9.760731	10.039433	10.116680	9.782986	9.401070	9.906208	8.900672	10.313564	10.274402	10.214264	10.838984	10.870351	10.762352	10.695283	9.850095	10.077125	9.748169	10.056200	10.382728	10.668712	10.265297	10.359182	9.088836	10.406073	10.072223	10.800421	10.604302	10.563528	10.734015	10.790142	9.982592	9.335281	10.918390	10.780871	10.307130	10.630112	10.542394	10.234480	9.709848	9.992511	9.873647	10.001482	10.454947	10.205670	10.103422	10.046625	10.640618	10.751903	9.772051	10.456649	10.671967	10.530696	10.947735	10.215648	10.608759	9.557714	10.541250	10.061198	10.544926	10.911612	10.677631	10.430011	10.565322	10.605791	10.286370	10.190413	10.251612	10.010929	10.253644	10.663859	11.107920	11.076924	10.479922	9.787425	10.162156	10.053909	9.696173	10.243220	9.870914	9.687451	9.842888	9.278901	9.901303	9.646619	9.916997	9.393746	10.602822	9.438669	9.595374	10.046469	9.891957	11.108217	10.514372	9.663882	9.826628	10.802840	9.556635	10.262831	10.636091	10.042830	9.189785	10.152953	10.301733	8.831940	9.978130	9.759792	10.817887	8.785471	10.136250	9.823884	9.979955	9.523327	10.259775	9.869932	9.033482	9.021275	8.912646	9.656115	10.430485	10.084130	8.995967	10.273491	10.791439	9.710473	9.256976	9.791757	10.045877	9.743792	9.795351	11.391155	11.835718	11.190467	11.735541	9.669262	10.385470	8.524770	9.761195	10.686753	11.838426	10.219870	9.625998	9.534750	10.983634	9.804849	10.251612	10.561314	11.766034	10.749156	11.066449	10.762650	9.045190	11.061554	11.560680	10.497395	11.184951	11.118709	8.976221	11.365654	10.181623	9.159233	10.819395	10.264395	10.104055	10.118547	9.935902	10.864843	10.911465	9.073756	10.305204	10.369561	10.069492	10.766127	9.890283	10.341269	10.127898	10.968006	10.111382	11.362109	10.954367	11.744200	10.251318	9.635213	11.189473	10.383819	10.195389	10.589660	10.665460	11.195637	10.578752	11.296440	11.757241	10.430330	9.413934	10.685697	10.885099	10.270574	9.803650	9.890146	9.411123	10.979516	9.767677	9.943197	9.526012	9.105810	9.080099	9.692841	10.188159	11.360153	9.406024	10.510068	9.269539	10.705507	10.474667	9.935596

CCDC121	10.282778	8.767294	8.184797	9.015016	9.114050	8.710550	9.647662	7.718569	6.903040	9.103050	9.168451	8.736729	9.525550	8.834192	8.853327	9.158942	8.827010	9.024612	7.797700	9.179234	8.127063	7.633902	8.573379	8.218403	9.068809	9.074192	9.462868	8.674991	8.619728	8.733953	8.759006	9.841361	9.820277	9.404032	9.781184	9.797503	10.037644	8.533168	8.088467	8.111918	8.957952	8.737472	8.122765	9.670699	9.856355	9.511815	8.437691	7.797769	9.149548	9.805636	9.626936	9.872877	9.669890	10.205513	8.216356	7.855750	10.212131	9.832155	8.959765	9.601437	9.150168	8.719507	8.137100	7.564875	8.220099	7.884890	9.037384	8.802888	9.076223	8.051848	9.674605	9.849917	8.626389	9.726848	9.792860	9.284296	10.133459	9.055523	9.876877	9.621360	10.135620	9.295221	10.186500	10.215794	10.211375	9.741831	9.835366	9.967417	8.981350	8.696372	9.042970	8.643164	8.828074	9.813229	10.225283	9.810261	9.041181	9.143350	9.380364	9.178480	7.307127	9.300486	9.076571	8.257083	7.121194	8.937800	6.242269	7.030795	6.787592	8.675628	8.720555	6.580790	7.229212	8.387288	8.010187	6.024627	9.113447	8.777808	8.318960	9.253455	8.426610	8.239783	8.268065	7.426963	8.351704	8.752132	9.389757	9.821926	9.146716	8.338861	9.539381	9.106595	8.421514	7.174321	9.305718	9.162133	8.242495	8.203158	10.868295	7.180642	7.202536	7.304277	9.005980	9.059743	9.057845	8.399107	8.168776	7.486779	8.404071	7.920144	8.330882	9.354532	8.160220	8.898856	9.870914	8.975910	7.736081	7.890146	8.039807	8.701221	8.302895	7.994267	8.534543	8.591289	8.930003	7.296370	9.712939	9.631674	9.458185	8.966798	9.756057	9.543959	9.620259	9.382862	9.046541	10.696919	9.280598	9.234252	9.055523	9.377775	8.570802	7.695178	6.815893	7.935134	8.248548	8.426298	8.171919	9.157875	7.959136	9.426037	9.241070	6.709432	9.056876	10.124419	9.458628	9.010103	9.511023	9.237341	9.785048	9.532917	9.690691	8.859264	9.212818	9.246132	8.990766	9.221273	10.331914	8.800815	7.481815	9.209310	8.432586	8.885279	8.328907	8.243837	8.743748	9.977842	8.455746	8.428035	10.189508	8.883900	10.498864	9.318981	8.008376	7.741802	8.020288	6.695877	8.385428	7.070379	7.294130	7.383782	6.355752	10.173663	7.562869	9.882077	8.086143	8.882957	8.764223	8.189568

GRASP	12.182211	13.253515	13.001582	13.348898	13.113930	12.304346	12.483749	12.151881	13.179963	12.441586	12.773621	12.579663	11.874166	11.264402	12.860767	12.834365	13.020295	11.921437	12.972576	12.940276	12.744556	12.396485	13.669109	12.920413	13.148421	12.471647	12.327331	13.434924	13.270823	13.066984	12.965824	13.212415	13.126344	11.801784	12.350062	12.359894	12.491562	12.714355	12.052817	12.401128	12.481464	12.777583	11.465697	11.117676	12.862587	11.296731	12.976037	12.360207	13.333932	11.236568	11.964164	11.894274	13.431034	12.449765	12.717483	12.840723	12.849310	13.550486	12.878188	12.500081	13.069657	12.867443	14.300954	12.779681	13.044091	13.267608	12.598661	12.655512	12.352099	12.742860	12.742860	12.670862	12.544041	12.386142	12.587474	12.912866	12.716248	12.630661	12.626503	13.306393	12.807530	13.162423	12.517113	12.838265	13.020101	13.006688	12.753452	12.636992	13.424093	13.106812	13.151060	13.121958	12.491075	12.122850	12.152049	11.987718	13.588376	12.810174	12.198594	13.508116	12.574922	13.046847	12.978391	12.846490	12.392195	12.543410	12.451713	12.254686	13.959948	12.734879	13.489283	13.059142	12.528093	12.349904	13.924569	13.732895	12.263875	13.300042	12.882408	11.397159	10.375445	10.528668	11.445261	11.343705	10.733261	10.667829	11.875845	9.877916	10.713006	10.490413	12.733221	11.028666	11.127384	11.255007	11.035832	9.963663	11.193393	12.550549	10.861007	11.065101	11.944227	12.283319	11.793617	11.870252	11.167837	12.021503	11.100158	11.071058	10.794399	10.957831	11.676671	11.434046	10.429581	10.192949	10.228061	10.834667	10.340048	10.489033	11.544245	9.956160	11.714242	13.398875	9.985494	10.639071	10.370786	10.058619	12.037614	10.311711	10.388230	10.367907	10.625346	10.351285	11.102683	11.042091	11.104426	12.170325	11.732575	11.439262	13.411878	11.131496	12.260507	11.136698	10.668285	11.245235	11.563886	10.450933	11.158915	10.099349	11.996703	10.117312	10.703888	11.096474	9.961205	10.815810	11.385706	10.301273	10.473309	10.690865	11.765403	10.909438	10.594702	10.324127	11.454008	10.500010	10.601358	10.525491	10.398534	12.242991	11.727379	11.601504	11.508636	10.671356	10.966241	10.650579	10.953621	11.123360	11.357963	10.483072	9.810561	10.382558	12.325875	11.027041	11.810691	11.505243	11.931067	10.550397	11.275334	11.910357	10.211224	13.264960	11.809762	12.298858	11.974354	10.361904	9.386992	11.607717	10.205059	10.470285

LY6G6E	4.426792	4.388470	5.120920	5.234617	6.830092	4.621436	4.444999	6.859613	5.471380	4.702350	5.243216	4.664747	5.681788	4.847214	4.662653	4.541102	5.956256	4.718963	5.220735	4.316491	5.410515	5.209030	4.003685	5.855821	4.921917	5.484060	4.934289	5.344189	4.625919	5.025068	5.850948	4.392767	5.033150	4.308576	4.568093	4.700271	4.865575	5.096222	4.735238	4.300684	4.832509	3.475268	4.683097	4.291112	3.890045	5.642536	5.273109	3.509038	5.515838	4.751098	4.052167	4.160159	4.390632	4.262886	4.715187	5.131601	4.234172	3.983281	3.938111	4.166251	4.886667	5.087179	5.214626	5.509173	4.829094	4.558305	5.371258	4.943402	5.311070	5.646889	4.590365	4.387386	5.225509	4.626760	4.457789	4.450065	4.990182	4.649826	4.691187	4.161178	4.239223	4.624205	4.807440	4.805579	5.311617	4.057171	4.031073	4.519767	4.736547	4.352665	4.669278	5.652204	4.970294	5.115570	5.087383	4.943641	3.887402	5.508384	6.325946	5.091838	6.707390	4.096007	5.101965	4.815819	5.399987	5.258293	5.438573	4.598089	6.303916	4.615319	6.055991	6.591326	6.323058	4.708061	5.622975	8.940204	6.134299	4.360033	5.125769	4.380590	4.527150	5.836808	5.227278	8.218871	4.913262	4.734206	5.837676	5.518583	5.109134	4.871035	4.726407	5.212013	5.334014	6.419483	4.606700	4.655551	5.928289	5.566754	3.857006	5.339523	5.858872	5.709005	6.667699	5.520989	4.990800	5.022770	6.386373	5.301629	5.391837	3.974872	4.907752	5.541274	5.273296	3.933266	5.071831	4.361307	4.181237	4.117437	5.757389	5.233225	3.121240	4.670157	4.926830	4.276377	5.257705	5.538641	3.570038	4.525222	4.388677	4.832062	6.023426	4.152302	4.446690	5.450854	5.274756	3.818032	3.097843	5.233633	4.505009	4.041017	5.652022	5.184812	5.269846	4.962938	4.598089	5.388158	5.247821	5.603982	5.421300	4.746162	5.007036	6.046918	4.233299	4.170305	4.872775	4.354817	4.325941	4.774707	5.430663	5.421462	4.917266	5.315035	4.667960	3.644627	5.789735	3.466476	3.476529	3.645616	4.043227	6.039453	5.257333	4.251298	4.516143	5.614247	4.766147	5.492415	6.423013	5.496067	5.133823	5.668508	6.011218	5.043683	4.920613	4.983789	5.483876	5.519352	4.420222	5.121515	5.201863	6.384819	5.506607	7.850493	5.671418	7.134838	7.788507	5.351089	6.251304	4.818345

AC022414.1	6.128661	3.326783	3.048272	4.011026	3.779768	5.268480	3.250993	3.778757	3.824156	3.376866	3.546976	3.962484	4.273115	4.463518	3.805556	3.705259	3.556216	3.300671	5.443514	3.655248	4.883876	2.880520	7.767780	2.954431	2.882920	3.774315	3.255150	4.227886	3.876112	3.521143	3.651791	3.869665	5.506022	3.388249	3.276359	4.852235	3.652414	3.214463	3.416712	3.087392	3.426736	3.003120	4.255015	3.595732	3.773875	5.639734	3.857006	5.223352	3.792920	3.677001	3.614957	3.505520	4.254350	4.667563	3.973522	3.332238	5.171712	2.949776	4.574373	3.859612	2.843897	3.885739	3.910773	3.658843	3.520242	3.518376	3.527928	3.348056	3.242102	2.809656	3.546557	3.796132	3.190001	3.236086	2.831611	3.360006	4.444999	4.001248	3.983064	3.833118	5.364822	4.084748	4.672733	4.621238	4.588197	3.632948	6.521545	3.298691	3.623070	3.686042	3.503315	4.571394	3.943719	3.238311	4.750429	4.884308	4.252167	4.409815	3.990914	4.543088	6.369725	3.998522	3.829935	3.537427	4.626946	3.841099	3.476529	3.550612	5.869386	3.630489	6.185209	3.183839	4.837863	4.116148	3.613360	8.967105	5.555164	3.567669	3.233635	3.458581	3.479650	3.195847	4.124030	3.738084	4.448486	5.322870	4.350904	3.648809	3.121823	3.321058	5.631190	5.145152	6.014517	3.367694	5.515838	3.440139	4.894383	6.608681	3.395752	4.716023	5.514453	5.568071	4.242508	5.232404	3.265442	6.023770	3.933031	5.997089	3.568264	4.449608	4.829094	4.773477	3.607655	4.156948	5.129592	3.901062	3.229919	3.420470	3.526862	4.755730	2.903488	4.780099	3.407300	4.280614	5.162655	3.851412	5.733384	4.099972	3.435083	3.435630	3.930246	4.159287	4.471446	4.977248	4.603341	6.302189	2.940730	4.419800	3.696522	2.934625	3.872419	3.999384	3.747084	3.829515	3.761124	2.921460	3.689738	3.372078	2.787050	4.053659	4.005218	3.186339	3.598578	4.712212	3.570590	3.995950	3.141186	3.808898	5.686968	3.833571	4.482648	3.822877	6.139271	4.604611	4.529240	4.472756	5.874764	3.525165	3.537834	4.806580	3.270657	3.707684	3.289532	3.386728	4.519116	4.502632	5.276751	4.664961	3.477266	3.843957	3.682453	4.689517	5.436481	5.030546	3.714240	3.241947	3.627415	3.339291	3.296822	6.296916	4.738874	6.662960	3.256748	4.138014	4.304427	3.231727	3.640119	3.369613

AC008581.2	6.128661	3.326783	3.048272	4.011026	3.779768	5.268480	3.250993	3.778757	3.824156	3.376866	3.546976	3.962484	4.273115	4.463518	3.805556	3.705259	3.556216	3.300671	5.443514	3.655248	4.883876	2.880520	7.767780	2.954431	2.882920	3.774315	3.255150	4.227886	3.876112	3.521143	3.651791	3.869665	5.506022	3.388249	3.276359	4.852235	3.652414	3.214463	3.416712	3.087392	3.426736	3.003120	4.255015	3.595732	3.773875	5.639734	3.857006	5.223352	3.792920	3.677001	3.614957	3.505520	4.254350	4.667563	3.973522	3.332238	5.171712	2.949776	4.574373	3.859612	2.843897	3.885739	3.910773	3.658843	3.520242	3.518376	3.527928	3.348056	3.242102	2.809656	3.546557	3.796132	3.190001	3.236086	2.831611	3.360006	4.444999	4.001248	3.983064	3.833118	5.364822	4.084748	4.672733	4.621238	4.588197	3.632948	6.521545	3.298691	3.623070	3.686042	3.503315	4.571394	3.943719	3.238311	4.750429	4.884308	4.252167	4.409815	3.990914	4.543088	6.369725	3.998522	3.829935	3.537427	4.626946	3.841099	3.476529	3.550612	5.869386	3.630489	6.185209	3.183839	4.837863	4.116148	3.613360	8.967105	5.555164	3.567669	3.233635	3.458581	3.479650	3.195847	4.124030	3.738084	4.448486	5.322870	4.350904	3.648809	3.121823	3.321058	5.631190	5.145152	6.014517	3.367694	5.515838	3.440139	4.894383	6.608681	3.395752	4.716023	5.514453	5.568071	4.242508	5.232404	3.265442	6.023770	3.933031	5.997089	3.568264	4.449608	4.829094	4.773477	3.607655	4.156948	5.129592	3.901062	3.229919	3.420470	3.526862	4.755730	2.903488	4.780099	3.407300	4.280614	5.162655	3.851412	5.733384	4.099972	3.435083	3.435630	3.930246	4.159287	4.471446	4.977248	4.603341	6.302189	2.940730	4.419800	3.696522	2.934625	3.872419	3.999384	3.747084	3.829515	3.761124	2.921460	3.689738	3.372078	2.787050	4.053659	4.005218	3.186339	3.598578	4.712212	3.570590	3.995950	3.141186	3.808898	5.686968	3.833571	4.482648	3.822877	6.139271	4.604611	4.529240	4.472756	5.874764	3.525165	3.537834	4.806580	3.270657	3.707684	3.289532	3.386728	4.519116	4.502632	5.276751	4.664961	3.477266	3.843957	3.682453	4.689517	5.436481	5.030546	3.714240	3.241947	3.627415	3.339291	3.296822	6.296916	4.738874	6.662960	3.256748	4.138014	4.304427	3.231727	3.640119	3.369613

METTL2A	12.567904	12.184406	12.300959	12.097568	12.503202	12.232669	12.211265	12.242069	12.283820	12.237422	12.251983	12.023112	12.439982	12.109967	12.239471	12.140038	12.404804	12.374349	12.890856	12.495360	12.698021	12.414309	13.228076	12.385972	12.162058	12.306066	12.725852	11.716891	12.104382	11.921125	12.476699	12.629358	12.295409	12.216379	12.354041	12.617130	12.443196	12.520730	12.287532	12.409034	12.289811	12.124346	11.959150	12.126733	11.926448	12.174511	12.025867	12.067345	12.179702	12.179402	12.259577	12.167248	12.283820	12.497333	12.529519	12.144213	12.380518	12.251358	12.113151	12.300500	12.620010	12.042411	12.552337	12.303753	12.476025	12.427666	12.305259	12.263875	12.117864	12.397534	12.073126	12.309356	12.281610	12.241297	12.306362	12.066729	12.527775	12.133719	12.179561	12.512588	12.421501	12.095947	12.317768	12.567105	12.354682	12.230488	12.080437	12.056520	12.618797	12.317768	12.118961	12.436133	11.980478	12.281610	12.147801	12.361628	12.386142	12.321603	12.323253	12.175621	12.554118	11.881091	12.252315	11.970649	13.216776	12.619822	12.322814	12.699525	13.433474	12.288638	13.066209	12.620992	12.361312	12.258961	12.145607	11.426495	12.192751	12.539964	12.708224	13.190470	12.487990	12.383064	12.253272	12.510794	12.428003	11.996843	13.065843	13.375856	12.872852	12.361312	12.728453	12.899561	12.344394	12.708762	12.408519	12.303440	13.432615	13.589555	12.616157	12.896890	13.079675	13.542577	12.609195	12.455171	12.464858	12.333192	13.154208	12.919349	12.452481	12.922049	11.856087	12.244367	12.769250	12.672419	13.183086	12.408694	12.557817	12.756165	12.601913	13.459822	13.205135	12.789777	12.947549	12.402762	12.820105	13.149535	12.644874	12.864593	13.076654	12.781892	12.819221	12.618446	12.869960	12.399470	12.396312	12.742336	13.067539	12.840549	12.650709	12.275923	12.012048	13.281802	13.050632	12.511756	12.649008	12.237262	12.382100	12.820775	12.577039	12.876066	12.817160	12.417829	12.549408	12.495856	12.530672	12.872501	12.596810	12.842092	12.095640	12.520730	12.728615	12.375466	12.209897	12.823209	12.597651	12.580507	12.468921	12.760314	12.019164	12.927582	13.233400	12.076508	12.694586	12.204356	12.935431	12.775154	12.971153	12.345318	12.756697	12.915163	12.693740	12.665471	12.092606	12.658466	12.777583	13.083113	13.082154	13.314357	12.210677	13.268237	12.099966	12.805797	12.085897	13.054365	12.648668	12.917111	12.444034	11.833001

OR9Q1	7.628448	7.318693	7.166091	7.563026	7.467854	8.358960	7.047214	8.011995	8.224190	7.491489	7.573258	7.498039	7.165196	6.957510	6.940135	7.481976	7.737275	7.017480	8.170959	7.158083	7.994418	7.749822	8.671736	7.348804	7.327054	7.763738	7.715828	8.672782	7.718727	7.310260	7.667444	6.358684	6.150420	6.920814	6.578323	6.940592	6.737942	6.864533	7.359173	7.308348	7.491660	7.106453	7.013769	7.264337	6.975741	6.655000	7.863395	7.004570	6.934730	6.786300	7.029566	6.588698	6.431908	6.519214	6.560538	7.914144	6.162362	6.777177	6.803991	6.602156	6.767805	7.477201	7.425999	7.493195	7.096562	6.972755	7.152007	7.358559	6.968555	7.554895	7.064759	7.203644	7.575603	6.954882	7.017298	6.937460	7.100031	6.766516	7.166260	6.326106	6.364856	6.511783	6.242766	6.155871	6.335850	6.850776	6.609648	6.388847	6.704931	6.804613	6.539864	7.325643	6.735632	6.739567	6.745450	6.616765	6.218038	7.532530	7.673529	7.715071	8.201070	6.850460	7.184736	7.558311	7.606389	7.567498	8.285318	7.504760	8.472319	7.782421	7.988952	7.908800	7.224803	8.582771	7.427269	10.095497	7.885821	7.247752	7.625330	7.064609	7.604209	7.393516	7.861985	8.390368	7.915822	8.297612	7.612498	7.754421	7.739929	7.663101	7.687159	8.598194	7.934018	8.312674	7.435375	7.837050	7.417717	8.122765	6.892688	8.052634	7.848996	8.284075	7.916471	8.337585	7.388883	7.841244	8.136005	8.055096	7.677784	7.854059	7.073851	7.970847	7.559819	6.976239	6.782610	7.029236	7.993626	8.206633	7.529332	8.404071	7.148656	6.903827	8.096606	7.887956	7.429167	8.253272	7.324856	7.418516	7.248072	7.196156	6.882462	6.414695	6.951242	6.668647	7.613888	6.618065	6.340875	6.837524	6.271204	7.326749	7.706992	7.331331	7.164871	7.394443	7.171477	7.405226	7.103438	7.308960	7.579796	7.147061	7.183448	8.633073	7.136713	7.057287	6.790858	7.195198	7.689354	7.143465	6.482390	6.031651	6.540422	6.688232	6.533404	6.599421	7.899114	6.386980	5.930750	7.112523	6.780704	6.834845	7.451363	6.901432	7.063346	6.850135	7.252149	7.756334	7.800830	8.885455	7.050856	7.756499	7.094649	7.523749	7.164719	6.886446	7.508297	8.117928	7.103438	7.479497	8.668823	8.304669	7.931121	10.388515	8.665154	7.951067	8.228265	7.570069	7.343187	7.998563

OR9I1	7.628448	7.318693	7.166091	7.563026	7.467854	8.358960	7.047214	8.011995	8.224190	7.491489	7.573258	7.498039	7.165196	6.957510	6.940135	7.481976	7.737275	7.017480	8.170959	7.158083	7.994418	7.749822	8.671736	7.348804	7.327054	7.763738	7.715828	8.672782	7.718727	7.310260	7.667444	6.358684	6.150420	6.920814	6.578323	6.940592	6.737942	6.864533	7.359173	7.308348	7.491660	7.106453	7.013769	7.264337	6.975741	6.655000	7.863395	7.004570	6.934730	6.786300	7.029566	6.588698	6.431908	6.519214	6.560538	7.914144	6.162362	6.777177	6.803991	6.602156	6.767805	7.477201	7.425999	7.493195	7.096562	6.972755	7.152007	7.358559	6.968555	7.554895	7.064759	7.203644	7.575603	6.954882	7.017298	6.937460	7.100031	6.766516	7.166260	6.326106	6.364856	6.511783	6.242766	6.155871	6.335850	6.850776	6.609648	6.388847	6.704931	6.804613	6.539864	7.325643	6.735632	6.739567	6.745450	6.616765	6.218038	7.532530	7.673529	7.715071	8.201070	6.850460	7.184736	7.558311	7.606389	7.567498	8.285318	7.504760	8.472319	7.782421	7.988952	7.908800	7.224803	8.582771	7.427269	10.095497	7.885821	7.247752	7.625330	7.064609	7.604209	7.393516	7.861985	8.390368	7.915822	8.297612	7.612498	7.754421	7.739929	7.663101	7.687159	8.598194	7.934018	8.312674	7.435375	7.837050	7.417717	8.122765	6.892688	8.052634	7.848996	8.284075	7.916471	8.337585	7.388883	7.841244	8.136005	8.055096	7.677784	7.854059	7.073851	7.970847	7.559819	6.976239	6.782610	7.029236	7.993626	8.206633	7.529332	8.404071	7.148656	6.903827	8.096606	7.887956	7.429167	8.253272	7.324856	7.418516	7.248072	7.196156	6.882462	6.414695	6.951242	6.668647	7.613888	6.618065	6.340875	6.837524	6.271204	7.326749	7.706992	7.331331	7.164871	7.394443	7.171477	7.405226	7.103438	7.308960	7.579796	7.147061	7.183448	8.633073	7.136713	7.057287	6.790858	7.195198	7.689354	7.143465	6.482390	6.031651	6.540422	6.688232	6.533404	6.599421	7.899114	6.386980	5.930750	7.112523	6.780704	6.834845	7.451363	6.901432	7.063346	6.850135	7.252149	7.756334	7.800830	8.885455	7.050856	7.756499	7.094649	7.523749	7.164719	6.886446	7.508297	8.117928	7.103438	7.479497	8.668823	8.304669	7.931121	10.388515	8.665154	7.951067	8.228265	7.570069	7.343187	7.998563

PPIAL4D	13.791984	14.400780	14.687871	14.828455	15.472304	15.056095	15.017982	15.738421	14.458184	14.437860	14.990649	15.144888	15.146873	15.284471	15.097561	14.935700	14.666375	15.029900	15.039969	14.436403	14.715882	14.402866	12.407456	14.913617	13.095970	14.639284	14.034368	14.225717	15.102359	14.496348	15.159483	15.321092	15.025188	14.206497	15.000739	15.050118	14.983442	14.835529	14.780447	14.754065	14.892277	14.376307	14.644889	14.072391	13.609930	14.190534	14.777592	14.649251	15.501368	14.125236	14.378847	14.332395	14.105943	14.086526	14.330841	14.838073	14.549736	14.219932	14.142712	14.464405	13.581820	14.976992	13.671534	14.256071	14.618223	14.344223	14.085269	14.833576	14.031176	14.310316	14.546394	14.879151	15.329439	14.671445	15.049528	14.082208	14.675489	13.555459	13.794844	14.446004	14.366636	13.612082	13.822206	14.854894	14.521823	14.507632	14.494741	14.940393	14.478214	15.147454	14.443066	13.537270	14.493555	13.806501	14.262005	14.803294	14.632630	15.545439	15.168793	14.196229	14.681437	14.986168	15.408414	14.838073	13.618283	15.473095	14.845730	14.098570	14.943708	15.262346	13.900462	14.910864	15.136479	15.448809	14.360460	13.841137	13.660563	15.021020	14.219249	15.528080	15.243980	14.828938	15.424214	15.050698	15.632923	15.456675	14.735321	15.676954	15.349378	15.531572	15.267207	15.460009	15.090679	15.163378	15.398455	15.622512	15.312332	14.928661	15.230037	14.707989	14.359688	14.356954	15.034636	15.669151	15.122219	15.278078	15.434895	15.352487	15.448092	15.058545	14.974820	15.310201	15.477212	15.036921	15.448809	14.802357	15.259618	15.365568	15.370133	15.662431	15.156920	14.366636	15.325061	14.625115	14.615271	15.547851	14.639680	14.832004	14.726053	15.102359	14.807128	14.596200	15.221141	15.195519	15.258866	15.069269	14.594501	14.814746	14.767548	15.043521	15.543645	15.049528	15.161343	15.028163	14.953863	15.294538	14.814746	15.003643	14.665858	15.126455	15.293162	15.159483	15.424214	14.996793	14.920608	14.992829	15.375547	15.197875	14.246613	14.611896	14.821349	14.903483	14.770475	15.552451	15.370867	15.183376	14.767089	14.915189	14.663179	14.925433	15.007673	14.925975	15.253922	15.028728	14.799897	15.101738	14.617785	15.396904	15.379850	15.769072	14.774671	14.920608	14.724263	14.512793	14.648445	15.211019	15.248690	14.699677	15.303728	13.811931	14.962238	15.187361	15.339763	15.365568	15.291679	15.105520	15.476405	15.746888

PPIAL4G	13.791984	14.400780	14.687871	14.828455	15.472304	15.056095	15.017982	15.738421	14.458184	14.437860	14.990649	15.144888	15.146873	15.284471	15.097561	14.935700	14.666375	15.029900	15.039969	14.436403	14.715882	14.402866	12.407456	14.913617	13.095970	14.639284	14.034368	14.225717	15.102359	14.496348	15.159483	15.321092	15.025188	14.206497	15.000739	15.050118	14.983442	14.835529	14.780447	14.754065	14.892277	14.376307	14.644889	14.072391	13.609930	14.190534	14.777592	14.649251	15.501368	14.125236	14.378847	14.332395	14.105943	14.086526	14.330841	14.838073	14.549736	14.219932	14.142712	14.464405	13.581820	14.976992	13.671534	14.256071	14.618223	14.344223	14.085269	14.833576	14.031176	14.310316	14.546394	14.879151	15.329439	14.671445	15.049528	14.082208	14.675489	13.555459	13.794844	14.446004	14.366636	13.612082	13.822206	14.854894	14.521823	14.507632	14.494741	14.940393	14.478214	15.147454	14.443066	13.537270	14.493555	13.806501	14.262005	14.803294	14.632630	15.545439	15.168793	14.196229	14.681437	14.986168	15.408414	14.838073	13.618283	15.473095	14.845730	14.098570	14.943708	15.262346	13.900462	14.910864	15.136479	15.448809	14.360460	13.841137	13.660563	15.021020	14.219249	15.528080	15.243980	14.828938	15.424214	15.050698	15.632923	15.456675	14.735321	15.676954	15.349378	15.531572	15.267207	15.460009	15.090679	15.163378	15.398455	15.622512	15.312332	14.928661	15.230037	14.707989	14.359688	14.356954	15.034636	15.669151	15.122219	15.278078	15.434895	15.352487	15.448092	15.058545	14.974820	15.310201	15.477212	15.036921	15.448809	14.802357	15.259618	15.365568	15.370133	15.662431	15.156920	14.366636	15.325061	14.625115	14.615271	15.547851	14.639680	14.832004	14.726053	15.102359	14.807128	14.596200	15.221141	15.195519	15.258866	15.069269	14.594501	14.814746	14.767548	15.043521	15.543645	15.049528	15.161343	15.028163	14.953863	15.294538	14.814746	15.003643	14.665858	15.126455	15.293162	15.159483	15.424214	14.996793	14.920608	14.992829	15.375547	15.197875	14.246613	14.611896	14.821349	14.903483	14.770475	15.552451	15.370867	15.183376	14.767089	14.915189	14.663179	14.925433	15.007673	14.925975	15.253922	15.028728	14.799897	15.101738	14.617785	15.396904	15.379850	15.769072	14.774671	14.920608	14.724263	14.512793	14.648445	15.211019	15.248690	14.699677	15.303728	13.811931	14.962238	15.187361	15.339763	15.365568	15.291679	15.105520	15.476405	15.746888

PPIAL4H	13.791984	14.400780	14.687871	14.828455	15.472304	15.056095	15.017982	15.738421	14.458184	14.437860	14.990649	15.144888	15.146873	15.284471	15.097561	14.935700	14.666375	15.029900	15.039969	14.436403	14.715882	14.402866	12.407456	14.913617	13.095970	14.639284	14.034368	14.225717	15.102359	14.496348	15.159483	15.321092	15.025188	14.206497	15.000739	15.050118	14.983442	14.835529	14.780447	14.754065	14.892277	14.376307	14.644889	14.072391	13.609930	14.190534	14.777592	14.649251	15.501368	14.125236	14.378847	14.332395	14.105943	14.086526	14.330841	14.838073	14.549736	14.219932	14.142712	14.464405	13.581820	14.976992	13.671534	14.256071	14.618223	14.344223	14.085269	14.833576	14.031176	14.310316	14.546394	14.879151	15.329439	14.671445	15.049528	14.082208	14.675489	13.555459	13.794844	14.446004	14.366636	13.612082	13.822206	14.854894	14.521823	14.507632	14.494741	14.940393	14.478214	15.147454	14.443066	13.537270	14.493555	13.806501	14.262005	14.803294	14.632630	15.545439	15.168793	14.196229	14.681437	14.986168	15.408414	14.838073	13.618283	15.473095	14.845730	14.098570	14.943708	15.262346	13.900462	14.910864	15.136479	15.448809	14.360460	13.841137	13.660563	15.021020	14.219249	15.528080	15.243980	14.828938	15.424214	15.050698	15.632923	15.456675	14.735321	15.676954	15.349378	15.531572	15.267207	15.460009	15.090679	15.163378	15.398455	15.622512	15.312332	14.928661	15.230037	14.707989	14.359688	14.356954	15.034636	15.669151	15.122219	15.278078	15.434895	15.352487	15.448092	15.058545	14.974820	15.310201	15.477212	15.036921	15.448809	14.802357	15.259618	15.365568	15.370133	15.662431	15.156920	14.366636	15.325061	14.625115	14.615271	15.547851	14.639680	14.832004	14.726053	15.102359	14.807128	14.596200	15.221141	15.195519	15.258866	15.069269	14.594501	14.814746	14.767548	15.043521	15.543645	15.049528	15.161343	15.028163	14.953863	15.294538	14.814746	15.003643	14.665858	15.126455	15.293162	15.159483	15.424214	14.996793	14.920608	14.992829	15.375547	15.197875	14.246613	14.611896	14.821349	14.903483	14.770475	15.552451	15.370867	15.183376	14.767089	14.915189	14.663179	14.925433	15.007673	14.925975	15.253922	15.028728	14.799897	15.101738	14.617785	15.396904	15.379850	15.769072	14.774671	14.920608	14.724263	14.512793	14.648445	15.211019	15.248690	14.699677	15.303728	13.811931	14.962238	15.187361	15.339763	15.365568	15.291679	15.105520	15.476405	15.746888

PPIAL4F	13.791984	14.400780	14.687871	14.828455	15.472304	15.056095	15.017982	15.738421	14.458184	14.437860	14.990649	15.144888	15.146873	15.284471	15.097561	14.935700	14.666375	15.029900	15.039969	14.436403	14.715882	14.402866	12.407456	14.913617	13.095970	14.639284	14.034368	14.225717	15.102359	14.496348	15.159483	15.321092	15.025188	14.206497	15.000739	15.050118	14.983442	14.835529	14.780447	14.754065	14.892277	14.376307	14.644889	14.072391	13.609930	14.190534	14.777592	14.649251	15.501368	14.125236	14.378847	14.332395	14.105943	14.086526	14.330841	14.838073	14.549736	14.219932	14.142712	14.464405	13.581820	14.976992	13.671534	14.256071	14.618223	14.344223	14.085269	14.833576	14.031176	14.310316	14.546394	14.879151	15.329439	14.671445	15.049528	14.082208	14.675489	13.555459	13.794844	14.446004	14.366636	13.612082	13.822206	14.854894	14.521823	14.507632	14.494741	14.940393	14.478214	15.147454	14.443066	13.537270	14.493555	13.806501	14.262005	14.803294	14.632630	15.545439	15.168793	14.196229	14.681437	14.986168	15.408414	14.838073	13.618283	15.473095	14.845730	14.098570	14.943708	15.262346	13.900462	14.910864	15.136479	15.448809	14.360460	13.841137	13.660563	15.021020	14.219249	15.528080	15.243980	14.828938	15.424214	15.050698	15.632923	15.456675	14.735321	15.676954	15.349378	15.531572	15.267207	15.460009	15.090679	15.163378	15.398455	15.622512	15.312332	14.928661	15.230037	14.707989	14.359688	14.356954	15.034636	15.669151	15.122219	15.278078	15.434895	15.352487	15.448092	15.058545	14.974820	15.310201	15.477212	15.036921	15.448809	14.802357	15.259618	15.365568	15.370133	15.662431	15.156920	14.366636	15.325061	14.625115	14.615271	15.547851	14.639680	14.832004	14.726053	15.102359	14.807128	14.596200	15.221141	15.195519	15.258866	15.069269	14.594501	14.814746	14.767548	15.043521	15.543645	15.049528	15.161343	15.028163	14.953863	15.294538	14.814746	15.003643	14.665858	15.126455	15.293162	15.159483	15.424214	14.996793	14.920608	14.992829	15.375547	15.197875	14.246613	14.611896	14.821349	14.903483	14.770475	15.552451	15.370867	15.183376	14.767089	14.915189	14.663179	14.925433	15.007673	14.925975	15.253922	15.028728	14.799897	15.101738	14.617785	15.396904	15.379850	15.769072	14.774671	14.920608	14.724263	14.512793	14.648445	15.211019	15.248690	14.699677	15.303728	13.811931	14.962238	15.187361	15.339763	15.365568	15.291679	15.105520	15.476405	15.746888

PPIAL4C	13.791984	14.400780	14.687871	14.828455	15.472304	15.056095	15.017982	15.738421	14.458184	14.437860	14.990649	15.144888	15.146873	15.284471	15.097561	14.935700	14.666375	15.029900	15.039969	14.436403	14.715882	14.402866	12.407456	14.913617	13.095970	14.639284	14.034368	14.225717	15.102359	14.496348	15.159483	15.321092	15.025188	14.206497	15.000739	15.050118	14.983442	14.835529	14.780447	14.754065	14.892277	14.376307	14.644889	14.072391	13.609930	14.190534	14.777592	14.649251	15.501368	14.125236	14.378847	14.332395	14.105943	14.086526	14.330841	14.838073	14.549736	14.219932	14.142712	14.464405	13.581820	14.976992	13.671534	14.256071	14.618223	14.344223	14.085269	14.833576	14.031176	14.310316	14.546394	14.879151	15.329439	14.671445	15.049528	14.082208	14.675489	13.555459	13.794844	14.446004	14.366636	13.612082	13.822206	14.854894	14.521823	14.507632	14.494741	14.940393	14.478214	15.147454	14.443066	13.537270	14.493555	13.806501	14.262005	14.803294	14.632630	15.545439	15.168793	14.196229	14.681437	14.986168	15.408414	14.838073	13.618283	15.473095	14.845730	14.098570	14.943708	15.262346	13.900462	14.910864	15.136479	15.448809	14.360460	13.841137	13.660563	15.021020	14.219249	15.528080	15.243980	14.828938	15.424214	15.050698	15.632923	15.456675	14.735321	15.676954	15.349378	15.531572	15.267207	15.460009	15.090679	15.163378	15.398455	15.622512	15.312332	14.928661	15.230037	14.707989	14.359688	14.356954	15.034636	15.669151	15.122219	15.278078	15.434895	15.352487	15.448092	15.058545	14.974820	15.310201	15.477212	15.036921	15.448809	14.802357	15.259618	15.365568	15.370133	15.662431	15.156920	14.366636	15.325061	14.625115	14.615271	15.547851	14.639680	14.832004	14.726053	15.102359	14.807128	14.596200	15.221141	15.195519	15.258866	15.069269	14.594501	14.814746	14.767548	15.043521	15.543645	15.049528	15.161343	15.028163	14.953863	15.294538	14.814746	15.003643	14.665858	15.126455	15.293162	15.159483	15.424214	14.996793	14.920608	14.992829	15.375547	15.197875	14.246613	14.611896	14.821349	14.903483	14.770475	15.552451	15.370867	15.183376	14.767089	14.915189	14.663179	14.925433	15.007673	14.925975	15.253922	15.028728	14.799897	15.101738	14.617785	15.396904	15.379850	15.769072	14.774671	14.920608	14.724263	14.512793	14.648445	15.211019	15.248690	14.699677	15.303728	13.811931	14.962238	15.187361	15.339763	15.365568	15.291679	15.105520	15.476405	15.746888

CCDC171	7.819040	6.928135	7.053815	7.169031	7.413210	8.037881	7.315588	8.274926	8.475899	7.735934	7.430625	7.935898	7.639694	7.358559	6.853335	7.995502	8.031179	7.319795	8.150964	7.310728	7.383159	7.852952	8.919357	7.485352	7.668497	8.143161	8.213459	8.133019	8.014777	7.599377	8.467193	6.384973	7.355178	7.783772	6.951856	6.933776	6.860916	6.860415	7.426641	7.661085	7.798320	7.361534	7.188249	7.412132	7.300310	7.197133	8.252957	7.771578	7.025384	7.198218	7.096114	6.635160	6.219047	6.683564	6.260824	7.927727	6.318752	7.075119	7.162049	6.825914	7.073851	7.792954	7.198379	7.576848	7.073236	7.304121	7.057440	6.946970	7.075905	7.802054	7.267734	7.434558	7.601841	7.025077	7.337505	7.383627	7.271730	6.993546	7.250407	6.980675	6.726582	6.864533	6.989679	6.989357	7.292246	6.762817	6.931597	7.111770	7.315270	7.301775	7.099873	7.681614	7.190616	7.086292	7.464195	7.585017	6.937142	7.684279	7.738668	7.377685	7.732870	7.358858	7.373269	7.079256	7.202384	7.506342	8.026826	7.516962	8.188791	7.719036	8.148380	7.568409	7.761592	8.299864	7.941332	9.278313	7.632690	7.307732	7.906643	7.915822	7.546612	7.329623	8.032084	8.522011	8.534684	8.119024	8.190228	8.433816	8.169238	8.010042	8.337585	8.435693	8.027797	7.974469	8.212820	8.019077	8.478049	8.545378	7.593360	8.366159	8.203755	8.718419	8.297298	8.028718	7.865882	8.088765	8.605743	8.515759	7.939788	9.178776	7.352520	8.065802	7.909740	7.374716	7.350831	7.283976	7.542477	8.353690	8.081629	8.913583	8.020574	7.536569	8.015866	7.481057	7.614639	8.909516	8.074558	7.514634	7.868330	7.832927	7.533448	6.904609	7.199002	7.143957	7.591030	6.930985	7.493966	7.778530	7.164871	7.784553	7.792804	7.863716	7.784873	7.951508	7.518657	7.751360	7.607143	7.818250	8.377069	7.667305	7.812489	9.017912	7.406485	7.519556	7.623954	7.693526	7.581018	7.659241	7.342076	7.242424	7.577460	7.832314	7.569464	7.663101	7.792182	7.850793	7.666067	7.798822	7.739929	7.829763	8.377215	7.660474	7.981300	8.000110	8.232389	7.663101	8.322153	7.917531	7.455081	8.273893	7.810948	8.128410	7.617231	7.658461	8.235110	8.302279	8.059157	8.207229	8.634112	8.270907	8.236375	8.254672	9.450439	8.260812	8.068759	7.875346	7.892168	8.282999

KIF21A	6.422080	6.246267	6.124959	6.929852	7.592865	7.137034	7.081292	6.949978	6.481749	7.902571	7.285979	6.683564	7.812790	7.099087	7.367347	7.199772	7.146904	7.633622	8.810751	7.465866	5.504117	7.288783	6.490462	7.512793	7.685481	7.147857	4.737391	7.785744	7.058706	5.290178	6.988738	4.625039	5.426670	5.613483	5.287363	5.336960	5.917082	6.448351	5.262672	5.809994	7.909583	5.268480	5.155213	5.748485	5.813341	4.606057	6.252157	4.434754	5.191120	5.716039	5.888681	5.473286	5.555822	6.202073	6.011588	7.352823	6.767147	5.976039	6.484786	4.970105	5.548432	7.020771	4.781346	6.686295	3.923084	4.967792	5.412057	6.365895	5.314218	4.672062	5.303814	5.910088	7.464349	5.232404	6.248802	4.383385	4.724322	4.692443	5.232618	6.132918	6.437280	6.367718	6.110060	6.682588	6.625166	6.682427	6.738272	6.156030	4.093091	5.682541	5.096594	4.115070	5.558298	5.731934	4.911116	6.760608	6.210682	7.946027	6.103756	7.949691	7.371725	7.428206	7.481214	6.635321	6.778314	6.774469	5.697884	6.473421	7.395714	8.098702	6.963876	6.998767	6.178333	8.489838	7.826623	4.551957	5.339915	6.742581	7.891070	6.147950	6.130691	4.460477	4.765468	7.513898	5.869213	6.240272	3.821455	6.248802	8.089401	6.940135	8.279787	4.963547	4.712842	4.236984	6.047988	5.072013	7.978000	5.392395	6.250316	6.694243	5.776908	4.990394	8.712362	5.532394	5.114185	4.732732	6.701120	6.874168	5.408333	3.984132	6.657110	6.108169	6.276382	4.176392	7.407121	4.224051	4.683553	4.066688	4.287426	6.104605	5.516654	5.384096	5.116018	6.062933	4.209499	6.750842	4.988939	6.832787	5.163669	4.261329	4.958737	6.013848	4.568743	5.235636	6.494926	4.746858	6.050288	6.024627	5.029742	5.393986	4.398284	5.428638	5.604154	4.034530	4.267396	5.218154	4.782618	4.893762	3.250421	6.102054	5.358153	6.225720	4.594262	4.037357	4.516358	4.444552	5.032560	5.129796	5.410318	6.820544	5.063876	5.341422	7.001942	4.922988	6.589328	5.185584	5.616676	4.583926	5.471598	5.217771	4.525882	4.905873	5.667961	5.349856	5.399206	4.840860	6.952479	6.220089	6.728831	4.739990	5.238860	6.334681	5.499091	5.173553	6.079720	6.485292	5.694139	6.991779	6.655338	6.145843	4.376736	4.127852	7.093223	6.584561	6.270170	4.679217	6.198966	6.653213

HINFP	11.275035	10.788398	10.502860	10.751617	10.789717	10.704762	10.874713	11.172088	10.894605	10.730598	10.608143	10.578284	10.330253	10.828144	10.980998	10.584473	10.890438	11.026307	11.743759	10.629643	11.185555	11.100018	11.952588	10.776235	10.953044	11.083497	10.400645	11.180812	10.373076	10.519232	10.757205	10.535147	10.939475	10.161998	10.701207	10.808753	10.705811	10.773135	11.204194	11.454892	10.557223	11.269090	10.540179	10.890293	10.596035	10.795715	10.447031	11.023071	11.215086	10.946533	10.973234	11.022131	10.979225	11.218335	10.872782	10.516841	10.977161	11.643282	11.432696	11.398667	11.765088	10.539729	11.739819	11.653482	11.441033	11.363130	11.328919	11.274151	10.998939	11.403818	10.710821	10.788244	10.456157	10.544160	10.622929	10.842557	10.818184	10.750537	11.106019	11.110014	10.998487	11.013251	10.813415	11.299815	10.830613	10.991194	10.885258	11.265593	11.546807	11.494786	11.246511	11.816305	11.114868	11.026892	10.788987	10.936234	10.885258	9.558784	10.084677	10.620579	10.123198	10.684205	10.308324	10.902781	11.366409	10.280952	10.879746	11.689126	11.558020	10.412086	11.776075	11.537619	11.427104	10.660719	10.734171	10.521111	11.533627	10.552938	10.097624	10.355971	10.650732	10.580549	10.398683	10.451937	10.925219	10.133459	10.544617	10.298493	10.147018	10.102338	10.328381	10.195828	10.256478	10.640300	10.202012	10.764309	10.096406	11.308236	10.761296	11.932304	11.455375	11.701300	11.082761	11.632620	10.750219	10.732241	11.094712	10.070706	10.829161	10.159237	9.986107	10.543736	10.593479	11.091462	11.386298	11.183767	11.081157	11.132135	11.783886	10.270875	11.582863	11.044735	11.320390	10.950866	11.002085	10.147018	11.445261	11.379010	11.117536	11.335682	10.954993	11.561285	11.069585	10.779258	10.231158	10.990030	12.202831	11.319554	11.356089	11.511703	10.900269	12.230324	11.835718	11.261380	11.699769	11.223249	11.058929	11.256480	11.932006	10.595584	10.597545	10.154056	10.430794	10.327332	10.820438	11.281507	10.632306	11.034586	10.765692	10.643569	11.197390	11.036123	10.732656	11.127669	11.444544	10.589350	10.976265	11.580987	11.213047	11.188596	12.071265	10.779119	11.027485	10.965807	10.444422	10.609923	9.382113	9.854297	10.726876	10.268358	11.332335	10.330253	10.732945	11.196939	10.027037	11.019905	11.942720	11.398352	10.414333	11.673261	11.171084	11.943486	10.479789	11.259741	10.352828	11.332335	11.017510	10.526109

KCNB1	7.732870	8.549523	8.628077	8.411096	8.529222	7.429167	9.738519	3.964673	3.322974	8.978787	8.282391	8.355695	8.965236	9.161081	8.156736	9.723772	7.886579	8.883590	7.262516	8.145954	7.835074	8.848337	10.288341	9.151110	10.594267	9.380364	10.332961	10.676730	9.542052	8.363315	8.936240	8.775061	8.560535	8.816517	9.302504	9.251342	9.681392	9.695415	9.874226	8.819333	8.721334	10.178735	9.049437	8.326196	9.636846	8.763320	9.775960	9.048228	7.655132	8.947521	8.219933	9.447171	9.204130	9.290980	8.770526	9.039311	10.001164	9.742277	9.135773	9.882077	10.138352	9.116189	11.087821	9.597565	8.772771	9.963816	9.671780	9.257116	8.303042	8.672782	9.374964	8.980147	9.138600	9.523811	9.267511	8.726175	9.537362	7.918437	8.394685	9.750211	9.153845	8.944146	9.366612	9.545320	10.259615	9.513223	9.248281	8.959458	9.855275	8.966352	10.069314	8.616784	8.917548	9.292527	8.384965	9.641622	8.499816	9.299855	9.028257	10.026726	8.577882	9.634626	8.377380	8.929239	6.597946	8.331700	9.170158	7.514207	8.151277	9.934635	7.254021	8.653270	7.761283	7.916120	6.020461	4.084550	7.053815	9.861442	9.599406	5.702406	6.215426	6.388007	7.032329	5.714931	4.195624	7.025699	4.040813	3.305388	5.397471	5.085506	6.652734	8.580730	6.007890	3.961596	9.252371	3.895449	5.108708	5.061210	7.203331	4.213858	4.340039	8.540524	5.913674	7.071171	5.693962	6.742265	6.974149	6.523859	5.783971	8.056042	6.246624	3.289377	6.436771	8.462198	6.365531	6.981481	6.428535	7.010741	6.062618	5.347748	7.312596	6.516905	5.101129	6.826564	5.951435	6.555657	7.617095	5.842965	6.510260	5.502582	9.385126	6.494756	7.322331	7.902878	8.562395	7.978000	6.837856	6.427331	7.255711	7.280042	9.946870	7.543733	6.651258	6.455588	8.051259	6.864858	6.935199	5.215220	5.206733	7.181248	5.600779	6.497857	6.708319	6.760278	6.923712	6.568705	6.201557	5.492415	9.779667	5.989717	6.479775	7.045786	6.269984	7.873058	6.478471	6.102897	7.122273	8.489838	8.945966	7.500701	5.841340	6.612494	6.409866	6.017083	6.767147	4.034749	5.337135	5.414924	6.863457	5.404268	6.980510	6.611503	6.358513	5.112773	6.701120	4.457789	4.884308	4.813729	6.763286	5.090658	4.130225	2.863017	5.909893	3.639909	3.336976	7.715237	6.893689	6.496679

KIAA0355	4.791226	5.132613	5.235235	5.183016	5.289796	5.262672	4.842988	5.895831	5.582483	5.146547	5.457696	5.044730	5.475224	4.965410	4.869995	5.331246	5.294069	5.248033	5.888505	5.147729	6.600558	5.805626	7.068891	5.531616	5.362147	4.776982	5.026830	5.419147	5.271843	5.149291	5.645625	4.991195	5.356854	5.449553	5.403667	4.440677	4.528825	4.967125	5.065719	4.922338	5.178363	5.240856	5.303814	4.718747	6.251142	5.317463	5.212774	5.503367	6.025527	6.804922	5.020938	4.986230	5.268266	5.113352	5.544882	4.749312	5.865783	4.694734	5.451858	5.422062	5.418743	5.690061	4.999198	5.212390	4.727660	5.593282	4.886667	5.134665	5.021782	5.046863	5.142569	5.595889	5.277375	5.177954	5.306921	5.276568	5.428231	5.267480	5.540532	4.733571	5.303625	4.740420	5.226103	5.215041	5.700463	5.437272	4.637149	5.624269	5.053435	5.050781	5.244418	5.580957	4.626946	5.575751	5.110560	5.465983	4.570033	4.848086	5.713823	5.793485	5.644266	5.484426	5.677707	5.468537	5.397646	5.706289	5.873510	5.391837	5.106656	5.567281	4.796678	6.148301	5.201486	5.751933	6.246787	6.524506	5.924523	4.880056	5.245814	4.643607	5.545078	5.594202	5.410127	5.941779	5.129796	5.741001	5.683615	5.335333	4.759964	5.843146	5.137208	5.426670	4.825211	4.485731	5.017104	5.619849	5.334938	6.409176	4.948949	6.055823	5.766833	6.554786	5.721773	5.804583	4.872577	5.235030	5.851124	5.467944	5.136441	5.344783	5.033150	5.207435	5.678637	5.548243	4.770546	4.202921	4.888354	5.391260	5.104831	5.821135	5.526878	4.924079	5.423847	5.871137	5.707221	5.657036	4.038891	5.210432	6.666542	4.327452	4.853109	4.851376	4.619929	4.063721	4.936406	5.766090	5.624075	5.151351	3.598390	5.136803	4.710518	5.964297	5.530117	4.925505	5.190523	5.343606	5.255133	5.586609	5.773122	5.236428	5.083258	5.786478	4.987082	5.061015	5.128988	5.329881	5.409725	5.310272	4.428056	5.250184	4.776361	5.440816	4.398066	5.556004	5.283372	4.852882	4.999641	5.140077	5.173168	4.865377	5.427255	4.906899	5.463646	4.626548	5.193075	4.970294	5.557747	5.871887	5.485387	5.480817	5.095809	4.721510	5.034193	5.430849	5.132404	5.640502	5.579093	5.456085	5.610492	5.285197	5.238461	5.093551	5.693593	5.320335	5.354082	5.598592	5.177346	4.681788

PAH	10.671356	11.134756	11.156631	11.671195	12.195864	12.283028	10.970588	13.072631	12.899183	11.747338	11.860081	11.664174	11.452540	11.123670	11.007562	12.395385	12.643737	10.907071	11.898092	12.437743	11.431952	12.183622	11.222825	11.297790	11.092378	12.222322	12.362103	12.238197	12.396622	11.914759	12.696982	10.329013	10.764768	10.814609	10.638627	11.050179	10.793806	11.018827	11.458612	11.385844	12.278505	10.982623	12.201238	12.259577	12.033077	12.364491	12.768169	12.556834	11.649323	14.069818	11.895762	11.769304	10.441249	11.143986	10.584342	12.418166	10.461290	10.982910	10.947866	10.407984	10.793216	12.053308	10.824286	11.641628	11.057484	11.546955	11.668318	11.574486	11.501190	12.034862	12.508996	12.795726	12.723828	11.950952	12.293882	11.171964	11.413703	11.155520	11.399700	10.497960	10.194616	10.230524	9.857107	10.710268	10.708724	10.975965	10.795127	11.027915	10.743352	11.318521	10.550997	11.208356	10.833585	11.352461	11.277499	10.773135	11.082337	11.779417	12.544739	12.316696	12.419600	11.376521	11.687212	11.581259	11.859051	12.069172	11.954526	11.598146	11.826393	12.650060	11.553440	12.010184	11.789113	12.924903	12.420264	13.673412	11.423450	11.676370	12.573686	10.740649	10.878998	10.759120	11.125198	13.067904	11.590536	11.811423	12.305101	11.778520	11.595768	11.498458	12.048760	11.832712	11.025247	11.655296	11.685883	11.114258	11.668177	11.386878	10.112915	10.825459	11.531011	10.290960	11.941813	10.890583	10.620713	11.908802	12.030200	12.535101	11.142070	12.398510	11.049601	11.009628	10.962648	10.351422	10.413303	10.460708	10.574665	10.873089	10.516242	12.454859	10.354019	10.827984	11.003940	10.677202	11.831962	12.665646	11.259428	10.156062	10.971775	10.620713	10.652063	9.925324	10.175781	9.885624	11.255319	9.542676	10.054496	10.436000	9.798436	10.121706	11.733026	10.350375	10.340199	10.113185	10.154668	10.466810	10.685697	10.652361	10.118700	10.728981	10.707338	13.105616	10.514059	10.459392	10.313861	10.440967	10.298823	10.817596	9.731236	9.466897	9.816047	10.043738	10.121857	9.610435	11.392179	9.592125	10.189508	10.178735	9.949662	10.887492	10.435682	10.115138	10.650447	10.758534	11.307552	11.330888	12.475223	11.465248	10.427019	11.957101	10.949348	11.889299	10.847688	10.725359	11.855196	11.298091	10.334168	10.405317	12.305577	10.650157	11.641628	12.847695	13.326618	12.389516	11.982747	10.392215	11.497421	11.612573

CASTOR2	9.067847	9.596795	10.796422	10.317267	9.931856	10.015078	10.675830	10.898787	10.497536	11.230200	10.901875	10.728828	10.907937	11.005994	9.251483	10.890293	9.823307	10.335092	10.745154	11.352031	10.652663	11.865849	11.082183	11.267197	10.882081	10.618351	9.914676	11.523953	9.893623	10.127268	10.052342	10.052635	10.688294	10.715070	10.793060	11.273685	11.490236	10.887345	10.984790	10.823244	10.002270	11.496506	10.725359	9.897656	11.196939	10.585651	10.629794	10.230046	11.502750	10.551284	10.225742	10.971775	10.672285	10.515470	10.711830	10.010785	11.163491	11.095867	11.231863	10.372426	11.585665	10.815069	10.876634	10.749026	10.414186	10.327943	11.609910	9.810408	10.397475	11.208956	10.527040	10.872332	10.902327	11.194445	11.366119	10.794845	11.082486	10.790437	10.163983	11.116371	9.888466	10.337046	10.535606	10.739298	11.310712	10.445286	12.455171	11.797787	11.124424	10.710117	10.852247	11.188160	10.326880	11.328919	10.147018	10.413149	10.345672	10.807365	10.519634	11.395344	10.531581	10.446580	10.551284	12.657642	11.258585	9.789131	10.463392	11.149291	9.915761	10.715826	11.356537	9.993695	10.398534	10.548745	10.754642	10.780002	9.765260	10.211375	10.703174	9.691309	9.381126	10.643878	10.230046	10.390339	9.287241	10.458023	11.624998	10.066161	11.208500	10.231920	10.418738	12.214583	9.340123	10.200381	10.808104	9.712195	9.931241	10.316237	10.745574	10.312288	10.830474	10.847104	10.350528	10.975234	10.121548	9.661074	11.022278	10.253789	9.772502	9.304406	10.505428	10.952431	10.963392	10.731781	11.151275	11.148990	10.115619	10.064990	10.944165	9.302664	11.738510	9.537522	10.583331	11.574486	10.591713	9.925599	10.806353	12.166633	11.103415	10.928007	10.549791	10.728085	10.757376	10.700452	9.477056	10.841568	11.463470	10.498577	10.770016	11.095424	10.573931	10.428979	10.226206	9.929076	10.389876	10.827827	10.742902	10.748889	11.287902	11.234915	10.970868	10.018168	11.259009	10.895178	10.693780	10.920184	11.460104	10.822371	10.958253	10.462035	10.945805	10.968317	10.551435	10.920323	11.084833	11.557743	11.431202	11.484876	11.016776	11.218772	11.644748	10.157125	11.324079	10.035004	10.265297	10.908532	10.873702	9.727754	11.583181	10.113467	10.137918	10.266592	11.827966	11.154511	10.037185	9.817564	10.458959	11.018364	9.951078	11.775503	9.053453	9.647368	9.675852	11.137872	10.437199	10.678664	10.995378	10.245587

C11orf96	15.423337	15.746888	15.129069	13.376280	12.649538	11.755427	13.624759	11.954825	11.882216	13.021558	12.047981	13.382177	12.642737	12.715232	14.884408	12.517113	14.697497	13.707975	12.344564	11.414134	12.684200	12.788897	15.076369	12.256640	13.853818	13.359821	14.558412	13.462463	14.224877	15.020432	12.626671	14.138248	13.295547	13.749075	13.749826	13.530118	13.360596	13.702665	13.652718	13.262596	13.534637	14.574718	14.690655	13.588617	16.128614	14.295679	13.301249	15.973135	16.114305	14.580994	14.231187	14.853259	14.794550	13.707975	15.204490	14.553109	14.902407	14.111066	13.163671	15.014487	13.320668	15.025759	16.387390	13.576482	14.668633	14.593195	12.993460	13.475325	12.627335	12.650060	14.193049	12.730828	13.624065	13.982948	12.416578	13.792498	12.832557	15.011645	13.386257	15.487850	13.784079	16.452541	13.593974	13.883013	13.281421	13.398018	12.795726	14.004466	13.844544	12.998772	13.015899	13.470952	14.422133	12.860392	14.302275	13.080043	15.472304	13.473987	13.121958	14.776105	13.791211	13.817328	14.524706	14.882312	13.453926	14.114654	14.590670	13.873120	14.413643	12.980619	13.310550	13.019739	12.486494	12.018432	13.238812	13.773212	15.158834	14.592389	13.756377	13.540385	13.641300	13.399764	13.004306	13.672956	11.855112	12.558148	13.809161	12.226768	12.090589	12.234346	12.659650	14.342484	13.068499	13.532157	12.969695	13.140875	12.718193	12.388737	10.822512	12.232212	13.234805	13.810879	12.242693	12.591334	14.840176	13.125419	11.850817	13.113930	13.365784	13.171908	14.681870	13.213003	12.928492	11.774910	12.157572	13.557270	13.113147	12.714534	14.312732	12.680252	14.454664	12.939558	13.143772	14.749262	13.296386	11.989104	15.242555	12.624160	14.187463	13.161096	13.594881	13.330907	14.350910	15.102359	13.171328	13.210738	14.350242	13.296386	13.106614	14.198488	14.956144	13.308350	12.110693	12.724823	14.390000	13.065843	13.198725	12.868696	13.164133	13.066209	13.992417	12.152235	13.054711	13.850714	13.820478	13.029714	13.709428	13.445662	13.512526	12.706541	13.216008	12.088879	14.185552	13.069482	12.500257	12.166337	13.464641	12.598484	14.836045	13.328825	12.789412	12.647261	14.622906	13.005406	13.839552	14.036135	13.132270	12.134507	12.902873	13.724675	13.678771	13.851451	14.726053	12.226923	13.824511	13.135455	11.609343	12.371963	12.829461	13.538127	14.392936	12.150502	11.092206	12.927582	11.960063	14.037568	11.911024	12.516134
[truncated: 42,051,511 more chars]
