# Supplementary material for: Immune-Related Long Non-coding RNA Signature and Clinical Nomogram to Evaluate Survival of Patients Suffering Esophageal Squamous Cell Carcinoma
Source: Front Cell Dev Biol. 2021 Mar 4;9:641960. doi: 10.3389/fcell.2021.641960 (PMC7969885; doi:10.3389/fcell.2021.641960)
Supplement: Supplementary Table 6 — mRNAs obtained by GSE53622 annotation. [file Table_6.docx]

id	GSM1297043	GSM1297041	GSM1297039	GSM1297037	GSM1297075	GSM1297073	GSM1297071	GSM1297069	GSM1297067	GSM1297065	GSM1297063	GSM1297061	GSM1297059	GSM1297057	GSM1297055	GSM1297053	GSM1297051	GSM1297049	GSM1297047	GSM1297045	GSM1297035	GSM1297033	GSM1297031	GSM1297029	GSM1297027	GSM1297025	GSM1297023	GSM1297021	GSM1297019	GSM1297017	GSM1297015	GSM1297013	GSM1297011	GSM1297009	GSM1297007	GSM1297005	GSM1297003	GSM1297001	GSM1296999	GSM1296997	GSM1296971	GSM1296969	GSM1296967	GSM1296965	GSM1296995	GSM1296993	GSM1296991	GSM1296989	GSM1296987	GSM1296985	GSM1296983	GSM1296981	GSM1296979	GSM1296977	GSM1296975	GSM1296973	GSM1296963	GSM1296961	GSM1296959	GSM1296957	GSM1297042	GSM1297040	GSM1297038	GSM1297036	GSM1297074	GSM1297072	GSM1297070	GSM1297068	GSM1297066	GSM1297064	GSM1297062	GSM1297060	GSM1297058	GSM1297056	GSM1297054	GSM1297052	GSM1297050	GSM1297048	GSM1297046	GSM1297044	GSM1297034	GSM1297032	GSM1297030	GSM1297028	GSM1297026	GSM1297024	GSM1297022	GSM1297020	GSM1297018	GSM1297016	GSM1297014	GSM1297012	GSM1297010	GSM1297008	GSM1297006	GSM1297004	GSM1297002	GSM1297000	GSM1296998	GSM1296996	GSM1296970	GSM1296968	GSM1296966	GSM1296964	GSM1296994	GSM1296992	GSM1296990	GSM1296988	GSM1296986	GSM1296984	GSM1296982	GSM1296980	GSM1296978	GSM1296976	GSM1296974	GSM1296972	GSM1296962	GSM1296960	GSM1296958	GSM1296956

CTNNA2	4.85146489166667	4.36901661666667	4.4718244	4.67674131666667	4.075702625	4.24013239166667	4.86289075	4.33133893333333	4.4159867	4.28110195833333	4.07775664166667	4.82904071666667	4.2231712	3.80218998333333	4.24335745833333	3.71657505833333	4.64847966666667	4.11685985	4.50430228333333	4.19346535	4.61520515	4.10621731666667	4.55568075	4.49216794166667	4.51475793333333	4.25290215833333	4.34074395833333	4.56487130833333	4.51179561666667	4.46597973333333	4.65793555	4.32946600833333	4.18714809166667	4.38005901666667	4.310185775	4.40382505833333	4.262047775	4.87503585	4.41297161666667	4.19234266666667	4.545583025	4.47861730833333	4.56487130833333	4.77601478333333	4.26652515833333	4.4619761	4.97490869166667	4.428719425	4.52055483333333	4.356950075	4.37710720833333	4.33043379166667	4.1095023	4.85037616666667	4.339553	4.47770515	4.282041725	4.26758835	4.63625878333333	4.328489775	3.83152513333333	4.30437938333333	3.86284045833333	4.27160165833333	4.02921226666667	3.80218998333333	3.99488165833333	4.28110195833333	3.80781481666667	4.1095023	3.96836553333333	4.19234266666667	4.39972780833333	4.16407313333333	4.1095023	3.905753025	3.75016011666667	3.87194265	4.1106042	3.75838870833333	4.72414796666667	3.93897055	4.2443259	4.6319392	3.84151389166667	4.08851591666667	4.11281023333333	4.421470575	4.15954296666667	4.17476265833333	3.8136599	4.3987315	4.09698049166667	3.92925663333333	3.95464144166667	3.8987918	4.040897575	4.15642159166667	3.838223825	4.567125	4.05319390833333	3.81605024166667	4.77272485	4.75143253333333	4.08433028333333	4.15745399166667	4.203896025	5.24289476666667	4.16299614166667	5.14089556666667	4.39039918333333	4.90871175	4.13438028333333	4.705709175	4.24718985833333	4.18081978333333	4.138474425	4.418789625	4.37489024166667	4.043026825

SNTG1	5.170393	5.41887256666667	4.98341465833333	6.27955291666667	5.90677250833333	6.2517663	7.02653026666667	5.81212459166667	5.89108584166667	5.69296955833333	5.84791786666667	6.38554745833333	5.55559675	5.65732808333333	6.138425225	4.71218959166667	5.88853345	6.74958845833333	6.107924175	5.62957723333333	5.27417344166667	6.181735025	4.61421155	5.95923255	5.35088976666667	4.71113178333333	5.3100359	5.17603111666667	5.59754975833333	5.546831425	5.68207400833333	6.2517663	5.50615049166667	6.32751184166667	5.45901654166667	5.52437941666667	6.2517663	5.53292085833333	5.760490375	5.13437999166667	5.32238468333333	5.58597898333333	5.69872159166667	5.63755270833333	4.97597943333333	4.25573735	3.95350946666667	5.8292169	5.24388391666667	5.367183075	4.96517085	5.65633813333333	4.86699436666667	6.50860958333333	5.9751687	5.33153680833333	5.58795861666667	5.83306814166667	5.43935509166667	5.32238468333333	6.17154675833333	6.31548078333333	6.06574364166667	5.79999791666667	6.47141914166667	6.44355459166667	5.303590975	6.59599640833333	6.160301025	6.560129475	6.56145719166667	5.8532158	6.5898034	6.12118446666667	6.40489274166667	5.2460388	5.922335025	5.89630836666667	5.79838183333333	5.948036925	6.05897065833333	5.94277896666667	5.90846126666667	6.141642375	5.49736965	5.98304395	6.601356975	6.41739583333333	6.42188529166667	5.52645741666667	6.10074335833333	5.43818919166667	5.92603411666667	5.76144219166667	5.979616925	5.64021978333333	5.20354711666667	6.35274666666667	5.75948789166667	5.52545381666667	5.69080398333333	5.97595255	5.94705471666667	5.47416700833333	5.591740875	6.23229269166667	5.83501515	4.64033936666667	5.00117389166667	4.60699911666667	5.482101875	5.53586285833333	7.50344081666667	4.32588960833333	5.82366751666667	5.81652036666667	5.78817238333333	5.68880476666667	5.61840579166667	5.6458775

CCDC7	10.1910863	9.056864675	9.43324608333333	9.67682161666667	9.3041126	8.8778449	9.91842590833333	9.22160494166667	9.106662475	9.60710205	9.19676581666667	10.3983831166667	10.2206735166667	9.298425025	9.70114725833333	9.388540625	9.48206465833333	9.557193025	8.83115135	8.85215985	9.24172186666667	8.39161398333333	9.22470684166667	9.311763425	9.41484764166667	9.75367038333333	9.22652003333333	9.16735169166667	8.46961650833333	9.08180965	8.83738886666667	9.22606134166667	9.19627585833333	9.32711606666667	9.48470895833333	9.68602584166667	9.037729975	9.08687805	8.9241622	9.18261260833333	8.72874058333333	9.58797373333333	8.641468925	8.57301913333333	9.62890875	8.86421215	9.0720726	9.1094042	8.081342075	9.47068484166667	9.028831875	9.22470684166667	9.330358125	9.462094325	9.94748681666667	10.1617358916667	9.35863609166667	9.04133171666667	8.92461889166667	9.27125073333333	9.62453731666667	9.38141521666667	9.50452185	9.34798774166667	9.41247076666667	9.11317438333333	8.78836945	9.62318166666667	8.29250424166667	9.73395598333333	9.00735385	8.92749955	9.899420875	9.24884609166667	9.31629211666667	9.3017146	8.70592035	9.40032650833333	8.71089086666667	8.50037185	8.709872025	10.1704848833333	8.75927773333333	8.57995885	9.01246160833333	8.619247325	9.524079925	9.76472428333333	8.92890170833333	8.96748466666667	8.64390555	9.01847910833333	8.39013935	8.4414262	8.64290230833333	8.81599143333333	9.06745036666667	8.97216300833333	8.98146720833333	9.10099604166667	8.647921775	9.22160494166667	8.413378975	8.525325	7.48985504166667	8.80139795	8.41202154166667	8.42476003333333	8.24270349166667	8.59302528333333	8.97266575	8.6667612	8.60593184166667	8.135196525	9.49953110833333	9.07434654166667	9.0540245	8.440899325	7.851546575	9.41715076666667

DNAJB1	14.2132241916667	14.4891443666667	13.96485035	14.0720390916667	14.0885897916667	13.1952399083333	14.236432725	14.4125419416667	14.1191611333333	13.93081475	14.0899292916667	12.8246255416667	14.5415836083333	14.5322324833333	14.26427225	13.7434058583333	13.1026328916667	15.048735725	14.37416785	14.2842943416667	14.371029675	14.7293250666667	14.9620915916667	14.427724725	13.7771113166667	14.0926224666667	14.003645225	14.2378149833333	13.9829878583333	14.5607495666667	14.1113310916667	14.082470775	14.2731900083333	12.1455145583333	14.2983229333333	12.3244889333333	14.105898725	14.4060458333333	13.9737942083333	14.2233219083333	14.847260375	14.4758644166667	14.3431664333333	14.1441992083333	13.46583445	14.120388025	13.9931917666667	14.265784475	14.0899292916667	14.5140672583333	14.99199845	14.2073641166667	14.4262315333333	14.0074276833333	14.0391426	13.57126835	14.6087993833333	14.12159925	14.0938637416667	13.7660959916667	13.645262425	13.4557593833333	13.7902025333333	13.6041330875	12.2396159	13.6812289916667	13.57301355	14.4025408	13.1946165416667	12.1807321916667	13.3193049583333	13.363866775	13.7940225416667	13.128643425	13.4298474333333	13.8405812166667	12.5372066583333	13.4586485833333	13.570433175	13.3256142916667	12.1741251583333	11.9566737416667	13.450467225	13.5180510583333	14.2610479166667	12.4015640666667	12.5696588166667	13.2460254166667	12.8802020166667	12.894282175	14.7572900333333	13.6858454833333	13.6356329916667	13.6866916666667	13.2587665833333	13.8199392	12.5690965416667	12.4114353583333	14.9779140333333	13.0620198083333	12.7564076083333	13.4943591	12.909394625	13.2128605083333	13.0536119916667	12.8543756916667	13.3530829583333	13.672832575	12.498597975	12.840994225	13.3675920333333	13.8614602166667	13.2799375583333	12.9139148666667	12.7738537166667	13.576244975	11.9455284	14.2460408916667	13.4649543916667	13.17270095

SHANK1	11.4601636916667	10.9436906083333	10.9021357916667	11.2395267333333	11.856139525	11.0142581583333	12.370296175	11.4108017166667	11.5323406833333	11.5034674333333	11.55164095	11.3154295916667	11.5111448666667	11.2327259583333	10.7812240416667	10.63254535	10.671542925	11.2495617333333	10.3721682083333	10.4819116333333	10.4822871416667	11.2233924	10.5322469083333	10.4453172666667	10.6797192416667	10.6757288666667	11.9263114333333	10.9182300166667	11.230835825	11.3320946416667	11.0999976083333	10.9353811	11.4387367	11.363901525	11.040528175	10.5639796666667	11.8265346166667	11.510754075	11.37567555	11.30822695	10.995163975	11.43469695	11.0454889833333	10.5512664916667	10.5983973083333	10.337717825	10.891242375	10.7885218833333	10.1899294916667	10.7325441416667	11.2997236583333	10.9157145083333	11.0146426833333	10.6558683583333	11.0604394416667	10.146512775	10.91013625	11.1288009583333	10.9153273416667	10.457410175	11.0938590416667	11.2801435833333	10.4413653083333	10.7740240666667	11.0808123083333	11.2157921833333	11.03980565	11.5961336833333	11.0182236333333	11.343036975	11.426992675	11.2018586833333	11.29542035	10.7782435583333	10.8850875833333	10.478660175	11.2251399	10.9440376833333	10.3945367666667	10.9061592666667	10.5307562833333	11.1238545166667	10.7767059333333	10.5794976583333	11.3115193	11.5534468416667	11.1790792916667	11.0826274416667	11.0175089583333	10.4958848833333	10.946906725	10.5512664916667	11.1046868	11.208942925	11.624532275	11.2406150083333	11.0507418833333	11.5287863916667	11.3934696166667	11.13577505	10.8239850833333	11.141230475	10.9665691416667	10.1770845916667	10.3641444833333	10.6511688416667	10.5659717333333	10.2478255083333	10.3825544833333	10.3864170083333	10.5509023833333	11.918121775	10.3388359916667	9.82078890833333	10.5056824166667	10.2001534166667	10.5444389083333	10.5868572833333	10.6293746333333	10.5983973083333

OBSL1	11.8074362166667	10.5775565833333	11.0870314666667	10.6352329833333	11.339247	11.3004748583333	10.878870175	10.8165123833333	10.852937175	10.9486958083333	10.9750833833333	11.6355627166667	11.179383775	10.436061525	10.599920275	10.80942375	11.460857775	11.4748997416667	10.960420475	10.5841235833333	10.6058164916667	10.6496091416667	10.6191395	10.8491534833333	11.143398475	11.5052707166667	10.6860936833333	10.9314304333333	11.1376399	10.8751259833333	11.1916460666667	10.5719557166667	11.08918345	11.392737725	11.8337195083333	11.8612030916667	11.2128973166667	10.8771107	11.0451598916667	11.1536039583333	10.6332993416667	10.683111325	10.3833420583333	10.511583325	10.878870175	10.7904047166667	10.3124336666667	10.36596635	10.4815083083333	11.005303025	10.9415949916667	10.7147398083333	10.9553221083333	10.7254659583333	10.5368715833333	10.6503573333333	10.6038899083333	10.7839123	11.19451335	10.409675875	12.1558313416667	11.1724975333333	11.0772433166667	11.7296484916667	12.3195462583333	11.9494775416667	11.4934089166667	11.4635169166667	11.34033385	11.410462825	11.5056318416667	11.9146982166667	11.3664862333333	11.75300075	11.0034620833333	10.93064125	11.3627772083333	11.3886248333333	10.90030275	10.991320675	12.1998168583333	12.196460925	11.2965765083333	11.2222793166667	11.93371625	11.74563025	11.9754521583333	11.5501880833333	11.9240142833333	11.1930897583333	11.42922935	11.297940475	11.2506923333333	11.2933100833333	11.7292748	10.99445015	11.1963735833333	12.04786795	11.311125675	11.4730116416667	11.9742969	10.9497712833333	11.0783715833333	10.4280419583333	10.217869775	11.2202347166667	11.1166050833333	11.2194717166667	11.0430757083333	11.1313582333333	11.3635795583333	10.7919885666667	11.3886248333333	11.8032520333333	10.6600165833333	11.2118401416667	12.3896401833333	11.1797537583333	10.9476724083333	10.5601410916667

FER	12.5048403	11.284173425	11.208942925	11.2527308083333	11.7703479083333	11.4971747583333	11.3242829	11.60398845	11.5578653583333	11.6297974583333	11.032984525	12.4447647416667	12.1652756583333	11.6694643083333	11.75788825	11.5618505	10.7485438416667	11.5926397083333	11.6077110916667	11.5618505	11.632505775	11.83053445	11.21761425	11.1993790583333	11.4559913916667	12.1807321916667	11.339247	11.5548527833333	11.8904865166667	11.291502775	11.3805222416667	11.3774736833333	11.21620365	10.7261291583333	11.9123960833333	12.501560375	11.8791305416667	11.5816748666667	10.9497712833333	11.75788825	11.4567547583333	11.51432045	11.671269575	11.5185086916667	11.7978122	11.5991025	11.6594960166667	11.824518825	11.0401699916667	11.1689128333333	10.9182300166667	11.5531172416667	11.8709570583333	12.1451222666667	11.75300075	11.8228949916667	11.74563025	11.6047856083333	11.8819037833333	11.7582463416667	10.9123611916667	11.0870314666667	9.86359230833333	10.5504732083333	11.4745197333333	11.9455284	10.782424925	11.39042995	11.46235385	11.0729886333333	10.4429923666667	11.14521145	12.4627799166667	11.30507995	11.2911160333333	11.18844885	11.4864514	10.7535022833333	11.5505559916667	10.7912120583333	11.9384515083333	12.0785704333333	11.4439013	10.9160737916667	11.658465575	11.444940575	11.2406150083333	10.8243180916667	10.1906714166667	11.2085666916667	11.1731632666667	10.8281377416667	11.1506157166667	11.13908545	12.064434275	9.84409466666667	10.934250825	11.4549596	11.4496634833333	11.0200205166667	11.5706660333333	10.4184334333333	11.6694643083333	10.86697045	11.66214955	10.8996373166667	11.73798855	11.3900978416667	12.1455145583333	11.9505991416667	10.5260512	11.1941109166667	11.9793031666667	11.11993505	11.6274751916667	11.1790792916667	11.5684552166667	10.724371075	11.1339689083333	11.6092244333333

HOXB6	8.60151801666667	5.9370347875	6.92467640833333	7.25390786666667	7.17233015	8.47715187083333	7.71651254583333	7.50651534583333	7.21779185	7.05869684583333	7.4303946125	10.2945564166667	6.57773980416667	6.15565492083333	6.2350072625	6.3295455875	9.68370060833333	6.19856041666667	7.34469012916667	6.74050210416667	6.242381975	7.2189387	6.51563115416667	7.5247492375	7.3613213125	10.4474545166667	6.56398677916667	6.67339843333333	6.3774526625	7.04410127916667	6.559749775	6.60102279583333	6.7412051375	9.18809195416667	11.8914215583333	6.98063093333333	6.82134760416667	7.360785575	6.54332606666667	6.47828504583333	8.0578331625	6.90155310833333	7.83543632083333	7.54373619583333	9.71635354583333	7.94409976666667	7.403491625	7.34640325416667	7.70339010416667	7.8246461125	7.26195752916667	6.49722507916667	7.41678227083333	6.54544535	7.1794299375	7.328870675	7.37388778333333	7.3613213125	7.50197858333333	8.01462670833333	8.0071378875	6.89165517083333	6.3738232375	7.54886728333333	7.53425402916667	7.227496475	8.68799105833333	7.8261324	7.65861262083333	7.85987569583333	7.76319464166667	7.670548075	8.31489272083333	8.32084152916667	7.63473664583333	7.29446565	8.22373200416667	6.70633356666667	7.48037515833333	6.43760715833333	5.95612899166667	5.83450005	7.62787378333333	8.2498534875	7.22445772083333	7.4765078625	6.79164088333333	6.85252214583333	6.97187277916667	7.51281844166667	8.0204521625	7.2509352	7.05869684583333	8.15589492083333	7.69293838333333	6.895626475	6.72342694583333	6.76413455833333	7.49017916666667	8.1240653875	8.59669108333333	7.23163065416667	6.22965119583333	7.6373746625	7.92361595833333	7.04235322916667	8.88239256666667	6.87817127083333	7.56407301666667	7.31461072083333	7.2172312625	6.72216503333333	8.58721974583333	8.84266431666667	7.68125437083333	6.59498996666667	9.09260140416667	6.84879797083333	7.04410127916667	7.79832082083333

HOXB3	8.60151801666667	5.9370347875	6.92467640833333	7.25390786666667	7.17233015	8.47715187083333	7.71651254583333	7.50651534583333	7.21779185	7.05869684583333	7.4303946125	10.2945564166667	6.57773980416667	6.15565492083333	6.2350072625	6.3295455875	9.68370060833333	6.19856041666667	7.34469012916667	6.74050210416667	6.242381975	7.2189387	6.51563115416667	7.5247492375	7.3613213125	10.4474545166667	6.56398677916667	6.67339843333333	6.3774526625	7.04410127916667	6.559749775	6.60102279583333	6.7412051375	9.18809195416667	11.8914215583333	6.98063093333333	6.82134760416667	7.360785575	6.54332606666667	6.47828504583333	8.0578331625	6.90155310833333	7.83543632083333	7.54373619583333	9.71635354583333	7.94409976666667	7.403491625	7.34640325416667	7.70339010416667	7.8246461125	7.26195752916667	6.49722507916667	7.41678227083333	6.54544535	7.1794299375	7.328870675	7.37388778333333	7.3613213125	7.50197858333333	8.01462670833333	8.0071378875	6.89165517083333	6.3738232375	7.54886728333333	7.53425402916667	7.227496475	8.68799105833333	7.8261324	7.65861262083333	7.85987569583333	7.76319464166667	7.670548075	8.31489272083333	8.32084152916667	7.63473664583333	7.29446565	8.22373200416667	6.70633356666667	7.48037515833333	6.43760715833333	5.95612899166667	5.83450005	7.62787378333333	8.2498534875	7.22445772083333	7.4765078625	6.79164088333333	6.85252214583333	6.97187277916667	7.51281844166667	8.0204521625	7.2509352	7.05869684583333	8.15589492083333	7.69293838333333	6.895626475	6.72342694583333	6.76413455833333	7.49017916666667	8.1240653875	8.59669108333333	7.23163065416667	6.22965119583333	7.6373746625	7.92361595833333	7.04235322916667	8.88239256666667	6.87817127083333	7.56407301666667	7.31461072083333	7.2172312625	6.72216503333333	8.58721974583333	8.84266431666667	7.68125437083333	6.59498996666667	9.09260140416667	6.84879797083333	7.04410127916667	7.79832082083333

SULT1C4	9.136523775	8.31466204166667	8.85087683333333	7.800043275	8.058553875	9.06551128333333	8.80586325	8.39869986666667	8.03117830833333	9.277313475	7.43179573333333	10.1713085416667	8.37303865833333	8.377106525	7.060215325	9.354988125	9.01297525833333	6.95221913333333	8.64093518333333	9.1530551	9.60791420833333	6.74410944166667	8.779206475	8.58856818333333	9.42072421666667	9.97501473333333	8.42171803333333	8.63681648333333	7.27773341666667	9.132749475	9.06167029166667	8.30336938333333	8.99070061666667	8.111985825	10.2481894083333	10.2654733916667	7.73803474166667	9.80448268333333	9.76310825	9.09553066666667	8.5994674	8.580396475	9.44058225	8.07839394166667	9.14782716666667	9.02034758333333	8.535292725	7.60239018333333	8.84563956666667	9.18457413333333	8.94143755	8.4773978	8.15758866666667	8.30746798333333	8.37508346666667	8.77298199166667	9.11993345	8.54360906666667	8.93806988333333	8.98146720833333	6.21600831666667	7.31196651666667	7.52729815	6.85814304166667	11.6540451166667	7.349518275	7.48698644166667	7.375280375	7.23609219166667	7.57783686666667	7.65388535833333	7.10317193333333	7.86223494166667	8.11825858333333	5.934751075	7.44607933333333	8.30591494166667	8.71475239166667	8.26156163333333	7.270544475	12.3314457	10.217869775	7.44317530833333	8.25885329166667	8.60539538333333	9.6592899	11.83053445	7.65174983333333	7.31981145833333	9.47542340833333	7.75332493333333	6.414216275	7.57344579166667	8.04450845	7.20693700833333	7.70367073333333	7.16232548333333	8.333019375	7.801197125	10.116767025	7.94435703333333	6.67856683333333	6.71730138333333	6.74610281666667	7.03502525	7.11478095	6.64834389166667	7.29365533333333	7.16852535833333	8.94186190833333	8.65418485	8.28581018333333	7.12725256666667	7.87768698333333	7.16852535833333	6.95358578333333	12.30344225	7.02704855833333	7.83894598333333	7.76084795833333

LNPK	8.84659819166667	8.73305545	8.52241499166667	8.59099210833333	9.579511975	9.2612694	8.022968975	8.81336174166667	9.21169858333333	9.19719063333333	8.4444208	7.63328161666667	8.46245245833333	8.9856801	9.68695163333333	8.31911250833333	9.78737088333333	9.83153578333333	9.352137375	8.62786819166667	8.52772891666667	9.86431620833333	8.582021475	8.34923416666667	9.34018350833333	8.99204639166667	8.95577006666667	8.8768317	9.74483329166667	8.91742895833333	9.19531680833333	8.71089086666667	8.80780606666667	9.15259453333333	8.97815918333333	9.24539353333333	9.62453731666667	9.611823	9.40861700833333	9.24318624166667	9.6592899	9.74996381666667	9.17867269166667	8.93668718333333	8.4773978	8.87183624166667	9.04179071666667	8.59397561666667	9.360413025	8.88122105	9.13460934166667	9.235078425	9.235078425	9.60830463333333	8.85393681666667	8.8319761	8.965634	9.34670505	9.19200934166667	8.98934508333333	8.68130785	8.360853775	8.711926	8.14799835	8.797202975	9.38580318333333	8.48950848333333	8.02242483333333	9.11993345	8.859115725	8.91690625833333	9.04937824166667	8.74913316666667	8.682286675	8.27384794166667	8.42983770833333	8.4773978	9.1029726	8.85215985	8.14607183333333	8.6667612	8.31364723333333	8.70798358333333	8.499322875	8.99987959166667	8.2675206	8.67789245	8.58441085833333	8.30336938333333	8.64337428333333	8.23128400833333	8.738873225	8.96748466666667	9.00964296666667	8.89405555	8.43351543333333	9.126505725	8.91410946666667	9.46919775	9.19764225833333	9.62759855	8.38422716666667	9.0540245	8.80139795	8.59990980833333	8.76654949166667	8.97126208333333	9.119480775	9.77352068333333	9.44516316666667	8.71089086666667	9.3836955	9.30028739166667	9.53443595	8.77966386666667	8.44745494166667	9.08869563333333	9.32939963333333	9.291151225	8.686777175

POPDC3	8.19543675833333	4.86085110833333	4.688902775	4.31217129166667	4.048345425	4.61955908333333	4.30917759166667	5.27629945	4.44254745	4.85659321666667	4.15845484166667	5.053877925	4.72324775	4.62161215833333	4.87091625	5.00439053333333	6.90123264166667	4.2939407	5.12814335833333	5.16938190833333	4.57718236666667	5.08997001666667	5.16081746666667	6.64623430833333	5.60487774166667	7.87768698333333	6.19129395	5.70649276666667	4.94355658333333	5.50436075	6.138425225	5.2543857	5.35088976666667	4.67877638333333	8.14240605833333	8.06709081666667	5.29020444166667	5.99939696666667	5.4137082	5.25334058333333	5.67425256666667	5.29020444166667	4.98651175	6.53273724166667	6.70726116666667	7.11340090833333	6.11631895833333	5.122114675	4.71113178333333	4.92770649166667	5.3439553	5.22716268333333	4.71793841666667	6.1186989	5.22492120833333	6.5862738	4.32588960833333	5.755893075	5.27417344166667	5.7745543	4.055304025	5.1863758	5.10174316666667	5.25334058333333	3.976955525	3.76208641666667	3.936908825	4.02921226666667	3.839428225	3.912461775	3.82170636666667	4.141758275	4.18298021666667	4.06699531666667	4.258212325	4.0917668	4.2443259	4.40083348333333	5.08890834166667	4.021539275	4.36495859166667	4.17889495	4.8912062	7.07292095833333	3.83152513333333	5.13437999166667	4.19044525833333	4.54864098333333	3.87726464166667	5.09317755833333	3.75125179166667	4.37388213333333	5.38157649166667	5.018425275	5.05900706666667	3.87078774166667	4.22594125	5.41985199166667	4.35594943333333	5.060028525	5.36511784166667	3.83041005	4.87292895	4.960685075	6.14334293333333	6.11297529166667	4.57030863333333	6.31952470833333	4.36096451666667	6.63574723333333	5.08571715833333	5.44873513333333	4.98892909166667	5.26783745	4.59576983333333	4.39039918333333	7.61232630833333	6.83545454166667	4.67674131666667	4.54233060833333

MAPKAP1	5.03726580833333	4.95627725833333	4.72615460833333	4.58777895833333	5.43596139166667	5.41246783333333	4.59179895833333	4.69863704166667	4.361998275	4.48239504166667	4.51179561666667	4.19234266666667	4.49712886666667	4.91325449166667	5.078596225	4.72414796666667	5.0486896	5.642111775	5.8386672	3.9861723	4.04503838333333	4.61010935833333	4.1481371	6.081909575	5.37341235	4.15642159166667	4.16407313333333	5.75400875	6.428668075	5.073382675	4.27700685	6.27095864166667	4.39670516666667	4.57030863333333	4.20479785833333	4.01435764166667	6.03236501666667	5.377778825	5.22820184166667	5.45060010833333	5.4308407	4.040897575	4.4047953	5.67617600833333	4.2379826	4.65900069166667	4.48717731666667	4.421470575	5.09864851666667	4.42570850833333	4.4159867	4.792098225	6.0079005	5.87782166666667	4.99463171666667	4.3934547	4.58881544166667	4.45416251666667	5.01427080833333	6.29443513333333	6.12364109166667	5.14829798333333	5.3439553	4.56186439166667	4.22787308333333	6.13511379166667	4.48239504166667	4.87006425833333	5.09217729166667	4.82580888333333	4.38635804166667	5.47416700833333	5.13336504166667	5.49526165833333	5.78817238333333	4.90369176666667	4.67067994166667	5.997550525	5.06786714166667	4.58556791666667	4.40903533333333	4.74548245833333	4.98009509166667	5.92854135	4.33428121666667	4.69963341666667	5.61354440833333	4.16522844166667	6.041758325	4.82135065	4.98009509166667	6.36396054166667	5.38659805833333	3.94341095833333	5.37570958333333	4.38726624166667	5.54491926666667	5.74704205	5.06903989166667	4.90147810833333	4.82796093333333	4.38097856666667	4.35186211666667	4.479514325	6.02896681666667	5.170393	5.79649798333333	5.11436066666667	4.82135065	5.37570958333333	4.37610179166667	4.93234401666667	6.42485146666667	5.69296955833333	5.82824421666667	4.2592488	3.79653381666667	5.71315170833333	5.256776325	4.94478124166667

FGD6	7.525066825	7.62037211666667	8.45352316666667	7.86670446666667	7.2960179	8.069486125	5.35692600833333	7.37359066666667	6.89400789166667	6.96657695833333	6.98530065833333	4.24826858333333	5.8174436	7.53349060833333	8.14849655833333	7.89296785	6.737597225	6.4975584	8.52061441666667	8.32106236666667	7.72354014166667	7.76455869166667	8.81108796666667	7.56091623333333	7.97923883333333	8.37811988333333	8.085928525	7.79244525833333	7.61520518333333	8.42983770833333	7.77602128333333	8.00359983333333	7.38291215	6.66802325833333	8.82067435833333	8.717376625	7.69103884166667	7.52443165	7.060215325	8.104851775	8.91506995	9.06886545833333	8.73259671666667	9.15935345833333	8.486570275	9.10051520833333	9.06791326666667	8.74913316666667	8.73207944166667	8.593472975	8.24215483333333	7.99508275	8.20882531666667	8.5671537	8.86467129166667	8.98237701666667	7.746730925	8.9217463	8.538352825	8.71688334166667	7.87562194166667	8.82572	8.67688486666667	8.703553525	7.435271425	8.73158135833333	9.203321075	8.448844675	9.03950406666667	8.4371155	7.687727325	7.44491683333333	8.555975275	8.37460103333333	9.09505498333333	8.612418275	8.66979525	7.92549585	9.32142165833333	8.64196594166667	8.08647256666667	7.84947208333333	9.69880400833333	9.34798774166667	8.759783975	7.0307229	8.99070061666667	7.95851929166667	8.472856875	9.02927973333333	8.30336938333333	9.03689006666667	9.28128599166667	8.90052724166667	9.02705223333333	8.91457238333333	8.09998893333333	8.95764171666667	8.83115135	10.319078025	10.4702277083333	9.84541846666667	9.81442805	9.679927325	9.35125324166667	9.94636165	10.2501973166667	10.1621245583333	10.0412389083333	9.59563205	10.0957326583333	9.275522425	10.0288478666667	10.1899294916667	9.663801325	9.68825589166667	8.69363141666667	9.69038334166667	9.19576385833333	9.38762769166667

RRM2B	10.7100606833333	9.21787520833333	9.346282425	9.15886451666667	9.047926775	9.07295375833333	9.028831875	9.82995544166667	9.14655231666667	9.23186806666667	9.2768977	9.24630831666667	9.50308771666667	9.579948275	9.47542340833333	9.47453338333333	9.71265901666667	9.722436925	9.66826963333333	9.51133800833333	9.60919951666667	9.86020724166667	10.1283050583333	9.389393125	9.70842734166667	9.95852463333333	9.49191133333333	9.596033825	9.75828255833333	9.37220353333333	9.83944360833333	9.53786854166667	9.13780860833333	9.03327476666667	9.721994775	9.67944865833333	9.16167278333333	9.7400082	9.41070553333333	9.85198440833333	9.52177871666667	9.40727955833333	9.22955863333333	9.64118481666667	9.25167913333333	9.71612960833333	9.51496844166667	8.67553405	9.431383225	9.57692496666667	9.05917338333333	9.645264775	8.256452925	9.12419129166667	9.10246554166667	8.67403964166667	9.70284449166667	9.21590233333333	9.54092946666667	9.50353634166667	9.549690725	9.37457540833333	10.347919	9.90731580833333	9.73142488333333	10.022234175	10.2237133416667	9.4233862	10.6068679083333	9.7100974	9.74607185833333	10.594157325	9.135601825	9.36666890833333	9.09553066666667	9.81608086666667	9.58391948333333	9.945018575	10.3574979	9.62453731666667	9.288795975	9.19160371666667	10.7201690666667	10.48105345	10.3466375416667	9.25209231666667	9.29384936666667	10.1440795833333	9.4968836	10.3462750333333	9.78059948333333	10.1054993333333	10.2539350666667	10.3366020166667	10.0976412833333	9.70331208333333	9.69707035833333	9.15645445833333	10.2621725583333	10.5748071083333	9.90565114166667	9.27035150833333	10.06035685	9.852861125	9.22249476666667	9.9560883	9.974167275	9.60093574166667	10.0903015	9.59906339166667	9.53179666666667	9.660226725	9.47257300833333	9.31502623333333	9.56828670833333	9.13172414166667	9.70886765833333	10.1505465666667	10.248995	9.62586958333333

ZNF7	10.9426936083333	10.3519044416667	10.7363658166667	10.404465475	11.62415315	10.7081516416667	11.4912151666667	10.7027965333333	11.1607791666667	11.3136753916667	10.692247	11.2907157	11.0772433166667	10.4112315166667	11.387566725	10.8401100166667	10.6971968666667	11.0863287333333	10.4131468	10.6263315083333	10.6603736666667	11.7513402	10.7197888416667	10.6887964	10.7309865083333	11.0491091083333	11.1203060666667	10.430465125	10.9754104083333	10.7463771	10.33090645	11.1117434416667	10.7990034333333	10.8447210916667	10.4958848833333	11.1937817916667	12.0368526666667	11.5618505	10.6550211916667	11.0390903416667	10.8608532	11.044463875	10.8461868083333	10.818385175	10.9969001083333	11.1473899333333	10.6173169916667	10.3455009333333	10.5739390666667	10.9430329583333	11.2025233	10.590660625	11.61832875	11.002804875	10.9215878333333	10.766208375	10.8368196833333	11.0380667083333	11.835989125	10.818385175	12.0001864416667	10.315132175	11.5754446583333	10.5444389083333	11.5174454333333	11.0560163166667	10.99552285	11.4982052833333	11.0031119583333	11.2696990833333	10.6683478	11.5371262416667	10.5601410916667	11.0013930666667	11.2861342166667	10.8277408416667	11.494176975	11.043790875	10.35233035	10.8941934333333	12.247084775	12.2710584083333	10.632187725	11.0975459833333	10.680077475	11.687529825	12.5395615083333	10.4819116333333	11.7194234416667	10.7268672916667	10.7736244333333	11.4133387916667	10.9512840333333	11.1506157166667	11.1926823916667	10.9073626	11.7613047833333	11.5436260583333	11.1039790666667	11.7019780416667	10.572725675	10.6865081333333	11.1630098	10.8412280166667	11.2864562916667	11.4904941666667	11.4971747583333	10.273326675	10.402059075	10.4024778916667	10.2206735166667	10.99445015	11.0938590416667	11.1405494333333	10.5876388833333	10.6377074083333	11.2997236583333	10.971536625	10.4153772	10.3745792583333

ANKRD1	7.61337419166667	4.64613160833333	6.841322025	4.73402499166667	5.03232479166667	4.535343	6.20814583333333	6.623007975	6.81976344166667	6.39845556666667	5.40750829166667	5.04230353333333	6.67710233333333	5.073382675	6.64623430833333	6.23950985833333	5.99598418333333	5.90321861666667	6.26211881666667	4.434278525	5.35882338333333	5.18519845833333	5.527437325	6.12364109166667	4.39039918333333	4.54020328333333	6.30781043333333	3.85170159166667	5.55559675	4.82998554166667	6.222261675	4.87091625	4.73832164166667	4.86699436666667	8.81418505	4.47770515	5.27529641666667	5.30061454166667	6.57365081666667	8.66441525833333	4.664314175	4.024477825	6.87986294166667	4.996867625	5.95490855	5.15046895833333	4.97812506666667	5.13847554166667	6.80918875	5.26895569166667	5.11568579166667	8.4304275	5.46375123333333	6.265839825	5.550710675	5.126264825	7.470826175	5.95574085833333	4.83989633333333	7.53401704166667	5.26673208333333	5.41985199166667	5.42082883333333	5.661700075	7.141526375	5.35794613333333	6.97843058333333	5.47315375	5.33648908333333	5.1863758	4.59576983333333	5.2815818	9.94886909166667	4.96638246666667	4.366029925	6.18568551666667	6.67239650833333	5.31421083333333	5.90677250833333	4.17980874166667	5.5907794	4.697573175	4.23000438333333	9.10015925	5.091030975	5.31824546666667	6.10873680833333	7.28012135	3.84051730833333	3.75838870833333	4.897128125	5.22265475	4.89595425	5.49035004166667	4.15642159166667	4.62587865833333	7.64166063333333	4.883731825	8.11940264166667	4.96517085	5.4892176	5.23035095	5.98649943333333	4.53433785	7.33974354166667	4.747391	4.127831	7.2266449	8.95895775	5.92603411666667	4.977020525	5.59857721666667	7.765634325	7.91685584166667	5.35794613333333	4.64133359166667	8.44937053333333	7.54177855833333	5.62140730833333	6.68987835833333

PACRG	5.46549339166667	4.90679766666667	5.33746485833333	5.27629945	5.30448685	5.99418615	4.4078674	4.86589074166667	4.490204975	5.27529641666667	4.48239504166667	4.42481975833333	5.0100817	4.48829116666667	5.02158241666667	3.946878775	6.48217831666667	5.514879125	5.94705471666667	5.4147768	4.55283246666667	5.575395825	5.02371664166667	6.05045175	4.88920265833333	4.16737369166667	5.28262243333333	5.66463360833333	5.895388975	5.46375123333333	5.64797583333333	5.77296926666667	4.78700018333333	6.04876668333333	4.84301391666667	5.32134071666667	6.25250383333333	5.980495875	5.64797583333333	5.56869470833333	5.68880476666667	4.56600969166667	5.04338205833333	6.503440675	5.28787750833333	5.29123589166667	5.72282524166667	4.955313625	4.30828979166667	5.95243336666667	6.19736666666667	4.75255506666667	4.894753975	6.23312839166667	4.7559661	5.6173492	4.26758835	4.909691175	6.31548078333333	4.32386374166667	4.81485295	5.44644633333333	5.70053848333333	6.36396054166667	5.20250346666667	6.64088086666667	5.06232444166667	5.3100359	5.6003113	4.26028479166667	4.768670525	5.207730075	4.83989633333333	4.65382089166667	4.822492325	4.42372025	5.03930840833333	5.09968536666667	5.07758634166667	4.644124975	4.879391675	6.83207999166667	4.17980874166667	5.575395825	4.96517085	5.06903989166667	5.82073376666667	4.58672223333333	4.39039918333333	4.61010935833333	4.89027461666667	5.54873173333333	6.32600435833333	4.75255506666667	5.42880058333333	6.89851890833333	6.39606678333333	6.2176337	5.12411041666667	6.16609928333333	4.690117325	4.85861940833333	4.73024025833333	5.5526373	6.48357195833333	5.37127428333333	5.42273213333333	6.70390775833333	4.6247727	6.36327274166667	4.49406044166667	7.06709683333333	5.67327496666667	6.08433614166667	5.12102319166667	4.52697115833333	6.20326725833333	4.93436960833333	5.91334729166667	4.60180214166667

SPEF2	12.5930323166667	11.47905225	13.2061458583333	14.07729275	8.364642925	16.0055115333333	11.5555906416667	11.9978846916667	14.0237926083333	12.93348965	15.1632730833333	14.8261234166667	11.44422215	11.4328736	10.11881175	13.2027218583333	14.341437425	10.0653276583333	10.0082740166667	10.9237681	11.7853110416667	12.4501055333333	12.5457898	14.989321275	14.94500215	14.5469723583333	12.2492084583333	12.6175954833333	11.0448237416667	11.9616964916667	11.5570626583333	11.545842925	15.873782975	14.178968975	13.6426254916667	13.799269175	10.7748204	13.4721219	13.9635675833333	11.9694157916667	13.9389915916667	11.6398002583333	13.312167125	13.8450858083333	14.5287330416667	12.03257945	11.4985882166667	11.56339385	11.36947175	15.5556811083333	14.4060458333333	13.3018009583333	11.8565277666667	10.8120670333333	11.8137761833333	12.966521825	11.6175120416667	12.3067398	12.6175954833333	13.264123675	12.4163683083333	13.4807854416667	11.6227232166667	12.557141575	13.14330865	11.8337195083333	13.7611314416667	14.1841737583333	12.6996624166667	13.8473484166667	12.7753567583333	13.1310777666667	12.6386311166667	13.9518039333333	12.533964725	14.3482967833333	12.9219389833333	12.2594871416667	12.082379725	12.7221714916667	12.2053036416667	14.3582404416667	13.3959354916667	14.9779140333333	14.9620915916667	13.741448775	11.178312475	14.2000424583333	12.3970415166667	14.400934925	13.8766797166667	11.8904865166667	12.4741889916667	14.5589896583333	13.1407286583333	12.7509478416667	12.4037688166667	12.5657560416667	14.1841737583333	12.8895588333333	13.8088736083333	13.073805875	12.3618830833333	10.7096961916667	11.75219825	12.968881	13.1071716666667	11.7757946333333	13.4752691916667	14.657054125	12.3542251583333	10.9419805083333	12.2210896416667	12.9836190416667	13.8821044333333	11.8220738583333	12.8192150333333	12.9278503083333	10.2853426583333	13.7017952166667

NFATC1	10.7725388333333	10.268273175	10.2690944333333	10.0885610583333	11.21761425	10.4755897583333	11.1057130583333	10.558939175	10.8349029416667	10.9505347083333	10.9136098583333	10.9571307916667	10.883221475	10.4843028583333	10.7743973416667	10.1242565166667	10.4838867083333	11.0709017666667	10.5456854416667	10.377533625	10.0321890166667	10.5930279833333	9.91032726666667	10.054135025	10.21041955	10.5509023833333	10.3265631083333	10.2367285833333	10.5523212166667	10.24386405	10.3442980083333	10.186667875	10.6527698	10.585362525	10.7665838833333	10.839776075	11.0611890333333	10.857474925	10.7608892083333	10.6621925666667	10.1770845916667	10.5810256583333	10.1957428083333	10.0587286583333	9.80492948333333	10.2442419583333	10.0412389083333	10.1840717583333	9.92873065833333	10.38024835	10.6511688416667	10.5482040916667	10.8090233916667	10.878488075	10.5441182916667	10.2991427166667	10.5639796666667	10.64844015	10.6285410083333	10.20309965	11.0924307333333	10.7414541583333	10.276116525	10.4958848833333	10.4668279916667	11.7019780416667	11.1154972666667	10.9123611916667	11.0315811583333	10.9783451666667	11.184429675	11.0950066583333	11.2247431833333	10.7774965	11.0079045333333	10.7081516416667	10.7268672916667	10.831890425	10.8824750083333	10.4895361083333	10.3975849666667	10.74562245	10.6865081333333	10.3362368833333	10.81171625	10.4875672	10.8025054583333	10.3610713833333	10.7258049	10.32163045	10.5596517833333	10.6659778416667	10.86914835	11.1405494333333	11.6425911333333	10.7885218833333	10.7990034333333	11.3183574	11.1637582666667	10.6266595583333	10.31869075	10.64420985	10.2900500333333	10.1299873833333	10.4779275	10.622992175	10.53724435	10.640008475	10.5631726083333	10.76764485	10.4895361083333	10.4224382166667	11.2032221333333	10.733288175	10.9259950416667	10.3987917	10.7399277916667	10.7463771	10.661840225	10.406884125

MINAR2	6.85538685833333	3.8371121	4.14274019166667	3.971604825	4.14371865833333	3.905753025	5.04338205833333	4.39258018333333	3.812622975	4.07882345	4.98106579166667	4.63093599166667	5.77716973333333	3.99268493333333	3.86613395833333	3.75350035833333	4.68477714166667	4.15432630833333	4.32670648333333	4.224150025	4.42481975833333	4.31516280833333	4.07457460833333	4.508464425	4.339553	4.75698904166667	4.1095023	4.035447825	4.1106042	4.00561259166667	4.21592141666667	4.16611878333333	3.557434975	3.84370838333333	3.92023596666667	5.97106113333333	4.27584504166667	4.91752975833333	4.17138054166667	6.258372575	4.5496205	4.05738384166667	4.41203210833333	5.01100034166667	4.33637641666667	4.54233060833333	5.84884216666667	4.350915225	4.233876925	4.4718244	4.480628975	4.30828979166667	4.506429475	5.92321303333333	4.097912125	4.30130591666667	4.56806040833333	5.153439775	3.98271365	3.931450225	3.90457709166667	4.55568075	4.92659856666667	5.21624118333333	4.21701204166667	4.02799813333333	3.67401930833333	5.17804041666667	4.46100053333333	4.55283246666667	4.62057545833333	4.45030740833333	6.00039116666667	3.936908825	4.3987315	3.8987918	3.62898665833333	4.02247413333333	4.263255	4.03445655833333	4.57030863333333	6.58691901666667	4.003549775	4.9195424	3.937874125	4.40284696666667	4.53925114166667	5.22716268333333	3.88647775833333	4.337410475	4.33043379166667	4.10724728333333	3.798771525	3.68338428333333	4.03764378333333	3.68736378333333	4.075702625	4.40903533333333	4.434278525	6.445141375	4.31414184166667	4.52610014166667	4.33328629166667	4.51773374166667	4.8072374	4.22787308333333	5.3343362	5.01735620833333	4.480628975	5.53996425	4.3987315	5.566976475	4.141758275	5.42880058333333	5.23035095	4.06900396666667	4.41506155	4.20571450833333	4.1018927	4.602780325

PRUNE2	7.53912439166667	4.21912249166667	5.36818925	4.30917759166667	5.13745513333333	4.55369980833333	4.73832164166667	5.60132373333333	4.666328775	4.54233060833333	4.02921226666667	6.92042344166667	6.71502808333333	4.4227704	5.34097245	4.35819395	4.76548715	7.139659575	5.485111725	4.71899701666667	5.68979354166667	5.84236155833333	4.7706093	5.70748306666667	4.77483110833333	7.687124975	6.297552775	5.17483658333333	4.79621500833333	4.64847966666667	5.37867954166667	4.57924936666667	4.71113178333333	5.1555947	7.9089283	7.56940810833333	4.690117325	5.99598418333333	5.09764620833333	4.76759788333333	5.23978975	4.62375229166667	4.98892909166667	5.71218308333333	4.537079425	5.18312655	5.328555675	5.08890834166667	4.65588558333333	4.98106579166667	5.127309825	5.41246783333333	4.19044525833333	5.56425255	4.80827406666667	4.9406758	4.24130721666667	4.93234401666667	5.661700075	4.97812506666667	3.76208641666667	4.64220138333333	4.87292895	5.20354711666667	3.69811276666667	3.92246294166667	3.95350946666667	4.84615233333333	3.72178564166667	3.86167775833333	4.127831	3.95899671666667	4.99894050833333	3.9661014	3.68608479166667	4.60485635	5.15454668333333	5.03622141666667	3.89460940833333	4.035447825	4.58881544166667	4.613234575	4.263255	4.350915225	4.13313858333333	4.52610014166667	3.88526006666667	4.53925114166667	4.89027461666667	5.255540825	4.15011734166667	4.43639113333333	4.268577875	4.683662975	3.98946053333333	3.932546125	4.10495005	4.45902586666667	4.25290215833333	5.08890834166667	4.47573676666667	4.31803169166667	4.92770649166667	5.20550439166667	4.52238	4.07678143333333	4.72324775	6.51078761666667	4.37710720833333	6.79131116666667	4.66519471666667	5.53793416666667	4.63723314166667	5.11328418333333	4.177708	4.48159451666667	4.26652515833333	4.67278611666667	5.74803075	4.90369176666667

GPR137B	11.9259261416667	11.55825445	11.7369473333333	11.088855975	11.1790792916667	12.7227603583333	11.3846584083333	11.7958947916667	10.84359385	11.8379474833333	12.3806821916667	12.7211045416667	11.7046170083333	11.3820783333333	11.1003561666667	12.430195425	12.1410311166667	10.9386855666667	11.5598018166667	11.9903497083333	12.0514729416667	11.4612426083333	12.3746631	11.9962714583333	11.4934089166667	12.03257945	11.6511098916667	11.7720983166667	10.99445015	11.40680705	11.6278737583333	11.9001097666667	11.495328725	12.6831265	11.626670975	12.23879665	11.4817372166667	11.7081843583333	12.2345932083333	11.5728018416667	11.929273675	11.50454985	11.9903497083333	11.9380168416667	12.3465970583333	11.9616964916667	12.1728373083333	11.8174744333333	12.0717905916667	11.66838785	11.7761557916667	11.8834269416667	11.8927815333333	11.6591054333333	11.8670714	12.05687395	11.913931575	11.654339925	12.105942725	11.9816922	13.18580525	12.4069098166667	12.3874454166667	12.5850501916667	12.9357780833333	11.8761205916667	12.36669285	12.39392575	12.47909375	12.8454667083333	12.3750783583333	13.16610495	12.3452729083333	12.9155862583333	13.2933426583333	12.8532684916667	12.5516382166667	12.4850765	11.9781875416667	12.5818258666667	13.160332775	13.9955171333333	12.2306593166667	12.6254772333333	13.577235475	12.9340515083333	12.1827137333333	13.002567275	13.203417575	13.011963125	12.8268431583333	12.18354635	12.452378375	12.5497662083333	12.8197190166667	12.40421355	12.6886469	12.60549005	12.6765518416667	12.9030126333333	12.2306593166667	12.5652457833333	13.0258011083333	12.6752219875	12.3422979833333	12.9357780833333	12.7976328583333	12.5865453166667	13.031934	13.193307375	13.2373203666667	11.656217175	12.8020915333333	12.9219389833333	13.03309195	12.4945779666667	13.5660586666667	12.3443503083333	11.5895178083333	12.588802475

LMNTD1	13.8376377666667	14.74733735	14.648708375	14.8548535916667	14.6325901333333	14.6430667333333	14.7055894833333	14.7572900333333	14.4495058333333	14.7034991833333	14.9094697583333	13.935575975	14.2671663083333	14.72730925	14.7572900333333	14.7547481083333	14.61397985	14.7205176666667	14.4226857416667	14.5020438083333	14.7686158333333	14.7143451416667	14.3514380333333	14.818607725	14.85238655	13.6840030333333	14.0490437833333	14.6239736916667	14.5607495666667	14.8842187583333	14.2145397416667	14.0503314	14.7205176666667	15.092462825	13.8667592	13.6894331166667	14.7547481083333	14.5038815833333	14.2842943416667	14.1500101916667	14.667083075	14.68415095	14.7077525	14.168404425	14.6527873333333	14.9978928	14.8842187583333	14.30297575	14.89930145	14.7754409333333	14.9148418416667	14.6904136083333	14.2204751583333	14.00243415	14.9094697583333	14.65081365	14.8110718166667	14.8336777583333	14.11256935	14.371029675	15.1104707416667	14.73390865	14.9020399416667	14.7226534166667	15.00595375	14.7920246166667	14.73390865	15.0769655666667	15.0427033083333	14.718655275	14.421103675	14.87708635	14.6628978166667	14.5038815833333	14.667083075	14.5550005583333	14.667083075	14.912278625	14.9645581833333	14.8794573583333	14.7731878916667	14.6549636583333	15.01216265	14.4739784	14.341437425	15.0831469166667	15.1833315916667	14.73869885	14.7143451416667	14.4850706333333	15.0276351833333	15.13692035	14.427724725	14.3076416166667	14.7948348083333	14.9507696666667	14.657054125	14.6067841833333	14.7802388333333	13.7930399666667	14.7974983916667	14.9620915916667	14.7316646166667	14.4908464833333	14.5664298833333	14.15404765	14.4361804666667	15.0703584333333	14.894107525	14.6758595916667	14.4397477583333	14.7293250666667	14.8794573583333	15.0306120416667	14.1886825416667	14.56279245	14.3943592583333	14.7754409333333	15.0306120416667	14.8136769166667

CLEC7A	6.864696	8.04016346666667	7.65827738333333	7.73803474166667	6.92501696666667	9.442472475	6.61689901666667	8.14192978333333	7.18643425	9.27461214166667	8.03538219166667	7.788158325	7.79298445	8.13040576666667	6.673017075	8.75371965	8.180440425	8.06026415833333	9.02175539166667	9.07164405	8.62596224166667	7.47562453333333	9.55990681666667	8.24602095	8.8039806	8.62290185833333	8.89405555	7.75064413333333	8.33702585833333	8.66201021666667	8.56867330833333	8.31466204166667	7.89296785	8.52826969166667	9.446490025	7.070041725	7.72520474166667	8.445841225	9.03372714166667	8.64638736666667	8.59785679166667	6.98158215833333	8.22157784166667	8.32905851666667	8.2675206	9.05602455	8.25292731666667	8.68375230833333	9.728395625	9.35995134166667	9.30818888333333	8.24163635833333	8.07674773333333	8.54169179166667	8.60280456666667	8.43902399166667	9.08180965	8.82318806666667	8.80780606666667	9.38762769166667	6.57166325	7.668390025	9.48834466666667	7.68648800833333	9.028831875	9.52087123333333	9.512679675	8.64290230833333	10.217869775	9.86020724166667	7.9814024	7.88978783333333	8.19808664166667	9.4277554	10.5983973083333	9.55621784166667	9.25719764166667	10.003538	10.8850875833333	9.250740625	6.91828460833333	8.21566409166667	8.83063586666667	10.2089178583333	9.68430186666667	8.0095181	7.1355085	9.1840561	7.33912221666667	10.160939175	9.06116049166667	9.80399703333333	8.910111275	10.9127535083333	9.114481175	9.19627585833333	8.40424725	8.90285985	9.55429406666667	9.38057958333333	8.68447144166667	7.69858204166667	11.602856675	8.1308782	8.95031519166667	11.4228997	10.3558299833333	10.2904392333333	9.75321625	10.503058125	10.7493099333333	9.47404223333333	10.4875672	8.86421215	10.982052575	9.10145314166667	8.94982423333333	10.2919749833333	9.750737375	9.92916715

MMACHC	10.946906725	10.3313573416667	10.2060439416667	10.27531875	10.1569732333333	10.03562315	9.60093574166667	9.585238675	9.687830775	10.1485742083333	9.42951640833333	9.71307581666667	9.76179089166667	10.0807009083333	9.95359933333333	10.384097475	11.3911680666667	9.45898255	10.2411048916667	10.2936263083333	10.4040896666667	10.1180361833333	10.173327525	10.2367285833333	10.3530735416667	11.1313582333333	10.268273175	10.0885610583333	10.0321890166667	10.5244636333333	9.89258960833333	9.94086763333333	10.0309458333333	10.7375745583333	11.0670126666667	11.3713269916667	10.3380831916667	10.1633244583333	9.37921308333333	9.84157025833333	10.26626505	10.2915805333333	10.3790883333333	10.485478825	10.46022315	10.5953164	10.6058164916667	10.6139707	10.73913815	10.4554224833333	10.2915805333333	9.861824025	10.3948990166667	10.7363658166667	10.07610735	10.4464281166667	10.2613668833333	10.528410425	10.2650558583333	10.8258465666667	9.35549675833333	9.70668029166667	10.8329994333333	10.0984817166667	11.2779260833333	10.1497399666667	10.9750833833333	9.53356816666667	10.5596517833333	10.027285575	8.984145025	8.85494786666667	10.4522980083333	9.77133523333333	9.15259453333333	10.5053205416667	10.58184315	10.115233725	10.090730825	10.0802966083333	11.41442885	10.0026032916667	10.018462175	10.81171625	10.2551392166667	10.740315675	10.5995079	10.159727225	10.0693001666667	11.0682286166667	10.7725388333333	9.148299925	9.603559825	10.7291696916667	10.76921485	10.4918133583333	11.3125544916667	10.6328931583333	10.114399525	11.1103347416667	10.402059075	9.60093574166667	10.103459075	11.19866735	10.5785258833333	10.0256584	10.306601825	10.5100494833333	10.440565075	10.7057641416667	10.748938275	9.88515190833333	10.669525325	11.5378634666667	10.3388359916667	10.1936096583333	11.605536575	10.5181477	10.6527698	10.3987917

POU6F2	6.00039116666667	7.71678190833333	8.67789245	7.33974354166667	8.535777725	11.1963735833333	9.0973311	7.9999538	8.41926360833333	8.41428779166667	8.84952995833333	8.813752575	8.55955131666667	7.55609866666667	8.0917251	8.03277863333333	7.72796763333333	7.97507355833333	7.90139170833333	7.5367911	7.9882338	7.17681736666667	7.98023625	7.788761475	6.62436875833333	6.692469175	6.56655971666667	7.23727353333333	7.54177855833333	7.2657012	6.57813499166667	6.51455034166667	7.372424975	7.61520518333333	6.27165035	5.64497814166667	7.31674675	7.874246775	6.5680586	6.92717863333333	6.30111015	6.08877931666667	6.914548675	6.89915848333333	5.42554189166667	6.85354335	6.4308335	5.24289476666667	6.23312839166667	7.663341325	7.33501970833333	8.354234475	8.53097458333333	7.39564355833333	8.05913828333333	6.32454565	7.882902775	7.74093078333333	7.03630040833333	6.44063283333333	11.4122968416667	10.0242607916667	10.4519100666667	10.409675875	12.1749483916667	11.352411175	9.09833678333333	9.31047634166667	6.85045274166667	7.4731488	9.39626775833333	11.1771749333333	10.2249992083333	9.23377270833333	11.683415075	9.173253675	7.2559939	7.47443041666667	8.02832288333333	10.382186225	11.5298755416667	12.3083565083333	10.5865107	9.6895289	11.3624169916667	11.4093544416667	9.59469621666667	8.26701150833333	10.0728614166667	8.26456484166667	9.32711606666667	8.641468925	11.0083196416667	8.113521575	6.914548675	10.1481495666667	9.04692176666667	10.8537358333333	8.28949861666667	11.7168402	11.4890705333333	9.79628703333333	9.24630831666667	8.956732575	7.28556258333333	9.222045675	7.299009625	10.5384115166667	9.60186775	9.08960063333333	10.9263073083333	11.1158888083333	9.92412455833333	7.56894295833333	10.0305098083333	7.2657012	12.1566548583333	7.95255818333333	10.5295700833333	8.2053418

CAPN10	8.24579634583333	8.82993020833333	8.61548956666667	8.33374056666667	8.49117349583333	9.95913398333333	7.25697813333333	8.02860902916667	8.14372690416667	8.13543918333333	7.77295430833333	7.31225579166667	8.28561088333333	8.51600551666667	9.41321142083333	8.32582187916667	12.3642732833333	8.61115745	8.20663983333333	8.59372429583333	7.96649823333333	8.42800134166667	8.56344345833333	8.72898039166667	8.66700444583333	8.51412932916667	8.35659556666667	8.65008565	8.94407867916667	8.52986056666667	8.78097914583334	8.26126812083333	8.032508475	11.9672191583333	7.67450238333333	7.935655425	8.96251699166667	8.93872759583333	8.83616149583333	8.0898001625	8.36402504583333	8.74980536666667	8.3103365125	8.31838739583333	8.0103730875	7.58377286666667	8.30107511666667	8.2995628375	7.83543632083333	8.15885914583333	8.120809225	8.15479616666667	8.41716786666667	9.11661512083333	8.62002858333333	8.09146820833333	8.06734486666667	8.65008565	8.67815312083333	8.40401039166667	8.45480330416667	7.01992537916667	8.73473254583333	8.96871966666667	7.10404392916667	8.24439640416667	6.7324261125	7.500213925	7.888993075	7.78463692916667	7.7249509375	7.6856722	7.03041839583333	7.2976049625	7.46717455	7.09328215	10.8008170708333	7.927862075	7.31890889583333	7.87636192916667	8.70569584583333	7.99966422916667	8.38636805	7.97216200833333	7.86308917083333	7.85177892083333	7.94144794166667	6.695335175	8.55672422916667	7.25569755833333	7.617860025	8.19266712916667	7.62680286666667	7.69455015833333	7.54045762916667	7.69075220833333	7.9298804625	8.15988661666667	7.7138230875	8.43323617916667	7.5584249	6.78006964166667	7.70339010416667	8.1932087875	9.4489161375	7.05618424166667	6.92611444166667	7.57430120416667	7.362420575	6.97245632083333	7.67628430833333	7.60626710416667	8.10927019166667	8.41456412083333	7.417938075	7.78192657083333	7.78354614583333	7.68907982083333	7.76267469583333	7.05804465416667

GPR35	8.24579634583333	8.82993020833333	8.61548956666667	8.33374056666667	8.49117349583333	9.95913398333333	7.25697813333333	8.02860902916667	8.14372690416667	8.13543918333333	7.77295430833333	7.31225579166667	8.28561088333333	8.51600551666667	9.41321142083333	8.32582187916667	12.3642732833333	8.61115745	8.20663983333333	8.59372429583333	7.96649823333333	8.42800134166667	8.56344345833333	8.72898039166667	8.66700444583333	8.51412932916667	8.35659556666667	8.65008565	8.94407867916667	8.52986056666667	8.78097914583334	8.26126812083333	8.032508475	11.9672191583333	7.67450238333333	7.935655425	8.96251699166667	8.93872759583333	8.83616149583333	8.0898001625	8.36402504583333	8.74980536666667	8.3103365125	8.31838739583333	8.0103730875	7.58377286666667	8.30107511666667	8.2995628375	7.83543632083333	8.15885914583333	8.120809225	8.15479616666667	8.41716786666667	9.11661512083333	8.62002858333333	8.09146820833333	8.06734486666667	8.65008565	8.67815312083333	8.40401039166667	8.45480330416667	7.01992537916667	8.73473254583333	8.96871966666667	7.10404392916667	8.24439640416667	6.7324261125	7.500213925	7.888993075	7.78463692916667	7.7249509375	7.6856722	7.03041839583333	7.2976049625	7.46717455	7.09328215	10.8008170708333	7.927862075	7.31890889583333	7.87636192916667	8.70569584583333	7.99966422916667	8.38636805	7.97216200833333	7.86308917083333	7.85177892083333	7.94144794166667	6.695335175	8.55672422916667	7.25569755833333	7.617860025	8.19266712916667	7.62680286666667	7.69455015833333	7.54045762916667	7.69075220833333	7.9298804625	8.15988661666667	7.7138230875	8.43323617916667	7.5584249	6.78006964166667	7.70339010416667	8.1932087875	9.4489161375	7.05618424166667	6.92611444166667	7.57430120416667	7.362420575	6.97245632083333	7.67628430833333	7.60626710416667	8.10927019166667	8.41456412083333	7.417938075	7.78192657083333	7.78354614583333	7.68907982083333	7.76267469583333	7.05804465416667

GAL3ST1	5.549659825	3.88526006666667	4.13109949166667	4.064898825	3.5947849	4.8072374	3.39853289166667	3.55885279166667	3.52884109166667	3.912461775	3.556104825	3.62393176666667	3.607366575	4.1106042	4.04622350833333	3.91700049166667	8.3657693	3.70182713333333	4.26445661666667	3.95777633333333	3.65945140833333	5.6003113	4.09698049166667	5.31119278333333	4.34795044166667	5.189937375	3.9147734	4.176778725	4.06079156666667	3.971604825	4.83765554166667	4.25488725	3.63279243333333	7.30913270833333	6.30572190833333	6.65223385	4.11987200833333	4.747391	4.73720489166667	4.86085110833333	5.15873735	4.41297161666667	4.54233060833333	5.40105285833333	4.75698904166667	4.77483110833333	4.86289075	4.812774675	4.161871975	4.879391675	4.47367345833333	4.26972968333333	3.7439275	6.01758168333333	4.79906281666667	4.63093599166667	3.86842555	4.92878054166667	4.78798888333333	4.41297161666667	4.24624565833333	3.65945140833333	4.67877638333333	4.2068357	4.7006097	3.65195390833333	3.82289100833333	3.39853289166667	3.71770076666667	3.46702925	3.38406459166667	3.50732393333333	3.36303483333333	3.607366575	3.396759825	3.66307870833333	3.40209168333333	3.695851625	3.67401930833333	3.54623583333333	3.75125179166667	3.477151725	4.031307325	4.17980874166667	3.65195390833333	3.42122610833333	3.59213996666667	4.048345425	3.511408425	3.76843148333333	4.05967894166667	4.21300761666667	3.90803829166667	3.905753025	3.62256374166667	3.81605024166667	3.85061613333333	4.41297161666667	3.7439275	4.91851714166667	4.15011734166667	3.45012391666667	4.52791141666667	4.90586546666667	4.12106518333333	3.91035815	4.46696494166667	6.02645373333333	4.453007975	5.623286525	4.41002290833333	4.85037616666667	4.31414184166667	5.27734721666667	4.02799813333333	4.30624979166667	6.91322913333333	5.57335978333333	4.42666711666667	3.5244499

ZNF521	6.31394556666667	4.656935525	4.81947958333333	4.67471653333333	5.9191077	5.01735620833333	5.14089556666667	4.83355489166667	4.67172261666667	4.83765554166667	4.75491586666667	5.485111725	4.84094744166667	4.768670525	5.13015945	4.86184074166667	5.99672560833333	5.000034475	5.46938143333333	5.966951625	5.12000276666667	4.645027875	5.6003113	5.000034475	5.13745513333333	4.91752975833333	5.553511425	4.72615460833333	5.596724675	5.201606925	5.09864851666667	5.328555675	4.366029925	4.2289531	4.39972780833333	4.69863704166667	5.93036776666667	5.73491465833333	5.50024889166667	5.07445225	5.10498871666667	4.73490975	5.451608675	5.64313004166667	4.88811624166667	5.12102319166667	5.29238863333333	5.157708775	4.63513973333333	4.91752975833333	4.88920265833333	4.56909456666667	5.21624118333333	6.12934905	4.44059075	5.07218190833333	5.72382095833333	4.85447411666667	4.753728625	6.36029920833333	6.34005863333333	5.608795625	4.70784294166667	4.57827949166667	4.611084475	5.550710675	4.42481975833333	4.69963341666667	3.795517075	5.002397475	4.33637641666667	5.390724	4.1095023	4.0917668	5.03120425833333	5.34862645833333	4.95627725833333	4.669520575	5.00768225	4.55466009166667	5.74803075	4.1683668	4.34901141666667	4.00561259166667	3.96359718333333	6.70532833333333	6.45250163333333	4.7006097	4.43531826666667	4.51475793333333	4.58982808333333	6.06147365	4.80827406666667	4.996867625	4.20011505833333	3.99064151666667	4.4462942	7.21861555833333	4.33428121666667	5.0100817	4.53824951666667	4.06593015	4.94355658333333	5.20060255	4.44822564166667	5.70649276666667	6.51237135	6.60007374166667	5.49632836666667	6.73065755833333	4.71445220833333	5.61448439166667	5.70932124166667	5.90065811666667	4.24914275	4.4159867	7.001733525	4.64734200833333	4.77813741666667	5.78626178333333

SMC5	9.36892748333333	8.99720454166667	8.975801525	9.291151225	9.37921308333333	9.44959046666667	8.39981014166667	8.369572325	8.149040725	8.33507211666667	8.60751143333333	9.05454125	9.13909698333333	8.7954535	9.3558778	8.98934508333333	7.58408555833333	9.84117550833333	9.11582745	8.73448420833333	8.81836040833333	9.88434308333333	8.222543225	9.15064904166667	9.440114675	9.23235029166667	9.36338105833333	10.033950425	9.66469236666667	9.15113736666667	8.81418505	9.31405536666667	9.43278838333333	8.03810015	8.96369055833333	8.93180466666667	9.80706423333333	9.060159775	9.10246554166667	8.83443758333333	8.57257758333333	8.9962514	8.87491901666667	8.55955131666667	8.20331464166667	8.16329085	8.457931075	8.8296979	9.13078204166667	9.16442515	9.51454425833333	9.251213625	9.7400082	9.71356900833333	8.984145025	8.5216034	8.63344703333333	8.53187930833333	9.41620731666667	9.11131928333333	9.68903575833333	9.01197323333333	9.6433763	9.95725063333333	8.41599213333333	9.99259065	8.2495222	9.288795975	8.82920276666667	9.10796010833333	9.133746725	8.76514685	9.14377724166667	8.92364006666667	8.319610775	9.18306429166667	8.86467129166667	9.75875961666667	9.30275641666667	8.9489102	9.40419091666667	9.5678162	9.14003079166667	8.45851334166667	9.60629726666667	9.28551639166667	9.71356900833333	8.79767885	10.6625963333333	9.33708134166667	9.38536081666667	9.699723	10.2658011666667	8.92461889166667	10.2166211083333	9.81818709166667	9.74389450833333	10.5322469083333	9.08299806666667	9.05965051666667	9.18595039166667	9.32615619166667	8.82372205833333	8.482136425	9.47208941666667	9.07252779166667	9.37821416666667	7.960228575	8.89076064166667	9.24318624166667	9.84332440833333	8.93359508333333	10.1804756333333	8.66580391666667	8.76514685	9.29432763333333	9.45898255	9.02562736666667	9.23973414166667	8.78836945

NDUFAF4	13.5157896583333	12.1071523416667	12.0917340666667	12.036426975	12.3079459333333	12.603650525	12.1279125583333	12.545304275	12.2236021833333	12.4903405333333	12.2687931833333	12.21725785	12.7021254833333	12.253276925	11.7494484416667	11.9040111333333	12.5406352083333	12.46107545	12.424331275	12.2341055916667	12.367659325	11.6234420916667	12.2679354916667	13.160332775	12.4225030833333	13.24529235	12.1279125583333	11.946316525	12.1865914583333	12.0993529333333	12.6744586916667	12.0997306916667	12.015272025	12.2964028333333	13.1197255166667	13.4550317916667	11.8477473833333	12.4105900333333	12.3771618166667	11.9409022083333	12.1133945666667	11.74563025	11.9297077583333	12.5912244	12.68421675	12.0040290833333	12.5264113	12.8010225583333	12.3374196	12.1612618833333	12.6270116666667	12.6566773416667	12.3378199666667	12.2248275416667	12.1325110916667	11.9804777666667	12.5939827166667	12.21502565	11.943975875	12.42337295	12.5667535083333	13.4974205	12.8171563916667	12.3435261333333	12.731184375	10.6872865583333	12.6312122083333	11.7902343416667	12.8679345583333	11.99474245	12.9842314416667	12.9400260666667	12.2326729333333	12.7284615	11.3150765	11.5887304416667	12.3662011583333	12.4510079166667	12.43647125	12.3824722	12.3152059916667	11.7384146416667	11.9629534916667	12.7468098833333	11.951715625	11.973231675	12.1217141333333	12.2024267333333	12.630223175	12.3952132583333	12.3233097166667	12.901864125	12.9610660166667	11.9813054166667	12.4737403583333	12.210372725	12.8493401833333	12.118621125	12.1951832916667	12.4456480416667	12.4474119833333	12.908165975	13.71577945	12.287955975	12.76127775	12.3025527416667	12.1640360666667	11.6393710083333	12.9634653	12.1745545	12.5989743166667	12.5539148333333	11.8815198166667	12.790749425	12.0893148833333	12.9007979916667	11.7853110416667	12.86014235	12.6991286666667	12.867358575

ARPC4	12.42138705	13.0807272166667	12.897697	12.8429528083333	13.0746740375	12.6993955416667	12.6803438666667	13.0654161458333	12.9668682625	12.9519194583333	12.94417525	12.6203779375	12.7286986875	12.9245033916667	13.0685362958333	13.108175075	12.9090764	12.9486188791667	12.7829432166667	12.8638697333333	13.2113158208333	12.8033610791667	12.7876536791667	13.2390115166667	12.9066727166667	12.496517025	12.9712877375	13.194928225	13.3932277541667	13.021992725	12.839552375	13.3325899791667	12.8147560583333	13.2788856291667	12.548043925	12.3796099666667	13.0310479208333	13.0225453583333	13.0813328708333	12.8715923291667	12.6139254875	12.8804655625	12.7408604208333	13.0942727041667	12.7920713708333	13.0527420125	12.7583293375	13.2577424916667	13.0729777291667	12.9131474	12.9222100416667	12.9770822541667	13.1068439625	13.0635869166667	13.0040984166667	12.8237470375	12.907240275	13.0254731791667	12.8993470541667	12.6672255708333	12.4454098333333	12.5143034291667	12.6554812666667	12.5782368166667	12.2613765875	12.2957742291667	12.6344124166667	12.5356054583333	13.1339977625	12.6358521375	12.5290985833333	12.4938953166667	12.5815748666667	12.6569464791667	12.8429528083333	12.8815228791667	12.7434371916667	12.4317909	12.7366517166667	12.7187737458333	12.5233989666667	12.272120675	12.7089232125	12.6818485416667	12.5885803333333	12.3945689083333	12.3691191166667	12.4716392	12.2217037541667	12.6603372208333	12.9969100458333	12.2481908375	11.9964806208333	12.5082367125	11.9046058541667	12.3193074083333	12.0394020333333	12.2165713875	12.2745830708333	11.8420650125	12.3604058291667	12.2664905416667	12.2542932208333	12.4712011875	12.30232945	12.4653075	12.6001910208333	12.4147945625	12.8071463583333	12.8401156041667	12.4716392	12.7033686291667	12.4441625791667	12.5541383208333	12.7403482083333	12.2324496625	11.77741555	12.3045117625	12.2677302791667	12.3544274791667

ARPC4-TTLL3	12.42138705	13.0807272166667	12.897697	12.8429528083333	13.0746740375	12.6993955416667	12.6803438666667	13.0654161458333	12.9668682625	12.9519194583333	12.94417525	12.6203779375	12.7286986875	12.9245033916667	13.0685362958333	13.108175075	12.9090764	12.9486188791667	12.7829432166667	12.8638697333333	13.2113158208333	12.8033610791667	12.7876536791667	13.2390115166667	12.9066727166667	12.496517025	12.9712877375	13.194928225	13.3932277541667	13.021992725	12.839552375	13.3325899791667	12.8147560583333	13.2788856291667	12.548043925	12.3796099666667	13.0310479208333	13.0225453583333	13.0813328708333	12.8715923291667	12.6139254875	12.8804655625	12.7408604208333	13.0942727041667	12.7920713708333	13.0527420125	12.7583293375	13.2577424916667	13.0729777291667	12.9131474	12.9222100416667	12.9770822541667	13.1068439625	13.0635869166667	13.0040984166667	12.8237470375	12.907240275	13.0254731791667	12.8993470541667	12.6672255708333	12.4454098333333	12.5143034291667	12.6554812666667	12.5782368166667	12.2613765875	12.2957742291667	12.6344124166667	12.5356054583333	13.1339977625	12.6358521375	12.5290985833333	12.4938953166667	12.5815748666667	12.6569464791667	12.8429528083333	12.8815228791667	12.7434371916667	12.4317909	12.7366517166667	12.7187737458333	12.5233989666667	12.272120675	12.7089232125	12.6818485416667	12.5885803333333	12.3945689083333	12.3691191166667	12.4716392	12.2217037541667	12.6603372208333	12.9969100458333	12.2481908375	11.9964806208333	12.5082367125	11.9046058541667	12.3193074083333	12.0394020333333	12.2165713875	12.2745830708333	11.8420650125	12.3604058291667	12.2664905416667	12.2542932208333	12.4712011875	12.30232945	12.4653075	12.6001910208333	12.4147945625	12.8071463583333	12.8401156041667	12.4716392	12.7033686291667	12.4441625791667	12.5541383208333	12.7403482083333	12.2324496625	11.77741555	12.3045117625	12.2677302791667	12.3544274791667

CRACR2A	8.898281125	8.58441085833333	8.763246775	8.96850814166667	8.9081181	9.16167278333333	9.3195178	8.47183739166667	9.24539353333333	8.83738886666667	8.77460095833333	9.445595775	8.85635194166667	8.33702585833333	8.30746798333333	8.16071089166667	9.02830098333333	9.0180261	8.072065075	8.24415698333333	8.60227746666667	8.66979525	8.291997825	9.02785883333333	8.42676914166667	8.338570375	8.199157725	8.41837	8.70798358333333	8.046473725	8.278086225	8.57834244166667	8.931271375	9.05965051666667	8.785427625	8.82473405	8.82022893333333	8.84333919166667	8.7532663	8.46245245833333	8.28340845833333	8.43095395833333	7.93387576666667	8.25189414166667	8.440899325	8.475275825	8.32310249166667	8.40516035	8.298377725	8.524299575	8.50130601666667	8.86953279166667	8.71144663333333	8.68971138333333	8.535777725	8.73019295833333	8.48060255833333	8.57208716666667	8.30641555	8.44937053333333	9.01197323333333	8.89652095833333	8.9925209	8.98837211666667	8.80050660833333	8.9939706	8.33655620833333	9.00735385	9.13602675	9.071149525	8.94761291666667	9.01425336666667	9.23835931666667	8.55907896666667	9.14279978333333	8.37061515	8.72089346666667	8.90619576666667	8.43197355833333	8.3471697	8.45201853333333	8.75743711666667	8.4371155	8.55105330833333	9.04457245	8.779206475	9.07955376666667	8.956732575	8.92602376666667	8.238616825	8.66828283333333	8.54699226666667	8.9384783	8.66247435	9.11902033333333	8.85215985	9.09600253333333	9.24884609166667	9.23186806666667	8.80540845	8.40717954166667	8.97076639166667	8.55492440833333	8.14607183333333	8.41926360833333	8.57257758333333	8.48112818333333	8.45304260833333	9.05868610833333	8.31911250833333	8.445841225	8.861626075	8.81154583333333	8.65559314166667	8.910111275	8.75658194166667	8.30283623333333	8.631828525	8.48261985	8.80244275

D2HGDH	10.9430329583333	11.31989795	11.2715325333333	11.4108017166667	11.2254735166667	10.3790883333333	10.7092769166667	10.8566946833333	10.4801853	10.776374675	10.4830527666667	10.17413685	10.3933623083333	11.1256433416667	11.3470919	10.9476724083333	10.9415949916667	11.4364638833333	11.471171625	11.2534278333333	10.9189114166667	11.30084235	11.1162112666667	11.2039860833333	11.3509805666667	10.2182610833333	11.032984525	11.3938737333333	11.6795803583333	11.6566056666667	11.105357075	10.9328378333333	10.4180178666667	10.2904392333333	10.4476616583333	10.18161685	11.3683256166667	10.6542751166667	10.7158953333333	10.7748204	10.622992175	11.48580155	10.91124625	10.9270024916667	10.9505347083333	10.9353811	10.7485438416667	10.8593336416667	11.1937817916667	11.1803917416667	10.99445015	10.546063825	11.4446261583333	11.6456087833333	11.2025233	11.0034620833333	10.572357075	10.6523425166667	11.3488624333333	10.8090233916667	11.2114428083333	9.74951903333333	10.58489685	10.8337493916667	10.433634375	9.99593061666667	11.1561366	10.4445170416667	9.81525473333333	10.971536625	10.2778151833333	11.235931225	10.5330763833333	9.97337968333333	9.93673649166667	10.7321313916667	10.4499117666667	10.4783084333333	10.94802585	10.8949867833333	11.9678169416667	10.1625186333333	10.2191020916667	10.399176825	11.1800718666667	10.6305289416667	9.43868498333333	10.5288525083333	11.5538304416667	10.8874095666667	9.909101975	10.0980477833333	9.460327375	11.024361475	10.1350682166667	9.82203395	10.496758625	10.9245036	10.4755897583333	10.4161036833333	10.3519044416667	10.7628088916667	10.7736244333333	10.5424705	10.706587775	10.8850875833333	10.3852198833333	11.5498379666667	10.4112315166667	10.6079735416667	10.8113265583333	9.41298314166667	10.8356737416667	11.7827675333333	10.3105309666667	11.0072108	10.3236813083333	9.83629188333333	11.3683256166667	11.0859242666667

SPPL3	8.445841225	9.82250195	10.35148425	9.67230795	9.58391948333333	8.35277756666667	9.72703330833333	9.852861125	10.0326667666667	9.10844005833333	9.41436835833333	8.68329548333333	8.82825418333333	9.55333915	9.86678629166667	9.31047634166667	8.89736060833333	9.76647539166667	9.96782080833333	9.67230795	9.307742	9.76432159166667	9.608728125	9.83237755833333	9.47897484166667	8.86366274166667	9.75787298333333	10.138226575	9.56407003333333	9.93835718333333	10.02346285	10.1737764166667	9.55812764166667	8.74224036666667	8.763246775	9.02562736666667	9.86102748333333	9.900175875	9.34064308333333	9.86766510833333	10.64807625	9.87207808333333	9.70714295	10.1437039833333	9.14003079166667	9.82778015833333	10.239159725	10.2554871833333	9.73788834166667	9.64383103333333	9.84994195833333	10.013853075	10.0915413	10.0069849166667	10.181229625	9.75828255833333	10.135458025	10.3637863083333	9.92375435	9.7553002	7.72354014166667	9.148762375	9.52642665	8.86760295	8.364240675	8.861626075	9.15935345833333	9.48743423333333	9.44337360833333	8.57348205	9.778305875	9.57510789166667	9.19484745	8.52966328333333	9.59692015833333	9.01197323333333	8.90962148333333	8.85110639166667	9.98633354166667	9.18016484166667	8.60177515	8.92461889166667	10.0864066083333	9.67544948333333	9.02512653333333	9.01009886666667	10.2621725583333	9.119480775	9.10051520833333	9.24031536666667	10.1160686416667	10.1927941666667	9.32000775	8.91506995	8.68624993333333	9.53265603333333	9.54277241666667	10.0382960666667	9.58437340833333	10.2355191666667	10.3603019333333	9.95935071666667	9.97706043333333	10.1262351166667	9.79628703333333	9.47542340833333	9.87116769166667	10.154948775	9.74483329166667	9.1906696	9.586175875	10.0489380083333	10.06569645	9.61633444166667	9.70584026666667	9.79676919166667	9.44337360833333	10.1601611333333	10.300361525	9.82822335

OR11G2	4.57137348333333	4.12229528333333	4.07882345	4.45115921666667	3.893426425	4.08851591666667	4.52898779166667	4.21912249166667	4.24826858333333	4.22505026666667	4.07134488333333	4.91442044166667	5.29123589166667	4.32386374166667	4.51370921666667	4.00999268333333	4.67787883333333	5.399029725	4.63305978333333	4.25488725	4.24914275	4.13109949166667	4.979047925	4.90147810833333	4.80316131666667	4.108372	5.12814335833333	5.74506199166667	4.82036908333333	4.64613160833333	5.26468096666667	4.68073703333333	3.84482813333333	4.62790769166667	4.47770515	4.359146625	4.66114760833333	5.49334536666667	4.71793841666667	4.76252700833333	5.16420199166667	4.59576983333333	4.99794086666667	5.72187083333333	4.19044525833333	5.14829798333333	5.31319136666667	5.18423503333333	4.58556791666667	4.89935284166667	4.87091625	4.54330015	4.161871975	5.550710675	4.83558559166667	4.87823230833333	4.51475793333333	4.92991125833333	4.480628975	4.75806056666667	3.86389350833333	4.19810324166667	4.61625569166667	5.6173492	3.93361386666667	4.509505	3.92496040833333	4.19234266666667	5.44644633333333	4.38097856666667	4.63093599166667	4.63723314166667	4.669520575	4.85037616666667	3.962526825	4.137419	4.59265796666667	4.32198345833333	4.18298021666667	4.58672223333333	4.111632325	3.99488165833333	4.61625569166667	4.90369176666667	3.931450225	4.39446349166667	4.45699755833333	4.82580888333333	5.40651104166667	4.11685985	5.19309993333333	4.56305699166667	4.09478471666667	4.24914275	4.45115921666667	4.02568050833333	4.23000438333333	4.9415684	5.51683608333333	5.08063435	4.46597973333333	4.54233060833333	5.000034475	5.32346678333333	4.41002290833333	4.49602461666667	4.78047113333333	6.74958845833333	4.339553	6.69314785	4.69863704166667	5.60229806666667	4.652676825	5.57926250833333	4.85974603333333	4.45602740833333	4.24718985833333	4.67787883333333	4.80433128333333	4.58241979166667

AKIRIN2	8.57348205	7.65774509166667	6.9563535	6.810578	8.31252919166667	8.616671525	6.26140625833333	9.29384936666667	7.61520518333333	8.62986514166667	6.91905025833333	6.188915175	8.53929365	7.5367911	7.81893236666667	8.38422716666667	8.91049593333333	10.7189561666667	8.22690084166667	8.59595736666667	7.314288875	8.06345484166667	9.10485650833333	8.751836375	7.82120025	7.17267591666667	7.057705175	8.149040725	8.34324809166667	8.001514325	9.931739675	8.51856025833333	7.64737070833333	9.45898255	7.83071075833333	8.03223831666667	8.08436514166667	8.58250536666667	8.92028675	8.46350844166667	8.6716691	8.11940264166667	8.401731375	9.26948546666667	6.998615075	8.02464245	8.38662095833333	8.278086225	8.96510433333333	8.1525602	9.63199205833333	8.52634974166667	7.48528250833333	8.0665366	7.55873083333333	7.39806645	9.0540245	7.93544106666667	7.32494479166667	8.54989949166667	8.862628675	8.18363113333333	7.96361378333333	9.13515515	8.327465575	10.2166211083333	7.90789741666667	9.04937824166667	9.08687805	7.90363365833333	7.65827738333333	8.5716089	8.30084284166667	8.277602525	7.32433510833333	7.56655451666667	8.14579476666667	9.496436825	8.63031905833333	8.119922525	8.18422995833333	7.11143919166667	8.10391784166667	8.159108975	8.612418275	7.91897891666667	8.61979589166667	7.78546004166667	6.37568504166667	8.5034354	9.14608020833333	9.95359933333333	7.82746845	8.440899325	6.60472939166667	8.24602095	8.10268665	8.74957956666667	8.40567906666667	8.32310249166667	8.71882579166667	7.25861729166667	8.894577375	8.39560916666667	8.439473525	8.00740176666667	7.89403294166667	7.10699465	8.83883193333333	7.31861950833333	8.89310475	9.15740435833333	8.15455800833333	8.478972275	7.67307831666667	7.95406095	8.67639044166667	8.7812519	8.13819046666667	8.256452925

RNF6	11.5738946	11.61112325	11.2363296083333	11.634515075	12.4123342916667	11.528423775	11.4022935333333	11.39940385	11.9804777666667	11.6659074916667	11.403040875	11.0663049916667	11.7513402	11.5994743583333	12.1895397416667	11.5941589083333	11.3025465416667	12.1138378333333	11.9546857416667	11.9289106916667	11.3339882	12.4087159666667	11.2911160333333	11.7731681916667	11.6940775166667	11.3072160833333	11.6077110916667	11.7887391333333	12.190259475	11.5895178083333	11.5461869416667	11.6216623	11.4262590333333	11.0633247666667	10.7904047166667	10.9969001083333	12.20367145	11.7644689416667	11.4214230083333	11.9521162083333	11.5291327083333	11.7109162916667	11.6898911833333	11.642205825	11.4843617833333	11.3817027833333	12.2844611166667	11.9220834083333	11.23046085	11.1166050833333	11.6198462166667	11.7648162666667	12.3645045916667	12.4261316583333	11.947152875	11.685253825	11.9009487833333	11.684519	11.81229275	11.9888038083333	12.616140275	11.7060618166667	11.437271475	11.920083225	11.0196702	12.3110445	11.6205609916667	11.7974234916667	11.414772875	11.39829615	11.9443641	12.150591475	11.7440902416667	12.1063614833333	12.3343950916667	11.4978397166667	11.8642752583333	11.9204648416667	11.523383625	11.8126730416667	11.3605041083333	12.1295438416667	11.4321000166667	11.502358525	11.9781875416667	11.2294360916667	10.6251996	11.3413304583333	12.8718544916667	11.185165725	11.6761092166667	11.9674169166667	12.0600286166667	11.6433290333333	11.5349136916667	11.5078323166667	10.81171625	11.0006123833333	12.1250050333333	11.3938737333333	11.7070775666667	11.9447346	11.9005377083333	11.3495641166667	11.01087085	12.0737471	11.4398783916667	11.3675917666667	11.50454985	11.364979325	12.370296175	11.6205609916667	11.9318404166667	10.950929275	11.4682426833333	11.1572399333333	10.9765277583333	11.484727	11.3801476916667	11.6006445416667

LYPD3	10.1725196416667	15.9960220916667	16.0142070583333	15.9237782	16.1520487333333	14.0062649583333	15.6711097083333	16.3137024	16.1520487333333	16.113934775	15.7697269333333	8.69082025	15.9064622666667	15.9315345	16.1772486333333	15.7338865416667	8.8072839	16.2193318666667	15.7066290583333	15.91498085	16.2495335833333	16.39493105	15.784983325	16.7226812583333	15.4537382666667	11.22444735	16.0258935166667	15.9582825916667	16.1391348	16.0791897833333	16.4447326166667	15.8653098666667	15.7486260916667	8.82067435833333	8.37361221666667	9.22016708333333	16.2495335833333	16.5317674916667	16.4447326166667	16.2339961416667	16.2042656333333	16.090774475	15.9582825916667	16.3470849416667	14.3482967833333	15.8383325666667	15.9859723583333	16.0691638333333	16.0374488333333	16.0374488333333	16.1391348	15.83077275	15.7697269333333	16.3470849416667	15.5512564416667	15.7697269333333	16.4447326166667	16.0791897833333	15.5221336416667	15.9673534416667	15.0703584333333	14.8966343583333	14.6284460166667	14.5469723583333	10.4902258833333	15.2193239916667	14.4633924916667	14.0734299166667	13.604496075	12.96407505	14.8629148	15.301852925	14.3908304333333	14.9391332833333	15.0615765666667	14.3350486166667	12.9756001333333	14.8087193833333	15.345488975	14.6949121583333	10.0611526083333	11.3813533416667	15.1336404666667	13.4481554583333	15.0733260666667	14.0707149083333	9.73622053333333	13.1980287833333	13.5694284666667	13.4634900333333	14.2160518416667	14.3757792583333	14.07729275	15.0615765666667	13.608143675	15.4243027666667	14.036419	13.66414075	14.3792394416667	13.44208435	12.5385984166667	13.2503335416667	12.521701325	14.4144157	15.5870133916667	14.4377971083333	14.7409853333333	15.0831469166667	14.4328794	14.3268677	13.934166025	15.2650881666667	15.406759425	12.7738537166667	15.508396775	15.039210975	10.5096862166667	13.9109585833333	15.290119725	10.5509023833333

MID1	4.60485635	4.2289531	4.46402134166667	3.98396318333333	4.285092425	4.81947958333333	3.64058418333333	3.88409523333333	3.91593421666667	4.6247727	3.86501250833333	3.4844003	3.62768649166667	4.09903998333333	4.83355489166667	4.23701905833333	4.42570850833333	4.64220138333333	4.617309275	3.77672200833333	4.221324425	4.77378615833333	4.62587865833333	5.44322508333333	4.4718244	4.061836425	3.854046525	4.128952175	4.666328775	3.973735825	5.23978975	4.41103275833333	3.770641325	4.04622350833333	4.41103275833333	4.85768484166667	5.02903219166667	5.57926250833333	5.09864851666667	5.4892176	5.54579601666667	4.43844	4.58140610833333	5.63053526666667	4.14585755833333	4.713205375	4.79906281666667	4.57240950833333	4.74930484166667	4.760314225	4.55971610833333	4.1882044	3.83595706666667	6.11958026666667	4.2231712	4.40083348333333	4.01435764166667	5.12102319166667	5.60680040833333	5.19536235833333	4.963016975	3.694768975	5.06351158333333	4.649483075	3.89679799166667	4.45115921666667	4.10289946666667	3.89227330833333	5.31119278333333	4.0078761	3.838223825	4.15845484166667	3.442586375	3.80438254166667	3.80684369166667	3.5935751	4.24826858333333	4.17889495	4.76661771666667	3.76596780833333	8.16329085	4.65900069166667	4.12677783333333	5.171525	3.764611825	3.909240625	3.51012440833333	3.9614432	4.31217129166667	4.08322809166667	4.17039641666667	4.60591755833333	4.30027178333333	4.879391675	6.17390685	4.024477825	4.251891475	6.56579801666667	4.73490975	5.54102934166667	4.2939407	3.560205975	4.49712886666667	4.72105203333333	4.33133893333333	3.88294669166667	4.44534256666667	5.68304789166667	5.43182890833333	6.05474264166667	4.38005901666667	4.685722125	5.58508399166667	6.1441534	3.99595204166667	4.15954296666667	4.117963675	4.53219865	4.53624929166667	4.51370921666667

EIF3B	12.548305425	12.8884463	12.7237683583333	12.649121475	12.6862224333333	13.0084425916667	12.5334882833333	12.68421675	12.521701325	12.865109125	12.93175565	12.244114325	12.706710675	13.0427266583333	12.978001475	12.2219012	12.391732275	12.685194075	13.0084425916667	12.7076553	12.7763784666667	13.1793538166667	12.8537857833333	13.0626219916667	12.5958960666667	12.5734875583333	12.5725102833333	12.7354192416667	12.7728759666667	12.8246255416667	12.763386175	12.763386175	12.6436968916667	12.5860556666667	12.4404061833333	12.3776096916667	12.8979356	13.1103883166667	12.8124184083333	12.813963275	12.7976328583333	12.8702352416667	12.7436904833333	12.83111185	12.5999690416667	12.8872860166667	12.7031196333333	13.1452170666667	12.9423278583333	12.6032318833333	12.5719766166667	12.9988931583333	13.016186275	12.993623425	12.8192150333333	13.0130175083333	12.964777525	12.9461435166667	12.9423278583333	12.905858725	13.484103425	13.7253859666667	13.67760105	13.8357694833333	13.28687105	13.8667592	13.695137875	12.905858725	13.6596320166667	13.1806770166667	13.0554708583333	13.66335695	13.9600657833333	13.174009975	13.1905828833333	14.3724417	13.1058939166667	13.1356932666667	13.5346846166667	13.8572965333333	13.2962436083333	13.1316052916667	13.7621985333333	13.3444534166667	13.4611203333333	13.2405472416667	13.05246665	13.1197255166667	13.2926640333333	13.1249649416667	13.3262903916667	13.9227796833333	13.5213793583333	13.6062775083333	13.7692653916667	13.42164015	13.7434058583333	13.10396855	13.0602371583333	13.306054325	14.0174773166667	13.6356329916667	13.3594846333333	14.0759487166667	13.9003358333333	13.812858	14.0813262416667	13.6605641666667	13.5100333	13.3481016166667	13.2725108916667	13.4807854416667	13.4760270333333	13.97057275	13.6434906916667	13.453508825	13.48163315	13.3839194083333	13.312167125	13.420218375

HLA-A	13.3053968166667	14.127909425	13.312913975	14.2671663083333	13.295486125	14.64497825	13.838661325	14.282826075	13.627458175	12.5349027	13.85816885	13.9065296166667	13.418751775	9.53223686666667	12.9988931583333	14.1554067916667	14.6738026916667	12.9773913166667	14.0800041333333	13.8720329083333	14.392674675	14.0012596166667	14.1343040416667	13.93081475	13.028241025	13.0536119916667	13.5668551916667	12.8426722583333	13.8307559916667	13.92725075	12.4141455666667	13.4042461583333	14.269993075	14.5984675666667	13.0368898	13.2622394166667	13.453508825	12.762350675	13.334230975	13.6514898166667	12.9388338	13.4966084666667	14.1384134166667	13.1980287833333	13.98513315	10.5085269083333	13.8405812166667	12.9995656666667	13.5876594833333	14.2000424583333	13.7611314416667	14.0301250916667	12.600413	13.6470212583333	13.2538590583333	13.5931197583333	12.9918297166667	12.8724674416667	13.784925425	13.4254222333333	14.912278625	14.3724417	14.99199845	14.0074276833333	15.1873199	14.4891443666667	14.0288199166667	14.5488647916667	14.5176714166667	15.0032595583333	13.8788907166667	13.3046967416667	14.1357234916667	10.99291925	14.73869885	14.0127035833333	14.7948348083333	15.0184028083333	14.68415095	14.0326619916667	14.274700225	13.8428912916667	14.87708635	14.8236926166667	14.9779140333333	14.6325901333333	14.1698024416667	14.8602831416667	13.09075125	14.74733735	14.6589883666667	14.71190555	13.9389915916667	14.2763428833333	14.341437425	14.4092298666667	13.7582839583333	13.6737470333333	14.259430575	14.7055894833333	14.7802388333333	14.1329416	14.0800041333333	14.190108625	14.0683132333333	11.2154273083333	14.577355375	14.9620915916667	13.7702194083333	14.515883675	15.0276351833333	13.9367207833333	15.009089275	13.4352543166667	14.0526804666667	14.4109315833333	14.3463851	14.9148418416667	14.5089658583333	14.6104738916667

NYAP1	7.798604875	6.25250383333333	5.94185621666667	6.11631895833333	6.86262681666667	7.416522875	6.36473330833333	5.91719066666667	6.55667590833333	6.79468460833333	6.4413796	7.08738564166667	6.57365081666667	6.28402719166667	7.09804688333333	6.43152844166667	6.90460845	7.92224445	6.3687361	6.25518099166667	6.5005932	7.3113529	6.54716043333333	7.43805644166667	6.79731146666667	7.53506526666667	6.64956780833333	6.48497233333333	6.8275595	6.95288179166667	7.4493567	6.19964000833333	6.53467245	6.58499179166667	7.34497685	6.90649953333333	7.10570254166667	7.17330060833333	7.246381175	7.07292095833333	7.29365533333333	6.69182425833333	6.70128395833333	6.52564285833333	6.421178475	6.62907710833333	5.91520025833333	6.56951250833333	7.12528415833333	6.79334741666667	7.46022033333333	6.53205264166667	4.883731825	7.06400038333333	5.68783094166667	5.96160064166667	5.52545381666667	6.44355459166667	7.11730268333333	6.74474600833333	7.246381175	6.64156519166667	7.04378355833333	7.28317230833333	7.0369697	7.22536585	6.18980128333333	6.3877628	7.198288025	6.60270946666667	6.380135525	7.02284831666667	6.68920345833333	6.51830799166667	6.75430906666667	6.65552751666667	7.65827738333333	6.80449616666667	6.3687361	6.52564285833333	8.19859208333333	8.59640985	6.97095796666667	7.31919828333333	6.699210125	6.862021425	5.89819270833333	5.39281689166667	7.70147536666667	6.97585598333333	7.24137955	6.69050035	7.00365334166667	6.65690114166667	8.38023938333333	6.87089175833333	7.21695988333333	7.25980210833333	7.340949	6.57952525	7.349518275	7.11920136666667	6.84200789166667	6.41191374166667	7.37766478333333	5.95490855	6.86394090833333	6.12847228333333	6.26211881666667	6.48785131666667	6.93484450833333	6.17470206666667	6.74888376666667	6.33047035833333	5.44873513333333	5.7901556	6.79588928333333	6.980300625	6.47456359166667	6.23229269166667

MRPL50	11.739559375	11.2922383666667	11.4574143583333	11.3594507166667	11.2399027333333	11.6452682	11.2895126416667	11.1291467583333	11.3187261333333	11.1231619583333	11.4774665416667	11.205753575	10.9230393083333	11.037692575	10.5096862166667	10.9600311833333	11.9479243416667	11.0747492583333	11.1572399333333	11.0659389916667	11.2035969416667	10.8976829666667	10.8706515666667	11.5479749166667	11.0625918083333	11.208942925	10.904663125	10.9497712833333	10.8450341833333	11.0845103416667	11.2530869333333	11.2740276	11.0625918083333	11.6617552333333	11.405960975	11.3239342333333	10.79558335	11.14521145	11.2043416583333	10.9219574	11.077953225	11.0999976083333	11.1756714833333	11.201526325	11.1908643083333	11.2172145	11.3441400333333	11.336792675	11.114752	11.002804875	11.4989285	11.2775845	10.8751259833333	10.9567732916667	11.1956589833333	10.96771795	11.133569275	11.159679825	10.9150352083333	11.1990339333333	11.2499394833333	12.461524275	11.9892168333333	11.5048915416667	11.5887304416667	10.7383339416667	11.6933169166667	11.4200132333333	11.5618505	11.8966407583333	11.4912151666667	11.496789525	11.7883410833333	11.708995875	11.60515845	11.6205609916667	11.0720193666667	11.0448237416667	11.28518	11.2258131916667	11.8390986666667	10.9160737916667	11.6492807916667	11.85040895	10.676538	11.5356555333333	11.8371529416667	11.3474088166667	12.3546298	11.5404299	12.207741075	11.063715325	11.95296625	11.2114428083333	11.1401896	11.5614234666667	11.3571575333333	11.1678068583333	11.024361475	11.2464355333333	11.4993303916667	11.8896284166667	11.4682426833333	11.4496634833333	11.7883410833333	11.4086625666667	11.136541175	11.4511549916667	10.8424532083333	11.523383625	11.1238545166667	11.7970628166667	11.638265575	11.62075455	11.118056575	11.744853675	11.7116683416667	11.9697901833333	11.5436260583333	11.590501375

UNC80	13.1152178	13.505263125	13.9153342666667	13.9749587833333	14.7429609666667	13.8635959083333	15.5407381333333	13.7168412916667	14.7143451416667	13.65238595	14.0275803333333	15.462044625	14.9507696666667	14.0578490916667	13.4142637	14.0163605916667	13.0913765666667	14.6023913166667	13.2373203666667	13.9318364083333	13.8376377666667	13.17465125	13.370423875	13.420218375	13.0276586166667	13.3682726083333	13.077594625	12.9715821666667	13.1525120666667	13.8788907166667	12.3837214916667	13.5610039666667	14.1927345333333	14.2969035	12.8438653416667	13.2855354	13.676743575	12.9230312916667	12.7468098833333	13.1058939166667	11.98404715	12.501560375	12.7216091916667	12.2998821166667	13.6840030333333	11.9455284	12.0368526666667	13.3383656916667	12.7733872	13.1905828833333	12.6422229833333	13.3567888916667	14.53565035	13.2662932833333	13.239306575	12.004788175	14.4177528166667	12.4063944666667	13.520514175	12.3874454166667	14.7429609666667	14.70147985	13.950667875	14.5140672583333	14.421103675	14.6046129083333	14.2810681833333	15.2328828083333	14.2503770416667	14.8313200916667	14.8578939833333	14.3977718916667	15.0865438916667	14.7293250666667	14.7948348083333	14.015040275	15.00595375	14.4043399	14.0618019166667	14.6161567166667	15.06705565	15.2120960833333	14.219141025	13.5502258583333	14.7664736333333	15.1336404666667	14.9949392	14.9507696666667	15.0335623833333	14.1256024333333	14.4633924916667	13.7361743	14.4328794	14.1045935166667	14.6798721333333	14.4809973583333	14.5550005583333	14.6798721333333	14.5984675666667	13.9411608583333	14.178968975	15.1602131166667	13.532131425	12.9444160916667	13.3875128	14.6325901333333	13.9796866	13.419543825	14.085975275	13.748287325	13.7434058583333	12.6944981166667	13.0403414916667	12.0440494333333	13.6293464	12.95462435	13.9469444833333	13.3278442333333	13.24746105	15.0153788083333

ELP2	13.661470025	12.6397345916667	13.0337121333333	12.9364464083333	12.6190854666667	12.9586834166667	12.6442138916667	12.4461573416667	12.7976328583333	12.660047425	13.0488654	13.3106692583333	12.9617333583333	12.7976328583333	12.4571981916667	13.05124085	12.972189625	12.576483025	12.6292285666667	12.7279204166667	12.4840896416667	12.4664163166667	12.6857168666667	12.8046597666667	12.7857802916667	13.2975446	12.6862224333333	12.7436904833333	12.5633422083333	12.7144138666667	12.6402462916667	12.7596522	12.856730075	12.8740952083333	13.134360625	13.2919593833333	12.562277225	12.4751035416667	12.6442138916667	12.7139152416667	12.540087475	12.742149725	12.9400260666667	12.7852137833333	13.1197255166667	12.8947866166667	12.909394625	12.5516382166667	12.790749425	12.958106125	12.724266575	12.744717925	12.642703875	12.500114075	12.7211045416667	12.8877934666667	12.5177924083333	12.6895936166667	12.7190348666667	12.8278480083333	12.787939025	13.3227764916667	12.6220126	13.0829572666667	13.1178746666667	12.3003464583333	13.19400885	12.4346028333333	12.539129975	12.6136777666667	12.5921909166667	12.240405375	13.0494144333333	12.8702352416667	12.1467410583333	12.626502475	12.6846804583333	12.6914097333333	12.7263840666667	12.4491875083333	13.3522548916667	12.9971815666667	12.9001276833333	13.2628733666667	12.5325556416667	13.0067659416667	13.5965748083333	12.5921909166667	12.4190092583333	12.7453059583333	12.9733229333333	12.6050803083333	12.247084775	12.8860467083333	13.3645725083333	12.7586192666667	12.7076553	13.1628836	12.576015775	13.2898598333333	13.0457183333333	12.8757636333333	12.6562254416667	12.8718544916667	12.6366518666667	12.5448126916667	12.5116969583333	12.228520475	13.0186633916667	12.4337570333333	12.9213977333333	12.315681175	12.9558687	12.9617333583333	12.6651447166667	12.7543063	13.2694207583333	12.1517212916667	12.9456067916667	12.7390522083333

LY6G6D	8.48542059166667	8.62110277916667	8.99790463333333	9.25881540833333	8.96536916666667	8.31126909583333	9.41225827916667	8.36008461666667	8.95334906666667	8.59669108333333	8.93204191666667	8.75634927916667	8.49074439166667	8.67003209583333	8.97241437916667	8.17429475833333	7.7332836125	8.83715562083333	8.4867831	8.3889163625	8.64519729583333	8.14472440833333	9.1358142875	8.74935636666667	8.46693841666667	8.49117349583333	8.8588863875	8.6577276625	8.64519729583333	8.73664734166667	9.07980542916667	8.8814398125	9.0756037875	8.0717973625	8.7469528375	8.5052777875	8.62002858333333	8.65865825	8.91716760833333	9.40137265833333	8.953804425	8.9629780875	8.75256491666667	8.758125975	8.4637836875	8.80475242083333	8.73134057083333	9.09806735	8.7266882375	8.53359925416667	9.02394957916667	9.03305895416667	9.13349055833333	9.43660667083333	8.90986637916667	8.89286615833333	8.76055524166667	9.0272632	8.7077469	8.51461460416667	8.64663457916667	8.80475242083333	8.36784924583333	8.75112461666667	8.22373200416667	8.7266882375	8.43827822916667	8.473578375	8.74980536666667	8.11856490833333	8.82650967083333	8.96631275833333	8.72995544166667	8.2227352625	9.01723890833333	8.07230863333333	7.98692124166667	7.8749512125	8.15376015416667	8.66750117083333	9.02537695	10.0105747791667	8.36059864166667	8.25216050833333	8.50774844166667	8.41031485833333	8.52799930416667	8.28025104583333	9.01588795833333	7.93198070416667	8.89943807916667	8.77895017083333	8.38344775416667	8.5240442875	8.94782725	8.77219244166667	8.29076422083333	8.20612785	9.25144637916667	8.99007008333333	8.05594164583333	9.08280208333333	8.4244895	8.534582525	8.79658383333333	8.93153802083333	8.25216050833333	8.29554877083333	8.12563023333333	7.3412549375	7.8837332375	8.87416970416667	8.52506772083333	9.08115096666667	8.6370671125	8.30830044583333	8.79928859166667	8.6056636125	8.68011325	8.26631075416667

LY6G6F-LY6G6D	8.48542059166667	8.62110277916667	8.99790463333333	9.25881540833333	8.96536916666667	8.31126909583333	9.41225827916667	8.36008461666667	8.95334906666667	8.59669108333333	8.93204191666667	8.75634927916667	8.49074439166667	8.67003209583333	8.97241437916667	8.17429475833333	7.7332836125	8.83715562083333	8.4867831	8.3889163625	8.64519729583333	8.14472440833333	9.1358142875	8.74935636666667	8.46693841666667	8.49117349583333	8.8588863875	8.6577276625	8.64519729583333	8.73664734166667	9.07980542916667	8.8814398125	9.0756037875	8.0717973625	8.7469528375	8.5052777875	8.62002858333333	8.65865825	8.91716760833333	9.40137265833333	8.953804425	8.9629780875	8.75256491666667	8.758125975	8.4637836875	8.80475242083333	8.73134057083333	9.09806735	8.7266882375	8.53359925416667	9.02394957916667	9.03305895416667	9.13349055833333	9.43660667083333	8.90986637916667	8.89286615833333	8.76055524166667	9.0272632	8.7077469	8.51461460416667	8.64663457916667	8.80475242083333	8.36784924583333	8.75112461666667	8.22373200416667	8.7266882375	8.43827822916667	8.473578375	8.74980536666667	8.11856490833333	8.82650967083333	8.96631275833333	8.72995544166667	8.2227352625	9.01723890833333	8.07230863333333	7.98692124166667	7.8749512125	8.15376015416667	8.66750117083333	9.02537695	10.0105747791667	8.36059864166667	8.25216050833333	8.50774844166667	8.41031485833333	8.52799930416667	8.28025104583333	9.01588795833333	7.93198070416667	8.89943807916667	8.77895017083333	8.38344775416667	8.5240442875	8.94782725	8.77219244166667	8.29076422083333	8.20612785	9.25144637916667	8.99007008333333	8.05594164583333	9.08280208333333	8.4244895	8.534582525	8.79658383333333	8.93153802083333	8.25216050833333	8.29554877083333	8.12563023333333	7.3412549375	7.8837332375	8.87416970416667	8.52506772083333	9.08115096666667	8.6370671125	8.30830044583333	8.79928859166667	8.6056636125	8.68011325	8.26631075416667

TPM2	16.6938615583333	14.36106535	14.0100072833333	12.7453059583333	13.647954325	14.8021338083333	14.0429420833333	13.9119804333333	14.241033875	14.1874223416667	13.5815251416667	16.3302858416667	15.85627565	13.8961212166667	14.3724417	14.047904	13.3952177083333	14.13703675	15.0459774	14.6862769916667	14.4328794	13.021150225	14.5089658583333	13.4752691916667	14.3253890083333	16.3302858416667	15.00595375	13.441409425	13.8261646416667	14.0074276833333	14.5984675666667	13.8961212166667	14.6284460166667	12.9284297083333	16.6392399583333	16.6649415583333	13.312913975	13.841729125	14.156645775	14.7572900333333	14.4527902	14.3792394416667	14.2548502583333	15.28644375	16.113934775	14.201540175	15.2120960833333	14.3139109083333	13.3875128	14.1033949916667	14.494740525	14.6968877083333	14.1471335166667	13.17270095	14.244370025	15.4276773083333	14.013877625	13.9785722666667	14.5570464083333	14.6023913166667	12.244114325	13.6969617	12.0062931166667	13.71864895	13.5124524166667	11.759780275	13.7542141333333	13.1085025666667	12.7676811833333	13.662514625	12.962321275	12.3110445	12.9306452333333	14.1113310916667	12.3275837583333	13.6320213916667	14.7572900333333	13.4864308583333	12.642703875	13.1926381	14.1329416	14.045461125	12.5628225833333	14.0991501333333	12.94284515	12.7374360833333	13.141294	14.18015695	12.488903425	14.4427443166667	13.3010495416667	13.0337121333333	12.2231616166667	13.66335695	12.516863425	11.7175871333333	13.0632773	13.1806770166667	12.7436904833333	12.7500199666667	13.6150235416667	13.7177649416667	12.1167008333333	12.3138792416667	11.9392227916667	13.332896575	14.0759487166667	12.6769899666667	13.541969925	14.0912434666667	13.5824136833333	11.5664864	13.23467165	14.2487820083333	13.3882606083333	11.946316525	13.5593280666667	13.3285445333333	11.7403808583333	13.66414075

AGA	10.2978868083333	12.273184075	12.19731135	12.3806821916667	12.3492861833333	11.0286702083333	11.7160036	11.4581724666667	12.025731925	11.9236129	11.8317467416667	10.1728980166667	11.3242829	12.2972775333333	12.3580122083333	11.674290525	12.0156772833333	12.2006641666667	12.0409411833333	12.2124743416667	11.6906956833333	12.1316746333333	12.0744704	11.5656597	12.13546815	10.035165975	11.8070215583333	11.6651451833333	12.3043299166667	12.744717925	11.8341069333333	11.951715625	11.5926397083333	11.55164095	9.54092946666667	10.3566062583333	11.6746522	12.2006641666667	11.5367767666667	11.7336295416667	11.9345074833333	11.9757989916667	12.0116503666667	11.7750094	11.7168402	11.8783734666667	11.9228391333333	11.8249375	12.0785704333333	12.3374196	12.0368526666667	11.8931595083333	11.4478274166667	11.9009487833333	11.8701716833333	11.390823425	12.0860846083333	11.8565277666667	12.2578527416667	11.7699299	10.6603736666667	10.81171625	11.8407167416667	10.322000975	12.5079805333333	11.1756714833333	10.8032590416667	10.3526835916667	12.1431003583333	11.2143531083333	10.9780057166667	11.5056318416667	11.7861402083333	11.5038172666667	11.761727725	10.8922670583333	10.5022046916667	11.8646362416667	11.5790815416667	10.7964692333333	11.178312475	11.9352971916667	11.846919075	10.1291621416667	11.072670925	11.0451598916667	11.426633325	11.2118401416667	10.6926124666667	11.1032271	11.0863287333333	11.0341045833333	10.35233035	11.237038075	10.1974084916667	11.1787139083333	10.2707893833333	11.4919381583333	11.3954232	11.4503715	11.0041932083333	11.45939305	11.8973789916667	11.3328774166667	11.1685570666667	12.265420025	10.6603736666667	11.6558642583333	11.7292748	11.6811126416667	11.5832083083333	11.3157970833333	11.5769076083333	11.660606675	10.9087616083333	11.545842925	11.9820605666667	10.96150755	11.4817372166667	11.4452785583333

CSDE1	8.96932985833333	8.46867076666667	7.80971700833333	7.83189280833333	8.41837	8.38960981666667	8.162750025	8.439937175	8.22207980833333	7.21926184166667	7.39164716666667	8.52878136666667	8.40817908333333	8.174528375	8.04016346666667	7.90410825	7.967895825	9.40292000833333	7.88077005	8.000994225	7.96301623333333	8.641468925	7.794082525	8.23567169166667	8.01434291666667	8.78070639166667	7.833966975	7.83016101666667	7.964195775	7.23553730833333	7.89979373333333	8.42476003333333	8.18315743333333	7.80828950833333	8.00740176666667	7.93695109166667	8.04704694166667	7.29418750833333	7.71624318333333	8.14137294166667	6.64088086666667	7.227214725	7.65774509166667	7.41997313333333	7.04755845833333	7.21504029166667	7.36707339166667	7.13033303333333	7.77550013333333	8.09695651666667	7.72023940833333	7.906291625	8.57007116666667	7.82279025833333	7.95962858333333	6.02387365	8.56322373333333	6.36793951666667	7.90941765	7.97812778333333	8.84471325833333	8.409150775	8.20227615	8.022968975	7.63385609166667	8.34324809166667	7.55016986666667	8.55288418333333	8.18100231666667	7.94647371666667	7.83348010833333	7.71472995833333	8.64496134166667	8.34324809166667	7.93123045833333	8.49699819166667	7.96240296666667	8.81191925833333	8.21929578333333	8.41428779166667	8.84758474166667	8.268113325	7.72905521666667	7.24828621666667	8.48950848333333	8.532903275	8.07106306666667	8.86211740833333	8.28495673333333	7.821740775	7.54021029166667	9.06791326666667	8.52772891666667	7.52443165	9.04369295	6.911150775	8.78991784166667	7.85906496666667	7.988718925	7.76403325833333	7.24396096666667	7.198288025	8.12540885833333	7.50852245833333	7.70147536666667	8.70400716666667	8.96182571666667	8.28445111666667	8.31409636666667	8.06709081666667	8.20781665833333	8.06129600833333	8.37811988333333	7.434165875	8.565739425	8.186266475	8.17835735	7.39806645	7.83944996666667	8.41972045

NEK5	5.65633813333333	7.33974354166667	6.88940976666667	7.12528415833333	7.61062251666667	6.63442134166667	7.47725403333333	7.253014775	7.013155025	7.41061439166667	7.06942048333333	6.724451125	6.9569431	7.0307229	7.458458425	7.296620525	6.07176163333333	7.23488161666667	7.09162461666667	7.321455625	6.955027575	7.67535224166667	7.50344081666667	7.93695109166667	6.88120535	6.34005863333333	7.147600275	6.67919664166667	7.61126938333333	6.89851890833333	7.24703090833333	7.48927714166667	7.03194930833333	7.8967227	5.966203775	7.04267660833333	7.44780359166667	7.64737070833333	7.93973534166667	7.659482025	7.43234464166667	6.96183981666667	7.35447710833333	7.1744649	6.80510874166667	7.210345825	7.12103848333333	7.17569580833333	7.392806175	7.16490981666667	7.3113529	6.88791735	6.51237135	7.47677956666667	6.53137754166667	6.95924915	6.35578110833333	6.93552516666667	6.94439705	7.62089398333333	8.958107775	7.32919075833333	6.9998061	7.08427549166667	6.46705624166667	6.39681019166667	6.75430906666667	7.32258515	6.12691128333333	6.65843384166667	6.81435373333333	6.88255923333333	7.52566054166667	7.305533825	6.50418884166667	6.5305636	7.16794871666667	8.13040576666667	6.4502938	7.0659289	6.70390775833333	6.20326725833333	6.56733945833333	6.72123403333333	6.66522548333333	6.37113890833333	6.54434775	7.05651791666667	6.62839033333333	6.58100214166667	6.85354335	6.39769980833333	6.29670533333333	5.87782166666667	7.07654913333333	7.851546575	6.62563488333333	6.91699638333333	7.19451305833333	7.31196651666667	6.70726116666667	7.18402409166667	8.00051745833333	7.08170128333333	6.27652200833333	6.93352621666667	6.53467245	6.36029920833333	7.52673725833333	6.633753725	7.09424611666667	6.11958026666667	6.33491514166667	6.32381556666667	7.67599419166667	6.39769980833333	6.323145675	6.19669175	6.4808852	7.502278225

PGLYRP4	5.127309825	9.79062058333333	9.78737088333333	8.7776576	9.75787298333333	9.92583431666667	9.19391603333333	9.11582745	9.90058610833333	8.925575825	8.9744767	5.997550525	7.51253281666667	9.565921775	10.9360928583333	8.92602376666667	5.171525	9.810895475	10.133368525	9.84117550833333	9.295759125	9.734421025	11.102867725	9.80755264166667	10.114399525	4.61010935833333	9.95359933333333	9.68734411666667	9.64871909166667	9.99593061666667	9.291151225	9.4181056	8.498393225	4.90679766666667	4.30917759166667	4.617309275	10.5837654416667	10.30932905	9.69439403333333	9.52177871666667	10.31123355	10.2699462416667	9.76267145833333	9.38807046666667	8.59302528333333	10.0167779583333	9.487900075	10.7020102333333	8.63080885833333	9.63714265	9.65529779166667	8.512878125	9.38096979166667	9.87739761666667	9.69619738333333	9.57179898333333	8.57995885	10.3015658	8.684237875	9.003775725	10.5053205416667	9.20464029166667	8.101615825	11.2114428083333	6.82167365	13.0149306583333	11.8658810166667	11.1249258333333	9.92916715	6.48357195833333	8.32264421666667	12.0804266666667	12.710817125	8.90434914166667	12.555705525	10.9123611916667	6.35051324166667	9.223397275	11.285520075	7.596603625	7.58577588333333	5.11328418333333	11.465822775	9.17280985	12.47909375	7.54125486666667	9.06973089166667	6.99155748333333	4.80433128333333	8.88732965833333	10.5902797083333	10.2959072166667	8.250184775	11.2431657916667	10.6757288666667	10.4112315166667	10.0480865166667	8.51622818333333	9.89341510833333	9.107046275	7.38984995833333	9.42859468333333	9.39983189166667	9.67544948333333	11.1132646583333	11.2650373666667	10.9196649333333	12.8101998166667	9.81442805	11.1099310583333	11.0064548166667	7.78927275	10.4542782	12.0409411833333	10.9640211333333	9.37548823333333	6.09754041666667	6.51830799166667	9.445595775	4.77930389166667

EDN1	11.0975459833333	11.6643648083333	12.9024490333333	12.0265627166667	12.803128575	13.5083984166667	12.2760053833333	11.7513402	12.500642075	12.3983699916667	12.5671498916667	12.3688898083333	12.9659499916667	12.2433310083333	12.4701553166667	11.4496634833333	12.4808505	11.46235385	12.1891609666667	12.0424744583333	12.0440494333333	12.4127680416667	11.51586705	12.0816579833333	12.7041276166667	10.5368715833333	11.5498379666667	12.8537857833333	12.8912643	11.3147571833333	12.4709919583333	12.1852414666667	11.9188907916667	13.0013137333333	11.9204648416667	11.1975258	12.1431003583333	12.225632925	13.864749275	12.9715821666667	11.7582463416667	10.840860225	13.439923175	11.626670975	13.068247425	12.4439491	12.762350675	11.0644262666667	11.9063380833333	12.615602	12.2281085833333	13.6443060416667	12.1258089083333	12.5221926333333	12.057317	12.063617625	14.1132226125	12.52409195	12.8751753583333	12.333509375	12.1237921916667	12.5202342666667	11.0968033583333	13.06700765	12.3331331166667	11.625582325	12.3199648916667	11.5809197583333	11.327862475	10.5404499166667	13.0506754833333	12.4261316583333	12.7061787	12.4682236083333	12.671473625	12.6975412416667	11.5150484916667	12.5958960666667	13.5781398666667	12.4279267416667	11.3065242916667	10.572357075	11.3642682416667	13.5150003833333	11.229748375	12.29141735	11.297225	11.6818913333333	10.9754104083333	11.7690899333333	12.500642075	10.9747290916667	13.4775815666667	11.3381769	11.6175120416667	12.5415392083333	11.8612030916667	11.5731480083333	12.1342141916667	12.710817125	11.9270898666667	12.4808505	10.9757948333333	12.8783966083333	12.2603464083333	11.6933169166667	13.5116191416667	12.140177075	11.5487335833333	12.7031196333333	13.3538490666667	12.461524275	11.927850575	12.3392091166667	12.8323870333333	11.708995875	10.9661834333333	12.813963275	14.354697075	10.5806393916667

PIPOX	6.77501233333333	6.46988230833333	6.265839825	6.32751184166667	6.67442865833333	6.76114361666667	6.82038475833333	5.96160064166667	6.63866813333333	6.66447699166667	7.3968942	6.86605038333333	5.94705471666667	6.46494565	7.18643425	6.67442865833333	6.637274925	6.73882945833333	6.769663225	6.320274625	5.95328179166667	6.49896905833333	6.31880616666667	6.32668329166667	6.61959393333333	6.931726825	6.55667590833333	6.6562921	7.1355085	6.891986525	7.09487224166667	6.76049560833333	6.78782379166667	7.20088341666667	7.01495000833333	7.415839475	7.15389984166667	7.04202985	6.929785275	7.21750264166667	6.82946754166667	6.78446141666667	7.04898169166667	6.53467245	6.19669175	6.53668581666667	6.81852565833333	6.507987025	6.29670533333333	6.520636925	6.7327851	6.9125939	6.64226356666667	7.2954331	7.08560825833333	6.869498575	6.4502938	7.187671825	6.88578850833333	7.18643425	7.25066114166667	6.96183981666667	7.58800115	6.72705409166667	7.24075795	7.134182075	6.559370075	6.32381556666667	7.70926514166667	7.00815195833333	7.15262315833333	7.46746676666667	6.421178475	6.64487866666667	6.80853135	6.91828460833333	7.17569580833333	6.95924915	6.74218866666667	6.6716762	6.11631895833333	7.41061439166667	6.8478471	6.653546475	6.79863740833333	6.80510874166667	8.002520925	6.083461775	7.32494479166667	6.59386139166667	6.64487866666667	7.06888018333333	7.44491683333333	7.238929725	7.989231475	7.51472684166667	7.21451231666667	7.72520474166667	7.23727353333333	7.50573916666667	6.97159590833333	6.88578850833333	7.02021258333333	6.86394090833333	6.72249205833333	6.39681019166667	6.46705624166667	6.60676565	6.68859711666667	6.79334741666667	6.90968315833333	7.08427549166667	7.619792375	7.5512356	7.16614005833333	7.13093330833333	7.18281396666667	7.148875425	7.35862363333333	7.07466381666667

JAK1	8.93073908333333	7.31803023333333	7.53069666666667	7.38582769166667	7.89510465833333	8.19498931666667	7.20023056666667	6.88314723333333	7.65225225	7.07118303333333	7.37327947916667	7.29788705833333	7.88130259166667	7.05585056666667	7.49390345833333	7.86115675833333	8.234157525	8.32696298333333	7.96301623333333	7.4911099	7.39869075	7.708818875	7.39975843333333	7.95464479166667	8.0665366	7.21098661666667	7.50966981666667	7.212178575	8.25990825	8.07784593333333	7.69858204166667	8.013764525	7.23785493333333	8.51622818333333	7.89832068333333	7.49164054166667	7.525066825	7.03011389166667	7.68436408333333	7.341560875	7.80873386666667	7.73582129166667	7.67253041666667	7.55551181666667	7.97557605833333	7.69651605833333	7.48416809166667	7.49332154166667	7.74896025833333	7.551793125	7.92549585	7.37811973333333	8.288511175	8.1308782	7.64610639166667	7.3675475	7.53624973333333	7.65827738333333	7.77321380833333	7.83189280833333	8.608522725	7.17914503333333	8.34422473333333	7.78978061666667	7.30019385833333	8.1565621	7.321455625	7.404937825	8.268113325	8.16329085	7.81246279166667	7.50852245833333	8.09223929166667	7.435836975	8.27907085	7.31316353333333	7.90206838333333	8.57560394166667	7.76938555	7.72686941666667	7.71914490833333	7.57783686666667	7.47258725833333	7.81610706666667	8.30804240833333	7.40837556666667	7.62534816666667	7.33857021666667	7.14272935833333	7.45614546666667	8.04234110833333	8.48321643333333	7.893532975	7.8449064	7.82958231666667	7.77487424166667	7.5385516	7.62534816666667	7.94435703333333	7.59600343333333	8.05973130833333	7.41303320833333	8.08865246666667	7.47443041666667	8.22546075833333	8.15455800833333	8.560933175	7.375280375	8.1308782	7.31674675	8.31619925	7.37928605	8.84758474166667	7.84154363333333	8.28095494166667	8.20175241666667	7.93123045833333	8.08335636666667	8.060776825	7.52566054166667

STIMATE-MUSTN1	12.8584129833333	11.8371529416667	12.118235175	11.12105475	11.5056318416667	11.686699925	11.6525927916667	11.0150144333333	11.2606101916667	12.5448126916667	11.1874035166667	12.764427075	12.0084826666667	11.6940775166667	11.612663925	12.0411497	10.2749442833333	11.930808275	11.5064020583333	12.2603464083333	12.4813575333333	11.1866301583333	11.0473653916667	10.92486485	11.9649178666667	11.8954895166667	12.4225030833333	11.6129963	12.1142465833333	11.5776190666667	12.226469725	11.2887921333333	12.2024267333333	11.4530731666667	11.5839614333333	11.34484365	12.046177825	12.5676300666667	12.0950052666667	12.562277225	11.113611625	11.7648162666667	11.8450362083333	11.5245993916667	13.1946165416667	12.0917340666667	12.29220925	11.1313582333333	11.7074261416667	11.3749692583333	11.7909812333333	11.9078069166667	12.2545127416667	12.39795185	11.5552373166667	12.8062943	12.39882355	12.0819963833333	12.50892495	12.5822395583333	10.79796285	9.56634321666667	9.43278838333333	9.67682161666667	9.478582775	10.05157835	9.092829475	8.98368761666667	9.27125073333333	9.69747909166667	9.68903575833333	9.60540180833333	9.58797373333333	9.498614875	9.766909275	8.9925209	9.83406541666667	10.3623207166667	9.47351875833333	9.45281165833333	9.15449361666667	9.360413025	9.49147149166667	8.84243295833333	9.75367038333333	9.65802264166667	9.11174605833333	10.7116237833333	9.23605358333333	10.3362368833333	9.54178184166667	9.19020434166667	9.52826018333333	10.0489380083333	9.524079925	9.82382424166667	9.61553475	9.557602025	10.0783158833333	9.36802801666667	9.32186685	9.16874195833333	9.46343676666667	9.79891768333333	9.17867269166667	9.51996269166667	10.039605625	9.10844005833333	9.497335375	9.58344561666667	9.754485575	9.10749988333333	9.932660725	8.52683058333333	10.0313344083333	9.40032650833333	10.220242425	9.46704600833333	9.50773276666667	9.51549753333333

AC006254.1	12.8584129833333	11.8371529416667	12.118235175	11.12105475	11.5056318416667	11.686699925	11.6525927916667	11.0150144333333	11.2606101916667	12.5448126916667	11.1874035166667	12.764427075	12.0084826666667	11.6940775166667	11.612663925	12.0411497	10.2749442833333	11.930808275	11.5064020583333	12.2603464083333	12.4813575333333	11.1866301583333	11.0473653916667	10.92486485	11.9649178666667	11.8954895166667	12.4225030833333	11.6129963	12.1142465833333	11.5776190666667	12.226469725	11.2887921333333	12.2024267333333	11.4530731666667	11.5839614333333	11.34484365	12.046177825	12.5676300666667	12.0950052666667	12.562277225	11.113611625	11.7648162666667	11.8450362083333	11.5245993916667	13.1946165416667	12.0917340666667	12.29220925	11.1313582333333	11.7074261416667	11.3749692583333	11.7909812333333	11.9078069166667	12.2545127416667	12.39795185	11.5552373166667	12.8062943	12.39882355	12.0819963833333	12.50892495	12.5822395583333	10.79796285	9.56634321666667	9.43278838333333	9.67682161666667	9.478582775	10.05157835	9.092829475	8.98368761666667	9.27125073333333	9.69747909166667	9.68903575833333	9.60540180833333	9.58797373333333	9.498614875	9.766909275	8.9925209	9.83406541666667	10.3623207166667	9.47351875833333	9.45281165833333	9.15449361666667	9.360413025	9.49147149166667	8.84243295833333	9.75367038333333	9.65802264166667	9.11174605833333	10.7116237833333	9.23605358333333	10.3362368833333	9.54178184166667	9.19020434166667	9.52826018333333	10.0489380083333	9.524079925	9.82382424166667	9.61553475	9.557602025	10.0783158833333	9.36802801666667	9.32186685	9.16874195833333	9.46343676666667	9.79891768333333	9.17867269166667	9.51996269166667	10.039605625	9.10844005833333	9.497335375	9.58344561666667	9.754485575	9.10749988333333	9.932660725	8.52683058333333	10.0313344083333	9.40032650833333	10.220242425	9.46704600833333	9.50773276666667	9.51549753333333

MUSTN1	12.8584129833333	11.8371529416667	12.118235175	11.12105475	11.5056318416667	11.686699925	11.6525927916667	11.0150144333333	11.2606101916667	12.5448126916667	11.1874035166667	12.764427075	12.0084826666667	11.6940775166667	11.612663925	12.0411497	10.2749442833333	11.930808275	11.5064020583333	12.2603464083333	12.4813575333333	11.1866301583333	11.0473653916667	10.92486485	11.9649178666667	11.8954895166667	12.4225030833333	11.6129963	12.1142465833333	11.5776190666667	12.226469725	11.2887921333333	12.2024267333333	11.4530731666667	11.5839614333333	11.34484365	12.046177825	12.5676300666667	12.0950052666667	12.562277225	11.113611625	11.7648162666667	11.8450362083333	11.5245993916667	13.1946165416667	12.0917340666667	12.29220925	11.1313582333333	11.7074261416667	11.3749692583333	11.7909812333333	11.9078069166667	12.2545127416667	12.39795185	11.5552373166667	12.8062943	12.39882355	12.0819963833333	12.50892495	12.5822395583333	10.79796285	9.56634321666667	9.43278838333333	9.67682161666667	9.478582775	10.05157835	9.092829475	8.98368761666667	9.27125073333333	9.69747909166667	9.68903575833333	9.60540180833333	9.58797373333333	9.498614875	9.766909275	8.9925209	9.83406541666667	10.3623207166667	9.47351875833333	9.45281165833333	9.15449361666667	9.360413025	9.49147149166667	8.84243295833333	9.75367038333333	9.65802264166667	9.11174605833333	10.7116237833333	9.23605358333333	10.3362368833333	9.54178184166667	9.19020434166667	9.52826018333333	10.0489380083333	9.524079925	9.82382424166667	9.61553475	9.557602025	10.0783158833333	9.36802801666667	9.32186685	9.16874195833333	9.46343676666667	9.79891768333333	9.17867269166667	9.51996269166667	10.039605625	9.10844005833333	9.497335375	9.58344561666667	9.754485575	9.10749988333333	9.932660725	8.52683058333333	10.0313344083333	9.40032650833333	10.220242425	9.46704600833333	9.50773276666667	9.51549753333333

HERC4	11.5824943666667	10.8120670333333	10.880387475	10.7581339416667	11.3820783333333	11.2018586833333	10.6092076916667	10.4480363583333	10.7919885666667	10.99905285	10.4975657166667	10.864263475	11.2402727333333	10.852937175	11.4474681583333	10.76764485	10.563601	11.3262989291667	11.4207192166667	11.0491091083333	10.980233275	11.3867866583333	11.0250402166667	11.4357643916667	11.1520832083333	10.7669402083333	11.1081520166667	11.040528175	11.7955610083333	10.86272805	11.030517625	10.9433662916667	10.766208375	10.5921937	10.9730535833333	10.6910121333333	11.0663049916667	10.97339425	10.97439825	11.0913458083333	10.8349029416667	11.2068826333333	11.2872125416667	11.0083196416667	10.9160737916667	10.8666003833333	11.0693836916667	11.1806950666667	10.8001996166667	10.8326576083333	11.08338295	11.3886248333333	11.426633325	11.4864514	11.0094185166667	11.0002353666667	11.0845103416667	11.0560163166667	11.220898225	11.0196702	10.683111325	10.5659717333333	10.8473279166667	11.5392863166667	10.4394323833333	11.9204648416667	10.7767059333333	10.627804175	10.8014508	10.7778847083333	10.2638424166667	11.1025248083333	11.9661980166667	10.826565775	11.4553193833333	11.0783715833333	11.41004175	11.2689689833333	11.2233924	10.7710861166667	10.51932965	11.0619074416667	11.5809197583333	10.6352329833333	11.4901622083333	11.2327259583333	10.7643055083333	10.4526526416667	10.5420798333333	10.76392445	11.0017295	10.9412405333333	11.049779175	11.1941109166667	11.119251825	10.501807225	11.034816875	10.9021357916667	11.3861262916667	10.546063825	11.44566875	10.6895391083333	11.3324973416667	11.2011511333333	10.9440376833333	11.3961375	11.3571575333333	11.2689689833333	11.368673525	11.4549596	10.9909672	10.6449643666667	11.7269658416667	11.2240675166667	11.14949005	10.9386855666667	10.4942942166667	11.1132646583333	10.938251225	11.0787308166667

MMP16	9.14426045	6.90123264166667	7.58800115	6.60826071666667	6.7503122	7.61062251666667	6.869498575	6.54365829166667	6.083461775	7.299009625	6.63942924166667	9.12794545833333	7.41352716666667	7.107578925	6.12934905	7.64552340833333	6.5005932	5.52437941666667	6.80853135	7.51472684166667	7.603571	5.79916375	7.366539375	7.20334935833333	7.28678616666667	8.64390555	7.47677956666667	6.99727938333333	6.21689609166667	7.05585056666667	7.17681736666667	6.5680586	7.102506125	5.70748306666667	8.51764245	8.54744824166667	6.21280073333333	7.49549410833333	7.58577588333333	8.10391784166667	7.57732030833333	7.10888616666667	7.83240795833333	7.41882361666667	7.906291625	7.27171180833333	8.07411434166667	6.58901686666667	7.57569364166667	7.821740775	7.39926451666667	6.10074335833333	7.41824386666667	7.49991619166667	7.05651791666667	8.07471265833333	7.76455869166667	7.72686941666667	7.52054051666667	7.69103884166667	6.93484450833333	7.62089398333333	6.72653388333333	8.61442265833333	11.9333501833333	7.57513376666667	7.81839644166667	7.238929725	7.48649204166667	6.99218681666667	6.09195375	7.48985504166667	8.140254425	7.71189573333333	6.76657179166667	8.817877875	6.62367098333333	6.92042344166667	7.9977282	6.311629325	11.2442138	9.09684721666667	8.00309365833333	9.1396151	9.058233875	8.42522444166667	6.58901686666667	8.55288418333333	6.16692735	8.65218976666667	8.61442265833333	6.755806875	7.173894	8.369572325	8.72229471666667	5.72573654166667	6.28336628333333	5.86905295	8.89174859166667	6.61484865833333	8.17505753333333	6.737597225	6.30861273333333	6.777132775	7.38123391666667	7.160095	8.594439725	7.16232548333333	7.85854243333333	8.90864591666667	6.37713004166667	5.77807068333333	8.608522725	8.36187725833333	7.65663390833333	6.33264478333333	8.816929025	8.56272661666667	6.179361325	6.20180194166667

AACS	9.57267214166667	12.0839641583333	11.9040111333333	12.19191635	11.656217175	11.0491091083333	11.512504775	11.84426255	11.8178724083333	11.5302262416667	11.3752975833333	9.11495396666667	10.885465	11.8209586416667	11.9455284	11.0826274416667	11.26859775	11.5538304416667	12.496314025	11.6587921	11.5847091416667	11.8997209666667	11.4137091166667	11.5162409083333	11.57134605	9.48031518333333	11.8422473333333	11.8085467416667	11.7063908333333	12.0322027833333	11.74982705	11.73400915	11.5027335583333	11.4133387916667	9.87345979166667	9.66826963333333	11.884179625	12.1096451083333	11.3406807666667	11.3664862333333	11.8642752583333	12.1525189	11.9785582833333	11.9267180916667	11.0625918083333	11.9697901833333	12.007698575	12.2943062333333	11.8194805166667	11.9475058416667	11.8431168166667	11.8772191666667	11.4574143583333	12.11543585	12.1737118583333	12.0934360416667	11.8220738583333	12.1308631333333	11.705716875	11.5873432	9.67854975	11.21653435	11.407195875	11.3096843916667	10.46219945	11.5606001833333	11.17067005	10.9483433833333	11.2043416583333	11.0038210166667	11.0487579166667	11.297940475	10.94470365	10.9021357916667	11.7266159583333	10.1529084083333	10.2579212583333	10.9314304333333	11.6496725666667	10.765064075	9.382290975	10.8450341833333	12.2366655666667	10.9147091916667	11.2986150666667	9.88515190833333	9.93042398333333	10.194475875	11.5961336833333	10.609691175	11.328911575	11.8848963583333	11.09094715	11.1975258	11.24709275	11.2043416583333	10.0898576	10.007360325	10.8480286083333	11.8461878583333	10.8721591833333	11.8379474833333	11.4597359083333	11.3817027833333	12.18354635	10.74562245	11.4096653083333	11.6569750083333	11.012770325	10.8650559083333	11.4822891291667	11.5363521916667	11.3539068166667	11.0761932833333	11.0556311333333	11.8348518583333	9.89010159166667	11.2157921833333	10.627440475	10.9987091583333

SULT1A3	12.1453184125	12.2278658583333	12.2221143375	12.3773857541667	12.2983697708333	13.1496249208333	12.1465210041667	11.9422276666667	11.4852863083333	12.73615865	13.1803611708333	13.0795498541667	12.2681612875	11.72800655	12.013867925	12.5508988791667	13.270839425	12.0675907583333	11.6869215791667	12.2208720375	12.4998470416667	11.9023237541667	12.0307609125	12.0866895125	12.1412178458333	12.3149814416667	12.0626018291667	11.9343038625	11.7923356291667	12.1642518458333	11.8444630125	12.319755575	12.2217037541667	13.0365685041667	11.627674475	11.860570525	11.9722772541667	11.3529603708333	11.6953843625	11.9814988083333	12.0315794416667	11.9718844333333	12.0346679458333	11.9287353375	12.8809936666667	12.0787689041667	11.8613787333333	11.8068282916667	12.3467918583333	12.4393159791667	12.0626018291667	11.8817118	12.0271688	12.0422718666667	11.5903534041667	11.7934537333333	11.855206375	11.7744663166667	12.1813216291667	12.1144265625	12.952459625	12.2842455916667	12.38181285	12.1165066666667	12.61493455	12.6697077541667	11.6250906083333	12.8237470375	12.000740025	13.211948475	12.5554961291667	12.3540191458333	11.8459880583333	12.4379884583333	12.1928543666667	11.6950002791667	12.8658737125	13.1121237333333	12.1971035	12.6434579833333	12.4983591291667	12.4735098708333	11.9129908166667	11.6711104833333	12.077992225	12.9555343666667	12.5708438291667	12.1247999541667	12.6439553916667	12.6158711375	12.1486557291667	13.4899359875	13.2224653291667	11.9869807083333	12.1445343583333	12.0094356791667	12.0011336333333	11.9643557083333	11.5179760333333	12.5762494	12.3260894583333	12.6942150375	13.933643525	12.299665725	12.812914675	12.3482054	12.4539039291667	12.475769975	13.0813328708333	11.9186783291667	12.7978924416667	11.8437140791667	11.56786105	11.6766534875	11.7577200708333	12.7819926916667	12.1606367208333	11.9984064583333	12.9561646125	12.810483825

SULT1A4	12.1453184125	12.2278658583333	12.2221143375	12.3773857541667	12.2983697708333	13.1496249208333	12.1465210041667	11.9422276666667	11.4852863083333	12.73615865	13.1803611708333	13.0795498541667	12.2681612875	11.72800655	12.013867925	12.5508988791667	13.270839425	12.0675907583333	11.6869215791667	12.2208720375	12.4998470416667	11.9023237541667	12.0307609125	12.0866895125	12.1412178458333	12.3149814416667	12.0626018291667	11.9343038625	11.7923356291667	12.1642518458333	11.8444630125	12.319755575	12.2217037541667	13.0365685041667	11.627674475	11.860570525	11.9722772541667	11.3529603708333	11.6953843625	11.9814988083333	12.0315794416667	11.9718844333333	12.0346679458333	11.9287353375	12.8809936666667	12.0787689041667	11.8613787333333	11.8068282916667	12.3467918583333	12.4393159791667	12.0626018291667	11.8817118	12.0271688	12.0422718666667	11.5903534041667	11.7934537333333	11.855206375	11.7744663166667	12.1813216291667	12.1144265625	12.952459625	12.2842455916667	12.38181285	12.1165066666667	12.61493455	12.6697077541667	11.6250906083333	12.8237470375	12.000740025	13.211948475	12.5554961291667	12.3540191458333	11.8459880583333	12.4379884583333	12.1928543666667	11.6950002791667	12.8658737125	13.1121237333333	12.1971035	12.6434579833333	12.4983591291667	12.4735098708333	11.9129908166667	11.6711104833333	12.077992225	12.9555343666667	12.5708438291667	12.1247999541667	12.6439553916667	12.6158711375	12.1486557291667	13.4899359875	13.2224653291667	11.9869807083333	12.1445343583333	12.0094356791667	12.0011336333333	11.9643557083333	11.5179760333333	12.5762494	12.3260894583333	12.6942150375	13.933643525	12.299665725	12.812914675	12.3482054	12.4539039291667	12.475769975	13.0813328708333	11.9186783291667	12.7978924416667	11.8437140791667	11.56786105	11.6766534875	11.7577200708333	12.7819926916667	12.1606367208333	11.9984064583333	12.9561646125	12.810483825

ANKRD30A	5.55862928333333	5.08252440833333	4.59997769166667	4.90586546666667	5.16314315	4.55184448333333	4.90586546666667	5.18312655	4.7771511	4.506429475	4.85550948333333	4.9195424	4.760314225	4.19434771666667	5.60319860833333	4.12229528333333	4.649483075	5.65521334166667	5.53996425	4.83765554166667	4.08433028333333	4.98219815	4.45602740833333	4.74641638333333	4.65070125	4.003549775	5.25133600833333	5.390724	5.14597308333333	5.02689723333333	7.03751019166667	4.695239575	4.87595029166667	4.93552183333333	4.576370125	4.44059075	5.0100817	5.57148620833333	5.14089556666667	4.97490869166667	5.125178525	4.46402134166667	4.832334525	5.50024889166667	4.2231712	5.04762459166667	4.98009509166667	6.96778875833333	4.59683404166667	5.07218190833333	4.73928888333333	6.04523669166667	5.47315375	5.388701925	4.87710051666667	5.37867954166667	5.21821305	6.57166325	5.06570035833333	4.563953975	7.24945885833333	4.53327731666667	4.90679766666667	4.4227704	4.125728225	4.58982808333333	4.55184448333333	4.91206978333333	4.32588960833333	4.37388213333333	4.62375229166667	5.363009525	4.28310633333333	3.80321476666667	4.24624565833333	4.33846348333333	4.125728225	5.01735620833333	5.170393	3.764611825	4.2289531	5.091030975	4.289092625	4.76141500833333	4.85447411666667	4.15011734166667	5.118961025	4.46516671666667	3.867233575	5.37341235	4.265394625	4.37610179166667	4.418789625	4.66519471666667	4.45205615	4.43313565833333	4.44822564166667	5.3343362	4.30437938333333	4.96401284166667	4.40687995833333	3.98827198333333	5.189937375	4.92557835833333	4.46696494166667	5.13437999166667	4.49216794166667	7.5324158	4.61625569166667	6.495300075	4.49216794166667	5.5319599	5.49736965	4.909691175	4.55466009166667	4.50011030833333	4.13540660833333	5.42880058333333	5.42554189166667	5.80728855

BRD3	12.6566773416667	11.244965975	11.39042995	11.3516750666667	11.1641077166667	12.0067796166667	11.1554142916667	11.7198061583333	11.5606001833333	11.0215105083333	11.3654035916667	12.0989443916667	11.6310321916667	11.3790430333333	11.60515845	11.66214955	11.6887018916667	11.174277875	11.4004623	11.4511549916667	11.5400258416667	11.7123616416667	11.15581865	11.48757865	11.4824869416667	12.5282693916667	11.3036645333333	11.4586326	11.1321175166667	11.5064020583333	11.3942748	11.69368565	11.3946703583333	12.0868849916667	12.3003464583333	12.6914097333333	11.4748997416667	11.1477174666667	11.5414769166667	11.7513402	11.3827745833333	11.545061825	11.6948221166667	11.9333501833333	12.479955875	12.1071523416667	11.85384455	11.3057739666667	11.3915472416667	11.432485775	11.0200205166667	11.1760549666667	11.6170945166667	11.6408490416667	12.1167008333333	12.07292235	11.4470684833333	11.655146	11.3539068166667	11.5103338083333	11.9670214	12.3126780333333	11.3976073583333	11.5118143666667	12.414568125	10.8337493916667	11.7081843583333	12.5102327333333	11.8051550333333	12.4265910916667	11.7494484416667	12.3258832166667	11.683415075	12.034117575	12.7170209166667	11.8943464416667	11.3360764166667	11.3231776	11.3206346333333	11.9509854083333	12.959876375	12.5448126916667	11.1383591416667	12.6956383666667	11.5645444166667	12.9871205833333	13.1622146833333	12.515033225	12.9012827916667	11.980095625	11.9368778583333	12.0140512666667	12.2107949833333	11.7459533166667	11.684519	11.44422215	11.29542035	13.0204814	11.8527274916667	12.840994225	12.317829175	12.1694652083333	11.7243383416667	11.69593475	12.0522158916667	11.9597302583333	11.8717593666667	12.8460196	11.6367008333333	11.9259261416667	11.1387134583333	11.947152875	11.9883632916667	11.606939225	11.62870925	11.9158492083333	12.5580702166667	12.1205448666667	11.63518155	11.9368778583333

ADAM7	8.03480338333333	7.715267425	8.1959042	7.52443165	8.28798768333333	8.03223831666667	8.2053418	7.57227085	7.900836625	7.85962578333333	7.50852245833333	7.9184414	7.75009851666667	6.94497745833333	7.33560889166667	7.496531175	8.105836875	8.159108975	7.88506155833333	7.75823035	6.59533788333333	7.019638175	7.6315346	7.22899075833333	7.47562453333333	7.57903273333333	7.47204458333333	7.70760366666667	8.4773978	7.65388535833333	7.960228575	8.002520925	7.64327454166667	8.76654949166667	8.1565621	8.31911250833333	8.113521575	8.39672325833333	7.681528525	8.35169179166667	7.65996385833333	8.11057064166667	7.29233335	8.084825575	7.31861950833333	7.78057345	7.88712554166667	7.56380908333333	7.55925806666667	7.597182875	8.47046444166667	8.26509473333333	8.472359075	8.40377353333333	8.27339385833333	8.030568675	7.75772005833333	8.16389916666667	8.15349528333333	7.657177025	7.79744025833333	7.93641930833333	7.69103884166667	7.5998906	7.900836625	7.583460175	7.26110544166667	7.655449725	7.01426715833333	7.68936630833333	7.81401100833333	7.767746725	7.57732030833333	7.198288025	7.35173706666667	7.079224475	7.48698644166667	7.079224475	7.49164054166667	7.21162745833333	7.3367108	6.92241575	6.627072075	7.43179573333333	7.44735373333333	6.65552751666667	8.05141264166667	7.56491295833333	8.47850595	7.40135389166667	7.456643225	7.96301623333333	7.68829090833333	8.21881454166667	7.93695109166667	7.61006516666667	7.57783686666667	8.11825858333333	7.53506526666667	7.8129644	7.52107438333333	7.160095	7.227214725	7.34497685	7.61062251666667	7.46243825	7.683805725	8.02019604166667	7.66558521666667	7.88712554166667	7.92964085	8.576058475	8.107904375	8.13617131666667	8.18315743333333	7.9291649	7.95359058333333	7.95194513333333	8.14297786666667	7.89196619166667

MOB3C	11.2956085666667	10.6650917208333	10.9118469166667	10.6829236833333	10.5415137166667	10.4730803333333	10.25729555	10.75957225	10.5792938041667	10.1491614458333	10.4817099708333	10.3676792708333	10.3605011416667	10.5187550375	10.8599199958333	10.8603023375	10.4255098291667	10.6583477291667	10.9675186041667	10.5697559625	10.8686330291667	11.002258125	11.1886201666667	11.4433037375	10.6081838666667	11.4595644791667	10.6897055666667	10.7195806916667	10.4828446375	10.8802133541667	10.9889485	10.6258028791667	10.5587544208333	10.4415480375	11.5138082166667	11.2232063458333	10.08921235	10.7156824958333	11.4362877666667	10.8565258291667	11.046035575	11.2332577458333	10.8955074291667	10.8309733083333	10.9323187791667	10.9778274875	10.986370725	10.8798397583333	10.7412825791667	10.8283137	10.1387853291667	11.1215877916667	10.7977804458333	10.9988810041667	11.1969501583333	11.0085124916667	10.8130023291667	10.6836843458333	10.569391625	10.926151175	11.2238904333333	10.8167097041667	11.2959993791667	10.9717516833333	10.4139942291667	12.4956599208333	10.4957078083333	11.231412525	11.0536536625	10.8080412541667	11.4800419958333	10.5989293166667	11.7626571833333	10.8636915208333	11.4234808458333	10.77382425	10.8275606916667	10.60333685	11.015195575	11.2091517833333	10.0191618083333	9.78759450833333	10.7837402125	11.2593356208333	10.506333375	10.2504299333333	10.0016218333333	10.5466440833333	10.2176713	11.2493932541667	11.0194946458333	10.8614308541667	11.1947079416667	10.8614308541667	11.2034095375	11.1882787958333	10.3869899	9.76029368333333	10.5985614375	10.992355675	11.0752782541667	11.1465160291667	11.1658038541667	11.3469230291667	11.5335937	11.9280116625	11.0624291166667	11.3073739666667	10.731189	11.3045319458333	10.5320614041667	11.3733746583333	11.4047692833333	11.0576385708333	11.298454325	11.5062034083333	10.0672366333333	11.2593356208333	11.8937645208333	11.1704907875

MKNK1	11.2956085666667	10.6650917208333	10.9118469166667	10.6829236833333	10.5415137166667	10.4730803333333	10.25729555	10.75957225	10.5792938041667	10.1491614458333	10.4817099708333	10.3676792708333	10.3605011416667	10.5187550375	10.8599199958333	10.8603023375	10.4255098291667	10.6583477291667	10.9675186041667	10.5697559625	10.8686330291667	11.002258125	11.1886201666667	11.4433037375	10.6081838666667	11.4595644791667	10.6897055666667	10.7195806916667	10.4828446375	10.8802133541667	10.9889485	10.6258028791667	10.5587544208333	10.4415480375	11.5138082166667	11.2232063458333	10.08921235	10.7156824958333	11.4362877666667	10.8565258291667	11.046035575	11.2332577458333	10.8955074291667	10.8309733083333	10.9323187791667	10.9778274875	10.986370725	10.8798397583333	10.7412825791667	10.8283137	10.1387853291667	11.1215877916667	10.7977804458333	10.9988810041667	11.1969501583333	11.0085124916667	10.8130023291667	10.6836843458333	10.569391625	10.926151175	11.2238904333333	10.8167097041667	11.2959993791667	10.9717516833333	10.4139942291667	12.4956599208333	10.4957078083333	11.231412525	11.0536536625	10.8080412541667	11.4800419958333	10.5989293166667	11.7626571833333	10.8636915208333	11.4234808458333	10.77382425	10.8275606916667	10.60333685	11.015195575	11.2091517833333	10.0191618083333	9.78759450833333	10.7837402125	11.2593356208333	10.506333375	10.2504299333333	10.0016218333333	10.5466440833333	10.2176713	11.2493932541667	11.0194946458333	10.8614308541667	11.1947079416667	10.8614308541667	11.2034095375	11.1882787958333	10.3869899	9.76029368333333	10.5985614375	10.992355675	11.0752782541667	11.1465160291667	11.1658038541667	11.3469230291667	11.5335937	11.9280116625	11.0624291166667	11.3073739666667	10.731189	11.3045319458333	10.5320614041667	11.3733746583333	11.4047692833333	11.0576385708333	11.298454325	11.5062034083333	10.0672366333333	11.2593356208333	11.8937645208333	11.1704907875

PILRB	10.91589415	11.3152530458333	12.2538754291667	11.9838354166667	11.6600748541667	10.5028481541667	11.8759321458333	11.3041958208333	11.5763517208333	10.8508691833333	11.4352354333333	10.7875918625	11.113085325	10.5270413458333	11.2253067083333	11.3156133375	10.2106099416667	11.3751334208333	10.2172480291667	11.1009079583333	10.11945695	10.711044925	10.6323665375	11.0070250291667	10.9885999458333	10.3716298916667	12.1348660958333	12.022040825	11.5058183	11.4051191333333	11.7166202666667	12.0162554833333	11.5489326166667	10.3504978541667	10.3042109291667	10.2361346333333	11.6312174625	11.7058893458333	11.3006586041667	11.7427551916667	11.3401629708333	11.5460149333333	11.2751551541667	10.91589415	10.8895121625	10.6594312291667	10.8159554791667	10.7996179	11.0806363458333	11.31969535	11.2622614208333	10.4047014541667	11.530403975	11.16800125	11.4341960125	10.8167097041667	10.8883688916667	10.9686903291667	11.3073739666667	10.47002835	12.0275758416667	10.6315499291667	11.2562632708333	10.743821075	11.0137151833333	10.8441540625	10.1672779333333	10.690430875	10.1306112708333	10.5611193583333	10.6315499291667	10.652148475	10.2440530041667	10.0935452083333	10.505920625	10.3612639166667	11.0245256375	10.9634574041667	9.99280202916667	10.2792433416667	11.3006586041667	11.3648148708333	10.0084946125	9.98053730833333	10.0605352083333	10.6124720583333	12.0472317	10.2041541041667	11.0216927166667	9.98656490833333	10.0448405	12.0354571708333	10.8000125916667	10.1627032583333	10.3998400708333	10.5737228125	10.5020059583333	11.535463975	10.4063314208333	10.6782925458333	10.8016198125	11.0058676	10.93956655	10.3299727041667	10.5645419458333	10.2078788125	9.8530618375	10.3367707791667	9.76533135833333	9.58198016666667	10.365440425	10.3885498041667	10.4039049166667	11.2170633625	10.37690845	10.9893273041667	10.4651231083333	11.4881485416667	11.3131039666667	10.8448776375

PILRA	10.91589415	11.3152530458333	12.2538754291667	11.9838354166667	11.6600748541667	10.5028481541667	11.8759321458333	11.3041958208333	11.5763517208333	10.8508691833333	11.4352354333333	10.7875918625	11.113085325	10.5270413458333	11.2253067083333	11.3156133375	10.2106099416667	11.3751334208333	10.2172480291667	11.1009079583333	10.11945695	10.711044925	10.6323665375	11.0070250291667	10.9885999458333	10.3716298916667	12.1348660958333	12.022040825	11.5058183	11.4051191333333	11.7166202666667	12.0162554833333	11.5489326166667	10.3504978541667	10.3042109291667	10.2361346333333	11.6312174625	11.7058893458333	11.3006586041667	11.7427551916667	11.3401629708333	11.5460149333333	11.2751551541667	10.91589415	10.8895121625	10.6594312291667	10.8159554791667	10.7996179	11.0806363458333	11.31969535	11.2622614208333	10.4047014541667	11.530403975	11.16800125	11.4341960125	10.8167097041667	10.8883688916667	10.9686903291667	11.3073739666667	10.47002835	12.0275758416667	10.6315499291667	11.2562632708333	10.743821075	11.0137151833333	10.8441540625	10.1672779333333	10.690430875	10.1306112708333	10.5611193583333	10.6315499291667	10.652148475	10.2440530041667	10.0935452083333	10.505920625	10.3612639166667	11.0245256375	10.9634574041667	9.99280202916667	10.2792433416667	11.3006586041667	11.3648148708333	10.0084946125	9.98053730833333	10.0605352083333	10.6124720583333	12.0472317	10.2041541041667	11.0216927166667	9.98656490833333	10.0448405	12.0354571708333	10.8000125916667	10.1627032583333	10.3998400708333	10.5737228125	10.5020059583333	11.535463975	10.4063314208333	10.6782925458333	10.8016198125	11.0058676	10.93956655	10.3299727041667	10.5645419458333	10.2078788125	9.8530618375	10.3367707791667	9.76533135833333	9.58198016666667	10.365440425	10.3885498041667	10.4039049166667	11.2170633625	10.37690845	10.9893273041667	10.4651231083333	11.4881485416667	11.3131039666667	10.8448776375

EFCAB11	9.91460061666667	10.2782382416667	10.2387377833333	10.5949115083333	10.0373878833333	9.85452464166667	10.2838388166667	10.4191933083333	10.5837654416667	10.2387377833333	10.7078134666667	9.79724158333333	9.65802264166667	10.3317121166667	10.621550725	9.85722324166667	9.93555589166667	10.342051525	10.1291621416667	10.2741449416667	10.237546325	10.6139707	10.21946315	10.1628878833333	10.115233725	10.3678756583333	10.276116525	10.3236813083333	9.73788834166667	10.225450075	10.0855958166667	10.1745820166667	9.87908794166667	9.91970055833333	10.66752385	10.63689965	9.98800139166667	10.1565645083333	9.8834328	10.226244575	10.2289590916667	10.051171975	9.82822335	10.2716281	9.88515190833333	10.095377	10.31123355	9.96018530833333	10.3305153666667	10.138226575	9.666049875	9.9799425	9.72795155833333	10.1009165916667	10.316356175	10.0167779583333	9.92747266666667	10.021871175	10.1978484	9.71356900833333	10.240361675	9.62925015833333	10.7470371333333	10.9419805083333	10.7217139416667	10.594157325	10.235923825	10.4958848833333	11.5610238416667	10.3667222333333	10.1770845916667	9.4134397	10.9812562333333	9.33838469166667	10.9328378333333	10.2092790583333	10.4698289916667	10.3373213583333	10.7601275	10.7851401916667	10.3702407083333	9.81305005833333	10.6842469	10.26626505	9.35167163333333	10.769931875	9.77571775833333	9.99343915	9.82339220833333	10.2427233083333	10.6934456416667	10.6068679083333	9.78567400833333	10.3171296666667	10.2865677	9.91930759166667	10.5112057333333	9.70796235833333	9.94294464166667	9.157883875	10.2658011666667	9.72795155833333	10.2431199666667	10.239159725	10.632187725	9.98633354166667	10.0937438833333	11.2933100833333	10.0524736083333	10.4417307666667	9.29384936666667	10.1808367916667	10.521634775	10.839776075	10.1397194333333	10.2728709416667	9.579948275	11.3886248333333	10.694500425	10.8870209166667

GSTM5	12.3919605166667	12.370533275	12.0442765708333	12.98920625	11.7154865958333	11.2532573833333	11.56786105	11.706226325	11.9953072583333	11.88323765	13.4987694916667	12.0671528625	11.9668296208333	11.5069188375	12.1626225916667	11.5126892291667	12.7507385583333	12.3193074083333	11.9511713541667	11.8330932333333	11.3655952	12.5678498041667	13.21913615	12.0374601958333	11.8451951541667	12.3773857541667	11.0536536625	12.103242775	10.7922036625	12.3915030625	12.2324496625	12.6424634291667	11.6501703	12.6643328208333	12.1966782875	11.8315480833333	11.5580599041667	12.1962582875	11.7766814125	12.4591427833333	12.112389675	11.8960909708333	11.7665731666667	11.3959616166667	11.8968226833333	12.0034735458333	11.7314116291667	12.1264399583333	10.9660080958333	12.3234787791667	11.8747674166667	11.627269825	11.9587602083333	11.8578608541667	12.3112413791667	10.9529550708333	10.6148944333333	11.1822871166667	11.0411335166667	11.9692107958333	12.7705166833333	15.3037720833333	12.7604893958333	11.9366819583333	11.8098353375	10.6106220083333	13.8620140625	12.5304303833333	12.3213974708333	10.6463560333333	14.5258868958333	14.2452054583333	11.5763517208333	14.231255875	12.1098623833333	13.2178216083333	9.9130955875	13.7022579333333	13.54982165	14.2786647875	10.8185713583333	12.2152361833333	12.2026550583333	14.1979037666667	10.5792938041667	11.2791978708333	10.1368662333333	9.80119757083333	14.4369887875	13.6556207416667	11.335569425	12.332498125	13.2396261041667	12.1318869708333	10.5366896916667	13.8153117666667	13.0164748458333	10.3165349541667	11.8755577875	10.7462083208333	9.84653941666667	14.231255875	9.10727307916667	11.7812801	9.77241673333333	12.6893793208333	11.6512910125	11.6423984791667	9.67434395	10.6908038625	12.1683832875	12.7952533708333	9.63365371666667	11.5300508916667	11.8839993208333	15.5287903666667	9.416443775	10.3238867833333	12.3599793541667	10.2180654291667

GSTM3	12.3919605166667	12.370533275	12.0442765708333	12.98920625	11.7154865958333	11.2532573833333	11.56786105	11.706226325	11.9953072583333	11.88323765	13.4987694916667	12.0671528625	11.9668296208333	11.5069188375	12.1626225916667	11.5126892291667	12.7507385583333	12.3193074083333	11.9511713541667	11.8330932333333	11.3655952	12.5678498041667	13.21913615	12.0374601958333	11.8451951541667	12.3773857541667	11.0536536625	12.103242775	10.7922036625	12.3915030625	12.2324496625	12.6424634291667	11.6501703	12.6643328208333	12.1966782875	11.8315480833333	11.5580599041667	12.1962582875	11.7766814125	12.4591427833333	12.112389675	11.8960909708333	11.7665731666667	11.3959616166667	11.8968226833333	12.0034735458333	11.7314116291667	12.1264399583333	10.9660080958333	12.3234787791667	11.8747674166667	11.627269825	11.9587602083333	11.8578608541667	12.3112413791667	10.9529550708333	10.6148944333333	11.1822871166667	11.0411335166667	11.9692107958333	12.7705166833333	15.3037720833333	12.7604893958333	11.9366819583333	11.8098353375	10.6106220083333	13.8620140625	12.5304303833333	12.3213974708333	10.6463560333333	14.5258868958333	14.2452054583333	11.5763517208333	14.231255875	12.1098623833333	13.2178216083333	9.9130955875	13.7022579333333	13.54982165	14.2786647875	10.8185713583333	12.2152361833333	12.2026550583333	14.1979037666667	10.5792938041667	11.2791978708333	10.1368662333333	9.80119757083333	14.4369887875	13.6556207416667	11.335569425	12.332498125	13.2396261041667	12.1318869708333	10.5366896916667	13.8153117666667	13.0164748458333	10.3165349541667	11.8755577875	10.7462083208333	9.84653941666667	14.231255875	9.10727307916667	11.7812801	9.77241673333333	12.6893793208333	11.6512910125	11.6423984791667	9.67434395	10.6908038625	12.1683832875	12.7952533708333	9.63365371666667	11.5300508916667	11.8839993208333	15.5287903666667	9.416443775	10.3238867833333	12.3599793541667	10.2180654291667

KRTAP10-1	10.904495125	10.6533575916667	10.7075994125	12.0903471208333	11.5576792291667	12.5109579416667	11.5666746083333	11.3792232291667	12.1780362291667	11.1918332083333	12.4134385791667	11.4962103791667	11.4483795708333	10.46240375	10.3881719166667	11.1086892666667	10.3943299	11.64872385	10.4435740291667	10.4583683333333	10.606680675	10.8805666833333	10.2260338333333	11.55504505	11.4980225	10.4738469083333	10.3033271208333	10.808435125	11.0724979916667	10.6643372291667	10.5866839916667	10.3509031625	11.9057636208333	11.0922553291667	10.4222240333333	10.6217312958333	11.7957279	11.336614125	11.540957775	11.0907714458333	11.6388650291667	10.66090735	10.33034285	10.3857734791667	10.5113945291667	10.0262798583333	10.086198475	10.8366353541667	11.3120093875	12.6947858458333	10.8096034958333	11.0382299708333	10.6778788875	10.4636324041667	10.3037668333333	9.83385274583333	10.8648502083333	11.1838672833333	10.4696731083333	10.5129243375	11.0436000125	11.0627641416667	10.3624811458333	11.0817075916667	10.6517710541667	11.7733518416667	10.7040820666667	11.3116791458333	11.2680548458333	11.1493422958333	11.2332577458333	10.9606005666667	11.1355991333333	10.5491867083333	10.7528626791667	10.2930278375	11.1984805708333	10.9460665375	10.5167858375	10.6467227083333	10.2751315166667	10.6900690916667	10.8076566208333	10.2303795958333	11.0449918166667	10.8548652666667	11.0054868083333	11.038901025	11.0672054791667	9.9878090125	10.7195806916667	10.5079540791667	11.0032870208333	10.5629625583333	11.53767785	10.9522035708333	11.2515910291667	11.0806363458333	11.4234808458333	10.5729337958333	10.60333685	11.0411335166667	10.3072506083333	10.3881719166667	10.3425891416667	10.2307778708333	10.3708343541667	10.5781711541667	10.7025997416667	10.7458309958333	10.4844841708333	10.730087725	10.4318495375	9.9130955875	10.5594606458333	9.8557309375	10.2751315166667	10.5951139541667	10.3935444541667	10.5160562583333

TSPEAR	10.904495125	10.6533575916667	10.7075994125	12.0903471208333	11.5576792291667	12.5109579416667	11.5666746083333	11.3792232291667	12.1780362291667	11.1918332083333	12.4134385791667	11.4962103791667	11.4483795708333	10.46240375	10.3881719166667	11.1086892666667	10.3943299	11.64872385	10.4435740291667	10.4583683333333	10.606680675	10.8805666833333	10.2260338333333	11.55504505	11.4980225	10.4738469083333	10.3033271208333	10.808435125	11.0724979916667	10.6643372291667	10.5866839916667	10.3509031625	11.9057636208333	11.0922553291667	10.4222240333333	10.6217312958333	11.7957279	11.336614125	11.540957775	11.0907714458333	11.6388650291667	10.66090735	10.33034285	10.3857734791667	10.5113945291667	10.0262798583333	10.086198475	10.8366353541667	11.3120093875	12.6947858458333	10.8096034958333	11.0382299708333	10.6778788875	10.4636324041667	10.3037668333333	9.83385274583333	10.8648502083333	11.1838672833333	10.4696731083333	10.5129243375	11.0436000125	11.0627641416667	10.3624811458333	11.0817075916667	10.6517710541667	11.7733518416667	10.7040820666667	11.3116791458333	11.2680548458333	11.1493422958333	11.2332577458333	10.9606005666667	11.1355991333333	10.5491867083333	10.7528626791667	10.2930278375	11.1984805708333	10.9460665375	10.5167858375	10.6467227083333	10.2751315166667	10.6900690916667	10.8076566208333	10.2303795958333	11.0449918166667	10.8548652666667	11.0054868083333	11.038901025	11.0672054791667	9.9878090125	10.7195806916667	10.5079540791667	11.0032870208333	10.5629625583333	11.53767785	10.9522035708333	11.2515910291667	11.0806363458333	11.4234808458333	10.5729337958333	10.60333685	11.0411335166667	10.3072506083333	10.3881719166667	10.3425891416667	10.2307778708333	10.3708343541667	10.5781711541667	10.7025997416667	10.7458309958333	10.4844841708333	10.730087725	10.4318495375	9.9130955875	10.5594606458333	9.8557309375	10.2751315166667	10.5951139541667	10.3935444541667	10.5160562583333

EPB41L1	12.7655438666667	12.940616175	12.7453059583333	13.057870725	12.7836657416667	12.4050809083333	12.6023058	12.3236478416667	12.9278503083333	12.1990202166667	12.5734875583333	12.5907074583333	12.6512308	12.916130525	12.8387693166667	12.8493401833333	12.9629737583333	12.6744586916667	12.6640954083333	12.552088775	13.002567275	13.1700826333333	13.3775064	12.5420281	12.6684769583333	12.4257259083333	12.9185113	12.60273605	12.53672075	13.0700559416667	12.84211235	13.000133375	12.4581088416667	13.0566076	12.7071557666667	12.644626475	12.3280561	12.6541819833333	12.6306873916667	12.7328907083333	12.2792629666667	12.644626475	12.7812528833333	12.9155862583333	12.6456361916667	12.7531374166667	12.8181395166667	12.4037688166667	13.1026328916667	12.6754546333333	12.812914675	12.659113	12.6975412416667	12.6397345916667	12.6704503833333	12.3780252416667	12.728935875	12.2747673416667	12.5641864083333	12.1728373083333	12.3152059916667	11.3068495166667	13.2983134833333	12.5543618083333	11.9616964916667	11.5574931	11.2624348416667	14.00243415	12.3101776166667	12.7128638833333	13.2266532083333	11.7035007416667	10.852937175	11.9236129	12.5406352083333	11.9116934166667	13.1973842833333	12.0577849333333	11.06152365	11.8811110166667	11.9274761416667	11.6401085333333	11.0560163166667	11.5060047583333	11.512504775	11.6655385333333	10.1725196416667	11.6062715833333	11.9123960833333	11.7905923666667	11.633311275	11.8519854833333	12.52885135	11.8004315583333	11.0648135916667	12.1170603	13.1197255166667	11.1767887583333	10.9174580583333	11.9989974	11.0534747416667	12.9053364166667	11.873004525	12.48596835	12.0710070666667	13.232552625	11.2783121416667	11.6440773083333	12.1170603	11.535988725	12.2808809166667	13.101295875	11.3605041083333	12.315681175	10.384887925	12.440827975	10.988072975	12.7295358083333	12.6857168666667	12.5685278666667

OPLAH	9.734421025	11.0312303833333	10.801788825	11.0507418833333	10.7034642666667	9.89982799166667	10.4590182666667	10.5659717333333	10.8743697916667	10.1222883166667	10.1725196416667	9.03641039166667	9.95725063333333	10.6699485666667	11.1383591416667	9.97301156666667	10.8759847166667	11.04340915	10.6948786416667	9.81305005833333	10.3019992	10.9408814333333	10.7800644666667	10.4895361083333	10.511583325	9.54880464166667	10.1489854166667	10.5534355833333	10.43166005	11.02981265	10.5829592166667	10.6527698	10.2609607666667	10.4815083083333	9.585238675	9.70284449166667	10.8067325916667	10.7096961916667	10.4037201666667	10.5365078	10.5088788416667	11.1003561666667	10.7287943416667	10.7919885666667	9.93515624166667	10.50459975	10.8893434416667	10.7501138916667	10.7368008833333	10.2912155166667	10.3074544166667	10.6742121583333	11.0670126666667	11.0101518666667	10.8824750083333	10.8537358333333	10.4127726666667	10.7877953583333	10.627804175	10.1509476166667	10.3610713833333	10.2912155166667	10.878488075	10.33090645	11.2319732666667	11.2693525666667	9.55244275833333	10.8778360416667	10.8165123833333	10.6887964	10.6003674833333	11.0072108	9.65845149166667	10.431190025	10.3329358333333	10.4705726	10.2005317833333	10.625983	10.216230425	10.0292860833333	10.7793443666667	9.54225544166667	10.390544	9.36084673333333	10.6910121333333	10.5794976583333	11.1623033333333	10.41347785	10.2051938916667	9.67096889166667	9.80625096666667	11.1350432333333	9.74483329166667	10.4682944	10.4779275	10.6930678666667	11.3242829	10.9317956333333	9.06745036666667	11.517039175	10.7804261416667	10.8361133666667	11.6084924916667	10.186667875	10.5979724666667	10.6173169916667	10.64420985	8.92651730833333	9.496026575	9.33883618333333	10.061572075	10.1274668666667	10.5318759	10.76543495	10.8443256416667	10.515047025	10.4115783666667	10.7940186083333	10.123786625	10.0360710333333

C16orf95	7.070041725	6.9393833	7.53018445	7.650678625	8.10268665	6.7730469	7.94541711666667	7.050862275	7.47951195833333	7.05455971666667	6.95288179166667	7.60888643333333	7.28990879166667	6.783129725	7.50463459166667	6.9563535	6.67096843333333	7.63210018333333	6.70465071666667	6.66317971666667	7.29474379166667	6.78180254166667	6.92575055833333	7.05521155	6.49981934166667	6.54872438333333	6.4502938	6.72055851666667	6.84325476666667	6.97340769166667	6.42188529166667	6.56145719166667	6.91035588333333	6.97585598333333	6.49247578333333	6.46494565	7.22358631666667	6.91578644166667	7.08933194166667	6.42033848333333	6.47574773333333	6.107924175	6.43572405833333	5.72076731666667	6.09355109166667	6.150357875	6.49896905833333	6.78180254166667	6.59815521666667	6.85216409166667	6.083461775	6.46988230833333	6.88386930833333	6.37113890833333	6.19669175	6.38240346666667	6.80714281666667	5.966203775	6.73629286666667	6.66522548333333	7.36211884166667	7.671444425	7.57569364166667	7.52107438333333	7.188269975	6.65690114166667	7.25120925833333	7.29788705833333	6.91634240833333	7.03878675833333	7.36048969166667	7.57961929166667	7.32036674166667	6.77501233333333	6.99346504166667	7.439305775	7.14458345	6.96778875833333	6.80377665833333	7.561568325	7.074128375	7.85689433333333	6.73694150833333	7.572832925	7.17330060833333	6.48928715	8.31058729166667	6.53954924166667	7.94435703333333	6.72055851666667	6.88120535	7.03811636666667	8.09695651666667	6.67856683333333	6.72055851666667	8.28678805833333	8.215089275	7.607704375	7.35119398333333	6.87089175833333	6.5898034	7.72294644166667	6.53273724166667	6.99282755	6.49247578333333	6.39152516666667	6.987286825	6.179361325	6.74410944166667	6.38476215833333	7.086730875	6.59052684166667	6.56431505	7.18505906666667	6.263501475	6.5931033	7.506757	6.36473330833333	7.63210018333333	7.004281725

PBXIP1	11.2373633083333	8.180440425	8.4800381	8.288511175	7.90206838333333	9.50452185	7.34718618333333	8.68775965833333	8.67210133333333	7.69103884166667	8.14799835	9.81941439166667	9.40207443333333	8.41299383333333	9.120869625	9.38762769166667	10.4061408166667	8.685246125	9.33838469166667	9.37548823333333	8.94233088333333	8.67403964166667	8.85215985	9.1447536	9.16216856666667	10.8688118916667	9.30543436666667	8.4444208	9.01009886666667	8.647921775	9.26988130833333	8.88930546666667	8.64447529166667	9.58573579166667	11.2179166166667	11.178312475	8.63293045833333	8.47690594166667	9.17368278333333	9.34261419166667	9.13323439166667	9.09007516666667	9.187397175	9.25947800833333	9.79724158333333	9.32897005833333	9.437687475	8.54744824166667	8.93317065	9.11317438333333	8.75515696666667	8.73305545	9.02466219166667	9.283130225	9.29065144166667	9.94886909166667	8.71647500833333	9.10749988333333	8.935118175	9.131237375	8.96144149166667	7.85807856666667	8.46459865	8.02464245	10.273326675	8.10691838333333	8.7160017	8.28000224166667	7.95082734166667	8.23613304166667	7.23193100833333	8.09695651666667	7.49500395	8.475275825	8.50396570833333	7.94017044166667	8.74719128333333	8.799065075	8.2549704	8.51713660833333	8.511336025	10.0744319916667	8.164333775	8.45605063333333	8.56529179166667	8.26606834166667	7.927602375	8.13716783333333	7.31739324166667	9.01246160833333	8.56818220833333	7.91465324166667	8.40234485833333	8.37811988333333	8.59030590416667	8.1963928	7.74398054166667	8.07784593333333	7.738684825	9.12794545833333	8.665331325	7.81610706666667	9.11582745	9.02371780833333	7.97397919166667	9.01380788333333	9.2998268	8.53097458333333	9.058233875	8.76079081666667	9.12326560833333	8.78443150833333	9.02785883333333	8.92511409166667	8.998536575	8.70751021666667	9.22470684166667	8.73399778333333	8.42676914166667	8.360853775

ATXN7L1	10.7119916833333	10.2096666583333	10.3783237666667	10.5735065583333	10.7336987	10.5933754916667	11.0264670833333	10.3721682083333	10.5504732083333	10.1953308333333	10.1009165916667	10.8949867833333	10.782424925	10.341641975	10.5138272833333	10.1633244583333	10.689167625	10.7158953333333	9.93630081666667	10.2567002166667	10.2650558583333	10.8570968916667	10.0989513583333	9.82907005833333	10.1970378083333	10.4958848833333	10.5663053083333	10.6111916083333	10.9607806583333	10.4417307666667	9.92093743333333	10.165808925	10.3135871416667	10.7718274166667	10.34716255	10.568795625	10.8454086666667	10.1569732333333	9.69581614166667	10.2001534166667	9.82203395	9.985532675	9.79446515833333	9.57179898333333	9.88883589166667	9.84589375833333	9.83237755833333	10.0247316333333	9.81728660833333	9.77392395833333	9.628525675	9.76954021666667	10.5456854416667	10.3313573416667	10.406522025	9.95643681666667	9.98032226666667	9.69581614166667	10.356255775	9.77312443333333	11.0655176333333	10.217472825	10.5794976583333	11.8642752583333	9.94677204166667	11.04340915	10.3983831166667	10.5865107	10.3758856666667	10.4127726666667	10.4843028583333	10.6872865583333	10.7624960333333	10.1324487166667	10.9512840333333	10.8368196833333	10.6546458583333	10.81281805	10.06195985	10.5185399	10.384887925	11.102188775	9.983868575	9.728395625	10.7684448166667	10.4830527666667	10.6111916083333	10.4176619666667	10.85184045	9.82203395	10.1409369083333	9.92703645833333	10.259584625	9.64871909166667	10.248995	11.7190625916667	10.766208375	10.3179044666667	10.173327525	10.4519100666667	10.135861875	10.4630196333333	9.777390875	10.1895353083333	10.164124825	9.97131721666667	9.882085	10.58489685	9.83319184166667	9.79810515833333	9.496026575	9.92747266666667	9.96356175	11.7589935083333	10.1283050583333	9.67274871666667	9.87484248333333	10.0769619583333	10.090730825	10.962553725

TDO2	5.20354711666667	7.58465230833333	8.25839510833333	7.82746845	6.75503975833333	9.66421665	7.38404451666667	7.94329386666667	6.503440675	8.488485525	8.40280550833333	7.551793125	8.472359075	7.54963650833333	6.75238478333333	9.9560883	6.61154091666667	7.884494275	7.634487825	8.99535305	8.7637709	7.67599419166667	9.146936475	8.69984448333333	7.906291625	5.46549339166667	7.84323385	7.65326568333333	8.36034350833333	8.14495316666667	8.65271366666667	7.561568325	6.49981934166667	9.219699425	6.05897065833333	4.443420775	8.12989700833333	8.682814325	8.05664168333333	9.00507715833333	8.335541375	7.650678625	7.65894785833333	8.0964417	8.38708958333333	9.74483329166667	7.49219983333333	8.14192978333333	9.50182605833333	9.03865570833333	8.37508346666667	7.32727508333333	8.71882579166667	8.41972045	7.6798049	8.07622910833333	8.89220491666667	9.15019934166667	8.4371155	8.40965201666667	8.956732575	10.3968247166667	10.8368196833333	10.107173075	10.9371792916667	10.3996095166667	13.0301413666667	9.96443173333333	11.032984525	10.6960220666667	9.27941565	9.42590730833333	10.8113265583333	10.4292877666667	10.2289590916667	11.4296055416667	8.50844495	11.6115347666667	12.2875140666667	9.79062058333333	7.72139515	7.726376575	11.4926248916667	12.1304018333333	11.4022935333333	11.1039790666667	7.523817425	9.97000988333333	9.19391603333333	10.6776751083333	12.24577265	10.1670505916667	9.64383103333333	11.028339325	10.181229625	9.67274871666667	11.0101518666667	8.47579976666667	12.3224578583333	8.135196525	11.3433838833333	10.0326667666667	11.568787	10.6266595583333	10.4488232	10.9567732916667	11.036287075	11.4485644833333	11.9785582833333	11.799113925	8.77579595833333	10.703101075	10.86914835	12.004381825	11.3961375	9.84919238333333	10.3439808916667	10.79830065	9.91080910833333	11.572420475

ZNF365	10.7014785416667	13.132282	13.751113025	13.4681552083333	14.2073641166667	9.24630831666667	14.0643996166667	13.158283725	12.4682236083333	13.4721219	12.6694372	10.2414708	13.838661325	12.7564076083333	14.2073641166667	13.4375531583333	8.83592169166667	13.4427852416667	13.2806732833333	13.40102065	11.9670214	12.611240425	11.56339385	11.4042087666667	13.65238595	9.693930075	14.082470775	14.2576986333333	13.9045177166667	13.08588685	12.6317327416667	12.6436968916667	12.5775136	8.736017975	9.34481250833333	10.4449298	14.7077525	14.2145397416667	13.1832185416667	13.711942125	13.4594952083333	14.1006630916667	13.2567523666667	10.9054446583333	11.5578653583333	14.0734299166667	13.1046602416667	13.1512038916667	10.9328378333333	12.0619908166667	13.0264905416667	13.2366721916667	13.4760270333333	13.5372319083333	13.16610495	13.2434965	12.909394625	14.1164578666667	13.7452882916667	12.4795096083333	9.64383103333333	9.393424375	9.79810515833333	11.1132646583333	9.05602455	8.99487275833333	9.80576564166667	10.7569232666667	7.97346688333333	11.0811600833333	9.98111111666667	10.4433912083333	11.2373633083333	10.4224382166667	10.3570715916667	11.1010834666667	8.74766549166667	9.43184414166667	10.7589898916667	9.19627585833333	8.333019375	8.33112569166667	11.2997236583333	11.1196222666667	11.1046868	9.071149525	9.10433055833333	10.4705726	9.14921909166667	9.99259065	10.2427233083333	9.723283875	9.30864065833333	10.3519044416667	8.49244585833333	9.20652433333333	10.2642520083333	9.84956451666667	9.86598649166667	8.76175106666667	9.35125324166667	9.66781955833333	9.04228765833333	9.83544501666667	10.356255775	9.97624684166667	11.45939305	10.1160686416667	10.2625794333333	10.225450075	9.87303573333333	10.9643625833333	11.5245993916667	9.81728660833333	9.82339220833333	11.9001097666667	9.945018575	10.6352329833333	10.324926675	9.497335375

POU5F1	8.18684245	7.68593156666667	7.85258474166667	7.681528525	8.149040725	7.715267425	8.05249740833333	7.57619343333333	7.96133965	8.13819046666667	7.88506155833333	8.01539524166667	7.49164054166667	6.980300625	7.496531175	7.609434275	7.69369399166667	8.00309365833333	7.99131585833333	8.31409636666667	7.30740175	8.25344699166667	7.64914819166667	7.64275718333333	7.75823035	8.1205116	7.838427925	7.84154363333333	8.335541375	8.05664168333333	7.98087950833333	8.2260001	8.28541158333333	8.64688179166667	8.54598220833333	8.48745835	8.47183739166667	8.0954509	8.09223929166667	8.03595288333333	7.596603625	7.79244525833333	7.687727325	7.86994016666667	7.62593523333333	7.726376575	7.70199845833333	7.80971700833333	7.671444425	7.90941765	7.92110699166667	8.24707035	8.738873225	8.65999509166667	8.478972275	7.97507355833333	8.21046635	7.93641930833333	8.24707035	7.82691680833333	7.547481675	7.75983114166667	7.88559756666667	7.75234836666667	7.88978783333333	7.66047423333333	7.933308825	7.53565475833333	7.966758925	8.758367425	7.92336901666667	7.96192095	7.55678278333333	7.15940084166667	7.94172535833333	7.50966981666667	7.69605179166667	7.6421897	7.882902775	8.046473725	7.48588683333333	7.745650475	7.97557605833333	7.35983868333333	7.96240296666667	7.84430019166667	8.31814086666667	7.72796763333333	8.10268665	7.61811995833333	8.028895175	7.92654975833333	8.26606834166667	8.12174676666667	8.68083718333333	7.9422621	7.85748985	8.27339385833333	8.318633925	8.21830426666667	8.24415698333333	7.884494275	8.060776825	7.66718561666667	7.81893236666667	8.0519544	8.11406380833333	7.571028	7.60297259166667	7.18705289166667	8.00462699166667	7.900836625	8.44287714166667	8.392597175	8.50286535	8.09066366666667	8.13871308333333	8.31305365833333	8.159108975	7.907346175

DDX19A	11.173905775	10.4732782833333	10.7907764333333	10.4634265083333	11.5483812666667	10.7684448166667	11.3047106916667	10.6956354083333	10.949025	10.7530924583333	10.7816314666667	11.2668058583333	10.7748204	10.6187719166667	10.5627525083333	10.7842899166667	10.5134429166667	11.3627772083333	11.29542035	11.0709017666667	10.8277408416667	10.53724435	10.7725388333333	11.1542967583333	10.684995425	11.39829615	10.5138272833333	10.4228179333333	10.523602425	11.230835825	10.667166075	10.8337493916667	10.706587775	10.8269705916667	11.230125425	11.3664862333333	10.5081553333333	10.7050450333333	10.7725388333333	10.7696102583333	11.0175089583333	10.7789681833333	10.9898443833333	11.1815244666667	11.1343238833333	10.992544275	11.0811600833333	11.401232775	11.5589969083333	11.1270209166667	11.205753575	11.544719375	10.806391425	10.8450341833333	10.51932965	10.489906475	11.407195875	11.3296296583333	11.14881755	11.07367765	11.5258171833333	11.1912222083333	11.3375225333333	10.6364744	11.4184966	11.4704736916667	11.5839614333333	11.2815281916667	11.48359205	11.1767887583333	11.2884114666667	11.388258375	11.6485244	11.5245993916667	11.31764545	11.3282347083333	11.1934390416667	11.3817027833333	11.6499693666667	11.7917720333333	11.028339325	10.9574882	11.4093544416667	11.062936475	11.276041	11.7269658416667	11.171236275	11.5345546416667	11.3257236	11.5660654416667	11.3624169916667	11.3531420583333	11.2356259583333	11.7699299	11.6103014166667	11.31764545	10.7820632416667	11.5668628166667	11.6310321916667	10.7485438416667	11.9633577833333	11.4277232	12.0020893083333	11.4485644833333	11.5954858333333	12.080029025	11.9554346166667	11.6404855583333	12.6050803083333	11.2341636166667	11.5645444166667	11.3534958083333	10.88109835	10.7631580166667	10.9831460083333	11.23807015	11.6025118833333	11.655146	11.6879616166667	11.7362421333333

BRICD5	9.9978779375	9.5393581125	9.53420808333333	9.58240151666667	9.4279507625	8.94025112083333	8.93204191666667	8.9904855	9.36226898333333	9.43749067083333	9.17346822916667	9.30841477083333	9.34965205	9.3110777875	9.1557932	9.42403702083333	9.44452947916667	9.50797694583333	9.99956941666667	9.44128464583333	9.3650416625	9.45348648333333	9.23258092083333	9.4849232375	8.84539823333333	9.60337965833333	9.36738271666667	9.3807746875	9.59126800416667	9.47519628333333	9.216632175	9.39415376666667	9.10727307916667	9.359312225	9.37481795416667	9.70215354583333	9.50749309166667	9.39319808333333	9.280178175	9.36226898333333	9.33205741666667	9.5061498	9.37291227916667	9.48185069166667	9.4618758375	9.53669334166667	9.11198694166667	9.25881540833333	9.27241964583333	9.27241964583333	9.30099607916667	9.51834335	9.53809290833333	9.49930952916667	9.2142473625	9.1362752625	9.00667181666667	9.48586814583333	9.4792129875	9.58730238333333	9.96932235833333	9.91352252916667	9.5518241375	10.0813282166667	9.16052910833333	9.67434395	9.2021296375	9.34234282916667	10.19722315	9.59166480833333	9.58954832916667	10.2409010208333	9.80119757083333	9.4484753375	9.16986652916667	9.7234989875	9.53019919166667	9.53201676666667	9.90796002916667	10.1132725125	9.51382987916667	9.6985889375	9.31384317083333	9.41597109583333	8.96251699166667	9.9999974625	10.0702336791667	9.2605920125	10.125238725	9.42486950416667	9.11925055416667	10.0358470916667	9.91563410833333	9.6516368375	9.4356869125	9.8649744875	9.7086475	9.98857891666667	9.70215354583333	9.92516419583333	9.73418850416667	10.0547175708333	10.3145290375	9.80987653333333	10.26768155	9.39367252916667	9.504275625	9.53669334166667	9.30147802916667	8.883311425	9.26104765833333	9.9982992375	9.71594050833333	9.70772395416667	9.2728688875	9.82405373333333	9.61965483333333	9.726360525	10.2668454416667	9.46230765833333

MLST8	9.9978779375	9.5393581125	9.53420808333333	9.58240151666667	9.4279507625	8.94025112083333	8.93204191666667	8.9904855	9.36226898333333	9.43749067083333	9.17346822916667	9.30841477083333	9.34965205	9.3110777875	9.1557932	9.42403702083333	9.44452947916667	9.50797694583333	9.99956941666667	9.44128464583333	9.3650416625	9.45348648333333	9.23258092083333	9.4849232375	8.84539823333333	9.60337965833333	9.36738271666667	9.3807746875	9.59126800416667	9.47519628333333	9.216632175	9.39415376666667	9.10727307916667	9.359312225	9.37481795416667	9.70215354583333	9.50749309166667	9.39319808333333	9.280178175	9.36226898333333	9.33205741666667	9.5061498	9.37291227916667	9.48185069166667	9.4618758375	9.53669334166667	9.11198694166667	9.25881540833333	9.27241964583333	9.27241964583333	9.30099607916667	9.51834335	9.53809290833333	9.49930952916667	9.2142473625	9.1362752625	9.00667181666667	9.48586814583333	9.4792129875	9.58730238333333	9.96932235833333	9.91352252916667	9.5518241375	10.0813282166667	9.16052910833333	9.67434395	9.2021296375	9.34234282916667	10.19722315	9.59166480833333	9.58954832916667	10.2409010208333	9.80119757083333	9.4484753375	9.16986652916667	9.7234989875	9.53019919166667	9.53201676666667	9.90796002916667	10.1132725125	9.51382987916667	9.6985889375	9.31384317083333	9.41597109583333	8.96251699166667	9.9999974625	10.0702336791667	9.2605920125	10.125238725	9.42486950416667	9.11925055416667	10.0358470916667	9.91563410833333	9.6516368375	9.4356869125	9.8649744875	9.7086475	9.98857891666667	9.70215354583333	9.92516419583333	9.73418850416667	10.0547175708333	10.3145290375	9.80987653333333	10.26768155	9.39367252916667	9.504275625	9.53669334166667	9.30147802916667	8.883311425	9.26104765833333	9.9982992375	9.71594050833333	9.70772395416667	9.2728688875	9.82405373333333	9.61965483333333	9.726360525	10.2668454416667	9.46230765833333

CTC1	10.5631726083333	9.35549675833333	9.3629448	9.16352800833333	9.11737229166667	8.67553405	8.77094225	9.14189448333333	9.32368785833333	8.85110639166667	8.54938428333333	10.0061679666667	9.47897484166667	9.15555419166667	9.277313475	9.357237425	9.21303188333333	8.99438999166667	9.52133895	9.29340571666667	9.33181315833333	9.81525473333333	9.32231320833333	9.3786963	9.38580318333333	10.3880000583333	9.41849085	9.25764194166667	9.59235401666667	8.90434914166667	9.06551128333333	9.85326255	9.36939973333333	9.69924190833333	9.68471804166667	10.1417124166667	9.54880464166667	9.59324718333333	9.22108949166667	9.495092775	9.10844005833333	9.462094325	9.84631699166667	9.45055241666667	9.64871909166667	9.24799294166667	9.438196975	9.21303188333333	9.22912686666667	8.85824333333333	9.17093331666667	9.0174923	9.40986274166667	9.58662811666667	9.30912254166667	9.74483329166667	9.11737229166667	9.16543	9.497335375	9.55058418333333	9.80096413333333	8.594439725	9.46165735	8.71285006666667	8.703122725	8.43902399166667	7.886143875	8.68375230833333	8.842895675	8.9929566	7.85748985	8.71647500833333	8.25189414166667	8.67939293333333	8.651213975	8.333491425	8.60897659166667	9.01425336666667	8.98004790833333	8.91410946666667	9.22423163333333	9.04505583333333	8.95854766666667	8.47144243333333	8.32062069166667	8.71039916666667	8.439937175	8.50037185	9.42072421666667	8.69791309166667	9.223397275	8.44993103333333	8.45509195	8.16228890833333	8.18838901666667	8.522033375	8.2549704	8.56867330833333	8.52583200833333	8.57995885	8.62834993333333	8.81515405833333	8.94804158333333	9.002830825	8.80925365833333	8.50451981666667	8.66056879166667	8.04234110833333	8.55750678333333	7.66108944166667	8.703122725	8.293030975	8.4304275	8.70798358333333	8.89652095833333	8.96893119166667	8.88020864166667	9.4946079	9.06270968333333	8.81880498333333

NNT	12.3234787791667	10.6603736666667	10.7658215916667	10.30932905	10.7189561666667	11.2696990833333	10.3293667083333	10.3864170083333	10.521634775	10.872956825	11.113611625	11.6230706833333	11.0633247666667	10.6566290833333	10.9537804833333	11.1125423333333	12.2524478333333	10.9167594166667	11.2986150666667	11.0312303833333	10.9035797416667	10.7696102583333	11.0953873333333	10.3941230333333	10.9321190083333	12.0305597416667	10.9824445416667	10.7851401916667	10.8763708666667	11.0094185166667	10.6289554166667	10.8487808833333	10.7673403166667	11.2334234166667	11.6826865166667	12.3443503083333	10.8105355416667	10.506944125	10.2654733916667	10.7557641416667	10.7793443666667	10.6926124666667	10.96963075	11.1036124166667	11.56491665	11.3047106916667	11.2627928333333	10.8778360416667	11.0221982916667	11.2391470583333	10.819511225	10.1696248333333	11.3746270333333	11.0041932083333	11.0520365916667	11.4843617833333	10.7189561666667	10.8150497416667	11.0924307333333	11.0837790083333	12.7170209166667	10.4409408666667	11.2283191166667	10.8673519333333	11.9575097333333	11.6591054333333	10.440565075	11.3183574	11.1455903583333	10.8465109833333	11.430726925	10.987327675	10.66752385	11.129529525	11.731200575	10.766208375	12.1480274583333	11.6077110916667	10.5573126666667	11.8096310916667	11.9297077583333	12.2994493333333	10.8386147583333	10.81908485	11.4391211083333	11.341686575	11.7347600333333	11.423284125	11.55164095	11.602097375	10.93251855	10.5416886583333	11.691783775	10.6780826666667	12.1815348916667	10.9987091583333	11.2495617333333	11.5858193083333	10.7526329	12.59948	11.8737640583333	11.4511549916667	12.5552867333333	10.9750833833333	11.21108835	11.99193365	11.74982705	11.7440902416667	11.4839876416667	10.9707232916667	13.5965748083333	10.9139727166667	10.8097832416667	11.0097711666667	11.4284677	11.5004634	12.4903405333333	11.037692575	10.5569098916667	11.4817372166667

RPL8	15.56568115	15.048735725	15.2502335583333	15.6410257833333	14.0746231666667	15.0276351833333	14.8287604166667	15.1264373	14.2810681833333	15.1728574583333	15.4672060583333	14.3204037083333	14.9978928	14.9978928	15.4400250416667	14.4667992416667	15.581383325	15.0246483416667	14.9094697583333	15.4276773083333	15.0306120416667	15.47595875	14.5570464083333	15.4985918916667	15.365952025	15.1139193916667	13.89505895	15.2328828083333	15.0831469166667	15.57601185	15.2687677	14.4758644166667	15.290119725	15.3093345916667	14.512293975	15.2981600916667	14.0526804666667	15.390335525	15.225946525	15.00595375	15.1661341083333	15.2328828083333	15.2193239916667	15.1763309166667	14.657054125	15.2650881666667	15.229747525	14.5073052333333	15.0894272416667	15.5870133916667	15.7066290583333	16.1274635083333	15.1336404666667	13.3241951083333	15.5221336416667	15.0032595583333	14.9702238166667	15.13692035	14.9279831333333	15.83077275	15.6824817166667	16.0055115333333	16.1520487333333	15.8904374666667	16.4187514166667	15.727394275	15.9237782	16.0055115333333	16.2339961416667	15.727394275	15.7138591583333	15.91498085	15.9315345	15.9406321083333	15.6896696083333	16.1274635083333	15.784983325	15.9673534416667	16.0691638333333	15.873782975	16.113934775	15.7066290583333	16.1910724916667	15.8653098666667	15.7198901333333	15.8653098666667	16.37080095	15.784983325	15.7486260916667	15.6355001416667	15.7414894	16.0691638333333	15.7198901333333	15.814756725	15.9582825916667	15.8653098666667	16.37080095	15.784983325	15.6896696083333	15.814756725	15.9960220916667	15.8990167	15.9960220916667	15.8065534666667	16.113934775	15.9582825916667	15.9960220916667	16.2042656333333	16.0573941333333	15.8219621166667	15.7559244416667	16.0374488333333	16.0142070583333	16.5882325166667	15.799582875	16.1520487333333	15.9767561166667	16.2495335833333	15.8990167	15.4316128083333

CEBPA	12.2341055916667	11.5630353166667	11.8670714	11.4559913916667	12.19191635	12.5010802333333	11.9962714583333	11.9903497083333	11.991564075	12.2747673416667	11.9345074833333	12.7955349083333	12.4751035416667	11.4273654166667	11.4883355166667	11.5645444166667	11.7324169333333	12.4718679833333	11.9188907916667	11.437625625	11.6989876416667	11.3470919	11.7753980416667	11.8337195083333	11.3664862333333	12.273184075	11.5824943666667	11.765185225	11.6775693083333	11.4708276916667	12.2085904583333	11.276041	11.8228949916667	11.54913165	12.9977920166667	11.3426956833333	11.9009487833333	11.9325731916667	12.2817357416667	11.7332325916667	11.4496634833333	11.2572113416667	11.04233505	10.96888565	11.185479075	10.7789681833333	10.6566290833333	10.9006915833333	11.500844625	11.9558859333333	12.4119267666667	11.9021431083333	11.43611165	10.834075425	10.7931763166667	10.500980975	12.12212835	11.55276425	11.6723181916667	11.2899480916667	12.0092346166667	11.7750094	11.9380168416667	11.4350551	11.6198462166667	12.648701825	12.3195462583333	12.23627775	11.9521162083333	11.9525962333333	12.2206544333333	12.1745545	11.742927775	11.7190625916667	11.7624702333333	11.5761677833333	12.3484591166667	12.345780775	12.1451222666667	12.1770216166667	10.98475965	11.7517355166667	12.202055425	11.451916725	12.9575419416667	11.3320946416667	11.0938590416667	11.9322368833333	11.6887018916667	12.1728373083333	11.8081782916667	11.6278737583333	12.02225065	11.6902780833333	11.9409022083333	12.4270361	11.8236908583333	11.8709570583333	12.3550491166667	12.4954559583333	11.48024075	11.47563195	11.6587921	11.3749692583333	11.3360764166667	11.7814654666667	11.1376399	11.4934089166667	12.2574255666667	12.195607525	12.1536913666667	12.038401575	11.2424523	11.5909354166667	11.205753575	11.0094185166667	11.1546552166667	11.6411895916667	11.6062715833333	11.6329290083333

ZEB2	15.0184028083333	14.29207405	14.657054125	13.7452882916667	13.2738329583333	14.3676663333333	14.1857941	14.3582404416667	14.3397031416667	15.1602131166667	14.2842943416667	15.9237782	14.9832780583333	14.3350486166667	13.9197186416667	15.3177335583333	14.6087993833333	13.5133528333333	13.887785	14.8287604166667	15.0643045166667	12.86014235	14.5886594916667	13.7621985333333	14.5232906166667	15.3056912416667	14.9645581833333	14.2548502583333	13.8010236666667	14.004911475	14.4963541416667	14.3692316583333	14.8699486666667	15.09815525	14.9620915916667	15.2576870916667	14.2936594416667	14.6798721333333	14.61397985	14.8602831416667	14.1033949916667	14.419240975	14.657054125	14.6862769916667	15.4897034083333	14.7802388333333	14.6628978166667	14.4077075916667	14.4547788916667	14.1486654166667	14.30297575	14.500239075	14.7640659	14.4459919583333	14.6304997416667	15.4400250416667	14.6798721333333	14.34486635	15.156314825	15.2399573083333	13.3778968666667	13.776083375	13.1980287833333	13.5494174416667	14.0912434666667	12.852746025	13.8001487666667	13.950667875	13.4550317916667	14.177662	13.3689321166667	13.2148779166667	13.0732507583333	14.5469723583333	13.5133528333333	14.1292641	14.0100072833333	14.211775675	13.2725108916667	13.7791494	13.3689321166667	13.7168412916667	13.321391725	14.9223639	13.9807861416667	13.9197186416667	12.5135886333333	14.4597852166667	13.0638965333333	14.7097912166667	14.0001337166667	13.321391725	13.2233931166667	13.7532714333333	13.4119895416667	13.3645725083333	13.950667875	13.1622146833333	13.5007024833333	13.16815045	13.479133875	13.1865714083333	13.6375130583333	13.2447546666667	12.6528003583333	13.7282923166667	14.083694575	13.1674768	13.7553717	14.1670824833333	13.741448775	12.3059641583333	13.1986854833333	13.14792425	14.259430575	13.38316305	13.7101806916667	12.8584129833333	12.4777234416667	14.046645825

GPR174	8.9131846	10.2210310333333	10.1601611333333	10.1895353083333	8.72644686666667	11.0451598916667	10.1982859833333	10.79558335	9.51896663333333	9.89687281666667	9.46252099166667	10.0430227416667	9.68602584166667	10.6719237833333	9.058233875	12.6220126	10.25066255	10.0852465083333	9.07816326666667	11.1176950333333	10.836451025	11.2247431833333	11.2887921333333	10.7306227416667	10.76392445	8.47579976666667	11.0689648166667	9.83764681666667	10.6499586916667	9.57132345	10.0712503	11.8171008	11.3579174083333	12.4474119833333	8.74620478333333	9.21787520833333	10.5034091416667	11.3546484166667	10.36070035	10.4076693916667	10.268273175	10.0960991583333	11.3705707416667	11.2100932	11.509666625	11.541832	10.8097832416667	10.1565645083333	11.56339385	10.5605583916667	10.7688463333333	9.71527460833333	11.0315811583333	10.55775005	10.775195775	10.960420475	10.2027121916667	10.6143770083333	10.5995079	12.31433185	9.52546253333333	9.68516599166667	11.3605041083333	7.80971700833333	9.96151823333333	8.09066366666667	9.29664133333333	10.8210096	9.80448268333333	12.1509618666667	8.66056879166667	8.026220775	7.715267425	10.9371792916667	10.1654121833333	9.7241175	10.329775075	12.3310232666667	9.32615619166667	10.418815025	9.00189508333333	10.9084288583333	10.1745820166667	7.29049713333333	10.8404768083333	9.6811178	10.651587675	11.4730116416667	8.71519911666667	10.0305098083333	10.8172624166667	10.014766425	9.30630015833333	9.59369666666667	9.131237375	10.1883097083333	9.985532675	10.680501275	9.31710229166667	9.70796235833333	9.94551363333333	10.39092635	11.879885975	9.93222460833333	9.25719764166667	10.43899855	8.9131846	8.368580775	10.6614548166667	10.1787390666667	10.3663602	10.1316676583333	10.4815083083333	9.01611445833333	11.9207928583333	11.7198061583333	8.36907771666667	10.5509023833333	9.71575140833333	12.672014175

ICA1L	11.5114672666667	8.7383879	8.748691075	8.68083718333333	9.01246160833333	8.85160831666667	8.23567169166667	8.80300369166667	8.69984448333333	8.77245620833333	9.73098034166667	10.8154489833333	10.1156550583333	8.889794925	9.349869075	8.860141825	8.99899866666667	9.50086338333333	9.26082591666667	8.99116335833333	9.114481175	9.26306665	9.16302515	9.35954015	9.587537375	11.8588339833333	9.431383225	9.349435025	9.20693985	9.02927973333333	9.18457413333333	9.22559156666667	9.15449361666667	8.709872025	11.48757865	11.7273945916667	8.86708225	8.850647275	9.13825335833333	9.42643701666667	8.85215985	8.46508935	8.72134320833333	9.01009886666667	9.52177871666667	8.860141825	9.65575276666667	8.60435615	8.80300369166667	9.12986750833333	8.67841379166667	9.39626775833333	9.838575025	9.28087420833333	9.19764225833333	9.313630975	9.04692176666667	8.75703444166667	9.349435025	9.16442515	10.3459231416667	8.019555675	10.5892734666667	8.58538408333333	9.53223686666667	7.50404556666667	8.17990050833333	8.77966386666667	8.04755969166667	8.48368870833333	9.914179275	9.45776886666667	7.93123045833333	8.90389271666667	9.003775725	7.90410825	9.47542340833333	9.66283631666667	7.71798951666667	8.54023758333333	9.14054775	10.02346285	8.02129649166667	8.965634	8.340733825	9.40207443333333	10.5360893083333	9.36122138333333	10.0412389083333	9.79933185833333	8.862628675	10.6887964	10.1910863	8.11454491666667	8.65999509166667	10.3353402416667	8.1346889	10.2136563	8.306964525	10.416539275	8.48368870833333	9.57510789166667	9.2436075	9.673127475	8.61623551666667	9.26082591666667	8.65745360833333	7.85748985	8.592518275	8.37604669166667	8.62547046666667	8.80677418333333	9.275964375	8.36669614166667	8.32463409166667	8.97354856666667	9.36621750833333	8.97216300833333	8.46720710833333	10.225450075

TMEM8B	10.7352154666667	8.781733725	8.98796298333333	8.785427625	8.44334104166667	8.860141825	8.333019375	8.8559255	8.50844495	8.486995925	8.63681648333333	9.808442425	9.08299806666667	9.12042280833333	8.63633005833333	9.006017175	9.62064585	8.58390675	8.82920276666667	8.763246775	9.02562736666667	8.63230339166667	9.07777099166667	9.23047039166667	8.71144663333333	10.776374675	8.64890015833333	8.33450988333333	7.846884125	8.49975086666667	9.13694515	8.65559314166667	8.99987959166667	9.61413186666667	10.7116237833333	10.4682944	7.98087950833333	8.76851021666667	9.00189508333333	8.88020864166667	9.51454425833333	8.88020864166667	9.16216856666667	9.26261210833333	9.66915371666667	9.35549675833333	9.42859468333333	8.53972030833333	8.72089346666667	9.82339220833333	9.07484345833333	8.46459865	8.850647275	8.36034350833333	8.440899325	9.35167163333333	8.479465175	8.76125596666667	8.99070061666667	9.03950406666667	7.82845124166667	9.07484345833333	8.34668346666667	8.672615075	9.56452501666667	8.15060805	9.23462919166667	8.46961650833333	7.88018380833333	9.22249476666667	8.60646375	8.63344703333333	8.98465464166667	8.81515405833333	7.74722135833333	8.33066458333333	9.08687805	8.42069139166667	7.35751986666667	8.19443521666667	9.80755264166667	9.116355225	8.32648980833333	9.311300325	8.35786781666667	9.633880775	9.7686764	8.26207273333333	8.50037185	9.07295375833333	8.42823383333333	8.4515305	7.32258515	8.60775270833333	8.28798768333333	7.71914490833333	8.186266475	8.77140460833333	8.03277863333333	9.579948275	8.89262756666667	9.422929875	8.76950824166667	8.00901856666667	7.50291813333333	8.92651730833333	8.82265860833333	8.02185345833333	8.45605063333333	8.25745250833333	9.544894	6.76905384166667	8.416908275	9.060159775	7.88018380833333	8.17090974166667	8.647921775	8.21721259166667	8.59397561666667	7.933308825

RAB5IF	10.992355675	13.1916232708333	13.1842985083333	13.5317364875	13.6789771166667	12.73615865	13.4501353416667	13.21913615	13.334591425	12.8676465666667	13.0099141833333	10.9565945666667	12.8638697333333	13.5359808208333	13.1896013625	12.4467832416667	11.94454935	13.5359808208333	13.3571749625	13.33805425	13.1997852708333	13.4461475875	13.5153950208333	13.3857100958333	13.361974125	11.4230919125	13.4756481125	13.3063785708333	13.514595325	13.6835765625	13.3605123583333	13.2930033458333	13.2289546708333	12.0516662625	11.5686211083333	11.2987971833333	13.4924465125	13.5690106083333	13.1175698833333	12.9613996875	13.4961894833333	13.5095875375	13.5193007041667	13.0545636208333	12.4956599208333	13.1043143958333	13.5988344166667	13.6742503416667	13.3311598416667	12.9320596125	13.2178216083333	13.2477640916667	13.2282872208333	13.4175359583333	13.1888928416667	12.7849248833333	13.3819621166667	12.8451935041667	13.1632069791667	12.7423894333333	12.9613996875	13.5120357791667	13.9019783458333	13.263808525	11.7245387291667	12.5703631583333	12.6525249708333	12.8412700083333	12.9543141708333	12.4080636208333	13.9824211625	13.1169148291667	12.7854970375	12.9697020875	12.9536648666667	13.1810200208333	12.36294925	12.0915241958333	12.6394427458333	13.2930033458333	12.050065475	11.5708365	12.0991486625	12.5815748666667	13.1055574916667	12.54460495	12.2622019	12.000740025	13.343372125	12.4815793708333	12.4938953166667	13.6909245916667	13.002249725	12.7105677625	12.3673816791667	13.16578005	12.577254275	11.9003237375	12.8627241666667	12.4245908541667	12.7120155125	13.1319436458333	13.0923946125	12.746570275	13.4191478	13.421942825	12.1953954083333	13.0146044041667	13.2165666458333	12.4285645458333	12.7230469375	12.701356375	12.6823465583333	12.4784149	12.2932457416667	13.5011571666667	11.8817118	13.8914119791667	13.0557783291667	12.2766047708333

SLA2	10.992355675	13.1916232708333	13.1842985083333	13.5317364875	13.6789771166667	12.73615865	13.4501353416667	13.21913615	13.334591425	12.8676465666667	13.0099141833333	10.9565945666667	12.8638697333333	13.5359808208333	13.1896013625	12.4467832416667	11.94454935	13.5359808208333	13.3571749625	13.33805425	13.1997852708333	13.4461475875	13.5153950208333	13.3857100958333	13.361974125	11.4230919125	13.4756481125	13.3063785708333	13.514595325	13.6835765625	13.3605123583333	13.2930033458333	13.2289546708333	12.0516662625	11.5686211083333	11.2987971833333	13.4924465125	13.5690106083333	13.1175698833333	12.9613996875	13.4961894833333	13.5095875375	13.5193007041667	13.0545636208333	12.4956599208333	13.1043143958333	13.5988344166667	13.6742503416667	13.3311598416667	12.9320596125	13.2178216083333	13.2477640916667	13.2282872208333	13.4175359583333	13.1888928416667	12.7849248833333	13.3819621166667	12.8451935041667	13.1632069791667	12.7423894333333	12.9613996875	13.5120357791667	13.9019783458333	13.263808525	11.7245387291667	12.5703631583333	12.6525249708333	12.8412700083333	12.9543141708333	12.4080636208333	13.9824211625	13.1169148291667	12.7854970375	12.9697020875	12.9536648666667	13.1810200208333	12.36294925	12.0915241958333	12.6394427458333	13.2930033458333	12.050065475	11.5708365	12.0991486625	12.5815748666667	13.1055574916667	12.54460495	12.2622019	12.000740025	13.343372125	12.4815793708333	12.4938953166667	13.6909245916667	13.002249725	12.7105677625	12.3673816791667	13.16578005	12.577254275	11.9003237375	12.8627241666667	12.4245908541667	12.7120155125	13.1319436458333	13.0923946125	12.746570275	13.4191478	13.421942825	12.1953954083333	13.0146044041667	13.2165666458333	12.4285645458333	12.7230469375	12.701356375	12.6823465583333	12.4784149	12.2932457416667	13.5011571666667	11.8817118	13.8914119791667	13.0557783291667	12.2766047708333

MTPAP	11.8931595083333	11.4364638833333	10.9972633583333	11.2352593083333	11.3813533416667	10.8666003833333	10.98475965	11.5805781333333	11.3774736833333	11.1419454583333	11.2982935833333	10.8916305083333	10.8090233916667	11.3169410666667	11.2097259666667	11.054552625	11.1997660583333	11.233793875	11.0315811583333	11.1332425666667	11.3433838833333	11.4631308333333	11.31949275	11.9100903833333	11.588386675	12.4395669416667	11.4083049	11.3624169916667	11.17758075	11.4639403833333	11.3849835833333	11.4704736916667	11.129529525	10.95605295	11.8032520333333	12.1922906583333	11.1828701833333	11.364979325	11.2693525666667	11.5093017916667	11.3144428833333	11.30185685	11.22125475	11.44422215	11.5404299	10.9707232916667	11.526510825	11.5030923083333	11.4527169333333	11.3849835833333	11.062936475	11.6895204333333	11.4096653083333	11.4431148583333	11.825765325	11.2172145	11.3605041083333	11.13908545	11.230125425	11.1832845666667	10.9835406583333	11.6709513916667	12.0124313166667	11.5276408333333	11.528423775	10.7205897	11.1325239666667	11.3047106916667	11.3805222416667	11.1302224166667	10.90507365	11.8163038583333	11.2858355	11.054552625	11.0416560583333	10.6496091416667	11.7373039666667	11.0466400166667	10.9661834333333	11.25880145	11.3571575333333	12.0761170583333	11.4284677	11.7081843583333	11.428103825	11.7373039666667	12.1071523416667	11.4715256833333	11.0352265083333	11.5618505	11.7239413833333	11.65549195	11.096135525	11.5664864	11.17143095	11.4063648666667	12.3003464583333	11.685558375	11.2911160333333	11.7160036	11.7601425083333	11.208942925	12.4545539666667	12.606535325	11.3063182333333	11.4586326	11.2944151416667	11.249224775	11.8815198166667	11.421792525	11.1824657666667	11.35644045	11.345193725	11.8403034416667	11.3320946416667	11.37567555	11.890067775	11.348473325	11.3216619833333	11.929273675

YJU2	11.4839876416667	11.958220325	11.88575805	11.9762173583333	11.5676618166667	11.4654444	11.3957857333333	11.8116053416667	11.7586025166667	11.9368778583333	11.825372775	11.2815281916667	11.3172677166667	12.05061705	11.825372775	11.5820604666667	12.198234625	11.904789825	11.951715625	11.7572174666667	11.6807143833333	11.9653249	11.7724295666667	11.946316525	11.8530999083333	11.5630353166667	11.6799309666667	11.7407727166667	11.496789525	11.86547315	11.7561182166667	11.9621161833333	11.7266159583333	11.8100395833333	11.22125475	11.3550156083333	11.7695141	12.0088601416667	11.221922825	11.6302237	11.971689825	11.9071135	12.017301325	12.280521525	12.0748320833333	12.1178108083333	12.2094988666667	11.9625539583333	12.2723412333333	12.089741475	11.8158651416667	11.8020861333333	12.1262500416667	12.2085904583333	12.0294077916667	12.2240198833333	11.918121775	12.1596044833333	12.0189741833333	12.2591093583333	11.7640863	11.423284125	11.9754521583333	11.535988725	11.3742720083333	11.3492073583333	11.4431148583333	11.421792525	11.5695230416667	11.3172677166667	11.8134267166667	11.4901622083333	11.0837790083333	11.5750241	11.3264911333333	11.4354157666667	11.6144877166667	11.4715256833333	11.5813004	11.48757865	11.53966185	10.9419805083333	11.5761677833333	11.624532275	11.164489475	11.129529525	11.5253735333333	11.686699925	12.1587642333333	11.4820913166667	11.3612411916667	11.2352593083333	11.4559913916667	11.8435483083333	11.13057255	11.7501757083333	12.3216200916667	11.0430757083333	11.8407167416667	12.075266	11.523383625	11.5945056583333	11.2428131833333	11.943975875	11.2576230916667	11.691783775	11.8100395833333	12.24842175	11.43798885	11.5495081166667	10.9761602583333	11.9263114333333	11.9994097833333	11.9100903833333	12.089741475	11.5495081166667	11.1721251583333	11.8081782916667	12.7602549166667	11.8666609583333

CAB39L	11.9836236833333	9.54225544166667	9.438196975	8.76898080833333	9.24980921666667	9.65575276666667	9.27365428333333	9.87070264166667	9.37177325833333	9.33321418333333	8.76654949166667	10.8980207583333	10.092918675	9.75408091666667	9.74305075	9.34438068333333	10.8953436916667	9.15991209166667	9.98838416666667	9.97833993333333	9.77312443333333	9.952361775	9.44516316666667	9.683521475	10.2458406333333	11.779499875	9.78382319166667	9.389870525	9.92453053333333	9.921332825	9.748599325	9.61091021666667	9.81007735833333	9.66915371666667	11.2872125416667	11.7255170416667	9.59369666666667	9.55812764166667	9.277313475	10.02519555	9.56548648333333	9.59023981666667	9.91673698333333	9.7105078	10.34241075	9.71527460833333	10.627440475	9.71142816666667	9.006017175	9.6347809	9.182097875	9.25253679166667	9.58976235	9.552037225	9.63156661666667	9.71785554166667	10.0364758583333	9.65621849166667	9.607505	9.911212725	9.20822946666667	9.50539336666667	9.02085209166667	8.73019295833333	10.3289721	9.52783803333333	9.57736215	8.67688486666667	8.87086751666667	8.62596224166667	8.4067182	8.39370431666667	8.54023758333333	9.80492948333333	7.76137635	9.28785676666667	9.21787520833333	9.177752275	8.73019295833333	8.68329548333333	9.79235871666667	9.53223686666667	8.748691075	9.53692870833333	9.852861125	10.0189542666667	8.54268981666667	9.176064475	8.327465575	10.1091856666667	9.21169858333333	9.18595039166667	9.82250195	9.311300325	8.6404919	8.44334104166667	8.02779786666667	8.64638736666667	8.08754240833333	9.23749938333333	8.931271375	7.66209683333333	9.60319949166667	9.2612694	7.928618975	9.223397275	9.34064308333333	10.0980477833333	8.59890789166667	9.36892748333333	10.2289590916667	8.31719399166667	8.58091206666667	9.22385636666667	9.22108949166667	9.21688079166667	9.97912404166667	8.84758474166667	8.58601561666667	8.759783975

CAAP1	5.073382675	4.64033936666667	4.04713170833333	4.08963481666667	5.06351158333333	6.15447426666667	4.25005865	4.84094744166667	4.36702491666667	4.45503961666667	4.84935865	4.25100265833333	4.418789625	4.60180214166667	5.4712492	5.56052278333333	4.74930484166667	4.816016475	5.33746485833333	5.211919525	4.15118036666667	5.58407845	3.909240625	6.11785979166667	5.780758525	4.90032935	5.74909436666667	6.1063381	5.52011276666667	5.56425255	4.6393107	5.377778825	4.82036908333333	4.86804739166667	4.67067994166667	5.5319599	5.95074298333333	5.778884625	5.32134071666667	5.31227555833333	4.16611878333333	4.99247835	5.018425275	4.33532039166667	3.711770875	4.74337093333333	4.418789625	4.656935525	5.873141325	6.43359675	4.822492325	5.05069551666667	3.694768975	5.33234265	3.86964848333333	4.055304025	4.52791141666667	4.04622350833333	5.22492120833333	5.49632836666667	4.87503585	4.27896138333333	5.78268739166667	4.09994219166667	4.35397924166667	4.649483075	4.00881020833333	4.611084475	5.14192449166667	4.4580822	5.2151974	4.432045325	3.94341095833333	4.27276220833333	4.35296875833333	4.44534256666667	6.23709786666667	6.64893395	5.197537	5.36922546666667	5.170393	5.61155829166667	5.171525	3.95017479166667	4.184996575	4.62889925	4.60088621666667	4.21192669166667	5.52920275833333	5.326621775	4.89935284166667	4.73928888333333	5.211919525	4.750433775	4.782758275	5.55170386666667	6.78446141666667	5.5022085	3.911419125	5.04966088333333	4.57343223333333	3.87078774166667	4.99894050833333	4.98771553333333	4.53925114166667	5.14089556666667	4.567125	4.70163844166667	3.92135188333333	5.88521655833333	5.45803265	4.21502835833333	4.812774675	4.10289946666667	3.87078774166667	4.1018927	5.13961736666667	5.8902603	6.06396311666667	4.125728225

TPT1	10.4890800583333	9.69038334166667	9.33579655	9.56124985	9.442472475	8.71235535	9.824283225	9.60186775	9.569144925	9.5928155	9.0180261	9.98470918333333	10.2587469666667	9.40252054166667	9.6811178	9.18832863333333	9.26082591666667	10.4826365083333	9.74045244166667	9.3434528	9.573560175	9.8834328	8.95991093333333	10.382913225	9.615115575	9.73577058333333	9.607505	9.85036418333333	10.1472782833333	9.74483329166667	10.24707065	9.84285273333333	9.579948275	9.39753216666667	9.79486831666667	9.9420301	10.2462958083333	9.33365533333333	9.50182605833333	9.52694540833333	9.04692176666667	9.73622053333333	9.60830463333333	9.818576775	9.93343423333333	9.22559156666667	9.84956451666667	10.23254885	9.44779018333333	9.32574429166667	9.721994775	9.49908795	9.984272	9.72484969166667	10.010773575	9.817741375	9.53692870833333	9.59023981666667	9.25209231666667	9.546304725	8.96987785	9.38006720833333	8.40965201666667	8.94478365	8.57649310833333	9.177752275	8.58906924166667	9.120869625	8.354234475	8.55543884166667	8.796402275	8.84055035	9.12557628333333	9.19531680833333	9.26773438333333	8.69504618333333	8.779206475	9.18880384166667	8.96893119166667	9.15991209166667	8.9939706	8.62834993333333	9.063171125	9.05125875	8.58441085833333	9.33537304166667	8.61623551666667	9.22249476666667	9.495092775	8.821700275	8.90099503333333	9.00551523333333	8.10391784166667	8.59154009166667	8.56421206666667	8.79951210833333	8.57454949166667	7.99615545	9.22912686666667	8.50943095833333	8.324127225	8.49188041666667	8.672615075	9.72531675833333	8.862628675	8.80967759166667	8.98986978333333	9.98075235	8.45954589166667	8.30283623333333	8.69939258333333	8.466669725	10.1059188	8.45451465833333	9.04320051666667	8.72037605	8.16529418333333	8.8319761	8.57995885	9.379662375

DPF3	7.27829234166667	5.32346678333333	5.85146426666667	5.94535455833333	5.17905539166667	6.38393616666667	5.53793416666667	5.69973553333333	5.628745675	5.550710675	6.45250163333333	6.09272539166667	5.71315170833333	5.04230353333333	4.91206978333333	5.20449400833333	4.99894050833333	5.6458775	6.495300075	4.90586546666667	5.69576305	5.51585541666667	6.4034157	6.03147445	5.426744575	6.47574773333333	6.20326725833333	5.27417344166667	5.7550021	6.112061375	6.30781043333333	5.62592511666667	6.27802068333333	5.56242626666667	6.64156519166667	7.02401584166667	5.451608675	6.7503122	6.13670685833333	6.18332735833333	6.34005863333333	7.12586193333333	6.28265325	6.72313661666667	5.50905394166667	5.327612425	6.19818808333333	5.64797583333333	4.768670525	5.792834125	6.74888376666667	6.169883075	5.270972825	6.24783014166667	5.8009379	6.35880866666667	4.8072374	5.78268739166667	5.79916375	5.52228829166667	4.71576270833333	5.51386359166667	5.63834938333333	5.60574740833333	5.1653561	5.575395825	6.06230976666667	5.67139935833333	4.83457635833333	5.77106630833333	5.90321861666667	5.88853345	5.71123295833333	4.669520575	5.61155829166667	4.72324775	4.830971775	4.87199023333333	5.35088976666667	4.957419525	5.12313813333333	5.018425275	5.31227555833333	5.6723045	4.70475344166667	4.81075010833333	4.55971610833333	4.88811624166667	5.2108879	5.28787750833333	5.68304789166667	5.4308407	4.37287369166667	4.89935284166667	5.53292085833333	5.44873513333333	5.39700335833333	5.78909119166667	5.303590975	4.93337844166667	5.01100034166667	6.527218175	5.5651756	4.71576270833333	5.00117389166667	5.23876719166667	5.03726580833333	6.0911181	4.22036293333333	6.50214656666667	5.04024386666667	5.52123825	5.26673208333333	6.00285793333333	5.05069551666667	4.58140610833333	4.69325549166667	5.89630836666667	5.28357275833333	4.91654405

BSX	9.33883618333333	9.462094325	9.422929875	9.83071770833333	10.2778151833333	9.39664281666667	10.4308569666667	9.35451149166667	10.0997208916667	9.65670091666667	9.73010673333333	10.1226723083333	9.74776665	8.99487275833333	9.00643414166667	8.69939258333333	9.11782889166667	9.99593061666667	8.75371965	8.69317739166667	8.95577006666667	9.349435025	8.83395916666667	8.61442265833333	9.20100326666667	9.09600253333333	9.0174923	9.21923738333333	9.62318166666667	9.39889275833333	9.10344201666667	9.25349141666667	9.64383103333333	9.88298375833333	9.32368785833333	9.39983189166667	10.225450075	9.88552295	9.737063275	9.75620250833333	8.73207944166667	9.04228765833333	8.81066338333333	8.57995885	8.85011799166667	8.59302528333333	8.434501875	8.74224036666667	9.10099604166667	8.83443758333333	8.97769849166667	9.189315625	9.47772424166667	9.546304725	9.22295939166667	8.53632365833333	9.24031536666667	9.17368278333333	9.33838469166667	8.87290028333333	9.4697267	9.74266033333333	9.22295939166667	10.013853075	9.06551128333333	9.70931241666667	9.07065694166667	9.51857009166667	9.76472428333333	9.78236806666667	10.0732835666667	9.52268223333333	9.79676919166667	9.121301075	9.23882716666667	9.4341767	9.6347809	9.168290925	9.15692076666667	9.07536643333333	8.836922375	9.33617749166667	9.39626775833333	8.66056879166667	9.80625096666667	9.555182225	9.66065478333333	9.68516599166667	9.95643681666667	9.12419129166667	9.46252099166667	9.45734620833333	9.891815375	9.67854975	10.19975295	10.03562315	10.0171966666667	10.0859903416667	10.2769692583333	9.60459400833333	9.20822946666667	9.51857009166667	9.08543134166667	8.60947813333333	9.12281406666667	9.20413733333333	8.97076639166667	9.19287955	9.23462919166667	8.80450975	9.55943838333333	8.75003116666667	9.31452535833333	9.04369295	9.21123668333333	8.895120775	8.97489020833333	9.47590374166667	9.186916425	9.60919951666667

PCDHA1	6.39152516666667	5.4789746	5.6411096	5.764437375	5.56425255	5.55953659166667	5.60229806666667	5.112251075	5.38469075833333	5.6241104	5.54392786666667	5.81478479166667	4.79411095833333	5.77106630833333	5.73682776666667	5.96958254166667	5.37452059166667	5.4137082	5.79649798333333	5.5927414	5.24195544166667	5.34196935833333	6.17154675833333	6.018516875	6.17390685	5.69296955833333	5.43710514166667	4.84935865	4.27798759166667	5.81212459166667	5.07218190833333	5.50615049166667	4.685722125	5.03726580833333	5.34097245	6.21191129166667	4.35819395	6.05045175	4.852466025	5.43818919166667	5.81652036666667	4.67674131666667	5.231579	5.064717325	4.51861790833333	4.67471653333333	5.31926836666667	4.55863708333333	5.95328179166667	4.535343	5.53102171666667	4.17476265833333	5.08252440833333	6.10873680833333	6.11703395833333	4.81947958333333	4.88478473333333	6.339217025	5.70053848333333	3.81605024166667	9.02273679166667	6.081909575	4.46877066666667	7.22899075833333	5.577321975	5.97595255	4.747391	4.92991125833333	4.4159867	4.41103275833333	4.936774575	4.51475793333333	5.53496716666667	6.85354335	6.15447426666667	4.73206480833333	7.27649536666667	4.01216488333333	4.62889925	5.16622001666667	6.73133699166667	7.444336	6.07673425	6.2176337	4.49312724166667	5.81909195	5.10930444166667	3.92815261666667	4.52238	5.106111275	4.60591755833333	5.157708775	4.68073703333333	4.64220138333333	5.25962125	4.33328629166667	3.88647775833333	6.54299384166667	4.68262605833333	5.455892825	5.83306814166667	4.57535033333333	4.41203210833333	5.56869470833333	5.14401780833333	4.44161573333333	5.02467455	5.51001904166667	4.043026825	5.78455018333333	4.96517085	4.55568075	4.19720234166667	5.23566219166667	4.53327731666667	4.602780325	4.62375229166667	6.13929583333333	4.49312724166667	5.02371664166667

PCDHA10	6.39152516666667	5.4789746	5.6411096	5.764437375	5.56425255	5.55953659166667	5.60229806666667	5.112251075	5.38469075833333	5.6241104	5.54392786666667	5.81478479166667	4.79411095833333	5.77106630833333	5.73682776666667	5.96958254166667	5.37452059166667	5.4137082	5.79649798333333	5.5927414	5.24195544166667	5.34196935833333	6.17154675833333	6.018516875	6.17390685	5.69296955833333	5.43710514166667	4.84935865	4.27798759166667	5.81212459166667	5.07218190833333	5.50615049166667	4.685722125	5.03726580833333	5.34097245	6.21191129166667	4.35819395	6.05045175	4.852466025	5.43818919166667	5.81652036666667	4.67674131666667	5.231579	5.064717325	4.51861790833333	4.67471653333333	5.31926836666667	4.55863708333333	5.95328179166667	4.535343	5.53102171666667	4.17476265833333	5.08252440833333	6.10873680833333	6.11703395833333	4.81947958333333	4.88478473333333	6.339217025	5.70053848333333	3.81605024166667	9.02273679166667	6.081909575	4.46877066666667	7.22899075833333	5.577321975	5.97595255	4.747391	4.92991125833333	4.4159867	4.41103275833333	4.936774575	4.51475793333333	5.53496716666667	6.85354335	6.15447426666667	4.73206480833333	7.27649536666667	4.01216488333333	4.62889925	5.16622001666667	6.73133699166667	7.444336	6.07673425	6.2176337	4.49312724166667	5.81909195	5.10930444166667	3.92815261666667	4.52238	5.106111275	4.60591755833333	5.157708775	4.68073703333333	4.64220138333333	5.25962125	4.33328629166667	3.88647775833333	6.54299384166667	4.68262605833333	5.455892825	5.83306814166667	4.57535033333333	4.41203210833333	5.56869470833333	5.14401780833333	4.44161573333333	5.02467455	5.51001904166667	4.043026825	5.78455018333333	4.96517085	4.55568075	4.19720234166667	5.23566219166667	4.53327731666667	4.602780325	4.62375229166667	6.13929583333333	4.49312724166667	5.02371664166667

PCDHA3	6.39152516666667	5.4789746	5.6411096	5.764437375	5.56425255	5.55953659166667	5.60229806666667	5.112251075	5.38469075833333	5.6241104	5.54392786666667	5.81478479166667	4.79411095833333	5.77106630833333	5.73682776666667	5.96958254166667	5.37452059166667	5.4137082	5.79649798333333	5.5927414	5.24195544166667	5.34196935833333	6.17154675833333	6.018516875	6.17390685	5.69296955833333	5.43710514166667	4.84935865	4.27798759166667	5.81212459166667	5.07218190833333	5.50615049166667	4.685722125	5.03726580833333	5.34097245	6.21191129166667	4.35819395	6.05045175	4.852466025	5.43818919166667	5.81652036666667	4.67674131666667	5.231579	5.064717325	4.51861790833333	4.67471653333333	5.31926836666667	4.55863708333333	5.95328179166667	4.535343	5.53102171666667	4.17476265833333	5.08252440833333	6.10873680833333	6.11703395833333	4.81947958333333	4.88478473333333	6.339217025	5.70053848333333	3.81605024166667	9.02273679166667	6.081909575	4.46877066666667	7.22899075833333	5.577321975	5.97595255	4.747391	4.92991125833333	4.4159867	4.41103275833333	4.936774575	4.51475793333333	5.53496716666667	6.85354335	6.15447426666667	4.73206480833333	7.27649536666667	4.01216488333333	4.62889925	5.16622001666667	6.73133699166667	7.444336	6.07673425	6.2176337	4.49312724166667	5.81909195	5.10930444166667	3.92815261666667	4.52238	5.106111275	4.60591755833333	5.157708775	4.68073703333333	4.64220138333333	5.25962125	4.33328629166667	3.88647775833333	6.54299384166667	4.68262605833333	5.455892825	5.83306814166667	4.57535033333333	4.41203210833333	5.56869470833333	5.14401780833333	4.44161573333333	5.02467455	5.51001904166667	4.043026825	5.78455018333333	4.96517085	4.55568075	4.19720234166667	5.23566219166667	4.53327731666667	4.602780325	4.62375229166667	6.13929583333333	4.49312724166667	5.02371664166667

PCDHA4	6.39152516666667	5.4789746	5.6411096	5.764437375	5.56425255	5.55953659166667	5.60229806666667	5.112251075	5.38469075833333	5.6241104	5.54392786666667	5.81478479166667	4.79411095833333	5.77106630833333	5.73682776666667	5.96958254166667	5.37452059166667	5.4137082	5.79649798333333	5.5927414	5.24195544166667	5.34196935833333	6.17154675833333	6.018516875	6.17390685	5.69296955833333	5.43710514166667	4.84935865	4.27798759166667	5.81212459166667	5.07218190833333	5.50615049166667	4.685722125	5.03726580833333	5.34097245	6.21191129166667	4.35819395	6.05045175	4.852466025	5.43818919166667	5.81652036666667	4.67674131666667	5.231579	5.064717325	4.51861790833333	4.67471653333333	5.31926836666667	4.55863708333333	5.95328179166667	4.535343	5.53102171666667	4.17476265833333	5.08252440833333	6.10873680833333	6.11703395833333	4.81947958333333	4.88478473333333	6.339217025	5.70053848333333	3.81605024166667	9.02273679166667	6.081909575	4.46877066666667	7.22899075833333	5.577321975	5.97595255	4.747391	4.92991125833333	4.4159867	4.41103275833333	4.936774575	4.51475793333333	5.53496716666667	6.85354335	6.15447426666667	4.73206480833333	7.27649536666667	4.01216488333333	4.62889925	5.16622001666667	6.73133699166667	7.444336	6.07673425	6.2176337	4.49312724166667	5.81909195	5.10930444166667	3.92815261666667	4.52238	5.106111275	4.60591755833333	5.157708775	4.68073703333333	4.64220138333333	5.25962125	4.33328629166667	3.88647775833333	6.54299384166667	4.68262605833333	5.455892825	5.83306814166667	4.57535033333333	4.41203210833333	5.56869470833333	5.14401780833333	4.44161573333333	5.02467455	5.51001904166667	4.043026825	5.78455018333333	4.96517085	4.55568075	4.19720234166667	5.23566219166667	4.53327731666667	4.602780325	4.62375229166667	6.13929583333333	4.49312724166667	5.02371664166667

PCDHA6	6.39152516666667	5.4789746	5.6411096	5.764437375	5.56425255	5.55953659166667	5.60229806666667	5.112251075	5.38469075833333	5.6241104	5.54392786666667	5.81478479166667	4.79411095833333	5.77106630833333	5.73682776666667	5.96958254166667	5.37452059166667	5.4137082	5.79649798333333	5.5927414	5.24195544166667	5.34196935833333	6.17154675833333	6.018516875	6.17390685	5.69296955833333	5.43710514166667	4.84935865	4.27798759166667	5.81212459166667	5.07218190833333	5.50615049166667	4.685722125	5.03726580833333	5.34097245	6.21191129166667	4.35819395	6.05045175	4.852466025	5.43818919166667	5.81652036666667	4.67674131666667	5.231579	5.064717325	4.51861790833333	4.67471653333333	5.31926836666667	4.55863708333333	5.95328179166667	4.535343	5.53102171666667	4.17476265833333	5.08252440833333	6.10873680833333	6.11703395833333	4.81947958333333	4.88478473333333	6.339217025	5.70053848333333	3.81605024166667	9.02273679166667	6.081909575	4.46877066666667	7.22899075833333	5.577321975	5.97595255	4.747391	4.92991125833333	4.4159867	4.41103275833333	4.936774575	4.51475793333333	5.53496716666667	6.85354335	6.15447426666667	4.73206480833333	7.27649536666667	4.01216488333333	4.62889925	5.16622001666667	6.73133699166667	7.444336	6.07673425	6.2176337	4.49312724166667	5.81909195	5.10930444166667	3.92815261666667	4.52238	5.106111275	4.60591755833333	5.157708775	4.68073703333333	4.64220138333333	5.25962125	4.33328629166667	3.88647775833333	6.54299384166667	4.68262605833333	5.455892825	5.83306814166667	4.57535033333333	4.41203210833333	5.56869470833333	5.14401780833333	4.44161573333333	5.02467455	5.51001904166667	4.043026825	5.78455018333333	4.96517085	4.55568075	4.19720234166667	5.23566219166667	4.53327731666667	4.602780325	4.62375229166667	6.13929583333333	4.49312724166667	5.02371664166667

PCDHA2	6.39152516666667	5.4789746	5.6411096	5.764437375	5.56425255	5.55953659166667	5.60229806666667	5.112251075	5.38469075833333	5.6241104	5.54392786666667	5.81478479166667	4.79411095833333	5.77106630833333	5.73682776666667	5.96958254166667	5.37452059166667	5.4137082	5.79649798333333	5.5927414	5.24195544166667	5.34196935833333	6.17154675833333	6.018516875	6.17390685	5.69296955833333	5.43710514166667	4.84935865	4.27798759166667	5.81212459166667	5.07218190833333	5.50615049166667	4.685722125	5.03726580833333	5.34097245	6.21191129166667	4.35819395	6.05045175	4.852466025	5.43818919166667	5.81652036666667	4.67674131666667	5.231579	5.064717325	4.51861790833333	4.67471653333333	5.31926836666667	4.55863708333333	5.95328179166667	4.535343	5.53102171666667	4.17476265833333	5.08252440833333	6.10873680833333	6.11703395833333	4.81947958333333	4.88478473333333	6.339217025	5.70053848333333	3.81605024166667	9.02273679166667	6.081909575	4.46877066666667	7.22899075833333	5.577321975	5.97595255	4.747391	4.92991125833333	4.4159867	4.41103275833333	4.936774575	4.51475793333333	5.53496716666667	6.85354335	6.15447426666667	4.73206480833333	7.27649536666667	4.01216488333333	4.62889925	5.16622001666667	6.73133699166667	7.444336	6.07673425	6.2176337	4.49312724166667	5.81909195	5.10930444166667	3.92815261666667	4.52238	5.106111275	4.60591755833333	5.157708775	4.68073703333333	4.64220138333333	5.25962125	4.33328629166667	3.88647775833333	6.54299384166667	4.68262605833333	5.455892825	5.83306814166667	4.57535033333333	4.41203210833333	5.56869470833333	5.14401780833333	4.44161573333333	5.02467455	5.51001904166667	4.043026825	5.78455018333333	4.96517085	4.55568075	4.19720234166667	5.23566219166667	4.53327731666667	4.602780325	4.62375229166667	6.13929583333333	4.49312724166667	5.02371664166667

PCDHA8	6.39152516666667	5.4789746	5.6411096	5.764437375	5.56425255	5.55953659166667	5.60229806666667	5.112251075	5.38469075833333	5.6241104	5.54392786666667	5.81478479166667	4.79411095833333	5.77106630833333	5.73682776666667	5.96958254166667	5.37452059166667	5.4137082	5.79649798333333	5.5927414	5.24195544166667	5.34196935833333	6.17154675833333	6.018516875	6.17390685	5.69296955833333	5.43710514166667	4.84935865	4.27798759166667	5.81212459166667	5.07218190833333	5.50615049166667	4.685722125	5.03726580833333	5.34097245	6.21191129166667	4.35819395	6.05045175	4.852466025	5.43818919166667	5.81652036666667	4.67674131666667	5.231579	5.064717325	4.51861790833333	4.67471653333333	5.31926836666667	4.55863708333333	5.95328179166667	4.535343	5.53102171666667	4.17476265833333	5.08252440833333	6.10873680833333	6.11703395833333	4.81947958333333	4.88478473333333	6.339217025	5.70053848333333	3.81605024166667	9.02273679166667	6.081909575	4.46877066666667	7.22899075833333	5.577321975	5.97595255	4.747391	4.92991125833333	4.4159867	4.41103275833333	4.936774575	4.51475793333333	5.53496716666667	6.85354335	6.15447426666667	4.73206480833333	7.27649536666667	4.01216488333333	4.62889925	5.16622001666667	6.73133699166667	7.444336	6.07673425	6.2176337	4.49312724166667	5.81909195	5.10930444166667	3.92815261666667	4.52238	5.106111275	4.60591755833333	5.157708775	4.68073703333333	4.64220138333333	5.25962125	4.33328629166667	3.88647775833333	6.54299384166667	4.68262605833333	5.455892825	5.83306814166667	4.57535033333333	4.41203210833333	5.56869470833333	5.14401780833333	4.44161573333333	5.02467455	5.51001904166667	4.043026825	5.78455018333333	4.96517085	4.55568075	4.19720234166667	5.23566219166667	4.53327731666667	4.602780325	4.62375229166667	6.13929583333333	4.49312724166667	5.02371664166667

PCDHA5	6.39152516666667	5.4789746	5.6411096	5.764437375	5.56425255	5.55953659166667	5.60229806666667	5.112251075	5.38469075833333	5.6241104	5.54392786666667	5.81478479166667	4.79411095833333	5.77106630833333	5.73682776666667	5.96958254166667	5.37452059166667	5.4137082	5.79649798333333	5.5927414	5.24195544166667	5.34196935833333	6.17154675833333	6.018516875	6.17390685	5.69296955833333	5.43710514166667	4.84935865	4.27798759166667	5.81212459166667	5.07218190833333	5.50615049166667	4.685722125	5.03726580833333	5.34097245	6.21191129166667	4.35819395	6.05045175	4.852466025	5.43818919166667	5.81652036666667	4.67674131666667	5.231579	5.064717325	4.51861790833333	4.67471653333333	5.31926836666667	4.55863708333333	5.95328179166667	4.535343	5.53102171666667	4.17476265833333	5.08252440833333	6.10873680833333	6.11703395833333	4.81947958333333	4.88478473333333	6.339217025	5.70053848333333	3.81605024166667	9.02273679166667	6.081909575	4.46877066666667	7.22899075833333	5.577321975	5.97595255	4.747391	4.92991125833333	4.4159867	4.41103275833333	4.936774575	4.51475793333333	5.53496716666667	6.85354335	6.15447426666667	4.73206480833333	7.27649536666667	4.01216488333333	4.62889925	5.16622001666667	6.73133699166667	7.444336	6.07673425	6.2176337	4.49312724166667	5.81909195	5.10930444166667	3.92815261666667	4.52238	5.106111275	4.60591755833333	5.157708775	4.68073703333333	4.64220138333333	5.25962125	4.33328629166667	3.88647775833333	6.54299384166667	4.68262605833333	5.455892825	5.83306814166667	4.57535033333333	4.41203210833333	5.56869470833333	5.14401780833333	4.44161573333333	5.02467455	5.51001904166667	4.043026825	5.78455018333333	4.96517085	4.55568075	4.19720234166667	5.23566219166667	4.53327731666667	4.602780325	4.62375229166667	6.13929583333333	4.49312724166667	5.02371664166667

PCDHA11	6.39152516666667	5.4789746	5.6411096	5.764437375	5.56425255	5.55953659166667	5.60229806666667	5.112251075	5.38469075833333	5.6241104	5.54392786666667	5.81478479166667	4.79411095833333	5.77106630833333	5.73682776666667	5.96958254166667	5.37452059166667	5.4137082	5.79649798333333	5.5927414	5.24195544166667	5.34196935833333	6.17154675833333	6.018516875	6.17390685	5.69296955833333	5.43710514166667	4.84935865	4.27798759166667	5.81212459166667	5.07218190833333	5.50615049166667	4.685722125	5.03726580833333	5.34097245	6.21191129166667	4.35819395	6.05045175	4.852466025	5.43818919166667	5.81652036666667	4.67674131666667	5.231579	5.064717325	4.51861790833333	4.67471653333333	5.31926836666667	4.55863708333333	5.95328179166667	4.535343	5.53102171666667	4.17476265833333	5.08252440833333	6.10873680833333	6.11703395833333	4.81947958333333	4.88478473333333	6.339217025	5.70053848333333	3.81605024166667	9.02273679166667	6.081909575	4.46877066666667	7.22899075833333	5.577321975	5.97595255	4.747391	4.92991125833333	4.4159867	4.41103275833333	4.936774575	4.51475793333333	5.53496716666667	6.85354335	6.15447426666667	4.73206480833333	7.27649536666667	4.01216488333333	4.62889925	5.16622001666667	6.73133699166667	7.444336	6.07673425	6.2176337	4.49312724166667	5.81909195	5.10930444166667	3.92815261666667	4.52238	5.106111275	4.60591755833333	5.157708775	4.68073703333333	4.64220138333333	5.25962125	4.33328629166667	3.88647775833333	6.54299384166667	4.68262605833333	5.455892825	5.83306814166667	4.57535033333333	4.41203210833333	5.56869470833333	5.14401780833333	4.44161573333333	5.02467455	5.51001904166667	4.043026825	5.78455018333333	4.96517085	4.55568075	4.19720234166667	5.23566219166667	4.53327731666667	4.602780325	4.62375229166667	6.13929583333333	4.49312724166667	5.02371664166667

PCDHA7	6.39152516666667	5.4789746	5.6411096	5.764437375	5.56425255	5.55953659166667	5.60229806666667	5.112251075	5.38469075833333	5.6241104	5.54392786666667	5.81478479166667	4.79411095833333	5.77106630833333	5.73682776666667	5.96958254166667	5.37452059166667	5.4137082	5.79649798333333	5.5927414	5.24195544166667	5.34196935833333	6.17154675833333	6.018516875	6.17390685	5.69296955833333	5.43710514166667	4.84935865	4.27798759166667	5.81212459166667	5.07218190833333	5.50615049166667	4.685722125	5.03726580833333	5.34097245	6.21191129166667	4.35819395	6.05045175	4.852466025	5.43818919166667	5.81652036666667	4.67674131666667	5.231579	5.064717325	4.51861790833333	4.67471653333333	5.31926836666667	4.55863708333333	5.95328179166667	4.535343	5.53102171666667	4.17476265833333	5.08252440833333	6.10873680833333	6.11703395833333	4.81947958333333	4.88478473333333	6.339217025	5.70053848333333	3.81605024166667	9.02273679166667	6.081909575	4.46877066666667	7.22899075833333	5.577321975	5.97595255	4.747391	4.92991125833333	4.4159867	4.41103275833333	4.936774575	4.51475793333333	5.53496716666667	6.85354335	6.15447426666667	4.73206480833333	7.27649536666667	4.01216488333333	4.62889925	5.16622001666667	6.73133699166667	7.444336	6.07673425	6.2176337	4.49312724166667	5.81909195	5.10930444166667	3.92815261666667	4.52238	5.106111275	4.60591755833333	5.157708775	4.68073703333333	4.64220138333333	5.25962125	4.33328629166667	3.88647775833333	6.54299384166667	4.68262605833333	5.455892825	5.83306814166667	4.57535033333333	4.41203210833333	5.56869470833333	5.14401780833333	4.44161573333333	5.02467455	5.51001904166667	4.043026825	5.78455018333333	4.96517085	4.55568075	4.19720234166667	5.23566219166667	4.53327731666667	4.602780325	4.62375229166667	6.13929583333333	4.49312724166667	5.02371664166667

PCDHA9	6.39152516666667	5.4789746	5.6411096	5.764437375	5.56425255	5.55953659166667	5.60229806666667	5.112251075	5.38469075833333	5.6241104	5.54392786666667	5.81478479166667	4.79411095833333	5.77106630833333	5.73682776666667	5.96958254166667	5.37452059166667	5.4137082	5.79649798333333	5.5927414	5.24195544166667	5.34196935833333	6.17154675833333	6.018516875	6.17390685	5.69296955833333	5.43710514166667	4.84935865	4.27798759166667	5.81212459166667	5.07218190833333	5.50615049166667	4.685722125	5.03726580833333	5.34097245	6.21191129166667	4.35819395	6.05045175	4.852466025	5.43818919166667	5.81652036666667	4.67674131666667	5.231579	5.064717325	4.51861790833333	4.67471653333333	5.31926836666667	4.55863708333333	5.95328179166667	4.535343	5.53102171666667	4.17476265833333	5.08252440833333	6.10873680833333	6.11703395833333	4.81947958333333	4.88478473333333	6.339217025	5.70053848333333	3.81605024166667	9.02273679166667	6.081909575	4.46877066666667	7.22899075833333	5.577321975	5.97595255	4.747391	4.92991125833333	4.4159867	4.41103275833333	4.936774575	4.51475793333333	5.53496716666667	6.85354335	6.15447426666667	4.73206480833333	7.27649536666667	4.01216488333333	4.62889925	5.16622001666667	6.73133699166667	7.444336	6.07673425	6.2176337	4.49312724166667	5.81909195	5.10930444166667	3.92815261666667	4.52238	5.106111275	4.60591755833333	5.157708775	4.68073703333333	4.64220138333333	5.25962125	4.33328629166667	3.88647775833333	6.54299384166667	4.68262605833333	5.455892825	5.83306814166667	4.57535033333333	4.41203210833333	5.56869470833333	5.14401780833333	4.44161573333333	5.02467455	5.51001904166667	4.043026825	5.78455018333333	4.96517085	4.55568075	4.19720234166667	5.23566219166667	4.53327731666667	4.602780325	4.62375229166667	6.13929583333333	4.49312724166667	5.02371664166667

SYNJ2	10.5244636333333	9.71265901666667	9.41120025	9.96522326666667	10.10216065	8.89736060833333	9.74820641666667	9.87303573333333	9.96954501666667	9.51223504166667	9.61984825833333	11.1473899333333	10.8778360416667	9.80448268333333	9.78692435833333	9.26488315	8.82519364166667	10.1350682166667	9.74607185833333	9.22061473333333	9.237031475	9.990452125	9.349435025	8.99164978333333	9.2408133	11.531296025	9.587537375	9.50586739166667	9.45194998333333	9.83900869166667	9.72449161666667	9.18115765833333	10.0578180083333	9.09007516666667	11.5349136916667	11.6468381291667	10.0924722666667	9.33537304166667	9.53398021666667	9.71828384166667	8.69791309166667	9.52546253333333	8.87238755	9.43234145	10.3015658	8.59640985	9.36572414166667	9.415734875	8.74272486666667	8.68624993333333	9.19340213333333	9.56036118333333	9.72484969166667	9.09684721666667	9.292040175	9.26855390833333	9.288795975	8.92461889166667	8.98004790833333	9.17867269166667	9.69126795833333	9.76267145833333	9.20872513333333	10.4581692833333	9.03641039166667	9.824283225	9.19340213333333	9.77352068333333	10.20226945	9.61680303333333	9.71612960833333	9.900995025	10.135861875	9.77090350833333	9.580833825	9.52804910833333	10.3119751583333	9.58344561666667	9.29664133333333	9.86266926666667	9.37126875	9.80664778333333	9.81525473333333	8.96609059166667	9.95767555	9.99723533333333	10.286132425	10.035165975	9.961096725	9.05125875	9.92213260833333	9.62318166666667	9.98225808333333	9.28041066666667	10.2330333333333	9.619021525	9.76819061666667	9.78481885	10.0506734583333	9.05355476666667	8.90197375	10.1316676583333	9.08047905833333	8.80450975	9.18546986666667	9.462094325	9.34207146666667	9.275522425	8.7532663	9.01566145833333	8.81971978333333	8.55955131666667	8.96273033333333	8.46720710833333	9.13078204166667	9.23929971666667	8.96322584166667	8.82873195	8.60751143333333	9.51175975

ACSL3	11.8845097083333	11.5223462	11.6566056666667	11.8048205583333	11.6095821583333	11.489428925	11.5912797083333	11.5824943666667	11.7720983166667	11.0075637333333	11.24459925	11.5505559916667	11.7545106083333	11.5226770083333	11.497488975	11.376411175	12.466874775	11.2579853916667	11.6198462166667	11.5691835	11.4460096583333	11.5132414833333	10.7325441416667	11.6985875333333	11.21039405	11.7019780416667	11.052772175	11.2312268666667	11.233092075	11.2315981833333	11.8171008	11.2478052	11.4317096083333	11.5407991083333	11.8074362166667	11.7081843583333	10.987327675	11.2047015416667	11.4688855083333	10.946906725	11.2215521833333	11.43436945	11.658465575	11.0934965333333	11.1419454583333	11.0507418833333	11.1313582333333	11.3934696166667	10.86771825	11.3946703583333	11.3657868083333	11.4485644833333	10.4910656666667	10.8510863583333	10.76764485	10.8585888666667	11.1800718666667	10.8172624166667	10.575694825	10.959358675	11.9935609583333	11.2312268666667	9.77475016666667	10.7565121333333	12.0205452666667	9.76554074166667	10.75497465	10.8484072166667	10.6523425166667	10.978750575	10.8996373166667	11.119251825	10.4040896666667	10.7846923083333	10.3996095166667	10.571508325	11.1380175916667	10.1862509833333	10.2769692583333	10.3656176	12.5594786833333	10.8650559083333	10.165808925	10.5899519833333	10.1134808166667	10.74562245	11.227651025	11.1313582333333	10.872956825	10.46219945	11.0487579166667	10.9061592666667	9.50586739166667	10.4413653083333	9.87070264166667	10.3380831916667	9.51633301666667	10.9091302666667	10.20226945	10.5049759833333	10.706587775	10.8198860166667	10.9303115416667	10.3225014083333	11.2606101916667	10.2186879083333	10.5748071083333	10.2399871166667	10.0855958166667	10.476356775	10.1982859833333	10.130420225	10.8228191416667	9.897727225	9.75492564166667	10.5022046916667	11.7568591416667	10.4922259333333	10.430465125	11.0182236333333

AFF3	9.29432763333333	9.2998268	9.62666130833333	9.70017130833333	10.1134808166667	9.51724313333333	10.430465125	9.22016708333333	9.84157025833333	9.478582775	9.651432225	10.189123775	9.79676919166667	9.06363208333333	8.986594625	8.72403179166667	9.07395639166667	9.76179089166667	8.97266575	8.975801525	8.91457238333333	9.07252779166667	8.87834883333333	8.74620478333333	8.748691075	9.06069035833333	9.0354605	9.14426045	9.42643701666667	9.23929971666667	9.29664133333333	9.36480266666667	9.557602025	9.91032726666667	9.32186685	9.21080935833333	9.42030009166667	9.51633301666667	9.10621828333333	9.22470684166667	8.94525708333333	9.17813545	8.954039625	9.05602455	8.81018689166667	8.53385956666667	8.69606343333333	9.19441289166667	8.79346453333333	8.894577375	9.30630015833333	9.15019934166667	9.71989020833333	9.41070553333333	9.39158905833333	8.82680535833333	9.275522425	9.073438725	8.99720454166667	8.51081288333333	9.458203675	9.489247625	9.13323439166667	9.86266926666667	9.063171125	9.59648643333333	9.32231320833333	9.72156395833333	9.34897708333333	9.55671014166667	9.84760208333333	9.56680634166667	10.0972464083333	8.871342075	9.50353634166667	9.02222115833333	9.41620731666667	8.95943646666667	8.88496090833333	9.15833918333333	9.22606134166667	8.88496090833333	8.93227916666667	8.615263775	9.462094325	9.45555605	9.57863910833333	9.4955569	9.68825589166667	8.66247435	9.49908795	9.27172153333333	9.31405536666667	9.14426045	9.95566975	9.557602025	9.33708134166667	9.46074875	9.77332255833333	9.136523775	9.04692176666667	10.0256584	9.24845199166667	8.73547365	8.91457238333333	9.11174605833333	8.966534925	9.14279978333333	8.82473405	8.39771189166667	9.04692176666667	9.09331330833333	8.984145025	9.15935345833333	9.2639574	8.907131825	8.78942864166667	9.08005709166667	8.927052675	9.33579655

ZNF563	7.25923676666667	7.492737425	6.77636395833333	6.68200588333333	7.206290275	6.61745720833333	6.69781155	5.858085325	6.90123264166667	6.041758325	6.56579801666667	6.86394090833333	7.00049489166667	6.96183981666667	7.45098580833333	7.25980210833333	7.31803023333333	6.08697085833333	7.0176409	7.124687125	6.64893395	7.54021029166667	7.2645801	7.26937910833333	7.07118303333333	7.28259465	7.314288875	7.18452301666667	6.97900894166667	7.65174983333333	6.48284681666667	7.40193138333333	6.80510874166667	7.09424611666667	7.09162461666667	7.70256570833333	6.68200588333333	7.05651791666667	7.134785875	7.057705175	7.38522528333333	7.286116125	7.36330638333333	6.95221913333333	7.18643425	7.456643225	7.39105859166667	7.254184275	7.40135389166667	7.35345289166667	6.98530065833333	6.97645464166667	7.404937825	7.79694136666667	7.1809933	7.655449725	6.84850089166667	6.45093723333333	7.392806175	7.23488161666667	6.58691901666667	6.769663225	7.10434548333333	6.17470206666667	7.13607696666667	6.57014316666667	5.997550525	5.773689175	6.2379381	6.67919664166667	6.07176163333333	6.19527208333333	6.8275595	5.73943181666667	6.43290905833333	6.82102155833333	6.64191437916667	6.70128395833333	6.20496000833333	6.47574773333333	6.36709734166667	6.19129395	6.493249575	6.67710233333333	6.15183188333333	6.141642375	6.38084359166667	6.03666274166667	7.06834181666667	7.12586193333333	5.85609921666667	6.03844185	6.81709465833333	6.94376375833333	6.49170240833333	6.39451754166667	6.42264453333333	6.79529673333333	5.69872159166667	6.438674975	7.26937910833333	6.80056770833333	6.15778135833333	6.45401484166667	6.37777528333333	6.04352600833333	6.53596566666667	6.602072175	6.78180254166667	6.82167365	6.72653388333333	6.02209645	7.01837270833333	7.26626465	6.73694150833333	6.5862738	6.51310794166667	6.510020875	7.12280756666667	6.61415903333333

CFAP65	4.60382705833333	4.88576699166667	4.2939407	4.638255275	5.34622214166667	4.09074995833333	6.21280073333333	6.075096075	5.78909119166667	4.68073703333333	4.666328775	5.1291031	5.74803075	6.0148863	4.80096826666667	5.60319860833333	4.67674131666667	5.80937455	4.60591755833333	6.18408195	6.89528771666667	4.15309530833333	4.84301391666667	4.52697115833333	4.740346475	5.31119278333333	5.07445225	5.04230353333333	4.79318954166667	4.3987315	4.75255506666667	4.6319392	5.41144461666667	4.17889495	4.48330838333333	4.42666711666667	4.638255275	4.95105651666667	4.6319392	4.8072374	5.23781710833333	4.92149175833333	5.03526733333333	5.7955502	4.18081978333333	5.18213341666667	5.367183075	5.212866025	4.52522428333333	4.830971775	4.83355489166667	4.510676125	4.031307325	5.58508399166667	4.49712886666667	4.93761881666667	4.82998554166667	4.90470955	5.41246783333333	4.65793555	4.02568050833333	4.63093599166667	4.37807450833333	5.455892825	4.91654405	5.16838866666667	6.390922	6.04876668333333	4.48239504166667	5.30151331666667	4.727091875	4.879391675	5.50436075	5.14931930833333	4.80433128333333	6.17470206666667	4.7847361	4.485186325	4.30437938333333	5.62775541666667	6.042706375	5.40932128333333	5.28357275833333	4.33637641666667	3.92023596666667	5.47028654166667	5.4137082	5.19860551666667	6.05225019166667	4.08963481666667	4.76141500833333	4.52522428333333	4.061836425	3.86842555	4.13005875833333	4.06079156666667	4.0798737	4.72615460833333	4.19346535	5.13961736666667	4.51657093333333	5.24195544166667	5.03526733333333	5.38372931666667	4.3987315	4.32386374166667	4.80096826666667	6.79923555	4.29716620833333	6.59464205	4.666328775	5.54873173333333	4.67877638333333	5.189937375	4.2422894	4.49904255833333	4.245391125	4.67380545833333	5.63053526666667	4.38097856666667

PTPMT1	12.1420289458333	12.050065475	12.2681612875	12.3389755458333	12.1863594833333	12.370533275	11.706226325	12.0866895125	11.9964806208333	12.0675907583333	12.3234787791667	11.7353263583333	11.7492646541667	12.047659675	12.1441510083333	12.1048843083333	12.9662359083333	11.7646426041667	12.2664905416667	12.1482289041667	12.2333947958333	12.0468052333333	12.4080636208333	12.3103859458333	12.2071828208333	12.0707912916667	12.0508487791667	12.1264399583333	11.8327205458333	12.1572977708333	12.0419021791667	12.2803156541667	11.9441699875	12.5031778708333	11.9441699875	12.2114186291667	11.8719919916667	11.943769	12.1073873291667	12.36294925	12.1730503625	12.0787689041667	12.1646637	12.3608148583333	12.2810876291667	12.4770061875	12.5119309541667	12.4335514375	12.6624101	12.4988424625	12.3560658833333	12.2953600083333	12.4107959333333	12.2572202	12.4979091291667	12.4600268041667	12.2526313625	12.52095865	12.2152361833333	12.5659956791667	12.33849745	12.4604436625	13.1882382291667	11.7599613916667	13.0010166416667	12.7089232125	13.0087094	12.2551972708333	13.0691136625	12.7631420958333	12.5369637041667	12.4498924125	12.6893793208333	12.7351672583333	11.768056675	12.5495127	12.0846036291667	12.3985967708333	12.1577392875	12.3201616916667	12.9152871083333	11.9846638916667	12.9939060666667	12.5427723	12.4236087416667	11.9607601916667	12.3260894583333	12.1988122958333	12.6834027	12.6747240166667	12.5528026458333	12.7631420958333	12.3577671833333	12.6082232625	12.3977067583333	11.8373385916667	12.5495127	13.4741426416667	12.1128089583333	12.0794824041667	12.597702825	12.6619101833333	13.077860625	12.9015734583333	13.2839047333333	12.8401156041667	12.5872332583333	12.73615865	12.1556081208333	12.0235911875	12.4595822625	12.0983293083333	12.5388641958333	12.9567449625	12.589520375	12.7048765958333	12.7726121208333	12.5499776333333	12.3608148583333	11.64610695

NDUFS3	12.1420289458333	12.050065475	12.2681612875	12.3389755458333	12.1863594833333	12.370533275	11.706226325	12.0866895125	11.9964806208333	12.0675907583333	12.3234787791667	11.7353263583333	11.7492646541667	12.047659675	12.1441510083333	12.1048843083333	12.9662359083333	11.7646426041667	12.2664905416667	12.1482289041667	12.2333947958333	12.0468052333333	12.4080636208333	12.3103859458333	12.2071828208333	12.0707912916667	12.0508487791667	12.1264399583333	11.8327205458333	12.1572977708333	12.0419021791667	12.2803156541667	11.9441699875	12.5031778708333	11.9441699875	12.2114186291667	11.8719919916667	11.943769	12.1073873291667	12.36294925	12.1730503625	12.0787689041667	12.1646637	12.3608148583333	12.2810876291667	12.4770061875	12.5119309541667	12.4335514375	12.6624101	12.4988424625	12.3560658833333	12.2953600083333	12.4107959333333	12.2572202	12.4979091291667	12.4600268041667	12.2526313625	12.52095865	12.2152361833333	12.5659956791667	12.33849745	12.4604436625	13.1882382291667	11.7599613916667	13.0010166416667	12.7089232125	13.0087094	12.2551972708333	13.0691136625	12.7631420958333	12.5369637041667	12.4498924125	12.6893793208333	12.7351672583333	11.768056675	12.5495127	12.0846036291667	12.3985967708333	12.1577392875	12.3201616916667	12.9152871083333	11.9846638916667	12.9939060666667	12.5427723	12.4236087416667	11.9607601916667	12.3260894583333	12.1988122958333	12.6834027	12.6747240166667	12.5528026458333	12.7631420958333	12.3577671833333	12.6082232625	12.3977067583333	11.8373385916667	12.5495127	13.4741426416667	12.1128089583333	12.0794824041667	12.597702825	12.6619101833333	13.077860625	12.9015734583333	13.2839047333333	12.8401156041667	12.5872332583333	12.73615865	12.1556081208333	12.0235911875	12.4595822625	12.0983293083333	12.5388641958333	12.9567449625	12.589520375	12.7048765958333	12.7726121208333	12.5499776333333	12.3608148583333	11.64610695

PLPP3	13.734666675	13.1134452125	13.0473086958333	12.7973892041667	11.8087504208333	13.167813625	12.9458751541667	12.9572856708333	12.6834027	13.6250524208333	12.4539039291667	14.0571831583333	13.4947244958333	13.2577424916667	12.3735275333333	13.794497375	14.1363801208333	12.1568664583333	12.38181285	13.3564324708333	13.0691136625	12.3437410791667	13.2851875333333	13.4138735791667	13.1790128291667	13.9137141375	12.7136661833333	12.9125891625	11.8660787416667	12.6968710791667	13.0064680041667	13.0527420125	13.1360170875	13.6129098916667	14.1248938833333	13.7864252541667	12.4192060458333	12.8558614458333	13.3084273833333	13.1875698791667	13.2851875333333	13.0122292208333	13.2106968458333	12.7555989166667	13.7935312541667	13.2078095708333	13.1716469375	12.9712877375	13.4093114166667	13.0795498541667	12.8451935041667	13.3491368416667	13.0563467	12.364713775	12.7772084333333	13.1397677541667	12.9131474	12.7854970375	13.5597488333333	13.4100577708333	11.7076157291667	11.8128624291667	12.2514934666667	11.9250271291667	13.1541556166667	11.4422512791667	12.1412178458333	12.1247999541667	11.9242314958333	11.9366819583333	12.6183459541667	11.7260778	11.6247308125	13.2542277375	11.7334310666667	12.3133205	12.2009012166667	12.64891165	11.6264903083333	12.0549168875	13.2456588833333	12.0750490416667	11.2924159458333	12.6153916958333	12.5834011	12.7423894333333	13.5039215708333	12.39063025	11.9378299833333	13.0752598625	11.9242314958333	11.7722639416667	11.5759936041667	11.9011514833333	11.2889731166667	12.4121305291667	12.0472317	13.4485668583333	11.7137084625	13.1319436458333	11.7965104583333	11.6038006333333	12.3919605166667	12.3380364458333	10.62246185	12.5550651208333	12.2368632208333	11.5564976583333	12.0975331166667	12.6243321125	13.6343167625	11.4040128333333	11.2893333708333	13.2832111125	12.0850507583333	12.0846036291667	13.9878920166667	11.6350057	11.7701389041667	12.3290660083333

AC119674.2	13.734666675	13.1134452125	13.0473086958333	12.7973892041667	11.8087504208333	13.167813625	12.9458751541667	12.9572856708333	12.6834027	13.6250524208333	12.4539039291667	14.0571831583333	13.4947244958333	13.2577424916667	12.3735275333333	13.794497375	14.1363801208333	12.1568664583333	12.38181285	13.3564324708333	13.0691136625	12.3437410791667	13.2851875333333	13.4138735791667	13.1790128291667	13.9137141375	12.7136661833333	12.9125891625	11.8660787416667	12.6968710791667	13.0064680041667	13.0527420125	13.1360170875	13.6129098916667	14.1248938833333	13.7864252541667	12.4192060458333	12.8558614458333	13.3084273833333	13.1875698791667	13.2851875333333	13.0122292208333	13.2106968458333	12.7555989166667	13.7935312541667	13.2078095708333	13.1716469375	12.9712877375	13.4093114166667	13.0795498541667	12.8451935041667	13.3491368416667	13.0563467	12.364713775	12.7772084333333	13.1397677541667	12.9131474	12.7854970375	13.5597488333333	13.4100577708333	11.7076157291667	11.8128624291667	12.2514934666667	11.9250271291667	13.1541556166667	11.4422512791667	12.1412178458333	12.1247999541667	11.9242314958333	11.9366819583333	12.6183459541667	11.7260778	11.6247308125	13.2542277375	11.7334310666667	12.3133205	12.2009012166667	12.64891165	11.6264903083333	12.0549168875	13.2456588833333	12.0750490416667	11.2924159458333	12.6153916958333	12.5834011	12.7423894333333	13.5039215708333	12.39063025	11.9378299833333	13.0752598625	11.9242314958333	11.7722639416667	11.5759936041667	11.9011514833333	11.2889731166667	12.4121305291667	12.0472317	13.4485668583333	11.7137084625	13.1319436458333	11.7965104583333	11.6038006333333	12.3919605166667	12.3380364458333	10.62246185	12.5550651208333	12.2368632208333	11.5564976583333	12.0975331166667	12.6243321125	13.6343167625	11.4040128333333	11.2893333708333	13.2832111125	12.0850507583333	12.0846036291667	13.9878920166667	11.6350057	11.7701389041667	12.3290660083333

IFT22	8.3851619	8.49794376666667	8.69504618333333	8.55492440833333	9.11541340833333	7.3367108	8.821200975	7.90528599166667	8.46720710833333	7.99232836666667	8.04450845	8.49648456666667	8.107904375	8.17505753333333	8.22981704166667	8.58349254166667	8.11504903333333	8.4444208	8.5676736	8.38611514166667	8.7217903	8.647921775	8.451050725	8.70080298333333	8.36807433333333	8.973998125	8.631828525	8.57301913333333	8.010141425	8.27857833333333	8.29147479166667	8.62194039166667	7.81401100833333	7.85364198333333	9.570506275	8.709872025	8.054062325	8.351126275	8.11940264166667	8.40965201666667	8.44745494166667	7.99186004166667	8.17090974166667	8.748691075	8.1277546	8.26207273333333	8.40614324166667	8.39013935	8.09798645	8.34473695	8.22690084166667	8.368580775	8.36713998333333	8.89174859166667	8.55645535833333	8.32106236666667	8.56044531666667	8.462988175	8.312026725	8.07357910833333	8.4304275	8.50797726666667	8.932734675	8.54122040833333	9.32939963333333	7.82539101666667	8.965634	7.76345526666667	7.964195775	7.88559756666667	8.31765478333333	8.81642101666667	8.41158014166667	7.49938943333333	7.88403631666667	8.709455975	8.06996655	8.37211495	8.13415403333333	8.15060805	9.64736674166667	9.63292783333333	8.294447975	8.996715525	7.76884748333333	8.97625954166667	9.921332825	8.69363141666667	9.1141243	8.24557174166667	8.283955225	9.817741375	7.7528692	7.72249438333333	9.02273679166667	8.70898641666667	8.2260001	9.661136725	8.8768317	9.14279978333333	9.14971231666667	8.96369055833333	8.757884525	9.34302553333333	8.1277546	7.731363575	7.92110699166667	7.99937465833333	7.95194513333333	7.68487656666667	7.99891026666667	8.43393876666667	8.23909885	9.90058610833333	7.87042711666667	8.3662572	9.166824	9.431383225	8.38865071666667	8.52772891666667

SYNM	16.2495335833333	13.9717922666667	12.8718544916667	11.931140425	13.1986854833333	14.515883675	13.7553717	13.6497739333333	14.2000424583333	12.9172670333333	12.92025315	15.873782975	15.7630766833333	13.9695187666667	13.99785205	13.016186275	12.0317754666667	13.5329794333333	14.8236926166667	14.3875977	13.9400026166667	12.3369699166667	14.0759487166667	13.2254010416667	13.8405812166667	16.2791471166667	14.8699486666667	12.972189625	13.0761975166667	13.4904111	14.36106535	13.7168412916667	14.4161239916667	11.5204866583333	16.4447326166667	16.613425425	13.011963125	13.3158362583333	13.4783310166667	14.3628995916667	13.9186404666667	13.9445631916667	13.479133875	14.5415836083333	15.6293082	13.2254010416667	14.85238655	13.5610039666667	12.181921	13.695137875	13.86774345	13.8766797166667	13.0816325916667	11.9697901833333	13.180045325	14.2936594416667	13.2560414333333	13.1919341833333	13.608143675	13.9389915916667	10.9673192583333	12.05687395	9.45055241666667	11.92557355	11.9093036166667	9.625456175	12.0612117666667	11.4567547583333	9.62318166666667	11.1674196	11.3499248083333	9.695341625	10.0103759833333	11.3646504166667	10.02019625	9.86359230833333	14.4361804666667	12.31311615	10.6905955916667	11.5479749166667	13.2648622166667	13.4579324833333	10.216230425	10.804132675	11.4086625666667	11.104988	11.6771659333333	13.2711737166667	10.252716075	13.6979142666667	10.0386893	9.478582775	11.0112232166667	11.9263114333333	11.414772875	11.088478625	10.0256584	11.5598018166667	9.615115575	10.5034091416667	11.9248644916667	11.6691177916667	10.647680825	11.1394283333333	11.341686575	10.6964442916667	11.5930630416667	9.73788834166667	10.4902258833333	12.2545127416667	12.478144425	10.8139547	9.64922493333333	8.89870729166667	9.862235575	10.1940041	13.5435554666667	9.88763298333333	9.80885460833333	12.9677883916667

NQO2	13.5468416333333	13.5171924333333	13.92725075	13.8778632416667	14.6549636583333	13.4119895416667	15.1077350416667	13.394322425	14.3531439916667	13.410428725	13.901442	14.7874202666667	14.282826075	13.3994919416667	13.0506754833333	13.0743927916667	12.7233335166667	14.127909425	13.1531334333333	13.1556465833333	13.2933426583333	13.20142475	13.0841853166667	13.570433175	13.4705605	13.661470025	13.4443089416667	13.3158362583333	13.2842694	13.9284348416667	12.9461435166667	13.66414075	14.1752017916667	13.9153342666667	13.289124025	13.5451377333333	14.044199175	13.1779047083333	13.4565571916667	13.7036621	13.1242463	13.0228260333333	13.1525120666667	13.0743927916667	13.483275325	12.3271580833333	12.338741975	13.1806770166667	13.6062775083333	13.933121025	13.5068025166667	13.9239245083333	14.2904660666667	13.823045075	13.181363025	12.3698220916667	13.88005245	13.1097329166667	13.933121025	13.0871432666667	14.5304022916667	14.667083075	14.3299457833333	14.5322324833333	14.26427225	14.65081365	14.5717037666667	14.73869885	14.227452975	14.7948348083333	15.039210975	14.15404765	14.577355375	14.3843059083333	14.282826075	13.6587931916667	14.8313200916667	14.4722450583333	13.6931782333333	13.85933545	14.0787258833333	14.5570464083333	14.044199175	13.2662932833333	14.9507696666667	14.7409853333333	14.818607725	14.512293975	14.8162748	14.2548502583333	14.4043399	14.1113310916667	14.6926031666667	14.295197475	14.9567111166667	14.7547481083333	14.912278625	15.1632730833333	14.6325901333333	14.3153773833333	14.4850706333333	14.8442556083333	13.96690645	13.6284294583333	13.7303452416667	14.1513249333333	13.6356329916667	13.08588685	14.3875977	14.0301250916667	14.4615780083333	13.6894331166667	13.5401929916667	13.066353125	13.4611203333333	13.4390716416667	14.0012596166667	14.236432725	14.1656626833333	14.7055894833333

DUSP3	14.8819536416667	13.5043698416667	13.320797425	13.0816325916667	12.8376576083333	12.9604577916667	13.1144791333333	13.410428725	13.2765049166667	13.047619075	13.5213793583333	14.4565233916667	13.7781265916667	13.44358445	13.462725925	13.334230975	13.5738180166667	12.643219075	13.8079143416667	13.9261802833333	13.782971725	13.3032240666667	13.2656354333333	13.4950898916667	13.4966084666667	15.3944873	13.7621985333333	13.400193425	13.2467446916667	13.5133528333333	13.672832575	13.735130625	13.6605641666667	13.6356329916667	15.390335525	15.4672060583333	13.3010495416667	13.4314411416667	13.5660586666667	13.9518039333333	13.8604046083333	13.90344385	13.9378093083333	13.6089687	14.3431664333333	13.735130625	14.036419	13.5843144666667	13.5859683083333	13.4649543916667	13.6675870333333	13.8221316	13.0343175416667	13.3667920416667	13.5443638333333	13.9762519833333	13.4274952333333	13.7630775	13.4289883	13.7224285916667	12.76127775	13.5435554666667	12.5609255833333	12.7441506916667	12.4368713	12.916130525	13.1420517833333	12.7048765958333	12.8537857833333	12.8317521333333	13.0307376	13.2168871166667	12.6944981166667	13.4260804333333	12.5102327333333	13.2861830833333	13.4579324833333	12.6175954833333	12.9417394416667	12.895307525	13.230641375	12.8317521333333	13.0457183333333	13.5494174416667	13.053017375	13.2815171083333	12.264576775	13.118520075	13.1550516583333	13.8908948916667	13.4611203333333	12.516863425	13.5387428916667	13.18580525	13.044583175	12.956460525	12.6640954083333	12.8426722583333	13.1085025666667	12.7671377333333	13.4602959916667	13.70272065	12.923601775	13.7272276083333	13.2898598333333	12.908758175	13.542740525	13.2799375583333	13.2968461416667	13.5494174416667	13.4935932	12.6754546333333	12.60273605	12.996638525	12.6195975333333	12.8718544916667	13.0271148666667	13.1229606833333	13.031934	13.10396855

CD4	12.3926407	11.6234420916667	12.05061705	12.050259975	13.3285445333333	11.7868322833333	13.62200835	11.69218085	12.7201162666667	11.7146176666667	12.2198129666667	13.1819427166667	12.4808505	11.5614234666667	11.4985882166667	11.5754446583333	11.03596855	13.068247425	11.683415075	11.7296484916667	11.6691177916667	11.4971747583333	11.2071862916667	11.6006445416667	11.81229275	11.9003237375	11.6522306833333	11.385364925	11.849231625	12.1891609666667	11.2128973166667	11.846919075	12.6881562083333	12.0551513666667	12.0388164916667	12.3160821666667	12.484600075	11.42922935	11.56524335	11.8861575583333	11.5078323166667	11.4654444	11.320943175	11.21620365	11.7509367	10.9084288583333	10.8908321583333	11.6489233	11.9361117583333	12.295556975	12.2198129666667	12.2578527416667	12.693462425	11.9625539583333	11.54260245	10.6649055833333	12.4283415	11.73400915	12.2835716083333	11.4708276916667	13.0392216583333	12.625004	12.2120592333333	12.531130425	12.343956025	12.5429966333333	12.7543063	12.5187542666667	12.4528342	12.487541925	12.6606270166667	12.2206544333333	12.5448126916667	12.1509618666667	12.1346602166667	11.5676618166667	13.28687105	12.91963445	11.9994097833333	12.3589004083333	12.2896477166667	12.5667535083333	11.9036209583333	11.4779366416667	12.8107678333333	12.6537086583333	12.9278503083333	12.3892279333333	12.81348825	12.1291180083333	12.3165034833333	12.247084775	12.8149887083333	12.338741975	13.1305878416667	13.0761975166667	12.907519675	13.0102233	12.6166077833333	12.3043299166667	12.7939716	12.7144138666667	12.000941175	11.5385948833333	11.825372775	12.4893471666667	11.9962714583333	11.5422054916667	12.5989743166667	12.367659325	12.6876920416667	12.0313834166667	11.9467504166667	11.155024025	11.770678525	11.7648162666667	12.0290333416667	12.3824722	12.251683475	12.8690686583333

SRPRB	10.8368196833333	12.3138792416667	12.4215906166667	12.540087475	12.7801271583333	12.462326175	11.8916056833333	12.4659800333333	12.4981202833333	12.3043299166667	12.7509478416667	11.3375225333333	11.9945651875	12.4563419583333	11.7336295416667	11.7227876083333	12.91963445	12.45136255	12.52409195	12.6975412416667	12.5116969583333	12.9876385	12.518275925	12.7011030333333	12.7368908416667	11.0670126666667	11.9678169416667	12.4225030833333	12.479955875	12.6660656916667	12.452378375	12.77853635	12.2137378333333	12.32117485	10.992544275	11.0341045833333	11.8853151666667	12.48596835	12.226469725	12.4813575333333	12.8549658583333	12.198234625	12.6586586666667	13.0294955083333	12.4315646666667	12.7687032	12.6270116666667	12.7201162666667	12.4709919583333	12.9352563083333	11.844663475	12.57979945	12.7122547666667	12.21502565	12.4723572833333	12.1320993083333	12.8964099833333	12.4571981916667	12.4082756583333	13.0483120583333	13.180045325	12.7586192666667	12.5725102833333	12.3724626666667	12.4377746666667	11.642205825	12.6391509	13.2806732833333	12.8969748916667	12.9745146166667	12.7071557666667	13.1885669333333	12.2755744916667	12.87462785	12.9773913166667	12.9954911666667	12.7061787	12.4836070666667	12.3869118416667	12.6442138916667	13.3515334583333	12.4532785	12.3733049416667	12.989519425	12.4069098166667	12.87636155	13.0714709916667	12.2619793166667	13.5617608833333	12.9960374833333	13.06700765	12.462326175	11.958220325	12.87462785	13.2711737166667	12.6956383666667	12.2990201416667	13.1980287833333	12.5984922	12.6914097333333	12.6980083833333	13.4634900333333	12.9853754833333	13.52385865	12.7227603583333	12.7812528833333	12.5076062916667	12.5543618083333	12.7170209166667	13.158283725	12.2906208	13.1569608666667	12.2675250666667	12.786823125	12.8278480083333	13.2279712833333	12.9172670333333	12.2311025	13.5100333	12.3798172166667

MYNN	11.179383775	11.30507995	10.91013625	11.1398416833333	10.5408711916667	11.189465025	10.6195893583333	11.1874035166667	10.7585831916667	10.7508267666667	10.8980207583333	11.1132646583333	10.57908995	11.30507995	11.3086201833333	11.0507418833333	11.7168402	10.9643625833333	11.0504310083333	11.4946024833333	11.1824657666667	11.33173475	11.568787	11.48359205	11.22659855	12.1435544583333	11.2616452583333	11.7160036	11.2664173916667	11.4786409333333	11.0323327416667	11.1975258	11.2258131916667	11.164489475	11.8954895166667	11.4682426833333	11.1095361333333	11.4627181416667	10.9730535833333	11.2729625666667	11.0655176333333	10.7421748083333	11.114752	11.351341375	11.0992950083333	10.8771107	11.1908643083333	11.0596766	11.0593297333333	11.328911575	11.122077	11.062936475	10.8938022916667	11.2753469583333	11.22691525	10.93390845	10.8476613083333	11.0975459833333	11.3025465416667	10.9483433833333	11.4045795333333	11.825372775	11.685558375	11.1912222083333	12.6566773416667	10.81908485	11.1901541416667	11.4718467833333	11.2032221333333	11.13908545	11.4162651666667	11.97355025	10.5549316666667	10.9965540083333	11.3132862083333	11.06860385	11.1117434416667	10.9643625833333	10.9537804833333	11.5710069666667	11.767528775	11.765950425	11.2323478	11.4000850916667	11.7035007416667	11.715671375	12.4456480416667	11.104988	11.7716953916667	11.47943685	12.6116906583333	11.8626743166667	10.7612989333333	11.233092075	10.6499586916667	12.4194028333333	11.2664173916667	11.4045795333333	10.96963075	12.140177075	11.2576230916667	11.94832135	11.170311525	12.0653086666667	11.1139507416667	11.0466400166667	10.51932965	10.7931763166667	10.8578576916667	11.2635210833333	12.1224925833333	11.3136753916667	11.6587921	11.3187261333333	11.1110336416667	11.3296296583333	12.1636527	11.1273741333333	11.5598018166667	11.605536575

PPA1	13.8983181916667	14.3908304333333	14.51071235	14.2610479166667	14.4077075916667	14.2610479166667	14.4758644166667	14.7499385916667	14.4459919583333	14.0514423083333	14.7640659	13.3816176916667	14.113875875	14.789742575	14.4963541416667	14.5865975583333	14.004911475	14.3860372083333	14.4109315833333	14.3692316583333	14.74733735	15.0306120416667	14.30615445	14.8794573583333	14.6430667333333	13.4950898916667	14.5886594916667	14.4309788083333	14.18015695	14.5646019166667	14.61397985	14.201540175	14.2998455083333	14.1752017916667	14.0353374833333	13.487174625	14.1086467166667	14.5433520583333	14.515883675	14.6738026916667	14.1427380833333	14.3076416166667	14.3381592083333	14.2533850083333	13.9989817	14.36106535	14.465199375	14.6611477166667	14.5089658583333	14.4043399	14.3236760583333	14.68415095	14.3236760583333	14.3076416166667	14.4328794	13.9055869333333	14.6430667333333	14.2671663083333	14.465199375	14.082470775	14.0012596166667	13.8473484166667	14.2904660666667	14.4443539833333	13.901442	14.1292641	13.6150235416667	14.30297575	14.1670824833333	14.9307420333333	14.1914740916667	14.0874428333333	14.818607725	14.0288199166667	14.3350486166667	13.838661325	14.4597852166667	14.1527055166667	14.2858034416667	14.295197475	13.527156225	13.9829878583333	14.7166750666667	13.8307559916667	13.6785048416667	14.5287330416667	13.9045177166667	14.274700225	14.1827283166667	13.913193025	14.349825825	14.6798721333333	14.1471335166667	14.1827283166667	13.5100333	14.15404765	14.6738026916667	14.312396275	14.2000424583333	13.67475365	14.6589883666667	14.5377311666667	14.6046129083333	14.2887769	14.2503770416667	13.93081475	13.9818544666667	14.0656333583333	13.8285300416667	14.4908464833333	14.0275803333333	14.21756365	14.1152258583333	14.277828625	13.8461367666667	14.371029675	13.3801175	14.4722450583333	14.0746231666667	14.5664298833333

EYA4	6.732067125	6.98853248333333	5.83208956666667	5.71882016666667	6.5862738	4.82796093333333	7.27359608333333	5.87100729166667	6.37568504166667	6.59176974166667	6.13424446666667	7.296620525	6.73419851666667	6.41270695833333	5.9751687	5.67810205833333	6.05392339166667	6.62969040833333	5.87944876666667	6.29822925	5.89449381666667	6.01386335	7.36156116666667	7.20211523333333	7.04503860833333	7.043221875	5.677007675	5.36615406666667	6.07673425	5.557624025	6.33415565833333	5.42082883333333	5.53996425	6.1936491	5.56869470833333	5.94277896666667	4.453007975	6.34302255833333	6.29358614166667	7.751162425	5.93306849166667	6.04082925833333	6.24107090833333	5.79916375	5.47990566666667	6.14881959166667	6.18568551666667	6.85745724166667	4.37610179166667	5.47616439166667	5.24388391666667	6.48645600833333	6.810578	7.38233871666667	5.88521655833333	5.13961736666667	6.35434874166667	6.12200050833333	6.11785979166667	6.73133699166667	5.87100729166667	6.43421143333333	5.078596225	5.899063175	5.34196935833333	6.45841340833333	4.36096451666667	5.780758525	4.76548715	4.92991125833333	6.021143325	5.61840579166667	7.48588683333333	5.623286525	5.98559190833333	4.09074995833333	4.74337093333333	4.15642159166667	6.5862738	4.07245948333333	6.15692685	6.11703395833333	6.06914035833333	5.38659805833333	5.53996425	6.03147445	4.669520575	4.68477714166667	5.13227493333333	4.87292895	6.013009825	5.29885645833333	6.92852376666667	5.326621775	4.45699755833333	3.723060125	4.692141475	4.816016475	5.88673599166667	5.81403274166667	6.25755120833333	5.743144625	4.54769205	4.9110181	5.55559675	4.515720925	5.81989971666667	6.408778075	5.03930840833333	6.50650844166667	4.44453341666667	5.360915675	4.88576699166667	6.00968073333333	5.44971561666667	4.53624929166667	3.87840355833333	4.39557236666667	4.53327731666667	6.169883075

TGM2	12.7564076083333	10.2888943333333	11.2100932	9.895935425	10.601543225	11.5319982916667	9.963087575	10.3686089583333	10.4300821583333	11.401582575	10.2786656166667	12.6180925666667	12.1608207083333	10.4963081083333	10.3591207	11.541832	10.8885586333333	10.8510863583333	11.166670375	11.2434998833333	11.63218295	9.40469768333333	11.0870314666667	10.384887925	11.1125423333333	12.6190854666667	10.5295700833333	10.0819455833333	10.4464281166667	10.5651423416667	10.6683478	10.2241250416667	10.53724435	10.4577793166667	12.5902222833333	12.87960645	10.741834125	10.8502772583333	11.709376075	11.4956432166667	10.6887964	10.91165365	10.7027965333333	10.2951456833333	11.9304447166667	11.2356259583333	11.1103347416667	11.3516750666667	11.030517625	10.7329204833333	10.5300186083333	11.25569785	10.972292975	10.8688118916667	10.9537804833333	11.43032555	10.9378957833333	11.2478052	11.0898799333333	11.9538193583333	11.2542786416667	11.13057255	10.4728823833333	10.8733577583333	10.271227125	10.37104885	10.7057641416667	10.9505347083333	9.83153578333333	11.4022935333333	10.971110725	11.0175089583333	8.79186565833333	11.884179625	11.3499248083333	10.257489575	13.3080939833333	11.25027585	9.73744423333333	11.235931225	10.7088791083333	11.3132862083333	9.87695954166667	11.1993790583333	11.0723250583333	11.3477921	9.86145205	11.8848963583333	9.76179089166667	12.6190854666667	9.38500366666667	11.4470684833333	10.6453411416667	10.5314888083333	10.66752385	10.4530959333333	9.726163425	10.39291705	9.565041475	10.4641916916667	11.4868726416667	11.1166050833333	11.2150743583333	10.4236603166667	11.7053666833333	11.189465025	11.8341069333333	10.0445903666667	11.3419959666667	11.86892365	12.1886556416667	10.0026032916667	11.1634048416667	9.63342665833333	11.174649725	10.884735425	10.8273805416667	10.882085925	9.96151823333333	12.3974616666667

SORD	9.31405536666667	8.199157725	7.7548948	7.76188296666667	7.66209683333333	6.76517564166667	8.05804941666667	8.43510350833333	8.1963928	8.13977138333333	8.905745925	7.46746676666667	8.05717868333333	8.48112818333333	8.63917994166667	7.80451180833333	9.687830775	7.28072111666667	8.59007210833333	8.1873246	7.99615545	8.19808664166667	7.833966975	8.162750025	8.104851775	8.927052675	7.56216311666667	7.56380908333333	7.28259465	8.10691838333333	8.518147825	8.37303865833333	7.521666525	8.925575825	9.26082591666667	8.91790284166667	7.29049713333333	8.000994225	7.826430025	8.11887123333333	9.20464029166667	8.4841298	8.15563305	8.619247325	7.55987390833333	8.67361984166667	8.80300369166667	8.083905375	8.15758866666667	7.78927275	7.650678625	8.08289571666667	8.413378975	7.8648195	8.37653824166667	8.67503203333333	8.33913670833333	8.41642395	8.01861064166667	8.26867154166667	7.53565475833333	9.01698551666667	7.270544475	6.97462729166667	7.415839475	8.21097586666667	8.30855848333333	7.95517108333333	6.26504835	7.75930169166667	8.33913670833333	7.09231525	9.39704936666667	6.90123264166667	6.98588438333333	8.07357910833333	7.19767501666667	6.57427455833333	7.746730925	6.82513060833333	7.56602129166667	6.78916241666667	8.48261985	8.475275825	7.54177855833333	7.41352716666667	7.94065678333333	5.84141815	8.36907771666667	7.96986838333333	7.62037211666667	7.57903273333333	7.72354014166667	7.75542415833333	6.00039116666667	6.225217	6.36709734166667	7.37296829166667	7.48861440833333	8.36243170833333	7.54963650833333	7.87562194166667	7.72354014166667	9.13780860833333	8.80499509166667	6.56951250833333	7.872646525	8.64638736666667	6.258372575	6.87783053333333	6.53273724166667	7.9846002	8.04016346666667	7.8129644	7.93641930833333	6.94690668333333	8.31911250833333	8.21622873333333	8.45999591666667	8.164333775

GABRB3	9.51175975	5.68689626666667	6.91905025833333	6.12282240833333	5.31319136666667	6.891986525	5.33648908333333	6.735668325	5.73297515	6.57952525	5.55559675	5.880482625	5.880482625	6.19527208333333	6.61221461666667	6.51830799166667	9.78918780833333	4.15118036666667	6.133346875	6.10234676666667	5.83110111666667	5.47990566666667	6.8275595	7.2266449	7.03138465	9.01566145833333	6.78180254166667	6.54933985	6.32106796666667	7.59020169166667	6.57014316666667	5.66650295	6.71367926666667	8.49291188333333	9.52783803333333	8.88546686666667	5.69679719166667	5.527437325	7.14272935833333	6.51382934166667	6.74888376666667	7.27471595833333	7.0168934	7.84845994166667	7.02467490833333	5.96077145833333	7.53185565833333	7.54228448333333	6.38084359166667	7.45275619166667	6.64016859166667	6.98215429166667	4.747391	5.62140730833333	6.08940326666667	7.18351369166667	6.08593745	6.38621459166667	6.365448375	6.91322913333333	8.38470655	4.683662975	4.946749425	6.11631895833333	8.31103703333333	4.18603395833333	4.127831	4.21192669166667	3.85883090833333	7.58577588333333	7.34718618333333	3.78548855	3.63279243333333	4.53008874166667	3.98827198333333	3.80900449166667	5.12102319166667	4.07678143333333	8.75743711666667	4.44923994166667	7.704155275	5.45060010833333	4.53824951666667	10.93251855	4.36495859166667	6.09669481666667	4.245391125	4.63513973333333	10.2741449416667	6.19129395	3.94580334166667	5.078596225	8.18100231666667	3.997050325	6.66175125	4.936774575	7.88506155833333	4.65900069166667	4.782758275	5.20250346666667	4.4462942	8.23365661666667	6.20496000833333	8.9604306	4.51773374166667	4.09074995833333	8.27071193333333	6.8752982	6.41270695833333	6.99096820833333	5.42554189166667	5.737720375	4.63093599166667	9.12281406666667	4.1882044	4.44453341666667	4.21701204166667	4.68681869166667	9.131237375	4.72414796666667

DLG1	4.97072769166667	4.06699531666667	3.80900449166667	4.70475344166667	6.56579801666667	7.2960179	4.52522428333333	3.890139475	3.73515468333333	4.49904255833333	4.98651175	4.41203210833333	4.46293625833333	4.35296875833333	5.25233420833333	3.95350946666667	4.72828980833333	6.27802068333333	6.411112525	4.54864098333333	4.19810324166667	5.77807068333333	4.75698904166667	5.09968536666667	5.35882338333333	3.810060775	5.399029725	5.735887375	7.06531028333333	4.68789791666667	4.59576983333333	5.56242626666667	4.302366825	3.98827198333333	4.32386374166667	4.43091141666667	6.94881511666667	4.85037616666667	4.67877638333333	5.46647429166667	5.24102825833333	4.576370125	4.72615460833333	4.759183025	4.24718985833333	5.05172549166667	4.26758835	4.383064975	5.11568579166667	4.57827949166667	4.91206978333333	5.75004875833333	4.20109206666667	4.87199023333333	3.997050325	4.20479785833333	4.15220664166667	4.56487130833333	5.565954175	4.65176085833333	7.69858204166667	4.01916375833333	6.5680586	5.824619025	5.8523182	8.698928125	5.0088772	6.11387184166667	5.19112474166667	5.14829798333333	5.153439775	6.64956780833333	4.453007975	4.58241979166667	5.3439553	5.26078876666667	6.21116124166667	6.970355725	6.187374325	4.77483110833333	4.85447411666667	5.38263135833333	5.78709845833333	4.83558559166667	5.16420199166667	4.922546525	5.61059491666667	4.428719425	6.25427673333333	4.76141500833333	5.13336504166667	6.52344795833333	7.61643631666667	4.98341465833333	6.46851783333333	7.19065496666667	6.54365829166667	5.97349889166667	6.75170075833333	6.26664835833333	6.15692685	5.24931593333333	6.01590590833333	4.98651175	5.843159875	5.95074298333333	5.87496216666667	5.126264825	5.1555947	4.576370125	5.891995425	6.05225019166667	6.30401625	4.41506155	4.82036908333333	4.816016475	7.00303549166667	6.98973956666667	7.16909093333333	4.50011030833333

AC067752.1	10.6111916083333	9.985532675	10.9378957833333	10.7511943	10.4449298	9.16874195833333	11.5483812666667	9.6705637	10.4195860916667	10.0048138083333	10.3380831916667	11.1369321416667	9.60581258333333	8.97168731666667	9.974596575	9.52316788333333	8.60593184166667	9.69308664166667	9.7815357	9.058233875	8.98368761666667	9.08180965	9.579511975	9.51896663333333	9.91503965833333	10.1804756333333	10.73913815	10.4231890083333	9.93304795	10.7123288	9.98470918333333	10.69756315	10.71663975	10.8337493916667	10.8893434416667	10.7073853583333	10.46260805	9.97215941666667	10.3718117166667	11.0116340666667	10.5096862166667	10.15852225	10.5973351833333	10.279032875	9.62193513333333	9.21923738333333	9.76267145833333	10.5334113083333	9.70017130833333	10.390544	10.6169276916667	10.9139727166667	10.1312217666667	11.1380175916667	11.0013930666667	9.78528060833333	10.3618816	10.6332993416667	10.9080821583333	9.83900869166667	10.5663053083333	10.384887925	10.8605112583333	10.7684448166667	9.650075475	9.844981225	10.3542362916667	10.1878607	9.86678629166667	10.2991427166667	10.6377074083333	9.98800139166667	9.76006771666667	9.3017146	10.4561678833333	9.722436925	9.43501909166667	8.80450975	9.620263	9.22016708333333	9.13172414166667	9.38580318333333	9.763939075	9.35863609166667	10.3721682083333	10.4752224083333	11.1491945416667	10.7789681833333	10.6158476666667	9.87164061666667	10.4971305166667	10.5553397083333	10.390544	10.362641575	11.1721251583333	10.6035239083333	10.0945926583333	10.61804865	10.6838734833333	10.5448322833333	10.8657939916667	10.3554429666667	10.696845925	10.2666506833333	9.31227445	9.91197805833333	10.308194675	10.19975295	9.97255511666667	10.1226723083333	10.5368715833333	10.30399485	9.62155398333333	9.86766510833333	10.599920275	10.3373213583333	9.91460061666667	10.6753741833333	10.30852645	10.4429923666667

ADH1A	13.5095875375	12.7083837541667	12.3095501125	12.2677302791667	10.5607363458333	12.6001910208333	13.0605105	12.602520925	12.6667265916667	13.7277599625	11.58452525	13.7516152333333	13.0503485833333	12.8844900416667	11.671431125	13.57422905	14.4319291041667	10.8617947375	11.6983973458333	13.0593609791667	13.2165666458333	10.2714276125	13.0394998791667	13.1134452125	13.5415145041667	13.5283333375	13.0034702208333	12.9256731666667	11.385548375	13.0127564125	12.4952454875	12.657935675	13.3842829125	14.0650164875	13.3043630333333	13.3118198541667	12.3867025333333	13.49397615	12.1203833208333	12.8915324583333	12.4938953166667	13.026145825	13.2471028708333	12.8303426583333	13.4803759291667	12.9578240333333	12.9536648666667	11.8789535625	13.2542277375	13.0892890666667	12.4103664166667	10.5625577333333	12.0866895125	11.0703135291667	12.3564776375	12.7069332208333	12.8643643875	12.7819926916667	13.4516026666667	13.691858525	9.11009815416667	7.33588769583333	11.5445453458333	7.68854212083333	12.0846036291667	6.45294472916667	9.08241002916667	9.109147725	5.6416106875	8.87020280416667	9.89918522083333	6.14291660833333	5.854640475	5.9699700375	6.95537499583333	6.26314775416667	10.5075527083333	11.1493422958333	5.768797825	8.40100307916667	6.36833780833333	6.9154881875	6.26102422083333	5.9458035375	9.17346822916667	10.0123057333333	7.25390786666667	8.77324584583333	9.35842125	9.8616380375	5.70013700833333	5.78668012083333	7.58827061666667	7.93306472083333	6.94217911666667	7.9486319	6.41388247916667	7.47593034583333	5.7554475875	9.98169012083333	7.89954760833333	6.8160263625	6.91224841666667	10.7083386958333	6.64925087916667	8.5316430625	8.14523543333333	7.91217235833333	7.13579273333333	8.9073765	10.654833525	8.69632065	5.21571929166667	6.03882597083333	8.55880545	11.6660926625	7.85177892083333	6.49648824583333	8.5771368	10.9893273041667

ADH1B	13.5095875375	12.7083837541667	12.3095501125	12.2677302791667	10.5607363458333	12.6001910208333	13.0605105	12.602520925	12.6667265916667	13.7277599625	11.58452525	13.7516152333333	13.0503485833333	12.8844900416667	11.671431125	13.57422905	14.4319291041667	10.8617947375	11.6983973458333	13.0593609791667	13.2165666458333	10.2714276125	13.0394998791667	13.1134452125	13.5415145041667	13.5283333375	13.0034702208333	12.9256731666667	11.385548375	13.0127564125	12.4952454875	12.657935675	13.3842829125	14.0650164875	13.3043630333333	13.3118198541667	12.3867025333333	13.49397615	12.1203833208333	12.8915324583333	12.4938953166667	13.026145825	13.2471028708333	12.8303426583333	13.4803759291667	12.9578240333333	12.9536648666667	11.8789535625	13.2542277375	13.0892890666667	12.4103664166667	10.5625577333333	12.0866895125	11.0703135291667	12.3564776375	12.7069332208333	12.8643643875	12.7819926916667	13.4516026666667	13.691858525	9.11009815416667	7.33588769583333	11.5445453458333	7.68854212083333	12.0846036291667	6.45294472916667	9.08241002916667	9.109147725	5.6416106875	8.87020280416667	9.89918522083333	6.14291660833333	5.854640475	5.9699700375	6.95537499583333	6.26314775416667	10.5075527083333	11.1493422958333	5.768797825	8.40100307916667	6.36833780833333	6.9154881875	6.26102422083333	5.9458035375	9.17346822916667	10.0123057333333	7.25390786666667	8.77324584583333	9.35842125	9.8616380375	5.70013700833333	5.78668012083333	7.58827061666667	7.93306472083333	6.94217911666667	7.9486319	6.41388247916667	7.47593034583333	5.7554475875	9.98169012083333	7.89954760833333	6.8160263625	6.91224841666667	10.7083386958333	6.64925087916667	8.5316430625	8.14523543333333	7.91217235833333	7.13579273333333	8.9073765	10.654833525	8.69632065	5.21571929166667	6.03882597083333	8.55880545	11.6660926625	7.85177892083333	6.49648824583333	8.5771368	10.9893273041667

TMTC2	6.15447426666667	7.55016986666667	6.59883068333333	6.93418845	8.37811988333333	8.16584395833333	7.71354716666667	7.72796763333333	7.38404451666667	7.41824386666667	7.68541283333333	8.15615679166667	7.50051165833333	7.31615383333333	8.54895103333333	7.51310406666667	7.31025495833333	9.22832763333333	8.13194826666667	8.149040725	7.66891875	9.295254925	7.06220724166667	7.198288025	7.87562194166667	6.93741035	7.86062830833333	8.55543884166667	9.26261210833333	7.81893236666667	7.02467490833333	7.31377455	7.9579863	6.78782379166667	6.68797494166667	6.5680586	8.56222751666667	7.90941765	7.746177925	7.762415375	6.83084248333333	7.20088341666667	6.87783053333333	6.90715841666667	6.524903425	6.493249575	6.55447500833333	7.09162461666667	7.79357515	7.9089283	7.20747220833333	7.87157458333333	6.8347138	7.6315346	6.028109975	5.64797583333333	8.17406114166667	6.86530675833333	8.22799306666667	7.609434275	6.780385325	7.10888616666667	8.123259225	8.019555675	6.03335128333333	8.34668346666667	7.013155025	7.47204458333333	5.30888879166667	7.40135389166667	7.30254744166667	7.2622557	7.15765674166667	7.704155275	8.06450121666667	7.37590343333333	8.84195500833333	8.72037605	7.82120025	7.086730875	6.80056770833333	7.97971059166667	6.9604848	6.61154091666667	7.63328161666667	6.1536546	7.0596334	6.09272539166667	9.74266033333333	7.893532975	6.44290830833333	7.45160013333333	8.86314575	7.15455295	8.37257153333333	7.37184780833333	8.3023979	7.66891875	7.77602128333333	8.155034325	6.14728135833333	7.06531028333333	6.63574723333333	6.74158396666667	7.79957504166667	7.81246279166667	7.65225225	5.89368925	6.67239650833333	7.286116125	7.435271425	6.99155748333333	5.2815818	6.29901051666667	4.76661771666667	4.48239504166667	6.393024275	7.52443165	7.60239018333333	7.650678625

FRMPD2	5.89368925	8.95577006666667	8.50894728333333	9.20693985	9.04457245	6.09754041666667	8.92840376666667	9.705378475	8.448844675	8.81018689166667	8.15860931666667	4.25005865	8.24116579166667	9.42590730833333	8.57783373333333	8.451050725	4.70475344166667	11.032984525	9.96481554166667	9.82860243333333	7.92167954166667	7.707009225	9.315501225	10.287024175	7.59366683333333	5.36208894166667	9.01655344166667	9.99851741666667	8.80499509166667	9.56172193333333	10.2904392333333	8.69939258333333	8.18475165	4.28809964166667	5.59377575	5.9323169	10.31750855	10.4260988583333	11.0142581583333	10.340493	10.6027208583333	9.79446515833333	8.86314575	8.75927773333333	7.89557763333333	9.42465609166667	9.04320051666667	10.9979551416667	8.33398970833333	8.3496737	8.154025025	10.3686089583333	8.94384210833333	9.7815357	9.895098225	9.19340213333333	9.59369666666667	10.3044270083333	7.9291649	9.39525429166667	5.89449381666667	7.69858204166667	5.892821925	7.59366683333333	4.91851714166667	10.662995525	7.4690921	7.224148725	5.64406685833333	5.78455018333333	6.04445175	5.8654886	8.641468925	5.38157649166667	5.6723045	7.41882361666667	5.00117389166667	7.81731503333333	9.945938775	6.50275845	4.19044525833333	5.00768225	10.5482040916667	8.16124230833333	6.65487665833333	4.46100053333333	5.70253175	4.81179368333333	5.22265475	6.23865941666667	7.23488161666667	5.778884625	8.77460095833333	6.93685505	6.72705409166667	6.93616958333333	7.193910325	7.37059144166667	9.23330131666667	6.84576470833333	6.31021560833333	5.48807850833333	5.642111775	7.97923883333333	8.98837211666667	4.85037616666667	9.50586739166667	10.546063825	7.70926514166667	6.96848423333333	5.858085325	9.96745814166667	9.03689006666667	7.4690921	9.87826785	8.42823383333333	5.27529641666667	6.633753725	8.20587135	4.67067994166667

FBXO21	6.85045274166667	6.87159964166667	6.62496390833333	6.47064316666667	6.131776975	6.06833519166667	5.949734275	6.542385675	6.3475359	6.408778075	5.735887375	5.9191077	5.36818925	6.72123403333333	6.54089473333333	6.48497233333333	8.01060475	5.18312655	7.47562453333333	6.056382625	6.31469075	6.94820169166667	7.46463336666667	7.244602025	6.7327851	6.87851200833333	7.31316353333333	6.880500475	6.50142603333333	6.83799996666667	7.20334935833333	6.70599545	6.230051875	6.80510874166667	7.35059130833333	6.93032724166667	6.18568551666667	6.86394090833333	6.33645039166667	6.70056215833333	6.739509575	7.1989222	6.7098751	7.416522875	6.48707709166667	7.14092606666667	7.305533825	6.3064348	6.771060625	6.24941475833333	5.8292169	6.67919664166667	5.5319599	6.22752675833333	6.76843211666667	7.13294301666667	6.446687025	6.83020955	6.449622025	6.777132775	5.84699576666667	6.01590590833333	6.03747638333333	5.47990566666667	7.24828621666667	6.16362138333333	6.322468275	5.19959509166667	6.62969040833333	5.51585541666667	5.71795834166667	6.90781049166667	4.55971610833333	6.057986625	6.16277881666667	5.17265765	5.9191077	5.30664471666667	6.74746250833333	5.90846126666667	5.43596139166667	6.19893275	7.37467966666667	6.85418244166667	4.873988275	5.622261375	5.75774213333333	4.62790769166667	6.65156973333333	6.43641949166667	6.82820395	5.68207400833333	5.76144219166667	6.37643818333333	6.1441534	5.96778360833333	5.95490855	6.28184195833333	5.873141325	6.70726116666667	5.48099319166667	6.35738701666667	6.473722825	7.05651791666667	6.53407053333333	5.65251761666667	6.44595796666667	6.25026105	6.81852565833333	6.46779858333333	6.05307568333333	6.3460148	5.7745543	6.813807925	4.45902586666667	6.09272539166667	5.984788925	5.02371664166667	7.01495000833333	5.26673208333333

RFPL3	9.126505725	9.03908018333333	9.24445845833333	9.23377270833333	9.65845149166667	9.18546986666667	10.20436555	9.283130225	9.7752348	9.02085209166667	9.55943838333333	9.985532675	9.54834169166667	8.709455975	8.75091850833333	8.4100903	8.96987785	9.37637135833333	8.8451569	8.78070639166667	8.75703444166667	8.81642101666667	8.61392724166667	8.56366318333333	9.06460029166667	9.12232943333333	8.73689844166667	9.12750715833333	8.86854605833333	8.90619576666667	8.85494786666667	8.620261275	9.16031618333333	8.91642435833333	9.312713675	9.090969825	9.39113676666667	9.02747416666667	8.81108796666667	8.96932985833333	8.54699226666667	8.40614324166667	8.67503203333333	8.57454949166667	8.26308525833333	7.98666509166667	8.29681115	8.26701150833333	8.65999509166667	8.64496134166667	8.738873225	8.9544846	8.486995925	8.482136425	8.711926	8.244635825	8.72739513333333	8.631828525	8.5716089	8.49188041666667	9.61946140833333	9.388540625	9.261730375	9.46790315833333	9.157883875	9.35634101666667	9.26988130833333	9.65222760833333	9.38622159166667	9.35167163333333	10.1254167416667	9.67274871666667	9.85036418333333	9.00964296666667	9.2408133	9.07536643333333	9.24172186666667	8.71144663333333	9.1094042	8.98368761666667	8.70400716666667	9.16167278333333	8.88496090833333	8.88831745833333	9.19118228333333	9.02705223333333	9.291151225	9.12842238333333	9.900175875	8.64585098333333	9.12842238333333	9.14518724166667	9.51041425833333	8.85728129166667	9.569144925	9.63199205833333	9.12794545833333	9.15064904166667	9.65222760833333	8.83154074166667	9.4094908	9.12557628333333	8.907131825	8.69127455833333	8.8830474	8.76125596666667	8.57208716666667	8.59007210833333	8.8778449	8.478972275	8.775178575	8.881658575	8.764687525	8.68035370833333	8.62786819166667	8.51027651666667	8.67553405	8.998094275	9.33838469166667	9.09237333333333

DDX19B	12.3848869916667	11.8699647166667	12.040720525	12.0587376333333	12.6519795833333	12.1801535291667	11.9707739	11.9865996	12.3273709208333	12.2855253875	12.5422880333333	12.2974883666667	12.270394675	12.1776165875	12.2988112125	12.0903471208333	12.8137257625	12.3014143166667	12.2920191583333	12.1577392875	12.1876295375	12.25639015	12.1148201541667	12.1473832125	12.3782635041667	12.609580675	12.3141055458333	12.0731303541667	12.4699238583333	12.3673816791667	12.1829263416667	12.2716997583333	12.0955577208333	12.3363630666667	12.5513897708333	12.631472475	12.3535720916667	12.1675238375	12.1776165875	12.3552471583333	12.0995418125	12.1889083041667	11.9639300166667	12.0742864708333	12.3941443708333	12.2009012166667	12.2266665833333	12.1247999541667	12.10859605	12.1207439458333	12.0549168875	12.0814532666667	12.6564513916667	12.5119309541667	12.4231826416667	12.1654811791667	12.2643684166667	12.2324496625	12.3455268416667	12.1456993916667	13.2701257958333	11.6685670791667	12.2864832	11.7492646541667	12.2733797791667	12.7920713708333	12.0086714041667	12.2643684166667	12.2542932208333	12.1526999916667	12.3730829583333	12.4272803791667	12.2208720375	12.2652138125	12.910849375	11.9481228458333	12.226260125	12.473964675	12.3826691625	12.4744167541667	11.8014838	12.4048502125	12.1829263416667	11.8222782541667	12.6203779375	12.3590963	11.3884416041667	12.6569464791667	12.6898184916667	12.370533275	11.8327205458333	11.7260778	12.3826691625	12.7297924625	12.4925165291667	11.8751626041667	11.9779870208333	12.0646527	12.4331374458333	11.7684538958333	12.8243259041667	11.8010624333333	12.6828642458333	11.8307415875	12.270394675	13.0064680041667	12.6383763666667	12.1863594833333	12.7819926916667	12.1441510083333	11.950792275	11.8851057625	12.3282505291667	11.9242314958333	12.4348346875	12.0255567416667	12.2200169458333	11.8377358625	12.0150895333333	12.04805665

AC012184.2	12.3848869916667	11.8699647166667	12.040720525	12.0587376333333	12.6519795833333	12.1801535291667	11.9707739	11.9865996	12.3273709208333	12.2855253875	12.5422880333333	12.2974883666667	12.270394675	12.1776165875	12.2988112125	12.0903471208333	12.8137257625	12.3014143166667	12.2920191583333	12.1577392875	12.1876295375	12.25639015	12.1148201541667	12.1473832125	12.3782635041667	12.609580675	12.3141055458333	12.0731303541667	12.4699238583333	12.3673816791667	12.1829263416667	12.2716997583333	12.0955577208333	12.3363630666667	12.5513897708333	12.631472475	12.3535720916667	12.1675238375	12.1776165875	12.3552471583333	12.0995418125	12.1889083041667	11.9639300166667	12.0742864708333	12.3941443708333	12.2009012166667	12.2266665833333	12.1247999541667	12.10859605	12.1207439458333	12.0549168875	12.0814532666667	12.6564513916667	12.5119309541667	12.4231826416667	12.1654811791667	12.2643684166667	12.2324496625	12.3455268416667	12.1456993916667	13.2701257958333	11.6685670791667	12.2864832	11.7492646541667	12.2733797791667	12.7920713708333	12.0086714041667	12.2643684166667	12.2542932208333	12.1526999916667	12.3730829583333	12.4272803791667	12.2208720375	12.2652138125	12.910849375	11.9481228458333	12.226260125	12.473964675	12.3826691625	12.4744167541667	11.8014838	12.4048502125	12.1829263416667	11.8222782541667	12.6203779375	12.3590963	11.3884416041667	12.6569464791667	12.6898184916667	12.370533275	11.8327205458333	11.7260778	12.3826691625	12.7297924625	12.4925165291667	11.8751626041667	11.9779870208333	12.0646527	12.4331374458333	11.7684538958333	12.8243259041667	11.8010624333333	12.6828642458333	11.8307415875	12.270394675	13.0064680041667	12.6383763666667	12.1863594833333	12.7819926916667	12.1441510083333	11.950792275	11.8851057625	12.3282505291667	11.9242314958333	12.4348346875	12.0255567416667	12.2200169458333	11.8377358625	12.0150895333333	12.04805665

PLCH1	4.44822564166667	5.43818919166667	5.15454668333333	4.70905974166667	5.34510054166667	4.81716043333333	6.14959958333333	6.42634760833333	5.87208589166667	4.963016975	5.14192449166667	4.853434925	6.28184195833333	6.21116124166667	4.99794086666667	4.51657093333333	5.98999241666667	4.70475344166667	4.61625569166667	6.05474264166667	5.47028654166667	4.61520515	4.72222786666667	4.80635555	4.15432630833333	4.55466009166667	4.66004348333333	3.7293304	4.12334434166667	3.9601097	5.28357275833333	4.003549775	4.67278611666667	5.2151974	4.36702491666667	4.09598105	4.2289531	4.42980535	4.879391675	4.25708764166667	4.79999713333333	4.258212325	4.68681869166667	5.23671275	4.125728225	4.78900190833333	4.79411095833333	4.210787125	4.26445661666667	4.56487130833333	4.557709	4.95627725833333	5.35983450833333	6.01047939166667	4.830971775	4.45416251666667	5.62592511666667	4.44534256666667	4.22036293333333	5.0953115	4.81179368333333	5.06351158333333	5.65344755	5.429887625	6.60007374166667	5.258661775	5.29450505	7.01371830833333	4.69863704166667	5.30888879166667	5.67327496666667	5.60487774166667	6.02974674166667	4.7006097	4.58241979166667	5.14718730833333	4.67787883333333	4.443420775	4.57137348333333	6.55061969166667	6.77777710833333	5.426744575	4.71113178333333	4.33133893333333	4.81179368333333	4.96739760833333	5.527437325	5.82824421666667	6.02292943333333	4.32198345833333	4.93761881666667	4.63093599166667	4.65588558333333	4.664314175	4.83989633333333	4.80635555	4.328489775	4.59071941666667	4.88478473333333	4.58445505833333	4.62375229166667	5.51386359166667	4.485186325	5.22594001666667	4.2949863	4.76349515	4.37489024166667	4.93234401666667	6.021143325	5.96958254166667	4.418789625	6.083461775	4.759183025	4.63723314166667	5.16081746666667	4.96739760833333	4.79716553333333	4.28809964166667	4.385351725	6.22368095

POLR2M	10.137056175	10.4464281166667	9.88726988333333	9.63613316666667	9.97215941666667	11.507509225	10.6233516833333	10.9809278083333	10.3864170083333	10.416539275	10.5651423416667	11.60398845	10.4037201666667	10.4702277083333	10.5711304666667	10.8180008083333	11.3183574	10.4883478333333	10.610413375	11.237038075	10.7485438416667	10.99445015	10.6542751166667	9.97542684166667	10.3027487416667	11.3713269916667	9.98075235	9.99216454166667	9.80755264166667	10.6600165833333	10.4606353416667	10.2462958083333	10.21004035	10.5108422833333	11.0301443333333	11.0409461	9.8294925	10.7004993416667	10.4606353416667	10.4072694666667	9.865582425	10.4613867166667	10.5833260083333	10.05827805	9.9420301	10.2266691833333	9.97873573333333	9.565041475	10.51584625	10.500980975	10.6336749583333	10.4308569666667	8.82211801666667	9.29664133333333	9.29252663333333	9.82029285833333	10.4691008666667	10.4983213833333	10.750505225	10.3968247166667	9.929600025	10.4149521166667	9.903525425	9.98470918333333	11.5479749166667	11.3539068166667	10.8968726333333	11.0866687083333	10.476761175	10.71663975	10.9917752916667	10.2974881833333	10.7631580166667	10.5573126666667	10.2932251333333	10.971536625	10.9189114166667	10.93064125	11.0733527583333	10.8499191666667	11.747396125	10.6971968666667	10.3400524166667	10.4570074666667	10.7224580666667	10.7771118333333	10.8608532	10.2810793	10.91052195	11.0982319416667	11.5769076083333	9.76136145	10.614717725	11.2740276	9.53997905833333	9.60581258333333	10.000563275	10.132057125	10.4145396416667	11.0596766	10.1489854166667	10.4895361083333	10.6445900666667	10.5388660583333	10.0933465333333	10.4641916916667	10.3277465	9.877817275	10.511583325	10.74285715	10.9809278083333	10.640008475	9.33496756666667	9.62318166666667	9.189315625	9.61413186666667	11.5185086916667	10.616218875	10.8608532	10.98655615

OR4C6	8.3151234	8.27440479166667	8.13819046666667	8.56867330833333	8.80870043333333	8.55955131666667	9.53875820833333	8.59785679166667	8.96748466666667	8.535777725	9.01009886666667	9.21835433333333	8.9081181	8.60080905833333	8.6774004	7.85633409166667	8.49442401666667	8.66880180833333	8.18891301666667	7.95631855833333	8.168322275	8.30178113333333	7.62202388333333	8.17090974166667	7.91577950833333	8.38960981666667	8.65318265	8.11887123333333	8.73689844166667	8.9489102	8.322152325	8.62834993333333	8.79439289166667	8.82873195	9.07683584166667	8.69317739166667	9.09833678333333	8.80499509166667	8.78836945	8.06345484166667	7.79694136666667	8.81744095	8.512323475	7.824397325	8.445841225	7.9882338	8.73305545	8.68035370833333	8.127296675	8.21929578333333	8.685246125	8.58808473333333	8.61712494166667	8.87883045833333	8.83738886666667	8.39672325833333	8.64585098333333	8.74913316666667	8.32905851666667	8.09897714166667	9.33883618333333	8.717376625	8.45745534166667	8.737919725	8.54360906666667	8.67210133333333	8.78635625	9.19764225833333	9.08644098333333	8.77094225	9.212117675	9.03950406666667	9.46343676666667	8.62290185833333	9.0180261	8.33450988333333	8.76421910833333	8.68470500833333	8.42018036666667	8.54169179166667	8.33259703333333	8.54598220833333	8.4178821	8.29681115	8.85676560833333	9.126505725	8.75473524166667	8.37107905	8.68822245833333	8.1873246	8.44491509166667	8.54315244166667	8.576058475	8.88592250833333	8.927052675	8.634032725	8.60593184166667	8.5994674	9.03950406666667	8.42618235833333	8.3316018	8.95218464166667	8.93317065	8.000994225	8.72037605	8.770019625	8.8768317	8.54023758333333	8.33913670833333	7.61865353333333	8.25546045	9.47772424166667	8.37604669166667	8.451050725	8.95943646666667	8.63773576666667	8.11250586666667	8.7292202	8.50236353333333	8.67598873333333

SLC25A37	10.950929275	10.3459231416667	10.6027208583333	10.5623629583333	10.3289721	11.3976073583333	10.1109480666667	10.614717725	10.2206735166667	10.6457263	10.9858189916667	10.8747226333333	10.8461868083333	10.1283050583333	10.436472775	10.672620875	10.6887964	11.088855975	10.6387912166667	10.6792669083333	10.5456854416667	10.7975980416667	10.4534831666667	11.3043532	10.616218875	11.5093017916667	11.19451335	10.8807458916667	10.9174580583333	10.3574979	11.1003561666667	10.4542782	10.6183847583333	10.5760591583333	11.6485244	10.8322571166667	10.8416766333333	10.6298019333333	11.22659855	10.647327775	10.7371678583333	10.29083355	10.6780826666667	10.6187719166667	10.6997392583333	10.31750855	10.18000125	10.8688118916667	10.566703425	11.3132862083333	11.1790792916667	11.03596855	10.931065425	10.7959852666667	10.5177006833333	10.6986549	10.7904047166667	10.723219775	10.4850893583333	10.4215953	11.372844325	10.96771795	11.004921375	11.0182236333333	11.712752375	11.6587921	11.142299625	11.4258523333333	11.9361117583333	11.7063908333333	10.1906714166667	10.4910656666667	11.4985882166667	10.91052195	11.9071135	11.1077951583333	10.8216281833333	10.8771107	11.0105391666667	10.7042731416667	11.1637582666667	10.79796285	10.3211494083333	12.0465984666667	11.0567325916667	11.3720648583333	11.7513402	10.8751259833333	11.4639403833333	11.0513895	11.659892375	12.04365515	10.1650146416667	10.8315442333333	10.931065425	11.2128973166667	12.393505075	10.6979535583333	11.0787308166667	11.2409567416667	10.9600311833333	10.6031497916667	11.832540575	10.736058575	11.0757994083333	11.104988	11.0394398083333	11.4559913916667	11.12105475	10.983890675	11.0355345916667	10.6407861166667	11.1277733333333	12.0518595833333	11.1325239666667	10.7050450333333	11.4971747583333	11.949852225	11.012449925	10.3546255166667

FMO5	8.24802405	9.31001445	8.8364013	9.3036884	8.31670425833333	11.4000850916667	8.982853175	9.27941565	9.51496844166667	9.03641039166667	9.76554074166667	9.89982799166667	8.61820814166667	9.35167163333333	8.7637709	9.44690350833333	14.2058121833333	9.12506650833333	8.21353541666667	9.09054048333333	9.35125324166667	9.85548545	9.6592899	10.0595403	9.57863910833333	8.93412065833333	8.92461889166667	9.05497765833333	8.24861051666667	9.05454125	8.28095494166667	8.62886449166667	9.34302553333333	13.166824125	8.52826969166667	8.26509473333333	8.87183624166667	8.69172925	8.61046613333333	8.3316018	8.661516725	9.24845199166667	9.13739526666667	9.36892748333333	9.168290925	9.07777099166667	9.39297179166667	8.38118433333333	8.98191765833333	9.700667025	8.398242975	8.76421910833333	9.151591475	9.56634321666667	8.86760295	9.47257300833333	8.85110639166667	7.89243905	9.24492166666667	9.16074203333333	8.96893119166667	8.34279256666667	9.55671014166667	8.19396290833333	9.01340034166667	8.25692778333333	8.54169179166667	8.57454949166667	7.21750264166667	8.19970503333333	8.63773576666667	8.027292325	9.18546986666667	8.66880180833333	8.8128727	8.51713660833333	8.52634974166667	8.17720695	8.10843584166667	8.894577375	8.87883045833333	9.286917675	10.028074675	7.60415978333333	9.60919951666667	8.046473725	7.448314425	8.59099210833333	7.578462725	8.62547046666667	7.61126938333333	6.65223385	7.92167954166667	9.146936475	8.14849655833333	6.3109681	8.094406525	6.87219209166667	8.24011166666667	8.35277756666667	8.635919475	8.338570375	7.35751986666667	9.73270225833333	6.53467245	8.60080905833333	8.28143178333333	8.03755788333333	8.18781211666667	8.45851334166667	8.70448370833333	6.903905225	7.17093558333333	8.174528375	7.86115675833333	8.31058729166667	7.45098580833333	5.897311975	8.14607183333333	8.76603770833333

C21orf58	7.95464479166667	8.67789245	8.29630441666667	8.75703444166667	9.182097875	8.60227746666667	9.35451149166667	8.42934195	8.486570275	8.4773978	8.95577006666667	9.15740435833333	8.97076639166667	8.67403964166667	8.52241499166667	8.2260001	8.113521575	8.89602301666667	7.8345672	7.99458134166667	8.51081288333333	9.25764194166667	8.26456484166667	8.00529461666667	8.01861064166667	7.99833741666667	8.17613399166667	8.52683058333333	8.046473725	8.4816187	8.36807433333333	8.26968861666667	8.44993103333333	8.49886293333333	7.98666509166667	7.90678598333333	8.61480576666667	7.79194111666667	8.01595076666667	8.07152965	7.79082046666667	8.28289991666667	7.9291649	7.74722135833333	8.3301117	8.20882531666667	7.668390025	7.92110699166667	8.49006413333333	7.81456171666667	7.774307975	8.06854219166667	8.41202154166667	8.73399778333333	8.13362299166667	7.76084795833333	7.94017044166667	7.91097105833333	8.45851334166667	7.729718875	9.949333375	9.20872513333333	9.02562736666667	9.07536643333333	9.73533868333333	9.06745036666667	8.37257153333333	8.82680535833333	8.82621398333333	8.85304851666667	9.15740435833333	8.886411025	9.19950909166667	8.59397561666667	9.20605604166667	8.97769849166667	9.062206575	9.47351875833333	8.295765375	9.23797865	9.38453646666667	9.826059425	8.89696199166667	8.84659819166667	8.38370515833333	9.91503965833333	9.81686935833333	8.85494786666667	10.5288525083333	8.16633889166667	8.70547134166667	8.78991784166667	9.63342665833333	8.36907771666667	9.40861700833333	9.19340213333333	8.6233491	9.63944066666667	9.17280985	8.04755969166667	8.18684245	9.4007109	9.39297179166667	8.634032725	8.95312890833333	8.59099210833333	8.32264421666667	8.69082025	8.24315470833333	8.51438241666667	7.7483928	8.47579976666667	9.114481175	8.65218976666667	8.78635625	9.19576385833333	9.660226725	8.49291188333333	8.93180466666667	8.64196594166667

LRCH1	14.5377311666667	12.8086026666667	12.829556475	12.3007469083333	13.0999579333333	13.1356932666667	12.6417017166667	12.9030126333333	12.946647375	12.7334106833333	12.5788865916667	14.1099143166667	13.96690645	12.815500575	12.8118807333333	13.2765049166667	13.7111367583333	13.1635303583333	13.1235892166667	13.2460254166667	12.81348825	12.8807291083333	12.6346883583333	12.7896506583333	12.9859501166667	14.6466555666667	13.2194882333333	12.603650525	13.078126625	12.5754789333333	13.1845722666667	12.8432333583333	13.1459482	13.71577945	14.5020438083333	14.9865439333333	13.138182175	13.0792819	12.98478995	13.3732212583333	12.5202342666667	12.93348965	13.1389082666667	13.4298474333333	13.8983181916667	12.8181395166667	13.1589580416667	12.6694372	12.6481258583333	12.7026115333333	12.9629737583333	13.2208402833333	13.3608559333333	13.2320017583333	13.1556465833333	13.6514898166667	13.028241025	12.7041276166667	13.068247425	13.19400885	13.371772075	13.053017375	12.6240804083333	13.4991752166667	13.0864977	13.1986854833333	13.0913765666667	13.5304589833333	13.089585075	13.248853375	12.7580394083333	13.1210793083333	13.1052210666667	13.2194882333333	13.118520075	13.136994025	13.4550317916667	13.4729544083333	12.9185113	13.3322833833333	13.7426466833333	13.488720775	13.0349568083333	13.3383656916667	13.6941290583333	13.239306575	13.057870725	13.7074542166667	13.2962436083333	13.17465125	12.9340515083333	13.0343175416667	12.8924470083333	12.9694275083333	12.847095325	12.2137378333333	12.6606270166667	12.9030126333333	12.9357780833333	12.9540039916667	13.3754869916667	12.3492861833333	12.8964099833333	12.7516120833333	12.1224925833333	13.64886915	13.5843144666667	13.6246233916667	13.49281735	13.4179054	13.5738180166667	11.8395390583333	13.7046873583333	12.9213977333333	13.3952177083333	12.4206839916667	13.0488654	12.844414625	12.4292757333333	13.4572533333333

PLA2G6	10.971110725	10.8176392916667	11.0196702	11.0992950083333	11.4140863666667	11.6425911333333	11.6591054333333	10.474031625	11.09447755	10.9545739416667	11.23807015	11.6974443166667	11.421792525	10.4895361083333	10.8056325083333	10.8900635166667	9.954379925	11.9962714583333	10.4288423083333	10.6788649916667	10.054920625	10.9486958083333	10.0736918083333	11.0250402166667	10.2687270166667	9.75747410833333	10.5311099	10.8056325083333	11.24459925	10.5949115083333	10.5322469083333	10.5154588666667	10.7042731416667	10.1670505916667	9.75787298333333	10.1405016833333	11.25569785	9.99086305833333	10.4822871416667	10.6195893583333	9.79235871666667	10.3105309666667	9.88681354166667	10.244614225	10.0740705333333	9.79362293333333	9.38096979166667	10.1226723083333	10.7812240416667	10.7816314666667	10.4503313916667	10.6905955916667	9.611823	9.41204579166667	9.80096413333333	8.83063586666667	10.4495527	9.852861125	10.5619901333333	10.0347123333333	11.8020861333333	11.1637582666667	10.9353811	11.17354635	11.0574717833333	11.6890849333333	10.244614225	11.1912222083333	10.8161170333333	10.8972744416667	11.0866687083333	11.17758075	11.1288009583333	10.8916305083333	11.46509415	10.6083941916667	11.2572113416667	11.6525927916667	10.4372971833333	11.2082269	10.7616896916667	11.0458413916667	10.7344399666667	10.4108585666667	10.6607214666667	10.8807458916667	10.8755795416667	10.7220730083333	11.1288009583333	10.1109480666667	11.0723250583333	11.2157921833333	11.0045884333333	11.1689128333333	10.706587775	10.9028824333333	11.2150743583333	10.9961958083333	10.6910121333333	10.2745932916667	10.1477211833333	10.2728709416667	10.4895361083333	10.3745792583333	9.83452378333333	10.222173225	10.6499586916667	10.6503573333333	10.7375745583333	10.3542362916667	10.2654733916667	10.235923825	9.94886909166667	8.52481044166667	9.48078894166667	8.9489102	11.0369926416667	10.022234175	10.4678765666667	10.3119751583333

PARVG	8.26867154166667	8.35277756666667	8.965634	8.8062671	9.23882716666667	8.93668718333333	9.64871909166667	9.09925841666667	9.066471675	8.89174859166667	8.69459336666667	9.42859468333333	9.23797865	8.50451981666667	8.247580125	9.96227999166667	7.91954875833333	9.30730685833333	8.71882579166667	8.8559255	9.19676581666667	9.04369295	8.60435615	9.4825512	8.79596970833333	8.77869386666667	8.80050660833333	8.73207944166667	9.19950909166667	8.53187930833333	8.86854605833333	9.478582775	9.04179071666667	9.77263848333333	9.112227825	7.73920148333333	9.11864413333333	9.126505725	9.237031475	8.381708525	8.47003455	8.62596224166667	8.49006413333333	8.340733825	8.96144149166667	8.868084675	8.127296675	8.39771189166667	8.486995925	8.9184083	8.42676914166667	8.403258675	9.51633301666667	9.329864925	8.66441525833333	8.1073987	8.752344475	8.69409343333333	9.11737229166667	8.81599143333333	8.69025928333333	8.62834993333333	9.0174923	8.59492328333333	8.59697231666667	9.25584964166667	8.66724769166667	9.50036891666667	8.84802724166667	10.057405675	8.50844495	8.74174598333333	9.01101026666667	9.01246160833333	8.98004790833333	9.00735385	9.46751014166667	9.861824025	8.72356944166667	8.898281125	9.349435025	9.40159784166667	8.72134320833333	8.875989475	9.33496756666667	9.05965051666667	8.92511409166667	9.62890875	8.60126088333333	9.10889125	9.26988130833333	8.662974625	8.45045408333333	8.7292202	9.66873480833333	8.59697231666667	8.92973970833333	9.438196975	9.28174641666667	7.992867725	8.268113325	9.14426045	9.54402950833333	8.382753925	8.0773066	8.89174859166667	8.84195500833333	8.60080905833333	8.67361984166667	9.002830825	8.40234485833333	8.05466113333333	9.09684721666667	7.78488271666667	9.16780343333333	9.04692176666667	7.95082734166667	8.335541375	7.93069544166667	9.42547525833333

BTRC	8.61979589166667	9.08915231666667	8.92651730833333	8.85865705	8.34866896666667	9.081377025	7.89930148333333	8.78991784166667	8.58695619166667	8.54218048333333	8.70212718333333	7.73639724166667	8.252426875	9.135601825	9.48206465833333	8.90197375	9.288795975	8.18100231666667	9.06925550833333	8.8334785	8.66724769166667	9.64074395833333	9.02222115833333	9.28405401666667	8.96414444166667	9.02273679166667	8.833014675	9.20920615	8.93953213333333	9.04133171666667	8.85304851666667	8.898281125	8.51578285	8.884079375	8.65999509166667	8.97861723333333	8.89220491666667	9.14921909166667	8.70898641666667	8.84055035	9.2056298	9.14608020833333	9.349869075	9.35954015	8.52110425	9.29702861666667	9.13909698333333	9.24172186666667	8.89967475833333	8.965634	8.52634974166667	9.04278656666667	8.57257758333333	8.99116335833333	9.35820640833333	9.19996488333333	8.5905397	9.18595039166667	8.84243295833333	8.886411025	8.65843663333333	8.17505753333333	8.94525708333333	8.49188041666667	8.4841298	9.39297179166667	7.92602010833333	8.52279583333333	9.24492166666667	8.7655611	8.19543675833333	8.813752575	9.39626775833333	8.19543675833333	9.02705223333333	8.54070765833333	8.64688179166667	8.07152965	8.74719128333333	8.053540525	8.69127455833333	8.50844495	8.5232739	8.60435615	8.18936749166667	8.76798363333333	9.04596135	8.14660076666667	8.016506325	8.46867076666667	8.39466414166667	9.16874195833333	7.92336901666667	8.8128727	8.2053418	8.23128400833333	9.495092775	8.697015525	8.18781211666667	9.0180261	9.32471271666667	8.15303908333333	8.7776576	9.40159784166667	9.452370525	8.23520641666667	8.8296979	8.98837211666667	8.66625304166667	8.70898641666667	7.88978783333333	8.89220491666667	9.61006324166667	9.4056037	8.250184775	9.186916425	8.56044531666667	8.90668976666667	8.68624993333333	8.87390605

ZSWIM5	9.77432975	9.51724313333333	8.85087683333333	9.37220353333333	8.76079081666667	9.67723733333333	8.93897689166667	8.79040525833333	8.96455003333333	8.86999053333333	9.19340213333333	9.686488825	9.056864675	9.44734175833333	9.38409195	9.06600838333333	9.13739526666667	8.954039625	9.46343676666667	9.19627585833333	9.21590233333333	9.73831540833333	9.48834466666667	8.48060255833333	9.39938098333333	10.1613409083333	9.12794545833333	9.198111125	8.62481299166667	8.98751653333333	8.87736359166667	9.50539336666667	8.884079375	8.96893119166667	9.99468328333333	10.1565645083333	9.23462919166667	8.86211740833333	8.26308525833333	8.98325939166667	9.133746725	9.34438068333333	9.48881261666667	9.40207443333333	9.29065144166667	9.18071311666667	9.549690725	8.68924809166667	9.0174923	9.17923419166667	8.69127455833333	8.251367875	9.01051185833333	8.72544860833333	8.88882345833333	9.29018041666667	8.70547134166667	8.89602301666667	9.003775725	9.30498321666667	9.83237755833333	8.54462835	8.22846261666667	8.054062325	9.16495244166667	9.22249476666667	7.41824386666667	8.19143718333333	9.13323439166667	8.38319035	8.89220491666667	7.90363365833333	8.55442540833333	7.89722555	8.54070765833333	7.937534725	9.27035150833333	8.73109978333333	7.75542415833333	9.498614875	8.64688179166667	8.61442265833333	10.0260539583333	8.18363113333333	7.22598754166667	9.0772993	7.61924733333333	8.08240869166667	8.3301117	8.328023075	8.81463643333333	8.81744095	7.96073305833333	8.445416025	8.51528900833333	9.34302553333333	7.36587165	8.061842725	8.07980334166667	8.028895175	8.66724769166667	8.36243170833333	8.43615038333333	9.170544975	7.079224475	8.842895675	7.39806645	9.97706043333333	8.19970503333333	8.535777725	8.32696298333333	9.36666890833333	8.07411434166667	7.46746676666667	7.166799725	8.3471697	9.151591475	8.52878136666667	9.86718390833333	8.08700735

NSFL1C	11.113611625	11.414772875	11.2079127916667	11.27642465	11.31764545	10.5291936916667	11.2258131916667	11.5614234666667	11.5754446583333	11.0581558166667	11.4597359083333	10.9597361583333	11.31764545	11.633311275	11.6659074916667	10.9965540083333	10.987327675	11.5291327083333	11.4635169166667	11.4879615666667	11.2826013166667	11.6906956833333	11.3871722333333	11.4364638833333	11.5305817083333	11.783111075	11.3979579666667	11.6456087833333	11.5030923083333	11.633311275	11.5316298	11.53378775	11.5272640666667	10.9440376833333	11.7898995	11.8635130416667	11.3997255833333	11.6610088416667	11.205405875	11.7509367	11.5742275583333	11.528423775	11.4446261583333	11.7251079666667	11.1018151	11.3709225333333	11.57874505	11.4221599083333	11.2283191166667	11.2790070666667	11.2319732666667	11.4919381583333	11.9645775083333	11.8070215583333	11.7955610083333	11.6359301083333	11.3339882	11.6252518666667	11.325017625	11.2729625666667	13.1210793083333	11.1325239666667	10.99905285	11.2664173916667	10.5592695083333	9.97706043333333	11.4570874916667	11.158996075	11.179383775	11.0693836916667	11.5945056583333	11.4752696416667	12.1861275083333	10.63689965	11.351341375	10.8210096	11.04233505	10.6422709916667	11.2194717166667	10.2136563	11.6159444083333	10.6880668	11.5461869416667	11.3467541583333	11.6540451166667	11.302972225	11.44422215	11.0957876916667	10.9574882	11.002804875	12.3496705916667	11.8635130416667	11.1575927916667	10.958948725	12.0265627166667	11.2653952	11.0116340666667	11.9994097833333	11.6785730958333	11.3797907833333	11.57356395	11.4258523333333	10.46022315	10.8032590416667	11.1656289166667	10.6373110916667	10.826565775	11.2291005583333	11.3664862333333	11.0394398083333	11.34033385	10.64807625	12.42337295	11.5589969083333	11.14267695	11.4669037583333	11.322793725	11.002443975	10.90507365	11.1095361333333

RRBP1	12.693462425	13.3501502333333	13.570433175	13.7393052083333	13.6711090583333	14.1357234916667	12.9634653	13.3200251	13.6411556333333	13.3241951083333	13.798249925	12.8080609833333	13.5738180166667	13.3630726416667	13.4999239583333	13.5108418166667	14.4615780083333	13.4729544083333	13.734202725	13.5931197583333	13.71864895	13.9378093083333	13.90344385	13.798249925	13.7372005583333	12.6297371916667	13.4026847	13.0798178083333	13.7621985333333	13.2628733666667	13.4481554583333	13.5511200333333	13.5213793583333	14.5664298833333	12.5372066583333	12.6586586666667	13.4481554583333	13.52214195	13.5593280666667	13.64886915	13.653352825	13.40344555	13.6202060083333	13.541969925	13.5230150833333	13.61251425	13.3645725083333	13.8766797166667	13.608143675	14.0847383833333	13.5843144666667	13.8366591583333	13.592329325	13.6894331166667	13.4950898916667	13.86774345	13.811693625	13.627458175	13.4752691916667	13.7443409166667	14.5488647916667	13.9796866	13.9217501083333	13.98513315	12.9552000333333	13.3356032833333	14.0353374833333	13.9635675833333	13.035605175	13.8919290666667	13.2968461416667	13.7393052083333	14.0353374833333	14.07729275	13.509141775	14.083694575	14.0720390916667	14.160512975	13.604496075	13.6822736083333	13.5295226416667	13.2227845833333	13.6338665166667	14.371029675	14.0377493833333	13.46583445	12.5567153083333	14.164370775	13.7204745333333	14.105898725	13.7750620916667	13.9829878583333	13.527156225	13.484103425	14.1241853333333	14.046645825	13.8137434916667	13.3106692583333	13.9119804333333	13.450467225	13.9097458666667	13.8843608583333	13.811693625	13.4950898916667	14.056517225	13.799269175	14.3153773833333	13.4298474333333	14.1086467166667	13.8625679083333	13.4705605	13.1609997	13.9737942083333	13.6666988416667	14.0926224666667	13.56859275	13.1190556333333	13.4729544083333	13.48560075	13.5124524166667

C3orf22	10.7970204708333	10.3785116416667	10.30913045	10.1563560291667	10.5618082166667	11.2217375041667	10.4604292458333	10.5607363458333	10.4596163	10.7349994083333	10.8780047041667	10.7129235458333	9.90932817083333	9.79301413333333	9.66260626666667	9.94995692083333	10.5709527833333	10.1615384	10.1280952583333	9.93106105	10.1339838291667	10.1880852041667	10.2134535666667	9.86041405	10.7133365125	10.9162533583333	10.4170692833333	10.47002835	10.4738469083333	10.2714276125	10.1587333333333	10.0959159083333	10.7599483291667	11.3995647166667	10.9041359541667	10.8591442208333	10.9656601333333	10.8366353541667	10.7645101666667	10.8448776375	10.7946407916667	10.2357214958333	9.72221585	10.2730988083333	10.2894769833333	10.3605011416667	9.8649744875	9.70604492916667	10.5954812708333	10.8849115041667	10.7385293833333	10.3560428791667	10.5839445125	10.5346935333333	10.2032919958333	10.039806225	10.3552119041667	10.682160525	10.4198102333333	10.5434653	11.53767785	11.0317580041667	11.079544475	10.7938133458333	12.0346679458333	11.5647305333333	11.8006469708333	11.1257960708333	11.6225412166667	11.3684995708333	11.0324977125	11.2824112125	10.9837156666667	10.9059969833333	10.5851296875	10.9800394125	11.0940759041667	11.3555018916667	10.9526005708333	10.8753527625	11.4117162666667	11.2002929041667	10.6056349583333	10.96531915	11.7763300666667	11.6334944791667	12.2957742291667	11.1134381416667	10.9953434125	11.3486678791667	10.7879800333333	10.9518189083333	11.2095433041667	11.55504505	11.8675931416667	11.1385363	11.2095433041667	12.0731303541667	11.3840803333333	11.7434952875	11.6161239458333	10.8696645208333	11.1417710291667	10.8809221208333	10.5517925	11.057303875	11.0107050083333	10.7133365125	11.7051731458333	12.0154746541667	11.8087504208333	11.2009752833333	11.4845443916667	11.266611625	11.3435939833333	10.5625577333333	12.4625530458333	11.2777552916667	10.8783307208333	10.9217726166667

CHST13	10.7970204708333	10.3785116416667	10.30913045	10.1563560291667	10.5618082166667	11.2217375041667	10.4604292458333	10.5607363458333	10.4596163	10.7349994083333	10.8780047041667	10.7129235458333	9.90932817083333	9.79301413333333	9.66260626666667	9.94995692083333	10.5709527833333	10.1615384	10.1280952583333	9.93106105	10.1339838291667	10.1880852041667	10.2134535666667	9.86041405	10.7133365125	10.9162533583333	10.4170692833333	10.47002835	10.4738469083333	10.2714276125	10.1587333333333	10.0959159083333	10.7599483291667	11.3995647166667	10.9041359541667	10.8591442208333	10.9656601333333	10.8366353541667	10.7645101666667	10.8448776375	10.7946407916667	10.2357214958333	9.72221585	10.2730988083333	10.2894769833333	10.3605011416667	9.8649744875	9.70604492916667	10.5954812708333	10.8849115041667	10.7385293833333	10.3560428791667	10.5839445125	10.5346935333333	10.2032919958333	10.039806225	10.3552119041667	10.682160525	10.4198102333333	10.5434653	11.53767785	11.0317580041667	11.079544475	10.7938133458333	12.0346679458333	11.5647305333333	11.8006469708333	11.1257960708333	11.6225412166667	11.3684995708333	11.0324977125	11.2824112125	10.9837156666667	10.9059969833333	10.5851296875	10.9800394125	11.0940759041667	11.3555018916667	10.9526005708333	10.8753527625	11.4117162666667	11.2002929041667	10.6056349583333	10.96531915	11.7763300666667	11.6334944791667	12.2957742291667	11.1134381416667	10.9953434125	11.3486678791667	10.7879800333333	10.9518189083333	11.2095433041667	11.55504505	11.8675931416667	11.1385363	11.2095433041667	12.0731303541667	11.3840803333333	11.7434952875	11.6161239458333	10.8696645208333	11.1417710291667	10.8809221208333	10.5517925	11.057303875	11.0107050083333	10.7133365125	11.7051731458333	12.0154746541667	11.8087504208333	11.2009752833333	11.4845443916667	11.266611625	11.3435939833333	10.5625577333333	12.4625530458333	11.2777552916667	10.8783307208333	10.9217726166667

ADAM29	7.16614005833333	7.27773341666667	7.691592325	7.92005815	7.884494275	7.76714046666667	8.30911568333333	7.06146445	7.56091623333333	7.61698044166667	7.77602128333333	7.634487825	6.98280363333333	7.34037416666667	6.78246663333333	6.931726825	7.00118948333333	7.5010982	6.7327851	7.02943565833333	6.98973956666667	6.616187575	7.09933909166667	6.80569213333333	7.650219825	7.26937910833333	7.45558410833333	7.153195325	7.82746845	7.066499225	7.16295023333333	6.83207999166667	7.505130225	7.37766478333333	7.62089398333333	7.36156116666667	7.76884748333333	7.70589544166667	7.663341325	7.02704855833333	7.26874934166667	7.14213880833333	6.87219209166667	6.8752982	6.4040341	6.97095796666667	6.46494565	6.84325476666667	7.238340075	6.78114418333333	7.37184780833333	7.435271425	8.001514325	7.38582769166667	7.22176664166667	7.36330638333333	7.666702425	7.188269975	7.03138465	6.903227175	7.70028855833333	7.74093078333333	7.596603625	7.78383044166667	7.48861440833333	8.234157525	7.672013725	7.49771558333333	7.931728325	7.824397325	7.98666509166667	7.82339403333333	7.72905521666667	7.31803023333333	7.06220724166667	7.14213880833333	7.060215325	7.5954683	7.0596334	6.764479675	7.32433510833333	7.27891691666667	7.456643225	6.74888376666667	7.707009225	7.497153825	8.11654126666667	7.69858204166667	7.414082675	7.15568314166667	7.04267660833333	6.61689901666667	7.826430025	7.35924831666667	7.82120025	7.65490436666667	7.79638631666667	8.08807009166667	7.9693647	7.51935500833333	7.78488271666667	7.02536274166667	7.31196651666667	6.91766443333333	7.11789028333333	7.21451231666667	6.723717275	7.10434548333333	7.41121605	6.93227245	7.68323405	7.28259465	7.80971700833333	7.671444425	7.263472875	7.378725775	7.38984995833333	7.07466381666667	7.65326568333333	7.523817425

TMEM52	8.50844495	8.33599743333333	8.38023938333333	8.67026894166667	8.10843584166667	8.081342075	7.54626845833333	7.66388121666667	8.28678805833333	8.595488075	7.78927275	7.9422621	8.08335636666667	7.641075725	8.40567906666667	8.29931580833333	9.70584026666667	8.66247435	8.604857825	8.55997810833333	8.08335636666667	8.286335875	7.96986838333333	8.33599743333333	8.369572325	8.10111541666667	7.9343368	8.403258675	7.798604875	8.01905775833333	8.37211495	8.31364723333333	7.84267175833333	9.052254	8.23217175	8.54796775833333	8.21248958333333	8.325164675	8.063977575	7.38640515833333	8.47144243333333	8.01119698333333	8.47431853333333	8.739401675	8.286335875	7.81893236666667	8.16584395833333	8.3547531	8.36187725833333	8.244635825	8.45693534166667	7.93223308333333	7.35226640833333	7.91046618333333	8.56913543333333	7.872646525	8.04080661666667	8.069486125	8.66580391666667	8.1205116	8.584858775	8.319610775	9.76089629166667	9.73914464166667	8.69459336666667	9.06886545833333	7.65774509166667	7.649654175	9.57817145833333	9.41436835833333	8.04498438333333	9.31001445	9.04505583333333	8.43758005833333	8.12933435833333	8.63633005833333	8.54699226666667	8.88122105	8.07572273333333	9.319021525	8.04865021666667	7.470286275	8.67361984166667	9.43547285833333	8.286335875	9.16396135833333	8.63917994166667	8.66828283333333	8.18781211666667	8.107904375	8.28095494166667	9.29933239166667	8.61046613333333	7.60136925	8.31058729166667	8.82825418333333	10.3825544833333	8.551483425	8.894577375	8.17720695	8.22690084166667	8.99943493333333	9.397948075	8.29739210833333	9.82295156666667	7.95134493333333	8.22099113333333	9.54880464166667	9.0180261	8.846155575	7.933308825	7.94329386666667	8.122791675	8.382753925	8.08700735	8.78635625	9.00507715833333	9.25028671666667	7.913004675	8.18315743333333

AFF2	7.794082525	7.62655825	8.379169075	8.03755788333333	8.38023938333333	8.96182571666667	8.548453575	8.37303865833333	7.82539101666667	8.00852153333333	10.4061408166667	8.28049985	9.148299925	8.584858775	8.18781211666667	8.89174859166667	7.49549410833333	8.75473524166667	8.053540525	8.592518275	7.57050700833333	7.80781901666667	8.06026415833333	7.35924831666667	6.8752982	8.11406380833333	7.76084795833333	7.45451809166667	8.27228149166667	6.58226158333333	8.013232325	8.10955528333333	7.81456171666667	7.69757114166667	7.35345289166667	6.9563535	7.55374699166667	7.31981145833333	7.89196619166667	8.05569140833333	7.85309088333333	7.34272655	8.488931425	7.782192425	7.25066114166667	7.299574675	7.650678625	7.09933909166667	7.82231499166667	7.89880223333333	6.90968315833333	6.60894191666667	7.788761475	6.66522548333333	6.59670515	7.59883135	8.39981014166667	8.38319035	7.79803676666667	7.74511231666667	9.56407003333333	9.31629211666667	9.04505583333333	9.337966225	10.931065425	8.986594625	9.92832075	9.66513115	7.82339403333333	8.200812875	10.6072418333333	9.76310825	8.68624993333333	9.54225544166667	8.512323475	7.70199845833333	8.41837	9.96267133333333	8.88592250833333	9.41892754166667	8.56529179166667	9.70796235833333	10.1148100333333	9.05497765833333	11.171764175	10.2478255083333	9.49057863333333	9.83629188333333	8.854456025	8.64244941666667	8.98934508333333	8.995827675	10.81468445	7.634487825	7.52624075833333	10.6979535583333	7.4656947	9.10796010833333	8.62685349166667	10.1687340166667	10.1337610833333	10.4581692833333	11.345193725	8.01539524166667	6.76843211666667	8.66481208333333	6.80918875	7.30864333333333	8.64006809166667	8.42877934166667	9.50452185	9.04644768333333	6.559370075	7.46514316666667	6.18247753333333	7.2401722	9.836699675	7.88871496666667	9.43636521666667	8.238616825

OTUB1	11.9274761416667	12.8901186583333	12.7536835	12.5376322583333	12.8532684916667	12.4447647416667	12.2453199833333	12.6857168666667	12.4908032583333	12.7103184	12.4265910916667	11.624532275	12.3956670583333	12.901864125	13.047619075	12.48982835	12.2867043083333	12.895840375	12.9726928583333	12.988893075	12.798152025	12.800460075	12.8454667083333	12.5585076166667	12.7591596666667	11.9690058	12.7221714916667	12.996638525	12.65465995	12.852746025	12.8532684916667	12.7912843416667	12.5633422083333	12.0277810916667	11.9670214	12.1711742666667	13.0798178083333	13.1085025666667	12.6287796916667	12.9063845583333	12.9527510916667	12.790749425	12.840994225	13.180045325	12.5652457833333	13.0560858	12.895307525	12.9552000333333	12.9346936833333	12.8823979	13.0457183333333	12.8261905083333	13.1622146833333	13.132282	12.8974584	13.0108192083333	13.00071955	13.05246665	12.989519425	12.9699766666667	12.1665042	12.1922906583333	12.692926575	13.0566076	11.6783737	13.2233931166667	12.52885135	12.8936439416667	12.9558687	12.9988931583333	12.912870175	12.7001469666667	13.1256286916667	12.6765518416667	11.8916056833333	13.0174115	12.4932078666667	12.559988525	13.3256142916667	12.6336537916667	12.3966157416667	12.403353825	12.99120565	12.3684936	12.718512625	12.5439252666667	12.3846618166667	12.2437326916667	12.916130525	12.57846135	12.8912643	12.8618738916667	12.92419445	14.269993075	12.8246255416667	12.467315075	12.844414625	12.940616175	13.2690950583333	13.608143675	12.9172670333333	12.5701103	12.350546075	12.2453199833333	12.7660601416667	12.8392807333333	13.028241025	13.1980287833333	12.8907368833333	12.972189625	12.7453059583333	12.9659499916667	13.199462625	13.3987592083333	12.7139152416667	12.9570294	12.580271675	13.1178746666667	13.1779047083333	12.9266166166667

RAP2B	9.309548625	10.7816314666667	10.1529084083333	10.2991427166667	10.4292877666667	9.97337968333333	10.0450906333333	10.93390845	10.2069205083333	10.390544	9.82822335	9.54311075	9.78104253333333	10.3207495083333	10.7585831916667	10.18000125	9.45055241666667	10.7287943416667	10.3510926083333	10.6641205583333	10.2720090666667	10.7463771	10.3317121166667	10.5760591583333	10.2900500333333	9.38807046666667	10.628160175	10.7375745583333	10.476356775	10.4819116333333	10.4590182666667	10.58883085	10.3510926083333	9.46790315833333	9.31710229166667	9.69308664166667	10.5448322833333	10.3578687916667	10.55650935	10.6934456416667	10.3887558333333	10.76211435	10.406884125	10.7589898916667	9.909962375	10.273326675	10.7050450333333	10.5631726083333	10.50459975	10.010773575	9.76647539166667	10.52201155	10.4324595166667	10.3362368833333	10.3053444333333	10.39251605	10.647680825	10.5181477	10.2547426166667	10.2928305416667	11.2982935833333	9.911212725	8.81971978333333	10.5639796666667	11.2107825666667	11.5118143666667	9.67192385833333	10.29990335	9.88386428333333	9.53179666666667	10.3400524166667	10.91013625	10.7782435583333	10.0197701416667	10.8224026333333	10.0134059083333	9.87695954166667	10.1324487166667	10.5244636333333	9.26306665	9.984272	9.41204579166667	11.9289106916667	10.5553397083333	10.120107375	9.81345976666667	10.1176213666667	9.599958075	9.45859685833333	10.528410425	10.5703833416667	10.0268881833333	9.686488825	10.7173608833333	9.30818888333333	10.4240891833333	8.28895966666667	8.85494786666667	11.3817027833333	9.46343676666667	10.1084049583333	10.4661023416667	9.55621784166667	9.40727955833333	10.747418225	10.0412389083333	10.3466375416667	10.3868161083333	9.7036749	10.1845142083333	9.9420301	10.1613409083333	10.9061592666667	9.76136145	10.6664473083333	10.9284040666667	9.34710834166667	10.2274517916667	10.4076693916667	9.75787298333333

NBPF11	9.2056298	8.67450711666667	8.3471697	8.8526135	9.06069035833333	8.27384794166667	8.94577069166667	8.35277756666667	8.685246125	8.434501875	8.50943095833333	9.21882504166667	9.51896663333333	8.53097458333333	8.36807433333333	8.251367875	7.89930148333333	9.15400360833333	8.16329085	7.89722555	8.48261985	8.64337428333333	8.24116579166667	8.7954535	8.37234324166667	8.499322875	8.48321643333333	8.5874833	8.60280456666667	8.620261275	8.41428779166667	8.186266475	8.40473328333333	8.19294795833333	8.228841125	8.30538075	8.92929650833333	8.03755788333333	8.34021045	8.07050834166667	7.73690679166667	8.67639044166667	7.62988515833333	7.58238585833333	8.56470943333333	7.51814899166667	7.53449101666667	8.5905397	7.801197125	7.45558410833333	7.428439975	7.825834775	8.530508075	8.19143718333333	8.149040725	7.42788356666667	7.44735373333333	7.61811995833333	7.930120075	7.55925806666667	9.34530801666667	8.953569225	8.458998275	8.650731025	8.25885329166667	8.930278925	8.333491425	8.796402275	8.60998564166667	8.29490485833333	8.538352825	8.9231771	8.975801525	8.6223815	8.595488075	8.39466414166667	8.67688486666667	8.488485525	8.30591494166667	8.686777175	8.65218976666667	8.35832260833333	8.52110425	8.64244941666667	8.80499509166667	8.42733516666667	8.930278925	8.47797495833333	8.82473405	7.97086885	8.60947813333333	8.53140681666667	8.5676736	7.9343368	8.69984448333333	8.35736911666667	8.71039916666667	8.54411631666667	8.86116145	8.09590623333333	8.475275825	8.97354856666667	8.51387624166667	7.87821420833333	8.3657693	8.24861051666667	8.36515990833333	8.4515305	7.5654861	7.19320405	7.26995246666667	7.67474618333333	8.82372205833333	8.08035505	7.45966298333333	8.010141425	8.054062325	7.90032115	8.20331464166667	7.83348010833333

DDX17	15.386313475	14.269993075	14.2378149833333	14.2000424583333	13.6426254916667	14.85238655	14.2305624666667	14.6046129083333	14.0746231666667	14.107174325	14.2763428833333	14.657054125	14.7034991833333	14.21756365	13.9227796833333	14.6992893416667	14.5089658583333	14.6389112166667	14.5415836083333	14.451178225	14.145758825	14.201540175	14.494740525	14.7429609666667	14.512293975	14.539541	14.2245923416667	14.3431664333333	14.274700225	14.0800041333333	14.6862769916667	14.2936594416667	14.354697075	14.6430667333333	14.8724941166667	14.7828660333333	14.2731900083333	14.241033875	14.4377971083333	14.4926359	14.2872888583333	14.274700225	14.6549636583333	14.6067841833333	14.7640659	14.3824525333333	14.5433520583333	14.37416785	14.37416785	14.4926359	14.4309788083333	14.3139109083333	14.31061245	14.8313200916667	14.4144157	14.56279245	14.2393514333333	14.3531439916667	14.2145397416667	14.4527902	14.364637875	14.4397477583333	14.5449579333333	14.4775829916667	15.0733260666667	14.236432725	14.539541	14.5415836083333	14.4177528166667	14.265784475	14.2073641166667	14.62202095	14.1827283166667	14.7522867083333	14.5849736666667	14.364637875	14.4226857416667	14.5322324833333	14.4397477583333	14.6239736916667	15.0615765666667	14.8842187583333	14.5089658583333	14.4025408	14.5984675666667	14.87708635	14.6410690916667	14.3204037083333	14.515883675	14.5530100166667	14.3253890083333	14.4244522916667	14.2904660666667	14.4926359	14.265784475	14.4615780083333	14.3221229083333	14.6104738916667	14.2763428833333	14.8442556083333	14.657054125	14.4705790833333	14.2518399916667	14.3236760583333	14.1152258583333	14.6992893416667	14.5753036916667	14.5415836083333	14.4025408	14.2474901166667	14.5830492	14.515883675	14.5020438083333	14.2349020166667	14.5488647916667	14.1709219416667	15.0335623833333	14.51071235	14.500239075	14.4495058333333

OTP	7.600828425	7.1989222	7.18584309166667	7.26110544166667	7.83518873333333	7.0659289	7.88559756666667	7.08487659166667	7.13667263333333	7.37811973333333	7.13033303333333	7.188269975	6.975151325	7.20911576666667	7.22598754166667	6.73002575	8.16877921666667	7.68541283333333	7.39869075	6.76114361666667	6.8444516	7.22964071666667	7.64610639166667	6.78246663333333	7.35627839166667	6.80056770833333	6.73133699166667	6.94376375833333	7.314288875	7.58577588333333	7.45786721666667	6.96254510833333	7.29843466666667	7.24514076666667	7.38404451666667	7.54125486666667	7.452175025	7.3361665	7.2162874	6.67856683333333	7.15765674166667	7.40837556666667	6.92785069166667	7.26049458333333	7.3477901	7.12659995	7.30133825	7.53449101666667	6.85418244166667	6.70918899166667	7.283866825	7.34551940833333	7.4784294	7.621493025	7.86609655833333	7.44780359166667	7.04568171666667	7.56433695	7.16614005833333	7.14581168333333	7.00630173333333	7.3019376	7.46688233333333	7.11020925833333	7.04378355833333	7.56491295833333	7.44780359166667	7.21861555833333	7.72085915833333	7.17569580833333	6.94497745833333	7.54177855833333	7.05156345	6.975151325	6.92717863333333	6.59052684166667	7.52225746666667	7.46414650833333	7.40135389166667	7.127807925	6.48284681666667	6.80377665833333	7.73471975833333	7.153195325	7.86164825833333	6.64487866666667	6.92374801666667	7.09804688333333	6.79994456666667	6.83868629166667	7.20508425	7.2395484	7.44317530833333	6.72653388333333	7.71409900833333	7.41466188333333	7.59253030833333	7.20568426666667	7.45966298333333	7.10123053333333	7.08354248333333	7.34440340833333	7.445489275	7.296620525	7.61520518333333	7.13667263333333	7.51310406666667	7.99131585833333	7.23080319166667	7.77550013333333	7.29788705833333	7.6225677	7.7799909	7.88927118333333	7.7483928	7.72413511666667	7.41303320833333	7.49879253333333	7.53126729166667	6.69715851666667

BRK1	14.2145397416667	14.2998455083333	14.2503770416667	14.1033949916667	13.7055820166667	13.4375531583333	14.0326619916667	14.1857941	13.75723885	14.2671663083333	13.8366591583333	13.9612443083333	13.9119804333333	14.29207405	14.277828625	14.1033949916667	13.91423525	13.9541211333333	14.421103675	14.1500101916667	14.105898725	13.9119804333333	13.771159075	13.7781265916667	13.9433845083333	13.8854083166667	14.0127035833333	14.2487820083333	13.85508265	14.0514423083333	13.9227796833333	14.3283993416667	13.89505895	14.0694923916667	14.1513249333333	14.0201742416667	14.0656333583333	14.1629431333333	13.8572965333333	14.3431664333333	14.177662	14.3139109083333	14.0847383833333	14.4294718916667	14.1927345333333	14.427724725	14.3153773833333	14.1500101916667	14.1306178166667	13.8494140916667	14.1384134166667	13.9796866	13.9737942083333	14.107174325	14.0991501333333	14.2686692416667	14.0503314	14.3892469416667	14.274700225	14.2936594416667	12.8171563916667	13.0727047	13.3193049583333	13.232552625	13.4752691916667	13.144627125	13.2622394166667	13.134360625	13.4974205	12.9733229333333	12.8936439416667	12.989519425	13.1980287833333	13.4156891583333	13.3053968166667	13.6284294583333	13.3270763416667	13.23467165	13.450467225	13.608143675	13.386735	13.2254010416667	13.8357694833333	13.5387428916667	13.1262418916667	13.3010495416667	13.3508640083333	13.1531334333333	12.497697975	13.4260804333333	13.986279475	12.7748629083333	12.8118807333333	13.1356932666667	12.803128575	13.232552625	13.2656354333333	13.1556465833333	13.1525120666667	13.14330865	13.2387164583333	13.505263125	12.60273605	13.6320213916667	12.9499592833333	13.2933426583333	13.8395483	13.6923255	13.3018009583333	13.50262465	13.3256142916667	13.4113073583333	13.100692275	13.4289883	13.452778075	13.6236897166667	13.4498034583333	13.40344555	13.6737470333333	13.2828821583333

F3	11.5731480083333	12.1320993083333	11.960165	12.8284427416667	13.0136394416667	13.400193425	12.2574255666667	13.1538383166667	12.6361256666667	11.7351274916667	11.8516230166667	12.0301876583333	12.370296175	12.786823125	11.8367863833333	12.05061705	12.5497662083333	12.7955349083333	11.3846584083333	13.0136394416667	12.8261905083333	12.2939030416667	11.3470919	12.5935190666667	12.533964725	10.6263315083333	11.5389498916667	11.5987696666667	12.4487345666667	11.3413304583333	12.3662011583333	12.5024686916667	12.3815660416667	12.59948	10.8251181583333	10.20731305	11.7557288583333	11.5302262416667	12.0017085416667	12.8498718833333	12.5488109	11.7459533166667	13.2480671333333	11.835989125	11.2775845	11.9420280583333	12.072543675	12.0593104333333	10.5065079166667	13.1376073666667	11.6989876416667	11.4236775666667	12.2528148916667	12.786823125	12.8757636333333	12.649121475	12.7441506916667	10.97439825	12.2821681166667	12.2293758416667	12.0677960666667	12.1308631333333	12.7031196333333	12.458934675	9.654799725	12.9063845583333	12.3851121666667	11.938838275	12.5079805333333	13.7781265916667	11.5858193083333	11.2082269	14.5232906166667	12.1669452	12.4772181916667	12.4078515833333	12.0201508916667	11.651839775	13.27588995	12.7543063	10.5553397083333	12.3396961416667	13.9250365	12.9253738	11.8154196416667	12.8783966083333	9.778305875	14.113875875	10.1678889166667	12.068210875	11.7895299333333	12.0185152166667	12.2723412333333	12.852746025	12.8268431583333	12.10672325	12.0877373333333	11.3336047583333	12.42337295	10.469517225	12.4005974416667	10.4887010333333	10.9189114166667	12.136374875	14.5195733916667	12.4341294166667	13.7621985333333	13.3018009583333	10.04725285	13.132282	12.5076062916667	11.49602405	14.1427380833333	11.9550582083333	12.3187215583333	12.4019704583333	11.3324973416667	14.3875977	13.8743989166667	9.97000988333333

GINS2	9.14518724166667	10.7907764333333	10.6905955916667	10.8326576083333	10.8224026333333	11.15136825	10.7581339416667	10.3913250333333	10.5549316666667	11.1343238833333	11.0462297583333	9.78329576666667	10.241903125	10.50924085	10.1862509833333	9.90608706666667	9.797655	10.4256891416667	10.418815025	10.433634375	10.4308569666667	10.2810793	10.7170255166667	10.30932905	10.5627525083333	9.15064904166667	9.98187775833333	10.2928305416667	10.534888275	10.5022046916667	10.0288478666667	10.49466965	10.2399871166667	10.5895974833333	9.35451149166667	9.35863609166667	10.988072975	10.3637863083333	10.1397194333333	9.99895085	10.0364758583333	9.98877366666667	10.1452230583333	11.1828701833333	9.98761663333333	10.30576505	10.6313385833333	11.0953873333333	11.233092075	10.3637863083333	10.74562245	10.1903114083333	11.0541887333333	10.90507365	10.5829592166667	10.4963081083333	10.3948990166667	10.8980207583333	10.79558335	10.6910121333333	11.8947334916667	12.06402615	12.608834775	11.3235379	11.5873432	10.7612989333333	12.11053915	12.161623975	12.4831684166667	11.9785582833333	12.5580702166667	11.765950425	11.9853022666667	12.1299872583333	11.3144428833333	12.1947789333333	10.98769475	12.4772181916667	12.6656204083333	12.7949718333333	12.3374196	12.1291180083333	11.7146176666667	11.0548999583333	10.9332106333333	11.7440902416667	12.6386311166667	11.421792525	12.763386175	11.2779260833333	11.5606001833333	12.837133975	12.161623975	12.01005715	11.5813004	12.0839641583333	11.5672571	13.2047745416667	12.409680475	11.1930897583333	12.8046597666667	12.2792629666667	12.9575419416667	11.7682562416667	11.9451089	12.4063944666667	11.6902780833333	11.938838275	11.7568591416667	11.0808123083333	11.051725325	12.4772181916667	11.6230706833333	12.5585076166667	12.21725785	12.616140275	12.725884075	12.7918033916667	12.153282325	12.5948964

KIF6	8.03117830833333	7.10317193333333	7.20334935833333	7.62307443333333	7.72249438333333	6.70056215833333	8.21881454166667	6.98911033333333	7.87562194166667	7.3968942	7.17198438333333	7.48416809166667	7.16490981666667	6.43572405833333	6.93803065	6.67096843333333	7.72739250833333	7.28864350833333	6.76313481666667	6.62496390833333	6.8275595	6.66522548333333	6.78180254166667	7.31552385	7.11547685	8.3048096	6.71367926666667	6.68797494166667	6.80124884166667	7.120419025	7.338007825	6.91190293333333	7.43234464166667	7.87369893333333	8.535777725	8.55543884166667	7.57050700833333	7.50167894166667	7.299574675	7.35119398333333	7.34669549166667	6.99155748333333	7.03384980833333	7.07528308333333	7.44041060833333	7.09424611666667	7.24396096666667	6.97900894166667	6.70056215833333	6.86668814166667	7.46243825	7.06220724166667	7.01264725833333	7.435271425	7.48927714166667	7.45909229166667	7.198288025	7.638193025	7.15514039166667	6.91578644166667	6.801867125	7.37766478333333	7.02401584166667	7.798604875	7.64794805833333	7.29049713333333	7.85854243333333	7.283866825	8.2229273	7.46352631666667	6.9475749	7.1508922	7.50051165833333	6.662431575	6.64358704166667	6.64358704166667	7.2843799	6.72123403333333	6.41504531666667	6.89400789166667	6.52209205	6.09436185833333	7.02284831666667	8.18475165	7.22835589166667	7.112144375	6.85745724166667	6.94497745833333	6.97957124166667	6.78990954166667	7.483605675	6.86328933333333	7.137920375	8.32264421666667	7.81401100833333	7.195709975	10.5775565833333	7.20393616666667	7.95194513333333	6.60956075833333	7.33375654166667	6.76114361666667	6.70918899166667	6.81118743333333	7.13168135	7.88712554166667	6.50580336666667	7.70256570833333	6.926478325	6.71655181666667	7.0469593	7.02401584166667	7.3675475	9.39158905833333	7.43805644166667	8.05296578333333	6.96657695833333	8.42069139166667	7.21808105833333	7.17507495

SLC7A14	10.5839445125	5.56825414583333	5.7223480375	5.4142425	5.42830382083333	6.4037249	6.34193792916667	6.13297264583333	5.852767	5.09368760416667	5.4746956625	7.384315675	8.316949125	6.02687620833333	5.11502322916667	5.27366625	6.71148608333333	5.7252025875	4.2626513875	5.1328199875	5.96820949166667	5.7630094875	6.25062844583333	5.7455653875	5.14989413333333	10.3429838208333	4.858152125	5.52176327083333	6.52240923333333	4.99948749166667	5.9665777	4.27526584166667	4.82951312916667	4.42821570416667	9.0898379	9.69512940416667	4.417387875	5.27579793333333	4.93814212083333	4.38473815833333	4.61573042083333	5.06058080833333	6.09474337916667	4.94836752083333	8.31987026666667	5.79422955416667	4.36749461666667	4.34014847916667	4.2680831125	4.96903827083333	4.48199477916667	4.7946488875	6.24739725833333	6.36361664166667	5.350279675	5.25090578333333	6.21316953333333	4.84767841666667	4.61678248333333	5.53149080833333	4.73067105	6.90878545416667	5.1731795375	6.16951285833333	6.67270679166667	5.7969617125	5.599895525	7.28528609583333	5.39018647916667	5.38917544166667	6.46389307916667	6.39343123333333	6.59144968333333	5.46506463333333	5.72424479583333	5.89409153333333	5.70103635	4.65533382083333	5.04398305416667	5.57393574583333	7.67743358333333	6.6432540375	4.85196545833333	5.43877214166667	5.81262865	6.81880792916667	6.35315391666667	5.60627390833333	6.98380058333333	6.56254219583333	6.1861432	5.21571929166667	4.5893217625	5.45213058333333	5.7581333875	5.78962339583333	5.18262998333333	5.892408675	5.61107660416667	4.55917659583333	5.053377375	7.50651534583333	4.60134417916667	4.49451613333333	4.84249479583333	5.65683310833333	4.85092052916667	5.00178568333333	4.49451613333333	5.6854500375	4.5748982375	4.9401554875	5.09050049583333	5.77332922083333	5.25090578333333	4.68126080416667	6.6274145125	5.12464447083333	5.34914802083333	6.37900670833333

AC026316.4	10.5839445125	5.56825414583333	5.7223480375	5.4142425	5.42830382083333	6.4037249	6.34193792916667	6.13297264583333	5.852767	5.09368760416667	5.4746956625	7.384315675	8.316949125	6.02687620833333	5.11502322916667	5.27366625	6.71148608333333	5.7252025875	4.2626513875	5.1328199875	5.96820949166667	5.7630094875	6.25062844583333	5.7455653875	5.14989413333333	10.3429838208333	4.858152125	5.52176327083333	6.52240923333333	4.99948749166667	5.9665777	4.27526584166667	4.82951312916667	4.42821570416667	9.0898379	9.69512940416667	4.417387875	5.27579793333333	4.93814212083333	4.38473815833333	4.61573042083333	5.06058080833333	6.09474337916667	4.94836752083333	8.31987026666667	5.79422955416667	4.36749461666667	4.34014847916667	4.2680831125	4.96903827083333	4.48199477916667	4.7946488875	6.24739725833333	6.36361664166667	5.350279675	5.25090578333333	6.21316953333333	4.84767841666667	4.61678248333333	5.53149080833333	4.73067105	6.90878545416667	5.1731795375	6.16951285833333	6.67270679166667	5.7969617125	5.599895525	7.28528609583333	5.39018647916667	5.38917544166667	6.46389307916667	6.39343123333333	6.59144968333333	5.46506463333333	5.72424479583333	5.89409153333333	5.70103635	4.65533382083333	5.04398305416667	5.57393574583333	7.67743358333333	6.6432540375	4.85196545833333	5.43877214166667	5.81262865	6.81880792916667	6.35315391666667	5.60627390833333	6.98380058333333	6.56254219583333	6.1861432	5.21571929166667	4.5893217625	5.45213058333333	5.7581333875	5.78962339583333	5.18262998333333	5.892408675	5.61107660416667	4.55917659583333	5.053377375	7.50651534583333	4.60134417916667	4.49451613333333	4.84249479583333	5.65683310833333	4.85092052916667	5.00178568333333	4.49451613333333	5.6854500375	4.5748982375	4.9401554875	5.09050049583333	5.77332922083333	5.25090578333333	4.68126080416667	6.6274145125	5.12464447083333	5.34914802083333	6.37900670833333

CLEC6A	4.303353375	4.61010935833333	4.89364545833333	4.63305978333333	4.73024025833333	6.603384975	5.30448685	5.92148618333333	4.48717731666667	5.02371664166667	4.17138054166667	5.96077145833333	4.95961636666667	4.91552208333333	4.29716620833333	5.23781710833333	4.337410475	4.656935525	4.89364545833333	5.23978975	4.509505	5.98649943333333	4.37388213333333	5.95243336666667	5.14292011666667	3.7942322	4.98771553333333	3.84584678333333	4.302366825	4.16737369166667	5.64694838333333	4.34995844166667	4.05627571666667	6.28660823333333	5.97595255	4.149223425	4.48829116666667	5.44971561666667	4.90032935	4.3549546	5.42780705833333	4.04622350833333	5.81313270833333	4.93337844166667	3.95350946666667	4.71218959166667	4.75255506666667	5.76251488333333	5.53004825	4.8072374	4.62889925	4.203896025	4.68477714166667	6.21191129166667	4.760314225	5.00117389166667	5.16420199166667	5.17804041666667	3.99930385833333	6.40265184166667	3.9147734	5.10498871666667	6.47216713333333	6.08271424166667	5.06903989166667	5.09419765	4.55283246666667	6.36248048333333	6.26738683333333	6.637274925	5.63053526666667	5.07656036666667	5.74130296666667	5.72686785	5.255540825	5.49229008333333	4.16522844166667	7.4493567	7.18035421666667	6.005441825	4.508464425	5.83953349166667	6.43934115	6.35578110833333	5.577321975	4.954343875	5.24289476666667	6.657721925	3.98396318333333	6.50275845	6.1441534	6.54872438333333	5.135343	4.14488695	4.62889925	6.70726116666667	5.53395658333333	5.10077205	4.55069105	4.52055483333333	6.87469296666667	5.19112474166667	8.28445111666667	4.43639113333333	7.18159225833333	6.25250383333333	6.21030875833333	5.81403274166667	6.62636613333333	7.17093558333333	5.30562465833333	5.622261375	7.50801045833333	6.345260775	8.36127639166667	5.94277896666667	6.23312839166667	7.41121605	4.42054164166667	8.15060805

MTPN	13.7721764083333	15.0831469166667	15.1174212666667	14.948326475	15.1077350416667	14.4926359	14.9752180916667	15.4575903	14.7166750666667	14.8895282083333	14.5530100166667	13.6246233916667	14.8110718166667	15.0733260666667	15.09815525	14.7166750666667	13.81891895	15.0217064166667	14.8136769166667	15.1973098583333	15.1430836166667	14.8865870416667	15.1833315916667	14.6304997416667	14.9197306916667	13.7101806916667	14.9045675833333	15.092462825	15.0184028083333	15.0615765666667	14.7097912166667	14.8362922416667	14.6067841833333	13.7234378333333	13.90344385	13.597492475	15.1139193916667	15.2502335583333	15.0032595583333	14.6589883666667	15.56568115	15.0306120416667	15.1048760583333	15.3579580416667	14.8236926166667	15.2328828083333	15.0831469166667	15.1800900333333	14.9620915916667	14.8794573583333	14.7252309	14.9674626083333	14.9865439333333	15.039210975	15.1302818083333	14.7974983916667	15.2468154916667	15.618710975	15.1207533083333	15.039210975	14.034006175	14.2233219083333	14.1268362333333	14.3283993416667	14.1317250833333	14.0991501333333	14.3170032666667	13.8898465333333	14.2810681833333	14.1357234916667	14.6239736916667	14.5664298833333	14.2219861583333	13.9541211333333	14.35633825	14.3013820416667	13.5601696	14.0991501333333	14.3076416166667	13.9207353166667	13.9261802833333	13.916524275	14.204528175	14.0979296916667	13.9967915416667	14.3045490583333	14.2289632916667	13.597492475	14.0759487166667	14.164370775	14.3676663333333	14.4226857416667	14.4411655333333	14.30297575	13.9445631916667	14.5195733916667	13.8337930083333	13.4246253333333	13.5626218333333	12.6939319583333	13.8720329083333	14.56279245	14.0163605916667	13.8296521	14.156645775	13.741448775	14.0694923916667	14.2763428833333	14.1241853333333	13.9955171333333	14.6904136083333	14.1256024333333	13.6551549916667	14.0288199166667	13.7681999916667	14.500239075	13.96485035	13.8720329083333	14.1618228083333	13.5083984166667

PAX9	9.56080151666667	15.7066290583333	14.127909425	15.0769655666667	15.3540364916667	12.9461435166667	14.6611477166667	15.2084540583333	15.1973098583333	14.5322324833333	14.0847383833333	9.24318624166667	14.789742575	15.5262258166667	15.5262258166667	14.9020399416667	7.9484088	14.9307420333333	15.5035464333333	14.70147985	14.9702238166667	15.3290216916667	14.8412522666667	14.6718137833333	15.2784907166667	10.5764502333333	15.06705565	15.1207533083333	15.2084540583333	15.1728574583333	15.3579580416667	15.2084540583333	14.9620915916667	7.81839644166667	7.96575633333333	9.51857009166667	15.1207533083333	15.4243027666667	15.1430836166667	14.156645775	15.1430836166667	14.7920246166667	15.1104707416667	13.9109585833333	14.3724417	14.9359352083333	15.3777313666667	14.9279831333333	14.9832780583333	15.0769655666667	15.1873199	15.436007925	15.7138591583333	15.5512564416667	15.0246483416667	15.4199685666667	15.2650881666667	15.444211725	15.345488975	14.847260375	12.2892396	11.2560704166667	15.3136672916667	12.4746445166667	10.8817964	12.43292905	11.680364275	11.600264425	11.6470358	13.7084619583333	13.065712375	12.7896506583333	9.62759855	11.633311275	13.0301413666667	11.3047106916667	13.0721123166667	13.8241318916667	12.3160821666667	14.4262315333333	11.9188907916667	9.891815375	12.4132063666667	10.913187075	10.1895353083333	12.1138378333333	12.2354776	10.051171975	13.5468416333333	11.9093036166667	12.355857375	12.5917146833333	11.2609645083333	9.91801125833333	12.790749425	13.3322833833333	15.0184028083333	12.7473030666667	11.4934089166667	14.6046129083333	10.490641975	12.7473030666667	11.0600457083333	15.1529307583333	11.5731480083333	11.3934696166667	11.9616964916667	12.2743988	9.76267145833333	10.706587775	11.460533275	13.7740900916667	12.7411170833333	13.3262903916667	11.3191493833333	13.1980287833333	10.1836960166667	12.6914097333333	13.4119895416667	12.77853635

RGMA	10.5987255666667	11.1934390416667	11.5237451916667	11.181831125	12.8317521333333	11.1074454416667	12.715938075	11.7613047833333	11.960165	12.4320171333333	11.9329100916667	11.7470404	11.89894165	11.39940385	11.357567525	10.909815425	10.0142807916667	11.8619112333333	11.1470598583333	11.343036975	11.46509415	11.1949025333333	10.8025054583333	10.8566946833333	11.0315811583333	11.0250402166667	11.9458994666667	11.6058721833333	11.685558375	11.21039405	11.2363296083333	10.9519987166667	11.7561182166667	10.5814300416667	11.0087053416667	10.8548652666667	12.2345932083333	11.7343681166667	11.28453495	11.6807143833333	11.6436590416667	11.2975735583333	10.857474925	10.7665838833333	10.4585673833333	10.9035797416667	10.1978484	10.5973351833333	10.8326576083333	10.96888565	11.8722246166667	11.1039790666667	10.9328378333333	11.221922825	11.062936475	10.259584625	11.43469695	11.50454985	10.9415949916667	10.670337625	11.40680705	11.2373633083333	10.7460395416667	11.3506069833333	10.6592419583333	12.1640360666667	12.0525821583333	11.339247	10.9640211333333	11.17354635	11.437271475	11.832540575	11.9093036166667	10.9805696916667	11.367946675	10.8243180916667	11.1862575	10.9790999666667	10.8589548	11.0087053416667	10.6809073166667	11.1815244666667	11.0038210166667	10.5400727416667	11.4737788083333	11.4971747583333	10.9360928583333	11.0341045833333	11.3413304583333	10.55775005	10.9553221083333	10.523220425	11.4904941666667	11.2826013166667	11.7190625916667	11.7513402	11.2756978833333	11.19866735	11.3698477166667	10.5295700833333	10.5841235833333	11.4496634833333	10.0989513583333	10.3119751583333	10.7228384083333	10.3953052083333	10.476356775	10.2741449416667	10.4581692833333	10.591824075	10.6403864583333	10.87179425	10.7907764333333	10.25189245	10.904663125	10.4558195166667	10.1691741416667	10.4895361083333	10.7702977583333	10.5330763833333

C9orf92	5.39281689166667	4.05417596666667	4.3276422	4.70163844166667	4.117963675	5.23671275	4.87292895	5.03930840833333	4.2379826	4.64033936666667	4.19623050833333	4.12106518333333	4.097912125	4.57827949166667	4.359146625	4.49312724166667	5.35692600833333	4.31116040833333	4.49712886666667	4.20479785833333	4.21395365833333	5.018425275	4.76349515	4.3987315	4.93552183333333	4.04713170833333	4.3934547	4.88576699166667	5.38659805833333	5.23876719166667	5.23461665	4.7006097	4.33133893333333	4.86479268333333	4.70675665	4.199161275	4.89827923333333	5.43488754166667	4.92770649166667	4.59265796666667	5.35273364166667	4.76455610833333	5.24102825833333	5.37226380833333	4.48432390833333	4.87091625	4.957419525	4.93234401666667	4.88920265833333	4.75491586666667	7.079224475	5.06232444166667	4.31295405833333	5.773689175	5.09968536666667	5.3960318	4.79109571666667	5.01530855833333	6.12040304166667	5.44533825	5.13745513333333	5.052876825	4.56487130833333	4.58881544166667	3.87506608333333	4.46516671666667	4.111632325	4.13438028333333	4.485186325	4.224150025	4.28008616666667	4.12677783333333	4.7847361	4.27160165833333	3.84051730833333	4.09598105	4.0322916	4.34607883333333	4.58777895833333	4.01810879166667	5.28464988333333	3.99930385833333	4.46516671666667	4.32094003333333	4.1683668	4.73402499166667	5.83674730833333	4.25005865	4.59179895833333	4.70371486666667	4.262047775	4.17138054166667	4.93436960833333	4.055304025	4.15745399166667	4.2379826	4.290980325	5.80356945	4.203896025	4.92055984166667	5.45375554166667	4.2860754	4.61625569166667	4.78586035833333	4.43091141666667	4.3934547	4.49312724166667	6.11047193333333	4.4580822	6.161212075	6.85418244166667	5.20550439166667	5.60680040833333	4.77813741666667	5.127309825	4.609167375	4.11480768333333	4.81075010833333	4.72615460833333	4.418789625

HS1BP3	12.5979621916667	10.6550211916667	10.61804865	10.5276582916667	10.7217139416667	10.8980207583333	10.3380831916667	10.4348946583333	10.8589548	10.6072418333333	10.7501138916667	11.9657351833333	11.2975735583333	10.6652778583333	10.9000188916667	10.83116835	11.7699299	10.1982859833333	10.9143108583333	10.82884605	10.572357075	10.2650558583333	10.2670402	10.2241250416667	10.6251996	12.1843992666667	10.91124625	10.3806930666667	11.1106962416667	10.7631580166667	10.72841425	10.6191395	10.5613244166667	11.1905443833333	12.31433185	12.80521305	10.9317956333333	10.1755237	9.96660331666667	10.356255775	10.6621925666667	10.678502425	10.5493449166667	10.392106425	11.276041	10.7718274166667	10.741834125	10.478998225	10.1974084916667	10.9136098583333	10.4998350333333	10.2282248666667	10.670739975	10.6038899083333	10.8476613083333	11.0394398083333	10.4914439416667	10.740315675	10.5307562833333	10.5655352666667	10.8465109833333	10.3526835916667	10.5787126833333	11.229748375	10.9028824333333	11.279776525	11.2783121416667	10.3542362916667	10.599920275	10.7092769166667	10.5764502333333	11.4669037583333	11.4166255166667	10.782424925	11.5268940083333	11.1043306833333	11.0589538416667	10.92486485	10.5968900416667	11.3861262916667	10.977260575	11.2132455833333	10.7702977583333	10.71312705	10.3313573416667	10.4931364166667	10.2940071	10.7688463333333	10.135458025	10.9505347083333	10.6340233	10.4115783666667	10.7081516416667	11.012449925	10.9831460083333	10.625983	10.6997392583333	11.20219855	10.3641444833333	10.35378905	10.40167215	10.2225656916667	10.527258325	10.978750575	10.42200985	10.9571307916667	10.4990563	11.2085666916667	10.9266566	11.2240675166667	10.9527927166667	10.6352329833333	11.23878845	10.6738245166667	10.99905285	10.2318091916667	11.122077	10.4297158833333	10.5731419166667	9.70194608333333

AL138752.2	11.94150785	11.7847643958333	11.45289505	11.4667271041667	11.5818676666667	11.444061725	11.4859582416667	11.6938815833333	11.4991294458333	11.4690894583333	11.1773778416667	11.4322928958333	11.7476190875	11.6018922416667	12.0516662625	11.4061629208333	11.49174465	11.5543050958333	11.9122100125	11.5307643416667	11.4370846166667	11.7676929416667	11.2245952666667	11.291698725	11.5658625708333	11.7873536416667	11.5010464625	11.7021580083333	11.8719919916667	11.6057043791667	11.5785509583333	11.5658625708333	11.4190066583333	11.055823725	11.6034198958333	11.7733518416667	11.8247281625	11.7580672958333	11.5006540125	11.5179760333333	11.48267635	11.8467476416667	11.7424091583333	11.667786	11.734564075	11.874384125	11.8789535625	11.682501525	11.463323875	11.216369	11.5179760333333	11.7796809125	12.0587376333333	12.0323911166667	11.8525516125	11.871553175	11.7151199333333	11.7294616458333	11.8479303875	11.5335937	11.4451095666667	11.93026545	11.1048374	11.9672191583333	10.9763440083333	11.7310213041667	11.8143751416667	11.4513483541667	11.9222641625	10.9293807916667	11.5628336416667	11.5852804125	11.6604320041667	11.5043603416667	11.2614781875	11.5043603416667	11.7987540875	11.1936104166667	11.3373480833333	11.4205536416667	12.1821248958333	11.4641289291667	11.9699928333333	11.8925964	11.2795826	11.8160845	11.3913576541667	11.171236275	12.1136162	11.5321694875	11.4315418958333	11.4777015916667	10.6984661666667	11.7551390333333	11.5718834375	11.1548396208333	12.0271688	11.7829393041667	10.9481846166667	11.3237360666667	12.1352700625	11.80345385	11.7573846791667	11.4603484833333	12.1679331375	11.0998077208333	11.5307643416667	11.7072518541667	11.5893216041667	11.9194704541667	11.2566504708333	12.1975363375	12.3678730208333	11.878575025	11.9102808958333	11.41938205	11.1882787958333	11.5050811291667	11.8192935083333	11.0342853666667

DCAF10	11.94150785	11.7847643958333	11.45289505	11.4667271041667	11.5818676666667	11.444061725	11.4859582416667	11.6938815833333	11.4991294458333	11.4690894583333	11.1773778416667	11.4322928958333	11.7476190875	11.6018922416667	12.0516662625	11.4061629208333	11.49174465	11.5543050958333	11.9122100125	11.5307643416667	11.4370846166667	11.7676929416667	11.2245952666667	11.291698725	11.5658625708333	11.7873536416667	11.5010464625	11.7021580083333	11.8719919916667	11.6057043791667	11.5785509583333	11.5658625708333	11.4190066583333	11.055823725	11.6034198958333	11.7733518416667	11.8247281625	11.7580672958333	11.5006540125	11.5179760333333	11.48267635	11.8467476416667	11.7424091583333	11.667786	11.734564075	11.874384125	11.8789535625	11.682501525	11.463323875	11.216369	11.5179760333333	11.7796809125	12.0587376333333	12.0323911166667	11.8525516125	11.871553175	11.7151199333333	11.7294616458333	11.8479303875	11.5335937	11.4451095666667	11.93026545	11.1048374	11.9672191583333	10.9763440083333	11.7310213041667	11.8143751416667	11.4513483541667	11.9222641625	10.9293807916667	11.5628336416667	11.5852804125	11.6604320041667	11.5043603416667	11.2614781875	11.5043603416667	11.7987540875	11.1936104166667	11.3373480833333	11.4205536416667	12.1821248958333	11.4641289291667	11.9699928333333	11.8925964	11.2795826	11.8160845	11.3913576541667	11.171236275	12.1136162	11.5321694875	11.4315418958333	11.4777015916667	10.6984661666667	11.7551390333333	11.5718834375	11.1548396208333	12.0271688	11.7829393041667	10.9481846166667	11.3237360666667	12.1352700625	11.80345385	11.7573846791667	11.4603484833333	12.1679331375	11.0998077208333	11.5307643416667	11.7072518541667	11.5893216041667	11.9194704541667	11.2566504708333	12.1975363375	12.3678730208333	11.878575025	11.9102808958333	11.41938205	11.1882787958333	11.5050811291667	11.8192935083333	11.0342853666667

C11orf54	12.3636460083333	11.3979579666667	10.8767468833333	10.9397461666667	11.06778735	11.2638907416667	11.0146426833333	11.118056575	10.946906725	11.1095361333333	10.4955307333333	11.585466225	11.1409117416667	10.8258465666667	11.3169410666667	11.2262281166667	12.0969790916667	10.8900635166667	11.3410012666667	10.9884425416667	11.270439275	11.363165975	11.484727	11.3455869583333	11.494176975	11.958220325	11.0729886333333	11.0927898583333	11.5483812666667	11.1498219083333	10.6865081333333	10.8032590416667	10.9737165166667	11.780675575	11.8154196416667	11.8646362416667	11.0870314666667	10.8600934166667	10.58448395	11.126694875	11.1912222083333	10.9757948333333	10.994796725	11.2546673083333	11.368673525	11.005303025	11.1546552166667	11.0964662083333	11.0312303833333	11.2154273083333	10.8082438916667	10.8131866083333	10.64844015	11.4166255166667	10.8386147583333	10.7820632416667	10.878488075	11.3433838833333	11.244965975	10.8143165583333	11.06152365	10.4480363583333	11.6818913333333	10.5493449166667	11.31013205	10.6842469	10.98875735	10.5130948833333	9.95891725	11.1430808166667	10.17413685	10.9204831	10.6395867	10.4495527	10.2434985083333	10.6219118666667	10.8329994333333	10.521634775	11.4586326	10.7317611916667	11.8970046083333	11.4771109	9.508670075	10.7971951666667	11.2671696833333	10.3694253333333	10.3105309666667	10.834075425	10.9505347083333	10.82884605	9.90608706666667	10.0189542666667	11.1380175916667	10.5173180916667	10.6507559	10.409675875	11.674290525	10.2009594166667	11.4908711166667	10.7885218833333	10.24386405	9.990066925	10.4348946583333	11.3268344833333	10.6542751166667	11.9135839833333	10.5692188416667	10.991320675	10.849584125	10.9824445416667	10.5162662666667	10.0636311333333	9.76089629166667	11.608877475	10.0043835333333	9.83719281666667	11.5544661666667	11.1085089666667	10.9408814333333	10.1437039833333

AP001273.2	12.3636460083333	11.3979579666667	10.8767468833333	10.9397461666667	11.06778735	11.2638907416667	11.0146426833333	11.118056575	10.946906725	11.1095361333333	10.4955307333333	11.585466225	11.1409117416667	10.8258465666667	11.3169410666667	11.2262281166667	12.0969790916667	10.8900635166667	11.3410012666667	10.9884425416667	11.270439275	11.363165975	11.484727	11.3455869583333	11.494176975	11.958220325	11.0729886333333	11.0927898583333	11.5483812666667	11.1498219083333	10.6865081333333	10.8032590416667	10.9737165166667	11.780675575	11.8154196416667	11.8646362416667	11.0870314666667	10.8600934166667	10.58448395	11.126694875	11.1912222083333	10.9757948333333	10.994796725	11.2546673083333	11.368673525	11.005303025	11.1546552166667	11.0964662083333	11.0312303833333	11.2154273083333	10.8082438916667	10.8131866083333	10.64844015	11.4166255166667	10.8386147583333	10.7820632416667	10.878488075	11.3433838833333	11.244965975	10.8143165583333	11.06152365	10.4480363583333	11.6818913333333	10.5493449166667	11.31013205	10.6842469	10.98875735	10.5130948833333	9.95891725	11.1430808166667	10.17413685	10.9204831	10.6395867	10.4495527	10.2434985083333	10.6219118666667	10.8329994333333	10.521634775	11.4586326	10.7317611916667	11.8970046083333	11.4771109	9.508670075	10.7971951666667	11.2671696833333	10.3694253333333	10.3105309666667	10.834075425	10.9505347083333	10.82884605	9.90608706666667	10.0189542666667	11.1380175916667	10.5173180916667	10.6507559	10.409675875	11.674290525	10.2009594166667	11.4908711166667	10.7885218833333	10.24386405	9.990066925	10.4348946583333	11.3268344833333	10.6542751166667	11.9135839833333	10.5692188416667	10.991320675	10.849584125	10.9824445416667	10.5162662666667	10.0636311333333	9.76089629166667	11.608877475	10.0043835333333	9.83719281666667	11.5544661666667	11.1085089666667	10.9408814333333	10.1437039833333

TAF1D	12.3636460083333	11.3979579666667	10.8767468833333	10.9397461666667	11.06778735	11.2638907416667	11.0146426833333	11.118056575	10.946906725	11.1095361333333	10.4955307333333	11.585466225	11.1409117416667	10.8258465666667	11.3169410666667	11.2262281166667	12.0969790916667	10.8900635166667	11.3410012666667	10.9884425416667	11.270439275	11.363165975	11.484727	11.3455869583333	11.494176975	11.958220325	11.0729886333333	11.0927898583333	11.5483812666667	11.1498219083333	10.6865081333333	10.8032590416667	10.9737165166667	11.780675575	11.8154196416667	11.8646362416667	11.0870314666667	10.8600934166667	10.58448395	11.126694875	11.1912222083333	10.9757948333333	10.994796725	11.2546673083333	11.368673525	11.005303025	11.1546552166667	11.0964662083333	11.0312303833333	11.2154273083333	10.8082438916667	10.8131866083333	10.64844015	11.4166255166667	10.8386147583333	10.7820632416667	10.878488075	11.3433838833333	11.244965975	10.8143165583333	11.06152365	10.4480363583333	11.6818913333333	10.5493449166667	11.31013205	10.6842469	10.98875735	10.5130948833333	9.95891725	11.1430808166667	10.17413685	10.9204831	10.6395867	10.4495527	10.2434985083333	10.6219118666667	10.8329994333333	10.521634775	11.4586326	10.7317611916667	11.8970046083333	11.4771109	9.508670075	10.7971951666667	11.2671696833333	10.3694253333333	10.3105309666667	10.834075425	10.9505347083333	10.82884605	9.90608706666667	10.0189542666667	11.1380175916667	10.5173180916667	10.6507559	10.409675875	11.674290525	10.2009594166667	11.4908711166667	10.7885218833333	10.24386405	9.990066925	10.4348946583333	11.3268344833333	10.6542751166667	11.9135839833333	10.5692188416667	10.991320675	10.849584125	10.9824445416667	10.5162662666667	10.0636311333333	9.76089629166667	11.608877475	10.0043835333333	9.83719281666667	11.5544661666667	11.1085089666667	10.9408814333333	10.1437039833333

TGFB2	14.3186975416667	11.8174744333333	12.251683475	11.554144025	11.2826013166667	13.0700559416667	11.5349136916667	12.0197364166667	11.9368778583333	12.0852755916667	13.4267583666667	13.3987592083333	13.053017375	11.8070215583333	11.5181590833333	12.064434275	11.4752696416667	11.6080957416667	11.7932878	11.9443641	12.2675250666667	11.2756978833333	12.004381825	12.3688898083333	12.2311025	14.12159925	12.3012111583333	11.6099448083333	12.0533566	11.6252518666667	11.8658810166667	10.9812562333333	11.7445138833333	11.5994743583333	13.8376377666667	13.99785205	11.244965975	11.8713469833333	11.73203235	12.4377746666667	12.0697739833333	11.80438315	12.0551513666667	12.1732634166667	13.3278442333333	12.10103115	12.6220126	11.9670214	12.1865914583333	12.2706199833333	12.50431935	12.576483025	11.5276408333333	11.5000912333333	11.1542967583333	11.92557355	11.3820783333333	11.6558642583333	11.9259261416667	12.4176758916667	12.5935190666667	13.5279403916667	12.5320694166667	13.812858	14.2858034416667	13.2848396666667	13.5593280666667	13.57979325	12.8026986916667	13.7312525583333	13.1465914416667	12.5921909166667	13.054278175	13.5746400833333	13.159645225	13.7204745333333	14.4411655333333	12.6739472	13.5789450333333	12.8817875333333	13.370423875	14.1164578666667	13.21226365	12.6552566916667	13.3370287666667	13.2962436083333	12.40421355	14.4309788083333	12.2951630416667	13.3696487833333	13.2785213333333	14.1927345333333	14.204528175	12.6862224333333	12.9300978916667	12.0761170583333	12.2615766416667	12.0486377333333	13.8357694833333	12.6442138916667	12.5850501916667	12.616140275	11.2627928333333	12.0189741833333	11.912794275	12.62011135	13.7007366166667	12.2679354916667	12.8057256416667	14.3892469416667	13.27588995	13.853956425	13.05124085	12.1640360666667	13.7491981833333	11.6418554833333	14.80004595	13.577235475	12.5576279333333	11.7861402083333

CKMT1A	9.18832863333333	13.841729125	14.015040275	13.1674768	12.622549625	12.7406037583333	13.4359788416667	13.6605641666667	13.484103425	13.748287325	13.3114725833333	8.80499509166667	13.0933531416667	13.8743989166667	13.8972210333333	12.6734606	13.31441505	13.4407112833333	13.776083375	13.7272276083333	13.2273523083333	14.0001337166667	13.3003482666667	13.483275325	13.4737954	9.44959046666667	13.9727862416667	13.9576902	13.5984028583333	14.3397031416667	13.5843144666667	14.107174325	12.9340515083333	13.2898598333333	7.80170391666667	9.08644098333333	13.4298474333333	14.2533850083333	13.4943591	13.7820292333333	13.2744587583333	13.751113025	13.6675870333333	13.9612443083333	12.8912643	13.7902025333333	14.046645825	14.26427225	14.5735643833333	13.6000874166667	13.9433845083333	13.18921875	14.18015695	13.668458425	13.9119804333333	13.8528809083333	14.1343040416667	14.2810681833333	12.9139148666667	13.5304589833333	12.5754789333333	13.7692653916667	12.247084775	13.6265166916667	7.11730268333333	13.077594625	12.0905289333333	13.3567888916667	11.612663925	12.4091926166667	13.3508640083333	12.7081294916667	13.4026847	12.3301864333333	12.287955975	11.5531172416667	12.8339629333333	12.6336537916667	13.3501502333333	13.5141902666667	8.45255091666667	10.5014025666667	13.1152178	13.3227764916667	11.99822175	10.49790835	8.910111275	11.6659074916667	13.0960317	12.496720025	13.0732507583333	13.2027218583333	13.3390509833333	13.3053968166667	9.797655	12.3489029833333	13.653352825	11.1934390416667	12.7827098416667	9.59518965833333	12.2553940416667	12.762350675	12.835491775	13.44358445	13.9400026166667	13.2320017583333	12.2236021833333	13.0841853166667	12.7692841333333	12.2990201416667	12.7873683333333	12.672014175	13.0933531416667	9.47351875833333	13.1137653	12.9527510916667	8.17720695	11.8383549666667	12.9433935166667	12.7359047083333

MYCBP	8.65947190833333	9.70796235833333	9.29796166666667	9.80350678333333	9.487900075	8.80540845	8.42983770833333	9.52826018333333	9.81608086666667	9.21080935833333	9.177752275	7.74951601666667	8.81971978333333	9.52745255833333	9.83629188333333	9.24445845833333	9.64650925	9.73142488333333	9.18457413333333	9.32806993333333	8.821700275	9.283130225	9.1473568	9.6185904	9.22385636666667	9.53592761666667	9.56259116666667	9.5678162	9.14189448333333	9.7100974	9.29065144166667	8.90389271666667	8.9544846	8.73988864166667	9.55104401666667	8.87974118333333	9.31670963333333	9.70017130833333	9.705378475	8.833014675	8.87930013333333	9.59782546666667	9.1840561	9.56680634166667	8.9384783	9.71442065	9.41620731666667	10.1982859833333	9.388540625	8.333491425	9.38096979166667	9.43089169166667	9.04505583333333	8.90005820833333	8.84333919166667	9.19576385833333	9.73622053333333	9.31001445	9.63806365	9.34897708333333	8.2204362	8.214548775	8.77815989166667	8.52966328333333	8.861626075	8.63917994166667	7.65894785833333	8.151087675	9.05965051666667	8.17990050833333	9.25440600833333	7.85577905833333	8.26655316666667	8.33450988333333	9.09331330833333	8.95718403333333	8.16983206666667	9.42163085833333	8.439937175	8.39209266666667	8.49593345833333	8.24270349166667	9.02085209166667	9.29384936666667	8.576058475	8.88688890833333	8.31765478333333	8.34422473333333	8.21153401666667	9.06838428333333	8.74819925	8.14799835	8.71882579166667	8.60126088333333	8.13819046666667	7.9977282	8.603908075	8.4841298	9.02747416666667	8.11250586666667	8.34021045	8.247580125	8.966534925	9.01698551666667	8.955351225	9.1237399	8.83443758333333	10.0321890166667	8.28289991666667	7.35447710833333	9.07065694166667	9.11317438333333	8.53187930833333	8.39419055833333	7.63210018333333	9.06745036666667	8.58538408333333	8.93461839166667	9.79628703333333	8.88831745833333

AC093155.3	8.18205496666667	7.698105675	7.967285625	8.06026415833333	8.08185559166667	8.10955528333333	8.31305365833333	7.48985504166667	8.475275825	7.9703466	8.38023938333333	8.44239195833333	8.364642925	7.90991645	7.649654175	7.74145285	7.86896935833333	8.1277546	7.67926596666667	7.71678190833333	7.7528692	7.55016986666667	7.25980210833333	7.84588375	7.441553775	7.70926514166667	7.737502925	7.66891875	7.93586978333333	8.08240869166667	7.56268353333333	7.86012560833333	8.631828525	8.13716783333333	8.20175241666667	7.97557605833333	7.73920148333333	7.99186004166667	7.920590025	7.60136925	7.46853124166667	7.5654861	7.40950549166667	7.80170391666667	7.80329328333333	7.09680668333333	7.51200123333333	7.874246775	7.25540121666667	7.93973534166667	8.1963928	7.72905521666667	7.72023940833333	7.92167954166667	7.9760917	7.3694039	7.51638066666667	7.85201126666667	7.92654975833333	7.544616	8.0773066	7.98087950833333	8.09223929166667	8.1439842	8.05619188333333	8.00412230833333	8.48368870833333	7.8961731	8.24315470833333	8.62084126666667	8.39869986666667	8.16584395833333	8.408638475	7.87964591666667	8.16633889166667	7.63498546666667	8.07622910833333	7.83618584166667	7.62307443333333	8.10330963333333	7.32089796666667	8.07050834166667	7.93544106666667	7.928618975	8.31619925	8.24163635833333	8.18527721666667	8.09750283333333	8.16633889166667	7.82231499166667	8.10111541666667	8.10268665	8.32264421666667	8.21304901666667	8.52731504166667	7.816692275	8.443897425	8.368580775	8.22099113333333	7.85633409166667	7.9999538	8.29681115	7.84267175833333	7.71678190833333	7.72249438333333	7.92224445	7.96526580833333	7.86062830833333	7.875176525	7.62534816666667	7.95255818333333	7.71354716666667	8.46350844166667	7.71409900833333	8.07622910833333	7.70256570833333	8.17406114166667	8.28495673333333	7.661614575	8.01595076666667

KHDC4	14.9148418416667	14.494740525	15.462044625	15.2784907166667	15.6293082	14.9045675833333	15.9859723583333	14.37416785	15.4985918916667	14.7596386666667	14.89930145	16.1274635083333	15.6652002083333	14.8578939833333	14.8919189333333	15.5221336416667	13.53134155	15.8383325666667	15.3982642333333	14.948326475	15.2399573083333	14.6367172916667	15.2045357166667	15.5603668666667	14.6527873333333	14.948326475	14.4791297416667	14.5810673083333	14.168404425	15.229747525	13.9400026166667	15.3056912416667	15.0615765666667	15.0803306416667	14.4262315333333	14.7166750666667	15.5975780166667	14.1099143166667	14.5214642083333	14.9752180916667	14.4983330666667	14.2160518416667	14.6883693	14.8548535916667	15.25397645	14.2334791	14.5195733916667	15.5458909083333	14.9359352083333	15.444211725	14.8865870416667	15.7414894	16.1648856916667	15.9673534416667	15.4712092083333	14.4328794	15.215611525	14.83890155	15.2687677	14.3283993416667	16.5882325166667	16.5621689666667	16.5621689666667	16.4447326166667	16.5621689666667	16.7607414083333	16.6938615583333	16.6392399583333	16.6649415583333	16.89429	16.7226812583333	16.6392399583333	16.9349993833333	16.5317674916667	16.6392399583333	16.6649415583333	16.8229118	17.1992909916667	16.5882325166667	16.6649415583333	16.5317674916667	16.5317674916667	16.5317674916667	16.4671769583333	16.3302858416667	16.49542085	16.4671769583333	16.39493105	16.5317674916667	16.4671769583333	16.5317674916667	16.37080095	16.4447326166667	16.39493105	16.5621689666667	16.49542085	16.8229118	16.5621689666667	16.2495335833333	16.39493105	16.613425425	16.5621689666667	16.4671769583333	16.2495335833333	16.2942187166667	16.5317674916667	16.4671769583333	16.0573941333333	16.5317674916667	16.39493105	16.4671769583333	16.2339961416667	16.2339961416667	16.1391348	16.1022656	16.3302858416667	16.37080095	16.6392399583333	16.613425425	16.5882325166667

CIRBP	5.81313270833333	7.212178575	6.46923394166667	6.24783014166667	7.74951601666667	6.485693525	7.49991619166667	6.44217180833333	7.173894	7.1744649	7.066499225	6.48151946666667	7.52953641666667	6.74346625833333	6.94312051666667	7.064683475	5.83208956666667	7.44607933333333	6.93287185833333	6.93352621666667	6.41270695833333	6.72653388333333	6.901873575	6.59815521666667	6.60068861666667	6.48785131666667	6.95156631666667	7.07528308333333	7.375280375	7.37059144166667	6.627072075	6.82240235833333	7.44317530833333	6.67096843333333	6.79468460833333	6.17635683333333	7.62089398333333	7.31025495833333	7.26626465	7.03569371666667	7.35564645	7.596603625	7.057705175	7.09162461666667	6.31021560833333	7.21451231666667	6.76187970833333	7.29788705833333	6.86012671666667	7.265200275	7.019638175	7.26747879166667	7.32974516666667	7.78276558333333	7.15020035833333	6.7130388	6.63866813333333	7.53069666666667	6.56579801666667	6.3460148	5.93036776666667	6.49247578333333	5.98999241666667	5.94625251666667	6.85677963333333	6.877100425	7.567156725	5.54779774166667	6.23159324166667	7.2313303	6.46705624166667	6.56292778333333	6.7503122	6.33714610833333	6.82946754166667	5.67897566666667	6.4808852	7.24193815	6.39152516666667	6.31952470833333	6.55223534166667	6.32988615	6.61809366666667	5.98559190833333	6.869498575	6.35274666666667	6.25518099166667	6.24858409166667	6.755806875	6.18247753333333	6.2858525	5.76923416666667	6.47216713333333	6.34005863333333	6.33645039166667	6.58499179166667	7.01901523333333	6.67919664166667	6.99155748333333	6.53809915833333	6.26211881666667	7.53624973333333	6.92168505833333	6.30714161666667	6.04700228333333	6.60761675	6.12040304166667	6.50650844166667	6.37713004166667	6.56292778333333	6.17390685	6.24783014166667	6.11047193333333	6.68200588333333	6.70667168333333	6.54933985	5.845937625	6.585636975	6.66175125	6.573073

MGST1	11.327862475	13.765060275	13.577235475	13.781003275	14.2503770416667	15.0306120416667	13.8983181916667	13.7361743	13.077594625	14.1927345333333	13.3179373333333	12.382866125	13.4999239583333	13.5410590833333	13.799269175	13.7093807	14.26265275	13.7074542166667	13.5076042	14.3992922416667	14.0377493833333	13.6098239583333	14.2936594416667	14.274700225	14.1737580083333	12.4567650666667	13.98513315	14.3628995916667	12.8218942166667	12.8107678333333	14.3692316583333	12.3122487083333	13.418751775	12.813963275	11.5305817083333	11.189465025	13.7443409166667	13.7452882916667	14.1578289583333	14.26427225	13.85816885	14.4633924916667	13.7781265916667	13.98513315	12.1861275083333	13.9197186416667	12.4922747583333	13.8778632416667	13.532131425	13.489460875	14.0734299166667	13.4449599	13.9433845083333	12.731765875	12.97999865	12.6297371916667	14.4775829916667	14.0899292916667	13.5363675083333	13.322101775	14.7874202666667	14.2219861583333	13.2975446	10.0189542666667	13.9717922666667	11.4115295083333	14.1256024333333	13.1204429833333	14.4109315833333	12.9842314416667	14.8236926166667	14.7143451416667	8.58808473333333	14.085975275	12.919046025	10.01936935	11.831349425	13.3623548	14.3514380333333	9.21398845	11.680364275	7.767746725	13.158283725	10.8491534833333	10.176297425	12.3794027166667	10.6860936833333	11.66214955	14.9045675833333	11.2442138	9.25672935833333	14.046645825	14.30297575	14.3582404416667	13.5401929916667	14.3860372083333	14.2000424583333	11.55593635	9.65184145	12.3983699916667	13.9818544666667	11.4195840166667	9.04742353333333	13.10396855	14.127909425	12.4922747583333	13.5535340083333	9.50539336666667	8.836922375	13.79195525	12.8387693166667	13.2545964166667	11.6425911333333	12.9444160916667	13.100692275	14.3045490583333	12.6528003583333	12.8817875333333	14.2842943416667	12.5676300666667

FAM78B	7.17914503333333	6.67377979166667	6.23620956666667	6.897275725	7.59083331666667	6.25099584166667	6.92717863333333	6.95860073333333	7.23609219166667	6.757758475	7.58465230833333	7.96301623333333	7.71798951666667	7.378725775	7.6798049	6.38240346666667	6.57427455833333	7.39450265833333	7.039355275	7.448314425	6.93287185833333	7.521666525	6.27482028333333	5.72573654166667	6.60826071666667	7.01553179166667	6.95358578333333	6.48217831666667	7.52896074166667	7.01495000833333	6.76843211666667	7.16232548333333	7.34718618333333	7.03253850833333	7.63328161666667	7.75009851666667	7.19697114166667	6.987286825	7.0369697	7.18281396666667	6.12040304166667	7.13728840833333	6.95288179166667	6.54638811666667	7.01211946666667	6.24607838333333	6.8478471	7.204487875	6.62907710833333	6.380135525	6.53809915833333	6.92785069166667	7.76512125	7.26626465	7.11920136666667	7.01211946666667	7.19137265833333	6.81508890833333	6.87589898333333	6.98280363333333	7.35396276666667	7.41466188333333	6.72705409166667	7.19951121666667	7.58465230833333	7.39926451666667	7.26626465	7.24887754166667	7.435271425	7.483605675	7.86012560833333	7.76293401666667	7.81246279166667	7.460763125	7.5090854	7.23028210833333	7.246381175	7.28500960833333	7.16422769166667	7.38522528333333	7.76660259166667	7.96885115833333	7.2162874	6.46055301666667	7.09043023333333	7.41942373333333	7.1809933	7.10123053333333	8.16389916666667	6.70390775833333	7.53185565833333	7.1355085	7.00049489166667	7.16358325	7.65663390833333	7.102506125	7.29788705833333	7.190008575	7.56380908333333	7.13850381666667	6.52781796666667	7.788158325	6.9953871	6.60270946666667	6.95924915	7.33375654166667	7.3019376	7.08866475	6.92317820833333	5.65251761666667	6.82038475833333	6.872774725	7.09162461666667	7.16232548333333	7.72905521666667	6.9569431	7.48184465	7.40898444166667	7.27891691666667	7.66443915

PLSCR4	13.0483120583333	11.7487231	11.6252518666667	11.3324973416667	11.4122968416667	13.16545515	12.3219919916667	12.386493225	12.206579925	12.4198120333333	12.0596630083333	13.6969617	12.938273025	12.386493225	11.4364638833333	12.87462785	12.1852414666667	11.5027335583333	12.1839395083333	12.6876920416667	12.5907074583333	11.544719375	12.5628225833333	12.0144513	12.10269775	13.4127020416667	12.5641864083333	11.7913817166667	11.2650373666667	11.917277775	12.0881187916667	11.8947334916667	12.4795096083333	11.5400258416667	13.2075024333333	13.05124085	11.5204866583333	11.941310725	12.2528148916667	12.24842175	11.8386923083333	11.7568591416667	11.8484879416667	12.063617625	12.202055425	12.0108213333333	11.918121775	10.478660175	11.683026175	12.4046195166667	12.0388164916667	11.328583825	10.9174580583333	10.8176392916667	11.0633247666667	11.4173578916667	11.767528775	11.4843617833333	12.2336399666667	12.0717905916667	12.6098037416667	10.8361133666667	10.9719667416667	10.976899425	13.0276586166667	11.6209481083333	11.1858855333333	12.212911825	10.36145645	12.503844475	11.83053445	10.043884875	10.4394323833333	12.4101428	12.552088775	12.229824125	12.6557058416667	12.0486377333333	11.588039275	11.6558642583333	12.5245839	12.5048403	10.6914536333333	11.2729625666667	11.5356555333333	12.416791375	10.7217139416667	12.2345932083333	11.7470404	13.0548490666667	11.6974443166667	10.6621925666667	11.634108225	12.0950052666667	10.1808367916667	10.4195860916667	9.72033719166667	10.1100737	10.5001925666667	10.4024778916667	12.05687395	11.63482985	11.8422473333333	11.4446261583333	11.9781875416667	11.4696992083333	11.631804775	9.6428903	10.9054446583333	11.96822585	11.8596015	12.48982835	9.98838416666667	8.82920276666667	10.5204657833333	10.1895353083333	11.9085787666667	11.9649178666667	11.6655385333333	12.4917329166667

PON2	12.5145301166667	11.37567555	11.2504840916667	11.2726256666667	11.63482985	12.265420025	10.6865081333333	11.3683256166667	11.2664173916667	11.6536243916667	11.8838190166667	11.9686139666667	11.827759775	11.2399027333333	11.33033725	11.502358525	10.8443256416667	11.5606001833333	11.2811678666667	11.4038169	11.6234420916667	11.0815307083333	11.8646362416667	11.3867866583333	11.6401085333333	12.4627799166667	11.3025465416667	11.268229675	11.9052300666667	11.3986748166667	11.3972232666667	11.1520832083333	11.1956589833333	11.2356259583333	12.3956670583333	12.5865453166667	11.7120196166667	11.36200305	11.49602405	11.5204866583333	11.1129059916667	11.2121826083333	10.674973575	11.3015343416667	11.6163034833333	11.3336047583333	11.5994743583333	11.5820604666667	11.4115295083333	11.3423558	11.0120115833333	11.1926823916667	12.04824535	11.3900978416667	11.3239342333333	11.2521123083333	10.5945390166667	11.1937817916667	11.3809445833333	11.1517300916667	13.6411556333333	12.0112152666667	12.1620485833333	12.8522002333333	12.6381216166667	12.4692673166667	12.8996243416667	11.4824869416667	11.7505625666667	11.9971131583333	12.3046936083333	12.988893075	11.6929484666667	11.9017197166667	12.8889747333333	12.8350142416667	12.4759771583333	12.145884225	11.824518825	11.6175120416667	12.19191635	14.31061245	12.2611765333333	12.7999144166667	11.4496634833333	12.4141455666667	12.202055425	12.237060875	12.135071975	12.1686155916667	12.0510805083333	13.441409425	12.5226496333333	12.2011382666667	12.4903405333333	12.1018588083333	11.4427661583333	12.6336537916667	12.4412440416667	13.5484254	12.7918033916667	12.210372725	14.0174773166667	12.4355627	11.7035007416667	11.63218295	12.1513649666667	12.0533566	12.1587642333333	12.762350675	12.6084193833333	11.9322368833333	11.6522306833333	13.9389915916667	12.5667535083333	12.37676025	14.8966343583333	12.3479516833333	11.88924055	11.9188907916667

NFKBIA	15.7486260916667	14.15404765	13.9197186416667	13.6133055333333	13.2905134666667	13.9433845083333	13.92725075	14.3992922416667	13.3853576833333	13.6019439833333	13.7093807	14.0087806166667	14.1698024416667	13.50161185	13.8060720916667	14.21037625	14.0950375583333	15.2611193416667	13.756296825	13.4565571916667	13.7372005583333	13.4950898916667	13.738346	14.2245923416667	14.1152258583333	13.6183502333333	13.2155282	13.98513315	13.620993375	13.9600657833333	14.6023913166667	14.0526804666667	13.9003358333333	14.2983229333333	14.6410690916667	13.5401929916667	13.238016175	13.4171665166667	14.0694923916667	11.6602573333333	14.364637875	14.4527902	14.6161567166667	13.002567275	13.5157896583333	13.4611203333333	13.3959354916667	13.6000874166667	14.0174773166667	14.102034475	14.5984675666667	14.2087842916667	14.4177528166667	12.7516120833333	13.8766797166667	13.9422518833333	14.036419	14.241033875	14.113875875	13.3437233083333	13.4974205	13.40102065	14.2000424583333	14.2145397416667	14.3431664333333	14.6883693	14.6161567166667	13.7940225416667	14.8699486666667	14.120388025	13.7404495916667	14.0669942916667	14.2260493166667	14.0540171833333	13.9518039333333	14.1329416	13.171325425	13.8992947833333	14.5449579333333	14.35633825	14.1427380833333	13.8898465333333	14.6611477166667	13.986279475	14.18015695	14.269993075	14.7166750666667	14.2533850083333	13.08103315	13.799269175	14.4791297416667	14.1709219416667	13.4366933666667	14.045461125	13.7771113166667	14.4479745916667	14.3676663333333	14.4377971083333	14.7293250666667	15.6355001416667	14.29207405	13.7640679416667	13.5851912666667	14.9727774916667	14.6284460166667	14.4633924916667	14.72730925	14.5735643833333	13.50262465	14.3514380333333	14.451178225	13.7553717	15.0306120416667	15.1800900333333	14.0734299166667	14.1764195333333	13.492075675	14.34486635	13.8357694833333	14.5753036916667

ILDR2	9.68825589166667	7.38699095	7.53449101666667	7.16490981666667	7.89243905	7.73803474166667	8.1873246	7.68541283333333	8.06812029166667	7.41352716666667	7.39222043333333	8.82920276666667	8.391097025	7.61865353333333	7.38233871666667	7.55609866666667	7.2162874	7.704155275	7.6157713	7.82845124166667	7.690465575	7.41243761666667	7.314288875	6.987286825	7.40950549166667	10.2609607666667	8.1455177	6.71502808333333	7.05585056666667	7.70926514166667	7.58854008333333	7.10054488333333	7.62307443333333	7.57673284166667	10.283076325	10.315132175	7.0596334	7.7783407	7.192539525	7.27593380833333	7.06888018333333	6.88940976666667	6.97159590833333	7.34385484166667	8.43143401666667	6.96906025833333	8.111985825	6.780385325	6.92916504166667	6.850945825	6.68859711666667	7.34210711666667	7.937534725	7.05651791666667	7.3032043	7.488079225	7.90528599166667	7.29474379166667	7.02704855833333	7.219834	8.98751653333333	8.35633766666667	6.679903875	8.06606233333333	9.446046	7.29233335	7.960228575	8.611944975	7.78606559166667	7.826430025	9.0826061	8.51081288333333	7.94491850833333	9.00507715833333	8.39013935	9.11174605833333	9.27125073333333	7.61062251666667	7.45098580833333	8.859115725	9.84242584166667	10.5573126666667	7.53565475833333	7.34892271666667	9.297473225	10.61002135	9.18880384166667	7.99131585833333	7.92964085	8.576058475	8.23767453333333	7.91046618333333	8.06812029166667	8.39869986666667	7.19320405	7.48698644166667	6.88458655833333	10.3972128	7.49219983333333	8.4178821	8.22981704166667	8.33655620833333	6.372760025	6.57427455833333	7.3019376	8.21622873333333	9.03950406666667	6.31469075	8.45556428333333	7.66500309166667	7.582967825	7.65996385833333	7.54282731666667	7.69651605833333	8.17555483333333	7.81947101666667	8.85110639166667	6.98410809166667	6.54514473333333	8.230344325

NAALADL1	9.93000265833333	8.53482063333333	8.647921775	8.29630441666667	8.23909885	8.35685346666667	8.09121131666667	8.355767175	8.48950848333333	8.88496090833333	8.83154074166667	8.77628200833333	8.15758866666667	8.351126275	8.63344703333333	8.722672	8.49496250833333	8.354234475	8.6667612	8.64006809166667	8.665331325	8.026220775	8.75743711666667	8.36515990833333	8.70547134166667	9.74305075	8.83829725	8.51528900833333	8.78264749166667	8.5034354	8.57940885	8.39419055833333	8.08807009166667	8.277602525	10.0226518416667	10.0171966666667	8.758367425	8.783965475	8.38470655	8.54268981666667	8.62834993333333	8.54547846666667	8.46771705	8.79293039166667	9.148762375	8.96455003333333	8.88882345833333	8.251367875	8.73019295833333	8.375514	9.2639574	8.19294795833333	8.48460361666667	8.27654828333333	8.6396011	9.1840561	8.21304901666667	8.551483425	8.89174859166667	8.91365106666667	8.4444208	8.17990050833333	8.13766550833333	8.15860931666667	7.78767925833333	8.351126275	8.64890015833333	8.56366318333333	8.14660076666667	8.36292435	8.86999053333333	8.30804240833333	7.71862859166667	8.779206475	8.2260001	8.80353201666667	8.16684950833333	8.67503203333333	8.2053418	8.17090974166667	7.68098021666667	7.35503378333333	8.33398970833333	9.30275641666667	8.25292731666667	7.45160013333333	7.17735376666667	8.31150115833333	6.99412739166667	9.09878833333333	8.21353541666667	7.801197125	8.12489341666667	8.18422995833333	7.37928605	7.33375654166667	8.00581294166667	7.83125643333333	7.7483928	7.967285625	8.013764525	7.441553775	8.094406525	7.93973534166667	7.87562194166667	8.72037605	9.12606195	7.85258474166667	8.709872025	8.432956925	8.69025928333333	8.377106525	8.19188733333333	8.30382858333333	8.511336025	8.07152965	8.51528900833333	8.48569109166667	7.964195775	8.27440479166667

LRRC4C	4.61010935833333	4.138474425	4.08322809166667	4.258212325	4.31516280833333	4.20479785833333	5.106111275	4.72324775	4.28423139166667	5.44644633333333	4.30828979166667	4.48330838333333	4.2360195	4.32198345833333	4.25387103333333	4.222209325	4.669520575	5.35983450833333	4.64133359166667	4.645027875	4.26972968333333	4.13540660833333	4.49602461666667	5.22594001666667	4.81947958333333	4.138474425	4.54769205	4.72414796666667	4.82464924166667	4.48717731666667	5.17603111666667	4.69425546666667	3.84700126666667	4.34698491666667	4.42980535	4.94778381666667	4.67067994166667	4.644124975	4.802114875	4.77272485	5.18519845833333	4.65793555	5.0088772	5.73491465833333	4.19810324166667	5.13634166666667	5.33234265	5.201606925	4.60382705833333	4.91206978333333	4.8912062	4.557709	3.93897055	5.557624025	4.46100053333333	4.883731825	4.73720489166667	4.9406758	4.48432390833333	4.95322815833333	3.881892575	3.976955525	4.39557236666667	4.10724728333333	3.84584678333333	3.899834175	4.128952175	4.490204975	3.76094594166667	4.03660776666667	4.12677783333333	4.003549775	3.92586006666667	3.74274349166667	3.644387875	4.087323525	3.94009973333333	4.06389798333333	3.85170159166667	3.98152069166667	4.32010620833333	3.85883090833333	4.480628975	4.37710720833333	3.867233575	3.612188525	3.824093125	4.68789791666667	4.37710720833333	4.13540660833333	3.9661014	4.576370125	4.09074995833333	3.82560103333333	3.975999075	4.01216488333333	4.07882345	4.38930708333333	4.19720234166667	5.09317755833333	4.47367345833333	3.534267775	5.018425275	5.329558875	4.4227704	4.062876875	4.78900190833333	6.769663225	4.36388495833333	6.72249205833333	4.71016199166667	5.63443499166667	4.67574021666667	5.14718730833333	4.22036293333333	4.46293625833333	4.23901671666667	4.68073703333333	4.81485295	3.88770253333333

CRYBG2	9.94294464166667	13.7949722083333	14.1152258583333	13.9625123916667	14.0174773166667	11.666692125	13.66335695	13.751113025	13.6338665166667	13.1896013625	12.8901186583333	10.46260805	12.928953425	14.1886825416667	14.1752017916667	12.937710825	9.54880464166667	14.0938637416667	14.1656626833333	13.8428912916667	13.5124524166667	13.711942125	13.4474018083333	14.0592017583333	13.591559975	10.0031143833333	13.823045075	13.453508825	13.4222455	13.9119804333333	14.2763428833333	13.66335695	13.3285445333333	10.20309965	9.74904354166667	9.79362293333333	13.7640679416667	14.1815548333333	14.3221229083333	13.5295226416667	13.9422518833333	14.3860372083333	14.045461125	14.0201742416667	13.0626219916667	14.044199175	14.236432725	14.5322324833333	13.8307559916667	13.3545785083333	13.9906723833333	14.6367172916667	13.9250365	14.277828625	13.7372005583333	14.0100072833333	14.419240975	14.0113488	14.0250042833333	13.6062775083333	11.4288195833333	10.988072975	12.095758	12.6008947166667	10.4348946583333	12.0072469333333	11.1284533666667	11.654339925	11.292593525	11.3889846416667	11.4887131666667	11.311125675	12.4390650166667	10.946906725	12.6402462916667	11.5432980083333	10.872956825	11.7928828083333	12.2947323416667	11.947152875	10.315132175	10.25189245	12.3351872666667	11.2907157	12.0017085416667	11.04233505	10.7383339416667	11.6675947833333	9.911212725	10.6465456666667	11.85384455	11.9793031666667	11.2707625416667	12.1291180083333	11.4052792333333	12.1071523416667	11.284173425	11.3642682416667	12.4163683083333	11.2247431833333	11.002443975	11.3896934	10.8243180916667	12.3347946666667	12.642703875	11.5188708083333	12.3236478416667	12.65465995	12.136374875	11.4934089166667	11.5710069666667	12.580271675	12.5344122083333	11.5483812666667	11.66214955	12.9876385	10.4664769916667	11.9804777666667	12.8454667083333	11.30084235

MANBA	13.3151588	12.53019765	12.587916575	12.3160821666667	13.2725108916667	12.7339168166667	12.5734875583333	12.7416307	12.5585076166667	12.9346936833333	13.0031865833333	13.248853375	13.0708076333333	12.6709351583333	13.05962405	12.57699495	12.9472642583333	12.9715821666667	13.18402475	12.798152025	12.9918297166667	12.5084928916667	12.8549658583333	12.8398240166667	13.0301413666667	13.42164015	12.837133975	12.9007979916667	13.0554708583333	12.8404071916667	12.865109125	12.5221926333333	12.9224811	12.9412313583333	13.2683617333333	13.5435554666667	12.799384025	12.792898125	12.761853925	12.6975412416667	12.9929816333333	12.6694372	12.6744586916667	13.08233985	13.057870725	12.7339168166667	12.9300978916667	12.7807006083333	12.7216091916667	13.0494144333333	12.3961090083333	12.9659499916667	12.8947866166667	12.9836190416667	12.8376576083333	12.8323870333333	12.87960645	12.8549658583333	13.2027218583333	13.00071955	13.3897269	13.67475365	13.71864895	13.26088015	12.9924504416667	13.3451920333333	12.7807006083333	13.2434965	13.7093807	13.4127020416667	14.1413614333333	13.5164110666667	13.9055869333333	12.7201162666667	12.9971815666667	13.5043698416667	13.230641375	12.8624048833333	12.8268431583333	12.648701825	13.171325425	12.703617625	12.4840896416667	13.2538590583333	13.52214195	13.20077725	13.0620198083333	12.9266166166667	13.4375531583333	13.263493375	12.9552000333333	13.0841853166667	13.2711737166667	13.1210793083333	13.6320213916667	12.92082965	13.7670768833333	12.6297371916667	13.264123675	13.5197355333333	13.0590979083333	13.6338665166667	12.9042883333333	12.910549725	12.9352563083333	13.2912832916667	13.0877248833333	12.6626629166667	13.5034733	13.229948375	12.6336537916667	13.3912051666667	13.1753140083333	13.1078475833333	12.9756001333333	12.0827580166667	13.3582007666667	12.9948642333333	12.9540039916667	12.695073575

SGSM2	11.1099310583333	10.66752385	10.6625963333333	10.7217139416667	10.4613867166667	10.0898576	9.81483478333333	10.2932251333333	10.4024778916667	10.128737475	10.3078222333333	10.315132175	10.1601611333333	10.8361133666667	10.7363658166667	10.3502819916667	10.6360463166667	10.6222826083333	10.8767468833333	10.3510926083333	10.51584625	11.4511549916667	10.615482725	10.66752385	10.2877771083333	11.14669605	10.5477462083333	10.6914536333333	10.9564158416667	10.8878262416667	10.8172624166667	10.4994687166667	10.4176619666667	9.99125556666667	10.9828230166667	11.1767887583333	10.5743323083333	10.627804175	10.6607214666667	10.4986956916667	10.5104189666667	10.8544918	10.743245175	10.500980975	10.7748204	10.9284040666667	10.8165123833333	10.6711164583333	10.6979535583333	10.8431589583333	10.1417124166667	10.3852198833333	10.962553725	11.2240675166667	10.9961958083333	10.7718274166667	10.6819563666667	10.5760591583333	10.7081516416667	10.4759657166667	10.6637664333333	9.96824564166667	10.93390845	10.7325441416667	10.2838388166667	10.1270651166667	10.1437039833333	9.59518965833333	10.6865081333333	10.2034843416667	9.81007735833333	9.80535213333333	9.96227999166667	10.043884875	9.439564175	10.0972464083333	10.46219945	10.27531875	11.0574717833333	9.64074395833333	11.0504310083333	11.1678068583333	10.1906714166667	9.95059544166667	9.78329576666667	10.2591668583333	10.9230393083333	10.1246349583333	10.3599013416667	10.7904047166667	10.5288525083333	9.81686935833333	9.95767555	10.0844426833333	10.2501973166667	9.35954015	11.395075525	10.264650175	10.1755237	10.2838388166667	10.2009594166667	9.7997523	9.84956451666667	10.4830527666667	11.0390903416667	10.1621245583333	11.4616143	10.054920625	10.1180361833333	10.24707065	10.6285410083333	9.661950625	10.7057641416667	10.87258615	10.3325548083333	9.97043543333333	10.501807225	11.62492935	10.9684950083333	9.864777125

ST8SIA4	4.383064975	4.03339724166667	4.061836425	4.19810324166667	3.644387875	4.03764378333333	3.442586375	3.82289100833333	3.5657117	3.7942322	3.56445749166667	3.4228733	3.63913164166667	4.23196318333333	4.149223425	4.44254745	4.611084475	3.842520875	4.57137348333333	4.383064975	3.82817183333333	3.96359718333333	4.30130591666667	4.84506578333333	5.60319860833333	5.0100817	4.685722125	4.55184448333333	4.506429475	4.245391125	5.231579	5.14401780833333	3.74515126666667	4.24624565833333	4.46293625833333	4.47474535	4.21395365833333	4.47861730833333	4.91851714166667	4.644124975	4.823565325	4.508464425	4.72828980833333	5.400096325	4.06389798333333	4.95322815833333	5.13961736666667	4.93436960833333	4.443420775	4.792098225	4.683662975	4.3987315	3.75232660833333	5.258661775	4.32198345833333	5.07445225	3.88647775833333	5.27629945	4.93552183333333	5.77716973333333	3.782834	3.630124675	4.64133359166667	3.971604825	3.92815261666667	3.71049449166667	3.92815261666667	3.44595958333333	3.77672200833333	4.108372	3.76208641666667	3.78824818333333	3.36709359166667	3.68857591666667	3.59213996666667	3.971604825	4.26116580833333	6.52344795833333	4.15011734166667	4.11987200833333	3.55452118333333	3.52884109166667	4.20571450833333	4.46516671666667	3.74515126666667	3.4640341	3.6774476	4.45902586666667	4.12106518333333	4.07245948333333	4.22505026666667	4.81716043333333	4.15011734166667	4.40687995833333	4.44161573333333	4.40180795833333	3.86501250833333	4.34795044166667	3.75459249166667	4.957419525	4.26116580833333	3.478643875	4.652676825	5.078596225	4.19346535	3.9661014	4.59683404166667	6.1536546	4.20479785833333	6.47456359166667	4.92991125833333	5.126264825	4.563953975	4.79318954166667	4.98106579166667	4.685722125	4.747391	4.81485295	4.740346475	4.53824951666667

FILIP1L	5.7886317875	6.43325290416667	7.36184000416667	6.24485660833333	7.49137522083333	6.82918142916667	8.06734486666667	7.5415167125	7.51338165833333	6.86986289166667	6.85069928333333	8.16556907083333	8.24391430833333	7.4530872	6.80886005	6.29861988333333	5.66998142083333	8.56934383333333	6.46389307916667	6.81942682083333	7.35596242083333	6.89232578333333	6.88285323333333	6.96685253333333	4.64673680833333	6.43049987083333	5.97304986666667	5.6794805	6.52029283333333	5.538481225	5.63795104583333	5.13486149583333	6.24150231666667	6.48607476666667	4.6116512375	4.08791972083333	5.26839657083333	5.350279675	5.7344837875	5.66792823333333	4.80584670833333	4.61678248333333	4.59733981666667	4.97227905833333	4.8734586125	5.6794805	5.26945706666667	6.29551622916667	5.40801585416667	5.17426900416667	4.57189149583333	5.96736761666667	7.8749512125	6.08645415416667	6.0695613	5.031764525	7.61729026666667	4.34746767916667	4.88865945	5.3271171	8.48978630833333	6.92818722916667	5.24653454583333	7.36787195833333	4.9754440625	7.96813411666667	7.46825643333333	8.03030704166667	5.6669615125	7.33289649166667	7.7663413125	7.98166808333333	8.1097805875	6.84606387916667	7.4303946125	7.47529534583333	6.93257215416667	7.29988426666667	7.2976049625	7.11179178333333	8.95240457916667	7.03166697916667	6.30447276666667	6.172701625	7.40107549166667	8.00877005	8.50678275	6.33304742083333	8.59076590416667	6.29318526666667	6.79094316666667	7.0715031875	5.31875691666667	6.63543665	7.9748274125	6.55103600416667	7.4936125	6.37073757083333	7.5551899	6.11000845	7.30464287916667	6.76013514166667	7.66361127083333	5.81783112083333	5.29832429583333	6.957264825	6.69679296666667	5.61689452916667	6.9525504625	5.46699008333333	5.05849976666667	5.49183984166667	5.72030935	6.2854684375	6.87817127083333	6.4318614125	6.20696075	6.44924722916667	5.79161227083333	7.47925860416667

CMSS1	5.7886317875	6.43325290416667	7.36184000416667	6.24485660833333	7.49137522083333	6.82918142916667	8.06734486666667	7.5415167125	7.51338165833333	6.86986289166667	6.85069928333333	8.16556907083333	8.24391430833333	7.4530872	6.80886005	6.29861988333333	5.66998142083333	8.56934383333333	6.46389307916667	6.81942682083333	7.35596242083333	6.89232578333333	6.88285323333333	6.96685253333333	4.64673680833333	6.43049987083333	5.97304986666667	5.6794805	6.52029283333333	5.538481225	5.63795104583333	5.13486149583333	6.24150231666667	6.48607476666667	4.6116512375	4.08791972083333	5.26839657083333	5.350279675	5.7344837875	5.66792823333333	4.80584670833333	4.61678248333333	4.59733981666667	4.97227905833333	4.8734586125	5.6794805	5.26945706666667	6.29551622916667	5.40801585416667	5.17426900416667	4.57189149583333	5.96736761666667	7.8749512125	6.08645415416667	6.0695613	5.031764525	7.61729026666667	4.34746767916667	4.88865945	5.3271171	8.48978630833333	6.92818722916667	5.24653454583333	7.36787195833333	4.9754440625	7.96813411666667	7.46825643333333	8.03030704166667	5.6669615125	7.33289649166667	7.7663413125	7.98166808333333	8.1097805875	6.84606387916667	7.4303946125	7.47529534583333	6.93257215416667	7.29988426666667	7.2976049625	7.11179178333333	8.95240457916667	7.03166697916667	6.30447276666667	6.172701625	7.40107549166667	8.00877005	8.50678275	6.33304742083333	8.59076590416667	6.29318526666667	6.79094316666667	7.0715031875	5.31875691666667	6.63543665	7.9748274125	6.55103600416667	7.4936125	6.37073757083333	7.5551899	6.11000845	7.30464287916667	6.76013514166667	7.66361127083333	5.81783112083333	5.29832429583333	6.957264825	6.69679296666667	5.61689452916667	6.9525504625	5.46699008333333	5.05849976666667	5.49183984166667	5.72030935	6.2854684375	6.87817127083333	6.4318614125	6.20696075	6.44924722916667	5.79161227083333	7.47925860416667

OR7D2	6.9475749	7.47788481666667	7.37184780833333	7.54177855833333	7.72294644166667	6.29510448333333	8.222543225	7.25001195833333	7.60193526666667	7.89832068333333	7.51524328333333	7.13850381666667	7.33857021666667	7.82793564166667	7.53297658333333	6.74082630833333	6.44290830833333	7.694854575	7.39105859166667	6.44887243333333	7.01495000833333	6.64016859166667	7.13667263333333	6.84260019166667	6.75898810833333	7.17914503333333	7.06834181666667	6.95758655	7.571028	7.35693584166667	7.21861555833333	7.10123053333333	7.57903273333333	7.11855360833333	7.54341655833333	7.16490981666667	7.81786669166667	8.08436514166667	7.72294644166667	7.597182875	7.41303320833333	8.13871308333333	7.422156975	7.13093330833333	6.91388091666667	7.99131585833333	7.40546740833333	7.41824386666667	7.050862275	7.2401722	7.634487825	7.89930148333333	7.51638066666667	7.698105675	8.05466113333333	7.44041060833333	7.095527	8.27440479166667	7.31254506666667	6.78381769166667	6.60603940833333	7.17851245	7.03502525	7.32036674166667	6.79468460833333	6.9475749	7.08048500833333	6.9569431	6.88718085	7.48476935	7.06709683333333	7.272898375	6.76517564166667	6.89915848333333	7.102506125	6.810578	6.40124803333333	6.54514473333333	6.9998061	6.675144925	6.49247578333333	6.780385325	6.78574375	6.66802325833333	6.61221461666667	6.79731146666667	6.85156043333333	6.33645039166667	7.171442675	6.58691901666667	6.86073455	6.98215429166667	6.54872438333333	6.72764310833333	7.41303320833333	7.08293825833333	6.41504531666667	7.4911099	7.18159225833333	6.49825018333333	6.61154091666667	7.568339425	7.27773341666667	5.92148618333333	6.618809175	7.43473675	7.11789028333333	6.61154091666667	6.66522548333333	6.73349835	6.99727938333333	7.32205540833333	6.93873875	7.414082675	7.349518275	7.33740245	6.53137754166667	7.44368639166667	6.99282755	7.224148725

TUBE1	10.612647525	10.145640125	9.212117675	9.66649425	10.8792631583333	10.116767025	9.661547425	9.69619738333333	9.856343425	9.16261181666667	9.77312443333333	10.7576799666667	9.80350678333333	9.58344561666667	10.51584625	9.7097404	10.9607806583333	10.7096961916667	10.2900500333333	10.0545145166667	9.45898255	10.45072255	9.05355476666667	10.381414675	10.5493449166667	10.400070625	9.990452125	10.44375685	10.94470365	9.97301156666667	9.51088574166667	9.95100449166667	10.0607135666667	9.63985794166667	9.27365428333333	10.3702407083333	9.7097404	9.585238675	9.68471804166667	9.69792198333333	8.85348764166667	9.59145404166667	9.17414999166667	8.53005785	9.51357516666667	8.85110639166667	9.39071778333333	9.23186806666667	9.53692870833333	10.0018247916667	9.651432225	9.737063275	9.93630081666667	10.02346285	9.4056037	8.74272486666667	9.515921025	8.88020864166667	9.73622053333333	9.30730685833333	11.03193485	9.89303093333333	9.890973325	9.64736674166667	9.86598649166667	9.75967308333333	9.62805526666667	9.51811660833333	9.58976235	10.0018247916667	9.826059425	9.89055345833333	8.873364875	9.85850786666667	10.167505275	9.99723533333333	10.5899519833333	11.02981265	9.64118481666667	10.30913045	11.19866735	11.12105475	9.20778985833333	9.68160001666667	10.6488037166667	10.1472782833333	10.754584225	9.43547285833333	10.0894400666667	10.264650175	9.76472428333333	10.372950875	10.7892430083333	9.47496915833333	10.217472825	9.56828670833333	10.31088965	10.0111258333333	10.000563275	10.5352797666667	10.090730825	9.902641975	9.89341510833333	9.14279978333333	8.95991093333333	10.0957326583333	9.88471656666667	9.18546986666667	9.97043543333333	9.73744423333333	10.5181477	9.41715076666667	9.51633301666667	9.05776881666667	8.986594625	9.54834169166667	10.26626505	9.79676919166667	9.30543436666667	9.60791420833333

JAKMIP3	8.28541158333333	7.91142259166667	7.99725846666667	8.875989475	8.82424165	7.71570814166667	9.349869075	8.199157725	8.631828525	8.42676914166667	8.47690594166667	9.08493245833333	8.4816187	7.937534725	8.17090974166667	8.05514178333333	7.737502925	8.86902108333333	7.9693647	7.8345672	8.05296578333333	7.82539101666667	7.8648195	7.84267175833333	7.58238585833333	7.933308825	8.03595288333333	7.96240296666667	8.10843584166667	8.17302170833333	7.92964085	7.964195775	8.07106306666667	8.10955528333333	7.86896935833333	7.71354716666667	7.99339445833333	7.82539101666667	7.98618585	7.80828950833333	7.60888643333333	7.85258474166667	7.586837175	7.64049128333333	7.64914819166667	7.97192683333333	7.22964071666667	7.73471975833333	7.53297658333333	7.77893200833333	7.88712554166667	7.84998155	7.67425858333333	7.35627839166667	7.33095725	7.17507495	7.466283575	7.90991645	7.54963650833333	7.36471060833333	8.391097025	8.486995925	8.15812938333333	8.92749955	8.54462835	8.61392724166667	8.67987279166667	8.665331325	8.77140460833333	8.49050600833333	8.58091206666667	8.78836945	9.22559156666667	8.13766550833333	8.61480576666667	8.31911250833333	8.80540845	8.14346960833333	8.68083718333333	8.53929365	7.960228575	8.019555675	9.14608020833333	8.35736911666667	8.37864189166667	8.56222751666667	8.48112818333333	8.61820814166667	8.36807433333333	7.59144205833333	8.39315135833333	9.223397275	7.85807856666667	7.81401100833333	8.47046444166667	8.11454491666667	8.0917251	8.07050834166667	8.89076064166667	7.58800115	7.663341325	8.44239195833333	8.036488875	7.45909229166667	8.33913670833333	7.649654175	7.62704748333333	7.44491683333333	8.062405875	7.439305775	7.49600651666667	7.84323385	9.251213625	7.28864350833333	8.532903275	7.35447710833333	7.46853124166667	7.84267175833333	7.78326185	7.52840544166667

FRZB	12.24577265	9.41030455	9.83629188333333	9.14003079166667	8.59743456666667	11.4140863666667	9.490132625	9.34157415833333	10.4292877666667	9.44151263333333	9.25028671666667	12.4443760583333	10.5768008666667	9.76136145	8.81108796666667	10.7793443666667	11.9036209583333	8.45404219166667	9.508221125	10.2951456833333	10.31285305	8.39672325833333	10.2966490166667	10.5300186083333	10.239527275	13.0554708583333	10.277413575	9.98920446666667	9.36892748333333	9.72964383333333	10.4468482583333	9.945938775	10.2128243166667	10.864263475	12.7976328583333	12.5245839	9.24318624166667	10.359500425	10.2877771083333	10.364544	9.86145205	9.824729	10.07610735	9.58840303333333	11.2696990833333	10.7511943	10.2458406333333	9.03818763333333	10.2289590916667	10.103849375	9.732285275	10.0911655583333	10.1214563916667	9.94164523333333	9.93304795	10.2478255083333	10.07610735	9.870256575	9.63944066666667	10.1804756333333	10.0623822333333	9.810895475	10.1804756333333	9.728395625	12.7807006083333	9.136523775	9.50182605833333	10.4280419583333	9.59145404166667	9.329864925	9.68034129166667	8.31911250833333	8.50894728333333	10.2289590916667	8.62429788333333	8.52110425	12.46107545	10.95605295	9.55671014166667	10.04638645	11.8954895166667	11.3078719166667	9.03233675	9.4056037	11.6163034833333	11.5598018166667	9.82651539166667	10.913187075	8.77676135833333	11.0924307333333	10.2535245166667	7.88178675	9.838575025	11.0625918083333	9.00460498333333	9.07777099166667	9.62407518333333	11.343036975	9.16442515	10.1109480666667	9.41849085	9.34438068333333	9.90518528333333	10.0498058583333	7.91097105833333	10.2498045833333	9.59949791666667	8.99535305	10.5300186083333	10.500555575	10.7254659583333	10.20436555	8.78307434166667	9.0973311	10.4744353333333	10.01207205	10.742514025	9.07536643333333	9.25858543333333	11.0876998333333

INKA2	11.605536575	11.0087053416667	10.8154489833333	10.6895391083333	11.0608057166667	10.5596517833333	11.379403425	10.6356893416667	11.18844885	10.751563075	10.6960220666667	12.1488811083333	11.363901525	10.6384159666667	10.5707751	10.2115540166667	10.5041729666667	10.8349029416667	10.4720948333333	10.5311099	10.7184951333333	10.628160175	10.151318175	10.3369395416667	10.3610713833333	11.2944151416667	10.8956711666667	10.3507137166667	10.8778360416667	10.750505225	10.5477462083333	10.313965725	11.0682286166667	10.4161036833333	11.1099310583333	11.70420215	10.9861853	10.73952485	10.6846028416667	10.8945637333333	10.7344399666667	10.23713875	10.2802737333333	10.3313573416667	10.558939175	10.4492048416667	10.382913225	10.5857428583333	10.1393440416667	10.214113	10.6035239083333	10.4456717166667	10.625026675	10.6581721666667	11.069747825	10.4955307333333	10.4661023416667	10.5987255666667	10.4975657166667	10.3027487416667	11.2989793	10.958948725	10.31950435	10.7298944083333	10.1299873833333	10.8702441	10.1775011166667	11.002072275	10.816907025	10.5651423416667	10.9408814333333	10.8326576083333	11.1840687083333	10.4641916916667	10.4755897583333	10.2642520083333	11.0487579166667	10.137056175	10.1716698166667	10.5464444833333	10.25147975	10.5162662666667	10.0915413	10.0163833	10.5810256583333	10.7116237833333	10.5450500208333	10.7948521166667	10.6905955916667	10.4244981833333	10.6237069166667	10.045510375	10.7688463333333	11.1256433416667	11.04233505	10.485478825	11.3746270333333	10.7034642666667	11.0480221416667	10.7224580666667	10.392106425	10.6003674833333	9.82250195	9.99259065	10.507752825	10.5138272833333	10.2009594166667	10.1923992666667	10.128737475	10.2191020916667	10.2822548833333	10.4356784583333	10.1755237	10.2865677	10.5223924	9.78382319166667	9.84589375833333	10.1633244583333	10.2511057916667	10.6407861166667

DNASE2B	4.52697115833333	4.383064975	4.535343	4.55466009166667	4.72324775	5.06570035833333	4.97072769166667	5.03726580833333	5.40105285833333	4.86184074166667	4.47573676666667	5.49526165833333	4.535343	4.19044525833333	4.50312849166667	4.63513973333333	8.68035370833333	4.66519471666667	4.36901661666667	4.74448875	4.02568050833333	4.371797775	5.39178135	6.02645373333333	4.36495859166667	4.08637315833333	4.00032819166667	4.21912249166667	4.54233060833333	5.0412383	5.4237743	4.27798759166667	3.890139475	4.33846348333333	4.27896138333333	4.09698049166667	4.62057545833333	4.68681869166667	5.21821305	5.6976834	5.10387183333333	4.47474535	5.95328179166667	5.77716973333333	4.21502835833333	4.94576598333333	5.86087000833333	4.79999713333333	4.55369980833333	4.6247727	4.63513973333333	4.28110195833333	4.68073703333333	5.608795625	4.38005901666667	4.69642039166667	4.30130591666667	6.00039116666667	4.30130591666667	4.56909456666667	3.81605024166667	4.82036908333333	4.23097901666667	4.11281023333333	4.202033325	3.81959511666667	4.332336275	5.0412383	4.05858813333333	3.96722695833333	4.33328629166667	4.74337093333333	5.14597308333333	4.38005901666667	4.15220664166667	6.31242605	4.07457460833333	4.39039918333333	7.112144375	4.234848125	4.53925114166667	4.08114406666667	4.95862358333333	4.71218959166667	7.75661866666667	4.03764378333333	3.58405050833333	4.92455391666667	3.842520875	6.329205025	4.45902586666667	4.1481371	4.494971825	3.87726464166667	3.931450225	4.08637315833333	4.73024025833333	4.22505026666667	4.19132811666667	5.58021739166667	4.41203210833333	4.177708	5.18759319166667	7.35983868333333	4.45115921666667	4.89027461666667	6.88512086666667	6.041758325	4.74930484166667	7.86543926666667	4.59265796666667	4.87595029166667	4.56305699166667	6.50142603333333	7.32919075833333	6.00123059166667	4.2860754	5.53496716666667	4.55283246666667	5.34196935833333

ACBD5	10.49790835	11.4904941666667	10.9780057166667	11.5323406833333	10.6233516833333	11.0673982916667	11.0487579166667	11.0394398083333	11.1162112666667	10.8284896583333	11.1582543083333	10.899244375	10.3741034916667	11.2872125416667	11.2226491666667	11.2884114666667	12.2503999916667	10.9600311833333	11.12105475	11.1847643166667	11.348473325	11.2664173916667	11.465822775	10.5829592166667	11.09094715	11.0704861666667	11.1532234833333	11.1502247166667	10.8874095666667	11.3371736333333	11.024361475	11.2950935333333	11.0873559916667	11.9108497083333	11.0167794916667	11.3282347083333	10.9065665083333	10.8743697916667	10.676538	11.0483413166667	11.3474088166667	11.4387367	11.44422215	11.3043532	11.0409461	11.248902775	11.3015343416667	11.2121826083333	11.747396125	11.2653952	11.0797164083333	11.1241958916667	10.6589017416667	10.783568125	11.285520075	10.7325441416667	11.1046868	11.2352593083333	11.1369321416667	11.1803917416667	9.78481885	10.8150497416667	12.1790995083333	10.1670505916667	10.259584625	10.5545011333333	10.6072418333333	10.5416886583333	10.3766923916667	10.286132425	10.5162662666667	10.8885586333333	10.4779275	10.6457263	10.5493449166667	10.20436555	11.2940625833333	10.609691175	10.5365078	10.8736803833333	10.2834956666667	11.3735584916667	11.0841148833333	10.9170947583333	11.1891169333333	10.5223924	10.4138270833333	11.0017295	10.3436245833333	11.0604394416667	10.4955307333333	11.1982937916667	10.859746575	11.005303025	10.1936096583333	10.362641575	11.0034620833333	10.5244636333333	10.667166075	11.184429675	11.2693525666667	11.034816875	11.7487231	12.386493225	10.5659717333333	10.9658327583333	10.7519521333333	10.789595925	10.7722019	10.5979724666667	11.6473588416667	11.1623033333333	10.5181477	10.58448395	10.43975535	11.7667849666667	10.6373110916667	11.56524335	11.28297055	11.6507569666667

LCE1B	4.996867625	5.9751687	6.40797193333333	7.39450265833333	7.28739089166667	5.41039171666667	7.23727353333333	6.15778135833333	6.01215780833333	6.90252933333333	6.69439601666667	7.3367108	7.12659995	6.8687758	6.12524645833333	5.43935509166667	6.03413668333333	7.07654913333333	6.00884046666667	6.4413796	5.978682025	6.04613815	6.56879458333333	5.83953349166667	5.30771575	6.1770551	6.44063283333333	6.54434775	6.14728135833333	7.1950993	6.30044350833333	6.03052256666667	6.25755120833333	6.28034841666667	5.477009275	5.20060255	6.862021425	6.449622025	6.165146375	5.39700335833333	7.62479258333333	6.69781155	6.33645039166667	6.2176337	7.15697099166667	6.80853135	5.64021978333333	6.52133365833333	5.5614942	5.33069273333333	5.775482875	6.393024275	6.57096464166667	6.346676525	6.63179246666667	5.96077145833333	6.00367545	6.70327563333333	6.18094188333333	5.75004875833333	6.33187936666667	7.3032043	6.68395904166667	7.39105859166667	6.93803065	7.20806453333333	7.02761371666667	6.975151325	8.155034325	6.85478974166667	7.12280756666667	7.59199554166667	8.30178113333333	7.1744649	6.54574596666667	5.94705471666667	6.98280363333333	6.02292943333333	7.55551181666667	6.53407053333333	6.87648061666667	6.84200789166667	8.39512661666667	7.24571956666667	8.536875925	7.02284831666667	7.22598754166667	6.65915351666667	6.511623825	6.164307025	6.946211725	6.26664835833333	6.637274925	8.6716691	7.21926184166667	6.87851200833333	7.04956788333333	6.22752675833333	8.50286535	7.45614546666667	5.89819270833333	7.349518275	6.33115975	6.45841340833333	7.137920375	7.32668075833333	6.47791706666667	6.74746250833333	7.434165875	6.8687758	6.71186961666667	6.32454565	7.42162103333333	6.94820169166667	5.88241164166667	8.50654274166667	6.41808758333333	5.8386672	6.48928715	6.42485146666667

CD99	8.703553525	7.960228575	7.90468865833333	8.05973130833333	8.500835825	7.63934964166667	7.73246671666667	7.57513376666667	8.215089275	7.68829090833333	7.91046618333333	8.560933175	8.445841225	7.7794798	8.50894728333333	7.5954683	7.334392275	8.737919725	8.38470655	8.01861064166667	7.78166071666667	8.82265860833333	7.32668075833333	8.24011166666667	8.28340845833333	8.32648980833333	8.03480338333333	8.322152325	8.12378493333333	7.967285625	7.74398054166667	8.24067144166667	8.17406114166667	6.958116025	8.7160017	8.11940264166667	8.552420375	7.77321380833333	7.99232836666667	8.069486125	7.93490190833333	8.17931615833333	7.95517108333333	7.726376575	7.44095513333333	7.48698644166667	7.2395484	7.81401100833333	7.687124975	7.88978783333333	7.738684825	8.01861064166667	8.37604669166667	8.312026725	7.85309088333333	7.586283275	7.84545261666667	7.60415978333333	8.47183739166667	7.73920148333333	7.89880223333333	7.36330638333333	7.48927714166667	7.71034295833333	7.224148725	8.28340845833333	7.44885958333333	7.27471595833333	7.98516725833333	7.45275619166667	8.17302170833333	7.73690679166667	8.4380593	7.49549410833333	7.52729815	7.34331021666667	7.306684875	8.63293045833333	7.25363145833333	7.60823079166667	8.180440425	7.964195775	7.36471060833333	7.80329328333333	7.51253281666667	7.9693647	8.18781211666667	7.91897891666667	8.09750283333333	7.55250805833333	7.56433695	10.2115540166667	7.07980634166667	7.56037049166667	8.32463409166667	7.83518873333333	7.263472875	8.80450975	8.41158014166667	8.1346889	7.378725775	7.88978783333333	6.95221913333333	6.90781049166667	7.262828725	7.01371830833333	7.80451180833333	6.54933985	7.92549585	7.086730875	7.76512125	7.38926633333333	7.42556758333333	7.04378355833333	7.27114775833333	7.91097105833333	7.916304775	7.71624318333333	8.1719332	7.6287103

ABI3BP	11.5067351333333	10.4585673833333	11.0761932833333	10.0197701416667	9.07584114166667	11.625582325	11.030517625	10.93251855	9.98075235	12.3780252416667	10.5609143	13.4957705	11.3997255833333	11.0337297583333	9.765121975	12.4306290916667	11.784585925	9.52177871666667	10.2806521916667	11.4715256833333	11.4996324916667	8.940002325	11.1532234833333	10.9843155	11.14669605	12.3267305	11.545468825	10.8721591833333	9.89055345833333	10.717740675	10.8165123833333	10.86914835	10.85523755	10.921206425	12.1807321916667	12.4549570666667	9.58437340833333	11.7262372833333	12.4333458416667	10.9423645833333	11.1524382	11.0232158833333	11.2431657916667	10.6332993416667	11.6761092166667	11.70420215	11.2950935333333	10.878488075	12.1193766916667	10.9443778666667	11.2495617333333	11.1166050833333	9.19287955	9.705378475	10.340493	10.90102425	11.8779799083333	11.1077951583333	11.586583875	11.7628441333333	8.328023075	9.4968836	8.682286675	8.03223831666667	10.4554224833333	6.737597225	10.0103759833333	10.3897471916667	6.61745720833333	11.0845103416667	9.189315625	7.29049713333333	7.23727353333333	9.28599946666667	6.94244640833333	7.913004675	10.7743973416667	11.0491091083333	8.18936749166667	9.77219498333333	8.8559255	10.2047893666667	7.378725775	9.25536705833333	8.68470500833333	10.430465125	8.70212718333333	10.9234198666667	7.75930169166667	11.5969108416667	8.04330091666667	7.35293989166667	6.862021425	9.51769735	8.318633925	7.96240296666667	8.84428041666667	7.65442175833333	8.603908075	7.97812778333333	9.98470918333333	8.94186190833333	8.07622910833333	8.71285006666667	7.198288025	8.48569109166667	8.78691484166667	8.20175241666667	8.76514685	10.4866818833333	10.054135025	9.34897708333333	7.76137635	5.83306814166667	9.21638355833333	9.69349788333333	10.461782	6.80246065833333	7.3817504	11.5544661666667

PLN	13.9995577083333	10.7040820666667	10.00078105	8.48086537083333	10.3279466083333	11.976008175	10.5607363458333	10.6583477291667	9.95746309166667	10.9348095208333	9.7364049375	13.680815075	12.5337265041667	10.6167578333333	10.7532973708333	10.8546785333333	11.7722639416667	10.7369843708333	11.7177601791667	11.5251794	11.7489019833333	8.856558775	10.3118103291667	9.68844351666667	10.971323675	14.0689028125	11.1319234083333	9.93324109166667	9.92113512916667	10.7006923125	10.9122006875	9.64136172916667	10.5214535583333	10.0207806125	14.0243984458333	14.1572373666667	9.27104185	10.9008579166667	10.9110678	11.5514691291667	9.91741172083333	10.0742512625	10.0384926833333	10.730804625	12.4489610375	10.0917749291667	11.4355900791667	9.69557888333333	9.983679525	9.97562940416667	10.1995539416667	10.4470478166667	9.89573559583333	9.93816075	10.1743594333333	11.1240252041667	10.6243094291667	10.33313495	10.7323377666667	11.1034197583333	8.14216792083333	8.8692769375	5.63005625	9.45172018333333	7.60974972083333	6.7228143375	9.057547175	8.25050160833333	5.07278229166667	9.12440923333333	8.12302545	6.10593456666667	4.993014125	10.8058029916667	6.50245250833333	6.54400302083333	11.667786	9.38119250416667	7.227496475	8.32827847083333	9.67877904583333	10.4322492708333	6.58193215833333	8.49162231666667	8.32538908333333	8.7318304	7.4878376375	10.6475043	6.99314629583333	11.7029008208333	6.76814893333333	6.40761060416667	6.89036172916667	9.64136172916667	8.2398398125	7.12014365833333	4.38585488333333	6.39122358333333	6.55257113333333	8.75494610416667	8.53312110833333	8.68600224583333	6.1484294125	8.40792234166667	5.95198251666667	8.68153127083333	8.91579113333333	6.8041364125	8.0462330125	10.2327910916667	9.25926169583333	6.2646626125	5.16144414583333	6.01258381666667	7.52648900833333	6.77142720833333	8.60202630833333	6.70359169583333	6.27205189583333	9.97933672916667

CEP85L	13.9995577083333	10.7040820666667	10.00078105	8.48086537083333	10.3279466083333	11.976008175	10.5607363458333	10.6583477291667	9.95746309166667	10.9348095208333	9.7364049375	13.680815075	12.5337265041667	10.6167578333333	10.7532973708333	10.8546785333333	11.7722639416667	10.7369843708333	11.7177601791667	11.5251794	11.7489019833333	8.856558775	10.3118103291667	9.68844351666667	10.971323675	14.0689028125	11.1319234083333	9.93324109166667	9.92113512916667	10.7006923125	10.9122006875	9.64136172916667	10.5214535583333	10.0207806125	14.0243984458333	14.1572373666667	9.27104185	10.9008579166667	10.9110678	11.5514691291667	9.91741172083333	10.0742512625	10.0384926833333	10.730804625	12.4489610375	10.0917749291667	11.4355900791667	9.69557888333333	9.983679525	9.97562940416667	10.1995539416667	10.4470478166667	9.89573559583333	9.93816075	10.1743594333333	11.1240252041667	10.6243094291667	10.33313495	10.7323377666667	11.1034197583333	8.14216792083333	8.8692769375	5.63005625	9.45172018333333	7.60974972083333	6.7228143375	9.057547175	8.25050160833333	5.07278229166667	9.12440923333333	8.12302545	6.10593456666667	4.993014125	10.8058029916667	6.50245250833333	6.54400302083333	11.667786	9.38119250416667	7.227496475	8.32827847083333	9.67877904583333	10.4322492708333	6.58193215833333	8.49162231666667	8.32538908333333	8.7318304	7.4878376375	10.6475043	6.99314629583333	11.7029008208333	6.76814893333333	6.40761060416667	6.89036172916667	9.64136172916667	8.2398398125	7.12014365833333	4.38585488333333	6.39122358333333	6.55257113333333	8.75494610416667	8.53312110833333	8.68600224583333	6.1484294125	8.40792234166667	5.95198251666667	8.68153127083333	8.91579113333333	6.8041364125	8.0462330125	10.2327910916667	9.25926169583333	6.2646626125	5.16144414583333	6.01258381666667	7.52648900833333	6.77142720833333	8.60202630833333	6.70359169583333	6.27205189583333	9.97933672916667

NFAM1	7.46352631666667	8.67639044166667	9.26442515	9.092829475	8.25885329166667	8.1346889	8.8778449	8.87974118333333	8.21405428333333	9.250740625	8.33913670833333	9.28128599166667	9.01521926666667	9.173253675	7.28739089166667	9.55898925	8.104851775	7.9291649	8.26555303333333	8.65887986666667	9.27172153333333	8.16633889166667	9.099726825	8.74671439166667	7.8345672	8.61342080833333	8.73158135833333	8.08035505	7.62988515833333	7.99891026666667	8.88122105	8.95031519166667	8.608522725	9.436848125	10.1529084083333	8.09066366666667	7.587363525	8.44690206666667	9.09925841666667	8.91049593333333	8.63773576666667	8.65466659166667	8.91224634166667	8.54360906666667	8.965634	9.27941565	8.80189605833333	8.84471325833333	9.11737229166667	8.33810663333333	9.063171125	7.77208035833333	7.729718875	7.600828425	8.47690594166667	9.133746725	9.11362989166667	8.813752575	8.28997516666667	9.46074875	7.04142870833333	8.79951210833333	8.78307434166667	8.5676736	8.69556865	8.09223929166667	8.41484045	8.78890531666667	8.5232739	10.0911655583333	8.505532025	7.99615545	8.6396011	9.27083296666667	8.635919475	8.92364006666667	8.61820814166667	8.40122860833333	9.07252779166667	8.82473405	8.081342075	8.62290185833333	8.48569109166667	8.4800381	8.4414262	8.46771705	9.309548625	9.24492166666667	8.44794389166667	8.85160831666667	9.045519875	8.60646375	7.964195775	7.7571807	8.15349528333333	8.36669614166667	8.565739425	8.14709130833333	8.328023075	9.55671014166667	8.23567169166667	9.21835433333333	9.13739526666667	8.759783975	8.211993425	8.76273385833333	8.64290230833333	7.928121775	8.69082025	8.55543884166667	9.99343915	7.73471975833333	8.07357910833333	8.52878136666667	9.55860505833333	9.25628595833333	8.81463643333333	8.46149996666667	7.86994016666667	9.661547425

COPS5	12.6029839666667	12.776111275	12.6554812666667	12.8933579375	12.5485581625	12.68596965	12.6643328208333	12.5639838666667	12.6048529833333	12.6942150375	12.7366517166667	12.1156685625	12.4595822625	12.8033610791667	12.6869957833333	12.5597336041667	12.8530072583333	12.5573847541667	12.64196235	12.7815107166667	12.8749016041667	12.6334257666667	12.9343725958333	12.7860295083333	12.6105355375	12.72038695	12.8074143333333	12.7876536791667	12.6752219875	12.9868350916667	12.716209925	12.9090764	12.6388910083333	12.4035613208333	12.6767709041667	12.701356375	12.6334257666667	12.7023685083333	12.6672255708333	12.3099794333333	12.8921238125	12.8029136333333	12.8638697333333	12.8462830375	12.6559656416667	12.7860295083333	12.9425865041667	12.872743225	12.9572856708333	12.8915324583333	12.7604893958333	12.7008644458333	12.3116354208333	12.4815793708333	12.4998470416667	12.3890185041667	12.6988560541667	12.9480679208333	12.6931945	12.6968710791667	12.7492394291667	13.2741458583333	13.4653944208333	12.7074055333333	12.8945343958333	12.868775825	12.6062789166667	12.7647025958333	13.1704084083333	12.95895185	12.764168225	13.1127745958333	12.6453797	13.0704317875	12.4578838291667	12.5602139791667	12.7120155125	13.0983049083333	13.5734157833333	13.260545875	13.1810200208333	12.7926187375	13.571708925	13.1476053375	13.4553955875	12.160227575	13.299250075	12.7120155125	13.038398325	13.22443745	13.2687283958333	12.5698845583333	13.4631079791667	13.1566431916667	13.0575032708333	13.1373006958333	13.2971953708333	12.7069332208333	12.3286515541667	13.1855235875	12.8530072583333	13.3289539083333	12.371453225	13.2396261041667	13.0898441583333	13.5647696666667	12.9142045041667	13.4741426416667	13.4356165791667	12.8018111958333	12.7621023	12.8401156041667	12.7434371916667	13.334591425	12.9702184208333	12.8982099916667	12.5678498041667	13.0679278416667	13.4257513333333	12.4707841208333

PPP1R42	12.6029839666667	12.776111275	12.6554812666667	12.8933579375	12.5485581625	12.68596965	12.6643328208333	12.5639838666667	12.6048529833333	12.6942150375	12.7366517166667	12.1156685625	12.4595822625	12.8033610791667	12.6869957833333	12.5597336041667	12.8530072583333	12.5573847541667	12.64196235	12.7815107166667	12.8749016041667	12.6334257666667	12.9343725958333	12.7860295083333	12.6105355375	12.72038695	12.8074143333333	12.7876536791667	12.6752219875	12.9868350916667	12.716209925	12.9090764	12.6388910083333	12.4035613208333	12.6767709041667	12.701356375	12.6334257666667	12.7023685083333	12.6672255708333	12.3099794333333	12.8921238125	12.8029136333333	12.8638697333333	12.8462830375	12.6559656416667	12.7860295083333	12.9425865041667	12.872743225	12.9572856708333	12.8915324583333	12.7604893958333	12.7008644458333	12.3116354208333	12.4815793708333	12.4998470416667	12.3890185041667	12.6988560541667	12.9480679208333	12.6931945	12.6968710791667	12.7492394291667	13.2741458583333	13.4653944208333	12.7074055333333	12.8945343958333	12.868775825	12.6062789166667	12.7647025958333	13.1704084083333	12.95895185	12.764168225	13.1127745958333	12.6453797	13.0704317875	12.4578838291667	12.5602139791667	12.7120155125	13.0983049083333	13.5734157833333	13.260545875	13.1810200208333	12.7926187375	13.571708925	13.1476053375	13.4553955875	12.160227575	13.299250075	12.7120155125	13.038398325	13.22443745	13.2687283958333	12.5698845583333	13.4631079791667	13.1566431916667	13.0575032708333	13.1373006958333	13.2971953708333	12.7069332208333	12.3286515541667	13.1855235875	12.8530072583333	13.3289539083333	12.371453225	13.2396261041667	13.0898441583333	13.5647696666667	12.9142045041667	13.4741426416667	13.4356165791667	12.8018111958333	12.7621023	12.8401156041667	12.7434371916667	13.334591425	12.9702184208333	12.8982099916667	12.5678498041667	13.0679278416667	13.4257513333333	12.4707841208333

MEF2C	9.59065653333333	8.91889295833333	9.18361099166667	8.91135703333333	9.549690725	8.97027915833333	9.70714295	8.7735097	9.10749988333333	8.94384210833333	9.00189508333333	9.80399703333333	9.29796166666667	8.68035370833333	9.26442515	8.80003964166667	8.49539040833333	9.56080151666667	8.87636833333333	8.76851021666667	8.94186190833333	9.03459930833333	8.72134320833333	8.72229471666667	9.304559675	9.40373885833333	9.60710205	9.41120025	9.70236100833333	9.27768001666667	8.93180466666667	9.311763425	9.549690725	9.54834169166667	9.40032650833333	9.66065478333333	9.87207808333333	9.37548823333333	9.35773556666667	9.31762243333333	8.53333894166667	9.18115765833333	8.92218960833333	8.842895675	8.50984575833333	8.67403964166667	8.60344725	8.84802724166667	8.78498449166667	9.10844005833333	9.14518724166667	9.18639844166667	9.55860505833333	9.272617125	9.29340571666667	8.74427688333333	8.536875925	8.96144149166667	9.40511030833333	8.61768925	9.53312831666667	9.230932875	9.42590730833333	8.783965475	8.435679275	9.346282425	8.95577006666667	9.26679426666667	8.817877875	9.214506275	9.2436075	9.1906696	9.083454025	9.11737229166667	9.32089804166667	8.8768317	9.17971915	9.38453646666667	8.92028675	9.01197323333333	8.662974625	8.9604306	8.81108796666667	8.98325939166667	9.62234490833333	9.01246160833333	9.17646485833333	9.106662475	9.70284449166667	9.10485650833333	8.79767885	9.44914495	9.5013607	9.11782889166667	9.93222460833333	9.633880775	9.445595775	9.73788834166667	9.7100974	8.83016251666667	8.82211801666667	9.31317535833333	9.30912254166667	8.94982423333333	8.69274164166667	9.14279978333333	9.02085209166667	8.64688179166667	8.89405555	8.93412065833333	9.09833678333333	8.90099503333333	8.94715431666667	8.64290230833333	9.41935561666667	9.00460498333333	8.68775965833333	9.32760176666667	9.42643701666667	8.88732965833333

NPIPA1	13.9153342666667	13.3293632833333	13.5230150833333	13.1806770166667	14.2000424583333	14.0201742416667	13.6996187916667	13.3522548916667	13.5746400833333	13.3278442333333	13.7940225416667	14.3482967833333	14.5195733916667	13.782971725	13.9458581083333	13.812858	13.3293632833333	14.241033875	13.7177649416667	14.160512975	13.5043698416667	13.8908948916667	13.7177649416667	14.26265275	14.0174773166667	14.36106535	13.913193025	13.9445631916667	13.8450858083333	13.8972210333333	13.8221316	13.3256142916667	13.6418962083333	13.6165726333333	14.0746231666667	14.4161239916667	14.1629431333333	13.4089360166667	13.8357694833333	13.5287262833333	13.2460254166667	13.4586485833333	13.5443638333333	13.7204745333333	13.9829878583333	13.2939983416667	13.3300671416667	13.2697464583333	13.7660959916667	14.164370775	13.49281735	14.0100072833333	13.9518039333333	14.1329416	13.487174625	13.3875128	13.238016175	13.2628733666667	14.0950375583333	14.0490437833333	14.71190555	13.8060720916667	13.7017952166667	14.0669942916667	13.9197186416667	14.3724417	13.6506232916667	14.1427380833333	14.282826075	14.3792394416667	14.11256935	14.4125419416667	13.8199392	14.1256024333333	14.013877625	13.8296521	14.6758595916667	14.4705790833333	13.9217501083333	14.1513249333333	14.6738026916667	14.5793502666667	13.3473938666667	13.3823065416667	14.4025408	14.6949121583333	14.427724725	13.8296521	14.9070025916667	14.1427380833333	14.0694923916667	14.6023913166667	14.95380875	14.34486635	14.5717037666667	14.2533850083333	14.494740525	14.7055894833333	14.1113310916667	14.1698024416667	14.3628995916667	14.0540171833333	14.4705790833333	13.8241318916667	13.9917082333333	14.74733735	14.4495058333333	13.9695187666667	14.5607495666667	13.9367207833333	13.9657662833333	13.6098239583333	13.9625123916667	12.9924504416667	13.66414075	13.6560864916667	14.66935455	14.0540171833333	13.935575975	14.3283993416667

AL109810.2	10.0714385291667	12.0212087541667	12.0496677208333	11.5674594583333	12.0991486625	10.4318495375	12.8094183875	12.7631420958333	12.4218064625	11.9335332166667	12.8368804875	9.429283125	11.5503720375	12.1215121083333	10.9649539375	11.0019008875	8.63896813333333	11.4286436416667	10.652148475	11.8168877791667	10.97708	11.2555266	10.915181275	10.0978445333333	12.8147560583333	9.92154557083333	12.6006538583333	12.3990288208333	11.7378158041667	11.1439293333333	11.6288916125	11.7555472791667	13.1213867958333	8.572332375	8.99418029583333	8.94259409583333	12.4779339333333	12.4008268	12.8457431541667	12.8892667833333	11.9411064666667	11.6494766791667	11.1875827708333	11.00364155	11.3411658625	10.6870947833333	10.7472276791667	11.8636887541667	10.88639095	11.133039425	12.5997245208333	11.3917230541667	12.7480855375	11.5837864833333	11.7755963375	11.983431475	12.3099794333333	12.528008925	11.2217375041667	10.5238263666667	8.56934383333333	9.2062901875	8.57374275	8.97650550416667	8.54821066666667	10.0646729166667	8.66650712083333	9.606054925	8.865815675	9.52292505833333	9.68135890833334	9.96087055	9.52568969166667	8.7667943	8.9742374125	8.76445331666667	10.0634214208333	8.71581307916667	8.701455975	8.62055127083333	8.69916035416667	9.30051904583333	8.834198375	8.4005478125	9.363637275	9.52719898333333	9.05007336666667	8.78663554583333	9.00529619583333	8.06578678333333	8.83568698333333	8.42647575	8.67864822916667	8.6496312375	9.14853115	10.3141512541667	8.81216085416667	9.46367207083333	9.44669676666667	8.67714263333333	8.28772449166667	9.2950347875	8.784708	8.5072711875	8.85613872083333	8.5771368	8.38144642916667	8.23839771666667	8.63473515833333	8.17275982083333	8.53950697916667	9.15860185	8.26181718333333	8.28824942916667	8.48542059166667	9.2025973375	7.9904921875	8.50678275	8.57533845416667	8.68107251666667

TRIM67	10.0714385291667	12.0212087541667	12.0496677208333	11.5674594583333	12.0991486625	10.4318495375	12.8094183875	12.7631420958333	12.4218064625	11.9335332166667	12.8368804875	9.429283125	11.5503720375	12.1215121083333	10.9649539375	11.0019008875	8.63896813333333	11.4286436416667	10.652148475	11.8168877791667	10.97708	11.2555266	10.915181275	10.0978445333333	12.8147560583333	9.92154557083333	12.6006538583333	12.3990288208333	11.7378158041667	11.1439293333333	11.6288916125	11.7555472791667	13.1213867958333	8.572332375	8.99418029583333	8.94259409583333	12.4779339333333	12.4008268	12.8457431541667	12.8892667833333	11.9411064666667	11.6494766791667	11.1875827708333	11.00364155	11.3411658625	10.6870947833333	10.7472276791667	11.8636887541667	10.88639095	11.133039425	12.5997245208333	11.3917230541667	12.7480855375	11.5837864833333	11.7755963375	11.983431475	12.3099794333333	12.528008925	11.2217375041667	10.5238263666667	8.56934383333333	9.2062901875	8.57374275	8.97650550416667	8.54821066666667	10.0646729166667	8.66650712083333	9.606054925	8.865815675	9.52292505833333	9.68135890833334	9.96087055	9.52568969166667	8.7667943	8.9742374125	8.76445331666667	10.0634214208333	8.71581307916667	8.701455975	8.62055127083333	8.69916035416667	9.30051904583333	8.834198375	8.4005478125	9.363637275	9.52719898333333	9.05007336666667	8.78663554583333	9.00529619583333	8.06578678333333	8.83568698333333	8.42647575	8.67864822916667	8.6496312375	9.14853115	10.3141512541667	8.81216085416667	9.46367207083333	9.44669676666667	8.67714263333333	8.28772449166667	9.2950347875	8.784708	8.5072711875	8.85613872083333	8.5771368	8.38144642916667	8.23839771666667	8.63473515833333	8.17275982083333	8.53950697916667	9.15860185	8.26181718333333	8.28824942916667	8.48542059166667	9.2025973375	7.9904921875	8.50678275	8.57533845416667	8.68107251666667

EIF4E	11.8959032583333	12.761853925	12.8936439416667	12.6562254416667	12.432506675	12.3657425166667	12.5780122833333	13.1394802583333	11.9781875416667	12.500114075	12.3244889333333	11.7259183166667	12.3152059916667	12.416791375	12.3763083916667	12.2453199833333	12.0084826666667	12.724847575	12.7227603583333	12.7602549166667	12.4377746666667	12.23627775	12.622549625	12.836627	12.5048403	11.8104289083333	12.479955875	12.6626629166667	12.8062943	12.8035935833333	12.7827098416667	12.4696924	12.1146065416667	11.798162425	11.8935481166667	11.7678571083333	12.5944621416667	12.7999144166667	12.7949718333333	12.4655499583333	12.8268431583333	12.8613107583333	12.6346883583333	12.7890792833333	12.2780305166667	12.7374360833333	12.6674936416667	13.2061458583333	12.772348275	12.4110018333333	12.811279575	12.909394625	12.1325110916667	12.3331331166667	12.293475975	12.3262957	12.8930719333333	13.0602371583333	12.5024686916667	12.3356581833333	10.5344987916667	11.94832135	12.3789904833333	12.0185152166667	12.038401575	11.5710069666667	12.3631593333333	12.3114382583333	12.1333790583333	11.6540451166667	11.84965965	11.8630874833333	12.01005715	11.5983924583333	11.6799309666667	12.3554452	11.2650373666667	11.9585723416667	12.533964725	12.1170603	11.7239413833333	10.85411215	12.907519675	11.904789825	12.07649975	12.0160472666667	12.424331275	11.8431168166667	11.3332438	11.95296625	12.2888330833333	11.875371875	12.2687931833333	12.03297995	11.8993453583333	11.876483925	11.731200575	11.8116053416667	12.1906758666667	12.333509375	12.3378199666667	12.3484591166667	12.0368526666667	12.0596630083333	12.1410311166667	12.5850501916667	12.3173301166667	12.5297533083333	12.3083565083333	12.2835716083333	12.3071456666667	12.5381389666667	12.1198078916667	11.987523	11.9612880416667	12.5979621916667	12.1189987083333	12.5415392083333	12.1455145583333	12.0253815583333

TRPA1	5.92509355833333	6.04700228333333	4.897128125	4.172665625	3.6774476	5.80937455	4.41297161666667	4.4473261	3.75570144166667	5.5614942	5.17905539166667	5.89449381666667	3.96359718333333	4.727091875	4.60485635	5.01427080833333	6.42485146666667	4.13438028333333	5.64497814166667	5.24102825833333	4.44161573333333	4.87503585	5.11568579166667	5.31614614166667	6.21116124166667	5.94013145833333	5.64021978333333	5.934751075	5.2482658	5.5614942	6.4413796	5.780758525	4.508464425	5.65913218333333	7.1508922	6.08790970833333	4.95322815833333	6.30781043333333	6.27872079166667	5.565954175	6.09195375	5.51803240833333	6.69991303333333	6.265839825	6.19454715	6.82820395	6.23620956666667	5.50123778333333	6.258372575	6.018516875	6.12282240833333	5.74606878333333	4.128952175	5.58597898333333	5.92321303333333	6.61415903333333	4.9406758	5.68979354166667	6.32527860833333	7.366539375	5.75852464166667	6.911150775	5.05069551666667	4.92455391666667	5.498293125	6.54933985	5.62775541666667	4.841975675	4.576370125	4.36901661666667	6.96848423333333	7.54070496666667	4.15642159166667	6.8478471	5.42470928333333	6.43799471666667	4.472817375	5.65913218333333	9.06745036666667	5.212866025	5.85993758333333	4.28809964166667	6.89400789166667	7.6157713	7.03011389166667	4.957419525	5.83017264166667	4.71218959166667	6.20040056666667	6.65984261666667	5.60680040833333	5.59754975833333	6.36248048333333	6.52344795833333	6.93803065	6.64358704166667	4.79808495	6.83400405	5.710337225	5.636456275	7.46463336666667	4.41103275833333	5.710337225	5.62775541666667	6.36170378333333	4.99894050833333	7.21281770833333	5.415860975	6.8190902	7.41526463333333	6.16763958333333	5.69483811666667	6.0167621	7.189475125	7.40322858333333	6.01758168333333	7.07794505	6.38846551666667	7.64737070833333	4.27160165833333

CYTH1	12.1342141916667	13.8366591583333	13.735130625	13.8635959083333	13.2806732833333	13.18580525	13.532131425	13.7820292333333	13.5552298333333	13.0337121333333	13.3809042	12.9352563083333	13.3921452083333	13.8688792083333	13.56515455	13.994455075	13.3959354916667	12.8984843833333	13.6140845916667	13.4943591	13.4337458083333	13.901442	13.7149280416667	13.8614602166667	13.3660384166667	12.320801975	13.2227845833333	13.4674284	13.253120425	13.3987592083333	13.7046873583333	13.9109585833333	13.5355941333333	13.8060720916667	12.4908032583333	11.8970046083333	13.3185547	13.5133528333333	13.8168544833333	13.4222455	13.82498885	13.711942125	13.9967915416667	13.668458425	13.38316305	13.8337930083333	13.6666988416667	13.64886915	13.7542141333333	13.5815251416667	13.27588995	13.6675870333333	13.6174285083333	13.7640679416667	13.8983181916667	13.7970969916667	13.3067028166667	13.7272276083333	13.4550317916667	13.5907625666667	12.58926255	13.4666571333333	13.3248386416667	13.0786865916667	12.8718544916667	12.8757636333333	13.0427266583333	13.2286031583333	13.057870725	13.5083984166667	13.000133375	13.128643425	12.324942025	13.14005525	12.744717925	13.028241025	12.74583335	13.205443325	12.8613107583333	13.1299690833333	13.2705051333333	13.1531334333333	13.0130175083333	12.833410325	13.1356932666667	12.9836190416667	13.3114725833333	12.9882248583333	12.9988931583333	12.9352563083333	12.7081294916667	12.4701553166667	13.14330865	12.5706160166667	13.452778075	13.2835400666667	13.489460875	13.1235892166667	13.2933426583333	13.5781398666667	13.0645149833333	12.847095325	13.2047745416667	13.3682726083333	12.7884927666667	13.7463275333333	13.0368898	12.966521825	12.7521459083333	13.068247425	13.6009954166667	12.5921909166667	13.1305878416667	13.5401929916667	13.2595614666667	13.2047745416667	14.2518399916667	13.2939983416667	13.3193049583333	13.5295226416667

RPL37A	6.65223385	7.11730268333333	7.127807925	7.04378355833333	7.19320405	6.81508890833333	7.603571	7.93069544166667	7.72139515	6.70200101666667	6.49608473333333	8.40122860833333	8.28143178333333	7.24396096666667	7.55873083333333	6.97900894166667	6.04876668333333	7.58408555833333	6.91699638333333	7.634487825	7.68209800833333	7.55746484166667	7.23371278333333	6.38554745833333	6.10553103333333	8.08952596666667	5.70053848333333	5.983976325	4.91325449166667	5.32557380833333	6.84576470833333	6.141642375	6.70726116666667	6.15183188333333	6.49689175833333	6.485693525	5.69973553333333	6.03747638333333	5.74506199166667	5.86812175833333	6.88940976666667	5.9294819	5.97106113333333	6.44595796666667	6.38316134166667	7.13728840833333	5.880482625	6.22304681666667	5.41697971666667	6.7401779	5.86905295	6.70796138333333	7.94647371666667	5.88521655833333	6.66383380833333	6.88386930833333	6.96330954166667	6.0911181	6.57365081666667	6.891986525	7.134785875	7.3007483	5.32557380833333	7.46463336666667	6.92168505833333	6.51830799166667	7.81456171666667	7.97971059166667	6.09195375	6.61154091666667	7.256627775	8.50130601666667	7.875176525	7.27714694166667	7.55551181666667	7.9184414	6.20814583333333	5.53102171666667	6.54574596666667	6.57014316666667	8.26606834166667	7.94435703333333	6.37643818333333	6.96183981666667	6.88314723333333	7.75234836666667	7.40193138333333	7.28803510833333	7.619792375	5.86905295	6.80853135	6.32751184166667	5.41985199166667	5.51915526666667	6.36327274166667	5.91139875	6.29057105	6.735668325	7.31316353333333	8.14082359166667	6.735668325	7.39621439166667	6.20969628333333	6.94120649166667	4.79318954166667	6.67021015	6.00968073333333	9.17867269166667	5.33069273333333	7.07528308333333	6.80310133333333	7.49600651666667	7.86994016666667	7.66108944166667	6.25427673333333	7.69319276666667	5.927679125	4.8440917	6.60270946666667	7.99833741666667

WASF2	5.79916375	6.35880866666667	6.14959958333333	6.50929938333333	6.57813499166667	6.6562921	6.060789425	6.399909175	6.64156519166667	6.31323984166667	6.26279403333333	5.6458775	6.493249575	5.6322886	7.42675208333333	6.013009825	4.98341465833333	8.77579595833333	6.47718199166667	6.19209213333333	5.97436386666667	7.188269975	5.511928275	7.17851245	7.378725775	5.85514745833333	6.84063886666667	7.24945885833333	6.82704920833333	6.58691901666667	6.48497233333333	6.73065755833333	6.21427064166667	5.49229008333333	5.65732808333333	5.79378330833333	6.61745720833333	6.813807925	6.68067039166667	6.46419815833333	6.33187936666667	6.38316134166667	5.54392786666667	5.86456365833333	5.37867954166667	6.1770551	5.67998533333333	6.45538780833333	6.16362138333333	6.82586836666667	6.79131116666667	6.0148863	6.71186961666667	6.84576470833333	6.45472701666667	6.11047193333333	6.36170378333333	5.91719066666667	7.305533825	5.32557380833333	7.02704855833333	6.42188529166667	6.76714710833333	6.78180254166667	6.12118446666667	6.94561610833333	6.76187970833333	6.08105208333333	6.07263301666667	5.73195755	6.41354868333333	6.44737514166667	6.74158396666667	5.61643985833333	6.295927975	6.27652200833333	5.84048410833333	6.83020955	6.8399633	6.285084375	5.65913218333333	6.41957618333333	6.328333625	6.58691901666667	6.55593524166667	5.78709845833333	6.67783206666667	6.4502938	5.93036776666667	6.161212075	6.14575641666667	6.772445575	6.9569431	6.06491893333333	7.14519241666667	6.55877498333333	6.996742575	6.4975584	6.28823875	6.682676825	5.52545381666667	6.14959958333333	6.47141914166667	5.98304395	6.17635683333333	6.559370075	6.20969628333333	5.98559190833333	6.00285793333333	6.56579801666667	6.00968073333333	6.478653025	6.26140625833333	5.24388391666667	6.09436185833333	6.42946571666667	5.779817875	6.04700228333333	7.171442675	5.934751075

C9orf153	4.46004483333333	4.92149175833333	5.22040228333333	4.24718985833333	5.14292011666667	4.52238	4.50312849166667	4.74930484166667	4.66334196666667	5.01216298333333	4.56071255833333	4.64033936666667	4.61421155	4.86184074166667	4.736033375	4.92149175833333	4.93234401666667	5.44533825	5.3397513	4.49406044166667	5.14292011666667	5.9930492625	4.15954296666667	4.873988275	4.39446349166667	4.68073703333333	5.46549339166667	4.41506155	5.2543857	4.37710720833333	5.55666916666667	5.3343362	5.060028525	4.2918764	4.76967493333333	5.52011276666667	4.72508045	5.15454668333333	5.75682274166667	4.617309275	4.61010935833333	5.30448685	4.472817375	5.31319136666667	4.05858813333333	4.68262605833333	4.44822564166667	4.76759788333333	4.245391125	4.51657093333333	6.060789425	4.94999915833333	4.06794450833333	5.07107728333333	5.054896275	4.62889925	4.00561259166667	4.509505	4.63723314166667	5.4147768	4.36796431666667	5.57148620833333	6.17154675833333	4.81840091666667	4.802114875	4.52327248333333	5.06351158333333	5.1598283	4.42666711666667	5.2891249	4.46004483333333	5.68499903333333	4.955313625	4.30624979166667	4.00881020833333	5.565954175	4.66727606666667	5.5651756	4.44254745	4.39446349166667	5.63834938333333	5.118961025	4.09478471666667	4.21912249166667	4.19434771666667	4.58140610833333	4.52155396666667	4.21809414166667	5.98737700833333	4.83989633333333	5.38469075833333	4.85037616666667	5.76836148333333	4.49904255833333	4.638255275	4.39557236666667	4.43313565833333	5.00344013333333	4.85974603333333	4.90768301666667	5.12411041666667	4.760314225	5.35882338333333	4.79999713333333	5.0412383	3.85292815833333	4.13540660833333	5.76251488333333	4.43737311666667	5.6695191	5.9191077	5.1863758	4.7815417	4.58982808333333	3.92246294166667	4.52791141666667	4.812774675	5.23035095	5.08252440833333	4.946749425

METTL23	11.002624425	10.8856558833333	10.9606005666667	11.0984247458333	11.026296375	11.7599613916667	10.6338491291667	11.0184106916667	10.9392302041667	10.9023129541667	11.1315437666667	11.0627641416667	10.9598836708333	11.0148285583333	11.0642577208333	11.2389677541667	11.8898480958333	11.2382642791667	11.1697849541667	11.3511609708333	11.1958353416667	11.1068966458333	11.4234808458333	11.00364155	11.1566631583333	10.7856948083333	10.77618975	10.8410441208333	11.060242575	11.0915226541667	10.9326781916667	11.1672319583333	11.0169557958333	11.6534580125	10.5939882375	10.902686275	10.99498035	10.9370095208333	10.8749243083333	10.7274249875	11.1864438291667	11.1160500375	11.11715415	11.2127485	11.1097335958333	11.3330606083333	11.076375075	11.0407371375	11.1400156416667	11.3077018833333	10.9885999458333	11.0081120875	11.4852863083333	11.2924159458333	11.2574172166667	11.0456651875	11.0303309791667	11.1428788833333	11.2440359583333	11.0310479208333	11.3944725791667	11.7555472791667	12.1211265541667	11.588212975	11.4286436416667	11.671431125	11.9234231958333	11.4208994333333	12.3090903083333	11.27133645	11.5616369833333	11.4404071	12.0327797	11.3921201125	11.7025253875	11.671431125	11.3529603708333	11.8640698625	11.7341886333333	11.8486849583333	11.5744282208333	11.671431125	11.5800244583333	11.419798625	11.5194583916667	11.1526365125	10.7776906041667	10.6814445541667	11.6357464125	11.4856267708333	10.9837156666667	10.5966848708333	11.2755224208333	11.212011375	12.2961971583333	11.2393368958333	12.0228148916667	11.878575025	11.4271790458333	11.3913576541667	11.8026393875	11.1105154916667	12.2097075375	11.8543975916667	12.0854924	11.8172876166667	11.72800655	11.8002520791667	11.8455711791667	11.6038006333333	11.350793775	11.4976643458333	11.6854061	12.5997245208333	11.6384698541667	11.6331201416667	11.4870554708333	11.8987356208333	11.7349437625	11.6373013375

AC005837.2	11.002624425	10.8856558833333	10.9606005666667	11.0984247458333	11.026296375	11.7599613916667	10.6338491291667	11.0184106916667	10.9392302041667	10.9023129541667	11.1315437666667	11.0627641416667	10.9598836708333	11.0148285583333	11.0642577208333	11.2389677541667	11.8898480958333	11.2382642791667	11.1697849541667	11.3511609708333	11.1958353416667	11.1068966458333	11.4234808458333	11.00364155	11.1566631583333	10.7856948083333	10.77618975	10.8410441208333	11.060242575	11.0915226541667	10.9326781916667	11.1672319583333	11.0169557958333	11.6534580125	10.5939882375	10.902686275	10.99498035	10.9370095208333	10.8749243083333	10.7274249875	11.1864438291667	11.1160500375	11.11715415	11.2127485	11.1097335958333	11.3330606083333	11.076375075	11.0407371375	11.1400156416667	11.3077018833333	10.9885999458333	11.0081120875	11.4852863083333	11.2924159458333	11.2574172166667	11.0456651875	11.0303309791667	11.1428788833333	11.2440359583333	11.0310479208333	11.3944725791667	11.7555472791667	12.1211265541667	11.588212975	11.4286436416667	11.671431125	11.9234231958333	11.4208994333333	12.3090903083333	11.27133645	11.5616369833333	11.4404071	12.0327797	11.3921201125	11.7025253875	11.671431125	11.3529603708333	11.8640698625	11.7341886333333	11.8486849583333	11.5744282208333	11.671431125	11.5800244583333	11.419798625	11.5194583916667	11.1526365125	10.7776906041667	10.6814445541667	11.6357464125	11.4856267708333	10.9837156666667	10.5966848708333	11.2755224208333	11.212011375	12.2961971583333	11.2393368958333	12.0228148916667	11.878575025	11.4271790458333	11.3913576541667	11.8026393875	11.1105154916667	12.2097075375	11.8543975916667	12.0854924	11.8172876166667	11.72800655	11.8002520791667	11.8455711791667	11.6038006333333	11.350793775	11.4976643458333	11.6854061	12.5997245208333	11.6384698541667	11.6331201416667	11.4870554708333	11.8987356208333	11.7349437625	11.6373013375

DCAF4	9.79933185833333	9.67274871666667	9.56548648333333	9.813959625	10.23098885	9.54178184166667	9.98596199166667	9.5928155	9.86517185	9.59742406666667	9.55429406666667	9.29481465	9.81525473333333	9.27222216666667	9.84409466666667	8.98618440833333	9.76911673333333	9.87739761666667	10.0026032916667	9.52642665	9.41162929166667	10.1417124166667	9.33964531666667	9.462094325	9.55990681666667	9.44826335	9.46252099166667	9.21732311666667	10.018462175	9.65973478333333	9.330358125	9.44337360833333	9.4936652	9.38667448333333	9.45734620833333	9.70748555	9.628525675	9.54402950833333	9.18071311666667	9.445595775	9.19576385833333	9.57903839166667	9.42992638333333	9.54277241666667	9.26306665	9.486953025	9.21787520833333	9.41484764166667	9.39704936666667	9.485645225	9.26354115	9.73533868333333	9.931739675	9.8597846	9.7400082	9.23882716666667	9.584765625	9.42465609166667	9.17867269166667	9.59108196666667	9.94127946666667	9.81176585	10.051171975	10.3462750333333	9.89553576666667	10.0753226583333	10.6691322083333	9.91842590833333	10.7855003666667	10.6256227583333	9.96782080833333	10.233851875	10.3674828833333	9.46074875	9.98838416666667	10.4138270833333	9.92832075	9.53875820833333	10.0807009083333	10.2411048916667	9.81899704166667	9.63156661666667	9.77613621666667	10.0937438833333	10.164580475	9.94976705	9.62110718333333	9.673127475	11.5630353166667	10.666810425	10.1919662416667	10.0915413	9.59518965833333	10.3610713833333	11.7376430583333	9.657103925	10.8502772583333	9.61984825833333	10.2511057916667	9.49908795	11.5765356583333	9.75367038333333	10.2978868083333	9.87739761666667	10.39291705	10.7295063583333	10.5806393916667	10.558939175	10.35378905	9.58797373333333	8.99346874166667	9.30630015833333	10.916432925	11.0034620833333	9.98877366666667	9.73188163333333	9.25394765	10.5748071083333	10.2060439416667	9.79676919166667

ICE2	8.52826969166667	7.692684	7.98087950833333	7.32789755	6.97957124166667	7.84376875833333	6.85677963333333	7.80873386666667	7.568339425	7.58017606666667	7.514206825	7.05399351666667	7.02653026666667	7.61288113333333	8.01539524166667	7.801197125	7.98666509166667	6.84711038333333	8.68971138333333	7.91142259166667	7.24263133333333	7.78166071666667	7.26874934166667	7.80971700833333	7.68879333333333	8.686777175	8.104851775	7.6071886	7.19137265833333	7.9238629	8.05761690833333	8.07674773333333	7.53964365	7.56433695	8.898281125	8.88357545	7.31615383333333	7.644918425	7.3968942	7.79194111666667	8.20882531666667	8.16633889166667	8.04234110833333	8.30283623333333	7.9977282	8.47850595	8.32463409166667	7.89455425833333	8.251367875	7.80067161666667	7.61924733333333	8.222543225	7.305533825	7.56894295833333	8.16124230833333	8.15812938333333	7.694854575	8.18363113333333	7.63385609166667	8.07622910833333	7.687124975	7.1989222	6.94690668333333	5.93821120833333	8.51387624166667	6.48217831666667	6.40124803333333	6.56365850833333	7.05225570833333	6.80310133333333	6.34005863333333	6.33115975	6.10954496666667	6.57734461666667	6.37568504166667	6.19129395	6.9393833	7.1508922	7.84267175833333	7.00118948333333	7.11855360833333	6.71442883333333	6.51685910833333	7.19320405	6.70667168333333	7.0596334	7.24263133333333	7.22299226666667	7.21861555833333	7.20568426666667	8.17249793333333	7.040686725	7.32611610833333	6.93091866666667	6.94497745833333	5.74803075	6.715741525	7.00499255	6.21116124166667	7.95904073333333	7.48649204166667	6.64956780833333	8.41599213333333	8.24557174166667	7.55551181666667	6.931726825	7.04503860833333	7.84051964166667	7.64856505833333	7.5954683	7.54021029166667	7.82279025833333	7.39869075	7.33740245	7.204487875	7.51200123333333	8.14849655833333	7.45786721666667	7.95359058333333	7.506757

SMAD4	13.3336208666667	12.4333458416667	12.2840300666667	12.2943062333333	11.6329290083333	12.7827098416667	12.0796485666667	12.4650650416667	12.3606021	12.0474514	12.2416674416667	13.047619075	12.2615766416667	12.2227697083333	12.098126675	12.402892875	12.6640954083333	11.8348518583333	12.0417350833333	12.247510175	12.11260975	12.2236021833333	12.1342141916667	12.1737118583333	12.3448355166667	13.0973659083333	12.06738545	12.0294077916667	11.7146176666667	12.253276925	12.1865914583333	12.06738545	12.237914025	12.524997525	12.7411170833333	13.0313582416667	11.980884225	12.0977041916667	12.021831	12.1711742666667	12.0305597416667	12.1608207083333	12.296807225	12.409680475	12.77853635	12.1732634166667	12.531130425	12.05687395	12.2603464083333	11.9975093	12.18354635	12.2429038916667	12.023387575	12.05687395	12.2358628083333	12.36669285	12.0843813333333	12.0108213333333	12.3262957	12.341378075	11.0950066583333	12.6517095833333	11.6591054333333	12.4063944666667	12.3878826083333	11.4779366416667	11.9384515083333	12.04365515	12.0356360333333	11.9467504166667	12.03257945	11.7823700916667	11.6787724916667	12.1422347916667	11.0548999583333	12.0977041916667	11.6198462166667	11.806635025	11.8884513166667	11.72023525	13.0548490666667	12.1480274583333	12.14843035	12.52885135	11.8580302916667	12.2812943416667	12.798152025	11.7895299333333	11.8884513166667	12.2503999916667	12.2658594166667	12.049870975	11.5606001833333	12.0697739833333	12.4082756583333	12.07649975	11.7259183166667	12.5939827166667	11.7871666333333	12.3258832166667	12.1295438416667	12.6185993416667	11.9052300666667	11.8920055333333	11.9931218416667	11.806635025	11.7109162916667	11.2743409666667	11.8141737416667	11.7699299	11.9975093	12.2817357416667	11.85083865	12.2727355333333	11.9978846916667	12.2133193833333	13.0288304083333	11.5991025	11.5746288833333	12.2306593166667

SHANK2	11.2711403666667	12.0893148833333	11.763655725	11.7425826083333	12.317829175	12.084825925	12.021831	12.1745545	11.9071135	11.8935481166667	11.9534028083333	11.9409022083333	12.0230159333333	12.1142465833333	12.2994493333333	11.927850575	11.3579174083333	12.7500199666667	12.239207225	12.1528810833333	11.85083865	12.1815348916667	11.7943808	11.5869361333333	11.7553657	11.19202035	11.4273654166667	11.9322368833333	12.1213100833333	11.7861402083333	11.9856427083333	11.987523	12.206981325	11.3467541583333	11.2114428083333	11.13908545	12.48982835	12.0040290833333	11.5816748666667	12.0277810916667	11.424045025	11.7198061583333	11.603226975	11.51000085	11.0333724416667	11.123513725	11.2453633166667	11.0751116833333	12.0273705916667	11.8619112333333	11.7008228166667	11.8085467416667	10.9854722416667	11.0950066583333	10.7088791083333	10.7839123	11.8174744333333	11.2576230916667	12.037261675	11.621317125	12.36669285	11.770678525	11.3923413583333	11.9860534666667	11.6879616166667	12.3896401833333	11.5772577666667	12.2293758416667	12.7536835	12.0977041916667	11.9796845666667	12.1130081666667	12.1163125	11.8775774416667	11.8317467416667	11.8717593666667	12.315681175	12.503844475	12.2244042583333	12.029837025	11.7467027166667	12.0392089666667	12.0025248166667	11.6525927916667	12.0717905916667	12.0449944666667	12.1447048	11.74982705	12.2582885416667	11.6263096416667	12.037993675	12.13848275	12.2909999333333	11.9935609583333	12.0245529166667	11.836605375	12.2107949833333	12.219394525	12.500642075	11.8261862166667	11.5869361333333	12.0036527416667	11.4715256833333	11.2675581916667	11.3118389916667	11.75219825	11.2125996833333	11.3441400333333	11.8726369583333	12.0273705916667	11.7951768416667	12.023387575	10.8702441	10.715058075	10.9987091583333	10.7001015833333	11.8500517083333	11.6429698666667	11.7440902416667	11.722386025

PATJ	10.1246349583333	11.4158566083333	10.9270024916667	11.17279555	11.512504775	10.489906475	10.9335275	10.7313914916667	11.1787139083333	10.5509023833333	10.946906725	9.95059544166667	10.2321384166667	11.0873559916667	11.3911680666667	10.8228191416667	10.6614548166667	11.3438040833333	11.6698378833333	11.004921375	10.9182300166667	11.4963967083333	10.747418225	10.959358675	10.9084288583333	10.3583509666667	11.0193190916667	11.0337297583333	11.22125475	11.3520380583333	10.7964692333333	10.7526329	11.164489475	10.15852225	10.3975849666667	10.322000975	11.0953873333333	11.220898225	10.7173608833333	10.713545975	11.341686575	11.244965975	11.1339689083333	11.136541175	10.7585831916667	10.9346168	11.2811678666667	11.5291327083333	11.1095361333333	11.1840687083333	11.1923605916667	11.4978397166667	11.012770325	11.5432980083333	10.8870209166667	10.7684448166667	11.1764115416667	11.6062715833333	11.345193725	10.736058575	10.0450906333333	10.9497712833333	10.2119906333333	10.137056175	11.0992950083333	11.0916995	10.2741449416667	9.71227291666667	10.5356874083333	9.60629726666667	11.5824943666667	10.485478825	10.6384159666667	9.64074395833333	10.3623207166667	10.1420474166667	10.1524914833333	10.616587975	11.320269075	10.3044270083333	10.741111	10.609691175	10.7820632416667	9.80448268333333	10.5639796666667	10.1887239416667	10.15852225	10.5037659416667	10.7329204833333	10.1862509833333	9.810895475	10.2995461416667	11.3470919	10.5134429166667	10.6918746916667	10.4256891416667	10.7220730083333	10.836451025	10.4846654833333	10.9905726333333	11.4126401666667	10.1444546916667	10.5295700833333	10.7088791083333	10.667166075	10.6503573333333	10.892685425	10.7452538416667	10.3933623083333	10.2874114666667	11.1354232166667	11.025429175	10.468685175	10.8232399083333	10.0815238083333	10.7684448166667	10.579886175	11.1227594166667	11.4616143	10.70852575

PDLIM3	12.6996624166667	10.1393440416667	9.98633354166667	8.60646375	9.16031618333333	9.76732071666667	8.91506995	9.939168625	9.69837386666667	10.3599013416667	9.562147725	11.8519854833333	11.0101518666667	9.42204840833333	9.43636521666667	10.1246349583333	10.4040896666667	9.87254320833333	10.257101525	9.7815357	10.5360893083333	8.80870043333333	9.13909698333333	10.5432527416667	10.6187719166667	12.9677883916667	10.1054993333333	9.57610176666667	9.95643681666667	9.71227291666667	10.5752416166667	9.389870525	9.765121975	8.887867925	13.2580457083333	12.7494695	9.38141521666667	10.2955209666667	9.70668029166667	11.0931527333333	9.84589375833333	9.37269289166667	9.92664269166667	10.474825675	11.23046085	9.124627175	10.3466375416667	10.377533625	9.091403125	9.983490475	9.379662375	10.958948725	10.0611526083333	10.1895353083333	9.94886909166667	11.1623033333333	10.520091075	10.1633244583333	9.81305005833333	10.0002116916667	9.765121975	9.92053275833333	7.89296785	9.97873573333333	9.251213625	8.14959635833333	9.4842707	9.599958075	8.03117830833333	9.24539353333333	9.47897484166667	9.07484345833333	9.08396963333333	10.3698580416667	8.27021601666667	10.45072255	10.2092790583333	9.24401314166667	9.36389349166667	9.00507715833333	9.23929971666667	9.20872513333333	9.03089319166667	10.741111	9.4946079	9.86640328333333	8.19143718333333	10.8113265583333	8.84952995833333	11.0401699916667	8.70898641666667	9.05355476666667	8.998536575	9.62759855	8.97625954166667	8.32359891666667	8.01806620833333	8.5671537	8.4564653	9.023211225	9.45595239166667	9.41484764166667	8.15860931666667	8.68130785	8.1205116	8.984145025	10.8431589583333	8.6984679	9.490132625	10.541338775	9.65575276666667	7.74398054166667	9.23281155	9.06925550833333	9.92625621666667	8.94100855	9.57817145833333	8.94982423333333	8.05619188333333	9.87164061666667

SH2D7	5.03930840833333	4.21192669166667	4.42372025	5.06113309166667	4.80533786666667	4.25573735	4.41782250833333	4.65070125	4.41962694166667	4.39446349166667	3.99595204166667	4.4462942	5.26078876666667	4.04952899166667	3.9614432	4.203896025	6.02558836666667	4.42054164166667	4.508464425	3.92246294166667	5.229288475	5.528242825	4.24718985833333	4.705709175	4.4462942	4.09478471666667	4.303353375	4.99463171666667	4.310185775	4.07775664166667	4.45602740833333	4.36901661666667	4.082140775	4.04191653333333	5.04458405	4.10390375833333	4.11281023333333	4.98009509166667	4.26445661666667	4.58241979166667	4.49712886666667	5.0100817	4.4078674	5.16735583333333	5.27315905833333	4.64220138333333	4.58241979166667	4.66519471666667	4.31116040833333	4.59576983333333	4.53327731666667	4.38097856666667	3.582549625	4.98009509166667	4.082140775	4.54020328333333	3.54623583333333	4.40382505833333	3.85170159166667	4.30027178333333	4.13647209166667	4.81947958333333	4.77813741666667	4.19346535	5.37570958333333	4.812774675	4.535343	4.71218959166667	4.46791538333333	4.24335745833333	4.22787308333333	4.52522428333333	5.01100034166667	4.25387103333333	4.29817321666667	4.31295405833333	4.57030863333333	4.08322809166667	3.75838870833333	4.23196318333333	4.05071508333333	4.99463171666667	4.15118036666667	4.64220138333333	4.12334434166667	4.57343223333333	4.221324425	4.40180795833333	4.62161215833333	3.82170636666667	4.67674131666667	4.25573735	3.65809486666667	3.67977121666667	4.69642039166667	4.10724728333333	4.23901671666667	4.2939407	3.82681465	4.85768484166667	4.040897575	5.055819225	4.30917759166667	4.736033375	4.49904255833333	4.01810879166667	4.19623050833333	5.47315375	4.128952175	5.92679064166667	5.1566631	5.03526733333333	4.024477825	4.49797680833333	4.37909631666667	4.20011505833333	3.81713595	4.26445661666667	3.84482813333333	3.52588423333333

TRIM29	8.4380593	15.7781512166667	15.386313475	15.4276773083333	15.2045357166667	14.1985925	15.406759425	15.4160185666667	15.4537382666667	14.9223639	14.894107525	7.9132956625	14.7316646166667	15.7781512166667	15.3415371166667	15.1234964083333	7.404332775	15.6293082	14.8966343583333	15.5923775583333	15.4022381166667	15.85627565	15.4985918916667	15.6410257833333	14.83890155	10.133368525	15.4575903	15.3375343833333	15.1873199	15.5603668666667	15.6585444166667	15.4985918916667	15.039210975	8.97266575	7.81893236666667	8.63681648333333	15.6235572	15.6293082	15.6896696083333	15.6355001416667	15.6087237833333	15.3777313666667	15.25397645	15.369521175	14.083694575	15.3290216916667	15.5362043833333	15.6135490416667	15.618710975	15.0525159083333	15.1940996583333	15.2120960833333	15.4243027666667	15.4316128083333	15.5458909083333	15.3415371166667	15.6896696083333	15.5313549166667	15.2084540583333	15.2784907166667	14.9422941416667	13.597492475	13.57126835	13.8461367666667	13.5108418166667	14.8819536416667	13.3100924166667	14.2872888583333	13.5721495	13.9284348416667	14.392674675	14.4377971083333	14.3892469416667	13.2725108916667	14.4871709833333	13.6822736083333	13.580759625	13.5907625666667	14.8087193833333	13.917581325	13.5876594833333	13.0933531416667	14.3775303333333	12.3899971916667	14.8750732583333	13.5694284666667	12.7122547666667	13.8438449333333	14.0514423083333	13.7253859666667	14.0391426	13.8516367666667	14.1241853333333	15.0306120416667	14.2073641166667	14.550749925	14.0074276833333	13.6470212583333	13.9045177166667	13.452778075	13.48163315	14.2289632916667	14.21037625	14.4926359	14.80004595	13.5279403916667	13.9119804333333	13.698785275	13.7263656333333	14.2858034416667	14.2763428833333	14.6992893416667	13.9588693833333	14.105898725	13.8438449333333	14.0979296916667	13.2187840666667	13.61575685	14.6122307083333	13.9422518833333

ZNF506	9.71442065	10.0445903666667	9.14145633333333	9.35451149166667	9.84631699166667	9.23005591666667	9.567278375	9.11993345	9.78692435833333	9.00643414166667	8.75611661666667	9.864777125	9.091403125	9.58706739166667	10.066608375	9.76911673333333	10.2282248666667	9.112227825	9.49191133333333	9.6433763	9.60963610833333	10.1054993333333	9.094155	9.945018575	9.39626775833333	10.2096666583333	9.76051965	9.92412455833333	9.463907375	9.250740625	9.41668023333333	10.1716698166667	9.90058610833333	9.78918780833333	9.870256575	10.2080941333333	9.91801125833333	9.72449161666667	9.61680303333333	9.64480661666667	9.74483329166667	9.76179089166667	9.9993556	9.86517185	9.52591685	9.50956853333333	9.99637683333333	9.42030009166667	9.56036118333333	9.47679953333333	9.32186685	9.62275734166667	9.84117550833333	10.2501973166667	9.88883589166667	10.092918675	9.35255276666667	9.10532413333333	9.7241175	9.54880464166667	9.29384936666667	9.33579655	8.40817908333333	8.199157725	10.2351017916667	9.91374403333333	8.61290266666667	9.0973311	8.6774004	8.478972275	8.35832260833333	8.90285985	9.51454425833333	9.39574425833333	8.99899866666667	9.14145633333333	10.0852465083333	9.09833678333333	8.8768317	8.78691484166667	8.488485525	9.91503965833333	8.23175398333333	9.67592103333333	9.11737229166667	9.28361398333333	10.216230425	9.70017130833333	9.44430623333333	9.7097404	8.80450975	9.34897708333333	9.983868575	10.013853075	10.0587286583333	8.582021475	9.41849085	8.42069139166667	8.61979589166667	9.51857009166667	10.0864066083333	9.48078894166667	9.524079925	10.0561858166667	9.59906339166667	9.39574425833333	9.98920446666667	7.639894925	9.32615619166667	9.21590233333333	9.41849085	9.748599325	9.77176594166667	10.0787243333333	9.875255275	9.36206593333333	9.02609295	8.78691484166667	9.44058225	9.75110441666667

SPDYE17	12.5175610208333	12.1949811125	11.9711152125	12.0134724791667	12.4825065333333	12.3345948791667	12.24230515	11.9595334208333	12.1829263416667	12.0707912916667	12.5752464166667	12.868775825	12.5143034291667	12.3411510125	12.6369091208333	12.1949811125	11.7535850958333	12.6993955416667	12.2191461041667	12.5040819125	12.3463767125	12.9033178875	12.0434464458333	12.0468052333333	12.717755275	12.4716392	12.123574975	12.5129160833333	12.5156938416667	12.24311745	12.0818271833333	12.4089542916667	12.0862893208333	12.1743398291667	12.2757899375	12.5408405083333	12.735661975	11.9987942833333	11.9398399875	12.370533275	12.0711996166667	12.2514934666667	12.1801535291667	11.9422276666667	12.3813371875	12.0818271833333	12.2283145291667	11.9768357291667	12.1445343583333	12.44373405	11.5612236541667	12.1515431291667	12.7038726208333	12.5095726	12.0975331166667	12.3112413791667	12.1606367208333	12.0342883	12.5713055708333	12.4716392	13.685371325	12.6916405416667	12.6057562791667	12.9373902791667	12.5393457416667	12.9730078958333	12.5432295791667	12.8732963625	12.3560658833333	12.4331374458333	12.9850827166667	13.2484602541667	12.2864832	12.2850650333333	13.0268027041667	12.857028075	13.2125620791667	13.5406260375	12.4960889541667	13.0497180583333	13.2611940708333	13.5811423833333	12.1264399583333	12.2156652541667	12.712559325	13.38500205	13.4970144833333	12.1132013666667	13.7438733875	12.5295495625	12.64196235	13.5802764375	13.3289539083333	12.6124692958333	13.1068439625	13.36929045	13.3656924875	13.7487427541667	12.7018676	13.0279498208333	12.6653825625	13.0497180583333	13.0034702208333	13.0291629583333	12.557849075	12.7028655833333	12.6834027	12.4684823416667	12.6574601625	12.371453225	12.8530072583333	12.1314747166667	12.2774123791667	12.8933579375	12.3924147291667	12.2538754291667	13.5072033583333	12.9045501375	12.226260125	12.7658020041667

SPDYE11	12.5175610208333	12.1949811125	11.9711152125	12.0134724791667	12.4825065333333	12.3345948791667	12.24230515	11.9595334208333	12.1829263416667	12.0707912916667	12.5752464166667	12.868775825	12.5143034291667	12.3411510125	12.6369091208333	12.1949811125	11.7535850958333	12.6993955416667	12.2191461041667	12.5040819125	12.3463767125	12.9033178875	12.0434464458333	12.0468052333333	12.717755275	12.4716392	12.123574975	12.5129160833333	12.5156938416667	12.24311745	12.0818271833333	12.4089542916667	12.0862893208333	12.1743398291667	12.2757899375	12.5408405083333	12.735661975	11.9987942833333	11.9398399875	12.370533275	12.0711996166667	12.2514934666667	12.1801535291667	11.9422276666667	12.3813371875	12.0818271833333	12.2283145291667	11.9768357291667	12.1445343583333	12.44373405	11.5612236541667	12.1515431291667	12.7038726208333	12.5095726	12.0975331166667	12.3112413791667	12.1606367208333	12.0342883	12.5713055708333	12.4716392	13.685371325	12.6916405416667	12.6057562791667	12.9373902791667	12.5393457416667	12.9730078958333	12.5432295791667	12.8732963625	12.3560658833333	12.4331374458333	12.9850827166667	13.2484602541667	12.2864832	12.2850650333333	13.0268027041667	12.857028075	13.2125620791667	13.5406260375	12.4960889541667	13.0497180583333	13.2611940708333	13.5811423833333	12.1264399583333	12.2156652541667	12.712559325	13.38500205	13.4970144833333	12.1132013666667	13.7438733875	12.5295495625	12.64196235	13.5802764375	13.3289539083333	12.6124692958333	13.1068439625	13.36929045	13.3656924875	13.7487427541667	12.7018676	13.0279498208333	12.6653825625	13.0497180583333	13.0034702208333	13.0291629583333	12.557849075	12.7028655833333	12.6834027	12.4684823416667	12.6574601625	12.371453225	12.8530072583333	12.1314747166667	12.2774123791667	12.8933579375	12.3924147291667	12.2538754291667	13.5072033583333	12.9045501375	12.226260125	12.7658020041667

ASTN1	10.3933623083333	6.93352621666667	7.206290275	7.34272655	7.913004675	7.137920375	8.19443521666667	8.17361143333333	8.16931455833333	6.138425225	7.21396461666667	9.10749988333333	9.73831540833333	7.65894785833333	6.57887239166667	7.5385516	8.07980334166667	6.80992164166667	6.89266504166667	7.49390345833333	7.60823079166667	6.85677963333333	6.896657025	6.45696331666667	6.49010539166667	9.25394765	5.44533825	5.73297515	4.21912249166667	5.75004875833333	5.11436066666667	4.939635175	6.55667590833333	7.366539375	9.26855390833333	11.8265346166667	4.2379826	5.91627995	4.93116945833333	6.14575641666667	5.95328179166667	5.54102934166667	5.73863274166667	4.91851714166667	7.72846560833333	6.47141914166667	6.40797193333333	4.57137348333333	6.58100214166667	4.47668578333333	5.90497220833333	5.56425255	6.97843058333333	5.88432855	5.26078876666667	5.528242825	7.129719725	5.79742544166667	5.10077205	5.858085325	5.97260084166667	7.9882338	3.98271365	6.48928715	9.30275641666667	6.56579801666667	8.291997825	7.94647371666667	5.13634166666667	7.36530371666667	7.68098021666667	7.83518873333333	8.11306225833333	7.16295023333333	7.59144205833333	7.36471060833333	7.177906325	5.63443499166667	6.15692685	6.926478325	10.0260539583333	8.95262451666667	6.74474600833333	6.25026105	7.42268463333333	7.040686725	6.57504109166667	7.48300389166667	7.6287103	7.03011389166667	6.246964375	4.0078761	3.40209168333333	5.78909119166667	6.13259841666667	7.16072213333333	5.42082883333333	6.73629286666667	6.58499179166667	4.76455610833333	5.27227235	6.64834389166667	4.18603395833333	4.356950075	4.35186211666667	6.72764310833333	5.52437941666667	4.78586035833333	7.09487224166667	5.56242626666667	6.841322025	5.12102319166667	4.36901661666667	7.30805265833333	5.118961025	4.003549775	7.29169275833333	4.02568050833333	3.899834175	7.55374699166667

DACH1	7.34611101666667	9.12042280833333	8.739401675	8.21622873333333	6.34302255833333	9.37269289166667	8.635919475	8.76175106666667	7.7548948	9.56828670833333	7.9693647	8.45304260833333	7.22835589166667	9.42163085833333	7.01264725833333	10.1250607083333	10.3321140166667	5.16735583333333	8.29681115	8.69747196666667	9.38667448333333	6.5479092	8.46771705	8.60080905833333	8.78355868333333	8.01905775833333	8.83968223333333	8.6396011	7.26808264166667	8.403258675	9.20373038333333	9.07434654166667	8.86062028333333	10.0095607333333	8.903371	9.22559156666667	7.2645801	9.1773608	8.91224634166667	9.259928725	8.76125596666667	8.1959042	9.33087654166667	8.72134320833333	8.87290028333333	9.645654625	8.75473524166667	8.56622091666667	9.20190699166667	8.67987279166667	8.40516035	7.48985504166667	7.62930498333333	7.911899075	9.15935345833333	9.36480266666667	9.38667448333333	8.74671439166667	8.92890170833333	9.15555419166667	7.9703466	9.58933430833333	7.95631855833333	6.77041611666667	9.76179089166667	7.435836975	7.09933909166667	6.91976564166667	7.517539575	7.21570014166667	9.611823	7.18402409166667	6.06396311666667	8.230344325	6.65223385	5.29965199166667	8.15959780833333	7.414082675	7.87717198333333	7.09424611666667	9.607505	11.0200205166667	7.37359066666667	7.47623615833333	7.62534816666667	9.77432975	7.093633575	7.452175025	8.611944975	9.4056037	6.15858731666667	5.95410824166667	10.8021252	7.41763228333333	4.82036908333333	7.87768698333333	7.57903273333333	7.62358238333333	5.56781358333333	8.325164675	8.122791675	7.86334303333333	6.17390685	8.11825858333333	7.187671825	7.78978061666667	6.76714710833333	7.98717739166667	6.603384975	8.604857825	9.33181315833333	8.3662572	6.31548078333333	7.19697114166667	6.64156519166667	7.96623754166667	11.2366875083333	6.74610281666667	8.9384783	7.95035304166667

CRLS1	11.6084924916667	11.081884475	11.5212430583333	11.39829615	11.1575927916667	11.2675581916667	11.2642606083333	11.4478274166667	11.1806950666667	11.4052792333333	11.597258825	11.0670126666667	10.8657939916667	11.181831125	11.1634048416667	11.270439275	13.214199825	10.992544275	11.5626319666667	11.4049590333333	11.3047106916667	11.123513725	11.4763556583333	11.49381285	11.234518675	11.5706660333333	11.233793875	11.2743409666667	11.0560163166667	11.46655045	11.3339882	11.2826013166667	11.025429175	12.50431935	11.4336132833333	11.6525927916667	11.0905957416667	11.4081227125	11.4188332333333	11.3147571833333	11.675724725	11.5093017916667	11.4500480416667	11.7936196666667	11.4704736916667	11.6944435416667	11.5027335583333	11.4752696416667	11.2753469583333	11.7219865583333	11.3132862083333	11.5994743583333	11.1441289083333	11.2861342166667	11.4908711166667	11.4439013	11.3774736833333	11.5392863166667	11.2154273083333	11.4336132833333	12.5775136	11.739559375	11.8908867916667	12.2461720416667	11.4915511416667	10.982052575	12.5202342666667	11.7288583666667	11.937226525	11.5034674333333	11.876483925	12.2981372583333	12.2951630416667	11.23878845	11.2254735166667	11.830137	11.5672571	11.2510757083333	11.715671375	11.4181579	11.8281920416667	11.3351005833333	11.4038169	11.9071135	11.005303025	11.4288195833333	12.2188976833333	11.913931575	11.22371335	11.8008623833333	12.0140512666667	12.478685375	11.3809445833333	11.2786369583333	12.4501055333333	11.4503715	11.6314027333333	11.3147571833333	11.5422054916667	11.9951551416667	12.0525821583333	12.4855430666667	11.6503712333333	11.3846584083333	12.2492084583333	11.0964662083333	11.7517355166667	12.3275837583333	12.0474514	11.545061825	11.5429763166667	11.102188775	11.7123616416667	12.4746445166667	11.5219495083333	11.7049796083333	12.187427525	11.86892365	11.3579174083333	11.460533275

PLEKHA7	7.85854243333333	12.3280561	12.2727355333333	12.4767941833333	11.7470404	10.3289721	11.8607926333333	11.7525932916667	11.8584258416667	10.7718274166667	11.0598611541667	8.126799325	11.197870325	12.5457898	11.7861402083333	11.2499394833333	11.2947092833333	11.43032555	12.1513649666667	11.8375242416667	12.1100796583333	12.5443972083333	11.4635169166667	11.2650373666667	11.742927775	8.6731133	11.9420280583333	11.7332325916667	11.9036209583333	12.226050525	11.7231538416667	12.3418182	11.6569750083333	11.0548999583333	8.4816187	7.99667675833333	12.13546815	11.9345074833333	11.471171625	11.3571575333333	11.8973789916667	12.2240198833333	12.1528810833333	12.055992875	11.2262281166667	11.7857517916667	12.0905289333333	12.43292905	12.2808809166667	10.96963075	11.6010090666667	11.811945375	11.5226770083333	12.0819963833333	12.02261385	11.8573117083333	11.9352971916667	11.5400258416667	11.8599637666667	11.3347189916667	7.94329386666667	9.00330966666667	10.9861853	9.87345979166667	11.2568448166667	8.736017975	8.90142945833333	9.716989375	7.55016986666667	10.0056583	9.20822946666667	8.49699819166667	8.60751143333333	9.21590233333333	9.65101713333333	9.11737229166667	10.3078222333333	9.98596199166667	9.03641039166667	10.3566062583333	10.382913225	9.776982075	9.82123129166667	8.67688486666667	9.6530765	8.85728129166667	8.93461839166667	9.4233862	9.21543308333333	9.06460029166667	8.64839240833333	8.953569225	9.41892754166667	8.35934713333333	7.70532880833333	9.84117550833333	10.08403975	8.62786819166667	8.61820814166667	9.04692176666667	8.1719332	9.21923738333333	8.97675146666667	10.7158953333333	9.80664778333333	9.11782889166667	9.81345976666667	9.91673698333333	8.647921775	8.228841125	9.97131721666667	9.82078890833333	8.89405555	9.22832763333333	9.696676675	10.4149521166667	11.2660555333333	8.739401675	10.3917443	9.370796075

TUBB2A	11.21907835	11.2911160333333	12.14843035	10.970347325	12.4110018333333	11.2775845	11.1836658583333	12.1198078916667	11.6363117083333	11.937226525	11.0419739083333	10.6050909833333	12.2227697083333	11.685253825	12.5921909166667	10.7900238833333	10.3844721666667	12.8715923291667	12.0181445166667	11.7613047833333	11.5902054333333	11.500844625	11.7750094	12.0388164916667	11.5196693333333	11.5392863166667	11.7373039666667	11.7227876083333	12.4510079166667	11.6444959666667	12.8776683083333	11.3997255833333	11.4904941666667	10.4037201666667	11.5150484916667	11.7324169333333	12.2959914833333	11.9458994666667	12.2743988	11.79216385	11.7262372833333	11.7418730833333	10.8326576083333	11.6448777666667	10.6649055833333	12.2024267333333	11.673083125	12.3824722	10.4072694666667	11.3782358083333	12.0301876583333	11.61191255	11.651839775	11.4726344	11.98404715	11.6890849333333	11.5728018416667	11.873004525	11.4083049	11.0083196416667	11.5742275583333	11.7008228166667	11.4296055416667	11.021874925	12.1922906583333	11.8089541	11.61832875	11.5330541666667	11.5895178083333	10.8239850833333	10.9564158416667	11.230125425	12.7594059333333	11.500844625	12.4536816333333	11.7278187083333	10.76392445	12.369348425	12.4981202833333	11.268229675	11.3001028166667	12.3580122083333	12.7206576333333	12.1213100833333	11.9032491333333	11.6726997416667	11.196768475	11.8293448166667	11.39829615	11.5626319666667	12.7681554	11.4996324916667	12.4795096083333	11.6536243916667	11.84387985	11.1874035166667	11.75788825	11.7239413833333	13.3032240666667	11.0924307333333	11.685558375	11.3268344833333	10.38024835	11.31764545	11.5185086916667	11.5389498916667	12.0860846083333	12.2006641666667	11.1162112666667	11.6025118833333	11.1039790666667	12.004381825	12.1478229208333	11.3212969833333	12.452378375	11.3047106916667	11.3954232	12.8256923916667	11.3934696166667	11.1003561666667

NBPF9	10.1489854166667	9.71737348333333	9.95359933333333	9.66065478333333	10.9647570833333	10.1113541833333	11.1539711166667	10.1080216833333	10.476761175	9.41668023333333	10.3519044416667	11.3306591916667	10.9567732916667	9.99767481666667	9.84876684166667	10.0502702166667	9.26809461666667	11.2032221333333	9.12986750833333	9.879959075	10.390544	10.1593240833333	9.55161105	10.2422793916667	10.3070468	10.558939175	10.257101525	9.78824573333333	9.7752348	9.49238265	9.82203395	10.1485742083333	10.216230425	9.29065144166667	10.0189542666667	10.4728823833333	9.97912404166667	9.00775005833333	9.56361585	9.27853935	8.54500405833333	9.14377724166667	8.68329548333333	8.462988175	9.26988130833333	8.27907085	8.61392724166667	8.61138693333333	9.32574429166667	9.75321625	9.49057863333333	9.4955569	10.2427233083333	9.19531680833333	8.90285985	8.23717023333333	9.52642665	8.36515990833333	9.74218338333333	9.14377724166667	11.6444959666667	10.5049759833333	10.3953052083333	10.6027208583333	10.5699475166667	11.0975459833333	10.7936080833333	10.7774965	10.8131866083333	10.5119603416667	11.0215105083333	10.7143764583333	11.1905443833333	11.1670443166667	10.7108576583333	10.9790999666667	10.8480286083333	10.7743973416667	9.81686935833333	10.6990064	11.11993505	11.7768584833333	10.7581339416667	10.3887558333333	10.7767059333333	10.7936080833333	11.8089541	10.3475834	11.30185685	10.5049759833333	10.362641575	11.7078053166667	10.534888275	9.98150248333333	11.67393075	11.2822211083333	11.3351005833333	11.464693675	10.7778847083333	9.844981225	9.9519621	10.151687475	10.040006825	8.69025928333333	9.59824975833333	10.2450271333333	10.0753226583333	9.24751750833333	9.84841021666667	10.0965198666667	10.2924466666667	8.486995925	10.0852465083333	9.12938660833333	9.670115925	9.51133800833333	10.3526835916667	9.59949791666667	9.16396135833333	9.92254819166667

SRRD	11.501628025	10.9790999666667	10.1687340166667	10.2060439416667	10.7407277583333	10.8616146666667	10.319917075	10.883221475	10.3698580416667	10.239159725	10.3530735416667	10.7722019	10.5775565833333	10.5703833416667	10.6038899083333	10.0819455833333	10.859746575	10.4112315166667	10.70240295	10.7235859166667	10.7696102583333	10.7508267666667	10.9259950416667	10.815793925	10.591411875	11.18844885	10.474825675	10.220242425	10.76392445	10.5291936916667	10.563601	10.8090233916667	10.2838388166667	10.541338775	10.7900238833333	11.0870314666667	10.4080745833333	10.8180008083333	10.2604506416667	10.880387475	10.920072175	10.577240575	10.524814775	10.8484072166667	10.6926124666667	10.6910121333333	10.874072375	10.8988570583333	10.621550725	10.585362525	10.6453411416667	10.5545011333333	10.3135871416667	10.457410175	10.1258089583333	10.1067055416667	10.8337493916667	10.7368008833333	10.5500822583333	10.7846923083333	11.3011728416667	11.4083049	10.8585888666667	10.970347325	11.721307075	11.401582575	11.6687463083333	10.8224026333333	11.3556571416667	10.4597952916667	10.8657939916667	10.7804261416667	11.5702459583333	10.8544918	11.376011025	11.5816748666667	11.2779260833333	10.715058075	11.7871666333333	10.5814300416667	11.4424334416667	11.1731632666667	11.121759625	11.233793875	10.815793925	11.351341375	10.5991330666667	11.3546484166667	10.4744353333333	11.2833533833333	11.1975258	10.4145396416667	10.2745932916667	11.5356555333333	11.0430757083333	10.991320675	11.1154972666667	10.4268652916667	11.047689175	11.339247	11.1113391916667	10.8743697916667	10.54728675	11.3954232	10.8581963333333	11.376411175	11.5828548666667	10.970347325	11.4545376666667	11.628296575	10.992544275	11.3282347083333	11.395075525	10.5260512	10.334173575	10.631761275	11.5813004	11.7868322833333	11.9108497083333	10.7616896916667

AC058822.1	7.69369399166667	7.66388121666667	7.85962578333333	7.619792375	7.86670446666667	7.76137635	8.55750678333333	8.328023075	8.08240869166667	8.0095181	7.88018380833333	7.92709596666667	7.67359318333333	7.58408555833333	7.32727508333333	7.02222090833333	7.57402961666667	7.6071886	7.2266449	7.004281725	7.672013725	7.14272935833333	7.746730925	7.12725256666667	7.47951195833333	7.70256570833333	7.978659025	7.70977168333333	7.50051165833333	7.84154363333333	7.948855	7.60888643333333	7.99458134166667	8.20377755	7.72354014166667	7.506757	7.74294895	7.90991645	7.99615545	8.27339385833333	8.11654126666667	7.838427925	7.93859618333333	7.91794835833333	7.7528692	7.42103133333333	7.91739750833333	8.20689531666667	7.84796001666667	7.49442741666667	8.27121185	8.14709130833333	7.93695109166667	7.92709596666667	8.23767453333333	7.72294644166667	7.8129644	8.27384794166667	7.76084795833333	7.458458425	7.33974354166667	7.77718458333333	7.74896025833333	7.48985504166667	7.9693647	7.87613041666667	8.05032904166667	7.95962858333333	8.013232325	7.83240795833333	8.17613399166667	8.11454491666667	7.54341655833333	7.22598754166667	7.9184414	7.75661866666667	7.379877425	7.33095725	7.43234464166667	7.15455295	7.58238585833333	7.23553730833333	7.55486798333333	7.69319276666667	7.833966975	7.89775408333333	8.3301117	7.76188296666667	7.47140944166667	7.6781919	7.726376575	7.79903776666667	8.34181428333333	7.69919105	7.88927118333333	7.9089283	7.900836625	8.05619188333333	8.28541158333333	8.036488875	8.00462699166667	8.472856875	8.18571946666667	8.01806620833333	7.56216311666667	7.81893236666667	7.452175025	7.5954683	7.36048969166667	7.41942373333333	7.71969125833333	8.530508075	7.868368375	8.07471265833333	7.93805580833333	7.72085915833333	7.84588375	8.13716783333333	8.22981704166667	7.74722135833333

DLX4	4.85659321666667	4.51953901666667	5.45375554166667	4.97177571666667	5.48616714166667	5.81563615833333	4.84094744166667	6.234662425	4.664314175	5.78709845833333	6.05561763333333	4.21592141666667	3.87406658333333	4.57444614166667	6.67783206666667	6.34302255833333	4.80316131666667	7.68436408333333	6.82240235833333	6.34225285833333	5.78160560833333	5.95243336666667	7.89243905	5.17804041666667	5.2108879	4.71016199166667	6.26738683333333	7.14336814166667	5.35794613333333	5.79203019166667	6.02558836666667	5.24931593333333	4.97812506666667	4.49216794166667	5.20060255	5.18759319166667	7.30431380833333	6.8347138	5.2108879	6.72705409166667	6.328333625	6.633116075	5.54779774166667	7.17093558333333	5.29123589166667	7.22238968333333	5.231579	3.86964848333333	6.83207999166667	6.23620956666667	7.14213880833333	5.58117110833333	5.35361239166667	7.12280756666667	6.060789425	6.62158961666667	4.89364545833333	7.02536274166667	6.66054216666667	5.6458775	7.41824386666667	5.98737700833333	7.01007860833333	8.560933175	8.73498088333333	9.11864413333333	8.104851775	6.311629325	8.48261985	5.79742544166667	5.94013145833333	6.914548675	8.95991093333333	6.23079429166667	8.06812029166667	7.82339403333333	5.78160560833333	6.46133099166667	9.24492166666667	6.159395425	10.1700709416667	9.41389706666667	8.635919475	7.27471595833333	6.31242605	8.82825418333333	9.119480775	7.12586193333333	8.001514325	6.780385325	7.22299226666667	7.466283575	7.55609866666667	6.95572241666667	7.927602375	7.004281725	7.58521366666667	9.630635475	8.42124595	7.37811973333333	9.40640285	6.77636395833333	6.93552516666667	7.2266449	9.07369755833333	7.4656947	8.66201021666667	9.93796431666667	9.03417474166667	8.36380941666667	7.10434548333333	8.854456025	10.6937764083333	8.92218960833333	8.37257153333333	6.93685505	11.1901541416667	8.482136425	7.729718875	3.99172610833333

FCGR1A	7.05900955833333	8.73547365	9.07021043333333	8.24802405	8.9217463	8.35736911666667	9.6185904	9.39938098333333	9.545824775	8.594439725	9.25394765	9.663801325	9.58222316666667	8.902412325	7.83726095833333	8.151087675	8.35934713333333	8.71144663333333	8.86314575	8.09007435833333	8.93461839166667	8.59302528333333	8.83115135	9.53443595	7.85051325833333	8.6223815	8.83783124166667	7.04755845833333	7.15697099166667	7.310825425	8.755680875	9.32329896666667	7.69541339166667	8.268113325	9.40597950833333	7.4784294	7.20568426666667	8.70212718333333	8.8559255	8.19700369166667	8.27907085	8.27954998333333	7.908404275	6.54299384166667	8.20587135	8.36292435	7.1989222	9.073438725	7.98193376666667	7.05455971666667	9.55671014166667	8.8319761	9.040322975	8.75371965	7.56216311666667	7.80329328333333	8.682814325	8.42476003333333	7.87369893333333	8.69459336666667	8.685246125	9.75665824166667	8.92364006666667	8.41742745833333	8.8526135	8.20990576666667	8.98237701666667	9.51896663333333	8.59201043333333	11.51000085	8.88496090833333	8.63681648333333	9.39113676666667	9.50725341666667	9.490132625	9.22160494166667	9.62969725	9.79486831666667	8.86366274166667	9.55472915	8.95031519166667	9.43184414166667	8.38817050833333	8.94233088333333	8.75882765	8.50751961666667	9.06551128333333	9.75194415	8.95490616666667	8.57649310833333	9.06069035833333	8.65367691666667	7.38755610833333	8.69317739166667	9.2612694	9.59742406666667	8.55804185	8.083905375	8.47850595	7.31919828333333	8.8778449	8.65999509166667	10.1042493666667	7.78767925833333	8.26308525833333	9.50264109166667	8.25344699166667	8.65367691666667	9.57464318333333	8.4380593	8.79596970833333	7.676574425	7.96361378333333	8.34866896666667	9.52456219166667	8.79346453333333	7.58961838333333	7.58465230833333	7.82958231666667	10.2442419583333

MUC6	11.0720193666667	9.10051520833333	8.31814086666667	9.55671014166667	9.36247203333333	18.0274730833333	9.72757016666667	8.77676135833333	7.960228575	8.65947190833333	8.97168731666667	10.5081553333333	10.235923825	8.920781025	7.95464479166667	8.9217463	17.1992909916667	9.57692496666667	8.29630441666667	7.91097105833333	8.91275618333333	9.28041066666667	8.10391784166667	8.84428041666667	8.05569140833333	9.226998075	8.439473525	8.93762295	9.608728125	8.91135703333333	8.9604306	7.49826404166667	8.38817050833333	17.4981071833333	7.78383044166667	8.37211495	8.74671439166667	7.74722135833333	7.84545261666667	8.02564620833333	7.36878105	9.69747909166667	8.269127475	7.434165875	9.49908795	7.85577905833333	7.86283530833333	8.4380593	7.33857021666667	7.03384980833333	7.21570014166667	7.28500960833333	8.74719128333333	8.618769925	7.81610706666667	6.524903425	8.24906243333333	7.72294644166667	8.32106236666667	7.40602305833333	8.83738886666667	9.121301075	8.7292202	9.81305005833333	9.00643414166667	14.5924675083333	8.06551123333333	9.62275734166667	9.489247625	8.99987959166667	8.97957231666667	9.26577935	10.9139727166667	9.75787298333333	8.19188733333333	7.745650475	14.648708375	8.21046635	7.6798049	8.41742745833333	12.1665042	8.60344725	8.49745698333333	8.43095395833333	9.83719281666667	9.56873605	10.9069463833333	9.1530551	8.89405555	7.68936630833333	9.37269289166667	7.65774509166667	8.351126275	15.169521525	8.536875925	7.79136785	8.1455177	9.44516316666667	8.3471697	7.8345672	8.27654828333333	10.689872025	8.5196153	7.77269480833333	8.99943493333333	8.46245245833333	8.21782325833333	8.123259225	7.83071075833333	6.70390775833333	9.50499393333333	6.2858525	7.76512125	8.94100855	7.61520518333333	7.39564355833333	7.62534816666667	7.32974516666667	7.28259465	7.36272230833333

AC004687.2	6.74574984583333	11.8038525208333	11.6877457208333	11.7729869875	11.1097335958333	10.7875918625	10.9071544916667	11.3152530458333	11.6004544833333	10.6085738375	11.0176813166667	6.0551801375	10.3283500958333	11.9087522458333	11.6527708166667	10.9172764083333	11.2878174625	10.98074875	11.2970477416667	11.4355900791667	11.0245256375	11.7087814458333	11.8042162625	9.89615510416667	11.0661219916667	7.4350040875	11.2949014083333	11.6117236583333	11.0252346958333	11.5752343791667	10.7354198833333	11.3522246166667	11.2361304166667	11.1257960708333	7.05368882916667	7.27385164583333	11.7068914541667	11.33833685	10.8174508541667	11.1805434041667	12.1399871	11.8214699833333	11.5029129333333	11.5373092375	10.77343325	11.9481228458333	11.6357464125	11.4422512791667	11.4404071	11.6847171458333	11.3637405416667	11.310311475	12.0086714041667	11.7547114875	12.015862275	12.0442765708333	11.3052728291667	11.7014141	11.8117753583333	11.2956085666667	10.3827338541667	9.67613483333333	11.1240252041667	11.9076306791667	13.1391942625	10.8772896791667	10.9377175083333	11.0792174166667	11.251242475	10.0759191291667	10.6870947833333	10.2106099416667	11.7832821416667	10.0461345	11.7133270291667	10.986370725	10.6021290208333	10.0522598458333	11.4171937291667	10.9507319916667	13.09365415	12.5688122041667	11.5511322541667	10.4723012166667	7.93779526666667	11.015543	12.3463767125	9.67076629583333	10.3197107125	10.730804625	10.2776143791667	11.3837239291667	12.0386090333333	10.4873511583333	10.9008579166667	11.4028483375	11.2655569916667	12.3642732833333	10.7754033458333	13.09365415	10.2512927708333	11.44690205	10.9852987875	11.7431122625	11.2684137125	11.4877701083333	11.2548487333333	12.2088101416667	10.9522035708333	11.4010477791667	12.1040788083333	11.4415189666667	11.1293381416667	12.1868104916667	11.3023735333333	11.3873694791667	13.0599306041667	12.0739248208333	11.7535850958333	11.5002773166667

RNF43	6.74574984583333	11.8038525208333	11.6877457208333	11.7729869875	11.1097335958333	10.7875918625	10.9071544916667	11.3152530458333	11.6004544833333	10.6085738375	11.0176813166667	6.0551801375	10.3283500958333	11.9087522458333	11.6527708166667	10.9172764083333	11.2878174625	10.98074875	11.2970477416667	11.4355900791667	11.0245256375	11.7087814458333	11.8042162625	9.89615510416667	11.0661219916667	7.4350040875	11.2949014083333	11.6117236583333	11.0252346958333	11.5752343791667	10.7354198833333	11.3522246166667	11.2361304166667	11.1257960708333	7.05368882916667	7.27385164583333	11.7068914541667	11.33833685	10.8174508541667	11.1805434041667	12.1399871	11.8214699833333	11.5029129333333	11.5373092375	10.77343325	11.9481228458333	11.6357464125	11.4422512791667	11.4404071	11.6847171458333	11.3637405416667	11.310311475	12.0086714041667	11.7547114875	12.015862275	12.0442765708333	11.3052728291667	11.7014141	11.8117753583333	11.2956085666667	10.3827338541667	9.67613483333333	11.1240252041667	11.9076306791667	13.1391942625	10.8772896791667	10.9377175083333	11.0792174166667	11.251242475	10.0759191291667	10.6870947833333	10.2106099416667	11.7832821416667	10.0461345	11.7133270291667	10.986370725	10.6021290208333	10.0522598458333	11.4171937291667	10.9507319916667	13.09365415	12.5688122041667	11.5511322541667	10.4723012166667	7.93779526666667	11.015543	12.3463767125	9.67076629583333	10.3197107125	10.730804625	10.2776143791667	11.3837239291667	12.0386090333333	10.4873511583333	10.9008579166667	11.4028483375	11.2655569916667	12.3642732833333	10.7754033458333	13.09365415	10.2512927708333	11.44690205	10.9852987875	11.7431122625	11.2684137125	11.4877701083333	11.2548487333333	12.2088101416667	10.9522035708333	11.4010477791667	12.1040788083333	11.4415189666667	11.1293381416667	12.1868104916667	11.3023735333333	11.3873694791667	13.0599306041667	12.0739248208333	11.7535850958333	11.5002773166667

TXNRD2	7.52054051666667	7.09487224166667	7.33740245	7.65774509166667	7.46514316666667	6.5574676	7.13728840833333	7.16614005833333	7.49991619166667	7.61760009166667	7.100006625	6.93418845	6.46055301666667	6.345260775	7.51365925	6.82038475833333	6.87917671666667	7.22176664166667	7.08738564166667	6.931726825	6.90460845	6.89400789166667	6.849792025	7.64049128333333	6.84200789166667	7.71409900833333	7.086730875	7.31919828333333	7.43130181666667	7.3968942	7.83348010833333	7.13168135	6.82167365	6.48217831666667	7.893532975	7.87369893333333	7.86062830833333	7.47900525	7.83894598333333	7.93805580833333	7.91142259166667	7.59310200833333	7.1508922	7.35627839166667	6.62436875833333	6.91634240833333	6.61484865833333	6.73694150833333	7.44368639166667	7.070041725	7.35503378333333	7.49332154166667	6.5479092	7.78057345	6.95924915	6.78246663333333	6.83400405	7.76403325833333	6.92717863333333	7.29732286666667	7.17735376666667	7.38404451666667	7.32668075833333	7.56268353333333	7.09933909166667	8.024066175	7.61006516666667	7.16232548333333	7.47900525	7.13168135	6.75303653333333	7.27649536666667	7.2266449	6.47541770416667	6.53273724166667	7.23429453333333	6.77501233333333	6.909113525	7.22176664166667	6.75303653333333	6.64088086666667	6.12452900833333	7.67088145	8.32012975833333	7.57619343333333	6.909113525	6.66175125	7.39745536666667	6.89400789166667	7.81088345	7.40026851666667	7.33095725	6.931726825	7.76345526666667	7.691592325	7.05838413333333	7.50291813333333	6.632460225	7.51872279166667	7.10505258333333	7.28500960833333	6.32751184166667	6.58035519166667	6.96657695833333	7.44735373333333	5.86087000833333	6.62436875833333	7.83894598333333	7.38755610833333	7.82958231666667	6.78642360833333	7.70471373333333	7.846884125	7.60297259166667	7.0176409	6.735668325	6.74958845833333	6.94820169166667	7.39975843333333	5.83306814166667

MUC2	6.65424311666667	5.40518485833333	5.10716860833333	4.87199023333333	5.81478479166667	6.36709734166667	4.89595425	4.98771553333333	5.90945328333333	6.1039579	4.58336269166667	5.04557781666667	4.35819395	4.736033375	5.40852341666667	4.262047775	6.04523669166667	4.72222786666667	5.93571900833333	5.24703029166667	4.97490869166667	5.57926250833333	5.21412320833333	4.98219815	5.64021978333333	5.54102934166667	5.37226380833333	5.61059491666667	5.95328179166667	5.52011276666667	6.08593745	5.10077205	5.33557505	6.393024275	5.99091528333333	6.00039116666667	6.23865941666667	6.44887243333333	6.872774725	5.31614614166667	6.44063283333333	7.198288025	6.39152516666667	6.04962533333333	5.49035004166667	5.7550021	5.71315170833333	5.41039171666667	5.71218308333333	6.013009825	6.21030875833333	5.824619025	5.63443499166667	6.245275725	6.04445175	6.58901686666667	5.108231475	6.24107090833333	5.99091528333333	5.980495875	5.44971561666667	5.30562465833333	5.72573654166667	5.270972825	4.90470955	5.79999791666667	5.42880058333333	5.13227493333333	7.79903776666667	6.62775695	4.86289075	5.34297193333333	4.310185775	4.54440353333333	4.33532039166667	4.19044525833333	8.62084126666667	4.79109571666667	4.72828980833333	5.42175125833333	4.816016475	4.00448406666667	5.49035004166667	5.40201090833333	4.8072374	4.52418780833333	4.82464924166667	4.05417596666667	4.89935284166667	4.99137435	5.07218190833333	5.58021739166667	5.57252066666667	5.59857721666667	5.38762225833333	4.72222786666667	5.64973066666667	6.14334293333333	6.52344795833333	5.72466863333333	5.64406685833333	5.06232444166667	5.37226380833333	5.26783745	5.68402035	4.46004483333333	5.32442271666667	5.17265765	4.86914890833333	6.20408345833333	5.14499941666667	5.67139935833333	5.895388975	7.63102280833333	5.33557505	5.48703414166667	5.000034475	5.81212459166667	6.03335128333333	4.472817375

ATMIN	12.0903471208333	13.0868204833333	13.0087094	12.9680656791667	12.5290985833333	12.2459723458333	12.7434371916667	13.0028769291667	12.819467025	12.7120155125	12.8033610791667	12.1128089583333	12.3027801833333	13.1360170875	13.1430271083333	12.7978924416667	12.4699238583333	12.5470533458333	13.22308885	12.9367580708333	12.9187786625	13.1803611708333	12.8524731291667	12.7870957291667	12.837906425	12.4476246041667	12.9497126916667	12.8484686083333	12.660880825	12.8993470541667	12.6584126541667	12.9898112958333	12.8088675833333	12.176399	12.2233819	12.2414428166667	12.908462075	12.9992294125	12.4561353291667	12.8179045583333	12.9015734583333	12.822684975	12.9176011666667	12.9216683583333	12.6869957833333	12.9314856625	13.0242482333333	13.1141222166667	12.9686119833333	12.7572069958333	12.6208630375	12.7398267458333	12.6902568875	13.107509625	13.0164748458333	12.8468209	12.8780324583333	13.1977065333333	12.9458751541667	12.8638697333333	11.3334242791667	12.3226681416667	12.0106203833333	11.8132392666667	12.3791966	11.3405073083333	12.2733797791667	12.0932310083333	11.6139392833333	11.8486849583333	12.3990288208333	12.8457431541667	11.91448655	12.4561353291667	11.5179760333333	12.3394526291667	11.5217724375	12.4825065333333	12.1486557291667	12.6988560541667	12.1988122958333	12.5055292	12.24230515	11.9992035916667	12.2974883666667	12.6272304208333	11.8781766875	12.160227575	12.1020933166667	12.4653075	12.3218060416667	12.0553632	12.0535542458333	12.6183459541667	11.6531202375	12.861592325	11.0627641416667	12.4192060458333	12.5133608291667	12.1859093958333	12.5040819125	12.1821248958333	12.5323125291667	12.4125511666667	12.322224925	12.6057562791667	12.4317909	11.8839993208333	12.4815793708333	12.1277070333333	12.4013101125	12.2737573	11.772617675	11.6285029125	12.1630015833333	12.810483825	12.7663321416667	11.3251647291667	12.2468516833333	12.3209884125

AC092718.8	12.0903471208333	13.0868204833333	13.0087094	12.9680656791667	12.5290985833333	12.2459723458333	12.7434371916667	13.0028769291667	12.819467025	12.7120155125	12.8033610791667	12.1128089583333	12.3027801833333	13.1360170875	13.1430271083333	12.7978924416667	12.4699238583333	12.5470533458333	13.22308885	12.9367580708333	12.9187786625	13.1803611708333	12.8524731291667	12.7870957291667	12.837906425	12.4476246041667	12.9497126916667	12.8484686083333	12.660880825	12.8993470541667	12.6584126541667	12.9898112958333	12.8088675833333	12.176399	12.2233819	12.2414428166667	12.908462075	12.9992294125	12.4561353291667	12.8179045583333	12.9015734583333	12.822684975	12.9176011666667	12.9216683583333	12.6869957833333	12.9314856625	13.0242482333333	13.1141222166667	12.9686119833333	12.7572069958333	12.6208630375	12.7398267458333	12.6902568875	13.107509625	13.0164748458333	12.8468209	12.8780324583333	13.1977065333333	12.9458751541667	12.8638697333333	11.3334242791667	12.3226681416667	12.0106203833333	11.8132392666667	12.3791966	11.3405073083333	12.2733797791667	12.0932310083333	11.6139392833333	11.8486849583333	12.3990288208333	12.8457431541667	11.91448655	12.4561353291667	11.5179760333333	12.3394526291667	11.5217724375	12.4825065333333	12.1486557291667	12.6988560541667	12.1988122958333	12.5055292	12.24230515	11.9992035916667	12.2974883666667	12.6272304208333	11.8781766875	12.160227575	12.1020933166667	12.4653075	12.3218060416667	12.0553632	12.0535542458333	12.6183459541667	11.6531202375	12.861592325	11.0627641416667	12.4192060458333	12.5133608291667	12.1859093958333	12.5040819125	12.1821248958333	12.5323125291667	12.4125511666667	12.322224925	12.6057562791667	12.4317909	11.8839993208333	12.4815793708333	12.1277070333333	12.4013101125	12.2737573	11.772617675	11.6285029125	12.1630015833333	12.810483825	12.7663321416667	11.3251647291667	12.2468516833333	12.3209884125

AKT3	12.3079459333333	10.0091086833333	9.72111820833333	8.3301117	9.32000775	10.6686985416667	9.990066925	10.1432492	9.88594978333333	10.6352329833333	10.6395867	11.8535038916667	11.2071862916667	9.92664269166667	9.69792198333333	10.3277465	9.94748681666667	9.346282425	10.27531875	10.6819563666667	10.6407861166667	8.52279583333333	9.94714974166667	9.68602584166667	10.04039335	12.39392575	10.528057425	9.62453731666667	8.68470500833333	10.1312217666667	10.49265135	9.56407003333333	10.2217906833333	9.98111111666667	12.5534552416667	12.6032318833333	9.145650675	10.2153563166667	10.6792669083333	10.5041729666667	10.0889846333333	10.400070625	10.3968247166667	10.520091075	11.348473325	10.2301923	11.0045884333333	9.81728660833333	10.064052975	9.95147331666667	10.182857025	10.123368025	9.05454125	9.31001445	9.70714295	10.4922259333333	10.4771670916667	10.0378686583333	10.4300821583333	10.625983	10.7069670333333	11.0369926416667	8.99771499166667	9.73788834166667	10.0343496666667	9.286424975	10.736058575	10.5276582916667	9.70796235833333	10.0061679666667	11.2602582666667	11.4883355166667	9.70748555	10.8581963333333	8.686777175	9.97752425	10.8001996166667	10.116767025	9.88515190833333	9.99216454166667	10.7665838833333	11.6628809833333	10.4080745833333	10.99552285	9.66513115	10.7631580166667	10.7635056583333	10.2543488416667	11.2205665583333	11.21620365	9.65399733333333	9.57222860833333	11.0826274416667	10.2699462416667	9.09505498333333	9.54834169166667	8.619247325	11.3332438	9.35995134166667	8.85962865	10.7944294666667	10.7706214833333	11.6179147583333	10.1156550583333	9.26988130833333	9.72531675833333	10.5821763	9.24539353333333	10.2060439416667	11.0666847416667	10.49466965	10.027285575	8.7812519	9.045519875	9.64383103333333	9.296236625	11.517039175	9.73577058333333	9.41668023333333	9.98596199166667

KIFC3	9.92375435	9.960644375	10.4519100666667	10.1737764166667	10.4963081083333	9.95147331666667	10.3182950083333	10.3362368833333	10.4253305166667	10.4240891833333	10.079544525	9.74483329166667	10.244614225	10.2511057916667	10.5441182916667	9.83237755833333	9.6530765	10.661840225	10.4843028583333	10.0260539583333	10.3901677	10.216230425	10.30932905	10.9073626	10.2206735166667	10.2318091916667	10.399176825	10.0061679666667	10.5833260083333	10.3305153666667	10.4990563	10.4634265083333	10.145640125	9.85368629166667	10.2282248666667	10.2096666583333	10.4204003083333	10.3345761083333	10.5166009333333	10.2881617	10.3427675333333	10.2703263916667	9.843730775	10.059122075	9.836699675	10.2765561833333	10.2450271333333	10.4661023416667	10.102550325	10.0163833	10.3427675333333	10.7808534	10.388343775	10.634390675	10.3119751583333	10.1254167416667	10.5268243666667	10.4204003083333	10.2769692583333	9.97833993333333	10.7959852666667	10.3459231416667	10.5204657833333	10.1226723083333	9.98509406666667	11.3911680666667	10.338453	10.3147212916667	10.632187725	10.46219945	10.0061679666667	10.63689965	11.0560163166667	10.3913250333333	10.5930279833333	10.6391956666667	10.3610713833333	10.8025054583333	11.0483413166667	10.2928305416667	9.89341510833333	10.6019249583333	10.7287943416667	10.3132196333333	10.400070625	10.4348946583333	10.3143367833333	9.86359230833333	10.259584625	10.326957425	10.7158953333333	10.7581339416667	10.3225014083333	11.2464355333333	11.0079045333333	10.5376179083333	10.39251605	10.6245043083333	10.86272805	10.0533049083333	10.5416886583333	10.20226945	10.5384115166667	10.0989513583333	10.6219118666667	10.4417307666667	11.1176950333333	10.909815425	10.3741034916667	10.4890800583333	10.173327525	10.2249992083333	11.4188332333333	10.4138270833333	10.7964692333333	10.36145645	9.876036325	10.720995375	10.7162804	10.0920085583333

HNRNPK	14.6904136083333	14.2563286	14.3186975416667	14.2936594416667	14.515883675	14.9223639	14.2998455083333	14.5530100166667	14.113875875	14.6549636583333	14.6527873333333	14.3908304333333	14.6430667333333	14.2936594416667	14.265784475	14.2686692416667	14.4427443166667	14.5701394583333	14.5469723583333	14.34486635	14.21037625	14.5249167833333	14.3350486166667	14.5865975583333	14.6346112833333	14.4244522916667	14.3843059083333	14.5089658583333	14.1827283166667	14.3139109083333	14.515883675	14.5089658583333	14.127909425	14.227452975	14.1554067916667	14.657054125	14.494740525	14.4346519833333	14.4581253166667	14.2731900083333	14.2610479166667	14.219141025	14.451178225	14.5449579333333	14.3908304333333	14.4479745916667	14.7409853333333	14.5607495666667	14.5717037666667	14.4025408	14.5377311666667	14.427724725	14.3013820416667	14.3661675333333	14.2305624666667	14.29207405	14.2610479166667	14.312396275	14.3860372083333	14.8412522666667	14.80427055	14.820747325	14.7077525	14.5849736666667	15.0276351833333	14.5753036916667	14.80427055	14.8895282083333	14.9279831333333	14.7572900333333	14.77778785	14.80004595	14.70147985	14.80004595	14.8236926166667	14.6304997416667	14.65081365	14.9307420333333	14.74733735	14.9567111166667	15.1174212666667	14.8724941166667	14.7731878916667	14.6798721333333	14.7754409333333	15.0459774	14.8578939833333	14.648708375	15.0733260666667	14.8497796833333	14.6628978166667	14.6430667333333	14.9507696666667	14.65081365	14.7920246166667	14.6389112166667	14.718655275	14.7948348083333	14.66935455	14.1670824833333	14.80427055	14.7499385916667	14.667083075	14.7166750666667	14.7055894833333	14.9674626083333	14.5232906166667	14.5701394583333	14.7499385916667	14.7754409333333	14.80004595	14.912278625	14.594415925	14.4963541416667	14.4581253166667	14.4832242916667	15.1174212666667	14.7143451416667	14.72730925	14.6758595916667

RHOH	7.59600343333333	8.053540525	8.757884525	8.63917994166667	7.30805265833333	9.73955326666667	7.45718173333333	8.488931425	7.95194513333333	8.46405893333333	8.55907896666667	7.458458425	8.377106525	8.73498088333333	7.01007860833333	10.694500425	9.002830825	7.39926451666667	7.875176525	9.05355476666667	9.32329896666667	9.08092490833333	9.776982075	9.80143100833333	9.29384936666667	7.29474379166667	8.6493973	8.8992014	8.87883045833333	8.1455177	9.16123660833333	9.91585179166667	8.78942864166667	11.2583720833333	7.51696985	7.17569580833333	8.16124230833333	9.14377724166667	9.30124145833333	8.44632826666667	9.286917675	8.36907771666667	9.85036418333333	9.62318166666667	9.53137905	10.151687475	9.06697433333333	9.07536643333333	9.77176594166667	9.75828255833333	9.37269289166667	8.15959780833333	9.04890688333333	8.33599743333333	9.00137649166667	8.93073908333333	9.10576088333333	9.47208941666667	8.98986978333333	9.50182605833333	7.84947208333333	8.98986978333333	10.0111258333333	8.1205116	8.05664168333333	8.21881454166667	8.78498449166667	8.329584675	10.9750833833333	9.64118481666667	8.15707283333333	7.39621439166667	8.37413723333333	9.81176585	7.597705475	8.78635625	8.72644686666667	11.1604297833333	9.777390875	9.19391603333333	7.435271425	8.03277863333333	9.05868610833333	11.5909354166667	8.79812115	8.0149105	8.28997516666667	9.81728660833333	8.57257758333333	9.116355225	9.27172153333333	11.1585997833333	8.34422473333333	8.68130785	7.31025495833333	10.568795625	8.288511175	8.54070765833333	8.751330725	8.63731774166667	9.26855390833333	8.95577006666667	10.6589017416667	9.33664844166667	9.84117550833333	9.77219498333333	8.46245245833333	9.135601825	9.41892754166667	9.61139459166667	9.55472915	9.33664844166667	8.63917994166667	8.86314575	10.6819563666667	9.58391948333333	8.80870043333333	9.3434528	9.170544975	10.64844015

ABHD8	14.0397911958333	13.3132823083333	12.747056475	13.352668925	13.5556246458333	13.5523247333333	13.299997275	13.5309002666667	13.51377155	13.31254055	13.6871703125	13.85677325	13.653779525	13.4348891541667	13.4241947291667	13.3252264666667	14.0508868541667	13.3498021	13.2715239541667	13.4386918041667	13.4523955833333	13.4607081625	13.1490032625	12.7151772666667	13.3176143291667	14.0599641708333	13.3842829125	13.2257349291667	13.3070790041667	13.2158871875	13.159989	13.4286188416667	13.2037064125	13.9995577083333	14.231255875	14.4519842125	13.314786925	13.277522225	12.8384622791667	12.9850827166667	12.8804655625	13.0473086958333	12.9555343666667	13.270839425	13.5979476666667	13.0070480375	13.2276617958333	13.0789842458333	13.2894919291667	13.1068439625	13.1684894166667	12.8363399291667	13.85875215	13.4022565166667	13.2402464375	13.1704084083333	13.0237329708333	13.1265533166667	12.910849375	13.1509038375	13.810344575	13.4485668583333	14.1183777583333	13.7239626375	13.3387083375	13.2674751208333	13.5251055541667	13.7051346875	12.9337705791667	13.2577424916667	13.5112304791667	13.6519378833333	13.1896013625	13.3842829125	13.4590718958333	13.7558342625	13.610305375	13.5934627166667	12.9480679208333	13.8225883375	13.1541556166667	13.2521434166667	12.8524731291667	13.51377155	12.8833095125	12.7777399	13.30142525	13.464599075	13.8153117666667	13.5911612708333	13.0170874583333	13.4310777166667	13.5299908125	13.268089225	13.2845545333333	13.1277167583333	13.8783769791667	13.1651370541667	13.4508297333333	13.8391048125	13.142398675	13.7487427541667	13.4764456583333	13.8133007458333	13.4038458541667	13.2296272791667	13.1541556166667	13.7577614041667	12.8142433416667	13.2438119875	13.3549065	13.5489214208333	13.2438119875	13.2810951958333	13.3297152125	13.3352775791667	12.82707195	13.462329075	14.0195210041667	13.5979476666667

MRPL34	14.0397911958333	13.3132823083333	12.747056475	13.352668925	13.5556246458333	13.5523247333333	13.299997275	13.5309002666667	13.51377155	13.31254055	13.6871703125	13.85677325	13.653779525	13.4348891541667	13.4241947291667	13.3252264666667	14.0508868541667	13.3498021	13.2715239541667	13.4386918041667	13.4523955833333	13.4607081625	13.1490032625	12.7151772666667	13.3176143291667	14.0599641708333	13.3842829125	13.2257349291667	13.3070790041667	13.2158871875	13.159989	13.4286188416667	13.2037064125	13.9995577083333	14.231255875	14.4519842125	13.314786925	13.277522225	12.8384622791667	12.9850827166667	12.8804655625	13.0473086958333	12.9555343666667	13.270839425	13.5979476666667	13.0070480375	13.2276617958333	13.0789842458333	13.2894919291667	13.1068439625	13.1684894166667	12.8363399291667	13.85875215	13.4022565166667	13.2402464375	13.1704084083333	13.0237329708333	13.1265533166667	12.910849375	13.1509038375	13.810344575	13.4485668583333	14.1183777583333	13.7239626375	13.3387083375	13.2674751208333	13.5251055541667	13.7051346875	12.9337705791667	13.2577424916667	13.5112304791667	13.6519378833333	13.1896013625	13.3842829125	13.4590718958333	13.7558342625	13.610305375	13.5934627166667	12.9480679208333	13.8225883375	13.1541556166667	13.2521434166667	12.8524731291667	13.51377155	12.8833095125	12.7777399	13.30142525	13.464599075	13.8153117666667	13.5911612708333	13.0170874583333	13.4310777166667	13.5299908125	13.268089225	13.2845545333333	13.1277167583333	13.8783769791667	13.1651370541667	13.4508297333333	13.8391048125	13.142398675	13.7487427541667	13.4764456583333	13.8133007458333	13.4038458541667	13.2296272791667	13.1541556166667	13.7577614041667	12.8142433416667	13.2438119875	13.3549065	13.5489214208333	13.2438119875	13.2810951958333	13.3297152125	13.3352775791667	12.82707195	13.462329075	14.0195210041667	13.5979476666667

CC2D2A	7.26626465	6.7730469	7.35924831666667	7.04142870833333	8.47579976666667	7.523817425	8.27907085	7.219834	7.23311056666667	7.63498546666667	7.11855360833333	7.84323385	7.219834	6.59234331666667	7.198288025	7.01426715833333	5.8532158	7.9760917	7.321455625	6.987286825	7.06888018333333	7.334392275	6.520636925	6.85045274166667	7.10054488333333	7.17851245	7.0633355	6.94439705	7.83518873333333	7.334392275	6.81709465833333	6.987286825	7.166799725	7.89930148333333	7.272898375	7.36048969166667	7.29843466666667	7.496531175	7.21750264166667	6.97095796666667	6.45767033333333	7.31981145833333	6.8732984	7.120419025	6.81852565833333	6.1112992	6.83931143333333	6.89851890833333	6.27955291666667	6.90781049166667	7.16232548333333	7.16358325	7.71354716666667	7.87821420833333	7.61126938333333	6.80310133333333	7.134785875	6.801867125	7.193910325	7.15020035833333	8.09282625833333	7.44667759166667	7.91954875833333	7.46514316666667	7.16614005833333	7.92549585	7.597182875	7.53018445	7.39975843333333	7.54282731666667	7.57783686666667	7.56037049166667	6.90252933333333	7.448314425	7.42619010833333	7.254184275	6.841322025	7.43011818333333	6.98973956666667	7.19065496666667	7.87098755833333	8.04755969166667	7.38051639166667	6.95924915	7.34272655	7.40135389166667	8.01539524166667	7.20023056666667	7.488079225	6.8732984	7.00693255	7.07722264166667	7.96986838333333	7.65121998333333	7.79586476666667	7.24263133333333	7.5654861	7.83189280833333	7.50463459166667	6.94376375833333	7.64914819166667	7.95082734166667	7.43473675	7.407222675	7.17033845	7.435836975	7.73082848333333	7.27114775833333	7.043221875	7.26995246666667	7.44735373333333	7.3314952	7.68323405	7.18705289166667	7.61062251666667	7.3675475	7.21926184166667	7.435836975	7.25120925833333	7.11986829166667

AHNAK	4.21592141666667	3.83152513333333	3.78410915833333	4.29605796666667	3.87937155833333	4.17373291666667	3.7304451	4.33846348333333	3.55885279166667	3.96722695833333	3.7439275	3.723060125	3.54887480833333	3.90095365833333	4.176778725	4.05071508333333	4.23196318333333	4.09698049166667	4.24013239166667	4.02247413333333	3.68857591666667	3.81123611666667	4.3249589	4.01916375833333	4.35819395	3.770641325	3.91035815	4.11480768333333	4.12677783333333	3.912461775	5.72282524166667	4.09478471666667	3.582549625	4.24335745833333	3.84944544166667	4.77930389166667	5.12102319166667	4.789978375	4.21502835833333	5.69385626666667	5.68304789166667	4.177708	4.79999713333333	4.88811624166667	3.98511664166667	4.79518681666667	4.35594943333333	4.31414184166667	4.27276220833333	5.39281689166667	4.42372025	4.747391	3.90354184166667	4.77813741666667	4.05417596666667	4.21701204166667	3.5947849	5.25962125	5.10387183333333	4.434278525	4.11883455833333	3.79653381666667	4.09994219166667	4.50544710833333	3.45326613333333	4.01107811666667	3.7439275	3.75570144166667	4.22505026666667	3.57639359166667	3.75717131666667	3.61637475833333	3.656722575	3.54051521666667	3.46854113333333	3.91593421666667	3.84584678333333	3.838223825	4.45902586666667	3.91035815	3.48293635833333	3.82681465	3.973735825	4.695239575	3.670269525	4.23901671666667	3.58562625	3.97069799166667	3.7942322	3.931450225	3.59972783333333	4.04503838333333	3.84817555	3.8987918	3.77426261666667	3.75016011666667	4.33428121666667	5.0343026	4.03968158333333	6.00123059166667	4.06079156666667	3.82817183333333	4.30828979166667	4.4718244	4.09478471666667	3.75838870833333	4.13218385833333	5.01530855833333	4.035447825	5.95074298333333	4.32946600833333	4.79906281666667	5.82555810833333	4.40581835	3.909240625	4.0322916	3.93897055	4.63625878333333	4.23901671666667	3.444174575

ADAMTS18	5.38659805833333	5.455892825	7.75234836666667	4.695239575	9.460327375	10.638037975	4.90032935	6.86736839166667	5.30562465833333	7.70471373333333	7.35396276666667	4.71016199166667	7.821740775	5.73297515	5.04667276666667	8.24215483333333	5.93821120833333	6.87219209166667	6.99282755	7.78927275	9.83153578333333	8.02464245	6.83799996666667	5.10716860833333	7.35564645	8.97266575	7.62815665833333	5.565954175	7.63210018333333	5.54873173333333	4.49602461666667	6.80449616666667	6.1777684	5.06903989166667	5.31319136666667	5.8174436	6.97214965	9.83406541666667	8.7143253	9.443857125	8.72787371666667	6.60956075833333	4.99137435	5.36415765833333	4.74337093333333	9.10532413333333	7.07794505	4.688902775	7.11789028333333	7.64327454166667	6.04876668333333	7.019638175	6.46494565	7.88761391666667	4.66334196666667	7.62988515833333	9.03327476666667	7.29365533333333	6.18660088333333	6.00367545	7.349518275	6.79403766666667	6.57887239166667	5.01216298333333	14.0800041333333	7.63604310833333	7.69858204166667	6.56215660833333	6.28660823333333	7.39621439166667	7.7682787	7.4784294	5.80528018333333	6.65690114166667	6.08877931666667	12.0997306916667	7.19451305833333	7.56037049166667	6.81253920833333	7.73027796666667	13.8754219166667	10.6742121583333	5.84963309166667	5.18519845833333	8.7735097	14.1343040416667	12.672014175	7.75390870833333	9.03459930833333	9.09553066666667	6.91578644166667	6.80377665833333	7.73358451666667	6.82383751666667	4.789978375	7.71354716666667	5.89449381666667	14.5322324833333	5.83398495	7.09617145833333	7.46964506666667	6.74346625833333	6.728950375	6.48497233333333	5.32238468333333	7.378725775	8.11761579166667	5.57060381666667	7.80930983333333	8.0519544	8.23662486666667	5.49632836666667	7.210345825	7.18884621666667	7.652791	7.192539525	11.60515845	4.968508325	6.4413796	7.19767501666667

ANKRD36C	13.2357553666667	13.0497180583333	12.6902568875	12.9320596125	13.0856200958333	15.1711894916667	13.3084273833333	13.1828778625	13.5639497916667	13.2577424916667	13.9768039541667	14.0484738916667	13.6352	12.5369637041667	12.4920038375	14.446983275	13.7097806958333	12.7684293	13.3721343291667	13.1391942625	12.7100716333333	13.2687283958333	13.8903707125	14.04483015	14.0294725041667	13.1861883291667	13.1187878541667	13.526776725	13.407763875	13.5681927791667	12.6687319583333	13.4439466958333	14.2452054583333	16.3589429458333	12.8743615291667	12.74869935	13.4198811	12.24230515	13.7606673	13.3028590416667	13.0880695375	12.7218903416667	12.8094183875	12.4595822625	14.1598025458333	12.7028655833333	12.8927594708333	12.4684823416667	12.7320818875	14.7398420916667	13.2450235083333	12.8998760125	13.4748795375	13.3612246916667	12.2643684166667	12.5951519583333	13.2755336666667	12.9050741791667	12.8773764583333	13.7258758	14.6315449375	13.9405817375	14.6748311416667	13.6645693416667	15.0443403541667	13.4108680416667	13.6269874333333	14.2526125	13.9159292708333	14.2371238541667	14.1795629625	13.9070501166667	13.1553491208333	14.9266002416667	13.8903707125	13.5161003625	14.1880524416667	15.5734739625	13.4662457916667	14.923790625	14.8782718541667	14.6399901541667	13.6909245916667	12.8558614458333	13.710658725	14.698088525	14.4696275583333	14.3684489958333	14.7304948416667	13.6361496791667	13.22443745	14.0423963083333	13.9405817375	13.2851875333333	14.3555176625	14.8953709416667	14.3603769166667	14.6893914541667	14.2168077458333	14.6728082375	13.7267966208333	14.3146441458333	15.1745941875	14.74197315	12.628535	14.6458169083333	13.7409491833333	12.6697077541667	15.0687070416667	14.1420497583333	14.5223774125	13.6397108125	13.3627137208333	13.0880695375	14.8457579916667	13.2865270666667	13.6936536458333	13.8302040458333	14.5518799708333	14.0423963083333

ANKRD36B	13.2357553666667	13.0497180583333	12.6902568875	12.9320596125	13.0856200958333	15.1711894916667	13.3084273833333	13.1828778625	13.5639497916667	13.2577424916667	13.9768039541667	14.0484738916667	13.6352	12.5369637041667	12.4920038375	14.446983275	13.7097806958333	12.7684293	13.3721343291667	13.1391942625	12.7100716333333	13.2687283958333	13.8903707125	14.04483015	14.0294725041667	13.1861883291667	13.1187878541667	13.526776725	13.407763875	13.5681927791667	12.6687319583333	13.4439466958333	14.2452054583333	16.3589429458333	12.8743615291667	12.74869935	13.4198811	12.24230515	13.7606673	13.3028590416667	13.0880695375	12.7218903416667	12.8094183875	12.4595822625	14.1598025458333	12.7028655833333	12.8927594708333	12.4684823416667	12.7320818875	14.7398420916667	13.2450235083333	12.8998760125	13.4748795375	13.3612246916667	12.2643684166667	12.5951519583333	13.2755336666667	12.9050741791667	12.8773764583333	13.7258758	14.6315449375	13.9405817375	14.6748311416667	13.6645693416667	15.0443403541667	13.4108680416667	13.6269874333333	14.2526125	13.9159292708333	14.2371238541667	14.1795629625	13.9070501166667	13.1553491208333	14.9266002416667	13.8903707125	13.5161003625	14.1880524416667	15.5734739625	13.4662457916667	14.923790625	14.8782718541667	14.6399901541667	13.6909245916667	12.8558614458333	13.710658725	14.698088525	14.4696275583333	14.3684489958333	14.7304948416667	13.6361496791667	13.22443745	14.0423963083333	13.9405817375	13.2851875333333	14.3555176625	14.8953709416667	14.3603769166667	14.6893914541667	14.2168077458333	14.6728082375	13.7267966208333	14.3146441458333	15.1745941875	14.74197315	12.628535	14.6458169083333	13.7409491833333	12.6697077541667	15.0687070416667	14.1420497583333	14.5223774125	13.6397108125	13.3627137208333	13.0880695375	14.8457579916667	13.2865270666667	13.6936536458333	13.8302040458333	14.5518799708333	14.0423963083333

UPRT	10.5428297916667	11.1477174666667	10.7994102333333	11.3661426916667	10.8964884916667	10.2427233083333	10.5760591583333	10.7975980416667	11.0451598916667	10.590660625	9.97501473333333	10.2485867583333	10.440565075	10.9061592666667	10.5119603416667	10.4846654833333	12.0525821583333	10.971110725	10.9987091583333	10.7581339416667	10.5872774916667	10.885465	10.5899519833333	10.6527698	10.689872025	10.5964797	10.612647525	10.6191395	10.638037975	11.1721251583333	10.9831460083333	11.028339325	10.7812240416667	11.3357126416667	10.4499117666667	10.3725905333333	10.4460825166667	10.6637664333333	10.8566946833333	10.4736621916667	10.9215878333333	11.1380175916667	11.2965765083333	10.8036860666667	10.2060439416667	10.568374825	10.7569232666667	10.5468436833333	11.0581558166667	10.9139727166667	10.859746575	10.9433662916667	9.79362293333333	10.3663602	10.189123775	10.2047893666667	10.826189675	10.8443256416667	10.7523474083333	10.5056824166667	10.390544	9.69219855833333	10.2318091916667	9.14279978333333	10.1278854583333	10.4775633333333	9.71864728333333	10.1148100333333	10.518970175	9.230932875	9.68992429166667	9.29664133333333	8.20281158333333	10.0095607333333	10.1176213666667	9.42904984166667	9.86359230833333	9.76219373333333	10.5468436833333	9.354988125	9.77312443333333	9.42424818333333	9.949333375	9.88594978333333	9.69349788333333	9.512679675	9.85677660833333	9.831945975	9.85161335833333	10.325378325	10.283076325	8.68329548333333	9.84994195833333	8.86508634166667	9.94976705	9.71612960833333	9.230932875	9.85113778333333	10.0318266833333	9.5013607	10.137056175	10.1637531083333	9.97873573333333	9.625456175	9.55671014166667	10.0296842916667	10.298281975	8.78307434166667	10.2111469	10.134682025	10.7658215916667	9.90955436666667	8.03595288333333	9.9229238	9.422929875	9.6811178	10.225450075	10.4449298	8.63825258333333	9.76732071666667

BAX	9.31047634166667	10.6686985416667	10.8368196833333	10.2108003333333	10.8600934166667	10.8150497416667	10.9157145083333	10.3353402416667	10.31869075	10.9371792916667	11.185479075	10.5692188416667	10.5041729666667	10.4755897583333	10.9747290916667	11.1575927916667	9.83111589166667	11.3191493833333	10.9564158416667	10.8045420416667	10.2289590916667	10.328553475	11.072670925	11.0385524708333	10.55650935	9.34018350833333	10.6187719166667	10.62485375	10.713545975	10.7184951333333	11.0625918083333	10.9160737916667	10.578003225	10.816907025	10.4986956916667	9.83982443333333	11.1398416833333	11.1803917416667	10.86914835	10.3913250333333	10.6699485666667	10.0868334916667	10.3061471666667	10.7375745583333	9.90816476666667	10.39092635	10.4131468	10.8706515666667	10.6068679083333	10.9869124833333	11.51000085	10.934250825	11.3635795583333	10.9219574	10.36070035	10.5910579333333	10.9215878333333	10.8120670333333	11.3282347083333	10.8056325083333	10.4249193833333	11.6006445416667	11.5352724166667	10.6233516833333	9.19860346666667	11.8293448166667	11.4635169166667	10.8900635166667	11.62415315	11.0373411416667	11.7216620916667	12.064871125	11.3495641166667	11.0670126666667	10.96150755	11.8873133333333	10.8086263583333	11.9392227916667	11.8337195083333	11.57134605	9.58391948333333	11.0534747416667	12.0404998666667	11.7470404	11.0312303833333	9.62193513333333	10.665646025	10.891978525	10.7371678583333	11.1779127416667	11.3413304583333	11.30507995	11.3624169916667	11.1196222666667	11.7917720333333	12.06402615	12.5362402583333	11.6386741333333	11.7713486916667	11.4496634833333	11.4879615666667	11.43798885	11.2312268666667	11.4864514	11.0466400166667	10.7116237833333	11.44422215	11.6306161333333	11.2226491666667	11.4779366416667	11.3605041083333	11.5523735416667	11.9773878833333	11.592340975	11.0193190916667	11.2922383666667	10.5314888083333	11.41442885	11.8415156583333	11.5941589083333

GFRAL	4.70905974166667	4.894753975	5.127309825	4.55069105	4.4227704	4.49797680833333	5.68499903333333	4.939635175	4.41392473333333	4.71793841666667	5.39804303333333	5.78268739166667	4.39039918333333	4.7847361	4.46100053333333	4.3934547	5.59857721666667	5.442194025	4.63410609166667	4.79621500833333	4.02352729166667	4.1756419	4.792098225	5.20882029166667	4.87823230833333	4.13218385833333	4.97177571666667	4.71445220833333	4.812774675	5.01629245833333	5.110215025	5.72466863333333	4.697573175	4.63093599166667	4.94576598333333	4.472817375	5.1566631	4.771681175	5.91139875	4.792098225	5.32134071666667	4.62587865833333	5.002397475	5.74909436666667	4.18191081666667	5.15246255833333	5.46375123333333	5.2151974	4.71899701666667	4.88687726666667	4.87595029166667	6.69500276666667	4.49797680833333	6.242830225	5.73297515	5.1566631	4.50112465833333	5.31227555833333	4.96739760833333	5.053877925	4.359146625	4.94355658333333	4.65588558333333	4.922546525	4.78381058333333	4.5496205	4.05627571666667	4.74219220833333	4.23901671666667	4.59683404166667	5.1598283	4.32946600833333	5.40105285833333	4.50751908333333	4.47668578333333	4.05319390833333	4.2860754	4.20999628333333	4.4159867	4.10390375833333	5.77716973333333	5.02269790833333	4.73490975	4.29817321666667	4.57240950833333	4.39972780833333	4.748342275	4.67471653333333	4.11480768333333	4.62161215833333	4.948951225	4.54020328333333	4.224150025	4.52898779166667	4.34995844166667	4.28008616666667	4.63305978333333	4.57535033333333	5.56242626666667	5.122114675	4.48330838333333	3.85641256666667	5.020575025	5.33746485833333	4.40083348333333	4.18191081666667	4.78586035833333	6.787125175	4.337410475	6.67021015	4.7006097	7.070041725	4.93234401666667	6.16829805	4.57137348333333	4.47089620833333	4.428719425	4.67574021666667	5.18312655	5.65064866666667

MRPL42	12.623024125	11.4507357333333	11.8947334916667	11.673519475	11.7589935083333	11.683415075	11.187762025	12.0785704333333	11.4133387916667	11.7974234916667	11.2341636166667	11.798162425	11.230835825	11.62415315	11.2936955416667	11.6297974583333	11.8576914166667	11.6363117083333	11.5809197583333	11.7186785333333	11.7898995	11.5552373166667	11.9871613166667	11.5078323166667	11.5869361333333	12.0088601416667	11.44169995	11.8081782916667	11.6871432333333	11.5356555333333	11.7932878	12.0230159333333	11.159679825	11.426633325	12.3820596583333	12.0424744583333	11.279388675	11.6632602416667	11.5916707666667	11.7463222583333	11.784585925	11.434022575	11.5150484916667	11.6791509083333	11.6006445416667	11.5374922333333	11.9063380833333	11.8749533333333	11.6895204333333	11.7231538416667	11.7594075916667	11.676506725	11.379403425	11.6503712333333	11.502358525	11.6166645833333	11.631804775	11.7690899333333	11.666692125	12.1133945666667	11.8943464416667	11.5512973083333	11.8285794166667	10.9284040666667	12.049870975	10.7143764583333	11.1445056833333	11.5672571	11.744853675	11.8646362416667	11.1227594166667	11.625582325	10.921206425	11.4669037583333	11.67393075	11.0775947083333	11.8596015	11.3375225333333	11.1536039583333	11.9409022083333	11.641524325	12.0072469333333	12.206981325	11.6198462166667	11.5200695166667	11.77986195	12.0368526666667	11.4350551	11.6558642583333	11.424045025	12.0301876583333	12.0432377416667	11.6103014166667	11.30185685	11.292593525	11.1520832083333	11.7671597083333	11.6006445416667	11.827759775	12.551141325	11.9228391333333	12.0705755166667	12.0585648916667	11.9951551416667	11.4956432166667	11.1869945583333	11.43032555	11.943975875	11.7035007416667	11.6883557166667	11.9455284	12.3396961416667	11.5538304416667	11.6129963	11.3968955416667	11.5820604666667	11.8341069333333	11.8261862166667	12.2500083166667	11.9796845666667

EVL	12.9998495208333	12.6777922541667	12.6198544416667	12.5455470375	11.9210111458333	12.4322619041667	12.1264399583333	12.64891165	12.2246159	12.3743981125	12.2762036708333	12.6936971916667	12.5966588791667	12.602520925	11.7188705625	13.3366821958333	12.99863245	12.0850507583333	12.1073873291667	12.4811040166667	12.9021565791667	12.4690041958333	12.9543141708333	12.5800355625	12.7235509375	12.9974867916667	12.5737467375	12.5100239583333	11.9881367583333	12.2097075375	12.4625530458333	13.021992725	12.7819926916667	13.6153901958333	13.2872654291667	13.1169148291667	12.283346	12.7647025958333	12.5919528	12.6193415	12.5261734041667	12.266896675	12.5247907125	12.6409877791667	12.9974867916667	13.0789842458333	12.4895877583333	12.5095726	13.3924972083333	12.631472475	12.7408604208333	12.2233819	12.7860295083333	12.5161854958333	12.6378885833333	12.7418902125	12.2737573	12.589520375	12.6687319583333	13.0466816458333	12.3498971916667	12.5541383208333	12.548043925	13.0679278416667	13.375928075	12.3890185041667	12.9662359083333	12.664857475	13.2309913833333	13.51680175	12.3826691625	12.3371947583333	12.5161854958333	13.344822725	12.5669517	13.1347044291667	12.7074055333333	13.1246056208333	12.3985967708333	12.8462830375	12.8484686083333	13.0503485833333	13.0304394833333	13.0460416541667	12.8653568166667	13.6049642125	13.3627137208333	13.5193007041667	12.04266845	13.2043848958333	13.0454130958333	12.6593439833333	11.50838115	12.7854970375	12.9992294125	12.5356054583333	13.1566431916667	12.5951519583333	13.3827347958333	12.6358521375	12.3494783875	12.8237470375	12.5682987041667	12.7809767458333	12.402219	12.6183459541667	13.230294875	12.5229004083333	12.8029136333333	12.6999046916667	12.9021565791667	11.3898956208333	12.6907177958333	12.18334265	13.1442826666667	12.8664265875	12.5427723	12.1511634166667	12.6514701916667	13.0724085083333

DEGS2	12.9998495208333	12.6777922541667	12.6198544416667	12.5455470375	11.9210111458333	12.4322619041667	12.1264399583333	12.64891165	12.2246159	12.3743981125	12.2762036708333	12.6936971916667	12.5966588791667	12.602520925	11.7188705625	13.3366821958333	12.99863245	12.0850507583333	12.1073873291667	12.4811040166667	12.9021565791667	12.4690041958333	12.9543141708333	12.5800355625	12.7235509375	12.9974867916667	12.5737467375	12.5100239583333	11.9881367583333	12.2097075375	12.4625530458333	13.021992725	12.7819926916667	13.6153901958333	13.2872654291667	13.1169148291667	12.283346	12.7647025958333	12.5919528	12.6193415	12.5261734041667	12.266896675	12.5247907125	12.6409877791667	12.9974867916667	13.0789842458333	12.4895877583333	12.5095726	13.3924972083333	12.631472475	12.7408604208333	12.2233819	12.7860295083333	12.5161854958333	12.6378885833333	12.7418902125	12.2737573	12.589520375	12.6687319583333	13.0466816458333	12.3498971916667	12.5541383208333	12.548043925	13.0679278416667	13.375928075	12.3890185041667	12.9662359083333	12.664857475	13.2309913833333	13.51680175	12.3826691625	12.3371947583333	12.5161854958333	13.344822725	12.5669517	13.1347044291667	12.7074055333333	13.1246056208333	12.3985967708333	12.8462830375	12.8484686083333	13.0503485833333	13.0304394833333	13.0460416541667	12.8653568166667	13.6049642125	13.3627137208333	13.5193007041667	12.04266845	13.2043848958333	13.0454130958333	12.6593439833333	11.50838115	12.7854970375	12.9992294125	12.5356054583333	13.1566431916667	12.5951519583333	13.3827347958333	12.6358521375	12.3494783875	12.8237470375	12.5682987041667	12.7809767458333	12.402219	12.6183459541667	13.230294875	12.5229004083333	12.8029136333333	12.6999046916667	12.9021565791667	11.3898956208333	12.6907177958333	12.18334265	13.1442826666667	12.8664265875	12.5427723	12.1511634166667	12.6514701916667	13.0724085083333

CMTM1	5.42727581666667	7.1782093875	7.5764631375	6.61187776666667	8.57533845416667	8.270463975	6.52877812083333	7.7327247125	7.2762145875	7.750371325	6.60102279583333	5.06411445416667	6.30447276666667	6.41151313333333	7.30345720416667	6.78747448333333	7.08899834583333	7.09709387083333	7.4371520375	7.39478122083333	6.458806375	7.40746268333333	8.75158355	7.331226225	6.92611444166667	5.0739174625	8.15836935	8.10299814166667	8.46746207916667	6.98063093333333	7.74010875833333	7.95382576666667	7.14304875	5.2716225875	6.3843491625	5.951137325	8.70823490416667	8.81811914166667	7.705612125	7.62680286666667	7.61951985416667	7.3931099125	7.42132618333333	7.93410628333333	6.11000845	8.38048105833333	7.4344513125	7.38019690833333	8.11629055833333	7.6357697	8.5912661	7.950087225	7.56969055416667	7.02852860416667	7.16577590833333	7.04172927916667	7.60014637916667	8.25163100833333	7.65094930416667	7.85778420833333	7.4547802	5.26728476666667	6.18935822916667	4.98058044166667	5.35932894583333	5.48660064166667	4.93719669583333	5.86502612916667	5.70393408333333	6.59016512083333	5.42325321666667	6.0216198875	4.9250661375	5.11626904583333	6.65058425	4.93494572083333	5.4564338875	6.68369220416667	5.3522813	5.91193464166667	4.6574355375	5.53744545	5.47658683333333	5.4978313875	4.65846812083333	5.95023862916667	5.80673922083333	4.67624076666667	5.73044050416667	5.2716225875	5.1379653375	6.50829830416667	6.83370947083333	4.94836752083333	5.66126460416667	5.40801585416667	6.0534995375	6.15319939583333	5.66897774583333	5.70304432916667	6.4274523625	4.8219214875	6.13379567083333	5.67755486666667	6.05003854166667	5.48864805416667	6.2139044875	6.5813024375	6.0730132375	6.61588425416667	6.49128917083333	9.65951234166667	7.0453601625	6.59566714583333	6.5030995625	6.4222649125	5.81262865	6.19856041666667	6.67602829583333	5.52694737083333

CKLF-CMTM1	5.42727581666667	7.1782093875	7.5764631375	6.61187776666667	8.57533845416667	8.270463975	6.52877812083333	7.7327247125	7.2762145875	7.750371325	6.60102279583333	5.06411445416667	6.30447276666667	6.41151313333333	7.30345720416667	6.78747448333333	7.08899834583333	7.09709387083333	7.4371520375	7.39478122083333	6.458806375	7.40746268333333	8.75158355	7.331226225	6.92611444166667	5.0739174625	8.15836935	8.10299814166667	8.46746207916667	6.98063093333333	7.74010875833333	7.95382576666667	7.14304875	5.2716225875	6.3843491625	5.951137325	8.70823490416667	8.81811914166667	7.705612125	7.62680286666667	7.61951985416667	7.3931099125	7.42132618333333	7.93410628333333	6.11000845	8.38048105833333	7.4344513125	7.38019690833333	8.11629055833333	7.6357697	8.5912661	7.950087225	7.56969055416667	7.02852860416667	7.16577590833333	7.04172927916667	7.60014637916667	8.25163100833333	7.65094930416667	7.85778420833333	7.4547802	5.26728476666667	6.18935822916667	4.98058044166667	5.35932894583333	5.48660064166667	4.93719669583333	5.86502612916667	5.70393408333333	6.59016512083333	5.42325321666667	6.0216198875	4.9250661375	5.11626904583333	6.65058425	4.93494572083333	5.4564338875	6.68369220416667	5.3522813	5.91193464166667	4.6574355375	5.53744545	5.47658683333333	5.4978313875	4.65846812083333	5.95023862916667	5.80673922083333	4.67624076666667	5.73044050416667	5.2716225875	5.1379653375	6.50829830416667	6.83370947083333	4.94836752083333	5.66126460416667	5.40801585416667	6.0534995375	6.15319939583333	5.66897774583333	5.70304432916667	6.4274523625	4.8219214875	6.13379567083333	5.67755486666667	6.05003854166667	5.48864805416667	6.2139044875	6.5813024375	6.0730132375	6.61588425416667	6.49128917083333	9.65951234166667	7.0453601625	6.59566714583333	6.5030995625	6.4222649125	5.81262865	6.19856041666667	6.67602829583333	5.52694737083333

SPATA5	8.03004540833333	7.55925806666667	7.60297259166667	7.14213880833333	7.04898169166667	8.23315546666667	5.80195006666667	7.375280375	7.15020035833333	7.84154363333333	7.36878105	7.100006625	7.16232548333333	7.49332154166667	7.321455625	7.57903273333333	8.47046444166667	6.7730469	8.00581294166667	7.657177025	7.78927275	7.678740925	8.33758601666667	7.81456171666667	7.78166071666667	8.11654126666667	7.52673725833333	7.683805725	7.794082525	7.348327125	7.86777288333333	7.90410825	6.81316333333333	8.14137294166667	8.23365661666667	8.332085025	7.23371278333333	7.76884748333333	6.6659887	7.348327125	7.88077005	7.55746484166667	8.01861064166667	8.30130739166667	7.51696985	8.11712540833333	8.03117830833333	7.6702147	8.08335636666667	8.12585160833333	7.29788705833333	7.65121998333333	6.7503122	7.393964525	7.62759090833333	7.89832068333333	7.58521366666667	7.98294754166667	7.39105859166667	8.08035505	7.85633409166667	7.35924831666667	8.12540885833333	6.89851890833333	9.03598665833333	7.14024473333333	7.58408555833333	7.00118948333333	8.78307434166667	7.07182334166667	7.066499225	7.36878105	7.11920136666667	7.31615383333333	6.632460225	7.47204458333333	7.193910325	7.92654975833333	8.33066458333333	8.5671537	8.41926360833333	8.00800819166667	8.74034393333333	7.49390345833333	6.53809915833333	8.05032904166667	8.391097025	7.11855360833333	6.65223385	8.04755969166667	7.4731488	7.66774384166667	6.84909505	7.928618975	7.34551940833333	7.53126729166667	7.96073305833333	8.234668275	7.28500960833333	7.54799789166667	8.709455975	7.833966975	7.87717198333333	8.448844675	8.199157725	7.88811874166667	7.687124975	7.372424975	8.22846261666667	8.1205116	8.15303908333333	8.7374204	7.89296785	8.68775965833333	7.90789741666667	8.59890789166667	9.09184225	8.69984448333333	8.71285006666667	8.5994674

SCAMP1	11.2642606083333	10.1940041	10.1126129583333	9.8813009	10.2111469	9.58131358333333	10.185845775	10.2145140083333	10.1250607083333	9.81132868333333	10.1246349583333	10.8605112583333	10.634390675	10.0644501166667	10.1750566166667	9.84409466666667	10.572357075	10.26626505	10.123368025	10.4184334333333	10.1312217666667	10.2084654083333	10.0002116916667	10.1432492	10.04638645	11.1756714833333	10.1210398166667	10.0018247916667	9.99468328333333	9.95100449166667	10.11881175	9.781927775	10.0627631	9.754485575	10.8861829666667	11.0337297583333	9.7036749	9.945938775	10.20309965	9.87563735	9.781927775	9.74218338333333	10.1836960166667	9.990452125	10.5768008666667	9.54925085	10.04588255	9.83452378333333	9.86431620833333	10.167505275	9.87826785	10.2434985083333	10.6407861166667	10.0039229916667	10.123786625	10.1308023166667	10.2249992083333	9.89553576666667	9.89141825	9.99509398333333	10.7313914916667	10.3277465	9.65621849166667	9.86020724166667	10.84359385	9.78781813333333	9.16588333333333	9.69176305833333	9.86854744166667	9.91585179166667	10.3281467166667	10.572357075	10.2900500333333	10.0103759833333	10.43166005	9.80802880833333	10.5177006833333	10.277413575	9.670115925	9.55288966666667	10.528410425	11.162651425	9.93343423333333	9.84285273333333	10.2096666583333	10.1546002	10.7907764333333	10.340914925	10.6111916083333	9.65101713333333	10.1605417333333	11.0845103416667	10.2286007833333	9.27172153333333	9.364389775	10.10216065	10.06569645	9.59324718333333	9.1141243	9.61006324166667	10.6496091416667	10.0753226583333	10.8465109833333	10.3305153666667	10.523602425	10.0565842333333	10.2822548833333	10.03562315	10.2126097875	10.3526835916667	9.68695163333333	9.22160494166667	10.806391425	10.1278854583333	9.7408584	9.579511975	9.39525429166667	10.4112315166667	10.451127	10.2241250416667

PRRT3	8.99771499166667	9.64610789166667	9.59065653333333	10.1883097083333	8.94761291666667	8.1873246	8.16071089166667	8.63825258333333	9.00460498333333	8.89405555	8.54169179166667	8.35883419166667	8.38960981666667	9.69038334166667	9.88298375833333	9.06745036666667	8.78942864166667	8.70751021666667	9.84456984166667	9.22108949166667	9.11737229166667	9.77049406666667	9.17971915	9.0174923	9.71227291666667	8.998094275	9.54277241666667	9.31452535833333	9.26082591666667	9.29796166666667	9.96954501666667	9.63944066666667	9.19719063333333	8.16684950833333	9.166824	9.309548625	9.341081375	8.59842283333333	8.58856818333333	8.83395916666667	9.63342665833333	9.88637849166667	9.766909275	9.11077986666667	9.08180965	9.401147475	10.2634356166667	9.85498678333333	9.17093331666667	9.09553066666667	9.10796010833333	9.16588333333333	9.7237141	9.69038334166667	9.54925085	10.4112315166667	9.04937824166667	9.57222860833333	8.97625954166667	9.24884609166667	7.7783407	7.64678326666667	9.3041126	8.23613304166667	8.91642435833333	7.58238585833333	8.13716783333333	7.50627369166667	9.2436075	7.62988515833333	7.91685584166667	8.13242494166667	6.89003731666667	7.95904073333333	8.4237182	8.22690084166667	8.27954998333333	8.00462699166667	8.62547046666667	9.56634321666667	8.20433166666667	8.92123313333333	9.18016484166667	8.79346453333333	7.69424574166667	7.79469890833333	8.29403910833333	7.99022335	7.652791	8.1205116	7.867210225	8.25692778333333	7.3314952	7.20023056666667	8.19700369166667	8.60227746666667	9.401147475	8.57400345	7.17198438333333	7.77602128333333	8.355193125	8.523789	8.60539538333333	9.177752275	9.02371780833333	8.45851334166667	8.89220491666667	8.54598220833333	9.54834169166667	8.62481299166667	8.00740176666667	9.10485650833333	7.84051964166667	9.42734543333333	8.38611514166667	8.83829725	9.037729975	8.17302170833333	8.770019625	7.30431380833333

TRAF3IP2	12.5457898	13.0167634166667	12.9494661	13.068247425	12.7081294916667	13.0732507583333	12.632188	12.8438653416667	12.8730190083333	12.8246255416667	12.9793317083333	11.8265346166667	12.809704275	13.0130175083333	13.0387188916667	12.4677654583333	13.248853375	12.938273025	12.8149887083333	12.8613107583333	12.7801271583333	13.0700559416667	13.0124953166667	13.1487099083333	12.6577047083333	11.8904865166667	12.7536835	12.8074143333333	12.7676811833333	12.7748629083333	13.1197255166667	12.9924504416667	12.8240262666667	13.394322425	12.1951832916667	11.8174744333333	12.9941887083333	12.8823979	13.06700765	12.79714555	12.769749725	12.7945001333333	12.9739422583333	12.9659499916667	12.5065921166667	12.7812528833333	12.8996243416667	12.686781525	12.96407505	12.8730190083333	13.0786865916667	13.6570629916667	12.937710825	13.1210793083333	13.09459025	13.00781875	13.035605175	12.7692841333333	13.0953722166667	12.7671377333333	13.001932175	12.3610276166667	11.9978846916667	12.4598136333333	13.3846464166667	12.5831450833333	13.171325425	12.2227697083333	13.0913765666667	12.9483493833333	12.4532785	12.9924504416667	13.542740525	12.8889747333333	12.9433935166667	13.56859275	12.5710716416667	12.76127775	12.9948642333333	12.7649781166667	13.627458175	12.9433935166667	13.5753810583333	13.1688283833333	13.0913765666667	12.8323870333333	13.3778968666667	12.8062943	12.2094988666667	12.8161131416667	13.2602116	13.3046967416667	13.0884141916667	13.0602371583333	13.4352543166667	12.8080609833333	13.0607838416667	12.9859501166667	13.784925425	12.5831450833333	13.7820292333333	12.3627391666667	12.4395669416667	12.772348275	12.521701325	12.4306290916667	12.944994825	13.2148779166667	13.1019491416667	13.61251425	12.98478995	13.2615079916667	14.0759487166667	12.792898125	13.1216942833333	12.8590238833333	12.7543063	12.9924504416667	12.6621572833333	12.6013272333333

COPZ2	12.5926289916667	10.5956461416667	10.839776075	10.4725076	10.07610735	10.5424705	10.2900500333333	10.3610713833333	10.4284899	11.0094185166667	10.7460395416667	12.280521525	11.5258171833333	10.61002135	10.5344987916667	10.4456717166667	10.1696248333333	10.2249992083333	10.7363658166667	10.916432925	11.0228735916667	9.92873065833333	11.017853675	10.64689975	10.744004	12.757496125	11.0369926416667	10.4441620166667	9.22606134166667	10.1091856666667	10.8778360416667	10.5549316666667	10.7409193791667	9.63714265	13.0645149833333	12.9456067916667	10.0171966666667	10.7601275	10.6301784666667	11.0989964083333	11.1227594166667	10.49466965	10.723966225	11.0593297333333	11.5555906416667	11.2032221333333	11.159679825	10.4184334333333	10.521634775	10.9537804833333	10.6757288666667	10.5311099	10.9021357916667	10.3671094333333	10.6652778583333	11.3426956833333	10.6982774333333	10.7478338666667	10.8654356416667	11.0430757083333	11.8399235416667	12.1778025833333	11.22125475	11.2902838583333	10.6038899083333	11.83053445	12.093025975	12.7061787	11.2613111166667	12.247510175	12.0893148833333	11.9888038083333	12.5865453166667	12.4190092583333	12.5425479666667	12.333509375	11.6709513916667	11.3968955416667	12.1325110916667	11.8356051666667	10.5448322833333	9.9039927	12.3046936083333	12.513133025	12.6640954083333	11.0366394333333	9.311763425	12.072543675	9.482987475	12.6656204083333	12.600413	12.247510175	11.1039790666667	12.1904676708333	12.1237921916667	11.868130425	12.264576775	11.0167794916667	11.2965765083333	11.89894165	12.7591596666667	12.8584129833333	11.2366875083333	11.641524325	12.0305597416667	11.6926020166667	12.769749725	11.8650417166667	11.9314967083333	13.0271148666667	11.3096843916667	12.1703301333333	12.4927583	12.488453925	12.539129975	12.2947323416667	10.8243180916667	11.6080957416667	10.8578576916667	11.7467027166667

USP6NL	10.6332993416667	12.3724626666667	12.2281085833333	11.709376075	12.45136255	11.0268698583333	12.5963701666667	12.5076062916667	12.576015775	11.9443641	11.930808275	10.4793722916667	11.8993453583333	12.8757636333333	12.815500575	11.75788825	10.924157925	12.9694275083333	13.2387164583333	12.8339629333333	12.280521525	12.7494695	12.2500083166667	12.5948964	12.8041062916667	10.7421748083333	12.5272702916667	12.5633422083333	12.769749725	12.672014175	12.8213874666667	12.9230312916667	12.3280561	11.2861342166667	10.6816359083333	10.7846923083333	12.6881562083333	12.6800815583333	12.1198078916667	12.4545539666667	12.672014175	12.5775136	12.543462525	12.1899139	11.43798885	12.13546815	12.547782425	12.659113	12.3418182	12.0819963833333	12.3538131333333	12.68421675	11.4038169	11.7304346833333	11.5078323166667	11.5836115333333	12.5439252666667	13.0708076333333	12.6512308	12.1770216166667	10.051171975	10.9035797416667	11.0811600833333	11.7407727166667	10.0740705333333	10.683111325	10.2084654083333	10.2018680083333	10.6251996	10.082743125	10.3758856666667	11.531296025	9.97000988333333	10.58448395	10.953117425	9.55009920833333	10.35233035	11.5385948833333	10.7336987	11.0013930666667	10.3690418416667	11.6933169166667	10.7936080833333	10.6819563666667	11.0419739083333	11.16005095	10.9750833833333	10.2919749833333	9.7815357	10.8889520833333	10.410088175	10.9021357916667	10.8930750916667	10.9153273416667	9.07536643333333	10.9430329583333	11.5731480083333	10.8792631583333	10.028074675	11.4170295666667	10.478998225	10.410088175	10.982052575	11.24605735	10.20731305	10.4671575083333	10.3718117166667	10.64689975	10.8480286083333	10.6169276916667	11.11213045	11.3738895833333	9.73914464166667	10.4284899	9.309548625	10.72804705	10.8454086666667	10.5802645333333	10.849584125	10.9405146

RCOR3	12.6754546333333	11.6302237	11.414772875	11.496789525	12.058910375	11.5472050833333	11.410462825	11.3624169916667	11.6209481083333	10.949373725	11.6974443166667	12.3021061583333	11.9750759916667	11.4184966	12.0088601416667	11.8904865166667	11.5012483	11.9486907833333	11.5544661666667	11.7849428666667	11.8051550333333	11.7753980416667	11.5509672	10.878870175	11.765185225	12.5344122083333	11.739559375	11.7182865833333	11.7782923583333	11.7791279583333	11.4200132333333	11.53339965	11.7004395833333	11.4420691166667	12.2981372583333	12.364041975	11.8290120833333	11.1203060666667	11.164489475	11.2657187833333	11.214698175	11.781905875	11.4534829083333	11.2711403666667	12.14843035	11.5601850333333	11.7160036	11.1455903583333	11.56420645	11.8435483083333	11.5738946	11.3849835833333	12.3318475166667	11.9674169166667	11.759780275	11.6179147583333	11.102188775	11.4748997416667	11.501628025	11.327151075	12.6151813916667	11.9777865	11.7262372833333	11.9165677	12.7468098833333	12.0432377416667	11.4503715	11.4446261583333	11.5809197583333	11.7881196708333	11.9052300666667	12.2755744916667	11.2312268666667	12.0619908166667	12.3592921916667	11.7699299	11.8701716833333	12.080029025	11.5676618166667	12.0533566	12.5696588166667	13.138182175	11.3047106916667	11.57803215	12.0160472666667	12.077364475	12.0237948	11.54913165	13.4572533333333	11.5103338083333	11.6496725666667	11.4383748083333	11.7105442333333	11.3690796916667	11.8527274916667	11.8666609583333	12.29141735	12.3501237916667	11.7958947916667	11.8465762083333	11.8709570583333	11.5114672666667	11.9082126416667	12.034459025	11.0931527333333	12.063617625	11.735525225	11.36160315	11.7487231	11.8726369583333	11.84426255	11.0873559916667	12.1333790583333	11.4798432416667	11.6378736916667	12.1690316666667	12.2658594166667	11.421792525	11.8658810166667	11.5684552166667

FAM193B	12.1677152916667	12.017301325	11.47905225	11.5103338083333	12.6151813916667	13.7930399666667	12.6461371833333	11.9240142833333	12.61272965	12.4333458416667	12.1943788416667	12.5831450833333	12.4995800083333	11.8403034416667	11.9100903833333	11.6302237	14.657054125	12.336155525	11.7564937416667	11.4481946583333	11.7012064	12.3038527583333	11.3934696166667	11.357567525	11.9534028083333	11.9777865	11.7425826083333	11.6823165333333	12.0388164916667	11.8241137083333	11.425117975	11.3339882	12.3860530083333	14.3792394416667	11.94832135	11.958948075	12.4447647416667	12.39392575	12.206981325	12.1329469916667	11.8646362416667	11.92817275	11.5945056583333	11.50818875	11.645956625	11.6058721833333	11.3328774166667	11.2071862916667	11.7545106083333	12.050259975	11.9259261416667	11.6496725666667	12.034117575	12.0164637	11.5578653583333	11.141230475	12.0546824083333	11.96822585	11.920083225	11.6485244	12.3943629916667	12.2658594166667	11.2872125416667	11.545842925	12.029837025	12.5497662083333	12.8329037166667	12.2289445583333	12.273184075	12.400136275	12.1847949166667	12.1686155916667	12.3983699916667	12.008092475	11.9486907833333	11.5858193083333	12.61272965	12.2099162083333	11.395075525	11.6433290333333	11.6485244	12.287955975	11.4978397166667	11.4083049	12.2574255666667	12.217702275	11.8078233833333	11.9333501833333	12.11260975	11.8096310916667	11.5958198083333	11.465822775	12.2053036416667	12.4346028333333	12.233149625	11.9585723416667	12.403353825	12.3883541166667	12.4346028333333	11.9697901833333	11.8337195083333	12.2565941333333	11.5645444166667	11.2950935333333	11.5440185916667	12.04365515	11.6408490416667	11.1582543083333	12.0713921666667	12.028642225	11.77793475	12.0577849333333	11.7046170083333	11.27496335	11.383891075	11.040528175	11.7663613666667	11.6408490416667	11.3357126416667	11.8000726

KCNN2	4.936774575	3.97264301666667	3.90803829166667	4.03968158333333	3.86842555	4.14585755833333	4.24624565833333	4.753728625	4.44059075	3.99817013333333	4.00244103333333	3.9614432	4.01568299166667	4.117963675	4.21300761666667	3.96490434166667	4.41297161666667	4.2231712	4.71445220833333	4.128952175	3.78548855	4.09903998333333	4.25488725	5.2151974	4.58881544166667	4.361998275	4.20479785833333	4.48159451666667	4.52155396666667	4.28809964166667	4.508464425	5.08458449166667	3.931450225	4.258212325	5.79649798333333	5.546831425	4.33043379166667	4.41506155	4.485186325	4.47367345833333	4.78798888333333	4.49602461666667	4.73402499166667	5.30888879166667	4.07882345	4.86085110833333	4.98106579166667	4.76661771666667	4.44534256666667	4.71445220833333	4.67471653333333	4.479514325	4.08114406666667	5.24102825833333	4.78900190833333	4.50751908333333	4.00032819166667	4.67380545833333	4.32670648333333	4.65588558333333	3.73259605	3.798771525	4.24718985833333	4.06900396666667	3.63546845	3.74900228333333	3.80684369166667	3.946878775	3.64669851666667	3.94580334166667	3.61894206666667	3.5947849	3.96951036666667	3.75971476666667	3.56307476666667	3.67515746666667	3.737761675	3.72442025833333	4.11883455833333	3.723060125	3.98511664166667	3.68999008333333	4.19434771666667	3.80438254166667	3.77909573333333	3.644387875	3.73388574166667	4.443420775	3.77426261666667	3.99930385833333	4.03968158333333	4.35594943333333	4.02352729166667	3.79653381666667	3.812622975	3.8371121	3.94009973333333	4.08851591666667	3.77553975	4.67172261666667	4.26028479166667	3.71049449166667	4.70784294166667	4.97812506666667	4.25488725	3.937874125	4.56806040833333	6.018516875	4.21912249166667	6.26140625833333	4.54440353333333	5.16735583333333	4.45030740833333	5.33234265	4.32094003333333	4.27798759166667	4.15118036666667	4.46402134166667	4.62375229166667	4.34795044166667

TMEM61	7.52896074166667	9.76350663333333	9.13602675	9.71485326666667	9.08299806666667	8.48614225	8.940002325	9.9441902	9.12419129166667	8.69984448333333	9.19287955	8.58538408333333	8.80586325	9.96018530833333	10.3980051666667	9.26722199166667	9.96699933333333	8.93317065	9.489247625	9.6385431	9.64610789166667	9.65101713333333	9.7570522	9.743497625	9.94164523333333	8.80189605833333	9.198111125	8.91224634166667	8.8039806	9.32044345833333	9.460327375	8.76751969166667	9.50996596666667	8.12489341666667	9.74951903333333	7.35564645	8.95624593333333	9.286424975	9.38715235833333	9.79573791666667	10.5825770333333	9.12842238333333	9.61633444166667	9.90608706666667	9.13825335833333	9.60046640833333	10.74441525	9.68903575833333	8.86467129166667	10.2387377833333	9.19531680833333	9.21688079166667	9.4510034	9.508670075	10.04588255	10.06035685	9.16074203333333	9.53957571666667	9.370796075	9.77432975	7.54963650833333	7.21098661666667	8.67210133333333	7.67926596666667	7.092930725	8.31252919166667	7.32789755	6.861457125	8.16584395833333	8.60998564166667	9.0354605	8.59640985	8.07255219166667	6.94690668333333	8.375514	6.69781155	8.52061441666667	7.62704748333333	8.19700369166667	7.06220724166667	6.414216275	7.04898169166667	8.89652095833333	8.916017775	8.05717868333333	7.54282731666667	7.3367108	7.52225746666667	7.55374699166667	7.72294644166667	7.40257545	7.65827738333333	6.897275725	8.01060475	8.02564620833333	7.69858204166667	7.11986829166667	7.283866825	7.2401722	9.058233875	7.762415375	8.08909971666667	7.45558410833333	7.89722555	8.19294795833333	6.52412431666667	7.17093558333333	8.44993103333333	7.25477635	7.49991619166667	8.42983770833333	6.891986525	7.66500309166667	7.26165935833333	7.34892271666667	7.84545261666667	7.12901885	7.537939	7.127807925	7.17033845

TRIP12	10.9632585583333	10.2865677	10.4053126416667	10.6523425166667	11.4872383	11.1252834	10.73913815	10.4204003083333	10.7470371333333	10.64689975	10.49466965	10.8710123583333	11.227651025	10.1899294916667	11.3601498333333	10.3261322166667	9.7105078	12.118621125	10.5945390166667	11.13577505	10.2865677	11.7113167916667	9.62453731666667	10.7460395416667	11.1689128333333	10.83116835	11.0301443333333	11.5237451916667	11.6408490416667	10.6422709916667	10.2318091916667	10.7774965	10.9091302666667	9.59418116666667	10.4590182666667	9.96522326666667	11.7533960083333	10.6187719166667	10.3265631083333	10.8028896	9.68261813333333	10.2241250416667	10.325378325	9.73489294166667	10.0679332666667	9.96868019166667	9.76647539166667	10.0159752583333	10.4292877666667	10.764714825	10.2874114666667	10.451127	11.5898474083333	11.5392863166667	10.4691008666667	10.0026032916667	10.670739975	9.354988125	11.588039275	10.31088965	12.4831684166667	10.1745820166667	10.4678765666667	11.4563744833333	10.233460875	11.544719375	10.8232399083333	10.74562245	10.5659717333333	11.14881755	10.5995079	11.0075637333333	11.0157092833333	10.990211025	10.7460395416667	11.13057255	11.3823997333333	11.5876922333333	10.6377074083333	10.5953164	11.4745197333333	11.6818913333333	10.7371678583333	9.83237755833333	11.5404299	10.9647570833333	11.5898474083333	10.7665838833333	11.7936196666667	10.5337610083333	10.5448322833333	10.552679575	10.6652778583333	10.8329994333333	11.33887195	10.6918746916667	11.28453495	11.0873559916667	11.0666847416667	10.4089152416667	10.9809278083333	9.61596005833333	10.5671190083333	9.97912404166667	10.3432001083333	12.0409411833333	11.1074454416667	9.906465775	10.8052677	10.4195860916667	10.7864881958333	9.875255275	11.4191800833333	10.1109480666667	10.5895974833333	10.6511688416667	10.5663053083333	10.35148425	10.7042731416667	10.5735065583333

NF1	6.90781049166667	6.68797494166667	7.04821105833333	6.57096464166667	7.9089283	7.148875425	7.20693700833333	6.58763124166667	6.71502808333333	7.18705289166667	8.21830426666667	6.633753725	6.49896905833333	6.16362138333333	7.55987390833333	6.67856683333333	6.96906025833333	6.79334741666667	7.02883426666667	6.850945825	6.84850089166667	7.98023625	7.10822425	7.29304450833333	6.9604848	7.37296829166667	7.46194565833333	7.38755610833333	7.134785875	7.34551940833333	7.08866475	7.15940084166667	7.40135389166667	6.65156973333333	7.060215325	7.551793125	7.21695988333333	7.191921025	7.18705289166667	7.137920375	7.422156975	6.91035588333333	7.00499255	7.09043023333333	6.26279403333333	6.86736839166667	7.64552340833333	6.77563099166667	7.42103133333333	7.23028210833333	7.07182334166667	7.01495000833333	7.26165935833333	7.25477635	6.78916241666667	7.23488161666667	7.582967825	7.07980634166667	7.75772005833333	7.49442741666667	7.644918425	7.81088345	7.48300389166667	7.64275718333333	6.8732984	7.66558521666667	7.12103848333333	6.8478471	7.14336814166667	6.975151325	7.78668815	6.73002575	7.87821420833333	6.7730469	7.187671825	6.70257901666667	7.54177855833333	7.29474379166667	7.43805644166667	6.84200789166667	7.15820169166667	8.05913828333333	7.43805644166667	7.9703466	6.68200588333333	6.89528771666667	6.96848423333333	6.03500149166667	7.74093078333333	7.5324158	7.15455295	7.67253041666667	8.54989949166667	7.06709683333333	7.81839644166667	7.66954886666667	8.05514178333333	8.07411434166667	7.01621956666667	7.22964071666667	8.44745494166667	6.77436175	7.85748985	7.24887754166667	7.08988083333333	6.764479675	7.55374699166667	7.00754390833333	7.678740925	7.37467966666667	8.319610775	7.153195325	8.03810015	7.171442675	7.09487224166667	7.53506526666667	9.112227825	8.29250424166667	7.70645875833333	7.73246671666667

ZIM2	14.512293975	14.3139109083333	14.7429609666667	14.274700225	14.3860372083333	13.6202060083333	14.50554015	12.7139152416667	14.1268362333333	15.1207533083333	13.6320213916667	15.156314825	14.87708635	12.6876920416667	13.4737954	13.134360625	14.6926031666667	13.7960609666667	13.4688309833333	13.4018283333333	13.9829878583333	12.1899139	13.4498034583333	14.2576986333333	13.4359788416667	15.33292745	15.01216265	14.5530100166667	14.21756365	14.105898725	13.570433175	13.9261802833333	15.0703584333333	13.9727862416667	15.9315345	15.386313475	14.0607265833333	14.371029675	14.282826075	13.668458425	13.7621985333333	13.9250365	13.827479275	14.003645225	14.3943592583333	12.488453925	12.1861275083333	13.4729544083333	13.2587665833333	14.5488647916667	14.818607725	14.9567111166667	11.61112325	10.706587775	11.1993790583333	11.4752696416667	14.0813262416667	13.6246233916667	14.4226857416667	15.0615765666667	15.7630766833333	14.9197306916667	15.5221336416667	15.4672060583333	14.80427055	15.5512564416667	15.5313549166667	15.7697269333333	15.618710975	15.2576870916667	15.7486260916667	15.4672060583333	15.3944873	15.8065534666667	15.6472384833333	15.91498085	15.3136672916667	15.8470782	15.6235572	15.7486260916667	14.6549636583333	14.73869885	14.894107525	14.9594767083333	15.4276773083333	14.657054125	13.9025146916667	14.72730925	14.2858034416667	15.2193239916667	15.5362043833333	15.2432312666667	14.380649825	15.1336404666667	13.407373775	14.85238655	15.290119725	15.33292745	15.0703584333333	15.651975575	14.4226857416667	14.76191125	14.9674626083333	14.0964870833333	13.60723025	14.3236760583333	14.9070025916667	13.670246725	15.7781512166667	15.236084325	15.0894272416667	15.0587922833333	12.0721549166667	12.6595749666667	12.9948642333333	12.0368526666667	15.1632730833333	15.2687677	14.8412522666667	13.9873624166667

LHPP	8.44287714166667	7.11789028333333	7.02883426666667	7.65442175833333	7.54341655833333	7.05156345	7.65894785833333	6.903905225	7.2843799	7.24828621666667	7.38984995833333	7.746177925	7.53126729166667	7.35396276666667	6.88578850833333	6.88578850833333	6.47291681666667	7.27714694166667	6.99412739166667	6.87022720833333	7.53964365	7.46514316666667	7.02467490833333	7.02284831666667	6.974033025	8.21622873333333	7.18452301666667	7.20806453333333	7.41526463333333	7.074128375	7.004281725	7.38984995833333	7.44203170833333	7.14519241666667	7.76345526666667	8.55907896666667	7.80067161666667	6.9604848	7.14822965	6.92374801666667	6.43219438333333	7.17093558333333	6.723717275	6.86328933333333	7.48416809166667	6.8528802	6.54365829166667	7.23609219166667	6.66054216666667	6.602072175	6.58763124166667	6.92916504166667	7.16541175833333	7.26808264166667	6.65690114166667	6.65156973333333	6.63442134166667	6.537297825	6.98410809166667	6.58226158333333	8.128317575	7.74398054166667	7.8248949	7.84154363333333	6.99282755	7.428439975	7.78668815	7.41526463333333	7.47496615833333	7.65774509166667	7.76403325833333	7.68098021666667	7.96133965	7.3361665	7.77602128333333	7.63604310833333	7.70199845833333	7.41824386666667	6.81976344166667	7.77775845	7.53349060833333	8.3316018	7.824397325	7.18226805	7.66718561666667	8.340733825	8.36334496666667	7.7794798	7.86012560833333	7.158761525	7.583460175	7.48011853333333	7.46964506666667	7.44368639166667	8.14240605833333	7.43289285	8.16931455833333	7.8129644	7.83240795833333	7.42268463333333	9.13909698333333	8.174528375	7.65225225	6.903227175	7.34272655	7.68263314166667	7.14822965	6.06574364166667	6.85677963333333	5.6003113	6.65223385	6.34978520833333	6.901873575	6.27004911666667	7.010804075	7.283866825	7.40546740833333	6.97843058333333	7.160095	7.06942048333333

PRIMA1	12.503422725	9.87207808333333	9.895935425	9.06886545833333	9.83982443333333	10.31595855	9.44105665833333	10.2108003333333	9.77883299166667	10.0831227083333	9.20235228333333	11.9352971916667	11.325017625	10.1413216916667	10.447247375	9.91197805833333	10.571508325	9.53443595	10.4429923666667	10.9094504083333	10.5806393916667	9.9519621	10.3483436583333	9.909962375	10.9160737916667	12.278442975	10.2874114666667	10.214113	10.257489575	10.4127726666667	10.2018680083333	9.93042398333333	10.2245747416667	9.42382585833333	12.4732793833333	12.3846618166667	10.388343775	10.5452677583333	9.62367923333333	10.7892430083333	10.364930325	10.0653276583333	10.4664769916667	10.5623629583333	11.2715325333333	10.1164178916667	10.7001015833333	9.6482902	9.90307260833333	10.3167137333333	9.89303093333333	9.14921909166667	10.1931783583333	10.1940041	10.4380961666667	10.4934979	10.04725285	10.1845142083333	11.2997236583333	10.6092076916667	10.6982774333333	7.87869541666667	9.51724313333333	9.77475016666667	10.05827805	9.876036325	9.19244325833333	8.53333894166667	10.021871175	8.67125495	7.3694039	7.97458126666667	6.64016859166667	7.52054051666667	9.48384479166667	9.843730775	9.4510034	9.97873573333333	9.16261181666667	8.75882765	10.4554224833333	9.08005709166667	9.515921025	8.03538219166667	7.21098661666667	10.0026032916667	10.4228179333333	9.13739526666667	7.41303320833333	9.83499924166667	8.500835825	9.85548545	6.61289744166667	8.61712494166667	9.4968836	9.10621828333333	10.9684950083333	9.4946079	7.82339403333333	10.3794789916667	8.8451569	9.21882504166667	8.51081288333333	9.09505498333333	7.54405583333333	7.23371278333333	9.19244325833333	7.001733525	8.168322275	9.57222860833333	8.34866896666667	9.67141531666667	9.93596328333333	10.3868161083333	9.91241780833333	8.71647500833333	9.96151823333333	8.10330963333333	8.45605063333333	11.5196693333333

MB21D2	9.47590374166667	7.85807856666667	8.3023979	7.96133965	6.87783053333333	9.97833993333333	6.87917671666667	7.98294754166667	7.18226805	8.162750025	6.82644443333333	8.1205116	7.066499225	7.906291625	7.64049128333333	8.38023938333333	9.18595039166667	6.602072175	8.51856025833333	7.93490190833333	8.0063663	6.849792025	8.77869386666667	8.42018036666667	7.967895825	9.3434528	7.79803676666667	8.74766549166667	7.3019376	8.122791675	8.57400345	7.66209683333333	7.58073043333333	8.580396475	9.7097404	9.05868610833333	7.38522528333333	8.32310249166667	8.23956795833333	8.3876511	8.82920276666667	8.30336938333333	8.635919475	9.04320051666667	8.52966328333333	8.80677418333333	8.58808473333333	8.9384783	8.57560394166667	8.53238421666667	8.97354856666667	8.33758601666667	7.53349060833333	8.49442401666667	7.94172535833333	8.78635625	7.97141813333333	9.083454025	8.283955225	8.619247325	7.4731488	8.57208716666667	7.90032115	9.721994775	10.5569098916667	10.2987266333333	9.71098871666667	8.604857825	8.52583200833333	8.333491425	8.61820814166667	9.44734175833333	9.07955376666667	8.80050660833333	7.72469713333333	9.93630081666667	7.7783407	7.9857199	9.72703330833333	7.93544106666667	9.52504286666667	8.28997516666667	9.66065478333333	9.48341896666667	8.327465575	8.3496737	9.47542340833333	9.41120025	8.00199818333333	9.41162929166667	10.2951456833333	8.13138714166667	9.71864728333333	9.08047905833333	9.59418116666667	9.25536705833333	8.92651730833333	8.22981704166667	9.852861125	9.2639574	9.24401314166667	10.035165975	8.10057743333333	9.49775068333333	9.47772424166667	8.8072839	9.31710229166667	9.983490475	8.958107775	10.5735065583333	9.86020724166667	9.99432204166667	10.5659717333333	10.0095607333333	9.67274871666667	9.116355225	10.1529084083333	10.3972128	9.23005591666667	8.67361984166667

PRKAR2A	12.1229210916667	12.608834775	12.8487753833333	12.5320694166667	12.9179353	11.8779799083333	12.43647125	12.6391509	12.516863425	12.3443503083333	12.05061705	11.8145765416667	12.5548435083333	12.716481775	13.1216942833333	12.3992340916667	12.4082756583333	12.3806821916667	13.0714709916667	12.8124184083333	12.7366517166667	12.8498718833333	12.3240568416667	12.608834775	12.0778101166667	12.1087888833333	12.4945779666667	12.5488109	13.38316305	12.796581575	12.6790614583333	12.4822872083333	12.40597585	12.0092346166667	12.1205448666667	12.2141453583333	12.764427075	12.737987825	12.372860975	13.1152178	12.191066075	12.6046256583333	12.4692673166667	12.8684829916667	12.478685375	12.8376576083333	12.8234678083333	13.01427815	12.531130425	12.217702275	12.2976992	12.6496634333333	12.932363575	13.0374763416667	12.5850501916667	12.7831765916667	12.7846359833333	12.963770175	12.4063944666667	12.4593508916667	12.4287875916667	11.9678169416667	11.5976176916667	11.8970046083333	11.4063648666667	12.2892396	12.063210775	11.742927775	11.86892365	11.86975775	11.3990716583333	12.2557740666667	11.9001097666667	11.763655725	12.1711742666667	11.9629534916667	11.3216619833333	11.9063380833333	12.3580122083333	12.2986022833333	11.646257275	11.6951784416667	12.352843675	11.9883632916667	11.742927775	11.5382360083333	11.0999976083333	11.6238039583333	11.2837838916667	11.9196580833333	11.7553657	12.1439381	11.6871432333333	11.6058721833333	11.7422357083333	11.8523757333333	12.247084775	11.7648162666667	11.3786114833333	11.464317475	12.0140512666667	11.5873432	12.0985319416667	12.3720643666667	12.222327475	11.8356051666667	12.6331977416667	11.7791279583333	12.1656867	12.1014528666667	11.702337975	12.1163125	12.1521118916667	11.9017197166667	12.3698220916667	11.8757437	11.3900978416667	11.6723181916667	11.8962786833333	11.5676618166667

PRAM1	9.68213623333333	9.877817275	10.081132625	9.92583431666667	10.5775565833333	9.20235228333333	10.1481495666667	9.573560175	10.4698289916667	9.85765425	9.73533868333333	9.60581258333333	9.69491718333333	9.39938098333333	9.41715076666667	9.448687325	9.135601825	9.67363538333333	9.446490025	9.1141243	9.349869075	9.148299925	9.189315625	9.17368278333333	9.7097404	9.508670075	9.71485326666667	9.51769735	9.83764681666667	9.60459400833333	9.6526333	9.686488825	9.92412455833333	9.6592899	10.2853426583333	9.71951955833333	10.3455009333333	9.69924190833333	9.89687281666667	9.52783803333333	10.0445903666667	9.65888631666667	9.52133895	9.34438068333333	9.08396963333333	9.43868498333333	9.17867269166667	9.54039348333333	9.65575276666667	9.54446325	9.96267133333333	9.77432975	9.607505	9.54880464166667	9.57414756666667	9.39843498333333	9.69439403333333	9.80625096666667	9.74904354166667	9.40861700833333	10.6079735416667	9.70931241666667	9.570506275	9.48609106666667	9.861824025	9.92453053333333	9.77049406666667	10.0877690666667	9.9338367	9.983868575	9.754485575	9.79856730833333	9.57093504166667	9.34018350833333	9.6482902	9.06167029166667	9.37031165833333	9.41715076666667	9.26988130833333	9.44516316666667	9.33365533333333	9.21590233333333	9.33275593333333	9.19441289166667	9.87650806666667	9.666049875	9.82029285833333	9.58173716666667	9.72964383333333	9.75367038333333	9.61006324166667	9.46521605833333	9.84075720833333	9.6705637	10.095377	9.7452797	10.0728614166667	9.9229238	9.93713581666667	9.58662811666667	9.674571275	9.55990681666667	9.68558669166667	9.35306623333333	9.5040294	9.176064475	9.46751014166667	9.16920365	9.65222760833333	9.777820325	9.85895400833333	9.60629726666667	9.71989020833333	9.21080935833333	9.73788834166667	9.56548648333333	9.49279765	9.52867283333333	9.90608706666667	9.683051225

DIS3L2	10.373735575	10.7034642666667	11.235931225	11.1821084666667	11.3520380583333	9.95100449166667	11.1113391916667	10.9328378333333	11.0682286166667	10.5655352666667	10.2904392333333	8.88450143333333	10.7732420666667	11.1350432333333	11.1699387666667	10.21946315	10.6926124666667	11.118464025	10.934250825	10.783186525	10.6019249583333	10.5185399	10.1953308333333	10.7104085166667	10.7306227416667	10.1573430916667	10.5181477	10.5322469083333	11.159679825	11.0038210166667	11.0733527583333	10.6649055833333	10.6387912166667	10.4634265083333	10.3293667083333	10.4195860916667	11.3172677166667	11.227651025	10.5519705833333	10.6488037166667	11.122077	11.104988	10.6309483083333	10.7414541583333	10.341641975	10.8713916666667	11.0366394333333	11.759780275	10.8506520083333	10.5639796666667	11.0837790083333	11.1459742833333	11.3216619833333	11.5429763166667	11.0470160416667	10.96963075	11.0250402166667	11.3567956166667	11.1063897	10.9673192583333	10.4380961666667	10.1130642083333	10.2695195083333	10.333754975	10.4698289916667	9.81132868333333	10.5768008666667	10.0949964416667	10.377533625	10.15852225	10.0720278166667	9.46343676666667	9.96660331666667	10.1927941666667	10.18161685	10.1424095333333	10.035165975	10.1808367916667	10.4975657166667	10.2687270166667	10.4815083083333	9.696676675	10.0533049083333	9.96443173333333	10.1378549916667	9.58706739166667	10.7565121333333	10.26748265	10.3475834	9.98961361666667	10.490641975	10.0859903416667	10.15376285	10.2857643416667	10.126633025	9.89341510833333	10.19975295	10.5034091416667	10.42200985	10.694500425	10.4526526416667	10.0305098083333	10.2535245166667	10.4040896666667	10.2450271333333	10.6340233	10.3987917	10.7189561666667	10.5244636333333	10.6305289416667	10.4958848833333	10.5910579333333	10.6960220666667	10.856356975	10.609691175	10.7092769166667	10.7628088916667	10.76921485	10.742514025	10.58448395

GYPC	13.0590979083333	11.2595088166667	11.31764545	11.1430808166667	10.2810793	11.04340915	10.4806526833333	10.6422709916667	10.723219775	11.5638114916667	10.8476613083333	12.60549005	11.9009487833333	11.0538325833333	10.8964884916667	11.8527274916667	10.70539815	11.4730116416667	11.60398845	11.5614234666667	11.4409493166667	11.483228	11.158996075	11.0390903416667	10.5428297916667	12.8376576083333	11.4052792333333	11.1343238833333	11.185479075	10.666810425	10.8889520833333	11.2464355333333	11.3797907833333	11.4181579	12.9284297083333	12.7896506583333	11.3332438	11.0747492583333	11.2506923333333	11.1787139083333	11.29542035	11.5327072416667	11.659892375	11.2609645083333	12.3219919916667	11.72023525	11.7509367	11.105357075	11.43611165	11.2428131833333	11.1498219083333	10.8908321583333	11.7105442333333	11.460533275	11.5645444166667	11.9093036166667	11.3567956166667	11.3206346333333	11.7883410833333	11.9055925	10.316356175	10.2330333333333	10.3097382833333	10.1046909416667	11.73400915	9.69126795833333	10.092918675	9.92832075	9.89982799166667	10.6139707	10.0972464083333	9.36939973333333	10.2501973166667	11.0581558166667	9.88726988333333	10.5631726083333	11.2538026583333	11.3597850833333	10.2857643416667	10.627440475	11.6529488416667	11.0491091083333	9.713775575	11.1898119916667	9.945938775	11.1003561666667	13.2815171083333	11.373190825	9.890973325	11.3957857333333	10.298281975	10.4284899	9.70495998333333	10.225450075	10.04588255	9.85722324166667	9.85113778333333	11.9621161833333	10.341641975	9.797655	10.6115680416667	10.8570968916667	10.990211025	10.2912155166667	9.56172193333333	10.5585696666667	11.1060515416667	9.69439403333333	11.197870325	11.2696990833333	10.6876859	10.003538	10.4522980083333	9.91842590833333	11.327862475	10.244614225	12.023387575	10.1001644	9.666049875	11.2233924

RDM1	7.02761371666667	5.68783094166667	6.69380246666667	7.28072111666667	7.06888018333333	6.841322025	6.4308335	6.35665519166667	7.10317193333333	6.74821494166667	6.81435373333333	6.61221461666667	6.86262681666667	6.20814583333333	6.86605038333333	6.50275845	6.53407053333333	6.63866813333333	7.392806175	7.195709975	6.95221913333333	7.56655451666667	7.00630173333333	6.95984925	7.27714694166667	6.51830799166667	7.49442741666667	7.15147738333333	7.53401704166667	7.0659289	6.880500475	7.17507495	6.35504728333333	7.14024473333333	6.77836881666667	6.72249205833333	7.52330799166667	6.70918899166667	6.69500276666667	7.134182075	6.36473330833333	6.61087155833333	6.92852376666667	6.70056215833333	6.64415971666667	6.764479675	6.99727938333333	6.258372575	6.67919664166667	6.52412431666667	6.51237135	6.68200588333333	6.12691128333333	6.94561610833333	6.77975395833333	6.62496390833333	6.40053460833333	6.24033626666667	7.30133825	7.22835589166667	8.22741431666667	6.58100214166667	8.73305545	7.65225225	7.55609866666667	7.124687125	7.47951195833333	6.69566758333333	8.90478388333333	7.270544475	6.97645464166667	7.21695988333333	8.28143178333333	6.12200050833333	6.86073455	7.42103133333333	7.10699465	7.87964591666667	7.46964506666667	7.45403938333333	9.02371780833333	7.428439975	7.630419825	6.82383751666667	6.4516666	8.05913828333333	9.11737229166667	6.91828460833333	8.32165043333333	6.96778875833333	7.35447710833333	7.67535224166667	7.19137265833333	6.86262681666667	7.54799789166667	7.48416809166667	8.30084284166667	8.81108796666667	7.00049489166667	6.83868629166667	6.88314723333333	7.306684875	7.989231475	6.4808852	7.60193526666667	7.06220724166667	6.8444516	6.29443513333333	7.43742328333333	7.19636080833333	7.84430019166667	8.16931455833333	6.65287900833333	9.037729975	7.931728325	8.21929578333333	8.87183624166667	8.09798645	7.72085915833333	7.0633355

C16orf54	7.23488161666667	9.96400001666667	10.0648957166667	9.12842238333333	7.72469713333333	9.16588333333333	9.21303188333333	9.63902631666667	9.02830098333333	9.313630975	8.38987458333333	9.32849245833333	8.15563305	9.42904984166667	7.43805644166667	11.0648135916667	10.3714480666667	7.76714046666667	8.10330963333333	9.78654094166667	9.80096413333333	8.03327618333333	9.34207146666667	9.79486831666667	9.31047634166667	8.85962865	9.4981546	8.42318403333333	7.855217475	8.21046635	9.34157415833333	10.1862509833333	9.53443595	11.1869945583333	9.481636725	8.069486125	8.07106306666667	9.32711606666667	9.545824775	9.40252054166667	9.674116625	8.88688890833333	9.94976705	10.0489380083333	9.95725063333333	10.451127	9.50452185	9.18880384166667	10.4705726	9.666049875	10.0360710333333	8.400318075	8.905745925	8.49794376666667	9.23648119166667	9.891815375	9.57414756666667	9.97912404166667	8.74620478333333	10.54728675	6.8399633	9.01425336666667	9.17414999166667	7.25799328333333	8.122791675	7.16490981666667	8.4414262	8.92973970833333	7.91794835833333	9.292974825	7.908404275	7.89243905	8.104851775	8.796402275	7.54626845833333	9.07816326666667	10.5945390166667	9.3750605	8.686777175	9.03372714166667	7.875176525	7.97346688333333	8.703553525	8.8319761	8.33599743333333	8.22500544166667	8.254482075	8.916017775	7.37707911666667	9.107046275	8.927052675	8.08240869166667	7.33026348333333	9.01101026666667	7.26937910833333	8.84099020833333	7.583460175	8.15812938333333	8.52634974166667	7.72796763333333	8.61290266666667	8.8364013	10.323288425	9.20045154166667	8.312026725	9.1029726	8.57063375	8.84333919166667	9.34392991666667	9.0772993	10.7414541583333	8.23717023333333	7.75661866666667	7.597182875	9.74128455	9.28405401666667	7.81456171666667	8.722672	7.99232836666667	10.4951144833333

ASB3	11.5004634	11.8477473833333	11.3086201833333	11.5664864	11.21039405	11.6982071583333	11.2428131833333	11.4556673333333	11.5630353166667	11.4402312666667	11.529525725	11.5501880833333	11.1641077166667	11.5181590833333	11.5586348333333	11.6187195083333	11.7445138833333	11.6107118416667	11.5226770083333	11.830137	11.6594960166667	12.140177075	11.9985911666667	11.0491091083333	11.605536575	11.811945375	11.2826013166667	11.8150676833333	11.43469695	11.7231538416667	11.270439275	11.49381285	11.7060618166667	11.6883557166667	11.5505559916667	11.7336295416667	11.7699299	11.4545376666667	11.285520075	11.4586326	11.7690899333333	11.7753980416667	11.545842925	11.759780275	11.9451089	11.7219865583333	11.80928975	11.5728018416667	11.66214955	11.7053666833333	11.3275163916667	11.14669605	11.6367008333333	11.6933169166667	11.7304346833333	11.7300854416667	11.2424523	11.846919075	12.0265627166667	11.5389498916667	12.091314325	11.9146982166667	11.8970046083333	11.8787765833333	12.7056843166667	12.0884912166667	11.9585723416667	11.6398002583333	11.929273675	11.8912374333333	11.9509854083333	11.586583875	11.5991025	12.196460925	11.846919075	11.5656597	11.9228391333333	11.658465575	11.5150484916667	12.0868849916667	13.9025146916667	12.6679927416667	11.683026175	11.8787765833333	11.8658810166667	12.5196766666667	12.7846359833333	11.6679772166667	12.6944981166667	12.2466185916667	11.3242829	11.4993303916667	12.2240198833333	11.7761557916667	12.3874454166667	11.7703479083333	11.6529488416667	13.0920574083333	11.7568591416667	12.3267305	12.0051849333333	11.8465762083333	11.9100903833333	12.3602095583333	11.687529825	12.1038489666667	11.53339965	11.6944435416667	12.0901653083333	11.799711325	12.317829175	11.6115347666667	11.4317096083333	12.52409195	11.8418826916667	11.8650417166667	13.1973842833333	11.75788825	12.1677152916667	11.7710225416667

TOGARAM2	8.75611661666667	7.84376875833333	7.745650475	8.324127225	8.16735940833333	7.45160013333333	8.46961650833333	8.06285705833333	8.32062069166667	7.56380908333333	7.893532975	8.53482063333333	8.19143718333333	7.46964506666667	7.48416809166667	7.8129644	7.340949	8.11603985	7.82339403333333	7.86609655833333	8.31305365833333	8.36243170833333	7.31316353333333	7.40375466666667	7.4250003	8.05761690833333	7.75176951666667	7.40193138333333	8.06812029166667	7.5998906	7.47562453333333	7.36471060833333	8.08754240833333	7.57457279166667	8.18205496666667	8.57940885	8.09385128333333	7.70147536666667	7.48063178333333	7.4656947	7.26995246666667	7.39745536666667	7.18351369166667	6.958116025	7.91739750833333	6.86809180833333	7.10888616666667	7.60040215833333	7.41526463333333	7.039355275	7.03878675833333	7.456643225	8.1089851	7.58800115	7.064683475	7.13168135	7.11986829166667	7.06220724166667	7.3675475	7.22964071666667	7.91358665	8.17885319166667	8.27706721666667	8.62084126666667	7.80672660833333	7.80930983333333	8.35934713333333	8.44491509166667	8.081342075	8.11603985	8.05514178333333	8.33655620833333	8.259343925	7.71914490833333	8.49188041666667	7.95575579166667	8.1777524	7.84998155	8.18315743333333	8.215089275	7.98516725833333	8.67077851666667	7.80873386666667	7.7783407	8.81154583333333	8.47690594166667	8.4564653	8.42522444166667	8.12632724166667	7.52729815	7.85689433333333	7.7587684	7.92005815	7.707009225	8.34923416666667	8.06551123333333	8.97769849166667	8.10843584166667	8.38118433333333	7.11478095	7.41303320833333	8.295765375	7.7113432	7.24075795	9.83629188333333	7.51524328333333	8.160175425	6.68200588333333	7.86670446666667	7.013155025	7.40375466666667	8.3496737	8.09798645	6.99800655	8.29053858333333	7.59600343333333	6.70056215833333	7.54963650833333	7.58238585833333	7.694854575

RHBDL2	6.15858731666667	11.4989285	11.1554142916667	11.60515845	11.341686575	10.3534141083333	10.7628088916667	10.1870376	10.9661834333333	10.2919749833333	9.9581283	7.35924831666667	9.993864975	11.12457335	11.5578653583333	10.5523212166667	10.337717825	11.1409117416667	11.6799309666667	10.98769475	10.934250825	11.320943175	11.0031119583333	10.7526329	10.9378957833333	7.15389984166667	10.9730535833333	10.757286575	11.28518	11.7628441333333	11.063715325	11.3698477166667	10.5276582916667	10.4208424916667	7.39450265833333	7.19320405	11.077953225	10.9263073083333	10.8187575416667	10.566703425	11.544719375	11.4946024833333	11.2929247083333	11.113611625	10.3714480666667	11.155024025	11.5843413583333	11.2349074416667	11.4879615666667	10.632187725	11.4350551	11.3583216333333	11.0845103416667	11.2595088166667	11.0916995	11.0538325833333	10.9196649333333	11.8137761833333	10.9740524416667	10.806391425	8.23567169166667	9.47021275	10.857474925	10.4983213833333	9.37637135833333	10.7553698833333	9.46704600833333	10.2865677	9.79016168333333	10.0360710333333	10.268273175	9.54880464166667	10.05531845	8.91742895833333	10.6038899083333	10.3143367833333	9.777820325	10.3690418416667	10.9204831	8.9198372	9.34847368333333	9.29162124166667	11.0625918083333	9.50452185	10.7001015833333	8.127296675	9.35995134166667	9.29065144166667	9.72795155833333	10.1775011166667	9.91673698333333	9.90137155	9.41620731666667	10.4713528083333	9.65101713333333	9.2768977	9.133746725	9.97301156666667	9.52783803333333	10.6596205	9.90307260833333	9.53786854166667	10.5360893083333	9.94840036666667	10.86272805	10.634390675	10.5223924	10.6722546333333	10.1906714166667	10.28186315	10.86697045	10.9153273416667	10.4212307583333	10.04638645	9.943781525	10.8706515666667	9.17515170833333	10.0937438833333	10.237546325	9.465645475

LRWD1	9.79810515833333	10.7535022833333	10.98475965	10.97439825	11.0315811583333	10.98875735	11.195266625	10.9160737916667	10.9809278083333	11.1670443166667	11.0942927666667	10.7535022833333	10.78588925	10.7643055083333	10.744004	10.6872865583333	10.7140047416667	10.7842899166667	11.0670126666667	10.558939175	10.796845775	10.4308569666667	10.8457886333333	11.2530869333333	10.91013625	10.6360463166667	10.599920275	10.8427945083333	10.5026381833333	10.7774965	10.7959852666667	10.5768008666667	10.8608532	11.1284533666667	10.7912120583333	10.7027965333333	11.052772175	10.9909672	11.071263875	10.6990064	11.1593417916667	10.7325441416667	10.6233516833333	11.0150144333333	10.8109394916667	10.8150497416667	10.7605131333333	11.5302262416667	10.90507365	10.625983	10.931065425	11.1169690833333	11.0079045333333	11.0953873333333	10.9127535083333	10.7535022833333	10.9961958083333	10.80942375	10.7740240666667	10.7088791083333	11.4244147	11.6016871083333	11.9479243416667	11.8344676416667	11.55164095	11.3871722333333	12.1304018333333	11.1277733333333	11.96822585	11.4328736	11.6425911333333	11.8004315583333	11.7810947333333	11.0567325916667	11.0946623333333	11.5912797083333	10.7855003666667	11.8838190166667	11.4904941666667	11.3934696166667	11.401232775	11.2895126416667	11.39042995	11.6481957333333	10.878870175	11.2521123083333	11.8511708666667	11.0167794916667	11.0733527583333	10.9861853	11.0811600833333	12.1217141333333	11.3499248083333	10.8870209166667	11.14881755	11.6230706833333	12.0309620833333	11.9770182833333	11.832540575	11.5371262416667	12.1393673833333	12.1217141333333	12.049870975	12.05687395	11.9297077583333	11.3915472416667	11.4394949083333	12.01005715	12.4627799166667	10.8733577583333	11.6047856083333	11.1394283333333	11.510754075	13.1674768	11.685558375	11.4151310833333	11.4854519916667	13.4282493833333	11.74563025	11.3180417666667

HOXA3	8.51438241666667	8.37970946666667	8.22099113333333	8.18100231666667	8.13766550833333	8.04179383333333	8.10391784166667	8.34473695	7.6702147	8.1777524	8.11098743333333	8.042852375	7.49938943333333	7.9484088	8.60177515	8.03175081666667	7.60297259166667	8.09798645	8.86999053333333	8.486570275	8.472856875	9.15886451666667	8.083905375	7.77660473333333	8.656035175	8.95854766666667	8.63230339166667	8.23520641666667	8.54938428333333	8.89870729166667	7.82845124166667	8.65318265	8.47579976666667	8.19970503333333	8.75003116666667	9.49413000833333	8.293030975	8.11000589166667	7.95904073333333	8.49699819166667	8.649865175	8.76421910833333	8.230344325	8.512323475	8.60799398333333	8.84195500833333	8.682814325	7.89510465833333	8.94982423333333	8.55853193333333	8.29490485833333	8.381708525	8.78498449166667	8.555975275	8.7143253	8.779206475	8.40516035	8.39981014166667	8.90052724166667	8.59640985	10.1696248333333	6.65487665833333	7.2960179	6.41739583333333	7.9343368	7.61062251666667	7.84845994166667	8.13923885	7.03751019166667	8.09852281666667	7.1508922	7.227778225	6.82644443333333	6.407249275	7.67769499166667	7.41526463333333	8.4178821	7.74783731666667	8.18151525833333	8.44491509166667	7.42048124166667	8.275500325	7.025978175	6.99282755	7.29049713333333	8.348200375	6.84576470833333	5.79838183333333	8.02564620833333	8.046473725	6.44217180833333	7.19951121666667	7.4656947	8.79489933333333	7.78927275	7.35751986666667	7.18281396666667	7.20911576666667	6.80992164166667	8.2549704	7.89132048333333	7.643842075	6.57734461666667	7.851546575	7.900836625	8.1976201	7.94597349166667	6.89785889166667	7.63549629166667	7.002378425	8.43246804166667	7.5324158	7.54626845833333	8.09998893333333	7.013155025	8.560933175	7.63498546666667	6.48707709166667	8.00051745833333	7.388741125

EPS15L1	10.3570715916667	10.631761275	10.507752825	10.5731419166667	10.287024175	9.97752425	10.1654121833333	10.8555951166667	10.1986602916667	10.6007887583333	10.1573430916667	9.75747410833333	10.451127	10.684995425	10.8021252	10.0226518416667	9.983490475	10.6596205	10.8353214	10.8210096	10.2625794333333	10.751563075	10.6072418333333	10.4168764916667	10.4661023416667	10.4503313916667	10.2650558583333	10.5973351833333	10.5100494833333	10.5123801583333	10.6364744	10.34241075	10.135458025	9.5928155	10.2342241333333	10.4850893583333	10.5631726083333	10.9497712833333	10.7344399666667	10.0933465333333	10.8105355416667	10.5631726083333	10.5926557	10.5953164	10.2034843416667	10.743245175	10.4866818833333	11.0600457083333	10.4145396416667	10.3871636916667	10.6305289416667	10.7756109166667	10.916432925	10.7658215916667	10.4215953	10.616218875	10.515047025	10.8713916666667	10.6952813416667	10.4534831666667	9.7036749	10.7193725416667	9.8597846	9.97542684166667	9.48078894166667	10.1541580083333	9.87345979166667	10.4249193833333	10.0611526083333	10.31285305	10.022234175	10.3618816	10.6664473083333	10.0226518416667	9.700667025	10.7340525583333	9.460327375	9.80967570833333	10.13859765	10.5204657833333	9.71398214166667	10.1242565166667	9.92175831666667	10.3733561083333	10.2286007833333	9.24845199166667	9.57007478333333	10.0296842916667	10.1242565166667	10.1931783583333	10.214113	10.0321890166667	10.0434280416667	10.1184175666667	10.3035388166667	10.3289721	10.783186525	9.79933185833333	10.3610713833333	10.58448395	9.7815357	9.90955436666667	10.1278854583333	10.6251996	10.7061988666667	9.92703645833333	10.2919749833333	9.876036325	9.98111111666667	9.93713581666667	10.0180243333333	10.9094504083333	10.340914925	10.7272324083333	10.167505275	10.73913815	9.93427139166667	9.858083725	10.4409408666667	10.6699485666667

ANKRD40CL	4.506429475	4.545583025	4.90369176666667	4.81840091666667	4.45205615	6.19736666666667	4.63513973333333	4.88811624166667	4.28008616666667	4.2360195	5.455892825	4.535343	4.46696494166667	4.86589074166667	4.563953975	4.45205615	5.88760754166667	5.10498871666667	4.46985861666667	4.62790769166667	4.4473261	4.73928888333333	4.59369346666667	4.31803169166667	5.110215025	3.89460940833333	4.366029925	5.18759319166667	4.41002290833333	4.65176085833333	5.29552266666667	4.954343875	4.56487130833333	6.45696331666667	4.51179561666667	4.263255	4.85861940833333	4.832334525	4.53824951666667	4.47861730833333	5.32557380833333	4.04952899166667	4.48717731666667	5.04338205833333	4.2939407	4.69325549166667	4.472817375	4.57343223333333	4.34281185	4.92659856666667	5.37867954166667	5.12000276666667	4.91654405	4.87292895	4.11480768333333	5.28357275833333	5.60680040833333	4.48717731666667	4.91206978333333	4.63093599166667	4.53219865	5.13634166666667	4.88920265833333	4.683662975	4.07775664166667	5.565954175	5.43488754166667	5.12102319166667	4.4619761	4.52238	4.69863704166667	4.96956821666667	4.54330015	3.86964848333333	4.18191081666667	4.80433128333333	4.33637641666667	4.71113178333333	4.161871975	4.39770036666667	4.57924936666667	4.92455391666667	4.80827406666667	4.99463171666667	4.56071255833333	4.26116580833333	5.09317755833333	4.31803169166667	4.609167375	4.06389798333333	4.6247727	5.90588820833333	4.92455391666667	4.617309275	4.76141500833333	5.02689723333333	5.17483658333333	4.957419525	5.106111275	4.669520575	4.57030863333333	4.55283246666667	4.46100053333333	4.55184448333333	5.24289476666667	4.41203210833333	4.14371865833333	5.22265475	4.12677783333333	6.02974674166667	5.09419765	5.15873735	5.44322508333333	4.509505	4.52610014166667	4.09074995833333	4.41695324166667	5.60406076666667	5.34097245	4.62270154166667

BRS3	7.95406095	8.71384840833333	8.32062069166667	8.600364325	9.01197323333333	7.09933909166667	9.34897708333333	8.11712540833333	8.81515405833333	8.752344475	8.37653824166667	8.22799306666667	9.30325005833333	9.02609295	8.81066338333333	7.62479258333333	7.66500309166667	8.686777175	8.17505753333333	7.908404275	8.12434584166667	8.60701585833333	7.9999538	7.442585225	8.17036706666667	7.74294895	9.48609106666667	8.07357910833333	8.39722928333333	8.75927773333333	8.61046613333333	8.0095181	8.86902108333333	7.91522786666667	7.39564355833333	7.41061439166667	8.870415075	9.16302515	8.72830366666667	8.38217830833333	8.57063375	9.023211225	8.96094025833333	8.20031584166667	8.18151525833333	8.29533216666667	8.9925209	8.54895103333333	7.886143875	7.94329386666667	8.91275618333333	8.52772891666667	8.432956925	8.359825725	8.39722928333333	7.8150656	8.45255091666667	8.995827675	8.45304260833333	8.1223269	7.393964525	8.30084284166667	7.44607933333333	7.846366025	7.73246671666667	7.964195775	7.9438425	8.25990825	7.64914819166667	8.11057064166667	8.2260001	7.99186004166667	9.50914073333333	8.21830426666667	8.234157525	7.79298445	7.5654861	7.35751986666667	7.56602129166667	7.54963650833333	7.60136925	7.80727978333333	9.17867269166667	7.61643631666667	7.92224445	8.19396290833333	8.34473695	8.0773066	7.84545261666667	7.38926633333333	7.99563946666667	7.296620525	7.49164054166667	8.25344699166667	7.68042426666667	7.69424574166667	7.68541283333333	7.82120025	8.518147825	7.428439975	7.941170525	8.425659175	7.91739750833333	7.62414805	7.72354014166667	7.91097105833333	8.26456484166667	7.87768698333333	7.35059130833333	7.23488161666667	7.59144205833333	9.32000775	7.55925806666667	7.46463336666667	7.4398224	7.36272230833333	7.00815195833333	8.06551123333333	7.74896025833333	8.5716089

NADK	11.848881975	11.3767696916667	11.83053445	11.75788825	11.9754521583333	12.2219012	11.2609645083333	11.770678525	11.715671375	11.8962786833333	11.8650417166667	11.9131873583333	11.9927367	11.671592675	11.9712775416667	12.2628607833333	13.509141775	11.602856675	12.0164637	11.856139525	11.835222575	11.5706660333333	11.5552373166667	11.8182827333333	11.5606001833333	11.95296625	11.6263096416667	11.1997660583333	12.03257945	11.1484272833333	11.9236129	11.8726369583333	11.494176975	12.9300978916667	12.1333790583333	11.7909812333333	11.7853110416667	11.7728057833333	11.94277775	12.1861275083333	11.590501375	11.6514721333333	12.211850075	11.73798855	11.9325731916667	12.118621125	11.6047856083333	11.9923578333333	11.9052300666667	11.6902780833333	11.6470358	11.8826326666667	11.8198860833333	12.064871125	12.0901653083333	12.0717905916667	11.8484879416667	11.9244487083333	11.7194234416667	11.9304447166667	11.918121775	11.884179625	12.4386321666667	11.6962930666667	12.04786795	11.585466225	11.9629534916667	12.0185152166667	11.9420280583333	12.2111963416667	11.6080957416667	11.85040895	11.8285794166667	11.8962786833333	12.1656867	12.1258089083333	12.251683475	12.4593508916667	11.904789825	12.786823125	11.597258825	11.2560704166667	11.5578653583333	12.075266	12.0733383583333	12.1549544416667	11.0430757083333	12.2723412333333	11.66717835	11.87418675	12.2163587	11.794746475	12.0619908166667	12.0020893083333	12.11260975	12.0564210833333	12.3815660416667	11.6473588416667	11.81229275	11.6425911333333	11.7985917083333	11.691783775	12.8161131416667	12.1087888833333	12.39795185	12.6069951916667	12.2163587	12.206579925	12.4265910916667	12.2116409166667	11.6837602666667	12.2120592333333	12.3750783583333	12.137649925	13.0174115	12.1765967416667	11.7012064	12.3160821666667	11.6270644583333	12.4105900333333

TBC1D8	6.511623825	7.06146445	7.40193138333333	7.04267660833333	7.07238718333333	6.55667590833333	7.39505978333333	7.416522875	7.17681736666667	6.623007975	7.02653026666667	7.60592470833333	7.378725775	6.79588928333333	7.212178575	7.2622557	6.19893275	7.31981145833333	6.78446141666667	7.0659289	7.48927714166667	7.05338414166667	7.01495000833333	6.39769980833333	6.66175125	6.66666621666667	6.79798854166667	6.87917671666667	6.97340769166667	6.511623825	6.70390775833333	6.80056770833333	6.62775695	6.980300625	6.59670515	6.82102155833333	6.92575055833333	6.76714710833333	7.11855360833333	6.89068614166667	6.84909505	6.53668581666667	6.54872438333333	6.92575055833333	6.13424446666667	6.52272641666667	6.60536920833333	6.58835050833333	6.48645600833333	6.60068861666667	7.04898169166667	6.36951041666667	7.54963650833333	6.6659887	6.9563535	6.65487665833333	6.80992164166667	6.62775695	6.76253231666667	6.52564285833333	7.29304450833333	7.548601625	7.190008575	7.137920375	6.96848423333333	7.139659575	7.70028855833333	7.32974516666667	6.53205264166667	6.91035588333333	7.46688233333333	7.92437408333333	7.726376575	6.526432	7.05156345	7.79244525833333	6.725831175	6.68532921666667	7.26747879166667	7.06834181666667	8.24802405	7.84106111666667	6.80714281666667	6.926478325	6.9998061	6.83931143333333	7.76137635	7.74722135833333	7.663341325	6.64415971666667	7.19767501666667	6.99282755	7.51872279166667	7.171442675	7.49826404166667	7.299574675	6.84325476666667	7.33560889166667	7.42447115	7.6702147	7.68263314166667	6.909113525	7.33560889166667	7.06531028333333	7.19697114166667	6.45250163333333	7.52953641666667	6.32751184166667	6.58428694166667	6.180201325	7.15020035833333	7.502278225	7.254184275	7.27829234166667	7.09933909166667	7.17198438333333	7.26690018333333	7.09102175833333	6.70599545	7.77149728333333

MAP4	14.8653905916667	13.48163315	13.784925425	13.2227845833333	12.9166928166667	13.3724965833333	13.3193049583333	13.5494174416667	13.4321406333333	13.5626218333333	13.174009975	14.5570464083333	13.7434058583333	13.4572533333333	13.4427852416667	13.4688309833333	13.371772075	13.0620198083333	13.322101775	13.489460875	13.3771159333333	12.7852137833333	12.856730075	13.2366721916667	13.295486125	14.7166750666667	13.5543953	13.4209498916667	13.10396855	13.18580525	13.4119895416667	13.3545785083333	13.4018283333333	13.2778653666667	14.9045675833333	14.9779140333333	13.1465914416667	13.4113073583333	13.149953225	13.505263125	13.4912514166667	13.3444534166667	13.400193425	13.471337675	13.811693625	13.4171665166667	13.532131425	13.2047745416667	13.1919341833333	13.0841853166667	13.4149817666667	13.342400775	13.2460254166667	12.87636155	13.27588995	13.7502246416667	13.3710865833333	13.371772075	13.42164015	13.5034733	12.996638525	13.3567888916667	12.181921	12.7713114916667	12.6759474583333	12.8667102583333	13.2595614666667	13.3100924166667	11.7757946333333	12.798152025	12.4945779666667	12.4903405333333	13.6461564833333	13.1576233666667	13.141294	13.0294955083333	12.9745146166667	12.6826019916667	12.9812481083333	12.4416866083333	12.8161131416667	12.4755627916667	13.6884802666667	13.4498034583333	13.002567275	12.552088775	12.539129975	12.9683429666667	11.8896284166667	13.14330865	13.3630726416667	12.77853635	12.9586834166667	12.9699766666667	12.4995800083333	12.091314325	12.3776096916667	12.8062943	12.5974434583333	12.47909375	13.2168871166667	12.728935875	12.5562191	12.2350239916667	12.8702352416667	11.9585723416667	13.6000874166667	12.615602	12.3496705916667	12.9213977333333	12.8509307083333	12.6694372	12.5576279333333	12.7201162666667	13.1026328916667	12.5334882833333	12.734915275	12.402892875	12.0977041916667	12.4225030833333

AKAP9	3.95464144166667	3.645575825	3.777965425	3.695851625	3.84584678333333	4.24826858333333	3.49827795	3.86167775833333	3.43601865	3.52588423333333	3.48892545	3.49827795	3.52588423333333	3.6774476	3.670269525	3.64669851666667	3.86284045833333	3.77553975	3.893426425	3.85519674166667	3.52588423333333	3.66804375833333	3.79308619166667	3.80218998333333	3.94456669166667	3.930358975	3.7815889	4.4580822	3.795517075	3.74146091666667	3.87078774166667	3.94341095833333	3.478643875	3.6378857	3.70714133333333	3.77178324166667	3.87726464166667	3.80321476666667	4.27700685	3.87726464166667	4.09994219166667	3.94139638333333	3.98827198333333	4.37610179166667	3.67271084166667	3.99488165833333	4.09598105	4.00881020833333	3.8291486	4.03339724166667	3.930358975	3.79182875833333	3.4801699	4.10621731666667	3.71049449166667	3.9614432	3.69811276666667	3.893426425	3.75125179166667	3.90457709166667	4.210787125	3.3931188	4.43639113333333	3.557434975	3.49992614166667	4.251891475	3.607366575	3.5935751	3.6378857	3.73894771666667	3.553038975	3.63673386666667	3.46854113333333	3.47399078333333	3.74027611666667	3.77553975	3.79182875833333	4.91654405	3.80008696666667	3.65809486666667	3.790613425	4.25100265833333	3.694768975	3.49066511666667	3.5947849	3.333600375	3.87506608333333	3.88526006666667	3.84151389166667	3.76944190833333	3.68464693333333	5.37226380833333	4.17889495	3.780421775	3.97925105	3.780421775	4.1481371	3.65195390833333	3.50128275833333	4.11281023333333	3.64190115	3.54337740833333	3.99817013333333	4.22036293333333	3.723060125	3.52588423333333	3.86389350833333	4.30527630833333	3.9614432	4.021539275	3.95899671666667	4.01810879166667	3.74768024166667	4.06389798333333	3.64321401666667	3.79308619166667	6.9125939	3.9147734	4.509505	3.90354184166667

NAPA	13.21039535	14.2145397416667	14.044199175	13.9239245083333	14.1268362333333	13.4649543916667	14.1957149333333	14.3397031416667	14.1914740916667	13.9250365	13.8148381	13.0186633916667	14.045461125	14.0174773166667	14.274700225	13.8428912916667	13.4768642833333	14.082470775	14.1045935166667	14.0656333583333	13.7168412916667	14.0607265833333	13.9075706166667	13.71864895	14.082470775	13.4314411416667	14.30615445	14.3204037083333	14.4633924916667	14.2319492833333	14.0926224666667	14.31061245	13.9480264583333	13.4171665166667	13.2602116	13.3459381666667	14.3892469416667	14.354697075	13.853956425	14.241033875	13.99785205	14.0618019166667	13.7443409166667	13.9065296166667	13.593805675	13.9250365	14.0874428333333	14.0938637416667	13.4807854416667	13.6284294583333	13.7321168833333	13.784925425	14.0113488	13.9884216166667	14.259430575	13.9261802833333	13.9284348416667	14.2378149833333	14.0062649583333	13.7426466833333	13.48560075	13.3786606333333	13.6401687916667	13.0714709916667	12.7463306666667	12.9477864583333	12.8339629333333	13.3032240666667	12.762350675	13.2405472416667	13.1986854833333	13.0960317	12.8930719333333	13.336335625	13.2983134833333	13.0901032416667	13.394322425	13.306054325	13.1616055833333	13.56515455	13.0325393583333	13.2751773833333	13.2215442333333	12.975052625	13.5694284666667	13.073805875	12.98478995	13.5171924333333	13.5150003833333	12.996638525	13.419543825	13.2168871166667	13.405024	13.3444534166667	13.1919341833333	13.482478725	13.2467446916667	13.306054325	13.6931782333333	13.627458175	13.1452170666667	12.7295358083333	12.9570294	12.8636072	13.1280018	13.3522548916667	13.057870725	13.134360625	13.398021325	13.2096652916667	13.1165645583333	13.7640679416667	13.67760105	12.452378375	13.7332085916667	13.6192792916667	12.7061787	12.6966269416667	13.0228260333333	13.5484254

TYROBP	12.5063802708333	12.5546026583333	13.1593016333333	12.5091274833333	11.5658625708333	13.002249725	12.3804621875	12.7647025958333	11.338684375	12.8462830375	12.4441625791667	13.9973217958333	12.9199438	12.4910338291667	11.0314057708333	13.8703988666667	12.7408604208333	11.4735889416667	11.613174325	12.0411497	13.1283226125	11.2999132375	13.0740993333333	12.7326443041667	12.0358296416667	12.6232900583333	12.6057562791667	11.867942	11.4906826416667	11.5837864833333	12.1547278791667	12.8221621708333	12.8760625916667	14.1506675625	13.5564185666667	12.4076008791667	11.5856427666667	12.8587184333333	13.57854245	13.0371830708333	13.2043848958333	12.5154422416667	12.9096864833333	12.6388910083333	13.7069897791667	13.6610170958333	12.5961331166667	13.2309913833333	13.6415259208333	12.6911874583333	13.2390115166667	12.3898186875	12.5499776333333	12.5490350458333	12.4707841208333	12.9514155541667	13.542355225	13.4046350791667	13.0146044041667	13.3789900708333	12.5942224291667	13.4233907958333	13.483689375	12.5470533458333	13.4123457916667	11.8022640041667	13.0485887291667	13.4424347958333	12.3981609208333	14.0207194083333	13.0807272166667	12.8298295541667	12.2916232083333	14.0689028125	13.1572921166667	13.8737710458333	13.5299908125	13.8342461416667	13.3387083375	13.9019783458333	12.8126665416667	13.0807272166667	13.1239177583333	13.75872835	14.0294725041667	13.2237284833333	12.6942150375	14.2418422166667	12.1984195	14.2976132166667	13.8433681125	13.3932277541667	12.9343725958333	13.2064901458333	13.1100606166667	13.4978921333333	13.6653701166667	13.3332587208333	13.1902834291667	13.51377155	13.61974265	13.9029792708333	14.4556511416667	13.4670427666667	13.1882382291667	14.4051928666667	13.7706892416667	13.5087700958333	13.7516152333333	14.1795629625	13.6112405041667	13.1088261625	13.2979290416667	13.5572154666667	14.7862955791667	14.2541176333333	13.5291244625	13.542355225	13.1956143583333	14.7176651708333

HCST	12.5063802708333	12.5546026583333	13.1593016333333	12.5091274833333	11.5658625708333	13.002249725	12.3804621875	12.7647025958333	11.338684375	12.8462830375	12.4441625791667	13.9973217958333	12.9199438	12.4910338291667	11.0314057708333	13.8703988666667	12.7408604208333	11.4735889416667	11.613174325	12.0411497	13.1283226125	11.2999132375	13.0740993333333	12.7326443041667	12.0358296416667	12.6232900583333	12.6057562791667	11.867942	11.4906826416667	11.5837864833333	12.1547278791667	12.8221621708333	12.8760625916667	14.1506675625	13.5564185666667	12.4076008791667	11.5856427666667	12.8587184333333	13.57854245	13.0371830708333	13.2043848958333	12.5154422416667	12.9096864833333	12.6388910083333	13.7069897791667	13.6610170958333	12.5961331166667	13.2309913833333	13.6415259208333	12.6911874583333	13.2390115166667	12.3898186875	12.5499776333333	12.5490350458333	12.4707841208333	12.9514155541667	13.542355225	13.4046350791667	13.0146044041667	13.3789900708333	12.5942224291667	13.4233907958333	13.483689375	12.5470533458333	13.4123457916667	11.8022640041667	13.0485887291667	13.4424347958333	12.3981609208333	14.0207194083333	13.0807272166667	12.8298295541667	12.2916232083333	14.0689028125	13.1572921166667	13.8737710458333	13.5299908125	13.8342461416667	13.3387083375	13.9019783458333	12.8126665416667	13.0807272166667	13.1239177583333	13.75872835	14.0294725041667	13.2237284833333	12.6942150375	14.2418422166667	12.1984195	14.2976132166667	13.8433681125	13.3932277541667	12.9343725958333	13.2064901458333	13.1100606166667	13.4978921333333	13.6653701166667	13.3332587208333	13.1902834291667	13.51377155	13.61974265	13.9029792708333	14.4556511416667	13.4670427666667	13.1882382291667	14.4051928666667	13.7706892416667	13.5087700958333	13.7516152333333	14.1795629625	13.6112405041667	13.1088261625	13.2979290416667	13.5572154666667	14.7862955791667	14.2541176333333	13.5291244625	13.542355225	13.1956143583333	14.7176651708333

RC3H2	11.824518825	11.6906956833333	11.57134605	11.5934022416667	11.7905923666667	11.7501757083333	11.43469695	11.8705505	11.6159444083333	11.2143531083333	11.3306591916667	11.5645444166667	11.5268940083333	11.4545376666667	11.596495275	11.7160036	11.4897906583333	11.5103338083333	11.5761677833333	11.57803215	11.8395390583333	11.9135839833333	11.7247391166667	11.8191065	11.7131247833333	12.207741075	11.631804775	11.651839775	11.5019499416667	11.7890978583333	11.6701974666667	11.6944435416667	11.696699	11.5750241	11.5664864	12.0546824083333	11.8481133916667	11.735525225	11.86232365	11.7016218	11.3986748166667	11.57874505	11.3965275083333	11.5858193083333	11.556290575	11.56420645	11.4722453166667	11.5951612	11.5422054916667	11.388258375	11.4203880666667	11.9055925	11.292593525	11.6016871083333	11.765185225	11.39940385	11.6780189666667	11.5223462	11.5128736833333	11.4244147	11.5436260583333	12.3021061583333	11.7582463416667	11.9207928583333	11.7887391333333	11.5626319666667	11.705716875	12.2771993416667	12.4908032583333	12.6013272333333	11.6628809833333	11.97391105	12.3305815833333	11.9502134083333	12.29141735	12.202055425	11.530946975	11.7105442333333	12.0209849	11.6594960166667	12.3892279333333	11.8163038583333	12.219394525	12.1575174833333	11.9931218416667	11.9816922	12.7976328583333	12.278442975	12.295556975	11.9825076333333	12.3820596583333	12.374133125	12.3101776166667	11.8783734666667	11.2007994333333	11.7605260333333	11.7834532083333	11.9074544416667	12.1435544583333	12.2116409166667	12.1745545	11.9907404666667	12.3794027166667	11.8166747583333	11.9082126416667	11.825765325	11.545061825	12.2528148916667	11.9274761416667	12.03602325	11.8557655416667	12.2528148916667	12.424331275	11.8407167416667	11.47943685	11.9585723416667	11.8873133333333	12.5628225833333	12.1843992666667	12.0193369

SPTB	4.60591755833333	5.4147768	5.60319860833333	4.95627725833333	5.7161104	4.093740425	6.32600435833333	5.79119435	5.83501515	5.36922546666667	4.74337093333333	6.07263301666667	6.1936491	4.92770649166667	4.98009509166667	4.830971775	4.77272485	5.892821925	4.65478205833333	5.41802705	5.73091026666667	4.612218	4.80827406666667	4.47668578333333	4.67674131666667	4.04402448333333	4.47668578333333	4.49406044166667	4.71793841666667	4.771681175	4.70475344166667	4.56071255833333	4.64133359166667	4.15845484166667	5.31614614166667	4.27700685	4.57827949166667	4.53433785	4.55069105	4.74448875	5.01735620833333	4.39670516666667	4.82580888333333	5.58988304166667	4.33532039166667	5.29450505	5.15246255833333	5.03823209166667	4.56186439166667	4.89827923333333	4.802114875	4.537079425	5.55559675	5.39700335833333	4.40284696666667	4.79518681666667	4.86289075	4.80635555	4.290980325	4.24718985833333	3.94901469166667	5.30151331666667	4.35819395	6.08593745	5.01216298333333	5.279548575	6.38240346666667	6.322468275	4.51953901666667	5.4789746	5.01427080833333	5.45375554166667	7.06400038333333	5.15454668333333	6.32381556666667	6.09588644166667	5.03120425833333	3.81819714166667	4.63513973333333	5.10930444166667	5.98559190833333	4.53925114166667	5.153439775	4.27160165833333	4.20999628333333	4.55069105	4.29017421666667	6.372760025	5.32346678333333	4.00561259166667	5.23361180833333	4.43313565833333	3.95130670833333	4.38635804166667	5.04024386666667	4.44822564166667	4.38412459166667	4.111632325	4.40903533333333	5.087869425	4.359146625	5.70431125833333	4.79906281666667	5.0343026	4.76141500833333	4.92149175833333	4.61010935833333	6.38846551666667	4.2949863	6.65915351666667	4.602780325	5.41887256666667	4.40382505833333	4.92360515	4.63723314166667	4.40284696666667	4.07245948333333	4.5803341	4.52055483333333	4.9406758

USP26	5.45265249166667	4.08114406666667	4.35998614166667	5.18213341666667	3.67401930833333	5.61547525833333	4.371797775	4.18081978333333	4.25005865	4.49312724166667	5.34510054166667	4.563953975	4.34514024166667	4.14488695	4.09074995833333	3.95777633333333	5.17697859166667	3.75570144166667	4.5496205	3.95130670833333	3.84370838333333	4.15432630833333	4.361998275	5.002397475	5.17905539166667	4.649483075	4.479514325	4.61830805833333	4.3549546	4.29017421666667	4.61520515	4.557709	5.997550525	4.83989633333333	4.9195424	5.399029725	4.344051675	5.2460388	5.53695673333333	4.740346475	6.01047939166667	5.00439053333333	4.85447411666667	5.44644633333333	4.78047113333333	4.83558559166667	4.88257656666667	4.98219815	4.42372025	5.866285325	6.386982725	4.666328775	4.08637315833333	5.29020444166667	4.33043379166667	4.75698904166667	4.13218385833333	5.327612425	4.86384488333333	5.76144219166667	4.207987625	4.03660776666667	4.34901141666667	4.24130721666667	3.99817013333333	3.98827198333333	4.617309275	4.51268873333333	4.370852175	4.40382505833333	4.1018927	4.40284696666667	5.477009275	4.65793555	3.50570625833333	4.125728225	3.76337073333333	3.931450225	4.18603395833333	3.77672200833333	3.66190675	4.03859033333333	4.59576983333333	4.67574021666667	4.27468664166667	4.001416775	4.51370921666667	5.11328418333333	3.586969725	4.97812506666667	5.59377575	4.55069105	3.6203186	5.09317755833333	5.02371664166667	4.7847361	4.2939407	4.822492325	5.557624025	5.2891249	5.11436066666667	4.28696936666667	4.48330838333333	5.05687025	4.1756419	3.89679799166667	4.77272485	6.17470206666667	4.67278611666667	6.56365850833333	4.82580888333333	5.33746485833333	4.99247835	4.70784294166667	6.10954496666667	4.339553	4.322875175	4.75143253333333	4.108372	5.11328418333333

EYS	4.62270154166667	4.13218385833333	3.98271365	4.23000438333333	3.66920745833333	4.082140775	3.44862535833333	3.80218998333333	3.556104825	3.77909573333333	3.550199675	3.32924500833333	3.64669851666667	4.09698049166667	4.03764378333333	3.84584678333333	4.494971825	3.782834	4.57240950833333	3.912461775	4.2231712	4.085428875	4.3276422	4.40284696666667	4.70371486666667	4.02799813333333	4.4078674	4.57924936666667	4.69425546666667	4.339553	4.62990135	4.48925233333333	3.75016011666667	4.04952899166667	4.108372	4.1095023	4.59784559166667	4.82688145833333	4.39039918333333	4.55466009166667	4.98892909166667	4.44059075	4.66334196666667	5.221531825	4.16299614166667	4.81485295	4.74123235833333	4.80096826666667	4.59265796666667	4.77813741666667	5.122114675	4.480628975	3.85519674166667	5.3343362	4.310185775	4.64133359166667	3.82070373333333	4.65900069166667	4.15118036666667	4.47089620833333	3.88294669166667	3.48170379166667	4.4078674	3.812622975	3.557434975	3.74027611666667	3.93897055	3.41442594166667	3.65432333333333	3.49384833333333	3.35566285	3.63145715833333	3.36913155	3.5947849	3.39122110833333	3.61363956666667	3.352012675	3.78410915833333	3.80781481666667	3.60366870833333	3.61363956666667	3.56445749166667	4.23901671666667	3.93585013333333	3.8291486	3.51873675	3.780421775	4.54133179166667	3.67271084166667	4.00999268333333	3.75232660833333	4.434278525	4.02568050833333	3.80900449166667	3.8957117	3.91700049166667	4.087323525	4.031307325	3.73894771666667	4.90256950833333	4.34698491666667	3.46108689166667	4.666328775	4.75698904166667	4.37287369166667	3.8957117	4.41392473333333	5.90154725	4.33428121666667	6.31469075	4.63093599166667	5.31824546666667	4.53105006666667	4.84301391666667	4.10495005	4.33637641666667	4.128952175	4.41392473333333	4.302366825	3.54887480833333

AC005324.3	7.3595435	9.28526889166667	9.0136041125	9.20755692083333	9.76843350833333	7.77403659583333	9.318816625	8.9639175	9.35190450416667	9.12675697083333	8.6791378	7.18674357083333	9.16374468333333	9.0652739875	10.3319130666667	8.1812587875	8.3829721375	9.7419680625	9.6640089875	9.16851644166667	9.20755692083333	10.4167078833333	8.64519729583333	9.5518241375	9.75344331666667	6.62938375833333	9.57883875	9.477501075	10.6235293	9.16098932083333	9.40619117916667	9.726360525	9.11760059166667	7.83372354166667	7.02051184583333	7.1782093875	10.17070275	8.83089360833333	8.51107445416667	8.44516555833333	9.62087651666667	9.6165687375	9.06042506666667	9.1463162625	8.49953687083333	8.8139688125	9.36018218333333	10.1368662333333	8.97241437916667	8.1036137375	9.3386104375	9.66260626666667	9.62296950416667	9.93936995833333	10.0367091916667	9.95832646666667	8.99371967083333	9.8780425625	9.40793799166667	8.94502036666667	8.89241624166667	10.0470248916667	9.836069525	10.1847114583333	6.29016404583333	9.1262838375	7.30640325833333	9.48102557916667	7.500213925	7.360785575	8.8444968375	9.43209279583333	9.2244692375	9.70646494166667	8.2429291	9.13395720833333	8.30721625416667	9.91396165416667	10.4378910625	8.00334674583333	8.37738923333333	6.42906689583333	9.41366838333333	7.89856145833333	9.79788007916667	7.77459110833333	7.5225274375	7.8749512125	10.6478785375	8.92771289583333	9.30477144583333	9.83564609166667	9.69902295833333	8.2951185125	7.1363748	11.535463975	7.098369425	7.18376889166667	8.55931514166667	8.09669910833333	8.9710142375	8.51361290833333	8.9596737	8.62216094583333	9.18809195416667	8.09774464166667	8.90502084583333	9.87140415416667	7.9471820625	9.30346922916667	7.94569530416667	9.25970336666667	9.64005285	8.41765477916667	9.42569128333333	10.1847114583333	7.4941654375	8.09469342083333	9.64943681666667	7.2293157375

CDRT1	7.3595435	9.28526889166667	9.0136041125	9.20755692083333	9.76843350833333	7.77403659583333	9.318816625	8.9639175	9.35190450416667	9.12675697083333	8.6791378	7.18674357083333	9.16374468333333	9.0652739875	10.3319130666667	8.1812587875	8.3829721375	9.7419680625	9.6640089875	9.16851644166667	9.20755692083333	10.4167078833333	8.64519729583333	9.5518241375	9.75344331666667	6.62938375833333	9.57883875	9.477501075	10.6235293	9.16098932083333	9.40619117916667	9.726360525	9.11760059166667	7.83372354166667	7.02051184583333	7.1782093875	10.17070275	8.83089360833333	8.51107445416667	8.44516555833333	9.62087651666667	9.6165687375	9.06042506666667	9.1463162625	8.49953687083333	8.8139688125	9.36018218333333	10.1368662333333	8.97241437916667	8.1036137375	9.3386104375	9.66260626666667	9.62296950416667	9.93936995833333	10.0367091916667	9.95832646666667	8.99371967083333	9.8780425625	9.40793799166667	8.94502036666667	8.89241624166667	10.0470248916667	9.836069525	10.1847114583333	6.29016404583333	9.1262838375	7.30640325833333	9.48102557916667	7.500213925	7.360785575	8.8444968375	9.43209279583333	9.2244692375	9.70646494166667	8.2429291	9.13395720833333	8.30721625416667	9.91396165416667	10.4378910625	8.00334674583333	8.37738923333333	6.42906689583333	9.41366838333333	7.89856145833333	9.79788007916667	7.77459110833333	7.5225274375	7.8749512125	10.6478785375	8.92771289583333	9.30477144583333	9.83564609166667	9.69902295833333	8.2951185125	7.1363748	11.535463975	7.098369425	7.18376889166667	8.55931514166667	8.09669910833333	8.9710142375	8.51361290833333	8.9596737	8.62216094583333	9.18809195416667	8.09774464166667	8.90502084583333	9.87140415416667	7.9471820625	9.30346922916667	7.94569530416667	9.25970336666667	9.64005285	8.41765477916667	9.42569128333333	10.1847114583333	7.4941654375	8.09469342083333	9.64943681666667	7.2293157375

UTP14A	11.8516230166667	11.4140863666667	11.5177929833333	11.11993505	12.1295438416667	12.1673323833333	11.2349074416667	11.6659074916667	11.6871432333333	11.88575805	11.7243383416667	11.5411164416667	11.5204866583333	11.5268940083333	11.5429763166667	11.4679092333333	11.856139525	11.8896284166667	11.8297435166667	11.6705910083333	11.5385948833333	11.7288583666667	11.4854519916667	12.30978125	11.8348518583333	11.6444959666667	11.48024075	11.5783568666667	11.8709570583333	11.8008623833333	11.5961336833333	11.6982071583333	11.4820913166667	11.739559375	11.7262372833333	11.913931575	11.9612880416667	11.846919075	11.5000912333333	11.720568625	11.471171625	11.615215075	11.655146	11.7667849666667	11.9455284	11.6687463083333	11.7113167916667	11.8912374333333	11.7720983166667	11.8171008	11.731200575	12.072543675	11.97355025	11.8584258416667	11.8950942583333	11.6951784416667	11.9112855083333	11.8461878583333	11.6274751916667	11.9052300666667	12.5098151833333	11.86892365	11.86232365	11.4434926166667	12.0189741833333	12.4110018333333	12.05687395	12.43595655	12.5780122833333	11.9975093	11.8145765416667	12.0778101166667	12.2801097833333	11.9447346	11.9985911666667	12.1724138083333	12.1258089083333	12.580271675	12.728935875	12.8091325	12.0905289333333	11.8367863833333	12.5010802333333	11.8599637666667	11.9593365833333	11.873004525	12.2276231333333	12.1852414666667	12.4487345666667	11.973231675	12.4461573416667	11.9927367	11.9860534666667	12.0808514833333	12.519265425	12.2871151416667	12.064434275	11.9686139666667	12.836627	11.9289106916667	12.30344225	11.5483812666667	12.2198129666667	12.6537086583333	12.244497975	12.2033105916667	11.8145765416667	12.9001276833333	12.5116969583333	12.310594275	11.6499693666667	12.219394525	12.1628118333333	12.7580394083333	11.9989974	11.963726125	11.9521162083333	12.5793612333333	12.5395615083333	12.372860975

DNAH7	7.30612164166667	7.35226640833333	7.36048969166667	7.26165935833333	7.73803474166667	7.002378425	7.54341655833333	8.57995885	7.41824386666667	6.88458655833333	7.54566714166667	8.16877921666667	8.523789	7.68436408333333	7.4911099	6.74410944166667	7.05278270833333	6.79798854166667	6.31702735	7.88077005	7.26626465	7.61643631666667	6.54365829166667	6.75303653333333	6.80310133333333	7.4250003	6.27095864166667	6.20969628333333	7.788761475	6.3794359	6.91190293333333	6.3378065	6.75170075833333	7.97141813333333	6.52133365833333	6.58226158333333	6.69314785	6.23865941666667	6.68920345833333	6.5680586	5.17804041666667	5.96342883333333	5.91816949166667	6.43572405833333	6.37713004166667	5.57926250833333	5.74130296666667	6.56292778333333	5.71315170833333	7.39975843333333	5.49035004166667	6.56951250833333	7.9760917	7.23429453333333	6.35578110833333	6.864696	8.29053858333333	5.32031858333333	6.36793951666667	5.60574740833333	6.68472555	7.582967825	6.96970946666667	8.36127639166667	8.08909971666667	6.76714710833333	7.54510475	8.32561349166667	6.57014316666667	7.50344081666667	8.05087028333333	7.33974354166667	8.47144243333333	7.74783731666667	7.57050700833333	7.75823035	7.23785493333333	6.00716590833333	6.528434875	5.60319860833333	9.401147475	7.57673284166667	6.52564285833333	7.10434548333333	7.74511231666667	7.80828950833333	7.47140944166667	7.90206838333333	8.07622910833333	7.17914503333333	6.96906025833333	5.31319136666667	6.864696	7.15697099166667	5.72282524166667	6.16609928333333	6.68342536666667	6.71442883333333	7.56775538333333	7.9882338	7.422156975	8.00359983333333	6.20326725833333	6.97340769166667	6.22446936666667	7.51995818333333	6.78446141666667	5.91627995	6.68067039166667	6.620339275	6.89003731666667	5.40852341666667	6.131776975	6.54574596666667	7.27773341666667	6.68131301666667	8.06345484166667	5.69872159166667	6.00968073333333	8.00199818333333

XRRA1	8.83063586666667	9.337966225	9.01246160833333	9.23186806666667	9.765982475	8.59640985	9.19244325833333	8.72692960833333	9.85113778333333	9.09505498333333	8.74370115833333	9.36987781666667	10.011606775	9.699723	9.85036418333333	8.518147825	7.78166071666667	10.08353915	9.41531880833333	9.51951014166667	9.13694515	10.4963081083333	8.647383475	8.90099503333333	9.58976235	8.68375230833333	10.1400969416667	9.77000968333333	10.0949964416667	8.9231771	9.0540245	9.830308425	9.24668224166667	7.60193526666667	8.57649310833333	8.35277756666667	10.8865989333333	9.07816326666667	8.36807433333333	8.755680875	8.1455177	9.32939963333333	8.793933	8.05466113333333	8.709872025	8.054062325	9.21350573333333	8.69082025	8.69606343333333	8.16684950833333	8.20031584166667	9.24539353333333	9.89894956666667	9.59824975833333	9.39438685	8.91224634166667	8.67841379166667	7.97458126666667	9.25303868333333	8.99116335833333	9.52133895	8.13415403333333	8.19970503333333	9.46477646666667	9.09007516666667	9.093743775	8.60701585833333	8.58390675	10.4402042083333	8.87930013333333	8.26039324166667	8.70635446666667	8.83592169166667	8.833014675	8.38565373333333	8.36187725833333	8.498393225	10.1148100333333	8.13766550833333	8.43902399166667	8.81515405833333	9.683521475	8.14495316666667	8.11151595833333	8.634032725	8.20930705	9.27125073333333	8.83493181666667	10.0960991583333	8.174528375	7.95464479166667	9.30864065833333	8.608522725	7.81786669166667	9.275964375	8.35169179166667	9.49325940833333	8.34021045	7.61698044166667	7.296620525	8.18891301666667	8.85160831666667	8.50451981666667	7.79298445	8.23520641666667	9.27941565	8.44239195833333	8.08289571666667	8.09590623333333	7.39564355833333	7.26165935833333	7.82691680833333	8.16484069166667	7.967895825	8.02512498333333	8.4067182	6.95288179166667	7.59600343333333	8.222543225	8.364642925

KCNMB1	15.8030681708333	12.6294828791667	12.4634935625	12.0791418083333	12.6898184916667	13.3656924875	12.828145375	12.6139254875	13.03777705	12.7679182916667	12.5114476	15.2244796125	14.6211147708333	12.9303715625	12.7920713708333	12.9572856708333	12.2778279666667	13.1929727375	13.6041330875	13.3311598416667	12.9862498583333	11.7500013791667	12.6687319583333	12.7621023	12.8287188166667	15.7958457083333	13.55799335	12.6643328208333	12.5857915625	12.8693564958333	13.2218451541667	12.7882158958333	13.0801195458333	12.077992225	15.7102441083333	16.0849821291667	12.9632195291667	13.024826375	13.196301825	13.6662205708333	13.268089225	12.9519194583333	12.2932457416667	13.0581849458333	14.488157675	12.557849075	13.352668925	13.2715239541667	11.8451951541667	12.6144304583333	12.8368804875	12.68596965	12.8530072583333	12.647362725	12.9758884125	13.6762941375	12.9933025291667	12.8982099916667	12.7588894666667	13.2218451541667	11.5006540125	12.0162554833333	10.4078719875	12.172215	11.2270935666667	12.2757899375	11.6457827041667	11.6512910125	10.2264568791667	11.3622100208333	11.7079948375	10.7787737	11.266611625	12.6399904416667	10.8610501208333	11.0414884958333	13.5184584666667	11.8283857291667	11.4698955333333	11.5172423041667	12.3040913375	12.4621215583333	12.1581685666667	11.2566504708333	11.6520352291667	11.4458392041667	12.4489610375	12.6124692958333	10.8332018125	13.0442801083333	10.7567177	10.6094494333333	11.2451646458333	12.0179299458333	11.5394740833333	11.0475272833333	10.768260425	12.6414595166667	11.5748264916667	11.0907714458333	11.3052728291667	11.9925472666667	10.4659000833333	10.823421975	10.9075519541667	11.4091800541667	12.2075626958333	12.172215	10.9871200791667	12.5427723	11.8271711958333	11.251242475	10.8279392916667	10.42387475	11.7095800291667	11.0828266875	10.9217726166667	10.4338554416667	10.5665043666667	12.1543163583333

KCNIP1	15.8030681708333	12.6294828791667	12.4634935625	12.0791418083333	12.6898184916667	13.3656924875	12.828145375	12.6139254875	13.03777705	12.7679182916667	12.5114476	15.2244796125	14.6211147708333	12.9303715625	12.7920713708333	12.9572856708333	12.2778279666667	13.1929727375	13.6041330875	13.3311598416667	12.9862498583333	11.7500013791667	12.6687319583333	12.7621023	12.8287188166667	15.7958457083333	13.55799335	12.6643328208333	12.5857915625	12.8693564958333	13.2218451541667	12.7882158958333	13.0801195458333	12.077992225	15.7102441083333	16.0849821291667	12.9632195291667	13.024826375	13.196301825	13.6662205708333	13.268089225	12.9519194583333	12.2932457416667	13.0581849458333	14.488157675	12.557849075	13.352668925	13.2715239541667	11.8451951541667	12.6144304583333	12.8368804875	12.68596965	12.8530072583333	12.647362725	12.9758884125	13.6762941375	12.9933025291667	12.8982099916667	12.7588894666667	13.2218451541667	11.5006540125	12.0162554833333	10.4078719875	12.172215	11.2270935666667	12.2757899375	11.6457827041667	11.6512910125	10.2264568791667	11.3622100208333	11.7079948375	10.7787737	11.266611625	12.6399904416667	10.8610501208333	11.0414884958333	13.5184584666667	11.8283857291667	11.4698955333333	11.5172423041667	12.3040913375	12.4621215583333	12.1581685666667	11.2566504708333	11.6520352291667	11.4458392041667	12.4489610375	12.6124692958333	10.8332018125	13.0442801083333	10.7567177	10.6094494333333	11.2451646458333	12.0179299458333	11.5394740833333	11.0475272833333	10.768260425	12.6414595166667	11.5748264916667	11.0907714458333	11.3052728291667	11.9925472666667	10.4659000833333	10.823421975	10.9075519541667	11.4091800541667	12.2075626958333	12.172215	10.9871200791667	12.5427723	11.8271711958333	11.251242475	10.8279392916667	10.42387475	11.7095800291667	11.0828266875	10.9217726166667	10.4338554416667	10.5665043666667	12.1543163583333

PTS	7.33061036666667	5.80987265416667	6.17741175	6.25796189166667	6.7279495625	6.79499067083333	6.22265424583333	6.02340154166667	6.05945884583333	6.2350072625	6.18698760416667	7.1151289	7.287713	5.94231759166667	7.1351471875	6.6856428375	6.40995777916667	7.14243408333333	6.36209213333333	6.59016512083333	5.58942329166667	7.27259884166667	5.10975973333333	6.18529087916667	6.97245632083333	6.39052361666667	6.7412051375	7.0111070875	7.7751871875	6.76685945	6.6112062375	7.252692975	6.34416159166667	5.852767	6.8002561375	5.26945706666667	7.1405854	6.06795135	6.06623247916667	6.99126284583333	6.31359270416667	6.0134365875	5.52962550416667	6.84541794166667	5.96118605	6.1091408875	5.9370347875	6.82918142916667	6.13467912916667	6.54544535	6.0370695625	5.84923762916667	6.71538480416667	7.74216273333333	6.252931525	6.4403204375	6.37155930833333	6.79499067083333	7.5528454625	6.8518622625	7.2514971375	4.86644255416667	6.15735410416667	6.01539610416667	5.18578712916667	7.13879529583333	5.56921560416667	6.6889002875	6.11827934583333	6.72216503333333	6.05945884583333	7.38204455833333	7.08643857916667	6.54332606666667	6.56398677916667	6.16872034583333	6.19928637916667	6.27050387916667	6.24820711666667	6.040429525	5.6036296875	6.2350072625	5.299254225	6.2399230625	6.21316953333333	5.2114037125	5.73635757083333	6.69148706666667	7.37443228333333	6.50615590416667	5.41844980833333	6.11088556666667	6.32491212916667	5.752604275	5.49980207083333	5.04284279583333	6.58256900416667	5.04508093333333	4.77872065416667	6.58323432916667	5.95369501666667	5.8735993375	4.4664723375	6.1051515875	5.83159534166667	6.60038117916667	6.0526629375	6.23425827916667	6.1522880375	6.57994022083333	6.1116802875	5.4203404125	5.9817968375	5.6027483375	6.32418060833333	5.3751150875	6.52240923333333	6.18779995416667	6.8041364125	4.9623927875

PLET1	7.33061036666667	5.80987265416667	6.17741175	6.25796189166667	6.7279495625	6.79499067083333	6.22265424583333	6.02340154166667	6.05945884583333	6.2350072625	6.18698760416667	7.1151289	7.287713	5.94231759166667	7.1351471875	6.6856428375	6.40995777916667	7.14243408333333	6.36209213333333	6.59016512083333	5.58942329166667	7.27259884166667	5.10975973333333	6.18529087916667	6.97245632083333	6.39052361666667	6.7412051375	7.0111070875	7.7751871875	6.76685945	6.6112062375	7.252692975	6.34416159166667	5.852767	6.8002561375	5.26945706666667	7.1405854	6.06795135	6.06623247916667	6.99126284583333	6.31359270416667	6.0134365875	5.52962550416667	6.84541794166667	5.96118605	6.1091408875	5.9370347875	6.82918142916667	6.13467912916667	6.54544535	6.0370695625	5.84923762916667	6.71538480416667	7.74216273333333	6.252931525	6.4403204375	6.37155930833333	6.79499067083333	7.5528454625	6.8518622625	7.2514971375	4.86644255416667	6.15735410416667	6.01539610416667	5.18578712916667	7.13879529583333	5.56921560416667	6.6889002875	6.11827934583333	6.72216503333333	6.05945884583333	7.38204455833333	7.08643857916667	6.54332606666667	6.56398677916667	6.16872034583333	6.19928637916667	6.27050387916667	6.24820711666667	6.040429525	5.6036296875	6.2350072625	5.299254225	6.2399230625	6.21316953333333	5.2114037125	5.73635757083333	6.69148706666667	7.37443228333333	6.50615590416667	5.41844980833333	6.11088556666667	6.32491212916667	5.752604275	5.49980207083333	5.04284279583333	6.58256900416667	5.04508093333333	4.77872065416667	6.58323432916667	5.95369501666667	5.8735993375	4.4664723375	6.1051515875	5.83159534166667	6.60038117916667	6.0526629375	6.23425827916667	6.1522880375	6.57994022083333	6.1116802875	5.4203404125	5.9817968375	5.6027483375	6.32418060833333	5.3751150875	6.52240923333333	6.18779995416667	6.8041364125	4.9623927875

NSF	11.3690796916667	11.48757865	12.1886556416667	12.5362402583333	12.7071557666667	12.3240568416667	11.6205609916667	12.2565941333333	13.021150225	12.5126991416667	12.4650650416667	12.1342141916667	12.5917146833333	12.3160821666667	12.3003464583333	12.521701325	11.7568591416667	12.5415392083333	11.57874505	12.2906208	11.7586025166667	12.9745146166667	12.2412181916667	12.0486377333333	11.8500517083333	11.9907404666667	12.9785766	11.5363521916667	12.52885135	12.1034309916667	12.14843035	12.6060225083333	11.685253825	12.6730092833333	11.297225	11.9694157916667	12.649121475	12.1631913333333	11.5177929833333	12.0989443916667	11.8666609583333	12.03257945	12.2821681166667	11.9005377083333	11.7004395833333	12.296807225	12.6512308	12.1579610916667	12.3110445	11.5983924583333	11.5684552166667	12.141404575	12.5662353166667	12.6317327416667	12.0623925666667	12.3623095583333	12.4101428	11.8386923083333	12.244497975	12.161623975	11.2707625416667	11.3817027833333	12.7521459083333	11.802441875	11.3705707416667	12.18783155	11.8573117083333	12.0112152666667	12.1513649666667	12.388809075	12.6336537916667	11.7564937416667	11.659892375	12.425293625	12.178702275	11.958220325	11.1099310583333	12.7902282666667	11.6363117083333	12.0796485666667	10.8980207583333	12.3356581833333	12.5334882833333	11.4679092333333	11.7160036	11.7324169333333	11.5356555333333	11.0264670833333	12.809704275	11.9513573	11.4798432416667	11.7695141	11.8158651416667	12.0140512666667	11.6219972416667	12.6278527916667	12.0713921666667	11.5574931	11.56420645	11.15581865	11.7440902416667	11.1607791666667	11.7190625916667	11.6761092166667	11.7012064	12.5506564333333	11.7487231	12.0769700833333	12.3327004	11.1771749333333	11.1881087416667	12.2416674416667	11.7864658083333	12.1656867	12.244114325	11.7620768166667	12.13546815	11.43798885	11.6429698666667	11.3657868083333

AC011462.1	8.487957325	8.36762415833333	8.35736911666667	8.42214336666667	8.03004540833333	7.82845124166667	7.48861440833333	8.14495316666667	7.9846002	8.269127475	8.39560916666667	7.48927714166667	7.370004375	8.09066366666667	8.07622910833333	8.07152965	8.47797495833333	7.99131585833333	8.83738886666667	7.91522786666667	8.64290230833333	8.75371965	8.65466659166667	8.07889375	8.11306225833333	8.200812875	8.09798645	8.40077755	8.13617131666667	8.332085025	8.63917994166667	7.8720714	7.68879333333333	8.36034350833333	8.11000589166667	7.91577950833333	8.15812938333333	8.22846261666667	7.94435703333333	8.375514	8.63633005833333	8.64447529166667	8.551994475	8.83395916666667	8.27706721666667	8.80870043333333	8.56322373333333	8.319610775	8.78836945	8.47850595	8.17668253333333	8.06551123333333	7.40193138333333	8.57257758333333	8.269127475	8.77460095833333	7.95035304166667	8.91410946666667	8.3301117	8.23662486666667	7.9422621	8.19143718333333	8.7637709	7.77893200833333	7.70589544166667	7.661614575	8.27654828333333	7.948855	8.22846261666667	8.02671881666667	7.91358665	8.09897714166667	7.68993234166667	8.04865021666667	7.32258515	7.39975843333333	8.43902399166667	7.86334303333333	8.24802405	8.03755788333333	7.47788481666667	7.49549410833333	8.32561349166667	8.701660275	7.650678625	7.26808264166667	7.60040215833333	8.71285006666667	7.88178675	8.27176984166667	8.01861064166667	7.7794798	7.99232836666667	7.85962578333333	7.44203170833333	8.28445111666667	7.56894295833333	7.47140944166667	8.23717023333333	9.357237425	8.35934713333333	8.54656363333333	8.61571535833333	8.96748466666667	8.70635446666667	7.84588375	8.275500325	8.93568545833333	8.28000224166667	9.14971231666667	8.298377725	9.1289299	8.06606233333333	8.432956925	8.54268981666667	8.70499479166667	8.13617131666667	8.50943095833333	8.66397386666667	8.72596560833333

B9D2	8.487957325	8.36762415833333	8.35736911666667	8.42214336666667	8.03004540833333	7.82845124166667	7.48861440833333	8.14495316666667	7.9846002	8.269127475	8.39560916666667	7.48927714166667	7.370004375	8.09066366666667	8.07622910833333	8.07152965	8.47797495833333	7.99131585833333	8.83738886666667	7.91522786666667	8.64290230833333	8.75371965	8.65466659166667	8.07889375	8.11306225833333	8.200812875	8.09798645	8.40077755	8.13617131666667	8.332085025	8.63917994166667	7.8720714	7.68879333333333	8.36034350833333	8.11000589166667	7.91577950833333	8.15812938333333	8.22846261666667	7.94435703333333	8.375514	8.63633005833333	8.64447529166667	8.551994475	8.83395916666667	8.27706721666667	8.80870043333333	8.56322373333333	8.319610775	8.78836945	8.47850595	8.17668253333333	8.06551123333333	7.40193138333333	8.57257758333333	8.269127475	8.77460095833333	7.95035304166667	8.91410946666667	8.3301117	8.23662486666667	7.9422621	8.19143718333333	8.7637709	7.77893200833333	7.70589544166667	7.661614575	8.27654828333333	7.948855	8.22846261666667	8.02671881666667	7.91358665	8.09897714166667	7.68993234166667	8.04865021666667	7.32258515	7.39975843333333	8.43902399166667	7.86334303333333	8.24802405	8.03755788333333	7.47788481666667	7.49549410833333	8.32561349166667	8.701660275	7.650678625	7.26808264166667	7.60040215833333	8.71285006666667	7.88178675	8.27176984166667	8.01861064166667	7.7794798	7.99232836666667	7.85962578333333	7.44203170833333	8.28445111666667	7.56894295833333	7.47140944166667	8.23717023333333	9.357237425	8.35934713333333	8.54656363333333	8.61571535833333	8.96748466666667	8.70635446666667	7.84588375	8.275500325	8.93568545833333	8.28000224166667	9.14971231666667	8.298377725	9.1289299	8.06606233333333	8.432956925	8.54268981666667	8.70499479166667	8.13617131666667	8.50943095833333	8.66397386666667	8.72596560833333

TMEM91	8.487957325	8.36762415833333	8.35736911666667	8.42214336666667	8.03004540833333	7.82845124166667	7.48861440833333	8.14495316666667	7.9846002	8.269127475	8.39560916666667	7.48927714166667	7.370004375	8.09066366666667	8.07622910833333	8.07152965	8.47797495833333	7.99131585833333	8.83738886666667	7.91522786666667	8.64290230833333	8.75371965	8.65466659166667	8.07889375	8.11306225833333	8.200812875	8.09798645	8.40077755	8.13617131666667	8.332085025	8.63917994166667	7.8720714	7.68879333333333	8.36034350833333	8.11000589166667	7.91577950833333	8.15812938333333	8.22846261666667	7.94435703333333	8.375514	8.63633005833333	8.64447529166667	8.551994475	8.83395916666667	8.27706721666667	8.80870043333333	8.56322373333333	8.319610775	8.78836945	8.47850595	8.17668253333333	8.06551123333333	7.40193138333333	8.57257758333333	8.269127475	8.77460095833333	7.95035304166667	8.91410946666667	8.3301117	8.23662486666667	7.9422621	8.19143718333333	8.7637709	7.77893200833333	7.70589544166667	7.661614575	8.27654828333333	7.948855	8.22846261666667	8.02671881666667	7.91358665	8.09897714166667	7.68993234166667	8.04865021666667	7.32258515	7.39975843333333	8.43902399166667	7.86334303333333	8.24802405	8.03755788333333	7.47788481666667	7.49549410833333	8.32561349166667	8.701660275	7.650678625	7.26808264166667	7.60040215833333	8.71285006666667	7.88178675	8.27176984166667	8.01861064166667	7.7794798	7.99232836666667	7.85962578333333	7.44203170833333	8.28445111666667	7.56894295833333	7.47140944166667	8.23717023333333	9.357237425	8.35934713333333	8.54656363333333	8.61571535833333	8.96748466666667	8.70635446666667	7.84588375	8.275500325	8.93568545833333	8.28000224166667	9.14971231666667	8.298377725	9.1289299	8.06606233333333	8.432956925	8.54268981666667	8.70499479166667	8.13617131666667	8.50943095833333	8.66397386666667	8.72596560833333

ATP5MD	14.908236175	15.0629405416667	14.4337656916667	14.762988575	14.6852139708333	14.0752859416667	14.7195864708333	15.1352804083333	14.4218947083333	14.8641526958333	14.572634075	14.223957125	14.44354915	14.8536200708333	14.8274419166667	14.5441549958333	14.9820552625	14.6336007083333	14.75111265	14.8590885625	14.9991992666667	14.8274419166667	14.9293625833333	14.6580212458333	14.6211147708333	14.8563737875	14.5441549958333	14.7176651708333	14.74197315	14.7934297125	14.6788435166667	14.5598696125	14.2912700583333	14.4992860708333	15.094005125	15.1745941875	14.6660938416667	14.7862955791667	14.4696275583333	14.6682188125	14.7176651708333	14.6249579291667	13.9824211625	14.74197315	14.5655159	14.7087718583333	14.8457579916667	15.1033136833333	14.5783528208333	14.410080725	14.4017378625	14.7108483833333	14.7815524333333	14.7815524333333	14.8174412625	14.420172325	14.7486379708333	14.8274419166667	14.6518004916667	14.5709216125	13.9560051208333	14.122239375	14.6788435166667	14.3131535916667	14.09445065	14.1013487833333	13.794497375	13.8065322333333	14.4642959333333	14.0120261916667	13.9606550458333	13.8391048125	14.482110825	13.7516152333333	14.0766207333333	13.7191029458333	14.0985399125	14.09445065	13.8183957375	13.7906263333333	13.6936536458333	13.7924976083333	14.0397911958333	13.9290111958333	14.0435706291667	13.9212427125	14.3868174541667	13.7635727208333	13.5988344166667	13.8998153083333	13.9170528	14.4117367625	14.1299409583333	14.1493378041667	13.296544875	13.8794715833333	14.1783154875	13.784440275	13.9973217958333	13.9846140041667	14.1758106625	14.1834510375	13.6871703125	14.2095802708333	14.0243984458333	13.9700457583333	13.6974379833333	13.8703988666667	14.300613775	14.0650164875	14.1145508666667	13.8014549166667	14.3440163916667	14.2944284583333	13.9973217958333	14.6399901541667	13.8033959875	13.9091580791667	14.0435706291667	14.3654027041667

TAF5	14.908236175	15.0629405416667	14.4337656916667	14.762988575	14.6852139708333	14.0752859416667	14.7195864708333	15.1352804083333	14.4218947083333	14.8641526958333	14.572634075	14.223957125	14.44354915	14.8536200708333	14.8274419166667	14.5441549958333	14.9820552625	14.6336007083333	14.75111265	14.8590885625	14.9991992666667	14.8274419166667	14.9293625833333	14.6580212458333	14.6211147708333	14.8563737875	14.5441549958333	14.7176651708333	14.74197315	14.7934297125	14.6788435166667	14.5598696125	14.2912700583333	14.4992860708333	15.094005125	15.1745941875	14.6660938416667	14.7862955791667	14.4696275583333	14.6682188125	14.7176651708333	14.6249579291667	13.9824211625	14.74197315	14.5655159	14.7087718583333	14.8457579916667	15.1033136833333	14.5783528208333	14.410080725	14.4017378625	14.7108483833333	14.7815524333333	14.7815524333333	14.8174412625	14.420172325	14.7486379708333	14.8274419166667	14.6518004916667	14.5709216125	13.9560051208333	14.122239375	14.6788435166667	14.3131535916667	14.09445065	14.1013487833333	13.794497375	13.8065322333333	14.4642959333333	14.0120261916667	13.9606550458333	13.8391048125	14.482110825	13.7516152333333	14.0766207333333	13.7191029458333	14.0985399125	14.09445065	13.8183957375	13.7906263333333	13.6936536458333	13.7924976083333	14.0397911958333	13.9290111958333	14.0435706291667	13.9212427125	14.3868174541667	13.7635727208333	13.5988344166667	13.8998153083333	13.9170528	14.4117367625	14.1299409583333	14.1493378041667	13.296544875	13.8794715833333	14.1783154875	13.784440275	13.9973217958333	13.9846140041667	14.1758106625	14.1834510375	13.6871703125	14.2095802708333	14.0243984458333	13.9700457583333	13.6974379833333	13.8703988666667	14.300613775	14.0650164875	14.1145508666667	13.8014549166667	14.3440163916667	14.2944284583333	13.9973217958333	14.6399901541667	13.8033959875	13.9091580791667	14.0435706291667	14.3654027041667

CCNL1	13.599265975	12.355857375	12.0309620833333	12.3750783583333	12.611240425	13.0877248833333	12.07292235	12.452378375	11.8666609583333	12.3618830833333	12.0953574416667	12.6669575	13.267133525	11.9554346166667	12.4461573416667	12.3653316083333	12.6971152166667	13.8939302166667	12.5439252666667	12.3275837583333	12.24082335	12.9172670333333	12.9259725333333	13.4058013416667	12.452378375	13.1427455666667	12.1295438416667	13.0457183333333	13.1268647416667	12.4995800083333	13.0217207666667	12.0537518916667	12.3997083083333	12.3128970916667	13.2662932833333	11.432485775	12.8667102583333	12.3331331166667	12.3780252416667	12.3698220916667	12.0149070416667	12.1109533333333	12.0067796166667	12.4545539666667	12.5272702916667	11.9318404166667	11.8055072	12.0857092083333	12.089741475	12.8166613583333	12.7463306666667	12.3606021	12.9941887083333	13.147286425	12.4046195166667	11.770678525	12.3435261333333	12.105099425	12.5788865916667	12.137649925	13.9796866	13.1550516583333	13.489460875	12.966521825	13.811693625	14.0874428333333	12.4091926166667	13.8528809083333	13.0676082583333	12.8187062583333	12.92419445	13.2332248583333	12.6679927416667	13.1707341833333	13.3390509833333	12.7237683583333	13.0913765666667	13.6183502333333	12.7021254833333	13.933121025	13.9818544666667	13.8854083166667	12.9756001333333	12.7076553	13.8168544833333	13.7312525583333	13.7820292333333	12.731765875	13.6804011583333	12.9063845583333	13.216246175	13.5668551916667	12.8702352416667	12.6645702333333	12.92419445	13.8450858083333	12.9527510916667	13.33077755	13.4058013416667	13.2320017583333	12.5939827166667	12.928953425	12.97999865	12.6046256583333	12.8460196	13.1172651	13.2884054666667	12.6923621166667	12.706710675	12.678040475	13.2027218583333	12.4827258583333	13.7670768833333	12.462326175	12.9924504416667	13.2822330583333	12.5719766166667	12.9806214166667	13.5387428916667	13.136994025

EXO1	9.06838428333333	10.4112315166667	10.4871351166667	10.2794538083333	10.94802585	11.2157921833333	10.9150352083333	10.3960554833333	10.9497712833333	10.892685425	11.4619760666667	10.1222883166667	10.465697825	10.4887010333333	10.3455009333333	9.46165735	9.373589925	10.3427675333333	10.279032875	9.97000988333333	10.3825544833333	10.2128243166667	10.313965725	10.136260225	10.15852225	9.3392826	10.1126129583333	10.014766425	10.48105345	10.5162662666667	9.869019275	10.2213761083333	9.93083546666667	9.882085	9.65222760833333	9.51088574166667	10.6293746333333	10.388343775	10.3690418416667	10.15376285	10.0627631	10.2924466666667	10.04039335	10.7612989333333	9.833640075	10.0778458	9.93083546666667	10.3975849666667	10.8684541666667	10.3124336666667	10.054135025	10.5452677583333	10.6519544333333	10.39092635	10.4783084333333	10.0205705916667	10.5509023833333	10.500980975	10.4127726666667	10.252716075	11.2413554333333	11.9120239416667	11.6010090666667	11.6205609916667	11.4993303916667	11.3749692583333	11.9409022083333	11.0409461	12.3347946666667	11.9762173583333	12.1163125	11.2428131833333	11.7273945916667	11.1231619583333	11.8059034833333	10.5699475166667	11.0859242666667	11.3086201833333	12.2687931833333	11.546539525	11.82248265	11.31013205	11.1767887583333	11.2891541	10.534129425	11.9883632916667	12.5930323166667	10.855999875	12.975052625	11.15136825	11.387566725	11.2428131833333	11.5695230416667	10.98475965	11.430726925	11.7324169333333	11.8397313	12.68421675	11.489428925	11.1949025333333	11.7097839833333	11.39829615	12.2024267333333	11.81229275	12.1541314	11.9123960833333	11.1699387666667	11.7182865833333	11.3320946416667	11.4313741833333	10.448395325	11.6452682	11.2247431833333	11.8154196416667	12.06079575	11.6470358	12.403353825	12.60273605	11.8580302916667	11.8134267166667

GMDS	7.34551940833333	6.45472701666667	6.18094188333333	6.84636305	7.87369893333333	7.68593156666667	6.43641949166667	6.24107090833333	6.91766443333333	6.29969763333333	6.27245344166667	6.59815521666667	7.177906325	5.81313270833333	6.65843384166667	5.54579601666667	7.78326185	9.50685594166667	7.51472684166667	6.75368535833333	6.31394556666667	7.76345526666667	6.478653025	7.77550013333333	7.42556758333333	6.33264478333333	6.73002575	6.59883068333333	8.35883419166667	7.03811636666667	7.60592470833333	7.28864350833333	6.66738975833333	7.6066095	6.72123403333333	6.56215660833333	7.80329328333333	7.04378355833333	7.59600343333333	6.50501645833333	7.3694039	7.63763161666667	7.070041725	6.30401625	7.04956788333333	6.58835050833333	7.244602025	7.78166071666667	7.198288025	7.13230843333333	8.20990576666667	7.50627369166667	7.24887754166667	7.57457279166667	7.6157713	6.9998061	7.20508425	8.093315875	7.48759605	7.52107438333333	8.52583200833333	6.63942924166667	8.026220775	6.51994874166667	6.5898034	7.35226640833333	6.407249275	6.507987025	7.124687125	6.465677275	6.06914035833333	6.54933985	7.28317230833333	6.5175539	6.99800655	5.464635875	7.6287103	7.83189280833333	6.94561610833333	6.97585598333333	6.76657179166667	5.90677250833333	6.903905225	7.166799725	6.894639675	7.56380908333333	7.2645801	5.89368925	6.77501233333333	6.43290905833333	7.044419	7.372424975	7.25477635	6.52912136666667	7.092930725	6.5574676	7.661614575	7.27829234166667	7.460763125	7.04956788333333	7.06834181666667	6.51382934166667	7.507361725	6.849792025	7.703109475	6.62367098333333	7.43234464166667	6.45767033333333	7.62358238333333	6.54514473333333	6.92096200833333	7.75823035	7.75930169166667	7.388741125	6.90715841666667	7.00118948333333	7.57569364166667	8.40567906666667	7.41824386666667	6.71243863333333

LYRM1	10.1505465666667	9.496026575	9.674571275	9.73744423333333	10.2466873083333	9.3750605	10.591824075	9.33838469166667	10.0265057583333	9.55943838333333	9.70194608333333	10.2987266333333	9.876036325	9.20464029166667	9.68075670833333	9.06116049166667	9.226998075	10.1497399666667	9.312713675	9.06791326666667	9.18261260833333	9.57132345	8.87636833333333	9.33181315833333	9.50308771666667	9.64247345	9.4181056	9.349869075	9.88045179166667	9.45990046666667	9.53738644166667	9.52087123333333	9.80802880833333	9.603559825	9.88845614166667	9.84631699166667	10.0898576	9.536457975	9.50086338333333	9.48743423333333	8.987001225	9.44105665833333	9.126505725	8.97675146666667	9.1773608	8.64688179166667	8.937187475	9.006017175	9.02175539166667	9.28405401666667	9.42734543333333	9.628525675	9.72111820833333	9.70236100833333	9.69308664166667	9.12606195	9.2998268	9.21638355833333	9.37728073333333	8.95895775	10.1274668666667	9.58391948333333	9.42904984166667	9.653557625	9.12042280833333	9.89687281666667	9.61946140833333	9.70668029166667	9.59906339166667	9.62586958333333	9.71356900833333	9.66421665	9.9441902	8.98986978333333	9.67770730833333	9.126505725	9.654799725	9.28551639166667	9.07777099166667	9.30221980833333	8.99204639166667	9.148299925	9.23235029166667	8.98837211666667	9.65973478333333	9.32711606666667	9.6482902	10.2450271333333	9.87484248333333	8.92218960833333	9.39438685	9.591875575	9.64610789166667	9.39525429166667	10.0997208916667	9.99125556666667	9.78236806666667	9.64610789166667	9.96782080833333	9.75492564166667	9.51896663333333	9.72574979166667	9.018981225	9.33838469166667	9.3832069	9.01847910833333	9.05732553333333	10.1670505916667	9.25628595833333	10.36596635	9.37313166666667	9.68034129166667	9.32186685	9.19244325833333	9.32760176666667	9.26948546666667	9.331319325	9.32329896666667	9.28361398333333	9.19860346666667

C3orf67	4.7815417	5.34734255	4.85861940833333	5.62034133333333	4.782758275	5.87782166666667	4.76455610833333	4.39156651666667	4.95206471666667	5.052876825	5.38762225833333	3.90803829166667	4.59897825	4.37287369166667	6.70532833333333	4.76455610833333	4.53008874166667	4.52697115833333	6.35201275833333	5.02689723333333	4.49312724166667	5.98915650833333	5.14597308333333	4.98545498333333	5.34297193333333	3.92023596666667	5.61354440833333	6.27245344166667	6.32454565	6.346676525	4.62889925	6.06998224166667	4.59683404166667	5.01735620833333	4.434278525	4.29916468333333	6.473722825	4.73720489166667	4.59576983333333	6.1536546	6.06147365	6.18332735833333	5.33867198333333	5.824619025	4.66727606666667	6.23620956666667	6.10002881666667	5.01629245833333	5.05799246666667	5.1598283	5.2108879	5.72076731666667	5.62034133333333	6.11542659166667	6.11047193333333	5.08458449166667	4.64847966666667	6.180201325	6.43016624166667	5.82073376666667	8.46917730833333	7.256627775	7.18705289166667	6.219278425	3.75016011666667	7.08805295	6.98158215833333	4.70270751666667	4.115796825	4.11987200833333	4.54330015	6.759774675	4.60485635	6.15447426666667	6.48928715	5.38469075833333	3.77553975	6.49010539166667	6.50860958333333	7.06146445	4.021539275	4.08963481666667	7.740357875	7.52729815	6.54365829166667	6.22835699166667	7.630419825	5.08458449166667	5.303590975	6.02209645	6.61959393333333	6.018516875	7.37359066666667	5.58212393333333	5.577321975	6.155322525	5.153439775	4.048345425	3.88876065	7.46514316666667	6.5175539	6.00367545	4.67972664166667	7.29365533333333	6.42264453333333	5.63755270833333	6.38621459166667	6.08877931666667	4.61520515	6.39534953333333	4.45602740833333	6.4808852	4.432045325	5.577321975	7.00566125833333	4.71113178333333	4.10724728333333	4.47474535	8.59099210833333	5.61840579166667

VRK2	6.603384975	6.79994456666667	6.59670515	6.73629286666667	6.54638811666667	6.56509979166667	7.64552340833333	6.91766443333333	6.20496000833333	6.22835699166667	6.38476215833333	8.31814086666667	7.101860325	6.76378944166667	6.32527860833333	6.47216713333333	6.27802068333333	5.764437375	4.98106579166667	6.31469075	6.63866813333333	6.824495175	5.70839040833333	6.64226356666667	5.96001181666667	6.585636975	6.40053460833333	6.02558836666667	6.82240235833333	6.14647701666667	6.14249028333333	6.406487225	6.13011929166667	6.64292103333333	6.3687361	6.31548078333333	6.76587266666667	7.075905	6.00285793333333	5.549659825	5.24388391666667	5.84048410833333	5.8902603	5.426744575	6.80124884166667	6.42485146666667	5.90065811666667	7.15765674166667	5.25334058333333	6.05561763333333	5.15046895833333	6.241933725	7.19320405	7.42162103333333	5.95243336666667	5.33557505	7.05900955833333	4.93552183333333	5.59947975	6.42634760833333	6.93552516666667	7.69319276666667	6.35356116666667	7.630419825	7.102506125	6.51078761666667	5.948036925	7.48300389166667	5.62775541666667	7.57673284166667	7.32205540833333	6.54365829166667	8.33066458333333	6.53668581666667	6.95984925	6.56509979166667	7.07654913333333	7.14024473333333	5.20060255	6.46988230833333	8.68329548333333	8.47382378333333	5.60487774166667	6.66738975833333	6.93091866666667	7.20911576666667	7.9977282	7.80563896666667	7.83125643333333	7.18159225833333	6.24033626666667	5.5651756	5.23671275	5.54579601666667	8.42934195	6.12118446666667	6.62907710833333	7.32258515	7.103742375	6.29901051666667	6.225217	7.58911965833333	7.29304450833333	6.13759665833333	5.76923416666667	7.428439975	7.04568171666667	6.64764070833333	6.77905188333333	6.70128395833333	6.03413668333333	6.277191075	6.77179379166667	6.2379381	7.09043023333333	6.17067161666667	6.74082630833333	5.770153075	5.37570958333333	8.002520925

LGI2	8.92364006666667	5.75213593333333	6.70390775833333	5.61643985833333	5.70839040833333	9.01380788333333	5.135343	6.112061375	4.46516671666667	7.502278225	7.21098661666667	9.961096725	7.989231475	5.53902828333333	5.92603411666667	7.88811874166667	5.92679064166667	5.743144625	7.40193138333333	7.34551940833333	8.51177801666667	5.15454668333333	7.2395484	6.31952470833333	6.459913	9.68903575833333	6.4516666	5.26362051666667	6.66866908333333	6.73882945833333	6.56365850833333	6.85045274166667	5.355721825	6.411112525	10.4971305166667	10.5314888083333	5.770153075	7.25540121666667	7.34718618333333	7.32789755	7.96301623333333	6.75898810833333	6.93091866666667	7.31981145833333	8.28242620833333	8.42421896666667	7.71354716666667	7.441553775	7.821740775	7.040686725	6.5607394	6.8732984	4.13109949166667	5.2543857	5.08997001666667	7.14637605	7.740357875	7.72573829166667	6.68532921666667	7.84051964166667	4.71218959166667	7.71624318333333	6.4040341	6.958116025	8.24163635833333	4.38097856666667	7.691592325	6.72519365833333	4.47367345833333	7.33974354166667	7.11617649166667	4.768670525	4.78047113333333	8.46826041666667	4.42570850833333	6.246964375	7.694854575	8.26701150833333	5.62034133333333	7.40602305833333	7.35119398333333	9.36892748333333	5.71218308333333	7.41882361666667	6.71995881666667	8.77815989166667	5.66082913333333	8.561306875	5.10387183333333	9.93796431666667	4.90147810833333	6.23620956666667	6.59599640833333	7.0609201	6.013009825	6.47718199166667	6.2858525	5.64313004166667	5.07558524166667	7.415839475	8.10440565833333	6.46988230833333	5.2891249	7.05225570833333	4.74548245833333	7.38582769166667	7.96240296666667	8.16178343333333	7.73358451666667	9.15113736666667	7.9882338	6.74474600833333	4.27276220833333	5.04338205833333	5.31926836666667	6.77905188333333	9.02418135	5.50436075	4.43639113333333	8.793933

TSHZ3	11.654339925	9.71356900833333	9.46121264166667	8.41299383333333	8.61623551666667	9.62805526666667	8.995827675	9.98312965833333	8.95172195833333	9.57510789166667	8.39209266666667	11.4635169166667	10.3825544833333	9.90816476666667	9.64383103333333	9.53914050833333	9.24884609166667	8.91790284166667	9.50182605833333	9.99808105833333	9.96565705833333	8.78942864166667	9.87345979166667	10.010773575	9.62586958333333	12.1258089083333	9.88925331666667	9.37269289166667	8.701251675	9.49191133333333	10.1836960166667	9.05454125	9.82078890833333	9.17867269166667	11.9629534916667	12.187029525	8.8902947	10.0757309083333	9.895935425	9.84841021666667	9.83071770833333	9.77090350833333	9.7452797	10.5041729666667	10.6527698	9.94294464166667	10.4076693916667	9.79676919166667	9.36084673333333	9.35679085833333	9.645264775	9.993864975	9.88045179166667	9.57692496666667	9.69219855833333	10.6387912166667	10.2442419583333	9.49953110833333	9.75408091666667	10.21004035	9.53043561666667	9.43184414166667	9.4936652	11.218700275	10.1046909416667	9.36715445	11.17758075	10.2191020916667	9.59418116666667	10.1906714166667	8.99535305	8.797202975	10.126633025	10.9979551416667	9.50685594166667	11.30753185	9.67141531666667	9.2741013	10.2621725583333	9.3434528	10.2782382416667	10.7228384083333	9.51454425833333	10.8608532	10.2991427166667	10.6183847583333	9.063171125	11.3187261333333	9.28551639166667	10.9979551416667	10.7508267666667	9.8694956	11.0487579166667	10.751563075	10.064052975	9.05965051666667	8.97675146666667	9.60791420833333	10.2904392333333	9.683051225	10.3078222333333	9.76136145	8.8830474	9.38536081666667	9.25167913333333	9.51811660833333	10.9665691416667	9.94339973333333	10.1948905833333	11.3957857333333	10.1444546916667	10.1895353083333	11.4173578916667	11.2743409666667	10.7116237833333	9.729222475	10.7862968083333	10.2769692583333	8.37653824166667	10.0949964416667

SENP1	6.75503975833333	6.62158961666667	6.31548078333333	7.17198438333333	6.79529673333333	6.17390685	7.27593380833333	6.861457125	6.901873575	6.14728135833333	6.37197970833333	6.89528771666667	7.04956788333333	6.53809915833333	6.71918628333333	6.53954924166667	6.65487665833333	7.07118303333333	5.99598418333333	6.74218866666667	6.73002575	6.33852943333333	6.42634760833333	6.87851200833333	6.82167365	6.66944055833333	6.869498575	6.29057105	6.316307375	6.15447426666667	5.82731045	7.14272935833333	6.50580336666667	5.94185621666667	6.21191129166667	6.872774725	6.69642741666667	6.45696331666667	6.40572345	6.12200050833333	5.78537223333333	6.46133099166667	6.39606678333333	6.42946571666667	6.21517435	6.43490204166667	6.48707709166667	6.2058813	7.05156345	6.11387184166667	6.503440675	6.50860958333333	7.139086775	6.70856058333333	6.39769980833333	6.64292103333333	6.717950125	5.57148620833333	6.50720644166667	6.68472555	7.134182075	6.48010203333333	7.04267660833333	6.50650844166667	5.41039171666667	6.70796138333333	6.230051875	6.88865130833333	6.80056770833333	6.40797193333333	6.78180254166667	6.64088086666667	7.07182334166667	6.43421143333333	7.050862275	6.4808852	5.86179054166667	7.05714315833333	5.74803075	6.929785275	6.77179379166667	6.89400789166667	6.99601435833333	6.495300075	6.62563488333333	6.70856058333333	8.360853775	6.62436875833333	6.806433125	6.35201275833333	6.36951041666667	7.38640515833333	6.88120535	5.68207400833333	6.63179246666667	7.03878675833333	7.32611610833333	7.74145285	6.64956780833333	6.71367926666667	6.57166325	6.65024493333333	7.05018306666667	6.164307025	6.56365850833333	6.62636613333333	6.38476215833333	6.32454565	6.675144925	6.1112992	6.85814304166667	6.28336628333333	6.54365829166667	6.52133365833333	6.43290905833333	7.06400038333333	6.77836881666667	6.15598731666667	6.81118743333333	6.86328933333333

ARHGDIA	13.8992947833333	14.3431664333333	14.77778785	14.4397477583333	14.89930145	14.648708375	14.0001337166667	14.6949121583333	14.2319492833333	14.6430667333333	14.3514380333333	14.0301250916667	14.30615445	14.7166750666667	14.6589883666667	14.2132241916667	14.2204751583333	14.4377971083333	14.789742575	14.7252309	14.9020399416667	14.7077525	14.6904136083333	14.6005448916667	14.6611477166667	14.4739784	14.92521735	14.7077525	14.667083075	14.4775829916667	14.7828660333333	15.0184028083333	14.312396275	14.3316746833333	13.5721495	13.9529996833333	14.6346112833333	14.9779140333333	14.5550005583333	14.5140672583333	14.6549636583333	14.6122307083333	14.5530100166667	14.8497796833333	14.2715663416667	14.6466555666667	14.5530100166667	14.9865439333333	14.7596386666667	14.2731900083333	14.8287604166667	14.74733735	14.8578939833333	14.9702238166667	14.7097912166667	15.1104707416667	14.9359352083333	14.7143451416667	14.8548535916667	14.9779140333333	15.06705565	15.095547425	14.4161239916667	14.9752180916667	13.809878875	15.0217064166667	14.8750732583333	14.80427055	14.89930145	15.0587922833333	15.06705565	15.03651825	15.1833315916667	14.7802388333333	14.8653905916667	15.2193239916667	14.7097912166667	15.1632730833333	14.9094697583333	14.9307420333333	14.3186975416667	14.6758595916667	15.169521525	15.25397645	15.1017513083333	14.5984675666667	14.2503770416667	14.77778785	14.9391332833333	15.0894272416667	14.8794573583333	14.3463851	14.948326475	14.948326475	15.4803730166667	15.009089275	15.1430836166667	14.9674626083333	15.406759425	15.0615765666667	15.039210975	14.6904136083333	15.1139193916667	15.229747525	15.01216265	15.169521525	15.4897034083333	15.03651825	15.0153788083333	15.406759425	15.1430836166667	15.2399573083333	15.4852249833333	15.2084540583333	15.2328828083333	14.9727774916667	14.6325901333333	14.99199845	14.8629148	15.0769655666667

ATP1A1	8.59397561666667	6.5175539	7.22964071666667	6.49896905833333	5.95490855	7.56216311666667	5.44322508333333	6.18408195	6.43490204166667	5.65344755	6.30781043333333	6.86012671666667	6.28109695833333	6.53137754166667	7.01837270833333	7.41882361666667	9.341081375	5.7161104	7.20859641666667	7.22598754166667	7.06146445	7.212178575	7.310825425	7.2162874	7.598264375	8.085928525	7.76990645	7.210345825	6.58100214166667	7.42619010833333	6.72653388333333	7.43011818333333	7.16232548333333	8.000994225	8.31103703333333	8.479465175	7.02943565833333	6.69500276666667	6.89851890833333	6.61289744166667	7.17033845	7.40322858333333	7.54341655833333	7.6421897	7.79803676666667	7.93695109166667	8.1073987	6.68859711666667	7.687727325	7.762415375	7.23080319166667	6.3687361	6.73002575	7.29106909166667	7.64678326666667	7.36530371666667	5.965264275	6.89596523333333	7.83726095833333	7.33912221666667	6.929785275	6.46988230833333	8.07839394166667	6.50418884166667	8.66108489166667	6.44290830833333	5.6173492	6.22675393333333	6.769663225	6.43290905833333	5.74413374166667	6.08271424166667	6.49981934166667	6.5479092	6.20262720833333	7.310825425	6.88578850833333	7.23488161666667	6.68131301666667	7.41007171666667	8.13304300833333	6.96590744166667	5.87944876666667	6.72825601666667	6.04002979166667	7.69605179166667	7.95517108333333	6.38846551666667	6.29278439166667	7.76938555	6.50275845	6.73133699166667	6.46133099166667	5.79119435	6.96657695833333	5.54199565833333	6.54716043333333	7.67359318333333	5.546831425	7.004281725	6.04613815	6.2353521	6.93484450833333	7.80329328333333	7.37059144166667	6.91828460833333	6.70856058333333	6.74158396666667	7.32205540833333	6.93685505	7.21861555833333	7.77718458333333	6.96254510833333	7.85469968333333	7.22110665	7.13168135	8.86366274166667	6.3378065	7.1762422	6.322468275

NEURL2	9.57863910833333	8.52279583333333	8.998536575	8.53385956666667	8.54360906666667	8.39771189166667	8.46459865	8.62136429166667	8.90962148333333	8.35883419166667	8.97076639166667	9.19160371666667	9.0174923	8.78070639166667	8.661516725	8.482136425	8.90142945833333	8.4178821	9.25764194166667	8.682286675	8.76603770833333	9.00735385	8.88122105	10.021871175	8.81880498333333	9.666049875	8.90619576666667	8.69459336666667	9.296236625	8.7954535	8.57560394166667	9.119480775	8.97266575	8.902412325	9.401147475	9.705378475	8.78443150833333	8.764687525	9.07777099166667	8.27706721666667	8.82265860833333	8.90525780833333	8.536875925	8.83783124166667	8.729717925	9.04320051666667	8.83115135	8.81971978333333	8.76603770833333	8.7812519	8.54699226666667	9.01197323333333	8.64890015833333	8.57783373333333	8.66247435	9.203321075	8.736017975	8.354234475	8.68775965833333	8.54411631666667	9.17368278333333	8.99987959166667	10.079145275	8.89220491666667	9.31452535833333	8.03595288333333	8.505532025	8.8364013	8.93953213333333	8.054062325	8.19808664166667	8.84099020833333	8.62938480833333	8.42018036666667	8.26606834166667	8.43662665833333	8.65800171666667	9.03689006666667	8.01434291666667	9.0180261	9.89383843333333	9.25303868333333	8.697015525	8.881658575	8.987001225	9.95359933333333	9.24864904166667	8.34473695	8.930278925	8.96369055833333	8.28193894166667	8.52966328333333	8.39981014166667	8.592518275	8.59640985	8.023521375	10.6143770083333	8.799065075	8.78942864166667	8.04498438333333	8.74322296666667	8.76751969166667	8.70635446666667	9.329864925	8.63126818333333	9.19860346666667	8.48614225	9.20284239166667	9.8269075	8.552420375	8.71235535	8.608522725	8.86116145	9.6496487	8.561783725	8.42776885	9.9229238	8.41104395833333	9.79062058333333	8.17302170833333

CFAP100	5.6173492	5.66283725	4.88811624166667	5.90415203333333	5.17603111666667	3.85883090833333	5.71985138333333	5.48616714166667	5.04557781666667	4.830971775	4.939635175	5.34966958333333	5.23361180833333	5.09217729166667	4.64847966666667	4.48239504166667	4.95105651666667	4.54330015	5.26995844166667	5.10077205	5.43182890833333	5.40105285833333	5.28688518333333	5.10174316666667	4.9195424	5.6322886	5.22040228333333	6.407249275	5.018425275	5.20550439166667	5.30562465833333	5.442194025	5.07000859166667	4.76548715	5.01530855833333	4.74930484166667	4.64613160833333	4.73206480833333	4.9406758	5.19421045833333	4.537079425	6.39012523333333	5.72382095833333	5.020575025	5.38762225833333	4.55184448333333	4.47770515	5.21920105	4.21395365833333	4.45602740833333	5.04338205833333	5.40405401666667	4.73024025833333	4.8072374	4.74448875	4.96638246666667	3.68464693333333	4.42372025	4.69963341666667	4.67674131666667	6.61154091666667	6.19129395	5.27227235	6.30401625	5.10716860833333	4.91752975833333	5.09764620833333	6.24941475833333	6.47574773333333	5.53102171666667	5.49526165833333	5.74037505	5.14718730833333	5.40750829166667	5.70649276666667	5.2617065	6.14334293333333	5.07445225	4.86479268333333	5.3343362	6.8275595	5.76923416666667	4.99463171666667	4.697573175	5.03526733333333	6.44595796666667	6.14494839166667	5.50905394166667	6.421178475	4.69425546666667	6.08593745	5.64797583333333	5.110215025	5.30151331666667	5.93821120833333	5.189937375	5.54779774166667	5.43488754166667	5.11118121666667	5.87496216666667	4.38726624166667	5.95832643333333	6.72953939166667	5.41246783333333	5.08571715833333	4.29916468333333	5.127309825	5.21821305	4.44822564166667	5.528242825	4.87710051666667	6.37113890833333	5.0088772	4.432045325	4.91752975833333	5.58212393333333	4.729262375	4.73720489166667	5.677007675	4.50430228333333

OSBPL11	11.48757865	11.6209481083333	11.8297435166667	11.7035007416667	11.4824869416667	11.2464355333333	11.4669037583333	11.5954858333333	11.4481946583333	11.0651712583333	11.605536575	11.3078719166667	11.1800718666667	11.4934089166667	11.3406807666667	11.8670714	10.7873883666667	11.3328774166667	11.603226975	11.489428925	11.6080957416667	11.2100932	11.9196580833333	11.5279761083333	11.6073227	11.586171175	11.6587921	11.8004315583333	11.3657868083333	11.5742275583333	11.531296025	11.6099448083333	11.5461869416667	11.1937817916667	11.575819425	11.6099448083333	11.2868731416667	11.2801435833333	11.4173578916667	11.4004623	11.82248265	11.6223592166667	11.3343049666667	11.66717835	11.5702459583333	11.7895299333333	11.5695230416667	11.529525725	11.5298755416667	11.7266159583333	11.5895178083333	11.4597359083333	11.2944151416667	11.3597850833333	11.7605260333333	11.529525725	11.5601850333333	11.8293448166667	11.3942748	11.3527786833333	11.7387926	11.4897906583333	11.51432045	11.6259515166667	10.7593753416667	10.5056824166667	11.6066366416667	11.430002575	11.8931595083333	11.744853675	11.3015343416667	11.873004525	11.2240675166667	11.4255179583333	11.572420475	11.625582325	11.4511549916667	11.0215105083333	11.302200525	11.3720648583333	11.994387925	11.2521123083333	11.3771067916667	11.5754446583333	11.8055072	11.4883355166667	11.4470684833333	11.3861262916667	11.328911575	11.66250505	11.8511708666667	11.7216620916667	11.189465025	11.47563195	11.4214230083333	11.1840687083333	11.2100932	11.75219825	11.4026558	11.9793031666667	12.0088601416667	11.434022575	12.4416866083333	11.5196693333333	11.4019322	11.3861262916667	11.0901980583333	10.8394387833333	11.4489345583333	11.5869361333333	11.3118389916667	11.683415075	11.14949005	11.3567956166667	11.624532275	12.3431207	10.8394387833333	11.7955610083333	11.9871613166667	11.696699

RAD51D	4.53766447083333	4.33378375416667	4.13266122083333	4.33689344583333	5.19910030416667	5.5655648875	4.77430863333333	5.12464447083333	5.00484438333333	4.37233573333333	5.4575038	4.97957150833333	4.36652742083333	4.10893715	5.4957950125	4.47136030416667	4.99525044166667	4.84878312083333	4.759748625	5.28113690833333	4.46052268333333	5.46144152083333	5.06172876666667	4.62532567916667	5.38816209166667	5.04917524166667	4.561288475	5.0070672625	4.96903827083333	4.13489344583333	4.95684839166667	5.35932894583333	4.858152125	4.5026770375	4.68524963333333	4.66680242083333	5.6140144	5.67376376666667	5.03282052916667	5.09916694166667	4.70729979583333	4.18448905	4.55517042083333	5.34147090416667	4.4525320625	4.84146155833333	4.81658845416667	4.77542294583333	4.9668900375	4.449773675	4.47719546666667	4.25053065416667	4.25641249583333	5.06622259166667	4.15590024583333	4.54499327916667	3.7370829125	4.6294003	5.24989574583333	5.00291880416667	5.82120886666667	5.44270955416667	5.719335775	4.90420065833333	4.20842273333333	5.43334969166667	5.5114587625	5.22874515833333	6.13218769583333	4.97227905833333	5.50387195416667	6.2000202875	4.94209515	4.9743854	4.8734586125	5.06411445416667	5.256158575	5.674742725	5.38018182083333	5.296139525	4.38768927083333	5.80046790833333	5.14141002916667	4.95684839166667	4.89770367916667	4.835080975	5.0739174625	4.76813420416667	6.1215924875	5.159282825	4.96903827083333	6.68305109583333	5.66039114166667	4.520046925	5.83063687916667	5.3176850875	5.89681017083333	5.46419355416667	5.55217058333333	4.86752087916667	5.12156893333333	5.7079367375	5.54919577916667	5.09368760416667	5.70103635	5.27046563333333	4.45651248333333	5.94489275	5.17854790416667	5.5007433375	4.42718955	4.8219214875	4.2805940625	5.43232302083333	4.02200670416667	4.2732531875	4.09324174166667	5.921093875	5.4481421875	4.56441264166667

AC004223.3	4.53766447083333	4.33378375416667	4.13266122083333	4.33689344583333	5.19910030416667	5.5655648875	4.77430863333333	5.12464447083333	5.00484438333333	4.37233573333333	5.4575038	4.97957150833333	4.36652742083333	4.10893715	5.4957950125	4.47136030416667	4.99525044166667	4.84878312083333	4.759748625	5.28113690833333	4.46052268333333	5.46144152083333	5.06172876666667	4.62532567916667	5.38816209166667	5.04917524166667	4.561288475	5.0070672625	4.96903827083333	4.13489344583333	4.95684839166667	5.35932894583333	4.858152125	4.5026770375	4.68524963333333	4.66680242083333	5.6140144	5.67376376666667	5.03282052916667	5.09916694166667	4.70729979583333	4.18448905	4.55517042083333	5.34147090416667	4.4525320625	4.84146155833333	4.81658845416667	4.77542294583333	4.9668900375	4.449773675	4.47719546666667	4.25053065416667	4.25641249583333	5.06622259166667	4.15590024583333	4.54499327916667	3.7370829125	4.6294003	5.24989574583333	5.00291880416667	5.82120886666667	5.44270955416667	5.719335775	4.90420065833333	4.20842273333333	5.43334969166667	5.5114587625	5.22874515833333	6.13218769583333	4.97227905833333	5.50387195416667	6.2000202875	4.94209515	4.9743854	4.8734586125	5.06411445416667	5.256158575	5.674742725	5.38018182083333	5.296139525	4.38768927083333	5.80046790833333	5.14141002916667	4.95684839166667	4.89770367916667	4.835080975	5.0739174625	4.76813420416667	6.1215924875	5.159282825	4.96903827083333	6.68305109583333	5.66039114166667	4.520046925	5.83063687916667	5.3176850875	5.89681017083333	5.46419355416667	5.55217058333333	4.86752087916667	5.12156893333333	5.7079367375	5.54919577916667	5.09368760416667	5.70103635	5.27046563333333	4.45651248333333	5.94489275	5.17854790416667	5.5007433375	4.42718955	4.8219214875	4.2805940625	5.43232302083333	4.02200670416667	4.2732531875	4.09324174166667	5.921093875	5.4481421875	4.56441264166667

CAPN14	7.40375466666667	14.6549636583333	13.9367207833333	15.0276351833333	14.0503314	10.8654356416667	13.9600657833333	13.386735	14.419240975	12.7071557666667	12.6790614583333	6.71502808333333	13.295486125	15.01216265	13.33077755	14.9832780583333	6.38316134166667	13.9625123916667	14.4077075916667	13.6758447	13.735130625	15.1207533083333	12.9230312916667	14.5589896583333	13.5884582166667	7.14822965	12.487541925	13.1452170666667	15.2576870916667	14.37416785	14.657054125	14.26265275	14.178968975	7.14399110833333	6.81976344166667	7.76345526666667	14.0746231666667	13.810810275	14.1857941	13.1806770166667	13.7393052083333	14.8021338083333	14.6904136083333	14.1164578666667	13.5643847833333	12.99120565	14.2715663416667	14.9832780583333	15.5458909083333	12.7423894333333	13.864749275	14.2349020166667	13.3786606333333	13.8614602166667	14.2518399916667	14.3204037083333	14.5195733916667	13.09459025	13.5660586666667	12.5259355083333	6.51915964166667	9.78329576666667	10.1084049583333	10.3754461833333	8.576058475	10.39291705	8.78586333333333	8.13242494166667	8.63126818333333	8.9544846	9.96660331666667	8.52634974166667	7.93387576666667	9.18306429166667	11.2711403666667	9.59949791666667	8.68775965833333	11.1291467583333	9.52364006666667	9.90955436666667	8.68130785	9.96481554166667	8.77579595833333	9.63156661666667	8.930278925	9.30682376666667	7.93695109166667	8.16877921666667	10.327316425	9.38006720833333	9.80967570833333	9.4233862	9.63985794166667	6.38476215833333	6.10713235	9.66649425	9.66649425	10.0823479583333	9.6895289	8.10330963333333	8.92461889166667	9.65670091666667	9.44293281666667	10.58612335	8.6716691	9.567278375	9.41298314166667	9.553861875	9.974167275	7.60823079166667	9.39392068333333	10.2695195083333	8.887867925	9.28405401666667	9.01611445833333	12.6816059583333	9.04505583333333	8.67639044166667	10.8499191666667	8.62084126666667

KMT2D	8.348200375	8.57560394166667	8.83443758333333	8.62734905	9.120869625	8.37107905	8.37653824166667	8.85215985	8.97532130833333	8.40817908333333	8.71940105833333	8.19143718333333	8.230344325	8.73305545	9.26261210833333	8.744772225	8.88546686666667	9.14655231666667	9.19764225833333	8.83016251666667	8.65418485	9.42030009166667	9.00690949166667	9.11034851666667	8.87883045833333	8.846155575	9.01566145833333	9.07434654166667	9.311763425	8.85393681666667	9.22606134166667	9.1237399	8.74370115833333	8.60998564166667	8.88930546666667	8.94804158333333	9.18361099166667	9.03417474166667	9.00964296666667	8.95218464166667	8.60344725	9.00690949166667	8.55543884166667	8.63344703333333	8.35071744166667	8.6716691	8.816929025	9.090969825	8.76751969166667	8.77966386666667	8.55543884166667	9.32471271666667	8.81599143333333	9.2741013	9.186916425	9.05125875	8.91690625833333	8.71235535	8.57649310833333	8.67403964166667	8.70592035	8.18684245	8.42618235833333	8.13819046666667	7.76293401666667	8.69939258333333	7.97507355833333	8.215089275	8.54744824166667	8.28997516666667	8.14449565	8.81744095	7.86609655833333	8.00199818333333	8.92218960833333	8.4484208	8.41742745833333	8.40377353333333	8.64290230833333	9.14327603333333	8.1073987	8.309637175	8.59890789166667	8.59990980833333	8.7954535	8.41428779166667	8.93073908333333	8.11887123333333	8.870415075	8.4100903	8.701251675	9.48384479166667	8.78498449166667	8.62886449166667	8.41878698333333	8.69606343333333	8.97126208333333	8.51713660833333	8.835452275	8.95624593333333	8.56955223333333	8.27954998333333	8.68035370833333	8.98191765833333	8.65745360833333	8.53333894166667	8.79240648333333	9.35075274166667	8.78836945	7.88228429166667	8.25344699166667	8.618769925	9.27172153333333	9.06270968333333	9.10099604166667	8.89560048333333	8.5676736	8.84952995833333	8.80780606666667	8.66397386666667

TULP4	9.9560883	10.29990335	10.7565121333333	10.2865677	9.743497625	10.9393869333333	10.0209906333333	10.585362525	10.1477211833333	10.1469040583333	10.3948990166667	10.79558335	9.80706423333333	10.7990034333333	10.45385095	10.0238668333333	10.7050450333333	10.0787243333333	10.35233035	10.7628088916667	10.5787126833333	10.590660625	10.39251605	9.86517185	9.960644375	10.0238668333333	10.4846654833333	10.1246349583333	10.0724257083333	10.176297425	10.8180008083333	10.7309865083333	10.000998825	10.3546255166667	10.2427233083333	10.3345761083333	10.55775005	10.8006177916667	10.1923992666667	10.8544918	10.7990034333333	11.0182236333333	10.490641975	10.966941925	10.045510375	10.754228175	10.5396655666667	10.3119751583333	10.8885586333333	10.7078134666667	10.5049759833333	11.0927898583333	9.86598649166667	10.241903125	10.286132425	10.3507137166667	10.9087616083333	10.9139727166667	10.406522025	10.5490285	8.92461889166667	10.1633244583333	9.77613621666667	10.591411875	11.401582575	10.8710123583333	10.689167625	10.0412389083333	10.4910656666667	9.93596328333333	9.565041475	10.7643055083333	10.2865677	10.21004035	10.5814300416667	10.3510926083333	9.97501473333333	10.0653276583333	10.4581692833333	10.5968900416667	11.3268344833333	10.3261322166667	10.2962241416667	10.3591207	10.51932965	10.5061588333333	10.7800644666667	10.000998825	8.74819925	10.4288423083333	10.372950875	10.5837654416667	9.961904575	10.7816314666667	10.4744353333333	10.1117927583333	10.992544275	10.3119751583333	10.1725196416667	10.4344952333333	11.1941109166667	10.5085269083333	10.7565121333333	11.0971520666667	10.7501138916667	10.30932905	10.7907764333333	10.72804705	10.8427945083333	10.640008475	10.6340233	10.9651507916667	11.2194717166667	10.4324595166667	9.7997523	10.5991330666667	12.0388164916667	10.8792631583333	10.3754461833333	10.64807625

COTL1	10.6191395	11.2668058583333	12.0245529166667	11.6252518666667	11.1971318416667	12.2939030416667	11.2514092416667	11.6914012333333	10.7100606833333	12.266695	11.5610238416667	12.0449944666667	11.5411164416667	11.4228997	10.3806930666667	13.373920475	13.5626218333333	11.3108007583333	11.6036128166667	12.1096451083333	12.4310799666667	11.1874035166667	12.5984922	12.149683875	11.687529825	11.4559913916667	11.722386025	11.6577322083333	11.743693825	11.53378775	11.97391105	12.4114353583333	12.0445037083333	13.9003358333333	12.077364475	11.48024075	11.5976176916667	12.1525189	12.5297533083333	12.1316746333333	12.37676025	11.8281920416667	12.1690316666667	12.196460925	12.4751035416667	13.129332175	11.6974443166667	12.487541925	13.1159098333333	12.5306631166667	12.8661429166667	11.765950425	11.9032491333333	11.739559375	11.8403034416667	12.0600286166667	12.6175954833333	12.7086380166667	12.1443639166667	12.9767731916667	11.8317467416667	12.1338172083333	12.9139148666667	12.3469866583333	12.017715375	11.8783734666667	13.2366721916667	12.3589004083333	12.7056843166667	13.3839194083333	12.3071456666667	12.388809075	12.9139148666667	12.4469679916667	11.976653175	12.584151375	12.5079805333333	13.203417575	12.9773913166667	13.0380777583333	11.918121775	12.5969475916667	12.462326175	12.762350675	12.199408325	12.403353825	12.2719001166667	13.1249649416667	12.089741475	13.1913123583333	12.7436904833333	12.857326075	12.552088775	12.7736204583333	12.7999144166667	12.6991286666667	13.2208402833333	13.3151588	13.1329842916667	11.7887391333333	13.016186275	13.1459482	13.2711737166667	12.6606270166667	12.139797125	12.7490093583333	13.5884582166667	13.0889930583333	13.1550516583333	13.5519530666667	13.0108192083333	12.5979621916667	12.7558773833333	13.0626219916667	13.19400885	13.1465914416667	12.036426975	13.02514525	12.1528810833333	13.4375531583333

ZNF451	11.0323327416667	9.85765425	9.37637135833333	9.44779018333333	10.2857643416667	11.2125996833333	8.92792624166667	9.91080910833333	9.54178184166667	10.0292860833333	9.6543889	9.900995025	9.84332440833333	9.726557625	9.62407518333333	9.88168326666667	9.83811425	10.167505275	10.264650175	10.26788045	10.0670141416667	10.63689965	10.145640125	10.5318759	10.3554429666667	10.2128243166667	9.7045424	9.84919238333333	10.25189245	9.96227999166667	9.84032644166667	9.96954501666667	9.890973325	9.70194608333333	9.90688865833333	9.77263848333333	10.325378325	10.1316676583333	9.64431238333333	10.237546325	9.557602025	9.850735	10.0260539583333	10.1214563916667	10.0095607333333	9.77090350833333	10.5041729666667	9.98715419166667	10.4215953	9.86718390833333	9.74655711666667	9.864777125	10.3301703333333	10.5573126666667	9.961904575	10.040796475	10.023053775	9.765982475	9.990452125	10.4994687166667	11.6006445416667	10.6550211916667	9.97954941666667	10.3833420583333	11.2602582666667	11.2028990666667	11.0651712583333	9.99808105833333	10.5337610083333	10.1541580083333	10.4072694666667	10.3852198833333	10.6633228083333	10.754228175	11.0916995	10.7493099333333	10.2034843416667	10.5768008666667	10.6263315083333	11.3805222416667	11.7750094	11.2442138	10.1529084083333	11.0673982916667	10.5138272833333	10.6558683583333	11.0927898583333	10.5065079166667	11.5710069666667	10.3507137166667	10.431190025	10.5663053083333	11.2363296083333	10.3656176	11.0017295	10.2919749833333	11.1039790666667	11.36200305	10.4554224833333	11.4590118416667	10.1577133666667	9.43324608333333	11.2631446833333	10.7576799666667	10.8001996166667	10.6348374333333	11.3072160833333	11.0822289416667	11.1154972666667	10.4492048416667	9.74045244166667	10.6309483083333	11.270077	10.6887964	10.0679332666667	10.6523425166667	10.9917752916667	11.9384515083333	11.3253118333333	11.1224187916667

CDH4	10.30932905	9.663801325	10.1704848833333	10.44375685	10.4526526416667	11.2711403666667	10.9426936083333	9.80576564166667	10.0442462083333	9.66334089166667	10.1278854583333	10.7765403041667	10.521634775	9.4134397	9.58131358333333	9.39525429166667	9.80802880833333	10.3329358333333	9.26988130833333	9.27035150833333	9.496026575	9.73142488333333	9.226998075	9.25303868333333	9.24539353333333	9.69707035833333	10.022234175	9.8269075	9.80056271666667	9.954379925	9.67770730833333	10.010773575	10.0331009416667	11.4854519916667	9.90731580833333	10.0412389083333	10.1862509833333	9.47496915833333	9.57414756666667	9.75747410833333	8.93227916666667	9.59742406666667	9.38182149166667	8.91790284166667	9.41935561666667	8.84011393333333	8.91135703333333	9.33087654166667	8.78586333333333	9.092829475	9.24884609166667	9.54225544166667	10.0648957166667	9.587537375	9.69924190833333	9.15064904166667	9.41761155833333	9.20872513333333	9.4594195	8.94384210833333	9.96565705833333	10.2658011666667	10.2434985083333	10.6195893583333	9.58222316666667	10.804132675	9.92093743333333	10.447247375	10.1316676583333	10.2857643416667	10.4671575083333	10.1601611333333	10.4990563	9.59324718333333	10.135861875	9.59782546666667	10.05531845	9.52087123333333	9.19905495833333	9.99125556666667	9.490132625	9.97337968333333	9.611823	9.33664844166667	10.225450075	10.5573126666667	10.8006177916667	10.4344952333333	10.3044270083333	9.32471271666667	10.0078625	10.2123952583333	10.0103759833333	9.67900834166667	10.41573205	10.1953308333333	10.146512775	10.3771245083333	10.364544	9.73788834166667	9.77176594166667	10.3074544166667	9.939168625	9.27768001666667	9.41030455	9.56548648333333	9.62496784166667	9.25167913333333	9.36084673333333	9.45374055	10.0972464083333	9.27172153333333	9.62712280833333	9.59648643333333	9.82029285833333	9.69176305833333	9.4337598	9.612283675	9.32231320833333	9.88726988333333

SLC7A11	7.57619343333333	9.6428903	10.023053775	8.95718403333333	11.4154857416667	10.8721591833333	10.4492048416667	11.0225675833333	9.79446515833333	11.1524382	13.66414075	8.69657786666667	9.84994195833333	8.82825418333333	9.41204579166667	9.1447536	12.545304275	9.64024775833333	10.4661023416667	10.4057146583333	8.93806988333333	9.26488315	11.712752375	11.4255179583333	10.1001644	6.05897065833333	10.2579212583333	10.44259115	10.35378905	9.52456219166667	9.90775529166667	9.42859468333333	8.60701585833333	11.4063648666667	7.12528415833333	8.12585160833333	9.748599325	10.392106425	8.975801525	9.85850786666667	10.4542782	8.97027915833333	9.06886545833333	10.0321890166667	8.02070828333333	10.3671094333333	10.7276175666667	10.8416766333333	9.10796010833333	9.54834169166667	8.68375230833333	11.2384584083333	8.6774004	8.82781555833333	8.85824333333333	8.69556865	10.0945926583333	11.7038872	10.1009165916667	9.810451275	12.9063845583333	14.6798721333333	10.432039025	12.240405375	9.33537304166667	11.1779127416667	14.5570464083333	10.7530924583333	10.736058575	9.50308771666667	14.648708375	14.204528175	10.6373110916667	14.5268570083333	10.874072375	13.99785205	11.6499693666667	12.47909375	13.827479275	12.3025527416667	8.76851021666667	8.45999591666667	11.233793875	13.0571358166667	11.1710416	8.594439725	9.70017130833333	8.79676539166667	13.9085702916667	12.6562254416667	9.23186806666667	13.0913765666667	12.8936439416667	11.0491091083333	10.4561678833333	13.668458425	10.248995	9.67230795	10.8638767916667	9.68075670833333	11.0068392583333	13.597492475	9.81941439166667	12.8218942166667	10.9256058083333	11.6640542416667	10.9754104083333	12.367659325	10.3578687916667	12.488903425	10.632187725	13.2648622166667	10.34716255	11.6073227	10.4453172666667	13.7224285916667	10.4519100666667	14.2349020166667	14.5020438083333	9.25719764166667

SLC16A1	7.36587165	8.29250424166667	8.20281158333333	8.03913049166667	8.08952596666667	9.986796275	7.52566054166667	7.741934525	8.21153401666667	7.81456171666667	7.36108145833333	6.71655181666667	7.84998155	7.97239718333333	8.53972030833333	7.17330060833333	14.7851708916667	9.00643414166667	8.4380593	8.06129600833333	7.76884748333333	8.39466414166667	7.70367073333333	8.26701150833333	7.75542415833333	7.25980210833333	8.26606834166667	8.02671881666667	8.0095181	8.11057064166667	8.29931580833333	8.10691838333333	7.6702147	13.6570629916667	7.33912221666667	7.10434548333333	8.1439842	8.11306225833333	7.882902775	7.67359318333333	7.77321380833333	8.29533216666667	8.25990825	8.04330091666667	7.16295023333333	8.0063663	7.941170525	8.06606233333333	7.92549585	7.61337419166667	8.38708958333333	8.2675206	8.06500796666667	8.53434441666667	7.801197125	7.838427925	8.017526525	8.00359983333333	8.2675206	7.16422769166667	7.38926633333333	7.36048969166667	10.333754975	7.85309088333333	7.3675475	7.803872825	7.81401100833333	8.07255219166667	8.03913049166667	8.05913828333333	6.68131301666667	7.11730268333333	8.35169179166667	6.95984925	7.4250003	7.59310200833333	8.76175106666667	8.21097586666667	8.66580391666667	8.87491901666667	7.13728840833333	7.05585056666667	8.96094025833333	7.25980210833333	7.7113432	7.75823035	7.35924831666667	6.6562921	6.75712434166667	7.74145285	7.12901885	7.99725846666667	7.84741366666667	8.706897975	8.3547531	6.70056215833333	9.68213623333333	7.71678190833333	7.825834775	6.95924915	7.76455869166667	6.70796138333333	7.0369697	8.62481299166667	8.7217903	8.466669725	8.425659175	7.46243825	8.30746798333333	7.27649536666667	8.379169075	8.08086618333333	7.67359318333333	10.35148425	8.954039625	7.46964506666667	7.52953641666667	7.78488271666667	8.39560916666667	7.40026851666667

CYP21A2	14.0932431041667	12.9514155541667	13.8478593291667	12.5393457416667	11.4135239541667	12.7679182916667	13.1100606166667	12.585288825	12.8518669958333	13.7567678375	12.9549121916667	15.0075215125	13.4803759291667	13.276842	12.4255097666667	14.2226540333333	12.2097075375	11.88751815	12.7100716333333	13.231671575	13.832273425	10.4860549416667	13.3564324708333	14.3068980333333	13.6955817416667	14.3473409416667	13.998416875	13.8826567958333	12.683947825	13.2916213375	13.1416728916667	12.9039557375	13.51377155	12.5383686916667	14.1623829708333	14.3799446333333	12.4304122583333	13.9080704541667	13.6706778916667	13.5398526291667	13.3352775791667	13.6574902458333	13.16578005	13.1333095958333	13.9405817375	13.9630399875	13.8837850083333	13.0963431125	13.295147525	13.4153354625	13.9394971041667	13.3542137875	13.2158871875	12.8373957916667	13.1855235875	13.9835413583333	13.7906263333333	13.196301825	13.6629357875	13.9313255791667	10.3213899291667	10.3021861375	9.72679546666667	9.6174537375	10.7822440833333	8.85283100833333	10.6572385166667	9.59126800416667	8.69581604166667	10.7704596208333	9.82536999166667	8.7687455125	8.703122725	10.1968151583333	8.8976024375	8.82993020833333	11.2835686375	11.9976969958333	9.26104765833333	10.1635387833333	8.98121555	10.0767594	8.93385787083333	8.81811914166667	9.676585125	11.2249415416667	9.65908810833333	11.064992425	9.10271907083333	12.2886149583333	9.09307139166667	9.01920487083333	9.10033722916667	8.95787474583333	9.11844430833333	9.51834335	9.14167540833333	9.51154887916667	9.34459659583333	9.76070797083333	10.4322492708333	9.58456951666667	8.95695830416667	10.7263164708333	9.31785807083333	9.33558479583333	10.0991418375	9.41914157916667	9.3039005	10.4572088208333	11.2973992791667	9.34690669583333	8.3439831125	8.50577863333333	11.3200835125	10.7742107041667	11.1563163958333	8.64220767916667	9.56058135	10.9304763958333

TNXB	14.0932431041667	12.9514155541667	13.8478593291667	12.5393457416667	11.4135239541667	12.7679182916667	13.1100606166667	12.585288825	12.8518669958333	13.7567678375	12.9549121916667	15.0075215125	13.4803759291667	13.276842	12.4255097666667	14.2226540333333	12.2097075375	11.88751815	12.7100716333333	13.231671575	13.832273425	10.4860549416667	13.3564324708333	14.3068980333333	13.6955817416667	14.3473409416667	13.998416875	13.8826567958333	12.683947825	13.2916213375	13.1416728916667	12.9039557375	13.51377155	12.5383686916667	14.1623829708333	14.3799446333333	12.4304122583333	13.9080704541667	13.6706778916667	13.5398526291667	13.3352775791667	13.6574902458333	13.16578005	13.1333095958333	13.9405817375	13.9630399875	13.8837850083333	13.0963431125	13.295147525	13.4153354625	13.9394971041667	13.3542137875	13.2158871875	12.8373957916667	13.1855235875	13.9835413583333	13.7906263333333	13.196301825	13.6629357875	13.9313255791667	10.3213899291667	10.3021861375	9.72679546666667	9.6174537375	10.7822440833333	8.85283100833333	10.6572385166667	9.59126800416667	8.69581604166667	10.7704596208333	9.82536999166667	8.7687455125	8.703122725	10.1968151583333	8.8976024375	8.82993020833333	11.2835686375	11.9976969958333	9.26104765833333	10.1635387833333	8.98121555	10.0767594	8.93385787083333	8.81811914166667	9.676585125	11.2249415416667	9.65908810833333	11.064992425	9.10271907083333	12.2886149583333	9.09307139166667	9.01920487083333	9.10033722916667	8.95787474583333	9.11844430833333	9.51834335	9.14167540833333	9.51154887916667	9.34459659583333	9.76070797083333	10.4322492708333	9.58456951666667	8.95695830416667	10.7263164708333	9.31785807083333	9.33558479583333	10.0991418375	9.41914157916667	9.3039005	10.4572088208333	11.2973992791667	9.34690669583333	8.3439831125	8.50577863333333	11.3200835125	10.7742107041667	11.1563163958333	8.64220767916667	9.56058135	10.9304763958333

ABI2	16.1274635083333	15.9859723583333	16.0374488333333	15.8383325666667	15.618710975	16.2193318666667	15.3944873	15.7921085416667	15.8219621166667	15.7921085416667	16.0374488333333	15.56568115	15.9493575916667	15.7066290583333	15.799582875	15.6135490416667	16.1274635083333	15.6824817166667	15.8383325666667	15.462044625	15.5556811083333	15.5313549166667	15.727394275	15.5556811083333	15.4985918916667	15.9493575916667	15.6824817166667	15.5870133916667	15.9064622666667	15.799582875	15.8904374666667	15.727394275	15.9064622666667	16.1648856916667	16.0142070583333	16.0691638333333	15.814756725	15.7198901333333	15.4672060583333	15.6896696083333	15.7697269333333	16.0258935166667	15.6410257833333	15.85627565	16.0055115333333	15.7414894	15.8470782	15.784983325	16.090774475	16.0691638333333	16.0142070583333	15.8904374666667	15.8653098666667	15.7630766833333	15.85627565	15.9859723583333	15.700189825	15.9673534416667	15.9406321083333	15.8383325666667	15.6135490416667	15.618710975	15.7486260916667	15.444211725	15.7697269333333	15.6585444166667	15.9767561166667	15.4942425166667	15.6472384833333	15.7066290583333	15.6896696083333	15.9237782	15.6037263583333	15.7486260916667	15.5975780166667	15.4942425166667	15.727394275	15.700189825	15.4112652583333	15.7138591583333	15.6711097083333	15.5603668666667	15.6087237833333	15.6087237833333	15.4852249833333	15.4852249833333	15.4160185666667	15.2981600916667	15.56568115	15.6824817166667	15.4243027666667	15.4276773083333	15.8904374666667	15.784983325	15.7138591583333	15.4852249833333	15.5870133916667	15.7781512166667	15.47595875	15.5870133916667	15.700189825	15.365952025	15.618710975	15.3777313666667	15.33292745	15.5870133916667	15.5923775583333	15.5975780166667	15.8470782	15.6711097083333	15.799582875	15.6769351666667	15.7338865416667	15.5313549166667	15.4942425166667	15.3214833666667	15.9673534416667	15.69535405	15.6824817166667	15.290119725

IGBP1	12.4046195166667	12.9726928583333	12.7046259583333	12.9659499916667	12.4474119833333	12.4337570333333	12.919046025	12.731184375	12.8256923916667	12.5159454416667	12.6810613833333	12.8398240166667	12.04824535	12.9364464083333	12.685194075	12.9300978916667	13.4345239916667	12.5173296333333	12.4202498166667	12.7263840666667	12.692926575	12.88540095	12.6306873916667	12.6991286666667	12.7939716	12.5740059166667	12.6537086583333	12.8046597666667	12.1843992666667	12.8268431583333	12.6660656916667	12.906960875	12.8010225583333	13.3538490666667	12.678040475	12.3921887583333	12.5552867333333	12.7468098833333	12.6481258583333	12.5107176416667	12.5874385	12.7602549166667	12.9117472333333	12.7139152416667	12.7500199666667	12.716481775	12.7596522	12.2302891583333	12.846546475	12.8035935833333	12.8696443333333	12.6923621166667	12.1109533333333	12.0356360333333	12.2227697083333	12.3443503083333	12.5907074583333	12.7001469666667	12.761853925	12.3331331166667	12.49908695	12.1669452	11.7053666833333	11.7568591416667	12.3645045916667	11.8997209666667	12.2429038916667	12.4050809083333	12.9733229333333	12.5472970333333	12.497697975	12.23627775	12.4532785	12.4496792916667	12.4705762833333	12.4917329166667	12.36147625	12.1193766916667	13.0483120583333	11.882283925	12.4840896416667	12.5734875583333	12.0889146833333	12.266695	12.3983699916667	12.0965982583333	12.5539148333333	12.5376322583333	12.6694372	12.4487345666667	13.2354463	12.7763784666667	11.94832135	12.4101428	12.1030545583333	13.016186275	12.2619793166667	11.67393075	12.4215906166667	12.3815660416667	12.440827975	12.4270361	12.0623925666667	12.5706160166667	12.1517212916667	11.9082126416667	11.67680025	12.4917329166667	12.2565941333333	12.5671498916667	12.8493401833333	12.0376587166667	11.5765356583333	12.9172670333333	11.7081843583333	12.072543675	11.4427661583333	12.4850765	12.3203584916667	12.5065921166667

LIPG	7.08170128333333	5.58407845	6.560129475	6.77975395833333	6.20884429166667	9.660226725	6.05307568333333	7.596603625	4.948951225	9.3832069	6.98474048333333	7.38984995833333	7.40837556666667	6.27652200833333	7.88228429166667	9.093743775	7.76512125	8.58441085833333	7.88178675	7.40602305833333	8.37604669166667	6.25755120833333	8.0294547	8.81191925833333	6.46419815833333	5.400096325	5.94185621666667	5.93821120833333	7.31493256666667	5.399029725	9.03034083333333	6.70856058333333	5.30771575	8.5905397	7.44607933333333	6.64834389166667	6.341623	8.312026725	9.30221980833333	7.38291215	8.67125495	5.50024889166667	6.738299725	7.676574425	7.09231525	8.32463409166667	6.30861273333333	9.76819061666667	8.80139795	7.514206825	8.18422995833333	9.9338367	6.82513060833333	7.39975843333333	5.03331626666667	7.13033303333333	8.57112621666667	8.46044338333333	6.53205264166667	7.71472995833333	7.659482025	9.945938775	7.13093330833333	10.1910863	8.19700369166667	10.1887239416667	9.557602025	6.8275595	10.8247142	9.615115575	7.8720714	8.50844495	11.3871722333333	8.28000224166667	6.14249028333333	7.404332775	7.98516725833333	9.23648119166667	11.5983924583333	8.56044531666667	11.0826274416667	7.26110544166667	8.2053418	8.32062069166667	7.42162103333333	9.05776881666667	10.2229494583333	7.90260113333333	5.30771575	8.67361984166667	8.821200975	8.52731504166667	8.6233491	9.12986750833333	10.1577133666667	8.33965076666667	10.4244981833333	8.85011799166667	8.63126818333333	8.79596970833333	9.00189508333333	7.66388121666667	7.35447710833333	8.024066175	11.7753980416667	7.20334935833333	9.71307581666667	9.05917338333333	8.07411434166667	10.2716281	8.584858775	7.99508275	11.0409461	8.24215483333333	8.29793538333333	8.46508935	8.937187475	7.65174983333333	9.59065653333333	8.80353201666667

RCAN3	11.951715625	12.8268431583333	12.6759474583333	12.7934592666667	12.7211045416667	12.0149070416667	12.539129975	12.5010802333333	12.895307525	12.2755744916667	12.222327475	11.13057255	12.0604174833333	12.762350675	13.00781875	12.62157875	12.3899971916667	12.7263840666667	12.9988931583333	12.857326075	12.6141732083333	12.809704275	12.5254900333333	12.2624244833333	12.8860467083333	11.671592675	12.8532684916667	12.6381216166667	13.251022725	12.9610660166667	12.3258832166667	13.2135649916667	12.5745387083333	12.6626629166667	11.2258131916667	11.9966897833333	12.6765518416667	12.8046597666667	12.3301864333333	12.547782425	12.833410325	12.8624048833333	12.8783966083333	12.533964725	12.4581088416667	12.9604577916667	13.15189735	12.7279204166667	12.4425276083333	12.6790614583333	12.5268295333333	12.840994225	12.9477864583333	13.0264905416667	12.964777525	13.0439770416667	12.692926575	12.91230815	12.8969748916667	12.9007979916667	11.2035969416667	12.0705755166667	12.9185113	12.6422229833333	12.6397345916667	11.8612030916667	12.5979621916667	11.71493805	11.6940775166667	12.3079459333333	11.2323478	12.1575174833333	12.2513034583333	11.3406807666667	12.53672075	12.7468098833333	11.4567547583333	12.4010561583333	12.0020893083333	12.5282693916667	12.5354003083333	11.5958198083333	12.2513034583333	12.5667535083333	12.1198078916667	11.8666609583333	10.8056325083333	12.425293625	11.844663475	11.48757865	11.83328595	12.2306593166667	13.0380777583333	12.100204125	12.7494695	12.1811083666667	13.2683617333333	11.3282347083333	12.1728373083333	12.8454667083333	12.2719001166667	11.4000850916667	11.9263114333333	12.584151375	11.9364860583333	12.0395951	12.042069275	11.5505559916667	12.2706199833333	12.0409411833333	12.7211045416667	12.0040290833333	12.6704503833333	12.97999865	12.0961761333333	11.958948075	13.0554708583333	11.7609104916667	12.57979945	11.9534028083333

ZNF773	10.6874862291667	10.1838838875	10.0470248916667	10.3719899625	10.4985085375	9.84137288333333	10.7868548458333	10.2776143791667	10.5366896916667	9.9198976	10.5705792208333	10.8595401083333	10.9448945083333	10.3122044125	10.3601016375	10.0613623416667	10.2872178208333	10.2685000958333	9.58017514166667	10.0427913833333	10.2784519291667	10.4559937	9.76157617083333	10.5129243375	10.11586185	10.4198102333333	10.0817346958333	10.0178064291667	10.2680768125	10.3704302833333	9.73725375416667	10.5454766	10.2332471041667	10.1399081875	9.96130747916667	10.5575313583333	10.2071167791667	9.88989740833333	10.0419486958333	10.187260375	9.12484684166667	9.8911957875	10.0236648416667	9.38691342083333	10.3919253625	9.90329901666667	10.0478853916667	9.741071475	9.86969750833333	9.6985889375	9.8983345125	10.1810332083333	10.4190041666667	10.5709527833333	10.1003480625	10.2751315166667	10.2771914166667	9.51333872083333	10.2436812791667	9.9633246625	11.1222478958333	10.2456491916667	10.0169873125	10.1938068791667	10.7003004625	10.1280952583333	10.220852275	10.716460075	10.3291694041667	10.2685000958333	10.4985085375	10.333964275	10.668915375	10.4575947458333	10.5175093875	10.1676970958333	10.5442786	10.1174184125	9.93244266666667	9.70260275	10.9814297625	11.0077341333333	9.61965483333333	10.4042775708333	10.4559937	10.3263476625	10.7690305916667	10.5458746333333	11.3877233291667	9.6378288875	9.69512940416667	9.0177592	10.3394319375	10.5587544208333	10.8167097041667	9.7201137	10.3226967125	10.582768125	10.5777799041667	10.3669158333333	10.6467227083333	10.6319745	9.86832960833333	10.1889238583333	9.93731792916667	10.3226967125	10.4051250375	10.0389253541667	10.9037622625	9.4393355875	9.95954340416667	10.3234848666667	10.6467227083333	9.30608285833333	10.0145236083333	10.5665043666667	9.89362677083333	9.9105681875	9.23116307916667	10.056385025

AC003005.1	10.6874862291667	10.1838838875	10.0470248916667	10.3719899625	10.4985085375	9.84137288333333	10.7868548458333	10.2776143791667	10.5366896916667	9.9198976	10.5705792208333	10.8595401083333	10.9448945083333	10.3122044125	10.3601016375	10.0613623416667	10.2872178208333	10.2685000958333	9.58017514166667	10.0427913833333	10.2784519291667	10.4559937	9.76157617083333	10.5129243375	10.11586185	10.4198102333333	10.0817346958333	10.0178064291667	10.2680768125	10.3704302833333	9.73725375416667	10.5454766	10.2332471041667	10.1399081875	9.96130747916667	10.5575313583333	10.2071167791667	9.88989740833333	10.0419486958333	10.187260375	9.12484684166667	9.8911957875	10.0236648416667	9.38691342083333	10.3919253625	9.90329901666667	10.0478853916667	9.741071475	9.86969750833333	9.6985889375	9.8983345125	10.1810332083333	10.4190041666667	10.5709527833333	10.1003480625	10.2751315166667	10.2771914166667	9.51333872083333	10.2436812791667	9.9633246625	11.1222478958333	10.2456491916667	10.0169873125	10.1938068791667	10.7003004625	10.1280952583333	10.220852275	10.716460075	10.3291694041667	10.2685000958333	10.4985085375	10.333964275	10.668915375	10.4575947458333	10.5175093875	10.1676970958333	10.5442786	10.1174184125	9.93244266666667	9.70260275	10.9814297625	11.0077341333333	9.61965483333333	10.4042775708333	10.4559937	10.3263476625	10.7690305916667	10.5458746333333	11.3877233291667	9.6378288875	9.69512940416667	9.0177592	10.3394319375	10.5587544208333	10.8167097041667	9.7201137	10.3226967125	10.582768125	10.5777799041667	10.3669158333333	10.6467227083333	10.6319745	9.86832960833333	10.1889238583333	9.93731792916667	10.3226967125	10.4051250375	10.0389253541667	10.9037622625	9.4393355875	9.95954340416667	10.3234848666667	10.6467227083333	9.30608285833333	10.0145236083333	10.5665043666667	9.89362677083333	9.9105681875	9.23116307916667	10.056385025

KDM4D	5.8893734	7.152063175	6.772445575	7.05225570833333	6.45472701666667	6.64559758333333	7.422156975	6.4034157	6.5305636	6.69566758333333	6.28184195833333	7.58238585833333	6.82240235833333	6.86328933333333	6.41504531666667	6.42781263333333	7.1283444	6.475087675	6.011301125	6.79131116666667	6.59744179166667	7.09617145833333	7.37123815833333	6.19454715	6.85354335	6.983493075	6.339217025	6.88184853333333	6.322468275	6.90460845	5.808344225	6.79798854166667	6.601356975	6.98280363333333	6.41191374166667	6.36029920833333	6.48357195833333	7.02653026666667	6.255916275	6.941911825	7.32319219166667	6.679903875	6.97714725	7.32668075833333	6.51382934166667	7.11789028333333	7.29732286666667	6.90649953333333	7.27829234166667	7.14272935833333	6.21280073333333	6.17226739166667	7.375280375	7.08866475	6.83400405	7.30968498333333	7.04142870833333	7.16072213333333	7.103742375	7.18452301666667	6.329205025	6.61019589166667	8.27606465	7.698105675	7.66500309166667	7.3675475	6.99033689166667	7.05585056666667	8.46196905	8.47850595	7.0469593	7.65663390833333	8.02185345833333	7.057705175	6.72519365833333	7.483605675	6.95435513333333	6.28898518333333	7.147600275	6.970355725	8.619247325	7.630419825	6.36103899166667	8.31058729166667	6.813807925	6.9998061	8.57348205	7.38522528333333	7.67359318333333	7.505130225	6.96970946666667	6.87469296666667	6.72519365833333	7.15940084166667	7.17330060833333	6.602072175	7.71472995833333	7.79244525833333	8.28445111666667	7.299574675	6.72123403333333	7.40135389166667	7.79903776666667	7.619792375	8.17555483333333	7.37059144166667	6.98280363333333	7.94329386666667	7.78057345	6.84909505	6.97095796666667	7.30805265833333	7.20911576666667	9.40373885833333	7.482471025	7.01371830833333	9.20513901666667	8.57348205	8.27954998333333	7.283866825

MAML3	5.826473825	5.73491465833333	5.50024889166667	4.822492325	6.169883075	5.28464988333333	6.65984261666667	4.70371486666667	6.4413796	5.85514745833333	5.1566631	6.06491893333333	6.23709786666667	4.79621500833333	6.8478471	4.67278611666667	5.11118121666667	6.80853135	6.70918899166667	5.50024889166667	6.68797494166667	7.44317530833333	5.173701425	4.73832164166667	6.10234676666667	4.84506578333333	6.04876668333333	6.42946571666667	7.0369697	5.30151331666667	4.65070125	5.591740875	5.51915526666667	4.59683404166667	5.46093680833333	5.68689626666667	7.31919828333333	5.29885645833333	4.936774575	6.056382625	6.07176163333333	5.5651756	6.056382625	5.29965199166667	4.6393107	5.56425255	5.14718730833333	4.80827406666667	5.67139935833333	6.33047035833333	5.01321970833333	5.78455018333333	4.55283246666667	6.29443513333333	4.99794086666667	4.59071941666667	4.73110184166667	5.14192449166667	5.764437375	5.77807068333333	5.08458449166667	4.74641638333333	5.90235153333333	5.1598283	5.07656036666667	5.62674555833333	6.55145231666667	4.05627571666667	4.24130721666667	4.90147810833333	4.736033375	5.57335978333333	4.89827923333333	4.28696936666667	5.51585541666667	4.77601478333333	5.53586285833333	5.84412998333333	4.85550948333333	5.40750829166667	6.33187936666667	6.83400405	4.62587865833333	4.768670525	5.84884216666667	4.77483110833333	4.95206471666667	4.47861730833333	6.16914264166667	5.5907794	4.506429475	5.49632836666667	4.61520515	4.62587865833333	5.24195544166667	5.03726580833333	5.1291031	5.70932124166667	4.26116580833333	5.27315905833333	4.1018927	4.428719425	4.93436960833333	5.01216298333333	4.16939595	5.45476871666667	5.843159875	5.9751687	4.14053975833333	6.63942924166667	4.74219220833333	5.25047555833333	4.2422894	5.1863758	4.332336275	4.268577875	4.97812506666667	4.39770036666667	4.49904255833333	5.67523288333333

L3MBTL3	4.42666711666667	4.048345425	3.99930385833333	4.15118036666667	3.5983881	4.18603395833333	4.48925233333333	3.61765856666667	3.7304451	3.81713595	3.82170636666667	3.37109355	3.64321401666667	4.17138054166667	4.222209325	3.8761543	4.41392473333333	3.764611825	4.546642225	3.87937155833333	3.87506608333333	3.9661014	4.31516280833333	4.41962694166667	4.67787883333333	4.06079156666667	4.30624979166667	4.55671806666667	4.65382089166667	4.685722125	4.60088621666667	4.46877066666667	3.867233575	4.08851591666667	4.38005901666667	4.08322809166667	4.45030740833333	4.75143253333333	4.52791141666667	4.48432390833333	6.07858055833333	4.55671806666667	4.81376534166667	5.38263135833333	4.30437938333333	4.9426219	5.09217729166667	4.82580888333333	4.54864098333333	4.79906281666667	4.747391	4.42666711666667	3.86842555	6.00459881666667	4.33328629166667	4.6247727	3.87726464166667	4.71899701666667	4.36796431666667	4.99137435	3.81123611666667	3.54762483333333	4.322875175	3.91806175	3.512837625	3.70325111666667	5.47616439166667	3.36303483333333	3.93478860833333	3.512837625	3.49683135	3.64795303333333	3.437766125	3.63546845	3.41750548333333	3.63913164166667	3.42434035833333	3.812622975	3.79653381666667	3.58562625	3.656722575	3.53753678333333	4.23701905833333	3.84584678333333	3.810060775	3.511408425	3.75717131666667	4.52697115833333	3.65195390833333	4.04622350833333	3.75717131666667	4.44254745	4.07353555833333	3.81819714166667	4.53824951666667	3.94009973333333	3.99064151666667	4.47089620833333	4.08637315833333	4.68681869166667	4.30027178333333	3.50417281666667	4.78586035833333	5.01629245833333	4.36388495833333	3.97264301666667	4.63625878333333	6.15447426666667	4.310185775	6.49390590833333	4.60485635	5.255540825	4.60382705833333	5.360915675	4.12677783333333	4.332336275	4.20571450833333	4.50312849166667	4.68681869166667	3.61894206666667

GLG1	6.77436175	6.32527860833333	6.558117975	6.323145675	6.62563488333333	6.225217	6.46851783333333	6.32668329166667	6.44430819166667	5.53695673333333	6.51078761666667	6.42033848333333	6.728950375	6.320274625	7.24330025	6.22080705	6.23229269166667	7.210345825	6.37568504166667	6.78381769166667	6.633116075	6.986635125	6.71047270833333	6.9604848	6.60536920833333	6.10954496666667	7.32376110833333	7.47725403333333	7.35924831666667	6.83868629166667	6.72953939166667	7.09804688333333	6.41504531666667	6.48357195833333	6.438674975	6.056382625	7.14581168333333	6.72653388333333	6.49247578333333	6.82704920833333	6.637274925	6.56145719166667	6.37643818333333	6.45472701666667	5.97682351666667	5.98999241666667	6.59052684166667	6.26504835	6.49608473333333	6.76378944166667	6.65156973333333	6.74821494166667	6.61415903333333	6.909113525	6.41739583333333	6.39012523333333	6.26279403333333	6.1777684	6.69439601666667	6.41577015833333	8.40817908333333	6.126012425	7.02148474166667	5.9191077	6.81852565833333	7.77550013333333	7.00693255	6.70796138333333	6.30861273333333	6.28034841666667	6.69642741666667	7.35924831666667	6.56733945833333	7.53565475833333	7.019638175	6.165146375	6.52564285833333	7.51310406666667	6.89003731666667	7.35983868333333	5.5651756	6.76587266666667	7.093633575	6.771060625	7.20747220833333	6.89915848333333	6.41739583333333	6.50860958333333	7.20393616666667	6.46133099166667	6.44430819166667	7.794082525	6.65984261666667	6.80449616666667	6.97276299166667	6.99727938333333	6.51310794166667	7.49442741666667	7.41061439166667	6.28034841666667	7.59420499166667	6.241933725	6.80918875	6.5607394	7.16614005833333	6.71655181666667	6.8190902	6.08509065	5.90321861666667	6.65156973333333	6.35132606666667	6.33264478333333	6.48785131666667	6.78446141666667	6.74746250833333	6.33852943333333	5.808344225	7.08487659166667	7.38522528333333	6.3064348

MYT1L	9.15449361666667	9.0772993	9.29702861666667	9.34481250833333	9.71188024166667	8.99720454166667	9.74218338333333	9.11737229166667	9.67544948333333	9.298425025	9.4687509	9.75110441666667	9.565921775	8.955351225	9.36848635	8.87883045833333	9.5040294	9.62712280833333	9.093743775	9.07865619166667	8.80300369166667	8.94049991666667	8.85676560833333	8.58601561666667	8.75703444166667	9.037729975	8.9489102	8.96700605833333	9.230932875	9.29702861666667	9.18016484166667	9.0180261	9.40511030833333	9.71864728333333	9.45194998333333	9.36572414166667	9.46919775	9.39113676666667	9.408133425	9.212117675	9.04890688333333	9.166824	8.94100855	8.97769849166667	8.8364013	8.91224634166667	8.96510433333333	9.20045154166667	8.82473405	8.98325939166667	9.26948546666667	9.286424975	9.44105665833333	9.37686731666667	9.46121264166667	9.16396135833333	9.235078425	9.41030455	9.11582745	8.8912541	9.46432354166667	9.41162929166667	9.20693985	9.51088574166667	9.21882504166667	9.46704600833333	9.69308664166667	9.42547525833333	9.69219855833333	9.54178184166667	9.65399733333333	9.86854744166667	9.810895475	9.17190161666667	9.62407518333333	9.43636521666667	9.311763425	9.29664133333333	9.189315625	9.29933239166667	9.00735385	9.03284314166667	9.090969825	8.91790284166667	9.22160494166667	9.26261210833333	9.3195178	9.2612694	9.43184414166667	8.849091575	9.3036884	9.439564175	9.45990046666667	9.43868498333333	9.92625621666667	9.23186806666667	9.497335375	9.83944360833333	9.84332440833333	9.295254925	9.36084673333333	9.49191133333333	9.45595239166667	9.06270968333333	9.250740625	9.126505725	9.20235228333333	9.25349141666667	9.166351	8.88592250833333	9.093743775	9.39025219166667	9.557602025	9.47404223333333	9.62064585	9.24712485	9.32526180833333	9.60540180833333	9.39392068333333	9.39158905833333

HGD	8.488931425	9.6433763	9.22874626666667	9.23462919166667	9.379662375	8.16931455833333	9.33496756666667	9.45374055	9.00093885833333	9.54731913333333	9.135601825	7.97719954166667	8.9131846	9.47351875833333	9.49325940833333	8.64093518333333	8.74130064166667	9.25394765	9.31809370833333	9.37588805	9.34018350833333	9.90137155	9.06886545833333	9.577771075	9.28361398333333	8.16735940833333	9.55288966666667	9.62367923333333	9.495092775	9.512679675	9.345810075	9.84631699166667	9.22470684166667	7.7783407	8.49244585833333	8.155034325	9.24218355833333	9.76179089166667	9.37177325833333	9.75367038333333	9.64783695	9.9399775	9.94164523333333	9.53356816666667	8.23520641666667	9.50539336666667	9.55333915	9.620263	9.23005591666667	8.65271366666667	9.89553576666667	9.748599325	8.55907896666667	9.19118228333333	9.80927415833333	9.52364006666667	9.513102275	9.813959625	9.175625125	9.26722199166667	7.30805265833333	7.95746913333333	8.45404219166667	8.83154074166667	6.74888376666667	9.97583196666667	9.66237621666667	8.31814086666667	9.43324608333333	8.73063873333333	8.3851619	8.3657693	9.70407686666667	8.5196153	9.20732398333333	9.53398021666667	8.486570275	8.86999053333333	9.446046	8.18205496666667	7.634487825	7.67307831666667	9.16074203333333	8.34324809166667	8.76421910833333	8.32603026666667	8.25081844166667	7.60592470833333	8.30178113333333	8.647921775	8.98146720833333	7.92437408333333	7.26626465	9.52364006666667	9.90608706666667	8.76751969166667	8.17302170833333	8.07572273333333	9.16495244166667	7.59366683333333	9.02175539166667	8.49794376666667	7.38123391666667	9.25764194166667	9.42643701666667	9.116355225	8.91506995	9.529086375	8.57301913333333	9.32231320833333	8.41428779166667	8.87543329166667	9.8813009	10.27531875	9.132749475	8.4237182	7.46688233333333	9.047926775	9.309548625	8.69791309166667

GPR82	8.59226435416667	8.61069705	8.29424354166667	8.39234492083333	8.68059544583333	8.75256491666667	7.4765078625	7.650219825	7.337056625	7.78411079166667	8.92293245	8.5780880875	8.2678169625	8.34101024166667	8.5270728125	9.55695158333333	8.712140675	9.43113745833333	8.94454945	8.90986637916667	9.242457125	9.14759198333334	9.6545943125	8.709664	9.06575983333333	8.67815312083333	9.35524244166667	9.39774012083333	9.40353525	9.19463017083333	8.1764082625	9.50935463333333	9.03572357916667	9.3169059625	8.9596737	9.1779438625	9.60433155833333	9.22582645416667	9.24339687083333	8.94075423333333	9.01920487083333	8.6300921	9.52480252916667	8.69632065	8.91110644583333	9.2819927625	9.0154403625	8.48978630833333	10.3110616	9.5518241375	9.27749674583333	8.69819049583333	9.11291737083333	8.7946461125	8.56247706666667	8.7469528375	8.88955019583333	9.4018361375	9.43301723333333	9.62475257916667	10.4754060833333	8.7727191	8.73620710833333	8.9026360875	7.9366852	9.6409643875	8.34692658333333	8.79571160416667	8.10663690416667	8.81355715833333	9.45398310416667	9.4663284375	7.90653880416667	9.10772999583333	8.07078570416667	9.25417682916667	10.2508841708333	10.1434765916667	8.95512869583333	9.21518684583333	8.011938625	8.70720409583333	9.046684725	8.19669824583333	9.38875120416666	8.22665592916667	9.77412685416667	9.29771744583333	10.4864721541667	9.88904460416667	8.9964834625	10.0212078833333	9.5393581125	9.4000792	11.2295922333333	10.7996179	10.3445012416667	10.5195056541667	8.56691205416667	9.24864904166667	9.187626225	8.71581307916667	9.97646281666667	8.28072739583333	9.42972139583333	10.1575282291667	9.1941644625	8.2826630625	10.0191618083333	9.6662720625	9.65908810833333	9.16468879583333	9.67076629583333	8.52222418333333	8.83989808333333	9.52064974583333	8.3420830875	9.76288985416667	9.557397525	9.47428780833333

CASK	8.59226435416667	8.61069705	8.29424354166667	8.39234492083333	8.68059544583333	8.75256491666667	7.4765078625	7.650219825	7.337056625	7.78411079166667	8.92293245	8.5780880875	8.2678169625	8.34101024166667	8.5270728125	9.55695158333333	8.712140675	9.43113745833333	8.94454945	8.90986637916667	9.242457125	9.14759198333334	9.6545943125	8.709664	9.06575983333333	8.67815312083333	9.35524244166667	9.39774012083333	9.40353525	9.19463017083333	8.1764082625	9.50935463333333	9.03572357916667	9.3169059625	8.9596737	9.1779438625	9.60433155833333	9.22582645416667	9.24339687083333	8.94075423333333	9.01920487083333	8.6300921	9.52480252916667	8.69632065	8.91110644583333	9.2819927625	9.0154403625	8.48978630833333	10.3110616	9.5518241375	9.27749674583333	8.69819049583333	9.11291737083333	8.7946461125	8.56247706666667	8.7469528375	8.88955019583333	9.4018361375	9.43301723333333	9.62475257916667	10.4754060833333	8.7727191	8.73620710833333	8.9026360875	7.9366852	9.6409643875	8.34692658333333	8.79571160416667	8.10663690416667	8.81355715833333	9.45398310416667	9.4663284375	7.90653880416667	9.10772999583333	8.07078570416667	9.25417682916667	10.2508841708333	10.1434765916667	8.95512869583333	9.21518684583333	8.011938625	8.70720409583333	9.046684725	8.19669824583333	9.38875120416666	8.22665592916667	9.77412685416667	9.29771744583333	10.4864721541667	9.88904460416667	8.9964834625	10.0212078833333	9.5393581125	9.4000792	11.2295922333333	10.7996179	10.3445012416667	10.5195056541667	8.56691205416667	9.24864904166667	9.187626225	8.71581307916667	9.97646281666667	8.28072739583333	9.42972139583333	10.1575282291667	9.1941644625	8.2826630625	10.0191618083333	9.6662720625	9.65908810833333	9.16468879583333	9.67076629583333	8.52222418333333	8.83989808333333	9.52064974583333	8.3420830875	9.76288985416667	9.557397525	9.47428780833333

PRODH	9.58706739166667	11.5954858333333	11.6902780833333	11.8418826916667	11.509666625	10.159727225	12.1847949166667	11.14881755	12.0088601416667	11.2527308083333	11.6190781833333	11.5656597	11.6092244333333	11.545842925	11.1949025333333	10.7971951666667	9.855976425	11.9653249	11.118464025	10.9430329583333	11.1343238833333	11.4897906583333	10.8646445083333	10.95605295	11.2254735166667	9.9162906	10.92809195	11.5089022	11.3043532	11.17143095	10.9157145083333	11.0744094166667	11.2833533833333	10.1409369083333	9.57903839166667	9.40419091666667	11.376011025	11.1821084666667	10.8382628916667	10.9854722416667	11.0072108	10.8666003833333	11.2534278333333	11.1866301583333	10.7383339416667	10.9028824333333	10.8657939916667	10.8232399083333	11.9228391333333	10.7743973416667	10.9433662916667	10.9618387333333	11.0510672583333	11.3846584083333	11.1724975333333	11.4354157666667	11.2085666916667	11.5352724166667	11.2198620833333	10.4713528083333	11.80928975	11.0491091083333	11.4549596	12.2840300666667	11.2815281916667	10.9567732916667	10.872956825	13.320797425	10.2974881833333	11.0556311333333	12.0261845416667	12.1367818666667	12.5696588166667	10.43975535	12.1690316666667	11.1866301583333	14.4705790833333	10.8499191666667	12.8187062583333	11.9085787666667	11.3957857333333	11.48024075	11.6951784416667	11.383891075	11.392737725	11.24709275	10.7748204	10.9545739416667	11.6294117916667	10.199031925	10.6507559	11.1277733333333	11.8772191666667	12.466874775	10.4806526833333	12.161623975	12.1488811083333	10.5692188416667	11.367946675	12.5145301166667	11.0261256666667	12.563781325	11.5601850333333	11.531296025	12.713417125	10.3975849666667	10.132057125	11.1477174666667	10.8243180916667	11.1752905	13.776083375	12.42337295	10.4340765083333	9.66558844166667	10.2822548833333	11.6883557166667	10.6395867	11.2646484083333	12.4069098166667	12.03297995

AC007326.4	9.58706739166667	11.5954858333333	11.6902780833333	11.8418826916667	11.509666625	10.159727225	12.1847949166667	11.14881755	12.0088601416667	11.2527308083333	11.6190781833333	11.5656597	11.6092244333333	11.545842925	11.1949025333333	10.7971951666667	9.855976425	11.9653249	11.118464025	10.9430329583333	11.1343238833333	11.4897906583333	10.8646445083333	10.95605295	11.2254735166667	9.9162906	10.92809195	11.5089022	11.3043532	11.17143095	10.9157145083333	11.0744094166667	11.2833533833333	10.1409369083333	9.57903839166667	9.40419091666667	11.376011025	11.1821084666667	10.8382628916667	10.9854722416667	11.0072108	10.8666003833333	11.2534278333333	11.1866301583333	10.7383339416667	10.9028824333333	10.8657939916667	10.8232399083333	11.9228391333333	10.7743973416667	10.9433662916667	10.9618387333333	11.0510672583333	11.3846584083333	11.1724975333333	11.4354157666667	11.2085666916667	11.5352724166667	11.2198620833333	10.4713528083333	11.80928975	11.0491091083333	11.4549596	12.2840300666667	11.2815281916667	10.9567732916667	10.872956825	13.320797425	10.2974881833333	11.0556311333333	12.0261845416667	12.1367818666667	12.5696588166667	10.43975535	12.1690316666667	11.1866301583333	14.4705790833333	10.8499191666667	12.8187062583333	11.9085787666667	11.3957857333333	11.48024075	11.6951784416667	11.383891075	11.392737725	11.24709275	10.7748204	10.9545739416667	11.6294117916667	10.199031925	10.6507559	11.1277733333333	11.8772191666667	12.466874775	10.4806526833333	12.161623975	12.1488811083333	10.5692188416667	11.367946675	12.5145301166667	11.0261256666667	12.563781325	11.5601850333333	11.531296025	12.713417125	10.3975849666667	10.132057125	11.1477174666667	10.8243180916667	11.1752905	13.776083375	12.42337295	10.4340765083333	9.66558844166667	10.2822548833333	11.6883557166667	10.6395867	11.2646484083333	12.4069098166667	12.03297995

DGCR6	9.58706739166667	11.5954858333333	11.6902780833333	11.8418826916667	11.509666625	10.159727225	12.1847949166667	11.14881755	12.0088601416667	11.2527308083333	11.6190781833333	11.5656597	11.6092244333333	11.545842925	11.1949025333333	10.7971951666667	9.855976425	11.9653249	11.118464025	10.9430329583333	11.1343238833333	11.4897906583333	10.8646445083333	10.95605295	11.2254735166667	9.9162906	10.92809195	11.5089022	11.3043532	11.17143095	10.9157145083333	11.0744094166667	11.2833533833333	10.1409369083333	9.57903839166667	9.40419091666667	11.376011025	11.1821084666667	10.8382628916667	10.9854722416667	11.0072108	10.8666003833333	11.2534278333333	11.1866301583333	10.7383339416667	10.9028824333333	10.8657939916667	10.8232399083333	11.9228391333333	10.7743973416667	10.9433662916667	10.9618387333333	11.0510672583333	11.3846584083333	11.1724975333333	11.4354157666667	11.2085666916667	11.5352724166667	11.2198620833333	10.4713528083333	11.80928975	11.0491091083333	11.4549596	12.2840300666667	11.2815281916667	10.9567732916667	10.872956825	13.320797425	10.2974881833333	11.0556311333333	12.0261845416667	12.1367818666667	12.5696588166667	10.43975535	12.1690316666667	11.1866301583333	14.4705790833333	10.8499191666667	12.8187062583333	11.9085787666667	11.3957857333333	11.48024075	11.6951784416667	11.383891075	11.392737725	11.24709275	10.7748204	10.9545739416667	11.6294117916667	10.199031925	10.6507559	11.1277733333333	11.8772191666667	12.466874775	10.4806526833333	12.161623975	12.1488811083333	10.5692188416667	11.367946675	12.5145301166667	11.0261256666667	12.563781325	11.5601850333333	11.531296025	12.713417125	10.3975849666667	10.132057125	11.1477174666667	10.8243180916667	11.1752905	13.776083375	12.42337295	10.4340765083333	9.66558844166667	10.2822548833333	11.6883557166667	10.6395867	11.2646484083333	12.4069098166667	12.03297995

MLIP	4.823565325	6.44595796666667	5.06351158333333	6.45250163333333	8.532903275	7.51089621666667	7.68593156666667	6.33047035833333	6.74158396666667	7.12901885	6.83020955	7.30133825	6.92168505833333	7.17735376666667	8.41158014166667	6.42634760833333	5.56781358333333	7.227778225	7.34669549166667	6.99033689166667	6.47216713333333	8.536875925	6.84260019166667	6.59883068333333	6.662431575	5.079599575	7.5998906	7.27410720833333	8.01905775833333	6.811853775	5.54102934166667	7.09617145833333	6.37197970833333	5.84884216666667	5.091030975	5.85609921666667	8.551483425	7.44885958333333	6.22001866666667	7.17735376666667	6.11703395833333	6.63866813333333	6.61558093333333	6.88314723333333	6.55061969166667	6.71655181666667	6.22149245833333	6.90123264166667	7.59600343333333	6.4975584	6.48151946666667	7.16232548333333	5.65913218333333	6.91388091666667	6.195909975	5.36511784166667	7.19636080833333	5.71218308333333	7.47258725833333	6.99282755	7.49549410833333	5.90945328333333	5.79119435	5.84513776666667	6.29822925	7.09424611666667	6.245275725	5.83589014166667	4.75698904166667	5.87100729166667	6.75503975833333	6.50142603333333	6.56431505	7.210345825	8.64585098333333	6.493249575	5.33329914166667	7.57457279166667	6.85478974166667	9.19244325833333	5.67523288333333	6.45093723333333	7.07980634166667	6.18408195	6.96399793333333	5.4913896	5.33648908333333	5.99418615	7.306684875	5.20882029166667	5.65064866666667	6.44430819166667	4.740346475	6.3378065	5.62674555833333	7.47384185833333	7.46746676666667	4.434278525	5.54491926666667	5.33746485833333	4.82464924166667	4.50222558333333	5.10290811666667	7.134785875	7.41526463333333	6.38084359166667	6.07858055833333	6.62969040833333	5.26362051666667	6.783129725	5.451608675	6.78114418333333	6.256698975	5.78909119166667	5.70932124166667	5.93571900833333	5.162070825	5.61547525833333	8.8559255	6.8752982

IGF2BP1	4.40581835	4.68477714166667	4.705709175	4.53219865	3.89132465	4.290980325	4.05627571666667	4.41002290833333	4.55568075	4.80533786666667	4.49312724166667	3.62256374166667	3.54762483333333	4.25573735	4.644124975	3.76094594166667	4.13313858333333	4.2592488	5.13745513333333	4.612218	4.05192228333333	4.17373291666667	4.48925233333333	3.66804375833333	5.47416700833333	4.27798759166667	5.39804303333333	5.50523408333333	4.91752975833333	5.20060255	5.72886108333333	4.98892909166667	4.79906281666667	4.535343	5.77716973333333	5.2891249	5.82555810833333	6.44063283333333	6.16277881666667	6.44000804166667	5.93571900833333	5.31319136666667	5.2617065	5.70253175	4.59997769166667	4.15432630833333	4.979047925	4.87710051666667	5.69385626666667	5.23259211666667	6.43016624166667	6.01215780833333	4.30917759166667	5.57834849166667	5.2151974	5.60487774166667	4.98771553333333	6.30572190833333	5.53102171666667	5.71882016666667	4.768670525	3.9601097	5.46278851666667	5.03526733333333	3.75125179166667	6.05474264166667	4.510676125	3.94456669166667	6.83341489166667	6.13259841666667	4.26116580833333	3.95252660833333	3.80554890833333	3.82170636666667	4.70905974166667	3.99064151666667	4.289092625	4.341768725	4.602780325	5.08063435	3.78548855	3.82070373333333	5.6322886	4.18191081666667	4.289092625	6.25335921666667	7.31739324166667	3.671417225	4.23701905833333	4.617309275	4.85550948333333	7.31254506666667	4.4619761	5.17905539166667	6.50650844166667	6.48862780833333	5.71985138333333	8.58140188333333	5.23566219166667	7.51310406666667	7.38233871666667	4.3276422	3.90095365833333	4.74930484166667	6.691149875	4.51861790833333	4.25005865	4.07678143333333	5.58795861666667	4.58881544166667	5.03010108333333	5.88673599166667	6.60603940833333	7.2313303	5.44029105833333	4.84506578333333	6.37113890833333	7.663341325	5.979616925	4.27374416666667

AP3M2	10.70466055	10.248995	10.6360463166667	10.4883478333333	10.2225656916667	10.2466873083333	10.44259115	10.0561858166667	10.239527275	9.40727955833333	10.971110725	10.5892734666667	10.2051938916667	10.4801853	10.035165975	10.5142106583333	10.2206735166667	9.56961935	10.3147212916667	10.35148425	10.4260988583333	9.87439875	10.4678765666667	10.29990335	10.133368525	10.5432527416667	10.2182610833333	9.77219498333333	9.20872513333333	10.1617358916667	9.824729	10.3534141083333	10.061572075	10.2108003333333	10.534888275	10.6686985416667	9.96824564166667	9.82651539166667	9.909962375	10.0557486666667	10.306601825	10.0039229916667	10.30576505	10.3388359916667	10.4975657166667	10.6816359083333	10.647327775	9.63342665833333	10.18161685	10.641902875	10.4384998666667	10.2786656166667	9.750361275	10.2579212583333	10.3542362916667	10.4819116333333	10.1775011166667	10.0627631	9.861824025	10.1553380833333	11.25880145	11.5349136916667	10.3983831166667	10.601173475	11.7262372833333	10.276116525	10.6600165833333	10.9647570833333	10.8861829666667	11.0301443333333	11.6252518666667	12.4015640666667	10.010773575	11.044463875	11.2464355333333	10.9516391	11.9116934166667	10.3817731416667	10.264650175	11.2627928333333	10.1678889166667	11.285520075	10.4550319166667	11.0075637333333	10.3745792583333	10.7673403166667	11.8453541	10.7104085166667	10.992544275	10.9393869333333	10.953117425	11.4115295083333	9.93000265833333	10.6111916083333	10.7812240416667	11.1046868	10.638037975	11.5776190666667	9.98715419166667	10.9961958083333	11.0390903416667	11.21620365	11.0992950083333	11.6084924916667	11.102188775	10.3698580416667	10.7885218833333	10.9987091583333	10.741834125	10.5902797083333	12.0604174833333	10.137471025	9.76219373333333	12.00595835	10.2567002166667	11.1014833833333	11.8228949916667	11.0863287333333	10.601543225	9.93555589166667

EPHA7	15.85627565	8.898281125	8.620261275	9.732285275	8.68329548333333	11.7985917083333	8.451050725	8.259343925	9.04596135	9.31762243333333	9.023211225	13.8635959083333	13.956539975	9.004183075	7.3361665	10.0382960666667	11.5887304416667	7.69541339166667	12.3562743916667	9.09460495	9.12506650833333	7.14399110833333	8.398242975	9.619021525	9.4687509	15.8470782	9.28405401666667	8.80003964166667	8.87930013333333	9.222045675	8.01539524166667	8.71940105833333	9.33708134166667	10.7470371333333	15.7697269333333	15.6585444166667	8.028895175	9.07021043333333	8.44287714166667	8.19188733333333	8.70212718333333	7.84998155	9.50499393333333	8.31561141666667	13.82498885	8.62547046666667	8.74620478333333	8.70034938333333	9.25349141666667	10.1974084916667	9.091403125	8.82781555833333	8.43351543333333	9.35167163333333	8.44239195833333	9.42244939166667	8.18684245	8.41428779166667	9.259928725	9.53043561666667	9.68734411666667	10.5022046916667	11.4474681583333	11.8947334916667	13.9045177166667	7.41243761666667	10.674973575	9.80187509166667	6.39918578333333	8.97354856666667	10.1063080916667	8.13819046666667	7.72686941666667	11.7016218	6.84200789166667	7.34669549166667	12.4377746666667	7.745650475	7.34892271666667	11.9597302583333	14.5089658583333	13.4383119666667	5.95923255	8.56622091666667	9.6433763	10.1316676583333	9.49413000833333	11.3378344416667	7.650219825	12.1130081666667	6.33852943333333	10.0924722666667	12.1451222666667	10.3211494083333	9.38622159166667	10.039605625	12.24842175	9.1396151	7.93918533333333	8.90005820833333	9.50224903333333	10.64689975	10.4292877666667	12.11543585	7.41942373333333	11.1166050833333	9.565041475	8.31561141666667	9.47351875833333	11.3683256166667	6.62102673333333	9.97912404166667	6.44355459166667	6.15113656666667	7.306684875	10.0778458	9.00551523333333	5.43388225	9.87864789166667	11.03193485

HS6ST3	4.86804739166667	6.26279403333333	5.53292085833333	5.74704205	6.49464226666667	4.234848125	7.314288875	6.85045274166667	6.757758475	4.98341465833333	5.897311975	7.08427549166667	7.040686725	5.88853345	6.00968073333333	5.53292085833333	5.38157649166667	6.05897065833333	4.31116040833333	5.98559190833333	5.85701861666667	4.95322815833333	5.08063435	4.649483075	4.13647209166667	5.15140121666667	4.8815265	3.971604825	4.64133359166667	4.537079425	4.50544710833333	4.84301391666667	5.3439553	5.25233420833333	4.344051675	4.83457635833333	4.602780325	4.54330015	4.26758835	3.82817183333333	4.48613384166667	4.792098225	4.251891475	5.29675638333333	4.210787125	4.28008616666667	4.31702865	4.19044525833333	4.36294506666667	4.27160165833333	4.199161275	4.28809964166667	5.11789035833333	4.54864098333333	4.39039918333333	4.21395365833333	6.71502808333333	4.13218385833333	4.149223425	4.21592141666667	4.86589074166667	6.03052256666667	4.4047953	6.58499179166667	5.20550439166667	5.16938190833333	6.88940976666667	7.3477901	4.30027178333333	6.01590590833333	6.89785889166667	5.64406685833333	7.41704166666667	5.99418615	6.861457125	6.62102673333333	5.12814335833333	4.32670648333333	6.4308335	5.03526733333333	8.903371	5.82555810833333	4.771681175	3.99930385833333	6.19129395	5.23035095	6.26140625833333	5.983976325	6.1039579	4.95105651666667	5.08458449166667	5.08161569166667	4.55863708333333	4.955313625	4.22036293333333	5.59479534166667	4.77601478333333	4.46293625833333	5.566976475	5.40852341666667	4.45602740833333	5.57834849166667	4.68073703333333	4.46100053333333	4.41962694166667	6.4413796	4.42666711666667	4.52327248333333	4.76349515	4.879391675	4.66334196666667	5.20995024166667	4.21300761666667	5.03120425833333	4.71793841666667	4.576370125	4.27374416666667	4.53105006666667	4.87595029166667	6.67377979166667

PLXNB2	12.8284427416667	13.570433175	13.420218375	13.5535340083333	13.3754869916667	13.26088015	12.9699766666667	12.9586834166667	13.3200251	13.1229606833333	13.3256142916667	12.8323870333333	13.2339414916667	13.7321168833333	13.5346846166667	13.2175064583333	13.917581325	13.2815171083333	13.6028841416667	13.3390509833333	13.28687105	13.7234378333333	13.44358445	13.1576233666667	13.4260804333333	13.1563255166667	13.5034733	13.4407112833333	13.6116942166667	13.5626218333333	13.541969925	13.5157896583333	13.2354463	13.4642437583333	12.8481618333333	13.2580457083333	13.4752691916667	13.3459381666667	13.4807854416667	13.2861830833333	13.3067028166667	13.4594952083333	13.3710865833333	13.4065910333333	13.2116333	13.4390716416667	13.5956278166667	13.518865875	13.8261646416667	13.2738329583333	13.309401	13.3645725083333	13.661470025	13.7532714333333	13.5034733	13.71864895	13.0584991666667	13.336335625	13.4321406333333	13.4474018083333	13.9445631916667	13.0743927916667	14.1099143166667	14.2998455083333	13.6202060083333	14.0263077083333	13.1525120666667	13.8983181916667	13.3356032833333	13.6551549916667	13.450467225	13.483275325	13.8832091583333	13.2738329583333	14.044199175	13.91423525	14.1857941	13.695137875	13.8983181916667	13.3508640083333	13.5213793583333	13.489460875	13.5781398666667	13.7168412916667	13.454214775	13.3689321166667	13.0626219916667	13.788062875	13.2135649916667	13.461932225	13.6418962083333	13.6737470333333	13.2254010416667	13.738346	13.7740900916667	13.916524275	13.60723025	13.8698539083333	13.2912832916667	14.1175943833333	13.913193025	13.462725925	13.798249925	13.3285445333333	13.9207353166667	13.8635959083333	13.9727862416667	13.3080939833333	13.9217501083333	14.451178225	13.9518039333333	14.312396275	13.7404495916667	13.5502258583333	13.90344385	13.4550317916667	14.4459919583333	13.6165726333333	13.576244975	13.71577945

TPST1	7.76608003333333	6.93227245	6.82383751666667	6.64834389166667	6.258372575	6.64764070833333	6.1186989	6.4516666	6.2073137	6.07085139166667	6.33714610833333	7.04821105833333	6.234662425	6.28336628333333	7.445489275	6.74474600833333	6.71995881666667	6.872774725	7.306684875	6.72653388333333	7.129719725	7.195709975	6.5766165	6.99155748333333	6.79529673333333	6.51382934166667	6.75170075833333	7.45614546666667	7.147600275	6.39606678333333	7.193910325	6.75503975833333	6.73133699166667	6.64226356666667	7.56216311666667	6.95984925	6.99601435833333	6.97340769166667	5.97260084166667	6.70667168333333	7.35983868333333	7.67307831666667	7.37590343333333	7.73298270833333	6.79468460833333	7.35226640833333	7.26165935833333	7.01426715833333	7.31377455	7.26808264166667	6.93227245	6.47718199166667	6.46923394166667	6.92501696666667	6.48645600833333	7.48985504166667	5.96077145833333	6.98588438333333	7.49164054166667	7.013155025	7.160095	7.72294644166667	7.246381175	6.987286825	6.810578	7.882902775	8.64496134166667	7.112144375	6.55593524166667	5.77633328333333	6.29057105	7.28556258333333	6.09195375	5.7745543	7.07353054166667	6.195909975	6.61154091666667	6.891986525	6.98215429166667	6.86262681666667	7.43473675	7.30497195	6.62158961666667	9.25719764166667	6.29278439166667	6.66317971666667	7.62704748333333	5.35088976666667	6.39681019166667	6.39918578333333	6.12282240833333	5.93923476666667	7.73639724166667	8.25189414166667	8.56272661666667	9.37921308333333	7.16614005833333	8.561306875	6.30714161666667	6.5005932	6.79923555	6.99033689166667	7.22238968333333	9.93343423333333	8.18151525833333	7.75983114166667	8.25292731666667	6.96330954166667	6.94120649166667	7.39105859166667	7.87659344166667	8.07889375	7.06834181666667	7.41352716666667	7.70199845833333	8.24367163333333	8.04234110833333	8.1455177	6.56951250833333	5.778884625

ASB11	8.25692778333333	8.02185345833333	8.21622873333333	7.851546575	7.978659025	7.53401704166667	7.80873386666667	7.75176951666667	7.73985964166667	8.001514325	8.061842725	8.68624993333333	8.10330963333333	7.507361725	7.88018380833333	7.49549410833333	11.9071135	8.87390605	7.525066825	7.03194930833333	7.51814899166667	7.51310406666667	7.60415978333333	7.8967227	8.026220775	8.13871308333333	8.457931075	8.24270349166667	7.63385609166667	7.92602010833333	7.44885958333333	7.51365925	7.59144205833333	9.55990681666667	7.61698044166667	7.67926596666667	8.119922525	7.72139515	8.15349528333333	7.54021029166667	6.87219209166667	7.34718618333333	7.6157713	7.23727353333333	6.93091866666667	6.983493075	6.86530675833333	7.32376110833333	7.0659289	7.803872825	7.30133825	8.13415403333333	7.931728325	7.1950993	7.46746676666667	6.850945825	6.48010203333333	7.147600275	8.03376271666667	7.11986829166667	8.548453575	8.60799398333333	8.50502355	8.13242494166667	7.90139170833333	8.61342080833333	7.5367911	8.23717023333333	7.816692275	8.33112569166667	8.499322875	8.40614324166667	8.13819046666667	8.101615825	8.55955131666667	8.00800819166667	8.97675146666667	8.54796775833333	7.908404275	7.71678190833333	7.58128390833333	7.77321380833333	8.19188733333333	7.75930169166667	8.38217830833333	8.22741431666667	8.15060805	8.38918200833333	8.82265860833333	7.84430019166667	7.782192425	8.48950848333333	7.798604875	7.51524328333333	8.53882584166667	8.160175425	8.14709130833333	8.38072273333333	8.335541375	7.933308825	7.89403294166667	8.08807009166667	8.43662665833333	7.873129975	7.59020169166667	7.57673284166667	7.933308825	7.62089398333333	8.02070828333333	8.30591494166667	8.36127639166667	7.76403325833333	7.80227559166667	7.04755845833333	8.327465575	7.933308825	7.58911965833333	8.17668253333333	8.17885319166667	8.03755788333333

IL13	6.04523669166667	3.87406658333333	4.34795044166667	3.88647775833333	3.63145715833333	3.99488165833333	3.508676875	6.31786493333333	3.5935751	4.82580888333333	3.971604825	3.98271365	3.70182713333333	4.14371865833333	4.14053975833333	4.37388213333333	4.303353375	3.65195390833333	4.33637641666667	3.76094594166667	3.95680781666667	3.5983881	3.97264301666667	8.08647256666667	4.43951955	5.71795834166667	4.233876925	5.33234265	4.29716620833333	3.98271365	6.44595796666667	4.08114406666667	3.82289100833333	4.01107811666667	7.09102175833333	5.743144625	4.45416251666667	4.73024025833333	4.50751908333333	4.24718985833333	4.98009509166667	4.506429475	4.87006425833333	5.22040228333333	4.172665625	4.823565325	5.00768225	7.54177855833333	6.50580336666667	4.69325549166667	6.057986625	4.061836425	3.842520875	5.14718730833333	4.2379826	4.57827949166667	4.669520575	4.66114760833333	4.303353375	4.08851591666667	3.82817183333333	3.5935751	4.64613160833333	3.81605024166667	3.6378857	3.85061613333333	4.385351725	4.43091141666667	3.997050325	3.62138225	3.83492170833333	4.12229528333333	3.37663846666667	3.63145715833333	3.50417281666667	3.60976025833333	3.66307870833333	4.07353555833333	4.11281023333333	3.71657505833333	3.49827795	3.39505000833333	3.86964848333333	4.4580822	3.64321401666667	3.80218998333333	4.146963325	3.89779840833333	3.76337073333333	3.74515126666667	4.224150025	4.73832164166667	4.11883455833333	3.74515126666667	3.6554436	4.29817321666667	4.73295375	4.76252700833333	4.19623050833333	4.28423139166667	4.328489775	3.62256374166667	5.43818919166667	4.792098225	4.86085110833333	3.93897055	4.52418780833333	6.57734461666667	5.16622001666667	6.15598731666667	4.2929499	4.418789625	4.48829116666667	4.7771511	4.771681175	5.74704205	4.75698904166667	5.11118121666667	4.85659321666667	4.41392473333333

TICRR	6.03961994166667	8.48345257083333	7.98997880833333	7.44520305416667	7.81584129583333	8.60512660416667	6.90220145416667	7.24296579166667	7.37269663333333	7.74216273333333	8.1275256375	6.12241145833333	6.14611671666667	7.8664005125	8.74891212083333	6.52171285416667	5.64066469166667	7.34922049583333	8.15279964166667	7.64461161666667	7.17708556666667	8.49074439166667	7.91383982083333	7.196665975	7.3258057	5.9458035375	7.41210829166667	8.23839771666667	7.648856625	8.0071378875	7.63684587916667	7.90762179583333	7.61364627083333	6.55707175416667	6.15406443333333	6.41231035	8.21072110833333	7.36364962916667	7.8744863375	7.4287504125	7.77920590416667	8.1127840625	7.97583387916667	8.49953687083333	7.2576608875	7.96331500833333	8.11329191666667	8.09412890416667	9.01825260416667	7.60689905	7.5099886125	7.234588075	8.30937642916667	8.64815709166667	7.92411849166667	8.4249922375	7.19921670833333	8.30058885	8.83223770833333	7.87128107083333	9.5101901125	8.29815655416667	8.74248261666667	8.77219244166667	7.84714889583333	9.76492312916667	7.61670837916667	7.531561475	8.4532828875	7.47231592083333	8.4317037875	7.57373770416667	7.81977565833333	7.65148490833333	8.43638852083333	7.44520305416667	6.7780729625	8.7415233125	9.02537695	9.33946395833333	7.72821662083333	7.2583052875	7.2625422125	8.63206595833333	7.16880814583333	10.0427913833333	9.053338675	7.56628790416667	8.49517645833333	7.62787378333333	8.881909775	8.214819025	9.71635354583333	7.7890171125	7.512267025	9.36226898333333	7.89856145833333	8.40401039166667	7.43098642916667	7.97997342083333	8.47073593333333	8.81441074166667	9.43396825	9.28526889166667	8.54243515	8.168080325	9.70646494166667	9.15530682083333	8.23240379583333	8.7974409125	6.85069928333333	8.31987026666667	9.43660667083333	9.60896382083333	7.97319919583333	9.23442450833333	8.92816500416667	9.53669334166667	9.76372285416667	8.099729775

KIF7	6.03961994166667	8.48345257083333	7.98997880833333	7.44520305416667	7.81584129583333	8.60512660416667	6.90220145416667	7.24296579166667	7.37269663333333	7.74216273333333	8.1275256375	6.12241145833333	6.14611671666667	7.8664005125	8.74891212083333	6.52171285416667	5.64066469166667	7.34922049583333	8.15279964166667	7.64461161666667	7.17708556666667	8.49074439166667	7.91383982083333	7.196665975	7.3258057	5.9458035375	7.41210829166667	8.23839771666667	7.648856625	8.0071378875	7.63684587916667	7.90762179583333	7.61364627083333	6.55707175416667	6.15406443333333	6.41231035	8.21072110833333	7.36364962916667	7.8744863375	7.4287504125	7.77920590416667	8.1127840625	7.97583387916667	8.49953687083333	7.2576608875	7.96331500833333	8.11329191666667	8.09412890416667	9.01825260416667	7.60689905	7.5099886125	7.234588075	8.30937642916667	8.64815709166667	7.92411849166667	8.4249922375	7.19921670833333	8.30058885	8.83223770833333	7.87128107083333	9.5101901125	8.29815655416667	8.74248261666667	8.77219244166667	7.84714889583333	9.76492312916667	7.61670837916667	7.531561475	8.4532828875	7.47231592083333	8.4317037875	7.57373770416667	7.81977565833333	7.65148490833333	8.43638852083333	7.44520305416667	6.7780729625	8.7415233125	9.02537695	9.33946395833333	7.72821662083333	7.2583052875	7.2625422125	8.63206595833333	7.16880814583333	10.0427913833333	9.053338675	7.56628790416667	8.49517645833333	7.62787378333333	8.881909775	8.214819025	9.71635354583333	7.7890171125	7.512267025	9.36226898333333	7.89856145833333	8.40401039166667	7.43098642916667	7.97997342083333	8.47073593333333	8.81441074166667	9.43396825	9.28526889166667	8.54243515	8.168080325	9.70646494166667	9.15530682083333	8.23240379583333	8.7974409125	6.85069928333333	8.31987026666667	9.43660667083333	9.60896382083333	7.97319919583333	9.23442450833333	8.92816500416667	9.53669334166667	9.76372285416667	8.099729775

ARHGEF16	7.91244564166667	9.87484248333333	9.99593061666667	10.4445170416667	9.31629211666667	10.0807009083333	9.25628595833333	9.223397275	9.524079925	9.53496325833333	9.557193025	7.11020925833333	8.482136425	9.60499546666667	9.8294925	9.35679085833333	10.406522025	9.66065478333333	10.1737764166667	9.69219855833333	9.826059425	9.580833825	10.15614755	9.57132345	9.446046	7.51524328333333	9.67544948333333	9.657103925	6.93616958333333	9.9519621	10.103654225	10.1226723083333	9.40333164166667	9.80274700833333	7.762415375	7.73191525	9.5453508	9.45636763333333	9.876036325	9.51549753333333	10.500555575	10.1246349583333	10.2136563	10.6711164583333	9.364389775	10.4942942166667	9.76472428333333	9.93555589166667	10.347919	10.1283050583333	10.0578180083333	9.85036418333333	9.63806365	9.98111111666667	10.2427233083333	10.012954475	9.22160494166667	10.138226575	9.914179275	9.36715445	8.8902947	8.9184083	10.6488037166667	10.3487595333333	8.93412065833333	8.58349254166667	11.2082269	8.82022893333333	9.77883299166667	9.32368785833333	9.03327476666667	9.298425025	9.6895289	10.0142807916667	9.56961935	10.4550319166667	9.12419129166667	9.08960063333333	9.75967308333333	9.01611445833333	9.2436075	8.23520641666667	9.45555605	9.3036884	8.93180466666667	7.992867725	8.75371965	8.89405555	5.85413349166667	9.18115765833333	8.32561349166667	9.72574979166667	10.1405016833333	8.15860931666667	8.18571946666667	9.59023981666667	9.446046	9.41892754166667	9.43636521666667	9.22295939166667	9.52694540833333	8.953569225	11.291502775	10.6211581166667	9.85498678333333	10.8243180916667	9.47068484166667	9.51496844166667	10.2458406333333	9.497335375	10.4994687166667	10.3844721666667	9.24401314166667	10.0256584	8.55955131666667	10.3733561083333	9.6185904	10.8457886333333	10.382913225	10.2987266333333

RET	8.785427625	6.87648061666667	6.41808758333333	7.04568171666667	7.09869196666667	6.9494722	6.11703395833333	6.386982725	6.29510448333333	6.411112525	6.34225285833333	7.3741849	6.0801823	5.97595255	6.55145231666667	6.76313481666667	8.821700275	6.10873680833333	6.5402188	6.824495175	6.15274419166667	7.25001195833333	6.29125269166667	6.0951249	7.0345033	9.00643414166667	6.84576470833333	6.96970946666667	6.76786575	6.59883068333333	6.64893395	7.127807925	6.68727483333333	6.63574723333333	8.56622091666667	9.25858543333333	6.78782379166667	6.64559758333333	6.698517925	7.24137955	6.58160273333333	6.31394556666667	6.777132775	6.71367926666667	7.71080830833333	7.49332154166667	6.90252933333333	6.5766165	6.575829775	7.39926451666667	6.77370956666667	6.90781049166667	6.7730469	6.54638811666667	6.98474048333333	7.434165875	6.72183800833333	6.738299725	7.02761371666667	7.13033303333333	7.69103884166667	6.88314723333333	6.975151325	6.84636305	6.495300075	7.72085915833333	5.90235153333333	6.78990954166667	7.05018306666667	6.699210125	6.57166325	7.72846560833333	8.74719128333333	6.66118995	6.27563768333333	6.66317971666667	7.58577588333333	6.780385325	6.8687758	6.542385675	5.74130296666667	6.99475883333333	6.78990954166667	6.31880616666667	7.00566125833333	7.03630040833333	6.975151325	6.880500475	6.50580336666667	6.94312051666667	7.444336	7.16358325	6.98973956666667	7.35059130833333	7.55873083333333	7.1809933	6.872774725	6.950931025	7.23488161666667	6.37777528333333	8.26356378333333	6.85745724166667	6.71047270833333	6.97714725	6.91322913333333	6.77501233333333	6.66383380833333	6.39534953333333	6.74346625833333	7.25477635	7.774307975	6.54434775	8.706897975	6.57952525	7.71798951666667	7.095527	6.47216713333333	7.41763228333333	6.71243863333333	6.74218866666667

C5AR1	9.69219855833333	8.91275618333333	10.4028540333333	7.99615545	8.11603985	9.89303093333333	9.234219825	9.97301156666667	7.6066095	11.1681956416667	8.67789245	11.7823700916667	9.72286421666667	9.39438685	6.79863740833333	11.4463806916667	9.72070379166667	8.382753925	9.32574429166667	9.175625125	9.98633354166667	7.07292095833333	9.87695954166667	11.61832875	9.17006050833333	10.9273818166667	9.87386503333333	9.52783803333333	6.82946754166667	9.81525473333333	10.054135025	9.24401314166667	8.93762295	9.98187775833333	12.478685375	9.87345979166667	7.82231499166667	9.44826335	11.6489233	10.461782	9.78329576666667	8.37864189166667	10.3466375416667	9.22016708333333	9.92832075	9.73914464166667	8.7776576	10.5926557	10.0611526083333	10.73952485	11.2822211083333	9.995492075	8.28997516666667	8.62886449166667	9.31452535833333	9.67501213333333	10.9719667416667	9.863143525	8.8039806	9.94794899166667	9.38807046666667	10.6115680416667	10.783568125	9.38252353333333	10.32163045	9.6592899	11.2409567416667	10.324926675	10.35378905	10.4890800583333	10.4127726666667	9.95522605	10.3101529333333	11.4682426833333	9.01246160833333	11.3015343416667	10.0557486666667	10.6139707	11.5765356583333	10.3754461833333	9.471590125	9.15064904166667	11.14521145	12.6904704083333	11.5930630416667	10.44375685	9.23281155	11.6705910083333	9.345810075	12.3169057833333	11.3900978416667	11.30753185	10.2051938916667	10.3483436583333	10.9386855666667	11.3749692583333	10.572357075	10.2330333333333	11.3809445833333	10.680501275	11.0567325916667	10.194475875	10.7944294666667	10.7581339416667	10.76211435	11.2975735583333	10.575694825	10.9226970166667	10.23713875	12.04786795	11.03980565	10.4236603166667	10.5311099	10.5960450416667	11.7925074083333	10.55775005	10.6876859	10.9505347083333	9.645264775	11.5078323166667

NUP153	10.5983973083333	11.6970555916667	11.7868322833333	11.6492807916667	11.3946703583333	11.99822175	11.6444959666667	11.9844494166667	11.0924307333333	11.2154273083333	11.1593417916667	10.7855003666667	11.0369926416667	11.4481946583333	11.3343049666667	11.4748997416667	11.0171321	11.7190625916667	12.11177165	11.784206175	11.867753575	11.9550582083333	12.253276925	12.5381389666667	11.5645444166667	11.4000850916667	11.5237451916667	11.7411507916667	11.5258171833333	11.9013541833333	12.2894436583333	11.7951768416667	11.4718467833333	11.0938590416667	11.3720648583333	10.9869124833333	11.603226975	11.8062314166667	11.830948725	11.7053666833333	11.7509367	11.602097375	11.520872575	11.8154196416667	11.3187261333333	11.4879615666667	11.6863511916667	11.960165	12.0395951	11.6425911333333	11.8212938166667	11.9502134083333	11.4752696416667	11.77986195	11.6511098916667	11.7186785333333	11.8297435166667	11.827759775	11.6077110916667	11.556290575	11.4350551	12.1575174833333	11.67393075	11.5038172666667	11.8626743166667	11.1302224166667	11.6837602666667	10.9794539833333	11.98404715	11.062936475	12.461524275	11.6095821583333	11.4810093416667	11.6659074916667	11.044463875	11.529525725	11.171764175	11.55164095	12.5501890583333	11.7993113833333	12.94284515	11.2884114666667	11.8749533333333	12.1367818666667	11.6073227	12.0808514833333	13.0889930583333	11.5754446583333	11.4934089166667	11.9505991416667	12.105099425	12.31352485	12.0555750333333	11.8337195083333	12.0214326083333	11.714258625	12.3284449583333	12.928953425	12.1631913333333	12.496720025	11.731200575	11.1517300916667	11.9123960833333	11.8422473333333	11.7414989583333	11.600264425	11.893980925	11.5998809	12.2675250666667	11.3115193	11.5765356583333	12.118621125	10.8506520083333	11.8666609583333	11.929273675	12.0413582166667	12.320801975	12.9461435166667	12.2461720416667	12.3422979833333

CCN6	4.58140610833333	4.75806056666667	4.07245948333333	5.80446058333333	6.30781043333333	8.81836040833333	5.91627995	6.17154675833333	5.41887256666667	4.55369980833333	5.75852464166667	5.56781358333333	4.21912249166667	4.46791538333333	5.00344013333333	5.03726580833333	5.06786714166667	4.15642159166667	4.59997769166667	4.38197743333333	4.128952175	6.64415971666667	6.66383380833333	6.692469175	6.65024493333333	5.36922546666667	4.73928888333333	4.21395365833333	4.53624929166667	4.45699755833333	4.82580888333333	4.490204975	5.70649276666667	4.645027875	4.282041725	4.16611878333333	5.04557781666667	4.85861940833333	5.37570958333333	4.36901661666667	6.081909575	4.43951955	4.750433775	5.57060381666667	4.853434925	5.01216298333333	5.192100225	4.963016975	4.71218959166667	7.02467490833333	6.04445175	5.23461665	4.05967894166667	5.40932128333333	4.59683404166667	5.091030975	4.86184074166667	4.72414796666667	4.2379826	5.46647429166667	4.41962694166667	4.322875175	4.57924936666667	3.70818398333333	9.579948275	7.29106909166667	4.10101340833333	7.39975843333333	6.44595796666667	7.6157713	5.2482658	4.823565325	5.217220275	4.39770036666667	8.18315743333333	4.67380545833333	6.57427455833333	4.07134488333333	3.946878775	7.334392275	9.85895400833333	8.603908075	9.4551338	3.94009973333333	7.96133965	4.70270751666667	8.1873246	7.51365925	3.80438254166667	8.05569140833333	3.91359475	4.36495859166667	4.62990135	4.609167375	4.39446349166667	3.89227330833333	9.106662475	4.08963481666667	7.1508922	9.38006720833333	8.71144663333333	3.76596780833333	4.736033375	7.77550013333333	4.29817321666667	8.34021045	7.598264375	6.14803923333333	7.50801045833333	6.82038475833333	5.75004875833333	6.68472555	4.36702491666667	10.3048656833333	7.40257545	4.30437938333333	4.03764378333333	4.52327248333333	4.41695324166667	5.6411096

ATXN3	9.44959046666667	8.96094025833333	8.26606834166667	8.66247435	7.80272595	7.98346735	8.247580125	8.73448420833333	8.52731504166667	8.01169885833333	8.29403910833333	8.751330725	7.99891026666667	8.55907896666667	8.72644686666667	8.92364006666667	8.697015525	8.283955225	8.84471325833333	8.78942864166667	8.771928675	8.91049593333333	8.931271375	9.11737229166667	8.90668976666667	9.49325940833333	8.76031966666667	9.06069035833333	8.309637175	8.90052724166667	8.73259671666667	9.00867418333333	8.48515009166667	8.17835735	9.72574979166667	9.95398405833333	8.67403964166667	8.66625304166667	8.34279256666667	8.72739513333333	8.82621398333333	9.06791326666667	8.937187475	8.79489933333333	8.73689844166667	8.763246775	8.97625954166667	8.55492440833333	8.708486225	8.48745835	8.58250536666667	8.78836945	8.14192978333333	8.51578285	8.76950824166667	8.87974118333333	8.69274164166667	8.53882584166667	8.49593345833333	8.548453575	7.64552340833333	7.92167954166667	8.41158014166667	8.28095494166667	9.16167278333333	8.53140681666667	8.67639044166667	8.15205105	8.49188041666667	8.02070828333333	7.9977282	7.68436408333333	8.4816187	7.83016101666667	7.99508275	8.26555303333333	7.95359058333333	8.199157725	8.89353219166667	8.18684245	8.99487275833333	8.43197355833333	8.74766549166667	8.46245245833333	7.9814024	8.53730979166667	7.85051325833333	8.010141425	8.14959635833333	8.53730979166667	8.70751021666667	8.56667040833333	7.8150656	8.62635114166667	8.53730979166667	7.86994016666667	9.08729039166667	8.09498031666667	7.15455295	8.12489341666667	8.22741431666667	7.62593523333333	8.41202154166667	8.48745835	9.16967255	8.29053858333333	8.51081288333333	8.14449565	8.381708525	8.08754240833333	8.042852375	8.34279256666667	8.34324809166667	9.08687805	7.93695109166667	8.20433166666667	8.70212718333333	8.72229471666667	9.05030123333333	8.11603985

PPFIA4	9.79486831666667	9.29481465	9.44337360833333	9.71098871666667	9.71612960833333	8.70034938333333	10.5569098916667	8.9939706	9.81985159166667	9.50725341666667	9.61596005833333	10.31869075	10.081132625	9.166351	9.07777099166667	8.97769849166667	9.02131511666667	9.9560883	8.78586333333333	8.68329548333333	9.29933239166667	9.15692076666667	8.88067333333333	8.85160831666667	8.79489933333333	9.34847368333333	8.95490616666667	9.040322975	9.06405529166667	9.31227445	9.28223910833333	9.2408133	9.52177871666667	9.64690625833333	9.47496915833333	9.46074875	9.2741013	9.02222115833333	9.13602675	9.10485650833333	8.40077755	9.02785883333333	8.80925365833333	8.584858775	8.98934508333333	8.54023758333333	8.78890531666667	8.82825418333333	8.41104395833333	8.82680535833333	8.81154583333333	8.97489020833333	9.28405401666667	9.037729975	9.46521605833333	8.87086751666667	8.95895775	8.86902108333333	9.15740435833333	8.75278535833333	9.41247076666667	9.86266926666667	9.30586555833333	10.154948775	9.20605604166667	9.5040294	9.41436835833333	9.826059425	9.91503965833333	9.68213623333333	9.85198440833333	9.65529779166667	10.1274668666667	9.497335375	9.77571775833333	9.24218355833333	9.76179089166667	9.20872513333333	9.01521926666667	9.440114675	9.51996269166667	9.30818888333333	9.22423163333333	8.862628675	9.77312443333333	9.81305005833333	9.88298375833333	9.71657748333333	9.815643425	8.94431525	9.612775675	9.36389349166667	9.46343676666667	9.17691023333333	9.89258960833333	9.36338105833333	9.52546253333333	9.47772424166667	9.91585179166667	9.00775005833333	8.909148575	10.0347123333333	9.28785676666667	8.72739513333333	9.16588333333333	9.07955376666667	9.289706825	8.910111275	8.738873225	8.462988175	8.69791309166667	9.05965051666667	9.037729975	8.99943493333333	9.341081375	9.18639844166667	8.75882765	9.15991209166667	9.14235506666667	9.437687475

TNKS1BP1	8.96230365	7.800043275	7.95194513333333	7.900836625	8.45745534166667	7.81893236666667	8.754236775	7.933308825	8.26097460833333	7.821740775	7.92167954166667	8.71144663333333	8.20377755	7.794082525	7.61391835	7.652791	7.59366683333333	8.8104251375	7.692112825	7.70977168333333	8.04704694166667	7.52107438333333	7.45098580833333	7.50852245833333	7.92437408333333	7.99186004166667	7.60888643333333	7.88018380833333	8.13415403333333	7.704155275	7.668390025	7.55925806666667	8.32106236666667	8.03431426666667	8.55105330833333	8.16178343333333	8.19036318333333	8.01217839166667	7.9846002	7.75234836666667	7.11143919166667	7.75390870833333	7.50966981666667	7.103742375	7.09162461666667	6.815678575	6.9393833	7.31919828333333	7.3367108	7.27410720833333	7.75602535833333	7.867210225	7.94435703333333	7.726376575	7.75234836666667	7.11789028333333	7.547481675	7.47677956666667	7.54913294166667	7.29304450833333	8.23662486666667	8.38960981666667	7.53912439166667	8.22846261666667	7.93490190833333	8.5232739	8.17668253333333	8.50602524166667	8.16684950833333	7.916304775	8.4841298	8.151552925	8.87290028333333	7.88978783333333	8.47690594166667	7.95962858333333	8.29931580833333	7.80971700833333	7.72354014166667	7.91097105833333	7.77602128333333	8.31911250833333	8.04330091666667	7.672013725	8.49136421666667	8.083905375	8.43246804166667	8.462988175	8.513349575	7.815575525	8.03755788333333	7.913004675	7.683805725	8.31150115833333	8.608522725	8.05514178333333	8.14346960833333	8.13415403333333	8.62886449166667	8.36807433333333	7.41824386666667	8.45556428333333	7.63269815833333	7.15262315833333	7.62704748333333	7.544616	7.65442175833333	8.13766550833333	7.609434275	8.03913049166667	7.92167954166667	7.69919105	7.9184414	7.81610706666667	7.78166071666667	7.54405583333333	7.55746484166667	7.949320675	7.97346688333333	8.03327618333333

RARB	11.3292622166667	6.41874811666667	5.42780705833333	4.62161215833333	6.27652200833333	7.03878675833333	6.7503122	6.510020875	6.028109975	5.53586285833333	5.764437375	6.46640339166667	9.59906339166667	5.86456365833333	7.34210711666667	5.87496216666667	6.34302255833333	6.87589898333333	8.294447975	5.88144885	6.10313131666667	6.58499179166667	6.178567625	4.202033325	6.61087155833333	10.7344399666667	7.824397325	5.933890025	7.46746676666667	6.46133099166667	5.279548575	5.22820184166667	5.255540825	5.49431639166667	10.0660963333333	11.062266425	6.21517435	5.68880476666667	4.30437938333333	5.53793416666667	6.35880866666667	5.3439553	4.4473261	6.32600435833333	7.18452301666667	4.66114760833333	6.849792025	5.20449400833333	4.28110195833333	6.09925825	6.0801823	4.45030740833333	6.161212075	5.77296926666667	5.87208589166667	6.60007374166667	5.72466863333333	4.38005901666667	5.47616439166667	4.97386210833333	6.65843384166667	5.23978975	4.01568299166667	4.79318954166667	4.258212325	4.021539275	5.09864851666667	5.360915675	3.96359718333333	5.12000276666667	6.21517435	4.93761881666667	5.46838549166667	4.22036293333333	5.390724	4.22678013333333	6.3460148	4.46004483333333	5.16838866666667	4.55971610833333	5.46278851666667	6.80714281666667	4.01339135833333	3.81959511666667	4.48239504166667	7.54177855833333	5.23566219166667	4.95206471666667	4.25290215833333	5.82555810833333	3.66190675	4.18714809166667	7.547481675	4.77601478333333	3.38406459166667	3.81123611666667	4.05858813333333	6.60472939166667	4.37388213333333	7.83997353333333	3.90095365833333	4.70784294166667	4.41782250833333	4.40284696666667	4.17039641666667	4.79999713333333	3.73894771666667	5.1291031	4.79518681666667	6.27563768333333	5.14931930833333	4.94478124166667	3.84817555	4.39670516666667	3.80554890833333	4.52327248333333	7.77045426666667	4.21502835833333	4.10495005	6.541621825

NPHP4	7.92279315	6.95924915	7.28317230833333	6.894639675	7.90941765	8.535292725	6.26921054166667	7.81344110833333	6.90781049166667	7.50463459166667	7.23080319166667	6.33187936666667	6.63179246666667	7.17093558333333	7.93490190833333	7.42906085	7.279554825	7.54282731666667	8.25794470833333	7.75390870833333	7.86334303333333	7.28198013333333	7.69319276666667	8.42823383333333	7.886143875	7.89722555	7.5385516	8.15060805	8.21721259166667	7.31316353333333	7.71914490833333	7.85906496666667	7.03751019166667	7.01553179166667	7.98346735	7.6315346	7.6287103	7.988718925	8.06500796666667	7.96474510833333	7.55873083333333	7.12224311666667	7.42556758333333	8.05717868333333	7.59600343333333	7.96623754166667	7.7445795	7.74783731666667	7.81401100833333	7.46294761666667	7.21861555833333	8.00581294166667	8.23717023333333	8.20781665833333	7.6315346	8.16389916666667	7.460763125	7.64430480833333	7.64914819166667	7.90584154166667	9.544894	7.7794798	8.268113325	8.996715525	8.582021475	8.706897975	8.28000224166667	7.67425858333333	8.86062028333333	8.0954509	7.08487659166667	8.78498449166667	7.782192425	7.544616	8.40614324166667	8.13617131666667	7.42447115	7.34497685	8.28445111666667	8.19498931666667	8.93359508333333	7.762415375	7.90260113333333	9.1029726	7.98516725833333	8.6716691	8.33398970833333	7.98346735	8.34422473333333	8.31150115833333	7.40375466666667	9.22016708333333	8.24509221666667	8.251367875	9.29252663333333	8.03595288333333	8.95991093333333	9.01380788333333	8.733549025	7.48123266666667	8.57995885	6.84260019166667	8.96273033333333	8.40614324166667	7.7548948	8.76851021666667	8.78070639166667	8.259343925	8.34324809166667	8.44794389166667	7.73471975833333	8.45352316666667	9.42643701666667	9.02131511666667	8.53434441666667	8.2260001	8.98237701666667	8.42124595	8.51081288333333	7.53624973333333

IQSEC1	5.98737700833333	5.00344013333333	4.87710051666667	5.7946758	6.15778135833333	5.93923476666667	4.84935865	5.49632836666667	5.13122064166667	5.28464988333333	5.35983450833333	4.94355658333333	5.04230353333333	4.31295405833333	5.5697365	4.92770649166667	6.44000804166667	5.596724675	5.23876719166667	4.81075010833333	4.90369176666667	5.25233420833333	4.72105203333333	4.90679766666667	5.31119278333333	5.326621775	5.4237743	5.31119278333333	5.565954175	5.04966088333333	5.35692600833333	4.74219220833333	5.22265475	5.85993758333333	6.22752675833333	5.01100034166667	5.99672560833333	4.963016975	5.71218308333333	5.054896275	6.11958026666667	5.14718730833333	5.508179525	5.6173492	4.51268873333333	5.11436066666667	5.78817238333333	5.04557781666667	5.23461665	5.27734721666667	5.99672560833333	6.1063381	4.55971610833333	5.58212393333333	4.90147810833333	6.24107090833333	4.12229528333333	5.39700335833333	5.26570160833333	5.196416425	5.35088976666667	4.74930484166667	6.07263301666667	5.36415765833333	6.60826071666667	4.822492325	4.9110181	5.03120425833333	5.8902603	5.326621775	4.88257656666667	5.44029105833333	4.61421155	4.5803341	4.84714924166667	5.162070825	5.88432855	6.06998224166667	4.80316131666667	5.48099319166667	3.95017479166667	4.683662975	4.768670525	5.677007675	5.000034475	4.39156651666667	5.24703029166667	6.37415488333333	5.16420199166667	5.33746485833333	5.31421083333333	6.32668329166667	5.11789035833333	4.51475793333333	6.131776975	5.8902603	5.97035753333333	5.451608675	5.060028525	5.94535455833333	5.54303723333333	4.66004348333333	5.80446058333333	5.40105285833333	4.823565325	5.19309993333333	4.82998554166667	7.06773185833333	4.82036908333333	7.147600275	5.59377575	7.15020035833333	6.02015538333333	5.44644633333333	5.60680040833333	6.66802325833333	6.47291681666667	5.72686785	5.622261375	4.515720925

NAAA	10.3690418416667	11.444940575	11.7004395833333	12.6391509	11.20219855	12.0835996583333	11.012449925	11.4420691166667	11.6536243916667	11.4214230083333	11.97355025	10.4131468	10.5428297916667	11.6489233	12.0819963833333	11.8576914166667	11.221922825	11.074065275	11.8568699583333	11.5898474083333	11.6043999833333	12.6991286666667	11.930808275	12.11543585	12.1439381	10.3530735416667	11.5934022416667	11.21761425	11.7898995	11.6577322083333	11.4350551	11.81120325	11.96822585	10.976899425	10.6773409416667	10.327316425	11.7324169333333	12.0428624416667	11.671269575	11.1916460666667	11.8924112666667	12.0486377333333	11.7259183166667	12.017715375	11.7671597083333	11.7304346833333	11.6107118416667	12.0901653083333	11.65733825	12.9745146166667	11.4049590333333	11.3001028166667	11.6496725666667	11.6783737	11.799711325	11.9633577833333	11.7000943333333	11.0315811583333	11.4985882166667	11.8701716833333	9.683051225	9.67682161666667	12.6704503833333	9.92334935	10.432039025	9.37313166666667	10.06035685	10.4453172666667	10.2690944333333	10.9643625833333	9.57692496666667	9.24884609166667	8.67789245	10.9454688666667	10.1546002	11.1681956416667	10.7718274166667	10.5814300416667	10.485478825	10.4914439416667	9.352137375	11.3590797	10.8032590416667	10.090730825	10.7855003666667	10.227841925	8.80540845	10.2822548833333	9.452370525	10.5497574833333	10.7012892333333	10.7162804	9.53312831666667	10.4744353333333	10.798668925	10.29990335	10.3462750333333	10.1853907583333	10.1650146416667	11.1656289166667	10.5108422833333	10.4678765666667	10.8378553416667	10.594157325	9.96267133333333	10.924157925	11.31617205	11.3410012666667	11.0670126666667	10.4613867166667	10.3758856666667	9.95891725	10.1222883166667	10.9816032916667	11.4063648666667	10.8198860166667	10.6827360416667	9.65529779166667	9.93835718333333	11.4688855083333

RXFP2	5.90945328333333	6.09195375	6.230051875	6.41191374166667	6.40572345	5.61354440833333	7.100006625	6.78916241666667	6.43219438333333	6.17067161666667	5.99501325833333	6.72055851666667	6.75097239166667	5.60487774166667	6.51600675833333	5.808344225	5.43818919166667	6.57952525	5.70253175	5.22040228333333	5.9294819	6.582876425	5.81989971666667	4.58881544166667	5.51098925	6.62102673333333	6.16829805	5.173701425	5.20658905833333	6.00884046666667	5.74909436666667	6.372760025	5.67139935833333	6.56879458333333	6.63660101666667	6.01590590833333	5.6322886	6.29057105	6.21280073333333	6.12691128333333	5.379722375	5.388701925	5.36615406666667	7.54510475	5.59947975	5.82168396666667	6.42033848333333	6.01047939166667	5.62674555833333	5.93923476666667	6.44063283333333	5.58021739166667	5.58021739166667	5.41985199166667	6.1777684	5.31119278333333	6.18568551666667	6.083461775	5.873141325	5.65427465	6.15183188333333	6.47291681666667	6.2058813	6.345260775	6.39534953333333	6.06396311666667	6.277191075	6.88120535	5.90497220833333	6.13670685833333	6.161212075	6.4040341	6.974033025	5.549659825	6.38165589166667	6.17313585833333	6.24858409166667	5.32238468333333	6.30111015	5.51291259166667	6.41504531666667	5.80356945	5.90755965	6.126012425	6.31702735	6.507987025	6.4413796	6.24443749166667	5.99672560833333	5.75112915833333	6.234662425	6.21689609166667	6.27314985	6.69715851666667	6.65223385	5.95574085833333	6.01215780833333	6.65552751666667	6.85156043333333	6.46988230833333	5.75400875	6.48862780833333	6.2066078	6.77436175	6.11047193333333	5.88595835	6.602072175	6.62158961666667	5.61643985833333	6.79057516666667	5.79838183333333	6.45250163333333	6.49825018333333	4.86589074166667	6.29510448333333	5.73682776666667	6.219278425	6.00629560833333	6.407249275	6.48497233333333

LRRC73	8.30130739166667	7.68209800833333	7.72195235	8.62938480833333	8.611944975	7.43805644166667	8.33259703333333	8.11761579166667	7.821740775	7.657177025	8.38217830833333	8.33758601666667	7.916304775	7.95311691666667	8.31103703333333	7.428439975	8.62635114166667	8.51669369166667	7.93695109166667	7.76293401666667	8.1073987	8.199157725	7.662739425	7.95359058333333	7.14092606666667	7.93805580833333	7.69369399166667	8.00412230833333	7.84796001666667	8.11654126666667	8.41104395833333	7.52566054166667	8.32696298333333	7.988718925	8.0773066	7.88077005	7.87042711666667	7.9977282	7.85748985	7.64327454166667	8.05087028333333	8.31561141666667	7.567156725	7.83671455833333	8.04704694166667	7.47951195833333	7.77775845	8.26308525833333	7.497153825	7.86670446666667	7.41061439166667	7.85309088333333	7.94541711666667	7.85309088333333	7.88811874166667	8.27495268333333	7.913004675	7.80563896666667	7.65663390833333	7.39564355833333	8.28000224166667	8.434501875	8.062405875	8.66441525833333	8.96510433333333	8.24163635833333	8.33810663333333	8.244635825	8.83115135	8.29681115	8.36907771666667	8.932734675	8.66880180833333	7.88343015833333	8.30746798333333	7.86543926666667	8.94186190833333	7.704155275	7.32919075833333	7.91685584166667	8.78635625	8.32905851666667	7.96073305833333	8.79861721666667	8.33913670833333	8.61092796666667	9.283130225	8.11250586666667	8.72356944166667	7.74783731666667	8.31364723333333	8.57400345	8.21405428333333	7.38233871666667	8.47382378333333	7.7794798	8.88450143333333	8.439937175	8.65649723333333	9.53738644166667	9.04278656666667	8.995827675	7.71034295833333	8.27495268333333	9.06460029166667	7.94065678333333	7.93918533333333	7.51089621666667	8.97027915833333	7.26937910833333	8.14495316666667	8.471007425	7.55746484166667	8.95079723333333	7.89132048333333	8.027292325	9.17190161666667	9.19627585833333	8.00199818333333	7.98717739166667

TRAPPC10	10.9133984666667	11.2429894875	11.4465581541667	11.2242574333333	11.1457823208333	11.2451646458333	11.0331784833333	11.2973992791667	11.2585867666667	10.6810802583333	11.1443172958333	10.5954812708333	10.6174893625	11.3020286875	11.2562632708333	11.2067215541667	11.3625971	10.7018390416667	11.4532780375	11.1889542083333	11.3558470416667	11.6464488666667	11.2687833666667	11.5608120125	11.3401629708333	10.9985310875	11.2052246833333	11.1414135375	11.2942388625	11.1965710291667	11.101283425	11.5050811291667	11.155977625	11.4750846916667	10.6728292875	10.6977583541667	11.2773858916667	11.2411560875	11.0695657583333	11.2551927541667	11.3469230291667	11.2073423208333	11.3013535916667	11.3070328	10.9460665375	11.4017573875	11.3715121708333	11.2781191125	11.4674169125	11.2132455833333	10.9008579166667	11.3174565833333	11.0817075916667	11.2931173958333	11.2995228083333	11.3059430708333	11.3469230291667	11.2249415416667	10.9330242333333	11.2963892416667	10.7331043291667	10.6486219333333	10.7551722666667	10.3815939083333	10.9129702916667	10.5035875416667	10.6295882833333	10.555538	10.8497516458333	10.7323377666667	10.5559350375	10.7814277541667	9.9789298875	10.5741356875	10.8837919166667	10.8118916416667	10.9464365791667	10.9103291	10.6882694666667	10.7090780125	10.9232295875	10.7913975708333	11.1355991333333	11.0364632541667	10.2926386041667	10.40949865	10.8286678541667	11.1609467625	11.3450186875	10.4988759958333	10.7018390416667	11.1058823	10.8214613708333	11.0375168583333	10.1522911708333	10.7853202791667	10.83769035	10.4318495375	10.4086865166667	10.7315763416667	11.3869794458333	10.5851296875	10.9939193416667	11.0350216916667	10.9059969833333	10.6239107333333	10.9977837208333	11.4081227125	10.9092903375	10.9885999458333	10.6999204208333	10.6851837208333	11.3566180333333	11.2817030416667	10.9598836708333	11.1961926416667	11.0274367958333	11.0346415125	10.8675350916667	11.0073872666667

FP565260.6	10.9133984666667	11.2429894875	11.4465581541667	11.2242574333333	11.1457823208333	11.2451646458333	11.0331784833333	11.2973992791667	11.2585867666667	10.6810802583333	11.1443172958333	10.5954812708333	10.6174893625	11.3020286875	11.2562632708333	11.2067215541667	11.3625971	10.7018390416667	11.4532780375	11.1889542083333	11.3558470416667	11.6464488666667	11.2687833666667	11.5608120125	11.3401629708333	10.9985310875	11.2052246833333	11.1414135375	11.2942388625	11.1965710291667	11.101283425	11.5050811291667	11.155977625	11.4750846916667	10.6728292875	10.6977583541667	11.2773858916667	11.2411560875	11.0695657583333	11.2551927541667	11.3469230291667	11.2073423208333	11.3013535916667	11.3070328	10.9460665375	11.4017573875	11.3715121708333	11.2781191125	11.4674169125	11.2132455833333	10.9008579166667	11.3174565833333	11.0817075916667	11.2931173958333	11.2995228083333	11.3059430708333	11.3469230291667	11.2249415416667	10.9330242333333	11.2963892416667	10.7331043291667	10.6486219333333	10.7551722666667	10.3815939083333	10.9129702916667	10.5035875416667	10.6295882833333	10.555538	10.8497516458333	10.7323377666667	10.5559350375	10.7814277541667	9.9789298875	10.5741356875	10.8837919166667	10.8118916416667	10.9464365791667	10.9103291	10.6882694666667	10.7090780125	10.9232295875	10.7913975708333	11.1355991333333	11.0364632541667	10.2926386041667	10.40949865	10.8286678541667	11.1609467625	11.3450186875	10.4988759958333	10.7018390416667	11.1058823	10.8214613708333	11.0375168583333	10.1522911708333	10.7853202791667	10.83769035	10.4318495375	10.4086865166667	10.7315763416667	11.3869794458333	10.5851296875	10.9939193416667	11.0350216916667	10.9059969833333	10.6239107333333	10.9977837208333	11.4081227125	10.9092903375	10.9885999458333	10.6999204208333	10.6851837208333	11.3566180333333	11.2817030416667	10.9598836708333	11.1961926416667	11.0274367958333	11.0346415125	10.8675350916667	11.0073872666667

CPEB4	11.7113167916667	11.5093017916667	11.6547489083333	11.6910443916667	11.9078069166667	11.3477921	11.8553966916667	11.97391105	11.8861575583333	11.2811678666667	11.0301443333333	10.7313914916667	11.6577322083333	11.6525927916667	11.8931595083333	11.492270725	11.4191800833333	12.2706199833333	12.0510805083333	11.9534028083333	11.7628441333333	11.8134267166667	12.063617625	11.3224431	11.4590118416667	11.6270644583333	11.4200132333333	11.4431148583333	11.51470825	11.920083225	12.239207225	11.7153018166667	11.5475917083333	11.0704861666667	12.5567153083333	11.2771872833333	11.3657868083333	11.6780189666667	11.742927775	11.430002575	12.1308631333333	12.16610385	11.421792525	11.468562175	10.9941240666667	11.4022935333333	11.1971318416667	11.6006445416667	11.1767887583333	11.465822775	11.654339925	11.655146	11.2868731416667	11.3357126416667	11.4394949083333	11.3324973416667	11.8959032583333	11.8422473333333	11.4045795333333	11.062266425	9.90816476666667	9.88045179166667	10.4276602833333	10.4292877666667	9.57093504166667	11.1166050833333	10.585362525	9.91080910833333	10.448395325	9.78567400833333	10.0748410916667	10.414161375	10.36145645	10.5173180916667	10.100531725	10.3637863083333	10.2865677	11.3191493833333	11.4177560583333	10.6585232916667	9.4551338	9.908637725	11.1699387666667	10.1242565166667	10.669525325	9.286424975	7.98023625	9.45595239166667	8.06904244166667	10.6360463166667	10.468685175	10.0787243333333	9.23605358333333	10.0142807916667	9.58131358333333	10.7702977583333	9.49279765	9.09460495	10.86697045	9.30730685833333	9.96267133333333	9.99170656666667	9.93750004166667	10.6403864583333	9.92703645833333	9.76819061666667	9.89383843333333	11.085607975	10.137056175	9.8972867	10.8052677	10.0712503	10.2936263083333	9.9039927	10.1853907583333	10.5609143	9.0791656	9.683521475	10.3745792583333	9.777390875

SLC9A1	6.897275725	6.77836881666667	6.72705409166667	6.45767033333333	7.23080319166667	6.4308335	6.8732984	6.59052684166667	7.54510475	6.91634240833333	6.93685505	7.59600343333333	7.82691680833333	5.99939696666667	6.85814304166667	6.15598731666667	7.227214725	7.392806175	7.092930725	6.880500475	6.50580336666667	7.102506125	6.63788805833333	6.06574364166667	6.83400405	6.80569213333333	6.601356975	6.54089473333333	6.87917671666667	7.102506125	7.210345825	7.45504230833333	7.28990879166667	7.34037416666667	7.66558521666667	7.561568325	7.23028210833333	7.38404451666667	7.01264725833333	7.04202985	6.96906025833333	6.50418884166667	6.53809915833333	6.75898810833333	5.99418615	7.192539525	7.195709975	7.05651791666667	6.94439705	7.05714315833333	7.15020035833333	7.40079709166667	7.502278225	7.80563896666667	7.767746725	7.60136925	7.60592470833333	7.3741849	7.05225570833333	7.04632523333333	7.212178575	7.12528415833333	7.00630173333333	6.74082630833333	6.51685910833333	7.14949741666667	7.01495000833333	6.758383225	7.2027431	7.06400038333333	6.77777710833333	7.5998906	7.3361665	6.06998224166667	6.89596523333333	7.07722264166667	6.70327563333333	6.69314785	6.9953871	6.81435373333333	6.25755120833333	6.47141914166667	6.23079429166667	6.526432	6.82644443333333	6.56431505	6.662431575	6.71186961666667	6.77836881666667	7.0176409	7.01901523333333	7.69541339166667	7.1809933	7.48528250833333	7.50852245833333	7.03253850833333	7.22536585	7.24765453333333	7.35564645	6.67096843333333	6.94497745833333	6.56292778333333	6.85156043333333	6.93091866666667	6.98911033333333	6.86809180833333	6.87851200833333	7.83568390833333	7.01007860833333	7.75064413333333	6.97843058333333	7.70926514166667	7.52840544166667	7.83671455833333	7.75064413333333	7.48184465	7.24945885833333	7.81610706666667	7.283866825	7.05455971666667

PRDX1	13.9227796833333	14.9223639	14.7851708916667	15.2722555666667	15.0865438916667	14.9832780583333	14.8336777583333	15.1873199	14.6346112833333	15.0615765666667	14.3482967833333	13.4864308583333	14.8362922416667	14.7754409333333	15.0643045166667	14.4294718916667	14.6284460166667	14.7143451416667	14.8629148	14.4397477583333	15.2940035083333	15.361847675	15.7486260916667	14.8110718166667	14.6862769916667	13.9378093083333	15.4022381166667	14.9336102833333	15.0557071416667	14.9223639	15.0153788083333	14.92521735	14.259430575	14.8110718166667	13.6174285083333	13.2201906833333	15.236084325	15.0525159083333	14.5810673083333	14.7974983916667	15.156314825	15.2650881666667	14.56279245	14.8629148	14.11256935	14.9307420333333	14.8087193833333	14.9752180916667	15.3579580416667	14.70147985	14.9359352083333	14.3582404416667	15.0525159083333	15.0643045166667	15.0733260666667	14.512293975	15.095547425	14.9070025916667	13.8843608583333	14.6549636583333	14.818607725	15.3177335583333	15.0865438916667	14.80427055	14.7143451416667	14.7547481083333	14.820747325	14.8653905916667	15.1973098583333	14.9645581833333	15.7697269333333	15.092462825	14.5717037666667	15.1139193916667	14.9197306916667	14.95380875	14.3582404416667	15.048735725	15.0246483416667	15.3498376083333	14.1357234916667	13.887785	14.5268570083333	14.9359352083333	14.894107525	14.5753036916667	13.8473484166667	14.2487820083333	14.847260375	14.8287604166667	14.7974983916667	15.0306120416667	15.345488975	14.5268570083333	15.0184028083333	15.2784907166667	14.6259421666667	14.3824525333333	14.8865870416667	14.6325901333333	14.8362922416667	14.847260375	15.3944873	15.1139193916667	15.2432312666667	15.2611193416667	14.9808324666667	15.039210975	14.3724417	14.7572900333333	14.596526825	15.406759425	14.5268570083333	14.8699486666667	15.1207533083333	15.444211725	14.4397477583333	15.1529307583333	15.5262258166667	14.9336102833333

PIK3C2B	6.06230976666667	4.20999628333333	4.57240950833333	4.35397924166667	4.16299614166667	4.792098225	4.14274019166667	4.63093599166667	4.67972664166667	4.57240950833333	4.809512175	4.58140610833333	4.12457974166667	4.268577875	4.81485295	4.10495005	4.69425546666667	5.45375554166667	5.055819225	6.00367545	3.975999075	4.27468664166667	4.51179561666667	4.57030863333333	4.85146489166667	4.713205375	5.47028654166667	5.000034475	4.84094744166667	4.832334525	4.85037616666667	5.02689723333333	4.37610179166667	4.92991125833333	5.18759319166667	5.66463360833333	5.020575025	5.22492120833333	5.99501325833333	4.71899701666667	5.11568579166667	4.692141475	5.19860551666667	5.67139935833333	4.56071255833333	5.07107728333333	5.1598283	5.09317755833333	4.649483075	4.93761881666667	5.26995844166667	4.9617686	4.61625569166667	5.53395658333333	4.43737311666667	4.90768301666667	4.30027178333333	5.22820184166667	4.85447411666667	5.19309993333333	4.58445505833333	4.53219865	4.85037616666667	4.73402499166667	4.82796093333333	4.50312849166667	4.664314175	4.359146625	4.74448875	4.33532039166667	4.55863708333333	5.018425275	4.27276220833333	4.64847966666667	4.537079425	4.59464514166667	4.48613384166667	4.802114875	4.16611878333333	6.33491514166667	4.69963341666667	4.432045325	4.41103275833333	5.2891249	4.44534256666667	4.0917668	5.57148620833333	4.747391	4.57343223333333	4.62889925	4.46877066666667	4.89827923333333	4.86589074166667	4.963016975	4.76967493333333	5.08063435	5.060028525	5.32134071666667	4.70371486666667	5.03930840833333	4.79518681666667	4.42481975833333	5.52437941666667	5.19309993333333	4.9426219	4.01435764166667	4.66334196666667	6.60068861666667	4.50222558333333	6.73694150833333	5.18213341666667	5.72997074166667	5.14192449166667	5.64797583333333	4.69642039166667	4.94576598333333	4.86384488333333	5.19860551666667	5.58407845	4.49904255833333

CENPH	9.32806993333333	10.740315675	10.4922259333333	10.1063080916667	10.417262075	10.5490285	10.0467969333333	10.3694253333333	10.2249992083333	11.1696311416667	10.5910579333333	8.90099503333333	9.97706043333333	10.4806526833333	10.643786575	10.0087152083333	9.24845199166667	10.4752224083333	10.6191395	10.3097382833333	10.15614755	10.0736918083333	10.6982774333333	10.4260988583333	10.1029681166667	9.13226131666667	10.30399485	10.1091856666667	10.1080216833333	10.0679332666667	10.4231890083333	10.4806526833333	9.71912328333333	9.79147918333333	9.15555419166667	8.99070061666667	10.279032875	10.21041955	10.0864066083333	10.2543488416667	10.1704848833333	9.86854744166667	10.0002116916667	10.8443256416667	9.98509406666667	10.1126129583333	10.36339005	10.474825675	10.97984555	10.1750566166667	10.1485742083333	10.3132196333333	10.4975657166667	10.37104885	10.1899294916667	10.1965925083333	10.5240503083333	10.6952813416667	10.3987917	10.661840225	11.4042087666667	11.6910443916667	11.4805776416667	11.0924307333333	11.3867866583333	10.81355685	11.017853675	10.3987917	11.9093036166667	10.9476724083333	11.2876344916667	10.9393869333333	11.76865155	10.7189561666667	11.4258523333333	11.081884475	11.170311525	11.33033725	11.42922935	11.3381769	11.99474245	11.6499693666667	11.4958336333333	11.2521123083333	10.25147975	12.2513034583333	13.1310777666667	10.5123801583333	10.6827360416667	10.500980975	11.7273945916667	11.0448237416667	11.7239413833333	9.415734875	9.729222475	10.613570925	11.625582325	11.8947334916667	10.2166211083333	10.4581692833333	11.8232517	11.6084924916667	12.2033105916667	11.2047015416667	11.6297974583333	10.6195893583333	10.7870301083333	11.052426775	11.686699925	11.1074454416667	10.9629037916667	11.5994743583333	10.7228384083333	11.9804777666667	11.2740276	10.738724825	12.239207225	11.7494484416667	11.2858355	11.7827675333333

SH3GL3	7.227214725	8.96609059166667	9.16735169166667	8.80450975	8.95943646666667	8.0459923	8.96510433333333	9.02830098333333	9.063171125	8.79812115	8.34279256666667	7.22835589166667	8.63917994166667	8.63031905833333	9.26306665	8.20031584166667	7.05018306666667	9.17006050833333	9.55244275833333	9.00690949166667	8.55955131666667	9.0791656	8.48614225	8.94623185833333	8.8319761	7.428439975	9.47641323333333	9.44959046666667	9.51549753333333	9.52133895	9.35357573333333	8.871342075	8.61623551666667	6.94561610833333	8.354234475	7.53624973333333	9.62805526666667	9.52783803333333	9.10796010833333	9.182097875	9.0498455	9.6811178	8.95172195833333	9.2612694	8.31409636666667	9.48743423333333	9.176064475	9.37821416666667	8.89696199166667	8.83395916666667	9.24318624166667	9.43045813333333	9.04937824166667	9.21732311666667	9.309548625	9.02418135	8.78443150833333	9.777390875	9.15645445833333	8.74427688333333	7.96885115833333	8.58808473333333	8.4841298	8.09798645	7.96301623333333	9.41849085	8.45304260833333	8.33965076666667	9.28502139166667	8.35319626666667	7.7799909	7.69319276666667	8.91224634166667	7.683805725	8.222543225	8.3715767	7.37928605	7.864271825	8.9544846	7.54177855833333	7.13294301666667	8.3662572	8.67598873333333	8.01861064166667	8.36187725833333	7.6798049	9.10099604166667	7.79469890833333	8.10843584166667	8.6774004	8.8364013	7.4731488	7.56380908333333	8.61342080833333	9.73744423333333	8.28000224166667	7.88871496666667	8.37970946666667	8.92651730833333	6.85607033333333	8.63080885833333	7.81137301666667	7.07722264166667	8.63449166666667	9.071149525	8.50751961666667	8.85215985	8.67789245	8.00412230833333	8.9493948	7.70256570833333	8.20587135	9.63659523333333	9.93427139166667	8.230344325	8.39869986666667	8.06450121666667	8.58091206666667	8.93762295	7.655449725

SRSF5	10.132057125	11.7470404	11.9162394	11.3675917666667	12.0189741833333	9.48078894166667	12.1681509833333	10.939073475	11.3886248333333	11.205405875	11.3560369416667	9.94714974166667	10.8028896	10.6173169916667	11.0968033583333	10.7725388333333	9.364389775	11.114387025	10.6328931583333	11.1302224166667	9.692647075	10.3641444833333	10.80487515	10.2414708	10.723219775	9.57093504166667	11.9554346166667	11.6302237	11.943562125	11.1934390416667	11.5139760666667	11.8182827333333	11.6926020166667	10.01207205	9.42951640833333	9.544894	11.5761677833333	12.1283236333333	11.6613946166667	12.0808514833333	11.049779175	11.4328736	11.2997236583333	10.9747290916667	10.2466873083333	10.9182300166667	10.6434054	11.1477174666667	10.8086263583333	11.155024025	12.2710584083333	9.71098871666667	11.4474681583333	10.9234198666667	11.4115295083333	10.2724454333333	10.8454086666667	11.1585997833333	11.2736929416667	10.5208646083333	10.182857025	9.86598649166667	10.5322469083333	10.199031925	9.67900834166667	9.7570522	9.850735	10.5743323083333	9.81899704166667	10.3116455	10.151687475	9.64610789166667	9.76267145833333	9.45055241666667	10.0031143833333	9.921332825	9.63566801666667	9.754485575	9.65222760833333	9.85239641666667	9.157883875	9.36572414166667	9.67501213333333	9.52642665	10.0716267583333	9.94086763333333	10.7235859166667	10.3171296666667	10.0915413	9.59145404166667	9.97752425	10.0318266833333	10.2186879083333	9.76310825	10.4089152416667	9.8942397	9.96868019166667	10.7485438416667	10.326957425	10.137056175	9.72286421666667	9.83629188333333	9.8597846	9.83719281666667	10.0489380083333	9.843730775	9.73831540833333	9.80535213333333	9.50586739166667	9.729222475	10.0632117083333	10.6592419583333	9.546304725	9.87386503333333	10.0087152083333	10.0696848333333	9.74820641666667	9.87826785	10.2237133416667	9.765982475

TTC4	9.67211590416667	9.95626255833333	10.0686200208333	10.2272632833333	10.40551365	10.6463560333333	9.63498063333333	10.2090984583333	10.223509175	10.333964275	10.6863009083333	9.520195475	9.520195475	10.2217906833333	10.2569008708333	10.0037304958333	10.2938167041667	10.307638325	10.6121103	10.3915346666667	10.1830521875	10.5870673875	10.6417281083333	10.8587718333333	9.85093639166667	10.1727088291667	10.1761008041667	10.38121515	10.4374915708333	10.2836672416667	10.1822655291667	10.1789626291667	9.68494201666667	9.75016254583333	9.90458776666667	10.0959159083333	10.2098535041667	10.39271655	10.2980843916667	10.1352631208333	10.1165924583333	10.1579110541667	10.0580480291667	10.6389934416667	10.1165924583333	10.4270534416667	10.4190041666667	10.6844248708333	10.2910245333333	10.0050292625	9.63542419166667	10.6893533666667	10.2058208458333	10.6137708125	10.4403846416667	10.378893925	10.1908788583333	10.4478490083333	10.1547744875	10.0970717166667	9.70260275	10.7610940708333	10.8790666666667	10.1718782083333	10.6709282166667	10.6295882833333	11.079544475	10.1583154958333	11.2109354583333	10.6049050875	11.7257176791667	10.7121602416667	11.3974153125	11.0809861958333	10.7810387208333	11.4761620708333	10.08921235	10.6246790291667	11.0684162333333	11.1624773791667	10.629165025	9.8329955625	11.2731355125	11.4381818291667	9.97278334166667	10.5536035458333	10.3122044125	10.7230290916667	9.75428324583333	10.808824875	10.5000138	10.7342462625	10.8636915208333	10.8508691833333	11.4694963083333	11.1439293333333	11.2156097458333	10.3145290375	11.0107050083333	11.0695657583333	10.8735190708333	9.957901925	10.8880026958333	10.9293807916667	11.7103631916667	11.2501076666667	11.060997375	11.5943322833333	11.1723113458333	11.0871937291667	9.68625733333333	11.2422697958333	11.3222447666667	11.2196669	11.4002736958333	11.2555266	10.9037622625	11.1504202166667	11.1690845375	10.8700404666667

MROH7-TTC4	9.67211590416667	9.95626255833333	10.0686200208333	10.2272632833333	10.40551365	10.6463560333333	9.63498063333333	10.2090984583333	10.223509175	10.333964275	10.6863009083333	9.520195475	9.520195475	10.2217906833333	10.2569008708333	10.0037304958333	10.2938167041667	10.307638325	10.6121103	10.3915346666667	10.1830521875	10.5870673875	10.6417281083333	10.8587718333333	9.85093639166667	10.1727088291667	10.1761008041667	10.38121515	10.4374915708333	10.2836672416667	10.1822655291667	10.1789626291667	9.68494201666667	9.75016254583333	9.90458776666667	10.0959159083333	10.2098535041667	10.39271655	10.2980843916667	10.1352631208333	10.1165924583333	10.1579110541667	10.0580480291667	10.6389934416667	10.1165924583333	10.4270534416667	10.4190041666667	10.6844248708333	10.2910245333333	10.0050292625	9.63542419166667	10.6893533666667	10.2058208458333	10.6137708125	10.4403846416667	10.378893925	10.1908788583333	10.4478490083333	10.1547744875	10.0970717166667	9.70260275	10.7610940708333	10.8790666666667	10.1718782083333	10.6709282166667	10.6295882833333	11.079544475	10.1583154958333	11.2109354583333	10.6049050875	11.7257176791667	10.7121602416667	11.3974153125	11.0809861958333	10.7810387208333	11.4761620708333	10.08921235	10.6246790291667	11.0684162333333	11.1624773791667	10.629165025	9.8329955625	11.2731355125	11.4381818291667	9.97278334166667	10.5536035458333	10.3122044125	10.7230290916667	9.75428324583333	10.808824875	10.5000138	10.7342462625	10.8636915208333	10.8508691833333	11.4694963083333	11.1439293333333	11.2156097458333	10.3145290375	11.0107050083333	11.0695657583333	10.8735190708333	9.957901925	10.8880026958333	10.9293807916667	11.7103631916667	11.2501076666667	11.060997375	11.5943322833333	11.1723113458333	11.0871937291667	9.68625733333333	11.2422697958333	11.3222447666667	11.2196669	11.4002736958333	11.2555266	10.9037622625	11.1504202166667	11.1690845375	10.8700404666667

CABYR	8.09852281666667	7.24193815	7.76403325833333	7.89028620833333	7.34497685	8.57995885	6.68595645833333	7.743439475	7.127807925	7.189475125	11.2240675166667	7.3361665	7.06279625833333	6.85607033333333	7.745650475	7.51472684166667	7.53185565833333	6.53137754166667	7.299009625	6.9998061	7.72796763333333	7.11617649166667	7.81456171666667	7.729718875	7.27535064166667	8.28193894166667	7.40898444166667	7.63210018333333	7.43067104166667	7.11278106666667	7.363992875	7.21451231666667	6.96183981666667	6.87851200833333	8.91224634166667	8.47333296666667	7.659482025	7.42906085	7.571028	7.867210225	8.06285705833333	7.46134775833333	7.59310200833333	7.68593156666667	7.63102280833333	8.228841125	8.36034350833333	6.51078761666667	7.80272595	7.9238629	7.18351369166667	6.92042344166667	7.64166063333333	6.657721925	7.52330799166667	8.09897714166667	7.34718618333333	7.81786669166667	8.04704694166667	7.34669549166667	11.4236775666667	12.2675250666667	9.24031536666667	9.471590125	11.114387025	7.97923883333333	10.8447210916667	7.92005815	8.7217903	8.582021475	12.8918006166667	12.049870975	7.846884125	9.97912404166667	7.79957504166667	8.74427688333333	7.85633409166667	7.77487424166667	8.472359075	8.84011393333333	10.8953436916667	9.29432763333333	9.47590374166667	10.72841425	8.25692778333333	9.182097875	12.0868849916667	8.39419055833333	12.518275925	9.47641323333333	7.77718458333333	10.0732835666667	10.5061588333333	8.71519911666667	8.69317739166667	11.3871722333333	8.42733516666667	10.548620125	7.76345526666667	9.50539336666667	9.24031536666667	8.55392016666667	9.02085209166667	9.71227291666667	9.17093331666667	9.47590374166667	9.41990620833333	8.054062325	9.49238265	7.59310200833333	9.68558669166667	8.910111275	7.99937465833333	9.71265901666667	9.442472475	11.6895204333333	10.7309865083333	8.52279583333333	11.7085670166667	7.414082675

TRDMT1	11.500844625	9.6347809	9.37588805	9.28551639166667	10.02519555	9.82651539166667	9.87164061666667	9.72484969166667	9.9560883	10.054135025	9.471100975	10.8416766333333	10.30576505	9.765982475	10.2741449416667	9.70495998333333	10.1713085416667	10.0765568416667	10.04039335	10.0984817166667	9.95100449166667	10.2904392333333	9.4551338	10.040006825	10.1628878833333	10.9483433833333	9.59742406666667	9.7815357	10.64689975	9.292040175	9.88434308333333	9.73142488333333	9.92792138333333	10.0765568416667	10.3530735416667	10.9069463833333	9.9338367	9.68558669166667	9.54834169166667	10.082743125	9.61456806666667	9.66513115	9.91801125833333	9.74568905833333	10.1870376	9.565921775	10.31750855	9.84075720833333	9.62496784166667	10.2145140083333	9.99593061666667	9.777390875	10.3671094333333	10.3578687916667	9.46432354166667	10.045510375	10.2494268583333	9.80755264166667	14.063152775	10.5619901333333	9.89687281666667	10.2485867583333	9.66513115	10.7631580166667	10.6328931583333	9.69747909166667	9.26809461666667	10.0226518416667	9.98800139166667	10.0343496666667	9.27461214166667	9.93673649166667	9.89894956666667	10.114399525	10.33090645	9.07777099166667	10.0031143833333	10.6111916083333	9.24884609166667	10.000563275	10.347919	10.9346168	9.53043561666667	9.911212725	9.96954501666667	11.1354232166667	10.6780826666667	9.81608086666667	9.16302515	9.78824573333333	9.66283631666667	10.44259115	9.67141531666667	10.0156224166667	9.85036418333333	9.44516316666667	10.4838867083333	10.48796815	9.8597846	10.1883097083333	9.93304795	9.79016168333333	10.6645539	9.70284449166667	9.45055241666667	9.88253726666667	10.1713085416667	9.573560175	10.390544	10.0533049083333	10.0069849166667	9.62666130833333	10.406884125	10.0984817166667	9.77571775833333	9.38057958333333	10.783568125	9.80625096666667	10.959358675	10.4713528083333

FGFR1	12.6141732083333	11.1790792916667	11.20219855	11.4096653083333	11.9184658666667	10.9450853666667	12.4598136333333	11.004921375	11.6359301083333	11.3835567833333	11.6452682	12.4701553166667	12.1447048	11.09861755	11.1441289083333	11.0333724416667	10.5408711916667	12.02261385	10.9245036	10.934250825	11.16005095	11.2568448166667	10.764714825	10.651587675	11.0596766	11.7838361	11.2872125416667	11.3426956833333	11.6499693666667	11.1941109166667	11.14949005	11.1815244666667	11.827360075	11.705716875	11.7699299	11.6485244	11.8341069333333	11.2968704833333	11.28518	11.4431148583333	10.6797192416667	11.2247431833333	10.9545739416667	10.63689965	11.0031119583333	10.3301703333333	10.5252205583333	10.76764485	10.5545011333333	10.9084288583333	11.2032221333333	11.1060515416667	11.4926248916667	11.4296055416667	11.25569785	10.6072418333333	10.9831460083333	10.782424925	11.234518675	10.7352154666667	11.9162394	11.7160036	10.91165365	11.4978397166667	11.14669605	11.6791509083333	11.5828548666667	11.51924745	11.1506157166667	11.629073975	11.799711325	11.5440185916667	11.6425911333333	11.1806950666667	11.6705910083333	11.064089175	11.8085467416667	11.30507995	10.7526329	11.279388675	11.4828657583333	12.0884912166667	11.1509851333333	10.6449643666667	11.717933225	12.1852414666667	12.919046025	11.7564937416667	11.9162394	10.9080821583333	11.2997236583333	11.3072160833333	11.4467356166667	11.4151310833333	12.1843992666667	11.9538193583333	11.6719609166667	12.731765875	12.443519	11.3713269916667	10.9397461666667	11.946316525	11.2100932	10.6507559	10.6637664333333	11.0221982916667	11.2319732666667	10.552679575	10.81468445	10.7643055083333	11.155024025	10.8972744416667	11.0905957416667	10.6139707	11.0239555333333	10.98875735	11.6974443166667	10.8960985416667	10.7722019	11.1771749333333

MOG	7.15389984166667	6.10313131666667	6.65915351666667	6.493249575	6.60761675	6.92575055833333	6.88578850833333	6.633753725	6.40124803333333	6.50580336666667	6.62636613333333	6.95358578333333	6.26211881666667	6.09669481666667	6.33645039166667	5.90588820833333	7.22238968333333	6.88512086666667	6.19129395	6.11785979166667	6.14647701666667	6.18660088333333	6.44217180833333	6.62102673333333	6.478653025	6.87469296666667	6.34302255833333	6.10156668333333	6.69050035	6.67856683333333	6.18408195	6.3687361	6.77436175	7.190008575	7.91577950833333	7.31196651666667	6.92096200833333	6.76187970833333	6.874007675	7.04503860833333	6.86736839166667	6.04445175	6.06574364166667	6.66522548333333	6.10873680833333	5.897311975	5.88595835	5.64406685833333	6.11387184166667	6.811853775	6.983493075	6.77563099166667	6.74474600833333	6.39012523333333	6.62102673333333	6.18489624166667	6.510020875	6.8399633	6.54365829166667	6.49608473333333	6.76905384166667	6.82586836666667	6.72519365833333	6.63179246666667	6.55593524166667	7.24887754166667	7.52673725833333	6.30714161666667	6.60956075833333	6.94820169166667	6.787125175	6.771060625	7.07292095833333	6.71243863333333	6.75097239166667	6.725831175	6.86530675833333	6.92852376666667	6.67783206666667	6.59464205	6.38621459166667	6.14803923333333	6.698517925	6.87022720833333	6.4308335	6.69182425833333	6.82704920833333	6.83084248333333	6.94881511666667	7.102506125	6.93227245	7.45403938333333	6.54638811666667	7.02822294166667	7.49991619166667	6.98280363333333	7.30497195	7.219834	7.010804075	7.08866475	6.89851890833333	6.69380246666667	6.98410809166667	6.70257901666667	6.75097239166667	6.72953939166667	6.61484865833333	6.89266504166667	6.69500276666667	7.08560825833333	6.56145719166667	6.85156043333333	7.551793125	7.15020035833333	7.13607696666667	6.56951250833333	6.6716762	7.851546575	6.90589670833333	6.88791735

HSPA8	13.160332775	10.2321384166667	9.6592899	10.2494268583333	10.289694575	10.9058347	9.131237375	9.93476125833333	10.4994687166667	10.0134059083333	9.28405401666667	10.4971305166667	11.4310404083333	10.0872885916667	9.15019934166667	10.0627631	10.2802737333333	13.1506037833333	10.0852465083333	10.2149556666667	9.6428903	10.7193725416667	9.61139459166667	10.3101529333333	10.66793845	11.327151075	10.0920085583333	10.0450906333333	10.7793443666667	9.62064585	10.7482129583333	10.680077475	10.7526329	10.418815025	10.1109480666667	10.7877953583333	10.6819563666667	9.34847368333333	10.5581448083333	11.0075637333333	9.87695954166667	10.783568125	10.80487515	9.6185904	10.6068679083333	9.81608086666667	10.6535513666667	10.8322571166667	10.8938022916667	9.77312443333333	13.408153975	10.3135871416667	11.2521123083333	10.8378553416667	10.84359385	10.30399485	9.949333375	10.20559775	10.2363454416667	11.1663193416667	13.7730636333333	11.7517355166667	11.6951784416667	11.94832135	9.67096889166667	11.9724754666667	9.96267133333333	12.5415392083333	11.608877475	10.3733561083333	10.749691325	11.1764115416667	12.1600024166667	12.164467625	10.2182610833333	11.9040111333333	11.4745197333333	12.92419445	10.9245036	11.2254735166667	10.9466012416667	9.68695163333333	9.83406541666667	10.7463771	13.1190556333333	10.4649425166667	11.6378736916667	12.689165025	13.05246665	10.500555575	12.2513034583333	12.7509478416667	11.4173578916667	11.3675917666667	13.0584991666667	10.0506734583333	10.6173169916667	10.4986956916667	13.11105415	10.501807225	9.91374403333333	11.4741238583333	12.0997306916667	9.95059544166667	11.2968704833333	11.0491091083333	11.9368778583333	11.2334234166667	11.17354635	11.5606001833333	11.7720983166667	10.5581448083333	11.5118143666667	11.5656597	10.7258049	11.9844494166667	10.9976123	12.770725525	10.6111916083333	10.6122965916667

TRPS1	6.94312051666667	5.22492120833333	5.892821925	6.955027575	8.28143178333333	7.87768698333333	4.62889925	6.04613815	6.6716762	7.20393616666667	5.65633813333333	4.36294506666667	6.35504728333333	5.38263135833333	8.33702585833333	5.48415848333333	4.51953901666667	8.793933	7.64430480833333	6.60826071666667	6.8528802	8.4100903	4.63305978333333	6.68395904166667	7.81246279166667	4.51953901666667	6.792709475	8.24861051666667	8.333491425	6.37349159166667	8.1898559	7.002378425	5.17603111666667	4.56806040833333	6.17555735	6.20262720833333	9.037331875	7.06400038333333	6.92042344166667	7.064683475	6.3378065	7.55016986666667	7.52729815	6.74746250833333	5.5633586	6.32668329166667	6.65552751666667	5.88521655833333	5.57451170833333	6.63574723333333	5.824619025	6.97159590833333	6.65552751666667	7.9977282	7.040686725	6.07429128333333	6.35201275833333	6.872774725	6.627072075	7.3741849	6.55667590833333	4.99794086666667	6.01215780833333	5.45375554166667	6.80124884166667	7.88811874166667	4.93234401666667	5.80528018333333	6.40489274166667	5.03930840833333	4.383064975	5.37452059166667	3.508676875	5.68304789166667	6.28034841666667	4.14274019166667	5.49431639166667	7.3367108	6.62563488333333	6.4034157	6.241933725	8.20331464166667	6.45538780833333	5.927679125	4.85146489166667	4.60485635	6.09754041666667	4.57827949166667	6.24607838333333	6.14881959166667	5.80937455	4.644124975	6.72055851666667	6.11387184166667	6.47930140833333	4.40903533333333	7.548601625	7.05838413333333	5.45901654166667	5.71416341666667	5.77198655	3.86613395833333	7.02222090833333	6.16277881666667	5.24195544166667	6.258372575	5.876765525	6.04962533333333	5.59754975833333	6.63866813333333	4.73206480833333	5.50024889166667	6.61809366666667	5.64021978333333	5.88432855	5.04557781666667	6.3378065	5.77106630833333	5.766309475	3.99930385833333

LAT2	13.913193025	13.7591727416667	14.2872888583333	14.3090437083333	15.0769655666667	13.9529996833333	15.3777313666667	13.89301535	14.66935455	13.853956425	14.2319492833333	15.1017513083333	14.70147985	13.6570629916667	13.484103425	14.1384134166667	13.0186633916667	14.594415925	13.8572965333333	13.7542141333333	14.0643996166667	13.7670768833333	13.7195569416667	14.0201742416667	13.89505895	13.977355925	13.8395483	13.8001487666667	13.66414075	14.127909425	13.24746105	13.8919290666667	14.419240975	14.2260493166667	13.4260804333333	13.6605641666667	14.371029675	13.2068344333333	13.6310840583333	13.7740900916667	13.08588685	13.2387164583333	12.5534552416667	12.895840375	13.935575975	12.8251494083333	12.8432333583333	13.622834375	13.5502258583333	13.9682216833333	13.5677928083333	14.0979296916667	14.618270675	14.047904	13.798249925	12.7016097166667	14.107174325	13.021150225	13.8395483	13.2227845833333	14.9507696666667	14.8724941166667	14.6862769916667	14.80624315	14.6758595916667	14.7802388333333	14.8412522666667	15.2650881666667	14.68415095	15.1234964083333	15.2784907166667	14.53565035	14.9020399416667	14.6087993833333	14.6758595916667	14.07729275	15.236084325	14.7034991833333	14.2563286	14.4809973583333	14.9359352083333	15.1800900333333	14.5176714166667	13.8972210333333	14.9594767083333	15.1430836166667	15.0865438916667	14.9752180916667	15.1460462916667	14.3582404416667	14.6883693	14.259430575	14.9045675833333	14.4377971083333	15.01216265	14.8578939833333	15.0894272416667	15.1973098583333	14.6778149	14.364637875	14.4109315833333	14.9422941416667	13.2181367583333	13.18921875	14.0578490916667	14.5073052333333	14.0301250916667	13.4642437583333	14.2245923416667	13.9695187666667	14.312396275	13.5643847833333	13.8317321333333	13.171325425	13.935575975	13.7420477291667	14.27950095	13.9554702666667	14.055300325	14.7293250666667

KIAA1257	9.661950625	9.478582775	8.92028675	8.24315470833333	8.821700275	9.66469236666667	9.227470725	9.20605604166667	9.59742406666667	8.92973970833333	10.257101525	10.534888275	8.48950848333333	9.38536081666667	10.1105163666667	9.39843498333333	10.0313344083333	9.29018041666667	9.297473225	9.929600025	9.38409195	8.846155575	9.24712485	8.74083754166667	9.53875820833333	10.0197701416667	10.3380831916667	8.76950824166667	9.3036884	9.50643220833333	9.64247345	9.17515170833333	9.27365428333333	8.60947813333333	10.2249992083333	9.77883299166667	9.45898255	8.94849429166667	8.11761579166667	9.49057863333333	9.84242584166667	9.22652003333333	9.5453508	10.5806393916667	9.6530765	9.44914495	9.66781955833333	8.77406471666667	8.28000224166667	8.95991093333333	9.20235228333333	8.706897975	9.64153864166667	9.177752275	9.41030455	10.1440795833333	8.8912541	8.97027915833333	9.570506275	9.67141531666667	9.352137375	9.277313475	9.3392826	9.88681354166667	9.11034851666667	7.96986838333333	8.578964875	9.237031475	9.777820325	9.939168625	10.9230393083333	11.73203235	9.22606134166667	8.62547046666667	10.6523425166667	10.0696848333333	8.80189605833333	10.3837830083333	8.09897714166667	10.6614548166667	9.82907005833333	9.88298375833333	9.06069035833333	9.6526333	8.39620023333333	9.9690997	9.0844393	7.87098755833333	9.75828255833333	9.11131928333333	8.07152965	9.70114725833333	8.71838005	8.23315546666667	8.87736359166667	10.1919662416667	10.3119751583333	10.8608532	9.34157415833333	8.74272486666667	8.52966328333333	9.36084673333333	8.74322296666667	12.0705755166667	11.2319732666667	9.003775725	8.37303865833333	8.74522520833333	8.94143755	8.3301117	8.39063175833333	8.488931425	7.9877223	7.691592325	9.35255276666667	9.24539353333333	9.52223368333333	8.60646375	12.908758175	8.18363113333333

EPOR	9.95973609166667	8.90197375	9.06697433333333	8.873364875	9.19676581666667	9.10621828333333	9.50586739166667	8.84428041666667	8.75703444166667	9.16216856666667	9.0115544	10.0774221416667	9.674571275	8.89174859166667	8.58958554166667	9.31762243333333	10.1887239416667	9.73098034166667	8.635462725	9.15259453333333	9.09642976666667	9.33181315833333	8.94577069166667	8.96322584166667	8.83783124166667	9.448687325	9.12606195	9.06791326666667	9.20190699166667	8.88592250833333	9.002830825	9.03503039166667	9.31227445	9.630635475	9.2768977	9.41849085	8.850647275	9.003775725	9.06503669166667	8.81971978333333	8.64839240833333	8.758367425	9.04133171666667	9.047926775	9.286424975	9.21835433333333	8.82424165	8.55955131666667	9.08180965	8.7292202	9.14327603333333	8.5671537	9.45323241666667	9.41531880833333	8.95262451666667	9.16216856666667	8.81066338333333	8.594439725	9.318611725	8.85215985	9.76350663333333	9.349869075	10.1569732333333	9.8269075	9.693930075	10.390544	9.56873605	9.21350573333333	9.68558669166667	10.30399485	9.29065144166667	9.59692015833333	10.1308023166667	9.44430623333333	9.544894	10.0292860833333	9.90137155	9.68863114166667	9.87254320833333	10.119247225	10.6061332083333	9.45472053333333	9.95359933333333	10.4280419583333	9.80492948333333	9.92916715	9.91930759166667	9.60319949166667	10.0111258333333	9.92254819166667	9.76954021666667	9.90518528333333	9.06838428333333	9.345810075	10.090730825	9.579948275	10.1625186333333	9.81007735833333	9.98509406666667	9.00330966666667	9.53542963333333	9.76219373333333	9.40727955833333	9.20100326666667	9.88045179166667	9.42590730833333	9.88434308333333	10.338453	9.37403768333333	9.95852463333333	9.09237333333333	9.50685594166667	10.302373075	9.98596199166667	9.99509398333333	9.15555419166667	9.8880168	10.7272324083333	9.88253726666667	9.44826335

FAM174C	10.866231375	11.8012624833333	11.9931218416667	12.017715375	11.8004315583333	11.8174744333333	11.6436590416667	11.66838785	11.660606675	11.946316525	11.875371875	10.7276175666667	11.2957967833333	11.7586025166667	11.6694643083333	11.5548527833333	11.6691177916667	11.4225410666667	12.023387575	11.5731480083333	11.824518825	11.61832875	11.7690899333333	11.7993113833333	11.5731480083333	10.58489685	11.7761557916667	11.6617552333333	11.685253825	11.8523757333333	11.4708276916667	11.7772182666667	11.403040875	11.7391389833333	10.7793443666667	10.8484072166667	11.7509367	11.5934022416667	11.545061825	11.7168402	11.74563025	11.5891254	11.7324169333333	11.6594960166667	11.3594507166667	11.848881975	11.7568591416667	12.029837025	11.7067053416667	11.4534829083333	11.6890849333333	11.7304346833333	11.8130518166667	11.8178724083333	11.713529275	11.86975775	11.5520144833333	11.8477473833333	11.4639403833333	11.7399779416667	12.156197075	12.247084775	12.1480274583333	12.0864940333333	11.61112325	11.4133387916667	12.078967375	11.66214955	11.9297077583333	11.76865155	12.2433310083333	12.04365515	11.4715256833333	11.8186960583333	11.4284677	11.56524335	11.5475917083333	12.0317754666667	11.8367863833333	11.8848963583333	11.2542786416667	10.7812240416667	12.033353625	12.2743988	11.8059034833333	11.1604297833333	11.7422357083333	11.7517355166667	12.0713921666667	11.8775774416667	11.4296055416667	11.8588339833333	12.1579610916667	11.9975093	11.5626319666667	12.000538875	11.960958325	11.2287441666667	11.4989285	11.8048205583333	11.6270644583333	11.8426813333333	11.7703479083333	12.118235175	10.6535513666667	11.401407675	11.7235508	12.265420025	11.7251079666667	11.5128736833333	11.544719375	11.8568699583333	11.0390903416667	11.7403808583333	11.6540451166667	11.832540575	11.2254735166667	11.4470684833333	12.4496792916667	11.6746522

FBXO41	9.446046	7.93695109166667	8.94330425	7.44735373333333	7.34272655	8.21405428333333	8.019555675	7.48476935	7.32258515	8.05804941666667	8.2260001	7.38818769166667	6.76253231666667	7.80451180833333	8.52772891666667	8.43662665833333	8.046473725	7.06942048333333	8.29403910833333	7.9291649	7.79957504166667	7.48759605	9.00735385	8.054062325	7.66558521666667	8.85824333333333	8.35071744166667	8.08335636666667	8.20831443333333	8.08185559166667	8.70034938333333	8.8778449	8.07839394166667	8.70400716666667	9.31809370833333	8.54699226666667	8.07622910833333	8.37811988333333	8.20331464166667	8.50984575833333	8.46826041666667	8.33398970833333	8.45999591666667	8.71882579166667	7.91097105833333	8.70080298333333	8.17720695	8.5034354	8.33702585833333	8.31670425833333	7.89243905	8.65559314166667	8.01700516666667	7.96073305833333	8.4067182	8.62886449166667	8.815557	8.45605063333333	7.85906496666667	8.4100903	8.83883193333333	9.569144925	8.1959042	9.119480775	9.31047634166667	8.7217903	10.4883478333333	8.9198372	9.9229238	9.39626775833333	9.08687805	10.2064993416667	8.67688486666667	9.0540245	8.02019604166667	10.2191020916667	7.46463336666667	9.777820325	9.61596005833333	9.36892748333333	9.69439403333333	8.97769849166667	8.937187475	9.98509406666667	7.08048500833333	8.8011824125	10.3277465	8.71647500833333	10.36145645	8.74766549166667	8.875989475	11.2975735583333	9.25858543333333	8.49648456666667	9.10015925	9.94677204166667	9.39478965833333	9.580833825	6.76517564166667	8.64839240833333	10.0082740166667	9.186916425	10.2481894083333	8.71562445833333	8.85011799166667	8.635462725	9.02609295	9.666961675	9.716989375	8.07938845	9.24712485	9.844981225	9.0791656	10.3369395416667	8.83249931666667	9.77219498333333	10.1713085416667	9.686488825	9.81261425833333	8.21672670833333

EIF1AY	10.5899519833333	9.30586555833333	9.16352800833333	8.86211740833333	8.14082359166667	8.59397561666667	8.83016251666667	9.14426045	8.97815918333333	3.71049449166667	7.73690679166667	8.51856025833333	3.57891131666667	4.03968158333333	9.04085205833333	8.94049991666667	9.59235401666667	8.817877875	9.54225544166667	9.43324608333333	9.14971231666667	8.55442540833333	9.22061473333333	9.25814955833333	9.203321075	10.1759041833333	4.16299614166667	9.12281406666667	4.24335745833333	9.07065694166667	4.383064975	9.06838428333333	8.70034938333333	9.06886545833333	10.789595925	10.5108422833333	8.51578285	8.903371	9.15645445833333	9.307742	9.471590125	9.44734175833333	4.55863708333333	9.17093331666667	3.92815261666667	9.47542340833333	4.729262375	4.77378615833333	9.307742	9.0540245	4.537079425	9.34261419166667	7.38755610833333	8.05913828333333	8.40817908333333	8.71562445833333	8.92792624166667	9.33664844166667	8.68624993333333	4.41782250833333	9.50725341666667	6.76253231666667	8.18684245	6.15778135833333	8.87636833333333	5.83674730833333	7.238340075	6.25518099166667	6.42946571666667	3.52754506666667	6.15447426666667	5.12313813333333	3.477151725	3.586969725	7.0307229	7.18351369166667	8.52772891666667	8.16684950833333	7.42103133333333	7.39926451666667	10.217472825	8.84659819166667	6.82704920833333	8.95991093333333	6.77905188333333	6.85216409166667	3.70714133333333	8.66441525833333	3.57121334166667	7.91142259166667	3.65945140833333	8.083905375	6.54574596666667	8.4484208	7.20859641666667	7.044419	5.89819270833333	6.81508890833333	6.07176163333333	10.10216065	7.54341655833333	6.44595796666667	4.57827949166667	7.20911576666667	4.04713170833333	7.43688079166667	4.4159867	5.873141325	9.1218467	7.44317530833333	4.46791538333333	9.65575276666667	5.72573654166667	7.03011389166667	6.734891125	8.15349528333333	8.656035175	8.66247435	8.95764171666667	3.52754506666667

SLC14A2	6.36170378333333	5.37867954166667	5.21624118333333	5.41802705	5.57335978333333	4.27798759166667	5.81563615833333	5.72686785	5.08659576666667	5.30664471666667	5.11789035833333	6.45472701666667	6.37643818333333	5.08997001666667	5.482101875	5.677007675	4.73928888333333	6.75238478333333	5.60132373333333	5.77807068333333	5.88241164166667	5.98737700833333	4.87503585	5.54102934166667	4.34698491666667	6.62563488333333	6.30492928333333	4.37287369166667	6.195909975	5.32134071666667	5.02689723333333	4.6622505	4.852466025	5.26362051666667	6.79403766666667	6.478653025	5.6695191	6.06230976666667	5.57148620833333	5.6488719	4.546642225	4.545583025	4.41695324166667	5.10077205	4.681784575	4.78586035833333	5.42470928333333	5.064717325	4.30624979166667	4.63513973333333	4.567125	5.03622141666667	5.498293125	5.18519845833333	4.77601478333333	4.50312849166667	5.57252066666667	4.4047953	4.84935865	4.909691175	5.26468096666667	5.87405735	4.207987625	6.17067161666667	5.0088772	5.3439553	6.2066078	5.38558591666667	4.34281185	5.57148620833333	5.21920105	5.88521655833333	6.22675393333333	5.135343	5.46647429166667	5.467505875	5.25183510833333	4.88576699166667	5.03010108333333	5.29779213333333	5.98737700833333	6.43016624166667	4.99794086666667	5.89630836666667	5.33069273333333	6.057986625	5.91520025833333	5.1653561	5.77633328333333	5.53102171666667	5.47616439166667	5.00768225	4.42481975833333	5.51098925	5.60406076666667	4.78700018333333	5.8625617	6.65984261666667	5.485111725	4.92770649166667	4.11987200833333	5.50024889166667	4.48829116666667	4.617309275	4.69642039166667	5.8532158	5.09317755833333	5.37452059166667	5.229288475	6.0148863	4.41695324166667	5.1291031	6.13011929166667	4.51953901666667	5.61840579166667	4.39557236666667	4.82998554166667	4.30027178333333	4.07457460833333	5.50701503333333

SHOX2	10.1713085416667	9.71485326666667	10.3427675333333	10.0498058583333	10.70166785	11.0534747416667	10.8681221916667	10.1214563916667	10.6058164916667	10.112191475	11.04340915	10.8349029416667	10.2478255083333	9.55671014166667	9.833640075	9.11266035833333	11.0116340666667	10.2912155166667	9.48384479166667	9.74045244166667	9.45281165833333	9.55566569166667	9.26809461666667	9.12842238333333	9.55333915	10.3101529333333	9.91374403333333	9.69837386666667	9.94127946666667	9.683051225	9.625456175	9.51811660833333	9.97174644166667	10.534129425	9.64431238333333	9.797655	10.5181477	10.20436555	10.1621245583333	10.064052975	9.2612694	9.32000775	9.11582745	9.27312065	9.41030455	8.96850814166667	8.89353219166667	9.3036884	8.96700605833333	9.145650675	9.49101396666667	9.61413186666667	10.2014148333333	9.728395625	9.6324307	9.13694515	9.61006324166667	9.64383103333333	9.57736215	8.88450143333333	11.8418826916667	10.9032780416667	10.904327125	10.7885218833333	9.97791586666667	10.7628088916667	10.70166785	11.8573117083333	10.2494268583333	10.2995461416667	10.9486958083333	10.6465456666667	11.3690796916667	11.632505775	10.4752224083333	11.6929484666667	10.6788649916667	10.9204831	10.5177006833333	11.320943175	10.26300475	10.82884605	10.7722019	12.6312122083333	12.13848275	10.1678889166667	10.48105345	11.2514092416667	11.5162409083333	10.9234198666667	11.1470598583333	10.1013192166667	11.48757865	10.86771825	10.7904047166667	11.5385948833333	10.2826800583333	11.352411175	11.4151310833333	9.97301156666667	9.95852463333333	11.5302262416667	10.2018680083333	11.284173425	11.2107825666667	10.7478338666667	10.6637664333333	10.696845925	10.1978484	11.357567525	10.601543225	11.3332438	11.336435575	11.2624348416667	10.8105355416667	10.5876388833333	9.70796235833333	10.23098885	10.931065425	10.1295520916667

KAT6B	14.1764195333333	12.278442975	12.145884225	11.987910225	12.367659325	12.3448355166667	11.8182827333333	12.0585648916667	12.4015640666667	12.1711742666667	12.4804102333333	13.394322425	12.9417394416667	12.440827975	12.762350675	12.3627391666667	12.7628980166667	12.6586586666667	12.5646791416667	12.5376322583333	12.531130425	12.8360528583333	12.1686155916667	11.7070775666667	12.6996624166667	13.9085702916667	12.7117762583333	12.6190854666667	12.7841531916667	12.5140767416667	12.5410458083333	12.3664470041667	12.5202342666667	12.3160821666667	13.4345239916667	13.6979142666667	12.6674936416667	12.3855581	11.5132414833333	12.6407582416667	12.414568125	12.6195975333333	13.1274317166667	12.9185113	13.2876598083333	12.3448355166667	13.20077725	12.208161275	12.20367145	12.2624244833333	12.141404575	11.893980925	13.1085025666667	12.8278480083333	12.923601775	13.4267583666667	12.400136275	12.3943629916667	13.077594625	13.0500216833333	12.31311615	11.575819425	11.9796845666667	11.7750094	11.7168402	12.208161275	11.173905775	11.94832135	11.5279761083333	11.61112325	11.432485775	11.8635130416667	11.51586705	11.6643648083333	12.3297195416667	11.21108835	12.3046936083333	12.2883968333333	11.3191493833333	12.0067796166667	12.0201508916667	13.289124025	11.189465025	11.7053666833333	12.1130081666667	12.6254772333333	11.827759775	11.6080957416667	11.9494775416667	11.8997209666667	11.4207192166667	11.8386923083333	12.3904471833333	11.7403808583333	11.2646484083333	11.5895178083333	12.7263840666667	12.0843813333333	11.2499394833333	12.222327475	11.9832392666667	12.0657212166667	11.97391105	12.1229210916667	11.1014833833333	12.0149070416667	11.987523	11.5898474083333	12.2399849666667	11.9657351833333	11.388258375	11.9146982166667	12.2906208	12.0577849333333	11.7281943916667	12.2939030416667	12.02225065	10.76764485	12.0020893083333	11.7078053166667

UROS	11.8017051166667	11.3774736833333	11.612663925	11.602856675	11.61191255	11.3351005833333	11.002804875	11.5574931	11.3749692583333	11.794746475	11.7594075916667	11.763655725	11.1756714833333	11.3683256166667	11.3675917666667	11.4904941666667	12.0660844416667	11.205753575	11.3328774166667	11.5432980083333	11.606939225	11.5422054916667	11.7553657	11.5212430583333	11.5684552166667	11.9793031666667	11.6047856083333	11.4985882166667	11.545468825	11.608877475	11.1288009583333	11.4612426083333	11.2222793166667	12.037993675	12.20488075	12.2006641666667	11.44169995	11.6066366416667	11.3474088166667	11.6163034833333	11.5761677833333	11.3144428833333	11.1369321416667	11.473399075	11.1685570666667	11.3661426916667	11.46509415	11.6754049666667	11.4963967083333	11.5212430583333	11.0905957416667	11.352411175	11.3224431	11.3246780083333	11.0693836916667	11.4413379833333	11.189465025	11.3767696916667	11.3438040833333	11.5188708083333	11.3477921	11.3343049666667	11.7418730833333	11.302972225	11.6587921	10.90507365	11.0996178333333	11.3965275083333	12.1250050333333	12.1372175166667	11.2822211083333	11.7487231	11.4570874916667	11.1361895666667	11.5166142666667	11.5552373166667	11.5656597	11.66838785	11.1328362833333	11.8171008	11.3842695916667	11.5162409083333	13.0271148666667	11.7027128	11.25569785	11.6929484666667	12.0917340666667	11.2876344916667	11.2899480916667	11.8873133333333	11.3954232	11.3140591833333	10.680077475	11.7917720333333	10.9571307916667	11.29542035	11.1032271	11.6496725666667	11.3096843916667	11.512504775	12.0368526666667	11.74784205	11.7761557916667	11.5038172666667	11.1277733333333	10.995163975	11.1982937916667	11.0157092833333	11.6841362916667	11.9542491166667	11.1536039583333	11.5574931	11.859248825	11.3801476916667	11.2018586833333	11.9248644916667	11.2011511333333	11.4534829083333	11.322793725	11.7440902416667

ZNF677	8.91556449166667	7.38640515833333	7.76032476666667	7.95746913333333	7.64610639166667	7.29474379166667	8.20781665833333	8.717376625	7.58408555833333	7.57457279166667	7.05225570833333	8.90668976666667	8.472856875	8.02464245	7.8961731	8.00359983333333	7.639894925	6.96254510833333	7.2132819	8.058553875	7.99339445833333	8.167838375	7.27114775833333	7.97192683333333	8.30804240833333	9.53738644166667	7.54125486666667	6.83682504166667	6.83020955	7.34331021666667	6.59815521666667	7.54689145	7.65225225	7.83894598333333	9.22794726666667	9.58797373333333	7.39450265833333	6.526432	6.30044350833333	6.70856058333333	7.58911965833333	7.434165875	7.75009851666667	7.90584154166667	8.20831443333333	7.598264375	8.05973130833333	7.8648195	7.57457279166667	7.252371175	7.36211884166667	7.13294301666667	7.83348010833333	7.28259465	7.52953641666667	8.09947061666667	7.87869541666667	7.06888018333333	7.1508922	7.78326185	6.84636305	7.03811636666667	6.51915964166667	6.86736839166667	6.36709734166667	7.03384980833333	6.49087593333333	8.3471697	5.80446058333333	6.55593524166667	7.0609201	6.50501645833333	7.33320358333333	7.40662056666667	7.2162874	7.28556258333333	7.07060166666667	6.28265325	6.31880616666667	6.24607838333333	7.90584154166667	6.79923555	5.56242626666667	7.11080571666667	6.45696331666667	6.715741525	5.72187083333333	6.54514473333333	6.07085139166667	7.35003230833333	6.67096843333333	5.77716973333333	6.02469836666667	7.21861555833333	6.02209645	5.661700075	6.14647701666667	5.71516535833333	6.35880866666667	5.3343362	6.56215660833333	6.37857751666667	5.08571715833333	5.931318725	7.86164825833333	6.39012523333333	6.71730138333333	6.79798854166667	6.10074335833333	6.78990954166667	5.303590975	5.66843639166667	4.82135065	5.61059491666667	6.445141375	5.72686785	5.90065811666667	6.09272539166667	5.35182895833333	7.39621439166667

TK2	9.98331006666667	10.3544309041667	10.219633475	10.3815939083333	9.36916360833333	9.96501940416667	9.75130850833333	10.033295875	9.800763425	9.82405373333333	9.74151864583333	10.6798983583333	9.2986405375	10.2759190375	10.1310120416667	10.1322529208333	10.67906595	8.53264374583333	10.0093347083333	9.8417894875	10.5649371	9.59211479583333	10.1518891666667	10.0024278083333	10.0509227166667	10.3601016375	10.2082797708333	9.9521619375	9.43479355	9.82583156666667	9.7946667375	10.039806225	9.96846291666667	10.1991934291667	10.2859483833333	10.2041541041667	9.4894736625	9.8911957875	10.0801011	9.676585125	10.7090780125	10.5016048958333	10.3885498041667	10.8174508541667	10.5312993541667	10.7420044666667	10.6579921291667	10.3055547416667	10.7517576041667	10.6133715458333	10.1555516625	10.2168221708333	9.72430455833333	10.1851497333333	10.5075527083333	10.5993204833333	10.1768852375	10.3835625333333	10.42512495	10.4742334791667	9.47089290833333	9.2614998875	9.27833025	8.97007850416667	9.88149208333333	9.9945026625	9.58368255	9.74675707083333	9.0300746375	9.74285554166667	9.10866565416667	10.0140669333333	9.15282481666667	10.1702779125	10.1364682583333	9.33252880416667	9.69512940416667	9.0295440875	9.2810801	9.72430455833333	9.37527436666667	9.00667181666667	9.73165325833333	9.88188413333333	9.9999974625	9.98206792083333	9.00622565833333	9.95379169583333	8.80375630833333	10.1880852041667	9.21472344166667	9.87760744583333	8.82398185416667	9.66719131666667	9.33205741666667	8.81311722083333	8.8366618375	9.04767515416667	8.84637688333333	8.8113169	10.2319738041667	9.769328475	9.81878690833333	10.1991934291667	9.459201025	9.60433155833333	10.7373712083333	9.990259525	10.4247087833333	9.955879025	10.17979995	9.79341725	9.56338085833333	9.4462680125	10.0651116875	10.0191618083333	9.475663575	9.50749309166667	9.65777431666667	10.00078105

AC010542.3	9.98331006666667	10.3544309041667	10.219633475	10.3815939083333	9.36916360833333	9.96501940416667	9.75130850833333	10.033295875	9.800763425	9.82405373333333	9.74151864583333	10.6798983583333	9.2986405375	10.2759190375	10.1310120416667	10.1322529208333	10.67906595	8.53264374583333	10.0093347083333	9.8417894875	10.5649371	9.59211479583333	10.1518891666667	10.0024278083333	10.0509227166667	10.3601016375	10.2082797708333	9.9521619375	9.43479355	9.82583156666667	9.7946667375	10.039806225	9.96846291666667	10.1991934291667	10.2859483833333	10.2041541041667	9.4894736625	9.8911957875	10.0801011	9.676585125	10.7090780125	10.5016048958333	10.3885498041667	10.8174508541667	10.5312993541667	10.7420044666667	10.6579921291667	10.3055547416667	10.7517576041667	10.6133715458333	10.1555516625	10.2168221708333	9.72430455833333	10.1851497333333	10.5075527083333	10.5993204833333	10.1768852375	10.3835625333333	10.42512495	10.4742334791667	9.47089290833333	9.2614998875	9.27833025	8.97007850416667	9.88149208333333	9.9945026625	9.58368255	9.74675707083333	9.0300746375	9.74285554166667	9.10866565416667	10.0140669333333	9.15282481666667	10.1702779125	10.1364682583333	9.33252880416667	9.69512940416667	9.0295440875	9.2810801	9.72430455833333	9.37527436666667	9.00667181666667	9.73165325833333	9.88188413333333	9.9999974625	9.98206792083333	9.00622565833333	9.95379169583333	8.80375630833333	10.1880852041667	9.21472344166667	9.87760744583333	8.82398185416667	9.66719131666667	9.33205741666667	8.81311722083333	8.8366618375	9.04767515416667	8.84637688333333	8.8113169	10.2319738041667	9.769328475	9.81878690833333	10.1991934291667	9.459201025	9.60433155833333	10.7373712083333	9.990259525	10.4247087833333	9.955879025	10.17979995	9.79341725	9.56338085833333	9.4462680125	10.0651116875	10.0191618083333	9.475663575	9.50749309166667	9.65777431666667	10.00078105

PTER	5.20250346666667	9.20190699166667	6.03581564166667	8.94623185833333	5.64973066666667	9.99086305833333	7.62089398333333	5.95923255	8.799065075	8.71940105833333	8.45745534166667	5.69973553333333	5.14931930833333	8.88592250833333	10.5895974833333	9.26988130833333	8.42934195	7.91465324166667	9.06791326666667	9.29252663333333	8.83063586666667	10.3714480666667	6.80784224166667	9.31405536666667	9.06116049166667	5.82073376666667	8.861626075	6.5574676	7.27471595833333	9.19287955	9.55898925	10.3240922583333	8.62986514166667	10.1936096583333	5.876765525	6.542385675	8.868084675	9.212117675	6.060789425	5.85413349166667	6.5175539	8.83783124166667	6.38554745833333	10.0607135666667	6.30401625	9.01655344166667	10.0247316333333	9.037729975	6.95984925	9.28929068333333	8.9231771	9.612775675	9.546304725	10.0502702166667	9.60963610833333	9.64074395833333	10.1067055416667	5.29238863333333	9.93555589166667	9.15019934166667	5.55953659166667	8.70400716666667	6.13670685833333	7.990761025	5.14292011666667	7.0114101	8.027292325	4.63723314166667	8.17406114166667	7.09680668333333	6.76786575	5.51291259166667	3.93361386666667	8.29147479166667	8.4100903	7.9693647	6.40797193333333	8.955351225	8.3547531	9.01521926666667	7.02148474166667	9.85113778333333	5.72466863333333	9.01655344166667	8.73259671666667	4.812774675	8.92028675	4.53327731666667	4.85447411666667	8.94049991666667	9.124627175	10.1808367916667	8.752344475	9.03183454166667	4.34795044166667	5.0258117	8.06904244166667	7.73358451666667	4.05967894166667	5.19959509166667	5.64313004166667	8.21405428333333	7.64275718333333	9.44105665833333	5.197537	8.3536777	9.28599946666667	8.21830426666667	6.178567625	8.36187725833333	10.2699462416667	10.241903125	9.04742353333333	9.831945975	9.68471804166667	9.94339973333333	9.99895085	5.091030975	9.68160001666667	9.51769735

HEY1	9.7515126	9.51041425833333	10.3132196333333	9.818576775	9.60406910833333	10.7189561666667	10.1205788333333	9.63156661666667	9.97255511666667	10.4004658833333	12.31311615	10.7990034333333	10.0844426833333	9.93796431666667	9.65053335	10.4112315166667	9.87164061666667	9.02371780833333	9.357237425	10.013853075	10.4112315166667	9.06745036666667	10.36526325	10.298281975	10.134682025	9.86395566666667	9.949333375	10.0056583	9.17368278333333	9.78329576666667	9.28087420833333	9.75787298333333	9.92873065833333	9.66961651666667	10.4084577916667	10.79830065	9.46608615833333	9.71989020833333	9.88083685	9.89226535833333	10.476761175	9.81728660833333	10.057405675	9.59782546666667	10.5601410916667	10.1059188	9.949333375	9.7237141	10.2728709416667	10.1936096583333	10.5647318583333	9.48206465833333	9.66513115	9.23462919166667	10.0309458333333	9.96356175	9.98312965833333	9.98032226666667	10.2355191666667	9.94677204166667	13.9682216833333	11.868130425	11.824518825	12.5680695416667	14.0526804666667	10.164580475	10.9139727166667	11.2018586833333	11.1321175166667	11.4679092333333	13.3594846333333	14.1709219416667	10.8439824833333	11.471171625	10.9809278083333	10.9443778666667	11.483228	11.586583875	10.0292860833333	13.7195569416667	12.7591596666667	13.9717922666667	10.6625963333333	12.7016097166667	10.976899425	14.0391426	14.1554067916667	12.3114382583333	11.9447346	12.1418231	11.23046085	12.644626475	12.6098037416667	10.7907764333333	11.2743409666667	13.171325425	13.180045325	13.627458175	10.97984555	11.9935609583333	11.1615262583333	12.685194075	11.8588339833333	11.2902838583333	9.74128455	11.45778115	11.44566875	10.8032590416667	10.99905285	11.483228	11.06152365	11.4405829333333	10.523602425	12.2116409166667	10.5783390833333	12.3343950916667	13.1026328916667	12.124594875	11.767528775	11.385731825

TBX18	12.3610276166667	10.4115783666667	11.0383932333333	10.0103759833333	10.276116525	8.58958554166667	11.054552625	10.87179425	10.5196816583333	11.5548527833333	9.9338367	11.9705949166667	11.7648162666667	10.8510863583333	11.86892365	11.0999976083333	7.80781901666667	11.0600457083333	10.776374675	11.1129059916667	11.73203235	9.315897875	11.545468825	10.2539350666667	10.8941934333333	12.2719001166667	12.0120395666667	11.0916995	10.6064934416667	10.9317956333333	10.99552285	10.6986549	11.2453633166667	7.73985964166667	11.5828548666667	12.21725785	10.5330763833333	11.33173475	10.54728675	10.742514025	10.2554871833333	11.0934965333333	10.5837654416667	10.5119603416667	11.3036645333333	11.297940475	11.3375225333333	10.751563075	10.3745792583333	10.8368196833333	10.960420475	10.2064993416667	11.1398416833333	10.8537358333333	11.0733527583333	11.62492935	10.7313914916667	10.3758856666667	11.0239555333333	11.3698477166667	13.1026328916667	11.3470919	9.05454125	10.3771245083333	11.2323478	13.6366663666667	10.6111916083333	12.7531374166667	8.53782911666667	10.69756315	11.0454889833333	9.68825589166667	12.6496634333333	11.9705949166667	12.8251494083333	11.21653435	12.115901275	10.4231890083333	12.8866499416667	10.1389730083333	11.0294181583333	12.3122487083333	12.5999690416667	12.637166375	12.5813238666667	10.892685425	9.09184225	12.285271325	13.01427815	13.1137653	12.19605565	10.040006825	13.0632773	13.0980169583333	10.1278854583333	9.490132625	9.31762243333333	12.1587642333333	12.2719001166667	9.908637725	12.7263840666667	12.1600024166667	12.6626629166667	9.06405529166667	13.4579324833333	12.2111963416667	12.815500575	9.74266033333333	11.3886248333333	13.1299690833333	12.0905289333333	8.38611514166667	10.5061588333333	9.98877366666667	10.7725388333333	10.2978868083333	9.40511030833333	9.49057863333333	8.656035175	12.0025248166667

PSMB9	8.59030590416667	8.96017076666667	9.42183963333333	8.88283577916667	8.17113494583333	9.95341705416667	8.27680775	9.68013430833333	8.5178951375	9.39319808333333	8.70720409583333	9.21055289166667	8.36784924583333	9.6703398125	8.3464457375	10.9767135916667	10.4026659625	8.42544180833333	8.8971613	9.78759450833333	9.92113512916667	9.750549325	10.3469000458333	9.81544907916667	9.8141938375	9.25836749583333	9.7277608625	9.70691162083333	9.03989521666667	9.58595583333333	8.99371967083333	10.2385425625	9.3056499625	11.3963325041667	9.59211479583333	8.856558775	8.83806424583333	10.0913534291667	9.4732666375	9.52432105833333	9.68328635	9.16468879583333	10.220852275	9.97480565416667	9.98697523333333	10.5924247	9.75130850833333	9.41141477083333	10.3173191083333	10.1318623916667	9.5980376125	9.26513190416667	9.53158785833333	9.4252790875	9.55879715416667	9.7184655625	9.89281027083333	10.1951107083333	9.9461502125	10.5136351	9.30752442916667	9.09482996666667	10.6085738375	8.03302740833333	9.85389634583333	9.054282875	9.07935968333333	9.6533170625	10.0789348041667	10.582768125	9.3514624375	7.43661437083333	9.17626466666667	10.4234246625	9.48144947083333	9.77547627916667	10.6089805875	10.7029488041667	10.2184744958333	10.4026659625	8.82650967083333	9.29090133333333	9.73076655833333	9.55836635	10.4608159916667	10.1988461083333	8.78916697916667	11.009244075	10.7583585666667	10.5591043416667	10.512566975	10.12561285	8.9007611375	10.4977370333333	9.7951035625	9.68283467916667	9.196978225	9.84137288333333	9.61160879583333	9.32595024166667	10.7769088833333	9.506644075	11.192886075	10.4684897875	10.8260181208333	11.1137811833333	9.541552575	9.6454597	10.3161573625	10.2788492458333	9.93535606666667	9.41095289166667	10.5446355958333	9.33946395833333	10.6299902	10.1611400416667	9.6689442625	10.673208	10.09479455	11.6660926625

TAP1	8.59030590416667	8.96017076666667	9.42183963333333	8.88283577916667	8.17113494583333	9.95341705416667	8.27680775	9.68013430833333	8.5178951375	9.39319808333333	8.70720409583333	9.21055289166667	8.36784924583333	9.6703398125	8.3464457375	10.9767135916667	10.4026659625	8.42544180833333	8.8971613	9.78759450833333	9.92113512916667	9.750549325	10.3469000458333	9.81544907916667	9.8141938375	9.25836749583333	9.7277608625	9.70691162083333	9.03989521666667	9.58595583333333	8.99371967083333	10.2385425625	9.3056499625	11.3963325041667	9.59211479583333	8.856558775	8.83806424583333	10.0913534291667	9.4732666375	9.52432105833333	9.68328635	9.16468879583333	10.220852275	9.97480565416667	9.98697523333333	10.5924247	9.75130850833333	9.41141477083333	10.3173191083333	10.1318623916667	9.5980376125	9.26513190416667	9.53158785833333	9.4252790875	9.55879715416667	9.7184655625	9.89281027083333	10.1951107083333	9.9461502125	10.5136351	9.30752442916667	9.09482996666667	10.6085738375	8.03302740833333	9.85389634583333	9.054282875	9.07935968333333	9.6533170625	10.0789348041667	10.582768125	9.3514624375	7.43661437083333	9.17626466666667	10.4234246625	9.48144947083333	9.77547627916667	10.6089805875	10.7029488041667	10.2184744958333	10.4026659625	8.82650967083333	9.29090133333333	9.73076655833333	9.55836635	10.4608159916667	10.1988461083333	8.78916697916667	11.009244075	10.7583585666667	10.5591043416667	10.512566975	10.12561285	8.9007611375	10.4977370333333	9.7951035625	9.68283467916667	9.196978225	9.84137288333333	9.61160879583333	9.32595024166667	10.7769088833333	9.506644075	11.192886075	10.4684897875	10.8260181208333	11.1137811833333	9.541552575	9.6454597	10.3161573625	10.2788492458333	9.93535606666667	9.41095289166667	10.5446355958333	9.33946395833333	10.6299902	10.1611400416667	9.6689442625	10.673208	10.09479455	11.6660926625

MPP3	6.57166325	6.47064316666667	4.94355658333333	5.922335025	5.415860975	5.388701925	4.99247835	5.055819225	4.91851714166667	5.10716860833333	6.230051875	5.41039171666667	5.467505875	6.00206103333333	6.95924915	5.20354711666667	4.97072769166667	6.14728135833333	7.99615545	6.49390590833333	5.37127428333333	7.341560875	6.50501645833333	7.598264375	6.42485146666667	6.54365829166667	6.49825018333333	6.71367926666667	6.96712810833333	6.52987506666667	6.06147365	6.24783014166667	5.67810205833333	5.07000859166667	6.88184853333333	6.46419815833333	5.30151331666667	6.42556058333333	6.30492928333333	6.07937631666667	5.92070156666667	6.33415565833333	6.29278439166667	6.3475359	6.64156519166667	6.72953939166667	6.815678575	6.45767033333333	5.80728855	6.17555735	5.62140730833333	6.33264478333333	5.72466863333333	6.45093723333333	5.98915650833333	6.62839033333333	6.41739583333333	6.51237135	6.78446141666667	6.01932009166667	7.816692275	7.34210711666667	6.38846551666667	5.91719066666667	4.86479268333333	6.59883068333333	6.46640339166667	5.18423503333333	5.84048410833333	5.75774213333333	7.78927275	7.95464479166667	5.59572529166667	6.41504531666667	6.13259841666667	6.65690114166667	5.16838866666667	7.103742375	7.96986838333333	6.155322525	5.88673599166667	4.68789791666667	6.225930275	8.37303865833333	4.56909456666667	5.2783407	4.41103275833333	4.96638246666667	7.90991645	5.94443094166667	5.30151331666667	7.60415978333333	7.16794871666667	6.38084359166667	6.59052684166667	7.64327454166667	7.0469593	6.526432	5.5633586	5.95243336666667	5.48415848333333	6.96778875833333	6.9494722	6.70327563333333	7.98346735	5.62592511666667	7.907346175	7.37590343333333	5.70053848333333	6.2058813	4.89027461666667	5.83110111666667	6.59599640833333	7.46352631666667	6.89400789166667	6.824495175	6.86668814166667	7.448314425	8.641468925	5.47616439166667

ATF6	12.6725434333333	12.1600024166667	12.279687775	12.0395951	11.912794275	12.7334106833333	12.1063614833333	12.5329767583333	12.6674936416667	12.3396961416667	12.901864125	13.000133375	12.3190685583333	12.3110445	12.3798172166667	12.5939827166667	13.0721123166667	12.2888330833333	12.5140767416667	12.7117762583333	12.4932078666667	12.5415392083333	12.2883968333333	12.8240262666667	12.74583335	12.8068783833333	12.252092225	12.552591775	12.2918290666667	12.1480274583333	12.4536816333333	12.4559287	12.105099425	12.5492591916667	13.0149306583333	12.9461435166667	12.3712167583333	12.4114353583333	12.7763784666667	12.3930672583333	12.4265910916667	12.1034309916667	12.4046195166667	12.5590435083333	12.5457898	12.4549570666667	12.2637428083333	12.2433310083333	12.1600024166667	12.5358106083333	12.3046936083333	12.4091926166667	12.478144425	12.033353625	12.2976992	12.4767941833333	12.533964725	12.1852414666667	12.4917329166667	12.4950350166667	13.208876725	11.85040895	11.114752	11.3590797	12.409680475	12.3392091166667	11.9066987333333	12.1130081666667	11.3735584916667	11.8709570583333	12.228520475	12.5126991416667	12.2028833833333	12.1100796583333	12.3122487083333	11.8693609416667	12.3606021	12.0989443916667	11.3502989166667	11.7728057833333	12.7227603583333	12.9370697333333	11.9325731916667	12.4181461583333	12.5506564333333	12.19689565	12.6704503833333	12.1861275083333	12.2141453583333	12.2578527416667	12.0457744	12.3992340916667	11.4008627833333	11.9657351833333	11.7695141	11.4872383	11.585466225	12.4903405333333	12.4813575333333	12.7531374166667	12.1367818666667	11.76324455	12.5362402583333	12.233149625	11.794058525	11.5680602833333	11.708995875	11.3282347083333	12.0564210833333	12.338741975	12.3688898083333	11.9892168333333	12.0985319416667	11.2118401416667	12.242499175	11.8473399333333	12.881258225	11.328583825	11.5869361333333	11.97391105

ERCC2	6.94468725416667	11.6752083458333	12.0553632	11.8295441666667	12.2673117083333	8.33576940416667	11.8979768416667	11.5903534041667	11.8711520208333	11.7111165416667	11.0742373458333	7.9155036875	11.0324977125	11.8388954875	12.3234787791667	10.9323187791667	7.45639434583333	11.9676169291667	12.0134724791667	11.1400156416667	10.3525069708333	11.3259151625	11.0749304708333	11.80345385	11.6589487666667	7.7751871875	12.2300566416667	12.0031035583333	12.1813216291667	12.0232017541667	11.9564880291667	11.523193525	11.2030606	7.7327247125	7.56917553333333	7.35722785416667	12.3932861666667	12.0825688708333	12.1065423666667	11.2766206	11.982670775	11.8668661791667	11.5335937	11.5179760333333	10.6037069083333	11.9647476875	12.3349909666667	12.602520925	10.9023129541667	11.1303974833333	12.1551696708333	12.3659718375	11.8263604166667	12.0723492958333	11.7233523208333	11.8995331625	11.3006586041667	12.7131405041667	11.8613787333333	11.6505641	10.1216595625	11.1541339375	10.9856456166667	12.15980345	9.53809290833333	11.4747097375	9.6431333	10.269732875	11.1918332083333	8.7211183375	11.2493932541667	12.6038906708333	11.5473983958333	10.50986785	10.823421975	10.9333690666667	9.37198839583333	10.1426412375	11.8755577875	10.5258450791667	8.291232325	9.4668083625	12.9331752041667	12.1040788083333	11.3581195208333	8.94782725	8.91388026666667	11.9418665166667	8.76976393333333	9.856159925	10.7754033458333	11.3428663291667	10.4773652125	11.6304199166667	10.9752468958333	11.6957625166667	12.2961971583333	10.5450500208333	11.8385236375	11.4124685041667	11.50364235	11.2853500375	8.75494610416667	10.4082661875	12.7033686291667	11.1795687666667	11.6865255583333	11.8759321458333	12.1437462791667	11.3837239291667	10.9514615666667	12.4432756083333	12.1751696208333	10.3621011583333	12.06022305	11.9362989083333	9.63317724583333	11.3880691541667	11.5235644083333	7.835934875

KLC3	6.94468725416667	11.6752083458333	12.0553632	11.8295441666667	12.2673117083333	8.33576940416667	11.8979768416667	11.5903534041667	11.8711520208333	11.7111165416667	11.0742373458333	7.9155036875	11.0324977125	11.8388954875	12.3234787791667	10.9323187791667	7.45639434583333	11.9676169291667	12.0134724791667	11.1400156416667	10.3525069708333	11.3259151625	11.0749304708333	11.80345385	11.6589487666667	7.7751871875	12.2300566416667	12.0031035583333	12.1813216291667	12.0232017541667	11.9564880291667	11.523193525	11.2030606	7.7327247125	7.56917553333333	7.35722785416667	12.3932861666667	12.0825688708333	12.1065423666667	11.2766206	11.982670775	11.8668661791667	11.5335937	11.5179760333333	10.6037069083333	11.9647476875	12.3349909666667	12.602520925	10.9023129541667	11.1303974833333	12.1551696708333	12.3659718375	11.8263604166667	12.0723492958333	11.7233523208333	11.8995331625	11.3006586041667	12.7131405041667	11.8613787333333	11.6505641	10.1216595625	11.1541339375	10.9856456166667	12.15980345	9.53809290833333	11.4747097375	9.6431333	10.269732875	11.1918332083333	8.7211183375	11.2493932541667	12.6038906708333	11.5473983958333	10.50986785	10.823421975	10.9333690666667	9.37198839583333	10.1426412375	11.8755577875	10.5258450791667	8.291232325	9.4668083625	12.9331752041667	12.1040788083333	11.3581195208333	8.94782725	8.91388026666667	11.9418665166667	8.76976393333333	9.856159925	10.7754033458333	11.3428663291667	10.4773652125	11.6304199166667	10.9752468958333	11.6957625166667	12.2961971583333	10.5450500208333	11.8385236375	11.4124685041667	11.50364235	11.2853500375	8.75494610416667	10.4082661875	12.7033686291667	11.1795687666667	11.6865255583333	11.8759321458333	12.1437462791667	11.3837239291667	10.9514615666667	12.4432756083333	12.1751696208333	10.3621011583333	12.06022305	11.9362989083333	9.63317724583333	11.3880691541667	11.5235644083333	7.835934875

POLR3G	9.78692435833333	9.82907005833333	9.84719424166667	9.84876684166667	10.5876388833333	9.91887936666667	10.3745792583333	10.5424705	9.95973609166667	10.4931364166667	10.2881617	9.70796235833333	9.98961361666667	9.97912404166667	10.103849375	9.68430186666667	9.02747416666667	10.337717825	10.32163045	10.1337610833333	10.102550325	9.95852463333333	10.0537308333333	9.824729	9.83982443333333	9.146936475	9.953234775	9.89258960833333	10.3996095166667	10.0386893	10.2249992083333	10.1148100333333	9.96400001666667	9.82860243333333	9.3832069	9.612283675	10.478998225	10.5659717333333	10.4380961666667	10.6492363416667	10.2543488416667	10.0260539583333	9.7100974	10.4115783666667	9.570506275	10.1350682166667	9.90438676666667	10.6652778583333	9.82203395	9.546304725	10.3353402416667	10.5424705	10.5973351833333	10.410088175	10.4340765083333	10.1853907583333	10.4577793166667	10.6926124666667	9.98761663333333	10.0528892666667	9.277313475	10.3436245833333	10.114399525	9.58797373333333	10.2551392166667	10.1080216833333	10.4542782	9.27643005833333	11.0787308166667	10.217472825	10.3475834	10.5037659416667	10.2535245166667	9.06600838333333	10.4215953	11.221922825	9.25672935833333	10.534888275	11.5691835	10.2535245166667	9.909101975	8.97905946666667	10.8861829666667	10.8028896	9.22249476666667	9.6482902	9.657103925	10.4449298	10.6038899083333	9.70194608333333	9.91801125833333	10.3798706916667	9.78018595	10.31869075	10.9607806583333	10.1783585083333	11.2616452583333	10.0802966083333	10.4445170416667	9.76554074166667	10.2047893666667	10.323288425	10.5420798333333	10.5987255666667	11.25027585	9.89258960833333	10.91052195	11.65809635	9.7686764	9.81648044166667	9.34302553333333	10.6664473083333	10.81281805	11.1641077166667	11.6643648083333	9.98761663333333	9.4094908	11.3474088166667	11.1524382	10.5976643083333

PPP1R3F	10.3462750333333	10.0632117083333	9.824283225	10.0434280416667	10.5743323083333	10.4153772	11.055301575	9.78737088333333	10.4674955583333	10.0769619583333	10.1617358916667	10.95823755	10.6550211916667	10.0018247916667	10.0920085583333	9.85452464166667	9.59145404166667	10.3388359916667	10.0578180083333	9.99723533333333	10.2485867583333	10.0960991583333	9.970867025	9.65973478333333	9.95398405833333	10.43166005	9.83544501666667	9.96018530833333	10.1936096583333	9.91970055833333	10.1808367916667	10.103459075	10.5973351833333	10.5509023833333	10.515047025	10.4963081083333	10.3975849666667	10.151687475	9.97255511666667	10.1389730083333	10.1029681166667	10.0728614166667	10.0305098083333	10.0421767666667	9.96151823333333	9.53542963333333	9.88726988333333	10.0134059083333	9.83811425	9.84760208333333	10.2249992083333	9.96660331666667	10.2900500333333	10.128737475	10.2080941333333	9.85326255	9.90438676666667	10.2363454416667	9.93596328333333	9.921332825	10.4590182666667	10.113947425	9.900175875	10.0214251333333	10.4108585666667	10.0031143833333	10.2128243166667	9.87886791666667	10.119247225	10.2765561833333	10.34919645	10.7593753416667	10.34989835	9.98838416666667	10.3007165083333	10.4709652	10.1337610833333	9.99216454166667	9.3590843	10.6699485666667	10.191495075	10.268273175	10.0142807916667	10.0226518416667	10.4640149958333	10.819511225	9.78781813333333	10.2387377833333	10.3427675333333	9.6895289	9.80576564166667	10.5504732083333	10.3228920166667	9.98633354166667	10.6245043083333	10.3897471916667	10.0296842916667	10.233460875	10.4542782	10.5441182916667	9.99509398333333	10.6122965916667	10.805973475	10.0945926583333	9.65184145	9.80664778333333	10.20309965	9.722436925	10.500980975	9.71527460833333	10.842072875	10.021871175	9.96481554166667	9.99808105833333	9.79724158333333	10.1291621416667	10.4417307666667	9.763939075	9.91241780833333	10.3459231416667

KCNJ9	5.87591940833333	6.04613815	5.8532158	6.40489274166667	6.00206103333333	6.74610281666667	6.24941475833333	5.75682274166667	5.29675638333333	6.23620956666667	6.31548078333333	5.75213593333333	6.386982725	6.28823875	5.633369575	5.82366751666667	6.013009825	5.91247053333333	5.61155829166667	5.58212393333333	6.60761675	7.73358451666667	6.322468275	5.6003113	5.64313004166667	6.18660088333333	6.3482401	5.99418615	6.10713235	6.150357875	6.56215660833333	6.528434875	6.41739583333333	6.11785979166667	5.8532158	5.93306849166667	6.021143325	5.93036776666667	7.02704855833333	5.92679064166667	6.339217025	5.74506199166667	6.36709734166667	6.59744179166667	6.005441825	5.48807850833333	7.54282731666667	6.39534953333333	7.68829090833333	5.49431639166667	6.25250383333333	6.08105208333333	6.53205264166667	6.38925563333333	6.41354868333333	6.01932009166667	5.48300426666667	5.76923416666667	6.2066078	6.02015538333333	6.12763226666667	6.04613815	6.31786493333333	5.80528018333333	6.25099584166667	5.95410824166667	5.13227493333333	5.26783745	5.99672560833333	6.92168505833333	6.264276875	5.73297515	6.42709209166667	5.50024889166667	6.011301125	5.485111725	6.00716590833333	5.81909195	5.44029105833333	5.91038576666667	5.83589014166667	7.17093558333333	5.677007675	5.59754975833333	4.96956821666667	6.37415488333333	6.21353833333333	5.36818925	6.55877498333333	5.19536235833333	6.30401625	5.91520025833333	6.161212075	5.69296955833333	6.1536546	6.08940326666667	6.31702735	6.13670685833333	6.63110269166667	5.16314315	5.508179525	6.320274625	6.77436175	6.99218681666667	5.76836148333333	5.43388225	6.37033623333333	6.04876668333333	4.61421155	6.12282240833333	5.72886108333333	6.70390775833333	6.633753725	6.47291681666667	5.74803075	5.92603411666667	5.6411096	6.73694150833333	6.81316333333333	5.67425256666667

RBM8A	12.3958880333333	13.2584061458333	13.2004425833333	13.1736569208333	13.1277167583333	12.8506666791667	13.0225453583333	13.1842985083333	13.1385452208333	12.8292756833333	13.0418693625	12.2502041541667	12.9736325958333	13.1246056208333	12.9712877375	12.7798671583333	12.2786661791667	13.5970336416667	13.3584952291667	13.1638337083333	13.1043143958333	13.0746740375	13.1547622875	13.5734157833333	13.2376682708333	12.2459723458333	12.9355171958333	13.21321275	13.3564324708333	13.3605123583333	13.4575929083333	13.55799335	13.2755336666667	12.5890325125	12.1713788833333	11.7904133541667	13.1983571333333	13.4553955875	13.0983049083333	13.1849070958333	13.361974125	13.5242665	13.4779562916667	13.4356165791667	12.9747836208333	13.2513884875	13.5681927791667	13.9824211625	13.211948475	12.897697	13.4424347958333	13.4205841333333	13.4123457916667	13.5072033583333	13.4477786333333	13.4022565166667	13.4477786333333	13.4302808625	13.4477786333333	13.2858592416667	13.2979290416667	13.034637175	13.0322366791667	12.8088675833333	13.1347044291667	12.7043767875	12.898777075	12.5313564	13.12327495	12.9803100333333	13.0610858166667	12.9326121666667	12.7566627375	13.0949812333333	13.0242482333333	13.0316461208333	12.5133608291667	12.8715923291667	12.8029136333333	13.3090808916667	13.3827347958333	13.3751226125	13.3916751875	12.9102640333333	12.8121495708333	13.0801195458333	13.1861883291667	12.6677431916667	13.0617038	12.9391256125	13.0563467	13.2667134041667	12.8418290708333	12.5914695416667	13.2251060458333	13.22308885	13.0904272458333	13.1155638166667	14.1285867625	13.0127564125	12.8809936666667	12.857028075	13.3939643625	13.55799335	13.0724085083333	13.0479655666667	13.0593609791667	12.7899394625	13.0359261916667	12.4853097833333	12.898777075	12.9879316791667	13.0856200958333	12.6737039	12.9833230958333	13.4590718958333	13.296544875	13.0838599291667	13.2017398541667	12.857028075

C14orf39	12.3958880333333	13.2584061458333	13.2004425833333	13.1736569208333	13.1277167583333	12.8506666791667	13.0225453583333	13.1842985083333	13.1385452208333	12.8292756833333	13.0418693625	12.2502041541667	12.9736325958333	13.1246056208333	12.9712877375	12.7798671583333	12.2786661791667	13.5970336416667	13.3584952291667	13.1638337083333	13.1043143958333	13.0746740375	13.1547622875	13.5734157833333	13.2376682708333	12.2459723458333	12.9355171958333	13.21321275	13.3564324708333	13.3605123583333	13.4575929083333	13.55799335	13.2755336666667	12.5890325125	12.1713788833333	11.7904133541667	13.1983571333333	13.4553955875	13.0983049083333	13.1849070958333	13.361974125	13.5242665	13.4779562916667	13.4356165791667	12.9747836208333	13.2513884875	13.5681927791667	13.9824211625	13.211948475	12.897697	13.4424347958333	13.4205841333333	13.4123457916667	13.5072033583333	13.4477786333333	13.4022565166667	13.4477786333333	13.4302808625	13.4477786333333	13.2858592416667	13.2979290416667	13.034637175	13.0322366791667	12.8088675833333	13.1347044291667	12.7043767875	12.898777075	12.5313564	13.12327495	12.9803100333333	13.0610858166667	12.9326121666667	12.7566627375	13.0949812333333	13.0242482333333	13.0316461208333	12.5133608291667	12.8715923291667	12.8029136333333	13.3090808916667	13.3827347958333	13.3751226125	13.3916751875	12.9102640333333	12.8121495708333	13.0801195458333	13.1861883291667	12.6677431916667	13.0617038	12.9391256125	13.0563467	13.2667134041667	12.8418290708333	12.5914695416667	13.2251060458333	13.22308885	13.0904272458333	13.1155638166667	14.1285867625	13.0127564125	12.8809936666667	12.857028075	13.3939643625	13.55799335	13.0724085083333	13.0479655666667	13.0593609791667	12.7899394625	13.0359261916667	12.4853097833333	12.898777075	12.9879316791667	13.0856200958333	12.6737039	12.9833230958333	13.4590718958333	13.296544875	13.0838599291667	13.2017398541667	12.857028075

TRIM46	8.84077027916667	8.21430152916667	8.51738952916667	8.91067589583333	8.65490165	7.7663413125	8.95919710833333	8.1338885125	8.17009956666667	8.11379269166667	8.5226054125	8.48921995416667	8.60103497083333	8.15733075	8.3015442625	8.00608962083333	7.4475786625	8.96482718333333	8.34945393333333	8.20254386666667	8.47407115833333	8.48978630833333	8.22013784166667	7.928370375	8.27096189166667	8.50577863333333	8.80326785416667	8.4406654875	8.40147999166667	8.290256875	8.91961008333333	9.10509032083333	8.72334106666667	8.59720344166667	8.42705215416667	8.79416294583333	8.57325059166667	7.86512938333333	8.5664456625	8.61503477083333	7.76319464166667	8.66373332916667	8.391853325	7.917126675	8.41318640416667	7.70339010416667	8.16759889166667	8.42148199166667	8.293281075	7.91980345416667	8.1036137375	8.41131205	8.7242805375	8.82849306666667	8.83223770833333	8.10609615	7.95992857916667	8.06734486666667	8.78813974583333	7.96500545833333	9.054282875	8.8814398125	8.50821110833333	9.03481485	8.29276760833333	8.6627244875	8.4219307	8.56446075	8.8139688125	8.72570710833333	8.73522726666667	8.75541892083333	8.78196994166667	8.293281075	8.613674025	8.2811933625	8.72995544166667	8.11578765	8.16855074583333	8.843587625	8.11125169583333	8.42293710833333	8.4716399125	7.67567321666667	8.9455138875	9.33343475833333	9.26239327916667	9.3056499625	9.18905973333333	8.04206747083333	9.101696925	8.88809269166667	8.81948160833333	8.59968860416667	9.40619117916667	8.57279835833333	8.43970535	8.86089086666667	9.2728688875	8.61548956666667	8.65442572083333	9.46274657916667	8.86632373333333	8.23839771666667	8.35711129166667	8.63103852083333	8.64471831666667	8.8900448125	8.207593975	8.61069705	9.17895344166667	8.25267709583333	8.4689240375	7.68680649166667	8.64363991666667	8.53505667916667	8.36008461666667	8.534582525	8.5006038375	8.36784924583333

AL713999.1	8.84077027916667	8.21430152916667	8.51738952916667	8.91067589583333	8.65490165	7.7663413125	8.95919710833333	8.1338885125	8.17009956666667	8.11379269166667	8.5226054125	8.48921995416667	8.60103497083333	8.15733075	8.3015442625	8.00608962083333	7.4475786625	8.96482718333333	8.34945393333333	8.20254386666667	8.47407115833333	8.48978630833333	8.22013784166667	7.928370375	8.27096189166667	8.50577863333333	8.80326785416667	8.4406654875	8.40147999166667	8.290256875	8.91961008333333	9.10509032083333	8.72334106666667	8.59720344166667	8.42705215416667	8.79416294583333	8.57325059166667	7.86512938333333	8.5664456625	8.61503477083333	7.76319464166667	8.66373332916667	8.391853325	7.917126675	8.41318640416667	7.70339010416667	8.16759889166667	8.42148199166667	8.293281075	7.91980345416667	8.1036137375	8.41131205	8.7242805375	8.82849306666667	8.83223770833333	8.10609615	7.95992857916667	8.06734486666667	8.78813974583333	7.96500545833333	9.054282875	8.8814398125	8.50821110833333	9.03481485	8.29276760833333	8.6627244875	8.4219307	8.56446075	8.8139688125	8.72570710833333	8.73522726666667	8.75541892083333	8.78196994166667	8.293281075	8.613674025	8.2811933625	8.72995544166667	8.11578765	8.16855074583333	8.843587625	8.11125169583333	8.42293710833333	8.4716399125	7.67567321666667	8.9455138875	9.33343475833333	9.26239327916667	9.3056499625	9.18905973333333	8.04206747083333	9.101696925	8.88809269166667	8.81948160833333	8.59968860416667	9.40619117916667	8.57279835833333	8.43970535	8.86089086666667	9.2728688875	8.61548956666667	8.65442572083333	9.46274657916667	8.86632373333333	8.23839771666667	8.35711129166667	8.63103852083333	8.64471831666667	8.8900448125	8.207593975	8.61069705	9.17895344166667	8.25267709583333	8.4689240375	7.68680649166667	8.64363991666667	8.53505667916667	8.36008461666667	8.534582525	8.5006038375	8.36784924583333

IGF2R	11.7861402083333	12.36147625	12.3974616666667	12.1922906583333	11.9701954833333	12.2341055916667	12.2624244833333	12.1770216166667	12.216784075	12.0237948	12.4554143666667	11.8281920416667	11.5000912333333	12.1823287916667	12.1312748	12.1451222666667	11.9431320666667	11.3976073583333	12.1084032166667	12.0525821583333	12.1640360666667	12.1711742666667	12.3921887583333	12.21725785	12.1620485833333	11.9409022083333	12.2141453583333	12.1774305916667	10.7247329333333	12.244497975	12.0317754666667	12.1620485833333	12.0690013166667	11.9165677	12.0909367583333	12.0926515916667	12.0901653083333	11.94277775	11.802441875	12.3088616416667	12.206981325	12.1656867	12.3126780333333	12.0909367583333	12.052988325	12.2755744916667	12.1525189	12.0555750333333	12.3754853666667	12.1295438416667	11.6227232166667	11.9244487083333	11.6623273	11.9554346166667	12.382866125	12.23183945	12.0510805083333	12.1367818666667	11.8924112666667	12.1084032166667	11.980884225	12.9144941416667	12.2085904583333	12.4091926166667	11.5523735416667	12.7354192416667	12.5921909166667	11.624532275	12.4202498166667	12.1258089083333	12.622549625	12.3623095583333	12.5254900333333	12.3811083333333	12.5729905	12.1205448666667	11.971689825	11.8473399333333	12.5939827166667	12.3815660416667	12.932363575	12.5349027	12.2901521833333	12.7113258083333	12.5344122083333	11.876483925	12.2231616166667	12.0701717333333	9.22515735833333	12.5264113	12.3698220916667	12.7655438666667	12.5710716416667	12.08124855	12.4917329166667	12.199408325	12.5334882833333	11.9550582083333	12.0209849	12.137649925	13.312913975	12.2727355333333	12.6900433666667	12.4341294166667	12.1922906583333	12.2710584083333	12.3746631	12.603650525	12.5516382166667	12.8532684916667	12.4958638833333	12.3310232666667	13.3987592083333	12.1852414666667	12.244497975	12.630223175	12.1553849	12.6397345916667	12.0337598083333	12.3318475166667

KCNA6	11.1816777958333	10.0063882458333	10.0478853916667	10.779156275	10.9701686625	8.90550186666667	11.3207889041667	10.4977370333333	10.6299902	9.97562940416667	10.3025609083333	10.9841030875	11.1465160291667	10.3029320833333	9.83879185833333	10.2565396625	8.7386305625	11.2585867666667	9.87047960833333	10.5621765458333	10.2549409166667	9.43613309166667	9.58323725	10.4628138416667	9.106854375	10.6928401666667	9.95079996666667	10.4136524666667	9.93244266666667	10.5475164791667	10.4286661041667	10.1567688708333	9.26923890416667	8.9909319875	9.504275625	10.7480234125	10.652148475	10.40551365	10.6167578333333	10.3989842625	10.729338025	10.2747687875	9.76372285416667	9.389631825	10.0551195375	9.53762749166667	9.101696925	10.4592277875	9.19365908333333	9.33598702083333	9.3860123875	10.2452424416667	10.0444182875	10.1132725125	10.1119921166667	9.66719131666667	10.1326635041667	10.4415480375	8.74935636666667	9.63317724583333	9.82800175416667	10.3429838208333	8.89241624166667	10.4777454166667	9.94272831666667	11.2232063458333	10.25291345	10.6185783375	9.8073084375	9.70731425	10.0384926833333	10.4490140208333	10.9675186041667	9.65645970416667	10.1418799166667	10.3307109083333	10.2223694583333	9.564297525	10.2291590125	10.1186146583333	9.88864601666667	14.4169384041667	10.7536663458333	9.9203137	10.1318623916667	11.2204006375	9.80906438333333	10.0491599041667	10.0804987583333	9.3700947375	9.8736624125	8.94164972916667	8.9392545125	9.59928065416667	10.1007241583333	9.890327525	9.30608285833333	9.44128464583333	10.436693475	8.26332452083333	9.037530925	10.5358883583333	8.63752675416667	8.8692769375	9.62214002083333	9.05582115833333	9.94481022083333	10.147935375	10.1631061708333	9.36761098333333	8.23839771666667	9.881068875	9.65419311666667	8.48591667083333	10.0922404125	9.35568727916667	10.2784519291667	8.58671785833333	9.06141539166667	9.442255925

GALNT8	11.1816777958333	10.0063882458333	10.0478853916667	10.779156275	10.9701686625	8.90550186666667	11.3207889041667	10.4977370333333	10.6299902	9.97562940416667	10.3025609083333	10.9841030875	11.1465160291667	10.3029320833333	9.83879185833333	10.2565396625	8.7386305625	11.2585867666667	9.87047960833333	10.5621765458333	10.2549409166667	9.43613309166667	9.58323725	10.4628138416667	9.106854375	10.6928401666667	9.95079996666667	10.4136524666667	9.93244266666667	10.5475164791667	10.4286661041667	10.1567688708333	9.26923890416667	8.9909319875	9.504275625	10.7480234125	10.652148475	10.40551365	10.6167578333333	10.3989842625	10.729338025	10.2747687875	9.76372285416667	9.389631825	10.0551195375	9.53762749166667	9.101696925	10.4592277875	9.19365908333333	9.33598702083333	9.3860123875	10.2452424416667	10.0444182875	10.1132725125	10.1119921166667	9.66719131666667	10.1326635041667	10.4415480375	8.74935636666667	9.63317724583333	9.82800175416667	10.3429838208333	8.89241624166667	10.4777454166667	9.94272831666667	11.2232063458333	10.25291345	10.6185783375	9.8073084375	9.70731425	10.0384926833333	10.4490140208333	10.9675186041667	9.65645970416667	10.1418799166667	10.3307109083333	10.2223694583333	9.564297525	10.2291590125	10.1186146583333	9.88864601666667	14.4169384041667	10.7536663458333	9.9203137	10.1318623916667	11.2204006375	9.80906438333333	10.0491599041667	10.0804987583333	9.3700947375	9.8736624125	8.94164972916667	8.9392545125	9.59928065416667	10.1007241583333	9.890327525	9.30608285833333	9.44128464583333	10.436693475	8.26332452083333	9.037530925	10.5358883583333	8.63752675416667	8.8692769375	9.62214002083333	9.05582115833333	9.94481022083333	10.147935375	10.1631061708333	9.36761098333333	8.23839771666667	9.881068875	9.65419311666667	8.48591667083333	10.0922404125	9.35568727916667	10.2784519291667	8.58671785833333	9.06141539166667	9.442255925

VPS8	11.233092075	10.99445015	11.1941109166667	11.2215521833333	11.4200132333333	11.3165296583333	11.043790875	11.0826274416667	11.24573245	11.2506923333333	11.2100932	10.6895391083333	11.1767887583333	11.1437297583333	11.2689689833333	11.052426775	11.5824943666667	11.5136403666667	11.33033725	11.6849152916667	11.1046868	11.6859465333333	11.4191800833333	10.971110725	11.28453495	11.4052792333333	11.2568448166667	11.6227232166667	11.6982071583333	11.3915472416667	11.14669605	11.1656289166667	10.750505225	11.0105391666667	11.1582543083333	11.2689689833333	11.044463875	11.0822289416667	10.9549623083333	11.343036975	11.17279555	11.2143531083333	11.03596855	11.230125425	10.9393869333333	11.3121797833333	11.2826013166667	11.4258523333333	10.9780057166667	11.3481355916667	11.0366394333333	10.9828230166667	11.230125425	11.4559913916667	11.53966185	11.2726256666667	10.9270024916667	11.1615262583333	11.3144428833333	10.8368196833333	11.8888404416667	11.4489345583333	11.78754065	11.184429675	12.196460925	11.6651451833333	11.1710416	11.770678525	11.6010090666667	11.638265575	11.2521123083333	12.4827258583333	10.9443778666667	11.3488624333333	11.2775845	11.4364638833333	11.7049796083333	11.5738946	11.3506069833333	11.3972232666667	11.6691177916667	11.8055072	11.28518	11.7035007416667	11.3871722333333	11.4669037583333	11.205753575	11.4912151666667	11.6155901666667	11.62870925	11.7445138833333	11.8341069333333	12.0432377416667	11.8228949916667	11.9032491333333	11.9612880416667	11.9712775416667	11.5253735333333	11.827360075	11.917277775	11.03446615	11.8297435166667	11.8074362166667	12.226050525	11.5684552166667	11.7955610083333	11.2922383666667	11.1670443166667	11.1623033333333	11.7247391166667	11.5000912333333	11.6628809833333	11.6709513916667	11.674290525	11.6914012333333	12.04824535	11.8550160583333	11.2420872916667	11.7951768416667	11.4759684833333

PCDHB15	11.1811375833333	9.24980921666667	9.50539336666667	9.03459930833333	9.63902631666667	9.61680303333333	10.0288478666667	9.72070379166667	10.0660963333333	10.1661675166667	8.77966386666667	11.2319732666667	10.8150497416667	9.49775068333333	9.40906840833333	9.45555605	9.70017130833333	9.73395598333333	9.289706825	9.86102748333333	10.2687270166667	8.868084675	9.25253679166667	9.27853935	9.21732311666667	11.237038075	9.35954015	9.25167913333333	8.39161398333333	8.862628675	9.58302888333333	9.02418135	9.6530765	9.27643005833333	11.043790875	10.9357322	8.96748466666667	9.34481250833333	8.775178575	9.77883299166667	8.87543329166667	9.24218355833333	9.746957025	9.00236645	10.1577133666667	9.38762769166667	9.07164405	9.09237333333333	9.16074203333333	8.99116335833333	9.71612960833333	8.88496090833333	9.84541846666667	8.8296979	9.18595039166667	9.27506685	9.73873335833333	9.29162124166667	9.55009920833333	9.69880400833333	9.90775529166667	9.31047634166667	8.59302528333333	9.45898255	9.33275593333333	9.83237755833333	9.36480266666667	9.81608086666667	8.62834993333333	9.22295939166667	8.90619576666667	9.52456219166667	9.80802880833333	10.816907025	9.45636763333333	9.32471271666667	9.82382424166667	8.90197375	9.14054775	9.15555419166667	9.72703330833333	10.7050450333333	9.46657071666667	9.61006324166667	9.810895475	10.2241250416667	8.956732575	10.2458406333333	8.608522725	9.99808105833333	9.52316788333333	8.32264421666667	10.0095607333333	10.2745932916667	9.34847368333333	8.322152325	8.093315875	8.51027651666667	8.9544846	8.28495673333333	8.84055035	9.66558844166667	8.00462699166667	8.551483425	10.2591668583333	8.2260001	9.524079925	8.907621175	8.71039916666667	10.133368525	9.64074395833333	8.82211801666667	10.5655352666667	8.79596970833333	8.54699226666667	10.1485742083333	8.34866896666667	8.79812115	7.89775408333333	9.80625096666667

MAP3K20	6.627072075	5.89449381666667	5.792834125	5.3439553	6.07339345833333	6.55593524166667	5.52645741666667	6.14249028333333	5.49229008333333	5.01100034166667	4.938665425	6.5680586	6.46133099166667	5.38157649166667	6.2379381	5.29552266666667	5.231579	6.09754041666667	5.59377575	5.8174436	5.4913896	5.11118121666667	4.80096826666667	6.57427455833333	5.055819225	6.4516666	5.29779213333333	5.29238863333333	5.74606878333333	5.36415765833333	5.36615406666667	5.65064866666667	5.46278851666667	6.19527208333333	6.68067039166667	6.05897065833333	5.23876719166667	7.56091623333333	5.90846126666667	5.40105285833333	5.426744575	5.01735620833333	7.71354716666667	6.12118446666667	5.755893075	5.53902828333333	5.97595255	5.221531825	5.18759319166667	5.303590975	4.9195424	5.566976475	6.10002881666667	6.57166325	5.965264275	5.09217729166667	6.1936491	5.53695673333333	5.75112915833333	5.00529823333333	6.473722825	5.18312655	5.02903219166667	6.06147365	5.25962125	8.63230339166667	6.39681019166667	5.71315170833333	5.665590775	5.582921425	6.08790970833333	5.878607175	6.90845738333333	5.70932124166667	5.60319860833333	6.20040056666667	5.400096325	6.715741525	8.23080535	5.04338205833333	7.06888018333333	6.06833519166667	5.80528018333333	5.41887256666667	6.297552775	5.76836148333333	5.23361180833333	5.53586285833333	5.73943181666667	5.596724675	6.15447426666667	6.68797494166667	6.19669175	6.6716762	5.17603111666667	5.40201090833333	7.27829234166667	7.45043895	6.45250163333333	5.11436066666667	6.80449616666667	4.37489024166667	7.72739250833333	5.62957723333333	7.34892271666667	7.06400038333333	7.30019385833333	6.81253920833333	5.54873173333333	6.74082630833333	4.690117325	5.79119435	7.33375654166667	6.84636305	7.31861950833333	5.08659576666667	5.46278851666667	6.84200789166667	5.69080398333333	6.2073137

SMCO4	7.28864350833333	7.87964591666667	8.09282625833333	8.90285985	7.70199845833333	8.8319761	6.74218866666667	8.4444208	8.06996655	7.99131585833333	7.92602010833333	6.00459881666667	6.5766165	8.09852281666667	8.28895966666667	8.03810015	10.1059188	7.32433510833333	8.391097025	8.09947061666667	8.61290266666667	7.71914490833333	8.82519364166667	8.62685349166667	8.03538219166667	6.82586836666667	7.98193376666667	7.76345526666667	8.439473525	8.09695651666667	8.512878125	7.97141813333333	7.83944996666667	9.33496756666667	7.38984995833333	7.2313303	7.95134493333333	8.34128665833333	8.488485525	7.803872825	8.95577006666667	8.54699226666667	8.77406471666667	8.68575455833333	8.0294547	8.45693534166667	8.72452928333333	8.8577648	8.63633005833333	9.066471675	8.83115135	8.27495268333333	7.90206838333333	8.35736911666667	8.49188041666667	8.817877875	8.28340845833333	9.24980921666667	8.00412230833333	8.89405555	8.82519364166667	7.52279740833333	8.432956925	8.39512661666667	8.06450121666667	6.78381769166667	7.86164825833333	8.26039324166667	8.72229471666667	8.4484208	7.56491295833333	8.5874833	6.09436185833333	8.440899325	6.85745724166667	8.6774004	7.20693700833333	8.07622910833333	8.127296675	7.594902375	7.65827738333333	7.77045426666667	7.35924831666667	8.9131846	7.930120075	7.2559939	6.69566758333333	8.88688890833333	8.0294547	8.55907896666667	8.20990576666667	8.07572273333333	8.78691484166667	7.99725846666667	7.947409975	8.03810015	8.74130064166667	8.04330091666667	8.69317739166667	8.58441085833333	8.16329085	8.67789245	10.0724257083333	8.56044531666667	8.63449166666667	8.401731375	9.26442515	7.82793564166667	9.56961935	8.83921701666667	8.60799398333333	8.55288418333333	8.57007116666667	8.71039916666667	8.77719965833333	9.17414999166667	8.821200975	8.57649310833333	7.746730925	9.25858543333333

KIF16B	10.500980975	11.3809445833333	11.1630098	10.934250825	10.5049759833333	10.464576475	11.0747492583333	11.2018586833333	10.5573126666667	10.9869124833333	10.805973475	10.402059075	9.84285273333333	11.02981265	10.9726481916667	10.9028824333333	10.27572155	10.831890425	11.3242829	11.1652493166667	10.9516391	11.1767887583333	11.2583720833333	11.0942927666667	10.913187075	11.21907835	10.874072375	10.9371792916667	10.8277408416667	11.2815281916667	10.7224580666667	11.021874925	10.7329204833333	10.0632117083333	10.9013899916667	10.999826875	10.5509023833333	11.357567525	11.2028990666667	10.4554224833333	11.1332425666667	11.2726256666667	11.0841148833333	11.3661426916667	10.8922670583333	11.3351005833333	11.1840687083333	11.24573245	11.3132862083333	11.1383591416667	10.9858189916667	11.4162651666667	10.6809073166667	11.1259488	10.901782425	10.9673192583333	11.2790070666667	11.4574143583333	11.31989795	10.7892430083333	12.244497975	11.0765568666667	10.7904047166667	12.5552867333333	10.04588255	10.834075425	12.11543585	11.331019575	11.0504310083333	10.8836008416667	11.6456087833333	12.7295358083333	11.9907404666667	10.70539815	10.2153563166667	11.8411407666667	10.7589898916667	10.6336749583333	12.0417350833333	10.622992175	10.3682517916667	10.7162804	11.2726256666667	10.798668925	11.17758075	10.0720278166667	9.9993556	11.04233505	10.092918675	11.034816875	11.1398416833333	11.44169995	11.013128925	11.9220834083333	12.5221926333333	10.98913965	11.94832135	10.3436245833333	11.5668628166667	10.987327675	11.0826274416667	11.6429698666667	11.0830259333333	11.3624169916667	11.2861342166667	11.2753469583333	11.6433290333333	11.3292622166667	11.9220834083333	11.1832845666667	11.2521123083333	10.7383339416667	12.0261845416667	12.2591093583333	11.152834825	10.9473452	10.4709652	11.0815307083333	11.9521162083333	11.0927898583333

CLASRP	11.6558642583333	11.1110336416667	11.0953873333333	11.1480921	11.9447346	11.2902838583333	11.5976176916667	11.0373411416667	11.5772577666667	11.2858355	11.1405494333333	11.5601850333333	11.779499875	10.8612470416667	11.4170295666667	10.91088935	11.5436260583333	11.8666609583333	11.08338295	10.960420475	10.95605295	11.3990716583333	10.852614925	11.2786369583333	11.2975735583333	11.3705707416667	10.9440376833333	10.9761602583333	11.7905923666667	11.343036975	11.0203771416667	11.044463875	11.292593525	11.5501880833333	11.4919381583333	11.4188332333333	11.6470358	11.1346623583333	11.1346623583333	11.19866735	11.0383932333333	11.291894675	10.9252447166667	10.86350625	11.2413554333333	11.0090696333333	10.882085925	10.9501588	10.7994102333333	10.98875735	11.15581865	11.0337297583333	11.602097375	11.500844625	11.27642465	10.82884605	11.14669605	11.0751116833333	11.1459742833333	10.6621925666667	12.2412181916667	11.6155901666667	11.5185086916667	11.3033163083333	11.4897906583333	11.3915472416667	11.615215075	11.3553466416667	11.5716921416667	11.248902775	11.3801476916667	11.554144025	11.1670443166667	11.2018586833333	11.4897906583333	10.934250825	11.5909354166667	11.4200132333333	10.8713916666667	11.4715256833333	11.51586705	11.6456087833333	11.297225	11.206560475	11.4771109	11.3539068166667	11.56339385	11.14949005	11.9025044	10.7804261416667	10.98655615	11.279776525	11.343036975	11.0845103416667	11.3657868083333	11.5407991083333	11.373190825	11.6636899333333	11.6895204333333	11.683026175	11.2957967833333	11.5461869416667	11.2733084583333	10.8953436916667	11.3509805666667	11.660606675	11.4639403833333	11.1710416	11.2899480916667	10.7736244333333	11.48138035	10.98875735	11.4108017166667	11.03596855	11.52618635	11.3423558	11.2481821666667	11.2940625833333	11.2746401666667	11.0916995

SLBP	11.2579853916667	12.1587642333333	11.980884225	12.046177825	11.7105442333333	12.9793317083333	11.8290120833333	12.0827580166667	11.588386675	12.199408325	12.373750125	11.4675918	11.49381285	12.2002559333333	12.0092346166667	11.9078069166667	12.013260375	11.7810947333333	11.848881975	12.108037425	12.0518595833333	12.02261385	12.420030925	12.4554143666667	12.10103115	11.7509367	11.9820605666667	12.0917340666667	11.86547315	12.2792629666667	11.9923578333333	12.3118325833333	11.7909812333333	12.0761170583333	11.83053445	11.7116683416667	11.9546857416667	12.1749483916667	12.0051849333333	12.0555750333333	11.9348926	12.0417350833333	11.8134267166667	12.6904704083333	12.0261845416667	12.244497975	12.2053036416667	12.3152059916667	12.559988525	11.9678169416667	12.209029825	12.0400324083333	12.0901653083333	11.9467504166667	12.0945968666667	11.8709570583333	12.0585648916667	12.1553849	12.1299872583333	12.0413582166667	12.3899971916667	12.3055239166667	12.32885815	12.2437326916667	12.4995800083333	11.9142748833333	12.6098037416667	12.3343950916667	13.1052210666667	12.4559287	12.7295358083333	12.4368713	12.5376322583333	12.2939030416667	12.1133945666667	12.5506564333333	11.7186785333333	12.2628607833333	12.4010561583333	12.4795096083333	13.487986925	12.856162525	12.840994225	12.3339445	12.3101776166667	12.9412313583333	13.6320213916667	12.5029330166667	12.3602095583333	12.3899971916667	12.6080271416667	12.6566773416667	13.4474018083333	12.660047425	12.6151813916667	12.4114353583333	12.9048119416667	12.811279575	12.5719766166667	12.0189741833333	12.909394625	12.7328907083333	13.834699275	12.082379725	12.88540095	12.50892495	12.4723572833333	12.3930672583333	12.430195425	12.5788865916667	12.5921909166667	13.0953722166667	12.3952132583333	12.3267305	12.5646791416667	12.840994225	13.1065162583333	12.8740952083333	12.3088616416667	13.1210793083333

C1orf167	9.41162929166667	9.45281165833333	9.497335375	9.36761098333333	10.0378686583333	9.68387974166667	9.90222311666667	9.11174605833333	9.699723	9.58391948333333	9.781927775	9.318611725	9.32574429166667	9.45149038333333	9.67900834166667	9.11131928333333	9.12326560833333	10.027285575	10.1117927583333	9.53312831666667	9.35451149166667	9.41120025	9.40159784166667	9.67634863333333	9.4277554	9.20045154166667	9.3036884	9.28128599166667	9.51408459166667	9.7408584	9.75828255833333	9.52996276666667	9.32897005833333	9.4825512	9.286424975	9.36247203333333	9.55244275833333	9.62925015833333	9.63292783333333	9.36480266666667	9.45055241666667	9.74128455	9.4981546	9.4936652	8.82424165	9.32711606666667	9.460327375	9.7686764	9.497335375	9.57556716666667	9.74045244166667	9.88726988333333	9.29702861666667	9.460327375	9.24539353333333	9.24445845833333	9.34530801666667	9.60093574166667	9.65973478333333	9.45149038333333	10.4550319166667	9.94976705	9.54225544166667	9.39392068333333	9.57222860833333	10.2450271333333	10.21041955	9.15351445833333	10.185845775	10.0026032916667	10.151318175	10.0043835333333	9.921332825	9.732285275	10.0960991583333	10.0933465333333	9.56361585	10.2587469666667	10.04725285	10.0318266833333	9.59906339166667	9.88168326666667	10.023053775	9.497335375	9.670115925	9.25394765	9.22160494166667	8.9489102	10.119247225	9.47351875833333	10.0226518416667	9.726163425	9.84332440833333	9.89531699583333	10.3155264416667	10.9332106333333	10.1791861916667	10.2241250416667	10.1541580083333	9.76219373333333	10.1759041833333	9.81261425833333	10.0498058583333	9.939168625	9.705378475	9.50643220833333	9.93222460833333	10.2018680083333	9.97954941666667	9.76647539166667	9.26442515	10.054135025	9.76179089166667	9.66283631666667	9.56873605	9.7100974	10.0720278166667	10.4682944	10.2745932916667	9.670115925

SEMA6D	10.7421748083333	9.09237333333333	9.42508291666667	9.41162929166667	8.37864189166667	10.1766858833333	9.52694540833333	9.7557679	9.33181315833333	9.810895475	10.151687475	11.9694157916667	10.0159752583333	9.19287955	8.8072839	10.054920625	10.7535022833333	9.04228765833333	9.15113736666667	10.090730825	10.24069715	8.35786781666667	9.55812764166667	9.59563205	9.76554074166667	11.1862575	9.393424375	9.38580318333333	8.46459865	9.23797865	9.44516316666667	9.203321075	9.55646399166667	10.2485867583333	10.78588925	11.0157092833333	9.250740625	9.50264109166667	9.40906840833333	9.79321156666667	9.80706423333333	9.17414999166667	9.921332825	9.32044345833333	10.4449298	9.9993556	9.7400082	9.49238265	10.0318266833333	9.97043543333333	9.48609106666667	9.33617749166667	9.23973414166667	9.17813545	9.24218355833333	10.1670505916667	9.42030009166667	9.86854744166667	9.89855505	10.1775011166667	12.0241448416667	11.6297974583333	9.95359933333333	10.114399525	11.081884475	9.234219825	11.9903497083333	9.56452501666667	9.32849245833333	8.81515405833333	11.5869361333333	11.1681956416667	10.0696848333333	10.7538304083333	11.83053445	10.8544918	9.7860847	9.599958075	9.58173716666667	11.6978649666667	10.9226970166667	11.638265575	8.580396475	11.5839614333333	9.95891725	10.6581721666667	10.720995375	10.1720866	10.9600311833333	11.6837602666667	9.86020724166667	10.464576475	11.8646362416667	13.1336349	11.0387117083333	10.528057425	13.0506754833333	10.4682944	10.7078134666667	13.0096050666667	8.99535305	11.91510375	8.54122040833333	10.96771795	7.147600275	11.5185086916667	9.939168625	9.40207443333333	9.02371780833333	10.7201690666667	10.37104885	9.0973311	11.0157092833333	10.21004035	9.126505725	9.63430166666667	12.1811083666667	7.35503378333333	10.8988570583333	9.64383103333333

TWIST1	11.8811110166667	11.032984525	11.71493805	10.94470365	11.0790622916667	11.1110336416667	11.102867725	11.4883355166667	11.158996075	12.0917340666667	11.3705707416667	11.411148125	11.9420280583333	11.7384146416667	10.995163975	11.3433838833333	10.2321384166667	11.1010834666667	10.9737165166667	11.6099448083333	11.43611165	10.0288478666667	11.2438581166667	11.57803215	11.31013205	11.1685570666667	11.9467504166667	11.1908643083333	10.82998625	11.3635795583333	11.4872383	10.8589548	11.5706660333333	9.83452378333333	11.3441400333333	11.7362421333333	11.0146426833333	11.2007994333333	12.1451222666667	12.0793162416667	11.0416560583333	11.5004634	11.0946623333333	11.4126401666667	11.7791279583333	11.7288583666667	11.761727725	11.1916460666667	10.5228331416667	11.2025233	11.9709528833333	11.51470825	11.3546484166667	10.7944294666667	11.372844325	11.8811110166667	11.5630353166667	11.0659389916667	11.4214230083333	12.0843813333333	12.0577849333333	11.1937817916667	11.638265575	12.1333790583333	11.9525962333333	12.2188976833333	12.8086026666667	12.3943629916667	11.517039175	11.6610088416667	11.7594075916667	11.12105475	13.2683617333333	12.9906493583333	11.452332325	12.1930875666667	11.50818875	12.0197364166667	12.7295358083333	11.6569750083333	12.2399849666667	11.937643125	11.94277775	13.3601687833333	12.8661429166667	12.4211834833333	11.4093544416667	12.30978125	11.8078233833333	13.043373375	12.5594786833333	11.8337195083333	11.4154857416667	11.6910443916667	11.502358525	11.3136753916667	10.8461868083333	11.84426255	12.948888375	11.312921725	12.23879665	11.3132862083333	11.99822175	11.1923605916667	11.1546552166667	11.5215953666667	12.4664163166667	11.517039175	12.00329435	13.199462625	13.09075125	10.7463771	12.496314025	12.2311025	12.8161131416667	11.46235385	12.5111982416667	12.05061705	10.8908321583333	11.7970628166667

CTDSP2	13.873143175	13.78007155	13.7463275333333	13.42164015	12.8783966083333	13.4572533333333	13.4634900333333	13.2595614666667	13.4688309833333	13.5068025166667	13.52467435	14.21037625	13.48560075	13.5738180166667	13.3912051666667	13.7361743	14.0938637416667	12.8213874666667	13.654206225	13.4611203333333	13.5815251416667	13.144627125	14.0656333583333	13.289124025	13.2332248583333	13.5867942083333	13.4611203333333	13.3179373333333	12.760723875	13.3227764916667	13.342400775	13.5295226416667	13.4983637666667	14.0288199166667	13.8572965333333	13.92725075	13.0186633916667	13.1825371833333	13.6785048416667	13.2587665833333	13.5468416333333	13.580759625	13.8060720916667	13.482478725	13.7443409166667	13.6969617	13.5859683083333	13.08103315	13.8395483	13.4119895416667	13.40344555	13.5255367583333	12.822430125	12.8947866166667	13.1589580416667	13.3193049583333	12.895840375	13.306054325	13.3653465583333	13.6434906916667	12.9941887083333	13.101295875	13.159645225	13.8473484166667	13.2988538666667	12.5448126916667	13.3682726083333	13.8461367666667	13.423764125	13.4768642833333	13.454214775	13.36159345	13.419543825	13.734202725	13.3875128	13.208876725	13.5459592333333	13.0877248833333	12.7586192666667	13.3732212583333	13.46583445	13.9318364083333	12.8218942166667	13.5535340083333	13.2648622166667	13.7949722083333	13.8657422166667	13.3018009583333	13.5346846166667	13.6384571083333	13.9153342666667	13.4729544083333	13.407373775	13.22481105	12.811279575	13.1980287833333	13.781003275	13.7443409166667	12.7400926583333	13.407373775	13.1550516583333	13.7084619583333	13.0403414916667	13.4999239583333	13.08233985	12.9352563083333	13.1274317166667	13.2412964416667	13.2027218583333	13.3067028166667	13.8148381	13.471337675	12.8641322666667	13.0749552833333	12.792898125	13.2155282	14.2858034416667	13.0694021583333	13.1946165416667	13.2738329583333

AQP6	8.93568545833333	7.51472684166667	7.34892271666667	8.162750025	8.3536777	7.20508425	8.37864189166667	7.88018380833333	8.16071089166667	7.99615545	7.76345526666667	8.79090379166667	8.32561349166667	7.38233871666667	7.35447710833333	7.71080830833333	7.53297658333333	7.95255818333333	7.3314952	7.27593380833333	7.95575579166667	7.687727325	7.90139170833333	7.57569364166667	7.40079709166667	8.82680535833333	7.57783686666667	7.63269815833333	7.61698044166667	7.89557763333333	7.72085915833333	7.47258725833333	8.21566409166667	7.70199845833333	9.408133425	7.89243905	8.08531809166667	7.78057345	7.68209800833333	7.95575579166667	7.72139515	8.36034350833333	7.56433695	7.57732030833333	8.024066175	6.94497745833333	7.20693700833333	7.59420499166667	7.7124456	7.212178575	8.11761579166667	7.45558410833333	7.668390025	7.219834	7.32258515	6.89132381666667	7.173894	7.35751986666667	7.46022033333333	7.38984995833333	8.06129600833333	8.53333894166667	7.7124456	8.49496250833333	7.91577950833333	7.95904073333333	9.42687719166667	8.27176984166667	8.7179342	8.04234110833333	8.12378493333333	8.59743456666667	8.56366318333333	8.030568675	8.18422995833333	8.26456484166667	8.33066458333333	7.86062830833333	7.666702425	8.06759891666667	7.279554825	7.36530371666667	8.20331464166667	8.28242620833333	8.27606465	7.93387576666667	8.33965076666667	8.10955528333333	8.482136425	7.60297259166667	7.84106111666667	7.94541711666667	7.92224445	7.70977168333333	8.48569109166667	7.80563896666667	8.65999509166667	8.28445111666667	8.28193894166667	8.15707283333333	7.153195325	8.62136429166667	7.80272595	7.600828425	8.13415403333333	7.58238585833333	7.46746676666667	7.08614628333333	7.44203170833333	6.841322025	7.60415978333333	7.74783731666667	8.1073987	7.44984575833333	7.41882361666667	7.506757	7.23371278333333	8.03376271666667	7.75064413333333	7.34892271666667

TET3	8.580396475	7.70645875833333	7.338007825	8.13242494166667	8.95764171666667	7.65774509166667	7.5471865625	8.3547531	8.234668275	7.86334303333333	8.01806620833333	8.13977138333333	8.53882584166667	8.037084725	8.1525602	7.677172175	6.07937631666667	8.92218960833333	8.31466204166667	7.9846002	8.20638435	9.30028739166667	7.59144205833333	8.27228149166667	8.234668275	7.35924831666667	8.4076656	8.32062069166667	8.65318265	7.906291625	7.57732030833333	8.24861051666667	8.21097586666667	6.56951250833333	7.00693255	6.9494722	9.02034758333333	7.89832068333333	7.815575525	8.00462699166667	6.90968315833333	8.293030975	7.41942373333333	7.09102175833333	7.97086885	7.08170128333333	7.25540121666667	7.64327454166667	7.34272655	6.49981934166667	7.544616	7.74398054166667	8.80540845	8.41742745833333	7.78110251666667	7.13850381666667	7.607704375	6.21517435	8.08436514166667	7.22899075833333	9.30818888333333	8.43662665833333	8.33450988333333	9.64922493333333	8.18475165	9.37821416666667	8.28242620833333	8.68575455833333	8.71562445833333	8.998094275	8.5569931	8.44239195833333	8.84243295833333	8.45509195	8.55907896666667	8.59201043333333	8.31058729166667	9.32000775	8.21046635	9.14279978333333	9.12750715833333	9.08396963333333	8.80870043333333	8.20175241666667	8.860141825	8.91181045	9.653557625	8.86421215	9.98715419166667	7.79638631666667	8.57301913333333	8.698928125	8.6404919	7.49991619166667	9.36939973333333	8.875989475	8.561783725	7.84545261666667	8.224517175	7.90032115	7.78978061666667	8.67639044166667	8.18315743333333	7.86164825833333	8.01273043333333	9.23005591666667	8.34620800833333	9.02512653333333	7.82793564166667	8.745766675	7.31919828333333	7.86670446666667	8.69747196666667	6.83084248333333	8.12540885833333	8.14449565	8.27228149166667	7.44984575833333	8.09282625833333	7.9343368

GET4	12.1793079791667	12.1268451041667	12.0903471208333	12.2398004333333	11.554659475	11.6681825333333	11.7547114875	11.6769830916667	11.8739754041667	10.9319573208333	11.8455711791667	11.5032798708333	11.4425998	12.1530817041667	12.2639514333333	11.52745245	12.2368632208333	11.2393368958333	11.8354138708333	11.4223504875	11.7166202666667	11.7812801	11.8613787333333	11.7748308	11.5856427666667	12.111577325	11.6991601	11.7755963375	11.7283608	11.8189012791667	11.4040128333333	12.1688236291667	11.8703610916667	12.0488412833333	12.0535542458333	12.2757899375	11.72800655	11.554659475	11.6479778	11.44114365	11.9929292708333	12.1730503625	11.8350372166667	11.5260017666667	11.9362989083333	11.8575015625	12.0042054541667	11.7934537333333	11.6434940375	11.7154865958333	11.5807489458333	11.86445575	11.8586299125	11.8675931416667	12.1211265541667	12.0617969541667	11.62075455	11.6869215791667	12.0292205666667	11.465269275	11.8295441666667	11.9110676083333	12.6777922541667	12.4761700833333	12.0854924	12.0895281791667	11.9050099458333	11.9794938666667	12.2459723458333	11.8664687125	11.5796281375	12.1511634166667	12.2745830708333	11.6067879333333	11.6789617	12.6374109625	11.6781963333333	11.6468381291667	11.249063775	12.0094356791667	11.9733909625	11.4002736958333	11.3428663291667	11.6619523916667	12.1285183833333	11.7825688125	11.8148221125	11.5369515041667	12.1191877	11.2773858916667	11.7688707416667	12.2979182291667	12.2815150416667	11.464505575	11.6079034166667	11.8789535625	12.1352700625	11.7118439791667	11.6582809625	11.8847030333333	12.2055252916667	12.1962582875	12.1243931875	12.4882160708333	12.5532343791667	11.974505925	11.9527812416667	11.59500295	12.2156652541667	11.360680725	12.4998470416667	11.8525516125	11.613174325	12.402219	11.66929105	12.1161068875	12.0642302125	11.44114365	11.8401134916667	11.6410193166667

SUN1	12.1793079791667	12.1268451041667	12.0903471208333	12.2398004333333	11.554659475	11.6681825333333	11.7547114875	11.6769830916667	11.8739754041667	10.9319573208333	11.8455711791667	11.5032798708333	11.4425998	12.1530817041667	12.2639514333333	11.52745245	12.2368632208333	11.2393368958333	11.8354138708333	11.4223504875	11.7166202666667	11.7812801	11.8613787333333	11.7748308	11.5856427666667	12.111577325	11.6991601	11.7755963375	11.7283608	11.8189012791667	11.4040128333333	12.1688236291667	11.8703610916667	12.0488412833333	12.0535542458333	12.2757899375	11.72800655	11.554659475	11.6479778	11.44114365	11.9929292708333	12.1730503625	11.8350372166667	11.5260017666667	11.9362989083333	11.8575015625	12.0042054541667	11.7934537333333	11.6434940375	11.7154865958333	11.5807489458333	11.86445575	11.8586299125	11.8675931416667	12.1211265541667	12.0617969541667	11.62075455	11.6869215791667	12.0292205666667	11.465269275	11.8295441666667	11.9110676083333	12.6777922541667	12.4761700833333	12.0854924	12.0895281791667	11.9050099458333	11.9794938666667	12.2459723458333	11.8664687125	11.5796281375	12.1511634166667	12.2745830708333	11.6067879333333	11.6789617	12.6374109625	11.6781963333333	11.6468381291667	11.249063775	12.0094356791667	11.9733909625	11.4002736958333	11.3428663291667	11.6619523916667	12.1285183833333	11.7825688125	11.8148221125	11.5369515041667	12.1191877	11.2773858916667	11.7688707416667	12.2979182291667	12.2815150416667	11.464505575	11.6079034166667	11.8789535625	12.1352700625	11.7118439791667	11.6582809625	11.8847030333333	12.2055252916667	12.1962582875	12.1243931875	12.4882160708333	12.5532343791667	11.974505925	11.9527812416667	11.59500295	12.2156652541667	11.360680725	12.4998470416667	11.8525516125	11.613174325	12.402219	11.66929105	12.1161068875	12.0642302125	11.44114365	11.8401134916667	11.6410193166667

REC8	9.55879715416667	8.22715757916666	8.50963835833333	8.13692505833333	6.99003822916667	8.90170160416667	5.8501225	7.89856145833333	7.52810476666667	8.806065175	7.8789277375	8.00175625416667	7.91019131666667	8.10462871666667	7.8461248875	8.8673426	11.9411064666667	8.22476130833333	8.40591115416667	8.1695733125	8.59468150416667	8.67188521666667	8.96586229583333	8.94738361666667	8.62264167916667	9.93021332083333	8.71860292083333	8.41358929583333	8.52222418333333	8.19010954166667	8.64815709166667	8.71408685416667	8.31335044583333	11.3016955958333	9.60810942083333	9.70349349166667	8.81489524583333	8.6240670625	8.48487685416667	8.72012047916667	9.09210779166667	8.59277177916667	9.15477653333333	8.93976722916667	9.39600600833333	9.80823561666667	8.8697616625	8.58624757083333	9.44081945416667	9.54380385	9.12484684166667	8.5226054125	8.38144642916667	8.98169243333333	9.35886019583333	9.17538841666667	8.18756835833333	8.60874965833333	8.79571160416667	9.403125825	7.94463777083333	7.7095184125	9.990259525	8.60874965833333	7.91819487916667	7.3697041375	7.3790059125	7.11705297916667	9.4983847375	9.40660512916667	7.500213925	7.28164165833333	5.09916694166667	8.88094719166667	8.39489537916667	8.48978630833333	9.53158785833333	9.1694381	9.7364049375	9.66581915833333	6.6479923	7.51281844166667	8.2010289625	8.50918912083333	8.15733075	7.86968296666667	7.42587884583333	9.3964552875	10.233656375	8.790654525	9.13349055833333	9.13846391666667	8.14876864166667	9.49389760416667	8.80754498333333	9.58198016666667	8.75494610416667	8.34553665833333	8.08507183333333	9.0856855375	8.9960395375	7.9192638375	9.09756450833333	8.93338286666667	9.6528549	10.3460990875	8.75766082083333	10.5971126125	8.71623835416667	8.46021965	8.4972275875	8.48032032916667	9.65951234166667	8.84493507916667	9.439839425	9.05582115833333	6.79164088333333	9.75945080416667	9.49930952916667	9.29090133333333

IPO4	9.55879715416667	8.22715757916666	8.50963835833333	8.13692505833333	6.99003822916667	8.90170160416667	5.8501225	7.89856145833333	7.52810476666667	8.806065175	7.8789277375	8.00175625416667	7.91019131666667	8.10462871666667	7.8461248875	8.8673426	11.9411064666667	8.22476130833333	8.40591115416667	8.1695733125	8.59468150416667	8.67188521666667	8.96586229583333	8.94738361666667	8.62264167916667	9.93021332083333	8.71860292083333	8.41358929583333	8.52222418333333	8.19010954166667	8.64815709166667	8.71408685416667	8.31335044583333	11.3016955958333	9.60810942083333	9.70349349166667	8.81489524583333	8.6240670625	8.48487685416667	8.72012047916667	9.09210779166667	8.59277177916667	9.15477653333333	8.93976722916667	9.39600600833333	9.80823561666667	8.8697616625	8.58624757083333	9.44081945416667	9.54380385	9.12484684166667	8.5226054125	8.38144642916667	8.98169243333333	9.35886019583333	9.17538841666667	8.18756835833333	8.60874965833333	8.79571160416667	9.403125825	7.94463777083333	7.7095184125	9.990259525	8.60874965833333	7.91819487916667	7.3697041375	7.3790059125	7.11705297916667	9.4983847375	9.40660512916667	7.500213925	7.28164165833333	5.09916694166667	8.88094719166667	8.39489537916667	8.48978630833333	9.53158785833333	9.1694381	9.7364049375	9.66581915833333	6.6479923	7.51281844166667	8.2010289625	8.50918912083333	8.15733075	7.86968296666667	7.42587884583333	9.3964552875	10.233656375	8.790654525	9.13349055833333	9.13846391666667	8.14876864166667	9.49389760416667	8.80754498333333	9.58198016666667	8.75494610416667	8.34553665833333	8.08507183333333	9.0856855375	8.9960395375	7.9192638375	9.09756450833333	8.93338286666667	9.6528549	10.3460990875	8.75766082083333	10.5971126125	8.71623835416667	8.46021965	8.4972275875	8.48032032916667	9.65951234166667	8.84493507916667	9.439839425	9.05582115833333	6.79164088333333	9.75945080416667	9.49930952916667	9.29090133333333

SETBP1	7.502278225	5.01530855833333	5.29779213333333	5.710337225	6.35356116666667	6.633116075	5.633369575	5.10387183333333	4.95627725833333	5.60132373333333	5.65427465	6.72953939166667	6.21600831666667	5.01427080833333	8.00740176666667	7.70760366666667	6.6562921	6.93287185833333	6.58499179166667	6.48217831666667	4.78586035833333	7.18159225833333	5.33329914166667	6.03335128333333	6.90649953333333	7.53401704166667	7.18351369166667	6.84850089166667	7.42675208333333	6.493249575	6.159395425	7.01553179166667	5.8625617	5.65427465	6.126012425	7.43868464166667	7.28990879166667	6.97159590833333	7.35564645	6.35132606666667	6.44290830833333	6.07429128333333	6.65156973333333	5.84884216666667	6.14647701666667	5.27417344166667	6.62969040833333	6.32600435833333	6.70796138333333	5.97682351666667	5.73195755	5.73943181666667	6.69781155	7.263472875	5.773689175	6.81508890833333	5.85701861666667	5.61354440833333	7.195709975	7.33560889166667	7.88018380833333	5.78909119166667	5.74506199166667	7.11340090833333	5.00529823333333	6.97843058333333	6.03581564166667	4.77813741666667	4.3934547	5.74704205	5.764437375	5.760490375	4.48613384166667	6.26064218333333	6.7730469	7.1355085	6.874007675	6.59670515	5.96001181666667	4.90586546666667	6.65424311666667	7.61391835	6.691149875	6.3922908	5.83110111666667	7.33202409166667	6.51382934166667	4.62057545833333	7.04755845833333	6.77370956666667	5.6322886	6.78513590833333	5.74909436666667	5.19421045833333	7.00303549166667	6.49170240833333	6.99475883333333	7.46463336666667	6.23865941666667	6.87022720833333	4.258212325	5.108231475	5.95243336666667	5.7901556	5.13015945	7.32727508333333	6.70667168333333	6.52344795833333	5.978682025	6.69500276666667	6.02209645	5.44429769166667	7.33501970833333	6.37643818333333	5.6976834	6.28823875	7.84899410833333	6.12691128333333	6.33714610833333	6.028109975

CCDC85B	12.7041276166667	11.6006445416667	12.0356360333333	11.684519	11.9570955666667	11.79216385	11.629073975	11.6529488416667	11.8205903833333	12.0269670083333	11.7153018166667	12.43647125	12.417235425	11.7958947916667	11.8783734666667	11.8137761833333	11.8051550333333	12.1410311166667	11.9521162083333	11.6062715833333	11.9285599833333	11.3339882	11.6179147583333	12.2508814916667	11.7710225416667	12.31433185	11.8036556666667	11.712752375	11.80438315	11.7216620916667	12.1690316666667	11.7053666833333	11.6902780833333	11.6013132583333	12.488903425	12.6060225083333	11.930808275	11.973231675	11.9274761416667	11.8096310916667	11.6547489083333	11.8638644666667	11.485120625	11.80928975	12.0816579833333	11.9694157916667	11.844663475	11.84965965	11.85040895	11.7194234416667	12.1346602166667	11.835222575	11.97471495	11.67680025	11.68154355	11.8403034416667	11.8182827333333	11.9935609583333	11.8070215583333	12.0985319416667	12.1608207083333	11.9728603333333	12.4645960083333	12.98302715	11.7470404	12.5594786833333	12.3216200916667	11.8779799083333	12.9739422583333	12.008092475	12.07292235	12.3305815833333	12.5939827166667	11.8557655416667	12.10269775	12.4474119833333	12.0909367583333	12.5202342666667	12.416791375	12.17539085	11.8819037833333	11.830948725	12.369348425	12.1018588083333	11.7644689416667	12.2107949833333	12.0440494333333	11.6433290333333	12.1843992666667	11.9985911666667	12.856162525	12.0144513	11.8877229666667	12.445171625	12.2701693666667	11.735525225	12.7841531916667	12.265420025	12.2947323416667	12.2276231333333	12.4283415	11.8970046083333	11.861554375	12.0051849333333	12.1087888833333	12.8974584	12.6517095833333	12.710817125	12.3956670583333	12.04824535	11.6259515166667	11.958220325	12.3195462583333	12.5676300666667	12.1694652083333	11.86232365	11.9333501833333	12.017715375	12.3698220916667	12.0537518916667

ARNTL	4.33428121666667	4.28809964166667	4.53105006666667	4.428719425	4.90147810833333	4.62161215833333	4.127831	4.51268873333333	3.660731325	4.22678013333333	4.50011030833333	4.21300761666667	4.08851591666667	3.99930385833333	3.99268493333333	5.4147768	4.3276422	6.48707709166667	6.44217180833333	5.10716860833333	4.74641638333333	5.170393	4.883731825	6.48010203333333	5.7901556	4.93234401666667	4.490204975	5.6488719	4.20885784166667	5.00117389166667	6.04002979166667	5.51915526666667	4.52898779166667	5.565954175	5.70355690833333	5.258661775	4.40284696666667	4.99586916666667	5.636456275	4.13313858333333	5.75948789166667	4.43531826666667	4.61955908333333	4.97072769166667	4.39670516666667	4.750433775	4.71793841666667	4.224150025	4.68789791666667	5.86087000833333	5.69080398333333	5.49431639166667	3.73515468333333	4.97597943333333	4.55568075	4.21192669166667	3.89460940833333	5.19536235833333	4.20885784166667	4.91206978333333	4.41297161666667	4.30130591666667	5.18519845833333	4.740346475	4.11883455833333	5.477009275	4.65070125	4.48925233333333	4.480628975	4.21502835833333	4.40284696666667	4.76967493333333	4.72324775	4.46597973333333	3.95017479166667	4.67972664166667	5.62957723333333	6.54574596666667	5.897311975	4.93234401666667	4.46791538333333	4.42771198333333	5.442194025	5.30448685	5.29675638333333	3.9601097	4.652676825	4.72007973333333	4.49904255833333	5.47315375	5.4913896	5.978682025	5.77716973333333	5.55666916666667	5.4308407	5.196416425	5.75400875	5.34734255	4.24130721666667	4.15845484166667	5.94013145833333	3.89679799166667	5.4308407	4.68477714166667	4.29716620833333	4.40083348333333	4.95862358333333	5.02371664166667	4.88920265833333	6.37491049166667	5.57451170833333	5.824619025	4.90147810833333	4.62161215833333	3.85170159166667	4.03968158333333	4.097912125	5.608795625	4.39770036666667	3.93361386666667

HNRNPA1P48	13.8317321333333	13.9807861416667	14.0683132333333	14.190108625	14.2393514333333	14.2810681833333	14.3204037083333	13.96690645	14.0720390916667	14.2763428833333	14.1113310916667	13.9917082333333	14.3139109083333	14.046645825	13.8832091583333	14.3013820416667	14.1384134166667	14.1554067916667	14.1427380833333	13.8168544833333	13.7263656333333	13.407373775	13.873143175	14.0113488	14.3013820416667	13.317291325	13.1952399083333	14.0607265833333	13.89301535	14.156645775	14.0912434666667	13.9931917666667	14.034006175	14.4077075916667	13.6666988416667	13.8168544833333	13.5610039666667	13.6923255	13.8018861666667	13.60723025	13.6904576333333	13.8528809083333	13.9075706166667	13.934166025	14.113875875	13.986279475	14.107174325	13.751113025	14.0734299166667	14.107174325	14.1427380833333	13.9518039333333	13.8337930083333	13.6931782333333	13.7111367583333	13.6858454833333	13.5617608833333	13.7750620916667	13.6694092583333	13.8843608583333	14.6651046083333	14.3792394416667	14.2319492833333	14.0746231666667	14.4244522916667	13.99785205	14.3860372083333	14.2563286	14.3775303333333	14.1886825416667	14.371029675	14.055300325	14.2576986333333	14.244370025	14.30615445	13.8854083166667	14.3299457833333	14.2393514333333	13.810810275	14.2936594416667	14.6628978166667	14.4983330666667	14.1590921166667	14.07729275	14.1113310916667	14.70147985	14.9020399416667	14.0377493833333	14.5089658583333	14.1441992083333	14.1175943833333	14.0746231666667	14.3943592583333	13.8889513416667	14.2305624666667	14.2260493166667	14.4758644166667	14.3186975416667	14.145758825	14.1164578666667	14.5701394583333	14.5589896583333	14.2503770416667	13.8698539083333	14.2000424583333	14.1972150333333	13.9967915416667	14.2487820083333	14.3316746833333	14.1578289583333	14.0618019166667	13.9955171333333	13.8688792083333	14.0490437833333	14.0301250916667	13.5475412583333	14.4775829916667	14.2969035	14.282826075	14.0100072833333

OR8H1	4.28696936666667	6.53596566666667	5.388701925	6.37349159166667	6.22675393333333	5.27529641666667	7.55318286666667	6.51237135	4.94778381666667	6.52912136666667	5.50338315833333	8.499322875	6.80853135	7.04378355833333	5.86812175833333	4.9195424	6.39383819166667	5.73491465833333	4.9406758	4.421470575	4.84935865	5.07558524166667	4.43737311666667	4.39670516666667	4.125728225	5.35983450833333	3.84944544166667	4.16522844166667	4.01339135833333	4.41506155	4.1018927	4.03859033333333	5.39492906666667	6.41270695833333	4.385351725	4.082140775	4.23901671666667	4.506429475	4.41203210833333	4.383064975	4.79808495	4.30721710833333	4.70784294166667	5.36615406666667	4.40581835	4.92055984166667	5.02467455	5.5697365	4.22594125	4.52898779166667	4.51657093333333	4.54233060833333	4.55671806666667	5.14192449166667	5.13634166666667	4.58777895833333	5.90677250833333	4.53433785	4.7815417	3.93585013333333	4.28310633333333	6.150357875	4.66334196666667	6.89596523333333	5.61255331666667	6.12691128333333	4.52418780833333	6.896657025	5.39178135	6.20969628333333	6.71367926666667	6.06574364166667	8.1963928	6.04082925833333	6.69314785	5.006452275	4.79999713333333	4.087323525	4.82904071666667	4.097912125	5.8386672	5.22368295	5.13961736666667	4.8072374	5.30448685	6.14494839166667	5.05799246666667	6.35578110833333	4.05967894166667	5.66378185833333	4.88576699166667	4.52055483333333	4.84094744166667	5.53102171666667	4.89027461666667	4.13109949166667	4.97597943333333	4.86804739166667	5.87405735	4.91206978333333	4.71218959166667	6.01590590833333	4.55671806666667	4.89935284166667	4.2929499	5.66463360833333	5.49334536666667	5.826473825	5.7550021	5.83017264166667	4.41782250833333	4.45416251666667	4.262047775	4.77378615833333	4.79906281666667	4.84820759166667	4.91654405	4.58140610833333	5.24388391666667	6.39681019166667

TARM1	8.44993103333333	7.33202409166667	7.73527455	8.47046444166667	7.78488271666667	7.74398054166667	7.89243905	7.34037416666667	7.63549629166667	7.448314425	7.80971700833333	8.1525602	7.61391835	7.40602305833333	7.2132819	7.46294761666667	7.363992875	7.76884748333333	7.4656947	7.05838413333333	7.299009625	7.6066095	7.3019376	7.51144511666667	7.03502525	7.1809933	7.27891691666667	7.586837175	7.33740245	7.92709596666667	8.140254425	7.97239718333333	8.10330963333333	7.816692275	7.3019376	7.873129975	8.00309365833333	7.51995818333333	7.88559756666667	7.6157713	7.10699465	7.76084795833333	7.32611610833333	7.35293989166667	7.54510475	7.29049713333333	7.36108145833333	7.86012560833333	7.34440340833333	7.1762422	7.025978175	7.61288113333333	7.94065678333333	7.77718458333333	7.75602535833333	7.57050700833333	7.42162103333333	7.78978061666667	8.51027651666667	7.6287103	8.1455177	8.36292435	8.11887123333333	8.69409343333333	7.63269815833333	8.44745494166667	10.3035388166667	8.11887123333333	8.60280456666667	7.75176951666667	8.25189414166667	8.462988175	8.23567169166667	7.85417920833333	7.788761475	7.65442175833333	8.24509221666667	7.71189573333333	7.63102280833333	7.933308825	8.13668228333333	7.195709975	7.872646525	7.64610639166667	8.03004540833333	7.60193526666667	9.182097875	8.36187725833333	7.98973426666667	7.607704375	8.60435615	8.64585098333333	8.466669725	7.81893236666667	7.88761391666667	8.28193894166667	8.9744767	8.416908275	8.63449166666667	8.64244941666667	7.80451180833333	8.07357910833333	7.99232836666667	7.65442175833333	7.85577905833333	7.782192425	7.88403631666667	8.33810663333333	7.85962578333333	7.73690679166667	7.56380908333333	7.72294644166667	8.4076656	8.08185559166667	7.74722135833333	8.11553545	8.04704694166667	8.32696298333333	8.66481208333333	8.00309365833333

CRAMP1	9.295997875	9.22893656666667	8.94502036666667	8.58624757083333	9.94771790416667	9.84308857083333	9.36738271666667	9.74023032083333	9.87278947083333	9.05665619166667	9.3249872625	9.22813745	8.87760424583333	9.35610940833333	9.90753555	9.3110777875	9.5775666125	9.04961187083333	10.2577054166667	9.59626012916667	9.42972139583333	9.93324109166667	9.70516922916667	9.21811477083333	9.11471757083333	9.4022974875	9.03886794583333	9.12208806666667	9.22893656666667	9.62343045	9.57331585416667	9.53875820833333	9.55311440833333	9.09621615	9.51747024166667	9.590448175	9.65731495833333	9.4462680125	9.383893725	9.3913629125	9.2012284625	9.6960067625	9.35568727916667	9.2705922375	8.55621531666667	9.42837040416667	9.34730499583333	8.8104251375	9.82583156666667	9.65823706666667	9.11661512083333	9.85917256666667	8.68202068333333	8.6426758625	9.04961187083333	9.17895344166667	9.286671325	9.42665710416667	8.7262062375	9.283834	8.87906529583333	8.76005182083333	8.01515287083333	8.6791378	9.99065759166667	10.6908038625	9.54294158333333	9.2527877375	9.71246596666667	9.22934275	9.0856855375	10.16435265	8.712140675	8.73664734166667	8.86444172083333	9.55786483333333	9.48363187916667	9.05246449166667	10.1280952583333	9.95379169583333	10.0402000875	9.73076655833333	9.1435266375	8.92951810833333	9.263749275	9.55836635	9.6640089875	7.91494055416667	9.3192696625	9.5526662125	9.33946395833333	9.98331006666667	9.77241673333333	9.6029844375	9.33946395833333	9.7955383625	9.22934275	9.81544907916667	9.30147802916667	9.97153182916667	10.2751315166667	8.85521000416667	9.51612702083333	9.41870919583333	9.40579160416667	9.04961187083333	9.23116307916667	9.26104765833333	9.70174240416667	8.9629780875	9.79213676666667	9.70174240416667	9.4668083625	9.10598958333333	8.81173254583333	9.0154403625	10.3327453208333	9.52245795833333	9.78504972916667	9.20673209166667

AL031708.1	9.295997875	9.22893656666667	8.94502036666667	8.58624757083333	9.94771790416667	9.84308857083333	9.36738271666667	9.74023032083333	9.87278947083333	9.05665619166667	9.3249872625	9.22813745	8.87760424583333	9.35610940833333	9.90753555	9.3110777875	9.5775666125	9.04961187083333	10.2577054166667	9.59626012916667	9.42972139583333	9.93324109166667	9.70516922916667	9.21811477083333	9.11471757083333	9.4022974875	9.03886794583333	9.12208806666667	9.22893656666667	9.62343045	9.57331585416667	9.53875820833333	9.55311440833333	9.09621615	9.51747024166667	9.590448175	9.65731495833333	9.4462680125	9.383893725	9.3913629125	9.2012284625	9.6960067625	9.35568727916667	9.2705922375	8.55621531666667	9.42837040416667	9.34730499583333	8.8104251375	9.82583156666667	9.65823706666667	9.11661512083333	9.85917256666667	8.68202068333333	8.6426758625	9.04961187083333	9.17895344166667	9.286671325	9.42665710416667	8.7262062375	9.283834	8.87906529583333	8.76005182083333	8.01515287083333	8.6791378	9.99065759166667	10.6908038625	9.54294158333333	9.2527877375	9.71246596666667	9.22934275	9.0856855375	10.16435265	8.712140675	8.73664734166667	8.86444172083333	9.55786483333333	9.48363187916667	9.05246449166667	10.1280952583333	9.95379169583333	10.0402000875	9.73076655833333	9.1435266375	8.92951810833333	9.263749275	9.55836635	9.6640089875	7.91494055416667	9.3192696625	9.5526662125	9.33946395833333	9.98331006666667	9.77241673333333	9.6029844375	9.33946395833333	9.7955383625	9.22934275	9.81544907916667	9.30147802916667	9.97153182916667	10.2751315166667	8.85521000416667	9.51612702083333	9.41870919583333	9.40579160416667	9.04961187083333	9.23116307916667	9.26104765833333	9.70174240416667	8.9629780875	9.79213676666667	9.70174240416667	9.4668083625	9.10598958333333	8.81173254583333	9.0154403625	10.3327453208333	9.52245795833333	9.78504972916667	9.20673209166667

GFPT1	10.0557486666667	10.1906714166667	10.0078625	10.96888565	9.94164523333333	10.9958406	9.763939075	10.2638424166667	10.3721682083333	10.15852225	10.80942375	10.7189561666667	10.1791861916667	10.186667875	9.9690997	10.3667222333333	11.8993453583333	9.64383103333333	9.7515126	9.97706043333333	10.2229494583333	10.21946315	10.5204657833333	11.1010834666667	10.97439825	10.743245175	9.96481554166667	9.93042398333333	9.83111589166667	10.0787243333333	9.7288377	9.97378895833333	10.6860936833333	11.2534278333333	10.6461664	10.82998625	9.93713581666667	10.135861875	10.2810793	9.33181315833333	10.4661023416667	9.813959625	10.028074675	10.0693001666667	10.3937266	9.59906339166667	9.94034049166667	10.5711304666667	9.77571775833333	11.1039790666667	9.98312965833333	10.2301923	9.82123129166667	9.906465775	9.78824573333333	9.98111111666667	10.0859903416667	9.80967570833333	9.79856730833333	10.5026381833333	10.8337493916667	10.191495075	9.63659523333333	9.97131721666667	10.8025054583333	10.3171296666667	9.95522605	10.1696248333333	9.295254925	9.70842734166667	10.123005125	10.1845142083333	9.58706739166667	10.001418875	9.45194998333333	10.2987266333333	10.1965925083333	9.77133523333333	9.40680740833333	10.0920085583333	11.3047106916667	10.08403975	9.56259116666667	10.36070035	10.3718117166667	10.2005317833333	10.9629037916667	9.79486831666667	10.8807458916667	9.974167275	9.64690625833333	10.86272805	10.1791861916667	9.35031244166667	9.990066925	10.6038899083333	10.040006825	11.1166050833333	8.94049991666667	9.599958075	10.2237133416667	10.176297425	10.3745792583333	10.3475834	10.713545975	9.705378475	9.59782546666667	9.34438068333333	9.88434308333333	9.73873335833333	10.6558683583333	10.2511057916667	9.33321418333333	10.4292877666667	9.945938775	9.68261813333333	11.1926823916667	10.1581087416667	10.2363454416667	10.253110825

WFDC11	4.77930389166667	4.58445505833333	4.16407313333333	4.33043379166667	4.61625569166667	4.44161573333333	5.02158241666667	4.19346535	3.936908825	5.56242626666667	5.83674730833333	4.18714809166667	4.177708	4.302366825	5.79742544166667	4.39670516666667	4.64220138333333	5.17804041666667	5.6241104	4.26116580833333	4.138474425	5.04024386666667	4.57924936666667	5.48703414166667	6.2353521	4.021539275	5.60971738333333	5.98220235	5.70053848333333	5.82271865	5.04762459166667	6.39012523333333	4.66727606666667	4.18191081666667	4.43091141666667	4.34514024166667	5.78709845833333	6.26827546666667	5.442194025	5.44429769166667	5.05172549166667	6.04352600833333	5.11118121666667	5.38064126666667	4.81179368333333	5.956517125	6.160301025	6.32454565	5.23876719166667	5.68304789166667	6.13011929166667	6.00367545	4.494971825	6.49896905833333	5.27529641666667	4.71793841666667	4.38097856666667	5.11568579166667	6.225217	7.07182334166667	7.87964591666667	5.81909195	5.628745675	6.64696773333333	3.84151389166667	6.150357875	4.86085110833333	5.57060381666667	4.97812506666667	4.617309275	5.34510054166667	5.93746846666667	6.14803923333333	5.01216298333333	5.45476871666667	5.3100359	4.09698049166667	4.823565325	6.00123059166667	5.06232444166667	4.93436960833333	5.14401780833333	5.23566219166667	5.576316275	5.67998533333333	5.51386359166667	4.78381058333333	4.66519471666667	5.25233420833333	4.816016475	5.3343362	7.173894	5.39804303333333	5.85514745833333	6.58901686666667	6.47456359166667	6.5479092	4.99463171666667	6.11462551666667	7.13230843333333	5.6488719	6.06230976666667	6.91578644166667	6.075096075	6.94439705	5.36511784166667	4.71686186666667	6.49390590833333	4.59683404166667	6.64559758333333	7.51365925	5.956517125	5.82824421666667	5.485111725	5.43596139166667	5.91334729166667	4.11987200833333	5.15246255833333	7.32089796666667	6.08105208333333

RPL31	15.4575903	15.0306120416667	15.290119725	15.2981600916667	14.2887769	15.2084540583333	15.28255625	15.5870133916667	15.2120960833333	15.1460462916667	15.325108875	14.9645581833333	14.7572900333333	15.3290216916667	15.33292745	14.9223639	15.3777313666667	15.25397645	15.2981600916667	15.2193239916667	14.1317250833333	15.2328828083333	15.169521525	15.2230127	15.4400250416667	15.1336404666667	14.211775675	15.2014589916667	14.66935455	15.2981600916667	15.13692035	15.0557071416667	15.1495015916667	15.3498376083333	15.3290216916667	14.7293250666667	14.8629148	15.1940996583333	15.325108875	14.9645581833333	15.1017513083333	15.2014589916667	15.5407381333333	15.3093345916667	15.0865438916667	15.37360755	14.8548535916667	14.8442556083333	14.9223639	15.325108875	15.28255625	15.4488835416667	15.25397645	14.156645775	15.4985918916667	15.1077350416667	14.7055894833333	15.2722555666667	14.3960605	15.1873199	14.7547481083333	14.9832780583333	14.89930145	14.9020399416667	15.4199685666667	15.0005057333333	14.9567111166667	14.87708635	14.8313200916667	14.8287604166667	14.9020399416667	14.8362922416667	14.9702238166667	14.6104738916667	14.6738026916667	14.8794573583333	14.9645581833333	14.9865439333333	14.77778785	14.9727774916667	15.581383325	15.0459774	14.8548535916667	14.7522867083333	14.7055894833333	15.0703584333333	15.4400250416667	14.7731878916667	15.1728574583333	14.8794573583333	14.8287604166667	14.9752180916667	14.7034991833333	14.718655275	14.5195733916667	14.9674626083333	15.1602131166667	14.7547481083333	14.5589896583333	14.847260375	15.1833315916667	15.0557071416667	14.6738026916667	14.6992893416667	14.9045675833333	15.0769655666667	15.1763309166667	15.1632730833333	15.25397645	14.77778785	14.7097912166667	14.4043399	14.7948348083333	15.2468154916667	14.4705790833333	14.7948348083333	15.009089275	14.8750732583333	15.095547425	14.5038815833333

ZNF613	8.76851021666667	9.13172414166667	9.20920615	9.10485650833333	7.89832068333333	8.24906243333333	8.40473328333333	9.002830825	8.8128727	8.16983206666667	8.12933435833333	8.50451981666667	8.001514325	9.11864413333333	8.86116145	9.063171125	9.08396963333333	7.70256570833333	9.02927973333333	8.898281125	9.03689006666667	9.045519875	9.26988130833333	9.28599946666667	9.09833678333333	9.17368278333333	9.15113736666667	9.114481175	8.47579976666667	9.19860346666667	9.15210589166667	9.45323241666667	8.74083754166667	8.94761291666667	9.1530551	9.45374055	8.57400345	9.20513901666667	9.39071778333333	9.16216856666667	9.04505583333333	9.35167163333333	9.3434528	9.23462919166667	9.19627585833333	9.32044345833333	9.56961935	9.60629726666667	9.36987781666667	8.92028675	8.729717925	9.34530801666667	8.45605063333333	9.10796010833333	9.52783803333333	9.166351	8.847054525	9.04369295	8.68971138333333	9.07434654166667	8.19036318333333	7.48985504166667	9.65888631666667	7.04378355833333	8.55750678333333	7.10570254166667	7.9846002	8.13194826666667	8.33758601666667	7.57732030833333	7.9857199	8.354234475	6.986635125	8.84155408333333	8.35071744166667	8.95312890833333	8.15011035	7.76403325833333	8.28340845833333	8.25398819166667	8.71235535	8.8902947	6.91905025833333	8.92840376666667	7.84051964166667	8.1277546	8.88122105	8.97126208333333	8.77719965833333	8.647383475	8.698928125	6.974033025	8.126799325	9.29885605	8.15860931666667	7.6798049	7.681528525	8.17835735	7.984047875	9.64247345	9.60581258333333	7.78978061666667	8.72544860833333	9.03327476666667	9.53312831666667	8.51908186666667	8.94100855	8.86553766666667	8.89560048333333	8.74174598333333	8.56913543333333	8.20175241666667	8.62786819166667	8.650731025	8.19188733333333	9.27812115	8.28798768333333	9.19905495833333	7.13728840833333	8.094406525

TMEM47	15.1940996583333	12.2760053833333	11.8158651416667	11.2222793166667	12.928953425	13.698785275	12.229824125	12.7801271583333	12.6800815583333	12.7939716	12.3523338166667	15.4316128083333	14.0746231666667	12.3038527583333	12.731765875	12.8035935833333	12.95462435	11.9212294333333	13.1268647416667	13.0602371583333	13.7640679416667	11.7030888416667	12.336155525	12.244497975	12.7681554	15.345488975	13.279249925	12.2760053833333	12.4723572833333	12.2764019583333	12.8690686583333	12.1652756583333	12.7836657416667	12.9726928583333	15.2468154916667	15.5556811083333	12.3301864333333	12.9388338	12.3606021	13.788062875	12.7144138666667	12.3662011583333	12.3798172166667	12.6566773416667	14.0237926083333	12.2637428083333	13.2447546666667	12.1084032166667	11.92557355	12.5872332583333	12.706710675	12.4682236083333	12.1737118583333	12.24577265	12.2053036416667	12.725374025	12.7547810833333	12.0409411833333	12.8376576083333	13.0427266583333	11.28518	12.2040899	10.7127200416667	12.178702275	11.765950425	11.3332438	12.1346602166667	12.247510175	11.085607975	11.88924055	12.11053915	11.4828657583333	11.6077110916667	13.0167634166667	11.492270725	12.5196766666667	13.3601687833333	12.3405472916667	11.4424334416667	12.2528148916667	12.5024686916667	12.6785589083333	11.608877475	12.8889747333333	12.2951630416667	12.910549725	11.017853675	12.555705525	11.3054657083333	13.541969925	12.1279125583333	11.7482758916667	11.2527308083333	12.0969790916667	11.0716307666667	10.5692188416667	11.141230475	11.121759625	12.0619908166667	11.98404715	11.6962930666667	12.0604174833333	10.8109394916667	11.0279781	10.4236603166667	11.9939589	12.8901186583333	10.8600934166667	12.10430865	12.7206576333333	12.7649781166667	10.6997392583333	10.7302810416667	11.0863287333333	12.0744704	10.5956461416667	11.9196580833333	10.96365625	10.749691325	12.1886556416667

TMEM191C	10.0349391541667	10.7837402125	10.6654619416667	10.9366493	9.86041405	10.1297697375	10.0857930791667	10.1748193166667	9.67076629583333	10.2082797708333	9.45447309583333	7.87539923333333	9.73465698333333	10.7506659958333	11.229268325	9.92812106666667	9.18283845	10.3213899291667	11.2041638708333	10.1190294875	10.2276468583333	10.1764916541667	10.0686200208333	9.983679525	10.4704001541667	8.71581307916667	10.8947752583333	10.3110616	10.1672779333333	10.1904914125	10.4746305041667	10.187260375	9.7376662875	8.612181625	9.16986652916667	10.0178064291667	10.0857930791667	10.4102852625	9.58548723333333	9.64943681666667	10.4139942291667	10.7226482375	10.4282659291667	10.6859179875	10.2184744958333	11.012949625	10.6763291791667	10.4063314208333	10.1368662333333	9.64670775416667	10.2231272333333	10.0804987583333	10.3528785666667	10.8271755666667	10.7032826708333	11.0209367666667	10.0829329166667	10.4497322333333	10.6900690916667	10.3564310166667	9.52846650833333	9.76711499583333	10.521068475	11.2393368958333	9.32258433333333	10.3460990875	9.29362754166667	9.6516368375	8.78967324166667	8.741993175	9.63221137916667	10.0887728458333	10.7571049208333	9.44712263333333	10.7544062	10.6505566166667	10.0187082208333	9.720911	11.210243625	10.13565995	9.52385999583333	8.67714263333333	9.99148106666667	10.0337206166667	9.4796625125	8.64220767916667	8.8613937625	9.174863825	9.05200975	9.27387779166667	9.34323916666667	9.50935463333333	9.61390410833333	9.83341595833333	8.39287426666667	10.056763975	10.7063933208333	8.8678438125	9.25926169583333	9.34086222916667	9.64005285	10.1395317375	9.46011392083333	10.0262798583333	11.7241398625	9.76372285416667	10.7327323125	9.89615510416667	9.74798653333333	9.57072065833333	9.71463695833333	10.8727714875	11.2009752833333	10.915520925	9.34041329583333	10.955859175	10.0780808416667	10.6420869333333	10.9660080958333	9.95954340416667

TMEM191B	10.0349391541667	10.7837402125	10.6654619416667	10.9366493	9.86041405	10.1297697375	10.0857930791667	10.1748193166667	9.67076629583333	10.2082797708333	9.45447309583333	7.87539923333333	9.73465698333333	10.7506659958333	11.229268325	9.92812106666667	9.18283845	10.3213899291667	11.2041638708333	10.1190294875	10.2276468583333	10.1764916541667	10.0686200208333	9.983679525	10.4704001541667	8.71581307916667	10.8947752583333	10.3110616	10.1672779333333	10.1904914125	10.4746305041667	10.187260375	9.7376662875	8.612181625	9.16986652916667	10.0178064291667	10.0857930791667	10.4102852625	9.58548723333333	9.64943681666667	10.4139942291667	10.7226482375	10.4282659291667	10.6859179875	10.2184744958333	11.012949625	10.6763291791667	10.4063314208333	10.1368662333333	9.64670775416667	10.2231272333333	10.0804987583333	10.3528785666667	10.8271755666667	10.7032826708333	11.0209367666667	10.0829329166667	10.4497322333333	10.6900690916667	10.3564310166667	9.52846650833333	9.76711499583333	10.521068475	11.2393368958333	9.32258433333333	10.3460990875	9.29362754166667	9.6516368375	8.78967324166667	8.741993175	9.63221137916667	10.0887728458333	10.7571049208333	9.44712263333333	10.7544062	10.6505566166667	10.0187082208333	9.720911	11.210243625	10.13565995	9.52385999583333	8.67714263333333	9.99148106666667	10.0337206166667	9.4796625125	8.64220767916667	8.8613937625	9.174863825	9.05200975	9.27387779166667	9.34323916666667	9.50935463333333	9.61390410833333	9.83341595833333	8.39287426666667	10.056763975	10.7063933208333	8.8678438125	9.25926169583333	9.34086222916667	9.64005285	10.1395317375	9.46011392083333	10.0262798583333	11.7241398625	9.76372285416667	10.7327323125	9.89615510416667	9.74798653333333	9.57072065833333	9.71463695833333	10.8727714875	11.2009752833333	10.915520925	9.34041329583333	10.955859175	10.0780808416667	10.6420869333333	10.9660080958333	9.95954340416667

TMEM18	9.06270968333333	9.227470725	9.35679085833333	9.36621750833333	9.478582775	9.03034083333333	9.41531880833333	9.02785883333333	9.47945113333333	9.05355476666667	9.11266035833333	9.567278375	9.092829475	9.24492166666667	9.45149038333333	9.370796075	9.87345979166667	8.895120775	9.14235506666667	9.41484764166667	9.74776665	9.3629448	9.14971231666667	9.562147725	9.37777211666667	9.51175975	9.106662475	9.07865619166667	8.46826041666667	9.645654625	9.175625125	9.41892754166667	9.44151263333333	9.88637849166667	9.45859685833333	9.45734620833333	9.203321075	9.39983189166667	8.52772891666667	9.15113736666667	9.11077986666667	9.10576088333333	9.43278838333333	9.43547285833333	9.3041126	9.24127241666667	9.38500366666667	8.87930013333333	9.15740435833333	9.34750165	8.79240648333333	9.05312258333333	9.03233675	9.30630015833333	9.38096979166667	9.57610176666667	9.66742095833333	8.95991093333333	9.436848125	9.13416769166667	8.74370115833333	9.53087495833333	8.472359075	9.03689006666667	10.4546419916667	9.06925550833333	8.66201021666667	9.41620731666667	8.80870043333333	9.44779018333333	9.33579655	9.40252054166667	9.32897005833333	9.3629448	10.130420225	9.4551338	9.33579655	8.76751969166667	9.18832863333333	9.42547525833333	11.8202425833333	9.833640075	9.42508291666667	9.1473568	9.23139328333333	10.1042493666667	10.5833260083333	9.099726825	9.09331330833333	9.40906840833333	9.42424818333333	9.458203675	9.44779018333333	9.552037225	9.309548625	9.10889125	9.22794726666667	11.528423775	8.58695619166667	9.22652003333333	9.57736215	9.77263848333333	9.38409195	9.55671014166667	9.15886451666667	9.16735169166667	9.1473568	8.95121126666667	8.95079723333333	8.973101525	8.74427688333333	8.79951210833333	9.54357819166667	9.54357819166667	9.17457594166667	9.235078425	10.6589017416667	8.53333894166667	9.65845149166667	9.57414756666667

MAEL	6.61484865833333	4.07882345	3.90223645833333	4.20999628333333	3.660731325	4.56806040833333	3.86167775833333	3.99268493333333	3.9147734	3.711770875	3.77672200833333	4.07134488333333	4.54440353333333	3.95777633333333	3.9479401	4.03968158333333	4.48432390833333	3.99817013333333	5.3960318	4.68681869166667	3.86613395833333	4.08963481666667	4.27700685	4.33846348333333	4.6319392	8.58140188333333	4.61955908333333	4.5496205	4.55863708333333	4.332336275	4.59997769166667	4.58336269166667	3.82817183333333	4.19234266666667	8.50984575833333	7.33974354166667	4.34607883333333	4.47089620833333	4.27374416666667	4.87091625	6.25335921666667	4.337410475	4.56600969166667	5.079599575	6.28184195833333	4.690117325	4.97072769166667	4.59897825	4.47089620833333	5.58117110833333	4.63625878333333	4.33133893333333	4.125728225	5.09968536666667	4.172665625	4.94778381666667	4.17476265833333	4.510676125	5.221531825	4.26652515833333	4.263255	3.60976025833333	4.23291245833333	4.69115821666667	5.81212459166667	3.70456366666667	3.86022540833333	4.20479785833333	3.7942322	3.45816426666667	4.07134488333333	4.20109206666667	3.82560103333333	4.00881020833333	3.74900228333333	3.9661014	4.001416775	3.76208641666667	3.7942322	7.72023940833333	4.337410475	4.82036908333333	4.224150025	3.92371073333333	3.80008696666667	4.37710720833333	3.81491895	4.51475793333333	3.656722575	4.322875175	4.1095023	7.92336901666667	9.48031518333333	3.78824818333333	3.98511664166667	3.695851625	3.95464144166667	5.948036925	3.74768024166667	6.723717275	12.5793612333333	3.54483786666667	4.60699911666667	4.66004348333333	4.25290215833333	3.85519674166667	5.554616075	5.47416700833333	4.54330015	6.407249275	4.52155396666667	6.47064316666667	4.74219220833333	4.7006097	4.04622350833333	4.20885784166667	5.44754924166667	4.359146625	4.33328629166667	4.021539275

TAF4B	8.49975086666667	9.73309245833333	9.332301675	9.173253675	8.44043165	9.02034758333333	8.78307434166667	9.557193025	9.27125073333333	9.03233675	9.349435025	8.29098985833333	7.94017044166667	8.99720454166667	9.28128599166667	9.46432354166667	8.791398425	9.96660331666667	9.87864789166667	9.38667448333333	9.354988125	9.645264775	9.82733675	10.5212723416667	9.442472475	9.026583775	9.47301451666667	9.59369666666667	9.02175539166667	9.7860847	10.2450271333333	9.86598649166667	9.18505965833333	9.77883299166667	9.617230275	9.09237333333333	8.781733725	9.777390875	9.781927775	9.6428903	9.67900834166667	9.42244939166667	9.58222316666667	9.7860847	9.26217445	9.52364006666667	9.69176305833333	9.18167015	9.52456219166667	9.452370525	9.445595775	10.0852465083333	9.08047905833333	9.4094908	9.42204840833333	9.214506275	9.911603525	9.50308771666667	9.38807046666667	9.45898255	8.57995885	10.3236813083333	8.80189605833333	9.14426045	9.261730375	8.56222751666667	10.0498058583333	8.44192248333333	9.900995025	9.297473225	8.72356944166667	10.7042731416667	8.62547046666667	8.08700735	7.23193100833333	8.71332470833333	8.09066366666667	9.47679953333333	9.79321156666667	9.512679675	10.3053444333333	8.26456484166667	9.9690997	9.9441902	7.737502925	9.89258960833333	10.9994499416667	8.13819046666667	8.29250424166667	9.61946140833333	10.0197701416667	10.5196816583333	9.60963610833333	9.15645445833333	10.2724454333333	9.47679953333333	9.24668224166667	10.6621925666667	10.0819455833333	10.5424705	9.721994775	9.5928155	10.2666506833333	9.71188024166667	9.71864728333333	10.0578180083333	9.39753216666667	8.89696199166667	9.09237333333333	8.95172195833333	9.15740435833333	9.00867418333333	9.444752725	10.4441620166667	9.22652003333333	9.58257986666667	10.689872025	9.03183454166667	10.199031925	8.65887986666667

CASC4	12.7175215083333	11.4919381583333	11.562248175	11.5443713166667	11.8104289083333	12.9929816333333	12.3435261333333	12.1389321	12.5701103	12.47909375	12.680606175	13.4688309833333	12.4908032583333	11.0659389916667	11.71493805	12.2396159	13.661470025	12.1665042	12.4306290916667	12.1620485833333	11.9348926	11.7171927333333	12.08124855	11.960958325	12.4315646666667	12.7081294916667	11.9212294333333	11.467242025	11.6404855583333	11.7682562416667	11.9329100916667	11.7146176666667	12.400136275	13.1622146833333	13.2467446916667	13.1506037833333	12.0036527416667	12.1342141916667	12.40421355	12.3378199666667	11.5980356083333	11.612663925	11.705716875	11.5684552166667	11.8523757333333	11.3846584083333	11.545061825	11.17279555	12.3680867166667	13.1865714083333	12.5855274583333	12.21725785	10.7561593416667	11.01352865	10.8763708666667	10.7302810416667	12.1451222666667	12.3501237916667	12.069365875	12.3275837583333	12.367659325	12.3190685583333	11.97391105	11.5501880833333	13.3410732083333	12.9042883333333	13.2387164583333	13.160332775	12.382866125	12.8181395166667	12.9340515083333	12.3554452	12.2240198833333	12.9617333583333	12.0905289333333	12.488453925	12.9477864583333	13.21039535	12.584589825	13.23467165	12.4238445333333	12.539129975	11.7541385166667	12.3365706083333	12.1882257583333	12.7026115333333	11.8545873833333	12.4917329166667	12.540087475	13.016186275	12.2683870833333	12.1389321	12.948888375	12.8930719333333	11.9491178333333	12.51216495	11.8950942583333	12.543462525	12.4755627916667	12.750529275	11.8290120833333	12.0432377416667	12.3943629916667	12.1130081666667	11.1113391916667	11.931140425	11.9994097833333	11.41004175	13.0804212833333	12.9439344083333	12.9785766	12.74583335	11.28453495	10.86272805	11.2352593083333	11.2635210833333	12.6689869583333	12.1229210916667	12.4759771583333	12.4132063666667

TOR1AIP2	11.7336295416667	12.500114075	12.4692673166667	12.5740059166667	12.524997525	11.9705949166667	12.2322263916667	12.2550005	12.4945779666667	12.04824535	11.8811110166667	11.6386741333333	12.2416674416667	12.4069098166667	12.2723412333333	11.9773878833333	12.3509430416667	12.108037425	12.4019704583333	12.497697975	12.16610385	12.484600075	12.2492084583333	12.4087159666667	12.48705105	11.4122968416667	12.454126225	12.2812943416667	12.5226496333333	12.4037688166667	12.3484591166667	12.6254772333333	12.219394525	12.1541314	11.4151310833333	11.3441400333333	12.43292905	12.3216200916667	12.3698220916667	11.9863991	12.4238445333333	12.8035935833333	12.6456361916667	12.0816579833333	12.1612618833333	12.4010561583333	12.7831765916667	12.7774843666667	12.04365515	11.8379474833333	12.3926407	12.4315646666667	12.2044709583333	12.3785017666667	12.2719001166667	12.4257259083333	12.4395669416667	12.548305425	12.382866125	12.1686155916667	11.980095625	11.99193365	11.686699925	11.6849152916667	12.3356581833333	12.217702275	11.8390986666667	11.7812801	11.9720790416667	11.2631446833333	11.8545873833333	11.8198860833333	12.3592921916667	12.2244042583333	12.3575221583333	12.226050525	11.4763556583333	11.4313741833333	11.9997560833333	11.7601425083333	12.2244042583333	12.2107949833333	12.4069098166667	12.1790995083333	12.4185466166667	12.0241448416667	11.825372775	11.7053666833333	12.317829175	11.802441875	12.1254015666667	11.9491178333333	11.9935609583333	12.3374196	11.8435483083333	11.8461878583333	11.54260245	11.938838275	12.2528148916667	11.7509367	12.118235175	11.6841362916667	11.7553657	11.9120239416667	11.7373039666667	11.9883632916667	12.244114325	12.0541727916667	12.5865453166667	11.9836236833333	12.075266	11.5702459583333	11.917277775	11.3938737333333	11.8553966916667	12.2188976833333	12.2662860833333	11.5567047416667	12.025731925	11.62415315

CNPPD1	11.1652493166667	12.43820225	12.2350239916667	12.252092225	12.0705755166667	11.2743409666667	11.9220834083333	11.7878982583333	12.1579610916667	11.6948221166667	11.4364638833333	11.645956625	11.908925725	12.6754546333333	12.5680695416667	11.6411895916667	11.5287863916667	11.9754521583333	12.43647125	11.97391105	11.6507569666667	11.8779799083333	11.4989285	11.7724295666667	12.0733383583333	11.6985875333333	12.1320993083333	11.9871613166667	11.8772191666667	12.1451222666667	12.058910375	12.2366655666667	12.0205452666667	11.7761557916667	11.6698378833333	11.7046170083333	12.252092225	12.347471575	11.8853151666667	11.7557288583333	12.1852414666667	12.3422979833333	12.253276925	11.7568591416667	11.388258375	11.9001097666667	12.2990201416667	11.7351274916667	11.8341069333333	11.6452682	11.875371875	12.3271580833333	12.1891609666667	12.531130425	12.0072469333333	12.468741075	12.3509430416667	11.83053445	12.2210896416667	11.6378736916667	10.7862968083333	10.9994499416667	10.9466012416667	11.66717835	11.3180417666667	11.6898911833333	11.2039860833333	11.520872575	11.0704861666667	11.4321000166667	11.4452785583333	11.7362421333333	11.3635795583333	11.31013205	11.352411175	11.6252518666667	11.1649238666667	11.426633325	11.3282347083333	10.9851253333333	11.541832	11.425117975	11.080269225	10.9680827416667	11.48138035	11.0056705916667	11.1398416833333	11.51586705	11.526510825	10.9230393083333	11.5858193083333	11.0394398083333	11.328583825	10.9895149583333	11.4262590333333	11.4861149333333	11.6993325583333	11.0619074416667	11.9641339083333	10.8996373166667	10.9450853666667	11.2424523	10.8751259833333	10.97339425	10.9684950083333	11.1125423333333	11.0211329583333	10.590660625	10.982052575	10.8322571166667	11.22444735	10.9629037916667	11.489428925	11.8896284166667	10.7912120583333	11.4679092333333	11.49602405	10.8953436916667	11.081884475	11.3735584916667

CYP1A2	9.32897005833333	9.42904984166667	9.00735385	9.17190161666667	10.966941925	9.637594125	10.9127535083333	9.96018530833333	10.3690418416667	9.62666130833333	9.86395566666667	10.8533803	10.5112057333333	9.54731913333333	9.579948275	8.97861723333333	9.028831875	10.852937175	9.03372714166667	9.76472428333333	9.64383103333333	9.99895085	8.849091575	9.01340034166667	9.62234490833333	9.39297179166667	9.189315625	9.42163085833333	9.577771075	9.11782889166667	8.62734905	8.96796725	9.99259065	9.35075274166667	8.93461839166667	9.31762243333333	10.1957428083333	9.292974825	9.29340571666667	9.27035150833333	8.440899325	8.60227746666667	8.56322373333333	8.53929365	8.63681648333333	8.04016346666667	8.1963928	8.76125596666667	9.21787520833333	9.06838428333333	9.01942851666667	9.1218467	9.51175975	8.97266575	8.44287714166667	7.9089283	10.1312217666667	8.37004933333333	9.29702861666667	9.0180261	10.2498045833333	9.60406910833333	9.03327476666667	9.536457975	9.52591685	10.6812532	9.683521475	10.4384998666667	9.70114725833333	10.390544	10.4191933083333	9.80143100833333	10.4744353333333	9.70931241666667	9.73955326666667	9.57093504166667	10.1573430916667	10.7313914916667	9.64024775833333	9.93596328333333	10.102550325	10.1254167416667	9.06116049166667	8.69317739166667	10.261767125	9.952361775	10.2034843416667	9.99808105833333	10.5743323083333	9.26627294166667	9.311300325	9.577771075	9.98150248333333	9.71227291666667	10.253110825	9.889693225	10.20226945	10.3281467166667	10.2149556666667	9.24218355833333	9.38715235833333	9.69747909166667	8.910111275	8.71039916666667	9.08729039166667	9.83111589166667	9.04179071666667	8.60701585833333	9.50452185	9.357237425	9.41120025	8.74174598333333	8.779206475	8.17668253333333	8.52731504166667	8.4515305	9.478582775	9.53223686666667	9.124627175	9.91970055833333

NPB	11.0940759041667	10.2705578875	10.3410778458333	10.41219155	11.1988506416667	10.5499198708333	10.526248225	10.1280952583333	10.347372975	10.327136925	10.366163275	10.8614308541667	10.9885999458333	10.0089119458333	10.8910372666667	10.1483618875	10.2714276125	11.8808967833333	10.4022684833333	10.4497322333333	9.99065759166667	11.0396227291667	9.687147875	10.1418799166667	10.8445233666667	10.6178551916667	10.915520925	10.606680675	11.8267584666667	10.3398532125	10.325930675	10.3911256916667	10.4151646583333	10.5426501458333	10.43626715	10.9679003458333	11.2755224208333	10.4290650375	10.3099456083333	10.3877990125	9.72467065416667	10.1162432666667	9.930629725	9.77372232083333	10.3889349333333	9.63041037916667	9.69902295833333	9.97646281666667	10.0698569125	10.1495387208333	10.1743594333333	10.1793924208333	10.9456650125	11.1432396458333	9.9554479	9.74023032083333	10.2176713	10.0207806125	10.7671402625	10.3188843875	10.7354198833333	11.0591417875	10.1061134458333	11.2924159458333	11.73712565	10.7297003833333	10.3180997375	10.6958287375	10.6509623708333	10.46699275	10.4812808791667	10.4501215791667	10.7397263208333	10.0161792791667	10.8531587375	10.5621765458333	10.9445407583333	10.7259670291667	10.4715232958333	10.4010588125	12.6509388208333	10.2348808958333	9.76330744166667	11.216369	10.7354198833333	11.2256433541667	13.1430271083333	10.1128385833333	11.4124685041667	9.86041405	10.2024908208333	10.5598964375	10.8738763791667	10.3500901708333	10.72704985	10.9485195958333	11.9054112833333	12.647880875	10.731189	12.3678730208333	10.2212035708333	10.3739195333333	9.89281027083333	9.92852570416667	11.2156097458333	9.83341595833333	10.3291694041667	10.5754682208333	10.6635446208333	9.9654401625	11.8979768416667	10.591617975	10.5418842458333	11.5989360833333	11.1647066708333	10.19012045	13.2383663166667	10.3222511916667	10.368063725	10.6378726916667

PCYT2	11.0940759041667	10.2705578875	10.3410778458333	10.41219155	11.1988506416667	10.5499198708333	10.526248225	10.1280952583333	10.347372975	10.327136925	10.366163275	10.8614308541667	10.9885999458333	10.0089119458333	10.8910372666667	10.1483618875	10.2714276125	11.8808967833333	10.4022684833333	10.4497322333333	9.99065759166667	11.0396227291667	9.687147875	10.1418799166667	10.8445233666667	10.6178551916667	10.915520925	10.606680675	11.8267584666667	10.3398532125	10.325930675	10.3911256916667	10.4151646583333	10.5426501458333	10.43626715	10.9679003458333	11.2755224208333	10.4290650375	10.3099456083333	10.3877990125	9.72467065416667	10.1162432666667	9.930629725	9.77372232083333	10.3889349333333	9.63041037916667	9.69902295833333	9.97646281666667	10.0698569125	10.1495387208333	10.1743594333333	10.1793924208333	10.9456650125	11.1432396458333	9.9554479	9.74023032083333	10.2176713	10.0207806125	10.7671402625	10.3188843875	10.7354198833333	11.0591417875	10.1061134458333	11.2924159458333	11.73712565	10.7297003833333	10.3180997375	10.6958287375	10.6509623708333	10.46699275	10.4812808791667	10.4501215791667	10.7397263208333	10.0161792791667	10.8531587375	10.5621765458333	10.9445407583333	10.7259670291667	10.4715232958333	10.4010588125	12.6509388208333	10.2348808958333	9.76330744166667	11.216369	10.7354198833333	11.2256433541667	13.1430271083333	10.1128385833333	11.4124685041667	9.86041405	10.2024908208333	10.5598964375	10.8738763791667	10.3500901708333	10.72704985	10.9485195958333	11.9054112833333	12.647880875	10.731189	12.3678730208333	10.2212035708333	10.3739195333333	9.89281027083333	9.92852570416667	11.2156097458333	9.83341595833333	10.3291694041667	10.5754682208333	10.6635446208333	9.9654401625	11.8979768416667	10.591617975	10.5418842458333	11.5989360833333	11.1647066708333	10.19012045	13.2383663166667	10.3222511916667	10.368063725	10.6378726916667

CA3	4.45503961666667	4.031307325	4.52418780833333	4.19234266666667	3.94009973333333	4.72105203333333	3.782834	4.138474425	3.80900449166667	4.12106518333333	4.453007975	4.46791538333333	4.05858813333333	4.39446349166667	4.69325549166667	4.31803169166667	5.91431983333333	4.59683404166667	4.44059075	4.84820759166667	4.03764378333333	4.040897575	4.263255	4.31217129166667	4.95206471666667	4.20011505833333	4.67674131666667	4.65176085833333	4.56909456666667	4.80433128333333	4.93436960833333	4.82904071666667	4.38197743333333	5.360915675	5.949734275	5.060028525	4.71113178333333	4.77930389166667	4.79411095833333	5.86727098333333	5.52011276666667	4.87595029166667	4.57030863333333	5.83110111666667	4.27798759166667	5.60487774166667	6.265839825	4.64734200833333	4.41695324166667	5.388701925	5.498293125	5.40852341666667	4.71899701666667	5.08458449166667	6.61745720833333	5.211919525	4.418789625	5.8292169	4.89935284166667	5.10387183333333	4.27160165833333	4.21701204166667	4.17980874166667	4.07678143333333	5.65913218333333	5.21920105	3.83595706666667	4.2289531	4.58445505833333	4.341768725	4.0322916	5.25962125	4.23000438333333	4.1095023	4.60485635	5.09317755833333	4.52522428333333	5.28688518333333	5.66082913333333	4.51475793333333	4.421470575	3.82289100833333	4.265394625	4.263255	4.84301391666667	4.480628975	4.19720234166667	4.48829116666667	4.46985861666667	6.386982725	5.07000859166667	5.54303723333333	4.42570850833333	6.73002575	5.78160560833333	5.01735620833333	4.70371486666667	4.82135065	4.13438028333333	4.91552208333333	5.94091113333333	4.00663116666667	5.3100359	5.04966088333333	4.740346475	5.12411041666667	4.79999713333333	5.58021739166667	4.303353375	6.25755120833333	4.87292895	5.42554189166667	5.65732808333333	5.303590975	6.46055301666667	5.76144219166667	4.91851714166667	5.212866025	5.16938190833333	4.73832164166667

CNGB3	6.112061375	6.74746250833333	7.18159225833333	6.10156668333333	6.83400405	6.33187936666667	7.79527465	6.263501475	6.9953871	6.84063886666667	6.65552751666667	7.24765453333333	7.05399351666667	6.59464205	6.51455034166667	6.85354335	7.18226805	7.20747220833333	6.60676565	7.28864350833333	6.50142603333333	6.2184742	6.64764070833333	5.50615049166667	6.54089473333333	6.155322525	6.99919410833333	5.81313270833333	6.91190293333333	6.34078100833333	6.96330954166667	7.17735376666667	6.84711038333333	7.15020035833333	7.04568171666667	7.05278270833333	6.2353521	6.10234676666667	5.8174436	7.040686725	6.51915964166667	5.2543857	5.86456365833333	6.60676565	5.90945328333333	6.40489274166667	6.841322025	7.16072213333333	6.53205264166667	6.81774981666667	6.98280363333333	6.88458655833333	7.55486798333333	7.09933909166667	7.41121605	7.20693700833333	7.43011818333333	7.07862004166667	7.27593380833333	6.507987025	8.211993425	7.95406095	10.3487595333333	8.10440565833333	7.12528415833333	9.7097404	8.063977575	7.73246671666667	7.85854243333333	7.441553775	7.782192425	7.92279315	7.46022033333333	7.29049713333333	8.59743456666667	8.381708525	8.151087675	8.36762415833333	9.00690949166667	7.56380908333333	7.64610639166667	7.39869075	9.07021043333333	7.39926451666667	7.81893236666667	6.01386335	7.50051165833333	7.893532975	8.99116335833333	8.08335636666667	9.51408459166667	7.14024473333333	8.60593184166667	9.990452125	9.071149525	8.09121131666667	7.24765453333333	6.31880616666667	7.7571807	6.987286825	8.31561141666667	7.38699095	6.15274419166667	9.112227825	7.299009625	9.12986750833333	7.34497685	10.4040896666667	8.22546075833333	8.38817050833333	7.67088145	8.33507211666667	9.52133895	7.89510465833333	8.4237182	9.09237333333333	6.77370956666667	8.41599213333333	9.1218467	7.46022033333333

TMEM178A	7.32036674166667	6.85538685833333	6.68859711666667	7.18452301666667	6.95156631666667	6.77636395833333	6.57813499166667	5.65251761666667	6.60603940833333	6.57096464166667	6.46494565	5.79119435	5.780758525	5.97184991666667	6.42485146666667	5.91719066666667	7.9977282	6.89596523333333	6.56365850833333	5.95490855	5.75400875	6.72519365833333	5.94358583333333	6.62969040833333	6.74218866666667	6.86530675833333	6.64559758333333	6.68920345833333	7.17971484166667	7.13093330833333	6.528434875	7.31919828333333	7.24396096666667	7.01621956666667	7.46463336666667	7.0307229	7.18159225833333	6.92168505833333	6.94820169166667	6.30714161666667	6.9998061	7.02536274166667	6.57952525	6.8478471	6.17067161666667	6.57365081666667	7.02401584166667	6.50501645833333	6.7327851	7.025978175	6.79588928333333	6.95221913333333	6.47574773333333	6.74958845833333	6.49087593333333	6.36029920833333	6.28402719166667	6.87648061666667	6.96712810833333	6.06998224166667	7.24075795	6.35051324166667	6.85745724166667	6.582876425	6.98215429166667	6.97276299166667	6.59386139166667	6.55373064166667	7.43805644166667	6.75503975833333	6.79731146666667	6.861457125	5.895388975	6.141642375	6.48357195833333	5.92509355833333	6.46133099166667	6.59234331666667	6.869498575	6.82644443333333	6.057986625	5.623286525	6.15692685	6.372760025	6.13259841666667	6.39918578333333	6.56431505	6.1777684	6.73694150833333	6.10873680833333	6.78246663333333	6.345260775	6.691149875	6.92096200833333	6.69991303333333	6.40489274166667	6.37033623333333	7.107578925	7.147600275	6.901873575	6.448169675	6.98215429166667	7.470826175	6.86736839166667	6.28109695833333	6.188915175	5.99181078333333	5.96342883333333	6.62158961666667	6.55520170833333	6.54514473333333	7.48927714166667	6.874007675	6.946211725	6.93873875	6.67919664166667	6.65487665833333	7.366539375	7.13230843333333	5.77716973333333

AL353588.1	5.092677425	5.9255638375	4.81228417916667	5.07809128333333	5.95369501666667	5.66039114166667	7.11822194583333	6.66280564583333	6.33304742083333	5.9440083875	5.82414327083333	7.0291349625	6.82674682083333	5.31569574583333	4.56246069166667	4.68735830416667	5.975560625	5.9817968375	4.7478666375	6.79499067083333	5.83821590833333	4.9260884625	6.23829875833333	4.30482784583333	4.20156269583333	6.07041681666667	4.61470835	5.123624275	4.39301744166667	4.583908875	4.3434317625	5.11626904583333	6.347888	5.50479741666667	5.094754575	5.00587525416667	4.33689344583333	4.96459184583333	4.2934453	4.61573042083333	4.73446737083333	4.15371080833333	4.51523942916667	5.42934410416667	4.2094270625	4.8897386375	4.75647757083333	4.82951312916667	5.15399322916667	4.468343025	4.4975528375	4.56441264166667	5.60081751666667	5.527840075	4.57876442916667	4.73446737083333	6.50245250833333	5.42830382083333	5.54919577916667	5.47268415416667	4.65221884166667	5.984382625	4.86752087916667	5.99383845	6.42372851666667	6.101956725	6.96152448333333	6.50020627083333	5.74363918333333	6.39343123333333	6.63212634583333	5.96820949166667	7.12753024583333	5.57973995	6.78543982916667	6.41073649166667	5.8809657375	4.83825977083333	5.47076787083333	5.98780749166667	6.9649669625	6.056382625	5.86217612083333	4.1475502125	6.64191437916667	5.54251644583333	6.19490961666667	6.30902656666667	7.021852825	4.91902977083333	6.1484294125	5.4142425	5.7750185875	5.83159534166667	5.97304986666667	5.58846107916667	5.30106392916667	5.085150825	5.89862794166667	4.96795296666667	5.37176904583333	5.593258575	4.96015072083333	4.86960658333333	4.93286122916667	6.0653312875	4.38359478333333	5.99045385	5.28309759583333	5.47747782083333	5.43133480416667	5.05849976666667	5.13486149583333	6.4845842875	6.32564148333333	5.3860919875	6.19702920833333	6.60793873333333	6.2893711125	7.47815710833333

SPATS1	5.092677425	5.9255638375	4.81228417916667	5.07809128333333	5.95369501666667	5.66039114166667	7.11822194583333	6.66280564583333	6.33304742083333	5.9440083875	5.82414327083333	7.0291349625	6.82674682083333	5.31569574583333	4.56246069166667	4.68735830416667	5.975560625	5.9817968375	4.7478666375	6.79499067083333	5.83821590833333	4.9260884625	6.23829875833333	4.30482784583333	4.20156269583333	6.07041681666667	4.61470835	5.123624275	4.39301744166667	4.583908875	4.3434317625	5.11626904583333	6.347888	5.50479741666667	5.094754575	5.00587525416667	4.33689344583333	4.96459184583333	4.2934453	4.61573042083333	4.73446737083333	4.15371080833333	4.51523942916667	5.42934410416667	4.2094270625	4.8897386375	4.75647757083333	4.82951312916667	5.15399322916667	4.468343025	4.4975528375	4.56441264166667	5.60081751666667	5.527840075	4.57876442916667	4.73446737083333	6.50245250833333	5.42830382083333	5.54919577916667	5.47268415416667	4.65221884166667	5.984382625	4.86752087916667	5.99383845	6.42372851666667	6.101956725	6.96152448333333	6.50020627083333	5.74363918333333	6.39343123333333	6.63212634583333	5.96820949166667	7.12753024583333	5.57973995	6.78543982916667	6.41073649166667	5.8809657375	4.83825977083333	5.47076787083333	5.98780749166667	6.9649669625	6.056382625	5.86217612083333	4.1475502125	6.64191437916667	5.54251644583333	6.19490961666667	6.30902656666667	7.021852825	4.91902977083333	6.1484294125	5.4142425	5.7750185875	5.83159534166667	5.97304986666667	5.58846107916667	5.30106392916667	5.085150825	5.89862794166667	4.96795296666667	5.37176904583333	5.593258575	4.96015072083333	4.86960658333333	4.93286122916667	6.0653312875	4.38359478333333	5.99045385	5.28309759583333	5.47747782083333	5.43133480416667	5.05849976666667	5.13486149583333	6.4845842875	6.32564148333333	5.3860919875	6.19702920833333	6.60793873333333	6.2893711125	7.47815710833333

SLC19A1	10.6616475208333	10.5517925	11.1958353416667	10.7199789541667	10.9008579166667	10.699561675	10.6946895333333	10.6505566166667	10.307638325	10.8354975708333	10.528233925	11.0088874875	11.4260556833333	10.7003004625	10.8156214541667	11.3127381083333	9.97562940416667	10.7810387208333	10.9539842208333	10.7480234125	11.2245952666667	10.0905161625	10.5928418416667	10.9137912875	10.3712484583333	10.5886354416667	10.805071425	10.8689801208333	10.9141417875	10.7141906	11.0288498208333	10.6505566166667	9.73682630833333	10.344903475	10.7571049208333	11.249063775	11.03538055	10.7487410583333	11.1300459916667	11.385548375	11.3181995833333	10.8030743208333	10.139158525	10.3846800458333	10.3931396791667	11.5168267208333	10.7623051916667	10.7837402125	11.0613563416667	10.9285779791667	10.8572859083333	10.8034725541667	10.6037069083333	10.5144123166667	10.4912548041667	10.9403216875	11.104154875	10.8798397583333	11.0198453583333	10.7067774041667	11.6097634833333	11.6243427125	11.456921125	11.9469516458333	11.5467043166667	11.1918332083333	11.1910432583333	10.9620127333333	11.0012122125	10.9176779916667	10.8819411625	10.9569520416667	11.8843446666667	11.8192935083333	11.4713486541667	11.7972431541667	11.5247923291667	11.1834752125	11.465269275	11.1296995458333	11.7154865958333	11.8424643333333	11.1910432583333	12.6388910083333	12.3599793541667	11.5670599583333	11.3988732375	12.5890325125	11.2574172166667	12.1572977708333	11.9552464125	11.2831619666667	10.0266969708333	11.7934537333333	11.2952569416667	10.8621601291667	11.5514691291667	11.5946751791667	11.7519668833333	11.5889279208333	11.8385236375	10.9573094958333	10.1818320958333	10.1830521875	10.6167578333333	10.8328285208333	12.2908103666667	11.7151199333333	11.82556905	12.2071828208333	11.449100825	10.5901158458333	11.8863356875	11.1222478958333	11.2378842875	10.8107375166667	11.8751626041667	11.5856427666667	10.9289703791667	10.5183438

COL18A1	10.6616475208333	10.5517925	11.1958353416667	10.7199789541667	10.9008579166667	10.699561675	10.6946895333333	10.6505566166667	10.307638325	10.8354975708333	10.528233925	11.0088874875	11.4260556833333	10.7003004625	10.8156214541667	11.3127381083333	9.97562940416667	10.7810387208333	10.9539842208333	10.7480234125	11.2245952666667	10.0905161625	10.5928418416667	10.9137912875	10.3712484583333	10.5886354416667	10.805071425	10.8689801208333	10.9141417875	10.7141906	11.0288498208333	10.6505566166667	9.73682630833333	10.344903475	10.7571049208333	11.249063775	11.03538055	10.7487410583333	11.1300459916667	11.385548375	11.3181995833333	10.8030743208333	10.139158525	10.3846800458333	10.3931396791667	11.5168267208333	10.7623051916667	10.7837402125	11.0613563416667	10.9285779791667	10.8572859083333	10.8034725541667	10.6037069083333	10.5144123166667	10.4912548041667	10.9403216875	11.104154875	10.8798397583333	11.0198453583333	10.7067774041667	11.6097634833333	11.6243427125	11.456921125	11.9469516458333	11.5467043166667	11.1918332083333	11.1910432583333	10.9620127333333	11.0012122125	10.9176779916667	10.8819411625	10.9569520416667	11.8843446666667	11.8192935083333	11.4713486541667	11.7972431541667	11.5247923291667	11.1834752125	11.465269275	11.1296995458333	11.7154865958333	11.8424643333333	11.1910432583333	12.6388910083333	12.3599793541667	11.5670599583333	11.3988732375	12.5890325125	11.2574172166667	12.1572977708333	11.9552464125	11.2831619666667	10.0266969708333	11.7934537333333	11.2952569416667	10.8621601291667	11.5514691291667	11.5946751791667	11.7519668833333	11.5889279208333	11.8385236375	10.9573094958333	10.1818320958333	10.1830521875	10.6167578333333	10.8328285208333	12.2908103666667	11.7151199333333	11.82556905	12.2071828208333	11.449100825	10.5901158458333	11.8863356875	11.1222478958333	11.2378842875	10.8107375166667	11.8751626041667	11.5856427666667	10.9289703791667	10.5183438

IZUMO1R	5.106111275	3.92496040833333	4.6393107	4.12229528333333	4.02676174166667	4.87710051666667	3.95777633333333	4.203896025	3.99930385833333	4.52791141666667	4.6622505	4.20571450833333	4.1481371	4.16522844166667	4.46402134166667	4.21300761666667	5.36615406666667	4.34514024166667	4.68073703333333	4.79808495	3.80218998333333	4.46293625833333	4.02921226666667	4.47573676666667	4.30721710833333	4.75143253333333	4.71016199166667	5.13122064166667	5.34196935833333	5.57252066666667	5.24102825833333	4.63093599166667	4.64311556666667	5.04667276666667	6.005441825	5.54303723333333	4.3934547	4.91552208333333	5.12411041666667	4.30027178333333	4.695239575	4.78900190833333	4.328489775	4.832334525	5.30888879166667	6.29278439166667	4.88037813333333	4.3987315	5.21920105	5.11436066666667	5.464635875	4.93337844166667	4.9727824	5.984788925	4.79518681666667	5.72686785	4.51773374166667	5.66843639166667	5.47028654166667	5.36511784166667	4.85447411666667	4.434278525	5.968635375	4.9195424	4.453007975	4.576370125	4.13943304166667	4.108372	4.8912062	4.5803341	4.32198345833333	4.58140610833333	3.84051730833333	4.06593015	3.84700126666667	4.048345425	4.428719425	4.50112465833333	4.23291245833333	4.42980535	4.075702625	5.24703029166667	4.48717731666667	4.62161215833333	4.20479785833333	4.01697755	5.38469075833333	4.03859033333333	4.92055984166667	4.63513973333333	4.26116580833333	4.30437938333333	5.30448685	4.70675665	5.37226380833333	4.84615233333333	5.07107728333333	5.25962125	5.1566631	4.43844	4.90871175	4.79808495	4.29017421666667	4.46696494166667	4.38197743333333	5.47416700833333	4.11385116666667	5.0835026	4.73295375	5.7672538	6.423307775	4.830971775	5.53496716666667	6.20884429166667	5.04762459166667	5.25334058333333	4.54133179166667	5.09217729166667	6.32600435833333	4.361998275

BOC	13.6150235416667	12.4528342	11.2442138	12.2341055916667	12.9007979916667	11.5765356583333	12.0961761333333	12.4005974416667	12.295556975	12.3733049416667	11.6016871083333	13.238016175	13.238016175	12.5676300666667	12.8802020166667	13.0816325916667	10.5049759833333	12.531130425	12.5259355083333	12.9024490333333	12.4850765	12.9144941416667	12.0857092083333	11.0239555333333	12.5385984166667	13.541969925	12.812914675	12.8984843833333	13.057870725	12.43595655	12.3921887583333	12.2416674416667	12.648701825	10.0256584	13.645262425	13.410428725	12.9172670333333	12.2437326916667	11.6775693083333	12.9306452333333	12.0525821583333	12.1741251583333	12.1865914583333	12.6331977416667	12.5362402583333	12.386493225	12.43647125	11.784206175	11.2982935833333	12.04824535	11.9534028083333	11.4467356166667	13.1572921166667	12.8203151333333	12.6980083833333	13.0506754833333	11.806635025	12.1915147666667	12.87960645	12.447837225	11.3025465416667	10.5388660583333	8.764687525	10.8356737416667	11.3972232666667	9.452370525	10.96150755	11.2093606416667	9.63613316666667	11.2568448166667	10.2806521916667	9.763939075	10.0915413	11.3746270333333	10.59381915	10.5081553333333	12.8074143333333	11.2579853916667	9.76954021666667	10.58883085	13.0708076333333	12.51216495	9.34302553333333	10.61002135	11.028339325	12.685194075	10.8874095666667	11.8607926333333	10.451127	12.2817357416667	10.1117927583333	9.66469236666667	10.31123355	11.2804803916667	10.7793443666667	9.7100974	12.266695	11.8008623833333	10.4272415916667	10.247459825	10.3281467166667	10.6979535583333	9.67141531666667	10.9597361583333	8.94049991666667	10.483424475	11.5219495083333	9.808442425	11.184429675	11.0534747416667	11.1937817916667	9.45323241666667	10.409675875	9.62586958333333	10.743245175	10.418815025	10.9611703916667	9.01009886666667	10.1919662416667	11.2613111166667

ZNF529	10.7948521166667	10.448395325	10.0260539583333	10.1448522166667	10.3466375416667	10.2119906333333	10.9284040666667	10.9065665083333	10.4449298	9.817741375	10.0197701416667	11.0225675833333	10.7100606833333	10.100531725	10.045510375	10.3207495083333	10.0125394166667	10.70852575	10.329775075	10.50924085	10.571508325	10.5041729666667	10.0623822333333	11.0480221416667	10.381015625	11.2035969416667	10.1222883166667	9.97174644166667	9.37031165833333	10.2005317833333	10.58184315	10.2047893666667	10.5049759833333	10.2642520083333	11.0193190916667	11.064089175	9.598664275	10.3392098666667	10.5976643083333	10.3459231416667	9.96481554166667	9.91080910833333	10.324926675	10.3027487416667	10.4208424916667	9.77049406666667	10.25066255	9.73142488333333	10.2314081833333	9.96522326666667	10.46022315	10.5464444833333	9.94677204166667	9.88515190833333	9.83584716666667	9.90955436666667	10.7796932083333	9.83811425	9.95935071666667	10.0296842916667	10.2363454416667	10.298281975	9.99086305833333	10.2563791083333	10.9519987166667	9.93796431666667	10.48796815	10.6664473083333	9.586175875	9.43636521666667	10.1870376	10.436061525	9.90307260833333	10.185845775	10.04851185	9.94714974166667	10.0078625	9.63944066666667	9.66649425	9.40333164166667	11.0513895	11.1158888083333	8.85547214166667	10.5748071083333	10.25066255	10.1405016833333	9.85452464166667	10.4783084333333	11.0171321	10.2877771083333	10.102550325	8.53434441666667	11.1923605916667	10.8412280166667	10.5902797083333	10.2794538083333	9.84242584166667	9.67192385833333	9.77000968333333	10.41048235	10.4887010333333	9.931739675	10.2330333333333	10.776004825	10.7224580666667	10.0917749291667	10.3825544833333	9.24318624166667	10.7050450333333	10.0949964416667	10.6722546333333	10.5441182916667	9.44734175833333	9.81305005833333	9.98225808333333	9.67944865833333	10.5884400333333	10.48796815	8.73109978333333	10.5609143

EMP3	8.19266712916667	6.179781325	5.88807049583333	6.8002561375	6.38515480833333	6.3191654375	6.35993101666667	6.49208909583333	6.10273904166667	6.74314556666667	6.44924722916667	6.77142720833333	6.54903211666667	6.33607361666667	7.2036427625	6.94408040416667	6.61320795	7.1351471875	6.95603795833333	6.26102422083333	7.5509531875	7.3919338	6.6092513375	6.54055676666667	6.27760587916667	6.9983108125	6.425206025	6.91797452083333	6.11913958333333	7.44125445416667	7.70790392083333	6.55483835833333	6.86361512083333	6.98942495	7.12692625833333	6.71273871666667	6.75204277083333	6.17352135416667	6.54125827916667	6.35395495416667	7.14428727916667	7.70904200833333	6.63278815	6.98559252083333	7.76111215416667	6.960167025	7.04294924166667	6.96877224583333	6.02687620833333	7.09648907083333	7.7327247125	6.11502605416667	6.74574984583333	6.469558125	6.7088747875	6.27607984583333	6.68028713333333	6.53632574166667	7.01044134166667	6.39190798333333	7.46049172916667	7.8723589625	7.8304358875	7.53425402916667	7.52137045416667	7.48037515833333	7.4463784625	7.43661437083333	8.349958025	6.8254994875	6.99950010416667	7.47925860416667	7.4293240625	8.01298137916667	6.87746547916667	6.73170205833333	7.37269663333333	7.40520261666667	7.40053280416667	8.33885354166667	7.22567669583333	6.99379621666667	7.32288867083333	7.46269293333333	6.9331990375	7.01460858333333	7.263762725	7.31890889583333	8.32386307083333	7.33470599166667	7.90815084583333	6.82674682083333	7.92575797916667	6.74314556666667	7.228673325	7.35596242083333	7.648856625	7.2671894875	8.8234550625	7.34301838333333	7.22567669583333	8.07757626666667	7.36731044583333	7.46717455	8.06879231666667	6.84098044583333	6.82730435416667	6.47103115416667	5.21349461666667	6.7780729625	6.24150231666667	7.399511475	8.10507432083333	6.87559859166667	6.49497117083333	7.4207562875	7.8932504125	6.99065255	7.44343085	5.93084324583333

CCDC114	8.19266712916667	6.179781325	5.88807049583333	6.8002561375	6.38515480833333	6.3191654375	6.35993101666667	6.49208909583333	6.10273904166667	6.74314556666667	6.44924722916667	6.77142720833333	6.54903211666667	6.33607361666667	7.2036427625	6.94408040416667	6.61320795	7.1351471875	6.95603795833333	6.26102422083333	7.5509531875	7.3919338	6.6092513375	6.54055676666667	6.27760587916667	6.9983108125	6.425206025	6.91797452083333	6.11913958333333	7.44125445416667	7.70790392083333	6.55483835833333	6.86361512083333	6.98942495	7.12692625833333	6.71273871666667	6.75204277083333	6.17352135416667	6.54125827916667	6.35395495416667	7.14428727916667	7.70904200833333	6.63278815	6.98559252083333	7.76111215416667	6.960167025	7.04294924166667	6.96877224583333	6.02687620833333	7.09648907083333	7.7327247125	6.11502605416667	6.74574984583333	6.469558125	6.7088747875	6.27607984583333	6.68028713333333	6.53632574166667	7.01044134166667	6.39190798333333	7.46049172916667	7.8723589625	7.8304358875	7.53425402916667	7.52137045416667	7.48037515833333	7.4463784625	7.43661437083333	8.349958025	6.8254994875	6.99950010416667	7.47925860416667	7.4293240625	8.01298137916667	6.87746547916667	6.73170205833333	7.37269663333333	7.40520261666667	7.40053280416667	8.33885354166667	7.22567669583333	6.99379621666667	7.32288867083333	7.46269293333333	6.9331990375	7.01460858333333	7.263762725	7.31890889583333	8.32386307083333	7.33470599166667	7.90815084583333	6.82674682083333	7.92575797916667	6.74314556666667	7.228673325	7.35596242083333	7.648856625	7.2671894875	8.8234550625	7.34301838333333	7.22567669583333	8.07757626666667	7.36731044583333	7.46717455	8.06879231666667	6.84098044583333	6.82730435416667	6.47103115416667	5.21349461666667	6.7780729625	6.24150231666667	7.399511475	8.10507432083333	6.87559859166667	6.49497117083333	7.4207562875	7.8932504125	6.99065255	7.44343085	5.93084324583333

PADI2	9.02747416666667	8.98191765833333	9.34157415833333	9.573560175	10.77146855	10.0831227083333	8.74427688333333	9.01986039166667	9.39478965833333	9.04742353333333	9.166824	8.44993103333333	8.62596224166667	9.32760176666667	8.51622818333333	9.990066925	8.548453575	8.87974118333333	9.062206575	8.86854605833333	10.2157667083333	9.23835931666667	9.50086338333333	10.0103759833333	9.146936475	9.47351875833333	8.52634974166667	8.8072839	9.29252663333333	9.06791326666667	9.47897484166667	9.15886451666667	9.25209231666667	9.14235506666667	9.954379925	8.594439725	8.82265860833333	10.3035388166667	9.25253679166667	9.45595239166667	9.47819235833333	8.62084126666667	9.5928155	8.70635446666667	9.42734543333333	9.291151225	9.4056037	9.62712280833333	9.68825589166667	9.7473541	10.3053444333333	8.89652095833333	9.41761155833333	9.80755264166667	8.66481208333333	9.599958075	9.3434528	9.61984825833333	9.09925841666667	9.14426045	8.298377725	8.51027651666667	9.38182149166667	8.24707035	13.913193025	9.55990681666667	8.34767179166667	7.9846002	8.49442401666667	9.78481885	8.29793538333333	7.59366683333333	8.2988733	9.29340571666667	8.8072839	8.90142945833333	9.584765625	9.17414999166667	8.9081181	8.61442265833333	13.6848971666667	9.17691023333333	8.435679275	9.37588805	8.72787371666667	8.88831745833333	9.28174641666667	9.72964383333333	7.80170391666667	10.05998855	9.71485326666667	9.166824	8.408638475	8.8526135	8.3316018	8.48745835	8.98096389166667	13.363866775	9.00735385	11.171764175	8.54218048333333	8.12110685	9.68471804166667	8.76031966666667	8.76031966666667	9.212117675	8.63344703333333	9.19996488333333	9.55244275833333	9.315897875	12.324942025	8.42476003333333	9.9229238	10.0103759833333	9.21350573333333	9.439564175	9.75787298333333	9.05075425833333	8.49886293333333	9.90222311666667

C2orf73	12.037261675	12.557141575	13.0488654	12.3698220916667	14.004911475	12.487541925	13.9785722666667	13.0392216583333	13.5363675083333	13.0992273666667	12.6422229833333	13.9529996833333	13.3416873833333	12.1270603333333	12.5293458166667	12.75532045	13.0102233	13.363866775	12.0112152666667	12.4194028333333	12.0409411833333	12.4425276083333	12.9172670333333	11.4321000166667	12.0973620416667	12.0136845833333	12.7216091916667	12.8624048833333	13.1190556333333	12.1096451083333	12.03602325	12.4350665416667	12.8197190166667	13.14005525	11.722386025	11.8530999083333	13.7452882916667	13.1973842833333	12.39882355	12.6470895583333	12.068210875	12.1935330916667	11.6129963	12.242111125	11.5414769166667	12.3750783583333	11.0056705916667	11.7589935083333	11.995459375	12.3469866583333	12.9412313583333	12.0514729416667	12.7807006083333	11.6511098916667	11.7936196666667	11.3320946416667	12.2488091166667	12.5268295333333	12.2227697083333	11.5253735333333	12.4461573416667	13.1065162583333	11.67680025	12.273184075	12.199408325	12.64663275	12.49723105	13.28687105	12.2901521833333	12.5463733	13.4775815666667	12.8251494083333	13.028241025	12.547782425	12.6674936416667	11.9071135	13.132282	12.2981372583333	11.8008623833333	12.190259475	12.3426613166667	12.7221714916667	11.8962786833333	11.3720648583333	12.64663275	12.8026986916667	12.547782425	12.6826019916667	12.49908695	11.392737725	12.0241448416667	11.3661426916667	12.1279125583333	12.53019765	12.4474119833333	12.2358628083333	12.5344122083333	12.487541925	12.9960374833333	11.3328774166667	11.545061825	12.6664956833333	10.8480286083333	10.9084288583333	11.385731825	11.6496725666667	11.0808123083333	10.9658327583333	11.5672571	11.4500480416667	11.7347600333333	12.9906493583333	11.7168402	10.8427945083333	11.42107965	11.3502989166667	11.1779127416667	11.51470825	11.5586348333333	12.23183945

CCDC9	16.4447326166667	16.2042656333333	16.5882325166667	16.4671769583333	16.0374488333333	16.3302858416667	16.6938615583333	15.6037263583333	16.2791471166667	15.9767561166667	16.1391348	17.35460805	17.0283161333333	15.7414894	15.7066290583333	16.2791471166667	15.1529307583333	16.8229118	16.2942187166667	15.7697269333333	16.0055115333333	15.5128524916667	16.090774475	16.0573941333333	15.3982642333333	16.2495335833333	16.1274635083333	16.1520487333333	15.83077275	16.5317674916667	16.0055115333333	16.6649415583333	15.7630766833333	16.2495335833333	16.1772486333333	16.4447326166667	16.3470849416667	15.0831469166667	15.9406321083333	16.0374488333333	15.69535405	15.6235572	15.8470782	16.1648856916667	16.980254275	15.7921085416667	16.2495335833333	17.0283161333333	16.6392399583333	16.89429	16.5621689666667	16.9349993833333	16.613425425	16.2791471166667	16.1022656	15.5221336416667	16.0691638333333	16.0055115333333	16.3470849416667	16.113934775	16.856940725	17.0283161333333	16.89429	16.7226812583333	16.613425425	16.4447326166667	16.49542085	16.7924633083333	16.613425425	16.5621689666667	16.49542085	16.613425425	16.4671769583333	16.6649415583333	16.856940725	16.5621689666667	17.0283161333333	16.5317674916667	16.2942187166667	16.980254275	16.5882325166667	16.5621689666667	16.4187514166667	16.5621689666667	16.39493105	16.5882325166667	16.7226812583333	16.6392399583333	16.7924633083333	16.7226812583333	16.6938615583333	16.6649415583333	16.5317674916667	16.5317674916667	16.7226812583333	16.6938615583333	16.89429	16.89429	16.4671769583333	16.613425425	16.8229118	16.5317674916667	16.7924633083333	16.5317674916667	16.4447326166667	17.0283161333333	17.2803091583333	16.89429	16.980254275	16.7607414083333	16.8229118	16.613425425	16.2193318666667	15.8065534666667	16.2495335833333	16.49542085	17.1320459	17.0842774333333	16.6938615583333	16.4187514166667

TRPV6	6.758383225	10.2900500333333	9.1218467	9.98920446666667	8.86999053333333	8.6731133	8.67688486666667	9.148762375	9.98032226666667	8.36034350833333	8.52878136666667	7.502278225	7.72294644166667	9.96481554166667	9.83452378333333	8.97027915833333	9.767731825	10.1063080916667	9.67770730833333	9.311300325	9.1237399	11.1923605916667	9.45374055	9.09007516666667	9.78918780833333	6.62367098333333	9.313630975	9.75239246666667	9.15692076666667	10.173327525	9.890973325	9.51896663333333	9.292040175	8.9489102	6.85045274166667	6.68920345833333	9.97833993333333	9.47641323333333	8.57783373333333	9.2998268	9.70584026666667	9.74904354166667	9.70017130833333	9.547789	9.34710834166667	9.14189448333333	9.95398405833333	9.03327476666667	9.877817275	9.62318166666667	9.21123668333333	8.21830426666667	10.054135025	9.861824025	10.067459125	9.81728660833333	9.05868610833333	9.59518965833333	9.41162929166667	9.09878833333333	9.26855390833333	7.33095725	8.24315470833333	8.05761690833333	9.56680634166667	6.179361325	6.35578110833333	7.61288113333333	9.05075425833333	7.523817425	8.39722928333333	8.93461839166667	7.16971491666667	6.37349159166667	7.66609071666667	6.48928715	8.850647275	7.16072213333333	7.09617145833333	6.78848231666667	8.1455177	8.22741431666667	6.77501233333333	7.62988515833333	6.633116075	7.652791	8.17249793333333	6.302455575	7.43688079166667	7.38522528333333	7.3817504	8.30641555	6.58901686666667	6.823097925	6.91322913333333	8.4564653	8.13871308333333	8.72874058333333	7.21808105833333	10.81468445	6.15274419166667	9.57222860833333	8.53882584166667	6.013009825	7.27535064166667	8.36515990833333	6.67442865833333	6.83799996666667	9.439107	6.87851200833333	10.4515253333333	7.51253281666667	6.34225285833333	6.99727938333333	6.98474048333333	9.062206575	10.5601410916667	6.165146375	8.63633005833333	6.67783206666667

ATP6V1B2	12.4292757333333	12.7896506583333	12.8432333583333	12.488453925	12.5567153083333	12.6136777666667	12.7536835	12.6795486	12.6412173166667	12.6528003583333	12.150591475	12.4461573416667	12.6407582416667	12.6660656916667	12.4927583	12.5516382166667	12.3025527416667	12.2909999333333	12.7526244083333	12.5948964	12.7796071583333	12.4283415	12.8679345583333	12.4850765	12.2867043083333	12.1656867	12.6481258583333	12.4050809083333	12.5429966333333	12.5187542666667	12.307547675	12.7374360833333	12.6180925666667	12.3961090083333	12.4456480416667	12.0440494333333	12.5264113	12.2867043083333	12.4456480416667	11.8908867916667	12.7195939833333	12.7113258083333	12.6171589666667	12.5070861083333	12.4650650416667	12.692926575	12.4655499583333	12.5818258666667	12.5604394333333	12.0921972916667	12.6872100416667	12.6287796916667	11.9762173583333	12.3904471833333	12.4181461583333	12.501560375	12.2835716083333	12.6640954083333	12.3147568916667	12.31352485	12.4619169416667	12.454126225	12.28577945	12.4755627916667	12.209029825	12.6013272333333	12.6704503833333	12.79233935	12.857326075	12.8192150333333	12.1843992666667	12.075266	12.0877373333333	12.98478995	12.7831765916667	12.6461371833333	12.1694652083333	12.3874454166667	12.724266575	12.4759771583333	12.3258832166667	12.5358106083333	12.0518595833333	12.9552000333333	12.8974584	12.373750125	12.7300491166667	12.958106125	12.287955975	12.713417125	13.18580525	12.3199648916667	11.6566056666667	12.3083565083333	11.8265346166667	12.5372066583333	12.8912643	13.3136506416667	12.1720161916667	11.8364243666667	12.1455145583333	12.6656204083333	12.5813238666667	12.150118875	11.95296625	12.6238182	12.372860975	12.4037688166667	12.5958960666667	12.3896401833333	12.2959914833333	12.0839641583333	12.0124313166667	12.4265910916667	12.6956383666667	12.2831203916667	12.075266	12.12212835	12.124594875	12.120221775

IL7R	8.403258675	9.13323439166667	10.5537715083333	9.482987475	8.42733516666667	10.966941925	9.932660725	10.1836960166667	9.63714265	9.35167163333333	9.4825512	8.83249931666667	9.69581614166667	10.0002116916667	8.33810663333333	11.796702325	9.65888631666667	8.76514685	9.63111984166667	11.1760549666667	10.7519521333333	10.1088055083333	10.5976643083333	11.2517728166667	9.9690997	8.698928125	10.3714480666667	10.2498045833333	10.5802645333333	9.11266035833333	10.8476613083333	11.7362421333333	10.8353214	12.244881075	9.438196975	8.43246804166667	9.92009464166667	10.5123801583333	11.1252834	9.22955863333333	10.1524914833333	9.26577935	11.129529525	10.9174580583333	10.713545975	11.6837602666667	10.02019625	10.000563275	11.0225675833333	10.6457263	10.511583325	10.0933465333333	10.55650935	10.0787243333333	10.528057425	10.2625794333333	10.1226723083333	10.5930279833333	9.99895085	11.0845103416667	9.12326560833333	10.5244636333333	11.367946675	10.6076090916667	8.68624993333333	9.76136145	12.2848587416667	11.2121826083333	11.171764175	10.9812562333333	10.3427675333333	11.4162651666667	10.9237681	12.14630095	8.96510433333333	12.1006163083333	9.8795664	11.48138035	11.2534278333333	10.80942375	8.08289571666667	9.16396135833333	11.3657868083333	13.08233985	11.0733527583333	10.3035388166667	8.74913316666667	11.7285272083333	9.04841031666667	11.5215953666667	12.0921972916667	11.36947175	10.239527275	10.8394387833333	10.5561337833333	10.866231375	10.4153772	10.3211494083333	10.9831460083333	10.2274517916667	11.0290294333333	10.85221535	11.4718467833333	10.6902661583333	10.5752416166667	10.9192867916667	11.832540575	11.7695141	11.5531172416667	12.244881075	11.3961375	10.4024778916667	12.1342141916667	11.1409117416667	12.1254015666667	10.9661834333333	9.93875319166667	11.34484365	9.721994775	11.2004569666667

COX6B2	5.54199565833333	7.06400038333333	6.958116025	7.751162425	6.69500276666667	7.13607696666667	6.93873875	9.84075720833333	6.75430906666667	6.43290905833333	8.3715767	6.97714725	7.39505978333333	7.21504029166667	7.51030740833333	7.45341820833333	6.996742575	8.28895966666667	7.2843799	6.76843211666667	7.875176525	7.45098580833333	7.34669549166667	7.92224445	6.824495175	6.33852943333333	8.062405875	7.33320358333333	6.620339275	6.65024493333333	7.82067988333333	9.13739526666667	6.85045274166667	6.44887243333333	6.724451125	6.32751184166667	7.28739089166667	6.772445575	7.53506526666667	8.269127475	7.12586193333333	6.48785131666667	6.52564285833333	7.8129644	7.98193376666667	7.21808105833333	7.15455295	7.567156725	8.30336938333333	7.649654175	8.79040525833333	7.74093078333333	7.03811636666667	7.20747220833333	7.662739425	6.96712810833333	7.70977168333333	7.93490190833333	7.238340075	6.5479092	6.46640339166667	7.83518873333333	8.07411434166667	8.49050600833333	6.3460148	8.17249793333333	6.8399633	10.988072975	8.30382858333333	7.24137955	7.94647371666667	7.71570814166667	9.02131511666667	7.370004375	7.064683475	6.80056770833333	8.6396011	7.74722135833333	8.017526525	7.340949	9.25440600833333	7.289187425	8.23520641666667	9.35357573333333	7.89510465833333	7.63657405	10.8176392916667	8.062405875	7.41704166666667	7.55987390833333	8.022968975	11.1901541416667	8.16983206666667	7.31316353333333	8.37864189166667	7.92964085	9.18361099166667	6.53883034166667	8.24861051666667	10.7846923083333	8.25546045	7.41526463333333	5.58699986666667	6.83084248333333	10.255903875	7.05018306666667	8.00901856666667	7.35564645	8.58601561666667	8.44043165	10.6905955916667	8.19188733333333	8.42124595	6.96970946666667	9.83279928333333	7.62815665833333	8.55750678333333	10.0708045166667	7.11278106666667	7.75542415833333

RFNG	10.7008852833333	10.7325441416667	10.9091302666667	10.9270024916667	11.0239555333333	10.3211494083333	11.4225410666667	10.90030275	10.8198860166667	10.7635056583333	10.5053205416667	11.0747492583333	11.0567325916667	10.8036860666667	10.9405146	10.4284899	10.9571307916667	11.0430757083333	10.8800392333333	10.6914536333333	10.6971968666667	11.1103347416667	10.7635056583333	10.6592419583333	10.6887964	10.7452538416667	10.6819563666667	10.7951957583333	10.720995375	10.90507365	10.8836008416667	10.74562245	10.7325441416667	10.6788649916667	10.744004	10.747418225	10.805973475	10.71663975	10.5396655666667	10.4550319166667	10.6589017416667	10.6289554166667	10.5692188416667	10.6364744	10.3388359916667	10.6496091416667	10.612647525	10.76392445	10.6531638166667	10.5181477	10.3714480666667	10.730804625	10.8228191416667	10.9192867916667	10.9058347	10.6523425166667	10.409321425	10.7181339666667	10.7399277916667	10.3362368833333	10.5902797083333	10.7605131333333	10.323288425	10.987327675	10.6058164916667	11.013128925	10.6592419583333	11.0487579166667	10.7538304083333	10.9858189916667	10.7302810416667	10.6050909833333	11.1321175166667	10.5979724666667	10.76764485	10.7452538416667	10.9357322	10.7862968083333	10.5592695083333	10.8243180916667	10.8533803	10.76921485	10.9564158416667	10.6827360416667	10.8465109833333	10.5041729666667	10.7291696916667	10.77146855	10.783568125	10.5991330666667	10.5651423416667	11.0520365916667	10.329775075	10.5899519833333	11.27496335	10.7940186083333	11.2722818166667	10.9094504083333	10.901782425	10.430465125	10.995163975	10.57908995	11.1173392166667	10.638037975	10.8424532083333	10.8296253416667	10.9497712833333	10.613570925	10.7658215916667	10.5983973083333	10.5130948833333	10.2411048916667	10.801788825	10.9934066	10.9440376833333	10.7530924583333	10.7900238833333	11.0859242666667	10.7189561666667	10.524814775

IFT122	10.86350625	9.87864789166667	10.035165975	10.2842200333333	10.36526325	10.2314081833333	9.80187509166667	10.0587286583333	10.1509476166667	9.32828119583333	9.94551363333333	10.5001925666667	10.436061525	10.1906714166667	10.4846654833333	10.1452230583333	10.5921937	10.8733577583333	10.259584625	10.340493	10.2186879083333	10.5876388833333	10.2853426583333	10.61804865	10.7658215916667	10.3459231416667	10.0607135666667	10.283076325	10.852614925	9.92873065833333	10.41573205	10.4910656666667	9.73955326666667	10.1633244583333	10.2206735166667	10.5295700833333	10.4049374333333	9.39753216666667	9.89141825	10.066608375	10.3035388166667	10.0095607333333	10.3674828833333	10.3317121166667	10.5609143	10.3427675333333	10.4613867166667	10.5295700833333	9.900995025	10.1485742083333	9.65845149166667	9.914179275	11.013128925	10.7123288	10.713545975	10.7885218833333	10.32163045	9.960644375	10.4032528583333	10.2301923	11.7909812333333	10.0989513583333	10.138226575	10.5240503083333	10.2974881833333	10.1700709416667	10.6990064	11.0416560583333	10.665646025	9.18361099166667	9.82860243333333	11.01087085	10.0765568416667	9.98596199166667	11.2572113416667	10.6699485666667	10.6607214666667	10.69756315	10.0238668333333	10.7885218833333	12.5969475916667	11.4919381583333	10.1073914791667	10.616218875	10.9167594166667	11.9742969	11.7913817166667	10.2778151833333	11.2226491666667	10.2634356166667	10.4822871416667	10.81171625	10.4570074666667	10.5739390666667	10.9234198666667	9.94460186666667	10.7900238833333	11.8535038916667	10.6395867	11.2726256666667	11.0094185166667	10.3817731416667	11.4170295666667	11.104988	10.7508267666667	11.01352865	10.5504732083333	10.7042731416667	10.6038899083333	10.5162662666667	10.1126129583333	10.5892734666667	10.7004993416667	11.01352865	11.3328774166667	11.28297055	11.4000850916667	10.5356874083333	10.7553698833333	10.5711304666667

BMP8A	12.4119267666667	11.7735354916667	11.7113167916667	12.0205452666667	12.1935330916667	10.8792631583333	12.956460525	11.5468691083333	11.74563025	12.0205452666667	11.9966897833333	12.9412313583333	12.946647375	13.1118234	11.8036556666667	11.720568625	11.5706660333333	12.217702275	10.863090975	10.9737165166667	11.4357643916667	11.92557355	11.1815244666667	11.2222793166667	10.8800392333333	11.9458994666667	13.6356329916667	11.5614234666667	12.0748320833333	11.9494775416667	11.660606675	10.934250825	11.6859465333333	11.7750094	11.8819037833333	11.987523	12.0017085416667	11.1898119916667	10.8028896	10.8747226333333	11.0800780666667	11.7487231	12.1304018333333	10.6457263	12.2901521833333	10.7371678583333	12.6018203833333	11.73203235	10.5979724666667	10.684995425	12.237914025	10.6415533416667	11.544719375	11.2660555333333	11.3534958083333	10.612647525	11.2478052	11.7171927333333	11.6115347666667	11.7219865583333	11.28453495	12.31352485	11.1930897583333	11.5858193083333	12.1030545583333	11.57134605	11.0146426833333	12.4279267416667	11.4601636916667	11.7387926	12.3851121666667	11.917277775	13.000133375	12.3461563666667	12.0237948	11.7030888416667	12.0934360416667	12.1681509833333	11.2047015416667	11.7761557916667	11.4688855083333	11.7620768166667	11.4470684833333	11.3300005666667	11.79216385	12.3584446833333	11.6698378833333	12.1757943166667	12.0973620416667	11.01087085	11.8186960583333	10.6348374333333	11.162651425	11.7557288583333	11.6141277333333	11.7285272083333	11.0642577208333	11.1139507416667	12.0819963833333	11.4207192166667	11.2438581166667	12.1790995083333	11.09094715	10.7470371333333	11.0116340666667	11.424045025	11.9204648416667	10.9726481916667	11.6982071583333	10.864263475	10.9661834333333	13.9227796833333	10.3880000583333	9.64383103333333	10.4698289916667	10.7251070916667	11.1630098	10.8224026333333	10.9979551416667	11.437271475

COA6	11.6985875333333	11.7909812333333	12.2410207708333	12.543462525	12.2831203916667	13.0798178083333	11.8004315583333	12.1367818666667	11.8947334916667	12.4425276083333	13.1046602416667	10.8996373166667	11.4019322	11.3553466416667	11.7857517916667	11.8403034416667	12.6739472	11.9997560833333	11.942427275	12.0756871	12.1229210916667	11.913931575	12.35974915	12.4443760583333	12.0827580166667	11.7418730833333	12.2821681166667	12.07649975	11.6036128166667	12.2053036416667	11.9467504166667	12.1694652083333	11.7259183166667	12.1312748	11.8658810166667	11.687529825	11.9204648416667	12.1799778583333	12.0062931166667	11.546539525	12.2116409166667	11.9629534916667	12.0604174833333	12.3896401833333	12.1393673833333	12.3733049416667	12.3374196	12.671473625	12.5126991416667	11.9868001	12.4123342916667	12.2723412333333	11.825372775	12.3496705916667	12.1640360666667	11.612663925	12.34092395	12.4751035416667	11.7791279583333	12.2073843166667	13.4289883	14.1827283166667	13.4142637	12.996638525	12.9739422583333	12.6684769583333	13.4991752166667	11.927850575	13.00071955	12.5902222833333	13.8766797166667	13.492075675	12.548305425	14.3775303333333	12.7201162666667	12.9278503083333	11.94832135	12.763386175	13.7940225416667	13.5116191416667	12.2628607833333	12.0205452666667	12.557141575	13.4744898833333	12.3961090083333	11.9994097833333	12.7468098833333	12.1608207083333	14.269993075	13.185241925	12.197761325	12.6775440333333	13.0554708583333	12.4163683083333	12.3182821416667	13.809878875	12.7728759666667	12.7016097166667	12.6008947166667	12.7339168166667	12.3645045916667	13.5867942083333	13.2313413916667	13.2806732833333	13.2233931166667	13.4127020416667	13.0174115	13.31441505	12.648701825	12.3789904833333	12.5685278666667	13.3087607833333	12.5740059166667	12.8696443333333	13.0638965333333	13.75723885	12.5969475916667	13.4314411416667	13.9695187666667	12.8702352416667

DHRS4	14.0514423083333	13.8148381	13.934166025	13.7771113166667	14.2518399916667	14.1317250833333	14.1500101916667	13.7046873583333	14.2204751583333	14.00243415	14.1152258583333	13.9097458666667	14.0683132333333	13.7902025333333	13.86774345	13.6411556333333	13.9003358333333	13.9250365	14.1554067916667	13.7630775	13.7869425833333	13.5133528333333	13.56515455	13.7253859666667	13.9284348416667	13.9097458666667	13.9469444833333	13.7940225416667	13.8972210333333	13.9727862416667	13.887785	13.8428912916667	13.9411608583333	14.1941852166667	14.0899292916667	14.1527055166667	14.0813262416667	14.0163605916667	13.9250365	13.8562499666667	14.0991501333333	13.9840948583333	13.6426254916667	13.7293441833333	13.65238595	13.7361743	13.5083984166667	13.6675870333333	13.599265975	13.9625123916667	14.1306178166667	14.0683132333333	13.6596320166667	13.9400026166667	13.7426466833333	13.56859275	13.886670625	14.034006175	13.7093807	13.662514625	13.9197186416667	13.7093807	14.211775675	13.4282493833333	13.8604046083333	13.9261802833333	14.044199175	13.6812289916667	13.9186404666667	13.8572965333333	14.004911475	13.88005245	13.441409425	13.8505836916667	13.8405812166667	13.654206225	13.9153342666667	13.9600657833333	14.1045935166667	13.9727862416667	13.661470025	13.5946453583333	14.2219861583333	13.6758447	13.6969617	13.61575685	13.6514898166667	13.5355941333333	14.6738026916667	13.6338665166667	13.533857925	13.5372319083333	13.9378093083333	14.0087806166667	14.236432725	13.934166025	13.9400026166667	13.8473484166667	13.9389915916667	13.798249925	13.6434906916667	13.8754219166667	13.441409425	13.4744898833333	13.57126835	13.765060275	13.3882606083333	13.3608559333333	13.6840030333333	13.6183502333333	13.6506232916667	13.913193025	13.4222455	13.527156225	13.662514625	13.4649543916667	13.5956278166667	13.6434906916667	13.8079143416667	13.2068344333333

BMP1	11.2534278333333	10.7900238833333	10.9869124833333	10.73952485	11.3878799333333	11.5048915416667	10.7295063583333	10.9556654	11.0419739083333	11.1731632666667	10.9335275	11.3381769	10.972292975	10.741834125	11.6367008333333	10.5123801583333	9.661547425	11.8126730416667	11.1847643166667	11.297940475	11.4184966	11.3455869583333	11.0440902666667	10.8755795416667	10.819511225	11.52420235	11.22659855	10.96771795	11.6898911833333	10.8976829666667	11.0101518666667	11.051725325	10.74363815	9.657103925	11.4567547583333	11.4692934083333	11.2891541	10.9136098583333	11.473399075	11.2132455833333	11.1670443166667	11.01087085	11.0190136	11.1491945416667	11.0593297333333	11.7008228166667	11.1214159583333	11.0826274416667	10.7855003666667	10.7940186083333	10.9147091916667	11.302200525	11.3413304583333	11.5656597	11.133569275	11.373190825	11.666692125	11.012770325	11.4824869416667	10.6309483083333	12.634136475	12.626502475	12.0253815583333	12.83111185	11.8484879416667	12.948888375	13.0343175416667	13.0067659416667	12.0313834166667	12.8930719333333	11.779499875	12.6046256583333	13.2128605083333	13.2075024333333	13.5443638333333	13.4119895416667	12.52409195	12.3101776166667	12.8713301666667	12.4777234416667	11.7109162916667	12.2137378333333	12.1367818666667	13.8572965333333	13.1786718416667	12.306357275	11.5495081166667	13.5443638333333	12.04537635	13.396630575	13.3515334583333	13.1733038666667	11.66838785	13.2751773833333	12.7831765916667	12.0205452666667	13.01427815	13.9422518833333	12.85786905	12.5604394333333	12.7822168333333	12.750529275	11.77986195	11.8619112333333	11.8635130416667	13.2480671333333	13.5171924333333	12.6278527916667	12.6476358916667	13.3732212583333	11.0373411416667	12.2185126	12.9793317083333	12.403353825	13.0084425916667	11.6190781833333	12.3589004083333	12.9284297083333	11.6643648083333	11.9903497083333

ITLN2	5.41039171666667	5.72076731666667	6.25427673333333	6.39606678333333	6.478653025	6.47216713333333	7.23193100833333	6.25250383333333	6.675144925	6.32175328333333	6.07858055833333	6.06998224166667	5.97106113333333	6.33187936666667	6.39152516666667	6.10156668333333	6.90781049166667	6.53596566666667	7.116803275	6.823097925	5.549659825	5.63053526666667	5.97260084166667	6.764479675	5.84141815	4.92770649166667	6.08697085833333	6.12282240833333	6.20326725833333	5.98304395	6.41036045833333	6.59670515	6.48357195833333	6.13424446666667	6.74158396666667	7.03630040833333	5.66378185833333	6.27482028333333	6.12282240833333	7.07980634166667	6.48151946666667	6.58835050833333	6.48217831666667	6.67783206666667	6.1039579	6.56431505	6.507987025	6.54933985	6.11631895833333	6.12364109166667	6.45696331666667	6.71502808333333	6.87219209166667	6.68131301666667	7.147600275	6.91035588333333	6.64226356666667	6.813807925	6.57096464166667	6.43290905833333	6.5607394	6.406487225	6.78381769166667	5.931318725	6.29443513333333	6.4975584	7.80727978333333	7.15697099166667	6.19527208333333	6.13511379166667	6.83744154166667	7.26110544166667	6.323145675	7.14458345	6.49981934166667	6.81976344166667	5.74037505	5.80195006666667	7.04202985	7.08111885833333	6.246964375	8.66481208333333	6.54574596666667	7.9999538	6.10002881666667	6.38846551666667	5.81821864166667	7.22598754166667	6.10954496666667	6.14334293333333	6.78180254166667	7.3741849	5.90846126666667	5.95328179166667	6.70726116666667	8.39560916666667	6.37415488333333	6.4516666	7.08738564166667	7.690465575	6.36793951666667	7.08560825833333	8.63230339166667	6.39451754166667	6.682676825	6.39769980833333	6.11297529166667	8.51856025833333	8.03595288333333	8.256452925	6.66666621666667	7.70760366666667	6.66944055833333	6.86328933333333	7.25001195833333	6.9393833	6.65156973333333	6.99282755	6.83207999166667	6.63942924166667

ARID4A	11.1014833833333	11.1117434416667	10.863090975	11.0087053416667	10.7228384083333	10.527258325	11.0380667083333	10.8025054583333	11.109201175	10.54728675	10.5995079	11.1477174666667	10.8960985416667	11.0221982916667	11.1480921	11.2121826083333	11.1154972666667	10.921206425	10.7184951333333	10.939073475	11.1975258	11.2657187833333	11.3216619833333	10.8537358333333	11.25027585	11.5836115333333	11.4446261583333	11.39940385	11.1771749333333	11.48024075	11.2598595	11.336435575	11.20749835	11.1081520166667	11.6205609916667	11.7053666833333	11.0142581583333	11.012449925	11.3292622166667	10.88817915	10.8510863583333	11.2366875083333	10.9123611916667	10.8404768083333	10.9640211333333	10.9476724083333	10.9647570833333	10.7936080833333	10.7927886583333	11.07367765	10.9021357916667	10.953117425	10.7743973416667	10.9084288583333	11.14521145	10.8368196833333	10.966941925	10.7900238833333	10.8506520083333	10.7344399666667	10.1820473416667	10.2237133416667	11.2936955416667	10.97439825	11.5374922333333	10.1878607	11.0175089583333	10.571508325	10.916432925	10.97439825	10.3530735416667	10.3147212916667	10.225450075	10.6776751083333	11.1150801666667	10.8165123833333	10.7940186083333	10.99552285	10.9153273416667	10.98875735	11.0811600833333	10.921206425	11.2811678666667	10.3678756583333	10.8337493916667	10.7235859166667	10.627440475	11.3893506666667	10.8001996166667	11.1113391916667	11.2693525666667	10.4546419916667	10.03562315	11.1173392166667	10.6993840916667	10.8025054583333	11.8484879416667	10.2554871833333	10.657432275	10.26626505	11.0560163166667	10.255903875	10.30932905	10.5142106583333	11.5920332333333	11.2258131916667	10.8143165583333	11.13577505	10.4994687166667	10.7624960333333	10.316356175	10.1820473416667	10.7272324083333	11.3257236	10.7158953333333	10.6956354083333	10.2928305416667	10.670739975	11.0286702083333	10.662995525

GSTA3	6.83682504166667	7.09933909166667	7.29106909166667	7.47725403333333	7.903152825	7.44095513333333	7.81786669166667	7.53069666666667	7.80971700833333	7.37642496666667	7.95684823333333	7.84106111666667	7.81088345	6.53407053333333	7.97971059166667	7.42788356666667	9.73350879166667	8.02779786666667	7.19451305833333	8.04234110833333	7.04267660833333	7.49600651666667	7.55486798333333	7.09043023333333	6.87783053333333	6.473722825	6.7730469	6.63110269166667	6.50275845	6.44063283333333	6.4516666	6.77041611666667	6.62563488333333	8.83154074166667	5.52228829166667	6.26921054166667	7.36048969166667	6.414216275	6.34384491666667	6.45472701666667	7.38123391666667	6.79994456666667	6.10313131666667	5.61155829166667	5.329558875	6.35434874166667	5.95574085833333	6.42033848333333	6.28336628333333	8.22981704166667	6.64088086666667	6.93552516666667	7.04821105833333	5.51001904166667	6.54299384166667	5.84141815	6.941911825	6.0801823	6.61087155833333	6.50214656666667	7.238929725	7.42556758333333	8.21881454166667	7.37467966666667	6.88512086666667	7.61232630833333	8.046473725	9.75967308333333	7.15624494166667	7.57961929166667	7.87098755833333	8.0964417	7.93544106666667	6.68987835833333	8.20737129166667	7.788761475	8.28495673333333	7.22899075833333	7.35119398333333	8.03175081666667	7.93805580833333	8.304316525	5.927679125	7.06773185833333	7.5512356	7.05900955833333	7.525066825	6.950931025	7.76938555	7.116803275	7.134182075	6.3378065	7.08738564166667	8.72356944166667	6.35201275833333	6.8687758	7.198288025	7.09804688333333	7.00815195833333	7.32036674166667	6.60956075833333	7.02943565833333	6.82038475833333	7.638193025	7.50852245833333	7.33202409166667	6.79131116666667	8.037084725	7.23488161666667	7.29365533333333	7.21570014166667	10.3078222333333	6.739509575	7.04503860833333	6.51382934166667	7.66609071666667	7.55811896666667	6.40489274166667	7.100006625	7.57050700833333

RPS9	9.80987653333333	9.80685600833333	11.1602403666667	9.25789575	9.12017812916667	10.4030534458333	9.3399144125	10.1698478875	8.9205338875	11.0543706791667	9.72430455833333	11.1903492625	10.6272505125	9.4849232375	8.18865101666667	11.6681825333333	9.64223322916667	9.7432741875	9.81628065416667	9.9037590625	10.3238867833333	8.29655778333333	10.4286661041667	11.2256433541667	9.50159337916667	11.0453244375	10.2405294125	9.4044443	8.94259409583333	9.02346451666667	10.4666524916667	10.0642515458333	9.74242185833333	9.77332255833333	12.2486154333333	9.8456561125	8.78916697916667	9.45492716666667	11.827559925	9.9599607	10.6786837083333	9.4393355875	10.6939694708333	9.65077524166667	10.3827338541667	10.7055811458333	9.71286741666667	11.2956085666667	11.0213217333333	10.4888905458333	11.1544759875	10.7524901541667	9.661749025	10.1376630083333	9.87413189166667	10.0813282166667	11.5183338875	10.9008579166667	9.8313258375	11.1958353416667	8.36832755416667	10.8508691833333	11.5412966791667	9.66719131666667	9.64223322916667	10.6782925458333	10.9690625666667	10.3815939083333	11.3511609708333	12.11842815	10.211772325	9.81628065416667	9.7845929625	11.2321605333333	10.0131801916667	11.4024746666667	10.3275314625	11.2310313458333	11.2134201291667	10.9781754416667	8.88283577916667	8.95100425	10.9228681625	12.4379884583333	11.3045319458333	10.1579110541667	9.28763400416667	11.5267024166667	10.0249635916667	11.6881586666667	11.235083375	11.3120093875	10.9348095208333	9.783050925	10.224786975	11.6097634833333	10.5332438458333	9.94995692083333	10.6999204208333	9.661749025	11.350793775	10.7434416625	11.9711152125	10.7629834541667	10.65907185	11.4796400458333	11.543822325	11.1781126083333	11.2525962333333	12.1453184125	11.0868500875	10.355636475	10.8880026958333	10.9763440083333	12.464830525	11.0505864458333	10.9194758625	11.015195575	10.3445012416667	12.1132013666667

LILRB3	9.80987653333333	9.80685600833333	11.1602403666667	9.25789575	9.12017812916667	10.4030534458333	9.3399144125	10.1698478875	8.9205338875	11.0543706791667	9.72430455833333	11.1903492625	10.6272505125	9.4849232375	8.18865101666667	11.6681825333333	9.64223322916667	9.7432741875	9.81628065416667	9.9037590625	10.3238867833333	8.29655778333333	10.4286661041667	11.2256433541667	9.50159337916667	11.0453244375	10.2405294125	9.4044443	8.94259409583333	9.02346451666667	10.4666524916667	10.0642515458333	9.74242185833333	9.77332255833333	12.2486154333333	9.8456561125	8.78916697916667	9.45492716666667	11.827559925	9.9599607	10.6786837083333	9.4393355875	10.6939694708333	9.65077524166667	10.3827338541667	10.7055811458333	9.71286741666667	11.2956085666667	11.0213217333333	10.4888905458333	11.1544759875	10.7524901541667	9.661749025	10.1376630083333	9.87413189166667	10.0813282166667	11.5183338875	10.9008579166667	9.8313258375	11.1958353416667	8.36832755416667	10.8508691833333	11.5412966791667	9.66719131666667	9.64223322916667	10.6782925458333	10.9690625666667	10.3815939083333	11.3511609708333	12.11842815	10.211772325	9.81628065416667	9.7845929625	11.2321605333333	10.0131801916667	11.4024746666667	10.3275314625	11.2310313458333	11.2134201291667	10.9781754416667	8.88283577916667	8.95100425	10.9228681625	12.4379884583333	11.3045319458333	10.1579110541667	9.28763400416667	11.5267024166667	10.0249635916667	11.6881586666667	11.235083375	11.3120093875	10.9348095208333	9.783050925	10.224786975	11.6097634833333	10.5332438458333	9.94995692083333	10.6999204208333	9.661749025	11.350793775	10.7434416625	11.9711152125	10.7629834541667	10.65907185	11.4796400458333	11.543822325	11.1781126083333	11.2525962333333	12.1453184125	11.0868500875	10.355636475	10.8880026958333	10.9763440083333	12.464830525	11.0505864458333	10.9194758625	11.015195575	10.3445012416667	12.1132013666667

TAC3	4.39039918333333	3.99595204166667	3.99595204166667	3.96951036666667	3.63546845	4.61520515	3.63145715833333	3.99064151666667	3.84482813333333	3.73894771666667	3.69375465	4.38197743333333	3.68223424166667	4.19765279166667	4.16407313333333	4.19434771666667	5.53004825	3.91359475	4.44822564166667	4.31803169166667	3.8136599	4.617309275	4.12677783333333	4.16522844166667	4.52697115833333	5.24195544166667	4.12334434166667	4.29916468333333	4.47668578333333	4.10289946666667	4.37287369166667	4.27160165833333	3.79308619166667	6.810578	4.20109206666667	4.67674131666667	4.4462942	4.40903533333333	4.51179561666667	4.9935499	5.84048410833333	4.960685075	4.6393107	5.28578914166667	4.58881544166667	6.14803923333333	5.03726580833333	4.86699436666667	6.43219438333333	4.71113178333333	4.58556791666667	4.21701204166667	3.84817555	5.15246255833333	4.29716620833333	5.0088772	4.01810879166667	4.68477714166667	4.948951225	4.23701905833333	3.780421775	3.764611825	4.28696936666667	3.64795303333333	3.51439604166667	4.480628975	3.832630725	3.83152513333333	3.9147734	3.71657505833333	3.68338428333333	3.61894206666667	3.63673386666667	3.81819714166667	3.51730585833333	3.63279243333333	4.894753975	4.172665625	3.73640415	4.54133179166667	3.7439275	3.86613395833333	3.99817013333333	3.70182713333333	3.68857591666667	3.86842555	4.08322809166667	4.04952899166667	3.83492170833333	3.79653381666667	3.87078774166667	4.09478471666667	4.01697755	3.737761675	3.86167775833333	3.812622975	3.975999075	4.177708	3.764611825	4.74930484166667	4.30721710833333	4.25100265833333	4.61955908333333	4.77813741666667	7.884494275	3.90095365833333	4.54133179166667	6.03500149166667	4.19623050833333	6.11631895833333	4.37287369166667	6.03335128333333	4.50544710833333	4.76548715	4.05417596666667	4.36901661666667	4.853434925	4.48239504166667	4.60088621666667	3.88526006666667

SCMH1	12.3292738666667	10.018462175	10.128737475	10.0636311333333	9.67274871666667	10.5833260083333	9.24630831666667	10.0670141416667	9.76472428333333	10.130420225	9.660226725	11.535988725	10.7669402083333	10.0268881833333	10.0524736083333	10.226244575	11.04233505	9.72070379166667	10.43166005	10.4698289916667	10.2001534166667	10.15614755	10.39291705	10.3806930666667	10.326957425	12.287955975	10.1927941666667	10.045510375	9.33838469166667	10.0632117083333	10.333754975	10.1218627333333	10.1054993333333	10.5477462083333	12.36147625	12.1636527	9.82651539166667	10.1299873833333	10.103849375	10.2634356166667	9.974167275	10.0442462083333	10.0565842333333	10.5509023833333	11.1975258	10.2810793	10.6076090916667	10.3466375416667	9.945938775	10.5516144166667	10.0898576	10.2501973166667	10.625983	10.2881617	10.2047893666667	10.9073626	10.0334908083333	10.0831227083333	10.384887925	10.319917075	10.2846004916667	10.3132196333333	10.0430227416667	10.2642520083333	11.535988725	10.6027208583333	10.3663602	9.61816694166667	10.6293746333333	10.1413216916667	10.3983831166667	9.67854975	10.0607135666667	10.3996095166667	9.28502139166667	10.4308569666667	10.4215953	11.0017295	9.81648044166667	10.0728614166667	11.3823997333333	10.6683478	9.84631699166667	10.6340233	10.36339005	10.9737165166667	10.662995525	10.4191933083333	9.75875961666667	10.8865989333333	10.4024778916667	11.348473325	9.70668029166667	10.1970378083333	11.0578053583333	9.50773276666667	10.5468436833333	10.5037659416667	10.0587286583333	10.6050909833333	10.7034642666667	9.91374403333333	9.83452378333333	10.5509023833333	10.0595403	9.97378895833333	10.5651423416667	9.64783695	10.5976643083333	10.6596205	10.7375745583333	10.20731305	10.4951144833333	11.0312303833333	10.447247375	10.440565075	11.0337297583333	10.43975535	9.73309245833333	10.5166009333333

WIPI1	11.4081227125	10.9634574041667	11.1868123583333	10.9977837208333	11.1076203	10.4129597333333	11.7957279	10.4615843583333	10.9348095208333	10.7678604416667	11.1664948583333	11.6769830916667	10.5470652166667	10.1693994875	10.8141356291667	11.3913576541667	10.4704001541667	11.0399878208333	11.3066869041667	11.4614284541667	11.1856823041667	10.9438641458333	11.1337690916667	12.3455268416667	11.038901025	11.2501076666667	10.979276975	11.0695657583333	11.3415085166667	11.2458949	10.8463488958333	11.1856823041667	11.7673442416667	11.3352633958333	10.9735553833333	11.4674169125	11.3204518541667	10.7274249875	11.2270935666667	11.2748017583333	10.7551722666667	10.5374311291667	10.7667620458333	10.9067564458333	11.170855825	10.629165025	11.4458392041667	11.0777739666667	11.2741842833333	11.5796281375	10.6576221833333	11.3859290583333	11.2429894875	11.0522316833333	10.9344338125	10.6432182708333	12.2803156541667	10.523411425	10.6145473666667	10.6999204208333	12.3445929125	11.2361304166667	11.1584270458333	10.9977837208333	9.88659601666667	11.33979215	11.6475593541667	10.9475088041667	9.63498063333333	11.3048953208333	11.5985810625	11.37003045	9.75553405	9.89281027083333	11.1119369458333	11.4994814416667	11.0364632541667	10.8459877208333	11.4135239541667	12.1933103291667	11.0328236041667	11.3818905583333	11.254472975	12.3932861666667	11.360680725	11.5267024166667	10.5575313583333	11.5318140458333	12.1165066666667	11.4525246291667	11.4973318666667	10.9198685541667	11.7957279	11.6331201416667	12.160227575	11.8582280666667	11.5073058833333	10.9539842208333	11.4429405083333	11.3152530458333	11.0418149833333	10.731189	10.968288875	11.0767288875	11.2738602708333	11.0173205291667	12.0891147833333	10.9735553833333	11.7547114875	11.7461377875	11.6541925208333	10.9759775458333	10.8324573625	10.40551365	10.7510105333333	10.6874862291667	12.335422725	10.5737228125	10.904495125	10.9316130333333

ARSG	11.4081227125	10.9634574041667	11.1868123583333	10.9977837208333	11.1076203	10.4129597333333	11.7957279	10.4615843583333	10.9348095208333	10.7678604416667	11.1664948583333	11.6769830916667	10.5470652166667	10.1693994875	10.8141356291667	11.3913576541667	10.4704001541667	11.0399878208333	11.3066869041667	11.4614284541667	11.1856823041667	10.9438641458333	11.1337690916667	12.3455268416667	11.038901025	11.2501076666667	10.979276975	11.0695657583333	11.3415085166667	11.2458949	10.8463488958333	11.1856823041667	11.7673442416667	11.3352633958333	10.9735553833333	11.4674169125	11.3204518541667	10.7274249875	11.2270935666667	11.2748017583333	10.7551722666667	10.5374311291667	10.7667620458333	10.9067564458333	11.170855825	10.629165025	11.4458392041667	11.0777739666667	11.2741842833333	11.5796281375	10.6576221833333	11.3859290583333	11.2429894875	11.0522316833333	10.9344338125	10.6432182708333	12.2803156541667	10.523411425	10.6145473666667	10.6999204208333	12.3445929125	11.2361304166667	11.1584270458333	10.9977837208333	9.88659601666667	11.33979215	11.6475593541667	10.9475088041667	9.63498063333333	11.3048953208333	11.5985810625	11.37003045	9.75553405	9.89281027083333	11.1119369458333	11.4994814416667	11.0364632541667	10.8459877208333	11.4135239541667	12.1933103291667	11.0328236041667	11.3818905583333	11.254472975	12.3932861666667	11.360680725	11.5267024166667	10.5575313583333	11.5318140458333	12.1165066666667	11.4525246291667	11.4973318666667	10.9198685541667	11.7957279	11.6331201416667	12.160227575	11.8582280666667	11.5073058833333	10.9539842208333	11.4429405083333	11.3152530458333	11.0418149833333	10.731189	10.968288875	11.0767288875	11.2738602708333	11.0173205291667	12.0891147833333	10.9735553833333	11.7547114875	11.7461377875	11.6541925208333	10.9759775458333	10.8324573625	10.40551365	10.7510105333333	10.6874862291667	12.335422725	10.5737228125	10.904495125	10.9316130333333

PLGLB1	9.716989375	9.8001132	9.10485650833333	9.71398214166667	9.22832763333333	9.49325940833333	9.88298375833333	9.732285275	8.97354856666667	8.19808664166667	10.3427675333333	9.99432204166667	9.86598649166667	9.562147725	9.74820641666667	9.64871909166667	9.45323241666667	9.35125324166667	9.71442065	10.1637531083333	9.47727790833333	10.5468436833333	9.234219825	9.25484538333333	9.68075670833333	9.76267145833333	9.90058610833333	9.84200871666667	9.67363538333333	9.7553002	8.97625954166667	9.07955376666667	10.128737475	8.90412092916667	9.71657748333333	10.3281467166667	10.30932905	9.76472428333333	9.23047039166667	9.47021275	8.88831745833333	10.1258089583333	9.54925085	7.96240296666667	9.51724313333333	9.34750165	8.815557	8.868084675	8.733549025	9.166351	8.91457238333333	10.015183425	10.1440795833333	9.70748555	9.388540625	8.03960018333333	8.49648456666667	7.544616	9.28502139166667	9.49147149166667	11.1169690833333	9.85498678333333	9.06503669166667	9.66961651666667	9.40597950833333	9.9441902	9.16495244166667	10.25147975	8.44491509166667	9.25764194166667	9.954843075	9.53043561666667	9.07536643333333	9.85722324166667	9.40159784166667	9.52087123333333	10.2018680083333	10.1210398166667	8.70547134166667	10.1766858833333	11.3172677166667	10.87179425	9.23749938333333	8.849091575	9.67363538333333	10.1878607	11.114387025	9.24630831666667	11.2560704166667	9.496026575	9.43456800833333	9.70748555	10.451127	8.7735097	10.1076098833333	10.2621725583333	9.862235575	10.96963075	8.98046293333333	9.30818888333333	9.730552775	9.66649425	10.3261322166667	8.00581294166667	9.20373038333333	9.65670091666667	9.19627585833333	8.24707035	9.13299193333333	9.17457594166667	9.22061473333333	8.391097025	9.05561776666667	8.83016251666667	8.81744095	8.40377353333333	9.357237425	8.86708225	9.00460498333333	8.87636833333333

RGPD1	9.716989375	9.8001132	9.10485650833333	9.71398214166667	9.22832763333333	9.49325940833333	9.88298375833333	9.732285275	8.97354856666667	8.19808664166667	10.3427675333333	9.99432204166667	9.86598649166667	9.562147725	9.74820641666667	9.64871909166667	9.45323241666667	9.35125324166667	9.71442065	10.1637531083333	9.47727790833333	10.5468436833333	9.234219825	9.25484538333333	9.68075670833333	9.76267145833333	9.90058610833333	9.84200871666667	9.67363538333333	9.7553002	8.97625954166667	9.07955376666667	10.128737475	8.90412092916667	9.71657748333333	10.3281467166667	10.30932905	9.76472428333333	9.23047039166667	9.47021275	8.88831745833333	10.1258089583333	9.54925085	7.96240296666667	9.51724313333333	9.34750165	8.815557	8.868084675	8.733549025	9.166351	8.91457238333333	10.015183425	10.1440795833333	9.70748555	9.388540625	8.03960018333333	8.49648456666667	7.544616	9.28502139166667	9.49147149166667	11.1169690833333	9.85498678333333	9.06503669166667	9.66961651666667	9.40597950833333	9.9441902	9.16495244166667	10.25147975	8.44491509166667	9.25764194166667	9.954843075	9.53043561666667	9.07536643333333	9.85722324166667	9.40159784166667	9.52087123333333	10.2018680083333	10.1210398166667	8.70547134166667	10.1766858833333	11.3172677166667	10.87179425	9.23749938333333	8.849091575	9.67363538333333	10.1878607	11.114387025	9.24630831666667	11.2560704166667	9.496026575	9.43456800833333	9.70748555	10.451127	8.7735097	10.1076098833333	10.2621725583333	9.862235575	10.96963075	8.98046293333333	9.30818888333333	9.730552775	9.66649425	10.3261322166667	8.00581294166667	9.20373038333333	9.65670091666667	9.19627585833333	8.24707035	9.13299193333333	9.17457594166667	9.22061473333333	8.391097025	9.05561776666667	8.83016251666667	8.81744095	8.40377353333333	9.357237425	8.86708225	9.00460498333333	8.87636833333333

RGPD2	9.716989375	9.8001132	9.10485650833333	9.71398214166667	9.22832763333333	9.49325940833333	9.88298375833333	9.732285275	8.97354856666667	8.19808664166667	10.3427675333333	9.99432204166667	9.86598649166667	9.562147725	9.74820641666667	9.64871909166667	9.45323241666667	9.35125324166667	9.71442065	10.1637531083333	9.47727790833333	10.5468436833333	9.234219825	9.25484538333333	9.68075670833333	9.76267145833333	9.90058610833333	9.84200871666667	9.67363538333333	9.7553002	8.97625954166667	9.07955376666667	10.128737475	8.90412092916667	9.71657748333333	10.3281467166667	10.30932905	9.76472428333333	9.23047039166667	9.47021275	8.88831745833333	10.1258089583333	9.54925085	7.96240296666667	9.51724313333333	9.34750165	8.815557	8.868084675	8.733549025	9.166351	8.91457238333333	10.015183425	10.1440795833333	9.70748555	9.388540625	8.03960018333333	8.49648456666667	7.544616	9.28502139166667	9.49147149166667	11.1169690833333	9.85498678333333	9.06503669166667	9.66961651666667	9.40597950833333	9.9441902	9.16495244166667	10.25147975	8.44491509166667	9.25764194166667	9.954843075	9.53043561666667	9.07536643333333	9.85722324166667	9.40159784166667	9.52087123333333	10.2018680083333	10.1210398166667	8.70547134166667	10.1766858833333	11.3172677166667	10.87179425	9.23749938333333	8.849091575	9.67363538333333	10.1878607	11.114387025	9.24630831666667	11.2560704166667	9.496026575	9.43456800833333	9.70748555	10.451127	8.7735097	10.1076098833333	10.2621725583333	9.862235575	10.96963075	8.98046293333333	9.30818888333333	9.730552775	9.66649425	10.3261322166667	8.00581294166667	9.20373038333333	9.65670091666667	9.19627585833333	8.24707035	9.13299193333333	9.17457594166667	9.22061473333333	8.391097025	9.05561776666667	8.83016251666667	8.81744095	8.40377353333333	9.357237425	8.86708225	9.00460498333333	8.87636833333333

USP27X	9.931739675	8.9384783	9.20464029166667	8.69657786666667	8.63230339166667	8.98237701666667	8.51528900833333	9.182097875	9.02512653333333	8.61138693333333	8.783965475	8.895120775	8.55907896666667	8.736017975	8.93180466666667	8.56044531666667	9.508221125	8.1525602	9.37548823333333	9.05454125	8.78498449166667	8.76654949166667	9.20100326666667	9.20732398333333	9.03089319166667	9.50725341666667	8.98368761666667	8.72311269166667	8.74083754166667	9.14655231666667	9.63430166666667	9.313630975	8.737919725	9.24712485	9.72484969166667	9.96443173333333	8.36187725833333	8.966534925	9.18880384166667	8.85304851666667	9.19020434166667	9.51088574166667	9.31001445	9.326671075	9.69176305833333	9.39158905833333	9.37728073333333	9.28785676666667	9.34157415833333	8.7637709	9.148762375	9.34750165	9.01380788333333	9.151591475	9.76089629166667	9.77352068333333	9.10796010833333	9.286917675	8.80925365833333	9.28599946666667	9.48881261666667	8.93073908333333	9.34018350833333	8.102103125	10.5300186083333	8.40424725	8.63230339166667	8.82572	9.12606195	8.49593345833333	9.01297525833333	9.138674475	7.56940810833333	8.83738886666667	7.93387576666667	8.34668346666667	8.62136429166667	7.967895825	8.54796775833333	8.1455177	9.37637135833333	9.25253679166667	8.42069139166667	9.10194070833333	7.73639724166667	9.71828384166667	8.72229471666667	9.0180261	9.18115765833333	8.9217463	8.28798768333333	10.001418875	8.14137294166667	8.697015525	8.445416025	8.62685349166667	8.21983948333333	9.286917675	9.16031618333333	9.07816326666667	9.093743775	9.18832863333333	9.92175831666667	8.90864591666667	8.52241499166667	9.15740435833333	9.00507715833333	8.95895775	9.332301675	8.66247435	9.729222475	8.46149996666667	8.61092796666667	8.836922375	8.82067435833333	9.15019934166667	9.0147203	8.92602376666667	8.35319626666667	8.95577006666667

CHP2	7.71914490833333	8.00199818333333	8.58695619166667	8.01861064166667	8.551483425	6.79658473333333	8.85304851666667	8.41878698333333	8.9544846	7.87869541666667	10.503058125	8.72452928333333	8.73063873333333	8.06812029166667	7.81088345	7.66609071666667	8.16983206666667	8.20482314166667	8.28340845833333	8.1205116	8.18838901666667	7.55811896666667	8.28143178333333	7.57513376666667	7.23311056666667	7.62704748333333	7.73803474166667	7.20334935833333	8.14192978333333	7.97557605833333	7.73639724166667	8.67210133333333	8.14754213333333	8.42069139166667	7.88712554166667	7.99615545	7.55873083333333	7.86115675833333	6.95984925	7.30371010833333	9.349869075	7.78057345	7.48861440833333	7.650219825	7.21304980416667	8.50702275833333	7.64794805833333	8.14607183333333	7.597182875	7.607704375	7.86012560833333	7.67926596666667	8.36034350833333	7.89775408333333	8.74083754166667	8.11553545	7.83240795833333	8.25398819166667	8.101615825	7.38926633333333	11.84965965	10.8893434416667	11.85384455	9.29481465	7.649654175	8.07305778333333	8.10529686666667	10.625983	8.08647256666667	8.08335636666667	12.034459025	11.3835567833333	9.07434654166667	9.80096413333333	10.0903015	8.69220983333333	11.7216620916667	8.06996655	11.1383591416667	9.26261210833333	8.338570375	8.22641101666667	10.528410425	8.9856801	9.77475016666667	8.38072273333333	7.89930148333333	9.40159784166667	9.80492948333333	7.58238585833333	9.86598649166667	12.3051209416667	8.92890170833333	12.0025248166667	9.64199300833333	10.0859903416667	7.596603625	7.61006516666667	9.28834158333333	8.27071193333333	9.15692076666667	9.34670505	10.1720866	7.767746725	8.616671525	10.23466	9.18639844166667	9.565921775	11.2132455833333	10.007360325	10.7892430083333	8.466090675	8.063977575	7.51089621666667	8.99771499166667	10.6838734833333	7.73690679166667	8.35319626666667	10.6719237833333	8.23217175

POLDIP3	12.0934360416667	12.4836070666667	12.5680695416667	12.5969475916667	11.7517355166667	11.88575805	12.2366655666667	12.0136845833333	12.3452729083333	12.0717905916667	11.7823700916667	12.2844611166667	11.921685825	12.5406352083333	12.2437326916667	12.1583760416667	12.78176855	11.2965765083333	12.4840896416667	12.4176758916667	12.2028833833333	12.1291180083333	12.150591475	12.32117485	12.1541314	12.3012111583333	12.5020083166667	12.3892279333333	12.0864940333333	12.4927583	12.4110018333333	12.4692673166667	12.303007625	12.3662011583333	11.9994097833333	12.191066075	11.9997560833333	12.1882257583333	12.137649925	12.16243335	12.3546298	12.53672075	12.6857168666667	12.4692673166667	12.4813575333333	12.551141325	12.6336537916667	12.3589004083333	12.7011030333333	12.3347946666667	12.0717905916667	12.2986022833333	12.0465984666667	12.3631593333333	12.4010561583333	12.5468096583333	11.8519854833333	12.2821681166667	12.093025975	12.2311025	11.691783775	12.3518594833333	12.1558313416667	12.3126780333333	13.3696487833333	12.0612117666667	12.2788893833333	12.3240568416667	12.0769700833333	12.2024267333333	12.2692837333333	12.0881187916667	12.105571225	12.3860530083333	12.1774305916667	12.1720161916667	12.0761170583333	11.8527274916667	12.7500199666667	11.8705505	13.0387188916667	12.212911825	12.2710584083333	12.2848587416667	11.8674327083333	12.49908695	12.7934592666667	12.3680867166667	12.3271580833333	12.6402462916667	12.1170603	12.0067796166667	11.973231675	11.9491178333333	11.63482985	12.3956670583333	11.7655518083333	12.2959914833333	11.7883410833333	12.4510079166667	12.3305815833333	12.7602549166667	12.0546824083333	12.4337570333333	12.5173296333333	12.501560375	12.3716896916667	11.9720790416667	12.202055425	12.7139152416667	12.3331331166667	13.0325393583333	12.1612618833333	12.2574255666667	12.4078515833333	12.3152059916667	13.2460254166667	12.1882257583333	12.2416674416667	12.287955975

NT5DC2	12.7390522083333	13.7820292333333	14.1228795	13.2711737166667	13.3601687833333	12.1174426416667	13.7332085916667	13.9840948583333	13.7434058583333	14.3045490583333	13.956539975	12.5939827166667	12.336155525	13.8754219166667	14.354697075	13.2266532083333	10.6263315083333	12.3327004	14.2487820083333	14.056517225	13.591559975	13.3732212583333	13.16815045	13.2553168666667	13.3747582333333	13.1695361416667	14.0503314	14.3076416166667	13.9796866	13.5526964	13.1459482	14.400934925	13.7263656333333	12.6050803083333	12.9683429666667	12.744717925	14.120388025	13.6551549916667	12.478685375	13.7730636333333	13.3608559333333	14.1292641	14.0618019166667	14.34486635	13.3886315583333	13.9989817	14.1164578666667	14.3221229083333	13.4383119666667	12.625945375	12.2687931833333	13.0847965333333	13.5157896583333	13.14005525	14.1590921166667	14.341437425	13.0816325916667	13.8473484166667	13.7740900916667	13.5931197583333	12.4425276083333	13.4579324833333	11.770678525	13.3936063	13.7393052083333	12.5107176416667	14.274700225	13.3501502333333	12.8912643	12.7295358083333	12.6146877083333	13.400193425	11.7239413833333	12.7955349083333	12.5793612333333	13.661470025	12.6734606	12.6744586916667	13.17196845	13.6140845916667	14.596526825	13.6401687916667	12.919046025	14.2533850083333	12.3523338166667	14.0885897916667	13.7443409166667	12.5865453166667	11.88068255	12.6795486	13.2628733666667	13.0620198083333	13.136994025	12.3003464583333	12.5662353166667	12.78176855	13.065712375	13.5815251416667	12.6041308166667	12.034459025	13.0410002916667	13.2020549583333	12.3059641583333	12.7328907083333	13.11105415	12.6412173166667	13.9422518833333	13.3875128	12.3869118416667	12.6366518666667	12.0748320833333	12.7671377333333	12.8256923916667	13.304029325	13.4267583666667	13.0560858	14.00243415	12.7960649666667	13.2545964166667	12.3649229583333

ADAMTS7	8.33450988333333	7.57050700833333	7.43130181666667	8.06500796666667	7.77376521666667	8.36380941666667	7.678740925	7.5010982	7.77487424166667	7.96837240833333	8.06551123333333	8.1959042	7.70199845833333	7.41243761666667	7.99937465833333	7.50463459166667	8.20227615	7.85417920833333	7.63604310833333	7.659482025	7.73471975833333	8.1439842	7.57569364166667	7.95134493333333	8.1719332	8.69317739166667	7.51995818333333	8.05761690833333	7.84154363333333	8.12632724166667	7.671444425	8.053540525	8.28997516666667	8.06026415833333	8.576058475	8.88688890833333	8.18891301666667	7.99937465833333	8.16983206666667	8.09798645	8.09750283333333	7.75930169166667	7.56602129166667	7.73920148333333	8.1089851	7.89832068333333	7.806190725	7.75661866666667	7.806190725	8.31409636666667	8.12933435833333	8.01700516666667	7.73082848333333	8.13040576666667	7.71678190833333	7.6225677	7.76293401666667	7.825834775	7.832972775	7.46414650833333	8.58906924166667	8.65698328333333	8.03755788333333	8.69363141666667	8.966534925	9.295254925	8.79812115	8.70798358333333	8.42269018333333	8.45605063333333	8.01539524166667	8.953569225	8.48569109166667	9.12042280833333	8.86508634166667	9.312713675	8.44794389166667	8.135196525	8.85110639166667	8.51484679166667	8.54360906666667	8.50751961666667	8.77245620833333	9.25904538333333	9.29065144166667	9.08180965	7.96575633333333	9.12700821666667	10.057405675	8.85962865	8.86314575	8.706897975	8.555975275	9.09237333333333	9.11077986666667	8.52878136666667	8.28242620833333	8.75658194166667	8.90864591666667	8.48321643333333	8.74272486666667	8.277602525	8.13415403333333	7.9814024	8.36292435	8.49496250833333	10.05531845	9.11902033333333	9.3242172	9.6433763	8.00529461666667	8.20482314166667	9.490132625	8.87238755	8.89353219166667	8.11712540833333	8.87736359166667	8.66056879166667	8.08289571666667	7.95134493333333

ZNF718	10.159727225	8.75927773333333	8.20831443333333	7.966758925	8.48112818333333	9.148762375	7.57513376666667	8.49593345833333	7.91465324166667	8.354234475	7.78439114166667	9.05561776666667	8.48060255833333	8.69459336666667	9.40469768333333	9.06551128333333	9.43501909166667	7.70471373333333	8.74083754166667	9.03865570833333	8.97769849166667	9.31809370833333	8.8062671	9.33275593333333	9.42465609166667	9.60629726666667	8.62734905	8.80050660833333	9.21923738333333	9.17923419166667	8.53782911666667	8.94049991666667	9.25584964166667	8.83115135	9.53398021666667	10.0537308333333	8.72497184166667	8.39560916666667	8.966534925	9.168290925	8.31765478333333	9.146936475	8.73305545	8.66349279166667	9.283130225	9.20464029166667	9.34392991666667	8.77815989166667	9.62925015833333	9.32089804166667	8.66775465	8.48112818333333	8.14959635833333	8.51713660833333	8.081342075	8.46196905	8.81463643333333	8.87974118333333	9.36572414166667	9.23005591666667	7.74951601666667	8.20031584166667	8.3471697	5.16081746666667	9.26948546666667	8.13923885	7.16541175833333	8.11603985	9.11737229166667	8.58808473333333	6.86809180833333	8.11250586666667	6.54638811666667	8.80967759166667	9.07683584166667	9.14608020833333	8.74671439166667	7.64552340833333	6.99800655	8.90619576666667	11.170311525	10.7321313916667	8.154025025	8.247580125	8.36292435	9.61367635	10.8356737416667	7.84796001666667	9.53179666666667	9.30543436666667	8.14959635833333	8.996715525	10.485478825	9.4594195	10.0125394166667	8.74957956666667	8.57834244166667	9.11317438333333	8.42522444166667	8.28242620833333	9.428146125	7.43742328333333	8.745766675	8.228841125	8.62786819166667	9.39438685	9.40640285	8.256452925	9.311763425	9.24799294166667	9.092829475	5.90235153333333	8.518147825	6.93803065	7.93586978333333	8.1923863	9.83071770833333	9.00867418333333	9.52694540833333	8.9226878

CAGE1	14.9407137125	15.3037720833333	15.5786975875	14.9407137125	12.9969100458333	14.7766143916667	14.7462478083333	15.094005125	12.8018111958333	14.8174412625	14.9906598625	14.3669169333333	14.5655159	15.3841055125	15.2138538041667	15.4179935666667	15.3037720833333	14.6768372458333	14.9879326041667	15.3963757666667	14.6249579291667	15.0572497125	14.7607749583333	15.0848454041667	15.4179935666667	14.96078415	14.5709216125	15.1477739416667	14.587628525	15.3232961208333	15.4964172041667	13.8977696125	15.2029973541667	15.4421183833333	14.7790133416667	14.8807055	14.4337656916667	15.199384425	15.4556642833333	15.3798144583333	14.7283171583333	15.1415803041667	15.3075129166667	15.6111364125	14.9495480708333	15.3196084625	15.2415942875	15.0786481041667	14.8563737875	15.6264327	15.4179935666667	15.6006521875	15.1924916125	14.1584605375	15.3559972666667	14.8123743666667	15.0751458166667	15.3559972666667	14.4068767125	15.4421183833333	14.9991992666667	15.0999532791667	14.7862955791667	15.4735839791667	15.9720547791667	15.3309745708333	15.4221356666667	15.1678278166667	16.1584672125	15.3841055125	15.2450233791667	15.315700425	15.33953575	15.0018826458333	14.8712213916667	14.7215855416667	14.9347727458333	15.4513109041667	15.0443403541667	14.8032021791667	15.5106246333333	15.2380208166667	15.199384425	15.1853257458333	14.9906598625	15.0629405416667	15.5198679791667	15.1711894916667	15.3037720833333	15.0473565625	14.8427539375	15.8106550958333	14.9580939125	15.0879855666667	15.1352804083333	15.0848454041667	15.4598174625	14.9849109958333	14.4714120708333	14.6172136958333	15.5152274041667	15.178210475	14.7003845958333	14.4841474625	15.1445649541667	15.1617431	15.07184225	14.9688432125	15.482799	14.6660938416667	15.2705116333333	14.7961666	15.0378646125	15.6441321333333	14.7283171583333	15.3270652833333	15.151216175	15.0656800833333	15.4598174625	14.5995062291667

SSR1	14.9407137125	15.3037720833333	15.5786975875	14.9407137125	12.9969100458333	14.7766143916667	14.7462478083333	15.094005125	12.8018111958333	14.8174412625	14.9906598625	14.3669169333333	14.5655159	15.3841055125	15.2138538041667	15.4179935666667	15.3037720833333	14.6768372458333	14.9879326041667	15.3963757666667	14.6249579291667	15.0572497125	14.7607749583333	15.0848454041667	15.4179935666667	14.96078415	14.5709216125	15.1477739416667	14.587628525	15.3232961208333	15.4964172041667	13.8977696125	15.2029973541667	15.4421183833333	14.7790133416667	14.8807055	14.4337656916667	15.199384425	15.4556642833333	15.3798144583333	14.7283171583333	15.1415803041667	15.3075129166667	15.6111364125	14.9495480708333	15.3196084625	15.2415942875	15.0786481041667	14.8563737875	15.6264327	15.4179935666667	15.6006521875	15.1924916125	14.1584605375	15.3559972666667	14.8123743666667	15.0751458166667	15.3559972666667	14.4068767125	15.4421183833333	14.9991992666667	15.0999532791667	14.7862955791667	15.4735839791667	15.9720547791667	15.3309745708333	15.4221356666667	15.1678278166667	16.1584672125	15.3841055125	15.2450233791667	15.315700425	15.33953575	15.0018826458333	14.8712213916667	14.7215855416667	14.9347727458333	15.4513109041667	15.0443403541667	14.8032021791667	15.5106246333333	15.2380208166667	15.199384425	15.1853257458333	14.9906598625	15.0629405416667	15.5198679791667	15.1711894916667	15.3037720833333	15.0473565625	14.8427539375	15.8106550958333	14.9580939125	15.0879855666667	15.1352804083333	15.0848454041667	15.4598174625	14.9849109958333	14.4714120708333	14.6172136958333	15.5152274041667	15.178210475	14.7003845958333	14.4841474625	15.1445649541667	15.1617431	15.07184225	14.9688432125	15.482799	14.6660938416667	15.2705116333333	14.7961666	15.0378646125	15.6441321333333	14.7283171583333	15.3270652833333	15.151216175	15.0656800833333	15.4598174625	14.5995062291667

PARD6G	9.07865619166667	8.10529686666667	8.13617131666667	8.51177801666667	7.98193376666667	8.12585160833333	7.99232836666667	7.77550013333333	7.90789741666667	8.584858775	8.29098985833333	9.33365533333333	8.65947190833333	8.01539524166667	7.98346735	8.15205105	8.164333775	8.08086618333333	7.47788481666667	7.73527455	8.21929578333333	8.32062069166667	7.72354014166667	7.84430019166667	7.933308825	9.49908795	7.71678190833333	8.06345484166667	7.84376875833333	8.094406525	8.14082359166667	7.893532975	7.69605179166667	8.16484069166667	9.16261181666667	8.81018689166667	8.13040576666667	7.84154363333333	7.79469890833333	7.788158325	7.908404275	7.74783731666667	8.03117830833333	7.5512356	8.10330963333333	7.502278225	7.85364198333333	7.95631855833333	7.51586044166667	7.94491850833333	7.68098021666667	8.228841125	7.84267175833333	8.14709130833333	8.09121131666667	7.98294754166667	7.61391835	7.428439975	7.96073305833333	9.41162929166667	7.62307443333333	8.36762415833333	8.67503203333333	8.31670425833333	10.023053775	7.82904454166667	7.537939	7.867210225	7.69541339166667	8.017526525	7.73803474166667	8.95490616666667	8.71519911666667	8.126799325	7.470826175	7.89832068333333	7.70256570833333	7.83726095833333	7.54689145	7.597182875	10.7519521333333	9.30275641666667	7.56433695	8.3496737	7.84154363333333	7.89832068333333	9.26538065833333	8.33450988333333	7.3968942	7.8961731	8.63825258333333	10.2943474166667	7.26049458333333	8.51438241666667	8.4067182	7.85577905833333	8.416908275	10.1405016833333	8.072065075	10.040006825	8.259343925	8.84563956666667	7.90139170833333	7.49164054166667	7.26626465	8.24067144166667	7.54070496666667	7.120419025	7.966758925	7.7794798	7.5512356	8.39672325833333	8.28495673333333	10.2654733916667	7.58017606666667	7.67253041666667	9.64024775833333	7.30497195	7.24330025	8.600364325

BAP1	10.0373878833333	10.3558299833333	10.4108585666667	10.3027487416667	10.4488232	10.1569732333333	9.607505	10.128737475	10.225450075	10.5930279833333	10.2318091916667	9.57863910833333	9.86854744166667	10.3203234833333	10.7004993416667	9.99851741666667	10.2001534166667	10.217869775	10.6838734833333	10.34716255	10.3179044666667	10.7069670333333	10.315132175	10.3570715916667	10.2802737333333	10.3182950083333	10.3549808416667	10.69756315	10.6780826666667	10.3321140166667	10.377533625	10.35378905	9.83499924166667	9.76089629166667	10.1324487166667	10.2494268583333	10.647327775	10.3852198833333	10.3956251833333	10.5388660583333	10.5930279833333	10.29990335	10.36596635	10.741111	10.167505275	10.52644525	10.4384998666667	10.6237069166667	10.5130948833333	10.1278854583333	9.915416425	10.3015658	10.6503573333333	10.6603736666667	10.66752385	10.5902797083333	10.0765568416667	10.4691008666667	10.3155264416667	10.29705995	10.7291696916667	10.2034843416667	10.6503573333333	10.3265631083333	9.64118481666667	10.631761275	10.9256058083333	10.02519555	10.0989513583333	10.3702407083333	10.5987255666667	10.7862968083333	10.4499117666667	10.4561678833333	10.2892593916667	11.3212969833333	9.723283875	10.2716281	11.1689128333333	10.8581963333333	9.93427139166667	10.4779275	11.1346623583333	10.7325441416667	9.283130225	9.80706423333333	10.7855003666667	10.0802966083333	10.3591207	10.6087534833333	10.2932251333333	10.2695195083333	10.8908321583333	10.3706198583333	10.3783237666667	10.8078386166667	10.86272805	10.2485867583333	10.2991427166667	10.952408425	10.3852198833333	10.40167215	10.23466	10.3913250333333	10.9219574	11.0851990416667	10.9512840333333	11.1641077166667	9.7100974	10.7732420666667	10.4024778916667	11.4559913916667	10.5404499166667	10.1824837166667	10.6499586916667	10.23466	10.24386405	10.6792669083333	10.884735425	10.1974084916667

BCAS3	11.7814654666667	11.5038172666667	11.9333501833333	11.96822585	11.1317293	11.4394949083333	11.5200695166667	11.7109162916667	11.5154734	11.631804775	11.798162425	11.249224775	11.229748375	11.8757437	11.66838785	11.9116934166667	11.6166645833333	11.4996324916667	11.73285025	11.4824869416667	11.5895178083333	11.50818875	12.0860846083333	11.5750241	11.411903025	11.6274751916667	11.7259183166667	11.6940775166667	10.90507365	11.546539525	11.714258625	11.6929484666667	11.55276425	11.6662778333333	11.4887131666667	11.5128736833333	11.3749692583333	11.30185685	11.520872575	11.5805781333333	12.178269875	11.6569750083333	11.859248825	11.8701716833333	11.7720983166667	12.1270603333333	11.6636899333333	11.1398416833333	11.735525225	11.8568699583333	11.6473588416667	11.411903025	11.494176975	11.86232365	11.960165	12.021831	11.4810093416667	11.5706660333333	11.6859465333333	11.3771067916667	10.9640211333333	11.61335235	12.057317	11.9009487833333	12.4598136333333	10.5557362916667	11.830137	11.5645444166667	11.4478274166667	11.0139017166667	11.6675947833333	11.5411164416667	10.7561593416667	11.95296625	11.1081520166667	11.085607975	11.2631446833333	11.1387134583333	11.04340915	11.37169735	11.9762173583333	12.3874454166667	11.7572174666667	11.1010834666667	10.6769119083333	11.7182865833333	11.6602573333333	11.3320946416667	11.214698175	11.530946975	11.2118401416667	10.2427233083333	11.8523757333333	11.1081520166667	12.077364475	11.4277232	11.3861262916667	12.52364675	11.216912225	12.4593508916667	12.0164637	11.9853022666667	11.3423558	12.1724138083333	10.9160737916667	11.4887131666667	11.6297974583333	11.5969108416667	11.4022935333333	11.244965975	12.1393673833333	11.8985295916667	10.9934066	12.1926211666667	11.5067351333333	11.731200575	11.9112855083333	11.320943175	11.70420215	11.7589935083333

AC087721.2	13.6310840583333	13.7791494	13.7204745333333	13.4666571333333	13.0980169583333	12.8930719333333	13.4565571916667	13.5610039666667	13.6183502333333	13.3080939833333	13.1959888083333	13.670246725	13.38316305	13.8450858083333	13.6320213916667	13.8720329083333	13.054278175	13.2168871166667	13.4666571333333	13.6028841416667	13.488720775	13.4943591	13.5721495	12.8618738916667	13.4274952333333	13.8766797166667	13.5867942083333	13.5502258583333	13.0397781	13.4864308583333	13.267133525	13.5876594833333	13.4443089416667	13.17196845	13.654206225	14.0429420833333	13.4474018083333	13.576244975	12.9806214166667	13.26088015	13.439923175	13.577235475	13.445727425	13.450467225	13.6649979333333	13.5459592333333	13.450467225	13.445727425	13.5519530666667	13.2771790833333	13.1216942833333	12.988893075	13.057870725	13.5355941333333	13.4904111	13.3248386416667	12.9733229333333	13.4474018083333	13.3410732083333	13.2828821583333	12.503422725	12.9773913166667	12.11543585	12.1198078916667	13.62200835	13.0271148666667	12.60549005	12.7822168333333	12.17951645	12.5048403	13.0798178083333	12.5671498916667	12.559988525	13.0798178083333	12.4078515833333	13.001932175	12.8645965083333	12.6537086583333	12.8197190166667	12.695073575	13.8667592	12.9505716416667	12.460239975	12.672014175	12.9694275083333	13.4807854416667	13.5851912666667	12.7344773416667	13.2738329583333	13.0798178083333	12.8454667083333	12.1250050333333	12.7411170833333	13.1700826333333	12.0168871666667	12.6163740291667	12.1325110916667	13.0613877916667	12.5334882833333	13.397359625	12.632709475	13.100692275	13.2785213333333	13.2962436083333	12.3794027166667	12.4746445166667	12.908758175	12.3401023416667	12.6240804083333	12.6412173166667	13.035605175	12.966521825	12.8901186583333	12.17539085	12.2662860833333	13.1622146833333	13.8049154	12.2341055916667	13.001932175	13.2215442333333

FGF14	10.7261291583333	9.29702861666667	9.777820325	9.6385431	10.5592695083333	9.59108196666667	10.3179044666667	9.5295644	10.1883097083333	10.186667875	9.611823	10.39092635	10.816907025	9.60629726666667	10.7673403166667	9.5888718	9.11737229166667	11.403040875	10.3035388166667	10.6122965916667	9.844981225	9.879959075	9.3392826	10.4123976	10.4308569666667	10.1176213666667	10.3167137333333	10.28850615	11.2546673083333	10.372950875	9.932660725	10.239527275	10.6918746916667	10.2229494583333	10.2802737333333	10.8150497416667	11.0556311333333	10.5945390166667	10.5291936916667	9.84032644166667	9.28041066666667	10.04638645	9.37777211666667	9.471100975	10.3667222333333	9.25719764166667	9.35167163333333	9.29018041666667	9.18974645	9.686488825	10.1849087083333	10.1700709416667	10.9740524416667	10.7743973416667	9.74568905833333	10.0769619583333	10.3132196333333	9.52745255833333	10.3105309666667	10.1617358916667	11.051725325	10.5651423416667	9.27312065	9.295254925	9.27853935	9.65101713333333	9.08092490833333	11.0473653916667	9.41620731666667	10.2145140083333	11.030517625	10.9073626	9.674116625	11.4434926166667	9.8001132	9.32711606666667	9.55058418333333	9.81176585	9.235078425	9.28502139166667	9.54225544166667	10.6384159666667	8.96748466666667	8.88020864166667	9.78781813333333	10.2136563	9.16967255	9.64431238333333	9.38807046666667	10.0421767666667	9.56124985	9.35255276666667	9.81899704166667	9.40727955833333	9.4134397	9.7570522	9.52223368333333	9.74483329166667	9.692647075	9.43636521666667	9.15935345833333	9.17867269166667	9.357237425	8.85348764166667	8.89076064166667	9.40159784166667	9.79235871666667	8.58140188333333	9.746957025	9.68387974166667	9.3036884	9.48078894166667	9.18115765833333	8.9925209	9.606709075	9.41849085	9.13033400833333	9.373589925	9.41070553333333	9.61984825833333

BCR	11.0848820333333	11.9158492083333	12.237914025	12.0400324083333	11.06860385	11.2936955416667	11.759780275	12.0305597416667	11.6910443916667	11.53966185	11.7605260333333	11.6636899333333	11.4763556583333	12.037261675	11.586583875	11.1749885333333	11.5916707666667	11.1604297833333	11.52618635	11.3470919	11.5389498916667	12.0514729416667	11.5174454333333	11.9158492083333	10.9765277583333	11.0953873333333	11.4854519916667	11.45939305	11.2997236583333	11.4115295083333	12.0036527416667	11.7324169333333	11.06860385	11.5991025	10.9415949916667	11.2660555333333	11.666692125	11.731200575	12.105571225	11.8779799083333	12.1636527	11.8527274916667	11.9633577833333	11.9055925	11.0913458083333	11.392737725	11.8450362083333	11.65549195	11.6933169166667	11.8166747583333	11.4993303916667	12.3479516833333	11.94052505	11.893980925	11.7030888416667	12.0685666833333	12.1803292	11.9100903833333	11.2887921333333	11.4296055416667	11.3861262916667	12.4598136333333	10.8172624166667	12.672014175	10.9607806583333	11.5067351333333	11.312921725	11.70420215	11.1036124166667	11.2753469583333	11.4971747583333	11.485120625	11.8048205583333	11.44422215	11.4262590333333	10.8778360416667	11.9892168333333	11.4704736916667	11.9431320666667	11.0604394416667	11.721307075	11.327862475	11.5166142666667	11.5341666	11.634515075	11.7537741833333	11.687529825	10.9204831	11.1129059916667	11.5253735333333	12.1372175166667	11.929273675	11.51470825	11.4446261583333	10.7008852833333	11.9923578333333	11.4090056666667	11.41004175	10.9979551416667	12.14843035	11.8297435166667	11.8070215583333	12.07292235	12.0149070416667	10.9321190083333	10.1254167416667	11.9220834083333	11.545468825	11.5468691083333	12.3405472916667	12.0839641583333	12.6107512666667	12.716481775	11.7768584833333	11.5363521916667	12.1084032166667	11.7791279583333	11.5790815416667	12.35333105	11.629073975

LRRC8C	12.4161375	11.2067215541667	11.8773983041667	10.6763291791667	10.5575313583333	12.0787689041667	10.7610940708333	11.2931173958333	10.636687025	11.7443020625	11.6685670791667	12.4900844416667	11.976008175	11.1472248958333	10.6673449625	11.9676169291667	10.9351916708333	10.8914364416667	11.2727941166667	12.0094356791667	11.8751626041667	10.9316130333333	11.53767785	11.8152436625	11.7877194541667	11.8719919916667	11.4859582416667	11.4656335875	11.1613203083333	10.4159178666667	11.8196833	11.9019314125	11.5062034083333	12.1200148333333	11.8724307875	11.62289695	11.3529603708333	11.4131664666667	11.9465334708333	12.0212087541667	11.457250925	11.0191663458333	11.9580160875	11.8675931416667	12.0430500916667	11.9830365916667	11.2893333708333	11.6850845583333	11.777038375	11.4595644791667	12.1195922916667	11.5406145041667	11.6261305791667	11.8156423916667	11.4877701083333	12.2283145291667	12.1408441583333	11.4186649166667	11.7551390333333	12.274965975	12.5152377333333	12.4035613208333	11.8222782541667	12.6414595166667	11.2393368958333	12.5143034291667	12.3477116291667	12.3278199291667	12.3124633708333	13.0497180583333	11.7461377875	12.4467832416667	13.6388549708333	12.5341884666667	12.6549583208333	12.9675015458333	12.5360254333333	12.6907177958333	12.7388022833333	12.0207650833333	11.6589487666667	12.1416138375	12.2034910208333	12.52386935	12.3999222916667	12.2842455916667	11.4656335875	12.5313564	11.7763300666667	12.73366375	12.6559656416667	13.3707552291667	12.7418902125	12.0854924	12.6988560541667	11.8299402583333	12.3398992416667	12.1412178458333	12.2401951708333	11.9787412125	12.0134724791667	12.0903471208333	12.6911874583333	11.5740610791667	12.4304122583333	12.3544274791667	12.8512322333333	12.3778174666667	11.6512910125	13.0322366791667	12.4699238583333	12.5022385041667	13.5104375583333	12.1966782875	13.1296506291667	12.2394115625	12.0134724791667	12.7947359833333	11.8617328041667	12.5247907125

AC093423.3	12.4161375	11.2067215541667	11.8773983041667	10.6763291791667	10.5575313583333	12.0787689041667	10.7610940708333	11.2931173958333	10.636687025	11.7443020625	11.6685670791667	12.4900844416667	11.976008175	11.1472248958333	10.6673449625	11.9676169291667	10.9351916708333	10.8914364416667	11.2727941166667	12.0094356791667	11.8751626041667	10.9316130333333	11.53767785	11.8152436625	11.7877194541667	11.8719919916667	11.4859582416667	11.4656335875	11.1613203083333	10.4159178666667	11.8196833	11.9019314125	11.5062034083333	12.1200148333333	11.8724307875	11.62289695	11.3529603708333	11.4131664666667	11.9465334708333	12.0212087541667	11.457250925	11.0191663458333	11.9580160875	11.8675931416667	12.0430500916667	11.9830365916667	11.2893333708333	11.6850845583333	11.777038375	11.4595644791667	12.1195922916667	11.5406145041667	11.6261305791667	11.8156423916667	11.4877701083333	12.2283145291667	12.1408441583333	11.4186649166667	11.7551390333333	12.274965975	12.5152377333333	12.4035613208333	11.8222782541667	12.6414595166667	11.2393368958333	12.5143034291667	12.3477116291667	12.3278199291667	12.3124633708333	13.0497180583333	11.7461377875	12.4467832416667	13.6388549708333	12.5341884666667	12.6549583208333	12.9675015458333	12.5360254333333	12.6907177958333	12.7388022833333	12.0207650833333	11.6589487666667	12.1416138375	12.2034910208333	12.52386935	12.3999222916667	12.2842455916667	11.4656335875	12.5313564	11.7763300666667	12.73366375	12.6559656416667	13.3707552291667	12.7418902125	12.0854924	12.6988560541667	11.8299402583333	12.3398992416667	12.1412178458333	12.2401951708333	11.9787412125	12.0134724791667	12.0903471208333	12.6911874583333	11.5740610791667	12.4304122583333	12.3544274791667	12.8512322333333	12.3778174666667	11.6512910125	13.0322366791667	12.4699238583333	12.5022385041667	13.5104375583333	12.1966782875	13.1296506291667	12.2394115625	12.0134724791667	12.7947359833333	11.8617328041667	12.5247907125

GRAP	8.94870224583333	8.5912661	9.03665022916667	8.38344775416667	8.80702904166667	9.20584292083333	8.73377340416667	8.31987026666667	8.7931974625	8.89536062916667	8.96677049166667	8.93204191666667	8.452284725	8.5052777875	7.96861178333333	10.1663620833333	8.69340440416667	8.22620555833333	8.4818775625	9.074595	9.00013413333333	8.9491525	9.05845999166667	8.87760424583333	9.36062987916667	8.7506974125	8.98638951666667	8.843587625	8.82596699166667	8.82190914583333	8.59226435416667	9.32022560416667	8.88094719166667	10.0613623416667	9.08752937083333	9.161920675	8.90938502916667	8.87952065833333	9.4462680125	8.9728836375	8.7401162875	8.60726364583333	8.91388026666667	8.53858933333333	9.05709510416667	9.4761584875	9.16851644166667	8.438760575	9.09756450833333	8.90938502916667	8.82496384583333	8.24579634583333	8.95695830416667	8.7474283875	8.9596737	9.072740775	8.54722025416667	9.2249321	8.80475242083333	9.24649527916667	8.94209639583333	8.80993224166667	9.19835729583333	8.01675574583333	8.92439054583333	9.23675633333333	9.30933558333333	9.13199272916667	8.92951810833333	8.9992168	8.4787391125	8.56344345833333	9.11244409166667	9.080268075	7.92361595833333	9.23485380833333	9.29041592916667	9.9554479	9.2456270375	9.036198525	9.10772999583333	8.17331657083333	9.00307024583333	9.36691167916667	9.12257175	9.08070198333333	8.78564547916667	8.96823769583333	8.3544937875	9.13935604166667	9.09856255833333	8.72808869166667	8.70523306666667	9.22934275	9.20352572916667	8.5311907	8.62165234166667	8.58569985	9.31569955	8.81311722083333	8.85419642083333	8.23742238333333	9.295997875	8.69819049583333	8.843587625	9.0300746375	9.37986479166667	8.852386675	8.81355715833333	9.52568969166667	9.2986405375	8.99228364583333	9.15811152916667	8.71623835416667	9.2244692375	9.14541895833333	8.92390113333333	8.9634582	8.91158374166667	9.11471757083333

GRAPL	8.94870224583333	8.5912661	9.03665022916667	8.38344775416667	8.80702904166667	9.20584292083333	8.73377340416667	8.31987026666667	8.7931974625	8.89536062916667	8.96677049166667	8.93204191666667	8.452284725	8.5052777875	7.96861178333333	10.1663620833333	8.69340440416667	8.22620555833333	8.4818775625	9.074595	9.00013413333333	8.9491525	9.05845999166667	8.87760424583333	9.36062987916667	8.7506974125	8.98638951666667	8.843587625	8.82596699166667	8.82190914583333	8.59226435416667	9.32022560416667	8.88094719166667	10.0613623416667	9.08752937083333	9.161920675	8.90938502916667	8.87952065833333	9.4462680125	8.9728836375	8.7401162875	8.60726364583333	8.91388026666667	8.53858933333333	9.05709510416667	9.4761584875	9.16851644166667	8.438760575	9.09756450833333	8.90938502916667	8.82496384583333	8.24579634583333	8.95695830416667	8.7474283875	8.9596737	9.072740775	8.54722025416667	9.2249321	8.80475242083333	9.24649527916667	8.94209639583333	8.80993224166667	9.19835729583333	8.01675574583333	8.92439054583333	9.23675633333333	9.30933558333333	9.13199272916667	8.92951810833333	8.9992168	8.4787391125	8.56344345833333	9.11244409166667	9.080268075	7.92361595833333	9.23485380833333	9.29041592916667	9.9554479	9.2456270375	9.036198525	9.10772999583333	8.17331657083333	9.00307024583333	9.36691167916667	9.12257175	9.08070198333333	8.78564547916667	8.96823769583333	8.3544937875	9.13935604166667	9.09856255833333	8.72808869166667	8.70523306666667	9.22934275	9.20352572916667	8.5311907	8.62165234166667	8.58569985	9.31569955	8.81311722083333	8.85419642083333	8.23742238333333	9.295997875	8.69819049583333	8.843587625	9.0300746375	9.37986479166667	8.852386675	8.81355715833333	9.52568969166667	9.2986405375	8.99228364583333	9.15811152916667	8.71623835416667	9.2244692375	9.14541895833333	8.92390113333333	8.9634582	8.91158374166667	9.11471757083333

TCEANC2	10.6199959166667	10.8755795416667	10.4691008666667	11.0038210166667	10.9084288583333	10.0043835333333	11.0041932083333	10.264650175	10.507752825	10.451127	10.20309965	10.3844721666667	10.7519521333333	11.0927898583333	11.1767887583333	10.31750855	9.62155398333333	10.4649425166667	10.7565121333333	10.239527275	10.5240503083333	11.3492073583333	10.4815083083333	9.39938098333333	11.30084235	10.4115783666667	10.3968247166667	10.359500425	11.2638907416667	11.3861262916667	10.3935444541667	10.7975980416667	10.9483433833333	9.67723733333333	10.5181477	10.356255775	10.9328378333333	10.987327675	10.7295063583333	11.19202035	10.4272415916667	11.1043306833333	10.4468482583333	10.4168764916667	10.173327525	10.64420985	11.0290294333333	10.3937266	10.6941625333333	10.5504732083333	9.82029285833333	10.5134429166667	10.4963081083333	11.2542786416667	11.452332325	10.8874095666667	10.6610932333333	10.8154489833333	11.196768475	10.7356243	9.30325005833333	9.2998268	9.98961361666667	9.07634685	9.81941439166667	9.562147725	9.45472053333333	9.46121264166667	9.80056271666667	9.63156661666667	9.74045244166667	9.54132330833333	9.651432225	9.47590374166667	9.98225808333333	9.815643425	9.68034129166667	9.56680634166667	9.95522605	10.123786625	9.868111775	9.59369666666667	9.75367038333333	9.62805526666667	9.87254320833333	9.94339973333333	9.330358125	9.64783695	9.637594125	9.75492564166667	9.29384936666667	9.4094908	9.71307581666667	9.817741375	10.0226518416667	9.68825589166667	10.1113541833333	9.68903575833333	10.123005125	9.83811425	9.876036325	9.63292783333333	9.838575025	10.0855958166667	10.30893185	9.900175875	10.0099661	10.1409369083333	9.911603525	9.80885460833333	9.32285545833333	9.78824573333333	10.1405016833333	10.1009165916667	10.1529084083333	10.0382960666667	10.340493	9.64118481666667	10.0595403	9.85198440833333

SLC25A48	5.41246783333333	6.230051875	6.48357195833333	6.31702735	6.53407053333333	5.59754975833333	5.992607775	5.40932128333333	5.66378185833333	6.18408195	6.23385413333333	5.57060381666667	5.02467455	6.28109695833333	6.66118995	6.19454715	5.575395825	6.94244640833333	7.04002458333333	6.32106796666667	6.71186961666667	6.58691901666667	6.56365850833333	5.8009379	6.573073	5.279548575	6.5862738	7.1674037	6.76114361666667	6.45841340833333	6.728950375	6.16763958333333	6.05994703333333	5.15246255833333	5.63144754166667	5.27734721666667	6.52133365833333	6.95435513333333	6.22080705	7.18402409166667	6.65156973333333	6.27872079166667	6.23950985833333	6.42264453333333	5.40852341666667	7.619792375	6.44000804166667	6.01386335	5.57834849166667	6.528434875	6.50214656666667	6.12118446666667	6.24033626666667	6.65487665833333	6.17555735	6.31469075	5.10174316666667	6.11462551666667	6.47930140833333	6.22752675833333	5.61354440833333	6.22752675833333	5.60680040833333	5.64497814166667	4.99137435	6.691149875	6.74958845833333	6.21517435	6.04523669166667	5.58407845	6.07429128333333	5.9323169	6.46494565	5.55953659166667	5.826473825	6.67239650833333	6.19818808333333	5.96423755	5.90321861666667	6.36473330833333	5.221531825	8.14959635833333	5.399029725	7.51638066666667	7.49771558333333	5.54102934166667	5.77106630833333	5.44122935	6.29358614166667	6.67783206666667	6.406487225	5.97106113333333	7.88018380833333	5.91247053333333	7.17033845	5.73297515	7.52896074166667	6.45472701666667	5.71707388333333	5.87100729166667	5.81120035833333	6.47930140833333	5.53695673333333	5.73943181666667	7.09043023333333	6.27401925833333	6.81253920833333	7.04503860833333	5.4137082	6.96399793333333	5.64797583333333	7.120419025	8.09998893333333	6.53273724166667	6.14881959166667	5.14292011666667	5.65824568333333	4.89254299166667	7.29233335	4.88257656666667

ATP6V0E2	7.57732030833333	6.24443749166667	5.6241104	5.6322886	6.64016859166667	6.68859711666667	6.0148863	5.7161104	6.41036045833333	6.10713235	6.42485146666667	5.61925595833333	5.80937455	6.80377665833333	6.3064348	5.68107735833333	7.30612164166667	6.90968315833333	6.62102673333333	5.80356945	6.09925825	5.6723045	6.63041895	6.35578110833333	6.73002575	7.04378355833333	5.93821120833333	5.79999791666667	6.9393833	6.65552751666667	6.84260019166667	7.46853124166667	6.53407053333333	6.42634760833333	6.80714281666667	7.88559756666667	6.29670533333333	6.57166325	6.99096820833333	6.56145719166667	6.35504728333333	6.691149875	6.234662425	6.180201325	6.72705409166667	7.38984995833333	6.75712434166667	6.88648389166667	6.70200101666667	7.06531028333333	7.01007860833333	6.50929938333333	5.70153421666667	6.83744154166667	6.75898810833333	6.618809175	6.1770551	6.874007675	6.96533775	6.806433125	7.45614546666667	6.08940326666667	7.36048969166667	6.58359223333333	6.914548675	6.62839033333333	5.90588820833333	5.7550021	7.407222675	6.22752675833333	7.02704855833333	6.43572405833333	5.75852464166667	6.47541770416667	5.775482875	5.81478479166667	6.79529673333333	6.76378944166667	6.08593745	6.58499179166667	6.02387365	5.979616925	6.24941475833333	6.06230976666667	5.8893734	6.59176974166667	6.76517564166667	4.792098225	6.89528771666667	6.1770551	6.242830225	6.70532833333333	6.69991303333333	6.32527860833333	6.68595645833333	6.739509575	6.42709209166667	7.31316353333333	5.99418615	6.57365081666667	6.3687361	6.311629325	6.87783053333333	6.27482028333333	6.47064316666667	6.82102155833333	5.8386672	6.715741525	6.8327755	6.68131301666667	7.84106111666667	6.42556058333333	6.94057503333333	6.72519365833333	6.903227175	6.58691901666667	6.90252933333333	7.2645801	7.37467966666667	6.48151946666667

KLRD1	7.71189573333333	5.05687025	6.03747638333333	5.44644633333333	6.70918899166667	6.54299384166667	6.24607838333333	5.31227555833333	4.535343	5.90945328333333	5.64406685833333	7.56091623333333	5.63144754166667	4.99137435	6.50142603333333	6.87783053333333	7.68593156666667	5.096408875	4.98892909166667	5.91334729166667	5.47522431666667	5.18759319166667	4.664314175	6.63179246666667	5.72187083333333	8.28242620833333	6.28975704166667	5.65995315	4.65176085833333	5.2108879	5.8625617	5.46549339166667	5.53695673333333	6.31786493333333	8.82680535833333	9.250740625	5.127309825	7.08614628333333	6.88940976666667	7.00365334166667	6.219278425	5.256776325	7.338007825	7.40602305833333	8.16735940833333	4.90256950833333	6.00039116666667	5.6003113	6.36103899166667	7.64552340833333	6.62158961666667	5.83953349166667	5.0343026	5.189937375	4.68477714166667	7.16295023333333	5.6003113	4.82688145833333	7.662739425	6.78574375	5.6003113	4.47668578333333	4.996867625	5.92854135	7.4250003	5.29020444166667	5.76923416666667	5.528242825	5.18112505	6.14803923333333	6.1770551	5.978682025	5.87944876666667	5.90945328333333	6.081909575	6.265839825	6.33852943333333	5.48703414166667	5.68689626666667	6.9125939	4.28423139166667	5.26995844166667	4.09698049166667	6.2379381	4.28110195833333	5.50436075	4.35397924166667	5.44754924166667	4.27374416666667	6.62839033333333	5.006452275	6.11542659166667	4.96956821666667	6.50580336666667	6.526432	6.59386139166667	4.39446349166667	4.3276422	6.51310794166667	7.62534816666667	6.53407053333333	5.40651104166667	7.33320358333333	5.81652036666667	4.922546525	4.80316131666667	5.32031858333333	5.92603411666667	6.941911825	7.75009851666667	5.78160560833333	5.30151331666667	6.03921009166667	4.81376534166667	4.50112465833333	4.968508325	5.415860975	5.35088976666667	5.64694838333333	5.60777716666667

VPS53	8.06345484166667	7.6157713	7.851546575	8.17505753333333	9.50182605833333	8.07674773333333	7.00566125833333	7.55016986666667	7.05225570833333	7.29365533333333	7.62202388333333	7.76084795833333	7.92110699166667	7.47384185833333	9.166824	7.2132819	7.093633575	9.10015925	7.82231499166667	7.84323385	8.028895175	9.19719063333333	7.65225225	8.15060805	8.47797495833333	7.70532880833333	7.466283575	9.04278656666667	8.64196594166667	7.74398054166667	7.641075725	8.16389916666667	7.788158325	7.38699095	7.54282731666667	7.90363365833333	8.4841298	8.167838375	8.04755969166667	8.31814086666667	8.01273043333333	8.09798645	8.21782325833333	7.227778225	7.51935500833333	7.57344579166667	7.655449725	7.68436408333333	7.94273865833333	7.92964085	7.51696985	8.11654126666667	9.066471675	8.80300369166667	7.87768698333333	7.99937465833333	7.978659025	7.29843466666667	8.30911568333333	7.91577950833333	9.341081375	7.71409900833333	8.38370515833333	8.97769849166667	8.61979589166667	8.277602525	7.68042426666667	7.51935500833333	7.264052575	7.73471975833333	8.07622910833333	8.14849655833333	8.24315470833333	7.75234836666667	7.8200803	8.224517175	8.277602525	8.48112818333333	8.21353541666667	7.65996385833333	7.598264375	8.48368870833333	8.58856818333333	7.794082525	8.39981014166667	7.49826404166667	8.65218976666667	7.54177855833333	8.70259450833333	8.37107905	8.13304300833333	9.30818888333333	8.535777725	8.37604669166667	8.82873195	8.512323475	8.88357545	8.54598220833333	8.75927773333333	8.16983206666667	8.6404919	8.24270349166667	8.34374149166667	7.57513376666667	8.46961650833333	8.56818220833333	8.05913828333333	7.3361665	7.816692275	7.67088145	8.27283913333333	7.44667759166667	8.81744095	7.67359318333333	8.2549704	8.02512498333333	7.94789065	8.71235535	8.462988175	7.928121775

RTKN2	5.42082883333333	10.313965725	9.87116769166667	10.1420474166667	10.1242565166667	8.75658194166667	9.47819235833333	9.856343425	9.89466155833333	8.96987785	9.97301156666667	6.941911825	8.32905851666667	10.6356893416667	10.4634265083333	9.439107	6.92785069166667	8.41878698333333	10.5719557166667	9.7408584	9.99170656666667	11.0982319416667	9.56828670833333	9.26899234166667	9.82295156666667	4.65382089166667	9.67141531666667	9.471100975	10.240361675	10.2289590916667	9.02131511666667	10.57908995	9.166351	8.00687400833333	4.82135065	5.57148620833333	9.81442805	10.04039335	9.943781525	9.49413000833333	10.239527275	10.3975849666667	10.26748265	10.0343496666667	9.68034129166667	9.95566975	10.3754461833333	10.6150711416667	10.9436906083333	9.25764194166667	9.72449161666667	10.199031925	9.85368629166667	10.4153772	10.39291705	9.85548545	9.97000988333333	10.0026032916667	9.61553475	9.69747909166667	10.0965198666667	8.93897689166667	9.83900869166667	9.32471271666667	9.74655711666667	8.457931075	8.50185603333333	9.78654094166667	7.51365925	10.5585696666667	8.391097025	8.84195500833333	9.41436835833333	9.157883875	9.6385431	9.57267214166667	9.24031536666667	10.01936935	8.375514	10.0595403	9.57464318333333	8.15959780833333	8.66056879166667	9.51408459166667	8.00199818333333	9.16261181666667	8.52110425	9.26442515	8.6667612	8.98520484166667	8.80677418333333	9.07634685	9.1038805	8.18475165	6.50720644166667	8.65418485	10.1840717583333	8.97126208333333	6.57427455833333	9.79062058333333	8.32359891666667	9.961096725	10.5623629583333	10.3554429666667	10.0334908083333	9.78104253333333	9.512679675	8.364642925	8.81880498333333	10.0318266833333	8.54699226666667	10.4123976	8.443897425	8.434501875	8.89310475	10.2014148333333	10.6607214666667	10.1593240833333	9.23462919166667	10.496758625

DHX8	7.3968942	7.76032476666667	7.75009851666667	7.98717739166667	7.7124456	7.11547685	8.01700516666667	7.64327454166667	8.06129600833333	7.49938943333333	7.47140944166667	7.90468865833333	8.135196525	7.61177998333333	7.596603625	7.2960179	7.78057345	7.609434275	7.238929725	7.246381175	7.644918425	7.61062251666667	7.10952	6.94690668333333	7.009464425	6.99096820833333	6.82038475833333	7.00693255	7.22964071666667	6.96970946666667	7.19451305833333	7.146985275	7.08235699166667	7.3314952	6.91578644166667	7.03502525	7.10888616666667	6.76905384166667	7.05900955833333	6.70327563333333	7.24703090833333	7.56602129166667	7.03138465	6.93091866666667	7.33501970833333	7.07118303333333	7.11547685	7.148875425	7.11920136666667	6.58691901666667	6.93287185833333	6.76253231666667	7.76990645	7.238929725	7.64049128333333	7.20508425	7.28500960833333	7.02761371666667	6.74746250833333	6.7327851	7.100006625	7.75602535833333	7.18402409166667	7.31615383333333	6.74746250833333	7.3367108	7.375280375	7.61643631666667	7.37123815833333	7.22899075833333	7.47623615833333	7.43742328333333	7.8967227	7.28072111666667	7.7548948	7.07238718333333	7.02021258333333	6.85954253333333	6.90968315833333	6.92916504166667	7.19767501666667	7.28990879166667	7.35693584166667	6.905223325	7.341560875	6.80714281666667	6.89266504166667	7.5654861	7.62759090833333	6.51600675833333	7.06279625833333	6.85478974166667	7.30497195	6.38165589166667	7.34210711666667	7.11617649166667	7.15147738333333	7.15820169166667	7.51586044166667	7.28012135	6.958116025	7.89979373333333	6.76253231666667	6.55223534166667	6.91634240833333	7.07794505	6.61959393333333	7.44735373333333	6.72249205833333	7.36471060833333	6.56951250833333	6.68067039166667	6.81118743333333	6.742824875	6.75303653333333	6.90968315833333	6.63942924166667	6.89851890833333	6.71655181666667	6.94497745833333

ANKRD34B	4.263255	3.83595706666667	3.976955525	3.976955525	3.737761675	4.245391125	3.41442594166667	4.97812506666667	3.536082325	3.78824818333333	3.88647775833333	3.46702925	3.60105128333333	4.16299614166667	4.06900396666667	3.88294669166667	4.26758835	3.670269525	4.40284696666667	3.81819714166667	3.71306755	4.2918764	4.46985861666667	4.46696494166667	4.36702491666667	3.8291486	3.98946053333333	5.08252440833333	4.161871975	4.064898825	4.203896025	4.19529993333333	3.72561649166667	4.02568050833333	4.24013239166667	4.04622350833333	4.11685985	4.39670516666667	4.38930708333333	5.112251075	4.76252700833333	4.45602740833333	4.67380545833333	5.26995844166667	4.07678143333333	4.77601478333333	4.99894050833333	3.746317775	4.32946600833333	4.04622350833333	4.638255275	3.78824818333333	3.69375465	5.0486896	4.07678143333333	4.46100053333333	3.86613395833333	4.55069105	4.22594125	4.1018927	4.0078761	3.59588780833333	4.29605796666667	3.79653381666667	3.607366575	3.65432333333333	4.01810879166667	4.12106518333333	3.63145715833333	3.46854113333333	3.60851389166667	3.67271084166667	3.36115875	3.63673386666667	3.69375465	3.64669851666667	3.572561225	3.94901469166667	3.73894771666667	3.81123611666667	5.6695191	3.78824818333333	3.976955525	3.6554436	3.610975325	3.60976025833333	3.57121334166667	6.4413796	3.59061676666667	3.842520875	3.57639359166667	5.3960318	3.98946053333333	3.76717175	4.01107811666667	4.472817375	3.81605024166667	4.52610014166667	3.78410915833333	6.42781263333333	4.44923994166667	3.4640341	4.515720925	5.773689175	4.24130721666667	3.905753025	4.48613384166667	7.85309088333333	5.6411096	8.499322875	6.72249205833333	6.582876425	4.25100265833333	4.70163844166667	4.27700685	4.177708	6.31323984166667	6.06230976666667	4.39039918333333	4.108372

TFEC	8.74034393333333	8.74083754166667	9.0115544	8.39620023333333	6.65156973333333	9.85850786666667	8.14709130833333	9.21350573333333	7.69605179166667	9.448687325	8.685246125	10.4429923666667	8.01806620833333	9.19441289166667	7.188269975	11.197870325	9.6428903	6.53407053333333	7.88343015833333	9.58437340833333	9.44914495	7.99563946666667	9.80187509166667	9.28502139166667	9.45194998333333	9.25303868333333	8.86854605833333	8.62136429166667	7.89775408333333	8.55105330833333	8.1346889	9.69491718333333	8.85348764166667	11.1060515416667	9.79147918333333	9.06270968333333	7.67425858333333	9.50725341666667	9.37637135833333	8.85635194166667	8.82372205833333	8.12378493333333	9.59145404166667	8.75278535833333	9.40252054166667	9.79101400833333	9.16031618333333	9.40727955833333	9.86517185	9.357237425	8.81018689166667	8.76703910833333	8.77815989166667	9.01101026666667	8.74174598333333	8.846155575	9.726557625	9.486953025	9.32044345833333	10.2108003333333	8.69274164166667	9.86359230833333	10.3261322166667	8.61820814166667	9.88845614166667	6.71850903333333	10.03562315	10.1589444166667	9.67544948333333	11.0567325916667	8.833014675	9.13226131666667	6.77370956666667	11.6511098916667	9.309548625	11.1756714833333	10.6842469	11.430726925	10.3074544166667	11.1003561666667	9.49953110833333	9.26035810833333	9.357237425	9.71989020833333	10.6003674833333	9.80310053333333	9.319021525	11.3587026083333	8.09947061666667	11.0279781	10.2991427166667	10.0256584	8.91275618333333	9.911603525	9.68213623333333	10.191495075	9.85161335833333	9.45281165833333	8.86854605833333	9.56828670833333	10.1840717583333	9.81305005833333	11.4115295083333	9.71307581666667	9.32142165833333	11.343036975	10.0242607916667	10.2728709416667	10.5303580166667	11.1139507416667	10.1176213666667	9.26082591666667	9.48384479166667	9.70194608333333	11.4779366416667	10.5392644333333	9.062206575	10.0321890166667	9.35679085833333	12.0997306916667

SRSF7	11.426633325	10.9757948333333	10.8131866083333	10.92486485	10.5464444833333	10.747418225	10.50459975	10.6523425166667	10.6846028416667	10.308194675	9.76472428333333	11.06860385	10.457410175	10.72134755	9.99259065	10.601173475	11.2334234166667	10.8404768083333	10.6169276916667	10.9501588	10.8284896583333	9.983490475	10.50459975	11.5067351333333	10.863090975	11.2312268666667	10.4522980083333	10.4417307666667	10.2318091916667	10.71312705	11.302200525	10.6050909833333	10.6422709916667	10.6072418333333	11.4718467833333	10.9185836833333	10.0778458	10.661840225	10.8165123833333	10.4324595166667	10.303115425	10.8078386166667	11.1803917416667	10.6222826083333	10.976899425	10.436472775	10.72134755	10.8861829666667	10.7767059333333	10.4460825166667	11.2179166166667	11.1176950333333	9.91970055833333	10.5561337833333	10.340914925	10.7665838833333	11.040528175	10.5868572833333	10.165808925	10.577240575	11.3817027833333	10.96365625	10.007360325	10.4759657166667	11.9686139666667	9.54925085	10.5699475166667	10.2928305416667	10.5983973083333	10.4449298	10.4380961666667	10.7855003666667	9.39025219166667	10.6503573333333	10.39092635	10.3466375416667	10.2229494583333	10.1417124166667	9.89341510833333	10.661840225	11.83328595	10.6997392583333	10.18324735	10.2955209666667	10.0877690666667	10.8375253583333	11.884179625	10.2749442833333	10.9707232916667	10.1350682166667	10.8045420416667	10.8180008083333	10.342051525	10.417262075	10.674973575	10.3321140166667	10.0844426833333	10.8389999833333	10.1687340166667	10.950929275	11.184429675	11.0808123083333	10.84359385	10.72134755	10.15614755	10.325378325	10.0924722666667	11.21907835	10.5872774916667	10.0997208916667	10.627804175	10.7940186083333	10.3289721	10.1927941666667	10.323288425	10.5196816583333	11.411148125	10.4597952916667	10.7452538416667	11.08918345

MMP14	11.5945056583333	11.0505864458333	11.2895126416667	11.8477473833333	11.621317125	11.1302224166667	12.4593508916667	11.0859242666667	11.3306591916667	11.2420872916667	11.8154196416667	12.2662860833333	12.3071456666667	12.0657212166667	11.1881087416667	11.1520832083333	10.96999	11.6418554833333	10.7371678583333	10.7608892083333	11.2114428083333	11.3499248083333	11.0352265083333	10.523220425	10.18748315	10.5334113083333	12.452378375	10.741834125	11.2868731416667	11.1993790583333	11.11213045	10.8904318	10.8972744416667	11.3520380583333	10.079544525	10.0268881833333	11.201526325	10.3179044666667	10.7340525583333	10.783568125	10.4918133583333	11.075444825	11.2198620833333	10.8416766333333	11.666692125	11.17143095	11.2233924	11.0483413166667	10.85523755	10.2634356166667	11.043790875	10.5276582916667	10.8638767916667	10.78588925	11.0504310083333	10.878870175	10.7460395416667	10.8600934166667	10.6453411416667	10.6730377	12.24577265	12.8979356	11.784206175	12.4941044416667	12.5813238666667	12.11260975	12.978001475	13.174009975	12.3496705916667	12.3262957	12.5429966333333	13.1986854833333	12.9709933083333	13.16545515	12.9812481083333	13.1946165416667	12.3479516833333	11.9762173583333	12.16610385	12.8802020166667	12.2844611166667	12.506168425	12.2524478333333	13.5543953	12.7655438666667	13.0228260333333	11.951715625	13.2387164583333	12.164859775	12.5633422083333	12.3662011583333	12.62157875	11.1898119916667	12.6606270166667	12.6195975333333	11.6980360625	11.73400915	11.9364860583333	12.8481618333333	12.1906758666667	12.4879782166667	12.587916575	11.6787724916667	11.3136753916667	11.5912797083333	12.5897782	13.5907625666667	12.734915275	12.9172670333333	12.87462785	11.5976176916667	13.8060720916667	12.6146877083333	12.0084826666667	12.7061787	11.4313741833333	12.098126675	12.837133975	10.5787126833333	11.9777865

NCSTN	13.7940225416667	13.8137434916667	13.4520130916667	14.0100072833333	13.3383656916667	14.4361804666667	13.7771113166667	14.3186975416667	14.1113310916667	14.1486654166667	14.3661675333333	13.70272065	13.8438449333333	13.8285300416667	13.71577945	14.0618019166667	14.7429609666667	13.6996187916667	14.1441992083333	14.2204751583333	14.1152258583333	13.3754869916667	14.0578490916667	14.0418505333333	13.9612443083333	13.7730636333333	13.9045177166667	13.9197186416667	14.0540171833333	13.834699275	13.765060275	14.0275803333333	13.8698539083333	14.392674675	13.4768642833333	13.454214775	13.956539975	13.676743575	13.8395483	13.6434906916667	13.91423525	14.1513249333333	13.8088736083333	13.9400026166667	14.2610479166667	14.2983229333333	14.201540175	14.0991501333333	13.798249925	14.3843059083333	13.8376377666667	13.4407112833333	13.8317321333333	14.1554067916667	14.1709219416667	14.044199175	13.3882606083333	13.834699275	14.2426505583333	14.1343040416667	14.4377971083333	14.265784475	14.4427443166667	13.8688792083333	14.70147985	14.5735643833333	13.916524275	14.1737580083333	14.364637875	13.7293441833333	14.0643996166667	14.3692316583333	14.4443539833333	14.1357234916667	14.718655275	14.1629431333333	13.827479275	14.2000424583333	14.1183777583333	14.1164578666667	14.6284460166667	14.989321275	14.18015695	14.2731900083333	14.5793502666667	14.2563286	13.92958755	13.887785	13.8307559916667	14.2872888583333	14.3366389666667	14.1241853333333	14.4722450583333	14.1427380833333	14.4705790833333	14.5089658583333	13.9695187666667	14.3366389666667	13.9749587833333	14.76191125	13.9695187666667	13.9762519833333	14.2998455083333	14.2686692416667	14.3381592083333	14.3013820416667	14.1752017916667	14.3908304333333	14.9949392	14.3960605	14.5469723583333	14.1164578666667	14.4758644166667	13.9480264583333	14.515883675	14.2474901166667	15.290119725	14.6992893416667	13.9967915416667	14.036419

ROBO1	5.06113309166667	4.567125	4.60180214166667	3.88876065	4.71576270833333	4.79999713333333	4.2939407	3.936908825	4.03968158333333	4.24335745833333	3.98271365	4.09698049166667	4.11987200833333	4.369907275	4.82796093333333	4.00032819166667	4.03660776666667	4.832334525	4.91851714166667	3.98729839166667	4.18298021666667	5.4137082	4.34698491666667	4.88257656666667	4.617309275	3.881892575	4.84820759166667	5.32134071666667	5.77716973333333	4.48925233333333	4.8440917	4.736033375	4.21300761666667	4.289092625	4.356950075	4.22787308333333	5.67523288333333	4.64613160833333	4.19044525833333	5.467505875	4.39156651666667	4.50544710833333	5.25768970833333	4.71793841666667	3.971604825	4.24335745833333	5.16938190833333	4.49797680833333	5.66463360833333	3.66190675	4.72508045	4.92659856666667	4.92455391666667	5.8523182	5.08063435	4.075702625	4.33043379166667	4.27374416666667	5.30448685	4.32588960833333	6.85418244166667	3.90803829166667	5.399029725	3.95252660833333	4.0322916	4.88920265833333	4.72105203333333	3.93585013333333	3.70932085	3.92135188333333	3.82681465	4.05417596666667	3.78410915833333	4.21502835833333	5.30061454166667	4.34281185	4.67674131666667	5.28578914166667	4.84094744166667	4.061836425	4.79318954166667	5.924123625	3.9479401	4.9617686	4.79109571666667	3.92135188333333	5.78537223333333	6.17313585833333	4.12677783333333	4.6319392	4.59997769166667	4.78047113333333	4.54330015	4.7771511	5.22716268333333	4.92149175833333	5.948036925	5.45901654166667	3.86613395833333	6.94244640833333	5.06351158333333	3.8730856	4.79109571666667	4.86804739166667	3.92691720833333	4.75491586666667	6.01215780833333	7.821740775	6.72953939166667	7.98618585	5.15873735	6.38240346666667	5.231579	4.22678013333333	5.18423503333333	4.46877066666667	5.86179054166667	4.557709	4.83989633333333	4.43313565833333

OST4	13.934871	14.2650283625	13.4717297875	14.0689028125	13.462329075	14.0243984458333	13.7089213291667	14.2990842208333	13.57854245	14.1770407666667	13.8883681708333	14.1893955833333	13.6964936541667	13.9571150875	13.8183957375	13.9868209458333	14.0333340833333	13.8204541583333	13.8703988666667	13.8312440625	14.1013487833333	14.231255875	14.0957623208333	13.7326627375	13.9417063708333	13.9222648958333	13.8411551708333	13.8556663083333	13.9114695083333	13.7645641083333	13.7906263333333	13.8883681708333	14.0423963083333	14.1106227041667	13.9560051208333	13.8703988666667	13.9973217958333	13.7209851375	13.6936536458333	14.0384459916667	14.0132906041667	13.7458079125	14.3308102333333	13.9594675833333	14.3083426625	14.2850488916667	14.0030396875	13.8444653708333	14.0932431041667	14.0793650083333	13.8714883666667	13.8204541583333	14.1449790166667	14.2418422166667	13.9949861041667	14.1262193333333	13.9722892541667	14.1770407666667	13.7567678375	14.2707797083333	13.896671125	13.8362143208333	14.8510831166667	14.4369887875	14.4451729708333	14.00939395	14.1106227041667	14.0132906041667	14.0571831583333	13.4294178666667	13.9949861041667	13.6742503416667	14.13117145	14.1979037666667	14.2739451166667	14.2770857541667	13.7706892416667	14.0676537625	14.09445065	14.3900386875	14.3669169333333	14.0358782416667	14.1285867625	14.2802845666667	13.9995577083333	14.1677434541667	14.028200125	14.0411451625	13.2165666458333	14.1964649833333	13.9324787166667	14.0972083875	13.5095875375	14.2022367625	13.8662507083333	14.0397911958333	13.6041330875	14.5011414416667	14.2990842208333	14.0985399125	14.0740265416667	14.3276335208333	13.653779525	14.435416225	13.8093762416667	13.9571150875	14.2481360625	14.5540052875	14.3178504041667	14.028200125	14.1584605375	14.0484738916667	14.2124999333333	14.3506319291667	14.0999066125	14.267917775	14.1795629625	13.9700457583333	14.2912700583333	13.9973217958333

AGBL5	13.934871	14.2650283625	13.4717297875	14.0689028125	13.462329075	14.0243984458333	13.7089213291667	14.2990842208333	13.57854245	14.1770407666667	13.8883681708333	14.1893955833333	13.6964936541667	13.9571150875	13.8183957375	13.9868209458333	14.0333340833333	13.8204541583333	13.8703988666667	13.8312440625	14.1013487833333	14.231255875	14.0957623208333	13.7326627375	13.9417063708333	13.9222648958333	13.8411551708333	13.8556663083333	13.9114695083333	13.7645641083333	13.7906263333333	13.8883681708333	14.0423963083333	14.1106227041667	13.9560051208333	13.8703988666667	13.9973217958333	13.7209851375	13.6936536458333	14.0384459916667	14.0132906041667	13.7458079125	14.3308102333333	13.9594675833333	14.3083426625	14.2850488916667	14.0030396875	13.8444653708333	14.0932431041667	14.0793650083333	13.8714883666667	13.8204541583333	14.1449790166667	14.2418422166667	13.9949861041667	14.1262193333333	13.9722892541667	14.1770407666667	13.7567678375	14.2707797083333	13.896671125	13.8362143208333	14.8510831166667	14.4369887875	14.4451729708333	14.00939395	14.1106227041667	14.0132906041667	14.0571831583333	13.4294178666667	13.9949861041667	13.6742503416667	14.13117145	14.1979037666667	14.2739451166667	14.2770857541667	13.7706892416667	14.0676537625	14.09445065	14.3900386875	14.3669169333333	14.0358782416667	14.1285867625	14.2802845666667	13.9995577083333	14.1677434541667	14.028200125	14.0411451625	13.2165666458333	14.1964649833333	13.9324787166667	14.0972083875	13.5095875375	14.2022367625	13.8662507083333	14.0397911958333	13.6041330875	14.5011414416667	14.2990842208333	14.0985399125	14.0740265416667	14.3276335208333	13.653779525	14.435416225	13.8093762416667	13.9571150875	14.2481360625	14.5540052875	14.3178504041667	14.028200125	14.1584605375	14.0484738916667	14.2124999333333	14.3506319291667	14.0999066125	14.267917775	14.1795629625	13.9700457583333	14.2912700583333	13.9973217958333

RAP2A	10.3203234833333	12.0781743333333	12.226469725	11.9494775416667	11.4496634833333	11.3567956166667	12.0905289333333	12.2972775333333	11.779499875	11.74329675	11.6444959666667	10.23466	11.2671696833333	12.115901275	12.2248275416667	11.9177333166667	10.49466965	11.4820913166667	12.3550491166667	12.1488811083333	11.7746522	11.407195875	11.4767310833333	11.7285272083333	11.8853151666667	11.2907157	12.105942725	12.1014528666667	11.8519854833333	12.5676300666667	12.17539085	11.9475058416667	11.761727725	10.435306725	11.425117975	11.311125675	12.0400324083333	12.4078515833333	12.013260375	11.8364243666667	12.0261845416667	12.3369699166667	11.9616964916667	11.8461878583333	11.3672205833333	12.2930155083333	11.7164003333333	12.2578527416667	11.7655518083333	11.79216385	11.8032520333333	11.98404715	10.6092076916667	11.7671597083333	11.7601425083333	11.5114672666667	11.5836115333333	12.4355627	11.7716953916667	11.5132414833333	10.4736621916667	11.78754065	10.6245043083333	12.2587377333333	14.0643996166667	10.7885218833333	11.4056072833333	11.20749835	10.6610932333333	10.4822871416667	11.341686575	10.4168764916667	10.3495871666667	11.569897	11.105357075	11.6170945166667	10.34919645	11.373190825	11.54913165	11.2534278333333	13.4649543916667	10.7927886583333	11.6511098916667	11.2527308083333	11.7243383416667	11.184429675	10.8292287166667	11.071263875	10.2494268583333	12.0149070416667	11.3979579666667	12.11177165	11.9357187583333	11.22125475	9.80187509166667	10.6352329833333	9.97667879166667	10.88143175	10.9549623083333	11.4679092333333	11.4574143583333	10.789595925	10.976899425	10.97984555	11.4273654166667	11.5691835	11.012770325	11.2399027333333	10.575694825	11.4534829083333	10.9794539833333	10.4732782833333	10.417262075	11.1470598583333	10.7904047166667	10.83116835	12.2289445583333	11.5961336833333	11.1847643166667	11.21761425

GTF2B	12.5468096583333	12.7687032	12.4430322166667	12.6769899666667	12.3580122083333	12.734915275	12.2206544333333	12.5196766666667	12.4549570666667	12.467315075	12.0737471	12.4078515833333	12.5344122083333	12.8789964583333	12.2760053833333	12.6412173166667	12.5680695416667	12.5729905	12.5725102833333	12.5883581916667	12.608834775	13.044583175	12.8498718833333	12.8613107583333	12.4855430666667	12.6528003583333	12.336155525	12.3671040333333	12.415021	12.372860975	12.7400926583333	12.5827163333333	12.3908133166667	12.87636155	12.644626475	12.0585648916667	12.1790995083333	12.644626475	12.447837225	12.0685666833333	12.515033225	12.6402462916667	12.7086380166667	12.6909651833333	12.5543618083333	12.6562254416667	12.9521681583333	12.7934592666667	12.620644525	12.306357275	12.7103184	12.3195462583333	12.811279575	12.8969748916667	12.5020083166667	12.7328907083333	12.5282693916667	12.7500199666667	12.5667535083333	12.5506564333333	12.0881187916667	12.1217141333333	12.369348425	12.0273705916667	12.6991286666667	12.1224925833333	11.7440902416667	12.4723572833333	12.6985834416667	12.6669575	11.7575518916667	11.876483925	12.5439252666667	12.6900433666667	12.4202498166667	12.4315646666667	12.2679354916667	12.4469679916667	12.4593508916667	12.7526244083333	12.3720643666667	12.2871151416667	12.4395669416667	11.9966897833333	12.8046597666667	12.1724138083333	11.683026175	12.2492084583333	11.8059034833333	12.4430322166667	12.2633103416667	11.7470404	12.5297533083333	12.2947323416667	12.3443503083333	12.1861275083333	12.2133193833333	12.036426975	12.5745387083333	12.9694275083333	12.0781743333333	12.11053915	12.8930719333333	12.4777234416667	12.3645045916667	13.1097329166667	12.5710716416667	12.3147568916667	12.3169057833333	12.4390650166667	12.6679927416667	12.4723572833333	12.5354003083333	12.4412440416667	12.9694275083333	13.0193242083333	12.8041062916667	12.5098151833333	12.5883581916667	12.7051272333333

UGDH	12.911149025	13.4282493833333	13.1872302333333	13.4729544083333	12.659113	12.5443972083333	12.6900433666667	12.7939716	13.6246233916667	13.18580525	13.6461564833333	12.285271325	12.6790614583333	13.2705051333333	13.0362472083333	12.8996243416667	13.933121025	13.6174285083333	12.7268539583333	13.2116333	13.0362472083333	13.16815045	13.0571358166667	13.100692275	13.1329842916667	12.6586586666667	12.8645965083333	13.1986854833333	12.5979621916667	13.1144791333333	12.8339629333333	13.4058013416667	12.8690686583333	12.9699766666667	13.2835400666667	12.2710584083333	12.9948642333333	13.0136394416667	13.505263125	12.800460075	13.3897269	12.8543756916667	13.462725925	12.8203151333333	12.8930719333333	12.8974584	13.1280018	13.1946165416667	13.2266532083333	13.0548490666667	12.731184375	12.7279204166667	12.9836190416667	12.9853754833333	13.7139558	13.1952399083333	12.8789964583333	12.8924470083333	12.895840375	12.9400260666667	13.3241951083333	13.6596320166667	12.813963275	11.48359205	13.3003482666667	12.080029025	12.996638525	14.550749925	11.1941109166667	12.498597975	13.9518039333333	13.593805675	12.50431935	13.741448775	12.2488091166667	12.7774843666667	12.7426291416667	12.540087475	12.11260975	12.6975412416667	12.7354192416667	12.8684829916667	11.84426255	13.3234625333333	11.9509854083333	12.1891609666667	12.524997525	12.7463306666667	13.9625123916667	12.8866499416667	12.370296175	13.0847965333333	13.4586485833333	12.931215675	12.19191635	13.5007024833333	12.105571225	12.247084775	12.3038527583333	12.0040290833333	12.6566773416667	14.1972150333333	11.7219865583333	12.21725785	13.2855354	13.16545515	12.7884927666667	12.8842279916667	11.9686139666667	12.7490093583333	12.3636460083333	14.1045935166667	12.126629875	12.3318475166667	12.3284449583333	13.199462625	12.5576279333333	12.5850501916667	13.016186275	13.1091497583333

PTK2	5.76836148333333	4.789978375	4.58556791666667	4.2949863	5.51098925	5.47315375	4.82464924166667	4.89595425	4.71445220833333	4.258212325	4.62161215833333	5.09764620833333	4.87199023333333	4.81485295	5.6411096	4.9406758	4.84615233333333	5.92509355833333	5.47416700833333	4.9415684	4.94999915833333	5.06113309166667	5.18312655	6.37857751666667	5.934751075	5.09864851666667	6.12524645833333	5.88853345	5.54779774166667	4.89827923333333	5.2108879	5.30061454166667	4.83765554166667	4.94355658333333	5.61155829166667	5.74803075	5.45901654166667	4.61955908333333	4.9426219	5.258661775	4.99019379166667	5.28262243333333	4.64033936666667	5.26362051666667	4.43091141666667	5.08458449166667	5.08997001666667	4.93761881666667	4.99894050833333	5.73491465833333	4.89254299166667	5.71516535833333	4.80533786666667	5.81403274166667	4.52155396666667	4.692141475	5.24931593333333	4.66727606666667	5.7550021	5.27315905833333	6.29670533333333	4.35819395	6.71502808333333	4.76252700833333	5.62957723333333	7.46853124166667	6.465677275	5.6488719	6.52412431666667	5.70748306666667	4.954343875	6.38621459166667	4.61830805833333	5.84141815	5.68304789166667	6.056382625	6.17555735	6.12847228333333	6.32175328333333	5.45901654166667	5.1863758	6.679903875	6.541621825	5.05900706666667	6.40265184166667	4.713205375	6.53809915833333	4.645027875	5.91247053333333	5.79119435	4.563953975	6.34447826666667	6.50418884166667	6.21191129166667	7.907346175	6.27955291666667	6.85045274166667	6.13259841666667	5.455892825	6.591129625	5.72686785	5.2108879	6.45401484166667	5.7745543	5.53902828333333	6.79057516666667	6.65843384166667	5.87405735	5.64694838333333	6.41666009166667	5.43818919166667	5.47616439166667	6.08697085833333	5.53004825	4.91552208333333	5.20060255	5.85609921666667	6.07673425	4.91552208333333	3.86842555

AIFM3	8.64290230833333	8.04755969166667	8.58808473333333	8.1873246	7.85906496666667	8.67503203333333	7.97768158333333	8.24415698333333	7.68593156666667	8.04545514166667	8.4237182	7.32258515	6.99412739166667	7.31861950833333	8.293531175	7.97397919166667	8.12489341666667	7.67926596666667	8.20990576666667	7.71354716666667	7.55925806666667	8.1455177	8.24163635833333	8.01119698333333	7.85854243333333	8.028895175	8.35277756666667	8.24163635833333	8.576935025	8.14607183333333	8.59842283333333	8.68971138333333	8.03810015	7.93586978333333	8.67210133333333	8.78635625	8.33507211666667	8.57940885	8.13362299166667	8.65745360833333	8.42823383333333	8.113521575	8.222543225	8.37107905	7.91897891666667	8.364240675	8.182610775	8.39466414166667	8.08240869166667	8.36669614166667	8.23613304166667	8.586479525	7.98087950833333	8.42522444166667	8.32603026666667	8.72134320833333	7.886143875	8.77245620833333	8.38072273333333	8.199157725	9.906465775	8.200812875	9.08869563333333	8.95031519166667	8.74671439166667	9.42590730833333	8.48745835	8.62290185833333	8.79489933333333	8.22799306666667	8.902412325	9.39025219166667	7.64610639166667	7.7483928	8.71519911666667	8.46044338333333	8.56366318333333	8.6223815	8.66349279166667	8.52683058333333	7.568339425	8.75371965	8.7143253	8.975801525	8.199157725	7.87869541666667	7.97141813333333	8.12933435833333	9.36528065833333	8.61480576666667	8.620261275	8.81154583333333	8.51027651666667	8.359825725	9.33579655	9.2408133	8.47629666666667	8.60126088333333	8.38918200833333	9.121301075	8.28289991666667	8.75047631666667	9.14426045	8.89405555	7.9579863	8.8334785	8.35832260833333	9.04369295	8.93180466666667	8.02832288333333	9.08493245833333	9.45687460833333	8.64447529166667	8.85304851666667	8.49886293333333	8.72403179166667	8.43902399166667	8.71882579166667	9.25028671666667	7.95311691666667

SOS2	12.703617625	12.8350142416667	12.393505075	12.5293458166667	12.1096451083333	12.1935330916667	12.4696924	12.6254772333333	12.5406352083333	12.08124855	12.1549544416667	12.4037688166667	12.2453199833333	12.5325556416667	12.3187215583333	12.8481618333333	12.603650525	11.9177333166667	12.4732793833333	12.7323979	12.7216091916667	12.7306165083333	12.5754789333333	12.48596835	12.5813238666667	13.1407286583333	12.6909651833333	12.5376322583333	12.4917329166667	12.6023058	12.39795185	12.6270116666667	12.7666041416667	12.3653316083333	13.0186633916667	13.129332175	12.5439252666667	12.4554143666667	12.3575221583333	12.5231511833333	12.2930155083333	12.6060225083333	12.5548435083333	12.3484591166667	12.3997083083333	12.3653316083333	12.533964725	12.1431003583333	12.4461573416667	12.4181461583333	12.0677960666667	12.552591775	12.212911825	12.3492861833333	12.615602	12.352843675	12.2587377333333	12.4238445333333	12.3698220916667	12.2780305166667	11.7713486916667	11.4971747583333	12.3860530083333	12.3314457	12.545304275	12.0541727916667	11.8523757333333	12.3780252416667	12.14630095	12.3926407	11.6754049666667	11.9259261416667	12.1034309916667	11.8553966916667	12.625945375	12.0067796166667	12.135071975	12.7987689166667	12.5860556666667	12.149683875	12.1673323833333	12.5534552416667	12.7945001333333	11.8815198166667	11.8032520333333	11.9285599833333	11.46509415	12.7753567583333	12.034117575	12.4912644	12.244497975	11.84426255	11.82164615	12.8823979	12.7395608333333	12.1815348916667	12.3837214916667	11.5790815416667	11.9155103583333	11.6148020166667	12.5902222833333	11.7525932916667	11.5287863916667	11.8415156583333	13.004985075	12.2776254166667	12.5325556416667	12.3297195416667	12.0245529166667	12.4315646666667	12.0600286166667	12.1389321	12.338252925	13.100692275	11.9013541833333	11.9028464416667	12.237914025	12.2358628083333	12.285271325	12.1839395083333

SH3GLB2	11.6613946166667	12.4581088416667	12.4482599083333	12.764427075	12.43820225	10.952408425	11.9701954833333	12.049870975	12.2085904583333	11.341686575	11.8386923083333	11.027599275	12.1308631333333	12.496314025	12.8211392791667	11.8573117083333	12.467315075	12.6709351583333	12.7233335166667	12.3067398	12.1146065416667	12.8208910916667	11.5404299	12.2002559333333	12.623024125	11.57874505	12.402892875	12.352843675	13.0853533416667	12.77853635	12.7912843416667	12.6909651833333	12.0067796166667	11.6170945166667	11.7008228166667	11.72023525	12.57846135	12.3443503083333	12.0036527416667	12.5609255833333	12.1698876833333	12.772348275	12.2875140666667	12.1815348916667	12.0474514	12.5662353166667	12.3203584916667	12.32117485	11.673519475	11.9025044	11.8791305416667	12.2683870833333	12.5107176416667	12.7128638833333	12.8101998166667	12.2561861666667	12.3824722	12.5530135166667	12.7733872	11.8431168166667	11.6473588416667	11.133569275	10.9640211333333	11.2118401416667	10.5212723416667	10.988072975	9.86020724166667	11.27496335	10.7034642666667	11.5004634	11.0090696333333	11.7791279583333	11.5341666	10.8172624166667	11.1473899333333	10.382913225	10.8828386666667	11.702337975	10.390544	11.1459742833333	11.2409567416667	10.7975980416667	11.2678800166667	11.0409461	11.3635795583333	11.1649238666667	11.7663613666667	11.33887195	11.452332325	10.738724825	10.9757948333333	11.2631446833333	10.5810256583333	10.5311099	10.9618387333333	10.8904318	10.8755795416667	11.7753980416667	10.8412280166667	11.54913165	10.8548652666667	10.8025054583333	11.5887304416667	10.9208636166667	11.3986748166667	11.2989793	11.027599275	11.60398845	10.81281805	10.749691325	10.578003225	11.0797164083333	11.8484879416667	10.7718274166667	10.8759847166667	11.6577322083333	10.489906475	11.4989285	11.674290525	11.0581558166667

KCTD9	10.7247329333333	10.227841925	9.93713581666667	10.1691741416667	10.3745792583333	9.89258960833333	10.0373878833333	10.0712503	10.0288478666667	9.547789	10.5166009333333	9.56361585	10.1413216916667	9.7815357	10.3725905333333	9.64431238333333	9.20732398333333	10.64689975	10.3483436583333	10.500980975	9.87303573333333	10.4759657166667	9.9229238	10.4253305166667	10.2286007833333	10.3733561083333	10.3313573416667	10.4819116333333	10.5244636333333	10.5456854416667	10.6391956666667	10.2258230916667	10.1130642083333	9.1773608	10.4682944	10.66793845	10.24386405	10.3964688	10.7302810416667	10.1509476166667	10.05998855	10.4826365083333	10.1088055083333	10.2853426583333	10.0595403	10.0026032916667	10.1262351166667	10.431190025	10.0242607916667	9.86020724166667	10.3317121166667	10.7470371333333	9.87908794166667	10.3257291333333	10.0997208916667	9.90222311666667	9.98961361666667	10.137056175	10.3293667083333	10.1725196416667	10.9219574	10.904663125	9.81525473333333	10.20309965	10.2695195083333	10.9013899916667	9.93713581666667	10.3549808416667	9.18261260833333	9.63342665833333	10.2217906833333	8.83063586666667	9.72795155833333	9.945018575	11.1862575	9.68075670833333	9.4551338	9.54225544166667	10.7581339416667	9.73350879166667	9.7928167	10.527258325	9.54132330833333	10.0889846333333	10.4759657166667	9.63659523333333	10.064052975	9.59824975833333	10.98769475	10.36339005	10.5719557166667	10.6910121333333	10.1063080916667	10.2060439416667	10.1613409083333	9.79101400833333	10.9869124833333	9.64431238333333	10.372950875	9.85198440833333	9.69308664166667	10.0467969333333	10.4720948333333	9.92175831666667	10.2846004916667	10.0848379583333	10.1246349583333	10.05157835	9.277313475	9.4134397	9.63806365	10.051171975	9.74996381666667	9.91374403333333	9.98150248333333	9.30864065833333	10.432039025	10.400070625	10.16655665	9.76136145

KRTAP12-1	6.11047193333333	5.221531825	4.98771553333333	6.65024493333333	6.4095551	5.08458449166667	6.329205025	6.241933725	6.37713004166667	5.57335978333333	5.948036925	5.95328179166667	6.23159324166667	6.33345005833333	4.62990135	5.49526165833333	5.03120425833333	6.30111015	5.29885645833333	6.1770551	6.815678575	5.82824421666667	5.83589014166667	5.51915526666667	4.84935865	5.45476871666667	5.6173492	5.51683608333333	5.196416425	5.99091528333333	5.23361180833333	5.20995024166667	6.44217180833333	5.667420075	5.18312655	4.9415684	6.732067125	5.35692600833333	6.45696331666667	4.946749425	5.429887625	7.6071886	5.02371664166667	5.61925595833333	6.2379381	5.08252440833333	5.127309825	5.65913218333333	4.713205375	4.86699436666667	5.19421045833333	4.69325549166667	6.03236501666667	5.73682776666667	4.80096826666667	7.16358325	6.50720644166667	4.81485295	4.9727824	4.82580888333333	5.18015243333333	5.64973066666667	5.73943181666667	5.98999241666667	6.29670533333333	5.83208956666667	6.40185915	6.20262720833333	6.39681019166667	6.47930140833333	5.75112915833333	5.88673599166667	6.39383819166667	6.36327274166667	5.97682351666667	5.50701503333333	6.13511379166667	5.78626178333333	5.43182890833333	5.93821120833333	5.557624025	6.59052684166667	6.58763124166667	4.88811624166667	5.52123825	6.42033848333333	8.82211801666667	6.27245344166667	6.33345005833333	4.823565325	6.2073137	4.59265796666667	5.35182895833333	5.74130296666667	5.82555810833333	5.47028654166667	6.15447426666667	5.667420075	6.72249205833333	6.9125939	4.96401284166667	7.81401100833333	5.24498075	5.15246255833333	6.21280073333333	5.77807068333333	5.32346678333333	6.59815521666667	4.356950075	6.60007374166667	4.67172261666667	5.63834938333333	4.883731825	5.02467455	4.63513973333333	7.47204458333333	4.76252700833333	5.09764620833333	4.97812506666667	5.1555947

ADGRE5	7.913004675	7.55987390833333	6.7401779	7.378725775	7.6702147	7.50966981666667	7.11020925833333	6.70200101666667	7.2954331	7.39505978333333	7.51253281666667	7.40837556666667	6.93685505	6.84380731666667	7.62414805	7.002378425	7.47140944166667	8.401731375	8.02019604166667	7.22476671666667	6.91190293333333	8.084825575	7.190008575	8.18781211666667	7.87717198333333	7.11617649166667	7.54070496666667	7.66609071666667	7.50852245833333	7.53018445	7.78488271666667	7.66954886666667	7.38522528333333	7.32919075833333	7.32789755	7.092930725	8.40614324166667	7.25363145833333	7.84267175833333	7.62593523333333	7.01495000833333	7.619792375	7.51200123333333	7.16614005833333	7.37928605	7.00566125833333	7.24703090833333	7.26874934166667	7.7587684	7.641075725	7.21750264166667	7.91409299166667	7.7483928	8.40280550833333	6.97781291666667	6.82889531666667	6.74082630833333	6.94057503333333	7.43011818333333	7.57457279166667	8.6984679	7.8129644	8.27907085	8.23365661666667	7.59937680833333	8.932734675	7.838427925	6.94561610833333	8.45201853333333	7.90468865833333	7.707009225	7.7794798	7.596603625	7.86334303333333	7.794082525	8.619247325	8.167838375	8.94804158333333	8.23767453333333	7.8345672	7.81731503333333	7.78057345	8.00852153333333	8.1455177	8.20781665833333	7.867210225	8.25546045	7.139659575	8.11454491666667	7.864271825	7.92005815	8.65318265	8.23263584166667	8.026220775	8.53782911666667	8.33507211666667	8.28193894166667	8.22546075833333	8.466090675	7.94491850833333	8.15455800833333	7.740357875	8.505532025	7.80170391666667	8.65887986666667	8.182610775	8.18475165	7.72085915833333	7.90941765	8.17036706666667	8.52583200833333	7.65774509166667	8.42522444166667	7.77893200833333	7.75772005833333	8.15060805	8.05569140833333	8.34021045	7.88559756666667	7.349518275

MAGI2	4.98545498333333	3.95899671666667	3.80218998333333	4.01435764166667	3.65066326666667	3.89779840833333	3.893426425	3.99930385833333	3.68464693333333	3.9661014	3.64190115	4.36702491666667	4.24130721666667	3.89679799166667	4.15642159166667	3.73259605	4.19044525833333	3.66682366666667	4.32386374166667	3.770641325	3.9614432	4.10724728333333	4.19529993333333	4.2939407	4.64847966666667	4.90768301666667	4.4619761	4.53433785	4.49216794166667	4.24013239166667	4.57343223333333	4.33637641666667	3.89227330833333	3.98152069166667	4.99894050833333	4.729262375	4.3276422	4.111632325	4.03764378333333	4.251891475	4.44161573333333	4.36294506666667	4.35819395	4.838864	4.01568299166667	4.35186211666667	4.40284696666667	4.38005901666667	4.27160165833333	4.41392473333333	4.46696494166667	4.26445661666667	4.03968158333333	4.81716043333333	4.01568299166667	4.44534256666667	4.00881020833333	4.27798759166667	4.19720234166667	4.06900396666667	3.88078246666667	3.91806175	4.062876875	3.839428225	3.6774476	3.84944544166667	3.8957117	3.899834175	3.78548855	3.8730856	3.99172610833333	3.55885279166667	4.265394625	4.02040568333333	3.78952711666667	3.976955525	3.97505105	3.90803829166667	3.90803829166667	3.55885279166667	5.09968536666667	5.2151974	4.087323525	4.15745399166667	3.912461775	4.043026825	4.15309530833333	4.4462942	3.82560103333333	4.383064975	3.83152513333333	4.2929499	4.3249589	3.78410915833333	3.62768649166667	3.746317775	3.99817013333333	4.149223425	4.1553789	4.3249589	3.90803829166667	4.184996575	4.62161215833333	4.56487130833333	4.02676174166667	4.39039918333333	4.509505	5.10716860833333	4.30828979166667	5.36922546666667	4.4047953	4.91752975833333	4.545583025	4.49113386666667	4.093740425	4.08114406666667	4.06900396666667	4.49602461666667	4.61955908333333	4.13540660833333

SNX22	7.2015586	7.754380925	7.3007483	8.062405875	7.81731503333333	8.29980986666667	6.71655181666667	7.74093078333333	6.67856683333333	7.43234464166667	6.84325476666667	6.75170075833333	7.16909093333333	7.72249438333333	7.40322858333333	9.73788834166667	9.20190699166667	7.91954875833333	7.619792375	8.59099210833333	7.96073305833333	8.56913543333333	8.1777524	8.09798645	8.86655378333333	6.36951041666667	7.57513376666667	8.101615825	8.64638736666667	8.14240605833333	7.51200123333333	8.83493181666667	7.657177025	10.328553475	6.4516666	6.57952525	7.81893236666667	8.77579595833333	7.86164825833333	7.74783731666667	8.32853386666667	7.9882338	8.634032725	8.21782325833333	8.62194039166667	8.90962148333333	8.15812938333333	7.47677956666667	8.821700275	8.52966328333333	7.88927118333333	6.98530065833333	8.37864189166667	7.80672660833333	7.34551940833333	8.03755788333333	7.62202388333333	8.67403964166667	8.89784426666667	8.30911568333333	7.34551940833333	7.52443165	8.17090974166667	7.22053391666667	13.0149306583333	7.01495000833333	5.84963309166667	6.93091866666667	7.5090854	7.56091623333333	6.30492928333333	6.9004841	6.53273724166667	6.89851890833333	6.73002575	6.64893395	7.61865353333333	10.18748315	7.10317193333333	7.85309088333333	12.442132175	7.16541175833333	7.15389984166667	7.06942048333333	7.244602025	7.38926633333333	7.41763228333333	7.18035421666667	6.87469296666667	7.11789028333333	7.0633355	7.47951195833333	7.0369697	6.77836881666667	6.58160273333333	7.256627775	7.279554825	7.349518275	6.78782379166667	9.18595039166667	7.08170128333333	7.51935500833333	7.937534725	8.09066366666667	7.966758925	6.71047270833333	7.14092606666667	6.93552516666667	7.55250805833333	7.55318286666667	7.39105859166667	8.21353541666667	7.079224475	6.520636925	7.85258474166667	7.90584154166667	8.72229471666667	7.11986829166667	7.60415978333333	8.63825258333333

ZNF254	8.22641101666667	9.4277554	8.80780606666667	8.50502355	9.17230494166667	8.69220983333333	9.32939963333333	9.80310053333333	9.43278838333333	8.98618440833333	8.2874613	8.615263775	7.77045426666667	9.445595775	9.65184145	9.18167015	10.461782	9.4999428	9.79486831666667	9.90307260833333	9.04369295	9.13602675	8.9241622	9.04742353333333	9.19118228333333	8.76079081666667	8.850647275	8.63230339166667	8.62194039166667	9.01942851666667	8.51908186666667	8.96850814166667	8.958107775	9.40597950833333	8.72229471666667	8.78836945	8.90285985	9.27994568333333	9.11993345	8.95121126666667	9.64871909166667	9.56314586666667	9.16920365	9.23377270833333	8.9925209	9.57267214166667	9.09684721666667	8.9744767	9.48126221666667	9.515921025	9.04410515833333	8.953569225	6.771060625	8.26308525833333	8.27440479166667	8.4414262	9.859391125	9.88515190833333	9.5888718	9.23929971666667	6.40124803333333	7.81456171666667	7.7113432	7.27714694166667	10.43975535	9.19627585833333	8.0519544	8.89405555	8.42934195	7.587363525	7.44041060833333	8.23263584166667	7.001733525	8.77245620833333	8.52878136666667	8.174528375	8.23613304166667	8.37061515	7.67359318333333	8.03960018333333	8.82424165	9.083454025	6.79131116666667	8.10057743333333	8.09066366666667	8.14799835	6.79798854166667	7.6702147	7.78383044166667	9.08644098333333	6.5862738	6.38084359166667	8.9544846	9.14971231666667	7.87369893333333	7.27593380833333	8.47144243333333	8.52826969166667	7.03138465	8.75927773333333	8.9217463	7.18035421666667	8.87443335833333	9.17144896666667	9.02785883333333	8.19443521666667	8.651213975	6.8347138	8.97815918333333	8.1777524	9.20652433333333	6.445141375	7.42326895833333	7.99339445833333	6.507987025	6.89851890833333	9.14106573333333	7.379877425	9.24273069166667	7.25799328333333

BMP7	8.256452925	10.6492363416667	10.8086263583333	10.6581721666667	10.818385175	10.0712503	10.0214251333333	10.5775565833333	10.9000188916667	10.5987255666667	10.9497712833333	6.92575055833333	9.45194998333333	10.9147091916667	11.013128925	10.2826800583333	8.55750678333333	11.072670925	11.173905775	10.83116835	10.527258325	11.04340915	11.3093260166667	10.04638645	10.7061988666667	8.61092796666667	11.0701408916667	11.3842695916667	10.6738245166667	11.235931225	10.796845775	11.1615262583333	10.0728614166667	8.62596224166667	9.1396151	9.09600253333333	11.2373633083333	10.616218875	9.734421025	11.1506157166667	10.4519100666667	10.7585831916667	10.5185399	11.1811375833333	9.84285273333333	11.1150801666667	10.8589548	10.7448403666667	10.3277465	11.002443975	10.632187725	10.4850893583333	11.1110336416667	10.9537804833333	11.1960117	10.9028824333333	10.0537308333333	10.7793443666667	11.097913375	10.1720866	11.414772875	11.7343681166667	12.5325556416667	12.058910375	12.280521525	11.18844885	10.7108576583333	11.7105442333333	11.7182865833333	8.937187475	11.3594507166667	11.4008627833333	10.159727225	10.003538	12.1757943166667	11.0670126666667	11.2100932	11.2442138	11.03980565	13.5150003833333	12.6292285666667	12.6351542583333	10.402059075	11.0593297333333	10.5014025666667	11.118056575	12.237060875	7.38233871666667	11.612663925	10.6691322083333	10.66793845	10.4530959333333	11.9621161833333	10.5876388833333	10.8533803	11.460857775	12.6422229833333	11.3986748166667	10.987327675	11.5279761083333	10.1440795833333	11.3809445833333	11.1430808166667	11.71493805	7.70199845833333	12.2545127416667	10.5592695083333	11.364979325	11.2517728166667	10.616218875	11.3072160833333	11.9661980166667	10.29083355	12.0600286166667	11.464693675	10.8113265583333	12.8381552416667	10.81355685	12.1410311166667	8.894577375

ANO5	4.2231712	5.13336504166667	3.8730856	4.63513973333333	3.75838870833333	4.25005865	5.18423503333333	4.371797775	3.83595706666667	4.33328629166667	4.43531826666667	5.04966088333333	5.12411041666667	4.30437938333333	4.49113386666667	4.09698049166667	4.48613384166667	4.06593015	4.98545498333333	4.41962694166667	4.57343223333333	4.02799813333333	4.23291245833333	4.28809964166667	4.40903533333333	5.217220275	4.369907275	4.44161573333333	4.44453341666667	4.24335745833333	4.789978375	4.70163844166667	4.74548245833333	4.61520515	4.94355658333333	4.78798888333333	6.01047939166667	4.94355658333333	4.83457635833333	5.5697365	4.65382089166667	4.289092625	4.58672223333333	5.33234265	3.95573993333333	4.76661771666667	4.72105203333333	4.79411095833333	4.93761881666667	4.56806040833333	4.909691175	4.8815265	4.13313858333333	5.0953115	4.63305978333333	4.5496205	5.32442271666667	4.79716553333333	5.07558524166667	4.258212325	3.91700049166667	4.66114760833333	4.19434771666667	4.55971610833333	3.70714133333333	4.35186211666667	4.258212325	4.31516280833333	4.76141500833333	4.508464425	4.29605796666667	4.4047953	5.21412320833333	3.73259605	3.6378857	3.90689708333333	4.09074995833333	5.1555947	5.126264825	3.97264301666667	5.07558524166667	3.68113930833333	4.25100265833333	3.89227330833333	5.73091026666667	7.875176525	4.42666711666667	4.37909631666667	3.92586006666667	4.361998275	4.29017421666667	4.4227704	8.14660076666667	4.40284696666667	4.48717731666667	4.040897575	4.789978375	4.72105203333333	5.34862645833333	4.948951225	4.18298021666667	4.04402448333333	4.638255275	4.83457635833333	4.07015480833333	4.22036293333333	4.418789625	5.90321861666667	5.50523408333333	5.79742544166667	4.50222558333333	5.9323169	4.85974603333333	5.20354711666667	4.64613160833333	4.28008616666667	4.40382505833333	4.85659321666667	5.51803240833333	4.27798759166667

PVR	11.208942925	9.17457594166667	9.51811660833333	9.090969825	9.92792138333333	9.90177325833333	8.93568545833333	9.94294464166667	9.26627294166667	9.58797373333333	9.778305875	9.7237141	9.859391125	8.9544846	9.77133523333333	9.25440600833333	10.5400727416667	10.4755897583333	9.93596328333333	9.49101396666667	9.94976705	9.19200934166667	9.49325940833333	10.7793443666667	9.743497625	10.667166075	9.415734875	9.49147149166667	9.80664778333333	9.126505725	10.2955209666667	9.43868498333333	8.8992014	9.92334935	11.3635795583333	10.4268652916667	9.57307153333333	9.250740625	10.6035239083333	9.657103925	9.58344561666667	9.34670505	9.52642665	9.69792198333333	9.60319949166667	9.4181056	9.35549675833333	10.448395325	9.49953110833333	9.51633301666667	9.512679675	10.9153273416667	9.69837386666667	9.869019275	9.30630015833333	9.52745255833333	9.77613621666667	9.46919775	9.47404223333333	9.15064904166667	11.2132455833333	10.971110725	11.1652493166667	10.8143165583333	10.1001644	11.2743409666667	11.1343238833333	10.3674828833333	10.9256058083333	10.2978868083333	10.3366020166667	10.9006915833333	10.6079735416667	10.6492363416667	10.2794538083333	10.738724825	9.750737375	10.5344987916667	11.1546552166667	10.98769475	9.58344561666667	10.1050880666667	10.7553698833333	10.9794539833333	9.71307581666667	9.44151263333333	10.1953308333333	10.5530348	10.41347785	10.014766425	10.6730377	11.606939225	10.6519544333333	10.5061588333333	11.5847091416667	11.1975258	11.1415966	9.889693225	10.7593753416667	10.8124241666667	10.5392644333333	10.021871175	10.2245747416667	10.0476842666667	11.5555906416667	10.2591668583333	11.113611625	10.8382628916667	11.421792525	11.1176950333333	10.84359385	10.605453425	11.6198462166667	10.5376179083333	10.90030275	10.0343496666667	10.3475834	11.4086625666667	10.4300821583333	9.1094042

CLEC4D	5.9573564	5.91247053333333	6.60603940833333	5.99181078333333	6.329205025	6.850945825	6.559370075	6.71442883333333	6.552906925	7.15389984166667	6.54933985	6.68200588333333	6.31548078333333	6.60472939166667	5.81478479166667	6.68200588333333	6.25518099166667	6.39845556666667	6.30319241666667	6.04002979166667	6.165146375	5.7955502	7.05278270833333	7.56602129166667	5.89368925	7.27471595833333	6.88578850833333	6.49170240833333	6.18822558333333	5.87100729166667	6.88940976666667	6.70327563333333	6.138425225	6.85954253333333	8.894577375	6.77836881666667	6.49608473333333	6.61745720833333	8.09385128333333	7.40837556666667	7.83780880833333	6.53407053333333	7.60823079166667	5.19536235833333	6.44595796666667	6.93352621666667	6.911150775	7.20747220833333	6.653546475	6.90845738333333	8.505532025	6.84200789166667	6.390922	6.811853775	6.42264453333333	6.85814304166667	7.85906496666667	7.45341820833333	6.806433125	7.966758925	5.933890025	6.60603940833333	7.42048124166667	6.67919664166667	5.93746846666667	6.64956780833333	7.28803510833333	6.5607394	7.5010982	7.54177855833333	6.723717275	5.44122935	6.998615075	6.339217025	5.92679064166667	5.36818925	6.05045175	6.80918875	7.656003025	6.27095864166667	5.52437941666667	6.22149245833333	6.72953939166667	8.92792624166667	6.1777684	6.46851783333333	6.603384975	7.04142870833333	6.259187975	7.37467966666667	6.7130388	7.21504029166667	6.80056770833333	6.657721925	6.85418244166667	7.21098661666667	6.81637415	7.23371278333333	7.35226640833333	6.62496390833333	7.838427925	6.35504728333333	7.821740775	6.673017075	6.692469175	7.0609201	6.6716762	7.09102175833333	6.56365850833333	8.02070828333333	7.74722135833333	7.2015586	7.16971491666667	6.99601435833333	7.992867725	6.62496390833333	7.34551940833333	7.75983114166667	6.76313481666667	8.10057743333333

FOXC1	10.5053205416667	10.1316676583333	9.9162906	9.86598649166667	8.78691484166667	10.448395325	9.37728073333333	10.5041729666667	10.5609143	10.72134755	10.1605417333333	9.53997905833333	10.268273175	9.547789	8.72403179166667	10.372950875	8.4371155	10.1366762916667	10.0276764666667	10.0894400666667	9.620263	10.5500822583333	9.74175274166667	10.7866795833333	10.2089178583333	10.59381915	9.25167913333333	9.60629726666667	9.22794726666667	9.48031518333333	10.3945367666667	9.3017146	10.435306725	8.1308782	11.0747492583333	10.757286575	8.81836040833333	9.524079925	10.7140047416667	9.911212725	10.5228331416667	10.05157835	10.1725196416667	9.65802264166667	10.2149556666667	9.32285545833333	9.38453646666667	9.06697433333333	10.2355191666667	11.5567047416667	10.5806393916667	9.92625621666667	8.40280550833333	8.512323475	8.72787371666667	9.48384479166667	10.2321384166667	9.76006771666667	9.98111111666667	10.2206735166667	9.39983189166667	9.28834158333333	8.51908186666667	9.99086305833333	12.8702352416667	10.0670141416667	9.96611523333333	8.64839240833333	8.490982775	9.2612694	8.77245620833333	9.02418135	9.6433763	9.85548545	9.83719281666667	8.82211801666667	9.94086763333333	9.41761155833333	9.96400001666667	7.990761025	11.7549123666667	7.56325655	9.277313475	10.0156224166667	10.2613668833333	12.4019704583333	11.03596855	11.24605735	7.116803275	10.0565842333333	9.66558844166667	9.74266033333333	8.61290266666667	8.74819925	7.38291215	8.22397875	8.85865705	11.46655045	8.71235535	10.253110825	8.57507296666667	8.4800381	8.90285985	8.4816187	10.0872885916667	6.66522548333333	9.46432354166667	8.8451569	10.79796285	9.2408133	11.4971747583333	9.4277554	7.54228448333333	10.0052447166667	7.607704375	9.80231140833333	12.1312748	11.60515845	9.882085	9.59108196666667

WFDC9	4.90768301666667	5.05900706666667	4.81485295	5.64973066666667	5.18519845833333	5.80195006666667	6.27563768333333	5.792834125	5.125178525	5.41144461666667	5.24931593333333	5.72382095833333	5.51386359166667	4.88687726666667	4.86479268333333	4.32588960833333	6.258372575	5.13122064166667	5.162070825	5.0088772	4.8072374	4.11685985	4.509505	4.05417596666667	5.3343362	5.58597898333333	6.40185915	5.30264448333333	5.18423503333333	5.39281689166667	5.23781710833333	5.57252066666667	5.67327496666667	6.51915964166667	5.85609921666667	5.24289476666667	6.187374325	5.006452275	5.667420075	5.42470928333333	4.760314225	5.87944876666667	5.4600655	4.82464924166667	4.82796093333333	4.617309275	6.33187936666667	5.808344225	4.22787308333333	5.79119435	5.13634166666667	5.565954175	5.71416341666667	5.13847554166667	6.02209645	5.90065811666667	5.62140730833333	6.52912136666667	5.9294819	5.12313813333333	4.64133359166667	6.00968073333333	5.17905539166667	7.050862275	5.68590104166667	5.70253175	6.27245344166667	6.328333625	6.31242605	5.92509355833333	5.14089556666667	5.82824421666667	7.66500309166667	5.74130296666667	5.33648908333333	5.106111275	5.27417344166667	5.0100817	5.82366751666667	5.18423503333333	5.528242825	4.95322815833333	5.8893734	5.33329914166667	5.54491926666667	6.66054216666667	6.90845738333333	5.96077145833333	5.57060381666667	4.95206471666667	5.93821120833333	5.19860551666667	5.36615406666667	5.514879125	5.54303723333333	5.29885645833333	5.14499941666667	4.88811624166667	5.79999791666667	4.55466009166667	5.11568579166667	5.88673599166667	5.03010108333333	4.32198345833333	5.49526165833333	5.93821120833333	5.05900706666667	5.49229008333333	4.54769205	6.08940326666667	4.946749425	5.04230353333333	5.85514745833333	5.68207400833333	5.90235153333333	5.18519845833333	5.67810205833333	5.74803075	5.84048410833333	5.8893734

CPM	10.9602258291667	11.7014141	11.6045927958333	11.6192539916667	11.6961139083333	11.93026545	11.3803349666667	11.6026842791667	11.6097634833333	11.5206796166667	11.5674594583333	11.1889542083333	11.4138977416667	11.4024746666667	11.3992377541667	11.4852863083333	11.1795687666667	11.871553175	11.7500013791667	11.5467043166667	11.6737251125	11.7233523208333	11.8906866541667	11.254472975	11.4359380208333	10.7988361791667	11.4429405083333	11.3799692375	11.4694963083333	11.8672520541667	11.5580599041667	11.457250925	11.2354426333333	10.9452771166667	11.0418149833333	10.7052215916667	11.513440925	11.5715190958333	11.6657230125	11.2791978708333	11.3588911541667	11.3917230541667	11.281006975	11.4859582416667	11.0998077208333	11.1584270458333	11.205579725	11.3358945291667	12.1340157	11.7619022708333	11.5511322541667	11.5591762666667	10.9576682583333	10.9289703791667	10.58122785	10.7145581333333	11.5811100791667	11.4667271041667	11.4021128666667	11.4429405083333	11.3599674583333	11.2204006375	11.7766814125	11.2278140708333	11.5852804125	11.3633727666667	11.0220366083333	11.1892909791667	11.9576607916667	11.2806632375	11.5514691291667	11.4502097708333	11.0706939666667	11.2196669	11.5539872333333	10.9529550708333	12.0739248208333	11.540957775	11.3566180333333	11.682501525	10.876558875	11.5935969083333	11.3751334208333	11.1443172958333	11.1879353833333	10.743821075	11.7029008208333	10.9282480083333	11.0738714625	11.35045295	13.1632069791667	12.2295999833333	11.5529407458333	10.7957843083333	10.366163275	12.3234787791667	11.5148783708333	11.3909957458333	10.791785825	12.3791966	13.1442826666667	11.6373013375	11.4028483375	11.64314945	11.35045295	11.1111864166667	10.7984847875	11.4763556583333	11.9287353375	11.4487495208333	12.3218060416667	11.7715220416667	10.6009811166667	10.3037668333333	10.5079540791667	11.1805434041667	11.6464488666667	11.4870554708333	11.8214699833333	11.3251647291667

MDM2	10.9602258291667	11.7014141	11.6045927958333	11.6192539916667	11.6961139083333	11.93026545	11.3803349666667	11.6026842791667	11.6097634833333	11.5206796166667	11.5674594583333	11.1889542083333	11.4138977416667	11.4024746666667	11.3992377541667	11.4852863083333	11.1795687666667	11.871553175	11.7500013791667	11.5467043166667	11.6737251125	11.7233523208333	11.8906866541667	11.254472975	11.4359380208333	10.7988361791667	11.4429405083333	11.3799692375	11.4694963083333	11.8672520541667	11.5580599041667	11.457250925	11.2354426333333	10.9452771166667	11.0418149833333	10.7052215916667	11.513440925	11.5715190958333	11.6657230125	11.2791978708333	11.3588911541667	11.3917230541667	11.281006975	11.4859582416667	11.0998077208333	11.1584270458333	11.205579725	11.3358945291667	12.1340157	11.7619022708333	11.5511322541667	11.5591762666667	10.9576682583333	10.9289703791667	10.58122785	10.7145581333333	11.5811100791667	11.4667271041667	11.4021128666667	11.4429405083333	11.3599674583333	11.2204006375	11.7766814125	11.2278140708333	11.5852804125	11.3633727666667	11.0220366083333	11.1892909791667	11.9576607916667	11.2806632375	11.5514691291667	11.4502097708333	11.0706939666667	11.2196669	11.5539872333333	10.9529550708333	12.0739248208333	11.540957775	11.3566180333333	11.682501525	10.876558875	11.5935969083333	11.3751334208333	11.1443172958333	11.1879353833333	10.743821075	11.7029008208333	10.9282480083333	11.0738714625	11.35045295	13.1632069791667	12.2295999833333	11.5529407458333	10.7957843083333	10.366163275	12.3234787791667	11.5148783708333	11.3909957458333	10.791785825	12.3791966	13.1442826666667	11.6373013375	11.4028483375	11.64314945	11.35045295	11.1111864166667	10.7984847875	11.4763556583333	11.9287353375	11.4487495208333	12.3218060416667	11.7715220416667	10.6009811166667	10.3037668333333	10.5079540791667	11.1805434041667	11.6464488666667	11.4870554708333	11.8214699833333	11.3251647291667

AC010422.8	11.4551394916667	11.1672319583333	11.2574172166667	11.3840803333333	11.4341960125	11.7584244291667	11.2016925041667	11.1145695125	11.4822891291667	11.171236275	11.59500295	11.1435641166667	11.1969501583333	11.202360925	11.2570280791667	11.1954628041667	11.7393491791667	11.4565646208333	11.4286436416667	10.9985310875	11.2738602708333	11.2368627916667	11.1443172958333	11.4106322708333	11.254472975	11.3450186875	11.1446797416667	11.1639329916667	11.28820595	11.3483044583333	11.3726601208333	11.0418149833333	11.2400877333333	11.3655952	11.360680725	11.5225116041667	11.1632073208333	11.1428788833333	11.1628306125	10.9814297625	11.2684137125	11.3751334208333	11.2087548083333	11.2400877333333	11.1903492625	11.2967234958333	11.2433328375	11.3585121208333	11.1785131916667	11.1090353708333	11.1613203083333	11.3006586041667	11.4073844333333	11.4551394916667	11.3428663291667	11.3200835125	11.0745793375	11.2741842833333	11.1388994541667	10.9963749083333	11.5293292166667	11.4190066583333	11.4021128666667	11.5172423041667	11.5244008708333	11.7036939708333	11.5148783708333	10.9927317625	11.5123202041667	12.103242775	11.559993425	11.335569425	11.3877233291667	11.0846961875	10.7256354291667	10.8342676041667	11.1439293333333	11.59782665	11.6041942166667	11.3711247625	11.3758432875	11.1303974833333	11.2386234291667	11.3548320125	11.3077018833333	10.583545725	11.1918332083333	11.018805675	11.5109494708333	11.378052025	11.0668487041667	11.192886075	11.8377358625	11.0583490583333	10.7830060541667	11.1457823208333	11.5010464625	10.8520279	11.150800425	11.3902638958333	11.4098535291667	11.09447755	11.1001768875	11.3106458291667	11.7676929416667	11.2278140708333	11.4138977416667	12.077992225	11.3465522083333	11.2200484	10.91255735	11.588212975	11.2519425625	11.9042165083333	11.7267909	12.2729598041667	11.2709514541667	12.0411497	12.0082875708333	11.202360925

DHPS	11.4551394916667	11.1672319583333	11.2574172166667	11.3840803333333	11.4341960125	11.7584244291667	11.2016925041667	11.1145695125	11.4822891291667	11.171236275	11.59500295	11.1435641166667	11.1969501583333	11.202360925	11.2570280791667	11.1954628041667	11.7393491791667	11.4565646208333	11.4286436416667	10.9985310875	11.2738602708333	11.2368627916667	11.1443172958333	11.4106322708333	11.254472975	11.3450186875	11.1446797416667	11.1639329916667	11.28820595	11.3483044583333	11.3726601208333	11.0418149833333	11.2400877333333	11.3655952	11.360680725	11.5225116041667	11.1632073208333	11.1428788833333	11.1628306125	10.9814297625	11.2684137125	11.3751334208333	11.2087548083333	11.2400877333333	11.1903492625	11.2967234958333	11.2433328375	11.3585121208333	11.1785131916667	11.1090353708333	11.1613203083333	11.3006586041667	11.4073844333333	11.4551394916667	11.3428663291667	11.3200835125	11.0745793375	11.2741842833333	11.1388994541667	10.9963749083333	11.5293292166667	11.4190066583333	11.4021128666667	11.5172423041667	11.5244008708333	11.7036939708333	11.5148783708333	10.9927317625	11.5123202041667	12.103242775	11.559993425	11.335569425	11.3877233291667	11.0846961875	10.7256354291667	10.8342676041667	11.1439293333333	11.59782665	11.6041942166667	11.3711247625	11.3758432875	11.1303974833333	11.2386234291667	11.3548320125	11.3077018833333	10.583545725	11.1918332083333	11.018805675	11.5109494708333	11.378052025	11.0668487041667	11.192886075	11.8377358625	11.0583490583333	10.7830060541667	11.1457823208333	11.5010464625	10.8520279	11.150800425	11.3902638958333	11.4098535291667	11.09447755	11.1001768875	11.3106458291667	11.7676929416667	11.2278140708333	11.4138977416667	12.077992225	11.3465522083333	11.2200484	10.91255735	11.588212975	11.2519425625	11.9042165083333	11.7267909	12.2729598041667	11.2709514541667	12.0411497	12.0082875708333	11.202360925

FDFT1	9.536457975	9.08729039166667	9.482987475	9.7752348	9.65802264166667	8.97625954166667	9.72111820833333	9.219699425	9.4341767	9.04841031666667	9.41935561666667	9.88594978333333	10.0391614083333	8.97076639166667	9.608728125	9.18361099166667	8.58958554166667	10.5655352666667	8.87390605	9.00867418333333	9.38096979166667	9.84919238333333	8.99487275833333	9.382290975	9.27312065	8.96455003333333	9.47351875833333	9.7241175	9.83237755833333	9.25253679166667	9.35031244166667	9.21080935833333	9.22061473333333	9.08396963333333	8.84155408333333	9.2639574	10.1054993333333	8.82022893333333	9.40207443333333	9.230932875	8.60947813333333	9.04596135	8.92929650833333	8.7160017	8.74130064166667	8.40516035	8.34181428333333	9.01297525833333	8.95312890833333	9.00643414166667	9.39664281666667	9.07295375833333	9.8795664	9.36761098333333	9.275522425	8.87238755	8.85635194166667	8.38470655	9.4056037	8.73498088333333	10.9303115416667	9.859391125	10.0043835333333	10.3317121166667	8.8334785	10.1274668666667	9.63806365	9.85368629166667	9.27812115	9.57007478333333	10.002252325	9.82651539166667	10.0061679666667	9.645264775	10.1485742083333	9.431383225	9.89637478333333	9.71098871666667	9.29933239166667	9.39664281666667	10.1716698166667	9.90438676666667	9.6428903	9.611823	9.94748681666667	9.76432159166667	11.0440902666667	9.784367075	10.406522025	9.44293281666667	9.73955326666667	9.92664269166667	9.69707035833333	9.25028671666667	10.0159752583333	10.0881734833333	10.3875979666667	9.9993556	10.027285575	9.55161105	9.39889275833333	9.82029285833333	9.69792198333333	9.16874195833333	9.633880775	9.65802264166667	9.67592103333333	8.92792624166667	9.17646485833333	8.757884525	9.15505945	9.20235228333333	9.848037125	8.65271366666667	9.56961935	9.47987389166667	9.28929068333333	9.67944865833333	9.62586958333333	9.33883618333333

TRIB3	8.51764245	11.426992675	9.96481554166667	10.7538304083333	11.7561182166667	9.81608086666667	11.4574143583333	11.3292622166667	11.5618505	10.079544525	10.628160175	7.69651605833333	10.3948990166667	10.6453411416667	11.1284533666667	9.25303868333333	9.55333915	10.8796402833333	10.7008852833333	10.2123952583333	10.3975849666667	10.836451025	10.2286007833333	10.248995	11.1154972666667	7.927602375	10.8006177916667	10.7873883666667	11.6687463083333	10.9332106333333	11.0017295	10.5204657833333	10.7904047166667	9.37269289166667	8.46508935	8.20433166666667	11.6841362916667	11.3224431	10.9654875083333	10.3806930666667	11.5363521916667	10.8767468833333	10.6621925666667	10.3583509666667	9.74128455	10.2621725583333	10.1581087416667	10.8247142	10.694500425	10.5344987916667	10.9790999666667	10.8870209166667	11.6456087833333	11.520872575	11.5200695166667	10.8828386666667	11.390823425	11.3212969833333	10.96150755	10.3281467166667	8.40424725	9.24401314166667	8.11250586666667	11.55593635	8.59397561666667	10.237546325	9.73533868333333	8.22207980833333	8.75371965	8.63449166666667	9.03089319166667	8.475275825	9.63018528333333	8.27495268333333	9.953234775	8.119922525	9.093743775	9.32574429166667	9.64783695	8.82372205833333	9.01611445833333	9.0354605	8.871342075	8.783965475	9.12557628333333	8.15812938333333	9.47897484166667	8.355767175	8.46350844166667	8.65271366666667	9.17230494166667	8.58808473333333	9.37177325833333	8.81744095	9.54880464166667	8.3876511	9.567278375	9.14003079166667	9.45422565833333	9.094155	8.56222751666667	8.02185345833333	8.51669369166667	9.12557628333333	9.20872513333333	8.634032725	9.05267498333333	10.363007875	8.85865705	9.82907005833333	9.51951014166667	9.34847368333333	10.4910656666667	9.36247203333333	9.59235401666667	9.4337598	8.90052724166667	9.498614875	9.50643220833333	7.95464479166667

ERC1	12.1608207083333	11.6910443916667	11.6989876416667	11.33887195	11.7452699833333	11.1579092583333	11.6613946166667	11.6787724916667	11.6780189666667	11.85384455	11.27642465	12.008092475	11.6687463083333	11.784206175	11.7735354916667	11.392737725	10.831890425	11.36200305	12.0088601416667	11.7601425083333	11.8565277666667	11.6010090666667	11.5512973083333	11.36947175	11.4129941416667	12.187427525	11.5578653583333	11.2517728166667	11.3438040833333	11.684519	11.5610238416667	11.3231776	11.3320946416667	10.74441525	12.2835716083333	12.2374670666667	11.4420691166667	11.6227232166667	11.3212969833333	11.659892375	11.8070215583333	11.8834269416667	11.74329675	12.10430865	11.8916056833333	11.89894165	11.8457882583333	11.83328595	12.0214326083333	11.7951768416667	11.6115347666667	11.9720790416667	11.1866301583333	11.3831501	11.259162425	11.5200695166667	12.217702275	11.88924055	11.7564937416667	11.9259261416667	11.291502775	10.4983213833333	10.7756109166667	11.5731480083333	11.3506069833333	11.9267180916667	11.867753575	12.0628110916667	12.4619169416667	11.4162651666667	11.37169735	11.7012064	11.7384146416667	11.2715325333333	12.0181445166667	11.037692575	11.155024025	11.0006123833333	12.1823287916667	11.48359205	10.7383339416667	12.3126780333333	11.6898911833333	11.2801435833333	11.3567956166667	11.1832845666667	10.717740675	11.21653435	10.6507559	11.6170945166667	11.79216385	11.270077	11.3375225333333	12.0040290833333	10.9835406583333	11.3587026083333	11.1561366	11.5523735416667	11.1779127416667	11.3426956833333	12.533964725	11.5606001833333	12.0156772833333	11.89894165	11.6799309666667	11.8557655416667	11.888073425	12.3244889333333	12.374133125	12.2429038916667	11.2125996833333	12.244497975	11.7887391333333	11.9357187583333	11.5570626583333	10.8865989333333	11.5998809	11.9720790416667	11.20219855	11.5816748666667

TSPYL4	12.222327475	11.291502775	11.9155103583333	11.1339689083333	10.946906725	11.3033163083333	11.25535535	11.633311275	11.6470358	11.5305817083333	11.1291467583333	11.6887018916667	11.3735584916667	11.1969501583333	11.673519475	11.1256433416667	12.0290333416667	11.1380175916667	11.8959032583333	11.4277232	10.9080821583333	11.5509672	11.1249258333333	11.4262590333333	11.1847643166667	12.209029825	11.633311275	11.588039275	11.1724975333333	11.234518675	11.7713486916667	11.4022935333333	11.3410012666667	11.102867725	11.7343681166667	12.1150337666667	10.8839829916667	11.4228997	10.9665691416667	11.3579174083333	11.6238039583333	12.0140512666667	12.0852755916667	10.527258325	11.5987696666667	11.6336776833333	11.7605260333333	11.82248265	11.1445056833333	11.2811678666667	11.6144877166667	11.1685570666667	11.4627181416667	11.597258825	11.9997560833333	12.3310232666667	11.4839876416667	11.579793675	11.84426255	11.61832875	10.3570715916667	10.6183847583333	10.4720948333333	11.1227594166667	12.1338172083333	9.2741013	10.5814300416667	10.6773409416667	10.8544918	10.1621245583333	10.55775005	10.6905955916667	10.0189542666667	11.13057255	10.406522025	11.052426775	10.4072694666667	10.3325548083333	10.2422793916667	11.09094715	12.786278725	11.1934390416667	9.99593061666667	11.1325239666667	10.74441525	11.3375225333333	11.3782358083333	11.06152365	10.435306725	11.0510672583333	10.5768008666667	9.47542340833333	10.4994687166667	10.6139707	10.21946315	9.37548823333333	9.67813213333333	10.6838734833333	11.6993325583333	11.2775845	11.6278737583333	10.7722019	11.0873559916667	10.114399525	9.49101396666667	11.0913458083333	11.2884114666667	11.407195875	11.4581724666667	10.8506520083333	11.4317096083333	10.3507137166667	11.2848715833333	10.5973351833333	10.2846004916667	10.8698368333333	11.4221599083333	10.3412407666667	10.2089178583333	10.605453425

FOXD3	10.76764485	9.90688865833333	9.92412455833333	9.89466155833333	10.6072418333333	10.1862509833333	11.6982071583333	10.116767025	10.4638383	10.3750050916667	10.6027208583333	11.8973789916667	11.3542758333333	10.015183425	9.619021525	9.55104401666667	9.95359933333333	10.7421748083333	9.227470725	9.57650310833333	10.0765568416667	10.05827805	9.596033825	9.60581258333333	9.33496756666667	10.6079735416667	9.72156395833333	9.50539336666667	9.47819235833333	9.76006771666667	9.46252099166667	9.0720726	10.1046909416667	10.0476842666667	10.2014148333333	10.590660625	9.74904354166667	9.47068484166667	9.53043561666667	9.61680303333333	8.80499509166667	9.645264775	8.81108796666667	8.85160831666667	9.76647539166667	8.81463643333333	8.987001225	9.21590233333333	9.124627175	9.38622159166667	9.32329896666667	9.4842707	9.54132330833333	8.74522520833333	9.16396135833333	8.75047631666667	9.25028671666667	8.86366274166667	9.214506275	9.22061473333333	10.6948786416667	11.0146426833333	10.676538	10.4716937833333	10.8889520833333	10.9750833833333	10.4215953	11.027599275	10.601543225	11.002804875	10.834075425	11.229748375	11.6398002583333	10.916432925	10.8710123583333	10.298281975	11.5706660333333	10.2481894083333	10.0125394166667	10.6857422916667	10.6496091416667	10.6169276916667	10.4057146583333	10.4971305166667	10.3257291333333	11.3968955416667	10.8139547	10.9375392333333	11.0968033583333	10.2092790583333	10.6600165833333	11.164489475	9.84285273333333	9.68863114166667	10.723966225	10.5605583916667	11.5983924583333	11.013128925	10.8032590416667	10.8154489833333	9.71864728333333	10.96365625	9.68695163333333	9.4999428	9.660226725	9.15991209166667	9.87345979166667	9.37313166666667	9.92254819166667	10.3388359916667	9.8972867	10.160939175	10.03562315	9.277313475	9.24799294166667	9.35167163333333	10.8161170333333	10.2431199666667	9.76310825	10.04725285

USP3	7.9184414	6.64226356666667	7.33501970833333	7.27171180833333	6.96848423333333	7.363992875	7.56775538333333	7.14458345	7.25923676666667	7.3477901	7.238340075	7.01837270833333	7.07182334166667	6.29443513333333	6.75503975833333	6.79994456666667	7.544616	6.64415971666667	7.11855360833333	6.49689175833333	6.66383380833333	7.366539375	6.97714725	7.40898444166667	6.69439601666667	6.93803065	6.37349159166667	6.64156519166667	7.306684875	6.48497233333333	7.23785493333333	6.87783053333333	6.82102155833333	7.41882361666667	7.11920136666667	7.2015586	7.36211884166667	6.758383225	7.48416809166667	7.009464425	7.10822425	6.9393833	6.81976344166667	6.85216409166667	6.60472939166667	6.54574596666667	6.51525555	6.85156043333333	6.83341489166667	7.404937825	7.07060166666667	7.009464425	5.75682274166667	6.70056215833333	6.75170075833333	6.633753725	6.38476215833333	6.8478471	6.66738975833333	6.81316333333333	7.42048124166667	7.46514316666667	7.51089621666667	7.37059144166667	6.99800655	7.29304450833333	7.83125643333333	7.49050329166667	7.66718561666667	7.06220724166667	7.11920136666667	7.48927714166667	7.31861950833333	6.864696	7.5010982	7.57903273333333	7.18584309166667	7.00566125833333	7.334392275	7.24945885833333	6.55145231666667	6.99033689166667	7.52896074166667	7.388741125	7.25001195833333	6.73133699166667	6.96657695833333	6.76517564166667	7.101860325	6.80784224166667	7.694854575	7.67769499166667	7.31803023333333	6.99412739166667	7.50167894166667	6.97159590833333	7.50627369166667	7.11406076666667	7.77775845	7.13351973333333	7.35862363333333	7.27471595833333	7.70926514166667	7.47496615833333	7.20911576666667	6.97095796666667	7.04267660833333	6.52781796666667	7.578462725	6.66522548333333	6.83400405	7.36108145833333	6.85814304166667	6.61809366666667	7.08427549166667	6.64487866666667	6.93352621666667	7.38818769166667	7.22964071666667	6.87589898333333

FAM155B	6.877100425	6.52272641666667	6.50580336666667	6.66738975833333	7.71354716666667	7.14822965	9.05454125	7.54228448333333	6.73419851666667	7.45451809166667	8.28000224166667	8.60998564166667	5.95243336666667	5.85993758333333	5.77807068333333	5.99349075	7.71296821666667	6.83799996666667	6.24443749166667	6.25099584166667	5.39492906666667	6.14959958333333	6.03747638333333	6.51310794166667	6.445141375	7.15820169166667	6.76049560833333	6.005441825	7.2960179	6.5994761	6.18332735833333	6.97159590833333	7.334392275	8.234157525	6.88718085	7.41466188333333	7.03194930833333	7.18452301666667	6.59815521666667	6.27802068333333	6.735668325	6.01047939166667	7.72520474166667	6.76517564166667	6.70200101666667	6.77905188333333	6.81637415	7.08427549166667	6.49170240833333	5.96423755	6.09272539166667	6.79131116666667	7.38818769166667	7.67599419166667	7.23371278333333	5.878607175	7.62358238333333	7.56655451666667	6.23229269166667	6.65424311666667	8.1223269	7.49500395	7.08235699166667	6.67572515	7.6798049	6.57242504166667	6.97340769166667	7.95575579166667	7.81344110833333	7.9484088	8.83249931666667	8.12989700833333	6.939986925	7.07238718333333	6.58035519166667	6.49464226666667	6.35504728333333	6.22368095	8.751836375	7.52225746666667	5.84236155833333	6.81852565833333	7.08614628333333	9.166824	7.06709683333333	7.24193815	7.147600275	6.295927975	8.46508935	7.224148725	6.9125939	9.06069035833333	6.14881959166667	7.50051165833333	7.32668075833333	8.27440479166667	8.97489020833333	7.16295023333333	7.92279315	7.02148474166667	8.27121185	7.24945885833333	8.52583200833333	7.505130225	8.00581294166667	7.43634795	7.77602128333333	9.006017175	6.87022720833333	6.2517663	7.22598754166667	7.33912221666667	6.42634760833333	8.140254425	8.42676914166667	6.5305636	7.17198438333333	7.73690679166667	6.79658473333333	8.31670425833333

PP2D1	5.2151974	4.28110195833333	4.30437938333333	3.85170159166667	4.6247727	5.39492906666667	4.60180214166667	7.49771558333333	4.06389798333333	4.15309530833333	4.4159867	4.44059075	4.202033325	4.55069105	6.59386139166667	4.3249589	4.262047775	4.72324775	4.29017421666667	4.33328629166667	4.35819395	4.73024025833333	4.85861940833333	4.36901661666667	5.22716268333333	4.41695324166667	4.535343	5.477009275	5.27315905833333	4.49797680833333	4.832334525	4.63625878333333	4.53219865	4.87595029166667	4.93761881666667	5.14192449166667	5.24102825833333	5.07000859166667	4.17980874166667	4.36495859166667	4.58982808333333	4.49312724166667	4.46877066666667	4.91442044166667	4.61625569166667	5.63053526666667	4.47861730833333	4.40083348333333	4.64613160833333	4.93552183333333	4.43639113333333	4.16611878333333	4.9406758	5.51683608333333	4.9195424	4.98545498333333	4.76967493333333	5.126264825	5.09317755833333	5.16420199166667	4.83355489166667	3.82070373333333	4.289092625	4.20109206666667	5.482101875	5.23035095	4.14274019166667	4.10621731666667	4.075702625	3.909240625	4.222209325	3.80781481666667	3.780421775	4.21912249166667	5.41802705	4.12334434166667	4.13943304166667	4.78047113333333	4.02040568333333	4.172665625	4.22678013333333	4.08637315833333	4.37489024166667	4.52791141666667	4.128952175	3.85292815833333	4.472817375	4.07245948333333	5.14089556666667	4.29916468333333	4.32588960833333	3.98271365	4.56909456666667	4.4227704	4.6622505	4.31516280833333	3.8371121	5.41887256666667	4.19529993333333	4.508464425	4.49904255833333	3.82560103333333	4.30527630833333	4.42980535	4.08114406666667	4.20011505833333	4.16737369166667	5.05687025	4.69863704166667	5.67425256666667	4.2918764	4.57137348333333	4.609167375	5.48300426666667	4.69963341666667	4.115796825	4.5496205	4.92360515	4.56186439166667	4.30130591666667

AGAP2	9.88763298333333	9.5040294	9.65101713333333	9.21923738333333	9.70194608333333	9.89341510833333	10.222173225	10.5953164	9.61946140833333	9.59906339166667	9.43045813333333	9.555182225	9.82907005833333	9.69126795833333	9.45472053333333	9.83071770833333	9.673127475	9.954843075	9.62890875	9.73270225833333	9.557193025	9.49413000833333	9.60319949166667	9.84242584166667	9.75492564166667	9.97255511666667	9.34157415833333	9.33087654166667	9.27125073333333	9.51175975	9.62759855	10.2919749833333	9.895935425	10.6387912166667	10.1637531083333	10.023053775	9.72531675833333	9.88845614166667	9.67813213333333	9.10145314166667	9.69619738333333	9.73489294166667	9.97752425	9.70714295	9.661950625	10.120107375	9.61047175833333	9.60406910833333	9.97131721666667	9.54092946666667	9.78236806666667	9.25349141666667	9.8541064	9.65184145	10.0018247916667	9.72531675833333	9.94164523333333	9.86062085833333	9.69176305833333	9.92375435	9.59235401666667	9.61413186666667	9.51896663333333	8.98889158333333	9.52456219166667	10.3147212916667	9.82860243333333	10.4914439416667	9.68825589166667	9.96745814166667	9.45010154166667	9.22606134166667	9.66513115	9.87484248333333	9.43729386666667	9.31629211666667	9.56452501666667	9.911212725	9.695341625	9.77571775833333	9.33964531666667	9.46608615833333	9.81899704166667	9.80399703333333	10.0783158833333	9.88594978333333	9.40906840833333	10.7088791083333	9.00822095	9.54225544166667	9.56080151666667	10.8101684583333	9.54446325	9.98032226666667	9.889693225	9.78872821666667	9.70114725833333	9.64153864166667	9.80187509166667	9.650075475	9.79235871666667	9.86431620833333	10.2462958083333	9.82339220833333	9.50725341666667	9.95852463333333	9.51951014166667	10.7352154666667	10.1105163666667	10.86350625	9.598664275	9.93750004166667	9.83111589166667	9.687830775	10.528057425	9.80274700833333	9.78967574166667	9.79362293333333	9.824729	10.20559775

SLC25A35	12.4299630791667	10.8535580666667	11.0436000125	10.521068475	10.6639434958333	11.1219183125	10.7191643541667	10.6970213958333	10.6137708125	11.1995725583333	10.58122785	11.7708505333333	11.3138672875	10.6829236833333	10.6009811166667	11.0653444458333	11.8218600041667	10.2656372791667	10.9981540791667	10.7129235458333	11.0346415125	10.1411293	10.943199625	10.7010872583333	10.8366353541667	11.9999712625	10.8279392916667	10.6209464958333	10.4856631416667	10.7727115375	10.7988361791667	10.8769287916667	10.79813175	11.9250271291667	12.2034910208333	12.3282505291667	10.6658119333333	10.7966575041667	10.63348715	10.6070548708333	10.9460665375	10.9882577583333	10.926151175	11.2295922333333	11.6560407166667	11.4223504875	11.157751025	11.0926102958333	11.0551007666667	10.8772896791667	11.1182603	10.6085738375	10.904495125	10.5594606458333	10.8610501208333	11.3722703875	10.75957225	11.1639329916667	10.9362758541667	11.3707466375	12.4882160708333	11.3558470416667	11.8263604166667	10.876558875	12.2022410791667	11.43744855	11.2034095375	11.3833534416667	12.0634142	11.3733746583333	11.3027593833333	11.1886201666667	11.9481228458333	11.9680213958333	10.8019570125	11.0861265	11.7083756875	11.5358221291667	11.6586288375	11.1988506416667	11.6681825333333	10.66090735	12.0438522916667	11.48889185	10.6897055666667	11.4160608875	11.955660275	11.2637059125	11.6402970458333	12.0810500166667	11.3181995833333	11.6805393291667	10.3585509	11.3877233291667	10.8482179125	10.8822804666667	10.2393435	11.5267024166667	11.3592652083333	10.5901158458333	11.655318975	11.8675931416667	12.2589235458333	11.0019008875	11.9069061166667	11.21377075	11.8214699833333	12.2992347375	11.174091825	11.2030606	11.2773858916667	10.8019570125	11.396711525	10.9623702291667	11.6015001833333	11.1254633708333	11.5321694875	12.8670344166667	11.9830365916667	10.8805666833333

RANGRF	12.4299630791667	10.8535580666667	11.0436000125	10.521068475	10.6639434958333	11.1219183125	10.7191643541667	10.6970213958333	10.6137708125	11.1995725583333	10.58122785	11.7708505333333	11.3138672875	10.6829236833333	10.6009811166667	11.0653444458333	11.8218600041667	10.2656372791667	10.9981540791667	10.7129235458333	11.0346415125	10.1411293	10.943199625	10.7010872583333	10.8366353541667	11.9999712625	10.8279392916667	10.6209464958333	10.4856631416667	10.7727115375	10.7988361791667	10.8769287916667	10.79813175	11.9250271291667	12.2034910208333	12.3282505291667	10.6658119333333	10.7966575041667	10.63348715	10.6070548708333	10.9460665375	10.9882577583333	10.926151175	11.2295922333333	11.6560407166667	11.4223504875	11.157751025	11.0926102958333	11.0551007666667	10.8772896791667	11.1182603	10.6085738375	10.904495125	10.5594606458333	10.8610501208333	11.3722703875	10.75957225	11.1639329916667	10.9362758541667	11.3707466375	12.4882160708333	11.3558470416667	11.8263604166667	10.876558875	12.2022410791667	11.43744855	11.2034095375	11.3833534416667	12.0634142	11.3733746583333	11.3027593833333	11.1886201666667	11.9481228458333	11.9680213958333	10.8019570125	11.0861265	11.7083756875	11.5358221291667	11.6586288375	11.1988506416667	11.6681825333333	10.66090735	12.0438522916667	11.48889185	10.6897055666667	11.4160608875	11.955660275	11.2637059125	11.6402970458333	12.0810500166667	11.3181995833333	11.6805393291667	10.3585509	11.3877233291667	10.8482179125	10.8822804666667	10.2393435	11.5267024166667	11.3592652083333	10.5901158458333	11.655318975	11.8675931416667	12.2589235458333	11.0019008875	11.9069061166667	11.21377075	11.8214699833333	12.2992347375	11.174091825	11.2030606	11.2773858916667	10.8019570125	11.396711525	10.9623702291667	11.6015001833333	11.1254633708333	11.5321694875	12.8670344166667	11.9830365916667	10.8805666833333

MBNL1	9.90307260833333	8.552420375	8.31911250833333	7.97719954166667	9.72703330833333	9.99593061666667	7.7794798	8.85962865	7.96474510833333	9.44959046666667	8.53385956666667	9.60140389166667	9.35995134166667	8.42776885	9.4687509	9.30682376666667	8.92973970833333	10.6289554166667	9.2639574	9.13780860833333	8.83883193333333	9.73098034166667	8.81836040833333	9.26035810833333	9.696676675	9.855976425	9.34302553333333	9.81818709166667	10.30399485	8.91275618333333	9.17691023333333	8.91457238333333	8.74766549166667	8.987001225	10.3007165083333	9.36480266666667	9.77352068333333	8.90619576666667	8.931271375	9.261730375	8.0917251	8.18100231666667	8.2988733	8.85728129166667	8.580396475	8.43758005833333	8.02564620833333	8.15303908333333	8.84243295833333	9.06886545833333	8.062405875	8.54218048333333	9.57692496666667	10.1059188	8.31150115833333	7.77718458333333	8.94715431666667	8.25794470833333	9.51223504166667	8.90619576666667	11.44422215	8.35071744166667	9.24218355833333	9.307742	10.0018247916667	10.81468445	8.486570275	9.41298314166667	9.19996488333333	9.93000265833333	9.28174641666667	9.15505945	8.99899866666667	9.176064475	10.1337610833333	9.06791326666667	10.1117927583333	10.7470371333333	9.60830463333333	8.94623185833333	9.99767481666667	10.1931783583333	9.05644770833333	8.8912541	9.984272	9.70017130833333	10.133368525	9.10015925	10.6834952083333	9.63342665833333	9.26261210833333	10.0720278166667	9.304559675	9.47208941666667	10.18161685	9.59782546666667	9.37637135833333	9.64690625833333	9.27643005833333	9.16302515	9.24586054166667	8.4076656	8.90389271666667	8.67888266666667	9.062206575	9.03327476666667	8.81744095	7.9238629	8.57560394166667	9.12042280833333	8.748691075	8.26867154166667	9.47496915833333	8.26207273333333	9.93630081666667	9.09642976666667	8.27654828333333	8.93953213333333	9.026583775	9.138674475

NOX1	7.53018445	9.46790315833333	8.70448370833333	9.37177325833333	7.27114775833333	7.78057345	6.87159964166667	8.15758866666667	7.886143875	8.49291188333333	8.325164675	6.78381769166667	6.80056770833333	9.11824448333333	8.62886449166667	8.25839510833333	8.11306225833333	7.61391835	9.03818763333333	8.96455003333333	9.41389706666667	9.9338367	9.26082591666667	8.67210133333333	8.854456025	8.9217463	8.92511409166667	9.05075425833333	7.94329386666667	9.54834169166667	9.00867418333333	9.20778985833333	8.5232739	7.73920148333333	8.44794389166667	8.28949861666667	8.4304275	9.04742353333333	7.91097105833333	8.081342075	9.16261181666667	8.631828525	9.1447536	9.48834466666667	8.61342080833333	9.09237333333333	9.212117675	9.56961935	9.33181315833333	8.69409343333333	8.86421215	8.57560394166667	8.759783975	9.1530551	9.08221395833333	9.17230494166667	9.07683584166667	9.38807046666667	8.99204639166667	9.0354605	8.38662095833333	9.80143100833333	8.88930546666667	7.69605179166667	7.190008575	7.442585225	7.45043895	8.28541158333333	7.97971059166667	8.615263775	7.746177925	8.80139795	7.46964506666667	8.02185345833333	7.833966975	8.24415698333333	8.23567169166667	7.48416809166667	8.93762295	8.21881454166667	8.53187930833333	7.52673725833333	8.53140681666667	8.38217830833333	8.2874613	7.78326185	9.28599946666667	7.28198013333333	8.48745835	8.7143253	10.2266691833333	8.79489933333333	8.3876511	8.57063375	8.03376271666667	9.14971231666667	7.66209683333333	9.028831875	8.95490616666667	9.36084673333333	8.28581018333333	8.18422995833333	8.93412065833333	10.2009594166667	8.27654828333333	7.63269815833333	7.58465230833333	9.44826335	7.95035304166667	8.14240605833333	9.53312831666667	9.40640285	7.72249438333333	9.24318624166667	7.596603625	8.48460361666667	8.4414262	9.31047634166667	9.51408459166667	8.53005785

KSR2	5.67327496666667	6.75097239166667	6.77777710833333	6.71186961666667	7.46243825	5.33746485833333	7.78718563333333	7.14092606666667	7.59199554166667	6.3877628	6.95758655	7.39042478333333	7.84267175833333	6.62158961666667	6.75170075833333	6.5994761	7.160095	6.96657695833333	6.34225285833333	6.541621825	7.46243825	7.3477901	6.54514473333333	6.2073137	6.18822558333333	6.61558093333333	5.8174436	6.081909575	6.26738683333333	6.01932009166667	6.53137754166667	6.41036045833333	6.507987025	6.60603940833333	6.2517663	6.32106796666667	5.79999791666667	6.09272539166667	5.70431125833333	5.26895569166667	6.47216713333333	6.40185915	5.9191077	6.465677275	5.70153421666667	6.12040304166667	5.49431639166667	6.29278439166667	5.74037505	5.79916375	5.39804303333333	5.71795834166667	6.59815521666667	6.17390685	6.5680586	6.17226739166667	6.64623430833333	5.84699576666667	5.51803240833333	5.84791786666667	6.47216713333333	6.905223325	5.68979354166667	6.14002625833333	6.94497745833333	6.06574364166667	7.26995246666667	7.340949	6.755806875	6.47216713333333	7.21926184166667	7.435271425	7.569973	6.26921054166667	7.441553775	6.70599545	7.03138465	6.18332735833333	6.69182425833333	7.78668815	7.0345033	7.806190725	6.32988615	5.99181078333333	6.54994171666667	7.07353054166667	6.520636925	6.58160273333333	7.254184275	5.31524535	6.478653025	5.85413349166667	6.43359675	5.737720375	6.24783014166667	6.4975584	6.44000804166667	5.710337225	6.4413796	5.63755270833333	5.7835982	7.42326895833333	7.02536274166667	6.55447500833333	5.97783559166667	5.98915650833333	4.94478124166667	6.393024275	5.575395825	6.45841340833333	4.88687726666667	6.075096075	5.66463360833333	6.78180254166667	6.20262720833333	6.075953425	6.81774981666667	6.00459881666667	6.637274925	6.64292103333333

RHCG	9.35451149166667	11.9479243416667	12.3851121666667	13.2628733666667	12.5917146833333	13.4267583666667	12.370770375	12.115901275	12.713417125	12.0657212166667	12.2272622416667	9.50353634166667	12.497697975	11.9935609583333	13.3293632833333	11.9939589	8.706897975	14.3661675333333	12.724847575	11.2950935333333	12.416791375	13.2128605083333	12.24842175	13.2799375583333	13.2412964416667	8.9384783	12.9983717416667	13.864749275	13.405024	12.8807291083333	12.911149025	12.5662353166667	12.803128575	9.312713675	8.81924343333333	9.09642976666667	14.0391426	12.3671040333333	12.8624048833333	12.2231616166667	12.4469679916667	13.488720775	12.5140767416667	11.8924112666667	12.9099783416667	11.5626319666667	11.4170295666667	12.8124184083333	12.3842104583333	13.450467225	12.7728759666667	12.8166613583333	13.3667920416667	13.2855354	13.398021325	12.105571225	12.5813238666667	12.2536771583333	13.4950898916667	11.8726369583333	10.667166075	10.36526325	10.011606775	10.0334908083333	9.08180965	11.9593365833333	10.93683975	10.2650558583333	10.1477211833333	9.700667025	10.0313344083333	10.834075425	11.28453495	9.84676184166667	11.343036975	9.58437340833333	9.24318624166667	11.62492935	12.1233577583333	10.0434280416667	9.000388675	9.42163085833333	11.2891541	9.41935561666667	11.8705505	9.462094325	9.15886451666667	9.88726988333333	9.74389450833333	9.36247203333333	10.4554224833333	9.61633444166667	10.8386147583333	10.3771245083333	11.2205665583333	12.7237683583333	9.80187509166667	10.1845142083333	12.3789904833333	9.22832763333333	9.57267214166667	9.93343423333333	9.50086338333333	9.80399703333333	11.606939225	9.903525425	11.8055072	10.7276175666667	9.74218338333333	10.145640125	9.83811425	10.4208424916667	11.4075729916667	10.1970378083333	10.6979535583333	10.9909672	8.8072839	9.62969725	11.0072108	9.10796010833333

UPP1	10.0688438166667	12.4469679916667	12.910549725	12.4528342	13.56515455	10.6641205583333	13.4744898833333	13.0387188916667	12.7284615	12.1553849	11.5534468416667	8.63917994166667	12.5264113	12.2687931833333	12.6254772333333	12.1170603	7.39869075	12.6656204083333	12.225632925	12.1359013166667	11.5958198083333	12.584589825	11.3742720083333	12.9521681583333	12.6730092833333	9.189315625	12.8984843833333	13.317291325	13.1065162583333	12.6862224333333	12.4763630083333	11.395075525	11.882283925	8.52455500833333	9.9338367	8.16584395833333	13.6384571083333	13.6347670083333	13.0518882	12.53672075	12.6616630833333	13.420218375	12.278442975	12.0510805083333	11.3516750666667	13.3024940166667	12.95462435	13.4026847	11.9927367	11.9071135	12.5883581916667	12.847095325	12.7071557666667	11.8970046083333	12.0445037083333	12.2990201416667	12.9988931583333	13.901442	12.5472970333333	11.4567547583333	10.5523212166667	11.7909812333333	10.5404499166667	11.3093260166667	8.128787725	14.0429420833333	11.6675947833333	10.6387912166667	12.7490093583333	11.2453633166667	10.4581692833333	11.6077110916667	13.2075024333333	12.0214326083333	11.2530869333333	13.068247425	9.84117550833333	11.0409461	13.2068344333333	10.5322469083333	8.1963928	8.001514325	12.678040475	12.0600286166667	12.0051849333333	9.11131928333333	8.199157725	9.49101396666667	10.018462175	10.978750575	11.0619074416667	11.8407167416667	11.1649238666667	10.3442980083333	11.6613946166667	11.143398475	10.5500822583333	10.2225656916667	12.19191635	8.39672325833333	9.890973325	9.96400001666667	9.57692496666667	11.0811600833333	13.950667875	12.1943788416667	12.3301864333333	12.4386321666667	10.9123611916667	11.23807015	10.5169707416667	11.5404299	12.150591475	11.4534829083333	12.5084928916667	11.4748997416667	9.28361398333333	11.6783737	11.4045795333333	10.327316425

USP6	9.5101901125	11.1617286625	10.9460665375	10.7319462916667	11.3063182333333	14.4068767125	11.2472751583333	11.1932644	11.2184992	10.66090735	10.9190991041667	10.1757139416667	11.1190696291667	11.7137084625	11.7405767875	10.4310234958333	13.397690475	10.946906725	10.8667854166667	11.26078735	11.00364155	12.2974883666667	10.6807042958333	10.6371053708333	10.3785116416667	9.27833025	11.015195575	10.7933922	11.47204605	11.2493932541667	11.2644545083333	11.3862814666667	11.1751395166667	14.8032021791667	8.9528767125	8.99321267083333	11.6817174416667	11.4142576083333	11.94492175	11.6135515916667	10.86253675	11.3211200791667	11.4179569791667	11.240785875	9.85093639166667	10.7637150541667	11.3992377541667	11.3851742541667	11.3330606083333	10.406703075	10.4628138416667	11.9214576291667	11.1048374	11.2878174625	11.3932914458333	10.865245775	11.3273337333333	11.0706939666667	10.58122785	10.8902476583333	10.9001608208333	10.2086916333333	9.67434395	10.8849115041667	9.22934275	11.7503691375	10.7674925833333	10.7420044666667	11.1229606875	10.791785825	10.7362121958333	10.969810375	10.5575313583333	9.96932235833333	10.5075527083333	10.2464915583333	11.0657283125	11.4249559291667	9.85262877083333	9.7715505875	9.42837040416667	9.61965483333333	10.4435740291667	9.8616380375	10.365791975	9.98656490833333	9.97397811666667	10.3585509	10.3624811458333	9.87932717083333	10.1040493708333	11.4694963083333	10.4941094833333	11.7427551916667	10.899244375	10.7133365125	10.3037668333333	9.99280202916667	10.4486092625	9.88864601666667	10.3989842625	10.7480234125	10.0702336791667	9.78504972916667	10.791785825	10.6950799916667	10.0328838541667	9.62296950416667	9.82183821666667	10.6855571541667	10.2155615125	10.4904339291667	10.2836672416667	10.1652134125	9.56236944583333	11.2009752833333	8.8900448125	10.2405294125	10.2632201833333	9.67293809583333

ZNF232	9.5101901125	11.1617286625	10.9460665375	10.7319462916667	11.3063182333333	14.4068767125	11.2472751583333	11.1932644	11.2184992	10.66090735	10.9190991041667	10.1757139416667	11.1190696291667	11.7137084625	11.7405767875	10.4310234958333	13.397690475	10.946906725	10.8667854166667	11.26078735	11.00364155	12.2974883666667	10.6807042958333	10.6371053708333	10.3785116416667	9.27833025	11.015195575	10.7933922	11.47204605	11.2493932541667	11.2644545083333	11.3862814666667	11.1751395166667	14.8032021791667	8.9528767125	8.99321267083333	11.6817174416667	11.4142576083333	11.94492175	11.6135515916667	10.86253675	11.3211200791667	11.4179569791667	11.240785875	9.85093639166667	10.7637150541667	11.3992377541667	11.3851742541667	11.3330606083333	10.406703075	10.4628138416667	11.9214576291667	11.1048374	11.2878174625	11.3932914458333	10.865245775	11.3273337333333	11.0706939666667	10.58122785	10.8902476583333	10.9001608208333	10.2086916333333	9.67434395	10.8849115041667	9.22934275	11.7503691375	10.7674925833333	10.7420044666667	11.1229606875	10.791785825	10.7362121958333	10.969810375	10.5575313583333	9.96932235833333	10.5075527083333	10.2464915583333	11.0657283125	11.4249559291667	9.85262877083333	9.7715505875	9.42837040416667	9.61965483333333	10.4435740291667	9.8616380375	10.365791975	9.98656490833333	9.97397811666667	10.3585509	10.3624811458333	9.87932717083333	10.1040493708333	11.4694963083333	10.4941094833333	11.7427551916667	10.899244375	10.7133365125	10.3037668333333	9.99280202916667	10.4486092625	9.88864601666667	10.3989842625	10.7480234125	10.0702336791667	9.78504972916667	10.791785825	10.6950799916667	10.0328838541667	9.62296950416667	9.82183821666667	10.6855571541667	10.2155615125	10.4904339291667	10.2836672416667	10.1652134125	9.56236944583333	11.2009752833333	8.8900448125	10.2405294125	10.2632201833333	9.67293809583333

FBF1	8.50477168333333	8.79416294583333	9.05665619166667	9.20072740416667	8.5801776625	8.82596699166667	8.488221425	8.2498534875	8.674769575	8.7376700625	8.49862807916667	8.205606575	8.41031485833333	8.6641945625	8.6240670625	8.34257222916667	7.99966422916667	8.96677049166667	8.84589757083333	8.67864822916667	8.54481620416667	9.22893656666667	9.268773125	8.632616925	8.72995544166667	8.54916765833333	8.58932739166667	8.7211183375	9.00439402916667	9.08752937083333	8.994631375	8.79618599166667	8.67101673333333	8.23742238333333	8.632616925	8.28873542083333	8.93338286666667	8.75256491666667	8.61952160833333	8.34692658333333	8.4877078375	8.88429040416667	8.82596699166667	8.82945033333333	8.5698117	8.55016788333333	8.66323370833333	8.7469528375	8.9286527375	8.65721844583333	8.71358655833333	8.4512906125	8.72063475833333	9.00755195416667	8.7318304	8.73522726666667	8.2658106875	8.72808869166667	8.69961853333333	8.64027999583333	8.99876762083333	8.73086925833333	9.74369606666667	8.94259409583333	8.31227795833333	9.48903012083333	8.79016155	8.70189372916667	9.3464937375	9.07980542916667	8.7931974625	9.14674439583333	9.57583446666667	8.8176594125	8.894849075	8.82496384583333	8.84931076666667	8.86340424583333	8.91297039166667	9.24690354583333	8.3108121625	9.8797627375	9.27197185	8.8971613	8.92439054583333	8.6041321125	8.61548956666667	8.7635088375	9.27666387916667	8.96871966666667	8.67188521666667	9.1143027375	8.85800406666667	8.55570705833333	10.0821467708333	8.92909910833333	9.75216830833333	9.1471466375	9.76112887083333	8.8971613	8.82093766666667	8.6036776625	9.390927275	9.10366125833333	9.36870691666667	8.77548726666667	8.810875675	9.60255508333333	8.94308077916667	8.716679175	8.59420767083333	9.1367344625	9.13717020833333	9.62005562916667	9.38161835416667	8.96960385416667	8.68202068333333	9.50203754583333	9.0300746375	8.53709285833333

AC087289.3	8.50477168333333	8.79416294583333	9.05665619166667	9.20072740416667	8.5801776625	8.82596699166667	8.488221425	8.2498534875	8.674769575	8.7376700625	8.49862807916667	8.205606575	8.41031485833333	8.6641945625	8.6240670625	8.34257222916667	7.99966422916667	8.96677049166667	8.84589757083333	8.67864822916667	8.54481620416667	9.22893656666667	9.268773125	8.632616925	8.72995544166667	8.54916765833333	8.58932739166667	8.7211183375	9.00439402916667	9.08752937083333	8.994631375	8.79618599166667	8.67101673333333	8.23742238333333	8.632616925	8.28873542083333	8.93338286666667	8.75256491666667	8.61952160833333	8.34692658333333	8.4877078375	8.88429040416667	8.82596699166667	8.82945033333333	8.5698117	8.55016788333333	8.66323370833333	8.7469528375	8.9286527375	8.65721844583333	8.71358655833333	8.4512906125	8.72063475833333	9.00755195416667	8.7318304	8.73522726666667	8.2658106875	8.72808869166667	8.69961853333333	8.64027999583333	8.99876762083333	8.73086925833333	9.74369606666667	8.94259409583333	8.31227795833333	9.48903012083333	8.79016155	8.70189372916667	9.3464937375	9.07980542916667	8.7931974625	9.14674439583333	9.57583446666667	8.8176594125	8.894849075	8.82496384583333	8.84931076666667	8.86340424583333	8.91297039166667	9.24690354583333	8.3108121625	9.8797627375	9.27197185	8.8971613	8.92439054583333	8.6041321125	8.61548956666667	8.7635088375	9.27666387916667	8.96871966666667	8.67188521666667	9.1143027375	8.85800406666667	8.55570705833333	10.0821467708333	8.92909910833333	9.75216830833333	9.1471466375	9.76112887083333	8.8971613	8.82093766666667	8.6036776625	9.390927275	9.10366125833333	9.36870691666667	8.77548726666667	8.810875675	9.60255508333333	8.94308077916667	8.716679175	8.59420767083333	9.1367344625	9.13717020833333	9.62005562916667	9.38161835416667	8.96960385416667	8.68202068333333	9.50203754583333	9.0300746375	8.53709285833333

ARRDC1	10.06195985	12.1295438416667	12.2453199833333	12.4549570666667	12.31433185	12.4350665416667	11.9903497083333	12.077364475	11.9762173583333	11.735525225	11.633311275	10.114399525	11.4828657583333	12.2040899	12.4087159666667	11.9025044	13.2988538666667	12.0309620833333	12.333509375	12.0067796166667	11.97471495	12.3203584916667	11.5185086916667	11.8927815333333	12.2776254166667	10.0859903416667	12.2892396	12.3484591166667	12.2210896416667	12.135071975	12.1130081666667	12.18783155	11.9538193583333	13.2480671333333	9.960644375	10.0783158833333	12.2574255666667	12.1541314	12.0525821583333	12.115901275	12.1333790583333	12.3921887583333	12.3961090083333	11.92817275	11.6010090666667	12.3860530083333	12.3518594833333	12.000941175	12.0465984666667	11.908925725	12.0705755166667	12.1926211666667	12.2557740666667	12.4655499583333	12.2964028333333	12.400136275	12.0977041916667	12.212911825	12.336155525	11.7407727166667	11.326106725	11.105357075	11.9270898666667	11.8321536333333	10.666810425	11.5891254	10.5695644083333	11.8626743166667	11.2579853916667	12.2137378333333	11.5185086916667	11.6849152916667	12.9300978916667	11.114752	12.149315325	11.4612426083333	11.2550301583333	11.5983924583333	11.8174744333333	11.5813004	10.4883478333333	10.3754461833333	11.7016218	11.3172677166667	11.0682286166667	10.6256227583333	10.7050450333333	12.1359013166667	11.876483925	11.017853675	11.620207125	11.8920055333333	11.8826326666667	11.9750759916667	11.7663613666667	11.5544661666667	10.4356784583333	11.27496335	11.2719127583333	11.428103825	11.473399075	11.8705505	11.5468691083333	11.3835567833333	12.1652756583333	11.500844625	11.6632602416667	12.8708264916667	10.782424925	11.8877229666667	11.3550156083333	12.4469679916667	12.3802421833333	11.5618505	11.181831125	12.0522158916667	10.4249193833333	12.0889146833333	12.0710070666667	11.7800805666667

SLC26A8	4.69642039166667	4.17980874166667	4.117963675	4.28310633333333	3.71420795	4.30027178333333	3.90223645833333	4.16299614166667	3.67639645	3.881892575	3.936908825	3.47399078333333	3.782834	4.31908713333333	4.23097901666667	3.98396318333333	4.64613160833333	4.67972664166667	4.67067994166667	3.973735825	3.96722695833333	4.160694875	4.52155396666667	5.02689723333333	4.85974603333333	4.54769205	4.996867625	4.77930389166667	4.838864	4.52898779166667	5.13745513333333	4.71576270833333	3.84051730833333	4.19529993333333	4.9426219	4.366029925	5.71315170833333	4.60382705833333	4.60485635	4.759183025	5.16314315	4.63513973333333	5.3439553	5.71123295833333	4.52610014166667	5.112251075	5.27417344166667	5.1555947	4.65176085833333	4.948951225	5.118961025	4.59997769166667	3.931450225	5.56869470833333	4.46696494166667	5.6411096	3.92586006666667	4.91851714166667	4.46004483333333	4.56071255833333	3.905753025	3.51873675	4.41962694166667	3.82289100833333	3.84817555	3.91806175	3.962526825	3.4844003	4.15118036666667	3.542001325	3.74768024166667	3.550199675	3.46702925	3.77426261666667	3.671417225	3.71537640833333	3.45816426666667	4.50430228333333	3.87506608333333	3.6378857	4.00881020833333	3.59213996666667	4.41506155	5.34622214166667	3.893426425	3.92496040833333	4.09903998333333	4.736033375	4.061836425	4.161871975	3.83376700833333	4.59683404166667	4.07245948333333	3.83376700833333	3.94456669166667	4.02676174166667	4.05967894166667	4.14488695	3.81819714166667	5.066744825	4.46100053333333	3.52884109166667	5.0088772	5.28262243333333	4.44923994166667	4.03968158333333	4.753728625	6.717950125	4.39446349166667	6.78114418333333	4.73928888333333	5.68880476666667	4.66004348333333	5.15046895833333	4.23097901666667	4.43844	4.24914275	4.67067994166667	4.80316131666667	3.59213996666667

KRT73	10.4344952333333	10.4276602833333	10.2462958083333	10.5794976583333	11.0689648166667	10.1923992666667	11.1067195416667	10.4284899	10.9237681	10.3591207	10.9466012416667	11.244965975	11.0822289416667	10.7363658166667	10.9230393083333	10.1105163666667	9.84032644166667	11.5177929833333	10.409321425	10.2587469666667	10.2567002166667	10.776004825	9.91241780833333	9.7686764	9.777390875	10.135861875	10.0524736083333	10.5404499166667	10.5326225666667	10.1581087416667	10.20559775	10.1156550583333	10.451127	9.68160001666667	10.1278854583333	10.2241250416667	10.9013899916667	10.340493	10.25066255	10.5240503083333	9.911603525	10.430465125	9.818576775	9.7290300875	9.72484969166667	9.42163085833333	9.50264109166667	9.6385431	9.92412455833333	9.78018595	9.92494756666667	10.164124825	9.92832075	9.63806365	9.40159784166667	9.0354605	10.5173180916667	10.185845775	10.3325548083333	9.77263848333333	10.7866795833333	10.3817731416667	10.0506734583333	10.9707232916667	9.77133523333333	11.4675918	10.252295925	10.8329994333333	11.0454889833333	10.6384159666667	11.0308654583333	10.5768008666667	11.2538026583333	10.5244636333333	11.1764115416667	10.3182950083333	10.9574882	10.6332993416667	10.4012553083333	10.5500822583333	9.666049875	10.6979535583333	10.1054993333333	9.57267214166667	10.72841425	10.5444389083333	10.2932251333333	10.7828255833333	11.19866735	9.72964383333333	10.7538304083333	10.4119855	10.7247329333333	11.0826274416667	11.2384584083333	10.5596517833333	10.29990335	10.364544	11.1797537583333	10.173327525	10.2286007833333	10.98655615	9.82123129166667	9.84589375833333	10.051171975	10.5760591583333	10.1046909416667	10.74667695	10.1270651166667	9.917241025	9.925380825	9.76219373333333	9.72449161666667	9.2741013	10.0989513583333	9.06405529166667	9.598664275	10.1728980166667	9.85452464166667	10.2153563166667

CUX1	12.2768075833333	10.81171625	10.7855003666667	9.82029285833333	10.6971968666667	11.262088	10.3277465	11.345193725	10.627440475	11.3683256166667	11.1641077166667	12.4261316583333	11.3550156083333	11.3264911333333	11.3300005666667	10.9519987166667	10.541338775	10.4441620166667	11.0401699916667	11.3771067916667	11.4075729916667	11.5291327083333	11.5824943666667	10.7197888416667	10.8143165583333	12.52885135	11.1241958916667	11.1173392166667	10.939073475	10.63254535	10.891242375	11.25535535	10.9215878333333	10.6422709916667	12.11260975	12.10672325	11.1369321416667	11.1291467583333	10.7336987	11.56339385	10.7924187583333	10.6691322083333	10.8984556583333	11.1607791666667	10.9994499416667	10.8616146666667	11.0585423	10.9869124833333	10.7100606833333	10.329775075	10.3061471666667	11.0538325833333	11.2079127916667	10.9812562333333	11.114752	11.4467356166667	11.0790622916667	10.8326576083333	10.9618387333333	11.2664173916667	11.5422054916667	12.43292905	10.585362525	12.4950350166667	11.8970046083333	13.336335625	12.8936439416667	11.221922825	11.1415966	10.7828255833333	11.9853022666667	12.4229923333333	11.3567956166667	11.7731681916667	12.2536771583333	12.4903405333333	11.0848820333333	11.3771067916667	12.0893148833333	11.9939589	12.1229210916667	12.17539085	11.4908711166667	12.0985319416667	11.8943464416667	11.8450362083333	11.0116340666667	11.1014833833333	12.0282380083333	11.9325731916667	12.064871125	12.4474119833333	12.4936861916667	11.7575518916667	11.7887391333333	12.5293458166667	12.7279204166667	11.1238545166667	11.385364925	12.178269875	12.7354192416667	12.895840375	10.2539350666667	12.7300491166667	11.0101518666667	10.92486485	11.696699	11.5219495083333	11.3169410666667	11.9059347416667	10.8778360416667	11.9554346166667	11.4820913166667	12.7195939833333	11.3583216333333	11.4696992083333	12.153282325	11.5772577666667	11.6194298	11.8411407666667

TNIK	10.5330763833333	8.05761690833333	7.86377196666667	7.35627839166667	8.94715431666667	9.45636763333333	8.30538075	9.31762243333333	9.13460934166667	9.18071311666667	8.64290230833333	9.20920615	8.70751021666667	7.68879333333333	8.873364875	8.14192978333333	9.68430186666667	9.20975130833333	8.662974625	8.62136429166667	8.228841125	8.82318806666667	7.79803676666667	7.66558521666667	8.64447529166667	10.3439808916667	8.71688334166667	8.76703910833333	9.15886451666667	8.65745360833333	8.448844675	8.33507211666667	8.75882765	9.74389450833333	10.0957326583333	10.4678765666667	8.751330725	9.02371780833333	8.68083718333333	9.42382585833333	8.14709130833333	8.46867076666667	8.37004933333333	8.49291188333333	8.52772891666667	8.45352316666667	8.38817050833333	7.597705475	8.45745534166667	8.4237182	8.594439725	8.54938428333333	7.83240795833333	8.31619925	7.16794871666667	8.00800819166667	8.82572	8.46350844166667	7.9999538	8.849091575	9.54682303333333	7.83240795833333	7.64327454166667	7.597182875	8.36713998333333	8.52009253333333	8.11250586666667	8.98004790833333	8.53929365	10.0292860833333	8.435679275	7.76884748333333	7.73527455	8.41837	7.89930148333333	8.53238421666667	9.18595039166667	9.0147203	7.70147536666667	9.7105078	7.855217475	9.949333375	8.58390675	8.2675206	8.60435615	9.01611445833333	8.03810015	9.50036891666667	8.72403179166667	8.861626075	9.11034851666667	8.78021899166667	9.18457413333333	8.88831745833333	10.1581087416667	9.50452185	8.69025928333333	7.74398054166667	8.264078275	9.92453053333333	7.9422621	9.62805526666667	7.63763161666667	9.797655	7.63498546666667	8.34235189166667	8.37460103333333	7.52729815	8.80870043333333	9.25394765	8.703553525	7.821740775	7.71080830833333	7.717344425	7.967285625	8.50894728333333	8.71285006666667	8.37508346666667	9.352137375	8.32853386666667

ASCC1	13.6493215416667	10.865245775	10.3735458416667	9.923136575	11.2426327416667	11.93941945	11.9591423291667	11.7151199333333	11.8886458791667	11.4436969583333	10.9485195958333	13.61974265	12.8773764583333	11.4595644791667	10.7795187875	11.0396227291667	10.9690625666667	10.9425290958333	11.2662364625	11.5911075625	11.3369831541667	10.5816365958333	11.0198453583333	10.5043863583333	11.75113845	13.9256083916667	12.1646637	11.0529591458333	10.1984731375	11.35045295	10.7822440833333	10.914510025	12.3371947583333	9.15089320416667	14.1119502208333	13.3162230166667	11.1845969958333	11.6471973208333	11.6961139083333	12.203880675	11.5286050833333	11.5953235166667	11.1443172958333	10.9591537	12.4467832416667	10.1627032583333	11.9087522458333	10.4022684833333	10.0963095125	11.1704907875	11.5814876333333	11.1097335958333	11.0922553291667	10.7587865416667	10.30136825	11.7454501166667	11.5235644083333	11.1167870833333	11.47924455	11.8234712791667	10.454064575	9.68013430833333	9.4685538875	10.5762546958333	9.34086222916667	9.92644945416667	10.2268719791667	10.6148944333333	9.56058135	10.1856182666667	10.2058208458333	9.10727307916667	9.1262838375	10.7499026083333	9.46813001666667	9.3807746875	12.0638218875	10.8822804666667	8.97883835	10.0410176916667	10.3263476625	11.5047206958333	9.24649527916667	9.251885725	10.4387492083333	10.8115214041667	10.325930675	11.1410711083333	10.0311401208333	11.4164453416667	8.9639175	9.87462061666667	10.1011179041667	9.05845999166667	10.1635387833333	9.33686489166667	9.84137288333333	10.4326486208333	9.87007799583333	10.1777151666667	9.98656490833333	10.425894	10.0054515083333	9.377526425	9.20538440833333	10.0024278083333	10.1842929833333	9.28290961666667	9.6106909875	11.1319234083333	10.7909942458333	9.08371182916667	9.22317833333333	9.24002475416667	9.06432779166667	9.68625733333333	10.2776143791667	9.3537972	9.12350275416667	10.7610940708333

ANAPC16	13.6493215416667	10.865245775	10.3735458416667	9.923136575	11.2426327416667	11.93941945	11.9591423291667	11.7151199333333	11.8886458791667	11.4436969583333	10.9485195958333	13.61974265	12.8773764583333	11.4595644791667	10.7795187875	11.0396227291667	10.9690625666667	10.9425290958333	11.2662364625	11.5911075625	11.3369831541667	10.5816365958333	11.0198453583333	10.5043863583333	11.75113845	13.9256083916667	12.1646637	11.0529591458333	10.1984731375	11.35045295	10.7822440833333	10.914510025	12.3371947583333	9.15089320416667	14.1119502208333	13.3162230166667	11.1845969958333	11.6471973208333	11.6961139083333	12.203880675	11.5286050833333	11.5953235166667	11.1443172958333	10.9591537	12.4467832416667	10.1627032583333	11.9087522458333	10.4022684833333	10.0963095125	11.1704907875	11.5814876333333	11.1097335958333	11.0922553291667	10.7587865416667	10.30136825	11.7454501166667	11.5235644083333	11.1167870833333	11.47924455	11.8234712791667	10.454064575	9.68013430833333	9.4685538875	10.5762546958333	9.34086222916667	9.92644945416667	10.2268719791667	10.6148944333333	9.56058135	10.1856182666667	10.2058208458333	9.10727307916667	9.1262838375	10.7499026083333	9.46813001666667	9.3807746875	12.0638218875	10.8822804666667	8.97883835	10.0410176916667	10.3263476625	11.5047206958333	9.24649527916667	9.251885725	10.4387492083333	10.8115214041667	10.325930675	11.1410711083333	10.0311401208333	11.4164453416667	8.9639175	9.87462061666667	10.1011179041667	9.05845999166667	10.1635387833333	9.33686489166667	9.84137288333333	10.4326486208333	9.87007799583333	10.1777151666667	9.98656490833333	10.425894	10.0054515083333	9.377526425	9.20538440833333	10.0024278083333	10.1842929833333	9.28290961666667	9.6106909875	11.1319234083333	10.7909942458333	9.08371182916667	9.22317833333333	9.24002475416667	9.06432779166667	9.68625733333333	10.2776143791667	9.3537972	9.12350275416667	10.7610940708333

GPATCH8	10.36339005	10.86234545	10.77146855	10.7302810416667	10.0799055916667	10.189123775	10.26748265	10.7170255166667	10.29705995	10.4755897583333	10.3758856666667	10.23254885	9.73350879166667	10.8220014333333	10.7012892333333	10.76392445	10.1895353083333	10.5127537916667	10.80942375	10.7421748083333	10.615482725	10.8850875833333	11.052772175	10.80942375	10.7078134666667	10.8781733666667	10.751563075	10.8404768083333	10.1887239416667	10.91013625	10.6251996	10.6971968666667	10.6150711416667	10.5983973083333	10.6332993416667	10.5841235833333	10.2794538083333	10.9673192583333	10.7421748083333	10.5679469416667	10.7862968083333	10.7971951666667	10.766208375	10.9937146166667	10.634390675	11.0257755833333	10.9273818166667	10.880387475	11.062266425	10.8097832416667	10.7804261416667	10.73952485	10.5388660583333	10.8212945583333	10.6511688416667	10.6115680416667	10.7800644666667	11.0769009083333	10.9440376833333	10.71312705	9.77571775833333	10.8673519333333	10.6108306416667	10.4184334333333	10.590660625	10.4445170416667	10.7523474083333	10.233460875	10.924157925	10.1088055083333	10.5707751	10.5061588333333	10.5444389083333	10.6364744	10.1389730083333	10.6430311416667	10.2249992083333	10.1477211833333	11.0541887333333	10.2018680083333	10.958948725	10.4264895833333	10.747418225	10.79830065	10.17413685	10.316356175	10.2170232333333	10.338453	10.6865081333333	10.891242375	10.601543225	10.4324595166667	10.417262075	10.9094504083333	10.3806930666667	10.5252205583333	10.6222826083333	10.6691322083333	10.4594373083333	11.0845103416667	10.855999875	10.5760591583333	10.8743697916667	10.9473452	10.9618387333333	10.736058575	10.7624960333333	10.9266566	10.6934456416667	10.76392445	10.5523212166667	10.6910121333333	10.6169276916667	11.1277733333333	10.316356175	10.8356737416667	11.3121797833333	11.4511549916667	11.052426775	11.0211329583333

MKI67	6.62839033333333	11.6129963	11.6058721833333	11.625582325	12.05687395	12.2764019583333	11.047689175	10.8721591833333	10.5743323083333	11.5188708083333	11.74563025	8.80003964166667	10.79830065	11.5509672	11.4872383	11.1536039583333	10.1148100333333	11.6719609166667	11.8791305416667	11.468562175	11.3399920916667	11.987910225	11.7746522	11.0264670833333	11.6297974583333	7.35173706666667	11.1409117416667	11.5761677833333	11.83053445	11.8477473833333	11.4166255166667	11.3968955416667	10.2794538083333	11.2254735166667	8.513349575	7.82339403333333	12.2358628083333	10.7605131333333	10.7684448166667	10.8638767916667	10.7100606833333	11.0013930666667	11.1281108083333	12.093789575	10.8394387833333	11.270077	11.3918988666667	11.081884475	12.2916232083333	11.12457335	10.9039447833333	10.6457263	11.0060646083333	11.2950935333333	11.1448538	11.024361475	10.406884125	11.2616452583333	11.7074261416667	11.502358525	13.3262903916667	12.18313895	13.6009954166667	13.2254010416667	12.4709919583333	13.8366591583333	13.390452325	12.102327825	14.004911475	13.5108418166667	12.8091325	13.2227845833333	13.9411608583333	12.082379725	13.8261646416667	13.7084619583333	13.3467056166667	13.263493375	13.187909525	13.6009954166667	12.620644525	12.6331977416667	13.57422905	12.6689869583333	12.51216495	14.3582404416667	14.6259421666667	13.2168871166667	12.9767731916667	11.6363117083333	12.4261316583333	13.698785275	12.9364464083333	13.0727047	12.4425276083333	12.5420281	13.2711737166667	14.4092298666667	12.8549658583333	12.2383566166667	13.1407286583333	12.68421675	12.907519675	12.5775136	13.5781398666667	12.9527510916667	12.637166375	12.13546815	13.203417575	13.4427852416667	11.3086201833333	12.7128638833333	13.7771113166667	13.1137653	13.0421333333333	13.1350482333333	13.8516367666667	13.1872302333333	12.5855274583333	13.556817675

ZNF500	6.59779850416667	6.04398887916667	5.14242230416667	5.50279582916667	5.892408675	5.23927847083333	4.39301744166667	5.0801169625	5.085150825	5.54631372083333	7.35031180833333	5.2254306125	4.37957766666667	5.6785388625	5.88717176666667	5.93523504166667	6.16075655	5.70484465	6.67819945	5.63885909583333	6.47754952916667	5.51743424583333	5.74083900833333	6.171907075	5.74083900833333	7.31346904166667	6.6050493	5.77247790833333	5.6992285625	6.45130191666667	6.17670596666667	6.18529087916667	5.33697697083333	5.7283823625	6.78006964166667	6.63212634583333	5.9458035375	6.27760587916667	5.76780764166667	6.14455089583333	6.46457190416667	6.17512970833333	6.23119376666667	6.33151955833333	6.10273904166667	6.30678820833333	6.39570815833333	5.7581333875	6.32564148333333	6.47828504583333	6.128052275	6.62938375833333	5.40057459166667	6.6908251125	6.35918574583333	6.69148706666667	5.58942329166667	6.7814733625	6.48184889166667	6.31359270416667	5.65778688333333	5.20938526666667	5.9396831125	5.340361875	6.59348234583333	5.41642034583333	5.01898222083333	4.66475444583333	5.605312575	5.7283823625	4.799529975	5.78118206666667	4.6064583375	5.487556325	4.60330369166667	5.5600296875	5.36358359166667	6.0345690875	5.27784395833333	6.10436502083333	5.93432055	5.22655135	5.76588905833333	6.13801094166667	5.2114037125	6.05677966666667	6.19246064583333	5.46506463333333	6.33816796666667	6.60304722083333	5.6785388625	6.59977492083333	5.97638803333333	5.84838001666667	6.31508576666667	6.2547288625	5.74268562083333	5.98519041666667	5.36974268333333	6.38354875416667	6.3577384125	5.19478640833333	6.0526629375	6.9761553125	6.4318614125	4.654301475	5.64066469166667	6.9621924625	6.56769902916667	7.20242916666667	5.98693822083333	7.02051184583333	6.12488773333333	6.96813649583333	5.98869724166667	6.76013514166667	6.93062295416667	6.3463456625	6.7398437375	5.54535764166667

C16orf71	6.59779850416667	6.04398887916667	5.14242230416667	5.50279582916667	5.892408675	5.23927847083333	4.39301744166667	5.0801169625	5.085150825	5.54631372083333	7.35031180833333	5.2254306125	4.37957766666667	5.6785388625	5.88717176666667	5.93523504166667	6.16075655	5.70484465	6.67819945	5.63885909583333	6.47754952916667	5.51743424583333	5.74083900833333	6.171907075	5.74083900833333	7.31346904166667	6.6050493	5.77247790833333	5.6992285625	6.45130191666667	6.17670596666667	6.18529087916667	5.33697697083333	5.7283823625	6.78006964166667	6.63212634583333	5.9458035375	6.27760587916667	5.76780764166667	6.14455089583333	6.46457190416667	6.17512970833333	6.23119376666667	6.33151955833333	6.10273904166667	6.30678820833333	6.39570815833333	5.7581333875	6.32564148333333	6.47828504583333	6.128052275	6.62938375833333	5.40057459166667	6.6908251125	6.35918574583333	6.69148706666667	5.58942329166667	6.7814733625	6.48184889166667	6.31359270416667	5.65778688333333	5.20938526666667	5.9396831125	5.340361875	6.59348234583333	5.41642034583333	5.01898222083333	4.66475444583333	5.605312575	5.7283823625	4.799529975	5.78118206666667	4.6064583375	5.487556325	4.60330369166667	5.5600296875	5.36358359166667	6.0345690875	5.27784395833333	6.10436502083333	5.93432055	5.22655135	5.76588905833333	6.13801094166667	5.2114037125	6.05677966666667	6.19246064583333	5.46506463333333	6.33816796666667	6.60304722083333	5.6785388625	6.59977492083333	5.97638803333333	5.84838001666667	6.31508576666667	6.2547288625	5.74268562083333	5.98519041666667	5.36974268333333	6.38354875416667	6.3577384125	5.19478640833333	6.0526629375	6.9761553125	6.4318614125	4.654301475	5.64066469166667	6.9621924625	6.56769902916667	7.20242916666667	5.98693822083333	7.02051184583333	6.12488773333333	6.96813649583333	5.98869724166667	6.76013514166667	6.93062295416667	6.3463456625	6.7398437375	5.54535764166667

EDA2R	9.82560370833333	6.44000804166667	7.17033845	6.40572345	5.62957723333333	6.51994874166667	4.546642225	6.739509575	5.41802705	6.68131301666667	5.48703414166667	7.16614005833333	6.316307375	6.62436875833333	7.31861950833333	7.2645801	6.1063381	4.76141500833333	7.572832925	6.83744154166667	6.63574723333333	7.12528415833333	7.13607696666667	7.21750264166667	7.32089796666667	9.36892748333333	7.89132048333333	7.8248949	6.23950985833333	6.83400405	7.39105859166667	7.60415978333333	5.88241164166667	5.82168396666667	9.442472475	9.36987781666667	6.70918899166667	7.45043895	6.759774675	7.67769499166667	7.121598725	6.84711038333333	8.31252919166667	7.02653026666667	8.322152325	7.941170525	7.537939	6.41191374166667	7.206290275	7.47562453333333	7.3367108	7.03138465	5.5651756	6.56431505	7.06773185833333	7.24396096666667	6.54365829166667	7.13728840833333	7.49050329166667	7.94647371666667	6.71655181666667	6.34302255833333	6.75712434166667	6.11047193333333	7.08866475	5.47794636666667	7.095527	5.87591940833333	6.96657695833333	6.71367926666667	4.55283246666667	6.22080705	6.40572345	7.31919828333333	5.97184991666667	7.657177025	5.75400875	6.62496390833333	7.299009625	5.87208589166667	6.769663225	6.38846551666667	7.30612164166667	8.66580391666667	7.54228448333333	7.27593380833333	8.12585160833333	7.263472875	5.12814335833333	7.83240795833333	7.47562453333333	7.27410720833333	5.157708775	6.80918875	7.22598754166667	5.74506199166667	5.64694838333333	6.528434875	6.264276875	5.99418615	6.24607838333333	5.73195755	7.45275619166667	5.50436075	6.38316134166667	6.181735025	8.00309365833333	6.65690114166667	7.87659344166667	7.20023056666667	6.60956075833333	5.69189906666667	7.08235699166667	7.54405583333333	7.4656947	6.011301125	6.79731146666667	6.43799471666667	6.19527208333333	6.10713235

TRIM15	7.24765453333333	7.63498546666667	6.5005932	7.37059144166667	7.80451180833333	6.77179379166667	7.49442741666667	7.86012560833333	6.60400856666667	7.71189573333333	7.03569371666667	8.61623551666667	9.02273679166667	7.41526463333333	6.76378944166667	6.897275725	8.13871308333333	7.7483928	6.68920345833333	6.50214656666667	6.37033623333333	6.7919706	6.18568551666667	6.573073	6.06672131666667	6.59533788333333	7.04821105833333	7.40837556666667	6.71110255	8.40077755	7.35003230833333	6.97462729166667	7.56268353333333	7.72905521666667	7.064683475	6.88791735	6.90123264166667	6.88120535	7.38291215	7.025978175	6.03052256666667	5.72076731666667	6.39681019166667	6.57096464166667	7.074128375	6.77436175	6.22835699166667	5.29450505	5.91520025833333	6.445141375	6.53273724166667	6.41504531666667	7.56380908333333	6.04799355833333	7.12345469166667	5.15873735	7.13168135	6.161212075	7.10317193333333	6.406487225	7.38291215	8.39370431666667	6.98215429166667	7.8150656	7.596603625	8.8912541	7.22358631666667	7.9693647	8.027292325	8.11454491666667	7.33740245	8.318633925	9.10844005833333	7.9760917	7.18035421666667	7.52443165	10.1650146416667	7.24703090833333	7.2622557	7.254184275	6.98973956666667	7.11730268333333	7.56325655	6.77436175	7.191921025	6.4413796	8.1976201	8.21405428333333	7.21750264166667	7.52330799166667	7.96623754166667	7.66388121666667	8.14240605833333	6.42634760833333	7.52780409166667	7.46853124166667	7.086730875	7.61520518333333	8.73689844166667	7.12401263333333	6.63660101666667	6.54365829166667	7.21396461666667	7.10317193333333	7.50627369166667	7.9484088	7.22176664166667	6.9953871	7.87369893333333	6.59386139166667	7.69605179166667	6.449622025	7.305533825	5.79838183333333	8.11250586666667	6.70327563333333	6.39383819166667	7.4690921	6.759774675	7.439305775

FGF13	8.88067333333333	5.24931593333333	4.67278611666667	3.890139475	4.328489775	5.498293125	4.34995844166667	4.67877638333333	4.177708	5.55170386666667	4.52522428333333	6.465677275	6.58835050833333	5.14597308333333	5.858085325	5.87944876666667	6.33491514166667	4.60180214166667	7.19767501666667	5.86347250833333	5.0412383	4.05858813333333	4.9935499	4.557709	6.26827546666667	8.59302528333333	6.159395425	5.197537	5.68880476666667	6.056382625	5.677007675	6.06833519166667	4.97386210833333	3.82560103333333	8.37460103333333	9.32849245833333	4.87006425833333	5.1555947	4.72615460833333	5.60574740833333	5.55953659166667	4.897128125	5.35361239166667	5.72686785	6.57365081666667	5.0258117	6.7130388	5.98559190833333	5.482101875	6.44595796666667	5.980495875	5.60487774166667	4.31414184166667	4.92360515	5.05900706666667	5.61448439166667	4.92659856666667	4.50011030833333	5.88853345	5.91988768333333	4.53433785	4.656935525	4.07775664166667	4.10621731666667	5.30061454166667	4.27700685	3.670269525	4.98219815	3.71657505833333	4.17039641666667	4.18191081666667	4.02921226666667	6.64956780833333	4.46516671666667	4.146963325	4.285092425	3.94456669166667	4.48925233333333	3.976955525	4.08322809166667	5.6723045	5.71707388333333	4.14585755833333	3.64321401666667	4.3276422	5.01953916666667	7.88018380833333	3.97505105	4.83765554166667	4.963016975	3.94009973333333	4.98892909166667	6.61087155833333	3.700710475	3.60605176666667	4.697573175	4.138474425	3.976955525	3.72694154166667	4.72007973333333	4.06389798333333	4.42372025	4.453007975	5.58597898333333	6.21191129166667	3.85883090833333	4.23291245833333	6.1063381	3.946878775	6.31880616666667	4.939635175	6.13929583333333	4.21395365833333	4.612218	3.9147734	4.108372	4.337410475	4.35594943333333	4.5496205	5.48300426666667

ZBP1	7.92110699166667	7.94982140833333	8.36292435	8.180440425	8.27907085	9.73577058333333	8.82825418333333	8.50602524166667	8.244635825	8.252426875	7.933308825	8.03861421666667	8.392597175	8.458998275	7.442585225	9.73744423333333	8.4564653	8.57560394166667	7.55678278333333	9.08915231666667	8.51856025833333	8.72692960833333	9.52591685	9.05917338333333	8.92218960833333	8.43849715833333	8.887867925	8.09121131666667	8.364642925	8.05913828333333	7.91142259166667	9.26773438333333	9.2355397	11.2082269	8.43849715833333	7.442585225	8.53385956666667	7.9999538	8.81240245	8.47431853333333	8.55392016666667	7.75064413333333	8.72497184166667	8.64244941666667	8.86467129166667	9.22061473333333	8.65887986666667	8.333491425	8.88688890833333	9.66649425	8.65418485	8.01217839166667	8.99027038333333	8.5716089	9.28785676666667	8.4484208	8.51387624166667	8.56222751666667	8.50894728333333	9.36987781666667	8.97489020833333	9.186916425	10.417262075	7.95962858333333	8.21622873333333	8.996715525	8.04865021666667	9.24539353333333	9.91032726666667	11.0060646083333	8.4100903	8.38708958333333	8.67403964166667	9.94251199166667	8.18527721666667	8.2988733	10.3167137333333	11.3646504166667	9.57736215	10.1088055083333	7.43688079166667	8.43143401666667	8.82067435833333	9.33365533333333	9.585238675	8.82920276666667	8.29098985833333	9.77090350833333	8.87390605	8.78836945	8.532903275	9.79676919166667	8.649865175	8.36127639166667	8.10955528333333	10.0949964416667	9.22559156666667	8.99204639166667	9.35167163333333	9.66513115	8.69274164166667	9.30630015833333	10.8930750916667	9.65973478333333	9.44058225	11.291894675	8.9544846	10.0636311333333	10.4433912083333	9.78382319166667	9.31001445	9.20100326666667	10.0957326583333	8.26509473333333	12.0564210833333	9.62586958333333	7.435836975	9.463907375	9.9039927	10.5493449166667

ZSCAN5A	9.13909698333333	9.36572414166667	8.910111275	9.29702861666667	10.15852225	8.98618440833333	10.4108585666667	9.1396151	9.72449161666667	9.29065144166667	9.32760176666667	10.1617358916667	9.75787298333333	8.90099503333333	8.884079375	8.77815989166667	8.76654949166667	10.0247316333333	8.96230365	8.76055524166667	9.099726825	9.25584964166667	8.70547134166667	8.61571535833333	8.94431525	9.198111125	9.312713675	9.05561776666667	9.64736674166667	9.20464029166667	8.5232739	9.16031618333333	9.46790315833333	10.1100737	9.23605358333333	9.38536081666667	9.80350678333333	8.80353201666667	8.91556449166667	8.797202975	8.44491509166667	8.78991784166667	8.70798358333333	8.44239195833333	8.64337428333333	8.52110425	8.616671525	8.708486225	8.58808473333333	8.763246775	8.94384210833333	8.998094275	9.56361585	9.32574429166667	9.17923419166667	8.44491509166667	9.186916425	8.86655378333333	9.19160371666667	8.482136425	9.67274871666667	9.66334089166667	8.90005820833333	9.40640285	9.373589925	9.633880775	9.20652433333333	10.16655665	9.23377270833333	9.92625621666667	9.90478876666667	9.82560370833333	9.77312443333333	9.17093331666667	9.63156661666667	9.36528065833333	9.891815375	9.36122138333333	9.11902033333333	9.34897708333333	9.67363538333333	9.90565114166667	9.19340213333333	9.490132625	9.963087575	10.000563275	9.95852463333333	9.78692435833333	10.5592695083333	9.14054775	9.26809461666667	9.36206593333333	9.311300325	9.78967574166667	9.71828384166667	10.7624960333333	9.69792198333333	9.78872821666667	9.90608706666667	9.45595239166667	9.114481175	9.81525473333333	9.26488315	8.70259450833333	8.83968223333333	9.17414999166667	9.29065144166667	8.94982423333333	9.00964296666667	8.8072839	9.112227825	9.68430186666667	8.873364875	8.7637709	9.33964531666667	8.935118175	9.295254925	9.28405401666667	9.389870525	9.53137905

C1orf52	11.5038172666667	10.7736244333333	11.133569275	11.133569275	11.411148125	11.2395267333333	11.3347189916667	10.9266566	11.2107825666667	11.158996075	11.036287075	11.3520380583333	11.3675917666667	10.9150352083333	11.221922825	11.0427571416667	11.6514721333333	11.9712775416667	11.162651425	11.201526325	11.0670126666667	11.17354635	11.1524382	11.9645775083333	11.437271475	11.4993303916667	11.3534958083333	11.5258171833333	11.53378775	11.4126401666667	11.7351274916667	11.3086201833333	11.5118143666667	11.0228735916667	11.3343049666667	11.4774665416667	11.2453633166667	11.1309543	11.39042995	11.3043532	11.1767887583333	11.156830125	11.0153767166667	11.3778682416667	11.174277875	11.0060646083333	11.1249258333333	11.3399920916667	10.9805696916667	11.3867866583333	11.3488624333333	11.4534829083333	11.562248175	11.3242829	11.2933100833333	10.8843423083333	11.2294360916667	11.3004748583333	11.36160315	11.1491945416667	11.5769076083333	11.118056575	11.95781185	11.4708276916667	11.6863511916667	11.0567325916667	11.3433838833333	11.0633247666667	11.8356051666667	11.4864514	11.14521145	11.2638907416667	11.7878982583333	11.411903025	12.047012	11.6294117916667	11.529525725	11.6787724916667	11.74329675	11.612299875	11.7336295416667	11.3224431	11.1085089666667	11.4737788083333	11.960958325	11.89894165	11.7403808583333	11.642205825	11.2822211083333	11.825765325	11.5196693333333	11.848881975	12.10269775	11.2790070666667	11.8137761833333	11.2154273083333	11.6944435416667	11.5279761083333	11.76324455	12.202055425	11.4075729916667	11.1862575	11.8178724083333	11.5089022	11.5606001833333	12.1698876833333	11.7582463416667	11.3343049666667	11.3231776	11.5969108416667	12.0096367416667	11.61335235	11.8371529416667	11.4492670916667	11.6944435416667	11.9220834083333	11.9028464416667	11.9825076333333	11.7030888416667	11.3534958083333

RHOT2	10.222173225	10.3442980083333	9.99259065	10.189123775	9.389870525	10.1497399666667	9.18071311666667	9.49101396666667	9.60319949166667	9.53265603333333	9.36939973333333	8.752344475	9.01425336666667	9.69439403333333	10.1481495666667	9.62234490833333	10.1704848833333	10.1164178916667	10.040006825	9.77613621666667	9.79362293333333	9.86989941666667	9.8597846	10.0378686583333	10.2217906833333	10.0125394166667	9.76472428333333	9.89982799166667	10.123005125	10.0156224166667	10.0636311333333	10.21946315	9.91887936666667	9.97000988333333	9.900995025	9.76472428333333	9.929600025	9.91374403333333	9.71442065	9.80231140833333	10.0095607333333	10.327316425	10.1845142083333	10.1613409083333	9.961904575	10.0347123333333	9.96356175	10.3901677	10.3964688	10.1172154583333	10.0980477833333	10.027285575	10.2932251333333	10.0783158833333	9.80056271666667	10.151687475	9.83237755833333	10.1986602916667	10.068396225	9.67854975	9.96400001666667	10.257101525	9.831945975	10.42200985	9.38580318333333	10.7478338666667	10.0693001666667	9.925380825	10.5260512	9.71737348333333	9.61456806666667	9.95725063333333	10.0855958166667	9.60540180833333	9.686488825	10.21041955	9.5040294	10.128737475	10.591411875	10.1704848833333	9.97954941666667	9.20822946666667	9.9039927	9.879959075	10.2724454333333	10.6971968666667	10.4713528083333	9.41849085	10.1923992666667	10.0623822333333	10.2699462416667	10.6853720166667	10.4922259333333	10.3558299833333	9.94460186666667	9.42424818333333	9.68471804166667	9.92792138333333	9.80967570833333	10.186667875	10.420034375	10.29705995	10.4053126416667	10.0660963333333	11.0913458083333	10.400070625	10.4826365083333	10.6699485666667	10.3817731416667	9.99593061666667	9.908637725	10.3475834	10.35148425	10.1601611333333	10.1270651166667	10.235923825	10.5833260083333	10.417262075	10.4053126416667	9.72703330833333

VNN2	4.285092425	4.282041725	4.74641638333333	4.33846348333333	3.81123611666667	7.2027431	4.95961636666667	6.6659887	4.37909631666667	5.16314315	4.92878054166667	5.70649276666667	4.30624979166667	4.70784294166667	4.00663116666667	6.81976344166667	7.03321648333333	4.289092625	4.92360515	5.948036925	5.49935525	4.67067994166667	6.79057516666667	7.28990879166667	4.29716620833333	5.31319136666667	6.76313481666667	4.20479785833333	4.06900396666667	4.00999268333333	6.503440675	6.53883034166667	5.06903989166667	9.106662475	8.59640985	5.59377575	4.10101340833333	5.23671275	7.31025495833333	5.327612425	7.86164825833333	4.98009509166667	7.811926125	6.01932009166667	4.96401284166667	7.36707339166667	4.87823230833333	6.256698975	6.80310133333333	6.64358704166667	8.29053858333333	4.77483110833333	3.644387875	5.15140121666667	4.115796825	4.63625878333333	7.305533825	6.16914264166667	4.085428875	6.66666621666667	4.177708	4.146963325	6.20969628333333	3.77909573333333	4.03445655833333	5.86456365833333	6.462885175	4.612218	6.56292778333333	5.020575025	4.78586035833333	3.95130670833333	4.41506155	5.26468096666667	4.29017421666667	4.62990135	4.65588558333333	7.76293401666667	6.77836881666667	5.83208956666667	4.91325449166667	3.962526825	4.79318954166667	8.88450143333333	4.66334196666667	4.44059075	4.23291245833333	4.0322916	3.87937155833333	4.49904255833333	4.9935499	7.14272935833333	5.108231475	5.62775541666667	3.5657117	6.37568504166667	4.90470955	4.21300761666667	5.24498075	4.688902775	7.02343930833333	4.88478473333333	6.88255923333333	6.88184853333333	4.39770036666667	5.17265765	4.42980535	6.13670685833333	4.87199023333333	6.56879458333333	6.99346504166667	6.4502938	4.15954296666667	8.74174598333333	4.86289075	7.166799725	5.80728855	6.04352600833333	4.42980535	6.05225019166667

BABAM2	12.212911825	12.47909375	11.9274761416667	12.3310232666667	12.6270116666667	12.2675250666667	12.2293758416667	12.516863425	12.2844611166667	12.626502475	12.7041276166667	12.3983699916667	12.4759771583333	12.4696924	12.4355627	12.4746445166667	12.6699783083333	12.62157875	12.440827975	12.5024686916667	12.3802421833333	12.6616630833333	12.6235559916667	12.3233097166667	12.333509375	12.5710716416667	12.57846135	12.6336537916667	12.76127775	12.5334882833333	12.2628607833333	12.2760053833333	12.17951645	12.3712167583333	12.4831684166667	12.6075148583333	12.6512308	12.324942025	12.3523338166667	12.65329555	12.2437326916667	12.35333105	12.2154467166667	12.2896477166667	12.2550005	12.5206883833333	12.4763630083333	12.3182821416667	12.2433310083333	12.40421355	12.301617475	12.2536771583333	12.7061787	12.6346883583333	12.4091926166667	12.3461563666667	12.2366655666667	12.5443972083333	12.3754853666667	12.212911825	12.4908032583333	12.05061705	14.0526804666667	12.5070861083333	12.5488109	13.016186275	12.0781743333333	12.86304345	12.3224578583333	12.2679354916667	12.1146065416667	12.4238445333333	13.0349568083333	12.552088775	12.9357780833333	12.7999144166667	12.4723572833333	12.682091125	12.7092084083333	13.0992273666667	12.5362402583333	13.054278175	12.9745146166667	12.6180925666667	13.1427455666667	12.5935190666667	12.217702275	12.487541925	11.7898995	12.7902282666667	12.51216495	12.8641322666667	12.409680475	12.989519425	12.105099425	12.4132063666667	12.2637428083333	13.48560075	13.0829572666667	12.1823287916667	12.8086026666667	12.2508814916667	12.4554143666667	13.0927318166667	12.0181445166667	12.9149879583333	12.6956383666667	12.6759474583333	13.3912051666667	12.5354003083333	12.7374360833333	12.725884075	13.1452170666667	12.86304345	12.6800815583333	12.6041308166667	12.7368908416667	12.2528148916667	12.7431839	12.1745545

TRIM3	10.4061408166667	8.63080885833333	8.88020864166667	9.02175539166667	9.43278838333333	9.5040294	8.45509195	8.920781025	8.92890170833333	8.94285730833333	8.55853193333333	9.19860346666667	8.94431525	8.56622091666667	9.50914073333333	8.96322584166667	10.0434280416667	9.35773556666667	9.31085525	8.98325939166667	8.882160975	9.18832863333333	8.8912541	9.60710205	9.38182149166667	10.2579212583333	9.26679426666667	9.00964296666667	9.86062085833333	9.05644770833333	9.226998075	8.966534925	8.9493948	9.284546375	10.06569645	10.2535245166667	9.298425025	9.00913584166667	8.89967475833333	9.06551128333333	8.81599143333333	8.99116335833333	8.99438999166667	9.10145314166667	9.352137375	8.99487275833333	9.20373038333333	9.09684721666667	8.91181045	9.43547285833333	8.996715525	8.91224634166667	9.20920615	9.36528065833333	9.18306429166667	9.288795975	8.93317065	8.96987785	9.29885605	8.9925209	9.78824573333333	8.76703910833333	9.48341896666667	9.3242172	9.08729039166667	9.91032726666667	9.41761155833333	9.12042280833333	8.98520484166667	9.05602455	8.51438241666667	9.37921308333333	8.89174859166667	9.28929068333333	9.06069035833333	9.3832069	9.61091021666667	8.92461889166667	9.08687805	8.79090379166667	9.05454125	8.91938296666667	9.28041066666667	10.035165975	9.55058418333333	8.751836375	8.535777725	9.19764225833333	9.34710834166667	9.26773438333333	9.10621828333333	9.24492166666667	8.93227916666667	9.31085525	9.8269075	8.45255091666667	9.44959046666667	8.68624993333333	9.02609295	8.91742895833333	9.43278838333333	8.89076064166667	9.02175539166667	8.92973970833333	8.66775465	9.34710834166667	10.0679332666667	9.42590730833333	9.51724313333333	9.62110718333333	8.84099020833333	8.59743456666667	10.176297425	9.20732398333333	9.48384479166667	8.7217903	8.88930546666667	9.36987781666667	8.64585098333333	8.48950848333333

SDR42E1	8.30591494166667	11.56491665	10.9215878333333	11.0625918083333	11.1604297833333	11.2950935333333	11.3605041083333	11.206560475	11.19202035	10.255903875	11.1926823916667	8.107904375	9.7452797	11.1309543	11.2671696833333	10.6143770083333	11.17354635	10.8759847166667	11.53339965	11.1824657666667	10.7669402083333	12.32117485	11.3942748	10.6558683583333	11.0992950083333	8.33702585833333	11.164489475	11.24573245	11.2417551	11.5056318416667	10.7189561666667	11.528423775	11.1252834	10.7452538416667	9.24884609166667	9.18115765833333	11.6456087833333	11.0101518666667	10.50924085	11.22125475	11.17758075	11.1298695666667	11.1912222083333	10.85411215	10.3721682083333	11.0971520666667	11.33887195	11.2150743583333	11.3509805666667	10.5545011333333	10.74363815	10.9730535833333	10.5049759833333	11.4556673333333	11.3015343416667	10.5711304666667	10.9150352083333	11.3360764166667	11.0815307083333	10.5291936916667	10.5022046916667	10.61804865	11.3893506666667	9.28268900833333	12.29141735	10.3754461833333	11.4460096583333	11.53966185	10.7684448166667	9.79891768333333	11.5374922333333	12.5667535083333	5.575395825	11.5702459583333	8.47382378333333	6.48645600833333	10.5396655666667	11.2753469583333	10.74363815	11.43469695	11.3438040833333	11.0670126666667	6.71502808333333	10.0331009416667	11.0010313583333	7.64275718333333	6.83341489166667	9.80492948333333	12.063617625	9.61323428333333	8.30033485833333	11.3664862333333	11.7403808583333	11.2864562916667	11.2363296083333	11.3072160833333	10.7112321916667	8.51177801666667	11.4887131666667	11.0079045333333	11.5710069666667	10.5337610083333	10.7900238833333	10.5775565833333	10.609691175	10.474031625	10.904327125	9.47301451666667	11.7970628166667	7.657177025	11.1491945416667	10.3373213583333	10.4698289916667	11.8390986666667	11.3239342333333	11.5567047416667	6.845071175	7.525066825	8.68083718333333	10.7162804

CPNE2	11.8981677666667	11.9165677	11.9169362583333	12.3716896916667	11.4971747583333	12.007698575	11.3587026083333	11.4431148583333	11.3360764166667	11.9082126416667	11.5185086916667	12.0761170583333	11.3813533416667	12.4091926166667	11.3239342333333	11.7209431166667	10.6313385833333	11.0355345916667	11.659892375	11.825372775	12.1827137333333	12.0156772833333	11.846919075	11.3675917666667	11.8089541	11.884179625	11.4170295666667	11.376411175	11.4402312666667	11.3553466416667	11.625582325	12.0432377416667	11.6795803583333	11.0200205166667	11.67393075	11.943975875	11.5443713166667	11.6095821583333	11.6205609916667	11.8422473333333	11.8395390583333	11.4682426833333	12.5468096583333	12.0793162416667	12.2553940416667	11.778723	11.9629534916667	12.0356360333333	12.39127385	11.7735354916667	11.686699925	11.4549596	12.5415392083333	12.1558313416667	11.9289106916667	12.2322263916667	12.0604174833333	11.62870925	12.2412181916667	11.6651451833333	13.0374763416667	12.4714104166667	12.8460196	11.7362421333333	12.3147568916667	12.938273025	12.5831450833333	12.2272622416667	11.4912151666667	12.4737403583333	12.3860530083333	13.1197255166667	12.4320171333333	12.5325556416667	12.2719001166667	12.6765518416667	11.7849428666667	12.7139152416667	12.706710675	11.9883632916667	12.0555750333333	13.0167634166667	11.641524325	12.3921887583333	11.95781185	12.5334882833333	12.1656867	12.1566548583333	12.7827098416667	12.8460196	12.39795185	13.2047745416667	12.9785766	12.8387693166667	12.6136777666667	12.1262500416667	12.2158837916667	12.6470895583333	12.4368713	12.3538131333333	13.20142475	12.907519675	12.3292738666667	12.5173296333333	12.2553940416667	12.5145301166667	13.2622394166667	12.2582885416667	12.4377746666667	12.8776683083333	12.0555750333333	12.5850501916667	12.4482599083333	12.9983717416667	12.643219075	12.4709919583333	13.1329842916667	12.3606021	12.135071975	12.6876920416667

STOML2	12.8974584	12.8543756916667	13.1648189583333	13.25175425	13.487986925	13.1046602416667	12.8807291083333	12.9726928583333	13.1052210666667	13.3018009583333	13.2116333	12.724847575	12.95116035	13.1531334333333	13.03309195	12.8964099833333	13.1628836	13.2332248583333	13.461932225	13.0271148666667	13.1635303583333	13.132282	12.69187135	13.22481105	13.1845722666667	13.174009975	13.1832185416667	13.129332175	13.2339414916667	13.2962436083333	13.065712375	13.1178746666667	12.844414625	12.8724674416667	12.8735737166667	13.2293061833333	12.9995656666667	13.09075125	13.0602371583333	12.9954911666667	12.8979356	13.05246665	12.9144941416667	13.4345239916667	12.86304345	13.2525325833333	13.342400775	13.7234378333333	13.3771159333333	13.09459025	13.22406385	13.089585075	13.4383119666667	13.24746105	13.2898598333333	13.25175425	12.7902282666667	13.2503335416667	13.1512038916667	13.370423875	13.1616055833333	14.1527055166667	13.2785213333333	13.1492966166667	12.9941887083333	13.1512038916667	13.7272276083333	13.0089762083333	13.712997875	13.986279475	13.4058013416667	12.9871205833333	13.4156891583333	13.2678167166667	12.6381216166667	12.8481618333333	13.7740900916667	13.1563255166667	13.3936063	13.3515334583333	13.3248386416667	12.932363575	13.3356032833333	13.6969617	12.4350665416667	13.3185547	13.147286425	12.731184375	13.7139558	13.3234625333333	13.16610495	13.2678167166667	12.3431207	13.44208435	12.9941887083333	12.9558687	13.6979142666667	13.189983975	12.7411170833333	13.19400885	13.7542141333333	13.901442	13.44358445	13.19400885	14.047904	13.62548145	13.0108192083333	13.52385865	13.6941290583333	13.4282493833333	12.7295358083333	13.9785722666667	13.9207353166667	14.2969035	13.199462625	13.089585075	14.5073052333333	13.9749587833333	13.8148381	12.910549725

PCDH11Y	8.894849075	6.80784224166667	7.28954810833333	6.388860575	5.844633875	8.07757626666667	5.5788055	6.5739626875	5.20302529166667	6.18698760416667	8.35548015	10.3839402416667	5.03877025	4.78328442916667	6.328769325	7.9366852	8.7727191	8.09358357916667	7.2563108375	7.19033177083333	6.127271775	6.24567705416667	6.45061551666667	7.6329898875	7.15354758333333	8.06833124166667	4.19390653333333	7.36364962916667	5.708855825	6.59498996666667	5.9255638375	6.40155359166667	5.52491661666667	8.488221425	7.92196199583333	7.46049172916667	7.35031180833333	7.41437227916667	6.556305575	6.45664654583333	6.18290244583333	7.34640325416667	6.17670596666667	6.92205040416667	6.64052472916667	7.34183399583333	5.27682333333333	6.13886052916667	7.74428002083333	7.41034305416667	6.56842659166667	7.4547802	6.89100497916667	7.6546630625	6.60640252916667	8.12656328333333	6.91733040833333	6.61450384583333	8.4637836875	6.19246064583333	5.42224169583333	5.44076020416667	5.57783523333333	4.77542294583333	4.70729979583333	6.21472249583333	4.64362027083333	4.6479108375	5.37176904583333	4.2304917	5.81949583333333	5.38816209166667	4.98956144166667	4.67624076666667	4.03811705833333	6.8518622625	4.87245959166667	7.33828902083333	5.79243215833333	6.39954747916667	7.30888802083333	9.5192383875	4.30775345	6.35702110416667	5.43653326666667	7.62332840833333	8.1983393625	6.49286267916667	4.21246715416667	7.2172312625	5.49980207083333	5.13904645416667	7.7563220125	6.18290244583333	4.86752087916667	6.53097057083333	5.47569435416667	4.97227905833333	5.948468475	6.20074723333333	5.07707335416667	5.07162959583333	5.40801585416667	6.37312580833333	5.03478496666667	5.462367375	4.28257402916667	7.50434007916667	7.88640297083333	7.04035565416667	4.25764998333333	7.97319919583333	3.93531937083333	6.69347515833333	6.3745326875	6.63278815	4.8037463	4.12046859583333	7.34922049583333	5.04398305416667

PCDH11X	8.894849075	6.80784224166667	7.28954810833333	6.388860575	5.844633875	8.07757626666667	5.5788055	6.5739626875	5.20302529166667	6.18698760416667	8.35548015	10.3839402416667	5.03877025	4.78328442916667	6.328769325	7.9366852	8.7727191	8.09358357916667	7.2563108375	7.19033177083333	6.127271775	6.24567705416667	6.45061551666667	7.6329898875	7.15354758333333	8.06833124166667	4.19390653333333	7.36364962916667	5.708855825	6.59498996666667	5.9255638375	6.40155359166667	5.52491661666667	8.488221425	7.92196199583333	7.46049172916667	7.35031180833333	7.41437227916667	6.556305575	6.45664654583333	6.18290244583333	7.34640325416667	6.17670596666667	6.92205040416667	6.64052472916667	7.34183399583333	5.27682333333333	6.13886052916667	7.74428002083333	7.41034305416667	6.56842659166667	7.4547802	6.89100497916667	7.6546630625	6.60640252916667	8.12656328333333	6.91733040833333	6.61450384583333	8.4637836875	6.19246064583333	5.42224169583333	5.44076020416667	5.57783523333333	4.77542294583333	4.70729979583333	6.21472249583333	4.64362027083333	4.6479108375	5.37176904583333	4.2304917	5.81949583333333	5.38816209166667	4.98956144166667	4.67624076666667	4.03811705833333	6.8518622625	4.87245959166667	7.33828902083333	5.79243215833333	6.39954747916667	7.30888802083333	9.5192383875	4.30775345	6.35702110416667	5.43653326666667	7.62332840833333	8.1983393625	6.49286267916667	4.21246715416667	7.2172312625	5.49980207083333	5.13904645416667	7.7563220125	6.18290244583333	4.86752087916667	6.53097057083333	5.47569435416667	4.97227905833333	5.948468475	6.20074723333333	5.07707335416667	5.07162959583333	5.40801585416667	6.37312580833333	5.03478496666667	5.462367375	4.28257402916667	7.50434007916667	7.88640297083333	7.04035565416667	4.25764998333333	7.97319919583333	3.93531937083333	6.69347515833333	6.3745326875	6.63278815	4.8037463	4.12046859583333	7.34922049583333	5.04398305416667

TRIM56	12.524997525	11.646257275	11.500844625	11.4090056666667	12.5084928916667	11.53339965	12.2578527416667	11.39829615	12.1856912833333	11.6263096416667	11.5064020583333	12.8251494083333	12.4567650666667	11.6577322083333	11.830948725	11.765185225	10.874072375	12.24577265	11.28518	11.6906956833333	11.8527274916667	11.9570955666667	11.2650373666667	11.4129941416667	11.6933169166667	12.1673323833333	11.7958947916667	11.7120196166667	12.2594871416667	11.8527274916667	11.23807015	11.628296575	12.0494644666667	11.54260245	11.8924112666667	12.2943062333333	12.1121696	11.6259515166667	11.5895178083333	11.8265346166667	11.3406807666667	11.5614234666667	11.6092244333333	11.195266625	11.87418675	11.1409117416667	11.74784205	11.0355345916667	11.3961375	11.4590118416667	11.3065242916667	11.3957857333333	12.2848587416667	11.7699299	11.61832875	11.3068495166667	11.6522306833333	11.4313741833333	11.5772577666667	11.784585925	13.1609997	11.798162425	11.214698175	11.495328725	11.8171008	12.05061705	11.4682426833333	11.9348926	11.6006445416667	11.743693825	11.7572174666667	11.5824943666667	12.0253815583333	11.8000726	11.7925074083333	11.7742804333333	12.2099162083333	12.0230159333333	10.8206218083333	11.7343681166667	12.1827137333333	12.93348965	11.2524616583333	11.3413304583333	11.8450362083333	12.632709475	12.1583760416667	11.6929484666667	12.5974434583333	11.5813004	11.5177929833333	11.6910443916667	11.8418826916667	11.4255179583333	12.0893148833333	11.7814654666667	11.6659074916667	12.5506564333333	11.8954895166667	11.4601636916667	11.55276425	12.0376587166667	11.73285025	11.043790875	10.9317956333333	11.5200695166667	11.685253825	10.6336749583333	11.5114672666667	11.4722453166667	11.884179625	10.859746575	11.4151310833333	10.5968900416667	10.9000188916667	11.0068392583333	11.4817372166667	11.4354157666667	11.0268698583333	11.6522306833333

PAGE2	4.98009509166667	6.27245344166667	5.50338315833333	6.23079429166667	5.72466863333333	5.43182890833333	7.209690675	5.628745675	6.02645373333333	6.62158961666667	6.159395425	6.84636305	7.71624318333333	7.286116125	5.981391325	5.74037505	5.49736965	5.73863274166667	4.84820759166667	6.16609928333333	5.65427465	5.71516535833333	5.63936880833333	4.957419525	4.80827406666667	4.96401284166667	5.77198655	4.75698904166667	5.171525	4.93337844166667	5.780758525	7.7682787	5.5907794	5.89991223333333	4.60591755833333	5.112251075	5.110215025	6.133346875	5.15246255833333	5.51001904166667	5.710337225	5.0835026	5.7901556	6.58035519166667	4.80635555	5.80195006666667	5.4600655	6.05045175	7.24396096666667	4.6622505	6.22080705	5.2460388	5.87003104166667	5.4308407	6.18822558333333	5.35088976666667	6.456329775	5.40105285833333	5.26259698333333	5.64973066666667	5.63936880833333	5.93821120833333	4.31116040833333	6.29201916666667	5.710337225	5.08890834166667	5.4237743	6.44217180833333	5.33867198333333	7.27593380833333	6.511623825	6.42556058333333	8.14082359166667	10.5601410916667	5.62592511666667	5.14089556666667	5.15246255833333	5.33069273333333	4.19623050833333	5.4789746	5.18213341666667	5.86347250833333	5.88673599166667	5.59377575	6.32600435833333	5.85146426666667	6.82586836666667	6.54638811666667	5.99181078333333	5.33069273333333	5.75852464166667	12.4598136333333	5.26570160833333	5.29552266666667	4.15309530833333	4.8912062	5.10498871666667	5.09968536666667	6.43934115	7.54021029166667	5.29123589166667	6.21427064166667	5.94091113333333	11.8341069333333	5.50523408333333	8.48950848333333	6.1536546	5.91431983333333	12.2840300666667	5.83674730833333	9.46521605833333	5.5022085	5.54579601666667	5.229288475	10.286132425	4.74930484166667	4.664314175	5.98999241666667	5.363009525	7.227778225

SPATA46	8.59618360833333	8.77943517083333	8.56741365	8.8104251375	8.53905974583333	8.5265901625	9.4727937625	8.57427647083333	9.33686489166667	8.80803470833333	9.03350095416667	9.071858325	9.1997369875	8.41812605	8.7707314375	8.1379279875	7.8100294875	9.08115096666667	8.60013706666667	8.18810056666667	8.7266882375	8.72995544166667	8.2180637625	7.88786632916667	8.32438065833333	8.1179371875	8.36008461666667	8.49267887083333	8.68600224583333	8.693862425	8.36490141666667	8.1513203	8.7974409125	8.19669824583333	8.32538908333333	8.23240379583333	9.01825260416667	8.74935636666667	8.9262705375	8.7376700625	8.3858844375	8.89052767083333	8.40792234166667	7.96048081666667	8.4219307	8.14985335416667	8.12656328333333	8.66750117083333	8.03403849166667	8.2797761125	8.55724994166667	8.60775270833333	8.51107445416667	8.521353825	8.44818234583333	7.84350130416667	8.42544180833333	8.68703624166667	8.5302829625	8.260683925	9.35332098333333	9.15624333333333	8.3318434125	8.896741475	7.8942936	9.612529675	9.094379975	9.26239327916667	9.2208521125	8.91202839583333	9.4462680125	9.37430654583333	9.38518224166667	8.843587625	9.418298225	8.71581307916667	8.9505562125	8.33530674583333	8.49028507083333	8.62760862083333	8.58369964583333	9.0103053625	8.804245175	8.33038814166667	9.16897280416667	8.8104251375	9.7619923125	8.769244525	9.159632775	8.2398398125	8.93693732916667	8.56446075	8.5419361375	8.81577421666667	9.63590059166667	9.06721235	9.42360602916667	10.1761008041667	9.33598702083333	8.74980536666667	8.23742238333333	9.77412685416667	9.05475945416667	8.27311649583333	8.9007611375	8.8900448125	8.63473515833333	8.5072711875	8.10556687083333	7.90815084583333	8.33885354166667	8.90691079583333	8.22715757916666	8.95832772083333	8.65342978333333	8.63103852083333	8.1202170625	8.669051975	8.92909910833333	8.94502036666667

AL512785.2	8.59618360833333	8.77943517083333	8.56741365	8.8104251375	8.53905974583333	8.5265901625	9.4727937625	8.57427647083333	9.33686489166667	8.80803470833333	9.03350095416667	9.071858325	9.1997369875	8.41812605	8.7707314375	8.1379279875	7.8100294875	9.08115096666667	8.60013706666667	8.18810056666667	8.7266882375	8.72995544166667	8.2180637625	7.88786632916667	8.32438065833333	8.1179371875	8.36008461666667	8.49267887083333	8.68600224583333	8.693862425	8.36490141666667	8.1513203	8.7974409125	8.19669824583333	8.32538908333333	8.23240379583333	9.01825260416667	8.74935636666667	8.9262705375	8.7376700625	8.3858844375	8.89052767083333	8.40792234166667	7.96048081666667	8.4219307	8.14985335416667	8.12656328333333	8.66750117083333	8.03403849166667	8.2797761125	8.55724994166667	8.60775270833333	8.51107445416667	8.521353825	8.44818234583333	7.84350130416667	8.42544180833333	8.68703624166667	8.5302829625	8.260683925	9.35332098333333	9.15624333333333	8.3318434125	8.896741475	7.8942936	9.612529675	9.094379975	9.26239327916667	9.2208521125	8.91202839583333	9.4462680125	9.37430654583333	9.38518224166667	8.843587625	9.418298225	8.71581307916667	8.9505562125	8.33530674583333	8.49028507083333	8.62760862083333	8.58369964583333	9.0103053625	8.804245175	8.33038814166667	9.16897280416667	8.8104251375	9.7619923125	8.769244525	9.159632775	8.2398398125	8.93693732916667	8.56446075	8.5419361375	8.81577421666667	9.63590059166667	9.06721235	9.42360602916667	10.1761008041667	9.33598702083333	8.74980536666667	8.23742238333333	9.77412685416667	9.05475945416667	8.27311649583333	8.9007611375	8.8900448125	8.63473515833333	8.5072711875	8.10556687083333	7.90815084583333	8.33885354166667	8.90691079583333	8.22715757916666	8.95832772083333	8.65342978333333	8.63103852083333	8.1202170625	8.669051975	8.92909910833333	8.94502036666667

GRM8	4.90871175	5.211919525	5.31824546666667	5.17697859166667	4.996867625	6.04799355833333	5.2891249	4.18926569166667	5.08161569166667	5.02903219166667	4.688902775	5.46938143333333	5.2543857	5.44029105833333	5.367183075	4.31414184166667	7.76084795833333	5.47794636666667	5.96342883333333	5.03726580833333	5.451608675	5.30264448333333	5.1863758	5.96001181666667	5.15873735	6.263501475	5.4789746	5.58896354166667	5.92854135	4.90147810833333	5.79119435	4.59265796666667	5.81652036666667	7.40546740833333	5.81120035833333	4.98444206666667	5.13847554166667	5.78537223333333	5.64021978333333	5.93571900833333	5.51803240833333	4.55283246666667	5.073382675	5.2543857	4.65176085833333	5.74506199166667	5.091030975	5.31421083333333	4.210787125	5.328555675	5.29020444166667	5.550710675	4.82796093333333	5.0835026	5.51386359166667	4.61955908333333	4.69115821666667	6.54994171666667	4.98009509166667	5.45265249166667	6.20814583333333	6.14334293333333	6.54365829166667	6.45472701666667	5.59754975833333	6.0167621	4.23097901666667	5.553511425	5.74037505	4.94478124166667	6.028109975	5.40518485833333	5.85609921666667	5.62140730833333	5.65824568333333	5.1168523	6.169883075	4.92055984166667	5.68499903333333	5.28262243333333	7.0114101	9.04085205833333	6.15447426666667	6.30044350833333	5.60777716666667	7.20393616666667	6.21427064166667	5.81652036666667	5.80528018333333	5.43935509166667	6.02645373333333	4.66004348333333	6.02558836666667	4.82904071666667	4.59997769166667	5.18885664166667	5.949734275	6.65424311666667	5.71985138333333	5.73297515	4.485186325	6.33264478333333	5.81821864166667	4.91654405	5.81120035833333	5.38263135833333	6.29969763333333	5.83208956666667	5.72382095833333	6.169883075	6.83744154166667	6.9125939	5.455892825	5.19860551666667	5.33329914166667	5.43596139166667	5.03823209166667	6.06147365	5.80937455	5.16314315

SDF2	11.9720790416667	12.075266	12.16610385	12.0282380083333	11.8186960583333	11.8962786833333	12.0388164916667	11.99193365	11.9605620583333	11.9177333166667	11.9207928583333	12.0001864416667	11.825765325	12.1951832916667	12.0744704	11.8779799083333	12.503844475	11.9860534666667	12.2848587416667	12.2792629666667	12.1549544416667	11.9728603333333	12.2163587	11.938838275	12.055992875	11.8059034833333	11.7861402083333	11.9825076333333	11.6761092166667	11.9318404166667	11.9697901833333	12.068210875	12.1703301333333	12.3378199666667	11.93371625	11.7347600333333	11.859248825	12.10672325	11.921685825	11.8321536333333	12.198604375	12.0721549166667	12.3484591166667	12.3083565083333	12.2116409166667	12.42337295	12.2210896416667	12.0616030916667	12.2964028333333	12.1998168583333	12.242111125	11.9616964916667	12.1312748	12.0872990166667	12.0881187916667	12.226050525	12.1480274583333	12.0889146833333	12.0945968666667	12.0417350833333	11.876483925	12.686781525	12.7743205	12.206579925	12.7873683333333	12.1636527	12.367659325	12.2867043083333	12.6050803083333	11.9036209583333	12.196460925	12.0067796166667	12.5662353166667	12.5780122833333	12.2374670666667	12.3518594833333	12.0793162416667	12.0555750333333	12.5226496333333	12.1254015666667	12.4593508916667	12.4248504333333	12.1229210916667	12.3571151666667	11.9868001	11.943975875	12.8213874666667	12.3763083916667	12.6046256583333	12.4443760583333	12.4082756583333	12.115901275	12.2311025	12.2755744916667	12.2611765333333	12.278442975	12.1549544416667	12.1270603333333	12.1217141333333	12.343956025	12.225259825	12.8213874666667	12.324942025	12.5780122833333	12.715938075	12.1711742666667	12.3966157416667	12.6966269416667	12.3492861833333	12.3820596583333	12.217702275	12.4496792916667	12.3310232666667	12.9012827916667	12.3896401833333	12.5506564333333	12.3698220916667	12.4945779666667	12.3815660416667	12.217702275

GALNT2	12.29141735	11.5656597	12.136374875	11.7336295416667	12.0164637	12.8234678083333	11.7452699833333	11.747396125	11.86975775	12.118621125	12.2918290666667	12.856162525	11.8530999083333	11.73798855	12.0756871	11.7644689416667	11.9616964916667	11.2254735166667	11.8947334916667	11.94052505	12.2570148333333	11.2521123083333	12.0697739833333	11.6433290333333	11.6025118833333	12.47909375	11.8348518583333	11.8709570583333	11.9513573	11.9285599833333	11.7175871333333	11.7564937416667	11.8523757333333	12.2771993416667	12.047012	12.649121475	12.2637428083333	11.8012624833333	11.8619112333333	12.0593104333333	11.6540451166667	11.759780275	11.5598018166667	11.8481133916667	11.8962786833333	11.8205903833333	11.5237451916667	11.7418730833333	11.5226770083333	11.830948725	11.666692125	11.8749533333333	11.6489233	11.8337195083333	12.0514729416667	11.6440773083333	11.6099448083333	11.6466404583333	11.7081843583333	11.5298755416667	12.3811083333333	12.539129975	12.2396159	13.257439275	12.6704503833333	12.287955975	13.363866775	12.3610276166667	12.9901031666667	13.2560414333333	12.4505576916667	12.4181461583333	12.5604394333333	12.4297307333333	12.813963275	12.867358575	12.136374875	12.3365706083333	12.929488325	12.5472970333333	12.460239975	13.4026847	12.2336399666667	12.5917146833333	12.1229210916667	12.3182821416667	12.7687032	13.0571358166667	12.672014175	12.5406352083333	12.857326075	12.6436968916667	12.9472642583333	12.6528003583333	13.6506232916667	12.216784075	12.0317754666667	13.316609775	12.2998821166667	12.909394625	12.9185113	12.10672325	12.2683870833333	11.9789241416667	11.8565277666667	12.837133975	12.442132175	12.1728373083333	12.5084928916667	12.8208910916667	12.4491875083333	11.917277775	13.011963125	12.4645960083333	13.0590979083333	12.1130081666667	12.5212289166667	12.7076553	11.6404855583333	12.1847949166667

AMZ1	8.316949125	8.23151899583333	8.1173706	8.25909860833333	9.33343475833333	8.25669035416667	9.40884270833333	8.629624975	9.40793799166667	8.64713263333333	8.81311722083333	9.45575422083333	9.37055386666667	8.5912661	8.5981398125	8.23151899583333	7.87636192916667	9.2986405375	8.05691018333333	8.166091425	8.82849306666667	8.92293245	8.26015074583333	8.32489938333333	8.0210023875	8.32932159583333	7.96216195833333	7.89220262083333	8.51361290833333	8.33576940416667	7.96861178333333	8.11856490833333	8.77166664166667	8.5888187125	8.445628625	8.3778959125	9.11562042916667	8.49909290416667	8.3858844375	8.45927208333333	8.03403849166667	8.1301513875	7.904987325	7.61610380833333	8.09669910833333	7.66416018333333	7.62063305	7.84714889583333	8.29554877083333	8.26940804583333	8.25619600416667	8.2667823375	8.589828825	8.0697263375	7.77920590416667	7.33943287916667	8.4877078375	7.94199372916667	8.16044315833333	7.8153205625	8.5240442875	8.7814928125	8.23493734583333	8.5917752625	8.6509725	9.32783585	8.7042454375	9.11009815416667	9.22893656666667	9.04058751666667	9.04389905416667	8.97007850416667	9.71120844166667	8.7979	8.98911833333333	8.68847175416667	8.816206225	8.63473515833333	8.39287426666667	8.60058669166667	8.94164972916667	9.2633039	8.852386675	8.44215722083333	8.84266431666667	8.637994175	8.79115110833333	8.74935636666667	8.99745976666667	8.18449080416667	8.581156975	8.5457303375	8.8366618375	8.57627579166667	9.1542486125	8.7539782125	9.295507025	9.00798550416667	9.16327657916667	8.63473515833333	8.77324584583333	8.98638951666667	8.32335070416667	7.88736972916667	8.5801776625	8.562975175	8.373874725	8.41456412083333	8.62264167916667	8.54821066666667	8.38685527083333	8.31887321666667	8.60312590833333	7.87288825	8.2010289625	7.97266434583333	8.26181718333333	8.6146142125	8.33424979583333	8.60254101666667

GNA12	8.316949125	8.23151899583333	8.1173706	8.25909860833333	9.33343475833333	8.25669035416667	9.40884270833333	8.629624975	9.40793799166667	8.64713263333333	8.81311722083333	9.45575422083333	9.37055386666667	8.5912661	8.5981398125	8.23151899583333	7.87636192916667	9.2986405375	8.05691018333333	8.166091425	8.82849306666667	8.92293245	8.26015074583333	8.32489938333333	8.0210023875	8.32932159583333	7.96216195833333	7.89220262083333	8.51361290833333	8.33576940416667	7.96861178333333	8.11856490833333	8.77166664166667	8.5888187125	8.445628625	8.3778959125	9.11562042916667	8.49909290416667	8.3858844375	8.45927208333333	8.03403849166667	8.1301513875	7.904987325	7.61610380833333	8.09669910833333	7.66416018333333	7.62063305	7.84714889583333	8.29554877083333	8.26940804583333	8.25619600416667	8.2667823375	8.589828825	8.0697263375	7.77920590416667	7.33943287916667	8.4877078375	7.94199372916667	8.16044315833333	7.8153205625	8.5240442875	8.7814928125	8.23493734583333	8.5917752625	8.6509725	9.32783585	8.7042454375	9.11009815416667	9.22893656666667	9.04058751666667	9.04389905416667	8.97007850416667	9.71120844166667	8.7979	8.98911833333333	8.68847175416667	8.816206225	8.63473515833333	8.39287426666667	8.60058669166667	8.94164972916667	9.2633039	8.852386675	8.44215722083333	8.84266431666667	8.637994175	8.79115110833333	8.74935636666667	8.99745976666667	8.18449080416667	8.581156975	8.5457303375	8.8366618375	8.57627579166667	9.1542486125	8.7539782125	9.295507025	9.00798550416667	9.16327657916667	8.63473515833333	8.77324584583333	8.98638951666667	8.32335070416667	7.88736972916667	8.5801776625	8.562975175	8.373874725	8.41456412083333	8.62264167916667	8.54821066666667	8.38685527083333	8.31887321666667	8.60312590833333	7.87288825	8.2010289625	7.97266434583333	8.26181718333333	8.6146142125	8.33424979583333	8.60254101666667

SYT6	6.05307568333333	5.83776461666667	6.13670685833333	5.591740875	8.28997516666667	5.1168523	6.4040341	6.53809915833333	6.903905225	5.91627995	6.36103899166667	6.96657695833333	7.16614005833333	6.29125269166667	7.04142870833333	5.710337225	6.99096820833333	6.19129395	4.979047925	6.526432	7.224148725	6.485693525	6.45841340833333	6.00206103333333	5.73491465833333	6.21191129166667	4.7847361	5.78626178333333	6.03335128333333	5.766309475	5.80264275	5.81212459166667	5.78537223333333	6.29901051666667	5.93746846666667	5.1555947	5.47616439166667	5.34196935833333	5.53292085833333	5.36511784166667	5.826473825	5.546831425	4.78700018333333	5.5907794	5.85413349166667	5.46647429166667	5.75213593333333	5.38263135833333	6.28265325	5.54102934166667	5.47315375	6.27652200833333	6.54089473333333	5.95243336666667	6.22080705	5.24931593333333	6.2379381	5.455892825	5.49334536666667	4.753728625	6.06574364166667	6.78642360833333	4.57343223333333	5.71882016666667	8.21353541666667	5.553511425	6.698517925	6.64893395	6.29278439166667	7.08111885833333	5.57060381666667	5.9573564	7.57402961666667	6.05897065833333	6.987286825	6.35578110833333	6.66522548333333	5.69973553333333	5.41802705	6.68532921666667	7.05521155	6.80992164166667	6.59052684166667	5.70153421666667	6.31323984166667	6.297552775	5.39281689166667	7.86994016666667	6.63442134166667	6.1777684	6.65843384166667	5.477009275	4.77272485	6.2176337	5.949734275	5.41246783333333	5.15246255833333	5.99418615	5.41246783333333	5.29238863333333	5.7672538	6.777132775	6.09669481666667	5.16081746666667	6.243623775	6.20496000833333	5.710337225	6.38393616666667	7.29788705833333	6.295927975	4.453007975	5.36922546666667	5.62957723333333	6.15183188333333	7.14519241666667	6.59599640833333	4.9195424	5.189937375	5.57252066666667	7.16971491666667

TMEM64	4.71218959166667	4.72105203333333	4.90679766666667	4.66334196666667	4.759183025	3.976955525	5.8386672	4.78700018333333	5.576316275	4.56806040833333	4.692141475	5.7745543	4.98771553333333	5.0100817	4.79716553333333	4.83355489166667	5.25047555833333	4.37388213333333	4.50112465833333	5.52011276666667	4.30917759166667	4.19529993333333	4.685722125	4.74219220833333	4.4473261	5.43935509166667	5.61840579166667	4.83558559166667	4.48159451666667	4.1683668	4.33428121666667	4.852466025	4.31516280833333	4.07678143333333	5.24195544166667	5.65633813333333	4.421470575	4.49113386666667	4.453007975	4.27468664166667	4.82580888333333	4.35819395	4.77483110833333	5.18015243333333	4.45030740833333	4.86479268333333	5.2783407	4.4227704	4.42481975833333	4.67278611666667	4.61830805833333	4.15432630833333	4.82580888333333	5.19309993333333	4.137419	4.40284696666667	4.89595425	4.576370125	4.45699755833333	4.62587865833333	4.49113386666667	5.46278851666667	4.16939595	5.628745675	5.88760754166667	4.66519471666667	4.41002290833333	6.54089473333333	5.39700335833333	4.92455391666667	4.46402134166667	5.4600655	5.8893734	4.98651175	4.48717731666667	5.53695673333333	4.73832164166667	4.92455391666667	5.37867954166667	4.177708	6.89596523333333	5.75852464166667	5.35182895833333	4.63625878333333	4.40180795833333	5.13015945	6.16763958333333	4.22787308333333	5.51291259166667	3.99817013333333	4.57718236666667	4.19346535	4.85447411666667	4.263255	4.59784559166667	4.35397924166667	5.229288475	4.9110181	4.50430228333333	4.36495859166667	4.86384488333333	5.59572529166667	4.93761881666667	4.873988275	4.92878054166667	4.54330015	4.78798888333333	5.42880058333333	5.06903989166667	6.17067161666667	4.49312724166667	4.78047113333333	4.67172261666667	5.69189906666667	3.98827198333333	4.18926569166667	5.04667276666667	4.4047953	4.57343223333333	4.54769205

AC012531.3	5.23829215	6.9578512875	6.76283356666667	7.2696657875	7.94144794166667	9.61965483333333	7.45188757916667	7.02852860416667	7.24228474166667	6.28224760416667	7.20940322083333	7.0225346125	5.5834999375	5.81440876666667	7.07892225833333	7.11582667083333	5.63700449166667	6.73034165416667	6.85386289583333	7.48834681666667	8.31936164166667	7.31346904166667	10.0951867208333	5.03774895	4.55817304166667	6.46389307916667	5.48660064166667	5.93616005833333	5.66897774583333	6.3546980125	6.0730132375	6.54903211666667	6.4688758875	5.78962339583333	6.08148082916667	5.7572824375	6.7240842	6.69748503333333	5.88558745416667	7.4371520375	5.929011625	6.0370695625	5.02107872083333	6.93005625833333	5.81783112083333	5.71266739583333	7.50138857083333	7.39897763333333	5.35524580416667	8.91961008333333	7.0453601625	7.11953482916667	6.63477370416667	6.42153188333333	5.82319308333333	6.42153188333333	7.269064225	5.65778688333333	4.90420065833333	5.126787325	9.8400754375	8.84827898333333	11.2242574333333	10.6840601916667	11.8121190625	11.0361278125	10.7776906041667	9.44936770833333	11.6888934125	11.6097634833333	6.91152685416667	10.9721298583333	10.5947252625	10.4761612458333	10.64594635	10.5071483583333	10.40949865	9.1941644625	11.2931173958333	11.9102808958333	10.9565945666667	11.1969501583333	11.060242575	4.96577665833333	8.7672794	11.7531983791667	10.9250547833333	10.5110240083333	6.76283356666667	10.39035585	9.95665007083333	10.9616731416667	5.492817725	10.4864721541667	11.1697849541667	10.6439982125	11.9350948958333	10.6490200291667	10.8211520791667	11.4190066583333	10.5737228125	11.0738714625	6.78608367916667	11.1254633708333	11.8613787333333	9.38298149583333	9.2335370125	11.75113845	9.39275522083333	10.926151175	9.648063575	8.97838820833333	11.7114925666667	11.5521940125	10.8860148666667	7.0715031875	12.004585	9.606054925	5.9378398375	5.84364492916667

HOXC10	5.23829215	6.9578512875	6.76283356666667	7.2696657875	7.94144794166667	9.61965483333333	7.45188757916667	7.02852860416667	7.24228474166667	6.28224760416667	7.20940322083333	7.0225346125	5.5834999375	5.81440876666667	7.07892225833333	7.11582667083333	5.63700449166667	6.73034165416667	6.85386289583333	7.48834681666667	8.31936164166667	7.31346904166667	10.0951867208333	5.03774895	4.55817304166667	6.46389307916667	5.48660064166667	5.93616005833333	5.66897774583333	6.3546980125	6.0730132375	6.54903211666667	6.4688758875	5.78962339583333	6.08148082916667	5.7572824375	6.7240842	6.69748503333333	5.88558745416667	7.4371520375	5.929011625	6.0370695625	5.02107872083333	6.93005625833333	5.81783112083333	5.71266739583333	7.50138857083333	7.39897763333333	5.35524580416667	8.91961008333333	7.0453601625	7.11953482916667	6.63477370416667	6.42153188333333	5.82319308333333	6.42153188333333	7.269064225	5.65778688333333	4.90420065833333	5.126787325	9.8400754375	8.84827898333333	11.2242574333333	10.6840601916667	11.8121190625	11.0361278125	10.7776906041667	9.44936770833333	11.6888934125	11.6097634833333	6.91152685416667	10.9721298583333	10.5947252625	10.4761612458333	10.64594635	10.5071483583333	10.40949865	9.1941644625	11.2931173958333	11.9102808958333	10.9565945666667	11.1969501583333	11.060242575	4.96577665833333	8.7672794	11.7531983791667	10.9250547833333	10.5110240083333	6.76283356666667	10.39035585	9.95665007083333	10.9616731416667	5.492817725	10.4864721541667	11.1697849541667	10.6439982125	11.9350948958333	10.6490200291667	10.8211520791667	11.4190066583333	10.5737228125	11.0738714625	6.78608367916667	11.1254633708333	11.8613787333333	9.38298149583333	9.2335370125	11.75113845	9.39275522083333	10.926151175	9.648063575	8.97838820833333	11.7114925666667	11.5521940125	10.8860148666667	7.0715031875	12.004585	9.606054925	5.9378398375	5.84364492916667

CCDC62	6.29125269166667	4.80316131666667	4.91654405	5.256776325	4.63723314166667	4.649483075	3.95017479166667	4.66004348333333	4.46597973333333	4.24013239166667	4.28008616666667	4.40083348333333	4.55971610833333	4.2379826	4.52238	5.98220235	5.58117110833333	4.285092425	5.94443094166667	5.192100225	4.082140775	5.55559675	5.11789035833333	4.73490975	5.118961025	4.96517085	4.359146625	4.22036293333333	4.81376534166667	4.80827406666667	5.31119278333333	4.69863704166667	4.1481371	4.45503961666667	5.2460388	5.37127428333333	4.76548715	5.10387183333333	4.740346475	5.0412383	4.93234401666667	5.122114675	4.75255506666667	5.89368925	4.83457635833333	5.54303723333333	5.09419765	5.47794636666667	5.04338205833333	5.59572529166667	4.65176085833333	6.67377979166667	5.091030975	5.1598283	4.81716043333333	6.423307775	4.681784575	5.4892176	6.258372575	5.71123295833333	4.43531826666667	4.79518681666667	5.40405401666667	5.42273213333333	4.546642225	5.14931930833333	4.49113386666667	4.27071034166667	5.33153680833333	4.51268873333333	4.064898825	5.41802705	4.58777895833333	5.44322508333333	4.557709	4.768670525	5.2108879	4.62057545833333	4.19434771666667	3.95680781666667	4.33532039166667	4.94355658333333	5.933890025	4.74123235833333	4.46516671666667	4.95961636666667	4.421470575	5.46278851666667	4.06389798333333	4.57343223333333	4.92991125833333	5.06570035833333	4.01810879166667	4.06900396666667	5.32031858333333	5.20882029166667	4.99894050833333	4.922546525	4.39446349166667	4.85659321666667	4.936774575	5.43710514166667	5.1598283	5.67327496666667	4.91552208333333	4.30527630833333	4.90147810833333	7.87869541666667	6.25099584166667	6.53536243333333	4.46696494166667	7.20088341666667	4.996867625	4.79621500833333	4.57827949166667	4.90470955	6.19818808333333	6.25026105	5.62674555833333	4.83558559166667

PLEKHM2	11.687529825	11.680364275	11.946316525	11.7190625916667	12.3610276166667	11.1993790583333	11.5487335833333	12.021831	12.004381825	11.98994525	11.7109162916667	11.6452682	11.9868001	11.7296484916667	12.5079805333333	11.8116053416667	11.4474681583333	12.0653086666667	12.2692837333333	11.9828339166667	12.0108213333333	12.341378075	12.0537518916667	11.9931218416667	11.8205903833333	11.7027128	12.3083565083333	12.1541314	12.3083565083333	11.875371875	12.06738545	12.00329435	11.5934022416667	11.327862475	11.56491665	11.55276425	12.461524275	12.187427525	12.2461720416667	12.1046691916667	12.0112152666667	12.126629875	11.6602573333333	11.8163038583333	11.8834269416667	12.2508814916667	11.8241137083333	12.0040290833333	11.686699925	11.5891254	11.77986195	12.2747673416667	12.161623975	12.120943025	12.2073843166667	12.1096451083333	11.9467504166667	12.1410311166667	11.4631308333333	11.7259183166667	12.049870975	11.2606101916667	12.671473625	12.3802421833333	11.770678525	12.3554452	11.722386025	11.5991025	12.0395951	11.9078069166667	11.2254735166667	11.75788825	11.930808275	11.626670975	12.0857092083333	12.2906208	11.5188708083333	12.2322263916667	12.301617475	12.5277484583333	11.671269575	11.6948221166667	11.9409022083333	12.2701693666667	12.3789904833333	12.0197364166667	11.4496634833333	12.04365515	11.2258131916667	12.1476183833333	12.2248275416667	12.2998821166667	11.937226525	11.9289106916667	12.812914675	12.0395951	12.2289445583333	12.0819963833333	12.19731135	11.24605735	11.8202425833333	11.2740276	12.2981372583333	12.0317754666667	11.848881975	12.3126780333333	12.4337570333333	12.0245529166667	12.8041062916667	12.285271325	11.796702325	12.1852414666667	12.6586586666667	12.2040899	12.4469679916667	12.247959925	11.84965965	12.2901521833333	11.0633247666667	11.61112325

TMEM185B	11.6799309666667	11.174277875	11.218298125	11.0139017166667	11.3957857333333	11.7369473333333	11.0964662083333	11.4890705333333	11.4737788083333	11.3861262916667	11.5895178083333	11.6492807916667	11.3957857333333	11.5223462	11.46509415	11.0538325833333	10.9654875083333	11.4126401666667	11.6726997416667	11.639055925	11.5367767666667	11.1277733333333	11.67393075	11.8220738583333	11.5223462	11.8924112666667	11.1256433416667	11.2715325333333	8.66880180833333	11.2050434916667	11.2895126416667	11.545468825	11.5150484916667	10.8258465666667	11.8607926333333	11.5589969083333	11.3043532	11.6687463083333	11.214698175	11.351341375	11.22125475	10.958948725	11.3805222416667	11.6170945166667	11.4463806916667	11.0644262666667	11.4879615666667	11.3015343416667	11.392737725	11.4214230083333	11.4409493166667	11.5676618166667	11.8356051666667	11.6887018916667	11.6329290083333	11.5731480083333	11.5382360083333	11.113611625	11.4368977583333	11.7324169333333	12.0445037083333	12.1262500416667	12.2002559333333	12.5671498916667	12.0917340666667	12.835491775	13.0307376	11.5926397083333	12.2835716083333	12.1130081666667	12.3343950916667	12.62011135	12.580271675	12.1342141916667	12.4461573416667	13.264123675	11.76324455	12.5788865916667	12.0392089666667	12.4903405333333	12.9036231416667	12.0116503666667	12.23183945	12.1720161916667	11.830948725	12.2302891583333	13.1569608666667	12.2215063083333	12.5963701666667	11.9165677	12.2268634416667	12.1891609666667	12.1237921916667	12.3631593333333	12.8718544916667	12.5706160166667	12.3351872666667	12.11260975	12.0376587166667	11.8186960583333	12.3571151666667	11.8232517	12.013260375	12.2875140666667	12.409680475	13.5593280666667	12.1895397416667	12.6785589083333	12.4105900333333	12.2383566166667	11.121759625	11.5132414833333	12.2322263916667	13.3430209416667	11.9593365833333	11.4527169333333	12.1790995083333	12.264576775	14.56279245	11.6787724916667

ZFP1	10.4775633333333	10.0903015	9.80576564166667	9.79062058333333	9.13460934166667	9.80576564166667	9.26082591666667	10.0924722666667	9.311300325	9.72070379166667	9.95147331666667	10.2583120916667	9.05561776666667	9.82733675	9.78918780833333	9.80143100833333	9.98312965833333	9.08960063333333	9.89466155833333	9.92093743333333	10.0965198666667	9.75620250833333	9.82203395	9.8972867	9.87303573333333	11.072670925	9.50685594166667	9.59418116666667	9.19200934166667	10.1366762916667	9.94794899166667	9.889693225	9.51223504166667	9.71227291666667	10.4480363583333	10.93064125	9.36987781666667	9.82295156666667	9.98187775833333	9.74820641666667	9.76350663333333	10.0778458	9.83764681666667	9.99259065	10.2478255083333	10.13859765	10.130420225	10.001418875	10.1870376	9.92053275833333	9.97215941666667	10.1413216916667	9.15886451666667	9.66513115	9.87254320833333	9.80885460833333	10.0048138083333	10.1601611333333	9.85368629166667	9.9229238	10.2330333333333	10.4228179333333	10.233851875	9.92703645833333	11.3597850833333	10.4204003083333	10.8071092416667	9.90438676666667	10.628160175	9.96660331666667	10.0815238083333	10.382913225	10.2919749833333	10.090730825	10.2466873083333	10.1849087083333	9.94460186666667	9.83584716666667	10.7220730083333	10.2351017916667	10.3656176	10.4204003083333	10.1156550583333	9.98150248333333	9.46521605833333	11.0390903416667	10.154948775	10.3603019333333	9.88298375833333	10.4004658833333	10.1262351166667	10.6079735416667	9.89226535833333	10.5104189666667	10.06195985	9.84200871666667	10.3293667083333	10.3519044416667	10.4887010333333	9.4968836	11.02981265	10.0247316333333	10.3317121166667	10.1953308333333	10.036942525	10.85221535	10.2924466666667	10.2962241416667	10.46260805	10.2213761083333	10.1910863	9.91887936666667	10.1428729416667	10.7519521333333	9.84676184166667	9.79101400833333	11.2356259583333	10.523220425	10.2092790583333	10.2111469

ST3GAL3	10.4634265083333	8.283955225	8.12933435833333	7.69757114166667	7.55551181666667	8.79951210833333	7.31674675	7.7483928	8.08289571666667	8.45999591666667	8.34668346666667	9.72574979166667	8.552420375	8.113521575	8.075186025	8.20331464166667	9.45194998333333	7.467981625	8.641468925	8.14449565	8.2229273	7.738684825	8.58808473333333	8.354234475	8.54218048333333	10.5360893083333	8.48569109166667	7.98243114166667	7.63657405	8.40567906666667	8.68775965833333	8.42171803333333	8.53140681666667	8.7735097	10.579886175	10.6691322083333	7.672013725	8.08807009166667	8.08865246666667	8.34128665833333	8.49975086666667	8.5994674	8.8334785	8.74174598333333	9.54132330833333	8.66930214166667	9.11077986666667	8.25990825	8.25745250833333	8.364240675	8.36762415833333	8.38817050833333	8.39209266666667	8.29147479166667	8.82318806666667	9.23929971666667	8.37604669166667	8.69317739166667	8.42214336666667	9.0772993	8.940002325	8.46508935	8.093315875	9.40511030833333	9.219699425	8.618769925	8.475275825	8.140254425	8.925575825	8.16735940833333	10.0309458333333	9.13033400833333	8.28495673333333	8.11712540833333	8.82621398333333	8.64543325	8.21881454166667	9.448687325	8.19143718333333	9.29702861666667	8.650306125	8.14660076666667	8.78635625	9.10485650833333	7.75542415833333	8.38319035	7.84588375	8.027292325	8.96273033333333	9.25672935833333	8.49745698333333	8.93897689166667	9.59563205	8.25990825	8.72311269166667	9.446046	9.37403768333333	8.0459923	8.14346960833333	9.22515735833333	9.10532413333333	8.74819925	8.15563305	8.76079081666667	8.94525708333333	9.32526180833333	8.68130785	9.39525429166667	8.41742745833333	8.45045408333333	9.60919951666667	8.90434914166667	8.96850814166667	9.6428903	9.55621784166667	9.55812764166667	9.4825512	9.47772424166667	10.1501004333333	8.96094025833333

OR6S1	5.14192449166667	5.07218190833333	5.38964895833333	5.85701861666667	5.67327496666667	4.062876875	6.57734461666667	6.25335921666667	5.661700075	5.9323169	6.68472555	7.06773185833333	6.61087155833333	6.08697085833333	6.31786493333333	6.41036045833333	5.10077205	5.99349075	5.550710675	6.48928715	6.58901686666667	6.10313131666667	6.19282915833333	5.34734255	4.59897825	5.80356945	5.78626178333333	6.2379381	6.73133699166667	6.527218175	5.68689626666667	5.58021739166667	5.40405401666667	5.6241104	6.17154675833333	5.01953916666667	4.49216794166667	5.47990566666667	5.14829798333333	4.97597943333333	4.73295375	7.27114775833333	4.58241979166667	5.50615049166667	5.16838866666667	5.34966958333333	5.57926250833333	5.5614942	4.31803169166667	5.02371664166667	4.57827949166667	4.59369346666667	5.41985199166667	5.34734255	5.97436386666667	4.873988275	5.5927414	4.63723314166667	6.26504835	5.81120035833333	7.040686725	6.322468275	5.221531825	6.52781796666667	5.18759319166667	4.695239575	5.92603411666667	6.50929938333333	6.862021425	6.438674975	6.93552516666667	6.84380731666667	6.57813499166667	6.17067161666667	6.88314723333333	7.05900955833333	5.98737700833333	5.51386359166667	6.29201916666667	6.79057516666667	6.3460148	6.46640339166667	5.35476978333333	5.67810205833333	5.75213593333333	5.75112915833333	5.6322886	7.0369697	8.38217830833333	5.50024889166667	7.04503860833333	5.72790364166667	4.98892909166667	6.811853775	5.95074298333333	5.60680040833333	5.05687025	4.97490869166667	6.38393616666667	5.06903989166667	4.82580888333333	7.58408555833333	4.58241979166667	5.122114675	5.78537223333333	5.922335025	5.933890025	6.34225285833333	4.39039918333333	5.92321303333333	4.41203210833333	5.24498075	5.20449400833333	6.075953425	5.83674730833333	5.54303723333333	5.2108879	4.94355658333333	5.33648908333333	6.12847228333333

SEM1	8.10691838333333	6.20884429166667	4.87091625	6.09436185833333	6.399909175	5.83589014166667	6.04082925833333	5.557624025	5.892821925	6.27004911666667	5.217220275	7.17198438333333	6.72313661666667	5.87782166666667	6.26504835	5.26259698333333	5.78160560833333	5.69296955833333	7.0114101	6.06230976666667	6.03052256666667	6.33187936666667	6.041758325	4.748342275	5.7745543	6.93616958333333	6.777132775	4.263255	6.36616499166667	6.38554745833333	5.67523288333333	5.661700075	6.633753725	6.135921175	6.61289744166667	7.82539101666667	5.71516535833333	5.54392786666667	6.26211881666667	5.91719066666667	5.68783094166667	6.00459881666667	5.73943181666667	6.87917671666667	7.52673725833333	7.03811636666667	7.53624973333333	6.50650844166667	6.0079005	5.79999791666667	6.72705409166667	6.542385675	6.438674975	6.39918578333333	6.53536243333333	7.019638175	6.97276299166667	6.47646620833333	6.28109695833333	7.33501970833333	9.02927973333333	6.79468460833333	6.10954496666667	7.14213880833333	5.75682274166667	5.73195755	6.60956075833333	5.96423755	4.832334525	5.6458775	6.49170240833333	6.18980128333333	5.95923255	6.07263301666667	7.40546740833333	6.297552775	6.69642741666667	7.64275718333333	7.11143919166667	6.59533788333333	7.43742328333333	8.48321643333333	6.02558836666667	4.47089620833333	6.43490204166667	7.96526580833333	7.0168934	5.37341235	6.63512606666667	6.811853775	5.514879125	8.72089346666667	8.164333775	7.02704855833333	5.30562465833333	6.74677720833333	5.29675638333333	6.53205264166667	6.64016859166667	6.79403766666667	6.891986525	6.675144925	7.458458425	6.41036045833333	6.9125939	7.908404275	6.77501233333333	7.67307831666667	6.25518099166667	6.060789425	7.57457279166667	5.99672560833333	6.74958845833333	6.09588644166667	6.78114418333333	7.49600651666667	9.29162124166667	7.44095513333333	5.845937625	7.20023056666667

RERE	6.18980128333333	4.16939595	4.760314225	4.25488725	5.10174316666667	5.279548575	4.25100265833333	4.32946600833333	4.210787125	4.83671265833333	4.341768725	4.30130591666667	4.33637641666667	3.98271365	4.83671265833333	3.98026660833333	4.792098225	5.1863758	6.421178475	4.86184074166667	4.98009509166667	5.6976834	5.02158241666667	5.106111275	5.20550439166667	5.10174316666667	5.40105285833333	5.34734255	5.26570160833333	5.01953916666667	6.21600831666667	5.4712492	5.0100817	5.19309993333333	6.91035588333333	5.6488719	5.70649276666667	5.808344225	5.43935509166667	6.08790970833333	6.14083579166667	5.38372931666667	5.75213593333333	5.34297193333333	5.845937625	5.63443499166667	6.03052256666667	4.80433128333333	5.826473825	5.38263135833333	5.6488719	6.05225019166667	4.67674131666667	5.05069551666667	4.93337844166667	6.075953425	5.45265249166667	5.873141325	5.51291259166667	5.5927414	4.79621500833333	4.23901671666667	5.81120035833333	4.809512175	4.71793841666667	4.98219815	4.86699436666667	4.4619761	5.91334729166667	4.56305699166667	3.9601097	4.64734200833333	4.048345425	4.33133893333333	4.1683668	4.4159867	4.957419525	5.43596139166667	5.363009525	4.75255506666667	4.3276422	4.097912125	4.62990135	5.14829798333333	4.74641638333333	4.39670516666667	4.35998614166667	4.366029925	4.20109206666667	5.04230353333333	4.82998554166667	5.63053526666667	5.18519845833333	4.62790769166667	6.27165035	5.03331626666667	6.04876668333333	5.12102319166667	5.108231475	5.21821305	4.894753975	4.28110195833333	5.256776325	5.54873173333333	5.50123778333333	4.74448875	4.92878054166667	5.89449381666667	5.12313813333333	5.99672560833333	5.73491465833333	5.7946758	5.19959509166667	4.68789791666667	4.82135065	5.48099319166667	4.71899701666667	4.96517085	5.96160064166667	4.2939407

NR1H3	10.0807009083333	9.93630081666667	10.4244981833333	9.91460061666667	9.32939963333333	10.4376859583333	9.73788834166667	9.64736674166667	9.71142816666667	10.3070468	9.700667025	11.0323327416667	9.50499393333333	9.8972867	9.332301675	10.6860936833333	11.6270644583333	9.37728073333333	9.46432354166667	9.890973325	10.0242607916667	9.67230795	10.1432492	10.0489380083333	9.686488825	10.0537308333333	9.728395625	9.74904354166667	9.59369666666667	9.87650806666667	9.70194608333333	9.78104253333333	9.8880168	11.8523757333333	10.500980975	10.222173225	9.661950625	9.81305005833333	10.1389730083333	9.8694956	10.214113	10.1472782833333	10.3754461833333	10.2786656166667	10.432837725	10.6683478	9.9799425	9.78967574166667	10.4123976	10.27572155	9.81899704166667	9.062206575	9.84589375833333	9.78872821666667	10.0434280416667	10.5490285	9.58840303333333	10.2798188416667	10.0296842916667	10.194475875	9.03598665833333	10.0533049083333	11.0845103416667	9.81132868333333	10.1601611333333	9.46432354166667	10.8228191416667	10.3894512416667	10.48105345	11.5352724166667	10.2450271333333	10.28850615	9.22249476666667	11.1376399	9.40032650833333	11.0333724416667	10.805973475	11.1982937916667	10.58883085	10.6964442916667	10.807474625	10.1978484	10.3714480666667	10.457410175	10.90102425	9.63156661666667	9.897727225	10.9740524416667	10.3758856666667	10.8032590416667	10.306601825	10.390544	10.252295925	10.2853426583333	9.223397275	10.2951456833333	10.776004825	10.4228179333333	9.19391603333333	9.30586555833333	11.3590797	10.8578576916667	11.9593365833333	10.8896808833333	10.4468482583333	11.6238039583333	10.6596205	11.4310404083333	11.07367765	11.201526325	10.051171975	9.83499924166667	10.73478335	11.7109162916667	11.489428925	11.385364925	9.68602584166667	10.988072975	10.0889846333333	12.247959925

TAT	7.3817504	7.04632523333333	6.51310794166667	6.675144925	8.10111541666667	6.62367098333333	7.97666008333333	7.51995818333333	7.5385516	6.88791735	7.44667759166667	7.2645801	7.91409299166667	6.85045274166667	6.591129625	6.448169675	6.465677275	7.80451180833333	6.021143325	6.40185915	6.91905025833333	7.24075795	7.05018306666667	6.5931033	6.06998224166667	6.61289744166667	6.87469296666667	6.35738701666667	7.00049489166667	7.38926633333333	6.41874811666667	6.61019589166667	7.27229930833333	6.97214965	6.7401779	6.76187970833333	7.22053391666667	6.75712434166667	6.80056770833333	6.88940976666667	6.42033848333333	7.36707339166667	6.31702735	5.876765525	7.32036674166667	7.21695988333333	6.24443749166667	7.08293825833333	5.98999241666667	6.801867125	6.39451754166667	6.51994874166667	7.8967227	7.16852535833333	7.19137265833333	5.59947975	7.02943565833333	6.98474048333333	6.83084248333333	6.33645039166667	6.950931025	7.61865353333333	6.61289744166667	6.87917671666667	7.30254744166667	8.298377725	6.93227245	7.66891875	7.59310200833333	7.40546740833333	7.27229930833333	7.49442741666667	8.25546045	7.25477635	7.740357875	7.019638175	7.53624973333333	6.54365829166667	7.35003230833333	6.86394090833333	6.82889531666667	7.663341325	7.93282061666667	6.9953871	7.58128390833333	7.91046618333333	7.4398224	7.31377455	8.0459923	6.64487866666667	7.02148474166667	7.81137301666667	7.34497685	7.22598754166667	7.50463459166667	7.11855360833333	7.671444425	7.99186004166667	8.4773978	6.34302255833333	7.24828621666667	7.77321380833333	7.03384980833333	6.473722825	7.81344110833333	7.506757	7.13351973333333	6.3109681	7.06400038333333	6.65424311666667	6.90781049166667	6.230051875	8.881658575	6.57096464166667	8.072065075	7.26747879166667	7.1283444	7.98087950833333	7.05018306666667	7.44041060833333

ATG16L1	10.5379957083333	10.15614755	10.4228179333333	10.6503573333333	10.7020102333333	10.4037201666667	11.0116340666667	10.5477462083333	10.7158953333333	9.88883589166667	10.2170232333333	10.7743973416667	10.7553698833333	10.0578180083333	10.4384998666667	10.0802966083333	10.384097475	10.613570925	10.4176619666667	10.217472825	10.3960554833333	10.4028540333333	10.2293589333333	10.9640211333333	10.1791861916667	10.6203644583333	10.018462175	10.217472825	10.6581721666667	10.0696848333333	10.1993549333333	10.0885610583333	10.457410175	10.2351017916667	10.2865677	10.44259115	10.3179044666667	9.92664269166667	10.2936263083333	9.96824564166667	9.82250195	9.57007478333333	9.78280608333333	9.875255275	9.86145205	9.73658934166667	9.8942397	10.1820473416667	9.65845149166667	10.096897025	9.61596005833333	10.0688438166667	10.5468436833333	10.4115783666667	10.3167137333333	9.877817275	9.96481554166667	9.73788834166667	10.0247316333333	9.71307581666667	10.9658327583333	10.6023330833333	10.30576505	11.1623033333333	10.02846355	10.3741034916667	11.221922825	10.601173475	10.001418875	10.4819116333333	10.577240575	10.6538835666667	10.8216281833333	10.120107375	10.798668925	11.0257755833333	10.4716937833333	10.2865677	10.41573205	10.6535513666667	10.8361133666667	10.7706214833333	10.9065665083333	10.6298019333333	10.2951456833333	10.7789681833333	10.6846028416667	10.7877953583333	10.9440376833333	9.85548545	10.5441182916667	9.95767555	9.84719424166667	10.3718117166667	10.4127726666667	10.0156224166667	10.1184175666667	9.983868575	10.9006915833333	10.2089178583333	9.90058610833333	10.0103759833333	9.954379925	10.501807225	10.4061408166667	10.689167625	10.2282248666667	10.0502702166667	9.882085	9.90222311666667	9.599958075	9.69581614166667	10.640008475	10.4918133583333	10.2258230916667	10.3570715916667	9.99851741666667	10.1836960166667	10.2485867583333	10.1424095333333

SVIL	7.671444425	7.44735373333333	8.28049985	8.05717868333333	8.04545514166667	7.33320358333333	8.18422995833333	7.55746484166667	7.35803011666667	7.92549585	7.39806645	8.39560916666667	7.95851929166667	7.460763125	7.60193526666667	8.64006809166667	7.02822294166667	8.49745698333333	7.77208035833333	7.37590343333333	8.31150115833333	8.113521575	8.037084725	8.75091850833333	7.6071886	7.9846002	7.99022335	8.05973130833333	8.3316018	7.88018380833333	7.73527455	8.44690206666667	7.74783731666667	8.682814325	8.12489341666667	7.95082734166667	8.053540525	8.17668253333333	8.27606465	8.168322275	7.70147536666667	7.46194565833333	7.86283530833333	7.3817504	7.64794805833333	8.19036318333333	7.497153825	7.76884748333333	8.46961650833333	8.19090655	8.01119698333333	8.01119698333333	8.07889375	8.19970503333333	7.97293150833333	7.62202388333333	7.62593523333333	7.86334303333333	7.73298270833333	8.46771705	8.65800171666667	8.06345484166667	7.44317530833333	7.85906496666667	7.51030740833333	7.41997313333333	7.86115675833333	7.692684	6.89266504166667	7.95575579166667	7.35564645	7.79803676666667	7.46194565833333	7.87821420833333	7.83894598333333	7.96575633333333	7.93282061666667	8.48321643333333	7.72195235	7.77718458333333	7.27593380833333	8.340733825	8.11098743333333	7.71409900833333	7.698105675	7.8449064	8.08865246666667	8.59697231666667	7.743439475	8.60799398333333	8.03277863333333	8.25593908333333	7.415839475	7.45614546666667	8.01905775833333	8.18891301666667	8.293531175	8.28541158333333	8.162750025	8.11887123333333	7.83518873333333	7.65326568333333	8.43095395833333	7.75176951666667	7.76455869166667	8.16071089166667	8.11825858333333	7.65490436666667	8.31150115833333	8.21097586666667	8.05804941666667	7.7783407	8.324127225	7.264052575	8.27339385833333	7.78978061666667	7.54070496666667	7.83016101666667	7.703109475	8.38422716666667

CCDC106	9.18167015	8.10268665	8.35234010833333	8.471007425	8.37107905	8.584858775	7.70645875833333	8.40817908333333	8.09998893333333	8.35071744166667	8.03960018333333	8.35736911666667	7.99937465833333	7.9291649	8.34021045	8.53385956666667	9.18071311666667	8.31058729166667	8.45201853333333	8.07622910833333	8.54070765833333	8.42676914166667	8.58906924166667	8.24657145833333	8.23520641666667	9.44734175833333	8.4237182	8.23175398333333	8.64688179166667	8.34021045	8.21929578333333	8.368580775	8.05569140833333	9.03284314166667	9.389870525	9.50308771666667	8.38072273333333	8.127296675	8.475275825	8.48321643333333	8.42877934166667	8.524299575	8.322152325	8.46867076666667	8.51027651666667	8.93412065833333	8.41484045	8.298377725	8.31364723333333	8.45451465833333	8.06759891666667	8.21929578333333	8.16633889166667	8.21830426666667	8.61480576666667	8.58538408333333	7.89243905	8.37811988333333	8.34473695	8.42069139166667	7.65663390833333	8.672615075	8.80050660833333	7.937534725	8.95218464166667	7.42675208333333	8.10440565833333	8.4100903	8.9198372	8.03755788333333	7.569973	8.04975595	7.01426715833333	8.16071089166667	8.1277546	7.92279315	8.48460361666667	8.52241499166667	7.38458683333333	8.14959635833333	8.16584395833333	8.14297786666667	8.92218960833333	8.93073908333333	8.17885319166667	7.71798951666667	8.26456484166667	7.98618585	7.45451809166667	8.36807433333333	7.80727978333333	7.45786721666667	8.23080535	7.51200123333333	7.79136785	8.37460103333333	9.393424375	8.07674773333333	7.73358451666667	8.709455975	8.70499479166667	8.20638435	7.88077005	8.40473328333333	7.547481675	8.16529418333333	7.88506155833333	7.6315346	9.08299806666667	8.561306875	8.2549704	9.25536705833333	8.238616825	8.22981704166667	8.75371965	8.333019375	9.20235228333333	8.062405875	8.10057743333333	7.43234464166667

SCNN1B	7.34385484166667	7.988718925	8.26039324166667	9.19905495833333	8.61768925	7.86164825833333	8.1976201	8.416908275	8.31150115833333	7.90991645	8.45556428333333	7.03384980833333	8.84952995833333	7.928618975	9.5928155	7.60592470833333	7.17569580833333	9.094155	8.25292731666667	7.90991645	8.27440479166667	10.1957428083333	7.40662056666667	8.11654126666667	8.55804185	7.02148474166667	8.9131846	8.98934508333333	9.22423163333333	8.703553525	8.80050660833333	8.931271375	7.86942576666667	7.51935500833333	7.5512356	7.36819641666667	9.9690997	8.92840376666667	9.98509406666667	8.73547365	8.48112818333333	8.78355868333333	8.20638435	7.85906496666667	7.2015586	8.24270349166667	8.123259225	7.833966975	8.13568184166667	8.39466414166667	8.47478683333333	9.21787520833333	8.44287714166667	8.8451569	8.953569225	8.13138714166667	8.41484045	7.94491850833333	9.49413000833333	7.45966298333333	7.62988515833333	7.29843466666667	9.00867418333333	7.92224445	7.14024473333333	9.81985159166667	7.41243761666667	8.06759891666667	7.42447115	7.71969125833333	7.97971059166667	8.09498031666667	7.86115675833333	6.777132775	7.868368375	7.91409299166667	7.746177925	7.81947101666667	8.86760295	7.69707313333333	7.62307443333333	7.75390870833333	7.56380908333333	6.72313661666667	7.690465575	7.73639724166667	7.04755845833333	7.652791	7.58465230833333	7.06709683333333	7.38582769166667	7.38984995833333	8.39722928333333	7.99891026666667	8.85160831666667	7.22598754166667	7.54070496666667	8.15860931666667	8.67888266666667	8.440899325	7.254184275	7.42103133333333	7.18035421666667	6.81435373333333	7.20334935833333	6.92042344166667	7.34551940833333	7.98346735	7.31919828333333	7.28012135	7.66388121666667	7.66558521666667	7.51310406666667	7.02883426666667	7.93123045833333	7.967895825	7.299574675	6.78381769166667	7.745650475	7.03569371666667

ZPR1	8.0149105	7.58187098333333	7.916304775	7.49164054166667	8.25794470833333	6.7503122	8.64196594166667	8.17090974166667	7.91522786666667	7.43634795	7.97923883333333	8.90142945833333	8.51027651666667	8.30178113333333	8.21405428333333	7.62655825	7.46414650833333	8.32561349166667	6.31880616666667	8.277602525	8.31103703333333	7.25732849166667	7.78546004166667	8.52878136666667	7.9846002	8.4444208	7.32036674166667	7.31254506666667	6.50650844166667	7.71409900833333	7.978659025	7.24330025	7.04568171666667	7.27714694166667	8.03960018333333	7.886143875	7.57673284166667	7.20693700833333	7.33320358333333	6.47064316666667	6.90460845	7.09738105833333	7.26049458333333	7.44780359166667	7.2657012	7.27229930833333	7.24330025	7.650678625	6.05994703333333	6.792709475	6.85745724166667	7.16541175833333	7.87613041666667	7.69541339166667	7.75064413333333	6.69500276666667	8.07938845	7.0168934	6.542385675	7.23028210833333	6.89266504166667	7.086730875	6.02469836666667	6.94561610833333	7.53964365	7.14272935833333	7.598264375	8.211993425	6.7130388	7.69369399166667	8.36292435	8.53434441666667	9.00189508333333	6.77905188333333	8.42421896666667	6.987286825	7.04898169166667	7.01837270833333	6.133346875	7.379877425	7.800043275	6.96183981666667	6.93741035	7.42906085	7.56602129166667	7.153195325	6.813807925	8.26655316666667	7.91794835833333	7.52896074166667	7.656003025	6.71995881666667	6.2176337	7.77208035833333	6.42709209166667	6.31469075	7.79957504166667	6.165146375	7.3019376	5.85993758333333	7.24137955	7.97923883333333	7.83071075833333	6.95924915	7.73471975833333	7.35693584166667	6.96590744166667	7.28130318333333	6.45250163333333	7.01495000833333	6.89851890833333	5.83398495	6.57166325	7.71034295833333	8.085928525	6.777132775	6.54574596666667	7.227778225	6.041758325	7.31981145833333

KRTAP1-3	14.4791297416667	14.2858034416667	14.1399349583333	14.1554067916667	14.4262315333333	13.7940225416667	14.4161239916667	14.2686692416667	14.2460408916667	14.3431664333333	14.3316746833333	14.8412522666667	14.4633924916667	14.5793502666667	14.1737580083333	14.2378149833333	14.1175943833333	14.5176714166667	14.1618228083333	14.3908304333333	13.977355925	13.7443409166667	14.354697075	13.89505895	13.7791494	14.70147985	14.2305624666667	14.120388025	14.282826075	13.9807861416667	14.1228795	13.8604046083333	14.3628995916667	13.4274952333333	14.4144157	13.8516367666667	14.4667992416667	14.364637875	13.9989817	14.282826075	14.419240975	14.0087806166667	14.1471335166667	13.8001487666667	13.6098239583333	13.97057275	13.8572965333333	14.0656333583333	13.956539975	14.2160518416667	14.4963541416667	13.8461367666667	13.85508265	13.8461367666667	13.7502246416667	13.4572533333333	14.0540171833333	14.1228795	13.7582839583333	14.2563286	14.00243415	14.277828625	14.0950375583333	14.3045490583333	13.7602031583333	14.265784475	14.1709219416667	14.4226857416667	14.2731900083333	14.0964870833333	14.2393514333333	14.3236760583333	14.3692316583333	13.8405812166667	14.2474901166667	14.1656626833333	14.1709219416667	14.0618019166667	14.1413614333333	13.9480264583333	13.771159075	14.0847383833333	14.013877625	13.88005245	14.2460408916667	14.0683132333333	13.9840948583333	14.0800041333333	13.9186404666667	13.82498885	14.4361804666667	13.809878875	14.190108625	14.77778785	14.26265275	14.4244522916667	14.3283993416667	14.2426505583333	14.4633924916667	14.371029675	13.52385865	14.0607265833333	13.5484254	13.6461564833333	14.594415925	13.7820292333333	13.8473484166667	13.9075706166667	14.0979296916667	13.9529996833333	14.0418505333333	14.30615445	14.2305624666667	13.6426254916667	13.7303452416667	13.312913975	13.672832575	13.6894331166667	13.7721764083333	13.864749275

C3	14.7499385916667	13.3552344916667	14.4092298666667	13.6560864916667	12.7453059583333	15.0005057333333	12.9853754833333	12.92419445	13.8148381	13.9493522333333	13.5346846166667	16.113934775	15.3982642333333	13.1695361416667	12.3720643666667	15.700189825	13.462725925	12.7912843416667	13.6098239583333	13.6858454833333	14.30615445	11.229748375	14.2260493166667	14.4791297416667	14.1985925	14.3843059083333	13.5660586666667	13.1026328916667	13.054278175	13.1316052916667	13.6140845916667	14.1413614333333	14.6304997416667	14.30615445	15.4316128083333	14.3253890083333	13.3545785083333	13.9378093083333	15.095547425	13.4246253333333	13.3285445333333	13.4579324833333	14.013877625	13.2279712833333	15.3498376083333	14.6367172916667	13.4267583666667	14.3299457833333	14.3283993416667	14.6046129083333	14.047904	13.8010236666667	14.6104738916667	13.0901032416667	13.92958755	14.8676166333333	13.916524275	14.265784475	14.648708375	14.4294718916667	13.9529996833333	13.44656775	12.3575221583333	12.1380810166667	13.711942125	12.555705525	14.063152775	14.76191125	12.2073843166667	14.5322324833333	12.9806214166667	13.0494144333333	12.1690316666667	14.4791297416667	11.9962714583333	13.229948375	14.2058121833333	13.9900642375	12.29141735	14.34486635	12.6966269416667	13.6605641666667	13.3882606083333	13.5956278166667	12.919046025	13.229948375	13.52385865	14.7596386666667	11.98994525	14.5469723583333	14.0991501333333	13.0560858	11.6906956833333	12.1034309916667	11.8884513166667	12.6175954833333	12.4005974416667	12.372860975	12.3347946666667	12.0921972916667	13.784925425	12.6312122083333	14.5415836083333	11.8426813333333	12.1827137333333	12.8684829916667	14.0787258833333	12.1943788416667	14.211775675	13.7074542166667	12.7660601416667	11.963726125	12.7649781166667	14.5195733916667	14.4527902	13.6246233916667	12.6581666416667	12.18783155	11.2833533833333	14.2487820083333

CCZ1	14.0880163125	14.04483015	13.6653701166667	13.9949861041667	14.1691034333333	14.3440163916667	13.5072033583333	14.0384459916667	13.6680227291667	13.8235884833333	14.0806651875	13.5690106083333	13.8924722083333	13.8944945833333	13.9606550458333	13.4250237833333	14.028200125	13.8914119791667	14.0370841916667	13.7805374125	14.1808558916667	14.1623829708333	14.1197745791667	14.0018468833333	13.8924722083333	13.8794715833333	14.0472749125	13.8133007458333	14.2802845666667	14.083082675	13.7487427541667	13.9630399875	13.7955165875	13.8652457458333	13.7815162541667	13.9170528	13.6232620458333	13.8534186666667	13.3491368416667	13.8235884833333	13.8760508166667	13.6762941375	13.9464012958333	13.7316847208333	14.04483015	13.6205996916667	14.0006966666667	14.231255875	13.8173635041667	13.7786379958333	13.734666675	13.7864252541667	14.4783563666667	14.1299409583333	14.1691034333333	14.1079105208333	13.9417063708333	13.998416875	14.0919329666667	14.0411451625	14.6192396333333	14.3228994833333	14.6172136958333	14.6808881666667	14.4487402125	14.3555176625	14.3589644625	14.0435706291667	14.0957623208333	13.8371484625	13.7115394416667	14.4714120708333	14.2198080916667	13.7906263333333	14.2168077458333	14.6788435166667	14.0030396875	14.3373990875	13.8652457458333	14.4573243541667	14.0867090541667	14.435416225	14.2418422166667	14.3083426625	14.0397911958333	14.0169189541667	13.5621913583333	14.1027147333333	14.2960504875	14.1027147333333	14.0853568291667	14.3212633083333	14.1921043125	14.0132906041667	14.2739451166667	14.1598025458333	14.0435706291667	13.675299175	13.9949861041667	14.4387724333333	14.40344035	14.0919329666667	14.04483015	14.5115031625	14.2168077458333	14.5840114333333	14.2385832083333	13.8400647583333	14.1377250833333	14.0999066125	14.1880524416667	14.1572373666667	13.9663363666667	14.2664753916667	14.3083426625	14.3291725625	14.2634625	13.7735768625	13.7567678375	13.9653083166667

CCZ1B	14.0880163125	14.04483015	13.6653701166667	13.9949861041667	14.1691034333333	14.3440163916667	13.5072033583333	14.0384459916667	13.6680227291667	13.8235884833333	14.0806651875	13.5690106083333	13.8924722083333	13.8944945833333	13.9606550458333	13.4250237833333	14.028200125	13.8914119791667	14.0370841916667	13.7805374125	14.1808558916667	14.1623829708333	14.1197745791667	14.0018468833333	13.8924722083333	13.8794715833333	14.0472749125	13.8133007458333	14.2802845666667	14.083082675	13.7487427541667	13.9630399875	13.7955165875	13.8652457458333	13.7815162541667	13.9170528	13.6232620458333	13.8534186666667	13.3491368416667	13.8235884833333	13.8760508166667	13.6762941375	13.9464012958333	13.7316847208333	14.04483015	13.6205996916667	14.0006966666667	14.231255875	13.8173635041667	13.7786379958333	13.734666675	13.7864252541667	14.4783563666667	14.1299409583333	14.1691034333333	14.1079105208333	13.9417063708333	13.998416875	14.0919329666667	14.0411451625	14.6192396333333	14.3228994833333	14.6172136958333	14.6808881666667	14.4487402125	14.3555176625	14.3589644625	14.0435706291667	14.0957623208333	13.8371484625	13.7115394416667	14.4714120708333	14.2198080916667	13.7906263333333	14.2168077458333	14.6788435166667	14.0030396875	14.3373990875	13.8652457458333	14.4573243541667	14.0867090541667	14.435416225	14.2418422166667	14.3083426625	14.0397911958333	14.0169189541667	13.5621913583333	14.1027147333333	14.2960504875	14.1027147333333	14.0853568291667	14.3212633083333	14.1921043125	14.0132906041667	14.2739451166667	14.1598025458333	14.0435706291667	13.675299175	13.9949861041667	14.4387724333333	14.40344035	14.0919329666667	14.04483015	14.5115031625	14.2168077458333	14.5840114333333	14.2385832083333	13.8400647583333	14.1377250833333	14.0999066125	14.1880524416667	14.1572373666667	13.9663363666667	14.2664753916667	14.3083426625	14.3291725625	14.2634625	13.7735768625	13.7567678375	13.9653083166667

PACSIN3	13.1786718416667	13.7272276083333	13.3936063	13.5475412583333	13.4611203333333	13.239306575	13.2001079166667	13.4407112833333	13.4634900333333	13.2061458583333	13.306054325	12.6050803083333	13.2233931166667	13.62200835	13.7452882916667	12.988893075	13.0096050666667	13.2615079916667	13.593805675	13.1071716666667	13.2765049166667	13.765060275	13.057870725	12.7884927666667	13.1913123583333	13.177155775	13.4096868166667	13.2587665833333	13.19400885	13.479133875	13.3724965833333	13.396630575	13.2128605083333	11.9207928583333	13.1779047083333	13.1144791333333	13.2718741916667	13.0727047	13.1628836	13.4407112833333	13.5287262833333	13.5451377333333	13.3278442333333	13.5535340083333	13.0985928583333	13.4298474333333	13.398021325	13.2975446	13.5660586666667	13.396630575	13.2861830833333	13.71864895	13.8241318916667	13.698785275	13.8635959083333	13.662514625	13.599265975	13.3793195083333	13.2313413916667	13.289124025	12.695073575	12.927195275	13.4864308583333	13.3522548916667	12.7390522083333	13.487174625	13.7553717	13.05962405	13.3451920333333	12.929488325	13.4768642833333	12.9370697333333	13.5843144666667	13.004985075	13.492075675	12.786823125	12.8607309666667	12.909394625	12.7416307	12.85786905	12.8624048833333	12.309318975	14.4161239916667	12.844414625	12.8641322666667	12.43820225	12.4950350166667	12.309318975	13.068247425	12.993623425	12.9812481083333	12.8268431583333	13.64886915	13.6310840583333	13.2876598083333	12.4751035416667	13.3778968666667	13.6375130583333	12.6506468416667	11.9616964916667	13.4282493833333	13.306054325	13.479133875	13.4375531583333	14.2715663416667	13.6979142666667	13.2480671333333	13.56859275	12.4127680416667	12.43820225	12.7955349083333	13.0089762083333	13.239306575	13.7532714333333	12.9253738	13.21039535	12.7216091916667	13.5443638333333	13.4775815666667	12.2289445583333

UBA1	13.5552298333333	14.1927345333333	14.37416785	14.2810681833333	14.371029675	13.873143175	14.1399349583333	14.2087842916667	14.3153773833333	14.341437425	14.1737580083333	13.33077755	14.07729275	14.259430575	14.3299457833333	14.1427380833333	13.44358445	13.9186404666667	14.4411655333333	14.1886825416667	14.1413614333333	13.239306575	14.3139109083333	14.0926224666667	14.26427225	13.6551549916667	14.5232906166667	14.1656626833333	14.6992893416667	14.3792394416667	14.29207405	14.512293975	14.500239075	13.7970969916667	13.3270763416667	13.6737470333333	14.6527873333333	14.20293335	13.8505836916667	14.0759487166667	14.2548502583333	14.1827283166667	14.127909425	14.3908304333333	13.9762519833333	14.2936594416667	14.4244522916667	14.718655275	14.0526804666667	14.1618228083333	14.0237926083333	14.3170032666667	14.244370025	14.6527873333333	14.3943592583333	14.3692316583333	14.4060458333333	14.3908304333333	14.7097912166667	14.1241853333333	14.1857941	14.31061245	14.62202095	13.809878875	14.0174773166667	13.7602031583333	13.9367207833333	14.1857941	13.7582839583333	14.4722450583333	14.0225032583333	14.1441992083333	14.11256935	14.5089658583333	14.036419	13.8788907166667	14.0315629333333	13.8473484166667	13.8528809083333	13.9829878583333	13.7065253416667	15.5221336416667	14.0926224666667	14.190108625	14.4479745916667	14.1033949916667	14.277828625	14.2378149833333	14.3170032666667	14.35633825	13.7582839583333	14.2000424583333	13.6666988416667	13.9749587833333	14.7409853333333	14.1886825416667	14.1554067916667	14.4565233916667	14.2858034416667	14.244370025	14.1033949916667	14.0720390916667	14.8110718166667	13.5610039666667	14.2233219083333	14.0225032583333	14.1618228083333	13.8832091583333	14.1268362333333	13.9967915416667	14.1384134166667	14.0188677666667	14.3316746833333	13.7452882916667	14.1554067916667	14.21756365	13.6737470333333	13.9261802833333	13.838661325	14.50554015

NR1H2	12.17539085	11.3328774166667	11.5378634666667	11.292593525	11.229748375	11.24709275	11.2646484083333	11.28518	11.3801476916667	11.3954232	10.98655615	11.8028369	11.4890705333333	11.4861149333333	11.465822775	11.3187261333333	11.31764545	11.2198620833333	11.8453541	11.3455869583333	11.248902775	10.8375253583333	11.3668652083333	11.4492670916667	11.3752975833333	11.8557655416667	11.832540575	11.3820783333333	11.2740276	11.6675947833333	11.675724725	11.196768475	11.5223462	11.002443975	12.5954075166667	12.6552566916667	11.3539068166667	11.47905225	11.7175871333333	11.844663475	11.637130675	11.5245993916667	11.6058721833333	11.5520144833333	11.5891254	11.6190781833333	11.5429763166667	11.4798432416667	11.25880145	11.31617205	11.5019499416667	11.6466404583333	11.1117434416667	11.2775845	11.2848715833333	11.4463806916667	11.3587026083333	11.6013132583333	11.4616143	11.434022575	11.1787139083333	11.45778115	11.069747825	10.9084288583333	10.229775825	10.5695644083333	10.8113265583333	10.7108576583333	10.75497465	11.1346623583333	11.297940475	10.9303115416667	10.5569098916667	11.1934390416667	10.73913815	10.51584625	11.062266425	10.91088935	11.1891169333333	10.9611703916667	10.3293667083333	10.96365625	10.9284040666667	11.2572113416667	10.9824445416667	10.1417124166667	10.7116237833333	11.2521123083333	11.1607791666667	11.4133387916667	11.071263875	11.118056575	11.1067195416667	11.1346623583333	10.6930678666667	11.3477921	10.852614925	10.5416886583333	11.0950066583333	11.4759684833333	11.3104909	11.06778735	11.0068392583333	10.7616896916667	10.9136098583333	10.44375685	10.8269705916667	10.8608532	11.379403425	10.8976829666667	10.9501588	11.6175120416667	10.672620875	10.3894512416667	10.8220014333333	11.3438040833333	10.42200985	10.6043334333333	10.9415949916667	10.94470365

GPR26	6.97900894166667	6.99475883333333	7.263472875	7.23193100833333	7.45614546666667	6.94690668333333	7.9089283	7.19767501666667	7.363992875	6.95435513333333	7.04378355833333	7.668390025	8.25593908333333	6.78990954166667	7.51144511666667	6.67919664166667	7.57732030833333	7.51935500833333	7.32549529166667	7.55609866666667	7.404332775	7.29732286666667	7.22053391666667	5.85413349166667	6.54365829166667	7.08560825833333	6.99601435833333	6.33714610833333	6.61154091666667	7.18884621666667	7.375280375	7.43179573333333	7.60592470833333	7.55609866666667	7.63711770833333	8.05804941666667	6.38554745833333	7.22899075833333	6.60761675	6.84636305	6.40572345	6.79403766666667	6.54514473333333	6.903905225	6.874007675	6.99346504166667	6.87851200833333	6.89266504166667	6.68131301666667	6.77975395833333	7.31254506666667	7.38458683333333	7.35059130833333	7.46688233333333	7.93123045833333	7.50573916666667	7.99833741666667	7.55678278333333	6.83744154166667	7.1674037	6.698517925	7.00630173333333	7.107578925	7.24514076666667	6.94120649166667	7.18643425	7.67088145	7.47900525	6.94497745833333	7.496531175	7.23488161666667	7.86896935833333	8.49539040833333	6.80992164166667	7.456643225	7.18884621666667	6.94820169166667	7.04821105833333	7.31493256666667	7.20568426666667	6.54514473333333	6.53536243333333	7.22238968333333	6.08877931666667	6.91388091666667	7.23727353333333	6.9494722	6.71502808333333	6.72313661666667	6.89596523333333	7.129719725	7.444336	7.139659575	7.07654913333333	7.09102175833333	7.13168135	7.15940084166667	7.206290275	6.618809175	6.5332879	6.986635125	7.609434275	7.002378425	6.97095796666667	6.99800655	6.54933985	6.74158396666667	7.21808105833333	6.63574723333333	6.54638811666667	7.36707339166667	7.44885958333333	7.45504230833333	9.157883875	7.97557605833333	7.29474379166667	7.94065678333333	7.73027796666667	7.36878105	7.81137301666667

GPSM1	11.75219825	11.8950942583333	11.7974234916667	11.676506725	12.6297371916667	11.7834532083333	12.8584129833333	12.0457744	12.3811083333333	12.1906758666667	12.30344225	12.6528003583333	12.3785017666667	11.5980356083333	12.023387575	11.3371736333333	11.3384968	12.5646791416667	11.4985882166667	11.4188332333333	11.65809635	11.8861575583333	11.1095361333333	11.2319732666667	11.8390986666667	11.6433290333333	11.6155901666667	11.8666609583333	11.9364860583333	11.7513402	11.51924745	11.3282347083333	12.2276231333333	11.55593635	11.4219762166667	11.56420645	12.6245838166667	12.3970415166667	12.16610385	11.980095625	11.270439275	11.654339925	11.2093606416667	11.0160885	11.2125996833333	11.028339325	10.7181339666667	11.072670925	11.3683256166667	11.5276408333333	11.8612030916667	11.7963185916667	11.6448777666667	11.3324973416667	11.0068392583333	10.6730377	11.929273675	11.7710225416667	11.9314967083333	11.3774736833333	12.296807225	12.1389321	11.343036975	12.1393673833333	11.8580302916667	12.424331275	11.7369473333333	12.4718679833333	12.2137378333333	12.50431935	12.4701553166667	12.2831203916667	12.4576588166667	12.0514729416667	12.0322027833333	11.6823165333333	12.445171625	11.804049375	11.5162409083333	11.8966407583333	12.2383566166667	12.5585076166667	11.6536243916667	11.7285272083333	12.3305815833333	12.6800815583333	12.3254389	12.1732634166667	12.3554452	11.7046170083333	12.0909367583333	11.9232334916667	12.16243335	11.8745815	12.4283415	11.9270898666667	12.1998168583333	12.7763784666667	12.6018203833333	12.20367145	11.492270725	12.237060875	11.0510672583333	10.689872025	11.430002575	11.328911575	11.57134605	10.99445015	11.9123960833333	11.5809197583333	11.592340975	11.195266625	11.5356555333333	10.804132675	11.3086201833333	10.96888565	11.9770182833333	11.7963185916667	11.185479075	11.7823700916667

SP140	11.9621161833333	11.364979325	11.5805781333333	11.92817275	12.118235175	12.81348825	11.4258523333333	11.5245993916667	11.9380168416667	11.6687463083333	11.7572174666667	12.2002559333333	12.3187215583333	11.88575805	11.2624348416667	12.9521681583333	11.6073227	12.0084826666667	11.7085670166667	12.33229585	12.3974616666667	12.5585076166667	12.3820596583333	12.3484591166667	12.3855581	11.8573117083333	11.8141737416667	11.4470684833333	12.3720643666667	12.0616030916667	11.36200305	12.4645960083333	12.1359013166667	13.3315421333333	11.798162425	11.8931595083333	11.9052300666667	12.01005715	11.8435483083333	11.63482985	12.1778025833333	11.7175871333333	12.648701825	11.9863991	12.800460075	12.369348425	12.31433185	11.832540575	12.6270116666667	12.5159454416667	11.8853151666667	12.0040290833333	12.7779954333333	12.9604577916667	12.5963701666667	12.5385984166667	12.5562191	11.99193365	12.3623095583333	12.63765555	13.6461564833333	11.3774736833333	12.7051272333333	12.3043299166667	11.88924055	12.8936439416667	12.4536816333333	12.846546475	12.1636527	13.3430209416667	11.590501375	12.2137378333333	12.8256923916667	12.8438653416667	12.5874385	13.0439770416667	13.48560075	13.69139155	12.9230312916667	13.4666571333333	12.5912244	13.5157896583333	12.8398240166667	12.1600024166667	13.1648189583333	12.822430125	13.5738180166667	13.216246175	13.0222646833333	12.454126225	12.8010225583333	12.1745545	12.4310799666667	13.057870725	13.2705051333333	12.7128638833333	11.6613946166667	12.1990202166667	13.0149306583333	12.6621572833333	12.939417425	12.6270116666667	13.461932225	13.537910275	12.8498718833333	14.3960605	13.2595614666667	13.604496075	13.52467435	13.0877248833333	12.8339629333333	12.2909999333333	13.4164430416667	12.3401023416667	13.44358445	13.0829572666667	10.8736803833333	13.180045325	12.7081294916667	13.9785722666667

AIFM2	10.0648957166667	10.3833420583333	9.80535213333333	9.92494756666667	10.000563275	10.31285305	9.7452797	10.5392644333333	10.0748410916667	10.3917443	10.4613867166667	8.85494786666667	9.53692870833333	10.75497465	10.287024175	9.90518528333333	8.9184083	10.5711304666667	10.651587675	10.44259115	10.416539275	10.7336987	10.5545011333333	10.123368025	10.4453172666667	10.3380831916667	9.96824564166667	10.1084049583333	10.0688438166667	10.4698289916667	10.373735575	10.154948775	9.89637478333333	9.05868610833333	10.521634775	10.4671575083333	10.0877690666667	10.6293746333333	10.2716281	10.4678765666667	10.4208424916667	10.4292877666667	10.4955307333333	10.8657939916667	10.1409369083333	10.447247375	10.6068679083333	10.72841425	11.1866301583333	10.614717725	10.7839123	10.35233035	9.83900869166667	10.2289590916667	9.74655711666667	9.93343423333333	10.8243180916667	10.9934066	10.6122965916667	10.5596517833333	9.890973325	9.88925331666667	10.5388660583333	10.340493	11.426992675	11.2965765083333	10.99445015	10.696845925	11.1188874333333	10.7038909916667	10.0976412833333	10.970347325	10.287024175	10.4409408666667	10.257101525	10.9776492583333	10.4709652	10.418815025	10.3436245833333	10.96888565	11.48580155	12.208161275	10.7379674916667	10.5825770333333	10.0611526083333	11.880304075	11.0228735916667	9.81132868333333	10.0391614083333	10.7078134666667	10.4215953	11.1067195416667	10.5490285	11.1176950333333	10.1755237	10.5185399	11.5436260583333	11.7890978583333	11.2215521833333	11.3040384416667	11.742927775	11.36160315	11.04340915	11.61832875	10.9527927166667	10.8980207583333	11.1821084666667	8.23567169166667	11.4296055416667	10.621550725	9.7686764	10.8938022916667	10.3179044666667	11.04340915	10.4503313916667	10.6792669083333	12.1757943166667	11.0064548166667	11.2837838916667	10.8751259833333

TNFSF12-TNFSF13	12.3428910083333	9.9186526375	10.361669025	9.429283125	8.23340604166667	10.1647975583333	9.8309168	9.7719804625	9.661749025	10.0315805458333	9.34132776666667	11.838151225	10.660195125	9.81963299166667	9.32873125833333	10.675551525	10.8910372666667	8.58512142916667	10.0663523541667	10.0913534291667	10.1415170541667	8.2424291625	10.1314447125	10.0037304958333	10.1822655291667	12.3718770291667	10.627622325	9.67119210416667	8.92243870416667	10.0324278916667	10.2508841708333	9.8780425625	9.94910123333333	10.4018656125	12.64196235	12.5059521125	8.75680819166667	10.0278755708333	9.85656001666667	10.2602231666667	10.4447234208333	10.5470652166667	10.4781179666667	10.4836555916667	11.4994814416667	10.8849115041667	10.9384683958333	9.88149208333333	10.5897747333333	10.3402727083333	10.5339452166667	9.8827605125	9.3450602625	9.2451576	10.1822655291667	10.8301832541667	10.1889238583333	10.2082797708333	10.3504978541667	10.8023153291667	10.4411530875	9.34369135833333	9.4827693375	8.92196795416667	10.4193897	7.4293240625	9.70646494166667	9.54380385	9.3724482125	9.77547627916667	9.5220062	8.98725887916667	7.6580112375	10.218895	8.65490165	9.90285729166667	10.0978445333333	9.90753555	9.2272344	9.88231113333333	9.84822367083333	9.3565659375	9.3234934125	9.806449375	9.377526425	9.69062886666667	8.57671406666667	10.3122044125	8.10556687083333	10.7381507166667	9.7525953	9.600701075	8.41228047083333	9.30477144583333	9.04961187083333	8.96960385416667	8.27096189166667	9.43749067083333	8.37280509583333	9.2043888125	9.93244266666667	9.738101875	10.1511328958333	9.63878470833333	8.99007008333333	9.84137288333333	10.3544309041667	9.44669676666667	10.2405294125	10.3493918083333	9.98656490833333	9.090307825	8.563937625	8.8229233375	10.0638420541667	9.91265397916667	9.8641359375	9.68013430833333	8.9505562125	10.1761008041667

TNFSF12	12.3428910083333	9.9186526375	10.361669025	9.429283125	8.23340604166667	10.1647975583333	9.8309168	9.7719804625	9.661749025	10.0315805458333	9.34132776666667	11.838151225	10.660195125	9.81963299166667	9.32873125833333	10.675551525	10.8910372666667	8.58512142916667	10.0663523541667	10.0913534291667	10.1415170541667	8.2424291625	10.1314447125	10.0037304958333	10.1822655291667	12.3718770291667	10.627622325	9.67119210416667	8.92243870416667	10.0324278916667	10.2508841708333	9.8780425625	9.94910123333333	10.4018656125	12.64196235	12.5059521125	8.75680819166667	10.0278755708333	9.85656001666667	10.2602231666667	10.4447234208333	10.5470652166667	10.4781179666667	10.4836555916667	11.4994814416667	10.8849115041667	10.9384683958333	9.88149208333333	10.5897747333333	10.3402727083333	10.5339452166667	9.8827605125	9.3450602625	9.2451576	10.1822655291667	10.8301832541667	10.1889238583333	10.2082797708333	10.3504978541667	10.8023153291667	10.4411530875	9.34369135833333	9.4827693375	8.92196795416667	10.4193897	7.4293240625	9.70646494166667	9.54380385	9.3724482125	9.77547627916667	9.5220062	8.98725887916667	7.6580112375	10.218895	8.65490165	9.90285729166667	10.0978445333333	9.90753555	9.2272344	9.88231113333333	9.84822367083333	9.3565659375	9.3234934125	9.806449375	9.377526425	9.69062886666667	8.57671406666667	10.3122044125	8.10556687083333	10.7381507166667	9.7525953	9.600701075	8.41228047083333	9.30477144583333	9.04961187083333	8.96960385416667	8.27096189166667	9.43749067083333	8.37280509583333	9.2043888125	9.93244266666667	9.738101875	10.1511328958333	9.63878470833333	8.99007008333333	9.84137288333333	10.3544309041667	9.44669676666667	10.2405294125	10.3493918083333	9.98656490833333	9.090307825	8.563937625	8.8229233375	10.0638420541667	9.91265397916667	9.8641359375	9.68013430833333	8.9505562125	10.1761008041667

AL662884.4	8.91865062916667	7.61729026666667	7.8566142125	7.71161946666667	7.62787378333333	7.917126675	7.3709148	7.35596242083333	6.74995032916667	7.54373619583333	7.22445772083333	6.73384843333333	7.0512128625	7.71057563333333	8.36647667083333	7.74371000833333	8.79416294583333	7.7332836125	8.41358929583333	7.8304358875	7.35722785416667	8.15733075	8.1076515375	7.84517950833333	7.841302375	8.73134057083333	8.23543905416667	8.3015442625	8.270463975	8.8852138875	8.43827822916667	7.81164957083333	7.73665201666667	8.49816849583333	8.770270125	8.809465625	8.11578765	7.82365227916667	7.7605863625	7.8528378125	8.3580952125	8.36106508333333	8.36932502083333	8.13741667083333	8.1251511375	8.422416775	7.9991424625	7.7159756625	7.96447044166667	8.27522650416667	8.04259674166667	7.8942936	8.68305490416667	8.46432879166667	8.4244895	8.48436670833333	7.56917553333333	8.07648842083333	8.5414561	7.9486319	8.10416175	6.9693848625	8.29225103333333	7.48037515833333	9.19180652916667	8.1179371875	6.93651231666667	6.52240923333333	7.87341445416667	6.71822957916667	6.89631112916667	6.47331982083333	8.12906104166667	7.617860025	7.291380925	6.4904906625	7.586560225	6.99570072916667	7.86037695833333	8.674769575	8.67864822916667	7.34524812916667	7.929402875	8.1932087875	7.45129297083333	8.38144642916667	8.669051975	7.04294924166667	8.89806269583333	8.87809686666667	7.18193015416667	8.17009956666667	7.41091522083333	6.73170205833333	7.85024740416667	7.31165970833333	8.3000723625	8.4689240375	7.7563220125	7.87341445416667	8.0082648625	7.28708852916667	7.94622360416667	8.25267709583333	8.21329221666667	8.22822784166667	7.52648900833333	8.68998533333333	7.8153205625	7.91494055416667	7.79612554166667	8.30310280833333	9.29643897916667	9.03524544583333	8.473578375	8.40591115416667	9.38691342083333	8.29378514166667	9.07369755833333	6.17670596666667

PRRT1	8.91865062916667	7.61729026666667	7.8566142125	7.71161946666667	7.62787378333333	7.917126675	7.3709148	7.35596242083333	6.74995032916667	7.54373619583333	7.22445772083333	6.73384843333333	7.0512128625	7.71057563333333	8.36647667083333	7.74371000833333	8.79416294583333	7.7332836125	8.41358929583333	7.8304358875	7.35722785416667	8.15733075	8.1076515375	7.84517950833333	7.841302375	8.73134057083333	8.23543905416667	8.3015442625	8.270463975	8.8852138875	8.43827822916667	7.81164957083333	7.73665201666667	8.49816849583333	8.770270125	8.809465625	8.11578765	7.82365227916667	7.7605863625	7.8528378125	8.3580952125	8.36106508333333	8.36932502083333	8.13741667083333	8.1251511375	8.422416775	7.9991424625	7.7159756625	7.96447044166667	8.27522650416667	8.04259674166667	7.8942936	8.68305490416667	8.46432879166667	8.4244895	8.48436670833333	7.56917553333333	8.07648842083333	8.5414561	7.9486319	8.10416175	6.9693848625	8.29225103333333	7.48037515833333	9.19180652916667	8.1179371875	6.93651231666667	6.52240923333333	7.87341445416667	6.71822957916667	6.89631112916667	6.47331982083333	8.12906104166667	7.617860025	7.291380925	6.4904906625	7.586560225	6.99570072916667	7.86037695833333	8.674769575	8.67864822916667	7.34524812916667	7.929402875	8.1932087875	7.45129297083333	8.38144642916667	8.669051975	7.04294924166667	8.89806269583333	8.87809686666667	7.18193015416667	8.17009956666667	7.41091522083333	6.73170205833333	7.85024740416667	7.31165970833333	8.3000723625	8.4689240375	7.7563220125	7.87341445416667	8.0082648625	7.28708852916667	7.94622360416667	8.25267709583333	8.21329221666667	8.22822784166667	7.52648900833333	8.68998533333333	7.8153205625	7.91494055416667	7.79612554166667	8.30310280833333	9.29643897916667	9.03524544583333	8.473578375	8.40591115416667	9.38691342083333	8.29378514166667	9.07369755833333	6.17670596666667

CCDC144NL	4.45030740833333	4.91552208333333	4.669520575	4.42980535	4.05858813333333	5.04024386666667	6.59670515	5.08458449166667	4.37710720833333	5.10174316666667	4.2918764	6.55145231666667	4.613234575	4.67972664166667	4.062876875	4.08114406666667	4.83558559166667	4.86479268333333	4.41103275833333	4.67674131666667	4.33428121666667	3.97925105	5.93746846666667	4.2379826	4.515720925	4.28809964166667	4.27700685	5.052876825	4.55568075	4.21809414166667	4.434278525	4.485186325	3.9147734	4.44254745	4.31116040833333	5.40852341666667	4.494971825	4.56600969166667	4.38197743333333	4.366029925	5.01216298333333	4.24130721666667	4.5496205	5.19860551666667	4.341768725	4.879391675	4.98341465833333	4.78700018333333	4.44059075	4.68789791666667	4.546642225	4.36096451666667	4.65478205833333	5.07656036666667	4.3249589	4.56600969166667	5.053877925	4.61955908333333	4.46985861666667	3.83492170833333	3.80218998333333	5.30448685	5.83306814166667	6.2066078	4.58556791666667	4.2231712	4.47089620833333	5.33153680833333	4.341768725	4.50011030833333	5.34622214166667	4.47573676666667	5.33746485833333	4.4462942	4.34074395833333	4.73928888333333	3.90457709166667	6.56215660833333	5.192100225	6.493249575	4.760314225	4.00881020833333	8.77406471666667	4.34281185	4.50544710833333	7.62815665833333	4.46985861666667	5.23361180833333	4.66519471666667	5.4147768	4.38635804166667	7.88506155833333	4.61625569166667	4.71576270833333	4.82580888333333	4.48829116666667	4.19132811666667	7.25120925833333	4.79906281666667	6.12763226666667	8.80139795	4.48432390833333	6.66944055833333	4.58140610833333	4.31516280833333	7.79357515	6.00884046666667	5.78160560833333	5.7745543	5.97436386666667	4.34074395833333	4.88257656666667	4.40083348333333	4.9406758	8.08952596666667	4.233876925	4.84714924166667	4.41962694166667	4.35998614166667	5.97783559166667

RYR1	12.206981325	14.1985925	14.2887769	14.4832242916667	15.1207533083333	12.8947866166667	14.99199845	14.2810681833333	14.56279245	13.9458581083333	13.9554702666667	12.1915147666667	14.8236926166667	14.1841737583333	14.4581253166667	14.11256935	11.912794275	14.5550005583333	14.282826075	14.3628995916667	14.1709219416667	14.2058121833333	13.6812289916667	14.1698024416667	14.4775829916667	11.9856427083333	14.5415836083333	14.0991501333333	14.451178225	14.4328794	14.6883693	14.6527873333333	14.3596884833333	12.2215063083333	11.8321536333333	11.7572174666667	14.912278625	13.7781265916667	14.18015695	14.371029675	14.4346519833333	14.4926359	14.4832242916667	13.8461367666667	13.783955125	13.8221316	13.6587931916667	12.9929816333333	13.698785275	13.8604046083333	14.2289632916667	14.1857941	14.0787258833333	13.185241925	13.31441505	13.8635959083333	14.4581253166667	13.8241318916667	13.5287262833333	13.6996187916667	12.1732634166667	11.705716875	12.0168871666667	12.1636527	11.592340975	13.147286425	12.0710070666667	12.1774305916667	11.6099448083333	11.7365773166667	12.1587642333333	12.5029330166667	11.9071135	11.4692934083333	12.3733049416667	11.720568625	12.2901521833333	12.4248504333333	11.8626743166667	12.2599545833333	11.1361895666667	11.546539525	12.1509618666667	11.6547489083333	12.2867043083333	11.9013541833333	11.3502989166667	11.3698477166667	11.2373633083333	11.3886248333333	11.798162425	11.7387926	12.3896401833333	12.5984922	12.1241915	12.6080271416667	11.8943464416667	11.8749533333333	12.0193369	11.7928828083333	11.6525927916667	11.5392863166667	11.7678571083333	12.2302891583333	12.3310232666667	11.55164095	11.4336132833333	11.3057739666667	12.0301876583333	11.8761205916667	11.9502134083333	12.2998821166667	11.7782923583333	11.47905225	11.451916725	12.0893148833333	11.3068495166667	11.4946024833333	11.9712775416667	11.012770325

CIT	9.27941565	8.92602376666667	8.97769849166667	8.98465464166667	10.0945926583333	8.97769849166667	10.3019992	9.13226131666667	9.86598649166667	9.27994568333333	9.3007507	9.43456800833333	9.80927415833333	8.986594625	9.286424975	8.69220983333333	8.8334785	9.80576564166667	9.15505945	8.76125596666667	9.16031618333333	9.047926775	8.703553525	8.7143253	8.74671439166667	8.58958554166667	8.75473524166667	8.61138693333333	9.02131511666667	8.93227916666667	9.173253675	8.9962514	9.14518724166667	8.97532130833333	9.05602455	8.93806988333333	9.22559156666667	8.88732965833333	8.86508634166667	8.67939293333333	8.83968223333333	9.11495396666667	8.709872025	8.31619925	8.214548775	8.38662095833333	8.560933175	8.60701585833333	8.42269018333333	8.5034354	8.76851021666667	9.07634685	8.90389271666667	8.96273033333333	8.84802724166667	8.49794376666667	8.70635446666667	8.69747196666667	8.50185603333333	8.20331464166667	9.26809461666667	8.91135703333333	8.523789	8.51622818333333	9.10485650833333	9.62275734166667	9.39938098333333	9.51724313333333	9.31762243333333	9.35451149166667	9.45323241666667	9.23797865	9.54178184166667	8.94668689166667	9.34392991666667	8.56044531666667	9.20464029166667	9.05965051666667	8.69409343333333	8.9081181	9.36848635	9.26773438333333	9.230932875	8.76703910833333	8.98046293333333	8.85348764166667	9.3558778	9.116355225	9.27222216666667	8.40567906666667	9.176064475	8.775178575	9.03417474166667	8.66625304166667	9.35995134166667	9.18880384166667	9.186916425	9.27899289166667	9.24492166666667	8.85110639166667	8.77094225	9.13694515	8.72692960833333	8.34576421666667	8.582021475	8.45404219166667	8.4444208	8.90434914166667	8.60280456666667	8.88262415833333	8.43351543333333	8.75703444166667	8.64585098333333	8.35786781666667	8.45304260833333	8.49886293333333	8.641468925	8.770520625	8.59492328333333	8.38023938333333

ARHGEF18	11.04340915	12.4795096083333	12.1509618666667	11.875371875	12.4732793833333	11.642205825	12.11177165	12.5448126916667	11.7463222583333	11.9155103583333	12.2844611166667	11.2481821666667	11.659892375	12.4772181916667	12.4559287	12.3518594833333	12.4091926166667	11.08338295	12.0249612083333	12.2976992	12.1604527333333	12.7934592666667	12.3662011583333	11.9888038083333	11.5961336833333	11.3769382416667	12.0993529333333	11.9641339083333	11.9570955666667	12.0623925666667	12.1803292	12.2293758416667	11.8048205583333	12.479955875	11.4704736916667	11.3672205833333	12.391732275	12.4917329166667	12.2771993416667	11.9923578333333	12.3046936083333	12.2771993416667	12.222327475	12.1517212916667	11.8666609583333	12.3110445	12.1673323833333	12.4619169416667	12.3602095583333	11.74784205	11.5920332333333	12.10672325	11.639055925	11.7568591416667	11.541832	11.94832135	12.1886556416667	12.6541819833333	11.84965965	11.8670714	11.0190136	11.5034674333333	11.6223592166667	12.0108213333333	10.8412280166667	12.1765967416667	11.6705910083333	12.1435544583333	11.464317475	12.025731925	11.5064020583333	11.7849428666667	11.345193725	11.6223592166667	11.36947175	11.5668628166667	11.52420235	11.497488975	11.642205825	11.8772191666667	10.73913815	10.7493099333333	11.5118143666667	11.835222575	11.1394283333333	10.86697045	11.0239555333333	12.2416674416667	10.7725388333333	11.60398845	11.351341375	11.5614234666667	11.9104714083333	11.37567555	11.341686575	11.8100395833333	11.89894165	11.020740575	11.2606101916667	11.7814654666667	11.717933225	11.5505559916667	11.1975258	12.1304018333333	12.462326175	11.4669037583333	11.6982071583333	12.7011030333333	10.9462719166667	11.5998809	11.4534829083333	12.1367818666667	11.4446261583333	11.4019322	11.546539525	11.3339882	10.6641205583333	12.5671498916667	12.1765967416667	11.7012064

BOLL	4.59784559166667	6.258372575	6.05307568333333	6.43290905833333	6.89322066666667	8.39370431666667	8.75371965	6.89980234166667	6.51685910833333	6.4808852	7.82904454166667	8.28095494166667	6.48151946666667	6.55593524166667	6.80449616666667	5.773689175	5.550710675	6.60676565	5.57834849166667	7.05156345	6.414216275	6.16197145833333	6.51685910833333	5.379722375	3.76208641666667	5.35182895833333	5.01530855833333	4.59464514166667	5.04667276666667	5.68499903333333	4.09598105	5.38064126666667	5.90321861666667	6.9475749	4.34901141666667	4.160694875	4.99019379166667	7.23371278333333	5.25233420833333	6.52987506666667	5.46093680833333	3.87937155833333	5.06786714166667	5.355721825	4.79518681666667	4.6622505	4.638255275	6.00716590833333	4.91752975833333	5.89368925	5.006452275	5.52011276666667	6.06230976666667	5.71416341666667	5.5697365	4.59265796666667	5.83674730833333	6.245275725	6.69642741666667	6.46055301666667	8.58601561666667	7.58128390833333	8.86366274166667	6.964596175	7.88927118333333	7.74145285	8.08289571666667	8.15060805	8.38662095833333	8.34324809166667	8.86655378333333	8.37061515	7.57457279166667	7.36156116666667	8.09998893333333	8.15303908333333	6.987814075	8.08700735	6.15778135833333	8.50286535	8.122791675	9.767731825	7.09738105833333	6.28402719166667	8.74034393333333	7.23429453333333	9.20464029166667	6.78782379166667	8.83783124166667	7.20334935833333	7.31196651666667	7.38755610833333	8.551994475	7.22964071666667	8.486995925	8.391097025	6.27095864166667	8.64688179166667	7.18705289166667	8.348200375	9.34897708333333	7.458458425	8.17613399166667	7.88130259166667	6.872774725	7.61126938333333	6.03581564166667	8.21782325833333	7.55987390833333	5.97035753333333	8.08240869166667	8.23263584166667	8.90099503333333	8.44192248333333	6.99033689166667	6.88458655833333	6.33645039166667	9.16967255	7.99725846666667	8.31466204166667

GSTCD	11.415671175	10.7626524625	10.6486219333333	10.9293807916667	10.81782005	10.7334934375	10.5943481708333	10.8000125916667	10.8990507166667	11.0133287875	10.3283500958333	10.6833032666667	10.9370095208333	10.7567177	10.5354835875	10.3593105625	10.6751738791667	10.8704478333333	10.625026675	10.4666524916667	10.6455337208333	10.9333690666667	10.7266855375	10.30872915	10.8396074291667	11.0587480708333	10.7102346	10.7006923125	10.9967270583333	10.690430875	10.743821075	10.6205496666667	10.4688930208333	10.5164336	11.0898799333333	11.2170633625	10.6315499291667	10.4544600958333	10.3141512541667	10.5591043416667	10.3099456083333	10.3589357666667	10.2875942875	10.6720892083333	10.4151646583333	10.6743836958333	10.9359125291667	10.7044668458333	10.65907185	10.2416869625	10.4900661791667	10.4086865166667	10.9732239166667	11.1068966458333	10.9645598333333	10.8955074291667	10.582768125	10.725286525	10.4953226083333	10.8069209166667	10.3429838208333	10.7118077333333	11.5640089708333	10.7430511625	11.0714473208333	10.6685231708333	11.3189377583333	10.83769035	11.9426025125	10.97708	10.8636915208333	11.0144504208333	11.2167232875	10.5055014791667	11.3077018833333	11.1363653708333	10.2164257666667	10.7996179	11.3977826625	10.8970735375	10.6217312958333	10.4559937	11.3056198375	10.5094635333333	10.8700404666667	11.1781126083333	11.8330932333333	10.904495125	10.3716298916667	10.3958403333333	10.8856558833333	10.7603203166667	10.5989293166667	11.1048374	11.0900389958333	10.7841011083333	10.9001608208333	11.1939463541667	11.0385524708333	11.3138672875	11.1918332083333	10.7922036625	10.9285779791667	10.5214535583333	11.1642985958333	10.8433764041667	10.7754033458333	11.0317580041667	10.6568369208333	10.8772896791667	11.0245256375	11.5935969083333	11.1563163958333	11.2758694416667	11.0468280291667	11.0687843333333	11.3138672875	11.3425257416667	10.731189	11.3073739666667

INTS12	11.415671175	10.7626524625	10.6486219333333	10.9293807916667	10.81782005	10.7334934375	10.5943481708333	10.8000125916667	10.8990507166667	11.0133287875	10.3283500958333	10.6833032666667	10.9370095208333	10.7567177	10.5354835875	10.3593105625	10.6751738791667	10.8704478333333	10.625026675	10.4666524916667	10.6455337208333	10.9333690666667	10.7266855375	10.30872915	10.8396074291667	11.0587480708333	10.7102346	10.7006923125	10.9967270583333	10.690430875	10.743821075	10.6205496666667	10.4688930208333	10.5164336	11.0898799333333	11.2170633625	10.6315499291667	10.4544600958333	10.3141512541667	10.5591043416667	10.3099456083333	10.3589357666667	10.2875942875	10.6720892083333	10.4151646583333	10.6743836958333	10.9359125291667	10.7044668458333	10.65907185	10.2416869625	10.4900661791667	10.4086865166667	10.9732239166667	11.1068966458333	10.9645598333333	10.8955074291667	10.582768125	10.725286525	10.4953226083333	10.8069209166667	10.3429838208333	10.7118077333333	11.5640089708333	10.7430511625	11.0714473208333	10.6685231708333	11.3189377583333	10.83769035	11.9426025125	10.97708	10.8636915208333	11.0144504208333	11.2167232875	10.5055014791667	11.3077018833333	11.1363653708333	10.2164257666667	10.7996179	11.3977826625	10.8970735375	10.6217312958333	10.4559937	11.3056198375	10.5094635333333	10.8700404666667	11.1781126083333	11.8330932333333	10.904495125	10.3716298916667	10.3958403333333	10.8856558833333	10.7603203166667	10.5989293166667	11.1048374	11.0900389958333	10.7841011083333	10.9001608208333	11.1939463541667	11.0385524708333	11.3138672875	11.1918332083333	10.7922036625	10.9285779791667	10.5214535583333	11.1642985958333	10.8433764041667	10.7754033458333	11.0317580041667	10.6568369208333	10.8772896791667	11.0245256375	11.5935969083333	11.1563163958333	11.2758694416667	11.0468280291667	11.0687843333333	11.3138672875	11.3425257416667	10.731189	11.3073739666667

NT5C3A	11.6910443916667	12.2181091583333	12.1241915	12.0514729416667	12.2040899	12.764427075	12.225632925	12.1724138083333	12.2599545833333	12.0921972916667	12.5226496333333	11.8869480833333	12.1596044833333	12.6739472	12.4545539666667	12.2412181916667	12.7195939833333	12.1150337666667	11.9104714083333	12.2503999916667	12.3216200916667	12.2396159	13.2227845833333	12.2500083166667	12.336155525	12.105942725	12.51216495	12.3305815833333	12.0025248166667	12.31352485	12.2776254166667	12.4818012083333	11.9724754666667	13.0488654	12.0120395666667	11.82248265	11.876483925	12.06402615	12.1553849	11.5316298	12.0424744583333	11.8845097083333	12.487541925	12.1620485833333	12.057317	11.6310321916667	12.1439381	11.8908867916667	12.3627391666667	12.2641600583333	12.5084928916667	12.042069275	12.0551513666667	12.2611765333333	12.479955875	12.136374875	12.1636527	12.2943062333333	12.1737118583333	12.0989443916667	12.7306165083333	12.7591596666667	13.1085025666667	12.3789904833333	12.762350675	12.7076553	13.2081167083333	12.4202498166667	12.9924504416667	13.214199825	12.9924504416667	12.5969475916667	12.8057256416667	12.3606021	12.7547810833333	13.1819427166667	13.57301355	12.5807864333333	13.0349568083333	12.9024490333333	11.8646362416667	12.9053364166667	12.787939025	12.3267305	13.08588685	12.8329037166667	12.2289445583333	12.9929816333333	12.798152025	12.5667535083333	12.4772181916667	13.1046602416667	13.0055645333333	12.196460925	12.2570148333333	13.3315421333333	13.1019491416667	12.4855430666667	12.4443760583333	11.9017197166667	13.1919341833333	12.85786905	12.2570148333333	13.489460875	13.2495669	13.8010236666667	12.2302891583333	13.3046967416667	12.4912644	13.077594625	12.7912843416667	12.543462525	12.6041308166667	13.0155242333333	13.542740525	12.6336537916667	12.19689565	13.232552625	12.8636072	13.1376073666667

ERAP2	8.11504903333333	11.1779127416667	11.651839775	11.392737725	10.6411546083333	11.9505991416667	7.224148725	11.49602405	11.0448237416667	12.141404575	11.1898119916667	11.67393075	10.9790999666667	8.50236353333333	10.6285410083333	13.1305878416667	11.5093017916667	11.4828657583333	8.22546075833333	12.2714994	8.45509195	11.2205665583333	11.9285599833333	8.43902399166667	8.46552205	11.02981265	8.5196153	8.65466659166667	10.7565121333333	11.37169735	7.61062251666667	11.6466404583333	11.3817027833333	12.3785017666667	11.3668652083333	11.19451335	8.04388136666667	8.44632826666667	8.38708958333333	10.126633025	11.4559913916667	12.2302891583333	11.9162394	8.6493973	9.07683584166667	9.51088574166667	8.9184083	11.9491178333333	12.2416674416667	11.7190625916667	12.6522495833333	11.9096928333333	10.971110725	10.96150755	12.461524275	12.2918290666667	8.52009253333333	8.96273033333333	11.7810947333333	9.43501909166667	7.77602128333333	10.7756109166667	10.8638767916667	9.70495998333333	11.4715256833333	8.69657786666667	7.30431380833333	10.8269705916667	11.7765043416667	13.2201906833333	11.1129059916667	9.85895400833333	11.7823700916667	8.20124505	10.1553380833333	12.47909375	12.7521459083333	12.7344773416667	8.046473725	12.3067398	7.7794798	11.1752905	11.9251897666667	7.83125643333333	8.13617131666667	11.625582325	8.97769849166667	8.83968223333333	9.06503669166667	11.0611890333333	8.55392016666667	12.3418182	9.341081375	11.4817372166667	9.87826785	10.7728842416667	7.31739324166667	8.24215483333333	7.05399351666667	11.3823997333333	11.0723250583333	12.8823979	12.46107545	8.29403910833333	10.1409369083333	9.06167029166667	8.21353541666667	12.3538131333333	11.5802552416667	12.693462425	12.5585076166667	11.6499693666667	12.1608207083333	11.25569785	12.7490093583333	12.3842104583333	8.06500796666667	9.133746725	11.233092075	9.96954501666667

RUNX2	7.964195775	9.28128599166667	10.18748315	10.036942525	9.67592103333333	11.216912225	9.56961935	10.647327775	10.3987917	10.0430227416667	10.3574979	10.6023330833333	8.889794925	9.73270225833333	8.80586325	11.4798432416667	10.534888275	8.66724769166667	9.722436925	10.32163045	10.807474625	10.9303115416667	11.2312268666667	11.0079045333333	10.3116455	8.66775465	10.0632117083333	10.4115783666667	9.88594978333333	9.28087420833333	10.1678889166667	11.1731632666667	10.8681221916667	12.9477864583333	9.6262034	8.457931075	9.865582425	9.97337968333333	11.1430808166667	9.93476125833333	10.7565121333333	9.7097404	10.6960220666667	11.1010834666667	10.4830527666667	11.1132646583333	9.98838416666667	10.0528892666667	11.047689175	11.559355625	10.9861853	10.012954475	11.1291467583333	10.1613409083333	10.743245175	10.2621725583333	10.468685175	10.3182950083333	10.3771245083333	10.7523474083333	13.011963125	11.5776190666667	11.2150743583333	12.4827258583333	11.6906956833333	11.7074261416667	12.907519675	12.1656867	11.523003425	12.2892396	11.5302262416667	12.0294077916667	12.07649975	12.6775440333333	12.278442975	13.0761975166667	11.25880145	12.2073843166667	11.4982052833333	11.6227232166667	11.9505991416667	12.6080271416667	12.214635575	13.6411556333333	12.78176855	12.345780775	11.7063908333333	12.6923621166667	11.0266684708333	12.9357780833333	12.13546815	12.5921909166667	11.2613111166667	12.4395669416667	12.1167008333333	11.2693525666667	12.4248504333333	11.1448538	11.9204648416667	12.6402462916667	12.6664956833333	11.197870325	11.6898911833333	11.14267695	10.4492048416667	12.3550491166667	13.2227845833333	13.244127475	13.3416873833333	12.6146877083333	12.4261316583333	10.0189542666667	12.4055209	11.8344676416667	12.4655499583333	12.0616030916667	11.9104714083333	12.7031196333333	11.0308654583333	11.9844494166667

TRABD2B	9.92873065833333	8.73019295833333	9.83719281666667	8.881658575	8.471007425	9.03327476666667	8.92218960833333	9.045519875	8.25398819166667	10.0434280416667	8.74913316666667	10.4534831666667	10.0802966083333	9.03284314166667	8.15455800833333	9.73533868333333	8.31765478333333	8.42214336666667	9.06886545833333	9.36715445	9.7452797	7.198288025	9.02927973333333	9.62275734166667	9.58391948333333	10.18000125	8.61480576666667	9.47404223333333	8.8296979	8.894577375	9.23797865	8.800966875	8.95991093333333	8.30336938333333	10.0688438166667	10.5300186083333	8.57834244166667	9.612283675	9.95566975	9.35357573333333	9.354988125	8.74322296666667	8.99027038333333	8.791398425	9.71188024166667	9.14279978333333	8.9384783	9.70236100833333	8.65218976666667	8.96510433333333	9.12042280833333	9.52364006666667	9.063171125	9.12232943333333	8.94525708333333	9.47496915833333	9.553861875	9.31047634166667	9.27083296666667	9.767731825	8.94478365	8.600364325	8.23217175	8.96369055833333	8.94186190833333	8.33965076666667	8.66247435	8.335541375	8.09066366666667	8.83016251666667	8.56529179166667	8.60799398333333	8.22846261666667	9.02785883333333	7.78488271666667	8.94715431666667	9.27941565	8.665331325	8.25398819166667	8.20882531666667	9.02175539166667	8.85635194166667	8.042852375	8.25292731666667	8.42124595	9.4936652	8.4076656	9.5453508	8.74957956666667	9.132749475	8.6984679	8.39620023333333	7.88871496666667	9.19719063333333	8.70751021666667	7.89510465833333	7.76884748333333	8.4564653	9.02512653333333	7.74722135833333	8.62136429166667	7.85748985	7.676574425	8.04234110833333	8.11712540833333	7.98717739166667	8.4237182	7.283866825	8.09498031666667	8.94525708333333	9.106662475	7.78439114166667	9.40597950833333	8.17036706666667	8.79861721666667	8.11406380833333	8.59842283333333	7.98516725833333	7.931728325	8.64244941666667

TYMS	10.0789348041667	9.33476645833333	8.097229675	9.7432741875	10.2082797708333	10.123577325	8.2586242	9.51333872083333	8.95648925416667	8.38048105833333	9.43749067083333	9.90886985	9.32735891666667	8.9272761125	9.46586581666667	9.05475945416667	9.70215354583333	10.5904701666667	9.6431333	9.67568525833333	9.021988275	10.5301883125	9.174863825	10.0854211625	10.325930675	10.2219819541667	9.75598520416667	9.9878090125	10.5293818875	9.69902295833333	9.29819334583333	9.04481414166667	9.0016357875	8.9714747	9.67211590416667	10.1984731375	10.1735519708333	8.82650967083333	9.13250539583333	9.6047947375	9.2569635	9.23258092083333	8.986797925	9.54201864166667	9.41141477083333	9.06294040416667	8.82190914583333	9.3807746875	8.85702345	9.88020543333333	8.856558775	9.43209279583333	9.52292505833333	9.86578445833333	9.4393355875	8.812637575	9.231630675	8.71013559583333	9.996153725	9.51698945416667	10.9392302041667	8.68059544583333	8.7876802625	9.67211590416667	9.5763024375	9.32395252916667	10.4063314208333	8.66373332916667	9.49973695416667	8.49674137916667	8.9639175	10.39035585	8.3298481875	8.834198375	10.6124720583333	10.1864594291667	9.363637275	10.6724377541667	9.49259015	9.00066376666667	9.85262877083333	10.4730803333333	9.58595583333333	9.53853774166667	9.415083225	10.8174508541667	10.8189211958333	9.5596726	10.223509175	9.62475257916667	8.703338125	9.23816898333333	9.43707099583333	9.42665710416667	10.8092235708333	9.40137265833333	10.0681647458333	10.17979995	8.9286527375	10.0024278083333	9.9461502125	9.35748649583333	8.87809686666667	11.025950625	10.0970717166667	10.5943481708333	8.99745976666667	10.4431917875	8.17429475833333	9.5775666125	8.14931854166667	9.49973695416667	9.24056433333333	9.6106909875	8.8011824125	8.98544247083333	10.0738811708333	9.56148589166667	10.3647371625	9.58368255

ENOSF1	10.0789348041667	9.33476645833333	8.097229675	9.7432741875	10.2082797708333	10.123577325	8.2586242	9.51333872083333	8.95648925416667	8.38048105833333	9.43749067083333	9.90886985	9.32735891666667	8.9272761125	9.46586581666667	9.05475945416667	9.70215354583333	10.5904701666667	9.6431333	9.67568525833333	9.021988275	10.5301883125	9.174863825	10.0854211625	10.325930675	10.2219819541667	9.75598520416667	9.9878090125	10.5293818875	9.69902295833333	9.29819334583333	9.04481414166667	9.0016357875	8.9714747	9.67211590416667	10.1984731375	10.1735519708333	8.82650967083333	9.13250539583333	9.6047947375	9.2569635	9.23258092083333	8.986797925	9.54201864166667	9.41141477083333	9.06294040416667	8.82190914583333	9.3807746875	8.85702345	9.88020543333333	8.856558775	9.43209279583333	9.52292505833333	9.86578445833333	9.4393355875	8.812637575	9.231630675	8.71013559583333	9.996153725	9.51698945416667	10.9392302041667	8.68059544583333	8.7876802625	9.67211590416667	9.5763024375	9.32395252916667	10.4063314208333	8.66373332916667	9.49973695416667	8.49674137916667	8.9639175	10.39035585	8.3298481875	8.834198375	10.6124720583333	10.1864594291667	9.363637275	10.6724377541667	9.49259015	9.00066376666667	9.85262877083333	10.4730803333333	9.58595583333333	9.53853774166667	9.415083225	10.8174508541667	10.8189211958333	9.5596726	10.223509175	9.62475257916667	8.703338125	9.23816898333333	9.43707099583333	9.42665710416667	10.8092235708333	9.40137265833333	10.0681647458333	10.17979995	8.9286527375	10.0024278083333	9.9461502125	9.35748649583333	8.87809686666667	11.025950625	10.0970717166667	10.5943481708333	8.99745976666667	10.4431917875	8.17429475833333	9.5775666125	8.14931854166667	9.49973695416667	9.24056433333333	9.6106909875	8.8011824125	8.98544247083333	10.0738811708333	9.56148589166667	10.3647371625	9.58368255

RUNX1	4.70784294166667	4.48717731666667	4.736033375	4.27374416666667	4.70675665	4.383064975	5.78709845833333	5.38263135833333	5.14292011666667	4.59179895833333	4.50751908333333	5.388701925	5.75112915833333	4.39770036666667	4.2592488	4.339553	4.77601478333333	4.740346475	4.71899701666667	4.28310633333333	5.08997001666667	4.33637641666667	4.47474535	4.54330015	4.79716553333333	4.1882044	4.841975675	4.63093599166667	4.77483110833333	4.41782250833333	4.73402499166667	4.65478205833333	4.03339724166667	5.29885645833333	4.41392473333333	4.33532039166667	4.67574021666667	4.6622505	4.82904071666667	4.853434925	5.18423503333333	4.59683404166667	4.939635175	5.72997074166667	4.207987625	5.19959509166667	5.30448685	5.15246255833333	4.63723314166667	4.97177571666667	4.90470955	4.58445505833333	3.911419125	5.54779774166667	4.48159451666667	4.93337844166667	4.82580888333333	4.94355658333333	4.39770036666667	4.453007975	3.88876065	4.1756419	4.42054164166667	3.84817555	4.87006425833333	4.30437938333333	4.54233060833333	4.47367345833333	3.71306755	4.02799813333333	4.26445661666667	4.14053975833333	4.602780325	3.74027611666667	3.89460940833333	3.832630725	4.40903533333333	3.97069799166667	3.899834175	3.8987918	4.23196318333333	3.78548855	4.33043379166667	4.00032819166667	3.83595706666667	3.91593421666667	4.69325549166667	5.23035095	4.149223425	4.04191653333333	3.74027611666667	4.47861730833333	4.05071508333333	3.9661014	4.031307325	3.99930385833333	4.15642159166667	4.18081978333333	3.98946053333333	5.19536235833333	4.45030740833333	3.91035815	4.90586546666667	5.23259211666667	4.43639113333333	4.07134488333333	4.72508045	6.66118995	4.34995844166667	6.81852565833333	4.69325549166667	5.577321975	4.61955908333333	5.07107728333333	4.21592141666667	4.49216794166667	4.19434771666667	4.68477714166667	4.70475344166667	4.27700685

CHD1L	10.503058125	9.46432354166667	9.15886451666667	9.16442515	10.3960554833333	10.3345761083333	8.77869386666667	9.57556716666667	9.03950406666667	9.75492564166667	9.97043543333333	9.90478876666667	9.99723533333333	9.43234145	9.683521475	9.44430623333333	9.763939075	10.7356243	9.98150248333333	9.7553002	9.71912328333333	10.0343496666667	9.74820641666667	10.0167779583333	10.1413216916667	9.64610789166667	9.3832069	9.630635475	10.4409408666667	9.57903839166667	9.55990681666667	9.95852463333333	9.11993345	9.34207146666667	9.21080935833333	9.90307260833333	9.96745814166667	9.62712280833333	9.49238265	9.579948275	8.80450975	9.516735775	9.83071770833333	9.633880775	9.8942397	9.70495998333333	10.040006825	10.149337475	9.552037225	9.73489294166667	9.452370525	9.79628703333333	10.7581339416667	10.418815025	9.553861875	10.1172154583333	10.0744319916667	9.51951014166667	9.83452378333333	9.974596575	12.4350665416667	10.2599956916667	10.410088175	10.326957425	10.8150497416667	11.2047015416667	10.5127537916667	9.79724158333333	10.5821763	10.384097475	10.2206735166667	11.1756714833333	10.0989513583333	10.8251181583333	10.490641975	10.23713875	9.88681354166667	10.8480286083333	10.1605417333333	10.6068679083333	10.568795625	10.7756109166667	10.5651423416667	10.4918133583333	10.4344952333333	10.6422709916667	10.72841425	9.62666130833333	11.0720193666667	10.1469040583333	9.91842590833333	11.47905225	10.31869075	9.810451275	11.1685570666667	10.216230425	10.4914439416667	11.3594507166667	10.579886175	9.88168326666667	10.2912155166667	9.98509406666667	10.9321190083333	10.666810425	10.3015658	10.7261291583333	10.62411455	10.6263315083333	10.1974084916667	10.0693001666667	9.69087439166667	9.7400082	10.6652778583333	10.2089178583333	10.384097475	10.4875672	11.0826274416667	10.3101529333333	10.4585673833333	9.76219373333333

IL4R	13.5230150833333	12.6517095833333	13.0566076	12.6572156166667	12.6512308	12.8618738916667	12.7934592666667	12.8086026666667	12.4737403583333	12.4518362583333	12.6656204083333	12.5297533083333	13.0313582416667	12.7385523583333	12.32885815	12.557141575	11.6536243916667	12.8656045083333	12.8376576083333	12.5111982416667	12.8251494083333	12.6013272333333	12.916130525	13.2047745416667	12.5710716416667	12.8240262666667	12.4822872083333	12.37676025	12.5917146833333	12.63765555	13.1779047083333	12.9977920166667	12.50892495	12.462326175	13.7463275333333	12.92082965	12.3190685583333	12.763386175	13.2412964416667	12.6759474583333	12.881258225	12.6961567333333	12.7046259583333	13.0626219916667	12.5963701666667	12.7902282666667	12.5652457833333	12.931215675	13.0694021583333	12.519265425	13.0301413666667	13.5668551916667	12.3874454166667	12.5020083166667	12.5268295333333	12.48982835	13.08588685	12.761853925	12.4287875916667	12.463267675	12.0697739833333	11.6429698666667	12.178702275	12.1270603333333	10.796845775	13.3100924166667	12.0832149333333	11.7699299	13.526397225	12.4220223083333	12.0062931166667	12.7263840666667	13.166824125	12.210372725	12.9610660166667	12.4337570333333	12.496314025	12.3297195416667	12.6765518416667	11.5941589083333	10.8254925166667	11.4108017166667	12.9444160916667	12.1536913666667	12.5576279333333	11.6558642583333	11.3015343416667	12.3461563666667	11.06778735	12.2461720416667	12.65465995	12.8398240166667	12.3716896916667	12.7681554	12.7763784666667	12.2120592333333	11.0279781	11.9361117583333	12.2057469416667	11.8705505	12.7306165083333	11.8527274916667	12.2578527416667	12.2412181916667	13.0174115	12.3051209416667	12.8020915333333	12.3169057833333	12.9941887083333	12.6312122083333	12.9172670333333	11.8658810166667	12.69187135	12.3720643666667	12.4283415	12.5516382166667	11.75788825	12.584589825	12.1823287916667	12.369348425

PDLIM7	14.4361804666667	11.61832875	11.6433290333333	10.7127200416667	11.4771109	11.8865138166667	11.07367765	11.53966185	11.2675581916667	12.068210875	11.0733527583333	13.8473484166667	13.043373375	11.62492935	12.015272025	11.9142748833333	11.4774665416667	11.6948221166667	11.6179147583333	12.052988325	12.3896401833333	11.3813533416667	11.739559375	11.9267180916667	12.126629875	14.8497796833333	12.761853925	11.4897906583333	12.098126675	11.69593475	11.75788825	11.3720648583333	11.5219495083333	10.831890425	14.7205176666667	14.9391332833333	11.49602405	11.7561182166667	11.5994743583333	12.4746445166667	11.8596015	11.5531172416667	11.1779127416667	12.0413582166667	13.1919341833333	11.827360075	12.4447647416667	11.7838361	11.2179166166667	11.526510825	11.6378736916667	11.721307075	11.646257275	11.4492670916667	11.6077110916667	12.65329555	11.7628441333333	11.5041708333333	12.0337598083333	12.1799778583333	12.6116906583333	11.9502134083333	10.8277408416667	12.226050525	11.4763556583333	11.6294117916667	12.1063614833333	11.8869480833333	12.2943062333333	11.9055925	11.1231619583333	11.83328595	12.0017085416667	12.9726928583333	11.6849152916667	12.6151813916667	12.8889747333333	11.7324169333333	11.0757994083333	11.6141277333333	12.0392089666667	12.24577265	12.2821681166667	12.7628980166667	11.8568699583333	11.7387926	11.3313733	13.1204429833333	11.114752	12.9988931583333	13.0651199166667	12.1545013166667	11.2880004333333	12.4105900333333	11.7594075916667	10.85184045	11.2876344916667	10.9080821583333	12.6131713166667	11.2291005583333	12.0214326083333	11.5276408333333	10.83116835	10.909815425	11.2982935833333	12.0997306916667	13.2254010416667	13.0451078583333	12.7190348666667	12.5921909166667	10.7919885666667	11.1088695666667	12.5831450833333	11.5411164416667	11.8701716833333	11.205405875	12.1389321	12.8718544916667	11.3212969833333	12.1178108083333

CACNA1G	9.43045813333333	10.0753226583333	10.0052447166667	9.81648044166667	10.6993840916667	10.0169873125	9.90438676666667	10.03562315	10.93683975	10.7170255166667	10.2064993416667	9.237031475	9.66237621666667	10.2485867583333	10.4671575083333	9.442039375	9.173253675	11.030517625	10.4998350333333	9.89894956666667	9.71785554166667	10.447247375	9.6347809	9.42951640833333	10.247459825	9.41761155833333	9.85161335833333	10.1919662416667	10.6761203583333	10.2947654166667	10.3686089583333	10.1970378083333	10.2051938916667	9.71575140833333	9.952821075	9.859391125	11.0775947083333	10.88817915	10.8874095666667	10.8980207583333	10.1625186333333	10.39251605	10.1350682166667	10.042560025	9.7557679	10.4180178666667	10.8378553416667	10.2915805333333	10.4480363583333	10.17959865	10.4846654833333	10.1824837166667	9.983868575	10.3412407666667	10.2089178583333	9.93630081666667	10.5519705833333	10.672620875	10.5743323083333	10.159727225	10.2947654166667	9.47496915833333	9.79016168333333	8.78635625	9.20513901666667	10.1397194333333	10.1923992666667	9.18071311666667	9.92583431666667	9.66334089166667	9.8541064	10.082743125	9.86359230833333	9.88552295	10.2149556666667	9.65053335	9.452370525	10.3599013416667	10.5073525916667	8.91457238333333	8.42476003333333	9.57556716666667	9.7557679	9.88925331666667	9.98838416666667	8.67077851666667	8.76031966666667	8.3851619	9.40207443333333	9.59648643333333	9.401147475	9.43868498333333	9.76179089166667	10.2225656916667	10.0696848333333	9.78236806666667	10.0524736083333	10.1299873833333	10.8220014333333	9.87345979166667	9.4697267	9.31670963333333	9.84456984166667	9.97583196666667	10.3362368833333	9.76732071666667	10.1389730083333	10.3392098666667	9.9162906	10.0103759833333	10.054920625	10.6507559	9.877817275	10.132057125	9.73744423333333	9.86145205	9.67723733333333	10.3442980083333	10.41048235	9.62496784166667

UNC5C	8.37460103333333	7.27359608333333	7.67425858333333	6.99475883333333	6.22080705	8.50702275833333	7.9846002	7.116803275	7.23429453333333	9.28361398333333	7.24330025	9.95767555	7.88018380833333	7.445489275	6.77563099166667	8.96094025833333	10.2241250416667	6.63574723333333	7.83780880833333	8.64290230833333	8.4444208	5.92509355833333	7.86223494166667	7.811926125	8.29630441666667	8.52634974166667	8.13819046666667	8.04450845	7.61643631666667	7.88506155833333	8.34128665833333	7.50463459166667	7.58854008333333	9.83279928333333	8.07622910833333	8.31150115833333	7.03751019166667	8.32106236666667	7.13850381666667	7.61643631666667	7.13230843333333	7.23609219166667	7.93123045833333	7.69707313333333	7.53449101666667	7.91897891666667	7.66558521666667	7.11920136666667	8.48614225	8.09590623333333	7.40898444166667	7.01007860833333	4.996867625	6.69991303333333	5.47616439166667	6.89980234166667	7.6066095	7.5954683	7.947409975	8.23080535	7.11278106666667	7.47562453333333	6.328333625	7.2559939	7.91244564166667	7.33375654166667	8.47797495833333	8.30178113333333	6.29510448333333	7.676574425	7.78439114166667	7.63210018333333	7.26747879166667	8.56818220833333	7.644918425	9.61091021666667	7.91685584166667	8.222543225	8.093315875	8.34620800833333	7.33560889166667	7.65121998333333	7.252371175	8.82372205833333	8.37061515	9.50353634166667	5.17265765	7.99339445833333	6.23865941666667	9.25303868333333	8.33702585833333	7.378725775	6.70257901666667	8.48614225	6.25427673333333	6.27314985	6.57096464166667	6.18332735833333	7.095527	7.55925806666667	7.95194513333333	6.692469175	6.06672131666667	6.61415903333333	8.791398425	7.63934964166667	8.44745494166667	7.08427549166667	8.058553875	8.9744767	8.91224634166667	7.07654913333333	6.241933725	7.06279625833333	6.75898810833333	5.13847554166667	6.70796138333333	7.3968942	5.53793416666667	8.20831443333333

ZNF846	9.78059948333333	7.43805644166667	7.71969125833333	7.103742375	6.01932009166667	7.206290275	5.19421045833333	6.4372196	6.1777684	6.13511379166667	6.225930275	7.8150656	5.80195006666667	5.5526373	7.53506526666667	7.85577905833333	6.71047270833333	6.18568551666667	8.23567169166667	7.52566054166667	7.28556258333333	8.28049985	8.09897714166667	7.87562194166667	7.95746913333333	8.70592035	8.05913828333333	7.81034196666667	7.94273865833333	7.52896074166667	7.8129644	7.50344081666667	7.50344081666667	6.55373064166667	8.82621398333333	9.22160494166667	7.44735373333333	7.55250805833333	6.74958845833333	8.08807009166667	7.87916005833333	7.53069666666667	8.38865071666667	8.017526525	8.06996655	8.11000589166667	9.05561776666667	7.23254185	7.88559756666667	8.07411434166667	7.42048124166667	6.76049560833333	7.04202985	8.26655316666667	8.16389916666667	8.35277756666667	7.171442675	7.52225746666667	7.8967227	8.360853775	7.0609201	5.5526373	7.6287103	6.09272539166667	7.05399351666667	5.922335025	4.78900190833333	5.57451170833333	4.81485295	6.2517663	4.73490975	6.23865941666667	5.229288475	5.24195544166667	5.73091026666667	6.3922908	7.09043023333333	6.14083579166667	7.25799328333333	6.46133099166667	5.83776461666667	6.5479092	6.65024493333333	7.264052575	5.69483811666667	6.013009825	5.5633586	6.05897065833333	7.120419025	7.46746676666667	5.91334729166667	6.23229269166667	7.43634795	7.05156345	7.76884748333333	6.49464226666667	6.75170075833333	6.164307025	5.8292169	7.77660473333333	5.633369575	5.52228829166667	6.29278439166667	8.234668275	6.83744154166667	7.0168934	7.206290275	6.73133699166667	6.31323984166667	6.91578644166667	7.12586193333333	7.23785493333333	6.47141914166667	8.662974625	5.83589014166667	6.95435513333333	6.246964375	6.348986575	7.884494275	5.88521655833333

PRELID2	7.548601625	6.97095796666667	6.69566758333333	6.74821494166667	7.13168135	6.903227175	7.05018306666667	7.02401584166667	7.0659289	6.97900894166667	6.62969040833333	7.5654861	6.92575055833333	6.33264478333333	6.7327851	6.46923394166667	6.98853248333333	7.019638175	6.79588928333333	6.302455575	6.390922	6.55145231666667	6.79131116666667	6.41874811666667	6.5332879	7.279554825	6.65156973333333	6.42485146666667	6.76587266666667	6.56215660833333	6.67919664166667	6.72055851666667	6.423307775	7.0307229	7.51638066666667	7.56602129166667	7.13667263333333	6.97843058333333	6.850945825	6.87917671666667	6.72183800833333	6.60676565	6.438674975	6.62907710833333	6.42556058333333	6.43572405833333	6.31323984166667	5.89991223333333	6.5574676	6.39012523333333	6.70390775833333	6.51830799166667	6.51455034166667	6.17226739166667	5.78455018333333	6.19736666666667	6.45767033333333	6.60536920833333	6.495300075	6.339217025	6.86073455	6.53273724166667	7.07654913333333	6.37568504166667	7.28803510833333	7.147600275	6.77179379166667	7.02943565833333	7.29365533333333	6.88940976666667	6.44737514166667	7.00049489166667	7.05399351666667	6.50650844166667	6.89400789166667	6.537297825	6.8732984	6.872774725	6.62907710833333	6.30401625	6.74346625833333	6.71850903333333	6.94881511666667	6.82704920833333	6.76657179166667	6.541621825	6.88578850833333	6.39918578333333	6.2066078	6.48419624166667	7.019638175	6.86809180833333	6.94561610833333	6.987286825	7.10822425	6.88940976666667	7.23488161666667	6.89596523333333	6.94244640833333	7.15624494166667	6.85216409166667	6.428668075	6.77975395833333	6.52987506666667	6.55447500833333	6.17154675833333	6.41504531666667	4.72105203333333	6.48707709166667	5.04024386666667	6.55520170833333	7.02822294166667	6.399909175	6.59815521666667	6.07768216666667	6.259187975	6.36951041666667	6.80449616666667	6.79863740833333	6.7730469

BMP15	4.45902586666667	4.02921226666667	4.11883455833333	4.10101340833333	4.89254299166667	4.56071255833333	4.1481371	4.30527630833333	4.108372	4.82904071666667	4.41203210833333	4.2422894	4.70371486666667	4.91442044166667	4.040897575	4.07678143333333	5.07107728333333	4.14488695	4.48925233333333	4.27584504166667	4.19623050833333	4.26652515833333	4.31618201666667	4.32588960833333	4.792098225	4.57535033333333	4.4227704	4.4473261	4.609167375	4.34901141666667	4.61010935833333	4.51179561666667	4.39770036666667	4.90586546666667	4.45902586666667	5.16081746666667	4.508464425	4.69863704166667	4.683662975	4.59576983333333	4.89595425	4.510676125	4.76455610833333	5.47522431666667	4.56305699166667	4.93552183333333	5.087869425	4.48432390833333	4.41782250833333	4.67877638333333	4.697573175	4.4718244	4.23701905833333	5.31926836666667	4.63723314166667	4.67574021666667	4.58672223333333	4.74219220833333	4.697573175	4.32198345833333	4.28809964166667	4.557709	4.91325449166667	4.172665625	4.43639113333333	4.51268873333333	4.28696936666667	4.27276220833333	4.649483075	4.656935525	4.33043379166667	4.81716043333333	5.6458775	5.201606925	3.937874125	4.05858813333333	4.234848125	4.33532039166667	4.341768725	4.39972780833333	3.91035815	3.85770428333333	4.24013239166667	3.94580334166667	4.32670648333333	4.263255	4.48239504166667	5.78268739166667	4.17373291666667	4.366029925	4.535343	4.443420775	4.812774675	4.33637641666667	4.59071941666667	4.87292895	4.58672223333333	5.16081746666667	4.45205615	5.33648908333333	4.74123235833333	4.7771511	4.75806056666667	5.08063435	4.99586916666667	4.27374416666667	4.98651175	7.83071075833333	4.84301391666667	6.864696	4.56909456666667	5.46938143333333	5.28464988333333	5.824619025	5.34966958333333	4.78900190833333	5.055819225	4.65588558333333	5.196416425	4.71686186666667

GABRD	9.0174923	7.94491850833333	7.8248949	7.741934525	8.35024235	7.691592325	8.703553525	7.69651605833333	8.13819046666667	8.22546075833333	8.08531809166667	8.32012975833333	8.93806988333333	8.17249793333333	7.72686941666667	7.41824386666667	7.7548948	8.40077755	7.71472995833333	7.79357515	7.96192095	8.21929578333333	7.95406095	7.50801045833333	7.53735195833333	8.41104395833333	7.46243825	7.51310406666667	7.84051964166667	8.43197355833333	7.80971700833333	7.81088345	8.535777725	8.084825575	8.56955223333333	9.02512653333333	8.36334496666667	7.9484088	7.58961838333333	7.825834775	7.34497685	8.05032904166667	7.551793125	7.49332154166667	8.164333775	7.908404275	7.66209683333333	7.80451180833333	7.470826175	7.78057345	7.85577905833333	7.76403325833333	8.24657145833333	7.88130259166667	8.159108975	7.82793564166667	8.26039324166667	8.02564620833333	7.851546575	7.708818875	8.32561349166667	8.381708525	8.26509473333333	8.74083754166667	8.488931425	8.41104395833333	8.26156163333333	8.022968975	8.745766675	8.91506995	8.18151525833333	8.3453091	9.8834328	8.21566409166667	7.81731503333333	8.18838901666667	8.269127475	7.74294895	8.16228890833333	8.04016346666667	8.81191925833333	8.98796298333333	8.0294547	8.93227916666667	7.9882338	8.59785679166667	8.06709081666667	8.140254425	8.52110425	7.93641930833333	8.351126275	8.07357910833333	8.45201853333333	8.1205116	8.560933175	8.1873246	8.401731375	8.4178821	8.59099210833333	7.88130259166667	8.92218960833333	8.43615038333333	8.17361143333333	7.5954683	8.612418275	8.4444208	8.06500796666667	7.87659344166667	8.434501875	7.788158325	8.02464245	8.1277546	7.64856505833333	7.93641930833333	8.60593184166667	8.09223929166667	8.21830426666667	8.43246804166667	7.94982140833333	8.391097025

IMPG2	5.90846126666667	5.94625251666667	5.69189906666667	5.20060255	5.83017264166667	6.63788805833333	5.98559190833333	5.58407845	5.86179054166667	5.258661775	5.84963309166667	4.83558559166667	5.79742544166667	5.31926836666667	5.68783094166667	4.472817375	5.21624118333333	5.73091026666667	5.73491465833333	4.92055984166667	4.84820759166667	6.07339345833333	5.37570958333333	5.50338315833333	5.58407845	5.81313270833333	5.03930840833333	5.79742544166667	5.43281713333333	6.181735025	5.18519845833333	5.90588820833333	5.6411096	6.27095864166667	5.73297515	6.50418884166667	5.71882016666667	5.90321861666667	5.22594001666667	5.15140121666667	5.68689626666667	5.35882338333333	5.38762225833333	5.85146426666667	5.23781710833333	5.31119278333333	5.71882016666667	5.091030975	5.582921425	5.53793416666667	6.601356975	4.91442044166667	5.508179525	5.44429769166667	4.948951225	5.40651104166667	5.38064126666667	6.48419624166667	5.57926250833333	5.25768970833333	5.81037075833333	6.164307025	5.377778825	5.52123825	5.256776325	6.53596566666667	5.28787750833333	5.97106113333333	5.42175125833333	5.70932124166667	5.59857721666667	5.826473825	5.52011276666667	5.53496716666667	5.52123825	5.46093680833333	5.45476871666667	5.118961025	4.87091625	5.51001904166667	6.36170378333333	4.99586916666667	4.72828980833333	5.85514745833333	5.67617600833333	6.1063381	5.429887625	5.442194025	5.73195755	4.97177571666667	5.677007675	5.33069273333333	5.47416700833333	5.6003113	6.302455575	5.000034475	5.2815818	6.29510448333333	6.01932009166667	5.96077145833333	5.710337225	5.81478479166667	5.661700075	5.76350409166667	5.779817875	4.9415684	5.59947975	6.61019589166667	5.087869425	6.4034157	6.18408195	5.377778825	5.20354711666667	5.32346678333333	5.4712492	6.12691128333333	6.04613815	5.83208956666667	6.09669481666667	5.96342883333333

BRF1	7.95631855833333	7.875176525	7.57732030833333	7.907346175	7.717344425	8.74130064166667	6.96657695833333	7.497153825	7.04956788333333	7.72294644166667	7.84430019166667	6.38925563333333	6.5332879	7.44607933333333	7.71409900833333	8.061842725	8.07572273333333	7.815575525	7.97666008333333	7.71969125833333	7.96526580833333	8.30084284166667	8.05913828333333	7.65388535833333	8.151552925	7.27829234166667	8.23263584166667	7.61337419166667	8.26968861666667	7.86994016666667	7.91739750833333	7.83780880833333	7.32433510833333	7.93859618333333	7.641075725	7.84947208333333	8.2988733	7.87042711666667	7.33375654166667	7.76714046666667	7.63604310833333	8.17406114166667	8.24906243333333	8.28895966666667	8.02671881666667	8.70034938333333	8.291997825	7.93805580833333	8.5216034	8.37004933333333	7.37359066666667	7.497153825	7.96240296666667	8.34422473333333	7.95684823333333	8.52683058333333	7.63711770833333	8.001514325	8.174528375	8.16329085	8.618769925	8.04330091666667	9.25584964166667	9.579511975	9.444752725	9.900175875	8.91224634166667	8.98934508333333	9.75620250833333	8.70259450833333	7.85469968333333	7.70532880833333	8.62429788333333	8.15205105	9.22160494166667	8.935118175	8.82372205833333	8.38817050833333	8.656035175	9.34018350833333	8.88831745833333	8.523789	8.79596970833333	9.20235228333333	8.472856875	9.587537375	9.61367635	7.765634325	9.18383354583333	8.60646375	8.22846261666667	9.1038805	8.460967325	9.33365533333333	9.145650675	8.41599213333333	10.6652778583333	8.56322373333333	7.80227559166667	8.20331464166667	8.35832260833333	8.466090675	8.95218464166667	10.24545775	9.996827975	9.92334935	9.45898255	8.98618440833333	9.485645225	8.20930705	8.13766550833333	9.24799294166667	9.7241175	9.32329896666667	8.81463643333333	8.49648456666667	9.05776881666667	9.64783695	9.59692015833333	9.05267498333333

UBAP1	12.7500199666667	13.26088015	13.321391725	13.1616055833333	13.487174625	12.643219075	13.0102233	13.1204429833333	12.9960374833333	12.8751753583333	12.5667535083333	12.4746445166667	13.1959888083333	13.390452325	13.5601696	12.8415457916667	12.5140767416667	13.5552298333333	13.580759625	13.2405472416667	13.044583175	13.526397225	12.6185993416667	12.9773913166667	13.1097329166667	12.8590238833333	13.1622146833333	13.2215442333333	13.113125125	13.1926381	13.2939983416667	13.2996462833333	12.9983717416667	12.1906758666667	13.05124085	12.4879782166667	13.3467056166667	13.6375130583333	13.748287325	13.1531334333333	13.419543825	13.4520130916667	13.4586485833333	13.3515334583333	12.750529275	13.4089360166667	13.4321406333333	13.488720775	13.3234625333333	12.9659499916667	13.5451377333333	13.5946453583333	12.9364464083333	13.50262465	12.8475893166667	13.1806770166667	13.672832575	13.7393052083333	13.810810275	13.021150225	12.48982835	12.881258225	11.98404715	12.2488091166667	11.7620768166667	13.2988538666667	12.8176696	12.310594275	12.5609255833333	12.9929816333333	12.7558773833333	12.26972435	13.019993025	12.8086026666667	12.240405375	12.2788893833333	12.5921909166667	12.2236021833333	12.8301026333333	12.296807225	12.0337598083333	11.9935609583333	12.8208910916667	12.632709475	12.4732793833333	12.2658594166667	11.27642465	12.1205448666667	12.6956383666667	12.8208910916667	13.7660959916667	12.1359013166667	12.3680867166667	13.22406385	12.2628607833333	12.0778101166667	12.634136475	11.8048205583333	12.3489029833333	12.164467625	12.8555603666667	13.035605175	11.9420280583333	12.4567650666667	12.9859501166667	12.9672147	12.93175565	12.7890792833333	12.5102327333333	13.2705051333333	12.7128638833333	13.4443089416667	13.6719295	12.5935190666667	12.0440494333333	12.317829175	12.1018588083333	12.625004	13.1305878416667	12.620644525

CRYAB	15.09815525	13.2835400666667	14.2245923416667	13.1427455666667	14.5249167833333	12.0665067916667	13.7869425833333	13.2580457083333	13.454214775	12.901864125	12.648701825	13.0853533416667	14.56279245	13.3262903916667	14.71190555	12.5443972083333	11.0931527333333	13.977355925	14.2983229333333	14.3977718916667	13.4737954	12.65329555	14.6549636583333	13.21226365	13.1407286583333	14.6389112166667	14.7851708916667	13.8337930083333	14.80427055	13.5560194583333	13.9153342666667	13.3430209416667	13.50262465	10.0696848333333	14.1737580083333	14.4262315333333	13.8821044333333	14.789742575	13.0761975166667	13.2697464583333	14.3824525333333	14.1317250833333	13.2254010416667	14.0275803333333	13.71577945	14.4060458333333	13.8473484166667	14.0250042833333	11.57134605	12.8438653416667	14.0201742416667	14.1752017916667	13.509141775	14.113875875	13.7253859666667	13.785907925	13.6202060083333	14.1329416	14.6611477166667	13.7791494	12.488903425	11.8407167416667	11.3231776	12.3378199666667	12.4545539666667	12.4306290916667	10.50459975	12.4461573416667	11.152834825	10.4032528583333	11.8028369	11.9625539583333	10.8150497416667	12.5974434583333	12.8532684916667	10.6387912166667	11.3724759166667	12.5268295333333	11.3328774166667	11.4556673333333	12.1006163083333	11.0193190916667	11.500844625	11.8633002625	13.312167125	11.696699	10.8067325916667	12.11260975	10.8988570583333	11.8403034416667	12.242111125	10.37104885	13.3100924166667	10.2567002166667	13.1733038666667	12.856162525	14.0607265833333	11.8178724083333	12.9977920166667	10.5444389083333	11.4188332333333	12.5780122833333	9.843730775	12.503844475	11.28518	11.43436945	11.9479243416667	12.3110445	10.6373110916667	11.48580155	12.0518595833333	13.4642437583333	11.3579174083333	11.2660555333333	13.6174285083333	13.4847749333333	10.6937764083333	10.5872774916667	12.4903405333333	11.1749885333333

RHPN2	7.833966975	9.84994195833333	10.1766858833333	10.7631580166667	9.79676919166667	10.5134429166667	10.18748315	10.5276582916667	10.0937438833333	9.55333915	10.5276582916667	8.55997810833333	9.06925550833333	10.033950425	9.19996488333333	10.0309458333333	12.341378075	11.2442138	10.5509023833333	10.5464444833333	9.53443595	9.4999428	10.082743125	11.306112175	9.77219498333333	8.20638435	10.1936096583333	10.7184951333333	9.746957025	10.3245220833333	11.0507418833333	10.342051525	9.93957129166667	12.7227603583333	7.638193025	8.07152965	10.6853720166667	11.040528175	11.6440773083333	11.12457335	11.136541175	10.3101529333333	10.0965198666667	10.057405675	9.51088574166667	9.570506275	9.87695954166667	11.510754075	9.58257986666667	10.8514713416667	10.2587469666667	10.880387475	9.80755264166667	10.2157667083333	10.41573205	10.0696848333333	10.5764502333333	10.980233275	9.106662475	10.271227125	9.716989375	9.88298375833333	11.3954232	10.04638645	10.1766858833333	10.6434054	9.25167913333333	9.33321418333333	9.75492564166667	10.457410175	10.03562315	9.46252099166667	10.3706198583333	8.72229471666667	9.18167015	9.60276938333333	10.9120401833333	9.64690625833333	10.9266566	9.833640075	10.3459231416667	8.873364875	11.312921725	10.577240575	8.90052724166667	8.68575455833333	8.665331325	10.2912155166667	9.24401314166667	8.89174859166667	10.0894400666667	10.6064934416667	10.7820632416667	10.4476616583333	8.50702275833333	9.67900834166667	10.3313573416667	9.17515170833333	10.2604506416667	10.145640125	10.0889846333333	9.57610176666667	11.7269658416667	9.949333375	10.91013625	8.79346453333333	10.7601275	11.3275163916667	10.1750566166667	10.1696248333333	12.04537635	10.7276175666667	10.2995461416667	9.51769735	10.3945367666667	10.2330333333333	10.0920085583333	11.6058721833333	9.97542684166667	9.04278656666667

SIPA1L1	11.7648162666667	12.3842104583333	12.3114382583333	12.369348425	12.6552566916667	11.7027128	12.038401575	12.220220925	12.2366655666667	12.2574255666667	11.942427275	11.4559913916667	11.8853151666667	12.26709835	12.6278527916667	11.763655725	12.084825925	12.4050809083333	12.2033105916667	12.407350175	12.3815660416667	12.370296175	12.5344122083333	12.03257945	12.3083565083333	11.9785582833333	12.2513034583333	12.135071975	12.682091125	12.3754853666667	12.0001864416667	12.06402615	12.1422347916667	11.7757946333333	11.8530999083333	11.7936196666667	12.4395669416667	12.338252925	11.9907404666667	12.0313834166667	12.244497975	12.3021061583333	12.0209849	12.14630095	11.960165	12.3712167583333	12.3542251583333	12.3435261333333	12.2466185916667	12.095758	12.1715835	12.149683875	12.3365706083333	12.2227697083333	12.0619908166667	12.037261675	12.2085904583333	12.4732793833333	12.391732275	11.97355025	12.7949718333333	12.2599545833333	11.9177333166667	11.660606675	11.2711403666667	11.9566737416667	12.3997083083333	12.178269875	12.9328607583333	12.233149625	11.8912374333333	12.3484591166667	12.8636072	11.8321536333333	12.5264113	12.5734875583333	12.0525821583333	12.0096367416667	13.230641375	11.8048205583333	11.4883355166667	11.7182865833333	12.4903405333333	12.3114382583333	12.3750783583333	11.98404715	12.1694652083333	11.9720790416667	11.7827675333333	12.0313834166667	12.8509307083333	13.0645149833333	12.2188976833333	12.0537518916667	12.6461371833333	12.4659800333333	12.191066075	12.1121696	12.46371945	11.739559375	12.4015640666667	12.1843992666667	12.3396961416667	12.6765518416667	12.004788175	11.7594075916667	12.0445037083333	12.2710584083333	12.623024125	12.58926255	12.28577945	12.2901521833333	12.4220223083333	13.2155282	12.120221775	12.198234625	11.1369321416667	12.7001469666667	11.8484879416667	12.3566808833333

DCP1A	11.7791279583333	11.7467027166667	11.5691835	11.0538325833333	10.474031625	10.6411546083333	10.9408814333333	11.4446261583333	11.0301443333333	10.9983530166667	10.8585888666667	11.761727725	11.2323478	11.6041942166667	11.297225	11.4034414833333	11.2312268666667	10.949373725	11.496789525	11.4296055416667	11.4277232	11.7445138833333	11.4559913916667	11.5578653583333	11.2198620833333	12.2990201416667	11.2922383666667	11.5150484916667	10.8491534833333	11.1912222083333	11.7324169333333	11.5371262416667	11.3675917666667	11.2495617333333	11.9728603333333	12.0092346166667	10.9021357916667	11.4350551	11.4154857416667	11.3827745833333	11.5483812666667	11.5432980083333	11.5253735333333	12.04824535	11.5850946	11.5067351333333	11.60515845	11.593791575	11.4288195833333	11.1764115416667	11.535988725	11.5706660333333	11.1699387666667	11.296201975	11.75219825	11.7955610083333	11.4700918583333	11.437625625	11.279388675	11.4049590333333	10.1919662416667	10.7804261416667	10.5436778583333	10.8447210916667	10.640008475	9.861824025	10.5764502333333	10.257489575	10.6581721666667	10.2258230916667	10.3741034916667	10.435306725	10.003538	10.7767059333333	10.464576475	10.7975980416667	10.2551392166667	10.1978484	10.8154489833333	11.20617	11.49381285	10.995163975	10.5627525083333	10.9153273416667	10.6219118666667	10.8150497416667	11.1554142916667	10.2342241333333	10.372950875	10.6993840916667	11.2202347166667	10.6499586916667	10.054920625	10.160939175	9.51357516666667	10.70240295	10.3534141083333	10.4332127666667	10.451127	8.4125394	10.85523755	10.7778847083333	11.1380175916667	10.6941625333333	10.73913815	10.754584225	10.7298944083333	10.9450853666667	11.0500837666667	10.6422709916667	11.3861262916667	10.9270024916667	10.6910121333333	10.9094504083333	10.9182300166667	10.940128775	11.121759625	11.0257755833333	10.86272805	10.6964442916667

TTLL11	8.83493181666667	6.46494565	6.28746714166667	6.07937631666667	6.931726825	6.88386930833333	5.79999791666667	6.618809175	6.90460845	7.11920136666667	6.71730138333333	7.24330025	6.99218681666667	6.849792025	7.00754390833333	6.96533775	7.16490981666667	6.57504109166667	7.39341365	6.59744179166667	7.21396461666667	7.23665485833333	6.70056215833333	7.101860325	7.13667263333333	8.84428041666667	6.81709465833333	7.321455625	7.171442675	6.77370956666667	7.16358325	6.59599640833333	6.462885175	6.69050035	8.682814325	8.887867925	7.34272655	7.28072111666667	6.76905384166667	7.444336	7.139659575	7.23080319166667	7.378725775	7.212178575	7.51524328333333	7.22835589166667	7.76714046666667	7.06220724166667	6.632460225	6.91388091666667	7.10630213333333	6.80569213333333	6.59464205	7.00754390833333	7.03138465	7.47900525	6.29125269166667	7.21570014166667	6.58035519166667	7.64552340833333	6.94376375833333	6.47791706666667	5.75112915833333	5.87496216666667	6.59176974166667	8.12174676666667	5.96423755	6.00039116666667	6.53273724166667	8.03960018333333	6.02974674166667	6.542385675	8.016506325	6.36103899166667	6.97095796666667	6.92852376666667	6.43641949166667	6.35808980833333	7.83071075833333	6.52781796666667	6.50501645833333	6.421178475	7.88077005	6.806433125	5.9323169	6.65156973333333	6.542385675	7.388741125	6.724451125	7.05838413333333	6.39918578333333	6.89785889166667	6.17555735	6.98530065833333	8.76851021666667	6.558117975	6.33569684166667	6.64415971666667	7.76512125	6.60472939166667	8.13415403333333	6.633116075	6.5898034	5.780758525	6.79529673333333	5.99843601666667	7.21926184166667	8.82572	5.96077145833333	6.65223385	6.169883075	6.178567625	10.6377074083333	5.642111775	7.80727978333333	6.90845738333333	6.35132606666667	6.769663225	5.84412998333333	5.65824568333333

MCTS1	10.138226575	9.17515170833333	9.36892748333333	9.2768977	9.60140389166667	8.9489102	9.44430623333333	8.84195500833333	9.24712485	8.67450711666667	9.283130225	9.48513751666667	9.5040294	9.24172186666667	9.08915231666667	9.312713675	9.393424375	9.09684721666667	9.35167163333333	9.25303868333333	9.36848635	9.49279765	9.07536643333333	9.51223504166667	9.51549753333333	10.2411048916667	9.47496915833333	9.567278375	9.01698551666667	9.40207443333333	9.36761098333333	9.71575140833333	9.516735775	9.39664281666667	9.8694956	10.3466375416667	9.20513901666667	9.17971915	9.36528065833333	9.330358125	9.10015925	9.33617749166667	9.26217445	9.10796010833333	9.090969825	8.91410946666667	9.55671014166667	9.13602675	9.106662475	9.33964531666667	9.16352800833333	9.22423163333333	9.55943838333333	9.53398021666667	9.19719063333333	9.292040175	9.25672935833333	9.1094042	9.28405401666667	9.17006050833333	10.1565645083333	8.95121126666667	9.54132330833333	8.61480576666667	9.1094042	8.45045408333333	8.78745048333333	9.18595039166667	8.886411025	8.86609368333333	9.091403125	8.538352825	8.99116335833333	9.28741124166667	8.94761291666667	9.10576088333333	9.12700821666667	9.40469768333333	9.24218355833333	9.34157415833333	9.45149038333333	9.35451149166667	9.14782716666667	8.97625954166667	8.91938296666667	9.50036891666667	9.34670505	9.46121264166667	9.82123129166667	9.170544975	9.74776665	9.3375044	9.19531680833333	9.08005709166667	9.25536705833333	9.84876684166667	8.66625304166667	9.39438685	9.23047039166667	8.982853175	9.30498321666667	9.06925550833333	9.36206593333333	9.22652003333333	8.34923416666667	8.523789	9.11687501666667	8.783965475	8.88882345833333	8.94049991666667	9.23835931666667	8.65559314166667	9.17691023333333	9.35954015	8.77406471666667	9.02609295	8.67026894166667	8.59697231666667	9.39071778333333	9.43278838333333

CLEC12B	7.29233335	9.126505725	8.76798363333333	9.49147149166667	9.124627175	9.03818763333333	9.50086338333333	9.83629188333333	9.63199205833333	9.29885605	9.70624959166667	8.711926	9.37548823333333	9.88253726666667	8.86999053333333	9.44105665833333	8.64337428333333	11.1797537583333	9.88726988333333	9.81941439166667	8.80540845	9.00330966666667	9.565041475	10.2314081833333	8.60799398333333	8.27339385833333	9.51769735	10.0569437166667	8.9493948	10.002252325	10.146034	9.536457975	9.60791420833333	8.30382858333333	8.550436275	8.10843584166667	10.2782382416667	10.783186525	11.0793725416667	10.3901677	10.8702441	10.176297425	9.723283875	9.37313166666667	8.75927773333333	9.55898925	9.52783803333333	11.3506069833333	8.80826335	9.346282425	8.96144149166667	10.2014148333333	9.30586555833333	10.0644501166667	10.1308023166667	9.48881261666667	10.1569732333333	10.7743973416667	8.4773978	9.78654094166667	7.23371278333333	8.438760575	7.40662056666667	8.45851334166667	7.80727978333333	10.4460825166667	9.11993345	9.05355476666667	8.3316018	8.55288418333333	8.25344699166667	7.89083754166667	9.5453508	9.1029726	8.83016251666667	9.19905495833333	8.532903275	8.74224036666667	10.0002116916667	8.04179383333333	7.42447115	7.927602375	10.8228191416667	9.19905495833333	9.45281165833333	7.85104381666667	7.607704375	8.785427625	7.44203170833333	8.81880498333333	9.16302515	7.95962858333333	9.116355225	9.15603220833333	8.416908275	8.70635446666667	8.49442401666667	9.04179071666667	10.420034375	9.07164405	8.99438999166667	8.24657145833333	8.61290266666667	8.91181045	9.03908018333333	9.44293281666667	10.13859765	10.9094504083333	8.577338575	8.90668976666667	7.525066825	9.87563735	9.30325005833333	8.65698328333333	10.5581448083333	8.87086751666667	7.619792375	8.61979589166667	8.36515990833333	8.89736060833333

DAO	9.2355397	9.69880400833333	9.439107	9.24668224166667	10.3825544833333	10.5509023833333	10.3694253333333	9.21638355833333	10.1172154583333	9.70668029166667	9.4936652	9.63902631666667	9.855976425	9.23929971666667	8.98986978333333	8.35736911666667	10.5651423416667	9.48649659166667	8.779206475	8.84011393333333	9.09600253333333	9.38453646666667	8.97675146666667	8.60539538333333	9.462094325	8.92840376666667	9.39938098333333	9.29065144166667	9.82651539166667	9.6433763	9.19340213333333	8.99487275833333	10.0039229916667	11.3642682416667	9.081377025	8.82680535833333	10.4554224833333	9.76911673333333	9.750361275	9.29432763333333	9.3836955	9.49101396666667	8.846155575	8.66625304166667	8.46720710833333	8.84333919166667	8.78021899166667	8.95262451666667	8.46720710833333	8.84802724166667	9.39938098333333	9.20190699166667	9.726163425	9.4594195	9.42859468333333	8.91790284166667	9.59418116666667	9.78059948333333	9.11174605833333	8.89784426666667	9.09779791666667	9.20975130833333	8.482136425	8.69556865	8.9929566	9.35773556666667	8.69606343333333	8.8451569	8.93619846666667	9.23973414166667	9.27812115	9.19441289166667	9.67096889166667	8.68035370833333	9.26988130833333	8.319610775	9.33964531666667	8.355193125	8.51578285	8.90099503333333	8.90389271666667	9.1141243	8.67403964166667	8.37004933333333	9.33883618333333	9.18361099166667	9.157883875	9.38096979166667	9.45281165833333	8.458998275	8.94623185833333	8.52110425	8.92218960833333	8.97489020833333	9.28741124166667	9.21494060833333	8.86366274166667	9.19287955	9.50452185	8.59302528333333	8.6716691	9.37220353333333	8.499322875	8.60126088333333	8.47579976666667	8.43849715833333	8.22981704166667	8.65513670833333	8.425659175	8.576935025	8.3851619	8.656035175	8.92792624166667	8.381708525	8.60227746666667	8.42318403333333	8.64890015833333	8.95121126666667	8.76751969166667	9.14655231666667

SPX	10.6195893583333	8.21248958333333	10.26748265	11.0808123083333	5.279548575	11.6929484666667	7.82958231666667	8.71882579166667	10.051171975	9.37457540833333	11.0341045833333	9.69707035833333	7.43742328333333	7.36819641666667	7.10952	8.35883419166667	10.7684448166667	6.68727483333333	7.68593156666667	7.33501970833333	6.95860073333333	7.310825425	8.51856025833333	8.34767179166667	8.93668718333333	10.5468436833333	9.442039375	8.57834244166667	7.32433510833333	7.448314425	8.05619188333333	8.45352316666667	11.213594675	9.891815375	10.6869030083333	10.7936080833333	7.46414650833333	10.3945367666667	10.0679332666667	8.65271366666667	9.487900075	7.99937465833333	9.62155398333333	9.70331208333333	9.62064585	8.09998893333333	7.81893236666667	7.93490190833333	7.729718875	9.56680634166667	10.63254535	9.81483478333333	8.39771189166667	7.95194513333333	7.55746484166667	9.54311075	8.06904244166667	8.74766549166667	9.63902631666667	8.61092796666667	10.4138270833333	9.80056271666667	9.148762375	9.89553576666667	10.18161685	8.02019604166667	10.9486958083333	10.176297425	9.76051965	10.3913250333333	8.92929650833333	8.1898559	8.224517175	9.460327375	8.70798358333333	9.58662811666667	8.17885319166667	6.958116025	8.23315546666667	7.12401263333333	6.91190293333333	8.76603770833333	9.114481175	8.24509221666667	8.53482063333333	8.98796298333333	8.51484679166667	10.017588525	7.61337419166667	8.98325939166667	9.92175831666667	9.37637135833333	8.22981704166667	11.19866735	10.0382960666667	9.13909698333333	8.98837211666667	9.19020434166667	10.8187575416667	9.78692435833333	9.33964531666667	8.592518275	8.62834993333333	8.14495316666667	7.71296821666667	8.19970503333333	9.06745036666667	8.31719399166667	8.93317065	8.64244941666667	9.13172414166667	8.36380941666667	9.45898255	10.1295520916667	9.8880168	8.783965475	9.10532413333333	9.63018528333333	7.86896935833333	8.2874613

RASA2	9.67544948333333	10.9851253333333	10.9277382833333	10.724371075	9.64610789166667	10.281533275	10.980233275	10.5523212166667	10.4752224083333	10.2974881833333	9.91970055833333	9.83237755833333	9.62110718333333	10.7096961916667	10.7892430083333	10.6578120916667	10.0445903666667	10.3953052083333	10.916432925	10.5408711916667	10.3948990166667	10.8605112583333	10.8694922083333	11.284173425	10.7235859166667	10.130420225	10.63254535	10.86771825	10.5592695083333	10.6340233	10.6546458583333	11.0458413916667	10.5477462083333	10.4653037	10.1870376	10.079544525	10.5134429166667	10.7643055083333	10.7605131333333	10.615482725	10.96888565	11.3165296583333	10.9091302666667	10.5573126666667	10.4453172666667	10.9185836833333	10.98769475	10.904327125	10.8916305083333	10.5585696666667	10.4057146583333	10.9412405333333	10.0430227416667	10.507752825	10.6027208583333	10.528057425	10.7193725416667	10.8071092416667	10.8968726333333	10.6683478	10.4480363583333	10.18324735	10.2233050083333	10.34241075	11.4177560583333	9.40419091666667	10.2900500333333	9.85677660833333	10.15614755	10.0720278166667	9.69087439166667	10.2888943333333	9.43868498333333	9.93427139166667	9.97000988333333	10.1836960166667	10.0700289916667	10.0815238083333	10.226244575	9.87695954166667	11.1409117416667	10.0318266833333	10.0720278166667	10.58883085	9.94840036666667	10.1246349583333	10.5300186083333	9.85036418333333	10.0430227416667	10.164580475	10.796845775	10.1222883166667	9.79628703333333	9.90518528333333	10.0334908083333	10.2485867583333	9.848037125	10.3686089583333	9.96227999166667	10.38024835	10.3702407083333	10.1779292166667	10.9450853666667	10.6691322083333	10.013853075	10.0993323166667	10.2080941333333	10.261767125	10.116767025	10.2027121916667	10.1737764166667	10.015183425	9.83319184166667	10.1808367916667	10.4272415916667	10.7287943416667	10.6038899083333	10.4914439416667	10.9006915833333	10.6914536333333

B3GNT10	10.338453	9.48126221666667	9.462094325	9.657103925	9.38896178333333	9.4968836	10.34919645	8.53632365833333	9.04320051666667	9.51951014166667	10.1613409083333	9.27941565	8.862628675	9.75321625	9.360413025	9.44151263333333	10.9405146	9.57610176666667	9.471590125	9.65802264166667	9.44337360833333	9.553861875	9.70284449166667	9.25349141666667	9.46252099166667	10.021871175	8.61979589166667	9.33321418333333	9.203321075	9.4825512	8.87491901666667	9.943781525	9.36848635	9.96018530833333	10.306601825	10.2991427166667	9.23377270833333	9.11495396666667	7.89510465833333	9.7452797	9.35634101666667	9.19244325833333	9.82203395	9.78872821666667	9.651432225	10.043884875	10.3833420583333	9.80350678333333	9.70668029166667	9.87484248333333	9.39297179166667	9.35075274166667	9.7557679	8.58349254166667	8.748691075	9.09779791666667	9.93796431666667	9.580833825	9.47641323333333	10.0364758583333	9.97954941666667	10.0911655583333	10.3035388166667	9.25028671666667	9.94164523333333	9.75321625	9.98920446666667	8.39722928333333	7.93490190833333	9.88253726666667	9.53223686666667	10.949373725	9.39438685	9.61139459166667	9.95147331666667	10.344704475	9.30543436666667	9.80310053333333	9.62193513333333	9.65101713333333	10.19975295	9.6526333	10.4176619666667	10.3868161083333	9.08180965	10.000998825	10.227074775	9.666049875	11.201526325	9.92375435	9.85765425	9.611823	10.0373878833333	10.1529084083333	10.590660625	9.59518965833333	8.69409343333333	10.2237133416667	8.90619576666667	9.50308771666667	9.481636725	8.74957956666667	10.283076325	10.342051525	10.7235859166667	10.3289721	9.19905495833333	10.5596517833333	10.2604506416667	10.2014148333333	8.89736060833333	10.7631580166667	10.5880287	9.92009464166667	9.373589925	9.295759125	10.9080821583333	10.58883085	9.90307260833333	9.73188163333333

FXYD4	6.43219438333333	6.50275845	6.78114418333333	7.13033303333333	6.783129725	7.06942048333333	6.824495175	6.84850089166667	6.87851200833333	6.83400405	6.679903875	6.79923555	6.65487665833333	6.28975704166667	6.39606678333333	6.58835050833333	11.4350551	6.29670533333333	5.824619025	5.80618989166667	6.7919706	6.77777710833333	6.93552516666667	6.29901051666667	5.42470928333333	6.11542659166667	6.19209213333333	6.263501475	6.08593745	6.97276299166667	6.31880616666667	5.94443094166667	6.14959958333333	8.413378975	5.71882016666667	5.59947975	6.0951249	5.50024889166667	6.02558836666667	5.35882338333333	5.73943181666667	6.56879458333333	6.53467245	6.22675393333333	6.90968315833333	5.5651756	5.83589014166667	6.34302255833333	5.6723045	5.355721825	6.051276375	5.38469075833333	5.20995024166667	5.582921425	5.88595835	5.81120035833333	5.32557380833333	5.57926250833333	5.49632836666667	5.62674555833333	6.52412431666667	7.173894	6.93032724166667	7.25923676666667	6.72183800833333	7.15262315833333	6.1536546	7.2645801	7.36330638333333	6.93685505	6.69182425833333	7.14336814166667	7.517539575	6.47646620833333	7.066499225	6.71110255	7.375280375	6.160301025	5.97106113333333	6.801867125	6.28109695833333	7.100006625	7.36530371666667	6.62839033333333	6.42556058333333	6.39451754166667	6.66054216666667	6.43421143333333	6.61484865833333	5.35983450833333	6.91905025833333	6.72825601666667	6.745396875	6.01047939166667	6.70200101666667	6.28336628333333	6.46219335	6.30401625	7.09933909166667	6.04523669166667	5.845937625	7.37059144166667	6.88512086666667	6.657721925	9.10344201666667	6.0148863	6.35665519166667	6.75368535833333	6.06672131666667	6.82513060833333	7.190008575	5.92509355833333	6.08433614166667	5.16735583333333	6.16362138333333	7.120419025	5.65427465	5.575395825	6.16277881666667	5.8386672

FANCL	12.306357275	12.013260375	12.025731925	11.8658810166667	12.57979945	12.52409195	12.075266	12.1749483916667	12.1138378333333	12.3401023416667	12.6831265	11.8749533333333	12.10269775	12.0149070416667	12.2311025	12.0112152666667	12.1167008333333	12.2603464083333	12.703617625	12.279687775	12.0864940333333	12.2964028333333	12.4154660666667	12.6956383666667	12.2141453583333	12.219394525	12.1178108083333	12.0997306916667	12.3926407	12.4716392	12.1575174833333	12.1435544583333	11.8658810166667	11.8004315583333	12.0428624416667	12.1096451083333	12.3554452	12.4346028333333	12.6876920416667	11.848881975	12.124594875	12.137649925	12.1715835	12.3855581	12.1549544416667	12.4154660666667	12.1488811083333	12.8268431583333	12.402892875	12.2276231333333	12.3088616416667	12.580271675	11.8134267166667	11.9345074833333	11.8078233833333	11.7369473333333	12.548305425	12.345780775	12.3653316083333	12.47909375	12.5212289166667	12.4795096083333	12.6412173166667	12.4275246583333	12.772348275	12.3343950916667	12.4141455666667	12.1150337666667	11.4752696416667	12.4430322166667	12.5807864333333	12.4350665416667	11.8630874833333	12.225632925	12.5268295333333	12.3199648916667	12.632188	12.7339168166667	12.196460925	12.7323979	13.089585075	12.43732	12.910549725	12.394774825	11.6787724916667	12.2557740666667	12.7836657416667	12.37676025	13.149953225	12.0657212166667	11.8962786833333	12.3716896916667	12.7884927666667	11.9777865	12.86014235	12.5618377166667	11.7160036	13.1973842833333	11.7772182666667	12.8947866166667	12.3566808833333	12.164467625	12.7500199666667	12.923601775	12.111383	12.18354635	12.370770375	12.2687931833333	12.145884225	12.2867043083333	13.24746105	12.1130081666667	11.7857517916667	11.8174744333333	12.017301325	12.21502565	12.74583335	12.5662353166667	12.6282903083333	12.811279575

ARMC8	6.5698278375	6.49497117083333	5.6018109	5.84923762916667	6.70566189166667	6.65255642916667	6.78815305416667	6.20772976666667	6.45208411666667	6.22714034583333	6.66280564583333	7.54373619583333	6.80480245416667	6.5859553875	6.82416634583333	5.77762020833333	6.2654440875	6.30902656666667	6.30902656666667	6.30678820833333	6.20294723333333	7.24363060833333	5.34914802083333	6.4904906625	6.63076082083333	6.96685253333333	6.74854935416667	6.69679296666667	7.21834830833333	5.88807049583333	6.3613713875	6.81942682083333	6.15645708333333	5.8754407875	6.5669495875	7.08141007083333	6.47103115416667	6.11424867916667	6.5054099125	6.59779850416667	5.84745681666667	6.05176328333333	6.712154125	5.51634575	5.24443233333333	5.99136303333333	5.59428554583333	5.73351403333333	7.1782093875	6.6334349	4.73348937083333	6.66770650833333	6.31059185416667	6.52812642083333	5.84188985416667	5.29072016666667	6.32348062083333	5.8268921375	6.472541975	6.82674682083333	8.03302740833333	6.31283294583333	6.30007057083333	6.10354460833333	5.18262998333333	5.6924343125	6.07977930833333	7.2036427625	6.49286267916667	6.85386289583333	6.56254219583333	6.66834617083333	6.53171509166667	5.91673530833333	6.64660102083333	6.40761060416667	6.62068300416667	6.39122358333333	5.7581333875	5.67278973333333	8.0098297625	7.2047860625	5.33697697083333	5.12156893333333	6.32418060833333	7.20242916666667	6.81152060416667	6.19169304166667	7.78138161666667	6.18133845416667	6.58323432916667	6.52171285416667	6.3606691	6.7240842	7.17652978333333	6.57850369166667	6.77003967083333	7.3907416875	6.601714575	6.8854546875	6.34041982083333	6.52240923333333	6.92279697916667	6.18133845416667	5.7136575625	6.3843491625	6.14206632916667	7.12131860416667	7.1151289	7.4293240625	5.91768007916667	5.61787749583333	5.563805575	5.45533077083333	5.78962339583333	6.18779995416667	6.50020627083333	6.00326669166667	7.05932147916667	6.2407035875

NME9	6.5698278375	6.49497117083333	5.6018109	5.84923762916667	6.70566189166667	6.65255642916667	6.78815305416667	6.20772976666667	6.45208411666667	6.22714034583333	6.66280564583333	7.54373619583333	6.80480245416667	6.5859553875	6.82416634583333	5.77762020833333	6.2654440875	6.30902656666667	6.30902656666667	6.30678820833333	6.20294723333333	7.24363060833333	5.34914802083333	6.4904906625	6.63076082083333	6.96685253333333	6.74854935416667	6.69679296666667	7.21834830833333	5.88807049583333	6.3613713875	6.81942682083333	6.15645708333333	5.8754407875	6.5669495875	7.08141007083333	6.47103115416667	6.11424867916667	6.5054099125	6.59779850416667	5.84745681666667	6.05176328333333	6.712154125	5.51634575	5.24443233333333	5.99136303333333	5.59428554583333	5.73351403333333	7.1782093875	6.6334349	4.73348937083333	6.66770650833333	6.31059185416667	6.52812642083333	5.84188985416667	5.29072016666667	6.32348062083333	5.8268921375	6.472541975	6.82674682083333	8.03302740833333	6.31283294583333	6.30007057083333	6.10354460833333	5.18262998333333	5.6924343125	6.07977930833333	7.2036427625	6.49286267916667	6.85386289583333	6.56254219583333	6.66834617083333	6.53171509166667	5.91673530833333	6.64660102083333	6.40761060416667	6.62068300416667	6.39122358333333	5.7581333875	5.67278973333333	8.0098297625	7.2047860625	5.33697697083333	5.12156893333333	6.32418060833333	7.20242916666667	6.81152060416667	6.19169304166667	7.78138161666667	6.18133845416667	6.58323432916667	6.52171285416667	6.3606691	6.7240842	7.17652978333333	6.57850369166667	6.77003967083333	7.3907416875	6.601714575	6.8854546875	6.34041982083333	6.52240923333333	6.92279697916667	6.18133845416667	5.7136575625	6.3843491625	6.14206632916667	7.12131860416667	7.1151289	7.4293240625	5.91768007916667	5.61787749583333	5.563805575	5.45533077083333	5.78962339583333	6.18779995416667	6.50020627083333	6.00326669166667	7.05932147916667	6.2407035875

ZNF391	11.9653249	10.7631580166667	10.72841425	10.3293667083333	10.26748265	11.4236775666667	10.6403864583333	10.4276602833333	11.20617	10.1400969416667	10.4008623166667	11.3849835833333	10.4843028583333	10.3913250333333	10.8202415916667	10.724371075	10.4975657166667	9.696676675	9.508670075	10.6578120916667	10.6461664	10.4264895833333	10.991320675	9.686488825	10.8210096	11.8873133333333	10.0305098083333	10.7877953583333	10.5987255666667	10.514613975	9.90438676666667	10.334173575	10.4429923666667	10.0757309083333	10.7998255666667	12.0893148833333	10.4004658833333	10.0696848333333	9.44734175833333	10.6496091416667	10.3583509666667	10.0265057583333	10.70466055	10.5616263	11.34484365	10.1903114083333	11.0873559916667	10.0787243333333	10.615482725	10.3945367666667	9.90438676666667	10.0256584	11.0190136	10.90102425	10.8473279166667	10.9629037916667	10.135861875	9.882085	10.856356975	10.6550211916667	11.856139525	10.9545739416667	11.4181579	10.6910121333333	12.7779954333333	11.2039860833333	11.06152365	9.42382585833333	11.1354232166667	10.616218875	10.30399485	10.7839123	10.7669402083333	10.6637664333333	11.3878799333333	10.1759041833333	11.2902838583333	9.87650806666667	9.437687475	10.23466	12.333509375	11.1196222666667	10.7123288	10.5739390666667	9.82560370833333	11.4026558	11.55825445	10.5711304666667	10.3901677	10.9486958083333	11.2936955416667	9.37403768333333	8.002520925	10.546063825	11.0666847416667	9.897727225	11.2198620833333	11.6566056666667	10.3733561083333	11.509666625	11.1025248083333	10.447247375	10.64689975	11.6602573333333	11.88068255	10.8172624166667	11.1302224166667	10.7842899166667	10.433634375	9.66469236666667	10.1728980166667	9.681868125	9.8269075	11.3861262916667	9.97000988333333	10.2355191666667	11.3154295916667	11.1670443166667	11.0876998333333	9.69837386666667

ARSJ	8.56272661666667	7.3325894	7.644918425	6.99800655	6.68859711666667	7.906291625	6.5402188	7.52054051666667	6.26827546666667	7.0609201	7.40602305833333	6.54299384166667	6.91190293333333	7.482471025	7.597705475	7.375280375	7.72905521666667	6.54872438333333	8.0149105	7.53912439166667	7.53912439166667	6.71850903333333	7.930120075	9.04841031666667	7.61454650833333	8.932734675	7.44885958333333	7.31861950833333	6.61959393333333	7.34385484166667	8.71940105833333	7.36819641666667	6.29901051666667	7.092930725	8.70448370833333	9.15064904166667	6.31021560833333	7.49826404166667	8.56222751666667	7.59937680833333	8.08952596666667	7.76293401666667	8.053540525	8.3151234	7.84106111666667	7.98973426666667	8.25344699166667	8.635919475	8.25593908333333	7.73082848333333	7.70589544166667	8.51027651666667	6.75646765833333	7.29049713333333	7.05838413333333	7.99131585833333	7.95406095	7.933308825	7.28317230833333	8.09947061666667	6.633753725	7.502278225	6.89528771666667	8.24602095	9.298425025	6.86394090833333	9.382290975	6.70532833333333	8.70259450833333	7.1053775625	6.91766443333333	7.086730875	6.67783206666667	7.89403294166667	8.01539524166667	9.20920615	5.18519845833333	6.88578850833333	9.58302888333333	5.94185621666667	7.01211946666667	8.81744095	8.95943646666667	9.17457594166667	6.32668329166667	8.487957325	8.46350844166667	7.8345672	6.4808852	7.82793564166667	9.106662475	7.95851929166667	7.64856505833333	8.053540525	7.6702147	6.9494722	7.08805295	8.882160975	8.93227916666667	8.97489020833333	7.964195775	7.35803011666667	6.08433614166667	8.07411434166667	8.14709130833333	8.2874613	8.79861721666667	9.62367923333333	6.80310133333333	9.38536081666667	8.3657693	9.27812115	9.485645225	9.97000988333333	8.488931425	7.38818769166667	8.932734675	9.66826963333333	8.79090379166667	8.30804240833333

PLA1A	9.24799294166667	7.71862859166667	9.08915231666667	7.35003230833333	7.52840544166667	10.0533049083333	7.22476671666667	8.451050725	8.18936749166667	8.20990576666667	9.331319325	8.55750678333333	10.164124825	7.29474379166667	7.73985964166667	8.96893119166667	10.5872774916667	7.00049489166667	9.11541340833333	9.80885460833333	8.71089086666667	8.182610775	8.498393225	7.71472995833333	8.38470655	8.167838375	7.21750264166667	6.88120535	6.78848231666667	7.76714046666667	7.5954683	8.107904375	7.45786721666667	9.35773556666667	7.43067104166667	7.433497275	9.458203675	8.56322373333333	9.14782716666667	9.34207146666667	8.89174859166667	8.0964417	8.50751961666667	7.48698644166667	8.68375230833333	9.62496784166667	8.77869386666667	8.401731375	9.98111111666667	7.53185565833333	7.99508275	8.7217903	9.13780860833333	9.86266926666667	8.3316018	8.60177515	8.52966328333333	9.50725341666667	8.55907896666667	9.289706825	8.479465175	8.43143401666667	8.70034938333333	7.984047875	9.39297179166667	7.17267591666667	7.23785493333333	8.65999509166667	7.10822425	8.78070639166667	7.204487875	7.703109475	6.48785131666667	8.154025025	7.433497275	7.10123053333333	11.6587921	8.95312890833333	7.73690679166667	9.05732553333333	10.4542782	10.4176619666667	6.77636395833333	6.59883068333333	8.57007116666667	8.584858775	8.90142945833333	8.8559255	4.93761881666667	8.26097460833333	8.06285705833333	6.74746250833333	8.83829725	6.64893395	7.20334935833333	8.02070828333333	7.36330638333333	7.659482025	6.939986925	7.715267425	8.00359983333333	8.13977138333333	8.98368761666667	7.96837240833333	7.7799909	7.803872825	7.61006516666667	7.16614005833333	8.91135703333333	8.71039916666667	8.6223815	7.92709596666667	7.967895825	6.81435373333333	8.672615075	8.57301913333333	7.98023625	8.0519544	7.49549410833333	9.12042280833333

AKAP8	9.18457413333333	8.0459923	7.9484088	8.18891301666667	8.13617131666667	8.69606343333333	6.38554745833333	7.56380908333333	6.8327755	7.16794871666667	8.215089275	8.02070828333333	7.41061439166667	7.69103884166667	8.84659819166667	8.76898080833333	8.475275825	9.19950909166667	8.744772225	8.56818220833333	8.3301117	9.45859685833333	8.1923863	8.925575825	8.68971138333333	8.91938296666667	8.4178821	8.577338575	6.65024493333333	8.618769925	8.11887123333333	8.49699819166667	8.09498031666667	7.7528692	8.62136429166667	9.14235506666667	8.43197355833333	8.16529418333333	7.86334303333333	8.26555303333333	7.82904454166667	8.43615038333333	8.47850595	7.707009225	8.98146720833333	8.47431853333333	8.37811988333333	8.490982775	8.62685349166667	8.44690206666667	8.37460103333333	8.68971138333333	8.74130064166667	9.08493245833333	8.425659175	8.43393876666667	7.92709596666667	8.24315470833333	8.51528900833333	8.37767194166667	9.28361398333333	7.77487424166667	8.5716089	8.58695619166667	8.23817860833333	9.099726825	7.740357875	7.762415375	8.02564620833333	7.81839644166667	7.56602129166667	8.46508935	6.82038475833333	8.11603985	8.45851334166667	8.27283913333333	8.7383879	8.92218960833333	8.3536777	8.91275618333333	7.8967227	8.15615679166667	7.95359058333333	8.3316018	8.86708225	7.73422946666667	8.14449565	7.83997353333333	5.5022085	8.54895103333333	7.78978061666667	9.02785883333333	9.03089319166667	8.61571535833333	8.62136429166667	8.8062671	8.745766675	7.63711770833333	8.0519544	8.68083718333333	8.27339385833333	7.77487424166667	8.71688334166667	8.75371965	8.84471325833333	8.69657786666667	8.92792624166667	9.01197323333333	8.48569109166667	8.38072273333333	7.89832068333333	8.59595736666667	8.97861723333333	8.71838005	8.56622091666667	8.69504618333333	7.46022033333333	8.222543225	9.05355476666667	7.68098021666667

CNTNAP5	7.32855059166667	5.97436386666667	5.8523182	5.32557380833333	5.32134071666667	7.33375654166667	5.37341235	4.695239575	5.30664471666667	4.57343223333333	5.0258117	7.50404556666667	5.85413349166667	5.415860975	5.44873513333333	5.01100034166667	6.50418884166667	5.37226380833333	5.94535455833333	4.822492325	5.81909195	6.92575055833333	4.812774675	4.92557835833333	4.59683404166667	8.27907085	5.24289476666667	4.65478205833333	4.86289075	5.360915675	6.37713004166667	4.656935525	6.37568504166667	4.852466025	6.39681019166667	8.24657145833333	4.812774675	4.85768484166667	4.57924936666667	5.10930444166667	7.02761371666667	5.02800883333333	4.936774575	7.45451809166667	5.38558591666667	5.78537223333333	5.29020444166667	5.06570035833333	6.230051875	5.073382675	4.80827406666667	5.50523408333333	4.68681869166667	5.48616714166667	4.40382505833333	4.82688145833333	5.68979354166667	4.85146489166667	6.54434775	5.48415848333333	10.5837654416667	8.62596224166667	7.01495000833333	5.5651756	5.63053526666667	7.52443165	4.83989633333333	5.57335978333333	5.4147768	4.90470955	8.21153401666667	5.899063175	6.316307375	6.32454565	5.207730075	5.8009379	5.557624025	5.087869425	4.72007973333333	5.67425256666667	7.48123266666667	12.1304018333333	4.812774675	4.125728225	5.56242626666667	5.15873735	6.411112525	4.59784559166667	5.79203019166667	6.71730138333333	9.954843075	5.7161104	10.9371792916667	5.24703029166667	5.934751075	7.18281396666667	5.19536235833333	5.06113309166667	6.04799355833333	5.09864851666667	9.51041425833333	6.58691901666667	5.56425255	6.66944055833333	5.018425275	6.05994703333333	5.367183075	6.46705624166667	9.35125324166667	6.7327851	5.59572529166667	5.467505875	5.44429769166667	5.01216298333333	5.53292085833333	4.41103275833333	6.31469075	5.2783407	6.446687025	6.36709734166667

AC005670.2	7.4530872	8.4512906125	7.89270345	7.91383982083333	7.75745037916667	6.33963782916667	7.71830905416667	7.20885609166667	8.74248261666667	6.72216503333333	7.18736235833333	7.19164684166667	7.04410127916667	6.8854546875	7.6010988375	6.41841785	8.36402504583333	7.67567321666667	7.6458149	7.51667525833333	6.7727462375	6.7608196125	6.50966012916667	6.91486930416667	7.47286802916667	6.53769849166667	7.11648988333333	7.12131860416667	7.0820291375	7.2047860625	7.53652041666667	7.950087225	8.37234324166667	6.71762575416667	7.29929215	7.3177117375	8.187083525	7.54538594583333	7.2392390625	7.07032169583333	7.42132618333333	7.71888675	8.02269690416667	6.81942682083333	6.6908251125	7.8214705125	7.81061270833333	7.8153205625	6.0695613	6.44177570416667	7.22082028333333	7.05932147916667	7.8214705125	6.8761898	8.05594164583333	8.92534495833333	7.35260315	7.62386521666667	8.62002858333333	7.09072599583333	7.9366852	7.5899100375	6.92279697916667	6.7398437375	6.91291151666667	7.06434192916667	6.9578512875	7.13130732916667	6.62333947916667	6.73856459166667	7.13579273333333	7.28288347916667	6.63543665	6.29318526666667	7.45246560833333	6.54903211666667	6.92467640833333	6.48607476666667	5.9255638375	6.704989525	6.09313824166667	5.95532470416667	6.66834617083333	6.59635077916667	6.51490294583333	7.20776837083333	6.60436897916667	6.97433015833333	7.585494775	5.96820949166667	7.1363748	7.16456875416667	7.51281844166667	6.40761060416667	7.13995215416667	6.97003259583333	6.87499558333333	6.81088271666667	7.15731386666667	7.3052528875	6.54055676666667	7.39897763333333	7.77018035833333	6.69347515833333	6.47897721666667	7.12753024583333	6.22187706666667	7.8794029875	6.574657825	7.1954046375	6.43118097083333	7.37842275416667	6.92132353333333	7.06183584583333	7.03723994583333	6.71148608333333	7.046003475	7.17002668333333	7.90762179583333	6.34193792916667

RPRML	7.4530872	8.4512906125	7.89270345	7.91383982083333	7.75745037916667	6.33963782916667	7.71830905416667	7.20885609166667	8.74248261666667	6.72216503333333	7.18736235833333	7.19164684166667	7.04410127916667	6.8854546875	7.6010988375	6.41841785	8.36402504583333	7.67567321666667	7.6458149	7.51667525833333	6.7727462375	6.7608196125	6.50966012916667	6.91486930416667	7.47286802916667	6.53769849166667	7.11648988333333	7.12131860416667	7.0820291375	7.2047860625	7.53652041666667	7.950087225	8.37234324166667	6.71762575416667	7.29929215	7.3177117375	8.187083525	7.54538594583333	7.2392390625	7.07032169583333	7.42132618333333	7.71888675	8.02269690416667	6.81942682083333	6.6908251125	7.8214705125	7.81061270833333	7.8153205625	6.0695613	6.44177570416667	7.22082028333333	7.05932147916667	7.8214705125	6.8761898	8.05594164583333	8.92534495833333	7.35260315	7.62386521666667	8.62002858333333	7.09072599583333	7.9366852	7.5899100375	6.92279697916667	6.7398437375	6.91291151666667	7.06434192916667	6.9578512875	7.13130732916667	6.62333947916667	6.73856459166667	7.13579273333333	7.28288347916667	6.63543665	6.29318526666667	7.45246560833333	6.54903211666667	6.92467640833333	6.48607476666667	5.9255638375	6.704989525	6.09313824166667	5.95532470416667	6.66834617083333	6.59635077916667	6.51490294583333	7.20776837083333	6.60436897916667	6.97433015833333	7.585494775	5.96820949166667	7.1363748	7.16456875416667	7.51281844166667	6.40761060416667	7.13995215416667	6.97003259583333	6.87499558333333	6.81088271666667	7.15731386666667	7.3052528875	6.54055676666667	7.39897763333333	7.77018035833333	6.69347515833333	6.47897721666667	7.12753024583333	6.22187706666667	7.8794029875	6.574657825	7.1954046375	6.43118097083333	7.37842275416667	6.92132353333333	7.06183584583333	7.03723994583333	6.71148608333333	7.046003475	7.17002668333333	7.90762179583333	6.34193792916667

SAMD14	8.08289571666667	6.46419815833333	7.2645801	7.01901523333333	7.59199554166667	7.23727353333333	7.94491850833333	6.9475749	6.880500475	7.13728840833333	7.51872279166667	7.84517950833333	8.022968975	7.02536274166667	6.99475883333333	7.60823079166667	7.28012135	7.51253281666667	6.81709465833333	6.73065755833333	7.97719954166667	6.72313661666667	6.8528802	6.90845738333333	7.57783686666667	7.48300389166667	7.62479258333333	6.95221913333333	6.77905188333333	7.41704166666667	6.77905188333333	7.02401584166667	7.09680668333333	7.3361665	7.84430019166667	7.41303320833333	7.18281396666667	7.378725775	7.415839475	7.63604310833333	7.39975843333333	7.71354716666667	6.7401779	7.07353054166667	7.283866825	7.81034196666667	7.47900525	7.08988083333333	7.48588683333333	7.24828621666667	7.01495000833333	6.9004841	6.861457125	7.21451231666667	7.72739250833333	7.299574675	6.91322913333333	7.39926451666667	7.0469593	7.04568171666667	7.941170525	7.66609071666667	7.68098021666667	7.659482025	7.550670775	7.98243114166667	7.49771558333333	7.59937680833333	7.58577588333333	7.31316353333333	7.40950549166667	7.45451809166667	8.35883419166667	7.9291649	7.594902375	7.65663390833333	8.25081844166667	7.147600275	7.3477901	7.39975843333333	8.28242620833333	7.75234836666667	7.61232630833333	8.37653824166667	7.88666206666667	7.91142259166667	8.24067144166667	7.363992875	7.17971484166667	7.91522786666667	7.62930498333333	7.48063178333333	7.30371010833333	7.691592325	8.05717868333333	7.65174983333333	7.456643225	8.19090655	8.16071089166667	6.93873875	7.61232630833333	8.41642395	7.40770269166667	6.93287185833333	7.81731503333333	7.913004675	8.12540885833333	7.44984575833333	7.61006516666667	7.52953641666667	7.94647371666667	7.13294301666667	7.79136785	7.444336	7.33912221666667	7.02148474166667	8.0063663	7.754380925	7.2027431	7.39105859166667

DLGAP4	10.9335275	11.0611890333333	11.2902838583333	11.3864366416667	11.8737640583333	10.6776751083333	11.8209586416667	11.2609645083333	11.6302237	11.3375225333333	11.3534958083333	11.0913458083333	11.34446325	11.1259488	11.3441400333333	10.9204831	10.2720090666667	11.937226525	11.311125675	11.25880145	10.9150352083333	10.9934066	10.4931364166667	11.1905443833333	10.9790999666667	10.571508325	11.328583825	11.6974443166667	11.3809445833333	11.3144428833333	11.4556673333333	11.3560369416667	11.2150743583333	10.6652778583333	10.9208636166667	10.90507365	11.85083865	11.44566875	11.4715256833333	11.634108225	11.5895178083333	11.4619760666667	11.0534747416667	10.643786575	10.1725196416667	10.8394387833333	11.7648162666667	10.9941240666667	10.6173169916667	10.86272805	11.234518675	11.2428131833333	10.8874095666667	11.30822695	11.1129059916667	10.6395867	11.1593417916667	11.5302262416667	10.9895149583333	10.8721591833333	11.376011025	10.9654875083333	10.8922670583333	11.0556311333333	10.6150711416667	11.8626743166667	11.16005095	10.9816032916667	11.0056705916667	10.8056325083333	11.2032221333333	10.934250825	11.3108007583333	10.6415533416667	11.1010834666667	10.8476613083333	10.7463771	11.1502247166667	11.166670375	10.7375745583333	10.5228331416667	10.8972744416667	11.30753185	10.684995425	11.1060515416667	10.5945390166667	10.75497465	10.8139547	10.7888543	10.4168764916667	10.8480286083333	10.782424925	11.1463360083333	10.9237681	11.3108007583333	11.2240675166667	11.0458413916667	11.09094715	11.2638907416667	10.87258615	10.4288423083333	10.8176392916667	10.4691008666667	10.6092076916667	10.5711304666667	10.255903875	10.8484072166667	10.8476613083333	10.6645539	10.7808534	10.5876388833333	11.09094715	10.8953436916667	10.165808925	10.8251181583333	10.5169707416667	10.406522025	10.764714825	10.8537358333333	10.27531875

ERGIC1	14.4891443666667	14.1752017916667	14.1874223416667	14.3892469416667	13.9239245083333	15.3375343833333	13.9717922666667	14.3299457833333	14.1099143166667	14.4633924916667	14.6549636583333	14.4722450583333	14.0813262416667	14.2319492833333	14.3843059083333	14.3463851	15.9859723583333	13.7084619583333	14.4963541416667	14.380649825	14.3139109083333	14.102034475	14.3892469416667	14.65081365	14.3943592583333	15.1017513083333	14.4908464833333	14.1927345333333	14.2763428833333	14.1427380833333	14.0669942916667	14.056517225	14.227452975	15.1833315916667	14.6798721333333	14.9645581833333	14.1857941	14.4025408	14.62202095	14.3186975416667	14.4963541416667	14.145758825	14.0592017583333	13.9389915916667	14.5089658583333	14.3090437083333	14.3757792583333	14.419240975	13.9695187666667	14.648708375	13.8854083166667	14.3268677	14.3186975416667	14.1086467166667	14.5304022916667	14.0885897916667	14.3892469416667	14.3676663333333	14.0618019166667	14.2686692416667	14.4850706333333	14.7077525	14.5377311666667	14.3992922416667	14.66935455	14.29207405	14.7754409333333	14.3139109083333	14.6466555666667	14.2000424583333	14.36106535	14.7974983916667	14.3661675333333	14.2245923416667	14.2289632916667	14.5415836083333	14.349825825	14.718655275	14.5924675083333	14.2983229333333	14.5589896583333	14.8676166333333	14.4309788083333	14.6389112166667	14.4397477583333	14.0100072833333	14.35633825	14.1815548333333	13.9445631916667	14.512293975	14.8895282083333	14.70147985	14.5073052333333	14.5020438083333	14.380649825	14.105898725	14.4043399	14.2887769	13.8317321333333	14.0592017583333	14.5550005583333	14.1113310916667	14.7731878916667	14.9020399416667	14.1268362333333	14.312396275	14.2305624666667	14.5020438083333	14.7205176666667	14.8602831416667	14.515883675	14.3514380333333	14.8136769166667	14.820747325	13.9737942083333	14.6904136083333	14.2349020166667	14.4361804666667	14.667083075	14.4109315833333

EFNB1	11.95296625	12.0769700833333	12.8679345583333	12.0628110916667	12.32117485	12.9117472333333	12.3267305	13.2020549583333	12.2227697083333	12.9659499916667	13.3860625083333	12.3461563666667	12.1852414666667	12.2771993416667	12.3698220916667	12.1604527333333	12.3422979833333	13.3882606083333	12.6098037416667	12.7001469666667	12.7295358083333	12.217702275	13.2447546666667	13.5304589833333	12.626502475	12.2374670666667	12.539129975	12.7531374166667	12.3236478416667	12.3339445	13.1316052916667	12.5159454416667	12.2354776	12.53019765	11.8985295916667	12.3071456666667	12.39795185	13.321391725	13.2175064583333	12.931215675	12.5372066583333	12.43595655	12.2817357416667	12.9172670333333	12.6517095833333	12.750529275	12.65329555	13.279249925	12.7395608333333	12.5734875583333	13.1506037833333	13.1019491416667	12.8661429166667	12.606535325	12.47909375	12.8208910916667	13.267133525	12.978001475	12.5254900333333	12.8339629333333	14.2686692416667	13.6587931916667	13.0645149833333	13.439923175	11.3384968	14.277828625	14.6628978166667	13.8614602166667	14.8548535916667	14.2842943416667	13.9217501083333	14.2998455083333	14.99199845	13.53134155	14.2305624666667	14.5607495666667	14.0885897916667	14.0225032583333	15.039210975	12.6914097333333	11.3720648583333	13.3114725833333	14.4328794	14.5550005583333	14.61397985	12.637166375	13.4156891583333	13.5643847833333	13.1959888083333	14.2671663083333	14.7596386666667	15.3093345916667	13.483275325	14.0813262416667	14.4443539833333	14.6778149	13.6570629916667	13.3377428083333	14.4397477583333	14.4633924916667	14.0694923916667	14.3045490583333	13.5443638333333	13.8395483	14.3076416166667	13.9588693833333	13.60723025	14.6104738916667	13.75723885	14.6087993833333	13.812858	13.334230975	14.5830492	14.8336777583333	14.8794573583333	14.4125419416667	12.7071557666667	14.68415095	13.22406385	12.26972435

CELA2B	7.68293359583333	7.22567669583333	7.65972294166667	8.45769320833333	8.05739779583333	7.80419231666667	8.22620555833333	8.32932159583333	8.16359500833333	7.36848873333333	7.86749155416667	8.30986145416667	8.58721974583333	7.417336975	7.3837288375	7.33230674583333	9.395499275	8.1416513625	7.38727352916667	7.44288026666667	8.20906618333333	7.64461161666667	7.46164670833333	6.58941013333333	6.67203635416667	7.53098197916667	7.21423846666667	7.31461072083333	6.69214671666667	7.21537021666667	7.6750492125	7.21779185	7.46323696666667	7.39655429583333	7.7653777875	7.35319639166667	7.18916067083333	7.16150515	7.09328215	7.1498488875	7.12252534166667	7.56864119166667	6.74250677083333	6.2646626125	6.72153602083333	6.96685253333333	6.81024982083333	7.12623094166667	7.23163065416667	6.91152685416667	7.28228739166667	7.02567045833333	6.8730365625	6.85448609166667	6.99250718333333	6.52812642083333	7.1363748	6.99890459166667	6.76482765833333	7.098369425	8.6245554375	7.8910790125	7.52080745	8.4337271	7.7316394125	7.6074464875	7.91217235833333	8.168080325	7.68800911666667	7.58882987083333	7.96103635416667	7.950087225	8.76150351666667	7.32063235416667	7.99429415	7.80591484583333	7.97839340416667	7.102183225	7.269064225	7.7459142	7.67567321666667	7.97167248333333	7.24670604166667	6.71017390416667	7.331226225	7.97266434583333	7.30834799583333	8.127048	7.48558467083333	6.68369220416667	7.94957104166667	7.31950487083333	7.06561959166667	6.65124665	7.73109602916667	7.17233015	7.1928717875	7.05553105833333	7.57024000416667	7.247970375	6.75204277083333	7.98797805	6.90085837083333	6.67058929166667	7.92467295833333	6.87746547916667	6.42906689583333	7.328870675	6.82481289166667	7.35660711666667	7.068611	7.3650071625	6.8808529125	7.61951985416667	7.06250175	7.01992537916667	6.73916951666667	6.8890305375	6.895626475	7.44179274166667

CELA2A	7.68293359583333	7.22567669583333	7.65972294166667	8.45769320833333	8.05739779583333	7.80419231666667	8.22620555833333	8.32932159583333	8.16359500833333	7.36848873333333	7.86749155416667	8.30986145416667	8.58721974583333	7.417336975	7.3837288375	7.33230674583333	9.395499275	8.1416513625	7.38727352916667	7.44288026666667	8.20906618333333	7.64461161666667	7.46164670833333	6.58941013333333	6.67203635416667	7.53098197916667	7.21423846666667	7.31461072083333	6.69214671666667	7.21537021666667	7.6750492125	7.21779185	7.46323696666667	7.39655429583333	7.7653777875	7.35319639166667	7.18916067083333	7.16150515	7.09328215	7.1498488875	7.12252534166667	7.56864119166667	6.74250677083333	6.2646626125	6.72153602083333	6.96685253333333	6.81024982083333	7.12623094166667	7.23163065416667	6.91152685416667	7.28228739166667	7.02567045833333	6.8730365625	6.85448609166667	6.99250718333333	6.52812642083333	7.1363748	6.99890459166667	6.76482765833333	7.098369425	8.6245554375	7.8910790125	7.52080745	8.4337271	7.7316394125	7.6074464875	7.91217235833333	8.168080325	7.68800911666667	7.58882987083333	7.96103635416667	7.950087225	8.76150351666667	7.32063235416667	7.99429415	7.80591484583333	7.97839340416667	7.102183225	7.269064225	7.7459142	7.67567321666667	7.97167248333333	7.24670604166667	6.71017390416667	7.331226225	7.97266434583333	7.30834799583333	8.127048	7.48558467083333	6.68369220416667	7.94957104166667	7.31950487083333	7.06561959166667	6.65124665	7.73109602916667	7.17233015	7.1928717875	7.05553105833333	7.57024000416667	7.247970375	6.75204277083333	7.98797805	6.90085837083333	6.67058929166667	7.92467295833333	6.87746547916667	6.42906689583333	7.328870675	6.82481289166667	7.35660711666667	7.068611	7.3650071625	6.8808529125	7.61951985416667	7.06250175	7.01992537916667	6.73916951666667	6.8890305375	6.895626475	7.44179274166667

DDR1	9.766909275	12.6254772333333	12.2574255666667	12.60549005	12.4257259083333	13.4366933666667	12.187029525	13.1793538166667	12.9477864583333	12.2603464083333	12.7822168333333	10.1650146416667	11.7853110416667	12.787939025	13.1465914416667	12.44001515	12.3566808833333	13.0769439666667	13.2467446916667	12.76127775	13.0973659083333	13.7434058583333	13.1919341833333	12.8301026333333	13.0584991666667	9.9039927	12.5010802333333	13.0500216833333	12.8181395166667	12.763909375	13.1190556333333	12.4404061833333	12.226469725	12.5297533083333	10.1113541833333	10.338453	13.18921875	12.161623975	12.8718544916667	13.0743927916667	12.9516707583333	12.724847575	13.2047745416667	13.1026328916667	12.762350675	12.5954075166667	12.7139152416667	12.4091926166667	12.8460196	12.4082756583333	12.43647125	12.6813336708333	13.1097329166667	13.3262903916667	13.1688283833333	13.2313413916667	12.5652457833333	12.6749893416667	12.1774305916667	12.093025975	13.9818544666667	12.678040475	12.149683875	12.5989743166667	13.505263125	13.6461564833333	11.6617552333333	13.2567523666667	13.1465914416667	11.9458994666667	13.3467056166667	13.3459381666667	12.944994825	12.6541819833333	13.7670768833333	11.7403808583333	12.8924470083333	12.627449175	12.8381552416667	12.7822168333333	13.756296825	13.541969925	12.7468098833333	12.4275246583333	12.8984843833333	13.27588995	13.9411608583333	12.7092084083333	13.5141902666667	12.3254389	12.6245838166667	13.181363025	13.8625679083333	13.0387188916667	13.887785	12.8329037166667	12.5813238666667	13.841729125	12.927195275	13.8889513416667	13.2615079916667	12.9036231416667	13.0451078583333	13.189983975	13.2068344333333	13.1310777666667	12.8884463	12.8041062916667	13.1452170666667	12.1827137333333	13.410428725	13.5451377333333	13.4260804333333	13.3410732083333	12.5420281	13.52467435	13.62200835	13.5502258583333	13.2828821583333	13.16815045

ADAM15	12.4225030833333	14.1033949916667	14.0656333583333	13.8049154	13.85508265	13.4579324833333	13.8778632416667	13.9884216166667	14.0847383833333	13.7781265916667	13.7692653916667	13.1465914416667	13.5601696	13.9445631916667	13.7502246416667	13.6694092583333	13.3608559333333	13.2460254166667	13.9884216166667	13.526397225	13.7282923166667	13.712997875	13.3293632833333	13.7902025333333	13.576244975	12.4945779666667	13.70272065	13.7074542166667	13.5789450333333	13.8168544833333	13.6941290583333	13.5387428916667	13.5502258583333	13.445727425	12.3832899166667	12.4593508916667	13.8562499666667	13.8199392	13.8049154	13.933121025	13.7611314416667	13.92958755	13.8010236666667	13.8221316	13.3575610333333	13.8428912916667	13.8992947833333	13.8473484166667	13.812858	13.5346846166667	13.248853375	13.994455075	13.823045075	13.771159075	13.8241318916667	13.853956425	13.9807861416667	13.7452882916667	13.38316305	13.8832091583333	13.8843608583333	12.7733872	12.988893075	13.3067028166667	12.7516120833333	13.1806770166667	13.1913123583333	12.948888375	12.8251494083333	13.5076042	12.8010225583333	13.1786718416667	12.7071557666667	12.7743205	13.6392528333333	13.1538383166667	12.7364125916667	13.16545515	12.96407505	12.88540095	12.0781743333333	12.4718679833333	13.4586485833333	13.1071716666667	12.8979356	12.497697975	12.21725785	12.908758175	12.519265425	12.737987825	12.7122547666667	13.9153342666667	12.5439252666667	12.8381552416667	13.4950898916667	13.3300671416667	13.2690950583333	12.77853635	12.78176855	12.905858725	12.993623425	12.8426722583333	13.14005525	13.2387164583333	13.5197355333333	12.552591775	13.113125125	13.6236897166667	12.7873683333333	12.9505716416667	13.01427815	12.8498718833333	13.149953225	12.7359047083333	13.2587665833333	13.62548145	12.4893471666667	12.910549725	13.0749552833333	12.6862224333333

IPO7	12.466874775	13.193307375	12.2764019583333	12.6616630833333	12.8068783833333	12.8836445083333	12.5306631166667	12.6131713166667	12.9172670333333	12.5429966333333	12.9715821666667	12.430195425	12.643219075	12.620644525	12.6041308166667	13.1641370583333	12.92082965	13.420218375	13.193307375	13.3936063	13.1058939166667	13.4688309833333	12.9516707583333	12.7692841333333	13.0877248833333	12.2111963416667	12.5652457833333	12.867358575	12.692926575	12.6361256666667	13.203417575	13.0113663	13.3300671416667	13.8148381	12.306357275	12.5065921166667	13.1628836	13.222146075	12.92082965	13.2848396666667	12.8964099833333	12.9456067916667	13.509141775	12.8086026666667	13.390452325	12.9472642583333	12.8057256416667	12.7483893416667	12.906960875	13.136994025	13.141294	13.174009975	13.3494539666667	13.19400885	13.1316052916667	13.4474018083333	13.068247425	13.1427455666667	13.144627125	12.99120565	13.6579175	12.9659499916667	13.2266532083333	13.5660586666667	13.9207353166667	13.933121025	12.9423278583333	13.8854083166667	13.2615079916667	13.1733038666667	13.0349568083333	13.8148381	13.2884054666667	13.4957705	13.00071955	12.7987689166667	13.7177649416667	13.418751775	12.8273007416667	13.3936063	13.604496075	13.2387164583333	12.8776683083333	13.0427266583333	13.6310840583333	13.4912514166667	13.67760105	13.6320213916667	13.9989817	12.8481618333333	13.3897269	13.8809609916667	13.5363675083333	13.405024	14.160512975	13.0571358166667	13.8766797166667	13.2467446916667	13.8307559916667	13.6657423	13.6000874166667	13.6140845916667	13.4089360166667	13.3390509833333	12.4341294166667	13.6150235416667	13.2731848666667	13.7084619583333	13.1506037833333	13.257439275	13.001932175	12.8074143333333	13.7869425833333	13.69139155	13.4565571916667	12.4275246583333	12.813963275	12.9516707583333	12.9346936833333	12.8918006166667

MCF2L	5.866285325	5.39178135	5.30061454166667	6.06230976666667	7.306684875	7.76188296666667	5.897311975	5.26078876666667	5.3100359	5.43818919166667	6.603384975	5.7946758	5.83674730833333	5.31421083333333	7.40837556666667	6.89596523333333	5.10387183333333	8.09998893333333	7.36330638333333	6.90781049166667	6.56879458333333	8.215089275	5.76144219166667	7.85807856666667	6.92916504166667	8.1455177	7.93695109166667	7.38123391666667	9.35634101666667	7.33974354166667	7.25363145833333	7.15020035833333	7.05156345	5.86347250833333	7.2954331	7.42162103333333	7.691592325	6.71110255	7.537939	7.31196651666667	6.987814075	8.04755969166667	7.314288875	6.339217025	7.11617649166667	7.13294301666667	5.82731045	7.30254744166667	6.348986575	7.14458345	7.11920136666667	6.601356975	7.5324158	7.873129975	6.57734461666667	6.264276875	5.80728855	4.85861940833333	6.18822558333333	6.181735025	7.11478095	6.3094404	6.55145231666667	6.787125175	7.87768698333333	5.92509355833333	5.557624025	6.03335128333333	5.56425255	5.54579601666667	5.72997074166667	6.28402719166667	5.207730075	5.88760754166667	6.328333625	5.6458775	6.5402188	5.73195755	4.92055984166667	6.72825601666667	8.80870043333333	6.438674975	5.0258117	6.225217	6.53536243333333	7.708204175	7.210345825	4.7815417	8.17931615833333	6.78782379166667	6.41504531666667	5.74506199166667	6.82167365	5.7955502	6.11387184166667	7.42731664166667	8.74174598333333	7.00566125833333	6.86073455	7.77269480833333	4.32198345833333	5.65995315	6.49825018333333	6.76517564166667	6.0079005	6.01932009166667	6.81774981666667	6.79588928333333	4.428719425	6.91905025833333	7.80828950833333	5.70431125833333	5.84236155833333	4.873988275	5.24703029166667	5.59754975833333	6.13424446666667	4.613234575	6.35201275833333	4.85768484166667

GNAO1	8.443897425	8.38370515833333	8.41599213333333	8.706897975	9.16495244166667	8.13304300833333	9.24586054166667	8.80244275	9.146936475	8.48745835	8.744772225	9.39025219166667	9.12938660833333	8.586479525	8.18571946666667	8.29403910833333	7.868368375	9.0354605	8.37211495	8.66247435	8.69082025	8.52878136666667	8.19443521666667	8.02564620833333	8.119922525	8.53434441666667	8.44334104166667	8.16178343333333	8.81154583333333	8.18151525833333	8.13716783333333	8.155034325	8.67450711666667	8.13923885	8.19036318333333	8.234157525	8.61290266666667	8.38319035	8.32012975833333	8.25839510833333	8.11553545	8.06450121666667	8.04131995833333	7.88761391666667	7.92654975833333	8.09947061666667	8.081342075	8.19036318333333	8.47046444166667	7.87157458333333	8.25189414166667	8.09947061666667	9.0498455	8.22099113333333	7.95406095	8.1205116	8.56867330833333	8.13923885	7.9877223	8.11887123333333	8.69082025	8.6667612	8.04234110833333	9.13323439166667	8.57257758333333	8.99070061666667	8.75473524166667	9.02609295	8.954039625	8.87834883333333	8.84383605833333	8.94715431666667	9.319021525	8.56044531666667	8.74766549166667	8.70212718333333	8.65698328333333	8.45201853333333	8.50943095833333	8.770019625	7.93223308333333	8.96987785	8.62938480833333	7.868368375	8.895120775	9.02371780833333	8.382753925	8.99346874166667	9.170544975	8.019555675	8.26356378333333	8.11940264166667	8.15812938333333	8.25745250833333	9.27994568333333	9.24712485	8.85494786666667	8.28678805833333	8.94285730833333	7.86942576666667	8.34279256666667	8.66201021666667	8.37460103333333	8.36713998333333	8.17990050833333	8.3851619	8.182610775	8.05664168333333	8.47431853333333	8.12540885833333	7.97086885	8.67361984166667	8.49745698333333	8.1277546	8.08754240833333	8.43095395833333	7.8150656	8.51578285	8.03960018333333	8.64006809166667

C14orf119	5.33111477083333	5.1509350875	4.12625302916667	4.60753944166667	4.42526413333333	4.514233575	3.6792171	3.76893669583333	3.84201738333333	4.677310075	3.93531937083333	4.3308863625	4.18873504583333	4.00401692083333	4.6792515125	5.3061346875	5.29832429583333	4.45953535	6.44632249583333	5.47173187916667	5.10975973333333	4.47420940416667	5.166787925	5.34914802083333	5.25283739583333	6.127271775	5.39229912083333	5.24879086666667	4.05255809583333	5.51437135833333	4.77872065416667	5.7223480375	5.13486149583333	5.69628012083333	4.92201914166667	5.5655648875	5.32609779166667	5.39752319583333	6.040429525	5.75635790833333	6.52751807083333	5.39435747083333	5.51743424583333	5.8160782625	5.66126460416667	6.09629062916667	6.82730435416667	5.83063687916667	5.46699008333333	6.262456425	6.22265424583333	5.88193024583333	3.4946012625	4.95482875	4.15169350416667	5.37076709166667	4.7722030125	6.0671444125	5.3137011	6.62466633333333	3.68796985	4.07513861666667	4.85395452083333	4.186591025	4.23243782083333	3.94847739583333	3.7971162625	3.6792171	3.86557323333333	4.5912591875	3.67805529166667	4.88315419583333	3.6661201875	4.30183637083333	3.63610115833333	5.15507069166667	4.4555335125	4.74278157083333	5.58747924166667	4.71269748333333	4.52953826666667	3.8511588625	4.44777587083333	5.03877025	4.36849046666667	3.8907320625	4.00834315416667	4.02200670416667	5.66897774583333	5.62825054583333	5.02419559583333	4.83825977083333	4.959119975	5.18162923333333	3.55378007916667	3.670843375	5.5655648875	4.28157184166667	5.00060418333333	5.36037509166667	7.1137308375	3.9743934375	4.20338963333333	5.299254225	5.113822425	4.834065625	5.10125760833333	5.296139525	3.99873699583333	6.19559102916667	4.20842273333333	5.16367257083333	4.5407675375	5.77675150833333	3.72374019166667	3.9877851875	4.45160768333333	6.10436502083333	5.4575038	4.552338475

LMLN2	5.33111477083333	5.1509350875	4.12625302916667	4.60753944166667	4.42526413333333	4.514233575	3.6792171	3.76893669583333	3.84201738333333	4.677310075	3.93531937083333	4.3308863625	4.18873504583333	4.00401692083333	4.6792515125	5.3061346875	5.29832429583333	4.45953535	6.44632249583333	5.47173187916667	5.10975973333333	4.47420940416667	5.166787925	5.34914802083333	5.25283739583333	6.127271775	5.39229912083333	5.24879086666667	4.05255809583333	5.51437135833333	4.77872065416667	5.7223480375	5.13486149583333	5.69628012083333	4.92201914166667	5.5655648875	5.32609779166667	5.39752319583333	6.040429525	5.75635790833333	6.52751807083333	5.39435747083333	5.51743424583333	5.8160782625	5.66126460416667	6.09629062916667	6.82730435416667	5.83063687916667	5.46699008333333	6.262456425	6.22265424583333	5.88193024583333	3.4946012625	4.95482875	4.15169350416667	5.37076709166667	4.7722030125	6.0671444125	5.3137011	6.62466633333333	3.68796985	4.07513861666667	4.85395452083333	4.186591025	4.23243782083333	3.94847739583333	3.7971162625	3.6792171	3.86557323333333	4.5912591875	3.67805529166667	4.88315419583333	3.6661201875	4.30183637083333	3.63610115833333	5.15507069166667	4.4555335125	4.74278157083333	5.58747924166667	4.71269748333333	4.52953826666667	3.8511588625	4.44777587083333	5.03877025	4.36849046666667	3.8907320625	4.00834315416667	4.02200670416667	5.66897774583333	5.62825054583333	5.02419559583333	4.83825977083333	4.959119975	5.18162923333333	3.55378007916667	3.670843375	5.5655648875	4.28157184166667	5.00060418333333	5.36037509166667	7.1137308375	3.9743934375	4.20338963333333	5.299254225	5.113822425	4.834065625	5.10125760833333	5.296139525	3.99873699583333	6.19559102916667	4.20842273333333	5.16367257083333	4.5407675375	5.77675150833333	3.72374019166667	3.9877851875	4.45160768333333	6.10436502083333	5.4575038	4.552338475

ZUP1	9.4968836	9.42508291666667	9.513102275	8.9939706	9.71828384166667	9.60830463333333	9.07584114166667	9.79628703333333	9.24218355833333	9.20822946666667	9.26948546666667	8.56421206666667	9.14235506666667	9.227470725	9.55058418333333	9.34302553333333	9.73142488333333	9.781927775	9.596033825	9.43547285833333	9.553861875	9.97912404166667	9.69087439166667	9.97833993333333	9.78329576666667	9.43501909166667	9.40861700833333	9.64783695	9.66558844166667	9.77090350833333	9.844981225	9.630635475	9.21350573333333	9.25858543333333	9.496026575	9.47542340833333	9.50353634166667	9.57692496666667	9.984272	9.95100449166667	9.42030009166667	9.36122138333333	9.045519875	9.58131358333333	9.471100975	9.80664778333333	9.78824573333333	10.0736918083333	9.51223504166667	9.42424818333333	9.70886765833333	9.818576775	9.70284449166667	9.84589375833333	10.1687340166667	9.59324718333333	9.4277554	9.62496784166667	9.36848635	9.65888631666667	10.0520460833333	9.852861125	9.393424375	9.468356875	9.42859468333333	9.01655344166667	9.81525473333333	9.14921909166667	10.002252325	9.57556716666667	9.705378475	9.94714974166667	9.699723	9.619021525	9.99125556666667	9.76006771666667	9.25764194166667	9.7557679	9.77000968333333	10.189123775	9.903525425	9.92213260833333	9.81648044166667	10.2379766333333	9.99637683333333	9.44105665833333	9.7105078	9.625456175	9.21080935833333	9.80576564166667	10.1063080916667	10.3313573416667	10.4732782833333	9.97215941666667	9.784367075	9.62805526666667	9.945938775	9.59906339166667	10.647680825	10.1481495666667	10.8254925166667	9.352137375	10.440565075	9.46165735	9.52042825833333	9.65222760833333	9.4697267	9.86598649166667	9.81941439166667	9.611823	9.56259116666667	10.0268881833333	10.5490285	9.956863325	9.96400001666667	10.0993323166667	9.2998268	10.136260225	9.47453338333333	9.870256575

DDIT4L	9.27035150833333	7.87098755833333	7.9438425	7.28259465	7.43634795	7.84430019166667	7.731363575	7.69369399166667	7.71862859166667	8.20587135	7.55551181666667	8.96893119166667	7.46463336666667	7.60040215833333	7.83071075833333	8.25344699166667	7.84430019166667	7.61232630833333	7.61520518333333	7.62930498333333	7.10434548333333	7.52840544166667	7.568339425	7.78110251666667	7.63711770833333	9.17813545	8.27021601666667	7.40257545	8.027292325	8.17885319166667	8.119922525	8.127296675	8.20689531666667	8.1976201	9.436848125	9.89341510833333	7.81839644166667	8.00740176666667	8.06996655	8.05032904166667	8.14754213333333	8.26308525833333	7.99186004166667	8.22799306666667	8.64196594166667	8.56955223333333	8.36127639166667	8.0294547	8.17302170833333	8.12933435833333	8.41537984166667	8.07980334166667	8.13304300833333	8.18891301666667	8.36187725833333	8.48261985	8.00901856666667	8.05141264166667	8.01700516666667	8.256452925	8.21983948333333	7.466283575	7.17569580833333	7.96240296666667	7.99186004166667	7.30371010833333	7.18884621666667	7.90260113333333	7.53912439166667	7.687124975	7.69424574166667	6.76253231666667	7.09617145833333	8.15707283333333	7.51935500833333	7.56433695	7.596603625	7.52225746666667	7.378725775	7.08427549166667	6.9393833	7.35564645	7.129719725	8.00359983333333	7.48927714166667	8.58856818333333	7.45718173333333	7.746730925	7.741934525	8.859115725	7.6421897	7.86334303333333	7.97507355833333	8.434501875	8.24067144166667	7.96885115833333	7.21504029166667	8.38023938333333	7.66443915	8.101615825	7.83568390833333	7.67926596666667	7.83568390833333	8.18571946666667	7.141526375	7.95517108333333	8.312026725	7.64737070833333	8.38662095833333	8.53929365	8.5216034	7.73527455	7.95851929166667	8.000994225	8.03376271666667	8.14240605833333	8.14495316666667	8.022968975	7.84376875833333	8.14959635833333

SPATA1	9.14327603333333	8.82680535833333	7.87042711666667	8.04388136666667	8.37061515	8.50797726666667	7.14272935833333	8.37811988333333	7.66388121666667	8.84195500833333	7.992867725	9.91801125833333	8.685246125	8.65318265	9.53443595	9.05561776666667	8.53782911666667	7.82067988333333	8.60177515	9.22295939166667	8.9939706	9.98920446666667	8.67503203333333	8.54656363333333	8.91506995	9.42118564166667	9.41935561666667	8.87834883333333	9.24492166666667	8.77579595833333	8.73109978333333	9.36848635	8.52241499166667	8.499322875	9.03183454166667	9.508221125	9.3558778	9.040322975	8.79346453333333	9.471100975	8.40965201666667	9.037729975	9.0498455	8.89602301666667	8.80780606666667	8.86314575	9.09054048333333	8.68175469166667	9.14145633333333	8.42823383333333	8.43662665833333	9.20145365833333	8.84428041666667	8.909148575	8.54547846666667	8.62136429166667	8.698928125	8.61623551666667	9.03865570833333	9.76954021666667	8.65175716666667	7.04002458333333	7.63102280833333	7.12401263333333	8.03117830833333	7.82793564166667	5.95243336666667	7.415839475	8.31058729166667	9.304559675	6.41808758333333	7.98666509166667	7.93282061666667	7.63328161666667	8.486570275	8.04080661666667	8.2260001	8.66481208333333	7.9760917	8.498393225	8.445416025	8.91642435833333	7.89028620833333	7.641075725	8.64543325	8.20689531666667	5.90677250833333	7.68323405	8.9929566	8.61046613333333	8.256452925	8.21046635	8.43197355833333	8.36762415833333	9.87484248333333	7.87562194166667	8.48745835	8.16633889166667	8.04755969166667	8.70499479166667	7.45403938333333	7.15624494166667	9.48834466666667	8.53097458333333	7.83125643333333	9.06460029166667	8.5034354	8.38817050833333	8.92651730833333	8.36713998333333	9.84456984166667	7.72739250833333	7.77602128333333	7.22110665	8.18527721666667	7.66954886666667	9.25814955833333	7.96192095	7.372424975	8.51669369166667

GGT5	9.86062085833333	9.77000968333333	10.7718274166667	10.2442419583333	9.633880775	10.8593336416667	10.4208424916667	10.2233050083333	9.46165735	10.2966490166667	9.95973609166667	11.9903497083333	10.4526526416667	9.865582425	9.730552775	11.4158566083333	10.801788825	9.24218355833333	9.8942397	10.4634265083333	11.04233505	9.275964375	10.1906714166667	10.1324487166667	10.1205788333333	10.3546255166667	10.4032528583333	9.93957129166667	9.74655711666667	9.80350678333333	10.6173169916667	10.2064993416667	10.4526526416667	10.6507559	10.4057146583333	10.012954475	10.514613975	10.1218627333333	10.8468748916667	10.744004	10.2411048916667	10.48105345	10.5134429166667	9.95767555	11.31949275	10.63254535	10.1432492	9.869019275	10.1546002	10.409675875	9.76267145833333	9.9420301	10.356255775	10.4942942166667	10.6745552333333	10.9234198666667	10.2314081833333	10.300361525	10.9790999666667	10.4515253333333	10.9196649333333	10.8916305083333	10.7485438416667	11.5103338083333	11.1498219083333	10.8389999833333	11.4549596	11.4759684833333	10.298281975	10.9512840333333	10.7971951666667	10.9273818166667	11.5902054333333	12.02225065	10.3682517916667	11.4767310833333	11.8116053416667	11.03980565	10.85221535	11.1313582333333	10.4284899	11.37567555	10.5605583916667	12.6397345916667	10.9483433833333	11.1025248083333	10.1745820166667	11.92557355	10.035165975	11.5706660333333	11.4704736916667	10.7247329333333	10.3656176	10.500555575	10.6952813416667	10.7258049	11.1771749333333	10.1226723083333	11.2341636166667	10.64807625	11.0380667083333	10.6535513666667	10.9157145083333	10.1437039833333	10.5872774916667	10.399176825	11.4696992083333	10.91088935	11.2711403666667	12.1030545583333	11.1696311416667	10.0894400666667	11.069747825	10.26748265	11.687529825	10.8980207583333	10.6776751083333	11.036287075	10.0984817166667	10.5545011333333

ZNF74	9.59563205	8.833014675	9.07065694166667	8.97076639166667	9.4337598	9.59418116666667	8.54462835	8.79861721666667	8.88262415833333	8.6774004	9.10984779166667	9.02085209166667	8.697015525	8.81463643333333	9.28502139166667	8.4371155	9.25947800833333	9.00690949166667	9.25584964166667	8.59640985	8.78586333333333	8.98618440833333	9.157883875	8.93227916666667	8.9544846	9.62275734166667	8.93461839166667	8.94849429166667	8.8912541	9.08593973333333	8.8451569	8.76798363333333	8.58808473333333	8.84011393333333	9.41484764166667	9.82651539166667	9.277313475	8.76125596666667	8.65318265	8.61979589166667	8.84011393333333	9.112227825	8.78070639166667	9.071149525	8.9217463	9.16588333333333	9.09878833333333	9.32089804166667	8.954039625	8.85728129166667	8.7532663	9.0844393	9.49775068333333	9.19996488333333	9.32526180833333	9.16123660833333	8.80139795	9.13780860833333	9.00551523333333	9.02222115833333	10.1766858833333	9.59824975833333	9.91197805833333	9.911603525	10.2547426166667	9.5928155	9.1038805	9.20920615	9.657103925	8.56366318333333	9.48649659166667	10.1910863	9.37269289166667	9.173253675	9.79573791666667	9.428146125	9.65621849166667	9.36892748333333	9.645264775	9.78824573333333	9.62064585	9.87345979166667	9.10485650833333	9.65529779166667	9.15210589166667	9.98877366666667	10.0171966666667	9.56828670833333	9.674571275	9.44826335	9.08047905833333	9.98187775833333	9.18832863333333	9.22470684166667	9.62496784166667	9.74218338333333	10.1605417333333	9.84456984166667	9.2741013	10.0134059083333	9.86640328333333	9.72111820833333	9.64610789166667	9.76051965	9.02980844166667	9.79016168333333	9.88168326666667	9.55671014166667	9.891815375	9.34018350833333	9.11582745	9.743497625	11.5512973083333	9.695341625	9.34302553333333	9.490132625	10.0078625	10.2018680083333	10.2802737333333	9.20975130833333

PROK1	4.63625878333333	4.1095023	3.95017479166667	4.1756419	3.700710475	4.07457460833333	3.57639359166667	3.77672200833333	3.56835755	3.6774476	3.542001325	3.74146091666667	3.62138225	4.093740425	4.00999268333333	3.83492170833333	4.59071941666667	4.19529993333333	4.51268873333333	3.92371073333333	3.912461775	4.04952899166667	4.27798759166667	4.37489024166667	4.65176085833333	3.96836553333333	4.34698491666667	5.32031858333333	4.59179895833333	4.15954296666667	4.53925114166667	4.69642039166667	3.74146091666667	4.024477825	4.09074995833333	4.11480768333333	4.6247727	4.34901141666667	4.383064975	4.56071255833333	5.05799246666667	4.32010620833333	5.30151331666667	5.20449400833333	4.17980874166667	4.72615460833333	4.649483075	4.822492325	4.57030863333333	4.73206480833333	4.617309275	4.43639113333333	4.05071508333333	5.25233420833333	4.289092625	4.63723314166667	3.80438254166667	4.66519471666667	4.04713170833333	4.37287369166667	3.8761543	3.74515126666667	4.37388213333333	3.746317775	3.84482813333333	3.893426425	3.91700049166667	3.68999008333333	4.12229528333333	4.44534256666667	3.44744255833333	3.854046525	3.74768024166667	4.07134488333333	3.64058418333333	3.92925663333333	3.77909573333333	3.76337073333333	3.77178324166667	3.88647775833333	3.60239741666667	3.65321091666667	4.1683668	3.893426425	3.79182875833333	3.79182875833333	3.94009973333333	4.42666711666667	3.64321401666667	3.8291486	3.66920745833333	4.26652515833333	4.03660776666667	3.92023596666667	3.83376700833333	3.87726464166667	4.09074995833333	4.01568299166667	3.71770076666667	4.89254299166667	4.37388213333333	3.88526006666667	5.20995024166667	4.656935525	4.39972780833333	3.867233575	4.3276422	5.85609921666667	4.30624979166667	6.10954496666667	4.46100053333333	5.157708775	4.494971825	4.713205375	4.061836425	4.29916468333333	4.20999628333333	4.38635804166667	4.81179368333333	3.76094594166667

IMMT	13.439923175	12.6131713166667	12.50892495	12.5534552416667	13.5287262833333	13.2495669	12.5439252666667	12.927195275	12.964777525	13.2467446916667	12.9456067916667	12.6991286666667	13.2075024333333	12.7769325	12.8667102583333	12.6254772333333	13.6000874166667	13.3959354916667	13.3270763416667	12.9012827916667	12.9527510916667	13.1952399083333	12.8776683083333	13.0518882	13.14330865	13.189983975	12.7912843416667	12.5372066583333	13.0325393583333	12.750529275	12.93175565	12.798152025	12.59948	13.1885669333333	13.0933531416667	13.6904576333333	13.0613877916667	12.4831684166667	12.798152025	12.9533257416667	12.3240568416667	12.6914097333333	12.5788865916667	12.901864125	12.8344857166667	12.8724674416667	12.8555603666667	13.20142475	12.8964099833333	12.8273007416667	12.8708264916667	12.9224811	13.1316052916667	13.1356932666667	12.584151375	12.9610660166667	12.8329037166667	12.7836657416667	12.9400260666667	12.7827098416667	13.7046873583333	13.8625679083333	13.244127475	13.6310840583333	13.487174625	13.6560864916667	13.7046873583333	13.6904576333333	13.4260804333333	13.6785048416667	13.320797425	13.3952177083333	13.3467056166667	13.6019439833333	13.518865875	13.5387428916667	13.410428725	13.8743989166667	13.4390716416667	14.1427380833333	13.9612443083333	13.317291325	13.462725925	13.6293464	13.5468416333333	13.5617608833333	14.1317250833333	13.0939551583333	13.9727862416667	13.2983134833333	13.1242463	13.986279475	13.712997875	13.4058013416667	14.0074276833333	13.44656775	13.3786606333333	13.86774345	13.738346	13.2387164583333	13.4721219	12.8836445083333	13.6019439833333	13.4768642833333	13.2313413916667	13.5475412583333	13.3623548	13.181363025	13.8572965333333	13.810810275	13.4058013416667	13.6434906916667	13.3522548916667	13.9065296166667	13.1576233666667	13.0102233	13.214199825	13.05962405	14.0656333583333	13.3278442333333

BLOC1S5-TXNDC5	4.51657093333333	5.197537	4.82135065	4.149223425	5.4600655	4.23291245833333	5.661700075	5.23829215	5.078596225	4.35594943333333	4.37807450833333	5.02903219166667	5.19112474166667	4.81716043333333	5.18112505	4.59576983333333	4.3881123	5.053877925	4.59683404166667	4.78900190833333	5.02371664166667	4.15011734166667	4.67972664166667	4.32946600833333	4.494971825	4.23097901666667	4.51861790833333	5.23259211666667	7.35119398333333	4.61010935833333	4.55568075	4.44059075	4.490204975	4.02921226666667	4.31414184166667	4.82904071666667	4.443420775	4.43531826666667	4.35186211666667	4.53105006666667	4.81840091666667	4.328489775	4.65176085833333	5.40518485833333	3.92023596666667	4.82135065	4.789978375	4.30624979166667	4.79109571666667	4.15954296666667	4.59179895833333	4.70675665	5.04458405	5.13634166666667	5.231579	4.64220138333333	4.35186211666667	4.65382089166667	4.385351725	4.59265796666667	4.76349515	4.47861730833333	4.16611878333333	3.95350946666667	4.69115821666667	4.31908713333333	4.55069105	4.64220138333333	4.52522428333333	4.51773374166667	3.8987918	4.30828979166667	4.58140610833333	4.58140610833333	4.43639113333333	4.55971610833333	4.53925114166667	5.10498871666667	4.94478124166667	3.92925663333333	5.096408875	4.32198345833333	4.18191081666667	3.92246294166667	4.57924936666667	4.64133359166667	4.89935284166667	6.33714610833333	4.117963675	4.86479268333333	4.16737369166667	4.61421155	4.32588960833333	4.2231712	5.03232479166667	4.72324775	5.07445225	4.59683404166667	4.14488695	5.75852464166667	5.44122935	3.94341095833333	4.68789791666667	4.91752975833333	4.90032935	4.18603395833333	4.46402134166667	7.81137301666667	5.53395658333333	7.77718458333333	4.63410609166667	6.11631895833333	4.79411095833333	4.78798888333333	4.03764378333333	4.30917759166667	5.5651756	5.162070825	4.36796431666667	4.65382089166667

KCNIP3	5.76144219166667	6.02645373333333	5.65344755	6.97843058333333	6.14959958333333	5.71985138333333	6.88865130833333	6.14575641666667	5.17483658333333	5.1291031	6.880500475	6.03335128333333	5.46375123333333	5.97682351666667	6.99919410833333	5.59754975833333	5.38263135833333	6.30492928333333	5.3439553	5.40518485833333	6.48645600833333	6.94120649166667	6.30714161666667	5.29885645833333	5.38372931666667	6.44290830833333	6.21517435	7.18643425	6.345260775	6.47456359166667	5.82073376666667	6.18408195	6.51455034166667	5.29356806666667	5.20658905833333	5.13122064166667	6.89132381666667	6.20408345833333	5.079599575	6.65984261666667	5.36922546666667	6.12763226666667	6.00039116666667	5.03010108333333	5.508179525	5.90154725	5.95243336666667	5.96077145833333	5.6488719	6.76905384166667	5.42175125833333	5.464635875	6.93616958333333	6.777132775	6.40797193333333	5.10716860833333	6.159395425	5.72790364166667	6.407249275	6.17226739166667	7.69424574166667	7.01901523333333	6.36170378333333	6.98973956666667	6.34978520833333	6.74158396666667	6.58160273333333	6.85878966666667	6.181735025	6.63660101666667	7.25732849166667	6.50580336666667	6.653546475	6.10002881666667	7.227778225	6.19818808333333	6.30177564166667	6.47064316666667	5.50338315833333	6.256698975	8.21153401666667	8.56421206666667	6.74677720833333	5.21920105	6.34384491666667	6.67572515	6.56733945833333	7.05225570833333	7.77208035833333	5.23978975	7.05585056666667	6.90781049166667	6.316307375	5.35273364166667	7.09617145833333	7.67599419166667	7.20747220833333	6.87589898333333	5.63053526666667	8.33758601666667	5.41985199166667	7.29169275833333	6.00285793333333	6.1441534	5.83306814166667	5.13745513333333	6.242830225	5.48099319166667	6.390922	6.49825018333333	6.996742575	5.22368295	7.101860325	4.27160165833333	6.04352600833333	6.37491049166667	6.70327563333333	6.2066078	5.89630836666667	5.27417344166667

NSD3	7.212178575	7.458458425	7.96240296666667	7.26626465	7.32611610833333	7.731363575	6.36709734166667	7.23727353333333	7.27891691666667	7.35983868333333	8.57995885	6.46494565	6.83400405	7.57569364166667	8.04450845	7.42268463333333	8.15060805	7.731363575	7.911899075	7.50627369166667	7.960228575	8.24116579166667	8.58441085833333	8.29147479166667	8.094406525	7.09680668333333	8.252426875	8.32853386666667	8.03538219166667	8.34767179166667	8.72544860833333	8.16735940833333	7.708818875	8.333019375	7.47677956666667	7.29365533333333	7.86777288333333	8.28949861666667	8.672615075	8.01273043333333	8.561306875	8.1898559	8.234157525	8.895120775	7.69651605833333	8.43197355833333	8.48745835	8.38708958333333	8.46459865	8.649865175	8.39315135833333	8.26207273333333	8.30084284166667	8.75658194166667	8.458998275	8.55750678333333	8.07839394166667	8.595488075	8.11553545	7.99131585833333	7.81893236666667	8.0063663	9.52591685	8.29053858333333	7.41942373333333	7.8248949	8.15959780833333	7.788761475	8.07050834166667	7.89880223333333	9.35306623333333	8.65175716666667	7.76455869166667	7.96575633333333	8.17090974166667	7.99232836666667	9.2436075	8.35071744166667	8.80780606666667	8.76654949166667	7.39975843333333	8.155034325	10.9415949916667	7.98023625	8.2495222	7.68323405	9.48609106666667	7.190008575	8.55750678333333	8.91085585833333	7.9977282	8.22546075833333	7.89196619166667	9.19244325833333	7.94065678333333	9.60963610833333	9.586175875	8.5905397	10.2241250416667	9.09833678333333	8.79767885	8.34324809166667	8.4484208	9.295759125	8.71940105833333	8.751330725	8.36907771666667	8.1565621	8.30336938333333	7.54626845833333	9.52783803333333	8.759783975	8.37508346666667	10.3182950083333	8.2988733	10.9189114166667	8.23956795833333	8.59785679166667	8.77140460833333	7.80563896666667

ZCCHC7	7.84998155	6.84063886666667	5.89991223333333	6.896657025	7.5954683	7.65388535833333	5.57252066666667	6.37491049166667	6.52912136666667	6.61484865833333	6.87589898333333	7.31493256666667	7.064683475	5.826473825	7.86164825833333	6.99346504166667	6.74958845833333	8.14660076666667	7.70589544166667	7.6157713	5.933890025	8.79240648333333	5.9573564	7.7483928	7.97666008333333	6.87022720833333	7.05585056666667	7.97239718333333	8.50130601666667	7.00049489166667	6.801867125	7.34272655	6.59670515	6.82102155833333	6.85814304166667	7.30805265833333	7.93123045833333	7.17681736666667	7.42326895833333	7.31919828333333	6.2010939	6.64764070833333	7.23193100833333	6.64834389166667	7.05838413333333	5.876765525	7.47623615833333	7.13093330833333	7.30968498333333	6.92433585	7.47140944166667	7.35059130833333	7.57569364166667	8.126799325	8.4125394	7.433497275	7.1744649	7.102506125	7.59366683333333	7.95517108333333	8.6731133	7.29418750833333	7.89455425833333	7.57344579166667	6.22675393333333	8.71089086666667	7.227214725	7.03502525	7.20859641666667	7.26995246666667	7.10054488333333	8.30178113333333	6.06756750833333	7.09804688333333	7.71034295833333	7.139659575	7.79694136666667	8.49050600833333	7.93805580833333	7.59420499166667	6.78642360833333	8.0459923	7.12901885	8.09897714166667	7.43067104166667	7.28012135	7.73803474166667	6.57014316666667	8.97168731666667	7.48123266666667	7.3675475	7.964195775	7.23311056666667	7.34611101666667	7.978659025	7.53449101666667	8.925575825	7.55318286666667	7.14092606666667	7.50404556666667	8.08531809166667	6.96848423333333	7.89083754166667	7.2622557	8.16329085	7.14519241666667	8.17249793333333	7.746730925	8.140254425	7.139086775	6.94439705	7.88927118333333	8.26257539166667	7.80930983333333	7.86994016666667	7.50966981666667	7.160095	8.35024235	8.1089851	7.27359608333333

CNOT3	9.87826785	9.48609106666667	9.17457594166667	9.40906840833333	10.0607135666667	9.00690949166667	10.0957326583333	9.42687719166667	9.97833993333333	9.43456800833333	9.68558669166667	10.2481894083333	9.914179275	9.18639844166667	9.17368278333333	8.70751021666667	8.5994674	10.1067055416667	9.20778985833333	8.87974118333333	9.313630975	9.663801325	8.67888266666667	9.34897708333333	9.18457413333333	9.65670091666667	8.90668976666667	9.09237333333333	9.58706739166667	9.17093331666667	8.91457238333333	8.889794925	9.59023981666667	9.03908018333333	9.29481465	9.48384479166667	9.69747909166667	9.230932875	9.41935561666667	9.21398845	8.72134320833333	9.03865570833333	8.6731133	8.6667612	8.90285985	8.43849715833333	8.43510350833333	8.75515696666667	8.93227916666667	9.19576385833333	9.037331875	9.35549675833333	9.25349141666667	9.309548625	8.81108796666667	8.25344699166667	9.000388675	8.70034938333333	8.96893119166667	8.72134320833333	9.62890875	9.88637849166667	8.89174859166667	9.64431238333333	9.6543889	9.88168326666667	9.38275609166667	9.82860243333333	9.72070379166667	9.824283225	9.85850786666667	9.654799725	9.97583196666667	9.51357516666667	9.512679675	9.24712485	10.0430227416667	9.25764194166667	9.05454125	9.45687460833333	10.2289590916667	10.1059188	9.32615619166667	8.84758474166667	9.52783803333333	9.41436835833333	9.96868019166667	9.61456806666667	10.0326667666667	9.063171125	9.34064308333333	9.41990620833333	9.51896663333333	9.34847368333333	9.817741375	9.41935561666667	10.0774221416667	9.94840036666667	9.80231140833333	8.98837211666667	9.230932875	9.76051965	8.80870043333333	8.62383624166667	9.12750715833333	9.026583775	9.32526180833333	8.43758005833333	9.33321418333333	8.935118175	9.04320051666667	8.93762295	9.45374055	8.4380593	8.7776576	8.78745048333333	9.39664281666667	9.10844005833333	8.7776576	9.10576088333333

METTL3	13.4160661	12.6203779375	12.3525887458333	12.4048502125	12.3743981125	12.5597336041667	12.4174556583333	12.4393159791667	12.4811040166667	12.0496677208333	12.3450542125	13.0273867416667	12.7434371916667	12.0924244416667	12.4061851583333	12.5987332583333	12.5504227458333	12.3507445583333	12.4290316625	12.5252437791667	12.4116810625	12.6153916958333	12.3808952625	12.73366375	12.6544209666667	13.4146227333333	12.6697077541667	12.6015738083333	12.3124633708333	12.5295495625	12.3858055541667	12.7990764708333	12.7518789958333	12.6353664333333	13.0942727041667	13.1704084083333	12.4484972375	12.304907275	12.2312907375	12.0919656791667	12.5308967708333	12.5417836541667	12.6593439833333	12.5450584833333	12.8018111958333	12.5693776791667	12.4427799125	12.31711795	12.396362375	12.4094365458333	12.1361380958333	12.4139081791667	12.5224211333333	12.3124633708333	12.5946792708333	12.6439553916667	12.4744167541667	12.2807012208333	12.5327662	12.4353146208333	13.0058673	12.207951175	12.5655009125	12.2498213041667	13.16578005	11.6038006333333	12.2983697708333	12.0535542458333	12.6911874583333	12.9061216416667	12.4960889541667	12.2725383833333	11.6612017291667	12.335422725	12.6907177958333	12.402219	12.6782996916667	12.4779339333333	12.5017843458333	12.963770175	13.2598865333333	13.0904272458333	12.2490087875	12.1622409666667	12.4222626958333	12.8850765208333	12.5323125291667	12.5129160833333	14.0435706291667	12.7876536791667	12.886348325	12.249421375	11.93026545	12.6993955416667	12.8793014541667	12.4838483541667	12.8648528166667	12.5495127	12.7297924625	12.8054693458333	11.9234231958333	12.5323125291667	12.0203480791667	12.2114186291667	12.7377119541667	12.6353664333333	12.2677302791667	12.8927594708333	12.1490982166667	12.1971035	11.9830365916667	11.6261305791667	12.7674094583333	12.5664944125	12.1604527333333	11.777038375	12.3303840083333	12.8265168333333	12.683947825	12.6086270791667

TOX4	13.4160661	12.6203779375	12.3525887458333	12.4048502125	12.3743981125	12.5597336041667	12.4174556583333	12.4393159791667	12.4811040166667	12.0496677208333	12.3450542125	13.0273867416667	12.7434371916667	12.0924244416667	12.4061851583333	12.5987332583333	12.5504227458333	12.3507445583333	12.4290316625	12.5252437791667	12.4116810625	12.6153916958333	12.3808952625	12.73366375	12.6544209666667	13.4146227333333	12.6697077541667	12.6015738083333	12.3124633708333	12.5295495625	12.3858055541667	12.7990764708333	12.7518789958333	12.6353664333333	13.0942727041667	13.1704084083333	12.4484972375	12.304907275	12.2312907375	12.0919656791667	12.5308967708333	12.5417836541667	12.6593439833333	12.5450584833333	12.8018111958333	12.5693776791667	12.4427799125	12.31711795	12.396362375	12.4094365458333	12.1361380958333	12.4139081791667	12.5224211333333	12.3124633708333	12.5946792708333	12.6439553916667	12.4744167541667	12.2807012208333	12.5327662	12.4353146208333	13.0058673	12.207951175	12.5655009125	12.2498213041667	13.16578005	11.6038006333333	12.2983697708333	12.0535542458333	12.6911874583333	12.9061216416667	12.4960889541667	12.2725383833333	11.6612017291667	12.335422725	12.6907177958333	12.402219	12.6782996916667	12.4779339333333	12.5017843458333	12.963770175	13.2598865333333	13.0904272458333	12.2490087875	12.1622409666667	12.4222626958333	12.8850765208333	12.5323125291667	12.5129160833333	14.0435706291667	12.7876536791667	12.886348325	12.249421375	11.93026545	12.6993955416667	12.8793014541667	12.4838483541667	12.8648528166667	12.5495127	12.7297924625	12.8054693458333	11.9234231958333	12.5323125291667	12.0203480791667	12.2114186291667	12.7377119541667	12.6353664333333	12.2677302791667	12.8927594708333	12.1490982166667	12.1971035	11.9830365916667	11.6261305791667	12.7674094583333	12.5664944125	12.1604527333333	11.777038375	12.3303840083333	12.8265168333333	12.683947825	12.6086270791667

FNBP1	14.2378149833333	12.34092395	12.2185126	11.4481946583333	11.1674196	11.675724725	11.9304447166667	12.0721549166667	11.8588339833333	12.0017085416667	11.1891169333333	13.4594952083333	13.103287325	12.3165034833333	11.9641339083333	12.5539148333333	11.020740575	11.5809197583333	12.4554143666667	12.3489029833333	12.0934360416667	11.645956625	12.229824125	11.7553657	12.0832149333333	14.4809973583333	12.623024125	11.7085670166667	11.546539525	12.0417350833333	12.3461563666667	12.2788893833333	12.341378075	12.1882257583333	14.6122307083333	14.5810673083333	11.6514721333333	12.11177165	11.8337195083333	12.1178108083333	12.0839641583333	12.2412181916667	12.1926211666667	12.552591775	13.1779047083333	12.4350665416667	12.6985834416667	12.2399849666667	12.2768075833333	11.641524325	12.008092475	12.1558313416667	12.042069275	11.6230706833333	12.2599545833333	12.856730075	12.164467625	12.1694652083333	12.0525821583333	12.5226496333333	10.5616263	10.8796402833333	11.0815307083333	11.0190136	10.7912120583333	9.7241175	10.7585831916667	10.8956711666667	10.4138270833333	11.4313741833333	10.5695644083333	9.96356175	9.78654094166667	11.5200695166667	10.1312217666667	10.5212723416667	12.2909999333333	11.6997095166667	10.7008852833333	11.1779127416667	11.5983924583333	11.4431148583333	10.6203644583333	11.1317293	10.7254659583333	10.6914536333333	11.1575927916667	11.71493805	10.1091856666667	11.9380168416667	10.86697045	10.3901677	10.5872774916667	10.8930750916667	10.079544525	10.859746575	9.90137155	10.7162804	10.191495075	11.206560475	10.9941240666667	11.19451335	10.9661834333333	10.9476724083333	10.4460825166667	10.949025	11.2880004333333	11.025429175	10.9087616083333	11.3579174083333	11.8426813333333	10.4421542833333	10.8889520833333	10.7265037833333	11.1696311416667	11.2230202916667	11.0196702	10.6823646833333	10.8067325916667	11.7883410833333

CCM2	10.8858467666667	10.384097475	10.855999875	10.7154696583333	10.0889846333333	11.0901980583333	10.0724257083333	10.173327525	10.3353402416667	10.7254659583333	10.2947654166667	9.21590233333333	10.0265057583333	10.5404499166667	10.3763105583333	10.5659717333333	10.9673192583333	9.70284449166667	10.5477462083333	10.381414675	10.5973351833333	10.0209906333333	10.7774965	10.2321384166667	10.252716075	10.461782	10.28186315	10.123786625	10.36070035	10.32163045	10.0787243333333	10.5041729666667	10.30576505	11.3235379	10.6834952083333	10.865245775	10.0493818	10.3530735416667	10.4649425166667	10.35148425	10.6391956666667	10.4822871416667	10.627804175	10.7356243	10.5631726083333	10.9972633583333	10.4671575083333	10.5933754916667	10.8836008416667	10.5119603416667	10.4581692833333	10.4004658833333	10.0740705333333	10.3228920166667	10.4671575083333	10.7313914916667	10.447247375	10.5204657833333	10.3871636916667	10.7975980416667	10.852937175	11.1536039583333	11.3801476916667	10.8945637333333	11.3419959666667	11.5064020583333	11.927850575	10.5549316666667	11.7168402	11.4467356166667	10.5735065583333	10.6788649916667	12.2231616166667	10.9219574	11.6092244333333	11.6775693083333	10.6076090916667	11.109201175	11.3579174083333	11.7016218	10.4606353416667	10.39251605	11.6444959666667	11.3823997333333	10.9256058083333	10.283076325	9.87207808333333	11.036287075	9.86640328333333	11.1328362833333	10.9263073083333	11.5128736833333	10.929974575	11.1231619583333	11.74982705	10.899244375	10.7608892083333	10.4771670916667	10.885465	10.8893434416667	12.161623975	11.1724975333333	11.2736929416667	11.2861342166667	11.9724754666667	11.6080957416667	11.541832	11.4244147	11.5291327083333	11.0373411416667	11.0060646083333	11.3140591833333	11.4590118416667	11.575819425	11.7917720333333	11.3360764166667	10.6838734833333	11.27642465	11.0087053416667	11.0934965333333

PICALM	11.5196693333333	10.7808534	10.8953436916667	10.4554224833333	10.884735425	11.069747825	10.7782435583333	11.1699387666667	10.7383339416667	11.0950066583333	10.6061332083333	11.4741238583333	10.9346168	10.806391425	10.8101684583333	10.9054446583333	10.91052195	11.0953873333333	11.0352265083333	11.0666847416667	11.1106962416667	10.9673192583333	11.178312475	11.2315981833333	11.0589538416667	11.1542967583333	10.9252447166667	10.572357075	10.6902661583333	10.7088791083333	11.1656289166667	10.7959852666667	10.5448322833333	10.7205897	11.4262590333333	11.136541175	10.6990064	10.8796402833333	11.12105475	10.9252447166667	11.248529425	11.1270209166667	10.9189114166667	11.054552625	10.9611703916667	11.0341045833333	10.819511225	11.1113391916667	11.4151310833333	10.983890675	11.3220464333333	11.296201975	10.3362368833333	10.4806526833333	10.3868161083333	10.2940071	11.4730116416667	11.0352265083333	10.99445015	11.013128925	10.418815025	10.962553725	10.5477462083333	11.0604394416667	11.0146426833333	11.6198462166667	11.0060646083333	11.1214159583333	10.939073475	11.3040384416667	11.0659389916667	11.0548999583333	11.43798885	11.3157970833333	10.960420475	10.9665691416667	10.8778360416667	10.5731419166667	10.9905726333333	10.4127726666667	11.0268698583333	10.999826875	10.5026381833333	11.2942388625	11.3011728416667	10.5995079	10.4280419583333	11.9005377083333	10.4460825166667	11.31013205	10.8344597833333	10.9405146	10.3698580416667	11.156830125	10.8224026333333	10.4542782	10.74441525	10.4914439416667	10.3455009333333	10.3119751583333	10.8176392916667	10.9958406	10.8605112583333	10.55650935	11.3771067916667	11.0337297583333	11.3360764166667	11.8290120833333	10.971110725	11.2861342166667	11.0272743166667	10.5420798333333	10.3502819916667	10.8865989333333	10.4127726666667	10.8646445083333	10.804132675	11.4478274166667	11.0466400166667	10.916432925

MACF1	5.980495875	5.36208894166667	6.01386335	6.03747638333333	5.78160560833333	6.591129625	5.94443094166667	5.05799246666667	5.60971738333333	5.88341255833333	5.78455018333333	6.64696773333333	6.622274	5.6976834	5.84513776666667	6.12691128333333	6.72249205833333	6.63942924166667	5.93923476666667	6.16277881666667	6.16362138333333	5.18885664166667	5.5526373	6.30401625	5.196416425	7.134182075	6.181735025	6.59533788333333	6.041758325	6.601356975	6.27314985	6.12452900833333	6.187374325	5.37127428333333	7.07353054166667	6.55447500833333	6.03666274166667	6.08105208333333	6.42264453333333	6.00039116666667	5.4712492	6.67633144166667	5.455892825	5.04557781666667	5.83953349166667	5.623286525	6.10074335833333	5.22716268333333	6.06833519166667	6.2176337	6.12524645833333	6.53668581666667	6.527218175	6.558117975	6.5305636	6.48645600833333	5.5651756	6.265839825	6.59386139166667	5.69483811666667	5.127309825	5.63053526666667	5.08252440833333	6.57166325	6.37033623333333	6.61351845833333	5.96077145833333	5.85146426666667	6.33415565833333	5.88673599166667	5.63144754166667	6.15447426666667	6.21030875833333	5.743144625	5.69679719166667	5.19309993333333	6.04523669166667	5.773689175	5.86087000833333	6.00285793333333	5.47990566666667	5.67044374166667	5.393785875	5.71416341666667	5.57926250833333	6.70465071666667	6.0951249	6.15447426666667	6.44887243333333	6.10074335833333	6.20496000833333	5.642111775	6.00968073333333	5.73863274166667	6.2058813	6.02209645	5.94013145833333	5.65064866666667	6.25099584166667	4.78798888333333	5.78626178333333	6.15778135833333	5.73195755	4.583908875	5.99843601666667	5.8009379	6.00285793333333	5.70153421666667	5.018425275	6.453387825	5.49632836666667	5.75774213333333	6.406487225	6.44063283333333	6.30781043333333	6.52344795833333	5.8174436	5.99418615	5.88760754166667	5.1566631

TENT4A	11.6871432333333	11.425117975	11.234518675	11.0427571416667	11.5680602833333	12.1422347916667	11.2576230916667	11.593791575	11.44422215	11.2467824083333	11.6955902833333	11.3509805666667	11.5132414833333	11.531296025	11.4745197333333	11.2775845	10.959358675	11.4188332333333	11.8036556666667	11.4154857416667	11.2474575666667	11.780675575	11.4915511416667	11.6566056666667	11.387566725	11.8713469833333	11.0866687083333	11.2373633083333	11.4786409333333	11.3567956166667	11.655146	11.2616452583333	11.3698477166667	11.1463360083333	11.468562175	11.8194805166667	11.50857355	11.1176950333333	11.6329290083333	11.2560704166667	11.5030923083333	11.6190781833333	11.206560475	11.74563025	11.8297435166667	11.6129963	11.6723181916667	11.612299875	11.6719609166667	11.5196693333333	11.152834825	11.859248825	11.687529825	11.6687463083333	11.8126730416667	11.4996324916667	11.47905225	11.471171625	11.1309543	11.6602573333333	13.2279712833333	11.81229275	11.714258625	11.8004315583333	12.0214326083333	12.3566808833333	11.96822585	12.2111963416667	12.3943629916667	11.8141737416667	11.8666609583333	11.946316525	11.6016871083333	11.5898474083333	12.3746631	11.5742275583333	12.0872990166667	11.9793031666667	11.7761557916667	11.7620768166667	12.1728373083333	11.9188907916667	10.8616146666667	12.7655438666667	12.643219075	11.9270898666667	12.0827580166667	11.2107825666667	12.00595835	11.904789825	11.8290120833333	12.1521118916667	12.6876920416667	11.927850575	12.9048119416667	11.9494775416667	12.023387575	12.959876375	12.3602095583333	13.0343175416667	12.7558773833333	12.3254389	13.2412964416667	12.1703301333333	11.913931575	12.5306631166667	12.632188	11.4137091166667	12.1839395083333	11.6525927916667	13.1926381	12.1342141916667	12.9400260666667	11.5030923083333	12.015272025	12.1865914583333	12.96407505	12.1770216166667	12.2154467166667	11.7695141

FCAR	7.92437408333333	8.1565621	8.79240648333333	8.01119698333333	7.76084795833333	9.63430166666667	9.03417474166667	8.42269018333333	8.88930546666667	10.6625963333333	8.06996655	8.75743711666667	9.12986750833333	8.33112569166667	6.558117975	8.71235535	8.026220775	9.41298314166667	8.13138714166667	8.49347309166667	7.9422621	7.01901523333333	8.50502355	10.8001996166667	7.98516725833333	10.1080216833333	8.66056879166667	8.20331464166667	7.20806453333333	8.04234110833333	8.39209266666667	9.06363208333333	8.15205105	7.84376875833333	12.9699766666667	9.03818763333333	7.50344081666667	7.46463336666667	9.439564175	8.20930705	8.64890015833333	7.30431380833333	8.87883045833333	7.40257545	8.36669614166667	7.882902775	7.32611610833333	8.94668689166667	8.30084284166667	9.30028739166667	10.0002116916667	8.661516725	8.25546045	8.94668689166667	8.06450121666667	8.23662486666667	10.16655665	7.886143875	7.6421897	9.42951640833333	7.42788356666667	8.82873195	9.7997523	9.0174923	7.7528692	10.4089152416667	9.74305075	8.551994475	10.5326225666667	10.134682025	8.55392016666667	8.39013935	9.24799294166667	9.25209231666667	7.254184275	8.62834993333333	8.05032904166667	10.5651423416667	10.2080941333333	8.49244585833333	7.73082848333333	7.98666509166667	9.15692076666667	12.4718679833333	10.1899294916667	8.72403179166667	9.65752599166667	8.57560394166667	8.81154583333333	9.61816694166667	8.71475239166667	10.9371792916667	9.35167163333333	8.88930546666667	11.5941589083333	10.7336987	8.68083718333333	8.42171803333333	9.46790315833333	8.45255091666667	9.49325940833333	8.41299383333333	9.39704936666667	8.47579976666667	9.47542340833333	8.91938296666667	8.71688334166667	8.84383605833333	9.72156395833333	10.921206425	10.48105345	9.19160371666667	8.551994475	10.7482129583333	9.74904354166667	8.37460103333333	9.92873065833333	8.97957231666667	8.298377725	10.2587469666667

NANP	7.27471595833333	7.72354014166667	7.72796763333333	6.85677963333333	6.45250163333333	6.84200789166667	6.46705624166667	7.95746913333333	7.15389984166667	7.72354014166667	7.908404275	6.39152516666667	6.56951250833333	7.88077005	8.250184775	7.10570254166667	7.22299226666667	6.93803065	7.80781901666667	7.51089621666667	7.740357875	8.50286535	7.97557605833333	8.17835735	7.46746676666667	7.78718563333333	8.02564620833333	7.75390870833333	7.14399110833333	7.71624318333333	8.36034350833333	7.988718925	7.254184275	6.69642741666667	7.87369893333333	8.07357910833333	7.37059144166667	7.82746845	8.17136015	8.1223269	8.21983948333333	7.771005975	7.94329386666667	8.39722928333333	7.62704748333333	8.23817860833333	8.50894728333333	8.17036706666667	8.17668253333333	8.07357910833333	7.99339445833333	8.09852281666667	7.57619343333333	7.603571	8.312026725	8.06285705833333	8.41926360833333	7.97192683333333	7.97397919166667	8.2549704	7.933308825	7.78110251666667	7.72520474166667	7.517539575	7.48123266666667	7.22964071666667	8.46771705	7.72796763333333	8.35319626666667	7.47951195833333	7.28500960833333	7.121598725	7.59199554166667	7.18584309166667	5.9751687	8.264078275	6.98530065833333	7.11986829166667	8.69274164166667	7.03751019166667	7.89930148333333	7.29304450833333	8.35832260833333	7.9238629	7.84106111666667	8.09998893333333	8.19396290833333	6.877100425	7.37811973333333	7.93805580833333	8.34374149166667	8.022968975	7.15262315833333	7.751162425	7.27714694166667	7.44317530833333	8.22690084166667	7.61924733333333	8.52110425	7.600828425	8.41926360833333	7.37928605	7.93918533333333	8.06606233333333	8.49188041666667	8.17406114166667	8.05569140833333	8.05717868333333	8.57112621666667	7.29304450833333	8.22799306666667	6.86262681666667	8.445416025	8.34374149166667	7.746730925	6.94820169166667	8.27339385833333	7.7682787	7.84741366666667	6.9604848

SNRPB2	12.5775136	12.307547675	12.3562743916667	12.4297307333333	13.18402475	12.9971815666667	12.315681175	12.5974434583333	12.4261316583333	12.8101998166667	12.8118807333333	12.5154422416667	12.5939827166667	12.4292757333333	12.6306873916667	12.5381389666667	12.8770846083333	12.7179890416667	12.7041276166667	12.7666041416667	12.5865453166667	12.5897782	12.83111185	12.9357780833333	12.6660656916667	12.837133975	12.4123342916667	12.350546075	12.8273007416667	12.4879782166667	12.338252925	12.3645045916667	12.32885815	12.8889747333333	12.4110018333333	12.8757636333333	12.3233097166667	12.3021061583333	12.2354776	12.3244889333333	12.4655499583333	12.2930155083333	12.3422979833333	12.57979945	12.626502475	12.5685278666667	12.6075148583333	12.7728759666667	12.39795185	12.5497662083333	12.5969475916667	12.6636318083333	12.7558773833333	12.5443972083333	12.4306290916667	12.3771618166667	12.425293625	12.8708264916667	12.4941044416667	12.728935875	14.4109315833333	13.977355925	13.506094125	13.6719295	12.856162525	13.3293632833333	13.5329794333333	13.6758447	13.6202060083333	13.5304589833333	13.33077755	13.66414075	13.9400026166667	13.3732212583333	12.96407505	13.3623548	13.6089687	13.592329325	13.2434965	12.9278503083333	13.4274952333333	13.4550317916667	12.92419445	13.420218375	13.3754869916667	12.9224811	13.5141902666667	13.2968461416667	13.4498034583333	13.0960317	13.0469983166667	13.6694092583333	12.8842279916667	12.8392807333333	13.9045177166667	13.144627125	13.9109585833333	13.2898598333333	13.7820292333333	13.4383119666667	13.2567523666667	13.400193425	13.3262903916667	12.5448126916667	13.2988538666667	12.856730075	12.9527510916667	12.750529275	13.9250365	12.9433935166667	12.744717925	12.7846359833333	13.3377428083333	13.1262418916667	13.1071716666667	12.7955349083333	13.24746105	13.01427815	13.1256286916667	12.940616175

TRIM55	7.87659344166667	9.17867269166667	9.116355225	9.53997905833333	9.63613316666667	7.7682787	10.1509476166667	9.040322975	9.66781955833333	9.05030123333333	9.10844005833333	8.930278925	9.379662375	8.91457238333333	8.70448370833333	8.69082025	8.3715767	9.047926775	8.44287714166667	8.71039916666667	8.63825258333333	8.60126088333333	8.42476003333333	8.4178821	8.40122860833333	8.01273043333333	9.120869625	8.93227916666667	9.354988125	9.20373038333333	9.14518724166667	8.958107775	8.99943493333333	8.66397386666667	7.62479258333333	7.630419825	9.53312831666667	8.82873195	8.63031905833333	8.97769849166667	8.986594625	9.33838469166667	8.884079375	8.63681648333333	8.21782325833333	8.52731504166667	8.47003455	8.75278535833333	8.60227746666667	8.328023075	9.296236625	8.87543329166667	8.50130601666667	8.525325	9.124627175	8.3048096	8.95121126666667	8.93227916666667	8.548453575	8.27121185	8.448844675	8.52583200833333	7.99725846666667	8.49794376666667	8.17613399166667	8.36034350833333	8.09998893333333	8.51578285	8.36515990833333	8.55804185	8.50984575833333	8.70751021666667	8.90478388333333	8.06854219166667	8.52279583333333	8.14709130833333	8.46826041666667	8.05514178333333	7.920590025	7.927602375	8.13819046666667	8.28678805833333	8.19808664166667	7.6421897	8.215089275	8.35786781666667	8.16484069166667	8.511336025	8.85110639166667	7.54282731666667	8.291997825	7.57513376666667	7.79031855833333	7.7113432	8.230344325	8.26701150833333	8.00199818333333	7.75176951666667	8.4841298	7.76990645	8.04330091666667	9.00189508333333	7.95406095	7.4731488	7.87098755833333	8.13138714166667	7.708818875	7.598264375	7.83726095833333	7.32376110833333	7.907346175	7.98973426666667	7.2401722	7.38051639166667	8.00412230833333	7.77893200833333	7.50463459166667	7.72573829166667	7.72354014166667	8.23662486666667

CENPI	8.26308525833333	8.96273033333333	9.17280985	8.67688486666667	8.75047631666667	9.25028671666667	8.33913670833333	8.52110425	8.54169179166667	9.34897708333333	9.442472475	7.79469890833333	8.894577375	8.81154583333333	9.06600838333333	8.927052675	7.96073305833333	9.0174923	9.26948546666667	8.99204639166667	8.83115135	9.34302553333333	9.471100975	8.80499509166667	8.54169179166667	7.788158325	8.57400345	8.615263775	8.74034393333333	8.81515405833333	8.47003455	8.72403179166667	7.754380925	8.11504903333333	7.63877955	7.72905521666667	8.92651730833333	8.43849715833333	7.51638066666667	8.20281158333333	8.18422995833333	8.18151525833333	8.84243295833333	9.71951955833333	8.83016251666667	8.82920276666667	9.24401314166667	9.18639844166667	9.46432354166667	8.40424725	8.33507211666667	8.91181045	9.357237425	9.61553475	8.95490616666667	9.04085205833333	8.39466414166667	9.173253675	9.00867418333333	9.08960063333333	11.7012064	11.14521145	12.0404998666667	10.620734875	9.17646485833333	10.43899855	10.9969001083333	10.5530348	11.4590118416667	10.7696102583333	10.46219945	10.239159725	10.9501588	9.36621750833333	11.2327259583333	11.24459925	10.1328782916667	11.1912222083333	11.3571575333333	11.5887304416667	9.33411035833333	9.73658934166667	10.0347123333333	10.1397194333333	9.591875575	11.1824657666667	11.7864658083333	10.5244636333333	10.8401100166667	9.85161335833333	10.988072975	11.2341636166667	10.5123801583333	9.68160001666667	11.1291467583333	10.826565775	9.67592103333333	10.91013625	10.63689965	9.73142488333333	10.5022046916667	10.289694575	10.864263475	11.0337297583333	10.478998225	9.82295156666667	10.4958848833333	10.88109835	9.60629726666667	10.5112057333333	9.487900075	11.119251825	10.9013899916667	11.14669605	10.0421767666667	10.5162662666667	9.83982443333333	11.4810093416667	11.0182236333333	10.9032780416667

GATAD2B	6.60894191666667	7.05278270833333	6.880500475	7.1674037	6.82889531666667	6.94312051666667	7.7113432	8.96893119166667	7.6225677	6.93352621666667	7.03751019166667	7.33026348333333	7.14519241666667	6.7919706	6.67377979166667	6.59176974166667	7.173894	7.826430025	6.27955291666667	6.66118995	7.42326895833333	7.21162745833333	7.01264725833333	6.92501696666667	5.47221455833333	6.94376375833333	6.22080705	6.64415971666667	6.777132775	6.88940976666667	6.15183188333333	6.18822558333333	7.02222090833333	7.11986829166667	6.89528771666667	6.49896905833333	6.95435513333333	6.423307775	6.40124803333333	5.93923476666667	6.10002881666667	6.50275845	6.22675393333333	5.73091026666667	6.48785131666667	6.717950125	6.22752675833333	6.84636305	6.64016859166667	6.465677275	6.43219438333333	6.16609928333333	6.68200588333333	6.57014316666667	6.346676525	6.34005863333333	6.61351845833333	6.41808758333333	5.94013145833333	5.8893734	6.69439601666667	6.737597225	6.22149245833333	6.90781049166667	6.49170240833333	7.67769499166667	7.35173706666667	8.85547214166667	6.46419815833333	7.18452301666667	7.04142870833333	6.50650844166667	7.07353054166667	6.68656881666667	7.41824386666667	6.82644443333333	6.85814304166667	6.62367098333333	6.653546475	7.21926184166667	7.89296785	7.71472995833333	6.83207999166667	6.7730469	6.0951249	7.198288025	7.458458425	7.22964071666667	6.96657695833333	6.4413796	7.06942048333333	6.423307775	6.75646765833333	6.66944055833333	7.0659289	6.4034157	6.65984261666667	6.98158215833333	7.16124751666667	6.01932009166667	6.40053460833333	7.630419825	6.95435513333333	6.56579801666667	6.48284681666667	7.28500960833333	6.19282915833333	6.17390685	6.08697085833333	6.285084375	5.279548575	5.95153166666667	6.423307775	6.59052684166667	6.57952525	6.63512606666667	5.8902603	6.72764310833333	6.39606678333333	7.34272655

APBA3	6.71335903333333	7.21066622083333	6.8317548	7.78354614583333	6.49569240416667	7.67743358333333	7.92575797916667	6.13631401666667	6.80278099583333	7.3662055125	7.2491682	6.5230871875	6.02687620833333	7.20183691666667	6.79959005833333	6.398820675	7.12252534166667	6.8875491	7.160984825	6.14766029583333	7.09196993333333	6.78279817916667	6.32564148333333	6.83242774583333	7.14918642083333	6.97680094583333	6.4838841	6.83963736666667	6.9824789625	6.91421479583333	7.0291349625	7.00661714166667	6.9787197625	7.44910814166667	6.41916215	6.34938589166667	7.22506628333333	6.7943611375	7.04035565416667	6.65190179166667	7.06679802916667	6.71957255	7.1466806625	6.65807788333333	6.98063093333333	6.6889002875	7.01800680416667	6.76685945	7.399511475	6.38811415833333	7.17417945	6.58067866666667	6.09068297083333	6.94217911666667	6.57919882083333	7.15234316666667	5.811662475	6.81152060416667	6.6545598875	6.43118097083333	6.88972354166667	7.652521625	7.03353314583333	7.11179178333333	7.2509352	7.8342670875	6.702927325	6.31989966666667	6.48533292916667	7.583214	7.17652978333333	6.70566189166667	7.0926229875	6.50100961666667	7.416181175	6.4154077375	6.7279495625	6.3345354	5.87154659166667	6.13218769583333	6.14455089583333	6.44323145	6.90220145416667	6.97433015833333	6.72978257083333	7.05742416666667	6.3191654375	7.18129277916667	6.7615116625	6.31833555	6.39417786666667	6.82416634583333	7.0680368375	6.64591594583333	6.55907252916667	6.38124974166667	6.93651231666667	6.6614706	7.330004325	7.33943287916667	6.75613726666667	6.75542331666667	6.9812717	6.71273871666667	6.90556001666667	5.56921560416667	7.15234316666667	7.4821578375	6.32211077916667	6.7539972125	6.50685744166667	6.87679052083333	7.1280761625	6.42595409583333	6.53699182083333	6.9693848625	6.3873727625	6.950589425	6.87248340833333	5.87634246666667

TJP3	6.71335903333333	7.21066622083333	6.8317548	7.78354614583333	6.49569240416667	7.67743358333333	7.92575797916667	6.13631401666667	6.80278099583333	7.3662055125	7.2491682	6.5230871875	6.02687620833333	7.20183691666667	6.79959005833333	6.398820675	7.12252534166667	6.8875491	7.160984825	6.14766029583333	7.09196993333333	6.78279817916667	6.32564148333333	6.83242774583333	7.14918642083333	6.97680094583333	6.4838841	6.83963736666667	6.9824789625	6.91421479583333	7.0291349625	7.00661714166667	6.9787197625	7.44910814166667	6.41916215	6.34938589166667	7.22506628333333	6.7943611375	7.04035565416667	6.65190179166667	7.06679802916667	6.71957255	7.1466806625	6.65807788333333	6.98063093333333	6.6889002875	7.01800680416667	6.76685945	7.399511475	6.38811415833333	7.17417945	6.58067866666667	6.09068297083333	6.94217911666667	6.57919882083333	7.15234316666667	5.811662475	6.81152060416667	6.6545598875	6.43118097083333	6.88972354166667	7.652521625	7.03353314583333	7.11179178333333	7.2509352	7.8342670875	6.702927325	6.31989966666667	6.48533292916667	7.583214	7.17652978333333	6.70566189166667	7.0926229875	6.50100961666667	7.416181175	6.4154077375	6.7279495625	6.3345354	5.87154659166667	6.13218769583333	6.14455089583333	6.44323145	6.90220145416667	6.97433015833333	6.72978257083333	7.05742416666667	6.3191654375	7.18129277916667	6.7615116625	6.31833555	6.39417786666667	6.82416634583333	7.0680368375	6.64591594583333	6.55907252916667	6.38124974166667	6.93651231666667	6.6614706	7.330004325	7.33943287916667	6.75613726666667	6.75542331666667	6.9812717	6.71273871666667	6.90556001666667	5.56921560416667	7.15234316666667	7.4821578375	6.32211077916667	6.7539972125	6.50685744166667	6.87679052083333	7.1280761625	6.42595409583333	6.53699182083333	6.9693848625	6.3873727625	6.950589425	6.87248340833333	5.87634246666667

MCM3AP	9.81483478333333	9.38896178333333	9.57903839166667	9.661950625	9.96443173333333	9.83111589166667	9.40032650833333	9.62234490833333	9.71442065	9.55860505833333	10.3587508333333	9.71657748333333	9.39210768333333	9.42859468333333	9.77930279166667	9.584765625	9.49101396666667	9.81899704166667	9.71227291666667	9.74175274166667	9.40597950833333	9.67274871666667	9.78781813333333	9.73270225833333	9.750361275	9.76136145	9.557193025	9.695341625	9.6433763	9.732285275	9.51175975	9.87303573333333	9.58344561666667	9.24295846666667	9.230932875	9.91241780833333	9.7928167	9.58573579166667	9.5040294	9.22295939166667	9.69924190833333	9.71098871666667	9.87439875	9.76350663333333	9.52826018333333	9.83900869166667	9.86678629166667	10.2060439416667	9.41531880833333	9.53398021666667	9.6811178	9.93427139166667	10.0587286583333	9.70017130833333	9.60896382083333	9.56634321666667	10.0056583	9.487900075	9.96356175	9.6592899	10.6143770083333	9.777390875	10.4460825166667	10.217472825	10.9958406	10.154948775	10.3667222333333	9.56634321666667	10.7612989333333	9.95398405833333	10.2318091916667	10.259584625	9.18306429166667	9.39478965833333	9.04644768333333	10.4561678833333	9.55812764166667	10.0644501166667	10.4554224833333	10.120107375	10.9972633583333	11.2395267333333	10.2466873083333	10.1485742083333	9.46790315833333	11.2771872833333	11.5809197583333	9.55860505833333	11.5945056583333	9.487900075	9.78567400833333	10.027285575	10.3852198833333	9.91032726666667	10.3442980083333	10.3610713833333	10.15852225	11.0264670833333	9.99216454166667	9.45149038333333	10.1895353083333	9.84956451666667	10.3369395416667	10.5892734666667	10.528057425	9.98225808333333	9.98225808333333	10.1824837166667	9.97833993333333	9.431383225	8.94049991666667	9.759228525	10.506944125	10.79558335	9.73831540833333	10.5085269083333	10.872956825	10.8254925166667	10.8097832416667	9.77930279166667

RPL13	13.741448775	13.5851912666667	13.6979142666667	13.812858	13.2678167166667	13.4935932	13.8337930083333	13.3067028166667	13.48163315	13.2412964416667	13.23467165	13.64886915	13.3501502333333	12.6795486	13.65238595	13.62548145	13.4611203333333	13.174009975	13.627458175	13.4966084666667	13.6174285083333	13.533857925	13.065712375	13.6443060416667	13.4991752166667	13.6356329916667	13.14330865	13.6246233916667	13.1427455666667	13.2068344333333	13.784925425	13.40344555	13.8376377666667	13.8516367666667	13.7244874416667	13.653352825	13.5843144666667	13.3383656916667	13.5329794333333	13.3437233083333	13.0613877916667	13.8698539083333	13.6587931916667	13.89301535	14.034006175	13.6302168166667	13.86774345	13.6028841416667	13.6302168166667	13.913193025	13.7670768833333	13.8688792083333	13.5484254	13.1926381	13.5355941333333	13.817872525	13.20077725	13.6320213916667	13.4113073583333	13.3928492083333	12.1084032166667	13.171325425	12.9960374833333	12.5979621916667	13.3860625083333	13.2279712833333	13.047619075	13.1641370583333	13.3185547	12.7406037583333	12.8020915333333	13.5494174416667	13.5255367583333	12.6939319583333	13.2835400666667	13.0786865916667	12.5140767416667	12.7170209166667	13.580759625	13.001932175	13.423764125	13.4042461583333	13.1118234	12.799384025	12.6391509	13.0439770416667	13.89505895	12.600413	12.8278480083333	12.4995800083333	12.8713301666667	14.2233219083333	12.6730092833333	13.5843144666667	12.7416307	12.30344225	13.5593280666667	13.1946165416667	13.3538490666667	12.23627775	13.1152178	13.3179373333333	12.8924470083333	12.9859501166667	12.9733229333333	13.001932175	13.2842694	13.482478725	13.7149280416667	12.799384025	12.7509478416667	13.1674768	12.7586192666667	13.5851912666667	13.147286425	13.2480671333333	13.1124240666667	12.7728759666667	13.2785213333333	12.7602549166667

ARHGAP21	10.4866818833333	9.79891768333333	9.743497625	9.730552775	10.2716281	10.27572155	10.2670402	9.69038334166667	10.1899294916667	9.89383843333333	9.84760208333333	10.4630196333333	10.7096961916667	9.41070553333333	9.908637725	9.186916425	10.7940186083333	10.7265037833333	9.952821075	9.23047039166667	9.38807046666667	10.2111469	9.09460495	10.1477211833333	9.77176594166667	9.79016168333333	9.9399775	10.064052975	10.328553475	9.79933185833333	9.732285275	9.48384479166667	9.99343915	10.11881175	9.60919951666667	9.98150248333333	10.4806526833333	9.68602584166667	10.159727225	9.78059948333333	9.07536643333333	9.21350573333333	9.04841031666667	8.682286675	9.1473568	8.82022893333333	9.02512653333333	8.84563956666667	9.33321418333333	9.6185904	9.42424818333333	9.81176585	10.2355191666667	10.0778458	9.41668023333333	9.12557628333333	9.58797373333333	9.19020434166667	9.68160001666667	9.12557628333333	11.181831125	10.323288425	10.6652778583333	10.6554394166667	9.97752425	10.980233275	9.76350663333333	10.4728823833333	10.337717825	10.3656176	10.2166211083333	10.6971968666667	10.7201690666667	10.3277465	10.6007887583333	10.02019625	10.8025054583333	10.8056325083333	10.2458406333333	9.99808105833333	10.0881734833333	10.6047191916667	10.2014148333333	10.216230425	10.6422709916667	10.2153563166667	10.50924085	10.0898576	10.5806393916667	10.261767125	10.2707893833333	11.3108007583333	11.158996075	10.2940071	10.7782435583333	10.3745792583333	10.8638767916667	10.7576799666667	10.8056325083333	9.84075720833333	10.1845142083333	9.985532675	10.0753226583333	9.21303188333333	10.035165975	10.6038899083333	10.4264895833333	9.43324608333333	9.84541846666667	9.75747410833333	10.04588255	9.87116769166667	10.839776075	9.69038334166667	9.81818709166667	10.0018247916667	10.1432492	10.4641916916667	10.079544525	10.3610713833333

FEZF1	6.60270946666667	5.54303723333333	5.927679125	4.9195424	6.05717670833333	6.86073455	5.97260084166667	5.328555675	6.00716590833333	6.00206103333333	6.88940976666667	5.57252066666667	5.47616439166667	5.58212393333333	5.53996425	5.44322508333333	7.67088145	5.94358583333333	6.18980128333333	6.07768216666667	5.54579601666667	6.230051875	5.92509355833333	6.71730138333333	5.65732808333333	5.59377575	5.6322886	7.127807925	6.07339345833333	5.5022085	6.59464205	5.64797583333333	5.755893075	7.23665485833333	6.65843384166667	6.25991309166667	6.78246663333333	6.72183800833333	6.380135525	5.71315170833333	5.75112915833333	5.70355690833333	5.74037505	5.546831425	5.75852464166667	5.72382095833333	5.51098925	5.57060381666667	6.5898034	6.28975704166667	5.6411096	6.65552751666667	5.35692600833333	6.4502938	5.00344013333333	5.90588820833333	5.96342883333333	6.21191129166667	5.67044374166667	5.84699576666667	7.72195235	5.84412998333333	7.44667759166667	6.03147445	6.08509065	6.30401625	6.48785131666667	7.74511231666667	6.62496390833333	5.90755965	7.10888616666667	7.470826175	5.99843601666667	6.37415488333333	6.04082925833333	6.180201325	6.399909175	5.63834938333333	5.37127428333333	8.39370431666667	6.1905363	7.4250003	5.7550021	6.65690114166667	6.423307775	6.792709475	6.41504531666667	7.59020169166667	6.61351845833333	6.2058813	6.22001866666667	6.08790970833333	7.23488161666667	6.259187975	6.43152844166667	6.46219335	7.75064413333333	5.792834125	6.43641949166667	6.73065755833333	5.7955502	6.99601435833333	5.948900025	7.801197125	7.72085915833333	5.126264825	5.949734275	7.811926125	6.7130388	7.94435703333333	6.43359675	8.26257539166667	6.734891125	5.59479534166667	5.29779213333333	6.83207999166667	7.16072213333333	5.88241164166667	6.946211725	5.74803075

DEDD2	11.3583216333333	11.4221599083333	11.739559375	11.9204648416667	11.4545376666667	10.9073626	11.3542758333333	11.213594675	11.61191255	11.092079925	11.28297055	10.667166075	11.3896934	11.407195875	11.2950935333333	11.291894675	11.5363521916667	11.6209481083333	11.62870925	11.0913458083333	11.1532234833333	11.089509125	11.6914012333333	11.3501118625	11.0747492583333	11.0841148833333	11.39940385	11.3406807666667	11.4052792333333	11.637472	11.9240142833333	11.5136403666667	11.270439275	11.4413379833333	11.0290294333333	10.7375745583333	11.1650865916667	11.1284533666667	11.6444959666667	10.8885586333333	11.659892375	11.8341069333333	11.50454985	11.0294181583333	10.98655615	11.6429698666667	11.3965275083333	11.30507995	11.4654444	11.2035969416667	12.31352485	11.5998809	11.2740276	11.1749885333333	11.1325239666667	11.5614234666667	10.949025	11.4763556583333	11.0538325833333	10.8555951166667	11.4225410666667	12.36147625	12.0685666833333	11.4200132333333	11.1971318416667	11.825372775	11.2899480916667	11.9486907833333	11.201526325	10.9084288583333	11.5920332333333	11.8924112666667	11.592340975	12.2844611166667	10.4053126416667	10.8097832416667	11.14949005	11.3470919	12.0305597416667	11.73798855	10.8506520083333	10.952408425	11.7772182666667	11.55825445	11.941704975	10.1557652416667	11.2334234166667	11.4090056666667	11.5920332333333	11.0873559916667	12.0084826666667	11.6890849333333	11.41004175	11.0483413166667	11.3187261333333	12.5506564333333	10.666810425	10.8666003833333	12.6278527916667	11.86547315	11.1383591416667	11.7425826083333	11.4619760666667	10.7804261416667	12.4114353583333	11.2696990833333	11.3842695916667	12.1063614833333	11.7951768416667	11.3096843916667	11.830137	12.0305597416667	11.4489345583333	10.7789681833333	11.7425826083333	11.4872383	10.86697045	11.4597359083333	10.8646445083333	10.6139707

ZDHHC11B	13.82498885	11.5501880833333	12.149315325	12.206579925	12.5818258666667	13.6969617	12.1652756583333	11.7243383416667	12.0901653083333	10.563601	13.1019491416667	13.312913975	11.778723	11.8232517	13.1538383166667	11.5174454333333	11.5215953666667	12.400136275	11.81229275	11.98956295	12.4447647416667	12.3899971916667	11.7074261416667	13.1103883166667	11.9542491166667	12.49723105	11.5676618166667	11.9935609583333	13.0804212833333	12.1703301333333	13.101295875	12.2057469416667	11.7097839833333	11.0863287333333	13.40102065	12.50892495	11.7012064	11.3778682416667	13.4375531583333	11.56524335	11.465822775	11.27496335	11.1067195416667	12.1224925833333	11.6080957416667	10.9360928583333	10.9393869333333	11.8954895166667	12.1229210916667	13.0847965333333	13.016186275	12.2727355333333	11.9563023166667	12.8015308583333	10.8721591833333	11.4364638833333	12.8543756916667	11.5071025416667	11.105357075	10.9270024916667	12.8057256416667	11.8646362416667	11.9455284	10.740315675	11.03446615	12.600413	11.102867725	12.4416866083333	10.8443256416667	10.6930678666667	12.2553940416667	11.8865138166667	10.8850875833333	11.4288195833333	12.3126780333333	9.95891725	11.7594075916667	12.3284449583333	9.606709075	11.0211329583333	11.1539711166667	12.7790607166667	9.84075720833333	11.3040384416667	12.9248123333333	11.0913458083333	11.2975735583333	11.0013930666667	13.5660586666667	11.407940525	10.8865989333333	11.1630098	12.7543063	10.414161375	11.3846584083333	12.209029825	10.859746575	10.6263315083333	11.1611143583333	10.97439825	10.4638383	12.089741475	12.6991286666667	13.066353125	10.2749442833333	9.94339973333333	10.372950875	12.1224925833333	10.5173180916667	11.166670375	11.1188874333333	11.2517728166667	10.3549808416667	10.34989835	9.86359230833333	11.5154734	9.68075670833333	10.3706198583333	11.1110336416667	12.5497662083333

CCDC25	10.6570447583333	10.240361675	10.2076634916667	10.3674828833333	9.59065653333333	9.661547425	9.915416425	10.0069849166667	10.0321890166667	10.0627631	10.3578687916667	10.1472782833333	9.78918780833333	10.1936096583333	10.05531845	9.97833993333333	11.0733527583333	9.81525473333333	10.3583509666667	10.2940071	10.100531725	10.1783585083333	10.176297425	9.98032226666667	10.237546325	10.9783451666667	10.2695195083333	10.3945367666667	10.227074775	10.4115783666667	10.322000975	10.2962241416667	9.831945975	10.4224382166667	10.96150755	10.9549623083333	9.94034049166667	10.1424095333333	9.94339973333333	10.0965198666667	10.5490285	10.2005317833333	10.3203234833333	10.3790883333333	10.0688438166667	10.5108422833333	10.3502819916667	10.2245747416667	10.3105309666667	10.1787390666667	9.77571775833333	9.985532675	9.75279813333333	9.81985159166667	10.1775011166667	10.298281975	9.7288377	10.2699462416667	9.900175875	10.0774221416667	9.22295939166667	10.5699475166667	9.68695163333333	9.75492564166667	10.0276764666667	9.836699675	9.996827975	10.3317121166667	9.22470684166667	9.721994775	9.7515126	9.05868610833333	10.2399871166667	10.1755237	10.4902258833333	9.43045813333333	9.79446515833333	9.48609106666667	10.5523212166667	9.55812764166667	10.2060439416667	8.9384783	9.58040200833333	9.487900075	9.96565705833333	9.899420875	11.336792675	9.9581283	9.66742095833333	10.6237069166667	11.3918988666667	9.57179898333333	9.19244325833333	9.776982075	8.59743456666667	9.637594125	10.9350022416667	9.31001445	8.83249931666667	10.3783237666667	9.62407518333333	10.16655665	9.78382319166667	10.364544	10.5360893083333	9.96018530833333	9.734421025	10.6391956666667	10.1389730083333	9.607505	8.9226878	10.0031143833333	8.7532663	10.2563791083333	10.05998855	9.1237399	10.1787390666667	9.56080151666667	10.0498058583333	9.23235029166667

TIAF1	10.0694925	11.3688766083333	11.3562386958333	11.3237360666667	10.6451527541667	9.76843350833333	10.5402613291667	10.92756005	10.6148944333333	10.6439982125	10.4042775708333	9.55646399166667	10.284029425	11.2570280791667	11.4896097916667	10.6246790291667	10.211772325	11.0081120875	11.5834099208333	11.0959616083333	10.8531587375	11.5900264208333	11.1149160833333	10.8118916416667	10.8260181208333	10.3525069708333	11.2306483375	11.1197786583333	11.4032411791667	11.3284092666667	11.2835686375	11.1424882875	10.6435959875	10.0742512625	10.3481313291667	10.0216481541667	11.3027593833333	11.5047206958333	11.0032870208333	10.76488945	11.1026962666667	11.5406145041667	11.3380056708333	11.3006586041667	10.4091183333333	11.59500295	11.5814876333333	11.8117753583333	11.0485496166667	10.7346116583333	10.8994408458333	11.3358945291667	11.4355900791667	11.6498209666667	11.4216077666667	11.6399543958333	11.1624773791667	11.4279135125	11.2274614541667	10.9133984666667	9.28246405833333	10.1951107083333	9.58017514166667	9.76492312916667	8.97377334583333	10.5398691541667	10.1280952583333	9.59443869166667	10.1851497333333	9.74798653333333	9.4356869125	9.74840287083333	10.7025997416667	10.0482991833333	9.98449059166667	10.3434123458333	10.1785487875	10.03200785	11.0509045708333	9.65419311666667	9.21518684583333	10.1434765916667	10.8196986208333	10.3548031791667	10.3650967875	9.0999430375	10.5563215666667	10.582768125	9.30933558333333	10.4673265333333	10.2520941875	10.0776339708333	9.92073509583333	10.582768125	10.7289820166667	10.3434123458333	9.84352759166667	10.3977950666667	10.539465	10.1522911708333	10.5000138	9.9646236375	10.3173191083333	10.499651875	10.7984847875	10.1951107083333	11.1296995458333	11.0478556583333	10.0987165375	10.4159178666667	10.1693994875	10.5415137166667	11.0456651875	11.0019008875	10.9774549166667	10.8735190708333	9.23951692916667	10.6338491291667	10.4382980166667	10.5113945291667

MYO18A	10.0694925	11.3688766083333	11.3562386958333	11.3237360666667	10.6451527541667	9.76843350833333	10.5402613291667	10.92756005	10.6148944333333	10.6439982125	10.4042775708333	9.55646399166667	10.284029425	11.2570280791667	11.4896097916667	10.6246790291667	10.211772325	11.0081120875	11.5834099208333	11.0959616083333	10.8531587375	11.5900264208333	11.1149160833333	10.8118916416667	10.8260181208333	10.3525069708333	11.2306483375	11.1197786583333	11.4032411791667	11.3284092666667	11.2835686375	11.1424882875	10.6435959875	10.0742512625	10.3481313291667	10.0216481541667	11.3027593833333	11.5047206958333	11.0032870208333	10.76488945	11.1026962666667	11.5406145041667	11.3380056708333	11.3006586041667	10.4091183333333	11.59500295	11.5814876333333	11.8117753583333	11.0485496166667	10.7346116583333	10.8994408458333	11.3358945291667	11.4355900791667	11.6498209666667	11.4216077666667	11.6399543958333	11.1624773791667	11.4279135125	11.2274614541667	10.9133984666667	9.28246405833333	10.1951107083333	9.58017514166667	9.76492312916667	8.97377334583333	10.5398691541667	10.1280952583333	9.59443869166667	10.1851497333333	9.74798653333333	9.4356869125	9.74840287083333	10.7025997416667	10.0482991833333	9.98449059166667	10.3434123458333	10.1785487875	10.03200785	11.0509045708333	9.65419311666667	9.21518684583333	10.1434765916667	10.8196986208333	10.3548031791667	10.3650967875	9.0999430375	10.5563215666667	10.582768125	9.30933558333333	10.4673265333333	10.2520941875	10.0776339708333	9.92073509583333	10.582768125	10.7289820166667	10.3434123458333	9.84352759166667	10.3977950666667	10.539465	10.1522911708333	10.5000138	9.9646236375	10.3173191083333	10.499651875	10.7984847875	10.1951107083333	11.1296995458333	11.0478556583333	10.0987165375	10.4159178666667	10.1693994875	10.5415137166667	11.0456651875	11.0019008875	10.9774549166667	10.8735190708333	9.23951692916667	10.6338491291667	10.4382980166667	10.5113945291667

MROH7	9.55472915	6.61558093333333	6.20262720833333	6.255916275	6.894639675	7.50051165833333	6.37713004166667	6.71243863333333	6.22368095	5.84513776666667	6.87089175833333	7.61760009166667	7.2027431	6.2058813	6.38240346666667	6.77975395833333	7.59600343333333	7.30913270833333	7.567156725	5.766309475	6.11631895833333	7.86377196666667	6.53205264166667	7.619792375	7.19951121666667	9.261730375	7.51524328333333	6.48645600833333	8.04704694166667	6.63442134166667	6.93484450833333	6.50650844166667	6.51310794166667	6.22675393333333	8.83592169166667	9.168290925	6.41191374166667	6.77436175	6.37777528333333	6.74158396666667	6.72764310833333	7.06942048333333	6.49896905833333	6.69642741666667	6.811853775	6.78180254166667	6.68342536666667	5.41887256666667	5.84791786666667	7.11617649166667	6.3064348	7.27171180833333	6.27563768333333	6.732067125	5.51803240833333	6.88648389166667	6.527218175	6.26211881666667	6.955027575	6.341623	7.33740245	5.79838183333333	5.51386359166667	5.06570035833333	5.81313270833333	7.20859641666667	6.04799355833333	6.31394556666667	6.42033848333333	6.45093723333333	6.86073455	5.63053526666667	6.24858409166667	5.99091528333333	5.24195544166667	6.65024493333333	7.09680668333333	6.09925825	5.27529641666667	5.86087000833333	7.16232548333333	6.61221461666667	7.698105675	5.582921425	6.55447500833333	7.51144511666667	7.765634325	5.31119278333333	5.67998533333333	7.19767501666667	5.45803265	6.45250163333333	6.54994171666667	6.47574773333333	6.36951041666667	6.66738975833333	5.49526165833333	6.72055851666667	6.075096075	7.20693700833333	6.05561763333333	5.68207400833333	6.692469175	6.85216409166667	6.54089473333333	6.60676565	5.49229008333333	5.74909436666667	5.57926250833333	6.66866908333333	7.70977168333333	6.32106796666667	6.55593524166667	5.956517125	5.84236155833333	6.320274625	8.77966386666667	5.94535455833333	6.66317971666667	5.22492120833333

ALDH6A1	13.9474854708333	13.2416404583333	12.9227561958333	13.3427108583333	12.8211392791667	13.1239177583333	12.9125891625	13.0772692958333	13.0081306708333	12.8029136333333	12.9753263791667	12.9776963958333	12.5786739708333	13.1308328041667	13.0285357166667	13.265248825	14.8536200708333	13.0758809791667	13.1664645375	12.9567449625	12.9885589666667	13.4333817625	13.1768570625	13.0826485583333	13.22308885	13.5690106083333	12.9303715625	12.7572069958333	12.7331506958333	13.0623209	13.1127745958333	12.8653568166667	13.0850749375	14.2835602083333	13.4787324458333	13.5556246458333	12.5900002416667	12.7870957291667	12.5378856125	13.0587985375	12.5587755625	12.9644262875	13.1016225083333	13.0527420125	13.3484606666667	12.610061775	12.9227561958333	12.2568044833333	12.9821300791667	13.3900896125	12.7497447333333	12.2498213041667	12.2798987791667	12.9724412416667	12.5308967708333	13.0279498208333	12.735661975	12.6424634291667	12.9320596125	12.6043782375	12.4657649958333	11.4747097375	13.3656924875	12.0883050041667	13.8268219583333	12.417911025	11.6520352291667	12.4539039291667	12.9309304541667	12.8693564958333	12.117626725	11.3784236458333	11.9519159166667	12.1352700625	12.7726121208333	11.868335125	12.8598411208333	12.3316466083333	11.8156423916667	13.2832111125	13.4684930958333	13.1259352916667	11.81360145	11.6531202375	11.8405100916667	12.6559656416667	12.5261734041667	11.5700714791667	13.0158552541667	12.5703631583333	12.1614429291667	12.0591104041667	12.64891165	11.7715220416667	12.1036399791667	11.8548017208333	13.5064483208333	12.1065423666667	11.1943121333333	11.661574925	12.3069427333333	12.3367702625	11.6338929541667	12.9039557375	11.7121906291667	12.2605422125	11.4830468791667	12.3890185041667	12.330802425	12.2498213041667	12.3367702625	12.24773505	10.8656148166667	13.5367997083333	11.6950002791667	12.308609075	12.7528809125	12.4703658	12.839025025	12.7751098333333

BBOF1	13.9474854708333	13.2416404583333	12.9227561958333	13.3427108583333	12.8211392791667	13.1239177583333	12.9125891625	13.0772692958333	13.0081306708333	12.8029136333333	12.9753263791667	12.9776963958333	12.5786739708333	13.1308328041667	13.0285357166667	13.265248825	14.8536200708333	13.0758809791667	13.1664645375	12.9567449625	12.9885589666667	13.4333817625	13.1768570625	13.0826485583333	13.22308885	13.5690106083333	12.9303715625	12.7572069958333	12.7331506958333	13.0623209	13.1127745958333	12.8653568166667	13.0850749375	14.2835602083333	13.4787324458333	13.5556246458333	12.5900002416667	12.7870957291667	12.5378856125	13.0587985375	12.5587755625	12.9644262875	13.1016225083333	13.0527420125	13.3484606666667	12.610061775	12.9227561958333	12.2568044833333	12.9821300791667	13.3900896125	12.7497447333333	12.2498213041667	12.2798987791667	12.9724412416667	12.5308967708333	13.0279498208333	12.735661975	12.6424634291667	12.9320596125	12.6043782375	12.4657649958333	11.4747097375	13.3656924875	12.0883050041667	13.8268219583333	12.417911025	11.6520352291667	12.4539039291667	12.9309304541667	12.8693564958333	12.117626725	11.3784236458333	11.9519159166667	12.1352700625	12.7726121208333	11.868335125	12.8598411208333	12.3316466083333	11.8156423916667	13.2832111125	13.4684930958333	13.1259352916667	11.81360145	11.6531202375	11.8405100916667	12.6559656416667	12.5261734041667	11.5700714791667	13.0158552541667	12.5703631583333	12.1614429291667	12.0591104041667	12.64891165	11.7715220416667	12.1036399791667	11.8548017208333	13.5064483208333	12.1065423666667	11.1943121333333	11.661574925	12.3069427333333	12.3367702625	11.6338929541667	12.9039557375	11.7121906291667	12.2605422125	11.4830468791667	12.3890185041667	12.330802425	12.2498213041667	12.3367702625	12.24773505	10.8656148166667	13.5367997083333	11.6950002791667	12.308609075	12.7528809125	12.4703658	12.839025025	12.7751098333333

AC244197.3	9.612529675	10.1991934291667	10.0755267833333	9.69151550833333	9.83694624583333	9.00576620416667	9.59626012916667	10.0195697458333	9.7290300875	9.8249326375	9.65645970416667	9.32735891666667	9.39684609166667	9.8245061125	10.2348808958333	9.5518241375	9.29909422083333	10.0050292625	10.218895	9.86290639583333	9.65598562916667	9.70260275	9.6188059625	9.75553405	9.83694624583333	9.3418228125	9.8911957875	9.62174455833333	10.30872915	9.78714762083333	10.2788492458333	10.5897747333333	9.31340316666667	9.10366125833333	9.9624756625	9.2757434	9.89076339166667	9.9521619375	10.0474685583333	9.69242281666667	10.4086865166667	10.3398532125	10.1942399875	9.91607119583333	9.2043888125	10.1399081875	10.1395317375	9.94227104583333	9.738939	9.51747024166667	9.87673380416667	10.3157424958333	9.390927275	9.77241673333333	9.81370969583333	9.85389634583333	9.95954340416667	10.2887002416667	9.96130747916667	9.66445450833333	8.69581604166667	9.1591089875	9.09352854166667	10.1248478333333	8.78813974583333	9.068148775	9.7277608625	10.0084946125	9.47378049583333	9.32828119583333	9.96130747916667	8.84311743333333	10.223509175	8.83089360833333	9.23399626666667	8.01622854583333	8.6056636125	9.88782489166667	10.252506	9.43844097916667	7.61094595	8.6761895875	10.187671925	9.07090323333333	9.39913687083333	8.32672639583333	8.49420905833333	9.52764529583333	8.171646675	9.16986652916667	10.1124022166667	10.1228387166667	9.42755041666667	8.6597335	9.48539137083333	9.45798627083333	8.17009956666667	8.04892494583333	9.8732477625	8.49862807916667	8.66373332916667	8.7266882375	9.77453995833333	9.8736624125	9.57156121666667	8.9021930375	9.96209228333333	9.30933558333333	9.25371953333333	9.85829579583333	10.0870610416667	10.1984731375	9.37986479166667	9.201680325	9.14586544166667	10.2268719791667	9.00713167083333	9.06862487083333	9.62005562916667	9.56384294166667

IDS	9.612529675	10.1991934291667	10.0755267833333	9.69151550833333	9.83694624583333	9.00576620416667	9.59626012916667	10.0195697458333	9.7290300875	9.8249326375	9.65645970416667	9.32735891666667	9.39684609166667	9.8245061125	10.2348808958333	9.5518241375	9.29909422083333	10.0050292625	10.218895	9.86290639583333	9.65598562916667	9.70260275	9.6188059625	9.75553405	9.83694624583333	9.3418228125	9.8911957875	9.62174455833333	10.30872915	9.78714762083333	10.2788492458333	10.5897747333333	9.31340316666667	9.10366125833333	9.9624756625	9.2757434	9.89076339166667	9.9521619375	10.0474685583333	9.69242281666667	10.4086865166667	10.3398532125	10.1942399875	9.91607119583333	9.2043888125	10.1399081875	10.1395317375	9.94227104583333	9.738939	9.51747024166667	9.87673380416667	10.3157424958333	9.390927275	9.77241673333333	9.81370969583333	9.85389634583333	9.95954340416667	10.2887002416667	9.96130747916667	9.66445450833333	8.69581604166667	9.1591089875	9.09352854166667	10.1248478333333	8.78813974583333	9.068148775	9.7277608625	10.0084946125	9.47378049583333	9.32828119583333	9.96130747916667	8.84311743333333	10.223509175	8.83089360833333	9.23399626666667	8.01622854583333	8.6056636125	9.88782489166667	10.252506	9.43844097916667	7.61094595	8.6761895875	10.187671925	9.07090323333333	9.39913687083333	8.32672639583333	8.49420905833333	9.52764529583333	8.171646675	9.16986652916667	10.1124022166667	10.1228387166667	9.42755041666667	8.6597335	9.48539137083333	9.45798627083333	8.17009956666667	8.04892494583333	9.8732477625	8.49862807916667	8.66373332916667	8.7266882375	9.77453995833333	9.8736624125	9.57156121666667	8.9021930375	9.96209228333333	9.30933558333333	9.25371953333333	9.85829579583333	10.0870610416667	10.1984731375	9.37986479166667	9.201680325	9.14586544166667	10.2268719791667	9.00713167083333	9.06862487083333	9.62005562916667	9.56384294166667

BNC2	6.96533775	4.02568050833333	4.2929499	4.08637315833333	4.9727824	4.63093599166667	4.73206480833333	4.1018927	3.88526006666667	4.46985861666667	3.67977121666667	5.76836148333333	5.33153680833333	4.210787125	4.38197743333333	3.937874125	4.753728625	4.58241979166667	5.514879125	4.82464924166667	4.12677783333333	4.36495859166667	4.339553	5.018425275	4.645027875	4.32588960833333	4.69325549166667	5.270972825	5.36415765833333	4.43531826666667	4.6319392	5.05172549166667	4.12457974166667	4.15011734166667	4.8440917	5.76251488333333	5.58508399166667	5.02903219166667	4.545583025	4.82036908333333	5.367183075	4.83989633333333	5.399029725	5.53292085833333	4.611084475	5.02158241666667	5.26362051666667	5.00529823333333	5.40201090833333	4.74548245833333	4.73206480833333	4.649483075	4.64033936666667	5.58896354166667	4.350915225	4.69115821666667	5.153439775	4.77601478333333	4.49312724166667	5.72686785	4.18191081666667	4.05967894166667	4.4580822	3.73388574166667	4.183981525	4.222209325	4.12677783333333	4.81716043333333	4.05071508333333	5.89108584166667	3.65432333333333	4.21701204166667	4.199161275	5.93036776666667	4.59784559166667	4.428719425	5.74803075	4.853434925	4.67380545833333	4.70371486666667	4.30527630833333	5.78537223333333	4.26028479166667	5.35088976666667	4.25708764166667	5.125178525	4.70675665	4.56487130833333	4.031307325	6.31394556666667	4.13438028333333	4.4473261	4.02247413333333	5.29675638333333	3.912461775	3.88409523333333	4.24130721666667	4.12106518333333	3.78824818333333	4.94355658333333	4.64133359166667	3.79308619166667	4.81947958333333	5.153439775	4.282041725	4.88576699166667	7.43473675	6.44290830833333	6.63179246666667	6.32106796666667	4.59576983333333	5.355721825	5.30448685	4.94778381666667	4.37710720833333	4.56909456666667	4.137419	4.54330015	4.70270751666667	3.90095365833333

RALGAPA1	9.52268223333333	9.05776881666667	8.6667612	8.7954535	9.57179898333333	9.250740625	9.57736215	8.88930546666667	9.14003079166667	8.68775965833333	8.472359075	9.899420875	10.0569437166667	8.72874058333333	9.21169858333333	8.67888266666667	8.10391784166667	10.3964688	8.4800381	8.90389271666667	8.9184083	9.88925331666667	8.23520641666667	8.78498449166667	8.93619846666667	8.89220491666667	8.440899325	9.136523775	9.99301340833333	8.686777175	8.55804185	8.582021475	8.60344725	7.34892271666667	8.304316525	8.535777725	9.41892754166667	7.82067988333333	8.60280456666667	8.61046613333333	7.89196619166667	8.42018036666667	8.329584675	8.319610775	8.06606233333333	7.64275718333333	7.99232836666667	8.1923863	8.27228149166667	8.59990980833333	8.89174859166667	8.364642925	9.53043561666667	9.28128599166667	8.2549704	8.083905375	8.79861721666667	7.78166071666667	8.75515696666667	8.58808473333333	9.6530765	8.37303865833333	9.553861875	9.567278375	8.60539538333333	9.52364006666667	8.82265860833333	9.0354605	9.357237425	9.4687509	9.2408133	9.157883875	8.93619846666667	9.30124145833333	9.579511975	9.187855275	9.16543	9.66237621666667	9.32329896666667	10.5181477	9.58662811666667	10.116767025	9.289706825	8.46771705	9.26577935	8.686777175	8.561783725	9.42030009166667	10.0043835333333	8.709455975	8.96455003333333	9.437687475	8.05717868333333	8.40077755	9.54834169166667	8.975801525	10.5360893083333	7.89403294166667	9.956863325	10.1862509833333	9.212117675	8.38319035	7.81456171666667	10.4191933083333	8.77676135833333	9.810451275	9.40032650833333	9.0826061	8.63080885833333	8.76703910833333	8.29053858333333	8.733549025	8.90005820833333	8.83249931666667	8.17361143333333	8.27907085	8.14660076666667	9.65973478333333	8.620261275	9.148299925

DTNBP1	11.9796845666667	10.66793845	10.904327125	10.72841425	10.8476613083333	11.4715256833333	10.63254535	10.70166785	10.76921485	11.06152365	10.5516144166667	11.01859775	11.0031119583333	10.88143175	10.5408711916667	10.9898443833333	11.6890849333333	10.7217139416667	10.9843155	10.91165365	10.8904318	10.9123611916667	11.376411175	11.15581865	11.0689648166667	12.1163125	11.1777467458333	10.9120401833333	10.8698368333333	11.0538325833333	10.9828230166667	10.8889520833333	10.7295063583333	11.5534468416667	12.1799778583333	12.178702275	10.6108306416667	10.7170255166667	10.8824750083333	10.9077413083333	11.1546552166667	10.6035239083333	10.676538	10.9189114166667	11.4042087666667	10.9423645833333	11.0239555333333	10.6111916083333	11.0494415166667	11.2743409666667	10.9295727166667	11.12457335	10.9611703916667	10.7994102333333	11.0308654583333	10.8416766333333	10.6797192416667	10.924157925	10.7631580166667	11.0225675833333	11.9494775416667	10.836451025	11.0931527333333	10.5276582916667	11.4586326	9.90816476666667	10.6348374333333	10.4661023416667	11.0531461166667	11.237038075	11.179383775	10.279032875	10.842072875	11.08338295	10.9189114166667	10.2959072166667	11.218700275	11.3132862083333	10.6211581166667	11.2352593083333	11.8293448166667	11.0971520666667	10.9740524416667	10.92809195	11.19866735	10.9843155	11.171764175	11.5219495083333	10.6465456666667	11.2997236583333	10.8930750916667	10.8097832416667	11.248902775	10.776374675	10.8900635166667	10.8052677	11.0964662083333	11.3499248083333	10.8578576916667	11.3954232	11.3635795583333	10.4040896666667	11.5987696666667	11.2822211083333	10.70539815	11.0797164083333	10.9013899916667	10.067459125	11.509666625	11.0150144333333	11.3938737333333	11.12457335	10.6430311416667	10.6233516833333	11.1634048416667	11.2018586833333	11.0761932833333	11.602097375	11.3467541583333	11.8407167416667

ZNF804B	5.17905539166667	6.36616499166667	4.84935865	6.699210125	5.96342883333333	5.09317755833333	6.894639675	5.118961025	4.51773374166667	5.31712470833333	5.367183075	7.101860325	5.73491465833333	5.65521334166667	4.617309275	5.91334729166667	5.891995425	5.16622001666667	4.612218	4.83355489166667	4.90256950833333	5.07218190833333	4.71445220833333	5.30448685	6.04445175	5.04230353333333	4.56806040833333	4.62691041666667	4.802114875	4.46402134166667	4.7771511	4.66004348333333	5.13745513333333	6.09355109166667	4.48925233333333	4.40581835	4.649483075	4.68789791666667	4.617309275	4.64220138333333	5.24703029166667	4.58140610833333	5.0486896	5.775482875	5.16938190833333	5.17905539166667	5.636456275	5.25047555833333	4.692141475	5.59857721666667	4.85146489166667	4.51475793333333	5.26468096666667	5.575395825	5.38659805833333	4.90871175	5.96778360833333	4.93436960833333	4.82580888333333	4.45602740833333	4.50112465833333	5.735887375	4.45205615	6.74218866666667	5.65732808333333	5.91627995	5.064717325	6.01215780833333	4.84714924166667	5.0258117	6.37491049166667	5.51098925	7.470286275	4.73402499166667	5.78909119166667	4.06079156666667	4.85447411666667	4.34514024166667	4.183981525	3.94456669166667	6.26140625833333	8.48261985	4.67067994166667	3.99488165833333	4.49904255833333	5.88673599166667	5.04458405	5.88521655833333	4.7771511	4.87006425833333	4.16299614166667	4.537079425	4.07775664166667	4.71016199166667	4.1683668	4.082140775	4.12457974166667	4.748342275	6.09588644166667	11.9232334916667	4.72324775	6.65915351666667	5.04557781666667	5.388701925	4.43951955	4.36294506666667	5.38558591666667	6.8190902	5.44873513333333	6.76187970833333	4.68073703333333	5.56425255	4.664314175	5.18519845833333	4.32386374166667	4.485186325	9.93343423333333	4.68262605833333	4.81716043333333	6.15778135833333

OLFM1	14.1815548333333	15.5923775583333	15.6235572	15.8219621166667	15.5870133916667	14.20293335	15.6355001416667	15.0587922833333	15.799582875	15.3982642333333	15.2045357166667	15.2328828083333	15.6235572	14.9594767083333	14.7920246166667	14.989321275	13.66335695	15.4276773083333	15.4112652583333	15.7781512166667	14.7451582666667	15.0032595583333	15.4537382666667	15.406759425	15.2193239916667	14.21037625	15.4316128083333	15.700189825	15.5975780166667	15.6235572	14.8794573583333	15.6711097083333	15.3214833666667	13.89505895	14.30297575	14.2487820083333	15.7138591583333	15.0217064166667	15.2784907166667	15.1430836166667	15.4852249833333	15.2432312666667	14.7754409333333	15.4852249833333	15.236084325	14.6968877083333	14.92521735	15.799582875	15.2576870916667	15.1908835666667	15.1264373	15.4022381166667	14.178968975	14.7166750666667	14.61397985	14.515883675	14.6758595916667	15.69535405	15.727394275	15.618710975	15.1234964083333	14.596526825	14.9223639	15.00595375	14.3824525333333	15.00595375	15.1430836166667	15.4160185666667	14.8602831416667	14.948326475	15.2576870916667	14.9359352083333	14.7686158333333	15.06705565	15.1207533083333	15.2784907166667	15.2784907166667	15.0459774	15.1017513083333	15.2328828083333	14.7572900333333	15.0587922833333	14.6284460166667	14.4092298666667	15.1661341083333	15.0557071416667	14.8442556083333	14.9020399416667	14.8136769166667	14.6926031666667	15.048735725	14.7293250666667	14.5415836083333	14.7097912166667	15.06705565	14.65081365	14.9094697583333	15.3982642333333	14.9307420333333	15.0831469166667	14.201540175	14.500239075	14.3860372083333	13.7970969916667	13.8625679083333	14.3482967833333	14.9223639	13.6587931916667	14.9727774916667	14.465199375	14.5249167833333	14.349825825	13.5668551916667	13.0217207666667	13.5387428916667	13.4337458083333	14.6949121583333	14.62202095	14.4758644166667	14.2058121833333

PARVB	7.644918425	7.338007825	7.11730268333333	7.13168135	8.96230365	7.01264725833333	7.53297658333333	7.07353054166667	7.57513376666667	7.668390025	7.48300389166667	7.26049458333333	7.3477901	6.84325476666667	7.146985275	6.99033689166667	8.31561141666667	7.72686941666667	7.32789755	7.31981145833333	6.9393833	7.35503378333333	7.28130318333333	7.38582769166667	7.08487659166667	7.76188296666667	7.195709975	7.45966298333333	7.80451180833333	7.63549629166667	7.94435703333333	7.668390025	7.60415978333333	7.81947101666667	7.99232836666667	7.692684	7.78488271666667	7.94435703333333	7.5954683	7.52896074166667	7.289187425	7.88666206666667	7.30019385833333	7.58911965833333	7.20088341666667	7.51472684166667	7.49771558333333	7.597182875	7.05585056666667	7.15624494166667	7.58187098333333	7.78718563333333	7.78668815	7.71862859166667	7.86543926666667	7.73920148333333	7.66209683333333	8.1963928	7.61924733333333	7.38755610833333	7.75390870833333	7.587363525	7.341560875	7.460763125	8.8912541	7.9882338	7.63328161666667	6.87469296666667	7.84741366666667	7.67474618333333	7.54228448333333	7.75332493333333	7.71034295833333	7.227214725	7.67535224166667	7.00566125833333	8.211993425	7.28198013333333	7.89722555	7.583460175	6.9475749	7.3817504	7.53624973333333	7.70256570833333	7.65442175833333	7.3694039	7.42731664166667	7.74896025833333	8.151552925	7.62202388333333	7.80170391666667	8.06606233333333	7.83997353333333	7.65996385833333	8.332085025	7.53018445	7.687727325	7.80067161666667	8.01861064166667	7.911899075	8.075186025	7.86777288333333	7.85962578333333	7.8648195	7.74951601666667	7.50344081666667	7.82067988333333	8.33112569166667	7.663341325	7.78718563333333	7.66718561666667	8.16684950833333	7.91244564166667	8.08335636666667	8.0459923	7.76512125	7.672013725	8.2260001	7.93918533333333	7.72905521666667

FAT4	6.90715841666667	5.06570035833333	5.44644633333333	5.37226380833333	5.66843639166667	5.934751075	4.56487130833333	4.59179895833333	4.35998614166667	5.58407845	5.59754975833333	6.12934905	5.69296955833333	5.760490375	5.7161104	6.64415971666667	6.71367926666667	5.70253175	7.458458425	5.85609921666667	4.56071255833333	5.95574085833333	5.02467455	6.38165589166667	6.72953939166667	8.44794389166667	6.3877628	6.27314985	6.18094188333333	5.99501325833333	5.93306849166667	6.45401484166667	5.53004825	5.54491926666667	7.61643631666667	8.04388136666667	6.459913	6.787125175	6.59533788333333	7.198288025	5.6411096	4.76548715	5.565954175	6.0801823	5.78537223333333	5.49526165833333	5.56781358333333	5.67998533333333	6.08790970833333	6.66666621666667	6.22675393333333	6.66054216666667	5.34510054166667	4.611084475	5.126264825	5.35476978333333	5.775482875	5.92603411666667	7.48528250833333	7.26995246666667	5.5651756	4.36901661666667	5.49229008333333	4.57030863333333	4.74123235833333	7.00693255	4.32670648333333	4.830971775	4.73720489166667	4.89027461666667	4.69642039166667	4.14488695	4.146963325	5.792834125	4.30624979166667	5.135343	6.49689175833333	6.3877628	5.30061454166667	4.8815265	3.711770875	5.84791786666667	5.2482658	4.838864	5.25233420833333	4.90470955	4.3276422	5.80446058333333	4.65793555	7.270544475	3.70818398333333	5.37341235	5.68107735833333	5.40518485833333	5.67998533333333	5.8009379	5.28578914166667	5.87003104166667	5.22594001666667	6.53407053333333	5.30061454166667	3.81123611666667	4.13109949166667	5.71882016666667	4.63093599166667	5.052876825	5.37127428333333	7.07528308333333	5.83953349166667	7.58911965833333	6.95358578333333	6.12763226666667	5.5927414	5.97595255	4.56600969166667	5.2783407	5.98915650833333	5.03526733333333	6.745396875	4.60088621666667

TUBB6	11.8232517	10.5318759	11.343036975	10.4276602833333	11.0225675833333	9.91673698333333	10.4028540333333	10.9497712833333	10.6652778583333	10.4115783666667	10.0636311333333	10.3147212916667	11.0225675833333	10.4244981833333	11.5695230416667	10.1524914833333	8.39919656666667	11.4405829333333	11.2118401416667	11.3867866583333	10.901782425	11.7216620916667	10.4577793166667	11.0006123833333	10.9466012416667	11.4515417166667	11.0387117083333	10.748938275	11.0301443333333	10.18000125	11.4503715	11.3509805666667	10.2266691833333	7.765634325	11.43032555	11.526510825	10.575694825	10.6652778583333	11.14881755	10.6176617333333	10.7287943416667	11.373190825	10.6607214666667	10.5699475166667	10.2670402	10.9393869333333	11.074065275	11.0090696333333	9.53443595	10.1029681166667	11.002072275	11.5498379666667	11.1611143583333	11.651839775	10.9353811	11.5012483	11.2205665583333	10.5420798333333	11.8356051666667	10.300361525	11.0797164083333	9.23835931666667	8.975801525	10.3101529333333	8.25794470833333	11.5041708333333	10.9941240666667	8.67450711666667	11.0120115833333	8.95854766666667	9.09007516666667	11.4586326	10.6589017416667	9.98715419166667	11.256456125	11.2568448166667	9.76089629166667	10.0708045166667	10.7291696916667	9.69087439166667	8.889794925	9.28041066666667	10.6683478	11.5934022416667	10.1804756333333	9.88552295	9.04890688333333	11.0290294333333	8.46459865	10.2153563166667	10.9462719166667	11.5790815416667	10.137471025	10.6809073166667	11.3720648583333	9.895935425	9.69581614166667	9.39525429166667	11.1473899333333	8.779206475	9.68034129166667	9.97706043333333	9.187397175	9.83237755833333	10.9091302666667	9.43729386666667	10.9987091583333	10.3345761083333	9.59418116666667	10.0868334916667	9.83153578333333	10.239159725	11.1398416833333	9.74951903333333	10.257489575	9.94636165	9.54682303333333	10.534129425	11.0462297583333	9.44293281666667

BCL2L14	14.6122307083333	14.5488647916667	14.71190555	14.5735643833333	13.9727862416667	15.6472384833333	13.8832091583333	14.6904136083333	14.3724417	14.7664736333333	14.4809973583333	14.1045935166667	14.5287330416667	14.4739784	14.3186975416667	14.26265275	14.7920246166667	14.34486635	14.6611477166667	14.5469723583333	14.0746231666667	14.156645775	14.8819536416667	14.70147985	14.5924675083333	13.97057275	13.489460875	14.2858034416667	14.2145397416667	14.7055894833333	14.5377311666667	14.577355375	14.6104738916667	14.5589896583333	14.6023913166667	14.29207405	14.4177528166667	14.2334791	14.4963541416667	14.1357234916667	14.6046129083333	14.5793502666667	14.2289632916667	14.494740525	14.6738026916667	14.6718137833333	14.80427055	15.0153788083333	15.1908835666667	14.5753036916667	14.6862769916667	14.6430667333333	14.5664298833333	14.004911475	14.5735643833333	13.90344385	13.7272276083333	14.667083075	14.2563286	14.515883675	14.8548535916667	14.9094697583333	14.948326475	15.09815525	15.2749915333333	14.30297575	15.4022381166667	14.9070025916667	14.9832780583333	15.0894272416667	15.0615765666667	14.9727774916667	14.7429609666667	15.009089275	14.8336777583333	15.0769655666667	14.9020399416667	15.0865438916667	14.7948348083333	15.0153788083333	15.2784907166667	15.039210975	14.72730925	14.8136769166667	14.7499385916667	15.2687677	15.4022381166667	15.225946525	14.9620915916667	14.9359352083333	14.89930145	14.80427055	14.6628978166667	14.80004595	14.596526825	14.9148418416667	14.8602831416667	15.2230127	14.83890155	15.1077350416667	15.1400769916667	15.0615765666667	15.38189755	14.8842187583333	14.85238655	15.1908835666667	14.9949392	14.8750732583333	14.6304997416667	14.9645581833333	15.28644375	15.092462825	14.92521735	15.2328828083333	14.8966343583333	15.215611525	15.4537382666667	14.9359352083333	15.1174212666667	15.1207533083333

PTMA	14.6122307083333	14.5488647916667	14.71190555	14.5735643833333	13.9727862416667	15.6472384833333	13.8832091583333	14.6904136083333	14.3724417	14.7664736333333	14.4809973583333	14.1045935166667	14.5287330416667	14.4739784	14.3186975416667	14.26265275	14.7920246166667	14.34486635	14.6611477166667	14.5469723583333	14.0746231666667	14.156645775	14.8819536416667	14.70147985	14.5924675083333	13.97057275	13.489460875	14.2858034416667	14.2145397416667	14.7055894833333	14.5377311666667	14.577355375	14.6104738916667	14.5589896583333	14.6023913166667	14.29207405	14.4177528166667	14.2334791	14.4963541416667	14.1357234916667	14.6046129083333	14.5793502666667	14.2289632916667	14.494740525	14.6738026916667	14.6718137833333	14.80427055	15.0153788083333	15.1908835666667	14.5753036916667	14.6862769916667	14.6430667333333	14.5664298833333	14.004911475	14.5735643833333	13.90344385	13.7272276083333	14.667083075	14.2563286	14.515883675	14.8548535916667	14.9094697583333	14.948326475	15.09815525	15.2749915333333	14.30297575	15.4022381166667	14.9070025916667	14.9832780583333	15.0894272416667	15.0615765666667	14.9727774916667	14.7429609666667	15.009089275	14.8336777583333	15.0769655666667	14.9020399416667	15.0865438916667	14.7948348083333	15.0153788083333	15.2784907166667	15.039210975	14.72730925	14.8136769166667	14.7499385916667	15.2687677	15.4022381166667	15.225946525	14.9620915916667	14.9359352083333	14.89930145	14.80427055	14.6628978166667	14.80004595	14.596526825	14.9148418416667	14.8602831416667	15.2230127	14.83890155	15.1077350416667	15.1400769916667	15.0615765666667	15.38189755	14.8842187583333	14.85238655	15.1908835666667	14.9949392	14.8750732583333	14.6304997416667	14.9645581833333	15.28644375	15.092462825	14.92521735	15.2328828083333	14.8966343583333	15.215611525	15.4537382666667	14.9359352083333	15.1174212666667	15.1207533083333

DTWD2	14.6122307083333	14.5488647916667	14.71190555	14.5735643833333	13.9727862416667	15.6472384833333	13.8832091583333	14.6904136083333	14.3724417	14.7664736333333	14.4809973583333	14.1045935166667	14.5287330416667	14.4739784	14.3186975416667	14.26265275	14.7920246166667	14.34486635	14.6611477166667	14.5469723583333	14.0746231666667	14.156645775	14.8819536416667	14.70147985	14.5924675083333	13.97057275	13.489460875	14.2858034416667	14.2145397416667	14.7055894833333	14.5377311666667	14.577355375	14.6104738916667	14.5589896583333	14.6023913166667	14.29207405	14.4177528166667	14.2334791	14.4963541416667	14.1357234916667	14.6046129083333	14.5793502666667	14.2289632916667	14.494740525	14.6738026916667	14.6718137833333	14.80427055	15.0153788083333	15.1908835666667	14.5753036916667	14.6862769916667	14.6430667333333	14.5664298833333	14.004911475	14.5735643833333	13.90344385	13.7272276083333	14.667083075	14.2563286	14.515883675	14.8548535916667	14.9094697583333	14.948326475	15.09815525	15.2749915333333	14.30297575	15.4022381166667	14.9070025916667	14.9832780583333	15.0894272416667	15.0615765666667	14.9727774916667	14.7429609666667	15.009089275	14.8336777583333	15.0769655666667	14.9020399416667	15.0865438916667	14.7948348083333	15.0153788083333	15.2784907166667	15.039210975	14.72730925	14.8136769166667	14.7499385916667	15.2687677	15.4022381166667	15.225946525	14.9620915916667	14.9359352083333	14.89930145	14.80427055	14.6628978166667	14.80004595	14.596526825	14.9148418416667	14.8602831416667	15.2230127	14.83890155	15.1077350416667	15.1400769916667	15.0615765666667	15.38189755	14.8842187583333	14.85238655	15.1908835666667	14.9949392	14.8750732583333	14.6304997416667	14.9645581833333	15.28644375	15.092462825	14.92521735	15.2328828083333	14.8966343583333	15.215611525	15.4537382666667	14.9359352083333	15.1174212666667	15.1207533083333

ITGA9	13.5535340083333	13.2068344333333	13.6411556333333	13.1165645583333	13.7791494	12.975052625	13.3538490666667	12.4598136333333	13.6694092583333	14.51071235	13.0362472083333	14.59067625	14.1099143166667	12.6689869583333	12.93348965	12.8080609833333	13.916524275	13.0727047	13.0877248833333	13.1242463	13.7224285916667	12.01005715	12.4563419583333	13.248853375	13.334951875	14.1241853333333	13.9119804333333	14.1841737583333	13.4443089416667	13.6979142666667	12.9370697333333	12.7846359833333	14.0001337166667	13.2560414333333	15.3579580416667	14.2872888583333	12.9977920166667	13.7263656333333	13.49281735	13.1506037833333	13.2373203666667	12.9444160916667	13.4847749333333	13.0721123166667	14.26427225	12.2706199833333	12.4287875916667	12.4741889916667	12.93175565	13.8405812166667	13.8029528	14.3757792583333	12.8836445083333	11.7168402	12.0051849333333	12.6355786083333	13.1305878416667	12.6971152166667	13.92958755	14.5681681583333	14.7664736333333	13.8788907166667	14.2233219083333	14.1033949916667	14.1306178166667	14.7293250666667	13.0645149833333	15.4316128083333	14.9702238166667	14.6992893416667	14.8819536416667	14.59067625	14.4125419416667	15.444211725	14.9808324666667	15.365952025	14.4791297416667	15.0153788083333	14.9702238166667	15.225946525	14.4025408	14.1914740916667	13.9635675833333	13.8483702416667	15.1940996583333	13.8528809083333	13.312167125	14.3283993416667	13.5443638333333	14.6968877083333	14.7166750666667	13.9554702666667	13.2926640333333	14.2842943416667	13.2725108916667	13.97057275	14.18015695	14.7166750666667	14.31061245	15.156314825	14.0592017583333	13.7910501333333	14.4309788083333	13.2405472416667	13.3010495416667	14.1241853333333	15.095547425	12.6985834416667	15.3579580416667	14.4963541416667	13.8854083166667	14.2904660666667	13.171325425	13.4634900333333	13.7149280416667	12.99120565	13.8018861666667	14.21037625	14.3992922416667	13.4721219

BARD1	11.152834825	11.6723181916667	11.5352724166667	11.4586326	12.0737471	11.6514721333333	11.5839614333333	11.7609104916667	11.3495641166667	11.4616143	11.3183574	10.754584225	11.4402312666667	11.4200132333333	11.606939225	11.0989964083333	10.18161685	12.0843813333333	12.0154746541667	11.4748997416667	11.8713469833333	11.8130518166667	11.218298125	11.3886248333333	11.958220325	10.7881647083333	11.3339882	11.7791279583333	12.139797125	11.8908867916667	12.1250050333333	11.7046170083333	11.12105475	10.3833420583333	11.1656289166667	11.1380175916667	11.5030923083333	11.2499394833333	11.6761092166667	11.3946703583333	11.3635795583333	11.3086201833333	11.57874505	11.8542078	11.3864366416667	11.34033385	11.4963967083333	11.279776525	11.9971131583333	11.74329675	11.7663613666667	11.4872383	11.3813533416667	12.0660844416667	11.5589969083333	11.5754446583333	11.2936955416667	11.7827675333333	11.3867866583333	10.9740524416667	11.780675575	10.9226970166667	11.9844494166667	11.6444959666667	11.6837602666667	10.591411875	11.7219865583333	10.8710123583333	10.7998255666667	11.2222793166667	11.1354232166667	10.6068679083333	10.58612335	10.6430311416667	11.4221599083333	11.651839775	10.971536625	11.2965765083333	11.4890705333333	11.181831125	12.30344225	11.531296025	10.9512840333333	10.9065665083333	10.5695644083333	12.082379725	12.109263175	11.586583875	12.1665042	10.5976643083333	11.1721251583333	11.2294360916667	11.2438581166667	10.2182610833333	10.5464444833333	11.02981265	11.0946623333333	11.6144877166667	10.90507365	11.0171321	10.866231375	10.6993840916667	11.7027128	11.5414769166667	10.9230393083333	11.8904865166667	11.5742275583333	11.20219855	10.66752385	11.4140863666667	10.8468748916667	11.4492670916667	10.8086263583333	12.461524275	11.1800718666667	11.9104714083333	12.3754853666667	11.5820604666667	11.34446325	11.8461878583333

CCM2L	15.2722555666667	14.8794573583333	14.9174232833333	14.7226534166667	14.8919189333333	14.820747325	14.83890155	14.9359352083333	14.8629148	15.1234964083333	15.1661341083333	14.9865439333333	14.95380875	14.80004595	14.7596386666667	14.7316646166667	14.820747325	14.9422941416667	14.8287604166667	14.8442556083333	14.8750732583333	14.6926031666667	14.6239736916667	14.4043399	14.618270675	15.462044625	14.89930145	15.0703584333333	14.8676166333333	14.6778149	14.7055894833333	14.7828660333333	14.74733735	14.65081365	15.4022381166667	15.290119725	15.048735725	15.048735725	14.8676166333333	14.9567111166667	14.7828660333333	14.6430667333333	14.74733735	14.80004595	14.0926224666667	14.6005448916667	14.5449579333333	14.6904136083333	14.65081365	14.92521735	14.74733735	14.7055894833333	14.6819042	14.8136769166667	14.4775829916667	14.4346519833333	14.6926031666667	14.8313200916667	15.0153788083333	14.89930145	14.99199845	15.0769655666667	15.0153788083333	15.1234964083333	14.8919189333333	15.1495015916667	15.1400769916667	15.1207533083333	15.2014589916667	15.1529307583333	15.2084540583333	15.1908835666667	15.0703584333333	15.2014589916667	15.0587922833333	14.894107525	15.4160185666667	15.225946525	15.229747525	15.1460462916667	14.7252309	15.0153788083333	14.80427055	14.667083075	15.3056912416667	14.9223639	14.8236926166667	15.0032595583333	14.9674626083333	14.9620915916667	15.0557071416667	14.894107525	15.2120960833333	15.1661341083333	15.1908835666667	15.0276351833333	15.3579580416667	15.25397645	15.1908835666667	15.3540364916667	14.8895282083333	14.9197306916667	14.9020399416667	14.8602831416667	15.4316128083333	14.7252309	14.8412522666667	14.7365031833333	15.2502335583333	15.0733260666667	15.1174212666667	15.1632730833333	14.7920246166667	14.4850706333333	14.4871709833333	14.2858034416667	14.9197306916667	14.8919189333333	14.95380875	15.00595375

PMP22	13.7332085916667	11.6993325583333	12.5725102833333	11.3668652083333	10.643786575	12.1807321916667	11.9804777666667	11.9155103583333	11.5327072416667	12.8656045083333	11.5188708083333	14.3463851	13.0037538583333	11.9112855083333	11.4056072833333	12.8381552416667	12.3418182	11.0470160416667	11.8650417166667	12.2719001166667	12.7580394083333	10.1029681166667	12.0388164916667	12.6730092833333	12.3443503083333	13.7214957416667	12.370770375	12.382866125	11.291502775	11.7292748	12.8197190166667	11.9542491166667	12.3244889333333	12.2840300666667	13.6514898166667	13.8049154	11.7097839833333	12.3956670583333	12.8964099833333	12.59948	12.296807225	12.4283415	12.7728759666667	12.4292757333333	13.5781398666667	12.8057256416667	12.563781325	12.6146877083333	12.49908695	12.24082335	13.0847965333333	12.8240262666667	12.0432377416667	11.8596015	12.239207225	13.068247425	12.956460525	12.3671040333333	12.7490093583333	13.000133375	11.4181579	12.5349027	11.66250505	12.3182821416667	12.4474119833333	10.59381915	12.693462425	12.5948964	11.3831501	12.1213100833333	11.9670214	11.5980356083333	11.7387926	13.4586485833333	11.460533275	13.1363409083333	12.3347946666667	12.105942725	11.951715625	12.3610276166667	12.3059641583333	12.1895397416667	11.7609104916667	13.3402522416667	12.3698220916667	12.693462425	11.5618505	13.3538490666667	10.9073626	13.5721495	12.616140275	12.2550005	11.1790792916667	11.9392227916667	11.4530731666667	11.61191255	11.61112325	11.7671597083333	12.244497975	11.74784205	12.39882355	12.8984843833333	11.9757989916667	11.9348926	11.674290525	12.5410458083333	13.3200251	12.0001864416667	12.3518594833333	13.15189735	12.503844475	11.279388675	12.19191635	11.6982071583333	13.1235892166667	12.4759771583333	12.5259355083333	12.31433185	11.3246780083333	12.7128638833333

PBX3	11.65809635	10.8143165583333	10.8180008083333	10.3019992	9.62925015833333	10.0373878833333	10.0632117083333	10.0980477833333	10.1824837166667	10.34919645	9.21494060833333	11.2775845	10.4115783666667	10.483424475	10.31950435	10.8082438916667	10.7722019	9.913301025	10.3546255166667	10.30893185	10.6226410916667	9.831945975	9.77432975	9.80096413333333	10.3682517916667	11.1010834666667	10.264650175	10.2387377833333	9.83719281666667	10.2501973166667	10.3901677	10.26788045	10.3048656833333	9.86854744166667	11.7494484416667	11.8126730416667	9.79891768333333	10.609691175	10.0740705333333	10.3236813083333	10.5061588333333	10.4709652	10.8506520083333	10.5956461416667	10.8616146666667	10.62411455	10.9335275	10.4089152416667	10.71312705	10.2034843416667	10.64689975	10.2153563166667	10.227074775	10.5073525916667	10.616218875	10.8228191416667	10.5659717333333	10.548620125	10.528410425	10.5326225666667	9.52364006666667	9.84876684166667	8.70751021666667	9.60186775	10.4752224083333	9.29065144166667	10.5014025666667	9.79676919166667	9.48513751666667	9.93555589166667	9.29384936666667	9.19676581666667	9.75875961666667	10.4421542833333	9.33365533333333	10.3833420583333	10.3261322166667	9.30818888333333	9.286424975	10.0321890166667	10.123368025	8.97027915833333	10.0506734583333	10.3147212916667	9.92792138333333	10.9573094958333	9.41715076666667	10.079145275	9.313630975	10.5764502333333	9.82907005833333	10.1126129583333	9.78872821666667	10.0708045166667	9.81132868333333	9.01340034166667	9.34710834166667	9.24127241666667	9.28041066666667	10.1887239416667	10.2170232333333	10.2153563166667	9.6385431	9.448687325	9.59235401666667	9.88434308333333	10.4819116333333	10.0769619583333	10.0716267583333	10.1184175666667	9.36122138333333	9.61984825833333	9.68213623333333	10.0318266833333	10.0205705916667	10.0578180083333	9.90688865833333	9.18595039166667	9.92747266666667	10.7057641416667

GID4	4.63093599166667	4.1018927	4.06900396666667	4.221324425	4.22787308333333	4.13943304166667	4.29017421666667	4.649483075	3.905753025	4.14488695	4.60807976666667	3.76337073333333	4.04503838333333	4.32010620833333	5.393785875	4.28310633333333	4.62375229166667	5.956517125	5.12411041666667	4.366029925	4.203896025	4.70675665	4.4473261	5.09217729166667	4.748342275	4.39446349166667	4.70163844166667	4.938665425	4.90369176666667	4.67972664166667	5.92321303333333	5.19309993333333	3.94901469166667	4.20999628333333	4.50222558333333	4.38635804166667	4.611084475	5.36818925	5.19959509166667	5.2543857	5.20449400833333	4.65176085833333	5.327612425	5.73943181666667	4.21809414166667	5.16314315	5.35983450833333	5.18213341666667	4.87091625	5.43182890833333	5.192100225	5.24388391666667	4.15118036666667	5.54873173333333	4.45115921666667	4.87006425833333	4.87710051666667	5.21624118333333	5.75774213333333	4.81840091666667	4.289092625	4.68789791666667	4.38005901666667	3.80554890833333	3.65066326666667	5.09968536666667	4.71016199166667	4.1683668	4.055304025	3.7439275	5.24703029166667	4.55069105	4.81075010833333	3.699437625	3.96490434166667	4.04191653333333	3.95464144166667	5.40651104166667	4.76455610833333	4.87823230833333	4.0917668	4.2918764	5.170393	4.56305699166667	3.83041005	3.8957117	4.67380545833333	4.60699911666667	4.93234401666667	4.954343875	4.41962694166667	5.52437941666667	4.90032935	4.58336269166667	4.36901661666667	4.2860754	4.05071508333333	4.960685075	4.10101340833333	5.06113309166667	4.59369346666667	3.9861723	5.0258117	5.35361239166667	5.28578914166667	4.24914275	4.812774675	6.76843211666667	4.65478205833333	6.734891125	4.67380545833333	5.60406076666667	4.67471653333333	5.1555947	4.20571450833333	4.45902586666667	4.32094003333333	5.327612425	5.94013145833333	3.95777633333333

IFI44L	11.218700275	9.203321075	10.4515253333333	8.65887986666667	8.41742745833333	11.4556673333333	7.54510475	9.66513115	7.81344110833333	8.87883045833333	9.36528065833333	10.5585696666667	12.4741889916667	9.47068484166667	8.01273043333333	9.58391948333333	9.311300325	9.88925331666667	9.24172186666667	9.70495998333333	9.650075475	8.33398970833333	10.4032528583333	9.06973089166667	9.445595775	10.9812562333333	9.49238265	8.709455975	8.92840376666667	8.88067333333333	9.26442515	9.292040175	9.27853935	9.42118564166667	11.6951784416667	10.2266691833333	9.32526180833333	9.26442515	8.105836875	9.59648643333333	10.0595403	9.16920365	10.7448403666667	9.07252779166667	10.6050909833333	11.9467504166667	9.870256575	9.13909698333333	10.364544	12.2399849666667	10.0732835666667	8.88546686666667	9.26722199166667	8.95624593333333	12.9149879583333	9.93596328333333	9.39525429166667	10.051171975	10.0957326583333	11.4722453166667	8.09223929166667	11.0462297583333	13.21039535	10.138226575	11.4779366416667	8.682814325	7.98243114166667	11.5976176916667	12.2727355333333	12.7902282666667	10.0852465083333	7.76345526666667	8.81191925833333	10.571508325	11.3801476916667	12.1198078916667	13.5746400833333	12.40597585	12.2808809166667	11.4500480416667	8.518147825	9.39889275833333	10.9805696916667	10.07610735	12.0067796166667	11.0083196416667	8.14297786666667	13.2731848666667	12.00329435	11.7738991416667	11.0483413166667	11.24459925	10.3207495083333	11.6492807916667	9.31047634166667	11.638265575	10.571508325	10.8843423083333	8.83063586666667	12.1749483916667	12.341378075	10.2951456833333	11.8285794166667	11.9361117583333	11.8202425833333	14.400934925	9.83279928333333	13.5984028583333	11.1325239666667	12.7400926583333	11.0811600833333	12.0600286166667	12.6790614583333	9.6324307	13.9217501083333	12.14843035	8.199157725	12.6595749666667	11.5052707166667	13.0700559416667

BAALC	8.25292731666667	6.060789425	6.96590744166667	6.4502938	5.68783094166667	7.21451231666667	6.33264478333333	5.83398495	5.73943181666667	6.53809915833333	7.10630213333333	7.54282731666667	6.222261675	6.541621825	6.27482028333333	7.0659289	7.36707339166667	5.72382095833333	7.74511231666667	5.73682776666667	5.866285325	5.710337225	6.4095551	6.09355109166667	6.35578110833333	8.29980986666667	6.698517925	5.98559190833333	5.4789746	6.53273724166667	6.399909175	5.44971561666667	5.36818925	6.15598731666667	9.08869563333333	8.738873225	6.133346875	6.26140625833333	6.69642741666667	6.86668814166667	7.48476935	6.5994761	6.97214965	7.02536274166667	7.35564645	6.52781796666667	7.53349060833333	6.179361325	7.23028210833333	6.89851890833333	5.94625251666667	5.84236155833333	6.322468275	6.19736666666667	6.40265184166667	7.31552385	6.48928715	6.13759665833333	6.5305636	7.21504029166667	8.729717925	6.30044350833333	7.21808105833333	7.81893236666667	8.57940885	6.92374801666667	6.64834389166667	6.37713004166667	7.30864333333333	6.777132775	7.190008575	7.9343368	5.96423755	7.15765674166667	8.17990050833333	8.33965076666667	7.14519241666667	6.37568504166667	6.23865941666667	7.10888616666667	7.11278106666667	7.66443915	7.65996385833333	7.60297259166667	7.87369893333333	7.02401584166667	6.59234331666667	8.40424725	5.54199565833333	8.05761690833333	7.10699465	6.691149875	6.72249205833333	8.35633766666667	8.552420375	6.98911033333333	6.051276375	6.62969040833333	7.4398224	8.69504618333333	8.02832288333333	6.55667590833333	7.45786721666667	7.38984995833333	4.97386210833333	7.7783407	6.970355725	9.18832863333333	7.86609655833333	6.601356975	6.83020955	5.978682025	8.98618440833333	7.64552340833333	6.44737514166667	7.30864333333333	7.35226640833333	7.0609201	6.72183800833333	5.760490375

BCKDHB	10.4793722916667	10.1164178916667	9.88552295	10.5356874083333	9.78824573333333	10.3798706916667	9.79446515833333	9.88045179166667	10.146034	9.81818709166667	9.995492075	9.66283631666667	9.57610176666667	10.0595403	9.9560883	9.72033719166667	11.3488624333333	9.7752348	9.62155398333333	9.73831540833333	9.58131358333333	9.99125556666667	9.79486831666667	10.0848379583333	10.404465475	11.0815307083333	9.94551363333333	10.2191020916667	9.899420875	9.97583196666667	10.191495075	9.89303093333333	10.107173075	10.3554429666667	11.2035969416667	11.2929247083333	9.70748555	9.81483478333333	9.97583196666667	9.67723733333333	10.0915413	10.017588525	9.79362293333333	9.906465775	9.96400001666667	9.9690997	10.0347123333333	9.863143525	9.57817145833333	10.548620125	9.67096889166667	9.73309245833333	9.70331208333333	9.79147918333333	10.18748315	10.1126129583333	9.56407003333333	9.722436925	9.66873480833333	9.47496915833333	9.683521475	9.16588333333333	10.5420798333333	9.4955569	10.5601410916667	8.95121126666667	9.900175875	9.148299925	9.234219825	9.11782889166667	9.36206593333333	8.37653824166667	8.987001225	8.95624593333333	8.755680875	9.03689006666667	9.58437340833333	9.04596135	8.79596970833333	8.488931425	10.3211494083333	9.92334935	9.15886451666667	9.14054775	8.99899866666667	9.148299925	10.571508325	9.21590233333333	9.06363208333333	9.51857009166667	9.22016708333333	9.553861875	10.775195775	9.15259453333333	8.74370115833333	8.45999591666667	9.26035810833333	9.6433763	8.7179342	9.24127241666667	9.29252663333333	9.60710205	9.61816694166667	9.31710229166667	8.98368761666667	9.33321418333333	9.52133895	9.40333164166667	8.907131825	9.345810075	9.74433669166667	9.683051225	8.000994225	10.2634356166667	8.86854605833333	9.57307153333333	10.2498045833333	9.24980921666667	8.73547365	9.10889125

NUB1	10.406703075	9.984901625	9.65203452916667	9.70387588333333	8.58275995416667	9.77111937083333	9.14899073333333	9.76977495	9.7845929625	9.383893725	9.590448175	9.9037590625	9.00066376666667	9.95832646666667	9.68283467916667	10.1352631208333	10.8245161458333	9.35009075833333	9.94481022083333	9.92273599583333	10.0298802041667	10.0118394125	10.1348751208333	9.93613205	9.8911957875	10.4848774208333	9.8141938375	9.66986622083333	9.24381032083333	10.0405949125	9.9873854125	10.0341500458333	9.890327525	10.629165025	10.6928401666667	10.5762546958333	9.4279507625	9.92812106666667	8.93827409166667	10.1086052333333	10.1061134458333	10.0951867208333	10.3218157125	10.0240638125	10.2808657458333	10.3477512	9.96171140416667	9.69286685833333	10.4684897875	10.30913045	9.97065122916667	9.9105681875	8.92293245	9.41141477083333	9.88745143333333	9.8975069625	9.84391272083333	9.96287945416667	10.0278755708333	10.3672961583333	9.58954832916667	9.62603649166667	9.51653439583333	8.7946461125	10.025426975	8.78520605833333	9.1557932	9.3710324125	9.3913629125	11.1355991333333	9.74882143333333	10.0518122166667	9.22134721666667	9.77676755416667	9.84221727916667	9.50705467916667	9.81504475833333	9.70475119166667	9.73249376666667	9.81283215833333	9.45077790833333	9.65419311666667	10.5543154708333	9.57072065833333	9.40353525	9.89204036666667	10.9004971666667	9.65866890416667	9.40619117916667	10.0195697458333	10.1904914125	9.88745143333333	9.8214368875	9.84822367083333	9.22039090833333	10.0216481541667	8.48436670833333	9.71286741666667	6.83713329166667	9.75685522083333	10.4222240333333	10.6509623708333	10.8208157041667	9.70731425	10.5171444166667	9.72177936666667	9.37336079583333	9.9873854125	9.83741981666667	9.87717857916667	10.450924775	9.85179888333333	9.35748649583333	9.68013430833333	9.75092089583333	9.8849342375	9.9910593125	10.3130363416667	9.62689205833333	10.3414413708333

WDR86	10.406703075	9.984901625	9.65203452916667	9.70387588333333	8.58275995416667	9.77111937083333	9.14899073333333	9.76977495	9.7845929625	9.383893725	9.590448175	9.9037590625	9.00066376666667	9.95832646666667	9.68283467916667	10.1352631208333	10.8245161458333	9.35009075833333	9.94481022083333	9.92273599583333	10.0298802041667	10.0118394125	10.1348751208333	9.93613205	9.8911957875	10.4848774208333	9.8141938375	9.66986622083333	9.24381032083333	10.0405949125	9.9873854125	10.0341500458333	9.890327525	10.629165025	10.6928401666667	10.5762546958333	9.4279507625	9.92812106666667	8.93827409166667	10.1086052333333	10.1061134458333	10.0951867208333	10.3218157125	10.0240638125	10.2808657458333	10.3477512	9.96171140416667	9.69286685833333	10.4684897875	10.30913045	9.97065122916667	9.9105681875	8.92293245	9.41141477083333	9.88745143333333	9.8975069625	9.84391272083333	9.96287945416667	10.0278755708333	10.3672961583333	9.58954832916667	9.62603649166667	9.51653439583333	8.7946461125	10.025426975	8.78520605833333	9.1557932	9.3710324125	9.3913629125	11.1355991333333	9.74882143333333	10.0518122166667	9.22134721666667	9.77676755416667	9.84221727916667	9.50705467916667	9.81504475833333	9.70475119166667	9.73249376666667	9.81283215833333	9.45077790833333	9.65419311666667	10.5543154708333	9.57072065833333	9.40353525	9.89204036666667	10.9004971666667	9.65866890416667	9.40619117916667	10.0195697458333	10.1904914125	9.88745143333333	9.8214368875	9.84822367083333	9.22039090833333	10.0216481541667	8.48436670833333	9.71286741666667	6.83713329166667	9.75685522083333	10.4222240333333	10.6509623708333	10.8208157041667	9.70731425	10.5171444166667	9.72177936666667	9.37336079583333	9.9873854125	9.83741981666667	9.87717857916667	10.450924775	9.85179888333333	9.35748649583333	9.68013430833333	9.75092089583333	9.8849342375	9.9910593125	10.3130363416667	9.62689205833333	10.3414413708333

LMOD3	9.83319184166667	6.56951250833333	6.27652200833333	5.28787750833333	6.14803923333333	6.87089175833333	5.5651756	6.92785069166667	6.408778075	6.35665519166667	6.38925563333333	6.72705409166667	7.289187425	6.60400856666667	6.28402719166667	6.63942924166667	6.46640339166667	6.32600435833333	5.29356806666667	7.59083331666667	8.09066366666667	5.87405735	6.96778875833333	7.13294301666667	7.93805580833333	10.0156224166667	7.23193100833333	6.179361325	5.27227235	7.06400038333333	6.80246065833333	6.33345005833333	7.112144375	5.37452059166667	9.81608086666667	10.1883097083333	6.11047193333333	7.29474379166667	6.769663225	7.37766478333333	6.79057516666667	7.09231525	6.77436175	7.02822294166667	7.69541339166667	6.90968315833333	7.78166071666667	7.41303320833333	6.633116075	6.98911033333333	7.31674675	6.52912136666667	7.29474379166667	6.66317971666667	7.35003230833333	7.209690675	7.56602129166667	7.41121605	6.911150775	7.731363575	6.8687758	5.60680040833333	5.35983450833333	4.62270154166667	4.79411095833333	4.30527630833333	4.52055483333333	5.112251075	4.22594125	5.15454668333333	5.24931593333333	4.960685075	5.30264448333333	4.83457635833333	5.1291031	5.65159290833333	6.70532833333333	5.82366751666667	4.328489775	5.58896354166667	6.40265184166667	4.82688145833333	5.90065811666667	4.736033375	5.192100225	7.482471025	5.35882338333333	5.482101875	4.59997769166667	6.09754041666667	4.59179895833333	4.366029925	5.4712492	4.695239575	7.04378355833333	7.80227559166667	4.485186325	4.92557835833333	4.546642225	5.13634166666667	5.09968536666667	5.37341235	4.7815417	5.19112474166667	6.09925825	4.759183025	6.18822558333333	6.78180254166667	5.04024386666667	6.89132381666667	5.77198655	5.5927414	6.06574364166667	4.90470955	5.15454668333333	5.16838866666667	5.40201090833333	4.87595029166667	4.71445220833333	6.86809180833333

IQCM	5.92321303333333	5.65159290833333	5.17804041666667	5.04458405	6.15113656666667	5.16735583333333	5.7550021	5.49035004166667	4.94355658333333	4.87503585	5.03823209166667	4.78900190833333	5.07218190833333	4.91654405	6.74346625833333	5.09317755833333	4.8912062	5.21624118333333	5.01530855833333	4.883731825	4.809512175	4.83457635833333	4.688902775	4.39972780833333	5.053877925	4.76967493333333	5.858085325	5.55953659166667	5.78817238333333	5.895388975	5.62674555833333	5.96077145833333	5.28357275833333	4.21809414166667	5.77633328333333	5.88595835	5.623286525	5.86087000833333	6.11703395833333	4.87710051666667	5.23035095	5.75682274166667	5.03331626666667	5.76546864166667	4.76349515	5.20354711666667	5.38469075833333	5.20658905833333	5.91719066666667	4.8912062	5.4892176	5.087869425	5.43388225	5.549659825	5.33746485833333	4.88478473333333	5.35794613333333	4.960685075	5.63755270833333	5.6250378	5.35182895833333	5.6322886	5.7946758	5.367183075	5.99501325833333	5.63443499166667	4.70905974166667	4.90470955	5.455892825	5.29123589166667	4.883731825	5.01735620833333	5.052876825	5.5022085	5.591740875	4.960685075	5.17265765	5.09864851666667	5.28262243333333	4.81840091666667	5.10387183333333	4.6319392	5.355721825	4.62790769166667	5.62140730833333	4.92557835833333	6.23865941666667	5.577321975	5.61840579166667	5.13847554166667	5.69296955833333	5.858085325	6.28184195833333	4.71793841666667	6.1936491	5.68304789166667	5.91431983333333	5.988237975	6.31323984166667	5.54199565833333	5.71516535833333	5.26078876666667	6.018516875	6.1770551	4.72105203333333	6.15692685	5.91038576666667	6.71655181666667	5.775482875	6.54716043333333	5.82271865	5.45803265	5.37867954166667	5.31524535	6.74888376666667	4.86914890833333	5.07107728333333	5.43710514166667	6.13259841666667	6.259187975

GIMAP8	10.6043334333333	10.1046909416667	10.5303580166667	9.72033719166667	9.35401866666667	10.5651423416667	10.6293746333333	10.05157835	10.2411048916667	10.534129425	10.6391956666667	11.586171175	10.0167779583333	10.4297158833333	9.44959046666667	11.213594675	10.2699462416667	8.49347309166667	9.61596005833333	10.50459975	10.6298019333333	8.97815918333333	10.7181339666667	10.436061525	10.5553397083333	10.9084288583333	10.36145645	9.80492948333333	9.48609106666667	10.0268881833333	10.18748315	10.8161170333333	10.5177006833333	11.3296296583333	10.5530348	11.2664173916667	9.55058418333333	10.58448395	10.883221475	11.052772175	10.601543225	10.3945367666667	10.5806393916667	10.1529084083333	11.4597359083333	10.9861853	10.6757288666667	10.2707893833333	11.1615262583333	10.6865081333333	10.4053126416667	10.372950875	9.06745036666667	8.55997810833333	9.869019275	10.011606775	11.1604297833333	10.6266595583333	10.335795025	11.460533275	8.58349254166667	9.67854975	9.650075475	8.135196525	9.89855505	10.64807625	9.77219498333333	9.45149038333333	9.38536081666667	10.9357322	9.86020724166667	8.1873246	8.02564620833333	10.1472782833333	8.74620478333333	9.90816476666667	10.9143108583333	10.2794538083333	9.84760208333333	10.35148425	9.925380825	8.86655378333333	9.50956853333333	9.34438068333333	9.81345976666667	9.862235575	9.27899289166667	10.239159725	8.16931455833333	10.5334113083333	9.76819061666667	9.00189508333333	8.97957231666667	9.37821416666667	9.97912404166667	9.77883299166667	9.07777099166667	9.36987781666667	8.77298199166667	9.19719063333333	10.0238668333333	9.88471656666667	10.500980975	9.85765425	9.36761098333333	9.88298375833333	9.485645225	9.53087495833333	10.1541580083333	10.7061988666667	10.5631726083333	9.34018350833333	8.39466414166667	8.230344325	9.73658934166667	10.3599013416667	9.50539336666667	10.0728614166667	9.70584026666667	11.306112175

AL049697.1	5.2011047375	5.7969617125	5.18698449583333	4.74171228333333	5.3271171	5.51338809166667	5.53149080833333	5.19161248333333	4.4703774125	7.44401119583333	5.88387055416667	6.0789784375	4.8037463	4.81327000833333	4.90633156666667	4.30775345	5.836318725	5.20938526666667	5.1969767125	5.69628012083333	4.9441689125	4.59027375	5.28411132083333	5.24443233333333	5.29072016666667	4.58725059583333	5.11502322916667	5.67570444583333	5.28113690833333	4.39501792916667	4.85196545833333	4.54385184166667	4.57876442916667	4.77872065416667	5.2040205625	5.22316885	4.52844960416667	5.159282825	4.72461420833333	5.5530743625	5.86954199583333	4.57084105833333	4.95802155416667	5.719335775	5.04714867916667	6.06036822916667	5.38711015833333	5.06730598333333	4.66059554583333	4.60134417916667	4.76086461666667	4.2304917	4.53273798333333	5.52491661666667	4.8953541125	5.01898222083333	4.3733779125	4.836149125	5.84838001666667	5.19053105833333	4.88749675416667	6.05176328333333	5.41936227916667	6.0216198875	8.62514172916667	5.7619785375	5.20302529166667	5.40892235	5.815210475	7.0839089875	5.13904645416667	5.11737132916667	7.11759648333333	5.12262640416667	4.21860831666667	4.84767841666667	6.30447276666667	5.09368760416667	5.20205519583333	4.2074116625	8.45769320833333	7.84237111666667	5.65474399583333	6.9427834625	6.46604033333333	5.78581700833333	7.3294679625	6.37155930833333	5.36563595416667	6.4632365875	4.9260884625	5.81783112083333	5.4698339875	5.708855825	5.7344837875	4.36047532916667	4.70112407083333	7.48388688333333	5.70593540416667	7.68907982083333	6.210735	5.28850120416667	5.63186807083333	5.908957275	4.40737367916667	5.72424479583333	5.38816209166667	6.61255602916667	5.68157568333333	7.46219195416667	7.66639657083333	6.253817975	4.60229123333333	5.94754582083333	4.42821570416667	4.44873279166667	7.23756423333333	5.96118605	5.78763542083333	5.70698791666667

CFAP206	5.2011047375	5.7969617125	5.18698449583333	4.74171228333333	5.3271171	5.51338809166667	5.53149080833333	5.19161248333333	4.4703774125	7.44401119583333	5.88387055416667	6.0789784375	4.8037463	4.81327000833333	4.90633156666667	4.30775345	5.836318725	5.20938526666667	5.1969767125	5.69628012083333	4.9441689125	4.59027375	5.28411132083333	5.24443233333333	5.29072016666667	4.58725059583333	5.11502322916667	5.67570444583333	5.28113690833333	4.39501792916667	4.85196545833333	4.54385184166667	4.57876442916667	4.77872065416667	5.2040205625	5.22316885	4.52844960416667	5.159282825	4.72461420833333	5.5530743625	5.86954199583333	4.57084105833333	4.95802155416667	5.719335775	5.04714867916667	6.06036822916667	5.38711015833333	5.06730598333333	4.66059554583333	4.60134417916667	4.76086461666667	4.2304917	4.53273798333333	5.52491661666667	4.8953541125	5.01898222083333	4.3733779125	4.836149125	5.84838001666667	5.19053105833333	4.88749675416667	6.05176328333333	5.41936227916667	6.0216198875	8.62514172916667	5.7619785375	5.20302529166667	5.40892235	5.815210475	7.0839089875	5.13904645416667	5.11737132916667	7.11759648333333	5.12262640416667	4.21860831666667	4.84767841666667	6.30447276666667	5.09368760416667	5.20205519583333	4.2074116625	8.45769320833333	7.84237111666667	5.65474399583333	6.9427834625	6.46604033333333	5.78581700833333	7.3294679625	6.37155930833333	5.36563595416667	6.4632365875	4.9260884625	5.81783112083333	5.4698339875	5.708855825	5.7344837875	4.36047532916667	4.70112407083333	7.48388688333333	5.70593540416667	7.68907982083333	6.210735	5.28850120416667	5.63186807083333	5.908957275	4.40737367916667	5.72424479583333	5.38816209166667	6.61255602916667	5.68157568333333	7.46219195416667	7.66639657083333	6.253817975	4.60229123333333	5.94754582083333	4.42821570416667	4.44873279166667	7.23756423333333	5.96118605	5.78763542083333	5.70698791666667

FAM153A	8.28242620833333	7.666702425	7.00693255	6.964596175	7.95134493333333	8.54122040833333	7.80329328333333	7.31316353333333	8.07050834166667	6.94120649166667	7.086730875	10.7317611916667	8.18151525833333	7.2843799	6.27401925833333	7.07118303333333	8.88882345833333	7.800043275	6.93227245	7.78668815	8.04450845	6.75712434166667	6.71730138333333	6.89980234166667	6.4975584	9.04890688333333	6.51455034166667	6.82102155833333	6.0801823	6.03147445	6.00367545	7.51310406666667	7.46514316666667	7.2622557	8.68924809166667	8.3471697	6.68656881666667	6.43152844166667	5.97595255	5.41802705	6.926478325	6.1770551	6.78916241666667	6.87851200833333	7.00499255	7.02704855833333	6.78782379166667	6.8687758	7.502278225	6.187374325	6.46640339166667	5.71123295833333	8.04016346666667	7.19451305833333	6.51600675833333	6.57887239166667	7.60888643333333	6.85607033333333	7.0307229	6.1441534	9.13515515	7.05018306666667	5.33648908333333	5.93571900833333	6.456329775	6.929785275	7.08487659166667	6.64226356666667	7.11920136666667	6.33491514166667	6.47930140833333	8.55804185	8.1873246	6.60676565	6.47141914166667	6.52987506666667	7.30019385833333	5.5633586	6.33569684166667	8.69409343333333	8.0954509	7.02081110833333	6.09195375	4.21395365833333	6.63866813333333	5.74130296666667	5.54199565833333	6.79334741666667	6.58359223333333	6.560129475	5.773689175	7.98087950833333	9.36987781666667	5.866285325	5.99939696666667	8.46405893333333	5.67425256666667	4.60699911666667	6.24033626666667	5.984788925	6.35201275833333	6.38846551666667	5.66463360833333	6.32527860833333	4.76455610833333	8.751836375	8.17136015	7.189475125	6.51600675833333	7.18035421666667	7.25120925833333	6.67572515	5.48616714166667	6.39451754166667	5.7946758	7.73422946666667	6.61809366666667	5.90846126666667	6.12847228333333	6.9393833

RBMS3	4.46459402916667	4.02400255833333	4.09538288333333	4.12515398333333	3.82349206666667	4.06023525416667	4.81894025	4.22460014583333	3.90159505833333	3.83434435833333	3.762728575	4.40737367916667	4.86752087916667	4.3280659875	4.20156269583333	3.95955320833333	4.5893217625	3.7948746375	4.62006727083333	3.91753112083333	4.07405508333333	3.956273875	4.25976679583333	4.2607253	4.60134417916667	3.9642507625	4.25764998333333	4.449773675	4.54913074166667	4.30673345	4.53273798333333	4.40737367916667	3.83653458333333	4.130579125	4.39501792916667	4.19390653333333	4.47906581666667	4.55917659583333	4.5893217625	4.61893357083333	5.04714867916667	4.57876442916667	4.8776664125	5.56921560416667	4.19183539166667	5.092677425	5.25386314166667	4.9260884625	4.5274412875	4.86336781666667	4.7798875125	6.210735	3.872514125	5.4025318875	4.36047532916667	4.74594942083333	3.90405946666667	4.79053704583333	4.4023274625	4.405306825	3.7948746375	3.597694175	4.38768927083333	3.72756861666667	3.73835469583333	3.71597573333333	3.87357609166667	3.8702181125	3.70519970416667	3.83434435833333	3.58189811666667	3.6792171	3.7124192125	3.6792171	3.61963033333333	3.6942618125	3.6953103	3.77853057916667	3.83096759166667	3.60172435	4.04247167916667	3.937391475	4.152650975	3.7971162625	3.73835469583333	3.45895165	3.670843375	4.30183637083333	3.61561769166667	3.98886625833333	3.67805529166667	4.2914283625	4.08907536666667	3.814289425	3.95955320833333	3.9384223375	4.00725363333333	4.15064885416667	3.8131414375	4.87881199166667	4.38359478333333	3.73961191666667	4.80584670833333	5.103389975	4.41153243333333	4.02507916666667	4.68627040833333	6.21964854583333	4.7062329125	6.64122302916667	4.57189149583333	6.80278099583333	4.54281537916667	4.984948525	4.09538288333333	4.38359478333333	4.22841309166667	4.5685774875	4.70112407083333	3.99761022916667

AC098650.1	4.46459402916667	4.02400255833333	4.09538288333333	4.12515398333333	3.82349206666667	4.06023525416667	4.81894025	4.22460014583333	3.90159505833333	3.83434435833333	3.762728575	4.40737367916667	4.86752087916667	4.3280659875	4.20156269583333	3.95955320833333	4.5893217625	3.7948746375	4.62006727083333	3.91753112083333	4.07405508333333	3.956273875	4.25976679583333	4.2607253	4.60134417916667	3.9642507625	4.25764998333333	4.449773675	4.54913074166667	4.30673345	4.53273798333333	4.40737367916667	3.83653458333333	4.130579125	4.39501792916667	4.19390653333333	4.47906581666667	4.55917659583333	4.5893217625	4.61893357083333	5.04714867916667	4.57876442916667	4.8776664125	5.56921560416667	4.19183539166667	5.092677425	5.25386314166667	4.9260884625	4.5274412875	4.86336781666667	4.7798875125	6.210735	3.872514125	5.4025318875	4.36047532916667	4.74594942083333	3.90405946666667	4.79053704583333	4.4023274625	4.405306825	3.7948746375	3.597694175	4.38768927083333	3.72756861666667	3.73835469583333	3.71597573333333	3.87357609166667	3.8702181125	3.70519970416667	3.83434435833333	3.58189811666667	3.6792171	3.7124192125	3.6792171	3.61963033333333	3.6942618125	3.6953103	3.77853057916667	3.83096759166667	3.60172435	4.04247167916667	3.937391475	4.152650975	3.7971162625	3.73835469583333	3.45895165	3.670843375	4.30183637083333	3.61561769166667	3.98886625833333	3.67805529166667	4.2914283625	4.08907536666667	3.814289425	3.95955320833333	3.9384223375	4.00725363333333	4.15064885416667	3.8131414375	4.87881199166667	4.38359478333333	3.73961191666667	4.80584670833333	5.103389975	4.41153243333333	4.02507916666667	4.68627040833333	6.21964854583333	4.7062329125	6.64122302916667	4.57189149583333	6.80278099583333	4.54281537916667	4.984948525	4.09538288333333	4.38359478333333	4.22841309166667	4.5685774875	4.70112407083333	3.99761022916667

AGBL1	4.6622505	4.64734200833333	5.5651756	5.41985199166667	6.19818808333333	4.70163844166667	6.35738701666667	5.53496716666667	5.71516535833333	4.62790769166667	5.04024386666667	5.4147768	6.15274419166667	5.50905394166667	4.802114875	4.92659856666667	4.59576983333333	4.76967493333333	4.55671806666667	5.13961736666667	5.9573564	6.14249028333333	5.329558875	4.62889925	4.47861730833333	5.33648908333333	4.15642159166667	4.117963675	4.65478205833333	4.47474535	4.64311556666667	4.39972780833333	5.1653561	4.77483110833333	5.055819225	4.84506578333333	5.10930444166667	4.62161215833333	4.87710051666667	4.46597973333333	5.10077205	4.55184448333333	4.93436960833333	5.53695673333333	4.59683404166667	5.12313813333333	5.258661775	4.883731825	4.51370921666667	4.838864	4.72615460833333	6.45250163333333	4.94355658333333	5.37570958333333	4.99894050833333	4.72414796666667	4.70163844166667	4.80316131666667	4.428719425	4.11987200833333	5.24498075	6.028109975	5.16938190833333	5.52920275833333	5.63538781666667	5.22820184166667	6.10074335833333	6.30177564166667	5.38157649166667	5.09317755833333	6.10074335833333	5.60680040833333	6.77905188333333	5.50905394166667	5.75307261666667	6.04613815	5.22716268333333	5.36208894166667	4.61830805833333	5.59857721666667	5.477009275	5.6723045	5.41144461666667	5.8902603	5.08161569166667	5.6173492	5.18213341666667	5.60132373333333	5.35182895833333	4.73832164166667	5.97106113333333	5.83306814166667	4.879391675	5.3343362	4.80433128333333	6.09272539166667	4.77272485	5.51803240833333	5.16735583333333	5.41697971666667	5.22492120833333	6.03052256666667	5.91431983333333	5.47616439166667	5.03120425833333	5.69385626666667	4.692141475	6.17635683333333	5.000034475	6.58901686666667	4.90871175	7.03502525	4.91442044166667	4.98771553333333	4.87595029166667	5.07758634166667	4.361998275	5.2891249	4.86289075	6.00123059166667

GPATCH2L	5.931318725	4.98771553333333	5.735887375	5.97783559166667	5.29885645833333	6.58100214166667	4.98545498333333	5.2543857	5.88144885	5.64406685833333	5.981391325	5.13336504166667	5.13336504166667	5.27734721666667	6.133346875	5.32442271666667	5.948900025	5.39492906666667	5.895388975	5.59947975	4.72615460833333	5.14192449166667	5.565954175	6.14881959166667	6.51600675833333	6.75097239166667	5.79649798333333	6.3064348	6.10234676666667	6.459913	6.52412431666667	6.45919934166667	6.44887243333333	6.33415565833333	6.97843058333333	7.05714315833333	6.234662425	6.46133099166667	6.49010539166667	6.179361325	6.29443513333333	5.78455018333333	6.2066078	6.65690114166667	6.25427673333333	6.54089473333333	6.40053460833333	6.42709209166667	6.0167621	6.32668329166667	6.63788805833333	6.71918628333333	6.40185915	6.50275845	6.15858731666667	6.88718085	5.93571900833333	6.9998061	6.21191129166667	6.62636613333333	6.3064348	4.97812506666667	6.277191075	6.73349835	5.60406076666667	6.93418845	6.28034841666667	5.00117389166667	6.50214656666667	5.8523182	5.72573654166667	5.8625617	5.80356945	6.09195375	6.08697085833333	5.69872159166667	5.7901556	6.16277881666667	6.042706375	6.19209213333333	5.06351158333333	5.01530855833333	6.08877931666667	6.255916275	5.46093680833333	5.60406076666667	5.74130296666667	4.81376534166667	6.11631895833333	6.20408345833333	7.12725256666667	6.27095864166667	7.44095513333333	6.783129725	6.28402719166667	5.97106113333333	6.03052256666667	6.24033626666667	6.00123059166667	6.042706375	6.13424446666667	5.46647429166667	6.341623	6.19964000833333	7.0469593	6.903905225	6.47646620833333	7.36211884166667	6.241933725	5.72466863333333	6.011301125	6.22304681666667	7.80930983333333	6.78513590833333	7.13093330833333	6.54872438333333	5.94185621666667	7.64049128333333	6.67096843333333	5.7835982

HEY2	9.09237333333333	9.00137649166667	9.38536081666667	9.29252663333333	7.286116125	9.35401866666667	9.34847368333333	8.905745925	8.97721753333333	10.6313385833333	8.47797495833333	9.995492075	8.99943493333333	9.24933856666667	8.34374149166667	9.813959625	9.31452535833333	8.51622818333333	8.68035370833333	9.77049406666667	9.877817275	7.61177998333333	9.15505945	9.21543308333333	9.67900834166667	9.66421665	9.53398021666667	9.439564175	6.41577015833333	9.272617125	9.26948546666667	8.8768317	9.437687475	9.44779018333333	8.83115135	9.42508291666667	9.27506685	9.64871909166667	7.846884125	9.08729039166667	9.177752275	9.25484538333333	9.78481885	9.47590374166667	10.2128243166667	9.67634863333333	9.70748555	9.10194070833333	10.0945926583333	9.50586739166667	9.4551338	8.416908275	9.170544975	9.094155	9.57307153333333	9.76819061666667	9.19244325833333	9.25789575	9.77219498333333	9.89010159166667	8.791398425	9.35954015	8.85676560833333	9.810895475	9.189315625	9.48078894166667	9.304559675	8.995827675	8.907131825	8.295765375	8.37303865833333	8.26655316666667	10.0528892666667	9.53087495833333	9.4825512	9.508221125	8.97216300833333	8.8128727	8.9604306	8.62481299166667	9.776982075	8.27228149166667	9.47351875833333	9.80927415833333	10.2166211083333	9.14921909166667	8.93227916666667	10.485478825	6.54514473333333	10.1063080916667	9.99637683333333	9.08180965	8.42171803333333	9.75747410833333	8.95121126666667	8.25344699166667	8.81744095	7.9438425	9.99509398333333	8.77406471666667	9.32939963333333	8.44794389166667	8.755680875	9.33883618333333	9.01009886666667	9.45687460833333	9.983868575	9.3036884	8.986594625	9.92494756666667	7.78439114166667	9.99468328333333	10.181229625	9.06503669166667	9.66237621666667	9.04841031666667	9.442472475	8.555975275	9.341081375	9.5453508

MCPH1	11.9678169416667	13.4149817666667	13.812858	13.3890025083333	14.5020438083333	11.6230706833333	14.2233219083333	13.5824136833333	13.7791494	13.533857925	13.2678167166667	12.3479516833333	13.2806732833333	13.4427852416667	13.4799664166667	13.3410732083333	11.4052792333333	13.9197186416667	13.3552344916667	13.132282	13.065712375	12.799384025	12.10103115	13.3501502333333	12.86014235	11.4255179583333	13.2815171083333	13.0271148666667	13.8049154	13.5329794333333	13.5124524166667	13.2842694	13.3158362583333	11.7209431166667	11.62870925	11.2510757083333	13.7692653916667	13.75723885	13.5956278166667	13.4089360166667	14.036419	13.756296825	13.0638965333333	12.5116969583333	12.0149070416667	13.8157854333333	12.7890792833333	13.6822736083333	12.9456067916667	13.129332175	13.698785275	13.3786606333333	13.0877248833333	13.0786865916667	12.958106125	12.68421675	13.66414075	13.4321406333333	13.253120425	13.132282	12.2033105916667	12.4950350166667	11.575819425	12.0282380083333	11.6155901666667	13.1525120666667	12.7390522083333	13.019993025	12.2570148333333	12.06079575	12.4015640666667	13.1492966166667	12.3589004083333	11.6675947833333	12.4430322166667	12.3426613166667	12.05061705	12.178269875	12.8537857833333	11.6628809833333	11.621317125	11.7537741833333	12.424331275	12.1669452	13.3074551916667	12.309318975	11.9479243416667	11.8477473833333	11.57803215	11.3235379	12.01005715	11.6503712333333	12.1295438416667	12.034117575	12.4840896416667	13.17465125	12.4571981916667	12.8990697666667	13.073805875	11.5154734	11.3125544916667	12.111383	11.3419959666667	11.218700275	12.96407505	11.879885975	12.0781743333333	12.644626475	13.3538490666667	12.1198078916667	11.7609104916667	12.710817125	12.4922747583333	11.2464355333333	12.6939319583333	11.8137761833333	11.2079127916667	10.9621867333333	11.8845097083333	11.3954232

CHM	7.9579863	6.38240346666667	6.66317971666667	7.05278270833333	6.93032724166667	7.88761391666667	6.09588644166667	6.39152516666667	5.59857721666667	6.950931025	6.82240235833333	6.08509065	5.88853345	7.092930725	6.987286825	6.81637415	6.757758475	6.45767033333333	7.89510465833333	6.65424311666667	6.528434875	6.68656881666667	6.23312839166667	7.62307443333333	6.97843058333333	6.6659887	7.05521155	6.91634240833333	7.244602025	7.02761371666667	6.74821494166667	6.73882945833333	6.20040056666667	6.07176163333333	7.4398224	7.08293825833333	6.98853248333333	7.39745536666667	7.16614005833333	7.10505258333333	7.3314952	7.253014775	7.44041060833333	7.9977282	7.47140944166667	8.37970946666667	7.70589544166667	7.61232630833333	7.78546004166667	6.54933985	7.75772005833333	7.855217475	4.70905974166667	7.94273865833333	6.97276299166667	7.55250805833333	7.0469593	8.04234110833333	7.35751986666667	7.56602129166667	8.53782911666667	6.99727938333333	8.21672670833333	7.61062251666667	6.27563768333333	8.6396011	8.298377725	6.633753725	7.68936630833333	7.29049713333333	6.57096464166667	7.59937680833333	7.17267591666667	7.97812778333333	7.93069544166667	8.21622873333333	6.60676565	7.703109475	8.87736359166667	6.463588	6.41666009166667	6.61351845833333	7.45504230833333	8.08035505	7.53349060833333	6.33415565833333	8.38960981666667	7.32789755	6.99800655	7.89775408333333	7.578462725	8.08086618333333	8.151087675	7.08427549166667	7.62930498333333	8.200812875	7.25799328333333	6.903905225	7.496531175	4.75143253333333	7.74239094166667	7.31803023333333	8.19188733333333	9.06973089166667	8.252426875	8.78264749166667	8.25081844166667	9.11495396666667	8.23613304166667	7.76660259166667	8.16178343333333	8.40516035	7.171442675	8.85547214166667	7.496531175	8.39981014166667	7.363992875	8.99116335833333	7.855217475	7.949320675

GTPBP10	12.5984922	12.4718679833333	12.498597975	12.60273605	12.4019704583333	12.4416866083333	12.587916575	12.5745387083333	12.4732793833333	12.2302891583333	12.7300491166667	12.7041276166667	12.140177075	12.2124743416667	12.4981202833333	12.6287796916667	12.5850501916667	12.2111963416667	12.208161275	12.62157875	12.587916575	12.425293625	12.3435261333333	12.4091926166667	12.7580394083333	12.85040265	12.6240804083333	12.464146975	12.2964028333333	12.6346883583333	12.1703301333333	12.7949718333333	12.7543063	12.8344857166667	12.5126991416667	12.6944981166667	12.4019704583333	12.21725785	12.2599545833333	12.2607380166667	12.2396159	12.1545013166667	12.4554143666667	12.352843675	12.6566773416667	12.4225030833333	12.6171589666667	12.4585418333333	12.2073843166667	12.6800815583333	12.2637428083333	12.3983699916667	12.912870175	12.7490093583333	12.643219075	12.6093576083333	11.8051550333333	12.2336399666667	12.5974434583333	12.496720025	13.6107867916667	12.9007979916667	13.0985928583333	12.4795096083333	13.0258011083333	12.7295358083333	13.2996462833333	12.6872100416667	12.2892396	12.8783966083333	12.847095325	13.14792425	12.4936861916667	12.0013260916667	13.398021325	12.725884075	13.1563255166667	13.317291325	12.2412181916667	12.7195939833333	12.9364464083333	13.8395483	12.4469679916667	12.7374360833333	12.2611765333333	13.0755644416667	13.2587665833333	12.4645960083333	13.3278442333333	12.4827258583333	12.3550491166667	13.66335695	12.7934592666667	12.4571981916667	12.5860556666667	12.7796071583333	12.910549725	13.3522548916667	12.8679345583333	13.6411556333333	13.334230975	12.7769325	13.5583734416667	13.57979325	12.9929816333333	12.2906208	12.1174426416667	12.3811083333333	12.531582375	13.0700559416667	12.7468098833333	12.5680695416667	12.4225030833333	13.6328876166667	13.3270763416667	12.5912244	14.818607725	13.620993375	12.61272965	13.4807854416667

ZNF33B	8.15060805	8.45745534166667	7.99833741666667	8.64585098333333	9.19200934166667	7.97293150833333	9.12042280833333	8.291997825	9.15259453333333	7.9814024	8.60177515	9.09184225	9.39574425833333	8.19808664166667	8.19808664166667	8.11887123333333	8.98368761666667	8.55288418333333	7.87157458333333	8.54938428333333	8.561306875	8.49442401666667	8.123259225	8.08240869166667	8.41837	8.23662486666667	8.04865021666667	8.12989700833333	7.729718875	8.1308782	7.56655451666667	8.19346961666667	8.62596224166667	8.90619576666667	8.03376271666667	8.335541375	7.96073305833333	7.967285625	7.743439475	7.378725775	7.98294754166667	8.02564620833333	7.75823035	7.73246671666667	8.82920276666667	8.23128400833333	8.66108489166667	7.84947208333333	8.111985825	8.19498931666667	7.638193025	7.66500309166667	9.33838469166667	8.745766675	8.98986978333333	8.535777725	8.49136421666667	7.737502925	8.31670425833333	8.22981704166667	9.45555605	8.27021601666667	8.12378493333333	9.25349141666667	9.82164248333333	9.349435025	8.354234475	8.68822245833333	8.47382378333333	8.94804158333333	10.0632117083333	8.52731504166667	9.289706825	7.88506155833333	9.57736215	8.02564620833333	8.45605063333333	9.22108949166667	7.63210018333333	8.05913828333333	8.84155408333333	9.6705637	8.22397875	7.51524328333333	8.69317739166667	9.8269075	10.4887010333333	8.87443335833333	9.58173716666667	7.96575633333333	8.09852281666667	7.02401584166667	8.91642435833333	9.17280985	7.54510475	8.84155408333333	9.00330966666667	9.99851741666667	8.00051745833333	9.49325940833333	7.864271825	8.62084126666667	7.84845994166667	8.07784593333333	7.96133965	8.13716783333333	7.76345526666667	9.56961935	8.09897714166667	6.88255923333333	7.272898375	7.40193138333333	9.210296425	7.62815665833333	8.0063663	8.69317739166667	7.798604875	8.22928075833333	9.72070379166667	9.84242584166667

PRAMEF14	4.750433775	7.25001195833333	7.05714315833333	7.51310406666667	7.86777288333333	5.42780705833333	8.70400716666667	7.69919105	7.65326568333333	6.92374801666667	7.238929725	8.821700275	9.05732553333333	8.50130601666667	6.93032724166667	6.717950125	5.4237743	7.84741366666667	5.84048410833333	7.12528415833333	8.1308782	7.07353054166667	7.05651791666667	6.2176337	5.32031858333333	6.48862780833333	6.07429128333333	5.78909119166667	5.42470928333333	6.16763958333333	5.67044374166667	5.74413374166667	7.272898375	7.06942048333333	5.58407845	5.00117389166667	5.80728855	5.41985199166667	5.04024386666667	4.96401284166667	5.08458449166667	5.84236155833333	5.9573564	5.15046895833333	6.23620956666667	6.78642360833333	4.838864	6.45250163333333	4.86699436666667	5.76836148333333	5.03726580833333	5.17265765	7.37928605	5.56242626666667	7.07528308333333	4.66004348333333	6.81316333333333	6.11297529166667	5.89368925	5.64313004166667	5.12411041666667	7.47258725833333	5.58988304166667	7.43234464166667	7.607704375	6.68595645833333	7.99563946666667	7.90789741666667	6.083461775	7.38755610833333	7.173894	7.30805265833333	9.43045813333333	7.97141813333333	8.149040725	7.35293989166667	6.277191075	5.68783094166667	6.65690114166667	7.63498546666667	7.933308825	7.677172175	7.04142870833333	5.992607775	6.322468275	7.039355275	6.41354868333333	7.64552340833333	7.03194930833333	5.35273364166667	6.4040341	4.92360515	5.01735620833333	6.67442865833333	6.17313585833333	6.02015538333333	5.992607775	5.57451170833333	7.05869684583333	5.393785875	5.91038576666667	7.75772005833333	5.87944876666667	4.750433775	6.00039116666667	7.17851245	6.79468460833333	6.43490204166667	6.03413668333333	6.48497233333333	4.43313565833333	5.33648908333333	5.13745513333333	5.18312655	7.60136925	5.97035753333333	4.76967493333333	4.57240950833333	5.04458405	7.56775538333333

RNF114	10.4519100666667	11.3047106916667	10.7050450333333	11.1328362833333	10.69756315	11.1188874333333	10.724371075	11.0938590416667	11.1630098	10.79830065	11.1284533666667	11.054552625	10.5902797083333	11.071263875	10.8922670583333	11.3118389916667	11.666692125	10.93683975	10.9726481916667	11.155024025	11.0419739083333	11.5139760666667	11.31617205	11.1106962416667	11.1806950666667	11.1401896	10.8454086666667	11.0017295	10.6869030083333	11.1539711166667	10.8646445083333	10.949373725	10.9979551416667	11.4908711166667	11.0982319416667	11.1731632666667	10.85184045	10.9618387333333	11.012449925	11.0153767166667	11.1441289083333	11.1470598583333	11.31764545	11.01087085	10.9987091583333	11.3997255833333	10.9353811	10.9692394833333	11.096135525	11.2004569666667	10.9139727166667	11.0257755833333	10.41347785	10.970347325	10.98655615	10.6332993416667	10.7767059333333	11.0483413166667	10.8447210916667	11.0999976083333	11.6536243916667	11.8670714	12.0474514	11.2733084583333	11.8783734666667	11.0999976083333	11.121759625	11.8612030916667	11.2100932	12.5667535083333	11.1828701833333	11.4946024833333	11.8191065	11.3767696916667	11.3997255833333	11.2154273083333	11.8962786833333	10.9230393083333	11.1291467583333	11.6444959666667	12.4154660666667	11.03596855	11.0811600833333	11.279388675	11.6525927916667	11.5472050833333	11.9678169416667	11.233793875	11.7300854416667	11.3742720083333	12.3657425166667	12.0945968666667	11.3086201833333	11.720568625	10.9851253333333	11.4511549916667	12.0612117666667	11.7123616416667	11.6223592166667	11.4982052833333	11.6811126416667	11.3275163916667	11.6481957333333	11.56420645	11.3831501	11.8853151666667	11.1832845666667	11.5121356333333	11.9071135	11.5461869416667	11.6811126416667	11.2319732666667	11.8565277666667	11.4574143583333	11.262088	10.7140047416667	12.643219075	11.804049375	11.3771067916667	11.86975775

PTPRG	6.874007675	6.316307375	6.295927975	5.70932124166667	7.11080571666667	7.422156975	6.77836881666667	6.71502808333333	6.58226158333333	6.07263301666667	6.07176163333333	7.656003025	7.64327454166667	6.449622025	6.69050035	6.759774675	6.242830225	7.23028210833333	6.69715851666667	6.75097239166667	6.56431505	6.79131116666667	5.677007675	6.77836881666667	6.42414925833333	6.68472555	6.58691901666667	7.158761525	6.43219438333333	6.051276375	5.99349075	6.7098751	6.67633144166667	5.980495875	6.99346504166667	7.14272935833333	6.95984925	6.18822558333333	7.11986829166667	7.06888018333333	6.408778075	6.14803923333333	6.637274925	6.27004911666667	5.94185621666667	5.1863758	6.627072075	5.48616714166667	6.26827546666667	6.73694150833333	6.01932009166667	6.408778075	7.23371278333333	7.00118948333333	7.03321648333333	6.46419815833333	6.68595645833333	6.32175328333333	7.492737425	6.31952470833333	8.88732965833333	6.18094188333333	6.63942924166667	6.734891125	6.05474264166667	8.20175241666667	6.88314723333333	6.38084359166667	5.42470928333333	6.30177564166667	5.99939696666667	6.73349835	6.38240346666667	7.35119398333333	6.7503122	7.521666525	6.950931025	6.14803923333333	7.42731664166667	5.65344755	7.24075795	7.95082734166667	6.46779858333333	7.40193138333333	6.8327755	6.34447826666667	6.150357875	6.23865941666667	6.79658473333333	7.77376521666667	6.03500149166667	6.063181275	6.29443513333333	7.29169275833333	6.37568504166667	5.63144754166667	7.78110251666667	6.411112525	6.52564285833333	4.94778381666667	6.75097239166667	5.81563615833333	6.25335921666667	5.51386359166667	6.164307025	6.929785275	7.89880223333333	5.824619025	6.34078100833333	6.68595645833333	6.94057503333333	6.11387184166667	7.43868464166667	6.92575055833333	7.23429453333333	6.00884046666667	6.27245344166667	6.8347138	5.76546864166667	6.68532921666667

SRP72	9.895935425	10.4838867083333	10.6068679083333	10.1529084083333	10.1378549916667	10.4300821583333	10.0744319916667	10.616218875	10.3702407083333	10.5482040916667	10.5448322833333	9.60459400833333	9.555182225	10.3941230333333	10.2442419583333	10.3011707	10.5968900416667	10.1214563916667	10.578003225	10.384887925	10.286132425	10.3116455	10.432837725	10.5240503083333	10.0360710333333	10.0937438833333	10.0712503	9.617230275	9.96660331666667	10.364930325	10.3794789916667	10.1316676583333	9.90816476666667	10.1974084916667	10.3599013416667	10.2379766333333	10.0476842666667	10.4147458791667	10.4184334333333	10.1923992666667	10.5276582916667	10.55650935	10.5707751	10.7340525583333	10.3007165083333	10.7839123	10.5519705833333	10.5490285	10.6757288666667	10.4558195166667	10.33090645	10.640008475	9.77613621666667	10.2051938916667	10.4280419583333	10.3015658	10.31750855	10.5802645333333	10.095377	10.4272415916667	8.94049991666667	9.929600025	10.229775825	9.20100326666667	10.8028896	9.45422565833333	9.84409466666667	9.74607185833333	10.6645539	10.1001644	9.92792138333333	9.66781955833333	10.0933465333333	10.18324735	9.94840036666667	10.4678765666667	9.71098871666667	9.82382424166667	10.4530959333333	9.63292783333333	10.4801853	9.16735169166667	10.7589898916667	10.4127726666667	9.39574425833333	9.76051965	10.558939175	9.45990046666667	8.91410946666667	10.176297425	10.2170232333333	9.56080151666667	9.74607185833333	10.3745792583333	9.96267133333333	9.61006324166667	9.79147918333333	9.89553576666667	9.77176594166667	10.71663975	10.8976829666667	10.4732782833333	10.2274517916667	10.0831227083333	10.2051938916667	9.96824564166667	10.2604506416667	10.457410175	10.1084049583333	10.3987917	10.0696848333333	10.4253305166667	10.1633244583333	10.2305668916667	10.0716267583333	10.3745792583333	10.02846355	10.783186525	9.98715419166667	10.4061408166667

KIF9	9.26488315	6.96906025833333	7.41882361666667	7.29169275833333	8.1346889	6.89596523333333	6.1936491	7.404332775	6.65487665833333	8.40965201666667	7.9422621	6.10477214166667	7.1674037	7.25732849166667	8.46720710833333	6.941911825	8.09498031666667	7.23080319166667	8.27606465	7.57402961666667	8.524299575	7.177906325	7.61811995833333	8.23909885	7.9882338	9.18639844166667	7.586837175	7.45403938333333	8.19543675833333	7.32494479166667	8.00901856666667	7.76032476666667	6.71918628333333	7.44667759166667	9.28128599166667	9.27035150833333	7.58187098333333	8.10529686666667	7.73298270833333	8.27121185	8.1277546	7.67088145	8.1963928	8.61768925	8.38865071666667	8.398242975	7.9882338	8.532903275	7.76608003333333	7.93918533333333	7.88130259166667	7.95517108333333	8.7812519	8.7292202	7.70256570833333	8.77966386666667	8.05249740833333	7.82279025833333	7.80227559166667	8.33450988333333	8.27654828333333	6.2066078	5.99672560833333	6.70918899166667	6.9604848	6.4372196	6.242830225	5.72686785	6.526432	6.12282240833333	5.48616714166667	6.950931025	5.20250346666667	6.1441534	6.4372196	6.31323984166667	6.65287900833333	7.47204458333333	6.92717863333333	5.81989971666667	8.60751143333333	6.32668329166667	4.83355489166667	6.70918899166667	6.55877498333333	5.8292169	5.328555675	7.42619010833333	6.07673425	7.65827738333333	6.71730138333333	7.20088341666667	6.39769980833333	6.47216713333333	6.36170378333333	6.31952470833333	6.03335128333333	7.25066114166667	6.02729868333333	8.38217830833333	7.91409299166667	4.81075010833333	8.551994475	8.79489933333333	8.291997825	6.986635125	7.44041060833333	9.16216856666667	7.82958231666667	8.72644686666667	6.88718085	7.26937910833333	8.58301454166667	8.41379961666667	7.314288875	7.05156345	7.678740925	7.35983868333333	7.62202388333333	5.90321861666667

WDR5B	10.2792433416667	9.06862487083333	9.26653360416667	8.8444968375	9.56102568333333	8.5650006125	9.42665710416667	9.523403975	9.56286851666667	9.03842167083333	9.23859324166667	10.1991934291667	9.47838756666667	9.18571012916667	9.63923349166667	8.9918480875	9.66044075416667	9.47877880833333	9.53669334166667	9.5775666125	9.16519122083333	9.24295846666667	9.0879957125	9.3399144125	9.4761584875	10.499651875	9.50475789166667	9.4141327125	9.229807275	9.782587075	9.45492716666667	9.7598704	9.40751105833333	9.27104185	10.499651875	10.7547794375	9.11291737083333	9.316500875	9.2986405375	10.0016218333333	9.69198080833333	9.62430625	9.14303790833333	9.1871568	9.04528785416667	9.29041592916667	9.34041329583333	9.20393385833333	8.87110479583333	9.63542419166667	9.06624002916667	10.0266969708333	8.23240379583333	9.08322604583333	8.68550034166667	8.52222418333333	9.64005285	8.79698418333333	9.35426507916667	9.2419527125	10.188516825	9.09856255833333	8.67052372916667	7.97997342083333	8.56068924583333	8.55126836666667	8.53709285833333	9.7938353875	8.6131617375	9.28994362083333	8.93153802083333	9.49930952916667	7.909172975	8.08159883333333	8.422416775	8.41318640416667	9.98206792083333	8.73664734166667	9.06141539166667	8.95240457916667	9.88989740833333	9.04717265	9.3039005	9.11291737083333	9.88947327083333	9.9599607	9.791697	8.5541727875	9.58595583333333	9.18283845	9.8166749	9.75300719166667	9.50203754583333	9.4685538875	9.846105375	9.91821858333333	8.48345257083333	9.8166749	9.7086475	10.3402727083333	9.48903012083333	9.5508141	9.27104185	9.32783585	8.5791868625	9.07415146666667	9.0276665	8.2398398125	8.90121224583333	9.22893656666667	9.27104185	10.0613623416667	7.822552625	8.70523306666667	7.9991424625	8.12352207916667	8.91297039166667	8.53950697916667	9.35974574583333	9.4462680125

FAM162A	10.2792433416667	9.06862487083333	9.26653360416667	8.8444968375	9.56102568333333	8.5650006125	9.42665710416667	9.523403975	9.56286851666667	9.03842167083333	9.23859324166667	10.1991934291667	9.47838756666667	9.18571012916667	9.63923349166667	8.9918480875	9.66044075416667	9.47877880833333	9.53669334166667	9.5775666125	9.16519122083333	9.24295846666667	9.0879957125	9.3399144125	9.4761584875	10.499651875	9.50475789166667	9.4141327125	9.229807275	9.782587075	9.45492716666667	9.7598704	9.40751105833333	9.27104185	10.499651875	10.7547794375	9.11291737083333	9.316500875	9.2986405375	10.0016218333333	9.69198080833333	9.62430625	9.14303790833333	9.1871568	9.04528785416667	9.29041592916667	9.34041329583333	9.20393385833333	8.87110479583333	9.63542419166667	9.06624002916667	10.0266969708333	8.23240379583333	9.08322604583333	8.68550034166667	8.52222418333333	9.64005285	8.79698418333333	9.35426507916667	9.2419527125	10.188516825	9.09856255833333	8.67052372916667	7.97997342083333	8.56068924583333	8.55126836666667	8.53709285833333	9.7938353875	8.6131617375	9.28994362083333	8.93153802083333	9.49930952916667	7.909172975	8.08159883333333	8.422416775	8.41318640416667	9.98206792083333	8.73664734166667	9.06141539166667	8.95240457916667	9.88989740833333	9.04717265	9.3039005	9.11291737083333	9.88947327083333	9.9599607	9.791697	8.5541727875	9.58595583333333	9.18283845	9.8166749	9.75300719166667	9.50203754583333	9.4685538875	9.846105375	9.91821858333333	8.48345257083333	9.8166749	9.7086475	10.3402727083333	9.48903012083333	9.5508141	9.27104185	9.32783585	8.5791868625	9.07415146666667	9.0276665	8.2398398125	8.90121224583333	9.22893656666667	9.27104185	10.0613623416667	7.822552625	8.70523306666667	7.9991424625	8.12352207916667	8.91297039166667	8.53950697916667	9.35974574583333	9.4462680125

CHRFAM7A	9.23210917916667	9.27876612083333	9.17895344166667	8.98911833333333	9.05100650416667	9.2819927625	9.05378963333333	9.4252790875	9.51570927916667	9.468974325	9.29319027083333	8.57184803333333	8.90170160416667	8.91531722083333	8.79016155	8.806065175	11.3970594041667	9.5455877875	8.986797925	8.83806424583333	8.5072711875	8.1941990625	8.73715942083333	8.68847175416667	9.06193843333333	8.9078696375	8.4931924875	8.68447144166667	8.95875270833333	8.78813974583333	9.83385274583333	8.80652064166667	8.50628399166667	10.72704985	9.37612970416667	9.1988292125	9.16986652916667	8.9491525	9.1160913375	9.263749275	9.17713551666667	8.716679175	9.51154887916667	8.82398185416667	8.67528304166667	8.99418029583333	8.76445331666667	9.06906048333333	9.1601141375	8.54338075416667	9.57797126666667	8.87660001666667	8.3066900375	8.454278425	8.43069072916667	8.30310280833333	8.87110479583333	8.936442825	8.82945033333333	8.66461367083333	9.73165325833333	8.82496384583333	9.661749025	8.78046269166667	9.97480565416667	9.03208564583333	8.67815312083333	9.22934275	9.48230792916667	9.0025986375	9.14541895833333	8.9374052125	9.3441553	8.84077027916667	8.89895434583333	8.9482679375	9.67252833333333	9.13250539583333	9.10598958333333	9.142124775	9.9054182125	9.66535979583333	8.9960395375	9.0457406125	8.61115745	10.33641945	9.91698900416667	7.9486319	8.6097318875	8.24091861666667	9.3514624375	9.66089575416667	9.22134721666667	8.42096867083333	8.80848189166667	9.39684609166667	9.24822246666667	9.92516419583333	8.72063475833333	8.80848189166667	8.87158915833333	8.1179371875	9.84697804166667	8.79884114583333	8.53905974583333	8.345023025	8.36784924583333	9.06042506666667	9.77547627916667	8.65442572083333	9.512890975	9.21835433333333	8.13692505833333	9.87007799583333	8.6577276625	8.14001290416667	9.856999925	8.83275699583333	9.16281848333333	9.80292377083333

CHRNA7	9.23210917916667	9.27876612083333	9.17895344166667	8.98911833333333	9.05100650416667	9.2819927625	9.05378963333333	9.4252790875	9.51570927916667	9.468974325	9.29319027083333	8.57184803333333	8.90170160416667	8.91531722083333	8.79016155	8.806065175	11.3970594041667	9.5455877875	8.986797925	8.83806424583333	8.5072711875	8.1941990625	8.73715942083333	8.68847175416667	9.06193843333333	8.9078696375	8.4931924875	8.68447144166667	8.95875270833333	8.78813974583333	9.83385274583333	8.80652064166667	8.50628399166667	10.72704985	9.37612970416667	9.1988292125	9.16986652916667	8.9491525	9.1160913375	9.263749275	9.17713551666667	8.716679175	9.51154887916667	8.82398185416667	8.67528304166667	8.99418029583333	8.76445331666667	9.06906048333333	9.1601141375	8.54338075416667	9.57797126666667	8.87660001666667	8.3066900375	8.454278425	8.43069072916667	8.30310280833333	8.87110479583333	8.936442825	8.82945033333333	8.66461367083333	9.73165325833333	8.82496384583333	9.661749025	8.78046269166667	9.97480565416667	9.03208564583333	8.67815312083333	9.22934275	9.48230792916667	9.0025986375	9.14541895833333	8.9374052125	9.3441553	8.84077027916667	8.89895434583333	8.9482679375	9.67252833333333	9.13250539583333	9.10598958333333	9.142124775	9.9054182125	9.66535979583333	8.9960395375	9.0457406125	8.61115745	10.33641945	9.91698900416667	7.9486319	8.6097318875	8.24091861666667	9.3514624375	9.66089575416667	9.22134721666667	8.42096867083333	8.80848189166667	9.39684609166667	9.24822246666667	9.92516419583333	8.72063475833333	8.80848189166667	8.87158915833333	8.1179371875	9.84697804166667	8.79884114583333	8.53905974583333	8.345023025	8.36784924583333	9.06042506666667	9.77547627916667	8.65442572083333	9.512890975	9.21835433333333	8.13692505833333	9.87007799583333	8.6577276625	8.14001290416667	9.856999925	8.83275699583333	9.16281848333333	9.80292377083333

CTBP1	8.62481299166667	8.29098985833333	8.46508935	9.09779791666667	9.09054048333333	8.27176984166667	9.70284449166667	8.54895103333333	9.00236645	8.31150115833333	9.28929068333333	9.12557628333333	8.99720454166667	8.757884525	8.67450711666667	8.512878125	7.44041060833333	9.695341625	8.08240869166667	8.56955223333333	9.18071311666667	9.64153864166667	8.377106525	9.40511030833333	8.81836040833333	8.729717925	9.43636521666667	9.187397175	8.86211740833333	9.10246554166667	8.44192248333333	9.04596135	9.4946079	8.304316525	8.83921701666667	8.88262415833333	9.579948275	8.439473525	8.64890015833333	8.02512498333333	7.37296829166667	8.75278535833333	8.34181428333333	8.02512498333333	8.529235525	8.07471265833333	8.222543225	8.39209266666667	8.65887986666667	8.61442265833333	8.304316525	8.94049991666667	9.26261210833333	8.651213975	8.49745698333333	7.93695109166667	8.80540845	7.36471060833333	8.709872025	8.382753925	8.83592169166667	8.46552205	8.70080298333333	9.04179071666667	8.22099113333333	8.847054525	8.816929025	8.9939706	9.003775725	8.76514685	9.16735169166667	8.434501875	9.1447536	8.91410946666667	8.70751021666667	8.54989949166667	8.8559255	9.29384936666667	8.23217175	8.96700605833333	8.90099503333333	9.90058610833333	8.84802724166667	8.757884525	8.98796298333333	8.98325939166667	10.0587286583333	9.20652433333333	9.187855275	8.65418485	9.11582745	8.631828525	9.80231140833333	8.69172925	10.1283050583333	9.166351	9.22016708333333	9.05497765833333	8.910111275	7.08427549166667	8.03861421666667	9.361656325	8.88592250833333	7.70199845833333	8.90197375	8.4414262	8.32012975833333	7.192539525	8.53187930833333	8.07674773333333	8.52772891666667	8.43510350833333	8.536875925	7.61454650833333	8.486570275	8.75047631666667	8.14799835	8.08289571666667	8.512878125	8.8526135

PPARGC1B	11.1477174666667	10.9556654	10.76392445	10.9054446583333	10.680077475	10.0623822333333	11.3212969833333	10.6430311416667	11.1129059916667	10.5837654416667	10.4526526416667	10.9673192583333	10.7104085166667	11.01859775	11.2121826083333	10.1903114083333	10.9174580583333	9.777390875	10.86272805	11.1623033333333	11.22371335	11.7490808666667	10.7643055083333	9.39704936666667	10.041720625	10.836451025	10.95605295	10.934250825	9.97542684166667	11.0593297333333	9.90137155	10.82998625	10.8296253416667	10.3953052083333	10.71663975	11.9907404666667	10.7971951666667	10.302373075	9.3017146	10.58184315	10.082743125	10.4558195166667	10.521634775	10.3702407083333	10.1628878833333	9.92093743333333	9.91289015	10.1720866	11.1430808166667	9.98111111666667	9.750361275	10.31869075	10.217869775	10.1720866	10.5477462083333	9.30682376666667	10.7057641416667	10.500555575	9.95059544166667	10.6819563666667	10.6087534833333	10.4534831666667	9.77432975	9.77930279166667	8.54023758333333	8.07980334166667	9.76006771666667	9.36666890833333	9.39626775833333	10.1428729416667	9.8880168	9.45194998333333	9.02085209166667	8.59201043333333	9.86640328333333	10.0653276583333	10.627804175	9.45990046666667	10.465697825	10.0679332666667	9.43547285833333	9.71737348333333	9.14235506666667	9.13825335833333	8.84471325833333	8.618769925	8.595488075	9.37821416666667	8.882160975	9.985532675	9.95891725	8.51027651666667	10.167505275	9.38500366666667	10.4638383	10.6047191916667	10.4649425166667	9.04320051666667	7.9846002	9.74266033333333	9.53312831666667	9.93673649166667	10.2014148333333	10.1682992333333	9.88515190833333	8.821200975	7.80781901666667	8.22799306666667	10.1953308333333	9.87563735	9.74218338333333	9.76647539166667	8.94577069166667	10.7725388333333	9.43729386666667	9.73577058333333	8.79090379166667	9.75787298333333	11.1057130583333	10.6219118666667

MS4A5	5.84236155833333	6.71730138333333	5.81563615833333	6.59464205	6.10954496666667	5.28578914166667	7.82845124166667	6.725831175	5.37226380833333	6.7919706	6.46419815833333	7.95684823333333	6.423307775	6.01386335	5.7955502	5.24931593333333	6.131776975	6.21427064166667	5.34622214166667	5.50123778333333	5.6488719	5.36922546666667	4.9426219	4.87823230833333	4.76141500833333	4.71445220833333	4.91752975833333	4.63625878333333	4.80096826666667	4.49113386666667	4.90470955	4.58672223333333	5.03622141666667	6.24607838333333	4.73832164166667	6.50860958333333	5.38064126666667	4.82796093333333	4.3881123	5.006452275	4.93761881666667	5.018425275	4.771681175	5.390724	4.302366825	4.963016975	4.91206978333333	5.58795861666667	5.06232444166667	4.83457635833333	4.79318954166667	4.53925114166667	5.485111725	5.62034133333333	5.29675638333333	5.2482658	4.789978375	4.92149175833333	5.08252440833333	4.19623050833333	4.60088621666667	6.02292943333333	4.59369346666667	6.26664835833333	4.70475344166667	5.76836148333333	5.03120425833333	6.95860073333333	6.11297529166667	6.23312839166667	6.510020875	6.70200101666667	8.00051745833333	6.24033626666667	6.32600435833333	5.59947975	4.946749425	4.031307325	4.72105203333333	4.43951955	6.27652200833333	5.51683608333333	5.326621775	4.977020525	4.356950075	5.61925595833333	4.77930389166667	5.71416341666667	4.97490869166667	5.11436066666667	5.1598283	4.52610014166667	4.199161275	6.30492928333333	4.59265796666667	4.97072769166667	4.936774575	4.91752975833333	5.18759319166667	4.90032935	4.32094003333333	6.411112525	4.792098225	4.88687726666667	4.34698491666667	5.04458405	4.957419525	6.29278439166667	4.370852175	6.5175539	4.64613160833333	5.50615049166667	4.4047953	4.94999915833333	4.62889925	4.33532039166667	4.51773374166667	4.4718244	4.563953975	5.51585541666667

EDNRA	8.500835825	9.35773556666667	10.5444389083333	9.57093504166667	9.02927973333333	9.27506685	9.60963610833333	9.62193513333333	9.20145365833333	9.66742095833333	8.98751653333333	10.9284040666667	9.22606134166667	9.57510789166667	8.71039916666667	10.0965198666667	9.75665824166667	8.52966328333333	8.76751969166667	9.71737348333333	9.78872821666667	8.83016251666667	9.573560175	9.49775068333333	9.05030123333333	8.97126208333333	9.765982475	9.40333164166667	8.40473328333333	9.12700821666667	9.56361585	9.599958075	9.442039375	9.23377270833333	8.96230365	8.6404919	9.38762769166667	9.36761098333333	9.71527460833333	9.78692435833333	9.34750165	9.53223686666667	9.75828255833333	9.50539336666667	9.487900075	10.3167137333333	9.52042825833333	9.295759125	9.23047039166667	9.04596135	9.465645475	9.98877366666667	9.43868498333333	9.21257606666667	9.67813213333333	10.2539350666667	9.38096979166667	9.45898255	9.61091021666667	9.69924190833333	9.145650675	10.119247225	9.92453053333333	10.4661023416667	10.4709652	9.53443595	10.4793722916667	10.1853907583333	9.60581258333333	9.46704600833333	9.081377025	8.98986978333333	11.0120115833333	10.7796932083333	9.62890875	10.2411048916667	10.4127726666667	9.37588805	9.87070264166667	9.45010154166667	10.328553475	10.2289590916667	10.3442980083333	11.3911680666667	10.7112321916667	10.6263315083333	9.7557679	10.5659717333333	8.68470500833333	10.621550725	10.36596635	9.20045154166667	8.87491901666667	10.2741449416667	10.315132175	8.9744767	9.97954941666667	8.998536575	10.4228179333333	9.22794726666667	10.2217906833333	9.4936652	8.846155575	9.07683584166667	9.59023981666667	9.56172193333333	10.5123801583333	10.4453172666667	9.22295939166667	10.4720948333333	9.64383103333333	8.86211740833333	10.9783451666667	9.60710205	9.69581614166667	9.50452185	9.651432225	9.49191133333333	8.80139795	9.09642976666667

BDNF	4.36901661666667	4.748342275	4.42980535	4.68262605833333	3.99595204166667	3.97264301666667	4.96956821666667	4.47474535	3.99268493333333	4.20571450833333	4.15011734166667	5.0088772	4.31217129166667	4.576370125	4.30437938333333	4.27374416666667	4.99794086666667	4.44822564166667	4.21701204166667	4.44534256666667	4.70163844166667	4.34901141666667	3.98946053333333	4.86085110833333	4.2379826	4.87503585	4.06389798333333	4.10621731666667	4.10495005	4.99794086666667	4.2068357	4.12229528333333	4.51268873333333	3.798771525	3.909240625	4.37489024166667	4.13943304166667	4.11385116666667	4.0798737	4.66727606666667	4.97490869166667	3.946878775	4.33133893333333	4.92455391666667	3.86167775833333	4.42372025	4.14053975833333	4.29817321666667	4.13438028333333	4.37610179166667	4.344051675	4.19623050833333	4.01916375833333	4.65588558333333	3.88876065	4.26758835	3.70818398333333	4.27700685	4.19234266666667	4.18191081666667	4.697573175	5.25233420833333	4.49216794166667	4.19234266666667	4.021539275	4.43091141666667	3.67271084166667	5.31319136666667	3.630124675	3.9147734	4.45602740833333	4.62990135	4.84615233333333	4.02676174166667	4.01216488333333	3.7304451	3.90803829166667	3.94580334166667	3.75570144166667	3.614860625	5.15873735	4.22787308333333	3.96359718333333	4.07457460833333	4.04622350833333	3.89679799166667	4.42372025	3.937874125	4.93552183333333	4.2068357	3.58947205833333	4.12106518333333	5.51291259166667	3.66190675	3.70583574166667	4.02247413333333	4.27071034166667	3.90095365833333	3.582549625	4.52697115833333	3.937874125	4.40581835	4.27374416666667	4.38635804166667	3.93585013333333	4.040897575	3.9661014	4.81376534166667	3.973735825	5.24498075	4.31217129166667	4.666328775	3.92586006666667	4.337410475	3.82070373333333	4.04503838333333	6.12200050833333	4.128952175	3.905753025	4.141758275

YPEL2	11.8772191666667	11.5710069666667	11.2997236583333	11.523383625	10.9436906083333	10.6869030083333	11.1298695666667	11.5253735333333	11.4387367	10.80942375	11.1361895666667	11.8329005166667	11.3268344833333	11.34484365	11.276041	11.545842925	12.2591093583333	10.392106425	11.292593525	11.4086625666667	11.5298755416667	11.1394283333333	11.3078719166667	10.5973351833333	10.9516391	12.1570780583333	11.3661426916667	10.7912120583333	10.5534355833333	11.270439275	11.31764545	11.3384968	11.2154273083333	11.3946703583333	12.0432377416667	12.3771618166667	10.8036860666667	10.6865081333333	11.5839614333333	11.2417551	11.629073975	11.554144025	11.1806950666667	11.0352265083333	11.2334234166667	12.367659325	11.31764545	10.4895361083333	11.2510757083333	11.1941109166667	11.3300005666667	11.0574717833333	10.4934979	10.8334041916667	11.339247	11.30185685	10.76764485	11.0666847416667	11.1699387666667	10.6169276916667	9.64074395833333	10.717740675	10.8600934166667	10.9567732916667	10.514613975	11.005303025	10.19975295	10.6391956666667	10.1432492	10.61804865	10.3313573416667	9.8880168	10.8251181583333	10.93645885	10.3754461833333	9.76819061666667	10.6263315083333	10.469517225	10.7718274166667	10.4372971833333	10.8949867833333	10.5516144166667	11.01352865	10.3369395416667	10.7189561666667	10.2745932916667	9.77475016666667	10.5752416166667	9.99086305833333	10.6391956666667	10.5605583916667	9.666049875	10.3526835916667	10.5902797083333	9.51811660833333	10.4671575083333	10.4850893583333	10.2567002166667	10.3960554833333	9.19441289166667	10.5400727416667	10.3105309666667	10.3228920166667	10.3663602	10.703101075	13.004442975	10.683111325	11.291502775	10.0480865166667	10.5783390833333	11.40680705	10.0069849166667	10.3745792583333	10.0595403	10.0095607333333	10.7736244333333	10.541338775	9.96565705833333	10.8904318	10.2974881833333

MEF2A	11.9599476291667	10.6517710541667	10.9774549166667	10.4761612458333	10.1124022166667	11.101283425	10.5144123166667	10.9012071208333	10.5000138	10.0995266041667	10.3647371625	11.5557634958333	11.1563163958333	10.4030534458333	10.5254297583333	10.8192980375	11.0436000125	10.1583154958333	10.5156525583333	10.922504475	10.7048527916667	10.0154029208333	11.0718250666667	10.7029488041667	10.6697369458333	12.0468052333333	10.7798788375	10.6544604875	10.1752901583333	10.5958455916667	10.7830060541667	10.72416865	10.6798983583333	10.854301975	12.2343494	11.9897541	10.3327453208333	10.5932017375	10.677126425	10.6268600541667	10.8974787041667	10.8906319791667	10.8794517208333	10.9752468958333	11.210243625	11.2274614541667	10.9935606083333	10.9421725458333	10.7495006291667	10.6594312291667	10.654833525	10.7868548458333	10.6981154958333	10.4738469083333	11.0339171708333	11.0595031666667	10.79813175	10.7694125541667	10.66773115	10.995681725	10.41822565	11.2059617875	9.8861641375	11.0439405708333	10.3230902208333	10.9796497666667	10.3192911875	10.17979995	10.2041541041667	9.93495875	10.9012071208333	10.4746305041667	10.3985874083333	10.6709282166667	10.0797250583333	10.4746305041667	10.693611025	10.3042109291667	10.7249200125	10.3445012416667	11.1454009041667	10.4769641333333	10.6085738375	10.3275314625	11.2770019166667	10.8256695416667	10.6102173625	10.8218148083333	10.3243071708333	10.8735190708333	10.7249200125	9.8797627375	10.6386035916667	10.8500982125	10.6829236833333	10.8463488958333	9.73465698333333	10.3201202791667	9.88864601666667	11.1197786583333	11.8533019	11.0543706791667	10.7641149791667	10.8504646333333	11.0587480708333	11.417556975	11.2589819375	10.8708319625	10.6747644041667	10.8039093708333	11.0991457083333	10.4632230708333	10.9464365791667	10.6693287666667	10.9511066541667	11.3002888375	11.1914341375	10.4486092625	11.0846961875	10.9495725041667

LYSMD4	11.9599476291667	10.6517710541667	10.9774549166667	10.4761612458333	10.1124022166667	11.101283425	10.5144123166667	10.9012071208333	10.5000138	10.0995266041667	10.3647371625	11.5557634958333	11.1563163958333	10.4030534458333	10.5254297583333	10.8192980375	11.0436000125	10.1583154958333	10.5156525583333	10.922504475	10.7048527916667	10.0154029208333	11.0718250666667	10.7029488041667	10.6697369458333	12.0468052333333	10.7798788375	10.6544604875	10.1752901583333	10.5958455916667	10.7830060541667	10.72416865	10.6798983583333	10.854301975	12.2343494	11.9897541	10.3327453208333	10.5932017375	10.677126425	10.6268600541667	10.8974787041667	10.8906319791667	10.8794517208333	10.9752468958333	11.210243625	11.2274614541667	10.9935606083333	10.9421725458333	10.7495006291667	10.6594312291667	10.654833525	10.7868548458333	10.6981154958333	10.4738469083333	11.0339171708333	11.0595031666667	10.79813175	10.7694125541667	10.66773115	10.995681725	10.41822565	11.2059617875	9.8861641375	11.0439405708333	10.3230902208333	10.9796497666667	10.3192911875	10.17979995	10.2041541041667	9.93495875	10.9012071208333	10.4746305041667	10.3985874083333	10.6709282166667	10.0797250583333	10.4746305041667	10.693611025	10.3042109291667	10.7249200125	10.3445012416667	11.1454009041667	10.4769641333333	10.6085738375	10.3275314625	11.2770019166667	10.8256695416667	10.6102173625	10.8218148083333	10.3243071708333	10.8735190708333	10.7249200125	9.8797627375	10.6386035916667	10.8500982125	10.6829236833333	10.8463488958333	9.73465698333333	10.3201202791667	9.88864601666667	11.1197786583333	11.8533019	11.0543706791667	10.7641149791667	10.8504646333333	11.0587480708333	11.417556975	11.2589819375	10.8708319625	10.6747644041667	10.8039093708333	11.0991457083333	10.4632230708333	10.9464365791667	10.6693287666667	10.9511066541667	11.3002888375	11.1914341375	10.4486092625	11.0846961875	10.9495725041667

CDH23	10.5041729666667	8.82067435833333	8.83115135	8.615263775	9.19020434166667	8.07471265833333	9.330358125	8.51528900833333	9.002830825	8.49794376666667	8.52481044166667	9.62407518333333	9.38807046666667	8.58601561666667	8.23767453333333	8.647921775	8.29098985833333	8.78635625	8.48614225	8.409150775	8.8768317	8.92749955	8.03810015	8.244635825	7.95134493333333	9.92453053333333	8.39370431666667	8.13362299166667	8.68375230833333	7.85258474166667	8.081342075	8.13242494166667	8.283955225	8.26097460833333	9.55288966666667	10.3852198833333	8.25344699166667	7.64610639166667	8.36127639166667	8.16931455833333	7.72573829166667	8.500835825	8.41428779166667	7.93544106666667	8.54656363333333	8.57940885	8.16329085	7.76660259166667	8.00800819166667	7.55925806666667	7.73422946666667	8.63773576666667	8.030568675	8.061842725	7.62479258333333	8.875989475	8.35685346666667	7.64856505833333	8.486995925	8.42823383333333	8.505532025	9.389393125	8.000994225	9.19676581666667	8.940002325	8.21830426666667	8.91938296666667	8.9131846	7.99937465833333	8.51908186666667	8.62596224166667	8.49347309166667	9.778305875	8.39672325833333	9.17691023333333	8.96322584166667	8.79240648333333	7.7587684	7.586837175	8.26207273333333	8.920781025	9.67544948333333	7.73639724166667	10.3817731416667	9.63430166666667	9.77176594166667	7.911899075	8.5569931	8.21782325833333	8.37604669166667	8.21566409166667	7.94065678333333	7.59310200833333	8.64839240833333	9.28551639166667	7.99131585833333	7.95035304166667	8.83493181666667	8.67553405	10.8702441	8.19970503333333	8.846155575	7.54405583333333	7.23193100833333	7.694854575	8.151087675	8.6404919	6.44430819166667	8.73448420833333	8.46405893333333	10.4815083083333	7.19636080833333	9.0174923	8.91457238333333	8.234668275	7.662739425	8.67077851666667	7.3477901	7.16490981666667	8.739401675

FAM178B	10.4503313916667	8.354234475	8.71838005	9.22423163333333	9.5013607	8.34473695	9.17646485833333	8.729717925	8.81836040833333	8.78745048333333	9.06503669166667	9.16074203333333	8.89262756666667	8.26039324166667	8.603908075	8.21566409166667	7.49390345833333	9.68863114166667	8.62834993333333	8.43902399166667	8.00462699166667	8.55543884166667	8.06551123333333	7.85201126666667	8.18838901666667	9.24933856666667	9.1237399	9.27853935	9.3434528	9.114481175	8.894577375	8.53238421666667	8.7532663	7.80329328333333	9.73955326666667	9.32044345833333	9.354988125	8.37864189166667	8.54656363333333	8.75743711666667	8.612418275	9.25628595833333	7.927602375	8.28242620833333	8.92602376666667	8.22928075833333	8.39560916666667	8.529235525	8.30033485833333	8.228841125	8.60751143333333	8.80050660833333	8.907621175	8.86999053333333	8.50451981666667	8.42733516666667	8.32062069166667	8.3151234	8.63497865	8.338570375	9.05030123333333	8.89220491666667	8.74671439166667	9.74433669166667	8.50702275833333	9.889693225	8.20781665833333	8.9217463	9.26773438333333	8.95262451666667	9.14921909166667	8.96414444166667	9.14608020833333	8.16633889166667	8.80540845	8.20331464166667	9.4999428	8.30641555	8.16178343333333	9.41436835833333	8.45404219166667	8.50130601666667	8.51177801666667	8.05717868333333	9.37548823333333	9.65621849166667	10.23098885	9.23605358333333	9.74433669166667	8.65513670833333	9.53738644166667	10.1009165916667	9.063171125	8.34620800833333	9.63806365	9.09878833333333	9.75787298333333	9.51408459166667	9.99125556666667	9.114481175	9.77049406666667	9.05075425833333	8.22099113333333	8.6774004	8.81971978333333	9.862235575	9.49101396666667	8.37653824166667	8.73109978333333	8.0294547	9.08543134166667	8.59154009166667	8.42983770833333	7.94622360416667	8.1963928	8.87183624166667	8.33965076666667	9.12326560833333	8.71647500833333	8.398242975

GPAT2	10.17959865	9.9420301	10.6846028416667	10.4910656666667	10.91124625	10.842072875	11.1611143583333	10.327316425	10.66752385	10.5631726083333	10.2943474166667	11.1869945583333	11.35644045	10.3070468	9.52504286666667	10.1176213666667	10.0242607916667	10.8232399083333	9.07777099166667	10.0171966666667	10.1497399666667	9.82860243333333	9.37637135833333	9.72156395833333	9.19576385833333	10.3396540083333	9.54178184166667	10.1076098833333	8.97675146666667	9.952821075	9.56036118333333	9.60319949166667	10.1029681166667	11.1003561666667	10.692247	10.4123976	10.1931783583333	10.0708045166667	9.76051965	9.70714295	9.26538065833333	10.0997208916667	9.79724158333333	9.88845614166667	10.1670505916667	8.7217903	8.73639624166667	9.43045813333333	8.70034938333333	9.89894956666667	9.619021525	9.83111589166667	10.225450075	9.18071311666667	9.71098871666667	8.81463643333333	9.85239641666667	8.80925365833333	9.063171125	9.187855275	10.3321140166667	10.80942375	10.220242425	10.5973351833333	10.6902661583333	10.3566062583333	10.35378905	11.0822289416667	10.2699462416667	10.3035388166667	10.6876859	10.638037975	11.3306591916667	10.674973575	10.5605583916667	10.4272415916667	10.3044270083333	10.0226518416667	9.53443595	10.507752825	10.8968726333333	10.4783084333333	9.59563205	9.925380825	10.485478825	10.7919885666667	10.490641975	11.178312475	10.620734875	10.1755237	10.2900500333333	9.53875820833333	9.63111984166667	9.80885460833333	10.3901677	10.341641975	9.98715419166667	10.1063080916667	10.1262351166667	9.67854975	9.882085	11.2646484083333	9.74483329166667	9.553861875	9.74951903333333	9.64922493333333	9.80755264166667	9.29796166666667	9.15505945	9.653557625	9.42687719166667	9.32368785833333	9.31762243333333	9.18071311666667	9.90608706666667	9.42204840833333	9.83452378333333	10.6538835666667	9.14921909166667	10.015183425

EVI5	11.1043306833333	10.9987091583333	11.1641077166667	10.874072375	10.5436778583333	11.104988	11.18844885	11.322793725	11.1901541416667	11.121759625	11.2406150083333	11.7403808583333	10.6637664333333	11.2858355	11.1332425666667	11.3567956166667	11.8407167416667	10.5979724666667	11.054552625	11.1678068583333	11.3140591833333	11.2413554333333	11.1554142916667	11.0689648166667	11.0116340666667	11.5253735333333	11.0416560583333	10.938251225	10.4783084333333	11.3047106916667	10.8491534833333	11.0394398083333	10.99291925	11.3246780083333	11.3567956166667	11.4879615666667	10.789595925	11.327862475	11.075444825	11.3527786833333	11.392737725	11.331019575	11.0390903416667	11.2050434916667	11.2240675166667	11.2230202916667	11.2481821666667	11.2417551	11.4154857416667	11.55164095	11.0279781	11.62870925	10.5783390833333	11.0804603833333	11.113611625	11.2464355333333	11.5983924583333	11.4752696416667	11.2947092833333	11.230125425	9.53914050833333	10.88143175	10.3293667083333	10.5208646083333	11.2251399	10.723219775	11.0002353666667	11.0190136	10.9869124833333	11.325017625	10.931065425	9.99851741666667	11.0239555333333	11.2722818166667	10.8220014333333	11.0793725416667	10.7767059333333	10.647680825	10.81908485	11.06778735	11.012449925	10.4153772	10.9196649333333	11.0200205166667	11.1203060666667	10.8187575416667	9.990452125	10.99291925	9.88168326666667	11.6647417666667	10.5173180916667	10.7940186083333	10.666810425	10.85184045	10.7004993416667	10.4858474583333	10.4858474583333	10.5643520333333	10.7684448166667	11.2550301583333	11.0589538416667	10.9946234375	10.523602425	11.0228735916667	10.992167075	10.9087616083333	11.2534278333333	10.680077475	10.6691322083333	11.5926397083333	11.2756978833333	11.0611890333333	10.678502425	10.5088788416667	11.017853675	11.1997660583333	11.2341636166667	11.1828701833333	10.8476613083333	10.8796402833333

ZNF582	8.0294547	6.622274	6.68067039166667	6.72123403333333	5.948900025	6.463588	5.99349075	6.70796138333333	6.44737514166667	5.87208589166667	6.07429128333333	6.92501696666667	6.26064218333333	6.25755120833333	5.91431983333333	6.92374801666667	6.49247578333333	5.61547525833333	6.72825601666667	6.73002575	6.70465071666667	6.29278439166667	6.51310794166667	7.24765453333333	8.05141264166667	7.9693647	7.548601625	7.06220724166667	6.698517925	6.56509979166667	6.245275725	6.4372196	6.53954924166667	6.49087593333333	7.80227559166667	7.90206838333333	6.68727483333333	7.02761371666667	6.8399633	7.05585056666667	6.90781049166667	6.32106796666667	7.17851245	6.90252933333333	6.89915848333333	6.98158215833333	7.05338414166667	6.28898518333333	6.96399793333333	6.79529673333333	6.27095864166667	6.44430819166667	5.94185621666667	6.560129475	6.88718085	6.745396875	6.12691128333333	6.94120649166667	6.75898810833333	7.189475125	6.20884429166667	6.32454565	6.60536920833333	6.311629325	6.160301025	6.013009825	6.4516666	6.575829775	5.83208956666667	6.10713235	6.24033626666667	6.36327274166667	4.91206978333333	6.23159324166667	5.50338315833333	6.258372575	6.20884429166667	5.892821925	5.6250378	5.88144885	5.70748306666667	5.997550525	5.43182890833333	6.72519365833333	6.11387184166667	5.71985138333333	6.602072175	6.49087593333333	6.00206103333333	7.16490981666667	5.485111725	6.19818808333333	6.81253920833333	6.27482028333333	6.08940326666667	6.14803923333333	6.50275845	6.1777684	6.32454565	6.408778075	7.414082675	5.760490375	6.50580336666667	6.38621459166667	6.92374801666667	6.00039116666667	6.465677275	6.43572405833333	6.256698975	6.49087593333333	6.54574596666667	6.21030875833333	5.58597898333333	6.20408345833333	6.931726825	5.84699576666667	5.192100225	6.94057503333333	6.18568551666667	6.23312839166667

NAA50	14.2686692416667	14.1384134166667	14.5984675666667	14.7034991833333	14.5377311666667	14.9020399416667	14.4581253166667	14.0643996166667	14.3960605	14.1175943833333	14.53402895	13.6000874166667	14.20293335	14.1384134166667	13.9493522333333	14.269993075	13.6328876166667	14.046645825	14.31061245	13.9625123916667	13.7055820166667	13.8854083166667	14.5140672583333	14.1957149333333	14.2936594416667	13.4042461583333	14.0964870833333	14.4547788916667	14.2858034416667	14.295197475	13.96485035	14.4043399	14.3236760583333	13.91423525	13.593805675	13.5876594833333	13.9657662833333	13.9873624166667	14.1737580083333	14.3724417	14.26265275	14.1175943833333	13.6666988416667	14.0012596166667	13.7902025333333	14.1527055166667	14.1500101916667	14.4615780083333	13.9576902	14.29207405	14.3283993416667	13.873143175	13.870943825	13.8088736083333	14.0669942916667	13.3416873833333	14.0301250916667	14.0950375583333	13.6884802666667	13.6737470333333	14.0800041333333	14.30615445	13.9727862416667	14.160512975	13.695137875	13.62200835	14.13703675	14.3381592083333	14.2334791	14.4092298666667	14.3013820416667	14.2563286	13.9250365	14.0113488	13.96485035	14.0188677666667	14.1471335166667	13.9469444833333	14.0113488	14.3013820416667	14.2936594416667	13.4058013416667	13.6737470333333	14.1590921166667	14.1698024416667	14.2610479166667	14.4060458333333	13.6996187916667	14.201540175	13.9445631916667	14.27950095	14.083694575	14.0847383833333	14.0899292916667	14.2378149833333	14.0979296916667	14.0540171833333	13.9411608583333	13.935575975	13.4366933666667	14.269993075	14.3381592083333	14.85238655	14.269993075	14.1228795	13.8494140916667	13.6133055333333	13.5668551916667	14.063152775	13.913193025	13.7949722083333	14.2872888583333	13.3839194083333	13.8743989166667	14.2219861583333	14.5038815833333	13.7007366166667	13.89301535	14.2548502583333	13.916524275

SEC14L6	7.65894785833333	6.64088086666667	6.715741525	7.42162103333333	6.90715841666667	6.76049560833333	7.460763125	7.17033845	6.98530065833333	6.63179246666667	6.81316333333333	8.11098743333333	7.39341365	7.10630213333333	6.40489274166667	7.681528525	8.466090675	7.28198013333333	6.657721925	6.76253231666667	8.04234110833333	6.86605038333333	6.70856058333333	7.746730925	6.32454565	7.652791	6.560129475	7.11730268333333	7.33202409166667	7.093633575	6.64559758333333	6.02015538333333	6.80784224166667	8.4939941	7.949320675	7.882902775	6.91578644166667	6.864696	6.49087593333333	6.15274419166667	6.2858525	7.58961838333333	6.80853135	6.59234331666667	7.78383044166667	7.06279625833333	6.5994761	6.46133099166667	6.475087675	6.85607033333333	6.02645373333333	5.58988304166667	6.84850089166667	6.64834389166667	6.97214965	6.99282755	6.57166325	6.46133099166667	6.70465071666667	6.26504835	6.85677963333333	7.075905	6.9393833	8.10330963333333	7.22110665	7.43011818333333	7.52279740833333	7.65326568333333	8.435679275	7.27359608333333	7.07292095833333	7.33560889166667	7.82793564166667	7.75332493333333	7.02704855833333	7.68042426666667	7.71678190833333	6.72953939166667	7.41121605	7.23254185	7.03384980833333	6.89980234166667	7.69651605833333	7.39450265833333	7.61062251666667	8.13242494166667	7.41942373333333	8.05249740833333	7.64430480833333	6.54716043333333	7.15389984166667	6.94497745833333	6.22368095	6.71918628333333	7.17093558333333	7.17971484166667	6.4975584	7.340949	7.363992875	6.69781155	6.27165035	7.717344425	6.77836881666667	6.62839033333333	6.72313661666667	7.35293989166667	7.38051639166667	7.37184780833333	7.548601625	7.76455869166667	6.22446936666667	6.39534953333333	7.43805644166667	7.01007860833333	6.72953939166667	6.37197970833333	6.99601435833333	6.78180254166667	6.258372575	6.880500475

DCDC1	4.11480768333333	3.92586006666667	4.27276220833333	4.05417596666667	5.37867954166667	3.88526006666667	6.01758168333333	4.40083348333333	5.45901654166667	4.47861730833333	4.61520515	3.911419125	3.97815159166667	4.8072374	4.56600969166667	4.160694875	4.87823230833333	5.70153421666667	4.31702865	5.11118121666667	3.98946053333333	4.021539275	4.77813741666667	4.18603395833333	4.41782250833333	3.89679799166667	4.337410475	4.33532039166667	5.327612425	4.1481371	4.41002290833333	4.332336275	5.38064126666667	4.73206480833333	4.082140775	4.08114406666667	4.73928888333333	4.3549546	4.23901671666667	4.46791538333333	4.59784559166667	4.27374416666667	4.48432390833333	5.127309825	3.92371073333333	4.64033936666667	4.51475793333333	4.54330015	4.27700685	4.515720925	4.53433785	4.32094003333333	5.92509355833333	4.93337844166667	4.66727606666667	4.41962694166667	5.23876719166667	4.58140610833333	4.12106518333333	4.03660776666667	3.75570144166667	4.79318954166667	5.58597898333333	4.99894050833333	5.43281713333333	5.03010108333333	5.54303723333333	5.11118121666667	4.16407313333333	4.955313625	5.12313813333333	3.62768649166667	3.84584678333333	4.75255506666667	5.29675638333333	4.19810324166667	4.81075010833333	4.262047775	5.61547525833333	5.45375554166667	6.04523669166667	4.56909456666667	4.25573735	3.81605024166667	4.32010620833333	3.90095365833333	5.53793416666667	4.26758835	5.508179525	4.160694875	4.681784575	4.285092425	3.67515746666667	5.04762459166667	4.322875175	4.99586916666667	4.602780325	4.54330015	4.63410609166667	4.70905974166667	4.86589074166667	3.63913164166667	4.480628975	4.67067994166667	4.02799813333333	4.25100265833333	4.27896138333333	5.390724	4.085428875	5.63443499166667	4.46004483333333	4.97490869166667	4.05192228333333	4.57535033333333	5.43935509166667	4.17980874166667	5.32557380833333	7.22598754166667	4.26116580833333	5.066744825

TMOD4	10.1096251916667	8.611944975	8.126799325	8.656035175	8.7374204	8.38319035	9.08180965	8.57007116666667	8.80300369166667	8.23613304166667	8.66247435	9.48341896666667	9.11266035833333	8.532903275	8.46405893333333	8.31670425833333	7.68829090833333	8.57257758333333	8.254482075	8.37361221666667	8.650306125	8.291997825	8.05717868333333	8.45954589166667	8.0063663	10.7258049	8.37211495	8.44794389166667	8.214548775	8.251367875	8.26308525833333	8.33810663333333	8.36762415833333	8.05141264166667	10.5581448083333	10.4176619666667	8.174528375	8.122791675	8.6774004	8.278086225	7.928121775	8.06551123333333	7.949320675	8.37653824166667	8.58808473333333	7.87717198333333	8.416908275	8.50037185	8.14754213333333	8.02070828333333	8.3023979	8.26097460833333	8.57783373333333	8.22928075833333	8.182610775	8.20831443333333	8.44491509166667	8.14799835	8.01539524166667	8.09223929166667	9.13226131666667	9.08687805	8.28445111666667	9.02927973333333	8.74766549166667	8.75927773333333	8.94761291666667	9.01521926666667	8.4841298	8.975801525	9.01566145833333	9.06886545833333	9.51088574166667	8.57301913333333	8.902412325	8.62383624166667	8.48112818333333	8.52826969166667	8.13668228333333	8.78942864166667	8.82920276666667	9.0517655	8.47382378333333	8.286335875	8.75703444166667	9.063171125	8.86508634166667	9.32615619166667	9.71307581666667	8.15349528333333	8.72356944166667	8.26606834166667	9.15064904166667	8.54656363333333	8.98465464166667	9.28087420833333	8.37767194166667	8.63449166666667	8.88020864166667	8.560933175	8.26655316666667	9.00690949166667	8.65466659166667	8.709455975	8.11940264166667	8.1073987	8.445841225	8.59397561666667	8.18422995833333	7.771005975	7.9977282	8.19543675833333	8.25081844166667	8.05032904166667	8.5196153	8.36807433333333	8.49539040833333	8.498393225	8.58808473333333	8.757884525

VPS72	10.1096251916667	8.611944975	8.126799325	8.656035175	8.7374204	8.38319035	9.08180965	8.57007116666667	8.80300369166667	8.23613304166667	8.66247435	9.48341896666667	9.11266035833333	8.532903275	8.46405893333333	8.31670425833333	7.68829090833333	8.57257758333333	8.254482075	8.37361221666667	8.650306125	8.291997825	8.05717868333333	8.45954589166667	8.0063663	10.7258049	8.37211495	8.44794389166667	8.214548775	8.251367875	8.26308525833333	8.33810663333333	8.36762415833333	8.05141264166667	10.5581448083333	10.4176619666667	8.174528375	8.122791675	8.6774004	8.278086225	7.928121775	8.06551123333333	7.949320675	8.37653824166667	8.58808473333333	7.87717198333333	8.416908275	8.50037185	8.14754213333333	8.02070828333333	8.3023979	8.26097460833333	8.57783373333333	8.22928075833333	8.182610775	8.20831443333333	8.44491509166667	8.14799835	8.01539524166667	8.09223929166667	9.13226131666667	9.08687805	8.28445111666667	9.02927973333333	8.74766549166667	8.75927773333333	8.94761291666667	9.01521926666667	8.4841298	8.975801525	9.01566145833333	9.06886545833333	9.51088574166667	8.57301913333333	8.902412325	8.62383624166667	8.48112818333333	8.52826969166667	8.13668228333333	8.78942864166667	8.82920276666667	9.0517655	8.47382378333333	8.286335875	8.75703444166667	9.063171125	8.86508634166667	9.32615619166667	9.71307581666667	8.15349528333333	8.72356944166667	8.26606834166667	9.15064904166667	8.54656363333333	8.98465464166667	9.28087420833333	8.37767194166667	8.63449166666667	8.88020864166667	8.560933175	8.26655316666667	9.00690949166667	8.65466659166667	8.709455975	8.11940264166667	8.1073987	8.445841225	8.59397561666667	8.18422995833333	7.771005975	7.9977282	8.19543675833333	8.25081844166667	8.05032904166667	8.5196153	8.36807433333333	8.49539040833333	8.498393225	8.58808473333333	8.757884525

SCNM1	10.1096251916667	8.611944975	8.126799325	8.656035175	8.7374204	8.38319035	9.08180965	8.57007116666667	8.80300369166667	8.23613304166667	8.66247435	9.48341896666667	9.11266035833333	8.532903275	8.46405893333333	8.31670425833333	7.68829090833333	8.57257758333333	8.254482075	8.37361221666667	8.650306125	8.291997825	8.05717868333333	8.45954589166667	8.0063663	10.7258049	8.37211495	8.44794389166667	8.214548775	8.251367875	8.26308525833333	8.33810663333333	8.36762415833333	8.05141264166667	10.5581448083333	10.4176619666667	8.174528375	8.122791675	8.6774004	8.278086225	7.928121775	8.06551123333333	7.949320675	8.37653824166667	8.58808473333333	7.87717198333333	8.416908275	8.50037185	8.14754213333333	8.02070828333333	8.3023979	8.26097460833333	8.57783373333333	8.22928075833333	8.182610775	8.20831443333333	8.44491509166667	8.14799835	8.01539524166667	8.09223929166667	9.13226131666667	9.08687805	8.28445111666667	9.02927973333333	8.74766549166667	8.75927773333333	8.94761291666667	9.01521926666667	8.4841298	8.975801525	9.01566145833333	9.06886545833333	9.51088574166667	8.57301913333333	8.902412325	8.62383624166667	8.48112818333333	8.52826969166667	8.13668228333333	8.78942864166667	8.82920276666667	9.0517655	8.47382378333333	8.286335875	8.75703444166667	9.063171125	8.86508634166667	9.32615619166667	9.71307581666667	8.15349528333333	8.72356944166667	8.26606834166667	9.15064904166667	8.54656363333333	8.98465464166667	9.28087420833333	8.37767194166667	8.63449166666667	8.88020864166667	8.560933175	8.26655316666667	9.00690949166667	8.65466659166667	8.709455975	8.11940264166667	8.1073987	8.445841225	8.59397561666667	8.18422995833333	7.771005975	7.9977282	8.19543675833333	8.25081844166667	8.05032904166667	8.5196153	8.36807433333333	8.49539040833333	8.498393225	8.58808473333333	8.757884525

GP1BB	10.6511688416667	12.0921972916667	12.0457744	11.6306161333333	11.8484879416667	9.94164523333333	11.5716921416667	11.8205903833333	11.608877475	11.460857775	10.776004825	11.4086625666667	11.48757865	11.605536575	11.9994097833333	10.8212945583333	9.66237621666667	11.6159444083333	11.8768607333333	11.5794626	11.216912225	11.2902838583333	11.251242475	11.1095361333333	11.4515417166667	10.4300821583333	11.9348926	11.770678525	11.9645775083333	11.63218295	11.8970046083333	11.6448777666667	11.2668058583333	9.22470684166667	11.0563524083333	10.7414541583333	12.120943025	12.2015812416667	11.714258625	11.4530731666667	11.8051550333333	11.9883632916667	11.5887304416667	11.856139525	10.971536625	11.8130518166667	11.8081782916667	12.1174426416667	10.5073525916667	10.6061332083333	11.3104909	11.6529488416667	12.0953574416667	11.7288583666667	11.7913817166667	11.7768584833333	11.5279761083333	11.798162425	11.5185086916667	11.3786114833333	10.15376285	11.9184658666667	10.0347123333333	12.3067398	11.6514721333333	10.9266566	11.1506157166667	9.51088574166667	10.3097382833333	9.71951955833333	11.7505625666667	10.9684950083333	10.4570074666667	10.959358675	10.216230425	10.7581339416667	8.8039806	9.58040200833333	11.43611165	9.67230795	10.9497712833333	10.5300186083333	10.082743125	11.1169690833333	11.0916995	11.0946623333333	10.5768008666667	10.5802645333333	10.6373110916667	10.2051938916667	10.2109736166667	10.5432527416667	9.90775529166667	10.4561678833333	10.6050909833333	11.2606101916667	9.47987389166667	11.5501880833333	10.3147212916667	10.8424532083333	9.956863325	10.9259950416667	8.38370515833333	9.86989941666667	10.181229625	9.114481175	10.5416886583333	10.0343496666667	10.59381915	10.3380831916667	9.92747266666667	9.82203395	11.717933225	9.48126221666667	10.1246349583333	11.9509854083333	11.5041708333333	10.220242425	12.1545013166667	8.635919475

SEPTIN5	10.6511688416667	12.0921972916667	12.0457744	11.6306161333333	11.8484879416667	9.94164523333333	11.5716921416667	11.8205903833333	11.608877475	11.460857775	10.776004825	11.4086625666667	11.48757865	11.605536575	11.9994097833333	10.8212945583333	9.66237621666667	11.6159444083333	11.8768607333333	11.5794626	11.216912225	11.2902838583333	11.251242475	11.1095361333333	11.4515417166667	10.4300821583333	11.9348926	11.770678525	11.9645775083333	11.63218295	11.8970046083333	11.6448777666667	11.2668058583333	9.22470684166667	11.0563524083333	10.7414541583333	12.120943025	12.2015812416667	11.714258625	11.4530731666667	11.8051550333333	11.9883632916667	11.5887304416667	11.856139525	10.971536625	11.8130518166667	11.8081782916667	12.1174426416667	10.5073525916667	10.6061332083333	11.3104909	11.6529488416667	12.0953574416667	11.7288583666667	11.7913817166667	11.7768584833333	11.5279761083333	11.798162425	11.5185086916667	11.3786114833333	10.15376285	11.9184658666667	10.0347123333333	12.3067398	11.6514721333333	10.9266566	11.1506157166667	9.51088574166667	10.3097382833333	9.71951955833333	11.7505625666667	10.9684950083333	10.4570074666667	10.959358675	10.216230425	10.7581339416667	8.8039806	9.58040200833333	11.43611165	9.67230795	10.9497712833333	10.5300186083333	10.082743125	11.1169690833333	11.0916995	11.0946623333333	10.5768008666667	10.5802645333333	10.6373110916667	10.2051938916667	10.2109736166667	10.5432527416667	9.90775529166667	10.4561678833333	10.6050909833333	11.2606101916667	9.47987389166667	11.5501880833333	10.3147212916667	10.8424532083333	9.956863325	10.9259950416667	8.38370515833333	9.86989941666667	10.181229625	9.114481175	10.5416886583333	10.0343496666667	10.59381915	10.3380831916667	9.92747266666667	9.82203395	11.717933225	9.48126221666667	10.1246349583333	11.9509854083333	11.5041708333333	10.220242425	12.1545013166667	8.635919475

AC000093.1	10.6511688416667	12.0921972916667	12.0457744	11.6306161333333	11.8484879416667	9.94164523333333	11.5716921416667	11.8205903833333	11.608877475	11.460857775	10.776004825	11.4086625666667	11.48757865	11.605536575	11.9994097833333	10.8212945583333	9.66237621666667	11.6159444083333	11.8768607333333	11.5794626	11.216912225	11.2902838583333	11.251242475	11.1095361333333	11.4515417166667	10.4300821583333	11.9348926	11.770678525	11.9645775083333	11.63218295	11.8970046083333	11.6448777666667	11.2668058583333	9.22470684166667	11.0563524083333	10.7414541583333	12.120943025	12.2015812416667	11.714258625	11.4530731666667	11.8051550333333	11.9883632916667	11.5887304416667	11.856139525	10.971536625	11.8130518166667	11.8081782916667	12.1174426416667	10.5073525916667	10.6061332083333	11.3104909	11.6529488416667	12.0953574416667	11.7288583666667	11.7913817166667	11.7768584833333	11.5279761083333	11.798162425	11.5185086916667	11.3786114833333	10.15376285	11.9184658666667	10.0347123333333	12.3067398	11.6514721333333	10.9266566	11.1506157166667	9.51088574166667	10.3097382833333	9.71951955833333	11.7505625666667	10.9684950083333	10.4570074666667	10.959358675	10.216230425	10.7581339416667	8.8039806	9.58040200833333	11.43611165	9.67230795	10.9497712833333	10.5300186083333	10.082743125	11.1169690833333	11.0916995	11.0946623333333	10.5768008666667	10.5802645333333	10.6373110916667	10.2051938916667	10.2109736166667	10.5432527416667	9.90775529166667	10.4561678833333	10.6050909833333	11.2606101916667	9.47987389166667	11.5501880833333	10.3147212916667	10.8424532083333	9.956863325	10.9259950416667	8.38370515833333	9.86989941666667	10.181229625	9.114481175	10.5416886583333	10.0343496666667	10.59381915	10.3380831916667	9.92747266666667	9.82203395	11.717933225	9.48126221666667	10.1246349583333	11.9509854083333	11.5041708333333	10.220242425	12.1545013166667	8.635919475

DEXI	11.7147778583333	10.4270534416667	10.8860148666667	10.611011125	11.1141688833333	10.9467539833333	10.6966451083333	10.611011125	10.729338025	10.6106220083333	10.7610940708333	11.5477833125	11.2773858916667	10.5859331041667	10.7342462625	10.5928418416667	10.9903918291667	10.7462083208333	10.9067564458333	10.7075994125	10.7898099041667	10.8159554791667	10.3892826375	10.5484121083333	10.5932017375	11.4862831666667	10.7476260458333	10.5191499125	11.6626930166667	10.6946895333333	10.7197888416667	10.6295882833333	10.4326486208333	10.636687025	11.4502097708333	11.9153070541667	10.9514615666667	10.8118916416667	10.7083386958333	10.8761777916667	10.8214613708333	10.8043373583333	10.3145290375	10.4784843041667	10.9023129541667	10.7868548458333	10.8275606916667	10.77343325	10.4346949458333	10.2264568791667	10.5603497416667	10.9330242333333	10.9781754416667	10.8222020333333	10.9362758541667	10.9118469166667	10.4781179666667	10.5028481541667	10.4575947458333	10.5525003958333	11.2578042416667	10.5908592791667	10.4988759958333	10.4039049166667	11.2245952666667	11.3131039666667	10.6264955333333	10.6643372291667	11.0496103458333	10.40551365	10.6932567541667	11.6300105791667	11.0285047666667	10.5110240083333	11.1785131916667	10.9096329166667	10.838438825	10.7765403041667	10.6268600541667	10.6893533666667	10.808435125	10.8047085958333	10.81782005	10.6009811166667	10.7879800333333	10.9217726166667	10.3572847458333	10.583545725	10.8243180916667	10.80618245	11.0411335166667	10.9728508875	10.4342858708333	11.080269225	11.4436969583333	10.7879800333333	10.6769119083333	11.1602403666667	10.9464365791667	11.0339171708333	11.1212353541667	10.7222655375	10.744209625	10.5993204833333	10.9649539375	10.8253053375	11.2080698458333	11.1617286625	11.1751395166667	10.1603514333333	10.1426412375	10.7641149791667	10.9213971291667	10.606680675	11.0687843333333	10.5762546958333	10.7547794375	10.4957078083333	10.6342069875	10.1806562125

CIITA	11.7147778583333	10.4270534416667	10.8860148666667	10.611011125	11.1141688833333	10.9467539833333	10.6966451083333	10.611011125	10.729338025	10.6106220083333	10.7610940708333	11.5477833125	11.2773858916667	10.5859331041667	10.7342462625	10.5928418416667	10.9903918291667	10.7462083208333	10.9067564458333	10.7075994125	10.7898099041667	10.8159554791667	10.3892826375	10.5484121083333	10.5932017375	11.4862831666667	10.7476260458333	10.5191499125	11.6626930166667	10.6946895333333	10.7197888416667	10.6295882833333	10.4326486208333	10.636687025	11.4502097708333	11.9153070541667	10.9514615666667	10.8118916416667	10.7083386958333	10.8761777916667	10.8214613708333	10.8043373583333	10.3145290375	10.4784843041667	10.9023129541667	10.7868548458333	10.8275606916667	10.77343325	10.4346949458333	10.2264568791667	10.5603497416667	10.9330242333333	10.9781754416667	10.8222020333333	10.9362758541667	10.9118469166667	10.4781179666667	10.5028481541667	10.4575947458333	10.5525003958333	11.2578042416667	10.5908592791667	10.4988759958333	10.4039049166667	11.2245952666667	11.3131039666667	10.6264955333333	10.6643372291667	11.0496103458333	10.40551365	10.6932567541667	11.6300105791667	11.0285047666667	10.5110240083333	11.1785131916667	10.9096329166667	10.838438825	10.7765403041667	10.6268600541667	10.6893533666667	10.808435125	10.8047085958333	10.81782005	10.6009811166667	10.7879800333333	10.9217726166667	10.3572847458333	10.583545725	10.8243180916667	10.80618245	11.0411335166667	10.9728508875	10.4342858708333	11.080269225	11.4436969583333	10.7879800333333	10.6769119083333	11.1602403666667	10.9464365791667	11.0339171708333	11.1212353541667	10.7222655375	10.744209625	10.5993204833333	10.9649539375	10.8253053375	11.2080698458333	11.1617286625	11.1751395166667	10.1603514333333	10.1426412375	10.7641149791667	10.9213971291667	10.606680675	11.0687843333333	10.5762546958333	10.7547794375	10.4957078083333	10.6342069875	10.1806562125

RBKS	11.057303875	11.3592652083333	11.4539903333333	12.2143904666667	11.8914215583333	10.2680768125	11.7733518416667	11.6350057	11.5946751791667	11.0749304708333	11.554659475	11.9265147625	11.8525516125	11.5168267208333	11.5361704583333	11.2652162833333	10.6102173625	12.0303737	10.9511066541667	11.1281108083333	11.6888934125	11.6146448666667	11.2472751583333	11.0464348875	10.8437881666667	10.6239107333333	11.0857661208333	11.3034904208333	11.519869425	11.4558293625	11.0595031666667	11.0926102958333	11.4859582416667	10.6401974666667	10.1402993125	10.0324278916667	11.5460149333333	10.7837402125	11.0165979833333	10.8572859083333	11.2027111833333	11.6843276458333	11.0029584166667	11.1421225416667	11.338684375	11.4070014625	11.1219183125	11.3167353625	10.92756005	11.0019008875	10.7067774041667	10.91144995	11.5686211083333	11.5518277166667	11.4505536166667	11.2469375791667	11.0364632541667	11.1182603	11.2574172166667	10.8107375166667	11.2329090166667	11.1591689333333	11.9122100125	11.7465124875	11.4804091958333	11.6004544833333	10.5971126125	11.9316685625	11.7233523208333	11.3077018833333	11.16873495	11.6399543958333	11.6353721333333	10.97708	12.0430500916667	10.6378726916667	11.6157672875	10.5920088875	10.8354975708333	11.6797556625	10.9085952333333	12.3445929125	11.6733013	10.413312325	11.2030606	11.4070014625	11.0191663458333	12.0342883	11.1093686541667	10.6759246125	11.3501118625	11.0184106916667	10.3425891416667	10.7462083208333	10.6798983583333	11.1279420708333	10.68787635	11.2673639375	11.7705132166667	10.3382680958333	11.4796400458333	11.7987540875	10.9960182041667	11.0951969958333	10.7950239375	11.45289505	11.015195575	10.9243307625	11.0237761875	10.4920196458333	10.7191643541667	10.8313562916667	11.1676132291667	11.0371668916667	11.5580599041667	11.0198453583333	10.7942240375	11.0756221166667	11.0371668916667	10.456786775

MRPL33	11.057303875	11.3592652083333	11.4539903333333	12.2143904666667	11.8914215583333	10.2680768125	11.7733518416667	11.6350057	11.5946751791667	11.0749304708333	11.554659475	11.9265147625	11.8525516125	11.5168267208333	11.5361704583333	11.2652162833333	10.6102173625	12.0303737	10.9511066541667	11.1281108083333	11.6888934125	11.6146448666667	11.2472751583333	11.0464348875	10.8437881666667	10.6239107333333	11.0857661208333	11.3034904208333	11.519869425	11.4558293625	11.0595031666667	11.0926102958333	11.4859582416667	10.6401974666667	10.1402993125	10.0324278916667	11.5460149333333	10.7837402125	11.0165979833333	10.8572859083333	11.2027111833333	11.6843276458333	11.0029584166667	11.1421225416667	11.338684375	11.4070014625	11.1219183125	11.3167353625	10.92756005	11.0019008875	10.7067774041667	10.91144995	11.5686211083333	11.5518277166667	11.4505536166667	11.2469375791667	11.0364632541667	11.1182603	11.2574172166667	10.8107375166667	11.2329090166667	11.1591689333333	11.9122100125	11.7465124875	11.4804091958333	11.6004544833333	10.5971126125	11.9316685625	11.7233523208333	11.3077018833333	11.16873495	11.6399543958333	11.6353721333333	10.97708	12.0430500916667	10.6378726916667	11.6157672875	10.5920088875	10.8354975708333	11.6797556625	10.9085952333333	12.3445929125	11.6733013	10.413312325	11.2030606	11.4070014625	11.0191663458333	12.0342883	11.1093686541667	10.6759246125	11.3501118625	11.0184106916667	10.3425891416667	10.7462083208333	10.6798983583333	11.1279420708333	10.68787635	11.2673639375	11.7705132166667	10.3382680958333	11.4796400458333	11.7987540875	10.9960182041667	11.0951969958333	10.7950239375	11.45289505	11.015195575	10.9243307625	11.0237761875	10.4920196458333	10.7191643541667	10.8313562916667	11.1676132291667	11.0371668916667	11.5580599041667	11.0198453583333	10.7942240375	11.0756221166667	11.0371668916667	10.456786775

PALMD	12.57846135	13.3100924166667	13.5832773166667	13.160332775	14.204528175	13.065712375	13.3473938666667	13.817872525	13.537910275	13.279249925	13.506094125	12.3592921916667	13.332896575	13.20399525	13.8983181916667	13.0245075	10.4641916916667	13.8010236666667	13.8562499666667	13.6310840583333	13.788062875	13.6579175	12.524997525	13.189983975	13.6202060083333	12.3501237916667	13.2313413916667	13.4042461583333	14.1343040416667	13.1700826333333	13.6174285083333	13.461932225	13.1439382083333	10.72841425	11.9228391333333	13.1197255166667	13.7553717	13.5171924333333	12.993623425	13.3921452083333	13.542740525	13.3416873833333	13.4337458083333	13.7065253416667	13.0792819	13.3087607833333	13.0457183333333	13.1229606833333	12.79233935	13.7940225416667	12.8020915333333	13.0792819	13.4427852416667	13.9458581083333	13.4254222333333	13.1966148416667	13.841729125	13.6418962083333	13.3074551916667	13.004985075	11.2868731416667	11.9856427083333	10.6395867	10.119666675	12.02225065	12.86014235	11.403040875	12.5349027	9.91585179166667	12.2094988666667	11.612299875	11.45939305	12.1346602166667	11.520872575	12.2124743416667	11.3057739666667	12.686781525	11.8426813333333	12.6080271416667	10.2498045833333	11.4042087666667	10.9328378333333	11.249224775	10.6027208583333	12.61272965	12.8718544916667	10.2111469	12.57699495	11.3371736333333	12.4536816333333	11.8399235416667	9.7686764	12.6116906583333	12.8515337583333	12.4981202833333	10.938251225	10.2478255083333	11.2722818166667	12.4751035416667	10.9291888666667	12.1882257583333	10.9549623083333	10.7306227416667	10.8694922083333	12.2959914833333	10.5314888083333	12.231478975	11.2068826333333	11.6940775166667	12.6522495833333	12.1737118583333	12.660047425	13.1427455666667	11.0901980583333	13.1688283833333	11.6103014166667	10.7519521333333	11.51432045	12.1741251583333	11.0670126666667

ZNF106	13.4579324833333	12.600413	12.442132175	12.2374670666667	11.8791305416667	12.13546815	12.2163587	12.4190092583333	12.352843675	12.4019704583333	12.1570780583333	13.3675920333333	12.9224811	12.5657560416667	11.9975093	12.62011135	12.5057358	11.4752696416667	12.4087159666667	12.3842104583333	12.6517095833333	12.3908133166667	12.543462525	12.1891609666667	12.1669452	13.4383119666667	12.2354776	12.0388164916667	12.0017085416667	12.1998168583333	12.3602095583333	12.2240198833333	12.5754789333333	12.5306631166667	13.8088736083333	13.6941290583333	12.1410311166667	12.0253815583333	12.233149625	11.835989125	12.402892875	12.3575221583333	12.5084928916667	12.4554143666667	12.8860467083333	12.5093300166667	12.4795096083333	12.19731135	12.40597585	12.2986022833333	11.691783775	12.050259975	11.632505775	11.7985917083333	12.2540737	12.3789904833333	11.951715625	12.2994493333333	11.9052300666667	12.3479516833333	11.6336776833333	12.324942025	11.4485644833333	11.60398845	12.488903425	12.0593104333333	12.1720161916667	11.6238039583333	12.1100796583333	12.03257945	11.9100903833333	11.42107965	11.8920055333333	11.9184658666667	11.6429698666667	11.7411507916667	12.0710070666667	11.5656597	12.04824535	12.0156772833333	12.3789904833333	11.7905923666667	11.5664864	12.0237948	11.5776190666667	11.9892168333333	12.4922747583333	11.7898995	11.4718467833333	12.26972435	11.6187195083333	11.1641077166667	12.0096367416667	12.02225065	11.2068826333333	11.5836115333333	12.0136845833333	12.4705762833333	11.3608573416667	11.5121356333333	11.5887304416667	11.8171008	12.4229923333333	12.5135886333333	12.0160472666667	11.5319982916667	12.1241915	11.3801476916667	11.9364860583333	11.759780275	12.3147568916667	11.7742804333333	11.6823165333333	11.6536243916667	11.4570874916667	11.9670214	12.993623425	11.55164095	12.1762012583333	12.118235175

CBWD5	12.852746025	13.066353125	13.2690950583333	13.3087607833333	13.5068025166667	13.3080939833333	13.3575610333333	13.5468416333333	13.2503335416667	13.1919341833333	13.4274952333333	12.3627391666667	13.1356932666667	13.1779047083333	13.216246175	13.42164015	13.3248386416667	13.4366933666667	13.6107867916667	13.016186275	13.2279712833333	13.181363025	12.8584129833333	13.527156225	13.4481554583333	13.0613877916667	13.046364975	13.053017375	13.0264905416667	13.3732212583333	13.199462625	12.9756001333333	13.09075125	12.8590238833333	13.0337121333333	12.9213977333333	12.8213874666667	13.1336349	13.2460254166667	12.8537857833333	12.9328607583333	13.0613877916667	12.8329037166667	13.370423875	12.94284515	13.2047745416667	13.1216942833333	13.44656775	13.593805675	13.2678167166667	13.14792425	13.2293061833333	12.9610660166667	13.11105415	13.216246175	12.5989743166667	13.2628733666667	12.91963445	13.0136394416667	13.0204814	13.3080939833333	13.647954325	13.418751775	13.0421333333333	13.4847749333333	12.5029330166667	13.5876594833333	13.4474018083333	13.2587665833333	12.4425276083333	13.4289883	13.418751775	13.0368898	13.1178746666667	13.0102233	13.1952399083333	13.3809042	13.2983134833333	13.0620198083333	13.1905828833333	13.4611203333333	13.4352543166667	12.8381552416667	13.0985928583333	13.0343175416667	13.28687105	13.1420517833333	12.7103184	13.676743575	12.99120565	13.410428725	12.9172670333333	13.4550317916667	13.100692275	13.0864977	13.398021325	12.836627	13.0889930583333	12.3365706083333	12.620644525	13.22481105	13.7949722083333	12.856162525	13.1262418916667	13.17465125	13.3293632833333	12.606535325	13.0877248833333	12.622549625	12.8317521333333	13.1674768	13.0602371583333	13.118520075	13.1165645583333	12.659113	13.2553168666667	12.8046597666667	12.932363575	13.289124025	12.8751753583333

ZNF28	10.5416886583333	10.85221535	10.440565075	10.520091075	10.8807458916667	10.418815025	11.2986150666667	10.7356243	10.88817915	10.3035388166667	10.2115540166667	11.483228	10.7050450333333	10.8476613083333	10.4558195166667	10.953117425	11.1373020916667	10.43975535	10.244614225	10.9321190083333	10.8930750916667	10.87258615	10.6819563666667	10.6050909833333	10.7272324083333	10.572357075	10.6585232916667	10.5181477	10.1299873833333	10.6884721333333	10.0565842333333	10.8045420416667	10.7927886583333	10.9077413083333	10.136260225	10.6453411416667	10.5400727416667	10.548620125	10.173327525	10.3894512416667	10.0391614083333	10.1029681166667	10.8870209166667	10.2206735166667	10.7569232666667	10.2865677	10.742514025	10.3844721666667	10.8337493916667	10.4476616583333	10.2853426583333	10.5169707416667	10.5814300416667	10.4955307333333	10.4998350333333	10.2634356166667	10.7565121333333	10.013853075	10.6047191916667	10.4123976	11.1085089666667	10.8473279166667	10.222173225	11.0010313583333	11.0190136	10.1059188	10.9621867333333	11.0859242666667	10.4752224083333	10.8922670583333	10.8439824833333	11.156830125	10.4324595166667	10.9632585583333	11.2409567416667	10.469517225	11.279388675	10.1820473416667	9.6176772	10.2587469666667	11.2929247083333	11.8848963583333	9.86020724166667	10.5162662666667	10.64807625	10.99445015	11.3115193	10.9909672	11.4489345583333	10.4664769916667	10.669525325	10.3345761083333	11.0851990416667	11.5012483	11.3553466416667	10.067459125	11.1975258	11.0394398083333	10.021871175	10.4736621916667	11.36160315	10.5829592166667	10.5884400333333	10.2108003333333	11.876483925	10.3333340666667	10.7306227416667	10.6997392583333	10.6941625333333	10.515047025	10.3859567666667	10.39251605	10.3698580416667	10.5169707416667	10.364544	10.1573430916667	10.9567732916667	10.91088935	10.5388660583333	10.7368008833333

ZNF468	10.5416886583333	10.85221535	10.440565075	10.520091075	10.8807458916667	10.418815025	11.2986150666667	10.7356243	10.88817915	10.3035388166667	10.2115540166667	11.483228	10.7050450333333	10.8476613083333	10.4558195166667	10.953117425	11.1373020916667	10.43975535	10.244614225	10.9321190083333	10.8930750916667	10.87258615	10.6819563666667	10.6050909833333	10.7272324083333	10.572357075	10.6585232916667	10.5181477	10.1299873833333	10.6884721333333	10.0565842333333	10.8045420416667	10.7927886583333	10.9077413083333	10.136260225	10.6453411416667	10.5400727416667	10.548620125	10.173327525	10.3894512416667	10.0391614083333	10.1029681166667	10.8870209166667	10.2206735166667	10.7569232666667	10.2865677	10.742514025	10.3844721666667	10.8337493916667	10.4476616583333	10.2853426583333	10.5169707416667	10.5814300416667	10.4955307333333	10.4998350333333	10.2634356166667	10.7565121333333	10.013853075	10.6047191916667	10.4123976	11.1085089666667	10.8473279166667	10.222173225	11.0010313583333	11.0190136	10.1059188	10.9621867333333	11.0859242666667	10.4752224083333	10.8922670583333	10.8439824833333	11.156830125	10.4324595166667	10.9632585583333	11.2409567416667	10.469517225	11.279388675	10.1820473416667	9.6176772	10.2587469666667	11.2929247083333	11.8848963583333	9.86020724166667	10.5162662666667	10.64807625	10.99445015	11.3115193	10.9909672	11.4489345583333	10.4664769916667	10.669525325	10.3345761083333	11.0851990416667	11.5012483	11.3553466416667	10.067459125	11.1975258	11.0394398083333	10.021871175	10.4736621916667	11.36160315	10.5829592166667	10.5884400333333	10.2108003333333	11.876483925	10.3333340666667	10.7306227416667	10.6997392583333	10.6941625333333	10.515047025	10.3859567666667	10.39251605	10.3698580416667	10.5169707416667	10.364544	10.1573430916667	10.9567732916667	10.91088935	10.5388660583333	10.7368008833333

ZNF320	10.5416886583333	10.85221535	10.440565075	10.520091075	10.8807458916667	10.418815025	11.2986150666667	10.7356243	10.88817915	10.3035388166667	10.2115540166667	11.483228	10.7050450333333	10.8476613083333	10.4558195166667	10.953117425	11.1373020916667	10.43975535	10.244614225	10.9321190083333	10.8930750916667	10.87258615	10.6819563666667	10.6050909833333	10.7272324083333	10.572357075	10.6585232916667	10.5181477	10.1299873833333	10.6884721333333	10.0565842333333	10.8045420416667	10.7927886583333	10.9077413083333	10.136260225	10.6453411416667	10.5400727416667	10.548620125	10.173327525	10.3894512416667	10.0391614083333	10.1029681166667	10.8870209166667	10.2206735166667	10.7569232666667	10.2865677	10.742514025	10.3844721666667	10.8337493916667	10.4476616583333	10.2853426583333	10.5169707416667	10.5814300416667	10.4955307333333	10.4998350333333	10.2634356166667	10.7565121333333	10.013853075	10.6047191916667	10.4123976	11.1085089666667	10.8473279166667	10.222173225	11.0010313583333	11.0190136	10.1059188	10.9621867333333	11.0859242666667	10.4752224083333	10.8922670583333	10.8439824833333	11.156830125	10.4324595166667	10.9632585583333	11.2409567416667	10.469517225	11.279388675	10.1820473416667	9.6176772	10.2587469666667	11.2929247083333	11.8848963583333	9.86020724166667	10.5162662666667	10.64807625	10.99445015	11.3115193	10.9909672	11.4489345583333	10.4664769916667	10.669525325	10.3345761083333	11.0851990416667	11.5012483	11.3553466416667	10.067459125	11.1975258	11.0394398083333	10.021871175	10.4736621916667	11.36160315	10.5829592166667	10.5884400333333	10.2108003333333	11.876483925	10.3333340666667	10.7306227416667	10.6997392583333	10.6941625333333	10.515047025	10.3859567666667	10.39251605	10.3698580416667	10.5169707416667	10.364544	10.1573430916667	10.9567732916667	10.91088935	10.5388660583333	10.7368008833333

CCDC121	9.76819061666667	9.19020434166667	8.68971138333333	8.81642101666667	7.70028855833333	8.5569931	7.79638631666667	8.09007435833333	8.283955225	8.028895175	8.18781211666667	8.82318806666667	7.58521366666667	8.83016251666667	8.6493973	8.63344703333333	10.1109480666667	6.47064316666667	8.754236775	8.75515696666667	8.64093518333333	8.304316525	9.03641039166667	8.03327618333333	8.43351543333333	9.91842590833333	8.886411025	8.39161398333333	7.95359058333333	8.833014675	7.64327454166667	8.74620478333333	8.57783373333333	8.91049593333333	9.379662375	10.1420474166667	8.126799325	8.10330963333333	7.24263133333333	8.02832288333333	8.821700275	9.07634685	9.1141243	8.95991093333333	9.42118564166667	9.23139328333333	9.44734175833333	8.78836945	9.35075274166667	8.835452275	8.87086751666667	8.1898559	7.75542415833333	8.168322275	8.55750678333333	9.1029726	8.440899325	8.975801525	8.9217463	9.157883875	8.122791675	7.662739425	10.7866795833333	8.34324809166667	9.91930759166667	8.27857833333333	8.25839510833333	8.14346960833333	7.47258725833333	7.68042426666667	7.63763161666667	7.79357515	7.74951601666667	7.86062830833333	8.60701585833333	8.42069139166667	7.99458134166667	8.07050834166667	8.11940264166667	8.91889295833333	9.78692435833333	8.96369055833333	8.5874833	7.77775845	7.73920148333333	8.93806988333333	8.797202975	8.11654126666667	6.45841340833333	8.488931425	7.87768698333333	7.93973534166667	8.97769849166667	8.24707035	7.26110544166667	8.364642925	7.53069666666667	9.72757016666667	7.95575579166667	9.09878833333333	7.95904073333333	8.75003116666667	8.49539040833333	9.82029285833333	8.53333894166667	8.91410946666667	8.66580391666667	8.7735097	9.06791326666667	8.31814086666667	9.39983189166667	8.771928675	7.47496615833333	9.04320051666667	7.7794798	8.15758866666667	10.2494268583333	8.30084284166667	9.19287955	8.79293039166667

GRASP	11.8573117083333	11.86892365	11.7936196666667	11.6259515166667	12.4270361	11.9971131583333	12.588802475	12.3606021	12.2701693666667	12.4932078666667	12.2133193833333	12.007698575	12.2926172833333	12.0040290833333	11.95781185	12.4283415	10.4119855	12.36147625	11.9686139666667	12.2959914833333	11.8705505	11.8588339833333	11.84965965	11.8638644666667	12.1715835	12.0953574416667	11.85384455	12.140177075	12.3396961416667	12.017301325	12.2437326916667	11.9093036166667	12.4865305583333	10.4638383	12.0832149333333	10.8176392916667	12.6496634333333	12.5690965416667	12.03297995	11.9903497083333	11.86975775	12.0424744583333	12.1774305916667	11.9923578333333	11.5538304416667	11.5212430583333	11.4262590333333	11.602856675	12.178702275	12.2801097833333	12.196460925	11.942427275	11.744853675	11.6473588416667	11.824518825	11.9678169416667	11.95296625	12.2133193833333	11.868130425	11.6263096416667	9.67592103333333	10.1134808166667	9.902641975	10.0382960666667	10.8980207583333	10.7616896916667	10.273326675	10.0965198666667	9.54311075	10.4445170416667	9.7860847	10.831890425	9.73622053333333	10.80101635	10.1524914833333	9.953234775	11.4545376666667	10.3549808416667	10.4204003083333	9.94551363333333	11.1162112666667	11.0507418833333	9.67096889166667	10.2363454416667	10.4272415916667	10.8657939916667	11.51470825	10.7368008833333	9.34157415833333	10.85184045	11.1506157166667	9.732285275	9.465645475	9.815643425	9.46074875	10.978750575	10.3694253333333	9.9338367	10.1164178916667	10.514613975	10.1978484	10.568374825	9.74904354166667	10.1246349583333	10.0167779583333	10.0744319916667	10.4838867083333	9.73744423333333	9.98800139166667	10.5228331416667	11.19866735	9.89637478333333	10.1437039833333	10.0489380083333	10.4570074666667	9.96824564166667	11.53339965	10.1328782916667	9.76647539166667	10.5987255666667

LY6G6E	5.636456275	6.473722825	6.47064316666667	7.147600275	7.3675475	5.04338205833333	7.51872279166667	7.75542415833333	7.22536585	6.49247578333333	6.80449616666667	7.88712554166667	7.97666008333333	7.4911099	6.69314785	6.63442134166667	5.89991223333333	7.439305775	4.67877638333333	6.06998224166667	7.98973426666667	6.77436175	6.77836881666667	6.25991309166667	5.14089556666667	6.23620956666667	5.859013175	5.98915650833333	5.162070825	5.8009379	5.02269790833333	5.58508399166667	6.42188529166667	5.70431125833333	4.52791141666667	5.20882029166667	5.08161569166667	5.02269790833333	5.60132373333333	5.2815818	5.39178135	6.823097925	5.2108879	5.667420075	5.981391325	5.36615406666667	5.6488719	5.53793416666667	5.528242825	4.9415684	5.38157649166667	6.11785979166667	6.05897065833333	5.4789746	6.30177564166667	5.40932128333333	5.3439553	4.88811624166667	5.1863758	5.40405401666667	6.35665519166667	7.10952	5.05900706666667	7.48123266666667	7.36156116666667	6.39534953333333	6.86530675833333	7.45786721666667	7.0168934	6.96657695833333	7.57569364166667	7.38640515833333	8.62834993333333	7.12280756666667	7.40950549166667	7.0307229	6.84636305	5.6003113	6.225930275	7.1283444	7.46688233333333	7.67535224166667	6.88578850833333	5.70932124166667	6.78642360833333	6.90649953333333	6.94561610833333	7.15697099166667	7.49050329166667	5.064717325	7.09617145833333	5.467505875	5.553511425	5.899063175	6.68920345833333	6.13011929166667	6.68727483333333	5.755893075	6.97585598333333	5.17265765	5.192100225	7.88228429166667	5.33069273333333	5.67617600833333	6.73002575	5.97260084166667	5.34622214166667	6.62839033333333	4.638255275	6.76378944166667	4.78798888333333	5.70153421666667	5.09968536666667	4.99794086666667	5.52123825	5.59857721666667	6.11631895833333	4.63093599166667	5.62957723333333	6.12200050833333

AC022414.1	4.74594942083333	7.34183399583333	7.04172927916667	6.63005467916667	7.53477814166667	6.24403063333333	8.59572272083333	7.2957255	7.71325769166667	7.234588075	6.34938589166667	8.80272322083333	8.171646675	7.4320701875	6.6226409875	7.13063317083333	6.82854963333333	7.58212842083333	4.65324885833333	6.5813024375	7.87397285416667	6.706966425	6.9787197625	5.9835101375	5.52491661666667	6.60640252916667	5.06622259166667	4.32146174583333	4.92407953333333	5.00291880416667	4.6116512375	4.53579614583333	6.6614706	6.24739725833333	5.05230115833333	4.86431878333333	4.87549307083333	4.6064583375	4.57677624583333	4.74882355833333	5.12051297916667	4.46245617916667	4.85605135	5.6272504875	4.73067105	5.11626904583333	5.28210211666667	5.07707335416667	5.0705429375	4.893094225	4.81013114166667	4.65533382083333	6.6856428375	5.43877214166667	5.45852459583333	4.80775573333333	6.853211775	4.86336781666667	5.09916694166667	4.05255809583333	5.37283807916667	6.8494435375	4.60134417916667	5.25817574166667	6.63693797083333	6.22880375416667	5.03282052916667	7.23282620833333	5.16367257083333	6.22407515833333	6.0671444125	6.17512970833333	8.1463363	5.89775234166667	7.14488793333333	7.0568305375	6.51955419166667	4.57677624583333	6.82674682083333	5.73727407083333	7.86037695833333	7.06306587916667	5.86502612916667	5.18759319166667	5.84745681666667	6.45585879166667	6.94788829583333	6.93451647916667	5.7223480375	6.04749792083333	5.52379521666667	4.38870969166667	5.36974268333333	5.27271570416667	4.654301475	5.01580050833333	5.7175161125	4.50593829166667	6.18290244583333	5.085150825	5.69434719166667	6.61383874583333	4.83047865833333	5.1561289	4.38051879166667	4.03602779583333	5.16032288333333	6.53367921666667	6.0611315375	6.686921825	4.61470835	5.34566134166667	4.4828517125	5.06520884166667	4.60229123333333	4.42100610833333	5.29501385833333	4.62840347083333	4.73776326666667	7.08587727083333

AC008581.2	4.74594942083333	7.34183399583333	7.04172927916667	6.63005467916667	7.53477814166667	6.24403063333333	8.59572272083333	7.2957255	7.71325769166667	7.234588075	6.34938589166667	8.80272322083333	8.171646675	7.4320701875	6.6226409875	7.13063317083333	6.82854963333333	7.58212842083333	4.65324885833333	6.5813024375	7.87397285416667	6.706966425	6.9787197625	5.9835101375	5.52491661666667	6.60640252916667	5.06622259166667	4.32146174583333	4.92407953333333	5.00291880416667	4.6116512375	4.53579614583333	6.6614706	6.24739725833333	5.05230115833333	4.86431878333333	4.87549307083333	4.6064583375	4.57677624583333	4.74882355833333	5.12051297916667	4.46245617916667	4.85605135	5.6272504875	4.73067105	5.11626904583333	5.28210211666667	5.07707335416667	5.0705429375	4.893094225	4.81013114166667	4.65533382083333	6.6856428375	5.43877214166667	5.45852459583333	4.80775573333333	6.853211775	4.86336781666667	5.09916694166667	4.05255809583333	5.37283807916667	6.8494435375	4.60134417916667	5.25817574166667	6.63693797083333	6.22880375416667	5.03282052916667	7.23282620833333	5.16367257083333	6.22407515833333	6.0671444125	6.17512970833333	8.1463363	5.89775234166667	7.14488793333333	7.0568305375	6.51955419166667	4.57677624583333	6.82674682083333	5.73727407083333	7.86037695833333	7.06306587916667	5.86502612916667	5.18759319166667	5.84745681666667	6.45585879166667	6.94788829583333	6.93451647916667	5.7223480375	6.04749792083333	5.52379521666667	4.38870969166667	5.36974268333333	5.27271570416667	4.654301475	5.01580050833333	5.7175161125	4.50593829166667	6.18290244583333	5.085150825	5.69434719166667	6.61383874583333	4.83047865833333	5.1561289	4.38051879166667	4.03602779583333	5.16032288333333	6.53367921666667	6.0611315375	6.686921825	4.61470835	5.34566134166667	4.4828517125	5.06520884166667	4.60229123333333	4.42100610833333	5.29501385833333	4.62840347083333	4.73776326666667	7.08587727083333

METTL2A	12.6611346333333	11.5858193083333	11.1692562416667	11.7004395833333	13.7224285916667	13.1268647416667	11.8530999083333	12.0905289333333	12.2540737	12.2947323416667	12.3339445	11.7027128	12.2768075833333	11.88924055	12.5264113	11.7667849666667	12.2453199833333	13.0037538583333	12.6381216166667	12.4091926166667	12.0593104333333	12.209029825	11.95781185	12.4655499583333	12.7564076083333	12.4461573416667	11.675724725	11.86975775	12.786823125	12.161623975	11.8048205583333	11.8709570583333	11.6577322083333	12.04365515	11.7004395833333	12.5780122833333	12.265420025	12.1109533333333	12.1827137333333	11.7738991416667	11.6010090666667	11.794058525	11.6099448083333	11.7724295666667	11.9575097333333	11.4996324916667	12.0619908166667	12.0657212166667	12.023387575	11.8375242416667	11.0648135916667	12.4206839916667	13.321391725	12.6754546333333	11.806635025	11.3474088166667	11.82164615	11.8209586416667	12.0282380083333	11.89894165	14.0429420833333	13.0013137333333	12.30978125	12.3452729083333	12.3305815833333	13.3667920416667	12.62011135	12.680606175	12.7046259583333	12.6386311166667	12.7543063	13.332896575	12.4696924	12.1886556416667	12.9954911666667	12.2771993416667	12.587916575	12.8823979	12.1521118916667	12.78176855	12.3592921916667	13.3675920333333	12.5395615083333	12.264576775	12.391732275	12.58926255	12.9906493583333	12.05687395	13.712997875	12.0832149333333	12.3780252416667	12.6632127083333	12.98478995	11.9994097833333	13.180045325	12.5754789333333	13.3410732083333	12.5706160166667	11.9575097333333	12.3169057833333	12.2926172833333	13.0626219916667	13.0155242333333	12.3724626666667	12.3173301166667	12.62157875	12.7671377333333	12.351346575	13.3234625333333	12.7354192416667	11.2786369583333	12.6790614583333	12.4069098166667	12.52364675	11.9962714583333	11.8845097083333	12.9400260666667	12.5590435083333	12.4105900333333	12.0551513666667

OR9Q1	7.96649823333333	7.8461248875	7.92084850833333	7.88426529583333	8.69104740416667	7.76319464166667	9.07891089583333	7.86866886666667	8.5240442875	8.013498425	8.18072137083333	8.92579979583333	8.72156675416667	7.80300961666667	8.07959589583333	7.696283925	7.46488826666667	8.42958982916667	7.7395305625	7.6458149	8.11966258333333	8.20806554583333	7.7884599	7.73499715416667	7.24363060833333	7.98268934166667	7.3294679625	7.46881167083333	8.1941990625	7.80145052083333	7.46049172916667	7.70443450416667	8.09093749166667	8.39894821666667	7.84714889583333	7.95382576666667	7.8085116875	7.62282106666667	7.5775785875	7.64940118333333	7.38900372916667	7.4063218125	7.1651607875	7.20055699166667	7.34922049583333	6.92818722916667	6.99187215	7.33828902083333	6.93651231666667	7.27259884166667	7.30996997083333	7.52356270833333	7.98744984583333	7.73776883333333	8.06371620833333	6.895626475	7.55042032083333	7.44520305416667	7.37498002083333	6.84353104166667	7.74371000833333	8.29403910833333	7.64019310416667	8.35659556666667	8.3464457375	8.379974425	8.66461367083333	8.37234324166667	8.05325315416667	8.17063840416667	8.46324830833333	8.32190137916667	8.8139688125	7.62680286666667	8.78610979166667	8.26726605416667	7.87128107083333	7.54829975833333	7.95658339583333	7.80250077083333	8.171646675	8.2010289625	8.11856490833333	7.71217066666667	8.25109315833333	8.179084675	8.62165234166667	9.49346230416667	8.31335044583333	7.61951985416667	7.9554634375	7.8304358875	7.77125162916667	8.10817010833333	8.59372429583333	8.28772449166667	7.81866440416667	8.30407255416667	8.29276760833333	8.83989808333333	8.1107790375	8.59572272083333	7.86968296666667	7.90339324166667	7.586560225	7.73665201666667	7.84350130416667	9.52064974583333	7.90706607916667	9.7817317375	8.04419490833333	8.44361923333333	7.7475293375	7.74064432916667	7.87182299166667	7.76007795416667	8.092532775	7.9225188	7.4350040875	8.1983393625

OR9I1	7.96649823333333	7.8461248875	7.92084850833333	7.88426529583333	8.69104740416667	7.76319464166667	9.07891089583333	7.86866886666667	8.5240442875	8.013498425	8.18072137083333	8.92579979583333	8.72156675416667	7.80300961666667	8.07959589583333	7.696283925	7.46488826666667	8.42958982916667	7.7395305625	7.6458149	8.11966258333333	8.20806554583333	7.7884599	7.73499715416667	7.24363060833333	7.98268934166667	7.3294679625	7.46881167083333	8.1941990625	7.80145052083333	7.46049172916667	7.70443450416667	8.09093749166667	8.39894821666667	7.84714889583333	7.95382576666667	7.8085116875	7.62282106666667	7.5775785875	7.64940118333333	7.38900372916667	7.4063218125	7.1651607875	7.20055699166667	7.34922049583333	6.92818722916667	6.99187215	7.33828902083333	6.93651231666667	7.27259884166667	7.30996997083333	7.52356270833333	7.98744984583333	7.73776883333333	8.06371620833333	6.895626475	7.55042032083333	7.44520305416667	7.37498002083333	6.84353104166667	7.74371000833333	8.29403910833333	7.64019310416667	8.35659556666667	8.3464457375	8.379974425	8.66461367083333	8.37234324166667	8.05325315416667	8.17063840416667	8.46324830833333	8.32190137916667	8.8139688125	7.62680286666667	8.78610979166667	8.26726605416667	7.87128107083333	7.54829975833333	7.95658339583333	7.80250077083333	8.171646675	8.2010289625	8.11856490833333	7.71217066666667	8.25109315833333	8.179084675	8.62165234166667	9.49346230416667	8.31335044583333	7.61951985416667	7.9554634375	7.8304358875	7.77125162916667	8.10817010833333	8.59372429583333	8.28772449166667	7.81866440416667	8.30407255416667	8.29276760833333	8.83989808333333	8.1107790375	8.59572272083333	7.86968296666667	7.90339324166667	7.586560225	7.73665201666667	7.84350130416667	9.52064974583333	7.90706607916667	9.7817317375	8.04419490833333	8.44361923333333	7.7475293375	7.74064432916667	7.87182299166667	7.76007795416667	8.092532775	7.9225188	7.4350040875	8.1983393625

PPIAL4D	13.4681552083333	13.9045177166667	13.8889513416667	14.3045490583333	14.1113310916667	14.427724725	14.0787258833333	14.4309788083333	13.7293441833333	14.400934925	14.3724417	13.3545785083333	13.5859683083333	14.1306178166667	14.21756365	13.1190556333333	13.834699275	14.4495058333333	13.6812289916667	13.916524275	14.2503770416667	14.27950095	14.0163605916667	14.1629431333333	13.8688792083333	13.1819427166667	14.2936594416667	14.211775675	14.1941852166667	14.1099143166667	13.771159075	13.806992375	13.62200835	13.5946453583333	12.901864125	13.3645725083333	13.9469444833333	14.227452975	13.698785275	13.8199392	13.604496075	13.672832575	13.6310840583333	13.5843144666667	13.0494144333333	13.7321168833333	13.6293464	13.4611203333333	14.1152258583333	13.7332085916667	13.97057275	13.62548145	13.71864895	13.2898598333333	13.3501502333333	13.2227845833333	14.15404765	14.168404425	13.6426254916667	13.89301535	14.4479745916667	14.7226534166667	14.5753036916667	14.2715663416667	14.9279831333333	14.5469723583333	14.989321275	14.5793502666667	14.9094697583333	14.56279245	14.618270675	14.3843059083333	14.87708635	14.2610479166667	14.539541	14.8261234166667	14.7409853333333	14.6346112833333	14.73390865	15.1048760583333	14.4686760333333	14.4581253166667	14.6949121583333	14.2686692416667	14.8162748	14.718655275	14.3268677	14.4459919583333	14.4739784	14.3892469416667	14.4527902	14.5701394583333	14.6259421666667	14.4025408	14.7596386666667	14.6611477166667	14.51071235	14.2842943416667	14.539541	14.3366389666667	14.7316646166667	14.6046129083333	14.2460408916667	14.4633924916667	14.4739784	14.5701394583333	14.4144157	14.465199375	14.3960605	14.5214642083333	14.648708375	14.718655275	14.1500101916667	14.2610479166667	14.2132241916667	14.0926224666667	14.1737580083333	14.2533850083333	14.5793502666667	14.4309788083333

PPIAL4G	13.4681552083333	13.9045177166667	13.8889513416667	14.3045490583333	14.1113310916667	14.427724725	14.0787258833333	14.4309788083333	13.7293441833333	14.400934925	14.3724417	13.3545785083333	13.5859683083333	14.1306178166667	14.21756365	13.1190556333333	13.834699275	14.4495058333333	13.6812289916667	13.916524275	14.2503770416667	14.27950095	14.0163605916667	14.1629431333333	13.8688792083333	13.1819427166667	14.2936594416667	14.211775675	14.1941852166667	14.1099143166667	13.771159075	13.806992375	13.62200835	13.5946453583333	12.901864125	13.3645725083333	13.9469444833333	14.227452975	13.698785275	13.8199392	13.604496075	13.672832575	13.6310840583333	13.5843144666667	13.0494144333333	13.7321168833333	13.6293464	13.4611203333333	14.1152258583333	13.7332085916667	13.97057275	13.62548145	13.71864895	13.2898598333333	13.3501502333333	13.2227845833333	14.15404765	14.168404425	13.6426254916667	13.89301535	14.4479745916667	14.7226534166667	14.5753036916667	14.2715663416667	14.9279831333333	14.5469723583333	14.989321275	14.5793502666667	14.9094697583333	14.56279245	14.618270675	14.3843059083333	14.87708635	14.2610479166667	14.539541	14.8261234166667	14.7409853333333	14.6346112833333	14.73390865	15.1048760583333	14.4686760333333	14.4581253166667	14.6949121583333	14.2686692416667	14.8162748	14.718655275	14.3268677	14.4459919583333	14.4739784	14.3892469416667	14.4527902	14.5701394583333	14.6259421666667	14.4025408	14.7596386666667	14.6611477166667	14.51071235	14.2842943416667	14.539541	14.3366389666667	14.7316646166667	14.6046129083333	14.2460408916667	14.4633924916667	14.4739784	14.5701394583333	14.4144157	14.465199375	14.3960605	14.5214642083333	14.648708375	14.718655275	14.1500101916667	14.2610479166667	14.2132241916667	14.0926224666667	14.1737580083333	14.2533850083333	14.5793502666667	14.4309788083333

PPIAL4H	13.4681552083333	13.9045177166667	13.8889513416667	14.3045490583333	14.1113310916667	14.427724725	14.0787258833333	14.4309788083333	13.7293441833333	14.400934925	14.3724417	13.3545785083333	13.5859683083333	14.1306178166667	14.21756365	13.1190556333333	13.834699275	14.4495058333333	13.6812289916667	13.916524275	14.2503770416667	14.27950095	14.0163605916667	14.1629431333333	13.8688792083333	13.1819427166667	14.2936594416667	14.211775675	14.1941852166667	14.1099143166667	13.771159075	13.806992375	13.62200835	13.5946453583333	12.901864125	13.3645725083333	13.9469444833333	14.227452975	13.698785275	13.8199392	13.604496075	13.672832575	13.6310840583333	13.5843144666667	13.0494144333333	13.7321168833333	13.6293464	13.4611203333333	14.1152258583333	13.7332085916667	13.97057275	13.62548145	13.71864895	13.2898598333333	13.3501502333333	13.2227845833333	14.15404765	14.168404425	13.6426254916667	13.89301535	14.4479745916667	14.7226534166667	14.5753036916667	14.2715663416667	14.9279831333333	14.5469723583333	14.989321275	14.5793502666667	14.9094697583333	14.56279245	14.618270675	14.3843059083333	14.87708635	14.2610479166667	14.539541	14.8261234166667	14.7409853333333	14.6346112833333	14.73390865	15.1048760583333	14.4686760333333	14.4581253166667	14.6949121583333	14.2686692416667	14.8162748	14.718655275	14.3268677	14.4459919583333	14.4739784	14.3892469416667	14.4527902	14.5701394583333	14.6259421666667	14.4025408	14.7596386666667	14.6611477166667	14.51071235	14.2842943416667	14.539541	14.3366389666667	14.7316646166667	14.6046129083333	14.2460408916667	14.4633924916667	14.4739784	14.5701394583333	14.4144157	14.465199375	14.3960605	14.5214642083333	14.648708375	14.718655275	14.1500101916667	14.2610479166667	14.2132241916667	14.0926224666667	14.1737580083333	14.2533850083333	14.5793502666667	14.4309788083333

PPIAL4F	13.4681552083333	13.9045177166667	13.8889513416667	14.3045490583333	14.1113310916667	14.427724725	14.0787258833333	14.4309788083333	13.7293441833333	14.400934925	14.3724417	13.3545785083333	13.5859683083333	14.1306178166667	14.21756365	13.1190556333333	13.834699275	14.4495058333333	13.6812289916667	13.916524275	14.2503770416667	14.27950095	14.0163605916667	14.1629431333333	13.8688792083333	13.1819427166667	14.2936594416667	14.211775675	14.1941852166667	14.1099143166667	13.771159075	13.806992375	13.62200835	13.5946453583333	12.901864125	13.3645725083333	13.9469444833333	14.227452975	13.698785275	13.8199392	13.604496075	13.672832575	13.6310840583333	13.5843144666667	13.0494144333333	13.7321168833333	13.6293464	13.4611203333333	14.1152258583333	13.7332085916667	13.97057275	13.62548145	13.71864895	13.2898598333333	13.3501502333333	13.2227845833333	14.15404765	14.168404425	13.6426254916667	13.89301535	14.4479745916667	14.7226534166667	14.5753036916667	14.2715663416667	14.9279831333333	14.5469723583333	14.989321275	14.5793502666667	14.9094697583333	14.56279245	14.618270675	14.3843059083333	14.87708635	14.2610479166667	14.539541	14.8261234166667	14.7409853333333	14.6346112833333	14.73390865	15.1048760583333	14.4686760333333	14.4581253166667	14.6949121583333	14.2686692416667	14.8162748	14.718655275	14.3268677	14.4459919583333	14.4739784	14.3892469416667	14.4527902	14.5701394583333	14.6259421666667	14.4025408	14.7596386666667	14.6611477166667	14.51071235	14.2842943416667	14.539541	14.3366389666667	14.7316646166667	14.6046129083333	14.2460408916667	14.4633924916667	14.4739784	14.5701394583333	14.4144157	14.465199375	14.3960605	14.5214642083333	14.648708375	14.718655275	14.1500101916667	14.2610479166667	14.2132241916667	14.0926224666667	14.1737580083333	14.2533850083333	14.5793502666667	14.4309788083333

PPIAL4C	13.4681552083333	13.9045177166667	13.8889513416667	14.3045490583333	14.1113310916667	14.427724725	14.0787258833333	14.4309788083333	13.7293441833333	14.400934925	14.3724417	13.3545785083333	13.5859683083333	14.1306178166667	14.21756365	13.1190556333333	13.834699275	14.4495058333333	13.6812289916667	13.916524275	14.2503770416667	14.27950095	14.0163605916667	14.1629431333333	13.8688792083333	13.1819427166667	14.2936594416667	14.211775675	14.1941852166667	14.1099143166667	13.771159075	13.806992375	13.62200835	13.5946453583333	12.901864125	13.3645725083333	13.9469444833333	14.227452975	13.698785275	13.8199392	13.604496075	13.672832575	13.6310840583333	13.5843144666667	13.0494144333333	13.7321168833333	13.6293464	13.4611203333333	14.1152258583333	13.7332085916667	13.97057275	13.62548145	13.71864895	13.2898598333333	13.3501502333333	13.2227845833333	14.15404765	14.168404425	13.6426254916667	13.89301535	14.4479745916667	14.7226534166667	14.5753036916667	14.2715663416667	14.9279831333333	14.5469723583333	14.989321275	14.5793502666667	14.9094697583333	14.56279245	14.618270675	14.3843059083333	14.87708635	14.2610479166667	14.539541	14.8261234166667	14.7409853333333	14.6346112833333	14.73390865	15.1048760583333	14.4686760333333	14.4581253166667	14.6949121583333	14.2686692416667	14.8162748	14.718655275	14.3268677	14.4459919583333	14.4739784	14.3892469416667	14.4527902	14.5701394583333	14.6259421666667	14.4025408	14.7596386666667	14.6611477166667	14.51071235	14.2842943416667	14.539541	14.3366389666667	14.7316646166667	14.6046129083333	14.2460408916667	14.4633924916667	14.4739784	14.5701394583333	14.4144157	14.465199375	14.3960605	14.5214642083333	14.648708375	14.718655275	14.1500101916667	14.2610479166667	14.2132241916667	14.0926224666667	14.1737580083333	14.2533850083333	14.5793502666667	14.4309788083333

CCDC171	7.90206838333333	7.20088341666667	7.83997353333333	7.66954886666667	7.51253281666667	7.05651791666667	7.46853124166667	7.18643425	7.224148725	7.22176664166667	7.252371175	7.66718561666667	7.08487659166667	7.24571956666667	7.55811896666667	6.87219209166667	7.070041725	7.67425858333333	7.35003230833333	7.39222043333333	7.05521155	7.57732030833333	7.100006625	7.27359608333333	7.27829234166667	7.51814899166667	7.59083331666667	7.4731488	7.583460175	7.58128390833333	7.586283275	7.7783407	7.71914490833333	7.442585225	7.72846560833333	7.73422946666667	7.81947101666667	7.42103133333333	7.40898444166667	7.4690921	6.97276299166667	7.09487224166667	7.32668075833333	7.262828725	7.54177855833333	6.77975395833333	7.139659575	7.195709975	7.17267591666667	7.20334935833333	7.45275619166667	7.60136925	7.80067161666667	7.886143875	7.89196619166667	7.2162874	7.30612164166667	7.54566714166667	7.21098661666667	7.404937825	8.037084725	7.875176525	8.3453091	8.06285705833333	7.422156975	7.37928605	7.97768158333333	7.8648195	7.97557605833333	7.48985504166667	7.671444425	7.94065678333333	7.29732286666667	7.127807925	7.49771558333333	7.650678625	7.59310200833333	7.9877223	7.67307831666667	7.842070475	8.049199675	8.07889375	7.83348010833333	7.66108944166667	7.74239094166667	7.92437408333333	8.550436275	7.62759090833333	7.82746845	7.61698044166667	8.019555675	8.32905851666667	8.54462835	7.71570814166667	8.312026725	7.96301623333333	8.107904375	8.30336938333333	8.11098743333333	7.90528599166667	7.79903776666667	7.7445795	8.30641555	7.68541283333333	8.07106306666667	7.53449101666667	7.70926514166667	7.079224475	7.7682787	6.57096464166667	7.75234836666667	8.03810015	8.27706721666667	8.03595288333333	8.04450845	7.907346175	8.107904375	8.069486125	8.061842725	8.04804935

KIF21A	7.69651605833333	7.62089398333333	6.88718085	8.48569109166667	8.24707035	7.41704166666667	7.08170128333333	7.717344425	7.91046618333333	6.79334741666667	6.95156631666667	6.131776975	6.91388091666667	7.34669549166667	8.51578285	6.84200789166667	7.11547685	8.00687400833333	7.916304775	6.97714725	6.44595796666667	9.112227825	6.59052684166667	7.48927714166667	7.94597349166667	6.028109975	8.45851334166667	8.52878136666667	8.67888266666667	7.67253041666667	7.61520518333333	8.30130739166667	7.35447710833333	5.98559190833333	6.63574723333333	6.83931143333333	9.93083546666667	7.99891026666667	6.61087155833333	7.99833741666667	6.97843058333333	8.35277756666667	7.72085915833333	6.653546475	6.36951041666667	7.42731664166667	7.51365925	7.551793125	7.23488161666667	6.95221913333333	6.316307375	7.27593380833333	6.89322066666667	7.97086885	7.79031855833333	6.911150775	7.33026348333333	6.15692685	8.22546075833333	7.15697099166667	7.62479258333333	6.44887243333333	7.12280756666667	8.3851619	5.76923416666667	7.69651605833333	5.53496716666667	7.6225677	6.81637415	6.35132606666667	7.28317230833333	7.07353054166667	6.178567625	7.16232548333333	8.1455177	5.66283725	6.61689901666667	7.467981625	7.340949	7.07118303333333	5.33746485833333	6.72123403333333	7.26937910833333	7.08293825833333	7.60888643333333	6.16829805	5.73405291666667	4.94999915833333	7.26874934166667	5.576316275	6.76714710833333	8.0149105	8.70592035	6.602072175	6.8752982	7.42788356666667	8.47850595	6.52272641666667	6.59670515	7.070041725	6.165146375	7.30254744166667	6.74410944166667	7.08560825833333	7.68487656666667	7.78166071666667	6.88458655833333	6.9475749	6.03747638333333	6.01386335	5.64313004166667	7.66609071666667	6.93352621666667	5.661700075	6.88791735	6.56292778333333	8.063977575	7.28317230833333	7.416522875	6.88184853333333

HINFP	10.2826800583333	9.939168625	9.97833993333333	10.0321890166667	9.7408584	9.74951903333333	9.77176594166667	9.80143100833333	9.939168625	9.53997905833333	9.84285273333333	10.045510375	9.76350663333333	9.91930759166667	10.1130642083333	9.71188024166667	10.2383473416667	9.97912404166667	10.1546002	9.7473541	9.85498678333333	9.95522605	9.890973325	9.63342665833333	9.99767481666667	10.2881617	9.899420875	9.82203395	9.84409466666667	10.0434280416667	9.98225808333333	9.90438676666667	9.88253726666667	10.1001644	10.5096862166667	10.6038899083333	10.0189542666667	9.91887936666667	9.96522326666667	9.96151823333333	10.0693001666667	10.0748410916667	9.84841021666667	9.84919238333333	10.068396225	10.1063080916667	10.0142807916667	9.98984027083333	9.810895475	9.97174644166667	9.95100449166667	10.2466873083333	10.26626505	10.2422793916667	10.4195860916667	10.2737682166667	9.77263848333333	10.1444546916667	10.0520460833333	9.79628703333333	10.3837830083333	10.043884875	9.90955436666667	9.512679675	9.46657071666667	10.082743125	9.66781955833333	9.84994195833333	10.1546002	10.2014148333333	9.995492075	10.362641575	10.079544525	10.0765568416667	9.765982475	9.73577058333333	9.69747909166667	9.57464318333333	10.1581087416667	9.67813213333333	10.4986956916667	9.32368785833333	9.58662811666667	9.87484248333333	10.0769619583333	9.54092946666667	10.0205705916667	10.165808925	10.2149556666667	10.0524736083333	9.92625621666667	10.0489380083333	10.27531875	9.96824564166667	10.1713085416667	9.986796275	10.5134429166667	9.89855505	9.99723533333333	10.0031143833333	9.92375435	10.1084049583333	10.2634356166667	9.921332825	10.5356874083333	9.91241780833333	9.79446515833333	10.514613975	9.900995025	10.42200985	9.88083685	10.0331009416667	10.0769619583333	11.109201175	10.1755237	10.2096666583333	10.096897025	10.457410175	10.4168764916667	9.94339973333333

KCNB1	10.70539815	7.91465324166667	7.55486798333333	6.57734461666667	6.42264453333333	6.311629325	6.68727483333333	7.40375466666667	7.81401100833333	6.66944055833333	6.97900894166667	7.74145285	7.75602535833333	7.84376875833333	7.77376521666667	7.88228429166667	8.03960018333333	4.77813741666667	8.45451465833333	8.17249793333333	7.79586476666667	7.05585056666667	7.404332775	7.10888616666667	8.82825418333333	11.3642682416667	8.90434914166667	7.57783686666667	7.7124456	8.1223269	7.75823035	8.312026725	8.27954998333333	5.212866025	11.311125675	11.2707625416667	7.013155025	7.388741125	6.26504835	8.35736911666667	8.66247435	8.44993103333333	8.42776885	8.60701585833333	9.33838469166667	7.98294754166667	9.54277241666667	7.48759605	6.79731146666667	8.19294795833333	7.48927714166667	6.507987025	6.82889531666667	6.96778875833333	8.08754240833333	9.15740435833333	6.67783206666667	7.70760366666667	7.32319219166667	8.86314575	6.89528771666667	5.37452059166667	4.64033936666667	3.930358975	4.08637315833333	6.17226739166667	5.42554189166667	4.84820759166667	4.759183025	4.137419	3.930358975	4.37909631666667	4.05417596666667	4.09994219166667	3.93478860833333	3.94901469166667	5.8893734	3.69375465	5.30771575	4.669520575	4.66334196666667	5.31614614166667	4.13109949166667	6.80310133333333	4.40581835	7.43688079166667	5.03010108333333	4.936774575	4.20999628333333	7.31861950833333	4.46004483333333	4.70270751666667	5.34622214166667	4.11385116666667	7.48416809166667	7.107578925	3.9601097	3.694768975	3.83041005	5.10174316666667	5.56869470833333	4.38635804166667	4.55971610833333	5.91139875	6.06491893333333	4.30828979166667	6.135921175	6.67377979166667	4.19234266666667	6.86736839166667	5.24388391666667	5.47315375	4.8912062	4.609167375	3.89679799166667	5.61840579166667	4.30527630833333	4.42570850833333	4.58140610833333	5.6976834

KIAA0355	5.94358583333333	5.88521655833333	5.93923476666667	6.41957618333333	5.95832643333333	5.931318725	5.878607175	5.48300426666667	6.18247753333333	5.67139935833333	5.79649798333333	6.26664835833333	6.14881959166667	5.41039171666667	6.75898810833333	5.25962125	5.948036925	6.16763958333333	5.86905295	5.70932124166667	6.08509065	5.67425256666667	5.079599575	6.70667168333333	6.558117975	5.89819270833333	6.90123264166667	6.4040341	5.72686785	6.20180194166667	6.33714610833333	6.465677275	6.12118446666667	6.28184195833333	6.18980128333333	5.83674730833333	6.88512086666667	6.11703395833333	6.08593745	5.89991223333333	5.79742544166667	5.399029725	6.65024493333333	5.135343	5.71218308333333	5.9751687	6.42556058333333	6.09669481666667	6.22446936666667	6.456329775	6.45401484166667	6.72183800833333	6.48357195833333	6.37857751666667	6.47791706666667	5.82271865	5.66378185833333	5.69080398333333	6.61689901666667	6.1770551	5.92148618333333	5.843159875	6.18094188333333	5.71218308333333	5.52011276666667	5.792834125	4.91654405	5.64313004166667	5.88595835	5.54303723333333	5.843159875	6.323145675	5.760490375	6.12364109166667	5.58896354166667	6.29670533333333	5.51915526666667	6.4308335	5.84963309166667	5.90154725	5.85146426666667	5.83110111666667	6.33569684166667	6.57504109166667	6.24443749166667	5.64313004166667	5.7955502	5.38964895833333	6.013009825	6.02292943333333	6.056382625	6.6562921	6.91976564166667	6.456329775	6.19736666666667	6.22304681666667	6.56509979166667	6.62636613333333	6.17313585833333	5.46549339166667	5.735887375	5.73682776666667	5.82073376666667	5.54779774166667	6.35356116666667	6.2353521	5.99349075	5.99501325833333	5.55666916666667	6.28823875	6.22835699166667	6.06672131666667	6.90252933333333	6.11958026666667	6.475087675	6.339217025	5.628745675	6.65487665833333	6.90649953333333	6.04799355833333

PAH	12.0705755166667	11.4574143583333	11.3292622166667	11.991564075	12.6220126	10.96771795	12.5822395583333	11.696699	12.170742575	11.7164003333333	12.1279125583333	12.6240804083333	12.6098037416667	11.7951768416667	11.5305817083333	11.41004175	10.7116237833333	12.562277225	11.2198620833333	11.1803917416667	11.5498379666667	11.8962786833333	10.99291925	10.992544275	11.0567325916667	11.3827745833333	11.9184658666667	11.452332325	11.6719609166667	11.9720790416667	11.437271475	11.1441289083333	11.9357187583333	11.155024025	11.036287075	11.2957967833333	12.118235175	11.5034674333333	11.489428925	11.3990716583333	10.8450341833333	11.8337195083333	11.0689648166667	10.48105345	11.5909354166667	10.3897471916667	10.9234198666667	11.1898119916667	10.5627525083333	10.7313914916667	11.069747825	11.0600457083333	11.4350551	10.7915830833333	11.0848820333333	10.10216065	11.18844885	10.8733577583333	11.27681655	10.8251181583333	12.0149070416667	12.0697739833333	11.1993790583333	11.9935609583333	11.5589969083333	12.4185466166667	11.376011025	12.24577265	12.10672325	11.9025044	12.07649975	12.195607525	12.0977041916667	11.8768607333333	11.824518825	11.27496335	12.5719766166667	12.034117575	11.2779260833333	12.1640360666667	11.8383549666667	12.2219012	11.424045025	11.13908545	12.0277810916667	12.2006641666667	12.2587377333333	11.8916056833333	12.5135886333333	10.94802585	11.8012624833333	11.3583216333333	11.7682562416667	11.2848715833333	12.1865914583333	11.8399235416667	12.2210896416667	12.1935330916667	12.0317754666667	11.189465025	11.1692562416667	12.23183945	11.185479075	10.5318759	11.07367765	11.3336047583333	11.1520832083333	10.7820632416667	10.7092769166667	10.64844015	10.9084288583333	11.4586326	10.4372971833333	9.84631699166667	10.4534831666667	10.7034642666667	10.81355685	11.3506069833333	10.7994102333333	11.2349074416667

CASTOR2	11.0221982916667	11.2534278333333	10.6607214666667	10.4149521166667	10.8401100166667	9.28361398333333	10.995163975	9.85326255	10.0156224166667	10.1755237	11.4741238583333	11.39829615	9.60319949166667	10.7635056583333	10.7336987	10.3015658	9.47641323333333	10.410088175	11.21108835	10.8934071416667	10.5034091416667	10.8307782666667	11.2082269	10.2826800583333	9.72156395833333	11.357567525	10.6550211916667	10.520091075	10.8984556583333	10.96771795	10.3228920166667	10.924157925	11.2001288416667	9.80755264166667	11.0666847416667	11.4511549916667	10.80101635	10.5276582916667	10.300361525	10.31285305	10.5783390833333	10.703101075	10.605453425	10.9909672	10.0111258333333	10.145640125	11.5060047583333	10.7702977583333	10.418815025	9.83764681666667	10.4441620166667	10.7793443666667	11.017853675	10.6195893583333	10.4858474583333	10.6031497916667	10.4585673833333	10.69756315	10.364930325	10.1042493666667	10.5872774916667	11.6651451833333	9.96151823333333	11.3786114833333	9.52177871666667	10.4228179333333	10.340914925	9.88883589166667	9.73395598333333	9.996827975	11.9525962333333	11.4096653083333	9.41484764166667	10.1713085416667	10.9549623083333	10.7816314666667	10.736058575	10.924157925	11.0466400166667	11.0246898	11.1166050833333	11.0927898583333	10.490641975	10.6108306416667	10.002252325	11.339247	10.9021357916667	10.137056175	10.7123288	11.2907157	10.5240503083333	10.563601	11.86892365	10.345102475	10.9737165166667	11.5638114916667	10.342051525	11.1284533666667	10.1704848833333	10.1569732333333	10.9291888666667	10.8202415916667	10.2838388166667	11.2911160333333	10.2642520083333	10.2834956666667	11.069747825	10.0957326583333	10.3623207166667	10.1770845916667	11.5291327083333	10.680501275	10.6360463166667	10.7020102333333	9.890973325	10.2670402	10.227074775	10.6019249583333	10.70466055	10.0276764666667

C11orf96	15.290119725	12.8829745166667	13.9840948583333	12.0616030916667	12.1488811083333	14.3350486166667	12.6397345916667	13.6302168166667	12.343956025	14.11256935	12.1886556416667	15.8653098666667	14.818607725	12.2570148333333	11.7351274916667	14.4615780083333	14.2983229333333	14.6549636583333	13.10396855	13.711942125	13.670246725	10.641902875	12.5443972083333	13.9807861416667	13.3151588	14.8442556083333	12.928953425	13.509141775	12.338252925	12.1014528666667	14.74733735	11.7247391166667	12.8907368833333	13.2919593833333	15.4672060583333	13.175966725	12.2416674416667	13.6560864916667	14.4397477583333	14.3514380333333	12.5534552416667	12.0001864416667	13.5519530666667	12.8010225583333	13.5694284666667	12.220220925	12.2040899	13.0136394416667	12.7857802916667	12.6557058416667	14.2132241916667	14.2671663083333	12.99120565	12.8493401833333	12.11543585	13.4760270333333	13.8788907166667	12.5775136	13.208876725	13.1178746666667	12.98478995	12.9954911666667	12.3776096916667	13.6866916666667	12.9793317083333	12.5552867333333	13.5372319083333	12.9677883916667	11.8865138166667	12.2780305166667	12.8645965083333	12.80521305	13.3018009583333	14.0100072833333	11.3441400333333	12.6297371916667	12.2028833833333	14.2145397416667	13.5511200333333	12.0926515916667	12.226469725	12.5831450833333	12.4082756583333	13.6831500916667	12.76127775	12.98478995	12.0013260916667	13.8328147166667	10.86697045	14.3366389666667	13.9612443083333	12.867358575	12.0546824083333	12.2594871416667	12.1100796583333	12.2240198833333	12.285271325	11.8877229666667	13.4752691916667	12.8537857833333	13.021150225	12.3284449583333	10.8964884916667	12.1389321	11.798162425	11.0041932083333	12.9617333583333	12.3003464583333	11.5728018416667	12.7769325	14.5377311666667	12.3374196	12.198604375	12.0249612083333	12.3405472916667	11.6378736916667	12.7676811833333	13.1733038666667	11.1103347416667	13.439923175

TIGD7	11.4483795708333	10.2648530166667	10.2295673791667	10.4864721541667	10.454064575	10.456786775	10.7663961291667	9.98940904166667	10.3621011583333	9.9840702875	10.1470911708333	11.0839469458333	10.6807042958333	10.2569008708333	10.1579110541667	10.1132725125	10.8780047041667	10.5446355958333	10.0487249291667	10.2656372791667	9.94731827916667	10.1040493708333	10.0349391541667	10.4326486208333	10.4129597333333	10.9867343166667	10.08378945	10.2541419541667	10.1019449333333	10.327136925	9.9752207875	10.218895	10.4278511208333	10.5750243625	10.9641918583333	11.0252346958333	10.0140669333333	9.84137288333333	9.63221137916667	10.1098494458333	9.99409350833333	9.99322627916667	10.1968151583333	9.93936995833333	10.5563215666667	10.0298802041667	10.1228387166667	9.6454597	9.751728375	10.2980843916667	9.88064432083333	9.9878090125	9.82972397083333	10.0825455416667	9.67252833333333	9.87413189166667	10.1599441791667	9.8641359375	10.0371652041667	9.68283467916667	11.0828266875	10.5874581875	10.9239630125	10.3005390166667	10.8516558958333	10.5974997458333	10.4784843041667	10.9133984666667	10.1224803125	10.589435475	10.5470652166667	10.8267681833333	10.3042109291667	10.7389314875	10.9675186041667	10.3998400708333	10.7491241041667	10.5932017375	9.90000193333333	10.7742107041667	10.602935325	11.4754507958333	9.789431775	10.62246185	10.2648530166667	11.1461551458333	10.9190991041667	11.0137151833333	11.2274614541667	10.6049050875	10.2788492458333	10.7708538	11.3533189333333	10.7036776291667	10.8317173291667	10.365440425	10.5859331041667	10.4415480375	10.2917777583333	11.1744638	10.5935973208333	10.4147458791667	10.6889820125	9.90329901666667	11.1961926416667	10.93956655	10.3126433583333	9.836069525	10.2828781916667	10.2082797708333	11.0718250666667	10.295333325	9.9936520625	10.69883065	10.1442671375	9.8347615125	10.5075527083333	10.7671402625	9.65908810833333	10.582768125

ZNF263	11.4483795708333	10.2648530166667	10.2295673791667	10.4864721541667	10.454064575	10.456786775	10.7663961291667	9.98940904166667	10.3621011583333	9.9840702875	10.1470911708333	11.0839469458333	10.6807042958333	10.2569008708333	10.1579110541667	10.1132725125	10.8780047041667	10.5446355958333	10.0487249291667	10.2656372791667	9.94731827916667	10.1040493708333	10.0349391541667	10.4326486208333	10.4129597333333	10.9867343166667	10.08378945	10.2541419541667	10.1019449333333	10.327136925	9.9752207875	10.218895	10.4278511208333	10.5750243625	10.9641918583333	11.0252346958333	10.0140669333333	9.84137288333333	9.63221137916667	10.1098494458333	9.99409350833333	9.99322627916667	10.1968151583333	9.93936995833333	10.5563215666667	10.0298802041667	10.1228387166667	9.6454597	9.751728375	10.2980843916667	9.88064432083333	9.9878090125	9.82972397083333	10.0825455416667	9.67252833333333	9.87413189166667	10.1599441791667	9.8641359375	10.0371652041667	9.68283467916667	11.0828266875	10.5874581875	10.9239630125	10.3005390166667	10.8516558958333	10.5974997458333	10.4784843041667	10.9133984666667	10.1224803125	10.589435475	10.5470652166667	10.8267681833333	10.3042109291667	10.7389314875	10.9675186041667	10.3998400708333	10.7491241041667	10.5932017375	9.90000193333333	10.7742107041667	10.602935325	11.4754507958333	9.789431775	10.62246185	10.2648530166667	11.1461551458333	10.9190991041667	11.0137151833333	11.2274614541667	10.6049050875	10.2788492458333	10.7708538	11.3533189333333	10.7036776291667	10.8317173291667	10.365440425	10.5859331041667	10.4415480375	10.2917777583333	11.1744638	10.5935973208333	10.4147458791667	10.6889820125	9.90329901666667	11.1961926416667	10.93956655	10.3126433583333	9.836069525	10.2828781916667	10.2082797708333	11.0718250666667	10.295333325	9.9936520625	10.69883065	10.1442671375	9.8347615125	10.5075527083333	10.7671402625	9.65908810833333	10.582768125

INSYN1	12.8305826833333	12.5420281	12.484600075	12.6297371916667	13.4065910333333	12.9024490333333	13.1506037833333	12.4320171333333	13.1085025666667	12.8181395166667	12.6769899666667	12.910549725	13.0469983166667	12.6826019916667	12.62157875	12.0713921666667	12.8166613583333	12.9053364166667	12.716481775	12.367659325	12.6971152166667	12.8171563916667	12.118621125	12.1926211666667	12.8836445083333	12.7477817333333	12.4705762833333	12.3855581	12.7769325	12.5212289166667	12.447837225	12.4132063666667	12.9960374833333	12.764427075	12.6180925666667	12.4619169416667	12.9400260666667	12.7179890416667	12.4447647416667	12.240405375	12.7216091916667	12.6407582416667	12.2906208	11.890067775	12.317829175	12.095758	12.1765967416667	12.206981325	12.4755627916667	12.5334882833333	12.8145234083333	12.6586586666667	12.9042883333333	12.5562191	12.48705105	12.1843992666667	12.50431935	12.5406352083333	12.2990201416667	11.9585723416667	12.856730075	12.4132063666667	11.94277775	12.229824125	12.7086380166667	13.12327495	12.8145234083333	12.464146975	12.770725525	12.64663275	12.7086380166667	12.5567153083333	12.5921909166667	12.543462525	12.1677152916667	11.655146	13.004442975	12.7175215083333	11.8407167416667	12.2496342916667	12.3509430416667	12.559988525	12.2641600583333	12.034459025	12.6862224333333	12.468741075	11.9712775416667	12.02225065	12.4692673166667	12.04537635	11.7985917083333	11.9396161083333	12.8475893166667	12.3422979833333	12.5259355083333	12.2210896416667	12.5488109	12.5145301166667	12.2496342916667	11.8962786833333	12.6013272333333	12.2188976833333	12.0778101166667	11.6982071583333	12.126629875	12.1217141333333	11.6694643083333	11.5664864	12.4709919583333	12.2896477166667	12.6470895583333	12.0909367583333	12.40421355	11.8576914166667	11.8947334916667	11.54260245	12.1951832916667	12.0348768666667	11.9329100916667	11.4737788083333

C1QTNF9B	9.10727307916667	9.88745143333333	9.90458776666667	9.600701075	9.98169012083333	8.89052767083333	10.1627032583333	10.2385425625	10.1647975583333	10.2648530166667	9.50111204166667	10.2256365833333	10.0228528083333	9.7364049375	10.3402727083333	9.17713551666667	8.7672794	9.9873854125	10.0634214208333	10.0080682583333	10.1387853291667	9.41870919583333	8.82849306666667	10.1086052333333	9.09352854166667	9.62566287916667	9.94863472916667	9.86788844166667	9.80292377083333	10.0963095125	9.43749067083333	9.31526372916667	8.8332465875	8.74980536666667	9.9272545625	9.67211590416667	9.57883875	9.80252920833333	9.12532139583333	9.72987528333333	9.5397773875	9.48230792916667	9.2806424375	10.11945695	9.6047947375	9.8249326375	9.71932142083333	9.51653439583333	8.89331847083333	9.96287945416667	9.24104285833333	10.0182432541667	9.78673265	9.642681875	9.62214002083333	9.79788007916667	10.0328838541667	9.92393945416667	9.78128911666667	9.06193843333333	8.03030704166667	9.6029844375	7.83372354166667	8.9918480875	8.99418029583333	8.06103641666667	8.58065427083333	9.67877904583333	7.90653880416667	8.497700375	8.9992168	8.77652168333333	10.0154029208333	9.16145469583333	8.8697616625	8.36008461666667	9.03208564583333	8.06156936666667	8.38095353333333	9.45878970416667	10.1627032583333	10.0909481916667	8.51461460416667	8.3439831125	8.82705755416667	9.83649577916667	8.69581604166667	8.56068924583333	9.01496978333333	9.05246449166667	8.76579940416667	9.37943772916667	8.18549834166667	9.0425371125	7.92084850833333	8.08265220416667	7.78301371666667	7.62959507083333	9.12532139583333	7.96447044166667	8.61848903333333	9.70215354583333	8.01462670833333	9.47134555	8.305095175	8.90550186666667	8.46173450833333	8.07331844583333	7.66472112083333	7.90439845416667	8.18756835833333	9.216632175	7.71941808333333	8.37033224166667	8.9519533	9.03572357916667	9.32209002916667	7.71888675	9.28994362083333	9.39913687083333

C1QTNF9	9.10727307916667	9.88745143333333	9.90458776666667	9.600701075	9.98169012083333	8.89052767083333	10.1627032583333	10.2385425625	10.1647975583333	10.2648530166667	9.50111204166667	10.2256365833333	10.0228528083333	9.7364049375	10.3402727083333	9.17713551666667	8.7672794	9.9873854125	10.0634214208333	10.0080682583333	10.1387853291667	9.41870919583333	8.82849306666667	10.1086052333333	9.09352854166667	9.62566287916667	9.94863472916667	9.86788844166667	9.80292377083333	10.0963095125	9.43749067083333	9.31526372916667	8.8332465875	8.74980536666667	9.9272545625	9.67211590416667	9.57883875	9.80252920833333	9.12532139583333	9.72987528333333	9.5397773875	9.48230792916667	9.2806424375	10.11945695	9.6047947375	9.8249326375	9.71932142083333	9.51653439583333	8.89331847083333	9.96287945416667	9.24104285833333	10.0182432541667	9.78673265	9.642681875	9.62214002083333	9.79788007916667	10.0328838541667	9.92393945416667	9.78128911666667	9.06193843333333	8.03030704166667	9.6029844375	7.83372354166667	8.9918480875	8.99418029583333	8.06103641666667	8.58065427083333	9.67877904583333	7.90653880416667	8.497700375	8.9992168	8.77652168333333	10.0154029208333	9.16145469583333	8.8697616625	8.36008461666667	9.03208564583333	8.06156936666667	8.38095353333333	9.45878970416667	10.1627032583333	10.0909481916667	8.51461460416667	8.3439831125	8.82705755416667	9.83649577916667	8.69581604166667	8.56068924583333	9.01496978333333	9.05246449166667	8.76579940416667	9.37943772916667	8.18549834166667	9.0425371125	7.92084850833333	8.08265220416667	7.78301371666667	7.62959507083333	9.12532139583333	7.96447044166667	8.61848903333333	9.70215354583333	8.01462670833333	9.47134555	8.305095175	8.90550186666667	8.46173450833333	8.07331844583333	7.66472112083333	7.90439845416667	8.18756835833333	9.216632175	7.71941808333333	8.37033224166667	8.9519533	9.03572357916667	9.32209002916667	7.71888675	9.28994362083333	9.39913687083333

LPIN1	10.1067055416667	11.2848715833333	11.4963967083333	11.39940385	12.068210875	8.61571535833333	10.6742121583333	10.3261322166667	10.7990034333333	10.2442419583333	9.92583431666667	8.1346889	11.0250402166667	10.8956711666667	11.8635130416667	10.7173608833333	8.58808473333333	11.012770325	11.2114428083333	11.2671696833333	9.767731825	11.7887391333333	10.7593753416667	10.1428729416667	11.8367863833333	10.1164178916667	11.4559913916667	11.2032221333333	11.63218295	10.99291925	10.7276175666667	11.484727	10.081132625	8.518147825	10.0915413	10.2720090666667	12.0062931166667	11.037692575	11.170311525	10.6535513666667	11.14669605	11.9612880416667	11.3627772083333	9.219699425	10.176297425	11.3078719166667	11.3889846416667	11.430726925	10.1720866	9.932660725	10.563601	10.9861853	11.3187261333333	10.782424925	10.9454688666667	11.528423775	10.78588925	11.7648162666667	11.6336776833333	10.5783390833333	8.33913670833333	8.04450845	9.06405529166667	8.94330425	8.615263775	9.21169858333333	7.42268463333333	7.66047423333333	7.89930148333333	8.59890789166667	8.40280550833333	7.72573829166667	6.97340769166667	8.92749955	8.32062069166667	7.88403631666667	8.14082359166667	8.50396570833333	8.60227746666667	10.1849087083333	9.77475016666667	8.028895175	8.59842283333333	8.081342075	8.081342075	9.30818888333333	8.4237182	8.295765375	8.403258675	8.74671439166667	8.53632365833333	9.12281406666667	8.33066458333333	8.04179383333333	7.74783731666667	8.68130785	7.886143875	10.5596517833333	8.35277756666667	9.21688079166667	8.40234485833333	8.706897975	9.30630015833333	8.52583200833333	9.34392991666667	8.47144243333333	8.96796725	7.28556258333333	9.037729975	8.4414262	9.21303188333333	9.01942851666667	9.0898379	8.39013935	8.57400345	9.7105078	8.68083718333333	8.16124230833333	9.05312258333333	8.31058729166667

AK2	13.5401929916667	14.0938637416667	13.9378093083333	14.1175943833333	13.8992947833333	14.4705790833333	13.864749275	14.1384134166667	13.8337930083333	13.7970969916667	14.0288199166667	13.8049154	13.5781398666667	14.107174325	13.7670768833333	14.1228795	14.3875977	13.9469444833333	14.0514423083333	13.8832091583333	14.0353374833333	13.9554702666667	14.105898725	14.1152258583333	14.1874223416667	13.810810275	13.7074542166667	14.11256935	13.788062875	13.90344385	13.6551549916667	14.0813262416667	13.82498885	13.9829878583333	13.5083984166667	13.8494140916667	13.7902025333333	13.7393052083333	14.1441992083333	13.8366591583333	13.9493522333333	13.823045075	14.021264575	13.7426466833333	13.9480264583333	13.8688792083333	13.9469444833333	14.11256935	14.1343040416667	14.1384134166667	13.5576132583333	14.2204751583333	14.0964870833333	14.0188677666667	14.063152775	13.577235475	13.785907925	13.8698539083333	13.8450858083333	14.021264575	13.9541211333333	14.034006175	14.1317250833333	14.1006630916667	13.91423525	13.306054325	14.1500101916667	14.0188677666667	14.3843059083333	13.8168544833333	13.748287325	13.5931197583333	14.2686692416667	13.9109585833333	13.6192792916667	13.986279475	14.0669942916667	14.015040275	13.6979142666667	14.046645825	14.3013820416667	13.7910501333333	14.1427380833333	13.935575975	13.9625123916667	14.1554067916667	14.2058121833333	13.7017952166667	13.8604046083333	13.99785205	14.236432725	13.9906723833333	14.4025408	14.1086467166667	13.88005245	13.7902025333333	14.1317250833333	13.9445631916667	13.668458425	13.96690645	13.3185547	13.488720775	13.5876594833333	14.0001337166667	13.6960256083333	14.0163605916667	13.7692653916667	13.3396866333333	13.7332085916667	13.75723885	14.5717037666667	13.5197355333333	13.5984028583333	14.1527055166667	13.654206225	14.241033875	14.1427380833333	13.4974205	13.9318364083333	13.48560075

HSPBP1	4.55971610833333	4.10724728333333	4.12457974166667	4.2379826	3.65945140833333	4.71113178333333	3.45642683333333	3.80684369166667	3.607366575	3.84584678333333	3.63418771666667	3.3314271	3.69247176666667	4.32946600833333	4.21912249166667	3.997050325	4.62057545833333	3.932546125	4.65176085833333	3.9614432	3.88526006666667	4.043026825	4.34995844166667	4.54233060833333	4.67172261666667	4.28696936666667	4.37610179166667	4.55369980833333	4.72414796666667	4.356950075	4.6247727	4.51370921666667	3.8957117	4.14053975833333	4.36096451666667	5.19860551666667	4.509505	4.55671806666667	5.060028525	4.69863704166667	5.0412383	4.58241979166667	4.87199023333333	5.60574740833333	4.17039641666667	5.11118121666667	5.229288475	4.99463171666667	4.73206480833333	4.90679766666667	4.80635555	4.48432390833333	3.85061613333333	5.42780705833333	4.383064975	4.789978375	3.87194265	4.809512175	4.3549546	4.63305978333333	3.832630725	3.65321091666667	4.44161573333333	3.80900449166667	3.53914015	4.01810879166667	3.95680781666667	3.3931188	3.70818398333333	3.534267775	3.53300575833333	3.5657117	3.3931188	3.70325111666667	3.46242046666667	3.72041143333333	3.50417281666667	4.29916468333333	3.86022540833333	3.70456366666667	3.588177475	3.553038975	4.24335745833333	3.96359718333333	3.7815889	3.58124660833333	3.73640415	4.45205615	3.80554890833333	4.021539275	4.085428875	4.385351725	4.51268873333333	3.81959511666667	3.9479401	4.97812506666667	4.268577875	4.14053975833333	3.80781481666667	5.02371664166667	4.371797775	3.536082325	4.82688145833333	5.11568579166667	4.369907275	4.0322916	4.67067994166667	6.3482401	4.66114760833333	6.715741525	4.61625569166667	5.31421083333333	4.510676125	4.97386210833333	4.12457974166667	4.40382505833333	4.19529993333333	4.58881544166667	4.62270154166667	3.54762483333333

TUBB	14.1709219416667	14.0874428333333	14.6466555666667	14.4758644166667	14.0100072833333	14.9336102833333	13.8614602166667	14.9197306916667	14.2378149833333	15.570936075	14.5287330416667	14.7293250666667	14.4809973583333	14.5020438083333	14.5232906166667	14.1306178166667	13.592329325	14.6239736916667	14.3824525333333	14.5322324833333	14.494740525	14.204528175	14.8110718166667	14.036419	14.6549636583333	14.50554015	14.3531439916667	14.4346519833333	14.8136769166667	14.2305624666667	14.3366389666667	14.53402895	13.8516367666667	13.7820292333333	14.341437425	14.77778785	14.5073052333333	14.2132241916667	14.2378149833333	14.3013820416667	14.113875875	14.6862769916667	14.044199175	14.4983330666667	14.1113310916667	14.9620915916667	14.0503314	14.68415095	14.4809973583333	14.5793502666667	14.789742575	13.8018861666667	14.5530100166667	14.4527902	14.66935455	14.4891443666667	13.7670768833333	13.520514175	14.4479745916667	14.15404765	15.6652002083333	15.581383325	15.799582875	15.4803730166667	15.7486260916667	15.56568115	15.7781512166667	15.3136672916667	15.6769351666667	15.33292745	15.6711097083333	15.386313475	15.7630766833333	15.6235572	15.5458909083333	15.6585444166667	15.700189825	15.5262258166667	15.7138591583333	15.700189825	15.700189825	15.6585444166667	15.5556811083333	15.7781512166667	15.369521175	15.700189825	15.7921085416667	15.7630766833333	15.4897034083333	15.6235572	15.5458909083333	15.436007925	15.7486260916667	15.462044625	15.5262258166667	15.1139193916667	15.69535405	15.8904374666667	15.700189825	15.7630766833333	15.8219621166667	15.5313549166667	15.784983325	15.7559244416667	15.651975575	15.6585444166667	15.9315345	15.5923775583333	15.7414894	15.6410257833333	15.5262258166667	15.8990167	15.8653098666667	15.7781512166667	15.6235572	15.5458909083333	16.0374488333333	16.1274635083333	15.7338865416667	15.7921085416667

ENPP3	11.1176950333333	8.91085585833333	8.755680875	9.19200934166667	9.8813009	9.37588805	9.70495998333333	9.00137649166667	9.222045675	9.13780860833333	8.79812115	10.8487808833333	10.7081516416667	9.20872513333333	9.35634101666667	9.5295644	10.277413575	10.6979535583333	8.94761291666667	9.24273069166667	9.283130225	10.591824075	8.78890531666667	9.08960063333333	9.31809370833333	10.4376859583333	9.65670091666667	9.43501909166667	10.7078134666667	9.16442515	9.40159784166667	8.73988864166667	9.22160494166667	9.439107	9.91032726666667	10.2724454333333	9.53786854166667	8.74083754166667	8.88546686666667	8.902412325	7.84430019166667	9.45281165833333	9.41761155833333	8.81191925833333	9.53786854166667	8.5716089	9.16302515	9.1773608	8.47431853333333	8.56421206666667	8.71688334166667	9.01340034166667	8.815557	8.85160831666667	8.684237875	8.50602524166667	9.26035810833333	8.21353541666667	9.06600838333333	9.05267498333333	10.1067055416667	8.83829725	8.631828525	8.90864591666667	8.490982775	9.16167278333333	8.16484069166667	9.30028739166667	8.90668976666667	8.82067435833333	8.79676539166667	9.25303868333333	9.7400082	9.45555605	9.408133425	8.21566409166667	10.26788045	9.047926775	8.023521375	8.83921701666667	9.17144896666667	10.0660963333333	8.650731025	8.36187725833333	9.17280985	9.56407003333333	9.146936475	9.31762243333333	8.93762295	9.05075425833333	8.87974118333333	8.45999591666667	8.37460103333333	8.18100231666667	8.8526135	8.12933435833333	8.42983770833333	9.14279978333333	8.954039625	9.01101026666667	8.24602095	9.70284449166667	8.39370431666667	7.90139170833333	8.03861421666667	8.96893119166667	9.50264109166667	8.40817908333333	8.27706721666667	8.359825725	8.34422473333333	7.53297658333333	8.05761690833333	6.85354335	7.37467966666667	8.10440565833333	8.68872105	7.65894785833333	7.78166071666667	8.82424165

NEK6	8.703122725	8.47183739166667	8.52061441666667	8.8451569	9.189315625	8.791398425	9.307742	8.43615038333333	8.91742895833333	8.62834993333333	9.12281406666667	9.59023981666667	9.05602455	8.54360906666667	8.5216034	9.04228765833333	8.04865021666667	9.4233862	7.86283530833333	8.04704694166667	8.43143401666667	8.63681648333333	7.93282061666667	8.77406471666667	8.66979525	8.74083754166667	8.3023979	8.22157784166667	8.59743456666667	8.5994674	8.487957325	8.0459923	9.02785883333333	8.6716691	8.83738886666667	8.79346453333333	8.70499479166667	8.35883419166667	8.5716089	8.398242975	7.57402961666667	7.6798049	8.26308525833333	7.88343015833333	8.498393225	8.010141425	7.656003025	8.31252919166667	8.154025025	8.20433166666667	8.14495316666667	8.338570375	8.524299575	8.54070765833333	7.46022033333333	7.639894925	8.53187930833333	8.17361143333333	8.19808664166667	8.19859208333333	9.11266035833333	8.95262451666667	9.11077986666667	8.8296979	8.76851021666667	9.63566801666667	8.56818220833333	9.21303188333333	9.41204579166667	9.13780860833333	9.41204579166667	9.16735169166667	9.4697267	9.48341896666667	9.72033719166667	9.765121975	9.38896178333333	9.03417474166667	8.51528900833333	8.89353219166667	8.222543225	8.916017775	8.4076656	8.83395916666667	10.0528892666667	9.13602675	9.43089169166667	9.3375044	9.85765425	9.66961651666667	9.0174923	8.661516725	9.6385431	8.76898080833333	9.187397175	8.30641555	9.18457413333333	9.02131511666667	9.29340571666667	8.61979589166667	8.29250424166667	8.910111275	9.04505583333333	8.80540845	8.8992014	9.42951640833333	9.39664281666667	9.04278656666667	8.74083754166667	9.4896997	8.05141264166667	8.069486125	8.871342075	8.36127639166667	9.26855390833333	9.06600838333333	8.55955131666667	8.80540845	8.318633925	9.19905495833333

TIMM8B	12.1165066666667	13.0285357166667	13.0413028416667	13.2438119875	13.0539450833333	12.3608148583333	12.910849375	12.6458866875	13.0424299958333	12.7689936666667	12.3188950583333	10.8410441208333	12.6006538583333	12.8993470541667	13.0122292208333	12.3686917041667	12.0891147833333	12.9414854	13.0146044041667	12.9050741791667	12.9453008083333	12.8524731291667	12.9567449625	12.7720962625	12.9447054583333	11.9153070541667	12.9572856708333	13.0629496458333	13.1410113291667	13.1730024083333	12.6757010458333	13.0183393541667	12.5727503916667	11.7957279	11.9407136291667	12.15939065	13.1155638166667	13.034637175	12.6463849666667	12.7695169291667	13.0673079541667	13.1121237333333	13.1749826291667	12.9010403916667	12.434366125	13.0491399166667	13.13537075	13.2748180708333	12.8638697333333	12.5036336	12.8982099916667	12.7834211666667	13.0666803875	12.9015734583333	12.9142045041667	12.9736325958333	12.84354935	13.1333095958333	12.6304552833333	12.7766554833333	12.0303737	12.04925465	12.3990288208333	11.7785076791667	11.7154865958333	12.0727330125	11.8342872875	12.1626225916667	12.4209337375	12.112389675	12.2992347375	11.8180775708333	12.1247999541667	12.3061607166667	11.6285029125	11.9276633583333	11.6128301125	11.91640355	12.6947858458333	12.1551696708333	12.3167046333333	11.0114286416667	11.8789535625	11.950406275	12.3968286291667	11.4517292208333	11.8259757708333	12.1490982166667	12.4833877416667	12.244689525	12.0883050041667	11.4124685041667	12.1933103291667	12.1301945458333	11.8207745125	11.9941734125	11.87703995	11.5300508916667	11.6717767958333	11.6413569583333	11.5109494708333	12.6257113041667	11.4709996583333	11.9659666	12.3932861666667	11.8475436583333	11.4536446875	11.9042165083333	11.4279135125	11.9222641625	11.5278084708333	12.2131156041667	11.4301640625	12.2300566416667	11.6491020458333	11.9441699875	11.210243625	11.5539872333333	12.4467832416667	11.3815280625

NKAPD1	12.1165066666667	13.0285357166667	13.0413028416667	13.2438119875	13.0539450833333	12.3608148583333	12.910849375	12.6458866875	13.0424299958333	12.7689936666667	12.3188950583333	10.8410441208333	12.6006538583333	12.8993470541667	13.0122292208333	12.3686917041667	12.0891147833333	12.9414854	13.0146044041667	12.9050741791667	12.9453008083333	12.8524731291667	12.9567449625	12.7720962625	12.9447054583333	11.9153070541667	12.9572856708333	13.0629496458333	13.1410113291667	13.1730024083333	12.6757010458333	13.0183393541667	12.5727503916667	11.7957279	11.9407136291667	12.15939065	13.1155638166667	13.034637175	12.6463849666667	12.7695169291667	13.0673079541667	13.1121237333333	13.1749826291667	12.9010403916667	12.434366125	13.0491399166667	13.13537075	13.2748180708333	12.8638697333333	12.5036336	12.8982099916667	12.7834211666667	13.0666803875	12.9015734583333	12.9142045041667	12.9736325958333	12.84354935	13.1333095958333	12.6304552833333	12.7766554833333	12.0303737	12.04925465	12.3990288208333	11.7785076791667	11.7154865958333	12.0727330125	11.8342872875	12.1626225916667	12.4209337375	12.112389675	12.2992347375	11.8180775708333	12.1247999541667	12.3061607166667	11.6285029125	11.9276633583333	11.6128301125	11.91640355	12.6947858458333	12.1551696708333	12.3167046333333	11.0114286416667	11.8789535625	11.950406275	12.3968286291667	11.4517292208333	11.8259757708333	12.1490982166667	12.4833877416667	12.244689525	12.0883050041667	11.4124685041667	12.1933103291667	12.1301945458333	11.8207745125	11.9941734125	11.87703995	11.5300508916667	11.6717767958333	11.6413569583333	11.5109494708333	12.6257113041667	11.4709996583333	11.9659666	12.3932861666667	11.8475436583333	11.4536446875	11.9042165083333	11.4279135125	11.9222641625	11.5278084708333	12.2131156041667	11.4301640625	12.2300566416667	11.6491020458333	11.9441699875	11.210243625	11.5539872333333	12.4467832416667	11.3815280625

SYT7	6.64415971666667	8.08531809166667	8.186266475	9.90731580833333	6.93091866666667	8.49745698333333	7.39975843333333	7.98243114166667	8.95262451666667	7.72520474166667	8.98146720833333	6.42485146666667	6.17154675833333	7.47443041666667	6.95221913333333	8.23315546666667	8.73207944166667	6.84063886666667	8.61290266666667	7.92497183333333	6.14249028333333	7.93859618333333	8.333491425	9.79533880833333	9.00964296666667	6.67633144166667	7.51638066666667	8.16124230833333	7.825834775	7.72294644166667	7.80930983333333	8.24163635833333	8.42823383333333	7.31493256666667	8.47478683333333	7.3741849	7.025978175	7.80505483333333	9.02222115833333	8.616671525	9.51408459166667	7.754380925	8.7532663	8.25292731666667	8.53882584166667	8.19970503333333	8.733549025	8.41642395	8.013764525	9.82907005833333	8.39672325833333	8.05913828333333	8.21983948333333	8.16983206666667	8.15563305	8.638756325	6.99800655	9.18261260833333	7.07182334166667	8.11603985	8.41158014166667	7.28678616666667	7.51872279166667	11.1298695666667	7.16072213333333	9.39071778333333	9.55429406666667	8.351126275	11.1241958916667	8.60646375	7.83568390833333	9.20822946666667	10.4866818833333	7.8747259	8.6223815	9.21688079166667	7.551793125	6.96533775	10.014766425	7.30968498333333	5.67327496666667	7.30913270833333	9.29933239166667	10.8284896583333	7.52107438333333	7.34611101666667	5.18312655	7.78439114166667	6.21116124166667	8.69657786666667	8.82318806666667	10.4594373083333	7.45341820833333	9.76350663333333	11.2467824083333	7.95962858333333	6.9563535	8.53005785	9.13739526666667	10.9835406583333	8.96700605833333	7.262828725	8.55105330833333	6.96970946666667	9.7860847	7.29843466666667	9.47641323333333	7.74398054166667	10.4061408166667	9.6324307	9.33087654166667	7.49050329166667	10.3105309666667	10.4606353416667	7.77718458333333	7.978659025	7.833966975	9.10889125	7.98973426666667	7.09102175833333

PTPRQ	6.96399793333333	8.584858775	7.80170391666667	7.470286275	7.691592325	6.241933725	8.29793538333333	7.42048124166667	7.42268463333333	7.68993234166667	7.62358238333333	7.97293150833333	7.41997313333333	7.54282731666667	6.98530065833333	6.03147445	6.28265325	6.777132775	7.09869196666667	6.78916241666667	6.408778075	6.449622025	6.58691901666667	5.30664471666667	7.11855360833333	7.72739250833333	7.378725775	7.661614575	6.1063381	6.95221913333333	6.46988230833333	5.59754975833333	7.676574425	6.30572190833333	6.66666621666667	8.43849715833333	7.60540448333333	7.09933909166667	6.914548675	6.81253920833333	6.53205264166667	6.15113656666667	7.33026348333333	7.004281725	7.5367911	5.30562465833333	6.71110255	5.45375554166667	6.86668814166667	6.29201916666667	6.48357195833333	5.9294819	6.637274925	6.169883075	6.74158396666667	5.7745543	7.99131585833333	6.03236501666667	6.61351845833333	6.76187970833333	7.83348010833333	7.02148474166667	5.467505875	5.79838183333333	6.08697085833333	6.13011929166667	5.13437999166667	7.07238718333333	5.2815818	5.95074298333333	7.97812778333333	6.24607838333333	6.05045175	6.19209213333333	7.63604310833333	4.289092625	7.98294754166667	5.21821305	4.245391125	4.3276422	5.17265765	7.31196651666667	4.203896025	4.258212325	6.399909175	7.37707911666667	5.866285325	7.08933194166667	6.28402719166667	5.93660110833333	8.00412230833333	4.49312724166667	6.06574364166667	7.52279740833333	6.44595796666667	6.17555735	5.211919525	6.2517663	6.75238478333333	6.51830799166667	6.04876668333333	5.14192449166667	7.82339403333333	7.72413511666667	4.421470575	4.95322815833333	4.84714924166667	6.77436175	6.31021560833333	6.769663225	10.5601410916667	6.49689175833333	4.361998275	4.87091625	5.27227235	8.23128400833333	7.20693700833333	4.94999915833333	7.21281770833333	9.53087495833333

DNPH1	12.7227603583333	12.9634653	12.9941887083333	12.939417425	12.5065921166667	12.4559287	12.4283415	12.7671377333333	12.5734875583333	12.7790607166667	12.8866499416667	12.4215906166667	12.4069098166667	12.9604577916667	12.989519425	12.6656204083333	13.1172651	12.5706160166667	12.9284297083333	12.8618738916667	12.7531374166667	12.7999144166667	12.796581575	12.644626475	12.7490093583333	12.763909375	12.5221926333333	12.7295358083333	8.39869986666667	12.703617625	12.4840896416667	12.8460196	12.6032318833333	13.2822330583333	12.6956383666667	12.7086380166667	12.7216091916667	12.8068783833333	12.6075148583333	12.7061787	12.8895588333333	12.8667102583333	12.9328607583333	12.9853754833333	12.689165025	13.1274317166667	12.9983717416667	13.0102233	12.9540039916667	12.8751753583333	12.74583335	12.478144425	13.0626219916667	12.9876385	12.7359047083333	12.7890792833333	12.7703078416667	12.671473625	12.6651447166667	12.786823125	12.6136777666667	12.6645702333333	12.6939319583333	12.3147568916667	13.5076042	12.6397345916667	11.8012624833333	11.6826865166667	13.17196845	12.3892279333333	12.7227603583333	12.1262500416667	12.9767731916667	12.7406037583333	12.2015812416667	12.2599545833333	12.501560375	12.8305826833333	12.731765875	13.0439770416667	12.909394625	12.1823287916667	12.761853925	12.23879665	12.5530135166667	12.6651447166667	12.8487753833333	12.4368713	7.66954886666667	12.8740952083333	12.7179890416667	12.4510079166667	11.8912374333333	12.799384025	12.8509307083333	12.24577265	12.959876375	12.837133975	12.600413	12.3896401833333	12.7144138666667	12.6581666416667	12.8912643	13.1256286916667	13.0973659083333	12.557141575	12.9853754833333	12.5831450833333	12.9259725333333	12.3618830833333	12.86304345	12.8979356	12.6679927416667	12.8509307083333	12.8684829916667	13.27588995	12.9929816333333	13.453508825	13.6876489583333	13.4321406333333

MDM4	12.9433935166667	12.3343950916667	12.2981372583333	11.9431320666667	12.1142465833333	12.4741889916667	12.1034309916667	12.3426613166667	12.4154660666667	12.19689565	12.2345932083333	13.0676082583333	12.5734875583333	12.320801975	12.496314025	12.939417425	13.1210793083333	12.5609255833333	12.1291180083333	12.5415392083333	12.2679354916667	12.3698220916667	12.2727355333333	12.9871205833333	12.6557058416667	13.3080939833333	12.6084193833333	12.6245838166667	8.15011035	12.4936861916667	12.1930875666667	12.600413	12.7790607166667	13.0343175416667	13.08233985	12.6975412416667	12.61272965	12.2281085833333	12.562277225	12.52364675	11.8344676416667	12.3542251583333	12.2867043083333	12.141404575	12.9461435166667	12.0889146833333	12.2981372583333	11.97355025	12.5334882833333	13.0607838416667	12.6909651833333	12.2939030416667	12.6971152166667	12.6481258583333	12.3195462583333	12.120221775	12.18313895	12.05061705	12.623024125	12.394774825	13.2525325833333	12.6185993416667	12.1389321	12.222327475	12.5580702166667	12.580271675	12.29141735	12.5448126916667	12.35974915	12.552088775	12.6914097333333	12.5420281	12.0713921666667	12.86014235	12.5381389666667	12.108037425	12.5196766666667	13.1905828833333	12.191066075	12.5729905	12.996638525	13.5526964	12.4746445166667	12.3443503083333	12.5963701666667	12.6346883583333	13.4594952083333	12.1170603	8.13617131666667	12.3798172166667	12.58926255	12.1839395083333	12.3374196	12.1856912833333	12.6632127083333	12.1150337666667	12.956460525	12.9773913166667	12.199408325	13.3793195083333	12.1250050333333	11.8530999083333	12.2322263916667	12.08124855	12.7113258083333	12.8432333583333	11.7351274916667	11.7589935083333	12.247084775	12.29141735	12.718512625	11.68154355	12.4087159666667	11.6496725666667	12.1084032166667	11.7046170083333	12.3832899166667	11.8000726	12.5719766166667	12.4154660666667

TUT4	6.64834389166667	4.48925233333333	5.34097245	5.880482625	6.057986625	6.34225285833333	4.685722125	5.14089556666667	5.15246255833333	5.66650295	5.66283725	5.06570035833333	5.557624025	5.18213341666667	5.0835026	6.12934905	5.399029725	7.69757114166667	6.35132606666667	5.891995425	4.77272485	5.966951625	5.12000276666667	6.15692685	6.13011929166667	6.230051875	6.73419851666667	6.81637415	7.02822294166667	6.36103899166667	6.59052684166667	6.04523669166667	6.69500276666667	5.30448685	6.61019589166667	6.30044350833333	6.195909975	6.510020875	7.00815195833333	6.75336094583333	6.265839825	6.37197970833333	6.22925051666667	6.0148863	4.93116945833333	5.86179054166667	5.94443094166667	5.58117110833333	6.71730138333333	7.06146445	7.842070475	6.57096464166667	6.74410944166667	7.11986829166667	5.69080398333333	5.66378185833333	6.36103899166667	6.32988615	6.61959393333333	6.34302255833333	6.59744179166667	4.28809964166667	6.85478974166667	4.82796093333333	5.20449400833333	7.299009625	5.97436386666667	4.95322815833333	6.44737514166667	5.65913218333333	7.28864350833333	4.736033375	6.13670685833333	6.37643818333333	5.28688518333333	5.82555810833333	6.08940326666667	7.58800115	7.12280756666667	5.554616075	5.90588820833333	6.25099584166667	6.028109975	6.164307025	6.69050035	5.1555947	6.372760025	5.51803240833333	5.83953349166667	6.61959393333333	6.05307568333333	7.23193100833333	6.181735025	5.9573564	7.52566054166667	7.06709683333333	6.96848423333333	7.52279740833333	6.37643818333333	7.086730875	7.15147738333333	4.53327731666667	6.77370956666667	5.53996425	7.21695988333333	6.264276875	6.63574723333333	6.246964375	6.83682504166667	6.755806875	7.58911965833333	7.17681736666667	7.38404451666667	7.19065496666667	6.14647701666667	6.75712434166667	6.85354335	7.76714046666667	7.11547685	5.50615049166667

APC	12.5671498916667	11.7216620916667	11.6473588416667	11.63482985	12.273184075	11.6628809833333	11.8935481166667	11.827360075	11.8435483083333	11.675011725	11.4745197333333	12.1608207083333	11.8386923083333	11.5371262416667	11.9570955666667	11.615215075	11.0504310083333	11.7943808	11.8158651416667	11.9686139666667	11.8020861333333	11.9112855083333	11.6329290083333	11.47905225	11.9082126416667	12.4279267416667	11.8826326666667	11.7572174666667	12.24082335	12.015272025	11.5512973083333	11.7525932916667	11.7470404	11.0733527583333	11.8500517083333	12.5958960666667	11.9096928333333	11.8198860833333	11.218700275	11.920083225	11.5038172666667	11.4288195833333	11.6525927916667	11.5432980083333	11.8395390583333	11.3608573416667	11.8904865166667	11.9259261416667	11.3534958083333	11.6511098916667	11.35644045	11.7194234416667	11.7517355166667	11.8705505	11.5684552166667	11.434022575	11.830137	11.7810947333333	11.6386741333333	11.8550160583333	11.4704736916667	11.1188874333333	10.8198860166667	11.0075637333333	11.6466404583333	11.6726997416667	11.7035007416667	12.5231511833333	12.042069275	10.992544275	10.929974575	11.4570874916667	10.7643055083333	11.0822289416667	11.735525225	11.4726344	11.39829615	11.6148020166667	11.31949275	11.7482758916667	11.47563195	12.139797125	11.405960975	11.3954232	11.5772577666667	11.5479749166667	10.834075425	11.30185685	11.6429698666667	11.4154857416667	11.326106725	11.0072108	11.47829265	10.8945637333333	11.6985875333333	10.5061588333333	12.2508814916667	10.959358675	11.4126401666667	11.156830125	11.4452785583333	10.5334113083333	11.1369321416667	11.3986748166667	11.2642606083333	11.5392863166667	11.0845103416667	10.8499191666667	11.4704736916667	11.83328595	10.5953164	12.3314457	10.9809278083333	11.3809445833333	11.1561366	10.7728842416667	11.0808123083333	10.6812532	11.4982052833333	11.1536039583333

LPA	4.63305978333333	5.35983450833333	5.8902603	6.485693525	6.46851783333333	4.40581835	6.95288179166667	6.1936491	6.55447500833333	5.464635875	6.29057105	7.07238718333333	6.53273724166667	6.09846643333333	5.44029105833333	5.0412383	5.60574740833333	6.54716043333333	4.99137435	6.20040056666667	6.37643818333333	6.42485146666667	6.35808980833333	4.27160165833333	4.77272485	4.535343	4.2443259	4.15642159166667	6.27482028333333	6.54716043333333	4.936774575	5.51585541666667	5.62775541666667	4.56071255833333	4.263255	4.26445661666667	4.91552208333333	4.40687995833333	4.19810324166667	4.49712886666667	4.6393107	5.19421045833333	5.94443094166667	5.20658905833333	4.55069105	4.82036908333333	4.41962694166667	4.74448875	6.67572515	4.78798888333333	4.45699755833333	4.344051675	5.876765525	4.98444206666667	4.90369176666667	4.4473261	6.02469836666667	4.52327248333333	4.72828980833333	4.16407313333333	7.57619343333333	6.560129475	6.37568504166667	5.40932128333333	6.55520170833333	6.112061375	5.956517125	6.64696773333333	6.24783014166667	5.79119435	6.27004911666667	6.63660101666667	7.05225570833333	5.22820184166667	6.20040056666667	5.30448685	5.98915650833333	6.1936491	5.0835026	5.83501515	6.37113890833333	7.84741366666667	6.09846643333333	5.329558875	5.40750829166667	5.91139875	6.02292943333333	5.98220235	7.08427549166667	5.24703029166667	5.90321861666667	5.270972825	4.7006097	5.442194025	5.71123295833333	5.29450505	4.82464924166667	4.695239575	5.983976325	4.71686186666667	5.514879125	6.26921054166667	4.563953975	4.78700018333333	4.383064975	5.59857721666667	4.909691175	5.70355690833333	4.98219815	5.61448439166667	4.46293625833333	4.99794086666667	5.03823209166667	4.613234575	4.56806040833333	4.64734200833333	4.79318954166667	4.70163844166667	4.89254299166667	5.10174316666667

CRELD1	8.91642435833333	8.33758601666667	8.20781665833333	8.08240869166667	8.238616825	8.013232325	7.26995246666667	7.97768158333333	7.88712554166667	7.94172535833333	7.650678625	8.017526525	7.75772005833333	8.07411434166667	8.69984448333333	8.25794470833333	8.65947190833333	8.113521575	8.86760295	8.355767175	8.1777524	8.403258675	8.19543675833333	8.17036706666667	8.32853386666667	8.77460095833333	7.9579863	8.79439289166667	8.66979525	8.40424725	8.3547531	8.027292325	8.21881454166667	8.439473525	9.234219825	9.33365533333333	8.35786781666667	8.14346960833333	8.06285705833333	8.42124595	8.27021601666667	8.55543884166667	8.60344725	8.0773066	8.70259450833333	8.59697231666667	8.42269018333333	8.47003455	8.29931580833333	8.30538075	8.106355425	8.05804941666667	8.58390675	9.07683584166667	8.40473328333333	8.86854605833333	7.788761475	8.43351543333333	8.25081844166667	8.14959635833333	9.44430623333333	7.91409299166667	8.00199818333333	8.60646375	9.38409195	8.89696199166667	7.57961929166667	7.80930983333333	9.04457245	8.084825575	6.96712810833333	8.59154009166667	6.93032724166667	8.075186025	8.0459923	8.128787725	8.61571535833333	8.093315875	8.11306225833333	7.97397919166667	8.78791004166667	7.63498546666667	8.329584675	8.70635446666667	8.1873246	8.164333775	9.10051520833333	7.638193025	7.60888643333333	8.46405893333333	7.73582129166667	7.548601625	7.341560875	8.28949861666667	8.91224634166667	7.801197125	8.4816187	7.743439475	7.9238629	8.08086618333333	7.70645875833333	8.123259225	9.06973089166667	7.75390870833333	9.14145633333333	8.5671537	9.51223504166667	9.05312258333333	8.63825258333333	7.47951195833333	8.37211495	7.82120025	8.92651730833333	8.84099020833333	8.9241622	8.35071744166667	8.86116145	8.27954998333333	8.54360906666667	7.73358451666667

SNRPC	8.81599143333333	8.89967475833333	8.87636833333333	9.03183454166667	9.40680740833333	8.56913543333333	9.37637135833333	9.28174641666667	9.292040175	9.26082591666667	9.13323439166667	9.071149525	9.27083296666667	9.03459930833333	8.72787371666667	8.81240245	9.01986039166667	9.06551128333333	8.75882765	8.821200975	9.08593973333333	8.80780606666667	9.01051185833333	8.80925365833333	8.80499509166667	9.237031475	8.8072839	8.86553766666667	8.83249931666667	8.97769849166667	8.83063586666667	9.01942851666667	9.00236645	8.98986978333333	9.07065694166667	9.21688079166667	9.052254	9.15886451666667	8.78691484166667	9.29065144166667	9.03598665833333	8.86655378333333	8.86609368333333	9.03089319166667	8.81108796666667	8.987001225	8.88262415833333	9.02034758333333	8.80189605833333	8.88020864166667	8.83395916666667	8.81108796666667	9.20822946666667	8.78836945	9.05312258333333	8.82621398333333	9.170544975	8.96609059166667	8.87183624166667	8.81880498333333	8.762233425	9.33456535	8.98237701666667	8.6984679	9.59742406666667	8.9962514	9.1447536	9.06270968333333	9.67141531666667	9.283130225	9.62234490833333	8.79346453333333	9.76911673333333	9.21303188333333	9.35451149166667	9.114481175	8.79489933333333	8.50130601666667	9.37269289166667	8.550436275	10.29990335	8.17249793333333	8.99720454166667	8.973998125	9.04505583333333	9.47496915833333	10.4308569666667	9.19860346666667	9.22016708333333	9.003775725	9.44058225	9.43324608333333	9.14377724166667	9.4094908	9.093743775	8.72311269166667	9.29340571666667	9.759228525	9.50499393333333	10.0578180083333	9.12281406666667	8.98520484166667	9.24172186666667	9.21169858333333	9.44779018333333	9.120869625	8.83016251666667	8.72497184166667	8.89870729166667	8.4515305	8.93806988333333	9.22470684166667	9.31710229166667	9.52316788333333	8.91889295833333	9.32471271666667	9.32329896666667	9.71657748333333	9.50086338333333	9.61139459166667

AICDA	4.18714809166667	4.26445661666667	3.83376700833333	3.96836553333333	4.06389798333333	4.809512175	4.0798737	5.52011276666667	4.14488695	6.04002979166667	3.95573993333333	4.45030740833333	3.78824818333333	3.79769870833333	3.711770875	5.258661775	4.383064975	4.290980325	4.61830805833333	4.20571450833333	3.67401930833333	5.020575025	5.514879125	4.41695324166667	5.48703414166667	4.30130591666667	4.10724728333333	4.894753975	4.936774575	4.59369346666667	5.47416700833333	7.21504029166667	4.18603395833333	7.11143919166667	4.67972664166667	4.683662975	4.31803169166667	4.14371865833333	4.82688145833333	4.35594943333333	4.40180795833333	4.02799813333333	4.39557236666667	5.00439053333333	5.229288475	4.54769205	5.62140730833333	5.4137082	4.69642039166667	4.41103275833333	5.20060255	4.79999713333333	7.20393616666667	4.83989633333333	4.58556791666667	6.30319241666667	4.57240950833333	6.717950125	4.98444206666667	5.10498871666667	4.19346535	4.15642159166667	4.81075010833333	3.75459249166667	3.68464693333333	4.161871975	4.32946600833333	5.67998533333333	4.234848125	5.992607775	4.23000438333333	4.9415684	4.31516280833333	3.74768024166667	4.67278611666667	4.54440353333333	4.1882044	10.0197701416667	5.207730075	6.06396311666667	3.881892575	4.567125	4.60485635	4.88257656666667	4.25573735	4.49406044166667	4.66114760833333	4.07015480833333	3.85883090833333	4.76252700833333	4.93116945833333	4.69642039166667	4.76455610833333	4.90256950833333	4.86085110833333	6.021143325	5.46549339166667	3.92496040833333	4.19044525833333	4.51861790833333	4.57827949166667	4.07457460833333	5.19309993333333	5.5614942	5.90065811666667	4.56600969166667	3.90095365833333	5.15873735	5.1168523	5.33069273333333	4.80827406666667	6.653546475	5.50523408333333	6.386982725	5.93746846666667	5.51683608333333	4.53105006666667	5.0835026	5.31227555833333	5.53793416666667

ERCC6L2	6.08509065	5.23978975	5.83306814166667	5.47221455833333	4.58336269166667	5.64406685833333	5.52123825	6.558117975	4.99463171666667	6.021143325	5.36615406666667	5.35476978333333	5.09764620833333	5.08252440833333	5.90154725	6.40265184166667	7.43289285	5.22594001666667	5.808344225	6.018516875	5.22594001666667	4.968508325	6.02292943333333	6.407249275	6.18408195	6.67096843333333	6.45538780833333	6.24607838333333	6.52564285833333	6.18408195	6.51915964166667	6.699210125	5.82555810833333	6.699210125	6.98215429166667	7.153195325	6.26921054166667	7.002378425	6.08790970833333	6.52412431666667	6.41504531666667	6.54994171666667	6.3482401	5.933890025	6.64292103333333	6.07673425	6.80056770833333	4.683662975	6.20180194166667	6.03052256666667	5.77807068333333	6.1186989	4.649483075	5.70649276666667	6.787125175	7.004281725	5.5319599	5.84884216666667	6.50860958333333	6.87589898333333	5.91627995	5.826473825	6.302455575	5.11118121666667	5.95328179166667	5.57926250833333	5.66378185833333	6.02015538333333	6.00968073333333	5.451608675	5.08659576666667	6.01386335	4.613234575	4.40083348333333	5.23876719166667	5.89991223333333	5.39178135	5.258661775	4.42980535	5.12102319166667	5.52228829166667	5.70748306666667	5.23876719166667	5.55862928333333	5.0835026	6.18247753333333	6.28265325	5.88432855	6.792709475	5.52228829166667	5.55953659166667	6.79994456666667	7.17569580833333	5.079599575	6.616187575	5.633369575	6.10313131666667	6.98911033333333	5.68107735833333	6.94497745833333	6.222261675	5.72466863333333	5.76251488333333	5.843159875	5.51386359166667	5.32557380833333	5.45803265	5.47616439166667	5.379722375	6.48284681666667	6.05994703333333	6.59815521666667	5.824619025	6.02015538333333	5.36818925	5.28357275833333	6.02896681666667	5.57060381666667	6.30177564166667	4.94355658333333

ENO1	13.916524275	14.657054125	14.421103675	14.61397985	14.2715663416667	15.325108875	14.1590921166667	14.9752180916667	13.5876594833333	15.00595375	14.5701394583333	13.253120425	14.0250042833333	14.8412522666667	14.59067625	13.8376377666667	13.5108418166667	14.577355375	14.4891443666667	14.2260493166667	14.3908304333333	15.1763309166667	14.83890155	14.5195733916667	14.8136769166667	13.6979142666667	14.9070025916667	14.7754409333333	15.25397645	14.8261234166667	14.5607495666667	14.85238655	14.0113488	13.0258011083333	13.4634900333333	13.789075975	14.7429609666667	14.3792394416667	14.4871709833333	15.1017513083333	14.5176714166667	14.9094697583333	14.1827283166667	14.7920246166667	13.9600657833333	14.80624315	14.2858034416667	15.0894272416667	14.6992893416667	14.2305624666667	14.6628978166667	14.53565035	14.1886825416667	14.2533850083333	14.7686158333333	14.4077075916667	13.5610039666667	14.3186975416667	14.3299457833333	14.7974983916667	14.7034991833333	15.215611525	15.5556811083333	15.3944873	15.6472384833333	14.8724941166667	15.4852249833333	15.1873199	15.7198901333333	15.28644375	15.0733260666667	15.2084540583333	15.700189825	14.8087193833333	15.236084325	15.5313549166667	14.9174232833333	15.3498376083333	15.390335525	15.618710975	15.57601185	14.2887769	15.4985918916667	15.5221336416667	15.2193239916667	15.4400250416667	15.1430836166667	15.2014589916667	14.7365031833333	15.2468154916667	15.345488975	15.215611525	15.4712092083333	15.436007925	15.436007925	15.4400250416667	15.4112652583333	15.6355001416667	15.4852249833333	14.7293250666667	15.386313475	15.03651825	15.7697269333333	15.390335525	15.4488835416667	15.4985918916667	15.6410257833333	15.3093345916667	15.38189755	15.4897034083333	15.1661341083333	15.5221336416667	15.6293082	15.7559244416667	15.5870133916667	15.6585444166667	15.9859723583333	15.6410257833333	15.38189755	15.2687677

SYN2	9.27833025	6.59016512083333	7.2841233625	6.47754952916667	4.93614820416667	8.44818234583333	6.12488773333333	6.89294285416667	6.3463456625	7.9587800125	7.49909098333333	10.6889820125	7.8784548125	6.24739725833333	7.5606433625	7.40520261666667	8.349958025	5.54348255	8.10507432083333	7.650449225	8.0410632875	5.43764716666667	7.56462495416667	8.10556687083333	7.52701770416667	10.7018390416667	8.39038555416667	7.85391059583333	6.47610697083333	7.3601641875	8.64519729583333	7.72222336666667	7.28101215	7.95772771666667	10.3696416875	10.4904339291667	7.06250175	8.08110412916667	8.69632065	8.1932087875	8.107904375	7.73835978333333	8.79618599166667	8.438760575	9.08070198333333	8.36601325	8.19117186666667	7.512267025	7.663040375	7.93306472083333	7.46488826666667	7.23223642916667	6.29712905416667	6.76283356666667	7.17538537916667	8.16151287083333	7.47815710833333	7.34524812916667	9.87886791666667	8.47122492916667	5.72030935	7.10088770833333	8.72570710833333	7.88103632083333	5.63186807083333	5.00291880416667	7.45531320833333	6.21805395	5.98604567083333	6.03374398333333	7.4919201875	5.98604567083333	4.08907536666667	6.80343899583333	4.2236606125	6.29240177916667	7.417938075	5.95023862916667	5.92366832916667	5.4153188875	4.99078407083333	5.47173187916667	5.94052129583333	8.3539560875	6.96685253333333	6.51273964583333	5.12862322916667	7.09015553333333	8.674769575	8.73377340416667	6.43681954583333	6.39417786666667	6.6788817375	5.96910895833333	6.43049987083333	6.87499558333333	4.668944475	4.10239608333333	5.18471674583333	4.96015072083333	6.62873372083333	6.916064425	4.4429841125	7.08324037083333	4.10893715	6.50685744166667	6.03285815	6.40446342083333	5.85655891666667	8.29710162916667	5.9396831125	6.60640252916667	4.1174117625	4.32336945833333	5.28309759583333	7.002055975	6.18133845416667	4.39098285	6.04657021666667	7.08457604166667

TIMP4	9.27833025	6.59016512083333	7.2841233625	6.47754952916667	4.93614820416667	8.44818234583333	6.12488773333333	6.89294285416667	6.3463456625	7.9587800125	7.49909098333333	10.6889820125	7.8784548125	6.24739725833333	7.5606433625	7.40520261666667	8.349958025	5.54348255	8.10507432083333	7.650449225	8.0410632875	5.43764716666667	7.56462495416667	8.10556687083333	7.52701770416667	10.7018390416667	8.39038555416667	7.85391059583333	6.47610697083333	7.3601641875	8.64519729583333	7.72222336666667	7.28101215	7.95772771666667	10.3696416875	10.4904339291667	7.06250175	8.08110412916667	8.69632065	8.1932087875	8.107904375	7.73835978333333	8.79618599166667	8.438760575	9.08070198333333	8.36601325	8.19117186666667	7.512267025	7.663040375	7.93306472083333	7.46488826666667	7.23223642916667	6.29712905416667	6.76283356666667	7.17538537916667	8.16151287083333	7.47815710833333	7.34524812916667	9.87886791666667	8.47122492916667	5.72030935	7.10088770833333	8.72570710833333	7.88103632083333	5.63186807083333	5.00291880416667	7.45531320833333	6.21805395	5.98604567083333	6.03374398333333	7.4919201875	5.98604567083333	4.08907536666667	6.80343899583333	4.2236606125	6.29240177916667	7.417938075	5.95023862916667	5.92366832916667	5.4153188875	4.99078407083333	5.47173187916667	5.94052129583333	8.3539560875	6.96685253333333	6.51273964583333	5.12862322916667	7.09015553333333	8.674769575	8.73377340416667	6.43681954583333	6.39417786666667	6.6788817375	5.96910895833333	6.43049987083333	6.87499558333333	4.668944475	4.10239608333333	5.18471674583333	4.96015072083333	6.62873372083333	6.916064425	4.4429841125	7.08324037083333	4.10893715	6.50685744166667	6.03285815	6.40446342083333	5.85655891666667	8.29710162916667	5.9396831125	6.60640252916667	4.1174117625	4.32336945833333	5.28309759583333	7.002055975	6.18133845416667	4.39098285	6.04657021666667	7.08457604166667

DISC1	4.41449314166667	4.1111182625	5.05535775	4.34014847916667	4.12168023333333	4.02971369583333	4.34746767916667	4.86960658333333	4.79762524166667	3.77121228333333	4.46148831666667	5.01478968333333	4.18348087083333	4.77430863333333	4.35138867083333	4.06023525416667	4.561288475	4.2680831125	4.55126776666667	4.6346229125	3.8702181125	4.1399864	4.37233573333333	4.44005515	4.63982503333333	4.23957455416667	5.57393574583333	5.64262090833333	4.7062329125	4.38768927083333	4.74498560416667	4.58191295	4.3586702875	4.08268443333333	4.371324975	4.23849965833333	4.56656734583333	4.44873279166667	4.41920828333333	4.63041867083333	4.87549307083333	4.38051879166667	4.79365025	5.5114587625	4.04563094583333	4.93814212083333	4.95378601666667	4.993014125	4.4723208875	4.75647757083333	4.72369785833333	4.49957643333333	4.4429841125	5.31061434166667	4.30576305	4.68126080416667	5.5834999375	4.73250927916667	4.29241315	4.66576174583333	3.84311462916667	4.48566008333333	4.24071980416667	4.9401554875	4.45160768333333	4.234362525	4.6792515125	5.0629180125	5.20009882083333	4.49549822083333	5.035744375	5.4251255875	4.19765279166667	4.37233573333333	4.38681214166667	5.40355344166667	4.4125018625	4.16989618333333	4.823028825	4.48007165	4.194823825	4.88425827916667	4.2976697125	4.22947874166667	4.91802345	4.36549425833333	4.70961086666667	4.65221884166667	4.7711452375	4.06646273333333	4.40028064583333	4.5037153875	4.22636069166667	3.802702375	5.12156893333333	4.29552213333333	4.83165315	4.07190218333333	4.88205153333333	4.97227905833333	4.29866895	4.44777587083333	4.80775573333333	5.05230115833333	4.19963816666667	4.30386637916667	4.56021433333333	6.33679825	4.2732531875	6.28623036666667	4.59416930416667	5.44589229166667	4.415524125	4.91383746666667	4.2934453	4.3456095375	4.01277812083333	4.50487469583333	4.518175825	4.2680831125

TSNAX-DISC1	4.41449314166667	4.1111182625	5.05535775	4.34014847916667	4.12168023333333	4.02971369583333	4.34746767916667	4.86960658333333	4.79762524166667	3.77121228333333	4.46148831666667	5.01478968333333	4.18348087083333	4.77430863333333	4.35138867083333	4.06023525416667	4.561288475	4.2680831125	4.55126776666667	4.6346229125	3.8702181125	4.1399864	4.37233573333333	4.44005515	4.63982503333333	4.23957455416667	5.57393574583333	5.64262090833333	4.7062329125	4.38768927083333	4.74498560416667	4.58191295	4.3586702875	4.08268443333333	4.371324975	4.23849965833333	4.56656734583333	4.44873279166667	4.41920828333333	4.63041867083333	4.87549307083333	4.38051879166667	4.79365025	5.5114587625	4.04563094583333	4.93814212083333	4.95378601666667	4.993014125	4.4723208875	4.75647757083333	4.72369785833333	4.49957643333333	4.4429841125	5.31061434166667	4.30576305	4.68126080416667	5.5834999375	4.73250927916667	4.29241315	4.66576174583333	3.84311462916667	4.48566008333333	4.24071980416667	4.9401554875	4.45160768333333	4.234362525	4.6792515125	5.0629180125	5.20009882083333	4.49549822083333	5.035744375	5.4251255875	4.19765279166667	4.37233573333333	4.38681214166667	5.40355344166667	4.4125018625	4.16989618333333	4.823028825	4.48007165	4.194823825	4.88425827916667	4.2976697125	4.22947874166667	4.91802345	4.36549425833333	4.70961086666667	4.65221884166667	4.7711452375	4.06646273333333	4.40028064583333	4.5037153875	4.22636069166667	3.802702375	5.12156893333333	4.29552213333333	4.83165315	4.07190218333333	4.88205153333333	4.97227905833333	4.29866895	4.44777587083333	4.80775573333333	5.05230115833333	4.19963816666667	4.30386637916667	4.56021433333333	6.33679825	4.2732531875	6.28623036666667	4.59416930416667	5.44589229166667	4.415524125	4.91383746666667	4.2934453	4.3456095375	4.01277812083333	4.50487469583333	4.518175825	4.2680831125

PABPC1L	8.67598873333333	9.22794726666667	9.05776881666667	9.03908018333333	9.55333915	9.37126875	9.16442515	8.94668689166667	9.10576088333333	9.03950406666667	9.07584114166667	8.98618440833333	8.19036318333333	8.27384794166667	9.00189508333333	9.002830825	8.33599743333333	9.50539336666667	8.70592035	8.85494786666667	8.44632826666667	9.38275609166667	9.12557628333333	9.59824975833333	9.07065694166667	8.4484208	9.08644098333333	9.25904538333333	9.39574425833333	9.20778985833333	8.75003116666667	8.88732965833333	9.05602455	8.8992014	8.69939258333333	8.54656363333333	9.2639574	9.09878833333333	8.97625954166667	8.82067435833333	8.75278535833333	8.66979525	8.90962148333333	9.09460495	8.87636833333333	8.89696199166667	8.30746798333333	9.04692176666667	9.22385636666667	9.32000775	9.00822095	8.88067333333333	9.67592103333333	9.17867269166667	8.67503203333333	8.35234010833333	9.138674475	8.77140460833333	9.471100975	8.65843663333333	11.42922935	10.390544	11.1887914833333	10.8322571166667	9.82295156666667	10.4332127666667	9.88637849166667	10.1759041833333	10.49790835	9.60406910833333	9.458203675	9.732285275	10.2157667083333	9.68863114166667	9.226998075	9.62759855	9.94714974166667	10.6792669083333	9.60319949166667	9.52745255833333	10.199031925	10.6905955916667	9.24172186666667	9.33964531666667	9.76472428333333	10.4476616583333	11.401232775	9.512679675	10.4914439416667	9.42382585833333	9.57736215	11.0848820333333	10.571508325	9.64783695	10.146512775	9.7237141	11.2481821666667	10.1783585083333	9.99301340833333	9.00690949166667	9.89383843333333	9.39071778333333	10.5464444833333	9.67501213333333	9.53914050833333	10.2115540166667	9.695341625	9.361656325	10.6263315083333	8.95031519166667	9.4181056	8.85547214166667	10.1728980166667	10.283076325	9.439107	9.71785554166667	10.31088965	10.8570968916667	10.20731305	9.34018350833333

ZNF268	10.4006641	9.3650416625	9.1694381	9.59972799583333	9.34132776666667	9.28575792916667	8.4532828875	9.12350275416667	9.102203125	9.0039794	8.96068542916667	9.06245812916667	8.99418029583333	9.23116307916667	9.23305643333333	9.20584292083333	9.68844351666667	9.32446495833333	9.52480252916667	9.3043361375	9.60116981666667	9.63542419166667	8.72334106666667	9.73418850416667	9.66804459583333	9.720911	9.17073914583333	8.96823769583333	9.17895344166667	9.27148613333333	9.04481414166667	9.57797126666667	9.199282025	8.953804425	9.34965205	9.80864851666667	9.19266140416667	8.8171849875	8.8976024375	9.58198016666667	9.28904332916667	9.196978225	9.61837867083333	9.24732117916667	9.42360602916667	9.44128464583333	9.88782489166667	9.52668602916667	9.27104185	9.09621615	8.92579979583333	8.68550034166667	9.43019225833333	9.88231113333333	9.76533135833333	9.779526275	9.4431532125	9.05709510416667	9.56657477916667	9.537157575	11.4061629208333	8.69530741666667	9.469969725	7.301637925	9.83963402083333	9.607303525	8.18810056666667	8.57627579166667	6.827881725	7.6085586125	8.67336657083333	9.17713551666667	8.69769252916667	9.03258994583333	10.374341375	9.10962599583333	9.47703872083333	8.78419849166667	8.36932502083333	8.55365765833333	8.65148557083333	9.5767140375	6.78815305416667	9.59000108333333	9.0089050125	8.40203811666667	8.26631075416667	8.4818775625	9.60896382083333	8.8804409875	7.8364502	8.45480330416667	8.71712998333333	8.9992168	10.1842929833333	8.11028826666667	6.71273871666667	8.1275256375	7.98797805	9.7441156	9.21472344166667	8.38246611666667	9.85179888333333	9.3964552875	9.934516325	9.19314084166667	8.32672639583333	10.1615384	9.68135890833334	9.15624333333333	8.1202170625	6.861739275	9.60210426666667	9.08322604583333	9.48812237083333	9.42360602916667	9.966801325	9.540661475	9.07609399583333	8.94738361666667

AC026786.1	10.4006641	9.3650416625	9.1694381	9.59972799583333	9.34132776666667	9.28575792916667	8.4532828875	9.12350275416667	9.102203125	9.0039794	8.96068542916667	9.06245812916667	8.99418029583333	9.23116307916667	9.23305643333333	9.20584292083333	9.68844351666667	9.32446495833333	9.52480252916667	9.3043361375	9.60116981666667	9.63542419166667	8.72334106666667	9.73418850416667	9.66804459583333	9.720911	9.17073914583333	8.96823769583333	9.17895344166667	9.27148613333333	9.04481414166667	9.57797126666667	9.199282025	8.953804425	9.34965205	9.80864851666667	9.19266140416667	8.8171849875	8.8976024375	9.58198016666667	9.28904332916667	9.196978225	9.61837867083333	9.24732117916667	9.42360602916667	9.44128464583333	9.88782489166667	9.52668602916667	9.27104185	9.09621615	8.92579979583333	8.68550034166667	9.43019225833333	9.88231113333333	9.76533135833333	9.779526275	9.4431532125	9.05709510416667	9.56657477916667	9.537157575	11.4061629208333	8.69530741666667	9.469969725	7.301637925	9.83963402083333	9.607303525	8.18810056666667	8.57627579166667	6.827881725	7.6085586125	8.67336657083333	9.17713551666667	8.69769252916667	9.03258994583333	10.374341375	9.10962599583333	9.47703872083333	8.78419849166667	8.36932502083333	8.55365765833333	8.65148557083333	9.5767140375	6.78815305416667	9.59000108333333	9.0089050125	8.40203811666667	8.26631075416667	8.4818775625	9.60896382083333	8.8804409875	7.8364502	8.45480330416667	8.71712998333333	8.9992168	10.1842929833333	8.11028826666667	6.71273871666667	8.1275256375	7.98797805	9.7441156	9.21472344166667	8.38246611666667	9.85179888333333	9.3964552875	9.934516325	9.19314084166667	8.32672639583333	10.1615384	9.68135890833334	9.15624333333333	8.1202170625	6.861739275	9.60210426666667	9.08322604583333	9.48812237083333	9.42360602916667	9.966801325	9.540661475	9.07609399583333	8.94738361666667

ERLEC1	7.84267175833333	7.68263314166667	7.32549529166667	7.04956788333333	8.13923885	9.04179071666667	7.821740775	7.62704748333333	8.01539524166667	7.56325655	7.91522786666667	8.25839510833333	8.1455177	7.73639724166667	8.22846261666667	8.12585160833333	7.27649536666667	8.23567169166667	8.09947061666667	7.55374699166667	7.63498546666667	8.84155408333333	6.96254510833333	8.17885319166667	8.62685349166667	7.978659025	7.48063178333333	7.65774509166667	7.56268353333333	7.48985504166667	7.28317230833333	8.15060805	7.70532880833333	7.32205540833333	6.43016624166667	7.72520474166667	8.24163635833333	7.29732286666667	8.15455800833333	7.630419825	6.50929938333333	7.00630173333333	6.80510874166667	6.84636305	7.31739324166667	6.524903425	6.55223534166667	7.095527	7.99891026666667	7.754380925	7.79082046666667	7.774307975	7.63549629166667	8.11553545	7.84267175833333	6.955027575	7.02021258333333	6.56292778333333	8.355767175	7.92497183333333	10.5420798333333	7.97346688333333	8.635462725	8.1873246	8.20689531666667	9.7408584	8.19808664166667	8.65947190833333	8.41972045	8.66775465	8.79767885	8.42069139166667	8.488931425	9.133746725	9.05561776666667	9.04692176666667	8.460967325	9.81305005833333	8.70635446666667	9.29481465	9.838575025	9.77974975833333	8.18151525833333	8.04804935	8.79861721666667	8.87736359166667	9.86989941666667	7.44780359166667	10.3933623083333	9.04179071666667	8.33112569166667	9.633880775	8.98191765833333	8.62986514166667	10.2563791083333	8.907621175	8.38611514166667	10.5428297916667	8.22348525833333	8.24067144166667	8.48112818333333	7.91097105833333	9.20693985	7.81947101666667	7.97346688333333	8.62547046666667	8.81515405833333	7.927602375	9.00775005833333	8.72089346666667	9.14971231666667	7.46194565833333	8.35024235	7.45098580833333	8.8526135	7.99232836666667	9.16967255	8.08865246666667	7.64327454166667	7.75983114166667

GOLGA4	11.0120115833333	11.8553966916667	11.6166645833333	11.4864514	12.064871125	11.320943175	12.1861275083333	12.2951630416667	12.3110445	12.0290333416667	12.0604174833333	11.7537741833333	11.5969108416667	12.091314325	11.994387925	11.8535038916667	12.3716896916667	12.2714994	12.4474119833333	12.6103198083333	12.0857092083333	11.958948075	11.7557288583333	11.941704975	11.920083225	11.638265575	11.7834532083333	11.94277775	11.8834269416667	12.1807321916667	12.050259975	11.7113167916667	11.8584258416667	11.4934089166667	11.8386923083333	11.7834532083333	11.7351274916667	12.220220925	11.9657351833333	12.0657212166667	11.8584258416667	12.17539085	11.7074261416667	11.8768607333333	10.8480286083333	11.6602573333333	11.5111448666667	11.7849428666667	11.8317467416667	12.0985319416667	11.9146982166667	12.1142465833333	10.1246349583333	10.670337625	10.8056325083333	10.6531638166667	12.1138378333333	12.1774305916667	11.8415156583333	11.7742804333333	10.0924722666667	10.6038899083333	10.3574979	10.8210096	11.1956589833333	11.3375225333333	11.3997255833333	11.4008627833333	11.2128973166667	11.0839469458333	11.492270725	11.0531461166667	11.2740276	10.7371678583333	10.8258465666667	11.3813533416667	11.2085666916667	11.1960117	12.1224925833333	11.6436590416667	11.0120115833333	11.0815307083333	11.7549123666667	11.09861755	11.0294181583333	10.6792669083333	10.5396655666667	10.9273818166667	10.5679469416667	11.1674196	11.3300005666667	10.5303580166667	10.253110825	11.174649725	10.6649055833333	11.4431148583333	11.2007994333333	10.806391425	11.1963735833333	10.9028824333333	10.8945637333333	11.0663049916667	10.8874095666667	11.2125996833333	11.1607791666667	10.7538304083333	11.1506157166667	11.2114428083333	11.2291005583333	11.41004175	11.52420235	10.9776492583333	9.48881261666667	10.0644501166667	10.20436555	10.1105163666667	11.1724975333333	11.496789525	11.1752905	10.8800392333333

SLC36A2	4.4047953	5.16622001666667	5.13015945	4.7847361	5.91520025833333	4.34514024166667	6.65092356666667	6.56431505	6.05717670833333	5.173701425	4.60485635	5.981391325	6.39918578333333	4.789978375	4.50544710833333	5.189937375	4.53327731666667	5.078596225	4.54020328333333	3.94580334166667	4.64847966666667	4.56186439166667	5.49632836666667	4.36702491666667	4.49712886666667	4.2939407	5.13847554166667	4.85861940833333	4.51773374166667	4.21701204166667	4.54330015	4.370852175	3.588177475	5.40750829166667	4.07678143333333	4.00448406666667	4.74930484166667	4.24335745833333	4.2422894	4.55369980833333	4.6622505	4.0917668	4.50112465833333	5.18112505	4.42771198333333	5.27529641666667	4.7771511	4.666328775	4.38635804166667	4.70784294166667	4.61955908333333	4.41103275833333	4.093740425	4.96739760833333	4.17039641666667	4.52697115833333	3.64058418333333	4.48239504166667	3.99595204166667	3.905753025	3.93585013333333	5.23259211666667	4.234848125	4.41103275833333	6.13011929166667	4.33133893333333	4.79109571666667	5.58021739166667	4.8815265	4.81716043333333	4.087323525	4.62057545833333	6.06147365	4.12229528333333	5.41985199166667	4.649483075	4.59576983333333	3.84700126666667	4.061836425	4.51370921666667	5.29238863333333	4.510676125	5.44533825	4.03445655833333	4.38930708333333	4.91552208333333	3.890139475	4.38726624166667	4.34795044166667	3.932546125	4.45416251666667	4.25488725	3.777965425	4.72414796666667	4.07245948333333	3.77426261666667	3.90689708333333	3.932546125	4.15954296666667	4.85447411666667	4.49216794166667	4.89364545833333	4.48925233333333	4.59897825	4.06593015	3.842520875	4.879391675	5.40105285833333	4.15745399166667	6.126012425	4.45699755833333	5.06786714166667	4.125728225	4.59683404166667	3.9861723	4.2379826	3.90223645833333	4.36901661666667	4.07353555833333	5.12102319166667

AC005726.1	10.9323187791667	11.3341465833333	11.3226184125	11.5350930541667	11.9429549083333	9.6516368375	10.6572385166667	10.5645419458333	10.801233575	11.5014381625	10.3593105625	10.768260425	11.2504840916667	11.6135515916667	11.6604320041667	11.2354426333333	11.7200207041667	12.0130447041667	11.4429405083333	11.37003045	11.0058676	11.7650007458333	11.5700714791667	10.7708538	11.4921044416667	11.2791978708333	11.2589819375	11.2991506291667	11.3648148708333	11.4754507958333	11.4081227125	11.0509045708333	10.9591537	10.8200638041667	10.8118916416667	10.854301975	11.6361209083333	11.4539903333333	11.3077018833333	10.8955074291667	10.993162925	11.4294174458333	11.6638720875	11.3891676541667	11.2087548083333	11.6471973208333	11.6380696333333	11.1566631583333	11.1182603	10.9359125291667	11.7968825708333	11.2751551541667	11.83465975	11.7653685166667	11.3483044583333	11.8735748416667	11.076375075	11.6041942166667	11.6728914333333	11.3450186875	11.0169557958333	12.0228148916667	11.336614125	11.5335937	11.0107050083333	12.1247999541667	11.1233378416667	11.4536446875	12.0821880541667	10.4761612458333	11.2515910291667	11.5921871041667	12.1539113833333	10.7491241041667	11.5420187458333	11.2662364625	9.94183766666667	10.8572859083333	8.76579940416667	10.80997585	7.91383982083333	10.8466929375	11.6766534875	11.4028483375	11.1472248958333	10.327136925	10.7315763416667	8.9045665125	11.6221782291667	10.629165025	10.6576221833333	11.1229606875	11.772617675	9.76889656666667	10.6156651958333	11.6173032791667	11.6850845583333	11.1454009041667	11.9515364625	11.5267024166667	11.5130575833333	11.93203865	10.3873808291667	10.365791975	12.1772261041667	11.1886201666667	11.7851269541667	11.5647305333333	11.1544759875	10.2176713	11.0256023791667	12.3722635166667	11.9366819583333	11.378052025	11.7465124875	11.88751815	10.8489671833333	10.8328285208333	12.0162554833333	10.4034865125

ALDOC	10.9323187791667	11.3341465833333	11.3226184125	11.5350930541667	11.9429549083333	9.6516368375	10.6572385166667	10.5645419458333	10.801233575	11.5014381625	10.3593105625	10.768260425	11.2504840916667	11.6135515916667	11.6604320041667	11.2354426333333	11.7200207041667	12.0130447041667	11.4429405083333	11.37003045	11.0058676	11.7650007458333	11.5700714791667	10.7708538	11.4921044416667	11.2791978708333	11.2589819375	11.2991506291667	11.3648148708333	11.4754507958333	11.4081227125	11.0509045708333	10.9591537	10.8200638041667	10.8118916416667	10.854301975	11.6361209083333	11.4539903333333	11.3077018833333	10.8955074291667	10.993162925	11.4294174458333	11.6638720875	11.3891676541667	11.2087548083333	11.6471973208333	11.6380696333333	11.1566631583333	11.1182603	10.9359125291667	11.7968825708333	11.2751551541667	11.83465975	11.7653685166667	11.3483044583333	11.8735748416667	11.076375075	11.6041942166667	11.6728914333333	11.3450186875	11.0169557958333	12.0228148916667	11.336614125	11.5335937	11.0107050083333	12.1247999541667	11.1233378416667	11.4536446875	12.0821880541667	10.4761612458333	11.2515910291667	11.5921871041667	12.1539113833333	10.7491241041667	11.5420187458333	11.2662364625	9.94183766666667	10.8572859083333	8.76579940416667	10.80997585	7.91383982083333	10.8466929375	11.6766534875	11.4028483375	11.1472248958333	10.327136925	10.7315763416667	8.9045665125	11.6221782291667	10.629165025	10.6576221833333	11.1229606875	11.772617675	9.76889656666667	10.6156651958333	11.6173032791667	11.6850845583333	11.1454009041667	11.9515364625	11.5267024166667	11.5130575833333	11.93203865	10.3873808291667	10.365791975	12.1772261041667	11.1886201666667	11.7851269541667	11.5647305333333	11.1544759875	10.2176713	11.0256023791667	12.3722635166667	11.9366819583333	11.378052025	11.7465124875	11.88751815	10.8489671833333	10.8328285208333	12.0162554833333	10.4034865125

EIF3I	10.5154588666667	9.25536705833333	9.40511030833333	9.67592103333333	9.440114675	9.93476125833333	8.57063375	8.57257758333333	9.0174923	9.32329896666667	8.973101525	10.7220730083333	9.13033400833333	8.551994475	9.47987389166667	10.0476842666667	10.1936096583333	9.17515170833333	9.565041475	9.4955569	9.41298314166667	9.759228525	9.36206593333333	9.77883299166667	10.0881734833333	10.1791861916667	9.46704600833333	9.94251199166667	10.1728980166667	9.41120025	9.66334089166667	9.08729039166667	9.69219855833333	9.55288966666667	10.19975295	10.30576505	9.54925085	9.08180965	8.458998275	9.54834169166667	9.04228765833333	9.900995025	9.463907375	9.13694515	10.1986602916667	9.61323428333333	9.39392068333333	8.52683058333333	9.30543436666667	9.50996596666667	9.19905495833333	8.98465464166667	9.80885460833333	9.61596005833333	9.12326560833333	9.68558669166667	8.71384840833333	9.16123660833333	9.985532675	9.08915231666667	8.72452928333333	8.48321643333333	8.69409343333333	8.74819925	11.1113391916667	9.04320051666667	8.4773978	9.52867283333333	8.41104395833333	9.35634101666667	7.45451809166667	7.920590025	7.272898375	9.67900834166667	8.08185559166667	9.30818888333333	9.18832863333333	9.67274871666667	8.40817908333333	9.14655231666667	9.478582775	9.21303188333333	8.57301913333333	9.04890688333333	9.27899289166667	8.8011824125	7.42788356666667	9.73188163333333	7.65174983333333	9.66558844166667	9.047926775	8.8128727	9.170544975	8.76851021666667	8.40614324166667	8.84428041666667	9.18880384166667	8.000994225	7.30968498333333	9.14377724166667	8.71940105833333	8.35277756666667	8.94668689166667	8.79812115	9.38453646666667	9.43278838333333	9.88515190833333	7.95134493333333	8.71562445833333	8.32648980833333	8.76851021666667	9.89855505	9.04644768333333	8.27228149166667	9.166351	9.04133171666667	10.1704848833333	9.326671075	8.41299383333333	9.80706423333333

HDGFL3	10.2058208458333	8.98816755	9.3169059625	9.0694932	9.27011640833333	9.48539137083333	9.36916360833333	9.0154403625	9.17668754583333	9.26558000416667	9.34604625	10.355636475	9.68758744583333	9.24822246666667	9.057547175	9.94731827916667	9.14899073333333	9.00576620416667	8.82803487083333	9.544678625	9.45734620833333	9.13846391666667	9.037530925	9.78547730833333	9.9019981875	10.0970717166667	9.60896382083333	9.78673265	9.32258433333333	9.24649527916667	9.60650317083333	9.4962317	9.55132753333333	9.56286851666667	10.218895	9.93613205	9.40535700416667	9.70819485	9.76576160833333	9.749741425	8.9596737	9.28478388333333	9.4458208875	9.17210327916667	9.544678625	9.26014341666667	9.19266140416667	8.969130525	9.40619117916667	9.12484684166667	9.71678342916667	9.23816898333333	9.4827693375	9.43161368333333	9.3120189375	9.389631825	9.4957917375	9.22317833333333	9.53669334166667	9.4723312125	9.60255508333333	9.03886794583333	8.71581307916667	8.521353825	8.8673426	8.6641945625	9.17626466666667	9.16851644166667	8.9500697125	10.1999531833333	8.84266431666667	8.7979	8.73715942083333	9.62947370416667	9.51570927916667	9.97974595833333	9.45575422083333	9.62603649166667	9.12017812916667	9.65504875833333	9.5220062	9.9203137	8.73377340416667	9.111049575	10.114173475	9.28763400416667	9.41785857916667	10.3985874083333	9.41552684166667	10.1293571166667	9.78355947916667	9.0699706625	9.37055386666667	9.2262906875	9.51199739583333	9.15811152916667	9.369638775	9.264191275	9.12440923333333	9.15530682083333	9.09856255833333	8.81355715833333	9.84308857083333	9.0756037875	8.41031485833333	9.54902774583333	9.5536005125	8.48032032916667	9.4039648875	9.54334447083333	9.32963227916667	8.42755200833333	8.94122305	8.9634582	10.0686200208333	9.29725092083333	9.04058751666667	8.90170160416667	8.6102258875	10.1499202

TM6SF1	10.2058208458333	8.98816755	9.3169059625	9.0694932	9.27011640833333	9.48539137083333	9.36916360833333	9.0154403625	9.17668754583333	9.26558000416667	9.34604625	10.355636475	9.68758744583333	9.24822246666667	9.057547175	9.94731827916667	9.14899073333333	9.00576620416667	8.82803487083333	9.544678625	9.45734620833333	9.13846391666667	9.037530925	9.78547730833333	9.9019981875	10.0970717166667	9.60896382083333	9.78673265	9.32258433333333	9.24649527916667	9.60650317083333	9.4962317	9.55132753333333	9.56286851666667	10.218895	9.93613205	9.40535700416667	9.70819485	9.76576160833333	9.749741425	8.9596737	9.28478388333333	9.4458208875	9.17210327916667	9.544678625	9.26014341666667	9.19266140416667	8.969130525	9.40619117916667	9.12484684166667	9.71678342916667	9.23816898333333	9.4827693375	9.43161368333333	9.3120189375	9.389631825	9.4957917375	9.22317833333333	9.53669334166667	9.4723312125	9.60255508333333	9.03886794583333	8.71581307916667	8.521353825	8.8673426	8.6641945625	9.17626466666667	9.16851644166667	8.9500697125	10.1999531833333	8.84266431666667	8.7979	8.73715942083333	9.62947370416667	9.51570927916667	9.97974595833333	9.45575422083333	9.62603649166667	9.12017812916667	9.65504875833333	9.5220062	9.9203137	8.73377340416667	9.111049575	10.114173475	9.28763400416667	9.41785857916667	10.3985874083333	9.41552684166667	10.1293571166667	9.78355947916667	9.0699706625	9.37055386666667	9.2262906875	9.51199739583333	9.15811152916667	9.369638775	9.264191275	9.12440923333333	9.15530682083333	9.09856255833333	8.81355715833333	9.84308857083333	9.0756037875	8.41031485833333	9.54902774583333	9.5536005125	8.48032032916667	9.4039648875	9.54334447083333	9.32963227916667	8.42755200833333	8.94122305	8.9634582	10.0686200208333	9.29725092083333	9.04058751666667	8.90170160416667	8.6102258875	10.1499202

CRISP2	4.705709175	9.59949791666667	7.17093558333333	10.0445903666667	6.59815521666667	8.535777725	7.64678326666667	6.45401484166667	7.27114775833333	6.33491514166667	7.598264375	6.61154091666667	7.31803023333333	8.40234485833333	8.48515009166667	6.97462729166667	4.74448875	5.35794613333333	9.21787520833333	8.32062069166667	6.28034841666667	8.665331325	6.346676525	9.36848635	9.4337598	4.61625569166667	8.94804158333333	9.47208941666667	8.75003116666667	7.5512356	6.31021560833333	8.86062028333333	8.26456484166667	4.1882044	4.322875175	4.47089620833333	10.7778847083333	9.32806993333333	6.0167621	7.01426715833333	6.759774675	8.59201043333333	7.84796001666667	6.02729868333333	7.348327125	6.33187936666667	7.37928605	8.013232325	6.99727938333333	8.74130064166667	7.65326568333333	7.21861555833333	6.728950375	7.75332493333333	8.04450845	7.05900955833333	5.40518485833333	6.633116075	8.9962514	8.76273385833333	3.93897055	5.85413349166667	4.359146625	5.7672538	4.26652515833333	4.77601478333333	3.905753025	5.577321975	3.95680781666667	4.90586546666667	5.18885664166667	4.18714809166667	6.64487866666667	5.61840579166667	4.37807450833333	3.74274349166667	4.645027875	3.810060775	5.948036925	3.63279243333333	5.363009525	10.363007875	4.31908713333333	4.07245948333333	4.06900396666667	4.59369346666667	4.06900396666667	4.78900190833333	3.65945140833333	3.95130670833333	4.00999268333333	4.31908713333333	4.46100053333333	4.18603395833333	3.92135188333333	3.9614432	4.221324425	3.936908825	4.21592141666667	5.221531825	4.34607883333333	4.289092625	4.852466025	5.05900706666667	4.42570850833333	4.55671806666667	4.50751908333333	6.84260019166667	4.35819395	6.996742575	5.29779213333333	5.76836148333333	4.40687995833333	4.90147810833333	4.13005875833333	4.3987315	4.86699436666667	4.57444614166667	4.68477714166667	4.92659856666667

RBBP4	10.2420912583333	9.34604625	9.32963227916667	9.04620451666667	9.09482996666667	10.4688930208333	9.2451576	9.54424637916667	9.27483949583333	9.26465415	9.439839425	10.1663620833333	9.42051215416667	9.44984600416667	9.67293809583333	9.56338085833333	9.84477553333333	9.014030625	9.67211590416667	9.48320322083333	9.32022560416667	9.30796544166667	9.7286166625	9.63964930416667	9.40051870416667	10.3347427041667	9.512890975	9.50015585833333	9.30933558333333	9.60210426666667	9.75428324583333	9.7955383625	9.56102568333333	9.76533135833333	9.92812106666667	10.4282659291667	9.39913687083333	9.66850222083333	9.32595024166667	9.41272695416667	9.65419311666667	9.31835271666667	9.33905939166667	9.904987025	9.4649962625	9.68237718333333	9.35474980833333	9.64630857083333	9.544678625	9.65243045416667	9.22182530833333	9.50111204166667	9.62603649166667	9.30477144583333	9.78587935416667	9.46011392083333	9.4489161375	9.61792207083333	9.38161835416667	9.4899161625	9.1160913375	9.27104185	9.2527877375	9.08845935416667	10.0349391541667	9.50203754583333	9.77372232083333	9.7090900375	9.54707108333333	9.263749275	9.0939493875	9.08420446666667	10.6598185416667	9.58107370416667	9.4849232375	9.44128464583333	9.0136041125	9.403125825	9.7492812875	10.1015242166667	10.3410778458333	9.24002475416667	9.8119573125	9.71165420416667	9.62343045	11.2184992	10.5071483583333	9.09663849166667	8.68399509166667	9.9203137	9.95341705416667	9.98574733333333	10.6174893625	9.41050504166667	9.0638436875	9.973195625	9.4306749125	9.64503569583333	9.61345531666667	9.89615510416667	9.32641363333333	8.99228364583333	9.97772005833333	9.8245061125	10.206709925	9.27104185	9.86082417083333	9.63041037916667	9.63878470833333	9.62005562916667	9.66445450833333	10.2381619875	9.2262906875	9.38252353333333	10.0240638125	9.1779438625	10.9176779916667	9.98531337083333	9.63964930416667	9.75016254583333

SYNC	10.2420912583333	9.34604625	9.32963227916667	9.04620451666667	9.09482996666667	10.4688930208333	9.2451576	9.54424637916667	9.27483949583333	9.26465415	9.439839425	10.1663620833333	9.42051215416667	9.44984600416667	9.67293809583333	9.56338085833333	9.84477553333333	9.014030625	9.67211590416667	9.48320322083333	9.32022560416667	9.30796544166667	9.7286166625	9.63964930416667	9.40051870416667	10.3347427041667	9.512890975	9.50015585833333	9.30933558333333	9.60210426666667	9.75428324583333	9.7955383625	9.56102568333333	9.76533135833333	9.92812106666667	10.4282659291667	9.39913687083333	9.66850222083333	9.32595024166667	9.41272695416667	9.65419311666667	9.31835271666667	9.33905939166667	9.904987025	9.4649962625	9.68237718333333	9.35474980833333	9.64630857083333	9.544678625	9.65243045416667	9.22182530833333	9.50111204166667	9.62603649166667	9.30477144583333	9.78587935416667	9.46011392083333	9.4489161375	9.61792207083333	9.38161835416667	9.4899161625	9.1160913375	9.27104185	9.2527877375	9.08845935416667	10.0349391541667	9.50203754583333	9.77372232083333	9.7090900375	9.54707108333333	9.263749275	9.0939493875	9.08420446666667	10.6598185416667	9.58107370416667	9.4849232375	9.44128464583333	9.0136041125	9.403125825	9.7492812875	10.1015242166667	10.3410778458333	9.24002475416667	9.8119573125	9.71165420416667	9.62343045	11.2184992	10.5071483583333	9.09663849166667	8.68399509166667	9.9203137	9.95341705416667	9.98574733333333	10.6174893625	9.41050504166667	9.0638436875	9.973195625	9.4306749125	9.64503569583333	9.61345531666667	9.89615510416667	9.32641363333333	8.99228364583333	9.97772005833333	9.8245061125	10.206709925	9.27104185	9.86082417083333	9.63041037916667	9.63878470833333	9.62005562916667	9.66445450833333	10.2381619875	9.2262906875	9.38252353333333	10.0240638125	9.1779438625	10.9176779916667	9.98531337083333	9.63964930416667	9.75016254583333

SPTSSB	6.97214965	7.76884748333333	7.5367911	8.00359983333333	8.010141425	7.50167894166667	7.09680668333333	7.05338414166667	7.482471025	7.55609866666667	7.79244525833333	7.1674037	6.77777710833333	7.33912221666667	8.15011035	7.16614005833333	8.87086751666667	7.45341820833333	7.8150656	7.35693584166667	7.02653026666667	7.2559939	7.83671455833333	7.21098661666667	7.37928605	6.39918578333333	7.72469713333333	8.023521375	7.8720714	7.77660473333333	7.97719954166667	7.07292095833333	6.9125939	8.04179383333333	7.03138465	6.84909505	8.167838375	7.68263314166667	7.23553730833333	7.83671455833333	7.95684823333333	8.07980334166667	7.31739324166667	7.79298445	7.48063178333333	7.72846560833333	7.644918425	7.88228429166667	7.20806453333333	7.596603625	7.99458134166667	7.68209800833333	7.8248949	8.03431426666667	8.186266475	8.16584395833333	7.91577950833333	8.04080661666667	8.00051745833333	7.227214725	6.59386139166667	6.90460845	9.508221125	7.37359066666667	10.9192867916667	7.502278225	6.79798854166667	7.70367073333333	7.86543926666667	8.59099210833333	7.47443041666667	7.52729815	6.41191374166667	6.46494565	6.64088086666667	5.70053848333333	8.34620800833333	6.3687361	6.02974674166667	7.91897891666667	7.51365925	7.435836975	6.71367926666667	6.27245344166667	7.75009851666667	7.641075725	7.39105859166667	7.16124751666667	6.905223325	6.39152516666667	7.04821105833333	6.87917671666667	7.433497275	7.79638631666667	7.265200275	7.21396461666667	7.81034196666667	7.2395484	6.82102155833333	7.89510465833333	6.79403766666667	7.36819641666667	6.955027575	8.013764525	7.15514039166667	6.69566758333333	6.82644443333333	7.58238585833333	6.74821494166667	7.24330025	8.15758866666667	8.61092796666667	7.10822425	9.1773608	7.21281770833333	7.32668075833333	7.98516725833333	7.3019376	7.53912439166667	7.85962578333333

PLEKHG4	10.29495555	10.1748193166667	10.4608159916667	10.6052722041667	11.1243846208333	10.9194758625	11.3453903416667	10.6137708125	11.1149160833333	10.5000138	10.98074875	11.2522869833333	10.856178425	10.0967084458333	10.0543247708333	9.80987653333333	10.1663620833333	10.9742253458333	9.91014482083333	9.9910593125	9.7715505875	10.0101710416667	9.58414644583333	9.96041484166667	10.4403846416667	9.95079996666667	10.187260375	10.2804629625	10.2607057041667	9.85093639166667	10.188516825	9.91265397916667	10.6417281083333	10.4808530666667	10.2836672416667	10.2276468583333	10.7814277541667	10.3516943458333	10.4374915708333	10.5148305	9.9431721875	9.8313258375	9.7273017375	9.60650317083333	9.46274657916667	9.4953248375	9.3724482125	9.2757434	9.76576160833333	10.056763975	10.1676970958333	10.1615384	9.95832646666667	9.67968799166667	9.4261721625	9.2619524125	10.0526814375	10.034531	9.82883624583333	9.72265057083333	10.64594635	10.5966848708333	10.252506	10.6264955333333	10.3846800458333	10.8632986125	10.374792175	11.0547262916667	10.8141356291667	10.8761777916667	11.0998077208333	10.8253053375	10.8682881791667	10.3083605625	10.5164336	9.96209228333333	10.7423444166667	10.6627959291667	9.952591425	10.4310234958333	9.90667721666667	10.2078788125	9.9177968375	9.49930952916667	10.8426238583333	10.6319745	10.6999204208333	10.7922036625	10.6295882833333	9.68883345	10.5390652458333	10.0663523541667	10.5011917708333	10.284029425	10.9826337791667	10.6705388	10.9738844791667	10.6631591666667	10.8309733083333	10.4820993875	9.96171140416667	10.4358699916667	10.0101710416667	9.6503044125	9.6445595	9.80864851666667	9.63087765833333	9.46367207083333	9.9910593125	9.75598520416667	10.0080682583333	10.1868527375	10.0817346958333	9.42665710416667	9.7201137	9.46274657916667	10.0105747791667	10.0371652041667	10.0702336791667	10.3966467583333

KCTD19	10.29495555	10.1748193166667	10.4608159916667	10.6052722041667	11.1243846208333	10.9194758625	11.3453903416667	10.6137708125	11.1149160833333	10.5000138	10.98074875	11.2522869833333	10.856178425	10.0967084458333	10.0543247708333	9.80987653333333	10.1663620833333	10.9742253458333	9.91014482083333	9.9910593125	9.7715505875	10.0101710416667	9.58414644583333	9.96041484166667	10.4403846416667	9.95079996666667	10.187260375	10.2804629625	10.2607057041667	9.85093639166667	10.188516825	9.91265397916667	10.6417281083333	10.4808530666667	10.2836672416667	10.2276468583333	10.7814277541667	10.3516943458333	10.4374915708333	10.5148305	9.9431721875	9.8313258375	9.7273017375	9.60650317083333	9.46274657916667	9.4953248375	9.3724482125	9.2757434	9.76576160833333	10.056763975	10.1676970958333	10.1615384	9.95832646666667	9.67968799166667	9.4261721625	9.2619524125	10.0526814375	10.034531	9.82883624583333	9.72265057083333	10.64594635	10.5966848708333	10.252506	10.6264955333333	10.3846800458333	10.8632986125	10.374792175	11.0547262916667	10.8141356291667	10.8761777916667	11.0998077208333	10.8253053375	10.8682881791667	10.3083605625	10.5164336	9.96209228333333	10.7423444166667	10.6627959291667	9.952591425	10.4310234958333	9.90667721666667	10.2078788125	9.9177968375	9.49930952916667	10.8426238583333	10.6319745	10.6999204208333	10.7922036625	10.6295882833333	9.68883345	10.5390652458333	10.0663523541667	10.5011917708333	10.284029425	10.9826337791667	10.6705388	10.9738844791667	10.6631591666667	10.8309733083333	10.4820993875	9.96171140416667	10.4358699916667	10.0101710416667	9.6503044125	9.6445595	9.80864851666667	9.63087765833333	9.46367207083333	9.9910593125	9.75598520416667	10.0080682583333	10.1868527375	10.0817346958333	9.42665710416667	9.7201137	9.46274657916667	10.0105747791667	10.0371652041667	10.0702336791667	10.3966467583333

GHRL	6.06833519166667	5.10174316666667	5.77198655	6.70327563333333	4.957419525	6.503440675	5.97682351666667	5.04024386666667	4.76967493333333	5.11328418333333	5.79838183333333	4.75255506666667	4.19132811666667	4.84615233333333	5.96160064166667	5.79649798333333	9.824729	5.229288475	6.38393616666667	5.95410824166667	5.33648908333333	6.32381556666667	6.44737514166667	6.18094188333333	6.60603940833333	5.90321861666667	6.421178475	7.12659995	6.65424311666667	5.95923255	6.37568504166667	6.810578	5.65521334166667	6.179361325	6.150357875	5.87003104166667	6.91828460833333	6.09024784166667	6.27802068333333	5.577321975	6.17154675833333	6.3922908	6.23709786666667	6.55447500833333	5.72076731666667	6.083461775	6.141642375	5.78626178333333	6.93352621666667	6.37349159166667	6.06914035833333	6.39918578333333	6.126012425	6.84200789166667	6.05307568333333	6.3687361	5.924123625	6.56431505	6.946211725	6.51830799166667	7.28198013333333	5.01427080833333	5.90154725	5.76546864166667	5.3702599	5.90588820833333	7.19065496666667	4.729262375	5.85413349166667	5.934751075	4.84820759166667	5.44429769166667	4.03339724166667	4.35998614166667	5.073382675	4.72222786666667	6.28660823333333	6.30111015	6.16197145833333	5.70748306666667	4.4718244	5.49334536666667	6.01590590833333	5.57926250833333	5.20354711666667	4.91442044166667	5.628745675	5.67425256666667	6.160301025	5.03726580833333	5.49035004166667	6.09355109166667	6.04613815	5.51803240833333	5.54392786666667	5.527437325	6.47646620833333	6.02292943333333	4.692141475	5.81652036666667	5.97436386666667	4.54133179166667	6.739509575	5.86347250833333	5.549659825	5.40518485833333	6.19209213333333	5.32557380833333	6.60400856666667	6.155322525	5.76923416666667	6.56365850833333	6.72953939166667	6.36616499166667	5.71123295833333	5.47794636666667	5.983976325	6.8687758	6.78642360833333	4.9415684

NLRC3	8.88450143333333	8.30178113333333	8.27606465	8.46552205	7.40193138333333	9.0115544	7.35693584166667	8.80499509166667	7.48649204166667	7.948855	7.6157713	7.70256570833333	6.528434875	8.79090379166667	7.48300389166667	10.1745820166667	8.63497865	7.678740925	7.85258474166667	9.22559156666667	9.06600838333333	8.59099210833333	9.56407003333333	9.11174605833333	9.01566145833333	8.97815918333333	9.15833918333333	8.4076656	7.93490190833333	8.36515990833333	8.58301454166667	9.92792138333333	9.02175539166667	10.4736621916667	8.86062028333333	9.01197323333333	8.327465575	9.09600253333333	8.78220615833333	8.5216034	9.0791656	8.74224036666667	9.23462919166667	9.57093504166667	9.5013607	9.93343423333333	9.01986039166667	8.5232739	10.308194675	9.26899234166667	8.85215985	8.28000224166667	8.59595736666667	8.30911568333333	8.83921701666667	9.028831875	8.168322275	9.16735169166667	8.84055035	9.94748681666667	7.911899075	8.1777524	9.24172186666667	7.26626465	8.291997825	7.12586193333333	8.001514325	8.800966875	7.948855	9.59648643333333	7.51310406666667	6.99919410833333	6.263501475	9.05561776666667	7.42326895833333	8.16684950833333	8.4484208	10.2119906333333	8.1525602	9.02175539166667	8.10843584166667	8.234157525	8.651213975	7.51472684166667	8.07357910833333	8.52826969166667	8.47579976666667	9.25672935833333	8.512878125	8.64839240833333	8.97126208333333	8.927052675	8.18781211666667	8.46044338333333	7.57513376666667	8.90005820833333	8.37303865833333	8.72311269166667	7.47204458333333	8.35736911666667	9.05312258333333	8.54023758333333	10.0493818	8.800966875	8.43662665833333	9.13780860833333	7.59020169166667	7.67926596666667	8.584858775	8.80300369166667	8.49975086666667	9.146936475	8.925575825	7.78166071666667	9.645654625	9.838575025	7.47140944166667	9.06973089166667	8.91938296666667	10.5416886583333

HAS1	9.295254925	3.9614432	6.78782379166667	4.08322809166667	3.98946053333333	6.493249575	4.23196318333333	7.24887754166667	4.17039641666667	7.96133965	4.035447825	7.0176409	8.3023979	3.84944544166667	4.12106518333333	6.64016859166667	6.05307568333333	6.47456359166667	5.59947975	3.97815159166667	6.02896681666667	4.02247413333333	4.873988275	8.53187930833333	6.16362138333333	9.68261813333333	4.36294506666667	6.8478471	4.515720925	4.35397924166667	8.04804935	4.43091141666667	3.95350946666667	4.61830805833333	11.113611625	5.528242825	4.46985861666667	6.8190902	7.07466381666667	6.81118743333333	4.93116945833333	4.58445505833333	5.17483658333333	5.76923416666667	5.743144625	4.56909456666667	4.748342275	7.64275718333333	5.37676219166667	4.44681015	9.24799294166667	5.57451170833333	4.24013239166667	6.075953425	4.21300761666667	4.36796431666667	6.22446936666667	5.20550439166667	4.055304025	3.67977121666667	5.59572529166667	5.415860975	5.88673599166667	5.079599575	6.73065755833333	6.8732984	8.85824333333333	5.4147768	5.71218308333333	4.44161573333333	4.30437938333333	4.52155396666667	7.41763228333333	9.10099604166667	4.19044525833333	4.75806056666667	4.21395365833333	5.43935509166667	8.96850814166667	4.546642225	4.24013239166667	4.57718236666667	5.51386359166667	8.401731375	6.864696	5.07107728333333	5.34622214166667	4.74219220833333	5.01530855833333	6.92852376666667	5.94358583333333	6.10873680833333	5.9191077	5.94625251666667	5.50523408333333	5.54392786666667	6.01590590833333	5.162070825	7.08354248333333	5.96001181666667	7.71034295833333	5.000034475	4.67471653333333	7.50573916666667	6.00629560833333	3.810060775	6.55061969166667	8.31252919166667	4.65070125	7.12725256666667	7.05838413333333	5.33329914166667	5.81652036666667	7.84899410833333	6.38084359166667	4.33043379166667	8.60998564166667	5.90154725	4.203896025	7.44984575833333

ATM	12.4037688166667	10.6880668	10.9761602583333	10.5743323083333	10.5493449166667	11.3470919	10.661840225	10.7565121333333	10.79830065	11.0387117083333	10.6384159666667	12.1380810166667	11.3683256166667	10.960420475	10.76764485	12.0411497	11.1542967583333	10.7793443666667	10.632187725	11.322793725	11.0556311333333	11.2811678666667	11.1749885333333	11.1771749333333	11.3560369416667	11.6252518666667	11.1689128333333	10.7463771	11.1779127416667	10.86697045	10.93645885	11.3125544916667	11.1840687083333	11.6473588416667	11.562248175	11.9032491333333	10.9230393083333	11.005303025	10.9215878333333	11.586583875	10.7635056583333	10.8684541666667	11.5323406833333	11.0953873333333	11.9832392666667	11.5606001833333	11.30753185	10.8996373166667	11.43798885	11.4052792333333	10.8480286083333	10.3566062583333	11.1196222666667	11.2467824083333	11.1346623583333	11.5570626583333	11.1858855333333	10.8996373166667	11.14521145	11.7853110416667	10.6773409416667	10.6691322083333	10.81908485	10.5100494833333	11.24709275	11.2121826083333	11.1923605916667	11.03980565	11.3915472416667	11.5630353166667	10.8105355416667	11.08338295	10.9757948333333	11.4581724666667	10.7597691583333	10.7565121333333	11.3827745833333	11.9844494166667	11.2517728166667	11.2568448166667	11.5887304416667	10.878488075	10.30932905	10.878870175	11.30185685	11.07367765	11.1670443166667	11.383891075	11.6386741333333	11.5567047416667	10.91052195	10.4590182666667	11.2132455833333	10.8389999833333	11.1990339333333	10.8239850833333	11.5761677833333	10.872956825	10.253110825	10.7276175666667	10.8356737416667	10.6542751166667	11.327151075	11.181831125	11.10073245	11.5128736833333	13.0488654	10.8807458916667	11.1674196	11.4195840166667	10.96963075	10.4138270833333	10.5739390666667	12.1389321	11.5404299	10.96150755	11.08918345	10.6664473083333	11.17279555	11.0448237416667

NAP1L1	4.62889925	4.161871975	4.11281023333333	4.15220664166667	3.971604825	5.29238863333333	3.4801699	3.86389350833333	3.64669851666667	3.83041005	3.60605176666667	3.58562625	3.73894771666667	4.061836425	3.93478860833333	3.95130670833333	4.802114875	6.38165589166667	4.822492325	4.31618201666667	3.7815889	4.34074395833333	4.22036293333333	4.57535033333333	4.77601478333333	4.245391125	4.48925233333333	4.71899701666667	4.75698904166667	4.322875175	4.602780325	4.59576983333333	4.07015480833333	4.06699531666667	5.01216298333333	4.57240950833333	4.62990135	4.51953901666667	4.62790769166667	4.58982808333333	4.77601478333333	4.41782250833333	4.67877638333333	5.4600655	3.9601097	4.729262375	4.89827923333333	4.88920265833333	4.61625569166667	5.18112505	4.62057545833333	4.45115921666667	3.764611825	5.27315905833333	4.29817321666667	4.82796093333333	3.81491895	4.61830805833333	4.24130721666667	4.48829116666667	4.54233060833333	3.57639359166667	4.64311556666667	4.04952899166667	3.71770076666667	6.63942924166667	4.22678013333333	3.8136599	5.19421045833333	4.21701204166667	4.00244103333333	4.33133893333333	3.95680781666667	4.1683668	4.08433028333333	4.23291245833333	5.9323169	6.25755120833333	4.546642225	4.17039641666667	3.80554890833333	5.171525	4.39557236666667	4.16407313333333	3.997050325	4.18081978333333	5.21412320833333	4.62990135	4.57137348333333	4.33043379166667	3.78952711666667	6.46705624166667	4.92770649166667	4.048345425	4.81840091666667	4.361998275	5.55170386666667	5.40201090833333	4.480628975	5.14931930833333	4.36096451666667	3.700710475	4.78700018333333	5.10930444166667	4.14053975833333	4.02040568333333	4.53925114166667	6.31323984166667	5.06570035833333	6.46133099166667	5.04667276666667	5.53695673333333	4.70371486666667	4.968508325	4.1106042	4.337410475	4.79999713333333	5.02158241666667	4.47770515	3.56307476666667

FEV	9.929600025	9.30028739166667	9.09642976666667	10.5659717333333	11.024361475	8.98796298333333	11.84387985	10.0180243333333	9.91374403333333	10.5053205416667	10.476356775	11.6025118833333	11.626670975	9.9441902	9.61984825833333	10.0450906333333	9.01566145833333	11.0203771416667	8.552420375	8.72403179166667	10.6131721666667	9.91289015	9.21543308333333	8.51387624166667	8.36034350833333	10.0815238083333	8.46350844166667	9.01101026666667	8.91158374166667	9.897727225	8.95943646666667	8.11406380833333	9.448687325	8.93073908333333	8.94623185833333	9.37777211666667	9.13078204166667	8.66828283333333	8.849091575	8.17505753333333	8.37604669166667	9.26488315	7.96361378333333	7.90991645	9.59369666666667	7.95359058333333	7.71862859166667	8.93568545833333	8.75371965	8.78691484166667	8.66625304166667	8.22641101666667	8.85728129166667	8.26555303333333	7.99891026666667	7.48063178333333	9.04179071666667	8.0964417	8.4304275	7.992867725	9.37588805	11.0574717833333	8.81599143333333	11.270439275	10.2321384166667	11.0072108	10.18000125	11.5843413583333	11.0072108	10.8774686583333	11.1241958916667	11.0520365916667	12.0628110916667	10.6683478	10.8828386666667	10.2206735166667	11.6408490416667	9.91801125833333	9.106662475	10.474825675	9.94127946666667	10.5276582916667	9.96782080833333	8.87238755	10.47977665	11.4492670916667	10.8150497416667	10.4372971833333	11.3328774166667	8.71039916666667	10.6733783	8.73448420833333	9.68558669166667	8.932734675	11.069747825	10.0480865166667	9.50264109166667	9.54225544166667	11.0811600833333	8.57400345	9.34750165	11.5664864	9.09007516666667	8.37767194166667	9.67192385833333	9.21732311666667	8.817877875	9.15886451666667	9.33456535	8.65271366666667	8.69459336666667	9.18261260833333	8.52009253333333	8.35277756666667	8.39063175833333	8.48569109166667	8.97354856666667	8.63031905833333	8.31466204166667	9.22606134166667

RFX1	10.0736918083333	10.7371678583333	10.7975980416667	10.4720948333333	10.4008623166667	10.1126129583333	9.722436925	10.8984556583333	10.1274668666667	10.3674828833333	10.3754461833333	9.76310825	9.817741375	10.315132175	10.32163045	10.2874114666667	10.1593240833333	10.29705995	11.0394398083333	10.4240891833333	10.7261291583333	10.6659778416667	10.40167215	10.4942942166667	10.7936080833333	9.86989941666667	10.0696848333333	10.6465456666667	10.528057425	10.4495527	11.1689128333333	10.303115425	10.2132508333333	10.5392644333333	10.217869775	10.0489380083333	10.3798706916667	10.7782435583333	10.6733783	10.4441620166667	10.7119916833333	10.807474625	10.88109835	10.8537358333333	10.4356784583333	10.7254659583333	10.6293746333333	10.7565121333333	10.85221535	10.7593753416667	10.683111325	10.5056824166667	10.6461664	11.09094715	10.4744353333333	10.9757948333333	10.9436906083333	10.81908485	10.5601410916667	10.46260805	9.700667025	9.7997523	10.0965198666667	10.2826800583333	10.3682517916667	10.469517225	10.028074675	10.1469040583333	10.1529084083333	10.257101525	9.9399775	10.3396540083333	10.1497399666667	10.3432001083333	10.08403975	10.4089152416667	9.78567400833333	10.0031143833333	10.4725076	10.2609607666667	10.58612335	9.98111111666667	10.0728614166667	10.5519705833333	10.6527698	10.2670402	10.49265135	10.130420225	10.3455009333333	10.6211581166667	10.2145140083333	10.2826800583333	10.5322469083333	10.523220425	10.0744319916667	10.1936096583333	10.018462175	10.257101525	10.2720090666667	10.2955209666667	10.363007875	10.18324735	10.5569098916667	10.863090975	10.8585888666667	10.4161036833333	10.9061592666667	10.703101075	10.4344952333333	10.431190025	10.7944294666667	10.6733783	10.5956461416667	10.8581963333333	10.6918746916667	10.85523755	10.5711304666667	10.2844102625	10.7796932083333	10.1962000083333

RRP12	6.66175125	5.7955502	6.15447426666667	5.72573654166667	6.02645373333333	6.58835050833333	5.77633328333333	7.43742328333333	6.48151946666667	5.968635375	6.51600675833333	6.011301125	5.845937625	5.19421045833333	7.0114101	5.91038576666667	5.3960318	6.95288179166667	6.92433585	6.42033848333333	5.86456365833333	6.35274666666667	5.9191077	6.896657025	7.67425858333333	7.35862363333333	6.87851200833333	6.45767033333333	6.724451125	7.09804688333333	6.76517564166667	6.78916241666667	6.82889531666667	5.83776461666667	7.46194565833333	7.92497183333333	7.22835589166667	7.55987390833333	7.49549410833333	6.603384975	7.11340090833333	7.17507495	7.21451231666667	7.05278270833333	6.50501645833333	6.2073137	7.16541175833333	6.36029920833333	6.85878966666667	7.120419025	7.67474618333333	7.53018445	7.025978175	7.40375466666667	7.15765674166667	7.77376521666667	7.21504029166667	7.43473675	7.44491683333333	7.28678616666667	6.72183800833333	6.15598731666667	6.41577015833333	5.58896354166667	5.80356945	7.31196651666667	6.28898518333333	7.33857021666667	6.66118995	5.58795861666667	6.6659887	6.00629560833333	6.80056770833333	6.159395425	5.91334729166667	6.51525555	6.37857751666667	6.96590744166667	5.40518485833333	6.1777684	5.73491465833333	5.792834125	5.775482875	6.52527314166667	6.68595645833333	7.50966981666667	7.121598725	5.14499941666667	6.50929938333333	6.3922908	5.743144625	7.31552385	7.22176664166667	7.30864333333333	6.79403766666667	6.70856058333333	6.49896905833333	7.20806453333333	6.65287900833333	6.246964375	6.19818808333333	6.25099584166667	6.98530065833333	6.31880616666667	6.57887239166667	5.6488719	6.53536243333333	6.28184195833333	6.45472701666667	6.64623430833333	6.92575055833333	7.04956788333333	7.29233335	7.10317193333333	6.48284681666667	7.34385484166667	6.71995881666667	6.25991309166667	7.91097105833333	6.48284681666667

FAM92A	11.8924112666667	9.81261425833333	9.83764681666667	9.445595775	10.3078222333333	10.5208646083333	10.276116525	10.0412389083333	10.1974084916667	10.5699475166667	10.9794539833333	11.5237451916667	11.0394398083333	9.78824573333333	9.51996269166667	10.1218627333333	10.34989835	9.876036325	9.78737088333333	10.0421767666667	10.3706198583333	9.61006324166667	10.5968900416667	9.906465775	10.2249992083333	11.88323765	10.10216065	9.825136275	9.94339973333333	9.97215941666667	9.85677660833333	9.6428903	9.99723533333333	10.3203234833333	11.5154734	11.9570955666667	10.2145140083333	10.0364758583333	10.0052447166667	9.81985159166667	9.78528060833333	9.49775068333333	9.71785554166667	9.93042398333333	10.6108306416667	10.0877690666667	10.112191475	10.095377	9.71356900833333	9.98800139166667	9.50996596666667	9.921332825	10.340914925	9.797655	9.903525425	9.80625096666667	10.01207205	9.73350879166667	9.95100449166667	10.130420225	11.9705949166667	11.5761677833333	11.0083196416667	11.7030888416667	11.74329675	11.237698425	11.9570955666667	12.0168871666667	11.1956589833333	11.42107965	11.3546484166667	11.8048205583333	11.025429175	11.7682562416667	11.4214230083333	11.2039860833333	11.7974234916667	10.6434054	10.0569437166667	10.6191395	11.7895299333333	12.1669452	11.1536039583333	12.5079805333333	10.8904318	11.372844325	12.1380810166667	12.2574255666667	11.7541385166667	11.8081782916667	10.7706214833333	11.6092244333333	11.43469695	10.9537804833333	11.8407167416667	11.1169690833333	11.1649238666667	12.382866125	11.5041708333333	12.3489029833333	11.497488975	11.6425911333333	11.341686575	11.0898799333333	11.428103825	11.6705910083333	11.2395267333333	11.7190625916667	10.5334113083333	11.0775947083333	10.874072375	10.723966225	11.2733084583333	12.1084032166667	10.9153273416667	11.2756978833333	11.2911160333333	12.050259975	11.2664173916667	10.9564158416667

GTPBP6	10.3313573416667	10.17413685	10.1054993333333	10.0924722666667	10.0807009083333	10.4986956916667	9.59023981666667	10.4288423083333	10.0095607333333	10.2314081833333	9.92703645833333	9.39253865	9.65973478333333	10.0855958166667	10.3143367833333	9.89553576666667	11.0094185166667	10.3733561083333	10.31123355	10.2955209666667	10.3459231416667	10.5493449166667	10.4480363583333	10.8416766333333	10.3396540083333	10.4123976	10.34241075	10.5344987916667	10.8684541666667	10.5416886583333	10.128737475	10.0331009416667	9.72111820833333	10.3240922583333	10.4072694666667	10.5344987916667	10.82998625	9.93875319166667	10.1042493666667	9.76819061666667	10.1903114083333	10.3171296666667	10.0163833	10.002252325	10.0997208916667	10.3901677	10.1755237	10.324926675	10.2092790583333	10.15852225	9.96954501666667	10.2213761083333	10.3427675333333	10.6449643666667	10.6926124666667	10.286132425	10.06195985	10.31950435	10.16655665	9.94086763333333	10.1205788333333	10.0819455833333	11.4413379833333	9.38182149166667	10.0774221416667	11.155024025	10.5444389083333	9.46751014166667	10.1919662416667	9.99767481666667	9.89982799166667	9.4999428	10.257489575	10.2842200333333	9.16442515	9.60540180833333	10.237546325	10.308194675	9.62318166666667	10.21946315	11.345193725	10.3546255166667	9.6176772	10.514613975	9.93515624166667	10.1682992333333	10.9054446583333	10.70539815	10.3483436583333	10.2554871833333	9.87207808333333	9.890973325	9.06167029166667	10.2442419583333	10.5240503083333	10.1541580083333	10.0557486666667	10.7038909916667	10.8900635166667	10.6256227583333	8.06551123333333	10.1883097083333	10.5825770333333	9.2639574	10.185845775	9.81176585	9.80056271666667	8.77815989166667	10.7808534	9.03503039166667	9.8942397	10.6076090916667	10.0889846333333	9.69491718333333	10.678502425	10.400070625	9.62496784166667	10.631761275	10.4664769916667	10.6387912166667

GOLGA7	12.634136475	12.4229923333333	12.644626475	12.6103198083333	11.946316525	12.105942725	12.4046195166667	12.2094988666667	12.3983699916667	11.8599637666667	12.247959925	11.86892365	11.9352971916667	12.2909999333333	11.99474245	12.2848587416667	12.503422725	11.941310725	12.301617475	12.075266	12.2085904583333	11.7482758916667	11.9994097833333	12.247084775	12.0917340666667	12.309318975	12.1325110916667	12.118621125	11.765185225	12.1604527333333	12.4292757333333	12.5381389666667	12.503844475	12.2185126	12.58926255	12.4087159666667	11.8403034416667	11.9534028083333	11.8981677666667	11.7861402083333	12.478144425	12.533964725	12.3802421833333	12.0905289333333	12.3763083916667	12.4840896416667	12.3688898083333	12.2826483583333	12.3240568416667	12.5029330166667	12.0604174833333	12.466874775	12.19191635	12.0020893083333	12.625004	12.2976992	12.02225065	12.2862620916667	12.1612618833333	12.0510805083333	12.1715835	12.5954075166667	12.3240568416667	12.1669452	13.4427852416667	12.19731135	12.1233577583333	12.5706160166667	12.2926172833333	12.8203151333333	12.6572156166667	13.193307375	12.05061705	12.3443503083333	12.7801271583333	12.1686155916667	13.2861830833333	11.5945056583333	11.9563023166667	12.2739391166667	13.1427455666667	12.4194028333333	12.6727763583333	12.0917340666667	12.4528342	11.88924055	12.8537857833333	12.4163683083333	12.0457744	12.273184075	12.3758585	12.0128290333333	11.4485644833333	12.716481775	12.4777234416667	12.6664956833333	12.7233335166667	12.40597585	12.8802020166667	11.9458994666667	12.7081294916667	12.4425276083333	12.4430322166667	12.9924504416667	12.5497662083333	12.21725785	12.2345932083333	12.5907074583333	12.2848587416667	12.08124855	13.6165726333333	12.1399871	11.659892375	12.9248123333333	12.1732634166667	12.6297371916667	13.08588685	12.1865914583333	12.2044709583333	11.8865138166667

DCST2	7.3477901	7.25799328333333	6.64415971666667	7.41942373333333	6.61415903333333	6.88791735	6.61019589166667	6.393024275	7.13230843333333	6.99601435833333	7.139086775	6.04613815	6.56292778333333	6.74677720833333	7.59420499166667	6.66054216666667	7.100006625	7.68263314166667	7.060215325	6.9953871	6.89068614166667	7.84376875833333	6.81709465833333	7.31739324166667	6.96183981666667	6.53137754166667	7.12528415833333	7.708204175	7.698105675	7.68098021666667	7.60297259166667	7.29365533333333	7.64430480833333	6.93803065	7.01211946666667	6.95924915	7.61520518333333	7.16358325	7.05225570833333	6.9953871	6.98158215833333	7.37642496666667	7.209690675	6.9475749	7.09738105833333	7.36048969166667	7.19065496666667	7.03751019166667	7.27891691666667	7.16072213333333	7.17267591666667	7.38522528333333	7.32789755	7.74951601666667	7.40602305833333	7.54510475	7.02536274166667	7.35003230833333	7.47788481666667	6.76049560833333	8.32853386666667	7.08354248333333	7.31552385	7.95134493333333	7.51365925	7.88403631666667	6.54299384166667	6.23159324166667	7.55873083333333	7.39869075	6.874007675	7.643842075	6.94312051666667	6.94376375833333	8.32853386666667	7.14519241666667	6.77041611666667	7.51995818333333	6.96533775	7.65663390833333	6.76905384166667	7.116803275	7.17735376666667	6.66175125	7.42103133333333	6.98280363333333	6.60676565	6.74346625833333	7.45504230833333	7.14519241666667	7.4250003	8.18475165	8.13617131666667	7.594902375	7.83125643333333	8.56421206666667	7.61337419166667	7.1809933	7.30254744166667	7.60297259166667	6.80714281666667	7.388741125	8.22690084166667	8.11098743333333	7.9484088	6.93418845	7.435271425	8.03538219166667	6.86012671666667	6.71442883333333	7.29106909166667	6.82889531666667	7.45341820833333	7.57732030833333	7.64327454166667	7.23609219166667	7.78110251666667	7.58187098333333	7.9238629	6.43490204166667

CYB5RL	7.1072867875	7.0111070875	6.67270679166667	6.49722507916667	6.3745326875	7.74119181666667	5.98262315	7.082647625	6.54474624166667	7.32822407083333	6.78747448333333	6.8174222375	5.32082965	7.46825643333333	6.61588425416667	7.5139330375	8.63056395833333	6.53501744166667	7.92196199583333	7.64355830833333	7.80591484583333	6.56842659166667	7.86308917083333	7.11759648333333	7.62386521666667	8.16458723333333	8.08931284166667	7.34005885416667	6.17512970833333	7.068611	7.21477630416667	6.99187215	6.61383874583333	7.2643163375	7.927862075	7.77238758333333	6.57055390416667	6.4245003625	5.483581375	5.9665777	7.5241245375	6.97812175	7.13130732916667	7.758499375	7.22445772083333	7.82874789166667	7.82038009166667	6.40837500416667	7.08771929583333	7.81920169166667	6.98942495	5.99713806666667	4.94726662083333	6.08389895833333	6.69956157916667	7.021852825	6.41388247916667	7.0084610875	6.93651231666667	7.8080542625	6.26701759583333	5.04284279583333	6.63543665	5.17750950416667	7.98692124166667	5.172091325	6.71957255	6.27522898333333	6.54964078333333	7.09771397083333	7.233411675	6.51643293333333	4.64898137083333	5.984382625	6.81285127083333	7.16263785833333	7.31054019166667	7.49017916666667	7.650449225	8.01726584583333	8.0410632875	7.05869684583333	5.93269269583333	6.8550883	6.04398887916667	7.44462641666667	7.8100294875	6.2255736375	5.54049679583333	6.63693797083333	6.44632249583333	6.674104225	7.12623094166667	6.7753216625	6.098003425	6.46457190416667	7.4202271875	6.95835837916667	5.43232302083333	7.63127870416667	6.84353104166667	5.59902848333333	7.34358252916667	7.88587072083333	7.56575369583333	6.52751807083333	6.24820711666667	6.85780014166667	6.21472249583333	6.96685253333333	7.09196993333333	7.22506628333333	5.78314279583333	7.39897763333333	6.07552475	6.75868566666667	8.81811914166667	7.21362325833333	7.652521625	5.7969617125

AL357673.1	7.1072867875	7.0111070875	6.67270679166667	6.49722507916667	6.3745326875	7.74119181666667	5.98262315	7.082647625	6.54474624166667	7.32822407083333	6.78747448333333	6.8174222375	5.32082965	7.46825643333333	6.61588425416667	7.5139330375	8.63056395833333	6.53501744166667	7.92196199583333	7.64355830833333	7.80591484583333	6.56842659166667	7.86308917083333	7.11759648333333	7.62386521666667	8.16458723333333	8.08931284166667	7.34005885416667	6.17512970833333	7.068611	7.21477630416667	6.99187215	6.61383874583333	7.2643163375	7.927862075	7.77238758333333	6.57055390416667	6.4245003625	5.483581375	5.9665777	7.5241245375	6.97812175	7.13130732916667	7.758499375	7.22445772083333	7.82874789166667	7.82038009166667	6.40837500416667	7.08771929583333	7.81920169166667	6.98942495	5.99713806666667	4.94726662083333	6.08389895833333	6.69956157916667	7.021852825	6.41388247916667	7.0084610875	6.93651231666667	7.8080542625	6.26701759583333	5.04284279583333	6.63543665	5.17750950416667	7.98692124166667	5.172091325	6.71957255	6.27522898333333	6.54964078333333	7.09771397083333	7.233411675	6.51643293333333	4.64898137083333	5.984382625	6.81285127083333	7.16263785833333	7.31054019166667	7.49017916666667	7.650449225	8.01726584583333	8.0410632875	7.05869684583333	5.93269269583333	6.8550883	6.04398887916667	7.44462641666667	7.8100294875	6.2255736375	5.54049679583333	6.63693797083333	6.44632249583333	6.674104225	7.12623094166667	6.7753216625	6.098003425	6.46457190416667	7.4202271875	6.95835837916667	5.43232302083333	7.63127870416667	6.84353104166667	5.59902848333333	7.34358252916667	7.88587072083333	7.56575369583333	6.52751807083333	6.24820711666667	6.85780014166667	6.21472249583333	6.96685253333333	7.09196993333333	7.22506628333333	5.78314279583333	7.39897763333333	6.07552475	6.75868566666667	8.81811914166667	7.21362325833333	7.652521625	5.7969617125

BCL2L13	12.0395951	11.8085467416667	11.9813054166667	11.7943808	11.7463222583333	11.3180417666667	11.179383775	12.0522158916667	11.628296575	11.5645444166667	11.6010090666667	11.593791575	11.63482985	11.7470404	12.16610385	11.721307075	11.5948447	11.7541385166667	11.8519854833333	11.9240142833333	12.07649975	12.4310799666667	11.8973789916667	11.8523757333333	12.0744704	12.2594871416667	12.0518595833333	11.844663475	12.1291180083333	11.8032520333333	11.9868001	12.2429038916667	11.6408490416667	11.4538064666667	11.9013541833333	12.2215063083333	12.0273705916667	11.868130425	11.632505775	12.10269775	11.9162394	11.9709528833333	12.3523338166667	12.1521118916667	12.0778101166667	12.1847949166667	12.4650650416667	12.18313895	11.7682562416667	11.8457882583333	11.868539825	11.8811110166667	12.4831684166667	12.496720025	12.4287875916667	12.59948	12.1815348916667	12.013260375	12.145884225	12.1612618833333	11.9678169416667	11.8154196416667	12.1287131333333	12.1170603	11.7895299333333	12.1943788416667	12.2650076	11.8390986666667	11.2499394833333	10.9854722416667	11.4045795333333	12.1087888833333	11.8116053416667	11.5994743583333	12.1612618833333	11.9028464416667	11.747396125	11.8516230166667	12.2488091166667	11.7440902416667	11.428103825	11.602097375	11.7861402083333	11.8662764666667	11.9135839833333	11.4214230083333	11.2474575666667	11.6473588416667	12.4430322166667	11.7369473333333	12.0525821583333	12.126629875	11.7085670166667	11.9236129	11.9078069166667	11.8154196416667	12.05687395	11.5219495083333	11.8166747583333	10.7816314666667	11.4737788083333	12.042069275	11.66250505	12.5293458166667	11.946316525	12.1620485833333	12.1100796583333	11.7624702333333	11.8104289083333	12.6956383666667	11.9348926	12.790749425	12.5173296333333	12.295556975	12.1283236333333	12.1541314	12.0593104333333	12.11543585	12.7531374166667	11.63518155

NLGN4Y	11.5912797083333	9.900175875	10.0844426833333	9.59824975833333	9.42687719166667	9.67813213333333	9.17414999166667	10.4004658833333	9.55566569166667	6.85156043333333	10.153311575	11.0733527583333	6.70257901666667	6.602072175	9.77049406666667	10.4040896666667	9.58344561666667	8.96094025833333	9.94794899166667	10.306601825	10.173327525	9.84994195833333	10.4394323833333	9.52996276666667	9.78692435833333	11.6401085333333	7.47951195833333	10.2621725583333	5.90065811666667	9.66558844166667	7.69103884166667	9.90775529166667	9.89982799166667	9.56961935	11.6536243916667	11.6675947833333	9.945938775	10.2051938916667	9.42734543333333	10.476761175	10.4348946583333	9.94551363333333	7.34497685	10.3887558333333	8.82572	10.87258615	8.04131995833333	7.30740175	10.090730825	10.2225656916667	8.10268665	9.55288966666667	10.0885610583333	10.0769619583333	10.2342241333333	10.5719557166667	10.199031925	10.46022315	10.5581448083333	7.91046618333333	10.662995525	9.05267498333333	10.2765561833333	8.775178575	11.2327259583333	8.52966328333333	9.99343915	10.0386893	9.64118481666667	7.05521155	10.2266691833333	7.93490190833333	7.87369893333333	9.10194070833333	9.496436825	9.777820325	10.647327775	9.10532413333333	9.813959625	9.12506650833333	12.1549544416667	11.7695141	8.998094275	10.5490285	10.13859765	10.0043835333333	4.480628975	9.73788834166667	10.76543495	10.883221475	8.04755969166667	10.0159752583333	8.76175106666667	10.750505225	8.35832260833333	9.50353634166667	9.05776881666667	8.52966328333333	9.09600253333333	11.6985875333333	9.80576564166667	9.911212725	8.53005785	8.73109978333333	5.96958254166667	9.40252054166667	9.06791326666667	6.68532921666667	11.005303025	10.5244636333333	8.9184083	10.7990034333333	9.2741013	9.62496784166667	10.3329358333333	10.5408711916667	10.8858467666667	10.0103759833333	10.2703263916667	7.54626845833333

AC132217.2	13.6297816083333	12.597195525	12.82707195	11.769722	11.254472975	12.4788895625	13.3969951	11.8617328041667	12.0814532666667	13.4371232625	12.0142512833333	14.1691034333333	13.3857100958333	12.5573847541667	12.0936128083333	12.6593439833333	12.5224211333333	11.9234231958333	12.6504125791667	12.788786025	13.0758809791667	10.3005390166667	12.4621215583333	13.4100577708333	12.8132014625	14.3952098791667	12.9868350916667	13.4508297333333	11.6681825333333	12.8065863416667	13.0880695375	12.1456993916667	13.2211922583333	12.6509388208333	14.4731117291667	14.1650167291667	11.9668296208333	13.2023884083333	12.6844486041667	13.0983049083333	12.4406170791667	12.5742723125	13.5793691416667	13.0832459041667	14.4117367625	12.5129160833333	12.8018111958333	12.2949476916667	12.6849372666667	12.7388022833333	13.15598605	13.0784066083333	13.2322771916667	12.1285183833333	12.4806303666667	13.9091580791667	12.9845106958333	12.4161375	13.4692580041667	13.905052325	13.5988344166667	12.5559623125	13.0485887291667	13.230294875	12.1008237291667	13.8872278125	14.058525425	14.2481360625	13.66374885	13.2528265041667	13.8122758125	13.4469847791667	13.689945375	14.5560234833333	13.3686023625	14.96078415	12.6569464791667	13.9756053833333	13.9594675833333	14.0689028125	13.6629357875	12.4327178625	13.36929045	13.6992020333333	14.6705841666667	13.505678625	11.7249235416667	13.399125575	12.0074727541667	13.9405817375	14.3868174541667	13.571708925	11.8409287541667	13.5383265833333	11.4592024458333	13.2908983791667	13.2839047333333	13.2282872208333	13.375928075	14.4487402125	12.7371634625	13.6474877916667	13.7239626375	12.8126665416667	13.1586208833333	14.2007913166667	15.3476632916667	12.6183459541667	14.2770857541667	13.4756481125	12.9561646125	12.9945264708333	13.3359694541667	14.3917525541667	14.6035021125	13.3939643625	14.1572373666667	13.8749104166667	13.1219985791667	12.5582889166667

IGF2	13.6297816083333	12.597195525	12.82707195	11.769722	11.254472975	12.4788895625	13.3969951	11.8617328041667	12.0814532666667	13.4371232625	12.0142512833333	14.1691034333333	13.3857100958333	12.5573847541667	12.0936128083333	12.6593439833333	12.5224211333333	11.9234231958333	12.6504125791667	12.788786025	13.0758809791667	10.3005390166667	12.4621215583333	13.4100577708333	12.8132014625	14.3952098791667	12.9868350916667	13.4508297333333	11.6681825333333	12.8065863416667	13.0880695375	12.1456993916667	13.2211922583333	12.6509388208333	14.4731117291667	14.1650167291667	11.9668296208333	13.2023884083333	12.6844486041667	13.0983049083333	12.4406170791667	12.5742723125	13.5793691416667	13.0832459041667	14.4117367625	12.5129160833333	12.8018111958333	12.2949476916667	12.6849372666667	12.7388022833333	13.15598605	13.0784066083333	13.2322771916667	12.1285183833333	12.4806303666667	13.9091580791667	12.9845106958333	12.4161375	13.4692580041667	13.905052325	13.5988344166667	12.5559623125	13.0485887291667	13.230294875	12.1008237291667	13.8872278125	14.058525425	14.2481360625	13.66374885	13.2528265041667	13.8122758125	13.4469847791667	13.689945375	14.5560234833333	13.3686023625	14.96078415	12.6569464791667	13.9756053833333	13.9594675833333	14.0689028125	13.6629357875	12.4327178625	13.36929045	13.6992020333333	14.6705841666667	13.505678625	11.7249235416667	13.399125575	12.0074727541667	13.9405817375	14.3868174541667	13.571708925	11.8409287541667	13.5383265833333	11.4592024458333	13.2908983791667	13.2839047333333	13.2282872208333	13.375928075	14.4487402125	12.7371634625	13.6474877916667	13.7239626375	12.8126665416667	13.1586208833333	14.2007913166667	15.3476632916667	12.6183459541667	14.2770857541667	13.4756481125	12.9561646125	12.9945264708333	13.3359694541667	14.3917525541667	14.6035021125	13.3939643625	14.1572373666667	13.8749104166667	13.1219985791667	12.5582889166667

PRR16	4.10495005	3.73640415	3.74515126666667	3.82681465	3.49384833333333	3.94247104166667	3.62640028333333	3.72041143333333	3.40733774166667	3.55885279166667	3.40381385	3.625169575	3.4228733	4.12106518333333	3.75838870833333	3.700710475	3.94247104166667	3.54887480833333	4.03445655833333	3.71420795	3.57891131666667	4.00999268333333	3.87937155833333	4.25488725	4.15309530833333	3.69811276666667	3.88526006666667	4.56806040833333	3.85292815833333	3.770641325	4.00999268333333	4.72615460833333	3.45816426666667	3.77178324166667	3.76596780833333	4.3549546	3.83041005	3.92371073333333	4.14488695	4.19720234166667	4.003549775	3.99817013333333	3.842520875	4.91325449166667	3.66417965833333	4.13109949166667	4.137419	4.2860754	3.86167775833333	4.17138054166667	3.997050325	4.04952899166667	3.477151725	4.30027178333333	3.78548855	4.06389798333333	3.82817183333333	4.035447825	3.84482813333333	4.15309530833333	4.546642225	3.76944190833333	4.12229528333333	3.54337740833333	3.59213996666667	5.17697859166667	3.6492844	3.41750548333333	3.80900449166667	3.74027611666667	3.45012391666667	3.553038975	3.534267775	4.003549775	3.50128275833333	3.656722575	3.62768649166667	3.77672200833333	3.87840355833333	3.9661014	3.62138225	3.86284045833333	4.1106042	4.12677783333333	3.57121334166667	3.78701804166667	3.57762789166667	4.03660776666667	3.50128275833333	3.68113930833333	3.612188525	4.57827949166667	3.64321401666667	3.64669851666667	4.07134488333333	3.65321091666667	4.37909631666667	3.79602544583333	3.49535419166667	4.35296875833333	3.78701804166667	3.465685875	3.99383243333333	4.33043379166667	3.58405050833333	3.88078246666667	3.93478860833333	4.60591755833333	3.85641256666667	4.6319392	3.99488165833333	4.57240950833333	3.98026660833333	4.17138054166667	3.695851625	3.92135188333333	3.97505105	4.52055483333333	3.82170636666667	3.42434035833333

PRMT2	13.19400885	12.8624048833333	12.9954911666667	13.20077725	12.8549658583333	12.4659800333333	12.6084193833333	12.606535325	12.77184425	12.6317327416667	12.672014175	12.7812528833333	12.8667102583333	12.852746025	12.7902282666667	12.7490093583333	12.686781525	12.7374360833333	12.964777525	13.002567275	12.9230312916667	12.6734606	12.57846135	12.6355786083333	12.91963445	13.0992273666667	13.0108192083333	12.923601775	13.0755644416667	13.0067659416667	12.8802020166667	13.1172651	12.6146877083333	12.004788175	13.0847965333333	13.3100924166667	12.7569178666667	12.5457898	12.4755627916667	12.83111185	12.8273007416667	12.8584129833333	12.812914675	12.682091125	12.9213977333333	12.91963445	12.9977920166667	12.958106125	12.6407582416667	12.6451232083333	12.6734606	12.731184375	12.724847575	12.9842314416667	13.0607838416667	13.0714709916667	12.632188	12.770725525	12.7655438666667	12.6506468416667	12.14630095	11.8364243666667	12.4767941833333	11.9055925	12.2888330833333	11.6910443916667	12.5111982416667	12.2336399666667	12.280521525	11.9494775416667	11.93371625	12.23879665	11.7153018166667	12.0055429333333	12.2326729333333	12.2628607833333	12.0965982583333	12.095758	12.307547675	12.0112152666667	12.0322027833333	12.35974915	13.2622394166667	12.4650650416667	12.0973620416667	12.0261845416667	11.8008623833333	12.49723105	12.6626629166667	12.2747673416667	11.9649178666667	12.3258832166667	11.9593365833333	12.3262957	12.265420025	12.06079575	11.8966407583333	11.5754446583333	11.8612030916667	11.155024025	12.1935330916667	11.7278187083333	11.9502134083333	12.7591596666667	11.9458994666667	12.1694652083333	12.7237683583333	12.025731925	12.6862224333333	12.1038489666667	11.8431168166667	12.1665042	12.3435261333333	12.4482599083333	12.3627391666667	12.57846135	11.763655725	11.9781875416667	12.1745545	11.7699299

CCDC32	14.89930145	14.004911475	13.91423525	14.2073641166667	13.3567888916667	14.0707149083333	13.7372005583333	13.9906723833333	13.6062775083333	14.259430575	13.627458175	14.9702238166667	13.9989817	13.7065253416667	13.40102065	14.4871709833333	15.4537382666667	13.5372319083333	14.27950095	14.00243415	14.3943592583333	14.0643996166667	14.4244522916667	14.0377493833333	14.190108625	14.5886594916667	14.085975275	13.99785205	13.2919593833333	13.61251425	14.0707149083333	14.13703675	14.30615445	15.1207533083333	15.156314825	14.4092298666667	13.5475412583333	13.8972210333333	14.3792394416667	13.8908948916667	14.36106535	13.7404495916667	14.1306178166667	14.1006630916667	14.89930145	14.4309788083333	14.4443539833333	13.7640679416667	14.512293975	14.5073052333333	13.5892432583333	14.1152258583333	14.4144157	14.0087806166667	14.0237926083333	14.312396275	14.0404397916667	14.2145397416667	14.3463851	14.4043399	13.9955171333333	14.2872888583333	14.5089658583333	14.0163605916667	14.3875977	13.9884216166667	14.3992922416667	14.4983330666667	13.6931782333333	14.0787258833333	14.168404425	13.7443409166667	14.34486635	14.68415095	13.3293632833333	14.539541	14.4346519833333	14.9045675833333	14.5717037666667	14.3186975416667	14.3221229083333	14.2969035	14.2460408916667	14.2474901166667	14.013877625	13.6858454833333	14.3943592583333	14.4891443666667	14.5550005583333	14.9174232833333	14.26427225	13.6675870333333	14.3350486166667	14.6202085916667	13.9075706166667	14.177662	13.61251425	14.4686760333333	13.8809609916667	14.8497796833333	14.18015695	14.400934925	14.7143451416667	14.5681681583333	13.9197186416667	14.5020438083333	14.5038815833333	13.3522548916667	14.71190555	14.7143451416667	14.9645581833333	14.62202095	14.1886825416667	14.0062649583333	14.4775829916667	14.4722450583333	14.30615445	14.127909425	14.4850706333333	14.4427443166667

PIN1	12.1947789333333	11.5961336833333	11.7932878	11.8269823166667	11.57356395	11.4170295666667	11.8096310916667	11.5951612	11.575819425	11.7027128	11.6811126416667	12.036426975	11.712752375	11.631804775	11.7105442333333	11.4527169333333	11.52618635	11.6511098916667	11.70420215	11.5869361333333	11.4398783916667	11.36947175	11.4431148583333	11.3986748166667	11.4258523333333	11.943562125	11.5809197583333	11.5980356083333	11.5323406833333	11.484727	11.5030923083333	11.5291327083333	11.3516750666667	11.4715256833333	11.9236129	12.0149070416667	11.61335235	11.4993303916667	11.379403425	11.4405829333333	11.7019780416667	11.5813004	11.6807143833333	11.7216620916667	11.634108225	11.81120325	11.7109162916667	11.7895299333333	11.5695230416667	11.4949493416667	11.5548527833333	11.545468825	11.7814654666667	11.8642752583333	11.8457882583333	11.8100395833333	11.50454985	11.806635025	11.7993113833333	11.6547489083333	11.7628441333333	11.8141737416667	11.9409022083333	11.765950425	11.5154734	11.4824869416667	11.6982071583333	11.6448777666667	11.1891169333333	12.3114382583333	11.9096928333333	11.7300854416667	11.73285025	11.5598018166667	11.57803215	11.5019499416667	11.6496725666667	11.7568591416667	11.55164095	11.69218085	11.5895178083333	11.4854519916667	11.49381285	11.4296055416667	11.3954232	11.3651914583333	11.6636899333333	11.75300075	11.8008623833333	11.67393075	11.8232517	11.4038169	11.61112325	11.4162651666667	11.4839876416667	11.6962930666667	11.8461878583333	11.430002575	11.9300861833333	11.8344676416667	11.825765325	11.960165	11.5121356333333	12.0881187916667	11.4741238583333	11.5638114916667	11.8713469833333	12.2206544333333	11.2572113416667	11.5805781333333	11.5483812666667	11.9052300666667	11.8550160583333	11.8285794166667	11.8048205583333	11.49381285	11.4763556583333	12.23627775	12.3059641583333	12.0309620833333

MPHOSPH6	9.39297179166667	10.49265135	10.0744319916667	10.29083355	10.3321140166667	10.916432925	10.2962241416667	10.409321425	10.06195985	10.468685175	10.3143367833333	9.119480775	9.56361585	10.43975535	9.77432975	9.91080910833333	9.92254819166667	10.7258049	10.849584125	10.7493099333333	10.6792669083333	10.7743973416667	11.233793875	11.0448237416667	10.4653037	9.743497625	10.7975980416667	10.4460825166667	10.5022046916667	10.7112321916667	10.7789681833333	10.6007887583333	10.2481894083333	9.746957025	10.3941230333333	9.69837386666667	10.02346285	10.5711304666667	10.625983	10.5643520333333	10.4678765666667	9.86640328333333	10.2481894083333	11.03193485	9.776982075	10.3667222333333	10.6083941916667	11.0232158833333	10.7407277583333	10.289694575	10.647327775	10.5388660583333	9.73142488333333	10.1962000083333	9.75408091666667	9.80096413333333	10.776374675	11.0272743166667	10.194475875	10.3578687916667	9.80056271666667	10.8996373166667	10.7964692333333	9.81176585	10.6035239083333	10.6550211916667	11.6404855583333	10.4433912083333	10.9692394833333	10.4249193833333	10.572357075	12.2383566166667	11.4485644833333	10.8807458916667	9.868111775	11.1847643166667	10.217472825	11.1752905	11.3556571416667	11.7109162916667	9.598664275	10.4149521166667	11.7027128	10.6880668	10.2621725583333	11.2312268666667	10.5108422833333	9.65670091666667	10.9087616083333	10.9350022416667	11.0898799333333	11.4919381583333	10.8747226333333	11.1611143583333	11.06860385	11.2082269	11.3025465416667	10.904663125	11.3313733	10.061572075	10.6019249583333	10.6340233	11.3579174083333	10.934250825	11.012449925	11.2442138	11.2929247083333	11.2940625833333	10.50924085	10.4191933083333	10.503058125	10.7839123	10.096897025	11.4824869416667	10.9556654	10.651587675	10.7964692333333	11.2107825666667	11.0975459833333	10.5497574833333

CYP24A1	5.3343362	7.99186004166667	8.498393225	7.72905521666667	9.093743775	11.4332575583333	7.801197125	9.19576385833333	7.15262315833333	8.54500405833333	9.08047905833333	5.99349075	11.1241958916667	7.57673284166667	6.679903875	7.6157713	5.23361180833333	10.0960991583333	8.66828283333333	8.57995885	9.71828384166667	7.55551181666667	12.4355627	11.401232775	9.92494756666667	4.79621500833333	7.38522528333333	6.83620095833333	10.40167215	6.79529673333333	10.6430311416667	7.93859618333333	9.50353634166667	4.57535033333333	5.51915526666667	7.057705175	7.05900955833333	10.253110825	11.17143095	8.76079081666667	9.59369666666667	6.76187970833333	7.99563946666667	10.8082438916667	9.62496784166667	8.487957325	7.3477901	10.3171296666667	9.55288966666667	9.40906840833333	10.9084288583333	10.1469040583333	6.96120915	7.93695109166667	7.78383044166667	9.09878833333333	9.14782716666667	9.34710834166667	8.167838375	10.3945367666667	7.59199554166667	10.0524736083333	14.6005448916667	12.6996624166667	6.7401779	10.1589444166667	9.77475016666667	12.5135886333333	14.1972150333333	13.0457183333333	8.211993425	10.15376285	11.8865138166667	11.39042995	11.428103825	9.23281155	12.6966269416667	10.6203644583333	10.4276602833333	7.65225225	8.63731774166667	12.0253815583333	11.9204648416667	10.4284899	10.342051525	11.13577505	13.9796866	11.0150144333333	10.15376285	10.3913250333333	11.545468825	11.6175120416667	8.97721753333333	8.98889158333333	11.5279761083333	9.52223368333333	10.1481495666667	13.93081475	13.3936063	9.68261813333333	10.4732782833333	9.0174923	8.08952596666667	9.64199300833333	10.647327775	11.66250505	11.6600748541667	9.446046	11.655146	12.1237921916667	13.000133375	10.469517225	13.4557593833333	13.9625123916667	11.7235508	11.249224775	12.59948	11.8150676833333	10.6384159666667	13.1085025666667

POLA1	4.79621500833333	4.1683668	5.073382675	5.00529823333333	4.727091875	6.32454565	4.13647209166667	3.604755325	4.24624565833333	4.70675665	4.67972664166667	3.82817183333333	4.74337093333333	4.36294506666667	5.53395658333333	5.32346678333333	4.64311556666667	5.13015945	5.97783559166667	5.17603111666667	4.86289075	5.75852464166667	4.92149175833333	5.91038576666667	6.52133365833333	4.97490869166667	6.28336628333333	6.51915964166667	6.5898034	5.467505875	4.54020328333333	6.63179246666667	4.67574021666667	5.965264275	4.54020328333333	5.4137082	6.764479675	5.26078876666667	6.042706375	5.68689626666667	5.14401780833333	5.86812175833333	4.76141500833333	6.03921009166667	4.812774675	4.960685075	5.45697495	4.98651175	5.377778825	6.31469075	4.67380545833333	5.61840579166667	5.367183075	5.95923255	4.509505	5.37676219166667	4.19810324166667	5.61255331666667	6.160301025	6.54716043333333	5.978682025	4.46985861666667	5.54392786666667	4.01216488333333	3.99268493333333	4.830971775	4.09994219166667	4.18714809166667	4.44923994166667	5.39178135	4.07882345	4.3549546	4.290980325	4.27896138333333	3.96836553333333	3.83492170833333	5.326621775	6.3794359	4.149223425	5.27529641666667	4.21701204166667	4.3881123	4.52697115833333	4.434278525	5.09968536666667	4.13218385833333	5.74506199166667	4.43531826666667	5.7946758	4.85037616666667	4.771681175	6.03147445	5.75948789166667	4.72007973333333	6.133346875	5.60971738333333	5.74506199166667	6.47718199166667	4.19234266666667	4.97072769166667	4.54864098333333	3.79653381666667	6.82704920833333	5.22716268333333	4.47367345833333	4.49113386666667	4.74337093333333	6.25335921666667	4.97597943333333	6.673017075	5.15246255833333	5.258661775	5.33557505	5.75682274166667	4.70270751666667	5.01427080833333	4.51475793333333	5.20060255	5.52920275833333	5.78455018333333

SCGB2B2	7.14336814166667	6.63942924166667	6.37857751666667	6.98158215833333	6.29901051666667	6.57014316666667	6.29901051666667	6.19893275	5.755893075	6.68472555	7.32611610833333	6.59670515	5.38659805833333	5.75774213333333	7.415839475	6.3064348	7.44667759166667	6.013009825	6.346676525	6.558117975	6.34978520833333	8.333491425	6.620339275	7.537939	7.55609866666667	7.72195235	7.65490436666667	7.97719954166667	6.99919410833333	7.6798049	6.38084359166667	7.22053391666667	7.08170128333333	6.40053460833333	7.20334935833333	7.872646525	7.43868464166667	6.485693525	6.46133099166667	7.20211523333333	7.057705175	7.62593523333333	7.25540121666667	6.70918899166667	6.01215780833333	6.88184853333333	6.84636305	6.478653025	8.04179383333333	7.88077005	6.39606678333333	6.66118995	7.262828725	7.41882361666667	7.16909093333333	7.35503378333333	5.94535455833333	6.43152844166667	7.60193526666667	7.19767501666667	6.777132775	6.78180254166667	8.09121131666667	7.5512356	6.63788805833333	6.28336628333333	5.87782166666667	6.372760025	7.1989222	6.524903425	6.92168505833333	6.728950375	5.59857721666667	6.018516875	5.24388391666667	7.00365334166667	7.43289285	7.492737425	6.56733945833333	6.76714710833333	7.55925806666667	7.80451180833333	6.242830225	7.12725256666667	6.9563535	8.53238421666667	7.782192425	6.449622025	7.52840544166667	7.78488271666667	6.92501696666667	6.4413796	9.01521926666667	6.56215660833333	7.35627839166667	6.87783053333333	7.50167894166667	7.04898169166667	6.27482028333333	6.77370956666667	7.348327125	6.70726116666667	7.39105859166667	7.50404556666667	7.09487224166667	6.61154091666667	7.116803275	6.5574676	7.35396276666667	6.65843384166667	8.11057064166667	7.12103848333333	7.745650475	6.897275725	6.95358578333333	7.224148725	7.99833741666667	6.47791706666667	7.66774384166667	5.984788925

DNAJC16	4.178301475	5.30106392916667	5.53949626666667	5.36974268333333	5.35838475833333	4.53766447083333	5.172091325	5.48044942916667	5.89775234166667	6.00753320416667	5.30013326666667	5.1796039125	5.43653326666667	5.564714075	5.78581700833333	5.20715956666667	4.1486802625	6.21964854583333	5.32609779166667	5.95877949166667	6.117446875	6.52877812083333	5.82969477083333	6.798312975	4.95482875	5.00178568333333	5.33111477083333	5.82969477083333	5.888953425	5.40801585416667	5.4303641625	6.15565492083333	5.8268921375	4.54816651666667	4.6263945375	5.67755486666667	5.77935125	6.6767168875	5.52694737083333	6.0508640625	5.56921560416667	5.17650485416667	6.0789784375	5.08404354583333	3.88709014583333	5.26621684583333	4.63143759583333	4.45753987916667	5.82414327083333	5.90801045833333	6.65321274166667	5.99989406666667	5.32082965	4.834065625	4.54499327916667	4.72056588333333	5.37727050833333	4.39922965416667	5.89681017083333	5.69628012083333	4.32629804583333	5.26215174166667	6.7727462375	6.07041681666667	4.49165090416667	4.63982503333333	5.90801045833333	5.20205519583333	5.7495715625	5.3762358875	4.804834575	5.36150230833333	5.4251255875	4.61059691666667	5.87154659166667	5.84838001666667	5.22874515833333	5.97070933333333	4.49264759166667	6.18370465416667	5.39330138333333	5.9647509125	5.51242043333333	5.42224169583333	4.9883223125	5.33193972916667	4.83047865833333	5.4153188875	5.54731458333333	5.5710450125	5.9699700375	5.59428554583333	4.7798875125	5.59713721666667	5.57017015833333	6.28146945833333	5.56739502916667	6.26783115	5.1368984	4.8219214875	4.8154347125	5.2254306125	6.3873727625	4.67426099583333	4.5089847125	5.33282089583333	4.30673345	5.36870735833333	5.23726492916667	6.03623919166667	6.42906689583333	5.0501782	6.03623919166667	5.08112502083333	5.299254225	4.7798875125	5.5114587625	4.6417674875	6.59272330833333	5.63005625

AGMAT	4.178301475	5.30106392916667	5.53949626666667	5.36974268333333	5.35838475833333	4.53766447083333	5.172091325	5.48044942916667	5.89775234166667	6.00753320416667	5.30013326666667	5.1796039125	5.43653326666667	5.564714075	5.78581700833333	5.20715956666667	4.1486802625	6.21964854583333	5.32609779166667	5.95877949166667	6.117446875	6.52877812083333	5.82969477083333	6.798312975	4.95482875	5.00178568333333	5.33111477083333	5.82969477083333	5.888953425	5.40801585416667	5.4303641625	6.15565492083333	5.8268921375	4.54816651666667	4.6263945375	5.67755486666667	5.77935125	6.6767168875	5.52694737083333	6.0508640625	5.56921560416667	5.17650485416667	6.0789784375	5.08404354583333	3.88709014583333	5.26621684583333	4.63143759583333	4.45753987916667	5.82414327083333	5.90801045833333	6.65321274166667	5.99989406666667	5.32082965	4.834065625	4.54499327916667	4.72056588333333	5.37727050833333	4.39922965416667	5.89681017083333	5.69628012083333	4.32629804583333	5.26215174166667	6.7727462375	6.07041681666667	4.49165090416667	4.63982503333333	5.90801045833333	5.20205519583333	5.7495715625	5.3762358875	4.804834575	5.36150230833333	5.4251255875	4.61059691666667	5.87154659166667	5.84838001666667	5.22874515833333	5.97070933333333	4.49264759166667	6.18370465416667	5.39330138333333	5.9647509125	5.51242043333333	5.42224169583333	4.9883223125	5.33193972916667	4.83047865833333	5.4153188875	5.54731458333333	5.5710450125	5.9699700375	5.59428554583333	4.7798875125	5.59713721666667	5.57017015833333	6.28146945833333	5.56739502916667	6.26783115	5.1368984	4.8219214875	4.8154347125	5.2254306125	6.3873727625	4.67426099583333	4.5089847125	5.33282089583333	4.30673345	5.36870735833333	5.23726492916667	6.03623919166667	6.42906689583333	5.0501782	6.03623919166667	5.08112502083333	5.299254225	4.7798875125	5.5114587625	4.6417674875	6.59272330833333	5.63005625

FAM161A	9.22794726666667	8.81515405833333	8.92973970833333	8.651213975	7.69319276666667	8.17931615833333	8.1346889	8.375514	8.16684950833333	8.975801525	8.54360906666667	8.32062069166667	7.01007860833333	8.576058475	8.833014675	8.60227746666667	10.798668925	8.21046635	9.15351445833333	8.89560048333333	8.67026894166667	8.335541375	9.1141243	8.63773576666667	8.73063873333333	9.09007516666667	8.79040525833333	8.8830474	8.07106306666667	8.94330425	9.1218467	8.50396570833333	8.42214336666667	8.87543329166667	9.00330966666667	9.19200934166667	8.31814086666667	8.68729530833333	7.95406095	8.391097025	8.7292202	8.873364875	8.81240245	9.35549675833333	8.71144663333333	9.38807046666667	9.01425336666667	8.48261985	8.759783975	8.759783975	8.96369055833333	8.68872105	7.806190725	8.47797495833333	8.228841125	8.83154074166667	8.45999591666667	8.817877875	8.910111275	8.68470500833333	8.16735940833333	8.7532663	9.55472915	9.28502139166667	10.2166211083333	8.85393681666667	9.73914464166667	8.33450988333333	9.166824	8.20689531666667	8.61342080833333	8.17036706666667	8.1898559	8.48460361666667	8.57112621666667	9.93476125833333	9.16074203333333	8.56529179166667	9.3036884	9.50643220833333	10.2535245166667	9.272617125	9.09925841666667	9.4842707	7.738684825	10.4356784583333	11.517039175	8.73639624166667	10.2132508333333	9.31502623333333	9.17368278333333	9.53356816666667	9.23929971666667	8.641468925	9.963087575	8.73063873333333	9.28041066666667	10.8861829666667	8.95991093333333	10.4280419583333	9.75321625	9.74483329166667	9.23882716666667	9.62586958333333	8.5196153	9.133746725	8.96893119166667	9.826059425	8.4076656	9.01655344166667	9.76350663333333	9.25028671666667	8.40077755	10.2170232333333	8.58091206666667	8.80353201666667	10.6289554166667	10.485478825	9.71864728333333	7.73471975833333

FGF18	4.30527630833333	4.979047925	4.750433775	5.11118121666667	5.88241164166667	4.1553789	6.00039116666667	5.20449400833333	5.70748306666667	4.92991125833333	4.54769205	5.14931930833333	5.19309993333333	5.29356806666667	4.63305978333333	4.52791141666667	4.89827923333333	5.73297515	4.27276220833333	4.99247835	5.13847554166667	4.506429475	4.95961636666667	4.36096451666667	4.087323525	4.939635175	4.30130591666667	4.68073703333333	4.65900069166667	5.2151974	4.83457635833333	4.68477714166667	5.86727098333333	5.44322508333333	4.71576270833333	4.350915225	4.55466009166667	4.46516671666667	4.27798759166667	4.31803169166667	4.47474535	4.51268873333333	4.37610179166667	4.782758275	5.2151974	4.34607883333333	4.44059075	5.57252066666667	4.13218385833333	4.108372	4.26445661666667	4.60485635	5.38372931666667	5.47028654166667	5.02467455	4.55283246666667	5.106111275	4.65478205833333	4.50312849166667	4.53327731666667	4.77272485	4.832334525	5.64973066666667	4.736033375	5.45697495	5.33069273333333	5.72466863333333	5.19536235833333	5.02269790833333	5.577321975	4.789978375	4.95105651666667	5.303590975	4.91442044166667	5.0088772	4.75255506666667	5.24195544166667	4.54020328333333	5.21412320833333	5.36208894166667	4.453007975	4.70371486666667	5.37570958333333	4.66334196666667	5.087869425	4.87091625	4.87503585	4.74123235833333	5.2617065	4.52697115833333	5.31119278333333	4.753728625	4.62057545833333	5.1598283	5.01100034166667	4.65478205833333	5.12000276666667	4.52055483333333	4.96739760833333	4.62691041666667	4.344051675	5.26995844166667	4.45030740833333	4.64613160833333	4.40581835	5.09419765	4.45205615	5.93036776666667	4.64311556666667	4.57030863333333	4.23000438333333	4.727091875	4.695239575	4.93116945833333	4.86184074166667	4.49797680833333	4.66334196666667	6.31880616666667	4.66004348333333	4.79518681666667

VMP1	11.0913458083333	9.3750605	9.55104401666667	8.49442401666667	9.71398214166667	9.04505583333333	8.84333919166667	9.72156395833333	8.63293045833333	9.45472053333333	9.17280985	9.82203395	11.709376075	9.060159775	9.03950406666667	10.3718117166667	10.237546325	12.4248504333333	10.806391425	10.3894512416667	10.3783237666667	10.8960985416667	10.115233725	11.1649238666667	8.42618235833333	10.5404499166667	9.7408584	10.2237133416667	10.7673403166667	9.03641039166667	11.5200695166667	9.48881261666667	10.4597952916667	9.818576775	10.54728675	8.884079375	9.82029285833333	9.24751750833333	11.0221982916667	10.112191475	8.78355868333333	8.55804185	9.76219373333333	9.98312965833333	9.345810075	8.72544860833333	9.08776835	10.128737475	9.61006324166667	10.0305098083333	10.5108422833333	10.1755237	10.939073475	10.6131721666667	10.079544525	8.96455003333333	9.9420301	7.798604875	7.66500309166667	9.17923419166667	11.7624702333333	9.93083546666667	9.544894	10.1824837166667	9.1218467	9.32142165833333	9.95359933333333	9.0815933375	9.89258960833333	10.6313385833333	9.345810075	10.0997208916667	10.6982774333333	9.628525675	10.2881617	9.78481885	10.501807225	11.1150801666667	10.5428297916667	9.83584716666667	9.64943681666667	11.783111075	9.84032644166667	11.0905957416667	8.48460361666667	10.457410175	8.13617131666667	11.5204866583333	11.1350432333333	10.9226970166667	11.21653435	9.315501225	10.74363815	9.36761098333333	10.4280419583333	10.43166005	9.27812115	8.99771499166667	10.2342241333333	8.35024235	8.13871308333333	9.663801325	9.42072421666667	10.52201155	10.2621725583333	9.61413186666667	10.090730825	11.9781875416667	9.22874626666667	9.22559156666667	10.4609966416667	8.40614324166667	11.5034674333333	11.5876922333333	10.1126129583333	9.77571775833333	8.35024235	8.4515305	7.58408555833333	9.57903839166667

RHOQ	11.2016925041667	8.25719014583333	8.53809097083333	7.45188757916667	8.32538908333333	8.716679175	7.65302834166667	8.3174243875	8.04474641666667	8.33280820416667	8.3708471	10.0793449	9.7239158	8.14372690416667	8.3174243875	8.6131617375	8.9821473375	8.4156859875	8.3549731125	8.66801874166667	8.658219175	7.98948287083333	8.6761895875	8.8113169	8.82190914583333	10.8459877208333	8.69679669583333	8.47122492916667	8.51155702083333	8.45580745833333	9.02905580416667	8.71581307916667	8.2623240625	8.75302582916667	11.1247495916667	10.9172764083333	8.55880545	8.54916765833333	8.88710928333333	8.79658383333333	8.7464595875	8.6041321125	8.80993224166667	8.83223770833333	9.6378288875	8.59968860416667	8.95469538333333	8.36738207083333	8.43271248333333	8.45532811666667	8.78286091666667	8.58932739166667	8.3373059375	8.43271248333333	8.41511014583333	9.20584292083333	8.51311385	8.371845825	8.5496418875	9.00213076666667	9.58595583333333	8.73282608333333	8.39038555416667	8.55828689166667	8.58932739166667	8.35047989583333	8.725210225	8.28656196666667	7.88477791666667	8.32386307083333	8.6245554375	8.42958982916667	8.09669910833333	8.86393744583333	8.13218660416667	8.47209823333333	9.414608	8.5496418875	8.13218660416667	8.02593349166667	9.30608285833333	8.7386305625	8.80803470833333	9.48903012083333	8.50628399166667	8.83089360833333	7.9587800125	8.3464457375	9.33905939166667	9.16519122083333	8.31590533333333	9.23116307916667	8.63056395833333	8.2227352625	8.39135550416667	8.1432237375	8.2344129	9.3482307125	8.01675574583333	8.535535225	8.92678499166667	8.0071378875	8.3393937375	8.38095353333333	8.4663802	8.4401844125	8.95240457916667	8.34155047083333	8.79618599166667	8.73086925833333	9.14541895833333	7.77295430833333	8.6245554375	8.0291749375	8.53505667916667	8.208569875	8.98725887916667	8.3459861125	8.68847175416667	8.73664734166667

PIGF	11.2016925041667	8.25719014583333	8.53809097083333	7.45188757916667	8.32538908333333	8.716679175	7.65302834166667	8.3174243875	8.04474641666667	8.33280820416667	8.3708471	10.0793449	9.7239158	8.14372690416667	8.3174243875	8.6131617375	8.9821473375	8.4156859875	8.3549731125	8.66801874166667	8.658219175	7.98948287083333	8.6761895875	8.8113169	8.82190914583333	10.8459877208333	8.69679669583333	8.47122492916667	8.51155702083333	8.45580745833333	9.02905580416667	8.71581307916667	8.2623240625	8.75302582916667	11.1247495916667	10.9172764083333	8.55880545	8.54916765833333	8.88710928333333	8.79658383333333	8.7464595875	8.6041321125	8.80993224166667	8.83223770833333	9.6378288875	8.59968860416667	8.95469538333333	8.36738207083333	8.43271248333333	8.45532811666667	8.78286091666667	8.58932739166667	8.3373059375	8.43271248333333	8.41511014583333	9.20584292083333	8.51311385	8.371845825	8.5496418875	9.00213076666667	9.58595583333333	8.73282608333333	8.39038555416667	8.55828689166667	8.58932739166667	8.35047989583333	8.725210225	8.28656196666667	7.88477791666667	8.32386307083333	8.6245554375	8.42958982916667	8.09669910833333	8.86393744583333	8.13218660416667	8.47209823333333	9.414608	8.5496418875	8.13218660416667	8.02593349166667	9.30608285833333	8.7386305625	8.80803470833333	9.48903012083333	8.50628399166667	8.83089360833333	7.9587800125	8.3464457375	9.33905939166667	9.16519122083333	8.31590533333333	9.23116307916667	8.63056395833333	8.2227352625	8.39135550416667	8.1432237375	8.2344129	9.3482307125	8.01675574583333	8.535535225	8.92678499166667	8.0071378875	8.3393937375	8.38095353333333	8.4663802	8.4401844125	8.95240457916667	8.34155047083333	8.79618599166667	8.73086925833333	9.14541895833333	7.77295430833333	8.6245554375	8.0291749375	8.53505667916667	8.208569875	8.98725887916667	8.3459861125	8.68847175416667	8.73664734166667

DOCK6	8.76421910833333	8.79346453333333	9.090969825	8.59154009166667	8.9925209	8.119922525	7.32319219166667	8.61820814166667	8.50602524166667	8.67361984166667	9.175625125	6.98096124166667	8.106355425	8.46196905	9.68471804166667	8.55853193333333	8.9544846	8.67639044166667	9.65184145	8.91049593333333	8.873364875	9.4968836	9.018981225	9.0973311	8.75278535833333	8.75743711666667	9.45898255	9.120869625	9.9441902	9.20513901666667	9.50725341666667	9.16495244166667	8.41299383333333	7.966758925	8.67888266666667	8.955351225	9.26627294166667	9.37403768333333	8.9929566	9.04890688333333	9.18505965833333	9.508221125	9.040322975	9.19244325833333	8.71039916666667	9.39626775833333	9.55472915	9.40333164166667	9.09878833333333	8.41428779166667	8.736017975	9.186916425	8.903371	9.20513901666667	9.51496844166667	9.315501225	8.56622091666667	9.36084673333333	8.84758474166667	8.80677418333333	9.15400360833333	8.462988175	9.20513901666667	9.16735169166667	8.135196525	9.87826785	8.67077851666667	7.341560875	8.00852153333333	8.821200975	8.14849655833333	9.05965051666667	7.76608003333333	8.62136429166667	8.83592169166667	8.90962148333333	8.64290230833333	8.61480576666667	9.37588805	9.40469768333333	8.26207273333333	8.022968975	9.62234490833333	9.33411035833333	8.63497865	8.354234475	7.803872825	7.88761391666667	8.603908075	8.94577069166667	8.59007210833333	9.24127241666667	8.44794389166667	8.81018689166667	8.89967475833333	8.74083754166667	9.34438068333333	8.06450121666667	8.50236353333333	8.295765375	8.9384783	7.35924831666667	8.44334104166667	9.70017130833333	8.53238421666667	8.63497865	9.28041066666667	9.87695954166667	8.38319035	8.51484679166667	8.34324809166667	8.47850595	9.77432975	9.114481175	8.87290028333333	8.73259671666667	8.000994225	9.42244939166667	9.90518528333333	6.59744179166667

OR2H1	5.859013175	4.84935865	4.51179561666667	4.50312849166667	5.135343	5.18015243333333	5.59377575	4.977020525	5.0343026	5.48703414166667	4.78798888333333	5.173701425	4.84714924166667	4.692141475	4.39039918333333	4.52055483333333	6.21427064166667	5.13336504166667	4.46696494166667	5.07758634166667	5.52011276666667	5.16838866666667	5.24289476666667	4.73024025833333	4.92557835833333	4.3987315	4.38726624166667	5.74909436666667	4.938665425	4.359146625	4.81840091666667	4.49712886666667	4.63723314166667	6.46419815833333	5.10077205	4.28310633333333	5.83306814166667	5.45265249166667	5.12313813333333	7.08293825833333	4.957419525	4.74219220833333	4.841975675	5.54102934166667	4.08963481666667	4.98651175	5.18213341666667	5.002397475	4.67380545833333	4.94778381666667	4.705709175	5.43388225	4.830971775	5.35182895833333	4.41782250833333	4.71016199166667	4.65382089166667	4.84094744166667	4.63513973333333	4.472817375	5.5614942	5.33867198333333	4.727091875	4.84301391666667	5.78537223333333	5.23781710833333	6.12282240833333	4.4462942	4.88687726666667	4.697573175	4.92659856666667	4.695239575	4.93761881666667	4.93436960833333	4.838864	4.77813741666667	4.71445220833333	4.3987315	4.66519471666667	4.3881123	5.16420199166667	4.51268873333333	4.78047113333333	4.82464924166667	4.78586035833333	4.172665625	4.90147810833333	5.70748306666667	4.95206471666667	4.57718236666667	4.61520515	4.47770515	4.87823230833333	5.2460388	5.58699986666667	4.98009509166667	5.45375554166667	5.87208589166667	4.841975675	6.84260019166667	4.78798888333333	5.279548575	4.82904071666667	5.17697859166667	4.645027875	4.09598105	4.67877638333333	6.46219335	5.01629245833333	6.31323984166667	4.576370125	6.311629325	4.508464425	4.96517085	4.58982808333333	5.08997001666667	5.066744825	4.909691175	4.88257656666667	4.59897825

CHD2	7.60136925	6.653546475	6.2176337	5.85993758333333	6.9569431	7.05338414166667	6.99346504166667	7.46514316666667	6.31702735	6.85216409166667	6.755806875	6.41354868333333	6.73065755833333	6.94244640833333	7.69541339166667	6.79863740833333	7.89243905	7.03630040833333	7.23553730833333	7.572832925	7.02536274166667	8.23175398333333	7.112144375	7.72354014166667	7.30968498333333	7.49164054166667	7.43179573333333	7.34611101666667	7.252371175	6.82038475833333	8.16529418333333	7.24396096666667	7.193910325	7.37123815833333	7.2132819	6.45093723333333	7.3113529	6.98158215833333	7.30913270833333	7.19137265833333	6.438674975	6.056382625	6.79588928333333	6.95156631666667	5.6173492	6.20180194166667	6.19893275	6.96712810833333	7.28012135	7.414082675	6.61087155833333	7.39869075	6.48419624166667	6.40489274166667	6.93873875	5.633369575	7.62037211666667	5.83501515	6.5766165	7.30371010833333	6.97957124166667	6.57813499166667	7.102506125	7.279554825	6.61415903333333	8.70751021666667	7.31981145833333	6.54299384166667	7.7783407	7.12659995	7.15389984166667	5.76251488333333	6.65915351666667	6.59464205	6.53205264166667	6.61289744166667	6.9475749	7.37811973333333	7.404332775	7.729718875	7.265200275	7.10505258333333	6.55061969166667	6.95435513333333	7.43011818333333	7.14399110833333	7.13093330833333	6.63660101666667	7.514206825	7.227214725	7.67769499166667	7.04267660833333	7.56325655	6.810578	7.11547685	7.4493567	6.81508890833333	6.771060625	6.80377665833333	6.65092356666667	7.25001195833333	5.859013175	6.61019589166667	7.07980634166667	6.67021015	7.85962578333333	6.76253231666667	6.77370956666667	6.98853248333333	7.22110665	7.2657012	6.39918578333333	7.75823035	6.38925563333333	7.428439975	6.018516875	7.11340090833333	7.05338414166667	7.36156116666667	7.33095725

ZNF235	10.1289498083333	9.50159337916667	9.229807275	9.59086925	10.0402000875	9.7150639375	9.9223404	9.52064974583333	10.11945695	9.72595660833333	9.94146235	10.2204579708333	9.89204036666667	9.40579160416667	9.77288145833333	9.4561600125	10.2553132	9.84822367083333	9.95913398333333	9.58017514166667	9.62782690833333	9.84352759166667	9.4966602125	9.467278075	10.4206214	10.19012045	9.52668602916667	9.55311440833333	10.0718272875	9.91563410833333	9.33598702083333	9.22675905416667	9.95832646666667	9.911408125	10.543898075	10.8856558833333	10.261163825	9.74369606666667	9.9203137	10.0278755708333	10.0045986708333	10.0216481541667	10.0478853916667	9.71165420416667	10.0423683958333	9.8827605125	10.125238725	9.83216176666667	10.025426975	10.1860483791667	9.99529302916667	10.1154443916667	9.968033225	10.0282691125	9.99193555416667	9.86742450833333	9.7376662875	9.87413189166667	9.85999592083333	9.927697025	10.0646729166667	9.5549556875	10.1048895041667	9.50378287083333	11.1344931208333	10.2344420666667	10.3899574458333	9.89531699583333	10.12561285	9.99065759166667	10.0931326041667	10.1044701541667	9.363637275	9.4805520625	9.68844351666667	9.49930952916667	10.09395745	10.1702779125	9.59166480833333	9.70090714166667	10.41219155	10.5971126125	9.41552684166667	9.70090714166667	9.90243254583333	9.8578689875	10.1615384	9.45032697916667	10.4193897	9.69512940416667	9.42837040416667	9.60770960416667	10.5539506583333	10.1268490708333	10.2597901583333	9.9654401625	10.4948920666667	10.370049375	9.8979206	10.7853202791667	10.415554625	9.93405404583333	10.0759191291667	10.07719205	10.1264340708333	10.1387853291667	10.0266969708333	9.98819277916667	10.63348715	10.087971275	10.4750240416667	10.0943818375	10.0178064291667	10.3608858666667	9.8245061125	9.66308860416667	10.3676792708333	10.25000095	9.810673375	9.53809290833333

ZNF233	10.1289498083333	9.50159337916667	9.229807275	9.59086925	10.0402000875	9.7150639375	9.9223404	9.52064974583333	10.11945695	9.72595660833333	9.94146235	10.2204579708333	9.89204036666667	9.40579160416667	9.77288145833333	9.4561600125	10.2553132	9.84822367083333	9.95913398333333	9.58017514166667	9.62782690833333	9.84352759166667	9.4966602125	9.467278075	10.4206214	10.19012045	9.52668602916667	9.55311440833333	10.0718272875	9.91563410833333	9.33598702083333	9.22675905416667	9.95832646666667	9.911408125	10.543898075	10.8856558833333	10.261163825	9.74369606666667	9.9203137	10.0278755708333	10.0045986708333	10.0216481541667	10.0478853916667	9.71165420416667	10.0423683958333	9.8827605125	10.125238725	9.83216176666667	10.025426975	10.1860483791667	9.99529302916667	10.1154443916667	9.968033225	10.0282691125	9.99193555416667	9.86742450833333	9.7376662875	9.87413189166667	9.85999592083333	9.927697025	10.0646729166667	9.5549556875	10.1048895041667	9.50378287083333	11.1344931208333	10.2344420666667	10.3899574458333	9.89531699583333	10.12561285	9.99065759166667	10.0931326041667	10.1044701541667	9.363637275	9.4805520625	9.68844351666667	9.49930952916667	10.09395745	10.1702779125	9.59166480833333	9.70090714166667	10.41219155	10.5971126125	9.41552684166667	9.70090714166667	9.90243254583333	9.8578689875	10.1615384	9.45032697916667	10.4193897	9.69512940416667	9.42837040416667	9.60770960416667	10.5539506583333	10.1268490708333	10.2597901583333	9.9654401625	10.4948920666667	10.370049375	9.8979206	10.7853202791667	10.415554625	9.93405404583333	10.0759191291667	10.07719205	10.1264340708333	10.1387853291667	10.0266969708333	9.98819277916667	10.63348715	10.087971275	10.4750240416667	10.0943818375	10.0178064291667	10.3608858666667	9.8245061125	9.66308860416667	10.3676792708333	10.25000095	9.810673375	9.53809290833333

SELENOT	14.1343040416667	14.0100072833333	13.4737954	13.7111367583333	13.653352825	14.0503314	13.8688792083333	13.71864895	13.2354463	13.789075975	14.27950095	14.2715663416667	14.2858034416667	13.9197186416667	13.735130625	13.6384571083333	14.3824525333333	14.0592017583333	13.6302168166667	14.015040275	14.2233219083333	13.8698539083333	14.21037625	14.4775829916667	13.9796866	14.648708375	13.7502246416667	14.26427225	13.89505895	13.57126835	13.3396866333333	13.7949722083333	14.1413614333333	14.1486654166667	13.6384571083333	14.371029675	14.1941852166667	14.1554067916667	13.7463275333333	13.9153342666667	14.2000424583333	13.6375130583333	13.8657422166667	14.219141025	13.838661325	14.0201742416667	14.392674675	14.044199175	13.89301535	13.956539975	14.055300325	13.6062775083333	14.380649825	14.596526825	13.783955125	13.8148381	14.37416785	14.0012596166667	14.0174773166667	14.3775303333333	15.092462825	14.5924675083333	15.225946525	14.8578939833333	15.0894272416667	15.1077350416667	14.400934925	15.009089275	15.2193239916667	15.0153788083333	14.9148418416667	14.8287604166667	14.7143451416667	14.9094697583333	15.1800900333333	15.009089275	14.83890155	14.9391332833333	15.048735725	14.9702238166667	14.7077525	15.1763309166667	14.9148418416667	14.282826075	15.229747525	14.6778149	14.7293250666667	14.7948348083333	15.0703584333333	14.9567111166667	15.0803306416667	14.80004595	14.8412522666667	14.9865439333333	15.169521525	14.7522867083333	14.7097912166667	14.7851708916667	15.156314825	15.2432312666667	14.648708375	14.9702238166667	15.0217064166667	14.8724941166667	15.0769655666667	14.9865439333333	14.6926031666667	15.0005057333333	15.0184028083333	15.1940996583333	14.8819536416667	15.09815525	15.1207533083333	14.6718137833333	15.0769655666667	15.0153788083333	14.3316746833333	14.6968877083333	14.73869885	14.6410690916667

SPDYE3	11.4026558	11.484727	11.0462297583333	11.5505559916667	11.8062314166667	11.8931595083333	11.17354635	11.0673982916667	11.5805781333333	11.1815244666667	11.9728603333333	11.9985911666667	11.74563025	11.5498379666667	12.0832149333333	11.6425911333333	11.0666847416667	12.02225065	11.6006445416667	11.912794275	11.8344676416667	12.3776096916667	11.348473325	11.5832083083333	12.2111963416667	11.6066366416667	11.385364925	12.080029025	12.2706199833333	11.61832875	11.3527786833333	11.9251897666667	11.410462825	11.2753469583333	11.423284125	11.835222575	12.3046936083333	11.3976073583333	11.2876344916667	11.6066366416667	11.4463806916667	11.7517355166667	11.56491665	11.114387025	11.7716953916667	11.2876344916667	11.4200132333333	11.3001028166667	11.61335235	12.0705755166667	11.0541887333333	11.7078053166667	12.2326729333333	12.1935330916667	11.6047856083333	11.5323406833333	11.3654035916667	11.0500837666667	12.0997306916667	11.7120196166667	13.4383119666667	12.046177825	12.1861275083333	12.6366518666667	11.5272640666667	12.6985834416667	11.8059034833333	11.943975875	11.8465762083333	11.5253735333333	12.3746631	12.6235559916667	11.74982705	11.7445138833333	12.39392575	12.5594786833333	12.796581575	13.14330865	11.9400638666667	12.5488109	12.7453059583333	13.2215442333333	11.767528775	11.5400258416667	12.2675250666667	12.8301026333333	12.7071557666667	11.2128973166667	13.6009954166667	12.029837025	12.13848275	13.1197255166667	12.8080609833333	11.9694157916667	12.9767731916667	12.9179353	12.966521825	13.5965748083333	12.242111125	12.5472970333333	12.0816579833333	12.382866125	12.68421675	12.4850765	12.3623095583333	12.3173301166667	12.0261845416667	11.8163038583333	11.9697901833333	12.0713921666667	12.5102327333333	11.3001028166667	11.9297077583333	12.3118325833333	11.9542491166667	11.6115347666667	12.5657560416667	12.1583760416667	11.4140863666667	12.0120395666667

SEMA6C	9.46751014166667	8.565739425	8.25593908333333	8.58441085833333	8.97769849166667	8.95218464166667	8.77676135833333	8.67841379166667	8.56913543333333	9.02131511666667	8.565739425	10.4076693916667	9.826059425	9.15935345833333	8.73305545	8.4237182	8.69409343333333	9.96356175	8.70499479166667	8.73988864166667	8.74224036666667	8.7179342	8.42069139166667	7.81344110833333	8.21929578333333	9.939168625	8.37811988333333	8.368580775	8.62886449166667	8.23767453333333	8.42476003333333	7.88506155833333	8.40567906666667	8.11151595833333	9.59369666666667	9.65101713333333	8.44937053333333	8.3453091	7.61454650833333	7.89979373333333	7.621493025	7.79136785	7.90363365833333	8.40567906666667	8.62886449166667	8.23717023333333	8.21097586666667	7.78383044166667	8.02464245	8.56366318333333	7.81137301666667	7.37359066666667	8.51622818333333	8.76514685	8.123259225	8.751330725	7.59020169166667	7.587363525	8.16633889166667	7.99615545	8.74766549166667	8.662974625	8.35934713333333	9.10433055833333	10.1258089583333	9.40032650833333	9.02609295	9.261730375	8.63344703333333	9.05454125	9.38715235833333	9.69792198333333	9.66961651666667	9.679927325	8.74083754166667	9.05312258333333	9.13078204166667	8.31008573333333	8.711926	8.95079723333333	10.017588525	10.149337475	8.59492328333333	8.82572	9.38500366666667	9.56452501666667	8.77815989166667	9.02371780833333	10.7309865083333	9.39478965833333	8.15758866666667	8.18100231666667	8.98520484166667	8.94431525	8.96796725	8.737919725	9.90307260833333	8.92890170833333	8.48460361666667	8.7374204	8.38865071666667	8.99535305	7.85104381666667	8.02185345833333	7.92110699166667	8.71039916666667	9.341081375	8.67403964166667	8.63681648333333	9.30682376666667	7.91244564166667	8.21046635	8.910111275	8.884079375	8.61046613333333	7.45718173333333	9.97706043333333	7.671444425	7.70532880833333	8.15011035

CAMK2G	6.941911825	8.42823383333333	8.5716089	9.01051185833333	9.86718390833333	6.84576470833333	10.0125394166667	9.26261210833333	9.55009920833333	8.36380941666667	8.49593345833333	9.90565114166667	9.858083725	8.77628200833333	8.017526525	8.04234110833333	6.85538685833333	9.3036884	7.08048500833333	7.84545261666667	9.39938098333333	8.48460361666667	8.3715767	7.16176278333333	6.52412431666667	7.76455869166667	6.66447699166667	6.32381556666667	7.12280756666667	6.95156631666667	6.53536243333333	7.28803510833333	8.327465575	7.47140944166667	7.68209800833333	6.75503975833333	7.2401722	6.93352621666667	6.69439601666667	6.57166325	6.59670515	6.84636305	5.95832643333333	5.60971738333333	7.102506125	6.01932009166667	6.083461775	6.98410809166667	7.64794805833333	7.18281396666667	6.89400789166667	6.72519365833333	7.62593523333333	6.448169675	6.6716762	5.74909436666667	7.30019385833333	6.2066078	6.66383380833333	6.169883075	7.50404556666667	9.02980844166667	6.73694150833333	8.44937053333333	8.59743456666667	8.37413723333333	8.635919475	9.41436835833333	7.83671455833333	8.4414262	8.67503203333333	8.83443758333333	10.1524914833333	8.05804941666667	9.0517655	8.4067182	8.50984575833333	7.2622557	7.54228448333333	8.83016251666667	8.20175241666667	10.4775633333333	8.24509221666667	7.18884621666667	7.61760009166667	8.8334785	7.59199554166667	8.66930214166667	8.931271375	7.27114775833333	7.990761025	6.97462729166667	7.48184465	7.27649536666667	8.17990050833333	7.64275718333333	8.03223831666667	7.46243825	8.20175241666667	7.20088341666667	7.07292095833333	9.20284239166667	6.83207999166667	6.47718199166667	7.13850381666667	7.37359066666667	7.2027431	7.32668075833333	7.39975843333333	7.874246775	6.93685505	6.801867125	6.77777710833333	6.06491893333333	6.08433614166667	6.97843058333333	6.85677963333333	6.64893395	6.78114418333333	7.77893200833333

OR1J2	4.9426219	4.81947958333333	4.366029925	5.064717325	4.78047113333333	4.15220664166667	4.747391	4.92455391666667	5.18759319166667	4.2422894	4.268577875	4.78586035833333	5.08252440833333	4.515720925	4.823565325	4.2918764	4.62889925	4.03660776666667	4.59784559166667	5.63053526666667	4.44254745	5.01321970833333	4.85447411666667	4.3934547	4.73402499166667	4.23291245833333	4.72222786666667	4.90871175	4.83765554166667	4.43844	4.753728625	5.97349889166667	4.50751908333333	4.160694875	4.7771511	5.30061454166667	4.62161215833333	4.664314175	4.60382705833333	4.65900069166667	5.112251075	4.57343223333333	4.97597943333333	5.67327496666667	4.19346535	5.10290811666667	5.28464988333333	5.74606878333333	4.557709	4.85768484166667	4.83558559166667	4.472817375	6.057986625	5.49334536666667	4.479514325	4.83457635833333	4.44254745	4.90147810833333	4.48159451666667	4.45205615	5.23566219166667	5.65344755	5.153439775	4.53433785	4.233876925	4.55369980833333	4.30828979166667	5.4913896	4.328489775	5.56242626666667	5.173701425	5.03622141666667	5.77633328333333	4.350915225	5.45265249166667	4.72414796666667	4.310185775	4.38412459166667	4.34698491666667	4.70784294166667	5.04024386666667	4.75255506666667	4.31414184166667	4.65382089166667	4.05738384166667	4.79621500833333	5.62775541666667	5.29965199166667	5.31319136666667	4.1882044	5.21920105	6.15274419166667	5.221531825	3.94139638333333	4.41695324166667	4.2443259	4.60382705833333	5.27315905833333	4.3934547	4.97812506666667	4.43531826666667	5.02467455	4.96739760833333	5.2891249	4.41506155	4.05071508333333	4.76141500833333	6.70796138333333	4.97812506666667	6.62907710833333	4.66727606666667	5.44322508333333	4.59071941666667	5.11436066666667	5.20995024166667	4.86289075	4.251891475	5.70153421666667	4.80096826666667	4.93116945833333

NOL7	12.4281341208333	15.0968513375	14.6229973208333	14.435416225	14.9375342458333	13.8456112875	14.4269781291667	14.8979679041667	14.4084687291667	14.3539205333333	13.7307989	12.9320596125	14.53483965	14.6640012125	14.834985	14.489995425	13.0623209	14.6294728791667	15.0200546125	14.8930132291667	14.0321124625	14.9991992666667	14.7908835958333	13.8988064875	14.7066709916667	13.0081306708333	14.6271940916667	14.7462478083333	15.199384425	14.7003845958333	14.6497610125	14.7327866333333	14.4714120708333	12.8336866291667	12.9320596125	12.6869957833333	15.1477739416667	15.0443403541667	14.44354915	14.4642959333333	14.7462478083333	15.07184225	14.7398420916667	14.2110759625	14.0546587541667	14.7987721708333	14.7934297125	14.97937325	14.5295676666667	14.3146441458333	14.4404566458333	14.6580212458333	14.7195864708333	14.8830862	14.4917411916667	14.3669169333333	14.5149754666667	15.400251175	15.2244796125	14.4369887875	12.66823485	13.1333095958333	12.717755275	13.2694207583333	13.1775302416667	12.6519795833333	12.6889059625	12.6086270791667	12.7418902125	12.808331825	13.3154975291667	12.8243259041667	12.6006538583333	13.1339977625	12.712559325	12.7146663083333	13.1698093875	13.0963431125	13.0208158125	12.9519194583333	13.1836216458333	12.6834027	13.0225453583333	13.2309913833333	13.3043630333333	13.1816528708333	12.44373405	12.9027308333333	12.2617779791667	13.0316461208333	12.6692120791667	12.9502654625	13.5112304791667	12.7772084333333	12.6439553916667	12.7772084333333	13.2825576083333	12.6378885833333	12.7240174666667	13.2513884875	12.8457431541667	12.4707841208333	12.8767230791667	13.3549065	13.0139587958333	12.8621393875	13.2322771916667	12.746570275	13.0304394833333	13.1277167583333	13.1373006958333	13.1855235875	12.5233989666667	12.9909275041667	12.8049364083333	13.5548125666667	13.1226317791667	13.0729777291667	13.0110927541667	13.0087094

RANBP9	12.4281341208333	15.0968513375	14.6229973208333	14.435416225	14.9375342458333	13.8456112875	14.4269781291667	14.8979679041667	14.4084687291667	14.3539205333333	13.7307989	12.9320596125	14.53483965	14.6640012125	14.834985	14.489995425	13.0623209	14.6294728791667	15.0200546125	14.8930132291667	14.0321124625	14.9991992666667	14.7908835958333	13.8988064875	14.7066709916667	13.0081306708333	14.6271940916667	14.7462478083333	15.199384425	14.7003845958333	14.6497610125	14.7327866333333	14.4714120708333	12.8336866291667	12.9320596125	12.6869957833333	15.1477739416667	15.0443403541667	14.44354915	14.4642959333333	14.7462478083333	15.07184225	14.7398420916667	14.2110759625	14.0546587541667	14.7987721708333	14.7934297125	14.97937325	14.5295676666667	14.3146441458333	14.4404566458333	14.6580212458333	14.7195864708333	14.8830862	14.4917411916667	14.3669169333333	14.5149754666667	15.400251175	15.2244796125	14.4369887875	12.66823485	13.1333095958333	12.717755275	13.2694207583333	13.1775302416667	12.6519795833333	12.6889059625	12.6086270791667	12.7418902125	12.808331825	13.3154975291667	12.8243259041667	12.6006538583333	13.1339977625	12.712559325	12.7146663083333	13.1698093875	13.0963431125	13.0208158125	12.9519194583333	13.1836216458333	12.6834027	13.0225453583333	13.2309913833333	13.3043630333333	13.1816528708333	12.44373405	12.9027308333333	12.2617779791667	13.0316461208333	12.6692120791667	12.9502654625	13.5112304791667	12.7772084333333	12.6439553916667	12.7772084333333	13.2825576083333	12.6378885833333	12.7240174666667	13.2513884875	12.8457431541667	12.4707841208333	12.8767230791667	13.3549065	13.0139587958333	12.8621393875	13.2322771916667	12.746570275	13.0304394833333	13.1277167583333	13.1373006958333	13.1855235875	12.5233989666667	12.9909275041667	12.8049364083333	13.5548125666667	13.1226317791667	13.0729777291667	13.0110927541667	13.0087094

PIGC	8.4156859875	7.82514295833333	7.66416018333333	7.93252685	7.36848873333333	7.42816177083333	7.6973221375	7.55899445	7.7605863625	7.35319639166667	7.4241413125	7.80300961666667	7.6470769875	7.99313109166667	7.87341445416667	7.23696419583333	8.86632373333333	6.92467640833333	7.76911651666667	7.65148490833333	7.5471865625	7.89803738333333	7.74867652916667	7.65690546666667	6.91421479583333	8.71358655833333	7.77178882083333	7.424735725	7.3613213125	7.77178882083333	7.80300961666667	7.23163065416667	7.78517137916667	8.0621243	8.46021965	8.57627579166667	7.2608000125	7.62680286666667	7.49137522083333	7.44230846666667	8.2127693	7.51338165833333	8.02975005416667	7.89748981666667	7.28164165833333	8.535535225	8.28922914166667	7.67953543333333	7.562423325	7.6851447	7.597444175	7.75146597083333	8.02646979583333	7.7095184125	8.07864384583333	8.1143043625	7.95772771666667	7.97790468333333	7.8583105	7.62680286666667	7.14488793333333	7.399511475	7.03723994583333	7.4593776375	8.88809269166667	7.602162725	7.6784664125	7.06366794166667	7.169402925	6.89165517083333	7.35777499166667	7.15484667083333	7.30888802083333	7.56864119166667	7.33943287916667	7.12753024583333	7.8075494	7.51612055416667	7.13879529583333	7.04235322916667	8.66130080833333	8.14472440833333	7.4293240625	8.013498425	6.9525504625	7.33943287916667	7.40746268333333	7.7605863625	7.10345715416667	8.10416175	7.21779185	6.79559300833333	7.6580112375	7.50259817916667	6.37900670833333	7.5951853375	7.4985282875	7.86402189583333	7.2036427625	8.23008068333333	8.3138718	6.71078762916667	7.9627096	8.16203617083333	6.57055390416667	7.73219098333333	8.06263146666667	7.99803280833333	7.73219098333333	8.1168333375	7.62447031666667	7.87341445416667	7.617860025	7.08080193333333	7.71441448333333	8.1650674375	7.67567321666667	7.01726715	7.26195752916667	7.34358252916667

DNM3	8.4156859875	7.82514295833333	7.66416018333333	7.93252685	7.36848873333333	7.42816177083333	7.6973221375	7.55899445	7.7605863625	7.35319639166667	7.4241413125	7.80300961666667	7.6470769875	7.99313109166667	7.87341445416667	7.23696419583333	8.86632373333333	6.92467640833333	7.76911651666667	7.65148490833333	7.5471865625	7.89803738333333	7.74867652916667	7.65690546666667	6.91421479583333	8.71358655833333	7.77178882083333	7.424735725	7.3613213125	7.77178882083333	7.80300961666667	7.23163065416667	7.78517137916667	8.0621243	8.46021965	8.57627579166667	7.2608000125	7.62680286666667	7.49137522083333	7.44230846666667	8.2127693	7.51338165833333	8.02975005416667	7.89748981666667	7.28164165833333	8.535535225	8.28922914166667	7.67953543333333	7.562423325	7.6851447	7.597444175	7.75146597083333	8.02646979583333	7.7095184125	8.07864384583333	8.1143043625	7.95772771666667	7.97790468333333	7.8583105	7.62680286666667	7.14488793333333	7.399511475	7.03723994583333	7.4593776375	8.88809269166667	7.602162725	7.6784664125	7.06366794166667	7.169402925	6.89165517083333	7.35777499166667	7.15484667083333	7.30888802083333	7.56864119166667	7.33943287916667	7.12753024583333	7.8075494	7.51612055416667	7.13879529583333	7.04235322916667	8.66130080833333	8.14472440833333	7.4293240625	8.013498425	6.9525504625	7.33943287916667	7.40746268333333	7.7605863625	7.10345715416667	8.10416175	7.21779185	6.79559300833333	7.6580112375	7.50259817916667	6.37900670833333	7.5951853375	7.4985282875	7.86402189583333	7.2036427625	8.23008068333333	8.3138718	6.71078762916667	7.9627096	8.16203617083333	6.57055390416667	7.73219098333333	8.06263146666667	7.99803280833333	7.73219098333333	8.1168333375	7.62447031666667	7.87341445416667	7.617860025	7.08080193333333	7.71441448333333	8.1650674375	7.67567321666667	7.01726715	7.26195752916667	7.34358252916667

DHRS7	13.3084273833333	12.9998495208333	13.1410113291667	13.314786925	12.8875397416667	12.6243321125	12.8638697333333	13.1929727375	12.9921400791667	13.1277167583333	13.0479655666667	13.1502785041667	12.5682987041667	13.231671575	13.0724085083333	13.2064901458333	15.1091028916667	12.8043830291667	12.3769610333333	13.2092710083333	13.2971953708333	13.1909476208333	13.12008425	13.4006070375	13.2845545333333	13.259164025	13.1162371958333	13.0880695375	8.79836918333333	13.1410113291667	13.0904272458333	13.0016229541667	13.1632069791667	13.9606550458333	12.8875397416667	13.2282872208333	12.9933025291667	13.1378947708333	13.2257349291667	13.038398325	12.9680656791667	13.2309913833333	13.2741458583333	13.29018665	13.3259523416667	13.5176217458333	12.9927160375	12.6584126541667	13.2282872208333	13.2631833708333	13.1977065333333	13.1936581125	13.1416728916667	13.002249725	13.2563969	13.0010166416667	13.2263610125	12.8484686083333	13.0539450833333	13.0605105	13.3998426833333	12.82594145	13.968870225	13.4123457916667	13.7041747291667	14.6958999333333	13.9802363708333	13.0917169875	13.6076869625	13.4582905333333	13.4278723083333	13.1730024083333	13.6680227291667	13.4795501458333	13.998416875	13.58805885	13.0310479208333	13.2463850541667	14.2976132166667	13.2158871875	12.577254275	12.7131405041667	14.1849839291667	13.6742503416667	13.0545636208333	12.9992294125	12.322224925	13.7697424	7.59573586666667	13.5291244625	13.324516875	13.7388256041667	13.0898441583333	13.4553955875	13.8225883375	13.1502785041667	13.5431479958333	12.6926443458333	12.9815301	12.5592610958333	13.8944945833333	12.8767230791667	12.3005466833333	12.8518669958333	13.1127745958333	13.5209467666667	13.8333038625	14.6893914541667	13.432579175	13.8411551708333	12.9536648666667	12.5616518208333	13.9547957	13.8872278125	13.21321275	13.5837958916667	12.6849372666667	13.6592126041667	13.5934627166667	12.9632195291667

PCNX4	13.3084273833333	12.9998495208333	13.1410113291667	13.314786925	12.8875397416667	12.6243321125	12.8638697333333	13.1929727375	12.9921400791667	13.1277167583333	13.0479655666667	13.1502785041667	12.5682987041667	13.231671575	13.0724085083333	13.2064901458333	15.1091028916667	12.8043830291667	12.3769610333333	13.2092710083333	13.2971953708333	13.1909476208333	13.12008425	13.4006070375	13.2845545333333	13.259164025	13.1162371958333	13.0880695375	8.79836918333333	13.1410113291667	13.0904272458333	13.0016229541667	13.1632069791667	13.9606550458333	12.8875397416667	13.2282872208333	12.9933025291667	13.1378947708333	13.2257349291667	13.038398325	12.9680656791667	13.2309913833333	13.2741458583333	13.29018665	13.3259523416667	13.5176217458333	12.9927160375	12.6584126541667	13.2282872208333	13.2631833708333	13.1977065333333	13.1936581125	13.1416728916667	13.002249725	13.2563969	13.0010166416667	13.2263610125	12.8484686083333	13.0539450833333	13.0605105	13.3998426833333	12.82594145	13.968870225	13.4123457916667	13.7041747291667	14.6958999333333	13.9802363708333	13.0917169875	13.6076869625	13.4582905333333	13.4278723083333	13.1730024083333	13.6680227291667	13.4795501458333	13.998416875	13.58805885	13.0310479208333	13.2463850541667	14.2976132166667	13.2158871875	12.577254275	12.7131405041667	14.1849839291667	13.6742503416667	13.0545636208333	12.9992294125	12.322224925	13.7697424	7.59573586666667	13.5291244625	13.324516875	13.7388256041667	13.0898441583333	13.4553955875	13.8225883375	13.1502785041667	13.5431479958333	12.6926443458333	12.9815301	12.5592610958333	13.8944945833333	12.8767230791667	12.3005466833333	12.8518669958333	13.1127745958333	13.5209467666667	13.8333038625	14.6893914541667	13.432579175	13.8411551708333	12.9536648666667	12.5616518208333	13.9547957	13.8872278125	13.21321275	13.5837958916667	12.6849372666667	13.6592126041667	13.5934627166667	12.9632195291667

AURKB	7.30497195	9.87826785	9.95147331666667	9.865582425	10.2794538083333	9.41030455	9.87254320833333	8.87736359166667	9.442472475	10.2076634916667	10.002252325	8.86366274166667	9.58573579166667	9.79676919166667	9.865582425	8.80780606666667	8.41837	10.04588255	10.01936935	9.76350663333333	9.663801325	9.73658934166667	10.2481894083333	9.36666890833333	9.60499546666667	7.30371010833333	9.25028671666667	9.34750165	6.90781049166667	9.64199300833333	9.653557625	9.7036749	8.905745925	9.28929068333333	7.45966298333333	7.4493567	9.94551363333333	8.99346874166667	9.11077986666667	8.8072839	9.31629211666667	9.13739526666667	9.458203675	10.4515253333333	9.29885605	9.830308425	9.695341625	9.7241175	10.4249193833333	9.4510034	9.73788834166667	9.471100975	10.112191475	9.915416425	9.80399703333333	10.0242607916667	9.099726825	9.76219373333333	10.103849375	9.87164061666667	12.6581666416667	11.634108225	12.4136707916667	10.909815425	10.1469040583333	10.987327675	11.2157921833333	11.0142581583333	11.8737640583333	11.1173392166667	11.1018151	11.1803917416667	11.5287863916667	10.8537358333333	11.0315811583333	10.9831460083333	10.8619748083333	11.7063908333333	11.8078233833333	11.5440185916667	10.7287943416667	10.2599956916667	11.8130518166667	11.08338295	10.3225014083333	11.5695230416667	12.6734606	11.34033385	7.14213880833333	10.885465	10.97984555	11.9158492083333	10.7774965	10.4212307583333	10.5743323083333	11.14267695	10.0537308333333	12.0128290333333	11.50454985	10.671542925	11.63482985	11.5048915416667	12.3216200916667	11.141230475	11.873385625	11.0387117083333	11.3455869583333	12.1915147666667	10.7478338666667	11.1797537583333	10.1759041833333	11.99822175	11.9781875416667	11.8924112666667	11.9409022083333	10.917897925	11.5912797083333	13.3300671416667	12.1770216166667	11.7895299333333

ITPR2	8.200812875	10.31123355	10.1180361833333	10.0442462083333	9.26855390833333	8.90285985	9.45281165833333	9.700667025	9.97912404166667	9.21350573333333	9.21543308333333	8.04016346666667	7.87369893333333	10.1910863	10.3690418416667	9.93083546666667	9.29340571666667	9.36122138333333	10.1489854166667	9.77883299166667	9.64650925	9.79362293333333	9.27461214166667	9.68992429166667	10.0960991583333	8.24657145833333	10.0334908083333	10.0421767666667	9.119480775	10.135458025	9.766909275	9.97667879166667	9.87864789166667	9.16780343333333	8.37413723333333	8.975801525	9.84956451666667	10.1477211833333	9.68602584166667	9.728395625	10.28850615	10.3694253333333	10.17959865	9.77432975	9.692647075	10.1849087083333	9.94794899166667	9.81608086666667	9.70886765833333	9.84117550833333	9.4007109	10.1728980166667	9.38500366666667	9.603559825	10.382186225	9.909101975	9.9162906	10.0039229916667	9.58222316666667	9.748599325	7.009464425	7.84947208333333	7.62759090833333	6.86394090833333	11.4317096083333	6.95758655	7.846884125	8.27121185	6.80784224166667	7.89722555	7.11478095	7.65326568333333	8.3851619	8.398242975	7.8648195	8.40234485833333	7.433497275	7.52780409166667	8.54744824166667	7.62534816666667	9.63292783333333	7.690465575	8.512323475	8.32310249166667	8.36334496666667	7.9999538	7.80067161666667	8.60897659166667	5.53292085833333	8.75371965	8.32062069166667	8.333019375	7.62815665833333	8.99535305	7.06709683333333	7.57164108333333	6.76657179166667	8.0964417	8.33398970833333	8.80450975	8.80925365833333	8.348200375	8.85676560833333	8.264078275	8.68035370833333	7.76345526666667	8.40567906666667	8.53385956666667	8.1439842	9.09833678333333	8.35277756666667	8.77460095833333	8.3715767	8.87491901666667	8.717376625	8.68775965833333	9.52268223333333	8.460967325	8.32012975833333	9.1773608

AMOTL1	7.80563896666667	4.39670516666667	4.55184448333333	3.98152069166667	6.346676525	5.17265765	4.65070125	4.146963325	5.16838866666667	4.82464924166667	4.9426219	5.30771575	5.661700075	4.58982808333333	5.26570160833333	4.88687726666667	4.30527630833333	6.49170240833333	4.90470955	4.98545498333333	4.55466009166667	6.462885175	5.108231475	4.59179895833333	6.05307568333333	6.10477214166667	5.07107728333333	5.40932128333333	6.241933725	4.68262605833333	5.99672560833333	5.066744825	5.46375123333333	4.58881544166667	6.94120649166667	6.74410944166667	5.46375123333333	4.54864098333333	5.112251075	5.54102934166667	5.34622214166667	5.4789746	5.256776325	4.262047775	4.62375229166667	4.65478205833333	5.72686785	4.199161275	6.32175328333333	4.789978375	6.60472939166667	5.76350409166667	6.05225019166667	6.42634760833333	4.96638246666667	6.23950985833333	5.08063435	5.18885664166667	5.5633586	6.616187575	4.337410475	3.90354184166667	5.66082913333333	4.84820759166667	4.66727606666667	7.171442675	4.36495859166667	4.7006097	5.71416341666667	5.197537	4.59179895833333	4.85861940833333	5.43182890833333	5.06113309166667	4.6622505	4.86085110833333	4.98545498333333	5.755893075	6.54089473333333	4.258212325	5.17603111666667	5.65521334166667	4.25488725	5.020575025	5.36511784166667	4.31116040833333	5.52545381666667	3.973735825	4.768670525	5.258661775	4.64613160833333	5.78268739166667	7.0307229	5.32134071666667	6.06833519166667	4.97490869166667	5.77807068333333	6.19964000833333	6.62496390833333	6.02558836666667	4.81075010833333	4.88576699166667	3.975999075	5.14718730833333	4.70371486666667	6.27314985	6.08509065	4.28008616666667	4.21300761666667	4.508464425	5.04458405	4.31295405833333	6.31702735	6.25427673333333	4.86589074166667	5.3960318	5.94277896666667	6.54716043333333	5.29356806666667	4.67471653333333

HBA2	12.4255097666667	11.0162524875	11.9125951791667	12.7235509375	10.0625726666667	10.5908592791667	10.7476260458333	11.7646426041667	12.3433234166667	14.3900386875	10.2776143791667	13.6771723125	13.8499988916667	10.555538	8.75256491666667	10.9849424916667	12.4427799125	11.877778675	11.6188988458333	11.6203840583333	10.3842848208333	11.8613787333333	11.0237761875	12.197997975	9.88188413333333	12.822684975	15.5241797291667	11.3711247625	11.0077341333333	9.94771790416667	12.0939926208333	10.532849475	10.4673265333333	11.1754809916667	14.0484738916667	12.33849745	10.8145005041667	11.3211200791667	11.5867600041667	13.7896392541667	10.9584088125	12.4406170791667	12.3090903083333	11.51451435	11.9265147625	12.3507445583333	12.0841727458333	10.7540292916667	11.5434620333333	12.7561424958333	12.1534868458333	11.4212513291667	12.5441612375	10.2369336666667	10.3110616	12.1428747541667	12.1486557291667	11.3807334125	10.5024214375	9.17073914583333	8.71358655833333	9.35053259166667	11.0169557958333	12.4099116375	9.48586814583333	10.0530970875	10.1571581625	10.4167078833333	11.7653685166667	9.58456951666667	9.72430455833333	9.2185896875	11.8983486791667	10.2660331083333	8.47309492083333	9.64005285	9.71932142083333	13.3649595333333	11.9327416416667	9.80685600833333	8.64220767916667	9.05892974583333	9.69015381666667	12.3835057041667	13.6250524208333	10.2029059208333	11.4969821416667	10.7297003833333	11.7181099041667	10.261163825	9.94359062916667	11.62075455	10.5032336333333	10.8629095125	10.46699275	10.8396074291667	9.21518684583333	9.48629382916667	12.9227561958333	11.0977296791667	10.4047014541667	10.0270868791667	9.72595660833333	11.0043908208333	10.3445012416667	11.0478556583333	10.0809167666667	10.25291345	9.12440923333333	12.8627241666667	11.097349025	14.4337656916667	9.88020543333333	11.2476313833333	9.36870691666667	10.3429838208333	10.2906363916667	11.4312072958333	11.2709514541667	8.7876802625

HBA1	12.4255097666667	11.0162524875	11.9125951791667	12.7235509375	10.0625726666667	10.5908592791667	10.7476260458333	11.7646426041667	12.3433234166667	14.3900386875	10.2776143791667	13.6771723125	13.8499988916667	10.555538	8.75256491666667	10.9849424916667	12.4427799125	11.877778675	11.6188988458333	11.6203840583333	10.3842848208333	11.8613787333333	11.0237761875	12.197997975	9.88188413333333	12.822684975	15.5241797291667	11.3711247625	11.0077341333333	9.94771790416667	12.0939926208333	10.532849475	10.4673265333333	11.1754809916667	14.0484738916667	12.33849745	10.8145005041667	11.3211200791667	11.5867600041667	13.7896392541667	10.9584088125	12.4406170791667	12.3090903083333	11.51451435	11.9265147625	12.3507445583333	12.0841727458333	10.7540292916667	11.5434620333333	12.7561424958333	12.1534868458333	11.4212513291667	12.5441612375	10.2369336666667	10.3110616	12.1428747541667	12.1486557291667	11.3807334125	10.5024214375	9.17073914583333	8.71358655833333	9.35053259166667	11.0169557958333	12.4099116375	9.48586814583333	10.0530970875	10.1571581625	10.4167078833333	11.7653685166667	9.58456951666667	9.72430455833333	9.2185896875	11.8983486791667	10.2660331083333	8.47309492083333	9.64005285	9.71932142083333	13.3649595333333	11.9327416416667	9.80685600833333	8.64220767916667	9.05892974583333	9.69015381666667	12.3835057041667	13.6250524208333	10.2029059208333	11.4969821416667	10.7297003833333	11.7181099041667	10.261163825	9.94359062916667	11.62075455	10.5032336333333	10.8629095125	10.46699275	10.8396074291667	9.21518684583333	9.48629382916667	12.9227561958333	11.0977296791667	10.4047014541667	10.0270868791667	9.72595660833333	11.0043908208333	10.3445012416667	11.0478556583333	10.0809167666667	10.25291345	9.12440923333333	12.8627241666667	11.097349025	14.4337656916667	9.88020543333333	11.2476313833333	9.36870691666667	10.3429838208333	10.2906363916667	11.4312072958333	11.2709514541667	8.7876802625

NRIP1	8.283955225	7.334392275	7.40257545	7.14458345	7.84741366666667	7.68936630833333	7.3032043	7.4784294	7.04898169166667	7.093633575	7.78546004166667	7.78488271666667	7.93282061666667	7.379877425	7.71969125833333	7.310825425	7.53912439166667	8.69363141666667	7.24765453333333	7.101860325	7.03502525	7.650219825	6.975151325	8.222543225	7.61865353333333	7.483605675	7.89979373333333	7.698105675	7.31316353333333	7.41243761666667	8.2549704	7.643842075	7.63934964166667	7.40375466666667	8.05804941666667	7.59420499166667	8.17036706666667	7.26808264166667	7.91465324166667	7.37359066666667	6.44355459166667	7.41007171666667	7.28864350833333	7.03569371666667	6.86328933333333	8.37211495	6.88940976666667	6.95860073333333	7.35862363333333	7.38341315833333	7.587363525	7.88761391666667	7.35396276666667	7.70589544166667	8.07938845	7.06834181666667	7.388741125	7.27114775833333	7.71472995833333	7.11789028333333	9.74433669166667	7.06834181666667	7.600828425	7.98717739166667	6.60761675	9.24586054166667	7.6315346	8.18891301666667	7.86334303333333	7.578462725	7.8648195	8.16735940833333	7.37359066666667	7.32668075833333	7.52566054166667	8.27954998333333	7.9760917	8.416908275	8.34923416666667	7.51696985	7.59366683333333	7.41824386666667	8.38422716666667	7.87869541666667	7.71570814166667	7.0633355	7.52107438333333	7.434165875	7.51995818333333	7.52566054166667	8.20227615	7.89028620833333	8.751330725	7.68042426666667	8.62734905	8.80050660833333	8.432956925	7.33026348333333	8.18422995833333	7.13033303333333	7.82958231666667	7.678740925	7.52330799166667	7.24330025	7.86670446666667	8.92218960833333	8.23767453333333	7.37467966666667	7.726376575	7.37296829166667	7.68993234166667	7.928121775	8.02019604166667	7.53069666666667	8.560933175	8.01861064166667	7.238929725	8.58441085833333	8.259343925	7.20747220833333

PARP16	10.3369395416667	10.0757309083333	9.921332825	10.1452230583333	9.39753216666667	9.72033719166667	9.85198440833333	9.612283675	10.066608375	9.71527460833333	9.53356816666667	10.05157835	9.67192385833333	10.1783585083333	10.0242607916667	9.889693225	10.8713916666667	8.97905946666667	9.82860243333333	9.77432975	9.84285273333333	10.116767025	10.092918675	9.35357573333333	9.6543889	10.5651423416667	9.7570522	9.81899704166667	6.69991303333333	10.2115540166667	9.57093504166667	10.02519555	9.75367038333333	10.1299873833333	10.6226410916667	10.5530348	9.38667448333333	9.361656325	9.19719063333333	9.53997905833333	9.810451275	9.899420875	9.93673649166667	10.04588255	9.95935071666667	10.3044270083333	10.01207205	9.49101396666667	10.0972464083333	9.91241780833333	9.33411035833333	9.82295156666667	9.59824975833333	9.32711606666667	9.984272	9.89226535833333	9.679927325	9.895935425	9.921332825	9.58797373333333	9.41162929166667	9.862235575	9.8834328	8.60947813333333	10.2399871166667	9.82250195	9.83153578333333	9.80350678333333	9.28405401666667	9.50353634166667	9.69707035833333	9.277313475	10.01936935	9.73395598333333	9.05497765833333	9.84332440833333	9.54092946666667	9.313630975	9.20284239166667	9.84919238333333	10.1448522166667	8.8778449	9.567278375	9.72574979166667	9.538317275	9.91585179166667	10.9393869333333	9.42590730833333	6.06914035833333	9.8942397	9.36892748333333	9.45472053333333	9.82860243333333	10.1100737	9.30028739166667	9.524079925	8.44794389166667	9.80231140833333	8.59201043333333	10.054920625	8.9493948	9.970867025	10.6076090916667	10.33090645	9.94794899166667	9.99259065	10.1405016833333	8.45556428333333	9.7400082	9.28785676666667	10.08353915	9.90222311666667	8.95718403333333	9.37921308333333	9.13226131666667	10.2919749833333	10.29990335	9.13172414166667	9.87739761666667	9.945018575

FCGR1B	5.31319136666667	9.37403768333333	9.44516316666667	8.907131825	9.71098871666667	8.44491509166667	9.6324307	8.89602301666667	9.437687475	8.82781555833333	8.35277756666667	7.652791	7.85104381666667	9.2436075	10.2186879083333	8.38817050833333	8.25546045	8.48368870833333	10.07610735	9.173253675	8.76421910833333	9.81483478333333	8.460967325	8.04804935	8.76898080833333	6.82704920833333	9.22061473333333	9.75110441666667	9.78737088333333	9.35773556666667	8.06759891666667	8.512323475	8.02070828333333	6.82820395	7.03630040833333	6.728950375	10.164124825	8.651213975	7.00303549166667	8.51528900833333	9.4955569	9.61367635	8.56272661666667	8.12110685	7.62037211666667	9.354988125	9.55566569166667	9.37269289166667	7.48927714166667	8.32463409166667	8.7143253	8.92929650833333	9.0772993	9.6705637	9.16352800833333	8.97905946666667	8.19808664166667	9.59782546666667	9.538317275	8.74370115833333	9.066471675	8.18527721666667	9.22061473333333	6.60761675	11.2668058583333	9.62407518333333	8.325164675	9.10576088333333	8.11057064166667	8.135196525	7.38341315833333	8.00359983333333	7.85689433333333	9.17515170833333	8.105836875	7.84323385	7.5512356	8.74620478333333	7.8345672	7.77208035833333	8.60435615	9.85326255	8.91556449166667	6.85814304166667	9.04692176666667	8.34473695	8.25794470833333	7.11080571666667	9.47257300833333	7.31803023333333	7.35173706666667	6.89003731666667	5.20354711666667	6.96848423333333	8.661516725	8.62596224166667	6.869498575	9.26899234166667	8.2549704	7.46194565833333	7.47677956666667	8.046473725	8.52683058333333	8.54656363333333	7.35862363333333	8.7954535	6.81976344166667	8.75047631666667	7.13667263333333	7.24945885833333	7.093633575	8.40567906666667	9.72286421666667	7.89557763333333	7.3694039	7.42447115	5.978682025	7.001733525	7.03751019166667	8.33702585833333

MZT2A	14.0707149083333	14.0759487166667	14.177662	14.3875977	13.65238595	13.864749275	14.00243415	13.977355925	14.1972150333333	13.9785722666667	14.0225032583333	13.487986925	13.9097458666667	14.1441992083333	14.204528175	13.6375130583333	14.4125419416667	13.8698539083333	14.2305624666667	13.9967915416667	13.7452882916667	14.0237926083333	14.156645775	13.756296825	14.274700225	13.7463275333333	13.6876489583333	13.9378093083333	13.7093807	14.0429420833333	13.9119804333333	13.9411608583333	13.99785205	14.0607265833333	13.9785722666667	13.8743989166667	14.1441992083333	13.934166025	14.0001337166667	13.96485035	14.120388025	13.8562499666667	14.0938637416667	14.2245923416667	14.0377493833333	14.1486654166667	14.2563286	13.89505895	14.190108625	13.9207353166667	14.0301250916667	13.7234378333333	14.364637875	13.8285300416667	14.3045490583333	14.021264575	13.8199392	14.0526804666667	13.57301355	14.0174773166667	13.9695187666667	14.2487820083333	13.8854083166667	13.8157854333333	14.500239075	14.015040275	14.1513249333333	13.7791494	13.8516367666667	14.045461125	14.2334791	14.145758825	13.811693625	13.9097458666667	14.0377493833333	14.29207405	12.9253738	13.9529996833333	13.7771113166667	14.1086467166667	14.4809973583333	13.450467225	13.8983181916667	13.9045177166667	13.3508640083333	13.782971725	14.3204037083333	13.747278475	14.004911475	13.6000874166667	14.0418505333333	14.0540171833333	13.439923175	13.5502258583333	13.6302168166667	14.0618019166667	13.6401687916667	13.9025146916667	13.9469444833333	13.6596320166667	13.986279475	14.1292641	14.1329416	13.6328876166667	13.79195525	13.9917082333333	13.6675870333333	13.838661325	13.934166025	13.2822330583333	13.2128605083333	13.9493522333333	13.1700826333333	14.2763428833333	13.4254222333333	14.4871709833333	13.9055869333333	14.2219861583333	14.27950095	13.834699275

EFNA1	8.20831443333333	6.42634760833333	7.81610706666667	9.01655344166667	6.53137754166667	10.51932965	7.57732030833333	8.17990050833333	9.67501213333333	8.21782325833333	9.931739675	8.38217830833333	7.20393616666667	6.051276375	7.393964525	7.24514076666667	8.15707283333333	11.4170295666667	7.177906325	5.92148618333333	7.338007825	7.21570014166667	6.82102155833333	8.35736911666667	9.62064585	8.28143178333333	6.78642360833333	8.99116335833333	7.43179573333333	7.75234836666667	7.569973	7.153195325	10.4292877666667	8.66481208333333	8.48569109166667	8.126799325	7.21926184166667	7.80067161666667	9.19576385833333	7.597705475	8.59201043333333	7.42048124166667	7.24263133333333	7.95255818333333	8.754236775	6.42781263333333	5.891995425	6.869498575	6.59464205	10.08403975	9.17190161666667	7.24514076666667	7.06888018333333	7.62704748333333	7.50801045833333	7.49826404166667	6.10156668333333	6.86736839166667	7.597182875	7.67474618333333	11.21620365	7.537939	8.151087675	8.76703910833333	8.4816187	10.8206218083333	10.2966490166667	9.52316788333333	9.16167278333333	8.95312890833333	9.699723	9.3375044	8.953569225	8.71882579166667	8.80300369166667	8.50286535	8.0773066	8.58538408333333	7.990761025	7.61126938333333	7.79244525833333	9.56407003333333	8.43351543333333	8.14849655833333	10.4008623166667	8.21304901666667	7.01007860833333	9.452370525	8.3876511	9.04278656666667	8.84195500833333	10.061572075	8.771928675	9.53443595	9.512679675	8.78890531666667	8.34279256666667	8.23217175	10.151318175	8.86902108333333	8.254482075	7.66500309166667	8.30804240833333	7.190008575	8.850647275	8.58349254166667	8.90864591666667	7.703109475	7.26690018333333	8.72403179166667	8.435679275	6.939986925	8.252426875	7.84376875833333	8.76751969166667	8.38960981666667	7.83348010833333	8.94143755	8.30538075	7.7445795

FAM91A1	9.98111111666667	9.79486831666667	9.778305875	10.0099661	9.899420875	9.53312831666667	8.97076639166667	9.94460186666667	9.40469768333333	9.38057958333333	9.812148775	9.5453508	9.76472428333333	9.69349788333333	9.876036325	10.0364758583333	9.80664778333333	10.1405016833333	9.859391125	9.661950625	10.1501004333333	10.0537308333333	10.306601825	10.4678765666667	9.97174644166667	9.6543889	9.71864728333333	9.91842590833333	10.1581087416667	10.2434985083333	10.0557486666667	10.0778458	9.337966225	9.40774255833333	9.21835433333333	9.43278838333333	9.611823	9.73142488333333	9.9519621	9.80802880833333	9.67854975	9.585238675	9.88083685	9.98715419166667	9.64383103333333	9.781927775	10.1910863	10.4272415916667	9.683521475	9.61596005833333	9.862235575	10.1779292166667	9.7928167	10.3007165083333	10.2494268583333	9.99468328333333	10.2547426166667	10.068396225	9.653557625	10.01936935	10.1593240833333	10.591824075	10.8468748916667	10.9328378333333	10.2850173	10.7706214833333	11.0491091083333	10.5268243666667	11.0880868	9.97583196666667	10.4215953	10.917897925	10.4456717166667	10.5512664916667	10.1919662416667	10.949025	10.32163045	10.5743323083333	10.9757948333333	10.6035239083333	10.6554394166667	10.8850875833333	11.152834825	11.1692562416667	11.0006123833333	10.1489854166667	10.4176619666667	11.096135525	10.2089178583333	10.5181477	10.7448403666667	11.3801476916667	10.3750050916667	10.42200985	10.8710123583333	10.5352797666667	10.613570925	9.482987475	10.8386147583333	10.6488037166667	10.98655615	9.76472428333333	11.0352265083333	10.45072255	10.4702277083333	11.0704861666667	10.7904047166667	11.0851990416667	10.64807625	10.575694825	9.93304795	10.2321384166667	11.3125544916667	11.233092075	11.2689689833333	10.6952813416667	9.75279813333333	11.3360764166667	11.22691525	9.97301156666667

CNOT1	6.83868629166667	7.00630173333333	7.55551181666667	7.30019385833333	7.90032115	7.42162103333333	6.986635125	6.76187970833333	7.09043023333333	6.73694150833333	7.18402409166667	6.71186961666667	7.32919075833333	6.9125939	7.80451180833333	6.93552516666667	6.241933725	8.59099210833333	7.03321648333333	7.51524328333333	7.01426715833333	8.42877934166667	6.99919410833333	7.71969125833333	7.83780880833333	6.60536920833333	8.09066366666667	7.76032476666667	8.230344325	7.53565475833333	7.4690921	8.03327618333333	7.442585225	7.265200275	7.15940084166667	6.80918875	8.44632826666667	7.505130225	7.83348010833333	7.25799328333333	7.23193100833333	7.12280756666667	7.16971491666667	6.94244640833333	6.46851783333333	6.45250163333333	6.19454715	6.89322066666667	7.27714694166667	7.57402961666667	7.34385484166667	7.18584309166667	7.765634325	8.06026415833333	7.20088341666667	6.91766443333333	7.655449725	6.90845738333333	7.39926451666667	6.94497745833333	8.20587135	7.04503860833333	7.71862859166667	6.91035588333333	6.68532921666667	8.511336025	6.5680586	7.23609219166667	6.22304681666667	7.24193815	7.05838413333333	7.48759605	7.09162461666667	7.38123391666667	7.13667263333333	7.191921025	7.14822965	8.37864189166667	7.452175025	7.15147738333333	6.258372575	7.79194111666667	7.00630173333333	6.877100425	7.94273865833333	7.08933194166667	7.28500960833333	7.08866475	7.95359058333333	7.173894	7.32376110833333	7.79527465	7.85469968333333	7.97141813333333	8.22500544166667	7.97557605833333	7.72139515	7.60888643333333	7.97812778333333	6.559370075	7.40662056666667	6.68342536666667	7.77660473333333	6.815678575	7.29843466666667	7.83671455833333	6.85478974166667	6.811853775	6.591129625	6.97585598333333	6.894639675	6.64292103333333	7.47900525	6.67442865833333	7.5998906	7.02536274166667	6.861457125	7.4493567	7.48476935	7.35627839166667

TLE4	6.698517925	5.94277896666667	4.84714924166667	5.23876719166667	7.20693700833333	6.98853248333333	6.27872079166667	5.10174316666667	6.17635683333333	7.05156345	6.50650844166667	6.582876425	5.201606925	5.6488719	6.20262720833333	6.456329775	7.21695988333333	7.98243114166667	6.85478974166667	6.39152516666667	6.50418884166667	7.51995818333333	6.02974674166667	7.0345033	7.204487875	7.03138465	7.3694039	5.95574085833333	6.96183981666667	6.98158215833333	6.45093723333333	6.90781049166667	7.55925806666667	6.998615075	6.49825018333333	6.75368535833333	7.31861950833333	6.823097925	6.65024493333333	6.99218681666667	5.91988768333333	5.892821925	6.11631895833333	5.92679064166667	6.28823875	6.91190293333333	6.75170075833333	5.97595255	7.83726095833333	7.11730268333333	7.18035421666667	6.73133699166667	6.47216713333333	6.86530675833333	6.73349835	5.5022085	6.013009825	6.4308335	7.71034295833333	7.60483248333333	7.02148474166667	6.86262681666667	6.74082630833333	5.48415848333333	6.21427064166667	7.17914503333333	6.74821494166667	6.17154675833333	6.03147445	6.662431575	6.43934115	6.675144925	5.23781710833333	6.41270695833333	6.83207999166667	6.5607394	6.777132775	7.95255818333333	7.348327125	6.76587266666667	5.71795834166667	5.83208956666667	6.64956780833333	6.66054216666667	6.30781043333333	5.88521655833333	5.978682025	5.79203019166667	6.585636975	7.2954331	6.66522548333333	6.96970946666667	7.13351973333333	7.02284831666667	7.39564355833333	6.54574596666667	7.59366683333333	6.75170075833333	6.83931143333333	5.34622214166667	7.07722264166667	6.47456359166667	7.2401722	6.61087155833333	6.74158396666667	6.66666621666667	6.87089175833333	7.48927714166667	6.86668814166667	7.36707339166667	7.04632523333333	6.772445575	7.97812778333333	6.10074335833333	5.72187083333333	6.5479092	6.256698975	8.18363113333333	7.66108944166667	6.61558093333333

EFNA5	7.84545261666667	8.18315743333333	8.555975275	8.00687400833333	9.39843498333333	8.34324809166667	8.87834883333333	8.16529418333333	8.42733516666667	8.50894728333333	8.21929578333333	8.0095181	8.551483425	8.49648456666667	9.131237375	7.72023940833333	8.06854219166667	9.3041126	8.873364875	8.518147825	8.01700516666667	9.34710834166667	7.517539575	8.268113325	8.861626075	7.137920375	8.61571535833333	9.10796010833333	10.0238668333333	8.22641101666667	8.20031584166667	8.66441525833333	8.19188733333333	7.73690679166667	7.88978783333333	7.95517108333333	10.0360710333333	8.65698328333333	8.07050834166667	8.4100903	8.15349528333333	8.45451465833333	8.028895175	7.92005815	7.771005975	8.01434291666667	8.40424725	8.7655611	8.03913049166667	8.306964525	8.2260001	8.87086751666667	9.35451149166667	9.5295644	9.01942851666667	8.57007116666667	8.18527721666667	8.604857825	8.7637709	8.31305365833333	8.745766675	7.99508275	8.52826969166667	7.06942048333333	7.466283575	9.32711606666667	8.44632826666667	7.48528250833333	8.409150775	7.75064413333333	8.20124505	8.31252919166667	7.7113432	7.79957504166667	8.05032904166667	7.95255818333333	8.36713998333333	8.62986514166667	8.43510350833333	8.37604669166667	7.338007825	8.13304300833333	6.98096124166667	7.54566714166667	8.701251675	7.8747259	7.36471060833333	8.00852153333333	9.46074875	7.639894925	7.93069544166667	8.33810663333333	8.53632365833333	7.984047875	8.764687525	7.3675475	8.05296578333333	8.57649310833333	7.9999538	7.77208035833333	7.95311691666667	6.62367098333333	7.38926633333333	7.967895825	7.832972775	8.60646375	8.04330091666667	8.55543884166667	8.59697231666667	8.20689531666667	7.55486798333333	8.57834244166667	8.41158014166667	8.62383624166667	8.11712540833333	8.64093518333333	7.794082525	8.31058729166667	8.800966875	8.25398819166667

MTRF1L	9.94551363333333	9.7097404	9.51996269166667	9.36715445	9.59108196666667	9.498614875	9.54039348333333	9.8795664	9.112227825	9.61413186666667	9.45859685833333	9.18071311666667	8.91742895833333	9.34207146666667	9.72033719166667	9.37457540833333	9.777390875	9.38500366666667	10.007360325	9.79573791666667	9.77049406666667	9.701538725	9.848037125	10.20226945	9.80448268333333	10.670739975	9.508670075	9.70842734166667	9.58840303333333	10.1295520916667	10.0736918083333	9.72531675833333	9.32760176666667	9.485645225	10.7001015833333	10.5330763833333	9.25814955833333	10.0197701416667	9.963087575	9.52364006666667	9.80625096666667	9.82203395	9.3832069	9.85113778333333	9.61323428333333	9.92583431666667	9.72795155833333	10.2383473416667	9.77090350833333	9.88925331666667	9.88925331666667	10.159727225	9.24845199166667	9.68558669166667	9.431383225	9.47987389166667	9.993864975	10.0095607333333	9.86598649166667	9.70584026666667	8.996715525	9.734421025	9.54880464166667	9.0180261	9.72703330833333	9.51088574166667	9.77312443333333	8.5232739	9.490132625	8.98889158333333	9.11864413333333	9.25440600833333	9.24273069166667	9.14145633333333	8.95218464166667	9.4999428	9.26261210833333	9.36621750833333	9.848037125	9.77974975833333	10.5477462083333	9.6176772	9.59108196666667	9.91460061666667	9.318611725	9.71356900833333	9.536457975	9.349435025	9.045519875	9.850735	9.468356875	9.51811660833333	9.855976425	9.50264109166667	9.460327375	9.20413733333333	9.93476125833333	9.15113736666667	9.67230795	9.587537375	10.1117927583333	9.14608020833333	10.1737764166667	9.81132868333333	9.57222860833333	9.63613316666667	9.50224903333333	10.3436245833333	9.62712280833333	10.1637531083333	9.34438068333333	10.0493818	10.4716937833333	10.054135025	9.23235029166667	9.57414756666667	9.98268700833333	10.2060439416667	9.75665824166667	9.96267133333333

GOLIM4	12.2901521833333	10.6425896916667	10.8113265583333	9.925380825	11.5071025416667	12.9527510916667	11.4207192166667	11.73798855	11.62870925	12.13546815	12.400136275	12.815500575	11.7878982583333	11.4188332333333	11.39829615	11.9467504166667	13.3732212583333	11.82248265	11.8815198166667	12.3292738666667	11.7849428666667	10.8109394916667	11.7928828083333	11.3275163916667	11.5056318416667	12.4350665416667	10.81355685	10.723966225	10.9824445416667	11.3534958083333	10.9571307916667	11.0968033583333	11.8519854833333	12.9423278583333	12.497697975	12.5516382166667	11.0390903416667	11.7097839833333	11.5345546416667	11.5305817083333	11.2689689833333	10.96888565	11.4586326	11.055301575	11.2858355	11.171764175	11.2808460833333	11.1132646583333	11.9621161833333	12.1843992666667	11.8422473333333	11.5716921416667	9.55244275833333	9.21835433333333	9.74951903333333	9.7815357	11.465822775	12.0490448333333	11.76865155	11.9212294333333	12.43292905	12.62157875	11.2675581916667	11.735525225	13.7820292333333	13.3114725833333	13.5884582166667	13.6392528333333	12.2433310083333	12.9876385	13.0325393583333	13.5150003833333	12.0356360333333	12.8256923916667	12.84211235	13.3682726083333	13.0960317	12.9677883916667	12.8515337583333	12.6581666416667	12.912870175	13.3594846333333	11.9513573	12.932363575	12.2896477166667	12.7558773833333	11.8888404416667	12.1367818666667	12.7263840666667	13.0613877916667	12.0265627166667	12.0518595833333	12.6709351583333	12.8415457916667	13.21039535	13.0089762083333	12.458934675	12.6391509	12.31433185	12.844414625	12.2844611166667	13.0276586166667	12.4333458416667	12.280521525	11.8209586416667	12.2723412333333	12.000941175	11.2858355	12.4903405333333	13.4164430416667	12.634136475	13.2828821583333	10.8566946833333	11.3968955416667	10.6550211916667	10.501807225	14.0074276833333	12.301617475	12.671473625	12.3785017666667

PHLDA1	9.07777099166667	11.9131873583333	11.5920332333333	11.7418730833333	11.80438315	11.0769009083333	11.6470358	12.0449944666667	11.4771109	11.7589935083333	10.8071092416667	9.89466155833333	11.5027335583333	11.7572174666667	11.5816748666667	12.0168871666667	10.9843155	12.029837025	12.4581088416667	12.1142465833333	11.6948221166667	12.0193369	12.6442138916667	12.4941044416667	12.1237921916667	9.37777211666667	11.8713469833333	12.1737118583333	12.0985319416667	11.9813054166667	12.2998821166667	11.8884513166667	11.5738946	10.4138270833333	10.2434985083333	8.615263775	11.7422357083333	11.8321536333333	12.0490448333333	11.6529488416667	11.9318404166667	11.9274761416667	12.1283236333333	11.9871613166667	10.62706055	11.4912151666667	11.6859465333333	11.684519	11.8062314166667	11.3115193	11.8265346166667	11.8017051166667	11.5468691083333	11.868130425	11.7765043416667	11.8186960583333	11.8865138166667	12.0245529166667	11.8717593666667	11.3590797	9.6428903	10.7940186083333	9.71307581666667	11.1858855333333	11.3968955416667	11.658465575	11.276041	9.76819061666667	10.3678756583333	10.1993549333333	10.36526325	10.7800644666667	11.784206175	11.3942748	10.9861853	10.88817915	10.7407277583333	11.0689648166667	10.978750575	9.98633354166667	9.95147331666667	11.07367765	12.008092475	11.9165677	10.610413375	11.3253118333333	9.6811178	10.4998350333333	10.1919662416667	11.1354232166667	10.5787126833333	10.3641444833333	10.8900635166667	10.1050880666667	10.5833260083333	10.8828386666667	10.345102475	10.3333340666667	11.8202425833333	10.006608525	10.6834952083333	11.0934965333333	8.7217903	10.7356243	10.5334113083333	10.4324595166667	10.5876388833333	11.9828339166667	9.78018595	11.3065242916667	10.880387475	11.5783568666667	11.9816922	11.1281108083333	11.22691525	11.5531172416667	10.98875735	10.7842899166667	10.9091302666667	10.83116835

ELOVL1	9.26261210833333	9.14106573333333	9.35031244166667	9.64153864166667	10.0933465333333	8.8577648	10.8206218083333	9.56314586666667	9.970867025	9.573560175	9.654799725	10.8694922083333	10.3044270083333	9.50956853333333	9.311763425	9.151591475	8.96414444166667	10.420034375	8.93619846666667	9.000388675	9.67274871666667	9.41761155833333	9.12938660833333	8.67553405	8.884079375	9.07683584166667	8.81018689166667	8.85494786666667	9.24445845833333	9.07683584166667	8.65649723333333	8.70898641666667	9.132749475	9.05732553333333	8.8830474	8.96273033333333	9.38182149166667	8.64839240833333	8.78021899166667	8.77406471666667	8.6984679	9.028831875	8.38960981666667	8.54169179166667	8.56421206666667	8.38960981666667	8.06551123333333	8.85304851666667	8.650306125	8.53882584166667	8.87390605	8.86609368333333	8.73109978333333	8.46826041666667	8.647383475	8.31466204166667	8.82372205833333	8.66775465	8.66441525833333	8.39771189166667	9.730552775	9.674571275	9.3836955	10.31869075	9.53087495833333	9.6385431	9.97301156666667	9.89010159166667	10.061572075	9.69837386666667	9.85036418333333	9.86766510833333	10.345102475	9.445595775	10.0989513583333	9.53738644166667	9.77219498333333	9.37313166666667	10.081132625	9.90816476666667	9.57817145833333	9.73489294166667	9.53914050833333	8.81018689166667	9.52591685	10.3686089583333	9.95643681666667	9.75828255833333	9.721994775	8.650731025	9.349869075	9.18546986666667	8.87636833333333	8.90389271666667	9.38536081666667	9.55058418333333	9.57464318333333	9.82733675	9.60540180833333	8.612418275	8.66625304166667	9.83111589166667	8.7954535	8.54315244166667	8.770520625	9.04692176666667	8.89696199166667	8.65418485	8.99535305	8.54598220833333	8.64890015833333	8.46196905	9.13780860833333	8.31364723333333	8.62886449166667	8.44632826666667	9.24218355833333	8.82519364166667	8.649865175	9.36206593333333

B3GNT4	8.82621398333333	8.97769849166667	8.75927773333333	9.02034758333333	8.47797495833333	8.729717925	8.72830366666667	9.05776881666667	8.52966328333333	8.61979589166667	8.69172925	8.51713660833333	8.518147825	8.71039916666667	8.83968223333333	8.63825258333333	8.7292202	8.72874058333333	8.96369055833333	8.67598873333333	9.11582745	8.93073908333333	8.92792624166667	9.00507715833333	8.68035370833333	8.86508634166667	8.561306875	8.64585098333333	8.78836945	8.85728129166667	9.01340034166667	8.75743711666667	8.398242975	8.69127455833333	8.43095395833333	9.028831875	8.682286675	8.7217903	8.796402275	8.955351225	8.882160975	9.19118228333333	9.11824448333333	9.29340571666667	8.97489020833333	9.18974645	9.11902033333333	9.37728073333333	9.319021525	8.836922375	9.15064904166667	8.85494786666667	8.84155408333333	9.22874626666667	9.12700821666667	9.23330131666667	8.982853175	9.13226131666667	8.90668976666667	9.124627175	8.7954535	9.12281406666667	9.33087654166667	9.00913584166667	8.90619576666667	9.33964531666667	9.40774255833333	8.717376625	9.3590843	8.80003964166667	9.023211225	9.33411035833333	9.00822095	9.10015925	9.75787298333333	8.80450975	8.770520625	8.364240675	9.1396151	9.53312831666667	8.78264749166667	9.17457594166667	9.50586739166667	9.361656325	8.72037605	8.93359508333333	9.12794545833333	8.57257758333333	9.529086375	8.577338575	9.28405401666667	9.94840036666667	8.98618440833333	8.8830474	9.25764194166667	8.91690625833333	9.25253679166667	8.37604669166667	9.6530765	9.555182225	9.415734875	9.611823	10.308194675	9.76157617083333	10.0799055916667	9.296236625	9.64690625833333	10.1557652416667	9.69087439166667	8.98796298333333	9.337966225	9.46608615833333	9.55333915	9.70931241666667	9.72574979166667	9.68903575833333	9.7097404	10.2844102625	9.60963610833333	9.67634863333333

DIABLO	8.82621398333333	8.97769849166667	8.75927773333333	9.02034758333333	8.47797495833333	8.729717925	8.72830366666667	9.05776881666667	8.52966328333333	8.61979589166667	8.69172925	8.51713660833333	8.518147825	8.71039916666667	8.83968223333333	8.63825258333333	8.7292202	8.72874058333333	8.96369055833333	8.67598873333333	9.11582745	8.93073908333333	8.92792624166667	9.00507715833333	8.68035370833333	8.86508634166667	8.561306875	8.64585098333333	8.78836945	8.85728129166667	9.01340034166667	8.75743711666667	8.398242975	8.69127455833333	8.43095395833333	9.028831875	8.682286675	8.7217903	8.796402275	8.955351225	8.882160975	9.19118228333333	9.11824448333333	9.29340571666667	8.97489020833333	9.18974645	9.11902033333333	9.37728073333333	9.319021525	8.836922375	9.15064904166667	8.85494786666667	8.84155408333333	9.22874626666667	9.12700821666667	9.23330131666667	8.982853175	9.13226131666667	8.90668976666667	9.124627175	8.7954535	9.12281406666667	9.33087654166667	9.00913584166667	8.90619576666667	9.33964531666667	9.40774255833333	8.717376625	9.3590843	8.80003964166667	9.023211225	9.33411035833333	9.00822095	9.10015925	9.75787298333333	8.80450975	8.770520625	8.364240675	9.1396151	9.53312831666667	8.78264749166667	9.17457594166667	9.50586739166667	9.361656325	8.72037605	8.93359508333333	9.12794545833333	8.57257758333333	9.529086375	8.577338575	9.28405401666667	9.94840036666667	8.98618440833333	8.8830474	9.25764194166667	8.91690625833333	9.25253679166667	8.37604669166667	9.6530765	9.555182225	9.415734875	9.611823	10.308194675	9.76157617083333	10.0799055916667	9.296236625	9.64690625833333	10.1557652416667	9.69087439166667	8.98796298333333	9.337966225	9.46608615833333	9.55333915	9.70931241666667	9.72574979166667	9.68903575833333	9.7097404	10.2844102625	9.60963610833333	9.67634863333333

AC048338.2	8.82621398333333	8.97769849166667	8.75927773333333	9.02034758333333	8.47797495833333	8.729717925	8.72830366666667	9.05776881666667	8.52966328333333	8.61979589166667	8.69172925	8.51713660833333	8.518147825	8.71039916666667	8.83968223333333	8.63825258333333	8.7292202	8.72874058333333	8.96369055833333	8.67598873333333	9.11582745	8.93073908333333	8.92792624166667	9.00507715833333	8.68035370833333	8.86508634166667	8.561306875	8.64585098333333	8.78836945	8.85728129166667	9.01340034166667	8.75743711666667	8.398242975	8.69127455833333	8.43095395833333	9.028831875	8.682286675	8.7217903	8.796402275	8.955351225	8.882160975	9.19118228333333	9.11824448333333	9.29340571666667	8.97489020833333	9.18974645	9.11902033333333	9.37728073333333	9.319021525	8.836922375	9.15064904166667	8.85494786666667	8.84155408333333	9.22874626666667	9.12700821666667	9.23330131666667	8.982853175	9.13226131666667	8.90668976666667	9.124627175	8.7954535	9.12281406666667	9.33087654166667	9.00913584166667	8.90619576666667	9.33964531666667	9.40774255833333	8.717376625	9.3590843	8.80003964166667	9.023211225	9.33411035833333	9.00822095	9.10015925	9.75787298333333	8.80450975	8.770520625	8.364240675	9.1396151	9.53312831666667	8.78264749166667	9.17457594166667	9.50586739166667	9.361656325	8.72037605	8.93359508333333	9.12794545833333	8.57257758333333	9.529086375	8.577338575	9.28405401666667	9.94840036666667	8.98618440833333	8.8830474	9.25764194166667	8.91690625833333	9.25253679166667	8.37604669166667	9.6530765	9.555182225	9.415734875	9.611823	10.308194675	9.76157617083333	10.0799055916667	9.296236625	9.64690625833333	10.1557652416667	9.69087439166667	8.98796298333333	9.337966225	9.46608615833333	9.55333915	9.70931241666667	9.72574979166667	9.68903575833333	9.7097404	10.2844102625	9.60963610833333	9.67634863333333

PRIM1	10.1955368208333	10.85127885	10.9362758541667	10.926151175	10.532849475	10.6620163958333	10.2718185833333	10.591617975	10.5974997458333	11.2080698458333	11.0653444458333	9.96588614583333	9.85656001666667	10.9254252625	10.7513786875	11.0551007666667	11.4621649583333	10.4924386416667	10.3540126708333	11.1515491708333	10.9464365791667	11.0991457083333	11.1639329916667	10.268910725	11.0411335166667	10.1376630083333	10.7826252541667	10.6311434458333	10.5777799041667	11.03538055	10.675551525	10.8675350916667	10.4230034708333	11.2274614541667	9.94359062916667	10.4026659625	10.8606822291667	10.6156651958333	9.74285554166667	10.7462083208333	10.4579743	10.5661385208333	10.9096329166667	11.2637059125	10.5657535	11.03538055	11.2382642791667	10.8982382083333	11.2866647166667	10.8422630416667	10.7773041666667	10.1458370625	11.2109354583333	10.9526005708333	11.0875279125	11.269525825	10.5016048958333	11.0227205875	10.9312479291667	11.0721722125	11.6300105791667	11.7420543958333	12.0086714041667	11.3837239291667	10.3188843875	10.6701430958333	11.1145695125	11.2253067083333	12.3586725458333	11.77741555	11.0882827125	10.979276975	12.0350775875	10.8305792625	11.5921871041667	11.1016492416667	12.0034735458333	11.4951390333333	11.4543559333333	12.1780362291667	11.3618031	11.8114042958333	11.4819142666667	11.0646199291667	10.922504475	11.6626930166667	12.2009012166667	11.002258125	11.2389677541667	11.101283425	11.076375075	11.2200484	11.9905450875	10.9598836708333	11.3207889041667	11.5811100791667	12.5867866666667	11.9635419541667	11.72760665	11.3614221708333	11.28820595	11.8094604208333	12.013867925	11.6520352291667	11.8124828958333	11.5740610791667	11.4032411791667	11.9362989083333	11.223552875	11.2945622125	10.7587865416667	11.5686211083333	11.6357464125	12.308609075	12.0370571708333	11.6995210375	11.6157672875	12.1988122958333	11.842899075	11.7642776208333

AC117378.1	10.1955368208333	10.85127885	10.9362758541667	10.926151175	10.532849475	10.6620163958333	10.2718185833333	10.591617975	10.5974997458333	11.2080698458333	11.0653444458333	9.96588614583333	9.85656001666667	10.9254252625	10.7513786875	11.0551007666667	11.4621649583333	10.4924386416667	10.3540126708333	11.1515491708333	10.9464365791667	11.0991457083333	11.1639329916667	10.268910725	11.0411335166667	10.1376630083333	10.7826252541667	10.6311434458333	10.5777799041667	11.03538055	10.675551525	10.8675350916667	10.4230034708333	11.2274614541667	9.94359062916667	10.4026659625	10.8606822291667	10.6156651958333	9.74285554166667	10.7462083208333	10.4579743	10.5661385208333	10.9096329166667	11.2637059125	10.5657535	11.03538055	11.2382642791667	10.8982382083333	11.2866647166667	10.8422630416667	10.7773041666667	10.1458370625	11.2109354583333	10.9526005708333	11.0875279125	11.269525825	10.5016048958333	11.0227205875	10.9312479291667	11.0721722125	11.6300105791667	11.7420543958333	12.0086714041667	11.3837239291667	10.3188843875	10.6701430958333	11.1145695125	11.2253067083333	12.3586725458333	11.77741555	11.0882827125	10.979276975	12.0350775875	10.8305792625	11.5921871041667	11.1016492416667	12.0034735458333	11.4951390333333	11.4543559333333	12.1780362291667	11.3618031	11.8114042958333	11.4819142666667	11.0646199291667	10.922504475	11.6626930166667	12.2009012166667	11.002258125	11.2389677541667	11.101283425	11.076375075	11.2200484	11.9905450875	10.9598836708333	11.3207889041667	11.5811100791667	12.5867866666667	11.9635419541667	11.72760665	11.3614221708333	11.28820595	11.8094604208333	12.013867925	11.6520352291667	11.8124828958333	11.5740610791667	11.4032411791667	11.9362989083333	11.223552875	11.2945622125	10.7587865416667	11.5686211083333	11.6357464125	12.308609075	12.0370571708333	11.6995210375	11.6157672875	12.1988122958333	11.842899075	11.7642776208333

RGL3	6.55593524166667	5.1598283	6.56292778333333	6.46133099166667	6.028109975	6.20408345833333	5.18759319166667	5.426744575	5.69385626666667	4.74448875	5.79999791666667	4.96401284166667	5.40405401666667	4.83765554166667	6.52564285833333	5.157708775	9.17230494166667	6.77836881666667	6.18568551666667	5.70355690833333	5.27529641666667	6.311629325	5.07218190833333	6.46988230833333	6.75646765833333	5.895388975	6.222261675	6.75097239166667	6.25427673333333	6.56145719166667	6.759774675	6.39383819166667	5.06351158333333	8.02512498333333	4.76141500833333	6.06230976666667	6.12364109166667	7.212178575	6.51600675833333	7.10822425	6.64292103333333	6.259187975	6.627072075	6.2073137	5.97595255	5.57060381666667	6.70532833333333	6.18822558333333	6.47646620833333	6.45919934166667	7.19320405	6.36616499166667	5.32557380833333	6.135921175	5.40105285833333	6.37113890833333	5.05687025	6.39606678333333	6.25427673333333	5.51291259166667	5.735887375	5.54199565833333	7.62930498333333	6.49825018333333	8.1277546	5.965264275	4.65176085833333	5.10498871666667	5.67998533333333	4.434278525	6.0148863	5.67617600833333	5.71707388333333	4.9935499	5.22492120833333	7.48011853333333	6.05717670833333	7.52279740833333	5.02903219166667	6.673017075	7.30019385833333	5.60406076666667	4.98444206666667	7.16909093333333	5.566976475	6.41270695833333	7.23785493333333	4.85447411666667	5.46647429166667	5.31824546666667	5.39492906666667	7.13667263333333	5.78455018333333	6.21427064166667	5.760490375	5.992607775	7.21926184166667	7.98666509166667	7.86670446666667	6.011301125	5.89108584166667	5.212866025	6.32988615	6.28336628333333	5.62957723333333	4.41695324166667	5.8532158	7.21695988333333	5.0835026	6.73419851666667	5.82168396666667	6.44887243333333	4.40903533333333	6.27095864166667	5.72886108333333	5.81037075833333	9.060159775	8.68329548333333	6.07176163333333	4.2918764

GAS6	10.1750566166667	8.90434914166667	8.88122105	8.94982423333333	8.21672670833333	8.97489020833333	9.11782889166667	9.01297525833333	7.86334303333333	7.46134775833333	9.09833678333333	7.868368375	8.30336938333333	7.79194111666667	8.91938296666667	7.84998155	8.02070828333333	8.46149996666667	8.252426875	8.34473695	7.73246671666667	8.74719128333333	7.88978783333333	8.286335875	8.57995885	9.84631699166667	9.24492166666667	9.018981225	8.3453091	8.18315743333333	8.44794389166667	9.28174641666667	8.46720710833333	10.4526526416667	9.85452464166667	10.194475875	8.80925365833333	8.478972275	8.43758005833333	9.32000775	8.33655620833333	8.68175469166667	8.71986490833333	8.4371155	8.31008573333333	8.79812115	8.57507296666667	8.72497184166667	8.513349575	8.6774004	8.27857833333333	8.41599213333333	8.61623551666667	8.55392016666667	8.86062028333333	8.47690594166667	7.96073305833333	8.24509221666667	8.67888266666667	8.14660076666667	9.06116049166667	7.0176409	7.56325655	7.38640515833333	6.79403766666667	7.966758925	6.69642741666667	7.97768158333333	6.54089473333333	6.950247825	8.595488075	7.1283444	7.04898169166667	9.47208941666667	8.01595076666667	9.24630831666667	9.002830825	8.78070639166667	7.66388121666667	7.582967825	7.71080830833333	8.256452925	9.546304725	7.05225570833333	8.9217463	7.61232630833333	7.11986829166667	6.891986525	7.81786669166667	8.27606465	9.18167015	8.82621398333333	10.4661023416667	8.9604306	7.7682787	9.70194608333333	6.96533775	8.07255219166667	7.429587275	8.37107905	9.41120025	7.690465575	7.98666509166667	8.06345484166667	9.02034758333333	9.53265603333333	7.56380908333333	8.99771499166667	8.84055035	7.25363145833333	9.56634321666667	7.75930169166667	9.43456800833333	8.03431426666667	7.97719954166667	7.06146445	7.59083331666667	7.24765453333333	7.198288025	8.754236775

PAX6	10.7959852666667	10.3155264416667	10.0002116916667	10.2136563	11.4567547583333	10.4057146583333	11.5363521916667	10.382913225	10.8984556583333	10.641902875	10.6233516833333	10.9600311833333	11.3612411916667	10.28850615	10.3798706916667	9.96699933333333	11.218298125	11.30185685	9.88386428333333	9.86359230833333	10.4084577916667	10.4268652916667	9.76350663333333	9.75665824166667	10.176297425	10.496758625	10.2321384166667	10.1440795833333	10.5956461416667	10.5537715083333	10.0696848333333	9.91758241666667	10.929974575	11.50454985	10.6570447583333	10.4231890083333	11.0633247666667	10.3733561083333	10.1258089583333	10.1452230583333	9.9441902	10.5208646083333	9.98111111666667	9.58222316666667	10.2145140083333	9.6185904	9.98187775833333	9.93042398333333	9.76089629166667	10.0103759833333	10.2547426166667	10.2080941333333	10.5420798333333	10.4858474583333	10.1993549333333	9.61006324166667	10.2458406333333	10.1409369083333	10.0569437166667	9.54132330833333	10.9553221083333	10.8484072166667	9.86062085833333	10.4942942166667	10.4264895833333	11.126694875	10.6503573333333	10.9335275	10.9143108583333	10.8105355416667	10.88109835	10.4858474583333	11.0633247666667	10.7096961916667	10.717740675	10.303115425	11.0716307666667	10.5623629583333	9.97791586666667	10.5731419166667	10.7375745583333	10.6809073166667	10.3439808916667	9.95522605	11.0589538416667	10.9393869333333	10.7684448166667	10.5910579333333	10.9816032916667	10.0156224166667	10.523220425	10.20731305	10.9754104083333	10.436061525	11.0913458083333	10.6019249583333	10.8468748916667	10.9647570833333	10.884735425	9.95398405833333	10.4691008666667	11.2222793166667	10.1755237	9.69880400833333	10.1931783583333	10.6581721666667	10.3897471916667	10.1084049583333	10.2286007833333	10.23713875	10.023053775	10.0156224166667	10.227074775	9.92334935	10.235923825	9.96565705833333	10.3366020166667	10.3455009333333	10.0855958166667	10.20436555

BRCA1	7.80067161666667	7.82691680833333	8.06606233333333	7.80971700833333	8.084825575	6.55373064166667	8.20331464166667	8.8992014	8.1089851	7.78110251666667	8.250184775	6.70918899166667	7.60540448333333	8.10057743333333	8.499322875	8.08086618333333	7.66388121666667	7.811926125	8.19859208333333	8.06026415833333	7.57513376666667	8.09998893333333	7.79469890833333	7.32727508333333	8.3023979	8.17931615833333	8.71144663333333	8.57454949166667	8.16584395833333	8.07411434166667	8.7179342	8.3536777	9.08687805	6.87159964166667	8.31619925	8.27228149166667	8.7292202	8.61820814166667	8.78355868333333	9.16167278333333	8.92973970833333	8.46552205	8.36713998333333	8.50451981666667	8.49291188333333	7.99339445833333	8.42421896666667	9.14145633333333	8.22846261666667	8.03913049166667	9.7570522	8.27857833333333	8.77719965833333	7.92964085	8.662974625	8.9217463	8.93568545833333	9.166824	8.21622873333333	8.04234110833333	6.99601435833333	7.56325655	7.1508922	6.735668325	5.76251488333333	6.8347138	6.15692685	6.6562921	6.76049560833333	6.4095551	7.153195325	7.16232548333333	5.50905394166667	6.411112525	6.63942924166667	6.53954924166667	7.6702147	7.08933194166667	7.27535064166667	7.02284831666667	6.75646765833333	6.92575055833333	6.3109681	6.475087675	7.01495000833333	6.998615075	6.909113525	6.53407053333333	8.37436913333333	7.07182334166667	7.1950993	6.80714281666667	7.67253041666667	6.757758475	7.02148474166667	7.86115675833333	7.29474379166667	7.27593380833333	7.35345289166667	7.20911576666667	7.14399110833333	7.35173706666667	8.03538219166667	7.43473675	6.93685505	6.89068614166667	6.88940976666667	8.05296578333333	7.05278270833333	7.51814899166667	7.07118303333333	7.85906496666667	6.85745724166667	6.89322066666667	6.46640339166667	8.244635825	7.68263314166667	6.87917671666667	7.76990645	6.57813499166667

MS4A4A	9.7432741875	7.88841685416667	8.79163204166667	8.1609766	8.33576940416667	8.64564211666667	9.04156121666667	8.26382102916667	8.33993060833333	8.39234492083333	7.91871015833333	10.0698569125	9.03572357916667	8.36313465833333	8.59572272083333	9.99409350833333	7.83157462083333	7.39013737083333	8.2811933625	9.1775565375	8.75766082083333	7.77920590416667	8.13064198333333	7.75309706666667	9.4039648875	8.98169243333333	9.42268963333333	8.56741365	7.9476503125	8.35047989583333	8.26015074583333	9.34459659583333	8.9455138875	8.05943479583333	8.81997435833333	8.6159754375	7.80145052083333	8.7294690625	8.66176347083333	8.54047262083333	7.98692124166667	8.65294815833333	8.3858844375	8.03030704166667	8.934369525	8.9500697125	8.43970535	7.967590725	8.581156975	8.562975175	7.97319919583333	7.81320275416667	9.16986652916667	9.25417682916667	8.5917752625	8.37484225	8.91067589583333	8.64071354166667	8.946459375	8.32239827083333	7.66694402083333	7.2902029625	6.9761553125	7.18193015416667	7.51612055416667	7.11759648333333	7.07032169583333	7.967590725	6.31059185416667	8.178054875	7.4475786625	7.0046371375	7.82667341666667	7.27024847083333	8.26382102916667	7.84460329583333	8.36784924583333	8.12563023333333	6.72216503333333	8.5126008	7.68854212083333	8.6924757375	6.798312975	5.4575038	7.88203552083333	7.5951853375	7.50651534583333	8.60825835416667	7.17708556666667	7.87539923333333	7.8707073375	7.2491682	7.00270695833333	8.1941990625	8.89331847083333	6.1632001	7.41034305416667	6.96152448333333	6.6928085125	7.68800911666667	7.53992697083333	7.5725518875	8.41949202916667	6.15645708333333	6.25062844583333	7.5172547125	7.28042123333333	6.720896275	8.12203683333333	6.9383847	7.26658241666667	6.04749792083333	6.38969043333333	7.24605037083333	8.78863738333333	8.43422032083333	6.895626475	6.09886234166667	6.71884765833333	8.1690468875

MS4A4E	9.7432741875	7.88841685416667	8.79163204166667	8.1609766	8.33576940416667	8.64564211666667	9.04156121666667	8.26382102916667	8.33993060833333	8.39234492083333	7.91871015833333	10.0698569125	9.03572357916667	8.36313465833333	8.59572272083333	9.99409350833333	7.83157462083333	7.39013737083333	8.2811933625	9.1775565375	8.75766082083333	7.77920590416667	8.13064198333333	7.75309706666667	9.4039648875	8.98169243333333	9.42268963333333	8.56741365	7.9476503125	8.35047989583333	8.26015074583333	9.34459659583333	8.9455138875	8.05943479583333	8.81997435833333	8.6159754375	7.80145052083333	8.7294690625	8.66176347083333	8.54047262083333	7.98692124166667	8.65294815833333	8.3858844375	8.03030704166667	8.934369525	8.9500697125	8.43970535	7.967590725	8.581156975	8.562975175	7.97319919583333	7.81320275416667	9.16986652916667	9.25417682916667	8.5917752625	8.37484225	8.91067589583333	8.64071354166667	8.946459375	8.32239827083333	7.66694402083333	7.2902029625	6.9761553125	7.18193015416667	7.51612055416667	7.11759648333333	7.07032169583333	7.967590725	6.31059185416667	8.178054875	7.4475786625	7.0046371375	7.82667341666667	7.27024847083333	8.26382102916667	7.84460329583333	8.36784924583333	8.12563023333333	6.72216503333333	8.5126008	7.68854212083333	8.6924757375	6.798312975	5.4575038	7.88203552083333	7.5951853375	7.50651534583333	8.60825835416667	7.17708556666667	7.87539923333333	7.8707073375	7.2491682	7.00270695833333	8.1941990625	8.89331847083333	6.1632001	7.41034305416667	6.96152448333333	6.6928085125	7.68800911666667	7.53992697083333	7.5725518875	8.41949202916667	6.15645708333333	6.25062844583333	7.5172547125	7.28042123333333	6.720896275	8.12203683333333	6.9383847	7.26658241666667	6.04749792083333	6.38969043333333	7.24605037083333	8.78863738333333	8.43422032083333	6.895626475	6.09886234166667	6.71884765833333	8.1690468875

ASIC1	9.03183454166667	8.46720710833333	8.95895775	9.02418135	9.15210589166667	8.66775465	8.98465464166667	8.7383879	8.927052675	8.55543884166667	8.80677418333333	8.8526135	8.92028675	7.933308825	8.74671439166667	7.984047875	8.74174598333333	9.28785676666667	8.40516035	8.62547046666667	8.234668275	8.32648980833333	8.46149996666667	8.6233491	8.22207980833333	8.4414262	8.53434441666667	8.81336174166667	9.1773608	8.68575455833333	8.68329548333333	8.49244585833333	8.89405555	8.67077851666667	8.80003964166667	8.68175469166667	9.08687805	8.35685346666667	8.71384840833333	8.5874833	8.17990050833333	8.435679275	8.20930705	7.91465324166667	8.18571946666667	7.663341325	7.89296785	8.46961650833333	8.03861421666667	8.52061441666667	8.45255091666667	8.72739513333333	8.42877934166667	8.78070639166667	8.45556428333333	7.61643631666667	8.4816187	8.11825858333333	8.37970946666667	7.61520518333333	8.81642101666667	8.93762295	8.86854605833333	9.073438725	8.65947190833333	9.41935561666667	8.48112818333333	8.99943493333333	9.30912254166667	8.77628200833333	8.965634	9.03908018333333	9.02830098333333	8.39869986666667	8.96144149166667	8.72497184166667	8.99164978333333	8.66441525833333	8.68130785	9.03989521666667	8.525325	8.47690594166667	8.71882579166667	8.35024235	8.94623185833333	8.64585098333333	9.17971915	9.01380788333333	9.25303868333333	8.06812029166667	8.98368761666667	8.85865705	8.83592169166667	8.64006809166667	9.319021525	8.935118175	9.06069035833333	8.751330725	9.46477646666667	8.52009253333333	8.85824333333333	8.7812519	8.67026894166667	8.13242494166667	8.348200375	8.24163635833333	8.42522444166667	8.66775465	8.466090675	8.3316018	8.40280550833333	8.53434441666667	8.580396475	8.47629666666667	8.9939706	8.105836875	8.22500544166667	8.61342080833333	8.48515009166667	8.43662665833333

FCHSD2	4.39770036666667	3.9861723	4.33428121666667	3.75838870833333	4.87503585	4.81485295	5.14499941666667	4.92659856666667	5.19536235833333	4.53925114166667	3.95680781666667	5.303590975	4.52055483333333	4.45902586666667	4.42666711666667	4.563953975	4.128952175	5.30151331666667	4.13647209166667	4.33133893333333	5.03120425833333	4.23701905833333	4.0917668	4.55283246666667	4.33846348333333	4.20885784166667	4.040897575	4.024477825	4.08322809166667	4.02352729166667	4.15642159166667	3.9861723	3.81959511666667	4.32946600833333	4.52697115833333	3.84700126666667	4.27071034166667	4.07882345	4.25100265833333	4.09274305833333	4.434278525	4.19810324166667	4.95206471666667	4.62270154166667	3.905753025	4.30721710833333	4.32094003333333	4.20288324166667	4.09903998333333	4.27798759166667	4.13438028333333	4.11281023333333	4.62587865833333	4.77483110833333	3.88409523333333	4.14053975833333	3.66804375833333	4.262047775	4.1553789	4.302366825	4.25573735	3.770641325	4.7006097	4.361998275	6.41666009166667	5.0953115	5.43388225	5.23671275	5.623286525	5.12102319166667	4.28423139166667	4.58241979166667	4.40581835	4.23291245833333	3.95350946666667	4.31702865	4.81179368333333	6.21116124166667	4.344051675	4.117963675	4.62057545833333	4.64220138333333	4.563953975	4.176778725	4.54133179166667	3.71770076666667	4.85659321666667	3.94580334166667	4.26028479166667	4.0917668	3.8987918	5.10930444166667	3.86842555	4.15642159166667	4.262047775	3.86022540833333	3.97264301666667	4.13943304166667	3.75570144166667	4.085428875	4.33846348333333	3.72041143333333	4.50312849166667	4.369907275	4.00881020833333	3.95130670833333	4.13647209166667	4.79999713333333	4.753728625	4.77272485	4.31295405833333	4.44453341666667	4.17373291666667	4.46100053333333	4.10621731666667	3.91035815	3.75016011666667	4.146963325	4.33532039166667	4.222209325

FAM20C	6.37713004166667	4.47861730833333	4.56806040833333	5.5526373	4.85146489166667	5.44429769166667	5.08659576666667	4.36796431666667	5.03622141666667	4.50544710833333	4.77601478333333	4.53433785	4.705709175	4.202033325	5.53496716666667	4.894753975	4.853434925	5.50523408333333	5.03120425833333	4.81716043333333	4.638255275	5.08063435	4.93552183333333	5.03622141666667	5.20250346666667	4.86914890833333	5.992607775	6.44290830833333	7.46022033333333	5.15246255833333	5.61643985833333	5.9573564	5.28688518333333	5.67998533333333	6.35051324166667	5.44644633333333	5.89630836666667	5.897311975	5.91334729166667	5.557624025	4.84615233333333	6.081909575	5.2783407	5.35983450833333	5.3397513	5.980495875	5.60574740833333	5.6411096	5.74704205	5.933890025	5.82168396666667	5.80937455	5.45060010833333	6.20814583333333	5.24498075	5.65732808333333	5.055819225	6.41270695833333	5.7835982	5.88521655833333	5.58508399166667	5.24931593333333	6.02387365	5.9623768	4.87710051666667	5.44429769166667	5.02467455	4.63410609166667	6.02292943333333	4.90679766666667	4.67877638333333	5.28357275833333	4.99586916666667	4.480628975	4.683662975	4.42481975833333	5.17697859166667	5.41985199166667	4.563953975	5.02158241666667	4.490204975	4.49312724166667	5.24703029166667	5.20449400833333	5.42470928333333	5.42554189166667	6.475087675	6.46779858333333	5.71985138333333	4.76455610833333	5.52011276666667	6.23385413333333	5.54779774166667	5.23461665	5.99418615	5.546831425	5.25768970833333	6.52209205	5.63538781666667	5.86456365833333	5.44029105833333	5.70053848333333	6.346676525	5.97436386666667	5.65344755	4.86479268333333	5.29885645833333	6.04352600833333	5.628745675	6.56509979166667	5.6322886	5.86905295	5.93821120833333	4.63305978333333	4.664314175	5.11436066666667	5.83953349166667	6.18980128333333	5.60574740833333	4.32386374166667

CHD6	11.1641077166667	10.9812562333333	10.5953164	10.6853720166667	10.4668279916667	10.9730535833333	10.5592695083333	11.0419739083333	10.7593753416667	10.6158476666667	11.1415966	11.044463875	10.4866818833333	11.2576230916667	11.1678068583333	11.4467356166667	11.1607791666667	10.7885218833333	10.9851253333333	11.2826013166667	10.972292975	11.502358525	11.2366875083333	10.6519544333333	10.962553725	11.6587921	10.7268672916667	10.97984555	10.3618816	11.1930897583333	10.91088935	10.8273805416667	10.8934071416667	10.7842899166667	11.5706660333333	11.5093017916667	10.8307782666667	11.0510672583333	10.6289554166667	11.0790622916667	11.0830259333333	10.9069463833333	11.1956589833333	11.174277875	11.1484272833333	11.21620365	11.052426775	10.5444389083333	11.1060515416667	11.4978397166667	10.3852198833333	10.3725905333333	10.8239850833333	10.8315442333333	11.23046085	10.9065665083333	10.9357322	10.9909672	11.2642606083333	11.2944151416667	10.7371678583333	11.713529275	11.62870925	11.4273654166667	11.702337975	10.5404499166667	10.97339425	11.2595088166667	11.5400258416667	11.2283191166667	11.0225675833333	11.302200525	11.004921375	11.1678068583333	10.558939175	10.8154489833333	11.3797907833333	10.9237681	10.7635056583333	11.5794626	12.0277810916667	11.7027128	9.93713581666667	10.94470365	10.4752224083333	11.48359205	11.4839876416667	10.5613244166667	11.8166747583333	11.120710425	10.4040896666667	11.4854519916667	11.1721251583333	11.0863287333333	10.7710861166667	11.034816875	11.675724725	10.9032780416667	10.9843155	12.1847949166667	10.8972744416667	10.9139727166667	11.0845103416667	11.259162425	10.8755795416667	11.3986748166667	10.7217139416667	10.8052677	11.512504775	11.037692575	11.4481946583333	10.9726481916667	10.9976123	11.3709225333333	10.7352154666667	10.3011707	12.0808514833333	11.28518	11.6492807916667	11.0394398083333

LGALS8	12.0122354416667	12.0691835958333	12.007013275	11.9500328166667	12.2886149583333	12.963770175	11.8941636833333	12.1361380958333	12.0488412833333	12.2174800625	12.7689936666667	12.0723492958333	12.1172514708333	12.2092643458333	11.8575015625	12.2762036708333	11.6685670791667	12.1340157	11.8691422958333	12.0472317	12.2097075375	12.2050921958333	12.4882160708333	12.6299801833333	12.2418892833333	12.1924559125	12.0086714041667	12.10859605	11.873195075	12.3205802333333	12.0434464458333	12.3175796458333	12.1132013666667	12.0354571708333	11.9083957041667	12.0061257333333	12.1801535291667	11.9980532208333	12.211850075	12.082986475	11.8267584666667	12.0122354416667	11.938234175	11.9987942833333	11.954871975	12.0098469458333	12.123574975	12.3120406458333	12.319755575	12.1416138375	12.0810500166667	12.7048765958333	12.3945689083333	12.1868104916667	12.1622409666667	12.1264399583333	12.2961971583333	11.9953072583333	12.1526999916667	12.2401951708333	13.2748180708333	13.4198811	12.7588894666667	13.1313415291667	13.0765707416667	12.8564463	13.2930033458333	12.4625530458333	13.1888928416667	13.2218451541667	13.3477477416667	13.1691822625	12.9536648666667	12.8715923291667	13.0509581666667	13.0593609791667	12.6290041291667	12.6247939083333	13.3176143291667	12.7777399	13.2721925416667	13.5234368666667	12.9414854	12.7120155125	12.7033686291667	13.1716469375	13.1373006958333	12.2936895083333	14.046053475	12.9027308333333	12.6963918375	12.7528809125	12.9742284375	12.8373957916667	12.8501372666667	13.0040984166667	13.29018665	13.2618737041667	12.6788101833333	13.1768570625	12.9963380041667	12.6931945	13.2145388708333	13.2701257958333	13.2178216083333	13.2017398541667	12.8804655625	12.94203365	13.038970275	12.6329536083333	12.7745917041667	12.4099116375	12.5129160833333	13.0724085083333	13.1593016333333	12.6898184916667	13.3319127583333	13.6844501	12.9497126916667	12.6869957833333

HEATR1	12.0122354416667	12.0691835958333	12.007013275	11.9500328166667	12.2886149583333	12.963770175	11.8941636833333	12.1361380958333	12.0488412833333	12.2174800625	12.7689936666667	12.0723492958333	12.1172514708333	12.2092643458333	11.8575015625	12.2762036708333	11.6685670791667	12.1340157	11.8691422958333	12.0472317	12.2097075375	12.2050921958333	12.4882160708333	12.6299801833333	12.2418892833333	12.1924559125	12.0086714041667	12.10859605	11.873195075	12.3205802333333	12.0434464458333	12.3175796458333	12.1132013666667	12.0354571708333	11.9083957041667	12.0061257333333	12.1801535291667	11.9980532208333	12.211850075	12.082986475	11.8267584666667	12.0122354416667	11.938234175	11.9987942833333	11.954871975	12.0098469458333	12.123574975	12.3120406458333	12.319755575	12.1416138375	12.0810500166667	12.7048765958333	12.3945689083333	12.1868104916667	12.1622409666667	12.1264399583333	12.2961971583333	11.9953072583333	12.1526999916667	12.2401951708333	13.2748180708333	13.4198811	12.7588894666667	13.1313415291667	13.0765707416667	12.8564463	13.2930033458333	12.4625530458333	13.1888928416667	13.2218451541667	13.3477477416667	13.1691822625	12.9536648666667	12.8715923291667	13.0509581666667	13.0593609791667	12.6290041291667	12.6247939083333	13.3176143291667	12.7777399	13.2721925416667	13.5234368666667	12.9414854	12.7120155125	12.7033686291667	13.1716469375	13.1373006958333	12.2936895083333	14.046053475	12.9027308333333	12.6963918375	12.7528809125	12.9742284375	12.8373957916667	12.8501372666667	13.0040984166667	13.29018665	13.2618737041667	12.6788101833333	13.1768570625	12.9963380041667	12.6931945	13.2145388708333	13.2701257958333	13.2178216083333	13.2017398541667	12.8804655625	12.94203365	13.038970275	12.6329536083333	12.7745917041667	12.4099116375	12.5129160833333	13.0724085083333	13.1593016333333	12.6898184916667	13.3319127583333	13.6844501	12.9497126916667	12.6869957833333

EBAG9	13.1336349	12.503422725	12.3653316083333	12.6481258583333	12.660047425	12.6397345916667	12.1488811083333	12.4755627916667	12.372860975	12.5140767416667	12.98478995	12.5999690416667	12.6190854666667	12.6734606	12.8041062916667	12.7441506916667	13.7681999916667	12.7426291416667	12.5448126916667	12.94284515	12.829556475	12.9906493583333	13.0288304083333	12.8317521333333	12.7852137833333	13.0337121333333	12.5944621416667	12.7822168333333	12.7453059583333	12.8166613583333	12.5048403	12.81348825	12.5907074583333	13.0999579333333	12.382866125	13.2628733666667	12.3874454166667	12.576015775	12.1591768166667	12.7463306666667	12.4536816333333	12.3824722	12.4141455666667	12.6961567333333	12.76127775	12.3147568916667	12.9172670333333	12.4377746666667	12.4368713	13.0055645333333	12.5429966333333	12.4198120333333	13.001932175	12.6366518666667	12.5745387083333	12.4229923333333	12.5701103	12.5667535083333	12.7092084083333	13.0816325916667	13.3793195083333	12.92419445	13.662514625	12.95462435	13.5213793583333	13.4065910333333	13.3763691583333	13.232552625	13.8908948916667	12.7918033916667	13.1178746666667	13.2061458583333	12.7086380166667	12.9818120916667	12.932363575	13.441409425	13.3114725833333	13.6506232916667	13.5965748083333	12.9941887083333	13.3010495416667	13.6848971666667	13.627458175	13.4783310166667	13.2835400666667	12.9219389833333	13.23467165	13.3444534166667	13.0816325916667	13.057870725	12.7323979	13.057870725	13.1952399083333	13.2975446	14.1152258583333	12.77184425	13.695137875	12.9785766	13.2158871875	13.3262903916667	13.1695361416667	12.9842314416667	13.782971725	13.1336349	13.0037538583333	13.6089687	13.3106692583333	13.1959888083333	13.3689321166667	13.0798178083333	12.424331275	12.9709933083333	13.0500216833333	13.7168412916667	13.4674284	13.031934	12.908165975	12.627449175	12.7441506916667	12.4714104166667

OPCML	4.33637641666667	5.12814335833333	5.88673599166667	5.5927414	5.14192449166667	4.98009509166667	6.926478325	6.27872079166667	5.80264275	5.13634166666667	4.96401284166667	5.464635875	6.64292103333333	5.92854135	4.25100265833333	4.53008874166667	4.47573676666667	5.20550439166667	4.28110195833333	4.69115821666667	5.01321970833333	5.06570035833333	6.44595796666667	4.09274305833333	4.224150025	4.42481975833333	5.13437999166667	3.99383243333333	4.332336275	3.94518501666667	4.221324425	4.06389798333333	4.38930708333333	4.59464514166667	4.21592141666667	3.867233575	4.71686186666667	4.09598105	4.18926569166667	4.28310633333333	4.64734200833333	4.0078761	4.32094003333333	4.80827406666667	3.96951036666667	5.76350409166667	4.43844	4.432045325	4.53008874166667	4.49113386666667	4.58241979166667	4.34698491666667	5.37867954166667	5.36511784166667	4.0798737	4.265394625	5.68783094166667	4.36096451666667	4.7847361	3.69375465	4.19044525833333	5.5022085	4.02799813333333	4.87006425833333	5.14829798333333	5.60777716666667	5.65427465	6.345260775	5.28262243333333	5.110215025	5.577321975	4.649483075	6.90968315833333	5.10174316666667	4.60180214166667	4.443420775	4.366029925	3.57639359166667	4.25488725	4.04713170833333	5.43488754166667	4.46516671666667	5.6250378	4.23196318333333	5.10174316666667	4.10289946666667	4.84094744166667	5.63443499166667	4.72414796666667	4.117963675	4.27374416666667	3.96359718333333	3.731508225	4.80316131666667	4.63723314166667	4.149223425	4.20479785833333	3.8957117	4.30130591666667	4.43737311666667	4.59265796666667	5.30151331666667	4.30721710833333	4.31803169166667	4.08322809166667	4.95961636666667	4.1553789	5.112251075	4.748342275	5.54873173333333	4.210787125	4.609167375	4.07015480833333	4.38197743333333	4.24013239166667	4.18298021666667	4.67172261666667	4.25488725	4.1553789	5.24931593333333

KCNIP4	6.33852943333333	5.30888879166667	5.665590775	5.897311975	6.328333625	4.60699911666667	7.32668075833333	5.50024889166667	6.524903425	5.76923416666667	6.3064348	7.02081110833333	6.2066078	5.4308407	5.575395825	4.89595425	4.68789791666667	5.88341255833333	6.29201916666667	5.824619025	5.002397475	4.782758275	5.05069551666667	4.68789791666667	4.87091625	5.824619025	5.16081746666667	5.16838866666667	4.93436960833333	5.6003113	5.775482875	4.88811624166667	6.135921175	5.87405735	5.39804303333333	5.91988768333333	5.44644633333333	5.29020444166667	5.45803265	4.894753975	4.979047925	5.4913896	5.33329914166667	4.84615233333333	5.5927414	4.979047925	5.67523288333333	5.485111725	5.1863758	5.2891249	5.2617065	5.858085325	6.528434875	5.66650295	5.87496216666667	5.54303723333333	5.98915650833333	5.23876719166667	5.97783559166667	5.125178525	5.88241164166667	6.64016859166667	5.12411041666667	6.18408195	5.71218308333333	5.16622001666667	6.950247825	7.193910325	7.05521155	5.56869470833333	7.83726095833333	8.54699226666667	5.72686785	6.55593524166667	5.53496716666667	5.125178525	5.477009275	5.91139875	6.23079429166667	5.94705471666667	6.155322525	6.21427064166667	4.5496205	5.577321975	5.229288475	5.74803075	5.58508399166667	5.05687025	6.463588	6.07937631666667	5.33069273333333	6.02015538333333	8.57507296666667	6.33187936666667	5.83589014166667	7.39505978333333	7.45718173333333	5.60971738333333	5.550710675	4.48829116666667	5.162070825	5.5697365	5.19959509166667	4.705709175	5.377778825	7.33857021666667	5.70932124166667	5.59947975	5.1863758	5.40932128333333	4.53327731666667	6.25991309166667	5.59857721666667	5.429887625	5.58988304166667	6.21600831666667	5.15873735	6.03500149166667	6.70667168333333	6.06998224166667

LIMS1	10.469517225	9.44959046666667	9.78329576666667	10.319078025	10.2749442833333	8.49975086666667	10.7736244333333	9.81176585	9.58933430833333	9.570506275	9.93630081666667	10.8394387833333	10.9680827416667	9.71989020833333	9.20284239166667	9.70017130833333	8.45045408333333	10.28186315	8.85728129166667	9.69747909166667	10.135458025	9.16167278333333	9.14279978333333	8.91224634166667	9.28599946666667	10.0391614083333	8.96700605833333	9.35401866666667	9.12506650833333	9.62890875	8.751330725	8.5905397	9.65670091666667	8.98465464166667	9.97043543333333	9.87908794166667	9.43868498333333	8.91506995	9.291151225	9.24127241666667	8.43902399166667	8.95624593333333	8.81744095	8.56421206666667	9.48031518333333	9.16031618333333	8.75091850833333	8.78498449166667	8.54070765833333	8.34620800833333	8.815557	8.92218960833333	9.33579655	9.0174923	9.04278656666667	8.97675146666667	9.07536643333333	8.82022893333333	8.81154583333333	8.79186565833333	9.7241175	10.2806521916667	9.31001445	10.1497399666667	9.87695954166667	9.77133523333333	10.1134808166667	10.2422793916667	9.304559675	9.89141825	10.1084049583333	10.1927941666667	10.6842469	9.921332825	10.0347123333333	10.13859765	9.86766510833333	9.53592761666667	9.50264109166667	10.0334908083333	10.134682025	10.2233050083333	10.0209906333333	9.95935071666667	10.05531845	10.2466873083333	10.2741449416667	10.5557362916667	10.3694253333333	9.11993345	9.81985159166667	8.84099020833333	8.87086751666667	9.40207443333333	10.054920625	9.36666890833333	9.4341767	9.09184225	10.1250607083333	8.77579595833333	9.16123660833333	10.1700709416667	8.85215985	8.3496737	9.27994568333333	9.51454425833333	9.40469768333333	8.80244275	9.01425336666667	9.22249476666667	8.42018036666667	8.72830366666667	9.80802880833333	8.775178575	8.82265860833333	8.821200975	8.71882579166667	9.39525429166667	8.81971978333333	9.1447536

LIMS3	10.469517225	9.44959046666667	9.78329576666667	10.319078025	10.2749442833333	8.49975086666667	10.7736244333333	9.81176585	9.58933430833333	9.570506275	9.93630081666667	10.8394387833333	10.9680827416667	9.71989020833333	9.20284239166667	9.70017130833333	8.45045408333333	10.28186315	8.85728129166667	9.69747909166667	10.135458025	9.16167278333333	9.14279978333333	8.91224634166667	9.28599946666667	10.0391614083333	8.96700605833333	9.35401866666667	9.12506650833333	9.62890875	8.751330725	8.5905397	9.65670091666667	8.98465464166667	9.97043543333333	9.87908794166667	9.43868498333333	8.91506995	9.291151225	9.24127241666667	8.43902399166667	8.95624593333333	8.81744095	8.56421206666667	9.48031518333333	9.16031618333333	8.75091850833333	8.78498449166667	8.54070765833333	8.34620800833333	8.815557	8.92218960833333	9.33579655	9.0174923	9.04278656666667	8.97675146666667	9.07536643333333	8.82022893333333	8.81154583333333	8.79186565833333	9.7241175	10.2806521916667	9.31001445	10.1497399666667	9.87695954166667	9.77133523333333	10.1134808166667	10.2422793916667	9.304559675	9.89141825	10.1084049583333	10.1927941666667	10.6842469	9.921332825	10.0347123333333	10.13859765	9.86766510833333	9.53592761666667	9.50264109166667	10.0334908083333	10.134682025	10.2233050083333	10.0209906333333	9.95935071666667	10.05531845	10.2466873083333	10.2741449416667	10.5557362916667	10.3694253333333	9.11993345	9.81985159166667	8.84099020833333	8.87086751666667	9.40207443333333	10.054920625	9.36666890833333	9.4341767	9.09184225	10.1250607083333	8.77579595833333	9.16123660833333	10.1700709416667	8.85215985	8.3496737	9.27994568333333	9.51454425833333	9.40469768333333	8.80244275	9.01425336666667	9.22249476666667	8.42018036666667	8.72830366666667	9.80802880833333	8.775178575	8.82265860833333	8.821200975	8.71882579166667	9.39525429166667	8.81971978333333	9.1447536

AC013271.1	10.469517225	9.44959046666667	9.78329576666667	10.319078025	10.2749442833333	8.49975086666667	10.7736244333333	9.81176585	9.58933430833333	9.570506275	9.93630081666667	10.8394387833333	10.9680827416667	9.71989020833333	9.20284239166667	9.70017130833333	8.45045408333333	10.28186315	8.85728129166667	9.69747909166667	10.135458025	9.16167278333333	9.14279978333333	8.91224634166667	9.28599946666667	10.0391614083333	8.96700605833333	9.35401866666667	9.12506650833333	9.62890875	8.751330725	8.5905397	9.65670091666667	8.98465464166667	9.97043543333333	9.87908794166667	9.43868498333333	8.91506995	9.291151225	9.24127241666667	8.43902399166667	8.95624593333333	8.81744095	8.56421206666667	9.48031518333333	9.16031618333333	8.75091850833333	8.78498449166667	8.54070765833333	8.34620800833333	8.815557	8.92218960833333	9.33579655	9.0174923	9.04278656666667	8.97675146666667	9.07536643333333	8.82022893333333	8.81154583333333	8.79186565833333	9.7241175	10.2806521916667	9.31001445	10.1497399666667	9.87695954166667	9.77133523333333	10.1134808166667	10.2422793916667	9.304559675	9.89141825	10.1084049583333	10.1927941666667	10.6842469	9.921332825	10.0347123333333	10.13859765	9.86766510833333	9.53592761666667	9.50264109166667	10.0334908083333	10.134682025	10.2233050083333	10.0209906333333	9.95935071666667	10.05531845	10.2466873083333	10.2741449416667	10.5557362916667	10.3694253333333	9.11993345	9.81985159166667	8.84099020833333	8.87086751666667	9.40207443333333	10.054920625	9.36666890833333	9.4341767	9.09184225	10.1250607083333	8.77579595833333	9.16123660833333	10.1700709416667	8.85215985	8.3496737	9.27994568333333	9.51454425833333	9.40469768333333	8.80244275	9.01425336666667	9.22249476666667	8.42018036666667	8.72830366666667	9.80802880833333	8.775178575	8.82265860833333	8.821200975	8.71882579166667	9.39525429166667	8.81971978333333	9.1447536

AC112229.3	10.469517225	9.44959046666667	9.78329576666667	10.319078025	10.2749442833333	8.49975086666667	10.7736244333333	9.81176585	9.58933430833333	9.570506275	9.93630081666667	10.8394387833333	10.9680827416667	9.71989020833333	9.20284239166667	9.70017130833333	8.45045408333333	10.28186315	8.85728129166667	9.69747909166667	10.135458025	9.16167278333333	9.14279978333333	8.91224634166667	9.28599946666667	10.0391614083333	8.96700605833333	9.35401866666667	9.12506650833333	9.62890875	8.751330725	8.5905397	9.65670091666667	8.98465464166667	9.97043543333333	9.87908794166667	9.43868498333333	8.91506995	9.291151225	9.24127241666667	8.43902399166667	8.95624593333333	8.81744095	8.56421206666667	9.48031518333333	9.16031618333333	8.75091850833333	8.78498449166667	8.54070765833333	8.34620800833333	8.815557	8.92218960833333	9.33579655	9.0174923	9.04278656666667	8.97675146666667	9.07536643333333	8.82022893333333	8.81154583333333	8.79186565833333	9.7241175	10.2806521916667	9.31001445	10.1497399666667	9.87695954166667	9.77133523333333	10.1134808166667	10.2422793916667	9.304559675	9.89141825	10.1084049583333	10.1927941666667	10.6842469	9.921332825	10.0347123333333	10.13859765	9.86766510833333	9.53592761666667	9.50264109166667	10.0334908083333	10.134682025	10.2233050083333	10.0209906333333	9.95935071666667	10.05531845	10.2466873083333	10.2741449416667	10.5557362916667	10.3694253333333	9.11993345	9.81985159166667	8.84099020833333	8.87086751666667	9.40207443333333	10.054920625	9.36666890833333	9.4341767	9.09184225	10.1250607083333	8.77579595833333	9.16123660833333	10.1700709416667	8.85215985	8.3496737	9.27994568333333	9.51454425833333	9.40469768333333	8.80244275	9.01425336666667	9.22249476666667	8.42018036666667	8.72830366666667	9.80802880833333	8.775178575	8.82265860833333	8.821200975	8.71882579166667	9.39525429166667	8.81971978333333	9.1447536

LIMS4	10.469517225	9.44959046666667	9.78329576666667	10.319078025	10.2749442833333	8.49975086666667	10.7736244333333	9.81176585	9.58933430833333	9.570506275	9.93630081666667	10.8394387833333	10.9680827416667	9.71989020833333	9.20284239166667	9.70017130833333	8.45045408333333	10.28186315	8.85728129166667	9.69747909166667	10.135458025	9.16167278333333	9.14279978333333	8.91224634166667	9.28599946666667	10.0391614083333	8.96700605833333	9.35401866666667	9.12506650833333	9.62890875	8.751330725	8.5905397	9.65670091666667	8.98465464166667	9.97043543333333	9.87908794166667	9.43868498333333	8.91506995	9.291151225	9.24127241666667	8.43902399166667	8.95624593333333	8.81744095	8.56421206666667	9.48031518333333	9.16031618333333	8.75091850833333	8.78498449166667	8.54070765833333	8.34620800833333	8.815557	8.92218960833333	9.33579655	9.0174923	9.04278656666667	8.97675146666667	9.07536643333333	8.82022893333333	8.81154583333333	8.79186565833333	9.7241175	10.2806521916667	9.31001445	10.1497399666667	9.87695954166667	9.77133523333333	10.1134808166667	10.2422793916667	9.304559675	9.89141825	10.1084049583333	10.1927941666667	10.6842469	9.921332825	10.0347123333333	10.13859765	9.86766510833333	9.53592761666667	9.50264109166667	10.0334908083333	10.134682025	10.2233050083333	10.0209906333333	9.95935071666667	10.05531845	10.2466873083333	10.2741449416667	10.5557362916667	10.3694253333333	9.11993345	9.81985159166667	8.84099020833333	8.87086751666667	9.40207443333333	10.054920625	9.36666890833333	9.4341767	9.09184225	10.1250607083333	8.77579595833333	9.16123660833333	10.1700709416667	8.85215985	8.3496737	9.27994568333333	9.51454425833333	9.40469768333333	8.80244275	9.01425336666667	9.22249476666667	8.42018036666667	8.72830366666667	9.80802880833333	8.775178575	8.82265860833333	8.821200975	8.71882579166667	9.39525429166667	8.81971978333333	9.1447536

ENC1	8.73019295833333	10.257101525	9.734421025	9.8942397	9.58257986666667	10.255903875	9.68516599166667	10.0852465083333	10.1067055416667	9.750361275	9.93515624166667	10.0815238083333	9.55990681666667	10.1577133666667	10.313965725	10.0728614166667	11.2327259583333	9.27125073333333	10.1156550583333	10.4883478333333	10.6122965916667	10.3301703333333	10.0061679666667	10.043884875	10.2959072166667	9.32000775	9.408133425	9.51088574166667	9.66873480833333	9.37126875	9.33964531666667	9.440114675	10.0305098083333	11.5323406833333	8.53882584166667	9.722436925	9.94636165	9.84876684166667	8.82621398333333	9.82733675	10.2342241333333	9.898113975	9.750361275	9.23835931666667	9.84032644166667	10.0807009083333	10.2462958083333	10.4376859583333	10.0774221416667	10.0205705916667	9.401147475	8.608522725	9.37637135833333	10.043884875	9.538317275	9.291151225	9.6324307	10.0864066083333	9.47208941666667	10.0265057583333	10.420034375	11.61112325	9.80664778333333	11.2790070666667	13.3601687833333	9.80231140833333	11.7365773166667	11.72023525	10.1940041	11.2467824083333	11.0534747416667	11.2929247083333	11.629073975	12.2901521833333	10.2543488416667	11.4470684833333	12.3874454166667	10.5764502333333	10.5162662666667	11.3253118333333	12.7021254833333	11.5716921416667	10.6823646833333	12.424331275	10.9054446583333	11.0182236333333	11.8228949916667	11.9666378416667	9.7570522	11.8947334916667	10.899244375	11.7605260333333	10.7189561666667	10.9120401833333	10.3053444333333	10.5545011333333	9.74305075	10.8431589583333	10.45385095	10.500980975	10.76392445	10.80942375	9.620263	9.67096889166667	10.1577133666667	9.97912404166667	11.8630874833333	10.7205897	11.3206346333333	11.9674169166667	10.465697825	9.73098034166667	10.4775633333333	10.7680760333333	10.96888565	9.64024775833333	13.7253859666667	10.7576799666667	9.00822095	11.4188332333333

ZCCHC10	9.33365533333333	9.33275593333333	9.54277241666667	9.37126875	9.00690949166667	8.51177801666667	9.09600253333333	8.77579595833333	8.89174859166667	8.95764171666667	8.74522520833333	7.63102280833333	8.17302170833333	9.10749988333333	8.96796725	8.1439842	8.69220983333333	9.56036118333333	9.70194608333333	8.91889295833333	8.79040525833333	9.11687501666667	9.42734543333333	9.14054775	9.214506275	8.19090655	8.93317065	9.138674475	9.19905495833333	9.30275641666667	9.35549675833333	9.22061473333333	8.800966875	8.74427688333333	8.67450711666667	8.14297786666667	9.17368278333333	9.2056298	9.10576088333333	9.01009886666667	9.50996596666667	9.182097875	9.15113736666667	9.4594195	8.460967325	9.26488315	9.170544975	9.628525675	9.22955863333333	8.84952995833333	9.32471271666667	9.20464029166667	9.52783803333333	9.3786963	9.1038805	9.37821416666667	9.14189448333333	9.5013607	9.39626775833333	8.90962148333333	8.95262451666667	9.0354605	9.27083296666667	9.18505965833333	8.35736911666667	9.00775005833333	9.05454125	8.2260001	9.4697267	8.37257153333333	8.74913316666667	8.58275995416667	8.33398970833333	8.69791309166667	8.51177801666667	8.84758474166667	8.20638435	9.11864413333333	9.23973414166667	8.66247435	8.34128665833333	8.50984575833333	9.62064585	8.833014675	9.006017175	7.9814024	8.67639044166667	8.5671537	8.97216300833333	8.7954535	9.37313166666667	8.620261275	9.07252779166667	8.87390605	9.38580318333333	8.931271375	8.77094225	9.02785883333333	9.091403125	8.96369055833333	9.22912686666667	8.3023979	9.13602675	9.1447536	8.86655378333333	8.87491901666667	8.91690625833333	8.68035370833333	8.96144149166667	8.80139795	8.88592250833333	8.90197375	9.6176772	9.18016484166667	8.87974118333333	9.10749988333333	8.62886449166667	9.09684721666667	9.41761155833333	8.66481208333333

RTN3	11.6771659333333	11.5215953666667	11.5606001833333	11.3809445833333	11.4908711166667	11.7936196666667	11.2067215541667	11.348473325	11.3144428833333	11.414772875	11.0075637333333	11.4000850916667	11.48580155	11.510754075	11.6675947833333	11.3542758333333	11.655146	11.7012064	11.8341069333333	11.5162409083333	11.36947175	11.634515075	11.43032555	11.2420872916667	11.5586348333333	11.5468691083333	11.4310404083333	11.4158566083333	11.393113275	11.3664862333333	11.8596015	11.608877475	11.3144428833333	11.6252518666667	11.520872575	11.6336776833333	11.702337975	11.4872383	11.25027585	11.425117975	11.4277232	11.2895126416667	11.43798885	11.33173475	11.4503715	11.28453495	11.48757865	11.3813533416667	11.1579092583333	11.52618635	11.489428925	11.497488975	11.71493805	11.6440773083333	11.562248175	11.8241137083333	11.3187261333333	11.5411164416667	11.4470684833333	11.35644045	10.9346168	11.1350432333333	11.3509805666667	12.4818012083333	11.0487579166667	11.7425826083333	11.1046868	11.4597359083333	12.445171625	11.4879615666667	11.66250505	11.4527169333333	11.421792525	11.3438040833333	11.0153767166667	11.77986195	11.1908643083333	11.47905225	11.8997209666667	11.3820783333333	11.8145765416667	11.4581724666667	11.4063648666667	11.5802552416667	11.3654035916667	11.9021431083333	11.0651712583333	11.1302224166667	11.8674327083333	11.465822775	11.2579853916667	12.1690316666667	12.1694652083333	13.20399525	11.5630353166667	11.218298125	12.2513034583333	11.5656597	12.13848275	12.120221775	11.22659855	11.0454889833333	10.74667695	11.129529525	11.1227594166667	11.2822211083333	11.1696311416667	11.5987696666667	11.7365773166667	11.7782923583333	11.3790430333333	11.4854519916667	11.884179625	12.1827137333333	11.3231776	11.7292748	11.7564937416667	11.3343049666667	11.5552373166667	11.19202035

SLC22A18AS	4.42771198333333	5.30771575	4.87595029166667	5.56052278333333	4.50011030833333	5.84141815	4.67972664166667	5.1291031	4.093740425	6.16277881666667	4.0078761	3.64058418333333	3.47399078333333	4.73402499166667	6.11958026666667	5.24498075	7.97923883333333	5.51915526666667	6.86736839166667	6.39606678333333	4.48613384166667	3.770641325	5.591740875	5.99598418333333	5.50123778333333	3.77178324166667	6.138425225	6.414216275	8.24367163333333	5.47990566666667	5.66283725	4.128952175	4.977020525	5.97783559166667	3.93478860833333	4.55369980833333	6.24941475833333	6.91828460833333	6.21689609166667	6.86073455	7.188269975	7.54070496666667	6.246964375	7.17330060833333	4.265394625	7.08738564166667	6.37857751666667	6.996742575	5.99843601666667	6.44000804166667	5.892821925	6.08877931666667	4.27160165833333	5.39804303333333	5.429887625	6.09355109166667	5.16735583333333	7.41942373333333	6.71655181666667	6.42485146666667	4.92149175833333	4.96956821666667	5.78709845833333	6.44000804166667	3.97505105	4.07134488333333	7.544616	4.94355658333333	7.44607933333333	6.30781043333333	4.802114875	6.40053460833333	5.07000859166667	4.161871975	5.85609921666667	7.10630213333333	6.06147365	4.58881544166667	8.379169075	6.380135525	3.45326613333333	3.465685875	4.57030863333333	5.43488754166667	5.764437375	3.396759825	4.00032819166667	4.149223425	8.24163635833333	5.565954175	4.841975675	5.23566219166667	6.60400856666667	5.90321861666667	4.13943304166667	5.51803240833333	5.06570035833333	5.171525	6.46419815833333	5.153439775	6.4516666	4.748342275	5.08063435	7.37707911666667	4.40180795833333	4.41506155	6.4095551	6.03052256666667	6.872774725	8.52772891666667	6.99282755	7.823910525	3.93361386666667	4.96638246666667	4.62790769166667	5.78160560833333	4.73832164166667	5.82168396666667	6.01932009166667	4.62161215833333

PLK3	11.3762111	11.0317580041667	10.9297736458333	10.9738844791667	11.4784667916667	13.4233907958333	11.5748264916667	12.0170942458333	11.4415189666667	11.2483557958333	11.0099615166667	11.0583490583333	11.5603926083333	10.5179241916667	11.1097335958333	10.2808657458333	14.488157675	13.3103808375	10.9456650125	11.1149160833333	10.9092903375	10.4466381875	11.4994814416667	10.9481846166667	10.8898722	10.9059969833333	11.1650865916667	11.3848209958333	11.6442866375	11.0446438083333	12.6468611541667	10.586317025	11.4242298625	14.1363801208333	11.3848209958333	10.8466929375	11.545265325	11.057303875	11.354091325	10.8271755666667	11.0875279125	10.8856558833333	10.3866165583333	11.251242475	10.1339838291667	9.930629725	10.3429838208333	10.45328955	10.2487908791667	11.7103631916667	11.8393188625	11.5441949541667	11.2755224208333	10.4010588125	10.2319738041667	10.0298802041667	11.3041958208333	11.0554663541667	10.8196986208333	10.4255098291667	11.2618666291667	10.9554937541667	10.6327192541667	10.9312479291667	10.6167578333333	13.0105212541667	11.8279759083333	11.3807334125	11.7599613916667	11.1186757291667	11.4238612958333	11.0489335125	12.2343494	10.6182167041667	11.0328236041667	10.5113945291667	11.6346724625	11.446195175	11.78439605	10.8895121625	10.3585509	10.2440530041667	11.1617286625	11.2748017583333	11.0554663541667	11.0277886875	11.0281587125	11.1591689333333	10.8572859083333	10.94352845	11.1697849541667	11.0169557958333	10.7342462625	11.0878933166667	11.1526365125	10.9410609833333	11.2451646458333	11.2404438708333	12.5625499041667	11.4301640625	10.425894	10.7059815041667	10.2113504583333	11.281006975	10.161930225	10.2726581875	10.8682881791667	10.9048683875	10.4707689	10.695458375	11.0661219916667	11.2931173958333	10.9860021458333	11.0742373458333	10.0131801916667	10.2452424416667	11.0162524875	12.2932457416667	11.6075168958333	10.9344338125

TCTEX1D4	11.3762111	11.0317580041667	10.9297736458333	10.9738844791667	11.4784667916667	13.4233907958333	11.5748264916667	12.0170942458333	11.4415189666667	11.2483557958333	11.0099615166667	11.0583490583333	11.5603926083333	10.5179241916667	11.1097335958333	10.2808657458333	14.488157675	13.3103808375	10.9456650125	11.1149160833333	10.9092903375	10.4466381875	11.4994814416667	10.9481846166667	10.8898722	10.9059969833333	11.1650865916667	11.3848209958333	11.6442866375	11.0446438083333	12.6468611541667	10.586317025	11.4242298625	14.1363801208333	11.3848209958333	10.8466929375	11.545265325	11.057303875	11.354091325	10.8271755666667	11.0875279125	10.8856558833333	10.3866165583333	11.251242475	10.1339838291667	9.930629725	10.3429838208333	10.45328955	10.2487908791667	11.7103631916667	11.8393188625	11.5441949541667	11.2755224208333	10.4010588125	10.2319738041667	10.0298802041667	11.3041958208333	11.0554663541667	10.8196986208333	10.4255098291667	11.2618666291667	10.9554937541667	10.6327192541667	10.9312479291667	10.6167578333333	13.0105212541667	11.8279759083333	11.3807334125	11.7599613916667	11.1186757291667	11.4238612958333	11.0489335125	12.2343494	10.6182167041667	11.0328236041667	10.5113945291667	11.6346724625	11.446195175	11.78439605	10.8895121625	10.3585509	10.2440530041667	11.1617286625	11.2748017583333	11.0554663541667	11.0277886875	11.0281587125	11.1591689333333	10.8572859083333	10.94352845	11.1697849541667	11.0169557958333	10.7342462625	11.0878933166667	11.1526365125	10.9410609833333	11.2451646458333	11.2404438708333	12.5625499041667	11.4301640625	10.425894	10.7059815041667	10.2113504583333	11.281006975	10.161930225	10.2726581875	10.8682881791667	10.9048683875	10.4707689	10.695458375	11.0661219916667	11.2931173958333	10.9860021458333	11.0742373458333	10.0131801916667	10.2452424416667	11.0162524875	12.2932457416667	11.6075168958333	10.9344338125

PRR14L	4.56071255833333	5.25962125	4.668368375	5.5319599	5.33069273333333	4.09274305833333	4.73720489166667	6.64191437916667	6.04799355833333	5.189937375	4.71113178333333	5.4308407	4.78586035833333	4.83355489166667	4.71899701666667	4.80316131666667	4.98444206666667	4.80635555	5.37867954166667	5.50338315833333	5.26078876666667	5.50024889166667	5.4147768	4.50751908333333	4.79411095833333	5.14718730833333	4.727091875	5.03726580833333	4.34698491666667	6.11047193333333	5.35882338333333	5.09968536666667	4.669520575	4.537079425	4.669520575	5.53793416666667	4.63305978333333	4.86699436666667	5.10174316666667	4.2939407	4.91752975833333	6.50860958333333	5.22040228333333	4.86804739166667	5.02467455	5.80728855	5.74506199166667	5.98304395	5.88144885	5.1653561	5.08571715833333	5.14718730833333	5.192100225	4.78381058333333	4.546642225	4.97490869166667	5.5633586	5.0486896	5.415860975	5.566976475	4.46696494166667	6.328333625	5.979616925	6.225930275	4.946749425	5.65732808333333	5.22492120833333	6.59670515	7.05455971666667	5.37341235	5.14401780833333	5.93571900833333	6.04523669166667	5.41144461666667	4.98892909166667	5.12000276666667	6.16763958333333	5.56242626666667	6.225217	6.09195375	4.82796093333333	5.192100225	5.88853345	5.14089556666667	5.06570035833333	5.054896275	4.697573175	3.96490434166667	5.64694838333333	5.57451170833333	5.43710514166667	5.2460388	5.0258117	5.053877925	5.34097245	5.38263135833333	4.84094744166667	4.61010935833333	5.06786714166667	4.44161573333333	6.23159324166667	6.75898810833333	5.53793416666667	5.30151331666667	6.03052256666667	5.40932128333333	5.23566219166667	5.84699576666667	4.90871175	5.7901556	5.19112474166667	6.31702735	5.927679125	5.54779774166667	4.99794086666667	5.390724	5.58508399166667	5.62957723333333	5.86727098333333	5.57926250833333

DDIAS	11.78439605	11.5781945083333	11.4862831666667	11.9668296208333	12.7583293375	11.5380497375	12.7545436916667	12.1507766708333	12.3668984416667	11.9611231833333	12.1845970916667	12.9567449625	12.6687319583333	11.55504505	12.2622019	11.4054432583333	10.2656372791667	12.7937154333333	11.4837898458333	11.5460149333333	11.1414135375	11.4694963083333	11.018805675	11.3684995708333	11.2562632708333	11.1197786583333	11.2361304166667	11.6645532875	11.7188705625	11.8510047583333	11.6645532875	11.1584270458333	11.8728207416667	10.9059969833333	11.1421225416667	10.9974378291667	11.9327416416667	11.3940742666667	11.9611231833333	11.4051191333333	10.4808530666667	11.1635815541667	10.6272505125	11.1079735875	10.9330242333333	9.6153251625	9.84858852916667	11.0792174166667	10.8418747541667	11.5380497375	11.5335937	11.6623273	10.8204317	10.6908038625	10.7018390416667	9.71286741666667	11.4939949125	10.5697559625	10.9518189083333	10.4082661875	11.709185975	12.0870920041667	11.0411335166667	12.1404171375	11.1762332541667	12.040720525	11.5477833125	12.5403613416667	11.9564880291667	11.8843446666667	12.3145443708333	12.3695852583333	12.2846599291667	11.7987540875	11.8964597208333	11.356976575	12.4076008791667	11.8026393875	11.7457917833333	12.0468052333333	11.45289505	11.3891676541667	11.0817075916667	10.9316130333333	12.0895281791667	11.7547114875	11.9791136541667	11.7547114875	12.3337269375	10.666212575	11.6395856333333	10.9935606083333	12.1801535291667	11.2339787458333	11.9937599291667	12.0850507583333	11.7930853041667	12.274965975	12.0015173166667	10.8313562916667	10.6239107333333	11.682501525	10.7102346	10.2938167041667	10.5346935333333	10.5974997458333	10.4888905458333	10.79813175	10.8362821958333	11.0230447375	10.8849115041667	11.2458949	10.0690719916667	9.4962317	10.6478785375	10.2898723041667	10.1676970958333	10.9452771166667	10.8000125916667	11.2059617875

PRCP	11.78439605	11.5781945083333	11.4862831666667	11.9668296208333	12.7583293375	11.5380497375	12.7545436916667	12.1507766708333	12.3668984416667	11.9611231833333	12.1845970916667	12.9567449625	12.6687319583333	11.55504505	12.2622019	11.4054432583333	10.2656372791667	12.7937154333333	11.4837898458333	11.5460149333333	11.1414135375	11.4694963083333	11.018805675	11.3684995708333	11.2562632708333	11.1197786583333	11.2361304166667	11.6645532875	11.7188705625	11.8510047583333	11.6645532875	11.1584270458333	11.8728207416667	10.9059969833333	11.1421225416667	10.9974378291667	11.9327416416667	11.3940742666667	11.9611231833333	11.4051191333333	10.4808530666667	11.1635815541667	10.6272505125	11.1079735875	10.9330242333333	9.6153251625	9.84858852916667	11.0792174166667	10.8418747541667	11.5380497375	11.5335937	11.6623273	10.8204317	10.6908038625	10.7018390416667	9.71286741666667	11.4939949125	10.5697559625	10.9518189083333	10.4082661875	11.709185975	12.0870920041667	11.0411335166667	12.1404171375	11.1762332541667	12.040720525	11.5477833125	12.5403613416667	11.9564880291667	11.8843446666667	12.3145443708333	12.3695852583333	12.2846599291667	11.7987540875	11.8964597208333	11.356976575	12.4076008791667	11.8026393875	11.7457917833333	12.0468052333333	11.45289505	11.3891676541667	11.0817075916667	10.9316130333333	12.0895281791667	11.7547114875	11.9791136541667	11.7547114875	12.3337269375	10.666212575	11.6395856333333	10.9935606083333	12.1801535291667	11.2339787458333	11.9937599291667	12.0850507583333	11.7930853041667	12.274965975	12.0015173166667	10.8313562916667	10.6239107333333	11.682501525	10.7102346	10.2938167041667	10.5346935333333	10.5974997458333	10.4888905458333	10.79813175	10.8362821958333	11.0230447375	10.8849115041667	11.2458949	10.0690719916667	9.4962317	10.6478785375	10.2898723041667	10.1676970958333	10.9452771166667	10.8000125916667	11.2059617875

PHIP	7.21695988333333	5.9323169	6.29443513333333	6.16829805	6.0951249	6.38084359166667	6.08509065	5.6003113	5.50615049166667	6.178567625	5.57335978333333	5.90321861666667	5.93306849166667	6.08105208333333	5.53102171666667	5.93036776666667	5.933890025	6.591129625	6.23620956666667	6.22835699166667	5.984788925	7.09487224166667	6.40124803333333	7.146985275	6.732067125	5.7955502	6.19736666666667	6.23229269166667	6.56509979166667	5.74130296666667	6.58428694166667	6.46055301666667	5.2815818	6.165146375	6.414216275	6.68656881666667	6.41504531666667	6.35356116666667	6.86809180833333	6.552906925	5.899063175	6.00285793333333	6.40185915	6.692469175	6.28660823333333	6.558117975	7.04898169166667	6.27652200833333	6.55520170833333	6.38925563333333	6.80449616666667	6.390922	6.04700228333333	6.98158215833333	6.27482028333333	6.662431575	6.43219438333333	6.70390775833333	6.4975584	6.742824875	7.40193138333333	5.81563615833333	7.79031855833333	7.044419	6.32175328333333	7.42619010833333	6.80784224166667	5.554616075	7.25799328333333	5.97184991666667	6.35808980833333	6.407249275	5.57451170833333	6.83868629166667	6.35356116666667	5.65427465	6.05392339166667	6.63574723333333	7.12345469166667	6.23950985833333	7.14822965	6.79798854166667	6.75430906666667	7.2313303	7.18351369166667	6.17067161666667	7.46352631666667	5.379722375	6.52564285833333	6.60400856666667	7.093633575	7.20747220833333	6.85677963333333	5.99349075	5.90415203333333	6.131776975	6.75646765833333	7.60239018333333	6.462885175	6.81435373333333	6.37491049166667	5.23259211666667	7.40898444166667	6.7503122	6.89266504166667	6.65156973333333	7.20806453333333	6.97781291666667	7.21570014166667	5.42082883333333	6.8732984	6.61689901666667	6.80377665833333	6.823097925	7.12401263333333	7.00303549166667	7.11547685	7.28130318333333	6.96330954166667	6.67633144166667

LINC02218	7.44491683333333	8.70635446666667	9.16588333333333	9.64610789166667	10.1148100333333	6.99601435833333	10.1477211833333	9.6895289	10.02519555	8.68575455833333	9.0517655	10.0260539583333	11.0480221416667	9.19576385833333	8.05087028333333	6.89003731666667	7.36530371666667	8.80139795	6.4095551	7.36530371666667	9.68602584166667	8.10529686666667	7.908404275	6.880500475	5.99939696666667	7.75602535833333	7.009464425	7.20911576666667	6.5175539	8.12434584166667	6.83020955	6.77501233333333	8.500835825	7.65996385833333	6.62367098333333	5.50123778333333	6.41957618333333	5.5022085	6.00123059166667	4.85768484166667	4.75806056666667	7.64327454166667	5.72790364166667	5.13961736666667	8.2204362	6.47930140833333	5.08161569166667	7.73027796666667	5.74222661666667	5.43281713333333	4.92991125833333	5.18112505	8.364240675	5.3960318	7.45786721666667	6.52272641666667	8.478972275	5.966951625	5.68304789166667	5.866285325	8.1976201	9.2768977	7.28500960833333	9.312713675	9.53356816666667	8.76514685	9.18880384166667	10.9223119333333	8.45745534166667	8.940002325	9.13825335833333	9.23462919166667	10.4732782833333	8.873364875	10.775195775	7.010804075	9.03372714166667	7.51696985	7.37123815833333	8.20737129166667	9.60140389166667	9.27812115	8.08289571666667	7.65663390833333	9.75665824166667	8.96932985833333	8.76898080833333	9.18115765833333	9.31809370833333	6.82102155833333	8.8334785	5.53695673333333	7.04956788333333	7.040686725	8.594439725	8.02464245	7.01553179166667	6.80449616666667	7.99022335	6.34978520833333	7.38051639166667	9.76310825	6.96120915	6.29670533333333	7.82845124166667	8.48261985	7.68936630833333	8.21097586666667	6.97095796666667	6.975151325	5.28464988333333	6.164307025	5.70649276666667	5.43596139166667	7.57903273333333	8.18781211666667	7.45043895	5.53586285833333	6.91035588333333	8.168322275

VAT1L	10.4530959333333	7.17198438333333	7.264052575	7.13351973333333	7.48476935	7.838427925	8.06450121666667	6.88718085	7.93490190833333	8.85865705	6.998615075	8.698928125	9.31085525	6.78381769166667	7.47204458333333	7.34551940833333	9.05965051666667	6.98096124166667	7.11278106666667	7.547481675	9.04841031666667	7.02536274166667	7.36048969166667	7.26110544166667	7.83125643333333	10.94802585	7.40837556666667	6.45841340833333	5.44754924166667	5.82073376666667	5.96958254166667	6.89596523333333	7.2015586	9.42859468333333	9.27643005833333	10.4986956916667	6.74888376666667	7.71189573333333	5.97106113333333	7.63549629166667	7.76032476666667	5.52437941666667	6.03581564166667	6.941911825	9.09054048333333	8.886411025	6.823097925	7.08560825833333	7.82958231666667	6.53407053333333	6.234662425	7.2401722	7.505130225	7.137920375	7.42731664166667	7.38123391666667	7.88018380833333	7.86377196666667	8.21248958333333	8.38422716666667	6.850945825	7.37467966666667	6.42781263333333	6.17635683333333	6.64292103333333	6.10002881666667	8.02185345833333	5.68979354166667	6.141642375	6.36396054166667	7.45614546666667	8.309637175	7.91465324166667	7.82958231666667	6.94120649166667	7.46352631666667	6.71047270833333	6.53205264166667	6.36248048333333	6.64226356666667	7.20911576666667	8.70592035	6.63942924166667	8.68375230833333	7.29233335	7.48985504166667	5.69576305	6.7730469	7.057705175	8.322152325	6.16277881666667	6.70667168333333	5.9623768	7.5010982	5.81652036666667	6.43359675	5.70932124166667	7.11730268333333	6.86668814166667	6.39012523333333	7.5998906	7.26626465	4.71899701666667	6.68859711666667	6.67919664166667	7.19951121666667	8.084825575	6.7327851	6.5332879	7.9760917	5.83398495	6.691149875	6.36616499166667	8.03004540833333	6.75712434166667	5.7835982	7.95255818333333	7.348327125	4.90256950833333	7.46746676666667

PRRT4	9.61413186666667	9.20284239166667	9.2436075	9.3195178	10.1328782916667	9.08960063333333	10.4344952333333	9.28551639166667	9.67592103333333	9.19676581666667	9.45555605	10.2442419583333	10.32163045	9.683051225	8.998094275	8.85635194166667	8.62685349166667	10.1409369083333	9.0498455	8.956732575	9.32471271666667	9.259928725	8.631828525	8.84952995833333	8.45851334166667	9.27365428333333	8.686777175	8.83443758333333	9.39938098333333	9.058233875	9.06405529166667	8.94100855	9.28929068333333	9.02705223333333	9.39574425833333	9.094155	9.3434528	8.98368761666667	9.09184225	8.79240648333333	8.635462725	8.9604306	8.61442265833333	8.259343925	8.71986490833333	8.62734905	8.717376625	9.34392991666667	8.391097025	8.51528900833333	8.57649310833333	8.94233088333333	9.16167278333333	9.12326560833333	8.50286535	8.686777175	8.76273385833333	8.38565373333333	8.79240648333333	8.70034938333333	9.766909275	9.79362293333333	8.74322296666667	9.585238675	9.34670505	9.78528060833333	9.55898925	9.64650925	9.58257986666667	9.55058418333333	9.76350663333333	9.729222475	10.5933754916667	9.54925085	9.95398405833333	9.35255276666667	9.929600025	8.96850814166667	9.04410515833333	9.33537304166667	9.33496756666667	10.0265057583333	9.51041425833333	9.28929068333333	9.40511030833333	9.75787298333333	9.63199205833333	9.77312443333333	9.94251199166667	8.77298199166667	9.36338105833333	9.3041126	9.01847910833333	9.06838428333333	9.74904354166667	9.34670505	9.56314586666667	9.62805526666667	9.96565705833333	8.99438999166667	8.97354856666667	10.2786656166667	8.78220615833333	8.3662572	9.43456800833333	8.85865705	9.00867418333333	9.63342665833333	8.83154074166667	8.615263775	8.4067182	8.565739425	9.18546986666667	8.36907771666667	8.31911250833333	8.54268981666667	8.99535305	9.03034083333333	8.59842283333333	9.277313475

ZDHHC23	9.674571275	9.4094908	9.53914050833333	10.0087152083333	9.766909275	9.04841031666667	10.02019625	9.17457594166667	9.56548648333333	9.17813545	9.42244939166667	9.66513115	9.29664133333333	8.93897689166667	9.092829475	8.67361984166667	9.52867283333333	9.48470895833333	9.13602675	9.38762769166667	8.5994674	8.77628200833333	8.91365106666667	8.8526135	9.14971231666667	9.37588805	9.47208941666667	9.36480266666667	9.34261419166667	9.309548625	9.47351875833333	9.63902631666667	9.71485326666667	9.80056271666667	10.033950425	9.96267133333333	9.49147149166667	9.37921308333333	9.50773276666667	9.64118481666667	9.0354605	9.28741124166667	9.28405401666667	9.31452535833333	8.995827675	8.95312890833333	9.21257606666667	9.19484745	8.859115725	9.060159775	9.21688079166667	9.70017130833333	9.60459400833333	9.72156395833333	9.64153864166667	9.47641323333333	9.360413025	9.54357819166667	9.469462225	8.97216300833333	9.859391125	9.41070553333333	9.52364006666667	10.4108585666667	9.32574429166667	9.354988125	9.35679085833333	9.80664778333333	9.33411035833333	9.51549753333333	9.96745814166667	9.53957571666667	9.388540625	9.10145314166667	9.51724313333333	8.98046293333333	9.57222860833333	9.25303868333333	9.17093331666667	9.379662375	10.0364758583333	8.56867330833333	8.88688890833333	8.898281125	9.628525675	9.40159784166667	9.92703645833333	9.360413025	9.95935071666667	9.028831875	9.52268223333333	9.67723733333333	9.86062085833333	9.38580318333333	9.91887936666667	9.382290975	9.471590125	9.81132868333333	9.579948275	9.57007478333333	9.55860505833333	9.53738644166667	9.81818709166667	9.496436825	9.133746725	9.36621750833333	9.18974645	9.35255276666667	9.166824	9.00093885833333	9.51175975	9.53179666666667	9.289706825	9.63902631666667	9.836699675	9.5453508	9.16588333333333	9.64247345	9.73350879166667	9.59648643333333

LTC4S	15.8653098666667	15.8653098666667	15.7559244416667	15.8817318166667	15.3290216916667	15.873782975	15.1495015916667	15.7338865416667	15.361847675	15.6652002083333	15.7198901333333	15.57601185	15.8383325666667	15.581383325	15.6235572	15.4803730166667	15.799582875	15.7630766833333	15.873782975	15.5870133916667	15.784983325	15.6824817166667	15.873782975	15.2502335583333	15.4316128083333	16.0258935166667	15.6711097083333	15.5603668666667	15.69535405	15.2749915333333	15.7338865416667	15.799582875	15.2687677	15.225946525	15.7414894	15.9315345	15.9859723583333	15.7697269333333	15.5512564416667	15.6585444166667	15.6355001416667	15.5176023166667	15.4276773083333	15.4276773083333	15.5870133916667	15.4803730166667	15.57601185	15.3177335583333	15.784983325	15.7138591583333	15.5458909083333	15.9960220916667	15.5923775583333	15.5923775583333	15.4160185666667	15.47595875	15.4712092083333	15.6355001416667	15.9237782	15.6037263583333	15.8219621166667	15.9960220916667	16.1648856916667	16.1910724916667	15.8904374666667	15.9960220916667	16.1772486333333	15.9960220916667	16.0258935166667	16.0142070583333	15.9960220916667	16.2339961416667	15.873782975	16.113934775	15.8990167	15.9673534416667	16.1648856916667	16.1022656	16.0258935166667	16.1022656	15.7781512166667	15.9767561166667	15.814756725	15.9582825916667	15.8904374666667	15.7559244416667	15.6585444166667	15.8817318166667	16.0142070583333	16.0055115333333	15.9582825916667	16.1022656	16.0573941333333	16.2042656333333	15.9064622666667	15.9767561166667	16.3470849416667	16.0691638333333	15.9064622666667	16.113934775	15.727394275	15.700189825	15.799582875	15.8904374666667	15.5556811083333	15.7559244416667	15.814756725	15.6652002083333	15.9493575916667	16.0374488333333	16.0485681333333	16.3470849416667	15.7414894	15.5407381333333	15.4400250416667	15.4022381166667	16.1274635083333	15.9960220916667	16.2042656333333	15.814756725

MMAA	9.49147149166667	9.57267214166667	9.40680740833333	9.60963610833333	9.20652433333333	9.06745036666667	8.21672670833333	9.250740625	9.14327603333333	9.10749988333333	8.903371	8.884079375	8.72089346666667	9.291151225	9.14426045	9.48078894166667	9.70495998333333	8.86211740833333	9.4134397	9.63156661666667	9.31405536666667	9.986796275	9.68034129166667	9.32368785833333	9.53738644166667	9.40159784166667	9.30221980833333	9.311763425	9.52996276666667	9.60093574166667	9.22061473333333	9.428146125	9.07865619166667	9.553861875	9.18361099166667	9.57179898333333	9.03327476666667	9.39574425833333	9.26488315	9.148762375	9.44293281666667	9.674571275	9.60234078333333	9.63430166666667	9.51454425833333	9.50914073333333	10.000563275	9.68213623333333	9.890973325	9.28834158333333	9.538317275	9.80350678333333	9.61323428333333	9.85498678333333	9.97706043333333	9.9519621	9.21835433333333	9.75665824166667	9.23797865	9.721994775	9.20605604166667	8.56044531666667	8.86953279166667	8.16931455833333	9.68992429166667	7.9184414	8.551483425	8.298377725	8.59785679166667	9.24031536666667	8.04179383333333	8.63917994166667	7.24330025	8.46245245833333	8.89652095833333	9.31001445	8.89870729166667	8.661516725	8.28242620833333	8.65367691666667	9.10015925	9.296236625	8.86760295	8.72229471666667	9.14189448333333	9.19531680833333	9.42465609166667	8.38960981666667	8.18684245	9.07484345833333	8.2549704	9.58662811666667	7.97086885	8.98325939166667	9.14003079166667	8.11761579166667	9.21923738333333	9.27035150833333	7.80563896666667	10.0326667666667	9.48206465833333	8.93461839166667	9.16442515	9.906465775	8.61342080833333	9.09007516666667	9.02927973333333	8.30591494166667	9.38536081666667	9.27035150833333	10.1350682166667	9.60186775	9.875255275	9.20652433333333	9.32231320833333	10.064052975	9.29481465	8.9384783	8.98096389166667	9.86517185

AKAP11	15.2576870916667	13.1531334333333	13.0313582416667	12.881258225	13.5484254	13.1229606833333	13.0645149833333	13.1946165416667	13.2427820583333	13.0571358166667	12.8392807333333	14.0950375583333	13.8376377666667	13.3675920333333	13.454214775	13.7234378333333	13.096654525	13.317291325	13.321391725	13.599265975	13.3882606083333	13.7372005583333	13.0089762083333	13.1959888083333	13.4847749333333	14.4060458333333	13.487986925	13.1872302333333	13.4864308583333	13.2360644333333	13.294808925	13.3459381666667	13.4096868166667	13.3285445333333	14.21756365	14.596526825	13.6320213916667	13.336335625	13.046364975	13.5832773166667	12.9761766916667	13.397359625	13.3682726083333	13.4565571916667	14.2132241916667	13.2538590583333	13.994455075	13.3100924166667	13.2135649916667	13.2560414333333	13.065712375	13.28687105	13.6969617	13.8049154	13.5213793583333	13.7234378333333	13.5068025166667	13.20399525	13.40102065	13.9250365	13.2731848666667	13.2373203666667	12.8836445083333	13.5346846166667	13.0469983166667	13.019993025	13.1865714083333	13.810810275	13.5116191416667	13.4807854416667	13.05246665	13.2354463	13.1793538166667	13.4164430416667	13.3193049583333	13.1078475833333	13.56515455	13.5610039666667	12.8690686583333	13.057870725	13.322101775	13.781003275	13.1216942833333	12.9954911666667	13.5329794333333	13.4520130916667	13.2061458583333	13.14792425	13.4579324833333	13.0349568083333	12.9756001333333	13.6236897166667	13.57979325	13.2933426583333	13.0518882	12.6297371916667	12.6775440333333	13.134360625	13.1531334333333	12.8817875333333	13.3508640083333	12.3688898083333	13.316609775	13.1733038666667	12.17539085	13.6497739333333	13.222146075	12.7655438666667	13.3270763416667	13.3771159333333	13.8473484166667	12.2396159	13.5660586666667	13.1753140083333	13.2765049166667	12.9012827916667	13.1249649416667	12.8549658583333	12.811279575	13.0877248833333

KCTD3	12.2528148916667	12.251683475	12.1312748	12.5539148333333	12.5492591916667	11.5174454333333	12.1891609666667	12.1435544583333	12.6481258583333	12.06079575	12.9042883333333	12.16243335	12.488903425	12.2185126	12.757496125	11.671592675	12.0092346166667	12.40597585	12.3509430416667	12.3244889333333	12.220220925	13.253120425	12.430195425	12.16610385	12.8537857833333	12.198234625	12.6800815583333	12.8101998166667	13.0645149833333	12.83111185	12.1728373083333	12.5349027	12.2998821166667	11.3612411916667	12.12212835	12.0685666833333	12.8208910916667	12.2111963416667	12.5897782	12.5098151833333	11.9236129	12.3780252416667	12.307547675	11.868539825	12.1237921916667	11.9240142833333	12.4069098166667	12.1439381	12.0322027833333	12.2141453583333	11.799711325	12.3750783583333	12.6407582416667	12.84211235	12.906960875	12.1861275083333	12.2289445583333	11.94832135	12.686781525	12.1130081666667	12.5329767583333	11.5983924583333	11.5019499416667	11.683026175	12.2972775333333	11.8116053416667	11.5404299	11.7209431166667	11.4715256833333	11.8819037833333	12.3815660416667	11.8126730416667	10.8934071416667	11.510754075	12.3318475166667	11.1346623583333	11.509666625	12.0457744	11.947152875	12.1087888833333	12.3961090083333	13.0276586166667	11.1901541416667	11.4188332333333	11.5761677833333	12.2848587416667	12.156197075	10.9965540083333	13.334230975	11.52420235	11.2950935333333	12.3284449583333	12.3952132583333	11.5223462	12.3240568416667	11.401232775	12.1882257583333	12.996638525	11.8293448166667	11.9392227916667	11.921685825	11.1821084666667	11.9762173583333	11.8317467416667	11.1941109166667	11.43469695	11.363165975	11.24605735	11.2391470583333	11.424045025	11.6175120416667	12.0827580166667	11.4415189666667	10.924157925	11.388258375	11.7351274916667	12.5813238666667	10.7812240416667	12.115901275	11.0673982916667

PURG	5.97349889166667	5.38659805833333	6.16692735	4.07775664166667	4.73720489166667	4.9727824	6.07339345833333	5.57834849166667	5.4147768	4.73402499166667	4.99137435	6.181735025	5.87591940833333	4.95627725833333	5.39700335833333	4.92991125833333	5.32031858333333	5.18112505	4.71016199166667	4.83671265833333	4.53824951666667	4.43091141666667	4.13647209166667	4.77930389166667	4.93337844166667	5.30888879166667	4.76252700833333	5.03930840833333	5.19309993333333	5.451608675	4.74930484166667	4.47089620833333	5.052876825	5.13336504166667	6.24107090833333	6.59234331666667	4.87710051666667	4.69642039166667	4.89027461666667	5.13227493333333	4.87823230833333	4.94478124166667	5.30562465833333	4.883731825	4.21192669166667	5.34862645833333	4.74930484166667	4.48330838333333	4.41297161666667	4.89595425	4.50222558333333	5.20449400833333	5.108231475	5.53292085833333	5.36511784166667	4.52898779166667	4.024477825	4.56909456666667	5.256776325	4.81485295	5.75213593333333	6.23950985833333	5.20250346666667	5.23035095	6.76786575	6.07768216666667	4.62161215833333	6.14803923333333	4.21395365833333	5.73943181666667	5.528242825	4.233876925	5.984788925	5.20658905833333	5.19860551666667	4.87091625	5.135343	4.7771511	5.20658905833333	4.49904255833333	6.82704920833333	6.37713004166667	4.750433775	5.13336504166667	5.35273364166667	5.76350409166667	4.91752975833333	4.73206480833333	4.977020525	5.82731045	5.74413374166667	5.577321975	5.09968536666667	5.49526165833333	4.62790769166667	4.58241979166667	4.66519471666667	5.78626178333333	4.93116945833333	5.14192449166667	5.49736965	5.01100034166667	4.89027461666667	4.57240950833333	4.2422894	5.28357275833333	5.57335978333333	5.40518485833333	4.81716043333333	5.87208589166667	5.44644633333333	5.07445225	5.464635875	5.2815818	5.79742544166667	5.38372931666667	5.363009525	6.14494839166667	5.36415765833333	4.28696936666667

GCNT3	4.72828980833333	12.2044709583333	11.09861755	12.2002559333333	11.9856427083333	11.5019499416667	9.63156661666667	10.1557652416667	12.0860846083333	9.75194415	12.3921887583333	4.27276220833333	9.64736674166667	11.5824943666667	10.8484072166667	9.81686935833333	10.388343775	10.5392644333333	11.3001028166667	10.2198038	11.673083125	13.1172651	10.1808367916667	11.233793875	12.1488811083333	4.443420775	9.97912404166667	11.50454985	11.3144428833333	10.5711304666667	10.5534355833333	12.4101428	12.0884912166667	12.0804266666667	4.78700018333333	4.59369346666667	11.586171175	11.062266425	11.19202035	10.264650175	11.1721251583333	11.5237451916667	12.2964028333333	11.0146426833333	11.4391211083333	10.5945390166667	12.1299872583333	11.327862475	11.9078069166667	11.8626743166667	11.012770325	10.7217139416667	11.6775693083333	11.6129963	11.7871666333333	11.7074261416667	10.9303115416667	11.4556673333333	11.8970046083333	11.2880004333333	8.107904375	7.89880223333333	11.5268940083333	9.15645445833333	4.85974603333333	8.38611514166667	4.853434925	9.74175274166667	5.192100225	7.72686941666667	8.293531175	9.42992638333333	4.38197743333333	11.2406150083333	8.53385956666667	5.34097245	13.5907625666667	8.72544860833333	8.06996655	11.3767696916667	4.748342275	4.60382705833333	9.373589925	6.76187970833333	10.9458611583333	6.67096843333333	4.84506578333333	5.020575025	5.50436075	5.78709845833333	6.52272641666667	6.64764070833333	8.16529418333333	7.6066095	5.18112505	7.77775845	8.478972275	7.1762422	5.8532158	7.88228429166667	4.40382505833333	6.50929938333333	7.05455971666667	11.868130425	11.6754049666667	9.44959046666667	9.21080935833333	7.95311691666667	8.19859208333333	6.83682504166667	9.26855390833333	10.9780057166667	9.00643414166667	6.26140625833333	6.8444516	10.8090233916667	4.53824951666667	6.8190902	11.028339325	6.39383819166667

GBF1	5.78626178333333	6.03335128333333	5.876765525	5.557624025	6.1905363	6.164307025	6.26827546666667	6.66522548333333	5.764437375	4.86804739166667	5.2815818	6.02974674166667	6.188915175	4.98009509166667	5.09764620833333	4.69425546666667	4.40382505833333	6.12364109166667	5.94185621666667	5.173701425	6.31702735	5.90235153333333	5.20550439166667	4.9426219	6.46988230833333	6.0148863	5.99418615	5.86812175833333	5.46938143333333	5.84513776666667	5.51098925	5.880482625	5.33867198333333	5.69189906666667	5.6723045	6.063181275	5.24703029166667	4.69425546666667	5.927679125	5.36818925	5.42082883333333	5.514879125	4.84714924166667	5.09217729166667	4.93761881666667	5.72573654166667	4.86804739166667	4.42570850833333	6.18568551666667	5.45697495	6.17154675833333	5.94277896666667	6.33264478333333	6.93552516666667	4.222209325	5.51098925	5.279548575	5.11118121666667	5.31119278333333	4.91752975833333	6.359562825	6.31021560833333	7.07238718333333	6.295927975	5.623286525	6.88648389166667	6.53407053333333	6.91578644166667	6.931726825	6.34078100833333	5.755893075	6.24107090833333	7.16909093333333	5.826473825	5.162070825	6.51382934166667	5.86727098333333	6.897275725	6.219278425	6.475087675	6.53407053333333	6.27165035	6.10156668333333	6.54933985	6.07768216666667	6.74610281666667	7.238929725	5.80264275	6.679903875	6.2379381	6.06914035833333	6.005441825	5.86347250833333	6.19893275	6.66118995	6.05994703333333	5.76923416666667	6.91634240833333	6.28265325	5.54303723333333	6.22304681666667	6.060789425	6.92042344166667	6.30861273333333	6.55223534166667	6.393024275	6.61689901666667	5.27629945	6.54514473333333	6.11703395833333	6.297552775	5.26783745	7.270544475	7.4784294	6.28184195833333	6.48497233333333	5.778884625	6.63574723333333	6.11462551666667	5.38558591666667

DELE1	13.0571358166667	12.2739391166667	12.4257259083333	12.3418182	12.3997083083333	12.17539085	11.9863991	12.3653316083333	12.2821681166667	12.214635575	12.226050525	12.9924504416667	12.5221926333333	12.5140767416667	12.5020083166667	12.6606270166667	12.6381216166667	12.8329037166667	12.5468096583333	12.8624048833333	12.52409195	12.5969475916667	12.4936861916667	12.5362402583333	12.92419445	13.294808925	12.4257259083333	12.7827098416667	12.4804102333333	12.24842175	12.533964725	12.7092084083333	12.6985834416667	12.8515337583333	13.08588685	13.3370287666667	12.7139152416667	12.3798172166667	12.4202498166667	12.40421355	12.4346028333333	12.2755744916667	12.443519	12.5552867333333	12.912870175	12.43292905	12.7279204166667	12.2416674416667	11.9777865	12.440827975	12.4333458416667	12.44001515	13.312167125	12.938273025	12.552088775	12.9388338	12.516863425	11.9204648416667	13.1268647416667	12.7494695	12.946647375	12.0185152166667	11.60515845	11.811945375	12.34092395	11.8166747583333	11.3813533416667	12.0368526666667	12.1895397416667	11.9304447166667	11.722386025	11.971689825	11.9082126416667	12.0237948	11.888073425	11.9781875416667	12.4101428	12.5212289166667	11.7422357083333	12.038401575	13.122302875	12.5939827166667	11.541832	11.7871666333333	12.2326729333333	12.3059641583333	11.8607926333333	12.0265627166667	12.2557740666667	12.1694652083333	11.8228949916667	12.57699495	12.0249612083333	12.0424744583333	13.0294955083333	11.3054657083333	11.55825445	11.8081782916667	11.912794275	11.4467356166667	11.8137761833333	11.22444735	12.5921909166667	11.2576230916667	11.7027128	11.8194805166667	12.2582885416667	11.25535535	12.1583760416667	12.05061705	11.6013132583333	9.890973325	12.3961090083333	11.9966897833333	11.7776128333333	12.0953574416667	11.7418730833333	10.9571307916667	12.4501055333333	11.9853022666667

NOP14	9.62367923333333	8.11454491666667	8.50236353333333	8.34128665833333	8.23217175	10.6542751166667	7.98087950833333	8.391097025	8.37970946666667	7.9089283	8.762233425	9.38762769166667	8.024066175	8.41158014166667	8.47579976666667	8.77579595833333	9.67723733333333	8.17555483333333	8.52583200833333	9.12506650833333	8.81515405833333	8.9131846	9.09779791666667	9.60830463333333	8.86508634166667	9.900995025	9.26261210833333	9.20373038333333	8.846155575	8.85348764166667	9.18974645	9.6347809	9.1289299	9.22912686666667	9.47542340833333	9.27899289166667	8.59697231666667	8.69984448333333	7.95359058333333	9.13033400833333	9.19860346666667	8.686777175	8.940002325	8.873364875	9.133746725	9.09833678333333	9.04505583333333	8.62136429166667	8.66349279166667	8.97625954166667	8.50984575833333	8.66481208333333	8.50185603333333	8.95218464166667	9.16780343333333	9.00093885833333	8.54122040833333	8.60646375	9.06838428333333	8.79439289166667	9.14782716666667	7.92110699166667	8.755680875	8.92123313333333	9.27994568333333	10.400070625	8.81418505	7.8129644	9.33838469166667	8.199157725	8.333019375	8.5232739	10.8566946833333	8.94982423333333	9.76219373333333	9.12557628333333	8.2874613	8.55392016666667	10.8120670333333	8.96987785	9.75620250833333	8.83154074166667	11.1398416833333	9.43868498333333	9.52504286666667	9.945938775	9.81985159166667	8.71519911666667	7.70471373333333	9.1473568	8.97168731666667	8.58856818333333	9.73270225833333	9.08869563333333	9.2056298	7.85807856666667	8.45693534166667	8.82211801666667	9.92916715	8.987001225	8.35633766666667	7.40770269166667	9.61047175833333	9.565041475	10.2567002166667	9.10485650833333	9.687830775	11.4056072833333	10.683111325	9.68992429166667	9.37177325833333	9.33708134166667	9.80664778333333	8.83783124166667	9.53179666666667	8.49006413333333	9.29664133333333	9.349869075	9.36892748333333	8.333491425

ADGRL1	9.7045424	9.485645225	9.31047634166667	9.47679953333333	10.83116835	9.55333915	10.9621867333333	9.90307260833333	10.8389999833333	9.78329576666667	10.0894400666667	10.9501588	10.5400727416667	9.54039348333333	9.35451149166667	8.87183624166667	8.49593345833333	10.4744353333333	8.88262415833333	9.14518724166667	9.61633444166667	9.85161335833333	8.90962148333333	9.119480775	9.60963610833333	9.74045244166667	9.51857009166667	9.382290975	9.79147918333333	9.45055241666667	8.80450975	9.17144896666667	10.1444546916667	9.73350879166667	9.51951014166667	9.68863114166667	10.1895353083333	9.428146125	9.56172193333333	9.4981546	9.04133171666667	9.22606134166667	8.57400345	8.59697231666667	9.08221395833333	8.3048096	8.16877921666667	9.00137649166667	9.21123668333333	9.3832069	9.32526180833333	9.48341896666667	10.3833420583333	9.50725341666667	9.14782716666667	8.364240675	9.954843075	9.00690949166667	9.36892748333333	8.97266575	10.4445170416667	10.0984817166667	9.360413025	9.88434308333333	9.66781955833333	10.6860936833333	9.88726988333333	10.5979724666667	10.4280419583333	10.4348946583333	10.5537715083333	10.1063080916667	10.5605583916667	9.869019275	9.81608086666667	9.25584964166667	10.0744319916667	9.76136145	9.17971915	9.63156661666667	9.99808105833333	10.4215953	9.4594195	8.91181045	10.39291705	10.2064993416667	10.5053205416667	10.1469040583333	10.6816359083333	9.5013607	9.59563205	9.673127475	10.3412407666667	9.96443173333333	10.6990064	10.240361675	10.6003674833333	10.5123801583333	10.527258325	9.81608086666667	9.92175831666667	10.36070035	9.15740435833333	8.95121126666667	9.497335375	9.63199205833333	9.25947800833333	9.35863609166667	9.6526333	9.88515190833333	9.612775675	9.291151225	9.85198440833333	8.76031966666667	9.44430623333333	9.13460934166667	9.65888631666667	9.67900834166667	9.46751014166667	9.824729

ETV1	11.68154355	8.78307434166667	9.54731913333333	11.5139760666667	9.4687509	11.5920332333333	8.50702275833333	9.76911673333333	11.013128925	9.67141531666667	11.9546857416667	11.0942927666667	10.08353915	9.145650675	9.14921909166667	9.71227291666667	9.96611523333333	8.47333296666667	9.693930075	9.3836955	9.42424818333333	10.47977665	9.24668224166667	11.84965965	11.7678571083333	11.496789525	9.297473225	8.931271375	10.0744319916667	8.91889295833333	8.703553525	9.00507715833333	10.9084288583333	8.595488075	11.1615262583333	11.6103014166667	8.70547134166667	9.21923738333333	10.1295520916667	9.40680740833333	11.012449925	9.06270968333333	10.2991427166667	10.2039426583333	10.8861829666667	8.96094025833333	9.93630081666667	9.765982475	8.97076639166667	12.857326075	10.41573205	10.1109480666667	9.53914050833333	9.38896178333333	9.38275609166667	9.28041066666667	9.39071778333333	9.32186685	10.0421767666667	10.4720948333333	10.0769619583333	11.5954858333333	8.38470655	9.67141531666667	12.0156772833333	8.95312890833333	10.3362368833333	10.9426936083333	9.24318624166667	10.4272415916667	11.30185685	10.0226518416667	9.72070379166667	10.92486485	10.3455009333333	11.2822211083333	11.42107965	9.82078890833333	9.48470895833333	11.13057255	10.3011707	12.1720161916667	9.87484248333333	11.143398475	11.4045795333333	11.9513573	9.382290975	10.1849087083333	10.9035797416667	11.22125475	9.96356175	9.7815357	11.4424334416667	10.5743323083333	9.17813545	9.90816476666667	9.645264775	8.8992014	9.296236625	10.5613244166667	9.86062085833333	10.3097382833333	10.0965198666667	10.5609143	8.98046293333333	10.28850615	11.055301575	9.750737375	11.3823997333333	10.9895149583333	10.33090645	10.6956354083333	9.869019275	9.993864975	10.333754975	9.9399775	11.7216620916667	10.2591668583333	8.882160975	9.74655711666667

TNFRSF1B	10.6610932333333	10.7680760333333	11.6841362916667	10.754228175	9.27506685	11.2719127583333	10.4445170416667	10.874072375	10.1324487166667	11.3239342333333	10.4609966416667	11.7362421333333	11.19866735	11.1960117	9.03598665833333	12.2570148333333	11.248529425	10.3061471666667	10.128737475	11.0682286166667	11.3720648583333	9.52042825833333	11.5531172416667	11.7757946333333	10.8953436916667	10.9571307916667	10.8916305083333	10.62411455	9.83319184166667	10.4049374333333	11.6155901666667	11.530946975	11.2576230916667	12.846546475	12.084825925	10.88817915	9.82382424166667	11.0261256666667	12.5719766166667	11.2434998833333	11.3169410666667	10.7732420666667	11.4126401666667	11.0272743166667	11.6016871083333	11.825765325	10.880387475	11.43469695	11.8500517083333	11.327862475	11.9860534666667	11.621317125	10.7061988666667	10.4297158833333	10.9869124833333	11.5060047583333	11.7782923583333	11.30753185	10.666810425	11.9705949166667	10.0716267583333	11.1343238833333	12.0249612083333	10.2511057916667	10.72841425	9.62712280833333	10.9123611916667	10.901782425	11.592340975	12.3479516833333	10.66793845	9.39574425833333	10.0167779583333	11.8317467416667	10.164124825	10.837130475	11.5645444166667	11.86975775	11.1930897583333	11.4446261583333	10.2857643416667	10.26748265	11.4221599083333	11.7834532083333	11.376411175	11.069747825	10.469517225	11.8166747583333	9.63902631666667	11.9078069166667	12.0282380083333	11.4679092333333	10.8045420416667	10.6211581166667	10.7306227416667	11.48138035	10.8187575416667	11.3121797833333	10.9629037916667	11.10707375	11.7589935083333	11.3720648583333	11.89894165	11.0394398083333	10.8401100166667	11.7362421333333	10.724371075	10.9073626	11.6701974666667	11.8383549666667	12.8702352416667	10.9170947583333	10.8401100166667	10.842072875	12.1406572	11.8096310916667	10.8759847166667	11.0142581583333	10.7407277583333	12.2508814916667

MTRF1	8.400318075	9.10576088333333	8.17613399166667	8.89967475833333	9.34750165	8.635462725	9.746957025	8.83592169166667	8.82372205833333	8.4444208	8.83395916666667	9.24845199166667	9.59108196666667	8.45352316666667	8.895120775	8.15060805	7.94982140833333	9.59906339166667	8.83883193333333	8.60539538333333	8.8577648	8.84563956666667	7.875176525	9.20513901666667	8.9925209	9.17515170833333	8.62635114166667	8.894577375	8.83783124166667	8.87636833333333	8.49539040833333	8.61820814166667	8.53730979166667	8.594439725	9.68034129166667	8.80050660833333	8.779206475	7.6781919	8.53730979166667	8.38072273333333	7.64552340833333	9.02371780833333	7.90410825	7.78439114166667	8.51764245	8.18363113333333	8.13304300833333	8.4304275	8.35319626666667	8.71940105833333	7.95575579166667	8.70592035	8.93227916666667	9.26261210833333	8.44043165	7.93223308333333	8.59990980833333	7.78767925833333	8.32062069166667	8.31252919166667	10.324926675	9.78481885	9.33321418333333	10.0524736083333	8.75515696666667	9.61456806666667	9.579511975	9.88471656666667	9.10433055833333	8.95991093333333	9.39938098333333	9.31629211666667	9.84760208333333	9.25349141666667	9.22955863333333	9.73744423333333	9.58706739166667	10.154948775	8.99987959166667	9.44734175833333	10.054135025	9.460327375	8.9217463	9.07777099166667	9.51549753333333	10.2089178583333	10.1682992333333	8.779206475	10.2342241333333	9.00330966666667	9.31405536666667	9.73350879166667	9.57903839166667	9.07683584166667	10.1477211833333	9.79191481666667	9.2408133	9.61367635	9.40774255833333	8.82424165	9.15645445833333	9.579511975	9.23579664166667	8.55105330833333	9.66513115	9.77571775833333	9.14189448333333	8.93897689166667	9.09054048333333	9.18306429166667	9.53957571666667	8.40280550833333	8.615263775	8.9231771	8.99027038333333	9.06791326666667	9.444752725	8.94431525	8.8062671	8.98986978333333

AC005041.1	9.65777431666667	8.8234550625	9.23305643333333	9.383893725	9.4261721625	8.6102258875	10.0016218333333	8.81902420833333	9.18997539583333	9.094379975	9.15764411666667	10.0767594	9.52245795833333	9.11291737083333	9.16374468333333	9.23859324166667	8.98544247083333	9.38431420833333	9.05200975	9.17073914583333	9.2043888125	9.45124689166667	9.18481689583333	8.71116875	8.82650967083333	9.66719131666667	9.46320446666667	9.21374709166667	9.73076655833333	9.01676947916667	9.1143027375	9.05623612916667	9.25650765833333	9.268773125	9.24056433333333	9.6188059625	9.40704348333333	8.78813974583333	8.70057618333333	9.15328477916667	8.95469538333333	9.2084773	8.82190914583333	8.93000931666667	9.23773901666667	9.04961187083333	9.0025986375	8.82448785	8.9742374125	9.13488224583333	8.64313829583333	8.72995544166667	9.2272344	9.28246405833333	9.0154403625	9.37612970416667	8.55265227916667	8.4716399125	9.03524544583333	8.96163360416667	9.19314084166667	9.520195475	9.25789575	10.3800595208333	10.1326635041667	9.62087651666667	10.1522911708333	9.966801325	9.26513190416667	9.47183977083333	9.512890975	9.83564609166667	10.6505566166667	9.54018627083333	10.3375195916667	10.1551434291667	9.72265057083333	9.201680325	9.20393385833333	10.8880026958333	10.2561414916667	10.7259670291667	9.48363187916667	9.83788053333333	9.35886019583333	9.63087765833333	10.0655120541667	9.63267926666667	9.88864601666667	9.5748755375	9.4966602125	9.36644320833333	9.19741644583333	9.90886985	10.0883672708333	10.0547175708333	10.1306112708333	10.1893295416667	9.095292825	9.28290961666667	9.5958329375	9.70475119166667	8.89197675416667	9.51065	9.87760744583333	10.60597485	9.7525953	10.1442671375	10.5851296875	9.15811152916667	9.216632175	9.62296950416667	9.70387588333333	10.3055547416667	10.2593757416667	9.00798550416667	10.6583477291667	9.383893725	9.04389905416667	9.11387709583333

C2orf81	9.65777431666667	8.8234550625	9.23305643333333	9.383893725	9.4261721625	8.6102258875	10.0016218333333	8.81902420833333	9.18997539583333	9.094379975	9.15764411666667	10.0767594	9.52245795833333	9.11291737083333	9.16374468333333	9.23859324166667	8.98544247083333	9.38431420833333	9.05200975	9.17073914583333	9.2043888125	9.45124689166667	9.18481689583333	8.71116875	8.82650967083333	9.66719131666667	9.46320446666667	9.21374709166667	9.73076655833333	9.01676947916667	9.1143027375	9.05623612916667	9.25650765833333	9.268773125	9.24056433333333	9.6188059625	9.40704348333333	8.78813974583333	8.70057618333333	9.15328477916667	8.95469538333333	9.2084773	8.82190914583333	8.93000931666667	9.23773901666667	9.04961187083333	9.0025986375	8.82448785	8.9742374125	9.13488224583333	8.64313829583333	8.72995544166667	9.2272344	9.28246405833333	9.0154403625	9.37612970416667	8.55265227916667	8.4716399125	9.03524544583333	8.96163360416667	9.19314084166667	9.520195475	9.25789575	10.3800595208333	10.1326635041667	9.62087651666667	10.1522911708333	9.966801325	9.26513190416667	9.47183977083333	9.512890975	9.83564609166667	10.6505566166667	9.54018627083333	10.3375195916667	10.1551434291667	9.72265057083333	9.201680325	9.20393385833333	10.8880026958333	10.2561414916667	10.7259670291667	9.48363187916667	9.83788053333333	9.35886019583333	9.63087765833333	10.0655120541667	9.63267926666667	9.88864601666667	9.5748755375	9.4966602125	9.36644320833333	9.19741644583333	9.90886985	10.0883672708333	10.0547175708333	10.1306112708333	10.1893295416667	9.095292825	9.28290961666667	9.5958329375	9.70475119166667	8.89197675416667	9.51065	9.87760744583333	10.60597485	9.7525953	10.1442671375	10.5851296875	9.15811152916667	9.216632175	9.62296950416667	9.70387588333333	10.3055547416667	10.2593757416667	9.00798550416667	10.6583477291667	9.383893725	9.04389905416667	9.11387709583333

SIK3	9.17457594166667	8.796402275	8.635919475	8.62547046666667	9.32711606666667	8.580396475	9.297473225	8.72739513333333	8.96932985833333	8.85824333333333	8.75371965	9.15603220833333	9.47351875833333	8.92028675	9.12326560833333	8.45352316666667	7.63210018333333	9.437687475	8.815557	8.52731504166667	8.65698328333333	9.06460029166667	8.66625304166667	8.65999509166667	8.80870043333333	9.673127475	8.875989475	8.66108489166667	8.9489102	8.81599143333333	8.97905946666667	8.72497184166667	9.28087420833333	8.70592035	9.56407003333333	9.63806365	8.99487275833333	8.86708225	9.16074203333333	9.12042280833333	8.50844495	8.87390605	8.67361984166667	8.67125495	8.375514	8.50844495	8.84563956666667	8.775178575	8.96414444166667	8.69791309166667	9.03417474166667	8.88930546666667	9.37403768333333	9.25904538333333	9.01340034166667	8.50894728333333	8.561783725	8.69363141666667	9.00460498333333	8.278086225	8.67789245	8.41104395833333	7.8150656	8.14240605833333	7.86115675833333	8.96932985833333	8.22799306666667	8.49886293333333	7.91465324166667	8.90005820833333	9.06973089166667	8.20689531666667	8.98889158333333	8.73305545	8.95312890833333	8.10691838333333	8.76273385833333	8.69747196666667	8.43902399166667	8.15563305	8.793933	8.50185603333333	8.448844675	7.91685584166667	9.222045675	8.53187930833333	8.649865175	8.54744824166667	9.22955863333333	8.662974625	8.222543225	9.26354115	8.8992014	9.0720726	8.882160975	8.89262756666667	8.854456025	8.89696199166667	8.62635114166667	8.21929578333333	8.15707283333333	8.796402275	8.98837211666667	8.34668346666667	9.11687501666667	8.57400345	8.49975086666667	7.75772005833333	8.4515305	8.32264421666667	8.611944975	8.3471697	8.85547214166667	7.99232836666667	8.66580391666667	7.86670446666667	8.09007435833333	8.8128727	8.11712540833333	8.60799398333333

HEXB	11.2073423208333	11.8735748416667	11.7957279	12.0562069791667	11.4709996583333	11.336614125	11.5101673291667	11.8102342458333	11.6946328291667	11.7393491791667	11.8038525208333	11.0418149833333	11.2052246833333	11.8506238	11.8377358625	11.5054512791667	12.1809202791667	11.3784236458333	11.5640089708333	11.75395635	11.722970725	11.6446868666667	11.8847030333333	11.8251551375	11.6728914333333	11.50364235	11.6239785541667	11.736762325	11.4870554708333	11.7099830666667	11.5054512791667	11.6196380083333	11.5561134625	12.1692484375	11.5616369833333	11.5595787208333	11.4667271041667	11.5640089708333	11.3469230291667	11.2479936833333	11.8168877791667	11.7949616583333	11.6053475125	11.7290665833333	11.4472683208333	11.7945636375	11.7036939708333	12.0739248208333	11.7158374875	11.7111165416667	11.3113224875	11.5744282208333	11.4903281875	11.7257176791667	11.7144381458333	11.5460149333333	11.5818676666667	11.7465124875	11.55504505	11.4256851458333	10.8910372666667	11.9909438125	11.4976643458333	11.1980820583333	12.2555840541667	10.6970213958333	10.5611193583333	10.5539506583333	11.4036291916667	11.5989360833333	10.9656601333333	11.1371171166667	11.5091019958333	11.3041958208333	11.2188893125	11.3977826625	11.2419211958333	10.9800394125	11.2977570166667	11.4047692833333	11.9433470958333	11.2581787375	11.5297006333333	11.3237360666667	11.012949625	11.2578042416667	11.7431122625	11.0759963458333	10.993162925	10.9638386916667	11.3851742541667	11.2347130583333	11.4312072958333	10.716460075	10.7540292916667	11.5251794	11.0991457083333	11.0502573875	10.823029525	11.5017889833333	11.7777737916667	11.4656335875	11.93097435	11.5190591291667	11.6769830916667	10.9410609833333	11.1275737333333	11.5811100791667	11.4308836666667	11.6556781041667	11.4614284541667	10.8047085958333	11.3906266875	11.6843276458333	11.1947079416667	11.1925214916667	11.4124685041667	11.7676929416667	11.5105439416667	11.6416899041667

GFM2	11.2073423208333	11.8735748416667	11.7957279	12.0562069791667	11.4709996583333	11.336614125	11.5101673291667	11.8102342458333	11.6946328291667	11.7393491791667	11.8038525208333	11.0418149833333	11.2052246833333	11.8506238	11.8377358625	11.5054512791667	12.1809202791667	11.3784236458333	11.5640089708333	11.75395635	11.722970725	11.6446868666667	11.8847030333333	11.8251551375	11.6728914333333	11.50364235	11.6239785541667	11.736762325	11.4870554708333	11.7099830666667	11.5054512791667	11.6196380083333	11.5561134625	12.1692484375	11.5616369833333	11.5595787208333	11.4667271041667	11.5640089708333	11.3469230291667	11.2479936833333	11.8168877791667	11.7949616583333	11.6053475125	11.7290665833333	11.4472683208333	11.7945636375	11.7036939708333	12.0739248208333	11.7158374875	11.7111165416667	11.3113224875	11.5744282208333	11.4903281875	11.7257176791667	11.7144381458333	11.5460149333333	11.5818676666667	11.7465124875	11.55504505	11.4256851458333	10.8910372666667	11.9909438125	11.4976643458333	11.1980820583333	12.2555840541667	10.6970213958333	10.5611193583333	10.5539506583333	11.4036291916667	11.5989360833333	10.9656601333333	11.1371171166667	11.5091019958333	11.3041958208333	11.2188893125	11.3977826625	11.2419211958333	10.9800394125	11.2977570166667	11.4047692833333	11.9433470958333	11.2581787375	11.5297006333333	11.3237360666667	11.012949625	11.2578042416667	11.7431122625	11.0759963458333	10.993162925	10.9638386916667	11.3851742541667	11.2347130583333	11.4312072958333	10.716460075	10.7540292916667	11.5251794	11.0991457083333	11.0502573875	10.823029525	11.5017889833333	11.7777737916667	11.4656335875	11.93097435	11.5190591291667	11.6769830916667	10.9410609833333	11.1275737333333	11.5811100791667	11.4308836666667	11.6556781041667	11.4614284541667	10.8047085958333	11.3906266875	11.6843276458333	11.1947079416667	11.1925214916667	11.4124685041667	11.7676929416667	11.5105439416667	11.6416899041667

DOCK9	12.5652457833333	12.857326075	12.715938075	12.5780122833333	12.894282175	11.4912151666667	12.7873683333333	12.5604394333333	12.8392807333333	12.462326175	12.4732793833333	11.6679772166667	12.7076553	12.894282175	12.919046025	12.4482599083333	10.2881617	12.9604577916667	12.8074143333333	12.813963275	12.6254772333333	13.3488197166667	12.111383	12.109263175	12.9439344083333	12.3970415166667	12.6552566916667	13.0374763416667	13.20077725	12.7796071583333	12.5676300666667	13.0228260333333	12.4840896416667	10.229775825	12.2739391166667	12.4275246583333	13.3067028166667	12.93348965	12.4932078666667	12.6131713166667	12.7890792833333	13.1407286583333	12.9472642583333	12.501560375	12.4927583	12.545304275	12.9745146166667	13.0714709916667	12.6402462916667	12.1178108083333	12.6674936416667	12.716481775	13.004985075	12.9977920166667	13.0013137333333	12.9144941416667	12.7912843416667	13.0743927916667	12.8708264916667	12.6734606	11.4188332333333	11.5598018166667	11.1099310583333	12.6146877083333	10.9747290916667	12.120221775	11.781905875	12.0741025416667	11.6754049666667	11.1615262583333	11.634515075	11.9184658666667	11.7014141	12.0785704333333	11.8873133333333	11.28297055	11.69368565	11.5520144833333	11.7541385166667	11.4284677	12.04365515	11.0167794916667	11.8418826916667	11.032984525	12.2268634416667	11.5041708333333	11.5249852666667	11.2657187833333	12.3837214916667	11.34033385	11.13057255	12.1847949166667	11.590501375	12.00329435	10.7258049	11.0585423	11.3797907833333	11.088855975	11.5139760666667	11.5850946	10.8514713416667	10.8032590416667	10.1670505916667	11.66250505	12.0124313166667	12.0893148833333	11.6436590416667	11.6314027333333	11.30084235	11.6746522	11.7004395833333	10.958948725	11.4350551	11.83328595	11.5958198083333	11.3813533416667	11.3467541583333	11.0747492583333	11.6103014166667	11.34446325

ZNF577	11.1506157166667	9.70842734166667	9.55472915	9.74568905833333	9.71265901666667	9.84032644166667	9.94086763333333	9.7237141	9.94840036666667	9.297473225	9.19118228333333	10.6411546083333	9.83111589166667	9.645654625	9.759228525	9.952361775	9.89341510833333	9.62890875	9.32186685	9.80755264166667	9.51496844166667	10.0382960666667	9.06460029166667	10.014766425	9.99808105833333	10.8424532083333	9.61139459166667	9.79404784166667	9.77219498333333	9.95852463333333	9.63018528333333	9.47987389166667	9.90438676666667	9.48513751666667	10.7501138916667	10.952408425	9.25764194166667	8.88930546666667	8.56421206666667	9.45734620833333	9.4946079	9.62712280833333	10.0296842916667	9.62453731666667	10.2943474166667	9.67944865833333	9.75665824166667	9.27506685	9.72757016666667	9.637594125	9.61367635	9.330358125	9.87739761666667	9.78737088333333	9.22061473333333	9.498614875	9.442472475	9.56548648333333	9.7752348	9.436848125	10.7842899166667	8.93568545833333	9.88434308333333	9.31317535833333	10.5775565833333	9.21543308333333	9.6530765	9.63902631666667	9.02512653333333	8.95943646666667	9.08869563333333	9.85498678333333	8.85160831666667	9.96481554166667	10.2511057916667	9.41668023333333	9.90307260833333	9.006017175	8.84758474166667	8.90525780833333	10.145640125	10.5169707416667	7.671444425	9.53398021666667	9.56828670833333	9.92747266666667	10.4826365083333	9.44293281666667	9.62890875	9.75875961666667	8.408638475	7.88761391666667	9.18639844166667	9.6496487	9.46121264166667	8.40234485833333	9.30682376666667	9.21169858333333	8.682814325	10.6865081333333	9.86266926666667	9.16352800833333	9.4936652	9.37821416666667	10.359500425	9.37177325833333	9.48384479166667	8.887867925	9.62275734166667	9.35549675833333	8.797202975	9.23139328333333	8.95943646666667	9.16074203333333	8.40717954166667	9.60963610833333	10.4413653083333	9.60093574166667	8.13242494166667	9.64650925

QTRT1	10.3953052083333	10.4526526416667	10.359500425	10.373735575	9.96151823333333	9.96782080833333	9.06405529166667	9.57414756666667	9.85722324166667	10.2834956666667	10.2749442833333	9.38580318333333	9.70931241666667	10.1501004333333	10.18161685	10.1986602916667	10.670337625	10.0078625	10.327316425	10.0209906333333	10.165808925	10.2399871166667	10.239159725	10.591824075	10.007360325	10.2551392166667	9.96522326666667	10.217869775	9.993864975	10.3694253333333	10.4253305166667	10.0247316333333	9.579511975	10.1546002	10.2591668583333	10.0595403	9.92494756666667	10.176297425	10.153311575	9.99259065	10.10216065	10.0957326583333	10.1009165916667	10.289694575	9.79533880833333	10.2924466666667	10.4678765666667	10.667166075	10.233851875	10.388343775	10.3281467166667	10.1759041833333	10.3558299833333	10.4409408666667	10.23098885	10.4515253333333	10.1927941666667	10.40167215	10.1557652416667	10.432039025	10.239527275	10.29705995	10.609691175	9.80274700833333	10.1088055083333	10.3362368833333	9.695341625	9.51175975	10.0524736083333	11.572420475	9.78280608333333	10.29083355	10.3228920166667	10.0167779583333	9.0973311	10.3245220833333	10.13859765	10.2802737333333	10.5334113083333	10.864263475	7.45098580833333	10.3933623083333	10.804132675	10.6430311416667	9.34064308333333	9.349435025	11.0534747416667	10.3466375416667	10.3960554833333	10.33090645	10.696845925	10.2411048916667	9.081377025	9.97706043333333	9.93128663333333	10.5260512	10.615482725	10.04588255	10.5256389583333	10.558939175	10.4145396416667	10.1067055416667	10.2458406333333	10.8025054583333	11.0326626833333	9.79404784166667	10.3996095166667	11.1862575	9.96481554166667	10.2039426583333	9.30028739166667	10.1716698166667	10.4089152416667	10.88143175	10.93064125	9.65399733333333	9.9162906	10.8176392916667	11.4225410666667	10.4394323833333

TWNK	9.198111125	9.722436925	10.0048138083333	9.80231140833333	9.44779018333333	10.182857025	9.80625096666667	10.3714480666667	9.77475016666667	9.58040200833333	10.1766858833333	8.561306875	8.99771499166667	9.66781955833333	9.81686935833333	9.34481250833333	8.785427625	9.41247076666667	10.30893185	9.68863114166667	9.72111820833333	9.166824	9.90955436666667	11.0290294333333	9.4968836	9.120869625	9.31809370833333	9.63613316666667	9.17190161666667	9.55058418333333	10.216230425	9.78918780833333	9.68387974166667	8.94668689166667	9.45281165833333	9.57222860833333	9.15603220833333	9.92375435	10.5108422833333	9.87439875	9.75239246666667	10.068396225	9.62110718333333	10.0815238083333	9.82295156666667	9.64650925	9.89982799166667	10.2687270166667	9.810451275	9.60919951666667	9.92375435	10.6237069166667	9.62496784166667	9.71912328333333	9.9560883	9.89258960833333	10.0506734583333	9.96227999166667	9.36528065833333	9.74433669166667	9.55009920833333	10.3483436583333	10.71312705	10.4089152416667	10.1755237	9.93957129166667	10.3678756583333	9.726163425	11.1771749333333	10.5318759	10.128737475	10.6941625333333	10.7688463333333	9.57179898333333	9.67723733333333	9.78737088333333	9.92703645833333	9.35634101666667	10.02346285	9.90731580833333	10.2305668916667	8.76654949166667	10.0373878833333	10.18000125	8.60080905833333	10.4308569666667	11.7768584833333	9.292040175	10.2305668916667	9.70668029166667	9.909962375	10.7265037833333	10.2728709416667	9.78382319166667	9.68992429166667	9.77571775833333	10.976899425	10.0533049083333	9.54682303333333	9.70194608333333	10.6846028416667	10.6686985416667	10.723219775	10.7743973416667	10.9597361583333	10.05998855	10.3603019333333	10.7907764333333	10.9360928583333	10.0688438166667	9.15400360833333	9.53043561666667	10.1205788333333	10.938251225	10.107173075	10.4292877666667	10.3968247166667	10.5464444833333	10.6637664333333	9.91241780833333

HIPK1	12.1521118916667	12.1480274583333	12.03257945	12.0249612083333	12.1513649666667	12.2215063083333	12.5917146833333	12.1295438416667	12.3469866583333	11.7648162666667	11.849231625	12.2701693666667	12.1521118916667	12.1447048	11.7175871333333	12.1998168583333	11.9158492083333	12.1939692166667	12.7344773416667	12.2637428083333	11.9021431083333	11.6775693083333	11.8749533333333	11.8457882583333	11.6514721333333	11.7513402	11.8783734666667	11.5319982916667	11.929273675	12.0124313166667	12.4705762833333	12.004381825	11.6780189666667	11.49602405	12.2466185916667	12.1553849	11.5400258416667	11.686699925	11.8607926333333	11.6058721833333	11.7505625666667	12.2675250666667	11.9911471583333	11.6929484666667	11.2982935833333	11.4971747583333	11.6386741333333	11.702337975	11.9566737416667	11.6569750083333	12.1316746333333	12.2662860833333	10.1687340166667	10.684995425	10.7201690666667	10.874072375	11.743693825	12.1762012583333	11.7753980416667	11.4872383	10.66752385	11.2402727333333	11.0673982916667	10.84359385	11.7609104916667	11.1524382	11.7667849666667	11.65549195	11.2822211083333	10.91124625	11.062266425	11.0337297583333	11.430726925	11.2790070666667	11.021874925	11.7070775666667	11.40680705	11.5668628166667	11.6456087833333	12.007698575	11.3520380583333	11.114752	11.4173578916667	11.0815307083333	10.86234545	11.088478625	10.6047191916667	11.2272718833333	9.86718390833333	11.3911680666667	11.2982935833333	11.2035969416667	11.602856675	10.880387475	11.0548999583333	10.6122965916667	11.1302224166667	11.0235968416667	11.1376399	11.20219855	10.610413375	10.8800392333333	10.6507559	11.47905225	10.9537804833333	10.938251225	11.1561366	11.1419454583333	10.9208636166667	11.0848820333333	11.8293448166667	11.4541742	9.43278838333333	9.974167275	10.119247225	10.5356874083333	11.6401085333333	11.2150743583333	11.5034674333333	11.3339882

DUSP26	11.6205609916667	9.92375435	10.066608375	10.1180361833333	10.3549808416667	9.74996381666667	10.999826875	9.65845149166667	9.99808105833333	10.4934979	9.99509398333333	11.6577322083333	10.7493099333333	10.744004	9.8001132	9.93222460833333	10.5519705833333	9.73831540833333	9.35075274166667	9.57222860833333	10.0980477833333	9.55058418333333	9.5013607	9.36206593333333	9.51633301666667	11.0427571416667	11.6647417666667	10.0708045166667	9.98312965833333	10.4024778916667	10.182857025	9.66469236666667	10.2782382416667	10.6937764083333	11.20617	10.692247	10.3758856666667	10.0378686583333	9.69792198333333	9.99593061666667	9.45281165833333	10.5212723416667	10.1878607	9.64783695	10.9219574	9.33496756666667	10.852614925	10.3124336666667	9.234219825	9.304559675	10.286132425	9.766909275	10.15376285	9.6482902	10.271227125	9.94551363333333	9.844981225	9.72033719166667	9.817741375	10.0933465333333	9.748599325	10.2604506416667	9.86517185	10.29990335	9.813959625	9.20652433333333	9.62925015833333	10.1481495666667	9.45734620833333	9.65621849166667	10.0434280416667	9.82822335	10.5973351833333	10.2919749833333	10.0972464083333	9.608728125	10.4131468	9.48126221666667	9.170544975	9.856343425	9.76732071666667	10.20226945	9.55104401666667	9.04841031666667	9.82339220833333	10.8322571166667	10.58184315	10.3432001083333	10.1931783583333	9.440114675	9.93083546666667	9.38896178333333	9.177752275	9.77219498333333	9.88045179166667	9.86266926666667	11.19866735	9.77219498333333	10.082743125	9.34157415833333	9.138674475	10.5049759833333	9.8694956	9.345810075	9.68695163333333	9.723283875	10.2241250416667	9.70194608333333	9.19391603333333	8.9226878	9.45898255	11.6879616166667	9.26627294166667	8.12378493333333	9.42118564166667	9.292974825	9.234219825	9.07395639166667	9.04841031666667	9.7570522

COMT	6.295927975	5.74803075	5.31524535	5.31421083333333	6.84325476666667	6.18247753333333	5.71416341666667	5.21624118333333	6.60761675	6.14494839166667	6.31323984166667	5.966951625	6.03747638333333	5.81989971666667	6.10002881666667	5.70932124166667	5.88673599166667	6.61745720833333	5.667420075	5.56052278333333	5.74037505	5.65824568333333	5.26362051666667	5.87496216666667	5.75213593333333	5.97595255	5.68783094166667	5.63053526666667	5.9191077	5.93821120833333	5.582921425	5.66843639166667	5.89819270833333	5.43281713333333	6.36793951666667	5.83953349166667	6.07768216666667	6.041758325	6.05717670833333	5.63834938333333	5.5927414	5.96077145833333	5.29675638333333	5.45697495	5.7161104	5.1555947	7.49164054166667	5.28464988333333	5.81120035833333	5.55666916666667	5.45476871666667	5.35692600833333	5.13634166666667	5.73943181666667	4.93116945833333	4.76759788333333	5.11328418333333	4.86589074166667	5.72886108333333	5.42780705833333	6.407249275	5.948900025	5.62592511666667	5.81120035833333	6.97900894166667	6.84636305	5.42175125833333	5.737720375	7.11617649166667	6.33569684166667	5.8523182	6.52209205	5.94185621666667	5.84791786666667	5.65251761666667	5.91038576666667	6.929785275	6.277191075	5.67523288333333	5.89368925	6.08509065	5.415860975	6.00716590833333	5.80264275	5.70649276666667	6.028109975	5.73091026666667	6.51237135	6.33115975	5.78160560833333	5.70253175	6.21427064166667	6.18568551666667	6.180201325	6.35356116666667	6.28975704166667	7.04755845833333	6.243623775	6.32106796666667	6.87851200833333	6.44430819166667	6.03844185	6.21517435	6.12200050833333	6.057986625	5.50615049166667	7.58073043333333	7.204487875	6.423307775	7.4250003	5.23361180833333	6.58763124166667	6.845071175	5.30151331666667	4.92557835833333	5.37226380833333	6.348986575	6.30572190833333	5.824619025	5.079599575

FOXN1	5.83017264166667	4.43639113333333	4.50751908333333	4.127831	4.34795044166667	4.79621500833333	4.2289531	4.63305978333333	4.91752975833333	6.83207999166667	6.69182425833333	4.25708764166667	3.87078774166667	4.13943304166667	5.31227555833333	4.61830805833333	5.52228829166667	5.83776461666667	6.17390685	4.97597943333333	5.171525	5.43935509166667	5.018425275	5.99418615	5.667420075	4.50112465833333	5.467505875	5.05687025	5.60132373333333	5.26259698333333	5.73943181666667	5.485111725	4.63305978333333	4.19132811666667	5.35273364166667	5.04557781666667	6.00629560833333	5.4600655	5.78537223333333	5.78537223333333	5.40852341666667	5.82168396666667	4.73295375	5.498293125	4.74123235833333	5.89991223333333	5.64694838333333	6.33115975	6.04002979166667	5.47221455833333	5.8893734	6.316307375	4.49602461666667	5.43596139166667	5.2617065	4.75806056666667	4.47474535	6.48010203333333	5.50905394166667	6.246964375	5.39492906666667	5.091030975	5.03526733333333	5.40405401666667	4.52155396666667	4.86589074166667	5.07107728333333	4.11281023333333	6.21116124166667	5.32031858333333	6.524903425	6.17390685	3.95350946666667	4.19132811666667	4.93116945833333	4.71899701666667	5.71516535833333	5.17265765	5.01530855833333	5.35794613333333	4.49712886666667	5.14192449166667	5.388701925	5.15454668333333	5.28578914166667	4.27276220833333	4.43091141666667	3.96951036666667	4.69963341666667	4.17039641666667	5.0486896	5.780758525	5.91719066666667	5.47616439166667	5.87405735	5.75213593333333	6.16829805	5.477009275	5.270972825	6.68656881666667	4.97490869166667	4.76141500833333	5.84699576666667	6.37568504166667	6.06914035833333	4.07882345	4.82904071666667	6.40797193333333	5.14292011666667	6.08271424166667	5.78709845833333	6.16362138333333	6.46219335	5.61255331666667	4.75806056666667	5.52011276666667	5.33867198333333	5.35476978333333	5.72573654166667	4.0917668

OR7G3	6.06396311666667	5.84699576666667	5.84791786666667	6.2073137	5.54199565833333	6.45696331666667	6.33187936666667	5.62592511666667	5.84236155833333	5.95074298333333	5.9191077	5.85146426666667	5.20060255	5.94443094166667	5.79119435	5.8532158	6.2184742	6.56509979166667	6.675144925	5.90497220833333	4.480628975	4.51657093333333	4.4462942	4.47089620833333	5.755893075	5.760490375	5.33867198333333	5.3439553	5.064717325	5.4237743	5.82824421666667	6.34447826666667	6.58160273333333	5.508179525	6.71850903333333	6.81508890833333	6.36248048333333	6.60826071666667	6.09024784166667	6.02645373333333	6.26504835	5.46838549166667	5.97783559166667	6.063181275	6.662431575	6.28898518333333	6.51915964166667	5.88673599166667	5.51291259166667	5.73405291666667	6.13759665833333	7.31981145833333	6.5766165	6.98474048333333	7.09043023333333	6.75238478333333	6.99919410833333	7.18035421666667	7.25799328333333	6.986635125	6.03335128333333	5.880482625	6.67239650833333	5.8654886	5.75307261666667	6.28034841666667	6.34302255833333	5.76836148333333	6.07085139166667	6.81435373333333	6.36709734166667	6.297552775	5.65064866666667	6.26827546666667	6.6716762	5.81563615833333	6.37491049166667	6.230051875	7.15455295	6.07263301666667	5.04338205833333	4.852466025	5.55862928333333	5.60319860833333	6.35578110833333	5.21624118333333	6.20408345833333	5.71985138333333	5.76546864166667	6.05717670833333	6.37113890833333	6.41808758333333	6.69500276666667	6.777132775	6.735668325	6.53273724166667	6.71918628333333	6.66944055833333	6.50275845	6.40489274166667	6.33187936666667	5.67897566666667	6.20262720833333	6.075953425	7.28864350833333	6.72519365833333	6.87022720833333	6.755806875	6.38476215833333	6.80918875	6.31786493333333	7.28990879166667	6.85677963333333	7.41466188333333	7.51696985	7.07794505	6.82586836666667	7.363992875	7.39564355833333	7.43130181666667

CLASP2	6.72653388333333	5.426744575	5.08252440833333	4.58982808333333	5.81313270833333	5.778884625	5.22492120833333	6.00123059166667	5.35088976666667	5.16622001666667	5.90235153333333	5.99939696666667	5.75774213333333	5.667420075	6.24607838333333	5.10498871666667	4.71793841666667	6.24607838333333	6.449622025	6.67239650833333	4.5803341	7.73639724166667	6.20326725833333	6.31323984166667	6.406487225	5.981391325	7.3367108	6.88865130833333	7.867210225	6.58359223333333	5.41039171666667	6.12763226666667	5.90235153333333	4.99019379166667	5.69483811666667	6.54994171666667	6.95572241666667	6.161212075	6.161212075	5.71516535833333	5.73091026666667	5.34734255	6.06230976666667	6.00716590833333	4.56600969166667	4.29817321666667	6.0911181	4.46791538333333	6.45696331666667	6.46851783333333	4.73720489166667	4.93552183333333	6.39012523333333	6.54434775	6.107924175	5.07558524166667	4.82904071666667	4.51657093333333	6.051276375	6.47291681666667	6.99727938333333	4.31702865	6.423307775	5.88241164166667	4.51861790833333	6.65287900833333	4.90147810833333	5.17483658333333	6.013009825	5.46838549166667	4.98771553333333	5.39700335833333	5.066744825	5.88144885	4.62375229166667	6.05392339166667	5.628745675	6.445141375	6.63574723333333	5.6488719	4.62375229166667	6.73349835	6.277191075	5.99501325833333	4.96401284166667	4.3249589	5.47028654166667	4.58556791666667	6.25335921666667	5.70839040833333	5.073382675	5.59572529166667	5.65159290833333	4.77813741666667	6.021143325	5.79742544166667	6.93287185833333	6.345260775	5.25133600833333	6.16362138333333	5.61255331666667	4.19044525833333	6.69380246666667	6.246964375	6.71918628333333	6.03500149166667	6.42781263333333	4.33428121666667	6.2073137	4.977020525	5.06113309166667	4.60088621666667	6.57952525	4.41962694166667	6.256698975	5.67327496666667	4.9406758	6.39681019166667	5.74413374166667	5.197537

PLEKHB2	13.870943825	12.1669452	11.5684552166667	11.2424523	11.9911471583333	12.8107678333333	11.868539825	11.21907835	11.8819037833333	12.4908032583333	10.5081553333333	14.00243415	12.4912644	11.102188775	10.796845775	12.962321275	14.5176714166667	11.9554346166667	12.0269670083333	12.6355786083333	12.0181445166667	10.9790999666667	11.4696992083333	11.8070215583333	11.7703479083333	14.0087806166667	12.3301864333333	12.0282380083333	8.82680535833333	11.590501375	11.6429698666667	11.4439013	12.251683475	13.806992375	13.776083375	12.3550491166667	11.8935481166667	11.9341002416667	11.3068495166667	11.6746522	11.0689648166667	11.3282347083333	11.666692125	11.3378344416667	12.4069098166667	11.173905775	11.013128925	11.1380175916667	11.9653249	12.1451222666667	12.6287796916667	10.222173225	11.3842695916667	10.6964442916667	10.9754104083333	10.7482129583333	11.634515075	11.4563744833333	11.784585925	11.1224187916667	10.9273818166667	11.0268698583333	10.49162865	10.3301703333333	11.3627772083333	10.7228384083333	11.22691525	11.2933100833333	10.41048235	11.1997660583333	10.7298944083333	10.2047893666667	10.5719557166667	11.62870925	10.2778151833333	10.7557641416667	11.2664173916667	12.1943788416667	10.9167594166667	11.0333724416667	10.5368715833333	10.3124336666667	10.5260512	11.0934965333333	10.8224026333333	10.9174580583333	10.6792669083333	12.0816579833333	7.36530371666667	12.2619793166667	11.1113391916667	10.75497465	10.433634375	10.4801853	10.082743125	10.8657939916667	10.4819116333333	10.8480286083333	10.9983530166667	10.6905955916667	10.5228331416667	10.3875979666667	10.1936096583333	10.6926124666667	9.77176594166667	10.3667222333333	10.7143764583333	9.55244275833333	10.93645885	10.9234198666667	10.6298019333333	10.5276582916667	10.123005125	9.69176305833333	9.91758241666667	9.90307260833333	10.9443778666667	10.8251181583333	9.95973609166667	11.8973789916667

TINAG	4.176778725	3.88409523333333	4.01107811666667	4.115796825	4.00663116666667	4.28809964166667	4.66004348333333	4.33428121666667	3.60239741666667	3.94580334166667	4.19234266666667	4.350915225	3.630124675	4.207987625	4.19234266666667	3.93897055	4.263255	3.79653381666667	4.34901141666667	3.824093125	3.8957117	3.85641256666667	4.0798737	4.09074995833333	4.45030740833333	4.137419	4.879391675	4.207987625	4.31217129166667	4.108372	4.290980325	4.268577875	4.05967894166667	4.20109206666667	4.48432390833333	4.258212325	4.371797775	4.51370921666667	4.55863708333333	4.55971610833333	4.92878054166667	4.56487130833333	5.968635375	5.57834849166667	4.13647209166667	4.98545498333333	5.162070825	4.84820759166667	4.428719425	4.61955908333333	4.60088621666667	4.57924936666667	3.86501250833333	5.37226380833333	4.26972968333333	4.748342275	3.89679799166667	4.68789791666667	4.36294506666667	4.79411095833333	3.76094594166667	3.92586006666667	4.31908713333333	3.711770875	4.302366825	3.87194265	3.798771525	4.00244103333333	3.699437625	3.67515746666667	4.15011734166667	3.63418771666667	4.32386374166667	4.075702625	3.54051521666667	3.71657505833333	3.75570144166667	3.72041143333333	4.05738384166667	3.66190675	4.50751908333333	3.92815261666667	4.030215125	4.09274305833333	3.69375465	3.607366575	3.87726464166667	4.09994219166667	3.909240625	3.92371073333333	3.656722575	4.265394625	4.05967894166667	3.80684369166667	5.43710514166667	4.00032819166667	4.82688145833333	4.15309530833333	4.366029925	4.782758275	4.31116040833333	3.550199675	5.87100729166667	5.13847554166667	4.33043379166667	4.00561259166667	4.63093599166667	6.17313585833333	4.25573735	6.00716590833333	4.48432390833333	4.83989633333333	4.48925233333333	4.98892909166667	4.07775664166667	4.37489024166667	4.21809414166667	4.67471653333333	5.14718730833333	4.55568075

CRHR2	11.556290575	11.81229275	11.53339965	12.0744704	12.762350675	11.2583720833333	12.88540095	11.562248175	12.8344857166667	11.87418675	11.5223462	12.093025975	12.0748320833333	11.3183574	11.1125423333333	11.0387117083333	10.3674828833333	12.1998168583333	10.952408425	11.1405494333333	10.6301784666667	10.8964884916667	10.9466012416667	11.31949275	12.4659800333333	10.9972633583333	11.9169362583333	11.7827675333333	10.7313914916667	12.642703875	11.2968704833333	11.8838190166667	12.3610276166667	11.4151310833333	10.9497712833333	11.0301443333333	12.6739472	12.206981325	12.1304018333333	11.804049375	11.5212430583333	11.7699299	11.3605041083333	11.5909354166667	11.4581724666667	10.9549623083333	10.9972633583333	11.94277775	11.4779366416667	11.5185086916667	11.9612880416667	11.8670714	11.98956295	12.4292757333333	12.2137378333333	11.3492073583333	11.1057130583333	12.394774825	11.556290575	11.9554346166667	11.7016218	11.5121356333333	11.123513725	11.5440185916667	11.2957967833333	11.8407167416667	11.06860385	11.7993113833333	11.4682426833333	11.74982705	11.6433290333333	11.10707375	11.339247	11.3654035916667	11.407940525	10.8836008416667	11.6694643083333	11.1477174666667	10.7736244333333	11.2602582666667	11.0153767166667	11.5790815416667	10.8006177916667	10.5976643083333	11.8553966916667	12.1410311166667	11.8954895166667	11.7164003333333	10.4153772	11.0772433166667	11.3605041083333	11.0112232166667	11.825765325	11.3033163083333	11.8285794166667	11.6477598666667	11.5472050833333	12.4447647416667	11.83328595	11.6783737	11.3157970833333	11.3886248333333	11.2356259583333	10.8487808833333	10.7816314666667	11.3033163083333	11.1473899333333	10.8067325916667	11.1173392166667	10.8878262416667	11.5895178083333	10.91088935	10.9000188916667	10.4372971833333	11.0390903416667	11.07367765	10.8416766333333	11.2975735583333	10.9941240666667	11.4481946583333

CHN1	12.336155525	9.88045179166667	10.3637863083333	9.33181315833333	9.93000265833333	11.0261256666667	9.929600025	10.2599956916667	10.134206575	10.5983973083333	9.97542684166667	11.824518825	11.4590118416667	10.216230425	9.721994775	10.35233035	9.72111820833333	9.41484764166667	10.29083355	10.5177006833333	10.7553698833333	9.39158905833333	10.432039025	9.7097404	9.98633354166667	11.6955902833333	10.1569732333333	9.75620250833333	7.17330060833333	9.82029285833333	10.2951456833333	10.0238668333333	10.308194675	10.3864170083333	12.219394525	12.4005974416667	10.1226723083333	9.98225808333333	10.15376285	10.7317611916667	10.222173225	9.79486831666667	10.2737682166667	10.4284899	10.98913965	10.9545739416667	10.36070035	10.0660963333333	10.3289721	10.04638645	10.2943474166667	9.81007735833333	9.96400001666667	10.0002116916667	10.0524736083333	10.4394323833333	10.430465125	10.2293589333333	10.26300475	10.23466	11.7278187083333	11.73400915	11.2858355	11.6970555916667	11.1074454416667	12.00595835	12.6572156166667	12.4211834833333	11.7081843583333	11.541832	11.50818875	11.2736929416667	11.8557655416667	12.846546475	11.9240142833333	13.14330865	11.7146176666667	12.033353625	12.247084775	12.1803292	11.24573245	11.5570626583333	12.2624244833333	13.591559975	13.2068344333333	12.5633422083333	10.28850615	11.5660654416667	11.047689175	12.2951630416667	12.6008947166667	11.9657351833333	11.464317475	12.5567153083333	11.4225410666667	11.3911680666667	12.18354635	12.9694275083333	12.4082756583333	11.6849152916667	12.4995800083333	11.73798855	11.6780189666667	10.9684950083333	11.5472050833333	12.2817357416667	12.630223175	12.0197364166667	12.0537518916667	12.6985834416667	12.136374875	11.1539711166667	12.0149070416667	12.2599545833333	12.1138378333333	10.9851253333333	11.6166645833333	12.333509375	10.9328378333333	11.7053666833333

SNRNP200	11.7046170083333	10.225450075	10.5902797083333	10.325378325	9.701538725	9.586175875	10.120107375	10.003538	10.364930325	9.7036749	9.62318166666667	10.93390845	10.58489685	10.185845775	9.89010159166667	10.2321384166667	10.4597952916667	9.61139459166667	10.1993549333333	10.2191020916667	10.0052447166667	9.96018530833333	9.91585179166667	10.4332127666667	10.0502702166667	11.4631308333333	10.2387377833333	10.0704383666667	7.03630040833333	10.1242565166667	10.3806930666667	10.4570074666667	10.1541580083333	9.990066925	11.60398845	11.4427661583333	9.63566801666667	9.94127946666667	9.81442805	9.876036325	10.356255775	10.2330333333333	10.384097475	10.3074544166667	10.6699485666667	10.24386405	10.4641916916667	9.89466155833333	9.929600025	9.98268700833333	10.043884875	10.0524736083333	10.0748410916667	9.93596328333333	9.71912328333333	10.81171625	10.1565645083333	9.98961361666667	9.96954501666667	10.31595855	9.37457540833333	9.856343425	8.94982423333333	8.94049991666667	10.1529084083333	9.437687475	9.848037125	9.28599946666667	8.64337428333333	9.081377025	9.36802801666667	9.02085209166667	10.018462175	9.22016708333333	9.95522605	9.67141531666667	9.35401866666667	9.09925841666667	9.59108196666667	8.89076064166667	11.7445138833333	9.38536081666667	9.44826335	9.61139459166667	9.237031475	9.72703330833333	11.0804603833333	9.332301675	5.82824421666667	9.586175875	9.705378475	10.406522025	10.0048138083333	9.116355225	9.07164405	8.84952995833333	8.63497865	9.87070264166667	8.37460103333333	9.70331208333333	9.89894956666667	9.21688079166667	9.78018595	9.96018530833333	9.85452464166667	9.13825335833333	10.007360325	8.97769849166667	8.88732965833333	9.04644768333333	9.46252099166667	9.40727955833333	9.448687325	10.7808534	7.83240795833333	9.92213260833333	10.4630196333333	10.0265057583333	9.87826785	10.067459125

COL22A1	9.42734543333333	6.063181275	6.13929583333333	6.1936491	6.65223385	7.158761525	6.95221913333333	6.06147365	5.53496716666667	5.3100359	5.31524535	7.224148725	5.26895569166667	5.550710675	5.28688518333333	7.578462725	6.99155748333333	6.05561763333333	6.39534953333333	7.74093078333333	6.4516666	7.70471373333333	7.07528308333333	6.8732984	7.08560825833333	8.9856801	5.70839040833333	6.37568504166667	5.78537223333333	5.75852464166667	7.09162461666667	4.92360515	6.09925825	7.63328161666667	9.00822095	7.62988515833333	6.27245344166667	6.90252933333333	6.0911181	7.8967227	6.258372575	5.30061454166667	6.47291681666667	6.47456359166667	7.81610706666667	7.08866475	6.92042344166667	5.52437941666667	7.666702425	7.43289285	7.09680668333333	5.303590975	7.7548948	6.81774981666667	6.459913	7.36819641666667	6.89596523333333	6.67633144166667	7.13850381666667	6.37491049166667	11.6948221166667	8.28798768333333	7.80873386666667	9.34207146666667	10.9969001083333	8.45509195	13.2939983416667	9.43636521666667	10.233460875	10.103459075	7.40602305833333	8.41537984166667	9.52042825833333	10.137056175	6.99601435833333	13.1707341833333	7.00877021666667	10.6083941916667	9.6592899	9.81648044166667	12.3921887583333	9.106662475	8.085928525	11.97471495	11.4688855083333	12.05687395	9.79446515833333	11.4391211083333	4.84935865	10.8254925166667	9.921332825	9.26488315	7.299574675	10.3913250333333	9.23835931666667	9.138674475	10.447247375	12.0555750333333	10.0700289916667	14.7316646166667	7.84051964166667	8.33810663333333	11.376411175	8.95172195833333	8.759783975	7.97768158333333	11.8920055333333	9.04644768333333	10.3790883333333	10.6691322083333	11.3441400333333	7.72023940833333	9.99851741666667	8.793933	9.5928155	12.1915147666667	12.3492861833333	9.57692496666667	7.45098580833333	7.7528692

ADH7	7.08805295	15.1234964083333	14.3943592583333	14.8653905916667	14.5449579333333	11.8241137083333	14.4177528166667	14.4443539833333	15.00595375	14.8136769166667	13.8992947833333	6.85418244166667	13.3875128	15.229747525	14.894107525	14.1698024416667	13.56859275	14.5865975583333	14.7731878916667	14.6819042	14.5793502666667	15.365952025	15.1264373	14.5469723583333	14.8699486666667	9.490132625	14.7686158333333	14.80004595	14.512293975	15.2014589916667	14.7572900333333	15.06705565	14.2426505583333	11.0571359666667	4.432045325	7.46352631666667	14.5793502666667	15.0335623833333	13.977355925	14.3482967833333	14.9702238166667	14.6651046083333	14.948326475	14.8313200916667	14.5717037666667	14.8629148	14.9422941416667	14.894107525	15.0865438916667	14.6778149	14.4244522916667	13.7692653916667	14.8162748	15.156314825	15.0615765666667	14.3628995916667	15.00595375	15.1336404666667	14.2969035	14.6883693	13.3501502333333	13.7721764083333	13.7084619583333	12.4645960083333	6.71918628333333	7.86670446666667	13.8428912916667	13.4065910333333	7.83944996666667	11.8453541	13.9045177166667	14.4908464833333	5.01953916666667	13.0864977	12.2281085833333	13.4179054	12.7884927666667	13.50161185	13.4254222333333	13.7253859666667	6.78279817916667	6.40053460833333	11.5432980083333	12.4154660666667	10.9972633583333	9.59692015833333	7.53185565833333	8.39919656666667	13.8461367666667	11.3709225333333	9.83584716666667	11.8228949916667	13.886670625	12.3331331166667	7.08293825833333	14.4581253166667	13.4345239916667	9.58131358333333	10.146512775	9.94127946666667	8.77579595833333	14.0174773166667	10.0799055916667	12.540087475	11.159679825	12.5268295333333	11.705716875	10.4558195166667	8.60799398333333	9.62193513333333	10.6686985416667	14.3316746833333	11.7085670166667	9.67274871666667	11.8182827333333	14.5865975583333	7.33375654166667	10.7027965333333	14.4346519833333	8.65698328333333

PLAAT5	12.3187215583333	10.0087152083333	10.0533049083333	9.36892748333333	9.953234775	10.023053775	10.2974881833333	10.135861875	9.93835718333333	10.5334113083333	9.38275609166667	12.190259475	11.1963735833333	10.2379766333333	9.67592103333333	10.3388359916667	12.1342141916667	9.93476125833333	9.911603525	9.77392395833333	10.1059188	9.09553066666667	9.63714265	9.861824025	10.337717825	12.5372066583333	10.6625963333333	9.6433763	9.364389775	9.84676184166667	9.68558669166667	10.0848379583333	10.1903114083333	9.34481250833333	12.1406572	12.622549625	9.826059425	9.630635475	9.88298375833333	9.808442425	10.2670402	10.253110825	10.225450075	9.7997523	11.092079925	10.137471025	10.4264895833333	9.750737375	9.70236100833333	9.8795664	9.27853935	9.20872513333333	10.3124336666667	9.79016168333333	10.0712503	10.70466055	10.55775005	9.497335375	10.0807009083333	10.4771670916667	8.15812938333333	9.3558778	8.14137294166667	10.5185399	8.94285730833333	8.56272661666667	8.8912541	11.4978397166667	8.34422473333333	8.93806988333333	9.23605358333333	8.234668275	9.26488315	8.80870043333333	8.5232739	8.17720695	11.0531461166667	8.35736911666667	8.55997810833333	8.31814086666667	9.75239246666667	8.83493181666667	8.13040576666667	7.95962858333333	8.736017975	9.428146125	9.17190161666667	9.71356900833333	9.04937824166667	9.71612960833333	8.66247435	8.08035505	9.02175539166667	10.1787390666667	8.8451569	9.006017175	9.84631699166667	9.76954021666667	9.14279978333333	9.91970055833333	8.73019295833333	9.03327476666667	7.87659344166667	8.33066458333333	8.27228149166667	9.77613621666667	8.73988864166667	7.61760009166667	8.50654274166667	8.306964525	12.5116969583333	7.659482025	7.20568426666667	7.49826404166667	8.771928675	8.16735940833333	8.97266575	8.15303908333333	7.96073305833333	9.83719281666667

MIA3	11.7561182166667	10.8807458916667	11.0160885	11.9550582083333	11.1025248083333	12.3426613166667	11.0483413166667	11.7667849666667	11.9975093	11.0957876916667	12.3114382583333	11.62415315	11.1847643166667	11.1321175166667	11.0701408916667	11.6238039583333	13.0560858	11.2826013166667	11.0470160416667	11.3078719166667	11.03193485	11.7255170416667	11.233092075	12.3422979833333	12.1690316666667	11.6429698666667	10.983890675	11.0772433166667	11.0513895	11.0585423	11.1517300916667	11.1554142916667	11.70420215	12.5202342666667	11.5027335583333	11.6841362916667	10.9737165166667	11.0793725416667	11.328911575	11.364979325	11.425117975	11.0772433166667	11.2517728166667	11.1498219083333	11.66717835	10.9139727166667	11.1699387666667	11.2891541	10.7112321916667	12.5376322583333	11.173905775	11.028339325	10.9861853	10.8326576083333	11.0380667083333	11.0146426833333	11.0171321	11.1259488	11.0116340666667	11.43611165	11.8364243666667	11.2675581916667	11.2018586833333	11.3455869583333	12.4181461583333	11.5980356083333	11.6632602416667	11.586171175	11.2911160333333	11.7738991416667	11.642205825	11.4140863666667	10.8506520083333	11.6791509083333	11.6198462166667	11.8051550333333	11.655146	11.5632145833333	11.7695141	11.637472	12.1367818666667	12.2433310083333	10.8934071416667	11.3805222416667	10.9651507916667	11.7731681916667	11.3096843916667	11.0491091083333	12.6909651833333	11.5555906416667	11.2826013166667	10.73913815	11.3886248333333	11.1908643083333	11.2664173916667	11.36200305	12.0413582166667	11.7243383416667	11.31617205	11.7074261416667	11.0905957416667	10.90507365	11.7168402	11.297940475	10.8206218083333	11.4049590333333	11.3371736333333	10.92486485	11.0504310083333	11.3455869583333	11.0851990416667	10.978750575	10.6838734833333	11.1663193416667	11.2349074416667	10.5424705	11.7391389833333	11.2514092416667	10.98913965	10.9013899916667

ZKSCAN7	8.770520625	7.63604310833333	7.838427925	8.30538075	8.06551123333333	8.02070828333333	8.4773978	7.85689433333333	7.87098755833333	8.06500796666667	7.967285625	8.854456025	7.82746845	7.61811995833333	7.69605179166667	8.35024235	8.65843663333333	7.873129975	7.33912221666667	7.98243114166667	8.19700369166667	8.36907771666667	8.295765375	8.123259225	8.09998893333333	8.99535305	8.41299383333333	8.43393876666667	7.57050700833333	8.488485525	7.321455625	7.87098755833333	8.35832260833333	8.35736911666667	8.41878698333333	9.02222115833333	7.93069544166667	7.87717198333333	7.3817504	8.030568675	8.00412230833333	7.708204175	8.111985825	8.00581294166667	8.222543225	8.78991784166667	7.9422621	8.04704694166667	8.11000589166667	8.08807009166667	7.53185565833333	7.68436408333333	7.90678598333333	8.06854219166667	8.23080535	8.34021045	8.05761690833333	8.22397875	8.293030975	8.18936749166667	8.24602095	8.338570375	8.228841125	7.9877223	9.26988130833333	8.26207273333333	8.30804240833333	8.26207273333333	8.211993425	7.89028620833333	8.24557174166667	8.09590623333333	8.74322296666667	8.29403910833333	8.07889375	8.43849715833333	8.08240869166667	8.47046444166667	7.39564355833333	8.21097586666667	8.63773576666667	9.203321075	7.683805725	8.99943493333333	8.32603026666667	8.3715767	9.0791656	8.62986514166667	8.59640985	8.68130785	8.09998893333333	7.79194111666667	8.11825858333333	8.41299383333333	8.61138693333333	8.06500796666667	7.79527465	7.88343015833333	7.81088345	7.69968070833333	8.51713660833333	8.23613304166667	8.026220775	7.46243825	7.35173706666667	8.482136425	8.39869986666667	7.3019376	8.6716691	7.79694136666667	7.47258725833333	7.3817504	7.8449064	8.584858775	8.093315875	7.67926596666667	8.95895775	7.655449725	7.54566714166667	7.99891026666667

CDC14B	6.386982725	5.52437941666667	5.73405291666667	4.759183025	5.97184991666667	6.75238478333333	4.33328629166667	4.37710720833333	4.91325449166667	4.771681175	5.37452059166667	4.310185775	5.17265765	4.1683668	4.8912062	5.20550439166667	5.81403274166667	7.28012135	6.11703395833333	4.77272485	5.41246783333333	6.12691128333333	5.060028525	5.511928275	5.70153421666667	6.26211881666667	5.97783559166667	6.47718199166667	6.65156973333333	5.3960318	6.107924175	5.06113309166667	5.14292011666667	4.2860754	7.127807925	5.978682025	6.43572405833333	5.622261375	5.826473825	5.5614942	4.968508325	5.68689626666667	6.41504531666667	5.367183075	4.72414796666667	5.03010108333333	5.36511784166667	4.96401284166667	6.159395425	6.20408345833333	6.82038475833333	6.872774725	5.75400875	5.5697365	4.303353375	5.2815818	4.68262605833333	5.21920105	5.53996425	5.72573654166667	6.78642360833333	5.229288475	7.496531175	6.32751184166667	4.897128125	7.22598754166667	4.67471653333333	5.52123825	5.96077145833333	5.880482625	6.256698975	5.399029725	5.33867198333333	6.3687361	4.21395365833333	5.231579	5.931318725	6.70327563333333	5.00344013333333	4.9406758	5.93571900833333	6.2184742	6.19893275	4.72007973333333	5.49035004166667	5.47221455833333	3.790613425	4.52238	8.027292325	7.09043023333333	4.79906281666667	5.88760754166667	7.45786721666667	5.28578914166667	6.05392339166667	6.15274419166667	6.75712434166667	7.44607933333333	5.52920275833333	5.73091026666667	6.26064218333333	4.80316131666667	4.71445220833333	5.00344013333333	7.38984995833333	5.65344755	5.31614614166667	6.13511379166667	4.2422894	6.72055851666667	7.65225225	7.05399351666667	6.475087675	6.33187936666667	4.07245948333333	5.04338205833333	6.11703395833333	6.83400405	6.08509065	4.11685985

PHACTR1	12.5506564333333	12.109263175	12.2998821166667	12.4501055333333	13.0258011083333	12.479955875	13.1973842833333	12.0564210833333	12.6195975333333	12.4087159666667	12.5710716416667	13.1733038666667	12.8398240166667	12.000941175	12.303007625	12.064871125	11.85040895	12.769749725	12.2302891583333	11.9044218833333	12.141404575	12.278442975	11.876483925	12.0522158916667	12.2154467166667	12.242499175	12.18354635	12.2679354916667	12.5883581916667	12.164859775	12.100204125	12.1895397416667	12.7216091916667	12.5344122083333	12.4425276083333	12.3952132583333	12.7846359833333	12.0677960666667	12.3025527416667	12.3908133166667	11.8705505	12.1224925833333	11.80438315	11.5443713166667	11.9240142833333	11.437271475	11.2957967833333	11.6791509083333	11.6540451166667	12.140177075	12.0961761333333	12.3492861833333	12.2466185916667	12.388809075	12.1146065416667	11.5769076083333	11.8565277666667	11.713529275	12.1316746333333	11.6726997416667	13.0031865833333	12.587916575	12.4069098166667	12.6790614583333	12.6346883583333	12.6032318833333	12.5989743166667	12.76127775	12.5107176416667	12.5788865916667	12.5860556666667	12.5902222833333	12.6240804083333	12.4932078666667	12.7564076083333	12.3110445	12.9417394416667	12.6754546333333	12.0852755916667	12.5221926333333	12.913424625	12.8783966083333	12.3152059916667	12.017301325	12.6971152166667	12.8770846083333	13.149953225	12.7368908416667	12.7569178666667	12.2524478333333	12.3067398	12.627449175	12.7774843666667	12.584151375	13.118520075	12.6966269416667	12.9117472333333	13.1316052916667	12.625004	12.237914025	12.2293758416667	12.6739472	12.1861275083333	11.6640542416667	11.713529275	12.303007625	12.1312748	11.6691177916667	12.1852414666667	12.118235175	12.1741251583333	12.0230159333333	11.973231675	11.94052505	11.9971131583333	11.9754521583333	12.3271580833333	12.3083565083333	11.8249375	12.0116503666667

MAP1LC3C	7.93490190833333	7.66500309166667	7.29418750833333	7.3361665	7.7445795	6.84636305	8.41926360833333	6.98530065833333	7.44041060833333	6.975151325	7.193910325	7.94541711666667	7.1355085	6.946211725	6.76714710833333	7.38818769166667	7.64049128333333	6.86736839166667	7.28556258333333	7.001733525	7.51253281666667	7.171442675	7.85051325833333	8.50185603333333	7.74239094166667	7.90410825	7.158761525	7.77893200833333	6.78114418333333	7.50051165833333	7.32855059166667	7.30612164166667	7.45451809166667	7.9999538	8.11603985	8.07411434166667	6.72705409166667	7.415839475	7.171442675	7.47443041666667	7.638193025	7.671444425	8.01119698333333	7.76884748333333	7.826430025	7.966758925	7.94435703333333	7.74511231666667	7.246381175	7.94017044166667	7.55811896666667	7.12224311666667	7.49050329166667	7.20334935833333	7.51030740833333	8.04179383333333	6.79131116666667	7.707009225	7.6225677	7.22176664166667	6.32751184166667	7.47725403333333	7.20334935833333	7.38926633333333	8.11000589166667	6.74218866666667	8.16178343333333	7.17507495	7.46022033333333	6.84909505	8.07305778333333	7.24571956666667	7.393964525	7.134785875	6.93091866666667	7.27891691666667	6.65843384166667	7.62815665833333	7.64737070833333	7.171442675	7.10699465	7.492737425	7.68593156666667	7.58521366666667	7.53506526666667	7.00365334166667	7.34718618333333	7.28072111666667	7.246381175	7.69103884166667	7.44885958333333	7.44317530833333	6.94439705	7.57164108333333	7.07980634166667	7.31316353333333	7.21162745833333	7.26626465	7.35396276666667	7.47562453333333	7.78439114166667	7.29365533333333	7.72520474166667	7.65174983333333	7.482471025	7.48588683333333	7.83240795833333	7.78606559166667	7.25732849166667	6.845071175	7.366539375	7.57164108333333	6.93484450833333	7.821740775	7.85469968333333	8.20990576666667	7.32727508333333	7.9877223	7.89979373333333	7.38051639166667

SH3KBP1	13.0186633916667	11.9575097333333	12.9672147	12.4714104166667	12.0514729416667	13.5401929916667	12.3842104583333	12.809704275	12.3169057833333	13.0607838416667	12.8613107583333	13.461932225	13.1913123583333	12.506168425	12.1998168583333	14.0847383833333	13.4775815666667	11.9364860583333	12.34092395	13.0816325916667	12.9767731916667	11.9923578333333	12.8086026666667	12.713417125	12.8740952083333	12.908758175	12.9954911666667	12.3584446833333	12.7763784666667	12.1521118916667	12.531130425	13.1350482333333	13.3032240666667	14.3757792583333	12.929488325	13.113125125	12.9592202833333	12.648701825	12.8613107583333	13.1019491416667	12.7857802916667	12.7206576333333	12.5754789333333	12.8438653416667	13.781003275	13.812858	12.8197190166667	12.21725785	12.972189625	13.0761975166667	13.0901032416667	12.3930672583333	13.1674768	12.8555603666667	12.7364125916667	12.9179353	12.3589004083333	13.17270095	13.1622146833333	13.5157896583333	13.5824136833333	13.439923175	13.078126625	12.5272702916667	14.3676663333333	13.175966725	13.4520130916667	13.1350482333333	13.6107867916667	13.9749587833333	12.6632127083333	13.6657423	13.1046602416667	13.79195525	12.8735737166667	12.7483893416667	13.7149280416667	14.1357234916667	13.0877248833333	13.4775815666667	13.9682216833333	13.645262425	13.6328876166667	13.6062775083333	14.1164578666667	13.9433845083333	13.2933426583333	13.9635675833333	12.659113	13.5643847833333	13.05246665	12.938273025	12.539129975	12.8080609833333	13.886670625	13.0755644416667	13.2081167083333	13.7332085916667	13.5576132583333	12.9653724416667	13.3234625333333	12.5948964	14.1709219416667	12.8289948916667	12.2835716083333	12.9284297083333	13.1648189583333	14.0315629333333	14.1857941	13.6719295	12.7339168166667	12.7738537166667	12.9694275083333	12.8026986916667	13.4752691916667	13.047619075	14.6346112833333	12.4696924	12.7591596666667	13.3763691583333

SEMG1	5.766309475	6.70056215833333	6.95758655	7.35693584166667	7.363992875	6.56215660833333	7.90032115	7.27359608333333	6.74346625833333	6.77041611666667	6.96657695833333	8.11504903333333	8.33066458333333	7.075905	6.345260775	6.63179246666667	6.32106796666667	7.321455625	5.78817238333333	6.4095551	6.91766443333333	6.46640339166667	5.89108584166667	6.88386930833333	5.75004875833333	7.16422769166667	6.27955291666667	6.1536546	5.94277896666667	7.14399110833333	6.67856683333333	6.58499179166667	6.55877498333333	6.8687758	6.627072075	6.738299725	6.4413796	6.82820395	6.021143325	6.45250163333333	5.81120035833333	6.64088086666667	5.78909119166667	5.62140730833333	7.24703090833333	5.933890025	5.88853345	6.26064218333333	6.16763958333333	6.07673425	6.29969763333333	6.68595645833333	7.58187098333333	6.61484865833333	7.597705475	6.38846551666667	7.48011853333333	7.04568171666667	6.328333625	6.662431575	6.34225285833333	8.29147479166667	7.314288875	8.9544846	6.8399633	7.09933909166667	6.29443513333333	8.25344699166667	8.04498438333333	7.53449101666667	7.33095725	7.37059144166667	9.15991209166667	7.52107438333333	6.91699638333333	7.08170128333333	7.6071886	6.07768216666667	7.127807925	6.62436875833333	8.06759891666667	6.3378065	6.42485146666667	6.769663225	7.32611610833333	8.14660076666667	7.85689433333333	7.35751986666667	6.98530065833333	6.67919664166667	7.39745536666667	6.7401779	6.225930275	6.50142603333333	6.86262681666667	7.09933909166667	6.91766443333333	6.9563535	7.45098580833333	5.6241104	6.8444516	7.583460175	6.49010539166667	5.826473825	7.35119398333333	7.39164716666667	6.60603940833333	6.3094404	6.85814304166667	6.62636613333333	6.89068614166667	7.0168934	6.61689901666667	7.43130181666667	7.3032043	6.85878966666667	6.75170075833333	7.299574675	6.39918578333333	7.66718561666667

ADPRHL1	10.2962241416667	9.14377724166667	9.51408459166667	9.85548545	10.2547426166667	9.960644375	10.8865989333333	9.45323241666667	10.0247316333333	9.72286421666667	9.734421025	10.302373075	10.6391956666667	9.126505725	9.64871909166667	8.90389271666667	10.1042493666667	9.95852463333333	9.74996381666667	8.54218048333333	9.31227445	9.52223368333333	8.96932985833333	9.515921025	9.431383225	9.97000988333333	9.877817275	9.81985159166667	9.87739761666667	9.393424375	9.30818888333333	9.53312831666667	9.56314586666667	10.0189542666667	9.50956853333333	10.9665691416667	10.2539350666667	9.53496325833333	9.612775675	9.529086375	8.916017775	9.16588333333333	8.73639624166667	8.61480576666667	9.4697267	8.74620478333333	8.91506995	9.03641039166667	8.88546686666667	9.64610789166667	9.24845199166667	9.9441902	9.0354605	9.15259453333333	9.21494060833333	8.82825418333333	9.309548625	9.00330966666667	9.15692076666667	9.20693985	10.1783585083333	9.92453053333333	9.6496487	10.23466	9.08869563333333	9.85239641666667	11.4244147	10.2210310333333	9.63292783333333	9.650075475	9.99086305833333	9.99808105833333	10.239527275	9.28551639166667	10.0467969333333	9.32044345833333	9.84760208333333	9.37269289166667	9.50956853333333	9.35357573333333	9.1473568	9.7570522	9.47068484166667	9.87345979166667	9.98920446666667	9.490132625	9.48743423333333	10.022234175	9.7860847	8.875989475	9.62712280833333	9.98920446666667	9.46297216666667	9.39626775833333	10.373735575	10.2258230916667	10.640008475	9.1218467	10.2745932916667	9.99432204166667	9.04596135	9.71989020833333	9.44914495	8.682814325	9.61946140833333	9.09779791666667	9.13694515	9.14327603333333	9.10099604166667	8.96510433333333	9.87650806666667	9.38762769166667	8.91938296666667	8.821200975	9.07021043333333	9.23047039166667	9.1218467	9.081377025	9.59518965833333	9.16352800833333

PNISR	14.0288199166667	12.2185126	12.0889146833333	12.1198078916667	12.8438653416667	13.5007024833333	12.8086026666667	12.6800815583333	12.7628980166667	10.842072875	12.4723572833333	13.5832773166667	13.3928492083333	12.2492084583333	12.7400926583333	13.02514525	12.8667102583333	13.5583734416667	13.0889930583333	13.0835345416667	12.3492861833333	12.6136777666667	12.506168425	12.92082965	12.928953425	13.304029325	12.3219919916667	12.5907074583333	13.3278442333333	12.8240262666667	12.6278527916667	12.6621572833333	12.8895588333333	12.7526244083333	13.073805875	13.5907625666667	13.000133375	12.3883541166667	12.4181461583333	12.6245838166667	12.2085904583333	12.3138792416667	12.576483025	12.2848587416667	12.8823979	12.2124743416667	12.44001515	11.9975093	12.4078515833333	12.88540095	12.3606021	12.440827975	12.649121475	12.5690965416667	12.1525189	12.08124855	12.7001469666667	12.3776096916667	12.912870175	12.908165975	13.2480671333333	12.3254389	12.2615766416667	12.0785704333333	14.1857941	12.16243335	12.3501237916667	12.4709919583333	12.095758	9.85198440833333	12.237060875	12.4482599083333	12.0697739833333	12.6795486	12.5293458166667	11.7827675333333	13.1512038916667	13.3912051666667	11.7243383416667	12.551141325	13.9114695083333	13.7970969916667	12.2553940416667	12.1283236333333	12.8273007416667	13.0985928583333	13.279249925	12.1575174833333	12.5344122083333	12.4822872083333	12.2466185916667	12.1591768166667	12.7328907083333	12.3763083916667	12.7086380166667	11.8662764666667	12.6046256583333	13.0067659416667	12.1018588083333	12.6023058	12.068210875	12.0020893083333	12.7960649666667	11.5728018416667	11.2922383666667	12.7206576333333	12.4767941833333	11.8638644666667	12.79714555	12.3343950916667	12.7139152416667	11.6425911333333	12.0395951	11.0454889833333	11.56339385	11.717933225	12.6476358916667	12.0985319416667	12.34092395	12.5282693916667

MYH8	4.611084475	5.42554189166667	4.57030863333333	4.49712886666667	4.95961636666667	4.03660776666667	6.48645600833333	5.82168396666667	5.40201090833333	4.613234575	4.38635804166667	6.28265325	5.58597898333333	4.69325549166667	4.04503838333333	4.98651175	4.50112465833333	5.0486896	4.506429475	4.23901671666667	4.97386210833333	4.936774575	4.17039641666667	4.28696936666667	4.54020328333333	3.97505105	4.310185775	4.19810324166667	4.76661771666667	4.26028479166667	4.55863708333333	4.35998614166667	4.06079156666667	4.683662975	4.207987625	4.09903998333333	4.49797680833333	4.35186211666667	4.3987315	4.48330838333333	5.002397475	4.19529993333333	4.617309275	5.25133600833333	4.13540660833333	4.83989633333333	4.88811624166667	4.83765554166667	4.48925233333333	4.73832164166667	4.58336269166667	4.371797775	4.146963325	5.170393	4.26445661666667	4.613234575	4.13438028333333	4.669520575	4.16737369166667	4.093740425	4.31116040833333	5.482101875	4.55369980833333	4.602780325	4.47861730833333	4.128952175	4.87091625	6.44887243333333	4.31516280833333	5.44122935	4.90032935	4.79621500833333	6.80510874166667	4.58445505833333	4.832334525	4.89935284166667	3.92371073333333	3.84051730833333	4.73295375	4.138474425	5.20060255	4.45030740833333	4.759183025	5.231579	4.64847966666667	4.42666711666667	4.37388213333333	6.28336628333333	4.97386210833333	4.89027461666667	4.87091625	4.202033325	3.9601097	4.727091875	4.22505026666667	3.83492170833333	4.2379826	4.17373291666667	5.13122064166667	4.713205375	5.3439553	5.86087000833333	4.62587865833333	4.649483075	4.46516671666667	4.42980535	5.23781710833333	5.83017264166667	4.39258018333333	6.12452900833333	4.36388495833333	5.69872159166667	5.018425275	5.74506199166667	4.72324775	4.4047953	5.3100359	4.97490869166667	5.02158241666667	4.62587865833333

GRAMD2A	4.93436960833333	8.36334496666667	8.60701585833333	8.36187725833333	8.99720454166667	10.4112315166667	7.56894295833333	9.07252779166667	8.18891301666667	8.11000589166667	8.68729530833333	7.23553730833333	8.160175425	8.2874613	9.00137649166667	8.5874833	8.15563305	7.89243905	8.932734675	8.65947190833333	8.435679275	9.08493245833333	8.79040525833333	8.058553875	8.38072273333333	6.64893395	8.25546045	8.48060255833333	8.25398819166667	8.31719399166667	8.036488875	8.462988175	8.20882531666667	7.03569371666667	6.2066078	5.84048410833333	8.24557174166667	8.49593345833333	7.48300389166667	8.6223815	8.30746798333333	8.44239195833333	8.43143401666667	8.98096389166667	8.128317575	8.97168731666667	8.4841298	8.51764245	8.52241499166667	8.61979589166667	8.02242483333333	8.56322373333333	9.3017146	8.953569225	8.91889295833333	9.59742406666667	8.28678805833333	8.52481044166667	8.64839240833333	8.37653824166667	8.6396011	8.68729530833333	7.83618584166667	7.32855059166667	9.68863114166667	11.0160885	7.81344110833333	9.13323439166667	9.023211225	9.20652433333333	9.4134397	9.11993345	8.733549025	8.576058475	9.41668023333333	8.69127455833333	7.56602129166667	9.10194070833333	9.18167015	8.74370115833333	8.75882765	9.00735385	8.48515009166667	9.45898255	10.10216065	9.54225544166667	6.459913	8.425659175	9.53496325833333	9.30818888333333	10.6003674833333	10.2213761083333	10.831890425	10.5142106583333	9.99895085	10.2915805333333	8.58301454166667	9.23330131666667	8.20433166666667	8.17505753333333	10.1529084083333	9.17006050833333	10.485478825	9.20464029166667	8.83493181666667	9.73873335833333	10.83116835	9.95522605	10.0111258333333	8.69082025	7.31674675	10.007360325	10.9328378333333	9.33838469166667	8.40234485833333	8.162750025	9.47068484166667	9.15259453333333	9.116355225	10.4449298

SRSF11	14.2426505583333	14.0127035833333	13.9518039333333	14.1815548333333	14.2474901166667	15.0459774	13.9840948583333	14.0912434666667	13.9433845083333	13.9119804333333	14.2378149833333	14.0734299166667	14.419240975	13.8720329083333	14.18015695	14.3170032666667	14.0759487166667	14.4397477583333	14.3139109083333	14.1099143166667	13.7312525583333	14.2260493166667	14.4377971083333	14.5607495666667	14.2858034416667	13.9541211333333	14.1413614333333	14.3463851	14.6325901333333	14.0899292916667	14.3153773833333	14.120388025	13.8562499666667	14.046645825	13.7681999916667	14.3139109083333	14.1554067916667	13.7930399666667	14.2132241916667	13.6979142666667	13.7195569416667	14.3204037083333	13.7272276083333	14.0720390916667	14.1874223416667	14.0288199166667	13.8667592	14.0163605916667	13.9657662833333	14.30297575	13.8614602166667	14.3221229083333	14.3596884833333	14.5089658583333	14.3316746833333	13.6605641666667	13.6804011583333	13.776083375	14.0578490916667	13.9894560916667	14.9832780583333	14.4775829916667	14.77778785	14.2904660666667	14.9674626083333	14.2576986333333	14.3139109083333	14.8602831416667	14.6778149	14.6046129083333	14.3268677	14.4891443666667	14.4983330666667	14.80427055	14.9094697583333	14.6005448916667	14.5810673083333	14.820747325	14.4495058333333	14.87708635	15.2014589916667	15.00595375	14.5433520583333	14.512293975	14.7948348083333	15.1207533083333	14.80004595	14.6589883666667	14.6005448916667	14.7034991833333	14.4963541416667	14.6161567166667	14.989321275	14.7851708916667	14.9174232833333	14.6466555666667	14.9174232833333	15.0246483416667	14.87708635	15.3056912416667	14.9020399416667	14.244370025	14.7077525	14.4125419416667	14.7077525	15.06705565	14.818607725	14.7252309	14.5664298833333	14.4722450583333	14.9620915916667	14.596526825	14.789742575	14.5020438083333	14.5322324833333	14.8629148	14.9620915916667	14.912278625	14.70147985	14.65081365

ZBTB46	7.263472875	7.803872825	7.26626465	6.76378944166667	6.15183188333333	6.92374801666667	6.560129475	6.90781049166667	6.63942924166667	7.70760366666667	6.89266504166667	6.64764070833333	5.53793416666667	6.51310794166667	6.950931025	7.49332154166667	7.30431380833333	6.3109681	7.54405583333333	7.16971491666667	6.986635125	6.52272641666667	7.219834	8.31008573333333	7.40898444166667	8.278086225	7.28012135	7.25980210833333	7.55678278333333	7.27229930833333	7.79031855833333	7.22110665	7.51696985	7.58961838333333	7.7799909	7.72573829166667	7.67307831666667	7.63763161666667	7.99667675833333	7.57673284166667	8.20331464166667	7.717344425	7.64552340833333	7.73422946666667	7.87562194166667	8.36034350833333	7.73920148333333	8.08909971666667	7.9484088	8.24315470833333	7.63269815833333	7.40770269166667	6.68656881666667	7.30497195	7.76137635	8.03480338333333	7.404332775	7.73803474166667	6.84200789166667	7.90139170833333	8.16178343333333	7.833966975	8.03861421666667	7.11617649166667	7.86334303333333	7.18159225833333	7.582967825	7.227214725	7.65121998333333	7.45558410833333	6.68920345833333	6.86012671666667	6.25991309166667	7.50852245833333	6.52209205	6.94057503333333	7.33501970833333	7.064683475	7.551793125	7.54021029166667	7.23553730833333	6.47216713333333	7.21504029166667	8.59640985	7.27891691666667	8.435679275	7.37642496666667	6.67710233333333	7.72905521666667	8.03538219166667	7.41352716666667	8.9226878	7.28500960833333	7.08866475	7.36211884166667	7.452175025	7.571028	7.07182334166667	7.6071886	7.56940810833333	7.98973426666667	7.41061439166667	7.37707911666667	7.58800115	8.439937175	7.39042478333333	8.25593908333333	8.13362299166667	8.107904375	7.85104381666667	9.235078425	7.63269815833333	7.65774509166667	8.309637175	7.874246775	8.25398819166667	8.511336025	9.02085209166667	7.40026851666667	7.47496615833333

GTF2A2	13.0676082583333	12.8176696	12.7580394083333	12.8624048833333	13.2628733666667	13.66335695	13.0307376	13.1492966166667	13.1058939166667	13.2399456333333	13.1865714083333	12.8101998166667	13.216246175	13.0337121333333	12.970460175	12.799384025	13.2495669	12.8708264916667	12.8493401833333	12.98478995	13.021150225	13.1959888083333	12.9785766	13.0108192083333	12.9773913166667	13.001932175	12.8246255416667	12.7201162666667	12.6862224333333	12.9024490333333	12.8161131416667	12.9709933083333	12.8213874666667	12.8522002333333	13.251022725	13.1973842833333	12.8618738916667	12.8829745166667	12.8171563916667	12.60273605	13.0536119916667	12.8889747333333	12.8381552416667	12.6895936166667	12.7873683333333	12.8107678333333	13.0313582416667	13.2339414916667	13.1197255166667	13.054278175	13.03309195	12.786823125	12.7195939833333	12.9224811	12.6739472	12.5268295333333	12.9166928166667	13.24529235	12.7836657416667	12.909394625	12.9144941416667	13.3936063	13.1986854833333	12.6517095833333	13.5372319083333	13.2835400666667	13.3538490666667	13.208876725	13.159645225	12.7006258583333	13.3262903916667	12.8041062916667	13.144627125	13.4321406333333	12.9099783416667	13.0096050666667	13.021150225	13.5076042	13.62548145	13.5083984166667	13.2215442333333	12.6166077833333	13.2266532083333	13.3771159333333	12.895307525	13.0374763416667	13.1249649416667	12.6517095833333	13.0294955083333	13.18580525	13.3410732083333	12.518275925	13.3193049583333	13.3010495416667	12.1583760416667	12.8532684916667	12.5818258666667	12.8392807333333	12.8595398916667	13.4298474333333	12.4763630083333	13.2181367583333	13.6434906916667	13.4649543916667	13.817872525	13.2828821583333	13.4209498916667	12.7195939833333	13.3151588	13.004442975	13.216246175	13.4737954	12.8702352416667	12.40597585	12.50892495	13.174009975	13.4407112833333	12.9521681583333	13.2155282	13.0337121333333

ATG7	9.61553475	10.25189245	9.99343915	9.6496487	10.1214563916667	10.1940041	9.48078894166667	10.5252205583333	9.94886909166667	10.23098885	10.191495075	9.8813009	9.686488825	10.334173575	10.402059075	10.1713085416667	10.255903875	10.164580475	10.20309965	10.1393440416667	10.4249193833333	10.7951957583333	10.2690944333333	10.534129425	9.9420301	10.068396225	10.1783585083333	10.1678889166667	10.469517225	10.30399485	10.1713085416667	10.191495075	9.94294464166667	10.3897471916667	10.0889846333333	9.79235871666667	10.3794789916667	10.625983	10.2707893833333	10.390544	10.2132508333333	10.1312217666667	9.98596199166667	10.4801853	9.722436925	10.36339005	9.67141531666667	10.0848379583333	10.8006177916667	10.1878607	10.040796475	10.1130642083333	9.05868610833333	9.68695163333333	9.27768001666667	9.58240151666667	10.406522025	10.3718117166667	10.3207495083333	10.04638645	9.86598649166667	9.65670091666667	10.0670141416667	9.547789	9.486953025	9.95725063333333	9.72574979166667	9.607505	10.74363815	10.0082740166667	9.64871909166667	9.73744423333333	9.90177325833333	10.1853907583333	10.1080216833333	10.6092076916667	10.3566062583333	9.90608706666667	10.8424532083333	10.2301923	9.45374055	9.86062085833333	10.227074775	10.1787390666667	10.151687475	9.79856730833333	9.176064475	9.37126875	8.662974625	10.3495871666667	9.96151823333333	9.90816476666667	9.67096889166667	10.2458406333333	9.40906840833333	9.80755264166667	10.1529084083333	9.54682303333333	9.77930279166667	9.79486831666667	10.2724454333333	9.62234490833333	9.83719281666667	10.5365078	10.059122075	10.46219945	10.1156550583333	9.91503965833333	10.6686985416667	10.7452538416667	9.97912404166667	10.028074675	9.107046275	10.4332127666667	9.64690625833333	9.37777211666667	9.65101713333333	10.2387377833333	10.31088965	10.4775633333333

TMEM183A	11.5961336833333	10.82998625	10.7265037833333	10.7948521166667	11.0905957416667	10.8273805416667	10.920072175	10.931065425	11.1821084666667	10.8870209166667	11.1912222083333	10.8109394916667	10.74285715	11.0394398083333	10.98475965	11.0068392583333	11.3583216333333	10.8807458916667	10.7272324083333	10.7538304083333	10.8431589583333	11.025429175	10.5605583916667	10.6666288666667	10.7855003666667	11.6234420916667	10.9574882	10.9618387333333	10.9371792916667	11.1018151	10.7092769166667	11.1373020916667	10.842072875	10.988072975	11.806635025	11.7384146416667	10.9909672	10.8733577583333	10.6237069166667	11.0787308166667	10.9750833833333	10.6934456416667	10.7816314666667	11.0548999583333	10.8465109833333	10.88817915	10.9136098583333	11.0589538416667	10.8394387833333	10.982052575	10.8960985416667	10.9673192583333	11.0279781	11.054552625	10.9466012416667	10.8202415916667	10.805973475	10.97984555	10.8424532083333	10.678502425	11.2082269	10.9317956333333	10.4971305166667	10.6664473083333	11.7035007416667	10.4664769916667	10.5108422833333	10.8681221916667	10.5895974833333	10.9574882	10.8131866083333	10.7612989333333	10.7851401916667	10.7217139416667	10.8368196833333	10.5833260083333	10.76921485	10.7463771	10.4340765083333	10.5616263	11.1787139083333	11.216912225	10.3948990166667	10.78588925	10.9147091916667	11.3108007583333	10.9501588	10.3487595333333	11.2047015416667	10.7665838833333	11.1214159583333	10.8427945083333	10.9501588	10.6233516833333	10.406884125	10.7519521333333	10.8473279166667	10.8781733666667	10.7940186083333	11.2230202916667	11.0211329583333	10.7004993416667	11.1685570666667	11.2583720833333	10.5639796666667	10.5256389583333	10.6816359083333	10.579886175	10.2912155166667	10.8984556583333	11.0608057166667	11.1815244666667	10.8258465666667	10.5162662666667	10.5065079166667	10.6256227583333	11.54260245	10.585362525	10.924157925	10.8612470416667
[truncated: 33,604,256 more chars]
